# MUC20 regulated by extrachromosomal circular DNA attenuates proteasome inhibitor resistance of multiple myeloma by modulating cuproptosis

**DOI:** 10.1186/s13046-024-02972-6

**Published:** 2024-03-05

**Authors:** Xiaobin Wang, Yingqing Shi, Hua Shi, Xiaoyu Liu, Aijun Liao, Zhuogang Liu, Robert Z. Orlowski, Rui Zhang, Huihan Wang

**Affiliations:** 1 Department of Hematology, Shengjing Hospital, China Medical University, Shenyang, China; 2 Center for Reproductive Medicine, Shengjing Hospital, China Medical University, Shenyang, China; 3 Department of Hematology, Daping Hospital, Chongqing, China; 4 Shenshan Medical Center, Memorial Hospital of Sun Yat-Sen University, Shanwei, China; 5 Departments of Lymphoma/Myeloma, The University of Texas MD Anderson Cancer Center, Houston, TX USA; 6 Department of Hematology, The First Affiliated Hospital, China Medical University, Shenyang, China

**Keywords:** Multiple myeloma, Proteasome inhibitor, Resistance, MUC20, Cuproptosis, EccDNA

## Abstract

**Background:**

Proteasome inhibitors (PIs) are one of the most important classes of drugs for the treatment of multiple myeloma (MM). However, almost all patients with MM develop PI resistance, resulting in therapeutic failure. Therefore, the mechanisms underlying PI resistance in MM require further investigation.

**Methods:**

We used several MM cell lines to establish PI-resistant MM cell lines. We performed RNA microarray and EccDNA-seq in MM cell lines and collected human primary MM samples to explore gene profiles. We evaluated the effect of MUC20 on cuproptosis of PI-resistant MM cells using Co-immunoprecipitation (Co-IP), Seahorse bioenergetic profiling and in vivo assay.

**Results:**

This study revealed that the downregulation of Mucin 20 (MUC20) could predict PI sensitivity and outcomes in MM patients. Besides, MUC20 attenuated PI resistance in MM cells by inducing cuproptosis via the inhibition of cyclin-dependent kinase inhibitor 2 A expression (CDKN2A), which was achieved by hindering MET proto-oncogene, receptor tyrosine kinase (MET) activation. Moreover, MUC20 suppressed MET activation by repressing insulin-like growth factor receptor-1 (IGF-1R) lactylation in PI-resistant MM cells. This study is the first to perform extrachromosomal circular DNA (eccDNA) sequencing for MM, and it revealed that eccDNA induced PI resistance by amplifying kinesin family member 3 C (KIF3C) to reduce MUC20 expression in MM.

**Conclusion:**

Our findings indicated that MUC20 regulated by eccDNA alleviates PI resistance of MM by modulating cuproptosis, which would provide novel strategies for the treatment of PI-resistant MM.

**Supplementary Information:**

The online version contains supplementary material available at 10.1186/s13046-024-02972-6.

## Background

Multiple myeloma (MM) is the second most common hematological malignancy and accounts for 10% of all hematologic malignancies [1, 2]. It is characterized by the infiltration of clonal plasma cells that produce monoclonal immunoglobulin into the bone marrow [3]. Significant therapeutic advances over the last two decades have improved MM patient outcomes. Proteasome inhibitors (PIs) are among the most efficient classes of drugs for the treatment of MM [4–6]. However, almost all patients with MM develop PI resistance, which results in therapeutic failure [7, 8]. Therefore, the mechanisms that induces PI resistance in MM must be further investigated to provide novel strategies for the treatment of PI-resistant MM.

Mucin 20 (MUC20) has been reported to play a role in cancer drug resistance, and MUC20 variant 2 has been shown to aggravate chemoresistance to cisplatin and paclitaxel in gastric cancer [9]. In contrast, our previous study revealed that MUC20 attenuates PI (carfilzomib, CFZ) resistance by regulating proteasome capacity via the MET proto-oncogene receptor, receptor tyrosine kinase (MET) pathway in mantle cell lymphoma [10]. Nonetheless, the effect of MUC20 on PI resistance in MM has not been elucidated.

Cuproptosis is a novel form of programmed cell death (PCD) that occurs by the direct binding of copper to lipoylated components involved in the tricarboxylic acid cycle, which results in the aggregation of lipoylated proteins and loss of iron-sulfur cluster proteins to induce proteotoxic stress and ultimately cell death [11]. A recent study indicated that the induction of cuproptosis by copper-organic complex-based nanosystems could reverse cisplatin chemotherapy resistance in non-small cell lung cancer (NSCLC) [12]. Moreover, cuproptosis can overcome chemotherapeutic resistance in prostate cancer by hindering autophagy [13]. However, the role of cuproptosis in PI resistance in MM remains largely unknown.

Cyclin-dependent kinase inhibitor 2 A (CDKN2A), a cuproptosis-related gene, suppresses cuproptosis of M2 macrophages [11, 14]. Bioinformatic studies have suggested that CDKN2A may be associated with drug resistance in MM [15–17]. However, the regulatory effects of CDKN2A on cuproptosis and the effects of MUC20 on CDKN2A in PI-resistant MM remain unclear.

Extrachromosomal circular DNA (eccDNA) or extrachromosomal DNA (ecDNA) are prevalent in various cancers and facilitate the high expression of oncogenes [18, 19]. Additionally, eccDNAs contribute to drug resistance in cancer [20, 21]. For example, eccDNA is involved in cisplatin resistance in hypopharyngeal squamous cell carcinoma by amplifying related genes [22]. Moreover, extrachromosomal mutant epidermal growth factor receptor (EGFR) DNA contributes to EGFR-tyrosine kinase inhibitor (TKI) resistance in glioblastoma [23]. However, the role of eccDNAs in PI resistance in MM has not been thoroughly studied.

Therefore, the primary aim of this study was to identify the roles of the encoding gene MUC20 in linear chromosomal DNA and eccDNA in the PI resistance of MM and investigate whether MUC20 and eccDNA regulate the PI resistance of MM by modulating cuproptosis via CDKN2A.

## Methods

### Cell culture

Drug-naïve OPM-2, ANBL-6, KAS-6/1, and U266 cells (PS) were purchased from the Cell Bank of the Chinese Academy of Sciences (Shanghai, China). PI-resistant OPM-2, ANBL-6, KAS-6/1, and U266 cells (PR) were generated as previously described [10]. Briefly, OPM-2, ANBL-6, KAS-6/1, and U266 cells were treated with CFZ (#S2853. Selleck Chemical, Houston, TX, USA) at 10% of the drug minimum inhibitory concentration (IC_10_) until they could be cultured in RPMI 1640 medium containing 20 nM CFZ. The cells were then routinely cultured in RPMI 1640 medium containing 10 nM CFZ. CFZ-resistant OPM-2, ANBL-6, KAS-6/1, and U266 cells were cultured in RPMI 1640 medium containing serial concentrations of BTZ. All in vitro experiments were performed after the exposure of PI-resistant cells to PI had stopped for at least 3 days. Moreover, all cultured cell lines were used within three months of resuscitation without microbial contamination.

### Cell counting Kit-8 (CCK-8) assay

CCK-8 assay was performed to detect the viability and proliferation of MM cells. First, 1 × 10^4^ MM cells were seeded per well in a 96-well plate in triplicate at each time point. Then 10 µL CCK-8 solution (#C0037, Beyotime Biotechnology, Shanghai, China) at a 1/10 dilution was added in each well to incubate MM cells for 2 h at 37 °C, and absorbance at 450 nm was assayed by Multiscan MK3 (Thermo Fisher Scientific, Waltham, MA, USA) to identify cell viability or proliferation rate. Cell proliferation was measured after 1–3 days, while cell viability was determined in 24 h after PI treatment, as previously described [24].

### Soft agar colony formation assay

Soft agar colony formation assay was performed using the soft agar colony formation assay kit (#CBA-150, Cell Biolabs, San Diego, CA, USA) as previously described [25].

### RNA microarray analysis

Total RNA was extracted from KAS-6/1 and U266 cells and sent to Aksomics (Shanghai, China) for RNA microarray analysis using the ArrayStar Gene Expression Array (Human, 4 × 44 K, Rockville, MD, USA). Image processing, data extraction, and analysis were performed according to the established protocols of ArrayStar. Subsequently, statistically significant differentially expressed RNAs were determined by fold-change filtering, and distinguishable RNA expression patterns between the two groups were presented using hierarchical clustering.

### Quantitative reverse transcription-PCR (qRT-PCR)

Total RNA from OPM-2, ANBL-6, KAS-6/1, and U266 cells was extracted using TRIzol reagent (#15,596,026; Invitrogen, Carlsbad, CA, USA). First-strand cDNA was prepared using the PrimeScript II 1st Strand cDNA Synthesis Kit (#6210A, Takara Biotechnology, Daliang, Liaoning, China). Next, qRT-PCR was performed with TB Green® Premix Ex Taq (Tli RNaseH Plus) (#RR420A, Takara Biotechnology). Then, the results of the target RNA were normalized to that of the internal control (GAPDH) and presented as 2^−△△Ct^ values relative to the control sample.

### Western blot (WB)

Total proteins were extracted from OPM-2, ANBL-6, KAS-6/1, and U266 cells using RIPA buffer (#9806, Cell Signaling Technology, Danvers, MA, USA). Equal amounts of protein were loaded and separated by SDS-polyacrylamide gel electrophoresis (SDS-PAGE) and then transferred onto a polyvinylidene fluoride (PVDF) membrane (#IPVH00010, Millipore, Billerica, MA, USA). Subsequently, the membranes were blocked with 5% non-fat milk for 1 h at room temperature (RT, 25℃) and then incubated with the primary antibody overnight at 4 °C. The following day, the membranes were washed with Tris-buffered saline containing 0.1% Tween 20 (TBST) and incubated with the corresponding secondary antibody at RT for 1 h. Finally, signals of the target proteins were detected using a Chemiluminescence Detection Kit (#P0018F, Beyotime Biotechnology). The primary antibodies used in this study included MUC20 antibody (1:300, #13380-1-AP, Proteintech, Rosemont, IL, USA), CDKN2A antibody (1:500, #ab270058, Abcam, Cambridge, UK), Met antibody (1:500, #ab216330, Abcam), Met (phospho Y1234 + Y1235) antibody (1:500, #ab278552, Abcam), CDKN2A (phospho Ser326) antibody (1:300, #GTX32378, GeneTex, Hsinchu City, Taiwan, China), ferredoxin 1 (FDX1) antibody (1:1000, #12592-1-AP, Proteintech), lipoyl synthase (LIAS) antibody (1:1000, #11577-1-AP, Proteintech), dihydrolipoamide S-acetyltransferase (DLAT) antibody (1:2000, #13426-1-AP, Proteintech), Metal response element binding transcription factor 1 (MTF1) antibody (1:500, #25383-1-AP, Proteintech), insulin-like growth factor receptor-1 (IGF-1R) antibody (1:1000, #ab182408, Abcam), His antibody (1:1000, #66005-1-Ig, Proteintech), kinesin family member 3 C (KIF3C) antibody (1:500, #ab236748, Abcam), and GAPDH (1:10,000, #KC-5G5, Aksomicks, Shanghai, China).

### Data collection

The MUC20 expression profiles of CD138 + bone marrow plasma cells in HDs and patients with PI-sensitive MM and PI-resistant MM were collected from the Gene Expression Omnibus (GEO) database (GSE5900, GSE16791, GSE31504, and GSE57317). To analyze the association between MUC20 expression and PI sensitivity, clinical data of 264 patients with MM were collected from the Millennium Pharmaceuticals GEP database and divided into high and low MUC20 expression groups. Moreover, clinical data of patients with MM who received dexamethasone treatment were collected from the Millennium Pharmaceuticals GEP database to validate whether the association between MUC20 expression and PI sensitivity and the outcome of MM was specific. In addition, MUC20 expression profiles of CD138 + bone marrow plasma cells in 538 patients with MM were collected from the GEO database (GSE4204) and divided into high and low MUC20 expression groups, followed by GSVA to identify differentially enriched pathways between the two groups.

### Collection of human primary MM samples

To further validate the MUC20 expression profile in patients with MM, fresh bone marrow aspirates from HDs (*n* = 17) and patients with NDMM (*n* = 53), PI-sensitive MM (tMM) receiving initial therapy with BTZ (*n* = 52), and RRMM receiving BTZ treatment (*n* = 40) were routinely collected from the Hematology Department of Shengjing Hospital. In addition, fresh bone marrow aspirates from 43 patients with NDMM and 61 patients with RRMM were collected at the hematology department of Shengjing Hospital to further confirm the association between MUC20 expression and PI sensitivity and the association between MUC20 expression with outcome of patients with MM were also collected at the Hematology Department of Shengjing Hospital. All human experimental procedures were approved by the Ethics Committee of Shengjing Hospital, China Medical University. Written informed consent was obtained from all participants recruited for the study. The MM cells were isolated using CD138 MicroBeads (Human) (#130-097-614, Miltenyi Biotec, Bergisch Gladbach, Germany).

### Flow cytometry analysis

To determine the MUC20 levels in CD138 + bone marrow plasma cells, bone marrow plasma cells were obtained using Ficoll-Paque gradient separation and then directly labeled with an CD138 APC-conjugated antibody (1:50, #FAB2780A, Bio-techne, Minneapolis, MN, USA) and an antibody against surface MUC20 (1:50, #PA5-14973, Thermo Fisher Scientific) followed by a PE-conjugated secondary antibody (1:100, #SA00008-2, Proteintech) for flow cytometry sorting. For detection of PCD in KAS-6/1 and U266 cells, cell suspensions were incubated with 10 µL Annexin V-FITC (#KGA106, Keygen, Nanjing, Jiangsu, China) and moderate propidium iodide (PI) (#P3566, Invitrogen, Carlsbad, CA, USA) at RT for 15 min. The cells were then analyzed using flow cytometry (FACSARIA, BD Biosciences, San Jose, CA, USA) according to standard protocols.

### Lentiviral transduction

Lentiviral constructs (pLVX-Puro (#QYV0008) for overexpression and pLKO.1-Puro (#QYV0024) for shRNA) were purchased from Qualityard Biotechnology (Beijing, China) and used for plasmid construction. They were co-transfected with pSPAX2 (packaging plasmid, #QYV0052, Qualityard Biotechnology) and pMD2.G (envelope plasmid, #QYV0053, Qualityard Biotechnology) into 293FT cells. Subsequently, viral supernatants were collected at 48 h post transfection and agitated at 4 °C overnight. The next day, viral particles were concentrated and resuspended in phosphate buffered saline (PBS). Then, KAS-6/1 and U266 cells were incubated with viral particles in the presence of 10 µg/mL polybrene (#H8761, Solarbio, Beijing, China) and then spin-infected for 1 h at 34 °C (900 g), followed by culturing with fresh RPMI 1640 medium for 48 h. Next, KAS-6/1 and U266 cells were selected with 2 µg/mL puromycin (#P8230, Solarbio) overnight.

### Cell treatments

To identify the effect of cuproptosis on KAS-6/1 and U266 cells, the cells were treated with 10, 50, and 100 nM TTM (#E1166, Selleck) or 50, 100, and 150 nM elesclomol (#S1052, Selleck). KAS-6/1 and U266 cells were treated with phosphatase inhibitor cocktail I (#HY-K0021, MedChemExpress, Monmouth Junction, NJ, USA) to determine the effect of phosphorylation on CDKN2A protein levels. KAS-6/1 and U266 cells were treated with sodium lactate (#HY-B2227B, MedChemExpress) to determine the effects of lactylation on IGF-1R levels. Additionally, 20 µg eccDNA from PI-resistant KAS-6/1 and U266 cells were transfected into PI-sensitive KAS-6/1 and U266 cells. Transfection was performed using Lipofectamine 2000 (#11,668,019, Thermo Fisher Scientific).

### Co-immunoprecipitation (Co-IP)

KAS-6/1 and U266 cells were lysed in a non-denaturing lysis buffer, and then the supernatant of the cell lysate was precleaned by protein A/G magnetic beads (Thermo Fisher Scientific) for 2 h at 4℃. Subsequently, approximately 300 µg of protein sample was incubated with 1 µg of MET antibody (#ab216330, Abcam), His antibody (#66005-1-Ig, Proteintech), or L-lactyl lysine antibody (#PTM-1401RM, PTM-BIO, Hangzhou, Zhejiang, China) and 30 µL of protein A/G magnetic beads overnight at 4 °C. The following day, the protein A/G magnetic beads were collected using a magnetic separation device (Thermo Fisher Scientific), and the precipitated complexes were cleaned. Finally, the bound proteins were analyzed by WB. Rabbit or Mouse IgG served as the negative controls.

### Construction of his-tagged expression vector

First, the wild-type (WT) MET open reading frame (ORF) was cloned into a His-tagged pcDNA3.2 expression vector (WT-MET). Then, codons for Y1234 and Y1235 were mutated into those for Phe (F) using a point mutation to generate the Y1234/Y1235 mutated His-tagged expression vector (MUT-MET).

### Seahorse bioenergetic profiling

OXPHOS and glycolysis in KAS-6/1 and U266 cells, which were treated as indicated, were detected using the Seahorse XF Cell Mito Stress Test Kit (#103015-100, Agilent, California, CA, USA) and Seahorse XF Cell Glycolysis Kit (#103020-100, Agilent), respectively, on a Seahorse XFe96 Bioanalyzer (Agilent) according to the manufacturer’s instructions [26]. Before detection, 25,000 KAS-6/1 and U266 cells/well were plated in Matrigel-coated 96-well Seahorse XF cell culture microplates.

### Detection of lactate level by enzyme-linked immunosorbent assay (ELISA)

The lactate level of KAS-6/1 and U266 cells was measured by ELISA using an EnzyChrom L-Lactate Assay Kit (#ECLC-100) obtained from BioAssay Systems (Hayward, CA, USA) according to the manufacturer’s protocol. Finally, the absorbance at 530 nm was immediately assayed using a Multiscan MK3 system (Thermo Fisher Scientific).

### In vivo assay

Animal protocols were approved by the Institutional Animal Care and Use Committee of Shengjing Hospital, China Medical University. Six-week-old male nude BALB/c mice were raised in a pathogen-free environment. PI-resistant KAS-6/1 and U266 cells were infected with lentiviruses containing the MUC20 expression vector (MUC20 OE), IGF-1R expression vector (IGF-1R OE), and CDKN2A expression vector (CDKN2A OE), whereas PI-sensitive KAS-6/1 and U266 cells were transfected with 20 µg eccDNA from PI-resistant MM cells. Xenograft tumor models were established by subcutaneously injecting 2 × 10^6^ KAS-6/1 or U266 cells into the right dorsal flank of nude mice. After tumor engraftment, nude mice were randomly assigned to receive CFZ (2 mg/kg) or a combination of CFZ and TTM/elesclomol (25 mg/kg) via intravenous injection twice a week. Treatments were performed for a week. The size of the xenograft tumors was measured every five days. At day 25 post-subcutaneous injection, the mice were sacrificed and the xenograft tumors were harvested to measure their size and weight.

### EccDNA-seq

Genomic DNA was isolated from KAS-6/1 and U266 cells using the QIAamp DNA Mini Kit (#51,304, Qiagen, Hilden, Germany). Each DNA sample was digested with PacI restriction enzyme to linearize the mitochondrial DNA and then treated with Plasmid Safe ATP-dependent DNase (#E3101K, NovoBiotec, Beijing, China) at 37 °C for 16 h to remove linear DNA. Subsequently, the samples were incubated at 70 °C for 30 min to inactivate the reaction. The DNA samples were cleaned using phenol/chloroform/isoamyl alcohol (PCI) solution (25:24:1) extraction and ethanol precipitation. Next, rolling circle amplification (RCA) was performed with phi29 DNA polymerase (#P7020-HC-L, Qiagen) in a 20 µL reaction volume at 30 °C for 14 h, followed by inactivation at 65 °C for 10 min. Subsequently, the amplified eccDNA was sheared to an average fragment size of 400 bp. The eccDNA-seq libraries were prepared using a NEBNext Ultra II DNA Library Prep Kit for Illumina (#E7645S, NEB, Ipswich, MA, USA). The prepared libraries were diluted to a final concentration of 8 pM and clustered on a flow cell with an Illumina cBot system using the NovaSeq 6000 S4 Reagent Kit (300 cycles) (#20,028,312, San Diego, CA, USA). Sequencing was performed on an Illumina NovaSeq 6000 using a NovaSeq 6000 S4 Reagent Kit (300 cycles) according to the manufacturer’s protocol.

### EccDNA-seq data analysis

The QC-filtered raw reads were trimmed and aligned to the human genome (UCSC HG38) using BWA software (V0.7.17). The alignment results were used for eccDNA calling using Circle-Map V1.1.4. Then, statistically significant eccDNA regions were identified at a “Split reads” threshold of 2. The eccDNAs were annotated with their overlapping cargo genes using the GENCODE (V42) database. GO and pathway enrichment analyses were also performed for the differentially expressed eccDNA-related genes.

### Statistical analyses

Statistical analyses were performed using SPSS software (version 21.0; IBM Corp., Armonk, NY, USA). All statistical tests were two-sided, and the standard deviation (SD) of the mean is represented as error bars. Comparisons of continuous variables between two groups were performed using the Mann-Whitney U test and Student’s *t* test, while statistical differences among three or more groups were examined using the Kruskal-Wallis test. In addition, multivariate logistic regression analysis was performed based on the progressive disease (PD) and MUC20 expression in patients with MM. Kaplan-Meier survival curves and log-rank tests were performed to determine the differences in PFS/OS between the two groups. Statistical significance was set at *P* < 0.05. significant.

## Results

### MUC20 is downregulated in PI-resistant MM cell lines and patients

First, PI-resistant MM lines were generated by continuous treatment with PI. Cell Counting Kit-8 (CCK-8) results showed that continuous treatment of PI-sensitive MM cell lines (PS) (OPM-2, ANBL-6, KAS-6/1, and U266) with CFZ induced resistance in these cell lines (Supplementary Figure S1A). Moreover, PI-resistant MM cell lines (PR) showed resistance to another PI, bortezomib (BTZ) (Supplementary Figure S1B), suggesting that the mechanisms underlying CFZ and BTZ resistance are identical.

**Fig. 1:**
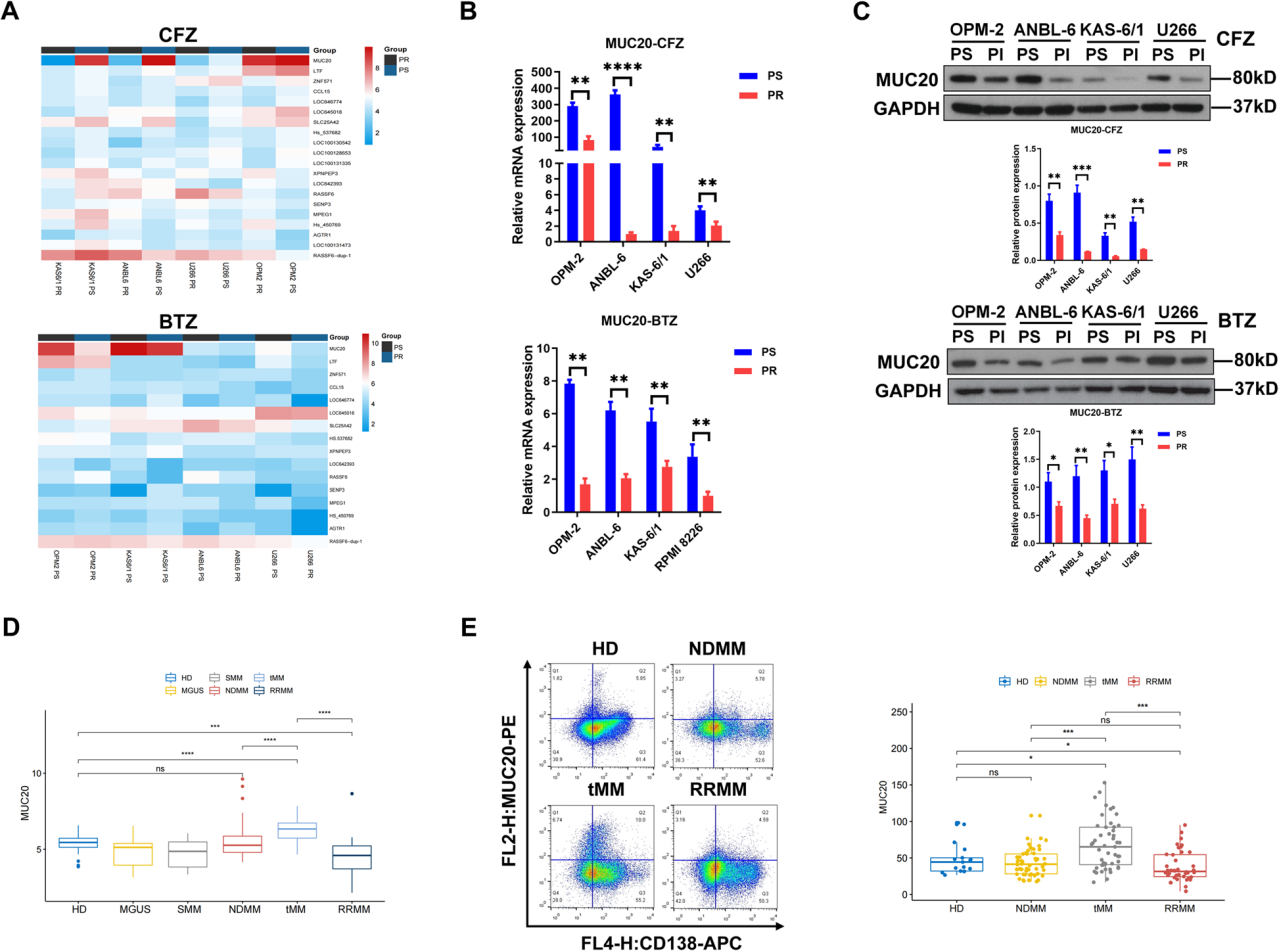
MUC20 is downregulated in proteasome inhibitor-resistant MM cell lines and patients. **A** Heatmap displaying differently expressed RNAs between PI-resistant MM cell lines treated with CFZ or BTZ and PI-sensitive MM cell lines. **B** MUC20 mRNA level detected in PI-resistant MM cell lines treated with CFZ or BTZ and PI-sensitive MM cell lines. **C** MUC20 protein level detected in PI-resistant MM cell lines treated with CFZ or BTZ and PI-sensitive MM cell lines. **D** MUC20 expression profiles of CD138 + bone marrow plasma cells in HDs and patients with MM collected from GEO. **E** Representative images of flow cytometric analysis for MUC20 and MUC20 expression profiles of CD138 + bone marrow plasma cells in HDs and patients with MM collected from GEO. PS: proteasome inhibitor-sensitive MM cells; PR: proteasome inhibitor-resistant MM cells; CFZ: carfilzomib; BTZ: bortezomib; HD: healthy donors; MGUS: monoclonal gammopathy of undetermined significance MM patients; SMM: smoldering MM patients; NDMM: newly diagnosed MM patients; tMM: PI-sensitive MM patients; VTD: thalidomide and dexamethasone; RRMM: relapsed/refractory MM patients. *P < 0.05, **P < 0.01, ***P < 0.001, ****P < 0.0001

To investigate the mechanisms regulating PI resistance in MM, an RNA microarray was performed to explore PI resistance-related genes. MUC20 mRNA levels were reduced in PI-resistant MM cell lines (PR) treated with CFZ or BTZ compared to those in PI-sensitive MM cell lines (PS) (Fig. 1A). QRT-PCR validated the data obtained from the RNA microarray (Fig. 1B). Moreover, WB revealed that MUC20 protein levels were decreased in PI-resistant MM cell lines treated with CFZ or BTZ compared to those in PI-sensitive MM cell lines (Fig. 1C). Thus, these results indicate that MUC20 is downregulated in PI-resistant MM cell lines.

In addition, MUC20 expression profiles of CD138 + bone marrow plasma cells from patients with MM were obtained from the GEO database. Analysis of the GEO data demonstrated that MUC20 expression in CD138 + bone marrow plasma cells was consistent among healthy donors (HDs) (GSE5900) and patients with monoclonal gammopathy of undetermined significance (MGUS) MM (GSE5900), smoldering MM (SMM) (GSE5900), and newly diagnosed MM (NDMM) (GSE16791) (Fig. 1D). In addition, MUC20 expression in the CD138 + bone marrow plasma cells of patients with PI-sensitive MM (tMM) receiving initial therapy with BTZ, thalidomide, and dexamethasone (VTD) (GSE31504) was higher than that in HDs and patients with MGUS MM, SMM, and NDMM (Fig. 1D). In contrast, MUC20 expression in CD138 + bone marrow plasma cells of patients with relapsed/refractory MM (RRMM) (GSE57317) was lower than that in HDs and patients with MGUS MM, SMM, and NDMM (Fig. 1D).

To further validate the MUC20 expression profile in patients with MM, we collected CD138 + bone marrow plasma cells from HDs (*n* = 17) and patients with NDMM (*n* = 53), tMM receiving initial therapy with BTZ (*n* = 52), and RRMM receiving BTZ treatment (*n* = 40) who were admitted to our hospital. The clinicopathological features of the HDs and patients with MM are listed in Supplementary Table S1. The MUC20 levels were determined using flow cytometry. The results showed that MUC20 levels in CD138 + bone marrow plasma cells in the tMM group were higher than those in the HD and NDMM groups, whereas the MUC20 levels in CD138 + bone marrow plasma cells in the RRMM group were lower than those in the HD, tMM, and NDMM groups (Fig. 1E). Taken together, these data suggest that MUC20 is downregulated in PI-resistant MM cell lines and patients.

### MUC20 predicts proteasome inhibitor sensitivity and outcomes of patients with MM

**Fig. 2:**
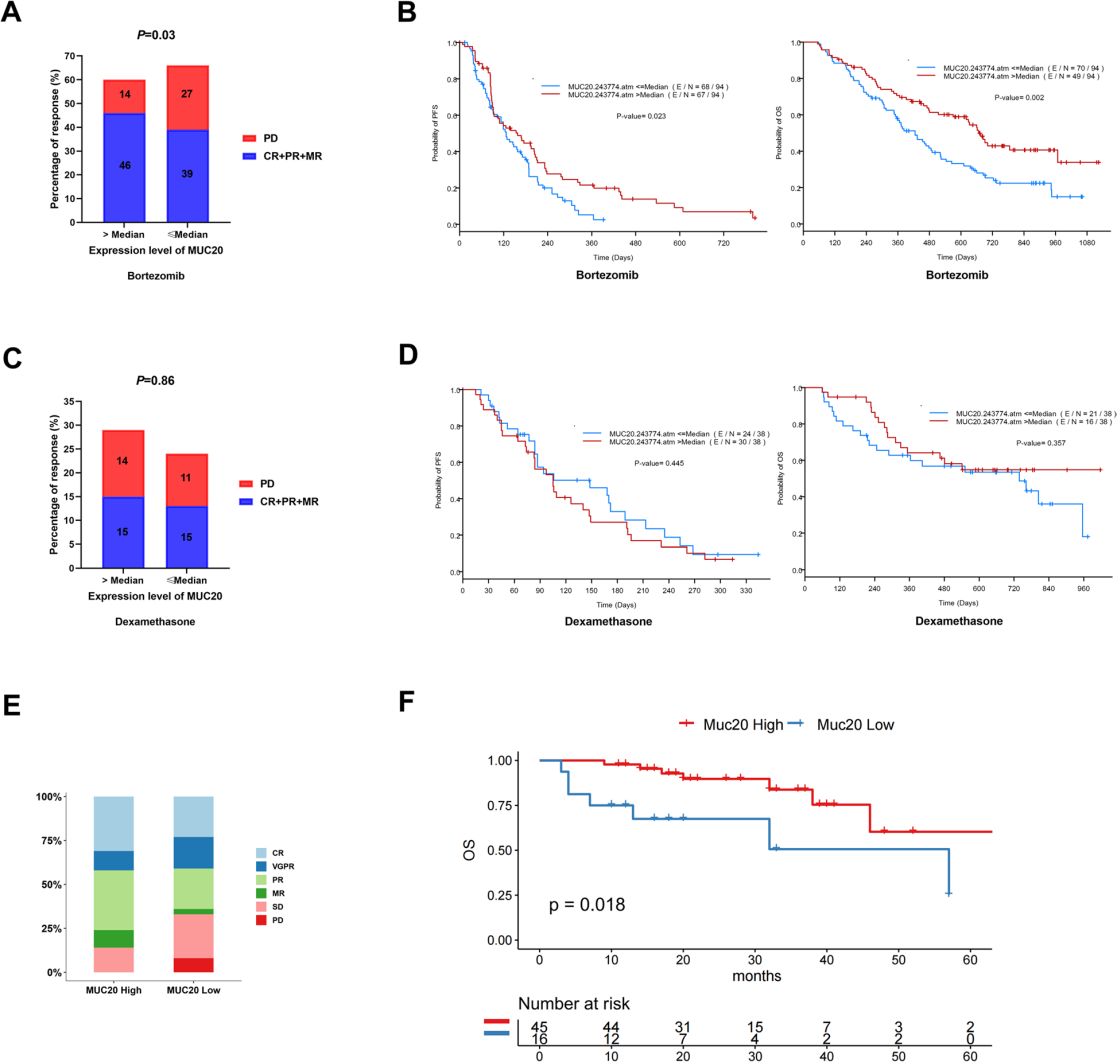
MUC20 levels are predictive of proteasome inhibitor sensitivity and outcome of MM patients. **A **Analysis of the ORR and PD in patients with MM with high and low MUC20 expression after BTZ treatment. **B** Kaplan–Meier survival analysis of PFS and OS in patients with MM with high and low MUC20 expression. **C** Analysis of ORR and PD in patients with MM with high and low MUC20 expression after dexamethasone treatment. **D** Kaplan–Meier survival analysis of PFS and OS in patients with MM high and low MUC20 expression after dexamethasone treatment. **E** Analysis of the ORR and PD in patients with NDMM with high and low MUC20 expression after BTZ treatment. **F** Kaplan–Meier survival analysis of the PFS and OS in patients with NDMM with high and low MUC20 expression after BTZ treatment. BTZ: bortezomib; ORR: overall response rate; PD: progressive disease; PFS: progression free survival; OS: overall survival

To analyze the association between MUC20 expression and PI sensitivity, clinical data of 264 patients with MM were collected from the Millennium Pharmaceuticals GEP database and divided into high and low MUC20 expression groups. The analysis indicated that PI (BZF) treatment led to a better overall response rate (ORR) (minor response (MR) + partial response (PR) + complete response (CR)) of 46% in the MUC20 group compared with that of 39% in the MUC20 group (Fig. 2A). The analysis also revealed that low expression of MUC20 was associated with PD (*P* = 0.03) (Fig. 2A). Kaplan–Meier survival analysis revealed that patients with MM with high MUC20 expression had longer median progression-free survival (PFS) and overall survival (OS) than those with low MUC20 expression after PI treatment (Fig. 2B).

To validate whether the association between MUC20 expression and PI sensitivity and the outcome of patients with MM was specific, clinical data of patients with MM receiving dexamethasone treatment were collected from the Millennium Pharmaceuticals GEP database and divided into high and low MUC20 expression groups. However, there was no difference in ORR (*P* = 0.86), PFS, or OS between the two groups after dexamethasone treatment (Fig. 2C and D). Therefore, these data suggest that MUC20 may be a biomarker for predicting PI sensitivity and outcomes in patients with MM.

We recruited 43 patients with NDMM admitted to our hospital to further confirm the association between MUC20 expression and PI sensitivity. The clinicopathological features of the patients are listed in Supplementary Table S2. Patients with NDMM were divided into high and low MUC20 expression groups. The analysis showed that PI treatment resulted in an ORR of 86% with 10% MR, 34% PR, 11% very good partial response (VGPR), and 31% CR with high expression in the MUC20 group, whereas it produced an ORR of 67% with 3% MR, 23% PR, 18% VGPR, and 23% CR in the low MUC20 expression group. These findings indicate that PI treatment resulted in a better ORR in patients with MM with high MUC20 expression (*P* < 0.05) (Fig. 2E).

Moreover, 61 patients with RRMM admitted to our hospital were enrolled for analysis, and the association between MUC20 expression and the outcome of patients with MM was analyzed. The clinicopathological features of the patients are listed in Supplementary Table S2. Patients with RRMM were divided into high MUC20 expression and low MUC20 expression groups. The results revealed that the OS of high MUC20 expression group was 22 months longer than that of low MUC20 expression group (*P* = 0.018) (Fig. 2F). Thus, these data revealed that MUC20 positively predicts PI sensitivity and outcomes in patients with MM.

### MUC20 attenuates proteasome inhibitor resistance of MM cells by inducing cuproptosis

The above studies indicate that MUC20 is positively associated with PI sensitivity in patients with MM. Besides, PI (10 nM CFZ) treatment increased MUC20 level in PI-sensitive KAS-6/1 and U266 cells but not in PI-resistant KAS-6/1 and U266 cells (Supplementary Figure S2). Thus, the effect of MUC20 on PI resistance in MM was determined.

**Fig. 3:**
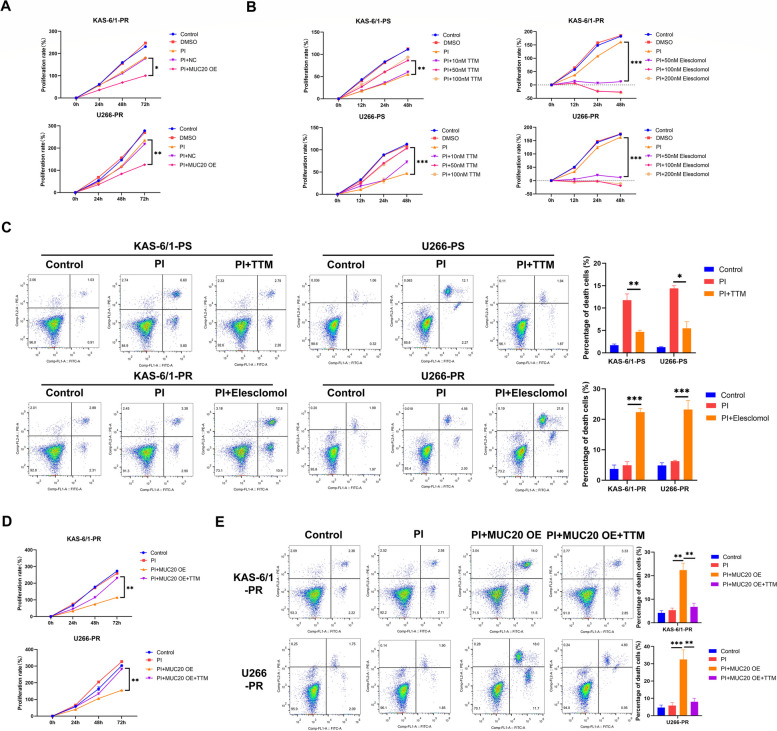
MUC20 attenuates proteasome inhibitor resistance of MM cells by inducing cuproptosis. **A** Proliferation rates of control vector and MUC20 OE-transfected PI-resistant KAS-6/1 and U266 cells treated with or without PI (CFZ). **B** Proliferation rates of PI-sensitive KAS-6/1 and U266 cells treated with or without TTM and PI-resistant KAS-6/1 and U266 cells treated with or without or elesclomol. **C** Representative images of the flow cytometric analysis for PCD in PI-sensitive KAS-6/1 and U266 cells treated with or without TTM and PI-resistant KAS-6/1 and U266 cells treated with or without or elesclomol. **D** Proliferation rates of MUC20 OE-transfected PI-resistant KAS-6/1 and U266 cells treated with or without PI (CFZ) and TTM. **E** Representative images of flow cytometric analysis for PCD in MUC20 OE-transfected PI-resistant KAS-6/1 and U266 cells treated with or without PI (CFZ) and TTM. PS: proteasome inhibitor-sensitive MM cells; PR: proteasome inhibitor-resistant MM cells; OE: overexpression; PI: proteasome inhibitor; TTM: tetrathiomolybdate. *P < 0.05, **P < 0.01, ***P < 0.001

CCK-8 and soft agar colony formation assay results showed that PI (10 nM CFZ) treatment had no significant effect on PI-resistant KAS-6/1 and U266 cell proliferation (Fig. 3A and Supplementary Figure S3A). In addition, MUC20 overexpression via transfection with a lentiviral vector carrying *MUC20* (Supplementary Figure S4A) enhanced the inhibitory effect of PI treatment on PI-resistant KAS-6/1 and U266 cell proliferation (Fig. 3A), suggesting that MUC20 alleviates PI resistance in MM cells.

Cuproptosis has been reported to reverse cisplatin chemotherapy resistance in NSCLC [12]. Cuproptosis of MM cells was detected after PI treatment. The CCK-8 and soft agar colony formation assay results indicated that the PI treatment suppressed PI-sensitive KAS-6/1 and U266 cell proliferation while chelation of copper with tetrathiomolybdate (TTM) reversed the inhibitory effect of the PI treatment on PI-sensitive KAS-6/1 and U266 cell proliferation in a dose-dependent manner (Fig. 3B and Supplementary Figure S3B). These findings suggest that PI induces cuproptosis in MM cells. In addition, treatment with the copper ionophore elesclomol facilitated the inhibitory effect of PI on PI-resistant KAS-6/1 and U266 cell proliferation in a dose-dependent manner (Fig. 3B and Supplementary Figure S3B). This result indicates that cuproptosis might be hindered in PI-resistant MM cells. In addition, 50 nM TTM and 50 nM elesclomol were utilized to treat MM cells in subsequent experiments as 50 nM TTM could effectively reverse the inhibitory effect of the PI treatment on PI-sensitive MM cells while 50 nM elesclomol could effectively facilitate the inhibitory effect of PI on PI-resistant MM cells.

To further identify the effect of PI on the cuproptosis of MM cells, flow cytometry was performed to detect PCD in MM cells after PI treatment. The results demonstrated that PI treatment for 48 h induced PCD at early and late stages in PI-sensitive KAS-6/1 and U266 cells while chelation of copper with TTM for 48 h abolished the promotional effect of PI treatment on PCD at early and late stages in PI-sensitive KAS-6/1 and U266 cells (Fig. 3C). Moreover, PI treatment had no significant effect on PCD at early and late stages in PI-resistant KAS-6/1 and U266 cells; however, treatment with elesclomol and PI for 48 h triggered PCD at early and late stages in PI-resistant KAS-6/1 and U266 cells (Fig. 3C). These results further indicate that cuproptosis is suppressed in PI-resistant MM cells.

The role of MUC20 in cuproptosis of PI-resistant MM cells was determined. The CCK-8 assay and soft agar colony formation assay revealed that MUC20 overexpression strengthened the inhibitory effect of PI treatment on PI-resistant KAS-6/1 and U266 cell proliferation. However, chelation of copper with TTM neutralized the effect of MUC20 overexpression on PI-resistant KAS-6/1 and U266 cell proliferation (Fig. 3D and Supplementary Figure S3C). In addition, flow cytometric analysis indicated that MUC20 overexpression for 48 h enhanced the promotional effect of PI treatment on PCD at early and late stages of PI-resistant KAS-6/1 and U266 cells while the TTM treatment reversed the effect of MUC20 overexpression on PCD at early and late stages of PI-resistant KAS-6/1 and U266 cells (Fig. 3E). These results suggest that MUC20 ameliorates PI resistance in MM cells by inducing cuproptosis.

### MUC20 triggers cuproptosis by inhibiting CDKN2A in proteasome inhibitor-resistant MM cells

**Fig. 4:**
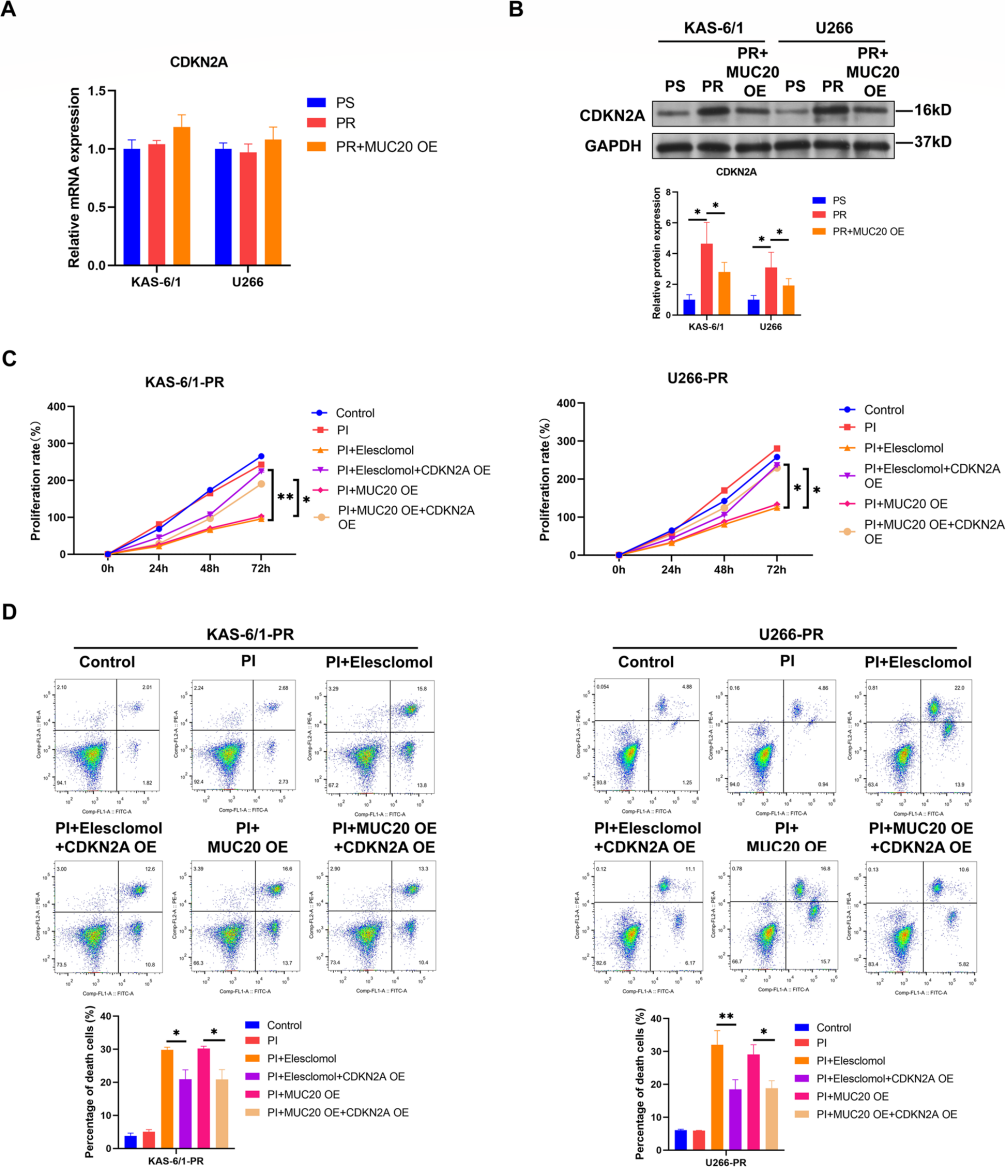
MUC20 induces cuproptosis through suppressing CDKN2A in proteasome inhibitor-resistant MM cells. **A** CDKN2A mRNA level in PI-sensitive, PI-resistant, and MUC20 OE-transfected PI-resistant KAS-6/1 and U266 cells. **B** CDKN2A protein level in PI-sensitive, PI-resistant, and MUC20 OE-transfected PI-resistant KAS-6/1 and U266 cells. **C** Proliferation rates of MUC20 OE or CDKN2A OE-transfected PI-resistant KAS-6/1 and U266 cells treated with or without PI (CFZ) and elesclomol. **D** Representative images of flow cytometric analysis for PCD in MUC20 OE or CDKN2A OE-transfected PI-resistant KAS-6/1 and U266 cells treated with or without PI (CFZ) and elesclomol. PS: proteasome inhibitor-sensitive MM cells; PR: proteasome inhibitor-resistant MM cells; OE: overexpression; PI: proteasome inhibitor. *P < 0.05, **P < 0.01

Next, the mechanism underlying the ability of MUC20 to induce cuproptosis in PI-resistant MM cells was investigated. CDKN2A has been reported to suppresses cuproptosis [11, 14]. In this study, qRT-PCR showed that CDKN2A mRNA levels in PI-resistant KAS-6/1 and U266 cells were consistent with those in PI-sensitive KAS-6/1 and U266 cells and that MUC20 overexpression had no effect on CDKN2A mRNA levels in PI-sensitive KAS-6/1 and U266 cells (Fig. 4A). In addition, WB analysis showed that CDKN2A protein levels in PI-resistant KAS-6/1 and U266 cells were higher than those in PI-sensitive KAS-6/1 and U266 cells and MUC20 overexpression reduced CDKN2A protein levels in PI-resistant KAS-6/1 and U266 cells (Fig. 4B). Besides, the levels of cuproptosis markers and downstream genes including FDX1, LIAS, DLAT, MTF1 were detected. It was found that the protein levels of FDX1, LIAS, DLAT except for MTF1 were also decreased in PI-resistant KAS-6/1 and U266 cells compared to those in PI-sensitive KAS-6/1 and U266 cells (Supplementary Figure S5A). More importantly, CDKN2A overexpression via transfection with a lentiviral vector carrying *CDKN2A* (Supplementary Figure S4B) decreased FDX1 protein levels in PI-resistant KAS-6/1 and U266 cells (Supplementary Figure S5B), suggesting that CDKN2A might suppress cuproptosis by downregulating FDX1 in PI-resistant MM cells.

Next, we investigated whether MUC20 induces cuproptosis in PI-resistant MM cells via CDKN2A. Both elesclomol treatment and MUC20 overexpression facilitated the inhibitory effect of PI on PI-resistant KAS-6/1 and U266 cell proliferation (Fig. 4C and Supplementary Figure S6) and the promotional effect of PI on the PCD of PI-resistant KAS-6/1 and U266 cells (Fig. 4D). However, CDKN2A overexpression for 48 h by transfection with lentiviral vectors carrying *CDKN2A* (Supplementary Figure S4B) reversed the effects of elesclomol treatment and MUC20 overexpression on the proliferation (Fig. 4C and Supplementary Figure S6) and PCD at early and late stages (Fig. 4D) of PI-resistant KAS-6/1 and U266 cells treated with PI. These results suggest that MUC20 induces cuproptosis by suppressing CDKN2A expression in PI-resistant MM cells.

### MUC20 reduces CDKN2A expression by hindering the activation of the MET proto-oncogene, receptor tyrosine kinase in proteasome inhibitor-resistant MM cells

**Fig. 5:**
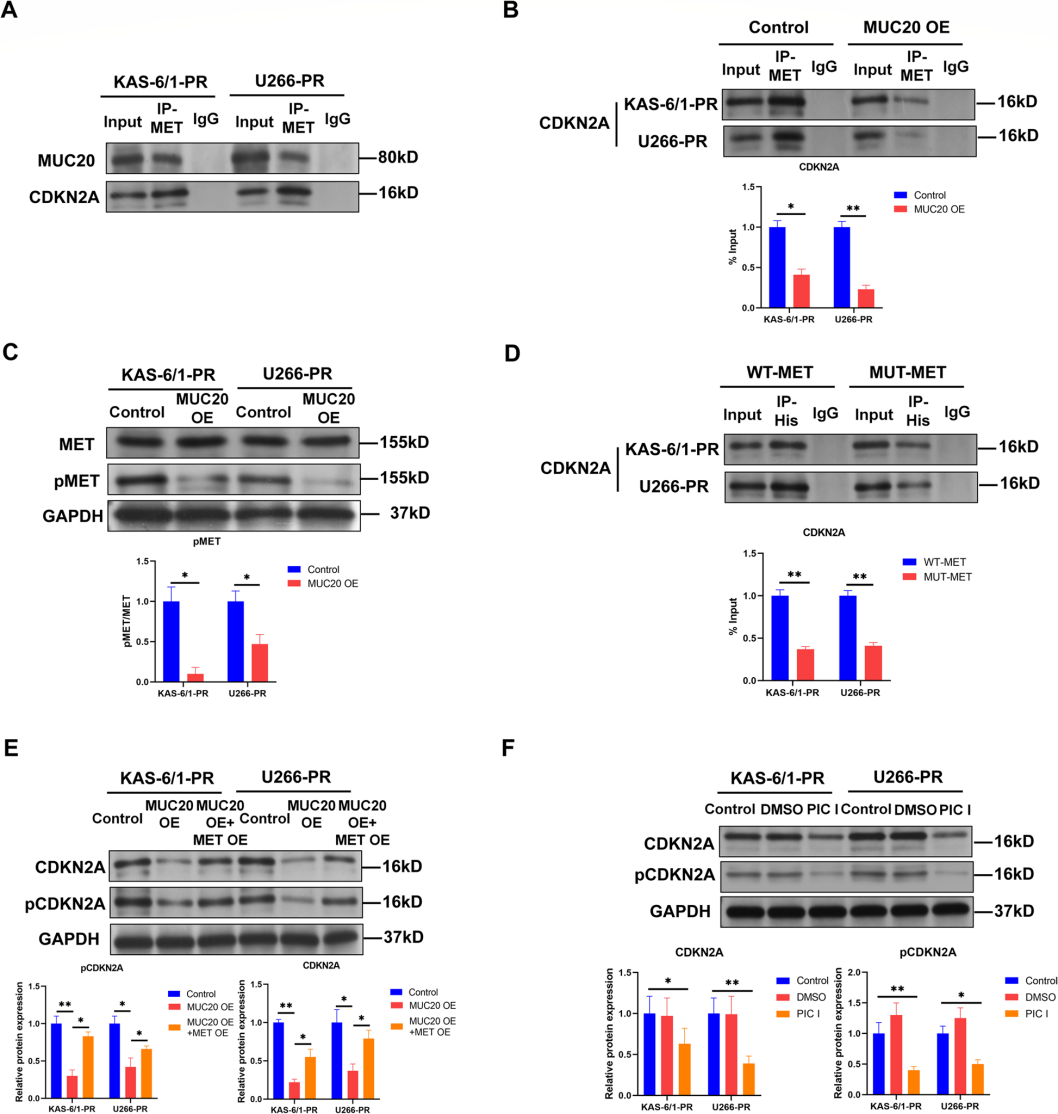
MUC20 increases CDKN2A expression through inhibiting MET activation in proteasome inhibitor-resistant MM cells. **A** Representative image of Co-IP using a MET antibody in PI-resistant KAS-6/1 and U266 cells. Rabbit IgG was used as negative control. **B** Representative image of Co-IP using a MET antibody in control or MUC20 OE-transfected PI-resistant KAS-6/1 and U266 cells. Rabbit IgG was used as negative control. **C** MET and pMET levels in control or MUC20 OE-transfected PI-resistant KAS-6/1 and U266 cells. **D** Representative image of Co-IP using a His antibody in WT-MET or MUT-MET-transfected PI-resistant KAS-6/1 and U266 cells. Rabbit IgG was used as negative control. **E** CDKN2A and pCDKN2A levels in control or MUC20 OE/CDKN2A OE-transfected PI-resistant KAS-6/1 and U266 cells. **F** CDKN2A and pCDKN2A levels in PI-resistant KAS-6/1 and U266 cells treated with or without phosphatase inhibitor cocktail I. PR: proteasome inhibitor-resistant MM cells; OE: overexpression; PIC I: phosphatase inhibitor cocktail I. *P < 0.05, **P < 0.01

Co-IP results showed that MUC20 could not bind with CDKN2A in PI-resistant KAS-6/1 and U266 cells (Supplementary Figure S7A and B), suggesting MUC20 should regulate CDKN2A expression by other proteins in MM cells. Previous studies have revealed that MUC20 associates with MET to regulate the phosphorylation and activation of MET downstream proteins [10, 27]. Co-IP results indicated that MUC20 was associated with MET in PI-resistant KAS-6/1 and U266 cells (Fig. 5A and Supplementary Figure S8A). In addition, CDKN2A binds with MET in PI-resistant KAS-6/1 and U266 cells (Fig. 5A). Moreover, MUC20 overexpression blocked the interaction between MET and CDKN2A in PI-resistant KAS-6/1 and U266 cells (Fig. 5B and Supplementary Figure S8B), indicating that MUC20 disrupted the interaction of MET and CDKN2A by binding with MET in PI-resistant MM cells.

Phosphorylation is critical for MET activation [28, 29], and decreased MUC20 expression is negatively associated with MET activation in PI-resistant mantle cell lymphoma [10]. Therefore, we evaluated the effect of MUC20 on MET activation in PI-resistant MM cells. The WB results demonstrated that MUC20 overexpression decreased phosphorylated MET (p-MET) levels in PI-resistant KAS-6/1 and U266 cells (Fig. 5C).

Next, the effect of phosphorylation on the interaction between MET and CDKN2A was determined, and His-tagged expression vectors containing the WT MET ORF or Y1234/Y1235-mutated (Y > F) MET ORF were constructed (Supplementary Figure S9). His-tag expression vectors were then transfected into PI-resistant KAS-6/1 and U266 cells, and Co-IP was performed using His antibody. The results revealed that mutations of phosphorylation sites (Y1234 and Y1235) disrupted the interaction between MET and CDKN2A in PI-resistant KAS-6/1 and U266 cells (Fig. 5D and Supplementary Figure S8C) but not affected MUC20 protein level (Supplementary Figure S10), suggesting that MUC20 should block the interaction between MET and CDKN2A by suppressing MET phosphorylation in PI-resistant MM cells.

In addition, MET can regulate downstream protein activation by modulating phosphorylation [30, 31], thus allowing for the determination of the effects of MUC20 and MET on CDKN2A phosphorylation. WB analysis indicated that MUC20 overexpression reduced phosphorylated CDKN2A levels in PI-resistant KAS-6/1 and U266 cells, whereas MET overexpression via transfection with a lentiviral vector carrying *MET* (Supplementary Figure S4C) abolished the effect of MUC20 overexpression on phosphorylated CDKN2A levels in PI-resistant KAS-6/1 and U266 cells (Fig. 5E). Besides, MUC20 overexpression decreased total CDKN2A levels in PI-resistant KAS-6/1 and U266 cells, whereas MET overexpression reversed the effect of MUC20 overexpression on total CDKN2A levels in PI-resistant KAS-6/1 and U266 cells (Fig. 5E). In addition, treatment with phosphatase inhibitor cocktail I significantly decreased CDKN2A and phosphorylated CDKN2A levels in PI-resistant KAS-6/1 and U266 cells (Fig. 5F), suggesting that phosphorylation increases CDKN2A expression in PI-resistant MM cells.

These data suggest that MUC20 decreases CDKN2A expression by inhibiting MET activation in PI-resistant MM cells.

### MUC20 suppresses MET activation by repressing insulin-like growth factor receptor-1 lactylation in proteasome inhibitor-resistant MM cells

**Fig. 6:**
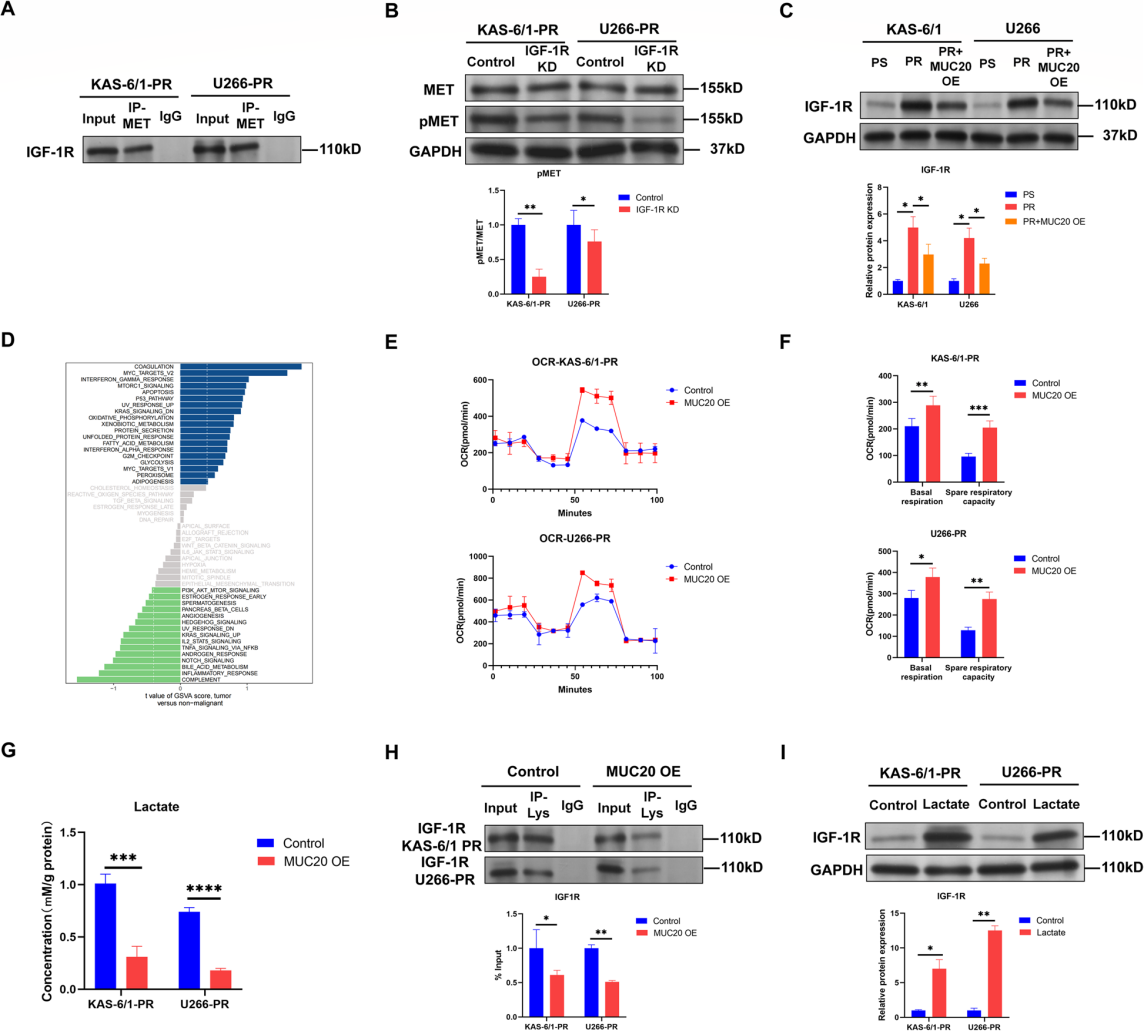
MUC20 hinders MET activation by repressing IGF-1R lactylation in proteasome inhibitor-resistant MM cells. **A** Representative image of Co-IP using a MET antibody in PI-resistant KAS-6/1 and U266 cells. Rabbit IgG was used as a negative control. **B** MET and pMET levels in control or IGF-1R KD-transfected PI-resistant KAS-6/1 and U266 cells. **C** IGF-1R level in PI-sensitive, PI-resistant and MUC20 OE-transfected PI-resistant KAS-6/1 and U266 cells. **D** Enriched pathways in patients with MM with high and low MUC20 expression. **E** OCR in control or MUC20 OE-transfected PI-resistant KAS-6/1 and U266 cells. **F** Basal respiration and spare respiratory capacity in control or MUC20 OE-transfected PI-resistant KAS-6/1 and U266 cells. **G** Lactate level in control or MUC20 OE-transfected PI-resistant KAS-6/1 and U266 cells. **H** Representative image of Co-IP using a L-lactyl lysine antibody in control or MUC20 OE-transfected PI-resistant KAS-6/1 and U266 cells. **I** IGF-1R level in PI-resistant KAS-6/1 and U266 cells treated with or without exogenous lactate (lactate sodium). PR: proteasome inhibitor-resistant MM cells; OE: overexpression; OCR: oxygen consumption rate. *P < 0.05, **P < 0.01, ***P < 0.001, ****P < 0.0001

IGF-1R has been reported to activate MET in prostate cancer cells [32]. IGF-1R is aberrantly overexpressed in MM with poor prognosis [33]. Therefore, we evaluated the effect of IGF-1R on MET activation in PI-resistant MM cells. Co-IP assays revealed that IGF-1R was associated with MET in PI-resistant KAS-6/1 and U266 cells (Fig. 6A and Supplementary Figure S8D). In addition, WB further found that knockdown of IGF-1R by shRNA (Supplementary Figure S4D) reduced phosphorylated MET levels in PI-resistant KAS-6/1 and U266 cells (Fig. 6B). Thus, IGF-1R activates MET in PI-resistant MM cells.

Moreover, the WB results revealed that IGF-1R levels were elevated in PI-resistant KAS-6/1 and U266 cells relative to PI-sensitive KAS-6/1 and U266 cells. In addition, MUC20 overexpression decreased IGF-1R levels in PI-resistant KAS-6/1 and U266 cells (Fig. 6C), suggesting that MUC20 might suppress MET activation by IGF-1R in PI-resistant MM cells.

MUC20 expression profiles of CD138 + bone marrow plasma cells in 538 patients with MM were collected from the GEO database (GSE4204) and divided into high and low MUC20 expression groups. Gene Set Variation Analysis (GSVA) was then used to identify differentially enriched pathways between the two groups. Analysis demonstrated that oxidative phosphorylation (OXPHOS) was activated in patients with MM with high MUC20 expression (Fig. 6D), and the Seahorse Mito Stress Test was performed to detect OXPHOS in PI-resistant MM cells. Measurement of the oxygen consumption rate (OCR) indicated that MUC20 overexpression significantly increased basal respiration and spare respiratory capacity in PI-resistant KAS-6/1 and U266 cells (Fig. 6E and F). In contrast, detection of the extracellular acidification rate (ECAR) using the Seahorse Glycolysis Stress Test revealed that MUC20 overexpression dramatically decreased glycolysis and the glycolytic capacity in PI-resistant KAS-6/1 and U266 cells (Supplementary Figure S11A and B). These findings suggest that MUC20 overexpression reversed the metabolic flux of glucose from glycolysis to OXPHOS in PI-resistant MM cells.

As MUC20 overexpression decreased glycolysis in PI-resistant MM cells, the lactate levels in PI-resistant MM cells were detected. Enzyme-linked immunosorbent assay (ELISA) results revealed that MUC20 overexpression reduced lactate levels in PI-resistant KAS-6/1 and U266 cells (Fig. 6G). Increasing evidence has shown that lactate promotes protein lactylation [34, 35]. Co-IP was then performed to detect IGF-1R lactylation using an antibody for L-lactyl lysine. The results revealed that MUC20 overexpression reduced IGF-1R lactylation in PI-resistant KAS-6/1 and U266 cells (Fig. 6H and Supplementary Figure S8E). Moreover, incubation with exogenous lactate (lactate sodium) elevated IGF-1R levels in PI-resistant KAS-6/1 and U266 cells (Fig. 6I), suggesting that MUC20 decreased IGF-1R levels by inhibiting IGF-1R lactylation by increasing OXPHOS in PI-resistant MM cells.

Taken together, these data suggest that MUC20 suppresses MET activation by repressing IGF-1R lactylation in PI-resistant MM cells.

### MUC20 overcomes proteasome inhibitor resistance in MM by inducing cuproptosis via inhibiting MET/CDKN2A pathway in vivo

To further confirm the effect and mechanism of MUC20 in decreasing PI resistance in MM in vivo, xenograft tumor mouse models were established by subcutaneously injecting PI-resistant KAS-6/1 and U266 cells into the right dorsal flanks of nude mice, which was followed by intraperitoneal injection of PI (CFZ), elesclomol, TTM, and lentivirus containing a lentiviral vector carrying the *MUC20/MET/CDKN2A* genes.

**Fig. 7:**
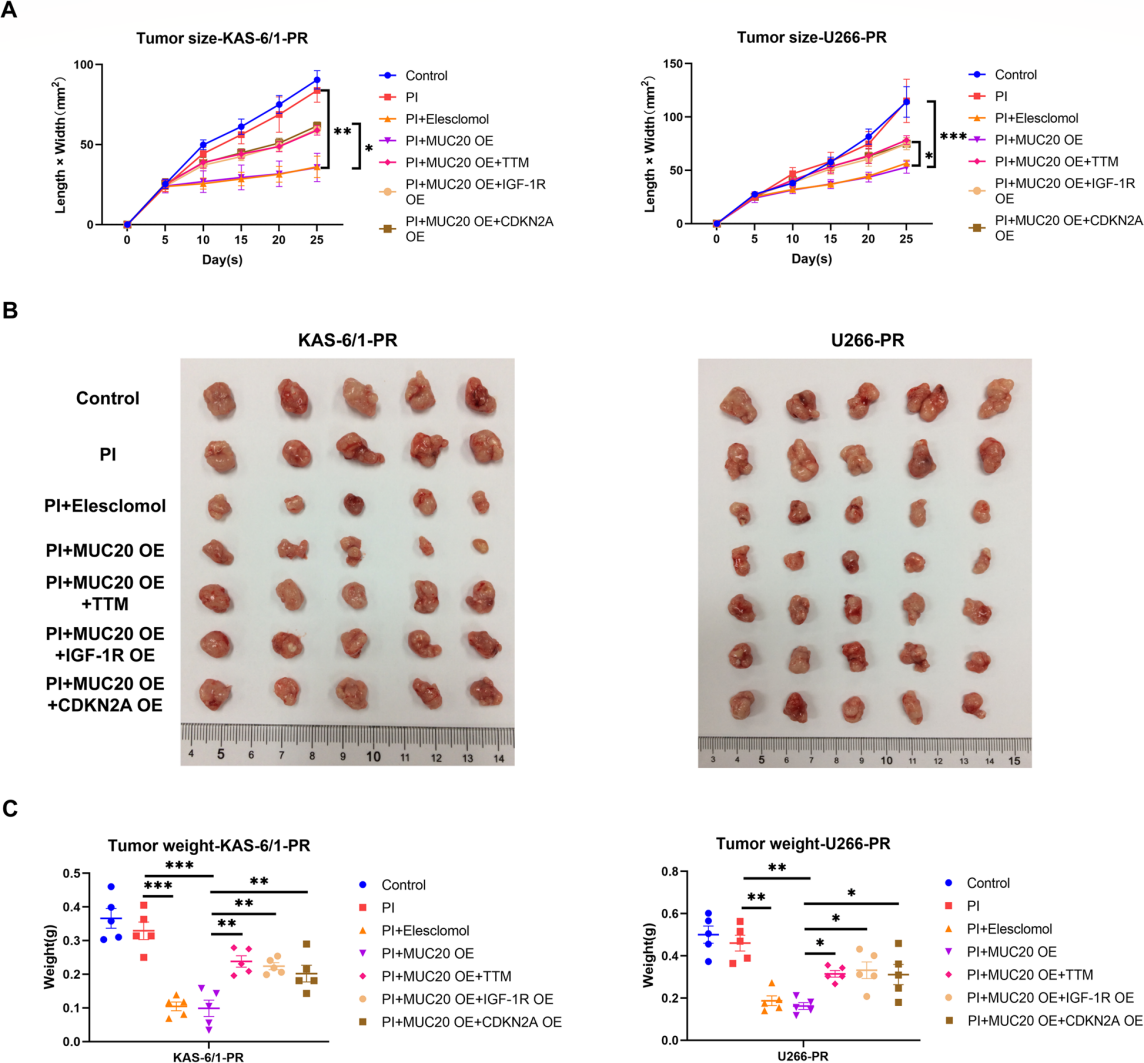
MUC20 attenuates proteasome inhibitor resistance in MM by triggering cuproptosis via inhibiting MET/CDKN2A pathway in vivo. **A** Quantification of the volume of xenograft tumors derived from control, MUC20 OE, MUC20 OE + IGF-1R OE, and MUC20 OE + CDKN2A OE PI-resistant KAS-6/1 and U266 cells in the presence and absence of PI (CFZ), TTM or elesclomol. **B** Images of xenograft tumors formed in nude mice injected with control, MUC20 OE, MUC20 OE + IGF-1R OE, and MUC20 OE + CDKN2A OE PI-resistant KAS-6/1 and U266 cells in the presence and absence of PI (CFZ), TTM or elesclomol. **C** Quantification of the weight of xenograft tumors formed in nude mice injected with control, MUC20 OE, MUC20 OE + IGF-1R OE, and MUC20 OE + CDKN2A OE PI-resistant KAS-6/1 and U266 cells in the presence and absence of PI (CFZ), TTM or elesclomol. PR: proteasome inhibitor-resistant MM cells; OE: overexpression; TTM: tetrathiomolybdate. *P < 0.05, **P < 0.01, ***P < 0.001

The results showed that PI injection had no significant effect on the size and weight of tumors developed from PI-resistant KAS-6/1 and U266 cells in mice (Fig. 7A-C and Supplementary Figure S6A). However, tumors developed from PI-resistant KAS-6/1 and U266 cells in mice injected with a combination of PI and elesclomol or a lentivirus-containing lentiviral vector carrying *MUC20* were much smaller and lighter than those developed from PI-resistant KAS-6/1 and U266 cells in control mice (Fig. 7A-C and Supplementary Figure S12A). Moreover, injection of TTM and a lentivirus containing a lentiviral vector carrying *MET* or *CDKN2A* abolished the inhibitory effect of MUC20 overexpression on tumors developed from PI-resistant KAS-6/1 and U266 cells in mice treated with PI (Fig. 7C and Supplementary Figure S12A). Combined with the above in vitro results, these data suggested that MUC20 alleviates PI resistance in MM by triggering cuproptosis by inhibiting the MET/CDKN2A pathway in vivo.

### Genome-wide detection of eccDNAs in proteasome inhibitor-resistant MM cells

Increasing evidence has shown that eccDNA contributes to drug resistance in cancer [20, 21]. To further elucidate the role of eccDNAs in PI-resistant MM cells, eccDNAs were isolated and subjected to eccDNA-Seq on a genomic scale (Supplementary Figure S13A). The results showed that 73,573, 113,090, 94,165, and 115,163 eccDNAs and 50,964, 47,109, 53,750, and 48,875 eccDNA-amplified genes were identified in the PI-resistant KAS-6/1, PI-sensitive KAS-6/1, PI-resistant U266, and PI-sensitive U266 cells, respectively (Supplementary Table S3-6). In addition, 73,320, 112,921, 93,810, and 114,986 microeccDNAs and 253, 169, 355, and 177 mega-eccDNAs were identified in the PI-resistant KAS-6/1, PI-sensitive KAS-6/1, PI-resistant U266, and PI-sensitive U266 cells, respectively (Supplementary Table S3-6). Further analysis confirmed that the size of eccDNAs among four cell lines showed a similar pattern and was distributed from 0.01 kb to 2500 kb, and it showed three distinctive peaks at 0.4, 06, and 0.8 kb (Supplementary Figure S13B). In addition, eccDNAs in PI-resistant KAS-6/1, PI-sensitive KAS-6/1, and PI-resistant U266 cells were distributed on all chromosomes except chromosome Y, whereas those in PI-sensitive U266 cells were distributed on all chromosomes except chromosomes X and Y (Supplementary Figure S13C).

### Identification and enrichment analysis of differentially expressed eccDNA-amplified encoding genes in MM cells

Differentially expressed eccDNA (DEED)-amplified encoding genes were identified based on the fragments per million (fpm) parameter. We identified 3112 upregulated and 2227 downregulated DEED-amplified encoding genes in PI-resistant KAS-6/1 cells compared to PI-sensitive KAS-6/1 cells and 3787 upregulated and 1954 downregulated DEED-amplified encoding genes in PI-resistant U266 cells compared to PI-sensitive U266 cells (Supplementary Figure S14A and B, Supplementary Table S7). Moreover, the most DEED-amplified encoding genes both in PI-resistant KAS-6/1 and U266 cells are listed in Supplementary Table S8.

Gene Ontology (GO) and Kyoto Encyclopedia of Genes and Genomes (KEGG) pathway enrichment analyses of DEED-amplified genes were performed. The results indicated that upregulated DEED-amplified encoding genes of PI-resistant KAS-6/1 and U266 cells were enriched in the biological process (BP) terms developmental process and anatomical structure development, cellular component (CC) terms membrane and cytoplasm, and molecular function (MF) terms protein binding and ion binding, while downregulated DEED-amplified encoding genes of PI-resistant KAS-6/1 and U266 cells were enriched in the BP terms response to stimulus and developmental process, CC terms signaling and membrane, and MF terms protein binding and catalytic activity (Supplementary Figure S15A). In addition, KEGG pathway enrichment analysis showed that upregulated DEED-amplified encoding genes of PI-resistant MM cells were involved in the TGF-beta and p53 signaling pathways (Supplementary Figure S15B); however, the downregulated DEED-amplified encoding genes of PI-resistant MM cells were involved in the platinum drug resistance and PPAR signaling pathways (Supplementary Figure S15B). All of these pathways played critical roles in drug resistance of cancers.

### EccDNA induces proteasome inhibitor resistance by amplifying kinesin family member 3 C to reduce MUC20 expression in MM

**Fig. 8:**
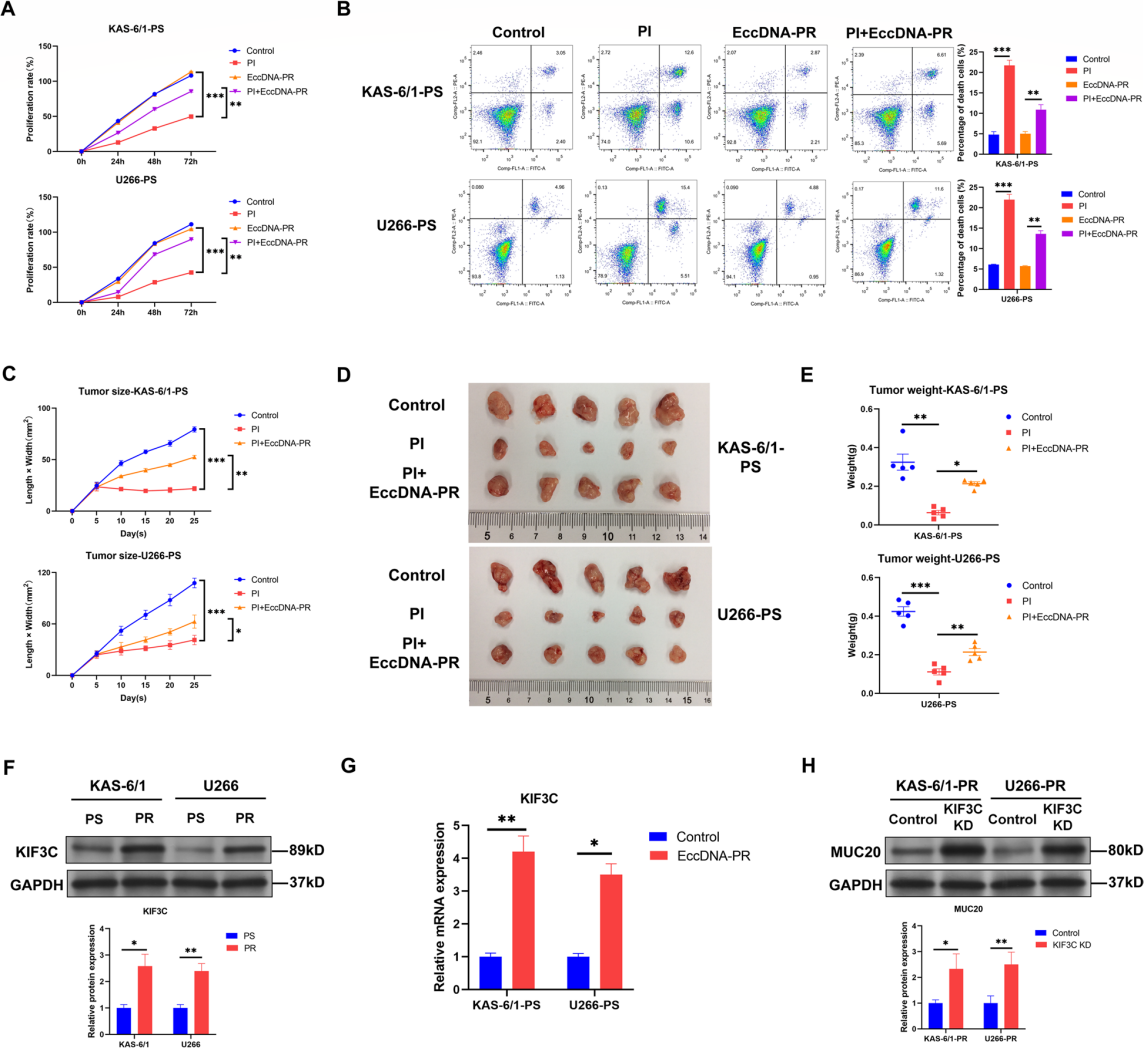
EccDNA induces proteasome inhibitor resistance by amplifying kinesin family member 3 C to decrease MUC20 expression in MM. **A** Proliferation rates of control and eccDNA-transfected PI-sensitive KAS-6/1 and U266 cells treated with or without PI (CFZ). **B** Representative images of flow cytometric analysis for PCD in control and eccDNA-transfected PI-sensitive KAS-6/1 and U266 cells treated with or without PI (CFZ). **C** Quantification of the volume of xenograft tumors derived from control and eccDNA-transfected PI-sensitive KAS-6/1 and U266 cells in the presence and absence of PI (CFZ). **D** Images of xenograft tumors formed in nude mice injected with control and eccDNA-transfected PI-sensitive KAS-6/1 and U266 cells in the presence and absence of PI (CFZ). **E** Quantification of the weights of xenograft tumors formed in nude mice injected with control and eccDNA-transfected PI-sensitive KAS-6/1 and U266 cells in the presence and absence of PI (CFZ). **F** KIF3C protein level in PI-sensitive and PI-resistant KAS-6/1 and U266 cells. **G** KIF3C mRNA level in control and eccDNA-transfected PI-sensitive KAS-6/1 and U266 cells. **H** MUC20 protein level in control and KIF3C-KD-transfected PI-resistant KAS-6/1 and U266 cells. PS: proteasome inhibitor-sensitive MM cells; PR: proteasome inhibitor-resistant MM cells; OE: overexpression; PI: proteasome inhibitor. *P < 0.05, **P < 0.01, ***P < 0.001

The association between eccDNA and PI resistance in MM was also explored. First, eccDNAs from PI-resistant KAS-6/1 and U266 cells were isolated (Supplementary Figure S13A) and transfected into PI-sensitive KAS-6/1 and U266 cells. The CCK-8 and soft agar colony formation assay results showed that transfection of eccDNAs from PI-resistant KAS-6/1 and U266 cells reversed the inhibitory effect of PI (CFZ) treatment on PI-sensitive KAS-6/1 and U266 cell proliferation (Fig. 8A and Supplementary Figure S16). Moreover, flow cytometric analysis demonstrated that transfection of eccDNAs from PI-resistant KAS-6/1 and U266 cells for 48 h decreased the PI-induced PCD at early and late stages of PI-sensitive KAS-6/1 and U266 cells (Fig. 8B).

Furthermore, xenograft tumor mouse models were established by subcutaneously injecting PI-sensitive KAS-6/1 and U266 cells and cells transfected with eccDNAs from PI-resistant MM cells into the right dorsal flanks of nude mice, followed by an intraperitoneal injection of PI (CFZ). The results showed that PI injection significantly reduced the size and weight of tumors developed from PI-sensitive KAS-6/1 and U266 cells in mice (Fig. 8C-E and Supplementary Figure S12B). In addition, the size and weight of tumors developed from PI-sensitive KAS-6/1 and U266 cells transfected with eccDNAs from PI-resistant MM cells were larger and heavier than those of tumors developed from PI-sensitive KAS-6/1 and U266 cells after intraperitoneal injection of PI (Fig. 8C-E and Supplementary Figure S12B). These results suggested that eccDNA induces PI resistance in MM cells.

In addition, the DEED-amplified encoding genes that contribute to PI resistance in MM were investigated. EccDNA-seq indicated that the *kinesin family member 3 C (KIF3C)* gene was absent in the eccDNAs of PI-sensitive KAS-6/1 and U266 cells (Supplementary Table S7 and S8). *KIF3C* is an oncogene [36, 37], and a previous study demonstrated that KIF3C overexpression leads to docetaxel resistance in breast cancer [38]. However, the effects of KIF3C on PI resistance in MM remain unclear. The qRT-PCR and WB results revealed that both KIF3C mRNA and protein levels were increased in PI-resistant KAS-6/1 and U266 cells compared to PI-sensitive KAS-6/1 and U266 cells (Supplementary Figure S17 and Fig. 8F), indicating that KIF3C should originate from the genomic DNA in PI-sensitive MM cells while originate from the genomic DNA and eccDNA in PI-resistant MM cells. Moreover, transfection with eccDNA from PI-resistant KAS-6/1 and U266 cells increased the KIF3C mRNA levels in PI-sensitive KAS-6/1 and U266 cells (Fig. 8G); knockdown of KIF3C using shRNA (Supplementary Figure S4E) increased MUC20 expression in PI-resistant KAS-6/1 and U266 cells (Fig. 8H), and knockdown of KIF3C for 48 h abolished the effect of eccDNA transfection from PI-resistant MM cells on proliferation and PCD at early and late stages in PI-sensitive KAS-6/1 and U266 cells (Supplementary Figure S18A-C). Therefore, the above data suggest that eccDNA induces PI resistance by amplifying KIF3C to decrease MUC20 expression in MM.

## Discussion

**Fig. 9:**
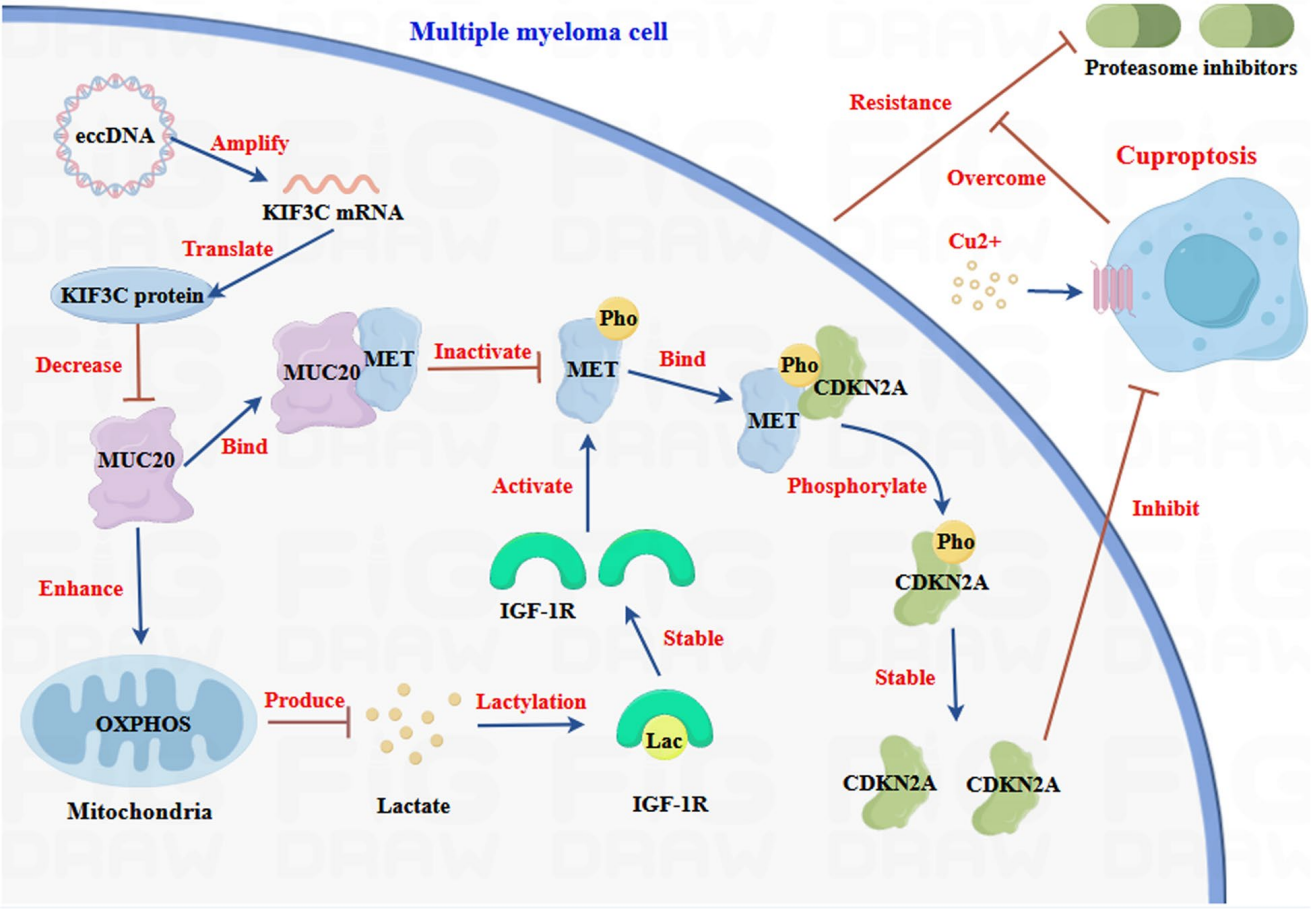
Schematic diagram of molecular mechanisms for the current study. Downregulation of MUC20 in PI-resistant MM cell lines and patients can predict the PI sensitivity and outcome of patients with MM. In addition, MUC20 attenuated PI resistance of MM cells by inducing cuproptosis in vitro and in vivo. Mechanistically, MUC20 triggered cuproptosis by inhibiting CDKN2A expression by hindering activation of MET in PI-resistant MM cells. Moreover, MUC20 suppressed MET activation by repressing IGF-1R lactylation in PI-resistant MM cells. In addition, eccDNA induced PI resistance by amplifying KIF3C to reduce MUC20 expression in MM.

The current study indicated that the downregulation of MUC20 in PI-resistant MM cell lines and patients could predict PI sensitivity and outcomes in patients with MM. In addition, MUC20 attenuated PI resistance of MM cells by inducing cuproptosis, both in vitro and in vivo. Mechanistically, MUC20 triggers cuproptosis by inhibiting CDKN2A expression and hindering the activation of MET in PI-resistant MM cells. Moreover, MUC20 suppresses MET activation by repressing IGF-1R lactylation in PI-resistant MM cells and eccDNA induced PI resistance by amplifying KIF3C to reduce MUC20 expression in MM cells (Fig. 9).

Several studies have shown that MUC20 is involved in drug resistance in different cancers [9, 10, 39, 40]. However, whether MUC20 regulates drug resistance in cancer by modulating PCD has not been reported. To date, only one study has shown that MUC20 regulates apoptosis and pyroptosis in signet ring cell carcinoma cells [9]. Therefore, our study revealed for the first time the mechanism by which MUC20 regulates drug resistance in cancers via a novel PCD (cuproptosis). This finding expands the knowledge of MUC20 in regulating PCD.

Our findings demonstrated that MUC20 hindered MET phosphorylation in PI-resistant MM cells. In contrast, a previous study revealed that MUC20 knockdown decreased MET phosphorylation in pancreatic ductal adenocarcinoma cells [27], suggesting that MUC20 might exert opposite effects on MET phosphorylation depending on the cancer type. In addition, phosphorylation is known to be important for protein-protein interactions [41]; however, the effect of phosphorylation on the association between MET and downstream proteins remains unknown. This study revealed for the first time the essential role of phosphorylation in the interaction between MET and downstream proteins.

In addition, this study revealed that MET-regulated phosphorylation increased CDKN2A expression. Numerous studies have shown that phosphorylation improves protein stability and expression. For instance, phosphorylation mediated by RNA-binding motif protein 45 (RBM45) enhances alanine-serine-cysteine transporter 2 (ASCT2) stability and expression in hepatocellular carcinoma [42]. Similarly, AKT serine/threonine kinase (AKT) facilitates zinc finger protein 322 (ZNF322) phosphorylation to improve its stability in lung cancer [43]. Thus, MET-mediated phosphorylation may increase CDKN2A expression by enhancing its stability in PI-resistant MM cells.

Moreover, the present study showed that MUC20 reduces IGF-1R expression by repressing lactylation in PI-resistant MM cells. A recent study has reported that hypoxia enhances β-catenin lactylation to improve its stability in colorectal cancer [44]. Decreased lactylation of lymphocyte cytosolic protein 1 (LCP1) caused by glycolysis inhibition reduces LCP1 stability during cerebral infarction progression [45]. Moreover, methyltransferase-like 3 (METTL3) lactylation enhances METTL 3 protein stability in intracerebral hemorrhage [46]. Therefore, MUC20 may decrease IGF-1R by reducing its stability and repressing lactylation via the inhibition of glycolysis.

To date, the eccDNA of MM cells has not been reported; therefore, this study reveals the characteristics of eccDNA in MM cells for the first time. Similar to a previous study [22], eccDNA induced drug resistance by amplifying a DEED-related encoding gene (*KIF3C*) in MM. *KIF3C* is an oncogene that contributes to drug resistance in cancer [38]. Our findings further revealed that DEED-amplified KIF3C may trigger PI resistance by reducing MUC20 expression in MM cells. KIF3C has been reported to enhance cancer progress by regulating the phosphatidylinositol 3-kinase (PI3K)/AKT pathway and transforming growth factor beta (TGF-β) pathway [36, 47, 48]. Thus, KIF3C might suppress MUC20 expression by PI3K/AKT pathway or TGF-β pathway in PI-resistant MM cells.

Additionally, this study found that MUC20 level of CD138 + bone marrow plasma cells in the tMM group is higher compared to the HD and NDMM groups. As DEED-amplified KIF3C could suppress MUC20 expression, the level of DEED-amplified KIF3C in CD138 + bone marrow plasma cells of the HD and NDMM groups might be higher than that of the tMM group.

Several limitations were noted in the current study. First, the effect of lactylation on IGF-1R stability and the mechanism by which KIF3C regulates MUC20 expression was not investigated. Second, the roles of eccDNA-amplified non-coding genes in PI resistance were not explored. Third, the detailed mechanisms underlying the differences in eccDNA between PI-resistant and PI-sensitive MM cells were not investigated. These issues remain to be further investigated.

## Conclusion

In summary, the current study revealed that MUC20 could predict PI sensitivity and outcomes in patients with MM. In addition, our findings suggest that MUC20 attenuates the PI resistance of MM cells by inducing cuproptosis by inhibiting CDKN2A expression and repressing IGF-1R lactylation to hinder the activation of MET. In addition, this study is the first to use eccDNA sequencing for MM, and the findings indicate that eccDNA induces PI resistance by amplifying KIF3C to reduce MUC20 expression in MM. These findings may provide novel strategies for the treatment of PI-resistant MM.

### Supplementary Information

**Additional file 1: Supplementary Figure S1.** Generate of PI-resistant MM lines. **Supplementary Figure S2.** PI treatment increases MUC20 level in PI-sensitive but not PI-resistant MM cells. **Supplementary Figure S3.** Clone formation of MM cells. **Supplementary Figure S4.** Efficiencies of overexpression or knockdown in MM cells. **Supplementary Figure S5.** The levels of cuproptosis markers and downstream genes in MM cells. **Supplementary Figure S6.** Clone formation of MM cells. **Supplementary Figure S7.** The association of MUC20 an CDKN2A in MM cells. **Supplementary Figure S8.** Representative images of silver staining following Co-IP. **Supplementary Figure S9.** Construction of His-tagged expression vectors containing the WT MET ORF or Y1234/Y1235-mutated (Y>F) MET ORF. **Supplementary Figure S10.** The effect of mutated MET of MUC20 expression in MM cells. **Supplementary Figure S11.** MUC20 overexpression reduces glycolysis and the glycolytic capacity in PI-resistant KAS-6/1 and U266 cells. **Supplementary Figure S12.** Representative images of nude mice subcutaneously injecting MM cells. **Supplementary Figure S13.** Characteristics of eccDNAs from PI-resistant or PI-sensitive MM cells. **Supplementary Figure S14.** Determination of DEED-amplified encoding genes between PI-resistant and PI-sensitive MM cells. **Supplementary Figure S15.** Enrichment analysis of DEED-amplified encoding genes between PI-resistant and PI-sensitive MM cells. **Supplementary Figure S16.** Clone formation of MM cells. **Supplementary Figure S17.** KIF3C mRNA level is upregulated in PI-resistant KAS-6/1 and U266 cells. **Supplementary Figure S18.** KIF3C knockdown abolishes the effect of eccDNA transfection from PI-resistant MM cells on proliferation and PCD in PI-sensitive MM cells.

**Additional file 2: Supplementary Table S1. **Demographic characteristics of HDs and patients with MM. **Supplementary Table S2. **Demographic characteristics of patients with NDMM and RRMM. **Supplementary Table S8. **DEED-amplified encoding genes both in PI-resistant KAS-6/1 and U266 cells.

**Additional file 3. **

## Data Availability

The datasets used and/or analyzed during the current study are available from the corresponding author on reasonable request. The original WB data are provided in the supplementary materials.

## Abbreviations

AKT: AKT serine/threonine kinase
BP: Biological process
BTZ: Bortezomib
CCK-8: Cell Counting Kit-8
CC: Cellular component
CDKN2A: Cyclin-dependent kinase inhibitor 2 A
CFZ: Carfilzomib
Co-IP: Co-immunoprecipitation
CR: Complete response
DEED: Differentially expressed eccDNA
eccDNA: Extrachromosomal circular DNA
EGFR: Epidermal growth factor receptor
ELISA: Enzyme-linked immunosorbent assay
fpm: Fragments per million
GEO: Gene Expression Omnibus
GO: Gene ontology
GSVA: Gene set variation analysis
HDs: Healthy donors
IGF-1R: Insulin-like growth factor receptor-1
KEGG: Kyoto Encyclopedia of Genes and Genomes
KIF3C: Kinesin family member 3 C
LCP1: Lymphocyte cytosolic protein 1
MET: MET proto-oncogene receptor, receptor tyrosine kinase
METTL3: Methyltransferase-like 3
MR: Minor response
MF: Molecular function
MGUS: Monoclonal gammopathy of undetermined significance
MUC20: Mucin 20
MM: Multiple myeloma
NDMM: Newly diagnosed MM
NSCLC: Non-small cell lung cancer
OCR: Oxygen consumption rate
ORF: Open reading frame
ORR: Overall response rate
OS: Overall survival
OXPHOS: Oxidative phosphorylation
PCD: Programmed cell death
PCI: Phenol/chloroform/isoamyl alcohol
PD: Progressive disease
PFS: Progression-free survival
PI: Proteasome inhibitor
PI3K: Phosphatidylinositol 3-kinase
p-MET: Phosphorylated MET
PR: Partial response
PVDF: Polyvinylidene fluoride
qRT-PCR: Quantitative reverse transcription-PCR
RBM45: RNA-binding motif protein 45
RCA: Rolling circle amplification
RRMM: Relapsed/refractory MM
SD: Standard deviation
SDS-PAGE: SDS-polyacrylamide gel electrophoresis
SMM: Smoldering MM
TBST: Tris-buffered saline containing 0.1% Tween 20
TGF-β: Transforming growth factor beta
TKI: Tyrosine kinase inhibitor
TTM: Tetrathiomolybdate
VGPR: Very good partial response
WB: Western blot
WT: Wild-type
ZNF322: Zinc finger protein 322
