# Supplementary material for: MUC20 regulated by extrachromosomal circular DNA attenuates proteasome inhibitor resistance of multiple myeloma by modulating cuproptosis
Source: J Exp Clin Cancer Res. 2024 Mar 5;43:68. doi: 10.1186/s13046-024-02972-6 (PMC10913264; doi:10.1186/s13046-024-02972-6)
Supplement: Supplementary file 1 — Additional file 1: Supplementary Figure S1. Generate of PI-resistant MM lines. Supplementary Figure S2. PI treatment increases MUC20 level in PI-sensitive but not PI-resistant MM cells. Supplementary Figure S3. Clone formation of MM cells. Supplementary Figure S4. Efficiencies of overexpression or knockdown in MM cells. Supplementary Figure S5. The levels of cuproptosis markers and downstream genes in MM cells. Supplementary Figure S6. Clone formation of MM cells. Supplementary Figure S7. The association of MUC20 an CDKN2A in MM cells. Supplementary Figure S8. Representative images of silver staining following Co-IP. Supplementary Figure S9. Construction of His-tagged expression vectors containing the WT MET ORF or Y1234/Y1235-mutated (Y>F) MET ORF. Supplementary Figure S10. The effect of mutated MET of MUC20 expression in MM cells. Supplementary Figure S11. MUC20 overexpression reduces glycolysis and the glycolytic capacity in PI-resistant KAS-6/1 and U266 cells. Supplementary Figure S12. Representative images of nude mice subcutaneously injecting MM cells. Supplementary Figure S13. Characteristics of eccDNAs from PI-resistant or PI-sensitive MM cells. Supplementary Figure S14. Determination of DEED-amplified encoding genes between PI-resistant and PI-sensitive MM cells. Supplementary Figure S15. Enrichment analysis of DEED-amplified encoding genes between PI-resistant and PI-sensitive MM cells. Supplementary Figure S16. Clone formation of MM cells. Supplementary Figure S17. KIF3C mRNA level is upregulated in PI-resistant KAS-6/1 and U266 cells. Supplementary Figure S18. KIF3C knockdown abolishes the effect of eccDNA transfection from PI-resistant MM cells on proliferation and PCD in PI-sensitive MM cells. [file 13046_2024_2972_MOESM1_ESM.docx]

**Supplementary Figures**


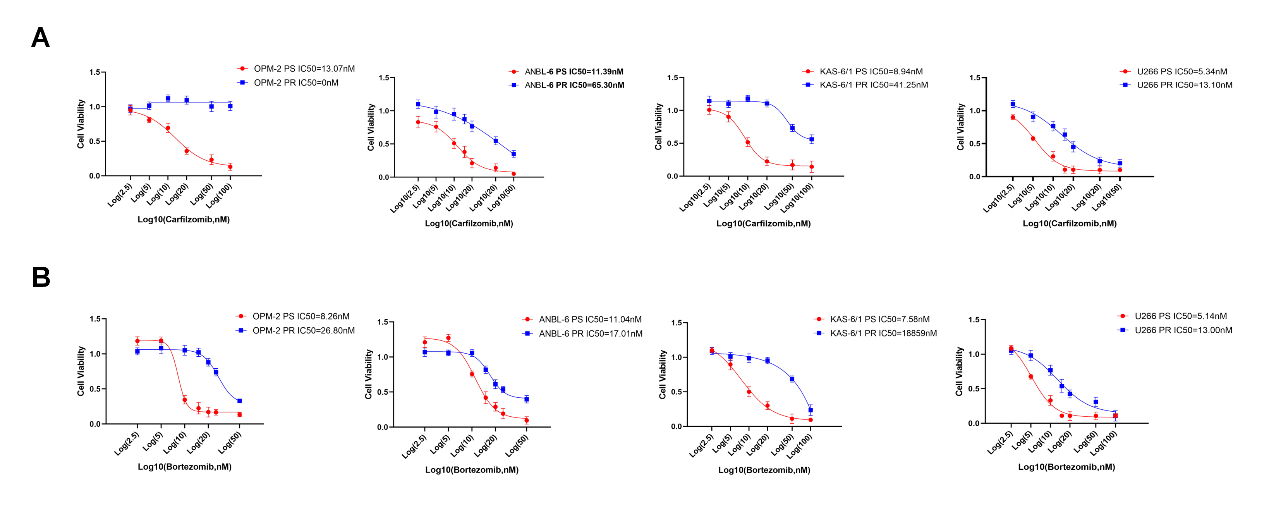


**Supplementary Figure S1 Generate of PI-resistant MM lines**. **A** Cell viability of OPM-2, ANBL-6, KAS-6/1, and U266 cells treated with or without carfilzomib. **B** Cell viability of OPM-2, ANBL-6, KAS-6/1, and U266 cells treated with or without bortezomib. PS: proteasome inhibitor-sensitive MM cells; PR: proteasome inhibitor-resistant MM cells.


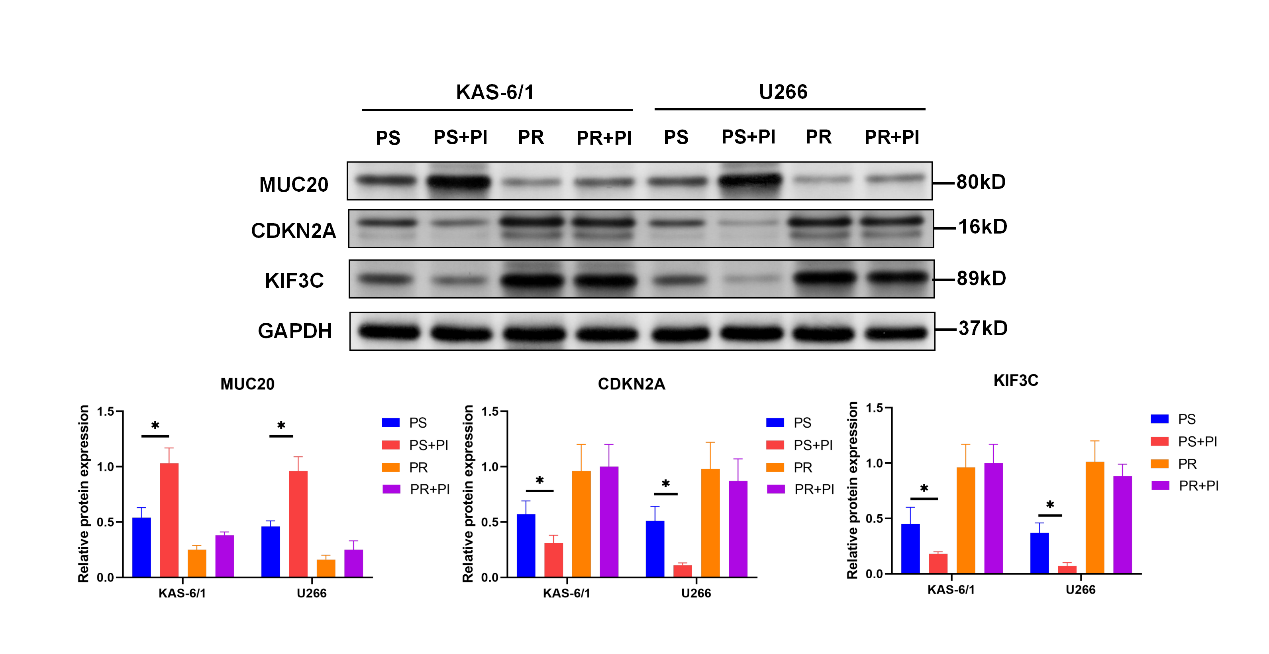


**Supplementary Figure S2 PI treatment increases MUC20 level in PI-sensitive but not PI-resistant MM cells.** Protein levels of MUC20, CDKN2A, KIF3C in PI-sensitive, PI-resistant KAS-6/1 and U266 cells treated with or without PI (CTZ). PS: proteasome inhibitor-sensitive MM cells; PR: proteasome inhibitor-resistant MM cells; PI: proteasome inhibitor. *P<0.05.


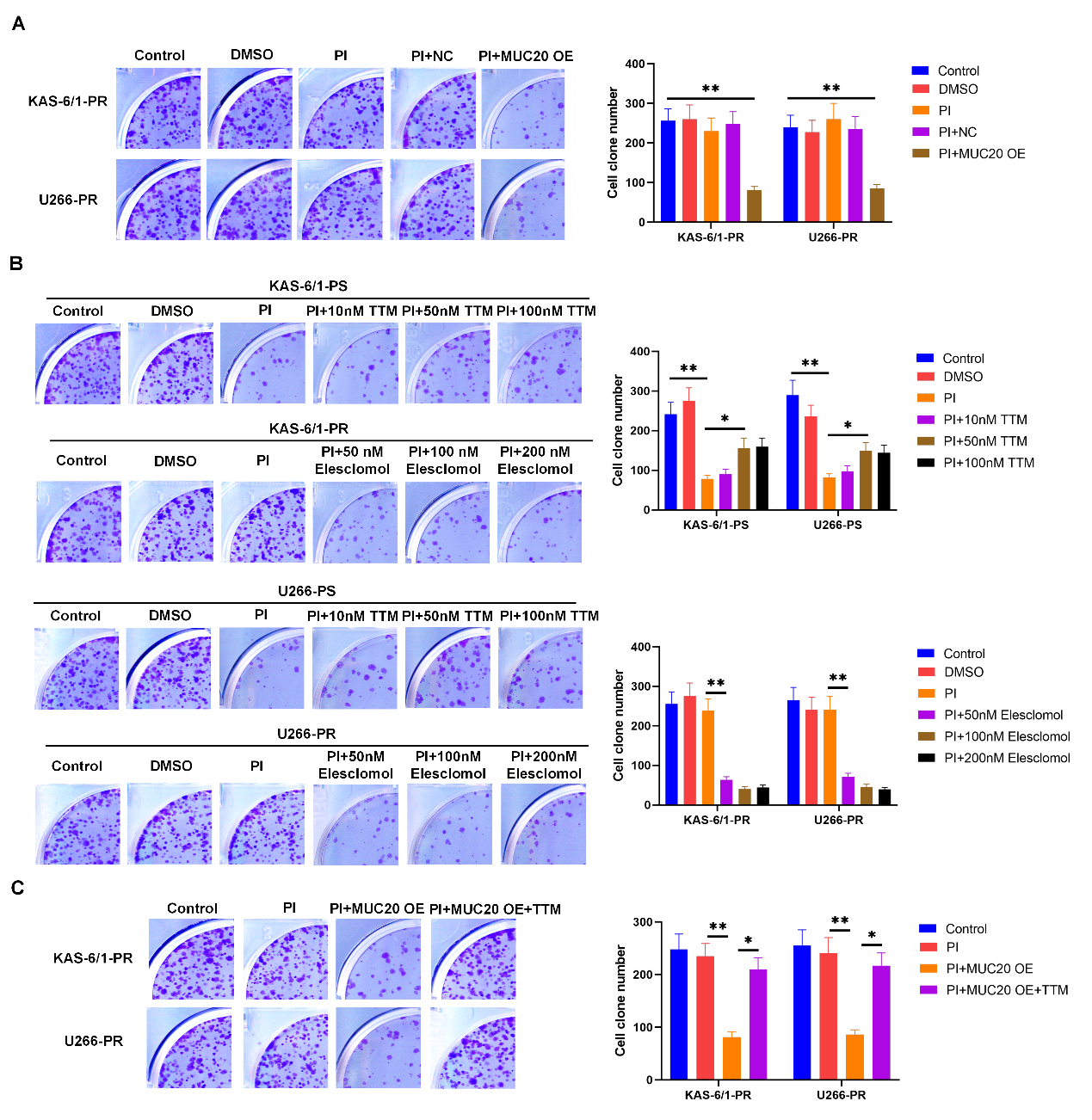


**Supplementary Figure S3 Clone formation of MM cells. A** Representative images of cell clones and quantification of cell clone number of control vector and MUC20 OE-transfected PI-resistant KAS-6/1 and U266 cells treated with or without PI (CFZ). **B** Representative images of cell clones and quantification of cell clone number of PI-sensitive KAS-6/1 and U266 cells treated with or without TTM and PI-resistant KAS-6/1 and U266 cells treated with or without or elesclomol. **D** Representative images of cell clones and quantification of cell clone number of MUC20 OE-transfected PI-resistant KAS-6/1 and U266 cells treated with or without PI (CFZ) and TTM. PS: proteasome inhibitor-sensitive MM cells; PR: proteasome inhibitor-resistant MM cells; OE: overexpression; PI: proteasome inhibitor; TTM: tetrathiomolybdate. *P<0.05, **P<0.01.


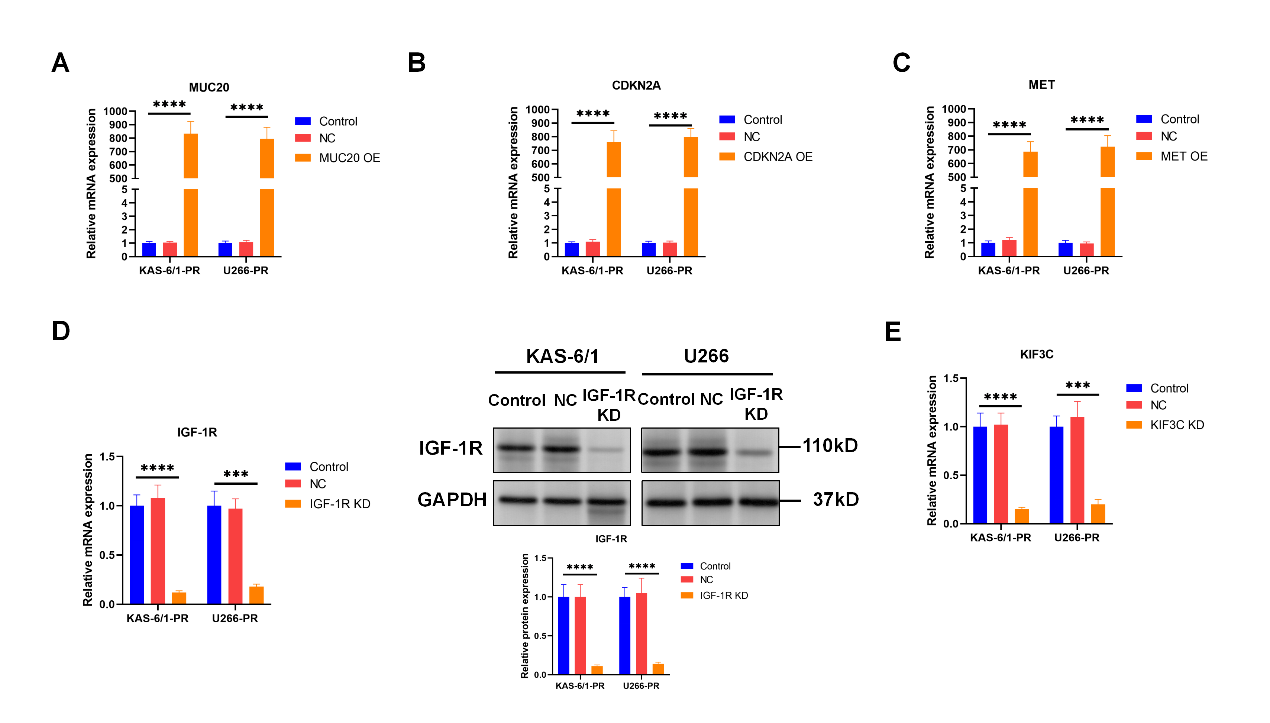


**Supplementary Figure S4 Efficiencies of overexpression or knockdown in MM cells. A** MUC20 mRNA level of control vector and MUC20 OE-transfected PI-resistant KAS-6/1 and U266 cells. **B** CDKN2A mRNA level of control vector and CDKN2A OE-transfected PI-resistant KAS-6/1 and U266 cells. **C** MET mRNA level of control vector and MET OE-transfected PI-resistant KAS-6/1 and U266 cells. **D** IGF-1R mRNA and protein level of control vector and IGF-1R KD-transfected PI-resistant KAS-6/1 and U266 cells. **E** KIF3C mRNA level of control vector and KIF3C KD-transfected PI-resistant KAS-6/1 and U266 cells. PR: proteasome inhibitor-resistant MM cells; OE: overexpression; KD: knockdown. ***P<0.001, ****P<0.0001.


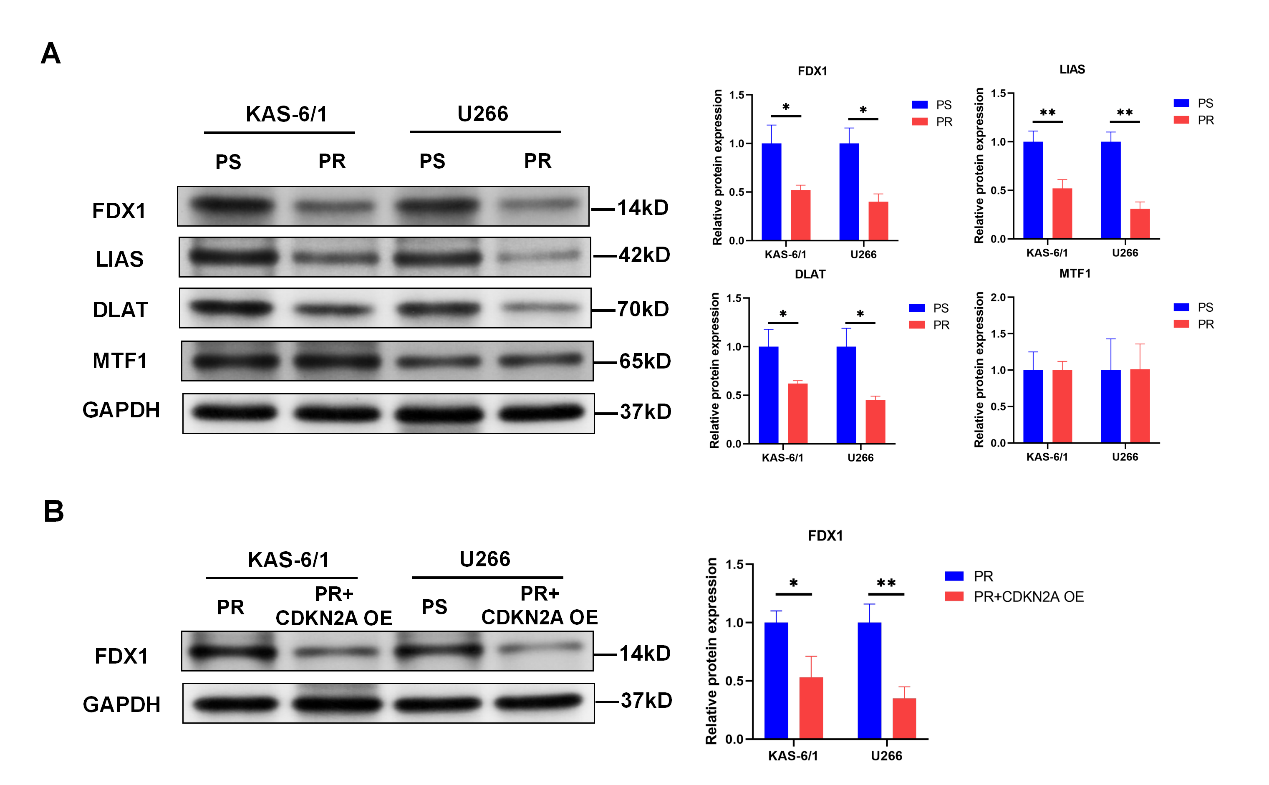


**Supplementary Figure S5 The levels of cuproptosis markers and downstream genes in MM cells. A** Protein levels of FDX1, LIAS, DLAT and MTF1 in PI-sensitive or PI-resistant KAS-6/1 and U266 cells. **B** Protein levels of FDX1 in CDKN2A OE-transfected PI-resistant KAS-6/1 and U266 cells. PS: proteasome inhibitor-sensitive MM cells; PR: proteasome inhibitor-resistant MM cells. *P<0.05, **P<0.01.


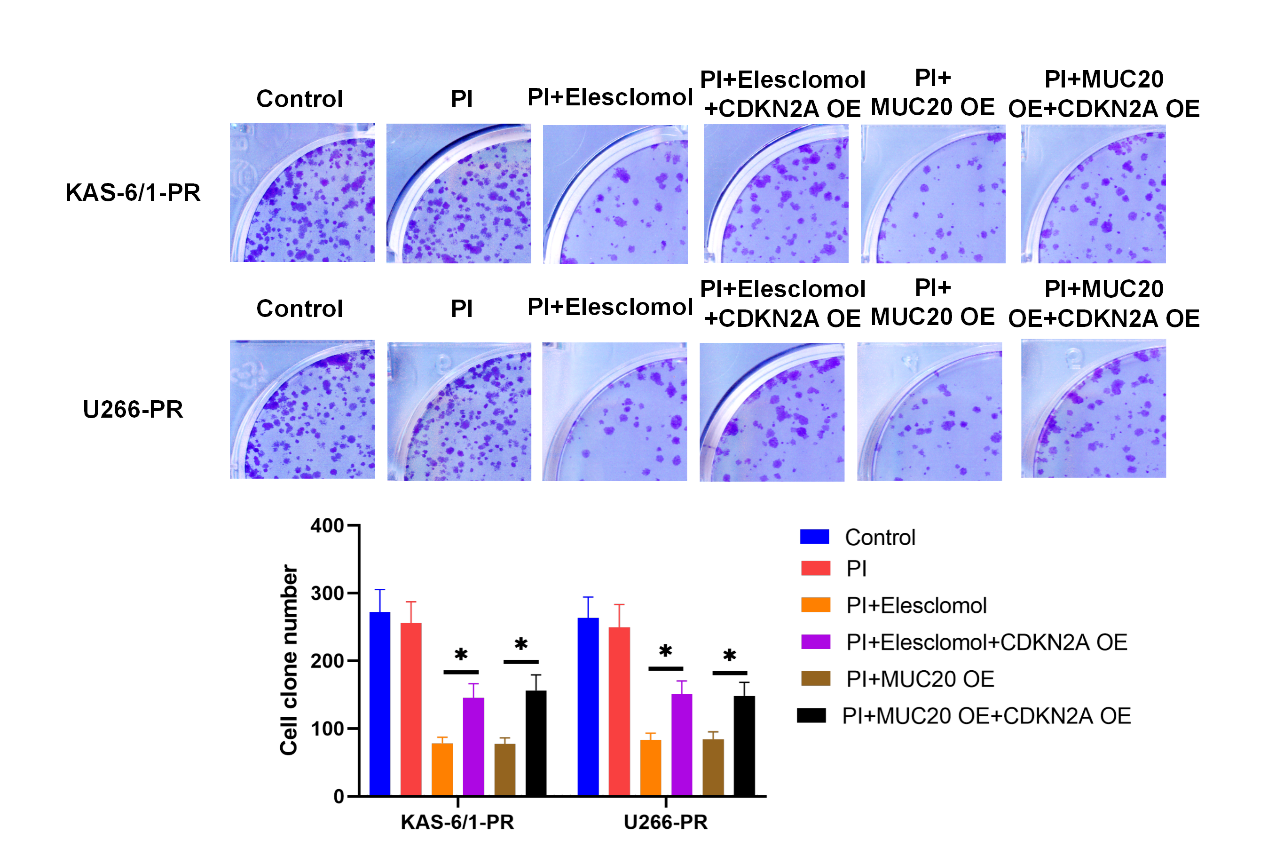


**Supplementary Figure S6 Clone formation of MM cells.** Representative images of cell clones and quantification of cell clone number of control vector, CDKN2A-OE or MUC20 OE-transfected PI-resistant KAS-6/1 and U266 cells treated with or without PI (CFZ) or elesclomol. PR: proteasome inhibitor-resistant MM cells; OE: overexpression; PI: proteasome inhibitor. *P<0.05, **P<0.01.


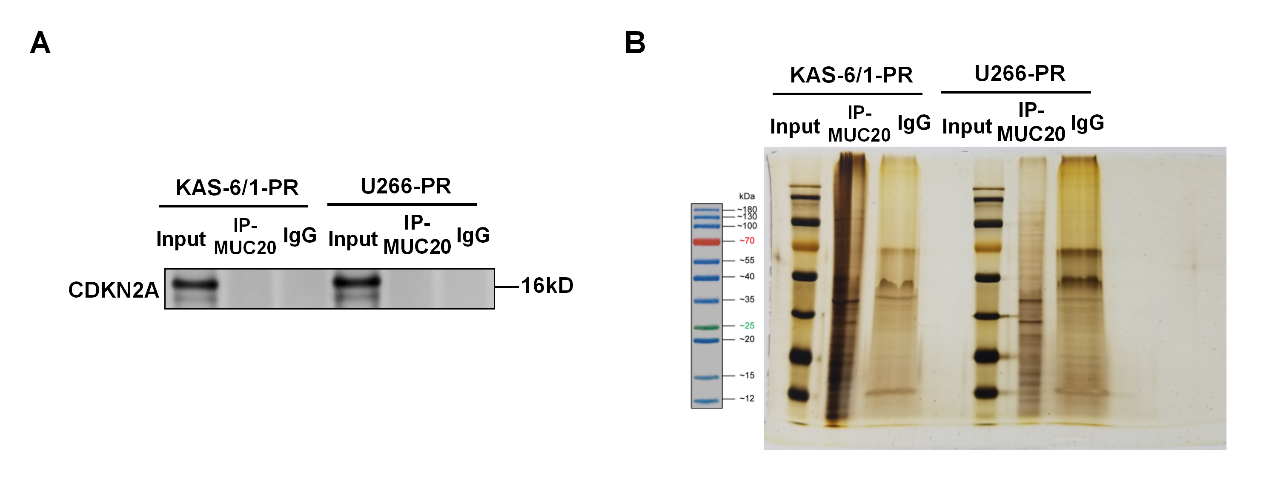


**Supplementary Figure S7 The association of MUC20 an CDKN2A in MM cells. A** Representative image of Co-IP using a MUC20 antibody in PI-resistant KAS-6/1 and U266 cells. Rabbit IgG was used as negative control. **B** Representative images of silver staining following Co-IP using a MUC20 antibody in PI-resistant KAS-6/1 and U266 cells. PR: proteasome inhibitor-resistant MM cells.


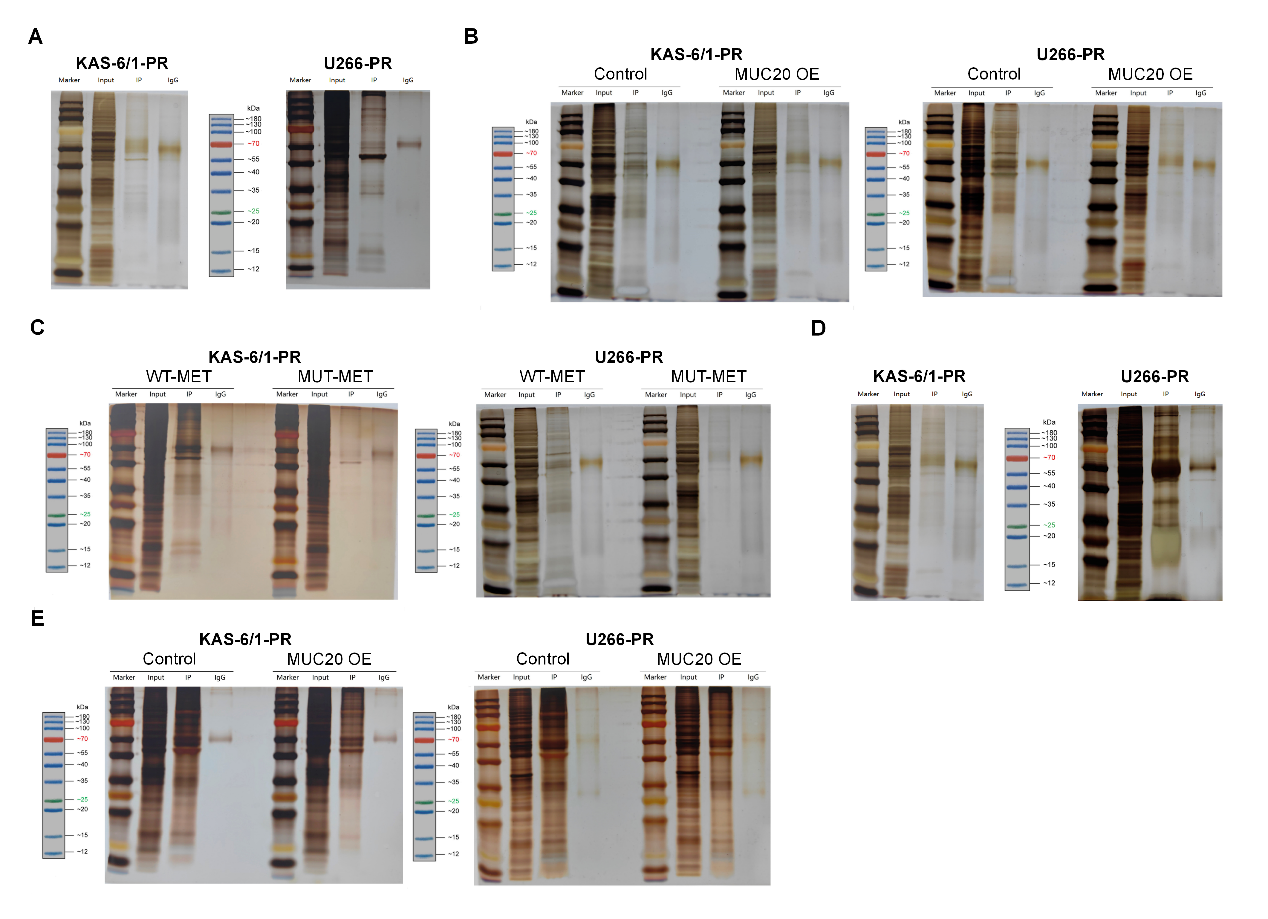


**Supplementary Figure S8 Representative images of silver staining following Co-IP. A** Representative images of silver staining following Co-IP using a MET antibody in PI-resistant KAS-6/1 and U266 cells. **B** Representative images of silver staining following Co-IP using a MET antibody in control or MUC20 OE-transfected PI-resistant KAS-6/1 and U266 cells. **C** Representative images of silver staining following Co-IP using a His antibody in WT-MET or MUT-MET-transfected PI-resistant KAS-6/1 and U266 cells. **D** Representative images of silver staining following Co-IP using a MET antibody in PI-resistant KAS-6/1 and U266 cells. **E** Representative images of silver staining following Co-IP using a L-lactyl lysine antibody in control or MUC20 OE-transfected PI-resistant KAS-6/1 and U266 cells. PR: proteasome inhibitor-resistant MM cells; OE: overexpression.


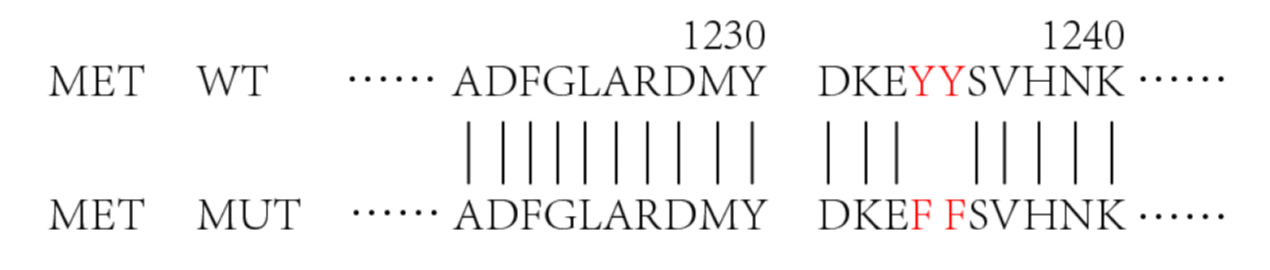


**Supplementary Figure S9 Construction of His-tagged expression vectors containing the WT MET ORF or Y1234/Y1235-mutated (Y>F) MET ORF.**


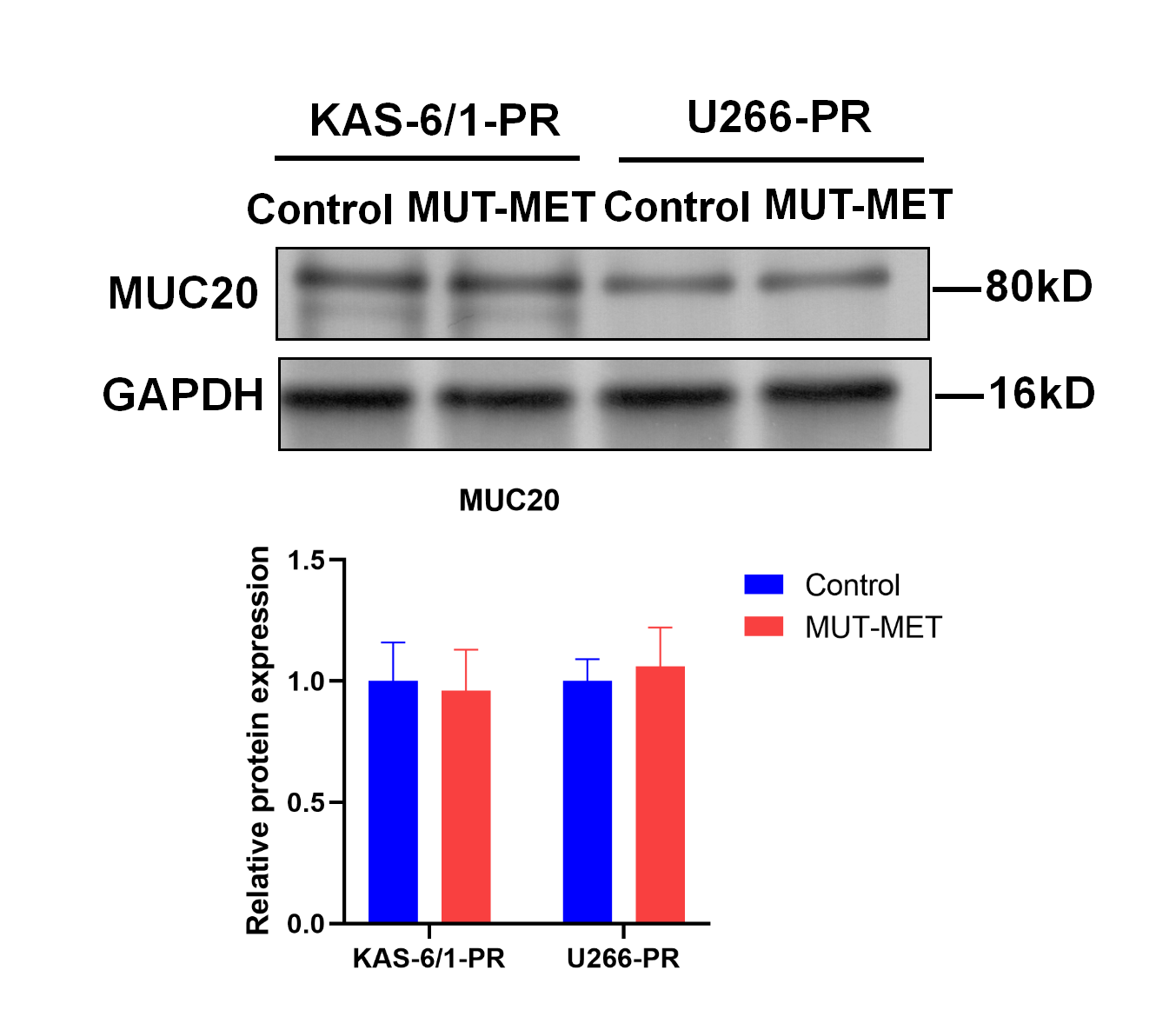


**Supplementary Figure S10 The effect of mutated MET of MUC20 expression in MM cells.** Protein levels of MUC20 in MUT-MET-transfected PI-sensitive or PI-resistant KAS-6/1 and U266 cells. PR: proteasome inhibitor-resistant MM cells.


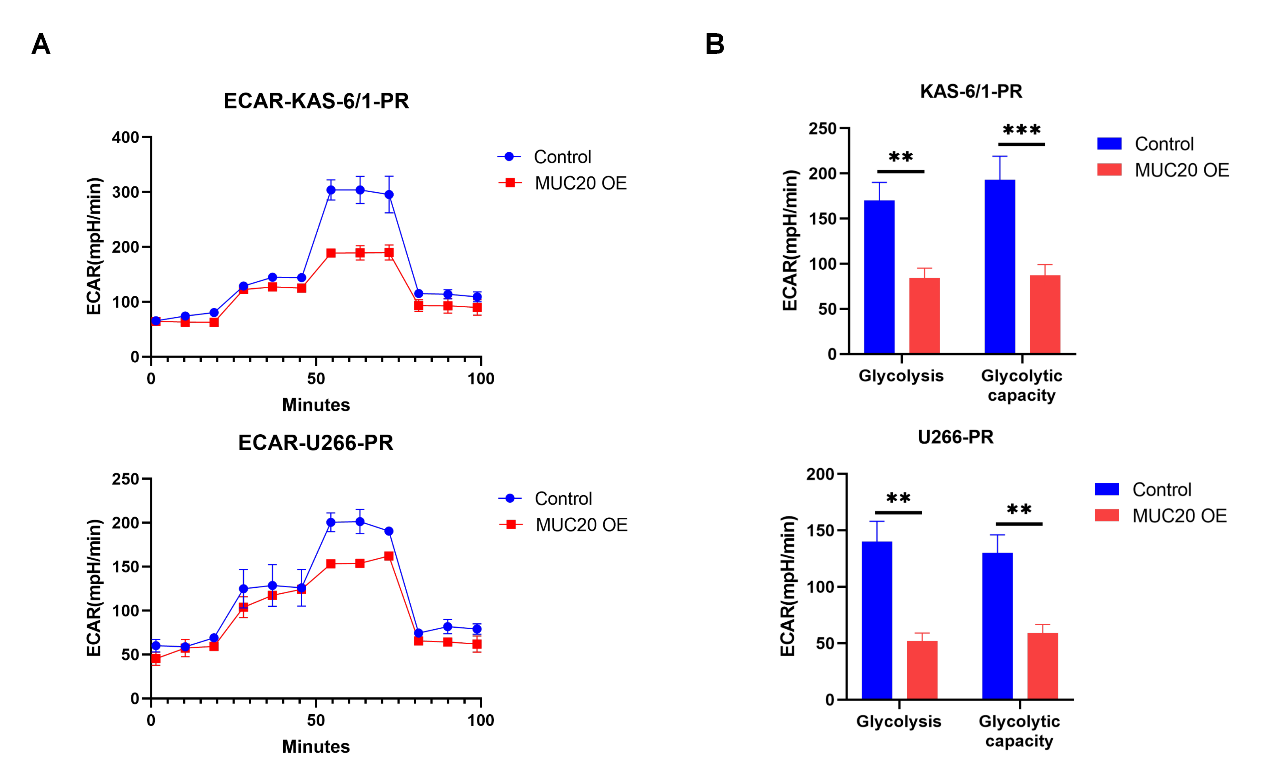


**Supplementary Figure S11 MUC20 overexpression reduces** **glycolysis and the** **glycolytic capacity in PI-resistant KAS-6/1 and U266 cells. A** ECAR in control or MUC20 OE-transfected PI-resistant KAS-6/1 and U266 cells. **B** Glycolysis and glycolytic capacity in control or MUC20 OE-transfected PI-resistant KAS-6/1 and U266 cells. ECAR: extracellular acidification rate; OE: overexpression. **P<0.01, ***P<0.001.


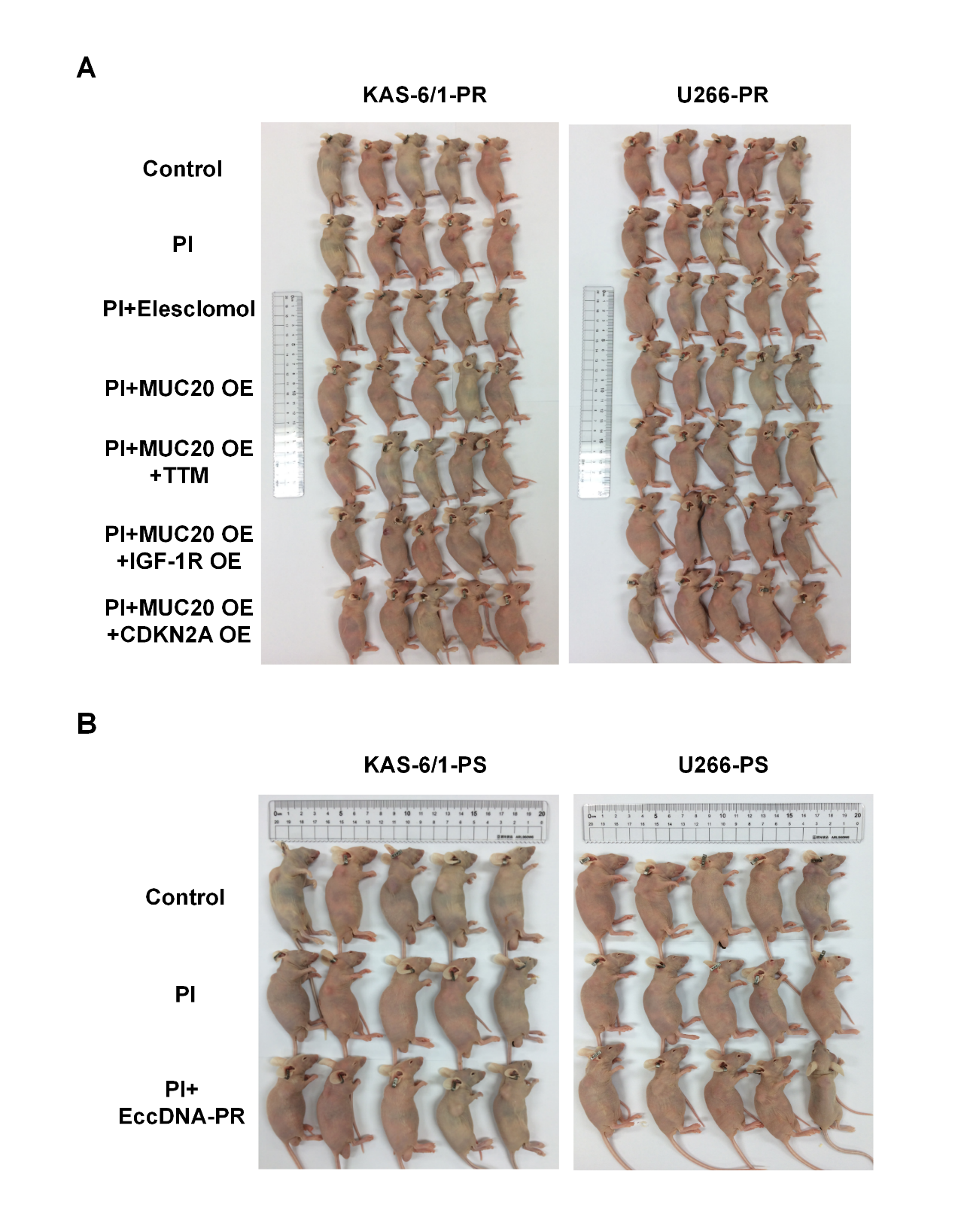


**Supplementary Figure S12** **Representative images of nude mice subcutaneously injecting MM cells. A** Representative images of nude mice subcutaneously injecting control, MUC20 OE, MUC20 OE+IGF-1R OE, and MUC20 OE+CDKN2A OE PI-resistant KAS-6/1 and U266 cells in the presence and absence of PI (CFZ), TTM or elesclomol. **B** Representative images of nude mice subcutaneously injecting control and eccDNA-transfected PI-sensitive KAS-6/1 and U266 cells in the presence and absence of PI (CFZ). PR: proteasome inhibitor-resistant MM cells; OE: overexpression; TTM: tetrathiomolybdate.


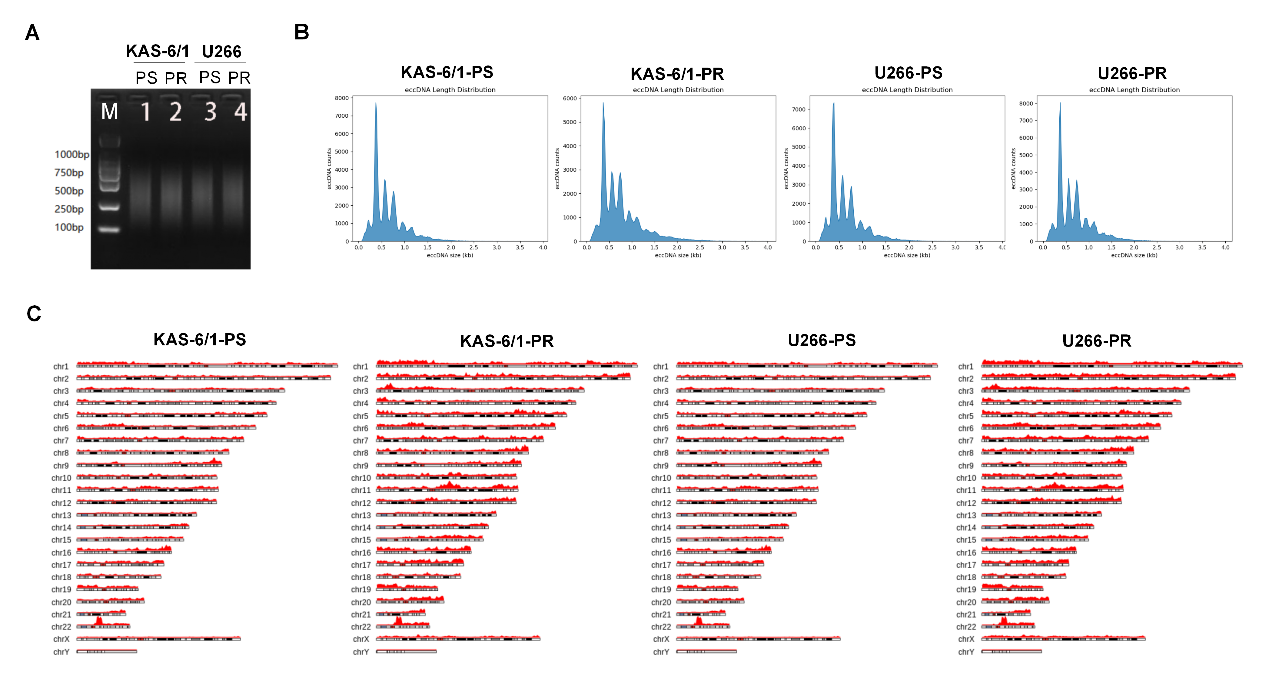


**Supplementary Figure S13 Characteristics of eccDNAs from PI-resistant or PI-sensitive MM cells. A** Representative image of isolated eccDNAs from PI-resistant or PI-sensitive KAS-6/1 and U266 cells. **B** The size of eccDNAs from PI-resistant or PI-sensitive KAS-6/1 and U266 cells. **C** Distribution of eccDNAs from PI-resistant or PI-sensitive KAS-6/1 and U266 cells on chromosomes. PS: proteasome inhibitor-sensitive MM cells; PR: proteasome inhibitor-resistant MM cells.


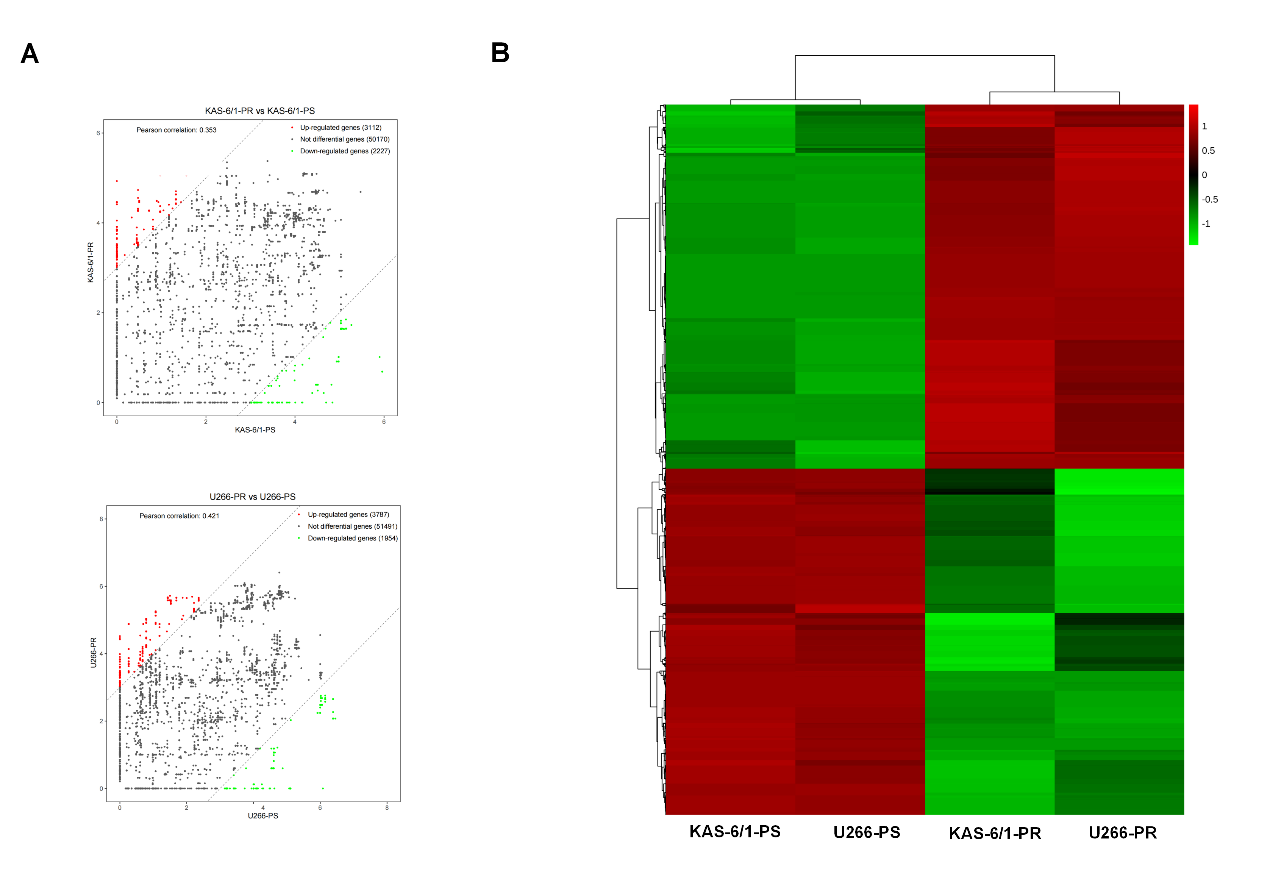


**Supplementary Figure S14 Determination of DEED-amplified encoding genes between PI-resistant and PI-sensitive MM cells. A** Overall DEED-amplified encoding genes between PI-resistant and PI-sensitive KAS-6/1 or U266 cells. **B** The heatmap for DEED-amplified encoding genes between PI-resistant and PI-sensitive KAS-6/1 or U266 cells. PS: proteasome inhibitor-sensitive MM cells; PR: proteasome inhibitor-resistant MM cells.


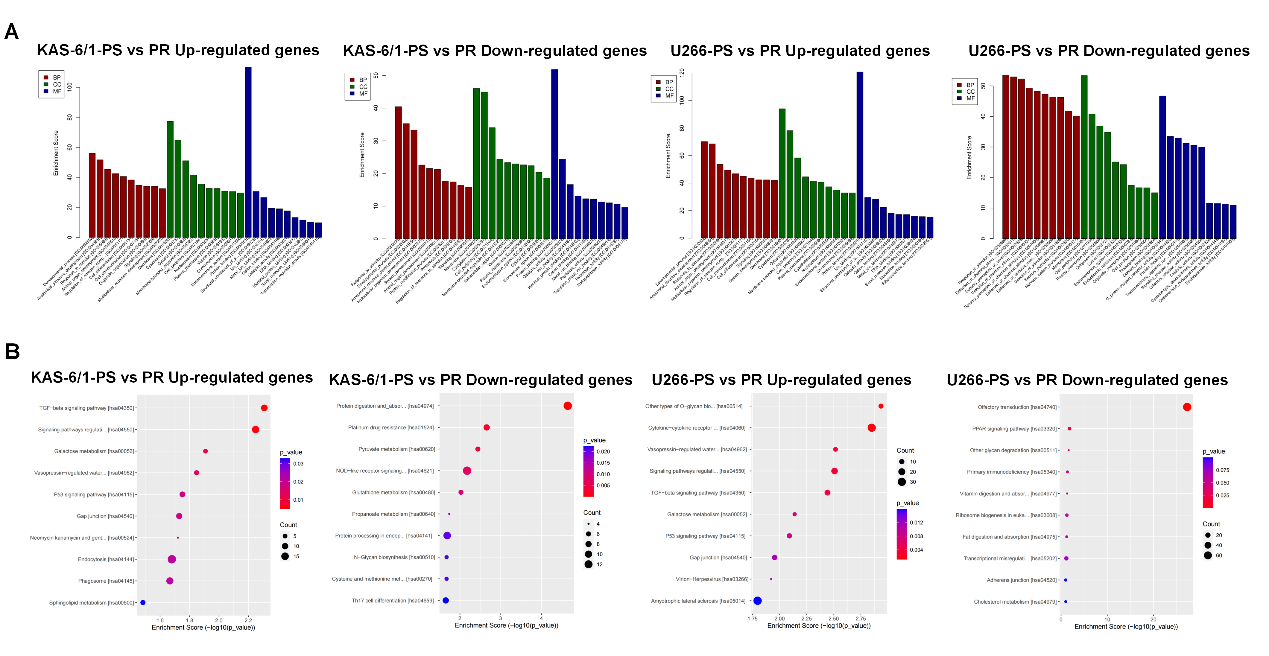


**Supplementary Figure S15 Enrichment analysis of DEED-amplified encoding genes between PI-resistant and PI-sensitive MM cells. A** GO enrichment analysis of DEED-amplified encoding genes between PI-resistant and PI-sensitive KAS-6/1 or U266 cells. **B** KEGG pathway enrichment analysis of DEED-amplified encoding genes between PI-resistant and PI-sensitive KAS-6/1 or U266 cells. PS: proteasome inhibitor-sensitive MM cells; PR: proteasome inhibitor-resistant MM cells.


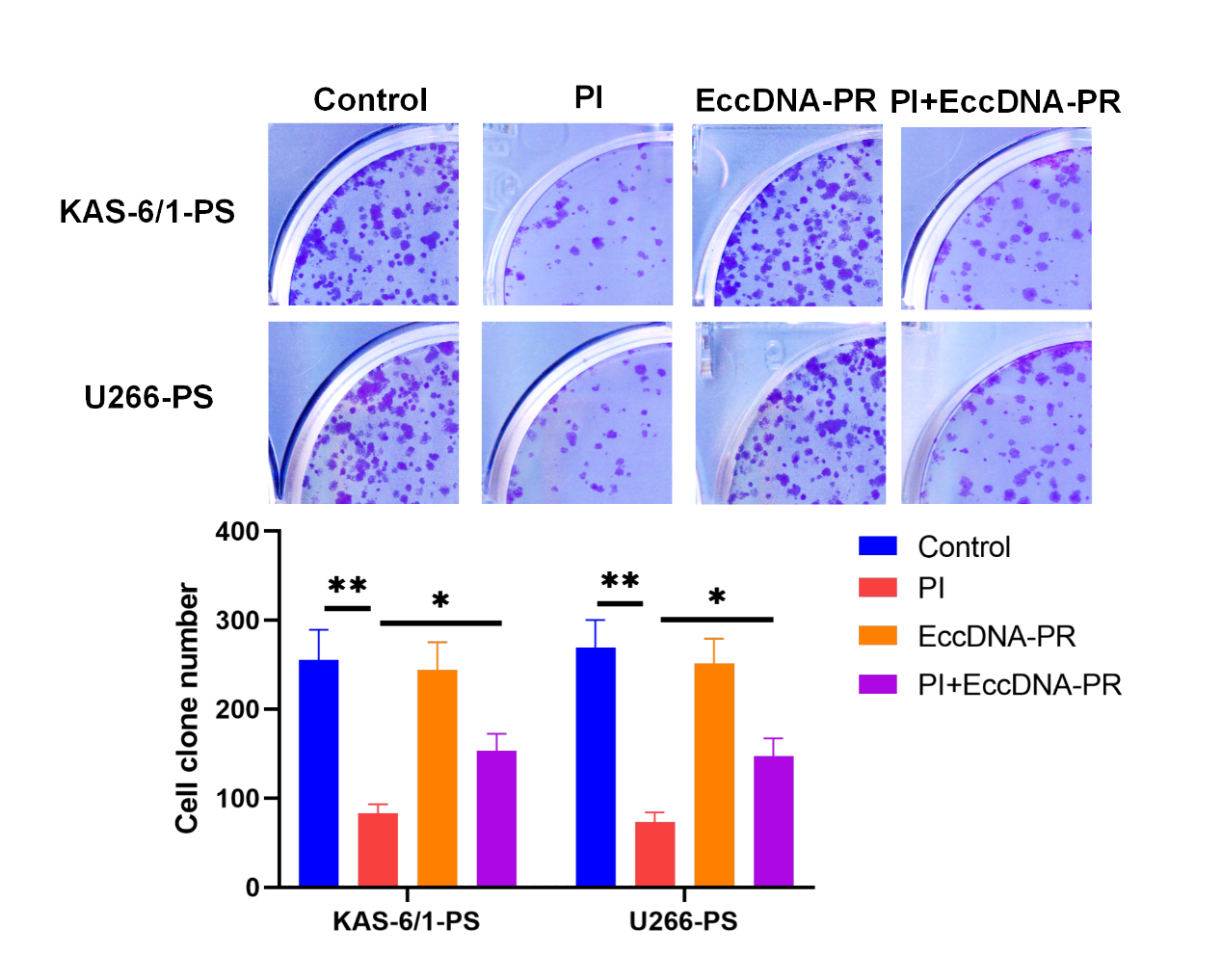


**Supplementary Figure S16 Clone formation of MM cells.** Representative images of cell clones and quantification of cell clone number of eccDNA from PI-resistant KAS-6/1 and U266 cells -transfected PI-sensitive KAS-6/1 and U266 cells treated with or without PI (CFZ). PS: proteasome inhibitor-sensitive MM cells; PR: proteasome inhibitor-resistant MM cells; PI: proteasome inhibitor. *P<0.05, **P<0.01.


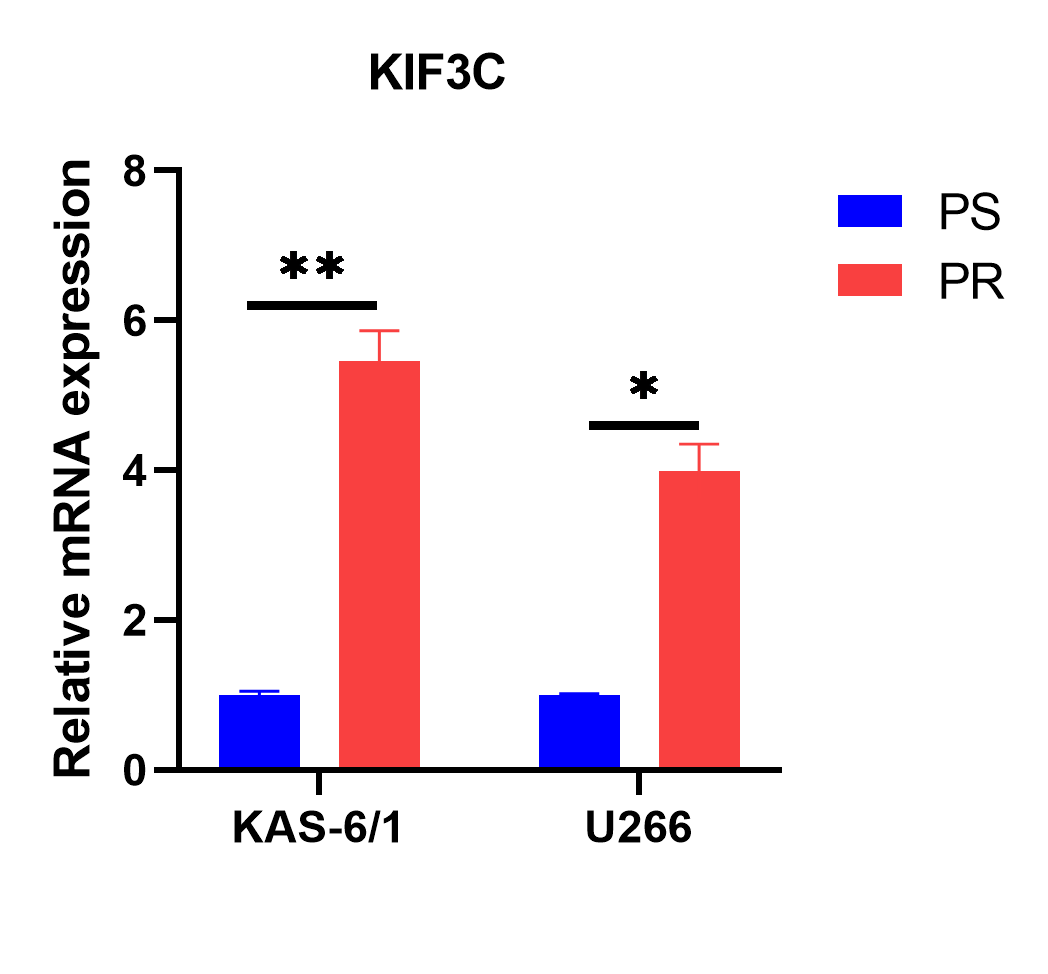


**Supplementary Figure S17 KIF3C mRNA level is upregulated in PI-resistant KAS-6/1 and U266 cells.** KIF3C mRNA level in PI-resistant or PI-sensitive KAS-6/1 and U266 cells. PS: proteasome inhibitor-sensitive MM cells; PR: proteasome inhibitor-resistant MM cells. *P<0.05, **P<0.01.


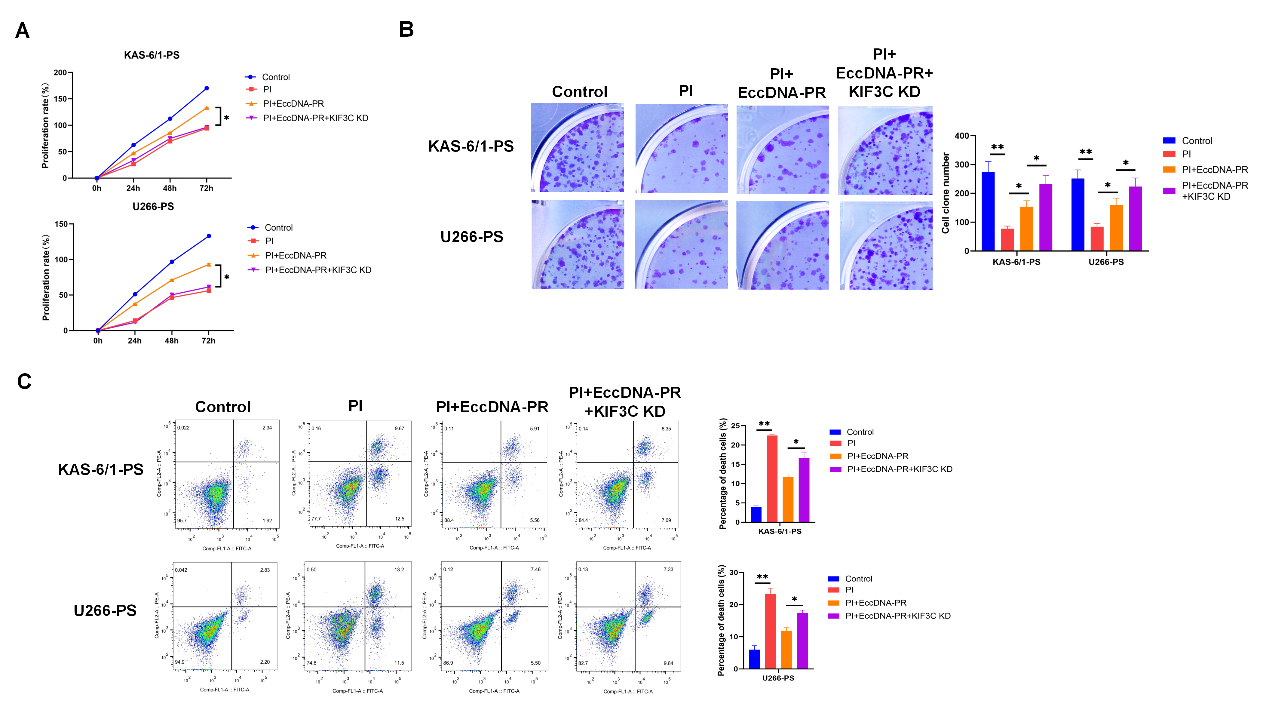


**Supplementary Figure S18 KIF3C knockdown abolishes the effect of eccDNA transfection from PI-resistant MM cells on proliferation and PCD in PI-sensitive MM cells. A** Proliferation rates of eccDNA from PI-resistant KAS-6/1 and U266 cells -transfected or KIF3C KD-transfected PI-sensitive KAS-6/1 and U266 cells treated with or without PI (CFZ). **B** Representative images of cell clones and quantification of cell clone number of eccDNA from PI-resistant KAS-6/1 and U266 cells -transfected or KIF3C KD-transfected PI-sensitive KAS-6/1 and U266 cells treated with or without PI (CFZ). **C** Representative images of flow cytometric analysis for PCD in eccDNA from PI-resistant KAS-6/1 and U266 cells-transfected or KIF3C KD-transfected PI- sensitive KAS-6/1 and U266 cells treated with or without PI (CFZ). PS: proteasome inhibitor-sensitive MM cells; PR: proteasome inhibitor-resistant MM cells; KD: knockdown. *P<0.05, **P<0.01.
