# Supplementary material for: MUC20 regulated by extrachromosomal circular DNA attenuates proteasome inhibitor resistance of multiple myeloma by modulating cuproptosis
Source: J Exp Clin Cancer Res. 2024 Mar 5;43:68. doi: 10.1186/s13046-024-02972-6 (PMC10913264; doi:10.1186/s13046-024-02972-6)
Supplement: Supplementary file 2 — Additional file 2: Supplementary Table S1. Demographic characteristics of HDs and patients with MM. Supplementary Table S2. Demographic characteristics of patients with NDMM and RRMM. Supplementary Table S8. DEED-amplified encoding genes both in PI-resistant KAS-6/1 and U266 cells. [file 13046_2024_2972_MOESM2_ESM.zip › Supplementary Table S1-2-8.docx]

**Supplementary Tables**

**Supplementary Table S1. Demographic** **characteristics of HDs and patients with MM**

| Parameter | HD (n=17) | NDMM (n=53) | tMM (n=52) | RRMM (n=40) |
| --- | --- | --- | --- | --- |
| Median age, years (range) | 65(38–78) | 69(45-80) | 70(51-82) | 72(61-85) |
| Female gender (%) | 8 (47%) | 18(33.9%) | 13(25%) | 12(30%) |
| R-ISS, I-II-III | - | 27-16-10 | 20-19-13 | 9-13-18 |

ISS, International Staging system.

**Supplementary Table S2. Demographic characteristics of patients with NDMM and RRMM**

| Parameter | NDMM (n=53) | RRMM (n=40) |
| --- | --- | --- |
| Median age, years (range) | 70(49-85) | 71(55-81) |
| Female gender (%) | 20(37.7%) | 15（37.5%） |
| R-ISS, I-II-III | 21-15-17 | 9-12-19 |

ISS, International Staging system.

**Supplementary Table S8. DEED-amplified encoding genes both in PI-resistant KAS-6/1 and U266 cells**

| Gene | KAS-6/1-PS | KAS-6/1-PR | Fold change (log2) | U266-PS | U266-PR | Fold change (log2) | Up/Down |
| --- | --- | --- | --- | --- | --- | --- | --- |
| KIF3C | 0 | 14.039 | 3.911 | 0.200 | 28.474 | 4.618 | Up |
| LINC00276 | 0 | 13.450 | 3.853 | 0.200 | 16.615 | 3.876 | Up |
| RN7SL674P | 0 | 15.579 | 4.051 | 0.200 | 13.623 | 3.607 | Up |
| E2F6 | 0 | 13.450 | 3.853 | 0.200 | 13.623 | 3.607 | Up |
| CYS1 | 0 | 13.450 | 3.853 | 0.200 | 12.663 | 3.509 | Up |
| MiR-4798 | 58.703 | 1.029 | -4.886 | 66.529 | 0 | -6.077 | Down |
| LINC01587 | 30.560 | 0.883 | -4.067 | 33.440 | 0 | -5.106 | Down |
| GRPEL1 | 30.560 | 0.883 | -4.067 | 33.440 | 0 | -5.106 | Down |
| MSX1 | 29.676 | 0.883 | -4.026 | 32.589 | 0 | -5.070 | Down |
| USP2-AS1 | 65.303 | 0.611 | -5.363 | 62.877 | 3.730 | -3.755 | Down |

PS: proteasome inhibitor-sensitive MM cells; PR: proteasome inhibitor-resistant MM cells.
