# Supplementary material for: MUC20 regulated by extrachromosomal circular DNA attenuates proteasome inhibitor resistance of multiple myeloma by modulating cuproptosis
Source: J Exp Clin Cancer Res. 2024 Mar 5;43:68. doi: 10.1186/s13046-024-02972-6 (PMC10913264; doi:10.1186/s13046-024-02972-6)
Supplement: Supplementary file 2 — Additional file 2: Supplementary Table S1. Demographic characteristics of HDs and patients with MM. Supplementary Table S2. Demographic characteristics of patients with NDMM and RRMM. Supplementary Table S8. DEED-amplified encoding genes both in PI-resistant KAS-6/1 and U266 cells. [file 13046_2024_2972_MOESM2_ESM.zip › Supplementary Table S4.pdf]

GeneID	bp_evid	ences_Nor	eccDNA_ID	ene_symbo	rDriverGe	ne_bioty	GeneLocus
ENSG0000(	1791	40.55517	chr2:8187	ENSG00000233105		Pseudoger	chr2:94964473-9496
ENSG0000(	1755	39.73999	chr5:4231	ENSG00000271196		Pseudoger	chr5:138454540-138
ENSG0000(	1599	36.20754	chr5:4231	Y_RNA		smallRNA	chr5:128371099-128
ENSG0000(	1562	35.36972	chr8:980	RN7SL826P		smallRNA	chr8:117163968-117
ENSG0000(	1511	34.21488	chr8:2747	ENSG00000253568		Pseudoger	chr8:86776479-8677
ENSG0000(	1496	33.87523	chr8:980	BAALC-AS2		lncRNA	chr8:103132959-103
ENSG0000(	1496	33.87523	chr8:980	ENSG00000287208		lncRNA	chr8:107186997-107
ENSG0000(	1496	33.87523	chr8:980	RNU6ATAC8P		smallRNA	chr8:102421197-102
ENSG0000(	1496	33.87523	chr8:980	AC012564.1		smallRNA	chr8:104755106-104
ENSG0000(	1496	33.87523	chr8:980	Y_RNA		smallRNA	chr8:105929126-105
ENSG0000(	1496	33.87523	chr8:980	ENSG00000289653		protein_c	chr8:102858046-102
ENSG0000(	1496	33.87523	chr8:980	OXR1-AS1		lncRNA	chr8:106520474-106
ENSG0000(	1496	33.87523	chr8:980	NDUFA5P2		Pseudoger	chr8:104566086-104
ENSG0000(	1496	33.87523	chr8:980	SLC16A14P1		Pseudoger	chr8:106215763-106
ENSG0000(	1496	33.87523	chr8:980	ENSG00000253477		lncRNA	chr8:103483398-103
ENSG0000(	1496	33.87523	chr8:980	ENSG00000254236		lncRNA	chr8:103020187-103
ENSG0000(	1496	33.87523	chr8:980	ENSG00000253923		Pseudoger	chr8:102417979-102
ENSG0000(	1496	33.87523	chr8:980	ENSG00000272037		lncRNA	chr8:102256392-102
ENSG0000(	1496	33.87523	chr8:980	ADI1P2		Pseudoger	chr8:102688317-102
ENSG0000(	1496	33.87523	chr8:980	AP003550.1		smallRNA	chr8:103092624-103
ENSG0000(	1496	33.87523	chr8:980	ENSG00000243939		Pseudoger	chr8:104981164-104
ENSG0000(	1496	33.87523	chr8:980	BAALC-AS1		lncRNA	chr8:103153394-103
ENSG0000(	1496	33.87523	chr8:980	ZFPM2	NCGv7	protein_c	chr8:104590733-105
ENSG0000(	1496	33.87523	chr8:980	AP003354.1		smallRNA	chr8:102960813-102
ENSG0000(	1496	33.87523	chr8:980	ENSG00000254041		lncRNA	chr8:105142860-105
ENSG0000(	1496	33.87523	chr8:980	UBR5-DT		lncRNA	chr8:102239386-102
ENSG0000(	1496	33.87523	chr8:980	TAGLN2P1		Pseudoger	chr8:106697427-106
ENSG0000(	1496	33.87523	chr8:980	AP003356.1		smallRNA	chr8:102733635-102
ENSG0000(	1496	33.87523	chr8:980	ENSG00000286766		lncRNA	chr8:107499773-107
ENSG0000(	1496	33.87523	chr8:980	PTMAP15		Pseudoger	chr8:103559099-103
ENSG0000(	1496	33.87523	chr8:980	Y_RNA		smallRNA	chr8:102950467-102
ENSG0000(	1496	33.87523	chr8:980	ENSG00000254492		Pseudoger	chr8:102154550-102
ENSG0000(	1496	33.87523	chr8:980	ENSG00000253420		lncRNA	chr8:105662352-105
ENSG0000(	1496	33.87523	chr8:980	MIR548A3		smallRNA	chr8:104484369-104
ENSG0000(	1496	33.87523	chr8:980	NRBF2P4		Pseudoger	chr8:107983116-107
ENSG0000(	1496	33.87523	chr8:980	RPL5P24		Pseudoger	chr8:102923371-102
ENSG0000(	1496	33.87523	chr8:980	ENSG00000243171		Pseudoger	chr8:105287830-105
ENSG0000(	1496	33.87523	chr8:980	AP003471.2		smallRNA	chr8:104425359-104
ENSG0000(	1496	33.87523	chr8:980	snoU13		smallRNA	chr8:103665295-103
ENSG0000(	1496	33.87523	chr8:980	MIR3151		smallRNA	chr8:103154614-103
ENSG0000(	1496	33.87523	chr8:980	ENSG00000240271		Pseudoger	chr8:105011107-105
ENSG0000(	1496	33.87523	chr8:980	SUMO2P19		Pseudoger	chr8:102242040-102
ENSG0000(	1496	33.87523	chr8:980	ANGPT1	NCGv7	protein_c	chr8:107249482-107
ENSG0000(	1496	33.87523	chr8:980	HSPE1P14		Pseudoger	chr8:102451763-102
ENSG0000(	1496	33.87523	chr8:980	ENSG00000286337		lncRNA	chr8:103319392-103
ENSG0000(	1496	33.87523	chr8:980	MTCO1P4		Pseudoger	chr8:103088796-103
ENSG0000(	1496	33.87523	chr8:980	ATP6V1C1	DriverDB	protein_c	chr8:103021063-103
ENSG0000(	1496	33.87523	chr8:980	HMGB1P46		Pseudoger	chr8:107173200-107
ENSG0000(	1496	33.87523	chr8:980	AZIN1	NCGv7	protein_c	chr8:102826111-102
ENSG0000(	1496	33.87523	chr8:980	RSPO2	NCGv7	protein_c	chr8:107899316-108

ENSG0000(	1496	33.87523	chr8:980E	ENSG00000285982		protein_c	chr8:103398658-103
ENSG0000(	1496	33.87523	chr8:980E	ABRA		protein_c	chr8:106759483-106
ENSG0000(	1496	33.87523	chr8:980E	NCALD		protein_c	chr8:101686542-102
ENSG0000(	1496	33.87523	chr8:980E	UBR5	NCGv7;AC	protein_c	chr8:102252273-102
ENSG0000(	1496	33.87523	chr8:980E	RNU6-1011P		smallRNA	chr8:103384961-103
ENSG0000(	1496	33.87523	chr8:980E	RPS12P15		Pseudoger	chr8:102503753-102
ENSG0000(	1496	33.87523	chr8:980E	RPL12P24		Pseudoger	chr8:105737280-105
ENSG0000(	1496	33.87523	chr8:980E	KLF10		protein_c	chr8:102648784-102
ENSG0000(	1496	33.87523	chr8:980E	LRP12		protein_c	chr8:104489231-104
ENSG0000(	1496	33.87523	chr8:980E	ENSG00000271830		lncRNA	chr8:103481266-103
ENSG0000(	1496	33.87523	chr8:980E	ENSG00000288945		lncRNA	chr8:103451131-103
ENSG0000(	1496	33.87523	chr8:980E	FZD6	DriverDBv	protein_c	chr8:103298433-103
ENSG0000(	1496	33.87523	chr8:980E	DCSTAMP	NCGv7	protein_c	chr8:104339087-104
ENSG0000(	1496	33.87523	chr8:980E	ENSG00000253851		lncRNA	chr8:103228425-103
ENSG0000(	1496	33.87523	chr8:980E	DCAF13	DriverDBv	protein_c	chr8:103414714-103
ENSG0000(	1496	33.87523	chr8:980E	SLC25A32		protein_c	chr8:103398635-103
ENSG0000(	1496	33.87523	chr8:980E	CTHRC1		protein_c	chr8:103371538-103
ENSG0000(	1496	33.87523	chr8:980E	ENSG00000261670		lncRNA	chr8:103464389-103
ENSG0000(	1496	33.87523	chr8:980E	BAALC	AC	protein_c	chr8:103140713-103
ENSG0000(	1496	33.87523	chr8:980E	DPYS	NCGv7	protein_c	chr8:104330324-104
ENSG0000(	1496	33.87523	chr8:980E	ENSG00000179577		lncRNA	chr8:104419512-104
ENSG0000(	1496	33.87523	chr8:980E	MAILR		lncRNA	chr8:102864271-103
ENSG0000(	1496	33.87523	chr8:980E	ENSG00000253526		lncRNA	chr8:105826570-105
ENSG0000(	1496	33.87523	chr8:980E	TMCC1P1		Pseudoger	chr8:105194990-105
ENSG0000(	1496	33.87523	chr8:980E	ENSG00000254141		lncRNA	chr8:105669991-105
ENSG0000(	1496	33.87523	chr8:980E	OXR1	DriverDBv	protein_c	chr8:106270144-106
ENSG0000(	1496	33.87523	chr8:980E	MIR5680		smallRNA	chr8:102125432-102
ENSG0000(	1496	33.87523	chr8:980E	ODF1		protein_c	chr8:102551589-102
ENSG0000(	1496	33.87523	chr8:980E	PDCL3P2		Pseudoger	chr8:101871830-101
ENSG0000(	1496	33.87523	chr8:980E	ENSG00000253263		lncRNA	chr8:102891876-102
ENSG0000(	1496	33.87523	chr8:980E	snoU13		smallRNA	chr8:102388526-102
ENSG0000(	1496	33.87523	chr8:980E	RNU6-1224P		smallRNA	chr8:102533241-102
ENSG0000(	1496	33.87523	chr8:980E	ENSG00000254644		Pseudoger	chr8:103568603-103
ENSG0000(	1496	33.87523	chr8:980E	ENSG00000254021		lncRNA	chr8:106265435-106
ENSG0000(	1496	33.87523	chr8:980E	AP000428.1		smallRNA	chr8:107254407-107
ENSG0000(	1496	33.87523	chr8:980E	GASAL1		lncRNA	chr8:102805517-102
ENSG0000(	1496	33.87523	chr8:980E	AP001572.1		smallRNA	chr8:103851599-103
ENSG0000(	1496	33.87523	chr8:980E	MTND1P5		Pseudoger	chr8:103085651-103
ENSG0000(	1496	33.87523	chr8:980E	ENSG00000253385		lncRNA	chr8:102854455-102
ENSG0000(	1496	33.87523	chr8:980E	ENSG00000239344		Pseudoger	chr8:103768281-103
ENSG0000(	1496	33.87523	chr8:980E	LINC01181		lncRNA	chr8:103121032-103
ENSG0000(	1496	33.87523	chr8:980E	POU5F1P2		Pseudoger	chr8:102621509-102
ENSG0000(	1496	33.87523	chr8:980E	RNA5SP275		Pseudoger	chr8:107884494-107
ENSG0000(	1496	33.87523	chr8:980E	RRM2B	DriverDBv	protein_c	chr8:102204502-102
ENSG0000(	1496	33.87523	chr8:980E	ENSG00000283959		lncRNA	chr8:102656241-102
ENSG0000(	1496	33.87523	chr8:980E	MTCO2P4		Pseudoger	chr8:103091263-103
ENSG0000(	1496	33.87523	chr8:980E	NPM1P52		Pseudoger	chr8:103016744-103
ENSG0000(	1496	33.87523	chr8:980E	ENSG00000283157		lncRNA	chr8:104699479-104
ENSG0000(	1496	33.87523	chr8:980E	LINC02933		lncRNA	chr8:103383078-103
ENSG0000(	1496	33.87523	chr8:980E	ENSG00000287289		lncRNA	chr8:107160354-107
ENSG0000(	1496	33.87523	chr8:980E	ENSG00000287949		lncRNA	chr8:107857589-107

ENSG0000(	1496	33.87523	chr8:9805	PGAM1P13		Pseudoger	chr8:107647279-107
ENSG0000(	1496	33.87523	chr8:9805	snoU13		smallRNA	chr8:101847256-101
ENSG0000(	1496	33.87523	chr8:9805	ENSG00000279138		TEC	chr8:104478539-104
ENSG0000(	1496	33.87523	chr8:9805	RIMS2	NCGv7	protein_c	chr8:103500610-104
ENSG0000(	1496	33.87523	chr8:9805	ENSG00000253633		lncRNA	chr8:102528740-102
ENSG0000(	1496	33.87523	chr8:9805	ENSG00000253629		lncRNA	chr8:101686547-101
ENSG0000(	1496	33.87523	chr8:9805	ZFPM2-AS1		lncRNA	chr8:105546089-106
ENSG0000(	1496	33.87523	chr8:9805	AP003471.3		smallRNA	chr8:104435453-104
ENSG0000(	1496	33.87523	chr8:9805	ENSG00000253997		Pseudoger	chr8:104990308-104
ENSG0000(	1496	33.87523	chr8:9805	RNU7-84P		smallRNA	chr8:106729795-106
ENSG0000(	1496	33.87523	chr8:9805	ENSG00000275905		Pseudoger	chr8:106980967-106
ENSG0000(	1475	33.3997	chr1:1021	PPIAL4E		protein_c	chr1:144372874-144
ENSG0000(	1475	33.3997	chr1:1021	ENSG00000227193		lncRNA	chr1:143905552-143
ENSG0000(	1475	33.3997	chr1:1021	NOTCH2NLC		protein_c	chr1:146148864-146
ENSG0000(	1475	33.3997	chr1:1021	NOTCH2NLB		protein_c	chr1:146149342-146
ENSG0000(	1475	33.3997	chr1:1021	FAM91A3P		Pseudoger	chr1:143766540-143
ENSG0000(	1475	33.3997	chr1:1021	FCGR1CP		Pseudoger	chr1:143874793-143
ENSG0000(	1475	33.3997	chr1:1021	CR812485.1		smallRNA	chr1:143828767-143
ENSG0000(	1475	33.3997	chr1:1021	RNVU1-11		smallRNA	chr1:148388490-148
ENSG0000(	1475	33.3997	chr1:1021	RNU1-120P		smallRNA	chr1:148263476-148
ENSG0000(	1475	33.3997	chr1:1021	ENSG00000271546		Pseudoger	chr1:148795796-148
ENSG0000(	1475	33.3997	chr1:1021	LSP1P5		lncRNA	chr1:143401427-143
ENSG0000(	1475	33.3997	chr1:1021	RN7SL261P		smallRNA	chr1:147689256-147
ENSG0000(	1475	33.3997	chr1:1021	RP11-277L2.3		lncRNA	chr1:149607448-149
ENSG0000(	1475	33.3997	chr1:1021	H3-7		protein_c	chr1:143894544-143
ENSG0000(	1475	33.3997	chr1:1021	RNU1-114P		smallRNA	chr1:143652050-143
ENSG0000(	1475	33.3997	chr1:1021	ABHD17AP2		Pseudoger	chr1:148146395-148
ENSG0000(	1475	33.3997	chr1:1021	SEC22B2P		Pseudoger	chr1:148772639-148
ENSG0000(	1475	33.3997	chr1:1021	RNU1-13P		smallRNA	chr1:148388490-148
ENSG0000(	1475	33.3997	chr1:1021	NUDT4P2		Pseudoger	chr1:148748952-148
ENSG0000(	1475	33.3997	chr1:1021	RNU1-122P		smallRNA	chr1:148334612-148
ENSG0000(	1475	33.3997	chr1:1021	RNVU1-8		smallRNA	chr1:147084616-147
ENSG0000(	1475	33.3997	chr1:1021	LINC01731		lncRNA	chr1:148271884-148
ENSG0000(	1475	33.3997	chr1:1021	RP11-403I13.9		lncRNA	chr1:143811359-143
ENSG0000(	1475	33.3997	chr1:1021	LINC01719		lncRNA	chr1:146052565-146
ENSG0000(	1475	33.3997	chr1:1021	RNVU1-7		smallRNA	chr1:145465617-145
ENSG0000(	1475	33.3997	chr1:1021	CHD1L	TAG;AC	protein_c	chr1:147242654-147
ENSG0000(	1475	33.3997	chr1:1021	ENSG00000272824		lncRNA	chr1:148358245-148
ENSG0000(	1475	33.3997	chr1:1021	LINC01691		lncRNA	chr1:121573946-121
ENSG0000(	1475	33.3997	chr1:1021	RP11-337C18.9		lncRNA	chr1:147175602-147
ENSG0000(	1475	33.3997	chr1:1021	PDIA3P1		Pseudoger	chr1:147172744-147
ENSG0000(	1475	33.3997	chr1:1021	SEC22B4P		Pseudoger	chr1:148772639-148
ENSG0000(	1475	33.3997	chr1:1021	ENSG00000227139		lncRNA	chr1:147697794-147
ENSG0000(	1475	33.3997	chr1:1021	RNVU1-17		smallRNA	chr1:143699456-143
ENSG0000(	1475	33.3997	chr1:1021	RNVU1-10		smallRNA	chr1:148362370-148
ENSG0000(	1475	33.3997	chr1:1021	ENSG00000223779		Pseudoger	chr1:143745249-143
ENSG0000(	1475	33.3997	chr1:1021	FCGR1BP		Pseudoger	chr1:143876113-143
ENSG0000(	1475	33.3997	chr1:1021	ENSG00000254539		lncRNA	chr1:149048575-149
ENSG0000(	1475	33.3997	chr1:1021	ENSG00000271644		Pseudoger	chr1:144965024-144
ENSG0000(	1475	33.3997	chr1:1021	PEX11B		protein_c	chr1:145911349-145
ENSG0000(	1475	33.3997	chr1:1021	ANKRD35		protein_c	chr1:145866559-145

ENSG0000(	1475	33.3997	chr1:1021	ENSG00000286185		protein_c	chr1:146069621-146
ENSG0000(	1475	33.3997	chr1:1021	RP11-439A17.10		lncRNA	chr1:143874925-143
ENSG0000(	1475	33.3997	chr1:1021	Y_RNA		smallRNA	chr1:148330271-148
ENSG0000(	1475	33.3997	chr1:1021	RNF115		protein_c	chr1:145738867-145
ENSG0000(	1475	33.3997	chr1:1021	RP11-14N7.2		lncRNA	chr1:143401429-143
ENSG0000(	1475	33.3997	chr1:1021	ENSG00000290705		lncRNA	chr1:147993862-148
ENSG0000(	1475	33.3997	chr1:1021	ENSG00000274415		lncRNA	chr1:147757185-147
ENSG0000(	1475	33.3997	chr1:1021	ENSG00000290735		lncRNA	chr1:143875171-143
ENSG0000(	1475	33.3997	chr1:1021	LIX1L		protein_c	chr1:145933422-145
ENSG0000(	1475	33.3997	chr1:1021	EMBP1		Pseudoger	chr1:121519345-121
ENSG0000(	1475	33.3997	chr1:1021	FMO5	DriverDB\	protein_c	chr1:147175351-147
ENSG0000(	1475	33.3997	chr1:1021	RPL7AP15		Pseudoger	chr1:147223554-147
ENSG0000(	1475	33.3997	chr1:1021	MTIF2P1		Pseudoger	chr1:121502344-121
ENSG0000(	1475	33.3997	chr1:1021	RNVU1-20		smallRNA	chr1:149636766-149
ENSG0000(	1475	33.3997	chr1:1021	ENSG00000225603		Pseudoger	chr1:147050817-147
ENSG0000(	1475	33.3997	chr1:1021	ENSG00000278431		lncRNA	chr1:145961387-145
ENSG0000(	1475	33.3997	chr1:1021	ENSG00000284964		Pseudoger	chr1:149607467-149
ENSG0000(	1475	33.3997	chr1:1021	BCL9	NCGv7;AC	protein_c	chr1:147541501-147
ENSG0000(	1475	33.3997	chr1:1021	HIST2H3DP1		Pseudoger	chr1:143905555-143
ENSG0000(	1475	33.3997	chr1:1021	SEC22B3P		Pseudoger	chr1:148772639-148
ENSG0000(	1475	33.3997	chr1:1021	CD160		protein_c	chr1:145719470-145
ENSG0000(	1475	33.3997	chr1:1021	GPR89A		protein_c	chr1:145607987-145
ENSG0000(	1475	33.3997	chr1:1021	RP11-666A1.5		lncRNA	chr1:144418122-144
ENSG0000(	1475	33.3997	chr1:1021	ENSG00000275129		Pseudoger	chr1:144965024-144
ENSG0000(	1475	33.3997	chr1:1021	GNRHR2		Pseudoger	chr1:145919012-145
ENSG0000(	1475	33.3997	chr1:1021	PDE4DIPP2		Pseudoger	chr1:148808504-149
ENSG0000(	1475	33.3997	chr1:1021	RP6-137J22.3		Pseudoger	chr1:145269604-145
ENSG0000(	1475	33.3997	chr1:1021	NBPF13P		Pseudoger	chr1:147099482-147
ENSG0000(	1475	33.3997	chr1:1021	KMT2CP1		Pseudoger	chr1:143461247-143
ENSG0000(	1475	33.3997	chr1:1021	AL109844.1		smallRNA	chr1:143828767-143
ENSG0000(	1475	33.3997	chr1:1021	RN7SKP88		smallRNA	chr1:148839482-148
ENSG0000(	1475	33.3997	chr1:1021	NKAIN1P1		Pseudoger	chr1:143487771-143
ENSG0000(	1475	33.3997	chr1:1021	ENSG00000237503		Pseudoger	chr1:143846097-143
ENSG0000(	1475	33.3997	chr1:1021	LINC00624		lncRNA	chr1:147258885-147
ENSG0000(	1475	33.3997	chr1:1021	FAM72D	NCGv7	protein_c	chr1:143955287-143
ENSG0000(	1475	33.3997	chr1:1021	RP11-353N4.3		Pseudoger	chr1:149693026-149
ENSG0000(	1475	33.3997	chr1:1021	RP6-206I17.2		lncRNA	chr1:148402453-148
ENSG0000(	1475	33.3997	chr1:1021	ENSG00000232721		lncRNA	chr1:143735983-143
ENSG0000(	1475	33.3997	chr1:1021	AL138796.1		smallRNA	chr1:149007076-149
ENSG0000(	1475	33.3997	chr1:1021	RNVU1-1		smallRNA	chr1:148362370-148
ENSG0000(	1475	33.3997	chr1:1021	NBPF9		protein_c	chr1:149054026-149
ENSG0000(	1475	33.3997	chr1:1021	PRKAB2	NCGv7	protein_c	chr1:147155106-147
ENSG0000(	1475	33.3997	chr1:1021	HJV		protein_c	chr1:146017467-146
ENSG0000(	1475	33.3997	chr1:1021	POLR3C		protein_c	chr1:145824052-145
ENSG0000(	1475	33.3997	chr1:1021	RNU1-137P		smallRNA	chr1:145431527-145
ENSG0000(	1475	33.3997	chr1:1021	ENSG00000288626		protein_c	chr1:147611590-147
ENSG0000(	1475	33.3997	chr1:1021	PDZK1P1		Pseudoger	chr1:147994301-148
ENSG0000(	1475	33.3997	chr1:1021	ENSG00000273059		lncRNA	chr1:148011799-148
ENSG0000(	1475	33.3997	chr1:1021	ENSG00000237188		lncRNA	chr1:147172755-147
ENSG0000(	1475	33.3997	chr1:1021	RNVU1-8		smallRNA	chr1:147079746-147
ENSG0000(	1475	33.3997	chr1:1021	NUDT17		protein_c	chr1:145845629-145

ENSG0000(	1475	33.3997	chr1:1021	RNVU1-12		smallRNA	chr1:148402715-148
ENSG0000(	1475	33.3997	chr1:1021	SRGAP2-AS1		lncRNA	chr1:121360156-121
ENSG0000(	1475	33.3997	chr1:1021	RP11-353N4.1		lncRNA	chr1:149621576-149
ENSG0000(	1475	33.3997	chr1:1021	KMT2CP3		Pseudoger	chr1:143461220-143
ENSG0000(	1475	33.3997	chr1:1021	XXyac-YX155B6.2		Pseudoger	chr1:148080598-148
ENSG0000(	1475	33.3997	chr1:1021	ENSG00000271439		Pseudoger	chr1:144401068-144
ENSG0000(	1475	33.3997	chr1:1021	GJA8		protein_c	chr1:147902795-147
ENSG0000(	1475	33.3997	chr1:1021	PFN1P6		Pseudoger	chr1:144442605-144
ENSG0000(	1475	33.3997	chr1:1021	RP11-439A17.7		lncRNA	chr1:143972630-143
ENSG0000(	1475	33.3997	chr1:1021	ENSG00000290999		lncRNA	chr1:148962571-149
ENSG0000(	1475	33.3997	chr1:1021	RNU6-1071P		smallRNA	chr1:148739378-148
ENSG0000(	1475	33.3997	chr1:1021	PIAS3		protein_c	chr1:145848521-145
ENSG0000(	1475	33.3997	chr1:1021	NOTCH2NLR		protein_c	chr1:146155128-146
ENSG0000(	1475	33.3997	chr1:1021	ENSG00000277702		Pseudoger	chr1:143419624-143
ENSG0000(	1475	33.3997	chr1:1021	POLR3GL		protein_c	chr1:145964689-145
ENSG0000(	1475	33.3997	chr1:1021	PFN1P3		Pseudoger	chr1:149084616-149
ENSG0000(	1475	33.3997	chr1:1021	PFN1P4		Pseudoger	chr1:148129497-148
ENSG0000(	1475	33.3997	chr1:1021	ENSG00000270962		Pseudoger	chr1:143784376-143
ENSG0000(	1475	33.3997	chr1:1021	ENSG00000213226		Pseudoger	chr1:147319110-147
ENSG0000(	1475	33.3997	chr1:1021	RNA5SP57		smallRNA	chr1:148193716-148
ENSG0000(	1475	33.3997	chr1:1021	NBPF10		protein_c	chr1:146075000-146
ENSG0000(	1475	33.3997	chr1:1021	RNU1-59P		smallRNA	chr1:149162782-149
ENSG0000(	1475	33.3997	chr1:1021	RP4-565E6.1		lncRNA	chr1:148162787-148
ENSG0000(	1475	33.3997	chr1:1021	SEC22B		protein_c	chr1:148770173-148
ENSG0000(	1475	33.3997	chr1:1021	SSBL4P		Pseudoger	chr1:147082338-147
ENSG0000(	1475	33.3997	chr1:1021	PFN1P5		Pseudoger	chr1:148129480-148
ENSG0000(	1475	33.3997	chr1:1021	ENSG00000224335		Pseudoger	chr1:148234273-148
ENSG0000(	1475	33.3997	chr1:1021	SRGAP2C		Pseudoger	chr1:121365263-121
ENSG0000(	1475	33.3997	chr1:1021	RP11-289H16.1		lncRNA	chr1:144917169-144
ENSG0000(	1475	33.3997	chr1:1021	ENSG00000227700		Pseudoger	chr1:148246169-148
ENSG0000(	1475	33.3997	chr1:1021	RNVU1-6		smallRNA	chr1:146052080-146
ENSG0000(	1475	33.3997	chr1:1021	ENSG00000224481		lncRNA	chr1:148295895-148
ENSG0000(	1475	33.3997	chr1:1021	ENSG00000227242		Pseudoger	chr1:147019656-147
ENSG0000(	1475	33.3997	chr1:1021	ACP6		protein_c	chr1:147629652-147
ENSG0000(	1475	33.3997	chr1:1021	RP11-289I10.3		Pseudoger	chr1:148511083-148
ENSG0000(	1475	33.3997	chr1:1021	TXNIP	NCGv7	protein_c	chr1:145992434-145
ENSG0000(	1475	33.3997	chr1:1021	RNA5SP58		smallRNA	chr1:148193716-148
ENSG0000(	1475	33.3997	chr1:1021	LINC02798		lncRNA	chr1:121396754-121
ENSG0000(	1475	33.3997	chr1:1021	Y_RNA		smallRNA	chr1:147420199-147
ENSG0000(	1475	33.3997	chr1:1021	LINC02804		lncRNA	chr1:148013203-148
ENSG0000(	1475	33.3997	chr1:1021	RP11-439A17.9		lncRNA	chr1:143877730-143
ENSG0000(	1475	33.3997	chr1:1021	OR13Z3P		Pseudoger	chr1:147482238-147
ENSG0000(	1475	33.3997	chr1:1021	ENSG00000230186		lncRNA	chr1:143905487-143
ENSG0000(	1475	33.3997	chr1:1021	AL590452.1		protein_c	chr1:148893126-148
ENSG0000(	1475	33.3997	chr1:1021	ENSG00000291232		lncRNA	chr1:148402516-148
ENSG0000(	1475	33.3997	chr1:1021	ENSG00000229002		Pseudoger	chr1:144472534-144
ENSG0000(	1475	33.3997	chr1:1021	LINC00869		Pseudoger	chr1:149655747-149
ENSG0000(	1475	33.3997	chr1:1021	RPL22P5		Pseudoger	chr1:143929994-143
ENSG0000(	1475	33.3997	chr1:1021	ENSG00000223612		Pseudoger	chr1:145233001-145
ENSG0000(	1475	33.3997	chr1:1021	ITGA10		protein_c	chr1:145891207-145
ENSG0000(	1475	33.3997	chr1:1021	ENSG00000275557		lncRNA	chr1:149607765-149

ENSG0000(	1475	33.3997	chr1:1021	RNVU1-3	smallRNA	chr1:148402715-148
ENSG0000(	1475	33.3997	chr1:1021	RP11-495P10.8	lncRNA	chr1:148290890-148
ENSG0000(	1475	33.3997	chr1:1021	RNU1-135P	smallRNA	chr1:148385829-148
ENSG0000(	1475	33.3997	chr1:1021	ENSG00000254913	lncRNA	chr1:149006308-149
ENSG0000(	1475	33.3997	chr1:1021	LINC02805	lncRNA	chr1:148156139-148
ENSG0000(	1475	33.3997	chr1:1021	RP11-495P10.7	lncRNA	chr1:148295792-148
ENSG0000(	1475	33.3997	chr1:1021	ENSG00000287374	lncRNA	chr1:145475606-145
ENSG0000(	1475	33.3997	chr1:1021	GPR89B	protein_c	chr1:147928393-147
ENSG0000(	1475	33.3997	chr1:1021	BX842679.1	protein_c	chr1:148159688-148
ENSG0000(	1475	33.3997	chr1:1021	EMBP1	lncRNA	chr1:121519103-121
ENSG0000(	1475	33.3997	chr1:1021	ENSG00000272755	lncRNA	chr1:148865453-148
ENSG0000(	1475	33.3997	chr1:1021	RP11-403I13.7	lncRNA	chr1:143790010-143
ENSG0000(	1475	33.3997	chr1:1021	RP11-343N15.5	lncRNA	chr1:121391395-121
ENSG0000(	1475	33.3997	chr1:1021	NBPF17P	Pseudoger	chr1:143595216-143
ENSG0000(	1475	33.3997	chr1:1021	RNU1-129P	smallRNA	chr1:148014417-148
ENSG0000(	1475	33.3997	chr1:1021	ANKRD34A	protein_c	chr1:145959440-145
ENSG0000(	1475	33.3997	chr1:1021	GJA5	protein_c	chr1:147756199-147
ENSG0000(	1475	33.3997	chr1:1021	RNA5SP59	Pseudoger	chr1:143439604-143
ENSG0000(	1475	33.3997	chr1:1021	RNVU1-18	smallRNA	chr1:143729407-143
ENSG0000(	1475	33.3997	chr1:1021	ENSG00000228626	Pseudoger	chr1:148288001-148
ENSG0000(	1475	33.3997	chr1:1021	PFN1P12	Pseudoger	chr1:143619994-143
ENSG0000(	1475	33.3997	chr1:1021	RP6-206I17.3	lncRNA	chr1:148435062-148
ENSG0000(	1475	33.3997	chr1:1021	CCT8P1	Pseudoger	chr1:147203276-147
ENSG0000(	1475	33.3997	chr1:1021	RP6-206I17.4	Pseudoger	chr1:148435103-148
ENSG0000(	1475	33.3997	chr1:1021	ENSG00000289642	lncRNA	chr1:149013782-149
ENSG0000(	1475	33.3997	chr1:1021	HYDIN2	Pseudoger	chr1:146875321-146
ENSG0000(	1475	33.3997	chr1:1021	XXyac-YX155B6.6	lncRNA	chr1:148162787-148
ENSG0000(	1475	33.3997	chr1:1021	RP11-666A1.3	Pseudoger	chr1:144418677-144
ENSG0000(	1475	33.3997	chr1:1021	PDE4DIP NCGv7;AC	protein_c	chr1:148808139-149
ENSG0000(	1475	33.3997	chr1:1021	NBPF25P	Pseudoger	chr1:149058924-149
ENSG0000(	1475	33.3997	chr1:1021	NOTCH2NLA	protein_c	chr1:146146202-146
ENSG0000(	1475	33.3997	chr1:1021	PFN1P8	Pseudoger	chr1:146957117-146
ENSG0000(	1475	33.3997	chr1:1021	RP11-277L2.4	lncRNA	chr1:149606196-149
ENSG0000(	1475	33.3997	chr1:1021	RNU1-92P	smallRNA	chr1:143720510-143
ENSG0000(	1475	33.3997	chr1:1021	ENSG00000223495	Pseudoger	chr1:143498784-143
ENSG0000(	1475	33.3997	chr1:1021	FAM72B	protein_c	chr1:143955289-143
ENSG0000(	1475	33.3997	chr1:1021	RNVU1-15	smallRNA	chr1:144412575-144
ENSG0000(	1475	33.3997	chr1:1021	ENSG00000235988	Pseudoger	chr1:148317683-148
ENSG0000(	1475	33.3997	chr1:1021	RP11-458D21.6	lncRNA	chr1:146237251-146
ENSG0000(	1475	33.3997	chr1:1021	ENSG00000264145	Pseudoger	chr1:143449570-143
ENSG0000(	1475	33.3997	chr1:1021	ENSG00000287190	lncRNA	chr1:146050440-146
ENSG0000(	1475	33.3997	chr1:1021	ENSG00000280778	protein_c	chr1:145927257-145
ENSG0000(	1475	33.3997	chr1:1021	RP11-277L2.5	lncRNA	chr1:149618320-149
ENSG0000(	1475	33.3997	chr1:1021	ENSG00000289318	lncRNA	chr1:143972669-143
ENSG0000(	1475	33.3997	chr1:1021	ENSG00000291233	lncRNA	chr1:148889429-148
ENSG0000(	1475	33.3997	chr1:1021	OR13Z2P	Pseudoger	chr1:147445579-147
ENSG0000(	1475	33.3997	chr1:1021	RNU2-38P	smallRNA	chr1:148939738-148
ENSG0000(	1475	33.3997	chr1:1021	GPR89C	protein_c	chr1:147953335-147
ENSG0000(	1475	33.3997	chr1:1021	FAM72C	protein_c	chr1:143944179-143
ENSG0000(	1475	33.3997	chr1:1021	ENSG00000289565	protein_c	chr1:145917713-145
ENSG0000(	1475	33.3997	chr1:1021	H2BP1	Pseudoger	chr1:143904287-143

ENSG0000(	1475	33.3997	chr1:1021	ENSG00000203825		Pseudoger	chr1:143541768-143
ENSG0000(	1475	33.3997	chr1:1021	ENSG00000234190		lncRNA	chr1:147777590-147
ENSG0000(	1475	33.3997	chr1:1021	H2BP1		lncRNA	chr1:143894527-143
ENSG0000(	1475	33.3997	chr1:1021	RNVU1-2		smallRNA	chr1:148385829-148
ENSG0000(	1475	33.3997	chr1:1021	ENSG00000223728		Pseudoger	chr1:147840962-147
ENSG0000(	1475	33.3997	chr1:1021	ABHD17AP1		Pseudoger	chr1:148146394-148
ENSG0000(	1475	33.3997	chr1:1021	PPIAL4G	NCGv7	protein_c	chr1:148482548-148
ENSG0000(	1475	33.3997	chr1:1021	PFN1P2		Pseudoger	chr1:149084616-149
ENSG0000(	1475	33.3997	chr1:1021	NBPF24		protein_c	chr1:148102151-148
ENSG0000(	1475	33.3997	chr1:1021	ENSG00000234225		lncRNA	chr1:147001931-147
ENSG0000(	1475	33.3997	chr1:1021	ENSG00000276509		lncRNA	chr1:146235805-146
ENSG0000(	1475	33.3997	chr1:1021	DRD5P2		Pseudoger	chr1:143449275-143
ENSG0000(	1475	33.3997	chr1:1021	ENSG00000228826		lncRNA	chr1:121494329-121
ENSG0000(	1475	33.3997	chr1:1021	LINC02806		lncRNA	chr1:148295180-148
ENSG0000(	1475	33.3997	chr1:1021	NUDT4B		protein_c	chr1:148748773-148
ENSG0000(	1475	33.3997	chr1:1021	RNVU1-16		smallRNA	chr1:145281115-145
ENSG0000(	1475	33.3997	chr1:1021	ENSG00000272583		lncRNA	chr1:121518365-121
ENSG0000(	1475	33.3997	chr1:1021	LINC02799		lncRNA	chr1:143499186-143
ENSG0000(	1475	33.3997	chr1:1021	ENSG00000255148		lncRNA	chr1:149018670-149
ENSG0000(	1475	33.3997	chr1:1021	AL732363.1		smallRNA	chr1:143541645-143
ENSG0000(	1475	33.3997	chr1:1021	RPL22P6		Pseudoger	chr1:143929994-143
ENSG0000(	1475	33.3997	chr1:1021	ENSG00000225871		Pseudoger	chr1:148435105-148
ENSG0000(	1475	33.3997	chr1:1021	ENSG00000289419		lncRNA	chr1:147608331-147
ENSG0000(	1475	33.3997	chr1:1021	RNVU1-9		smallRNA	chr1:148038753-148
ENSG0000(	1475	33.3997	chr1:1021	PDZK1		protein_c	chr1:145670851-145
ENSG0000(	1475	33.3997	chr1:1021	WI2-925H4.1		lncRNA	chr1:145601945-145
ENSG0000(	1475	33.3997	chr1:1021	LIX1L-AS1		lncRNA	chr1:145926589-145
ENSG0000(	1475	33.3997	chr1:1021	ENSG00000244619		lncRNA	chr1:145892846-145
ENSG0000(	1475	33.3997	chr1:1021	PDE4DIPP6		Pseudoger	chr1:148415258-148
ENSG0000(	1475	33.3997	chr1:1021	OR13Z1P		Pseudoger	chr1:147419053-147
ENSG0000(	1475	33.3997	chr1:1021	RNU1-143P		smallRNA	chr1:143791542-143
ENSG0000(	1475	33.3997	chr1:1021	RBM8A	NCGv7	protein_c	chr1:145921555-145
ENSG0000(	1463	33.12798	chr1:1021	RNY4P25		smallRNA	chr1:151439000-151
ENSG0000(	1463	33.12798	chr1:1021	SELENBP1		protein_c	chr1:151364304-151
ENSG0000(	1463	33.12798	chr1:1021	CA14		protein_c	chr1:150257251-150
ENSG0000(	1463	33.12798	chr1:1021	SCNM1		protein_c	chr1:151156664-151
ENSG0000(	1463	33.12798	chr1:1021	TMOD4		protein_c	chr1:151169986-151
ENSG0000(	1463	33.12798	chr1:1021	BNIPL	DriverDB\	protein_c	chr1:151036321-151
ENSG0000(	1463	33.12798	chr1:1021	LYSMD1		protein_c	chr1:151159748-151
ENSG0000(	1463	33.12798	chr1:1021	VPS72		protein_c	chr1:151176304-151
ENSG0000(	1463	33.12798	chr1:1021	RN7SL473P		smallRNA	chr1:150566564-150
ENSG0000(	1463	33.12798	chr1:1021	TNFAIP8L2		protein_c	chr1:151156649-151
ENSG0000(	1463	33.12798	chr1:1021	ANXA9		protein_c	chr1:150982249-150
ENSG0000(	1463	33.12798	chr1:1021	RFX5-AS1		lncRNA	chr1:151346938-151
ENSG0000(	1463	33.12798	chr1:1021	ADAMTSL4		protein_c	chr1:150549369-150
ENSG0000(	1463	33.12798	chr1:1021	C1orf54		protein_c	chr1:150268200-150
ENSG0000(	1463	33.12798	chr1:1021	PI4KB		protein_c	chr1:151291797-151
ENSG0000(	1463	33.12798	chr1:1021	APH1A		protein_c	chr1:150265399-150
ENSG0000(	1463	33.12798	chr1:1021	CERS2		protein_c	chr1:150960583-150
ENSG0000(	1463	33.12798	chr1:1021	RFX5		protein_c	chr1:151340640-151
ENSG0000(	1463	33.12798	chr1:1021	PIP5K1A		protein_c	chr1:151197949-151

ENSG0000(	1463	33.12798	chr1:1021	CTSK		protein_c	chr1:150794880-150
ENSG0000(	1463	33.12798	chr1:1021	UBE2D3P3		Pseudoger	chr1:150800473-150
ENSG0000(	1463	33.12798	chr1:1021	CTXND2		protein_c	chr1:150887136-150
ENSG0000(	1463	33.12798	chr1:1021	Y_RNA		smallRNA	chr1:150882451-150
ENSG0000(	1463	33.12798	chr1:1021	ENSG00000224645		lncRNA	chr1:151340648-151
ENSG0000(	1463	33.12798	chr1:1021	RN7SL480P		smallRNA	chr1:150211632-150
ENSG0000(	1463	33.12798	chr1:1021	GOLPH3L		protein_c	chr1:150646230-150
ENSG0000(	1463	33.12798	chr1:1021	RNU2-17P		smallRNA	chr1:150236967-150
ENSG0000(	1463	33.12798	chr1:1021	ENSG00000259357		lncRNA	chr1:150965245-150
ENSG0000(	1463	33.12798	chr1:1021	RN7SL600P		smallRNA	chr1:150568973-150
ENSG0000(	1463	33.12798	chr1:1021	MIR4257		smallRNA	chr1:150551929-150
ENSG0000(	1463	33.12798	chr1:1021	SNORA40		smallRNA	chr1:150600539-150
ENSG0000(	1463	33.12798	chr1:1021	MCL1	NCGv7;AC	protein_c	chr1:150560895-150
ENSG0000(	1463	33.12798	chr1:1021	PRPF3	NCGv7	protein_c	chr1:150321479-150
ENSG0000(	1463	33.12798	chr1:1021	ENSG00000289288		lncRNA	chr1:151146793-151
ENSG0000(	1463	33.12798	chr1:1021	ANP32E		protein_c	chr1:150218417-150
ENSG0000(	1463	33.12798	chr1:1021	ADAMTSL4-AS2		lncRNA	chr1:150548562-150
ENSG0000(	1463	33.12798	chr1:1021	MINDY1		protein_c	chr1:150996549-151
ENSG0000(	1463	33.12798	chr1:1021	RNU6-884P		smallRNA	chr1:151022746-151
ENSG0000(	1463	33.12798	chr1:1021	HORMAD1	NCGv7	protein_c	chr1:150698060-150
ENSG0000(	1463	33.12798	chr1:1021	SETDB1	NCGv7;AC	protein_c	chr1:150926263-150
ENSG0000(	1463	33.12798	chr1:1021	ENSG00000276110		lncRNA	chr1:150255095-150
ENSG0000(	1463	33.12798	chr1:1021	ENSG00000261168		lncRNA	chr1:151130075-151
ENSG0000(	1463	33.12798	chr1:1021	ENSG00000288880		lncRNA	chr1:150629814-150
ENSG0000(	1463	33.12798	chr1:1021	CIART		protein_c	chr1:150282543-150
ENSG0000(	1463	33.12798	chr1:1021	ENSG00000231073		lncRNA	chr1:150973123-150
ENSG0000(	1463	33.12798	chr1:1021	ARNT	NCGv7;AC	protein_c	chr1:150809713-150
ENSG0000(	1463	33.12798	chr1:1021	POGZ		protein_c	chr1:151402724-151
ENSG0000(	1463	33.12798	chr1:1021	TARS2		protein_c	chr1:150487414-150
ENSG0000(	1463	33.12798	chr1:1021	GABPB2		protein_c	chr1:151070578-151
ENSG0000(	1463	33.12798	chr1:1021	C1orf56		protein_c	chr1:151047751-151
ENSG0000(	1463	33.12798	chr1:1021	CDC42SE1		protein_c	chr1:151050971-151
ENSG0000(	1463	33.12798	chr1:1021	RPS29P29		Pseudoger	chr1:151111912-151
ENSG0000(	1463	33.12798	chr1:1021	RPS27AP6		Pseudoger	chr1:150881236-150
ENSG0000(	1463	33.12798	chr1:1021	MRPS21	NCGv7	protein_c	chr1:150293861-150
ENSG0000(	1463	33.12798	chr1:1021	CYCSP51		Pseudoger	chr1:150903896-150
ENSG0000(	1463	33.12798	chr1:1021	RNU6-1309P		smallRNA	chr1:150812591-150
ENSG0000(	1463	33.12798	chr1:1021	RNU6-1042P		smallRNA	chr1:150701866-150
ENSG0000(	1463	33.12798	chr1:1021	ENSG00000236713		Pseudoger	chr1:150780272-150
ENSG0000(	1463	33.12798	chr1:1021	FALEC		lncRNA	chr1:150515757-150
ENSG0000(	1463	33.12798	chr1:1021	ENSG00000289041		lncRNA	chr1:150281114-150
ENSG0000(	1463	33.12798	chr1:1021	ADAMTSL4-AS1		lncRNA	chr1:150560202-150
ENSG0000(	1463	33.12798	chr1:1021	ZNF687-AS1		lncRNA	chr1:151279678-151
ENSG0000(	1463	33.12798	chr1:1021	ENSG00000290074		lncRNA	chr1:150579917-150
ENSG0000(	1463	33.12798	chr1:1021	LINC02988		lncRNA	chr1:150173049-150
ENSG0000(	1463	33.12798	chr1:1021	RPRD2		protein_c	chr1:150363091-150
ENSG0000(	1463	33.12798	chr1:1021	MLLT11	DriverDBv	protein_c	chr1:151060397-151
ENSG0000(	1463	33.12798	chr1:1021	PRUNE1		protein_c	chr1:151008420-151
ENSG0000(	1463	33.12798	chr1:1021	snoU13		smallRNA	chr1:150261694-150
ENSG0000(	1463	33.12798	chr1:1021	AL356356.1		protein_c	chr1:150549421-150
ENSG0000(	1463	33.12798	chr1:1021	CTSS	NCGv7	protein_c	chr1:150730079-150

ENSG0000(	1463	33.12798	chr1:1021	ENSG00000273481		lncRNA	chr1:151327949-151
ENSG0000(	1463	33.12798	chr1:1021	PSMB4		protein_c	chr1:151399560-151
ENSG0000(	1463	33.12798	chr1:1021	PSMD4		protein_c	chr1:151254709-151
ENSG0000(	1463	33.12798	chr1:1021	ENSA		protein_c	chr1:150600851-150
ENSG0000(	1463	33.12798	chr1:1021	SEMA6C		protein_c	chr1:151131685-151
ENSG0000(	1463	33.12798	chr1:1021	ENSG00000289457		lncRNA	chr1:150561466-150
ENSG0000(	1463	33.12798	chr1:1021	ZNF687	NCGv7	protein_c	chr1:151281618-151
ENSG0000(	1463	33.12798	chr1:1021	RN7SL444P		smallRNA	chr1:151300667-151
ENSG0000(	1463	33.12798	chr1:1021	ECM1		protein_c	chr1:150508062-150
ENSG0000(	1462	33.10533	chr1:1021	ENSG00000290790		lncRNA	chr1:149676889-149
ENSG0000(	1462	33.10533	chr1:1021	ENSG00000290791		lncRNA	chr1:149842875-149
ENSG0000(	1462	33.10533	chr1:1021	SNORA40		smallRNA	chr1:117688621-117
ENSG0000(	1462	33.10533	chr1:1021	MIR942		smallRNA	chr1:117094643-117
ENSG0000(	1462	33.10533	chr1:1021	TAFA3		protein_c	chr1:112718905-112
ENSG0000(	1462	33.10533	chr1:1021	DENND2C		protein_c	chr1:114582848-114
ENSG0000(	1462	33.10533	chr1:1021	SIKE1		protein_c	chr1:114769479-114
ENSG0000(	1462	33.10533	chr1:1021	ENSG00000239216		lncRNA	chr1:119000618-119
ENSG0000(	1462	33.10533	chr1:1021	AL355794.1		smallRNA	chr1:116592106-116
ENSG0000(	1462	33.10533	chr1:1021	H4C14		protein_c	chr1:149832657-149
ENSG0000(	1462	33.10533	chr1:1021	MIR320B1		smallRNA	chr1:116671749-116
ENSG0000(	1462	33.10533	chr1:1021	RNA5SP55		Pseudoger	chr1:116962347-116
ENSG0000(	1462	33.10533	chr1:1021	H2BC21		protein_c	chr1:149884459-149
ENSG0000(	1462	33.10533	chr1:1021	NAP1L4P1		Pseudoger	chr1:116532936-116
ENSG0000(	1462	33.10533	chr1:1021	ENSG00000229911		Pseudoger	chr1:118712999-118
ENSG0000(	1462	33.10533	chr1:1021	RP11-196G18.23		lncRNA	chr1:149831312-149
ENSG0000(	1462	33.10533	chr1:1021	DCLRE1B		protein_c	chr1:113904619-113
ENSG0000(	1462	33.10533	chr1:1021	RLIMP2		Pseudoger	chr1:113125321-113
ENSG0000(	1462	33.10533	chr1:1021	ENSG00000279513		TEC	chr1:117493515-117
ENSG0000(	1462	33.10533	chr1:1021	LINC01525		lncRNA	chr1:117272182-117
ENSG0000(	1462	33.10533	chr1:1021	PKMP1		Pseudoger	chr1:114535995-114
ENSG0000(	1462	33.10533	chr1:1021	CD101-AS1		lncRNA	chr1:117025482-117
ENSG0000(	1462	33.10533	chr1:1021	RPS15AP9		Pseudoger	chr1:117138229-117
ENSG0000(	1462	33.10533	chr1:1021	CASQ2		protein_c	chr1:115700021-115
ENSG0000(	1462	33.10533	chr1:1021	Y_RNA		smallRNA	chr1:116452536-116
ENSG0000(	1462	33.10533	chr1:1021	PHGDH	NCGv7	protein_c	chr1:119648411-119
ENSG0000(	1462	33.10533	chr1:1021	TBX15		protein_c	chr1:118883046-118
ENSG0000(	1462	33.10533	chr1:1021	ENSG00000232499		Pseudoger	chr1:113449700-113
ENSG0000(	1462	33.10533	chr1:1021	WDR3		protein_c	chr1:117929720-117
ENSG0000(	1462	33.10533	chr1:1021	MIR548AC		smallRNA	chr1:116560024-116
ENSG0000(	1462	33.10533	chr1:1021	RNVU1-5		smallRNA	chr1:120942599-120
ENSG0000(	1462	33.10533	chr1:1021	RP6-42F4.1		lncRNA	chr1:120150758-120
ENSG0000(	1462	33.10533	chr1:1021	PFN1P9		Pseudoger	chr1:119853316-119
ENSG0000(	1462	33.10533	chr1:1021	H2AC21		protein_c	chr1:149887469-149
ENSG0000(	1462	33.10533	chr1:1021	H2AC20	NCGv7	protein_c	chr1:149886918-149
ENSG0000(	1462	33.10533	chr1:1021	ENSG00000232895		lncRNA	chr1:114206427-114
ENSG0000(	1462	33.10533	chr1:1021	AL358813.2		protein_c	chr1:149704411-149
ENSG0000(	1462	33.10533	chr1:1021	ENSG00000271143		Pseudoger	chr1:115916497-115
ENSG0000(	1462	33.10533	chr1:1021	CTTNBP2NL		protein_c	chr1:112396214-112
ENSG0000(	1462	33.10533	chr1:1021	SNORA42		smallRNA	chr1:115621872-115
ENSG0000(	1462	33.10533	chr1:1021	AL512638.1		smallRNA	chr1:115485651-115
ENSG0000(	1462	33.10533	chr1:1021	ENSG00000228040		Pseudoger	chr1:112890767-112

ENSG0000(	1462	33.10533	chr1:1021	CNOT7P2		Pseudoger	chr1:115564601-115
ENSG0000(	1462	33.10533	chr1:1021	NGF-AS1		lncRNA	chr1:115283034-115
ENSG0000(	1462	33.10533	chr1:1021	HIPK1-AS1		lncRNA	chr1:113924000-113
ENSG0000(	1462	33.10533	chr1:1021	AKR7A2P1		Pseudoger	chr1:112923423-112
ENSG0000(	1462	33.10533	chr1:1021	LINC01779		lncRNA	chr1:116164209-116
ENSG0000(	1462	33.10533	chr1:1021	TENT5C-DT		lncRNA	chr1:117596832-117
ENSG0000(	1462	33.10533	chr1:1021	NR1H5P		Pseudoger	chr1:114837227-114
ENSG0000(	1462	33.10533	chr1:1021	LINC01762		lncRNA	chr1:116423724-116
ENSG0000(	1462	33.10533	chr1:1021	ENSG00000275296		Pseudoger	chr1:149754303-149
ENSG0000(	1462	33.10533	chr1:1021	ENSG00000233030		lncRNA	chr1:149785659-149
ENSG0000(	1462	33.10533	chr1:1021	H2AC19		protein_c	chr1:149851061-149
ENSG0000(	1462	33.10533	chr1:1021	Y_RNA		smallRNA	chr1:114727720-114
ENSG0000(	1462	33.10533	chr1:1021	LINC01357		lncRNA	chr1:112849821-112
ENSG0000(	1462	33.10533	chr1:1021	AL358813.3		smallRNA	chr1:149737309-149
ENSG0000(	1462	33.10533	chr1:1021	GAPDHP33		Pseudoger	chr1:119596167-119
ENSG0000(	1462	33.10533	chr1:1021	ENSG00000272715		lncRNA	chr1:116909149-116
ENSG0000(	1462	33.10533	chr1:1021	ELOCP20		Pseudoger	chr1:115556826-115
ENSG0000(	1462	33.10533	chr1:1021	ENSG00000236887		Pseudoger	chr1:113198825-113
ENSG0000(	1462	33.10533	chr1:1021	RBMX2P3		Pseudoger	chr1:119084998-119
ENSG0000(	1462	33.10533	chr1:1021	WARS2-IT1		lncRNA	chr1:119047405-119
ENSG0000(	1462	33.10533	chr1:1021	RP11-277L2.6		Pseudoger	chr1:120861539-120
ENSG0000(	1462	33.10533	chr1:1021	ZNF697	NCGv7	protein_c	chr1:119619377-119
ENSG0000(	1462	33.10533	chr1:1021	REG4		protein_c	chr1:119794017-119
ENSG0000(	1462	33.10533	chr1:1021	IGSF3		protein_c	chr1:116574399-116
ENSG0000(	1462	33.10533	chr1:1021	H3C14		protein_c	chr1:149840687-149
ENSG0000(	1462	33.10533	chr1:1021	LINC01649		lncRNA	chr1:115904855-115
ENSG0000(	1462	33.10533	chr1:1021	MAGI3		protein_c	chr1:113390515-113
ENSG0000(	1462	33.10533	chr1:1021	RSBN1	NCGv7	protein_c	chr1:113761832-113
ENSG0000(	1462	33.10533	chr1:1021	RP11-353N4.4		lncRNA	chr1:149701425-149
ENSG0000(	1462	33.10533	chr1:1021	HSD3BP3		Pseudoger	chr1:119538509-119
ENSG0000(	1462	33.10533	chr1:1021	HIST2H2BC		Pseudoger	chr1:149850193-149
ENSG0000(	1462	33.10533	chr1:1021	H3C15		protein_c	chr1:149852608-149
ENSG0000(	1462	33.10533	chr1:1021	HSD3BP4		Pseudoger	chr1:119564066-119
ENSG0000(	1462	33.10533	chr1:1021	HSD3B1		protein_c	chr1:119507198-119
ENSG0000(	1462	33.10533	chr1:1021	HSD3BP2		Pseudoger	chr1:119439001-119
ENSG0000(	1462	33.10533	chr1:1021	FCGR1A		protein_c	chr1:149782671-149
ENSG0000(	1462	33.10533	chr1:1021	GAPDHP23		Pseudoger	chr1:119462029-119
ENSG0000(	1462	33.10533	chr1:1021	HSD3B2		protein_c	chr1:119414931-119
ENSG0000(	1462	33.10533	chr1:1021	ENSG00000230381		lncRNA	chr1:116429049-116
ENSG0000(	1462	33.10533	chr1:1021	LINC02868		lncRNA	chr1:116694112-116
ENSG0000(	1462	33.10533	chr1:1021	ATP1A1-AS1		lncRNA	chr1:116378437-116
ENSG0000(	1462	33.10533	chr1:1021	RPS3AP12		Pseudoger	chr1:119126539-119
ENSG0000(	1462	33.10533	chr1:1021	ENSG00000287103		lncRNA	chr1:115356664-115
ENSG0000(	1462	33.10533	chr1:1021	ENSG00000271810		protein_c	chr1:112702614-112
ENSG0000(	1462	33.10533	chr1:1021	ENSG00000290792		lncRNA	chr1:149844498-149
ENSG0000(	1462	33.10533	chr1:1021	PNRC2P1		Pseudoger	chr1:117778087-117
ENSG0000(	1462	33.10533	chr1:1021	snoU13		smallRNA	chr1:112652588-112
ENSG0000(	1462	33.10533	chr1:1021	GAPDHP74		Pseudoger	chr1:119434166-119
ENSG0000(	1462	33.10533	chr1:1021	ENSG00000232450		Pseudoger	chr1:113698884-113
ENSG0000(	1462	33.10533	chr1:1021	ENSG00000271427		lncRNA	chr1:117364899-117
ENSG0000(	1462	33.10533	chr1:1021	snoU13		smallRNA	chr1:112371004-112

ENSG0000(	1462	33.10533	chr1:1021	VTCN1		protein_c	chr1:117143587-117
ENSG0000(	1462	33.10533	chr1:1021	NEFHP1		Pseudoger	chr1:116739981-116
ENSG0000(	1462	33.10533	chr1:1021	CD101		protein_c	chr1:117001750-117
ENSG0000(	1462	33.10533	chr1:1021	RNA5SP56		Pseudoger	chr1:118264372-118
ENSG0000(	1462	33.10533	chr1:1021	GAPDHP27		Pseudoger	chr1:119558755-119
ENSG0000(	1462	33.10533	chr1:1021	TRIM45		protein_c	chr1:117111060-117
ENSG0000(	1462	33.10533	chr1:1021	NOTCH2	NCGv7;AC	protein_c	chr1:119911553-120
ENSG0000(	1462	33.10533	chr1:1021	ADAM30		protein_c	chr1:119893533-119
ENSG0000(	1462	33.10533	chr1:1021	RNU6-465P		smallRNA	chr1:120126974-120
ENSG0000(	1462	33.10533	chr1:1021	VANGL1	NCGv7	protein_c	chr1:115641970-115
ENSG0000(	1462	33.10533	chr1:1021	PTGFRN	NCGv7	protein_c	chr1:116909916-116
ENSG0000(	1462	33.10533	chr1:1021	WNT2B		protein_c	chr1:112466541-112
ENSG0000(	1462	33.10533	chr1:1021	PTPN22	NCGv7	protein_c	chr1:113813811-113
ENSG0000(	1462	33.10533	chr1:1021	ENSG00000287217		lncRNA	chr1:116289237-116
ENSG0000(	1462	33.10533	chr1:1021	U3		smallRNA	chr1:116278606-116
ENSG0000(	1462	33.10533	chr1:1021	HMGCS2		protein_c	chr1:119748002-119
ENSG0000(	1462	33.10533	chr1:1021	MAB21L3		protein_c	chr1:116111399-116
ENSG0000(	1462	33.10533	chr1:1021	NHLH2		protein_c	chr1:115836377-115
ENSG0000(	1462	33.10533	chr1:1021	SYT6		protein_c	chr1:114089291-114
ENSG0000(	1462	33.10533	chr1:1021	Y_RNA		smallRNA	chr1:114490724-114
ENSG0000(	1462	33.10533	chr1:1021	H2BC19P		Pseudoger	chr1:149843041-149
ENSG0000(	1462	33.10533	chr1:1021	TSHB		protein_c	chr1:115029826-115
ENSG0000(	1462	33.10533	chr1:1021	TSPAN2		protein_c	chr1:115048011-115
ENSG0000(	1462	33.10533	chr1:1021	NGF		protein_c	chr1:115285904-115
ENSG0000(	1462	33.10533	chr1:1021	HIPK1		protein_c	chr1:113929324-113
ENSG0000(	1462	33.10533	chr1:1021	AP4B1		protein_c	chr1:113894194-113
ENSG0000(	1462	33.10533	chr1:1021	PHTF1		protein_c	chr1:113696831-113
ENSG0000(	1462	33.10533	chr1:1021	ENSG00000271419		Pseudoger	chr1:114353986-114
ENSG0000(	1462	33.10533	chr1:1021	RP3-328E19.4		Pseudoger	chr1:120844645-120
ENSG0000(	1462	33.10533	chr1:1021	CAPZA1		protein_c	chr1:112619805-112
ENSG0000(	1462	33.10533	chr1:1021	AL157904.1		smallRNA	chr1:116905150-116
ENSG0000(	1462	33.10533	chr1:1021	BOLA1		protein_c	chr1:149887890-149
ENSG0000(	1462	33.10533	chr1:1021	ENSG00000227712		lncRNA	chr1:119230313-119
ENSG0000(	1462	33.10533	chr1:1021	RN7SL420P		smallRNA	chr1:115606471-115
ENSG0000(	1462	33.10533	chr1:1021	AMPD1		protein_c	chr1:114673090-114
ENSG0000(	1462	33.10533	chr1:1021	BCAS2		protein_c	chr1:114567557-114
ENSG0000(	1462	33.10533	chr1:1021	OLFML3		protein_c	chr1:113979391-114
ENSG0000(	1462	33.10533	chr1:1021	CD58	NCGv7	protein_c	chr1:116514534-116
ENSG0000(	1462	33.10533	chr1:1021	snoU13		smallRNA	chr1:119259041-119
ENSG0000(	1462	33.10533	chr1:1021	CD2		protein_c	chr1:116754430-116
ENSG0000(	1462	33.10533	chr1:1021	TTF2		protein_c	chr1:117060326-117
ENSG0000(	1462	33.10533	chr1:1021	WARS2		protein_c	chr1:119031216-119
ENSG0000(	1462	33.10533	chr1:1021	HAO2		protein_c	chr1:119368779-119
ENSG0000(	1462	33.10533	chr1:1021	ENSG00000282048		lncRNA	chr1:114032377-114
ENSG0000(	1462	33.10533	chr1:1021	TENT5C	NCGv7;AC	protein_c	chr1:117606048-117
ENSG0000(	1462	33.10533	chr1:1021	MRPL53P1		Pseudoger	chr1:112625906-112
ENSG0000(	1462	33.10533	chr1:1021	HIST2H2AA3		protein_c	chr1:149841933-149
ENSG0000(	1462	33.10533	chr1:1021	ATP1A1	NCGv7;AC	protein_c	chr1:116372668-116
ENSG0000(	1462	33.10533	chr1:1021	SLC22A15		protein_c	chr1:115976513-116
ENSG0000(	1462	33.10533	chr1:1021	H3C13		protein_c	chr1:149813225-149
ENSG0000(	1462	33.10533	chr1:1021	H2BC18		protein_c	chr1:149782689-149

ENSG0000(	1462	33.10533	chr1:1021	MTND5P20		Pseudoger	chr1:113576757-113
ENSG0000(	1462	33.10533	chr1:1021	ENSG00000273483		lncRNA	chr1:112517799-112
ENSG0000(	1462	33.10533	chr1:1021	AL358813.1		Pseudoger	chr1:149717832-149
ENSG0000(	1462	33.10533	chr1:1021	HSD3BP5		Pseudoger	chr1:119601340-119
ENSG0000(	1462	33.10533	chr1:1021	SLC16A1-AS1		lncRNA	chr1:112956415-113
ENSG0000(	1462	33.10533	chr1:1021	ENSG00000286276		lncRNA	chr1:116493350-116
ENSG0000(	1462	33.10533	chr1:1021	LRIG2-DT		lncRNA	chr1:113011687-113
ENSG0000(	1462	33.10533	chr1:1021	ENSG00000226984		Pseudoger	chr1:114459934-114
ENSG0000(	1462	33.10533	chr1:1021	GAPDHP32		Pseudoger	chr1:119533749-119
ENSG0000(	1462	33.10533	chr1:1021	NUTF2P4		Pseudoger	chr1:112748095-112
ENSG0000(	1462	33.10533	chr1:1021	RNU7-70P		smallRNA	chr1:112634719-112
ENSG0000(	1462	33.10533	chr1:1021	NOTCH2P1		Pseudoger	chr1:119886304-119
ENSG0000(	1462	33.10533	chr1:1021	VPS25P1		Pseudoger	chr1:117549415-117
ENSG0000(	1462	33.10533	chr1:1021	CSDE1	NCGv7	protein_c	chr1:114716913-114
ENSG0000(	1462	33.10533	chr1:1021	BCL2L15		protein_c	chr1:113876816-113
ENSG0000(	1462	33.10533	chr1:1021	TXNP3		Pseudoger	chr1:112363281-112
ENSG0000(	1462	33.10533	chr1:1021	AP4B1-AS1		lncRNA	chr1:113856635-113
ENSG0000(	1462	33.10533	chr1:1021	ENSG00000226973		Pseudoger	chr1:115471941-115
ENSG0000(	1462	33.10533	chr1:1021	ENSG00000270719		Pseudoger	chr1:117700029-117
ENSG0000(	1462	33.10533	chr1:1021	RNVU1-19		smallRNA	chr1:120850819-120
ENSG0000(	1462	33.10533	chr1:1021	ENSG00000274468		Pseudoger	chr1:115479428-115
ENSG0000(	1462	33.10533	chr1:1021	ENSG00000273406		lncRNA	chr1:120076616-120
ENSG0000(	1462	33.10533	chr1:1021	ENSG00000270631		Pseudoger	chr1:115577229-115
ENSG0000(	1462	33.10533	chr1:1021	ENSG00000225075		lncRNA	chr1:112693688-112
ENSG0000(	1462	33.10533	chr1:1021	HSD3BP1		Pseudoger	chr1:119467221-119
ENSG0000(	1462	33.10533	chr1:1021	ENSG00000237993		lncRNA	chr1:116013813-116
ENSG0000(	1462	33.10533	chr1:1021	LINC01780		lncRNA	chr1:119327399-119
ENSG0000(	1462	33.10533	chr1:1021	FTH1P22		Pseudoger	chr1:116775104-116
ENSG0000(	1462	33.10533	chr1:1021	MAN1A2	NCGv7	protein_c	chr1:117367449-117
ENSG0000(	1462	33.10533	chr1:1021	RN7SL432P		smallRNA	chr1:114697629-114
ENSG0000(	1462	33.10533	chr1:1021	ENSG00000231128		lncRNA	chr1:113812379-113
ENSG0000(	1462	33.10533	chr1:1021	RNU6-817P		smallRNA	chr1:116413766-116
ENSG0000(	1462	33.10533	chr1:1021	MRPL57P1		Pseudoger	chr1:114279011-114
ENSG0000(	1462	33.10533	chr1:1021	AL136376.1		protein_c	chr1:116373268-116
ENSG0000(	1462	33.10533	chr1:1021	SPAG17	NCGv7	protein_c	chr1:117953590-118
ENSG0000(	1462	33.10533	chr1:1021	ENSG00000287980		lncRNA	chr1:118185349-118
ENSG0000(	1462	33.10533	chr1:1021	WARS2-AS1		lncRNA	chr1:119140391-119
ENSG0000(	1462	33.10533	chr1:1021	PSMC1P12		Pseudoger	chr1:118614333-118
ENSG0000(	1462	33.10533	chr1:1021	GDAP2		protein_c	chr1:117863485-117
ENSG0000(	1462	33.10533	chr1:1021	LINC00622		lncRNA	chr1:119597702-119
ENSG0000(	1462	33.10533	chr1:1021	ENSG00000226172		lncRNA	chr1:119000344-119
ENSG0000(	1462	33.10533	chr1:1021	RP11-763B22.10		Pseudoger	chr1:120861539-120
ENSG0000(	1462	33.10533	chr1:1021	RNVU1-13		smallRNA	chr1:120850818-120
ENSG0000(	1462	33.10533	chr1:1021	RPL6P2		Pseudoger	chr1:119219314-119
ENSG0000(	1462	33.10533	chr1:1021	ENSG00000270780		Pseudoger	chr1:114001433-114
ENSG0000(	1462	33.10533	chr1:1021	PDE4DIPP4		lncRNA	chr1:149677473-149
ENSG0000(	1462	33.10533	chr1:1021	RP3-328E19.5		Pseudoger	chr1:120861538-120
ENSG0000(	1462	33.10533	chr1:1021	GAPDHP58		Pseudoger	chr1:119495836-119
ENSG0000(	1462	33.10533	chr1:1021	EIF2S2P5		Pseudoger	chr1:114468315-114
ENSG0000(	1462	33.10533	chr1:1021	SLC16A1		protein_c	chr1:112911847-112
ENSG0000(	1462	33.10533	chr1:1021	PPM1J	NCGv7	protein_c	chr1:112709994-112

ENSG0000(	1462	33.10533	chr1:1021	RNU1-68P		smallRNA	chr1:149700151-149
ENSG0000(	1462	33.10533	chr1:1021	ENSG00000224950		lncRNA	chr1:116493016-116
ENSG0000(	1462	33.10533	chr1:1021	RNU1-75P		smallRNA	chr1:119331397-119
ENSG0000(	1462	33.10533	chr1:1021	ENSG00000285698		lncRNA	chr1:115270767-115
ENSG0000(	1462	33.10533	chr1:1021	RHOC	AC	protein_c	chr1:112701127-112
ENSG0000(	1462	33.10533	chr1:1021	GAPDHP64		Pseudoger	chr1:116713833-116
ENSG0000(	1462	33.10533	chr1:1021	MOV10	NCGv7	protein_c	chr1:112673141-112
ENSG0000(	1462	33.10533	chr1:1021	LINC01765		lncRNA	chr1:115099580-115
ENSG0000(	1462	33.10533	chr1:1021	H4C15		protein_c	chr1:149854045-149
ENSG0000(	1462	33.10533	chr1:1021	NRAS	NCGv7;AC	protein_c	chr1:114704469-114
ENSG0000(	1462	33.10533	chr1:1021	ENSG00000233839		Pseudoger	chr1:113168994-113
ENSG0000(	1462	33.10533	chr1:1021	ENSG00000287807		lncRNA	chr1:112978610-112
ENSG0000(	1462	33.10533	chr1:1021	HAO2-IT1		lncRNA	chr1:119368946-119
ENSG0000(	1462	33.10533	chr1:1021	HNRNPA1P43		Pseudoger	chr1:115856910-115
ENSG0000(	1462	33.10533	chr1:1021	ENSG00000226500		Pseudoger	chr1:149754301-149
ENSG0000(	1462	33.10533	chr1:1021	TRIM33	NCGv7;AC	protein_c	chr1:114392790-114
ENSG0000(	1462	33.10533	chr1:1021	LRIG2	NCGv7	protein_c	chr1:113073198-113
ENSG0000(	1462	33.10533	chr1:1021	ST7L		protein_c	chr1:112523514-112
ENSG0000(	1462	33.10533	chr1:1021	LINC01356		lncRNA	chr1:112820170-112
ENSG0000(	1462	33.10533	chr1:1021	NBPF7P		Pseudoger	chr1:119834870-119
ENSG0000(	1462	33.10533	chr1:1021	PPM1J-DT		lncRNA	chr1:112715672-112
ENSG0000(	1462	33.10533	chr1:1021	SYCP1		protein_c	chr1:114854863-114
ENSG0000(	1462	33.10533	chr1:1021	VDAC2P3		Pseudoger	chr1:117640812-117
ENSG0000(	1462	33.10533	chr1:1021	RP5-1042I8.7		lncRNA	chr1:119909255-119
ENSG0000(	1451	32.85625	chr8:980Ę	ERICH5		protein_c	chr8:98064522-9809
ENSG0000(	1451	32.85625	chr8:980Ę	ENSG00000253740		lncRNA	chr8:100618581-100
ENSG0000(	1451	32.85625	chr8:980Ę	ENSG00000253468		Pseudoger	chr8:98412882-9841
ENSG0000(	1451	32.85625	chr8:980Ę	GAPDHP62		Pseudoger	chr8:100550724-100
ENSG0000(	1451	32.85625	chr8:980Ę	ZNF706	DriverDB\	protein_c	chr8:101177878-101
ENSG0000(	1451	32.85625	chr8:980Ę	ENSG00000289048		lncRNA	chr8:101491259-101
ENSG0000(	1451	32.85625	chr8:980Ę	FLJ42969		lncRNA	chr8:101034868-101
ENSG0000(	1451	32.85625	chr8:980Ę	RIDA	DriverDB\	protein_c	chr8:98102344-9811
ENSG0000(	1451	32.85625	chr8:980Ę	SNORD77		smallRNA	chr8:100004583-100
ENSG0000(	1451	32.85625	chr8:980Ę	RN7SKP85		smallRNA	chr8:98921233-9892
ENSG0000(	1451	32.85625	chr8:980Ę	ENSG00000253824		lncRNA	chr8:100380486-100
ENSG0000(	1451	32.85625	chr8:980Ę	VPS13B	NCGv7	protein_c	chr8:99013266-9987
ENSG0000(	1451	32.85625	chr8:980Ę	ENSG00000287952		lncRNA	chr8:99893373-9989
ENSG0000(	1451	32.85625	chr8:980Ę	RGS22		protein_c	chr8:99960936-1001
ENSG0000(	1451	32.85625	chr8:980Ę	ENSG00000254024		lncRNA	chr8:101461177-101
ENSG0000(	1451	32.85625	chr8:980Ę	ENSG00000253217		lncRNA	chr8:100337595-100
ENSG0000(	1451	32.85625	chr8:980Ę	ENSG00000253942		Pseudoger	chr8:101223941-101
ENSG0000(	1451	32.85625	chr8:980Ę	UFM1P3		Pseudoger	chr8:100165226-100
ENSG0000(	1451	32.85625	chr8:980Ę	ENSG00000237704		Pseudoger	chr8:99340305-9934
ENSG0000(	1451	32.85625	chr8:980Ę	AP001205.1		smallRNA	chr8:100707878-100
ENSG0000(	1451	32.85625	chr8:980Ę	LINC02844		lncRNA	chr8:101261541-101
ENSG0000(	1451	32.85625	chr8:980Ę	ENSG00000253415		Pseudoger	chr8:98904603-9890
ENSG0000(	1451	32.85625	chr8:980Ę	ENSG00000236583		Pseudoger	chr8:99233048-9923
ENSG0000(	1451	32.85625	chr8:980Ę	ENSG00000271930		lncRNA	chr8:98943595-9894
ENSG0000(	1451	32.85625	chr8:980Ę	ENSG00000260368		lncRNA	chr8:100913247-100
ENSG0000(	1451	32.85625	chr8:980Ę	ENSG00000287479		lncRNA	chr8:101275425-101
ENSG0000(	1451	32.85625	chr8:980Ę	ZNNT1		lncRNA	chr8:101166805-101

ENSG0000(	1451	32.85625	chr8:980[illegible]	YWHAZ	DriverDB\	protein_c	chr8:100916523-100
ENSG0000(	1451	32.85625	chr8:980[illegible]	OSR2	NCGv7	protein_c	chr8:98944403-9895
ENSG0000(	1451	32.85625	chr8:980[illegible]	ANKRD46	DriverDB\	protein_c	chr8:100509752-100
ENSG0000(	1451	32.85625	chr8:980[illegible]	ENSG00000253532		lncRNA	chr8:99119352-9912
ENSG0000(	1451	32.85625	chr8:980[illegible]	COX6C	DriverDB\	protein_c	chr8:99873200-9989
ENSG0000(	1451	32.85625	chr8:980[illegible]	NACA4P		Pseudoger	chr8:101361794-101
ENSG0000(	1451	32.85625	chr8:980[illegible]	RNF19A	DriverDB\	protein_c	chr8:100257060-100
ENSG0000(	1451	32.85625	chr8:980[illegible]	ENSG00000253842		lncRNA	chr8:100427559-100
ENSG0000(	1451	32.85625	chr8:980[illegible]	ENSG00000271882		lncRNA	chr8:101208148-101
ENSG0000(	1451	32.85625	chr8:980[illegible]	ENSG00000253737		lncRNA	chr8:101139665-101
ENSG0000(	1451	32.85625	chr8:980[illegible]	ENSG00000253666		lncRNA	chr8:100475667-100
ENSG0000(	1451	32.85625	chr8:980[illegible]	Y_RNA		smallRNA	chr8:100185138-100
ENSG0000(	1451	32.85625	chr8:980[illegible]	ENSG00000253355		lncRNA	chr8:101128987-101
ENSG0000(	1451	32.85625	chr8:980[illegible]	VPS13B-DT		lncRNA	chr8:98957568-9901
ENSG0000(	1451	32.85625	chr8:980[illegible]	GRHL2	NCGv7	protein_c	chr8:101492439-101
ENSG0000(	1451	32.85625	chr8:980[illegible]	MRPL57P7		Pseudoger	chr8:98906260-9890
ENSG0000(	1451	32.85625	chr8:980[illegible]	MIR1273A		smallRNA	chr8:100023982-100
ENSG0000(	1451	32.85625	chr8:980[illegible]	Y_RNA		smallRNA	chr8:100962935-100
ENSG0000(	1451	32.85625	chr8:980[illegible]	RN7SL685P		smallRNA	chr8:100957883-100
ENSG0000(	1451	32.85625	chr8:980[illegible]	MIR875		smallRNA	chr8:99536786-9953
ENSG0000(	1451	32.85625	chr8:980[illegible]	ENSG00000253395		lncRNA	chr8:101122145-101
ENSG0000(	1451	32.85625	chr8:980[illegible]	RPL19P14		Pseudoger	chr8:98782055-9878
ENSG0000(	1451	32.85625	chr8:980[illegible]	Y_RNA		smallRNA	chr8:99527826-9952
ENSG0000(	1451	32.85625	chr8:980[illegible]	ENSG00000289284		lncRNA	chr8:100688004-100
ENSG0000(	1451	32.85625	chr8:980[illegible]	RNU6-1092P		smallRNA	chr8:100683488-100
ENSG0000(	1451	32.85625	chr8:980[illegible]	SPAG1	DriverDB\	protein_c	chr8:100157906-100
ENSG0000(	1451	32.85625	chr8:980[illegible]	SNX31	NCGv7	protein_c	chr8:100572889-100
ENSG0000(	1451	32.85625	chr8:980[illegible]	ENSG00000253562		lncRNA	chr8:99091738-9909
ENSG0000(	1451	32.85625	chr8:980[illegible]	FBXO43	DriverDB\	protein_c	chr8:100133351-100
ENSG0000(	1451	32.85625	chr8:980[illegible]	ENSG00000253180		Pseudoger	chr8:99003407-9900
ENSG0000(	1451	32.85625	chr8:980[illegible]	RN7SKP249		smallRNA	chr8:101137959-101
ENSG0000(	1451	32.85625	chr8:980[illegible]	KCNS2		protein_c	chr8:98426958-9843
ENSG0000(	1451	32.85625	chr8:980[illegible]	RNU6ATAC41P		smallRNA	chr8:100739802-100
ENSG0000(	1451	32.85625	chr8:980[illegible]	ENSG00000225017		Pseudoger	chr8:99346052-9934
ENSG0000(	1451	32.85625	chr8:980[illegible]	Y_RNA		smallRNA	chr8:100158868-100
ENSG0000(	1451	32.85625	chr8:980[illegible]	RNU4-83P		smallRNA	chr8:100831728-100
ENSG0000(	1451	32.85625	chr8:980[illegible]	RN7SL563P		smallRNA	chr8:101397635-101
ENSG0000(	1451	32.85625	chr8:980[illegible]	STK3		protein_c	chr8:98371228-9894
ENSG0000(	1451	32.85625	chr8:980[illegible]	ENSG00000271560		Pseudoger	chr8:101337496-101
ENSG0000(	1451	32.85625	chr8:980[illegible]	ENSG00000253912		Pseudoger	chr8:99804159-9980
ENSG0000(	1451	32.85625	chr8:980[illegible]	ENSG00000253911		lncRNA	chr8:98603253-9860
ENSG0000(	1451	32.85625	chr8:980[illegible]	NIPAL2	DriverDB\	protein_c	chr8:98189826-9829
ENSG0000(	1451	32.85625	chr8:980[illegible]	MIR4471		smallRNA	chr8:100382763-100
ENSG0000(	1451	32.85625	chr8:980[illegible]	ENSG00000254112		Pseudoger	chr8:98633547-9863
ENSG0000(	1451	32.85625	chr8:980[illegible]	AC104986.1		smallRNA	chr8:98999284-9899
ENSG0000(	1451	32.85625	chr8:980[illegible]	ENSG00000253282		lncRNA	chr8:101287445-101
ENSG0000(	1451	32.85625	chr8:980[illegible]	Y_RNA		smallRNA	chr8:100755101-100
ENSG0000(	1451	32.85625	chr8:980[illegible]	POP1		protein_c	chr8:98117293-9815
ENSG0000(	1451	32.85625	chr8:980[illegible]	LINC02845		lncRNA	chr8:101387299-101
ENSG0000(	1451	32.85625	chr8:980[illegible]	ENSG00000254364		lncRNA	chr8:100492528-100
ENSG0000(	1451	32.85625	chr8:980[illegible]	ENSG00000253153		Pseudoger	chr8:101562008-101

ENSG0000C	1451	32.85625	chr8:9805	ENSG00000254084		lncRNA	chr8:101411873-101
ENSG0000C	1451	32.85625	chr8:9805	ENSG00000272321		lncRNA	chr8:98436669-9843
ENSG0000C	1451	32.85625	chr8:9805	RN7SL350P		smallRNA	chr8:99613786-9961
ENSG0000C	1451	32.85625	chr8:9805	ENSG00000253991		Pseudoger	chr8:101528723-101
ENSG0000C	1451	32.85625	chr8:9805	PABPC1	NCGv7	protein_c	chr8:100685816-100
ENSG0000C	1451	32.85625	chr8:9805	RNU7-67P		smallRNA	chr8:101307367-101
ENSG0000C	1451	32.85625	chr8:9805	DUXAP2		Pseudoger	chr8:101366417-101
ENSG0000C	1451	32.85625	chr8:9805	POLR2K	DriverDBv	protein_c	chr8:100150623-100
ENSG0000C	1451	32.85625	chr8:9805	ENSG00000278914		TEC	chr8:98391401-9839
ENSG0000C	1451	32.85625	chr8:9805	ENSG00000253539		lncRNA	chr8:99796615-9979
ENSG0000C	1451	32.85625	chr8:9805	RPS20P23		Pseudoger	chr8:100776512-100
ENSG0000C	1451	32.85625	chr8:9805	MIR599		smallRNA	chr8:99536636-9953
ENSG0000C	1451	32.85625	chr8:9805	RPS26P6		Pseudoger	chr8:100895771-100
ENSG0000C	1451	32.85625	chr8:9805	RNU6-914P		smallRNA	chr8:98192205-9819
ENSG0000C	1451	32.85625	chr8:9805	ENSG00000229625		lncRNA	chr8:98961428-9896
ENSG0000C	1451	32.85625	chr8:9805	RNU6-748P		smallRNA	chr8:98462944-9846
ENSG0000C	1451	32.85625	chr8:9805	ENSG00000235683		Pseudoger	chr8:99695957-9969
ENSG0000C	1450	32.83361	chr1:1021	MTMR11		protein_c	chr1:149928651-149
ENSG0000C	1450	32.83361	chr1:1021	OTUD7B		protein_c	chr1:149937812-150
ENSG0000C	1450	32.83361	chr1:1021	VPS45	NCGv7	protein_c	chr1:150067279-150
ENSG0000C	1450	32.83361	chr1:1021	SV2A		protein_c	chr1:149903318-149
ENSG0000C	1450	32.83361	chr1:1021	ENSG00000285184		lncRNA	chr1:150045660-150
ENSG0000C	1450	32.83361	chr1:1021	PLEKHO1		protein_c	chr1:150149183-150
ENSG0000C	1450	32.83361	chr1:1021	SF3B4		protein_c	chr1:149923317-149
ENSG0000C	1450	32.83361	chr1:1021	RPL6P31		Pseudoger	chr1:150053864-150
ENSG0000C	1436	32.51659	chr1:1021	LINC01750		lncRNA	chr1:111989770-111
ENSG0000C	1436	32.51659	chr1:1021	snoU13		smallRNA	chr1:112195502-112
ENSG0000C	1436	32.51659	chr1:1021	KCND3-IT1		lncRNA	chr1:111853762-111
ENSG0000C	1436	32.51659	chr1:1021	KCND3-AS1		lncRNA	chr1:111909336-111
ENSG0000C	1436	32.51659	chr1:1021	LINC02884		lncRNA	chr1:112176836-112
ENSG0000C	1430	32.38073	chr8:9805	ENSG00000254339		lncRNA	chr8:114282037-114
ENSG0000C	1430	32.38073	chr8:9805	KCNV1		protein_c	chr8:109963636-109
ENSG0000C	1430	32.38073	chr8:9805	ENY2	DriverDBv	protein_c	chr8:109334324-109
ENSG0000C	1430	32.38073	chr8:9805	RAD21-AS1		lncRNA	chr8:116874424-116
ENSG0000C	1430	32.38073	chr8:9805	EBAG9		protein_c	chr8:109539711-109
ENSG0000C	1430	32.38073	chr8:9805	NUDCD1	DriverDBv	protein_c	chr8:109240919-109
ENSG0000C	1430	32.38073	chr8:9805	CSMD3	NCGv7	protein_c	chr8:112222928-113
ENSG0000C	1430	32.38073	chr8:9805	EEF1A1P37		Pseudoger	chr8:111225585-111
ENSG0000C	1430	32.38073	chr8:9805	UTP23	DriverDBv	protein_c	chr8:116766505-116
ENSG0000C	1430	32.38073	chr8:9805	RPSAP48		Pseudoger	chr8:110105076-110
ENSG0000C	1430	32.38073	chr8:9805	ENSG00000287819		lncRNA	chr8:113432224-113
ENSG0000C	1430	32.38073	chr8:9805	H2AZP7		Pseudoger	chr8:111621458-111
ENSG0000C	1430	32.38073	chr8:9805	MED30		protein_c	chr8:117520713-117
ENSG0000C	1430	32.38073	chr8:9805	ENSG00000253717		lncRNA	chr8:110766863-110
ENSG0000C	1430	32.38073	chr8:9805	SLC30A8		protein_c	chr8:116950273-117
ENSG0000C	1430	32.38073	chr8:9805	LINC02237		lncRNA	chr8:111376639-111
ENSG0000C	1430	32.38073	chr8:9805	ENSG00000287826		lncRNA	chr8:116289622-116
ENSG0000C	1430	32.38073	chr8:9805	snoU13		smallRNA	chr8:112563875-112
ENSG0000C	1430	32.38073	chr8:9805	SYBU-AS1		lncRNA	chr8:109644115-109
ENSG0000C	1430	32.38073	chr8:9805	ENSG00000286308		lncRNA	chr8:117265272-117
ENSG0000C	1430	32.38073	chr8:9805	EIF3E	NCGv7;AC	protein_c	chr8:108162787-108

ENSG0000(	1430	32.38073	chr8:980E	AC103863.1		smallRNA	chr8:116136773-116
ENSG0000(	1430	32.38073	chr8:980E	AC107890.1		smallRNA	chr8:113407527-113
ENSG0000(	1430	32.38073	chr8:980E	TRPS1	NCGv7	protein_c	chr8:115408496-115
ENSG0000(	1430	32.38073	chr8:980E	LINC01608		lncRNA	chr8:110899996-111
ENSG0000(	1430	32.38073	chr8:980E	SYBU		protein_c	chr8:109573978-109
ENSG0000(	1430	32.38073	chr8:980E	TRHR		protein_c	chr8:109086585-109
ENSG0000(	1430	32.38073	chr8:980E	TRMT10BP1		Pseudoger	chr8:108652373-108
ENSG0000(	1430	32.38073	chr8:980E	EIF3H		protein_c	chr8:116642130-116
ENSG0000(	1430	32.38073	chr8:980E	ENSG00000255402		lncRNA	chr8:109973943-109
ENSG0000(	1430	32.38073	chr8:980E	ENSG00000241385		Pseudoger	chr8:109899246-109
ENSG0000(	1430	32.38073	chr8:980E	RNA5SP276		Pseudoger	chr8:116022686-116
ENSG0000(	1430	32.38073	chr8:980E	TMEM74		protein_c	chr8:108606850-108
ENSG0000(	1430	32.38073	chr8:980E	MAPK6P5		Pseudoger	chr8:109470131-109
ENSG0000(	1430	32.38073	chr8:980E	EMC2	DriverDB\	protein_c	chr8:108443601-108
ENSG0000(	1430	32.38073	chr8:980E	ENSG00000289767		protein_c	chr8:109539726-109
ENSG0000(	1430	32.38073	chr8:980E	ENSG00000289094		lncRNA	chr8:108195494-108
ENSG0000(	1430	32.38073	chr8:980E	RPS17P14		Pseudoger	chr8:108378831-108
ENSG0000(	1430	32.38073	chr8:980E	ENSG00000227514		Pseudoger	chr8:113376669-113
ENSG0000(	1430	32.38073	chr8:980E	LINC00536		lncRNA	chr8:115950511-116
ENSG0000(	1430	32.38073	chr8:980E	ENSG00000253207		lncRNA	chr8:113600310-113
ENSG0000(	1430	32.38073	chr8:980E	RAD21	NCGv7;AC	protein_c	chr8:116845934-116
ENSG0000(	1430	32.38073	chr8:980E	LINC01609		lncRNA	chr8:111093263-111
ENSG0000(	1430	32.38073	chr8:980E	RPL30P16		Pseudoger	chr8:112538889-112
ENSG0000(	1430	32.38073	chr8:980E	ENSG00000253949		lncRNA	chr8:108581062-108
ENSG0000(	1430	32.38073	chr8:980E	ENSG00000253107		Pseudoger	chr8:110555408-110
ENSG0000(	1430	32.38073	chr8:980E	MIR2053		smallRNA	chr8:112643493-112
ENSG0000(	1430	32.38073	chr8:980E	ENSG00000286937		lncRNA	chr8:113438334-113
ENSG0000(	1430	32.38073	chr8:980E	ENSG00000287942		lncRNA	chr8:111699519-111
ENSG0000(	1430	32.38073	chr8:980E	RNU4-37P		smallRNA	chr8:112148742-112
ENSG0000(	1430	32.38073	chr8:980E	RN7SL228P		smallRNA	chr8:117017946-117
ENSG0000(	1430	32.38073	chr8:980E	ENSG00000286946		lncRNA	chr8:112446575-112
ENSG0000(	1430	32.38073	chr8:980E	TRPS1-AS1		lncRNA	chr8:115509602-115
ENSG0000(	1430	32.38073	chr8:980E	ENSG00000250267		lncRNA	chr8:109298598-109
ENSG0000(	1430	32.38073	chr8:980E	ENSG00000253122		lncRNA	chr8:110609241-110
ENSG0000(	1430	32.38073	chr8:980E	ENSG00000253271		Pseudoger	chr8:117055818-117
ENSG0000(	1430	32.38073	chr8:980E	RPS10P16		Pseudoger	chr8:117523271-117
ENSG0000(	1430	32.38073	chr8:980E	ENSG00000253499		Pseudoger	chr8:114318400-114
ENSG0000(	1430	32.38073	chr8:980E	AARD	DriverDB\	protein_c	chr8:116938207-116
ENSG0000(	1430	32.38073	chr8:980E	ENSG00000253657		Pseudoger	chr8:108572643-108
ENSG0000(	1430	32.38073	chr8:980E	ENSG00000253956		lncRNA	chr8:110334743-110
ENSG0000(	1430	32.38073	chr8:980E	Y_RNA		smallRNA	chr8:113950886-113
ENSG0000(	1430	32.38073	chr8:980E	CARS1P2		Pseudoger	chr8:114791653-114
ENSG0000(	1430	32.38073	chr8:980E	ENSG00000253754		lncRNA	chr8:108226200-108
ENSG0000(	1430	32.38073	chr8:980E	SERPINA15P		Pseudoger	chr8:111380603-111
ENSG0000(	1430	32.38073	chr8:980E	ENSG00000253489		lncRNA	chr8:111179193-111
ENSG0000(	1430	32.38073	chr8:980E	ENSG00000255099		Pseudoger	chr8:109777912-109
ENSG0000(	1430	32.38073	chr8:980E	ENSG00000253796		lncRNA	chr8:108871128-109
ENSG0000(	1430	32.38073	chr8:980E	AURKBP1		Pseudoger	chr8:108131100-108
ENSG0000(	1430	32.38073	chr8:980E	NDUFB9P3		Pseudoger	chr8:110809394-110
ENSG0000(	1430	32.38073	chr8:980E	SNORD112		smallRNA	chr8:109596243-109
ENSG0000(	1430	32.38073	chr8:980E	PKHD1L1	NCGv7	protein_c	chr8:109362461-109

ENSG0000(	1430	32.38073	chr8:980[illegible]	SNORA31		smallRNA	chr8:117313528-117
ENSG0000(	1430	32.38073	chr8:980[illegible]	AF130343.1		smallRNA	chr8:116287351-116
ENSG0000(	1430	32.38073	chr8:980[illegible]	RNU2-67P		smallRNA	chr8:109873913-109
ENSG0000(	1430	32.38073	chr8:980[illegible]	MIR3610		smallRNA	chr8:116874728-116
ENSG0000(	1430	32.38073	chr8:980[illegible]	AC024996.1		smallRNA	chr8:112475601-112
ENSG0000(	1430	32.38073	chr8:980[illegible]	ENSG00000253622		lncRNA	chr8:117128455-117
ENSG0000(	1430	32.38073	chr8:980[illegible]	MTCO1P47		Pseudoger	chr8:110933263-110
ENSG0000(	1430	32.38073	chr8:980[illegible]	ENSG00000289382		lncRNA	chr8:116652014-116
ENSG0000(	1430	32.38073	chr8:980[illegible]	ENSG00000253672		lncRNA	chr8:116402543-116
ENSG0000(	1425	32.26751	chr1:1021	Y_RNA		smallRNA	chr1:151841736-151
ENSG0000(	1420	32.15429	chr1:1021	CELF3		protein_c	chr1:151700058-151
ENSG0000(	1420	32.15429	chr1:1021	TDRKH	DriverDB\	protein_c	chr1:151770107-151
ENSG0000(	1420	32.15429	chr1:1021	ENSG00000232536		lncRNA	chr1:151540516-151
ENSG0000(	1420	32.15429	chr1:1021	THEM5	DriverDB\	protein_c	chr1:151847101-151
ENSG0000(	1420	32.15429	chr1:1021	AL589765.1		protein_c	chr1:151710433-151
ENSG0000(	1420	32.15429	chr1:1021	ENSG00000227045		lncRNA	chr1:151701026-151
ENSG0000(	1420	32.15429	chr1:1021	ENSG00000269489		lncRNA	chr1:151798054-151
ENSG0000(	1420	32.15429	chr1:1021	SNORA44		smallRNA	chr1:151527831-151
ENSG0000(	1420	32.15429	chr1:1021	RNU6-662P		smallRNA	chr1:151747597-151
ENSG0000(	1420	32.15429	chr1:1021	ENSG00000232937		lncRNA	chr1:151765709-151
ENSG0000(	1420	32.15429	chr1:1021	ENSG00000236940		Pseudoger	chr1:151757659-151
ENSG0000(	1420	32.15429	chr1:1021	RNU6-1062P		smallRNA	chr1:151629324-151
ENSG0000(	1420	32.15429	chr1:1021	TDRKH-AS1		lncRNA	chr1:151790804-151
ENSG0000(	1420	32.15429	chr1:1021	RIIAD1		protein_c	chr1:151710433-151
ENSG0000(	1420	32.15429	chr1:1021	OAZ3		protein_c	chr1:151762899-151
ENSG0000(	1420	32.15429	chr1:1021	MIR554		smallRNA	chr1:151545796-151
ENSG0000(	1420	32.15429	chr1:1021	ENSG00000223861		Pseudoger	chr1:151557446-151
ENSG0000(	1420	32.15429	chr1:1021	LINGO4		protein_c	chr1:151800264-151
ENSG0000(	1420	32.15429	chr1:1021	ENSG00000249602		lncRNA	chr1:151763384-151
ENSG0000(	1420	32.15429	chr1:1021	RORC	DriverDB\	protein_c	chr1:151806071-151
ENSG0000(	1420	32.15429	chr1:1021	C2CD4D	DriverDB\	protein_c	chr1:151837818-151
ENSG0000(	1420	32.15429	chr1:1021	C2CD4D-AS1		lncRNA	chr1:151841877-151
ENSG0000(	1420	32.15429	chr1:1021	ENSG00000268288		lncRNA	chr1:151766486-151
ENSG0000(	1420	32.15429	chr1:1021	MRPL9		protein_c	chr1:151759647-151
ENSG0000(	1420	32.15429	chr1:1021	ENSG00000250734		lncRNA	chr1:151612038-151
ENSG0000(	1420	32.15429	chr1:1021	CGN	DriverDB\	protein_c	chr1:151510510-151
ENSG0000(	1420	32.15429	chr1:1021	TUFT1		protein_c	chr1:151540305-151
ENSG0000(	1420	32.15429	chr1:1021	SNX27		protein_c	chr1:151612006-151
ENSG0000(	1420	32.15429	chr1:1021	ENSG00000269621		lncRNA	chr1:151755541-151
ENSG0000(	1414	32.01843	chr5:4231	RNU6-47P		smallRNA	chr5:109014834-109
ENSG0000(	1414	32.01843	chr5:4231	ENSG00000253613		lncRNA	chr5:111076921-111
ENSG0000(	1414	32.01843	chr5:4231	RNU6-334P		smallRNA	chr5:104780288-104
ENSG0000(	1414	32.01843	chr5:4231	PSMC1P5		Pseudoger	chr5:107195156-107
ENSG0000(	1414	32.01843	chr5:4231	AC008967.1		smallRNA	chr5:111555895-111
ENSG0000(	1414	32.01843	chr5:4231	EIF3KP1		Pseudoger	chr5:103032376-103
ENSG0000(	1414	32.01843	chr5:4231	ENSG00000244245		Pseudoger	chr5:108593609-108
ENSG0000(	1414	32.01843	chr5:4231	ENSG00000248428		lncRNA	chr5:110970951-111
ENSG0000(	1414	32.01843	chr5:4231	ENSG00000289260		lncRNA	chr5:108727866-108
ENSG0000(	1414	32.01843	chr5:4231	ENSG00000248268		lncRNA	chr5:111277517-111
ENSG0000(	1414	32.01843	chr5:4231	PJA2		protein_c	chr5:109334713-109
ENSG0000(	1414	32.01843	chr5:4231	ENSG00000251099		Pseudoger	chr5:111572236-111

ENSG0000(	1414	32.01843	chr5:4231	RN7SKP122		smallRNA	chr5:107810629-107
ENSG0000(	1414	32.01843	chr5:4231	ENSG00000249476		lncRNA	chr5:109237120-109
ENSG0000(	1414	32.01843	chr5:4231	ENSG00000250728		Pseudoger	chr5:111155244-111
ENSG0000(	1414	32.01843	chr5:4231	ENSG00000278958		TEC	chr5:104434772-104
ENSG0000(	1414	32.01843	chr5:4231	ENSG00000253584		lncRNA	chr5:104917492-105
ENSG0000(	1414	32.01843	chr5:4231	ENSG00000248827		Pseudoger	chr5:107724961-107
ENSG0000(	1414	32.01843	chr5:4231	ENSG00000288965		lncRNA	chr5:111238810-111
ENSG0000(	1414	32.01843	chr5:4231	NREP		protein_c	chr5:111662621-111
ENSG0000(	1414	32.01843	chr5:4231	ENSG00000251574		lncRNA	chr5:104383298-105
ENSG0000(	1414	32.01843	chr5:4231	LINC02115		lncRNA	chr5:103528434-103
ENSG0000(	1414	32.01843	chr5:4231	HMGN1P14		Pseudoger	chr5:111846050-111
ENSG0000(	1414	32.01843	chr5:4231	LINC01023		lncRNA	chr5:108725707-108
ENSG0000(	1414	32.01843	chr5:4231	TMEM232	DriverDBv	protein_c	chr5:110289233-110
ENSG0000(	1414	32.01843	chr5:4231	ENSG00000253776		lncRNA	chr5:104773641-104
ENSG0000(	1414	32.01843	chr5:4231	ENSG00000250441		Pseudoger	chr5:108894347-108
ENSG0000(	1414	32.01843	chr5:4231	ENSG00000251204		Pseudoger	chr5:106415576-106
ENSG0000(	1414	32.01843	chr5:4231	RNA5SP189		Pseudoger	chr5:105922994-105
ENSG0000(	1414	32.01843	chr5:4231	LINC01848		lncRNA	chr5:109883182-109
ENSG0000(	1414	32.01843	chr5:4231	ENSG00000250383		lncRNA	chr5:108818041-108
ENSG0000(	1414	32.01843	chr5:4231	RNU1-140P		smallRNA	chr5:104098874-104
ENSG0000(	1414	32.01843	chr5:4231	ENSG00000270779		Pseudoger	chr5:110908284-110
ENSG0000(	1414	32.01843	chr5:4231	ENSG00000286503		lncRNA	chr5:108382156-108
ENSG0000(	1414	32.01843	chr5:4231	GJA1P1		Pseudoger	chr5:109051315-109
ENSG0000(	1414	32.01843	chr5:4231	MACIR		protein_c	chr5:103258763-103
ENSG0000(	1414	32.01843	chr5:4231	FER	TAG	protein_c	chr5:108747841-109
ENSG0000(	1414	32.01843	chr5:4231	WDR36		protein_c	chr5:111092321-111
ENSG0000(	1414	32.01843	chr5:4231	HMGN1P13		Pseudoger	chr5:111572102-111
ENSG0000(	1414	32.01843	chr5:4231	SLC25A46		protein_c	chr5:110738136-110
ENSG0000(	1414	32.01843	chr5:4231	STARD4		protein_c	chr5:111496033-111
ENSG0000(	1414	32.01843	chr5:4231	MIR548F3		smallRNA	chr5:110513829-110
ENSG0000(	1414	32.01843	chr5:4231	ENSG00000249318		lncRNA	chr5:111265809-111
ENSG0000(	1414	32.01843	chr5:4231	RN7SKP230		smallRNA	chr5:109699500-109
ENSG0000(	1414	32.01843	chr5:4231	ENSG00000286882		lncRNA	chr5:109165289-109
ENSG0000(	1414	32.01843	chr5:4231	ENSG00000251627		Pseudoger	chr5:110983563-110
ENSG0000(	1414	32.01843	chr5:4231	NIHCOLE		lncRNA	chr5:104079847-104
ENSG0000(	1414	32.01843	chr5:4231	AC012603.1		smallRNA	chr5:109685580-109
ENSG0000(	1414	32.01843	chr5:4231	MAN2A1	NCGv7	protein_c	chr5:109689927-109
ENSG0000(	1414	32.01843	chr5:4231	NUDT12	DriverDBv	protein_c	chr5:103548855-103
ENSG0000(	1414	32.01843	chr5:4231	KRT18P42		Pseudoger	chr5:109588338-109
ENSG0000(	1414	32.01843	chr5:4231	BCLAF1P1		Pseudoger	chr5:110948029-110
ENSG0000(	1414	32.01843	chr5:4231	RPS3AP21		Pseudoger	chr5:111192226-111
ENSG0000(	1414	32.01843	chr5:4231	PPIP5K2		protein_c	chr5:103120149-103
ENSG0000(	1414	32.01843	chr5:4231	MAN2A1-DT		lncRNA	chr5:109687802-109
ENSG0000(	1414	32.01843	chr5:4231	RN7SL782P		smallRNA	chr5:107734775-107
ENSG0000(	1414	32.01843	chr5:4231	RACK1P1		Pseudoger	chr5:108784098-108
ENSG0000(	1414	32.01843	chr5:4231	RAB9BP1		Pseudoger	chr5:105099473-105
ENSG0000(	1414	32.01843	chr5:4231	ENSG00000249068		Pseudoger	chr5:109840128-109
ENSG0000(	1414	32.01843	chr5:4231	EFNA5		protein_c	chr5:107376894-107
ENSG0000(	1414	32.01843	chr5:4231	ENSG00000250567		lncRNA	chr5:103408941-103
ENSG0000(	1414	32.01843	chr5:4231	SNORA51		smallRNA	chr5:110684794-110
ENSG0000(	1414	32.01843	chr5:4231	GIN1		protein_c	chr5:103086000-103

ENSG0000(	1414	32.01843	chr5:4231	ENSG00000250145		Pseudoger	chr5:106543066-106
ENSG0000(	1414	32.01843	chr5:4231	ENSG00000251367		lncRNA	chr5:109467353-109
ENSG0000(	1414	32.01843	chr5:4231	ENSG00000283462		lncRNA	chr5:103880129-103
ENSG0000(	1414	32.01843	chr5:4231	RN7SKP57		smallRNA	chr5:111719769-111
ENSG0000(	1414	32.01843	chr5:4231	PDZPH1P		Pseudoger	chr5:103430406-103
ENSG0000(	1414	32.01843	chr5:4231	ENSG00000248440		lncRNA	chr5:109497877-109
ENSG0000(	1414	32.01843	chr5:4231	ENSG00000248867		Pseudoger	chr5:109448349-109
ENSG0000(	1414	32.01843	chr5:4231	CAMK4		protein_c	chr5:111223653-111
ENSG0000(	1414	32.01843	chr5:4231	ENSG00000248203		lncRNA	chr5:103246048-103
ENSG0000(	1414	32.01843	chr5:4231	TSLP		protein_c	chr5:111070062-111
ENSG0000(	1414	32.01843	chr5:4231	PGAM5P1		Pseudoger	chr5:109884610-109
ENSG0000(	1414	32.01843	chr5:4231	ENSG00000248876		lncRNA	chr5:109470843-109
ENSG0000(	1414	32.01843	chr5:4231	Y_RNA		smallRNA	chr5:108891437-108
ENSG0000(	1414	32.01843	chr5:4231	ENSG00000249959		lncRNA	chr5:107699392-107
ENSG0000(	1414	32.01843	chr5:4231	SNORA31		smallRNA	chr5:106546665-106
ENSG0000(	1414	32.01843	chr5:4231	FBXL17	NCGv7	protein_c	chr5:107859035-108
ENSG0000(	1414	32.01843	chr5:4231	RN7SL255P		smallRNA	chr5:104260533-104
ENSG0000(	1414	32.01843	chr5:4231	LINC01950		lncRNA	chr5:106815197-107
ENSG0000(	1414	32.01843	chr5:4231	STARD4-AS1		lncRNA	chr5:111510396-111
ENSG0000(	1381	31.27118	chr5:4231	ENSG00000279691		TEC	chr5:133052769-133
ENSG0000(	1343	30.41071	chr2:8187	AC017084.1		smallRNA	chr2:70455530-7045
ENSG0000(	1303	29.50496	chr1:1021	ENSG00000272161		lncRNA	chr1:2326201-23266
ENSG0000(	1301	29.45967	chr5:4231	Y_RNA		smallRNA	chr5:132993099-132
ENSG0000(	1301	29.45967	chr5:4231	AFF4	NCGv7	protein_c	chr5:132875395-132
ENSG0000(	1301	29.45967	chr5:4231	ENSG00000272203		lncRNA	chr5:132817248-132
ENSG0000(	1301	29.45967	chr5:4231	AFF4-DT		lncRNA	chr5:132963770-132
ENSG0000(	1301	29.45967	chr5:4231	ZCCHC10		protein_c	chr5:132996985-133
ENSG0000(	1301	29.45967	chr5:4231	EEF1A1P50		Pseudoger	chr5:132942932-132
ENSG0000(	1301	29.45967	chr5:4231	IL13		protein_c	chr5:132656263-132
ENSG0000(	1301	29.45967	chr5:4231	ENSG00000286408		lncRNA	chr5:133051065-133
ENSG0000(	1301	29.45967	chr5:4231	IL5		protein_c	chr5:132541445-132
ENSG0000(	1301	29.45967	chr5:4231	RAD50		protein_c	chr5:132556019-132
ENSG0000(	1301	29.45967	chr5:4231	ENSG00000283782		protein_c	chr5:132410832-132
ENSG0000(	1301	29.45967	chr5:4231	ENSG00000229738		Pseudoger	chr5:132987652-132
ENSG0000(	1301	29.45967	chr5:4231	ENSG00000251616		lncRNA	chr5:133111055-133
ENSG0000(	1301	29.45967	chr5:4231	IL4		protein_c	chr5:132673986-132
ENSG0000(	1301	29.45967	chr5:4231	Y_RNA		smallRNA	chr5:132945342-132
ENSG0000(	1301	29.45967	chr5:4231	IRF1-AS1		lncRNA	chr5:132410636-132
ENSG0000(	1301	29.45967	chr5:4231	ENSG00000248648		Pseudoger	chr5:133003119-133
ENSG0000(	1301	29.45967	chr5:4231	CCNI2		protein_c	chr5:132747426-132
ENSG0000(	1301	29.45967	chr5:4231	IRF1	NCGv7	protein_c	chr5:132440440-132
ENSG0000(	1301	29.45967	chr5:4231	RNA5SP192		Pseudoger	chr5:132848577-132
ENSG0000(	1301	29.45967	chr5:4231	TH2LCRR		lncRNA	chr5:132630589-132
ENSG0000(	1301	29.45967	chr5:4231	UQCRQ	DriverDB\	protein_c	chr5:132866630-132
ENSG0000(	1301	29.45967	chr5:4231	ATP6V0E1P1		Pseudoger	chr5:132948493-132
ENSG0000(	1301	29.45967	chr5:4231	SOWAHA		protein_c	chr5:132813302-132
ENSG0000(	1301	29.45967	chr5:4231	SEPTIN8		protein_c	chr5:132750819-132
ENSG0000(	1301	29.45967	chr5:4231	ENSG00000287054		lncRNA	chr5:133025847-133
ENSG0000(	1301	29.45967	chr5:4231	ENSG00000230612		lncRNA	chr5:132688681-132
ENSG0000(	1301	29.45967	chr5:4231	KIF3A		protein_c	chr5:132692628-132
ENSG0000(	1301	29.45967	chr5:4231	SHROOM1		protein_c	chr5:132822141-132

ENSG0000(	1301	29.45967	chr5:4231	RPL6P15		Pseudoger	chr5:133172218-133
ENSG0000(	1301	29.45967	chr5:4231	GDF9		protein_c	chr5:132861181-132
ENSG0000(	1301	29.45967	chr5:4231	LEAP2		protein_c	chr5:132873444-132
ENSG0000(	1301	29.45967	chr5:4231	Y_RNA		smallRNA	chr5:132468147-132
ENSG0000(	1301	29.45967	chr5:4231	HSPA4	NCGv7;AC	protein_c	chr5:133052013-133
ENSG0000(	1301	29.45967	chr5:4231	LINC02863		lncRNA	chr5:132419416-132
ENSG0000(	1290	29.21059	chr1:373	AL590683.2		smallRNA	chr1:24279767-2427
ENSG0000(	1283	29.05208	chr5:4231	ENSG00000281938		protein_c	chr5:131807143-132
ENSG0000(	1283	29.05208	chr5:4231	ENSG00000287390		lncRNA	chr5:130655383-130
ENSG0000(	1283	29.05208	chr5:4231	ACSL6	NCGv7	protein_c	chr5:131949973-132
ENSG0000(	1283	29.05208	chr5:4231	MIR4460		smallRNA	chr5:129397062-129
ENSG0000(	1283	29.05208	chr5:4231	ENSG00000279584		TEC	chr5:131797415-131
ENSG0000(	1283	29.05208	chr5:4231	ADAMTS19	NCGv7	protein_c	chr5:129460281-129
ENSG0000(	1283	29.05208	chr5:4231	ADAMTS19-AS1		lncRNA	chr5:129424782-129
ENSG0000(	1283	29.05208	chr5:4231	ENSG00000249418		Pseudoger	chr5:130598348-130
ENSG0000(	1283	29.05208	chr5:4231	HINT1		protein_c	chr5:131155383-131
ENSG0000(	1283	29.05208	chr5:4231	FNIP1		protein_c	chr5:131641714-131
ENSG0000(	1283	29.05208	chr5:4231	ACTBP4		Pseudoger	chr5:131659046-131
ENSG0000(	1283	29.05208	chr5:4231	AC004769.1		smallRNA	chr5:130277625-130
ENSG0000(	1283	29.05208	chr5:4231	MINAR2		protein_c	chr5:129748094-129
ENSG0000(	1283	29.05208	chr5:4231	CDC42SE2		protein_c	chr5:131245493-131
ENSG0000(	1283	29.05208	chr5:4231	ENSG00000288697		Pseudoger	chr5:128932070-128
ENSG0000(	1283	29.05208	chr5:4231	RNA5SP191		Pseudoger	chr5:130114409-130
ENSG0000(	1283	29.05208	chr5:4231	ACSL6-AS1		lncRNA	chr5:132003592-132
ENSG0000(	1283	29.05208	chr5:4231	ISOC1		protein_c	chr5:129094749-129
ENSG0000(	1283	29.05208	chr5:4231	RAPGEF6		protein_c	chr5:131423921-131
ENSG0000(	1283	29.05208	chr5:4231	ENSG00000248634		lncRNA	chr5:128663978-128
ENSG0000(	1283	29.05208	chr5:4231	ENSG00000273217		protein_c	chr5:131425891-131
ENSG0000(	1283	29.05208	chr5:4231	RNU6ATAC10P		smallRNA	chr5:129974891-129
ENSG0000(	1283	29.05208	chr5:4231	ENSG00000279370		TEC	chr5:131261321-131
ENSG0000(	1283	29.05208	chr5:4231	ENSG00000234758		lncRNA	chr5:131944408-131
ENSG0000(	1283	29.05208	chr5:4231	ENSG00000286274		lncRNA	chr5:129150677-129
ENSG0000(	1283	29.05208	chr5:4231	MIR4633		smallRNA	chr5:129097688-129
ENSG0000(	1283	29.05208	chr5:4231	ENSG00000244192		Pseudoger	chr5:130989897-130
ENSG0000(	1283	29.05208	chr5:4231	ENSG00000231585		lncRNA	chr5:132011448-132
ENSG0000(	1283	29.05208	chr5:4231	RNU7-53P		smallRNA	chr5:130386621-130
ENSG0000(	1283	29.05208	chr5:4231	ENSG00000290020		lncRNA	chr5:131210048-131
ENSG0000(	1283	29.05208	chr5:4231	ENSG00000251680		lncRNA	chr5:129500361-129
ENSG0000(	1283	29.05208	chr5:4231	ARL2BPP4		Pseudoger	chr5:130529419-130
ENSG0000(	1283	29.05208	chr5:4231	ENSG00000248955		Pseudoger	chr5:131008583-131
ENSG0000(	1283	29.05208	chr5:4231	ENSG00000250405		Pseudoger	chr5:130994253-130
ENSG0000(	1283	29.05208	chr5:4231	HSPA8P4		Pseudoger	chr5:130140031-130
ENSG0000(	1283	29.05208	chr5:4231	CHSY3		protein_c	chr5:129904465-130
ENSG0000(	1283	29.05208	chr5:4231	MEIKIN		protein_c	chr5:131806990-131
ENSG0000(	1283	29.05208	chr5:4231	LYRM7		protein_c	chr5:131170944-131
ENSG0000(	1268	28.71242	chr5:4231	PPP2CA		protein_c	chr5:134194035-134
ENSG0000(	1268	28.71242	chr5:4231	SEC24A		protein_c	chr5:134648785-134
ENSG0000(	1268	28.71242	chr5:4231	ENSG00000271737		lncRNA	chr5:134004834-134
ENSG0000(	1268	28.71242	chr5:4231	C5orf15		protein_c	chr5:133955510-133
ENSG0000(	1268	28.71242	chr5:4231	ENSG00000250409		lncRNA	chr5:133387773-133
ENSG0000(	1268	28.71242	chr5:4231	RN7SL541P		smallRNA	chr5:134517810-134

ENSG0000(	1268	28.71242	chr5:4231	RNU6-456P		smallRNA	chr5:134500178-134
ENSG0000(	1268	28.71242	chr5:4231	CAMLG		protein_c	chr5:134738495-134
ENSG0000(	1268	28.71242	chr5:4231	SKP1		protein_c	chr5:134148935-134
ENSG0000(	1268	28.71242	chr5:4231	ENSG00000243648		Pseudoger	chr5:134422838-134
ENSG0000(	1268	28.71242	chr5:4231	ENSG00000272772		protein_c	chr5:134157088-134
ENSG0000(	1268	28.71242	chr5:4231	RNU6-1311P		smallRNA	chr5:134634839-134
ENSG0000(	1268	28.71242	chr5:4231	LINC01843		lncRNA	chr5:134506552-134
ENSG0000(	1268	28.71242	chr5:4231	TCF7	NCGv7	protein_c	chr5:134114681-134
ENSG0000(	1268	28.71242	chr5:4231	LINC02999		lncRNA	chr5:134429051-134
ENSG0000(	1268	28.71242	chr5:4231	ENSG00000251243		Pseudoger	chr5:133720495-133
ENSG0000(	1268	28.71242	chr5:4231	PPP2CA-DT		lncRNA	chr5:134226410-134
ENSG0000(	1268	28.71242	chr5:4231	ENSG00000248245		lncRNA	chr5:133160438-133
ENSG0000(	1268	28.71242	chr5:4231	ENSG00000239615		Pseudoger	chr5:134267421-134
ENSG0000(	1268	28.71242	chr5:4231	ENSG00000279197		TEC	chr5:133574829-133
ENSG0000(	1268	28.71242	chr5:4231	ENSG00000250244		lncRNA	chr5:133256492-133
ENSG0000(	1268	28.71242	chr5:4231	CDKL3		protein_c	chr5:134286350-134
ENSG0000(	1268	28.71242	chr5:4231	ENSG00000280104		TEC	chr5:133457805-133
ENSG0000(	1268	28.71242	chr5:4231	RNU6-757P		smallRNA	chr5:134715483-134
ENSG0000(	1268	28.71242	chr5:4231	VDAC1		protein_c	chr5:133971871-134
ENSG0000(	1268	28.71242	chr5:4231	RNU6-1164P		smallRNA	chr5:134701172-134
ENSG0000(	1268	28.71242	chr5:4231	ENSG00000279469		TEC	chr5:134394360-134
ENSG0000(	1268	28.71242	chr5:4231	ENSG00000250994		lncRNA	chr5:134436686-134
ENSG0000(	1268	28.71242	chr5:4231	UBE2B		protein_c	chr5:134371184-134
ENSG0000(	1268	28.71242	chr5:4231	MIR1289-2		smallRNA	chr5:133427596-133
ENSG0000(	1268	28.71242	chr5:4231	FSTL4		protein_c	chr5:133196455-133
ENSG0000(	1268	28.71242	chr5:4231	ENSG00000273345		protein_c	chr5:134196905-134
ENSG0000(	1268	28.71242	chr5:4231	SAR1B		protein_c	chr5:134601149-134
ENSG0000(	1268	28.71242	chr5:4231	MIR3661		smallRNA	chr5:134225757-134
ENSG0000(	1268	28.71242	chr5:4231	ENSG00000248559		lncRNA	chr5:134399495-134
ENSG0000(	1268	28.71242	chr5:4231	JADE2	DriverDBv	protein_c	chr5:134524312-134
ENSG0000(	1268	28.71242	chr5:4231	AC005195.1		smallRNA	chr5:133625845-133
ENSG0000(	1268	28.71242	chr5:4231	ENSG00000288758		lncRNA	chr5:133239988-133
ENSG0000(	1268	28.71242	chr5:4231	WSPAR		lncRNA	chr5:133913677-133
ENSG0000(	1268	28.71242	chr5:4231	ENSG00000271128		Pseudoger	chr5:134081914-134
ENSG0000(	1268	28.71242	chr5:4231	snoU13		smallRNA	chr5:133850857-133
ENSG0000(	1268	28.71242	chr5:4231	CDKN2AIPNL		protein_c	chr5:134402065-134
ENSG0000(	1268	28.71242	chr5:4231	ENSG00000280420		TEC	chr5:134522613-134
ENSG0000(	1268	28.71242	chr5:4231	ENSG00000249478		lncRNA	chr5:133243764-133
ENSG0000(	1267	28.68978	chr5:4231	MIR3936HG		lncRNA	chr5:132311285-132
ENSG0000(	1267	28.68978	chr5:4231	ENSG00000224431		Pseudoger	chr5:132199456-132
ENSG0000(	1267	28.68978	chr5:4231	IL3		protein_c	chr5:132060655-132
ENSG0000(	1267	28.68978	chr5:4231	snoZ6		smallRNA	chr5:132080066-132
ENSG0000(	1267	28.68978	chr5:4231	CSF2		protein_c	chr5:132073789-132
ENSG0000(	1267	28.68978	chr5:4231	SLC22A5	DriverDBv	protein_c	chr5:132369710-132
ENSG0000(	1267	28.68978	chr5:4231	PDLIM4		protein_c	chr5:132257696-132
ENSG0000(	1267	28.68978	chr5:4231	SLC22A4		protein_c	chr5:132294394-132
ENSG0000(	1267	28.68978	chr5:4231	ENSG00000235775		Pseudoger	chr5:132163603-132
ENSG0000(	1267	28.68978	chr5:4231	P4HA2		protein_c	chr5:132190147-132
ENSG0000(	1267	28.68978	chr5:4231	MIR3936		smallRNA	chr5:132365490-132
ENSG0000(	1267	28.68978	chr5:4231	ENSG00000227616		Pseudoger	chr5:132147491-132
ENSG0000(	1267	28.68978	chr5:4231	ENSG00000224015		lncRNA	chr5:132179234-132

ENSG0000(	1267	28.68978	chr5:4231	P4HA2-AS1	lncRNA	chr5:132184876-132
ENSG0000(	1263	28.5992	chr5:4231	ENSG00000249950	Pseudoger	chr5:125862746-125
ENSG0000(	1263	28.5992	chr5:4231	C5orf63	protein_c	chr5:127045235-127
ENSG0000(	1263	28.5992	chr5:4231	ENSG00000249261	lncRNA	chr5:124868972-124
ENSG0000(	1263	28.5992	chr5:4231	ENSG00000287794	Pseudoger	chr5:122373512-122
ENSG0000(	1263	28.5992	chr5:4231	ENSG00000248752	lncRNA	chr5:125333369-126
ENSG0000(	1263	28.5992	chr5:4231	RP11-395P13.6	lncRNA	chr5:125395379-125
ENSG0000(	1263	28.5992	chr5:4231	ENSG00000248853	Pseudoger	chr5:121059244-121
ENSG0000(	1263	28.5992	chr5:4231	snoU13	smallRNA	chr5:123009542-123
ENSG0000(	1263	28.5992	chr5:4231	RN7SL711P	smallRNA	chr5:123070782-123
ENSG0000(	1263	28.5992	chr5:4231	GRAMD2B	protein_c	chr5:126360132-126
ENSG0000(	1263	28.5992	chr5:4231	ENSG00000289458	lncRNA	chr5:124497294-124
ENSG0000(	1263	28.5992	chr5:4231	LINC02240	lncRNA	chr5:124808981-125
ENSG0000(	1263	28.5992	chr5:4231	HMGB1P22	Pseudoger	chr5:125265528-125
ENSG0000(	1263	28.5992	chr5:4231	CUL1P1	Pseudoger	chr5:127784618-127
ENSG0000(	1263	28.5992	chr5:4231	ENSG00000213661	Pseudoger	chr5:121616247-121
ENSG0000(	1263	28.5992	chr5:4231	LINC02039	lncRNA	chr5:126179565-126
ENSG0000(	1263	28.5992	chr5:4231	ENSG00000288008	lncRNA	chr5:124993408-124
ENSG0000(	1263	28.5992	chr5:4231	snoU13	smallRNA	chr5:122796125-122
ENSG0000(	1263	28.5992	chr5:4231	ENSG00000229855	lncRNA	chr5:121164687-121
ENSG0000(	1263	28.5992	chr5:4231	RP11-395P13.3	lncRNA	chr5:125370165-125
ENSG0000(	1263	28.5992	chr5:4231	ENSG00000288766	lncRNA	chr5:123511177-123
ENSG0000(	1263	28.5992	chr5:4231	RNU6-963P	smallRNA	chr5:126553302-126
ENSG0000(	1263	28.5992	chr5:4231	PRRC1	protein_c	chr5:127517640-127
ENSG0000(	1263	28.5992	chr5:4231	CCDC192	protein_c	chr5:127703391-127
ENSG0000(	1263	28.5992	chr5:4231	RNU6-752P	smallRNA	chr5:126755316-126
ENSG0000(	1263	28.5992	chr5:4231	ENSG00000250603	lncRNA	chr5:127838486-127
ENSG0000(	1263	28.5992	chr5:4231	ENSG00000213655	Pseudoger	chr5:123236079-123
ENSG0000(	1263	28.5992	chr5:4231	ENSG00000250803	protein_c	chr5:122129622-122
ENSG0000(	1263	28.5992	chr5:4231	ENSG00000271766	lncRNA	chr5:127478295-127
ENSG0000(	1263	28.5992	chr5:4231	ENSG00000248443	lncRNA	chr5:124829472-124
ENSG0000(	1263	28.5992	chr5:4231	ENSG00000248799	lncRNA	chr5:127651693-127
ENSG0000(	1263	28.5992	chr5:4231	ENSG00000286111	lncRNA	chr5:125493261-125
ENSG0000(	1263	28.5992	chr5:4231	ENSG00000251135	Pseudoger	chr5:121040090-121
ENSG0000(	1263	28.5992	chr5:4231	ENSG00000249916	lncRNA	chr5:122369762-122
ENSG0000(	1263	28.5992	chr5:4231	RPSAP37	Pseudoger	chr5:125966777-125
ENSG0000(	1263	28.5992	chr5:4231	ENSG00000249904	Pseudoger	chr5:125886285-125
ENSG0000(	1263	28.5992	chr5:4231	ENSG00000286615	lncRNA	chr5:127231959-127
ENSG0000(	1263	28.5992	chr5:4231	AC093267.1	smallRNA	chr5:122776431-122
ENSG0000(	1263	28.5992	chr5:4231	KRT18P16	Pseudoger	chr5:123636110-123
ENSG0000(	1263	28.5992	chr5:4231	ENSG00000289054	lncRNA	chr5:122326610-122
ENSG0000(	1263	28.5992	chr5:4231	ENSG00000249112	lncRNA	chr5:124707827-124
ENSG0000(	1263	28.5992	chr5:4231	PRDM6-AS1	lncRNA	chr5:123087248-123
ENSG0000(	1263	28.5992	chr5:4231	RN7SL689P	smallRNA	chr5:123022489-123
ENSG0000(	1263	28.5992	chr5:4231	ENSG00000283897	lncRNA	chr5:127215159-127
ENSG0000(	1263	28.5992	chr5:4231	LMNB1-DT	lncRNA	chr5:126751963-126
ENSG0000(	1263	28.5992	chr5:4231	HSPE1P10	Pseudoger	chr5:126737438-126
ENSG0000(	1263	28.5992	chr5:4231	RP11-395P13.2	lncRNA	chr5:125368568-125
ENSG0000(	1263	28.5992	chr5:4231	CEP120	protein_c	chr5:123344890-123
ENSG0000(	1263	28.5992	chr5:4231	PRELID3BP8	Pseudoger	chr5:120658077-120
ENSG0000(	1263	28.5992	chr5:4231	ENSG00000230929	Pseudoger	chr5:126628019-126

ENSG0000(	1263	28.5992	chr5:4231	ENSG00000248296		lncRNA	chr5:124492775-124
ENSG0000(	1263	28.5992	chr5:4231	ZNF608	NCGv7	protein_c	chr5:124636913-124
ENSG0000(	1263	28.5992	chr5:4231	ENSG00000250602		lncRNA	chr5:126372477-126
ENSG0000(	1263	28.5992	chr5:4231	RP11-395P13.5		lncRNA	chr5:125382859-125
ENSG0000(	1263	28.5992	chr5:4231	MEGF10	DriverDB\	protein_c	chr5:127290796-127
ENSG0000(	1263	28.5992	chr5:4231	POGLUT2P1		Pseudoger	chr5:128109486-128
ENSG0000(	1263	28.5992	chr5:4231	MGC32805		lncRNA	chr5:122436497-122
ENSG0000(	1263	28.5992	chr5:4231	BOLA3P3		Pseudoger	chr5:126663337-126
ENSG0000(	1263	28.5992	chr5:4231	ALDH7A1		protein_c	chr5:126531200-126
ENSG0000(	1263	28.5992	chr5:4231	LINC02201		lncRNA	chr5:122628952-122
ENSG0000(	1263	28.5992	chr5:4231	PHAX	NCGv7	protein_c	chr5:126600925-126
ENSG0000(	1263	28.5992	chr5:4231	AC011416.1		smallRNA	chr5:127260936-127
ENSG0000(	1263	28.5992	chr5:4231	FBN2	NCGv7	protein_c	chr5:128257909-128
ENSG0000(	1263	28.5992	chr5:4231	PPIC		protein_c	chr5:123023250-123
ENSG0000(	1263	28.5992	chr5:4231	ENSG00000242814		Pseudoger	chr5:124808828-124
ENSG0000(	1263	28.5992	chr5:4231	LINC01170		lncRNA	chr5:124059794-124
ENSG0000(	1263	28.5992	chr5:4231	SLC12A2-DT		lncRNA	chr5:127939152-128
ENSG0000(	1263	28.5992	chr5:4231	LOX	IntOGen-I	protein_c	chr5:122063195-122
ENSG0000(	1263	28.5992	chr5:4231	ENSG00000282925		lncRNA	chr5:127588746-127
ENSG0000(	1263	28.5992	chr5:4231	CTXN3		protein_c	chr5:127649082-127
ENSG0000(	1263	28.5992	chr5:4231	RNU4-69P		smallRNA	chr5:120710698-120
ENSG0000(	1263	28.5992	chr5:4231	SRFBP1		protein_c	chr5:121961975-122
ENSG0000(	1263	28.5992	chr5:4231	RPL23AP44		Pseudoger	chr5:121575848-121
ENSG0000(	1263	28.5992	chr5:4231	ENSG00000272139		lncRNA	chr5:122311297-122
ENSG0000(	1263	28.5992	chr5:4231	ENSG00000279118		TEC	chr5:126496279-126
ENSG0000(	1263	28.5992	chr5:4231	ARGFXP1		Pseudoger	chr5:122675795-122
ENSG0000(	1263	28.5992	chr5:4231	ENSG00000250080		Pseudoger	chr5:127170535-127
ENSG0000(	1263	28.5992	chr5:4231	RN7SKP117		smallRNA	chr5:125350868-125
ENSG0000(	1263	28.5992	chr5:4231	FTMT		protein_c	chr5:121851882-121
ENSG0000(	1263	28.5992	chr5:4231	TEX43		protein_c	chr5:126631705-126
ENSG0000(	1263	28.5992	chr5:4231	HMGB3P17		Pseudoger	chr5:123468781-123
ENSG0000(	1263	28.5992	chr5:4231	ENSG00000249577		Pseudoger	chr5:127465822-127
ENSG0000(	1263	28.5992	chr5:4231	SELENOTP2		Pseudoger	chr5:127176696-127
ENSG0000(	1263	28.5992	chr5:4231	ENSG00000250194		Pseudoger	chr5:125074665-125
ENSG0000(	1263	28.5992	chr5:4231	ENSG00000251214		lncRNA	chr5:124459912-124
ENSG0000(	1263	28.5992	chr5:4231	SNX2		protein_c	chr5:122775079-122
ENSG0000(	1263	28.5992	chr5:4231	ENSG00000250847		Pseudoger	chr5:121195980-121
ENSG0000(	1263	28.5992	chr5:4231	ENSG00000249600		Pseudoger	chr5:121671373-121
ENSG0000(	1263	28.5992	chr5:4231	SLC12A2		protein_c	chr5:128083766-128
ENSG0000(	1263	28.5992	chr5:4231	HNRNPKP1		Pseudoger	chr5:127511464-127
ENSG0000(	1263	28.5992	chr5:4231	SLC27A6		protein_c	chr5:128538013-129
ENSG0000(	1263	28.5992	chr5:4231	ENSG00000279772		TEC	chr5:123508736-123
ENSG0000(	1263	28.5992	chr5:4231	ENSG00000279446		TEC	chr5:127073587-127
ENSG0000(	1263	28.5992	chr5:4231	MRPS5P3		Pseudoger	chr5:127143082-127
ENSG0000(	1263	28.5992	chr5:4231	LMNB1		protein_c	chr5:126776623-126
ENSG0000(	1263	28.5992	chr5:4231	AC010235.1		smallRNA	chr5:126196835-126
ENSG0000(	1263	28.5992	chr5:4231	MARCHF3		protein_c	chr5:126867714-127
ENSG0000(	1263	28.5992	chr5:4231	HMGB1P29		Pseudoger	chr5:124220579-124
ENSG0000(	1263	28.5992	chr5:4231	SNX24		protein_c	chr5:122843439-123
ENSG0000(	1263	28.5992	chr5:4231	ENSG00000288890		lncRNA	chr5:123619519-123
ENSG0000(	1263	28.5992	chr5:4231	Y_RNA		smallRNA	chr5:123510436-123

ENSG0000(	1263	28.5992	chr5:4231	ENSG00000248927		lncRNA	chr5:120781218-120
ENSG0000(	1263	28.5992	chr5:4231	SNCAIP		protein_c	chr5:122311354-122
ENSG0000(	1263	28.5992	chr5:4231	RP11-395P13.4		lncRNA	chr5:125376834-125
ENSG0000(	1263	28.5992	chr5:4231	CSNK1G3		protein_c	chr5:123512177-123
ENSG0000(	1263	28.5992	chr5:4231	ENSG00000247311		lncRNA	chr5:122114598-122
ENSG0000(	1263	28.5992	chr5:4231	ENSG00000251456		lncRNA	chr5:124734618-124
ENSG0000(	1263	28.5992	chr5:4231	ENSG00000248600		lncRNA	chr5:124452277-124
ENSG0000(	1263	28.5992	chr5:4231	ENSG00000251421		lncRNA	chr5:124395603-124
ENSG0000(	1263	28.5992	chr5:4231	ZNF474-AS1		lncRNA	chr5:122154496-122
ENSG0000(	1263	28.5992	chr5:4231	ENSG00000279860		TEC	chr5:121363002-121
ENSG0000(	1263	28.5992	chr5:4231	ENSG00000249621		lncRNA	chr5:122321291-122
ENSG0000(	1263	28.5992	chr5:4231	ENSG00000250438		lncRNA	chr5:124469591-124
ENSG0000(	1263	28.5992	chr5:4231	ENSG00000260686		lncRNA	chr5:122832356-122
ENSG0000(	1263	28.5992	chr5:4231	PRDM6	AC	protein_c	chr5:123089241-123
ENSG0000(	1263	28.5992	chr5:4231	ENSG00000250956		Pseudoger	chr5:127179756-127
ENSG0000(	1263	28.5992	chr5:4231	PPIC-AS1		lncRNA	chr5:123036271-123
ENSG0000(	1263	28.5992	chr5:4231	KRT8P33		Pseudoger	chr5:123400922-123
ENSG0000(	1263	28.5992	chr5:4231	ZNF474		protein_c	chr5:122129546-122
ENSG0000(	1261	28.55392	chr16:23(	ENSG00000260472		Pseudoger	chr16:31697397-316
ENSG0000(	1251	28.32748	chr5:4231	SRP19		protein_c	chr5:112861188-112
ENSG0000(	1251	28.32748	chr5:4231	AC008536.1		smallRNA	chr5:112905638-112
ENSG0000(	1251	28.32748	chr5:4231	EPB41L4A		protein_c	chr5:112142441-112
ENSG0000(	1251	28.32748	chr5:4231	ENSG00000258864		protein_c	chr5:112827213-112
ENSG0000(	1251	28.32748	chr5:4231	APC	NCGv7;AC	protein_c	chr5:112707498-112
ENSG0000(	1251	28.32748	chr5:4231	ENSG00000251187		lncRNA	chr5:112192020-112
ENSG0000(	1251	28.32748	chr5:4231	ENSG00000232633		lncRNA	chr5:113323028-113
ENSG0000(	1251	28.32748	chr5:4231	ENSG00000251014		Pseudoger	chr5:112546973-112
ENSG0000(	1251	28.32748	chr5:4231	ENSG00000248350		Pseudoger	chr5:112363456-112
ENSG0000(	1251	28.32748	chr5:4231	KCNN2	NCGv7	protein_c	chr5:114055926-114
ENSG0000(	1251	28.32748	chr5:4231	LINC02200		lncRNA	chr5:112628436-112
ENSG0000(	1251	28.32748	chr5:4231	RNU6-482P		smallRNA	chr5:112778363-112
ENSG0000(	1251	28.32748	chr5:4231	EPB41L4A-AS1		lncRNA	chr5:112160526-112
ENSG0000(	1251	28.32748	chr5:4231	ENSG00000286839		lncRNA	chr5:113738106-113
ENSG0000(	1251	28.32748	chr5:4231	ENSG00000250882		lncRNA	chr5:112173570-112
ENSG0000(	1251	28.32748	chr5:4231	ENSG00000251076		lncRNA	chr5:112228283-112
ENSG0000(	1251	28.32748	chr5:4231	ZRSR2P1		Pseudoger	chr5:112891610-112
ENSG0000(	1251	28.32748	chr5:4231	REEP5	NCGv7	protein_c	chr5:112876385-112
ENSG0000(	1251	28.32748	chr5:4231	NREP-AS1		lncRNA	chr5:111912508-112
ENSG0000(	1251	28.32748	chr5:4231	MCC	AC	protein_c	chr5:113022106-113
ENSG0000(	1251	28.32748	chr5:4231	HMGB3P16		Pseudoger	chr5:112452703-112
ENSG0000(	1251	28.32748	chr5:4231	ENSG00000290027		lncRNA	chr5:112521511-112
ENSG0000(	1251	28.32748	chr5:4231	YTHDC2		protein_c	chr5:113513694-113
ENSG0000(	1251	28.32748	chr5:4231	RNU4ATAC13P		smallRNA	chr5:113493835-113
ENSG0000(	1251	28.32748	chr5:4231	LINC01957		lncRNA	chr5:114576041-114
ENSG0000(	1251	28.32748	chr5:4231	TSSK1B		protein_c	chr5:113432553-113
ENSG0000(	1251	28.32748	chr5:4231	XBP1P1		Pseudoger	chr5:112885094-112
ENSG0000(	1251	28.32748	chr5:4231	RN7SKP89		smallRNA	chr5:114253513-114
ENSG0000(	1251	28.32748	chr5:4231	CBX3P3		Pseudoger	chr5:112777630-112
ENSG0000(	1251	28.32748	chr5:4231	ENSG00000246316		lncRNA	chr5:114475339-114
ENSG0000(	1251	28.32748	chr5:4231	SNORA13		smallRNA	chr5:112161485-112
ENSG0000(	1251	28.32748	chr5:4231	EPB41L4A-DT		lncRNA	chr5:112419583-112

ENSG0000(	1251	28.32748	chr5:4231	DCP2		protein_c	chr5:112976702-113
ENSG0000(	1247	28.2369	chr5:4231	TRIM36		protein_c	chr5:115124762-115
ENSG0000(	1247	28.2369	chr5:4231	LINC02215		lncRNA	chr5:118596188-118
ENSG0000(	1247	28.2369	chr5:4231	LAMTOR3P2		Pseudoger	chr5:119132651-119
ENSG0000(	1247	28.2369	chr5:4231	RNU6-644P		smallRNA	chr5:116188455-116
ENSG0000(	1247	28.2369	chr5:4231	ARL14EPL		protein_c	chr5:116032324-116
ENSG0000(	1247	28.2369	chr5:4231	PRR16		protein_c	chr5:120464300-120
ENSG0000(	1247	28.2369	chr5:4231	ENSG00000286745		lncRNA	chr5:118581242-118
ENSG0000(	1247	28.2369	chr5:4231	ENSG00000272265		lncRNA	chr5:116078110-116
ENSG0000(	1247	28.2369	chr5:4231	DMXL1	NCGv7	protein_c	chr5:119037772-119
ENSG0000(	1247	28.2369	chr5:4231	ENSG00000251132		lncRNA	chr5:115031273-115
ENSG0000(	1247	28.2369	chr5:4231	AC034236.2		smallRNA	chr5:116030018-116
ENSG0000(	1247	28.2369	chr5:4231	PTMAP2		Pseudoger	chr5:118973796-118
ENSG0000(	1247	28.2369	chr5:4231	SEMA6A	NCGv7	protein_c	chr5:116443555-116
ENSG0000(	1247	28.2369	chr5:4231	snoU13		smallRNA	chr5:119069297-119
ENSG0000(	1247	28.2369	chr5:4231	RPL35AP15		Pseudoger	chr5:117301104-117
ENSG0000(	1247	28.2369	chr5:4231	SEPTIN7P10		Pseudoger	chr5:119126782-119
ENSG0000(	1247	28.2369	chr5:4231	snoU13		smallRNA	chr5:119468959-119
ENSG0000(	1247	28.2369	chr5:4231	HMGN1P15		Pseudoger	chr5:115289036-115
ENSG0000(	1247	28.2369	chr5:4231	ENSG00000213663		Pseudoger	chr5:119547131-119
ENSG0000(	1247	28.2369	chr5:4231	RPS17P2		Pseudoger	chr5:116716243-116
ENSG0000(	1247	28.2369	chr5:4231	ENSG00000249944		Pseudoger	chr5:115489634-115
ENSG0000(	1247	28.2369	chr5:4231	CDO1		protein_c	chr5:115804733-115
ENSG0000(	1247	28.2369	chr5:4231	ENSG00000271918		lncRNA	chr5:116083807-116
ENSG0000(	1247	28.2369	chr5:4231	AP3S1		protein_c	chr5:115841592-115
ENSG0000(	1247	28.2369	chr5:4231	TNFAIP8	NCGv7	protein_c	chr5:119268692-119
ENSG0000(	1247	28.2369	chr5:4231	AC034236.1		smallRNA	chr5:116043171-116
ENSG0000(	1247	28.2369	chr5:4231	CTNNA1P1		Pseudoger	chr5:115389643-115
ENSG0000(	1247	28.2369	chr5:4231	LVRN		protein_c	chr5:115962454-116
ENSG0000(	1247	28.2369	chr5:4231	TUBAP15		Pseudoger	chr5:119652523-119
ENSG0000(	1247	28.2369	chr5:4231	ENSG00000251477		Pseudoger	chr5:117719240-117
ENSG0000(	1247	28.2369	chr5:4231	ENSG00000249021		lncRNA	chr5:115691462-115
ENSG0000(	1247	28.2369	chr5:4231	RNU6-718P		smallRNA	chr5:120337549-120
ENSG0000(	1247	28.2369	chr5:4231	snoU13		smallRNA	chr5:119610202-119
ENSG0000(	1247	28.2369	chr5:4231	ENSG00000250015		lncRNA	chr5:116302354-116
ENSG0000(	1247	28.2369	chr5:4231	LINC02208		lncRNA	chr5:118000253-118
ENSG0000(	1247	28.2369	chr5:4231	ENSG00000250242		lncRNA	chr5:115087892-115
ENSG0000(	1247	28.2369	chr5:4231	FABP5P6		Pseudoger	chr5:119555250-119
ENSG0000(	1247	28.2369	chr5:4231	LINCADL		lncRNA	chr5:115956571-115
ENSG0000(	1247	28.2369	chr5:4231	FEM1C		protein_c	chr5:115520908-115
ENSG0000(	1247	28.2369	chr5:4231	MIR5706		smallRNA	chr5:119154637-119
ENSG0000(	1247	28.2369	chr5:4231	DMXL1-DT		lncRNA	chr5:119006347-119
ENSG0000(	1247	28.2369	chr5:4231	ENSG00000249150		lncRNA	chr5:116819220-116
ENSG0000(	1247	28.2369	chr5:4231	TMED7-TI(	DriverDB\	protein_c	chr5:115578642-115
ENSG0000(	1247	28.2369	chr5:4231	RPL7L1P4		Pseudoger	chr5:117778826-117
ENSG0000(	1247	28.2369	chr5:4231	ENSG00000249426		lncRNA	chr5:118760474-118
ENSG0000(	1247	28.2369	chr5:4231	SEMA6A-AS1		lncRNA	chr5:116447547-116
ENSG0000(	1247	28.2369	chr5:4231	ENSG00000243304		Pseudoger	chr5:115264669-115
ENSG0000(	1247	28.2369	chr5:4231	RNU7-34P		smallRNA	chr5:118758211-118
ENSG0000(	1247	28.2369	chr5:4231	AK3P4		Pseudoger	chr5:115402380-115
ENSG0000(	1247	28.2369	chr5:4231	ENSG00000250949		lncRNA	chr5:117031200-117

ENSG0000(	1247	28.2369	chr5:4231	AC027320.1		smallRNA	chr5:119257340-119
ENSG0000(	1247	28.2369	chr5:4231	CCT5P1		Pseudoger	chr5:115512077-115
ENSG0000(	1247	28.2369	chr5:4231	LINC02147		lncRNA	chr5:117730515-118
ENSG0000(	1247	28.2369	chr5:4231	ENSG00000261036		lncRNA	chr5:120345907-120
ENSG0000(	1247	28.2369	chr5:4231	ENSG00000249791		lncRNA	chr5:115188563-115
ENSG0000(	1247	28.2369	chr5:4231	H3P24		Pseudoger	chr5:115770586-115
ENSG0000(	1247	28.2369	chr5:4231	RPS14P8		Pseudoger	chr5:116562562-116
ENSG0000(	1247	28.2369	chr5:4231	ENSG00000250650		Pseudoger	chr5:117019548-117
ENSG0000(	1247	28.2369	chr5:4231	RN7SL174P		smallRNA	chr5:119306344-119
ENSG0000(	1247	28.2369	chr5:4231	RNU2-49P		smallRNA	chr5:115774319-115
ENSG0000(	1247	28.2369	chr5:4231	LINC02214		lncRNA	chr5:116742991-116
ENSG0000(	1247	28.2369	chr5:4231	LINC00992		lncRNA	chr5:117415509-117
ENSG0000(	1247	28.2369	chr5:4231	HSD17B4		protein_c	chr5:119452465-119
ENSG0000(	1247	28.2369	chr5:4231	SEMA6A-AS2		lncRNA	chr5:116574482-116
ENSG0000(	1247	28.2369	chr5:4231	ATG12		protein_c	chr5:115828200-115
ENSG0000(	1247	28.2369	chr5:4231	ENSG00000234259		Pseudoger	chr5:119680197-119
ENSG0000(	1247	28.2369	chr5:4231	TMED7		protein_c	chr5:115613210-115
ENSG0000(	1247	28.2369	chr5:4231	LINC02216		lncRNA	chr5:118575575-118
ENSG0000(	1247	28.2369	chr5:4231	ENSG00000289497		lncRNA	chr5:115364197-115
ENSG0000(	1247	28.2369	chr5:4231	ENSG00000250928		Pseudoger	chr5:119011471-119
ENSG0000(	1247	28.2369	chr5:4231	COMMD10		protein_c	chr5:116085016-116
ENSG0000(	1247	28.2369	chr5:4231	ENSG00000185641		Pseudoger	chr5:116051917-116
ENSG0000(	1247	28.2369	chr5:4231	FAM170A	DriverDBv	protein_c	chr5:119629558-119
ENSG0000(	1247	28.2369	chr5:4231	ENSG00000271797		lncRNA	chr5:115262505-115
ENSG0000(	1247	28.2369	chr5:4231	HMGN2P27		Pseudoger	chr5:116218026-116
ENSG0000(	1247	28.2369	chr5:4231	RNU6-373P		smallRNA	chr5:119007571-119
ENSG0000(	1247	28.2369	chr5:4231	RNA5SP190		Pseudoger	chr5:119445716-119
ENSG0000(	1247	28.2369	chr5:4231	RNU6-701P		smallRNA	chr5:119138859-119
ENSG0000(	1247	28.2369	chr5:4231	CCDC112		protein_c	chr5:115267190-115
ENSG0000(	1247	28.2369	chr5:4231	LINC02148		lncRNA	chr5:118282575-118
ENSG0000(	1247	28.2369	chr5:4231	ENSG00000248709		lncRNA	chr5:115738978-115
ENSG0000(	1247	28.2369	chr5:4231	DTWD2	NCGv7	protein_c	chr5:118836074-118
ENSG0000(	1247	28.2369	chr5:4231	ENSG00000285809		lncRNA	chr5:117313085-117
ENSG0000(	1247	28.2369	chr5:4231	DDX43P1		Pseudoger	chr5:115974513-115
ENSG0000(	1247	28.2369	chr5:4231	TICAM2		protein_c	chr5:115578496-115
ENSG0000(	1247	28.2369	chr5:4231	PGGT1B		protein_c	chr5:115204012-115
ENSG0000(	1247	28.2369	chr5:4231	ENSG00000250678		Pseudoger	chr5:119010060-119
ENSG0000(	1247	28.2369	chr5:4231	ENSG00000251293		lncRNA	chr5:120245448-120
ENSG0000(	1247	28.2369	chr5:4231	TICAM2-AS1		lncRNA	chr5:115602057-115
ENSG0000(	1247	28.2369	chr5:4231	TRIM36-IT1		lncRNA	chr5:115148764-115
ENSG0000(	1247	28.2369	chr5:4231	ENSG00000249433		Pseudoger	chr5:115493557-115
ENSG0000(	1234	27.94253	chr5:4231	MIR4461		smallRNA	chr5:134928039-134
ENSG0000(	1234	27.94253	chr5:4231	ENSG00000270892		Pseudoger	chr5:134863091-134
ENSG0000(	1234	27.94253	chr5:4231	MTND3P25		Pseudoger	chr5:134928393-134
ENSG0000(	1234	27.94253	chr5:4231	MTND4LP30		Pseudoger	chr5:134928030-134
ENSG0000(	1234	27.94253	chr5:4231	MTCYBP18		Pseudoger	chr5:134923303-134
ENSG0000(	1234	27.94253	chr5:4231	MTND4P12		Pseudoger	chr5:134926660-134
ENSG0000(	1234	27.94253	chr5:4231	TXNDC15		protein_c	chr5:134874371-134
ENSG0000(	1234	27.94253	chr5:4231	DDX46		protein_c	chr5:134758771-134
ENSG0000(	1234	27.94253	chr5:4231	C5orf24		protein_c	chr5:134845680-134
ENSG0000(	1234	27.94253	chr5:4231	CATSPER3		protein_c	chr5:134967907-135

ENSG00000	1234	27.94253	chr5:4231	ENSG00000277619		lncRNA	chr5:135034521-135
ENSG00000	1234	27.94253	chr5:4231	MTND5P11		Pseudogen	chr5:134924648-134
ENSG00000	1234	27.94253	chr5:4231	PITX1		protein_c	chr5:135027734-135
ENSG00000	1234	27.94253	chr5:4231	ENSG00000279799		TEC	chr5:134905235-134
ENSG00000	1234	27.94253	chr5:4231	EPIST		lncRNA	chr5:135038831-135
ENSG00000	1234	27.94253	chr5:4231	PCBD2		protein_c	chr5:134905120-135
ENSG00000	1234	27.94253	chr5:4231	MTND6P4		Pseudogen	chr5:134924123-134
ENSG00000	1234	27.94253	chr5:4231	ENSG00000241179		Pseudogen	chr5:134804778-134
ENSG00000	1226	27.76138	chr5:4231	LINC02900		lncRNA	chr5:135236234-135
ENSG00000	1226	27.76138	chr5:4231	ENSG00000249639		lncRNA	chr5:135450613-135
ENSG00000	1226	27.76138	chr5:4231	TIFAB		protein_c	chr5:135444226-135
ENSG00000	1226	27.76138	chr5:4231	NEUROG1		protein_c	chr5:135534282-135
ENSG00000	1226	27.76138	chr5:4231	DCANP1		protein_c	chr5:135444214-135
ENSG00000	1226	27.76138	chr5:4231	ENSG00000250167		lncRNA	chr5:135476913-135
ENSG00000	1226	27.76138	chr5:4231	CXCL14		protein_c	chr5:135570679-135
ENSG00000	1226	27.76138	chr5:4231	ENSG00000248753		lncRNA	chr5:135120526-135
ENSG00000	1226	27.76138	chr5:4231	MACROH2A1		protein_c	chr5:135334381-135
ENSG00000	1226	27.76138	chr5:4231	PITX1-AS1		lncRNA	chr5:135033280-135
ENSG00000	1226	27.76138	chr5:4231	ENSG00000248482		lncRNA	chr5:135124380-135
ENSG00000	1226	27.76138	chr5:4231	ENSG00000270021		lncRNA	chr5:135399280-135
ENSG00000	1226	27.76138	chr5:4231	SLC25A48-AS1		lncRNA	chr5:135648584-135
ENSG00000	1170	26.49332	chr5:4231	RAB5CP2		Pseudogen	chr5:91476382-9147
ENSG00000	1129	25.56493	chr12:624	RPL31P50		Pseudogen	chr12:31597114-315
ENSG00000	1122	25.40642	chr1:9219	RNU6-210P		smallRNA	chr1:93010257-9301
ENSG00000	1122	25.40642	chr1:9219	ENSG00000229052		Pseudogen	chr1:92930696-9293
ENSG00000	1122	25.40642	chr1:9219	SNORA66		smallRNA	chr1:92838018-9283
ENSG00000	1122	25.40642	chr1:9219	RN7SKP123		smallRNA	chr1:93026252-9302
ENSG00000	1122	25.40642	chr1:9219	ENSG00000225505		Pseudogen	chr1:92732000-9273
ENSG00000	1122	25.40642	chr1:9219	AC093577.1		smallRNA	chr1:92982237-9298
ENSG00000	1122	25.40642	chr1:9219	SNORA51		smallRNA	chr1:92846059-9284
ENSG00000	1122	25.40642	chr1:9219	SNORA66		smallRNA	chr1:92840719-9284
ENSG00000	1122	25.40642	chr1:9219	ACTBP12		Pseudogen	chr1:92229018-9222
ENSG00000	1122	25.40642	chr1:9219	Y_RNA		smallRNA	chr1:93027410-9302
ENSG00000	1122	25.40642	chr1:9219	AL451010.1		smallRNA	chr1:92229256-9222
ENSG00000	1122	25.40642	chr1:9219	DIPK1A		protein_c	chr1:92832737-9296
ENSG00000	1122	25.40642	chr1:9219	SNORD21		smallRNA	chr1:92837289-9283
ENSG00000	1122	25.40642	chr1:9219	RNU6-970P		smallRNA	chr1:92969604-9296
ENSG00000	1122	25.40642	chr1:9219	RPAP2		protein_c	chr1:92299059-9240
ENSG00000	1122	25.40642	chr1:9219	ENSG00000287797		lncRNA	chr1:92978265-9298
ENSG00000	1122	25.40642	chr1:9219	GFI1	TAG;AC	protein_c	chr1:92473043-9248
ENSG00000	1122	25.40642	chr1:9219	EVI5	AC	protein_c	chr1:92508696-9279
ENSG00000	1122	25.40642	chr1:9219	ENSG00000223787		Pseudogen	chr1:92580476-9258
ENSG00000	1122	25.40642	chr1:9219	H3P3		Pseudogen	chr1:92749175-9274
ENSG00000	1122	25.40642	chr1:9219	GLMN		protein_c	chr1:92246402-9229
ENSG00000	1122	25.40642	chr1:9219	RPL5	NCGv7;AC	protein_c	chr1:92832013-9284
ENSG00000	1122	25.40642	chr1:9219	ENSG00000226773		Pseudogen	chr1:92203148-9220
ENSG00000	1122	25.40642	chr1:9219	RN7SL824P		smallRNA	chr1:92402391-9240
ENSG00000	1122	25.40642	chr1:9219	RNU4-59P		smallRNA	chr1:92700819-9270
ENSG00000	1122	25.40642	chr1:9219	RN7SL692P		smallRNA	chr1:92974829-9297
ENSG00000	1122	25.40642	chr1:9219	C1orf146		protein_c	chr1:92217915-9224
ENSG00000	1122	25.40642	chr1:9219	ENSG00000289544		lncRNA	chr1:92961858-9296

ENSG0000(	1122	25.40642	chr1:921(	HMGB3P9		Pseudoger	chr1:92647048-9264
ENSG0000(	1122	25.40642	chr1:921(	CCNJP2		Pseudoger	chr1:92755794-9275
ENSG0000(	1121	25.38378	chr1:1021	RNU7-122P		smallRNA	chr1:109207794-109
ENSG0000(	1108	25.0894	chr1:1021	SETSIP		protein_c	chr1:92074533-9207
ENSG0000(	1108	25.0894	chr1:1021	GAPDHP46		Pseudoger	chr1:92114803-9211
ENSG0000(	1108	25.0894	chr1:1021	ENSG00000273487		lncRNA	chr1:92189237-9219
ENSG0000(	1108	25.0894	chr1:1021	BTBD8		protein_c	chr1:92080305-9218
ENSG0000(	1108	25.0894	chr1:1021	EPHX4		protein_c	chr1:92029985-9206
ENSG0000(	1108	25.0894	chr1:1021	PRKAR1AP1		Pseudoger	chr1:92125301-9212
ENSG0000(	1108	25.0894	chr1:1021	ENSG00000289483		lncRNA	chr1:92028938-9202
ENSG0000(	1108	25.0894	chr1:1021	LPCAT2BP		Pseudoger	chr1:92066306-9206
ENSG0000(	1107	25.06676	chr4:253(	Y_RNA		smallRNA	chr4:140700835-140
ENSG0000(	1095	24.79503	chr1:1021	FAM102B		protein_c	chr1:108560089-108
ENSG0000(	1095	24.79503	chr1:1021	ENSG00000289612		lncRNA	chr1:107140235-107
ENSG0000(	1095	24.79503	chr1:1021	ENSG00000289192		lncRNA	chr1:101964793-101
ENSG0000(	1095	24.79503	chr1:1021	ENSG00000232952		Pseudoger	chr1:105891739-105
ENSG0000(	1095	24.79503	chr1:1021	LINC01768		lncRNA	chr1:109828355-109
ENSG0000(	1095	24.79503	chr1:1021	ENSG00000260879		lncRNA	chr1:108199926-108
ENSG0000(	1095	24.79503	chr1:1021	CHIAP3		Pseudoger	chr1:111353275-111
ENSG0000(	1095	24.79503	chr1:1021	RNU6-965P		smallRNA	chr1:101728642-101
ENSG0000(	1095	24.79503	chr1:1021	ENSG00000270066		lncRNA	chr1:109100193-109
ENSG0000(	1095	24.79503	chr1:1021	DENND2D		protein_c	chr1:111185969-111
ENSG0000(	1095	24.79503	chr1:1021	LINC01160		lncRNA	chr1:111599655-111
ENSG0000(	1095	24.79503	chr1:1021	PRPF38B	NCGv7	protein_c	chr1:108692310-108
ENSG0000(	1095	24.79503	chr1:1021	OLFM3	NCGv7	protein_c	chr1:101802560-101
ENSG0000(	1095	24.79503	chr1:1021	AKNAD1		protein_c	chr1:108815898-108
ENSG0000(	1095	24.79503	chr1:1021	ENSG00000229283		lncRNA	chr1:111317600-111
ENSG0000(	1095	24.79503	chr1:1021	GSTM1	DriverDB\	protein_c	chr1:109687814-109
ENSG0000(	1095	24.79503	chr1:1021	GNAT2		protein_c	chr1:109603091-109
ENSG0000(	1095	24.79503	chr1:1021	ENSG00000232971		lncRNA	chr1:108734256-108
ENSG0000(	1095	24.79503	chr1:1021	CEPT1		protein_c	chr1:111139479-111
ENSG0000(	1095	24.79503	chr1:1021	LINC01661		lncRNA	chr1:106818224-106
ENSG0000(	1095	24.79503	chr1:1021	DRAM2		protein_c	chr1:111117163-111
ENSG0000(	1095	24.79503	chr1:1021	HIGD1AP12		Pseudoger	chr1:111380291-111
ENSG0000(	1095	24.79503	chr1:1021	MTATP6P14		Pseudoger	chr1:106802755-106
ENSG0000(	1095	24.79503	chr1:1021	ENSG00000280186		TEC	chr1:108200413-108
ENSG0000(	1095	24.79503	chr1:1021	GPR61		protein_c	chr1:109539872-109
ENSG0000(	1095	24.79503	chr1:1021	NTNG1		protein_c	chr1:107140007-107
ENSG0000(	1095	24.79503	chr1:1021	ENSG00000289355		lncRNA	chr1:101150560-101
ENSG0000(	1095	24.79503	chr1:1021	KCNC4-DT		lncRNA	chr1:110208834-110
ENSG0000(	1095	24.79503	chr1:1021	SLC6A17-AS1		lncRNA	chr1:110165948-110
ENSG0000(	1095	24.79503	chr1:1021	ENSG00000230864		lncRNA	chr1:102763322-102
ENSG0000(	1095	24.79503	chr1:1021	ATXN7L2		protein_c	chr1:109483479-109
ENSG0000(	1095	24.79503	chr1:1021	RPL7P8		Pseudoger	chr1:109651370-109
ENSG0000(	1095	24.79503	chr1:1021	ALX3		protein_c	chr1:110059870-110
ENSG0000(	1095	24.79503	chr1:1021	GSTM3		protein_c	chr1:109733932-109
ENSG0000(	1095	24.79503	chr1:1021	LAMTOR5	AC	protein_c	chr1:110401249-110
ENSG0000(	1095	24.79503	chr1:1021	LINC01397		lncRNA	chr1:110082651-110
ENSG0000(	1095	24.79503	chr1:1021	ENSG00000244716		Pseudoger	chr1:108992282-108
ENSG0000(	1095	24.79503	chr1:1021	AMY1A		protein_c	chr1:103655760-103
ENSG0000(	1095	24.79503	chr1:1021	CHIAP1		Pseudoger	chr1:111250254-111

ENSG0000(	1095	24.79503	chr1:1021	CDK4P1		Pseudoger	chr1:105433994-105
ENSG0000(	1095	24.79503	chr1:1021	ENSG00000283999		lncRNA	chr1:110473756-110
ENSG0000(	1095	24.79503	chr1:1021	ENSG00000228665		Pseudoger	chr1:109030067-109
ENSG0000(	1095	24.79503	chr1:1021	ACTG1P4		Pseudoger	chr1:103569553-103
ENSG0000(	1095	24.79503	chr1:1021	RNU6-792P		smallRNA	chr1:111490317-111
ENSG0000(	1095	24.79503	chr1:1021	GSTM5	NCGv7	protein_c	chr1:109711780-109
ENSG0000(	1095	24.79503	chr1:1021	RP11-347K2.2		lncRNA	chr1:103418079-103
ENSG0000(	1095	24.79503	chr1:1021	ENSG00000273221		lncRNA	chr1:111184415-111
ENSG0000(	1095	24.79503	chr1:1021	ENSG00000254942		lncRNA	chr1:109539906-109
ENSG0000(	1095	24.79503	chr1:1021	DNAJA1P5		Pseudoger	chr1:101893105-101
ENSG0000(	1095	24.79503	chr1:1021	NBPF4		protein_c	chr1:108222464-108
ENSG0000(	1095	24.79503	chr1:1021	ENSG00000228399		Pseudoger	chr1:101256274-101
ENSG0000(	1095	24.79503	chr1:1021	COL11A1	NCGv7	protein_c	chr1:102876467-103
ENSG0000(	1095	24.79503	chr1:1021	HENMT1		protein_c	chr1:108648290-108
ENSG0000(	1095	24.79503	chr1:1021	ENSG00000225113		lncRNA	chr1:109596225-109
ENSG0000(	1095	24.79503	chr1:1021	ENSG00000270976		Pseudoger	chr1:106780223-106
ENSG0000(	1095	24.79503	chr1:1021	RNPC3		protein_c	chr1:103525691-103
ENSG0000(	1095	24.79503	chr1:1021	VAV3	TAG;AC	protein_c	chr1:107571161-107
ENSG0000(	1095	24.79503	chr1:1021	NRBF2P3		Pseudoger	chr1:110848077-110
ENSG0000(	1095	24.79503	chr1:1021	CHIA		protein_c	chr1:111290851-111
ENSG0000(	1095	24.79503	chr1:1021	SNORA25		smallRNA	chr1:110272484-110
ENSG0000(	1095	24.79503	chr1:1021	KCNA3		protein_c	chr1:110653560-110
ENSG0000(	1095	24.79503	chr1:1021	snoU13		smallRNA	chr1:101228664-101
ENSG0000(	1095	24.79503	chr1:1021	ENSG00000237349		Pseudoger	chr1:108986963-108
ENSG0000(	1095	24.79503	chr1:1021	ENSG00000228703		lncRNA	chr1:109628417-109
ENSG0000(	1095	24.79503	chr1:1021	Y_RNA		smallRNA	chr1:111446798-111
ENSG0000(	1095	24.79503	chr1:1021	CHIAP2		lncRNA	chr1:111280059-111
ENSG0000(	1095	24.79503	chr1:1021	RBM15	NCGv7;AC	protein_c	chr1:110338506-110
ENSG0000(	1095	24.79503	chr1:1021	SORT1		protein_c	chr1:109309568-109
ENSG0000(	1095	24.79503	chr1:1021	FTLP17		Pseudoger	chr1:104153306-104
ENSG0000(	1095	24.79503	chr1:1021	ENSG00000233359		lncRNA	chr1:102199739-102
ENSG0000(	1095	24.79503	chr1:1021	RNU6V		smallRNA	chr1:109591534-109
ENSG0000(	1095	24.79503	chr1:1021	ENSG00000226483		Pseudoger	chr1:108508574-108
ENSG0000(	1095	24.79503	chr1:1021	PGBP		Pseudoger	chr1:111382860-111
ENSG0000(	1095	24.79503	chr1:1021	ENSG00000237897		Pseudoger	chr1:105890693-105
ENSG0000(	1095	24.79503	chr1:1021	PSRC1		protein_c	chr1:109279556-109
ENSG0000(	1095	24.79503	chr1:1021	ENSG00000230932		Pseudoger	chr1:106080801-106
ENSG0000(	1095	24.79503	chr1:1021	ENSG00000258634		lncRNA	chr1:110058340-110
ENSG0000(	1095	24.79503	chr1:1021	ENSG00000235005		lncRNA	chr1:109884176-109
ENSG0000(	1095	24.79503	chr1:1021	KCNA2		protein_c	chr1:110519837-110
ENSG0000(	1095	24.79503	chr1:1021	LINC02785		lncRNA	chr1:108040263-108
ENSG0000(	1095	24.79503	chr1:1021	SPATA42		lncRNA	chr1:108857217-108
ENSG0000(	1095	24.79503	chr1:1021	AMY1B		protein_c	chr1:103687415-103
ENSG0000(	1095	24.79503	chr1:1021	STRIP1		protein_c	chr1:110031577-110
ENSG0000(	1095	24.79503	chr1:1021	ELAPOR1		protein_c	chr1:109113679-109
ENSG0000(	1095	24.79503	chr1:1021	CFAP276		protein_c	chr1:109105951-109
ENSG0000(	1095	24.79503	chr1:1021	AMPD2		protein_c	chr1:109616104-109
ENSG0000(	1095	24.79503	chr1:1021	KCNC4		protein_c	chr1:110210314-110
ENSG0000(	1095	24.79503	chr1:1021	TMEM167B		protein_c	chr1:109090764-109
ENSG0000(	1095	24.79503	chr1:1021	WDR77		protein_c	chr1:111439890-111
ENSG0000(	1095	24.79503	chr1:1021	ENSG00000285923		lncRNA	chr1:108661533-108

ENSG0000(	1095	24.79503	chr1:1021	RANP5		Pseudoger	chr1:109046828-109
ENSG0000(	1095	24.79503	chr1:1021	LINC01677		lncRNA	chr1:105927620-106
ENSG0000(	1095	24.79503	chr1:1021	AMYP1		Pseudoger	chr1:103713723-103
ENSG0000(	1095	24.79503	chr1:1021	NDUFA5P10		Pseudoger	chr1:109810642-109
ENSG0000(	1095	24.79503	chr1:1021	ATP5PB		protein_c	chr1:111448864-111
ENSG0000(	1095	24.79503	chr1:1021	RAP1A	AC	protein_c	chr1:111542218-111
ENSG0000(	1095	24.79503	chr1:1021	KCNA10		protein_c	chr1:110517217-110
ENSG0000(	1095	24.79503	chr1:1021	ENSG00000288803		lncRNA	chr1:110680508-110
ENSG0000(	1095	24.79503	chr1:1021	KRT18P57		Pseudoger	chr1:111648291-111
ENSG0000(	1095	24.79503	chr1:1021	STXBP3		protein_c	chr1:108746674-108
ENSG0000(	1095	24.79503	chr1:1021	AMIGO1		protein_c	chr1:109504178-109
ENSG0000(	1095	24.79503	chr1:1021	MIR197		smallRNA	chr1:109598893-109
ENSG0000(	1095	24.79503	chr1:1021	ENSG00000241720		lncRNA	chr1:109725820-109
ENSG0000(	1095	24.79503	chr1:1021	SLC25A24		protein_c	chr1:108134043-108
ENSG0000(	1095	24.79503	chr1:1021	AMY2A		protein_c	chr1:103617427-103
ENSG0000(	1095	24.79503	chr1:1021	OVGP1		protein_c	chr1:111414319-111
ENSG0000(	1095	24.79503	chr1:1021	WDR47		protein_c	chr1:108970214-109
ENSG0000(	1095	24.79503	chr1:1021	PSMA5		protein_c	chr1:109399042-109
ENSG0000(	1095	24.79503	chr1:1021	CYB561D1		protein_c	chr1:109494052-109
ENSG0000(	1095	24.79503	chr1:1021	GSTM2		protein_c	chr1:109668022-109
ENSG0000(	1095	24.79503	chr1:1021	ADORA3		protein_c	chr1:111499429-111
ENSG0000(	1095	24.79503	chr1:1021	LINC01307		lncRNA	chr1:101323337-101
ENSG0000(	1095	24.79503	chr1:1021	FNDC7		protein_c	chr1:108712908-108
ENSG0000(	1095	24.79503	chr1:1021	ENSG00000237480		lncRNA	chr1:105956694-106
ENSG0000(	1095	24.79503	chr1:1021	ENSG00000270342		Pseudoger	chr1:106544342-106
ENSG0000(	1095	24.79503	chr1:1021	PPIAP7		Pseudoger	chr1:101270875-101
ENSG0000(	1095	24.79503	chr1:1021	ENSG00000260948		lncRNA	chr1:111431046-111
ENSG0000(	1095	24.79503	chr1:1021	ENSG00000285981		lncRNA	chr1:104998406-105
ENSG0000(	1095	24.79503	chr1:1021	SLC25A24P2		Pseudoger	chr1:108383736-108
ENSG0000(	1095	24.79503	chr1:1021	ENSG00000238122		lncRNA	chr1:108261196-108
ENSG0000(	1095	24.79503	chr1:1021	CCNT2P1		Pseudoger	chr1:111007700-111
ENSG0000(	1095	24.79503	chr1:1021	ENSG00000234441		Pseudoger	chr1:103668071-103
ENSG0000(	1095	24.79503	chr1:1021	KCND3		protein_c	chr1:111770662-111
ENSG0000(	1095	24.79503	chr1:1021	AMY1C		protein_c	chr1:103745323-103
ENSG0000(	1095	24.79503	chr1:1021	ENSG00000271277		Pseudoger	chr1:101882516-101
ENSG0000(	1095	24.79503	chr1:1021	PRMT6		protein_c	chr1:107056674-107
ENSG0000(	1095	24.79503	chr1:1021	SOD2P1		Pseudoger	chr1:103100143-103
ENSG0000(	1095	24.79503	chr1:1021	RP11-347K2.1		lncRNA	chr1:103414879-103
ENSG0000(	1095	24.79503	chr1:1021	RPSAP19		Pseudoger	chr1:101786340-101
ENSG0000(	1095	24.79503	chr1:1021	ENSG00000260246		lncRNA	chr1:109693117-109
ENSG0000(	1095	24.79503	chr1:1021	SCARNA16		smallRNA	chr1:101133153-101
ENSG0000(	1095	24.79503	chr1:1021	NDUFA4P1		Pseudoger	chr1:107505203-107
ENSG0000(	1095	24.79503	chr1:1021	ENSG00000283354		Pseudoger	chr1:108495475-108
ENSG0000(	1095	24.79503	chr1:1021	RNA5SP54		Pseudoger	chr1:111041834-111
ENSG0000(	1095	24.79503	chr1:1021	AC114491.1		smallRNA	chr1:107448469-107
ENSG0000(	1095	24.79503	chr1:1021	INKA2		protein_c	chr1:111680630-111
ENSG0000(	1095	24.79503	chr1:1021	ENSG00000270380		lncRNA	chr1:110456505-110
ENSG0000(	1095	24.79503	chr1:1021	TAF13		protein_c	chr1:109062496-109
ENSG0000(	1095	24.79503	chr1:1021	ENSG00000290126		lncRNA	chr1:108690627-108
ENSG0000(	1095	24.79503	chr1:1021	CHIAP2		Pseudoger	chr1:111280060-111
ENSG0000(	1095	24.79503	chr4:253(	AC105252.1		smallRNA	chr4:133560856-133

ENSG0000(	1095	24.79503	chr1:1021	ENSG00000272982		lncRNA	chr1:111181374-111
ENSG0000(	1095	24.79503	chr1:1021	SARS1		protein_c	chr1:109213918-109
ENSG0000(	1095	24.79503	chr1:1021	ENSG00000273010		lncRNA	chr1:110963302-110
ENSG0000(	1095	24.79503	chr1:1021	ENSG00000228076		Pseudoger	chr1:108766841-108
ENSG0000(	1095	24.79503	chr1:1021	SLC16A4-AS1		lncRNA	chr1:110370154-110
ENSG0000(	1095	24.79503	chr1:1021	SYPL2		protein_c	chr1:109466546-109
ENSG0000(	1095	24.79503	chr1:1021	TMEM167B-DT		lncRNA	chr1:109087971-109
ENSG0000(	1095	24.79503	chr1:1021	ENSG00000290117		lncRNA	chr1:109546610-109
ENSG0000(	1095	24.79503	chr1:1021	C1orf162		protein_c	chr1:111473792-111
ENSG0000(	1095	24.79503	chr1:1021	CYMP-AS1		lncRNA	chr1:110487680-110
ENSG0000(	1095	24.79503	chr1:1021	CD53		protein_c	chr1:110871188-110
ENSG0000(	1095	24.79503	chr1:1021	MTCO1P14		Pseudoger	chr1:106804474-106
ENSG0000(	1095	24.79503	chr1:1021	LRIF1		protein_c	chr1:110947190-110
ENSG0000(	1095	24.79503	chr1:1021	ENSG00000243960		lncRNA	chr1:111438638-111
ENSG0000(	1095	24.79503	chr1:1021	UBL4B		protein_c	chr1:110112443-110
ENSG0000(	1095	24.79503	chr1:1021	PIFO		protein_c	chr1:111346600-111
ENSG0000(	1095	24.79503	chr1:1021	ENSG00000232240		Pseudoger	chr1:111323833-111
ENSG0000(	1095	24.79503	chr1:1021	ENSG00000235526		lncRNA	chr1:110177643-110
ENSG0000(	1095	24.79503	chr1:1021	UBE2FP3		Pseudoger	chr1:111437514-111
ENSG0000(	1095	24.79503	chr1:1021	AHCYL1	AC	protein_c	chr1:109984765-110
ENSG0000(	1095	24.79503	chr1:1021	OR11I1P		Pseudoger	chr1:110853939-110
ENSG0000(	1095	24.79503	chr1:1021	PGBP		lncRNA	chr1:111384519-111
ENSG0000(	1095	24.79503	chr1:1021	TMIGD3		protein_c	chr1:111483348-111
ENSG0000(	1095	24.79503	chr1:1021	SLC25A24P1		Pseudoger	chr1:108273139-108
ENSG0000(	1095	24.79503	chr1:1021	CLCC1		protein_c	chr1:108881885-108
ENSG0000(	1095	24.79503	chr1:1021	GPSM2		protein_c	chr1:108875350-108
ENSG0000(	1095	24.79503	chr1:1021	NBPF5P		Pseudoger	chr1:108376119-108
ENSG0000(	1095	24.79503	chr1:1021	GNAI3		protein_c	chr1:109548615-109
ENSG0000(	1095	24.79503	chr1:1021	INKA2-AS1		lncRNA	chr1:111739579-111
ENSG0000(	1095	24.79503	chr1:1021	SLC6A17		protein_c	chr1:110150494-110
ENSG0000(	1095	24.79503	chr1:1021	VAV3-AS1		lncRNA	chr1:107964443-107
ENSG0000(	1095	24.79503	chr1:1021	LAMTOR5-AS1		lncRNA	chr1:110347116-110
ENSG0000(	1095	24.79503	chr1:1021	Y_RNA		smallRNA	chr1:110764408-110
ENSG0000(	1095	24.79503	chr1:1021	ENSG00000224698		lncRNA	chr1:108420689-108
ENSG0000(	1095	24.79503	chr1:1021	RBM15-AS1		lncRNA	chr1:110286375-110
ENSG0000(	1095	24.79503	chr1:1021	CSF1	AC	protein_c	chr1:109910242-109
ENSG0000(	1095	24.79503	chr1:1021	DDX20	NCGv7	protein_c	chr1:111754832-111
ENSG0000(	1095	24.79503	chr1:1021	AL390036.1		smallRNA	chr1:108018653-108
ENSG0000(	1095	24.79503	chr1:1021	ST13P21		Pseudoger	chr1:108502358-108
ENSG0000(	1095	24.79503	chr1:1021	RN7SKP285		smallRNA	chr1:103523562-103
ENSG0000(	1095	24.79503	chr1:1021	AMY2B	NCGv7	protein_c	chr1:103553815-103
ENSG0000(	1095	24.79503	chr1:1021	NBPF6		protein_c	chr1:108450282-108
ENSG0000(	1095	24.79503	chr1:1021	CHI3L2		protein_c	chr1:111200771-111
ENSG0000(	1095	24.79503	chr1:1021	GSTM4		protein_c	chr1:109656099-109
ENSG0000(	1095	24.79503	chr1:1021	AL591042.1		smallRNA	chr1:107776174-107
ENSG0000(	1095	24.79503	chr1:1021	EPS8L3		protein_c	chr1:109750080-109
ENSG0000(	1095	24.79503	chr1:1021	ENSG00000232811		lncRNA	chr1:110943467-110
ENSG0000(	1095	24.79503	chr1:1021	S1PR1	NCGv7	protein_c	chr1:101236865-101
ENSG0000(	1095	24.79503	chr1:1021	PROK1		protein_c	chr1:110451149-110
ENSG0000(	1095	24.79503	chr1:1021	RNU6-352P		smallRNA	chr1:101859851-101
ENSG0000(	1095	24.79503	chr1:1021	RNPC3-DT		lncRNA	chr1:103415980-103

ENSG0000(	1095	24.79503	chr1:1021	ENSG00000261654		lncRNA	chr1:110936369-110
ENSG0000(	1095	24.79503	chr1:1021	CELSR2		protein_c	chr1:109249539-109
ENSG0000(	1095	24.79503	chr1:1021	RNU6-151P		smallRNA	chr1:111650431-111
ENSG0000(	1095	24.79503	chr1:1021	ENSG00000282852		Pseudoger	chr1:110256754-110
ENSG0000(	1095	24.79503	chr1:1021	MYBPHL		protein_c	chr1:109292365-109
ENSG0000(	1095	24.79503	chr1:1021	S1PR1-DT		lncRNA	chr1:101234555-101
ENSG0000(	1095	24.79503	chr1:1021	SEPTIN2P1		Pseudoger	chr1:105698039-105
ENSG0000(	1095	24.79503	chr1:1021	ENSG00000290552		lncRNA	chr1:108375838-108
ENSG0000(	1095	24.79503	chr1:1021	AL365361.1		smallRNA	chr1:110652942-110
ENSG0000(	1095	24.79503	chr1:1021	ENSG00000215869		Pseudoger	chr1:104072983-104
ENSG0000(	1095	24.79503	chr1:1021	ENSG00000261055		lncRNA	chr1:109895973-109
ENSG0000(	1095	24.79503	chr1:1021	LINC01676		lncRNA	chr1:105587575-105
ENSG0000(	1095	24.79503	chr1:1021	ENSG00000251484		Pseudoger	chr1:109103535-109
ENSG0000(	1095	24.79503	chr1:1021	ENSG00000284830		lncRNA	chr1:111745299-111
ENSG0000(	1095	24.79503	chr1:1021	ENSG00000282887		lncRNA	chr1:110472543-110
ENSG0000(	1095	24.79503	chr1:1021	CYMP		Pseudoger	chr1:110480752-110
ENSG0000(	1095	24.79503	chr1:1021	SLC16A4	NCGv7	protein_c	chr1:110362851-110
ENSG0000(	1095	24.79503	chr1:1021	ENSG00000230759		lncRNA	chr1:103414879-103
ENSG0000(	1095	24.79503	chr1:1021	ENSG00000225191		Pseudoger	chr1:103926567-103
ENSG0000(	1095	24.79503	chr1:1021	ENSG00000290547		lncRNA	chr1:108272943-108
ENSG0000(	1095	24.79503	chr1:1021	ENSG00000271578		Pseudoger	chr1:101190520-101
ENSG0000(	1095	24.79503	chr1:1021	LINC01709		lncRNA	chr1:101639509-101
ENSG0000(	1080	24.45538	chr1:9219	RWDD3		protein_c	chr1:95234210-9524
ENSG0000(	1080	24.45538	chr1:9219	AL356479.1		smallRNA	chr1:95504976-9550
ENSG0000(	1080	24.45538	chr1:9219	ALG14-AS1		lncRNA	chr1:95061596-9506
ENSG0000(	1080	24.45538	chr1:9219	ENSG00000288736		lncRNA	chr1:94541937-9455
ENSG0000(	1080	24.45538	chr1:9219	SLC44A3-AS1		Pseudoger	chr1:94613814-9485
ENSG0000(	1080	24.45538	chr1:9219	GCLM		protein_c	chr1:93885199-9390
ENSG0000(	1080	24.45538	chr1:9219	LINC01650		lncRNA	chr1:95351251-9535
ENSG0000(	1080	24.45538	chr1:9219	ABCA4		protein_c	chr1:93992834-9412
ENSG0000(	1080	24.45538	chr1:9219	MTATP6P13		Pseudoger	chr1:93925406-9392
ENSG0000(	1080	24.45538	chr1:9219	LINC01761		lncRNA	chr1:95474737-9547
ENSG0000(	1080	24.45538	chr1:9219	ENSG00000232918		Pseudoger	chr1:94406395-9440
ENSG0000(	1080	24.45538	chr1:9219	ENSG00000233129		Pseudoger	chr1:93934479-9393
ENSG0000(	1080	24.45538	chr1:9219	ARHGAP29-AS1		lncRNA	chr1:94247819-9441
ENSG0000(	1080	24.45538	chr1:9219	CNN3-DT		lncRNA	chr1:94927361-9496
ENSG0000(	1080	24.45538	chr1:9219	snoU13		smallRNA	chr1:94151418-9415
ENSG0000(	1080	24.45538	chr1:9219	ENSG00000286692		lncRNA	chr1:94417743-9441
ENSG0000(	1080	24.45538	chr1:9219	CNN3		protein_c	chr1:94896949-9492
ENSG0000(	1080	24.45538	chr1:9219	MTCO2P21		Pseudoger	chr1:93926615-9392
ENSG0000(	1080	24.45538	chr1:9219	ENSG00000223675		lncRNA	chr1:94585556-9459
ENSG0000(	1080	24.45538	chr1:9219	MTCO1P21		Pseudoger	chr1:93927714-9392
ENSG0000(	1080	24.45538	chr1:9219	SLC44A3	NCGv7	protein_c	chr1:94820342-9489
ENSG0000(	1080	24.45538	chr1:9219	ARHGAP29		protein_c	chr1:94148988-9427
ENSG0000(	1080	24.45538	chr1:9219	ENSG00000236098		lncRNA	chr1:94318479-9432
ENSG0000(	1080	24.45538	chr1:9219	AC092812.1		smallRNA	chr1:95886540-9588
ENSG0000(	1080	24.45538	chr1:9219	ENSG00000237954		lncRNA	chr1:95356229-9538
ENSG0000(	1080	24.45538	chr1:9219	ENSG00000250890		Pseudoger	chr1:93926032-9392
ENSG0000(	1080	24.45538	chr1:9219	ENSG00000228852		lncRNA	chr1:95243167-9527
ENSG0000(	1080	24.45538	chr1:9219	ENSG00000260464		lncRNA	chr1:93847174-9384
ENSG0000(	1080	24.45538	chr1:9219	KATNBL1P2		Pseudoger	chr1:94650544-9465

ENSG0000(	1080	24.45538	chr1:921(	RN7SL440P		smallRNA	chr1:94150738-9415
ENSG0000(	1080	24.45538	chr1:921(	F3		protein_c	chr1:94529173-9454
ENSG0000(	1080	24.45538	chr1:921(	ABCD3		protein_c	chr1:94418389-9451
ENSG0000(	1080	24.45538	chr1:921(	MIR760		smallRNA	chr1:93846832-9384
ENSG0000(	1080	24.45538	chr1:921(	ALG14		protein_c	chr1:94974405-9507
ENSG0000(	1080	24.45538	chr1:921(	MIR378G		smallRNA	chr1:94745860-9474
ENSG0000(	1080	24.45538	chr1:921(	GAPDHP29		Pseudoger	chr1:94302038-9430
ENSG0000(	1080	24.45538	chr1:921(	LINC02607		lncRNA	chr1:95510059-9578
ENSG0000(	1080	24.45538	chr1:921(	TLCD4-RWDD3		protein_c	chr1:95117923-9524
ENSG0000(	1080	24.45538	chr1:921(	ENSG00000233482		lncRNA	chr1:94145111-9414
ENSG0000(	1080	24.45538	chr1:921(	MTND4P11		Pseudoger	chr1:93922574-9392
ENSG0000(	1080	24.45538	chr1:921(	RWDD3-DT		lncRNA	chr1:95161676-9523
ENSG0000(	1080	24.45538	chr1:921(	CHCHD2P5		Pseudoger	chr1:93921268-9392
ENSG0000(	1080	24.45538	chr1:921(	MTND3P21		Pseudoger	chr1:93924386-9392
ENSG0000(	1080	24.45538	chr1:921(	DNTTIP2		protein_c	chr1:93866284-9387
ENSG0000(	1080	24.45538	chr1:921(	LINC01760		lncRNA	chr1:95310928-9531
ENSG0000(	1080	24.45538	chr1:921(	MTCO3P21		Pseudoger	chr1:93924743-9392
ENSG0000(	1080	24.45538	chr1:921(	ENSG00000287919		lncRNA	chr1:95282233-9528
ENSG0000(	1080	24.45538	chr1:921(	ENSG00000231992		lncRNA	chr1:95120147-9513
ENSG0000(	1080	24.45538	chr1:921(	Y_RNA		smallRNA	chr1:95125511-9512
ENSG0000(	1080	24.45538	chr1:921(	TLCD4		protein_c	chr1:95117355-9519
ENSG0000(	1080	24.45538	chr1:921(	ENSG00000271252		lncRNA	chr1:95743096-9575
ENSG0000(	1079	24.43273	chr1:1021	SLC27A3		protein_c	chr1:153774354-153
ENSG0000(	1079	24.43273	chr1:1021	RNU6-239P		smallRNA	chr1:154295503-154
ENSG0000(	1079	24.43273	chr1:1021	JTB		protein_c	chr1:153974269-153
ENSG0000(	1079	24.43273	chr1:1021	UBE2Q1-AS1		lncRNA	chr1:154553609-154
ENSG0000(	1079	24.43273	chr1:1021	CRNN		protein_c	chr1:152409243-152
ENSG0000(	1079	24.43273	chr1:1021	S100A7	NCGv7;AC	protein_c	chr1:153457744-153
ENSG0000(	1079	24.43273	chr1:1021	ENSG00000285641		protein_c	chr1:153975850-153
ENSG0000(	1079	24.43273	chr1:1021	KCNN3	NCGv7	protein_c	chr1:154697455-154
ENSG0000(	1079	24.43273	chr1:1021	JTB-DT		lncRNA	chr1:153977727-153
ENSG0000(	1079	24.43273	chr1:1021	ENSG00000229699		lncRNA	chr1:153174518-153
ENSG0000(	1079	24.43273	chr1:1021	RAB13		protein_c	chr1:153981617-153
ENSG0000(	1079	24.43273	chr1:1021	NPR1		protein_c	chr1:153678688-153
ENSG0000(	1079	24.43273	chr1:1021	FLG-AS1		lncRNA	chr1:152168125-152
ENSG0000(	1079	24.43273	chr1:1021	UBAP2L		protein_c	chr1:154220179-154
ENSG0000(	1079	24.43273	chr1:1021	FLG	NCGv7	protein_c	chr1:152302165-152
ENSG0000(	1079	24.43273	chr1:1021	SPRR1B		protein_c	chr1:153031203-153
ENSG0000(	1079	24.43273	chr1:1021	Y_RNA		smallRNA	chr1:153785720-153
ENSG0000(	1079	24.43273	chr1:1021	AQP10		protein_c	chr1:154321090-154
ENSG0000(	1079	24.43273	chr1:1021	ENSG00000238279		lncRNA	chr1:153533430-153
ENSG0000(	1079	24.43273	chr1:1021	RNU7-57P		smallRNA	chr1:154338743-154
ENSG0000(	1079	24.43273	chr1:1021	ENSG00000285779		protein_c	chr1:153959151-153
ENSG0000(	1079	24.43273	chr1:1021	RN7SL44P		smallRNA	chr1:153500467-153
ENSG0000(	1079	24.43273	chr1:1021	SPRR2C		Pseudoger	chr1:153140491-153
ENSG0000(	1079	24.43273	chr1:1021	CREB3L4	NCGv7	protein_c	chr1:153967534-153
ENSG0000(	1079	24.43273	chr1:1021	LCEP1		Pseudoger	chr1:152744299-152
ENSG0000(	1079	24.43273	chr1:1021	SPTLC1P4		Pseudoger	chr1:152077952-152
ENSG0000(	1079	24.43273	chr1:1021	C1orf43		protein_c	chr1:154206696-154
ENSG0000(	1079	24.43273	chr1:1021	SNAPIN		protein_c	chr1:153658703-153
ENSG0000(	1079	24.43273	chr1:1021	MIR190B		smallRNA	chr1:154193665-154

ENSG0000(	1079	24.43273	chr1:1021	LCE1D	NCGv7	protein_c	chr1:152796721-152
ENSG0000(	1079	24.43273	chr1:1021	ENSG00000273110		lncRNA	chr1:154480012-154
ENSG0000(	1079	24.43273	chr1:1021	ENSG00000237920		Pseudoger	chr1:154376966-154
ENSG0000(	1079	24.43273	chr1:1021	ENSG00000229021		lncRNA	chr1:151994531-152
ENSG0000(	1079	24.43273	chr1:1021	LAPTM4BP1		Pseudoger	chr1:153379821-153
ENSG0000(	1079	24.43273	chr1:1021	ENSG00000289062		lncRNA	chr1:152897800-152
ENSG0000(	1079	24.43273	chr1:1021	SLC39A1		protein_c	chr1:153959099-153
ENSG0000(	1079	24.43273	chr1:1021	RNU6-121P		smallRNA	chr1:154297650-154
ENSG0000(	1079	24.43273	chr1:1021	ENSG00000273026		lncRNA	chr1:153966516-153
ENSG0000(	1079	24.43273	chr1:1021	TPM3	NCGv7;AC	protein_c	chr1:154155308-154
ENSG0000(	1079	24.43273	chr1:1021	LCEP2		Pseudoger	chr1:152737518-152
ENSG0000(	1079	24.43273	chr1:1021	MIR5698		smallRNA	chr1:154104521-154
ENSG0000(	1079	24.43273	chr1:1021	snoU13		smallRNA	chr1:153754124-153
ENSG0000(	1079	24.43273	chr1:1021	ATP8B2	NCGv7	protein_c	chr1:154325525-154
ENSG0000(	1079	24.43273	chr1:1021	RNU6-160P		smallRNA	chr1:153331622-153
ENSG0000(	1079	24.43273	chr1:1021	NUP210L	NCGv7	protein_c	chr1:153992685-154
ENSG0000(	1079	24.43273	chr1:1021	SPRR1A		protein_c	chr1:152984081-152
ENSG0000(	1079	24.43273	chr1:1021	FLG2		protein_c	chr1:152348735-152
ENSG0000(	1079	24.43273	chr1:1021	ENSG00000285651		lncRNA	chr1:151885251-151
ENSG0000(	1079	24.43273	chr1:1021	RPTN		protein_c	chr1:152153595-152
ENSG0000(	1079	24.43273	chr1:1021	CKS1B	AC	protein_c	chr1:154974653-154
ENSG0000(	1079	24.43273	chr1:1021	CRCT1		protein_c	chr1:152514482-152
ENSG0000(	1079	24.43273	chr1:1021	LINC02962		lncRNA	chr1:152205858-152
ENSG0000(	1079	24.43273	chr1:1021	LCEP3		Pseudoger	chr1:152656332-152
ENSG0000(	1079	24.43273	chr1:1021	SHE	NCGv7	protein_c	chr1:154469772-154
ENSG0000(	1079	24.43273	chr1:1021	INTS3	AC	protein_c	chr1:153728050-153
ENSG0000(	1079	24.43273	chr1:1021	ILF2		protein_c	chr1:153661788-153
ENSG0000(	1079	24.43273	chr1:1021	GATAD2B		protein_c	chr1:153789030-153
ENSG0000(	1079	24.43273	chr1:1021	HAX1	NCGv7;AC	protein_c	chr1:154272355-154
ENSG0000(	1079	24.43273	chr1:1021	S100A8	NCGv7;AC	protein_c	chr1:153390032-153
ENSG0000(	1079	24.43273	chr1:1021	LCE3D		protein_c	chr1:152579381-152
ENSG0000(	1079	24.43273	chr1:1021	LCE1F		protein_c	chr1:152775140-152
ENSG0000(	1079	24.43273	chr1:1021	LCEP4		Pseudoger	chr1:152644393-152
ENSG0000(	1079	24.43273	chr1:1021	ENSG00000271853		lncRNA	chr1:153626332-153
ENSG0000(	1079	24.43273	chr1:1021	LCE7A		protein_c	chr1:152859996-152
ENSG0000(	1079	24.43273	chr1:1021	ENSG00000278694		Pseudoger	chr1:154312462-154
ENSG0000(	1079	24.43273	chr1:1021	S100A1	NCGv7	protein_c	chr1:153627926-153
ENSG0000(	1079	24.43273	chr1:1021	CHTOP		protein_c	chr1:153633982-153
ENSG0000(	1079	24.43273	chr1:1021	ENSG00000284738		lncRNA	chr1:153923284-153
ENSG0000(	1079	24.43273	chr1:1021	SHC1		protein_c	chr1:154962298-154
ENSG0000(	1079	24.43273	chr1:1021	ADAR		protein_c	chr1:154581695-154
ENSG0000(	1079	24.43273	chr1:1021	NBPF18P		Pseudoger	chr1:152018662-152
ENSG0000(	1079	24.43273	chr1:1021	UBE2Q1		protein_c	chr1:154548577-154
ENSG0000(	1079	24.43273	chr1:1021	ENSG00000287064		lncRNA	chr1:154671593-154
ENSG0000(	1079	24.43273	chr1:1021	CHRNB2	NCGv7	protein_c	chr1:154567778-154
ENSG0000(	1079	24.43273	chr1:1021	ENSG00000289935		lncRNA	chr1:153945551-153
ENSG0000(	1079	24.43273	chr1:1021	KPRP		protein_c	chr1:152758025-152
ENSG0000(	1079	24.43273	chr1:1021	SPRR2E		protein_c	chr1:153093135-153
ENSG0000(	1079	24.43273	chr1:1021	LCE1E		protein_c	chr1:152786214-152
ENSG0000(	1079	24.43273	chr1:1021	U3		smallRNA	chr1:153998041-153
ENSG0000(	1079	24.43273	chr1:1021	SNORD59		smallRNA	chr1:154288460-154

ENSG0000(	1079	24.43273	chr1:1021	PRR9		protein_c	chr1:153217584-153
ENSG0000(	1079	24.43273	chr1:1021	LCE6A		protein_c	chr1:152842856-152
ENSG0000(	1079	24.43273	chr1:1021	LCE3B		protein_c	chr1:152613811-152
ENSG0000(	1079	24.43273	chr1:1021	LCE2D		protein_c	chr1:152663380-152
ENSG0000(	1079	24.43273	chr1:1021	LCE2C		protein_c	chr1:152675279-152
ENSG0000(	1079	24.43273	chr1:1021	LCE2A		protein_c	chr1:152698345-152
ENSG0000(	1079	24.43273	chr1:1021	LCE4A	NCGv7	protein_c	chr1:152708160-152
ENSG0000(	1079	24.43273	chr1:1021	PSMD8P1		Pseudoger	chr1:154414369-154
ENSG0000(	1079	24.43273	chr1:1021	RPSAP17		Pseudoger	chr1:154378207-154
ENSG0000(	1079	24.43273	chr1:1021	DENND4B	NCGv7	protein_c	chr1:153929501-153
ENSG0000(	1079	24.43273	chr1:1021	C1orf68		protein_c	chr1:152719522-152
ENSG0000(	1079	24.43273	chr1:1021	ENSG00000223599		Pseudoger	chr1:153852106-153
ENSG0000(	1079	24.43273	chr1:1021	THEM4		protein_c	chr1:151870866-151
ENSG0000(	1079	24.43273	chr1:1021	ENSG00000272030		lncRNA	chr1:153631438-153
ENSG0000(	1079	24.43273	chr1:1021	TCHH	NCGv7	protein_c	chr1:152106317-152
ENSG0000(	1079	24.43273	chr1:1021	LCE1A		protein_c	chr1:152827473-152
ENSG0000(	1079	24.43273	chr1:1021	LCE2B		protein_c	chr1:152686123-152
ENSG0000(	1079	24.43273	chr1:1021	SPRR2G		protein_c	chr1:153149582-153
ENSG0000(	1079	24.43273	chr1:1021	PGLYRP3		protein_c	chr1:153297116-153
ENSG0000(	1079	24.43273	chr1:1021	LELP1		protein_c	chr1:153203430-153
ENSG0000(	1079	24.43273	chr1:1021	LORICRIN		protein_c	chr1:153259687-153
ENSG0000(	1079	24.43273	chr1:1021	ENSG00000243613		lncRNA	chr1:153746851-153
ENSG0000(	1079	24.43273	chr1:1021	SMCP		protein_c	chr1:152878322-152
ENSG0000(	1079	24.43273	chr1:1021	SNORA58		smallRNA	chr1:154259727-154
ENSG0000(	1079	24.43273	chr1:1021	IVL		protein_c	chr1:152908546-152
ENSG0000(	1079	24.43273	chr1:1021	SPRR3		protein_c	chr1:153001747-153
ENSG0000(	1079	24.43273	chr1:1021	SPRR2D		protein_c	chr1:153039732-153
ENSG0000(	1079	24.43273	chr1:1021	ENSG00000233222		lncRNA	chr1:153750983-153
ENSG0000(	1079	24.43273	chr1:1021	PGLYRP4		protein_c	chr1:153330120-153
ENSG0000(	1079	24.43273	chr1:1021	ENSG00000271380		lncRNA	chr1:154961825-154
ENSG0000(	1079	24.43273	chr1:1021	S100A9		protein_c	chr1:153357854-153
ENSG0000(	1079	24.43273	chr1:1021	S100A12		protein_c	chr1:153373711-153
ENSG0000(	1079	24.43273	chr1:1021	TDRD10	NCGv7	protein_c	chr1:154502219-154
ENSG0000(	1079	24.43273	chr1:1021	CFAP141		protein_c	chr1:154199085-154
ENSG0000(	1079	24.43273	chr1:1021	PMVK		protein_c	chr1:154924740-154
ENSG0000(	1079	24.43273	chr1:1021	PBXIP1		protein_c	chr1:154944076-154
ENSG0000(	1079	24.43273	chr1:1021	ENSG00000282386		lncRNA	chr1:153964361-153
ENSG0000(	1079	24.43273	chr1:1021	SPRR2A		protein_c	chr1:153056120-153
ENSG0000(	1079	24.43273	chr1:1021	PYGO2		protein_c	chr1:154957026-154
ENSG0000(	1079	24.43273	chr1:1021	HMGN3P1		Pseudoger	chr1:152399577-152
ENSG0000(	1079	24.43273	chr1:1021	TCHHL1		protein_c	chr1:152084141-152
ENSG0000(	1079	24.43273	chr1:1021	S100A11		protein_c	chr1:152032506-152
ENSG0000(	1079	24.43273	chr1:1021	S100A7P1		Pseudoger	chr1:153427020-153
ENSG0000(	1079	24.43273	chr1:1021	LINC01527		lncRNA	chr1:152930040-152
ENSG0000(	1079	24.43273	chr1:1021	CRTC2		protein_c	chr1:153947669-153
ENSG0000(	1079	24.43273	chr1:1021	KRT8P28		Pseudoger	chr1:151949523-151
ENSG0000(	1079	24.43273	chr1:1021	LCE5A		protein_c	chr1:152510803-152
ENSG0000(	1079	24.43273	chr1:1021	Y_RNA		smallRNA	chr1:153726252-153
ENSG0000(	1079	24.43273	chr1:1021	LCE3E		protein_c	chr1:152565654-152
ENSG0000(	1079	24.43273	chr1:1021	LCE3A		protein_c	chr1:152622834-152
ENSG0000(	1079	24.43273	chr1:1021	ENSG00000231827		Pseudoger	chr1:153795173-153

ENSG0000(	1079	24.43273	chr1:1021	RPS7P2		Pseudoger	chr1:154078866-154
ENSG0000(	1079	24.43273	chr1:1021	SPRR5		protein_c	chr1:152947206-152
ENSG0000(	1079	24.43273	chr1:1021	RN7SL372P		smallRNA	chr1:153704088-153
ENSG0000(	1079	24.43273	chr1:1021	RNU6-179P		smallRNA	chr1:154039916-154
ENSG0000(	1079	24.43273	chr1:1021	GEMIN2P1		Pseudoger	chr1:153717303-153
ENSG0000(	1079	24.43273	chr1:1021	IL6R-AS1		lncRNA	chr1:154402328-154
ENSG0000(	1079	24.43273	chr1:1021	S100A15A		Pseudoger	chr1:153396591-153
ENSG0000(	1079	24.43273	chr1:1021	S100A7A	NCGv7	protein_c	chr1:153416520-153
ENSG0000(	1079	24.43273	chr1:1021	RPLP0P4		Pseudoger	chr1:153225080-153
ENSG0000(	1079	24.43273	chr1:1021	SPRR4		protein_c	chr1:152970648-152
ENSG0000(	1079	24.43273	chr1:1021	RPS27	NCGv7	protein_c	chr1:153990762-153
ENSG0000(	1079	24.43273	chr1:1021	IL6R		protein_c	chr1:154405193-154
ENSG0000(	1079	24.43273	chr1:1021	ENSG00000291199		lncRNA	chr1:153793937-153
ENSG0000(	1079	24.43273	chr1:1021	RN7SL431P		smallRNA	chr1:154166247-154
ENSG0000(	1079	24.43273	chr1:1021	LCE3C		protein_c	chr1:152600234-152
ENSG0000(	1079	24.43273	chr1:1021	S100A2		protein_c	chr1:153561108-153
ENSG0000(	1079	24.43273	chr1:1021	HRNR	NCGv7	protein_c	chr1:152212076-152
ENSG0000(	1079	24.43273	chr1:1021	MIR4258		smallRNA	chr1:154975693-154
ENSG0000(	1079	24.43273	chr1:1021	SPRR2B	NCGv7	protein_c	chr1:153070226-153
ENSG0000(	1079	24.43273	chr1:1021	ENSG00000270361		lncRNA	chr1:154937370-154
ENSG0000(	1079	24.43273	chr1:1021	S100A10		protein_c	chr1:151982915-151
ENSG0000(	1079	24.43273	chr1:1021	AL590431.1		smallRNA	chr1:154254445-154
ENSG0000(	1079	24.43273	chr1:1021	SPRR2F		protein_c	chr1:153112121-153
ENSG0000(	1079	24.43273	chr1:1021	S100A3		protein_c	chr1:153547329-153
ENSG0000(	1079	24.43273	chr1:1021	ENSG00000286581		lncRNA	chr1:151944890-151
ENSG0000(	1079	24.43273	chr1:1021	LCE1B		protein_c	chr1:152811971-152
ENSG0000(	1079	24.43273	chr1:1021	ENSG00000285867		lncRNA	chr1:153586813-153
ENSG0000(	1079	24.43273	chr1:1021	ENSG00000236327		Pseudoger	chr1:153890595-153
ENSG0000(	1079	24.43273	chr1:1021	ENSG00000286391		lncRNA	chr1:154564855-154
ENSG0000(	1079	24.43273	chr1:1021	S100A13		protein_c	chr1:153618787-153
ENSG0000(	1079	24.43273	chr1:1021	S100A5		protein_c	chr1:153537147-153
ENSG0000(	1079	24.43273	chr1:1021	S100A7L2		Pseudoger	chr1:153437058-153
ENSG0000(	1079	24.43273	chr1:1021	PUDPP2		Pseudoger	chr1:152124016-152
ENSG0000(	1079	24.43273	chr1:1021	ENSG00000233875		lncRNA	chr1:154579065-154
ENSG0000(	1079	24.43273	chr1:1021	S100A14		protein_c	chr1:153614255-153
ENSG0000(	1079	24.43273	chr1:1021	ENSG00000234262		Pseudoger	chr1:153023962-153
ENSG0000(	1079	24.43273	chr1:1021	S100A6		protein_c	chr1:153534599-153
ENSG0000(	1079	24.43273	chr1:1021	ENSG00000226716		lncRNA	chr1:152122534-152
ENSG0000(	1079	24.43273	chr1:1021	S100A16		protein_c	chr1:153606886-153
ENSG0000(	1079	24.43273	chr1:1021	S100A4	AC	protein_c	chr1:153543613-153
ENSG0000(	1079	24.43273	chr1:1021	ENSG00000231416		Pseudoger	chr1:153995632-153
ENSG0000(	1079	24.43273	chr1:1021	SNORA31		smallRNA	chr1:153012482-153
ENSG0000(	1079	24.43273	chr1:1021	AL606500.1		protein_c	chr1:154612591-154
ENSG0000(	1079	24.43273	chr1:1021	ENSG00000285818		Pseudoger	chr1:152079557-152
ENSG0000(	1079	24.43273	chr1:1021	LCE1C		protein_c	chr1:152804832-152
ENSG0000(	1078	24.41009	chr1:1021	RPL23AP90		Pseudoger	chr1:100196816-100
ENSG0000(	1078	24.41009	chr1:1021	ENSG00000288810		lncRNA	chr1:98047173-9804
ENSG0000(	1078	24.41009	chr1:1021	LINC01776		lncRNA	chr1:98210747-9827
ENSG0000(	1078	24.41009	chr1:1021	ENSG00000228084		lncRNA	chr1:99968382-9996
ENSG0000(	1078	24.41009	chr1:1021	RTCA-AS1		lncRNA	chr1:100251528-100
ENSG0000(	1078	24.41009	chr1:1021	LINC01349		lncRNA	chr1:100627049-100

ENSG0000(	1078	24.41009	chr1:1021	ENSG00000233983		Pseudoger	chr1:99464378-9946
ENSG0000(	1078	24.41009	chr1:1021	ENSG00000228086		lncRNA	chr1:100462399-100
ENSG0000(	1078	24.41009	chr1:1021	BRI3P1		Pseudoger	chr1:100213293-100
ENSG0000(	1078	24.41009	chr1:1021	CDC14A		protein_c	chr1:100345001-100
ENSG0000(	1078	24.41009	chr1:1021	ENSG00000283761		protein_c	chr1:99970011-1000
ENSG0000(	1078	24.41009	chr1:1021	AL592205.2		smallRNA	chr1:96935545-9693
ENSG0000(	1078	24.41009	chr1:1021	ENSG00000230287		Pseudoger	chr1:100249090-100
ENSG0000(	1078	24.41009	chr1:1021	DPH5		protein_c	chr1:100989623-101
ENSG0000(	1078	24.41009	chr1:1021	DPYD	NCGv7	protein_c	chr1:97077743-9799
ENSG0000(	1078	24.41009	chr1:1021	ENSG00000215871		Pseudoger	chr1:100331804-100
ENSG0000(	1078	24.41009	chr1:1021	ENSG00000231996		Pseudoger	chr1:99842610-9984
ENSG0000(	1078	24.41009	chr1:1021	ENSG00000223906		lncRNA	chr1:100344477-100
ENSG0000(	1078	24.41009	chr1:1021	RNU6-750P		smallRNA	chr1:99978939-9997
ENSG0000(	1078	24.41009	chr1:1021	AGL		protein_c	chr1:99850361-9992
ENSG0000(	1078	24.41009	chr1:1021	AL592205.1		smallRNA	chr1:96902699-9690
ENSG0000(	1078	24.41009	chr1:1021	VCAM1		protein_c	chr1:100719742-100
ENSG0000(	1078	24.41009	chr1:1021	AC104457.1		smallRNA	chr1:100378682-100
ENSG0000(	1078	24.41009	chr1:1021	SNX7	NCGv7	protein_c	chr1:98661701-9876
ENSG0000(	1078	24.41009	chr1:1021	EXTL2		protein_c	chr1:100872372-100
ENSG0000(	1078	24.41009	chr1:1021	Y_RNA		smallRNA	chr1:99791662-9979
ENSG0000(	1078	24.41009	chr1:1021	RNU6-1318P		smallRNA	chr1:100000637-100
ENSG0000(	1078	24.41009	chr1:1021	SLC30A7		protein_c	chr1:100896076-100
ENSG0000(	1078	24.41009	chr1:1021	ENSG00000270911		Pseudoger	chr1:97855575-9785
ENSG0000(	1078	24.41009	chr1:1021	ENSG00000241073		lncRNA	chr1:100057990-100
ENSG0000(	1078	24.41009	chr1:1021	AL451051.1		smallRNA	chr1:99829465-9982
ENSG0000(	1078	24.41009	chr1:1021	HNRNPA1P68		Pseudoger	chr1:100941017-100
ENSG0000(	1078	24.41009	chr1:1021	RPL36AP12		Pseudoger	chr1:100651947-100
ENSG0000(	1078	24.41009	chr1:1021	DPH5-DT		lncRNA	chr1:101025844-101
ENSG0000(	1078	24.41009	chr1:1021	SLC35A3		protein_c	chr1:99969351-1000
ENSG0000(	1078	24.41009	chr1:1021	ENSG00000288826		lncRNA	chr1:100036632-100
ENSG0000(	1078	24.41009	chr1:1021	GPR88		protein_c	chr1:100538139-100
ENSG0000(	1078	24.41009	chr1:1021	PLPPR4		protein_c	chr1:99264292-9930
ENSG0000(	1078	24.41009	chr1:1021	RPL26P9		Pseudoger	chr1:97585862-9758
ENSG0000(	1078	24.41009	chr1:1021	SEC63P1		Pseudoger	chr1:97545701-9754
ENSG0000(	1078	24.41009	chr1:1021	ENSG00000273204		lncRNA	chr1:100894928-100
ENSG0000(	1078	24.41009	chr1:1021	PLPPR5-AS1		lncRNA	chr1:99004276-9924
ENSG0000(	1078	24.41009	chr1:1021	ENSG00000227034		Pseudoger	chr1:99008218-9900
ENSG0000(	1078	24.41009	chr1:1021	PLPPR5		protein_c	chr1:98890245-9922
ENSG0000(	1078	24.41009	chr1:1021	MIR553		smallRNA	chr1:100281241-100
ENSG0000(	1078	24.41009	chr1:1021	ENSG00000285922		lncRNA	chr1:98052077-9805
ENSG0000(	1078	24.41009	chr1:1021	NFU1P2		Pseudoger	chr1:98077000-9807
ENSG0000(	1078	24.41009	chr1:1021	ENSG00000226952		Pseudoger	chr1:100099239-100
ENSG0000(	1078	24.41009	chr1:1021	DPYD-IT1		lncRNA	chr1:97394154-9742
ENSG0000(	1078	24.41009	chr1:1021	HMGB3P10		Pseudoger	chr1:99698242-9969
ENSG0000(	1078	24.41009	chr1:1021	ENSG00000235795		lncRNA	chr1:100995473-100
ENSG0000(	1078	24.41009	chr1:1021	PALMD		protein_c	chr1:99646113-9969
ENSG0000(	1078	24.41009	chr1:1021	ENSG00000259946		lncRNA	chr1:97967005-9796
ENSG0000(	1078	24.41009	chr1:1021	DPYD-AS2		lncRNA	chr1:97796921-9779
ENSG0000(	1078	24.41009	chr1:1021	ENSG00000280040		TEC	chr1:98660388-9866
ENSG0000(	1078	24.41009	chr1:1021	MFSD14A		protein_c	chr1:100038095-100
ENSG0000(	1078	24.41009	chr1:1021	MIR137HG		lncRNA	chr1:97933474-9804

ENSG0000(	1078	24.41009	chr1:1021	SASS6		protein_c	chr1:100083563-100
ENSG0000(	1078	24.41009	chr1:1021	LINC01708		lncRNA	chr1:99472332-9960
ENSG0000(	1078	24.41009	chr1:1021	AC093157.1		protein_c	chr1:100990205-100
ENSG0000(	1078	24.41009	chr1:1021	AL160056.1		smallRNA	chr1:98373385-9837
ENSG0000(	1078	24.41009	chr1:1021	ENSG00000285525		lncRNA	chr1:100628230-100
ENSG0000(	1078	24.41009	chr1:1021	DPYD-AS1		lncRNA	chr1:97095923-9732
ENSG0000(	1078	24.41009	chr1:1021	RNU4-75P		smallRNA	chr1:99784740-9978
ENSG0000(	1078	24.41009	chr1:1021	ENSG00000285530		lncRNA	chr1:100220488-100
ENSG0000(	1078	24.41009	chr1:1021	ENSG00000230718		Pseudoger	chr1:97774669-9777
ENSG0000(	1078	24.41009	chr1:1021	DBT		protein_c	chr1:100186919-100
ENSG0000(	1078	24.41009	chr1:1021	RTCA	NCGv7	protein_c	chr1:100266216-100
ENSG0000(	1078	24.41009	chr1:1021	BCAS2P2		Pseudoger	chr1:100393033-100
ENSG0000(	1078	24.41009	chr1:1021	RPL7AP17		Pseudoger	chr1:100586649-100
ENSG0000(	1078	24.41009	chr1:1021	TRMT13		protein_c	chr1:100133150-100
ENSG0000(	1078	24.41009	chr1:1021	LRRC39		protein_c	chr1:100148448-100
ENSG0000(	1078	24.41009	chr1:1021	FRRS1		protein_c	chr1:99703970-9976
ENSG0000(	1069	24.20629	chr1:9219	FNBP1L	NCGv7	protein_c	chr1:93448118-9355
ENSG0000(	1069	24.20629	chr1:9219	ENSG00000229635		Pseudoger	chr1:93384487-9338
ENSG0000(	1069	24.20629	chr1:9219	CCDC18		protein_c	chr1:93179919-9327
ENSG0000(	1069	24.20629	chr1:9219	ENSG00000225297		Pseudoger	chr1:93199755-9319
ENSG0000(	1069	24.20629	chr1:9219	BCAR3-AS1		lncRNA	chr1:93591966-9361
ENSG0000(	1069	24.20629	chr1:9219	ENSG00000229567		Pseudoger	chr1:93278961-9327
ENSG0000(	1069	24.20629	chr1:9219	RPL36AP11		Pseudoger	chr1:93190740-9319
ENSG0000(	1069	24.20629	chr1:9219	CCDC18-AS1		lncRNA	chr1:93262186-9334
ENSG0000(	1069	24.20629	chr1:9219	BCAR3		protein_c	chr1:93561741-9384
ENSG0000(	1069	24.20629	chr1:9219	DR1		protein_c	chr1:93345907-9336
ENSG0000(	1069	24.20629	chr1:9219	MTF2		protein_c	chr1:93079235-9313
ENSG0000(	1069	24.20629	chr1:9219	Y_RNA		smallRNA	chr1:93385711-9338
ENSG0000(	1069	24.20629	chr1:9219	RNA5SP53		Pseudoger	chr1:93488333-9348
ENSG0000(	1069	24.20629	chr1:9219	TMED5		protein_c	chr1:93149742-9318
ENSG0000(	1066	24.13836	chr1:1021	RNU1-130P		smallRNA	chr1:96225901-9622
ENSG0000(	1066	24.13836	chr1:1021	LINC01787		lncRNA	chr1:96254069-9637
ENSG0000(	1066	24.13836	chr1:1021	NDUFS5P2		Pseudoger	chr1:96584422-9658
ENSG0000(	1066	24.13836	chr1:1021	RPL7P9		Pseudoger	chr1:96678874-9667
ENSG0000(	1066	24.13836	chr1:1021	ENSG00000225923		Pseudoger	chr1:96390652-9639
ENSG0000(	1066	24.13836	chr1:1021	RN7SKP270		smallRNA	chr1:96695856-9669
ENSG0000(	1066	24.13836	chr1:1021	LINC02790		lncRNA	chr1:95937901-9602
ENSG0000(	1066	24.13836	chr1:1021	EEF1A1P11		Pseudoger	chr1:96446930-9644
ENSG0000(	1066	24.13836	chr1:1021	UBE2WP1		Pseudoger	chr1:96418594-9641
ENSG0000(	1066	24.13836	chr1:1021	PTBP2		protein_c	chr1:96721665-9682
ENSG0000(	1066	24.13836	chr1:1021	RN7SL831P		smallRNA	chr1:96583209-9658
ENSG0000(	1057	23.93457	chr5:4231	PSME2P1		Pseudoger	chr5:98213402-9821
ENSG0000(	1052	23.82135	chr4:253(	RPL14P3		Pseudoger	chr4:140366240-140
ENSG0000(	1052	23.82135	chr4:253(	snoU13		smallRNA	chr4:141297774-141
ENSG0000(	1052	23.82135	chr4:253(	ELF2		protein_c	chr4:139028112-139
ENSG0000(	1052	23.82135	chr4:253(	MGARP	DriverDB\	protein_c	chr4:139266165-139
ENSG0000(	1052	23.82135	chr4:253(	NDUFC1		protein_c	chr4:139266880-139
ENSG0000(	1052	23.82135	chr4:253(	TBC1D9		protein_c	chr4:140620782-140
ENSG0000(	1052	23.82135	chr4:253(	UCP1		protein_c	chr4:140559431-140
ENSG0000(	1052	23.82135	chr4:253(	ZNF330		protein_c	chr4:141220887-141
ENSG0000(	1052	23.82135	chr4:253(	RNU6-506P		smallRNA	chr4:139231026-139

ENSG0000(	1052	23.82135	chr4:253(	CLGN		protein_c	chr4:140388453-140
ENSG0000(	1052	23.82135	chr4:253(	SCOC		protein_c	chr4:140257286-140
ENSG0000(	1052	23.82135	chr4:253(	ENSG00000248863		Pseudoger	chr4:139426299-139
ENSG0000(	1052	23.82135	chr4:253(	Y_RNA		smallRNA	chr4:140779039-140
ENSG0000(	1052	23.82135	chr4:253(	ENSG00000250698		lncRNA	chr4:140128015-140
ENSG0000(	1052	23.82135	chr4:253(	RN7SL311P		smallRNA	chr4:139185441-139
ENSG0000(	1052	23.82135	chr4:253(	RN7SL152P		smallRNA	chr4:140577925-140
ENSG0000(	1052	23.82135	chr4:253(	RN7SKP237		smallRNA	chr4:139685127-139
ENSG0000(	1052	23.82135	chr4:253(	SNORD112		smallRNA	chr4:140275041-140
ENSG0000(	1052	23.82135	chr4:253(	NOCT		protein_c	chr4:139015781-139
ENSG0000(	1052	23.82135	chr4:253(	FTH1P24		Pseudoger	chr4:139546266-139
ENSG0000(	1052	23.82135	chr4:253(	ENSG00000288785		lncRNA	chr4:139177456-139
ENSG0000(	1052	23.82135	chr4:253(	ENSG00000287449		lncRNA	chr4:140577845-140
ENSG0000(	1052	23.82135	chr4:253(	TNRC18P1		Pseudoger	chr4:140641840-140
ENSG0000(	1052	23.82135	chr4:253(	RN7SL382P		smallRNA	chr4:139100377-139
ENSG0000(	1052	23.82135	chr4:253(	ENSG00000273472		lncRNA	chr4:140756410-140
ENSG0000(	1052	23.82135	chr4:253(	ENSG00000248621		Pseudoger	chr4:140600401-140
ENSG0000(	1052	23.82135	chr4:253(	ENSG00000272717		lncRNA	chr4:139556799-139
ENSG0000(	1052	23.82135	chr4:253(	SCOC-AS1		lncRNA	chr4:140283724-140
ENSG0000(	1052	23.82135	chr4:186(	ENSG00000270669		Pseudoger	chr4:77216416-7721
ENSG0000(	1052	23.82135	chr4:253(	ACA64		smallRNA	chr4:139436368-139
ENSG0000(	1052	23.82135	chr4:253(	RAB33B-AS1		lncRNA	chr4:139411927-139
ENSG0000(	1052	23.82135	chr4:253(	RNF150		protein_c	chr4:140859807-141
ENSG0000(	1052	23.82135	chr4:253(	Y_RNA		smallRNA	chr4:139200395-139
ENSG0000(	1052	23.82135	chr4:253(	AC093602.1		smallRNA	chr4:139017493-139
ENSG0000(	1052	23.82135	chr4:253(	RNU6-531P		smallRNA	chr4:139055475-139
ENSG0000(	1052	23.82135	chr4:253(	MGST2		protein_c	chr4:139665768-139
ENSG0000(	1052	23.82135	chr4:253(	NDUFB4P9		Pseudoger	chr4:140314163-140
ENSG0000(	1052	23.82135	chr4:253(	ELMOD2		protein_c	chr4:140524168-140
ENSG0000(	1052	23.82135	chr4:253(	RAB33B		protein_c	chr4:139453232-139
ENSG0000(	1052	23.82135	chr4:253(	ENSG00000250577		lncRNA	chr4:138923930-138
ENSG0000(	1052	23.82135	chr4:253(	SETD7	NCGv7	protein_c	chr4:139495941-139
ENSG0000(	1052	23.82135	chr4:253(	ENSG00000272632		lncRNA	chr4:141430831-141
ENSG0000(	1052	23.82135	chr4:253(	AC093766.1		smallRNA	chr4:138811678-138
ENSG0000(	1052	23.82135	chr4:253(	ENSG00000280262		TEC	chr4:140498402-140
ENSG0000(	1052	23.82135	chr4:253(	RNU6-1074P		smallRNA	chr4:139256657-139
ENSG0000(	1052	23.82135	chr4:253(	ENSG00000286320		lncRNA	chr4:139763053-139
ENSG0000(	1052	23.82135	chr4:253(	ENSG00000290022		lncRNA	chr4:139841168-139
ENSG0000(	1052	23.82135	chr4:253(	MGAT4D		protein_c	chr4:140442262-140
ENSG0000(	1052	23.82135	chr4:253(	LINC02276		lncRNA	chr4:141569931-141
ENSG0000(	1052	23.82135	chr4:253(	RNU6-1214P		smallRNA	chr4:139318378-139
ENSG0000(	1052	23.82135	chr4:253(	LINC02432		lncRNA	chr4:141302910-141
ENSG0000(	1052	23.82135	chr4:253(	QKILA		lncRNA	chr4:139564298-139
ENSG0000(	1052	23.82135	chr4:253(	RN7SKP253		smallRNA	chr4:139794125-139
ENSG0000(	1052	23.82135	chr4:253(	MAML3	NCGv7	protein_c	chr4:139716753-140
ENSG0000(	1052	23.82135	chr4:253(	ENSG00000250189		Pseudoger	chr4:141405172-141
ENSG0000(	1052	23.82135	chr4:253(	ENSG00000248747		lncRNA	chr4:141240739-141
ENSG0000(	1052	23.82135	chr4:253(	ENSG00000286896		lncRNA	chr4:139559283-139
ENSG0000(	1052	23.82135	chr4:253(	NAA15		protein_c	chr4:139301446-139
ENSG0000(	1052	23.82135	chr4:253(	ENSG00000250195		lncRNA	chr4:138819954-139
ENSG0000(	1052	23.82135	chr4:253(	IL15		protein_c	chr4:141636583-141

ENSG0000C	1052	23.82135	chr4:253(	ENSG00000248335		lncRNA	chr4:140712168-140
ENSG0000C	1052	23.82135	chr4:253(	ENSG00000250501		lncRNA	chr4:138773537-138
ENSG0000C	1052	23.82135	chr4:253(	H3P16		Pseudoger	chr4:139698144-139
ENSG0000C	1052	23.82135	chr4:253(	PPP1R14BP3		Pseudoger	chr4:139114930-139
ENSG0000C	1040	23.54962	chr5:4231	ENSG00000251314		lncRNA	chr5:95962001-9663
ENSG0000C	1040	23.54962	chr5:4231	ENSG00000251361		lncRNA	chr5:93019663-9306
ENSG0000C	1040	23.54962	chr5:4231	TTC37		protein_c	chr5:95461755-9555
ENSG0000C	1040	23.54962	chr5:4231	GLRX		protein_c	chr5:95751319-9582
ENSG0000C	1040	23.54962	chr5:4231	ENSG00000286121		lncRNA	chr5:91642643-9179
ENSG0000C	1040	23.54962	chr5:4231	ENSG00000286509		lncRNA	chr5:93088856-9309
ENSG0000C	1040	23.54962	chr5:4231	MTCYBP35		Pseudoger	chr5:94569540-9457
ENSG0000C	1040	23.54962	chr5:4231	ENSG00000278905		TEC	chr5:93600357-9360
ENSG0000C	1040	23.54962	chr5:4231	AC093311.1		smallRNA	chr5:94578769-9457
ENSG0000C	1040	23.54962	chr5:4231	RPL7P18		Pseudoger	chr5:94825961-9482
ENSG0000C	1040	23.54962	chr5:4231	NR2F1-AS1		lncRNA	chr5:93360779-9358
ENSG0000C	1040	23.54962	chr5:4231	HSPD1P11		Pseudoger	chr5:95768999-9577
ENSG0000C	1040	23.54962	chr5:4231	PCBP2P3		Pseudoger	chr5:91843687-9184
ENSG0000C	1040	23.54962	chr5:4231	POU5F2		protein_c	chr5:93733220-9374
ENSG0000C	1040	23.54962	chr5:4231	ENSG00000248734		lncRNA	chr5:96784777-9678
ENSG0000C	1040	23.54962	chr5:4231	ENSG00000270133		lncRNA	chr5:94611906-9461
ENSG0000C	1040	23.54962	chr5:4231	ELL2		protein_c	chr5:95885098-9596
ENSG0000C	1040	23.54962	chr5:4231	FAM81B		protein_c	chr5:95391366-9545
ENSG0000C	1040	23.54962	chr5:4231	CAST	NCGv7	protein_c	chr5:96525267-9677
ENSG0000C	1040	23.54962	chr5:4231	LDHBP3		Pseudoger	chr5:92840807-9284
ENSG0000C	1040	23.54962	chr5:4231	ENSG00000250955		lncRNA	chr5:95964999-9598
ENSG0000C	1040	23.54962	chr5:4231	RNU6-308P		smallRNA	chr5:95521369-9552
ENSG0000C	1040	23.54962	chr5:4231	Y_RNA		smallRNA	chr5:96962656-9696
ENSG0000C	1040	23.54962	chr5:4231	KIAA0825		protein_c	chr5:94150851-9461
ENSG0000C	1040	23.54962	chr5:4231	ENSG00000286577		lncRNA	chr5:94111720-9417
ENSG0000C	1040	23.54962	chr5:4231	SPATA9		protein_c	chr5:95652181-9569
ENSG0000C	1040	23.54962	chr5:4231	ENSG00000251409		lncRNA	chr5:95835521-9585
ENSG0000C	1040	23.54962	chr5:4231	SETP22		Pseudoger	chr5:97056402-9705
ENSG0000C	1040	23.54962	chr5:4231	GGCTP1		Pseudoger	chr5:95834424-9583
ENSG0000C	1040	23.54962	chr5:4231	RTRAFP2		Pseudoger	chr5:95440044-9544
ENSG0000C	1040	23.54962	chr5:4231	ENSG00000248588		lncRNA	chr5:92823935-9284
ENSG0000C	1040	23.54962	chr5:4231	ENSG00000232578		Pseudoger	chr5:94592771-9459
ENSG0000C	1040	23.54962	chr5:4231	LINC02058		lncRNA	chr5:92907180-9293
ENSG0000C	1040	23.54962	chr5:4231	ENSG00000251023		lncRNA	chr5:93860669-9386
ENSG0000C	1040	23.54962	chr5:4231	AC020900.1		smallRNA	chr5:96590192-9659
ENSG0000C	1040	23.54962	chr5:4231	LIX1		protein_c	chr5:97091867-9714
ENSG0000C	1040	23.54962	chr5:4231	ENSG00000249169		lncRNA	chr5:92654848-9267
ENSG0000C	1040	23.54962	chr5:4231	POLD2P1		Pseudoger	chr5:93267429-9326
ENSG0000C	1040	23.54962	chr5:4231	FABP5P5		Pseudoger	chr5:95973041-9597
ENSG0000C	1040	23.54962	chr5:4231	ENSG00000247121		lncRNA	chr5:96814028-9693
ENSG0000C	1040	23.54962	chr5:4231	NR2F1		protein_c	chr5:93583222-9359
ENSG0000C	1040	23.54962	chr5:4231	SLF1		protein_c	chr5:94618669-9473
ENSG0000C	1040	23.54962	chr5:4231	ENSG00000227836		Pseudoger	chr5:96942299-9694
ENSG0000C	1040	23.54962	chr5:4231	MIR583HG		lncRNA	chr5:96050115-9621
ENSG0000C	1040	23.54962	chr5:4231	CCT7P2		Pseudoger	chr5:92889387-9289
ENSG0000C	1040	23.54962	chr5:4231	ARSK		protein_c	chr5:95555101-9560
ENSG0000C	1040	23.54962	chr5:4231	AC026781.1		smallRNA	chr5:92717432-9271

ENSG0000(	1040	23.54962	chr5:4231	ENSG00000287180		lncRNA	chr5:93621683-9367
ENSG0000(	1040	23.54962	chr5:4231	AC120120.1		smallRNA	chr5:92405946-9240
ENSG0000(	1040	23.54962	chr5:4231	ENSG00000249776		lncRNA	chr5:92410256-9266
ENSG0000(	1040	23.54962	chr5:4231	AC020900.2		smallRNA	chr5:96694531-9669
ENSG0000(	1040	23.54962	chr5:4231	ENSG00000249746		lncRNA	chr5:96213263-9621
ENSG0000(	1040	23.54962	chr5:4231	NPM1P27		Pseudoger	chr5:93682838-9368
ENSG0000(	1040	23.54962	chr5:4231	LINC01554		lncRNA	chr5:95838245-9586
ENSG0000(	1040	23.54962	chr5:4231	MIR2277		smallRNA	chr5:93620696-9362
ENSG0000(	1040	23.54962	chr5:4231	ENSG00000272406		lncRNA	chr5:93741640-9374
ENSG0000(	1040	23.54962	chr5:4231	PCSK1		protein_c	chr5:96390333-9643
ENSG0000(	1040	23.54962	chr5:4231	MCTP1		protein_c	chr5:94703690-9528
ENSG0000(	1040	23.54962	chr5:4231	ERAP2		protein_c	chr5:96875986-9691
ENSG0000(	1040	23.54962	chr5:4231	ENSG00000289274		lncRNA	chr5:93598593-9360
ENSG0000(	1040	23.54962	chr5:4231	ERAP1		protein_c	chr5:96760810-9680
ENSG0000(	1040	23.54962	chr5:4231	ENSG00000289337		lncRNA	chr5:96165522-9617
ENSG0000(	1040	23.54962	chr5:4231	RNU6-524P		smallRNA	chr5:96210121-9621
ENSG0000(	1040	23.54962	chr5:4231	RHOBTB3		protein_c	chr5:95713522-9582
ENSG0000(	1040	23.54962	chr5:4231	RFESD		protein_c	chr5:95646754-9568
ENSG0000(	1040	23.54962	chr5:4231	ENSG00000250240		lncRNA	chr5:95701249-9573
ENSG0000(	1040	23.54962	chr5:4231	ENSG00000250362		lncRNA	chr5:95861786-9587
ENSG0000(	1040	23.54962	chr5:4231	MTND6P3		Pseudoger	chr5:94568929-9456
ENSG0000(	1040	23.54962	chr5:4231	MCTP1-AS1		lncRNA	chr5:94979151-9498
ENSG0000(	1040	23.54962	chr5:4231	MIR583		smallRNA	chr5:96079138-9607
ENSG0000(	1040	23.54962	chr5:4231	FAM172A		protein_c	chr5:93617725-9411
ENSG0000(	1040	23.54962	chr5:4231	LNPEP		protein_c	chr5:96935394-9703
ENSG0000(	1040	23.54962	chr5:4231	ENSG00000272021		lncRNA	chr5:95849309-9584
ENSG0000(	1040	23.54962	chr5:4231	ENSG00000272109		lncRNA	chr5:96803688-9680
ENSG0000(	1040	23.54962	chr5:4231	ENSG00000250158		lncRNA	chr5:96247776-9627
ENSG0000(	1040	23.54962	chr5:4231	GPR150		protein_c	chr5:95620087-9562
ENSG0000(	1040	23.54962	chr5:4231	MTND5P12		Pseudoger	chr5:94567461-9456
ENSG0000(	1040	23.54962	chr5:4231	ENSG00000287447		lncRNA	chr5:93790505-9380
ENSG0000(	1040	23.54962	chr5:4231	ENSG00000249984		lncRNA	chr5:92675956-9268
ENSG0000(	1040	23.54962	chr5:4231	ENSG00000249180		lncRNA	chr5:96741079-9674
ENSG0000(	1040	23.54962	chr5:4231	ENSG00000249175		lncRNA	chr5:94788789-9479
ENSG0000(	1026	23.23261	chr1:1021	MIR30C1		smallRNA	chr1:40757284-4075
ENSG0000(	1017	23.02881	chr2:2577	ENSG00000270460		lncRNA	chr2:173811076-173
ENSG0000(	1013	22.93824	chr2:8187	AC007040.3		smallRNA	chr2:71013089-7101
ENSG0000(	1003	22.7118	chrX:1578	SNORA11		smallRNA	chrX:54814370-5481
ENSG0000(	994	22.508	chr12:624	RNA5SP356		Pseudoger	chr12:30306431-303
ENSG0000(	993	22.48536	chr5:4231	SLCO4C1		protein_c	chr5:102233986-102
ENSG0000(	993	22.48536	chr5:4231	CHD1	NCGv7	protein_c	chr5:98853985-9892
ENSG0000(	993	22.48536	chr5:4231	LINC02113		lncRNA	chr5:99549432-9957
ENSG0000(	993	22.48536	chr5:4231	OR7H2P		Pseudoger	chr5:101816475-101
ENSG0000(	993	22.48536	chr5:4231	RNU6-1119P		smallRNA	chr5:100153672-100
ENSG0000(	993	22.48536	chr5:4231	RIOK2	NCGv7	protein_c	chr5:97160867-9718
ENSG0000(	993	22.48536	chr5:4231	Y_RNA		smallRNA	chr5:98936638-9893
ENSG0000(	993	22.48536	chr5:4231	AC022142.1		smallRNA	chr5:98639097-9863
ENSG0000(	993	22.48536	chr5:4231	RNU1-73P		smallRNA	chr5:97175950-9717
ENSG0000(	993	22.48536	chr5:4231	LINC01340		lncRNA	chr5:97504663-9769
ENSG0000(	993	22.48536	chr5:4231	RN7SKP68		smallRNA	chr5:102302504-102
ENSG0000(	993	22.48536	chr5:4231	RGMB-AS1		lncRNA	chr5:98769618-9877

ENSG0000(	993	22.48536	chr5:4231	CSNK1A1P3		Pseudoger	chr5:98833382-9883
ENSG0000(	993	22.48536	chr5:4231	LINC00492		lncRNA	chr5:102581368-102
ENSG0000(	993	22.48536	chr5:4231	RGMB		protein_c	chr5:98768650-9879
ENSG0000(	993	22.48536	chr5:4231	FAM174A	DriverDBv	protein_c	chr5:100535374-100
ENSG0000(	993	22.48536	chr5:4231	GUSBP19		Pseudoger	chr5:100375700-100
ENSG0000(	993	22.48536	chr5:4231	MTND4P35		Pseudoger	chr5:100048988-100
ENSG0000(	993	22.48536	chr5:4231	ENSG00000273957		Pseudoger	chr5:100388853-100
ENSG0000(	993	22.48536	chr5:4231	ENSG00000286953		lncRNA	chr5:97799404-9788
ENSG0000(	993	22.48536	chr5:4231	ENSG00000248758		lncRNA	chr5:97188090-9720
ENSG0000(	993	22.48536	chr5:4231	PAM	DriverDBv	protein_c	chr5:102753981-103
ENSG0000(	993	22.48536	chr5:4231	ENSG00000249787		lncRNA	chr5:100399047-100
ENSG0000(	993	22.48536	chr5:4231	ENSG00000286828		lncRNA	chr5:97183827-9721
ENSG0000(	993	22.48536	chr5:4231	MTCO2P22		Pseudoger	chr5:100052859-100
ENSG0000(	993	22.48536	chr5:4231	ENSG00000248236		Pseudoger	chr5:99594329-9959
ENSG0000(	993	22.48536	chr5:4231	ENSG00000248261		lncRNA	chr5:102141893-102
ENSG0000(	993	22.48536	chr5:4231	LINC02234		lncRNA	chr5:97840912-9792
ENSG0000(	993	22.48536	chr5:4231	GUSBP7		Pseudoger	chr5:100062275-100
ENSG0000(	993	22.48536	chr5:4231	ST8SIA4		protein_c	chr5:100806933-100
ENSG0000(	993	22.48536	chr5:4231	MTCO3P22		Pseudoger	chr5:100051133-100
ENSG0000(	993	22.48536	chr5:4231	KRT8P32		Pseudoger	chr5:98392070-9839
ENSG0000(	993	22.48536	chr5:4231	AC113407.1		smallRNA	chr5:100050365-100
ENSG0000(	993	22.48536	chr5:4231	MTCO2P24		Pseudoger	chr5:98409675-9841
ENSG0000(	993	22.48536	chr5:4231	ENSG00000249977		Pseudoger	chr5:97449358-9744
ENSG0000(	993	22.48536	chr5:4231	CTBP2P4		Pseudoger	chr5:98576341-9857
ENSG0000(	993	22.48536	chr5:4231	EEF1A1P20		Pseudoger	chr5:99996547-9999
ENSG0000(	993	22.48536	chr5:4231	MTND4LP5		Pseudoger	chr5:100050359-100
ENSG0000(	993	22.48536	chr5:4231	ENSG00000286338		lncRNA	chr5:102664610-102
ENSG0000(	993	22.48536	chr5:4231	RN7SKP62		smallRNA	chr5:100733058-100
ENSG0000(	993	22.48536	chr5:4231	MTATP6P2		Pseudoger	chr5:100051920-100
ENSG0000(	993	22.48536	chr5:4231	ENSG00000206356		Pseudoger	chr5:99522311-9952
ENSG0000(	993	22.48536	chr5:4231	Y_RNA		smallRNA	chr5:100021804-100
ENSG0000(	993	22.48536	chr5:4231	ENSG00000251054		lncRNA	chr5:97223371-9722
ENSG0000(	993	22.48536	chr5:4231	ENSG00000289912		lncRNA	chr5:100903394-100
ENSG0000(	993	22.48536	chr5:4231	CHD1-DT		lncRNA	chr5:98929163-9899
ENSG0000(	993	22.48536	chr5:4231	ENSG00000249761		Pseudoger	chr5:97336858-9733
ENSG0000(	993	22.48536	chr5:4231	FAM174A-DT		lncRNA	chr5:100428124-100
ENSG0000(	993	22.48536	chr5:4231	YTHDF1P1		Pseudoger	chr5:97368776-9737
ENSG0000(	993	22.48536	chr5:4231	MTCYBP22		Pseudoger	chr5:100045928-100
ENSG0000(	993	22.48536	chr5:4231	ENSG00000247402		lncRNA	chr5:102605635-102
ENSG0000(	993	22.48536	chr5:4231	MTND5P10		Pseudoger	chr5:100046977-100
ENSG0000(	993	22.48536	chr5:4231	MTND6P22		Pseudoger	chr5:100046450-100
ENSG0000(	993	22.48536	chr5:4231	RNA5SP188		Pseudoger	chr5:102131007-102
ENSG0000(	993	22.48536	chr5:4231	ENSG00000249495		Pseudoger	chr5:101976296-101
ENSG0000(	993	22.48536	chr5:4231	SLCO6A1		protein_c	chr5:102371774-102
ENSG0000(	993	22.48536	chr5:4231	ENSG00000251193		Pseudoger	chr5:97737894-9773
ENSG0000(	993	22.48536	chr5:4231	MTCYBP40		Pseudoger	chr5:97677547-9767
ENSG0000(	993	22.48536	chr5:4231	ENSG00000176183		Pseudoger	chr5:98338744-9833
ENSG0000(	993	22.48536	chr5:4231	MIR548P		smallRNA	chr5:100816482-100
ENSG0000(	993	22.48536	chr5:4231	MTCO1P22		Pseudoger	chr5:100053686-100
ENSG0000(	993	22.48536	chr5:4231	DDX18P4		Pseudoger	chr5:98679402-9868
ENSG0000(	993	22.48536	chr5:4231	ENSG00000241597		Pseudoger	chr5:98954394-9895

ENSG0000(	993	22.48536	chr5:4231	ENSG00000248928		Pseudoger	chr5:99594880-9959
ENSG0000(	993	22.48536	chr5:4231	ENSG00000290580		lncRNA	chr5:99489559-9949
ENSG0000(	993	22.48536	chr5:4231	LINC01846		lncRNA	chr5:98085866-9816
ENSG0000(	993	22.48536	chr5:4231	MTCO1P24		Pseudoger	chr5:98410235-9841
ENSG0000(	993	22.48536	chr5:4231	RNU6-402P		smallRNA	chr5:98889731-9888
ENSG0000(	993	22.48536	chr5:4231	ENSG00000249101		Pseudoger	chr5:98025965-9802
ENSG0000(	993	22.48536	chr5:4231	ENSG00000249444		Pseudoger	chr5:99499070-9949
ENSG0000(	993	22.48536	chr5:4231	MTND3P19		Pseudoger	chr5:100050719-100
ENSG0000(	993	22.48536	chr5:4231	LINC00491		lncRNA	chr5:102604220-102
ENSG0000(	993	22.48536	chr5:4231	MRPS35P2		Pseudoger	chr5:98402542-9840
ENSG0000(	993	22.48536	chr5:4231	CRLF3P2		Pseudoger	chr5:99948295-9994
ENSG0000(	993	22.48536	chr5:4231	ENSG00000279232		lncRNA	chr5:98792861-9879
ENSG0000(	993	22.48536	chr5:4231	ENSG00000250806		lncRNA	chr5:100654112-100
ENSG0000(	993	22.48536	chr5:4231	ENSG00000249017		lncRNA	chr5:102500541-102
ENSG0000(	993	22.48536	chr5:4231	ENSG00000249135		Pseudoger	chr5:98203059-9820
ENSG0000(	993	22.48536	chr5:4231	LIX1-AS1		lncRNA	chr5:97089075-9743
ENSG0000(	993	22.48536	chr5:4231	RN7SL802P		smallRNA	chr5:101581830-101
ENSG0000(	993	22.48536	chr5:4231	GUSBP8		Pseudoger	chr5:99532628-9953
ENSG0000(	979	22.16835	chr2:8187	RPSAP28		Pseudoger	chr2:73370019-7337
ENSG0000(	975	22.07777	chr4:253(	SNORD73A		smallRNA	chr4:151103827-151
ENSG0000(	975	22.07777	chr4:253(	SNORD73		smallRNA	chr4:151102057-151
ENSG0000(	975	22.07777	chr4:253(	RPS3A	NCGv7	protein_c	chr4:151099624-151
ENSG0000(	975	22.07777	chr4:253(	AK4P6		Pseudoger	chr4:151027090-151
ENSG0000(	972	22.00984	chr4:253(	SERF1AP1		Pseudoger	chr4:137301001-137
ENSG0000(	972	22.00984	chr4:253(	ENSG00000287144		lncRNA	chr4:137943022-138
ENSG0000(	972	22.00984	chr4:253(	AC060835.1		smallRNA	chr4:137228128-137
ENSG0000(	972	22.00984	chr4:253(	PCDH18	NCGv7	protein_c	chr4:137518918-137
ENSG0000(	972	22.00984	chr2:2577	AC016706.1		smallRNA	chr2:142312940-142
ENSG0000(	972	22.00984	chr4:253(	ENSG00000250977		lncRNA	chr4:138664725-138
ENSG0000(	972	22.00984	chr4:253(	ENSG00000249091		lncRNA	chr4:138277115-138
ENSG0000(	972	22.00984	chr4:253(	ENSG00000279749		TEC	chr4:136553236-136
ENSG0000(	972	22.00984	chr4:253(	LINC00499		lncRNA	chr4:138309613-138
ENSG0000(	972	22.00984	chr4:253(	LINC00500		lncRNA	chr4:138425523-138
ENSG0000(	972	22.00984	chr4:253(	ENSG00000250777		lncRNA	chr4:137690477-137
ENSG0000(	972	22.00984	chr4:253(	LINC00498		lncRNA	chr4:138298856-138
ENSG0000(	972	22.00984	chr4:253(	ENSG00000287441		lncRNA	chr4:137751029-137
ENSG0000(	972	22.00984	chr4:253(	SLC7A11-AS1		lncRNA	chr4:138057464-138
ENSG0000(	972	22.00984	chr4:253(	ENSG00000248305		Pseudoger	chr4:138481255-138
ENSG0000(	972	22.00984	chr4:253(	RPS23P2		Pseudoger	chr4:137448087-137
ENSG0000(	972	22.00984	chr4:253(	ENSG00000250126		lncRNA	chr4:137645265-137
ENSG0000(	972	22.00984	chr4:253(	ENSG00000280352		TEC	chr4:137601899-137
ENSG0000(	972	22.00984	chr4:253(	LINC02510		lncRNA	chr4:137193756-137
ENSG0000(	972	22.00984	chr4:253(	STMN1P2		Pseudoger	chr4:137425426-137
ENSG0000(	972	22.00984	chr4:253(	SLC7A11		protein_c	chr4:138164097-138
ENSG0000(	972	22.00984	chr4:253(	LINC02172		lncRNA	chr4:137545731-137
ENSG0000(	972	22.00984	chr4:253(	LINC00616		lncRNA	chr4:137978547-138
ENSG0000(	972	22.00984	chr4:253(	ENSG00000248795		Pseudoger	chr4:138560704-138
ENSG0000(	972	22.00984	chr4:253(	ENSG00000250034		lncRNA	chr4:137807706-137
ENSG0000(	972	22.00984	chr4:253(	AC093875.1		smallRNA	chr4:136707178-136
ENSG0000(	972	22.00984	chr4:253(	LINC02511		lncRNA	chr4:136795919-137
ENSG0000(	971	21.98719	chr4:253(	EDNRA		protein_c	chr4:147480917-147

ENSG0000(	971	21.98719	chr4:253(	NR3C2	NCGv7	protein_c	chr4:148078762-148
ENSG0000(	971	21.98719	chr4:253(	ENSG00000260303		lncRNA	chr4:146052604-146
ENSG0000(	971	21.98719	chr4:253(	ARHGAP10		protein_c	chr4:147732063-148
ENSG0000(	971	21.98719	chr4:253(	ENSG00000286420		lncRNA	chr4:145444899-145
ENSG0000(	971	21.98719	chr4:253(	RPSAP36		Pseudoger	chr4:143424753-143
ENSG0000(	971	21.98719	chr4:253(	ENSG00000248346		lncRNA	chr4:146957438-146
ENSG0000(	971	21.98719	chr4:253(	HSPD1P5		Pseudoger	chr4:144845625-144
ENSG0000(	971	21.98719	chr4:253(	LINC02430		lncRNA	chr4:149147793-149
ENSG0000(	971	21.98719	chr4:253(	MIR548G		smallRNA	chr4:147344629-147
ENSG0000(	971	21.98719	chr4:253(	GYPE		protein_c	chr4:143870864-143
ENSG0000(	971	21.98719	chr4:253(	Y_RNA		smallRNA	chr4:145958128-145
ENSG0000(	971	21.98719	chr4:253(	GAB1		protein_c	chr4:143336762-143
ENSG0000(	971	21.98719	chr4:253(	POU4F2		protein_c	chr4:146638893-146
ENSG0000(	971	21.98719	chr4:253(	INPP4B	NCGv7	protein_c	chr4:142023160-142
ENSG0000(	971	21.98719	chr4:253(	RNA5SP165		Pseudoger	chr4:147745303-147
ENSG0000(	971	21.98719	chr4:253(	ZNF827		protein_c	chr4:145757627-145
ENSG0000(	971	21.98719	chr4:253(	ENSG00000249806		lncRNA	chr4:142566019-142
ENSG0000(	971	21.98719	chr4:253(	ENSG00000261129		lncRNA	chr4:144505900-144
ENSG0000(	971	21.98719	chr4:253(	MMAA		protein_c	chr4:145599042-145
ENSG0000(	971	21.98719	chr4:253(	ENSG00000285713		protein_c	chr4:144210701-145
ENSG0000(	971	21.98719	chr4:253(	LINC02355		lncRNA	chr4:149154279-149
ENSG0000(	971	21.98719	chr4:253(	SMAD1		protein_c	chr4:145481194-145
ENSG0000(	971	21.98719	chr4:253(	RNU1-44P		smallRNA	chr4:146580048-146
ENSG0000(	971	21.98719	chr4:253(	ENSG00000248356		lncRNA	chr4:145642336-145
ENSG0000(	971	21.98719	chr4:253(	ENSG00000228981		Pseudoger	chr4:143349275-143
ENSG0000(	971	21.98719	chr4:253(	C4orf51		protein_c	chr4:145680146-145
ENSG0000(	971	21.98719	chr4:253(	ENSG00000278943		TEC	chr4:145376909-145
ENSG0000(	971	21.98719	chr4:253(	NCOA4P3		Pseudoger	chr4:145624159-145
ENSG0000(	971	21.98719	chr4:253(	ENSG00000249317		lncRNA	chr4:148940941-148
ENSG0000(	971	21.98719	chr4:253(	ENSG00000251300		Pseudoger	chr4:143045086-143
ENSG0000(	971	21.98719	chr4:253(	RTN3P1		Pseudoger	chr4:145375543-145
ENSG0000(	971	21.98719	chr4:253(	ENSG00000250406		lncRNA	chr4:144851572-144
ENSG0000(	971	21.98719	chr4:253(	Y_RNA		smallRNA	chr4:145149537-145
ENSG0000(	971	21.98719	chr4:253(	ENSG00000250540		Pseudoger	chr4:143497412-143
ENSG0000(	971	21.98719	chr4:253(	TMEM184C-DT		lncRNA	chr4:147609552-147
ENSG0000(	971	21.98719	chr4:253(	ENSG00000279845		TEC	chr4:146180851-146
ENSG0000(	971	21.98719	chr4:253(	ENSG00000280219		TEC	chr4:147487971-147
ENSG0000(	971	21.98719	chr4:253(	LINC02266		lncRNA	chr4:145335263-145
ENSG0000(	971	21.98719	chr4:253(	RN7SL254P		smallRNA	chr4:147808926-147
ENSG0000(	971	21.98719	chr4:253(	ENSG00000270387		Pseudoger	chr4:147916529-147
ENSG0000(	971	21.98719	chr4:253(	ENSG00000279376		TEC	chr4:147025914-147
ENSG0000(	971	21.98719	chr4:253(	ENSG00000279016		TEC	chr4:148819351-148
ENSG0000(	971	21.98719	chr4:253(	KRT18P51		Pseudoger	chr4:144572238-144
ENSG0000(	971	21.98719	chr4:253(	FREM3		protein_c	chr4:143577302-143
ENSG0000(	971	21.98719	chr4:253(	SMAD1-AS2		lncRNA	chr4:145497073-145
ENSG0000(	971	21.98719	chr4:253(	ENSG00000260090		lncRNA	chr4:147005492-147
ENSG0000(	971	21.98719	chr4:253(	ENSG00000285783		lncRNA	chr4:144111039-144
ENSG0000(	971	21.98719	chr4:253(	TTC29		protein_c	chr4:146706617-146
ENSG0000(	971	21.98719	chr4:253(	RNU7-197P		smallRNA	chr4:148991572-148
ENSG0000(	971	21.98719	chr4:253(	RPS23P4		Pseudoger	chr4:145269810-145
ENSG0000(	971	21.98719	chr4:253(	ASS1P8		Pseudoger	chr4:148526493-148

ENSG0000(	971	21.98719	chr4:253(	HHIP	protein_c	chr4:144646156-144
ENSG0000(	971	21.98719	chr4:253(	USP38-DT	lncRNA	chr4:142933195-143
ENSG0000(	971	21.98719	chr4:253(	RPL5P13	Pseudoger	chr4:142286917-142
ENSG0000(	971	21.98719	chr4:253(	ABCE1	protein_c	chr4:145098288-145
ENSG0000(	971	21.98719	chr4:253(	ENSG00000250969	lncRNA	chr4:143286293-143
ENSG0000(	971	21.98719	chr4:253(	ENSG00000289314	lncRNA	chr4:144687750-144
ENSG0000(	971	21.98719	chr4:253(	OTUD4	protein_c	chr4:145110838-145
ENSG0000(	971	21.98719	chr4:253(	LSM6	protein_c	chr4:146175703-146
ENSG0000(	971	21.98719	chr4:253(	TMEM184C	protein_c	chr4:147617386-147
ENSG0000(	971	21.98719	chr4:253(	PRMT9	protein_c	chr4:147637785-147
ENSG0000(	971	21.98719	chr4:253(	GUSBP5	Pseudoger	chr4:143559472-143
ENSG0000(	971	21.98719	chr4:253(	Y_RNA	smallRNA	chr4:143309466-143
ENSG0000(	971	21.98719	chr4:253(	RNA5SP166	Pseudoger	chr4:148557947-148
ENSG0000(	971	21.98719	chr4:253(	GYPA	protein_c	chr4:144109303-144
ENSG0000(	971	21.98719	chr4:253(	NMNAT1P4	Pseudoger	chr4:145456005-145
ENSG0000(	971	21.98719	chr4:253(	GYPB	protein_c	chr4:143996104-144
ENSG0000(	971	21.98719	chr4:253(	ENSG00000250357	lncRNA	chr4:148693032-148
ENSG0000(	971	21.98719	chr4:253(	REELD1	protein_c	chr4:146214515-146
ENSG0000(	971	21.98719	chr4:253(	MIR3139	smallRNA	chr4:143343460-143
ENSG0000(	971	21.98719	chr4:253(	ENSG00000251248	lncRNA	chr4:142514446-142
ENSG0000(	971	21.98719	chr4:253(	SMARCA5	protein_c	chr4:143513702-143
ENSG0000(	971	21.98719	chr4:253(	ENSG00000250354	lncRNA	chr4:148146471-148
ENSG0000(	971	21.98719	chr4:253(	SMAD1-AS1	lncRNA	chr4:145514615-145
ENSG0000(	971	21.98719	chr4:253(	ENSG00000241281	Pseudoger	chr4:147425974-147
ENSG0000(	971	21.98719	chr4:253(	HHIP-AS1	lncRNA	chr4:144642919-144
ENSG0000(	971	21.98719	chr4:253(	USP38	protein_c	chr4:143184917-143
ENSG0000(	971	21.98719	chr4:253(	LINC01095	lncRNA	chr4:146077717-146
ENSG0000(	971	21.98719	chr4:253(	ENSG00000251452	Pseudoger	chr4:144920713-144
ENSG0000(	971	21.98719	chr4:253(	GTF2F2P1	Pseudoger	chr4:147506091-147
ENSG0000(	971	21.98719	chr4:253(	AC097372.1	smallRNA	chr4:146122204-146
ENSG0000(	971	21.98719	chr4:253(	ENSG00000248924	Pseudoger	chr4:143555082-143
ENSG0000(	971	21.98719	chr4:253(	AC093887.1	smallRNA	chr4:146639261-146
ENSG0000(	971	21.98719	chr4:253(	ENSG00000248764	lncRNA	chr4:146934001-146
ENSG0000(	971	21.98719	chr4:253(	LSM3P4	Pseudoger	chr4:142317238-142
ENSG0000(	971	21.98719	chr4:253(	RN7SKP235	smallRNA	chr4:145035132-145
ENSG0000(	971	21.98719	chr4:253(	SMARCA5-AS1	lncRNA	chr4:143513472-143
ENSG0000(	971	21.98719	chr4:253(	ENSG00000287292	lncRNA	chr4:148445703-149
ENSG0000(	971	21.98719	chr4:253(	ENSG00000286371	lncRNA	chr4:147060613-147
ENSG0000(	971	21.98719	chr4:253(	SLC10A7	protein_c	chr4:146253975-146
ENSG0000(	971	21.98719	chr4:253(	ENSG00000249752	lncRNA	chr4:149027445-149
ENSG0000(	971	21.98719	chr4:253(	ENSG00000250345	Pseudoger	chr4:143760399-143
ENSG0000(	971	21.98719	chr4:253(	ENSG00000249741	Pseudoger	chr4:143911514-143
ENSG0000(	971	21.98719	chr4:253(	LINC02507	lncRNA	chr4:147567606-147
ENSG0000(	971	21.98719	chr4:253(	ENSG00000288998	lncRNA	chr4:146522425-146
ENSG0000(	971	21.98719	chr4:253(	ENSG00000286771	lncRNA	chr4:143351515-143
ENSG0000(	971	21.98719	chr4:253(	ATP5MGP4	Pseudoger	chr4:148703526-148
ENSG0000(	971	21.98719	chr4:253(	ENSG00000251687	lncRNA	chr4:145833118-145
ENSG0000(	971	21.98719	chr4:253(	ENSG00000251010	lncRNA	chr4:146241806-146
ENSG0000(	971	21.98719	chr4:253(	ANAPC10	protein_c	chr4:144831908-145
ENSG0000(	971	21.98719	chr4:253(	ENSG00000286055	lncRNA	chr4:144010237-144
ENSG0000(	971	21.98719	chr4:253(	ENSG00000287565	lncRNA	chr4:149377257-149

ENSG0000(	971	21.98719	chr4:253(	ENSG00000251600		lncRNA	chr4:143559457-144
ENSG0000(	971	21.98719	chr4:253(	MIR4799		smallRNA	chr4:147782595-147
ENSG0000(	971	21.98719	chr4:253(	PRMT5P1		Pseudoger	chr4:147458724-147
ENSG0000(	971	21.98719	chr4:253(	ENSG00000279481		TEC	chr4:145830456-145
ENSG0000(	969	21.94191	chr4:253(	ENSG00000251199		lncRNA	chr4:134026665-134
ENSG0000(	969	21.94191	chr4:253(	RNU1-89P		smallRNA	chr4:135995929-135
ENSG0000(	969	21.94191	chr4:253(	ENSG00000241411		Pseudoger	chr4:134952259-134
ENSG0000(	969	21.94191	chr4:253(	ENSG00000249000		lncRNA	chr4:134759399-134
ENSG0000(	969	21.94191	chr4:253(	ENSG00000251228		Pseudoger	chr4:134944275-134
ENSG0000(	969	21.94191	chr4:253(	TERF1P3		Pseudoger	chr4:136355110-136
ENSG0000(	969	21.94191	chr4:253(	TARS2P1		Pseudoger	chr4:135565117-135
ENSG0000(	969	21.94191	chr4:253(	KRT18P54		Pseudoger	chr4:135371177-135
ENSG0000(	969	21.94191	chr4:253(	ENSG00000251388		lncRNA	chr4:133871246-134
ENSG0000(	969	21.94191	chr4:253(	ENSG00000261166		lncRNA	chr4:136097797-136
ENSG0000(	969	21.94191	chr4:253(	PABPC4L	NCGv7	protein_c	chr4:134196333-134
ENSG0000(	969	21.94191	chr4:253(	ENSG00000250191		Pseudoger	chr4:133574566-133
ENSG0000(	969	21.94191	chr4:253(	EEF1A1P35		Pseudoger	chr4:135045464-135
ENSG0000(	969	21.94191	chr4:253(	ENSG00000250865		lncRNA	chr4:135792384-135
ENSG0000(	969	21.94191	chr4:253(	ENSG00000249847		lncRNA	chr4:134932889-134
ENSG0000(	969	21.94191	chr4:253(	RNU6-569P		smallRNA	chr4:135315418-135
ENSG0000(	969	21.94191	chr4:253(	ENSG00000250665		lncRNA	chr4:136125909-136
ENSG0000(	969	21.94191	chr4:253(	AC096763.1		smallRNA	chr4:134043467-134
ENSG0000(	969	21.94191	chr4:253(	LINC00613		lncRNA	chr4:135866983-135
ENSG0000(	969	21.94191	chr4:253(	LINC02462		lncRNA	chr4:134383467-134
ENSG0000(	969	21.94191	chr4:253(	AC108867.1		smallRNA	chr4:135371590-135
ENSG0000(	969	21.94191	chr4:253(	LINC02485		lncRNA	chr4:135112905-135
ENSG0000(	969	21.94191	chr4:253(	ENSG00000286253		lncRNA	chr4:134057468-134
ENSG0000(	969	21.94191	chr4:253(	ENSG00000248434		lncRNA	chr4:135067233-135
ENSG0000(	969	21.94191	chr4:253(	PES1P1		Pseudoger	chr4:134327063-134
ENSG0000(	969	21.94191	chr4:253(	ENSG00000251492		Pseudoger	chr4:136004509-136
ENSG0000(	969	21.94191	chr4:253(	ENSG00000251567		lncRNA	chr4:136118675-136
ENSG0000(	968	21.91926	chr4:253(	RNU6-1230P		smallRNA	chr4:149934376-149
ENSG0000(	968	21.91926	chr4:253(	DCLK2		protein_c	chr4:150078445-150
ENSG0000(	968	21.91926	chr4:253(	ENSG00000250905		lncRNA	chr4:149666057-149
ENSG0000(	968	21.91926	chr4:253(	RNA5SP167		Pseudoger	chr4:149907560-149
ENSG0000(	968	21.91926	chr4:253(	ZBTB8OSP1		Pseudoger	chr4:150720981-150
ENSG0000(	968	21.91926	chr4:253(	AC110813.1		smallRNA	chr4:150639756-150
ENSG0000(	968	21.91926	chr4:253(	ENSG00000248521		Pseudoger	chr4:150422645-150
ENSG0000(	968	21.91926	chr4:253(	RNU7-194P		smallRNA	chr4:150209706-150
ENSG0000(	968	21.91926	chr4:253(	ENSG00000249690		lncRNA	chr4:150579089-150
ENSG0000(	968	21.91926	chr4:253(	ENSG00000250704		lncRNA	chr4:149956208-149
ENSG0000(	968	21.91926	chr4:253(	LRBA		protein_c	chr4:150264435-151
ENSG0000(	968	21.91926	chr4:253(	ENSG00000286711		lncRNA	chr4:150047004-150
ENSG0000(	968	21.91926	chr4:253(	RNA5SP168		Pseudoger	chr4:150970671-150
ENSG0000(	968	21.91926	chr4:253(	IQCM		protein_c	chr4:149351709-149
ENSG0000(	968	21.91926	chr4:253(	AKIRIN2P1		Pseudoger	chr4:149789952-149
ENSG0000(	968	21.91926	chr4:253(	MAB21L2		protein_c	chr4:150582151-150
ENSG0000(	967	21.89662	chrX:478(	U3		smallRNA	chrX:72726123-7272
ENSG0000(	954	21.60225	chr2:2577	ARHGAP15		protein_c	chr2:143091362-143
ENSG0000(	954	21.60225	chr2:2577	RNU6-904P		smallRNA	chr2:141167257-141
ENSG0000(	954	21.60225	chr2:2577	Y_RNA		smallRNA	chr2:143897263-143

ENSG0000(	954	21.60225	chr2:2577	MTND5P24		Pseudoger	chr2:143094789-143
ENSG0000(	954	21.60225	chr2:2577	ARHGAP15-AS1		lncRNA	chr2:143629133-143
ENSG0000(	954	21.60225	chr2:2577	MTCO2P5		Pseudoger	chr2:143100930-143
ENSG0000(	954	21.60225	chr2:2577	KYNU		protein_c	chr2:142877657-143
ENSG0000(	954	21.60225	chr2:2577	ENSG00000244125		lncRNA	chr2:142131178-142
ENSG0000(	954	21.60225	chr2:2577	ENSG00000228655		lncRNA	chr2:143295421-143
ENSG0000(	954	21.60225	chr2:2577	UBE2V1P14		Pseudoger	chr2:142543418-142
ENSG0000(	954	21.60225	chr2:2577	AC012003.1		smallRNA	chr2:141997377-141
ENSG0000(	954	21.60225	chr2:2577	Y_RNA		smallRNA	chr2:140992543-140
ENSG0000(	954	21.60225	chr2:2577	RPS16P3		Pseudoger	chr2:141208082-141
ENSG0000(	954	21.60225	chr2:2577	ENSG00000283994		lncRNA	chr2:143601517-143
ENSG0000(	954	21.60225	chr2:2577	MTND4LP12		Pseudoger	chr2:143098123-143
ENSG0000(	954	21.60225	chr2:2577	MTCYBP11		Pseudoger	chr2:143093056-143
ENSG0000(	954	21.60225	chr2:2577	MTATP6P5		Pseudoger	chr2:143099936-143
ENSG0000(	954	21.60225	chr2:2577	MTND4P22		Pseudoger	chr2:143096839-143
ENSG0000(	954	21.60225	chr2:2577	ENSG00000236356		lncRNA	chr2:143799467-143
ENSG0000(	954	21.60225	chr2:2577	AC013444.2		smallRNA	chr2:143005700-143
ENSG0000(	954	21.60225	chr2:2577	MTND3P9		Pseudoger	chr2:143098484-143
ENSG0000(	954	21.60225	chr2:2577	ENSG00000257284		lncRNA	chr2:143162078-143
ENSG0000(	954	21.60225	chr2:2577	LRRC57P1		Pseudoger	chr2:140898423-140
ENSG0000(	954	21.60225	chr2:2577	RRN3P4		Pseudoger	chr2:142854095-142
ENSG0000(	954	21.60225	chr2:2577	MTCO3P5		Pseudoger	chr2:143098898-143
ENSG0000(	954	21.60225	chr2:2577	ENSG00000257226		lncRNA	chr2:143765940-143
ENSG0000(	954	21.60225	chr2:2577	MTND6P11		Pseudoger	chr2:143094233-143
ENSG0000(	954	21.60225	chr2:2577	ENSG00000257277		lncRNA	chr2:143674315-143
ENSG0000(	954	21.60225	chr2:2577	SFXN4P1		Pseudoger	chr2:142947300-142
ENSG0000(	954	21.60225	chr2:2577	AC012353.1		smallRNA	chr2:141638844-141
ENSG0000(	953	21.57961	chr4:253(	ENSG00000249463		lncRNA	chr4:132027175-132
ENSG0000(	953	21.57961	chr4:253(	ENSG00000251598		lncRNA	chr4:131978485-132
ENSG0000(	953	21.57961	chr4:253(	PCDH10	NCGv7	protein_c	chr4:133149294-133
ENSG0000(	953	21.57961	chr4:253(	R3HDM2P1		Pseudoger	chr4:133124104-133
ENSG0000(	953	21.57961	chr4:253(	ENSG00000248369		lncRNA	chr4:132435802-132
ENSG0000(	953	21.57961	chr4:253(	PCDH10-DT		lncRNA	chr4:133075311-133
ENSG0000(	953	21.57961	chr4:253(	ENSG00000251488		lncRNA	chr4:132836772-132
ENSG0000(	953	21.57961	chr4:253(	ENSG00000249513		lncRNA	chr4:132985510-132
ENSG0000(	953	21.57961	chr4:253(	AARS1P1		Pseudoger	chr4:132592084-132
ENSG0000(	953	21.57961	chr4:253(	ENSG00000286467		lncRNA	chr4:133261022-133
ENSG0000(	953	21.57961	chr4:253(	ELL2P2		Pseudoger	chr4:132367786-132
ENSG0000(	953	21.57961	chr4:253(	LINC01256		lncRNA	chr4:132591064-132
ENSG0000(	947	21.44374	chr10:817	SHTN1		protein_c	chr10:116881477-11
ENSG0000(	947	21.44374	chr10:817	ENSG00000277687		lncRNA	chr10:118692361-11
ENSG0000(	947	21.44374	chr10:817	SLC18A2		protein_c	chr10:117241093-11
ENSG0000(	947	21.44374	chr10:817	LDHAP5		Pseudoger	chr10:118932674-11
ENSG0000(	947	21.44374	chr10:817	SLC25A18P1		Pseudoger	chr10:118572871-11
ENSG0000(	947	21.44374	chr10:817	ENSG00000263041		lncRNA	chr10:117825894-11
ENSG0000(	947	21.44374	chr10:817	AL356865.2		smallRNA	chr10:118546094-11
ENSG0000(	947	21.44374	chr10:817	ENSG00000235198		lncRNA	chr10:117562084-11
ENSG0000(	947	21.44374	chr10:817	LINC03036		lncRNA	chr10:118784563-11
ENSG0000(	947	21.44374	chr10:817	U3		smallRNA	chr10:118785752-11
ENSG0000(	947	21.44374	chr10:817	CACUL1		protein_c	chr10:118674167-11
ENSG0000(	947	21.44374	chr10:817	ENSG00000287655		lncRNA	chr10:117005462-11

ENSG0000(	947	21.44374	chr10:817	EMX2		protein_c	chr10:117542445-11
ENSG0000(	947	21.44374	chr10:817	AL356865.1		smallRNA	chr10:118630923-11
ENSG0000(	947	21.44374	chr10:817	VAX1		protein_c	chr10:117128521-11
ENSG0000(	947	21.44374	chr10:817	ENSG00000231104		lncRNA	chr10:118017487-11
ENSG0000(	947	21.44374	chr10:817	CASC2		lncRNA	chr10:118046279-11
ENSG0000(	947	21.44374	chr10:817	KCNK18		protein_c	chr10:117197489-11
ENSG0000(	947	21.44374	chr10:817	ENSG00000277879		lncRNA	chr10:117267116-11
ENSG0000(	947	21.44374	chr10:817	ENSG00000258114		lncRNA	chr10:117425194-11
ENSG0000(	947	21.44374	chr10:817	RAB11FIP2		protein_c	chr10:118004916-11
ENSG0000(	947	21.44374	chr10:817	RPL12P26		Pseudoger	chr10:117196168-11
ENSG0000(	947	21.44374	chr10:817	PDZD8		protein_c	chr10:117277274-11
ENSG0000(	947	21.44374	chr10:817	FAM204A		protein_c	chr10:118297925-11
ENSG0000(	947	21.44374	chr10:817	ENSG00000235742		Pseudoger	chr10:117023674-11
ENSG0000(	947	21.44374	chr10:817	MIR3663		smallRNA	chr10:117167678-11
ENSG0000(	947	21.44374	chr10:817	ENO4		protein_c	chr10:116849499-11
ENSG0000(	947	21.44374	chr10:817	LINC00867		lncRNA	chr10:118341437-11
ENSG0000(	947	21.44374	chr10:817	LINC02944		lncRNA	chr10:118241468-11
ENSG0000(	947	21.44374	chr10:817	SLC18A2-AS1		lncRNA	chr10:117238762-11
ENSG0000(	947	21.44374	chr10:817	TOMM22P5		Pseudoger	chr10:118643760-11
ENSG0000(	947	21.44374	chr10:817	PRLHR		protein_c	chr10:118589997-11
ENSG0000(	947	21.44374	chr10:817	RPL17P36		Pseudoger	chr10:118872054-11
ENSG0000(	947	21.44374	chr10:817	ENSG00000288172		lncRNA	chr10:117579854-11
ENSG0000(	947	21.44374	chr10:817	AL359836.1		smallRNA	chr10:117358356-11
ENSG0000(	947	21.44374	chr10:817	AL157788.1		smallRNA	chr10:119005790-11
ENSG0000(	947	21.44374	chr10:817	MIR3663HG		lncRNA	chr10:117144564-11
ENSG0000(	947	21.44374	chr10:817	EMX2OS		lncRNA	chr10:117473215-11
ENSG0000(	947	21.44374	chr10:817	LINC02674		lncRNA	chr10:117734510-11
ENSG0000(	939	21.26259	chr2:2577	AC009480.1		smallRNA	chr2:147940980-147
ENSG0000(	937	21.2173	chr2:2577	LINC02631		lncRNA	chr2:138601599-138
ENSG0000(	937	21.2173	chr2:2577	LINC01853		lncRNA	chr2:140103583-140
ENSG0000(	937	21.2173	chr2:2577	RNF14P1		Pseudoger	chr2:138412066-138
ENSG0000(	937	21.2173	chr2:2577	SPOPL-DT		lncRNA	chr2:138418284-138
ENSG0000(	937	21.2173	chr2:2577	AC078851.2		smallRNA	chr2:139826692-139
ENSG0000(	937	21.2173	chr2:2577	Y_RNA		smallRNA	chr2:137715332-137
ENSG0000(	937	21.2173	chr2:2577	AHCYP4		Pseudoger	chr2:138901433-138
ENSG0000(	937	21.2173	chr2:2577	HNMT		protein_c	chr2:137964020-138
ENSG0000(	937	21.2173	chr2:2577	RPL9P13		Pseudoger	chr2:139732708-139
ENSG0000(	937	21.2173	chr2:2577	MRPS18BP2		Pseudoger	chr2:139668547-139
ENSG0000(	937	21.2173	chr2:2577	YWHAEP5		Pseudoger	chr2:138288029-138
ENSG0000(	937	21.2173	chr2:2577	ENSG00000260059		lncRNA	chr2:138599663-138
ENSG0000(	937	21.2173	chr2:2577	ENSG00000286778		lncRNA	chr2:139323921-139
ENSG0000(	937	21.2173	chr2:2577	LRP1B	NCGv7	protein_c	chr2:140231423-142
ENSG0000(	937	21.2173	chr2:2577	ENSG00000287147		lncRNA	chr2:138917204-138
ENSG0000(	937	21.2173	chr2:2577	MTND1P27		Pseudoger	chr2:140220584-140
ENSG0000(	937	21.2173	chr2:2577	ENSG00000229131		lncRNA	chr2:139469775-139
ENSG0000(	937	21.2173	chr2:2577	RPL15P5		Pseudoger	chr2:138278574-138
ENSG0000(	937	21.2173	chr2:2577	RN7SKP286		smallRNA	chr2:138863597-138
ENSG0000(	937	21.2173	chr2:2577	COPRSP1		Pseudoger	chr2:140683360-140
ENSG0000(	937	21.2173	chr2:2577	ENSG00000226939		lncRNA	chr2:139366165-139
ENSG0000(	937	21.2173	chr2:2577	LINC01832		lncRNA	chr2:138101878-138
ENSG0000(	937	21.2173	chr2:2577	YY1P2		Pseudoger	chr2:138897151-138

ENSG0000(	937	21.2173	chr2:2577	MTCO1P44		Pseudoger	chr2:140216997-140
ENSG0000(	937	21.2173	chr2:2577	ENSG00000223554		lncRNA	chr2:139824896-139
ENSG0000(	937	21.2173	chr2:2577	AC020601.1		lncRNA	chr2:137878754-137
ENSG0000(	937	21.2173	chr2:2577	RNA5SP105		Pseudoger	chr2:137512098-137
ENSG0000(	937	21.2173	chr2:2577	NXPH2		protein_c	chr2:138669157-138
ENSG0000(	937	21.2173	chr2:2577	RN7SL283P		smallRNA	chr2:139982684-139
ENSG0000(	937	21.2173	chr2:2577	MTND2P19		Pseudoger	chr2:140217651-140
ENSG0000(	937	21.2173	chr2:2577	IDI1P1		Pseudoger	chr2:138307319-138
ENSG0000(	937	21.2173	chr2:2577	SNORA72		smallRNA	chr2:139511424-139
ENSG0000(	937	21.2173	chr2:2577	ENSG00000241772		lncRNA	chr2:138569090-138
ENSG0000(	937	21.2173	chr2:2577	SPOPL		protein_c	chr2:138501770-138
ENSG0000(	937	21.2173	chr2:2577	ENSG00000230569		lncRNA	chr2:138470656-138
ENSG0000(	934	21.14937	chrX:1578	SNORD112		smallRNA	chrX:55903587-5590
ENSG0000(	934	21.14937	chrX:1578	RNU6-434P		smallRNA	chrX:54343546-5434
ENSG0000(	934	21.14937	chrX:1578	SPIN2B		protein_c	chrX:57118551-5712
ENSG0000(	934	21.14937	chrX:1578	SPIN4		protein_c	chrX:63347228-6335
ENSG0000(	934	21.14937	chrX:1578	U3		smallRNA	chrX:54064845-5406
ENSG0000(	934	21.14937	chrX:1578	ENSG00000288661		protein_c	chrX:63754485-6375
ENSG0000(	934	21.14937	chrX:1578	ZXDA		protein_c	chrX:57905430-5791
ENSG0000(	934	21.14937	chrX:1578	PFN5P		Pseudoger	chrX:64405473-6440
ENSG0000(	934	21.14937	chrX:1578	NLRP2B		protein_c	chrX:57677067-5768
ENSG0000(	934	21.14937	chrX:1578	MTRNR2L10		protein_c	chrX:55181391-5518
ENSG0000(	934	21.14937	chrX:1578	ENSG00000287370		lncRNA	chrX:64205974-6423
ENSG0000(	934	21.14937	chrX:1578	KLF8	AC	protein_c	chrX:56232356-5629
ENSG0000(	934	21.14937	chrX:1578	MDH1P1		Pseudoger	chrX:57766320-5776
ENSG0000(	934	21.14937	chrX:1578	hsa-mir-4536-2		smallRNA	chrX:55451495-5545
ENSG0000(	934	21.14937	chrX:1578	MTCO1P52		Pseudoger	chrX:55178207-5517
ENSG0000(	934	21.14937	chrX:1578	MAGED2		protein_c	chrX:54807599-5481
ENSG0000(	934	21.14937	chrX:1578	MTND2P25		Pseudoger	chrX:62843697-6284
ENSG0000(	934	21.14937	chrX:1578	SPIN2A		protein_c	chrX:57134530-5713
ENSG0000(	934	21.14937	chrX:1578	ITIH6		protein_c	chrX:54748918-5479
ENSG0000(	934	21.14937	chrX:1578	ENSG00000186678		Pseudoger	chrX:55172717-5517
ENSG0000(	934	21.14937	chrX:1578	WNK3		protein_c	chrX:54192823-5435
ENSG0000(	934	21.14937	chrX:1578	HDGFL3P1		Pseudoger	chrX:55054945-5505
ENSG0000(	934	21.14937	chrX:1578	FGD1		protein_c	chrX:54445454-5449
ENSG0000(	934	21.14937	chrX:1578	ENSG00000236571		Pseudoger	chrX:53337783-5333
ENSG0000(	934	21.14937	chrX:1578	RIBC1		protein_c	chrX:53422690-5343
ENSG0000(	934	21.14937	chrX:1578	RNA5SP505		Pseudoger	chrX:53909054-5390
ENSG0000(	934	21.14937	chrX:1578	MTND2P24		Pseudoger	chrX:55179194-5517
ENSG0000(	934	21.14937	chrX:1578	ALAS2		protein_c	chrX:55009055-5503
ENSG0000(	934	21.14937	chrX:1578	ENSG00000226110		Pseudoger	chrX:53768986-5376
ENSG0000(	934	21.14937	chrX:1578	PGAM4P1		Pseudoger	chrX:54671985-5467
ENSG0000(	934	21.14937	chrX:1578	TSR2		protein_c	chrX:54440404-5444
ENSG0000(	934	21.14937	chrX:1578	MIR1468		smallRNA	chrX:63786002-6378
ENSG0000(	934	21.14937	chrX:1578	PAGE3		protein_c	chrX:55258415-5526
ENSG0000(	934	21.14937	chrX:1578	HUWE1	NCGv7	protein_c	chrX:53532096-5368
ENSG0000(	934	21.14937	chrX:1578	MRPS18CP7		Pseudoger	chrX:53825887-5382
ENSG0000(	934	21.14937	chrX:1578	Z97054.1		smallRNA	chrX:53486672-5348
ENSG0000(	934	21.14937	chrX:1578	FOXR2	NCGv7	protein_c	chrX:55623400-5562
ENSG0000(	934	21.14937	chrX:1578	PHF8		protein_c	chrX:53936676-5404
ENSG0000(	934	21.14937	chrX:1578	PAGE5		protein_c	chrX:55220346-5522

ENSG0000(	934	21.14937	chrX:1578	ENSG00000224735		Pseudoger	chrX:53759026-5375
ENSG0000(	934	21.14937	chrX:1578	ENSG00000233250		lncRNA	chrX:53432722-5343
ENSG0000(	934	21.14937	chrX:1578	CBX1P1		Pseudoger	chrX:63299247-6329
ENSG0000(	934	21.14937	chrX:1578	ARHGEF9-IT1		lncRNA	chrX:63670196-6367
ENSG0000(	934	21.14937	chrX:1578	MTHFD1P1		Pseudoger	chrX:57392646-5739
ENSG0000(	934	21.14937	chrX:1578	ENSG00000276929		Pseudoger	chrX:55137930-5514
ENSG0000(	934	21.14937	chrX:1578	PSMA5P1		Pseudoger	chrX:55599663-5560
ENSG0000(	934	21.14937	chrX:1578	MIR98		smallRNA	chrX:53556223-5355
ENSG0000(	934	21.14937	chrX:1578	TRO	NCGv7	protein_c	chrX:54920462-5493
ENSG0000(	934	21.14937	chrX:1578	MTND1P31		Pseudoger	chrX:62842542-6284
ENSG0000(	934	21.14937	chrX:1578	NBDY		protein_c	chrX:56729241-5681
ENSG0000(	934	21.14937	chrX:1578	SPIN3		protein_c	chrX:56818298-5699
ENSG0000(	934	21.14937	chrX:1578	PFKFB1		protein_c	chrX:54932961-5499
ENSG0000(	934	21.14937	chrX:1578	RRAGB		protein_c	chrX:55717749-5575
ENSG0000(	934	21.14937	chrX:1578	KRT8P17		Pseudoger	chrX:57984686-5798
ENSG0000(	934	21.14937	chrX:1578	ENSG00000278283		Pseudoger	chrX:55044749-5504
ENSG0000(	934	21.14937	chrX:1578	KDM5C	NCGv7;AC	protein_c	chrX:53176283-5322
ENSG0000(	934	21.14937	chrX:1578	AL353698.1		protein_c	chrX:56074324-5607
ENSG0000(	934	21.14937	chrX:1578	KDM5C-IT1		lncRNA	chrX:53212408-5321
ENSG0000(	934	21.14937	chrX:1578	ENSG00000286977		lncRNA	chrX:57222706-5722
ENSG0000(	934	21.14937	chrX:1578	ASB12		protein_c	chrX:64224194-6423
ENSG0000(	934	21.14937	chrX:1578	MAGEH1		protein_c	chrX:55452127-5545
ENSG0000(	934	21.14937	chrX:1578	MPV17L2P1		Pseudoger	chrX:53340986-5334
ENSG0000(	934	21.14937	chrX:1578	ENSG00000277499		Pseudoger	chrX:63087071-6308
ENSG0000(	934	21.14937	chrX:1578	MTND1P30		Pseudoger	chrX:55180377-5518
ENSG0000(	934	21.14937	chrX:1578	IQSEC2		protein_c	chrX:53225828-5332
ENSG0000(	934	21.14937	chrX:1578	FAM120C		protein_c	chrX:54068324-5418
ENSG0000(	934	21.14937	chrX:1578	ENSG00000275387		Pseudoger	chrX:54842014-5484
ENSG0000(	934	21.14937	chrX:1578	ARHGEF9		protein_c	chrX:63634967-6380
ENSG0000(	934	21.14937	chrX:1578	ENSG00000227329		Pseudoger	chrX:53164391-5316
ENSG0000(	934	21.14937	chrX:1578	BTF3P8		Pseudoger	chrX:63766875-6376
ENSG0000(	934	21.14937	chrX:1578	ENSG00000227486		lncRNA	chrX:55908123-5620
ENSG0000(	934	21.14937	chrX:1578	ZXDB	IntOGen-I	protein_c	chrX:57592011-5759
ENSG0000(	934	21.14937	chrX:1578	ZFRP1		Pseudoger	chrX:62878811-6288
ENSG0000(	934	21.14937	chrX:1578	ENSG00000230105		lncRNA	chrX:56618391-5662
ENSG0000(	934	21.14937	chrX:1578	ENSG00000224799		lncRNA	chrX:56973510-5697
ENSG0000(	934	21.14937	chrX:1578	MIRLET7F2		smallRNA	chrX:53557192-5355
ENSG0000(	934	21.14937	chrX:1578	RPL37P24		Pseudoger	chrX:54147372-5414
ENSG0000(	934	21.14937	chrX:1578	FAAH2	NCGv7	protein_c	chrX:57286706-5748
ENSG0000(	934	21.14937	chrX:1578	RPSAP62		Pseudoger	chrX:53322990-5332
ENSG0000(	934	21.14937	chrX:1578	PAGE2		protein_c	chrX:55089018-5509
ENSG0000(	934	21.14937	chrX:1578	UBQLN2		protein_c	chrX:56563627-5656
ENSG0000(	934	21.14937	chrX:1578	TIPINP1		Pseudoger	chrX:53456273-5345
ENSG0000(	934	21.14937	chrX:1578	MYCLP2		Pseudoger	chrX:57933837-5793
ENSG0000(	934	21.14937	chrX:1578	ENSG00000290748		lncRNA	chrX:55281371-5528
ENSG0000(	934	21.14937	chrX:1578	SSBL2P		Pseudoger	chrX:62779250-6278
ENSG0000(	934	21.14937	chrX:1578	AMER1	NCGv7	protein_c	chrX:64185117-6420
ENSG0000(	934	21.14937	chrX:1578	ENSG00000232765		Pseudoger	chrX:55279839-5528
ENSG0000(	934	21.14937	chrX:1578	ENSG00000288739		lncRNA	chrX:55488883-5549
ENSG0000(	934	21.14937	chrX:1578	ENSG00000226310		lncRNA	chrX:57121572-5713
ENSG0000(	934	21.14937	chrX:1578	HNRNPDP1		Pseudoger	chrX:64044305-6404

ENSG0000(	934	21.14937	chrX:1578	VTRNA3-1P		smallRNA	chrX:53462209-5346
ENSG0000(	934	21.14937	chrX:1578	RN7SL799P		smallRNA	chrX:64210690-6421
ENSG0000(	934	21.14937	chrX:1578	APEX2		protein_c	chrX:55000363-5500
ENSG0000(	934	21.14937	chrX:1578	GNL3L		protein_c	chrX:54530183-5462
ENSG0000(	934	21.14937	chrX:1578	USP51	NCGv7	protein_c	chrX:55484616-5548
ENSG0000(	934	21.14937	chrX:1578	FAM104B		protein_c	chrX:55143102-5516
ENSG0000(	934	21.14937	chrX:1578	ENSG00000169164		Pseudoger	chrX:55654709-5565
ENSG0000(	934	21.14937	chrX:1578	LINC01278		lncRNA	chrX:63222993-6356
ENSG0000(	934	21.14937	chrX:1578	Y_RNA		smallRNA	chrX:55582733-5558
ENSG0000(	934	21.14937	chrX:1578	ENSG00000278319		Pseudoger	chrX:55101637-5510
ENSG0000(	934	21.14937	chrX:1578	SMC1A	NCGv7	protein_c	chrX:53374149-5342
ENSG0000(	934	21.14937	chrX:1578	Y_RNA		smallRNA	chrX:53324562-5332
ENSG0000(	934	21.14937	chrX:1578	ENSG00000290520		lncRNA	chrX:55654721-5565
ENSG0000(	934	21.14937	chrX:1578	ENSG00000277516		Pseudoger	chrX:55520382-5552
ENSG0000(	934	21.14937	chrX:1578	MRPL32P2		Pseudoger	chrX:53807776-5380
ENSG0000(	934	21.14937	chrX:1578	SPIN2P1		Pseudoger	chrX:57068914-5706
ENSG0000(	934	21.14937	chrX:1578	SPIN4-AS1		lncRNA	chrX:63349646-6335
ENSG0000(	934	21.14937	chrX:1578	PAGE2B		protein_c	chrX:55075030-5507
ENSG0000(	934	21.14937	chrX:1578	GOT2P6		Pseudoger	chrX:55961208-5596
ENSG0000(	934	21.14937	chrX:1578	SNORA11		smallRNA	chrX:54927305-5492
ENSG0000(	934	21.14937	chrX:1578	HSD17B10		protein_c	chrX:53431258-5343
ENSG0000(	934	21.14937	chrX:1578	KPNA4P1		Pseudoger	chrX:64305047-6430
ENSG0000(	934	21.14937	chrX:1578	ENSG00000229594		Pseudoger	chrX:55633031-5563
ENSG0000(	934	21.14937	chrX:1578	MTMR8	NCGv7	protein_c	chrX:64268081-6439
ENSG0000(	934	21.14937	chrX:1578	RPL23AP83		Pseudoger	chrX:56242937-5624
ENSG0000(	934	21.14937	chrX:1578	RPL7AP71		Pseudoger	chrX:54223324-5422
ENSG0000(	934	21.14937	chrX:1578	UQCRBP1		Pseudoger	chrX:56737242-5673
ENSG0000(	934	21.14937	chrX:1578	PPP1R11P2		Pseudoger	chrX:57229034-5722
ENSG0000(	934	21.14937	chrX:1578	ENSG00000226010		Pseudoger	chrX:64144872-6414
ENSG0000(	929	21.03615	chr10:817	LINC02666		lncRNA	chr10:129784983-12
ENSG0000(	929	21.03615	chr10:817	ADAM12		protein_c	chr10:126012381-12
ENSG0000(	929	21.03615	chr10:817	ENSG00000258539		protein_c	chr10:124617080-12
ENSG0000(	929	21.03615	chr10:817	RPS8P4		Pseudoger	chr10:119638456-11
ENSG0000(	929	21.03615	chr10:817	ENSG00000286114		Pseudoger	chr10:122657498-12
ENSG0000(	929	21.03615	chr10:817	IKZF5		protein_c	chr10:122990807-12
ENSG0000(	929	21.03615	chr10:817	WDR11		protein_c	chr10:120851305-12
ENSG0000(	929	21.03615	chr10:817	INSYN2A		protein_c	chr10:127135426-12
ENSG0000(	929	21.03615	chr10:817	ENSG00000232935		lncRNA	chr10:127013501-12
ENSG0000(	929	21.03615	chr10:817	DMBT1L1		Pseudoger	chr10:122729429-12
ENSG0000(	929	21.03615	chr10:817	CUZD1		protein_c	chr10:122832158-12
ENSG0000(	929	21.03615	chr10:817	DENND10		protein_c	chr10:119104086-11
ENSG0000(	929	21.03615	chr10:817	ENSG00000232259		lncRNA	chr10:127929376-12
ENSG0000(	929	21.03615	chr10:817	RGS10		protein_c	chr10:119499817-11
ENSG0000(	929	21.03615	chr10:817	SFXN4	NCGv7	protein_c	chr10:119140767-11
ENSG0000(	929	21.03615	chr10:817	Y_RNA		smallRNA	chr10:130505106-13
ENSG0000(	929	21.03615	chr10:817	ENSG00000287361		lncRNA	chr10:128965406-12
ENSG0000(	929	21.03615	chr10:817	C10orf88		protein_c	chr10:122930901-12
ENSG0000(	929	21.03615	chr10:817	HTRA1	NCGv7	protein_c	chr10:122458551-12
ENSG0000(	929	21.03615	chr10:817	AL360176.1		smallRNA	chr10:125842944-12
ENSG0000(	929	21.03615	chr10:817	ENSG00000231138		lncRNA	chr10:123776670-12
ENSG0000(	929	21.03615	chr10:817	LINC02646		lncRNA	chr10:130213488-13

ENSG0000(	929	21.03615	chr10:817	RN7SKP167		smallRNA	chr10:121410916-12
ENSG0000(	929	21.03615	chr10:817	BTBD16		protein_c	chr10:122271296-12
ENSG0000(	929	21.03615	chr10:817	ENSG00000227076		lncRNA	chr10:127934698-12
ENSG0000(	929	21.03615	chr10:817	ARMS2		protein_c	chr10:122454653-12
ENSG0000(	929	21.03615	chr10:817	GNG10P1		Pseudoger	chr10:125960208-12
ENSG0000(	929	21.03615	chr10:817	ENSG00000282413		Pseudoger	chr10:119818866-11
ENSG0000(	929	21.03615	chr10:817	RPS26P39		Pseudoger	chr10:123171535-12
ENSG0000(	929	21.03615	chr10:817	RNA5SP328		Pseudoger	chr10:126145474-12
ENSG0000(	929	21.03615	chr10:817	C10orf88B		Pseudoger	chr10:122879681-12
ENSG0000(	929	21.03615	chr10:817	ENSG00000289087		lncRNA	chr10:129235841-12
ENSG0000(	929	21.03615	chr10:817	ENSG00000234134		lncRNA	chr10:125718771-12
ENSG0000(	929	21.03615	chr10:817	AL583860.1		smallRNA	chr10:126556963-12
ENSG0000(	929	21.03615	chr10:817	PPIAP32		Pseudoger	chr10:130131770-13
ENSG0000(	929	21.03615	chr10:817	RPL19P16		Pseudoger	chr10:121133090-12
ENSG0000(	929	21.03615	chr10:817	ENSG00000286135		protein_c	chr10:122672850-12
ENSG0000(	929	21.03615	chr10:817	ENSG00000280561		lncRNA	chr10:125707416-12
ENSG0000(	929	21.03615	chr10:817	ENSG00000227374		lncRNA	chr10:129768844-12
ENSG0000(	929	21.03615	chr10:817	PHACTR2P1		Pseudoger	chr10:119793589-11
ENSG0000(	929	21.03615	chr10:817	LINC02641		lncRNA	chr10:123356450-12
ENSG0000(	929	21.03615	chr10:817	SNORA19		smallRNA	chr10:119060011-11
ENSG0000(	929	21.03615	chr10:817	TXNP1		Pseudoger	chr10:119683127-11
ENSG0000(	929	21.03615	chr10:817	MKI67	NCGv7	protein_c	chr10:128096659-12
ENSG0000(	929	21.03615	chr10:817	Y_RNA		smallRNA	chr10:124814958-12
ENSG0000(	929	21.03615	chr10:817	HMX3		protein_c	chr10:123135970-12
ENSG0000(	929	21.03615	chr10:817	ENSG00000275327		lncRNA	chr10:129837505-12
ENSG0000(	929	21.03615	chr10:817	FAM53B-AS1		lncRNA	chr10:124703625-12
ENSG0000(	929	21.03615	chr10:817	NANOS1	NCGv7	protein_c	chr10:119029714-11
ENSG0000(	929	21.03615	chr10:817	AC009987.1		smallRNA	chr10:123734785-12
ENSG0000(	929	21.03615	chr10:817	ENSG00000287609		lncRNA	chr10:126393489-12
ENSG0000(	929	21.03615	chr10:817	OAT		protein_c	chr10:124397303-12
ENSG0000(	929	21.03615	chr10:817	ENSG00000288757		lncRNA	chr10:123997144-12
ENSG0000(	929	21.03615	chr10:817	AL353664.1		smallRNA	chr10:120124132-12
ENSG0000(	929	21.03615	chr10:817	AC068058.1		smallRNA	chr10:123911479-12
ENSG0000(	929	21.03615	chr10:817	ENSG00000286344		lncRNA	chr10:123560326-12
ENSG0000(	929	21.03615	chr10:817	ENSG00000227307		lncRNA	chr10:120925547-12
ENSG0000(	929	21.03615	chr10:817	TEX36		protein_c	chr10:125576522-12
ENSG0000(	929	21.03615	chr10:817	DMBT1		protein_c	chr10:122560665-12
ENSG0000(	929	21.03615	chr10:817	ENSG00000289400		lncRNA	chr10:129035305-12
ENSG0000(	929	21.03615	chr10:817	AL355531.1		smallRNA	chr10:129487869-12
ENSG0000(	929	21.03615	chr10:817	RNU2-42P		smallRNA	chr10:125890566-12
ENSG0000(	929	21.03615	chr10:817	ENSG00000285615		lncRNA	chr10:129372194-12
ENSG0000(	929	21.03615	chr10:817	BUB1P1		Pseudoger	chr10:127867881-12
ENSG0000(	929	21.03615	chr10:817	HMX2		protein_c	chr10:123148136-12
ENSG0000(	929	21.03615	chr10:817	ENSG00000236426		lncRNA	chr10:119330233-11
ENSG0000(	929	21.03615	chr10:817	SNORA19		smallRNA	chr10:119060983-11
ENSG0000(	929	21.03615	chr10:817	ENSG00000237224		lncRNA	chr10:129693722-12
ENSG0000(	929	21.03615	chr10:817	ENSG00000275005		lncRNA	chr10:129845328-12
ENSG0000(	929	21.03615	chr10:817	LINC00601		lncRNA	chr10:126413869-12
ENSG0000(	929	21.03615	chr10:817	ENSG00000287427		lncRNA	chr10:128026878-12
ENSG0000(	929	21.03615	chr10:817	ENSG00000289280		lncRNA	chr10:124915447-12
ENSG0000(	929	21.03615	chr10:817	ENSG00000285529		lncRNA	chr10:130525712-13

ENSG0000(	929	21.03615	chr10:817	MCMBP	NCGv7	protein_c	chr10:119829404-11
ENSG0000(	929	21.03615	chr10:817	ENSG00000287466		lncRNA	chr10:129621988-12
ENSG0000(	929	21.03615	chr10:817	FGFR2	NCGv7;AC	protein_c	chr10:121478332-12
ENSG0000(	929	21.03615	chr10:817	AL391005.1		Pseudoger	chr10:127736059-12
ENSG0000(	929	21.03615	chr10:817	RPL21P16		Pseudoger	chr10:120354701-12
ENSG0000(	929	21.03615	chr10:817	ENSG00000282787		lncRNA	chr10:125162379-12
ENSG0000(	929	21.03615	chr10:817	CTBP2	AC	protein_c	chr10:124984317-12
ENSG0000(	929	21.03615	chr10:817	ENSG00000289126		lncRNA	chr10:119080929-11
ENSG0000(	929	21.03615	chr10:817	UROS		protein_c	chr10:125784980-12
ENSG0000(	929	21.03615	chr10:817	ENSG00000232334		Pseudoger	chr10:124004304-12
ENSG0000(	929	21.03615	chr10:817	EDRF1-AS1		lncRNA	chr10:125725634-12
ENSG0000(	929	21.03615	chr10:817	TACC2		protein_c	chr10:121989163-12
ENSG0000(	929	21.03615	chr10:817	LINC01163		lncRNA	chr10:128181032-12
ENSG0000(	929	21.03615	chr10:817	SEC23IP		protein_c	chr10:119892707-11
ENSG0000(	929	21.03615	chr10:817	ATE1		protein_c	chr10:121709393-12
ENSG0000(	929	21.03615	chr10:817	NSMCE4A		protein_c	chr10:121957091-12
ENSG0000(	929	21.03615	chr10:817	PLEKHA1		protein_c	chr10:122374696-12
ENSG0000(	929	21.03615	chr10:817	CLRN3		protein_c	chr10:127877841-12
ENSG0000(	929	21.03615	chr10:817	FANK1	NCGv7	protein_c	chr10:125896564-12
ENSG0000(	929	21.03615	chr10:817	ENSG00000287326		lncRNA	chr10:126425930-12
ENSG0000(	929	21.03615	chr10:817	EEF1AKMT2		protein_c	chr10:124748149-12
ENSG0000(	929	21.03615	chr10:817	FAM24A		protein_c	chr10:122910610-12
ENSG0000(	929	21.03615	chr10:817	PLPP4		protein_c	chr10:120457227-12
ENSG0000(	929	21.03615	chr10:817	ENSG00000225424		lncRNA	chr10:129278251-12
ENSG0000(	929	21.03615	chr10:817	EDRF1-DT		lncRNA	chr10:125700436-12
ENSG0000(	929	21.03615	chr10:817	FOXI2		protein_c	chr10:127737185-12
ENSG0000(	929	21.03615	chr10:817	ENSG00000285955		lncRNA	chr10:122435924-12
ENSG0000(	929	21.03615	chr10:817	ENSG00000224250		lncRNA	chr10:121736303-12
ENSG0000(	929	21.03615	chr10:817	ENSG00000273599		lncRNA	chr10:124996064-12
ENSG0000(	929	21.03615	chr10:817	YBX2P1		Pseudoger	chr10:123991910-12
ENSG0000(	929	21.03615	chr10:817	ENSG00000277218		lncRNA	chr10:130104569-13
ENSG0000(	929	21.03615	chr10:817	ENSG00000234640		lncRNA	chr10:128316282-12
ENSG0000(	929	21.03615	chr10:817	FAM53B		protein_c	chr10:124619292-12
ENSG0000(	929	21.03615	chr10:817	ENSG00000285715		lncRNA	chr10:122017982-12
ENSG0000(	929	21.03615	chr10:817	RPS27P18		Pseudoger	chr10:125473686-12
ENSG0000(	929	21.03615	chr10:817	GRK5		protein_c	chr10:119207571-11
ENSG0000(	929	21.03615	chr10:817	ACADSB		protein_c	chr10:123008979-12
ENSG0000(	929	21.03615	chr10:817	LHPP		protein_c	chr10:124461823-12
ENSG0000(	929	21.03615	chr10:817	EDRF1		protein_c	chr10:125719515-12
ENSG0000(	929	21.03615	chr10:817	ENSG00000273891		lncRNA	chr10:121965764-12
ENSG0000(	929	21.03615	chr10:817	ENSG00000285973		lncRNA	chr10:122145106-12
ENSG0000(	929	21.03615	chr10:817	EIF3A	NCGv7	protein_c	chr10:119033670-11
ENSG0000(	929	21.03615	chr10:817	AC073587.1		smallRNA	chr10:120463834-12
ENSG0000(	929	21.03615	chr10:817	ENSG00000228021		lncRNA	chr10:125683229-12
ENSG0000(	929	21.03615	chr10:817	MIR4681		smallRNA	chr10:119377972-11
ENSG0000(	929	21.03615	chr10:817	EBF3-AS1		lncRNA	chr10:129857638-12
ENSG0000(	929	21.03615	chr10:817	RAD1P1		Pseudoger	chr10:119621068-11
ENSG0000(	929	21.03615	chr10:817	GPR26		protein_c	chr10:123666355-12
ENSG0000(	929	21.03615	chr10:817	ENSG00000234542		lncRNA	chr10:123574227-12
ENSG0000(	929	21.03615	chr10:817	ENSG00000234522		Pseudoger	chr10:120434983-12
ENSG0000(	929	21.03615	chr10:817	BUB3		protein_c	chr10:123154402-12

ENSG0000(	929	21.03615	chr10:817	LINC01153		lncRNA	chr10:121178700-12
ENSG0000(	929	21.03615	chr10:817	AC012391.1		smallRNA	chr10:123216102-12
ENSG0000(	929	21.03615	chr10:817	ENSG00000279725		TEC	chr10:127734562-12
ENSG0000(	929	21.03615	chr10:817	MMP21		protein_c	chr10:125753580-12
ENSG0000(	929	21.03615	chr10:817	C10orf90	NCGv7	protein_c	chr10:126424997-12
ENSG0000(	929	21.03615	chr10:817	NPM1P31		Pseudoger	chr10:124867510-12
ENSG0000(	929	21.03615	chr10:817	ENSG00000278831		lncRNA	chr10:124623353-12
ENSG0000(	929	21.03615	chr10:817	ENSG00000270902		Pseudoger	chr10:123323422-12
ENSG0000(	929	21.03615	chr10:817	Y_RNA		smallRNA	chr10:126920807-12
ENSG0000(	929	21.03615	chr10:817	MIR3941		smallRNA	chr10:122416965-12
ENSG0000(	929	21.03615	chr10:817	SNORD60		smallRNA	chr10:126779702-12
ENSG0000(	929	21.03615	chr10:817	CTAGE7P		Pseudoger	chr10:130106046-13
ENSG0000(	929	21.03615	chr10:817	ENSG00000271670		lncRNA	chr10:120879256-12
ENSG0000(	929	21.03615	chr10:817	RNU6-728P		smallRNA	chr10:122345690-12
ENSG0000(	929	21.03615	chr10:817	ENSG00000286088		protein_c	chr10:122832167-12
ENSG0000(	929	21.03615	chr10:817	CHST15		protein_c	chr10:124007668-12
ENSG0000(	929	21.03615	chr10:817	TEX36-AS1		lncRNA	chr10:125574371-12
ENSG0000(	929	21.03615	chr10:817	ENSG00000226899		lncRNA	chr10:124945204-12
ENSG0000(	929	21.03615	chr10:817	MIR4682		smallRNA	chr10:119958513-11
ENSG0000(	929	21.03615	chr10:817	ENSG00000289966		lncRNA	chr10:119883898-11
ENSG0000(	929	21.03615	chr10:817	NACAP2		Pseudoger	chr10:119997086-11
ENSG0000(	929	21.03615	chr10:817	BCCIP		protein_c	chr10:125823546-12
ENSG0000(	929	21.03615	chr10:817	MIR4297		smallRNA	chr10:129843299-12
ENSG0000(	929	21.03615	chr10:817	CPXM2	NCGv7	protein_c	chr10:123706207-12
ENSG0000(	929	21.03615	chr10:817	C10orf143		protein_c	chr10:130020025-13
ENSG0000(	929	21.03615	chr10:817	PSTK		protein_c	chr10:122954381-12
ENSG0000(	929	21.03615	chr10:817	TIAL1	NCGv7	protein_c	chr10:119571802-11
ENSG0000(	929	21.03615	chr10:817	MGMT	NCGv7	protein_c	chr10:129467190-12
ENSG0000(	929	21.03615	chr10:817	Y_RNA		smallRNA	chr10:124766196-12
ENSG0000(	929	21.03615	chr10:817	ENSG00000276742		lncRNA	chr10:121956782-12
ENSG0000(	929	21.03615	chr10:817	ENSG00000249456		lncRNA	chr10:124917143-12
ENSG0000(	929	21.03615	chr10:817	BAG3		protein_c	chr10:119651380-11
ENSG0000(	929	21.03615	chr10:817	ENSG00000223455		Pseudoger	chr10:120069980-12
ENSG0000(	929	21.03615	chr10:817	DHX32		protein_c	chr10:125836337-12
ENSG0000(	929	21.03615	chr10:817	ENSG00000278484		lncRNA	chr10:120984966-12
ENSG0000(	929	21.03615	chr10:817	WDR11-DT		lncRNA	chr10:120759898-12
ENSG0000(	929	21.03615	chr10:817	ENSG00000223432		lncRNA	chr10:121615425-12
ENSG0000(	929	21.03615	chr10:817	FANK1-AS1		lncRNA	chr10:125972188-12
ENSG0000(	929	21.03615	chr10:817	Y_RNA		smallRNA	chr10:121846600-12
ENSG0000(	929	21.03615	chr10:817	MIR4296		smallRNA	chr10:125032783-12
ENSG0000(	929	21.03615	chr10:817	ENSG00000232985		lncRNA	chr10:128958772-12
ENSG0000(	929	21.03615	chr10:817	ABRAXAS2		protein_c	chr10:124801819-12
ENSG0000(	929	21.03615	chr10:817	RPS10P18		Pseudoger	chr10:124489823-12
ENSG0000(	929	21.03615	chr10:817	LINC01561		lncRNA	chr10:120597949-12
ENSG0000(	929	21.03615	chr10:817	PRDX3		protein_c	chr10:119167720-11
ENSG0000(	929	21.03615	chr10:817	RN7SL846P		smallRNA	chr10:119768247-11
ENSG0000(	929	21.03615	chr10:817	C10orf120		protein_c	chr10:122697709-12
ENSG0000(	929	21.03615	chr10:817	GRK5-IT1		lncRNA	chr10:119208531-11
ENSG0000(	929	21.03615	chr10:817	ENSG00000276375		Pseudoger	chr10:130282445-13
ENSG0000(	929	21.03615	chr10:817	PTPRE		protein_c	chr10:127907103-12
ENSG0000(	929	21.03615	chr10:817	RP13-238F13.3		lncRNA	chr10:124447713-12

ENSG0000(	929	21.03615	chr10:817	ZRANB1	protein_c	chr10:124942123-12
ENSG0000(	929	21.03615	chr10:817	ENSG00000287768	lncRNA	chr10:123943255-12
ENSG0000(	929	21.03615	chr10:817	MRPS21P6	Pseudoger	chr10:125166677-12
ENSG0000(	929	21.03615	chr10:817	ENSG00000271353	Pseudoger	chr10:119562101-11
ENSG0000(	929	21.03615	chr10:817	RN7SL749P	smallRNA	chr10:119212857-11
ENSG0000(	929	21.03615	chr10:817	SAR1AP2	Pseudoger	chr10:126272818-12
ENSG0000(	929	21.03615	chr10:817	NKX1-2	protein_c	chr10:124445243-12
ENSG0000(	929	21.03615	chr10:817	LINC02930	lncRNA	chr10:120608580-12
ENSG0000(	929	21.03615	chr10:817	FAM24B	protein_c	chr10:122849078-12
ENSG0000(	929	21.03615	chr10:817	MIR4484	smallRNA	chr10:125819740-12
ENSG0000(	929	21.03615	chr10:817	ENSG00000287936	lncRNA	chr10:120842521-12
ENSG0000(	929	21.03615	chr10:817	ATE1-AS1	Pseudoger	chr10:121928056-12
ENSG0000(	929	21.03615	chr10:817	INPP5F	protein_c	chr10:119726042-11
ENSG0000(	929	21.03615	chr10:817	ENSG00000234677	lncRNA	chr10:123913574-12
ENSG0000(	929	21.03615	chr10:817	LINC02667	lncRNA	chr10:128912810-12
ENSG0000(	929	21.03615	chr10:817	ENSG00000271343	Pseudoger	chr10:119093140-11
ENSG0000(	929	21.03615	chr10:817	ENSG00000225152	lncRNA	chr10:123425713-12
ENSG0000(	929	21.03615	chr10:817	RPS15AP5	Pseudoger	chr10:121713607-12
ENSG0000(	929	21.03615	chr10:817	ENSG00000223528	lncRNA	chr10:126988095-12
ENSG0000(	929	21.03615	chr10:817	NPS	protein_c	chr10:127549309-12
ENSG0000(	929	21.03615	chr10:817	GLRX3	protein_c	chr10:130136391-13
ENSG0000(	929	21.03615	chr10:817	EBF3	protein_c	chr10:129835233-12
ENSG0000(	929	21.03615	chr10:817	ALDOAP2	Pseudoger	chr10:125666875-12
ENSG0000(	927	20.99086	chr2:8187	AC074391.2	smallRNA	chr2:66041250-6604
ENSG0000(	917	20.76443	chr2:2577	ENSG00000235435	lncRNA	chr2:145294277-145
ENSG0000(	917	20.76443	chr2:2577	ENSG00000281772	TEC	chr2:148062154-148
ENSG0000(	917	20.76443	chr2:2577	RND3	protein_c	chr2:150468195-150
ENSG0000(	917	20.76443	chr2:2577	METAP2P1	Pseudoger	chr2:145826285-145
ENSG0000(	917	20.76443	chr2:2577	RNU6-715P	smallRNA	chr2:147495438-147
ENSG0000(	917	20.76443	chr2:2577	ORC4	protein_c	chr2:147930396-148
ENSG0000(	917	20.76443	chr2:2577	USP12P2	Pseudoger	chr2:148295656-148
ENSG0000(	917	20.76443	chr2:2577	RN7SL124P	smallRNA	chr2:151372003-151
ENSG0000(	917	20.76443	chr2:2577	ENSG00000271583	Pseudoger	chr2:146833501-146
ENSG0000(	917	20.76443	chr2:2577	LINC01931	lncRNA	chr2:149745648-149
ENSG0000(	917	20.76443	chr2:2577	ENSG00000236885	lncRNA	chr2:151114811-151
ENSG0000(	917	20.76443	chr2:2577	snoU13	smallRNA	chr2:147323971-147
ENSG0000(	917	20.76443	chr2:2577	ENSG00000281469	TEC	chr2:148044380-148
ENSG0000(	917	20.76443	chr2:2577	LINC02612	lncRNA	chr2:150612381-150
ENSG0000(	917	20.76443	chr2:2577	Y_RNA	smallRNA	chr2:147472133-147
ENSG0000(	917	20.76443	chr2:2577	UBBP3	Pseudoger	chr2:148863564-148
ENSG0000(	917	20.76443	chr2:2577	LYPD6	protein_c	chr2:149329985-149
ENSG0000(	917	20.76443	chr2:2577	RPL17P13	Pseudoger	chr2:149439897-149
ENSG0000(	917	20.76443	chr2:2577	LINC01911	lncRNA	chr2:146682289-146
ENSG0000(	917	20.76443	chr2:2577	ENSG00000225107	lncRNA	chr2:145569294-145
ENSG0000(	917	20.76443	chr2:2577	RPS29P8	Pseudoger	chr2:148595158-148
ENSG0000(	917	20.76443	chr2:2577	RNA5SP106	Pseudoger	chr2:147713455-147
ENSG0000(	917	20.76443	chr2:2577	ENSG00000289474	lncRNA	chr2:148881726-148
ENSG0000(	917	20.76443	chr2:2577	EPC2	protein_c	chr2:148644440-148
ENSG0000(	917	20.76443	chr2:2577	RNU7-2P	smallRNA	chr2:146145156-146
ENSG0000(	917	20.76443	chr2:2577	RPL17P12	Pseudoger	chr2:146194296-146
ENSG0000(	917	20.76443	chr2:2577	PABPC1P2	Pseudoger	chr2:146587506-146

ENSG0000(	917	20.76443	chr2:2577	KIF5C-AS1		lncRNA	chr2:148866470-148
ENSG0000(	917	20.76443	chr2:2577	ENSG00000286225		lncRNA	chr2:145572662-145
ENSG0000(	917	20.76443	chr2:2577	MIR4773-1		smallRNA	chr2:151368334-151
ENSG0000(	917	20.76443	chr2:2577	ACVR2A	NCGv7	protein_c	chr2:147844517-147
ENSG0000(	917	20.76443	chr2:2577	ENSG00000279876		TEC	chr2:145874520-145
ENSG0000(	917	20.76443	chr2:2577	LINC01920		lncRNA	chr2:150552532-150
ENSG0000(	917	20.76443	chr2:2577	TXNP5		Pseudoger	chr2:149068596-149
ENSG0000(	917	20.76443	chr2:2577	MBD5		protein_c	chr2:148021011-148
ENSG0000(	917	20.76443	chr2:2577	LINC01818		lncRNA	chr2:150169474-150
ENSG0000(	917	20.76443	chr2:2577	RPS20P13		Pseudoger	chr2:148870024-148
ENSG0000(	917	20.76443	chr2:2577	RNU6-601P		smallRNA	chr2:149610668-149
ENSG0000(	917	20.76443	chr2:2577	ENSG00000228064		lncRNA	chr2:150982506-151
ENSG0000(	917	20.76443	chr2:2577	RNU6-692P		smallRNA	chr2:147408342-147
ENSG0000(	917	20.76443	chr2:2577	FAM8A3P		Pseudoger	chr2:149320593-149
ENSG0000(	917	20.76443	chr2:2577	ENSG00000223911		lncRNA	chr2:147899401-147
ENSG0000(	917	20.76443	chr2:2577	ENSG00000286081		lncRNA	chr2:150150633-150
ENSG0000(	917	20.76443	chr2:2577	ENSG00000286335		lncRNA	chr2:149170561-149
ENSG0000(	917	20.76443	chr2:2577	ENSG00000222031		lncRNA	chr2:151001220-151
ENSG0000(	917	20.76443	chr2:2577	LYPD6B		protein_c	chr2:149038107-149
ENSG0000(	917	20.76443	chr2:2577	ENSG00000232359		lncRNA	chr2:150595102-150
ENSG0000(	917	20.76443	chr2:2577	RIF1		protein_c	chr2:151409883-151
ENSG0000(	917	20.76443	chr2:2577	MMADHC-DT		lncRNA	chr2:149587196-150
ENSG0000(	917	20.76443	chr2:2577	snR65		smallRNA	chr2:145335070-145
ENSG0000(	917	20.76443	chr2:2577	LINC02993		lncRNA	chr2:144523541-144
ENSG0000(	917	20.76443	chr2:2577	SGCEP1		Pseudoger	chr2:144754776-144
ENSG0000(	917	20.76443	chr2:2577	LINC01412		lncRNA	chr2:144521868-144
ENSG0000(	917	20.76443	chr2:2577	RNU2-9P		smallRNA	chr2:148825691-148
ENSG0000(	917	20.76443	chr2:2577	RNU6-1275P		smallRNA	chr2:147877422-147
ENSG0000(	917	20.76443	chr2:2577	ZEB2-AS1		lncRNA	chr2:144517978-144
ENSG0000(	917	20.76443	chr2:2577	ENSG00000286167		lncRNA	chr2:148909406-148
ENSG0000(	917	20.76443	chr2:2577	RBM43		protein_c	chr2:151247940-151
ENSG0000(	917	20.76443	chr2:2577	FABP5P10		Pseudoger	chr2:151186188-151
ENSG0000(	917	20.76443	chr2:2577	AC105402.1		smallRNA	chr2:148891878-148
ENSG0000(	917	20.76443	chr2:2577	AC013406.1		smallRNA	chr2:147466630-147
ENSG0000(	917	20.76443	chr2:2577	USP8P2		Pseudoger	chr2:148872253-148
ENSG0000(	917	20.76443	chr2:2577	SNORA48		smallRNA	chr2:148260574-148
ENSG0000(	917	20.76443	chr2:2577	ENSG00000226218		lncRNA	chr2:145600907-145
ENSG0000(	917	20.76443	chr2:2577	ENSG00000237220		lncRNA	chr2:150566134-150
ENSG0000(	917	20.76443	chr2:2577	ENSG00000229143		Pseudoger	chr2:147810346-147
ENSG0000(	917	20.76443	chr16:231	ENSG00000260438		Pseudoger	chr16:57713782-577
ENSG0000(	917	20.76443	chr2:2577	MMADHC		protein_c	chr2:149569637-149
ENSG0000(	917	20.76443	chr2:2577	TNFAIP6	NCGv7	protein_c	chr2:151357592-151
ENSG0000(	917	20.76443	chr2:2577	NMI		protein_c	chr2:151270470-151
ENSG0000(	917	20.76443	chr2:2577	TEX41		lncRNA	chr2:144666312-145
ENSG0000(	917	20.76443	chr2:2577	OTX2P2		Pseudoger	chr2:146477427-146
ENSG0000(	917	20.76443	chr2:2577	LINC01966		lncRNA	chr2:144877734-144
ENSG0000(	917	20.76443	chr2:2577	RPL6P5		Pseudoger	chr2:145337230-145
ENSG0000(	917	20.76443	chr2:2577	ENSG00000233842		lncRNA	chr2:145872209-145
ENSG0000(	917	20.76443	chr2:2577	LINC01817		lncRNA	chr2:150234892-150
ENSG0000(	917	20.76443	chr2:2577	ENSG00000287847		lncRNA	chr2:147150707-147
ENSG0000(	917	20.76443	chr2:2577	ENSG00000234940		lncRNA	chr2:144688413-144

ENSG0000(	913	20.67385	chrX:904	ENSG00000203262	Pseudoger	chrX:96070669-9607
ENSG0000(	913	20.67385	chrX:904	EIF4A1P10	Pseudoger	chrX:92113246-9211
ENSG0000(	913	20.67385	chrX:904	TUSC2P2	Pseudoger	chrX:92770855-9277
ENSG0000(	913	20.67385	chrX:904	DIAPH2-AS1	lncRNA	chrX:97431286-9764
ENSG0000(	913	20.67385	chrX:904	ENSG00000289479	lncRNA	chrX:92100276-9210
ENSG0000(	913	20.67385	chrX:904	BRDTP1	Pseudoger	chrX:96337236-9633
ENSG0000(	913	20.67385	chrX:904	ENSG00000236413	Pseudoger	chrX:91422309-9142
ENSG0000(	913	20.67385	chrX:904	PAICSP7	Pseudoger	chrX:94636663-9463
ENSG0000(	913	20.67385	chrX:904	RPS7P13	Pseudoger	chrX:95679130-9567
ENSG0000(	913	20.67385	chrX:904	RNU2-26P	smallRNA	chrX:91798093-9179
ENSG0000(	913	20.67385	chrX:904	RN7SL379P	smallRNA	chrX:96023360-9602
ENSG0000(	913	20.67385	chrX:904	RPS29P28	Pseudoger	chrX:96195477-9619
ENSG0000(	913	20.67385	chrX:904	AL121869.1	smallRNA	chrX:92460452-9246
ENSG0000(	913	20.67385	chrX:904	AL390966.1	smallRNA	chrX:95134279-9513
ENSG0000(	913	20.67385	chrX:904	PCDH11X NCGv7	protein_c	chrX:91779261-9262
ENSG0000(	913	20.67385	chrX:904	PABPC5-AS1	lncRNA	chrX:91414878-9143
ENSG0000(	913	20.67385	chrX:904	NAP1L3	protein_c	chrX:93670930-9367
ENSG0000(	913	20.67385	chrX:904	PABPC5	protein_c	chrX:91434595-9143
ENSG0000(	913	20.67385	chrX:904	SKP2P1	Pseudoger	chrX:96620654-9662
ENSG0000(	913	20.67385	chrX:904	AP2B1P1	Pseudoger	chrX:93222220-9322
ENSG0000(	913	20.67385	chrX:904	KAT7P1	Pseudoger	chrX:95973328-9597
ENSG0000(	913	20.67385	chrX:904	Y_RNA	smallRNA	chrX:96701507-9670
ENSG0000(	913	20.67385	chrX:904	AL591708.1	smallRNA	chrX:94893501-9489
ENSG0000(	913	20.67385	chrX:904	DLGAP5P2	Pseudoger	chrX:93042490-9304
ENSG0000(	913	20.67385	chrX:904	RPL26P36	Pseudoger	chrX:92676519-9267
ENSG0000(	913	20.67385	chrX:904	RPL6P29	Pseudoger	chrX:98251679-9825
ENSG0000(	913	20.67385	chrX:904	HNRNPDLP3	Pseudoger	chrX:95610578-9561
ENSG0000(	913	20.67385	chrX:904	NT5DC1P1	Pseudoger	chrX:93388075-9338
ENSG0000(	913	20.67385	chrX:904	ENSG00000233887	Pseudoger	chrX:97847263-9784
ENSG0000(	913	20.67385	chrX:904	KRT18P11	Pseudoger	chrX:92459670-9246
ENSG0000(	913	20.67385	chrX:904	SERBP1P4	Pseudoger	chrX:91560436-9156
ENSG0000(	913	20.67385	chrX:904	USP37P1	Pseudoger	chrX:93776911-9377
ENSG0000(	913	20.67385	chrX:904	ENSG00000270583	Pseudoger	chrX:95720243-9572
ENSG0000(	913	20.67385	chrX:904	CCNB1IP1P3	Pseudoger	chrX:94776739-9477
ENSG0000(	913	20.67385	chrX:904	KPNB1P1	Pseudoger	chrX:97975878-9797
ENSG0000(	913	20.67385	chrX:904	RN7SL74P	smallRNA	chrX:97341046-9734
ENSG0000(	913	20.67385	chrX:904	RPA4	protein_c	chrX:96883908-9688
ENSG0000(	913	20.67385	chrX:904	RNA5SP510	Pseudoger	chrX:95582064-9558
ENSG0000(	913	20.67385	chrX:904	NDUFB5P2	Pseudoger	chrX:96953630-9695
ENSG0000(	913	20.67385	chrX:904	FAM133A NCGv7	protein_c	chrX:93674013-9371
ENSG0000(	913	20.67385	chrX:904	VDAC1P3	Pseudoger	chrX:91982655-9198
ENSG0000(	913	20.67385	chrX:904	GTF3C6P1	Pseudoger	chrX:95584303-9558
ENSG0000(	913	20.67385	chrX:904	RPL7P55	Pseudoger	chrX:93573834-9357
ENSG0000(	913	20.67385	chrX:904	RNU6-332P	smallRNA	chrX:94003815-9400
ENSG0000(	913	20.67385	chrX:904	Y_RNA	smallRNA	chrX:94178005-9417
ENSG0000(	913	20.67385	chrX:904	CALM1P1	Pseudoger	chrX:95499604-9550
ENSG0000(	913	20.67385	chrX:904	MIR548M	smallRNA	chrX:95063141-9506
ENSG0000(	913	20.67385	chrX:904	ENSG00000206062	lncRNA	chrX:91307781-9130
ENSG0000(	913	20.67385	chrX:904	ST13P18	Pseudoger	chrX:93287733-9328
ENSG0000(	913	20.67385	chrX:904	RN7SKP194	smallRNA	chrX:96410432-9641
ENSG0000(	913	20.67385	chrX:904	NCKAP1P1	Pseudoger	chrX:97906644-9790

ENSG0000(	913	20.67385	chrX:904:	TUBB4BP8		Pseudoger	chrX:94319038-9432
ENSG0000(	913	20.67385	chrX:904:	HNRNPDLP1		Pseudoger	chrX:95638375-9563
ENSG0000(	913	20.67385	chrX:904:	ENSG00000286523		lncRNA	chrX:96127747-9637
ENSG0000(	913	20.67385	chrX:904:	SNX3P1X		Pseudoger	chrX:92515323-9251
ENSG0000(	913	20.67385	chrX:904:	ENSG00000271094		Pseudoger	chrX:95453369-9545
ENSG0000(	913	20.67385	chrX:904:	RNU6-555P		smallRNA	chrX:90597235-9059
ENSG0000(	913	20.67385	chrX:904:	DIAPH2	NCGv7	protein_c	chrX:96684712-9760
ENSG0000(	912	20.65121	chr4:253(	RN7SKP35		smallRNA	chr4:151576766-151
ENSG0000(	908	20.56063	chrX:478(	MIR325		smallRNA	chrX:77005404-7700
ENSG0000(	908	20.56063	chrX:478(	RPS23P8		Pseudoger	chrX:70962964-7096
ENSG0000(	908	20.56063	chrX:478(	AWAT2		protein_c	chrX:70040542-7004
ENSG0000(	908	20.56063	chrX:478(	RPS6P26		Pseudoger	chrX:74376125-7437
ENSG0000(	908	20.56063	chrX:478(	AWAT1		protein_c	chrX:70234655-7024
ENSG0000(	908	20.56063	chrX:478(	CXorf65		protein_c	chrX:71103889-7110
ENSG0000(	908	20.56063	chrX:478(	JPX		lncRNA	chrX:73944182-7407
ENSG0000(	908	20.56063	chrX:478(	NHSL2		protein_c	chrX:71910845-7216
ENSG0000(	908	20.56063	chrX:478(	NAP1L6P		Pseudoger	chrX:73126037-7312
ENSG0000(	908	20.56063	chrX:478(	UHRF2P1		Pseudoger	chrX:74105572-7410
ENSG0000(	908	20.56063	chrX:478(	CHIC1	AC	protein_c	chrX:73563197-7368
ENSG0000(	908	20.56063	chrX:478(	BMP2KL		Pseudoger	chrX:74185929-7418
ENSG0000(	908	20.56063	chrX:478(	KIF4CP		Pseudoger	chrX:79323446-7932
ENSG0000(	908	20.56063	chrX:478(	RPL7P54		Pseudoger	chrX:78763226-7876
ENSG0000(	908	20.56063	chr2:2577	NUDCP1		Pseudoger	chr2:152389937-152
ENSG0000(	908	20.56063	chrX:478(	P2RY10BP		Pseudoger	chrX:79084936-7908
ENSG0000(	908	20.56063	chrX:478(	TPT1P15		Pseudoger	chrX:72159845-7216
ENSG0000(	908	20.56063	chrX:478(	RTL3		protein_c	chrX:78656068-7865
ENSG0000(	908	20.56063	chrX:478(	RNA5SP507		Pseudoger	chrX:70253042-7025
ENSG0000(	908	20.56063	chrX:478(	TERF1P7		Pseudoger	chrX:75326772-7532
ENSG0000(	908	20.56063	chr2:2577	ENSG00000283228		protein_c	chr2:151802651-151
ENSG0000(	908	20.56063	chrX:478(	ATRX	NCGv7;AC	protein_c	chrX:77504880-7778
ENSG0000(	908	20.56063	chrX:478(	GJB1		protein_c	chrX:71212811-7122
ENSG0000(	908	20.56063	chr2:2577	MTND6P9		Pseudoger	chr2:155313440-155
ENSG0000(	908	20.56063	chr2:2577	MTND5P30		Pseudoger	chr2:155311697-155
ENSG0000(	908	20.56063	chrX:478(	ERCC6L		protein_c	chrX:72204657-7223
ENSG0000(	908	20.56063	chrX:478(	ENSG00000285171		protein_c	chrX:71103987-7111
ENSG0000(	908	20.56063	chrX:478(	PKMP2		Pseudoger	chrX:66497748-6649
ENSG0000(	908	20.56063	chrX:478(	UBE2DNL		Pseudoger	chrX:84934113-8493
ENSG0000(	908	20.56063	chrX:478(	CITED1		protein_c	chrX:72301638-7230
ENSG0000(	908	20.56063	chr2:2577	CDK7P1		Pseudoger	chr2:157210847-157
ENSG0000(	908	20.56063	chrX:478(	CAPZA1P1		Pseudoger	chrX:88096393-8809
ENSG0000(	908	20.56063	chrX:478(	PABPC1L2B-AS1		lncRNA	chrX:72998388-7300
ENSG0000(	908	20.56063	chrX:478(	MKRN5P		Pseudoger	chrX:74161062-7416
ENSG0000(	908	20.56063	chrX:478(	ATXN7L3P1		Pseudoger	chrX:66055587-6605
ENSG0000(	908	20.56063	chrX:478(	HNRNPH3P1		Pseudoger	chrX:80529037-8053
ENSG0000(	908	20.56063	chrX:478(	RNU6-1225P		smallRNA	chrX:68102068-6810
ENSG0000(	908	20.56063	chr2:2577	NEB	NCGv7	protein_c	chr2:151485336-151
ENSG0000(	908	20.56063	chrX:478(	SOCS6P1		Pseudoger	chrX:71527814-7153
ENSG0000(	908	20.56063	chrX:478(	TLE1P1		Pseudoger	chrX:65408084-6540
ENSG0000(	908	20.56063	chrX:478(	RNU4-81P		smallRNA	chrX:70450879-7045
ENSG0000(	908	20.56063	chrX:478(	XIST		lncRNA	chrX:73820649-7385
ENSG0000(	908	20.56063	chrX:478(	LINC00269		lncRNA	chrX:69179557-6920

ENSG0000(	908	20.56063	chrX:478(	ENSG00000228160		lncRNA	chrX:69569635-6957
ENSG0000(	908	20.56063	chr2:2577	ATP5F1AP2		Pseudoger	chr2:155269633-155
ENSG0000(	908	20.56063	chrX:478(	KRT8P27		Pseudoger	chrX:64623117-6462
ENSG0000(	908	20.56063	chr2:2577	ENSG00000287085		lncRNA	chr2:152450162-152
ENSG0000(	908	20.56063	chrX:478(	SFR1P2		Pseudoger	chrX:85849410-8585
ENSG0000(	908	20.56063	chr2:2577	DNAJA1P2		Pseudoger	chr2:153761450-153
ENSG0000(	908	20.56063	chrX:478(	HNRNPA1P25		Pseudoger	chrX:74473017-7447
ENSG0000(	908	20.56063	chr2:2577	AC009951.2		Pseudoger	chr2:144450668-144
ENSG0000(	908	20.56063	chrX:478(	ENSG00000274398		Pseudoger	chrX:76250183-7625
ENSG0000(	908	20.56063	chrX:478(	ENSG00000284391		lncRNA	chrX:70427450-7043
ENSG0000(	908	20.56063	chrX:478(	CPXCR1		protein_c	chrX:88747225-8875
ENSG0000(	908	20.56063	chrX:478(	DLG3	NCGv7	protein_c	chrX:70444835-7050
ENSG0000(	908	20.56063	chrX:478(	CXCR3		protein_c	chrX:71615916-7161
ENSG0000(	908	20.56063	chrX:478(	SPRYD7P1		Pseudoger	chrX:77374212-7738
ENSG0000(	908	20.56063	chrX:478(	SAR1AP4		Pseudoger	chrX:75884546-7588
ENSG0000(	908	20.56063	chrX:478(	MAGEE1		protein_c	chrX:76427710-7643
ENSG0000(	908	20.56063	chrX:478(	ENSG00000226870		Pseudoger	chrX:71719142-7172
ENSG0000(	908	20.56063	chrX:478(	MORF4L1P5		Pseudoger	chrX:65327988-6533
ENSG0000(	908	20.56063	chrX:478(	DMRTC1B		protein_c	chrX:72776890-7284
ENSG0000(	908	20.56063	chrX:478(	TGIF2LX		protein_c	chrX:89921908-8992
ENSG0000(	908	20.56063	chrX:478(	PABPN1P1		Pseudoger	chrX:71420083-7142
ENSG0000(	908	20.56063	chrX:478(	P2RY4		protein_c	chrX:70258166-7026
ENSG0000(	908	20.56063	chrX:478(	ENSG00000291017		lncRNA	chrX:71662991-7166
ENSG0000(	908	20.56063	chrX:478(	STAU2P1		Pseudoger	chrX:90039213-9003
ENSG0000(	908	20.56063	chrX:478(	HDX		protein_c	chrX:84317874-8450
ENSG0000(	908	20.56063	chrX:478(	BRWD3		protein_c	chrX:80669503-8080
ENSG0000(	908	20.56063	chrX:478(	NLGN3		protein_c	chrX:71144821-7117
ENSG0000(	908	20.56063	chrX:478(	HEPH		protein_c	chrX:66162671-6626
ENSG0000(	908	20.56063	chrX:478(	COX7B		protein_c	chrX:77899440-7790
ENSG0000(	908	20.56063	chrX:478(	SH3BGRL		protein_c	chrX:81202102-8129
ENSG0000(	908	20.56063	chrX:478(	SATL1		protein_c	chrX:85092284-8524
ENSG0000(	908	20.56063	chrX:478(	RPL7P53		Pseudoger	chrX:73535503-7353
ENSG0000(	908	20.56063	chrX:478(	DMRTC1		protein_c	chrX:72872025-7294
ENSG0000(	908	20.56063	chrX:478(	SLC7A3		protein_c	chrX:70925579-7093
ENSG0000(	908	20.56063	chr2:2577	ENSG00000270776		Pseudoger	chr2:152539197-152
ENSG0000(	908	20.56063	chrX:478(	ENSG00000276689		Pseudoger	chrX:65786763-6578
ENSG0000(	908	20.56063	chrX:478(	U3		smallRNA	chrX:69692956-6969
ENSG0000(	908	20.56063	chrX:478(	UBE2V1P7		Pseudoger	chrX:78554412-7855
ENSG0000(	908	20.56063	chrX:478(	EFNB1		protein_c	chrX:68829021-6884
ENSG0000(	908	20.56063	chrX:478(	FAM236D		protein_c	chrX:72807425-7280
ENSG0000(	908	20.56063	chr2:2577	RPRM		protein_c	chr2:153477338-153
ENSG0000(	908	20.56063	chrX:478(	RN7SL790P		smallRNA	chrX:74390692-7439
ENSG0000(	908	20.56063	chrX:478(	KIF4A		protein_c	chrX:70290104-7042
ENSG0000(	908	20.56063	chrX:478(	AL158069.1		smallRNA	chrX:69504705-6950
ENSG0000(	908	20.56063	chrX:478(	RNU6-1078P		smallRNA	chrX:71965972-7196
ENSG0000(	908	20.56063	chr2:2577	LINC01958		lncRNA	chr2:156655224-156
ENSG0000(	908	20.56063	chrX:478(	TBX22	NCGv7	protein_c	chrX:80014753-8003
ENSG0000(	908	20.56063	chr2:2577	RNA5SP107		Pseudoger	chr2:154590407-154
ENSG0000(	908	20.56063	chrX:478(	CCT4P2		Pseudoger	chrX:65270913-6527
ENSG0000(	908	20.56063	chrX:478(	RP3-326L13.3		lncRNA	chrX:83511296-8351
ENSG0000(	908	20.56063	chr2:2577	ENSG00000286530		lncRNA	chr2:157033837-157

ENSG0000(	908	20.56063	chrX:478(	NANOGP9		Pseudoger	chrX:65772741-6577
ENSG0000(	908	20.56063	chrX:478(	SHC1P1		Pseudoger	chrX:64432401-6443
ENSG0000(	908	20.56063	chr2:2577	MTCO1P45		Pseudoger	chr2:155263458-155
ENSG0000(	908	20.56063	chrX:478(	ENSG00000290713		lncRNA	chrX:71719925-7172
ENSG0000(	908	20.56063	chrX:478(	ATP7A		protein_c	chrX:77910690-7805
ENSG0000(	908	20.56063	chrX:478(	PGK1P1		Pseudoger	chrX:68070520-6807
ENSG0000(	908	20.56063	chrX:478(	HMGN1P35		Pseudoger	chrX:69174124-6917
ENSG0000(	908	20.56063	chrX:478(	FNDC3CP		Pseudoger	chrX:78165696-7817
ENSG0000(	908	20.56063	chrX:478(	TPMTP4		Pseudoger	chrX:86128893-8612
ENSG0000(	908	20.56063	chrX:478(	AL590763.1		smallRNA	chrX:71625753-7162
ENSG0000(	908	20.56063	chrX:478(	ENSG00000284618		lncRNA	chrX:81000150-8100
ENSG0000(	908	20.56063	chrX:478(	TRAPPC2LP1		Pseudoger	chrX:70361486-7036
ENSG0000(	908	20.56063	chrX:478(	RNU6-493P		smallRNA	chrX:80900757-8090
ENSG0000(	908	20.56063	chr2:2577	ENSG00000286679		lncRNA	chr2:155525424-155
ENSG0000(	908	20.56063	chrX:478(	ENSG00000276892		Pseudoger	chrX:85851575-8585
ENSG0000(	908	20.56063	chrX:478(	RPL21P134		Pseudoger	chrX:75384346-7538
ENSG0000(	908	20.56063	chrX:478(	RPSAP15		Pseudoger	chrX:87703343-8770
ENSG0000(	908	20.56063	chrX:478(	ENSG00000283599		protein_c	chrX:71667542-7167
ENSG0000(	908	20.56063	chrX:478(	RNU1-56P		smallRNA	chrX:71020275-7102
ENSG0000(	908	20.56063	chrX:478(	THAP12P1		Pseudoger	chrX:74396871-7439
ENSG0000(	908	20.56063	chrX:478(	Y_RNA		smallRNA	chrX:71491066-7149
ENSG0000(	908	20.56063	chrX:478(	RNA5SP509		Pseudoger	chrX:77066709-7706
ENSG0000(	908	20.56063	chr2:2577	ENSG00000224675		lncRNA	chr2:154435853-154
ENSG0000(	908	20.56063	chrX:478(	snoU13		smallRNA	chrX:71440517-7144
ENSG0000(	908	20.56063	chrX:478(	ENSG00000226854		lncRNA	chrX:75903105-7590
ENSG0000(	908	20.56063	chrX:478(	RNU6-995P		smallRNA	chrX:80936434-8093
ENSG0000(	908	20.56063	chrX:478(	ENSG00000290714		lncRNA	chrX:71760764-7176
ENSG0000(	908	20.56063	chrX:478(	ENSG00000224523		Pseudoger	chrX:81408938-8142
ENSG0000(	908	20.56063	chrX:478(	RP11-262D11.1		Pseudoger	chrX:72132150-7213
ENSG0000(	908	20.56063	chrX:478(	ACA64		smallRNA	chrX:88148792-8814
ENSG0000(	908	20.56063	chrX:478(	MIR223HG		lncRNA	chrX:66015414-6602
ENSG0000(	908	20.56063	chr2:2577	ARL6IP6		protein_c	chr2:152717647-152
ENSG0000(	908	20.56063	chrX:478(	FABP5P15		Pseudoger	chrX:77727867-7772
ENSG0000(	908	20.56063	chrX:478(	PABPC1P3		Pseudoger	chrX:74583088-7458
ENSG0000(	908	20.56063	chrX:478(	IGBP1		protein_c	chrX:70133447-7016
ENSG0000(	908	20.56063	chrX:478(	NEXMIF		protein_c	chrX:74732856-7492
ENSG0000(	908	20.56063	chrX:478(	ENSG00000234442		Pseudoger	chrX:71761561-7176
ENSG0000(	908	20.56063	chrX:478(	CXorf49		protein_c	chrX:71714371-7171
ENSG0000(	908	20.56063	chrX:478(	COPS8P1		Pseudoger	chrX:86798598-8679
ENSG0000(	908	20.56063	chr2:2577	RPL23AP29		Pseudoger	chr2:153370612-153
ENSG0000(	908	20.56063	chrX:478(	POF1B	NCGv7	protein_c	chrX:85277396-8537
ENSG0000(	908	20.56063	chrX:478(	NAP1L2	NCGv7	protein_c	chrX:73212299-7321
ENSG0000(	908	20.56063	chrX:478(	BLOC1S2P1		Pseudoger	chrX:64726748-6472
ENSG0000(	908	20.56063	chrX:478(	AL590764.1		smallRNA	chrX:71141185-7114
ENSG0000(	908	20.56063	chrX:478(	ENSG00000226515		Pseudoger	chrX:74342713-7434
ENSG0000(	908	20.56063	chrX:478(	SNORA4		smallRNA	chrX:82561201-8256
ENSG0000(	908	20.56063	chrX:478(	ENSG00000232576		Pseudoger	chrX:88908403-8890
ENSG0000(	908	20.56063	chrX:478(	PRXL2CP1		Pseudoger	chrX:65356890-6535
ENSG0000(	908	20.56063	chr2:2577	ATP5PBP4		Pseudoger	chr2:153158938-153
ENSG0000(	908	20.56063	chrX:478(	ENSG00000237971		Pseudoger	chrX:65185018-6518
ENSG0000(	908	20.56063	chrX:478(	EIF3JP1		Pseudoger	chrX:82497669-8249

ENSG0000(	908	20.56063	chrX:478(	ENSG00000279437		TEC	chrX:83506023-8350
ENSG0000(	908	20.56063	chrX:478(	WBP11P3		Pseudoger	chrX:80560146-8056
ENSG0000(	908	20.56063	chr2:2577	ENSG00000225214		lncRNA	chr2:152098328-152
ENSG0000(	908	20.56063	chrX:478(	FXYD6P3		Pseudoger	chrX:73875068-7387
ENSG0000(	908	20.56063	chrX:478(	AARSD1P1		Pseudoger	chrX:74069276-7407
ENSG0000(	908	20.56063	chr2:2577	ENSG00000287900		lncRNA	chr2:154688296-154
ENSG0000(	908	20.56063	chr2:2577	SNORD56		smallRNA	chr2:153446813-153
ENSG0000(	908	20.56063	chrX:478(	ENSG00000271589		Pseudoger	chrX:74769639-7476
ENSG0000(	908	20.56063	chrX:478(	SNX12		protein_c	chrX:71056332-7107
ENSG0000(	908	20.56063	chr2:2577	HEBP2P1		Pseudoger	chr2:156033247-156
ENSG0000(	908	20.56063	chrX:478(	ITGB1BP2		protein_c	chrX:71301750-7130
ENSG0000(	908	20.56063	chrX:478(	LAS1L		protein_c	chrX:65438549-6553
ENSG0000(	908	20.56063	chrX:478(	PPATP2		Pseudoger	chrX:78699384-7869
ENSG0000(	908	20.56063	chrX:478(	RNU6-562P		smallRNA	chrX:75202703-7520
ENSG0000(	908	20.56063	chrX:478(	RN7SL746P		smallRNA	chrX:71084489-7108
ENSG0000(	908	20.56063	chr2:2577	ENSG00000238004		lncRNA	chr2:154459857-154
ENSG0000(	908	20.56063	chrX:478(	YIPF6		protein_c	chrX:68498562-6853
ENSG0000(	908	20.56063	chrX:478(	RN7SL648P		smallRNA	chrX:74242610-7424
ENSG0000(	908	20.56063	chrX:478(	MTND4P31		Pseudoger	chrX:70124972-7012
ENSG0000(	908	20.56063	chrX:478(	ENSG00000271457		Pseudoger	chrX:87143854-8714
ENSG0000(	908	20.56063	chrX:478(	LRRFIP2P1		Pseudoger	chrX:73237647-7323
ENSG0000(	908	20.56063	chrX:478(	Y_RNA		smallRNA	chrX:86019608-8601
ENSG0000(	908	20.56063	chrX:478(	DACH2	NCGv7	protein_c	chrX:86148451-8683
ENSG0000(	908	20.56063	chrX:478(	VSIG4		protein_c	chrX:66021738-6604
ENSG0000(	908	20.56063	chrX:478(	UBE2V1P9		Pseudoger	chrX:90289536-9028
ENSG0000(	908	20.56063	chrX:478(	ENSG00000260118		lncRNA	chrX:68013470-6801
ENSG0000(	908	20.56063	chrX:478(	VDAC1P1		Pseudoger	chrX:80929500-8093
ENSG0000(	908	20.56063	chrX:478(	MAP2K4P1		Pseudoger	chrX:73524275-7356
ENSG0000(	908	20.56063	chrX:478(	ENSG00000274022		Pseudoger	chrX:90208895-9020
ENSG0000(	908	20.56063	chrX:478(	PDZD11		protein_c	chrX:70281118-7029
ENSG0000(	908	20.56063	chrX:478(	MORF4L1P6		Pseudoger	chrX:73474563-7347
ENSG0000(	908	20.56063	chrX:478(	C4orf46P2		Pseudoger	chrX:77910682-7796
ENSG0000(	908	20.56063	chrX:478(	PABPC1L2A		protein_c	chrX:73077276-7307
ENSG0000(	908	20.56063	chrX:478(	TRAPPC13P1		Pseudoger	chrX:76655498-7665
ENSG0000(	908	20.56063	chrX:478(	OTUD6A		protein_c	chrX:70062457-7006
ENSG0000(	908	20.56063	chrX:478(	RNU6-867P		smallRNA	chrX:76244968-7624
ENSG0000(	908	20.56063	chrX:478(	ENSG00000235461		lncRNA	chrX:85210684-8522
ENSG0000(	908	20.56063	chr2:2577	MTND2P20		Pseudoger	chr2:155264163-155
ENSG0000(	908	20.56063	chr2:2577	ENSG00000234584		lncRNA	chr2:157725708-157
ENSG0000(	908	20.56063	chrX:478(	DDX3P2		Pseudoger	chrX:74133004-7413
ENSG0000(	908	20.56063	chr2:2577	CACNB4		protein_c	chr2:151832771-152
ENSG0000(	908	20.56063	chrX:478(	TSIX		lncRNA	chrX:73792205-7382
ENSG0000(	908	20.56063	chrX:478(	FRMD8P1		Pseudoger	chrX:65550898-6555
ENSG0000(	908	20.56063	chrX:478(	ENSG00000230478		Pseudoger	chrX:87425583-8742
ENSG0000(	908	20.56063	chrX:478(	ETF1P3		Pseudoger	chrX:65794345-6579
ENSG0000(	908	20.56063	chrX:478(	CHM		protein_c	chrX:85861180-8604
ENSG0000(	908	20.56063	chrX:478(	SRIP2		Pseudoger	chrX:89367435-8936
ENSG0000(	908	20.56063	chrX:478(	ENSG00000271533		lncRNA	chrX:74209976-7421
ENSG0000(	908	20.56063	chrX:478(	ARR3		protein_c	chrX:70268305-7028
ENSG0000(	908	20.56063	chr2:2577	ENSG00000230991		lncRNA	chr2:154964308-154
ENSG0000(	908	20.56063	chrX:478(	TEX11		protein_c	chrX:70528940-7090

ENSG0000(	908	20.56063	chrX:478(	ZCRB1P1		Pseudoger	chrX:71314912-7131
ENSG0000(	908	20.56063	chrX:478(	ABCB7		protein_c	chrX:75051048-7515
ENSG0000(	908	20.56063	chr2:2577	TUBAP13		Pseudoger	chr2:153420677-153
ENSG0000(	908	20.56063	chrX:478(	GEMIN8P3		Pseudoger	chrX:86304367-8630
ENSG0000(	908	20.56063	chrX:478(	CNOT7P1		Pseudoger	chrX:69937166-6993
ENSG0000(	908	20.56063	chrX:478(	RPL22P22		Pseudoger	chrX:82506434-8250
ENSG0000(	908	20.56063	chrX:478(	ENSG00000231875		Pseudoger	chrX:81363268-8136
ENSG0000(	908	20.56063	chrX:478(	SHISA5P2		Pseudoger	chrX:74066083-7406
ENSG0000(	908	20.56063	chrX:478(	MTCYBP31		Pseudoger	chrX:70120353-7012
ENSG0000(	908	20.56063	chr2:2577	ENSG00000286234		lncRNA	chr2:152176020-152
ENSG0000(	908	20.56063	chrX:478(	FAM236C		protein_c	chrX:72912615-7291
ENSG0000(	908	20.56063	chrX:478(	IGBP1-AS1		lncRNA	chrX:70163842-7016
ENSG0000(	908	20.56063	chrX:478(	TOMM20P4		Pseudoger	chrX:73223124-7322
ENSG0000(	908	20.56063	chrX:478(	MAGEE2		protein_c	chrX:75782987-7578
ENSG0000(	908	20.56063	chrX:478(	ENSG00000288059		lncRNA	chrX:81475532-8149
ENSG0000(	908	20.56063	chrX:478(	CYLC1	NCGv7	protein_c	chrX:83861126-8388
ENSG0000(	908	20.56063	chrX:478(	OPHN1		protein_c	chrX:67949349-6843
ENSG0000(	908	20.56063	chrX:478(	MIR325HG		lncRNA	chrX:76656866-7701
ENSG0000(	908	20.56063	chrX:478(	GCNA		protein_c	chrX:71578437-7161
ENSG0000(	908	20.56063	chrX:478(	EEF1A1P29		Pseudoger	chrX:86160264-8616
ENSG0000(	908	20.56063	chrX:478(	ENSG00000229601		Pseudoger	chrX:71413834-7141
ENSG0000(	908	20.56063	chrX:478(	TERF1P4		Pseudoger	chrX:83748825-8374
ENSG0000(	908	20.56063	chr2:2577	ENSG00000287048		lncRNA	chr2:156788731-156
ENSG0000(	908	20.56063	chr2:2577	ENSG00000288066		lncRNA	chr2:151796688-151
ENSG0000(	908	20.56063	chrX:478(	TTC3P1		Pseudoger	chrX:75740831-7574
ENSG0000(	908	20.56063	chr2:2577	NR4A2	NCGv7	protein_c	chr2:156324437-156
ENSG0000(	908	20.56063	chrX:478(	COX6CP12		Pseudoger	chrX:68645326-6864
ENSG0000(	908	20.56063	chr2:2577	PHB1P4		Pseudoger	chr2:154286589-154
ENSG0000(	908	20.56063	chrX:478(	MIR361		smallRNA	chrX:85903636-8590
ENSG0000(	908	20.56063	chrX:478(	SNORD45		smallRNA	chrX:87146733-8714
ENSG0000(	908	20.56063	chrX:478(	AKIRIN1P2		Pseudoger	chrX:68132868-6813
ENSG0000(	908	20.56063	chrX:478(	CXorf49B		protein_c	chrX:71763349-7176
ENSG0000(	908	20.56063	chr2:2577	ENSG00000286889		lncRNA	chr2:156803339-156
ENSG0000(	908	20.56063	chr2:2577	LINC01850		lncRNA	chr2:153337705-153
ENSG0000(	908	20.56063	chrX:478(	ZNF711	NCGv7	protein_c	chrX:85243991-8527
ENSG0000(	908	20.56063	chrX:478(	ENSG00000271693		Pseudoger	chrX:88369343-8836
ENSG0000(	908	20.56063	chr2:2577	RPLP0P7		Pseudoger	chr2:156777706-156
ENSG0000(	908	20.56063	chrX:478(	PJA1		protein_c	chrX:69160851-6916
ENSG0000(	908	20.56063	chrX:478(	MIR545		smallRNA	chrX:74287104-7428
ENSG0000(	908	20.56063	chr2:2577	ENSG00000234932		Pseudoger	chr2:152850653-152
ENSG0000(	908	20.56063	chrX:478(	LINC00891		lncRNA	chrX:71697196-7170
ENSG0000(	908	20.56063	chrX:478(	CDX4	NCGv7	protein_c	chrX:73447053-7345
ENSG0000(	908	20.56063	chrX:478(	BUD31P2		Pseudoger	chrX:75201491-7520
ENSG0000(	908	20.56063	chrX:478(	ENSG00000233484		Pseudoger	chrX:84403898-8440
ENSG0000(	908	20.56063	chrX:478(	WASHC3P1		Pseudoger	chrX:70857163-7085
ENSG0000(	908	20.56063	chrX:478(	ENSG00000237717		Pseudoger	chrX:71744828-7174
ENSG0000(	908	20.56063	chr2:2577	AC008166.1		smallRNA	chr2:154146105-154
ENSG0000(	908	20.56063	chrX:478(	RPS6KA6		protein_c	chrX:84058346-8420
ENSG0000(	908	20.56063	chrX:478(	SERBP1P1		Pseudoger	chrX:68783472-6878
ENSG0000(	908	20.56063	chrX:478(	CYCSP43		Pseudoger	chrX:69485343-6948
ENSG0000(	908	20.56063	chr2:2577	FAM133DP		Pseudoger	chr2:157379724-157

ENSG0000(	908	20.56063	chrX:478(	RHOG2P		Pseudoger	chrX:71352418-7135
ENSG0000(	908	20.56063	chrX:478(	RLIM	NCGv7	protein_c	chrX:74582976-7461
ENSG0000(	908	20.56063	chrX:478(	CAPZA1P3		Pseudoger	chrX:72727977-7272
ENSG0000(	908	20.56063	chrX:478(	MIR374A		smallRNA	chrX:74287286-7428
ENSG0000(	908	20.56063	chrX:478(	MIR421		smallRNA	chrX:74218377-7421
ENSG0000(	908	20.56063	chrX:478(	RPS7P14		Pseudoger	chrX:74409518-7441
ENSG0000(	908	20.56063	chrX:478(	FTX		lncRNA	chrX:73940435-7429
ENSG0000(	908	20.56063	chrX:478(	P2RY10		protein_c	chrX:78945332-7896
ENSG0000(	908	20.56063	chrX:478(	ITM2A		protein_c	chrX:79360384-7936
ENSG0000(	908	20.56063	chrX:478(	IL2RG	NCGv7	protein_c	chrX:71107404-7111
ENSG0000(	908	20.56063	chrX:478(	DLG3-AS1		lncRNA	chrX:70452958-7045
ENSG0000(	908	20.56063	chrX:478(	AL451105.1		Pseudoger	chrX:75899804-7589
ENSG0000(	908	20.56063	chrX:478(	LDHBP2		Pseudoger	chrX:76334841-7633
ENSG0000(	908	20.56063	chr2:2577	CYTIP		protein_c	chr2:157414619-157
ENSG0000(	908	20.56063	chr2:2577	GPD2		protein_c	chr2:156435290-156
ENSG0000(	908	20.56063	chrX:478(	ENSG00000286077		lncRNA	chrX:70037840-7003
ENSG0000(	908	20.56063	chr2:2577	STAM2	NCGv7	protein_c	chr2:152116801-152
ENSG0000(	908	20.56063	chrX:478(	INGX		Pseudoger	chrX:71491682-7149
ENSG0000(	908	20.56063	chrX:478(	STIP1P3		Pseudoger	chrX:86084716-8608
ENSG0000(	908	20.56063	chrX:478(	ENSG00000230781		Pseudoger	chrX:84973217-8497
ENSG0000(	908	20.56063	chrX:478(	FOXO4	NCGv7	protein_c	chrX:71095851-7110
ENSG0000(	908	20.56063	chrX:478(	MIR548I4		smallRNA	chrX:84225752-8422
ENSG0000(	908	20.56063	chrX:478(	ENSG00000230187		Pseudoger	chrX:70672895-7067
ENSG0000(	908	20.56063	chr2:2577	MTCYBP9		Pseudoger	chr2:155314066-155
ENSG0000(	908	20.56063	chr2:2577	Y_RNA		smallRNA	chr2:152284350-152
ENSG0000(	908	20.56063	chrX:478(	YWHAZP7		Pseudoger	chrX:64612632-6461
ENSG0000(	908	20.56063	chrX:478(	RNU6-330P		smallRNA	chrX:74680053-7468
ENSG0000(	908	20.56063	chrX:478(	AL445523.1		smallRNA	chrX:65215237-6521
ENSG0000(	908	20.56063	chrX:478(	YWHAZP8		Pseudoger	chrX:73274785-7327
ENSG0000(	908	20.56063	chrX:478(	ENSG00000271205		Pseudoger	chrX:87723089-8772
ENSG0000(	908	20.56063	chrX:478(	PBDC1		protein_c	chrX:76173040-7617
ENSG0000(	908	20.56063	chrX:478(	NDUFA5P7		Pseudoger	chrX:86066227-8606
ENSG0000(	908	20.56063	chrX:478(	ENSG00000230934		Pseudoger	chrX:71691932-7169
ENSG0000(	908	20.56063	chrX:478(	RBMXP5		Pseudoger	chrX:65956289-6595
ENSG0000(	908	20.56063	chrX:478(	TAF1	NCGv7;AC	protein_c	chrX:71366222-7153
ENSG0000(	908	20.56063	chrX:478(	ENSG00000271199		lncRNA	chrX:73958059-7396
ENSG0000(	908	20.56063	chrX:478(	ZDHHC15		protein_c	chrX:75368427-7552
ENSG0000(	908	20.56063	chrX:478(	FAM226B		lncRNA	chrX:72777608-7277
ENSG0000(	908	20.56063	chrX:478(	SETP4		Pseudoger	chrX:84755136-8475
ENSG0000(	908	20.56063	chr2:2577	AC011308.1		protein_c	chr2:156496085-156
ENSG0000(	908	20.56063	chr2:2577	RN7SKP281		smallRNA	chr2:157298807-157
ENSG0000(	908	20.56063	chr2:2577	ERMN		protein_c	chr2:157318631-157
ENSG0000(	908	20.56063	chr2:2577	GALNT5		protein_c	chr2:157257705-157
ENSG0000(	908	20.56063	chrX:478(	ENSG00000231963		lncRNA	chrX:73080167-7308
ENSG0000(	908	20.56063	chrX:478(	PHKA1		protein_c	chrX:72578814-7271
ENSG0000(	908	20.56063	chrX:478(	ARL5AP5		Pseudoger	chrX:76029895-7603
ENSG0000(	908	20.56063	chrX:478(	POU3F4		protein_c	chrX:83508290-8351
ENSG0000(	908	20.56063	chr2:2577	CBX3P6		Pseudoger	chr2:154940541-154
ENSG0000(	908	20.56063	chrX:478(	EDA2R		protein_c	chrX:66595637-6663
ENSG0000(	908	20.56063	chrX:478(	CTHRC1P1		Pseudoger	chrX:79177200-7917
ENSG0000(	908	20.56063	chr2:2577	ENSG00000283118		lncRNA	chr2:144444848-144

ENSG0000(	908	20.56063	chrX:478(	HMGN1P34		Pseudoger	chrX:78519593-7852
ENSG0000(	908	20.56063	chrX:478(	ACTR3P2		Pseudoger	chrX:68771322-6877
ENSG0000(	908	20.56063	chrX:478(	RNU6-974P		smallRNA	chrX:82001220-8200
ENSG0000(	908	20.56063	chrX:478(	EIF4BP9		Pseudoger	chrX:66074835-6607
ENSG0000(	908	20.56063	chrX:478(	AL590762.1		Pseudoger	chrX:71300730-7130
ENSG0000(	908	20.56063	chrX:478(	MIR223		smallRNA	chrX:66018870-6601
ENSG0000(	908	20.56063	chrX:478(	FAM236B		protein_c	chrX:72781865-7278
ENSG0000(	908	20.56063	chrX:478(	EDA		protein_c	chrX:69616067-7003
ENSG0000(	908	20.56063	chrX:478(	RNU2-68P		smallRNA	chrX:72376979-7237
ENSG0000(	908	20.56063	chrX:478(	TMEM184CP1		Pseudoger	chrX:88061590-8806
ENSG0000(	908	20.56063	chr2:2577	snoU13		smallRNA	chr2:156410144-156
ENSG0000(	908	20.56063	chr2:2577	UBQLN4P2		Pseudoger	chr2:152876820-152
ENSG0000(	908	20.56063	chr2:2577	RPL30P2		Pseudoger	chr2:152131555-152
ENSG0000(	908	20.56063	chrX:478(	RNU1-112P		smallRNA	chrX:72740706-7274
ENSG0000(	908	20.56063	chrX:478(	RN7SL460P		smallRNA	chrX:77885377-7788
ENSG0000(	908	20.56063	chrX:478(	RPS4X		protein_c	chrX:72255679-7227
ENSG0000(	908	20.56063	chrX:478(	ENSG00000237265		Pseudoger	chrX:71663276-7166
ENSG0000(	908	20.56063	chrX:478(	ENSG00000280375		TEC	chrX:74122134-7412
ENSG0000(	908	20.56063	chrX:478(	ENSG00000225012		lncRNA	chrX:89423738-8944
ENSG0000(	908	20.56063	chrX:478(	GPR174	NCGv7	protein_c	chrX:79144688-7917
ENSG0000(	908	20.56063	chrX:478(	MMADHCP1		Pseudoger	chrX:76222637-7622
ENSG0000(	908	20.56063	chr2:2577	ENSG00000227400		lncRNA	chr2:153421616-153
ENSG0000(	908	20.56063	chrX:478(	RNU6-394P		smallRNA	chrX:66676258-6667
ENSG0000(	908	20.56063	chrX:478(	EIF3MP1		Pseudoger	chrX:82875649-8287
ENSG0000(	908	20.56063	chrX:478(	PSMA1P1		Pseudoger	chrX:80709928-8071
ENSG0000(	908	20.56063	chrX:478(	PDK1P2		Pseudoger	chrX:81600072-8160
ENSG0000(	908	20.56063	chrX:478(	ENSG00000237311		lncRNA	chrX:65925836-6600
ENSG0000(	908	20.56063	chrX:478(	RAB41		protein_c	chrX:70282093-7028
ENSG0000(	908	20.56063	chrX:478(	MAGT1		protein_c	chrX:77825747-7789
ENSG0000(	908	20.56063	chr2:2577	ACVR1C		protein_c	chr2:157526767-157
ENSG0000(	908	20.56063	chrX:478(	FAM236A		protein_c	chrX:72938163-7293
ENSG0000(	908	20.56063	chrX:478(	PGK1		protein_c	chrX:77910739-7812
ENSG0000(	908	20.56063	chrX:478(	ZCCHC13		protein_c	chrX:74304180-7430
ENSG0000(	908	20.56063	chrX:478(	MATR3P1		Pseudoger	chrX:72660470-7266
ENSG0000(	908	20.56063	chrX:478(	ENSG00000229030		Pseudoger	chrX:71848775-7184
ENSG0000(	908	20.56063	chrX:478(	MRPS22P1		Pseudoger	chrX:87807549-8780
ENSG0000(	908	20.56063	chrX:478(	ENSG00000289038		lncRNA	chrX:68498309-6849
ENSG0000(	908	20.56063	chrX:478(	Y_RNA		smallRNA	chrX:72284945-7228
ENSG0000(	908	20.56063	chrX:478(	ZMYM3	NCGv7	protein_c	chrX:71239624-7125
ENSG0000(	908	20.56063	chrX:478(	RPL31P63		Pseudoger	chrX:68600099-6860
ENSG0000(	908	20.56063	chrX:478(	ZC3H12B		protein_c	chrX:65034788-6550
ENSG0000(	908	20.56063	chrX:478(	BX276092.1		smallRNA	chrX:71759385-7175
ENSG0000(	908	20.56063	chr2:2577	ENSG00000286207		lncRNA	chr2:152635268-152
ENSG0000(	908	20.56063	chr2:2577	FMNL2		protein_c	chr2:152335174-152
ENSG0000(	908	20.56063	chrX:478(	RNU6-1044P		smallRNA	chrX:73397531-7339
ENSG0000(	908	20.56063	chrX:478(	PABPC1L2B		protein_c	chrX:73002939-7300
ENSG0000(	908	20.56063	chrX:478(	USP12PX		Pseudoger	chrX:90112650-9011
ENSG0000(	908	20.56063	chrX:478(	ENSG00000225925		Pseudoger	chrX:68474710-6847
ENSG0000(	908	20.56063	chrX:478(	PCNPP4		Pseudoger	chrX:75537547-7553
ENSG0000(	908	20.56063	chrX:478(	FOXN3P2		Pseudoger	chrX:75888700-7588
ENSG0000(	908	20.56063	chrX:478(	SEPHS1P4		Pseudoger	chrX:73769248-7377

ENSG00000	908	20.56063	chrX:4786	CCNYL5		Pseudoger	chrX:65821255-6582
ENSG00000	908	20.56063	chr2:2577	GALNT13	NCGv7	protein_c	chr2:153871922-154
ENSG00000	908	20.56063	chrX:4786	ENSG00000224617		Pseudoger	chrX:71789386-7178
ENSG00000	908	20.56063	chrX:4786	AL161723.1		smallRNA	chrX:89790335-8979
ENSG00000	908	20.56063	chr2:2577	ENSG00000282440		lncRNA	chr2:156305108-156
ENSG00000	908	20.56063	chrX:4786	GRPEL2P2		Pseudoger	chrX:64697878-6469
ENSG00000	908	20.56063	chr2:2577	MTND4P28		Pseudoger	chr2:155311023-155
ENSG00000	908	20.56063	chrX:4786	ENSG00000271157		Pseudoger	chrX:88827554-8882
ENSG00000	908	20.56063	chrX:4786	BMI1P1		Pseudoger	chrX:67791955-6779
ENSG00000	908	20.56063	chrX:4786	RP13-216E22.4		lncRNA	chrX:73948973-7394
ENSG00000	908	20.56063	chrX:4786	ENSG00000233710		Pseudoger	chrX:67533163-6753
ENSG00000	908	20.56063	chrX:4786	RTL5		protein_c	chrX:72127110-7213
ENSG00000	908	20.56063	chrX:4786	ATG4AP1		Pseudoger	chrX:82998699-8299
ENSG00000	908	20.56063	chrX:4786	MSN	NCGv7;AC	protein_c	chrX:65588377-6574
ENSG00000	908	20.56063	chrX:4786	ENSG00000282914		lncRNA	chrX:87707579-8775
ENSG00000	908	20.56063	chrX:4786	HDAC8		protein_c	chrX:72329516-7257
ENSG00000	908	20.56063	chrX:4786	PIN4		protein_c	chrX:72181353-7230
ENSG00000	908	20.56063	chrX:4786	CORO1CP1		Pseudoger	chrX:79369364-7937
ENSG00000	908	20.56063	chrX:4786	KLHL4		protein_c	chrX:87517409-8767
ENSG00000	908	20.56063	chrX:4786	RPS26P11		Pseudoger	chrX:72044545-7204
ENSG00000	908	20.56063	chrX:4786	PGAM4		protein_c	chrX:77967949-7796
ENSG00000	908	20.56063	chrX:4786	CYSLTR1		protein_c	chrX:78271468-7832
ENSG00000	908	20.56063	chrX:4786	ACA64		smallRNA	chrX:80857076-8085
ENSG00000	908	20.56063	chrX:4786	ZC4H2	NCGv7	protein_c	chrX:64915802-6503
ENSG00000	908	20.56063	chrX:4786	ENSG00000289132		lncRNA	chrX:80810091-8081
ENSG00000	908	20.56063	chrX:4786	SLC16A2		protein_c	chrX:74421493-7453
ENSG00000	908	20.56063	chrX:4786	PHKA1-AS1		lncRNA	chrX:72688950-7271
ENSG00000	908	20.56063	chrX:4786	IGBP1-AS2		lncRNA	chrX:70148582-7014
ENSG00000	908	20.56063	chrX:4786	ENSG00000237182		Pseudoger	chrX:71736454-7173
ENSG00000	908	20.56063	chrX:4786	ENSG00000285547		protein_c	chrX:72301691-7257
ENSG00000	908	20.56063	chrX:4786	ATP5MKP1		Pseudoger	chrX:74173890-7417
ENSG00000	908	20.56063	chrX:4786	HMGN5	AC	protein_c	chrX:81113699-8120
ENSG00000	908	20.56063	chr2:2577	ENSG00000224612		lncRNA	chr2:153168399-153
ENSG00000	908	20.56063	chrX:4786	OGT		protein_c	chrX:71533087-7157
ENSG00000	908	20.56063	chrX:4786	LPAR4	NCGv7	protein_c	chrX:78747709-7875
ENSG00000	908	20.56063	chrX:4786	POMPP1		Pseudoger	chrX:83559896-8356
ENSG00000	908	20.56063	chrX:4786	FCF1P9		Pseudoger	chrX:86481532-8648
ENSG00000	908	20.56063	chrX:4786	DGAT2L6	NCGv7	protein_c	chrX:70177483-7020
ENSG00000	908	20.56063	chrX:4786	RNY4P23		smallRNA	chrX:70396279-7039
ENSG00000	908	20.56063	chrX:4786	UPRT		protein_c	chrX:75156388-7530
ENSG00000	908	20.56063	chrX:4786	APOOL		protein_c	chrX:85003877-8509
ENSG00000	908	20.56063	chr2:2577	AC108057.1		smallRNA	chr2:157015172-157
ENSG00000	908	20.56063	chrX:4786	ENSG00000236190		Pseudoger	chrX:86888586-8688
ENSG00000	908	20.56063	chrX:4786	AC003001.1		smallRNA	chrX:85244095-8524
ENSG00000	908	20.56063	chrX:4786	AR	NCGv7;AC	protein_c	chrX:67544021-6773
ENSG00000	908	20.56063	chrX:4786	RAB11FIP1P1		Pseudoger	chrX:74202834-7420
ENSG00000	908	20.56063	chrX:4786	MTFR1P1		Pseudoger	chrX:66360766-6636
ENSG00000	908	20.56063	chrX:4786	ENSG00000289575		lncRNA	chrX:87723928-8772
ENSG00000	908	20.56063	chrX:4786	RPSAP14		Pseudoger	chrX:74031462-7403
ENSG00000	908	20.56063	chrX:4786	AL357115.1		smallRNA	chrX:81240820-8124
ENSG00000	908	20.56063	chrX:4786	ENSG00000226280		Pseudoger	chrX:67373573-6737

ENSG0000(	908	20.56063	chrX:478(	MED12	NCGv7;AC	protein_c	chrX:71118543-7114
ENSG0000(	908	20.56063	chrX:478(	BRAFP1		Pseudoger	chrX:75582676-7558
ENSG0000(	908	20.56063	chr2:2577	RNU6-546P		smallRNA	chr2:155330421-155
ENSG0000(	908	20.56063	chrX:478(	ENSG00000283446		Pseudoger	chrX:85143427-8515
ENSG0000(	908	20.56063	chrX:478(	RN7SL581P		smallRNA	chrX:70222008-7022
ENSG0000(	908	20.56063	chrX:478(	BX119917.1		smallRNA	chrX:72152336-7215
ENSG0000(	908	20.56063	chrX:478(	AP1M2P1		Pseudoger	chrX:65469087-6547
ENSG0000(	908	20.56063	chrX:478(	TAF9B		protein_c	chrX:78129748-7813
ENSG0000(	908	20.56063	chrX:478(	ENSG00000228427		lncRNA	chrX:71183382-7119
ENSG0000(	908	20.56063	chr2:2577	RNU6-1001P		smallRNA	chr2:155089439-155
ENSG0000(	908	20.56063	chrX:478(	Y_RNA		smallRNA	chrX:68361075-6836
ENSG0000(	908	20.56063	chr2:2577	ENSG00000226338		Pseudoger	chr2:153208086-153
ENSG0000(	908	20.56063	chrX:478(	RPL34P36		Pseudoger	chrX:87806433-8780
ENSG0000(	908	20.56063	chrX:478(	RN7SL264P		smallRNA	chrX:72223244-7222
ENSG0000(	908	20.56063	chrX:478(	RN7SL388P		smallRNA	chrX:72198712-7219
ENSG0000(	908	20.56063	chrX:478(	5S_rRNA		smallRNA	chrX:69672479-6967
ENSG0000(	908	20.56063	chrX:478(	FGF16		protein_c	chrX:77454157-7745
ENSG0000(	908	20.56063	chr2:2577	ENSG00000279166		TEC	chr2:144494265-144
ENSG0000(	908	20.56063	chr2:2577	PRPF40A		protein_c	chr2:152651593-152
ENSG0000(	908	20.56063	chrX:478(	STARD8		protein_c	chrX:68647666-6872
ENSG0000(	908	20.56063	chr2:2577	LINC01876		lncRNA	chr2:156011530-156
ENSG0000(	908	20.56063	chrX:478(	NALF2		protein_c	chrX:69504326-6953
ENSG0000(	908	20.56063	chrX:478(	ENSG00000283178		Pseudoger	chrX:71869763-7187
ENSG0000(	908	20.56063	chr2:2577	AC009951.1		Pseudoger	chr2:144461112-144
ENSG0000(	908	20.56063	chrX:478(	GDPD2		protein_c	chrX:70423031-7043
ENSG0000(	908	20.56063	chrX:478(	ENSG00000276391		Pseudoger	chrX:85207187-8521
ENSG0000(	908	20.56063	chrX:478(	CHMP1B2P		Pseudoger	chrX:80228489-8033
ENSG0000(	908	20.56063	chrX:478(	U3		smallRNA	chrX:70846081-7084
ENSG0000(	908	20.56063	chrX:478(	NUTF2P7		Pseudoger	chrX:71016531-7101
ENSG0000(	908	20.56063	chrX:478(	ENSG00000226820		Pseudoger	chrX:66437345-6644
ENSG0000(	908	20.56063	chrX:478(	ENSG00000270745		Pseudoger	chrX:88982215-8898
ENSG0000(	908	20.56063	chrX:478(	SOCS5P4		Pseudoger	chrX:71043214-7104
ENSG0000(	908	20.56063	chrX:478(	RNA5SP508		Pseudoger	chrX:76655009-7665
ENSG0000(	908	20.56063	chrX:478(	DDX3P1		Pseudoger	chrX:74121012-7413
ENSG0000(	908	20.56063	chrX:478(	TENT5D	NCGv7	protein_c	chrX:80335504-8044
ENSG0000(	908	20.56063	chrX:478(	RNU6-854P		smallRNA	chrX:77837289-7783
ENSG0000(	908	20.56063	chrX:478(	MIR374B		smallRNA	chrX:74218547-7421
ENSG0000(	908	20.56063	chr2:2577	snoU13		smallRNA	chr2:156422195-156
ENSG0000(	908	20.56063	chrX:478(	HK2P1		Pseudoger	chrX:80571871-8057
ENSG0000(	908	20.56063	chrX:478(	RNF19BPX		Pseudoger	chrX:90209796-9021
ENSG0000(	908	20.56063	chrX:478(	NONO	IntOGen-I	protein_c	chrX:71254814-7130
ENSG0000(	908	20.56063	chr2:2577	KCNJ3		protein_c	chr2:154697855-154
ENSG0000(	908	20.56063	chr2:2577	AC019201.1		smallRNA	chr2:157441550-157
ENSG0000(	908	20.56063	chr2:2577	AC009297.1		smallRNA	chr2:154231718-154
ENSG0000(	908	20.56063	chrX:478(	MIR676		smallRNA	chrX:70022857-7002
ENSG0000(	908	20.56063	chrX:478(	Z95400.1		smallRNA	chrX:87485879-8748
ENSG0000(	908	20.56063	chrX:478(	RNU6-245P		smallRNA	chrX:68539443-6853
ENSG0000(	908	20.56063	chr2:2577	ARL5A		protein_c	chr2:151788984-151
ENSG0000(	908	20.56063	chrX:478(	RN7SL641P		smallRNA	chrX:74453623-7445
ENSG0000(	904	20.47006	chr2:2577	EDDM3CP		Pseudoger	chr2:134319654-134
ENSG0000(	904	20.47006	chr2:2577	AC013718.1		Pseudoger	chr2:134456196-134

ENSG0000(	902	20.42477	chr4:253(	RNU6-1282P		smallRNA	chr4:151300518-151
ENSG0000(	902	20.42477	chr4:253(	ENSG00000249012		Pseudoger	chr4:151259503-151
ENSG0000(	902	20.42477	chr4:253(	PRSS48		protein_c	chr4:151277161-151
ENSG0000(	902	20.42477	chr4:253(	ENSG00000286761		lncRNA	chr4:151883711-151
ENSG0000(	902	20.42477	chr4:253(	ENSG00000248991		lncRNA	chr4:151912853-151
ENSG0000(	902	20.42477	chr4:253(	ENSG00000249708		lncRNA	chr4:151887543-151
ENSG0000(	902	20.42477	chr4:253(	ENSG00000270681		lncRNA	chr4:151139991-151
ENSG0000(	902	20.42477	chr4:253(	FHIP1A-DT		lncRNA	chr4:151407551-151
ENSG0000(	902	20.42477	chr4:253(	ENSG00000251249		lncRNA	chr4:151955991-151
ENSG0000(	902	20.42477	chr4:253(	AC097455.1		smallRNA	chr4:151565723-151
ENSG0000(	902	20.42477	chr4:253(	RNA5SP169		Pseudoger	chr4:151971497-151
ENSG0000(	902	20.42477	chr4:253(	ENSG00000286066		lncRNA	chr4:151949338-151
ENSG0000(	902	20.42477	chr4:253(	ENSG00000249818		lncRNA	chr4:151904932-151
ENSG0000(	902	20.42477	chr4:253(	FHIP1A		protein_c	chr4:151409176-151
ENSG0000(	902	20.42477	chr4:253(	ENSG00000251455		lncRNA	chr4:151674483-151
ENSG0000(	902	20.42477	chr4:253(	ENSG00000249184		lncRNA	chr4:151798917-151
ENSG0000(	902	20.42477	chr4:253(	ENSG00000270265		lncRNA	chr4:151333775-151
ENSG0000(	902	20.42477	chr4:253(	SH3D19		protein_c	chr4:151102751-151
ENSG0000(	902	20.42477	chr4:253(	ENSG00000250350		Pseudoger	chr4:151279030-151
ENSG0000(	902	20.42477	chr4:253(	GATB		protein_c	chr4:151670504-151
ENSG0000(	900	20.37948	chr2:8187	ENSG00000287771		lncRNA	chr2:102037963-102
ENSG0000(	900	20.37948	chr2:8187	RFX8		protein_c	chr2:101397359-101
ENSG0000(	900	20.37948	chr2:8187	RNF149		protein_c	chr2:101271219-101
ENSG0000(	900	20.37948	chr2:8187	LINC01127		lncRNA	chr2:101962052-101
ENSG0000(	900	20.37948	chr2:8187	IL1R2		protein_c	chr2:101991960-102
ENSG0000(	900	20.37948	chr2:8187	CNOT11		protein_c	chr2:101252886-101
ENSG0000(	900	20.37948	chr2:8187	ENSG00000286737		lncRNA	chr2:100739958-100
ENSG0000(	900	20.37948	chr2:8187	LINC01868		lncRNA	chr2:100669892-100
ENSG0000(	900	20.37948	chr1:3733	ENSG00000285794		Pseudoger	chr1:22030527-2203
ENSG0000(	900	20.37948	chr2:8187	ENSG00000223947		lncRNA	chr2:100993676-101
ENSG0000(	900	20.37948	chr2:8187	ENSG00000289167		lncRNA	chr2:101961603-101
ENSG0000(	900	20.37948	chr2:8187	ENSG00000228488		lncRNA	chr2:100722221-100
ENSG0000(	900	20.37948	chr2:8187	ENSG00000289077		lncRNA	chr2:100818723-100
ENSG0000(	900	20.37948	chr2:8187	ENSG00000286047		lncRNA	chr2:101535601-101
ENSG0000(	900	20.37948	chr2:8187	ENSG00000232034		lncRNA	chr2:100822661-100
ENSG0000(	900	20.37948	chr2:8187	PRCPP1		Pseudoger	chr2:101520834-101
ENSG0000(	900	20.37948	chr2:8187	NPAS2		protein_c	chr2:100820139-100
ENSG0000(	900	20.37948	chr2:8187	NPAS2-AS1		lncRNA	chr2:100970767-100
ENSG0000(	900	20.37948	chr2:8187	SNORD89		smallRNA	chr2:101272936-101
ENSG0000(	900	20.37948	chr2:8187	MAP4K4		protein_c	chr2:101696850-101
ENSG0000(	900	20.37948	chr2:8187	FLJ20373		protein_c	chr2:101892493-101
ENSG0000(	900	20.37948	chr2:8187	RPS6P3		Pseudoger	chr2:101509530-101
ENSG0000(	900	20.37948	chr2:8187	BBIP1P1		Pseudoger	chr2:101068378-101
ENSG0000(	900	20.37948	chr2:8187	RPL31	NCGv7	protein_c	chr2:101002229-101
ENSG0000(	900	20.37948	chr2:8187	TBC1D8-AS1		lncRNA	chr2:101151660-101
ENSG0000(	900	20.37948	chr2:8187	CREG2		protein_c	chr2:101345550-101
ENSG0000(	900	20.37948	chr2:8187	ENSG00000226058		Pseudoger	chr2:101375587-101
ENSG0000(	900	20.37948	chr2:8187	LINC01870		lncRNA	chr2:101479390-101
ENSG0000(	900	20.37948	chr2:8187	MIR5696		smallRNA	chr2:101309450-101
ENSG0000(	900	20.37948	chr2:8187	TBC1D8		protein_c	chr2:101007228-101
ENSG0000(	900	20.37948	chr2:8187	ENSG00000288948		lncRNA	chr2:101797890-101

ENSG0000(	900	20.37948	chr2:8187	RN7SL548P		smallRNA	chr2:101093569-101
ENSG0000(	900	20.37948	chr2:8187	ENSG00000286101		lncRNA	chr2:101551592-101
ENSG0000(	899	20.35684	chr1:1021	SNORD46		smallRNA	chr1:44776490-4477
ENSG0000(	899	20.35684	chr2:8187	RPS12P3		Pseudoger	chr2:60938204-6093
ENSG0000(	899	20.35684	chr1:1021	SNORD55		smallRNA	chr1:44775864-4477
ENSG0000(	898	20.33419	chr2:2577	ZEB2		protein_c	chr2:144364364-144
ENSG0000(	898	20.33419	chr2:2577	ENSG00000232377		lncRNA	chr2:143937073-143
ENSG0000(	898	20.33419	chr2:2577	GTDC1		protein_c	chr2:143938068-144
ENSG0000(	897	20.31155	chr10:11(	TCERG1L-AS1		lncRNA	chr10:131095218-13
ENSG0000(	897	20.31155	chr10:11(	AL607076.1		smallRNA	chr10:130995306-13
ENSG0000(	897	20.31155	chr10:11(	MIR378C		smallRNA	chr10:130962588-13
ENSG0000(	897	20.31155	chr10:11(	TCERG1L		protein_c	chr10:131092391-13
ENSG0000(	892	20.19833	chr2:8187	MIR4432		smallRNA	chr2:60387362-6038
ENSG0000(	892	20.19833	chr2:8187	RN7SL361P		smallRNA	chr2:60640705-6064
ENSG0000(	892	20.19833	chr2:8187	RNA5SP94		Pseudoger	chr2:59694762-5969
ENSG0000(	892	20.19833	chr2:8187	ENSG00000233953		lncRNA	chr2:60495686-6049
ENSG0000(	892	20.19833	chr2:8187	IFITM3P9		Pseudoger	chr2:60682873-6068
ENSG0000(	892	20.19833	chr2:8187	AC007131.3		smallRNA	chr2:59241621-5924
ENSG0000(	892	20.19833	chr2:8187	ENSG00000231815		lncRNA	chr2:59434552-5944
ENSG0000(	892	20.19833	chr2:8187	ENSG00000287640		lncRNA	chr2:60383141-6038
ENSG0000(	892	20.19833	chr2:8187	RNU6-508P		smallRNA	chr2:59647621-5964
ENSG0000(	892	20.19833	chr2:8187	MIR4432HG		lncRNA	chr2:60336446-6043
ENSG0000(	892	20.19833	chr2:8187	RNU1-32P		smallRNA	chr2:60384605-6038
ENSG0000(	892	20.19833	chr2:8187	ENSG00000270447		Pseudoger	chr2:59514890-5951
ENSG0000(	892	20.19833	chr2:8187	ENSG00000271955		lncRNA	chr2:59218680-6010
ENSG0000(	892	20.19833	chr2:8187	ENSG00000233891		lncRNA	chr2:59238703-5973
ENSG0000(	892	20.19833	chr2:8187	AC007179.2		smallRNA	chr2:59532984-5953
ENSG0000(	892	20.19833	chr2:8187	ENSG00000285611		lncRNA	chr2:60057601-6007
ENSG0000(	892	20.19833	chr2:8187	ENSG00000285673		lncRNA	chr2:59014354-5927
ENSG0000(	892	20.19833	chr2:8187	LINC01793		lncRNA	chr2:59217708-5927
ENSG0000(	892	20.19833	chr2:8187	BCL11A	NCGv7;AC	protein_c	chr2:60450520-6055
ENSG0000(	892	20.19833	chr2:8187	ENSG00000286604		lncRNA	chr2:59778685-5979
ENSG0000(	891	20.17569	chr2:8187	RPS28P5		Pseudoger	chr2:74754670-7475
ENSG0000(	891	20.17569	chr8:2747	RNU7-177P		smallRNA	chr8:97773978-9777
ENSG0000(	884	20.01718	chr2:8187	ENSG00000279181		TEC	chr2:73998337-7399
ENSG0000(	879	19.90396	chr2:8187	Y_RNA		smallRNA	chr2:55286018-5528
ENSG0000(	877	19.85867	chr2:8187	REL	NCGv7;AC	protein_c	chr2:60881491-6093
ENSG0000(	877	19.85867	chr2:8187	ACTG2		protein_c	chr2:73892314-7391
ENSG0000(	877	19.85867	chr2:8187	ENSG00000271452		lncRNA	chr2:75669989-7567
ENSG0000(	877	19.85867	chr2:8187	RN7SL635P		smallRNA	chr2:65545403-6554
ENSG0000(	877	19.85867	chr2:8187	PUS10		protein_c	chr2:60940222-6101
ENSG0000(	877	19.85867	chr2:8187	LINC01798		lncRNA	chr2:66574030-6673
ENSG0000(	877	19.85867	chr2:8187	DYSF	NCGv7	protein_c	chr2:71453561-7168
ENSG0000(	877	19.85867	chr2:8187	SPR		protein_c	chr2:72887382-7289
ENSG0000(	877	19.85867	chr2:8187	CLEC4F		protein_c	chr2:70808643-7082
ENSG0000(	877	19.85867	chr2:8187	ENSG00000286045		lncRNA	chr2:75710782-7572
ENSG0000(	877	19.85867	chr2:8187	CCDC142		protein_c	chr2:74471986-7448
ENSG0000(	877	19.85867	chr2:8187	RNA5SP98		Pseudoger	chr2:76772909-7677
ENSG0000(	877	19.85867	chr2:8187	MXD1		protein_c	chr2:69897688-6994
ENSG0000(	877	19.85867	chr2:8187	ENSG00000284902		lncRNA	chr2:73469525-7347
ENSG0000(	877	19.85867	chr2:8187	RNA5SP97		Pseudoger	chr2:73968123-7396

ENSG0000(	877	19.85867	chr2:8187	MTHFD2	NCGv7	protein_c	chr2:74186172-7421
ENSG0000(	877	19.85867	chr2:8187	RPS10P9		Pseudoger	chr2:64665607-6466
ENSG0000(	877	19.85867	chr2:8187	ENSG00000286202		lncRNA	chr2:76691007-7669
ENSG0000(	877	19.85867	chr2:8187	SMYD5		protein_c	chr2:73214222-7322
ENSG0000(	877	19.85867	chr2:8187	PEX13		protein_c	chr2:61017225-6105
ENSG0000(	877	19.85867	chr2:8187	Y_RNA		smallRNA	chr2:64834056-6483
ENSG0000(	877	19.85867	chr2:8187	MIR3126		smallRNA	chr2:69103682-6910
ENSG0000(	877	19.85867	chr2:8187	RNU6-105P		smallRNA	chr2:71379755-7137
ENSG0000(	877	19.85867	chr2:8187	AC016727.1		smallRNA	chr2:61555360-6155
ENSG0000(	877	19.85867	chr2:8187	ALMS1	NCGv7	protein_c	chr2:73385758-7362
ENSG0000(	877	19.85867	chr2:8187	BRD7P6		Pseudoger	chr2:70353010-7035
ENSG0000(	877	19.85867	chr2:8187	ALMS1P1		Pseudoger	chr2:73671030-7368
ENSG0000(	877	19.85867	chr2:8187	TGFA-IT1		lncRNA	chr2:70467385-7046
ENSG0000(	877	19.85867	chr2:8187	ENSG00000279485		TEC	chr2:63517892-6351
ENSG0000(	877	19.85867	chr2:8187	TGFA	NCGv7	protein_c	chr2:70447284-7055
ENSG0000(	877	19.85867	chr2:8187	AC007040.1		smallRNA	chr2:71017024-7101
ENSG0000(	877	19.85867	chr2:8187	LGALSL		protein_c	chr2:64453969-6446
ENSG0000(	877	19.85867	chr2:8187	ENSG00000287026		lncRNA	chr2:76633365-7667
ENSG0000(	877	19.85867	chr2:8187	ENSG00000234624		Pseudoger	chr2:61416887-6141
ENSG0000(	877	19.85867	chr2:8187	AC007365.2		smallRNA	chr2:64776104-6477
ENSG0000(	877	19.85867	chr2:8187	LINC01293		lncRNA	chr2:74940258-7494
ENSG0000(	877	19.85867	chr2:8187	NECAP1P2		Pseudoger	chr2:74350069-7435
ENSG0000(	877	19.85867	chr2:8187	RN7SKP203		smallRNA	chr2:76445079-7644
ENSG0000(	877	19.85867	chr2:8187	ENSG00000235035		lncRNA	chr2:70687142-7069
ENSG0000(	877	19.85867	chr2:8187	FNBP1P1		Pseudoger	chr2:74120680-7412
ENSG0000(	877	19.85867	chr2:8187	ENSG00000278060		lncRNA	chr2:72932974-7293
ENSG0000(	877	19.85867	chr2:8187	PRADC1		protein_c	chr2:73228010-7323
ENSG0000(	877	19.85867	chr2:8187	RPL11P1		Pseudoger	chr2:64960053-6496
ENSG0000(	877	19.85867	chr2:8187	Y_RNA		smallRNA	chr2:62726636-6272
ENSG0000(	877	19.85867	chr2:8187	RPL23AP92		Pseudoger	chr2:69873565-6987
ENSG0000(	877	19.85867	chr2:8187	RNU6-100P		smallRNA	chr2:64578892-6457
ENSG0000(	877	19.85867	chr2:8187	LBX2-AS1		lncRNA	chr2:74502552-7450
ENSG0000(	877	19.85867	chr2:8187	FIGLA		protein_c	chr2:70777310-7079
ENSG0000(	877	19.85867	chr2:8187	AFTPH		protein_c	chr2:64524299-6459
ENSG0000(	877	19.85867	chr2:8187	ENSG00000270996		lncRNA	chr2:75719120-7572
ENSG0000(	877	19.85867	chr2:8187	ENSG00000286883		lncRNA	chr2:74465339-7447
ENSG0000(	877	19.85867	chr2:8187	USP21P2		Pseudoger	chr2:76260755-7626
ENSG0000(	877	19.85867	chr2:8187	AC007040.2		smallRNA	chr2:71012331-7101
ENSG0000(	877	19.85867	chr2:8187	ENSG00000286979		lncRNA	chr2:70629657-7064
ENSG0000(	877	19.85867	chr2:8187	LRRTM4-AS1		lncRNA	chr2:76985965-7700
ENSG0000(	877	19.85867	chr2:8187	DBIL5P2		Pseudoger	chr2:63117851-6311
ENSG0000(	877	19.85867	chr2:8187	GKN2		protein_c	chr2:68945232-6895
ENSG0000(	877	19.85867	chr2:8187	ALMS1P1		lncRNA	chr2:73641083-7369
ENSG0000(	877	19.85867	chr2:8187	ENSG00000284932		Pseudoger	chr2:68117026-6811
ENSG0000(	877	19.85867	chr2:8187	ARHGAP25		protein_c	chr2:68679601-6882
ENSG0000(	877	19.85867	chr2:8187	CNRIP1		protein_c	chr2:68284171-6832
ENSG0000(	877	19.85867	chr2:8187	RSL24D1P2		Pseudoger	chr2:62561058-6256
ENSG0000(	877	19.85867	chr2:8187	FBXO41		protein_c	chr2:73254682-7328
ENSG0000(	877	19.85867	chr2:8187	RPL31P30		Pseudoger	chr2:61856695-6185
ENSG0000(	877	19.85867	chr2:8187	ENSG00000227293		lncRNA	chr2:66235377-6623
ENSG0000(	877	19.85867	chr2:8187	ENSG00000271146		Pseudoger	chr2:61249780-6125

ENSG0000(	877	19.85867	chr2:8187	MEIS1-AS2	lncRNA	chr2:66439088-6644
ENSG0000(	877	19.85867	chr2:8187	RAB11FIP5	protein_c	chr2:73073382-7315
ENSG0000(	877	19.85867	chr2:8187	ENSG00000270696	lncRNA	chr2:75660462-7566
ENSG0000(	877	19.85867	chr2:8187	MIR5192	smallRNA	chr2:62205826-6220
ENSG0000(	877	19.85867	chr2:8187	LINC00309	lncRNA	chr2:64185078-6420
ENSG0000(	877	19.85867	chr2:8187	RNU4-51P	smallRNA	chr2:60911303-6091
ENSG0000(	877	19.85867	chr2:8187	EGR4	protein_c	chr2:73290929-7329
ENSG0000(	877	19.85867	chr2:8187	ENSG00000286244	lncRNA	chr2:73834422-7385
ENSG0000(	877	19.85867	chr2:8187	SANBR	protein_c	chr2:61065871-6113
ENSG0000(	877	19.85867	chr2:8187	ENSG00000281920	lncRNA	chr2:65623272-6562
ENSG0000(	877	19.85867	chr2:8187	ENSG00000290118	lncRNA	chr2:64275361-6427
ENSG0000(	877	19.85867	chr2:8187	ENSG00000287025	lncRNA	chr2:76893894-7689
ENSG0000(	877	19.85867	chr2:8187	BOLA3	protein_c	chr2:74135400-7414
ENSG0000(	877	19.85867	chr2:8187	ATP6V1B1	protein_c	chr2:70935900-7096
ENSG0000(	877	19.85867	chr2:8187	RPS24P7	Pseudoger	chr2:61803143-6180
ENSG0000(	877	19.85867	chr2:8187	USP34-DT	lncRNA	chr2:61471188-6148
ENSG0000(	877	19.85867	chr2:8187	OR7E62P	Pseudoger	chr2:71055527-7105
ENSG0000(	877	19.85867	chr2:8187	SEMA4F	protein_c	chr2:74654228-7468
ENSG0000(	877	19.85867	chr2:8187	RPSAP26	Pseudoger	chr2:62146413-6214
ENSG0000(	877	19.85867	chr2:8187	ENSG00000230968	lncRNA	chr2:77672215-7767
ENSG0000(	877	19.85867	chr2:8187	BMP10	protein_c	chr2:68860909-6887
ENSG0000(	877	19.85867	chr2:8187	RN7SL632P	smallRNA	chr2:60831665-6083
ENSG0000(	877	19.85867	chr2:8187	ENSG00000271243	Pseudoger	chr2:61575774-6157
ENSG0000(	877	19.85867	chr2:8187	AC007389.4	smallRNA	chr2:65667256-6566
ENSG0000(	877	19.85867	chr2:8187	RN7SL18P	smallRNA	chr2:62491178-6249
ENSG0000(	877	19.85867	chr2:8187	CCT7	protein_c	chr2:73233420-7325
ENSG0000(	877	19.85867	chr2:8187	MIR4778	smallRNA	chr2:66358249-6635
ENSG0000(	877	19.85867	chr2:8187	VAX2	protein_c	chr2:70900576-7096
ENSG0000(	877	19.85867	chr2:8187	RN7SL470P	smallRNA	chr2:70075018-7007
ENSG0000(	877	19.85867	chr2:8187	LINC01812	lncRNA	chr2:67796054-6782
ENSG0000(	877	19.85867	chr2:8187	ENSG00000279070	lncRNA	chr2:74196698-7419
ENSG0000(	877	19.85867	chr2:8187	AC017083.3	smallRNA	chr2:68273104-6827
ENSG0000(	877	19.85867	chr2:8187	DUSP11	protein_c	chr2:73761782-7378
ENSG0000(	877	19.85867	chr2:8187	AFTPH-DT	lncRNA	chr2:64522187-6452
ENSG0000(	877	19.85867	chr2:8187	GKN3P	Pseudoger	chr2:68921248-6892
ENSG0000(	877	19.85867	chr2:8187	DQX1	protein_c	chr2:74518131-7452
ENSG0000(	877	19.85867	chr2:8187	TEX261	protein_c	chr2:70968325-7099
ENSG0000(	877	19.85867	chr2:8187	ENSG00000286360	lncRNA	chr2:61878940-6188
ENSG0000(	877	19.85867	chr2:8187	SFXN5	protein_c	chr2:72942036-7307
ENSG0000(	877	19.85867	chr2:8187	C1D	protein_c	chr2:68041130-6811
ENSG0000(	877	19.85867	chr2:8187	EXOC6B	protein_c	chr2:72175984-7282
ENSG0000(	877	19.85867	chr2:8187	NAT8	protein_c	chr2:73640723-7364
ENSG0000(	877	19.85867	chr2:8187	ENSG00000288869	lncRNA	chr2:69789544-6979
ENSG0000(	877	19.85867	chr2:8187	TPRKB	protein_c	chr2:73729104-7373
ENSG0000(	877	19.85867	chr2:8187	snoU13	smallRNA	chr2:62264995-6226
ENSG0000(	877	19.85867	chr2:8187	ANKRD53	protein_c	chr2:70978380-7098
ENSG0000(	877	19.85867	chr2:8187	RPL21P33	Pseudoger	chr2:60852260-6085
ENSG0000(	877	19.85867	chr2:8187	MIR1285-2	smallRNA	chr2:70252918-7025
ENSG0000(	877	19.85867	chr2:8187	SNRPG	protein_c	chr2:70281362-7029
ENSG0000(	877	19.85867	chr2:8187	RPL38P2	Pseudoger	chr2:77788382-7779
ENSG0000(	877	19.85867	chr2:8187	ENSG00000273064	lncRNA	chr2:68252870-6825

ENSG0000(	877	19.85867	chr2:8187	ENSG00000217702		lncRNA	chr2:74123965-7413
ENSG0000(	877	19.85867	chr2:8187	ENSG00000287687		lncRNA	chr2:74723873-7477
ENSG0000(	877	19.85867	chr2:8187	COMMD1		protein_c	chr2:61888724-6214
ENSG0000(	877	19.85867	chr2:8187	ENSG00000237217		Pseudoger	chr2:64450096-6445
ENSG0000(	877	19.85867	chr2:8187	HNRNPA1P66		Pseudoger	chr2:63751697-6375
ENSG0000(	877	19.85867	chr2:8187	B3GALNT1P1		Pseudoger	chr2:69597353-6959
ENSG0000(	877	19.85867	chr2:8187	AHSA2P		Pseudoger	chr2:61177418-6119
ENSG0000(	877	19.85867	chr2:8187	LINC01797		lncRNA	chr2:66696190-6670
ENSG0000(	877	19.85867	chr2:8187	RPS20P9		Pseudoger	chr2:62939916-6294
ENSG0000(	877	19.85867	chr2:8187	ALMS1-IT1		lncRNA	chr2:73456764-7345
ENSG0000(	877	19.85867	chr2:8187	AC096664.3		smallRNA	chr2:63695393-6369
ENSG0000(	877	19.85867	chr2:8187	AC096546.1		smallRNA	chr2:73488579-7348
ENSG0000(	877	19.85867	chr2:8187	SLC4A5	NCGv7	protein_c	chr2:74216242-7434
ENSG0000(	877	19.85867	chr2:8187	ANXA4		protein_c	chr2:69644425-6982
ENSG0000(	877	19.85867	chr2:8187	snoU13		smallRNA	chr2:64908587-6490
ENSG0000(	877	19.85867	chr2:8187	snoU13		smallRNA	chr2:69667359-6966
ENSG0000(	877	19.85867	chr2:8187	MEIS1	TAG;AC	protein_c	chr2:66433452-6657
ENSG0000(	877	19.85867	chr2:8187	ETAA1		protein_c	chr2:67397322-6741
ENSG0000(	877	19.85867	chr2:8187	ENSG00000236469		lncRNA	chr2:71002531-7106
ENSG0000(	877	19.85867	chr2:8187	ENSG00000236605		lncRNA	chr2:67324627-6732
ENSG0000(	877	19.85867	chr2:8187	snoU13		smallRNA	chr2:75635141-7563
ENSG0000(	877	19.85867	chr2:8187	ENSG00000280037		TEC	chr2:73761269-7376
ENSG0000(	877	19.85867	chr2:8187	HK2-DT		lncRNA	chr2:74832655-7483
ENSG0000(	877	19.85867	chr2:8187	ENSG00000289156		lncRNA	chr2:68251603-6834
ENSG0000(	877	19.85867	chr2:8187	ENSG00000272702		lncRNA	chr2:73113018-7311
ENSG0000(	877	19.85867	chr2:8187	ENSG00000287474		lncRNA	chr2:75799974-7620
ENSG0000(	877	19.85867	chr2:8187	AC007881.1		smallRNA	chr2:71125523-7112
ENSG0000(	877	19.85867	chr2:8187	ACA59		smallRNA	chr2:63883249-6388
ENSG0000(	877	19.85867	chr2:8187	ENSG00000289176		lncRNA	chr2:64337103-6434
ENSG0000(	877	19.85867	chr2:8187	C2orf78		protein_c	chr2:73784183-7381
ENSG0000(	877	19.85867	chr2:8187	snoU13		smallRNA	chr2:70930340-7093
ENSG0000(	877	19.85867	chr2:8187	snoU13		smallRNA	chr2:69955645-6995
ENSG0000(	877	19.85867	chr2:8187	CSP1		Pseudoger	chr2:63717122-6371
ENSG0000(	877	19.85867	chr2:8187	CD207		protein_c	chr2:70830211-7083
ENSG0000(	877	19.85867	chr2:8187	ENSG00000289247		lncRNA	chr2:61854376-6185
ENSG0000(	877	19.85867	chr2:8187	ENSG00000287435		lncRNA	chr2:73305941-7330
ENSG0000(	877	19.85867	chr2:8187	ENSG00000289250		lncRNA	chr2:69663299-6966
ENSG0000(	877	19.85867	chr2:8187	MTFR2P1		Pseudoger	chr2:63232453-6323
ENSG0000(	877	19.85867	chr2:8187	ENSG00000270354		Pseudoger	chr2:64208498-6420
ENSG0000(	877	19.85867	chr2:8187	ENSG00000275381		Pseudoger	chr2:69844509-6984
ENSG0000(	877	19.85867	chr2:8187	ENSG00000225815		lncRNA	chr2:66327349-6632
ENSG0000(	877	19.85867	chr2:8187	RPL27P5		Pseudoger	chr2:63108118-6310
ENSG0000(	877	19.85867	chr2:8187	ENSG00000288932		lncRNA	chr2:64273709-6429
ENSG0000(	877	19.85867	chr2:8187	PELI1		protein_c	chr2:64092652-6414
ENSG0000(	877	19.85867	chr2:8187	VPS54		protein_c	chr2:63892146-6401
ENSG0000(	877	19.85867	chr2:8187	EVA1A-AS		lncRNA	chr2:75524068-7554
ENSG0000(	877	19.85867	chr2:8187	WDPCP		protein_c	chr2:63119559-6382
ENSG0000(	877	19.85867	chr2:8187	U3		smallRNA	chr2:75627953-7562
ENSG0000(	877	19.85867	chr2:8187	LINC01829		lncRNA	chr2:67123357-6739
ENSG0000(	877	19.85867	chr2:8187	MIR5000		smallRNA	chr2:75090812-7509
ENSG0000(	877	19.85867	chr2:8187	ENSG00000288986		lncRNA	chr2:64765463-6476

ENSG0000(	877	19.85867	chr2:8187	ENSG00000226756	lncRNA	chr2:64644612-6464
ENSG0000(	877	19.85867	chr2:8187	ENSG00000286347	lncRNA	chr2:69030042-6903
ENSG0000(	877	19.85867	chr2:8187	ENSG00000225889	lncRNA	chr2:64143239-6425
ENSG0000(	877	19.85867	chr2:8187	RNU6-111P	smallRNA	chr2:73298683-7329
ENSG0000(	877	19.85867	chr2:8187	ENSG00000270335	Pseudoger	chr2:62348948-6235
ENSG0000(	877	19.85867	chr2:8187	ENSG00000272735	lncRNA	chr2:71067519-7106
ENSG0000(	877	19.85867	chr2:8187	PPP3R1	protein_c	chr2:68178857-6825
ENSG0000(	877	19.85867	chr2:8187	WDR54	protein_c	chr2:74421678-7442
ENSG0000(	877	19.85867	chr2:8187	RPS4XP5	Pseudoger	chr2:63642455-6364
ENSG0000(	877	19.85867	chr2:8187	ENSG00000229229	lncRNA	chr2:70402934-7042
ENSG0000(	877	19.85867	chr2:8187	STAMBP	protein_c	chr2:73828916-7387
ENSG0000(	877	19.85867	chr2:8187	ENSG00000233849	lncRNA	chr2:70301451-7030
ENSG0000(	877	19.85867	chr2:8187	ASPRV1	protein_c	chr2:69960089-6996
ENSG0000(	877	19.85867	chr2:8187	ENSG00000288065	lncRNA	chr2:71112877-7111
ENSG0000(	877	19.85867	chr2:8187	UGP2	protein_c	chr2:63840952-6389
ENSG0000(	877	19.85867	chr2:8187	C2orf74	protein_c	chr2:61145068-6116
ENSG0000(	877	19.85867	chr2:8187	RPL26P13	Pseudoger	chr2:60711484-6071
ENSG0000(	877	19.85867	chr2:8187	KRT18P26	Pseudoger	chr2:74306728-7430
ENSG0000(	877	19.85867	chr2:8187	ENSG00000213486	Pseudoger	chr2:61710076-6171
ENSG0000(	877	19.85867	chr2:8187	LINC02245	lncRNA	chr2:64901840-6505
ENSG0000(	877	19.85867	chr2:8187	ENSG00000233870	Pseudoger	chr2:71178587-7117
ENSG0000(	877	19.85867	chr2:8187	APLF	protein_c	chr2:68467572-6865
ENSG0000(	877	19.85867	chr2:8187	TOR1BP1	Pseudoger	chr2:74618856-7462
ENSG0000(	877	19.85867	chr2:8187	PROKR1	protein_c	chr2:68643579-6865
ENSG0000(	877	19.85867	chr2:8187	GKN1	protein_c	chr2:68974573-6898
ENSG0000(	877	19.85867	chr2:8187	ANTXR1	protein_c	chr2:69013176-6924
ENSG0000(	877	19.85867	chr2:8187	NFU1	protein_c	chr2:69396113-6943
ENSG0000(	877	19.85867	chr2:8187	RN7SL211P	smallRNA	chr2:64906865-6490
ENSG0000(	877	19.85867	chr2:8187	ENSG00000273763	Pseudoger	chr2:65203502-6522
ENSG0000(	877	19.85867	chr2:8187	NAGK	protein_c	chr2:71064344-7107
ENSG0000(	877	19.85867	chr2:8187	ELOCP21	Pseudoger	chr2:70955899-7095
ENSG0000(	877	19.85867	chr2:8187	MCEE	protein_c	chr2:71109684-7113
ENSG0000(	877	19.85867	chr2:8187	DGUOK-AS1	lncRNA	chr2:73947322-7398
ENSG0000(	877	19.85867	chr2:8187	B3GNT2	protein_c	chr2:62196115-6222
ENSG0000(	877	19.85867	chr2:8187	INO80B-WBP1	protein_c	chr2:74455088-7446
ENSG0000(	877	19.85867	chr2:8187	AC007386.4	lncRNA	chr2:65030727-6503
ENSG0000(	877	19.85867	chr2:8187	RPL39P15	Pseudoger	chr2:70253855-7025
ENSG0000(	877	19.85867	chr2:8187	FAM161A	protein_c	chr2:61824848-6185
ENSG0000(	877	19.85867	chr2:8187	ENSG00000280257	TEC	chr2:65790039-6579
ENSG0000(	877	19.85867	chr2:8187	DNAJB12P1	Pseudoger	chr2:65500993-6550
ENSG0000(	877	19.85867	chr2:8187	LINC01143	lncRNA	chr2:70887871-7088
ENSG0000(	877	19.85867	chr2:8187	NOTO	protein_c	chr2:73202574-7321
ENSG0000(	877	19.85867	chr2:8187	ENSG00000214525	Pseudoger	chr2:68528241-6852
ENSG0000(	877	19.85867	chr2:8187	ENSG00000238012	lncRNA	chr2:64330481-6433
ENSG0000(	877	19.85867	chr2:8187	KRT18P33	Pseudoger	chr2:65666695-6566
ENSG0000(	877	19.85867	chr2:8187	DCTN1-AS1	lncRNA	chr2:74385474-7439
ENSG0000(	877	19.85867	chr2:8187	MPHOSPH10	protein_c	chr2:71130310-7115
ENSG0000(	877	19.85867	chr2:8187	SNRNP27	protein_c	chr2:69893956-6990
ENSG0000(	877	19.85867	chr2:8187	PAIP2B	protein_c	chr2:71182738-7122
ENSG0000(	877	19.85867	chr2:8187	ENSG00000229831	Pseudoger	chr2:61820208-6182
ENSG0000(	877	19.85867	chr2:8187	ENSG00000279201	TEC	chr2:74191388-7419

ENSG0000(	877	19.85867	chr2:8187	LINC01888		lncRNA	chr2:68832014-6883
ENSG0000(	877	19.85867	chr2:8187	PCBP1	NCGv7	protein_c	chr2:70087477-7008
ENSG0000(	877	19.85867	chr2:8187	ENSG00000226622		lncRNA	chr2:62533681-6266
ENSG0000(	877	19.85867	chr2:8187	snoU109		smallRNA	chr2:75489576-7548
ENSG0000(	877	19.85867	chr2:8187	RPS20P10		Pseudoger	chr2:71984182-7198
ENSG0000(	877	19.85867	chr2:8187	LINC01628		lncRNA	chr2:66921510-6692
ENSG0000(	877	19.85867	chr2:8187	ENSG00000232693		lncRNA	chr2:65373700-6538
ENSG0000(	877	19.85867	chr2:8187	SUPT4H1P1		Pseudoger	chr2:75651288-7565
ENSG0000(	877	19.85867	chr2:8187	RN7SL604P		smallRNA	chr2:69516751-6951
ENSG0000(	877	19.85867	chr2:8187	snoU13		smallRNA	chr2:69912695-6991
ENSG0000(	877	19.85867	chr2:8187	ENSG00000274769		lncRNA	chr2:61115787-6116
ENSG0000(	877	19.85867	chr2:8187	AC079117.2		smallRNA	chr2:77020336-7702
ENSG0000(	877	19.85867	chr2:8187	snoU13		smallRNA	chr2:75418846-7541
ENSG0000(	877	19.85867	chr2:8187	ENSG00000237293		lncRNA	chr2:75474453-7548
ENSG0000(	877	19.85867	chr2:8187	AC017083.1		smallRNA	chr2:68229926-6823
ENSG0000(	877	19.85867	chr2:8187	LINC01816		lncRNA	chr2:70124034-7013
ENSG0000(	877	19.85867	chr2:8187	GCFC2		protein_c	chr2:75652000-7571
ENSG0000(	877	19.85867	chr2:8187	AC079117.3		smallRNA	chr2:77041778-7704
ENSG0000(	877	19.85867	chr2:8187	VDAC2P5		Pseudoger	chr2:65205108-6520
ENSG0000(	877	19.85867	chr2:8187	WDR4P2		Pseudoger	chr2:68445710-6844
ENSG0000(	877	19.85867	chr2:8187	ENSG00000226605		lncRNA	chr2:62826064-6285
ENSG0000(	877	19.85867	chr2:8187	RNU6-548P		smallRNA	chr2:64994746-6499
ENSG0000(	877	19.85867	chr2:8187	ENSG00000273302		lncRNA	chr2:61199979-6120
ENSG0000(	877	19.85867	chr2:8187	ENSG00000229839		lncRNA	chr2:62069447-6214
ENSG0000(	877	19.85867	chr2:8187	ENSG00000273398		protein_c	chr2:68131238-6826
ENSG0000(	877	19.85867	chr2:8187	RPL21P37		Pseudoger	chr2:62532583-6253
ENSG0000(	877	19.85867	chr2:8187	C2orf74-DT		lncRNA	chr2:61141592-6114
ENSG0000(	877	19.85867	chr2:8187	ENSG00000286480		lncRNA	chr2:63106879-6319
ENSG0000(	877	19.85867	chr2:8187	C2orf81		protein_c	chr2:74414176-7442
ENSG0000(	877	19.85867	chr2:8187	NONOP2		Pseudoger	chr2:60936819-6093
ENSG0000(	877	19.85867	chr2:8187	CYP26B1		protein_c	chr2:72129238-7214
ENSG0000(	877	19.85867	chr2:8187	SNORD78		smallRNA	chr2:72760534-7276
ENSG0000(	877	19.85867	chr2:8187	ENSG00000238201		lncRNA	chr2:64338067-6434
ENSG0000(	877	19.85867	chr2:8187	ENSG00000273275		lncRNA	chr2:68179833-6818
ENSG0000(	877	19.85867	chr2:8187	TVP23BP2		Pseudoger	chr2:74628328-7462
ENSG0000(	877	19.85867	chr2:8187	MOB4P1		Pseudoger	chr2:70810798-7081
ENSG0000(	877	19.85867	chr2:8187	ENSG00000287749		lncRNA	chr2:76197855-7625
ENSG0000(	877	19.85867	chr2:8187	ENSG00000273245		lncRNA	chr2:73750256-7375
ENSG0000(	877	19.85867	chr2:8187	ENSG00000286524		lncRNA	chr2:62817764-6281
ENSG0000(	877	19.85867	chr2:8187	ENSG00000236498		lncRNA	chr2:61868432-6188
ENSG0000(	877	19.85867	chr2:8187	RNA5SP95		Pseudoger	chr2:60998752-6099
ENSG0000(	877	19.85867	chr2:8187	RNU2-39P		smallRNA	chr2:72721806-7272
ENSG0000(	877	19.85867	chr2:8187	ENSG00000271657		Pseudoger	chr2:62168862-6217
ENSG0000(	877	19.85867	chr2:8187	ENSG00000223859		lncRNA	chr2:67040546-6704
ENSG0000(	877	19.85867	chr2:8187	ATP1B3P1		Pseudoger	chr2:60734895-6073
ENSG0000(	877	19.85867	chr2:8187	LINC01805		lncRNA	chr2:64486353-6450
ENSG0000(	877	19.85867	chr2:8187	ENSG00000230355		Pseudoger	chr2:66881087-6688
ENSG0000(	877	19.85867	chr2:8187	OR7E46P		Pseudoger	chr2:71037685-7103
ENSG0000(	877	19.85867	chr2:8187	RPL27P6		Pseudoger	chr2:63684305-6368
ENSG0000(	877	19.85867	chr2:8187	ENSG00000203395		lncRNA	chr2:68361214-6836
ENSG0000(	877	19.85867	chr2:8187	ENSG00000228079		lncRNA	chr2:64086353-6408

ENSG0000(	877	19.85867	chr2:8187	ENSG00000277498		Pseudoger	chr2:61858137-6186
ENSG0000(	877	19.85867	chr2:8187	PCBP1-AS1		lncRNA	chr2:69960104-7010
ENSG0000(	877	19.85867	chr2:8187	LGALSL-DT		lncRNA	chr2:64395220-6445
ENSG0000(	877	19.85867	chr2:8187	ENSG00000270571		lncRNA	chr2:75154366-7518
ENSG0000(	877	19.85867	chr2:8187	INO80B		protein_c	chr2:74455087-7445
ENSG0000(	877	19.85867	chr2:8187	MOGS		protein_c	chr2:74461057-7446
ENSG0000(	877	19.85867	chr2:8187	TTC31		protein_c	chr2:74483073-7449
ENSG0000(	877	19.85867	chr2:8187	PCGF1		protein_c	chr2:74505043-7450
ENSG0000(	877	19.85867	chr2:8187	TLX2		protein_c	chr2:74513463-7451
ENSG0000(	877	19.85867	chr2:8187	AUP1		protein_c	chr2:74526645-7452
ENSG0000(	877	19.85867	chr2:8187	RTKN		protein_c	chr2:74425835-7444
ENSG0000(	877	19.85867	chr2:8187	MOB1A		protein_c	chr2:74152528-7417
ENSG0000(	877	19.85867	chr2:8187	DGUOK		protein_c	chr2:73926826-7395
ENSG0000(	877	19.85867	chr2:8187	ENSG00000285068		lncRNA	chr2:73352610-7338
ENSG0000(	877	19.85867	chr2:8187	ENSG00000271889		lncRNA	chr2:61151433-6116
ENSG0000(	877	19.85867	chr2:8187	SERTAD2	TAG;AC	protein_c	chr2:64631621-6475
ENSG0000(	877	19.85867	chr2:8187	HK2		protein_c	chr2:74834127-7489
ENSG0000(	877	19.85867	chr2:8187	ENSG00000287130		lncRNA	chr2:76684975-7669
ENSG0000(	877	19.85867	chr2:8187	ENSG00000287123		lncRNA	chr2:64836985-6484
ENSG0000(	877	19.85867	chr2:8187	ENSG00000289855		lncRNA	chr2:61764544-6176
ENSG0000(	877	19.85867	chr2:8187	XPO1	NCGv7;AC	protein_c	chr2:61476032-6153
ENSG0000(	877	19.85867	chr2:8187	ENSG00000233060		lncRNA	chr2:70089721-7009
ENSG0000(	877	19.85867	chr2:8187	PNPP1		Pseudoger	chr2:76258034-7625
ENSG0000(	877	19.85867	chr2:8187	BOLA3-DT		lncRNA	chr2:74148007-7415
ENSG0000(	877	19.85867	chr2:8187	ENSG00000235725		lncRNA	chr2:65589566-6564
ENSG0000(	877	19.85867	chr2:8187	RPS15AP15		Pseudoger	chr2:65511771-6551
ENSG0000(	877	19.85867	chr2:8187	RPS29P10		Pseudoger	chr2:61589498-6158
ENSG0000(	877	19.85867	chr2:8187	PSAT1P2		Pseudoger	chr2:62552463-6255
ENSG0000(	877	19.85867	chr2:8187	MRPL36P1		Pseudoger	chr2:70102551-7010
ENSG0000(	877	19.85867	chr2:8187	ENSG00000289943		lncRNA	chr2:63839866-6384
ENSG0000(	877	19.85867	chr2:8187	SUCLA2P2		Pseudoger	chr2:76106016-7610
ENSG0000(	877	19.85867	chr2:8187	HTRA2	IntOGen-I	protein_c	chr2:74529596-7453
ENSG0000(	877	19.85867	chr2:8187	LOXL3		protein_c	chr2:74532258-7455
ENSG0000(	877	19.85867	chr2:8187	DOK1		protein_c	chr2:74549026-7455
ENSG0000(	877	19.85867	chr2:8187	LINC01800		lncRNA	chr2:64846130-6486
ENSG0000(	877	19.85867	chr2:8187	Y_RNA		smallRNA	chr2:69334600-6933
ENSG0000(	877	19.85867	chr2:8187	ZNF638	NCGv7	protein_c	chr2:71276561-7143
ENSG0000(	877	19.85867	chr2:8187	RN7SL160P		smallRNA	chr2:70965571-7096
ENSG0000(	877	19.85867	chr2:8187	ENSG00000286002		lncRNA	chr2:67677499-6768
ENSG0000(	877	19.85867	chr2:8187	ENSG00000271536		Pseudoger	chr2:73877566-7387
ENSG0000(	877	19.85867	chr2:8187	DNMT3AP1		Pseudoger	chr2:66820684-6682
ENSG0000(	877	19.85867	chr2:8187	HMGA1P8		Pseudoger	chr2:74418122-7441
ENSG0000(	877	19.85867	chr2:8187	RNU6-1216P		smallRNA	chr2:69182877-6918
ENSG0000(	877	19.85867	chr2:8187	SLC1A4		protein_c	chr2:64988477-6502
ENSG0000(	877	19.85867	chr2:8187	PNO1		protein_c	chr2:68157888-6817
ENSG0000(	877	19.85867	chr2:8187	PLEK		protein_c	chr2:68365282-6839
ENSG0000(	877	19.85867	chr2:8187	LINC01799		lncRNA	chr2:66904436-6697
ENSG0000(	877	19.85867	chr2:8187	AAK1		protein_c	chr2:69457997-6967
ENSG0000(	877	19.85867	chr2:8187	C2orf42		protein_c	chr2:70149885-7024
ENSG0000(	877	19.85867	chr2:8187	TIA1	NCGv7	protein_c	chr2:70209444-7024
ENSG0000(	877	19.85867	chr2:8187	PCYOX1		protein_c	chr2:70257386-7028

ENSG0000(	877	19.85867	chr2:8187	LINC02831		lncRNA	chr2:67562067-6762
ENSG0000(	877	19.85867	chr2:8187	FAM136A		protein_c	chr2:70295975-7030
ENSG0000(	877	19.85867	chr2:8187	ENSG00000235495		lncRNA	chr2:67565604-6768
ENSG0000(	877	19.85867	chr2:8187	POLE4		protein_c	chr2:74958643-7497
ENSG0000(	877	19.85867	chr2:8187	ADD2	NCGv7	protein_c	chr2:70607618-7076
ENSG0000(	877	19.85867	chr2:8187	TACR1		protein_c	chr2:75046463-7519
ENSG0000(	877	19.85867	chr2:8187	ENSG00000234521		Pseudoger	chr2:74407797-7441
ENSG0000(	877	19.85867	chr2:8187	EVA1A		protein_c	chr2:75469302-7556
ENSG0000(	877	19.85867	chr2:8187	MRPL19		protein_c	chr2:75646783-7569
ENSG0000(	877	19.85867	chr2:8187	ENSG00000281195		lncRNA	chr2:71373938-7137
ENSG0000(	877	19.85867	chr2:8187	PAPOLG		protein_c	chr2:60756253-6080
ENSG0000(	877	19.85867	chr2:8187	RNU6-542P		smallRNA	chr2:74319399-7431
ENSG0000(	877	19.85867	chr2:8187	ENSG00000271597		Pseudoger	chr2:69251818-6925
ENSG0000(	877	19.85867	chr2:8187	USP34		protein_c	chr2:61187463-6147
ENSG0000(	877	19.85867	chr2:8187	CCT4		protein_c	chr2:61868085-6188
ENSG0000(	877	19.85867	chr2:8187	EHBP1		protein_c	chr2:62673851-6304
ENSG0000(	877	19.85867	chr2:8187	OTX1		protein_c	chr2:63050057-6305
ENSG0000(	877	19.85867	chr2:8187	ENSG00000286739		lncRNA	chr2:74715269-7480
ENSG0000(	877	19.85867	chr2:8187	HMGN2P21		Pseudoger	chr2:70803157-7080
ENSG0000(	877	19.85867	chr2:8187	OR7E91P		lncRNA	chr2:71024127-7102
ENSG0000(	877	19.85867	chr2:8187	ENSG00000230477		Pseudoger	chr2:75598071-7559
ENSG0000(	877	19.85867	chr2:8187	ENSG00000235499		lncRNA	chr2:73985132-7398
ENSG0000(	877	19.85867	chr2:8187	LINC01828		lncRNA	chr2:67086446-6731
ENSG0000(	877	19.85867	chr2:8187	LINC01873		lncRNA	chr2:66383306-6639
ENSG0000(	877	19.85867	chr2:8187	ENSG00000287172		lncRNA	chr2:76185020-7639
ENSG0000(	877	19.85867	chr2:8187	ENSG00000285857		lncRNA	chr2:61527340-6152
ENSG0000(	877	19.85867	chr2:8187	RPL37P13		Pseudoger	chr2:62507545-6250
ENSG0000(	877	19.85867	chr2:8187	ENSG00000289463		lncRNA	chr2:71419974-7142
ENSG0000(	877	19.85867	chr2:8187	PPIAP64		Pseudoger	chr2:68125265-6812
ENSG0000(	877	19.85867	chr2:8187	Vault		smallRNA	chr2:65555432-6555
ENSG0000(	877	19.85867	chr2:8187	LINC02579		lncRNA	chr2:64606975-6461
ENSG0000(	877	19.85867	chr2:8187	RPS15AP13		Pseudoger	chr2:72288785-7228
ENSG0000(	877	19.85867	chr2:8187	ENSG00000236209		lncRNA	chr2:74919555-7492
ENSG0000(	877	19.85867	chr2:8187	ENSG00000228541		lncRNA	chr2:62296246-6246
ENSG0000(	877	19.85867	chr2:8187	AC007881.2		smallRNA	chr2:71039307-7103
ENSG0000(	877	19.85867	chr2:8187	MDH1		protein_c	chr2:63588609-6360
ENSG0000(	877	19.85867	chr2:8187	GAPDHP57		Pseudoger	chr2:75455994-7545
ENSG0000(	877	19.85867	chr2:8187	ENSG00000270437		Pseudoger	chr2:63083008-6308
ENSG0000(	877	19.85867	chr2:8187	RPL23AP37		Pseudoger	chr2:64347193-6434
ENSG0000(	877	19.85867	chr2:8187	RN7SKP164		smallRNA	chr2:76595413-7659
ENSG0000(	877	19.85867	chr2:8187	MEIS1-AS3		lncRNA	chr2:66426735-6643
ENSG0000(	877	19.85867	chr2:8187	SPRED2		protein_c	chr2:65310851-6543
ENSG0000(	877	19.85867	chr2:8187	ENSG00000231024		lncRNA	chr2:69700192-6971
ENSG0000(	877	19.85867	chr2:8187	REL-DT		lncRNA	chr2:60823069-6088
ENSG0000(	877	19.85867	chr2:8187	ENSG00000264324		protein_c	chr2:74211604-7436
ENSG0000(	877	19.85867	chr2:8187	LRRTM4	NCGv7	protein_c	chr2:76747685-7759
ENSG0000(	877	19.85867	chr2:8187	ENSG00000258881		protein_c	chr2:70939318-7099
ENSG0000(	877	19.85867	chr2:8187	MIR4433		smallRNA	chr2:64340759-6434
ENSG0000(	877	19.85867	chr2:8187	DNAAF10		protein_c	chr2:68122936-6815
ENSG0000(	877	19.85867	chr2:8187	AC118345.2		smallRNA	chr2:66239500-6623
ENSG0000(	877	19.85867	chr2:8187	SNORA36C		smallRNA	chr2:69520043-6952

ENSG0000(	877	19.85867	chr2:8187	TAF13P2		Pseudoger	chr2:74351134-7435
ENSG0000(	877	19.85867	chr2:8187	ENSG00000234255		lncRNA	chr2:65439838-6545
ENSG0000(	877	19.85867	chr2:8187	ENSG00000289327		lncRNA	chr2:71453081-7145
ENSG0000(	877	19.85867	chr2:8187	RNU6-1145P		smallRNA	chr2:61605616-6160
ENSG0000(	877	19.85867	chr2:8187	RNA5SP96		Pseudoger	chr2:69181897-6918
ENSG0000(	877	19.85867	chr2:8187	ENSG00000286623		lncRNA	chr2:74415422-7441
ENSG0000(	877	19.85867	chr2:8187	RAB1A		protein_c	chr2:65070696-6513
ENSG0000(	877	19.85867	chr2:8187	SNORA70B		smallRNA	chr2:61417244-6141
ENSG0000(	877	19.85867	chr2:8187	ENSG00000267520		lncRNA	chr2:60925909-6093
ENSG0000(	877	19.85867	chr2:8187	ACTR2		protein_c	chr2:65227788-6527
ENSG0000(	877	19.85867	chr2:8187	CEP68		protein_c	chr2:65056354-6508
ENSG0000(	877	19.85867	chr2:8187	ENSG00000289410		lncRNA	chr2:61728808-6176
ENSG0000(	877	19.85867	chr2:8187	TET3	NCGv7	protein_c	chr2:73984910-7410
ENSG0000(	877	19.85867	chr2:8187	ATP6V1B1-AS1		lncRNA	chr2:70941817-7094
ENSG0000(	877	19.85867	chr2:8187	ENSG00000289533		lncRNA	chr2:67331881-6734
ENSG0000(	877	19.85867	chr2:8187	GFPT1		protein_c	chr2:69319780-6938
ENSG0000(	877	19.85867	chr2:8187	LINC02934		lncRNA	chr2:65436711-6620
ENSG0000(	877	19.85867	chr2:8187	DCTN1	NCGv7	protein_c	chr2:74361154-7439
ENSG0000(	877	19.85867	chr2:8187	RN7SL51P		smallRNA	chr2:62262389-6226
ENSG0000(	877	19.85867	chr2:8187	EMX1		protein_c	chr2:72916260-7293
ENSG0000(	877	19.85867	chr2:8187	FBXO48		protein_c	chr2:68459422-6846
ENSG0000(	877	19.85867	chr2:8187	FBXL12P1		Pseudoger	chr2:68023694-6802
ENSG0000(	877	19.85867	chr2:8187	GMCL1		protein_c	chr2:69829660-6988
ENSG0000(	877	19.85867	chr2:8187	NAT8B		protein_c	chr2:73700576-7370
ENSG0000(	877	19.85867	chr2:8187	RPL36AP16		Pseudoger	chr2:69594741-6959
ENSG0000(	877	19.85867	chr2:8187	ENSG00000159239		protein_c	chr2:74393836-7442
ENSG0000(	877	19.85867	chr2:8187	RN7SL341P		smallRNA	chr2:64817378-6481
ENSG0000(	877	19.85867	chr2:8187	MRPL53		protein_c	chr2:74471982-7447
ENSG0000(	877	19.85867	chr2:8187	LINC01291		lncRNA	chr2:74918148-7493
ENSG0000(	877	19.85867	chr2:8187	SNORA74		smallRNA	chr2:65158662-6515
ENSG0000(	877	19.85867	chr2:8187	M1AP		protein_c	chr2:74557883-7464
ENSG0000(	877	19.85867	chr2:8187	RNU6-612P		smallRNA	chr2:60719640-6071
ENSG0000(	877	19.85867	chr2:8187	TMEM17		protein_c	chr2:62500218-6251
ENSG0000(	877	19.85867	chr2:8187	LBX2		protein_c	chr2:74497517-7450
ENSG0000(	877	19.85867	chr2:8187	ENSG00000272183		lncRNA	chr2:74501717-7450
ENSG0000(	877	19.85867	chr2:8187	ENSG00000286932		lncRNA	chr2:77210587-7732
ENSG0000(	877	19.85867	chr2:8187	PRELID1P6		Pseudoger	chr2:63622178-6362
ENSG0000(	877	19.85867	chr2:8187	WBP1		protein_c	chr2:74458400-7446
ENSG0000(	877	19.85867	chr2:8187	ENSG00000228384		lncRNA	chr2:70994510-7100
ENSG0000(	877	19.85867	chr2:8187	ENSG00000287250		lncRNA	chr2:73823154-7382
ENSG0000(	877	19.85867	chr2:8187	EHBP1-AS1		lncRNA	chr2:62957326-6304
ENSG0000(	877	19.85867	chr2:8187	OR7E91P		Pseudoger	chr2:71029028-7102
ENSG0000(	877	19.85867	chr2:8187	LINC01890		lncRNA	chr2:68822855-6883
ENSG0000(	877	19.85867	chr2:8187	ENSG00000270462		lncRNA	chr2:75697583-7569
ENSG0000(	877	19.85867	chr2:8187	ENSG00000289615		lncRNA	chr2:72144651-7214
ENSG0000(	875	19.81338	chr2:8187	MIR216A		smallRNA	chr2:55988950-5598
ENSG0000(	873	19.7681	chr1:1021	RN7SKP272		smallRNA	chr1:89987713-8998
ENSG0000(	872	19.74545	chr5:4231	LINC01339		lncRNA	chr5:90153052-9029
ENSG0000(	872	19.74545	chr5:4231	ENSG00000248195		lncRNA	chr5:86335024-8633
ENSG0000(	872	19.74545	chr5:4231	TMEM161B-DT		lncRNA	chr5:88268864-8843
ENSG0000(	872	19.74545	chr5:4231	MEF2C-AS1		lncRNA	chr5:88883328-8946

ENSG0000(	872	19.74545	chr5:4231	ENSG00000234292		lncRNA	chr5:91280097-9128
ENSG0000(	872	19.74545	chr5:4231	ENSG00000248363		lncRNA	chr5:86353803-8636
ENSG0000(	872	19.74545	chr5:4231	MEF2C-AS2		lncRNA	chr5:88676014-8877
ENSG0000(	872	19.74545	chr5:4231	RNU4-90P		smallRNA	chr5:91270727-9127
ENSG0000(	872	19.74545	chr5:4231	ENSG00000248667		lncRNA	chr5:85420028-8542
ENSG0000(	872	19.74545	chr5:4231	MIR3607		smallRNA	chr5:86620506-8662
ENSG0000(	872	19.74545	chr5:4231	ENSG00000255647		lncRNA	chr5:90410000-9041
ENSG0000(	872	19.74545	chr5:4231	ENSG00000248170		Pseudoger	chr5:85237681-8523
ENSG0000(	872	19.74545	chr5:4231	LINC00461		lncRNA	chr5:88507546-8869
ENSG0000(	872	19.74545	chr5:4231	RNU6-448P		smallRNA	chr5:84196883-8419
ENSG0000(	872	19.74545	chr5:4231	ENSG00000286638		lncRNA	chr5:91280229-9128
ENSG0000(	872	19.74545	chr5:4231	ENSG00000248701		lncRNA	chr5:86797685-8680
ENSG0000(	872	19.74545	chr5:4231	LUCAT1		lncRNA	chr5:91054834-9131
ENSG0000(	872	19.74545	chr5:4231	LINC02144		lncRNA	chr5:87665345-8773
ENSG0000(	872	19.74545	chr5:4231	RBBP4P6		Pseudoger	chr5:85190629-8519
ENSG0000(	872	19.74545	chr5:4231	RPL5P17		Pseudoger	chr5:85464434-8546
ENSG0000(	872	19.74545	chr5:4231	AC008394.1		protein_c	chr5:87216606-8723
ENSG0000(	872	19.74545	chr5:4231	POLR3G		protein_c	chr5:90471748-9051
ENSG0000(	872	19.74545	chr5:4231	NBPF22P		Pseudoger	chr5:86282766-8629
ENSG0000(	872	19.74545	chr5:4231	ENSG00000250253		Pseudoger	chr5:85210060-8521
ENSG0000(	872	19.74545	chr5:4231	ENSG00000250306		Pseudoger	chr5:88382948-8838
ENSG0000(	872	19.74545	chr5:4231	RN7SKP34		smallRNA	chr5:87102603-8710
ENSG0000(	872	19.74545	chr5:4231	EDIL3-DT		lncRNA	chr5:84382424-8449
ENSG0000(	872	19.74545	chr5:4231	ENSG00000249061		lncRNA	chr5:86967321-8713
ENSG0000(	872	19.74545	chr5:4231	ENSG00000289462		lncRNA	chr5:88140403-8814
ENSG0000(	872	19.74545	chr5:4231	MIR3660		smallRNA	chr5:90016621-9001
ENSG0000(	872	19.74545	chr5:4231	RPS2P25		Pseudoger	chr5:85762559-8576
ENSG0000(	872	19.74545	chr5:4231	PTP4A1P4		Pseudoger	chr5:86087526-8608
ENSG0000(	872	19.74545	chr5:4231	MBLAC2	DriverDBv	protein_c	chr5:90458209-9047
ENSG0000(	872	19.74545	chr5:4231	ENSG00000249023		lncRNA	chr5:85429702-8543
ENSG0000(	872	19.74545	chr5:4231	ENSG00000250377		lncRNA	chr5:88691757-8869
ENSG0000(	872	19.74545	chr5:4231	LYSMD3		protein_c	chr5:90515611-9052
ENSG0000(	872	19.74545	chr5:4231	COX7C	NCGv7	protein_c	chr5:86617928-8662
ENSG0000(	872	19.74545	chr5:4231	SNORA70		smallRNA	chr5:88382772-8838
ENSG0000(	872	19.74545	chr5:4231	LINC02161		lncRNA	chr5:89581209-8967
ENSG0000(	872	19.74545	chr5:4231	RNU6-804P		smallRNA	chr5:86663144-8666
ENSG0000(	872	19.74545	chr5:4231	RNA5SP187		Pseudoger	chr5:88274258-8827
ENSG0000(	872	19.74545	chr5:4231	ENSG00000289184		lncRNA	chr5:88143177-8814
ENSG0000(	872	19.74545	chr5:4231	ENSG00000280009		TEC	chr5:86305233-8630
ENSG0000(	872	19.74545	chr5:4231	LINC02059		lncRNA	chr5:86746818-8674
ENSG0000(	872	19.74545	chr5:4231	ENSG00000249153		lncRNA	chr5:85848502-8584
ENSG0000(	872	19.74545	chr5:4231	MIR4280		smallRNA	chr5:87114879-8711
ENSG0000(	872	19.74545	chr5:4231	ENSG00000289008		lncRNA	chr5:88685191-8868
ENSG0000(	872	19.74545	chr5:4231	ARRDC3		protein_c	chr5:91368631-9138
ENSG0000(	872	19.74545	chr5:4231	RN7SL629P		smallRNA	chr5:87294191-8729
ENSG0000(	872	19.74545	chr5:4231	AC010595.1		smallRNA	chr5:85634412-8563
ENSG0000(	872	19.74545	chr5:4231	TMEM251P1		Pseudoger	chr5:91030990-9103
ENSG0000(	872	19.74545	chr5:4231	ENSG00000241059		lncRNA	chr5:91355380-9135
ENSG0000(	872	19.74545	chr5:4231	ARRDC3-AS1		lncRNA	chr5:91380349-9161
ENSG0000(	872	19.74545	chr5:4231	LINC02488		lncRNA	chr5:87662040-8770
ENSG0000(	872	19.74545	chr5:4231	EDIL3		protein_c	chr5:83940554-8438

ENSG0000(	872	19.74545	chr5:4231	ENSG00000249349		lncRNA	chr5:85112342-8511
ENSG0000(	872	19.74545	chr5:4231	TMEM161B		protein_c	chr5:88189633-8826
ENSG0000(	872	19.74545	chr5:4231	ADGRV1	NCGv7	protein_c	chr5:90529344-9116
ENSG0000(	872	19.74545	chr5:4231	MEF2C	NCGv7;AC	protein_c	chr5:88717117-8890
ENSG0000(	872	19.74545	chr5:4231	ENSG00000249792		lncRNA	chr5:85212958-8521
ENSG0000(	872	19.74545	chr5:4231	ENSG00000249842		lncRNA	chr5:85446974-8544
ENSG0000(	872	19.74545	chr5:4231	ENSG00000271904		lncRNA	chr5:88433892-8849
ENSG0000(	872	19.74545	chr5:4231	ENSG00000285190		lncRNA	chr5:87412342-8749
ENSG0000(	872	19.74545	chr5:4231	ENSG00000240388		Pseudoger	chr5:91354478-9135
ENSG0000(	872	19.74545	chr5:4231	CCNH		protein_c	chr5:87318416-8741
ENSG0000(	872	19.74545	chr5:4231	ZP3P1		Pseudoger	chr5:83875166-8387
ENSG0000(	872	19.74545	chr5:4231	RPS3AP22		Pseudoger	chr5:88381957-8838
ENSG0000(	872	19.74545	chr5:4231	ENSG00000250124		lncRNA	chr5:86380660-8638
ENSG0000(	872	19.74545	chr5:4231	LINC02060		lncRNA	chr5:88408982-8843
ENSG0000(	872	19.74545	chr5:4231	ENSG00000250555		lncRNA	chr5:88692651-8869
ENSG0000(	872	19.74545	chr5:4231	ENSG00000290550		lncRNA	chr5:86282460-8629
ENSG0000(	872	19.74545	chr5:4231	ENSG00000285618		lncRNA	chr5:91132303-9114
ENSG0000(	872	19.74545	chr5:4231	AC026700.1		smallRNA	chr5:85528044-8552
ENSG0000(	872	19.74545	chr5:4231	ENSG00000251585		Pseudoger	chr5:86402465-8640
ENSG0000(	872	19.74545	chr5:4231	ENSG00000288740		lncRNA	chr5:89301289-8930
ENSG0000(	872	19.74545	chr5:4231	ENSG00000251093		lncRNA	chr5:91226475-9122
ENSG0000(	872	19.74545	chr5:4231	ENSG00000287783		lncRNA	chr5:87767608-8777
ENSG0000(	872	19.74545	chr5:4231	ENSG00000212930		lncRNA	chr5:91223419-9122
ENSG0000(	872	19.74545	chr5:4231	ENSG00000251066		Pseudoger	chr5:85549409-8554
ENSG0000(	872	19.74545	chr5:4231	CETN3		protein_c	chr5:90392257-9040
ENSG0000(	872	19.74545	chr5:4231	ENSG00000214942		lncRNA	chr5:89900664-8999
ENSG0000(	872	19.74545	chr5:4231	RASA1	NCGv7	protein_c	chr5:87267883-8739
ENSG0000(	872	19.74545	chr5:4231	RNU6-727P		smallRNA	chr5:87420341-8742
ENSG0000(	872	19.74545	chr5:4231	ENSG00000253321		lncRNA	chr5:90353037-9035
ENSG0000(	872	19.74545	chr5:4231	PPIAP79		Pseudoger	chr5:84999812-8500
ENSG0000(	872	19.74545	chr5:4231	ENSG00000260871		lncRNA	chr5:90388468-9038
ENSG0000(	872	19.74545	chr5:4231	ENSG00000250831		lncRNA	chr5:89466537-8947
ENSG0000(	872	19.74545	chr5:4231	ENSG00000287862		lncRNA	chr5:87863703-8814
ENSG0000(	872	19.74545	chr5:4231	RNU6-606P		smallRNA	chr5:87299687-8729
ENSG0000(	872	19.74545	chr5:4231	ENSG00000250874		lncRNA	chr5:85663232-8566
ENSG0000(	872	19.74545	chr5:4231	ENSG00000242477		Pseudoger	chr5:86884231-8688
ENSG0000(	872	19.74545	chr5:4231	AC109496.1		smallRNA	chr5:83973796-8397
ENSG0000(	872	19.74545	chr5:4231	H3P23		Pseudoger	chr5:88602780-8860
ENSG0000(	871	19.72281	chr8:9805	AC011652.2		smallRNA	chr8:129496222-129
ENSG0000(	871	19.72281	chr8:9805	AC011652.1		smallRNA	chr8:129496512-129
ENSG0000(	871	19.72281	chr8:9805	ENSG00000253926		lncRNA	chr8:129415949-129
ENSG0000(	871	19.72281	chr8:9805	hsa-mir-3669		smallRNA	chr8:129497348-129
ENSG0000(	871	19.72281	chr8:9805	AC016178.2		smallRNA	chr8:129497587-129
ENSG0000(	871	19.72281	chr8:9805	MIR3686		smallRNA	chr8:129484057-129
ENSG0000(	871	19.72281	chr8:9805	AC016178.1		smallRNA	chr8:129496970-129
ENSG0000(	871	19.72281	chr8:9805	hsa-mir-3673		smallRNA	chr8:129495842-129
ENSG0000(	849	19.22464	chr5:4231	RNU6-236P		smallRNA	chr5:139717985-139
ENSG0000(	849	19.22464	chr5:4231	ENSG00000249526		lncRNA	chr5:139684645-139
ENSG0000(	846	19.15671	chr2:2577	ENSG00000228098		Pseudoger	chr2:134816362-134
ENSG0000(	846	19.15671	chr2:2577	VDAC2P4		Pseudoger	chr2:134797169-134
ENSG0000(	846	19.15671	chr2:2577	MANEALP1		Pseudoger	chr2:135897955-135

ENSG0000(	846	19.15671	chr2:2577	DARS1		protein_c	chr2:135905881-135
ENSG0000(	846	19.15671	chr2:2577	LCT		protein_c	chr2:135787850-135
ENSG0000(	846	19.15671	chr2:2577	AC010146.2		smallRNA	chr2:136544371-136
ENSG0000(	846	19.15671	chr2:2577	RAB3GAP1		protein_c	chr2:135052289-135
ENSG0000(	846	19.15671	chr2:2577	CCNT2-AS1		lncRNA	chr2:134735464-134
ENSG0000(	846	19.15671	chr2:2577	MCM6		protein_c	chr2:135839626-135
ENSG0000(	846	19.15671	chr2:2577	ENSG00000237262		lncRNA	chr2:136077892-136
ENSG0000(	846	19.15671	chr2:2577	DARS1-AS1		lncRNA	chr2:135985124-136
ENSG0000(	846	19.15671	chr2:2577	snoU13		smallRNA	chr2:135065632-135
ENSG0000(	846	19.15671	chr2:2577	CCNT2		protein_c	chr2:134918235-134
ENSG0000(	846	19.15671	chr2:2577	SMC4P1		Pseudoger	chr2:136544712-136
ENSG0000(	846	19.15671	chr2:2577	ENSG00000289974		lncRNA	chr2:136119456-136
ENSG0000(	846	19.15671	chr2:2577	G3BP1P1		Pseudoger	chr2:135510546-135
ENSG0000(	846	19.15671	chr2:2577	Y_RNA		smallRNA	chr2:135810169-135
ENSG0000(	846	19.15671	chr2:2577	ZRANB3	NCGv7	protein_c	chr2:135136916-135
ENSG0000(	846	19.15671	chr2:2577	RNU6-512P		smallRNA	chr2:135656477-135
ENSG0000(	846	19.15671	chr2:2577	SNORA40		smallRNA	chr2:135136628-135
ENSG0000(	846	19.15671	chr2:2577	MIR5590		smallRNA	chr2:134857820-134
ENSG0000(	846	19.15671	chr2:2577	LCT-AS1		lncRNA	chr2:135820191-135
ENSG0000(	846	19.15671	chr2:2577	HNRNPKP2		Pseudoger	chr2:136199114-136
ENSG0000(	846	19.15671	chr2:2577	R3HDM1		protein_c	chr2:135531455-135
ENSG0000(	846	19.15671	chr2:2577	CXCR4	NCGv7;AC	protein_c	chr2:136114349-136
ENSG0000(	846	19.15671	chr2:2577	UBXN4		protein_c	chr2:135741734-135
ENSG0000(	846	19.15671	chr2:2577	THSD7B	NCGv7	protein_c	chr2:136765545-137
ENSG0000(	846	19.15671	chr2:2577	MAP3K19	NCGv7	protein_c	chr2:134964485-135
ENSG0000(	846	19.15671	chr2:2577	UBBP1		Pseudoger	chr2:136329441-136
ENSG0000(	846	19.15671	chr2:2577	ENSG00000287463		lncRNA	chr2:134718188-134
ENSG0000(	846	19.15671	chr2:2577	MIR128-1		smallRNA	chr2:135665397-135
ENSG0000(	846	19.15671	chr2:2577	RN7SKP141		smallRNA	chr2:136390332-136
ENSG0000(	846	19.15671	chr2:2577	ENSG00000279024		TEC	chr2:136230753-136
ENSG0000(	846	19.15671	chr2:2577	ACMSD		protein_c	chr2:134838616-134
ENSG0000(	846	19.15671	chr2:2577	AC092786.1		smallRNA	chr2:136645108-136
ENSG0000(	846	19.15671	chr2:2577	TMEM163		protein_c	chr2:134455759-134
ENSG0000(	845	19.13407	chr1:1021	AL137855.1		smallRNA	chr1:57757084-5775
ENSG0000(	845	19.13407	chr1:1021	AL161740.1		smallRNA	chr1:56966280-5696
ENSG0000(	845	19.13407	chr1:1021	FYB2	NCGv7	protein_c	chr1:56718789-5681
ENSG0000(	845	19.13407	chr1:1021	DAB1-AS1		lncRNA	chr1:57860532-5788
ENSG0000(	845	19.13407	chr1:1021	ENSG00000227935		lncRNA	chr1:57386576-5738
ENSG0000(	845	19.13407	chr1:1021	RPS20P5		Pseudoger	chr1:57605847-5760
ENSG0000(	845	19.13407	chr1:1021	RPL21P23		Pseudoger	chr1:56538452-5653
ENSG0000(	845	19.13407	chr1:1021	RN7SL713P		smallRNA	chr1:58565629-5856
ENSG0000(	845	19.13407	chr1:1021	ENSG00000229913		lncRNA	chr1:56823679-5682
ENSG0000(	845	19.13407	chr1:1021	ENSG00000284686		protein_c	chr1:56173433-5652
ENSG0000(	845	19.13407	chr1:1021	ENSG00000236341		lncRNA	chr1:56963886-5699
ENSG0000(	845	19.13407	chr1:1021	ENSG00000260971		lncRNA	chr1:56154545-5647
ENSG0000(	845	19.13407	chr1:1021	ENSG00000270209		Pseudoger	chr1:58552913-5855
ENSG0000(	845	19.13407	chr1:1021	DAB1	NCGv7	protein_c	chr1:56994778-5854
ENSG0000(	845	19.13407	chr1:1021	C8A		protein_c	chr1:56854768-5691
ENSG0000(	845	19.13407	chr1:1021	AC119674.1		smallRNA	chr1:56377333-5637
ENSG0000(	845	19.13407	chr1:1021	ENSG00000225475		Pseudoger	chr1:56619409-5661
ENSG0000(	845	19.13407	chr1:1021	RPSAP20		Pseudoger	chr1:56207567-5620

ENSG0000(	845	19.13407	chr1:1021	HNRNPA1P6		Pseudoger	chr1:58048175-5804
ENSG0000(	845	19.13407	chr1:1021	RPS26P15		Pseudoger	chr1:58056133-5805
ENSG0000(	845	19.13407	chr1:1021	C8B		protein_c	chr1:56929207-5697
ENSG0000(	845	19.13407	chr1:1021	ENSG00000230546		Pseudoger	chr1:58084419-5808
ENSG0000(	845	19.13407	chr1:1021	LINC01767		lncRNA	chr1:56414918-5641
ENSG0000(	845	19.13407	chr1:1021	ENSG00000290536		lncRNA	chr1:58047889-5804
ENSG0000(	845	19.13407	chr1:1021	OMA1	NCGv7	protein_c	chr1:58415384-5854
ENSG0000(	845	19.13407	chr1:1021	TACSTD2	AC	protein_c	chr1:58575433-5857
ENSG0000(	845	19.13407	chr1:1021	RPL23AP85		Pseudoger	chr1:56585612-5658
ENSG0000(	845	19.13407	chr1:1021	PRKAA2		protein_c	chr1:56645314-5671
ENSG0000(	845	19.13407	chr1:1021	ENSG00000286918		lncRNA	chr1:58546168-5856
ENSG0000(	845	19.13407	chr1:1021	ENSG00000185839		Pseudoger	chr1:58630841-5863
ENSG0000(	845	19.13407	chr1:1021	PLPP3		protein_c	chr1:56494761-5664
ENSG0000(	845	19.13407	chr1:1021	ENSG00000235038		lncRNA	chr1:58060139-5808
ENSG0000(	845	19.13407	chr1:1021	ENSG00000233216		Pseudoger	chr1:58228682-5822
ENSG0000(	839	18.9982	chr22:22(	IGLVVI-25-1		Pseudoger	chr22:22679097-226
ENSG0000(	836	18.93027	chr2:8187	ENSG00000270569		Pseudoger	chr2:57429548-5743
ENSG0000(	836	18.93027	chr2:8187	ENSG00000289529		lncRNA	chr2:58429434-5847
ENSG0000(	836	18.93027	chr2:8187	FANCL		protein_c	chr2:58159243-5824
ENSG0000(	836	18.93027	chr2:8187	ACTG1P22		Pseudoger	chr2:57755428-5776
ENSG0000(	836	18.93027	chr12:624	ENSG00000271018		Pseudoger	chr12:50756892-507
ENSG0000(	836	18.93027	chr2:8187	ENSG00000231043		Pseudoger	chr2:58460292-5846
ENSG0000(	836	18.93027	chr2:8187	ENSG00000285755		lncRNA	chr2:57289648-5738
ENSG0000(	836	18.93027	chr2:8187	VRK2		protein_c	chr2:57907629-5815
ENSG0000(	836	18.93027	chr2:8187	AC073215.1		smallRNA	chr2:58062581-5806
ENSG0000(	836	18.93027	chr2:8187	EIF3FP3		Pseudoger	chr2:58251440-5825
ENSG0000(	836	18.93027	chr2:8187	LINC01122		lncRNA	chr2:58427738-5906
ENSG0000(	836	18.93027	chr2:8187	LINC01795		lncRNA	chr2:58275532-5829
ENSG0000(	836	18.93027	chr2:8187	ENSG00000273063		lncRNA	chr2:58241349-5824
ENSG0000(	836	18.93027	chr2:8187	SNORD78		smallRNA	chr2:57544535-5754
ENSG0000(	836	18.93027	chr2:8187	EIF2S2P7		Pseudoger	chr2:57048350-5704
ENSG0000(	836	18.93027	chr2:8187	PPIAP63		Pseudoger	chr2:56750300-5675
ENSG0000(	836	18.93027	chr2:8187	snoU13		smallRNA	chr2:57016061-5701
ENSG0000(	836	18.93027	chr2:8187	ENSG00000287875		lncRNA	chr2:58040211-5804
ENSG0000(	835	18.90763	chr2:8187	REG1B		protein_c	chr2:79085023-7908
ENSG0000(	835	18.90763	chr2:8187	ENSG00000234877		lncRNA	chr2:78597911-7859
ENSG0000(	835	18.90763	chr2:8187	ENSG00000290771		lncRNA	chr2:79135503-7913
ENSG0000(	835	18.90763	chr2:8187	LINC01851		lncRNA	chr2:77915870-7791
ENSG0000(	835	18.90763	chr2:8187	RNU6-812P		smallRNA	chr2:78882628-7888
ENSG0000(	835	18.90763	chr2:8187	CYCSP6		Pseudoger	chr2:78412793-7841
ENSG0000(	835	18.90763	chr2:8187	ENSG00000286260		lncRNA	chr2:78880713-7893
ENSG0000(	835	18.90763	chr2:8187	REG3G		protein_c	chr2:79025686-7902
ENSG0000(	835	18.90763	chr2:8187	ENSG00000270470		Pseudoger	chr2:78437850-7843
ENSG0000(	835	18.90763	chr2:8187	ENSG00000229494		lncRNA	chr2:78088729-7812
ENSG0000(	835	18.90763	chr2:8187	REG1CP		Pseudoger	chr2:79135701-7913
ENSG0000(	835	18.90763	chr2:8187	RNU6-827P		smallRNA	chr2:78882447-7888
ENSG0000(	835	18.90763	chr2:8187	REG1A	NCGv7	protein_c	chr2:79120362-7912
ENSG0000(	835	18.90763	chr2:8187	ENSG00000227088		lncRNA	chr2:77652025-7829
ENSG0000(	835	18.90763	chr2:8187	ENSG00000224879		lncRNA	chr2:79158374-7918
ENSG0000(	835	18.90763	chr2:8187	REG3A		protein_c	chr2:79157003-7915
ENSG0000(	827	18.72648	chr1:1021	ENSG00000272864		lncRNA	chr1:74698769-7469

ENSG0000(	827	18.72648	chr1:1021	AL354978.1	smallRNA	chr1:66730967-6673
ENSG0000(	827	18.72648	chr1:1021	RNU7-80P	smallRNA	chr1:67772593-6777
ENSG0000(	827	18.72648	chr1:1021	RABGGTB	protein_c	chr1:75786197-7579
ENSG0000(	827	18.72648	chr1:1021	SNORA31	smallRNA	chr1:67102645-6710
ENSG0000(	827	18.72648	chr1:1021	Y_RNA	smallRNA	chr1:64066640-6406
ENSG0000(	827	18.72648	chr1:1021	MIR3116-1	smallRNA	chr1:62078786-6207
ENSG0000(	827	18.72648	chr1:1021	SGIP1	protein_c	chr1:66533267-6675
ENSG0000(	827	18.72648	chr1:1021	ZRANB2-AS1	lncRNA	chr1:71048855-7106
ENSG0000(	827	18.72648	chr1:1021	ENSG00000235055	Pseudoger	chr1:68043330-6804
ENSG0000(	827	18.72648	chr1:1021	RNU6-414P	smallRNA	chr1:61816419-6181
ENSG0000(	827	18.72648	chr1:1021	RNA5SP50	Pseudoger	chr1:73749517-7374
ENSG0000(	827	18.72648	chr1:1021	ENSG00000231252	lncRNA	chr1:60659631-6086
ENSG0000(	827	18.72648	chr1:1021	CASP3P1	Pseudoger	chr1:70660657-7066
ENSG0000(	827	18.72648	chr1:1021	RNU6-1031P	smallRNA	chr1:67541127-6754
ENSG0000(	827	18.72648	chr1:1021	ENSG00000288804	lncRNA	chr1:65067808-6506
ENSG0000(	827	18.72648	chr1:1021	ANKRD13C	protein_c	chr1:70258999-7035
ENSG0000(	827	18.72648	chr1:1021	ROR1	protein_c	chr1:63774017-6418
ENSG0000(	827	18.72648	chr1:1021	RPL31P12	Pseudoger	chr1:72301472-7230
ENSG0000(	827	18.72648	chr1:1021	MIR186	smallRNA	chr1:71067631-7106
ENSG0000(	827	18.72648	chr1:1021	LINC02777	lncRNA	chr1:58882868-5893
ENSG0000(	827	18.72648	chr1:1021	HNRNPCP9	Pseudoger	chr1:67660155-6766
ENSG0000(	827	18.72648	chr1:1021	LINC01788	lncRNA	chr1:70706441-7078
ENSG0000(	827	18.72648	chr1:1021	MIR4711	smallRNA	chr1:59733227-5973
ENSG0000(	827	18.72648	chr1:1021	AL136985.1	smallRNA	chr1:58764849-5876
ENSG0000(	827	18.72648	chr1:1021	NEGR1-IT1	lncRNA	chr1:71794232-7183
ENSG0000(	827	18.72648	chr1:1021	LINC02797	lncRNA	chr1:72793104-7285
ENSG0000(	827	18.72648	chr1:1021	FOXD3-AS1	lncRNA	chr1:63320878-6332
ENSG0000(	827	18.72648	chr1:1021	RN7SKP19	smallRNA	chr1:73191604-7319
ENSG0000(	827	18.72648	chr1:1021	LINC02791	lncRNA	chr1:69215835-6924
ENSG0000(	827	18.72648	chr1:1021	Y_RNA	smallRNA	chr1:63338263-6333
ENSG0000(	827	18.72648	chr1:1021	DNAI4	protein_c	chr1:66812885-6692
ENSG0000(	827	18.72648	chr1:1021	HHLA3	lncRNA	chr1:70354786-7038
ENSG0000(	827	18.72648	chr1:1021	RNU6-622P	smallRNA	chr1:75183045-7518
ENSG0000(	827	18.72648	chr1:1021	GNG12-AS1	lncRNA	chr1:67832293-6820
ENSG0000(	827	18.72648	chr1:1021	RNU2-15P	smallRNA	chr1:65415816-6541
ENSG0000(	827	18.72648	chr1:1021	DYNLT5	protein_c	chr1:66752459-6677
ENSG0000(	827	18.72648	chr1:1021	AL360297.1	smallRNA	chr1:71141975-7114
ENSG0000(	827	18.72648	chr1:1021	ENSG00000289394	lncRNA	chr1:66925327-6692
ENSG0000(	827	18.72648	chr1:1021	RN7SL488P	smallRNA	chr1:63529617-6352
ENSG0000(	827	18.72648	chr1:1021	ENSG00000235215	lncRNA	chr1:59055999-5907
ENSG0000(	827	18.72648	chr1:1021	PIGPP2	Pseudoger	chr1:62189131-6218
ENSG0000(	827	18.72648	chr1:1021	Y_RNA	smallRNA	chr1:58722279-5872
ENSG0000(	827	18.72648	chr1:1021	ENSG00000287453	lncRNA	chr1:69551848-6956
ENSG0000(	827	18.72648	chr1:1021	ENSG00000285778	lncRNA	chr1:73787370-7391
ENSG0000(	827	18.72648	chr1:1021	AL109843.1	smallRNA	chr1:67239440-6723
ENSG0000(	827	18.72648	chr1:1021	snoU13	smallRNA	chr1:65571549-6557
ENSG0000(	827	18.72648	chr1:1021	ENSG00000236674	Pseudoger	chr1:63359823-6336
ENSG0000(	827	18.72648	chr1:1021	RNU7-123P	smallRNA	chr1:63536711-6353
ENSG0000(	827	18.72648	chr1:1021	SNORD45A	smallRNA	chr1:75787889-7578
ENSG0000(	827	18.72648	chr1:1021	COX6CP13	Pseudoger	chr1:65298755-6529
ENSG0000(	827	18.72648	chr1:1021	PDE4B	protein_c	chr1:65792514-6637

ENSG0000(	827	18.72648	chr1:1021	LAMTOR5P1		Pseudoger	chr1:62038842-6203
ENSG0000(	827	18.72648	chr1:1021	MSH4		protein_c	chr1:75796882-7591
ENSG0000(	827	18.72648	chr1:1021	SLC44A5	NCGv7	protein_c	chr1:75202129-7561
ENSG0000(	827	18.72648	chr1:1021	ENSG00000228988		lncRNA	chr1:70218589-7022
ENSG0000(	827	18.72648	chr1:1021	RNU6-809P		smallRNA	chr1:64028894-6402
ENSG0000(	827	18.72648	chr1:1021	ENSG00000275678		lncRNA	chr1:67121605-6712
ENSG0000(	827	18.72648	chr1:1021	LINC01702		lncRNA	chr1:67522299-6753
ENSG0000(	827	18.72648	chr1:1021	RPS15AP7		Pseudoger	chr1:62190522-6219
ENSG0000(	827	18.72648	chr1:1021	ENSG00000229943		lncRNA	chr1:74963314-7496
ENSG0000(	827	18.72648	chr1:1021	ENSG00000286863		lncRNA	chr1:72283170-7275
ENSG0000(	827	18.72648	chr1:1021	ENSG00000233894		lncRNA	chr1:74468195-7446
ENSG0000(	827	18.72648	chr1:1021	IL23R		protein_c	chr1:67138907-6725
ENSG0000(	827	18.72648	chr1:1021	LINC01748		lncRNA	chr1:60515716-6064
ENSG0000(	827	18.72648	chr1:1021	LINC01739		lncRNA	chr1:62975751-6302
ENSG0000(	827	18.72648	chr1:1021	ENSG00000233877		Pseudoger	chr1:64941979-6494
ENSG0000(	827	18.72648	chr1:1021	AL138847.1		protein_c	chr1:62607766-6260
ENSG0000(	827	18.72648	chr1:1021	DIRAS3		protein_c	chr1:68045886-6805
ENSG0000(	827	18.72648	chr1:1021	RN7SL242P		smallRNA	chr1:70180146-7018
ENSG0000(	827	18.72648	chr1:1021	LINC02778		lncRNA	chr1:60114875-6014
ENSG0000(	827	18.72648	chr1:1021	ENSG00000286455		lncRNA	chr1:63011197-6301
ENSG0000(	827	18.72648	chr1:1021	NEGR1		protein_c	chr1:71395943-7228
ENSG0000(	827	18.72648	chr1:1021	Y_RNA		smallRNA	chr1:62211557-6221
ENSG0000(	827	18.72648	chr1:1021	GNG12		protein_c	chr1:67701475-6783
ENSG0000(	827	18.72648	chr1:1021	INSL5		protein_c	chr1:66797740-6680
ENSG0000(	827	18.72648	chr1:1021	RNU6-1246P		smallRNA	chr1:72717663-7271
ENSG0000(	827	18.72648	chr1:1021	RNU6-1177P		smallRNA	chr1:61852499-6185
ENSG0000(	827	18.72648	chr1:1021	FGGY		protein_c	chr1:59296638-5981
ENSG0000(	827	18.72648	chr1:1021	ELOCP18		Pseudoger	chr1:68375327-6837
ENSG0000(	827	18.72648	chr1:1021	ENSG00000238139		Pseudoger	chr1:67561073-6756
ENSG0000(	827	18.72648	chr1:1021	C1orf87		protein_c	chr1:59987269-6007
ENSG0000(	827	18.72648	chr1:1021	NFIA-AS2		lncRNA	chr1:60912675-6105
ENSG0000(	827	18.72648	chr1:1021	NFIA-AS1		lncRNA	chr1:61248945-6125
ENSG0000(	827	18.72648	chr1:1021	ENSG00000237852		lncRNA	chr1:65486406-6549
ENSG0000(	827	18.72648	chr1:1021	ENSG00000286429		lncRNA	chr1:63487957-6350
ENSG0000(	827	18.72648	chr1:1021	ENSG00000269933		lncRNA	chr1:71005854-7100
ENSG0000(	827	18.72648	chr1:1021	LINC01758		lncRNA	chr1:69433255-6943
ENSG0000(	827	18.72648	chr1:1021	KRT8P21		Pseudoger	chr1:73104792-7310
ENSG0000(	827	18.72648	chr1:1021	LRRC7-AS1		lncRNA	chr1:70013982-7003
ENSG0000(	827	18.72648	chr1:1021	RAVER2		protein_c	chr1:64745075-6483
ENSG0000(	827	18.72648	chr1:1021	ENSG00000213579		Pseudoger	chr1:75582099-7558
ENSG0000(	827	18.72648	chr1:1021	SLC2A3P2		Pseudoger	chr1:64984608-6498
ENSG0000(	827	18.72648	chr1:1021	ENSG00000261213		lncRNA	chr1:75122518-7512
ENSG0000(	827	18.72648	chr1:1021	ZRANB2		protein_c	chr1:71063291-7108
ENSG0000(	827	18.72648	chr1:1021	ENSG00000213703		Pseudoger	chr1:62641122-6264
ENSG0000(	827	18.72648	chr1:1021	AK4		protein_c	chr1:65147549-6523
ENSG0000(	827	18.72648	chr1:1021	RN7SL392P		smallRNA	chr1:67656833-6765
ENSG0000(	827	18.72648	chr1:1021	LEPROT		protein_c	chr1:65420587-6543
ENSG0000(	827	18.72648	chr1:1021	RNU6-1176P		smallRNA	chr1:65022968-6502
ENSG0000(	827	18.72648	chr1:1021	ENSG00000226324		Pseudoger	chr1:71367054-7136
ENSG0000(	827	18.72648	chr1:1021	MIR1262		smallRNA	chr1:68183518-6818
ENSG0000(	827	18.72648	chr1:1021	DEPDC1-AS1		lncRNA	chr1:68496676-6853

ENSG0000(	827	18.72648	chr1:1021	ENSG00000229225		lncRNA	chr1:63078081-6307
ENSG0000(	827	18.72648	chr1:1021	ENSG00000237227		Pseudoger	chr1:62208136-6220
ENSG0000(	827	18.72648	chr1:1021	FPGT		protein_c	chr1:74198238-7423
ENSG0000(	827	18.72648	chr1:1021	PATJ	NCGv7	protein_c	chr1:61742477-6217
ENSG0000(	827	18.72648	chr1:1021	RPS7P4		Pseudoger	chr1:68242474-6824
ENSG0000(	827	18.72648	chr1:1021	KANK4		protein_c	chr1:62236165-6231
ENSG0000(	827	18.72648	chr1:1021	ANGPTL3		protein_c	chr1:62597520-6260
ENSG0000(	827	18.72648	chr1:1021	ZRANB2-DT		lncRNA	chr1:71081324-7148
ENSG0000(	827	18.72648	chr1:1021	ENSG00000226208		lncRNA	chr1:70715933-7072
ENSG0000(	827	18.72648	chr1:1021	AL157407.1		smallRNA	chr1:68058177-6805
ENSG0000(	827	18.72648	chr1:1021	ENSG00000237163		Pseudoger	chr1:62905180-6290
ENSG0000(	827	18.72648	chr1:1021	HHLA3-AS1		lncRNA	chr1:70359562-7036
ENSG0000(	827	18.72648	chr1:1021	RNU7-62P		smallRNA	chr1:64384398-6438
ENSG0000(	827	18.72648	chr1:1021	ENSG00000230027		lncRNA	chr1:76041691-7606
ENSG0000(	827	18.72648	chr1:1021	PTGER3		protein_c	chr1:70852353-7104
ENSG0000(	827	18.72648	chr1:1021	ENSG00000230863		Pseudoger	chr1:75641178-7572
ENSG0000(	827	18.72648	chr1:1021	LHX8		protein_c	chr1:75128434-7516
ENSG0000(	827	18.72648	chr1:1021	TYW3		protein_c	chr1:74733152-7476
ENSG0000(	827	18.72648	chr1:1021	RN7SL130P		smallRNA	chr1:63655743-6365
ENSG0000(	827	18.72648	chr1:1021	GDI2P2		Pseudoger	chr1:72274552-7227
ENSG0000(	827	18.72648	chr1:1021	RP4-535B20.1		lncRNA	chr1:65066627-6506
ENSG0000(	827	18.72648	chr1:1021	ENSG00000229440		Pseudoger	chr1:68381441-6838
ENSG0000(	827	18.72648	chr1:1021	LINC01360		lncRNA	chr1:73305609-7335
ENSG0000(	827	18.72648	chr1:1021	PIN1P1		Pseudoger	chr1:69919322-6992
ENSG0000(	827	18.72648	chr1:1021	ENSG00000280317		TEC	chr1:72979014-7297
ENSG0000(	827	18.72648	chr1:1021	NFIA		protein_c	chr1:60865259-6146
ENSG0000(	827	18.72648	chr1:1021	PGBD4P8		Pseudoger	chr1:60097415-6009
ENSG0000(	827	18.72648	chr1:1021	LINC01358		lncRNA	chr1:58933643-5924
ENSG0000(	827	18.72648	chr1:1021	LRRC53		protein_c	chr1:74469376-7451
ENSG0000(	827	18.72648	chr1:1021	MYSM1		protein_c	chr1:58643440-5870
ENSG0000(	827	18.72648	chr1:1021	ENSG00000237324		lncRNA	chr1:74341579-7437
ENSG0000(	827	18.72648	chr1:1021	TM2D1		protein_c	chr1:61681046-6172
ENSG0000(	827	18.72648	chr1:1021	ENSG00000229294		lncRNA	chr1:65279456-6530
ENSG0000(	827	18.72648	chr1:1021	USP1		protein_c	chr1:62436297-6245
ENSG0000(	827	18.72648	chr1:1021	AC096534.1		smallRNA	chr1:61083455-6108
ENSG0000(	827	18.72648	chr1:1021	LRRIQ3	NCGv7	protein_c	chr1:74026015-7419
ENSG0000(	827	18.72648	chr1:1021	FPGT-TNNI3K		protein_c	chr1:74198235-7454
ENSG0000(	827	18.72648	chr1:1021	JAK1	NCGv7;AC	protein_c	chr1:64833223-6506
ENSG0000(	827	18.72648	chr1:1021	RNU4ATAC4P		smallRNA	chr1:67267601-6726
ENSG0000(	827	18.72648	chr1:1021	ENSG00000224493		Pseudoger	chr1:75521562-7552
ENSG0000(	827	18.72648	chr1:1021	SRSF11		protein_c	chr1:70205682-7025
ENSG0000(	827	18.72648	chr1:1021	ENSG00000241042		lncRNA	chr1:59054397-5905
ENSG0000(	827	18.72648	chr1:1021	CTH	DriverDB\	protein_c	chr1:70411218-7043
ENSG0000(	827	18.72648	chr1:1021	CTBP2P8		Pseudoger	chr1:68161761-6816
ENSG0000(	827	18.72648	chr1:1021	ENSG00000271992		lncRNA	chr1:70445071-7044
ENSG0000(	827	18.72648	chr1:1021	COX6B1P7		Pseudoger	chr1:68282388-6828
ENSG0000(	827	18.72648	chr1:1021	ENSG00000290013		lncRNA	chr1:58784270-5878
ENSG0000(	827	18.72648	chr1:1021	RPS29P7		Pseudoger	chr1:65154480-6515
ENSG0000(	827	18.72648	chr1:1021	DOCK7		protein_c	chr1:62454298-6268
ENSG0000(	827	18.72648	chr1:1021	LINC01707		lncRNA	chr1:69055838-6922
ENSG0000(	827	18.72648	chr1:1021	ERICH3		protein_c	chr1:74568117-7467

ENSG0000(	827	18.72648	chr1:1021	TNNI3K		protein_c	chr1:74235387-7454
ENSG0000(	827	18.72648	chr1:1021	MIR101-1		smallRNA	chr1:65058434-6505
ENSG0000(	827	18.72648	chr1:1021	SGO1P1		Pseudoger	chr1:69606855-6960
ENSG0000(	827	18.72648	chr1:1021	DLEU2L		lncRNA	chr1:63547082-6355
ENSG0000(	827	18.72648	chr1:1021	MRPS21P1		Pseudoger	chr1:65092392-6509
ENSG0000(	827	18.72648	chr1:1021	DOCK7-DT		lncRNA	chr1:62688482-6271
ENSG0000(	827	18.72648	chr1:1021	CHORDC1P5		Pseudoger	chr1:70530526-7053
ENSG0000(	827	18.72648	chr1:1021	ERICH3-AS1		lncRNA	chr1:74577430-7462
ENSG0000(	827	18.72648	chr1:1021	AL445464.1		smallRNA	chr1:75842632-7584
ENSG0000(	827	18.72648	chr1:1021	LINC02796		lncRNA	chr1:72765031-7279
ENSG0000(	827	18.72648	chr1:1021	RN7SL475P		smallRNA	chr1:59974759-5997
ENSG0000(	827	18.72648	chr1:1021	ALG6		protein_c	chr1:63367575-6343
ENSG0000(	827	18.72648	chr1:1021	CRYZ		protein_c	chr1:74705482-7473
ENSG0000(	827	18.72648	chr1:1021	SNORD45B		smallRNA	chr1:75789477-7578
ENSG0000(	827	18.72648	chr1:1021	LINC02238		lncRNA	chr1:73635216-7371
ENSG0000(	827	18.72648	chr1:1021	FOXD3		protein_c	chr1:63322567-6332
ENSG0000(	827	18.72648	chr1:1021	ENSG00000284928		lncRNA	chr1:64186791-6419
ENSG0000(	827	18.72648	chr1:1021	RPE65		protein_c	chr1:68428822-6844
ENSG0000(	827	18.72648	chr1:1021	WLS	NCGv7	protein_c	chr1:68098473-6823
ENSG0000(	827	18.72648	chr1:1021	ENSG00000231080		lncRNA	chr1:66826942-6682
ENSG0000(	827	18.72648	chr1:1021	ENSG00000248458		lncRNA	chr1:66665864-6667
ENSG0000(	827	18.72648	chr1:1021	LEPR	NCGv7	protein_c	chr1:65420652-6564
ENSG0000(	827	18.72648	chr1:1021	PHB1P3		Pseudoger	chr1:58999676-5900
ENSG0000(	827	18.72648	chr1:1021	DNAJC6		protein_c	chr1:65248219-6541
ENSG0000(	827	18.72648	chr1:1021	ENSG00000285079		lncRNA	chr1:65703962-6571
ENSG0000(	827	18.72648	chr1:1021	IL12RB2		protein_c	chr1:67307364-6739
ENSG0000(	827	18.72648	chr1:1021	PGM1		protein_c	chr1:63593411-6366
ENSG0000(	827	18.72648	chr1:1021	ENSG00000226883		lncRNA	chr1:59754747-5978
ENSG0000(	827	18.72648	chr1:1021	ENSG00000203605		lncRNA	chr1:63139250-6316
ENSG0000(	827	18.72648	chr1:1021	L1TD1		protein_c	chr1:62194849-6221
ENSG0000(	827	18.72648	chr1:1021	ITGB3BP		protein_c	chr1:63440770-6359
ENSG0000(	827	18.72648	chr1:1021	CYP2J2		protein_c	chr1:59893308-5992
ENSG0000(	827	18.72648	chr1:1021	SLC35D1		protein_c	chr1:66999350-6705
ENSG0000(	827	18.72648	chr1:1021	HOOK1		protein_c	chr1:59814786-5987
ENSG0000(	827	18.72648	chr1:1021	GADD45A		protein_c	chr1:67685201-6768
ENSG0000(	827	18.72648	chr1:1021	TXNP2		Pseudoger	chr1:68514375-6851
ENSG0000(	827	18.72648	chr1:1021	SERBP1		protein_c	chr1:67407810-6743
ENSG0000(	827	18.72648	chr1:1021	C1orf141		protein_c	chr1:67092165-6723
ENSG0000(	827	18.72648	chr1:1021	EFCAB7		protein_c	chr1:63523372-6357
ENSG0000(	827	18.72648	chr1:1021	LRRC7	NCGv7	protein_c	chr1:69567922-7015
ENSG0000(	827	18.72648	chr1:1021	LINC01135		lncRNA	chr1:58784384-5890
ENSG0000(	827	18.72648	chr1:1021	ENSG00000270549		Pseudoger	chr1:62530636-6253
ENSG0000(	827	18.72648	chr1:1021	RPSAP65		Pseudoger	chr1:63262113-6326
ENSG0000(	827	18.72648	chr1:1021	ENSG00000285041		lncRNA	chr1:68633701-6864
ENSG0000(	827	18.72648	chr1:1021	ENSG00000235782		lncRNA	chr1:70947379-7095
ENSG0000(	827	18.72648	chr1:1021	ENSG00000235804		Pseudoger	chr1:65077413-6507
ENSG0000(	827	18.72648	chr1:1021	DEPDC1	DriverDB\	protein_c	chr1:68474152-6849
ENSG0000(	827	18.72648	chr1:1021	CFL1P3		Pseudoger	chr1:63843196-6384
ENSG0000(	827	18.72648	chr1:1021	RN7SL180P		smallRNA	chr1:62072448-6207
ENSG0000(	827	18.72648	chr1:1021	LINC01359		lncRNA	chr1:64972225-6500
ENSG0000(	827	18.72648	chr1:1021	RNU4ATAC8P		smallRNA	chr1:73883713-7388

ENSG0000(	827	18.72648	chr1:1021	ENSG00000223920		Pseudoger	chr1:61654194-6165
ENSG0000(	827	18.72648	chr1:1021	MIR3117		smallRNA	chr1:66628440-6662
ENSG0000(	827	18.72648	chr1:1021	ENSG00000234318		lncRNA	chr1:62896009-6290
ENSG0000(	827	18.72648	chr1:1021	ENSG00000287283		lncRNA	chr1:69706950-6971
ENSG0000(	827	18.72648	chr1:1021	SNORD45C		smallRNA	chr1:75787072-7578
ENSG0000(	827	18.72648	chr1:1021	RNA5SP49		Pseudoger	chr1:63186336-6318
ENSG0000(	827	18.72648	chr1:1021	PDE4B-AS1		lncRNA	chr1:66042500-6605
ENSG0000(	827	18.72648	chr1:1021	ARL5AP3		Pseudoger	chr1:68049360-6804
ENSG0000(	827	18.72648	chr1:1021	Y_RNA		smallRNA	chr1:156484098-156
ENSG0000(	827	18.72648	chr1:1021	RNU4-88P		smallRNA	chr1:66094461-6609
ENSG0000(	827	18.72648	chr1:1021	RNU6-503P		smallRNA	chr1:75538015-7553
ENSG0000(	827	18.72648	chr1:1021	ENSG00000271618		Pseudoger	chr1:71738173-7173
ENSG0000(	827	18.72648	chr1:1021	ENSG00000290094		lncRNA	chr1:65310302-6531
ENSG0000(	827	18.72648	chr1:1021	ENSG00000231985		lncRNA	chr1:71570956-7157
ENSG0000(	827	18.72648	chr1:1021	MIER1		protein_c	chr1:66924895-6698
ENSG0000(	827	18.72648	chr1:1021	ENSG00000285407		lncRNA	chr1:68679202-6894
ENSG0000(	827	18.72648	chr1:1021	DNAJB6P4		Pseudoger	chr1:67278052-6727
ENSG0000(	827	18.72648	chr1:1021	ENSG00000224127		lncRNA	chr1:75127830-7513
ENSG0000(	827	18.72648	chr1:1021	ENSG00000224149		lncRNA	chr1:75129974-7513
ENSG0000(	827	18.72648	chr1:1021	FGGY-DT		lncRNA	chr1:59131932-5929
ENSG0000(	827	18.72648	chr1:1021	PATJ-DT		lncRNA	chr1:61741998-6174
ENSG0000(	827	18.72648	chr1:1021	RNU6-371P		smallRNA	chr1:62298149-6229
ENSG0000(	827	18.72648	chr1:1021	ENSG00000285473		lncRNA	chr1:68974010-6902
ENSG0000(	827	18.72648	chr1:1021	LRRC40		protein_c	chr1:70144805-7020
ENSG0000(	827	18.72648	chr1:1021	CACHD1	NCGv7	protein_c	chr1:64470129-6469
ENSG0000(	827	18.72648	chr1:1021	ENSG00000225605		lncRNA	chr1:75926454-7601
ENSG0000(	827	18.72648	chr1:1021	ENSG00000272506		lncRNA	chr1:65003470-6500
ENSG0000(	827	18.72648	chr1:1021	ENSG00000234784		Pseudoger	chr1:64918443-6491
ENSG0000(	827	18.72648	chr1:1021	RN7SL854P		smallRNA	chr1:65761060-6576
ENSG0000(	827	18.72648	chr1:1021	ASB17		protein_c	chr1:75918873-7593
ENSG0000(	827	18.72648	chr1:1021	ENSG00000228734		lncRNA	chr1:63249920-6325
ENSG0000(	827	18.72648	chr1:1021	MIR4794		smallRNA	chr1:64579847-6457
ENSG0000(	827	18.72648	chr1:1021	ENSG00000278967		TEC	chr1:62607766-6260
ENSG0000(	827	18.72648	chr1:1021	ENSG00000287224		lncRNA	chr1:61588049-6158
ENSG0000(	827	18.72648	chr1:1021	ENSG00000283445		lncRNA	chr1:58715609-5877
ENSG0000(	827	18.72648	chr1:1021	ENSG00000225087		lncRNA	chr1:72636547-7289
ENSG0000(	827	18.72648	chr1:1021	ENSG00000233589		lncRNA	chr1:68479129-6848
ENSG0000(	827	18.72648	chr1:1021	ENSG00000224570		Pseudoger	chr1:65576129-6557
ENSG0000(	827	18.72648	chr1:1021	MIR3671		smallRNA	chr1:65057755-6505
ENSG0000(	827	18.72648	chr1:1021	ENSG00000270457		lncRNA	chr1:59289303-5928
ENSG0000(	827	18.72648	chr1:1021	ENSG00000272226		lncRNA	chr1:58812808-5881
ENSG0000(	827	18.72648	chr1:1021	DLSTP1		Pseudoger	chr1:75743423-7574
ENSG0000(	827	18.72648	chr1:1021	ENSG00000231740		lncRNA	chr1:58838448-5885
ENSG0000(	827	18.72648	chr1:1021	ROR1-AS1		lncRNA	chr1:64094379-6417
ENSG0000(	827	18.72648	chr1:1021	JUN	NCGv7;AC	protein_c	chr1:58776845-5878
ENSG0000(	827	18.72648	chr1:1021	ACADM		protein_c	chr1:75724431-7578
ENSG0000(	827	18.72648	chr1:1021	ENSG00000227485		lncRNA	chr1:63024207-6302
ENSG0000(	827	18.72648	chr1:1021	RN7SL538P		smallRNA	chr1:69879592-6987
ENSG0000(	827	18.72648	chr1:1021	ENSG00000177452		Pseudoger	chr1:63788721-6378
ENSG0000(	827	18.72648	chr1:1021	LINC00466		lncRNA	chr1:63159083-6331
ENSG0000(	827	18.72648	chr1:1021	RNU6-586P		smallRNA	chr1:67196140-6719

ENSG0000(	827	18.72648	chr1:1021	AC099791.1		smallRNA	chr1:61629031-6162
ENSG0000(	827	18.72648	chr1:1021	UBE2U		protein_c	chr1:64203623-6426
ENSG0000(	827	18.72648	chr1:1021	ENSG00000284808		lncRNA	chr1:61481087-6153
ENSG0000(	827	18.72648	chr1:1021	RNU6-387P		smallRNA	chr1:67417214-6741
ENSG0000(	827	18.72648	chr1:1021	ATG4C		protein_c	chr1:62784132-6286
ENSG0000(	826	18.70383	chr1:3733	RN7SL165P		smallRNA	chr1:26814822-2681
ENSG0000(	821	18.59061	chr1:1021	BCAN		protein_c	chr1:156641390-156
ENSG0000(	821	18.59061	chr1:1021	MIR9-1HG		lncRNA	chr1:156404250-156
ENSG0000(	821	18.59061	chr1:1021	RAB25		protein_c	chr1:156061160-156
ENSG0000(	821	18.59061	chr1:1021	NES	NCGv7	protein_c	chr1:156668763-156
ENSG0000(	821	18.59061	chr4:253(	USP53		protein_c	chr4:119212587-119
ENSG0000(	821	18.59061	chr1:1021	ARHGEF11	NCGv7	protein_c	chr1:156934840-157
ENSG0000(	821	18.59061	chr1:3733	PRDX3P2		Pseudoger	chr1:28526318-2852
ENSG0000(	821	18.59061	chr1:1021	HAPLN2		protein_c	chr1:156619331-156
ENSG0000(	821	18.59061	chr1:3733	ENSG00000229820		Pseudoger	chr1:28453541-2845
ENSG0000(	821	18.59061	chr1:1021	KHDC4		protein_c	chr1:155913045-155
ENSG0000(	821	18.59061	chr4:253(	C4orf33	DriverDBv	protein_c	chr4:129093317-129
ENSG0000(	821	18.59061	chr1:3733	LINC01756		lncRNA	chr1:29329620-2935
ENSG0000(	821	18.59061	chr1:1021	ENSG00000285677		lncRNA	chr1:156001953-156
ENSG0000(	821	18.59061	chr1:1021	ASH1L-IT1		lncRNA	chr1:155396010-155
ENSG0000(	821	18.59061	chr1:1021	ENSG00000286005		Pseudoger	chr1:157437484-157
ENSG0000(	821	18.59061	chr4:253(	METTL14-DT		lncRNA	chr4:118664087-118
ENSG0000(	821	18.59061	chr4:253(	TRPC3		protein_c	chr4:121874481-121
ENSG0000(	821	18.59061	chr4:253(	SEC24D		protein_c	chr4:118722823-118
ENSG0000(	821	18.59061	chr4:253(	ANKRD50	NCGv7	protein_c	chr4:124664048-124
ENSG0000(	821	18.59061	chr1:3733	DNAJC8		protein_c	chr1:28199456-2823
ENSG0000(	821	18.59061	chr4:253(	SCLT1		protein_c	chr4:128864921-129
ENSG0000(	821	18.59061	chr4:253(	ENSG00000286251		lncRNA	chr4:126431694-126
ENSG0000(	821	18.59061	chr4:253(	SLC25A31		protein_c	chr4:127730400-127
ENSG0000(	821	18.59061	chr1:1021	RHBG	NCGv7	protein_c	chr1:156369211-156
ENSG0000(	821	18.59061	chr4:253(	LARP1B		protein_c	chr4:128061286-128
ENSG0000(	821	18.59061	chr1:1021	ENSG00000232519		lncRNA	chr1:155609776-155
ENSG0000(	821	18.59061	chr4:253(	IL21-AS1		lncRNA	chr4:122618983-122
ENSG0000(	821	18.59061	chr4:253(	LINC02264		lncRNA	chr4:117834145-117
ENSG0000(	821	18.59061	chr4:253(	AC021205.1		smallRNA	chr4:122827014-122
ENSG0000(	821	18.59061	chr4:253(	PRDM5		protein_c	chr4:120684919-120
ENSG0000(	821	18.59061	chr1:3733	ENSG00000279443		TEC	chr1:28544460-2854
ENSG0000(	821	18.59061	chr1:1021	DAP3		protein_c	chr1:155687960-155
ENSG0000(	821	18.59061	chr4:253(	PDE5A		protein_c	chr4:119494397-119
ENSG0000(	821	18.59061	chr4:253(	ENSG00000286241		lncRNA	chr4:119686538-119
ENSG0000(	821	18.59061	chr1:1021	ENSG00000286073		Pseudoger	chr1:157287267-157
ENSG0000(	821	18.59061	chr1:1021	MSTO1		protein_c	chr1:155563235-155
ENSG0000(	821	18.59061	chr4:253(	ENSG00000282855		Pseudoger	chr4:127809170-127
ENSG0000(	821	18.59061	chr4:253(	METTL14	NCGv7	protein_c	chr4:118685392-118
ENSG0000(	821	18.59061	chr4:253(	PP12613		lncRNA	chr4:121764585-121
ENSG0000(	821	18.59061	chr1:1021	CRABP2		protein_c	chr1:156699606-156
ENSG0000(	821	18.59061	chr1:1021	THBS3-AS1		lncRNA	chr1:155194996-155
ENSG0000(	821	18.59061	chr1:1021	ENSG00000285570		lncRNA	chr1:156689676-156
ENSG0000(	821	18.59061	chr1:1021	MEX3A	DriverDBv	protein_c	chr1:156072013-156
ENSG0000(	821	18.59061	chr1:1021	HDGF		protein_c	chr1:156742109-156
ENSG0000(	821	18.59061	chr1:3733	RP1-212P9.2		lncRNA	chr1:28867575-2887

ENSG0000(	821	18.59061	chr4:253(	NDUFS5P5		Pseudoger	chr4:118400514-118
ENSG0000(	821	18.59061	chr4:253(	JADRR		lncRNA	chr4:128768228-128
ENSG0000(	821	18.59061	chr1:1021	HCN3		protein_c	chr1:155277463-155
ENSG0000(	821	18.59061	chr1:1021	PKLR		protein_c	chr1:155289293-155
ENSG0000(	821	18.59061	chr1:1021	RIT1	NCGv7;AC	protein_c	chr1:155897808-155
ENSG0000(	821	18.59061	chr1:3733	TMEM200B		protein_c	chr1:29119429-2912
ENSG0000(	821	18.59061	chr4:253(	ENSG00000280059		TEC	chr4:130808507-130
ENSG0000(	821	18.59061	chr4:253(	CCNA2	TAG	protein_c	chr4:121816444-121
ENSG0000(	821	18.59061	chr1:3733	MED18		protein_c	chr1:28329002-2833
ENSG0000(	821	18.59061	chr4:253(	LINC02263		lncRNA	chr4:117360596-117
ENSG0000(	821	18.59061	chr1:3733	ATP5IF1		protein_c	chr1:28236109-2824
ENSG0000(	821	18.59061	chr4:253(	GTF2IP12		Pseudoger	chr4:119440561-119
ENSG0000(	821	18.59061	chr1:3733	SESN2	NCGv7	protein_c	chr1:28259518-2828
ENSG0000(	821	18.59061	chr1:3733	ENSG00000233427		lncRNA	chr1:28870483-2887
ENSG0000(	821	18.59061	chr1:3733	ENSG00000228943		Pseudoger	chr1:28109739-2811
ENSG0000(	821	18.59061	chr1:1021	EFNA3		protein_c	chr1:155078837-155
ENSG0000(	821	18.59061	chr4:253(	RPL21P50		Pseudoger	chr4:123745750-123
ENSG0000(	821	18.59061	chr1:3733	ENSG00000284676		lncRNA	chr1:29755175-2979
ENSG0000(	821	18.59061	chr1:3733	SPCS2P4		Pseudoger	chr1:28095742-2809
ENSG0000(	821	18.59061	chr1:3733	RNU6-176P		smallRNA	chr1:28142737-2814
ENSG0000(	821	18.59061	chr1:1021	ISG20L2		protein_c	chr1:156721891-156
ENSG0000(	821	18.59061	chr1:1021	ENSG00000286151		Pseudoger	chr1:157400927-157
ENSG0000(	821	18.59061	chr1:1021	ENSG00000228239		lncRNA	chr1:157232231-157
ENSG0000(	821	18.59061	chr1:1021	MRPL24		protein_c	chr1:156737303-156
ENSG0000(	821	18.59061	chr1:1021	DCST1-AS1		lncRNA	chr1:155045191-155
ENSG0000(	821	18.59061	chr4:253(	KIAA1109		protein_c	chr4:122152331-122
ENSG0000(	821	18.59061	chr4:253(	FABP2		protein_c	chr4:119317250-119
ENSG0000(	821	18.59061	chr4:253(	CEP170P1		Pseudoger	chr4:118467590-118
ENSG0000(	821	18.59061	chr1:1021	SYT11		protein_c	chr1:155859567-155
ENSG0000(	821	18.59061	chr4:253(	SPATA5		protein_c	chr4:122923070-123
ENSG0000(	821	18.59061	chr4:253(	ENSG00000286269		lncRNA	chr4:117648832-117
ENSG0000(	821	18.59061	chr4:253(	BBS7		protein_c	chr4:121824329-121
ENSG0000(	821	18.59061	chr1:3733	TAF12-DT		lncRNA	chr1:28643228-2864
ENSG0000(	821	18.59061	chr4:253(	FGF2	NCGv7	protein_c	chr4:122826708-122
ENSG0000(	821	18.59061	chr4:253(	IL21		protein_c	chr4:122610108-122
ENSG0000(	821	18.59061	chr1:1021	ENSG00000227673		lncRNA	chr1:155710098-155
ENSG0000(	821	18.59061	chr4:253(	PLK4		protein_c	chr4:127880893-127
ENSG0000(	821	18.59061	chr4:253(	ENSG00000226655		lncRNA	chr4:123744923-123
ENSG0000(	821	18.59061	chr1:1021	AL589685.1		Pseudoger	chr1:156323511-156
ENSG0000(	821	18.59061	chr1:3733	ENSG00000233372		lncRNA	chr1:30140263-3014
ENSG0000(	821	18.59061	chr1:3733	ENSG00000228176		lncRNA	chr1:29708851-2970
ENSG0000(	821	18.59061	chr1:1021	ENSG00000229953		lncRNA	chr1:156646507-156
ENSG0000(	821	18.59061	chr1:1021	ENSG00000229961		Pseudoger	chr1:157182860-157
ENSG0000(	821	18.59061	chr1:3733	LINC01648		lncRNA	chr1:30013952-3003
ENSG0000(	821	18.59061	chr1:3733	RP11-242O24.5		lncRNA	chr1:29152489-2915
ENSG0000(	821	18.59061	chr4:253(	ENSG00000253825		lncRNA	chr4:120035033-120
ENSG0000(	821	18.59061	chr1:1021	PRCC	AC	protein_c	chr1:156750610-156
ENSG0000(	821	18.59061	chr1:1021	ETV3L		protein_c	chr1:157092043-157
ENSG0000(	821	18.59061	chr1:1021	METTL25B		protein_c	chr1:156728442-156
ENSG0000(	821	18.59061	chr1:1021	ADAM15		protein_c	chr1:155050566-155
ENSG0000(	821	18.59061	chr1:1021	ENSG00000284592		Pseudoger	chr1:157204779-157

ENSG0000(	821	18.59061	chr1:1021	GON4L		protein_c	chr1:155749659-155
ENSG0000(	821	18.59061	chr4:253(	MFSD8		protein_c	chr4:127917799-127
ENSG0000(	821	18.59061	chr4:253(	ADAD1		protein_c	chr4:122378966-122
ENSG0000(	821	18.59061	chr4:253(	SMIM43		protein_c	chr4:121758881-121
ENSG0000(	821	18.59061	chr4:253(	ANXA5		protein_c	chr4:121667946-121
ENSG0000(	821	18.59061	chr1:1021	SNORA26		smallRNA	chr1:156192063-156
ENSG0000(	821	18.59061	chr4:253(	MAD2L1		protein_c	chr4:120055623-120
ENSG0000(	821	18.59061	chr4:253(	NDST3		protein_c	chr4:118033618-118
ENSG0000(	821	18.59061	chr4:253(	PRSS12		protein_c	chr4:118280038-118
ENSG0000(	821	18.59061	chr4:253(	C4orf3		protein_c	chr4:119296419-119
ENSG0000(	821	18.59061	chr4:253(	TUBB4BP5		Pseudoger	chr4:121451541-121
ENSG0000(	821	18.59061	chr4:253(	ABHD18		protein_c	chr4:127965306-128
ENSG0000(	821	18.59061	chr4:253(	HSPA4L		protein_c	chr4:127781821-127
ENSG0000(	821	18.59061	chr1:1021	ARHGEF2-AS1		lncRNA	chr1:155991390-156
ENSG0000(	821	18.59061	chr4:253(	INTU		protein_c	chr4:127623271-127
ENSG0000(	821	18.59061	chr4:253(	SPRY1		protein_c	chr4:123396795-123
ENSG0000(	821	18.59061	chr1:373:	PTPRU	NCGv7	protein_c	chr1:29236516-2932
ENSG0000(	821	18.59061	chr4:253(	PGRMC2		protein_c	chr4:128269237-128
ENSG0000(	821	18.59061	chr4:253(	Y_RNA		smallRNA	chr4:119947131-119
ENSG0000(	821	18.59061	chr1:373:	ENSG00000270927		Pseudoger	chr1:29904865-2990
ENSG0000(	821	18.59061	chr1:373:	ENSG00000237934		lncRNA	chr1:29223933-2922
ENSG0000(	821	18.59061	chr4:253(	ENSG00000248802		lncRNA	chr4:128582999-128
ENSG0000(	821	18.59061	chr1:1021	ENSG00000272405		lncRNA	chr1:156641666-156
ENSG0000(	821	18.59061	chr4:253(	ENSG00000250322		Pseudoger	chr4:123259949-123
ENSG0000(	821	18.59061	chr4:253(	PGBD4P4		Pseudoger	chr4:130494673-130
ENSG0000(	821	18.59061	chr1:1021	BGLAP		protein_c	chr1:156242184-156
ENSG0000(	821	18.59061	chr4:253(	ENSG00000271053		Pseudoger	chr4:116858093-116
ENSG0000(	821	18.59061	chr4:253(	TRMT112P1		Pseudoger	chr4:116378919-116
ENSG0000(	821	18.59061	chr1:1021	ENSG00000251246		protein_c	chr1:155063748-155
ENSG0000(	821	18.59061	chr4:253(	ENSG00000287924		Pseudoger	chr4:130963925-130
ENSG0000(	821	18.59061	chr4:253(	ENSG00000287101		lncRNA	chr4:127228519-127
ENSG0000(	821	18.59061	chr4:253(	ENSG00000249002		Pseudoger	chr4:119006032-119
ENSG0000(	821	18.59061	chr4:253(	RPL7AP28		Pseudoger	chr4:131857343-131
ENSG0000(	821	18.59061	chr4:253(	ENSG00000250213		Pseudoger	chr4:131112748-131
ENSG0000(	821	18.59061	chr1:1021	ENSG00000289593		lncRNA	chr1:156504660-156
ENSG0000(	821	18.59061	chr1:373:	SNHG3		lncRNA	chr1:28505980-2851
ENSG0000(	821	18.59061	chr1:1021	CYCSP52		Pseudoger	chr1:157128362-157
ENSG0000(	821	18.59061	chr1:1021	SMU1P1		Pseudoger	chr1:157059232-157
ENSG0000(	821	18.59061	chr4:253(	ENSG00000287951		lncRNA	chr4:123349250-123
ENSG0000(	821	18.59061	chr1:1021	GBA		protein_c	chr1:155234452-155
ENSG0000(	821	18.59061	chr4:253(	ENSG00000237868		Pseudoger	chr4:122077840-122
ENSG0000(	821	18.59061	chr1:1021	POU5F1P4		Pseudoger	chr1:155433178-155
ENSG0000(	821	18.59061	chr1:1021	ENSG00000290592		lncRNA	chr1:157171116-157
ENSG0000(	821	18.59061	chr4:253(	RNU6-948P		smallRNA	chr4:121192797-121
ENSG0000(	821	18.59061	chr4:253(	LTV1P1		Pseudoger	chr4:120091046-120
ENSG0000(	821	18.59061	chr1:373:	ENSG00000289576		lncRNA	chr1:28116812-2812
ENSG0000(	821	18.59061	chr4:253(	ENSG00000251195		Pseudoger	chr4:129177386-129
ENSG0000(	821	18.59061	chr1:1021	RN7SL612P		smallRNA	chr1:156985757-156
ENSG0000(	821	18.59061	chr4:253(	ZSWIM5P3		Pseudoger	chr4:129135926-129
ENSG0000(	821	18.59061	chr1:373:	Y_RNA		smallRNA	chr1:28985710-2898
ENSG0000(	821	18.59061	chr4:253(	BBS7-DT		lncRNA	chr4:121870583-121

ENSG0000(	821	18.59061	chr4:253(	RBM48P1		Pseudoger	chr4:126563509-126
ENSG0000(	821	18.59061	chr4:253(	ENSG00000261668		lncRNA	chr4:127840198-127
ENSG0000(	821	18.59061	chr4:253(	NT5C3AP1		Pseudoger	chr4:117574512-117
ENSG0000(	821	18.59061	chr4:253(	ENSG00000213480		Pseudoger	chr4:121369433-121
ENSG0000(	821	18.59061	chr1:1021	MUC1	DriverDB\	protein_c	chr1:155185824-155
ENSG0000(	821	18.59061	chr1:1021	ETV3	NCGv7;AC	protein_c	chr1:157121191-157
ENSG0000(	821	18.59061	chr1:1021	NAXE		protein_c	chr1:156591756-156
ENSG0000(	821	18.59061	chr1:1021	YY1AP1	AC	protein_c	chr1:155659443-155
ENSG0000(	821	18.59061	chr1:1021	DCST1	DriverDB\	protein_c	chr1:155033824-155
ENSG0000(	821	18.59061	chr1:1021	DCST2	DriverDB\	protein_c	chr1:155018520-155
ENSG0000(	821	18.59061	chr1:1021	LENEP	DriverDB\	protein_c	chr1:154993586-154
ENSG0000(	821	18.59061	chr4:253(	Y_RNA		smallRNA	chr4:127028107-127
ENSG0000(	821	18.59061	chr4:253(	KRT18P21		Pseudoger	chr4:115920707-115
ENSG0000(	821	18.59061	chr1:373:	OPRD1	NCGv7	protein_c	chr1:28812170-2887
ENSG0000(	821	18.59061	chr4:253(	ENSG00000287021		lncRNA	chr4:127532828-127
ENSG0000(	821	18.59061	chr1:373:	Y_RNA		smallRNA	chr1:28881726-2888
ENSG0000(	821	18.59061	chr4:253(	LINC02502		lncRNA	chr4:119939540-119
ENSG0000(	821	18.59061	chr1:373:	ENSG00000238231		Pseudoger	chr1:27990158-2799
ENSG0000(	821	18.59061	chr1:373:	AL139151.1		smallRNA	chr1:29432481-2943
ENSG0000(	821	18.59061	chr4:253(	KLHL2P1		Pseudoger	chr4:119334329-119
ENSG0000(	821	18.59061	chr4:253(	H3P15		Pseudoger	chr4:126106139-126
ENSG0000(	821	18.59061	chr4:253(	MYOZ2		protein_c	chr4:119135832-119
ENSG0000(	821	18.59061	chr4:253(	SYNPO2		protein_c	chr4:118850688-119
ENSG0000(	821	18.59061	chr4:253(	RNU6-550P		smallRNA	chr4:120810469-120
ENSG0000(	821	18.59061	chr1:1021	TRIM46	DriverDB\	protein_c	chr1:155173787-155
ENSG0000(	821	18.59061	chr1:1021	KRTCAP2		protein_c	chr1:155169408-155
ENSG0000(	821	18.59061	chr4:253(	ACTN4P1		Pseudoger	chr4:116598161-116
ENSG0000(	821	18.59061	chr1:1021	TSACC		protein_c	chr1:156337314-156
ENSG0000(	821	18.59061	chr1:373:	SRSF4		protein_c	chr1:29147743-2918
ENSG0000(	821	18.59061	chr1:373:	MECR		protein_c	chr1:29192657-2923
ENSG0000(	821	18.59061	chr4:253(	ENSG00000248715		Pseudoger	chr4:131540281-131
ENSG0000(	821	18.59061	chr1:1021	CLK2		protein_c	chr1:155262868-155
ENSG0000(	821	18.59061	chr1:1021	SCAMP3		protein_c	chr1:155255979-155
ENSG0000(	821	18.59061	chr1:1021	ASH1L	NCGv7	protein_c	chr1:155335268-155
ENSG0000(	821	18.59061	chr4:253(	SEPTIN14P4		Pseudoger	chr4:118640673-118
ENSG0000(	821	18.59061	chr1:1021	ARHGEF2	AC	protein_c	chr1:155946851-156
ENSG0000(	821	18.59061	chr1:1021	LAMTOR2		protein_c	chr1:156054782-156
ENSG0000(	821	18.59061	chr1:1021	MEF2D	NCGv7	protein_c	chr1:156463727-156
ENSG0000(	821	18.59061	chr4:253(	NUDT6	DriverDB\	protein_c	chr4:122888697-122
ENSG0000(	821	18.59061	chr4:253(	NDNF-AS1		lncRNA	chr4:121071429-121
ENSG0000(	821	18.59061	chr1:373:	Y_RNA		smallRNA	chr1:28422555-2842
ENSG0000(	821	18.59061	chr4:253(	TNIP3		protein_c	chr4:121131408-121
ENSG0000(	821	18.59061	chr4:253(	ENSG00000273716		Pseudoger	chr4:118460224-118
ENSG0000(	821	18.59061	chr4:253(	RNU6-224P		smallRNA	chr4:131273242-131
ENSG0000(	821	18.59061	chr1:373:	SNORA73B		smallRNA	chr1:28508559-2850
ENSG0000(	821	18.59061	chr1:1021	KRT8P45		Pseudoger	chr1:157073257-157
ENSG0000(	821	18.59061	chr1:1021	SSR2		protein_c	chr1:156009048-156
ENSG0000(	821	18.59061	chr1:1021	TMEM79		protein_c	chr1:156282935-156
ENSG0000(	821	18.59061	chr1:1021	CCT3		protein_c	chr1:156308968-156
ENSG0000(	821	18.59061	chr4:253(	ENSG00000287845		lncRNA	chr4:116714844-116
ENSG0000(	821	18.59061	chr1:1021	ENSG00000287839		lncRNA	chr1:155626755-155

ENSG00000	821	18.59061	chr4:2530	ENSG00000289342		lncRNA	chr4:121950963-121
ENSG00000	821	18.59061	chr1:1021	MSTO2P		Pseudogen	chr1:155745829-155
ENSG00000	821	18.59061	chr4:2530	ENSG00000289869		lncRNA	chr4:126776969-126
ENSG00000	821	18.59061	chr4:2530	LINC02379		lncRNA	chr4:126064128-126
ENSG00000	821	18.59061	chr4:2530	SNHG27		lncRNA	chr4:131727587-131
ENSG00000	821	18.59061	chr4:2530	ENSG00000249837		lncRNA	chr4:124149377-124
ENSG00000	821	18.59061	chr4:2530	ENSG00000249795		Pseudogen	chr4:127498327-127
ENSG00000	821	18.59061	chr1:1021	MTX1P1		Pseudogen	chr1:155230975-155
ENSG00000	821	18.59061	chr4:2530	ENSG00000287520		lncRNA	chr4:116720123-116
ENSG00000	821	18.59061	chr4:2530	AC109357.1		smallRNA	chr4:123007572-123
ENSG00000	821	18.59061	chr1:1021	MIR92B		smallRNA	chr1:155195177-155
ENSG00000	821	18.59061	chr1:3733	RN7SL559P		smallRNA	chr1:28031886-2803
ENSG00000	821	18.59061	chr1:1021	LINC02772		lncRNA	chr1:157273760-157
ENSG00000	821	18.59061	chr4:2530	JADE1		protein_c	chr4:128809700-128
ENSG00000	821	18.59061	chr1:1021	AL713999.1		smallRNA	chr1:155236749-155
ENSG00000	821	18.59061	chr4:2530	ENSG00000240775		Pseudogen	chr4:122840343-122
ENSG00000	821	18.59061	chr4:2530	BBS12		protein_c	chr4:122732702-122
ENSG00000	821	18.59061	chr4:2530	ENSG00000275647		Pseudogen	chr4:128944628-128
ENSG00000	821	18.59061	chr4:2530	NUP58P1		Pseudogen	chr4:125681177-125
ENSG00000	821	18.59061	chr1:1021	RNU7-150P		smallRNA	chr1:155143271-155
ENSG00000	821	18.59061	chr4:2530	LINC02615		lncRNA	chr4:128292751-128
ENSG00000	821	18.59061	chr1:1021	ENSG00000237390		lncRNA	chr1:156388226-156
ENSG00000	821	18.59061	chr4:2530	LINC02465		lncRNA	chr4:129771596-129
ENSG00000	821	18.59061	chr4:2530	RNU6-583P		smallRNA	chr4:127891991-127
ENSG00000	821	18.59061	chr1:3733	RCC1	NCGv7	protein_c	chr1:28505943-2853
ENSG00000	821	18.59061	chr1:3733	AL645859.1		smallRNA	chr1:29253542-2925
ENSG00000	821	18.59061	chr4:2530	FKBP4P1		Pseudogen	chr4:118193405-118
ENSG00000	821	18.59061	chr1:3733	PHACTR4		protein_c	chr1:28369582-2850
ENSG00000	821	18.59061	chr1:1021	ENSG00000260460		lncRNA	chr1:156509854-156
ENSG00000	821	18.59061	chr4:2530	ENSG00000260404		Pseudogen	chr4:118595912-118
ENSG00000	821	18.59061	chr4:2530	IL2	AC	protein_c	chr4:122451470-122
ENSG00000	821	18.59061	chr4:2530	ENSG00000178636		Pseudogen	chr4:119192773-119
ENSG00000	821	18.59061	chr1:3733	AC092265.1		smallRNA	chr1:29644545-2964
ENSG00000	821	18.59061	chr4:2530	LINC02435		lncRNA	chr4:123505279-123
ENSG00000	821	18.59061	chr1:3733	RNU6ATAC27P		smallRNA	chr1:28481362-2848
ENSG00000	821	18.59061	chr4:2530	ENSG00000251555		lncRNA	chr4:131930031-131
ENSG00000	821	18.59061	chr4:2530	SNORA11		smallRNA	chr4:119569699-119
ENSG00000	821	18.59061	chr1:1021	PMF1-BGLAP		protein_c	chr1:156212982-156
ENSG00000	821	18.59061	chr1:1021	BCAN-AS1		lncRNA	chr1:156637783-156
ENSG00000	821	18.59061	chr4:2530	AC093816.1		smallRNA	chr4:121402587-121
ENSG00000	821	18.59061	chr1:1021	ENSG00000287624		lncRNA	chr1:156768105-156
ENSG00000	821	18.59061	chr1:1021	DPM3		protein_c	chr1:155139891-155
ENSG00000	821	18.59061	chr4:2530	ENSG00000260091		lncRNA	chr4:119409333-119
ENSG00000	821	18.59061	chr4:2530	LINC01378		lncRNA	chr4:117406166-117
ENSG00000	821	18.59061	chr4:2530	SETP12		Pseudogen	chr4:120895494-120
ENSG00000	821	18.59061	chr4:2530	ENSG00000287692		lncRNA	chr4:122700445-122
ENSG00000	821	18.59061	chr4:2530	LINC02377		lncRNA	chr4:131379717-131
ENSG00000	821	18.59061	chr1:3733	TRNAU1AP		protein_c	chr1:28553085-2857
ENSG00000	821	18.59061	chr1:3733	SNORD99		smallRNA	chr1:28578749-2857
ENSG00000	821	18.59061	chr1:1021	AL355388.1		smallRNA	chr1:156077373-156
ENSG00000	821	18.59061	chr4:2530	ENSG00000250149		lncRNA	chr4:125676718-126

ENSG0000(	821	18.59061	chr4:253(	GAPDHP56		Pseudoger	chr4:130503357-130
ENSG0000(	821	18.59061	chr1:1021	HMGN2P18		Pseudoger	chr1:155148544-155
ENSG0000(	821	18.59061	chr4:253(	ENSG00000249259		Pseudoger	chr4:115922015-116
ENSG0000(	821	18.59061	chr1:1021	ENSG00000237588		lncRNA	chr1:156687695-156
ENSG0000(	821	18.59061	chr4:253(	ENSG00000249244		Pseudoger	chr4:119391831-119
ENSG0000(	821	18.59061	chr4:253(	ENSG00000241651		Pseudoger	chr4:128069014-128
ENSG0000(	821	18.59061	chr1:1021	ENSG00000223356		lncRNA	chr1:156712212-156
ENSG0000(	821	18.59061	chr4:253(	ENSG00000271302		Pseudoger	chr4:121118299-121
ENSG0000(	821	18.59061	chr4:253(	LINC02479		lncRNA	chr4:130376229-130
ENSG0000(	821	18.59061	chr1:1021	AL365181.1		smallRNA	chr1:156587856-156
ENSG0000(	821	18.59061	chr4:253(	LINC02516		lncRNA	chr4:124499940-124
ENSG0000(	821	18.59061	chr1:1021	ENSG00000271267		Pseudoger	chr1:155566050-155
ENSG0000(	821	18.59061	chr4:253(	SNORA70		smallRNA	chr4:131768140-131
ENSG0000(	821	18.59061	chr4:253(	ENSG00000250183		Pseudoger	chr4:119937557-119
ENSG0000(	821	18.59061	chr4:253(	RPSAP35		Pseudoger	chr4:117413462-117
ENSG0000(	821	18.59061	chr4:253(	Y_RNA		smallRNA	chr4:125362819-125
ENSG0000(	821	18.59061	chr4:253(	ENSG00000249125		lncRNA	chr4:123490242-123
ENSG0000(	821	18.59061	chr1:1021	ENSG00000223503		Pseudoger	chr1:155590601-155
ENSG0000(	821	18.59061	chr4:253(	ENSG00000250193		lncRNA	chr4:129299175-129
ENSG0000(	821	18.59061	chr1:373:	ENSG00000214812		Pseudoger	chr1:28120449-2812
ENSG0000(	821	18.59061	chr4:253(	RNU4-33P		smallRNA	chr4:119367562-119
ENSG0000(	821	18.59061	chr4:253(	ENSG00000223605		Pseudoger	chr4:117249924-117
ENSG0000(	821	18.59061	chr1:1021	IQGAP3	DriverDBv	protein_c	chr1:156525405-156
ENSG0000(	821	18.59061	chr1:1021	ASH1L-AS1		lncRNA	chr1:155562026-155
ENSG0000(	821	18.59061	chr4:253(	RNU6-1054P		smallRNA	chr4:118606450-118
ENSG0000(	821	18.59061	chr4:253(	CDRT15P11		Pseudoger	chr4:129133106-129
ENSG0000(	821	18.59061	chr4:253(	RNU6-1217P		smallRNA	chr4:119434005-119
ENSG0000(	821	18.59061	chr4:253(	ENSG00000287290		lncRNA	chr4:116751473-116
ENSG0000(	821	18.59061	chr4:253(	RN7SL335P		smallRNA	chr4:122087506-122
ENSG0000(	821	18.59061	chr1:373:	AL645944.1		smallRNA	chr1:29884902-2988
ENSG0000(	821	18.59061	chr4:253(	TMEM248P1		Pseudoger	chr4:125723267-125
ENSG0000(	821	18.59061	chr1:373:	AL353354.1		protein_c	chr1:28200502-2820
ENSG0000(	821	18.59061	chr4:253(	RNY5P4		smallRNA	chr4:129860422-129
ENSG0000(	821	18.59061	chr1:1021	ENSG00000236263		lncRNA	chr1:155211151-155
ENSG0000(	821	18.59061	chr1:1021	THBS3		protein_c	chr1:155195588-155
ENSG0000(	821	18.59061	chr1:1021	SLC50A1		protein_c	chr1:155135344-155
ENSG0000(	821	18.59061	chr1:1021	EFNA1		protein_c	chr1:155127876-155
ENSG0000(	821	18.59061	chr1:1021	Y_RNA		smallRNA	chr1:155120490-155
ENSG0000(	821	18.59061	chr4:253(	LINC01091		lncRNA	chr4:123539026-123
ENSG0000(	821	18.59061	chr4:253(	RN7SKP137		smallRNA	chr4:121061172-121
ENSG0000(	821	18.59061	chr4:253(	ENSG00000249416		Pseudoger	chr4:131768233-131
ENSG0000(	821	18.59061	chr4:253(	ENSG00000249409		lncRNA	chr4:120639090-120
ENSG0000(	821	18.59061	chr4:253(	ENSG00000287748		lncRNA	chr4:129362911-129
ENSG0000(	821	18.59061	chr4:253(	RN7SL205P		smallRNA	chr4:131720262-131
ENSG0000(	821	18.59061	chr4:253(	RNU6-119P		smallRNA	chr4:116839279-116
ENSG0000(	821	18.59061	chr1:373:	PTAFR		protein_c	chr1:28147166-2819
ENSG0000(	821	18.59061	chr1:373:	ENSG00000271398		lncRNA	chr1:28247144-2824
ENSG0000(	821	18.59061	chr1:373:	ENSG00000290123		lncRNA	chr1:28234080-2823
ENSG0000(	821	18.59061	chr1:1021	RNU4-19P		smallRNA	chr1:155894281-155
ENSG0000(	821	18.59061	chr1:373:	RNU7-29P		smallRNA	chr1:28144156-2814
ENSG0000(	821	18.59061	chr1:1021	RP11-85G21.2		lncRNA	chr1:157287703-157

ENSG0000(	821	18.59061	chr4:253(	ENSG00000273007		lncRNA	chr4:122879778-122
ENSG0000(	821	18.59061	chr4:253(	PPIAP76		Pseudoger	chr4:124062462-124
ENSG0000(	821	18.59061	chr1:373:	ENSG00000225750		lncRNA	chr1:29144494-2914
ENSG0000(	821	18.59061	chr4:253(	snoU13		smallRNA	chr4:128756026-128
ENSG0000(	821	18.59061	chr1:1021	SMG5	NCGv7	protein_c	chr1:156249224-156
ENSG0000(	821	18.59061	chr4:253(	QRFPR		protein_c	chr4:121328642-121
ENSG0000(	821	18.59061	chr4:253(	SAR1AP3		Pseudoger	chr4:120342056-120
ENSG0000(	821	18.59061	chr4:253(	FOSL1P1		Pseudoger	chr4:128155389-128
ENSG0000(	821	18.59061	chr1:373:	SCARNA24		smallRNA	chr1:28689665-2868
ENSG0000(	821	18.59061	chr4:253(	ENSG00000286637		lncRNA	chr4:116754453-116
ENSG0000(	821	18.59061	chr1:1021	MTX1		protein_c	chr1:155208695-155
ENSG0000(	821	18.59061	chr1:1021	ENSG00000288835		lncRNA	chr1:156503083-156
ENSG0000(	821	18.59061	chr1:373:	ENSG00000225616		Pseudoger	chr1:28982278-2898
ENSG0000(	821	18.59061	chr1:1021	EFNA4		protein_c	chr1:155063737-155
ENSG0000(	821	18.59061	chr1:1021	DAP3P1		Pseudoger	chr1:155586644-155
ENSG0000(	821	18.59061	chr1:1021	GLMP		protein_c	chr1:156290089-156
ENSG0000(	821	18.59061	chr4:253(	ENSG00000250791		lncRNA	chr4:116489364-116
ENSG0000(	821	18.59061	chr1:1021	LRRC71		protein_c	chr1:156920632-156
ENSG0000(	821	18.59061	chr1:1021	INSRR		protein_c	chr1:156840063-156
ENSG0000(	821	18.59061	chr4:253(	ENSG00000291201		lncRNA	chr4:118591719-118
ENSG0000(	821	18.59061	chr1:1021	ENSG00000246203		Pseudoger	chr1:155614726-155
ENSG0000(	821	18.59061	chr1:1021	MIR765		smallRNA	chr1:156936131-156
ENSG0000(	821	18.59061	chr4:253(	ENSG00000244083		Pseudoger	chr4:122815771-122
ENSG0000(	821	18.59061	chr1:373:	ENSG00000225011		Pseudoger	chr1:29488193-2949
ENSG0000(	821	18.59061	chr4:253(	LINC02466		lncRNA	chr4:129612330-129
ENSG0000(	821	18.59061	chr4:253(	EEF1GP8		Pseudoger	chr4:129903010-129
ENSG0000(	821	18.59061	chr1:1021	RUSC1-AS1		lncRNA	chr1:155316863-155
ENSG0000(	821	18.59061	chr1:373:	XKR8		protein_c	chr1:27959588-2796
ENSG0000(	821	18.59061	chr1:1021	SCARNA4		smallRNA	chr1:155925958-155
ENSG0000(	821	18.59061	chr1:373:	ENSG00000270605		lncRNA	chr1:28239509-2824
ENSG0000(	821	18.59061	chr4:253(	ENSG00000291202		lncRNA	chr4:119405523-119
ENSG0000(	821	18.59061	chr1:373:	EYA3		protein_c	chr1:27970344-2808
ENSG0000(	821	18.59061	chr4:253(	ENSG00000244021		Pseudoger	chr4:127812720-127
ENSG0000(	821	18.59061	chr1:1021	RXFP4		protein_c	chr1:155941638-155
ENSG0000(	821	18.59061	chr1:1021	snoU13		smallRNA	chr1:155415558-155
ENSG0000(	821	18.59061	chr4:253(	ENSG00000291203		lncRNA	chr4:119454762-119
ENSG0000(	821	18.59061	chr1:373:	ENSG00000289291		lncRNA	chr1:28736044-2873
ENSG0000(	821	18.59061	chr4:253(	ENSG00000250458		Pseudoger	chr4:123861860-123
ENSG0000(	821	18.59061	chr1:1021	GPATCH4	NCGv7	protein_c	chr1:156594301-156
ENSG0000(	821	18.59061	chr1:373:	EPB41	NCGv7	protein_c	chr1:28887091-2912
ENSG0000(	821	18.59061	chr1:1021	UBQLN4		protein_c	chr1:156035299-156
ENSG0000(	821	18.59061	chr1:1021	TTC24		protein_c	chr1:156579723-156
ENSG0000(	821	18.59061	chr1:1021	NTRK1	NCGv7;AC	protein_c	chr1:156815640-156
ENSG0000(	821	18.59061	chr4:253(	CUL4AP1		Pseudoger	chr4:116425983-116
ENSG0000(	821	18.59061	chr1:1021	RNU6-1297P		smallRNA	chr1:155419397-155
ENSG0000(	821	18.59061	chr4:253(	ENSG00000286700		lncRNA	chr4:124025295-124
ENSG0000(	821	18.59061	chr1:1021	GBAP1		lncRNA	chr1:155213821-155
ENSG0000(	821	18.59061	chr4:253(	NDNF		protein_c	chr4:121035613-121
ENSG0000(	821	18.59061	chr1:1021	ENSG00000272971		lncRNA	chr1:156614742-156
ENSG0000(	821	18.59061	chr4:253(	ENSG00000248187		lncRNA	chr4:128567972-128
ENSG0000(	821	18.59061	chr4:253(	LINC01365		lncRNA	chr4:119799089-119

ENSG0000(	821	18.59061	chr1:1021	PEAR1	NCGv7	protein_c	chr1:156893698-156
ENSG0000(	821	18.59061	chr1:1021	MIR9-1		smallRNA	chr1:156420331-156
ENSG0000(	821	18.59061	chr4:253(	CICP16		Pseudoger	chr4:118635970-118
ENSG0000(	821	18.59061	chr4:253(	Y_RNA		smallRNA	chr4:121329069-121
ENSG0000(	821	18.59061	chr1:1021	ARHGEF2-AS2		lncRNA	chr1:155978799-155
ENSG0000(	821	18.59061	chr1:373:	SNHG12		lncRNA	chr1:28578538-2858
ENSG0000(	821	18.59061	chr4:253(	ENSG00000248209		Pseudoger	chr4:127493505-127
ENSG0000(	821	18.59061	chr4:253(	ENSG00000286734		lncRNA	chr4:124324973-124
ENSG0000(	821	18.59061	chr4:253(	ENSG00000250503		lncRNA	chr4:130881027-130
ENSG0000(	821	18.59061	chr1:1021	MIR555		smallRNA	chr1:155346350-155
ENSG0000(	821	18.59061	chr1:373:	YTHDF2	NCGv7	protein_c	chr1:28736621-2876
ENSG0000(	821	18.59061	chr1:1021	LMNA		protein_c	chr1:156082573-156
ENSG0000(	821	18.59061	chr1:1021	SLC25A44		protein_c	chr1:156193932-156
ENSG0000(	821	18.59061	chr1:1021	PMF1		protein_c	chr1:156212993-156
ENSG0000(	821	18.59061	chr1:1021	PAQR6		protein_c	chr1:156243320-156
ENSG0000(	821	18.59061	chr1:1021	FAM189B	AC	protein_c	chr1:155247205-155
ENSG0000(	821	18.59061	chr1:1021	GBAP1		Pseudoger	chr1:155214368-155
ENSG0000(	821	18.59061	chr1:1021	RUSC1		protein_c	chr1:155320894-155
ENSG0000(	821	18.59061	chr4:253(	RPF2P2		Pseudoger	chr4:115628812-115
ENSG0000(	821	18.59061	chr1:1021	FDPS	AC	protein_c	chr1:155308748-155
ENSG0000(	821	18.59061	chr1:373:	RAB42	NCGv7	protein_c	chr1:28592200-2859
ENSG0000(	821	18.59061	chr1:373:	TAF12		protein_c	chr1:28587829-2864
ENSG0000(	821	18.59061	chr1:1021	FLAD1		protein_c	chr1:154983338-154
ENSG0000(	821	18.59061	chr1:1021	ZBTB7B	NCGv7	protein_c	chr1:155002630-155
ENSG0000(	821	18.59061	chr1:1021	RNU6-106P		smallRNA	chr1:155358712-155
ENSG0000(	821	18.59061	chr4:253(	ENSG00000289243		lncRNA	chr4:128653873-128
ENSG0000(	821	18.59061	chr1:373:	snoU13		smallRNA	chr1:28517476-2851
ENSG0000(	821	18.59061	chr1:1021	SH2D2A		protein_c	chr1:156806243-156
ENSG0000(	821	18.59061	chr1:1021	SEMA4A		protein_c	chr1:156147366-156
ENSG0000(	821	18.59061	chr4:253(	PGAM4P2		Pseudoger	chr4:115764099-115
ENSG0000(	821	18.59061	chr1:1021	ENSG00000234937		Pseudoger	chr1:155845367-155
ENSG0000(	821	18.59061	chr4:253(	EXOSC9		protein_c	chr4:121801318-121
ENSG0000(	821	18.59061	chr4:253(	ENSG00000288921		lncRNA	chr4:117783097-117
ENSG0000(	821	18.59061	chr4:253(	TTC39CP1		Pseudoger	chr4:116193997-116
ENSG0000(	821	18.59061	chr4:253(	ENSG00000250950		lncRNA	chr4:119456350-119
ENSG0000(	821	18.59061	chr1:373:	AL353354.2		protein_c	chr1:28200559-2820
ENSG0000(	821	18.59061	chr4:253(	MAD2L1-DT		lncRNA	chr4:120066904-120
ENSG0000(	821	18.59061	chr1:1021	snoU13		smallRNA	chr1:156529330-156
ENSG0000(	821	18.59061	chr1:373:	GMEB1		protein_c	chr1:28668778-2871
ENSG0000(	821	18.59061	chr4:253(	ENSG00000248491		lncRNA	chr4:127094410-127
ENSG0000(	821	18.59061	chr1:1021	SNORA42		smallRNA	chr1:155919909-155
ENSG0000(	821	18.59061	chr1:1021	ENSG00000289103		lncRNA	chr1:155000799-155
ENSG0000(	821	18.59061	chr4:253(	EIF3KP3		Pseudoger	chr4:116043407-116
ENSG0000(	821	18.59061	chr4:253(	LINC02262		lncRNA	chr4:117314597-117
ENSG0000(	821	18.59061	chr1:373:	ENSG00000270103		lncRNA	chr1:28648600-2864
ENSG0000(	821	18.59061	chr4:253(	SNHG8		lncRNA	chr4:118278703-118
ENSG0000(	821	18.59061	chr1:1021	ENSG00000273088		protein_c	chr1:155169409-155
ENSG0000(	821	18.59061	chr4:253(	TECRP2		Pseudoger	chr4:124462863-124
ENSG0000(	821	18.59061	chr4:253(	ENSG00000273077		lncRNA	chr4:128552590-128
ENSG0000(	821	18.59061	chr1:1021	VHLL		protein_c	chr1:156298624-156
ENSG0000(	821	18.59061	chr4:253(	CETN4P		Pseudoger	chr4:122728532-122

ENSG0000(	821	18.59061	chr4:253(	TRAM1L1	DriverDB\	protein_c	chr4:117083554-117
ENSG0000(	821	18.59061	chr4:253(	ENSG00000250945		lncRNA	chr4:127043430-127
ENSG0000(	821	18.59061	chr4:253(	SEPTIN7P14		Pseudoger	chr4:119457968-119
ENSG0000(	821	18.59061	chr4:253(	TUBAP10		Pseudoger	chr4:124435750-124
ENSG0000(	821	18.59061	chr4:253(	FAT4	NCGv7	protein_c	chr4:125314918-125
ENSG0000(	818	18.52268	chr12:62	MIR4698		smallRNA	chr12:47187812-471
ENSG0000(	817	18.50004	chr1:1021	BRDT	NCGv7	protein_c	chr1:91949343-9201
ENSG0000(	817	18.50004	chr1:1021	ENSG00000237568		lncRNA	chr1:89260582-8926
ENSG0000(	817	18.50004	chr1:1021	KYAT3		protein_c	chr1:88935773-8899
ENSG0000(	817	18.50004	chr2:8187	PPP4R3B		protein_c	chr2:55547292-5561
ENSG0000(	817	18.50004	chr1:1021	GTF2B	NCGv7	protein_c	chr1:88852633-8889
ENSG0000(	817	18.50004	chr2:8187	EML6-AS1		lncRNA	chr2:54747103-5475
ENSG0000(	817	18.50004	chr1:1021	RNA5SP51		Pseudoger	chr1:85883680-8588
ENSG0000(	817	18.50004	chr2:8187	ENSG00000289606		lncRNA	chr2:55235605-5523
ENSG0000(	817	18.50004	chr2:2577	OR7E89P		Pseudoger	chr2:158853755-158
ENSG0000(	817	18.50004	chr1:1021	ENSG00000229505		Pseudoger	chr1:86029854-8603
ENSG0000(	817	18.50004	chr1:1021	TTLL7		protein_c	chr1:83865024-8399
ENSG0000(	817	18.50004	chr1:1021	RPL5P6		Pseudoger	chr1:91023919-9102
ENSG0000(	817	18.50004	chr1:1021	CLCA4-AS1		lncRNA	chr1:86569024-8670
ENSG0000(	817	18.50004	chr1:1021	PKN2-AS1		lncRNA	chr1:87620803-8868
ENSG0000(	817	18.50004	chr2:8187	MTIF2		protein_c	chr2:55236595-5526
ENSG0000(	817	18.50004	chr1:1021	ENSG00000229486		Pseudoger	chr1:84015865-8401
ENSG0000(	817	18.50004	chr1:1021	ENSG00000225568		Pseudoger	chr1:87045875-8704
ENSG0000(	817	18.50004	chr2:8187	RPS27A	NCGv7	protein_c	chr2:55231903-5523
ENSG0000(	817	18.50004	chr1:1021	ENSG00000271949		protein_c	chr1:89633140-8993
ENSG0000(	817	18.50004	chr1:1021	ENSG00000267734		lncRNA	chr1:86932199-8693
ENSG0000(	817	18.50004	chr1:1021	ENSG00000237076		lncRNA	chr1:83766417-8380
ENSG0000(	817	18.50004	chr1:1021	AL035706.1		smallRNA	chr1:83793877-8379
ENSG0000(	817	18.50004	chr1:1021	ENSG00000285325		lncRNA	chr1:84785427-8478
ENSG0000(	817	18.50004	chr1:1021	AL606519.1		smallRNA	chr1:80329379-8032
ENSG0000(	817	18.50004	chr1:1021	LINC02609		lncRNA	chr1:90769086-9085
ENSG0000(	817	18.50004	chr1:1021	ENSG00000289712		Pseudoger	chr1:89236843-8923
ENSG0000(	817	18.50004	chr2:8187	BTF3P5		Pseudoger	chr2:55435156-5543
ENSG0000(	817	18.50004	chr1:1021	CLCA2		protein_c	chr1:86424171-8645
ENSG0000(	817	18.50004	chr1:1021	ENSG00000282057		lncRNA	chr1:85482281-8557
ENSG0000(	817	18.50004	chr1:1021	LINC01725		lncRNA	chr1:83575776-8386
ENSG0000(	817	18.50004	chr1:1021	ENSG00000260322		lncRNA	chr1:80114943-8011
ENSG0000(	817	18.50004	chr1:1021	LINC02788		lncRNA	chr1:90835660-9084
ENSG0000(	817	18.50004	chr1:1021	ENSG00000272094		lncRNA	chr1:90860550-9086
ENSG0000(	817	18.50004	chr1:1021	TGFBR3	NCGv7	protein_c	chr1:91680343-9190
ENSG0000(	817	18.50004	chr1:1021	LRRC8C-DT		lncRNA	chr1:89581291-8963
ENSG0000(	817	18.50004	chr1:1021	LINC01763		lncRNA	chr1:90851122-9085
ENSG0000(	817	18.50004	chr1:1021	AL139139.1		smallRNA	chr1:87151298-8715
ENSG0000(	817	18.50004	chr1:1021	GBP1P1		Pseudoger	chr1:89410319-8942
ENSG0000(	817	18.50004	chr1:1021	GBP6		protein_c	chr1:89364059-8938
ENSG0000(	817	18.50004	chr1:1021	ENSG00000249237		Pseudoger	chr1:84344678-8434
ENSG0000(	817	18.50004	chr1:1021	SELENOF		protein_c	chr1:86862445-8691
ENSG0000(	817	18.50004	chr1:1021	ENSG00000226394		Pseudoger	chr1:89661212-8966
ENSG0000(	817	18.50004	chr1:1021	ENSG00000286548		lncRNA	chr1:89427533-8952
ENSG0000(	817	18.50004	chr1:1021	LINC02801		lncRNA	chr1:87212669-8726
ENSG0000(	817	18.50004	chr2:8187	AC019198.1		smallRNA	chr2:55472744-5547

ENSG0000(	817	18.50004	chr1:1021	Y_RNA		smallRNA	chr1:85264296-8526
ENSG0000(	817	18.50004	chr2:2577	OR7E28P		Pseudoger	chr2:158862311-158
ENSG0000(	817	18.50004	chr1:1021	ENSG00000225598		lncRNA	chr1:80373364-8037
ENSG0000(	817	18.50004	chr1:1021	LINC02787		lncRNA	chr1:90510910-9053
ENSG0000(	817	18.50004	chr1:1021	LINC02795		lncRNA	chr1:86288704-8632
ENSG0000(	817	18.50004	chr2:8187	RN7SKP208		smallRNA	chr2:55951654-5595
ENSG0000(	817	18.50004	chr1:1021	BARHL2		protein_c	chr1:90711539-9071
ENSG0000(	817	18.50004	chr1:1021	SAMD13		protein_c	chr1:84298366-8438
ENSG0000(	817	18.50004	chr1:1021	LRRC8B		protein_c	chr1:89524829-8959
ENSG0000(	817	18.50004	chr1:1021	LMO4	AC	protein_c	chr1:87328880-8734
ENSG0000(	817	18.50004	chr2:8187	ENSG00000272180		lncRNA	chr2:55952158-5618
ENSG0000(	817	18.50004	chr1:1021	ENSG00000287406		lncRNA	chr1:90242088-9028
ENSG0000(	817	18.50004	chr1:1021	ENSG00000267561		protein_c	chr1:86993009-8716
ENSG0000(	817	18.50004	chr1:1021	Y_RNA		smallRNA	chr1:89020246-8902
ENSG0000(	817	18.50004	chr1:1021	LINC01781		lncRNA	chr1:80535755-8064
ENSG0000(	817	18.50004	chr1:1021	PRKACB		protein_c	chr1:84078062-8423
ENSG0000(	817	18.50004	chr1:1021	CCN1		protein_c	chr1:85580761-8558
ENSG0000(	817	18.50004	chr1:1021	HMGB1P18		Pseudoger	chr1:80283352-8028
ENSG0000(	817	18.50004	chr1:1021	BCL10	NCGv7;AC	protein_c	chr1:85265776-8527
ENSG0000(	817	18.50004	chr1:1021	AL356270.1		smallRNA	chr1:86606270-8660
ENSG0000(	817	18.50004	chr1:1021	HSP90B3P		Pseudoger	chr1:91642516-9164
ENSG0000(	817	18.50004	chr1:1021	ENSG00000289881		lncRNA	chr1:84614068-8462
ENSG0000(	817	18.50004	chr1:1021	UOX		Pseudoger	chr1:84363706-8439
ENSG0000(	817	18.50004	chr1:1021	PHKA1P1		Pseudoger	chr1:90892992-9089
ENSG0000(	817	18.50004	chr1:1021	GNG5		protein_c	chr1:84498323-8450
ENSG0000(	817	18.50004	chr1:1021	ENSG00000285782		lncRNA	chr1:83397555-8342
ENSG0000(	817	18.50004	chr2:8187	ENSG00000203327		lncRNA	chr2:55214387-5521
ENSG0000(	817	18.50004	chr1:1021	ENSG00000285361		lncRNA	chr1:84477039-8447
ENSG0000(	817	18.50004	chr1:1021	ENSG00000236676		lncRNA	chr1:81585941-8162
ENSG0000(	817	18.50004	chr1:1021	LINC01555		lncRNA	chr1:84628230-8463
ENSG0000(	817	18.50004	chr1:1021	ENSG00000231349		Pseudoger	chr1:86404176-8640
ENSG0000(	817	18.50004	chr1:1021	ENSG00000230285		lncRNA	chr1:85599131-8560
ENSG0000(	817	18.50004	chr1:1021	ENSG00000272691		lncRNA	chr1:85578500-8557
ENSG0000(	817	18.50004	chr1:1021	ENSG00000287372		lncRNA	chr1:90388193-9042
ENSG0000(	817	18.50004	chr1:1021	PRKACB-DT		lncRNA	chr1:84076331-8407
ENSG0000(	817	18.50004	chr2:8187	ENSG00000289627		lncRNA	chr2:54581577-5458
ENSG0000(	817	18.50004	chr1:1021	ENSG00000285201		lncRNA	chr1:84038529-8406
ENSG0000(	817	18.50004	chr1:1021	ENSG00000285179		lncRNA	chr1:81209834-8122
ENSG0000(	817	18.50004	chr1:1021	RN7SKP247		smallRNA	chr1:81251789-8125
ENSG0000(	817	18.50004	chr2:8187	MIR217		smallRNA	chr2:55982967-5598
ENSG0000(	817	18.50004	chr1:1021	ENSG00000234108		Pseudoger	chr1:80495903-8049
ENSG0000(	817	18.50004	chr1:1021	ENSG00000229067		Pseudoger	chr1:91600171-9160
ENSG0000(	817	18.50004	chr2:8187	ENSG00000271894		lncRNA	chr2:56147630-5638
ENSG0000(	817	18.50004	chr1:1021	ODF2L		protein_c	chr1:86346824-8639
ENSG0000(	817	18.50004	chr2:8187	ENSG00000240401		lncRNA	chr2:55282350-5534
ENSG0000(	817	18.50004	chr1:1021	ENSG00000272672		lncRNA	chr1:89939601-8994
ENSG0000(	817	18.50004	chr1:1021	ENSG00000285851		lncRNA	chr1:84498350-8455
ENSG0000(	817	18.50004	chr1:1021	WDR82P2		Pseudoger	chr1:91534666-9153
ENSG0000(	817	18.50004	chr1:1021	FEN1P1		Pseudoger	chr1:91328369-9132
ENSG0000(	817	18.50004	chr1:1021	ENSG00000284637		Pseudoger	chr1:89203280-8920
ENSG0000(	817	18.50004	chr1:1021	CAPNS1P1		Pseudoger	chr1:89394033-8939

ENSG0000(	817	18.50004	chr2:8187	MIR217HG		lncRNA	chr2:55963191-5604
ENSG0000(	817	18.50004	chr1:1021	SPATA1		protein_c	chr1:84506300-8456
ENSG0000(	817	18.50004	chr2:8187	ENSG00000290071		lncRNA	chr2:55137264-5513
ENSG0000(	817	18.50004	chr1:1021	ZNF644		protein_c	chr1:90915298-9102
ENSG0000(	817	18.50004	chr1:1021	ST13P20		Pseudoger	chr1:81721693-8172
ENSG0000(	817	18.50004	chr1:1021	ENSG00000280099		TEC	chr1:85152487-8515
ENSG0000(	817	18.50004	chr1:1021	RNU6-125P		smallRNA	chr1:88816779-8881
ENSG0000(	817	18.50004	chr1:1021	LINC01712		lncRNA	chr1:83445967-8348
ENSG0000(	817	18.50004	chr1:1021	LRRC8D-DT		lncRNA	chr1:89820174-8982
ENSG0000(	817	18.50004	chr1:1021	LINC01361		lncRNA	chr1:82970820-8298
ENSG0000(	817	18.50004	chr1:1021	AC104169.1		smallRNA	chr1:84793068-8479
ENSG0000(	817	18.50004	chr1:1021	DNASE2B		protein_c	chr1:84398484-8441
ENSG0000(	817	18.50004	chr1:1021	C1orf52		protein_c	chr1:85249953-8525
ENSG0000(	817	18.50004	chr1:1021	ELOCP19		Pseudoger	chr1:88829102-8882
ENSG0000(	817	18.50004	chr1:1021	CDCA4P2		Pseudoger	chr1:86552625-8655
ENSG0000(	817	18.50004	chr1:1021	TXN2P1		Pseudoger	chr1:84085741-8408
ENSG0000(	817	18.50004	chr1:1021	HNRNPA3P14		Pseudoger	chr1:81426456-8142
ENSG0000(	817	18.50004	chr1:1021	RBMXL1		protein_c	chr1:88979456-8899
ENSG0000(	817	18.50004	chr1:1021	ENSG00000227062		Pseudoger	chr1:80464301-8046
ENSG0000(	817	18.50004	chr2:8187	RNU6-433P		smallRNA	chr2:55014418-5501
ENSG0000(	817	18.50004	chr1:1021	GBP7		protein_c	chr1:89131742-8917
ENSG0000(	817	18.50004	chr1:1021	snoU13		smallRNA	chr1:89768212-8976
ENSG0000(	817	18.50004	chr1:1021	ENSG00000235308		Pseudoger	chr1:88923370-8892
ENSG0000(	817	18.50004	chr1:1021	ENSG00000284734		lncRNA	chr1:89198714-8920
ENSG0000(	817	18.50004	chr1:1021	ENSG00000233290		lncRNA	chr1:82212413-8284
ENSG0000(	817	18.50004	chr2:8187	RNA5SP93		Pseudoger	chr2:56235217-5623
ENSG0000(	817	18.50004	chr2:8187	RNU7-81P		smallRNA	chr2:54850289-5485
ENSG0000(	817	18.50004	chr2:8187	LINC01813		lncRNA	chr2:56077417-5609
ENSG0000(	817	18.50004	chr1:1021	Y_RNA		smallRNA	chr1:85435175-8543
ENSG0000(	817	18.50004	chr1:1021	SH3GLB1	NCGv7	protein_c	chr1:86704570-8674
ENSG0000(	817	18.50004	chr2:8187	ENSG00000233251		lncRNA	chr2:56173534-5618
ENSG0000(	817	18.50004	chr1:1021	HNRNPA1P64		Pseudoger	chr1:80451083-8045
ENSG0000(	817	18.50004	chr1:1021	MED28P8		Pseudoger	chr1:81557121-8155
ENSG0000(	817	18.50004	chr1:1021	MCOLN2		protein_c	chr1:84925583-8499
ENSG0000(	817	18.50004	chr2:8187	RNU6-775P		smallRNA	chr2:55451004-5545
ENSG0000(	817	18.50004	chr1:1021	ENSG00000224326		lncRNA	chr1:80534978-8058
ENSG0000(	817	18.50004	chr1:1021	RN7SL583P		smallRNA	chr1:88477831-8847
ENSG0000(	817	18.50004	chr1:1021	RN7SL235P		smallRNA	chr1:91939269-9193
ENSG0000(	817	18.50004	chr2:8187	SPTBN1-AS2		lncRNA	chr2:54661011-5468
ENSG0000(	817	18.50004	chr1:1021	RNA5SP52		Pseudoger	chr1:87453240-8745
ENSG0000(	817	18.50004	chr1:1021	HS2ST1	NCGv7	protein_c	chr1:86914635-8710
ENSG0000(	817	18.50004	chr1:1021	ENSG00000284882		lncRNA	chr1:84574114-8458
ENSG0000(	817	18.50004	chr1:1021	ENSG00000230721		Pseudoger	chr1:86784913-8678
ENSG0000(	817	18.50004	chr1:1021	ENSG00000288629		protein_c	chr1:89579592-8957
ENSG0000(	817	18.50004	chr1:1021	ENSG00000233235		Pseudoger	chr1:89324522-8933
ENSG0000(	817	18.50004	chr1:1021	ENSG00000238081		Pseudoger	chr1:89289676-8929
ENSG0000(	817	18.50004	chr1:1021	RPL7P10		Pseudoger	chr1:81098267-8109
ENSG0000(	817	18.50004	chr1:1021	DDAH1		protein_c	chr1:85318481-8557
ENSG0000(	817	18.50004	chr1:1021	Y_RNA		smallRNA	chr1:91261625-9126
ENSG0000(	817	18.50004	chr2:8187	RNU6-221P		smallRNA	chr2:55456106-5545
ENSG0000(	817	18.50004	chr1:1021	ADGRL2	NCGv7	protein_c	chr1:81306147-8199

ENSG0000(	817	18.50004	chr1:1021	LRRC8C		protein_c	chr1:89633072-8976
ENSG0000(	817	18.50004	chr1:1021	RPF1		protein_c	chr1:84479259-8449
ENSG0000(	817	18.50004	chr1:1021	ENSG00000287015		lncRNA	chr1:90045998-9004
ENSG0000(	817	18.50004	chr1:1021	ENSG00000273264		lncRNA	chr1:85467295-8546
ENSG0000(	817	18.50004	chr1:1021	LINC01364		lncRNA	chr1:87353521-8737
ENSG0000(	817	18.50004	chr2:8187	RNU6-634P		smallRNA	chr2:55499950-5550
ENSG0000(	817	18.50004	chr1:1021	ARID3BP1		Pseudoger	chr1:81501794-8150
ENSG0000(	817	18.50004	chr2:8187	SNORA12		smallRNA	chr2:55565703-5556
ENSG0000(	817	18.50004	chr1:1021	RNU6-695P		smallRNA	chr1:90253456-9025
ENSG0000(	817	18.50004	chr2:8187	PPP4R3B-DT		lncRNA	chr2:55617869-5561
ENSG0000(	817	18.50004	chr1:1021	ENSG00000284846		lncRNA	chr1:86821558-8683
ENSG0000(	817	18.50004	chr1:1021	U3		smallRNA	chr1:90657750-9065
ENSG0000(	817	18.50004	chr1:1021	ENSG00000235089		Pseudoger	chr1:81208568-8120
ENSG0000(	817	18.50004	chr1:1021	HFM1		protein_c	chr1:91260766-9140
ENSG0000(	817	18.50004	chr1:1021	ZNF326		protein_c	chr1:89995110-9003
ENSG0000(	817	18.50004	chr1:1021	GBP4		protein_c	chr1:89181144-8919
ENSG0000(	817	18.50004	chr1:1021	GBP2		protein_c	chr1:89106132-8915
ENSG0000(	817	18.50004	chr1:1021	ENSG00000234953		lncRNA	chr1:81513880-8155
ENSG0000(	817	18.50004	chr2:8187	ENSG00000234943		lncRNA	chr2:54545368-5454
ENSG0000(	817	18.50004	chr1:1021	SYDE2		protein_c	chr1:85156889-8520
ENSG0000(	817	18.50004	chr1:1021	GBP1		protein_c	chr1:89051882-8906
ENSG0000(	817	18.50004	chr1:1021	CTBS		protein_c	chr1:84549611-8457
ENSG0000(	817	18.50004	chr1:1021	SSX2IP		protein_c	chr1:84643706-8469
ENSG0000(	817	18.50004	chr1:1021	ZNHIT6	AC	protein_c	chr1:85649417-8570
ENSG0000(	817	18.50004	chr1:1021	LRRC8D		protein_c	chr1:89821014-8993
ENSG0000(	817	18.50004	chr1:1021	SNORA2		smallRNA	chr1:84277321-8427
ENSG0000(	817	18.50004	chr1:1021	GBP3		protein_c	chr1:89006679-8902
ENSG0000(	817	18.50004	chr1:1021	SNORD81		smallRNA	chr1:85592280-8559
ENSG0000(	817	18.50004	chr1:1021	CDC7		protein_c	chr1:91500851-9152
ENSG0000(	817	18.50004	chr1:1021	COL24A1		protein_c	chr1:85729233-8615
ENSG0000(	817	18.50004	chr1:1021	LPAR3	NCGv7	protein_c	chr1:84811602-8489
ENSG0000(	817	18.50004	chr1:1021	ENSG00000235251		Pseudoger	chr1:87044935-8704
ENSG0000(	817	18.50004	chr2:8187	CFAP36		protein_c	chr2:55519604-5554
ENSG0000(	817	18.50004	chr2:8187	PRORSD1P		Pseudoger	chr2:55282319-5528
ENSG0000(	817	18.50004	chr2:8187	CLHC1		protein_c	chr2:55172547-5523
ENSG0000(	817	18.50004	chr2:8187	MIR216B		smallRNA	chr2:56000714-5600
ENSG0000(	817	18.50004	chr1:1021	CLCA3P		Pseudoger	chr1:86634276-8665
ENSG0000(	817	18.50004	chr2:8187	CCDC88A	NCGv7	protein_c	chr2:55287842-5541
ENSG0000(	817	18.50004	chr1:1021	GEMIN8P4		Pseudoger	chr1:89993593-8999
ENSG0000(	817	18.50004	chr1:1021	DNAI3		protein_c	chr1:84999147-8513
ENSG0000(	817	18.50004	chr1:1021	MTND2P30		Pseudoger	chr1:81080790-8108
ENSG0000(	817	18.50004	chr1:1021	GBP1P1		lncRNA	chr1:89407679-8942
ENSG0000(	817	18.50004	chr1:1021	PKN2		protein_c	chr1:88684222-8883
ENSG0000(	817	18.50004	chr1:1021	ENSG00000279778		TEC	chr1:87805286-8780
ENSG0000(	817	18.50004	chr1:1021	ENSG00000227960		lncRNA	chr1:81505099-8150
ENSG0000(	817	18.50004	chr1:1021	ENSG00000234683		Pseudoger	chr1:81596157-8159
ENSG0000(	817	18.50004	chr2:8187	ENSG00000285519		lncRNA	chr2:54768492-5480
ENSG0000(	817	18.50004	chr1:1021	LINC01362		lncRNA	chr1:82903183-8316
ENSG0000(	817	18.50004	chr1:1021	ENSG00000235756		Pseudoger	chr1:80092103-8009
ENSG0000(	817	18.50004	chr2:8187	EFEMP1		protein_c	chr2:55865967-5592
ENSG0000(	817	18.50004	chr1:1021	ENSG00000232622		Pseudoger	chr1:84636158-8463

ENSG0000(	817	18.50004	chr1:1021	TTLL7-IT1		lncRNA	chr1:83979118-8398
ENSG0000(	817	18.50004	chr2:8187	RTN4	NCGv7	protein_c	chr2:54972187-5511
ENSG0000(	817	18.50004	chr1:1021	ENSG00000287076		lncRNA	chr1:90719576-9072
ENSG0000(	817	18.50004	chr1:1021	RN7SL653P		smallRNA	chr1:91829776-9183
ENSG0000(	817	18.50004	chr1:1021	NEDD8P1		Pseudoger	chr1:84244334-8424
ENSG0000(	817	18.50004	chr2:8187	RPL23AP32		Pseudoger	chr2:54529343-5452
ENSG0000(	817	18.50004	chr1:1021	ENSG00000286758		lncRNA	chr1:88462936-8846
ENSG0000(	817	18.50004	chr1:1021	ENSG00000289582		lncRNA	chr1:89127160-8912
ENSG0000(	817	18.50004	chr1:1021	ENSG00000231613		lncRNA	chr1:89788914-8979
ENSG0000(	817	18.50004	chr2:8187	SPTBN1-AS1		lncRNA	chr2:54516048-5454
ENSG0000(	817	18.50004	chr1:1021	CLCA4		protein_c	chr1:86547078-8658
ENSG0000(	817	18.50004	chr1:1021	CLCA1		protein_c	chr1:86468368-8650
ENSG0000(	817	18.50004	chr1:1021	ENSG00000230053		Pseudoger	chr1:88498309-8849
ENSG0000(	817	18.50004	chr1:1021	LINC01140		lncRNA	chr1:87129765-8716
ENSG0000(	817	18.50004	chr2:8187	AC093165.1		smallRNA	chr2:55108710-5510
ENSG0000(	817	18.50004	chr1:1021	CLCA3P		lncRNA	chr1:86634273-8665
ENSG0000(	817	18.50004	chr2:8187	PNPT1		protein_c	chr2:55634061-5569
ENSG0000(	817	18.50004	chr1:1021	AL590113.1		smallRNA	chr1:85284610-8528
ENSG0000(	817	18.50004	chr1:1021	ENSG00000285409		lncRNA	chr1:79967733-8005
ENSG0000(	817	18.50004	chr1:1021	ENSG00000230735		lncRNA	chr1:89629725-8967
ENSG0000(	817	18.50004	chr1:1021	RP4-604K5.2		lncRNA	chr1:86943685-8694
ENSG0000(	817	18.50004	chr1:1021	RPL36AP10		Pseudoger	chr1:88577880-8857
ENSG0000(	817	18.50004	chr1:1021	PTGES3P1		Pseudoger	chr1:89104285-8910
ENSG0000(	817	18.50004	chr2:8187	CDPF1P1		Pseudoger	chr2:55224280-5522
ENSG0000(	817	18.50004	chr2:8187	EML6		protein_c	chr2:54723499-5497
ENSG0000(	817	18.50004	chr1:1021	MCOLN3		protein_c	chr1:85018082-8504
ENSG0000(	817	18.50004	chr2:8187	CCDC85A		protein_c	chr2:56183990-5638
ENSG0000(	817	18.50004	chr1:1021	MIR4423		smallRNA	chr1:85133794-8513
ENSG0000(	817	18.50004	chr1:1021	ENSG00000277670		Pseudoger	chr1:80124004-8012
ENSG0000(	817	18.50004	chr1:1021	GBP5		protein_c	chr1:89256189-8927
ENSG0000(	817	18.50004	chr1:1021	ENSG00000286802		lncRNA	chr1:89128432-8914
ENSG0000(	817	18.50004	chr1:1021	ENSG00000270507		Pseudoger	chr1:88313153-8831
ENSG0000(	817	18.50004	chr1:1021	BCL10-AS1		lncRNA	chr1:85276388-8544
ENSG0000(	817	18.50004	chr1:1021	ENSG00000285374		lncRNA	chr1:84607099-8461
ENSG0000(	814	18.43211	chrX:1578	MIR362		smallRNA	chrX:50008964-5000
ENSG0000(	813	18.40946	chr5:4231	HSPD1P18		Pseudoger	chr5:136381268-136
ENSG0000(	812	18.38682	chr12:624	RN7SL744P		smallRNA	chr12:54344610-543
ENSG0000(	812	18.38682	chr12:624	ENSG00000289310		lncRNA	chr12:40229956-402
ENSG0000(	812	18.38682	chr12:624	DNAJC22		protein_c	chr12:49346888-493
ENSG0000(	812	18.38682	chr12:624	RESF1	NCGv7	protein_c	chr12:31959370-319
ENSG0000(	812	18.38682	chr12:624	RNU6-574P		smallRNA	chr12:51987657-519
ENSG0000(	812	18.38682	chr12:624	ENSG00000285517		lncRNA	chr12:30795458-308
ENSG0000(	812	18.38682	chr12:624	Y_RNA		smallRNA	chr12:42454720-424
ENSG0000(	812	18.38682	chr12:624	FAM242C		lncRNA	chr12:54085132-541
ENSG0000(	812	18.38682	chr1:1021	ENSG00000289212		lncRNA	chr1:77219520-7722
ENSG0000(	812	18.38682	chr12:624	MIR4701		smallRNA	chr12:48771975-487
ENSG0000(	812	18.38682	chr12:624	ENSG00000289229		lncRNA	chr12:45728313-457
ENSG0000(	812	18.38682	chr12:624	SNORA2B		smallRNA	chr12:48667457-486
ENSG0000(	812	18.38682	chr12:624	COX14		protein_c	chr12:50112082-501
ENSG0000(	812	18.38682	chr12:624	ENSG00000205537		lncRNA	chr12:47882649-479
ENSG0000(	812	18.38682	chr12:624	MUC19		protein_c	chr12:40393395-405

ENSG0000(	812	18.38682	chr12:624	ENSG00000289046		lncRNA	chr12:45990924-459
ENSG0000(	812	18.38682	chr12:624	ENSG00000289695		protein_c	chr12:50924969-509
ENSG0000(	812	18.38682	chr1:1021	AC104458.1		smallRNA	chr1:76718140-7671
ENSG0000(	812	18.38682	chr12:624	CBX5		protein_c	chr12:54230942-542
ENSG0000(	812	18.38682	chr12:624	RNU6-769P		smallRNA	chr12:50633273-506
ENSG0000(	812	18.38682	chr12:624	C12orf54		protein_c	chr12:48482498-484
ENSG0000(	812	18.38682	chr12:624	snoU13		smallRNA	chr12:31450170-314
ENSG0000(	812	18.38682	chr12:624	SLC38A2		protein_c	chr12:46358188-463
ENSG0000(	812	18.38682	chr12:624	ENSG00000272368		lncRNA	chr12:50112197-501
ENSG0000(	812	18.38682	chr12:624	TMEM106C		protein_c	chr12:47963569-479
ENSG0000(	812	18.38682	chr12:624	ARF3		protein_c	chr12:48935723-489
ENSG0000(	812	18.38682	chr12:624	FKBP11		protein_c	chr12:48921518-489
ENSG0000(	812	18.38682	chr12:624	PPHLN1		protein_c	chr12:42238447-424
ENSG0000(	812	18.38682	chr12:624	ENSG00000272369		lncRNA	chr12:46537502-466
ENSG0000(	812	18.38682	chr1:1021	LINC02792		lncRNA	chr1:79325008-7934
ENSG0000(	812	18.38682	chr12:624	ALG10B		protein_c	chr12:38316762-383
ENSG0000(	812	18.38682	chr12:624	ASB8		protein_c	chr12:48147789-481
ENSG0000(	812	18.38682	chr12:624	DNM1L		protein_c	chr12:32679200-327
ENSG0000(	812	18.38682	chr12:624	HOXC-AS1		lncRNA	chr12:53999022-540
ENSG0000(	812	18.38682	chr12:624	PRR13		protein_c	chr12:53441678-534
ENSG0000(	812	18.38682	chr12:624	AAAS		protein_c	chr12:53307456-533
ENSG0000(	812	18.38682	chr12:624	CCDC184		protein_c	chr12:48183644-481
ENSG0000(	812	18.38682	chr12:624	ANO6		protein_c	chr12:45215987-454
ENSG0000(	812	18.38682	chr12:624	PLEKHA8P1		Pseudoger	chr12:45139782-452
ENSG0000(	812	18.38682	chr12:624	KRT81		protein_c	chr12:52285913-522
ENSG0000(	812	18.38682	chr12:624	HOXC-AS2		lncRNA	chr12:53993810-539
ENSG0000(	812	18.38682	chr12:624	RNU6-472P		smallRNA	chr12:33923374-339
ENSG0000(	812	18.38682	chr12:624	ENSG00000177359		Pseudoger	chr12:31112236-312
ENSG0000(	812	18.38682	chr12:624	ENSG00000289154		lncRNA	chr12:54278612-542
ENSG0000(	812	18.38682	chr12:624	RNA5SP357		Pseudoger	chr12:34205699-342
ENSG0000(	812	18.38682	chr12:624	RNU6-671P		smallRNA	chr12:45554590-455
ENSG0000(	812	18.38682	chr12:624	FLJ13224		lncRNA	chr12:31324316-313
ENSG0000(	812	18.38682	chr12:624	RPL32P27		Pseudoger	chr12:48903418-489
ENSG0000(	812	18.38682	chr12:624	IPO8	DriverDB\	protein_c	chr12:30628988-306
ENSG0000(	812	18.38682	chr12:624	ENSG00000239397		Pseudoger	chr12:46004038-460
ENSG0000(	812	18.38682	chr12:624	AC046130.1		smallRNA	chr12:34027268-340
ENSG0000(	812	18.38682	chr12:624	Y_RNA		smallRNA	chr12:32396050-323
ENSG0000(	812	18.38682	chr12:624	KRT6A		protein_c	chr12:52487176-524
ENSG0000(	812	18.38682	chr12:624	SNORA2A		smallRNA	chr12:48656648-486
ENSG0000(	812	18.38682	chr12:624	SMAGP		protein_c	chr12:51244558-512
ENSG0000(	812	18.38682	chr12:624	ATP5MC2		protein_c	chr12:53632726-536
ENSG0000(	812	18.38682	chr12:624	U6		smallRNA	chr12:51014134-510
ENSG0000(	812	18.38682	chr12:624	YARS2		protein_c	chr12:32727490-327
ENSG0000(	812	18.38682	chr12:624	PRICKLE1		protein_c	chr12:42456757-425
ENSG0000(	812	18.38682	chr12:624	TMEM117		protein_c	chr12:43835967-443
ENSG0000(	812	18.38682	chr12:624	ZCRB1	NCGv7	protein_c	chr12:42312086-423
ENSG0000(	812	18.38682	chr12:624	SNORA22		smallRNA	chr12:39819750-398
ENSG0000(	812	18.38682	chr12:624	ETFBKMT		protein_c	chr12:31647160-316
ENSG0000(	812	18.38682	chr12:624	SNORA64		smallRNA	chr12:47346166-473
ENSG0000(	812	18.38682	chr12:624	SINHCAF		protein_c	chr12:31280584-313
ENSG0000(	812	18.38682	chr12:624	RNA5SP361		Pseudoger	chr12:45117771-451

ENSG0000(	812	18.38682	chr12:62⁄	ALG10	DriverDB\	protein_c	chr12:34022468-340
ENSG0000(	812	18.38682	chr12:62⁄	SMIM41		protein_c	chr12:52079704-521
ENSG0000(	812	18.38682	chr12:62⁄	FGD4		protein_c	chr12:32399558-326
ENSG0000(	812	18.38682	chr12:62⁄	CPNE8		protein_c	chr12:38646822-389
ENSG0000(	812	18.38682	chr12:62⁄	LIMA1		protein_c	chr12:50175788-502
ENSG0000(	812	18.38682	chr12:62⁄	KIF21A	NCGv7	protein_c	chr12:39293228-394
ENSG0000(	812	18.38682	chr12:62⁄	HOXC8		protein_c	chr12:54008985-540
ENSG0000(	812	18.38682	chr1:1021	ZZZ3		protein_c	chr1:77562416-7768
ENSG0000(	812	18.38682	chr12:62⁄	TMDD1		protein_c	chr12:51813921-518
ENSG0000(	812	18.38682	chr12:62⁄	RNU6-600P		smallRNA	chr12:48892130-488
ENSG0000(	812	18.38682	chr12:62⁄	KRT3		protein_c	chr12:52789685-527
ENSG0000(	812	18.38682	chr12:62⁄	OR8S1		protein_c	chr12:48525632-485
ENSG0000(	812	18.38682	chr12:62⁄	RN7SL519P		smallRNA	chr12:50841500-508
ENSG0000(	812	18.38682	chr12:62⁄	TMPRSS12		protein_c	chr12:50842920-508
ENSG0000(	812	18.38682	chr12:62⁄	RNU6-834P		smallRNA	chr12:49593104-495
ENSG0000(	812	18.38682	chr12:62⁄	ENSG00000290959		lncRNA	chr12:43739635-437
ENSG0000(	812	18.38682	chr12:62⁄	KRT5		protein_c	chr12:52514575-525
ENSG0000(	812	18.38682	chr12:62⁄	KRT73		protein_c	chr12:52607570-526
ENSG0000(	812	18.38682	chr12:62⁄	ENSG00000248576		lncRNA	chr12:54163139-541
ENSG0000(	812	18.38682	chr12:62⁄	ENSG00000270718		Pseudoger	chr12:39170646-391
ENSG0000(	812	18.38682	chr12:62⁄	NPFF		protein_c	chr12:53506688-535
ENSG0000(	812	18.38682	chr12:62⁄	KRT6B		protein_c	chr12:52446651-524
ENSG0000(	812	18.38682	chr12:62⁄	GPR84		protein_c	chr12:54362445-543
ENSG0000(	812	18.38682	chr12:62⁄	ACVRL1		protein_c	chr12:51906908-519
ENSG0000(	812	18.38682	chr12:62⁄	DHH		protein_c	chr12:49086656-490
ENSG0000(	812	18.38682	chr12:62⁄	TARBP2		protein_c	chr12:53500921-535
ENSG0000(	812	18.38682	chr12:62⁄	CCDC65		protein_c	chr12:48904110-489
ENSG0000(	812	18.38682	chr12:62⁄	SP1		protein_c	chr12:53380176-534
ENSG0000(	812	18.38682	chr12:62⁄	DBX2		protein_c	chr12:45014672-450
ENSG0000(	812	18.38682	chr12:62⁄	KRT79		protein_c	chr12:52821408-528
ENSG0000(	812	18.38682	chr12:62⁄	ENSG00000270766		Pseudoger	chr12:31244440-312
ENSG0000(	812	18.38682	chr1:1021	RNU6-161P		smallRNA	chr1:76753135-7675
ENSG0000(	812	18.38682	chr12:62⁄	RNU6-1273P		smallRNA	chr12:51037499-510
ENSG0000(	812	18.38682	chr12:62⁄	RNU6-249P		smallRNA	chr12:42398550-423
ENSG0000(	812	18.38682	chr12:62⁄	RNA5SP358		Pseudoger	chr12:38161466-381
ENSG0000(	812	18.38682	chr12:62⁄	ANP32D	AC	protein_c	chr12:48472559-484
ENSG0000(	812	18.38682	chr12:62⁄	RNU6-199P		smallRNA	chr12:51162901-511
ENSG0000(	812	18.38682	chr12:62⁄	COL2A1	NCGv7	protein_c	chr12:47972967-480
ENSG0000(	812	18.38682	chr12:62⁄	SCAF11		protein_c	chr12:45919131-459
ENSG0000(	812	18.38682	chr12:62⁄	AMIGO2		protein_c	chr12:47075707-470
ENSG0000(	812	18.38682	chr12:62⁄	Y_RNA		smallRNA	chr12:50939927-509
ENSG0000(	812	18.38682	chr12:62⁄	SLC38A4		protein_c	chr12:46764761-468
ENSG0000(	812	18.38682	chr12:62⁄	FAM186A	IntOGen-I	protein_c	chr12:50326230-503
ENSG0000(	812	18.38682	chr12:62⁄	OR7E47P		lncRNA	chr12:52084744-521
ENSG0000(	812	18.38682	chr12:62⁄	KRT87P		lncRNA	chr12:52250544-522
ENSG0000(	812	18.38682	chr12:62⁄	MIR148B		smallRNA	chr12:54337216-543
ENSG0000(	812	18.38682	chr12:62⁄	ENSG00000270273		Pseudoger	chr12:39539581-395
ENSG0000(	812	18.38682	chr12:62⁄	PCED1B-AS1		lncRNA	chr12:47205893-472
ENSG0000(	812	18.38682	chr12:62⁄	H3-5	NCGv7	protein_c	chr12:31791185-317
ENSG0000(	812	18.38682	chr12:62⁄	ENSG00000291180		lncRNA	chr12:54076838-540
ENSG0000(	812	18.38682	chr12:62⁄	PCBP2	NCGv7	protein_c	chr12:53452102-534

ENSG0000(	812	18.38682	chr12:62⁴	ENSG00000270175		lncRNA	chr12:53500151-535
ENSG0000(	812	18.38682	chr12:62⁴	SCN8A	DriverDB\	protein_c	chr12:51590266-518
ENSG0000(	812	18.38682	chr12:62⁴	ENSG00000246331		lncRNA	chr12:30755074-307
ENSG0000(	812	18.38682	chr12:62⁴	LRRK2	NCGv7	protein_c	chr12:40196744-403
ENSG0000(	812	18.38682	chr12:62⁴	RPAP3		protein_c	chr12:47661249-477
ENSG0000(	812	18.38682	chr12:62⁴	ENSG00000291250		lncRNA	chr12:31111651-311
ENSG0000(	812	18.38682	chr12:62⁴	ENSG00000291251		lncRNA	chr12:31158897-311
ENSG0000(	812	18.38682	chr12:62⁴	ENSG00000291252		lncRNA	chr12:31172906-312
ENSG0000(	812	18.38682	chr12:62⁴	ENSG00000291253		lncRNA	chr12:43736628-437
ENSG0000(	812	18.38682	chr12:62⁴	ENSG00000244266		Pseudoger	chr12:50365652-503
ENSG0000(	812	18.38682	chr12:62⁴	DDX11-AS1		lncRNA	chr12:31019434-310
ENSG0000(	812	18.38682	chr12:62⁴	ENSG00000245482		lncRNA	chr12:34022281-340
ENSG0000(	812	18.38682	chr12:62⁴	ARID2	NCGv7;AC	protein_c	chr12:45729706-459
ENSG0000(	812	18.38682	chr12:62⁴	ENSG00000269514		lncRNA	chr12:48198357-482
ENSG0000(	812	18.38682	chr12:62⁴	ENSG00000244436		Pseudoger	chr12:31897820-318
ENSG0000(	812	18.38682	chr12:62⁴	KRT77		protein_c	chr12:52689626-527
ENSG0000(	812	18.38682	chr12:62⁴	RPL35AP27		Pseudoger	chr12:32863118-328
ENSG0000(	812	18.38682	chr12:62⁴	RPL35AP29		Pseudoger	chr12:51106817-511
ENSG0000(	812	18.38682	chr12:62⁴	ENSG00000291295		lncRNA	chr12:49293252-492
ENSG0000(	812	18.38682	chr12:62⁴	ENSG00000268069		lncRNA	chr12:47784923-477
ENSG0000(	812	18.38682	chr12:62⁴	ENSG00000283536		protein_c	chr12:53241900-532
ENSG0000(	812	18.38682	chr12:62⁴	BCDIN3D		protein_c	chr12:49836043-498
ENSG0000(	812	18.38682	chr12:62⁴	ENSG00000197376		Pseudoger	chr12:48507354-485
ENSG0000(	812	18.38682	chr12:62⁴	ENSG00000290967		lncRNA	chr12:52344219-523
ENSG0000(	812	18.38682	chr12:62⁴	C1QL4		protein_c	chr12:49332409-493
ENSG0000(	812	18.38682	chr12:62⁴	OR5BS1P		protein_c	chr12:48559882-485
ENSG0000(	812	18.38682	chr12:62⁴	ATF7-NPFF		protein_c	chr12:53506691-536
ENSG0000(	812	18.38682	chr12:62⁴	CNTN1	NCGv7	protein_c	chr12:40692439-410
ENSG0000(	812	18.38682	chr12:62⁴	H1-7		protein_c	chr12:48328980-483
ENSG0000(	812	18.38682	chr1:1021	RN7SL370P		smallRNA	chr1:77645324-7764
ENSG0000(	812	18.38682	chr12:62⁴	YAF2		protein_c	chr12:42157104-422
ENSG0000(	812	18.38682	chr12:62⁴	DDX11		protein_c	chr12:31073860-311
ENSG0000(	812	18.38682	chr12:62⁴	HOXC4	DriverDB\	protein_c	chr12:54016931-540
ENSG0000(	812	18.38682	chr12:62⁴	ENSG00000243517		Pseudoger	chr12:31251992-312
ENSG0000(	812	18.38682	chr12:62⁴	ENSG00000291098		lncRNA	chr12:31131455-311
ENSG0000(	812	18.38682	chr12:62⁴	FLJ12825		lncRNA	chr12:54058254-541
ENSG0000(	812	18.38682	chr12:62⁴	CALCOCO1	NCGv7	protein_c	chr12:53708517-537
ENSG0000(	812	18.38682	chr12:62⁴	KNOP1P2		Pseudoger	chr12:45880950-458
ENSG0000(	812	18.38682	chr12:62⁴	IRAK4	NCGv7	protein_c	chr12:43758944-437
ENSG0000(	812	18.38682	chr12:62⁴	ENSG00000284682		Pseudoger	chr12:30306065-303
ENSG0000(	812	18.38682	chr12:62⁴	MREGP1		Pseudoger	chr12:31254361-312
ENSG0000(	812	18.38682	chr12:62⁴	MCRS1	NCGv7	protein_c	chr12:49556544-495
ENSG0000(	812	18.38682	chr12:62⁴	HOXC6	NCGv7	protein_c	chr12:53990624-540
ENSG0000(	812	18.38682	chr1:1021	IFI44		protein_c	chr1:78649796-7866
ENSG0000(	812	18.38682	chr1:1021	GIPC2		protein_c	chr1:77979542-7813
ENSG0000(	812	18.38682	chr1:1021	IFI44L	NCGv7	protein_c	chr1:78619902-7864
ENSG0000(	812	18.38682	chr12:62⁴	CELA1	NCGv7	protein_c	chr12:51328442-513
ENSG0000(	812	18.38682	chr12:62⁴	KANSL2		protein_c	chr12:48653211-486
ENSG0000(	812	18.38682	chr12:62⁴	MIR4494		smallRNA	chr12:47364186-473
ENSG0000(	812	18.38682	chr12:62⁴	Y_RNA		smallRNA	chr12:48927939-489
ENSG0000(	812	18.38682	chr1:1021	RNA5SP22		Pseudoger	chr1:78094807-7809

ENSG0000(	812	18.38682	chr12:624	Y_RNA		smallRNA	chr12:54099191-540
ENSG0000(	812	18.38682	chr12:624	ENSG00000271547		Pseudoger	chr12:48800576-488
ENSG0000(	812	18.38682	chr12:624	RPL7P41		Pseudoger	chr12:52885429-528
ENSG0000(	812	18.38682	chr12:624	ENSG00000271508		Pseudoger	chr12:43633093-436
ENSG0000(	812	18.38682	chr12:624	ENSG00000241251		Pseudoger	chr12:43560784-435
ENSG0000(	812	18.38682	chr12:624	ENSG00000271490		Pseudoger	chr12:50806335-508
ENSG0000(	812	18.38682	chr12:624	MFSD5		protein_c	chr12:53251251-532
ENSG0000(	812	18.38682	chr12:624	ENSG00000249388		lncRNA	chr12:54081736-541
ENSG0000(	812	18.38682	chr12:624	Y_RNA		smallRNA	chr12:44880868-448
ENSG0000(	812	18.38682	chr12:624	RNA5SP359		Pseudoger	chr12:38163403-381
ENSG0000(	812	18.38682	chr12:624	AC078864.2		smallRNA	chr12:52113925-521
ENSG0000(	812	18.38682	chr12:624	LETMD1	AC	protein_c	chr12:51047962-510
ENSG0000(	812	18.38682	chr12:624	Y_RNA		smallRNA	chr12:33982465-339
ENSG0000(	812	18.38682	chr1:1021	HSPE1P25		Pseudoger	chr1:77853355-7785
ENSG0000(	812	18.38682	chr12:624	ENSG00000271379		Pseudoger	chr12:42069935-420
ENSG0000(	812	18.38682	chr12:624	Y_RNA		smallRNA	chr12:32705403-327
ENSG0000(	812	18.38682	chr12:624	ENSG00000271357		Pseudoger	chr12:32100485-321
ENSG0000(	812	18.38682	chr12:624	DAZAP2		protein_c	chr12:51238724-512
ENSG0000(	812	18.38682	chr12:624	RN7SL10P		smallRNA	chr12:42242215-422
ENSG0000(	812	18.38682	chr12:624	ENSG00000271272		Pseudoger	chr12:42929848-429
ENSG0000(	812	18.38682	chr12:624	RN7SL390P		smallRNA	chr12:54255631-542
ENSG0000(	812	18.38682	chr12:624	KRT88P		Pseudoger	chr12:52263948-522
ENSG0000(	812	18.38682	chr12:624	Y_RNA		smallRNA	chr12:31506303-315
ENSG0000(	812	18.38682	chr12:624	PRKAG1	NCGv7	protein_c	chr12:49002274-490
ENSG0000(	812	18.38682	chr1:1021	USP33		protein_c	chr1:77695987-7775
ENSG0000(	812	18.38682	chr12:624	ENSG00000271596		Pseudoger	chr12:50949117-509
ENSG0000(	812	18.38682	chr12:624	ENSG00000271621		Pseudoger	chr12:50847925-508
ENSG0000(	812	18.38682	chr12:624	AQP6		protein_c	chr12:49967194-499
ENSG0000(	812	18.38682	chr12:624	AC078864.1		smallRNA	chr12:52110634-521
ENSG0000(	812	18.38682	chr12:624	AC023158.1		smallRNA	chr12:33412530-334
ENSG0000(	812	18.38682	chr12:624	AC107016.2		smallRNA	chr12:52951708-529
ENSG0000(	812	18.38682	chr12:624	RN7SL246P		smallRNA	chr12:45874029-458
ENSG0000(	812	18.38682	chr12:624	ENSG00000240399		Pseudoger	chr12:48054813-480
ENSG0000(	812	18.38682	chr12:624	PCED1B	NCGv7	protein_c	chr12:47079603-472
ENSG0000(	812	18.38682	chr12:624	RPS10P20		Pseudoger	chr12:48487946-484
ENSG0000(	812	18.38682	chr12:624	C12orf40		protein_c	chr12:39626167-399
ENSG0000(	812	18.38682	chr12:624	ENSG00000289854		lncRNA	chr12:54358489-543
ENSG0000(	812	18.38682	chr1:1021	PIGK		protein_c	chr1:77088989-7721
ENSG0000(	812	18.38682	chr1:1021	MIGA1		protein_c	chr1:77779624-7787
ENSG0000(	812	18.38682	chr12:624	ENSG00000285102		Pseudoger	chr12:52275821-522
ENSG0000(	812	18.38682	chr12:624	RPS27P21		Pseudoger	chr12:42678873-426
ENSG0000(	812	18.38682	chr12:624	HOXC9		protein_c	chr12:53994895-540
ENSG0000(	812	18.38682	chr12:624	HOXC10		protein_c	chr12:53985065-539
ENSG0000(	812	18.38682	chr12:624	SENP1		protein_c	chr12:48042897-481
ENSG0000(	812	18.38682	chr12:624	RAPGEF3	NCGv7	protein_c	chr12:47734363-477
ENSG0000(	812	18.38682	chr12:624	MIR3198-2		smallRNA	chr12:54231397-542
ENSG0000(	812	18.38682	chr12:624	ENSG00000203437		Pseudoger	chr12:31465062-314
ENSG0000(	812	18.38682	chr12:624	MARK3P1		Pseudoger	chr12:46682071-466
ENSG0000(	812	18.38682	chr12:624	HOXC13-AS		lncRNA	chr12:53935328-539
ENSG0000(	812	18.38682	chr12:624	DDN		protein_c	chr12:48995149-489
ENSG0000(	812	18.38682	chr1:1021	ST6GALNA(	NCGv7	protein_c	chr1:76074746-7663

ENSG0000(	812	18.38682	chr12:624	RPL35AP28		Pseudoger	chr12:49863173-498
ENSG0000(	812	18.38682	chr12:624	AC026357.1		smallRNA	chr12:33041725-330
ENSG0000(	812	18.38682	chr12:624	RPL12P32		Pseudoger	chr12:31948334-319
ENSG0000(	812	18.38682	chr12:624	ENSG00000270926		Pseudoger	chr12:31105105-311
ENSG0000(	812	18.38682	chr12:624	PKP2	DriverDBv	protein_c	chr12:32790745-328
ENSG0000(	812	18.38682	chr1:1021	AC093430.1		smallRNA	chr1:79135133-7913
ENSG0000(	812	18.38682	chr12:624	Y_RNA		smallRNA	chr12:50743568-507
ENSG0000(	812	18.38682	chr12:624	RNU6-618P		smallRNA	chr12:31396390-313
ENSG0000(	812	18.38682	chr12:624	KRT76		protein_c	chr12:52768155-527
ENSG0000(	812	18.38682	chr12:624	Y_RNA		smallRNA	chr12:49132853-491
ENSG0000(	812	18.38682	chr12:624	RNU6-940P		smallRNA	chr12:48975543-489
ENSG0000(	812	18.38682	chr12:624	AC023157.1		smallRNA	chr12:31713750-317
ENSG0000(	812	18.38682	chr12:624	ZNF740		protein_c	chr12:53180704-531
ENSG0000(	812	18.38682	chr12:624	RNU6-238P		smallRNA	chr12:50656973-506
ENSG0000(	812	18.38682	chr12:624	KRT71		protein_c	chr12:52543909-525
ENSG0000(	812	18.38682	chr12:624	METTL7A	NCGv7	protein_c	chr12:50923472-509
ENSG0000(	812	18.38682	chr12:624	TMBIM6	NCGv7	protein_c	chr12:49707725-497
ENSG0000(	812	18.38682	chr12:624	MYG1		protein_c	chr12:53299695-533
ENSG0000(	812	18.38682	chr12:624	ENSG00000270807		Pseudoger	chr12:33717853-337
ENSG0000(	812	18.38682	chr12:624	LMBR1L		protein_c	chr12:49097136-491
ENSG0000(	812	18.38682	chr12:624	CSAD		protein_c	chr12:53157663-531
ENSG0000(	812	18.38682	chr12:624	GALNT6		protein_c	chr12:51351247-513
ENSG0000(	812	18.38682	chr12:624	ITGB7	NCGv7	protein_c	chr12:53191323-532
ENSG0000(	812	18.38682	chr12:624	MAP3K12	NCGv7	protein_c	chr12:53479669-535
ENSG0000(	812	18.38682	chr12:624	SLC4A8		protein_c	chr12:51391317-515
ENSG0000(	812	18.38682	chr12:624	CERS5		protein_c	chr12:50129289-501
ENSG0000(	812	18.38682	chr12:624	AC117372.1		smallRNA	chr12:38321566-383
ENSG0000(	812	18.38682	chr12:624	NELL2		protein_c	chr12:44508275-449
ENSG0000(	812	18.38682	chr12:624	SMARCD1	NCGv7	protein_c	chr12:50085200-501
ENSG0000(	812	18.38682	chr12:624	HDAC7	NCGv7	protein_c	chr12:47782722-478
ENSG0000(	812	18.38682	chr12:624	PRPH		protein_c	chr12:49295147-492
ENSG0000(	812	18.38682	chr12:624	DIP2B		protein_c	chr12:50504985-507
ENSG0000(	812	18.38682	chr12:624	AMHR2		protein_c	chr12:53423855-534
ENSG0000(	812	18.38682	chr1:1021	ENSG00000282898		lncRNA	chr1:79323769-7932
ENSG0000(	812	18.38682	chr12:624	ENSG00000290531		lncRNA	chr12:30978308-310
ENSG0000(	812	18.38682	chr12:624	FAM186B	DriverDBv	protein_c	chr12:49582885-496
ENSG0000(	812	18.38682	chr12:624	Y_RNA		smallRNA	chr12:51156868-511
ENSG0000(	812	18.38682	chr12:624	POU6F1		protein_c	chr12:51186936-512
ENSG0000(	812	18.38682	chr12:624	KRT85		protein_c	chr12:52360006-523
ENSG0000(	812	18.38682	chr12:624	PPP1R1A		protein_c	chr12:54575387-545
ENSG0000(	812	18.38682	chr12:624	TROAP		protein_c	chr12:49323236-493
ENSG0000(	812	18.38682	chr1:1021	PSAT1P3		Pseudoger	chr1:79054945-7905
ENSG0000(	812	18.38682	chr12:624	PCBP2-OT1		lncRNA	chr12:53464468-534
ENSG0000(	812	18.38682	chr12:624	TFCP2	AC	protein_c	chr12:51093656-511
ENSG0000(	812	18.38682	chr12:624	FAIM2		protein_c	chr12:49866896-499
ENSG0000(	812	18.38682	chr12:624	SLC4A8-AS1		lncRNA	chr12:51421956-514
ENSG0000(	812	18.38682	chr12:624	ESPL1	NCGv7;AC	protein_c	chr12:53268299-532
ENSG0000(	812	18.38682	chr12:624	KRT87P		Pseudoger	chr12:52250466-522
ENSG0000(	812	18.38682	chr12:624	KRT7		protein_c	chr12:52232520-522
ENSG0000(	812	18.38682	chr12:624	HNRNPA1	AC	protein_c	chr12:54280193-542
ENSG0000(	812	18.38682	chr12:624	ACVR1B	NCGv7	protein_c	chr12:51951699-519

ENSG0000(	812	18.38682	chr12:62⁊	EIF4B		protein_c	chr12:53006282-530
ENSG0000(	812	18.38682	chr12:62⁊	KCNH3		protein_c	chr12:49539030-495
ENSG0000(	812	18.38682	chr12:62⁊	RNU6-950P		smallRNA	chr12:54317781-543
ENSG0000(	812	18.38682	chr12:62⁊	SYT10	NCGv7	protein_c	chr12:33374238-334
ENSG0000(	812	18.38682	chr12:62⁊	ENSG00000272822		protein_c	chr12:48903503-489
ENSG0000(	812	18.38682	chr12:62⁊	ENSG00000257848		Pseudoger	chr12:48171590-481
ENSG0000(	812	18.38682	chr12:62⁊	LINC02400		lncRNA	chr12:41764144-417
ENSG0000(	812	18.38682	chr12:62⁊	EIF4A1P4		Pseudoger	chr12:53153272-531
ENSG0000(	812	18.38682	chr12:62⁊	BICD1	DriverDB\	protein_c	chr12:32106835-323
ENSG0000(	812	18.38682	chr12:62⁊	AMN1		protein_c	chr12:31671142-317
ENSG0000(	812	18.38682	chr12:62⁊	OR5BJ1P		Pseudoger	chr12:48394510-483
ENSG0000(	812	18.38682	chr12:62⁊	RP11-125N22.4		Pseudoger	chr12:37542012-375
ENSG0000(	812	18.38682	chr12:62⁊	ENSG00000257807		Pseudoger	chr12:47484689-474
ENSG0000(	812	18.38682	chr12:62⁊	ENSG00000257808		lncRNA	chr12:53159586-531
ENSG0000(	812	18.38682	chr12:62⁊	ENSG00000257813		Pseudoger	chr12:43569769-435
ENSG0000(	812	18.38682	chr12:62⁊	ENSG00000257824		lncRNA	chr12:54543111-545
ENSG0000(	812	18.38682	chr12:62⁊	ENSG00000257829		lncRNA	chr12:52306616-523
ENSG0000(	812	18.38682	chr12:62⁊	ENSG00000257830		lncRNA	chr12:52274647-522
ENSG0000(	812	18.38682	chr12:62⁊	KRT90P		Pseudoger	chr12:52411742-524
ENSG0000(	812	18.38682	chr12:62⁊	ENSG00000286069		lncRNA	chr12:53739611-538
ENSG0000(	812	18.38682	chr12:62⁊	GXYLT1	NCGv7	protein_c	chr12:42081845-421
ENSG0000(	812	18.38682	chr12:62⁊	ENSG00000257849		lncRNA	chr12:42966122-429
ENSG0000(	812	18.38682	chr12:62⁊	HNRNPA3P10		Pseudoger	chr12:51712469-517
ENSG0000(	812	18.38682	chr12:62⁊	ENSG00000257852		Pseudoger	chr12:32072734-320
ENSG0000(	812	18.38682	chr1:1021	ENSG00000235400		Pseudoger	chr1:78749073-7875
ENSG0000(	812	18.38682	chr12:62⁊	MTND1P24		Pseudoger	chr12:41699391-417
ENSG0000(	812	18.38682	chr12:62⁊	ENSG00000257864		Pseudoger	chr12:44248432-442
ENSG0000(	812	18.38682	chr12:62⁊	PHB1P18		Pseudoger	chr12:48168847-481
ENSG0000(	812	18.38682	chr12:62⁊	ENSG00000257896		Pseudoger	chr12:43738829-437
ENSG0000(	812	18.38682	chr12:62⁊	RP11-125N22.3		Pseudoger	chr12:37544285-375
ENSG0000(	812	18.38682	chr12:62⁊	ENSG00000257905		Pseudoger	chr12:47593208-475
ENSG0000(	812	18.38682	chr12:62⁊	LINC02156		lncRNA	chr12:47377638-474
ENSG0000(	812	18.38682	chr12:62⁊	ENSG00000277672		Pseudoger	chr12:49075039-490
ENSG0000(	812	18.38682	chr12:62⁊	EEF1A1P17		Pseudoger	chr12:43659827-436
ENSG0000(	812	18.38682	chr12:62⁊	ENSG00000278126		lncRNA	chr12:51201684-512
ENSG0000(	812	18.38682	chr12:62⁊	GLYCAM1		Pseudoger	chr12:54608187-546
ENSG0000(	812	18.38682	chr12:62⁊	LINC02395		lncRNA	chr12:49900311-499
ENSG0000(	812	18.38682	chr12:62⁊	OR5BK1P		Pseudoger	chr12:48355792-483
ENSG0000(	812	18.38682	chr12:62⁊	ENSG00000257645		Pseudoger	chr12:38532895-385
ENSG0000(	812	18.38682	chr12:62⁊	RNU6-1069P		smallRNA	chr12:31802958-318
ENSG0000(	812	18.38682	chr12:62⁊	METTL7AP1		Pseudoger	chr12:52229093-522
ENSG0000(	812	18.38682	chr12:62⁊	ENSG00000257653		lncRNA	chr12:48766194-487
ENSG0000(	812	18.38682	chr12:62⁊	ENSG00000257657		lncRNA	chr12:45475520-456
ENSG0000(	812	18.38682	chr12:62⁊	OR11M1P		Pseudoger	chr12:48627879-486
ENSG0000(	812	18.38682	chr12:62⁊	ADCY6-DT		lncRNA	chr12:48789147-487
ENSG0000(	812	18.38682	chr12:62⁊	ENSG00000257663		lncRNA	chr12:52076841-520
ENSG0000(	812	18.38682	chr12:62⁊	RNU5F-4P		smallRNA	chr12:31594093-315
ENSG0000(	812	18.38682	chr12:62⁊	ENSG00000278385		lncRNA	chr12:47905122-479
ENSG0000(	812	18.38682	chr12:62⁊	ENSG00000278351		lncRNA	chr12:45256473-452
ENSG0000(	812	18.38682	chr12:62⁊	KRT7-AS		lncRNA	chr12:52245048-522
ENSG0000(	812	18.38682	chr12:62⁊	RNA5SP360		Pseudoger	chr12:41658676-416

ENSG0000(	812	18.38682	chr12:624	ENSG00000257674		Pseudoger	chr12:42286911-422
ENSG0000(	812	18.38682	chr12:624	BTBD10P1		Pseudoger	chr12:52748776-527
ENSG0000(	812	18.38682	chr1:1021	RNFT1P2		Pseudoger	chr1:78170481-7817
ENSG0000(	812	18.38682	chr12:624	ENSG00000257680		Pseudoger	chr12:40728811-407
ENSG0000(	812	18.38682	chr12:624	ENSG00000257687		Pseudoger	chr12:42627008-426
ENSG0000(	812	18.38682	chr12:624	ENSG00000257700		lncRNA	chr12:52692605-526
ENSG0000(	812	18.38682	chr12:624	PFKM		protein_c	chr12:48105139-481
ENSG0000(	812	18.38682	chr12:624	CPNE8-AS1		lncRNA	chr12:38906451-389
ENSG0000(	812	18.38682	chr1:1021	ST6GALNAC5		protein_c	chr1:76867480-7706
ENSG0000(	812	18.38682	chr12:624	LSM6P2		Pseudoger	chr12:49770960-497
ENSG0000(	812	18.38682	chr12:624	ENSG00000257735		lncRNA	chr12:48350945-484
ENSG0000(	812	18.38682	chr12:624	ENSG00000257738		Pseudoger	chr12:45200817-452
ENSG0000(	812	18.38682	chr1:1021	ENSG00000227556		Pseudoger	chr1:78317157-7831
ENSG0000(	812	18.38682	chr12:624	LINC02386		lncRNA	chr12:30230779-302
ENSG0000(	812	18.38682	chr12:624	TWF1		protein_c	chr12:43793723-438
ENSG0000(	812	18.38682	chr12:624	SLC2A13	NCGv7	protein_c	chr12:39755025-401
ENSG0000(	812	18.38682	chr1:1021	ENSG00000235011		lncRNA	chr1:79323769-7932
ENSG0000(	812	18.38682	chr12:624	GPR84-AS1		lncRNA	chr12:54353661-544
ENSG0000(	812	18.38682	chr12:624	ENSG00000258101		lncRNA	chr12:49232790-492
ENSG0000(	812	18.38682	chr12:624	HIGD1AP9		Pseudoger	chr12:49619334-496
ENSG0000(	812	18.38682	chr12:624	IFITM3P2		Pseudoger	chr12:31754720-317
ENSG0000(	812	18.38682	chr12:624	PPIAP45		Pseudoger	chr12:47341614-473
ENSG0000(	812	18.38682	chr1:1021	ENSG00000228187		Pseudoger	chr1:77194825-7719
ENSG0000(	812	18.38682	chr12:624	PPIAP44		Pseudoger	chr12:30861921-308
ENSG0000(	812	18.38682	chr12:624	KRT128P		Pseudoger	chr12:52630339-526
ENSG0000(	812	18.38682	chr12:624	ENSG00000258121		lncRNA	chr12:48483287-485
ENSG0000(	812	18.38682	chr12:624	ENSG00000258134		Pseudoger	chr12:32227410-322
ENSG0000(	812	18.38682	chr12:624	ENSG00000277173		lncRNA	chr12:47768529-477
ENSG0000(	812	18.38682	chr12:624	LINC02396		lncRNA	chr12:49908882-499
ENSG0000(	812	18.38682	chr12:624	ENSG00000279840		TEC	chr12:47960497-479
ENSG0000(	812	18.38682	chr1:1021	ENSG00000285928		lncRNA	chr1:78022565-7802
ENSG0000(	812	18.38682	chr12:624	LINC00941		lncRNA	chr12:30755167-308
ENSG0000(	812	18.38682	chr12:624	DDN-AS1		lncRNA	chr12:48998367-490
ENSG0000(	812	18.38682	chr1:1021	NEXN-AS1		lncRNA	chr1:77881348-7788
ENSG0000(	812	18.38682	chr12:624	LINC02406		lncRNA	chr12:39087347-391
ENSG0000(	812	18.38682	chr12:624	ENSG00000258167		lncRNA	chr12:40395853-404
ENSG0000(	812	18.38682	chr12:624	CCNT1		protein_c	chr12:48688458-487
ENSG0000(	812	18.38682	chr12:624	PUS7L		protein_c	chr12:43718992-437
ENSG0000(	812	18.38682	chr12:624	ENSG00000276900		lncRNA	chr12:31729117-317
ENSG0000(	812	18.38682	chr12:624	ENSG00000258181		lncRNA	chr12:47248124-472
ENSG0000(	812	18.38682	chr12:624	ENSG00000276814		lncRNA	chr12:48039784-480
ENSG0000(	812	18.38682	chr12:624	ENSG00000258202		Pseudoger	chr12:30454919-304
ENSG0000(	812	18.38682	chr12:624	ENSG00000258203		lncRNA	chr12:48005277-480
ENSG0000(	812	18.38682	chr12:624	ZNF75BP		Pseudoger	chr12:44008620-440
ENSG0000(	812	18.38682	chr12:624	ENSG00000276691		lncRNA	chr12:47788426-477
ENSG0000(	812	18.38682	chr12:624	ENSG00000258232		lncRNA	chr12:49265156-492
ENSG0000(	812	18.38682	chr12:624	SLC38A2-AS1		lncRNA	chr12:46371463-463
ENSG0000(	812	18.38682	chr12:624	ENSG00000277342		lncRNA	chr12:32109076-321
ENSG0000(	812	18.38682	chr12:624	GPR84-AS1		lncRNA	chr12:54353792-544
ENSG0000(	812	18.38682	chr12:624	VTI1BP3		Pseudoger	chr12:53131350-531
ENSG0000(	812	18.38682	chr12:624	LINC02416		lncRNA	chr12:47353754-473

ENSG0000(	812	18.38682	chr12:624	ENSG00000257925		lncRNA	chr12:47237734-472
ENSG0000(	812	18.38682	chr12:624	MRPS36P5		Pseudoger	chr12:43054089-430
ENSG0000(	812	18.38682	chr12:624	ENSG00000257932		lncRNA	chr12:30320834-303
ENSG0000(	812	18.38682	chr12:624	RP11-125N22.1		Pseudoger	chr12:37544607-375
ENSG0000(	812	18.38682	chr12:624	ENSG00000257947		lncRNA	chr12:44244394-442
ENSG0000(	812	18.38682	chr12:624	ENSG00000257954		Pseudoger	chr12:49389516-493
ENSG0000(	812	18.38682	chr12:624	ENSG00000257955		lncRNA	chr12:48019771-480
ENSG0000(	812	18.38682	chr12:624	ENSG00000257964		lncRNA	chr12:49576840-495
ENSG0000(	812	18.38682	chr12:624	ENSG00000257985		lncRNA	chr12:48011304-480
ENSG0000(	812	18.38682	chr12:624	TEX49		protein_c	chr12:48727435-487
ENSG0000(	812	18.38682	chr12:624	ENSG00000257989		lncRNA	chr12:52092485-521
ENSG0000(	812	18.38682	chr12:624	HIGD1AP1		Pseudoger	chr12:53142053-531
ENSG0000(	812	18.38682	chr12:624	ENSG00000258017		lncRNA	chr12:49127782-491
ENSG0000(	812	18.38682	chr12:624	LINC02471		lncRNA	chr12:40156113-402
ENSG0000(	812	18.38682	chr1:1021	ENSG00000223905		Pseudoger	chr1:76353583-7635
ENSG0000(	812	18.38682	chr12:624	ENSG00000258021		Pseudoger	chr12:51900565-519
ENSG0000(	812	18.38682	chr12:624	OR5BT1P		Pseudoger	chr12:48385417-483
ENSG0000(	812	18.38682	chr12:624	SNORA25		smallRNA	chr12:31811088-318
ENSG0000(	812	18.38682	chr12:624	KRT125P		Pseudoger	chr12:52729517-527
ENSG0000(	812	18.38682	chr1:1021	NSRP1P1		Pseudoger	chr1:77847110-7784
ENSG0000(	812	18.38682	chr12:624	ENSG00000258051		Pseudoger	chr12:48160245-481
ENSG0000(	812	18.38682	chr12:624	ENSG00000287051		lncRNA	chr12:52298856-523
ENSG0000(	812	18.38682	chr12:624	BCDIN3D-AS1		lncRNA	chr12:49827913-498
ENSG0000(	812	18.38682	chr1:1021	ENSG00000233099		lncRNA	chr1:77346046-7734
ENSG0000(	812	18.38682	chr12:624	ENSG00000258068		lncRNA	chr12:42485353-424
ENSG0000(	812	18.38682	chr12:624	ARL2BPP2		Pseudoger	chr12:52763405-527
ENSG0000(	812	18.38682	chr12:624	ENSG00000257643		Pseudoger	chr12:32692504-326
ENSG0000(	812	18.38682	chr1:1021	ADGRL4		protein_c	chr1:78889764-7928
ENSG0000(	812	18.38682	chr12:624	ENSG00000258234		lncRNA	chr12:48231098-482
ENSG0000(	812	18.38682	chr1:1021	PTGFR	NCGv7	protein_c	chr1:78303884-7854
ENSG0000(	812	18.38682	chr12:624	NCKAP1L		protein_c	chr12:54497752-545
ENSG0000(	812	18.38682	chr12:624	ATF1	NCGv7;AC	protein_c	chr12:50763710-508
ENSG0000(	812	18.38682	chr12:624	ENSG00000286591		lncRNA	chr12:41951493-420
ENSG0000(	812	18.38682	chr12:624	ENSG00000257094		Pseudoger	chr12:32007127-320
ENSG0000(	812	18.38682	chr12:624	LINC02450		lncRNA	chr12:42687195-427
ENSG0000(	812	18.38682	chr12:624	KRT127P		Pseudoger	chr12:52751978-527
ENSG0000(	812	18.38682	chr12:624	ENSG00000257128		Pseudoger	chr12:41911256-419
ENSG0000(	812	18.38682	chr12:624	LINC02874		lncRNA	chr12:52205392-522
ENSG0000(	812	18.38682	chr12:624	AC067735.1		smallRNA	chr12:38913240-389
ENSG0000(	812	18.38682	chr12:624	LINC02451		lncRNA	chr12:42646583-426
ENSG0000(	812	18.38682	chr12:624	AK6P2		Pseudoger	chr12:38078529-380
ENSG0000(	812	18.38682	chr12:624	DENND5B-AS1		lncRNA	chr12:31589923-316
ENSG0000(	812	18.38682	chr12:624	WNT1	TAG;AC	protein_c	chr12:48978322-489
ENSG0000(	812	18.38682	chr12:624	ENSG00000257225		lncRNA	chr12:42459366-424
ENSG0000(	812	18.38682	chr12:624	DBX2-AS1		lncRNA	chr12:45050901-451
ENSG0000(	812	18.38682	chr12:624	ENSG00000257228		lncRNA	chr12:41409467-414
ENSG0000(	812	18.38682	chr12:624	Y_RNA		smallRNA	chr12:38274448-382
ENSG0000(	812	18.38682	chr12:624	ENSG00000257237		Pseudoger	chr12:39058028-390
ENSG0000(	812	18.38682	chr12:624	ENSG00000286671		lncRNA	chr12:44499961-445
ENSG0000(	812	18.38682	chr12:624	ENSG00000257239		lncRNA	chr12:41829898-419
ENSG0000(	812	18.38682	chr12:624	PARK7P1		Pseudoger	chr12:49595148-495

ENSG0000(	812	18.38682	chr12:62<	PHB1P19		Pseudoger	chr12:51124099-511
ENSG0000(	812	18.38682	chr12:62<	ENSG00000257253		lncRNA	chr12:49861207-498
ENSG0000(	812	18.38682	chr12:62<	ENSG00000257256		lncRNA	chr12:50219604-502
ENSG0000(	812	18.38682	chr12:62<	SLC38A4-AS1		lncRNA	chr12:46383433-468
ENSG0000(	812	18.38682	chr12:62<	ENSG00000257262		lncRNA	chr12:30200983-302
ENSG0000(	812	18.38682	chr12:62<	ENSG00000257292		Pseudoger	chr12:38205098-382
ENSG0000(	812	18.38682	chr12:62<	ENSG00000257298		lncRNA	chr12:50185580-501
ENSG0000(	812	18.38682	chr12:62<	PFDN5		protein_c	chr12:53295291-532
ENSG0000(	812	18.38682	chr12:62<	SPATS2		protein_c	chr12:49366584-495
ENSG0000(	812	18.38682	chr12:62<	NR4A1		protein_c	chr12:52022832-520
ENSG0000(	812	18.38682	chr12:62<	PDE1B		protein_c	chr12:54549601-545
ENSG0000(	812	18.38682	chr12:62<	ENSG00000256159		Pseudoger	chr12:31477506-314
ENSG0000(	812	18.38682	chr12:62<	LINC02387		lncRNA	chr12:31363481-313
ENSG0000(	812	18.38682	chr12:62<	SNORA74		smallRNA	chr12:42347061-423
ENSG0000(	812	18.38682	chr12:62<	ENSG00000226472		Pseudoger	chr12:31005777-310
ENSG0000(	812	18.38682	chr1:1021	TPI1P1		Pseudoger	chr1:76699789-7670
ENSG0000(	812	18.38682	chr12:62<	ENSG00000256372		Pseudoger	chr12:31853021-318
ENSG0000(	812	18.38682	chr12:62<	OR8T1P		Pseudoger	chr12:48442030-484
ENSG0000(	812	18.38682	chr12:62<	ENSG00000256465		Pseudoger	chr12:31893370-318
ENSG0000(	812	18.38682	chr12:62<	ENSG00000256070		Pseudoger	chr12:33454345-334
ENSG0000(	812	18.38682	chr12:62<	ENSG00000256538		lncRNA	chr12:34037232-340
ENSG0000(	812	18.38682	chr12:62<	ENSG00000286576		lncRNA	chr12:39447404-394
ENSG0000(	812	18.38682	chr12:62<	AK6P1		Pseudoger	chr12:34249481-342
ENSG0000(	812	18.38682	chr12:62<	ENSG00000226138		Pseudoger	chr12:48085062-480
ENSG0000(	812	18.38682	chr1:1021	ENSG00000226084		Pseudoger	chr1:77129114-7712
ENSG0000(	812	18.38682	chr12:62<	ENSG00000256843		Pseudoger	chr12:31748100-317
ENSG0000(	812	18.38682	chr12:62<	ENSG00000256984		Pseudoger	chr12:31012503-310
ENSG0000(	812	18.38682	chr12:62<	TUBA1B		protein_c	chr12:49127782-491
ENSG0000(	812	18.38682	chr12:62<	SMUG1	NCGv7	protein_c	chr12:54121277-541
ENSG0000(	812	18.38682	chr12:62<	ENSG00000256986		Pseudoger	chr12:34162477-341
ENSG0000(	812	18.38682	chr12:62<	AC121334.1		smallRNA	chr12:39443048-394
ENSG0000(	812	18.38682	chr12:62<	RPL7AP74		Pseudoger	chr12:39466442-394
ENSG0000(	812	18.38682	chr12:62<	HOXC12		protein_c	chr12:53954903-539
ENSG0000(	812	18.38682	chr12:62<	NFE2		protein_c	chr12:54292111-543
ENSG0000(	812	18.38682	chr12:62<	ATG101		protein_c	chr12:52069246-520
ENSG0000(	812	18.38682	chr12:62<	HOXC11	NCGv7;AC	protein_c	chr12:53973126-539
ENSG0000(	812	18.38682	chr12:62<	ENSG00000256987		Pseudoger	chr12:31953889-319
ENSG0000(	812	18.38682	chr12:62<	HOXC13	NCGv7;AC	protein_c	chr12:53938831-539
ENSG0000(	812	18.38682	chr12:62<	ASS1P14		Pseudoger	chr12:32985838-329
ENSG0000(	812	18.38682	chr12:62<	RACGAP1P1		Pseudoger	chr12:45063473-450
ENSG0000(	812	18.38682	chr1:1021	DNAJB4		protein_c	chr1:77979175-7801
ENSG0000(	812	18.38682	chr12:62<	ENSG00000257534		lncRNA	chr12:54162065-541
ENSG0000(	812	18.38682	chr12:62<	ITGA5		protein_c	chr12:54395261-544
ENSG0000(	812	18.38682	chr12:62<	ZNF385A		protein_c	chr12:54369133-543
ENSG0000(	812	18.38682	chr12:62<	LINC02402		lncRNA	chr12:42615221-426
ENSG0000(	812	18.38682	chr12:62<	FMNL3	NCGv7	protein_c	chr12:49636499-497
ENSG0000(	812	18.38682	chr12:62<	AQP5		protein_c	chr12:49961872-499
ENSG0000(	812	18.38682	chr12:62<	RACGAP1		protein_c	chr12:49976923-500
ENSG0000(	812	18.38682	chr12:62<	LARP4		protein_c	chr12:50392383-504
ENSG0000(	812	18.38682	chr12:62<	TAMALIN		protein_c	chr12:52006946-520
ENSG0000(	812	18.38682	chr12:62<	KRT84	NCGv7	protein_c	chr12:52377812-523

ENSG0000(	812	18.38682	chr12:62<	KRT82		protein_c	chr12:52393931-524
ENSG0000(	812	18.38682	chr12:62<	ENSG00000257511		Pseudoger	chr12:32726383-327
ENSG0000(	812	18.38682	chr12:62<	BICD1-AS1		lncRNA	chr12:32104117-321
ENSG0000(	812	18.38682	chr12:62<	ENSG00000257531		Pseudoger	chr12:50217662-502
ENSG0000(	812	18.38682	chr12:62<	OR7E47P		Pseudoger	chr12:52107278-521
ENSG0000(	812	18.38682	chr12:62<	LINC02422		lncRNA	chr12:31876969-319
ENSG0000(	812	18.38682	chr12:62<	ENSG00000257550		lncRNA	chr12:53513891-535
ENSG0000(	812	18.38682	chr12:62<	ENSG00000257570		Pseudoger	chr12:49709237-497
ENSG0000(	812	18.38682	chr12:62<	SSBL3P		Pseudoger	chr12:45071769-450
ENSG0000(	812	18.38682	chr12:62<	ENSG00000257586		Pseudoger	chr12:38994803-389
ENSG0000(	812	18.38682	chr12:62<	AQP5-AS1		lncRNA	chr12:49951512-499
ENSG0000(	812	18.38682	chr12:62<	SCAT2		lncRNA	chr12:54262615-542
ENSG0000(	812	18.38682	chr12:62<	MYG1-AS1		lncRNA	chr12:53298655-533
ENSG0000(	812	18.38682	chr12:62<	ENSG00000257616		Pseudoger	chr12:52730425-527
ENSG0000(	812	18.38682	chr12:62<	ENSG00000286950		lncRNA	chr12:32755510-327
ENSG0000(	812	18.38682	chr12:62<	ENSG00000278475		lncRNA	chr12:45789189-457
ENSG0000(	812	18.38682	chr1:1021	FUBP1	NCGv7;AC	protein_c	chr1:77944055-7797
ENSG0000(	812	18.38682	chr1:1021	NEXN		protein_c	chr1:77888513-7794
ENSG0000(	812	18.38682	chr12:62<	AK4P3		protein_c	chr12:31615023-316
ENSG0000(	812	18.38682	chr12:62<	ENSG00000257500		lncRNA	chr12:52407580-524
ENSG0000(	812	18.38682	chr12:62<	ENSG00000257496		lncRNA	chr12:46384233-464
ENSG0000(	812	18.38682	chr1:1021	AK5		protein_c	chr1:77282019-7755
ENSG0000(	812	18.38682	chr12:62<	KRT73-AS1		lncRNA	chr12:52601467-526
ENSG0000(	812	18.38682	chr12:62<	TNS2-AS1		lncRNA	chr12:53012884-530
ENSG0000(	812	18.38682	chr12:62<	ANKRD49P2		Pseudoger	chr12:31534715-315
ENSG0000(	812	18.38682	chr12:62<	ENSG00000278896		TEC	chr12:46404644-464
ENSG0000(	812	18.38682	chr12:62<	LRRK2-DT		lncRNA	chr12:40186009-402
ENSG0000(	812	18.38682	chr12:62<	ENSG00000257343		Pseudoger	chr12:52746596-527
ENSG0000(	812	18.38682	chr12:62<	ENSG00000257346		lncRNA	chr12:49090208-490
ENSG0000(	812	18.38682	chr12:62<	ENSG00000257368		Pseudoger	chr12:45444766-454
ENSG0000(	812	18.38682	chr12:62<	ENSG00000257373		lncRNA	chr12:42979254-429
ENSG0000(	812	18.38682	chr12:62<	ENSG00000278842		Pseudoger	chr12:50286244-502
ENSG0000(	812	18.38682	chr12:62<	ENSG00000257376		Pseudoger	chr12:42431665-424
ENSG0000(	812	18.38682	chr12:62<	ENSG00000257378		lncRNA	chr12:49954639-499
ENSG0000(	812	18.38682	chr12:62<	RN7SKP289		smallRNA	chr12:53816185-538
ENSG0000(	812	18.38682	chr12:62<	ENSG00000257379		lncRNA	chr12:53441741-534
ENSG0000(	812	18.38682	chr12:62<	ENSG00000257389		Pseudoger	chr12:52837403-528
ENSG0000(	812	18.38682	chr12:62<	RNU6-494P		smallRNA	chr12:32477382-324
ENSG0000(	812	18.38682	chr12:62<	KRT126P		Pseudoger	chr12:52715567-527
ENSG0000(	812	18.38682	chr12:62<	ENSG00000257404		Pseudoger	chr12:52782650-527
ENSG0000(	812	18.38682	chr12:62<	ENSG00000257405		Pseudoger	chr12:42636815-426
ENSG0000(	812	18.38682	chr12:62<	RPAP3-DT		lncRNA	chr12:47706058-477
ENSG0000(	812	18.38682	chr12:62<	ENSG00000257435		lncRNA	chr12:32988763-329
ENSG0000(	812	18.38682	chr12:62<	LINC02963		lncRNA	chr12:34190471-342
ENSG0000(	812	18.38682	chr12:62<	AC023050.1		smallRNA	chr12:31915636-319
ENSG0000(	812	18.38682	chr12:62<	ENSG00000257464		Pseudoger	chr12:49442424-494
ENSG0000(	812	18.38682	chr12:62<	ENSG00000257475		lncRNA	chr12:53012104-530
ENSG0000(	812	18.38682	chr12:62<	LINC01154		lncRNA	chr12:54428303-544
ENSG0000(	812	18.38682	chr12:62<	ENSG00000255628		lncRNA	chr12:34149839-341
ENSG0000(	812	18.38682	chr12:62<	LINC02354		lncRNA	chr12:47826854-478
ENSG0000(	812	18.38682	chr12:62<	ENSG00000279875		TEC	chr12:48189349-481

ENSG0000(	812	18.38682	chr12:624	ENSG00000258119		lncRNA	chr12:38544177-385
ENSG0000(	812	18.38682	chr12:624	MTND2P17		Pseudoger	chr12:41698495-416
ENSG0000(	812	18.38682	chr12:624	NCKAP5L		protein_c	chr12:49791146-498
ENSG0000(	812	18.38682	chr1:1021	ENSG00000213560		Pseudoger	chr1:78091499-7809
ENSG0000(	812	18.38682	chr1:1021	ENSG00000213561		Pseudoger	chr1:78043383-7804
ENSG0000(	812	18.38682	chr12:624	KMT2D	NCGv7	protein_c	chr12:49018975-490
ENSG0000(	812	18.38682	chr12:624	ENSG00000261586		lncRNA	chr12:51817899-518
ENSG0000(	812	18.38682	chr12:624	ENSG00000273765		lncRNA	chr12:48360920-483
ENSG0000(	812	18.38682	chr12:624	RHEBL1		protein_c	chr12:49064676-490
ENSG0000(	812	18.38682	chr12:624	TUBA1A		protein_c	chr12:49184686-491
ENSG0000(	812	18.38682	chr12:624	HOXC-AS3		lncRNA	chr12:53981509-539
ENSG0000(	812	18.38682	chr12:624	TUBA1C	NCGv7	protein_c	chr12:49188736-492
ENSG0000(	812	18.38682	chr12:624	AQP2		protein_c	chr12:49950737-499
ENSG0000(	812	18.38682	chr12:624	CACNB3		protein_c	chr12:48813794-488
ENSG0000(	812	18.38682	chr12:624	GPD1		protein_c	chr12:50103982-501
ENSG0000(	812	18.38682	chr12:624	ANKRD33		protein_c	chr12:51888009-518
ENSG0000(	812	18.38682	chr12:624	ENSG00000275286		lncRNA	chr12:44498616-444
ENSG0000(	812	18.38682	chr12:624	KRT80		protein_c	chr12:52168996-521
ENSG0000(	812	18.38682	chr12:624	KRT1		protein_c	chr12:52674736-526
ENSG0000(	812	18.38682	chr12:624	SPRYD3		protein_c	chr12:53064316-530
ENSG0000(	812	18.38682	chr12:624	IGFBP6		protein_c	chr12:53097436-531
ENSG0000(	812	18.38682	chr1:1021	ENSG00000238015		Pseudoger	chr1:78666272-7866
ENSG0000(	812	18.38682	chr12:624	ENSG00000273853		lncRNA	chr12:33432311-334
ENSG0000(	812	18.38682	chr12:624	ENSG00000213470		Pseudoger	chr12:53632788-536
ENSG0000(	812	18.38682	chr12:624	LALBA		protein_c	chr12:48567684-485
ENSG0000(	812	18.38682	chr12:624	ENSG00000260122		lncRNA	chr12:51809705-518
ENSG0000(	812	18.38682	chr12:624	ASIC1		protein_c	chr12:50057548-500
ENSG0000(	812	18.38682	chr12:624	TNS2		protein_c	chr12:53046969-530
ENSG0000(	812	18.38682	chr12:624	KRT18		protein_c	chr12:52948871-529
ENSG0000(	812	18.38682	chr12:624	ENSG00000260030		lncRNA	chr12:53754358-537
ENSG0000(	812	18.38682	chr12:624	MIR1293		smallRNA	chr12:50234142-502
ENSG0000(	812	18.38682	chr12:624	BIN2		protein_c	chr12:51281038-513
ENSG0000(	812	18.38682	chr12:624	CSRNP2		protein_c	chr12:51061205-510
ENSG0000(	812	18.38682	chr12:624	SLC11A2		protein_c	chr12:50979401-510
ENSG0000(	812	18.38682	chr12:624	KRT86		protein_c	chr12:52249300-523
ENSG0000(	812	18.38682	chr12:624	CAPRIN2		protein_c	chr12:30709552-308
ENSG0000(	812	18.38682	chr12:624	snoU13		smallRNA	chr12:48960738-489
ENSG0000(	812	18.38682	chr12:624	ZNF641		protein_c	chr12:48337180-483
ENSG0000(	812	18.38682	chr12:624	PRPF40B	NCGv7	protein_c	chr12:49568218-496
ENSG0000(	812	18.38682	chr12:624	SNORA34		smallRNA	chr12:48654382-486
ENSG0000(	812	18.38682	chr1:1021	RNA5SP23		Pseudoger	chr1:78375164-7837
ENSG0000(	812	18.38682	chr12:624	RNU6-400P		smallRNA	chr12:33576095-335
ENSG0000(	812	18.38682	chr12:624	RNU6-1093P		smallRNA	chr12:50256238-502
ENSG0000(	812	18.38682	chr1:1021	ENSG00000288543		lncRNA	chr1:77067920-7707
ENSG0000(	812	18.38682	chr12:624	SNORA75		smallRNA	chr12:31076867-310
ENSG0000(	812	18.38682	chr1:1021	ENSG00000273338		lncRNA	chr1:78004346-7800
ENSG0000(	812	18.38682	chr12:624	OR8S21P		Pseudoger	chr12:48417150-484
ENSG0000(	812	18.38682	chr12:624	SOAT2		protein_c	chr12:53103486-531
ENSG0000(	812	18.38682	chr12:624	ENSG00000258253		lncRNA	chr12:52380460-524
ENSG0000(	812	18.38682	chr1:1021	AC104837.1		smallRNA	chr1:78687060-7868
ENSG0000(	812	18.38682	chr12:624	HIGD1C		protein_c	chr12:50952538-509

ENSG0000(	812	18.38682	chr1:1021	ACTG1P21		Pseudoger	chr1:77773865-7777
ENSG0000(	812	18.38682	chr12:62⁄	ENSG00000274591		lncRNA	chr12:46239106-462
ENSG0000(	812	18.38682	chr12:62⁄	KRT72		protein_c	chr12:52585589-526
ENSG0000(	812	18.38682	chr12:62⁄	KRT74		protein_c	chr12:52565782-525
ENSG0000(	812	18.38682	chr12:62⁄	LINC02555		lncRNA	chr12:40140926-401
ENSG0000(	812	18.38682	chr12:62⁄	KRT4		protein_c	chr12:52806549-528
ENSG0000(	812	18.38682	chr1:1021	LINC02567		lncRNA	chr1:76758124-7677
ENSG0000(	812	18.38682	chr12:62⁄	WNT10B	AC	protein_c	chr12:48965340-489
ENSG0000(	812	18.38682	chr1:1021	ENSG00000287870		lncRNA	chr1:77248633-7725
ENSG0000(	812	18.38682	chr12:62⁄	ENSG00000274156		lncRNA	chr12:50934942-509
ENSG0000(	812	18.38682	chr1:1021	MGC27382		lncRNA	chr1:78229599-7836
ENSG0000(	812	18.38682	chr12:62⁄	ENSG00000274124		lncRNA	chr12:48152817-481
ENSG0000(	812	18.38682	chr12:62⁄	ENSG00000274105		lncRNA	chr12:32728169-327
ENSG0000(	812	18.38682	chr12:62⁄	FIGNL2		protein_c	chr12:51817899-518
ENSG0000(	812	18.38682	chr12:62⁄	SP7		protein_c	chr12:53326575-533
ENSG0000(	812	18.38682	chr12:62⁄	KRT8		protein_c	chr12:52897187-529
ENSG0000(	812	18.38682	chr12:62⁄	KRT78		protein_c	chr12:52837804-528
ENSG0000(	812	18.38682	chr12:62⁄	KRT6C		protein_c	chr12:52468516-524
ENSG0000(	812	18.38682	chr12:62⁄	ENSG00000285692		lncRNA	chr12:53531752-536
ENSG0000(	812	18.38682	chr12:62⁄	DENND5B		protein_c	chr12:31382226-315
ENSG0000(	812	18.38682	chr1:1021	ADH5P2		Pseudoger	chr1:79521080-7952
ENSG0000(	812	18.38682	chr12:62⁄	ENSG00000274682		lncRNA	chr12:40978744-409
ENSG0000(	812	18.38682	chr12:62⁄	ENSG00000275228		lncRNA	chr12:48327942-483
ENSG0000(	812	18.38682	chr12:62⁄	ENSG00000274976		lncRNA	chr12:32820142-328
ENSG0000(	812	18.38682	chr1:1021	ENSG00000287647		lncRNA	chr1:77431314-7743
ENSG0000(	812	18.38682	chr12:62⁄	ENSG00000275097		lncRNA	chr12:31280422-312
ENSG0000(	812	18.38682	chr12:62⁄	ENSG00000260470		lncRNA	chr12:54145069-541
ENSG0000(	812	18.38682	chr12:62⁄	ATF7		protein_c	chr12:53507856-536
ENSG0000(	812	18.38682	chr12:62⁄	ENSG00000260473		lncRNA	chr12:51815043-518
ENSG0000(	812	18.38682	chr12:62⁄	CISTR		lncRNA	chr12:53746337-537
ENSG0000(	812	18.38682	chr1:1021	ENSG00000219201		Pseudoger	chr1:77810861-7781
ENSG0000(	812	18.38682	chr12:62⁄	GTSF1		protein_c	chr12:54455950-544
ENSG0000(	812	18.38682	chr12:62⁄	ENSG00000260597		lncRNA	chr12:54019910-540
ENSG0000(	812	18.38682	chr12:62⁄	ENSG00000274964		lncRNA	chr12:32339368-323
ENSG0000(	812	18.38682	chr12:62⁄	ENSG00000274723		lncRNA	chr12:46970504-469
ENSG0000(	812	18.38682	chr12:62⁄	ENSG00000274943		lncRNA	chr12:42361267-423
ENSG0000(	812	18.38682	chr12:62⁄	ENSG00000256176		Pseudoger	chr12:31311617-313
ENSG0000(	812	18.38682	chr12:62⁄	KRT89P		Pseudoger	chr12:52341197-523
ENSG0000(	812	18.38682	chr12:62⁄	ENSG00000274902		lncRNA	chr12:47731908-477
ENSG0000(	812	18.38682	chr12:62⁄	KRT83		protein_c	chr12:52314301-523
ENSG0000(	812	18.38682	chr12:62⁄	ENSG00000274797		lncRNA	chr12:50953924-509
ENSG0000(	812	18.38682	chr12:62⁄	ENSG00000274737		lncRNA	chr12:47817451-478
ENSG0000(	812	18.38682	chr12:62⁄	ENSG00000285732		lncRNA	chr12:39611819-396
ENSG0000(	812	18.38682	chr12:62⁄	AC079603.1		smallRNA	chr12:43291495-432
ENSG0000(	812	18.38682	chr1:1021	AC095030.1		smallRNA	chr1:77391422-7739
ENSG0000(	812	18.38682	chr12:62⁄	TSPAN11	DriverDB\	protein_c	chr12:30926428-309
ENSG0000(	812	18.38682	chr12:62⁄	ENSG00000275481		lncRNA	chr12:46388856-463
ENSG0000(	812	18.38682	chr1:1021	ENSG00000288822		lncRNA	chr1:79488644-7949
ENSG0000(	812	18.38682	chr12:62⁄	IFITM3P6		Pseudoger	chr12:47135498-471
ENSG0000(	812	18.38682	chr12:62⁄	NF1P12		Pseudoger	chr12:38206231-382
ENSG0000(	812	18.38682	chr12:62⁄	ZNF970P		Pseudoger	chr12:37575587-375

ENSG0000(	812	18.38682	chr12:624	ADI1P3		Pseudoger	chr12:47463087-474
ENSG0000(	812	18.38682	chr12:624	PDZRN4	NCGv7	protein_c	chr12:41188320-415
ENSG0000(	812	18.38682	chr12:624	ENSG00000276148		lncRNA	chr12:32725248-327
ENSG0000(	812	18.38682	chr12:624	ENSG00000276136		lncRNA	chr12:32000375-320
ENSG0000(	812	18.38682	chr12:624	DUX4L27		Pseudoger	chr12:34208415-342
ENSG0000(	812	18.38682	chr12:624	AC023513.1		smallRNA	chr12:42079761-420
ENSG0000(	812	18.38682	chr12:624	HOTAIR	AC	lncRNA	chr12:53962308-539
ENSG0000(	812	18.38682	chr12:624	ENSG00000273046		lncRNA	chr12:54017110-540
ENSG0000(	812	18.38682	chr12:624	OR7A19P		Pseudoger	chr12:46592573-465
ENSG0000(	812	18.38682	chr1:1021	AC096951.1		smallRNA	chr1:77007744-7700
ENSG0000(	812	18.38682	chr12:624	LINC02381		lncRNA	chr12:54126019-541
ENSG0000(	812	18.38682	chr12:624	ENSG00000276115		lncRNA	chr12:32352349-323
ENSG0000(	812	18.38682	chr1:1021	RNA5SP21		Pseudoger	chr1:77779904-7777
ENSG0000(	812	18.38682	chr12:624	ENSG00000287386		lncRNA	chr12:45164186-451
ENSG0000(	812	18.38682	chr12:624	RNU6-333P		smallRNA	chr12:53138545-531
ENSG0000(	812	18.38682	chr12:624	LINC00938		lncRNA	chr12:45718046-457
ENSG0000(	812	18.38682	chr12:624	snoU13		smallRNA	chr12:31303717-313
ENSG0000(	812	18.38682	chr12:624	MIR615		smallRNA	chr12:54033950-540
ENSG0000(	812	18.38682	chr12:624	ENSG00000276390		lncRNA	chr12:47699401-476
ENSG0000(	812	18.38682	chr12:624	AC121336.1		smallRNA	chr12:39807095-398
ENSG0000(	812	18.38682	chr12:624	ENSG00000258344		lncRNA	chr12:54276631-543
ENSG0000(	812	18.38682	chr1:1021	ENSG00000272855		lncRNA	chr1:76636877-7663
ENSG0000(	812	18.38682	chr12:624	ENSG00000258273		Pseudoger	chr12:48333755-483
ENSG0000(	812	18.38682	chr12:624	CLUHP8		Pseudoger	chr12:38082046-380
ENSG0000(	812	18.38682	chr12:624	Y_RNA		smallRNA	chr12:48809911-488
ENSG0000(	812	18.38682	chr12:624	LINC00592		lncRNA	chr12:52164115-522
ENSG0000(	812	18.38682	chr12:624	DDX23	NCGv7	protein_c	chr12:48829756-488
ENSG0000(	812	18.38682	chr12:624	ADCY6		protein_c	chr12:48766194-487
ENSG0000(	812	18.38682	chr12:624	ENSG00000258283		lncRNA	chr12:48995150-489
ENSG0000(	812	18.38682	chr12:624	POLR2KP1		Pseudoger	chr12:49594091-495
ENSG0000(	812	18.38682	chr12:624	SNORD81		smallRNA	chr12:53791305-537
ENSG0000(	812	18.38682	chr12:624	RNU6-318P		smallRNA	chr12:31366288-313
ENSG0000(	812	18.38682	chr12:624	RNU6-713P		smallRNA	chr12:40554161-405
ENSG0000(	812	18.38682	chr1:1021	RNU6-1102P		smallRNA	chr1:78088988-7808
ENSG0000(	812	18.38682	chr12:624	ENSG00000250654		Pseudoger	chr12:54079267-540
ENSG0000(	812	18.38682	chr12:624	ENSG00000285870		lncRNA	chr12:30696121-307
ENSG0000(	812	18.38682	chr12:624	LINC02461		lncRNA	chr12:43155315-431
ENSG0000(	812	18.38682	chr12:624	ENSG00000276454		lncRNA	chr12:47265665-472
ENSG0000(	812	18.38682	chr12:624	ENSG00000276431		Pseudoger	chr12:30169881-301
ENSG0000(	812	18.38682	chr12:624	TROAP-AS1		lncRNA	chr12:49292631-493
ENSG0000(	812	18.38682	chr12:624	STMN1P1		Pseudoger	chr12:31715342-317
ENSG0000(	812	18.38682	chr12:624	MIR196A2		smallRNA	chr12:53991738-539
ENSG0000(	812	18.38682	chr12:624	ENSG00000288710		protein_c	chr12:49002274-490
ENSG0000(	812	18.38682	chr12:624	ENSG00000280054		TEC	chr12:47728151-477
ENSG0000(	812	18.38682	chr12:624	ENSG00000273049		protein_c	chr12:53985845-540
ENSG0000(	812	18.38682	chr12:624	ADAMTS20	NCGv7	protein_c	chr12:43353866-435
ENSG0000(	812	18.38682	chr12:624	SLC48A1		protein_c	chr12:47753916-477
ENSG0000(	812	18.38682	chr12:624	FIGNL2-DT		lncRNA	chr12:51848223-518
ENSG0000(	812	18.38682	chr12:624	KRT2		protein_c	chr12:52644558-526
ENSG0000(	812	18.38682	chr12:624	COPZ1		protein_c	chr12:54301202-543
ENSG0000(	812	18.38682	chr12:624	VDR		protein_c	chr12:47841537-479

ENSG0000(	812	18.38682	chr12:62<	ENDOU		protein_c	chr12:47709734-477
ENSG0000(	812	18.38682	chr12:62<	RARG		protein_c	chr12:53210567-532
ENSG0000(	812	18.38682	chr12:62<	ENSG00000259937		lncRNA	chr12:33404872-334
ENSG0000(	812	18.38682	chr12:62<	ABCD2		protein_c	chr12:39550033-396
ENSG0000(	812	18.38682	chr12:62<	RPL30P13		Pseudoger	chr12:40068243-400
ENSG0000(	812	18.38682	chr12:62<	SLC38A1		protein_c	chr12:46183063-462
ENSG0000(	812	18.38682	chr12:62<	HOXC5		protein_c	chr12:54033050-540
ENSG0000(	812	18.38682	chr12:62<	OR10AD1		protein_c	chr12:48202339-482
ENSG0000(	812	18.38682	chr12:62<	ENSG00000229899		Pseudoger	chr12:40286289-402
ENSG0000(	812	18.38682	chr12:62<	TUBB8P5		Pseudoger	chr12:38201566-382
ENSG0000(	812	18.38682	chr12:62<	RND1	TAG	protein_c	chr12:48857145-488
ENSG0000(	812	18.38682	chr12:62<	ENSG00000287537		lncRNA	chr12:49536677-495
ENSG0000(	812	18.38682	chr12:62<	ENSG00000275488		Pseudoger	chr12:53574632-535
ENSG0000(	812	18.38682	chr12:62<	ENSG00000288663		lncRNA	chr12:53295492-533
ENSG0000(	812	18.38682	chr12:62<	KRT75		protein_c	chr12:52424070-524
ENSG0000(	812	18.38682	chr12:62<	TUBB8P4		Pseudoger	chr12:34164773-341
ENSG0000(	812	18.38682	chr12:62<	SNORD112		smallRNA	chr12:33362583-333
ENSG0000(	812	18.38682	chr1:1021	RNU7-8P		smallRNA	chr1:77420325-7742
ENSG0000(	812	18.38682	chr12:62<	RNU6-1203P		smallRNA	chr12:48081319-480
ENSG0000(	812	18.38682	chr12:62<	ENSG00000275854		lncRNA	chr12:32736930-327
ENSG0000(	812	18.38682	chr1:1021	RNA5SP20		Pseudoger	chr1:77614869-7761
ENSG0000(	812	18.38682	chr12:62<	NR4A1AS		lncRNA	chr12:52058459-520
ENSG0000(	812	18.38682	chr12:62<	ENSG00000287442		lncRNA	chr12:38155476-381
ENSG0000(	812	18.38682	chr12:62<	ENSG00000275769		lncRNA	chr12:31443792-314
ENSG0000(	806	18.25096	chr12:301	ENSG00000257342		lncRNA	chr12:57694132-577
ENSG0000(	806	18.25096	chr12:301	CNPY2-AS1		lncRNA	chr12:56300075-563
ENSG0000(	806	18.25096	chr12:301	RPS26		protein_c	chr12:56041351-560
ENSG0000(	806	18.25096	chr12:301	RN7SL312P		smallRNA	chr12:57513917-575
ENSG0000(	806	18.25096	chr12:301	OR6C6		protein_c	chr12:55293988-552
ENSG0000(	806	18.25096	chr12:301	SPRYD4		protein_c	chr12:56468578-564
ENSG0000(	806	18.25096	chr12:301	MIR616		smallRNA	chr12:57519163-575
ENSG0000(	806	18.25096	chr12:301	OR9K1P		Pseudoger	chr12:55113077-551
ENSG0000(	806	18.25096	chr12:301	OR6C75		protein_c	chr12:55362975-553
ENSG0000(	806	18.25096	chr12:301	PYM1		protein_c	chr12:55901413-559
ENSG0000(	806	18.25096	chr12:301	ENSG00000257384		Pseudoger	chr12:55732366-557
ENSG0000(	806	18.25096	chr12:301	MUCL1		protein_c	chr12:54830518-548
ENSG0000(	806	18.25096	chr12:301	ENSG00000257390		protein_c	chr12:55757275-558
ENSG0000(	806	18.25096	chr12:301	RNU1-69P		smallRNA	chr12:55874678-558
ENSG0000(	806	18.25096	chr12:301	SNORD59A		smallRNA	chr12:56645027-566
ENSG0000(	806	18.25096	chr12:301	SNORA48		smallRNA	chr12:56861375-568
ENSG0000(	806	18.25096	chr12:301	OR9K2	NCGv7	protein_c	chr12:55126406-551
ENSG0000(	806	18.25096	chr12:301	DDIT3	NCGv7;AC	protein_c	chr12:57516588-575
ENSG0000(	806	18.25096	chr12:301	STAT2		protein_c	chr12:56341597-563
ENSG0000(	806	18.25096	chr12:301	OR10P1		protein_c	chr12:55636892-556
ENSG0000(	806	18.25096	chr12:301	AGAP2-AS1		lncRNA	chr12:57726271-577
ENSG0000(	806	18.25096	chr12:301	ENSG00000273973		lncRNA	chr12:55929170-559
ENSG0000(	806	18.25096	chr12:301	OR6U2P		Pseudoger	chr12:55611623-556
ENSG0000(	806	18.25096	chr12:301	ENSG00000257350		Pseudoger	chr12:55122668-551
ENSG0000(	806	18.25096	chr12:301	OR6C69P		Pseudoger	chr12:55262539-552
ENSG0000(	806	18.25096	chr12:301	PRIM1		protein_c	chr12:56731296-567
ENSG0000(	806	18.25096	chr12:301	ENSG00000273536		Pseudoger	chr12:56840973-568

ENSG0000(	806	18.25096	chr12:301	ENSG00000273890		lncRNA	chr12:56010091-560
ENSG0000(	806	18.25096	chr12:301	ENSG00000144785		protein_c	chr12:56285916-563
ENSG0000(	806	18.25096	chr12:301	INHBC		protein_c	chr12:57434784-574
ENSG0000(	806	18.25096	chr12:301	AC023237.1		smallRNA	chr12:57121662-571
ENSG0000(	806	18.25096	chr12:301	NACA	AC	protein_c	chr12:56712305-567
ENSG0000(	806	18.25096	chr12:301	APOF		protein_c	chr12:56360568-563
ENSG0000(	806	18.25096	chr12:301	PA2G4	AC	protein_c	chr12:56104537-561
ENSG0000(	806	18.25096	chr12:301	DCTN2		protein_c	chr12:57529633-575
ENSG0000(	806	18.25096	chr12:301	OR6C74		protein_c	chr12:55247198-552
ENSG0000(	806	18.25096	chr12:301	MYL6B		protein_c	chr12:56152256-561
ENSG0000(	806	18.25096	chr12:301	MIR1228		smallRNA	chr12:57194504-571
ENSG0000(	806	18.25096	chr12:301	METTL7B		protein_c	chr12:55681736-556
ENSG0000(	806	18.25096	chr12:301	OR6C5P		Pseudoger	chr12:55311835-553
ENSG0000(	806	18.25096	chr12:301	ENSG00000258921		lncRNA	chr12:55638912-556
ENSG0000(	806	18.25096	chr12:301	R3HDM2-DT		lncRNA	chr12:57431116-574
ENSG0000(	806	18.25096	chr12:301	ENSG00000259099		Pseudoger	chr12:56380361-563
ENSG0000(	806	18.25096	chr12:301	LRP1-AS		lncRNA	chr12:57144620-571
ENSG0000(	806	18.25096	chr12:301	OLA1P3		Pseudoger	chr12:55870048-558
ENSG0000(	806	18.25096	chr12:301	GPR182		protein_c	chr12:56994492-569
ENSG0000(	806	18.25096	chr12:301	ZBTB39		protein_c	chr12:56998836-570
ENSG0000(	806	18.25096	chr12:301	TAC3		protein_c	chr12:57010000-570
ENSG0000(	806	18.25096	chr12:301	MYO1A		protein_c	chr12:57028517-570
ENSG0000(	806	18.25096	chr12:301	NEMP1		protein_c	chr12:57055643-570
ENSG0000(	806	18.25096	chr12:301	PSMB3P1		Pseudoger	chr12:55654558-556
ENSG0000(	806	18.25096	chr12:301	OR6C70		protein_c	chr12:55469200-554
ENSG0000(	806	18.25096	chr12:301	NAB2	NCGv7	protein_c	chr12:57089043-570
ENSG0000(	806	18.25096	chr12:301	STAT6	NCGv7	protein_c	chr12:57095408-571
ENSG0000(	806	18.25096	chr12:301	ENSG00000257870		lncRNA	chr12:55009746-550
ENSG0000(	806	18.25096	chr12:301	VDAC1P5		Pseudoger	chr12:54802746-548
ENSG0000(	806	18.25096	chr12:301	ANKRD52		protein_c	chr12:56237807-562
ENSG0000(	806	18.25096	chr12:301	PIP4K2C		protein_c	chr12:57591174-576
ENSG0000(	806	18.25096	chr12:301	MARS1		protein_c	chr12:57475445-575
ENSG0000(	806	18.25096	chr12:301	MBD6	NCGv7	protein_c	chr12:57520710-575
ENSG0000(	806	18.25096	chr12:301	RNF41		protein_c	chr12:56202179-562
ENSG0000(	806	18.25096	chr12:301	ENSG00000258830		protein_c	chr12:57249609-572
ENSG0000(	806	18.25096	chr12:301	ENSG00000257411		protein_c	chr12:56101331-561
ENSG0000(	806	18.25096	chr12:301	TMEM198B		Pseudoger	chr12:55829608-558
ENSG0000(	806	18.25096	chr12:301	ENSG00000258260		lncRNA	chr12:56267793-562
ENSG0000(	806	18.25096	chr12:301	ENSG00000276727		lncRNA	chr12:57229498-572
ENSG0000(	806	18.25096	chr12:301	AC009779.1		smallRNA	chr12:55554995-555
ENSG0000(	806	18.25096	chr12:301	ENSG00000258199		lncRNA	chr12:56162359-561
ENSG0000(	806	18.25096	chr12:301	RN7SL770P		smallRNA	chr12:56195147-561
ENSG0000(	806	18.25096	chr12:301	ENSG00000258311		protein_c	chr12:55716036-557
ENSG0000(	806	18.25096	chr12:301	RNU6-879P		smallRNA	chr12:57415296-574
ENSG0000(	806	18.25096	chr12:301	RNU6-343P		smallRNA	chr12:56588518-565
ENSG0000(	806	18.25096	chr12:301	ENSG00000258317		lncRNA	chr12:56120033-561
ENSG0000(	806	18.25096	chr12:301	ENSG00000258345		lncRNA	chr12:56118968-561
ENSG0000(	806	18.25096	chr12:301	ENSG00000258816		Pseudoger	chr12:56855694-568
ENSG0000(	806	18.25096	chr12:301	OR10AE3P		Pseudoger	chr12:55646195-556
ENSG0000(	806	18.25096	chr12:301	ENSG00000276272		lncRNA	chr12:56411944-564
ENSG0000(	806	18.25096	chr12:301	ENSG00000258554		lncRNA	chr12:55966838-559

ENSG0000(	806	18.25096	chr12:301	ENSG00000258679		Pseudoger	chr12:56822985-568
ENSG0000(	806	18.25096	chr12:301	ENSG00000258763		lncRNA	chr12:55434734-555
ENSG0000(	806	18.25096	chr12:301	NXPH4		protein_c	chr12:57216794-572
ENSG0000(	806	18.25096	chr12:301	CD63-AS1		lncRNA	chr12:55729094-557
ENSG0000(	806	18.25096	chr12:301	Y_RNA		smallRNA	chr12:57227974-572
ENSG0000(	806	18.25096	chr12:301	SHMT2		protein_c	chr12:57229573-572
ENSG0000(	806	18.25096	chr12:301	ESYT1		protein_c	chr12:56118250-561
ENSG0000(	806	18.25096	chr12:301	MYL6B-AS1		lncRNA	chr12:56150796-561
ENSG0000(	806	18.25096	chr12:301	SMARCC2		protein_c	chr12:56162359-561
ENSG0000(	806	18.25096	chr12:301	GSTP1P1		Pseudoger	chr12:55900300-559
ENSG0000(	806	18.25096	chr12:301	RPL41		protein_c	chr12:56116590-561
ENSG0000(	806	18.25096	chr12:301	DTX3		protein_c	chr12:57604622-576
ENSG0000(	806	18.25096	chr12:301	ENSG00000257634		lncRNA	chr12:54682973-546
ENSG0000(	806	18.25096	chr12:301	SARNP		protein_c	chr12:55752463-558
ENSG0000(	806	18.25096	chr12:301	OR6C68		protein_c	chr12:55492378-554
ENSG0000(	806	18.25096	chr12:301	OR6C65		protein_c	chr12:55400529-554
ENSG0000(	806	18.25096	chr12:301	OR6C3		protein_c	chr12:55330043-553
ENSG0000(	806	18.25096	chr12:301	HSPD1P4		Pseudoger	chr12:56511002-565
ENSG0000(	806	18.25096	chr12:301	OR6C1		protein_c	chr12:55314343-553
ENSG0000(	806	18.25096	chr12:301	ENSG00000257553		lncRNA	chr12:56104614-561
ENSG0000(	806	18.25096	chr12:301	NABP2		protein_c	chr12:56222015-562
ENSG0000(	806	18.25096	chr12:301	APONP		Pseudoger	chr12:56376818-563
ENSG0000(	806	18.25096	chr12:301	OR6C72P		Pseudoger	chr12:55283750-552
ENSG0000(	806	18.25096	chr12:301	ENSG00000257509		lncRNA	chr12:55761550-557
ENSG0000(	806	18.25096	chr12:301	ENSG00000274569		Pseudoger	chr12:56413647-564
ENSG0000(	806	18.25096	chr12:301	DCD	AC	protein_c	chr12:54644589-546
ENSG0000(	806	18.25096	chr12:301	ENSG00000257449		lncRNA	chr12:56029649-560
ENSG0000(	806	18.25096	chr12:301	SDR9C7		protein_c	chr12:56923133-569
ENSG0000(	806	18.25096	chr12:301	ENSG00000270458		Pseudoger	chr12:55662337-556
ENSG0000(	806	18.25096	chr12:301	OR6C73P		Pseudoger	chr12:55396340-553
ENSG0000(	806	18.25096	chr12:301	ENSG00000278400		Pseudoger	chr12:56941174-569
ENSG0000(	806	18.25096	chr12:301	ENSG00000278399		Pseudoger	chr12:56981211-569
ENSG0000(	806	18.25096	chr12:301	ENSG00000224713		lncRNA	chr12:57612118-576
ENSG0000(	806	18.25096	chr12:301	MARCHF9		protein_c	chr12:57755103-577
ENSG0000(	806	18.25096	chr12:301	OR6C7P		Pseudoger	chr12:55342841-553
ENSG0000(	806	18.25096	chr12:301	OR10A7	NCGv7	protein_c	chr12:55221025-552
ENSG0000(	806	18.25096	chr12:301	R3HDM2		protein_c	chr12:57253762-574
ENSG0000(	806	18.25096	chr12:301	ENSG00000257740		lncRNA	chr12:56308868-563
ENSG0000(	806	18.25096	chr12:301	STAC3		protein_c	chr12:57243453-572
ENSG0000(	806	18.25096	chr12:301	RDH16		protein_c	chr12:56951431-569
ENSG0000(	806	18.25096	chr12:301	PHC1P1		Pseudoger	chr12:55411727-554
ENSG0000(	806	18.25096	chr12:301	SLC39A5		protein_c	chr12:56230049-562
ENSG0000(	806	18.25096	chr12:301	SUOX		protein_c	chr12:55997180-560
ENSG0000(	806	18.25096	chr12:301	CNPY2		protein_c	chr12:56309842-563
ENSG0000(	806	18.25096	chr12:301	NDUFA4L2		protein_c	chr12:57234903-572
ENSG0000(	806	18.25096	chr12:301	OR6C2		protein_c	chr12:55444069-554
ENSG0000(	806	18.25096	chr12:301	PMEL		protein_c	chr12:55954105-559
ENSG0000(	806	18.25096	chr12:301	OR10U1P		Pseudoger	chr12:55193882-551
ENSG0000(	806	18.25096	chr12:301	OR6C4		protein_c	chr12:55549602-555
ENSG0000(	806	18.25096	chr12:301	OR9R1P		Pseudoger	chr12:55158847-551
ENSG0000(	806	18.25096	chr12:301	OR2AP1		protein_c	chr12:55572468-555

ENSG0000(	806	18.25096	chr12:301	OR6C76		protein_c	chr12:55426254-554
ENSG0000(	806	18.25096	chr12:301	INHBE		protein_c	chr12:57452323-574
ENSG0000(	806	18.25096	chr12:301	KIF5A		protein_c	chr12:57546026-575
ENSG0000(	806	18.25096	chr12:301	OR6C71P		Pseudoger	chr12:55376824-553
ENSG0000(	806	18.25096	chr12:301	ENSG00000287200		lncRNA	chr12:57457596-574
ENSG0000(	806	18.25096	chr12:301	HSD17B6		protein_c	chr12:56752161-567
ENSG0000(	806	18.25096	chr12:301	COQ10A		protein_c	chr12:56266890-562
ENSG0000(	806	18.25096	chr12:301	TIMELESS		protein_c	chr12:56416363-564
ENSG0000(	806	18.25096	chr12:301	ENSG00000285528		protein_c	chr12:56449700-564
ENSG0000(	806	18.25096	chr12:301	PAN2		protein_c	chr12:56316223-563
ENSG0000(	806	18.25096	chr12:301	ZC3H10		protein_c	chr12:56118260-561
ENSG0000(	806	18.25096	chr12:301	RAB5B		protein_c	chr12:55973913-559
ENSG0000(	806	18.25096	chr12:301	NEUROD4		protein_c	chr12:55019974-550
ENSG0000(	806	18.25096	chr12:301	SLC26A10		Pseudoger	chr12:57619527-576
ENSG0000(	806	18.25096	chr12:301	snoU13		smallRNA	chr12:57668944-576
ENSG0000(	806	18.25096	chr12:301	ATP5F1B	NCGv7	protein_c	chr12:56638175-566
ENSG0000(	806	18.25096	chr12:301	OS9	NCGv7	protein_c	chr12:57693841-577
ENSG0000(	806	18.25096	chr12:301	MIP		protein_c	chr12:56449502-564
ENSG0000(	806	18.25096	chr12:301	ENSG00000285133		protein_c	chr12:57517712-575
ENSG0000(	806	18.25096	chr12:301	ENSG00000290754		lncRNA	chr12:55608043-556
ENSG0000(	806	18.25096	chr12:301	TSPAN31	DriverDB\	protein_c	chr12:57738013-577
ENSG0000(	806	18.25096	chr12:301	GLI1	NCGv7;AC	protein_c	chr12:57459785-574
ENSG0000(	806	18.25096	chr12:301	ENSG00000285625		protein_c	chr12:56714612-567
ENSG0000(	806	18.25096	chr12:301	ENSG00000287908		protein_c	chr12:57615280-576
ENSG0000(	806	18.25096	chr12:301	ARHGEF25	NCGv7	protein_c	chr12:57610180-576
ENSG0000(	806	18.25096	chr12:301	ENSG00000289965		lncRNA	chr12:55709985-557
ENSG0000(	806	18.25096	chr12:301	ENSG00000237493		Pseudoger	chr12:55980432-559
ENSG0000(	806	18.25096	chr12:301	RNU7-40P		smallRNA	chr12:56352387-563
ENSG0000(	806	18.25096	chr12:301	ENSG00000287715		lncRNA	chr12:56987734-569
ENSG0000(	806	18.25096	chr12:301	PTGES3		protein_c	chr12:56663341-566
ENSG0000(	806	18.25096	chr12:301	ERBB3	NCGv7;AC	protein_c	chr12:56076799-561
ENSG0000(	806	18.25096	chr12:301	DGKA	NCGv7	protein_c	chr12:55927319-559
ENSG0000(	806	18.25096	chr12:301	OR6C66P		Pseudoger	chr12:55388445-553
ENSG0000(	806	18.25096	chr12:301	IL23A		protein_c	chr12:56334174-563
ENSG0000(	806	18.25096	chr12:301	B4GALNT1		protein_c	chr12:57623409-576
ENSG0000(	806	18.25096	chr12:301	CS		protein_c	chr12:56271699-563
ENSG0000(	806	18.25096	chr12:301	ITGA7		protein_c	chr12:55684568-557
ENSG0000(	806	18.25096	chr12:301	OR6C64P		Pseudoger	chr12:55522593-555
ENSG0000(	806	18.25096	chr12:301	GDF11		protein_c	chr12:55743122-557
ENSG0000(	806	18.25096	chr12:301	IKZF4	DriverDB\	protein_c	chr12:56007659-560
ENSG0000(	806	18.25096	chr12:301	RBMS2		protein_c	chr12:56521820-565
ENSG0000(	806	18.25096	chr12:301	GLS2	NCGv7	protein_c	chr12:56470944-564
ENSG0000(	806	18.25096	chr12:301	CD63		protein_c	chr12:55725323-557
ENSG0000(	806	18.25096	chr12:301	DNAJC14		protein_c	chr12:55820960-558
ENSG0000(	806	18.25096	chr12:301	RPL13AP23		Pseudoger	chr12:57674665-576
ENSG0000(	806	18.25096	chr12:301	BAZ2A	NCGv7	protein_c	chr12:56595596-566
ENSG0000(	806	18.25096	chr12:301	MMP19		protein_c	chr12:55835433-558
ENSG0000(	806	18.25096	chr12:301	ORMDL2		protein_c	chr12:55818041-558
ENSG0000(	806	18.25096	chr12:301	LACRT		protein_c	chr12:54630811-546
ENSG0000(	806	18.25096	chr12:301	CDK2		protein_c	chr12:55966781-559
ENSG0000(	806	18.25096	chr12:301	RN7SL809P		smallRNA	chr12:56670452-566

ENSG0000(	806	18.25096	chr12:301	LRP1	NCGv7	protein_c	chr12:57128483-572
ENSG0000(	806	18.25096	chr12:301	RNU6-594P		smallRNA	chr12:57511064-575
ENSG0000(	806	18.25096	chr12:301	RDH5		protein_c	chr12:55720367-557
ENSG0000(	806	18.25096	chr12:301	AGAP2	AC	protein_c	chr12:57723761-577
ENSG0000(	806	18.25096	chr12:301	MYL6		protein_c	chr12:56158346-561
ENSG0000(	806	18.25096	chr12:301	CDK4	NCGv7;AC	protein_c	chr12:57747727-577
ENSG0000(	806	18.25096	chr12:301	ARHGAP9		protein_c	chr12:57472264-574
ENSG0000(	806	18.25096	chr12:301	ENSG00000290813		lncRNA	chr12:55376742-553
ENSG0000(	806	18.25096	chr12:301	ENSG00000290814		lncRNA	chr12:55385837-553
ENSG0000(	806	18.25096	chr12:301	TESPA1		protein_c	chr12:54948015-549
ENSG0000(	806	18.25096	chr12:301	BLOC1S1		protein_c	chr12:55716038-557
ENSG0000(	805	18.22831	chr5:4231	snoU13		smallRNA	chr5:138115202-138
ENSG0000(	805	18.22831	chr5:4231	MIR3655		smallRNA	chr5:140647844-140
ENSG0000(	805	18.22831	chr5:4231	RNU5B-4P		smallRNA	chr5:139447907-139
ENSG0000(	805	18.22831	chr5:4231	ENSG00000287067		Pseudoger	chr5:137755740-137
ENSG0000(	805	18.22831	chr5:4231	ENSG00000232174		Pseudoger	chr5:138620329-138
ENSG0000(	805	18.22831	chr5:4231	RPL36P11		Pseudoger	chr5:140609085-140
ENSG0000(	805	18.22831	chr5:4231	ENSG00000251387		lncRNA	chr5:139772528-139
ENSG0000(	805	18.22831	chr5:4231	STING1		protein_c	chr5:139475533-139
ENSG0000(	805	18.22831	chr5:4231	ENSG00000289155		lncRNA	chr5:138543464-138
ENSG0000(	805	18.22831	chr5:4231	ENSG00000278946		lncRNA	chr5:140867513-140
ENSG0000(	805	18.22831	chr5:4231	WNT8A		protein_c	chr5:138083990-138
ENSG0000(	805	18.22831	chr5:4231	CTNNA1	NCGv7	protein_c	chr5:138610967-138
ENSG0000(	805	18.22831	chr5:4231	RPS27AP18		Pseudoger	chr5:138266996-138
ENSG0000(	805	18.22831	chr5:4231	SMIM32		protein_c	chr5:136191468-136
ENSG0000(	805	18.22831	chr5:4231	VTRNA1-1		smallRNA	chr5:140711275-140
ENSG0000(	805	18.22831	chr5:4231	PURA		protein_c	chr5:140107777-140
ENSG0000(	805	18.22831	chr5:4231	ANKRD49P3		Pseudoger	chr5:136857628-136
ENSG0000(	805	18.22831	chr5:4231	ENSG00000279028		lncRNA	chr5:140966212-140
ENSG0000(	805	18.22831	chr5:4231	PCDHB1-AS1		lncRNA	chr5:141046260-141
ENSG0000(	805	18.22831	chr5:4231	RPL12P21		Pseudoger	chr5:139035148-139
ENSG0000(	805	18.22831	chr5:4231	ENSG00000223539		Pseudoger	chr5:135742462-135
ENSG0000(	805	18.22831	chr5:4231	SNORD45		smallRNA	chr5:140527352-140
ENSG0000(	805	18.22831	chr5:4231	ENSG00000250515		Pseudoger	chr5:140732290-140
ENSG0000(	805	18.22831	chr5:4231	SMAD5		protein_c	chr5:136132845-136
ENSG0000(	805	18.22831	chr5:4231	ENSG00000253965		lncRNA	chr5:140347868-140
ENSG0000(	805	18.22831	chr5:4231	REEP2		protein_c	chr5:138439057-138
ENSG0000(	805	18.22831	chr5:4231	RNU6-888P		smallRNA	chr5:138123011-138
ENSG0000(	805	18.22831	chr5:4231	PCDHB16		protein_c	chr5:141182387-141
ENSG0000(	805	18.22831	chr5:4231	SMAD5-AS1		lncRNA	chr5:136129507-136
ENSG0000(	805	18.22831	chr5:4231	SNHG4		lncRNA	chr5:139274102-139
ENSG0000(	805	18.22831	chr5:4231	SNORA27		smallRNA	chr5:140579667-140
ENSG0000(	805	18.22831	chr5:4231	AC106753.1		smallRNA	chr5:137980461-137
ENSG0000(	805	18.22831	chr5:4231	PCDHA12		protein_c	chr5:140875302-141
ENSG0000(	805	18.22831	chr5:4231	CDC25C	NCGv7;AC	protein_c	chr5:138285269-138
ENSG0000(	805	18.22831	chr5:4231	FBXL21P		Pseudoger	chr5:135930317-135
ENSG0000(	805	18.22831	chr5:4231	NRG2	NCGv7	protein_c	chr5:139846779-140
ENSG0000(	805	18.22831	chr5:4231	CTNNA1-AS1		lncRNA	chr5:138744434-138
ENSG0000(	805	18.22831	chr5:4231	NCOA4P4		Pseudoger	chr5:139494256-139
ENSG0000(	805	18.22831	chr5:4231	RN7SL682P		smallRNA	chr5:138279094-138
ENSG0000(	805	18.22831	chr5:4231	VTRNA1-3		smallRNA	chr5:140726158-140

ENSG0000(	805	18.22831	chr5:4231	ENSG00000249689		Pseudoger	chr5:139492538-139
ENSG0000(	805	18.22831	chr5:4231	ETF1		protein_c	chr5:138506095-138
ENSG0000(	805	18.22831	chr5:4231	PCDHB12		protein_c	chr5:141208697-141
ENSG0000(	805	18.22831	chr5:4231	PKD2L2	DriverDBv	protein_c	chr5:137887968-137
ENSG0000(	805	18.22831	chr5:4231	MIR5692C1		smallRNA	chr5:135802985-135
ENSG0000(	805	18.22831	chr5:4231	SLC35A4		protein_c	chr5:140564446-140
ENSG0000(	805	18.22831	chr5:4231	PCDHB14		protein_c	chr5:141223343-141
ENSG0000(	805	18.22831	chr5:4231	PCDHB10		protein_c	chr5:141192353-141
ENSG0000(	805	18.22831	chr5:4231	Y_RNA		smallRNA	chr5:140019492-140
ENSG0000(	805	18.22831	chr5:4231	PCDHB8	NCGv7	protein_c	chr5:141177790-141
ENSG0000(	805	18.22831	chr5:4231	RNA5SP194		smallRNA	chr5:139012329-139
ENSG0000(	805	18.22831	chr5:4231	ENSG00000253927		lncRNA	chr5:135900353-135
ENSG0000(	805	18.22831	chr5:4231	WDR55		protein_c	chr5:140664868-140
ENSG0000(	805	18.22831	chr5:4231	PCDHA11	NCGv7	protein_c	chr5:140868183-141
ENSG0000(	805	18.22831	chr5:4231	ANKHD1-DT		lncRNA	chr5:140370891-140
ENSG0000(	805	18.22831	chr5:4231	CYSTM1	DriverDBv	protein_c	chr5:140175156-140
ENSG0000(	805	18.22831	chr5:4231	CXXC5-AS1		lncRNA	chr5:139648999-139
ENSG0000(	805	18.22831	chr5:4231	ENSG00000249593		lncRNA	chr5:139012647-139
ENSG0000(	805	18.22831	chr5:4231	RPL7P19		Pseudoger	chr5:138473744-138
ENSG0000(	805	18.22831	chr5:4231	ENSG00000288892		lncRNA	chr5:141039993-141
ENSG0000(	805	18.22831	chr5:4231	AC011431.2		smallRNA	chr5:135801229-135
ENSG0000(	805	18.22831	chr5:4231	VTRNA1-2		smallRNA	chr5:140718925-140
ENSG0000(	805	18.22831	chr5:4231	RNU6-572P		smallRNA	chr5:139221758-139
ENSG0000(	805	18.22831	chr5:4231	ENSG00000250692		lncRNA	chr5:139848290-139
ENSG0000(	805	18.22831	chr5:4231	PCDHA14		Pseudoger	chr5:140861224-140
ENSG0000(	805	18.22831	chr5:4231	snoU13		smallRNA	chr5:138623479-138
ENSG0000(	805	18.22831	chr5:4231	RN7SL867P		smallRNA	chr5:138764220-138
ENSG0000(	805	18.22831	chr5:4231	TGFBI		protein_c	chr5:136028988-136
ENSG0000(	805	18.22831	chr5:4231	SNORA74		smallRNA	chr5:139276180-139
ENSG0000(	805	18.22831	chr5:4231	ENSG00000278907		lncRNA	chr5:140849105-140
ENSG0000(	805	18.22831	chr5:4231	ENSG00000271792		lncRNA	chr5:139775305-139
ENSG0000(	805	18.22831	chr5:4231	PCDHA6		protein_c	chr5:140827978-141
ENSG0000(	805	18.22831	chr5:4231	PCDHB4		protein_c	chr5:141121818-141
ENSG0000(	805	18.22831	chr5:4231	ENSG00000249803		lncRNA	chr5:136466645-136
ENSG0000(	805	18.22831	chr5:4231	AC114296.1		smallRNA	chr5:135804505-135
ENSG0000(	805	18.22831	chr5:4231	ENSG00000278936		lncRNA	chr5:141183401-141
ENSG0000(	805	18.22831	chr5:4231	ENSG00000280336		lncRNA	chr5:141168231-141
ENSG0000(	805	18.22831	chr5:4231	MIR874		smallRNA	chr5:137647572-137
ENSG0000(	805	18.22831	chr5:4231	SNORA74A		smallRNA	chr5:139278781-139
ENSG0000(	805	18.22831	chr5:4231	HAUS1P1		Pseudoger	chr5:140581504-140
ENSG0000(	805	18.22831	chr5:4231	ENSG00000272742		lncRNA	chr5:139364677-139
ENSG0000(	805	18.22831	chr5:4231	ENSG00000290893		lncRNA	chr5:141155996-141
ENSG0000(	805	18.22831	chr5:4231	AC005609.1		protein_c	chr5:140860756-140
ENSG0000(	805	18.22831	chr5:4231	TRPC7	NCGv7	protein_c	chr5:136212745-136
ENSG0000(	805	18.22831	chr5:4231	ENSG00000254239		lncRNA	chr5:135897637-135
ENSG0000(	805	18.22831	chr5:4231	ENSG00000278901		lncRNA	chr5:140878073-140
ENSG0000(	805	18.22831	chr5:4231	EGR1	NCGv7	protein_c	chr5:138465479-138
ENSG0000(	805	18.22831	chr5:4231	ENSG00000277795		Pseudoger	chr5:141259235-141
ENSG0000(	805	18.22831	chr5:4231	SMIM33		protein_c	chr5:139471004-139
ENSG0000(	805	18.22831	chr5:4231	KDM3B	IntOGen-I	protein_c	chr5:138352685-138
ENSG0000(	805	18.22831	chr5:4231	MYOT		protein_c	chr5:137867858-137

ENSG0000(	805	18.22831	chr5:4231	ENSG00000254363		lncRNA	chr5:140157319-140
ENSG0000(	805	18.22831	chr5:4231	PAIP2		protein_c	chr5:139341587-139
ENSG0000(	805	18.22831	chr5:4231	SIL1		protein_c	chr5:138946724-139
ENSG0000(	805	18.22831	chr5:4231	PSD2-AS1		lncRNA	chr5:139740951-139
ENSG0000(	805	18.22831	chr5:4231	FAM53C		protein_c	chr5:138331935-138
ENSG0000(	805	18.22831	chr5:4231	ENSG00000278915		lncRNA	chr5:140875346-140
ENSG0000(	805	18.22831	chr5:4231	PCDHB6	NCGv7	protein_c	chr5:141150057-141
ENSG0000(	805	18.22831	chr5:4231	ENSG00000270697		lncRNA	chr5:137761546-137
ENSG0000(	805	18.22831	chr5:4231	PCDHB11		protein_c	chr5:141199610-141
ENSG0000(	805	18.22831	chr5:4231	CD14		protein_c	chr5:140631728-140
ENSG0000(	805	18.22831	chr5:4231	CXXC5	NCGv7	protein_c	chr5:139647299-139
ENSG0000(	805	18.22831	chr5:4231	SNORD63		smallRNA	chr5:138558970-138
ENSG0000(	805	18.22831	chr5:4231	HARS1		protein_c	chr5:140673035-140
ENSG0000(	805	18.22831	chr5:4231	Y_RNA		smallRNA	chr5:139554900-139
ENSG0000(	805	18.22831	chr5:4231	ENSG00000279558		lncRNA	chr5:141078076-141
ENSG0000(	805	18.22831	chr5:4231	PCDHB2		protein_c	chr5:141094607-141
ENSG0000(	805	18.22831	chr5:4231	ENSG00000272255		lncRNA	chr5:139644460-139
ENSG0000(	805	18.22831	chr5:4231	PCDHB1	NCGv7	protein_c	chr5:141051374-141
ENSG0000(	805	18.22831	chr5:4231	ENSG00000248106		Pseudoger	chr5:140764110-140
ENSG0000(	805	18.22831	chr5:4231	HNRNPA0		protein_c	chr5:137745651-137
ENSG0000(	805	18.22831	chr5:4231	HARS2		protein_c	chr5:140691430-140
ENSG0000(	805	18.22831	chr5:4231	PCDHB17P		Pseudoger	chr5:141155996-141
ENSG0000(	805	18.22831	chr5:4231	DNAJC18		protein_c	chr5:139408588-139
ENSG0000(	805	18.22831	chr5:4231	ENSG00000279472		lncRNA	chr5:141177849-141
ENSG0000(	805	18.22831	chr5:4231	NME5	DriverDB\	protein_c	chr5:138115175-138
ENSG0000(	805	18.22831	chr5:4231	BRD8	NCGv7	protein_c	chr5:138139770-138
ENSG0000(	805	18.22831	chr5:4231	PCDHB9		protein_c	chr5:141187127-141
ENSG0000(	805	18.22831	chr5:4231	ENSG00000283155		lncRNA	chr5:140562443-140
ENSG0000(	805	18.22831	chr5:4231	H3P25		Pseudoger	chr5:139612002-139
ENSG0000(	805	18.22831	chr5:4231	KIF20A		protein_c	chr5:138178719-138
ENSG0000(	805	18.22831	chr5:4231	PCDHB18P		lncRNA	chr5:141234333-141
ENSG0000(	805	18.22831	chr5:4231	PCDHA10	NCGv7	protein_c	chr5:140855883-141
ENSG0000(	805	18.22831	chr5:4231	ENSG00000289267		lncRNA	chr5:138463990-138
ENSG0000(	805	18.22831	chr5:4231	ENSG00000279375		lncRNA	chr5:141191599-141
ENSG0000(	805	18.22831	chr5:4231	ENSG00000272154		lncRNA	chr5:141100242-141
ENSG0000(	805	18.22831	chr5:4231	SPOCK1		protein_c	chr5:136975298-137
ENSG0000(	805	18.22831	chr5:4231	PCDHA3		protein_c	chr5:140801028-141
ENSG0000(	805	18.22831	chr5:4231	KLHL3	DriverDB\	protein_c	chr5:137617500-137
ENSG0000(	805	18.22831	chr5:4231	UBE2D2	IntOGen-I	protein_c	chr5:139526431-139
ENSG0000(	805	18.22831	chr5:4231	PCDHA13	NCGv7	protein_c	chr5:140882124-141
ENSG0000(	805	18.22831	chr5:4231	ENSG00000250378		lncRNA	chr5:135812667-135
ENSG0000(	805	18.22831	chr5:4231	ENSG00000280029		lncRNA	chr5:141136683-141
ENSG0000(	805	18.22831	chr5:4231	NDUFA2	DriverDB\	protein_c	chr5:140638740-140
ENSG0000(	805	18.22831	chr5:4231	CDC23	NCGv7	protein_c	chr5:138187650-138
ENSG0000(	805	18.22831	chr5:4231	ANKHD1		protein_c	chr5:140401814-140
ENSG0000(	805	18.22831	chr5:4231	HNRNPA1P13		Pseudoger	chr5:136428892-136
ENSG0000(	805	18.22831	chr5:4231	MALINC1		lncRNA	chr5:140071312-140
ENSG0000(	805	18.22831	chr5:4231	ENSG00000279983		protein_c	chr5:141223372-141
ENSG0000(	805	18.22831	chr5:4231	SRA1		protein_c	chr5:140537340-140
ENSG0000(	805	18.22831	chr5:4231	PROB1		protein_c	chr5:139390592-139
ENSG0000(	805	18.22831	chr5:4231	ANKHD1-EIF4EBP3		protein_c	chr5:140401908-140

ENSG0000(	805	18.22831	chr5:4231	RNA5SP193		Pseudoger	chr5:136969200-136
ENSG0000(	805	18.22831	chr5:4231	SNORD63		smallRNA	chr5:138561043-138
ENSG0000(	805	18.22831	chr5:4231	MATR3		protein_c	chr5:139273752-139
ENSG0000(	805	18.22831	chr5:4231	RNU4-14P		smallRNA	chr5:140397323-140
ENSG0000(	805	18.22831	chr5:4231	AC138517.1		protein_c	chr5:139472523-139
ENSG0000(	805	18.22831	chr5:4231	SLC23A1	DriverDBv	protein_c	chr5:139367196-139
ENSG0000(	805	18.22831	chr5:4231	ENSG00000250284		lncRNA	chr5:136734830-136
ENSG0000(	805	18.22831	chr5:4231	VTRNA2-1		smallRNA	chr5:136080470-136
ENSG0000(	805	18.22831	chr5:4231	FAM13B-AS1		lncRNA	chr5:138032774-138
ENSG0000(	805	18.22831	chr5:4231	ENSG00000279726		lncRNA	chr5:140785698-140
ENSG0000(	805	18.22831	chr5:4231	RNU6-1148P		smallRNA	chr5:138069355-138
ENSG0000(	805	18.22831	chr5:4231	MZB1		protein_c	chr5:139387467-139
ENSG0000(	805	18.22831	chr5:4231	ENSG00000250260		lncRNA	chr5:138039199-138
ENSG0000(	805	18.22831	chr5:4231	SPATA24		protein_c	chr5:139396563-139
ENSG0000(	805	18.22831	chr5:4231	RN7SKP64		smallRNA	chr5:139333038-139
ENSG0000(	805	18.22831	chr5:4231	PKD2L2-DT		lncRNA	chr5:137809780-137
ENSG0000(	805	18.22831	chr5:4231	HSPA9		protein_c	chr5:138553756-138
ENSG0000(	805	18.22831	chr5:4231	TRPC7-AS2		lncRNA	chr5:136303757-136
ENSG0000(	805	18.22831	chr5:4231	PCDHAC1		protein_c	chr5:140926299-141
ENSG0000(	805	18.22831	chr5:4231	AC009009.1		smallRNA	chr5:135713119-135
ENSG0000(	805	18.22831	chr5:4231	SLC4A9	DriverDBv	protein_c	chr5:140360194-140
ENSG0000(	805	18.22831	chr5:4231	APBB3		protein_c	chr5:140558268-140
ENSG0000(	805	18.22831	chr5:4231	GFRA3		protein_c	chr5:138252380-138
ENSG0000(	805	18.22831	chr5:4231	IK		protein_c	chr5:140647058-140
ENSG0000(	805	18.22831	chr5:4231	IL9		protein_c	chr5:135892246-135
ENSG0000(	805	18.22831	chr5:4231	DND1		protein_c	chr5:140670794-140
ENSG0000(	805	18.22831	chr5:4231	SLC25A48		protein_c	chr5:135476913-135
ENSG0000(	805	18.22831	chr5:4231	PCDHAC2	NCGv7	protein_c	chr5:140966470-141
ENSG0000(	805	18.22831	chr5:4231	ENSG00000249971		lncRNA	chr5:138347027-138
ENSG0000(	805	18.22831	chr5:4231	LECT2		protein_c	chr5:135922279-135
ENSG0000(	805	18.22831	chr5:4231	PCDHB19P		Pseudoger	chr5:141239923-141
ENSG0000(	805	18.22831	chr5:4231	FAM13B		protein_c	chr5:137937960-138
ENSG0000(	805	18.22831	chr5:4231	PCDHB3		protein_c	chr5:141100473-141
ENSG0000(	805	18.22831	chr5:4231	NPY6R		Pseudoger	chr5:137801193-137
ENSG0000(	805	18.22831	chr5:4231	PCDHB5		protein_c	chr5:141135206-141
ENSG0000(	805	18.22831	chr5:4231	PCDHB7		protein_c	chr5:141172644-141
ENSG0000(	805	18.22831	chr5:4231	EIF4EBP3		protein_c	chr5:140547662-140
ENSG0000(	805	18.22831	chr5:4231	ENSG00000279068		lncRNA	chr5:141182409-141
ENSG0000(	805	18.22831	chr5:4231	PCDHB15		protein_c	chr5:141245395-141
ENSG0000(	805	18.22831	chr5:4231	RNA5SP195		Pseudoger	chr5:139299852-139
ENSG0000(	805	18.22831	chr5:4231	AC106775.1		smallRNA	chr5:137533454-137
ENSG0000(	805	18.22831	chr5:4231	ENSG00000290895		lncRNA	chr5:141240121-141
ENSG0000(	805	18.22831	chr5:4231	ENSG00000289752		Pseudoger	chr5:141087470-141
ENSG0000(	805	18.22831	chr5:4231	TMCO6		protein_c	chr5:140639435-140
ENSG0000(	805	18.22831	chr5:4231	ENSG00000283602		Pseudoger	chr5:140569962-140
ENSG0000(	805	18.22831	chr5:4231	PCDHA8		protein_c	chr5:140841187-141
ENSG0000(	805	18.22831	chr5:4231	TRPC7-AS1		lncRNA	chr5:136214048-136
ENSG0000(	805	18.22831	chr5:4231	ZMAT2		protein_c	chr5:140698680-140
ENSG0000(	805	18.22831	chr5:4231	LRRTM2		protein_c	chr5:138868921-138
ENSG0000(	805	18.22831	chr5:4231	PSD2		protein_c	chr5:139795808-139
ENSG0000(	805	18.22831	chr5:4231	PCDHB18P		Pseudoger	chr5:141234551-141

ENSG0000(	805	18.22831	chr5:4231	PFDN1		protein_c	chr5:140245035-140
ENSG0000(	805	18.22831	chr5:4231	PCDHA1		protein_c	chr5:140786136-141
ENSG0000(	805	18.22831	chr5:4231	PCDHA2		protein_c	chr5:140794852-141
ENSG0000(	805	18.22831	chr5:4231	AC011379.1		smallRNA	chr5:140195702-140
ENSG0000(	805	18.22831	chr5:4231	PCDHA4		protein_c	chr5:140807074-141
ENSG0000(	805	18.22831	chr5:4231	PCDHA5		protein_c	chr5:140821604-141
ENSG0000(	805	18.22831	chr5:4231	MATR3		protein_c	chr5:139293674-139
ENSG0000(	805	18.22831	chr5:4231	PCDHB13		protein_c	chr5:141213919-141
ENSG0000(	805	18.22831	chr5:4231	IGIP		protein_c	chr5:140125937-140
ENSG0000(	805	18.22831	chr5:4231	ENSG00000279240		lncRNA	chr5:136376348-136
ENSG0000(	805	18.22831	chr5:4231	ENSG00000286403		lncRNA	chr5:139503242-139
ENSG0000(	805	18.22831	chr5:4231	RNU6-460P		smallRNA	chr5:138116573-138
ENSG0000(	805	18.22831	chr5:4231	ENSG00000250069		lncRNA	chr5:140200163-140
ENSG0000(	805	18.22831	chr5:4231	ENSG00000272108		lncRNA	chr5:141118680-141
ENSG0000(	805	18.22831	chr5:4231	PCDHA9		protein_c	chr5:140847772-141
ENSG0000(	805	18.22831	chr5:4231	HBEGF		protein_c	chr5:140332843-140
ENSG0000(	799	18.09245	chr8:980	AC011626.1		smallRNA	chr8:121069388-121
ENSG0000(	799	18.09245	chr8:980	ENSG00000272502		lncRNA	chr8:120812219-120
ENSG0000(	799	18.09245	chr8:980	SNORA32		smallRNA	chr8:119388348-119
ENSG0000(	799	18.09245	chr8:980	DSCC1	DriverDB\	protein_c	chr8:119833976-119
ENSG0000(	799	18.09245	chr8:980	EXT1	NCGv7;AC	protein_c	chr8:117794490-118
ENSG0000(	799	18.09245	chr8:980	ENSG00000254037		Pseudoger	chr8:119480279-119
ENSG0000(	799	18.09245	chr8:980	CYCSP23		Pseudoger	chr8:119618162-119
ENSG0000(	799	18.09245	chr8:980	SAMD12		protein_c	chr8:118189455-118
ENSG0000(	799	18.09245	chr8:980	NCAPGP1		Pseudoger	chr8:120635558-120
ENSG0000(	799	18.09245	chr8:980	ENSG00000254247		Pseudoger	chr8:118982579-118
ENSG0000(	799	18.09245	chr8:980	RNA5SP277		Pseudoger	chr8:119891132-119
ENSG0000(	799	18.09245	chr8:980	RPL35AP19		Pseudoger	chr8:121380137-121
ENSG0000(	799	18.09245	chr8:980	RNU6-12P		smallRNA	chr8:118976513-118
ENSG0000(	799	18.09245	chr8:980	RN7SKP153		smallRNA	chr8:119711830-119
ENSG0000(	799	18.09245	chr8:980	TNFRSF11I	DriverDB\	protein_c	chr8:118923557-118
ENSG0000(	799	18.09245	chr8:980	ENSG00000253619		lncRNA	chr8:120913065-121
ENSG0000(	799	18.09245	chr8:980	AC027238.1		smallRNA	chr8:121186758-121
ENSG0000(	799	18.09245	chr8:980	MAL2-AS1		lncRNA	chr8:119214625-119
ENSG0000(	799	18.09245	chr8:980	HAS2		protein_c	chr8:121612116-121
ENSG0000(	799	18.09245	chr8:980	ENSG00000254278		lncRNA	chr8:119062942-119
ENSG0000(	799	18.09245	chr8:980	ENPP2		protein_c	chr8:119557086-119
ENSG0000(	799	18.09245	chr8:980	SAMD12-AS1		lncRNA	chr8:118620498-118
ENSG0000(	799	18.09245	chr8:980	COLEC10		protein_c	chr8:118995452-119
ENSG0000(	799	18.09245	chr8:980	ENSG00000225885		lncRNA	chr8:118282139-118
ENSG0000(	799	18.09245	chr8:980	RN7SL396P		smallRNA	chr8:119862662-119
ENSG0000(	799	18.09245	chr8:980	ENSG00000286362		lncRNA	chr8:119832875-119
ENSG0000(	799	18.09245	chr8:980	ENSG00000248318		lncRNA	chr8:120761253-120
ENSG0000(	799	18.09245	chr8:980	MRPL13	DriverDB\	protein_c	chr8:120380761-120
ENSG0000(	799	18.09245	chr8:980	ENSG00000271219		Pseudoger	chr8:121113358-121
ENSG0000(	799	18.09245	chr8:980	MTBP	DriverDB\	protein_c	chr8:120445400-120
ENSG0000(	799	18.09245	chr8:980	ENSG00000279347		TEC	chr8:119838736-119
ENSG0000(	799	18.09245	chr8:980	SNTB1	NCGv7	protein_c	chr8:120535745-120
ENSG0000(	799	18.09245	chr8:980	MAL2	DriverDB\	protein_c	chr8:119165034-119
ENSG0000(	799	18.09245	chr8:980	HAS2-AS1		lncRNA	chr8:121639293-121
ENSG0000(	799	18.09245	chr8:980	LINC02855		lncRNA	chr8:121668934-121

ENSG0000(	799	18.09245	chr8:980	AC022909.1		smallRNA	chr8:121542897-121
ENSG0000(	799	18.09245	chr8:980	DEPTOR	DriverDB	protein_c	chr8:119873717-120
ENSG0000(	799	18.09245	chr8:980	ENSG00000286282		lncRNA	chr8:119350291-119
ENSG0000(	799	18.09245	chr8:980	TAF2	DriverDB	protein_c	chr8:119730774-119
ENSG0000(	799	18.09245	chr8:980	CCN3	AC	protein_c	chr8:119416446-119
ENSG0000(	799	18.09245	chr8:980	RPS26P35		Pseudoger	chr8:118761856-118
ENSG0000(	799	18.09245	chr8:980	DEPTOR-AS1		lncRNA	chr8:119867419-119
ENSG0000(	799	18.09245	chr8:980	ENSG00000254343		lncRNA	chr8:120052180-120
ENSG0000(	799	18.09245	chr8:980	ENSG00000253398		lncRNA	chr8:119419910-119
ENSG0000(	799	18.09245	chr8:980	COL14A1	NCGv7	protein_c	chr8:120059780-120
ENSG0000(	791	17.9113	chr8:980	SQLE	DriverDB	protein_c	chr8:124998497-125
ENSG0000(	791	17.9113	chr8:980	LINC00824		lncRNA	chr8:128405269-128
ENSG0000(	791	17.9113	chr8:980	ENSG00000286034		lncRNA	chr8:128323131-128
ENSG0000(	791	17.9113	chr8:980	ENSG00000287785		lncRNA	chr8:129241220-129
ENSG0000(	791	17.9113	chr8:980	ENSG00000237586		Pseudoger	chr8:124301315-124
ENSG0000(	791	17.9113	chr8:980	ZHX1		protein_c	chr8:123248451-123
ENSG0000(	791	17.9113	chr8:980	CASC8		lncRNA	chr8:127289808-127
ENSG0000(	791	17.9113	chr8:980	RNY4P5		smallRNA	chr8:123044717-123
ENSG0000(	791	17.9113	chr8:980	SOD1P3		Pseudoger	chr8:125951861-125
ENSG0000(	791	17.9113	chr8:980	ENSG00000287781		lncRNA	chr8:127253213-127
ENSG0000(	791	17.9113	chr8:980	WASHC5	DriverDB	protein_c	chr8:125024260-125
ENSG0000(	791	17.9113	chr8:980	ENSG00000286010		lncRNA	chr8:127663280-127
ENSG0000(	791	17.9113	chr8:980	ENSG00000263443		lncRNA	chr8:122700269-122
ENSG0000(	791	17.9113	chr8:980	ENSG00000254431		lncRNA	chr8:125348196-125
ENSG0000(	791	17.9113	chr8:980	TMEM65	DriverDB	protein_c	chr8:124306189-124
ENSG0000(	791	17.9113	chr8:980	ENSG00000255313		lncRNA	chr8:124936328-124
ENSG0000(	791	17.9113	chr8:980	RFPL4AP5		Pseudoger	chr8:126174186-126
ENSG0000(	791	17.9113	chr8:980	FER1L6-AS2		lncRNA	chr8:124044395-124
ENSG0000(	791	17.9113	chr8:980	RN7SL590P		smallRNA	chr8:125444565-125
ENSG0000(	791	17.9113	chr8:980	ENSG00000253530		lncRNA	chr8:126506326-126
ENSG0000(	791	17.9113	chr8:980	ENSG00000253410		Pseudoger	chr8:123192875-123
ENSG0000(	791	17.9113	chr8:980	MIR4663		smallRNA	chr8:123215788-123
ENSG0000(	791	17.9113	chr8:980	CCAT2		lncRNA	chr8:127400399-127
ENSG0000(	791	17.9113	chr8:980	ENSG00000253220		lncRNA	chr8:126497385-126
ENSG0000(	791	17.9113	chr8:980	PVT1	TAG;AC	lncRNA	chr8:127794526-128
ENSG0000(	791	17.9113	chr8:980	MRS2P1		Pseudoger	chr8:124920428-124
ENSG0000(	791	17.9113	chr8:980	ENSG00000254249		lncRNA	chr8:124036010-124
ENSG0000(	791	17.9113	chr8:980	ENSG00000255325		lncRNA	chr8:122485515-122
ENSG0000(	791	17.9113	chr8:980	CASC19		lncRNA	chr8:127072694-127
ENSG0000(	791	17.9113	chr8:980	ENSG00000253372		lncRNA	chr8:122807746-122
ENSG0000(	791	17.9113	chr8:980	ENSG00000253111		lncRNA	chr8:125466939-125
ENSG0000(	791	17.9113	chr8:980	LINC01151		lncRNA	chr8:122485194-122
ENSG0000(	791	17.9113	chr8:980	ENSG00000253470		lncRNA	chr8:125749055-125
ENSG0000(	791	17.9113	chr8:980	RNU1-106P		smallRNA	chr8:127999131-127
ENSG0000(	791	17.9113	chr8:980	POU5F1B		protein_c	chr8:127322183-127
ENSG0000(	791	17.9113	chr8:980	MRPS36P3		Pseudoger	chr8:122091480-122
ENSG0000(	791	17.9113	chr8:980	UBA52P5		Pseudoger	chr8:123236852-123
ENSG0000(	791	17.9113	chr8:980	ENSG00000254000		lncRNA	chr8:123924759-123
ENSG0000(	791	17.9113	chr8:980	FER1L6		protein_c	chr8:123851987-124
ENSG0000(	791	17.9113	chr8:980	ENSG00000286266		lncRNA	chr8:127578473-127
ENSG0000(	791	17.9113	chr8:980	ENSG00000214803		lncRNA	chr8:124192671-124

ENSG0000(	791	17.9113	chr8:980!	MIR1205		smallRNA	chr8:127960633-127
ENSG0000(	791	17.9113	chr8:980!	HMGB1P19		Pseudoger	chr8:123123106-123
ENSG0000(	791	17.9113	chr8:980!	ENSG00000255491		lncRNA	chr8:124811042-124
ENSG0000(	791	17.9113	chr8:980!	LINC02964		lncRNA	chr8:125648327-126
ENSG0000(	791	17.9113	chr8:980!	WASHC5-AS1		lncRNA	chr8:125040684-125
ENSG0000(	791	17.9113	chr8:980!	TBC1D31		protein_c	chr8:123041968-123
ENSG0000(	791	17.9113	chr8:980!	snoU13		smallRNA	chr8:123266642-123
ENSG0000(	791	17.9113	chr8:980!	NSMCE2	NCGv7	protein_c	chr8:125091679-125
ENSG0000(	791	17.9113	chr8:980!	ANXA13	DriverDB\	protein_c	chr8:123680794-123
ENSG0000(	791	17.9113	chr8:980!	FBXO32	NCGv7	protein_c	chr8:123497889-123
ENSG0000(	791	17.9113	chr8:980!	ENSG00000253225		Pseudoger	chr8:129250967-129
ENSG0000(	791	17.9113	chr8:980!	NTAQ1		protein_c	chr8:123416726-123
ENSG0000(	791	17.9113	chr8:980!	ENSG00000272043		lncRNA	chr8:122780302-122
ENSG0000(	791	17.9113	chr8:980!	FAM83A-AS1		lncRNA	chr8:123193645-123
ENSG0000(	791	17.9113	chr8:980!	ZHX2	NCGv7	protein_c	chr8:122781655-122
ENSG0000(	791	17.9113	chr8:980!	SMILR		lncRNA	chr8:122414332-122
ENSG0000(	791	17.9113	chr8:980!	RNU6-869P		smallRNA	chr8:126504763-126
ENSG0000(	791	17.9113	chr8:980!	AC090573.1		smallRNA	chr8:126141676-126
ENSG0000(	791	17.9113	chr8:980!	ENSG00000253841		Pseudoger	chr8:126073046-126
ENSG0000(	791	17.9113	chr8:980!	RNU11-4P		smallRNA	chr8:126671522-126
ENSG0000(	791	17.9113	chr8:980!	ATAD2	NCGv7	protein_c	chr8:123319850-123
ENSG0000(	791	17.9113	chr8:980!	PCAT1		lncRNA	chr8:126556323-127
ENSG0000(	791	17.9113	chr8:980!	DUTP2		Pseudoger	chr8:123352181-123
ENSG0000(	791	17.9113	chr8:980!	ENSG00000287657		lncRNA	chr8:123157729-123
ENSG0000(	791	17.9113	chr8:980!	MIR1207		smallRNA	chr8:128049152-128
ENSG0000(	791	17.9113	chr8:980!	ZHX1-C8or	DriverDB\	protein_c	chr8:123226189-123
ENSG0000(	791	17.9113	chr8:980!	FAM83A	DriverDB\	protein_c	chr8:123178960-123
ENSG0000(	791	17.9113	chr8:980!	MIR1208		smallRNA	chr8:128150116-128
ENSG0000(	791	17.9113	chr8:980!	SQLE-DT		lncRNA	chr8:124996985-124
ENSG0000(	791	17.9113	chr8:980!	AC068570.1		smallRNA	chr8:128819794-128
ENSG0000(	791	17.9113	chr8:980!	RNF139	DriverDB\	protein_c	chr8:124474880-124
ENSG0000(	791	17.9113	chr8:980!	snoU13		smallRNA	chr8:122671291-122
ENSG0000(	791	17.9113	chr8:980!	LINC02912		lncRNA	chr8:127946559-127
ENSG0000(	791	17.9113	chr8:980!	RNU6-628P		smallRNA	chr8:123262843-123
ENSG0000(	791	17.9113	chr8:980!	ENSG00000254227		lncRNA	chr8:125998994-126
ENSG0000(	791	17.9113	chr8:980!	IMPDH1P6		Pseudoger	chr8:123400582-123
ENSG0000(	791	17.9113	chr8:980!	RNU6-756P		smallRNA	chr8:124260646-124
ENSG0000(	791	17.9113	chr8:980!	RN7SKP155		smallRNA	chr8:123588759-123
ENSG0000(	791	17.9113	chr8:980!	ENSG00000253286		lncRNA	chr8:123614219-123
ENSG0000(	791	17.9113	chr8:980!	RNU4-25P		smallRNA	chr8:128010382-128
ENSG0000(	791	17.9113	chr8:980!	TATDN1		protein_c	chr8:124488485-124
ENSG0000(	791	17.9113	chr8:980!	ENSG00000253573		lncRNA	chr8:126766196-126
ENSG0000(	791	17.9113	chr8:980!	NDUFB9	NCGv7	protein_c	chr8:124539101-124
ENSG0000(	791	17.9113	chr8:980!	MIR4662B		smallRNA	chr8:124821978-124
ENSG0000(	791	17.9113	chr8:980!	DERL1	NCGv7	protein_c	chr8:123013170-123
ENSG0000(	791	17.9113	chr8:980!	ENSG00000286955		lncRNA	chr8:124940041-124
ENSG0000(	791	17.9113	chr8:980!	ENSG00000271509		Pseudoger	chr8:127292233-127
ENSG0000(	791	17.9113	chr8:980!	RN7SKP226		smallRNA	chr8:128220504-128
ENSG0000(	791	17.9113	chr8:980!	MIR548AA1		smallRNA	chr8:123348034-123
ENSG0000(	791	17.9113	chr8:980!	ENSG00000254303		lncRNA	chr8:121954640-122
ENSG0000(	791	17.9113	chr8:980!	MYC	NCGv7;AC	protein_c	chr8:127735434-127

ENSG0000(	791	17.9113	chr8:980	CASC11		lncRNA	chr8:127686343-127
ENSG0000(	791	17.9113	chr8:980	ENSG00000259631		lncRNA	chr8:122977026-122
ENSG0000(	791	17.9113	chr8:980	ENSG00000250727		Pseudoger	chr8:124141888-124
ENSG0000(	791	17.9113	chr8:980	ENSG00000224722		Pseudoger	chr8:127086263-127
ENSG0000(	791	17.9113	chr8:980	CDK5P1		Pseudoger	chr8:122769274-122
ENSG0000(	791	17.9113	chr8:980	ENSG00000253336		Pseudoger	chr8:123288355-123
ENSG0000(	791	17.9113	chr8:980	C8orf76	DriverDB	protein_c	chr8:123219967-123
ENSG0000(	791	17.9113	chr8:980	ENSG00000253227		lncRNA	chr8:124271667-124
ENSG0000(	791	17.9113	chr8:980	ENSG00000253543		Pseudoger	chr8:126582631-126
ENSG0000(	791	17.9113	chr8:980	ENSG00000278275		Pseudoger	chr8:128009551-128
ENSG0000(	791	17.9113	chr8:980	RNF139-DT		lncRNA	chr8:124462485-124
ENSG0000(	791	17.9113	chr8:980	ENSG00000253106		lncRNA	chr8:124488510-124
ENSG0000(	791	17.9113	chr8:980	LINC00964		lncRNA	chr8:124823702-124
ENSG0000(	791	17.9113	chr8:980	ARF1P3		Pseudoger	chr8:124151923-124
ENSG0000(	791	17.9113	chr8:980	ENSG00000272384		lncRNA	chr8:122779971-122
ENSG0000(	791	17.9113	chr8:980	RN7SL329P		smallRNA	chr8:125270604-125
ENSG0000(	791	17.9113	chr8:980	AC016405.2		smallRNA	chr8:122846691-122
ENSG0000(	791	17.9113	chr8:980	U3		smallRNA	chr8:123180311-123
ENSG0000(	791	17.9113	chr8:980	RN7SKP206		smallRNA	chr8:129290983-129
ENSG0000(	791	17.9113	chr8:980	PRNCR1		lncRNA	chr8:127079874-127
ENSG0000(	791	17.9113	chr8:980	LINC00861		lncRNA	chr8:125859721-126
ENSG0000(	791	17.9113	chr8:980	TRIB1	AC	protein_c	chr8:125430358-125
ENSG0000(	791	17.9113	chr8:980	KNOP1P5		Pseudoger	chr8:126072587-126
ENSG0000(	791	17.9113	chr8:980	ENSG00000253607		lncRNA	chr8:123002560-123
ENSG0000(	791	17.9113	chr8:980	RNU6-875P		smallRNA	chr8:123342414-123
ENSG0000(	791	17.9113	chr8:980	FER1L6-AS1		lncRNA	chr8:123984138-124
ENSG0000(	791	17.9113	chr8:980	MTSS1	DriverDB	protein_c	chr8:124550784-124
ENSG0000(	791	17.9113	chr8:980	ZNF572	NCGv7	protein_c	chr8:124973295-124
ENSG0000(	791	17.9113	chr8:980	LRATD2		protein_c	chr8:126552443-126
ENSG0000(	791	17.9113	chr8:980	ENSG00000254113		lncRNA	chr8:123563070-123
ENSG0000(	791	17.9113	chr8:980	KLHL38	DriverDB	protein_c	chr8:123644442-123
ENSG0000(	791	17.9113	chr8:980	ENSG00000253427		lncRNA	chr8:126474189-126
ENSG0000(	791	17.9113	chr8:980	RNU6-442P		smallRNA	chr8:125900951-125
ENSG0000(	791	17.9113	chr8:980	FAM83A-AS2		lncRNA	chr8:123181638-123
ENSG0000(	791	17.9113	chr8:980	FAM91A1	DriverDB	protein_c	chr8:123768439-123
ENSG0000(	791	17.9113	chr8:980	TRMT12		protein_c	chr8:124450820-124
ENSG0000(	791	17.9113	chr8:980	ENSG00000244791		lncRNA	chr8:126325454-126
ENSG0000(	790	17.88866	chr1:373	ENSG00000272478		lncRNA	chr1:25831913-2583
ENSG0000(	790	17.88866	chr1:373	Y_RNA		smallRNA	chr1:27255464-2725
ENSG0000(	786	17.79808	chr1:373	H3P1		Pseudoger	chr1:23949016-2394
ENSG0000(	783	17.73015	chr4:253(	ELOVL6		protein_c	chr4:110045846-110
ENSG0000(	783	17.73015	chr4:253(	GSTCD-AS1		lncRNA	chr4:105746245-105
ENSG0000(	783	17.73015	chr4:253(	ENSG00000251170		lncRNA	chr4:104230380-104
ENSG0000(	783	17.73015	chr4:253(	Y_RNA		smallRNA	chr4:112274072-112
ENSG0000(	783	17.73015	chr4:253(	RN7SL184P		smallRNA	chr4:113419840-113
ENSG0000(	783	17.73015	chr4:253(	ENSG00000251126		lncRNA	chr4:112880298-112
ENSG0000(	783	17.73015	chr4:253(	RAC1P5		Pseudoger	chr4:107203349-107
ENSG0000(	783	17.73015	chr4:253(	ENSG00000273447		lncRNA	chr4:109692004-109
ENSG0000(	783	17.73015	chr4:253(	OSTCP4		Pseudoger	chr4:112685055-112
ENSG0000(	783	17.73015	chr4:253(	ENSG00000251259		lncRNA	chr4:105137280-105
ENSG0000(	783	17.73015	chr4:253(	LEF1-AS1		lncRNA	chr4:108167525-108

ENSG0000(	783	17.73015	chr4:253(	TACR3-AS1		lncRNA	chr4:103548745-103
ENSG0000(	783	17.73015	chr4:253(	NPNT	NCGv7	protein_c	chr4:105894775-106
ENSG0000(	783	17.73015	chr4:253(	ENSG00000251473		Pseudoger	chr4:105102891-105
ENSG0000(	783	17.73015	chr4:253(	FAM241A	NCGv7	protein_c	chr4:112145454-112
ENSG0000(	783	17.73015	chr4:253(	LYPLA1P2		Pseudoger	chr4:110945109-110
ENSG0000(	783	17.73015	chr4:253(	LARP7	NCGv7	protein_c	chr4:112637077-112
ENSG0000(	783	17.73015	chr4:253(	ENSG00000251572		Pseudoger	chr4:102461250-102
ENSG0000(	783	17.73015	chr4:253(	snoU13		smallRNA	chr4:102859393-102
ENSG0000(	783	17.73015	chr4:253(	UGT8		protein_c	chr4:114598770-114
ENSG0000(	783	17.73015	chr4:253(	RNF14P2		Pseudoger	chr4:110264865-110
ENSG0000(	783	17.73015	chr4:253(	DDX3P3		Pseudoger	chr4:103572089-103
ENSG0000(	783	17.73015	chr4:253(	GAR1-DT		lncRNA	chr4:109815047-109
ENSG0000(	783	17.73015	chr4:253(	RN7SL275P		smallRNA	chr4:110117736-110
ENSG0000(	783	17.73015	chr4:253(	MIR576		smallRNA	chr4:109488698-109
ENSG0000(	783	17.73015	chr4:253(	TET2-AS1		lncRNA	chr4:105171354-105
ENSG0000(	783	17.73015	chr4:253(	PABPC1P7		Pseudoger	chr4:102896725-102
ENSG0000(	783	17.73015	chr4:253(	ENSG00000288913		lncRNA	chr4:110197678-110
ENSG0000(	783	17.73015	chr4:253(	ENSG00000260651		lncRNA	chr4:102500841-102
ENSG0000(	783	17.73015	chr4:253(	AP1AR-DT		lncRNA	chr4:112229561-112
ENSG0000(	783	17.73015	chr4:253(	ENSG00000250670		lncRNA	chr4:104556960-104
ENSG0000(	783	17.73015	chr4:253(	ENSG00000250740		lncRNA	chr4:105927060-105
ENSG0000(	783	17.73015	chr4:253(	MIR577		smallRNA	chr4:114656759-114
ENSG0000(	783	17.73015	chr4:253(	MIR302A		smallRNA	chr4:112648183-112
ENSG0000(	783	17.73015	chr4:253(	NFKB1		protein_c	chr4:102501330-102
ENSG0000(	783	17.73015	chr4:253(	MANBA		protein_c	chr4:102630770-102
ENSG0000(	783	17.73015	chr4:253(	GAR1		protein_c	chr4:109815510-109
ENSG0000(	783	17.73015	chr4:253(	UBE2D3		protein_c	chr4:102794383-102
ENSG0000(	783	17.73015	chr4:253(	RPL34	NCGv7	protein_c	chr4:108620569-108
ENSG0000(	783	17.73015	chr4:253(	RPL7L1P13		Pseudoger	chr4:110399773-110
ENSG0000(	783	17.73015	chr4:253(	TET2	NCGv7;AC	protein_c	chr4:105145875-105
ENSG0000(	783	17.73015	chr4:253(	ARHGEF38		protein_c	chr4:105552620-105
ENSG0000(	783	17.73015	chr4:253(	ENSG00000250511		lncRNA	chr4:110512488-110
ENSG0000(	783	17.73015	chr4:253(	ENSG00000251081		lncRNA	chr4:107258700-107
ENSG0000(	783	17.73015	chr4:253(	ENSG00000251288		Pseudoger	chr4:102751401-102
ENSG0000(	783	17.73015	chr4:253(	TACR3		protein_c	chr4:103586031-103
ENSG0000(	783	17.73015	chr4:253(	EXOC7P1		Pseudoger	chr4:108417705-108
ENSG0000(	783	17.73015	chr4:253(	ZNF969P		Pseudoger	chr4:110415898-110
ENSG0000(	783	17.73015	chr4:253(	MRPS33P3		Pseudoger	chr4:115142982-115
ENSG0000(	783	17.73015	chr4:253(	H3P14		Pseudoger	chr4:112564988-112
ENSG0000(	783	17.73015	chr4:253(	ENSG00000250500		Pseudoger	chr4:112797050-112
ENSG0000(	783	17.73015	chr4:253(	ENSG00000251309		lncRNA	chr4:101976894-102
ENSG0000(	783	17.73015	chr4:253(	ENSG00000251312		Pseudoger	chr4:111063583-111
ENSG0000(	783	17.73015	chr4:253(	RNU6-205P		smallRNA	chr4:110278185-110
ENSG0000(	783	17.73015	chr4:253(	ENSG00000288781		lncRNA	chr4:114281113-114
ENSG0000(	783	17.73015	chr4:253(	ENSG00000250920		lncRNA	chr4:103550586-103
ENSG0000(	783	17.73015	chr4:253(	AF213884.3		smallRNA	chr4:102565875-102
ENSG0000(	783	17.73015	chr4:253(	CXXC4	NCGv7	protein_c	chr4:104468308-104
ENSG0000(	783	17.73015	chr4:253(	ENSG00000250855		lncRNA	chr4:111802795-111
ENSG0000(	783	17.73015	chr4:253(	ENSG00000250522		lncRNA	chr4:105540190-105
ENSG0000(	783	17.73015	chr4:253(	ENSG00000288685		lncRNA	chr4:111959584-111
ENSG0000(	783	17.73015	chr4:253(	ENSG00000288691		lncRNA	chr4:111847565-111

ENSG0000(	783	17.73015	chr4:253(	ENSG00000288692		lncRNA	chr4:110876410-111
ENSG0000(	783	17.73015	chr4:253(	MIR297		smallRNA	chr4:110860582-110
ENSG0000(	783	17.73015	chr4:253(	RTEL1P1		Pseudoger	chr4:112356135-112
ENSG0000(	783	17.73015	chr4:253(	AC004052.1		smallRNA	chr4:104278107-104
ENSG0000(	783	17.73015	chr4:253(	CCDC34P1		Pseudoger	chr4:112068010-112
ENSG0000(	783	17.73015	chr4:253(	ENSG00000285330		protein_c	chr4:109713916-109
ENSG0000(	783	17.73015	chr4:253(	PIMREGP2		Pseudoger	chr4:105526596-105
ENSG0000(	783	17.73015	chr4:253(	RNU6-35P		smallRNA	chr4:109992325-109
ENSG0000(	783	17.73015	chr4:253(	ENSG00000289096		lncRNA	chr4:110619749-110
ENSG0000(	783	17.73015	chr4:253(	ATP5F1EP1		Pseudoger	chr4:105532475-105
ENSG0000(	783	17.73015	chr4:253(	ARSJ		protein_c	chr4:113900284-113
ENSG0000(	783	17.73015	chr4:253(	AC093680.1		smallRNA	chr4:106415706-106
ENSG0000(	783	17.73015	chr4:253(	ACTR6P1		Pseudoger	chr4:106836498-106
ENSG0000(	783	17.73015	chr4:253(	RPL7AP30		Pseudoger	chr4:112788083-112
ENSG0000(	783	17.73015	chr4:253(	AC084209.1		smallRNA	chr4:108577072-108
ENSG0000(	783	17.73015	chr4:253(	RPL34-DT		lncRNA	chr4:108538190-108
ENSG0000(	783	17.73015	chr4:253(	ENSG00000241981		Pseudoger	chr4:102662611-102
ENSG0000(	783	17.73015	chr4:253(	RNU6-289P		smallRNA	chr4:111331412-111
ENSG0000(	783	17.73015	chr4:253(	RNU6-553P		smallRNA	chr4:105406997-105
ENSG0000(	783	17.73015	chr4:253(	KRT8P46		Pseudoger	chr4:102728746-102
ENSG0000(	783	17.73015	chr4:253(	RNU7-151P		smallRNA	chr4:102837047-102
ENSG0000(	783	17.73015	chr4:253(	RN7SL728P		smallRNA	chr4:102348394-102
ENSG0000(	783	17.73015	chr4:253(	TBCK		protein_c	chr4:106041599-106
ENSG0000(	783	17.73015	chr4:253(	CAMK2D		protein_c	chr4:113418054-113
ENSG0000(	783	17.73015	chr4:253(	CISD2		protein_c	chr4:102868974-102
ENSG0000(	783	17.73015	chr4:253(	ANK2	NCGv7	protein_c	chr4:112818032-113
ENSG0000(	783	17.73015	chr4:253(	TIFA		protein_c	chr4:112272968-112
ENSG0000(	783	17.73015	chr4:253(	HIGD1AP14		Pseudoger	chr4:109673843-109
ENSG0000(	783	17.73015	chr4:253(	ENSG00000248778		Pseudoger	chr4:105679050-105
ENSG0000(	783	17.73015	chr4:253(	RPL36AP23		Pseudoger	chr4:111551854-111
ENSG0000(	783	17.73015	chr4:253(	LRIT3		protein_c	chr4:109848107-109
ENSG0000(	783	17.73015	chr4:253(	ENSG00000249257		Pseudoger	chr4:108773613-108
ENSG0000(	783	17.73015	chr4:253(	EEF1A1P9		Pseudoger	chr4:105484698-105
ENSG0000(	783	17.73015	chr4:253(	RNU6-635P		smallRNA	chr4:103924540-103
ENSG0000(	783	17.73015	chr4:253(	RNU6-551P		smallRNA	chr4:107435118-107
ENSG0000(	783	17.73015	chr4:253(	RBMXP4		Pseudoger	chr4:109346326-109
ENSG0000(	783	17.73015	chr4:253(	ENSG00000234841		Pseudoger	chr4:112826307-112
ENSG0000(	783	17.73015	chr4:253(	ALPK1	NCGv7	protein_c	chr4:112285509-112
ENSG0000(	783	17.73015	chr4:253(	ZACNP1		Pseudoger	chr4:108415220-108
ENSG0000(	783	17.73015	chr4:253(	AP1AR		protein_c	chr4:112231740-112
ENSG0000(	783	17.73015	chr4:253(	ZGRF1		protein_c	chr4:112539333-112
ENSG0000(	783	17.73015	chr4:253(	NDST4	NCGv7	protein_c	chr4:114827763-115
ENSG0000(	783	17.73015	chr4:253(	ENSG00000249373		lncRNA	chr4:113031287-113
ENSG0000(	783	17.73015	chr4:253(	SETP20		Pseudoger	chr4:109553243-109
ENSG0000(	783	17.73015	chr4:253(	SNORD112		smallRNA	chr4:107117332-107
ENSG0000(	783	17.73015	chr4:253(	RNU1-138P		smallRNA	chr4:113420323-113
ENSG0000(	783	17.73015	chr4:253(	ENSG00000249304		lncRNA	chr4:113943256-113
ENSG0000(	783	17.73015	chr4:253(	ACTR3BP4		Pseudoger	chr4:102961956-102
ENSG0000(	783	17.73015	chr4:253(	LINC02428		lncRNA	chr4:103255822-103
ENSG0000(	783	17.73015	chr4:253(	ENSG00000248716		Pseudoger	chr4:114103938-114
ENSG0000(	783	17.73015	chr4:253(	TOX4P1		Pseudoger	chr4:112455924-112

ENSG0000(	783	17.73015	chr4:253(	ENSG00000286291		lncRNA	chr4:103871890-103
ENSG0000(	783	17.73015	chr4:253(	ANK2-AS1		lncRNA	chr4:112973272-113
ENSG0000(	783	17.73015	chr4:253(	ENSG00000286242		lncRNA	chr4:102814252-102
ENSG0000(	783	17.73015	chr4:253(	SEC24B-AS1		lncRNA	chr4:109347475-109
ENSG0000(	783	17.73015	chr4:253(	ENSG00000286147		lncRNA	chr4:106525401-106
ENSG0000(	783	17.73015	chr4:253(	ENSG00000286136		lncRNA	chr4:108669949-108
ENSG0000(	783	17.73015	chr4:253(	ENSG00000286124		lncRNA	chr4:101640946-101
ENSG0000(	783	17.73015	chr4:253(	COL25A1	NCGv7	protein_c	chr4:108808725-109
ENSG0000(	783	17.73015	chr4:253(	COL25A1-DT		lncRNA	chr4:109303035-109
ENSG0000(	783	17.73015	chr4:253(	UBE2D3-AS1		lncRNA	chr4:102827611-102
ENSG0000(	783	17.73015	chr4:253(	ENSG00000196656		Pseudoger	chr4:113214046-113
ENSG0000(	783	17.73015	chr4:253(	MCUB		protein_c	chr4:109560209-109
ENSG0000(	783	17.73015	chr4:253(	CXXC4-AS1		lncRNA	chr4:104490849-104
ENSG0000(	783	17.73015	chr4:253(	CYP2U1-AS1		lncRNA	chr4:107863473-107
ENSG0000(	783	17.73015	chr4:253(	RPL6P14		Pseudoger	chr4:104886118-104
ENSG0000(	783	17.73015	chr4:253(	ENSG00000228814		Pseudoger	chr4:112881718-112
ENSG0000(	783	17.73015	chr4:253(	ENSG00000248161		lncRNA	chr4:102418602-102
ENSG0000(	783	17.73015	chr4:253(	ENSG00000248200		Pseudoger	chr4:110146374-110
ENSG0000(	783	17.73015	chr4:253(	ENSG00000248656		lncRNA	chr4:111516117-111
ENSG0000(	783	17.73015	chr4:253(	ENSG00000248242		lncRNA	chr4:104653874-104
ENSG0000(	783	17.73015	chr4:253(	snoU2_19		smallRNA	chr4:110433109-110
ENSG0000(	783	17.73015	chr4:253(	GET1P1		Pseudoger	chr4:112693459-112
ENSG0000(	783	17.73015	chr4:253(	RNU6-462P		smallRNA	chr4:101723876-101
ENSG0000(	783	17.73015	chr4:253(	PLA2G12A		protein_c	chr4:109709989-109
ENSG0000(	783	17.73015	chr4:253(	MIR367		smallRNA	chr4:112647874-112
ENSG0000(	783	17.73015	chr4:253(	MIR302D		smallRNA	chr4:112648004-112
ENSG0000(	783	17.73015	chr4:253(	MIR302C		smallRNA	chr4:112648363-112
ENSG0000(	783	17.73015	chr4:253(	RN7SL55P		smallRNA	chr4:109450775-109
ENSG0000(	783	17.73015	chr4:253(	RPL23AP94		Pseudoger	chr4:112530371-112
ENSG0000(	783	17.73015	chr4:253(	RPL36AP19		Pseudoger	chr4:112171215-112
ENSG0000(	783	17.73015	chr4:253(	OSTC		protein_c	chr4:108650585-108
ENSG0000(	783	17.73015	chr4:253(	ENSG00000248373		lncRNA	chr4:104900125-105
ENSG0000(	783	17.73015	chr4:253(	RN7SL89P		smallRNA	chr4:105293658-105
ENSG0000(	783	17.73015	chr4:253(	CYP2U1	DriverDB\	protein_c	chr4:107931549-107
ENSG0000(	783	17.73015	chr4:253(	DKK2		protein_c	chr4:106921802-107
ENSG0000(	783	17.73015	chr4:253(	NEUROG2-AS1		lncRNA	chr4:112515385-112
ENSG0000(	783	17.73015	chr4:253(	MTND5P5		Pseudoger	chr4:101972423-101
ENSG0000(	783	17.73015	chr4:253(	LINC01438		lncRNA	chr4:110794403-110
ENSG0000(	783	17.73015	chr4:253(	ENSG00000250046		lncRNA	chr4:112693047-112
ENSG0000(	783	17.73015	chr4:253(	SLC9B1		protein_c	chr4:102885048-103
ENSG0000(	783	17.73015	chr4:253(	SLC9B2		protein_c	chr4:103019868-103
ENSG0000(	783	17.73015	chr4:253(	BDH2		protein_c	chr4:103077592-103
ENSG0000(	783	17.73015	chr4:253(	RN7SL808P		smallRNA	chr4:114799331-114
ENSG0000(	783	17.73015	chr4:253(	ETNPPL		protein_c	chr4:108742048-108
ENSG0000(	783	17.73015	chr4:253(	PITX2	AC	protein_c	chr4:110617423-110
ENSG0000(	783	17.73015	chr4:253(	LINC02945		lncRNA	chr4:111804418-112
ENSG0000(	783	17.73015	chr4:253(	RRH		protein_c	chr4:109827972-109
ENSG0000(	783	17.73015	chr4:253(	MIR302CHG		lncRNA	chr4:112646476-112
ENSG0000(	783	17.73015	chr4:253(	ENSG00000240459		Pseudoger	chr4:112116510-112
ENSG0000(	783	17.73015	chr4:253(	SNORA31		smallRNA	chr4:105105987-105
ENSG0000(	783	17.73015	chr4:253(	ENSG00000276992		Pseudoger	chr4:104996900-104

ENSG0000(	783	17.73015	chr4:253(	CDC42P4		Pseudoger	chr4:109555170-109
ENSG0000(	783	17.73015	chr4:253(	AC093628.1		smallRNA	chr4:104490876-104
ENSG0000(	783	17.73015	chr4:253(	NEUROG2	NCGv7	protein_c	chr4:112513516-112
ENSG0000(	783	17.73015	chr4:253(	AIMP1		protein_c	chr4:106315544-106
ENSG0000(	783	17.73015	chr4:253(	PANCR		lncRNA	chr4:110595504-110
ENSG0000(	783	17.73015	chr4:253(	RPSAP34		Pseudoger	chr4:108407843-108
ENSG0000(	783	17.73015	chr4:253(	RCC2P8		Pseudoger	chr4:108788745-108
ENSG0000(	783	17.73015	chr4:253(	CFI		protein_c	chr4:109731008-109
ENSG0000(	783	17.73015	chr4:253(	CIR1P2		Pseudoger	chr4:114713579-114
ENSG0000(	783	17.73015	chr4:253(	ENSG00000250229		Pseudoger	chr4:113840050-113
ENSG0000(	783	17.73015	chr4:253(	RNU6-351P		smallRNA	chr4:104974662-104
ENSG0000(	783	17.73015	chr4:253(	RNU6-431P		smallRNA	chr4:108652150-108
ENSG0000(	783	17.73015	chr4:253(	GIMD1		protein_c	chr4:106357392-106
ENSG0000(	783	17.73015	chr4:253(	ZBED1P1		Pseudoger	chr4:110291644-110
ENSG0000(	783	17.73015	chr4:253(	TUBB8P3		Pseudoger	chr4:112077598-112
ENSG0000(	783	17.73015	chr4:253(	RNU6-733P		smallRNA	chr4:107867807-107
ENSG0000(	783	17.73015	chr4:253(	AC024198.1		smallRNA	chr4:110853376-110
ENSG0000(	783	17.73015	chr4:253(	HSBP1P2		Pseudoger	chr4:110251871-110
ENSG0000(	783	17.73015	chr4:253(	SGMS2		protein_c	chr4:107824563-107
ENSG0000(	783	17.73015	chr4:253(	ARHGEF38-IT1		lncRNA	chr4:105561591-105
ENSG0000(	783	17.73015	chr4:253(	ENSG00000249604		lncRNA	chr4:107936031-107
ENSG0000(	783	17.73015	chr4:253(	LINC02503		lncRNA	chr4:103961616-104
ENSG0000(	783	17.73015	chr4:253(	EGF		protein_c	chr4:109912883-110
ENSG0000(	783	17.73015	chr4:253(	LEF1	NCGv7;AC	protein_c	chr4:108047545-108
ENSG0000(	783	17.73015	chr4:253(	PAPSS1		protein_c	chr4:107590276-107
ENSG0000(	783	17.73015	chr4:253(	SEC24B		protein_c	chr4:109433772-109
ENSG0000(	783	17.73015	chr4:253(	ENSG00000224207		Pseudoger	chr4:102734358-102
ENSG0000(	783	17.73015	chr4:253(	CASP6		protein_c	chr4:109688622-109
ENSG0000(	783	17.73015	chr4:253(	ENPEP	IntOGen-I	protein_c	chr4:110365733-110
ENSG0000(	783	17.73015	chr4:253(	INTS12	NCGv7	protein_c	chr4:105682627-105
ENSG0000(	783	17.73015	chr4:253(	SLC39A8		protein_c	chr4:102251080-102
ENSG0000(	783	17.73015	chr4:253(	LRRC37A15P		Pseudoger	chr4:102727274-102
ENSG0000(	783	17.73015	chr4:253(	RPL32P13		Pseudoger	chr4:112738672-112
ENSG0000(	783	17.73015	chr4:253(	GSTCD		protein_c	chr4:105708778-105
ENSG0000(	783	17.73015	chr4:253(	LINC02173		lncRNA	chr4:106433489-106
ENSG0000(	783	17.73015	chr4:253(	CENPE		protein_c	chr4:103105349-103
ENSG0000(	783	17.73015	chr4:253(	HADH		protein_c	chr4:107989714-108
ENSG0000(	783	17.73015	chr4:253(	ENSG00000249635		lncRNA	chr4:106003317-106
ENSG0000(	783	17.73015	chr4:253(	KRT19P3		Pseudoger	chr4:109879070-109
ENSG0000(	783	17.73015	chr4:253(	Y_RNA		smallRNA	chr4:112755817-112
ENSG0000(	783	17.73015	chr4:253(	AC097473.1		smallRNA	chr4:108789200-108
ENSG0000(	783	17.73015	chr4:253(	PPA2		protein_c	chr4:105369077-105
ENSG0000(	782	17.7075	chrX:1578	CENPVP2		Pseudoger	chrX:51682067-5168
ENSG0000(	782	17.7075	chrX:1578	ENSG00000236576		Pseudoger	chrX:51856968-5185
ENSG0000(	782	17.7075	chrX:1578	AKAP4		protein_c	chrX:50190777-5020
ENSG0000(	782	17.7075	chrX:1578	ENSG00000229151		lncRNA	chrX:51190598-5139
ENSG0000(	782	17.7075	chrX:1578	ENSG00000228827		Pseudoger	chrX:51903338-5190
ENSG0000(	782	17.7075	chrX:1578	ENSG00000276474		Pseudoger	chrX:52326166-5232
ENSG0000(	782	17.7075	chrX:1578	RBM22P10		Pseudoger	chrX:52485045-5248
ENSG0000(	782	17.7075	chrX:1578	ENSG00000227058		Pseudoger	chrX:52545151-5254
ENSG0000(	782	17.7075	chrX:1578	ENSG00000228771		Pseudoger	chrX:52583989-5258

ENSG0000(	782	17.7075	chrX:1578	ENSG00000234792		Pseudoger	chrX:52612656-5261
ENSG0000(	782	17.7075	chrX:1578	BMP15		protein_c	chrX:50910735-5091
ENSG0000(	782	17.7075	chrX:1578	RBM22P8		Pseudoger	chrX:52527284-5252
ENSG0000(	782	17.7075	chrX:1578	ENSG00000227493		Pseudoger	chrX:51030422-5103
ENSG0000(	782	17.7075	chrX:1578	IPO7P1		Pseudoger	chrX:51921864-5192
ENSG0000(	782	17.7075	chrX:1578	ENSG00000237926		Pseudoger	chrX:51162864-5116
ENSG0000(	782	17.7075	chrX:1578	RBM22P6		Pseudoger	chrX:52485046-5248
ENSG0000(	782	17.7075	chrX:1578	RP11-472D17.3		Pseudoger	chrX:52421452-5242
ENSG0000(	782	17.7075	chrX:1578	RNA5SP504		Pseudoger	chrX:52665231-5266
ENSG0000(	782	17.7075	chrX:1578	ENSG00000228354		Pseudoger	chrX:52655207-5265
ENSG0000(	782	17.7075	chrX:1578	MAGED4		protein_c	chrX:52184876-5219
ENSG0000(	782	17.7075	chrX:1578	RNU6-935P		smallRNA	chrX:50649641-5064
ENSG0000(	782	17.7075	chrX:1578	ENSG00000279750		lncRNA	chrX:52053176-5205
ENSG0000(	782	17.7075	chrX:1578	SSXP5		Pseudoger	chrX:52672718-5267
ENSG0000(	782	17.7075	chrX:1578	RP11-472D17.2		Pseudoger	chrX:52448586-5245
ENSG0000(	782	17.7075	chrX:1578	H3P44		Pseudoger	chrX:50905438-5090
ENSG0000(	782	17.7075	chrX:1578	UQCR10P1		Pseudoger	chrX:51923552-5192
ENSG0000(	782	17.7075	chrX:1578	ENSG00000224556		Pseudoger	chrX:52422069-5245
ENSG0000(	782	17.7075	chrX:1578	ENSG00000225397		Pseudoger	chrX:52722338-5272
ENSG0000(	782	17.7075	chrX:1578	S100A11P10		Pseudoger	chrX:52687363-5268
ENSG0000(	782	17.7075	chrX:1578	CENPVL3		protein_c	chrX:51617020-5161
ENSG0000(	782	17.7075	chrX:1578	DGKK		protein_c	chrX:50365409-5047
ENSG0000(	782	17.7075	chrX:1578	NUDT10	NCGv7	protein_c	chrX:51332231-5133
ENSG0000(	782	17.7075	chrX:1578	ENSG00000229826		Pseudoger	chrX:52735901-5273
ENSG0000(	782	17.7075	chrX:1578	SNORA11D		smallRNA	chrX:52190621-5219
ENSG0000(	782	17.7075	chrX:1578	ENSG00000278160		Pseudoger	chrX:52481515-5248
ENSG0000(	782	17.7075	chrX:1578	RP11-472D17.1		Pseudoger	chrX:52458937-5246
ENSG0000(	782	17.7075	chrX:1578	SHROOM4		protein_c	chrX:50586796-5081
ENSG0000(	782	17.7075	chrX:1578	ENSG00000223958		Pseudoger	chrX:52707602-5270
ENSG0000(	782	17.7075	chrX:1578	SSX8P		Pseudoger	chrX:52624998-5263
ENSG0000(	782	17.7075	chrX:1578	ENSG00000287767		lncRNA	chrX:51325790-5133
ENSG0000(	782	17.7075	chrX:1578	LINC01496		lncRNA	chrX:51498490-5151
ENSG0000(	782	17.7075	chrX:1578	TPMTP3		Pseudoger	chrX:51979223-5198
ENSG0000(	782	17.7075	chrX:1578	ENSG00000225957		Pseudoger	chrX:52382053-5238
ENSG0000(	782	17.7075	chrX:1578	ENSG00000277289		Pseudoger	chrX:52531709-5253
ENSG0000(	782	17.7075	chrX:1578	CENPVL1		protein_c	chrX:51710512-5171
ENSG0000(	782	17.7075	chrX:1578	ENSG00000276897		Pseudoger	chrX:52409772-5241
ENSG0000(	782	17.7075	chrX:1578	ENSG00000287215		lncRNA	chrX:52195836-5226
ENSG0000(	782	17.7075	chrX:1578	RBM22P7		Pseudoger	chrX:52509902-5251
ENSG0000(	782	17.7075	chrX:1578	RP11-204I15.1		Pseudoger	chrX:52448587-5245
ENSG0000(	782	17.7075	chrX:1578	ENSG00000278358		Pseudoger	chrX:52480836-5248
ENSG0000(	782	17.7075	chrX:1578	ENSG00000226530		lncRNA	chrX:51395915-5146
ENSG0000(	782	17.7075	chrX:1578	SNORA11E		smallRNA	chrX:52063347-5206
ENSG0000(	782	17.7075	chrX:1578	XAGE2		protein_c	chrX:52369021-5237
ENSG0000(	782	17.7075	chrX:1578	RNU6-504P		smallRNA	chrX:51870706-5187
ENSG0000(	782	17.7075	chrX:1578	RBM22P9		Pseudoger	chrX:52509904-5251
ENSG0000(	782	17.7075	chrX:1578	HMGB1P15		Pseudoger	chrX:50931114-5093
ENSG0000(	782	17.7075	chrX:1578	Y_RNA		smallRNA	chrX:50171197-5017
ENSG0000(	782	17.7075	chrX:1578	ENSG00000288783		lncRNA	chrX:50161928-5016
ENSG0000(	782	17.7075	chrX:1578	MIR188		smallRNA	chrX:50003503-5000
ENSG0000(	782	17.7075	chrX:1578	ENSG00000182776		lncRNA	chrX:52050860-5205

ENSG0000(	782	17.7075	chrX:1578	MIR500A		smallRNA	chrX:50008431-5000
ENSG0000(	782	17.7075	chrX:1578	EZHIP		protein_c	chrX:51406948-5140
ENSG0000(	782	17.7075	chrX:1578	ENSG00000290686		lncRNA	chrX:52622935-5263
ENSG0000(	782	17.7075	chrX:1578	SSX2	NCGv7;AC	protein_c	chrX:52696896-5270
ENSG0000(	782	17.7075	chrX:1578	AL121865.1		smallRNA	chrX:50645118-5064
ENSG0000(	782	17.7075	chrX:1578	SSX7		protein_c	chrX:52644061-5265
ENSG0000(	782	17.7075	chrX:1578	AF222686.1		smallRNA	chrX:50006656-5000
ENSG0000(	782	17.7075	chrX:1578	MIR660		smallRNA	chrX:50013241-5001
ENSG0000(	782	17.7075	chrX:1578	XAGE1C		protein_c	chrX:52512076-5251
ENSG0000(	782	17.7075	chrX:1578	RNU6-421P		smallRNA	chrX:49945336-4994
ENSG0000(	782	17.7075	chrX:1578	XAGE1E		protein_c	chrX:52495667-5250
ENSG0000(	782	17.7075	chrX:1578	XAGE1D		protein_c	chrX:52495667-5250
ENSG0000(	782	17.7075	chrX:1578	XAGE1A		protein_c	chrX:52512077-5251
ENSG0000(	782	17.7075	chrX:1578	XAGE1B		protein_c	chrX:52492086-5250
ENSG0000(	782	17.7075	chrX:1578	RP11-472D17.4		Pseudoger	chrX:52382052-5238
ENSG0000(	782	17.7075	chrX:1578	MAGED1	NCGv7	protein_c	chrX:51803007-5190
ENSG0000(	782	17.7075	chrX:1578	RP11-472D17.6		Pseudoger	chrX:52409771-5241
ENSG0000(	782	17.7075	chrX:1578	MIR501		smallRNA	chrX:50009722-5000
ENSG0000(	782	17.7075	chrX:1578	ENSG00000179028		lncRNA	chrX:52199840-5220
ENSG0000(	782	17.7075	chrX:1578	MIR502		smallRNA	chrX:50014598-5001
ENSG0000(	782	17.7075	chrX:1578	ENSG00000250084		Pseudoger	chrX:52448587-5245
ENSG0000(	782	17.7075	chrX:1578	SSXP4		Pseudoger	chrX:52598494-5260
ENSG0000(	782	17.7075	chrX:1578	SSXP1		Pseudoger	chrX:52606535-5261
ENSG0000(	782	17.7075	chrX:1578	CENPVL2		protein_c	chrX:51681212-5168
ENSG0000(	782	17.7075	chrX:1578	ENSG00000231593		Pseudoger	chrX:52561769-5256
ENSG0000(	782	17.7075	chrX:1578	MIR532		smallRNA	chrX:50003148-5000
ENSG0000(	782	17.7075	chrX:1578	ENSG00000230926		Pseudoger	chrX:51082818-5109
ENSG0000(	782	17.7075	chrX:1578	CLCN5		protein_c	chrX:49922596-5009
ENSG0000(	782	17.7075	chrX:1578	NUDT11	NCGv7	protein_c	chrX:51490011-5149
ENSG0000(	782	17.7075	chrX:1578	MAGED4B		protein_c	chrX:52061827-5206
ENSG0000(	782	17.7075	chrX:1578	RBM22P11		Pseudoger	chrX:52526971-5252
ENSG0000(	782	17.7075	chrX:1578	MIR500B		smallRNA	chrX:50010671-5001
ENSG0000(	782	17.7075	chrX:1578	GSPT2		protein_c	chrX:51743442-5174
ENSG0000(	782	17.7075	chrX:1578	PYY3		Pseudoger	chrX:50156159-5015
ENSG0000(	782	17.7075	chrX:1578	LINC01284		lncRNA	chrX:51095836-5122
ENSG0000(	779	17.63957	chr1:1021	C1orf50-AS1		lncRNA	chr1:42775813-4277
ENSG0000(	778	17.61693	chr1:3733	WASF2		protein_c	chr1:27404230-2749
ENSG0000(	778	17.61693	chr1:3733	SNRPFP2		Pseudoger	chr1:25887360-2588
ENSG0000(	778	17.61693	chr1:3733	UBXN11		protein_c	chr1:26281328-2631
ENSG0000(	778	17.61693	chr1:3733	Y_RNA		smallRNA	chr1:24625411-2462
ENSG0000(	778	17.61693	chr1:3733	ENSG00000290006		lncRNA	chr1:26692586-2669
ENSG0000(	778	17.61693	chr1:3733	MIR4425		smallRNA	chr1:25023503-2502
ENSG0000(	778	17.61693	chr1:3733	SNRPEP7		Pseudoger	chr1:27211265-2721
ENSG0000(	778	17.61693	chr1:3733	CRYBG2		protein_c	chr1:26321698-2636
ENSG0000(	778	17.61693	chr1:3733	TENT5B		protein_c	chr1:27005020-2701
ENSG0000(	778	17.61693	chr1:3733	ENSG00000272432		lncRNA	chr1:25247837-2524
ENSG0000(	778	17.61693	chr1:3733	AL031284.1		smallRNA	chr1:25406231-2540
ENSG0000(	778	17.61693	chr1:3733	Y_RNA		smallRNA	chr1:26593940-2659
ENSG0000(	778	17.61693	chr1:3733	ZNF593OS		protein_c	chr1:26169516-2617
ENSG0000(	778	17.61693	chr1:3733	NCMAP-DT		lncRNA	chr1:24538802-2455
ENSG0000(	778	17.61693	chr1:3733	ENSG00000225643		lncRNA	chr1:25581478-2559

ENSG0000(	778	17.61693	chr1:373:	TMEM222		protein_c	chr1:27322145-2733
ENSG0000(	778	17.61693	chr1:373:	ENSG00000259984		Pseudoger	chr1:25336429-2533
ENSG0000(	778	17.61693	chr1:373:	RNU6-424P		smallRNA	chr1:27693731-2769
ENSG0000(	778	17.61693	chr1:373:	C1orf232		protein_c	chr1:26164101-2616
ENSG0000(	778	17.61693	chr1:373:	ENSG00000270733		Pseudoger	chr1:26263041-2626
ENSG0000(	778	17.61693	chr1:373:	ZNF683		protein_c	chr1:26361634-2637
ENSG0000(	778	17.61693	chr1:373:	EXTL1		protein_c	chr1:26019884-2603
ENSG0000(	778	17.61693	chr1:373:	SLC30A2		protein_c	chr1:26037252-2604
ENSG0000(	778	17.61693	chr1:373:	RNU6-48P		smallRNA	chr1:27325219-2732
ENSG0000(	778	17.61693	chr1:373:	ENSG00000231953		lncRNA	chr1:25208139-2520
ENSG0000(	778	17.61693	chr1:373:	ENSG00000289452		lncRNA	chr1:26620707-2662
ENSG0000(	778	17.61693	chr1:373:	PAFAH2		protein_c	chr1:25959767-2599
ENSG0000(	778	17.61693	chr1:373:	ENSG00000236528		lncRNA	chr1:25859580-2586
ENSG0000(	778	17.61693	chr1:373:	TRIM63		protein_c	chr1:26051301-2606
ENSG0000(	778	17.61693	chr1:373:	LDLRAP1		protein_c	chr1:25543606-2556
ENSG0000(	778	17.61693	chr1:373:	RNU6-949P		smallRNA	chr1:27675603-2767
ENSG0000(	778	17.61693	chr1:373:	RN7SL857P		smallRNA	chr1:24529455-2452
ENSG0000(	778	17.61693	chr1:373:	ENSG00000225854		Pseudoger	chr1:26326688-2632
ENSG0000(	778	17.61693	chr1:373:	ENSG00000225886		lncRNA	chr1:27669468-2770
ENSG0000(	778	17.61693	chr1:373:	SFN		protein_c	chr1:26863149-2686
ENSG0000(	778	17.61693	chr1:373:	KDF1		protein_c	chr1:26949562-2696
ENSG0000(	778	17.61693	chr1:373:	DHDDS-AS1		lncRNA	chr1:26462756-2646
ENSG0000(	778	17.61693	chr1:373:	ENSG00000289554		lncRNA	chr1:26881109-2688
ENSG0000(	778	17.61693	chr1:373:	SCARNA18		smallRNA	chr1:26006216-2600
ENSG0000(	778	17.61693	chr1:373:	PIGV		protein_c	chr1:26787054-2680
ENSG0000(	778	17.61693	chr1:373:	RPA2		protein_c	chr1:27891524-2791
ENSG0000(	778	17.61693	chr1:373:	SELENON		protein_c	chr1:25800193-2581
ENSG0000(	778	17.61693	chr11:14	RNU6-1175P		smallRNA	chr11:70075363-700
ENSG0000(	778	17.61693	chr1:373:	ENSG00000229985		Pseudoger	chr1:27176751-2717
ENSG0000(	778	17.61693	chr1:373:	ENSG00000235069		Pseudoger	chr1:26647447-2664
ENSG0000(	778	17.61693	chr1:373:	ENSG00000241169		lncRNA	chr1:27457198-2745
ENSG0000(	778	17.61693	chr1:373:	DPPA2P2		Pseudoger	chr1:26519354-2652
ENSG0000(	778	17.61693	chr1:373:	SDHDP6		Pseudoger	chr1:25294164-2529
ENSG0000(	778	17.61693	chr1:373:	AL391650.1		smallRNA	chr1:26071578-2607
ENSG0000(	778	17.61693	chr1:373:	STX12		protein_c	chr1:27773219-2782
ENSG0000(	778	17.61693	chr1:373:	PPP1R8		protein_c	chr1:27830782-2785
ENSG0000(	778	17.61693	chr1:373:	ARID1A	NCGv7;AC	protein_c	chr1:26693236-2678
ENSG0000(	778	17.61693	chr1:373:	ZPLD2P		Pseudoger	chr1:26209741-2622
ENSG0000(	778	17.61693	chr1:373:	WDTC1-DT		lncRNA	chr1:27229106-2723
ENSG0000(	778	17.61693	chr1:373:	DHDDS		protein_c	chr1:26432282-2647
ENSG0000(	778	17.61693	chr1:373:	RPS6KA1		protein_c	chr1:26529761-2657
ENSG0000(	778	17.61693	chr1:373:	MAN1C1		protein_c	chr1:25616791-2578
ENSG0000(	778	17.61693	chr1:373:	MTFR1L	NCGv7	protein_c	chr1:25818640-2583
ENSG0000(	778	17.61693	chr1:373:	GPR3		protein_c	chr1:27392622-2739
ENSG0000(	778	17.61693	chr1:373:	STMN1	AC	protein_c	chr1:25884181-2590
ENSG0000(	778	17.61693	chr1:373:	RSRP1		protein_c	chr1:25242249-2533
ENSG0000(	778	17.61693	chr1:373:	SYF2	NCGv7	protein_c	chr1:25222276-2523
ENSG0000(	778	17.61693	chr1:373:	RCAN3		protein_c	chr1:24502351-2454
ENSG0000(	778	17.61693	chr1:373:	RPL32P6		Pseudoger	chr1:26983628-2698
ENSG0000(	778	17.61693	chr1:373:	RNU6-1171P		smallRNA	chr1:25340971-2534
ENSG0000(	778	17.61693	chr1:373:	ENSG00000278572		Pseudoger	chr1:26218581-2622

ENSG0000(	778	17.61693	chr1:373:	PAQR7		protein_c	chr1:25861484-2587
ENSG0000(	778	17.61693	chr1:373:	RNU6-1245P		smallRNA	chr1:27824538-2782
ENSG0000(	778	17.61693	chr1:373:	NUDC		protein_c	chr1:26900238-2694
ENSG0000(	778	17.61693	chr1:373:	SRRM1		protein_c	chr1:24631716-2467
ENSG0000(	778	17.61693	chr1:373:	SLC9A1		protein_c	chr1:27098809-2716
ENSG0000(	778	17.61693	chr1:373:	ENSG00000287244		lncRNA	chr1:27724822-2772
ENSG0000(	778	17.61693	chr1:373:	NCMAP		protein_c	chr1:24556087-2460
ENSG0000(	778	17.61693	chr1:373:	RPEP3		Pseudoger	chr1:27739954-2774
ENSG0000(	778	17.61693	chr1:373:	PDIK1L		protein_c	chr1:26111165-2612
ENSG0000(	778	17.61693	chr1:373:	RN7SL501P		smallRNA	chr1:26763624-2676
ENSG0000(	778	17.61693	chr1:373:	TMEM50A		protein_c	chr1:25338317-2536
ENSG0000(	778	17.61693	chr1:373:	OSTCP2		Pseudoger	chr1:26985897-2698
ENSG0000(	778	17.61693	chr1:373:	ENSG00000284699		lncRNA	chr1:24704894-2471
ENSG0000(	778	17.61693	chr1:373:	ENSG00000223583		Pseudoger	chr1:26454653-2645
ENSG0000(	778	17.61693	chr1:373:	RN7SL490P		smallRNA	chr1:26348465-2634
ENSG0000(	778	17.61693	chr1:373:	ENSG00000235912		Pseudoger	chr1:27649419-2764
ENSG0000(	778	17.61693	chr1:373:	ENSG00000223624		Pseudoger	chr1:25888970-2588
ENSG0000(	778	17.61693	chr1:373:	ACTG1P20		Pseudoger	chr1:27325329-2732
ENSG0000(	778	17.61693	chr1:373:	NPM1P39		Pseudoger	chr1:27206930-2720
ENSG0000(	778	17.61693	chr1:373:	MACO1		protein_c	chr1:25430858-2550
ENSG0000(	778	17.61693	chr1:373:	SCARNA17		smallRNA	chr1:26006006-2600
ENSG0000(	778	17.61693	chr1:373:	ZDHHC18		protein_c	chr1:26826688-2685
ENSG0000(	778	17.61693	chr1:373:	LINC02574		lncRNA	chr1:27660328-2766
ENSG0000(	778	17.61693	chr1:373:	AL033528.1		smallRNA	chr1:25911749-2591
ENSG0000(	778	17.61693	chr1:373:	AHDC1		protein_c	chr1:27534035-2760
ENSG0000(	778	17.61693	chr1:373:	ENSG00000261025		lncRNA	chr1:24968423-2497
ENSG0000(	778	17.61693	chr1:373:	NR0B2		protein_c	chr1:26911489-2691
ENSG0000(	778	17.61693	chr1:373:	LIN28A	AC	protein_c	chr1:26410817-2642
ENSG0000(	778	17.61693	chr1:373:	ENSG00000228172		lncRNA	chr1:25816749-2582
ENSG0000(	778	17.61693	chr1:373:	ENSG00000284309		lncRNA	chr1:26046665-2604
ENSG0000(	778	17.61693	chr1:373:	IFI6		protein_c	chr1:27666064-2767
ENSG0000(	778	17.61693	chr1:373:	CD52		protein_c	chr1:26317958-2632
ENSG0000(	778	17.61693	chr1:373:	ENSG00000261349		Pseudoger	chr1:25266102-2526
ENSG0000(	778	17.61693	chr1:373:	FCN3		protein_c	chr1:27369110-2737
ENSG0000(	778	17.61693	chr1:373:	IFITM3P7		Pseudoger	chr1:25125053-2512
ENSG0000(	778	17.61693	chr1:373:	ENSG00000227050		lncRNA	chr1:27938875-2796
ENSG0000(	778	17.61693	chr1:373:	ENSG00000233478		lncRNA	chr1:25644544-2565
ENSG0000(	778	17.61693	chr1:373:	FAM76A		protein_c	chr1:27725961-2776
ENSG0000(	778	17.61693	chr1:373:	LINC02793		lncRNA	chr1:25041136-2504
ENSG0000(	778	17.61693	chr1:373:	ZNF593		protein_c	chr1:26169908-2617
ENSG0000(	778	17.61693	chr1:373:	MAP3K6	NCGv7	protein_c	chr1:27355184-2736
ENSG0000(	778	17.61693	chr1:373:	FAM110D		protein_c	chr1:26159079-2616
ENSG0000(	778	17.61693	chr1:373:	MIR1976		smallRNA	chr1:26554542-2655
ENSG0000(	778	17.61693	chr1:373:	snoU13		smallRNA	chr1:26642286-2664
ENSG0000(	778	17.61693	chr1:373:	ENSG00000287810		Pseudoger	chr1:26817300-2681
ENSG0000(	778	17.61693	chr1:373:	AUNIP		protein_c	chr1:25831913-2585
ENSG0000(	778	17.61693	chr1:373:	CHCHD3P3		Pseudoger	chr1:27200834-2720
ENSG0000(	778	17.61693	chr1:373:	ENSG00000229247		Pseudoger	chr1:26640377-2664
ENSG0000(	778	17.61693	chr1:373:	ENSG00000255054		protein_c	chr1:25811470-2582
ENSG0000(	778	17.61693	chr1:373:	ENSG00000270031		lncRNA	chr1:27819983-2782
ENSG0000(	778	17.61693	chr1:373:	WDTC1	NCGv7	protein_c	chr1:27234632-2730

ENSG0000(	778	17.61693	chr1:3733	GPN2		protein_c	chr1:26876132-2689
ENSG0000(	778	17.61693	chr1:3733	SYTL1	NCGv7	protein_c	chr1:27342020-2735
ENSG0000(	778	17.61693	chr1:3733	ENSG00000227312		Pseudoger	chr1:24563627-2456
ENSG0000(	778	17.61693	chr1:3733	ENSG00000237429		lncRNA	chr1:27525805-2753
ENSG0000(	778	17.61693	chr1:3733	TRNP1		protein_c	chr1:26993692-2700
ENSG0000(	778	17.61693	chr1:3733	RN7SL679P		smallRNA	chr1:26593246-2659
ENSG0000(	778	17.61693	chr1:3733	ENSG00000231344		Pseudoger	chr1:27739091-2773
ENSG0000(	778	17.61693	chr1:3733	ENSG00000284657		lncRNA	chr1:25239494-2524
ENSG0000(	778	17.61693	chr1:3733	CLIC4		protein_c	chr1:24745382-2484
ENSG0000(	778	17.61693	chr1:3733	AL109927.1		protein_c	chr1:27850574-2785
ENSG0000(	778	17.61693	chr1:3733	SCARNA1		smallRNA	chr1:27834401-2783
ENSG0000(	778	17.61693	chr1:3733	CATSPER4		protein_c	chr1:26190561-2620
ENSG0000(	778	17.61693	chr1:3733	FGR	TAG;AC	protein_c	chr1:27612064-2763
ENSG0000(	778	17.61693	chr1:3733	RNU6-110P		smallRNA	chr1:25964197-2596
ENSG0000(	778	17.61693	chr1:3733	CD164L2		protein_c	chr1:27379176-2738
ENSG0000(	778	17.61693	chr1:3733	RPL12P13		Pseudoger	chr1:26980165-2698
ENSG0000(	778	17.61693	chr1:3733	GPATCH3		protein_c	chr1:26890488-2690
ENSG0000(	778	17.61693	chr1:3733	ENSG00000284602		lncRNA	chr1:25232586-2523
ENSG0000(	778	17.61693	chr1:3733	snoU13		smallRNA	chr1:24666983-2466
ENSG0000(	778	17.61693	chr1:3733	RHCE		protein_c	chr1:25362249-2543
ENSG0000(	778	17.61693	chr1:3733	MIR3917		smallRNA	chr1:25906362-2590
ENSG0000(	778	17.61693	chr1:3733	Y_RNA		smallRNA	chr1:25877496-2587
ENSG0000(	778	17.61693	chr1:3733	ENSG00000269971		lncRNA	chr1:27773858-2777
ENSG0000(	778	17.61693	chr1:3733	ENSG00000286433		lncRNA	chr1:27827812-2783
ENSG0000(	778	17.61693	chr1:3733	RHD	DriverDBv	protein_c	chr1:25272393-2533
ENSG0000(	778	17.61693	chr1:3733	THEMIS2		protein_c	chr1:27872543-2788
ENSG0000(	778	17.61693	chr1:3733	RNU6-1208P		smallRNA	chr1:24777873-2477
ENSG0000(	778	17.61693	chr1:3733	ENSG00000238084		Pseudoger	chr1:25398721-2539
ENSG0000(	778	17.61693	chr1:3733	RUNX3	AC	protein_c	chr1:24899511-2496
ENSG0000(	778	17.61693	chr1:3733	CHMP1AP1		Pseudoger	chr1:27686810-2768
ENSG0000(	778	17.61693	chr1:3733	snoU13		smallRNA	chr1:25346274-2534
ENSG0000(	778	17.61693	chr1:3733	ENSG00000226698		lncRNA	chr1:26876133-2687
ENSG0000(	778	17.61693	chr1:3733	ENSG00000233755		lncRNA	chr1:25043707-2511
ENSG0000(	778	17.61693	chr1:3733	SMPDL3B		protein_c	chr1:27935000-2795
ENSG0000(	778	17.61693	chr1:3733	CEP85		protein_c	chr1:26234200-2627
ENSG0000(	778	17.61693	chr1:3733	RUNX3-AS1		lncRNA	chr1:24961345-2496
ENSG0000(	778	17.61693	chr1:3733	SH3BGRL3		protein_c	chr1:26280086-2628
ENSG0000(	778	17.61693	chr1:3733	HMGN2		protein_c	chr1:26472440-2647
ENSG0000(	778	17.61693	chr1:3733	CNKSR1	NCGv7	protein_c	chr1:26177484-2618
ENSG0000(	778	17.61693	chr1:3733	ENSG00000243659		Pseudoger	chr1:27311240-2731
ENSG0000(	778	17.61693	chr1:3733	ENSG00000231207		Pseudoger	chr1:27389468-2739
ENSG0000(	778	17.61693	chr1:3733	ENSG00000260063		lncRNA	chr1:26692132-2669
ENSG0000(	773	17.50371	chrX:1578	RNU6-707P		smallRNA	chrX:48153980-4815
ENSG0000(	773	17.50371	chrX:1578	MAGIX		protein_c	chrX:49162987-4916
ENSG0000(	773	17.50371	chrX:1578	ENSG00000290184		protein_c	chrX:49258343-4927
ENSG0000(	773	17.50371	chrX:1578	ENSG00000233139		Pseudoger	chrX:48153868-4815
ENSG0000(	773	17.50371	chrX:1578	ENSG00000286031		lncRNA	chrX:49279677-4928
ENSG0000(	773	17.50371	chrX:1578	GAGE2A		protein_c	chrX:49589496-4959
ENSG0000(	773	17.50371	chrX:1578	S100A11P8		Pseudoger	chrX:52770809-5277
ENSG0000(	773	17.50371	chrX:1578	ENSG00000279155		TEC	chrX:48939992-4894
ENSG0000(	773	17.50371	chrX:1578	ENSG00000234780		Pseudoger	chrX:48197221-4819

ENSG0000(	773	17.50371	chrX:1578	ENSG00000228343		lncRNA	chrX:48579774-4858
ENSG0000(	773	17.50371	chrX:1578	S100A11P6		Pseudoger	chrX:48274904-4827
ENSG0000(	773	17.50371	chrX:1578	ENSG00000232828		lncRNA	chrX:48698963-4873
ENSG0000(	773	17.50371	chrX:1578	ZNF182		protein_c	chrX:47974851-4800
ENSG0000(	773	17.50371	chrX:1578	ENSG00000280116		Pseudoger	chrX:49155242-4915
ENSG0000(	773	17.50371	chrX:1578	ENSG00000291285		lncRNA	chrX:48107992-4811
ENSG0000(	773	17.50371	chrX:1578	ENSG00000235224		Pseudoger	chrX:53113018-5311
ENSG0000(	773	17.50371	chrX:1578	SSX4B		protein_c	chrX:48402082-4841
ENSG0000(	773	17.50371	chr2:8187	FABP7P2		Pseudoger	chr2:95368507-9536
ENSG0000(	773	17.50371	chrX:1578	USP27X		protein_c	chrX:49879484-4988
ENSG0000(	773	17.50371	chrX:1578	ENSG00000270012		lncRNA	chrX:49273054-4927
ENSG0000(	773	17.50371	chrX:1578	EIF4A2P4		Pseudoger	chrX:52832704-5283
ENSG0000(	773	17.50371	chrX:1578	ENSG00000241207		Pseudoger	chrX:48276704-4827
ENSG0000(	773	17.50371	chrX:1578	GAGE12H		protein_c	chrX:49579949-4958
ENSG0000(	773	17.50371	chrX:1578	ENSG00000196395		Pseudoger	chrX:52824269-5282
ENSG0000(	773	17.50371	chrX:1578	ENSG00000225055		Pseudoger	chrX:48357125-4835
ENSG0000(	773	17.50371	chrX:1578	SSXP3		Pseudoger	chrX:48156367-4816
ENSG0000(	773	17.50371	chrX:1578	ENSG00000286118		lncRNA	chrX:52925956-5292
ENSG0000(	773	17.50371	chrX:1578	ENSG00000231489		Pseudoger	chrX:48423342-4842
ENSG0000(	773	17.50371	chrX:1578	PAGE1		protein_c	chrX:49687447-4969
ENSG0000(	773	17.50371	chrX:1578	SSX9P		lncRNA	chrX:48301550-4830
ENSG0000(	773	17.50371	chrX:1578	ENSG00000290734		lncRNA	chrX:48117084-4812
ENSG0000(	773	17.50371	chrX:1578	SSX4	NCGv7;AC	protein_c	chrX:48383516-4839
ENSG0000(	773	17.50371	chrX:1578	ENSG00000226971		Pseudoger	chrX:48446893-4844
ENSG0000(	773	17.50371	chrX:1578	AF196972.2		Pseudoger	chrX:48412303-4841
ENSG0000(	773	17.50371	chrX:1578	ACAA2P1		Pseudoger	chrX:48775644-4877
ENSG0000(	773	17.50371	chrX:1578	ENSG00000286268		protein_c	chrX:48521806-4854
ENSG0000(	773	17.50371	chrX:1578	ENSG00000226867		Pseudoger	chrX:52781452-5278
ENSG0000(	773	17.50371	chrX:1578	ENSG00000229662		Pseudoger	chrX:48244894-4824
ENSG0000(	773	17.50371	chrX:1578	ERAS	AC	protein_c	chrX:48826513-4883
ENSG0000(	773	17.50371	chrX:1578	PRICKLE3	DriverDB\	protein_c	chrX:49174802-4918
ENSG0000(	773	17.50371	chrX:1578	WAS	NCGv7;AC	protein_c	chrX:48676596-4869
ENSG0000(	773	17.50371	chrX:1578	PPP1R3F		protein_c	chrX:49269793-4930
ENSG0000(	773	17.50371	chrX:1578	SLC38A5		protein_c	chrX:48458537-4847
ENSG0000(	773	17.50371	chrX:1578	FOXP3		protein_c	chrX:49250438-4926
ENSG0000(	773	17.50371	chrX:1578	S100A11P7		Pseudoger	chrX:48228833-4822
ENSG0000(	773	17.50371	chrX:1578	S100A11P9		Pseudoger	chrX:48336899-4833
ENSG0000(	773	17.50371	chrX:1578	AL139396.1		smallRNA	chrX:53143034-5314
ENSG0000(	773	17.50371	chrX:1578	ENSG00000270497		Pseudoger	chrX:53065053-5306
ENSG0000(	773	17.50371	chrX:1578	AF196779.1		smallRNA	chrX:49203242-4920
ENSG0000(	773	17.50371	chrX:1578	KANTR		protein_c	chrX:53094142-5317
ENSG0000(	773	17.50371	chrX:1578	RNA5SP503		Pseudoger	chrX:48206258-4820
ENSG0000(	773	17.50371	chrX:1578	ENSG00000234019		lncRNA	chrX:53093710-5309
ENSG0000(	773	17.50371	chrX:1578	SSX1	NCGv7;AC	protein_c	chrX:48255392-4826
ENSG0000(	773	17.50371	chrX:1578	ENSG00000286181		lncRNA	chrX:49262866-4927
ENSG0000(	773	17.50371	chrX:1578	TIMM17B		protein_c	chrX:48893447-4889
ENSG0000(	773	17.50371	chrX:1578	PRAF2	DriverDB\	protein_c	chrX:49071161-4907
ENSG0000(	773	17.50371	chrX:1578	WDR45	NCGv7	protein_c	chrX:49074433-4910
ENSG0000(	773	17.50371	chrX:1578	FTSJ1	DriverDB\	protein_c	chrX:48476021-4848
ENSG0000(	773	17.50371	chrX:1578	GRIPAP1		protein_c	chrX:48973720-4900
ENSG0000(	773	17.50371	chrX:1578	GPKOW	DriverDB\	protein_c	chrX:49113407-4912

ENSG0000(	773	17.50371	chrX:1578	TBC1D25		protein_c	chrX:48539714-4856
ENSG0000(	773	17.50371	chrX:1578	TFE3	NCGv7;AC	protein_c	chrX:49028726-4904
ENSG0000(	773	17.50371	chrX:1578	OTUD5	DriverDBv	protein_c	chrX:48922024-4895
ENSG0000(	773	17.50371	chrX:1578	ACTG1P10		Pseudoger	chrX:53142832-5314
ENSG0000(	773	17.50371	chrX:1578	GAGE12D		protein_c	chrX:49541733-4954
ENSG0000(	773	17.50371	chrX:1578	SSX2B	AC	protein_c	chrX:52751132-5279
ENSG0000(	773	17.50371	chrX:1578	ENSG00000234448		Pseudoger	chrX:48371905-4837
ENSG0000(	773	17.50371	chrX:1578	ENSG00000279528		TEC	chrX:48580741-4858
ENSG0000(	773	17.50371	chrX:1578	ENSG00000229885		Pseudoger	chrX:52801400-5280
ENSG0000(	773	17.50371	chrX:1578	SALL1P1		Pseudoger	chrX:49664844-4966
ENSG0000(	773	17.50371	chrX:1578	ENSG00000234391		Pseudoger	chrX:48306769-4830
ENSG0000(	773	17.50371	chrX:1578	GPR173		protein_c	chrX:53048789-5308
ENSG0000(	773	17.50371	chrX:1578	USP27X-DT		lncRNA	chrX:49876724-4987
ENSG0000(	773	17.50371	chrX:1578	FAM156A		protein_c	chrX:52926402-5299
ENSG0000(	773	17.50371	chrX:1578	TSPYL2	NCGv7	protein_c	chrX:53082367-5308
ENSG0000(	773	17.50371	chrX:1578	SSXP9		Pseudoger	chrX:48322349-4832
ENSG0000(	773	17.50371	chrX:1578	AF207550.1		Pseudoger	chrX:48939992-4894
ENSG0000(	773	17.50371	chrX:1578	RP11-344N17.12		Pseudoger	chrX:48380150-4838
ENSG0000(	773	17.50371	chrX:1578	S100A11P5		Pseudoger	chrX:48177731-4817
ENSG0000(	773	17.50371	chrX:1578	EBP	DriverDBv	protein_c	chrX:48521799-4852
ENSG0000(	773	17.50371	chrX:1578	RNU7-37P		smallRNA	chrX:53130481-5313
ENSG0000(	773	17.50371	chrX:1578	RNU6-1056P		smallRNA	chrX:48724455-4872
ENSG0000(	773	17.50371	chrX:1578	HDAC6		protein_c	chrX:48801377-4882
ENSG0000(	773	17.50371	chrX:1578	PCSK1N		protein_c	chrX:48831096-4883
ENSG0000(	773	17.50371	chrX:1578	GATA1	NCGv7;AC	protein_c	chrX:48786540-4879
ENSG0000(	773	17.50371	chrX:1578	PORCN	NCGv7	protein_c	chrX:48508959-4852
ENSG0000(	773	17.50371	chrX:1578	RBM3	TAG;AC	protein_c	chrX:48574449-4858
ENSG0000(	773	17.50371	chrX:1578	ENSG00000287757		lncRNA	chrX:48071223-4807
ENSG0000(	773	17.50371	chrX:1578	GAGE1		protein_c	chrX:49599020-4960
ENSG0000(	773	17.50371	chrX:1578	GAGE2E		protein_c	chrX:49331626-4933
ENSG0000(	773	17.50371	chrX:1578	SSX3		protein_c	chrX:48346427-4835
ENSG0000(	773	17.50371	chrX:1578	SSX5		protein_c	chrX:48186220-4819
ENSG0000(	773	17.50371	chrX:1578	snoU13		smallRNA	chrX:48081767-4808
ENSG0000(	773	17.50371	chrX:1578	RNU6-722P		smallRNA	chrX:48959179-4895
ENSG0000(	773	17.50371	chrX:1578	SSX9P		Pseudoger	chrX:48296816-4830
ENSG0000(	773	17.50371	chrX:1578	ENSG00000204620		lncRNA	chrX:48568014-4857
ENSG0000(	773	17.50371	chrX:1578	GAGE10		protein_c	chrX:49303646-4931
ENSG0000(	773	17.50371	chrX:1578	FAM156B		protein_c	chrX:52891306-5290
ENSG0000(	773	17.50371	chrX:1578	RN7SL262P		smallRNA	chrX:49152651-4915
ENSG0000(	773	17.50371	chrX:1578	GAGE12G		protein_c	chrX:49570400-4957
ENSG0000(	773	17.50371	chrX:1578	ENSG00000230100		lncRNA	chrX:48333675-4833
ENSG0000(	773	17.50371	chrX:1578	ENSG00000204368		Pseudoger	chrX:48212791-4821
ENSG0000(	773	17.50371	chrX:1578	SPANXN5		protein_c	chrX:52796144-5279
ENSG0000(	773	17.50371	chrX:1578	GAGE12C		protein_c	chrX:49532177-4953
ENSG0000(	773	17.50371	chrX:1578	CCDC120	NCGv7	protein_c	chrX:49053572-4906
ENSG0000(	773	17.50371	chrX:1578	GAGE12F		protein_c	chrX:49551278-4956
ENSG0000(	773	17.50371	chrX:1578	ENSG00000237345		Pseudoger	chrX:48279423-4828
ENSG0000(	773	17.50371	chrX:1578	VN1R110P		Pseudoger	chrX:48636165-4863
ENSG0000(	773	17.50371	chrX:1578	PQBP1		protein_c	chrX:48890197-4890
ENSG0000(	773	17.50371	chrX:1578	MRPL32P1		Pseudoger	chrX:48583093-4858
ENSG0000(	773	17.50371	chrX:1578	CPSF1P2		Pseudoger	chrX:49911620-4991

ENSG0000(	773	17.50371	chrX:1578	ENSG00000288908		lncRNA	chrX:48958643-4895
ENSG0000(	773	17.50371	chrX:1578	WDR13	DriverDBv	protein_c	chrX:48590042-4860
ENSG0000(	773	17.50371	chrX:1578	RNU6-29P		smallRNA	chrX:48776965-4877
ENSG0000(	773	17.50371	chrX:1578	SUV39H1		protein_c	chrX:48695554-4870
ENSG0000(	773	17.50371	chrX:1578	PAGE4		protein_c	chrX:49829260-4983
ENSG0000(	773	17.50371	chrX:1578	CCDC22		protein_c	chrX:49235470-4925
ENSG0000(	773	17.50371	chrX:1578	snoU13		smallRNA	chrX:48561702-4856
ENSG0000(	773	17.50371	chrX:1578	CACNA1F		protein_c	chrX:49205063-4923
ENSG0000(	773	17.50371	chrX:1578	SYP		protein_c	chrX:49187815-4920
ENSG0000(	773	17.50371	chrX:1578	PLP2	DriverDBv	protein_c	chrX:49171898-4917
ENSG0000(	773	17.50371	chrX:1578	RN7SL139P		smallRNA	chrX:49007559-4900
ENSG0000(	773	17.50371	chrX:1578	GAGE12B		protein_c	chrX:49529869-4952
ENSG0000(	773	17.50371	chrX:1578	ENSG00000289245		lncRNA	chrX:49155862-4915
ENSG0000(	773	17.50371	chrX:1578	GAGE12E		protein_c	chrX:49551289-4955
ENSG0000(	773	17.50371	chrX:1578	KCND1		protein_c	chrX:48961378-4897
ENSG0000(	773	17.50371	chrX:1578	ZNF630		protein_c	chrX:47983356-4807
ENSG0000(	773	17.50371	chrX:1578	RNU4-52P		smallRNA	chrX:49082028-4908
ENSG0000(	773	17.50371	chrX:1578	PIM2	DriverDBv	protein_c	chrX:48913182-4891
ENSG0000(	773	17.50371	chrX:1578	ENSG00000230241		Pseudoger	chrX:48135658-4813
ENSG0000(	773	17.50371	chrX:1578	SYP-AS1		lncRNA	chrX:49198966-4920
ENSG0000(	773	17.50371	chrX:1578	SLC35A2	DriverDBv	protein_c	chrX:48903180-4891
ENSG0000(	773	17.50371	chrX:1578	ZNF630-AS1		lncRNA	chrX:48056310-4806
ENSG0000(	773	17.50371	chrX:1578	HSPB1P2		Pseudoger	chrX:49233956-4923
ENSG0000(	773	17.50371	chrX:1578	VDAC1P2		Pseudoger	chrX:49632500-4963
ENSG0000(	773	17.50371	chrX:1578	SSX6P		Pseudoger	chrX:48109981-4811
ENSG0000(	773	17.50371	chrX:1578	XAGE3		protein_c	chrX:52862525-5286
ENSG0000(	773	17.50371	chrX:1578	ENSG00000288053		protein_c	chrX:49071470-4907
ENSG0000(	773	17.50371	chrX:1578	GLOD5		protein_c	chrX:48761747-4877
ENSG0000(	773	17.50371	chrX:1578	XAGE5		protein_c	chrX:52811287-5281
ENSG0000(	773	17.50371	chrX:1578	SPACA5		protein_c	chrX:48004336-4800
ENSG0000(	773	17.50371	chrX:1578	ENSG00000235350		Pseudoger	chrX:48433201-4844
ENSG0000(	773	17.50371	chrX:1578	PORCN-DT		lncRNA	chrX:48506523-4850
ENSG0000(	773	17.50371	chrX:1578	SPACA5B		protein_c	chrX:48130626-4813
ENSG0000(	773	17.50371	chrX:1578	RP11-552J9.15		Pseudoger	chrX:52748497-5275
ENSG0000(	770	17.43578	chr4:1865	ENSG00000250677		Pseudoger	chr4:83237303-8323
ENSG0000(	767	17.36785	chr1:3733	RPL11		protein_c	chr1:23691742-2369
ENSG0000(	767	17.36785	chr2:8187	AC008064.1		smallRNA	chr2:53651401-5365
ENSG0000(	767	17.36785	chr1:3733	ASAP3		protein_c	chr1:23428563-2348
ENSG0000(	767	17.36785	chr1:3733	PNRC2		protein_c	chr1:23956839-2396
ENSG0000(	767	17.36785	chr1:3733	RP5-886K2.1		Pseudoger	chr1:23705801-2370
ENSG0000(	767	17.36785	chr1:3733	RCAN3AS		lncRNA	chr1:24496254-2453
ENSG0000(	767	17.36785	chr1:3733	ENSG00000285802		lncRNA	chr1:23576436-2359
ENSG0000(	767	17.36785	chr1:3733	CNR2	AC	protein_c	chr1:23870515-2391
ENSG0000(	767	17.36785	chr1:3733	MIR378F		smallRNA	chr1:23929070-2392
ENSG0000(	767	17.36785	chr1:3733	ENSG00000225315		lncRNA	chr1:24040835-2408
ENSG0000(	767	17.36785	chr1:3733	MYOM3	NCGv7	protein_c	chr1:24056035-2411
ENSG0000(	767	17.36785	chr1:3733	IL22RA1		protein_c	chr1:24119771-2414
ENSG0000(	767	17.36785	chr1:3733	MYOM3-AS1		lncRNA	chr1:24066774-2408
ENSG0000(	767	17.36785	chr1:3733	ENSG00000232557		lncRNA	chr1:23907111-2390
ENSG0000(	767	17.36785	chr1:3733	ELOA-AS1		lncRNA	chr1:23706901-2377
ENSG0000(	767	17.36785	chr1:3733	SRSF10		protein_c	chr1:23964347-2398

ENSG0000(	767	17.36785	chr1:373:	LINC02800		lncRNA	chr1:24200240-2421
ENSG0000(	767	17.36785	chr1:373:	RPL36P5		Pseudoger	chr1:24007881-2400
ENSG0000(	767	17.36785	chr1:373:	RN7SL24P		smallRNA	chr1:23881794-2388
ENSG0000(	767	17.36785	chr1:373:	NIPAL3		protein_c	chr1:24415802-2447
ENSG0000(	767	17.36785	chr1:373:	GRHL3		protein_c	chr1:24199558-2436
ENSG0000(	767	17.36785	chr1:373:	IFNLR1		protein_c	chr1:24154168-2418
ENSG0000(	767	17.36785	chr1:373:	STPG1		protein_c	chr1:24356999-2441
ENSG0000(	767	17.36785	chr1:373:	GRHL3-AS1		lncRNA	chr1:24307556-2432
ENSG0000(	767	17.36785	chr1:373:	PITHD1		protein_c	chr1:23778418-2378
ENSG0000(	767	17.36785	chr1:373:	snoU13		smallRNA	chr1:23882255-2388
ENSG0000(	767	17.36785	chr1:373:	E2F2	NCGv7	protein_c	chr1:23506438-2353
ENSG0000(	767	17.36785	chr1:373:	FUCA1		protein_c	chr1:23845077-2386
ENSG0000(	767	17.36785	chr1:373:	GALE		protein_c	chr1:23795599-2380
ENSG0000(	767	17.36785	chr1:373:	ENSG00000271420		lncRNA	chr1:23378380-2337
ENSG0000(	767	17.36785	chr1:373:	ENSG00000232482		Pseudoger	chr1:23410832-2341
ENSG0000(	767	17.36785	chr1:373:	ENSG00000289835		lncRNA	chr1:23790609-2379
ENSG0000(	767	17.36785	chr1:373:	EEF1A1P48		Pseudoger	chr1:23670294-2368
ENSG0000(	767	17.36785	chr1:373:	HMGCL		protein_c	chr1:23801885-2383
ENSG0000(	767	17.36785	chr1:373:	ENSG00000288982		lncRNA	chr1:24476362-2447
ENSG0000(	767	17.36785	chr1:373:	ENSG00000235052		lncRNA	chr1:23549139-2355
ENSG0000(	767	17.36785	chr1:373:	AL590683.1		smallRNA	chr1:24227917-2422
ENSG0000(	767	17.36785	chr1:373:	LYPLA2		protein_c	chr1:23791145-2379
ENSG0000(	767	17.36785	chr1:373:	MDS2	AC	lncRNA	chr1:23581495-2364
ENSG0000(	767	17.36785	chr1:373:	TCEA3		protein_c	chr1:23380909-2342
ENSG0000(	767	17.36785	chr1:373:	ID3	NCGv7	protein_c	chr1:23557926-2355
ENSG0000(	767	17.36785	chr1:373:	RN7SL532P		smallRNA	chr1:23736610-2373
ENSG0000(	767	17.36785	chr1:373:	BTBD6P1		Pseudoger	chr1:23901471-2390
ENSG0000(	767	17.36785	chr1:373:	ELOA		protein_c	chr1:23743448-2376
ENSG0000(	765	17.32256	chr2:257:	RNA5SP108		Pseudoger	chr2:161409554-161
ENSG0000(	763	17.27727	chr1:373:	ENSG00000231251		Pseudoger	chr1:30226523-3022
ENSG0000(	762	17.25463	chr4:253(	ENSG00000286150		lncRNA	chr4:100421655-100
ENSG0000(	762	17.25463	chr4:253(	ENSG00000254044		lncRNA	chr4:97334635-9763
ENSG0000(	762	17.25463	chr4:253(	DDIT4L-AS1		lncRNA	chr4:100190033-100
ENSG0000(	762	17.25463	chr4:253(	ENSG00000251523		lncRNA	chr4:98496364-9850
ENSG0000(	762	17.25463	chr4:253(	ADH4		protein_c	chr4:99123657-9915
ENSG0000(	762	17.25463	chr4:253(	LAMTOR3		protein_c	chr4:99878336-9989
ENSG0000(	762	17.25463	chr4:253(	C4orf17		protein_c	chr4:99511012-9954
ENSG0000(	762	17.25463	chr4:253(	CRYZP2		Pseudoger	chr4:97916353-9791
ENSG0000(	762	17.25463	chr4:253(	ENSG00000250300		Pseudoger	chr4:99469598-9949
ENSG0000(	762	17.25463	chr4:253(	PPP3CA	NCGv7	protein_c	chr4:101023409-101
ENSG0000(	762	17.25463	chr4:253(	AC121157.1		smallRNA	chr4:100129945-100
ENSG0000(	762	17.25463	chr4:253(	ADH5		protein_c	chr4:99070978-9908
ENSG0000(	762	17.25463	chr4:253(	C4orf54		protein_c	chr4:99636529-9965
ENSG0000(	762	17.25463	chr4:253(	ENSG00000248676		lncRNA	chr4:99594799-9962
ENSG0000(	762	17.25463	chr4:253(	ENSG00000250403		Pseudoger	chr4:99990737-9999
ENSG0000(	762	17.25463	chr4:253(	LINC02267		lncRNA	chr4:96310701-9681
ENSG0000(	762	17.25463	chr4:253(	ABT1P1		Pseudoger	chr4:99022311-9902
ENSG0000(	762	17.25463	chr4:253(	COX7A2P2		Pseudoger	chr4:96902801-9690
ENSG0000(	762	17.25463	chr4:253(	METAP1		protein_c	chr4:98995659-9906
ENSG0000(	762	17.25463	chr4:253(	STPG2		protein_c	chr4:97184093-9814
ENSG0000(	762	17.25463	chr4:253(	LINC01218		lncRNA	chr4:100812255-100

ENSG0000(	762	17.25463	chr4:253(	ENSG00000279098		TEC	chr4:99942081-9994
ENSG0000(	762	17.25463	chr4:253(	STPG2-AS1		lncRNA	chr4:97366681-9749
ENSG0000(	762	17.25463	chr4:253(	ENSG00000272777		lncRNA	chr4:99067256-9906
ENSG0000(	762	17.25463	chr4:253(	ENSG00000263923		lncRNA	chr4:98928897-9899
ENSG0000(	762	17.25463	chr4:253(	MTTP		protein_c	chr4:99564081-9962
ENSG0000(	762	17.25463	chr4:253(	MIR1255A		smallRNA	chr4:101330302-101
ENSG0000(	762	17.25463	chr4:253(	TSPAN5		protein_c	chr4:98470367-9865
ENSG0000(	762	17.25463	chr4:253(	BTF3P13		Pseudoger	chr4:98740742-9874
ENSG0000(	762	17.25463	chr4:253(	TSPAN5-DT		lncRNA	chr4:98658894-9866
ENSG0000(	762	17.25463	chr4:253(	RPL5P12		Pseudoger	chr4:98025390-9802
ENSG0000(	762	17.25463	chr4:253(	ADH1C		protein_c	chr4:99336497-9935
ENSG0000(	762	17.25463	chr4:253(	ENSG00000287512		lncRNA	chr4:98961083-9896
ENSG0000(	762	17.25463	chr4:253(	ENSG00000250655		Pseudoger	chr4:96212279-9621
ENSG0000(	762	17.25463	chr4:253(	ENSG00000249764		Pseudoger	chr4:98713804-9871
ENSG0000(	762	17.25463	chr4:253(	DYNLL1P6		Pseudoger	chr4:100041841-100
ENSG0000(	762	17.25463	chr4:253(	BANK1		protein_c	chr4:101411286-102
ENSG0000(	762	17.25463	chr4:253(	EMCN		protein_c	chr4:100395341-100
ENSG0000(	762	17.25463	chr4:253(	DNAJB14		protein_c	chr4:99896248-9994
ENSG0000(	762	17.25463	chr4:253(	FLJ20021		lncRNA	chr4:101347752-101
ENSG0000(	762	17.25463	chr4:253(	ENSG00000246090		lncRNA	chr4:99088805-9930
ENSG0000(	762	17.25463	chr4:253(	TBCAP3		Pseudoger	chr4:98909537-9890
ENSG0000(	762	17.25463	chr4:253(	PCNAP1		Pseudoger	chr4:99160514-9916
ENSG0000(	762	17.25463	chr4:253(	AC034154.1		smallRNA	chr4:97486806-9748
ENSG0000(	762	17.25463	chr4:253(	LINC01217		lncRNA	chr4:100778582-100
ENSG0000(	762	17.25463	chr4:253(	EIF4E	AC	protein_c	chr4:98879276-9892
ENSG0000(	762	17.25463	chr4:253(	ADH1B	NCGv7	protein_c	chr4:99304971-9935
ENSG0000(	762	17.25463	chr4:253(	ENSG00000214559		lncRNA	chr4:98251688-9826
ENSG0000(	762	17.25463	chr4:253(	H2AZ1		protein_c	chr4:99948086-9995
ENSG0000(	762	17.25463	chr4:253(	AC019131.1		smallRNA	chr4:98929923-9892
ENSG0000(	762	17.25463	chr4:253(	ADH1A		protein_c	chr4:99276369-9929
ENSG0000(	762	17.25463	chr4:253(	DAPP1		protein_c	chr4:99816827-9987
ENSG0000(	762	17.25463	chr4:253(	ADH7		protein_c	chr4:99412261-9943
ENSG0000(	762	17.25463	chr4:253(	RN7SKP28		smallRNA	chr4:96348738-9634
ENSG0000(	762	17.25463	chr4:253(	DUTP8		Pseudoger	chr4:98048555-9804
ENSG0000(	762	17.25463	chr4:253(	MIR3684		smallRNA	chr4:98997387-9899
ENSG0000(	762	17.25463	chr4:253(	RNU7-149P		smallRNA	chr4:98966815-9896
ENSG0000(	762	17.25463	chr4:253(	RNU6-34P		smallRNA	chr4:96152297-9615
ENSG0000(	762	17.25463	chr4:253(	H2AZ1-DT		lncRNA	chr4:99950006-1001
ENSG0000(	762	17.25463	chr4:253(	FAM177A1P1		Pseudoger	chr4:98955982-9895
ENSG0000(	762	17.25463	chr4:253(	ENSG00000287841		lncRNA	chr4:96841995-9685
ENSG0000(	762	17.25463	chr4:253(	ENSG00000279913		TEC	chr4:99844138-9984
ENSG0000(	762	17.25463	chr4:253(	Y_RNA		smallRNA	chr4:98173784-9817
ENSG0000(	762	17.25463	chr4:253(	ENSG00000253170		lncRNA	chr4:97120701-9713
ENSG0000(	762	17.25463	chr4:253(	NDUFS5P4		Pseudoger	chr4:98976800-9897
ENSG0000(	762	17.25463	chr4:253(	RAP1GDS1	NCGv7;AC	protein_c	chr4:98261384-9844
ENSG0000(	762	17.25463	chr4:253(	ENSG00000242318		Pseudoger	chr4:98409290-9840
ENSG0000(	762	17.25463	chr4:253(	DDIT4L		protein_c	chr4:100185870-100
ENSG0000(	762	17.25463	chr4:253(	LINC01216		lncRNA	chr4:100660279-100
ENSG0000(	762	17.25463	chr4:253(	ENSG00000289532		lncRNA	chr4:98259390-9826
ENSG0000(	762	17.25463	chr4:253(	TRMT10A		protein_c	chr4:99546709-9956
ENSG0000(	762	17.25463	chr4:253(	ADH6		protein_c	chr4:99202638-9921

ENSG00000	760	17.20934	chr1:1534	AL591806.1		protein_c	chr1:161065865-161
ENSG00000	760	17.20934	chr1:1534	SMIM42		protein_c	chr1:158127287-158
ENSG00000	760	17.20934	chr1:1534	CFAP126		protein_c	chr1:161364733-161
ENSG00000	760	17.20934	chr1:1534	ENSG00000237409		Pseudoger	chr1:160020300-160
ENSG00000	760	17.20934	chr1:1534	ENSG00000288670		lncRNA	chr1:161368022-161
ENSG00000	760	17.20934	chr1:1534	SNORD64		smallRNA	chr1:159851906-159
ENSG00000	760	17.20934	chr1:1534	ATF6-DT		lncRNA	chr1:161749452-161
ENSG00000	760	17.20934	chr1:1534	ENSG00000228560		lncRNA	chr1:159346166-159
ENSG00000	760	17.20934	chr1:1534	CFAP45	NCGv7	protein_c	chr1:159872364-159
ENSG00000	760	17.20934	chr1:1534	DCAF8-DT		lncRNA	chr1:160261731-160
ENSG00000	760	17.20934	chr1:1534	RPL31P11		Pseudoger	chr1:161683695-161
ENSG00000	760	17.20934	chr1:1534	ENSG00000213080		Pseudoger	chr1:160894980-160
ENSG00000	760	17.20934	chr1:1534	ELL2P1		Pseudoger	chr1:158175850-158
ENSG00000	760	17.20934	chr1:1534	FCGR2C		protein_c	chr1:161581339-161
ENSG00000	760	17.20934	chr1:1534	OR6N2		protein_c	chr1:158774222-158
ENSG00000	760	17.20934	chr1:1534	GLRX5P2		Pseudoger	chr1:161034834-161
ENSG00000	760	17.20934	chr1:1534	NECTIN4-AS1		lncRNA	chr1:161084465-161
ENSG00000	760	17.20934	chr1:1534	AL590560.1		protein_c	chr1:159910094-159
ENSG00000	760	17.20934	chr1:1534	AL121985.1		Pseudoger	chr1:160697970-160
ENSG00000	760	17.20934	chr1:1534	MIR5187		smallRNA	chr1:161227186-161
ENSG00000	760	17.20934	chr1:1534	ENSG00000273933		Pseudoger	chr1:159972548-159
ENSG00000	760	17.20934	chr1:1534	ENSG00000288093		lncRNA	chr1:161399998-161
ENSG00000	760	17.20934	chr1:1534	AL590714.1		protein_c	chr1:161220370-161
ENSG00000	760	17.20934	chr1:1534	ENSG00000270149		protein_c	chr1:160997957-161
ENSG00000	760	17.20934	chr1:1534	RP11-122G18.8		lncRNA	chr1:161374762-161
ENSG00000	760	17.20934	chr1:1534	ENSG00000228863		lncRNA	chr1:160670778-160
ENSG00000	760	17.20934	chr1:1534	OR6K2		protein_c	chr1:158699678-158
ENSG00000	760	17.20934	chr1:1534	ENSG00000273112		lncRNA	chr1:161513176-161
ENSG00000	760	17.20934	chr1:1534	OR6N1		protein_c	chr1:158747814-158
ENSG00000	760	17.20934	chr1:1534	HSPA6		protein_c	chr1:161524540-161
ENSG00000	760	17.20934	chr1:1534	RP11-226L15.5		lncRNA	chr1:160024953-160
ENSG00000	760	17.20934	chr1:1534	OR6Y1		protein_c	chr1:158544550-158
ENSG00000	760	17.20934	chr1:1534	ENSG00000227741		lncRNA	chr1:160202199-160
ENSG00000	760	17.20934	chr1:1534	ENSG00000215840		Pseudoger	chr1:161406068-161
ENSG00000	760	17.20934	chr1:1534	RAD1P2		Pseudoger	chr1:159081133-159
ENSG00000	760	17.20934	chr1:1534	OR10K1		protein_c	chr1:158461574-158
ENSG00000	760	17.20934	chr1:1534	TSTD1	DriverDBv	protein_c	chr1:161037631-161
ENSG00000	760	17.20934	chr1:1534	MPTX1		Pseudoger	chr1:159276503-159
ENSG00000	760	17.20934	chr1:1534	OR10R1P		Pseudoger	chr1:158514785-158
ENSG00000	760	17.20934	chr1:1534	OR10J3		protein_c	chr1:159313720-159
ENSG00000	760	17.20934	chr1:1534	ACKR1		protein_c	chr1:159203307-159
ENSG00000	760	17.20934	chr1:1534	ENSG00000274562		Pseudoger	chr1:160776975-160
ENSG00000	760	17.20934	chr1:1534	OR10J1		protein_c	chr1:159437845-159
ENSG00000	760	17.20934	chr1:1534	ENSG00000279430		TEC	chr1:159910094-159
ENSG00000	760	17.20934	chr1:1534	NHLH1		protein_c	chr1:160367071-160
ENSG00000	760	17.20934	chr1:1534	OR6K5P		Pseudoger	chr1:158742146-158
ENSG00000	760	17.20934	chr1:1534	SNHG28		lncRNA	chr1:159834480-159
ENSG00000	760	17.20934	chr1:1534	FCRL6		protein_c	chr1:159800511-159
ENSG00000	760	17.20934	chr1:1534	ENSG00000288775		lncRNA	chr1:159776325-159
ENSG00000	760	17.20934	chr1:1534	RNU4-42P		smallRNA	chr1:160392768-160
ENSG00000	760	17.20934	chr1:1534	OR10T1P		Pseudoger	chr1:158445068-158

ENSG0000(	760	17.20934	chr1:1534	OR10J4		protein_c	chr1:159432204-159
ENSG0000(	760	17.20934	chr1:1534	OR10K2		protein_c	chr1:158418210-158
ENSG0000(	760	17.20934	chr1:1534	DUSP12	AC	protein_c	chr1:161749758-161
ENSG0000(	760	17.20934	chr1:1534	LINC02819		lncRNA	chr1:159466321-159
ENSG0000(	760	17.20934	chr1:1534	RN7SL466P		smallRNA	chr1:161735808-161
ENSG0000(	760	17.20934	chr1:1534	ENSG00000224985		lncRNA	chr1:161153760-161
ENSG0000(	760	17.20934	chr1:1534	OR6K4P		Pseudoger	chr1:158724113-158
ENSG0000(	760	17.20934	chr1:1534	ENSG00000290105		lncRNA	chr1:159890207-159
ENSG0000(	760	17.20934	chr1:1534	OR6K6		protein_c	chr1:158754720-158
ENSG0000(	760	17.20934	chr1:1534	FCGR2B	NCGv7;AC	protein_c	chr1:161663143-161
ENSG0000(	760	17.20934	chr1:1534	OR10AA1P		Pseudoger	chr1:158808399-158
ENSG0000(	760	17.20934	chr1:1534	ENSG00000290115		lncRNA	chr1:161165695-161
ENSG0000(	760	17.20934	chr1:1534	ITLN1	NCGv7	protein_c	chr1:160876540-160
ENSG0000(	760	17.20934	chr1:1534	COPA		protein_c	chr1:160288594-160
ENSG0000(	760	17.20934	chr1:1534	OR10AE1P		Pseudoger	chr1:159581620-159
ENSG0000(	760	17.20934	chr1:1534	OR10J9P		Pseudoger	chr1:159405423-159
ENSG0000(	760	17.20934	chr1:1534	CRPP1		Pseudoger	chr1:159704983-159
ENSG0000(	760	17.20934	chr1:1534	HSPA7		Pseudoger	chr1:161606291-161
ENSG0000(	760	17.20934	chr1:1534	FCER1A	NCGv7	protein_c	chr1:159289714-159
ENSG0000(	760	17.20934	chr1:1534	ENSG00000289768		protein_c	chr1:161544807-161
ENSG0000(	760	17.20934	chr1:1534	IGSF9	DriverDB\	protein_c	chr1:159927039-159
ENSG0000(	760	17.20934	chr1:1534	ENSG00000225279		lncRNA	chr1:160062461-160
ENSG0000(	760	17.20934	chr1:1534	CD84		protein_c	chr1:160541095-160
ENSG0000(	760	17.20934	chr1:1534	ENSG00000276632		Pseudoger	chr1:159649151-159
ENSG0000(	760	17.20934	chr1:1534	PYDC5		protein_c	chr1:158999971-159
ENSG0000(	760	17.20934	chr1:1534	OR10J5		protein_c	chr1:159535078-159
ENSG0000(	760	17.20934	chr1:1534	ENSG00000234425		lncRNA	chr1:160537073-160
ENSG0000(	760	17.20934	chr1:1534	Y_RNA		smallRNA	chr1:160326104-160
ENSG0000(	760	17.20934	chr1:1534	SUMO1P3		Pseudoger	chr1:160317403-160
ENSG0000(	760	17.20934	chr1:1534	CADM3		protein_c	chr1:159171609-159
ENSG0000(	760	17.20934	chr1:1534	SLAMF9		protein_c	chr1:159951492-159
ENSG0000(	760	17.20934	chr1:1534	SLAMF1	NCGv7	protein_c	chr1:160608106-160
ENSG0000(	760	17.20934	chr1:1534	CD48		protein_c	chr1:160678746-160
ENSG0000(	760	17.20934	chr1:1534	ENSG00000224515		lncRNA	chr1:161556290-161
ENSG0000(	760	17.20934	chr1:1534	SPTA1	NCGv7	protein_c	chr1:158610704-158
ENSG0000(	760	17.20934	chr1:1534	MNDA		protein_c	chr1:158831351-158
ENSG0000(	760	17.20934	chr1:1534	PYHIN1		protein_c	chr1:158930796-158
ENSG0000(	760	17.20934	chr1:1534	IFI16		protein_c	chr1:158999968-159
ENSG0000(	760	17.20934	chr1:1534	AIM2		protein_c	chr1:159061599-159
ENSG0000(	760	17.20934	chr1:1534	ENSG00000287040		lncRNA	chr1:159900475-159
ENSG0000(	760	17.20934	chr1:1534	ENSG00000277882		Pseudoger	chr1:161411597-161
ENSG0000(	760	17.20934	chr1:1534	RPSAP18		Pseudoger	chr1:160266340-160
ENSG0000(	760	17.20934	chr1:1534	LINC01133		lncRNA	chr1:159958035-159
ENSG0000(	760	17.20934	chr1:1534	ENSG00000235226		Pseudoger	chr1:159759170-159
ENSG0000(	760	17.20934	chr1:1534	RPS23P10		Pseudoger	chr1:161536571-161
ENSG0000(	760	17.20934	chr1:1534	FCGR3A	NCGv7	protein_c	chr1:161541759-161
ENSG0000(	760	17.20934	chr1:1534	AL359753.1		smallRNA	chr1:159059249-159
ENSG0000(	760	17.20934	chr1:1534	OR6K3		protein_c	chr1:158716327-158
ENSG0000(	760	17.20934	chr1:1534	KLHDC9	DriverDB\	protein_c	chr1:161098361-161
ENSG0000(	760	17.20934	chr1:1534	FCGR3B		protein_c	chr1:161623196-161
ENSG0000(	760	17.20934	chr1:1534	FCRLB	DriverDB\	protein_c	chr1:161721544-161

ENSG0000(	760	17.20934	chr1:153⁴	SLAMF6		protein_c	chr1:160485030-160
ENSG0000(	760	17.20934	chr1:153⁴	VANGL2		protein_c	chr1:160400564-160
ENSG0000(	760	17.20934	chr1:153⁴	NCSTN	NCGv7	protein_c	chr1:160343294-160
ENSG0000(	760	17.20934	chr1:153⁴	PEX19		protein_c	chr1:160276807-160
ENSG0000(	760	17.20934	chr1:153⁴	SETP9		Pseudoger	chr1:160670148-160
ENSG0000(	760	17.20934	chr1:153⁴	IGSF8		protein_c	chr1:160091340-160
ENSG0000(	760	17.20934	chr1:153⁴	KCNJ9	NCGv7	protein_c	chr1:160081538-160
ENSG0000(	760	17.20934	chr1:153⁴	KCNJ10		protein_c	chr1:159998651-160
ENSG0000(	760	17.20934	chr1:153⁴	CD244		protein_c	chr1:160830160-160
ENSG0000(	760	17.20934	chr1:153⁴	ENSG00000233691		Pseudoger	chr1:160935537-160
ENSG0000(	760	17.20934	chr1:153⁴	ENSG00000272668		lncRNA	chr1:159854870-159
ENSG0000(	760	17.20934	chr1:153⁴	AL138930.2		smallRNA	chr1:160545648-160
ENSG0000(	760	17.20934	chr1:153⁴	RNU6-481P		smallRNA	chr1:161401289-161
ENSG0000(	760	17.20934	chr1:153⁴	ENSG00000236656		lncRNA	chr1:158474454-158
ENSG0000(	760	17.20934	chr1:153⁴	OR10J2P		Pseudoger	chr1:159279041-159
ENSG0000(	760	17.20934	chr1:153⁴	ENSG00000289273		lncRNA	chr1:161518705-161
ENSG0000(	760	17.20934	chr1:153⁴	AL121987.1		smallRNA	chr1:160205377-160
ENSG0000(	760	17.20934	chr1:153⁴	Y_RNA		smallRNA	chr1:161699506-161
ENSG0000(	760	17.20934	chr1:153⁴	OR10T2		protein_c	chr1:158398522-158
ENSG0000(	760	17.20934	chr1:153⁴	OR10J8P		Pseudoger	chr1:159366161-159
ENSG0000(	760	17.20934	chr1:153⁴	PPIAP37		Pseudoger	chr1:160848010-160
ENSG0000(	760	17.20934	chr1:153⁴	PCP4L1		protein_c	chr1:161258745-161
ENSG0000(	760	17.20934	chr1:153⁴	OR10X1		protein_c	chr1:158578919-158
ENSG0000(	760	17.20934	chr1:153⁴	OR6P1		protein_c	chr1:158560606-158
ENSG0000(	760	17.20934	chr1:153⁴	ARHGAP30		protein_c	chr1:161046946-161
ENSG0000(	760	17.20934	chr1:153⁴	LY9		protein_c	chr1:160796074-160
ENSG0000(	760	17.20934	chr1:153⁴	OR10J7P		Pseudoger	chr1:159351093-159
ENSG0000(	760	17.20934	chr1:153⁴	OR10Z1		protein_c	chr1:158605268-158
ENSG0000(	760	17.20934	chr1:153⁴	ENSG00000289141		lncRNA	chr1:161389547-161
ENSG0000(	760	17.20934	chr1:153⁴	OR10R2	NCGv7	protein_c	chr1:158472220-158
ENSG0000(	760	17.20934	chr1:153⁴	SLAMF7		protein_c	chr1:160739057-160
ENSG0000(	760	17.20934	chr1:153⁴	VSIG8		protein_c	chr1:159854316-159
ENSG0000(	760	17.20934	chr1:153⁴	ENSG00000289121		lncRNA	chr1:161046027-161
ENSG0000(	760	17.20934	chr1:153⁴	ENSG00000289106		lncRNA	chr1:161364221-161
ENSG0000(	760	17.20934	chr1:153⁴	ACA64		smallRNA	chr1:161141208-161
ENSG0000(	760	17.20934	chr1:153⁴	OR10R3P		Pseudoger	chr1:158491219-158
ENSG0000(	760	17.20934	chr1:153⁴	ATP1A2		protein_c	chr1:160115759-160
ENSG0000(	760	17.20934	chr1:153⁴	ENSG00000256029		protein_c	chr1:159834474-159
ENSG0000(	760	17.20934	chr1:153⁴	ENSG00000198358		lncRNA	chr1:160932465-160
ENSG0000(	760	17.20934	chr1:153⁴	ENSG00000176320		lncRNA	chr1:158197922-158
ENSG0000(	760	17.20934	chr1:153⁴	RRM2P2		Pseudoger	chr1:161378707-161
ENSG0000(	760	17.20934	chr1:153⁴	ENSG00000289484		lncRNA	chr1:159501664-159
ENSG0000(	760	17.20934	chr1:153⁴	RNA5SP60		Pseudoger	chr1:159178473-159
ENSG0000(	760	17.20934	chr1:153⁴	MPZ		protein_c	chr1:161304735-161
ENSG0000(	760	17.20934	chr1:153⁴	TOMM40L		protein_c	chr1:161225939-161
ENSG0000(	760	17.20934	chr1:153⁴	APOA2		protein_c	chr1:161222292-161
ENSG0000(	760	17.20934	chr1:153⁴	FCER1G		protein_c	chr1:161215234-161
ENSG0000(	760	17.20934	chr1:153⁴	NDUFS2		protein_c	chr1:161197104-161
ENSG0000(	760	17.20934	chr1:153⁴	ADAMTS4		protein_c	chr1:161184302-161
ENSG0000(	760	17.20934	chr1:153⁴	B4GALT3	NCGv7	protein_c	chr1:161171310-161
ENSG0000(	760	17.20934	chr1:153⁴	DEDD	DriverDB\	protein_c	chr1:161120974-161

ENSG0000(	760	17.20934	chr1:153⁴	NIT1		protein_c	chr1:161118086-161
ENSG0000(	760	17.20934	chr1:153⁴	USF1		protein_c	chr1:161039251-161
ENSG0000(	760	17.20934	chr1:153⁴	F11R	DriverDB\	protein_c	chr1:160995211-161
ENSG0000(	760	17.20934	chr1:153⁴	ITLN2		protein_c	chr1:160945025-160
ENSG0000(	760	17.20934	chr1:153⁴	OR10J6P		Pseudoger	chr1:159598298-159
ENSG0000(	760	17.20934	chr1:153⁴	DUSP23	DriverDB\	protein_c	chr1:159780932-159
ENSG0000(	760	17.20934	chr1:153⁴	SLAMF8		protein_c	chr1:159826811-159
ENSG0000(	760	17.20934	chr1:153⁴	AL138930.1		smallRNA	chr1:160503376-160
ENSG0000(	760	17.20934	chr1:153⁴	ENSG00000258465		protein_c	chr1:160216800-160
ENSG0000(	760	17.20934	chr1:153⁴	TAGLN2		protein_c	chr1:159918107-159
ENSG0000(	760	17.20934	chr1:153⁴	EI24P2		Pseudoger	chr1:158454198-158
ENSG0000(	760	17.20934	chr1:153⁴	CADM3-AS1		lncRNA	chr1:159194325-159
ENSG0000(	760	17.20934	chr1:153⁴	CD1E		protein_c	chr1:158353696-158
ENSG0000(	760	17.20934	chr1:153⁴	CD1B	NCGv7	protein_c	chr1:158327951-158
ENSG0000(	760	17.20934	chr1:153⁴	ENSG00000234211		lncRNA	chr1:161671978-161
ENSG0000(	760	17.20934	chr1:153⁴	CD1C		protein_c	chr1:158289923-158
ENSG0000(	760	17.20934	chr1:153⁴	CD1A	NCGv7	protein_c	chr1:158254424-158
ENSG0000(	760	17.20934	chr1:153⁴	CD1D	NCGv7	protein_c	chr1:158178030-158
ENSG0000(	760	17.20934	chr1:153⁴	hsa-mir-4259		smallRNA	chr1:159899979-159
ENSG0000(	760	17.20934	chr1:153⁴	PEA15		protein_c	chr1:160205380-160
ENSG0000(	760	17.20934	chr1:153⁴	ENSG00000275801		Pseudoger	chr1:160775954-160
ENSG0000(	760	17.20934	chr1:153⁴	ENSG00000283360		lncRNA	chr1:161403409-161
ENSG0000(	760	17.20934	chr1:153⁴	ENSG00000232188		lncRNA	chr1:160931739-160
ENSG0000(	760	17.20934	chr1:153⁴	FCRLA		protein_c	chr1:161706972-161
ENSG0000(	760	17.20934	chr1:153⁴	ENSG00000231100		Pseudoger	chr1:159557368-159
ENSG0000(	760	17.20934	chr1:153⁴	ENSG00000283696		lncRNA	chr1:161399409-161
ENSG0000(	760	17.20934	chr1:153⁴	UFC1	DriverDB\	protein_c	chr1:161152776-161
ENSG0000(	760	17.20934	chr1:153⁴	LINC01704		lncRNA	chr1:158131983-158
ENSG0000(	760	17.20934	chr1:153⁴	ATP1A4		protein_c	chr1:160151586-160
ENSG0000(	760	17.20934	chr1:153⁴	PYHIN5P		Pseudoger	chr1:158878746-158
ENSG0000(	760	17.20934	chr1:153⁴	NECTIN4	DriverDB\	protein_c	chr1:161070998-161
ENSG0000(	760	17.20934	chr1:153⁴	PIGM	DriverDB\	protein_c	chr1:160024953-160
ENSG0000(	760	17.20934	chr1:153⁴	CRP	NCGv7	protein_c	chr1:159712289-159
ENSG0000(	760	17.20934	chr1:153⁴	HMGN1P5		Pseudoger	chr1:158266753-158
ENSG0000(	760	17.20934	chr1:153⁴	RPS23P9		Pseudoger	chr1:161617992-161
ENSG0000(	760	17.20934	chr1:153⁴	APCS		protein_c	chr1:159587826-159
ENSG0000(	760	17.20934	chr1:153⁴	OR2AQ1P		Pseudoger	chr1:158796014-158
ENSG0000(	760	17.20934	chr1:153⁴	CASQ1		protein_c	chr1:160190575-160
ENSG0000(	760	17.20934	chr1:153⁴	PPOX	DriverDB\	protein_c	chr1:161166056-161
ENSG0000(	760	17.20934	chr1:153⁴	ENSG00000283317		lncRNA	chr1:161433444-161
ENSG0000(	760	17.20934	chr1:153⁴	HSP90AA3P		Pseudoger	chr1:158523672-158
ENSG0000(	760	17.20934	chr1:153⁴	NR1I3	DriverDB\	protein_c	chr1:161229666-161
ENSG0000(	760	17.20934	chr1:153⁴	FCGR2A		protein_c	chr1:161505430-161
ENSG0000(	760	17.20934	chr1:153⁴	OR6K1P		Pseudoger	chr1:158694539-158
ENSG0000(	760	17.20934	chr1:153⁴	ENSG00000229808		Pseudoger	chr1:161890833-161
ENSG0000(	760	17.20934	chr1:153⁴	SDHC	NCGv7;AC	protein_c	chr1:161314381-161
ENSG0000(	760	17.20934	chr1:153⁴	PFDN2	DriverDB\	protein_c	chr1:161100556-161
ENSG0000(	760	17.20934	chr1:153⁴	DCAF8		protein_c	chr1:160215715-160
ENSG0000(	760	17.20934	chr1:153⁴	USP21	DriverDB\	protein_c	chr1:161159450-161
ENSG0000(	760	17.20934	chr1:153⁴	ENSG00000229914		Pseudoger	chr1:158195633-158
ENSG0000(	759	17.1867	chr1:1021	AL359473.1		smallRNA	chr1:45033969-4503

ENSG0000(	756	17.11876	chr1:373:	ENSG00000240553		lncRNA	chr1:23020147-2308
ENSG0000(	756	17.11876	chr1:373:	HNRNPR		protein_c	chr1:23303771-2334
ENSG0000(	756	17.11876	chr1:373:	ZNF436-AS1		lncRNA	chr1:23368939-2337
ENSG0000(	756	17.11876	chr1:373:	ENSG00000285959		lncRNA	chr1:21983606-2203
ENSG0000(	756	17.11876	chr1:373:	ENSG00000225952		lncRNA	chr1:22835713-2283
ENSG0000(	756	17.11876	chr1:373:	PPIAP34		Pseudoger	chr1:22322840-2232
ENSG0000(	756	17.11876	chr1:373:	MIR4419A		smallRNA	chr1:23057858-2305
ENSG0000(	756	17.11876	chr1:373:	ENSG00000289014		lncRNA	chr1:23167098-2316
ENSG0000(	756	17.11876	chr1:373:	ZBTB40		protein_c	chr1:22428838-2253
ENSG0000(	756	17.11876	chr1:373:	C1QA		protein_c	chr1:22635077-2263
ENSG0000(	756	17.11876	chr1:373:	ZBTB40-IT1		lncRNA	chr1:22517474-2251
ENSG0000(	756	17.11876	chr1:373:	LUZP1	NCGv7	protein_c	chr1:23084030-2317
ENSG0000(	756	17.11876	chr1:373:	ZNF436		protein_c	chr1:23359448-2336
ENSG0000(	756	17.11876	chr1:373:	RN7SL186P		smallRNA	chr1:22010650-2201
ENSG0000(	756	17.11876	chr1:373:	RP1-224A6.9		lncRNA	chr1:22100613-2210
ENSG0000(	756	17.11876	chr1:373:	LINC01635		lncRNA	chr1:22023990-2202
ENSG0000(	756	17.11876	chr1:373:	ENSG00000229010		Pseudoger	chr1:23140325-2314
ENSG0000(	756	17.11876	chr1:373:	ENSG00000232037		Pseudoger	chr1:21908098-2190
ENSG0000(	756	17.11876	chr1:373:	RN7SL768P		smallRNA	chr1:22003585-2200
ENSG0000(	756	17.11876	chr1:373:	MIR4684		smallRNA	chr1:22719517-2271
ENSG0000(	756	17.11876	chr1:373:	C1QB		protein_c	chr1:22652762-2266
ENSG0000(	756	17.11876	chr1:373:	ENSG00000285873		lncRNA	chr1:22142850-2215
ENSG0000(	756	17.11876	chr1:373:	LDLRAD2		protein_c	chr1:21812265-2182
ENSG0000(	756	17.11876	chr1:373:	MIR4418		smallRNA	chr1:22266239-2226
ENSG0000(	756	17.11876	chr1:373:	ENSG00000215381		Pseudoger	chr1:23244765-2324
ENSG0000(	756	17.11876	chr1:373:	ENSG00000284726		lncRNA	chr1:23297797-2330
ENSG0000(	756	17.11876	chr1:373:	LINC00339		lncRNA	chr1:22024558-2203
ENSG0000(	756	17.11876	chr1:373:	WNT4		protein_c	chr1:22117313-2214
ENSG0000(	756	17.11876	chr1:373:	CELA3B		protein_c	chr1:21977022-2199
ENSG0000(	756	17.11876	chr1:373:	ENSG00000283234		Pseudoger	chr1:21950679-2195
ENSG0000(	756	17.11876	chr1:373:	RNU6-514P		smallRNA	chr1:23162704-2316
ENSG0000(	756	17.11876	chr1:373:	RNU6-135P		smallRNA	chr1:23163953-2316
ENSG0000(	756	17.11876	chr1:373:	Y_RNA		smallRNA	chr1:23370254-2337
ENSG0000(	756	17.11876	chr1:373:	ENSG00000231978		Pseudoger	chr1:21768269-2176
ENSG0000(	756	17.11876	chr1:373:	ENSG00000289694		protein_c	chr1:22025142-2209
ENSG0000(	756	17.11876	chr1:373:	ENSG00000279625		TEC	chr1:22364630-2236
ENSG0000(	756	17.11876	chr1:373:	RN7SL421P		smallRNA	chr1:21978951-2197
ENSG0000(	756	17.11876	chr1:373:	RN7SL386P		smallRNA	chr1:21987481-2198
ENSG0000(	756	17.11876	chr1:373:	RNU6-1022P		smallRNA	chr1:21987816-2198
ENSG0000(	756	17.11876	chr1:373:	MPHOSPH6P1		Pseudoger	chr1:22068340-2206
ENSG0000(	756	17.11876	chr1:373:	ENSG00000289692		protein_c	chr1:22636506-2264
ENSG0000(	756	17.11876	chr1:373:	USP48		protein_c	chr1:21678298-2178
ENSG0000(	756	17.11876	chr1:373:	MIR4253		smallRNA	chr1:22863159-2286
ENSG0000(	756	17.11876	chr1:373:	KDM1A	NCGv7	protein_c	chr1:23019443-2308
ENSG0000(	756	17.11876	chr1:373:	TEX46		protein_c	chr1:23010834-2301
ENSG0000(	756	17.11876	chr1:373:	HTR1D		protein_c	chr1:23191895-2321
ENSG0000(	756	17.11876	chr1:373:	LINC01355		lncRNA	chr1:23281307-2328
ENSG0000(	756	17.11876	chr1:373:	CDC42	NCGv7	protein_c	chr1:22052627-2210
ENSG0000(	756	17.11876	chr1:373:	CELA3A		protein_c	chr1:22001657-2201
ENSG0000(	756	17.11876	chr1:373:	CDC42-AS1		lncRNA	chr1:22028317-2205
ENSG0000(	756	17.11876	chr1:373:	HSPG2	NCGv7	protein_c	chr1:21822244-2193

ENSG0000(	756	17.11876	chr1:373:	AL611946.1		smallRNA	chr1:22748817-2274
ENSG0000(	756	17.11876	chr1:373:	MIR3115		smallRNA	chr1:23044305-2304
ENSG0000(	756	17.11876	chr1:373:	CDC42-IT1		lncRNA	chr1:22059197-2206
ENSG0000(	756	17.11876	chr1:373:	EPHB2		protein_c	chr1:22710839-2292
ENSG0000(	756	17.11876	chr1:373:	C1QC		protein_c	chr1:22643014-2264
ENSG0000(	756	17.11876	chr1:373:	EPHA8		protein_c	chr1:22563489-2260
ENSG0000(	756	17.11876	chr1:373:	RNU6-776P		smallRNA	chr1:22010985-2201
ENSG0000(	756	17.11876	chr1:373:	LACTBL1		protein_c	chr1:22953043-2297
ENSG0000(	754	17.07348	chr8:274'	AC116154.1		smallRNA	chr8:95678838-9567
ENSG0000(	753	17.05083	chr8:274'	MIR4661		smallRNA	chr8:91205485-9120
ENSG0000(	753	17.05083	chr8:274'	CPP		Pseudoger	chr8:91157285-9115
ENSG0000(	751	17.00554	chr2:2577	RMRPP3		smallRNA	chr2:160396829-160
ENSG0000(	751	17.00554	chr2:2577	CD302		protein_c	chr2:159768628-159
ENSG0000(	751	17.00554	chr2:2577	ITGB6		protein_c	chr2:160099667-160
ENSG0000(	751	17.00554	chr2:2577	GSTM3P2		Pseudoger	chr2:159056005-159
ENSG0000(	751	17.00554	chr2:2577	RN7SL423P		smallRNA	chr2:160877547-160
ENSG0000(	751	17.00554	chr2:2577	PLA2R1	NCGv7	protein_c	chr2:159932006-160
ENSG0000(	751	17.00554	chr2:2577	RPEP5		Pseudoger	chr2:161931199-161
ENSG0000(	751	17.00554	chr2:2577	ENSG00000236841		lncRNA	chr2:162159762-162
ENSG0000(	751	17.00554	chr2:2577	ENSG00000287091		lncRNA	chr2:159797288-159
ENSG0000(	751	17.00554	chr2:2577	CAPZA1P2		Pseudoger	chr2:159462417-159
ENSG0000(	751	17.00554	chr2:2577	ENSG00000223642		lncRNA	chr2:159386367-159
ENSG0000(	751	17.00554	chr2:2577	MXRA7P1		Pseudoger	chr2:161340816-161
ENSG0000(	751	17.00554	chr2:2577	ENSG00000228586		lncRNA	chr2:158685903-158
ENSG0000(	751	17.00554	chr2:2577	ENSG00000285155		lncRNA	chr2:160141981-160
ENSG0000(	751	17.00554	chr2:2577	KCNH7-AS1		lncRNA	chr2:162768936-162
ENSG0000(	751	17.00554	chr2:2577	RNU6-627P		smallRNA	chr2:163288995-163
ENSG0000(	751	17.00554	chr2:2577	ENSG00000251621		lncRNA	chr2:161424015-161
ENSG0000(	751	17.00554	chr2:2577	ENSG00000225813		Pseudoger	chr2:161281902-161
ENSG0000(	751	17.00554	chr2:2577	snoU13		smallRNA	chr2:161558544-161
ENSG0000(	751	17.00554	chr2:2577	ENSG00000288091		lncRNA	chr2:161577192-161
ENSG0000(	751	17.00554	chr2:2577	FAP		protein_c	chr2:162170684-162
ENSG0000(	751	17.00554	chr2:2577	ENSG00000231675		Pseudoger	chr2:158703287-158
ENSG0000(	751	17.00554	chr2:2577	ENSG00000227055		Pseudoger	chr2:159812234-159
ENSG0000(	751	17.00554	chr2:2577	LY75-CD3(	IntOGen-I	protein_c	chr2:159771851-159
ENSG0000(	751	17.00554	chr2:2577	DPP4		protein_c	chr2:161992245-162
ENSG0000(	751	17.00554	chr2:2577	TIMM8AP1		Pseudoger	chr2:162077357-162
ENSG0000(	751	17.00554	chr2:2577	RPL7AP22		Pseudoger	chr2:158548425-158
ENSG0000(	751	17.00554	chr2:2577	RNU6-580P		smallRNA	chr2:159027036-159
ENSG0000(	751	17.00554	chr2:2577	snoZ5		smallRNA	chr2:158534200-158
ENSG0000(	751	17.00554	chr2:2577	ENSG00000286621		lncRNA	chr2:158847619-158
ENSG0000(	751	17.00554	chr2:2577	LINC01806		lncRNA	chr2:161244720-161
ENSG0000(	751	17.00554	chr2:2577	GCA		protein_c	chr2:162318840-162
ENSG0000(	751	17.00554	chr2:2577	Y_RNA		smallRNA	chr2:159536244-159
ENSG0000(	751	17.00554	chr2:2577	RPL7P61		Pseudoger	chr2:163152251-163
ENSG0000(	751	17.00554	chr2:2577	ENSG00000233397		lncRNA	chr2:162092232-162
ENSG0000(	751	17.00554	chr2:2577	RN7SL393P		smallRNA	chr2:158447164-158
ENSG0000(	751	17.00554	chr2:2577	CCDC148		protein_c	chr2:158171073-158
ENSG0000(	751	17.00554	chr2:2577	SLC4A10	NCGv7	protein_c	chr2:161424332-161
ENSG0000(	751	17.00554	chr2:2577	ENSG00000270557		lncRNA	chr2:157877683-157
ENSG0000(	751	17.00554	chr2:2577	KRT18P46		Pseudoger	chr2:161580072-161

ENSG0000(	751	17.00554	chr2:2577	MIR4785		smallRNA	chr2:160407810-160
ENSG0000(	751	17.00554	chr2:2577	EIF3EP2		Pseudoger	chr2:162088259-162
ENSG0000(	751	17.00554	chr2:2577	PSMD14		protein_c	chr2:161308425-161
ENSG0000(	751	17.00554	chr2:2577	AC009299.1		smallRNA	chr2:161350217-161
ENSG0000(	751	17.00554	chr2:2577	RBMS1		protein_c	chr2:160272151-160
ENSG0000(	751	17.00554	chr2:2577	TANK		protein_c	chr2:161136908-161
ENSG0000(	751	17.00554	chr2:2577	AC092841.1		smallRNA	chr2:161918782-161
ENSG0000(	751	17.00554	chr2:2577	ENSG00000271320		lncRNA	chr2:157917712-157
ENSG0000(	751	17.00554	chr2:2577	RNU2-21P		smallRNA	chr2:158935798-158
ENSG0000(	751	17.00554	chr2:2577	ENSG00000226946		Pseudoger	chr2:161568502-161
ENSG0000(	751	17.00554	chr2:2577	CCDC148-AS1		lncRNA	chr2:158166650-158
ENSG0000(	751	17.00554	chr2:2577	GCG		protein_c	chr2:162142882-162
ENSG0000(	751	17.00554	chr2:2577	ENSG00000279844		TEC	chr2:160849313-160
ENSG0000(	751	17.00554	chr2:2577	PSMD14-DT		lncRNA	chr2:161222785-161
ENSG0000(	751	17.00554	chr2:2577	LINC02478		lncRNA	chr2:160257716-160
ENSG0000(	751	17.00554	chr2:2577	IFIH1		protein_c	chr2:162267074-162
ENSG0000(	751	17.00554	chr2:2577	TBR1	NCGv7	protein_c	chr2:161416297-161
ENSG0000(	751	17.00554	chr2:2577	ENSG00000232337		Pseudoger	chr2:161048606-161
ENSG0000(	751	17.00554	chr2:2577	BTF3L4P2		Pseudoger	chr2:159003975-159
ENSG0000(	751	17.00554	chr2:2577	ACVR1	NCGv7	protein_c	chr2:157736251-157
ENSG0000(	751	17.00554	chr2:2577	KCNH7		protein_c	chr2:162371407-162
ENSG0000(	751	17.00554	chr2:2577	MARCHF7		protein_c	chr2:159712457-159
ENSG0000(	751	17.00554	chr2:2577	MTA3P1		Pseudoger	chr2:157961410-157
ENSG0000(	751	17.00554	chr2:2577	ENSG00000270893		Pseudoger	chr2:159444117-159
ENSG0000(	751	17.00554	chr2:2577	TANK-AS1		lncRNA	chr2:161096231-161
ENSG0000(	751	17.00554	chr2:2577	LY75		protein_c	chr2:159803355-159
ENSG0000(	751	17.00554	chr2:2577	RNU6-932P		smallRNA	chr2:157985789-157
ENSG0000(	751	17.00554	chr2:2577	AC013731.1		smallRNA	chr2:157855270-157
ENSG0000(	751	17.00554	chr2:2577	BAZ2B-AS1		lncRNA	chr2:159615292-159
ENSG0000(	751	17.00554	chr2:2577	PTP4A1P1		Pseudoger	chr2:158065545-158
ENSG0000(	751	17.00554	chr2:2577	ENSG00000289488		lncRNA	chr2:158545074-158
ENSG0000(	751	17.00554	chr2:2577	TANC1		protein_c	chr2:158968640-159
ENSG0000(	751	17.00554	chr2:2577	PKP4-AS1		lncRNA	chr2:158658337-158
ENSG0000(	751	17.00554	chr2:2577	HNRNPDLP2		Pseudoger	chr2:158419955-158
ENSG0000(	751	17.00554	chr2:2577	RNU6-436P		smallRNA	chr2:157912772-157
ENSG0000(	751	17.00554	chr2:2577	RPS3AP13		Pseudoger	chr2:159689217-159
ENSG0000(	751	17.00554	chr2:2577	ENSG00000224076		lncRNA	chr2:161422659-161
ENSG0000(	751	17.00554	chr2:2577	UPP2		protein_c	chr2:157876702-158
ENSG0000(	751	17.00554	chr2:2577	DPP4-DT		lncRNA	chr2:162073256-162
ENSG0000(	751	17.00554	chr2:2577	DAPL1		protein_c	chr2:158795317-158
ENSG0000(	751	17.00554	chr2:2577	PKP4	NCGv7	protein_c	chr2:158456952-158
ENSG0000(	751	17.00554	chr2:2577	UPP2-IT1		lncRNA	chr2:158127957-158
ENSG0000(	751	17.00554	chr2:2577	WDSUB1		protein_c	chr2:159235798-159
ENSG0000(	751	17.00554	chr2:2577	RNA5SP109		Pseudoger	chr2:162496936-162
ENSG0000(	751	17.00554	chr2:2577	ENSG00000226266		lncRNA	chr2:159670708-159
ENSG0000(	751	17.00554	chr2:2577	BAZ2B	NCGv7	protein_c	chr2:159318979-159
ENSG0000(	751	17.00554	chr2:2577	OR7E90P		Pseudoger	chr2:158874642-158
ENSG0000(	751	17.00554	chr2:2577	AHCTF1P1		Pseudoger	chr2:161500885-161
ENSG0000(	751	17.00554	chr2:2577	ENSG00000280105		TEC	chr2:161433874-161
ENSG0000(	750	16.9829	chr4:1865	ENSG00000250214		Pseudoger	chr4:77864508-7786
ENSG0000(	749	16.96026	chr2:2577	SNORA51		smallRNA	chr2:169727872-169

ENSG0000(	748	16.93761	chr1:2449	SRP14P4	Pseudoger	chr1:171345105-171
ENSG0000(	747	16.91497	chr2:8187	IGKV3D-15	protein_c	chr2:90114838-9011
ENSG0000(	743	16.82439	chr2:8187	IGKV1D-16	protein_c	chr2:90100236-9010
ENSG0000(	743	16.82439	chr2:8187	LSP1P4	Pseudoger	chr2:91636684-9165
ENSG0000(	743	16.82439	chr2:8187	LONRF2	protein_c	chr2:100271875-100
ENSG0000(	743	16.82439	chr2:8187	ANKRD36C NCGv7	protein_c	chr2:95836919-9599
ENSG0000(	743	16.82439	chr2:8187	IGKV2-18	Pseudoger	chr2:89128724-8912
ENSG0000(	743	16.82439	chr2:8187	ENSG00000286654	lncRNA	chr2:96912549-9691
ENSG0000(	743	16.82439	chr2:8187	IGKV3D-11	protein_c	chr2:90172802-9017
ENSG0000(	743	16.82439	chr2:8187	IGKV3D-25	Pseudoger	chr2:89989987-8999
ENSG0000(	743	16.82439	chr2:8187	IGKV2-24	protein_c	chr2:89176328-8917
ENSG0000(	743	16.82439	chr2:8187	IGKV6D-41	protein_c	chr2:90069662-9007
ENSG0000(	743	16.82439	chr2:8187	KMT2CP5	Pseudoger	chr2:91696452-9171
ENSG0000(	743	16.82439	chr2:8187	IGKV3D-20	protein_c	chr2:90038848-9003
ENSG0000(	743	16.82439	chr2:8187	AC159540.1	lncRNA	chr2:97415474-9743
ENSG0000(	743	16.82439	chr2:8187	IGKV1D-42	protein_c	chr2:90190193-9019
ENSG0000(	743	16.82439	chr2:8187	ENSG00000275490	Pseudoger	chr2:90309229-9030
ENSG0000(	743	16.82439	chr2:8187	IGKV2D-36	Pseudoger	chr2:89887022-8988
ENSG0000(	743	16.82439	chr1:1534	RNA5SP61	Pseudoger	chr1:162338643-162
ENSG0000(	743	16.82439	chr2:8187	ENSG00000272913	lncRNA	chr2:95524873-9552
ENSG0000(	743	16.82439	chr2:8187	ENSG00000273305	lncRNA	chr2:95537969-9553
ENSG0000(	743	16.82439	chr2:8187	ARID5A	protein_c	chr2:96536743-9655
ENSG0000(	743	16.82439	chr2:8187	ENSG00000290846	lncRNA	chr2:91617160-9166
ENSG0000(	743	16.82439	chr2:8187	ITPRIPL1	protein_c	chr2:96325317-9633
ENSG0000(	743	16.82439	chr2:8187	ZNF514	protein_c	chr2:95122087-9516
ENSG0000(	743	16.82439	chr2:8187	IGKV3-7	protein_c	chr2:88978468-8897
ENSG0000(	743	16.82439	chr2:8187	IGKV2-40	protein_c	chr2:89330116-8933
ENSG0000(	743	16.82439	chr2:8187	TRIM43B	protein_c	chr2:95477008-9548
ENSG0000(	743	16.82439	chr2:8187	IGKV3D-7	protein_c	chr2:90234812-9023
ENSG0000(	743	16.82439	chr2:8187	SNX18P14	Pseudoger	chr2:94786688-9478
ENSG0000(	743	16.82439	chr2:8187	STARD7	protein_c	chr2:96184859-9620
ENSG0000(	743	16.82439	chr2:8187	ENSG00000222000	lncRNA	chr2:98331389-9835
ENSG0000(	743	16.82439	chr2:8187	HMGN2P22	Pseudoger	chr2:100591150-100
ENSG0000(	743	16.82439	chr2:8187	GXYLT1P7	Pseudoger	chr2:94734655-9473
ENSG0000(	743	16.82439	chr2:8187	IGKV2-4	Pseudoger	chr2:88931666-8893
ENSG0000(	743	16.82439	chr2:8187	IGKV1-13	Pseudoger	chr2:89045995-8904
ENSG0000(	743	16.82439	chr2:8187	IGKV3D-34	Pseudoger	chr2:89906757-8990
ENSG0000(	743	16.82439	chr2:8187	FAM95A	lncRNA	chr2:94755326-9479
ENSG0000(	743	16.82439	chr1:1534	OLFML2B	protein_c	chr1:161983192-162
ENSG0000(	743	16.82439	chr2:8187	ENSG00000275497	Pseudoger	chr2:95633850-9563
ENSG0000(	743	16.82439	chr2:8187	CYCSP7	Pseudoger	chr2:100370255-100
ENSG0000(	743	16.82439	chr2:8187	ENSG00000236750	Pseudoger	chr2:95641634-9564
ENSG0000(	743	16.82439	chr2:8187	NANOGNBP1	Pseudoger	chr2:100660199-100
ENSG0000(	743	16.82439	chr2:8187	IGKV1-35	Pseudoger	chr2:89286689-8928
ENSG0000(	743	16.82439	chr2:8187	ENSG00000287607	Pseudoger	chr2:91578478-9157
ENSG0000(	743	16.82439	chr2:8187	IGKV1-17	protein_c	chr2:89117342-8911
ENSG0000(	743	16.82439	chr2:8187	ENSG00000236847	Pseudoger	chr2:97035461-9703
ENSG0000(	743	16.82439	chr2:8187	IGKV1OR1-1	Pseudoger	chr2:91486789-9148
ENSG0000(	743	16.82439	chr2:8187	ENSG00000235186	Pseudoger	chr2:94991880-9499
ENSG0000(	743	16.82439	chr1:1534	ATF6	protein_c	chr1:161766298-161
ENSG0000(	743	16.82439	chr2:8187	ENSG00000235833	Pseudoger	chr2:97523949-9752

ENSG0000(	743	16.82439	chr2:8187	AC113612.1	Pseudoger	chr2:90261265-9026
ENSG0000(	743	16.82439	chr2:8187	ENSG00000233275	Pseudoger	chr2:95434759-9543
ENSG0000(	743	16.82439	chr2:8187	ENSG00000234837	Pseudoger	chr2:94750582-9475
ENSG0000(	743	16.82439	chr2:8187	RNA5SP101	Pseudoger	chr2:96956708-9695
ENSG0000(	743	16.82439	chr2:8187	ENSG00000291024	lncRNA	chr2:94811046-9481
ENSG0000(	743	16.82439	chr2:8187	PGBD4P5	Pseudoger	chr2:88922328-8892
ENSG0000(	743	16.82439	chr2:8187	MRPL30	protein_c	chr2:99181152-9919
ENSG0000(	743	16.82439	chr2:8187	IGKV3-31	Pseudoger	chr2:89252211-8925
ENSG0000(	743	16.82439	chr2:8187	snoU13	smallRNA	chr2:96981282-9698
ENSG0000(	743	16.82439	chr2:8187	IGKV2OR2-2	Pseudoger	chr2:97050729-9705
ENSG0000(	743	16.82439	chr2:8187	ENSG00000230083	Pseudoger	chr2:95590969-9559
ENSG0000(	743	16.82439	chr2:8187	ATP5F1BP1	Pseudoger	chr2:98206151-9820
ENSG0000(	743	16.82439	chr2:8187	IGKV2OR2-7	Pseudoger	chr2:97372532-9737
ENSG0000(	743	16.82439	chr2:8187	IGKV2D-30	protein_c	chr2:89936859-8993
ENSG0000(	743	16.82439	chr2:8187	MGAT4A	protein_c	chr2:98619106-9873
ENSG0000(	743	16.82439	chr2:8187	IGKV1D-17	protein_c	chr2:90082635-9008
ENSG0000(	743	16.82439	chr2:8187	ENSG00000283214	Pseudoger	chr2:91736724-9173
ENSG0000(	743	16.82439	chr2:8187	ABCD1P5	Pseudoger	chr2:91840601-9184
ENSG0000(	743	16.82439	chr2:8187	TRIM64FP	Pseudoger	chr2:95514827-9552
ENSG0000(	743	16.82439	chr2:8187	SLC9B1P2	Pseudoger	chr2:91883076-9193
ENSG0000(	743	16.82439	chr2:8187	ENSG00000288960	lncRNA	chr2:95074906-9507
ENSG0000(	743	16.82439	chr2:8187	ENSG00000224719	Pseudoger	chr2:95502791-9550
ENSG0000(	743	16.82439	chr2:8187	IGKV1D-13	protein_c	chr2:90154073-9015
ENSG0000(	743	16.82439	chr2:8187	IGKV2D-26	protein_c	chr2:89985922-8998
ENSG0000(	743	16.82439	chr2:8187	AC018696.5	Pseudoger	chr2:91544070-9154
ENSG0000(	743	16.82439	chr2:8187	MRPS5	protein_c	chr2:95085369-9512
ENSG0000(	743	16.82439	chr2:8187	ENSG00000235147	Pseudoger	chr2:92006680-9200
ENSG0000(	743	16.82439	chr2:8187	IGKV1D-22	Pseudoger	chr2:90010741-9001
ENSG0000(	743	16.82439	chr2:8187	IGKV6-21	protein_c	chr2:89159751-8916
ENSG0000(	743	16.82439	chr2:8187	IGKV3-11	protein_c	chr2:89027171-8902
ENSG0000(	743	16.82439	chr2:8187	PABPC1P6	Pseudoger	chr2:91877969-9188
ENSG0000(	743	16.82439	chr2:8187	ENSG00000236026	Pseudoger	chr2:95606924-9560
ENSG0000(	743	16.82439	chr2:8187	ENSG00000277852	Pseudoger	chr2:89203509-8920
ENSG0000(	743	16.82439	chr2:8187	ENSG00000270699	Pseudoger	chr2:100377421-100
ENSG0000(	743	16.82439	chr2:8187	ENSG00000232594	Pseudoger	chr2:94961047-9496
ENSG0000(	743	16.82439	chr2:8187	RNU4-8P	smallRNA	chr2:97664591-9766
ENSG0000(	743	16.82439	chr2:8187	AC159540.3	lncRNA	chr2:97404351-9740
ENSG0000(	743	16.82439	chr2:8187	SNRNP200 NCGv7	protein_c	chr2:96274338-9632
ENSG0000(	743	16.82439	chr2:8187	CNNM3-DT	lncRNA	chr2:96812239-9681
ENSG0000(	743	16.82439	chr2:8187	ENSG00000274028	Pseudoger	chr2:95588149-9558
ENSG0000(	743	16.82439	chr2:8187	IGKV2D-40	protein_c	chr2:89851791-8985
ENSG0000(	743	16.82439	chr2:8187	CRACDL	protein_c	chr2:98793846-9893
ENSG0000(	743	16.82439	chr2:8187	COX5B	protein_c	chr2:97646062-9764
ENSG0000(	743	16.82439	chr2:8187	ENSG00000273306	lncRNA	chr2:99405218-9940
ENSG0000(	743	16.82439	chr2:8187	ENSG00000287362	lncRNA	chr2:95067074-9507
ENSG0000(	743	16.82439	chr2:8187	EIF5B	protein_c	chr2:99337371-9940
ENSG0000(	743	16.82439	chr2:8187	ENSG00000277095	Pseudoger	chr2:91589464-9162
ENSG0000(	743	16.82439	chr2:8187	ENSG00000281904	lncRNA	chr2:90365736-9036
ENSG0000(	743	16.82439	chr2:8187	IGKV1-8	protein_c	chr2:88992409-8899
ENSG0000(	743	16.82439	chr2:8187	IGKV1-16	protein_c	chr2:89099859-8910
ENSG0000(	743	16.82439	chr1:153	SPATA46	protein_c	chr1:162373203-162

ENSG0000(	743	16.82439	chr2:8187	IGKV2-36		Pseudoger	chr2:89295233-8929
ENSG0000(	743	16.82439	chr2:8187	IGKV2OR2-8		Pseudoger	chr2:97376674-9737
ENSG0000(	743	16.82439	chr2:8187	ENSG00000236431		Pseudoger	chr2:95536117-9553
ENSG0000(	743	16.82439	chr2:8187	AC018696.1		Pseudoger	chr2:91443388-9144
ENSG0000(	743	16.82439	chr2:8187	ENSG00000276118		Pseudoger	chr2:91578478-9157
ENSG0000(	743	16.82439	chr2:8187	IGKV1OR2-3		Pseudoger	chr2:97060128-9706
ENSG0000(	743	16.82439	chr2:8187	RNU7-46P		smallRNA	chr2:98840675-9884
ENSG0000(	743	16.82439	chr2:8187	ENSG00000235959		Pseudoger	chr2:95640181-9564
ENSG0000(	743	16.82439	chr2:8187	RN7SL210P		smallRNA	chr2:96004565-9600
ENSG0000(	743	16.82439	chr2:8187	NKAIN1P2		Pseudoger	chr2:91723023-9172
ENSG0000(	743	16.82439	chr2:8187	TXNDC9		protein_c	chr2:99318982-9934
ENSG0000(	743	16.82439	chr2:8187	ANKRD36	NCGv7	protein_c	chr2:97113153-9726
ENSG0000(	743	16.82439	chr2:8187	ENSG00000273155		protein_c	chr2:99154998-9919
ENSG0000(	743	16.82439	chr2:8187	ENSG00000234903		Pseudoger	chr2:95468863-9546
ENSG0000(	743	16.82439	chr2:8187	IGKV3OR2-5		Pseudoger	chr2:97348898-9734
ENSG0000(	743	16.82439	chr2:8187	ASTL		protein_c	chr2:96122818-9613
ENSG0000(	743	16.82439	chr2:8187	ENSG00000230343		Pseudoger	chr2:97034442-9703
ENSG0000(	743	16.82439	chr2:8187	ENSG00000273634		Pseudoger	chr2:97008368-9700
ENSG0000(	743	16.82439	chr2:8187	STARD7-AS1		lncRNA	chr2:96208389-9624
ENSG0000(	743	16.82439	chr2:8187	CYP4F32P		Pseudoger	chr2:94759259-9476
ENSG0000(	743	16.82439	chr2:8187	IGKV1D-12		protein_c	chr2:90159680-9016
ENSG0000(	743	16.82439	chr2:8187	MITD1		protein_c	chr2:99161427-9918
ENSG0000(	743	16.82439	chr2:8187	UBTFL3		Pseudoger	chr2:95625213-9562
ENSG0000(	743	16.82439	chr2:8187	IGKV2OR2-10		Pseudoger	chr2:97331533-9733
ENSG0000(	743	16.82439	chr2:8187	FER1L5		protein_c	chr2:96642737-9670
ENSG0000(	743	16.82439	chr2:8187	UBTFL5		Pseudoger	chr2:95450310-9545
ENSG0000(	743	16.82439	chr2:8187	LINC01104		lncRNA	chr2:100208254-100
ENSG0000(	743	16.82439	chr2:8187	ENSG00000233757		protein_c	chr2:95207521-9525
ENSG0000(	743	16.82439	chr2:8187	IGKV1-27		protein_c	chr2:89213423-8921
ENSG0000(	743	16.82439	chr2:8187	LINC02611		lncRNA	chr2:98761098-9877
ENSG0000(	743	16.82439	chr2:8187	IGKV2D-23		Pseudoger	chr2:90009402-9000
ENSG0000(	743	16.82439	chr2:8187	AC073416.1		Pseudoger	chr2:90099156-9009
ENSG0000(	743	16.82439	chr2:8187	AC073464.11		Pseudoger	chr2:94795808-9480
ENSG0000(	743	16.82439	chr2:8187	ENSG00000230964		Pseudoger	chr2:91578478-9157
ENSG0000(	743	16.82439	chr2:8187	ANKRD39		protein_c	chr2:96836611-9685
ENSG0000(	743	16.82439	chr2:8187	CNN2P8		Pseudoger	chr2:94737337-9473
ENSG0000(	743	16.82439	chr2:8187	PROM2		protein_c	chr2:95274449-9529
ENSG0000(	743	16.82439	chr2:8187	RN7SL575P		smallRNA	chr2:95003547-9500
ENSG0000(	743	16.82439	chr2:8187	RNU6-1320P		smallRNA	chr2:94846533-9484
ENSG0000(	743	16.82439	chr2:8187	DUSP2	NCGv7	protein_c	chr2:96143169-9614
ENSG0000(	743	16.82439	chr2:8187	KMT5AP2		Pseudoger	chr2:91747940-9174
ENSG0000(	743	16.82439	chr2:8187	PDCL3		protein_c	chr2:100562993-100
ENSG0000(	743	16.82439	chr2:8187	ENSG00000229689		Pseudoger	chr2:95525345-9552
ENSG0000(	743	16.82439	chr2:8187	RNA5SP100		Pseudoger	chr2:91674857-9167
ENSG0000(	743	16.82439	chr2:8187	CHST10	NCGv7	protein_c	chr2:100391860-100
ENSG0000(	743	16.82439	chr2:8187	ENSG00000277701		lncRNA	chr2:97281356-9729
ENSG0000(	743	16.82439	chr2:8187	ENSG00000230747		lncRNA	chr2:96307263-9632
ENSG0000(	743	16.82439	chr2:8187	CHEK2P3		Pseudoger	chr2:91957436-9196
ENSG0000(	743	16.82439	chr1:1534	ENSG00000227094		lncRNA	chr1:162316852-162
ENSG0000(	743	16.82439	chr2:8187	ENSG00000275767		Pseudoger	chr2:91589464-9162
ENSG0000(	743	16.82439	chr2:8187	IGKV2-19		Pseudoger	chr2:89134975-8913

ENSG0000(	743	16.82439	chr2:8187	IGKV2D-29		protein_c	chr2:89947512-8994
ENSG0000(	743	16.82439	chr2:8187	UNC50		protein_c	chr2:98608579-9861
ENSG0000(	743	16.82439	chr2:8187	TSGA10		protein_c	chr2:98997261-9915
ENSG0000(	743	16.82439	chr2:8187	ENSG00000248134		Pseudoger	chr2:97018343-9701
ENSG0000(	743	16.82439	chr2:8187	RALBP1P2		Pseudoger	chr2:100425348-100
ENSG0000(	743	16.82439	chr2:8187	REV1		protein_c	chr2:99400475-9949
ENSG0000(	743	16.82439	chr2:8187	ENSG00000235480		lncRNA	chr2:96527940-9653
ENSG0000(	743	16.82439	chr2:8187	ENSG00000277747		Pseudoger	chr2:95460144-9546
ENSG0000(	743	16.82439	chr2:8187	INPP4A	NCGv7	protein_c	chr2:98444854-9859
ENSG0000(	743	16.82439	chr2:8187	AC027612.2		smallRNA	chr2:91763925-9176
ENSG0000(	743	16.82439	chr2:8187	ENSG00000261600		lncRNA	chr2:91580336-9158
ENSG0000(	743	16.82439	chr2:8187	ENSG00000227120		Pseudoger	chr2:95436133-9543
ENSG0000(	743	16.82439	chr2:8187	LYG2		protein_c	chr2:99242246-9925
ENSG0000(	743	16.82439	chr2:8187	IGKV1OR2-6		Pseudoger	chr2:97355058-9735
ENSG0000(	743	16.82439	chr2:8187	ENSG00000273825		Pseudoger	chr2:91589469-9162
ENSG0000(	743	16.82439	chr2:8187	ENSG00000259848		Pseudoger	chr2:94886861-9489
ENSG0000(	743	16.82439	chr2:8187	CNNM4		protein_c	chr2:96760902-9681
ENSG0000(	743	16.82439	chr2:8187	IGKV2-23		Pseudoger	chr2:89172022-8917
ENSG0000(	743	16.82439	chr1:1534	NOS1AP		protein_c	chr1:162069691-162
ENSG0000(	743	16.82439	chr2:8187	IGKV1-12		protein_c	chr2:89040224-8904
ENSG0000(	743	16.82439	chr2:8187	TMEM131		protein_c	chr2:97756333-9799
ENSG0000(	743	16.82439	chr2:8187	YWHAQP5		Pseudoger	chr2:98694109-9869
ENSG0000(	743	16.82439	chr2:8187	CIAO1		protein_c	chr2:96266159-9627
ENSG0000(	743	16.82439	chr2:8187	SLC2AXP1		Pseudoger	chr2:95196449-9519
ENSG0000(	743	16.82439	chr2:8187	ENSG00000233447		Pseudoger	chr2:94974544-9497
ENSG0000(	743	16.82439	chr2:8187	AC018690.1		smallRNA	chr2:99520103-9952
ENSG0000(	743	16.82439	chr2:8187	ENSG00000233850		lncRNA	chr2:95025193-9502
ENSG0000(	743	16.82439	chr2:8187	IGKV1D-37		protein_c	chr2:89884740-8988
ENSG0000(	743	16.82439	chr2:8187	NMS		protein_c	chr2:100470482-100
ENSG0000(	743	16.82439	chr2:8187	MTCO3P45		Pseudoger	chr2:94900990-9490
ENSG0000(	743	16.82439	chr2:8187	SOWAHCP5		Pseudoger	chr2:94861362-9486
ENSG0000(	743	16.82439	chr2:8187	ENSG00000230393		lncRNA	chr2:100104919-100
ENSG0000(	743	16.82439	chr2:8187	ARPP19P2		Pseudoger	chr2:100509277-100
ENSG0000(	743	16.82439	chr1:1534	ENSG00000285636		lncRNA	chr1:162146709-162
ENSG0000(	743	16.82439	chr2:8187	KMT2CP2		Pseudoger	chr2:91696435-9171
ENSG0000(	743	16.82439	chr2:8187	IGKV2-26		Pseudoger	chr2:89196096-8919
ENSG0000(	743	16.82439	chr2:8187	TMEM127	NCGv7	protein_c	chr2:96248514-9626
ENSG0000(	743	16.82439	chr2:8187	ACTR3BP2		Pseudoger	chr2:91940668-9194
ENSG0000(	743	16.82439	chr2:8187	IGKV2-30		protein_c	chr2:89244781-8924
ENSG0000(	743	16.82439	chr2:8187	IGKV2D-38		Pseudoger	chr2:89872463-8987
ENSG0000(	743	16.82439	chr2:8187	GPAT2		protein_c	chr2:96021946-9603
ENSG0000(	743	16.82439	chr2:8187	IGKV2-28		protein_c	chr2:89221698-8922
ENSG0000(	743	16.82439	chr2:8187	SMC3P1		Pseudoger	chr2:99102018-9910
ENSG0000(	743	16.82439	chr2:8187	ANKRD36B		protein_c	chr2:97492663-9758
ENSG0000(	743	16.82439	chr2:8187	TRIM43		protein_c	chr2:95592001-9559
ENSG0000(	743	16.82439	chr2:8187	snoU13		smallRNA	chr2:100577469-100
ENSG0000(	743	16.82439	chr2:8187	IGKV2OR2-1		Pseudoger	chr2:97046588-9704
ENSG0000(	743	16.82439	chr2:8187	MAL		protein_c	chr2:95025677-9505
ENSG0000(	743	16.82439	chr2:8187	ENSG00000238162		Pseudoger	chr2:95485541-9548
ENSG0000(	743	16.82439	chr2:8187	LINC01849		lncRNA	chr2:100603752-100
ENSG0000(	743	16.82439	chr2:8187	ENSG00000223917		Pseudoger	chr2:95486480-9548

ENSG0000(	743	16.82439	chr2:8187	IGKV1D-43		protein_c	chr2:90209873-9021
ENSG0000(	743	16.82439	chr2:8187	IGKV3-25		Pseudoger	chr2:89192500-8919
ENSG0000(	743	16.82439	chr2:8187	IGKV1D-39		protein_c	chr2:89862482-8986
ENSG0000(	743	16.82439	chr2:8187	IGKV2D-18		Pseudoger	chr2:90052581-9005
ENSG0000(	743	16.82439	chr2:8187	IGKV3-34		Pseudoger	chr2:89275298-8927
ENSG0000(	743	16.82439	chr2:8187	DRD5P1		Pseudoger	chr2:91684447-9168
ENSG0000(	743	16.82439	chr2:8187	IGKV1D-32		Pseudoger	chr2:89928422-8992
ENSG0000(	743	16.82439	chr2:8187	RNU7-96P		smallRNA	chr2:97913054-9791
ENSG0000(	743	16.82439	chr2:8187	IGKV7-3		Pseudoger	chr2:88915081-8891
ENSG0000(	743	16.82439	chr2:8187	ENSG00000289685		protein_c	chr2:95085391-9516
ENSG0000(	743	16.82439	chr2:8187	ENSG00000231062		lncRNA	chr2:95051395-9505
ENSG0000(	743	16.82439	chr2:8187	IGKV1D-35		Pseudoger	chr2:89895502-8989
ENSG0000(	743	16.82439	chr2:8187	FAHD2A		protein_c	chr2:95402708-9541
ENSG0000(	743	16.82439	chr2:8187	CNNM3		protein_c	chr2:96816245-9683
ENSG0000(	743	16.82439	chr2:8187	KCNIP3	DriverDB\	protein_c	chr2:95297327-9538
ENSG0000(	743	16.82439	chr2:8187	ENSG00000291126		lncRNA	chr2:95526651-9553
ENSG0000(	743	16.82439	chr2:8187	TEKT4		protein_c	chr2:94871430-9487
ENSG0000(	743	16.82439	chr2:8187	IGKV1-37		protein_c	chr2:89297264-8929
ENSG0000(	743	16.82439	chr2:8187	SEMA4C		protein_c	chr2:96859718-9687
ENSG0000(	743	16.82439	chr2:8187	FAM178B		protein_c	chr2:96875882-9698
ENSG0000(	743	16.82439	chr2:8187	CNGA3		protein_c	chr2:98346188-9839
ENSG0000(	743	16.82439	chr2:8187	ENSG00000228873		lncRNA	chr2:96145602-9614
ENSG0000(	743	16.82439	chr2:8187	IGKV2-38		Pseudoger	chr2:89309898-8931
ENSG0000(	743	16.82439	chr2:8187	IGKV2-10		Pseudoger	chr2:89019992-8902
ENSG0000(	743	16.82439	chr2:8187	ENSG00000237085		Pseudoger	chr2:91859384-9185
ENSG0000(	743	16.82439	chr2:8187	KANSL3		protein_c	chr2:96593170-9664
ENSG0000(	743	16.82439	chr2:8187	MTCO1P48		Pseudoger	chr2:94899566-9490
ENSG0000(	743	16.82439	chr2:8187	ENSG00000283196		Pseudoger	chr2:91654920-9165
ENSG0000(	743	16.82439	chr2:8187	IGKV1-5		protein_c	chr2:88947301-8894
ENSG0000(	743	16.82439	chr2:8187	LSP1P5		lncRNA	chr2:91587019-9166
ENSG0000(	743	16.82439	chr2:8187	ENSG00000227987		lncRNA	chr2:98346995-9835
ENSG0000(	743	16.82439	chr2:8187	ENSG00000231331		Pseudoger	chr2:94953161-9495
ENSG0000(	743	16.82439	chr2:8187	AC113612.2		Pseudoger	chr2:90255285-9025
ENSG0000(	743	16.82439	chr2:8187	FAHD2CP		Pseudoger	chr2:96013730-9602
ENSG0000(	743	16.82439	chr2:8187	ENSG00000248821		Pseudoger	chr2:95413456-9541
ENSG0000(	743	16.82439	chr2:8187	IGKV1-39		protein_c	chr2:89319625-8932
ENSG0000(	743	16.82439	chr2:8187	ENSG00000279791		TEC	chr2:97094935-9709
ENSG0000(	743	16.82439	chr2:8187	IGKV1-9		protein_c	chr2:89009982-8901
ENSG0000(	743	16.82439	chr2:8187	COA5		protein_c	chr2:98599314-9860
ENSG0000(	743	16.82439	chr2:8187	ENSG00000278766		lncRNA	chr2:97421075-9743
ENSG0000(	743	16.82439	chr1:1534	MIR556		smallRNA	chr1:162342546-162
ENSG0000(	743	16.82439	chr2:8187	FAHD2B	NCGv7	protein_c	chr2:97083583-9709
ENSG0000(	743	16.82439	chr2:8187	RN7SL611P		smallRNA	chr2:100556050-100
ENSG0000(	743	16.82439	chr2:8187	ENSG00000283427		Pseudoger	chr2:91607493-9162
ENSG0000(	743	16.82439	chr2:8187	IGKV1-6		protein_c	chr2:88966262-8896
ENSG0000(	743	16.82439	chr2:8187	ENSG00000286698		lncRNA	chr2:91759462-9176
ENSG0000(	743	16.82439	chr2:8187	ENSG00000233037		Pseudoger	chr2:95542730-9554
ENSG0000(	743	16.82439	chr2:8187	HMGN1P36		Pseudoger	chr2:97827248-9782
ENSG0000(	743	16.82439	chr2:8187	IGKV3-20		protein_c	chr2:89142574-8914
ENSG0000(	743	16.82439	chr2:8187	RNU4-84P		smallRNA	chr2:98782410-9878
ENSG0000(	743	16.82439	chr2:8187	ENSG00000271003		Pseudoger	chr2:95607073-9561

ENSG0000(	743	16.82439	chr2:8187	ENSG00000289135		lncRNA	chr2:96815093-9681
ENSG0000(	743	16.82439	chr2:8187	ENSG00000275094		Pseudoger	chr2:97000436-9700
ENSG0000(	743	16.82439	chr2:8187	LIPT1		protein_c	chr2:99154955-9916
ENSG0000(	743	16.82439	chr2:8187	TRIM51JP		Pseudoger	chr2:95574901-9558
ENSG0000(	743	16.82439	chr2:8187	IGKV2-29		Pseudoger	chr2:89234174-8923
ENSG0000(	743	16.82439	chr1:1534	C1orf226		protein_c	chr1:162378841-162
ENSG0000(	743	16.82439	chr2:8187	TRIM43CP		Pseudoger	chr2:97025981-9703
ENSG0000(	743	16.82439	chr2:8187	GPAT2P2		Pseudoger	chr2:97081098-9708
ENSG0000(	743	16.82439	chr2:8187	ENSG00000290565		lncRNA	chr2:91686102-9171
ENSG0000(	743	16.82439	chr2:8187	ENSG00000275075		Pseudoger	chr2:91578478-9157
ENSG0000(	743	16.82439	chr2:8187	C2orf92		protein_c	chr2:97664217-9770
ENSG0000(	743	16.82439	chr2:8187	IGKV3D-31		Pseudoger	chr2:89929701-8993
ENSG0000(	743	16.82439	chr2:8187	IGKV1-22		Pseudoger	chr2:89170775-8917
ENSG0000(	743	16.82439	chr2:8187	IGKV1-33		protein_c	chr2:89268001-8926
ENSG0000(	743	16.82439	chr2:8187	GPAT2P1		Pseudoger	chr2:95792220-9580
ENSG0000(	743	16.82439	chr2:8187	ENSG00000291176		lncRNA	chr2:96010526-9602
ENSG0000(	743	16.82439	chr1:1534	MIR4654		smallRNA	chr1:162157107-162
ENSG0000(	743	16.82439	chr2:8187	LINC00342		lncRNA	chr2:95807052-9583
ENSG0000(	743	16.82439	chr2:8187	IGKV1D-33		protein_c	chr2:89913982-8991
ENSG0000(	743	16.82439	chr2:8187	ENSG00000241962		protein_c	chr2:99141485-9932
ENSG0000(	743	16.82439	chr2:8187	UBTFL6		Pseudoger	chr2:97636780-9763
ENSG0000(	743	16.82439	chr2:8187	C2orf15		protein_c	chr2:99141707-9915
ENSG0000(	743	16.82439	chr2:8187	IGKV2-14		Pseudoger	chr2:89078010-8907
ENSG0000(	743	16.82439	chr2:8187	IGKV1OR2-2		Pseudoger	chr2:92034522-9203
ENSG0000(	743	16.82439	chr2:8187	ENSG00000270193		Pseudoger	chr2:95616492-9561
ENSG0000(	743	16.82439	chr2:8187	AFF3	NCGv7;AC	protein_c	chr2:99545419-1001
ENSG0000(	743	16.82439	chr2:8187	IGKV2D-10		Pseudoger	chr2:90179889-9018
ENSG0000(	743	16.82439	chr2:8187	VWA3B		protein_c	chr2:98087116-9831
ENSG0000(	743	16.82439	chr2:8187	IGKV2OR2-7D		Pseudoger	chr2:97335671-9733
ENSG0000(	743	16.82439	chr2:8187	ZNF2		protein_c	chr2:95165432-9518
ENSG0000(	743	16.82439	chr2:8187	KMT2CP1		lncRNA	chr2:91686102-9171
ENSG0000(	743	16.82439	chr2:8187	ZAP70		protein_c	chr2:97713576-9773
ENSG0000(	743	16.82439	chr2:8187	NCAPH		protein_c	chr2:96335766-9637
ENSG0000(	743	16.82439	chr2:8187	IGKV1D-27		Pseudoger	chr2:89968867-8996
ENSG0000(	743	16.82439	chr2:8187	ENSG00000235584		lncRNA	chr2:95660588-9566
ENSG0000(	743	16.82439	chr2:8187	NEURL3		protein_c	chr2:96497646-9650
ENSG0000(	743	16.82439	chr2:8187	IGKV1OR2-118		Pseudoger	chr2:90315365-9031
ENSG0000(	743	16.82439	chr2:8187	ANKRD23		protein_c	chr2:96824526-9685
ENSG0000(	743	16.82439	chr2:8187	ENSG00000290575		lncRNA	chr2:91892472-9191
ENSG0000(	743	16.82439	chr2:8187	IGKV2D-14		Pseudoger	chr2:90121786-9012
ENSG0000(	743	16.82439	chr2:8187	IGKV3-15		protein_c	chr2:89085177-8908
ENSG0000(	743	16.82439	chr2:8187	ACTR1B		protein_c	chr2:97655939-9766
ENSG0000(	743	16.82439	chr2:8187	IGKV1D-8		protein_c	chr2:90220727-9022
ENSG0000(	743	16.82439	chr2:8187	IGKV2D-28		protein_c	chr2:89959979-8996
ENSG0000(	743	16.82439	chr2:8187	ENSG00000290897		lncRNA	chr2:95789654-9579
ENSG0000(	743	16.82439	chr2:8187	IGSF3P2		Pseudoger	chr2:91736726-9176
ENSG0000(	743	16.82439	chr2:8187	LYG1		protein_c	chr2:99284238-9930
ENSG0000(	743	16.82439	chr2:8187	IGKV1OR2-11		Pseudoger	chr2:97322137-9732
ENSG0000(	743	16.82439	chr2:8187	ENSG00000232502		Pseudoger	chr2:94760774-9476
ENSG0000(	743	16.82439	chr2:8187	IGKV6D-21		protein_c	chr2:90021567-9002
ENSG0000(	743	16.82439	chr2:8187	RN7SL360P		smallRNA	chr2:100600866-100

ENSG0000(	743	16.82439	chr2:8187	KMT2CP4		Pseudoger	chr2:91696451-9171
ENSG0000(	743	16.82439	chr2:8187	GGT8P		Pseudoger	chr2:91775944-9178
ENSG0000(	743	16.82439	chr2:8187	IGKV2D-19		Pseudoger	chr2:90046796-9004
ENSG0000(	743	16.82439	chr2:8187	IGKV2D-24		protein_c	chr2:90004797-9000
ENSG0000(	743	16.82439	chr1:1534	ENSG00000227818		lncRNA	chr1:162039016-162
ENSG0000(	743	16.82439	chr2:8187	LMAN2L		protein_c	chr2:96705929-9674
ENSG0000(	743	16.82439	chr2:8187	ENSG00000291025		lncRNA	chr2:94867486-9494
ENSG0000(	743	16.82439	chr2:8187	ENSG00000287832		lncRNA	chr2:100642444-100
ENSG0000(	743	16.82439	chr1:1534	ENSG00000254706		protein_c	chr1:162365407-162
ENSG0000(	743	16.82439	chr2:8187	IGKV1OR2-1		Pseudoger	chr2:91817771-9181
ENSG0000(	743	16.82439	chr2:8187	IGKV1OR2-9		Pseudoger	chr2:97386082-9738
ENSG0000(	743	16.82439	chr2:8187	ENSG00000278131		Pseudoger	chr2:91589494-9162
ENSG0000(	743	16.82439	chr2:8187	CNN2P11		Pseudoger	chr2:94725674-9472
ENSG0000(	743	16.82439	chr2:8187	MIR3127		smallRNA	chr2:96798278-9679
ENSG0000(	743	16.82439	chr2:8187	RPS24P6		Pseudoger	chr2:94912432-9491
ENSG0000(	743	16.82439	chr2:8187	ENSG00000224585		Pseudoger	chr2:94971954-9497
ENSG0000(	743	16.82439	chr2:8187	IGKV1-32		Pseudoger	chr2:89253571-8925
ENSG0000(	743	16.82439	chr2:8187	ANKRD20A8P		Pseudoger	chr2:94791103-9485
ENSG0000(	743	16.82439	chr2:8187	ENSG00000265897		Pseudoger	chr2:90359808-9036
ENSG0000(	743	16.82439	chr2:8187	AC018696.7		Pseudoger	chr2:91561304-9156
ENSG0000(	743	16.82439	chr2:8187	ENSG00000286036		lncRNA	chr2:97618638-9763
ENSG0000(	743	16.82439	chr2:8187	RN7SL313P		smallRNA	chr2:97100584-9710
ENSG0000(	743	16.82439	chr2:8187	OR7E102P		Pseudoger	chr2:95546531-9554
ENSG0000(	743	16.82439	chr2:8187	ENSG00000237308		Pseudoger	chr2:95496022-9549
ENSG0000(	743	16.82439	chr2:8187	ADRA2B		protein_c	chr2:96112876-9611
ENSG0000(	743	16.82439	chr2:8187	AC092675.1		smallRNA	chr2:98296938-9829
ENSG0000(	743	16.82439	chr2:8187	ENSG00000289370		lncRNA	chr2:95206349-9520
ENSG0000(	741	16.77911	chr2:2577	TTC21B	NCGv7	protein_c	chr2:165857475-165
ENSG0000(	741	16.77911	chr2:2577	ITGA6		protein_c	chr2:172427354-172
ENSG0000(	741	16.77911	chr2:2577	DLX2		protein_c	chr2:172099438-172
ENSG0000(	741	16.77911	chr2:2577	FASTKD1		protein_c	chr2:169528508-169
ENSG0000(	741	16.77911	chr2:2577	RNU6-1006P		smallRNA	chr2:170119996-170
ENSG0000(	741	16.77911	chr2:2577	KLHL41		protein_c	chr2:169509702-169
ENSG0000(	741	16.77911	chr2:2577	CYP2C56P		Pseudoger	chr2:164321933-164
ENSG0000(	741	16.77911	chr2:2577	PHF5GP		Pseudoger	chr2:168231921-168
ENSG0000(	741	16.77911	chr2:2577	ENSG00000236283		lncRNA	chr2:164840661-165
ENSG0000(	741	16.77911	chr2:2577	RNA5SP110		Pseudoger	chr2:164858432-164
ENSG0000(	741	16.77911	chr2:2577	AC110086.1		smallRNA	chr2:164423525-164
ENSG0000(	741	16.77911	chr2:2577	ENSG00000235934		lncRNA	chr2:170814686-170
ENSG0000(	741	16.77911	chr2:2577	B3GALT1-AS1		lncRNA	chr2:167814757-167
ENSG0000(	741	16.77911	chr2:2577	SLC38A11		protein_c	chr2:164894354-164
ENSG0000(	741	16.77911	chr2:2577	METAP1D		protein_c	chr2:171999943-172
ENSG0000(	741	16.77911	chr2:2577	PPIG	NCGv7	protein_c	chr2:169584342-169
ENSG0000(	741	16.77911	chr2:2577	snoU13		smallRNA	chr2:171146330-171
ENSG0000(	741	16.77911	chr2:2577	RPL21P38		Pseudoger	chr2:171587093-171
ENSG0000(	741	16.77911	chr2:2577	ITGA6-AS1		lncRNA	chr2:172464262-172
ENSG0000(	741	16.77911	chr2:2577	DCAF17		protein_c	chr2:171434217-171
ENSG0000(	741	16.77911	chr2:2577	MYO3B		protein_c	chr2:170178145-170
ENSG0000(	741	16.77911	chr2:2577	SCN1A-AS1		lncRNA	chr2:165957188-166
ENSG0000(	741	16.77911	chr2:2577	RNU6-182P		smallRNA	chr2:171856566-171
ENSG0000(	741	16.77911	chr2:2577	ERICH2-DT		lncRNA	chr2:170700368-170

ENSG0000(	741	16.77911	chr2:2577	ENSG00000277998		Pseudoger	chr2:164573741-164
ENSG0000(	741	16.77911	chr2:2577	ENSG00000226963		lncRNA	chr2:172427774-172
ENSG0000(	741	16.77911	chr2:2577	RN7SKP152		smallRNA	chr2:166180652-166
ENSG0000(	741	16.77911	chr2:2577	Y_RNA		smallRNA	chr2:170783167-170
ENSG0000(	741	16.77911	chr2:2577	ENSG00000288089		lncRNA	chr2:165433438-165
ENSG0000(	741	16.77911	chr2:2577	ENSG00000229827		Pseudoger	chr2:169577371-169
ENSG0000(	741	16.77911	chr2:2577	ENSG00000235192		lncRNA	chr2:165794851-165
ENSG0000(	741	16.77911	chr2:2577	DYNC1I2		protein_c	chr2:171687409-171
ENSG0000(	741	16.77911	chr2:2577	RN7SL95P		smallRNA	chr2:168440906-168
ENSG0000(	741	16.77911	chr2:2577	CERS6-AS1		lncRNA	chr2:168771951-168
ENSG0000(	741	16.77911	chr2:2577	STK39		protein_c	chr2:167954020-168
ENSG0000(	741	16.77911	chr2:2577	UBE2V1P6		Pseudoger	chr2:169115195-169
ENSG0000(	741	16.77911	chr2:2577	SCN1A		protein_c	chr2:165984641-166
ENSG0000(	741	16.77911	chr2:2577	CFAP210		protein_c	chr2:169645425-169
ENSG0000(	741	16.77911	chr2:2577	SSB	NCGv7	protein_c	chr2:169791933-169
ENSG0000(	741	16.77911	chr2:2577	ENSG00000288048		lncRNA	chr2:172234230-172
ENSG0000(	741	16.77911	chr2:2577	METTL5		protein_c	chr2:169810081-169
ENSG0000(	741	16.77911	chr2:2577	ENSG00000228222		lncRNA	chr2:167293171-167
ENSG0000(	741	16.77911	chr2:2577	XIRP2	NCGv7	protein_c	chr2:166888480-167
ENSG0000(	741	16.77911	chr2:2577	CSRNP3		protein_c	chr2:165469647-165
ENSG0000(	741	16.77911	chr2:2577	U3		smallRNA	chr2:169816279-169
ENSG0000(	741	16.77911	chr2:2577	MAPRE1P3		Pseudoger	chr2:165285253-165
ENSG0000(	741	16.77911	chr2:2577	NOSTRIN		protein_c	chr2:168786539-168
ENSG0000(	741	16.77911	chr2:2577	TTC21B-AS1		lncRNA	chr2:165933857-165
ENSG0000(	741	16.77911	chr2:2577	RN7SL455P		smallRNA	chr2:165149195-165
ENSG0000(	741	16.77911	chr2:2577	GORASP2		protein_c	chr2:170928464-170
ENSG0000(	741	16.77911	chr2:2577	SCN7A		protein_c	chr2:166403573-166
ENSG0000(	741	16.77911	chr2:2577	ENSG00000278924		TEC	chr2:172423807-172
ENSG0000(	741	16.77911	chr2:2577	CYB5AP2		Pseudoger	chr2:169790093-169
ENSG0000(	741	16.77911	chr2:2577	ENSG00000232555		lncRNA	chr2:172137345-172
ENSG0000(	741	16.77911	chr2:2577	RNU7-148P		smallRNA	chr2:167631480-167
ENSG0000(	741	16.77911	chr2:2577	BBS5		protein_c	chr2:169479480-169
ENSG0000(	741	16.77911	chr2:2577	CYBRD1		protein_c	chr2:171522247-171
ENSG0000(	741	16.77911	chr2:2577	RN7SL813P		smallRNA	chr2:168451324-168
ENSG0000(	741	16.77911	chr2:2577	ENSG00000234061		Pseudoger	chr2:171492905-171
ENSG0000(	741	16.77911	chr2:2577	ENSG00000286611		lncRNA	chr2:170820066-170
ENSG0000(	741	16.77911	chr2:2577	RNA5SP111		Pseudoger	chr2:164895677-164
ENSG0000(	741	16.77911	chr2:2577	COBLL1		protein_c	chr2:164653624-164
ENSG0000(	741	16.77911	chr2:2577	GAD1		protein_c	chr2:170813213-170
ENSG0000(	741	16.77911	chr2:2577	ENSG00000232411		lncRNA	chr2:165833048-165
ENSG0000(	741	16.77911	chr2:2577	TLK1		protein_c	chr2:170990823-171
ENSG0000(	741	16.77911	chr2:2577	G6PC2		protein_c	chr2:168901291-168
ENSG0000(	741	16.77911	chr2:2577	SLC25A12		protein_c	chr2:171783405-171
ENSG0000(	741	16.77911	chr2:2577	GRB14		protein_c	chr2:164492417-164
ENSG0000(	741	16.77911	chr2:2577	RPS26P20		Pseudoger	chr2:171374931-171
ENSG0000(	741	16.77911	chr2:2577	DLX2-DT		lncRNA	chr2:172103006-172
ENSG0000(	741	16.77911	chr2:2577	UBR3	NCGv7	protein_c	chr2:169827454-170
ENSG0000(	741	16.77911	chr2:2577	PTCHD3P2		Pseudoger	chr2:169767185-169
ENSG0000(	741	16.77911	chr2:2577	SCN2A		protein_c	chr2:165194993-165
ENSG0000(	741	16.77911	chr2:2577	AC009495.1		smallRNA	chr2:165814215-165
ENSG0000(	741	16.77911	chr2:2577	PHOSPHO2		protein_c	chr2:169694454-169

ENSG0000(	741	16.77911	chr2:2577	SPC25		protein_c	chr2:168834132-168
ENSG0000(	741	16.77911	chr2:2577	ENSG00000286557		lncRNA	chr2:172323260-172
ENSG0000(	741	16.77911	chr2:2577	ENSG00000237844		lncRNA	chr2:163743913-164
ENSG0000(	741	16.77911	chr2:2577	SNORA70F		smallRNA	chr2:164687643-164
ENSG0000(	741	16.77911	chr2:2577	HMGB1P4		Pseudoger	chr2:170601662-170
ENSG0000(	741	16.77911	chr2:2577	snoU13		smallRNA	chr2:166358730-166
ENSG0000(	741	16.77911	chr2:2577	snoU13		smallRNA	chr2:172156029-172
ENSG0000(	741	16.77911	chr2:2577	B3GALT1		protein_c	chr2:167293001-167
ENSG0000(	741	16.77911	chr2:2577	CERS6		protein_c	chr2:168456249-168
ENSG0000(	741	16.77911	chr2:2577	SP5		protein_c	chr2:170715337-170
ENSG0000(	741	16.77911	chr2:2577	ERICH2		protein_c	chr2:170766878-170
ENSG0000(	741	16.77911	chr2:2577	MIR4774		smallRNA	chr2:168582943-168
ENSG0000(	741	16.77911	chr2:2577	DAP3P2		Pseudoger	chr2:171491422-171
ENSG0000(	741	16.77911	chr2:2577	ENSG00000289413		lncRNA	chr2:172091379-172
ENSG0000(	741	16.77911	chr2:2577	EIF2S2P4		Pseudoger	chr2:170751805-170
ENSG0000(	741	16.77911	chr2:2577	PRPS1P1		Pseudoger	chr2:164213539-164
ENSG0000(	741	16.77911	chr2:2577	FIGN		protein_c	chr2:163593396-163
ENSG0000(	741	16.77911	chr2:2577	ENSG00000288958		lncRNA	chr2:172081990-172
ENSG0000(	741	16.77911	chr2:2577	NSA2P5		Pseudoger	chr2:170077224-170
ENSG0000(	741	16.77911	chr2:2577	SCN9A	NCGv7	protein_c	chr2:166195185-166
ENSG0000(	741	16.77911	chr2:2577	ABCB11		protein_c	chr2:168915498-169
ENSG0000(	741	16.77911	chr2:2577	RNU6-766P		smallRNA	chr2:168606390-168
ENSG0000(	741	16.77911	chr2:2577	SCN3A	NCGv7	protein_c	chr2:165087526-165
ENSG0000(	741	16.77911	chr2:2577	ENSG00000225182		Pseudoger	chr2:166889502-166
ENSG0000(	741	16.77911	chr2:2577	DHRS9		protein_c	chr2:169064789-169
ENSG0000(	741	16.77911	chr2:2577	ENSG00000224331		Pseudoger	chr2:164687287-164
ENSG0000(	741	16.77911	chr2:2577	LINC01124		lncRNA	chr2:170712451-170
ENSG0000(	741	16.77911	chr2:2577	RPS15P4		Pseudoger	chr2:171517270-171
ENSG0000(	741	16.77911	chr2:2577	CTAGE14P		Pseudoger	chr2:167713663-167
ENSG0000(	741	16.77911	chr2:2577	ENSG00000235321		lncRNA	chr2:169100743-169
ENSG0000(	741	16.77911	chr2:2577	METTL8		protein_c	chr2:171315746-171
ENSG0000(	741	16.77911	chr2:2577	KLHL23		protein_c	chr2:169694488-169
ENSG0000(	741	16.77911	chr2:2577	ENSG00000229195		lncRNA	chr2:165794857-165
ENSG0000(	741	16.77911	chr2:2577	ENSG00000251569		protein_c	chr2:169479480-169
ENSG0000(	741	16.77911	chr2:2577	ENSG00000213981		lncRNA	chr2:170640374-170
ENSG0000(	741	16.77911	chr2:2577	ENSG00000283657		Pseudoger	chr2:166414489-166
ENSG0000(	741	16.77911	chr2:2577	ENSG00000286115		lncRNA	chr2:170890393-170
ENSG0000(	741	16.77911	chr2:2577	MYO3B-AS1		lncRNA	chr2:170332085-170
ENSG0000(	741	16.77911	chr2:2577	ENSG00000226072		Pseudoger	chr2:169658132-169
ENSG0000(	741	16.77911	chr2:2577	GALNT3		protein_c	chr2:165747588-165
ENSG0000(	741	16.77911	chr2:2577	Y_RNA		smallRNA	chr2:166123494-166
ENSG0000(	741	16.77911	chr2:2577	AC010092.1		smallRNA	chr2:170916209-170
ENSG0000(	741	16.77911	chr2:2577	XIRP2-AS1		lncRNA	chr2:167123904-167
ENSG0000(	741	16.77911	chr2:2577	LRP2		protein_c	chr2:169127109-169
ENSG0000(	741	16.77911	chr2:2577	DLX1		protein_c	chr2:172084740-172
ENSG0000(	740	16.75646	chr1:3733	ENSG00000226487		lncRNA	chr1:20412304-2041
ENSG0000(	740	16.75646	chr2:8187	LINC00954		lncRNA	chr2:19868860-1988
ENSG0000(	740	16.75646	chr2:8187	WDR35		protein_c	chr2:19910263-1999
ENSG0000(	740	16.75646	chr2:8187	DRG1P1		Pseudoger	chr2:20239892-2024
ENSG0000(	740	16.75646	chr2:8187	TTC32		protein_c	chr2:19896631-1990
ENSG0000(	740	16.75646	chr2:8187	RN7SL140P		smallRNA	chr2:20175346-2017

ENSG0000(	740	16.75646	chr2:8187	MATN3		protein_c	chr2:19992052-2001
ENSG0000(	740	16.75646	chr2:8187	AC007041.1		smallRNA	chr2:20353642-2035
ENSG0000(	740	16.75646	chr2:8187	LINC01808		lncRNA	chr2:19468997-1952
ENSG0000(	740	16.75646	chr2:8187	SDC1		protein_c	chr2:20200797-2022
ENSG0000(	740	16.75646	chr2:8187	CISD1P1		Pseudoger	chr2:19826249-1982
ENSG0000(	740	16.75646	chr2:8187	ENSG00000235911		lncRNA	chr2:19711715-1971
ENSG0000(	740	16.75646	chr2:8187	TTC32-DT		lncRNA	chr2:19902022-1990
ENSG0000(	740	16.75646	chr2:8187	RNU7-113P		smallRNA	chr2:20246326-2024
ENSG0000(	740	16.75646	chr2:8187	WDR35-DT		lncRNA	chr2:19990209-2000
ENSG0000(	740	16.75646	chr2:8187	ENSG00000234378		lncRNA	chr2:20063856-2010
ENSG0000(	740	16.75646	chr2:8187	LAPTM4A	NCGv7	protein_c	chr2:20032650-2005
ENSG0000(	740	16.75646	chr2:8187	RPS16P2		Pseudoger	chr2:20155618-2015
ENSG0000(	740	16.75646	chr2:8187	ENSG00000234597		lncRNA	chr2:19458220-1946
ENSG0000(	740	16.75646	chr2:8187	RNU6-961P		smallRNA	chr2:20175805-2017
ENSG0000(	740	16.75646	chr2:8187	LAPTM4A-DT		lncRNA	chr2:20052114-2005
ENSG0000(	740	16.75646	chr2:8187	PUM2		protein_c	chr2:20248691-2035
ENSG0000(	737	16.68853	chr1:3733	ENSG00000284640		lncRNA	chr1:35141515-3514
ENSG0000(	737	16.68853	chr1:3733	ENSG00000236274		Pseudoger	chr1:35509742-3551
ENSG0000(	737	16.68853	chr1:3733	ENSG00000270241		Pseudoger	chr1:34276859-3427
ENSG0000(	737	16.68853	chr1:3733	CSMD2	NCGv7	protein_c	chr1:33513998-3416
ENSG0000(	737	16.68853	chr1:3733	GJB5		protein_c	chr1:34755047-3475
ENSG0000(	737	16.68853	chr1:3733	SMIM12		protein_c	chr1:34712737-3485
ENSG0000(	737	16.68853	chr1:3733	ZMYM6		protein_c	chr1:34986165-3503
ENSG0000(	737	16.68853	chr1:3733	ENSG00000284773		protein_c	chr1:34974356-3498
ENSG0000(	737	16.68853	chr1:3733	ENSG00000255811		lncRNA	chr1:34761426-3478
ENSG0000(	737	16.68853	chr1:3733	RN7SL503P		smallRNA	chr1:35292200-3529
ENSG0000(	737	16.68853	chr1:3733	SNORA62		smallRNA	chr1:35310274-3531
ENSG0000(	737	16.68853	chr1:3733	C1orf94		protein_c	chr1:34166883-3421
ENSG0000(	737	16.68853	chr1:3733	MIR3605		smallRNA	chr1:33332393-3333
ENSG0000(	737	16.68853	chr1:3733	PHC2-AS1		lncRNA	chr1:33350352-3336
ENSG0000(	737	16.68853	chr1:3733	TFAP2E-AS1		lncRNA	chr1:35569807-3557
ENSG0000(	737	16.68853	chr1:3733	SNORD112		smallRNA	chr1:34943756-3494
ENSG0000(	737	16.68853	chr1:3733	ENSG00000278997		TEC	chr1:33141871-3314
ENSG0000(	737	16.68853	chr1:3733	A3GALT2		protein_c	chr1:33306766-3332
ENSG0000(	737	16.68853	chr1:3733	RN7SL281P		smallRNA	chr1:35706025-3570
ENSG0000(	737	16.68853	chr1:3733	DLGAP3		protein_c	chr1:34865436-3492
ENSG0000(	737	16.68853	chr1:3733	GJA4		protein_c	chr1:34792999-3479
ENSG0000(	737	16.68853	chr1:3733	RNY5P1		smallRNA	chr1:35427816-3542
ENSG0000(	737	16.68853	chr1:3733	ENSG00000235907		Pseudoger	chr1:33512008-3351
ENSG0000(	737	16.68853	chr1:3733	TMEM35B		protein_c	chr1:34981380-3498
ENSG0000(	737	16.68853	chr1:3733	ENSG00000271741		protein_c	chr1:34981533-3503
ENSG0000(	737	16.68853	chr1:3733	ENSG00000232335		lncRNA	chr1:35739389-3574
ENSG0000(	737	16.68853	chr1:3733	ENSG00000284721		lncRNA	chr1:33194788-3320
ENSG0000(	737	16.68853	chr1:3733	RPL12P45		Pseudoger	chr1:35053468-3505
ENSG0000(	737	16.68853	chr1:3733	AL138837.1		smallRNA	chr1:33442025-3344
ENSG0000(	737	16.68853	chr1:3733	NCDN		protein_c	chr1:35557473-3556
ENSG0000(	737	16.68853	chr1:3733	EFCAB14P1		Pseudoger	chr1:35122022-3512
ENSG0000(	737	16.68853	chr1:3733	PHC2	NCGv7	protein_c	chr1:33323623-3343
ENSG0000(	737	16.68853	chr1:3733	ZMYM4		protein_c	chr1:35268709-3542
ENSG0000(	737	16.68853	chr1:3733	CLSPN		protein_c	chr1:35720218-3576
ENSG0000(	737	16.68853	chr1:3733	KIAA0319L		protein_c	chr1:35393883-3555

ENSG00000	737	16.68853	chr1:3733	AL121988.1		smallRNA	chr1:34778561-3477
ENSG00000	737	16.68853	chr1:3733	TRIM62		protein_c	chr1:33145399-3318
ENSG00000	737	16.68853	chr1:3733	AL513327.1		Pseudogen	chr1:33299374-3330
ENSG00000	737	16.68853	chr1:3733	CSMD2-AS1		lncRNA	chr1:33868953-3389
ENSG00000	737	16.68853	chr1:3733	ZMYM4-AS1		lncRNA	chr1:35358822-3536
ENSG00000	737	16.68853	chr1:3733	Y_RNA		smallRNA	chr1:35195969-3519
ENSG00000	737	16.68853	chr1:3733	C1orf216		protein_c	chr1:35713877-3571
ENSG00000	737	16.68853	chr1:3733	AZIN2		protein_c	chr1:33081104-3312
ENSG00000	737	16.68853	chr1:3733	ZMYM1		protein_c	chr1:35032172-3511
ENSG00000	737	16.68853	chr1:3733	HSPD1P14		Pseudogen	chr1:33838523-3384
ENSG00000	737	16.68853	chr1:3733	ENSG00000225313		lncRNA	chr1:33307348-3334
ENSG00000	737	16.68853	chr1:3733	ENSG00000270115		lncRNA	chr1:33261212-3326
ENSG00000	737	16.68853	chr1:3733	RN7SL136P		smallRNA	chr1:35264222-3526
ENSG00000	737	16.68853	chr1:3733	GJB4		protein_c	chr1:34759740-3476
ENSG00000	737	16.68853	chr1:3733	AC115286.1		smallRNA	chr1:34175866-3417
ENSG00000	737	16.68853	chr1:3733	SFPQ	NCGv7;AC	protein_c	chr1:35176378-3519
ENSG00000	737	16.68853	chr1:3733	RN7SKP16		smallRNA	chr1:33336566-3333
ENSG00000	737	16.68853	chr1:3733	ENSG00000230163		lncRNA	chr1:34850694-3485
ENSG00000	737	16.68853	chr1:3733	TLR12P		Pseudogen	chr1:33466249-3346
ENSG00000	737	16.68853	chr1:3733	RNA5SP42		Pseudogen	chr1:34112949-3411
ENSG00000	737	16.68853	chr1:3733	TFAP2E		protein_c	chr1:35573314-3559
ENSG00000	737	16.68853	chr1:3733	PSMB2		protein_c	chr1:35599541-3564
ENSG00000	737	16.68853	chr1:3733	HMGB4		protein_c	chr1:33860475-3386
ENSG00000	737	16.68853	chr1:3733	GJB3		protein_c	chr1:34781214-3478
ENSG00000	737	16.68853	chr1:3733	MIR552		smallRNA	chr1:34669599-3466
ENSG00000	737	16.68853	chr1:3733	ENSG00000287703		lncRNA	chr1:34640157-3468
ENSG00000	737	16.68853	chr1:3733	ZSCAN20		protein_c	chr1:33472645-3350
ENSG00000	737	16.68853	chr1:3733	ENSG00000279179		TEC	chr1:33162851-3316
ENSG00000	737	16.68853	chr1:3733	ZNF362	NCGv7	protein_c	chr1:33256492-3330
ENSG00000	737	16.68853	chr1:3733	RPL5P4		Pseudogen	chr1:35350722-3535
ENSG00000	737	16.68853	chr1:3733	GPR199P		Pseudogen	chr1:34975699-3497
ENSG00000	736	16.66589	chr4:1865	CXCL2	AC	protein_c	chr4:74097040-7409
ENSG00000	736	16.66589	chr4:1865	MRPL1		protein_c	chr4:77862830-7795
ENSG00000	736	16.66589	chr4:1865	AFP		protein_c	chr4:73431138-7345
ENSG00000	736	16.66589	chr4:1865	IGBP1P4		Pseudogen	chr4:82401578-8240
ENSG00000	736	16.66589	chr4:1865	RN7SL127P		smallRNA	chr4:78898855-7889
ENSG00000	736	16.66589	chr4:1865	MIR548AH		smallRNA	chr4:76575551-7657
ENSG00000	736	16.66589	chr4:1865	PPBPP2		lncRNA	chr4:74054038-7405
ENSG00000	736	16.66589	chr4:1865	ENSG00000289379		lncRNA	chr4:77820363-7782
ENSG00000	736	16.66589	chr4:1865	COX5BP1		Pseudogen	chr4:81919995-8192
ENSG00000	736	16.66589	chr4:1865	SNORD50		smallRNA	chr4:76402076-7640
ENSG00000	736	16.66589	chr4:1865	ENSG00000289515		lncRNA	chr4:76305887-7630
ENSG00000	736	16.66589	chr4:1865	ENSG00000289186		lncRNA	chr4:82566385-8257
ENSG00000	736	16.66589	chr4:1865	ENSG00000251017		Pseudogen	chr4:74085995-7408
ENSG00000	736	16.66589	chr4:1865	HNRNPA1P55		Pseudogen	chr4:73938604-7393
ENSG00000	736	16.66589	chr4:1865	ABRAXAS1		protein_c	chr4:83459517-8352
ENSG00000	736	16.66589	chr4:1865	BIN2P1		Pseudogen	chr4:82275071-8227
ENSG00000	736	16.66589	chr4:1865	RPL30P5		Pseudogen	chr4:83502699-8350
ENSG00000	736	16.66589	chr4:1865	PRKG2-AS1		lncRNA	chr4:81164922-8119
ENSG00000	736	16.66589	chr4:1865	ENSG00000289496		lncRNA	chr4:77076049-7707
ENSG00000	736	16.66589	chr4:1865	ENSG00000287037		lncRNA	chr4:73997933-7400

ENSG0000(	736	16.66589	chr4:1865	snoR442	smallRNA	chr4:82949168-8294
ENSG0000(	736	16.66589	chr4:1865	NPM1P41	Pseudoger	chr4:82010665-8201
ENSG0000(	736	16.66589	chr4:1865	ENSG00000248646	lncRNA	chr4:75361207-7543
ENSG0000(	736	16.66589	chr4:1865	HNRNPD-DT	lncRNA	chr4:82374142-8238
ENSG0000(	736	16.66589	chr4:1865	TXNP6	Pseudoger	chr4:76958860-7695
ENSG0000(	736	16.66589	chr4:1865	ENSG00000250315	Pseudoger	chr4:75101477-7510
ENSG0000(	736	16.66589	chr4:1865	FAM47E-STBD1	protein_c	chr4:76251721-7631
ENSG0000(	736	16.66589	chr4:1865	ENSG00000248128	Pseudoger	chr4:78003143-7800
ENSG0000(	736	16.66589	chr4:1865	PARM1 AC	protein_c	chr4:74933095-7505
ENSG0000(	736	16.66589	chr4:1865	LINC01094	lncRNA	chr4:78638780-7868
ENSG0000(	736	16.66589	chr4:1865	ENSG00000270244	Pseudoger	chr4:76886029-7688
ENSG0000(	736	16.66589	chr4:1865	ENSG00000251454	lncRNA	chr4:75341279-7535
ENSG0000(	736	16.66589	chr4:1865	THAP9-AS1	lncRNA	chr4:82893009-8290
ENSG0000(	736	16.66589	chr4:1865	LINC00575	lncRNA	chr4:82610974-8262
ENSG0000(	736	16.66589	chr4:1865	HNRNPA1P56	Pseudoger	chr4:77987860-7798
ENSG0000(	736	16.66589	chr4:1865	HIGD1AP13	Pseudoger	chr4:78648954-7864
ENSG0000(	736	16.66589	chr4:1865	MRPS18C	protein_c	chr4:83455932-8346
ENSG0000(	736	16.66589	chr4:1865	HELQ	protein_c	chr4:83407343-8345
ENSG0000(	736	16.66589	chr4:1865	ENSG00000248831	Pseudoger	chr4:77350370-7735
ENSG0000(	736	16.66589	chr4:1865	ENSG00000248113	Pseudoger	chr4:82580117-8258
ENSG0000(	736	16.66589	chr4:1865	ENSG00000273156	lncRNA	chr4:82344876-8234
ENSG0000(	736	16.66589	chr4:1865	PPEF2	protein_c	chr4:75859864-7590
ENSG0000(	736	16.66589	chr4:1865	ENSG00000249976	Pseudoger	chr4:73337233-7333
ENSG0000(	736	16.66589	chr4:1865	ART3	protein_c	chr4:76011184-7611
ENSG0000(	736	16.66589	chr4:1865	ENSG00000251399	lncRNA	chr4:79596542-7959
ENSG0000(	736	16.66589	chr4:1865	CXCL13	protein_c	chr4:77511753-7761
ENSG0000(	736	16.66589	chr4:1865	ENSG00000248926	Pseudoger	chr4:77958000-7795
ENSG0000(	736	16.66589	chr4:1865	MIR5096	smallRNA	chr4:78820752-7882
ENSG0000(	736	16.66589	chr4:1865	SHROOM3-AS1	lncRNA	chr4:76708853-7680
ENSG0000(	736	16.66589	chr4:1865	NAA11	protein_c	chr4:79225694-7932
ENSG0000(	736	16.66589	chr4:1865	RNU6-1000P	smallRNA	chr4:76356610-7635
ENSG0000(	736	16.66589	chr4:1865	SNX5P1	Pseudoger	chr4:76344550-7634
ENSG0000(	736	16.66589	chr4:1865	ANTXR2	protein_c	chr4:79901146-8012
ENSG0000(	736	16.66589	chr4:1865	CXCL11	protein_c	chr4:76033682-7604
ENSG0000(	736	16.66589	chr4:1865	CXCL10	protein_c	chr4:76021118-7602
ENSG0000(	736	16.66589	chr4:1865	ENSG00000250532	lncRNA	chr4:74418917-7444
ENSG0000(	736	16.66589	chr4:1865	ENSG00000286490	lncRNA	chr4:82612184-8261
ENSG0000(	736	16.66589	chr4:1865	ENSG00000239793	Pseudoger	chr4:78768499-7876
ENSG0000(	736	16.66589	chr4:1865	PPBPP2	Pseudoger	chr4:74054041-7405
ENSG0000(	736	16.66589	chr4:1865	ENSG00000250735	lncRNA	chr4:75401195-7542
ENSG0000(	736	16.66589	chr4:1865	ENSG00000249970	Pseudoger	chr4:73543822-7354
ENSG0000(	736	16.66589	chr4:1865	SNORA75	smallRNA	chr4:79843102-7984
ENSG0000(	736	16.66589	chr4:1865	RPL36AP18	Pseudoger	chr4:76401251-7640
ENSG0000(	736	16.66589	chr4:1865	PAQR3	protein_c	chr4:78887127-7893
ENSG0000(	736	16.66589	chr4:1865	RN7SL218P	smallRNA	chr4:74011578-7401
ENSG0000(	736	16.66589	chr4:1865	Y_RNA	smallRNA	chr4:82944738-8294
ENSG0000(	736	16.66589	chr4:1865	SOWAHB	protein_c	chr4:76894152-7689
ENSG0000(	736	16.66589	chr4:1865	SDAD1-AS1	lncRNA	chr4:75980790-7600
ENSG0000(	736	16.66589	chr4:1865	LINC02469	lncRNA	chr4:79663761-7969
ENSG0000(	736	16.66589	chr4:1865	SDAD1	protein_c	chr4:75940950-7599
ENSG0000(	736	16.66589	chr4:1865	ENOPH1	protein_c	chr4:82430590-8246

ENSG0000(	736	16.66589	chr4:1865	ENSG00000248401		Pseudoger	chr4:83247179-8324
ENSG0000(	736	16.66589	chr4:1865	MTHFD2L		protein_c	chr4:74114174-7430
ENSG0000(	736	16.66589	chr4:1865	RPL7P17		Pseudoger	chr4:77082403-7708
ENSG0000(	736	16.66589	chr4:1865	ENSG00000227304		Pseudoger	chr4:82494786-8249
ENSG0000(	736	16.66589	chr4:1865	PF4		protein_c	chr4:73980811-7398
ENSG0000(	736	16.66589	chr4:1865	HNRNPDL	NCGv7	protein_c	chr4:82422565-8243
ENSG0000(	736	16.66589	chr4:1865	BMP3		protein_c	chr4:81030708-8105
ENSG0000(	736	16.66589	chr4:1865	PRDM8	AC	protein_c	chr4:80183879-8020
ENSG0000(	736	16.66589	chr4:1865	PPBP		protein_c	chr4:73986439-7398
ENSG0000(	736	16.66589	chr4:1865	AC104687.1		smallRNA	chr4:76853964-7685
ENSG0000(	736	16.66589	chr4:1865	PLAC8	AC	protein_c	chr4:83090048-8313
ENSG0000(	736	16.66589	chr4:1865	SCD5		protein_c	chr4:82629539-8279
ENSG0000(	736	16.66589	chr4:1865	CXCL5		protein_c	chr4:73995642-7399
ENSG0000(	736	16.66589	chr4:1865	ENSG00000249171		lncRNA	chr4:83668510-8373
ENSG0000(	736	16.66589	chr4:1865	ENSG00000287632		lncRNA	chr4:78669690-7869
ENSG0000(	736	16.66589	chr4:1865	NAAA	AC	protein_c	chr4:75913660-7594
ENSG0000(	736	16.66589	chr4:1865	PCAT4		lncRNA	chr4:79827471-7987
ENSG0000(	736	16.66589	chr4:1865	NUP54	NCGv7	protein_c	chr4:76107562-7614
ENSG0000(	736	16.66589	chr4:1865	ALB	NCGv7	protein_c	chr4:73397114-7342
ENSG0000(	736	16.66589	chr4:1865	ENSG00000250006		Pseudoger	chr4:77311397-7731
ENSG0000(	736	16.66589	chr4:1865	LINC02562		lncRNA	chr4:75081702-7508
ENSG0000(	736	16.66589	chr4:1865	ENSG00000249072		Pseudoger	chr4:78008512-7800
ENSG0000(	736	16.66589	chr4:1865	SCARB2		protein_c	chr4:76158737-7623
ENSG0000(	736	16.66589	chr4:1865	RPSAP39		Pseudoger	chr4:80161129-8016
ENSG0000(	736	16.66589	chr4:1865	CCDC158		protein_c	chr4:76312997-7642
ENSG0000(	736	16.66589	chr4:1865	FRAS1		protein_c	chr4:78057323-7854
ENSG0000(	736	16.66589	chr4:1865	AC093897.1		smallRNA	chr4:78188336-7818
ENSG0000(	736	16.66589	chr4:1865	SEPTIN11		protein_c	chr4:76949751-7704
ENSG0000(	736	16.66589	chr4:1865	RCHY1		protein_c	chr4:75479033-7551
ENSG0000(	736	16.66589	chr4:1865	MTCYBP44		Pseudoger	chr4:81733385-8173
ENSG0000(	736	16.66589	chr4:1865	CXCL1	TAG;AC	protein_c	chr4:73869393-7387
ENSG0000(	736	16.66589	chr4:1865	G3BP2	NCGv7	protein_c	chr4:75641849-7572
ENSG0000(	736	16.66589	chr4:1865	MIR4450		smallRNA	chr4:76573568-7657
ENSG0000(	736	16.66589	chr4:1865	BMP2K		protein_c	chr4:78776342-7891
ENSG0000(	736	16.66589	chr4:1865	CXCL9		protein_c	chr4:76001275-7600
ENSG0000(	736	16.66589	chr4:1865	CXCL3	AC	protein_c	chr4:74036589-7403
ENSG0000(	736	16.66589	chr4:1865	RPL7AP26		Pseudoger	chr4:82490823-8249
ENSG0000(	736	16.66589	chr4:1865	SNORD75		smallRNA	chr4:77702746-7770
ENSG0000(	736	16.66589	chr4:1865	RNU5A-2P		smallRNA	chr4:81334303-8133
ENSG0000(	736	16.66589	chr4:1865	COPS4		protein_c	chr4:83034447-8307
ENSG0000(	736	16.66589	chr4:1865	BTC		protein_c	chr4:74744759-7479
ENSG0000(	736	16.66589	chr4:1865	ENSG00000274154		Pseudoger	chr4:83369416-8336
ENSG0000(	736	16.66589	chr4:1865	PRDM8-AS1		lncRNA	chr4:80182637-8019
ENSG0000(	736	16.66589	chr4:1865	ODAPH		protein_c	chr4:75556048-7556
ENSG0000(	736	16.66589	chr4:1865	THAP6		protein_c	chr4:75513946-7555
ENSG0000(	736	16.66589	chr4:1865	RNU6-774P		smallRNA	chr4:84233657-8423
ENSG0000(	736	16.66589	chr4:1865	Y_RNA		smallRNA	chr4:75662121-7566
ENSG0000(	736	16.66589	chr4:1865	COQ2		protein_c	chr4:83261536-8328
ENSG0000(	736	16.66589	chr4:1865	RNU6-1187P		smallRNA	chr4:77150328-7715
ENSG0000(	736	16.66589	chr4:1865	Y_RNA		smallRNA	chr4:83636196-8363
ENSG0000(	736	16.66589	chr4:1865	ENSG00000269559		lncRNA	chr4:74156511-7415

ENSG0000(	736	16.66589	chr4:1865	LINC01088		lncRNA	chr4:78939485-7930
ENSG0000(	736	16.66589	chr4:1865	HPSE		protein_c	chr4:83292461-8333
ENSG0000(	736	16.66589	chr4:1865	ENSG00000286074		lncRNA	chr4:76148561-7620
ENSG0000(	736	16.66589	chr4:1865	ENSG00000249278		Pseudoger	chr4:76509284-7650
ENSG0000(	736	16.66589	chr4:1865	CFAP299		protein_c	chr4:80335730-8096
ENSG0000(	736	16.66589	chr4:1865	HNRNPD	NCGv7	protein_c	chr4:82352498-8237
ENSG0000(	736	16.66589	chr4:1865	LINC02499		lncRNA	chr4:73508803-7353
ENSG0000(	736	16.66589	chr4:1865	PRKG2		protein_c	chr4:81087370-8121
ENSG0000(	736	16.66589	chr4:1865	SNORA31		smallRNA	chr4:81928313-8192
ENSG0000(	736	16.66589	chr4:1865	AC112719.1		smallRNA	chr4:76095408-7609
ENSG0000(	736	16.66589	chr4:1865	GK2		protein_c	chr4:79406361-7940
ENSG0000(	736	16.66589	chr4:1865	ENSG00000251185		lncRNA	chr4:75269068-7536
ENSG0000(	736	16.66589	chr4:1865	EPGN		protein_c	chr4:74308470-7431
ENSG0000(	736	16.66589	chr4:1865	ENSG00000288796		protein_c	chr4:73981074-7398
ENSG0000(	736	16.66589	chr4:1865	MICOS10P4		Pseudoger	chr4:78379197-7837
ENSG0000(	736	16.66589	chr4:1865	FAM47E		protein_c	chr4:76214040-7628
ENSG0000(	736	16.66589	chr4:1865	GPAT3		protein_c	chr4:83535914-8360
ENSG0000(	736	16.66589	chr4:1865	HNRNPA3P13		Pseudoger	chr4:82128535-8212
ENSG0000(	736	16.66589	chr4:1865	TMEM150C		protein_c	chr4:82483170-8256
ENSG0000(	736	16.66589	chr4:1865	ENSG00000251647		Pseudoger	chr4:83377363-8337
ENSG0000(	736	16.66589	chr4:1865	ENSG00000286035		lncRNA	chr4:83075957-8308
ENSG0000(	736	16.66589	chr4:1865	FGF5	NCGv7;AC	protein_c	chr4:80266639-8033
ENSG0000(	736	16.66589	chr4:1865	SEC31A		protein_c	chr4:82818509-8290
ENSG0000(	736	16.66589	chr4:1865	RASGEF1B		protein_c	chr4:81426393-8204
ENSG0000(	736	16.66589	chr4:1865	AC097470.1		smallRNA	chr4:74326886-7432
ENSG0000(	736	16.66589	chr4:1865	CCNG2		protein_c	chr4:77157207-7743
ENSG0000(	736	16.66589	chr4:1865	ENSG00000270842		Pseudoger	chr4:82775461-8277
ENSG0000(	736	16.66589	chr4:1865	BMP2K-DT		lncRNA	chr4:78773654-7877
ENSG0000(	736	16.66589	chr4:1865	VAMP9P		Pseudoger	chr4:82284971-8234
ENSG0000(	736	16.66589	chr4:1865	CXCL8		protein_c	chr4:73740519-7374
ENSG0000(	736	16.66589	chr4:1865	RASSF6	NCGv7	protein_c	chr4:73571550-7362
ENSG0000(	736	16.66589	chr4:1865	ENSG00000289241		lncRNA	chr4:74099403-7409
ENSG0000(	736	16.66589	chr4:1865	PF4V1		protein_c	chr4:73853296-7385
ENSG0000(	736	16.66589	chr4:1865	RNU2-16P		smallRNA	chr4:75829454-7582
ENSG0000(	736	16.66589	chr4:1865	ENSG00000271676		Pseudoger	chr4:77112495-7711
ENSG0000(	736	16.66589	chr4:1865	AREG	NCGv7	protein_c	chr4:74445136-7445
ENSG0000(	736	16.66589	chr4:1865	Y_RNA		smallRNA	chr4:78632273-7863
ENSG0000(	736	16.66589	chr4:1865	ENSG00000289480		lncRNA	chr4:82348252-8234
ENSG0000(	736	16.66589	chr4:1865	LIN54		protein_c	chr4:82909973-8301
ENSG0000(	736	16.66589	chr4:1865	CNOT6L		protein_c	chr4:77713387-7781
ENSG0000(	736	16.66589	chr4:1865	OR7E94P		Pseudoger	chr4:79587757-7958
ENSG0000(	736	16.66589	chr4:1865	ENSG00000270480		Pseudoger	chr4:82691737-8269
ENSG0000(	736	16.66589	chr4:1865	RNU6-499P		smallRNA	chr4:82174547-8217
ENSG0000(	736	16.66589	chr4:1865	PPBPP1		Pseudoger	chr4:73847866-7384
ENSG0000(	736	16.66589	chr4:1865	ENSG00000289530		lncRNA	chr4:73706323-7370
ENSG0000(	736	16.66589	chr4:1865	ENSG00000289586		lncRNA	chr4:76908814-7694
ENSG0000(	736	16.66589	chr4:1865	SNORD42		smallRNA	chr4:82402638-8240
ENSG0000(	736	16.66589	chr4:1865	ENSG00000288888		lncRNA	chr4:77056585-7705
ENSG0000(	736	16.66589	chr4:1865	snoU13		smallRNA	chr4:78640100-7864
ENSG0000(	736	16.66589	chr4:1865	LINC02483		lncRNA	chr4:75354076-7536
ENSG0000(	736	16.66589	chr4:1865	ENSG00000287375		lncRNA	chr4:74881174-7488

ENSG0000(	736	16.66589	chr4:1865	RNU6-615P		smallRNA	chr4:83003412-8300
ENSG0000(	736	16.66589	chr4:1865	ENSG00000229717		lncRNA	chr4:75822966-7583
ENSG0000(	736	16.66589	chr4:1865	HMGB1P44		Pseudoger	chr4:77963940-7796
ENSG0000(	736	16.66589	chr4:1865	RPL36P8		Pseudoger	chr4:76036750-7603
ENSG0000(	736	16.66589	chr4:1865	PARM1-AS1		lncRNA	chr4:74955974-7497
ENSG0000(	736	16.66589	chr4:1865	AFM	NCGv7	protein_c	chr4:73481745-7350
ENSG0000(	736	16.66589	chr4:1865	ENSG00000242727		Pseudoger	chr4:76891034-7689
ENSG0000(	736	16.66589	chr4:1865	ENSG00000232327		Pseudoger	chr4:80386178-8038
ENSG0000(	736	16.66589	chr4:1865	ENSG00000287401		lncRNA	chr4:76240740-7631
ENSG0000(	736	16.66589	chr4:1865	LINC00989		lncRNA	chr4:79491802-7962
ENSG0000(	736	16.66589	chr4:1865	CXCL6		protein_c	chr4:73836640-7384
ENSG0000(	736	16.66589	chr4:1865	EREG		protein_c	chr4:74365145-7438
ENSG0000(	736	16.66589	chr4:1865	CXCL1P1		Pseudoger	chr4:73944011-7394
ENSG0000(	736	16.66589	chr4:1865	ENSG00000249960		Pseudoger	chr4:82571137-8257
ENSG0000(	736	16.66589	chr4:1865	MIR575		smallRNA	chr4:82753337-8275
ENSG0000(	736	16.66589	chr4:1865	SERBP1P5		Pseudoger	chr4:78180866-7818
ENSG0000(	736	16.66589	chr4:1865	THAP9		protein_c	chr4:82900684-8291
ENSG0000(	736	16.66589	chr4:1865	ENSG00000288019		lncRNA	chr4:74076533-7407
ENSG0000(	736	16.66589	chr4:1865	CDKL2		protein_c	chr4:75576496-7563
ENSG0000(	736	16.66589	chr4:1865	UMLILO		lncRNA	chr4:73710302-7371
ENSG0000(	736	16.66589	chr4:1865	RNU6-145P		smallRNA	chr4:76532222-7653
ENSG0000(	736	16.66589	chr4:1865	ENSG00000290776		lncRNA	chr4:79587302-7958
ENSG0000(	736	16.66589	chr4:1865	SHROOM3	NCGv7	protein_c	chr4:76435229-7678
ENSG0000(	736	16.66589	chr4:1865	ENSG00000224218		lncRNA	chr4:76758554-7680
ENSG0000(	736	16.66589	chr4:1865	ENSG00000250560		Pseudoger	chr4:75194867-7519
ENSG0000(	736	16.66589	chr4:1865	ENSG00000250057		lncRNA	chr4:83233512-8324
ENSG0000(	736	16.66589	chr4:1865	STBD1		protein_c	chr4:76306733-7631
ENSG0000(	736	16.66589	chr4:1865	AC112249.1		smallRNA	chr4:76493247-7649
ENSG0000(	736	16.66589	chr4:1865	KPNA2P1		Pseudoger	chr4:80079532-8008
ENSG0000(	736	16.66589	chr4:1865	HSPE1P23		Pseudoger	chr4:74917822-7491
ENSG0000(	736	16.66589	chr4:1865	ENSG00000249051		Pseudoger	chr4:73777636-7377
ENSG0000(	736	16.66589	chr4:1865	SLC25A14P1		Pseudoger	chr4:83477524-8347
ENSG0000(	736	16.66589	chr4:1865	ANXA3		protein_c	chr4:78551747-7861
ENSG0000(	736	16.66589	chr4:1865	CCNI	NCGv7	protein_c	chr4:77047155-7707
ENSG0000(	736	16.66589	chr4:1865	USO1		protein_c	chr4:75724577-7581
ENSG0000(	736	16.66589	chr4:1865	ENSG00000289443		lncRNA	chr4:77048964-7705
ENSG0000(	736	16.66589	chr4:1865	ENSG00000248165		lncRNA	chr4:74993877-7503
ENSG0000(	736	16.66589	chr4:1865	ENSG00000249036		lncRNA	chr4:77394491-7749
ENSG0000(	735	16.64324	chr8:2747	MIR378D2HG		lncRNA	chr8:93915734-9391
ENSG0000(	735	16.64324	chr8:2747	TP53INP1	NCGv7	protein_c	chr8:94925972-9494
ENSG0000(	735	16.64324	chr8:2747	CIBAR1-DT		lncRNA	chr8:93213302-9370
ENSG0000(	735	16.64324	chr8:2747	ENSG00000243979		Pseudoger	chr8:94891239-9489
ENSG0000(	735	16.64324	chr8:2747	TRIQK		protein_c	chr8:92883532-9301
ENSG0000(	735	16.64324	chr8:2747	MYL12AP1		Pseudoger	chr8:93849561-9385
ENSG0000(	735	16.64324	chr8:2747	ENSG00000272249		lncRNA	chr8:97132835-9713
ENSG0000(	735	16.64324	chr8:2747	Y_RNA		smallRNA	chr8:96309221-9630
ENSG0000(	735	16.64324	chr8:2747	INTS8	AC	protein_c	chr8:94813311-9488
ENSG0000(	735	16.64324	chr8:2747	ENSG00000253576		lncRNA	chr8:91975908-9197
ENSG0000(	735	16.64324	chr8:2747	ENSG00000254020		lncRNA	chr8:92723024-9272
ENSG0000(	735	16.64324	chr8:2747	ENSG00000253634		lncRNA	chr8:92460539-9265
ENSG0000(	735	16.64324	chr8:2747	ENSG00000271971		lncRNA	chr8:93715378-9371

ENSG0000(	735	16.64324	chr8:2747	TMEM67		protein_c	chr8:93754844-9381
ENSG0000(	735	16.64324	chr8:2747	PDP1		protein_c	chr8:93857807-9392
ENSG0000(	735	16.64324	chr8:2747	GEM	DriverDBv	protein_c	chr8:94249253-9426
ENSG0000(	735	16.64324	chr8:2747	VIRMA	NCGv7	protein_c	chr8:94487689-9455
ENSG0000(	735	16.64324	chr8:2747	Y_RNA		smallRNA	chr8:94713715-9471
ENSG0000(	735	16.64324	chr8:2747	AC105081.1		smallRNA	chr8:93973854-9397
ENSG0000(	735	16.64324	chr8:2747	RNU6-703P		smallRNA	chr8:98048856-9804
ENSG0000(	735	16.64324	chr8:2747	ENSG00000254283		Pseudoger	chr8:94594411-9459
ENSG0000(	735	16.64324	chr8:2747	SUMO2P18		Pseudoger	chr8:97858049-9785
ENSG0000(	735	16.64324	chr8:2747	ENSG00000288752		lncRNA	chr8:98045653-9804
ENSG0000(	735	16.64324	chr8:2747	MATN2		protein_c	chr8:97868840-9803
ENSG0000(	735	16.64324	chr8:2747	ENSG00000253577		lncRNA	chr8:92765651-9285
ENSG0000(	735	16.64324	chr8:2747	CIBAR1		protein_c	chr8:93698561-9373
ENSG0000(	735	16.64324	chr8:2747	snoU13		smallRNA	chr8:94906017-9490
ENSG0000(	735	16.64324	chr8:2747	ENSG00000254248		lncRNA	chr8:95071732-9508
ENSG0000(	735	16.64324	chr8:2747	RNU6-1172P		smallRNA	chr8:96431054-9643
ENSG0000(	735	16.64324	chr8:2747	RBM12B-AS1		TEC	chr8:93740121-9374
ENSG0000(	735	16.64324	chr8:2747	ENSG00000254089		lncRNA	chr8:93259667-9329
ENSG0000(	735	16.64324	chr8:2747	ENSG00000213750		Pseudoger	chr8:97624203-9762
ENSG0000(	735	16.64324	chr8:2747	ENSG00000253749		lncRNA	chr8:92420850-9242
ENSG0000(	735	16.64324	chr8:2747	LAPTM4B	NCGv7;AC	protein_c	chr8:97775057-9785
ENSG0000(	735	16.64324	chr8:2747	ENSG00000254315		lncRNA	chr8:94533628-9453
ENSG0000(	735	16.64324	chr8:2747	ENSG00000272509		lncRNA	chr8:94884609-9488
ENSG0000(	735	16.64324	chr8:2747	ENSG00000253945		Pseudoger	chr8:95403802-9540
ENSG0000(	735	16.64324	chr8:2747	ENSG00000253901		lncRNA	chr8:91542924-9190
ENSG0000(	735	16.64324	chr8:2747	UQCRB-AS1		lncRNA	chr8:96235427-9623
ENSG0000(	735	16.64324	chr8:2747	SDC2		protein_c	chr8:96493813-9661
ENSG0000(	735	16.64324	chr8:2747	CPQ	NCGv7	protein_c	chr8:96645242-9714
ENSG0000(	735	16.64324	chr8:2747	ENSG00000270077		lncRNA	chr8:97144170-9714
ENSG0000(	735	16.64324	chr8:2747	RNU6-690P		smallRNA	chr8:95599114-9559
ENSG0000(	735	16.64324	chr8:2747	RPL6P23		Pseudoger	chr8:94201913-9420
ENSG0000(	735	16.64324	chr8:2747	ENSG00000233778		Pseudoger	chr8:92144088-9214
ENSG0000(	735	16.64324	chr8:2747	TSPYL5	DriverDBv	protein_c	chr8:97273488-9727
ENSG0000(	735	16.64324	chr8:2747	ENSG00000270131		lncRNA	chr8:96140572-9614
ENSG0000(	735	16.64324	chr8:2747	ENSG00000287654		lncRNA	chr8:97643366-9764
ENSG0000(	735	16.64324	chr8:2747	ENSG00000253118		Pseudoger	chr8:92351912-9235
ENSG0000(	735	16.64324	chr8:2747	LINC02894		lncRNA	chr8:94637285-9463
ENSG0000(	735	16.64324	chr8:2747	ENSG00000253854		lncRNA	chr8:93719574-9372
ENSG0000(	735	16.64324	chr8:2747	DPY19L4		protein_c	chr8:94719703-9479
ENSG0000(	735	16.64324	chr8:2747	NDUFAF6	DriverDBv	protein_c	chr8:94895533-9511
ENSG0000(	735	16.64324	chr8:2747	CDH17	NCGv7;AC	protein_c	chr8:94127162-9421
ENSG0000(	735	16.64324	chr8:2747	RUNX1T1	NCGv7;AC	protein_c	chr8:91954967-9210
ENSG0000(	735	16.64324	chr8:2747	CFAP418-AS1		lncRNA	chr8:95204456-9581
ENSG0000(	735	16.64324	chr8:2747	ENSG00000253105		lncRNA	chr8:96370800-9638
ENSG0000(	735	16.64324	chr8:2747	ENSG00000253682		lncRNA	chr8:92402920-9241
ENSG0000(	735	16.64324	chr8:2747	RBM12B		protein_c	chr8:93728155-9374
ENSG0000(	735	16.64324	chr8:2747	ENSG00000241003		Pseudoger	chr8:94688228-9468
ENSG0000(	735	16.64324	chr8:2747	RPL30-AS1		lncRNA	chr8:98041726-9804
ENSG0000(	735	16.64324	chr8:2747	Y_RNA		smallRNA	chr8:97772313-9777
ENSG0000(	735	16.64324	chr8:2747	RNA5SP274		Pseudoger	chr8:93441277-9344
ENSG0000(	735	16.64324	chr8:2747	ENSG00000270682		Pseudoger	chr8:97788210-9778

ENSG0000(	735	16.64324	chr8:2747	ENSG00000254190		Pseudoger	chr8:94590115-9459
ENSG0000(	735	16.64324	chr8:2747	ENSG00000254057		lncRNA	chr8:93834454-9384
ENSG0000(	735	16.64324	chr8:2747	SRSF3P2		Pseudoger	chr8:95947781-9594
ENSG0000(	735	16.64324	chr8:2747	AC091096.1		smallRNA	chr8:92635335-9263
ENSG0000(	735	16.64324	chr8:2747	LINC02906		lncRNA	chr8:93134095-9316
ENSG0000(	735	16.64324	chr8:2747	RPS4XP10		Pseudoger	chr8:94352923-9435
ENSG0000(	735	16.64324	chr8:2747	ENSG00000288005		lncRNA	chr8:92687222-9269
ENSG0000(	735	16.64324	chr8:2747	ENSG00000253722		lncRNA	chr8:93733216-9373
ENSG0000(	735	16.64324	chr8:2747	PRR13P7		Pseudoger	chr8:91909738-9191
ENSG0000(	735	16.64324	chr8:2747	ENSG00000253197		lncRNA	chr8:92882984-9296
ENSG0000(	735	16.64324	chr8:2747	IRF5P1		Pseudoger	chr8:92972575-9297
ENSG0000(	735	16.64324	chr8:2747	RAD54B	DriverDB\	protein_c	chr8:94371960-9447
ENSG0000(	735	16.64324	chr8:2747	ESRP1		protein_c	chr8:94641074-9470
ENSG0000(	735	16.64324	chr8:2747	FSBP		protein_c	chr8:94378377-9443
ENSG0000(	735	16.64324	chr8:2747	GAPDHP30		Pseudoger	chr8:96068729-9606
ENSG0000(	735	16.64324	chr8:2747	snoU13		smallRNA	chr8:91464035-9146
ENSG0000(	735	16.64324	chr8:2747	ENSG00000254307		lncRNA	chr8:94791643-9479
ENSG0000(	735	16.64324	chr8:2747	RBM12B-DT		lncRNA	chr8:93741193-9374
ENSG0000(	735	16.64324	chr8:2747	CFAP418		protein_c	chr8:95244913-9526
ENSG0000(	735	16.64324	chr8:2747	ENSG00000271349		Pseudoger	chr8:96126967-9612
ENSG0000(	735	16.64324	chr8:2747	PTDSS1	NCGv7	protein_c	chr8:96261902-9633
ENSG0000(	735	16.64324	chr8:2747	RPL30	DriverDB\	protein_c	chr8:98024851-9804
ENSG0000(	735	16.64324	chr8:2747	ZNF317P1		Pseudoger	chr8:93646066-9364
ENSG0000(	735	16.64324	chr8:2747	ENSG00000253585		lncRNA	chr8:94097764-9410
ENSG0000(	735	16.64324	chr8:2747	LINC01298		lncRNA	chr8:95206876-9521
ENSG0000(	735	16.64324	chr8:2747	VIRMA-DT		lncRNA	chr8:94553668-9457
ENSG0000(	735	16.64324	chr8:2747	PLEKHF2		protein_c	chr8:95115642-9515
ENSG0000(	735	16.64324	chr8:2747	FLJ46284		lncRNA	chr8:92699742-9287
ENSG0000(	735	16.64324	chr8:2747	ENSG00000253960		lncRNA	chr8:93215925-9323
ENSG0000(	735	16.64324	chr8:2747	RPS23P1		Pseudoger	chr8:97865054-9786
ENSG0000(	735	16.64324	chr8:2747	PSMA2P2		Pseudoger	chr8:93939364-9394
ENSG0000(	735	16.64324	chr8:2747	UQCRB		protein_c	chr8:96222947-9623
ENSG0000(	735	16.64324	chr8:2747	ENSG00000253177		lncRNA	chr8:92668660-9268
ENSG0000(	735	16.64324	chr8:2747	ENSG00000253429		Pseudoger	chr8:94708952-9470
ENSG0000(	735	16.64324	chr8:2747	ENSG00000268080		Pseudoger	chr8:93229659-9322
ENSG0000(	735	16.64324	chr8:2747	MTDH	DriverDB\	protein_c	chr8:97644184-9773
ENSG0000(	735	16.64324	chr8:2747	SLC26A7		protein_c	chr8:91209494-9139
ENSG0000(	735	16.64324	chr8:2747	ENSG00000253872		lncRNA	chr8:95505406-9552
ENSG0000(	735	16.64324	chr8:2747	RNU6-1209P		smallRNA	chr8:95055214-9505
ENSG0000(	735	16.64324	chr8:2747	ENSG00000248762		lncRNA	chr8:95986108-9599
ENSG0000(	735	16.64324	chr8:2747	ENSG00000253175		Pseudoger	chr8:94565036-9456
ENSG0000(	735	16.64324	chr8:2747	RN7SKP231		smallRNA	chr8:91594965-9159
ENSG0000(	735	16.64324	chr8:2747	MTERF3	NCGv7	protein_c	chr8:96239398-9626
ENSG0000(	735	16.64324	chr8:2747	CCNE2	DriverDB\	protein_c	chr8:94879770-9489
ENSG0000(	735	16.64324	chr8:2747	MRPS16P1		Pseudoger	chr8:91917507-9191
ENSG0000(	735	16.64324	chr8:2747	ENSG00000275386		Pseudoger	chr8:93974671-9397
ENSG0000(	735	16.64324	chr8:2747	SNORA72		smallRNA	chr8:98042086-9804
ENSG0000(	735	16.64324	chr8:2747	ENSG00000270861		Pseudoger	chr8:97853021-9785
ENSG0000(	735	16.64324	chr8:2747	ENSG00000260640		lncRNA	chr8:97241742-9724
ENSG0000(	735	16.64324	chr8:2747	GDF6		protein_c	chr8:96142333-9616
ENSG0000(	735	16.64324	chr8:2747	ENSG00000253528		Pseudoger	chr8:94974573-9497

ENSG0000(	735	16.64324	chr8:2747	ENSG00000287322		lncRNA	chr8:95039617-9504
ENSG0000(	735	16.64324	chr8:2747	U3		smallRNA	chr8:97358265-9735
ENSG0000(	735	16.64324	chr8:2747	RPL34P18		Pseudoger	chr8:93957338-9395
ENSG0000(	735	16.64324	chr8:2747	MIR3150BHG		lncRNA	chr8:95066808-9507
ENSG0000(	730	16.53002	chr2:8187	RNU6-997P		smallRNA	chr2:53570374-5357
ENSG0000(	728	16.48474	chr4:253(	AC098870.1		smallRNA	chr4:85910478-8591
ENSG0000(	727	16.46209	chr8:2747	ENSG00000254219		lncRNA	chr8:91042690-9104
ENSG0000(	727	16.46209	chr8:2747	ENSG00000253358		lncRNA	chr8:91016588-9101
ENSG0000(	727	16.46209	chr8:2747	AF070718.1		smallRNA	chr8:90463327-9046
ENSG0000(	727	16.46209	chr8:2747	ENSG00000254251		lncRNA	chr8:90806474-9085
ENSG0000(	727	16.46209	chr8:2747	ENSG00000254182		Pseudoger	chr8:90701082-9070
ENSG0000(	727	16.46209	chr8:2747	RN7SL777P		smallRNA	chr8:91139261-9113
ENSG0000(	727	16.46209	chr8:2747	ENSG00000254180		lncRNA	chr8:90198389-9062
ENSG0000(	727	16.46209	chr8:2747	ENSG00000289502		lncRNA	chr8:90957029-9095
ENSG0000(	727	16.46209	chr8:2747	RNU6-925P		smallRNA	chr8:89900721-8990
ENSG0000(	727	16.46209	chr8:2747	LINC00534		lncRNA	chr8:90221341-9068
ENSG0000(	727	16.46209	chr8:2747	LINC01030		lncRNA	chr8:90592804-9060
ENSG0000(	727	16.46209	chr8:2747	NECAB1	DriverDB\	protein_c	chr8:90791741-9095
ENSG0000(	727	16.46209	chr8:2747	TMEM64	DriverDB\	protein_c	chr8:90621995-9079
ENSG0000(	727	16.46209	chr8:2747	PIP4P2		protein_c	chr8:90993802-9104
ENSG0000(	727	16.46209	chr8:2747	DECR1	DriverDB\	protein_c	chr8:90001405-9005
ENSG0000(	727	16.46209	chr8:2747	C8orf88		protein_c	chr8:90958471-9098
ENSG0000(	727	16.46209	chr8:2747	RNA5SP273		Pseudoger	chr8:90511128-9051
ENSG0000(	727	16.46209	chr8:2747	LRRC69	DriverDB\	protein_c	chr8:91101832-9121
ENSG0000(	727	16.46209	chr8:2747	ENSG00000246792		lncRNA	chr8:90646420-9067
ENSG0000(	727	16.46209	chr8:2747	OTUD6B-AS1		lncRNA	chr8:91059318-9107
ENSG0000(	727	16.46209	chr8:2747	NBN	NCGv7	protein_c	chr8:89924515-9000
ENSG0000(	727	16.46209	chr8:2747	OSGIN2		protein_c	chr8:89901849-8992
ENSG0000(	727	16.46209	chr8:2747	OTUD6B	DriverDB\	protein_c	chr8:91070196-9108
ENSG0000(	727	16.46209	chr8:2747	CALB1		protein_c	chr8:90058608-9009
ENSG0000(	725	16.4168	chr1:1534	FCRL3		protein_c	chr1:157674321-157
ENSG0000(	725	16.4168	chr1:1534	ENSG00000236957		Pseudoger	chr1:157925065-157
ENSG0000(	725	16.4168	chr1:1534	ENSG00000236731		Pseudoger	chr1:157629939-157
ENSG0000(	725	16.4168	chr1:1534	MRPS21P2		Pseudoger	chr1:157861197-157
ENSG0000(	725	16.4168	chr1:1534	ENSG00000227217		lncRNA	chr1:157691762-157
ENSG0000(	725	16.4168	chr1:1534	FCRL2		protein_c	chr1:157745733-157
ENSG0000(	725	16.4168	chr1:1534	CD5L		protein_c	chr1:157830911-157
ENSG0000(	725	16.4168	chr1:1534	ENSG00000233712		Pseudoger	chr1:157636300-157
ENSG0000(	725	16.4168	chr1:1534	ENSG00000231700		Pseudoger	chr1:157709086-157
ENSG0000(	725	16.4168	chr1:1534	FCRL4	NCGv7	protein_c	chr1:157573747-157
ENSG0000(	725	16.4168	chr1:1534	KIRREL1		protein_c	chr1:157993273-158
ENSG0000(	725	16.4168	chr1:1534	FCRL5		protein_c	chr1:157513377-157
ENSG0000(	725	16.4168	chr1:1534	ENSG00000291226		lncRNA	chr1:157925974-157
ENSG0000(	725	16.4168	chr1:1534	KIRREL1-IT1		lncRNA	chr1:158025550-158
ENSG0000(	725	16.4168	chr1:1534	VDAC1P9		Pseudoger	chr1:157724180-157
ENSG0000(	725	16.4168	chr1:1534	FCRL1	NCGv7	protein_c	chr1:157794403-157
ENSG0000(	724	16.39416	chr20:291	SRMP1		Pseudoger	chr20:47859788-478
ENSG0000(	722	16.34887	chr8:2747	AC103816.1		smallRNA	chr8:84296003-8429
ENSG0000(	722	16.34887	chr1:3733	ENSG00000272482		lncRNA	chr1:12618900-1261
ENSG0000(	719	16.28094	chr1:3733	SNORD85		smallRNA	chr1:30968164-3096
ENSG0000(	719	16.28094	chr1:3733	MIR5585		smallRNA	chr1:32086949-3208

ENSG0000(	719	16.28094	chr1:3733	ENSG00000229167		lncRNA	chr1:31571585-3157
ENSG0000(	719	16.28094	chr1:3733	LINC01226		lncRNA	chr1:31506226-3158
ENSG0000(	719	16.28094	chr1:3733	HDAC1	NCGv7;AC	protein_c	chr1:32292083-3233
ENSG0000(	719	16.28094	chr1:3733	ENSG00000287691		lncRNA	chr1:32925454-3295
ENSG0000(	719	16.28094	chr1:3733	S100PBP		protein_c	chr1:32816767-3285
ENSG0000(	719	16.28094	chr1:3733	SERINC2		protein_c	chr1:31409565-3143
ENSG0000(	719	16.28094	chr1:3733	TMEM39B		protein_c	chr1:32072031-3210
ENSG0000(	719	16.28094	chr1:3733	ENSG00000217644		Pseudoger	chr1:32979947-3298
ENSG0000(	719	16.28094	chr1:3733	SNORD103A		smallRNA	chr1:30935688-3093
ENSG0000(	719	16.28094	chr1:3733	SNRNP40		protein_c	chr1:31259568-3129
ENSG0000(	719	16.28094	chr1:3733	ENSG00000264078		lncRNA	chr1:31644694-3164
ENSG0000(	719	16.28094	chr1:3733	ENSG00000291132		lncRNA	chr1:32231656-3224
ENSG0000(	719	16.28094	chr1:3733	RNU6-40P		smallRNA	chr1:31497577-3149
ENSG0000(	719	16.28094	chr1:3733	RN7SL122P		smallRNA	chr1:32457835-3245
ENSG0000(	719	16.28094	chr1:3733	AL031602.1		smallRNA	chr1:32926294-3292
ENSG0000(	719	16.28094	chr1:3733	EEF1A1P46		Pseudoger	chr1:31487589-3148
ENSG0000(	719	16.28094	chr1:3733	ENSG00000229044		lncRNA	chr1:31333067-3134
ENSG0000(	719	16.28094	chr1:3733	TMEM54		protein_c	chr1:32894594-3290
ENSG0000(	719	16.28094	chr1:3733	HPCA		protein_c	chr1:32885994-3289
ENSG0000(	719	16.28094	chr1:3733	SELENOWP1		Pseudoger	chr1:31094987-3109
ENSG0000(	719	16.28094	chr1:3733	Y_RNA		smallRNA	chr1:32639951-3264
ENSG0000(	719	16.28094	chr1:3733	ENSG00000232768		Pseudoger	chr1:31050872-3105
ENSG0000(	719	16.28094	chr1:3733	ENSG00000237329		Pseudoger	chr1:31036734-3103
ENSG0000(	719	16.28094	chr1:3733	GAPDHP20		Pseudoger	chr1:32402109-3240
ENSG0000(	719	16.28094	chr1:3733	FABP3		protein_c	chr1:31365253-3137
ENSG0000(	719	16.28094	chr1:3733	RP11-439L8.3		lncRNA	chr1:31506240-3150
ENSG0000(	719	16.28094	chr1:3733	FNDC5		protein_c	chr1:32862268-3287
ENSG0000(	719	16.28094	chr1:3733	LRRC37A12P		Pseudoger	chr1:32423214-3242
ENSG0000(	719	16.28094	chr1:3733	PTP4A2	AC	protein_c	chr1:31906421-3194
ENSG0000(	719	16.28094	chr1:3733	ENSG00000250135		lncRNA	chr1:32170733-3217
ENSG0000(	719	16.28094	chr1:3733	ENSG00000224409		lncRNA	chr1:32717734-3272
ENSG0000(	719	16.28094	chr1:3733	SPOCD1		protein_c	chr1:31790422-3181
ENSG0000(	719	16.28094	chr1:3733	TINAGL1		protein_c	chr1:31576485-3158
ENSG0000(	719	16.28094	chr1:3733	PUM1		protein_c	chr1:30931506-3106
ENSG0000(	719	16.28094	chr1:3733	FAM167B		protein_c	chr1:32247222-3224
ENSG0000(	719	16.28094	chr1:3733	ADGRB2		protein_c	chr1:31727117-3176
ENSG0000(	719	16.28094	chr1:3733	ENSG00000288678		protein_c	chr1:31919563-3191
ENSG0000(	719	16.28094	chr1:3733	ENSG00000269967		lncRNA	chr1:31851913-3192
ENSG0000(	719	16.28094	chr1:3733	HCRTR1		protein_c	chr1:31617686-3163
ENSG0000(	719	16.28094	chr1:3733	ENSG00000284702		lncRNA	chr1:31972189-3198
ENSG0000(	719	16.28094	chr1:3733	MTMR9LP		Pseudoger	chr1:32231847-3224
ENSG0000(	719	16.28094	chr1:3733	ENSG00000270850		Pseudoger	chr1:32421979-3242
ENSG0000(	719	16.28094	chr1:3733	CCDC28B		protein_c	chr1:32200595-3220
ENSG0000(	719	16.28094	chr1:3733	ZCCHC17		protein_c	chr1:31296982-3136
ENSG0000(	719	16.28094	chr1:3733	IQCC		protein_c	chr1:32205671-3220
ENSG0000(	719	16.28094	chr1:3733	TMEM234		protein_c	chr1:32214472-3222
ENSG0000(	719	16.28094	chr1:3733	BSDC1		protein_c	chr1:32364633-3239
ENSG0000(	719	16.28094	chr1:3733	ZBTB8A		protein_c	chr1:32539427-3260
ENSG0000(	719	16.28094	chr1:3733	YARS1		protein_c	chr1:32775237-3281
ENSG0000(	719	16.28094	chr1:3733	ENSG00000228634		lncRNA	chr1:31933020-3193
ENSG0000(	719	16.28094	chr1:3733	RNF19B		protein_c	chr1:32936445-3296

ENSG0000(	719	16.28094	chr1:3733	KHDRBS1		protein_c	chr1:32013868-3206
ENSG0000(	719	16.28094	chr1:3733	PEF1-AS1		lncRNA	chr1:31644049-3166
ENSG0000(	719	16.28094	chr1:3733	LINC01778		lncRNA	chr1:30824217-3083
ENSG0000(	719	16.28094	chr1:3733	MARCKSL1		protein_c	chr1:32333839-3233
ENSG0000(	719	16.28094	chr1:3733	SNORD103B		smallRNA	chr1:30949117-3094
ENSG0000(	719	16.28094	chr1:3733	DCDC2B		protein_c	chr1:32209089-3221
ENSG0000(	719	16.28094	chr1:3733	KPNA6		protein_c	chr1:32108056-3217
ENSG0000(	719	16.28094	chr1:3733	ENSG00000290045		lncRNA	chr1:32240526-3224
ENSG0000(	719	16.28094	chr1:3733	ENSG00000231949		lncRNA	chr1:30415825-3042
ENSG0000(	719	16.28094	chr1:3733	ENSG00000236065		lncRNA	chr1:32987075-3303
ENSG0000(	719	16.28094	chr1:3733	ZBTB8OS		protein_c	chr1:32600172-3265
ENSG0000(	719	16.28094	chr1:3733	ENSG00000233775		lncRNA	chr1:32349194-3235
ENSG0000(	719	16.28094	chr1:3733	Y_RNA		smallRNA	chr1:32286452-3228
ENSG0000(	719	16.28094	chr1:3733	AL136115.1		protein_c	chr1:31913573-3191
ENSG0000(	719	16.28094	chr1:3733	ENSG00000229607		lncRNA	chr1:30810378-3081
ENSG0000(	719	16.28094	chr1:3733	FKSG48		protein_c	chr1:32973667-3297
ENSG0000(	719	16.28094	chr1:3733	TSSK3		protein_c	chr1:32351521-3236
ENSG0000(	719	16.28094	chr1:3733	KIAA1522		protein_c	chr1:32741830-3277
ENSG0000(	719	16.28094	chr1:3733	ENSG00000203325		lncRNA	chr1:32052291-3207
ENSG0000(	719	16.28094	chr1:3733	RBBP4		protein_c	chr1:32651142-3268
ENSG0000(	719	16.28094	chr1:3733	SYNC		protein_c	chr1:32679906-3270
ENSG0000(	719	16.28094	chr1:3733	PEF1		protein_c	chr1:31629866-3164
ENSG0000(	719	16.28094	chr1:3733	SDC3		protein_c	chr1:30869466-3090
ENSG0000(	719	16.28094	chr1:3733	LAPTM5		protein_c	chr1:30732469-3075
ENSG0000(	719	16.28094	chr1:3733	MATN1		protein_c	chr1:30711277-3072
ENSG0000(	719	16.28094	chr1:3733	ENSG00000236335		lncRNA	chr1:30409560-3041
ENSG0000(	719	16.28094	chr1:3733	ENSG00000203620		lncRNA	chr1:31842019-3185
ENSG0000(	719	16.28094	chr1:3733	RN7SKP91		smallRNA	chr1:30843823-3084
ENSG0000(	719	16.28094	chr1:3733	MIR4254		smallRNA	chr1:31758660-3175
ENSG0000(	719	16.28094	chr1:3733	FAM229A		protein_c	chr1:32361270-3236
ENSG0000(	719	16.28094	chr1:3733	ENSG00000289710		protein_c	chr1:30576655-3057
ENSG0000(	719	16.28094	chr1:3733	AC114494.1		protein_c	chr1:31429345-3142
ENSG0000(	719	16.28094	chr1:3733	ENSG00000287510		lncRNA	chr1:30731693-3073
ENSG0000(	719	16.28094	chr1:3733	ENSG00000224066		lncRNA	chr1:32204769-3220
ENSG0000(	719	16.28094	chr1:3733	MATN1-AS1		lncRNA	chr1:30718504-3072
ENSG0000(	719	16.28094	chr1:3733	ENSG00000278966		TEC	chr1:32973553-3297
ENSG0000(	719	16.28094	chr1:3733	ZBTB8B		protein_c	chr1:32465072-3249
ENSG0000(	719	16.28094	chr1:3733	ENSG00000239670		Pseudoger	chr1:32986952-3298
ENSG0000(	719	16.28094	chr1:3733	LCK	NCGv7;AC	protein_c	chr1:32251244-3228
ENSG0000(	719	16.28094	chr1:3733	COL16A1		protein_c	chr1:31652263-3170
ENSG0000(	719	16.28094	chr1:3733	MIR4420		smallRNA	chr1:30739156-3073
ENSG0000(	719	16.28094	chr1:3733	AK2		protein_c	chr1:33007986-3308
ENSG0000(	719	16.28094	chr1:3733	NKAIN1		protein_c	chr1:31179745-3123
ENSG0000(	719	16.28094	chr1:3733	ENSG00000254553		protein_c	chr1:32465057-3260
ENSG0000(	719	16.28094	chr1:3733	ENSG00000235143		lncRNA	chr1:30858158-3086
ENSG0000(	719	16.28094	chr1:3733	TXLNA		protein_c	chr1:32179675-3219
ENSG0000(	719	16.28094	chr1:3733	ENSG00000254545		lncRNA	chr1:31789130-3179
ENSG0000(	719	16.28094	chr1:3733	ENSG00000225142		Pseudoger	chr1:31108188-3110
ENSG0000(	719	16.28094	chr1:3733	ENSG00000229447		Pseudoger	chr1:31263245-3126
ENSG0000(	719	16.28094	chr1:3733	EIF3I	AC	protein_c	chr1:32221077-3224
ENSG0000(	712	16.12243	chr1:1021	RNU6-969P		smallRNA	chr1:52805108-5280

ENSG0000(	712	16.12243	chr1:1021	P3R3URF-PIK3R3		protein_c	chr1:46043661-4617
ENSG0000(	712	16.12243	chr1:1021	PPP1R8P1		Pseudoger	chr1:48325080-4832
ENSG0000(	712	16.12243	chr1:1021	LEXM		protein_c	chr1:54806063-5484
ENSG0000(	712	16.12243	chr1:1021	LINC01398		lncRNA	chr1:46446600-4645
ENSG0000(	712	16.12243	chr1:1021	RNU6-723P		smallRNA	chr1:48344209-4834
ENSG0000(	712	16.12243	chr1:1021	BSND		protein_c	chr1:54998933-5501
ENSG0000(	712	16.12243	chr1:1021	USP24		protein_c	chr1:55066359-5521
ENSG0000(	712	16.12243	chr1:1021	CMPK1		protein_c	chr1:47333790-4739
ENSG0000(	712	16.12243	chr1:1021	ENSG00000272371		lncRNA	chr1:52554818-5255
ENSG0000(	712	16.12243	chr1:1021	PARS2		protein_c	chr1:54756898-5476
ENSG0000(	712	16.12243	chr1:1021	AGBL4		protein_c	chr1:48532854-5002
ENSG0000(	712	16.12243	chr1:1021	AKR1A1		protein_c	chr1:45550543-4557
ENSG0000(	712	16.12243	chr1:1021	ENSG00000225028		lncRNA	chr1:47818066-4782
ENSG0000(	712	16.12243	chr1:1021	PODN		protein_c	chr1:53062052-5308
ENSG0000(	712	16.12243	chr1:1021	ZSWIM5		protein_c	chr1:45016399-4530
ENSG0000(	712	16.12243	chr1:1021	SNORD112		smallRNA	chr1:54525386-5452
ENSG0000(	712	16.12243	chr1:1021	RNU6-1026P		smallRNA	chr1:50582404-5058
ENSG0000(	712	16.12243	chr1:1021	RNU7-95P		smallRNA	chr1:53688749-5368
ENSG0000(	712	16.12243	chr1:1021	BEND5		protein_c	chr1:48727519-4877
ENSG0000(	712	16.12243	chr1:1021	ELAVL4	NCGv7	protein_c	chr1:50024029-5020
ENSG0000(	712	16.12243	chr1:1021	COA7		protein_c	chr1:52684449-5269
ENSG0000(	712	16.12243	chr1:1021	ZYG11B		protein_c	chr1:52726453-5282
ENSG0000(	712	16.12243	chr1:1021	PDZK1IP1	AC	protein_c	chr1:47183582-4719
ENSG0000(	712	16.12243	chr1:1021	CYP4A22		protein_c	chr1:47137435-4714
ENSG0000(	712	16.12243	chr1:1021	GLIS1		protein_c	chr1:53506237-5373
ENSG0000(	712	16.12243	chr1:1021	ENSG00000232027		Pseudoger	chr1:51372270-5137
ENSG0000(	712	16.12243	chr1:1021	snoU13		smallRNA	chr1:52411442-5241
ENSG0000(	712	16.12243	chr1:1021	DMBX1		protein_c	chr1:46489836-4651
ENSG0000(	712	16.12243	chr1:1021	SLC1A7		protein_c	chr1:53087179-5314
ENSG0000(	712	16.12243	chr1:1021	CZIB		protein_c	chr1:53214099-5322
ENSG0000(	712	16.12243	chr1:1021	FAAHP1		Pseudoger	chr1:46432129-4644
ENSG0000(	712	16.12243	chr1:1021	ELAVL4-AS1		lncRNA	chr1:50174306-5017
ENSG0000(	712	16.12243	chr1:1021	ACOT11		protein_c	chr1:54542257-5463
ENSG0000(	712	16.12243	chr1:1021	FAM151A		protein_c	chr1:54609181-5462
ENSG0000(	712	16.12243	chr1:1021	TAL1	NCGv7;AC	protein_c	chr1:47216290-4723
ENSG0000(	712	16.12243	chr1:1021	PLA2G12AP1		Pseudoger	chr1:52368677-5236
ENSG0000(	712	16.12243	chr1:1021	PHB1P12		Pseudoger	chr1:50780340-5078
ENSG0000(	712	16.12243	chr1:1021	LINC00853		lncRNA	chr1:47179250-4718
ENSG0000(	712	16.12243	chr1:1021	TSEN15P2		Pseudoger	chr1:51859778-5186
ENSG0000(	712	16.12243	chr1:1021	ENSG00000225183		Pseudoger	chr1:54089856-5409
ENSG0000(	712	16.12243	chr1:1021	IPP		protein_c	chr1:45694324-4575
ENSG0000(	712	16.12243	chr1:1021	ENSG00000235563		lncRNA	chr1:53114576-5311
ENSG0000(	712	16.12243	chr1:1021	TEX38		protein_c	chr1:46668855-4667
ENSG0000(	712	16.12243	chr1:1021	ENSG00000272491		lncRNA	chr1:48227888-4822
ENSG0000(	712	16.12243	chr1:1021	ENSG00000235105		Pseudoger	chr1:48435967-4843
ENSG0000(	712	16.12243	chr1:1021	TCEANC2		protein_c	chr1:54053584-5411
ENSG0000(	712	16.12243	chr1:1021	PDCL3P6		Pseudoger	chr1:52179848-5218
ENSG0000(	712	16.12243	chr1:1021	snoU13		smallRNA	chr1:49151392-4915
ENSG0000(	712	16.12243	chr1:1021	ENSG00000230728		lncRNA	chr1:54621477-5462
ENSG0000(	712	16.12243	chr1:1021	ENSG00000289407		lncRNA	chr1:45583238-4558
ENSG0000(	712	16.12243	chr1:1021	TMEM59		protein_c	chr1:54026681-5405

ENSG0000(	712	16.12243	chr1:1021	TMA16P2		Pseudoger	chr1:45846994-4584
ENSG0000(	712	16.12243	chr1:1021	TUBAP9		Pseudoger	chr1:47074778-4707
ENSG0000(	712	16.12243	chr1:1021	LRRC42		protein_c	chr1:53946085-5396
ENSG0000(	712	16.12243	chr1:1021	MIR4781		smallRNA	chr1:54054079-5405
ENSG0000(	712	16.12243	chr1:1021	SLC5A9		protein_c	chr1:48222685-4824
ENSG0000(	712	16.12243	chr1:1021	OSBPL9		protein_c	chr1:51577179-5179
ENSG0000(	712	16.12243	chr1:1021	TXNDC12		protein_c	chr1:52020131-5205
ENSG0000(	712	16.12243	chr1:1021	ENSG00000281825		Pseudoger	chr1:45605657-4560
ENSG0000(	712	16.12243	chr1:1021	snoU13		smallRNA	chr1:45358652-4535
ENSG0000(	712	16.12243	chr1:1021	CYP4Z1	NCGv7	protein_c	chr1:47067231-4711
ENSG0000(	712	16.12243	chr1:1021	SCP2		protein_c	chr1:52927276-5305
ENSG0000(	712	16.12243	chr1:1021	MRPL37		protein_c	chr1:54184041-5422
ENSG0000(	712	16.12243	chr1:1021	AL162430.2		smallRNA	chr1:51188463-5118
ENSG0000(	712	16.12243	chr1:1021	KNCN		protein_c	chr1:46545641-4655
ENSG0000(	712	16.12243	chr1:1021	PRDX1		protein_c	chr1:45510914-4554
ENSG0000(	712	16.12243	chr1:1021	ENSG00000234578		lncRNA	chr1:53267935-5326
ENSG0000(	712	16.12243	chr1:1021	UQCRH		protein_c	chr1:46303698-4631
ENSG0000(	712	16.12243	chr1:1021	PIK3R3	NCGv7	protein_c	chr1:46040140-4613
ENSG0000(	712	16.12243	chr1:1021	ENSG00000234810		lncRNA	chr1:55329288-5607
ENSG0000(	712	16.12243	chr1:1021	RN7SL290P		smallRNA	chr1:51995740-5199
ENSG0000(	712	16.12243	chr1:1021	TSPAN1	AC	protein_c	chr1:46175073-4618
ENSG0000(	712	16.12243	chr1:1021	RNU6-877P		smallRNA	chr1:51382308-5138
ENSG0000(	712	16.12243	chr1:1021	GPX7		protein_c	chr1:52602371-5260
ENSG0000(	712	16.12243	chr1:1021	P3R3URF		protein_c	chr1:46175486-4617
ENSG0000(	712	16.12243	chr1:1021	FAAH		protein_c	chr1:46394317-4641
ENSG0000(	712	16.12243	chr1:1021	NSUN4		protein_c	chr1:46340789-4636
ENSG0000(	712	16.12243	chr1:1021	LINC01144		lncRNA	chr1:45303910-4530
ENSG0000(	712	16.12243	chr1:1021	ENSG00000291138		lncRNA	chr1:46433827-4648
ENSG0000(	712	16.12243	chr1:1021	CYP4X1		protein_c	chr1:47023669-4705
ENSG0000(	712	16.12243	chr1:1021	TMEM275		protein_c	chr1:46532166-4654
ENSG0000(	712	16.12243	chr1:1021	DHCR24		protein_c	chr1:54849627-5488
ENSG0000(	712	16.12243	chr1:1021	RN7SL62P		smallRNA	chr1:52714399-5271
ENSG0000(	712	16.12243	chr1:1021	AL049745.1		smallRNA	chr1:53828792-5382
ENSG0000(	712	16.12243	chr1:1021	MAGOH		protein_c	chr1:53226900-5323
ENSG0000(	712	16.12243	chr1:1021	ENSG00000280378		TEC	chr1:54033126-5403
ENSG0000(	712	16.12243	chr1:1021	ENSG00000290081		lncRNA	chr1:47180175-4718
ENSG0000(	712	16.12243	chr1:1021	AL645730.1		smallRNA	chr1:49982215-4998
ENSG0000(	712	16.12243	chr1:1021	SHISAL2A		protein_c	chr1:52633168-5266
ENSG0000(	712	16.12243	chr1:1021	Y_RNA		smallRNA	chr1:50499758-5049
ENSG0000(	712	16.12243	chr1:1021	RPL21P24		Pseudoger	chr1:47497894-4749
ENSG0000(	712	16.12243	chr1:1021	LINC01753		lncRNA	chr1:55915603-5594
ENSG0000(	712	16.12243	chr1:1021	TRABD2B		protein_c	chr1:47760528-4799
ENSG0000(	712	16.12243	chr1:1021	PIGQP1		Pseudoger	chr1:55938714-5593
ENSG0000(	712	16.12243	chr1:1021	ANAPC10P1		Pseudoger	chr1:52253621-5225
ENSG0000(	712	16.12243	chr1:1021	CYP4A26P		Pseudoger	chr1:46967679-4696
ENSG0000(	712	16.12243	chr1:1021	ENSG00000233406		Pseudoger	chr1:51250603-5125
ENSG0000(	712	16.12243	chr1:1021	ENSG00000233407		lncRNA	chr1:50206084-5022
ENSG0000(	712	16.12243	chr1:1021	EPS15-AS1		lncRNA	chr1:51518288-5156
ENSG0000(	712	16.12243	chr1:1021	ENSG00000280836		Pseudoger	chr1:45581219-4558
ENSG0000(	712	16.12243	chr1:1021	HECTD3		protein_c	chr1:45002540-4501
ENSG0000(	712	16.12243	chr1:1021	UROD		protein_c	chr1:45010950-4501

ENSG0000(	712	16.12243	chr1:1021	ENSG00000236723		lncRNA	chr1:53209783-5321
ENSG0000(	712	16.12243	chr1:1021	AL592294.1		smallRNA	chr1:45232218-4523
ENSG0000(	712	16.12243	chr1:1021	GAPDHP51		Pseudoger	chr1:51707138-5170
ENSG0000(	712	16.12243	chr1:1021	PRPF38A		protein_c	chr1:52404602-5242
ENSG0000(	712	16.12243	chr1:1021	AL591415.1		smallRNA	chr1:47621093-4762
ENSG0000(	712	16.12243	chr1:1021	MIR4422		smallRNA	chr1:55225641-5522
ENSG0000(	712	16.12243	chr1:1021	MIR1273G		smallRNA	chr1:52940314-5294
ENSG0000(	712	16.12243	chr1:1021	HIGD1AP11		Pseudoger	chr1:53073110-5307
ENSG0000(	712	16.12243	chr1:1021	AL589663.1		smallRNA	chr1:51900920-5190
ENSG0000(	712	16.12243	chr1:1021	CYP4A44P		Pseudoger	chr1:47002995-4700
ENSG0000(	712	16.12243	chr1:1021	ENSG00000233271		lncRNA	chr1:54980950-5499
ENSG0000(	712	16.12243	chr1:1021	PPIAP36		Pseudoger	chr1:45415020-4541
ENSG0000(	712	16.12243	chr1:1021	ENSG00000287582		lncRNA	chr1:54514417-5451
ENSG0000(	712	16.12243	chr1:1021	ENSG00000232762		lncRNA	chr1:53304536-5330
ENSG0000(	712	16.12243	chr1:1021	AL162430.1		smallRNA	chr1:51190883-5119
ENSG0000(	712	16.12243	chr1:1021	ENSG00000290102		lncRNA	chr1:50967883-5096
ENSG0000(	712	16.12243	chr1:1021	KTI12		protein_c	chr1:52032103-5203
ENSG0000(	712	16.12243	chr1:1021	HNRNPA1P63		Pseudoger	chr1:54536796-5453
ENSG0000(	712	16.12243	chr1:1021	ENSG00000232245		lncRNA	chr1:54416256-5442
ENSG0000(	712	16.12243	chr1:1021	MIR4421		smallRNA	chr1:51059837-5105
ENSG0000(	712	16.12243	chr1:1021	SPATA6	NCGv7	protein_c	chr1:48295373-4847
ENSG0000(	712	16.12243	chr1:1021	CZIB-DT		lncRNA	chr1:53220663-5322
ENSG0000(	712	16.12243	chr1:1021	FOXE3		protein_c	chr1:47416285-4741
ENSG0000(	712	16.12243	chr1:1021	RRAS2P1		Pseudoger	chr1:52993201-5299
ENSG0000(	712	16.12243	chr1:1021	AC099796.1		smallRNA	chr1:54504668-5450
ENSG0000(	712	16.12243	chr1:1021	TTC39A-AS1		lncRNA	chr1:51329654-5133
ENSG0000(	712	16.12243	chr1:1021	ENSG00000279324		TEC	chr1:49994318-4999
ENSG0000(	712	16.12243	chr1:1021	MAGOH-DT		lncRNA	chr1:53238550-5324
ENSG0000(	712	16.12243	chr1:1021	MKNK1	NCGv7	protein_c	chr1:46557407-4661
ENSG0000(	712	16.12243	chr1:1021	MROH7-TTC4		protein_c	chr1:54641786-5474
ENSG0000(	712	16.12243	chr1:1021	MRPS17P1		Pseudoger	chr1:44988705-4499
ENSG0000(	712	16.12243	chr1:1021	EEF1GP7		Pseudoger	chr1:52573114-5257
ENSG0000(	712	16.12243	chr1:1021	RP5-850O15.4		lncRNA	chr1:50437028-5043
ENSG0000(	712	16.12243	chr1:1021	ENSG00000230953		Pseudoger	chr1:52920422-5292
ENSG0000(	712	16.12243	chr1:1021	SLC25A3P1		Pseudoger	chr1:53413149-5344
ENSG0000(	712	16.12243	chr1:1021	HSPB11		protein_c	chr1:53916574-5394
ENSG0000(	712	16.12243	chr1:1021	ENSG00000281112		Pseudoger	chr1:45592722-4559
ENSG0000(	712	16.12243	chr1:1021	LDLRAD1	NCGv7	protein_c	chr1:54007298-5401
ENSG0000(	712	16.12243	chr1:1021	ENSG00000229687		Pseudoger	chr1:53841547-5384
ENSG0000(	712	16.12243	chr1:1021	LINC01562		lncRNA	chr1:51195095-5123
ENSG0000(	712	16.12243	chr1:1021	NDUFS5P3		Pseudoger	chr1:52709122-5270
ENSG0000(	712	16.12243	chr1:1021	ENSG00000226938		lncRNA	chr1:53348488-5334
ENSG0000(	712	16.12243	chr1:1021	LRRC41		protein_c	chr1:46261196-4630
ENSG0000(	712	16.12243	chr1:1021	CYP4Z2P		Pseudoger	chr1:46843178-4690
ENSG0000(	712	16.12243	chr1:1021	CC2D1B		protein_c	chr1:52345723-5236
ENSG0000(	712	16.12243	chr1:1021	ENSG00000226957		lncRNA	chr1:46046818-4604
ENSG0000(	712	16.12243	chr1:1021	CYP4A27P		Pseudoger	chr1:47000898-4700
ENSG0000(	712	16.12243	chr1:1021	LURAP1		protein_c	chr1:46203334-4622
ENSG0000(	712	16.12243	chr1:1021	ENSG00000236476		Pseudoger	chr1:46742329-4674
ENSG0000(	712	16.12243	chr1:1021	AL353898.2		Pseudoger	chr1:54033143-5403
ENSG0000(	712	16.12243	chr1:1021	CFL1P2		Pseudoger	chr1:51157788-5115

ENSG0000(	712	16.12243	chr1:1021	MTND2P29		Pseudoger	chr1:50017092-5001
ENSG0000(	712	16.12243	chr1:1021	ENSG00000236434		lncRNA	chr1:51264916-5126
ENSG0000(	712	16.12243	chr1:1021	ENSG00000238140		lncRNA	chr1:51461721-5146
ENSG0000(	712	16.12243	chr1:1021	NRDC		protein_c	chr1:51789191-5187
ENSG0000(	712	16.12243	chr1:1021	NENFP1		Pseudoger	chr1:46665910-4666
ENSG0000(	712	16.12243	chr1:1021	EIF2B3		protein_c	chr1:44850522-4498
ENSG0000(	712	16.12243	chr1:1021	DMRTA2		protein_c	chr1:50417550-5042
ENSG0000(	712	16.12243	chr1:1021	ZYG11A		protein_c	chr1:52842511-5289
ENSG0000(	712	16.12243	chr1:1021	ZNF859P		Pseudoger	chr1:49841821-4984
ENSG0000(	712	16.12243	chr1:1021	RPS13P2		Pseudoger	chr1:52772194-5277
ENSG0000(	712	16.12243	chr1:1021	ENSG00000237453		lncRNA	chr1:54792885-5479
ENSG0000(	712	16.12243	chr1:1021	GYG1P3		Pseudoger	chr1:55222379-5522
ENSG0000(	712	16.12243	chr1:1021	ENSG00000228838		lncRNA	chr1:53288024-5328
ENSG0000(	712	16.12243	chr1:1021	MIR4422HG		lncRNA	chr1:55217645-5532
ENSG0000(	712	16.12243	chr1:1021	ENSG00000284601		lncRNA	chr1:54974900-5498
ENSG0000(	712	16.12243	chr1:1021	ENSG00000237478		Pseudoger	chr1:49691262-4969
ENSG0000(	712	16.12243	chr1:1021	RNA5SP48		Pseudoger	chr1:51973410-5197
ENSG0000(	712	16.12243	chr1:1021	MIR5095		smallRNA	chr1:52934930-5293
ENSG0000(	712	16.12243	chr1:1021	MROH7		protein_c	chr1:54641754-5471
ENSG0000(	712	16.12243	chr1:1021	HNRNPA3P12		Pseudoger	chr1:53974969-5397
ENSG0000(	712	16.12243	chr1:1021	ENSG00000285839		protein_c	chr1:52020153-5203
ENSG0000(	712	16.12243	chr1:1021	MKNK1-AS1		lncRNA	chr1:46538611-4657
ENSG0000(	712	16.12243	chr1:1021	SNORA58		smallRNA	chr1:53771018-5377
ENSG0000(	712	16.12243	chr1:1021	CYP46A4P		Pseudoger	chr1:48089368-4808
ENSG0000(	712	16.12243	chr1:1021	CYB5RL		protein_c	chr1:54169651-5420
ENSG0000(	712	16.12243	chr1:1021	Y_RNA		smallRNA	chr1:51107222-5110
ENSG0000(	712	16.12243	chr1:1021	TUT4		protein_c	chr1:52408282-5255
ENSG0000(	712	16.12243	chr1:1021	SKINT1L		Pseudoger	chr1:48161799-4817
ENSG0000(	712	16.12243	chr1:1021	H3P2		Pseudoger	chr1:52943536-5294
ENSG0000(	712	16.12243	chr1:1021	PCSK9		protein_c	chr1:55039447-5506
ENSG0000(	712	16.12243	chr1:1021	ENSG00000287724		lncRNA	chr1:54874672-5488
ENSG0000(	712	16.12243	chr1:1021	ENSG00000242396		lncRNA	chr1:54886812-5497
ENSG0000(	712	16.12243	chr1:1021	ENSG00000242391		lncRNA	chr1:52881216-5288
ENSG0000(	712	16.12243	chr1:1021	FOXD2-AS1		lncRNA	chr1:47432133-4743
ENSG0000(	712	16.12243	chr1:1021	YIPF1		protein_c	chr1:53851719-5388
ENSG0000(	712	16.12243	chr1:1021	NDC1		protein_c	chr1:53765478-5383
ENSG0000(	712	16.12243	chr1:1021	RAB3B		protein_c	chr1:51907956-5199
ENSG0000(	712	16.12243	chr1:1021	MTCO2P34		Pseudoger	chr1:55372710-5537
ENSG0000(	712	16.12243	chr1:1021	AL357673.1		protein_c	chr1:54169660-5417
ENSG0000(	712	16.12243	chr1:1021	SLC25A6P3		Pseudoger	chr1:51709062-5170
ENSG0000(	712	16.12243	chr1:1021	ENSG00000229032		Pseudoger	chr1:51980473-5198
ENSG0000(	712	16.12243	chr1:1021	CYP4A11	NCGv7	protein_c	chr1:46929177-4694
ENSG0000(	712	16.12243	chr1:1021	AL136380.1		smallRNA	chr1:44913068-4491
ENSG0000(	712	16.12243	chr1:1021	ENSG00000228407		Pseudoger	chr1:52160261-5216
ENSG0000(	712	16.12243	chr1:1021	DMRTB1		protein_c	chr1:53459399-5346
ENSG0000(	712	16.12243	chr1:1021	ENSG00000231413		lncRNA	chr1:48078787-4808
ENSG0000(	712	16.12243	chr1:1021	SSBP3-AS1		lncRNA	chr1:54236440-5423
ENSG0000(	712	16.12243	chr1:1021	LINC02808		lncRNA	chr1:50229662-5032
ENSG0000(	712	16.12243	chr1:1021	ENSG00000284700		lncRNA	chr1:50423609-5042
ENSG0000(	712	16.12243	chr1:1021	RN7SL788P		smallRNA	chr1:52150105-5215
ENSG0000(	712	16.12243	chr1:1021	HMGB1P45		Pseudoger	chr1:50398825-5039

ENSG0000(	712	16.12243	chr1:1021	LINC01738		lncRNA	chr1:47688463-4770
ENSG0000(	712	16.12243	chr1:1021	ATP6V0E1P4		Pseudoger	chr1:47550196-4755
ENSG0000(	712	16.12243	chr1:1021	ENSG00000227857		lncRNA	chr1:46134531-4613
ENSG0000(	712	16.12243	chr1:1021	BTF3L4		protein_c	chr1:52056199-5209
ENSG0000(	712	16.12243	chr1:1021	AL645730.2		protein_c	chr1:49994318-4999
ENSG0000(	712	16.12243	chr1:1021	AL356458.1		smallRNA	chr1:47502504-4750
ENSG0000(	712	16.12243	chr1:1021	CYP4A43P		Pseudoger	chr1:46994382-4699
ENSG0000(	712	16.12243	chr1:1021	DHCR24-DT		lncRNA	chr1:54887563-5488
ENSG0000(	712	16.12243	chr1:1021	TESK2		protein_c	chr1:45343883-4549
ENSG0000(	712	16.12243	chr1:1021	ENSG00000271355		Pseudoger	chr1:47483698-4748
ENSG0000(	712	16.12243	chr1:1021	CALR4P		Pseudoger	chr1:51561866-5159
ENSG0000(	712	16.12243	chr1:1021	ENSG00000233114		Pseudoger	chr1:46104950-4610
ENSG0000(	712	16.12243	chr1:1021	AL109659.1		protein_c	chr1:48103634-4810
ENSG0000(	712	16.12243	chr1:1021	DNAJC19P7		Pseudoger	chr1:52252062-5225
ENSG0000(	712	16.12243	chr1:1021	TMEM61		protein_c	chr1:54980628-5499
ENSG0000(	712	16.12243	chr1:1021	TXNDC12-AS1		lncRNA	chr1:52050918-5205
ENSG0000(	712	16.12243	chr1:1021	ENSG00000237173		Pseudoger	chr1:54524824-5452
ENSG0000(	712	16.12243	chr1:1021	AL353898.3		Pseudoger	chr1:54099968-5410
ENSG0000(	712	16.12243	chr1:1021	ENSG00000290466		lncRNA	chr1:48096092-4816
ENSG0000(	712	16.12243	chr1:1021	ENSG00000232993		lncRNA	chr1:53069938-5308
ENSG0000(	712	16.12243	chr1:1021	ENSG00000287661		lncRNA	chr1:48552991-4855
ENSG0000(	712	16.12243	chr1:1021	CYP4Z2P		lncRNA	chr1:46843095-4690
ENSG0000(	712	16.12243	chr1:1021	ENSG00000285954		lncRNA	chr1:53344031-5336
ENSG0000(	712	16.12243	chr1:1021	CYP4B1	NCGv7	protein_c	chr1:46757838-4681
ENSG0000(	712	16.12243	chr1:1021	FAF1		protein_c	chr1:50437028-5096
ENSG0000(	712	16.12243	chr1:1021	EFCAB14-AS1		lncRNA	chr1:46674036-4669
ENSG0000(	712	16.12243	chr1:1021	MOB3C		protein_c	chr1:46607719-4661
ENSG0000(	712	16.12243	chr1:1021	MRPS6P2		Pseudoger	chr1:50846468-5084
ENSG0000(	712	16.12243	chr1:1021	LINC01755		lncRNA	chr1:55868254-5595
ENSG0000(	712	16.12243	chr1:1021	FLJ00388		protein_c	chr1:47761307-4776
ENSG0000(	712	16.12243	chr1:1021	RNA5SP47		Pseudoger	chr1:44932323-4493
ENSG0000(	712	16.12243	chr1:1021	ENSG00000279214		TEC	chr1:48262230-4826
ENSG0000(	712	16.12243	chr1:1021	ENSG00000229846		lncRNA	chr1:49025595-4918
ENSG0000(	712	16.12243	chr1:1021	ENSG00000225632		lncRNA	chr1:54285404-5428
ENSG0000(	712	16.12243	chr1:1021	ORC1		protein_c	chr1:52372829-5240
ENSG0000(	712	16.12243	chr1:1021	ATPAF1		protein_c	chr1:46632737-4667
ENSG0000(	712	16.12243	chr1:1021	ENSG00000279096		TEC	chr1:48102068-4810
ENSG0000(	712	16.12243	chr1:1021	STIL	NCGv7;AC	protein_c	chr1:47250139-4731
ENSG0000(	712	16.12243	chr1:1021	ENSG00000223429		Pseudoger	chr1:52162186-5216
ENSG0000(	712	16.12243	chr1:1021	FOXD2		protein_c	chr1:47438044-4744
ENSG0000(	712	16.12243	chr1:1021	EPS15	NCGv7;AC	protein_c	chr1:51354263-5151
ENSG0000(	712	16.12243	chr1:1021	ENSG00000223390		lncRNA	chr1:52033391-5204
ENSG0000(	712	16.12243	chr1:1021	ENSG00000234080		Pseudoger	chr1:50326131-5032
ENSG0000(	712	16.12243	chr1:1021	TTC39A		protein_c	chr1:51287258-5134
ENSG0000(	712	16.12243	chr1:1021	LINC02794		lncRNA	chr1:48050659-4809
ENSG0000(	712	16.12243	chr1:1021	AGBL4-IT1		lncRNA	chr1:49374201-4947
ENSG0000(	712	16.12243	chr1:1021	ENSG00000272100		lncRNA	chr1:52353487-5235
ENSG0000(	712	16.12243	chr1:1021	ENSG00000232514		Pseudoger	chr1:48497263-4849
ENSG0000(	712	16.12243	chr1:1021	GOT2P1		Pseudoger	chr1:55367466-5536
ENSG0000(	712	16.12243	chr1:1021	OSTCP5		Pseudoger	chr1:45069977-4507
ENSG0000(	712	16.12243	chr1:1021	SSBP3		protein_c	chr1:54225433-5441

ENSG0000(	712	16.12243	chr1:1021	TUBBP10		Pseudoger	chr1:52994726-5299
ENSG0000(	712	16.12243	chr1:1021	MMACHC	NCGv7	protein_c	chr1:45500300-4551
ENSG0000(	712	16.12243	chr1:1021	CDCP2		protein_c	chr1:54132687-5415
ENSG0000(	712	16.12243	chr1:1021	Y_RNA		smallRNA	chr1:55484871-5548
ENSG0000(	712	16.12243	chr1:1021	RPL7AP16		Pseudoger	chr1:45651039-4565
ENSG0000(	712	16.12243	chr1:1021	LINC02812		lncRNA	chr1:53366656-5336
ENSG0000(	712	16.12243	chr1:1021	CPT2		protein_c	chr1:53196792-5321
ENSG0000(	712	16.12243	chr1:1021	ENSG00000291246		lncRNA	chr1:48164710-4818
ENSG0000(	712	16.12243	chr1:1021	ENSG00000280425		lncRNA	chr1:54137746-5414
ENSG0000(	712	16.12243	chr1:1021	FCF1P6		Pseudoger	chr1:50405430-5040
ENSG0000(	712	16.12243	chr1:1021	MTND1P34		Pseudoger	chr1:47164510-4716
ENSG0000(	712	16.12243	chr1:1021	ENSG00000279049		Pseudoger	chr1:54099968-5410
ENSG0000(	712	16.12243	chr1:1021	Y_RNA		smallRNA	chr1:51865633-5186
ENSG0000(	712	16.12243	chr1:1021	C1orf185	NCGv7	protein_c	chr1:51102221-5114
ENSG0000(	712	16.12243	chr1:1021	RP5-1024G6.8		lncRNA	chr1:53242364-5324
ENSG0000(	712	16.12243	chr1:1021	ENSG00000286597		lncRNA	chr1:48926021-4893
ENSG0000(	712	16.12243	chr1:1021	RNF11		protein_c	chr1:51236273-5127
ENSG0000(	712	16.12243	chr1:1021	CDKN2C	NCGv7;AC	protein_c	chr1:50960745-5097
ENSG0000(	712	16.12243	chr1:1021	AGBL4-AS1		lncRNA	chr1:49257411-4926
ENSG0000(	712	16.12243	chr1:1021	RNU6-1281P		smallRNA	chr1:51538625-5153
ENSG0000(	712	16.12243	chr1:1021	MIR761		smallRNA	chr1:51836341-5183
ENSG0000(	712	16.12243	chr1:1021	RNU2-30P		smallRNA	chr1:52754322-5275
ENSG0000(	712	16.12243	chr1:1021	ENSG00000225779		Pseudoger	chr1:46370586-4637
ENSG0000(	712	16.12243	chr1:1021	ENSG00000236004		Pseudoger	chr1:52189916-5219
ENSG0000(	712	16.12243	chr1:1021	MIR1273F		smallRNA	chr1:52928674-5292
ENSG0000(	712	16.12243	chr1:1021	LRP8-DT		lncRNA	chr1:53328233-5333
ENSG0000(	712	16.12243	chr1:1021	MAST2		protein_c	chr1:45786987-4603
ENSG0000(	712	16.12243	chr1:1021	FAF1-AS1		lncRNA	chr1:50461469-5047
ENSG0000(	712	16.12243	chr1:1021	LINC01389		lncRNA	chr1:47380928-4740
ENSG0000(	712	16.12243	chr1:1021	RAD54L		protein_c	chr1:46246461-4627
ENSG0000(	712	16.12243	chr1:1021	CCNB1IP1P1		Pseudoger	chr1:44958557-4495
ENSG0000(	712	16.12243	chr1:1021	POMGNT1		protein_c	chr1:46188683-4622
ENSG0000(	712	16.12243	chr1:1021	RNU6-1253P		smallRNA	chr1:50750296-5075
ENSG0000(	712	16.12243	chr1:1021	AL353898.1		Pseudoger	chr1:54015654-5401
ENSG0000(	712	16.12243	chr1:1021	TTC22		protein_c	chr1:54779712-5480
ENSG0000(	712	16.12243	chr1:1021	RN7SKP291		smallRNA	chr1:55376526-5537
ENSG0000(	712	16.12243	chr1:1021	RP4-758J24.5		lncRNA	chr1:54026683-5402
ENSG0000(	712	16.12243	chr1:1021	ENSG00000231866		Pseudoger	chr1:52925249-5292
ENSG0000(	712	16.12243	chr1:1021	LINC02784		lncRNA	chr1:54516412-5451
ENSG0000(	712	16.12243	chr1:1021	LRP8		protein_c	chr1:53242364-5332
ENSG0000(	712	16.12243	chr1:1021	snoU13		smallRNA	chr1:51231253-5123
ENSG0000(	712	16.12243	chr1:1021	RPS15AP10		Pseudoger	chr1:45645816-4564
ENSG0000(	712	16.12243	chr1:1021	GPBP1L1		protein_c	chr1:45627304-4568
ENSG0000(	712	16.12243	chr1:1021	TMEM69		protein_c	chr1:45688181-4569
ENSG0000(	712	16.12243	chr1:1021	DIO1		protein_c	chr1:53891239-5391
ENSG0000(	712	16.12243	chr1:1021	ENSG00000277397		Pseudoger	chr1:53180921-5318
ENSG0000(	712	16.12243	chr1:1021	EFCAB14		protein_c	chr1:46674659-4671
ENSG0000(	712	16.12243	chr1:1021	CYP4A22-AS1		lncRNA	chr1:47096653-4717
ENSG0000(	712	16.12243	chr1:1021	PPIAP35		Pseudoger	chr1:44988234-4498
ENSG0000(	712	16.12243	chr1:1021	ENSG00000256407		protein_c	chr1:54132686-5420
ENSG0000(	712	16.12243	chr1:1021	ECHDC2		protein_c	chr1:52895910-5292

ENSG0000(	712	16.12243	chr1:1021	NASP		protein_c	chr1:45583846-4561
ENSG0000(	712	16.12243	chr1:1021	RNU6-830P		smallRNA	chr1:55398514-5539
ENSG0000(	712	16.12243	chr1:1021	SNORA26		smallRNA	chr1:51724775-5172
ENSG0000(	712	16.12243	chr1:1021	ENSG00000223814		lncRNA	chr1:47761132-4776
ENSG0000(	712	16.12243	chr1:1021	RPL6P1		Pseudoger	chr1:45781277-4578
ENSG0000(	712	16.12243	chr1:1021	RNU4-61P		smallRNA	chr1:48447936-4844
ENSG0000(	712	16.12243	chr1:1021	ENSG00000281133		Pseudoger	chr1:45580892-4558
ENSG0000(	712	16.12243	chr1:1021	ENSG00000288208		protein_c	chr1:45329262-4549
ENSG0000(	712	16.12243	chr1:1021	MUTYH	NCGv7;AC	protein_c	chr1:45329163-4534
ENSG0000(	712	16.12243	chr1:1021	ENSG00000235612		lncRNA	chr1:56145721-5615
ENSG0000(	712	16.12243	chr1:1021	ENSG00000230828		Pseudoger	chr1:50114937-5011
ENSG0000(	712	16.12243	chr1:1021	TUBAP8		Pseudoger	chr1:46891639-4689
ENSG0000(	712	16.12243	chr1:1021	CCDC17		protein_c	chr1:45620044-4562
ENSG0000(	712	16.12243	chr1:1021	ENSG00000284645		lncRNA	chr1:50252569-5025
ENSG0000(	712	16.12243	chr1:1021	ENSG00000272175		lncRNA	chr1:51801028-5180
ENSG0000(	712	16.12243	chr1:1021	TOE1		protein_c	chr1:45340052-4534
ENSG0000(	712	16.12243	chr1:1021	CCDC163		protein_c	chr1:45493866-4550
ENSG0000(	712	16.12243	chr1:1021	ENSG00000266993		lncRNA	chr1:51793934-5179
ENSG0000(	712	16.12243	chr1:1021	ENSG00000287078		lncRNA	chr1:52365443-5236
ENSG0000(	712	16.12243	chr1:1021	HMGB1P48		Pseudoger	chr1:45530927-4553
ENSG0000(	712	16.12243	chr1:1021	ENSG00000288527		Pseudoger	chr1:53441268-5344
ENSG0000(	712	16.12243	chr1:1021	ENSG00000226252		lncRNA	chr1:47225797-4723
ENSG0000(	712	16.12243	chr1:1021	HPDL		protein_c	chr1:45326895-4532
ENSG0000(	712	16.12243	chr1:1021	ZFYVE9		protein_c	chr1:52142089-5234
ENSG0000(	712	16.12243	chr1:1021	ENSG00000230896		lncRNA	chr1:45694684-4569
ENSG0000(	712	16.12243	chr1:1021	ENSG00000223720		lncRNA	chr1:48172972-4820
ENSG0000(	712	16.12243	chr1:1021	TTC4	NCGv7	protein_c	chr1:54715861-5474
ENSG0000(	711	16.09979	chr1:373:	ENSG00000236073		Pseudoger	chr1:21073639-2107
ENSG0000(	710	16.07715	chr7:251(	ENSG00000259294		lncRNA	chr7:101822247-101
ENSG0000(	709	16.0545	chr8:2747	RPL36AP31		Pseudoger	chr8:86016819-8601
ENSG0000(	709	16.0545	chr8:2747	RPL32P4		Pseudoger	chr8:86043102-8604
ENSG0000(	709	16.0545	chr8:2747	SOX5P1		Pseudoger	chr8:87788562-8778
ENSG0000(	709	16.0545	chr8:2747	ENSG00000275103		Pseudoger	chr8:89826593-8983
ENSG0000(	709	16.0545	chr8:2747	REXO1L1		protein_c	chr8:85656466-8566
ENSG0000(	709	16.0545	chr8:2747	E2F5-DT		lncRNA	chr8:85172077-8517
ENSG0000(	709	16.0545	chr8:2747	ENSG00000253699		lncRNA	chr8:86180418-8621
ENSG0000(	709	16.0545	chr8:2747	REXO1L5P		Pseudoger	chr8:85814767-8581
ENSG0000(	709	16.0545	chr8:2747	WWP1	NCGv7	protein_c	chr8:86342547-8647
ENSG0000(	709	16.0545	chr8:2747	ENSG00000253171		lncRNA	chr8:87974165-8801
ENSG0000(	709	16.0545	chr8:2747	CA13		protein_c	chr8:85220587-8528
ENSG0000(	709	16.0545	chr8:2747	WWP1-AS1		lncRNA	chr8:86333274-8634
ENSG0000(	709	16.0545	chr8:2747	ENSG00000254115		lncRNA	chr8:86707547-8676
ENSG0000(	709	16.0545	chr8:2747	PSKH2		protein_c	chr8:86047109-8608
ENSG0000(	709	16.0545	chr8:2747	GOLGA2P1		Pseudoger	chr8:86657388-8666
ENSG0000(	709	16.0545	chr8:2747	ENSG00000253283		Pseudoger	chr8:85086615-8508
ENSG0000(	709	16.0545	chr8:2747	snoU13		smallRNA	chr8:84919818-8491
ENSG0000(	709	16.0545	chr8:2747	SLC7A13	NCGv7	protein_c	chr8:86214063-8632
ENSG0000(	709	16.0545	chr8:2747	MIOXP1		Pseudoger	chr8:86593755-8659
ENSG0000(	709	16.0545	chr8:2747	RPSAP74		Pseudoger	chr8:89243401-8924
ENSG0000(	709	16.0545	chr8:2747	CA3		protein_c	chr8:85373436-8544
ENSG0000(	709	16.0545	chr8:2747	COX6B1P6		Pseudoger	chr8:89823548-8982

ENSG0000(	709	16.0545	chr8:2747	REXO1L2P		Pseudoger	chr8:85826902-8582
ENSG0000(	709	16.0545	chr8:2747	ATP6V0D2		protein_c	chr8:85987323-8615
ENSG0000(	709	16.0545	chr8:2747	REXO1L8P		Pseudoger	chr8:85642777-8564
ENSG0000(	709	16.0545	chr8:2747	IARS2P1		Pseudoger	chr8:87598462-8760
ENSG0000(	709	16.0545	chr8:2747	ENSG00000253778		lncRNA	chr8:86765935-8681
ENSG0000(	709	16.0545	chr8:2747	ENSG00000254380		lncRNA	chr8:85495693-8554
ENSG0000(	709	16.0545	chr8:2747	ENSG00000271156		lncRNA	chr8:88808318-8880
ENSG0000(	709	16.0545	chr8:2747	AC013751.1		smallRNA	chr8:86657870-8665
ENSG0000(	709	16.0545	chr8:2747	LINC02849		lncRNA	chr8:85851446-8586
ENSG0000(	709	16.0545	chr8:2747	REXO1L9P		Pseudoger	chr8:85762909-8576
ENSG0000(	709	16.0545	chr8:2747	ENSG00000253154		lncRNA	chr8:85833377-8595
ENSG0000(	709	16.0545	chr8:2747	ENSG00000287927		lncRNA	chr8:85973793-8597
ENSG0000(	709	16.0545	chr8:2747	REXO1L11P		Pseudoger	chr8:85733419-8573
ENSG0000(	709	16.0545	chr8:2747	DCAF4L2	NCGv7	protein_c	chr8:87870747-8787
ENSG0000(	709	16.0545	chr8:2747	REXO1L12P		Pseudoger	chr8:85714922-8571
ENSG0000(	709	16.0545	chr8:2747	ACTBP6		Pseudoger	chr8:84948585-8494
ENSG0000(	709	16.0545	chr8:2747	REXO1L10P		Pseudoger	chr8:85744543-8574
ENSG0000(	709	16.0545	chr8:2747	ENSG00000253675		lncRNA	chr8:86098965-8615
ENSG0000(	709	16.0545	chr8:2747	ENSG00000273978		Pseudoger	chr8:87867601-8786
ENSG0000(	709	16.0545	chr8:2747	REXO1L3P		Pseudoger	chr8:85654684-8565
ENSG0000(	709	16.0545	chr8:2747	NTAN1P2		Pseudoger	chr8:86481754-8648
ENSG0000(	709	16.0545	chr8:2747	CPNE3		protein_c	chr8:86514435-8656
ENSG0000(	709	16.0545	chr8:2747	CA1		protein_c	chr8:85327608-8537
ENSG0000(	709	16.0545	chr8:2747	E2F5	DriverDB\	protein_c	chr8:85177154-8521
ENSG0000(	709	16.0545	chr8:2747	JCHAINP1		Pseudoger	chr8:84980073-8498
ENSG0000(	709	16.0545	chr8:2747	ENSG00000254208		lncRNA	chr8:85177522-8517
ENSG0000(	709	16.0545	chr8:2747	ENSG00000286984		lncRNA	chr8:88809949-8881
ENSG0000(	709	16.0545	chr8:2747	ENSG00000253553		lncRNA	chr8:88326836-8888
ENSG0000(	709	16.0545	chr8:2747	RBIS		protein_c	chr8:85214048-8522
ENSG0000(	709	16.0545	chr8:2747	CA3-AS1		lncRNA	chr8:85440596-8546
ENSG0000(	709	16.0545	chr8:2747	RIPK2-DT		lncRNA	chr8:89545424-8975
ENSG0000(	709	16.0545	chr8:2747	LRRCC1		protein_c	chr8:85107215-8514
ENSG0000(	709	16.0545	chr8:2747	ENSG00000258256		lncRNA	chr8:85222446-8524
ENSG0000(	709	16.0545	chr8:2747	KRT8P4		Pseudoger	chr8:89412621-8941
ENSG0000(	709	16.0545	chr8:2747	ENSG00000253680		Pseudoger	chr8:85617344-8561
ENSG0000(	709	16.0545	chr8:2747	ENSG00000253500		lncRNA	chr8:87540835-8775
ENSG0000(	709	16.0545	chr8:2747	SLC2A3P4		Pseudoger	chr8:86503591-8650
ENSG0000(	709	16.0545	chr8:2747	ENSG00000257962		lncRNA	chr8:85246295-8524
ENSG0000(	709	16.0545	chr8:2747	CNBD1	NCGv7	protein_c	chr8:86866415-8761
ENSG0000(	709	16.0545	chr8:2747	UBE2Q2P10		Pseudoger	chr8:86660525-8666
ENSG0000(	709	16.0545	chr8:2747	RIPK2		protein_c	chr8:89757806-8979
ENSG0000(	709	16.0545	chr8:2747	CA2		protein_c	chr8:85463968-8548
ENSG0000(	709	16.0545	chr8:2747	RMDN1	NCGv7	protein_c	chr8:86468257-8651
ENSG0000(	709	16.0545	chr8:2747	VTA1P2		Pseudoger	chr8:87575598-8757
ENSG0000(	709	16.0545	chr8:2747	MMP16		protein_c	chr8:88032011-8832
ENSG0000(	709	16.0545	chr8:2747	RNA5SP272		Pseudoger	chr8:88396437-8839
ENSG0000(	709	16.0545	chr8:2747	CNGB3	DriverDB\	protein_c	chr8:86553977-8674
ENSG0000(	709	16.0545	chr8:2747	ENSG00000250962		Pseudoger	chr8:88485110-8848
ENSG0000(	709	16.0545	chr8:2747	ENSG00000254212		Pseudoger	chr8:84864668-8486
ENSG0000(	708	16.03186	chr1:2337	RNU4-27P		smallRNA	chr1:36402721-3640
ENSG0000(	708	16.03186	chr1:2337	GRIK3	NCGv7	protein_c	chr1:36795527-3703

ENSG0000(	708	16.03186	chr1:2337	ZC3H12A	NCGv7	protein_c	chr1:37474580-3748
ENSG0000(	708	16.03186	chr4:253(	ENSG00000284968		lncRNA	chr4:86924630-8693
ENSG0000(	708	16.03186	chr4:253(	ENSG00000272856		lncRNA	chr4:87460807-8746
ENSG0000(	708	16.03186	chr4:253(	ENSG00000277695		lncRNA	chr4:89747802-8975
ENSG0000(	708	16.03186	chr1:2337	C1orf109		protein_c	chr1:37681570-3769
ENSG0000(	708	16.03186	chr1:2337	COL8A2		protein_c	chr1:36095239-3612
ENSG0000(	708	16.03186	chr4:253(	IBSP		protein_c	chr4:87799554-8781
ENSG0000(	708	16.03186	chr4:253(	ENSG00000251095		lncRNA	chr4:89410960-8972
ENSG0000(	708	16.03186	chr4:253(	PIGY-DT		lncRNA	chr4:88523826-8852
ENSG0000(	708	16.03186	chr1:2337	MIR5581		smallRNA	chr1:37500935-3750
ENSG0000(	708	16.03186	chr1:2337	MEAF6		protein_c	chr1:37489993-3751
ENSG0000(	708	16.03186	chr4:253(	RPL3P13		Pseudoger	chr4:84544304-8454
ENSG0000(	708	16.03186	chr1:2337	SNIP1		protein_c	chr1:37534449-3755
ENSG0000(	708	16.03186	chr4:253(	RN7SKP244		smallRNA	chr4:88583666-8858
ENSG0000(	708	16.03186	chr4:253(	TMSB4XP8		Pseudoger	chr4:90838903-9083
ENSG0000(	708	16.03186	chr4:253(	ENSG00000287392		lncRNA	chr4:92183235-9226
ENSG0000(	708	16.03186	chr4:253(	ENSG00000248196		Pseudoger	chr4:87166844-8716
ENSG0000(	708	16.03186	chr4:253(	ENSG00000251401		Pseudoger	chr4:90682996-9068
ENSG0000(	708	16.03186	chr1:2337	RSPO1		protein_c	chr1:37611350-3763
ENSG0000(	708	16.03186	chr1:2337	ENSG00000235673		Pseudoger	chr1:37840986-3784
ENSG0000(	708	16.03186	chr4:253(	ATOH1		protein_c	chr4:93828753-9383
ENSG0000(	708	16.03186	chr1:2337	ADPRS		protein_c	chr1:36088892-3609
ENSG0000(	708	16.03186	chr4:253(	ENSG00000251411		Pseudoger	chr4:86913266-8691
ENSG0000(	708	16.03186	chr1:2337	MAP7D1		protein_c	chr1:36155579-3618
ENSG0000(	708	16.03186	chr4:253(	ENSG00000276542		lncRNA	chr4:89748283-8974
ENSG0000(	708	16.03186	chr4:253(	ENSG00000287239		lncRNA	chr4:89995223-8999
ENSG0000(	708	16.03186	chr1:2337	OSCP1		protein_c	chr1:36415827-3645
ENSG0000(	708	16.03186	chr1:2337	ENSG00000223944		lncRNA	chr1:37154761-3732
ENSG0000(	708	16.03186	chr1:2337	RNU6-510P		smallRNA	chr1:37991462-3799
ENSG0000(	708	16.03186	chr1:2337	MRPS15		protein_c	chr1:36455718-3646
ENSG0000(	708	16.03186	chr1:2337	DNALI1		protein_c	chr1:37556919-3756
ENSG0000(	708	16.03186	chr4:253(	snoU13		smallRNA	chr4:87768485-8776
ENSG0000(	708	16.03186	chr4:253(	ENSG00000289034		lncRNA	chr4:88006143-8800
ENSG0000(	708	16.03186	chr4:253(	MAPK10		protein_c	chr4:85990007-8659
ENSG0000(	708	16.03186	chr1:2337	FTLP18		Pseudoger	chr1:36630335-3663
ENSG0000(	708	16.03186	chr4:253(	RNU6-33P		smallRNA	chr4:88684848-8868
ENSG0000(	708	16.03186	chr1:2337	GNL2		protein_c	chr1:37566816-3759
ENSG0000(	708	16.03186	chr4:253(	HERC3		protein_c	chr4:88523810-8870
ENSG0000(	708	16.03186	chr4:253(	RPL6P13		Pseudoger	chr4:86870191-8687
ENSG0000(	708	16.03186	chr4:253(	ENSG00000287552		lncRNA	chr4:94315486-9434
ENSG0000(	708	16.03186	chr4:253(	PPM1K		protein_c	chr4:88257620-8828
ENSG0000(	708	16.03186	chr1:2337	AGO4	NCGv7	protein_c	chr1:35808016-3585
ENSG0000(	708	16.03186	chr4:253(	ENSG00000288563		lncRNA	chr4:89582507-8970
ENSG0000(	708	16.03186	chr1:2337	RNU6-636P		smallRNA	chr1:37203610-3720
ENSG0000(	708	16.03186	chr4:253(	HMGB3P15		Pseudoger	chr4:94195940-9419
ENSG0000(	708	16.03186	chr4:253(	ENSG00000250908		lncRNA	chr4:93318623-9331
ENSG0000(	708	16.03186	chr4:253(	ENSG00000274238		lncRNA	chr4:89743792-8974
ENSG0000(	708	16.03186	chr1:2337	ACTN4P2		Pseudoger	chr1:37776670-3777
ENSG0000(	708	16.03186	chr1:2337	SH3D21		protein_c	chr1:36306368-3632
ENSG0000(	708	16.03186	chr1:2337	EVA1B		protein_c	chr1:36322030-3632
ENSG0000(	708	16.03186	chr4:253(	WDFY3-AS1		lncRNA	chr4:84796614-8481

ENSG0000(	708	16.03186	chr1:2337	AL929472.1		protein_c	chr1:37826560-3782
ENSG0000(	708	16.03186	chr4:253(	AFF1	IntOGen-I	protein_c	chr4:86935002-8714
ENSG0000(	708	16.03186	chr4:253(	RNU6ATAC31P		smallRNA	chr4:88206427-8820
ENSG0000(	708	16.03186	chr1:2337	ENSG00000237749		Pseudoger	chr1:37556247-3755
ENSG0000(	708	16.03186	chr1:2337	ENSG00000223589		Pseudoger	chr1:38080572-3808
ENSG0000(	708	16.03186	chr4:253(	RNU6-907P		smallRNA	chr4:89130852-8913
ENSG0000(	708	16.03186	chr4:253(	RNU6-1298P		smallRNA	chr4:88226729-8822
ENSG0000(	708	16.03186	chr4:253(	ENSG00000214980		Pseudoger	chr4:84244003-8424
ENSG0000(	708	16.03186	chr4:253(	ENSG00000251285		Pseudoger	chr4:88220569-8822
ENSG0000(	708	16.03186	chr1:2337	RPS29P6		Pseudoger	chr1:37330852-3733
ENSG0000(	708	16.03186	chr4:253(	NUDT9		protein_c	chr4:87422573-8745
ENSG0000(	708	16.03186	chr4:253(	LINC02994		lncRNA	chr4:83796436-8429
ENSG0000(	708	16.03186	chr4:253(	ENSG00000287181		lncRNA	chr4:88352222-8835
ENSG0000(	708	16.03186	chr4:253(	RN7SKP248		smallRNA	chr4:90370123-9037
ENSG0000(	708	16.03186	chr1:2337	RNU6-584P		smallRNA	chr1:37885023-3788
ENSG0000(	708	16.03186	chr4:253(	GAPDHP60		Pseudoger	chr4:87207092-8720
ENSG0000(	708	16.03186	chr1:2337	snoU13		smallRNA	chr1:37750202-3775
ENSG0000(	708	16.03186	chr4:253(	ENSG00000248511		lncRNA	chr4:92297251-9230
ENSG0000(	708	16.03186	chr4:253(	ENSG00000250572		lncRNA	chr4:87261931-8726
ENSG0000(	708	16.03186	chr4:253(	AC083829.1		smallRNA	chr4:88493933-8849
ENSG0000(	708	16.03186	chr4:253(	Y_RNA		smallRNA	chr4:88330176-8833
ENSG0000(	708	16.03186	chr4:253(	NKX6-1		protein_c	chr4:84491985-8449
ENSG0000(	708	16.03186	chr4:253(	CDS1		protein_c	chr4:84583127-8465
ENSG0000(	708	16.03186	chr4:253(	WDFY3	NCGv7	protein_c	chr4:84668765-8496
ENSG0000(	708	16.03186	chr4:253(	MIR5705		smallRNA	chr4:87300495-8730
ENSG0000(	708	16.03186	chr4:253(	PTPN13	NCGv7	protein_c	chr4:86594315-8681
ENSG0000(	708	16.03186	chr4:253(	RNU6-469P		smallRNA	chr4:84886386-8488
ENSG0000(	708	16.03186	chr4:253(	HSD17B11		protein_c	chr4:87336515-8739
ENSG0000(	708	16.03186	chr4:253(	Y_RNA		smallRNA	chr4:87412228-8741
ENSG0000(	708	16.03186	chr4:253(	C4orf36		protein_c	chr4:86876205-8689
ENSG0000(	708	16.03186	chr1:2337	FTH1P1		Pseudoger	chr1:37544763-3754
ENSG0000(	708	16.03186	chr4:253(	ENSG00000285458		protein_c	chr4:86886472-8693
ENSG0000(	708	16.03186	chr4:253(	HSD17B13		protein_c	chr4:87303789-8732
ENSG0000(	708	16.03186	chr1:2337	CDCA8		protein_c	chr1:37692481-3770
ENSG0000(	708	16.03186	chr1:2337	C1orf122		protein_c	chr1:37806979-3780
ENSG0000(	708	16.03186	chr1:2337	MANEAL		protein_c	chr1:37793847-3780
ENSG0000(	708	16.03186	chr4:253(	KRT19P6		Pseudoger	chr4:91885046-9188
ENSG0000(	708	16.03186	chr4:253(	ENSG00000249755		Pseudoger	chr4:88527160-8852
ENSG0000(	708	16.03186	chr4:253(	SMARCAD1-DT		lncRNA	chr4:94117792-9420
ENSG0000(	708	16.03186	chr4:253(	CHCHD2P7		Pseudoger	chr4:87785920-8778
ENSG0000(	708	16.03186	chr4:253(	PPM1K-DT		lncRNA	chr4:88284507-8834
ENSG0000(	708	16.03186	chr4:253(	ENSG00000249951		lncRNA	chr4:94675245-9470
ENSG0000(	708	16.03186	chr4:253(	NCOA4P2		Pseudoger	chr4:88508591-8851
ENSG0000(	708	16.03186	chr1:2337	ENSG00000271914		lncRNA	chr1:35929720-3593
ENSG0000(	708	16.03186	chr1:2337	POU3F1		protein_c	chr1:38043829-3804
ENSG0000(	708	16.03186	chr1:2337	INPP5B		protein_c	chr1:37860697-3794
ENSG0000(	708	16.03186	chr4:253(	TIGD2		protein_c	chr4:89111533-8911
ENSG0000(	708	16.03186	chr1:2337	MTF1		protein_c	chr1:37809574-3785
ENSG0000(	708	16.03186	chr1:2337	ENSG00000230955		lncRNA	chr1:37860697-3786
ENSG0000(	708	16.03186	chr1:2337	ENSG00000286379		lncRNA	chr1:36329630-3633
ENSG0000(	708	16.03186	chr4:253(	ENSG00000286618		lncRNA	chr4:87974385-8800

ENSG0000(	708	16.03186	chr4:253(	WDFY3-AS2		lncRNA	chr4:84965534-8501
ENSG0000(	708	16.03186	chr1:2337	ENSG00000233728		lncRNA	chr1:37799720-3780
ENSG0000(	708	16.03186	chr4:253(	Y_RNA		smallRNA	chr4:87743952-8774
ENSG0000(	708	16.03186	chr1:2337	ENSG00000284748		lncRNA	chr1:37596126-3760
ENSG0000(	708	16.03186	chr1:2337	ZC3H12A-DT		lncRNA	chr1:37350934-3747
ENSG0000(	708	16.03186	chr4:253(	RN7SL681P		smallRNA	chr4:87386886-8738
ENSG0000(	708	16.03186	chr4:253(	ENSG00000255723		lncRNA	chr4:87317170-8734
ENSG0000(	708	16.03186	chr1:2337	LSM10		protein_c	chr1:36391238-3639
ENSG0000(	708	16.03186	chr4:253(	BMPR1B-DT		lncRNA	chr4:94743668-9475
ENSG0000(	708	16.03186	chr1:2337	AGO3		protein_c	chr1:35930718-3607
ENSG0000(	708	16.03186	chr4:253(	RPL35AP11		Pseudoger	chr4:94369833-9437
ENSG0000(	708	16.03186	chr4:253(	ENSG00000248627		lncRNA	chr4:92833685-9283
ENSG0000(	708	16.03186	chr1:2337	ENSG00000284650		lncRNA	chr1:37133489-3713
ENSG0000(	708	16.03186	chr4:253(	ENSG00000286189		lncRNA	chr4:84970180-8497
ENSG0000(	708	16.03186	chr4:253(	NAP1L5		protein_c	chr4:88695913-8869
ENSG0000(	708	16.03186	chr4:253(	ENSG00000286978		lncRNA	chr4:88358945-8836
ENSG0000(	708	16.03186	chr4:253(	PKD2	NCGv7	protein_c	chr4:88007635-8807
ENSG0000(	708	16.03186	chr4:253(	ABCG2	NCGv7	protein_c	chr4:88090150-8823
ENSG0000(	708	16.03186	chr4:253(	SPP1		protein_c	chr4:87975667-8798
ENSG0000(	708	16.03186	chr4:253(	RN7SL552P		smallRNA	chr4:84687728-8468
ENSG0000(	708	16.03186	chr1:2337	ENSG00000286899		lncRNA	chr1:35908980-3591
ENSG0000(	708	16.03186	chr1:2337	CSF3R	NCGv7;AC	protein_c	chr1:36466043-3648
ENSG0000(	708	16.03186	chr1:2337	TEKT2		protein_c	chr1:36084094-3608
ENSG0000(	708	16.03186	chr1:2337	AGO1		protein_c	chr1:35869808-3593
ENSG0000(	708	16.03186	chr4:253(	SNCA-AS1		lncRNA	chr4:89836408-8984
ENSG0000(	708	16.03186	chr4:253(	RPL30P6		Pseudoger	chr4:95644952-9564
ENSG0000(	708	16.03186	chr1:2337	RN7SL131P		smallRNA	chr1:36191915-3619
ENSG0000(	708	16.03186	chr1:2337	UBE2V2P4		Pseudoger	chr1:36241898-3624
ENSG0000(	708	16.03186	chr4:253(	ENSG00000250202		Pseudoger	chr4:86876338-8687
ENSG0000(	708	16.03186	chr1:2337	MIR4255		smallRNA	chr1:37161563-3716
ENSG0000(	708	16.03186	chr1:2337	ENSG00000234481		lncRNA	chr1:36769812-3677
ENSG0000(	708	16.03186	chr4:253(	ENSG00000270720		lncRNA	chr4:89119284-8911
ENSG0000(	708	16.03186	chr4:253(	RN7SKP96		smallRNA	chr4:86336318-8633
ENSG0000(	708	16.03186	chr4:253(	SLC10A6		protein_c	chr4:86823468-8684
ENSG0000(	708	16.03186	chr4:253(	KLHL8	NCGv7	protein_c	chr4:87160103-8724
ENSG0000(	708	16.03186	chr4:253(	SNCA		protein_c	chr4:89700345-8983
ENSG0000(	708	16.03186	chr4:253(	PYURF		protein_c	chr4:88520998-8852
ENSG0000(	708	16.03186	chr4:253(	MAPK10-AS1		lncRNA	chr4:86117912-8621
ENSG0000(	708	16.03186	chr4:253(	ENSG00000279013		TEC	chr4:91603275-9160
ENSG0000(	708	16.03186	chr1:2337	ENSG00000271554		lncRNA	chr1:35992109-3601
ENSG0000(	708	16.03186	chr1:2337	MIR3659		smallRNA	chr1:38089231-3808
ENSG0000(	708	16.03186	chr4:253(	TECRP1		Pseudoger	chr4:86949669-8695
ENSG0000(	708	16.03186	chr1:2337	STK40		protein_c	chr1:36339624-3638
ENSG0000(	708	16.03186	chr1:2337	ENSG00000284705		lncRNA	chr1:36703953-3671
ENSG0000(	708	16.03186	chr1:2337	SF3A3		protein_c	chr1:37956975-3799
ENSG0000(	708	16.03186	chr1:2337	UTP11		protein_c	chr1:38009258-3802
ENSG0000(	708	16.03186	chr4:253(	LNCPRESS2		lncRNA	chr4:92268767-9227
ENSG0000(	708	16.03186	chr4:253(	ENSG00000241853		Pseudoger	chr4:88163579-8816
ENSG0000(	708	16.03186	chr4:253(	ENSG00000249052		lncRNA	chr4:91887886-9190
ENSG0000(	708	16.03186	chr4:253(	ENSG00000249049		lncRNA	chr4:91319034-9132
ENSG0000(	708	16.03186	chr1:2337	CFAP97P1		Pseudoger	chr1:35873270-3587

ENSG0000(	708	16.03186	chr4:253(	PIGY	NCGv7	protein_c	chr4:88520998-8852
ENSG0000(	708	16.03186	chr4:253(	ENSG00000249001		lncRNA	chr4:87568035-8773
ENSG0000(	708	16.03186	chr4:253(	ENSG00000248984		lncRNA	chr4:91108023-9111
ENSG0000(	708	16.03186	chr4:253(	CCSER1		protein_c	chr4:90127394-9160
ENSG0000(	708	16.03186	chr1:2337	SNORA63		smallRNA	chr1:36418450-3641
ENSG0000(	708	16.03186	chr1:2337	TRAPPC3		protein_c	chr1:36136570-3615
ENSG0000(	708	16.03186	chr4:253(	ENSG00000280056		TEC	chr4:92260367-9226
ENSG0000(	708	16.03186	chr1:2337	ENSG00000232862		Pseudoger	chr1:36080066-3608
ENSG0000(	708	16.03186	chr4:253(	RNU6-1059P		smallRNA	chr4:95868667-9586
ENSG0000(	708	16.03186	chr1:2337	THRAP3	NCGv7;AC	protein_c	chr1:36224432-3630
ENSG0000(	708	16.03186	chr4:253(	PMPCAP1		Pseudoger	chr4:92182477-9218
ENSG0000(	708	16.03186	chr4:253(	RNU6-112P		smallRNA	chr4:88275205-8827
ENSG0000(	708	16.03186	chr1:2337	Y_RNA		smallRNA	chr1:37737955-3773
ENSG0000(	708	16.03186	chr4:253(	MIR4451		smallRNA	chr4:85722468-8572
ENSG0000(	708	16.03186	chr4:253(	ENSG00000248749		lncRNA	chr4:84371393-8438
ENSG0000(	708	16.03186	chr4:253(	ENSG00000248750		Pseudoger	chr4:92884663-9288
ENSG0000(	708	16.03186	chr1:2337	FHL3		protein_c	chr1:37996770-3800
ENSG0000(	708	16.03186	chr1:2337	EPHA10	DriverDB\	protein_c	chr1:37713880-3776
ENSG0000(	708	16.03186	chr4:253(	UNC5C	NCGv7	protein_c	chr4:95162504-9554
ENSG0000(	708	16.03186	chr4:253(	UNC5C-AS1		lncRNA	chr4:95549129-9555
ENSG0000(	708	16.03186	chr1:2337	ENSG00000284720		lncRNA	chr1:36768122-3676
ENSG0000(	708	16.03186	chr4:253(	MMRN1		protein_c	chr4:89879532-8995
ENSG0000(	708	16.03186	chr4:253(	BMPR1B		protein_c	chr4:94757955-9515
ENSG0000(	708	16.03186	chr4:253(	RNU1-36P		smallRNA	chr4:88000237-8800
ENSG0000(	708	16.03186	chr4:253(	GPRIN3		protein_c	chr4:89236383-8930
ENSG0000(	708	16.03186	chr4:253(	RN7SKP48		smallRNA	chr4:85100496-8510
ENSG0000(	708	16.03186	chr4:253(	MEPE		protein_c	chr4:87821398-8784
ENSG0000(	708	16.03186	chr4:253(	DMP1		protein_c	chr4:87650280-8766
ENSG0000(	708	16.03186	chr4:253(	DSPP	NCGv7	protein_c	chr4:87608529-8761
ENSG0000(	708	16.03186	chr4:253(	SPARCL1		protein_c	chr4:87473335-8753
ENSG0000(	708	16.03186	chr1:2337	ENSG00000227416		Pseudoger	chr1:37782457-3778
ENSG0000(	708	16.03186	chr4:253(	GRID2		protein_c	chr4:92303966-9381
ENSG0000(	708	16.03186	chr4:253(	ENSG00000248725		Pseudoger	chr4:85246157-8524
ENSG0000(	708	16.03186	chr4:253(	HERC5	DriverDB\	protein_c	chr4:88457119-8850
ENSG0000(	708	16.03186	chr4:253(	HERC6		protein_c	chr4:88378739-8844
ENSG0000(	708	16.03186	chr4:253(	HERC3		protein_c	chr4:88592434-8870
ENSG0000(	708	16.03186	chr1:2337	YRDC		protein_c	chr1:37802945-3780
ENSG0000(	708	16.03186	chr4:253(	RNU6-818P		smallRNA	chr4:88201703-8820
ENSG0000(	708	16.03186	chr4:253(	FAM13A		protein_c	chr4:88725955-8911
ENSG0000(	708	16.03186	chr4:253(	ARHGAP24		protein_c	chr4:85475150-8600
ENSG0000(	708	16.03186	chr4:253(	MIR4452		smallRNA	chr4:86542482-8654
ENSG0000(	708	16.03186	chr4:253(	ENSG00000249262		Pseudoger	chr4:87410644-8741
ENSG0000(	708	16.03186	chr4:253(	HSP90AB3P		Pseudoger	chr4:87891843-8789
ENSG0000(	708	16.03186	chr4:253(	RACK1P3		Pseudoger	chr4:92821986-9282
ENSG0000(	708	16.03186	chr1:2337	SNORA63		smallRNA	chr1:37884237-3788
ENSG0000(	708	16.03186	chr4:253(	MTND1P19		Pseudoger	chr4:92702345-9270
ENSG0000(	708	16.03186	chr4:253(	PDLIM5	NCGv7	protein_c	chr4:94451857-9466
ENSG0000(	708	16.03186	chr4:253(	RNA5SP164		Pseudoger	chr4:93820171-9382
ENSG0000(	708	16.03186	chr4:253(	PDHA2		protein_c	chr4:95840093-9584
ENSG0000(	708	16.03186	chr1:2337	RNA5SP43		Pseudoger	chr1:37264677-3726
ENSG0000(	708	16.03186	chr4:253(	SMARCAD1		protein_c	chr4:94207611-9429

ENSG0000(	708	16.03186	chr4:253(	HPGDS		protein_c	chr4:94298535-9434
ENSG0000(	708	16.03186	chr4:253(	FAM13A-AS1		lncRNA	chr4:88709298-8873
ENSG0000(	705	15.96393	chr8:2747	HNRNPA1P36		Pseudoger	chr8:81807772-8180
ENSG0000(	705	15.96393	chr8:2747	ENSG00000241746		Pseudoger	chr8:80265528-8026
ENSG0000(	705	15.96393	chr8:2747	ENSG00000280193		TEC	chr8:81696368-8169
ENSG0000(	705	15.96393	chr8:2747	ENSG00000286763		lncRNA	chr8:80894016-8093
ENSG0000(	705	15.96393	chr8:2747	RALYL	NCGv7	protein_c	chr8:84182787-8492
ENSG0000(	705	15.96393	chr8:2747	ENSG00000272264		lncRNA	chr8:80032724-8003
ENSG0000(	705	15.96393	chr8:2747	ENSG00000254288		lncRNA	chr8:74052340-7409
ENSG0000(	705	15.96393	chr8:2747	ENSG00000254162		lncRNA	chr8:80535006-8053
ENSG0000(	705	15.96393	chr8:2747	C4orf46P3		Pseudoger	chr8:78723796-7872
ENSG0000(	705	15.96393	chr8:2747	ENSG00000272254		lncRNA	chr8:73042910-7304
ENSG0000(	705	15.96393	chr8:2747	ENSG00000288756		lncRNA	chr8:77000170-7700
ENSG0000(	705	15.96393	chr8:2747	RPS26P34		Pseudoger	chr8:81627269-8162
ENSG0000(	705	15.96393	chr8:2747	ENSG00000254001		lncRNA	chr8:78148101-7815
ENSG0000(	705	15.96393	chr8:2747	RNU6-1213P		smallRNA	chr8:80405516-8040
ENSG0000(	705	15.96393	chr8:2747	DSTNP3		Pseudoger	chr8:73984493-7398
ENSG0000(	705	15.96393	chr8:2747	PAG1	NCGv7	protein_c	chr8:80967810-8111
ENSG0000(	705	15.96393	chr8:2747	AC016194.1		smallRNA	chr8:75786365-7578
ENSG0000(	705	15.96393	chr8:2747	VENTXP6		Pseudoger	chr8:73651289-7365
ENSG0000(	705	15.96393	chr8:2747	ENSG00000253235		lncRNA	chr8:73326991-7337
ENSG0000(	705	15.96393	chr8:2747	RNU105C		smallRNA	chr8:54330687-5433
ENSG0000(	705	15.96393	chr8:2747	RPS20P21		Pseudoger	chr8:73982125-7398
ENSG0000(	705	15.96393	chr8:2747	TPD52	DriverDB\	protein_c	chr8:80034745-8023
ENSG0000(	705	15.96393	chr8:2747	ENSG00000254366		lncRNA	chr8:77399072-7753
ENSG0000(	705	15.96393	chr8:2747	RNU6-1197P		smallRNA	chr8:74079537-7407
ENSG0000(	705	15.96393	chr8:2747	CRISPLD1	NCGv7	protein_c	chr8:74984505-7503
ENSG0000(	705	15.96393	chr8:2747	LINC01109		lncRNA	chr8:76404058-7640
ENSG0000(	705	15.96393	chr8:2747	IMPA1	DriverDB\	protein_c	chr8:81656914-8168
ENSG0000(	705	15.96393	chr8:2747	AC105242.1		smallRNA	chr8:77286816-7728
ENSG0000(	705	15.96393	chr8:2747	PKIA-AS1		lncRNA	chr8:78268637-7855
ENSG0000(	705	15.96393	chr8:2747	ENSG00000253836		lncRNA	chr8:82862779-8295
ENSG0000(	705	15.96393	chr8:2747	PKMP4		Pseudoger	chr8:75376709-7537
ENSG0000(	705	15.96393	chr8:2747	ENSG00000286113		lncRNA	chr8:73879046-7388
ENSG0000(	705	15.96393	chr8:2747	RN7SL107P		smallRNA	chr8:80613544-8061
ENSG0000(	705	15.96393	chr8:2747	IMPA1P1		Pseudoger	chr8:81605569-8162
ENSG0000(	705	15.96393	chr8:2747	KCNB2	NCGv7	protein_c	chr8:72537225-7293
ENSG0000(	705	15.96393	chr8:2747	ENSG00000254205		lncRNA	chr8:80122852-8012
ENSG0000(	705	15.96393	chr8:2747	HAUS1P3		Pseudoger	chr8:72618722-7261
ENSG0000(	705	15.96393	chr8:2747	hsa-mir-3149		smallRNA	chr8:76966768-7696
ENSG0000(	705	15.96393	chr8:2747	HIGD1AP6		Pseudoger	chr8:75302296-7530
ENSG0000(	705	15.96393	chr8:2747	CASC9		lncRNA	chr8:75120409-7535
ENSG0000(	705	15.96393	chr8:2747	ENSG00000253726		lncRNA	chr8:72732045-7275
ENSG0000(	705	15.96393	chr8:2747	HMGB1P41		Pseudoger	chr8:80812142-8081
ENSG0000(	705	15.96393	chr8:2747	ENSG00000276418		protein_c	chr8:79918860-8008
ENSG0000(	705	15.96393	chr8:2747	LINC02986		lncRNA	chr8:80541300-8054
ENSG0000(	705	15.96393	chr8:2747	ENSG00000254014		Pseudoger	chr8:81244156-8124
ENSG0000(	705	15.96393	chr8:2747	ENSG00000253681		lncRNA	chr8:76576551-7657
ENSG0000(	705	15.96393	chr8:2747	ENSG00000253581		Pseudoger	chr8:82979411-8297
ENSG0000(	705	15.96393	chr8:2747	ENSG00000237061		lncRNA	chr8:73083787-7308
ENSG0000(	705	15.96393	chr8:2747	C8orf89		protein_c	chr8:73241331-7325

ENSG0000(	705	15.96393	chr8:2747	ENSG00000253503		lncRNA	chr8:82514568-8267
ENSG0000(	705	15.96393	chr8:2747	ZFHX4	NCGv7	protein_c	chr8:76681239-7686
ENSG0000(	705	15.96393	chr8:2747	HNRNPA1P4		Pseudoger	chr8:82291624-8229
ENSG0000(	705	15.96393	chr8:2747	ENSG00000254347		lncRNA	chr8:72462311-7247
ENSG0000(	705	15.96393	chr8:2747	ENSG00000253416		lncRNA	chr8:76904591-7691
ENSG0000(	705	15.96393	chr8:2747	PSMC2P2		Pseudoger	chr8:84678798-8467
ENSG0000(	705	15.96393	chr8:2747	MIR2052HG		lncRNA	chr8:74599775-7482
ENSG0000(	705	15.96393	chr8:2747	RDH10	DriverDBv	protein_c	chr8:73294602-7332
ENSG0000(	705	15.96393	chr2:2577	MIR1258		smallRNA	chr2:179860836-179
ENSG0000(	705	15.96393	chr8:2747	ENSG00000254080		lncRNA	chr8:74609698-7463
ENSG0000(	705	15.96393	chr8:2747	RDH10-AS1		lncRNA	chr8:73297711-7335
ENSG0000(	705	15.96393	chr8:2747	FABP5	DriverDBv	protein_c	chr8:81280536-8128
ENSG0000(	705	15.96393	chr8:2747	ENSG00000253238		lncRNA	chr8:80265907-8048
ENSG0000(	705	15.96393	chr8:2747	ENSG00000253636		lncRNA	chr8:73052178-7306
ENSG0000(	705	15.96393	chr8:2747	CHMP4C	DriverDBv	protein_c	chr8:81732448-8175
ENSG0000(	705	15.96393	chr8:2747	NIPA2P4		Pseudoger	chr8:81617220-8161
ENSG0000(	705	15.96393	chr8:2747	ENSG00000270866		lncRNA	chr8:76403998-7640
ENSG0000(	705	15.96393	chr8:2747	LINC01607		lncRNA	chr8:79768110-7980
ENSG0000(	705	15.96393	chr8:2747	MIR5681A		smallRNA	chr8:74548543-7454
ENSG0000(	705	15.96393	chr8:2747	LINC01419		lncRNA	chr8:83403758-8340
ENSG0000(	705	15.96393	chr8:2747	HNF4G	DriverDBv	protein_c	chr8:75407914-7556
ENSG0000(	705	15.96393	chr8:2747	PEX2	NCGv7	protein_c	chr8:76980258-7700
ENSG0000(	705	15.96393	chr8:2747	ENSG00000253983		lncRNA	chr8:74093545-7420
ENSG0000(	705	15.96393	chr8:2747	Y_RNA		smallRNA	chr8:73979784-7397
ENSG0000(	705	15.96393	chr8:2747	STAU2		protein_c	chr8:73420369-7374
ENSG0000(	705	15.96393	chr8:2747	SBSPON		protein_c	chr8:73064543-7312
ENSG0000(	705	15.96393	chr2:2577	SNORA43		smallRNA	chr2:179934401-179
ENSG0000(	705	15.96393	chr8:2747	ENSG00000289242		lncRNA	chr8:81161397-8122
ENSG0000(	705	15.96393	chr8:2747	ENSG00000254394		lncRNA	chr8:82033533-8296
ENSG0000(	705	15.96393	chr2:2577	ENSG00000237477		lncRNA	chr2:179273831-179
ENSG0000(	705	15.96393	chr8:2747	MIR5708		smallRNA	chr8:80241389-8024
ENSG0000(	705	15.96393	chr8:2747	ENSG00000253115		lncRNA	chr8:74103516-7410
ENSG0000(	705	15.96393	chr2:2577	ZNF385B		protein_c	chr2:179441982-179
ENSG0000(	705	15.96393	chr8:2747	ENSG00000253273		Pseudoger	chr8:73831143-7383
ENSG0000(	705	15.96393	chr8:2747	ENSG00000253374		lncRNA	chr8:81521618-8153
ENSG0000(	705	15.96393	chr8:2747	ENSG00000253564		Pseudoger	chr8:73215929-7321
ENSG0000(	705	15.96393	chr8:2747	ENSG00000213791		Pseudoger	chr8:80300869-8030
ENSG0000(	705	15.96393	chr8:2747	ENSG00000253760		Pseudoger	chr8:80568050-8056
ENSG0000(	705	15.96393	chr8:2747	ZNF704	DriverDBv	protein_c	chr8:80628451-8087
ENSG0000(	705	15.96393	chr8:2747	HEY1	NCGv7;AC	protein_c	chr8:79762371-7976
ENSG0000(	705	15.96393	chr8:2747	ENSG00000286675		lncRNA	chr8:78626775-7866
ENSG0000(	705	15.96393	chr8:2747	ENSG00000260398		lncRNA	chr8:78605952-7860
ENSG0000(	705	15.96393	chr8:2747	STAU2-AS1		lncRNA	chr8:73420004-7344
ENSG0000(	705	15.96393	chr8:2747	RNU2-71P		smallRNA	chr8:80585585-8058
ENSG0000(	705	15.96393	chr8:2747	RN7SL308P		smallRNA	chr8:80776666-8077
ENSG0000(	705	15.96393	chr2:2577	ACA59		smallRNA	chr2:179023257-179
ENSG0000(	705	15.96393	chr8:2747	ENSG00000287052		lncRNA	chr8:79747351-7974
ENSG0000(	705	15.96393	chr8:2747	ENSG00000254273		Pseudoger	chr8:73511276-7351
ENSG0000(	705	15.96393	chr8:2747	ENSG00000253237		Pseudoger	chr8:80351132-8035
ENSG0000(	705	15.96393	chr8:2747	ENSG00000253706		lncRNA	chr8:74798784-7486
ENSG0000(	705	15.96393	chr8:2747	RNU2-54P		smallRNA	chr8:76265543-7626

ENSG0000(	705	15.96393	chr8:2747	PKIA		protein_c	chr8:78516340-7860
ENSG0000(	705	15.96393	chr8:2747	ENSG00000254538		lncRNA	chr8:73670441-7373
ENSG0000(	705	15.96393	chr8:2747	FABP12		protein_c	chr8:81524981-8159
ENSG0000(	705	15.96393	chr8:2747	RNU11-6P		smallRNA	chr8:80749561-8074
ENSG0000(	705	15.96393	chr8:2747	SLC10A5P1		Pseudoger	chr8:81634357-8163
ENSG0000(	705	15.96393	chr8:2747	LINC02839		lncRNA	chr8:82098451-8216
ENSG0000(	705	15.96393	chr8:2747	AC009941.1		smallRNA	chr8:79097363-7909
ENSG0000(	705	15.96393	chr8:2747	RPS3AP32		Pseudoger	chr8:74055882-7405
ENSG0000(	705	15.96393	chr8:2747	LINC02235		lncRNA	chr8:81841952-8192
ENSG0000(	705	15.96393	chr2:2577	snoU13		smallRNA	chr2:179125536-179
ENSG0000(	705	15.96393	chr2:2577	RAD52P1		Pseudoger	chr2:179399728-179
ENSG0000(	705	15.96393	chr8:2747	snoU13		smallRNA	chr8:75892800-7589
ENSG0000(	705	15.96393	chr8:2747	ZBTB10	DriverDB\	protein_c	chr8:80485619-8052
ENSG0000(	705	15.96393	chr8:2747	FABP9		protein_c	chr8:81458253-8146
ENSG0000(	705	15.96393	chr8:2747	THAP12P7		Pseudoger	chr8:78760142-7876
ENSG0000(	705	15.96393	chr8:2747	ENSG00000253859		lncRNA	chr8:81439436-8152
ENSG0000(	705	15.96393	chr8:2747	RNU6-1300P		smallRNA	chr8:74048677-7404
ENSG0000(	705	15.96393	chr8:2747	ENSG00000288897		lncRNA	chr8:84227057-8422
ENSG0000(	705	15.96393	chr8:2747	ENSG00000248978		Pseudoger	chr8:84828573-8482
ENSG0000(	705	15.96393	chr8:2747	FABP4		protein_c	chr8:81478419-8148
ENSG0000(	705	15.96393	chr8:2747	JPH1	DriverDB\	protein_c	chr8:74234700-7432
ENSG0000(	705	15.96393	chr2:2577	TXNL4AP1		Pseudoger	chr2:179814251-179
ENSG0000(	705	15.96393	chr8:2747	RPL3P9		Pseudoger	chr8:79571153-7957
ENSG0000(	705	15.96393	chr8:2747	ENSG00000254177		lncRNA	chr8:81058523-8106
ENSG0000(	705	15.96393	chr8:2747	RN7SL41P		smallRNA	chr8:80204606-8020
ENSG0000(	705	15.96393	chr8:2747	ENSG00000287352		lncRNA	chr8:76162119-7630
ENSG0000(	705	15.96393	chr8:2747	MRPS28	DriverDB\	protein_c	chr8:79918717-8003
ENSG0000(	705	15.96393	chr8:2747	LINC01111		lncRNA	chr8:76406654-7652
ENSG0000(	705	15.96393	chr8:2747	LINC02886		lncRNA	chr8:75167704-7517
ENSG0000(	705	15.96393	chr8:2747	ENSG00000253214		lncRNA	chr8:81149107-8116
ENSG0000(	705	15.96393	chr8:2747	LNMICC		lncRNA	chr8:81279871-8128
ENSG0000(	705	15.96393	chr8:2747	ENSG00000254060		lncRNA	chr8:81036964-8104
ENSG0000(	705	15.96393	chr8:2747	RPSAP47		Pseudoger	chr8:80558870-8055
ENSG0000(	705	15.96393	chr8:2747	PMP2		protein_c	chr8:81440326-8144
ENSG0000(	705	15.96393	chr8:2747	PRXL2AP2		Pseudoger	chr8:73277364-7327
ENSG0000(	705	15.96393	chr8:2747	ENSG00000253334		lncRNA	chr8:81791823-8181
ENSG0000(	705	15.96393	chr8:2747	SLC10A5		protein_c	chr8:81693631-8169
ENSG0000(	705	15.96393	chr8:2747	UBE2W	DriverDB\	protein_c	chr8:73780097-7387
ENSG0000(	705	15.96393	chr8:2747	ENSG00000253659		lncRNA	chr8:79259402-7931
ENSG0000(	705	15.96393	chr8:2747	RNU6-1040P		smallRNA	chr8:84665759-8466
ENSG0000(	705	15.96393	chr8:2747	SLC25A51P3		Pseudoger	chr8:80594815-8059
ENSG0000(	705	15.96393	chr8:2747	ZFHX4-AS1		lncRNA	chr8:76491200-7668
ENSG0000(	705	15.96393	chr8:2747	RNU7-85P		smallRNA	chr8:79800133-7980
ENSG0000(	705	15.96393	chr8:2747	ENSG00000253423		Pseudoger	chr8:83200952-8320
ENSG0000(	705	15.96393	chr8:2747	UBE2HP1		Pseudoger	chr8:81254870-8125
ENSG0000(	705	15.96393	chr8:2747	SNX16	NCGv7	protein_c	chr8:81799581-8184
ENSG0000(	705	15.96393	chr2:2577	CWC22	NCGv7	protein_c	chr2:179944876-180
ENSG0000(	705	15.96393	chr8:2747	CKS1BP7		Pseudoger	chr8:80644939-8064
ENSG0000(	705	15.96393	chr8:2747	ENSG00000249328		lncRNA	chr8:79769372-7987
ENSG0000(	705	15.96393	chr8:2747	ENSG00000278193		Pseudoger	chr8:72660584-7266
ENSG0000(	705	15.96393	chr8:2747	Y_RNA		smallRNA	chr8:74376687-7437

ENSG0000(	705	15.96393	chr8:2747	ENSG00000253391		Pseudoger	chr8:79520145-7952
ENSG0000(	705	15.96393	chr8:2747	ENSG00000254189		Pseudoger	chr8:83099976-8310
ENSG0000(	705	15.96393	chr8:2747	HIGD1AP18		Pseudoger	chr8:77013751-7701
ENSG0000(	705	15.96393	chr8:2747	LINC02605		lncRNA	chr8:78835307-7884
ENSG0000(	705	15.96393	chr8:2747	RNU6-1220P		smallRNA	chr8:78398515-7839
ENSG0000(	705	15.96393	chr8:2747	Metazoa_SRP		smallRNA	chr8:73833775-7383
ENSG0000(	705	15.96393	chr8:2747	STMN2		protein_c	chr8:79611117-7966
ENSG0000(	705	15.96393	chr2:2577	SESTD1		protein_c	chr2:179101678-179
ENSG0000(	705	15.96393	chr8:2747	ENSG00000253383		Pseudoger	chr8:73590574-7359
ENSG0000(	705	15.96393	chr8:2747	TPM3P3		Pseudoger	chr8:84162118-8416
ENSG0000(	705	15.96393	chr8:2747	ENSG00000260838		lncRNA	chr8:72947150-7295
ENSG0000(	705	15.96393	chr8:2747	GYG1P1		Pseudoger	chr8:73905357-7391
ENSG0000(	705	15.96393	chr8:2747	ENSG00000258677		protein_c	chr8:73688691-7387
ENSG0000(	705	15.96393	chr8:2747	IL7		protein_c	chr8:78675743-7880
ENSG0000(	705	15.96393	chr8:2747	MRPL9P1		Pseudoger	chr8:76603240-7660
ENSG0000(	705	15.96393	chr8:2747	ENSG00000253339		lncRNA	chr8:73358663-7336
ENSG0000(	705	15.96393	chr8:2747	SNORA20		smallRNA	chr8:80316939-8031
ENSG0000(	705	15.96393	chr8:2747	ENSG00000254202		lncRNA	chr8:83912713-8414
ENSG0000(	705	15.96393	chr8:2747	RNU6-285P		smallRNA	chr8:73018945-7301
ENSG0000(	705	15.96393	chr8:2747	TMEM70	DriverDB\	protein_c	chr8:73972437-7398
ENSG0000(	705	15.96393	chr8:2747	ZFAND1		protein_c	chr8:81701334-8173
ENSG0000(	705	15.96393	chr8:2747	PI15		protein_c	chr8:74824534-7485
ENSG0000(	705	15.96393	chr8:2747	ENSG00000253596		lncRNA	chr8:74347757-7435
ENSG0000(	705	15.96393	chr8:2747	ENSG00000290914		lncRNA	chr8:81626057-8163
ENSG0000(	705	15.96393	chr8:2747	ENSG00000285758		lncRNA	chr8:79891553-7993
ENSG0000(	705	15.96393	chr8:2747	RPL7	DriverDB\	protein_c	chr8:73290242-7329
ENSG0000(	705	15.96393	chr8:2747	GDAP1		protein_c	chr8:74320613-7451
ENSG0000(	705	15.96393	chr8:2747	PCBP2P2		Pseudoger	chr8:74603244-7460
ENSG0000(	705	15.96393	chr8:2747	ENSG00000253777		Pseudoger	chr8:74804287-7480
ENSG0000(	705	15.96393	chr8:2747	ENSG00000272518		lncRNA	chr8:79956465-7995
ENSG0000(	705	15.96393	chr8:2747	ENSG00000254238		lncRNA	chr8:75026428-7502
ENSG0000(	705	15.96393	chr8:2747	FTH1P11		Pseudoger	chr8:81521682-8152
ENSG0000(	705	15.96393	chr8:2747	ENSG00000274116		Pseudoger	chr8:83293925-8329
ENSG0000(	705	15.96393	chr8:2747	TERF1	DriverDB\	protein_c	chr8:73008856-7304
ENSG0000(	705	15.96393	chr8:2747	ENSG00000253362		Pseudoger	chr8:84163449-8416
ENSG0000(	705	15.96393	chr8:2747	ENSG00000253784		lncRNA	chr8:72874859-7288
ENSG0000(	705	15.96393	chr8:2747	LY96		protein_c	chr8:73991392-7402
ENSG0000(	705	15.96393	chr8:2747	ELOC	NCGv7	protein_c	chr8:73939169-7397
ENSG0000(	705	15.96393	chr8:2747	ZC2HC1A		protein_c	chr8:78666050-7871
ENSG0000(	705	15.96393	chr8:2747	ENSG00000251867		lncRNA	chr8:80484561-8048
ENSG0000(	705	15.96393	chr8:2747	ENSG00000285744		lncRNA	chr8:78805293-7895
ENSG0000(	705	15.96393	chr8:2747	ENSG00000272425		lncRNA	chr8:81275399-8127
ENSG0000(	705	15.96393	chr8:2747	ENSG00000254043		lncRNA	chr8:74891151-7489
ENSG0000(	704	15.94128	chr8:2747	ENSG00000254051		Pseudoger	chr8:68176768-6817
ENSG0000(	703	15.91864	chr1:3733	ENSG00000287192		lncRNA	chr1:21177054-2117
ENSG0000(	703	15.91864	chr1:3733	ECE1-AS1		lncRNA	chr1:21293290-2129
ENSG0000(	703	15.91864	chr1:3733	ENSG00000235432		Pseudoger	chr1:20692734-2069
ENSG0000(	703	15.91864	chr1:3733	RPS4XP4		Pseudoger	chr1:20525989-2052
ENSG0000(	703	15.91864	chr1:3733	ALPL		protein_c	chr1:21509397-2157
ENSG0000(	703	15.91864	chr1:3733	EIF4G3		protein_c	chr1:20806292-2117
ENSG0000(	703	15.91864	chr1:3733	CAMK2N1		protein_c	chr1:20482391-2048

ENSG0000(	703	15.91864	chr1:373:	CROCCP5	Pseudoger	chr1:21434318-2143
ENSG0000(	703	15.91864	chr1:373:	ENSG00000289715	protein_c	chr1:21547404-2155
ENSG0000(	703	15.91864	chr1:373:	RPS15AP6	Pseudoger	chr1:21003550-2100
ENSG0000(	703	15.91864	chr1:373:	SH2D5	protein_c	chr1:20719731-2073
ENSG0000(	703	15.91864	chr1:373:	ENSG00000289402	lncRNA	chr1:20486358-2048
ENSG0000(	703	15.91864	chr1:373:	RP5-930J4.4	lncRNA	chr1:20742987-2074
ENSG0000(	703	15.91864	chr1:373:	DDOST	protein_c	chr1:20651767-2066
ENSG0000(	703	15.91864	chr1:373:	AL359815.1	smallRNA	chr1:21592411-2159
ENSG0000(	703	15.91864	chr1:373:	PFN1P10	Pseudoger	chr1:21459756-2146
ENSG0000(	703	15.91864	chr1:373:	FAM43B	protein_c	chr1:20552573-2055
ENSG0000(	703	15.91864	chr1:373:	ECE1	protein_c	chr1:21217247-2134
ENSG0000(	703	15.91864	chr1:373:	PINK1	protein_c	chr1:20633458-2065
ENSG0000(	703	15.91864	chr1:373:	AL031005.1	smallRNA	chr1:21176566-2117
ENSG0000(	703	15.91864	chr1:373:	CDA	protein_c	chr1:20589086-2061
ENSG0000(	703	15.91864	chr1:373:	VWA5B1	protein_c	chr1:20290875-2035
ENSG0000(	703	15.91864	chr1:373:	AL391357.1	Pseudoger	chr1:20650363-2065
ENSG0000(	703	15.91864	chr1:373:	ENSG00000236009	lncRNA	chr1:21415898-2141
ENSG0000(	703	15.91864	chr1:373:	HSPE1P27	Pseudoger	chr1:21161475-2116
ENSG0000(	703	15.91864	chr1:373:	NBPF3	protein_c	chr1:21440128-2148
ENSG0000(	703	15.91864	chr1:373:	MIR1256	smallRNA	chr1:20988314-2098
ENSG0000(	703	15.91864	chr1:373:	PINK1-AS	lncRNA	chr1:20642657-2065
ENSG0000(	703	15.91864	chr1:373:	PDE4DIPP10	Pseudoger	chr1:21411460-2141
ENSG0000(	703	15.91864	chr1:373:	LINC01141	lncRNA	chr1:20360579-2043
ENSG0000(	703	15.91864	chr1:373:	LINC02596	lncRNA	chr1:21586472-2159
ENSG0000(	703	15.91864	chr1:373:	ENSG00000236936	lncRNA	chr1:21266082-2126
ENSG0000(	703	15.91864	chr1:373:	ENSG00000233069	lncRNA	chr1:20732880-2073
ENSG0000(	703	15.91864	chr1:373:	NBPF2P	Pseudoger	chr1:21424625-2142
ENSG0000(	703	15.91864	chr1:373:	KIF17	protein_c	chr1:20664014-2071
ENSG0000(	703	15.91864	chr1:373:	PPP1R11P1	Pseudoger	chr1:21397987-2139
ENSG0000(	703	15.91864	chr1:373:	MUL1	protein_c	chr1:20499448-2050
ENSG0000(	703	15.91864	chr1:373:	ENSG00000284641	lncRNA	chr1:20476222-2047
ENSG0000(	703	15.91864	chr1:373:	RAP1GAP	protein_c	chr1:21596221-2166
ENSG0000(	703	15.91864	chr1:373:	HP1BP3	protein_c	chr1:20740266-2078
ENSG0000(	703	15.91864	chr1:373:	HS6ST1P1	Pseudoger	chr1:21428303-2142
ENSG0000(	703	15.91864	chr1:373:	RNU7-200P	smallRNA	chr1:20841241-2084
ENSG0000(	703	15.91864	chr1:373:	ENSG00000284743	lncRNA	chr1:20478779-2048
ENSG0000(	703	15.91864	chr1:373:	ENSG00000226664	lncRNA	chr1:20294211-2032
ENSG0000(	698	15.80542	chr2:257	YWHAZP2	Pseudoger	chr2:126557435-126
ENSG0000(	697	15.78278	chr1:1021	HYI	protein_c	chr1:43450989-4345
ENSG0000(	697	15.78278	chr1:1021	KRT8P47	Pseudoger	chr1:44103306-4410
ENSG0000(	697	15.78278	chr1:1021	ENSG00000238186	lncRNA	chr1:40515754-4051
ENSG0000(	697	15.78278	chr1:1021	ENSG00000273637	lncRNA	chr1:38839333-3887
ENSG0000(	697	15.78278	chr1:1021	ENSG00000260920	lncRNA	chr1:40464319-4046
ENSG0000(	697	15.78278	chr1:1021	ENSG00000285649	lncRNA	chr1:43968351-4397
ENSG0000(	697	15.78278	chr1:1021	LINC02811	lncRNA	chr1:39801414-3981
ENSG0000(	697	15.78278	chr1:1021	GTF2F2P2	Pseudoger	chr1:40593633-4059
ENSG0000(	697	15.78278	chr1:1021	Y_RNA	smallRNA	chr1:39881566-3988
ENSG0000(	697	15.78278	chr1:1021	TMCO2	protein_c	chr1:40245947-4025
ENSG0000(	697	15.78278	chr1:1021	MYCL-AS1	lncRNA	chr1:39897745-3989
ENSG0000(	697	15.78278	chr1:1021	CTPS1	protein_c	chr1:40979300-4101
ENSG0000(	697	15.78278	chr1:1021	SLFNL1	protein_c	chr1:41015589-4102

ENSG0000(	697	15.78278	chr1:1021	ENSG00000283580		protein_c	chr1:42767292-4279
ENSG0000(	697	15.78278	chr1:1021	AL451006.1		smallRNA	chr1:41759141-4175
ENSG0000(	697	15.78278	chr1:1021	ENSG00000226438		lncRNA	chr1:39249838-3925
ENSG0000(	697	15.78278	chr1:1021	ENSG00000233514		Pseudoger	chr1:44122153-4412
ENSG0000(	697	15.78278	chr1:1021	BEST4		protein_c	chr1:44783585-4478
ENSG0000(	697	15.78278	chr1:1021	RNU6-605P		smallRNA	chr1:38926870-3892
ENSG0000(	697	15.78278	chr1:1021	RNA5SP44		Pseudoger	chr1:39154164-3915
ENSG0000(	697	15.78278	chr1:1021	MIR5584		smallRNA	chr1:44545493-4454
ENSG0000(	697	15.78278	chr1:1021	HPCAL4		protein_c	chr1:39678648-3969
ENSG0000(	697	15.78278	chr1:1021	KLF17		protein_c	chr1:44118821-4413
ENSG0000(	697	15.78278	chr1:1021	ENSG00000233674		Pseudoger	chr1:43743471-4374
ENSG0000(	697	15.78278	chr1:1021	B4GALT2		protein_c	chr1:43978943-4399
ENSG0000(	697	15.78278	chr1:1021	ENSG00000236180		Pseudoger	chr1:42570970-4257
ENSG0000(	697	15.78278	chr1:1021	KDM4A-AS1		lncRNA	chr1:43685123-4370
ENSG0000(	697	15.78278	chr1:1021	RNU6-870P		smallRNA	chr1:43023549-4302
ENSG0000(	697	15.78278	chr1:1021	FOXO6		protein_c	chr1:41361922-4138
ENSG0000(	697	15.78278	chr1:1021	MIR30E		smallRNA	chr1:40754355-4075
ENSG0000(	697	15.78278	chr1:1021	BMP8B-AS1		lncRNA	chr1:39779969-3978
ENSG0000(	697	15.78278	chr1:1021	SLFNL1-AS1		lncRNA	chr1:41014590-4104
ENSG0000(	697	15.78278	chr1:1021	PPIH		protein_c	chr1:42658335-4267
ENSG0000(	697	15.78278	chr1:1021	RHBDL2		protein_c	chr1:38885807-3894
ENSG0000(	697	15.78278	chr1:1021	ENSG00000233708		Pseudoger	chr1:42886597-4288
ENSG0000(	697	15.78278	chr1:1021	ERI3		protein_c	chr1:44221070-4435
ENSG0000(	697	15.78278	chr1:1021	PTCH2	AC	protein_c	chr1:44819844-4484
ENSG0000(	697	15.78278	chr1:1021	ENSG00000229213		Pseudoger	chr1:39795843-3979
ENSG0000(	697	15.78278	chr1:1021	ENSG00000230615		lncRNA	chr1:44030414-4411
ENSG0000(	697	15.78278	chr1:1021	TMEM269		protein_c	chr1:42784991-4281
ENSG0000(	697	15.78278	chr1:1021	ENSG00000227163		Pseudoger	chr1:44087958-4408
ENSG0000(	697	15.78278	chr1:1021	RNU6-880P		smallRNA	chr1:42991438-4299
ENSG0000(	697	15.78278	chr1:1021	KIF2C		protein_c	chr1:44739818-4476
ENSG0000(	697	15.78278	chr1:1021	UBE2V1P8		Pseudoger	chr1:40942251-4094
ENSG0000(	697	15.78278	chr1:1021	RNU5E-6P		smallRNA	chr1:44819883-4481
ENSG0000(	697	15.78278	chr1:1021	RNF220		protein_c	chr1:44405194-4465
ENSG0000(	697	15.78278	chr1:1021	MKRN8P		Pseudoger	chr1:42891094-4289
ENSG0000(	697	15.78278	chr1:1021	KDM4A		protein_c	chr1:43650149-4370
ENSG0000(	697	15.78278	chr1:1021	ST3GAL3		protein_c	chr1:43705824-4393
ENSG0000(	697	15.78278	chr1:1021	RPS3AP11		Pseudoger	chr1:42491739-4249
ENSG0000(	697	15.78278	chr1:1021	TIE1		protein_c	chr1:43300982-4332
ENSG0000(	697	15.78278	chr1:1021	LINC01685		lncRNA	chr1:38474825-3851
ENSG0000(	697	15.78278	chr1:1021	EXO5-DT		lncRNA	chr1:40493157-4050
ENSG0000(	697	15.78278	chr1:1021	ENSG00000230881		lncRNA	chr1:41535443-4153
ENSG0000(	697	15.78278	chr1:1021	RRAGC-DT		lncRNA	chr1:38859912-3896
ENSG0000(	697	15.78278	chr1:1021	OOSP1P1		Pseudoger	chr1:44155028-4415
ENSG0000(	697	15.78278	chr1:1021	ENSG00000213172		Pseudoger	chr1:40364766-4036
ENSG0000(	697	15.78278	chr1:1021	RPS15AP11		Pseudoger	chr1:44780331-4478
ENSG0000(	697	15.78278	chr1:1021	PTPRF		protein_c	chr1:43525187-4362
ENSG0000(	697	15.78278	chr1:1021	COL9A2	NCGv7	protein_c	chr1:40300489-4031
ENSG0000(	697	15.78278	chr1:1021	SCMH1-DT		lncRNA	chr1:41242373-4128
ENSG0000(	697	15.78278	chr1:1021	ENSG00000230638		Pseudoger	chr1:41542069-4154
ENSG0000(	697	15.78278	chr1:1021	TMEM125		protein_c	chr1:43269983-4327
ENSG0000(	697	15.78278	chr1:1021	ENSG00000284677		lncRNA	chr1:40436199-4045

ENSG0000(	697	15.78278	chr1:1021	ENSG00000228477		Pseudoger	chr1:39962680-3996
ENSG0000(	697	15.78278	chr1:1021	ZNF691-DT		lncRNA	chr1:42832522-4284
ENSG0000(	697	15.78278	chr1:1021	RNA5SP46		Pseudoger	chr1:43196417-4319
ENSG0000(	697	15.78278	chr1:1021	YBX1	AC	protein_c	chr1:42682418-4270
ENSG0000(	697	15.78278	chr1:1021	TMEM53		protein_c	chr1:44635238-4467
ENSG0000(	697	15.78278	chr1:1021	ENSG00000236505		Pseudoger	chr1:40563534-4056
ENSG0000(	697	15.78278	chr1:1021	ENSG00000287422		lncRNA	chr1:39226670-3923
ENSG0000(	697	15.78278	chr1:1021	ENSG00000287400		lncRNA	chr1:41241772-4133
ENSG0000(	697	15.78278	chr1:1021	ENSG00000261798		lncRNA	chr1:39788976-3979
ENSG0000(	697	15.78278	chr1:1021	ENSG00000226499		Pseudoger	chr1:44843921-4484
ENSG0000(	697	15.78278	chr1:1021	RRAGC	IntOGen-I	protein_c	chr1:38838198-3885
ENSG0000(	697	15.78278	chr1:1021	OAZ1P1		Pseudoger	chr1:40132764-4013
ENSG0000(	697	15.78278	chr1:1021	RPS8		protein_c	chr1:44775251-4477
ENSG0000(	697	15.78278	chr1:1021	ELOVL1		protein_c	chr1:43363398-4336
ENSG0000(	697	15.78278	chr1:1021	OXCT2P1		Pseudoger	chr1:39514956-3951
ENSG0000(	697	15.78278	chr1:1021	ENSG00000225903		lncRNA	chr1:39633416-3963
ENSG0000(	697	15.78278	chr1:1021	SMAP2		protein_c	chr1:40344850-4042
ENSG0000(	697	15.78278	chr1:1021	PPIE		protein_c	chr1:39692182-3976
ENSG0000(	697	15.78278	chr1:1021	ERI3-IT1		lncRNA	chr1:44243408-4424
ENSG0000(	697	15.78278	chr1:1021	NT5C1A		protein_c	chr1:39651229-3967
ENSG0000(	697	15.78278	chr1:1021	ZMPSTE24		protein_c	chr1:40258041-4029
ENSG0000(	697	15.78278	chr1:1021	ENSG00000290041		lncRNA	chr1:44807524-4480
ENSG0000(	697	15.78278	chr1:1021	ENSG00000286552		lncRNA	chr1:38149544-3816
ENSG0000(	697	15.78278	chr1:1021	ZMYND12		protein_c	chr1:42430329-4245
ENSG0000(	697	15.78278	chr1:1021	CITED4		protein_c	chr1:40861054-4086
ENSG0000(	697	15.78278	chr1:1021	MFSD2A		protein_c	chr1:39955112-3996
ENSG0000(	697	15.78278	chr1:1021	NFYC		protein_c	chr1:40691648-4077
ENSG0000(	697	15.78278	chr1:1021	BMP8A		protein_c	chr1:39491636-3952
ENSG0000(	697	15.78278	chr1:1021	RIMS3		protein_c	chr1:40620680-4066
ENSG0000(	697	15.78278	chr1:1021	ENSG00000227278		lncRNA	chr1:40514461-4051
ENSG0000(	697	15.78278	chr1:1021	ENSG00000227994		Pseudoger	chr1:44172506-4417
ENSG0000(	697	15.78278	chr1:1021	ATP6V1E1P1		Pseudoger	chr1:42903232-4290
ENSG0000(	697	15.78278	chr1:1021	ENSG00000277513		Pseudoger	chr1:43104086-4310
ENSG0000(	697	15.78278	chr1:1021	ST3GAL3-AS1		lncRNA	chr1:43709392-4372
ENSG0000(	697	15.78278	chr1:1021	CDC20-DT		lncRNA	chr1:43354684-4335
ENSG0000(	697	15.78278	chr1:1021	ENSG00000237899		lncRNA	chr1:40669089-4068
ENSG0000(	697	15.78278	chr1:1021	MYCBP		protein_c	chr1:38862493-3887
ENSG0000(	697	15.78278	chr1:1021	PLK3		protein_c	chr1:44800377-4480
ENSG0000(	697	15.78278	chr1:1021	EIF1P2		Pseudoger	chr1:38958275-3895
ENSG0000(	697	15.78278	chr1:1021	MED8-AS1		lncRNA	chr1:43385113-4338
ENSG0000(	697	15.78278	chr1:1021	RNU5D-1		smallRNA	chr1:44731055-4473
ENSG0000(	697	15.78278	chr1:1021	RN7SL326P		smallRNA	chr1:40804846-4080
ENSG0000(	697	15.78278	chr1:1021	RNA5SP45		Pseudoger	chr1:41466937-4146
ENSG0000(	697	15.78278	chr1:1021	C1orf210		protein_c	chr1:43281877-4328
ENSG0000(	697	15.78278	chr1:1021	RIMKLA	DriverDB\	protein_c	chr1:42380792-4242
ENSG0000(	697	15.78278	chr1:1021	KLF18		protein_c	chr1:44137821-4414
ENSG0000(	697	15.78278	chr1:1021	ENSG00000227527		lncRNA	chr1:42335386-4233
ENSG0000(	697	15.78278	chr1:1021	FOXJ3		protein_c	chr1:42176539-4233
ENSG0000(	697	15.78278	chr1:1021	SLC2A1-DT		lncRNA	chr1:42959049-4299
ENSG0000(	697	15.78278	chr1:1021	CDC20	NCGv7	protein_c	chr1:43358981-4336
ENSG0000(	697	15.78278	chr1:1021	ENSG00000274944		protein_c	chr1:38864501-3888

ENSG0000(	697	15.78278	chr1:1021	MPL	NCGv7;AC	protein_c	chr1:43337818-4335
ENSG0000(	697	15.78278	chr1:1021	MACF1	NCGv7	protein_c	chr1:39081316-3948
ENSG0000(	697	15.78278	chr1:1021	ARMH1		protein_c	chr1:44674692-4472
ENSG0000(	697	15.78278	chr1:1021	EDN2		protein_c	chr1:41478775-4148
ENSG0000(	697	15.78278	chr1:1021	HSPA5P1		Pseudoger	chr1:38708931-3871
ENSG0000(	697	15.78278	chr1:1021	IPO13		protein_c	chr1:43946950-4396
ENSG0000(	697	15.78278	chr1:1021	CCDC30		protein_c	chr1:42463221-4265
ENSG0000(	697	15.78278	chr1:1021	MYCL	NCGv7;AC	protein_c	chr1:39895426-3990
ENSG0000(	697	15.78278	chr1:1021	ENSG00000225333		Pseudoger	chr1:39718028-3971
ENSG0000(	697	15.78278	chr1:1021	DYNLT4		protein_c	chr1:44805893-4480
ENSG0000(	697	15.78278	chr1:1021	RNU6-1058P		smallRNA	chr1:43716467-4371
ENSG0000(	697	15.78278	chr1:1021	FOXO6-AS1		lncRNA	chr1:41375004-4137
ENSG0000(	697	15.78278	chr1:1021	MIR3659HG		lncRNA	chr1:38047314-3811
ENSG0000(	697	15.78278	chr1:1021	PPIEL		lncRNA	chr1:39522280-3955
ENSG0000(	697	15.78278	chr1:1021	ARTN		protein_c	chr1:43933320-4393
ENSG0000(	697	15.78278	chr1:1021	GUCA2B		protein_c	chr1:42153410-4215
ENSG0000(	697	15.78278	chr1:1021	PABPC4	NCGv7	protein_c	chr1:39560709-3957
ENSG0000(	697	15.78278	chr1:1021	ENSG00000231296		Pseudoger	chr1:40262672-4026
ENSG0000(	697	15.78278	chr1:1021	ENSG00000288772		lncRNA	chr1:43368180-4336
ENSG0000(	697	15.78278	chr1:1021	ENSG00000225721		lncRNA	chr1:44759037-4477
ENSG0000(	697	15.78278	chr1:1021	TRIT1		protein_c	chr1:39838110-3988
ENSG0000(	697	15.78278	chr1:1021	HNRNPFP1		Pseudoger	chr1:42040597-4204
ENSG0000(	697	15.78278	chr1:1021	RNU6-608P		smallRNA	chr1:39120940-3912
ENSG0000(	697	15.78278	chr1:1021	SLC6A9	NCGv7	protein_c	chr1:43991500-4403
ENSG0000(	697	15.78278	chr1:1021	SZT2-AS1		lncRNA	chr1:43447776-4344
ENSG0000(	697	15.78278	chr1:1021	ATP6V0CP4		Pseudoger	chr1:42952202-4295
ENSG0000(	697	15.78278	chr1:1021	RLF		protein_c	chr1:40161387-4024
ENSG0000(	697	15.78278	chr1:1021	ENSG00000271329		Pseudoger	chr1:44187943-4418
ENSG0000(	697	15.78278	chr1:1021	LINC01343		lncRNA	chr1:38209034-3821
ENSG0000(	697	15.78278	chr1:1021	ZNF684		protein_c	chr1:40531573-4054
ENSG0000(	697	15.78278	chr1:1021	GJA9		protein_c	chr1:38874069-3888
ENSG0000(	697	15.78278	chr1:1021	ENSG00000235002		Pseudoger	chr1:42412398-4241
ENSG0000(	697	15.78278	chr1:1021	CAP1		protein_c	chr1:40040233-4007
ENSG0000(	697	15.78278	chr1:1021	PPT1		protein_c	chr1:40072710-4009
ENSG0000(	697	15.78278	chr1:1021	TMSB4XP1		Pseudoger	chr1:42500205-4250
ENSG0000(	697	15.78278	chr1:1021	AKIRIN1	DriverDB\	protein_c	chr1:38991276-3900
ENSG0000(	697	15.78278	chr1:1021	Y_RNA		smallRNA	chr1:39944890-3994
ENSG0000(	697	15.78278	chr1:1021	KCNQ4	DriverDB\	protein_c	chr1:40783787-4084
ENSG0000(	697	15.78278	chr1:1021	GUCA2A		protein_c	chr1:42162690-4216
ENSG0000(	697	15.78278	chr1:1021	HYI-AS1		lncRNA	chr1:43453927-4345
ENSG0000(	697	15.78278	chr1:1021	SHMT1P1		Pseudoger	chr1:43850300-4385
ENSG0000(	697	15.78278	chr1:1021	FAM183A		protein_c	chr1:43145153-4315
ENSG0000(	697	15.78278	chr1:1021	RNU7-121P		smallRNA	chr1:39723566-3972
ENSG0000(	697	15.78278	chr1:1021	HEYL		protein_c	chr1:39623435-3963
ENSG0000(	697	15.78278	chr1:1021	SNORD38B		smallRNA	chr1:44778390-4477
ENSG0000(	697	15.78278	chr1:1021	ENSG00000279667		TEC	chr1:40473055-4047
ENSG0000(	697	15.78278	chr1:1021	SLC2A1		protein_c	chr1:42925353-4295
ENSG0000(	697	15.78278	chr1:1021	RNU6-1237P		smallRNA	chr1:40177843-4017
ENSG0000(	697	15.78278	chr1:1021	ENSG00000275350		Pseudoger	chr1:38383838-3838
ENSG0000(	697	15.78278	chr1:1021	EBNA1BP2		protein_c	chr1:43164175-4327
ENSG0000(	697	15.78278	chr1:1021	ENSG00000286838		lncRNA	chr1:40559666-4058

ENSG0000(	697	15.78278	chr1:1021	ENSG00000288955	lncRNA	chr1:42924460-4292
ENSG0000(	697	15.78278	chr1:1021	RN7SL479P	smallRNA	chr1:44117100-4411
ENSG0000(	697	15.78278	chr1:1021	ENSG00000290111	lncRNA	chr1:40394763-4039
ENSG0000(	697	15.78278	chr1:1021	Y_RNA	smallRNA	chr1:38950825-3895
ENSG0000(	697	15.78278	chr1:1021	ZNF691	protein_c	chr1:42846573-4285
ENSG0000(	697	15.78278	chr1:1021	ENSG00000291157	lncRNA	chr1:41302911-4130
ENSG0000(	697	15.78278	chr1:1021	ERMAP	protein_c	chr1:42817122-4284
ENSG0000(	697	15.78278	chr1:1021	C1orf50	protein_c	chr1:42767245-4277
ENSG0000(	697	15.78278	chr1:1021	CLDN19	protein_c	chr1:42733093-4274
ENSG0000(	697	15.78278	chr1:1021	EXO5	protein_c	chr1:40508741-4051
ENSG0000(	697	15.78278	chr1:1021	ENSG00000234917	lncRNA	chr1:42678735-4268
ENSG0000(	697	15.78278	chr1:1021	ENSG00000287587	lncRNA	chr1:42036143-4205
ENSG0000(	697	15.78278	chr1:1021	P3H1	protein_c	chr1:42746335-4276
ENSG0000(	697	15.78278	chr1:1021	SNORA55	smallRNA	chr1:39567374-3956
ENSG0000(	697	15.78278	chr1:1021	PPCS	protein_c	chr1:42456117-4247
ENSG0000(	697	15.78278	chr1:1021	ENSG00000226804	Pseudoger	chr1:44150594-4415
ENSG0000(	697	15.78278	chr1:1021	ENSG00000287113	lncRNA	chr1:43348288-4334
ENSG0000(	697	15.78278	chr1:1021	SNORD38A	smallRNA	chr1:44777843-4477
ENSG0000(	697	15.78278	chr1:1021	RP11-7O11.3	lncRNA	chr1:43944370-4394
ENSG0000(	697	15.78278	chr1:1021	ENSG00000287743	lncRNA	chr1:40659848-4066
ENSG0000(	697	15.78278	chr1:1021	ENSG00000229528	lncRNA	chr1:40863914-4087
ENSG0000(	697	15.78278	chr1:1021	DMAP1	protein_c	chr1:44213455-4422
ENSG0000(	697	15.78278	chr1:1021	Y_RNA	smallRNA	chr1:44153385-4415
ENSG0000(	697	15.78278	chr1:1021	MED8	protein_c	chr1:43383917-4338
ENSG0000(	697	15.78278	chr1:1021	ENSG00000289711	Pseudoger	chr1:39733327-3973
ENSG0000(	697	15.78278	chr1:1021	HSPE1P8	Pseudoger	chr1:39304294-3930
ENSG0000(	697	15.78278	chr1:1021	ENSG00000287987	lncRNA	chr1:38193619-3821
ENSG0000(	697	15.78278	chr1:1021	NFYC-AS1	lncRNA	chr1:40690380-4069
ENSG0000(	697	15.78278	chr1:1021	PABPC4-AS1	lncRNA	chr1:39565052-3957
ENSG0000(	697	15.78278	chr1:1021	ENSG00000284989	protein_c	chr1:43650149-4393
ENSG0000(	697	15.78278	chr1:1021	NDUFS5	protein_c	chr1:39026318-3903
ENSG0000(	697	15.78278	chr1:1021	RNU6-536P	smallRNA	chr1:42569033-4256
ENSG0000(	697	15.78278	chr1:1021	ZFP69B	protein_c	chr1:40450102-4046
ENSG0000(	697	15.78278	chr1:1021	CFAP57	protein_c	chr1:43172330-4325
ENSG0000(	697	15.78278	chr1:1021	RNU6-369P	smallRNA	chr1:44390722-4439
ENSG0000(	697	15.78278	chr1:1021	RPL36AP9	Pseudoger	chr1:41264550-4126
ENSG0000(	697	15.78278	chr1:1021	ENSG00000228776	Pseudoger	chr1:42140635-4214
ENSG0000(	697	15.78278	chr1:1021	SCMH1	protein_c	chr1:41027202-4124
ENSG0000(	697	15.78278	chr1:1021	ENSG00000284719	lncRNA	chr1:39799419-3980
ENSG0000(	697	15.78278	chr1:1021	ENSG00000283973	lncRNA	chr1:42959065-4296
ENSG0000(	697	15.78278	chr1:1021	ENSG00000228940	Pseudoger	chr1:40938104-4093
ENSG0000(	697	15.78278	chr1:1021	DPH2	protein_c	chr1:43970000-4397
ENSG0000(	697	15.78278	chr1:1021	ENSG00000285728	lncRNA	chr1:42658687-4268
ENSG0000(	697	15.78278	chr1:1021	CCDC24	protein_c	chr1:43991359-4399
ENSG0000(	697	15.78278	chr1:1021	ZFP69	protein_c	chr1:40477290-4049
ENSG0000(	697	15.78278	chr1:1021	ENSG00000227311	Pseudoger	chr1:40333078-4033
ENSG0000(	697	15.78278	chr1:1021	BMP8B	protein_c	chr1:39757182-3978
ENSG0000(	697	15.78278	chr1:1021	HIVEP3	protein_c	chr1:41506365-4203
ENSG0000(	697	15.78278	chr1:1021	BTBD19	protein_c	chr1:44808523-4481
ENSG0000(	697	15.78278	chr1:1021	PPIEL	Pseudoger	chr1:39531838-3955
ENSG0000(	697	15.78278	chr1:1021	ENSG00000284895	protein_c	chr1:41585306-4162

ENSG0000(	697	15.78278	chr1:1021	ENSG00000286640		Pseudoger	chr1:41302938-4130
ENSG0000(	697	15.78278	chr1:1021	RPL23AP17		Pseudoger	chr1:41098638-4109
ENSG0000(	697	15.78278	chr1:1021	SVBP		protein_c	chr1:42807052-4281
ENSG0000(	697	15.78278	chr1:1021	ENSG00000284632		lncRNA	chr1:38754216-3881
ENSG0000(	697	15.78278	chr1:1021	ENSG00000286668		lncRNA	chr1:40939294-4094
ENSG0000(	697	15.78278	chr1:1021	SZT2		protein_c	chr1:43389882-4345
ENSG0000(	697	15.78278	chr1:1021	RNU6-753P		smallRNA	chr1:38396659-3839
ENSG0000(	697	15.78278	chr1:1021	ATP6V0B	NCGv7	protein_c	chr1:43974487-4397
ENSG0000(	697	15.78278	chr1:1021	LINC02786		lncRNA	chr1:38129464-3814
ENSG0000(	697	15.78278	chr1:1021	AL390776.1		smallRNA	chr1:44333105-4433
ENSG0000(	697	15.78278	chr1:1021	OXCT2	NCGv7	protein_c	chr1:39769523-3977
ENSG0000(	697	15.78278	chr1:1021	RNU5F-1		smallRNA	chr1:44721786-4472
ENSG0000(	697	15.78278	chr1:1021	ZMPSTE24-DT		lncRNA	chr1:40256333-4025
ENSG0000(	696	15.76013	chr7:2516	ENSG00000272219		lncRNA	chr7:101960116-101
ENSG0000(	696	15.76013	chr6:9221	ENSG00000219575		Pseudoger	chr6:71550958-7155
ENSG0000(	694	15.71484	chr16:239	RP11-293B20.3		Pseudoger	chr16:33577495-335
ENSG0000(	693	15.6922	chr1:2449	MIR488		smallRNA	chr1:177029363-177
ENSG0000(	693	15.6922	chr5:4231	RN7SL378P		smallRNA	chr5:82078427-8207
ENSG0000(	690	15.62427	chr12:201	AC144522.1		smallRNA	chr12:110632604-11
ENSG0000(	688	15.57898	chr1:2449	ENSG00000225591		Pseudoger	chr1:173741674-173
ENSG0000(	688	15.57898	chr1:2449	snoU13		smallRNA	chr1:173660097-173
ENSG0000(	688	15.57898	chr1:2449	RABGAP1L-IT1		lncRNA	chr1:174896958-174
ENSG0000(	688	15.57898	chr1:2449	RABGAP1L		protein_c	chr1:174159410-174
ENSG0000(	688	15.57898	chr1:2449	RPS29P5		Pseudoger	chr1:175921975-175
ENSG0000(	688	15.57898	chr1:2449	snoU13		smallRNA	chr1:174200129-174
ENSG0000(	688	15.57898	chr1:2449	ENSG00000228686		lncRNA	chr1:176017277-176
ENSG0000(	688	15.57898	chr1:2449	LINC01657		lncRNA	chr1:175877343-175
ENSG0000(	688	15.57898	chr1:2449	ENSG00000227815		Pseudoger	chr1:176272483-176
ENSG0000(	688	15.57898	chr1:2449	RPL30P1		Pseudoger	chr1:174090136-174
ENSG0000(	688	15.57898	chr1:2449	ENTR1P2		Pseudoger	chr1:175044626-175
ENSG0000(	688	15.57898	chr1:2449	ENSG00000237317		Pseudoger	chr1:174367105-174
ENSG0000(	688	15.57898	chr1:2449	PAPPA2		protein_c	chr1:176463171-176
ENSG0000(	688	15.57898	chr1:2449	ENSG00000289425		lncRNA	chr1:174967328-174
ENSG0000(	688	15.57898	chr1:2449	ENSG00000289426		lncRNA	chr1:173637713-173
ENSG0000(	688	15.57898	chr1:2449	LINC02803		lncRNA	chr1:175904762-175
ENSG0000(	688	15.57898	chr1:2449	AL021398.1		smallRNA	chr1:177186360-177
ENSG0000(	688	15.57898	chr1:2449	RABGAP1L-AS1		lncRNA	chr1:174934947-174
ENSG0000(	688	15.57898	chr1:2449	CACYBP		protein_c	chr1:174999163-175
ENSG0000(	688	15.57898	chr1:2449	ENSG00000235869		Pseudoger	chr1:174922107-174
ENSG0000(	688	15.57898	chr1:2449	GPR52		protein_c	chr1:174447964-174
ENSG0000(	688	15.57898	chr1:2449	TNR	NCGv7	protein_c	chr1:175315194-175
ENSG0000(	688	15.57898	chr1:2449	DARS2		protein_c	chr1:173824653-173
ENSG0000(	688	15.57898	chr1:2449	BRINP2		protein_c	chr1:177170958-177
ENSG0000(	688	15.57898	chr1:2449	RNU2-12P		smallRNA	chr1:176243862-176
ENSG0000(	688	15.57898	chr1:2449	SERPINC1		protein_c	chr1:173903800-173
ENSG0000(	688	15.57898	chr1:2449	Y_RNA		smallRNA	chr1:173808489-173
ENSG0000(	688	15.57898	chr1:2449	ENSG00000232463		Pseudoger	chr1:176231200-176
ENSG0000(	688	15.57898	chr1:2449	GAS5-AS1		lncRNA	chr1:173862473-173
ENSG0000(	688	15.57898	chr1:2449	RC3H1-DT		lncRNA	chr1:174022509-174
ENSG0000(	688	15.57898	chr1:2449	BANF1P4		Pseudoger	chr1:174756850-174
ENSG0000(	688	15.57898	chr1:2449	ENSG00000286754		lncRNA	chr1:176829128-176

ENSG0000(	688	15.57898	chr1:244{	CENPL	DriverDB\	protein_c	chr1:173799550-173
ENSG0000(	688	15.57898	chr1:244{	COP1		protein_c	chr1:175944831-176
ENSG0000(	688	15.57898	chr2:818{	RN7SL674P		smallRNA	chr2:11584773-1158
ENSG0000(	688	15.57898	chr1:244{	ENSG00000285777		protein_c	chr1:173596060-173
ENSG0000(	688	15.57898	chr1:244{	ENSG00000231020		Pseudoger	chr1:176305672-176
ENSG0000(	688	15.57898	chr1:244{	ENSG00000230687		lncRNA	chr1:175203228-175
ENSG0000(	688	15.57898	chr1:244{	ENSG00000287697		lncRNA	chr1:174998353-174
ENSG0000(	688	15.57898	chr1:244{	SCARNA3		smallRNA	chr1:175968398-175
ENSG0000(	688	15.57898	chr1:244{	RABGAP1L-DT		lncRNA	chr1:174110268-174
ENSG0000(	688	15.57898	chr1:244{	ENSG00000237249		Pseudoger	chr1:174892417-174
ENSG0000(	688	15.57898	chr1:244{	COP1-DT		lncRNA	chr1:176207646-176
ENSG0000(	688	15.57898	chr1:244{	RPS29P4		Pseudoger	chr1:175297080-175
ENSG0000(	688	15.57898	chr1:244{	RC3H1		protein_c	chr1:173931084-174
ENSG0000(	688	15.57898	chr1:244{	RN7SKP160		smallRNA	chr1:173791548-173
ENSG0000(	688	15.57898	chr1:244{	TNN	NCGv7	protein_c	chr1:175067833-175
ENSG0000(	688	15.57898	chr1:244{	MRPS14	NCGv7	protein_c	chr1:175010789-175
ENSG0000(	688	15.57898	chr1:244{	PTP4A1P7		Pseudoger	chr1:176616273-176
ENSG0000(	688	15.57898	chr1:244{	NDUFAF4P4		Pseudoger	chr1:174849667-174
ENSG0000(	688	15.57898	chr1:244{	RNA5SP68		Pseudoger	chr1:173969318-173
ENSG0000(	688	15.57898	chr1:244{	RC3H1-IT1		lncRNA	chr1:174009267-174
ENSG0000(	688	15.57898	chr1:244{	TEX50		protein_c	chr1:173635338-173
ENSG0000(	688	15.57898	chr1:244{	ASTN1	NCGv7	protein_c	chr1:176857302-177
ENSG0000(	688	15.57898	chr1:244{	Y_RNA		smallRNA	chr1:175022479-175
ENSG0000(	688	15.57898	chr1:244{	ZBTB37	DriverDB\	protein_c	chr1:173868082-173
ENSG0000(	688	15.57898	chr1:244{	TNR-IT1		lncRNA	chr1:175538775-175
ENSG0000(	688	15.57898	chr1:244{	KIAA0040		protein_c	chr1:175156986-175
ENSG0000(	688	15.57898	chr1:244{	ENSG00000260990		lncRNA	chr1:175307218-175
ENSG0000(	688	15.57898	chr1:244{	ANKRD45		protein_c	chr1:173608336-173
ENSG0000(	688	15.57898	chr1:244{	MORF4L1P7		Pseudoger	chr1:176367699-176
ENSG0000(	688	15.57898	chr1:244{	SNORD78		smallRNA	chr1:173865622-173
ENSG0000(	688	15.57898	chr1:244{	RNA5SP67		Pseudoger	chr1:173921070-173
ENSG0000(	688	15.57898	chr1:244{	KLHL20		protein_c	chr1:173714941-173
ENSG0000(	688	15.57898	chr1:244{	AL022400.1		smallRNA	chr1:174348265-174
ENSG0000(	688	15.57898	chr1:244{	RNU6-307P		smallRNA	chr1:174996524-174
ENSG0000(	688	15.57898	chr1:244{	GAS5		lncRNA	chr1:173858559-173
ENSG0000(	686	15.53369	chr7:251(	POLR2J3		protein_c	chr7:102562133-102
ENSG0000(	686	15.53369	chr7:251(	ENSG00000279724		Pseudoger	chr7:102188599-102
ENSG0000(	686	15.53369	chr7:251(	MIR5480		smallRNA	chr7:102405742-102
ENSG0000(	686	15.53369	chr7:251(	ENSG00000261535		lncRNA	chr7:102153355-102
ENSG0000(	686	15.53369	chr7:251(	Y_RNA		smallRNA	chr7:103433461-103
ENSG0000(	686	15.53369	chr7:251(	RELN	NCGv7	protein_c	chr7:103471381-103
ENSG0000(	686	15.53369	chr7:251(	ENSG00000279482		TEC	chr7:103161947-103
ENSG0000(	686	15.53369	chr7:251(	RPL7AP39		Pseudoger	chr7:102755146-102
ENSG0000(	686	15.53369	chr7:251(	RN7SKP86		smallRNA	chr7:103484208-103
ENSG0000(	686	15.53369	chr7:251(	ENSG00000224415		Pseudoger	chr7:103141349-103
ENSG0000(	686	15.53369	chr7:251(	POLR2J		protein_c	chr7:102473128-102
ENSG0000(	686	15.53369	chr7:251(	AC073127.1		smallRNA	chr7:103014256-103
ENSG0000(	686	15.53369	chr7:251(	POLR2J2		protein_c	chr7:102665368-102
ENSG0000(	686	15.53369	chr7:251(	RPS29P16		Pseudoger	chr7:103348601-103
ENSG0000(	686	15.53369	chr7:251(	SLC26A5		protein_c	chr7:103352730-103
ENSG0000(	686	15.53369	chr7:251(	DNAJC2		protein_c	chr7:103312289-103

ENSG0000(	686	15.53369	chr7:251(	DPY19L2P2		Pseudoger	chr7:103175343-103
ENSG0000(	686	15.53369	chr7:251(	ARMC10		protein_c	chr7:103074881-103
ENSG0000(	686	15.53369	chr7:251(	NFE4		lncRNA	chr7:102973483-102
ENSG0000(	686	15.53369	chr7:251(	ENSG00000170409		Pseudoger	chr7:102327256-102
ENSG0000(	686	15.53369	chr7:251(	RASA4DP		Pseudoger	chr7:102681836-102
ENSG0000(	686	15.53369	chr7:251(	PRKRIP1		protein_c	chr7:102363872-102
ENSG0000(	686	15.53369	chr7:251(	PMPCB		protein_c	chr7:103297435-103
ENSG0000(	686	15.53369	chr7:251(	RASA4B		protein_c	chr7:102479976-102
ENSG0000(	686	15.53369	chr7:251(	RASA4	DriverDBv	protein_c	chr7:102579646-102
ENSG0000(	686	15.53369	chr7:251(	RPL23AP95		Pseudoger	chr7:103152007-103
ENSG0000(	686	15.53369	chr7:251(	SPDYE6		protein_c	chr7:102345746-102
ENSG0000(	686	15.53369	chr7:251(	LRRC17	NCGv7	protein_c	chr7:102913000-102
ENSG0000(	686	15.53369	chr7:251(	FAM185A		protein_c	chr7:102748971-102
ENSG0000(	686	15.53369	chr7:251(	UPK3BL1		protein_c	chr7:102637025-102
ENSG0000(	686	15.53369	chr7:251(	S100A11P1		Pseudoger	chr7:103262000-103
ENSG0000(	686	15.53369	chr7:251(	Y_RNA		smallRNA	chr7:102336869-102
ENSG0000(	686	15.53369	chr7:251(	ENSG00000280004		Pseudoger	chr7:102186819-102
ENSG0000(	686	15.53369	chr7:251(	ORC5	DriverDBv	protein_c	chr7:104126341-104
ENSG0000(	686	15.53369	chr7:251(	ENSG00000286830		lncRNA	chr7:102699228-102
ENSG0000(	686	15.53369	chr7:251(	Y_RNA		smallRNA	chr7:103434994-103
ENSG0000(	686	15.53369	chr7:251(	DPY19L2P2		lncRNA	chr7:103175133-103
ENSG0000(	686	15.53369	chr7:251(	ENSG00000278586		Pseudoger	chr7:102264706-102
ENSG0000(	686	15.53369	chr7:251(	MIR5090		smallRNA	chr7:102465742-102
ENSG0000(	686	15.53369	chr7:251(	PMS2P12		Pseudoger	chr7:102337316-102
ENSG0000(	686	15.53369	chr7:251(	AC005086.1		Pseudoger	chr7:102161120-102
ENSG0000(	686	15.53369	chr7:251(	SLC26A5-AS1		lncRNA	chr7:103445207-103
ENSG0000(	686	15.53369	chr7:251(	AC093668.1		smallRNA	chr7:102507203-102
ENSG0000(	686	15.53369	chr7:251(	CUX1	NCGv7;AC	protein_c	chr7:101815904-102
ENSG0000(	686	15.53369	chr7:251(	RN7SKP198		smallRNA	chr7:102857450-102
ENSG0000(	686	15.53369	chr7:251(	SNORA48		smallRNA	chr7:102194076-102
ENSG0000(	686	15.53369	chr7:251(	ENSG00000290830		lncRNA	chr7:102364162-102
ENSG0000(	686	15.53369	chr7:251(	AC093668.2		Pseudoger	chr7:102479732-102
ENSG0000(	686	15.53369	chr7:251(	ENSG00000239486		Pseudoger	chr7:102380465-102
ENSG0000(	686	15.53369	chr7:251(	ENSG00000239480		lncRNA	chr7:102426818-102
ENSG0000(	686	15.53369	chr7:251(	MIR4467		smallRNA	chr7:102471469-102
ENSG0000(	686	15.53369	chr7:251(	PSMC2		protein_c	chr7:103328570-103
ENSG0000(	686	15.53369	chr7:251(	POLR2J3		protein_c	chr7:102537918-102
ENSG0000(	686	15.53369	chr7:251(	ORAI2		protein_c	chr7:102433106-102
ENSG0000(	686	15.53369	chr7:251(	SPDYE2B		protein_c	chr7:102650319-102
ENSG0000(	686	15.53369	chr7:251(	RNU6-1136P		smallRNA	chr7:102834605-102
ENSG0000(	686	15.53369	chr7:251(	AC005088.1		smallRNA	chr7:102238321-102
ENSG0000(	686	15.53369	chr7:251(	MIR4285		smallRNA	chr7:102293103-102
ENSG0000(	686	15.53369	chr7:251(	ENSG00000280404		Pseudoger	chr7:102161120-102
ENSG0000(	686	15.53369	chr7:251(	ENSG00000267645		protein_c	chr7:102637049-102
ENSG0000(	686	15.53369	chr7:251(	AC005086.3		Pseudoger	chr7:102162296-102
ENSG0000(	686	15.53369	chr7:251(	ENSG00000289613		lncRNA	chr7:103315169-103
ENSG0000(	686	15.53369	chr7:251(	ALKBH4		protein_c	chr7:102456238-102
ENSG0000(	686	15.53369	chr7:251(	NAPEPLD		protein_c	chr7:103099776-103
ENSG0000(	686	15.53369	chr7:251(	SH2B2		protein_c	chr7:102285091-102
ENSG0000(	686	15.53369	chr7:251(	ENSG00000239969		Pseudoger	chr7:102375808-102
ENSG0000(	686	15.53369	chr7:251(	AC005086.4		Pseudoger	chr7:102188599-102

ENSG0000(	686	15.53369	chr7:2516	UPK3BL2		protein_c	chr7:102537919-102
ENSG0000(	686	15.53369	chr7:2516	LRWD1	NCGv7	protein_c	chr7:102464956-102
ENSG0000(	686	15.53369	chr7:2516	SPDYE2		protein_c	chr7:102551226-102
ENSG0000(	686	15.53369	chr7:2516	ENSG00000205236		protein_c	chr7:102582523-102
ENSG0000(	686	15.53369	chr7:2516	FBXL13		protein_c	chr7:102812838-103
ENSG0000(	686	15.53369	chr7:2516	ENSG00000279168		lncRNA	chr7:102579104-102
ENSG0000(	686	15.53369	chr7:2516	ENSG00000270249		protein_c	chr7:102541501-102
ENSG0000(	686	15.53369	chr7:2516	ENSG00000289956		lncRNA	chr7:103152899-103
ENSG0000(	686	15.53369	chr7:2516	AC105052.1		smallRNA	chr7:102606366-102
ENSG0000(	686	15.53369	chr7:2516	AC005086.2		Pseudoger	chr7:102186819-102
ENSG0000(	686	15.53369	chr7:2516	ENSG00000236226		lncRNA	chr7:103030104-103
ENSG0000(	686	15.53369	chr7:2516	CRYZP1		Pseudoger	chr7:103088664-103
ENSG0000(	686	15.53369	chr7:2516	AC093668.3		smallRNA	chr7:102480080-102
ENSG0000(	682	15.44312	chr1:3733	ENSG00000271732		lncRNA	chr1:16617391-1661
ENSG0000(	680	15.39783	chr1:3733	FAM131C2P		Pseudoger	chr1:16035178-1604
ENSG0000(	680	15.39783	chr1:3733	CLCNKB		protein_c	chr1:16040252-1605
ENSG0000(	677	15.3299	chr1:2449	ENSG00000225545		lncRNA	chr1:170587249-170
ENSG0000(	677	15.3299	chr1:2449	RN7SL425P		smallRNA	chr1:171492411-171
ENSG0000(	677	15.3299	chr1:2449	MYOC		protein_c	chr1:171635417-171
ENSG0000(	677	15.3299	chr1:2449	RN7SL269P		smallRNA	chr1:169957944-169
ENSG0000(	677	15.3299	chr1:2449	DNM3OS		lncRNA	chr1:172138397-172
ENSG0000(	677	15.3299	chr1:2449	ENSG00000226552		Pseudoger	chr1:171083565-171
ENSG0000(	677	15.3299	chr1:2449	GOT2P2		Pseudoger	chr1:173141100-173
ENSG0000(	677	15.3299	chr1:2449	CYCSP53		Pseudoger	chr1:171444699-171
ENSG0000(	677	15.3299	chr1:2449	ENSG00000271459		Pseudoger	chr1:171755803-171
ENSG0000(	677	15.3299	chr1:2449	SELE		protein_c	chr1:169722640-169
ENSG0000(	677	15.3299	chr1:2449	ENSG00000224228		lncRNA	chr1:172775905-173
ENSG0000(	677	15.3299	chr1:2449	ENSG00000224000		lncRNA	chr1:172906900-172
ENSG0000(	677	15.3299	chr1:2449	FMO3		protein_c	chr1:171090901-171
ENSG0000(	677	15.3299	chr1:2449	snoU13		smallRNA	chr1:173281077-173
ENSG0000(	677	15.3299	chr1:2449	METTL18		protein_c	chr1:169792529-169
ENSG0000(	677	15.3299	chr1:2449	ENSG00000231424		lncRNA	chr1:170748573-171
ENSG0000(	677	15.3299	chr1:2449	RNU6-290P		smallRNA	chr1:171418644-171
ENSG0000(	677	15.3299	chr1:2449	ENSG00000230704		lncRNA	chr1:169762929-169
ENSG0000(	677	15.3299	chr1:2449	MIR3119-2		smallRNA	chr1:170151378-170
ENSG0000(	677	15.3299	chr1:2449	C1orf112	DriverDBv	protein_c	chr1:169662007-169
ENSG0000(	677	15.3299	chr1:2449	ENSG00000271811		lncRNA	chr1:170667381-170
ENSG0000(	677	15.3299	chr1:2449	AL354732.1		smallRNA	chr1:170370213-170
ENSG0000(	677	15.3299	chr1:2449	Y_RNA		smallRNA	chr1:171814512-171
ENSG0000(	677	15.3299	chr1:2449	RNU6-773P		smallRNA	chr1:171519816-171
ENSG0000(	677	15.3299	chr1:2449	SLC9C2	NCGv7	protein_c	chr1:173500460-173
ENSG0000(	677	15.3299	chr1:2449	ENSG00000232751		Pseudoger	chr1:173351689-173
ENSG0000(	677	15.3299	chr1:2449	HMGB1P11		Pseudoger	chr1:171270954-171
ENSG0000(	677	15.3299	chr1:2449	ENSG00000237707		lncRNA	chr1:169104124-169
ENSG0000(	677	15.3299	chr1:2449	ENSG00000232261		Pseudoger	chr1:171751543-171
ENSG0000(	677	15.3299	chr1:2449	ENSG00000225243		lncRNA	chr1:171199244-171
ENSG0000(	677	15.3299	chr1:2449	ENSG00000234604		Pseudoger	chr1:169474060-169
ENSG0000(	677	15.3299	chr1:2449	RNA5SP66		Pseudoger	chr1:169067264-169
ENSG0000(	677	15.3299	chr1:2449	PRRC2C	DriverDBv	protein_c	chr1:171485530-171
ENSG0000(	677	15.3299	chr1:2449	MIR214		smallRNA	chr1:172138798-172
ENSG0000(	677	15.3299	chr1:2449	PFN1P1		Pseudoger	chr1:171670517-171

ENSG0000(	677	15.3299	chr1:2449	GORAB	DriverDBv	protein_c	chr1:170531819-170
ENSG0000(	677	15.3299	chr1:2449	TNFSF18		protein_c	chr1:173039202-173
ENSG0000(	677	15.3299	chr1:2449	MRPS10P1		Pseudoger	chr1:169990067-169
ENSG0000(	677	15.3299	chr1:2449	C1orf105		protein_c	chr1:172420685-172
ENSG0000(	677	15.3299	chr1:2449	SELL		protein_c	chr1:169690665-169
ENSG0000(	677	15.3299	chr1:2449	MIR1295A		smallRNA	chr1:171101728-171
ENSG0000(	677	15.3299	chr1:2449	MIR199A2		smallRNA	chr1:172144535-172
ENSG0000(	677	15.3299	chr1:2449	FMO6P		Pseudoger	chr1:171137740-171
ENSG0000(	677	15.3299	chr1:2449	ENSG00000235575		lncRNA	chr1:169310665-169
ENSG0000(	677	15.3299	chr1:2449	PRDX6-AS1		lncRNA	chr1:173417793-173
ENSG0000(	677	15.3299	chr1:2449	MYOCOS		protein_c	chr1:171600621-171
ENSG0000(	677	15.3299	chr1:2449	NTMT2		protein_c	chr1:170145959-170
ENSG0000(	677	15.3299	chr1:2449	VAMP4	NCGv7	protein_c	chr1:171700160-171
ENSG0000(	677	15.3299	chr1:2449	GM2AP2		Pseudoger	chr1:171392229-171
ENSG0000(	677	15.3299	chr1:2449	ENSG00000224600		Pseudoger	chr1:171824610-171
ENSG0000(	677	15.3299	chr1:2449	SCYL3		protein_c	chr1:169849631-169
ENSG0000(	677	15.3299	chr1:2449	ENSG00000231615		Pseudoger	chr1:173362397-173
ENSG0000(	677	15.3299	chr1:2449	RNU6-693P		smallRNA	chr1:172613428-172
ENSG0000(	677	15.3299	chr1:2449	DNM3-IT1		lncRNA	chr1:171864187-171
ENSG0000(	677	15.3299	chr1:2449	FMO2		protein_c	chr1:171185249-171
ENSG0000(	677	15.3299	chr1:2449	BLZF1	DriverDBv	protein_c	chr1:169367970-169
ENSG0000(	677	15.3299	chr1:2449	CCDC181		protein_c	chr1:169394870-169
ENSG0000(	677	15.3299	chr1:2449	SUCO		protein_c	chr1:172532349-172
ENSG0000(	677	15.3299	chr1:2449	FMO4		protein_c	chr1:171314183-171
ENSG0000(	677	15.3299	chr1:2449	ENSG00000238272		lncRNA	chr1:173555251-173
ENSG0000(	677	15.3299	chr1:2449	SCARNA20		smallRNA	chr1:171768070-171
ENSG0000(	677	15.3299	chr1:2449	ISCUP1		Pseudoger	chr1:170211010-170
ENSG0000(	677	15.3299	chr1:2449	RN7SL333P		smallRNA	chr1:169859756-169
ENSG0000(	677	15.3299	chr1:2449	ENSG00000235303		lncRNA	chr1:170598854-170
ENSG0000(	677	15.3299	chr1:2449	LINC01681		lncRNA	chr1:170173865-170
ENSG0000(	677	15.3299	chr1:2449	SNORD112		smallRNA	chr1:172348143-172
ENSG0000(	677	15.3299	chr1:2449	ENSG00000288139		Pseudoger	chr1:169915004-169
ENSG0000(	677	15.3299	chr1:2449	F5	NCGv7	protein_c	chr1:169511951-169
ENSG0000(	677	15.3299	chr1:2449	SLC19A2	DriverDBv	protein_c	chr1:169463909-169
ENSG0000(	677	15.3299	chr1:2449	DNM3		protein_c	chr1:171817887-172
ENSG0000(	677	15.3299	chr1:2449	GORAB-AS1		lncRNA	chr1:170460453-170
ENSG0000(	677	15.3299	chr1:2449	ATP1B1		protein_c	chr1:169105697-169
ENSG0000(	677	15.3299	chr1:2449	RPL4P3		Pseudoger	chr1:171683128-171
ENSG0000(	677	15.3299	chr1:2449	ENSG00000226375		lncRNA	chr1:173174300-173
ENSG0000(	677	15.3299	chr1:2449	HAUS4P1		Pseudoger	chr1:170369223-170
ENSG0000(	677	15.3299	chr1:2449	ENSG00000289466		TEC	chr1:169112164-169
ENSG0000(	677	15.3299	chr1:2449	ENSG00000287282		lncRNA	chr1:169059576-169
ENSG0000(	677	15.3299	chr1:2449	PRRX1	NCGv7	protein_c	chr1:170662728-170
ENSG0000(	677	15.3299	chr1:2449	NME7		protein_c	chr1:169132531-169
ENSG0000(	677	15.3299	chr1:2449	MROH9		protein_c	chr1:170935526-171
ENSG0000(	677	15.3299	chr1:2449	FASLG		protein_c	chr1:172659103-172
ENSG0000(	677	15.3299	chr1:2449	ENSG00000279061		TEC	chr1:172752586-172
ENSG0000(	677	15.3299	chr1:2449	ENSG00000236741		Pseudoger	chr1:171762074-171
ENSG0000(	677	15.3299	chr1:2449	RNU6-157P		smallRNA	chr1:172366540-172
ENSG0000(	677	15.3299	chr1:2449	Y_RNA		smallRNA	chr1:171253906-171
ENSG0000(	677	15.3299	chr1:2449	SLC25A38P1		Pseudoger	chr1:172748560-172

ENSG0000(	677	15.3299	chr1:2449	FMO1		protein_c	chr1:171248471-171
ENSG0000(	677	15.3299	chr1:2449	SIGLEC30P		Pseudoger	chr1:170115636-170
ENSG0000(	677	15.3299	chr1:2449	SELP	NCGv7	protein_c	chr1:169588849-169
ENSG0000(	677	15.3299	chr1:2449	LINC01142		lncRNA	chr1:170271395-170
ENSG0000(	677	15.3299	chr1:2449	ENSG00000213062		lncRNA	chr1:169486076-169
ENSG0000(	677	15.3299	chr1:2449	PRDX6		protein_c	chr1:173477330-173
ENSG0000(	677	15.3299	chr1:2449	snoU13		smallRNA	chr1:171481907-171
ENSG0000(	677	15.3299	chr1:2449	ENSG00000213060		Pseudoger	chr1:171803517-171
ENSG0000(	677	15.3299	chr1:2449	ENSG00000287336		lncRNA	chr1:172210711-172
ENSG0000(	677	15.3299	chr1:2449	KIFAP3		protein_c	chr1:169921326-170
ENSG0000(	677	15.3299	chr1:2449	ENSG00000232959		lncRNA	chr1:170024077-170
ENSG0000(	677	15.3299	chr1:2449	METTL13		protein_c	chr1:171781660-171
ENSG0000(	677	15.3299	chr1:2449	AIMP1P2		Pseudoger	chr1:172885947-172
ENSG0000(	677	15.3299	chr1:2449	TNFSF4		protein_c	chr1:173183731-173
ENSG0000(	677	15.3299	chr1:2449	PIGC	NCGv7	protein_c	chr1:172370189-172
ENSG0000(	677	15.3299	chr1:2449	BX284613.1		smallRNA	chr1:171041347-171
ENSG0000(	674	15.26197	chr1:2449	ENSG00000228687		Pseudoger	chr1:192796533-192
ENSG0000(	672	15.21668	chr2:8187	snoU13		smallRNA	chr2:88307360-8830
ENSG0000(	672	15.21668	chr2:8187	ENSG00000290104		lncRNA	chr2:88864876-8886
ENSG0000(	672	15.21668	chr2:8187	THNSL2		protein_c	chr2:88170295-8818
ENSG0000(	672	15.21668	chr2:8187	WBP1P2		Pseudoger	chr2:87972656-8797
ENSG0000(	672	15.21668	chr2:8187	RPL38P6		Pseudoger	chr2:88428078-8842
ENSG0000(	672	15.21668	chr2:8187	SMYD1		protein_c	chr2:88067825-8811
ENSG0000(	672	15.21668	chr2:8187	FOXI3		protein_c	chr2:88446787-8845
ENSG0000(	672	15.21668	chr8:2747	ENSG00000233170		Pseudoger	chr8:37747338-3774
ENSG0000(	672	15.21668	chr2:8187	PAFAH1B1P1		Pseudoger	chr2:87565828-8756
ENSG0000(	672	15.21668	chr2:8187	RNU6-1007P		smallRNA	chr2:88414898-8841
ENSG0000(	672	15.21668	chr2:8187	AC012671.3		smallRNA	chr2:88294592-8829
ENSG0000(	672	15.21668	chr2:8187	ANKRD36BP2		Pseudoger	chr2:88782712-8880
ENSG0000(	672	15.21668	chr2:8187	ENSG00000289429		lncRNA	chr2:87659372-8768
ENSG0000(	672	15.21668	chr2:8187	IGKC		protein_c	chr2:88857161-8885
ENSG0000(	672	15.21668	chr2:8187	AC096579.1		smallRNA	chr2:88767318-8876
ENSG0000(	672	15.21668	chr2:8187	IGKV5-2		protein_c	chr2:88897232-8889
ENSG0000(	672	15.21668	chr2:8187	ANAPC1P4		Pseudoger	chr2:87700984-8773
ENSG0000(	672	15.21668	chr2:8187	CYTOR		lncRNA	chr2:87454781-8763
ENSG0000(	672	15.21668	chr2:8187	ENSG00000284879		lncRNA	chr2:87455476-8776
ENSG0000(	672	15.21668	chr2:8187	RPIA	NCGv7	protein_c	chr2:88691673-8875
ENSG0000(	672	15.21668	chr2:8187	RPS14P5		Pseudoger	chr2:87654890-8765
ENSG0000(	672	15.21668	chr2:8187	MIR4780		smallRNA	chr2:88082519-8808
ENSG0000(	672	15.21668	chr2:8187	EIF2AK3-DT		lncRNA	chr2:88627539-8863
ENSG0000(	672	15.21668	chr2:8187	AC012671.1		smallRNA	chr2:88462767-8846
ENSG0000(	672	15.21668	chr2:8187	MTATP8P2		Pseudoger	chr2:87824942-8782
ENSG0000(	672	15.21668	chr2:8187	ENSG00000288437		Pseudoger	chr2:88003008-8800
ENSG0000(	672	15.21668	chr2:8187	MIR4436A		smallRNA	chr2:88812370-8881
ENSG0000(	672	15.21668	chr2:8187	KRCC1	NCGv7	protein_c	chr2:88027205-8806
ENSG0000(	672	15.21668	chr2:8187	TEX37		protein_c	chr2:88524649-8852
ENSG0000(	672	15.21668	chr2:8187	MIR4435-1		smallRNA	chr2:87629755-8762
ENSG0000(	672	15.21668	chr2:8187	EIF2AK3	NCGv7	protein_c	chr2:88556741-8869
ENSG0000(	672	15.21668	chr2:8187	PLGLB2		protein_c	chr2:87748087-8775
ENSG0000(	672	15.21668	chr2:8187	ENSG00000273445		lncRNA	chr2:87477495-8747
ENSG0000(	672	15.21668	chr2:8187	RGPD2		protein_c	chr2:87755960-8782

ENSG0000(	672	15.21668	chr2:8187	RNU6-568P		smallRNA	chr2:88367793-8836
ENSG0000(	672	15.21668	chr2:8187	FABP1		protein_c	chr2:88122982-8812
ENSG0000(	672	15.21668	chr2:8187	ENSG00000290802		lncRNA	chr2:88811186-8882
ENSG0000(	672	15.21668	chr2:8187	MALLP2		Pseudoger	chr2:88811633-8881
ENSG0000(	672	15.21668	chr2:8187	RNU2-63P		smallRNA	chr2:88016354-8801
ENSG0000(	672	15.21668	chr2:8187	ANAPC1P5		Pseudoger	chr2:87980714-8799
ENSG0000(	672	15.21668	chr2:8187	RNU6-1168P		smallRNA	chr2:88383494-8838
ENSG0000(	672	15.21668	chr2:8187	ENSG00000225420		lncRNA	chr2:88538720-8857
ENSG0000(	672	15.21668	chr2:8187	IGKJ4		protein_c	chr2:88860886-8886
ENSG0000(	672	15.21668	chr2:8187	ENSG00000240040		lncRNA	chr2:88811186-8886
ENSG0000(	672	15.21668	chr2:8187	ENSG00000287670		lncRNA	chr2:88016780-8802
ENSG0000(	672	15.21668	chr2:8187	snoU13		smallRNA	chr2:88011102-8801
ENSG0000(	672	15.21668	chr2:8187	ENSG00000288734		lncRNA	chr2:88136919-8813
ENSG0000(	672	15.21668	chr2:8187	NDUFB4P7		Pseudoger	chr2:87968609-8796
ENSG0000(	672	15.21668	chr2:8187	IGKV4-1		protein_c	chr2:88885397-8888
ENSG0000(	672	15.21668	chr2:8187	MRPL45P1		Pseudoger	chr2:88364695-8836
ENSG0000(	672	15.21668	chr2:8187	IGKJ3		protein_c	chr2:88861221-8886
ENSG0000(	672	15.21668	chr2:8187	ANKRD36BP2		lncRNA	chr2:88765807-8880
ENSG0000(	672	15.21668	chr2:8187	AC012671.4		smallRNA	chr2:88374139-8837
ENSG0000(	672	15.21668	chr2:8187	RNY4P15		smallRNA	chr2:88229569-8822
ENSG0000(	672	15.21668	chr2:8187	IGKJ1		protein_c	chr2:88861886-8886
ENSG0000(	672	15.21668	chr2:8187	IGKJ5		protein_c	chr2:88860568-8886
ENSG0000(	663	15.01288	chr4:1865	COX18		protein_c	chr4:73052362-7306
ENSG0000(	663	15.01288	chr4:1865	HMGA1P2		Pseudoger	chr4:73098822-7309
ENSG0000(	663	15.01288	chr4:1865	ANKRD17	NCGv7	protein_c	chr4:73073376-7325
ENSG0000(	663	15.01288	chr4:1865	SNORA3		smallRNA	chr4:73263960-7326
ENSG0000(	663	15.01288	chr4:1865	ANKRD17-DT		lncRNA	chr4:73259209-7331
ENSG0000(	663	15.01288	chr4:1865	ENSG00000250877		lncRNA	chr4:72323028-7233
ENSG0000(	663	15.01288	chr4:1865	HNRNPA1P67		Pseudoger	chr4:72807267-7280
ENSG0000(	663	15.01288	chr4:1865	RNU6ATAC5P		smallRNA	chr4:73026748-7302
ENSG0000(	663	15.01288	chr4:1865	ADAMTS3	NCGv7	protein_c	chr4:72280969-7256
ENSG0000(	663	15.01288	chr4:1865	RNU4ATAC9P		smallRNA	chr4:72965178-7296
ENSG0000(	653	14.78645	chr4:1865	ENSG00000249235		Pseudoger	chr4:68861332-6886
ENSG0000(	645	14.60529	chr1:3733	IFFO2		protein_c	chr1:18904280-1895
ENSG0000(	645	14.60529	chr1:3733	SDHB	NCGv7;AC	protein_c	chr1:17018722-1705
ENSG0000(	645	14.60529	chr1:3733	EPHA2-AS1		lncRNA	chr1:16155176-1615
ENSG0000(	645	14.60529	chr1:3733	PDE4DIPP9		Pseudoger	chr1:16855407-1685
ENSG0000(	645	14.60529	chr1:3733	SRARP		protein_c	chr1:16004236-1600
ENSG0000(	645	14.60529	chr1:3733	ANO7L1		Pseudoger	chr1:16216469-1622
ENSG0000(	645	14.60529	chr1:3733	AL121992.1		smallRNA	chr1:15684472-1568
ENSG0000(	645	14.60529	chr1:3733	ENSG00000233078		lncRNA	chr1:16006160-1600
ENSG0000(	645	14.60529	chr1:3733	ENSG00000237301		lncRNA	chr1:15586136-1560
ENSG0000(	645	14.60529	chr1:3733	DYNLL1P3		Pseudoger	chr1:18513118-1851
ENSG0000(	645	14.60529	chr1:3733	SZRD1		protein_c	chr1:16352575-1639
ENSG0000(	645	14.60529	chr1:3733	OTUD3		protein_c	chr1:19882395-1991
ENSG0000(	645	14.60529	chr1:3733	AL137127.1		smallRNA	chr1:19083552-1908
ENSG0000(	645	14.60529	chr1:3733	CROCCP2		Pseudoger	chr1:16618969-1665
ENSG0000(	645	14.60529	chr1:3733	MFAP2		protein_c	chr1:16974502-1698
ENSG0000(	645	14.60529	chr1:3733	MRTO4		protein_c	chr1:19251805-1926
ENSG0000(	645	14.60529	chr1:3733	ENSG00000261135		lncRNA	chr1:16514645-1651
ENSG0000(	645	14.60529	chr1:3733	PADI2	AC	protein_c	chr1:17066761-1711

ENSG0000(	645	14.60529	chr1:3733	RP13-279N23.2		protein_c	chr1:18849273-1892
ENSG0000(	645	14.60529	chr1:3733	MFFP1		Pseudoger	chr1:15191828-1519
ENSG0000(	645	14.60529	chr1:3733	ACTL8		protein_c	chr1:17755333-1782
ENSG0000(	645	14.60529	chr1:3733	CELA2B		protein_c	chr1:15465909-1549
ENSG0000(	645	14.60529	chr1:3733	ENSG00000280114		Pseudoger	chr1:16681097-1668
ENSG0000(	645	14.60529	chr1:3733	RSC1A1		protein_c	chr1:15659713-1566
ENSG0000(	645	14.60529	chr1:3733	NBPF1	NCGv7	protein_c	chr1:16562319-1661
ENSG0000(	645	14.60529	chr1:3733	RNU6-1099P		smallRNA	chr1:19305076-1930
ENSG0000(	645	14.60529	chr1:3733	SPEN	NCGv7	protein_c	chr1:15836095-1594
ENSG0000(	645	14.60529	chr1:3733	ENSG00000228549		lncRNA	chr1:16870945-1688
ENSG0000(	645	14.60529	chr1:3733	ENSG00000285853		lncRNA	chr1:16515034-1652
ENSG0000(	645	14.60529	chr1:3733	LINC01757		lncRNA	chr1:20243095-2024
ENSG0000(	645	14.60529	chr1:3733	PLA2G2D		protein_c	chr1:20111939-2011
ENSG0000(	645	14.60529	chr1:3733	IGSF21		protein_c	chr1:18107798-1837
ENSG0000(	645	14.60529	chr1:3733	CROCCP2		lncRNA	chr1:16618253-1664
ENSG0000(	645	14.60529	chr1:3733	SPATA21		protein_c	chr1:16387117-1643
ENSG0000(	645	14.60529	chr1:3733	ENSG00000287756		lncRNA	chr1:14774469-1477
ENSG0000(	645	14.60529	chr1:3733	AKR7A2		protein_c	chr1:19303965-1931
ENSG0000(	645	14.60529	chr1:3733	EFHD2-AS1		lncRNA	chr1:15402979-1540
ENSG0000(	645	14.60529	chr1:3733	MICOS10-DT		lncRNA	chr1:19591802-1959
ENSG0000(	645	14.60529	chr1:3733	ZBTB17		protein_c	chr1:15941869-1597
ENSG0000(	645	14.60529	chr1:3733	EMC1-AS1		lncRNA	chr1:19210348-1924
ENSG0000(	645	14.60529	chr1:3733	ENSG00000178715		Pseudoger	chr1:15828232-1582
ENSG0000(	645	14.60529	chr1:3733	ENSG00000272084		lncRNA	chr1:19072110-1907
ENSG0000(	645	14.60529	chr1:3733	PDE4DIPP8		Pseudoger	chr1:16548651-1655
ENSG0000(	645	14.60529	chr1:3733	SLC66A1		protein_c	chr1:19312326-1932
ENSG0000(	645	14.60529	chr1:3733	NBL1	NCGv7	protein_c	chr1:19596979-1965
ENSG0000(	645	14.60529	chr1:3733	HTR6		protein_c	chr1:19664875-1968
ENSG0000(	645	14.60529	chr1:3733	PLA2G2F		protein_c	chr1:20139323-2015
ENSG0000(	645	14.60529	chr1:3733	MIR3972		smallRNA	chr1:17277889-1727
ENSG0000(	645	14.60529	chr1:3733	ENSG00000223643		lncRNA	chr1:16851257-1685
ENSG0000(	645	14.60529	chr1:3733	TBCAP2		Pseudoger	chr1:14692129-1469
ENSG0000(	645	14.60529	chr1:3733	ENSG00000288636		protein_c	chr1:17005068-1701
ENSG0000(	645	14.60529	chr1:3733	ENSG00000290849		lncRNA	chr1:16687339-1669
ENSG0000(	645	14.60529	chr1:3733	PADI4		protein_c	chr1:17308195-1736
ENSG0000(	645	14.60529	chr1:3733	ENSG00000225478		Pseudoger	chr1:18595414-1859
ENSG0000(	645	14.60529	chr1:3733	ATP13A2		protein_c	chr1:16985958-1701
ENSG0000(	645	14.60529	chr1:3733	ALDH4A1		protein_c	chr1:18871430-1890
ENSG0000(	645	14.60529	chr1:3733	AL021920.1		smallRNA	chr1:16681255-1668
ENSG0000(	645	14.60529	chr1:3733	ENSG00000290850		lncRNA	chr1:16740280-1675
ENSG0000(	645	14.60529	chr1:3733	AKR7L		protein_c	chr1:19265982-1927
ENSG0000(	645	14.60529	chr1:3733	ENSG00000270620		Pseudoger	chr1:15917698-1591
ENSG0000(	645	14.60529	chr1:3733	ARHGEF19-AS1		lncRNA	chr1:16197854-1619
ENSG0000(	645	14.60529	chr1:3733	UQCRHL		protein_c	chr1:15807169-1580
ENSG0000(	645	14.60529	chr1:3733	CROCCP3		lncRNA	chr1:16467436-1649
ENSG0000(	645	14.60529	chr1:3733	MICOS10-NBL1		protein_c	chr1:19597067-1965
ENSG0000(	645	14.60529	chr1:3733	ENSG00000279151		lncRNA	chr1:16701546-1670
ENSG0000(	645	14.60529	chr1:3733	LINC02783		lncRNA	chr1:17189783-1719
ENSG0000(	645	14.60529	chr1:3733	ENSG00000236045		lncRNA	chr1:15334166-1533
ENSG0000(	645	14.60529	chr1:3733	SPEN-AS1		lncRNA	chr1:15834474-1584
ENSG0000(	645	14.60529	chr1:3733	C1orf134		protein_c	chr1:16228873-1622

ENSG0000(	645	14.60529	chr1:373:	NECAP2		protein_c	chr1:16440721-1646
ENSG0000(	645	14.60529	chr1:373:	TMEM51-AS2		lncRNA	chr1:15164344-1517
ENSG0000(	645	14.60529	chr1:373:	ENSG00000224621		lncRNA	chr1:16159266-1616
ENSG0000(	645	14.60529	chr1:373:	LINC01772		lncRNA	chr1:16460948-1646
ENSG0000(	645	14.60529	chr1:373:	AL355149.2		protein_c	chr1:16539066-1653
ENSG0000(	645	14.60529	chr1:373:	PADI6		protein_c	chr1:17372196-1740
ENSG0000(	645	14.60529	chr1:373:	RCC2		protein_c	chr1:17406760-1743
ENSG0000(	645	14.60529	chr1:373:	RCC2-AS1		lncRNA	chr1:17406760-1740
ENSG0000(	645	14.60529	chr1:373:	KLHDC7A		protein_c	chr1:18480930-1848
ENSG0000(	645	14.60529	chr1:373:	TAS1R2		protein_c	chr1:18839599-1885
ENSG0000(	645	14.60529	chr1:373:	ENSG00000231353		Pseudoger	chr1:15988182-1598
ENSG0000(	645	14.60529	chr1:373:	CLCNKA		protein_c	chr1:16018875-1603
ENSG0000(	645	14.60529	chr1:373:	RNF186		protein_c	chr1:19814029-1981
ENSG0000(	645	14.60529	chr1:373:	ENSG00000290851		lncRNA	chr1:16754910-1677
ENSG0000(	645	14.60529	chr1:373:	ENSG00000270728		Pseudoger	chr1:19297080-1929
ENSG0000(	645	14.60529	chr1:373:	ENSG00000225387		lncRNA	chr1:18385829-1838
ENSG0000(	645	14.60529	chr1:373:	ENSG00000284653		lncRNA	chr1:18015712-1804
ENSG0000(	645	14.60529	chr1:373:	RNU1-4		smallRNA	chr1:16740516-1674
ENSG0000(	645	14.60529	chr1:373:	MST1P2		Pseudoger	chr1:16645622-1665
ENSG0000(	645	14.60529	chr1:373:	FBXO42		protein_c	chr1:16246840-1635
ENSG0000(	645	14.60529	chr1:373:	SLC25A34		protein_c	chr1:15736258-1574
ENSG0000(	645	14.60529	chr1:373:	ENSG00000224174		lncRNA	chr1:16520694-1652
ENSG0000(	645	14.60529	chr1:373:	AKR7A3		protein_c	chr1:19282573-1928
ENSG0000(	645	14.60529	chr1:373:	PAX7	AC	protein_c	chr1:18630846-1874
ENSG0000(	645	14.60529	chr1:373:	TMCO4		protein_c	chr1:19682240-1979
ENSG0000(	645	14.60529	chr1:373:	UBXN10		protein_c	chr1:20186096-2019
ENSG0000(	645	14.60529	chr1:373:	AC004824.1		smallRNA	chr1:17413631-1741
ENSG0000(	645	14.60529	chr1:373:	RNF186-AS1		lncRNA	chr1:19814367-1981
ENSG0000(	645	14.60529	chr1:373:	DNAJC16		protein_c	chr1:15526813-1559
ENSG0000(	645	14.60529	chr1:373:	ENSG00000282843		lncRNA	chr1:17193232-1720
ENSG0000(	645	14.60529	chr1:373:	PLA2G2A		protein_c	chr1:19975431-1998
ENSG0000(	645	14.60529	chr1:373:	CHCHD2P6		Pseudoger	chr1:15604597-1560
ENSG0000(	645	14.60529	chr1:373:	ENSG00000272510		lncRNA	chr1:15565611-1556
ENSG0000(	645	14.60529	chr1:373:	ENSG00000282740		lncRNA	chr1:16739938-1675
ENSG0000(	645	14.60529	chr1:373:	RNU7-179P		smallRNA	chr1:15608078-1560
ENSG0000(	645	14.60529	chr1:373:	IGSF21-AS1		lncRNA	chr1:18166929-1817
ENSG0000(	645	14.60529	chr1:373:	TMEM82		protein_c	chr1:15742499-1574
ENSG0000(	645	14.60529	chr1:373:	FBLIM1		protein_c	chr1:15756607-1578
ENSG0000(	645	14.60529	chr1:373:	CTRC		protein_c	chr1:15438442-1544
ENSG0000(	645	14.60529	chr1:373:	RPL12P14		Pseudoger	chr1:15792796-1579
ENSG0000(	645	14.60529	chr1:373:	ENSG00000235241		Pseudoger	chr1:16889095-1688
ENSG0000(	645	14.60529	chr1:373:	Y_RNA		smallRNA	chr1:17158197-1715
ENSG0000(	645	14.60529	chr1:373:	MICOS10		protein_c	chr1:19484403-1962
ENSG0000(	645	14.60529	chr1:373:	RNU1-2		smallRNA	chr1:16895980-1689
ENSG0000(	645	14.60529	chr1:373:	HSPB7		protein_c	chr1:16014028-1601
ENSG0000(	645	14.60529	chr1:373:	ENSG00000234607		Pseudoger	chr1:15969632-1597
ENSG0000(	645	14.60529	chr1:373:	MIR3675		smallRNA	chr1:16858949-1685
ENSG0000(	645	14.60529	chr1:373:	snoU13		smallRNA	chr1:15910897-1591
ENSG0000(	645	14.60529	chr1:373:	PLA2G2C		protein_c	chr1:20161253-2018
ENSG0000(	645	14.60529	chr1:373:	DDI2		protein_c	chr1:15617458-1566
ENSG0000(	645	14.60529	chr1:373:	RNU1-1		smallRNA	chr1:16514122-1651

ENSG0000(	645	14.60529	chr1:373:	CPLANE2		protein_c	chr1:16231692-1623
ENSG0000(	645	14.60529	chr1:373:	RNU1-3		smallRNA	chr1:16666785-1666
ENSG0000(	645	14.60529	chr1:373:	CASP9	NCGv7	protein_c	chr1:15490832-1552
ENSG0000(	645	14.60529	chr1:373:	TBC1D3P6		Pseudoger	chr1:15989871-1599
ENSG0000(	645	14.60529	chr1:373:	ZBTB2P1		Pseudoger	chr1:15226373-1522
ENSG0000(	645	14.60529	chr1:373:	ENSG00000272426		lncRNA	chr1:16904339-1690
ENSG0000(	645	14.60529	chr1:373:	ENSG00000286898		lncRNA	chr1:16976302-1697
ENSG0000(	645	14.60529	chr1:373:	AL355149.1		smallRNA	chr1:16548914-1654
ENSG0000(	645	14.60529	chr1:373:	ENSG00000282143		lncRNA	chr1:16656879-1666
ENSG0000(	645	14.60529	chr1:373:	FAM131C		protein_c	chr1:16057769-1607
ENSG0000(	645	14.60529	chr1:373:	CELA2A		protein_c	chr1:15456728-1547
ENSG0000(	645	14.60529	chr1:373:	ENSG00000237938		lncRNA	chr1:15720312-1573
ENSG0000(	645	14.60529	chr1:373:	EIF1AXP1		Pseudoger	chr1:16685621-1668
ENSG0000(	645	14.60529	chr1:373:	ENSG00000275503		Pseudoger	chr1:15989140-1598
ENSG0000(	645	14.60529	chr1:373:	FHAD1	DriverDB\	protein_c	chr1:15236521-1540
ENSG0000(	645	14.60529	chr1:373:	PADI3		protein_c	chr1:17249098-1728
ENSG0000(	645	14.60529	chr1:373:	ARHGEF10I	NCGv7	protein_c	chr1:17539698-1769
ENSG0000(	645	14.60529	chr1:373:	LINC02810		lncRNA	chr1:17717625-1774
ENSG0000(	645	14.60529	chr1:373:	MIR4695		smallRNA	chr1:18883202-1888
ENSG0000(	645	14.60529	chr1:373:	ENSG00000227066		lncRNA	chr1:20154171-2016
ENSG0000(	645	14.60529	chr1:373:	EPHA2	NCGv7;AC	protein_c	chr1:16124337-1615
ENSG0000(	645	14.60529	chr1:373:	LINC01783		lncRNA	chr1:16533886-1653
ENSG0000(	645	14.60529	chr1:373:	ENSG00000283773		Pseudoger	chr1:16642767-1664
ENSG0000(	645	14.60529	chr1:373:	FHAD1-AS1		lncRNA	chr1:15326680-1534
ENSG0000(	645	14.60529	chr1:373:	CD24P1		Pseudoger	chr1:15614643-1561
ENSG0000(	645	14.60529	chr1:373:	MST1L		Pseudoger	chr1:16757232-1676
ENSG0000(	645	14.60529	chr1:373:	CAPZB		protein_c	chr1:19338775-1948
ENSG0000(	645	14.60529	chr1:373:	RN7SL304P		smallRNA	chr1:19970969-1997
ENSG0000(	645	14.60529	chr1:373:	ENSG00000280222		TEC	chr1:18109389-1811
ENSG0000(	645	14.60529	chr1:373:	PADI1		protein_c	chr1:17205128-1724
ENSG0000(	645	14.60529	chr1:373:	ARHGEF19		protein_c	chr1:16197854-1621
ENSG0000(	645	14.60529	chr1:373:	SCARNA21		smallRNA	chr1:15542165-1554
ENSG0000(	645	14.60529	chr1:373:	AC004824.2		protein_c	chr1:17329012-1734
ENSG0000(	645	14.60529	chr1:373:	PLEKHM2		protein_c	chr1:15684320-1573
ENSG0000(	645	14.60529	chr1:373:	ENSG00000286064		Pseudoger	chr1:19260521-1926
ENSG0000(	645	14.60529	chr1:373:	RNU4-28P		smallRNA	chr1:19510593-1951
ENSG0000(	645	14.60529	chr1:373:	CROCCP4		Pseudoger	chr1:16750233-1675
ENSG0000(	645	14.60529	chr1:373:	ENSG00000290122		lncRNA	chr1:16905199-1690
ENSG0000(	645	14.60529	chr1:373:	ENSG00000291077		lncRNA	chr1:16215907-1621
ENSG0000(	645	14.60529	chr1:373:	AGMAT		protein_c	chr1:15571699-1558
ENSG0000(	645	14.60529	chr1:373:	snoU13		smallRNA	chr1:19532170-1953
ENSG0000(	645	14.60529	chr1:373:	ENSG00000284710		lncRNA	chr1:20272018-2027
ENSG0000(	645	14.60529	chr1:373:	snoU13		smallRNA	chr1:17449763-1744
ENSG0000(	645	14.60529	chr1:373:	AL137798.1		protein_c	chr1:16673003-1667
ENSG0000(	645	14.60529	chr1:373:	ENSG00000290096		lncRNA	chr1:17439186-1743
ENSG0000(	645	14.60529	chr1:373:	SLC25A34-AS1		lncRNA	chr1:15740048-1574
ENSG0000(	645	14.60529	chr1:373:	UBR4		protein_c	chr1:19074510-1921
ENSG0000(	645	14.60529	chr1:373:	PLA2G5		protein_c	chr1:20028179-2009
ENSG0000(	645	14.60529	chr1:373:	EMC1		protein_c	chr1:19215660-1925
ENSG0000(	645	14.60529	chr1:373:	EFHD2	NCGv7	protein_c	chr1:15409888-1543
ENSG0000(	645	14.60529	chr1:373:	MIR1290		smallRNA	chr1:18897071-1889

ENSG0000(	645	14.60529	chr1:373:	AL021920.2		protein_c	chr1:16733952-1673
ENSG0000(	645	14.60529	chr1:373:	ENSG00000226526		lncRNA	chr1:16978926-1700
ENSG0000(	645	14.60529	chr1:373:	MT1XP1		Pseudoger	chr1:16241213-1624
ENSG0000(	645	14.60529	chr1:373:	ENSG00000238142		lncRNA	chr1:16887577-1688
ENSG0000(	645	14.60529	chr1:373:	TMEM51-AS1		lncRNA	chr1:15111815-1515
ENSG0000(	645	14.60529	chr1:373:	ENSG00000226396		Pseudoger	chr1:19608114-1960
ENSG0000(	645	14.60529	chr1:373:	RN7SL277P		smallRNA	chr1:19424384-1942
ENSG0000(	645	14.60529	chr1:373:	TMEM51		protein_c	chr1:15152532-1522
ENSG0000(	645	14.60529	chr1:373:	ENSG00000288398		lncRNA	chr1:16228674-1623
ENSG0000(	645	14.60529	chr1:373:	LINC01654		lncRNA	chr1:18065657-1807
ENSG0000(	645	14.60529	chr1:373:	PLA2G2E		protein_c	chr1:19920009-1992
ENSG0000(	645	14.60529	chr1:373:	CROCCP3		Pseudoger	chr1:16474396-1649
ENSG0000(	645	14.60529	chr1:373:	ENSG00000271742		lncRNA	chr1:15682873-1568
ENSG0000(	645	14.60529	chr1:373:	RPL22P3		Pseudoger	chr1:16369150-1636
ENSG0000(	645	14.60529	chr1:373:	RN7SL85P		smallRNA	chr1:19319805-1932
ENSG0000(	645	14.60529	chr1:373:	ESPNP		Pseudoger	chr1:16692280-1672
ENSG0000(	644	14.58265	chr8:2747	IDO2		protein_c	chr8:39934614-4001
ENSG0000(	644	14.58265	chr8:2747	ENSG00000253790		Pseudoger	chr8:39997869-3999
ENSG0000(	644	14.58265	chr8:2747	RNU6-596P		smallRNA	chr8:57289552-5728
ENSG0000(	642	14.53736	chr6:1979	RN7SL415P		smallRNA	chr6:91739609-9173
ENSG0000(	640	14.49207	chr8:2747	AC084262.3		smallRNA	chr8:29323264-2932
ENSG0000(	639	14.46943	chr8:2747	VCPIP1	NCGv7	protein_c	chr8:66628487-6666
ENSG0000(	639	14.46943	chr1:2449	GPR161		protein_c	chr1:168079542-168
ENSG0000(	639	14.46943	chr8:2747	C8orf34-AS1		lncRNA	chr8:68302246-6833
ENSG0000(	639	14.46943	chr8:2747	ENSG00000254031		lncRNA	chr8:71155454-7120
ENSG0000(	639	14.46943	chr8:2747	ARFGEF1-DT		lncRNA	chr8:67343975-6734
ENSG0000(	639	14.46943	chr8:2747	ENSG00000212989		Pseudoger	chr8:72429620-7243
ENSG0000(	639	14.46943	chr1:2449	ENSG00000241666		lncRNA	chr1:167627385-167
ENSG0000(	639	14.46943	chr8:2747	U8		smallRNA	chr8:71815494-7181
ENSG0000(	639	14.46943	chr8:2747	LINC01592		lncRNA	chr8:68880139-6910
ENSG0000(	639	14.46943	chr8:2747	ENSG00000288966		lncRNA	chr8:70405438-7040
ENSG0000(	639	14.46943	chr8:2747	ENSG00000250979		Pseudoger	chr8:72202751-7225
ENSG0000(	639	14.46943	chr8:2747	EYA1	NCGv7	protein_c	chr8:71197433-7159
ENSG0000(	639	14.46943	chr8:2747	TCF24	DriverDB\	protein_c	chr8:66946501-6696
ENSG0000(	639	14.46943	chr1:2449	CREG1	DriverDB\	protein_c	chr1:167529117-167
ENSG0000(	639	14.46943	chr8:2747	LINC03020		lncRNA	chr8:70471134-7048
ENSG0000(	639	14.46943	chr1:2449	RNU6-1310P		smallRNA	chr1:168263375-168
ENSG0000(	639	14.46943	chr1:2449	DCAF6		protein_c	chr1:167935783-168
ENSG0000(	639	14.46943	chr1:2449	RCSD1		protein_c	chr1:167630093-167
ENSG0000(	639	14.46943	chr8:2747	MYBL1	NCGv7	protein_c	chr8:66562175-6661
ENSG0000(	639	14.46943	chr8:2747	RNU6-1324P		smallRNA	chr8:66501205-6650
ENSG0000(	639	14.46943	chr1:2449	MPC2	DriverDB\	protein_c	chr1:167916675-167
ENSG0000(	639	14.46943	chr1:2449	ANKRD36BP1		Pseudoger	chr1:168245565-168
ENSG0000(	639	14.46943	chr8:2747	ARFGEF1	DriverDB\	protein_c	chr8:67173511-6734
ENSG0000(	639	14.46943	chr1:2449	ENSG00000287831		lncRNA	chr1:168898633-168
ENSG0000(	639	14.46943	chr8:2747	NCOA2	NCGv7;AC	protein_c	chr8:70109782-7040
ENSG0000(	639	14.46943	chr1:2449	TIPRL		protein_c	chr1:168178962-168
ENSG0000(	639	14.46943	chr8:2747	VXN	NCGv7	protein_c	chr8:66492326-6651
ENSG0000(	639	14.46943	chr8:2747	RNU1-101P		smallRNA	chr8:70077906-7007
ENSG0000(	639	14.46943	chr8:2747	SUMO2P20		Pseudoger	chr8:69987415-6998
ENSG0000(	639	14.46943	chr8:2747	BTF3P12		Pseudoger	chr8:70273369-7027

ENSG0000(	639	14.46943	chr8:2747	RRS1	NCGv7	protein_c	chr8:66429014-6643
ENSG0000(	639	14.46943	chr1:2449	ENSG00000232194		lncRNA	chr1:167820406-167
ENSG0000(	639	14.46943	chr8:2747	PREX2	NCGv7	protein_c	chr8:67952046-6823
ENSG0000(	639	14.46943	chr1:2449	ENSG00000283255		lncRNA	chr1:168401483-168
ENSG0000(	639	14.46943	chr8:2747	TRAM1	DriverDBv	protein_c	chr8:70573218-7060
ENSG0000(	639	14.46943	chr8:2747	PPP1R42		protein_c	chr8:66964099-6705
ENSG0000(	639	14.46943	chr8:2747	ENSG00000241961		Pseudoger	chr8:67406515-6740
ENSG0000(	639	14.46943	chr8:2747	RPS20P20		Pseudoger	chr8:71895991-7189
ENSG0000(	639	14.46943	chr1:2449	QRSL1P1		Pseudoger	chr1:168449672-168
ENSG0000(	639	14.46943	chr8:2747	C8orf44		lncRNA	chr8:66667615-6668
ENSG0000(	639	14.46943	chr8:2747	ENSG00000250869		Pseudoger	chr8:67133554-6713
ENSG0000(	639	14.46943	chr8:2747	RNA5SP269		Pseudoger	chr8:68695643-6869
ENSG0000(	639	14.46943	chr1:2449	LINC00970		lncRNA	chr1:168903905-169
ENSG0000(	639	14.46943	chr8:2747	MCMDC2	DriverDBv	protein_c	chr8:66870749-6692
ENSG0000(	639	14.46943	chr8:2747	Y_RNA		smallRNA	chr8:70357347-7035
ENSG0000(	639	14.46943	chr8:2747	AC109335.1		smallRNA	chr8:67058906-6705
ENSG0000(	639	14.46943	chr8:2747	TRPA1	NCGv7	protein_c	chr8:72019917-7207
ENSG0000(	639	14.46943	chr8:2747	ENSG00000253623		Pseudoger	chr8:66480648-6648
ENSG0000(	639	14.46943	chr5:4231	MIR378H		smallRNA	chr5:154829458-154
ENSG0000(	639	14.46943	chr1:2449	SFT2D2		protein_c	chr1:168225938-168
ENSG0000(	639	14.46943	chr1:2449	ENSG00000231029		Pseudoger	chr1:167591392-167
ENSG0000(	639	14.46943	chr8:2747	ENSG00000241204		Pseudoger	chr8:70424125-7042
ENSG0000(	639	14.46943	chr8:2747	C8orf44-S	NCGv7	protein_c	chr8:66667596-6686
ENSG0000(	639	14.46943	chr8:2747	ENSG00000271113		Pseudoger	chr8:69794062-6979
ENSG0000(	639	14.46943	chr8:2747	RN7SL675P		smallRNA	chr8:69922754-6992
ENSG0000(	639	14.46943	chr8:2747	PTTG3P		Pseudoger	chr8:66767400-6676
ENSG0000(	639	14.46943	chr8:2747	Y_RNA		smallRNA	chr8:66653469-6665
ENSG0000(	639	14.46943	chr8:2747	RNU7-102P		smallRNA	chr8:69110513-6911
ENSG0000(	639	14.46943	chr8:2747	AC104012.1		smallRNA	chr8:71832604-7183
ENSG0000(	639	14.46943	chr8:2747	snoU13		smallRNA	chr8:70604532-7060
ENSG0000(	639	14.46943	chr8:2747	ENSG00000241105		Pseudoger	chr8:70506251-7050
ENSG0000(	639	14.46943	chr1:2449	MIR557		smallRNA	chr1:168375524-168
ENSG0000(	639	14.46943	chr8:2747	MSC		protein_c	chr8:71841560-7184
ENSG0000(	639	14.46943	chr8:2747	SGK3	DriverDBv	protein_c	chr8:66712734-6686
ENSG0000(	639	14.46943	chr8:2747	CSPP1	DriverDBv	protein_c	chr8:67062417-6719
ENSG0000(	639	14.46943	chr1:2449	RPL29P7		Pseudoger	chr1:168938467-168
ENSG0000(	639	14.46943	chr8:2747	PDE7A-DT		lncRNA	chr8:65842138-6584
ENSG0000(	639	14.46943	chr1:2449	ENSG00000250762		Pseudoger	chr1:167819898-167
ENSG0000(	639	14.46943	chr8:2747	RPL31P40		Pseudoger	chr8:68293446-6829
ENSG0000(	639	14.46943	chr1:2449	ENSG00000237131		Pseudoger	chr1:168215405-168
ENSG0000(	639	14.46943	chr8:2747	SNHG6		lncRNA	chr8:66920561-6692
ENSG0000(	639	14.46943	chr8:2747	DNAJC5B		protein_c	chr8:66021553-6610
ENSG0000(	639	14.46943	chr8:2747	ENSG00000287127		lncRNA	chr8:66363774-6643
ENSG0000(	639	14.46943	chr1:2449	RPL7AP21		Pseudoger	chr1:168578653-168
ENSG0000(	639	14.46943	chr8:2747	ENSG00000285655		protein_c	chr8:66430185-6644
ENSG0000(	639	14.46943	chr8:2747	SLCO5A1-AS1		lncRNA	chr8:69834087-6985
ENSG0000(	639	14.46943	chr8:2747	CPA6		protein_c	chr8:67422038-6774
ENSG0000(	639	14.46943	chr1:2449	ENSG00000227722		Pseudoger	chr1:168317497-168
ENSG0000(	639	14.46943	chr8:2747	H2AZP2		Pseudoger	chr8:70103798-7010
ENSG0000(	639	14.46943	chr8:2747	C8orf34		protein_c	chr8:68330955-6881
ENSG0000(	639	14.46943	chr8:2747	LACTB2-AS1		lncRNA	chr8:70608577-7066

ENSG0000(	639	14.46943	chr1:2449	ENSG00000228697		lncRNA	chr1:168400829-168
ENSG0000(	639	14.46943	chr8:2747	RNA5SP271		Pseudoger	chr8:72357728-7235
ENSG0000(	639	14.46943	chr1:2449	ADCY10	DriverDBv	protein_c	chr1:167809386-167
ENSG0000(	639	14.46943	chr8:2747	ENSG00000287214		lncRNA	chr8:67313043-6731
ENSG0000(	639	14.46943	chr8:2747	CRH		protein_c	chr8:66176376-6617
ENSG0000(	639	14.46943	chr8:2747	ENSG00000254277		lncRNA	chr8:71675300-7175
ENSG0000(	639	14.46943	chr8:2747	TRIM55		protein_c	chr8:66126896-6617
ENSG0000(	639	14.46943	chr1:2449	MIR1255B2		smallRNA	chr1:167998660-167
ENSG0000(	639	14.46943	chr8:2747	ADHFE1		protein_c	chr8:66432492-6646
ENSG0000(	639	14.46943	chr1:2449	AL049798.1		smallRNA	chr1:168747796-168
ENSG0000(	639	14.46943	chr8:2747	LACTB2	DriverDBv	protein_c	chr8:70635318-7066
ENSG0000(	639	14.46943	chr8:2747	ENSG00000253143		lncRNA	chr8:70471112-7048
ENSG0000(	639	14.46943	chr8:2747	PRDM14	AC	protein_c	chr8:70051651-7007
ENSG0000(	639	14.46943	chr8:2747	AC099805.1		smallRNA	chr8:71449695-7144
ENSG0000(	639	14.46943	chr1:2449	ENSG00000235736		lncRNA	chr1:168763365-168
ENSG0000(	639	14.46943	chr1:2449	LINC00626		lncRNA	chr1:168784012-168
ENSG0000(	639	14.46943	chr8:2747	Y_RNA		smallRNA	chr8:68223318-6822
ENSG0000(	639	14.46943	chr8:2747	ENSG00000254337		lncRNA	chr8:68848737-6885
ENSG0000(	639	14.46943	chr8:2747	ENSG00000254332		Pseudoger	chr8:69129598-6913
ENSG0000(	639	14.46943	chr8:2747	ENSG00000253190		lncRNA	chr8:66112667-6612
ENSG0000(	639	14.46943	chr8:2747	SLCO5A1	DriverDBv	protein_c	chr8:69667046-6983
ENSG0000(	639	14.46943	chr8:2747	ENSG00000255206		lncRNA	chr8:68082204-6809
ENSG0000(	639	14.46943	chr8:2747	RN7SL19P		smallRNA	chr8:70654580-7065
ENSG0000(	639	14.46943	chr8:2747	SULF1	NCGv7	protein_c	chr8:69466624-6966
ENSG0000(	639	14.46943	chr8:2747	ENSG00000280393		TEC	chr8:69938168-6993
ENSG0000(	639	14.46943	chr8:2747	RNA5SP268		Pseudoger	chr8:67099302-6709
ENSG0000(	639	14.46943	chr8:2747	ENSG00000253216		Pseudoger	chr8:69446057-6944
ENSG0000(	639	14.46943	chr1:2449	RPL34P1		Pseudoger	chr1:168210616-168
ENSG0000(	639	14.46943	chr1:2449	ENSG00000285622		lncRNA	chr1:168695874-169
ENSG0000(	639	14.46943	chr8:2747	ENSG00000253223		Pseudoger	chr8:66984135-6698
ENSG0000(	639	14.46943	chr8:2747	SDCBPP2		Pseudoger	chr8:69942948-6994
ENSG0000(	639	14.46943	chr8:2747	COPS5	DriverDBv	protein_c	chr8:67043079-6708
ENSG0000(	639	14.46943	chr8:2747	ENSG00000255130		lncRNA	chr8:67732587-6773
ENSG0000(	639	14.46943	chr8:2747	ENSG00000285579		lncRNA	chr8:70669369-7122
ENSG0000(	639	14.46943	chr8:2747	ENSG00000255107		Pseudoger	chr8:69859264-6986
ENSG0000(	639	14.46943	chr1:2449	RPL7AP19		Pseudoger	chr1:168542737-168
ENSG0000(	639	14.46943	chr8:2747	MSC-AS1		lncRNA	chr8:71828167-7211
ENSG0000(	639	14.46943	chr1:2449	AKR1D1P1		Pseudoger	chr1:167519536-167
ENSG0000(	639	14.46943	chr8:2747	RNA5SP270		Pseudoger	chr8:69929905-6993
ENSG0000(	639	14.46943	chr1:2449	GCSHP5		Pseudoger	chr1:168055901-168
ENSG0000(	639	14.46943	chr1:2449	XCL2	NCGv7	protein_c	chr1:168540768-168
ENSG0000(	639	14.46943	chr1:2449	SUMO1P2		Pseudoger	chr1:168898136-168
ENSG0000(	639	14.46943	chr8:2747	RPS15AP25		Pseudoger	chr8:68525517-6852
ENSG0000(	639	14.46943	chr8:2747	AC022861.1		smallRNA	chr8:67711632-6771
ENSG0000(	639	14.46943	chr1:2449	TBX19		protein_c	chr1:168280877-168
ENSG0000(	639	14.46943	chr8:2747	RN7SKP29		smallRNA	chr8:69700224-6970
ENSG0000(	639	14.46943	chr8:2747	ENSG00000285791		protein_c	chr8:66432486-6651
ENSG0000(	639	14.46943	chr8:2747	RRS1-DT		lncRNA	chr8:66419589-6642
ENSG0000(	639	14.46943	chr1:2449	MPZL1		protein_c	chr1:167721192-167
ENSG0000(	639	14.46943	chr8:2747	XKR9	DriverDBv	protein_c	chr8:70669339-7079
ENSG0000(	639	14.46943	chr8:2747	LINC00967		lncRNA	chr8:66192093-6619

ENSG0000(	639	14.46943	chr1:2449	XCL1	DriverDB\	protein_c	chr1:168576605-168
ENSG0000(	639	14.46943	chr8:2747	LINC01603		lncRNA	chr8:69424849-6944
ENSG0000(	639	14.46943	chr8:2747	ENSG00000253287		lncRNA	chr8:71779605-7179
ENSG0000(	639	14.46943	chr8:2747	NACAP10		Pseudoger	chr8:67569416-6757
ENSG0000(	639	14.46943	chr8:2747	Y_RNA		smallRNA	chr8:70780914-7078
ENSG0000(	639	14.46943	chr8:2747	ENSG00000253373		lncRNA	chr8:69175579-6917
ENSG0000(	639	14.46943	chr8:2747	ENSG00000287230		lncRNA	chr8:70525053-7053
ENSG0000(	639	14.46943	chr8:2747	TRAPPC2P2		Pseudoger	chr8:71448811-7144
ENSG0000(	639	14.46943	chr8:2747	ENSG00000254557		lncRNA	chr8:69713390-6971
ENSG0000(	639	14.46943	chr8:2747	ENSG00000253317		lncRNA	chr8:72196334-7220
ENSG0000(	639	14.46943	chr1:2449	DPT		protein_c	chr1:168695468-168
ENSG0000(	639	14.46943	chr8:2747	NDUFS5P6		Pseudoger	chr8:67849044-6784
ENSG0000(	637	14.42414	chr5:4231	LARS1		protein_c	chr5:146110566-146
ENSG0000(	637	14.42414	chr5:4231	ENSG00000248493		Pseudoger	chr5:145997218-145
ENSG0000(	637	14.42414	chr5:4231	ENSG00000213488		Pseudoger	chr5:144522140-144
ENSG0000(	637	14.42414	chr5:4231	ENSG00000288095		lncRNA	chr5:141320910-141
ENSG0000(	637	14.42414	chr5:4231	ENSG00000248125		lncRNA	chr5:145429811-145
ENSG0000(	637	14.42414	chr5:4231	ENSG00000273557		lncRNA	chr5:141468465-141
ENSG0000(	637	14.42414	chr5:4231	ENSG00000279855		lncRNA	chr5:141427295-141
ENSG0000(	637	14.42414	chr5:4231	ENSG00000287527		lncRNA	chr5:141822076-141
ENSG0000(	637	14.42414	chr5:4231	PCDHGA5		protein_c	chr5:141364162-141
ENSG0000(	637	14.42414	chr5:4231	PRELID2		protein_c	chr5:145471799-145
ENSG0000(	637	14.42414	chr5:4231	RNA5SP196		Pseudoger	chr5:146586099-146
ENSG0000(	637	14.42414	chr5:4231	PCDHGA11		protein_c	chr5:141421047-141
ENSG0000(	637	14.42414	chr5:4231	PCDHGB6		protein_c	chr5:141408021-141
ENSG0000(	637	14.42414	chr5:4231	MIR5197		smallRNA	chr5:143679860-143
ENSG0000(	637	14.42414	chr5:4231	ENSG00000275740		protein_c	chr5:146203550-146
ENSG0000(	637	14.42414	chr5:4231	ENSG00000289478		lncRNA	chr5:141682325-141
ENSG0000(	637	14.42414	chr5:4231	STK32A-AS1		lncRNA	chr5:147180204-147
ENSG0000(	637	14.42414	chr5:4231	ENSG00000250347		Pseudoger	chr5:142036707-142
ENSG0000(	637	14.42414	chr5:4231	RN7SL87P		smallRNA	chr5:144140879-144
ENSG0000(	637	14.42414	chr5:4231	ENSG00000285605		lncRNA	chr5:144439204-144
ENSG0000(	637	14.42414	chr5:4231	RPL35AP17		Pseudoger	chr5:146138771-146
ENSG0000(	637	14.42414	chr5:4231	PCDHGA10		protein_c	chr5:141412987-141
ENSG0000(	637	14.42414	chr5:4231	PCDHGA6		protein_c	chr5:141373891-141
ENSG0000(	637	14.42414	chr5:4231	ENSG00000251556		lncRNA	chr5:146099406-146
ENSG0000(	637	14.42414	chr5:4231	PCDHGA3		protein_c	chr5:141343829-141
ENSG0000(	637	14.42414	chr5:4231	ENSG00000289306		lncRNA	chr5:142149435-142
ENSG0000(	637	14.42414	chr5:4231	PCDHGB2		protein_c	chr5:141359994-141
ENSG0000(	637	14.42414	chr5:4231	RPL7P21		Pseudoger	chr5:143332605-143
ENSG0000(	637	14.42414	chr5:4231	KCTD16	DriverDB\	protein_c	chr5:144170873-144
ENSG0000(	637	14.42414	chr5:4231	RN7SL68P		smallRNA	chr5:141479316-141
ENSG0000(	637	14.42414	chr5:4231	NDFIP1		protein_c	chr5:142108779-142
ENSG0000(	637	14.42414	chr5:4231	PCDHGC4		protein_c	chr5:141484997-141
ENSG0000(	637	14.42414	chr5:4231	FGF1		protein_c	chr5:142592178-142
ENSG0000(	637	14.42414	chr5:4231	PCDH12		protein_c	chr5:141943581-141
ENSG0000(	637	14.42414	chr5:4231	GNPDA1		protein_c	chr5:141991749-142
ENSG0000(	637	14.42414	chr5:4231	FCHSD1		protein_c	chr5:141639302-141
ENSG0000(	637	14.42414	chr5:4231	CKS1BP5		Pseudoger	chr5:144439206-144
ENSG0000(	637	14.42414	chr5:4231	RNF14		protein_c	chr5:141958328-141
ENSG0000(	637	14.42414	chr5:4231	ENSG00000251205		lncRNA	chr5:143531331-143

ENSG0000(	637	14.42414	chr5:4231	RNU7-180P		smallRNA	chr5:146245191-146
ENSG0000(	637	14.42414	chr5:4231	PCDHGB8P		Pseudoger	chr5:141426286-141
ENSG0000(	637	14.42414	chr5:4231	PCDHGA9		protein_c	chr5:141402778-141
ENSG0000(	637	14.42414	chr5:4231	ENSG00000228737		lncRNA	chr5:141618414-141
ENSG0000(	637	14.42414	chr5:4231	ENSG00000285366		TEC	chr5:142946569-142
ENSG0000(	637	14.42414	chr5:4231	DIAPH1	NCGv7	protein_c	chr5:141515016-141
ENSG0000(	637	14.42414	chr5:4231	PCDHGB9P		Pseudoger	chr5:141439853-141
ENSG0000(	637	14.42414	chr5:4231	ENSG00000251031		lncRNA	chr5:144856890-145
ENSG0000(	637	14.42414	chr5:4231	PCDHGB5		protein_c	chr5:141397947-141
ENSG0000(	637	14.42414	chr5:4231	SPRY4-AS1		lncRNA	chr5:142325209-142
ENSG0000(	637	14.42414	chr5:4231	PCDHGB3	DriverDB\	protein_c	chr5:141370242-141
ENSG0000(	637	14.42414	chr5:4231	ENSG00000280026		lncRNA	chr5:141350109-141
ENSG0000(	637	14.42414	chr5:4231	AC132803.1		smallRNA	chr5:145197775-145
ENSG0000(	637	14.42414	chr5:4231	ENSG00000254099		lncRNA	chr5:141970637-141
ENSG0000(	637	14.42414	chr5:4231	PLAC8L1		protein_c	chr5:146084313-146
ENSG0000(	637	14.42414	chr5:4231	GPR151		protein_c	chr5:146513144-146
ENSG0000(	637	14.42414	chr5:4231	PCDHGB7		protein_c	chr5:141417645-141
ENSG0000(	637	14.42414	chr5:4231	ENSG00000248842		lncRNA	chr5:146400981-146
ENSG0000(	637	14.42414	chr5:4231	LINC01844		lncRNA	chr5:142716229-142
ENSG0000(	637	14.42414	chr5:4231	RELL2		protein_c	chr5:141636950-141
ENSG0000(	637	14.42414	chr5:4231	ENSG00000250842		lncRNA	chr5:145337932-145
ENSG0000(	637	14.42414	chr5:4231	HDAC3		protein_c	chr5:141620876-141
ENSG0000(	637	14.42414	chr5:4231	DPYSL3		protein_c	chr5:147390808-147
ENSG0000(	637	14.42414	chr5:4231	SPRY4		protein_c	chr5:142310427-142
ENSG0000(	637	14.42414	chr5:4231	TCERG1	NCGv7	protein_c	chr5:146447311-146
ENSG0000(	637	14.42414	chr5:4231	PCDHGA4		protein_c	chr5:141355021-141
ENSG0000(	637	14.42414	chr5:4231	ENSG00000261757		lncRNA	chr5:142703782-142
ENSG0000(	637	14.42414	chr5:4231	NR3C1	NCGv7	protein_c	chr5:143277931-143
ENSG0000(	637	14.42414	chr5:4231	ENSG00000287203		lncRNA	chr5:142732704-142
ENSG0000(	637	14.42414	chr5:4231	ENSG00000250407		lncRNA	chr5:146563226-146
ENSG0000(	637	14.42414	chr5:4231	ENSG00000226040		Pseudoger	chr5:141896168-141
ENSG0000(	637	14.42414	chr5:4231	ENSG00000249881		lncRNA	chr5:143605628-143
ENSG0000(	637	14.42414	chr5:4231	PCDHGA12		protein_c	chr5:141430507-141
ENSG0000(	637	14.42414	chr5:4231	ENSG00000271871		lncRNA	chr5:141952419-141
ENSG0000(	637	14.42414	chr5:4231	ARHGAP26	NCGv7	protein_c	chr5:142770377-143
ENSG0000(	637	14.42414	chr5:4231	ARHGAP26-AS1		lncRNA	chr5:142859604-142
ENSG0000(	637	14.42414	chr5:4231	YIPF5		protein_c	chr5:144158162-144
ENSG0000(	637	14.42414	chr5:4231	ENSG00000279130		TEC	chr5:143406959-143
ENSG0000(	637	14.42414	chr5:4231	RN7SKP246		smallRNA	chr5:144535354-144
ENSG0000(	637	14.42414	chr5:4231	AC005215.1		smallRNA	chr5:142534255-142
ENSG0000(	637	14.42414	chr5:4231	DIAPH1-AS1		lncRNA	chr5:141558311-141
ENSG0000(	637	14.42414	chr5:4231	ENSG00000225869		Pseudoger	chr5:143752090-143
ENSG0000(	637	14.42414	chr5:4231	ENSG00000250025		lncRNA	chr5:146307098-146
ENSG0000(	637	14.42414	chr5:4231	PCDHGC3		protein_c	chr5:141475947-141
ENSG0000(	637	14.42414	chr5:4231	TAF7		protein_c	chr5:141259884-141
ENSG0000(	637	14.42414	chr5:4231	ENSG00000272070		lncRNA	chr5:141326210-141
ENSG0000(	637	14.42414	chr5:4231	HMHB1		protein_c	chr5:143812161-143
ENSG0000(	637	14.42414	chr5:4231	RNA5SP196		smallRNA	chr5:146586099-146
ENSG0000(	637	14.42414	chr5:4231	PCDHGA7		protein_c	chr5:141382739-141
ENSG0000(	637	14.42414	chr5:4231	GRXCR2		protein_c	chr5:145858521-145
ENSG0000(	637	14.42414	chr5:4231	PCDHGA1		protein_c	chr5:141330514-141

ENSG0000(	637	14.42414	chr5:4231	STK32A	DriverDB\	protein_c	chr5:147234963-147
ENSG0000(	637	14.42414	chr5:4231	PPP2R2B	DriverDB\	protein_c	chr5:146580742-147
ENSG0000(	637	14.42414	chr5:4231	ENSG00000278925		lncRNA	chr5:141825708-141
ENSG0000(	637	14.42414	chr5:4231	SH3RF2		protein_c	chr5:145936578-146
ENSG0000(	637	14.42414	chr5:4231	RN7SL791P		smallRNA	chr5:146656403-146
ENSG0000(	637	14.42414	chr5:4231	PPP2R2B-IT1		lncRNA	chr5:146914207-146
ENSG0000(	637	14.42414	chr5:4231	ENSG00000249429		lncRNA	chr5:143489855-143
ENSG0000(	637	14.42414	chr5:4231	ENSG00000249605		lncRNA	chr5:144369334-144
ENSG0000(	637	14.42414	chr5:4231	RPS27AP10		Pseudoger	chr5:141588935-141
ENSG0000(	637	14.42414	chr5:4231	AC005618.1		Pseudoger	chr5:141320094-141
ENSG0000(	637	14.42414	chr5:4231	RNU7-156P		smallRNA	chr5:143542514-143
ENSG0000(	637	14.42414	chr5:4231	PCDHGB1	NCGv7	protein_c	chr5:141350099-141
ENSG0000(	637	14.42414	chr5:4231	ENSG00000287726		lncRNA	chr5:141882815-141
ENSG0000(	637	14.42414	chr5:4231	TRPC6P2		Pseudoger	chr5:145728360-145
ENSG0000(	637	14.42414	chr5:4231	ENSG00000271724		lncRNA	chr5:146375591-146
ENSG0000(	637	14.42414	chr5:4231	PCDHGC5	NCGv7	protein_c	chr5:141489081-141
ENSG0000(	637	14.42414	chr5:4231	AC005370.1		smallRNA	chr5:142650000-142
ENSG0000(	637	14.42414	chr5:4231	DELE1		protein_c	chr5:141923855-141
ENSG0000(	637	14.42414	chr5:4231	PCDHGA2	DriverDB\	protein_c	chr5:141338760-141
ENSG0000(	637	14.42414	chr5:4231	ENSG00000279679		TEC	chr5:143375628-143
ENSG0000(	637	14.42414	chr5:4231	PCDHGB4		protein_c	chr5:141387698-141
ENSG0000(	637	14.42414	chr5:4231	ENSG00000286468		lncRNA	chr5:147093259-147
ENSG0000(	637	14.42414	chr5:4231	PCDH1	DriverDB\	protein_c	chr5:141853090-141
ENSG0000(	637	14.42414	chr5:4231	AC011357.1		smallRNA	chr5:146734835-146
ENSG0000(	637	14.42414	chr5:4231	ENSG00000280047		TEC	chr5:142165767-142
ENSG0000(	637	14.42414	chr5:4231	NAMPTP2		Pseudoger	chr5:145001853-145
ENSG0000(	637	14.42414	chr5:4231	PCDHGA8	NCGv7	protein_c	chr5:141390157-141
ENSG0000(	637	14.42414	chr5:4231	POU4F3		protein_c	chr5:146338839-146
ENSG0000(	637	14.42414	chr5:4231	ENSG00000286736		lncRNA	chr5:141718711-141
ENSG0000(	637	14.42414	chr5:4231	SLC25A2		protein_c	chr5:141302635-141
ENSG0000(	637	14.42414	chr5:4231	ENSG00000272239		lncRNA	chr5:147401760-147
ENSG0000(	637	14.42414	chr5:4231	RPL35AP16		Pseudoger	chr5:146137755-146
ENSG0000(	637	14.42414	chr5:4231	RPS12P10		Pseudoger	chr5:142566700-142
ENSG0000(	637	14.42414	chr5:4231	KRT8P48		Pseudoger	chr5:146706381-146
ENSG0000(	637	14.42414	chr5:4231	ASS1P10		Pseudoger	chr5:145228811-145
ENSG0000(	637	14.42414	chr5:4231	RBM27	NCGv7	protein_c	chr5:146203605-146
ENSG0000(	637	14.42414	chr5:4231	ARAP3		protein_c	chr5:141653401-141
ENSG0000(	637	14.42414	chr5:4231	ARHGAP26-IT1		lncRNA	chr5:143192500-143
ENSG0000(	635	14.37886	chr2:8187	ENSG00000290110		lncRNA	chr2:85328084-8532
ENSG0000(	635	14.37886	chr2:8187	CD8B		protein_c	chr2:86815339-8686
ENSG0000(	635	14.37886	chr2:8187	GPR160P1		Pseudoger	chr2:85686053-8568
ENSG0000(	635	14.37886	chr2:8187	Y_RNA		smallRNA	chr2:86159956-8616
ENSG0000(	635	14.37886	chr2:8187	ANAPC1P1		Pseudoger	chr2:86871301-8691
ENSG0000(	635	14.37886	chr2:8187	CENPNP1		Pseudoger	chr2:87221113-8722
ENSG0000(	635	14.37886	chr2:8187	ANAPC1P2		Pseudoger	chr2:87031815-8705
ENSG0000(	635	14.37886	chr2:8187	ENSG00000271014		Pseudoger	chr2:85360798-8536
ENSG0000(	635	14.37886	chr2:8187	AC015971.1		smallRNA	chr2:86586140-8658
ENSG0000(	635	14.37886	chr2:8187	ENSG00000276362		Pseudoger	chr2:84850711-8485
ENSG0000(	635	14.37886	chr2:8187	VAMP8		protein_c	chr2:85561562-8558
ENSG0000(	635	14.37886	chr2:8187	SNORA19		smallRNA	chr2:86364136-8636
ENSG0000(	635	14.37886	chr2:8187	ENSG00000230975		lncRNA	chr2:80699388-8087

ENSG0000(	635	14.37886	chr2:8187	MTND5P27		Pseudoger	chr2:82815809-8281
ENSG0000(	635	14.37886	chr2:8187	MRPL35		protein_c	chr2:86199355-8621
ENSG0000(	635	14.37886	chr2:8187	AC068279.2		Pseudoger	chr2:87359054-8735
ENSG0000(	635	14.37886	chr2:8187	LINC01943		lncRNA	chr2:87439523-8745
ENSG0000(	635	14.37886	chr2:8187	FUNDC2P2		Pseudoger	chr2:84290683-8429
ENSG0000(	635	14.37886	chr2:8187	RNU1-38P		smallRNA	chr2:85728194-8572
ENSG0000(	635	14.37886	chr2:8187	MTND4P25		Pseudoger	chr2:82814984-8281
ENSG0000(	635	14.37886	chr2:8187	CHMP4AP1		Pseudoger	chr2:81418723-8141
ENSG0000(	635	14.37886	chr2:8187	ENSG00000224731		lncRNA	chr2:80028012-8003
ENSG0000(	635	14.37886	chr2:8187	RN7SL830P		smallRNA	chr2:85532344-8553
ENSG0000(	635	14.37886	chr2:8187	RNF103	NCGv7	protein_c	chr2:86603398-8662
ENSG0000(	635	14.37886	chr2:8187	RETSAT		protein_c	chr2:85341955-8535
ENSG0000(	635	14.37886	chr2:8187	RBM7P1		Pseudoger	chr2:80162428-8016
ENSG0000(	635	14.37886	chr2:8187	IMMT		protein_c	chr2:86143932-8619
ENSG0000(	635	14.37886	chr2:8187	RP11-685N3.1		protein_c	chr2:87338477-8733
ENSG0000(	635	14.37886	chr2:8187	ST6GALNAC2P1		Pseudoger	chr2:84039885-8404
ENSG0000(	635	14.37886	chr2:8187	PTCD3		protein_c	chr2:86106223-8614
ENSG0000(	635	14.37886	chr2:8187	ENSG00000229498		lncRNA	chr2:85815130-8582
ENSG0000(	635	14.37886	chr2:8187	LYARP1		Pseudoger	chr2:82268427-8226
ENSG0000(	635	14.37886	chr2:8187	RMND5A		protein_c	chr2:86720291-8677
ENSG0000(	635	14.37886	chr2:8187	CD8A		protein_c	chr2:86784610-8680
ENSG0000(	635	14.37886	chr2:8187	ENSG00000286011		lncRNA	chr2:85418504-8542
ENSG0000(	635	14.37886	chr2:8187	ENSG00000213605		Pseudoger	chr2:86885075-8688
ENSG0000(	635	14.37886	chr2:8187	RNU7-162P		smallRNA	chr2:85373454-8537
ENSG0000(	635	14.37886	chr2:8187	ENSG00000290085		lncRNA	chr2:84969747-8497
ENSG0000(	635	14.37886	chr2:8187	ENSG00000286211		lncRNA	chr2:82831234-8286
ENSG0000(	635	14.37886	chr2:8187	SNRPEP11		Pseudoger	chr2:85262144-8526
ENSG0000(	635	14.37886	chr2:8187	ENSG00000233444		Pseudoger	chr2:81666477-8166
ENSG0000(	635	14.37886	chr2:8187	ENSG00000286227		lncRNA	chr2:79269905-7929
ENSG0000(	635	14.37886	chr2:8187	RNU6-674P		smallRNA	chr2:85204926-8520
ENSG0000(	635	14.37886	chr2:8187	RPL12P18		Pseudoger	chr2:84874696-8487
ENSG0000(	635	14.37886	chr2:8187	RPS2P17		Pseudoger	chr2:84915868-8491
ENSG0000(	635	14.37886	chr2:8187	Y_RNA		smallRNA	chr2:85367585-8536
ENSG0000(	635	14.37886	chr2:8187	AC068279.1		Pseudoger	chr2:87369233-8736
ENSG0000(	635	14.37886	chr2:8187	KCMF1		protein_c	chr2:84971093-8505
ENSG0000(	635	14.37886	chr2:8187	SH2D6		protein_c	chr2:85418714-8543
ENSG0000(	635	14.37886	chr2:8187	TGOLN2		protein_c	chr2:85318027-8532
ENSG0000(	635	14.37886	chr2:8187	TCF7L1	NCGv7	protein_c	chr2:85133392-8531
ENSG0000(	635	14.37886	chr2:8187	RGPD1		protein_c	chr2:86913783-8701
ENSG0000(	635	14.37886	chr2:8187	DUXAP1		Pseudoger	chr2:84750769-8475
ENSG0000(	635	14.37886	chr2:8187	RN7SL126P		smallRNA	chr2:85567664-8556
ENSG0000(	635	14.37886	chr2:8187	RPSAP22		Pseudoger	chr2:85490930-8549
ENSG0000(	635	14.37886	chr2:8187	ANAPC1P2		lncRNA	chr2:87030675-8707
ENSG0000(	635	14.37886	chr2:8187	RBX1P1		Pseudoger	chr2:82609652-8260
ENSG0000(	635	14.37886	chr2:8187	ENSG00000272564		lncRNA	chr2:85904279-8590
ENSG0000(	635	14.37886	chr2:8187	MIR4779		smallRNA	chr2:86193026-8619
ENSG0000(	635	14.37886	chr2:8187	U8		smallRNA	chr2:86347062-8634
ENSG0000(	635	14.37886	chr2:8187	LRRTM1	NCGv7	protein_c	chr2:80288351-8030
ENSG0000(	635	14.37886	chr2:8187	MTND6P7		Pseudoger	chr2:82817538-8281
ENSG0000(	635	14.37886	chr2:8187	ENSG00000266931		Pseudoger	chr2:87055658-8707
ENSG0000(	635	14.37886	chr2:8187	TMSB10		protein_c	chr2:84905656-8490

ENSG0000(	635	14.37886	chr2:8187	WBP1P1		Pseudoger	chr2:86930250-8693
ENSG0000(	635	14.37886	chr2:8187	LINC01955		lncRNA	chr2:87249095-8725
ENSG0000(	635	14.37886	chr2:8187	ENSG00000246575		Pseudoger	chr2:85315041-8531
ENSG0000(	635	14.37886	chr2:8187	LSM3P3		Pseudoger	chr2:85102389-8510
ENSG0000(	635	14.37886	chr2:8187	ENSG00000204745		Pseudoger	chr2:87125198-8719
ENSG0000(	635	14.37886	chr2:8187	AC096753.1		smallRNA	chr2:79794270-7979
ENSG0000(	635	14.37886	chr2:8187	SNORD94		smallRNA	chr2:86135870-8613
ENSG0000(	635	14.37886	chr2:8187	CTNNA2	NCGv7	protein_c	chr2:79185231-8064
ENSG0000(	635	14.37886	chr2:8187	RNU7-64P		smallRNA	chr2:85441916-8544
ENSG0000(	635	14.37886	chr2:8187	CHMP3-AS1		lncRNA	chr2:86562070-8661
ENSG0000(	635	14.37886	chr2:8187	ENSG00000289076		lncRNA	chr2:84459572-8446
ENSG0000(	635	14.37886	chr2:8187	CRLF3P3		Pseudoger	chr2:84031140-8403
ENSG0000(	635	14.37886	chr2:8187	DNAH6		protein_c	chr2:84516455-8481
ENSG0000(	635	14.37886	chr2:8187	ELMOD3		protein_c	chr2:85354394-8539
ENSG0000(	635	14.37886	chr2:8187	GGCX		protein_c	chr2:85544720-8556
ENSG0000(	635	14.37886	chr2:8187	MIR4771-2		smallRNA	chr2:87194786-8719
ENSG0000(	635	14.37886	chr2:8187	ENSG00000223977		Pseudoger	chr2:83218890-8321
ENSG0000(	635	14.37886	chr2:8187	ENSG00000288858		lncRNA	chr2:85594864-8559
ENSG0000(	635	14.37886	chr2:8187	GNLY		protein_c	chr2:85685175-8569
ENSG0000(	635	14.37886	chr2:8187	ST3GAL5		protein_c	chr2:85837120-8590
ENSG0000(	635	14.37886	chr2:8187	KDM3A		protein_c	chr2:86440647-8649
ENSG0000(	635	14.37886	chr2:8187	ENSG00000228272		lncRNA	chr2:84315108-8435
ENSG0000(	635	14.37886	chr2:8187	CHMP3		protein_c	chr2:86503430-8656
ENSG0000(	635	14.37886	chr2:8187	LINC01815		lncRNA	chr2:81461358-8146
ENSG0000(	635	14.37886	chr2:8187	ENSG00000235463		Pseudoger	chr2:85068809-8506
ENSG0000(	635	14.37886	chr2:8187	MTCYBP7		Pseudoger	chr2:82818131-8281
ENSG0000(	635	14.37886	chr2:8187	ST3GAL5-AS1		lncRNA	chr2:85889151-8589
ENSG0000(	635	14.37886	chr2:8187	ANKRD11P1		Pseudoger	chr2:81194337-8120
ENSG0000(	635	14.37886	chr2:8187	RN7SL113P		smallRNA	chr2:85368282-8536
ENSG0000(	635	14.37886	chr2:8187	ENSG00000273080		lncRNA	chr2:86195154-8619
ENSG0000(	635	14.37886	chr2:8187	SNORD112		smallRNA	chr2:83858823-8385
ENSG0000(	635	14.37886	chr2:8187	IGKV3OR2-268		protein_c	chr2:87338511-8733
ENSG0000(	635	14.37886	chr2:8187	PLGLB1		protein_c	chr2:87002559-8702
ENSG0000(	635	14.37886	chr2:8187	Y_RNA		smallRNA	chr2:82307067-8230
ENSG0000(	635	14.37886	chr2:8187	CAPG	AC	protein_c	chr2:85394753-8541
ENSG0000(	635	14.37886	chr2:8187	POLR1A		protein_c	chr2:86020216-8610
ENSG0000(	635	14.37886	chr2:8187	RNF103-CH	DriverDBv	protein_c	chr2:86505668-8672
ENSG0000(	635	14.37886	chr2:8187	RNU6-685P		smallRNA	chr2:82268612-8226
ENSG0000(	635	14.37886	chr2:8187	ENSG00000273196		lncRNA	chr2:85387074-8538
ENSG0000(	635	14.37886	chr2:8187	PARTICL		lncRNA	chr2:85537462-8553
ENSG0000(	635	14.37886	chr2:8187	REEP1		protein_c	chr2:86213993-8633
ENSG0000(	635	14.37886	chr2:8187	CTNNA2-AS1		lncRNA	chr2:79492704-7951
ENSG0000(	635	14.37886	chr2:8187	ANAPC1P3		Pseudoger	chr2:87118534-8712
ENSG0000(	635	14.37886	chr2:8187	RNU6-1312P		smallRNA	chr2:83657735-8365
ENSG0000(	635	14.37886	chr2:8187	Y_RNA		smallRNA	chr2:85332895-8533
ENSG0000(	635	14.37886	chr2:8187	RPL37P10		Pseudoger	chr2:83594956-8359
ENSG0000(	635	14.37886	chr2:8187	SUCLG1		protein_c	chr2:84423528-8446
ENSG0000(	635	14.37886	chr2:8187	Y_RNA		smallRNA	chr2:85460144-8546
ENSG0000(	635	14.37886	chr2:8187	ENSG00000231781		lncRNA	chr2:81983272-8200
ENSG0000(	635	14.37886	chr2:8187	RNU6-561P		smallRNA	chr2:79636862-7963
ENSG0000(	635	14.37886	chr2:8187	RNA5SP99		Pseudoger	chr2:81496214-8149

ENSG0000(	635	14.37886	chr2:8187	NDUFB4P5		Pseudoger	chr2:86934462-8693
ENSG0000(	635	14.37886	chr2:8187	RN7SKP83		smallRNA	chr2:85820435-8582
ENSG0000(	635	14.37886	chr2:8187	Y_RNA		smallRNA	chr2:85434507-8543
ENSG0000(	635	14.37886	chr2:8187	RN7SL251P		smallRNA	chr2:85442495-8544
ENSG0000(	635	14.37886	chr2:8187	DBF4P3		Pseudoger	chr2:87301658-8730
ENSG0000(	635	14.37886	chr2:8187	RN7SL201P		smallRNA	chr2:81967079-8196
ENSG0000(	635	14.37886	chr2:8187	LINC01809		lncRNA	chr2:83522814-8352
ENSG0000(	635	14.37886	chr2:8187	ENSG00000277171		Pseudoger	chr2:79547151-7954
ENSG0000(	635	14.37886	chr2:8187	LDHAP7		Pseudoger	chr2:84777259-8477
ENSG0000(	635	14.37886	chr2:8187	ENSG00000224881		Pseudoger	chr2:87379880-8738
ENSG0000(	635	14.37886	chr2:8187	ENSG00000287931		lncRNA	chr2:87075653-8711
ENSG0000(	635	14.37886	chr2:8187	TCF7L1-IT1		lncRNA	chr2:85186409-8518
ENSG0000(	635	14.37886	chr2:8187	MAT2A		protein_c	chr2:85539168-8554
ENSG0000(	635	14.37886	chr2:8187	VAMP5		protein_c	chr2:85584431-8559
ENSG0000(	635	14.37886	chr2:8187	ENSG00000287625		lncRNA	chr2:84926019-8496
ENSG0000(	635	14.37886	chr2:8187	RNF181		protein_c	chr2:85595725-8559
ENSG0000(	635	14.37886	chr2:8187	TMEM150A		protein_c	chr2:85598547-8560
ENSG0000(	635	14.37886	chr2:8187	C2orf68		protein_c	chr2:85605254-8561
ENSG0000(	635	14.37886	chr2:8187	ENSG00000287763		lncRNA	chr2:87311460-8734
ENSG0000(	635	14.37886	chr2:8187	DHFRP3		Pseudoger	chr2:82856826-8285
ENSG0000(	635	14.37886	chr2:8187	TRABD2A		protein_c	chr2:84821650-8490
ENSG0000(	635	14.37886	chr2:8187	PEBP1P2		Pseudoger	chr2:85341281-8534
ENSG0000(	635	14.37886	chr2:8187	SFTPB		protein_c	chr2:85657314-8566
ENSG0000(	635	14.37886	chr2:8187	AC233263.2		Pseudoger	chr2:87369232-8736
ENSG0000(	635	14.37886	chr2:8187	AC016670.1		smallRNA	chr2:80244623-8024
ENSG0000(	635	14.37886	chr2:8187	ATOH8		protein_c	chr2:85751344-8579
ENSG0000(	635	14.37886	chr2:8187	AC093162.1		smallRNA	chr2:85299034-8529
ENSG0000(	635	14.37886	chr2:8187	LINC01964		lncRNA	chr2:85061213-8506
ENSG0000(	635	14.37886	chr2:8187	GNA13P1		Pseudoger	chr2:79573764-7957
ENSG0000(	635	14.37886	chr2:8187	RNU6-640P		smallRNA	chr2:86515204-8651
ENSG0000(	635	14.37886	chr2:8187	ENSG00000287628		lncRNA	chr2:85934535-8593
ENSG0000(	635	14.37886	chr2:8187	USP39		protein_c	chr2:85602856-8564
ENSG0000(	635	14.37886	chr2:8187	AC233263.1		Pseudoger	chr2:87344236-8734
ENSG0000(	635	14.37886	chr2:8187	ENSG00000291013		lncRNA	chr2:86861825-8689
ENSG0000(	635	14.37886	chr2:8187	ENSG00000237031		lncRNA	chr2:80572681-8061
ENSG0000(	635	14.37886	chr2:8187	ENSG00000237498		lncRNA	chr2:82476825-8253
ENSG0000(	635	14.37886	chr2:8187	MIR4264		smallRNA	chr2:79649294-7964
ENSG0000(	632	14.31092	chr1:3733	VPS13D		protein_c	chr1:12230030-1251
ENSG0000(	632	14.31092	chr1:3733	PDPN		protein_c	chr1:13583465-1361
ENSG0000(	632	14.31092	chr1:3733	LINC01647		lncRNA	chr1:11609468-1161
ENSG0000(	632	14.31092	chr1:3733	AL359771.1		smallRNA	chr1:13623902-1362
ENSG0000(	632	14.31092	chr1:3733	MIIP		protein_c	chr1:12019466-1203
ENSG0000(	632	14.31092	chr1:3733	ENSG00000237445		lncRNA	chr1:13657311-1365
ENSG0000(	632	14.31092	chr1:3733	PRAMEF1		protein_c	chr1:12791397-1279
ENSG0000(	632	14.31092	chr1:3733	LRRC38		protein_c	chr1:13474973-1351
ENSG0000(	632	14.31092	chr1:3733	PRAMEF12		protein_c	chr1:12773738-1277
ENSG0000(	632	14.31092	chr1:3733	PRDM2	NCGv7	protein_c	chr1:13700188-1382
ENSG0000(	632	14.31092	chr1:3733	RNU6-1265P		smallRNA	chr1:14124233-1412
ENSG0000(	632	14.31092	chr1:3733	RNA5SP41		Pseudoger	chr1:13623184-1362
ENSG0000(	632	14.31092	chr1:3733	KAZN		protein_c	chr1:13892792-1511
ENSG0000(	632	14.31092	chr1:3733	DHRS3		protein_c	chr1:12567910-1261

ENSG0000(	632	14.31092	chr11:14	ENSG00000256220		lncRNA	chr11:61227168-612
ENSG0000(	632	14.31092	chr1:373	MFN2		protein_c	chr1:11980181-1201
ENSG0000(	632	14.31092	chr1:373	KIAA2013		protein_c	chr1:11919591-1192
ENSG0000(	632	14.31092	chr1:373	PRAMEF35P		Pseudoger	chr1:13104403-1310
ENSG0000(	632	14.31092	chr11:14	PGA4		protein_c	chr11:61222347-612
ENSG0000(	632	14.31092	chr1:373	DRAXIN		protein_c	chr1:11691710-1172
ENSG0000(	632	14.31092	chr1:373	SNORA70		smallRNA	chr1:12221148-1222
ENSG0000(	632	14.31092	chr1:373	FBXO6		protein_c	chr1:11664200-1167
ENSG0000(	632	14.31092	chr1:373	ENSG00000231606		lncRNA	chr1:14221887-1430
ENSG0000(	632	14.31092	chr1:373	FBXO2		protein_c	chr1:11637018-1165
ENSG0000(	632	14.31092	chr1:373	ENSG00000284708		lncRNA	chr1:11623558-1164
ENSG0000(	632	14.31092	chr1:373	SBF1P2		Pseudoger	chr1:11877770-1188
ENSG0000(	632	14.31092	chr1:373	TNFRSF1B	NCGv7;AC	protein_c	chr1:12166991-1220
ENSG0000(	632	14.31092	chr1:373	PRAMEF20		protein_c	chr1:13410450-1342
ENSG0000(	632	14.31092	chr1:373	PLOD1		protein_c	chr1:11934205-1197
ENSG0000(	632	14.31092	chr1:373	ENSG00000285646		lncRNA	chr1:11907940-1191
ENSG0000(	632	14.31092	chr1:373	PRAMEF30P		Pseudoger	chr1:12838125-1284
ENSG0000(	632	14.31092	chr1:373	PRAMEF17		protein_c	chr1:13389632-1339
ENSG0000(	632	14.31092	chr1:373	PRAMEF19		protein_c	chr1:13368431-1337
ENSG0000(	632	14.31092	chr1:373	PRAMEF29P		Pseudoger	chr1:12926162-1292
ENSG0000(	632	14.31092	chr1:373	PRAMEF14		protein_c	chr1:13341892-1334
ENSG0000(	632	14.31092	chr1:373	ENSG00000287384		lncRNA	chr1:11979533-1198
ENSG0000(	632	14.31092	chr1:373	ENSG00000270914		Pseudoger	chr1:12017216-1201
ENSG0000(	632	14.31092	chr1:373	CFAP107		protein_c	chr1:12746200-1276
ENSG0000(	632	14.31092	chr1:373	PRAMEF21		protein_c	chr1:13410450-1342
ENSG0000(	632	14.31092	chr1:373	PRAMEF16		protein_c	chr1:13389628-1339
ENSG0000(	632	14.31092	chr1:373	ENSG00000285604		lncRNA	chr1:12088441-1209
ENSG0000(	632	14.31092	chr1:373	SNORA59A	AC	smallRNA	chr1:12507246-1250
ENSG0000(	632	14.31092	chr1:373	ENSG00000226166		Pseudoger	chr1:12692909-1269
ENSG0000(	632	14.31092	chr1:373	PRAMEF28P		Pseudoger	chr1:13342528-1334
ENSG0000(	632	14.31092	chr1:373	PRAMEF4	NCGv7	protein_c	chr1:12879212-1288
ENSG0000(	632	14.31092	chr1:373	PRAMEF18		protein_c	chr1:13369067-1337
ENSG0000(	632	14.31092	chr1:373	PRAMEF9		protein_c	chr1:13315581-1332
ENSG0000(	632	14.31092	chr1:373	PRAMEF5		protein_c	chr1:13254198-1326
ENSG0000(	632	14.31092	chr1:373	RNU6ATAC18P		smallRNA	chr1:12569972-1257
ENSG0000(	632	14.31092	chr1:373	ENSG00000259961		lncRNA	chr1:13513220-1351
ENSG0000(	632	14.31092	chr1:373	PRAMEF7		protein_c	chr1:12916610-1292
ENSG0000(	632	14.31092	chr1:373	PRAMEF11		protein_c	chr1:12824610-1283
ENSG0000(	632	14.31092	chr1:373	PRAMEF13		protein_c	chr1:13341907-1334
ENSG0000(	632	14.31092	chr1:373	NPPA		protein_c	chr1:11845709-1184
ENSG0000(	632	14.31092	chr1:373	RP13-221M14.3		Pseudoger	chr1:13095174-1309
ENSG0000(	632	14.31092	chr1:373	AADACL4	NCGv7	protein_c	chr1:12644085-1266
ENSG0000(	632	14.31092	chr1:373	HNRNPCL1	NCGv7	protein_c	chr1:12847377-1284
ENSG0000(	632	14.31092	chr1:373	XX-FW84067D5.2		Pseudoger	chr1:13305955-1330
ENSG0000(	632	14.31092	chr1:373	MAD2L2		protein_c	chr1:11658918-1169
ENSG0000(	632	14.31092	chr1:373	ENSG00000289380		lncRNA	chr1:13757778-1375
ENSG0000(	632	14.31092	chr1:373	RP11-248D7.2		Pseudoger	chr1:13245863-1324
ENSG0000(	632	14.31092	chr1:373	PRAMEF2		protein_c	chr1:12857086-1286
ENSG0000(	632	14.31092	chr1:373	TNFRSF8	NCGv7	protein_c	chr1:12063303-1214
ENSG0000(	632	14.31092	chr1:373	RP13-221M14.2		lncRNA	chr1:13079329-1307
ENSG0000(	632	14.31092	chr1:373	NPPB		protein_c	chr1:11857464-1185

ENSG00000	632	14.31092	chr1:3733	RPL23AP89		Pseudoger	chr1:12080293-1208
ENSG00000	632	14.31092	chr1:3733	Y_RNA		smallRNA	chr1:12024012-1202
ENSG00000	632	14.31092	chr1:3733	RNU5E-4P		smallRNA	chr1:11909808-1190
ENSG00000	632	14.31092	chr1:3733	RNU6-1072P		smallRNA	chr1:12922554-1292
ENSG00000	632	14.31092	chr1:3733	RNU6-771P		smallRNA	chr1:13279125-1327
ENSG00000	632	14.31092	chr1:3733	PRAMEF10		protein_c	chr1:12892896-1289
ENSG00000	632	14.31092	chr1:3733	MIR4632		smallRNA	chr1:12191713-1219
ENSG00000	632	14.31092	chr1:3733	PRAMEF32P		Pseudoger	chr1:13273539-1327
ENSG00000	632	14.31092	chr1:3733	ENSG00000288927		lncRNA	chr1:12618389-1261
ENSG00000	632	14.31092	chr1:3733	RNU6-777P		smallRNA	chr1:12077881-1207
ENSG00000	632	14.31092	chr1:3733	FBXO44		protein_c	chr1:11654375-1166
ENSG00000	632	14.31092	chr1:3733	RN7SL649P		smallRNA	chr1:12036742-1203
ENSG00000	632	14.31092	chr1:3733	PRAMEF26		protein_c	chr1:13148905-1315
ENSG00000	632	14.31092	chr1:3733	AADACL3		protein_c	chr1:12716110-1272
ENSG00000	632	14.31092	chr1:3733	SCARNA11		smallRNA	chr1:13696070-1369
ENSG00000	632	14.31092	chr1:3733	AGTRAP		protein_c	chr1:11736084-1175
ENSG00000	632	14.31092	chr1:3733	LINC01784		lncRNA	chr1:12822686-1282
ENSG00000	632	14.31092	chr1:3733	PRAMEF6		protein_c	chr1:12938472-1294
ENSG00000	632	14.31092	chr1:3733	HNRNPCL2		protein_c	chr1:13115488-1311
ENSG00000	632	14.31092	chr1:3733	CLCN6		protein_c	chr1:11806096-1184
ENSG00000	632	14.31092	chr1:3733	KAZN-AS1		lncRNA	chr1:14338825-1441
ENSG00000	632	14.31092	chr1:3733	BRWD1P1		Pseudoger	chr1:13555001-1355
ENSG00000	632	14.31092	chr1:3733	PRAMEF8		protein_c	chr1:13281035-1328
ENSG00000	632	14.31092	chr1:3733	C1orf167		protein_c	chr1:11761787-1178
ENSG00000	632	14.31092	chr1:3733	C1orf167-AS1		lncRNA	chr1:11777077-1177
ENSG00000	632	14.31092	chr1:3733	RP11-219C24.10		lncRNA	chr1:13324039-1332
ENSG00000	632	14.31092	chr1:3733	LINC02766		lncRNA	chr1:12525716-1253
ENSG00000	632	14.31092	chr1:3733	PRAMEF34P		Pseudoger	chr1:13095179-1309
ENSG00000	632	14.31092	chr1:3733	RNU5E-1		smallRNA	chr1:11908152-1190
ENSG00000	632	14.31092	chr1:3733	snoU13		smallRNA	chr1:12739736-1273
ENSG00000	632	14.31092	chr1:3733	RPL10P17		Pseudoger	chr1:12220794-1222
ENSG00000	632	14.31092	chr1:3733	MTHFR	NCGv7	protein_c	chr1:11785723-1180
ENSG00000	632	14.31092	chr1:3733	RP11-219C24.6		Pseudoger	chr1:13305955-1330
ENSG00000	631	14.28828	chr8:2747	ENSG00000254309		Pseudoger	chr8:36121398-3612
ENSG00000	631	14.28828	chr8:2747	ENSG00000254306		lncRNA	chr8:37600537-3762
ENSG00000	631	14.28828	chr8:2747	ENSG00000253800		Pseudoger	chr8:64570913-6457
ENSG00000	631	14.28828	chr8:2747	RPL36AP32		Pseudoger	chr8:28810103-2881
ENSG00000	631	14.28828	chr8:2747	MTND1P6		Pseudoger	chr8:33014081-3301
ENSG00000	631	14.28828	chr8:2747	MAPK6P1		Pseudoger	chr8:53537060-5354
ENSG00000	631	14.28828	chr8:2747	SNORA3		smallRNA	chr8:56121242-5612
ENSG00000	631	14.28828	chr8:2747	RPL31P41		Pseudoger	chr8:65155407-6515
ENSG00000	631	14.28828	chr8:2747	ENSG00000253604		lncRNA	chr8:29905735-2990
ENSG00000	631	14.28828	chr8:2747	RNU6-533P		smallRNA	chr8:36309584-3630
ENSG00000	631	14.28828	chr8:2747	RNU6-356P		smallRNA	chr8:41032892-4103
ENSG00000	631	14.28828	chr8:2747	IFITM8P		Pseudoger	chr8:63409064-6340
ENSG00000	631	14.28828	chr8:2747	ENSG00000286804		lncRNA	chr8:28414499-2841
ENSG00000	631	14.28828	chr8:2747	RNU6-656P		smallRNA	chr8:46831050-4683
ENSG00000	631	14.28828	chr8:2747	XKR4		protein_c	chr8:55102028-5554
ENSG00000	631	14.28828	chr8:2747	AC026241.1		smallRNA	chr8:47085352-4708
ENSG00000	631	14.28828	chr8:2747	U3		smallRNA	chr8:43378298-4337
ENSG00000	631	14.28828	chr8:2747	SNX18P27		Pseudoger	chr8:43513643-4351

ENSG0000(	631	14.28828	chr8:2747	ENSG00000272343		lncRNA	chr8:56222688-5622
ENSG0000(	631	14.28828	chr8:2747	RPS29P2		Pseudoger	chr8:41271431-4127
ENSG0000(	631	14.28828	chr8:2747	ENSG00000253843		Pseudoger	chr8:48193850-4819
ENSG0000(	631	14.28828	chr8:2747	ENSG00000234713		Pseudoger	chr8:43276270-4327
ENSG0000(	631	14.28828	chr8:2747	ENSG00000272338		lncRNA	chr8:33360839-3336
ENSG0000(	631	14.28828	chr8:2747	ENSG00000285601		lncRNA	chr8:29351292-2936
ENSG0000(	631	14.28828	chr8:2747	SCARA3		protein_c	chr8:27633868-2767
ENSG0000(	631	14.28828	chr8:2747	ENSG00000254314		lncRNA	chr8:52150820-5215
ENSG0000(	631	14.28828	chr8:2747	RP11-1002K11.1		lncRNA	chr8:32766125-3276
ENSG0000(	631	14.28828	chr8:2747	MAP2K1P1		Pseudoger	chr8:30027713-3002
ENSG0000(	631	14.28828	chr8:2747	CERNA3		lncRNA	chr8:56074592-5607
ENSG0000(	631	14.28828	chr8:2747	SNORA51		smallRNA	chr8:59137372-5913
ENSG0000(	631	14.28828	chr8:2747	MAPK6P4		Pseudoger	chr8:46972476-4697
ENSG0000(	631	14.28828	chr8:2747	RNU6-895P		smallRNA	chr8:41298163-4129
ENSG0000(	631	14.28828	chr8:2747	ENSG00000254316		Pseudoger	chr8:30249502-3025
ENSG0000(	631	14.28828	chr8:2747	SNAI2		protein_c	chr8:48917598-4892
ENSG0000(	631	14.28828	chr8:2747	INTS9-AS1		lncRNA	chr8:28798142-2880
ENSG0000(	631	14.28828	chr8:2747	ENSG00000254033		lncRNA	chr8:34229294-3424
ENSG0000(	631	14.28828	chr8:2747	ENSG00000272375		lncRNA	chr8:30197404-3019
ENSG0000(	631	14.28828	chr8:2747	ENSG00000253186		Pseudoger	chr8:43253732-4325
ENSG0000(	631	14.28828	chr8:2747	MRPL15	DriverDB\	protein_c	chr8:54135241-5414
ENSG0000(	631	14.28828	chr8:2747	KCNU1	NCGv7	protein_c	chr8:36784324-3693
ENSG0000(	631	14.28828	chr8:2747	MIR4470		smallRNA	chr8:61714788-6171
ENSG0000(	631	14.28828	chr8:2747	TCIM		protein_c	chr8:40153482-4015
ENSG0000(	631	14.28828	chr8:2747	BRIX1P1		Pseudoger	chr8:51435602-5143
ENSG0000(	631	14.28828	chr8:2747	ENSG00000254342		Pseudoger	chr8:43372559-4337
ENSG0000(	631	14.28828	chr8:2747	ENSG00000254048		lncRNA	chr8:58991720-5899
ENSG0000(	631	14.28828	chr8:2747	TTPA		protein_c	chr8:63048553-6308
ENSG0000(	631	14.28828	chr8:2747	GTF2E2		protein_c	chr8:30578318-3065
ENSG0000(	631	14.28828	chr8:2747	ENSG00000286837		lncRNA	chr8:42842989-4284
ENSG0000(	631	14.28828	chr8:2747	RPL23P10		Pseudoger	chr8:36581180-3658
ENSG0000(	631	14.28828	chr8:2747	ASNSP1		lncRNA	chr8:46579213-4663
ENSG0000(	631	14.28828	chr8:2747	ENSG00000253834		lncRNA	chr8:61821481-6182
ENSG0000(	631	14.28828	chr8:2747	ENSG00000286878		lncRNA	chr8:48818293-4882
ENSG0000(	631	14.28828	chr8:2747	SNORA70		smallRNA	chr8:33540040-3354
ENSG0000(	631	14.28828	chr8:2747	RN7SKP294		smallRNA	chr8:49330210-4933
ENSG0000(	631	14.28828	chr8:2747	ENSG00000289514		lncRNA	chr8:41797777-4179
ENSG0000(	631	14.28828	chr8:2747	LINC01288		lncRNA	chr8:34784028-3486
ENSG0000(	631	14.28828	chr8:2747	ENSG00000253690		lncRNA	chr8:28250063-2833
ENSG0000(	631	14.28828	chr8:2747	ENSG00000253711		lncRNA	chr8:61300164-6130
ENSG0000(	631	14.28828	chr8:2747	PBK	AC	protein_c	chr8:27809624-2783
ENSG0000(	631	14.28828	chr8:2747	GGH	DriverDB\	protein_c	chr8:63014881-6303
ENSG0000(	631	14.28828	chr8:2747	ENSG00000254320		Pseudoger	chr8:36321039-3632
ENSG0000(	631	14.28828	chr8:2747	SMIM19	DriverDB\	protein_c	chr8:42541155-4255
ENSG0000(	631	14.28828	chr8:2747	RN7SL781P		smallRNA	chr8:29142693-2914
ENSG0000(	631	14.28828	chr8:2747	ENSG00000254321		lncRNA	chr8:38699274-3870
ENSG0000(	631	14.28828	chr8:2747	IGLV8OR8-1		Pseudoger	chr8:47202444-4720
ENSG0000(	631	14.28828	chr8:2747	ENSG00000254325		lncRNA	chr8:55893595-5589
ENSG0000(	631	14.28828	chr8:2747	ENSG00000272479		lncRNA	chr8:40519565-4052
ENSG0000(	631	14.28828	chr8:2747	GULOP		Pseudoger	chr8:27560274-2758
ENSG0000(	631	14.28828	chr8:2747	ENSG00000272457		lncRNA	chr8:53876150-5387

ENSG0000(	631	14.28828	chr8:2747	SCARA5		protein_c	chr8:27869883-2799
ENSG0000(	631	14.28828	chr8:2747	ENSG00000254038		lncRNA	chr8:37067441-3706
ENSG0000(	631	14.28828	chr8:2747	CLVS1		protein_c	chr8:61057158-6150
ENSG0000(	631	14.28828	chr8:2747	UBXN2B		protein_c	chr8:58411359-5845
ENSG0000(	631	14.28828	chr8:2747	PNOC		protein_c	chr8:28316986-2834
ENSG0000(	631	14.28828	chr8:2747	ENSG00000285992		lncRNA	chr8:47129262-4713
ENSG0000(	631	14.28828	chr8:2747	LINC00588		lncRNA	chr8:57279543-5728
ENSG0000(	631	14.28828	chr8:2747	ENSG00000254330		lncRNA	chr8:64703774-6473
ENSG0000(	631	14.28828	chr8:2747	RPL10AP3		Pseudoger	chr8:34322992-3432
ENSG0000(	631	14.28828	chr8:2747	ENSG00000278428		Pseudoger	chr8:59893856-5989
ENSG0000(	631	14.28828	chr8:2747	SARAF		protein_c	chr8:30063003-3008
ENSG0000(	631	14.28828	chr8:2747	ENSG00000254302		lncRNA	chr8:34174886-3420
ENSG0000(	631	14.28828	chr8:2747	ENSG00000289104		lncRNA	chr8:38612986-3861
ENSG0000(	631	14.28828	chr8:2747	RNU6-665P		smallRNA	chr8:47667771-4766
ENSG0000(	631	14.28828	chr8:2747	ENSG00000253356		lncRNA	chr8:38148741-3816
ENSG0000(	631	14.28828	chr8:2747	LYN	NCGv7	protein_c	chr8:55879835-5601
ENSG0000(	631	14.28828	chr8:2747	RNU6-178P		smallRNA	chr8:28416448-2841
ENSG0000(	631	14.28828	chr8:2747	RN7SL135P		smallRNA	chr8:63234637-6323
ENSG0000(	631	14.28828	chr8:2747	KRT8P3		Pseudoger	chr8:61578220-6157
ENSG0000(	631	14.28828	chr8:2747	RPS20	DriverDBv	protein_c	chr8:56067254-5607
ENSG0000(	631	14.28828	chr8:2747	ENSG00000253803		Pseudoger	chr8:46870902-4687
ENSG0000(	631	14.28828	chr8:2747	SIRLNT		lncRNA	chr8:40298697-4035
ENSG0000(	631	14.28828	chr8:2747	ADAM3A		Pseudoger	chr8:39451045-3952
ENSG0000(	631	14.28828	chr8:2747	ENSG00000253723		Pseudoger	chr8:59821124-5982
ENSG0000(	631	14.28828	chr8:2747	RNA5SP262		Pseudoger	chr8:32192028-3219
ENSG0000(	631	14.28828	chr8:2747	ENSG00000253844		lncRNA	chr8:51961458-5202
ENSG0000(	631	14.28828	chr8:2747	ENSG00000254086		lncRNA	chr8:29864644-2987
ENSG0000(	631	14.28828	chr8:2747	SNORD112		smallRNA	chr8:42043504-4204
ENSG0000(	631	14.28828	chr8:2747	ENSG00000254287		lncRNA	chr8:39918076-3992
ENSG0000(	631	14.28828	chr8:2747	CHRNA6		protein_c	chr8:42752620-4279
ENSG0000(	631	14.28828	chr8:2747	LINC01299		lncRNA	chr8:65526738-6556
ENSG0000(	631	14.28828	chr8:2747	ZNF703	DriverDBv	protein_c	chr8:37695782-3770
ENSG0000(	631	14.28828	chr8:2747	ENSG00000289095		lncRNA	chr8:49908959-4991
ENSG0000(	631	14.28828	chr8:2747	ENSG00000254290		lncRNA	chr8:37597480-3759
ENSG0000(	631	14.28828	chr8:2747	SBF1P1		Pseudoger	chr8:55449509-5545
ENSG0000(	631	14.28828	chr8:2747	ENSG00000255166		Pseudoger	chr8:43247191-4324
ENSG0000(	631	14.28828	chr8:2747	ENSG00000253369		lncRNA	chr8:53393133-5348
ENSG0000(	631	14.28828	chr8:2747	ENSG00000254070		Pseudoger	chr8:46671839-4667
ENSG0000(	631	14.28828	chr8:2747	SNORD65		smallRNA	chr8:41426655-4142
ENSG0000(	631	14.28828	chr8:2747	ENSG00000253583		lncRNA	chr8:63586000-6358
ENSG0000(	631	14.28828	chr8:2747	MTND2P32		Pseudoger	chr8:33015261-3301
ENSG0000(	631	14.28828	chr8:2747	RN7SL323P		smallRNA	chr8:56022021-5602
ENSG0000(	631	14.28828	chr8:2747	ENSG00000253509		lncRNA	chr8:41275115-4127
ENSG0000(	631	14.28828	chr8:2747	ENSG00000253233		Pseudoger	chr8:39867436-3986
ENSG0000(	631	14.28828	chr8:2747	ENSG00000285880		protein_c	chr8:37934340-3796
ENSG0000(	631	14.28828	chr8:2747	ENSG00000287844		lncRNA	chr8:63651457-6369
ENSG0000(	631	14.28828	chr8:2747	PLPBP	DriverDBv	protein_c	chr8:37762595-3777
ENSG0000(	631	14.28828	chr8:2747	TMEM68	DriverDBv	protein_c	chr8:55696424-5577
ENSG0000(	631	14.28828	chr8:2747	CYP7A1		protein_c	chr8:58490178-5850
ENSG0000(	631	14.28828	chr8:2747	TOX-DT		lncRNA	chr8:59119040-5912
ENSG0000(	631	14.28828	chr8:2747	ENSG00000253232		Pseudoger	chr8:33523154-3352

ENSG0000(	631	14.28828	chr8:2747	LSM12P1		Pseudoger	chr8:35525176-3552
ENSG0000(	631	14.28828	chr8:2747	RPL30P10		Pseudoger	chr8:57392546-5739
ENSG0000(	631	14.28828	chr8:2747	COX6CP8		Pseudoger	chr8:64331927-6433
ENSG0000(	631	14.28828	chr8:2747	STAR	DriverDB\	protein_c	chr8:38142700-3815
ENSG0000(	631	14.28828	chr8:2747	ENSG00000270328		Pseudoger	chr8:56776283-5677
ENSG0000(	631	14.28828	chr8:2747	ENSG00000255101		lncRNA	chr8:42705583-4272
ENSG0000(	631	14.28828	chr8:2747	HMGB1P23		Pseudoger	chr8:29218905-2921
ENSG0000(	631	14.28828	chr8:2747	ASNSP1		Pseudoger	chr8:46579213-4661
ENSG0000(	631	14.28828	chr8:2747	MTFR1	DriverDB\	protein_c	chr8:65644734-6577
ENSG0000(	631	14.28828	chr8:2747	ENSG00000250637		Pseudoger	chr8:43442902-4345
ENSG0000(	631	14.28828	chr8:2747	ENSG00000288964		lncRNA	chr8:29529299-2952
ENSG0000(	631	14.28828	chr8:2747	SRPK2P		Pseudoger	chr8:62863082-6286
ENSG0000(	631	14.28828	chr8:2747	ENSG00000253354		lncRNA	chr8:40369981-4040
ENSG0000(	631	14.28828	chr8:2747	Y_RNA		smallRNA	chr8:47805079-4780
ENSG0000(	631	14.28828	chr8:2747	ENSG00000254095		lncRNA	chr8:31339197-3134
ENSG0000(	631	14.28828	chr8:2747	ENSG00000254069		Pseudoger	chr8:43493937-4349
ENSG0000(	631	14.28828	chr8:2747	ENSG00000253361		lncRNA	chr8:38543276-3856
ENSG0000(	631	14.28828	chr8:2747	ENSG00000260253		lncRNA	chr8:29352420-2935
ENSG0000(	631	14.28828	chr8:2747	ENSG00000253206		lncRNA	chr8:49536386-4955
ENSG0000(	631	14.28828	chr8:2747	ENSG00000233109		Pseudoger	chr8:48913916-4891
ENSG0000(	631	14.28828	chr8:2747	AC022616.1		smallRNA	chr8:43366713-4336
ENSG0000(	631	14.28828	chr8:2747	ENSG00000255201		lncRNA	chr8:38421889-3842
ENSG0000(	631	14.28828	chr8:2747	Y_RNA		smallRNA	chr8:41803349-4180
ENSG0000(	631	14.28828	chr8:2747	ENSG00000254055		lncRNA	chr8:56638894-5665
ENSG0000(	631	14.28828	chr8:2747	ENSG00000289275		lncRNA	chr8:48318504-4831
ENSG0000(	631	14.28828	chr8:2747	HMBOX1		protein_c	chr8:28890395-2906
ENSG0000(	631	14.28828	chr8:2747	NPM1P21		Pseudoger	chr8:56101199-5610
ENSG0000(	631	14.28828	chr8:2747	TRMT112P7		Pseudoger	chr8:54479597-5447
ENSG0000(	631	14.28828	chr8:2747	RN7SKP135		smallRNA	chr8:63609354-6360
ENSG0000(	631	14.28828	chr8:2747	RNU6-1218P		smallRNA	chr8:30043801-3004
ENSG0000(	631	14.28828	chr8:2747	CCDC25		protein_c	chr8:27733316-2777
ENSG0000(	631	14.28828	chr8:2747	RPL5P22		Pseudoger	chr8:28299683-2830
ENSG0000(	631	14.28828	chr8:2747	ENSG00000289323		lncRNA	chr8:27508941-2750
ENSG0000(	631	14.28828	chr8:2747	ENSG00000242970		Pseudoger	chr8:58588420-5858
ENSG0000(	631	14.28828	chr8:2747	ENSG00000253319		Pseudoger	chr8:43511820-4351
ENSG0000(	631	14.28828	chr8:2747	ENSG00000289334		lncRNA	chr8:30094324-3009
ENSG0000(	631	14.28828	chr8:2747	MTND1P7		Pseudoger	chr8:46828117-4682
ENSG0000(	631	14.28828	chr8:2747	ENSG00000254050		lncRNA	chr8:62473217-6247
ENSG0000(	631	14.28828	chr8:2747	RPS20P22		Pseudoger	chr8:38434347-3843
ENSG0000(	631	14.28828	chr8:2747	RPL17P33		Pseudoger	chr8:29632091-2963
ENSG0000(	631	14.28828	chr8:2747	ENSG00000274310		Pseudoger	chr8:54467208-5447
ENSG0000(	631	14.28828	chr8:2747	RNU6-13P		smallRNA	chr8:56917084-5691
ENSG0000(	631	14.28828	chr8:2747	NRG1-IT3		lncRNA	chr8:32440704-3244
ENSG0000(	631	14.28828	chr8:2747	SNORA7		smallRNA	chr8:51015021-5101
ENSG0000(	631	14.28828	chr8:2747	ENSG00000274295		Pseudoger	chr8:53394215-5339
ENSG0000(	631	14.28828	chr8:2747	LSM1	DriverDB\	protein_c	chr8:38163335-3817
ENSG0000(	631	14.28828	chr8:2747	CHRNB3		protein_c	chr8:42697366-4273
ENSG0000(	631	14.28828	chr8:2747	ENSG00000253205		lncRNA	chr8:63384839-6347
ENSG0000(	631	14.28828	chr8:2747	ENSG00000254067		Pseudoger	chr8:39522976-3958
ENSG0000(	631	14.28828	chr8:2747	NDUFA5P12		Pseudoger	chr8:47164870-4716
ENSG0000(	631	14.28828	chr8:2747	ENSG00000254065		Pseudoger	chr8:47592147-4759

ENSG0000(	631	14.28828	chr8:2747	SDCBP		protein_c	chr8:58552924-5858
ENSG0000(	631	14.28828	chr8:2747	AC134698.1		smallRNA	chr8:43560668-4356
ENSG0000(	631	14.28828	chr8:2747	SNORD38		smallRNA	chr8:39018615-3901
ENSG0000(	631	14.28828	chr8:2747	HSPA8P13		Pseudoger	chr8:46549089-4655
ENSG0000(	631	14.28828	chr8:2747	SNORD13		smallRNA	chr8:33513475-3351
ENSG0000(	631	14.28828	chr8:2747	ENSG00000250516		Pseudoger	chr8:28225929-2822
ENSG0000(	631	14.28828	chr8:2747	NUGGC		protein_c	chr8:28021964-2808
ENSG0000(	631	14.28828	chr8:2747	YTHDF3-DT		lncRNA	chr8:63167725-6316
ENSG0000(	631	14.28828	chr8:2747	ENSG00000253204		Pseudoger	chr8:30921060-3092
ENSG0000(	631	14.28828	chr8:2747	ENSG00000253586		lncRNA	chr8:38799643-3880
ENSG0000(	631	14.28828	chr8:2747	ENSG00000254061		Pseudoger	chr8:33436268-3343
ENSG0000(	631	14.28828	chr8:2747	ENSG00000285971		lncRNA	chr8:62191088-6224
ENSG0000(	631	14.28828	chr8:2747	ENSG00000253198		Pseudoger	chr8:43674270-4367
ENSG0000(	631	14.28828	chr8:2747	RPL29P19		Pseudoger	chr8:48384590-4838
ENSG0000(	631	14.28828	chr8:2747	TGS1	NCGv7	protein_c	chr8:55773446-5582
ENSG0000(	631	14.28828	chr8:2747	RNU4-50P		smallRNA	chr8:58814412-5881
ENSG0000(	631	14.28828	chr8:2747	MIR4469		smallRNA	chr8:42896197-4289
ENSG0000(	631	14.28828	chr8:2747	ENSG00000253195		Pseudoger	chr8:43542076-4354
ENSG0000(	631	14.28828	chr8:2747	RN7SL149P		smallRNA	chr8:41840062-4184
ENSG0000(	631	14.28828	chr8:2747	ENSG00000250494		Pseudoger	chr8:53162339-5316
ENSG0000(	631	14.28828	chr8:2747	ENSG00000253907		Pseudoger	chr8:28238430-2823
ENSG0000(	631	14.28828	chr8:2747	ENSG00000287788		lncRNA	chr8:48925917-4893
ENSG0000(	631	14.28828	chr8:2747	MIR124-2HG		lncRNA	chr8:64373105-6438
ENSG0000(	631	14.28828	chr8:2747	ENSG00000248585		Pseudoger	chr8:38201699-3820
ENSG0000(	631	14.28828	chr8:2747	RPL5P23		Pseudoger	chr8:42341767-4234
ENSG0000(	631	14.28828	chr8:2747	KAT6A	NCGv7;AC	protein_c	chr8:41929479-4205
ENSG0000(	631	14.28828	chr8:2747	LINC01301		lncRNA	chr8:60289928-6051
ENSG0000(	631	14.28828	chr8:2747	MIR4288		smallRNA	chr8:28505116-2850
ENSG0000(	631	14.28828	chr8:2747	ENSG00000253330		Pseudoger	chr8:47511034-4751
ENSG0000(	631	14.28828	chr8:2747	ENSG00000286471		lncRNA	chr8:54381698-5439
ENSG0000(	631	14.28828	chr8:2747	ADAM9	DriverDB	protein_c	chr8:38996754-3910
ENSG0000(	631	14.28828	chr8:2747	ADAM18		protein_c	chr8:39584489-3973
ENSG0000(	631	14.28828	chr8:2747	LINC02599		lncRNA	chr8:48590401-4859
ENSG0000(	631	14.28828	chr8:2747	OPRK1	NCGv7	protein_c	chr8:53225724-5325
ENSG0000(	631	14.28828	chr8:2747	RPL34P17		Pseudoger	chr8:52313154-5231
ENSG0000(	631	14.28828	chr8:2747	ENSG00000255361		Pseudoger	chr8:42725547-4272
ENSG0000(	631	14.28828	chr8:2747	ENSG00000253838		lncRNA	chr8:39914229-3991
ENSG0000(	631	14.28828	chr8:2747	ENSG00000255366		lncRNA	chr8:47190772-4719
ENSG0000(	631	14.28828	chr8:2747	ENSG00000289921		lncRNA	chr8:41651070-4165
ENSG0000(	631	14.28828	chr8:2747	RNA5SP264		Pseudoger	chr8:36764445-3676
ENSG0000(	631	14.28828	chr8:2747	RPL6P22		Pseudoger	chr8:33859480-3386
ENSG0000(	631	14.28828	chr8:2747	IDO1		protein_c	chr8:39902275-3992
ENSG0000(	631	14.28828	chr8:2747	RANP9		Pseudoger	chr8:33039523-3303
ENSG0000(	631	14.28828	chr8:2747	ENSG00000253664		lncRNA	chr8:51257688-5132
ENSG0000(	631	14.28828	chr8:2747	LINC02866		lncRNA	chr8:40114651-4012
ENSG0000(	631	14.28828	chr8:2747	ASNSP4		Pseudoger	chr8:46697630-4669
ENSG0000(	631	14.28828	chr8:2747	ENSG00000279302		TEC	chr8:27701194-2770
ENSG0000(	631	14.28828	chr8:2747	BHLHE22		protein_c	chr8:64580365-6458
ENSG0000(	631	14.28828	chr8:2747	RPL37P6		Pseudoger	chr8:56588385-5658
ENSG0000(	631	14.28828	chr8:2747	ENSG00000254222		lncRNA	chr8:61264624-6129
ENSG0000(	631	14.28828	chr8:2747	ENSG00000253667		Pseudoger	chr8:53971231-5397

ENSG0000(	631	14.28828	chr8:2747	RN7SL806P		smallRNA	chr8:42881236-4288
ENSG0000(	631	14.28828	chr8:2747	ENSG00000289853		lncRNA	chr8:40372572-4045
ENSG0000(	631	14.28828	chr8:2747	NARS1P2		Pseudoger	chr8:62110524-6211
ENSG0000(	631	14.28828	chr8:2747	ENSG00000286484		lncRNA	chr8:46810696-4681
ENSG0000(	631	14.28828	chr8:2747	EXTL3-AS1		lncRNA	chr8:28690215-2870
ENSG0000(	631	14.28828	chr8:2747	ENSG00000253975		Pseudoger	chr8:62482198-6248
ENSG0000(	631	14.28828	chr8:2747	ENSG00000253396		lncRNA	chr8:41435298-4144
ENSG0000(	631	14.28828	chr8:2747	RBPMS		protein_c	chr8:30384511-3057
ENSG0000(	631	14.28828	chr8:2747	FNTA		protein_c	chr8:43034194-4308
ENSG0000(	631	14.28828	chr8:2747	ENSG00000286578		lncRNA	chr8:58269979-5827
ENSG0000(	631	14.28828	chr8:2747	ENSG00000253322		lncRNA	chr8:57594166-5780
ENSG0000(	631	14.28828	chr8:2747	NRG1-IT1		lncRNA	chr8:32026210-3213
ENSG0000(	631	14.28828	chr8:2747	ENSG00000253524		lncRNA	chr8:36004316-3609
ENSG0000(	631	14.28828	chr8:2747	RPL26P26		Pseudoger	chr8:58113189-5811
ENSG0000(	631	14.28828	chr8:2747	PDCL3P1		Pseudoger	chr8:60387262-6038
ENSG0000(	631	14.28828	chr8:2747	ENSG00000260588		lncRNA	chr8:41828165-4182
ENSG0000(	631	14.28828	chr8:2747	ENSG00000285669		lncRNA	chr8:30192429-3020
ENSG0000(	631	14.28828	chr8:2747	ADRB3	NCGv7	protein_c	chr8:37962990-3796
ENSG0000(	631	14.28828	chr8:2747	ENSG00000253523		lncRNA	chr8:58031334-5803
ENSG0000(	631	14.28828	chr8:2747	ENSG00000286554		lncRNA	chr8:46814925-4681
ENSG0000(	631	14.28828	chr8:2747	ENSG00000253961		lncRNA	chr8:31167078-3117
ENSG0000(	631	14.28828	chr8:2747	RPS27AP13		Pseudoger	chr8:53887008-5388
ENSG0000(	631	14.28828	chr8:2747	LINC01605		lncRNA	chr8:37421341-3755
ENSG0000(	631	14.28828	chr8:2747	BAG4	NCGv7	protein_c	chr8:38176533-3821
ENSG0000(	631	14.28828	chr8:2747	RPS15AP24		Pseudoger	chr8:30117384-3011
ENSG0000(	631	14.28828	chr8:2747	UNC5D	NCGv7	protein_c	chr8:35235475-3579
ENSG0000(	631	14.28828	chr8:2747	SLC20A2		protein_c	chr8:42416475-4254
ENSG0000(	631	14.28828	chr8:2747	RAB11FIP1	DriverDBv	protein_c	chr8:37858618-3789
ENSG0000(	631	14.28828	chr8:2747	IDI1P2		Pseudoger	chr8:48294026-4829
ENSG0000(	631	14.28828	chr8:2747	DCTN6-DT		lncRNA	chr8:30155828-3015
ENSG0000(	631	14.28828	chr8:2747	ENSG00000253160		Pseudoger	chr8:39309909-3931
ENSG0000(	631	14.28828	chr8:2747	ENSG00000253668		Pseudoger	chr8:53712697-5371
ENSG0000(	631	14.28828	chr8:2747	RNA5SP267		Pseudoger	chr8:59455885-5945
ENSG0000(	631	14.28828	chr8:2747	ENSG00000286348		lncRNA	chr8:55067585-5508
ENSG0000(	631	14.28828	chr8:2747	ENSG00000253376		lncRNA	chr8:57888975-5793
ENSG0000(	631	14.28828	chr8:2747	ENSG00000253688		lncRNA	chr8:48551527-4855
ENSG0000(	631	14.28828	chr8:2747	RN7SL709P		smallRNA	chr8:37908741-3790
ENSG0000(	631	14.28828	chr8:2747	ENSG00000253380		lncRNA	chr8:49496763-4951
ENSG0000(	631	14.28828	chr8:2747	ENSG00000290028		lncRNA	chr8:47369674-4736
ENSG0000(	631	14.28828	chr8:2747	MTND5P41		Pseudoger	chr8:36279491-3627
ENSG0000(	631	14.28828	chr8:2747	ENSG00000253135		Pseudoger	chr8:42128774-4212
ENSG0000(	631	14.28828	chr8:2747	ENSG00000253377		lncRNA	chr8:31275542-3139
ENSG0000(	631	14.28828	chr8:2747	RNU6-1276P		smallRNA	chr8:27773645-2777
ENSG0000(	631	14.28828	chr8:2747	AC098612.1		smallRNA	chr8:34970206-3497
ENSG0000(	631	14.28828	chr8:2747	ENSG00000254216		Pseudoger	chr8:56252960-5625
ENSG0000(	631	14.28828	chr8:2747	AC104037.1		smallRNA	chr8:33195837-3319
ENSG0000(	631	14.28828	chr8:2747	ENSG00000271555		lncRNA	chr8:52722903-5272
ENSG0000(	631	14.28828	chr8:2747	GPAT4-AS1		lncRNA	chr8:41509593-4157
ENSG0000(	631	14.28828	chr8:2747	FAM110B	DriverDBv	protein_c	chr8:57994523-5820
ENSG0000(	631	14.28828	chr8:2747	THAP1		protein_c	chr8:42836674-4284
ENSG0000(	631	14.28828	chr8:2747	ENSG00000253340		Pseudoger	chr8:50859530-5086

ENSG0000(	631	14.28828	chr8:2747	ENSG00000253108		lncRNA	chr8:34228439-3434
ENSG0000(	631	14.28828	chr8:2747	ENSG00000253112		lncRNA	chr8:30596914-3059
ENSG0000(	631	14.28828	chr8:2747	BUD31P1		Pseudoger	chr8:33641581-3364
ENSG0000(	631	14.28828	chr8:2747	ENSG00000253114		Pseudoger	chr8:54696398-5469
ENSG0000(	631	14.28828	chr8:2747	ENSG00000253116		lncRNA	chr8:58091442-5810
ENSG0000(	631	14.28828	chr8:2747	ENSG00000255497		Pseudoger	chr8:43313948-4331
ENSG0000(	631	14.28828	chr8:2747	ENSG00000253121		lncRNA	chr8:63024372-6302
ENSG0000(	631	14.28828	chr8:2747	ENSG00000255487		lncRNA	chr8:38275888-3827
ENSG0000(	631	14.28828	chr8:2747	ENSG00000253123		lncRNA	chr8:37326575-3733
ENSG0000(	631	14.28828	chr8:2747	ENSG00000286131		protein_c	chr8:32647202-3264
ENSG0000(	631	14.28828	chr8:2747	ENSG00000244235		Pseudoger	chr8:42101318-4210
ENSG0000(	631	14.28828	chr8:2747	PLAG1	NCGv7;AC	protein_c	chr8:56160909-5621
ENSG0000(	631	14.28828	chr8:2747	KRT18P37		Pseudoger	chr8:41511240-4151
ENSG0000(	631	14.28828	chr8:2747	ENSG00000290005		lncRNA	chr8:28540998-2854
ENSG0000(	631	14.28828	chr8:2747	TUBBP1		Pseudoger	chr8:30351873-3035
ENSG0000(	631	14.28828	chr8:2747	PENK		protein_c	chr8:56436674-5644
ENSG0000(	631	14.28828	chr8:2747	ADGRA2		protein_c	chr8:37784191-3784
ENSG0000(	631	14.28828	chr8:2747	RPL7AP80		Pseudoger	chr8:39860219-3986
ENSG0000(	631	14.28828	chr8:2747	ALKAL1		protein_c	chr8:52534037-5256
ENSG0000(	631	14.28828	chr8:2747	LINC02947		lncRNA	chr8:48515852-4851
ENSG0000(	631	14.28828	chr8:2747	VDAC3	DriverDB\	protein_c	chr8:42391624-4240
ENSG0000(	631	14.28828	chr8:2747	AC018607.1		smallRNA	chr8:55908961-5590
ENSG0000(	631	14.28828	chr8:2747	ENSG00000253750		Pseudoger	chr8:64554236-6455
ENSG0000(	631	14.28828	chr8:2747	NASPP1		Pseudoger	chr8:60937705-6093
ENSG0000(	631	14.28828	chr8:2747	ENSG00000253762		lncRNA	chr8:63703077-6375
ENSG0000(	631	14.28828	chr8:2747	GOT1L1		protein_c	chr8:37934281-3794
ENSG0000(	631	14.28828	chr8:2747	RPL10AP2		Pseudoger	chr8:47157118-4715
ENSG0000(	631	14.28828	chr8:2747	RNA5SP259		Pseudoger	chr8:28530411-2853
ENSG0000(	631	14.28828	chr8:2747	Y_RNA		smallRNA	chr8:42949388-4294
ENSG0000(	631	14.28828	chr8:2747	POTEA		protein_c	chr8:43292483-4336
ENSG0000(	631	14.28828	chr8:2747	RN7SL798P		smallRNA	chr8:55980242-5598
ENSG0000(	631	14.28828	chr8:2747	ENSG00000253389		lncRNA	chr8:41660991-4166
ENSG0000(	631	14.28828	chr8:2747	ENSG00000253139		lncRNA	chr8:56536758-5654
ENSG0000(	631	14.28828	chr8:2747	ENSG00000240915		lncRNA	chr8:63013068-6301
ENSG0000(	631	14.28828	chr8:2747	ENSG00000240919		Pseudoger	chr8:53532908-5353
ENSG0000(	631	14.28828	chr8:2747	ENSG00000253388		lncRNA	chr8:37480089-3748
ENSG0000(	631	14.28828	chr8:2747	ENSG00000253793		Pseudoger	chr8:30983459-3098
ENSG0000(	631	14.28828	chr8:2747	UBE2V2	DriverDB\	protein_c	chr8:48008415-4806
ENSG0000(	631	14.28828	chr8:2747	ENSG00000253381		lncRNA	chr8:40104169-4010
ENSG0000(	631	14.28828	chr8:2747	FGFR1	NCGv7;AC	protein_c	chr8:38400215-3846
ENSG0000(	631	14.28828	chr8:2747	YTHDF3	DriverDB\	protein_c	chr8:63168553-6321
ENSG0000(	631	14.28828	chr8:2747	ENSG00000255321		lncRNA	chr8:60551957-6055
ENSG0000(	631	14.28828	chr8:2747	ENSG00000254348		Pseudoger	chr8:47189305-4718
ENSG0000(	631	14.28828	chr8:2747	NRG1	NCGv7	protein_c	chr8:31639222-3285
ENSG0000(	631	14.28828	chr8:2747	POLB	DriverDB\	protein_c	chr8:42338454-4237
ENSG0000(	631	14.28828	chr8:2747	VN1R46P		Pseudoger	chr8:43219960-4322
ENSG0000(	631	14.28828	chr8:2747	ENSG00000248347		Pseudoger	chr8:47089572-4719
ENSG0000(	631	14.28828	chr8:2747	ADAM32	DriverDB\	protein_c	chr8:39106990-3928
ENSG0000(	631	14.28828	chr8:2747	PCMTD1	NCGv7	protein_c	chr8:51817575-5189
ENSG0000(	631	14.28828	chr8:2747	ENSG00000286701		lncRNA	chr8:49046652-4905
ENSG0000(	631	14.28828	chr8:2747	RNU6-295P		smallRNA	chr8:48307927-4830

ENSG0000C	631	14.28828	chr8:2747	ENSG00000253181		lncRNA	chr8:37717579-3773
ENSG0000C	631	14.28828	chr8:2747	RPL10P18		Pseudoger	chr8:33540185-3354
ENSG0000C	631	14.28828	chr8:2747	ENSG00000240015		lncRNA	chr8:38123274-3812
ENSG0000C	631	14.28828	chr8:2747	LINC01602		lncRNA	chr8:57855500-5798
ENSG0000C	631	14.28828	chr8:2747	ENSG00000253614		lncRNA	chr8:57261226-5726
ENSG0000C	631	14.28828	chr8:2747	ENSG00000286693		lncRNA	chr8:35672195-3570
ENSG0000C	631	14.28828	chr8:2747	ENSG00000272159		lncRNA	chr8:38408048-3840
ENSG0000C	631	14.28828	chr8:2747	ENSG00000254383		lncRNA	chr8:40900016-4090
ENSG0000C	631	14.28828	chr8:2747	ENSG00000272155		lncRNA	chr8:65714334-6571
ENSG0000C	631	14.28828	chr8:2747	ENSG00000188512		Pseudoger	chr8:33969567-3396
ENSG0000C	631	14.28828	chr8:2747	MTND6P19		Pseudoger	chr8:36278960-3627
ENSG0000C	631	14.28828	chr8:2747	PDE7A		protein_c	chr8:65714334-6584
ENSG0000C	631	14.28828	chr8:2747	POMK	DriverDBv	protein_c	chr8:43093498-4313
ENSG0000C	631	14.28828	chr8:2747	ENSG00000286677		lncRNA	chr8:38082122-3809
ENSG0000C	631	14.28828	chr8:2747	RNU6-528P		smallRNA	chr8:33338867-3333
ENSG0000C	631	14.28828	chr8:2747	AP3M2	DriverDBv	protein_c	chr8:42152946-4217
ENSG0000C	631	14.28828	chr8:2747	ENSG00000247134		lncRNA	chr8:32927913-3304
ENSG0000C	631	14.28828	chr8:2747	ATP6V1H		protein_c	chr8:53715543-5384
ENSG0000C	631	14.28828	chr8:2747	ENSG00000253993		Pseudoger	chr8:33226848-3322
ENSG0000C	631	14.28828	chr8:2747	ENSG00000254392		Pseudoger	chr8:50490795-5049
ENSG0000C	631	14.28828	chr8:2747	ENSG00000255289		lncRNA	chr8:60660820-6066
ENSG0000C	631	14.28828	chr8:2747	ENSG00000253610		Pseudoger	chr8:36308245-3630
ENSG0000C	631	14.28828	chr8:2747	ENSG00000288818		lncRNA	chr8:52714605-5271
ENSG0000C	631	14.28828	chr8:2747	MBOAT4		protein_c	chr8:30131671-3014
ENSG0000C	631	14.28828	chr8:2747	HOOK3	DriverDBv	protein_c	chr8:42896946-4303
ENSG0000C	631	14.28828	chr8:2747	ENSG00000264578		lncRNA	chr8:41609692-4162
ENSG0000C	631	14.28828	chr8:2747	MIR486		smallRNA	chr8:41660441-4166
ENSG0000C	631	14.28828	chr8:2747	ENSG00000272256		lncRNA	chr8:30082758-3008
ENSG0000C	631	14.28828	chr8:2747	XKR4-AS1		lncRNA	chr8:55517240-5552
ENSG0000C	631	14.28828	chr8:2747	ENSG00000254006		lncRNA	chr8:64801236-6481
ENSG0000C	631	14.28828	chr8:2747	CYCSP22		Pseudoger	chr8:50762460-5076
ENSG0000C	631	14.28828	chr8:2747	XRCC6P4		Pseudoger	chr8:62855068-6285
ENSG0000C	631	14.28828	chr8:2747	AC136365.1		smallRNA	chr8:39756082-3975
ENSG0000C	631	14.28828	chr8:2747	RN7SKP201		smallRNA	chr8:36266485-3626
ENSG0000C	631	14.28828	chr8:2747	ENSG00000260955		lncRNA	chr8:54042989-5404
ENSG0000C	631	14.28828	chr8:2747	ENSG00000253782		lncRNA	chr8:46930604-4695
ENSG0000C	631	14.28828	chr8:2747	DUSP4		protein_c	chr8:29333064-2935
ENSG0000C	631	14.28828	chr8:2747	ENSG00000254370		lncRNA	chr8:28415524-2842
ENSG0000C	631	14.28828	chr8:2747	CLU		protein_c	chr8:27596917-2761
ENSG0000C	631	14.28828	chr8:2747	ENSG00000253924		lncRNA	chr8:52294455-5230
ENSG0000C	631	14.28828	chr8:2747	RP11-503E24.3		lncRNA	chr8:42554790-4255
ENSG0000C	631	14.28828	chr8:2747	EPHX2		protein_c	chr8:27490781-2754
ENSG0000C	631	14.28828	chr8:2747	NKAIN3	DriverDBv	protein_c	chr8:62248591-6301
ENSG0000C	631	14.28828	chr8:2747	ENSG00000289603		lncRNA	chr8:42140453-4214
ENSG0000C	631	14.28828	chr8:2747	RNF170		protein_c	chr8:42849637-4289
ENSG0000C	631	14.28828	chr8:2747	AFG3L2P1		Pseudoger	chr8:43270198-4327
ENSG0000C	631	14.28828	chr8:2747	LYPLA1	NCGv7	protein_c	chr8:54046367-5410
ENSG0000C	631	14.28828	chr8:2747	ENSG00000260949		lncRNA	chr8:38062881-3806
ENSG0000C	631	14.28828	chr8:2747	LINC02948		lncRNA	chr8:29548171-2956
ENSG0000C	631	14.28828	chr8:2747	ENSG00000253608		lncRNA	chr8:48551567-4869
ENSG0000C	631	14.28828	chr8:2747	LINC01605		lncRNA	chr8:37516399-3752

ENSG0000(	631	14.28828	chr8:2747	ENSG00000272128		lncRNA	chr8:38099471-3809
ENSG0000(	631	14.28828	chr8:2747	ENSG00000253745		lncRNA	chr8:46922561-4693
ENSG0000(	631	14.28828	chr8:2747	ENSG00000253976		lncRNA	chr8:55135203-5514
ENSG0000(	631	14.28828	chr8:2747	ENSG00000183154		lncRNA	chr8:37734761-3773
ENSG0000(	631	14.28828	chr8:2747	ENSG00000272024		lncRNA	chr8:51950284-5195
ENSG0000(	631	14.28828	chr8:2747	ENSG00000279041		TEC	chr8:30552345-3055
ENSG0000(	631	14.28828	chr8:2747	ENSG00000272010		lncRNA	chr8:65591850-6559
ENSG0000(	631	14.28828	chr8:2747	RN7SL250P		smallRNA	chr8:54420468-5442
ENSG0000(	631	14.28828	chr8:2747	TPT1P8		Pseudoger	chr8:36887456-3689
ENSG0000(	631	14.28828	chr8:2747	ENSG00000274489		Pseudoger	chr8:36795255-3679
ENSG0000(	631	14.28828	chr8:2747	LINC00968		lncRNA	chr8:56494309-5655
ENSG0000(	631	14.28828	chr8:2747	CYP4F44P		Pseudoger	chr8:43539973-4354
ENSG0000(	631	14.28828	chr8:2747	AC009800.1		smallRNA	chr8:52995362-5299
ENSG0000(	631	14.28828	chr8:2747	AC144573.1		smallRNA	chr8:37949736-3794
ENSG0000(	631	14.28828	chr8:2747	IFITM3P8		Pseudoger	chr8:60906059-6090
ENSG0000(	631	14.28828	chr8:2747	ENSG00000253642		lncRNA	chr8:33604856-3403
ENSG0000(	631	14.28828	chr8:2747	RN7SL621P		smallRNA	chr8:33580212-3358
ENSG0000(	631	14.28828	chr8:2747	ENSG00000260484		lncRNA	chr8:53388701-5339
ENSG0000(	631	14.28828	chr8:2747	ENSG00000279099		TEC	chr8:35254344-3525
ENSG0000(	631	14.28828	chr8:2747	ENSG00000253645		lncRNA	chr8:38970360-3897
ENSG0000(	631	14.28828	chr8:2747	ENSG00000253708		lncRNA	chr8:30176554-3018
ENSG0000(	631	14.28828	chr8:2747	DDHD2	DriverDB\	protein_c	chr8:38225218-3827
ENSG0000(	631	14.28828	chr8:2747	ENSG00000253397		lncRNA	chr8:27904481-2791
ENSG0000(	631	14.28828	chr8:2747	ENSG00000253174		lncRNA	chr8:41540381-4154
ENSG0000(	631	14.28828	chr8:2747	ENSG00000271938		lncRNA	chr8:42139461-4213
ENSG0000(	631	14.28828	chr8:2747	SMARCE1P4		Pseudoger	chr8:37095150-3709
ENSG0000(	631	14.28828	chr8:2747	SEC11B		Pseudoger	chr8:54522799-5452
ENSG0000(	631	14.28828	chr8:2747	ENSG00000253840		Pseudoger	chr8:54247633-5424
ENSG0000(	631	14.28828	chr8:2747	MIR3622A		smallRNA	chr8:27701673-2770
ENSG0000(	631	14.28828	chr8:2747	ENSG00000254432		lncRNA	chr8:60808735-6080
ENSG0000(	631	14.28828	chr8:2747	BTF3P1		Pseudoger	chr8:51721670-5172
ENSG0000(	631	14.28828	chr8:2747	ENSG00000253615		Pseudoger	chr8:27903566-2790
ENSG0000(	631	14.28828	chr8:2747	PPDPFL		protein_c	chr8:49054311-4907
ENSG0000(	631	14.28828	chr8:2747	RNU6-1202P		smallRNA	chr8:60485182-6048
ENSG0000(	631	14.28828	chr8:2747	ENSG00000253413		Pseudoger	chr8:59760047-5976
ENSG0000(	631	14.28828	chr8:2747	PRKDC	NCGv7	protein_c	chr8:47773111-4796
ENSG0000(	631	14.28828	chr8:2747	CA8		protein_c	chr8:60185412-6028
ENSG0000(	631	14.28828	chr8:2747	MIR3148		smallRNA	chr8:29957272-2995
ENSG0000(	631	14.28828	chr8:2747	AC131254.1		smallRNA	chr8:29928605-2992
ENSG0000(	631	14.28828	chr8:2747	ENSG00000251354		Pseudoger	chr8:47546804-4754
ENSG0000(	631	14.28828	chr8:2747	ENSG00000253707		Pseudoger	chr8:43284626-4328
ENSG0000(	631	14.28828	chr8:2747	GPAT4	NCGv7	protein_c	chr8:41577187-4162
ENSG0000(	631	14.28828	chr8:2747	ENSG00000286648		lncRNA	chr8:31827238-3184
ENSG0000(	631	14.28828	chr8:2747	ENSG00000272092		lncRNA	chr8:38382364-3838
ENSG0000(	631	14.28828	chr8:2747	AC025674.1		smallRNA	chr8:57183634-5718
ENSG0000(	631	14.28828	chr8:2747	ENSG00000285632		lncRNA	chr8:38223957-3823
ENSG0000(	631	14.28828	chr8:2747	MIR4287		smallRNA	chr8:27886039-2788
ENSG0000(	631	14.28828	chr8:2747	ENSG00000253632		lncRNA	chr8:29527312-2953
ENSG0000(	631	14.28828	chr8:2747	ENSG00000250031		Pseudoger	chr8:58424588-5842
ENSG0000(	631	14.28828	chr8:2747	ENSG00000253176		Pseudoger	chr8:27838477-2784
ENSG0000(	631	14.28828	chr8:2747	ENSG00000253363		lncRNA	chr8:36378069-3677

ENSG0000(	631	14.28828	chr8:2747	ENSG00000272076		lncRNA	chr8:51810110-5181
ENSG0000(	631	14.28828	chr8:2747	ENSG00000253939		lncRNA	chr8:39903775-3999
ENSG0000(	631	14.28828	chr8:2747	PLEKHA2		protein_c	chr8:38901235-3897
ENSG0000(	631	14.28828	chr8:2747	IKBKB-DT		lncRNA	chr8:42233674-4227
ENSG0000(	631	14.28828	chr8:2747	HTRA4		protein_c	chr8:38974228-3898
ENSG0000(	631	14.28828	chr8:2747	TM2D2	DriverDBv	protein_c	chr8:38988808-3899
ENSG0000(	631	14.28828	chr8:2747	ENSG00000253746		lncRNA	chr8:37405439-3740
ENSG0000(	631	14.28828	chr8:2747	ENSG00000253871		lncRNA	chr8:57341021-5736
ENSG0000(	631	14.28828	chr8:2747	ERLIN2	DriverDBv	protein_c	chr8:37736601-3775
ENSG0000(	631	14.28828	chr8:2747	Y_RNA		smallRNA	chr8:38744020-3874
ENSG0000(	631	14.28828	chr8:2747	ENSG00000254198		Pseudoger	chr8:43125995-4312
ENSG0000(	631	14.28828	chr8:2747	EIF4EBP1	DriverDBv	protein_c	chr8:38030534-3806
ENSG0000(	631	14.28828	chr8:2747	WRN	NCGv7;AC	protein_c	chr8:31033788-3117
ENSG0000(	631	14.28828	chr8:2747	NSMAF	NCGv7	protein_c	chr8:58583508-5865
ENSG0000(	631	14.28828	chr8:2747	MTCYBP20		Pseudoger	chr8:46837578-4683
ENSG0000(	631	14.28828	chr8:2747	ENSG00000287998		lncRNA	chr8:64798804-6486
ENSG0000(	631	14.28828	chr8:2747	PPIAP86		Pseudoger	chr8:65377592-6537
ENSG0000(	631	14.28828	chr8:2747	LINC02209		lncRNA	chr8:29920560-2995
ENSG0000(	631	14.28828	chr8:2747	PPIAP84		Pseudoger	chr8:30349866-3035
ENSG0000(	631	14.28828	chr8:2747	RNU6-819P		smallRNA	chr8:47131781-4713
ENSG0000(	631	14.28828	chr8:2747	SNORD54		smallRNA	chr8:56073835-5607
ENSG0000(	631	14.28828	chr8:2747	ENSG00000236814		Pseudoger	chr8:56050038-5605
ENSG0000(	631	14.28828	chr8:2747	ENSG00000254898		lncRNA	chr8:38335981-3833
ENSG0000(	631	14.28828	chr8:2747	ENSG00000253455		lncRNA	chr8:48620465-4862
ENSG0000(	631	14.28828	chr8:2747	TDGF1P5		Pseudoger	chr8:54073114-5407
ENSG0000(	631	14.28828	chr8:2747	SDR16C6P		Pseudoger	chr8:56373064-5639
ENSG0000(	631	14.28828	chr8:2747	RNU6-663P		smallRNA	chr8:32911493-3291
ENSG0000(	631	14.28828	chr8:2747	KIF13B		protein_c	chr8:29067278-2926
ENSG0000(	631	14.28828	chr8:2747	ST18		protein_c	chr8:52110838-5246
ENSG0000(	631	14.28828	chr8:2747	RNA5SP266		Pseudoger	chr8:57126655-5712
ENSG0000(	631	14.28828	chr8:2747	RGS20		protein_c	chr8:53851795-5395
ENSG0000(	631	14.28828	chr8:2747	C8orf86	DriverDBv	lncRNA	chr8:38510834-3856
ENSG0000(	631	14.28828	chr8:2747	LINC02842		lncRNA	chr8:61785047-6194
ENSG0000(	631	14.28828	chr8:2747	ENSG00000248964		lncRNA	chr8:29815004-2985
ENSG0000(	631	14.28828	chr8:2747	LINC02155		lncRNA	chr8:61889839-6189
ENSG0000(	631	14.28828	chr8:2747	ENSG00000253275		lncRNA	chr8:31497423-3149
ENSG0000(	631	14.28828	chr8:2747	ENSG00000237810		Pseudoger	chr8:62143311-6214
ENSG0000(	631	14.28828	chr8:2747	TEX15	NCGv7	protein_c	chr8:30831544-3091
ENSG0000(	631	14.28828	chr8:2747	LINC02984		lncRNA	chr8:53493523-5352
ENSG0000(	631	14.28828	chr8:2747	ENSG00000287204		lncRNA	chr8:31536630-3154
ENSG0000(	631	14.28828	chr8:2747	snoU13		smallRNA	chr8:27755032-2775
ENSG0000(	631	14.28828	chr8:2747	RNU6-1331P		smallRNA	chr8:53914719-5391
ENSG0000(	631	14.28828	chr8:2747	ENSG00000253346		Pseudoger	chr8:30331961-3033
ENSG0000(	631	14.28828	chr8:2747	AC100817.1		smallRNA	chr8:55677233-5567
ENSG0000(	631	14.28828	chr8:2747	snoU13		smallRNA	chr8:42225633-4222
ENSG0000(	631	14.28828	chr8:2747	DUSP26	TAG;AC	protein_c	chr8:33591330-3360
ENSG0000(	631	14.28828	chr8:2747	ENSG00000254687		lncRNA	chr8:53177571-5324
ENSG0000(	631	14.28828	chr8:2747	ENSG00000228862		lncRNA	chr8:60629662-6065
ENSG0000(	631	14.28828	chr8:2747	ENSG00000287301		lncRNA	chr8:57420834-5742
ENSG0000(	631	14.28828	chr8:2747	ASPH		protein_c	chr8:61500556-6171
ENSG0000(	631	14.28828	chr8:2747	ENSG00000243181		Pseudoger	chr8:34874159-3487

ENSG0000(	631	14.28828	chr8:2747	TOX	NCGv7	protein_c	chr8:58805412-5911
ENSG0000(	631	14.28828	chr8:2747	ASH2L	DriverDB\	protein_c	chr8:38105493-3814
ENSG0000(	631	14.28828	chr8:2747	ADAM5		Pseudoger	chr8:39314591-3941
ENSG0000(	631	14.28828	chr8:2747	ENSG00000288735		lncRNA	chr8:29288506-2932
ENSG0000(	631	14.28828	chr8:2747	TTI2		protein_c	chr8:33473386-3351
ENSG0000(	631	14.28828	chr8:2747	ENSG00000251142		Pseudoger	chr8:57240057-5724
ENSG0000(	631	14.28828	chr8:2747	RNU6-607P		smallRNA	chr8:37668898-3766
ENSG0000(	631	14.28828	chr8:2747	TCEA1	DriverDB\	protein_c	chr8:53966552-5402
ENSG0000(	631	14.28828	chr8:2747	RPL12P48		Pseudoger	chr8:37983070-3798
ENSG0000(	631	14.28828	chr8:2747	LINC00251		lncRNA	chr8:65161145-6518
ENSG0000(	631	14.28828	chr8:2747	RFPL4AP7		Pseudoger	chr8:49352630-4935
ENSG0000(	631	14.28828	chr8:2747	RBPMS-AS1		lncRNA	chr8:30382119-3038
ENSG0000(	631	14.28828	chr8:2747	PXDNL	NCGv7	protein_c	chr8:51319577-5180
ENSG0000(	631	14.28828	chr8:2747	ENSG00000254111		lncRNA	chr8:37559992-3756
ENSG0000(	631	14.28828	chr8:2747	ENSG00000269924		lncRNA	chr8:47527397-4752
ENSG0000(	631	14.28828	chr8:2747	ENSG00000253281		lncRNA	chr8:58255771-5827
ENSG0000(	631	14.28828	chr8:2747	PCMTD1-DT		lncRNA	chr8:51899268-5194
ENSG0000(	631	14.28828	chr8:2747	CHCHD7	DriverDB\	protein_c	chr8:56211686-5621
ENSG0000(	631	14.28828	chr8:2747	RB1CC1	NCGv7	protein_c	chr8:52622458-5274
ENSG0000(	631	14.28828	chr8:2747	ZNF395		protein_c	chr8:28345590-2840
ENSG0000(	631	14.28828	chr8:2747	ENSG00000276605		Pseudoger	chr8:54354168-5435
ENSG0000(	631	14.28828	chr8:2747	AC123767.1		smallRNA	chr8:39560937-3956
ENSG0000(	631	14.28828	chr8:2747	PENK-AS1		lncRNA	chr8:56445807-5655
ENSG0000(	631	14.28828	chr8:2747	SNTG1		protein_c	chr8:49909789-5079
ENSG0000(	631	14.28828	chr8:2747	SMIM18		protein_c	chr8:30638580-3064
ENSG0000(	631	14.28828	chr8:2747	LINC01414		lncRNA	chr8:63687179-6436
ENSG0000(	631	14.28828	chr8:2747	ENSG00000254129		lncRNA	chr8:29110573-2914
ENSG0000(	631	14.28828	chr8:2747	ENSG00000243503		Pseudoger	chr8:36987977-3698
ENSG0000(	631	14.28828	chr8:2747	RNF122		protein_c	chr8:33547754-3356
ENSG0000(	631	14.28828	chr8:2747	RPL3P10		Pseudoger	chr8:39157538-3915
ENSG0000(	631	14.28828	chr8:2747	SEPTIN10P1		Pseudoger	chr8:56476826-5647
ENSG0000(	631	14.28828	chr8:2747	PURG		protein_c	chr8:30995802-3103
ENSG0000(	631	14.28828	chr8:2747	VENTXP5		Pseudoger	chr8:33722305-3372
ENSG0000(	631	14.28828	chr8:2747	ENSG00000253560		Pseudoger	chr8:27697217-2769
ENSG0000(	631	14.28828	chr8:2747	LINC03018		lncRNA	chr8:57746129-5775
ENSG0000(	631	14.28828	chr8:2747	NKX6-3		protein_c	chr8:41645177-4165
ENSG0000(	631	14.28828	chr8:2747	RNU6-104P		smallRNA	chr8:43303376-4330
ENSG0000(	631	14.28828	chr8:2747	ZMAT4		protein_c	chr8:40530590-4089
ENSG0000(	631	14.28828	chr8:2747	ENSG00000270980		Pseudoger	chr8:54191582-5419
ENSG0000(	631	14.28828	chr8:2747	LETM2	DriverDB\	protein_c	chr8:38386207-3840
ENSG0000(	631	14.28828	chr8:2747	ENSG00000253260		lncRNA	chr8:59561324-5960
ENSG0000(	631	14.28828	chr8:2747	KCTD9P6		Pseudoger	chr8:31219894-3122
ENSG0000(	631	14.28828	chr8:2747	LINC02847		lncRNA	chr8:48597458-4862
ENSG0000(	631	14.28828	chr8:2747	MIR548AO		smallRNA	chr8:41271048-4127
ENSG0000(	631	14.28828	chr8:2747	ENSG00000253884		Pseudoger	chr8:43246755-4324
ENSG0000(	631	14.28828	chr8:2747	SOX17	NCGv7	protein_c	chr8:54457935-5446
ENSG0000(	631	14.28828	chr8:2747	CYP7B1	NCGv7	protein_c	chr8:64587763-6479
ENSG0000(	631	14.28828	chr8:2747	TACC1	NCGv7	protein_c	chr8:38728186-3885
ENSG0000(	631	14.28828	chr8:2747	AC084262.2		smallRNA	chr8:29313756-2931
ENSG0000(	631	14.28828	chr8:2747	ENSG00000253894		lncRNA	chr8:63465831-6347
ENSG0000(	631	14.28828	chr8:2747	TRIM60P15		Pseudoger	chr8:46792065-4679

ENSG0000(	631	14.28828	chr8:2747	NPBWR1		protein_c	chr8:52939182-5294
ENSG0000(	631	14.28828	chr8:2747	ENSG00000254136		lncRNA	chr8:51996210-5200
ENSG0000(	631	14.28828	chr8:2747	RNU7-174P		smallRNA	chr8:57556585-5755
ENSG0000(	631	14.28828	chr8:2747	ENSG00000253879		lncRNA	chr8:59601319-5962
ENSG0000(	631	14.28828	chr8:2747	ENSG00000253567		lncRNA	chr8:28447264-2845
ENSG0000(	631	14.28828	chr8:2747	ENSG00000254673		protein_c	chr8:43018424-4307
ENSG0000(	631	14.28828	chr8:2747	LINC01606		lncRNA	chr8:57142659-5724
ENSG0000(	631	14.28828	chr8:2747	SPIDR	DriverDB\	protein_c	chr8:47260878-4773
ENSG0000(	631	14.28828	chr8:2747	AC084262.1		smallRNA	chr8:29269974-2927
ENSG0000(	631	14.28828	chr8:2747	CLXN		protein_c	chr8:48710789-4873
ENSG0000(	631	14.28828	chr8:2747	FUT10		protein_c	chr8:33370824-3347
ENSG0000(	631	14.28828	chr8:2747	ENSG00000280147		TEC	chr8:29587001-2958
ENSG0000(	631	14.28828	chr8:2747	NSD3	NCGv7;AC	protein_c	chr8:38269704-3838
ENSG0000(	631	14.28828	chr8:2747	ESCO2		protein_c	chr8:27771949-2781
ENSG0000(	631	14.28828	chr8:2747	ENSG00000253845		Pseudoger	chr8:43672823-4367
ENSG0000(	631	14.28828	chr8:2747	ENSG00000254131		Pseudoger	chr8:42484600-4248
ENSG0000(	631	14.28828	chr8:2747	GINS4	DriverDB\	protein_c	chr8:41529218-4154
ENSG0000(	631	14.28828	chr8:2747	ENSG00000253452		lncRNA	chr8:35862123-3619
ENSG0000(	631	14.28828	chr8:2747	ENSG00000253821		lncRNA	chr8:57492884-5759
ENSG0000(	631	14.28828	chr8:2747	snoU13		smallRNA	chr8:58662241-5866
ENSG0000(	631	14.28828	chr8:2747	ENSG00000254150		Pseudoger	chr8:59557528-5955
ENSG0000(	631	14.28828	chr8:2747	SLC2A13P1		Pseudoger	chr8:60141821-6014
ENSG0000(	631	14.28828	chr8:2747	ENSG00000254145		Pseudoger	chr8:43539137-4353
ENSG0000(	631	14.28828	chr8:2747	RNF5P1		Pseudoger	chr8:38600661-3860
ENSG0000(	631	14.28828	chr8:2747	ENSG00000287590		lncRNA	chr8:30279549-3028
ENSG0000(	631	14.28828	chr8:2747	ENSG00000254143		lncRNA	chr8:40161458-4017
ENSG0000(	631	14.28828	chr8:2747	TARDBPP4		Pseudoger	chr8:63136223-6313
ENSG0000(	631	14.28828	chr8:2747	ENSG00000253892		lncRNA	chr8:48657260-4865
ENSG0000(	631	14.28828	chr8:2747	ENSG00000253817		Pseudoger	chr8:47198032-4719
ENSG0000(	631	14.28828	chr8:2747	PLPP5	DriverDB\	protein_c	chr8:38263130-3826
ENSG0000(	631	14.28828	chr8:2747	RN7SKP32		smallRNA	chr8:47068021-4706
ENSG0000(	631	14.28828	chr8:2747	ENSG00000261542		lncRNA	chr8:63215981-6321
ENSG0000(	631	14.28828	chr8:2747	HIKESHIP3		Pseudoger	chr8:30754236-3075
ENSG0000(	631	14.28828	chr8:2747	AC103686.1		smallRNA	chr8:47890058-4789
ENSG0000(	631	14.28828	chr8:2747	HGSNAT		protein_c	chr8:43140464-4320
ENSG0000(	631	14.28828	chr8:2747	ENSG00000287105		lncRNA	chr8:62658343-6269
ENSG0000(	631	14.28828	chr8:2747	MOS	NCGv7;AC	protein_c	chr8:56112942-5611
ENSG0000(	631	14.28828	chr8:2747	AC090186.1		protein_c	chr8:51817583-5181
ENSG0000(	631	14.28828	chr8:2747	GOLGA7	DriverDB\	protein_c	chr8:41488200-4151
ENSG0000(	631	14.28828	chr8:2747	ENSG00000287615		lncRNA	chr8:64456198-6446
ENSG0000(	631	14.28828	chr8:2747	MTCYBP19		Pseudoger	chr8:36277763-3627
ENSG0000(	631	14.28828	chr8:2747	ENSG00000253290		Pseudoger	chr8:27903229-2790
ENSG0000(	631	14.28828	chr8:2747	MTND6P20		Pseudoger	chr8:46838788-4683
ENSG0000(	631	14.28828	chr8:2747	SDR16C5		protein_c	chr8:56300005-5632
ENSG0000(	631	14.28828	chr8:2747	ELP3		protein_c	chr8:28089673-2819
ENSG0000(	631	14.28828	chr8:2747	ENSG00000287975		lncRNA	chr8:60876969-6090
ENSG0000(	631	14.28828	chr8:2747	ENSG00000261449		lncRNA	chr8:42151772-4215
ENSG0000(	631	14.28828	chr8:2747	ENSG00000228984		Pseudoger	chr8:46784800-4678
ENSG0000(	631	14.28828	chr8:2747	ENSG00000248911		Pseudoger	chr8:52975111-5297
ENSG0000(	631	14.28828	chr8:2747	HMBOX1-IT1		lncRNA	chr8:28949676-2895
ENSG0000(	631	14.28828	chr8:2747	MAK16		protein_c	chr8:33485182-3350

ENSG0000(	631	14.28828	chr8:2747	RAB2A	DriverDB\	protein_c	chr8:60516936-6062
ENSG0000(	631	14.28828	chr8:2747	DKK4		protein_c	chr8:42374063-4237
ENSG0000(	631	14.28828	chr8:2747	MIR124-2		smallRNA	chr8:64379149-6437
ENSG0000(	631	14.28828	chr8:2747	ENSG00000287535		lncRNA	chr8:41566858-4156
ENSG0000(	631	14.28828	chr8:2747	PLAT		protein_c	chr8:42174718-4220
ENSG0000(	631	14.28828	chr8:2747	IKBKB	NCGv7	protein_c	chr8:42271302-4233
ENSG0000(	631	14.28828	chr8:2747	AC016113.1		smallRNA	chr8:53037629-5303
ENSG0000(	631	14.28828	chr8:2747	FBXO16		protein_c	chr8:28348287-2849
ENSG0000(	631	14.28828	chr8:2747	ENSG00000254775		lncRNA	chr8:60046755-6013
ENSG0000(	631	14.28828	chr8:2747	SFRP1		protein_c	chr8:41261962-4130
ENSG0000(	631	14.28828	chr8:2747	Y_RNA		smallRNA	chr8:65592731-6559
ENSG0000(	631	14.28828	chr8:2747	ENSG00000288761		lncRNA	chr8:48921503-4892
ENSG0000(	631	14.28828	chr8:2747	ANK1	NCGv7	protein_c	chr8:41653220-4189
ENSG0000(	631	14.28828	chr8:2747	BPNT2		protein_c	chr8:56957931-5699
ENSG0000(	631	14.28828	chr8:2747	ENSG00000254777		lncRNA	chr8:60910053-6096
ENSG0000(	631	14.28828	chr8:2747	CEBPD	DriverDB\	protein_c	chr8:47736913-4773
ENSG0000(	631	14.28828	chr8:2747	INTS9		protein_c	chr8:28767661-2889
ENSG0000(	631	14.28828	chr8:2747	RNA5SP260		Pseudoger	chr8:29048494-2904
ENSG0000(	631	14.28828	chr8:2747	HSPA8P11		Pseudoger	chr8:30237382-3024
ENSG0000(	631	14.28828	chr8:2747	RNU6-1086P		smallRNA	chr8:27679832-2767
ENSG0000(	631	14.28828	chr8:2747	RN7SKP41		smallRNA	chr8:43381644-4338
ENSG0000(	631	14.28828	chr8:2747	LINC01289		lncRNA	chr8:63769428-6381
ENSG0000(	631	14.28828	chr8:2747	RN7SKP97		smallRNA	chr8:61631680-6163
ENSG0000(	631	14.28828	chr8:2747	ENSG00000253312		Pseudoger	chr8:43251711-4325
ENSG0000(	631	14.28828	chr8:2747	RN7SL457P		smallRNA	chr8:33715784-3371
ENSG0000(	631	14.28828	chr8:2747	CHD7	NCGv7	protein_c	chr8:60678740-6086
ENSG0000(	631	14.28828	chr8:2747	ENSG00000259366		lncRNA	chr8:29055929-2905
ENSG0000(	631	14.28828	chr8:2747	RNA5SP261		Pseudoger	chr8:31598099-3159
ENSG0000(	631	14.28828	chr8:2747	ENSG00000254204		lncRNA	chr8:53515170-5351
ENSG0000(	631	14.28828	chr8:2747	PSAT1P1		Pseudoger	chr8:49740216-4974
ENSG0000(	631	14.28828	chr8:2747	C1GALT1P3		Pseudoger	chr8:61909984-6191
ENSG0000(	631	14.28828	chr8:2747	ADAM2	NCGv7	protein_c	chr8:39743735-3983
ENSG0000(	631	14.28828	chr8:2747	RNU6-519P		smallRNA	chr8:47979414-4797
ENSG0000(	631	14.28828	chr8:2747	MCM4	DriverDB\	protein_c	chr8:47960185-4797
ENSG0000(	631	14.28828	chr8:2747	ENSG00000280064		TEC	chr8:37858949-3786
ENSG0000(	631	14.28828	chr8:2747	PPP2CB		protein_c	chr8:30774457-3081
ENSG0000(	631	14.28828	chr8:2747	UBXN8		protein_c	chr8:30729131-3076
ENSG0000(	631	14.28828	chr8:2747	ARMC1	DriverDB\	protein_c	chr8:65602458-6563
ENSG0000(	631	14.28828	chr8:2747	GSR		protein_c	chr8:30678066-3072
ENSG0000(	631	14.28828	chr8:2747	ENSG00000253551		lncRNA	chr8:52194362-5219
ENSG0000(	631	14.28828	chr8:2747	ENSG00000251127		lncRNA	chr8:61759094-6176
ENSG0000(	631	14.28828	chr8:2747	SUMO2P16		Pseudoger	chr8:31131539-3113
ENSG0000(	631	14.28828	chr8:2747	DCTN6		protein_c	chr8:30156319-3018
ENSG0000(	631	14.28828	chr8:2747	LEPROTL1	NCGv7	protein_c	chr8:30095408-3017
ENSG0000(	631	14.28828	chr8:2747	ENSG00000279881		TEC	chr8:48428143-4843
ENSG0000(	631	14.28828	chr8:2747	ENSG00000254194		lncRNA	chr8:33973701-3400
ENSG0000(	631	14.28828	chr8:2747	RNU6ATAC32P		smallRNA	chr8:54152807-5415
ENSG0000(	631	14.28828	chr8:2747	ENSG00000253493		Pseudoger	chr8:64973488-6497
ENSG0000(	631	14.28828	chr8:2747	ENSG00000253875		lncRNA	chr8:27732915-2775
ENSG0000(	631	14.28828	chr8:2747	LINC02099		lncRNA	chr8:29748309-2979
ENSG0000(	631	14.28828	chr8:2747	FZD3	NCGv7	protein_c	chr8:28494205-2857

ENSG0000(	631	14.28828	chr8:2747	ENSG00000287152		lncRNA	chr8:35080592-3509
ENSG0000(	631	14.28828	chr8:2747	EXTL3		protein_c	chr8:28600469-2875
ENSG0000(	631	14.28828	chr8:2747	RNA5SP265		Pseudoger	chr8:55745232-5574
ENSG0000(	631	14.28828	chr8:2747	ENSG00000253849		lncRNA	chr8:50381015-5038
ENSG0000(	631	14.28828	chr8:2747	ENSG00000259607		lncRNA	chr8:29067279-2906
ENSG0000(	631	14.28828	chr8:2747	ENSG00000254165		lncRNA	chr8:42537529-4253
ENSG0000(	631	14.28828	chr8:2747	ENSG00000271148		lncRNA	chr8:49906863-4990
ENSG0000(	631	14.28828	chr8:2747	SNORA1		smallRNA	chr8:55902723-5590
ENSG0000(	631	14.28828	chr8:2747	ENSG00000288041		lncRNA	chr8:58227578-5824
ENSG0000(	631	14.28828	chr8:2747	ENSG00000253857		lncRNA	chr8:55161446-5516
ENSG0000(	631	14.28828	chr8:2747	ENSG00000253475		lncRNA	chr8:51895957-5189
ENSG0000(	631	14.28828	chr8:2747	PPIAP85		Pseudoger	chr8:58503588-5850
ENSG0000(	631	14.28828	chr8:2747	ATP6V1G1P2		Pseudoger	chr8:47194002-4719
ENSG0000(	631	14.28828	chr8:2747	ENSG00000254802		lncRNA	chr8:60965802-6096
ENSG0000(	631	14.28828	chr8:2747	ENSG00000253829		lncRNA	chr8:38844866-3884
ENSG0000(	631	14.28828	chr8:2747	NPM1P6		Pseudoger	chr8:61202350-6120
ENSG0000(	631	14.28828	chr8:2747	RNA5SP263		Pseudoger	chr8:32256496-3225
ENSG0000(	631	14.28828	chr8:2747	BHLHE22-AS1		lncRNA	chr8:64574306-6458
ENSG0000(	631	14.28828	chr8:2747	ENSG00000253344		lncRNA	chr8:37626015-3767
ENSG0000(	631	14.28828	chr8:2747	MTND2P38		Pseudoger	chr8:46827228-4682
ENSG0000(	631	14.28828	chr8:2747	ENSG00000253484		lncRNA	chr8:49817586-4982
ENSG0000(	631	14.28828	chr8:2747	LINC00589		lncRNA	chr8:29673922-2974
ENSG0000(	631	14.28828	chr8:2747	ENSG00000253474		lncRNA	chr8:49086301-4922
ENSG0000(	631	14.28828	chr8:2747	LINC00293		lncRNA	chr8:46822174-4690
ENSG0000(	631	14.28828	chr8:2747	RNU1-124P		smallRNA	chr8:43073474-4307
ENSG0000(	631	14.28828	chr8:2747	RP1		protein_c	chr8:54509422-5487
ENSG0000(	631	14.28828	chr8:2747	BRF2	DriverDBv	protein_c	chr8:37843268-3784
ENSG0000(	631	14.28828	chr8:2747	ENSG00000287959		lncRNA	chr8:47941426-4794
ENSG0000(	630	14.26564	chr5:4231	ENSG00000243385		Pseudoger	chr5:83201229-8320
ENSG0000(	630	14.26564	chr5:4231	VCAN	NCGv7	protein_c	chr5:83471618-8358
ENSG0000(	630	14.26564	chr5:4231	RNU6-620P		smallRNA	chr5:83703448-8370
ENSG0000(	630	14.26564	chr5:4231	FTH1P9		Pseudoger	chr5:83426676-8342
ENSG0000(	630	14.26564	chr5:4231	VCAN-AS1		lncRNA	chr5:83531352-8358
ENSG0000(	630	14.26564	chr5:4231	COQ10BP2		Pseudoger	chr5:83279921-8328
ENSG0000(	630	14.26564	chr5:4231	HAPLN1	NCGv7	protein_c	chr5:83637805-8372
ENSG0000(	630	14.26564	chr5:4231	RNU4-11P		smallRNA	chr5:83803554-8380
ENSG0000(	630	14.26564	chr5:4231	RN7SKP295		smallRNA	chr5:83685866-8368
ENSG0000(	630	14.26564	chr5:4231	ENSG00000242858		Pseudoger	chr5:83746388-8374
ENSG0000(	629	14.24299	chr2:2577	RNU5E-9P		smallRNA	chr2:178142548-178
ENSG0000(	629	14.24299	chr2:2577	ENSG00000271401		lncRNA	chr2:178644717-178
ENSG0000(	629	14.24299	chr2:2577	SP3		protein_c	chr2:173880850-173
ENSG0000(	629	14.24299	chr2:2577	HOXD1		protein_c	chr2:176188668-176
ENSG0000(	629	14.24299	chr2:2577	PRKRA		protein_c	chr2:178431292-178
ENSG0000(	629	14.24299	chr2:2577	ENSG00000290018		lncRNA	chr2:174719821-174
ENSG0000(	629	14.24299	chr2:2577	ENSG00000229779		lncRNA	chr2:175904371-175
ENSG0000(	629	14.24299	chr2:2577	Y_RNA		smallRNA	chr2:175176499-175
ENSG0000(	629	14.24299	chr2:2577	PDK1		protein_c	chr2:172555373-172
ENSG0000(	629	14.24299	chr2:2577	SP9		protein_c	chr2:174334954-174
ENSG0000(	629	14.24299	chr2:2577	TTN		protein_c	chr2:178525989-178
ENSG0000(	629	14.24299	chr2:2577	ENSG00000218175		Pseudoger	chr2:176200908-176
ENSG0000(	629	14.24299	chr2:2577	RBM45		protein_c	chr2:178112424-178

ENSG0000(	629	14.24299	chr2:2577	ENSG00000237655	lncRNA	chr2:177603089-177
ENSG0000(	629	14.24299	chr2:2577	RNU6-187P	smallRNA	chr2:176929985-176
ENSG0000(	629	14.24299	chr2:2577	AC009336.1	smallRNA	chr2:176108643-176
ENSG0000(	629	14.24299	chr2:2577	ALDH7A1P2	Pseudoger	chr2:172893763-172
ENSG0000(	629	14.24299	chr2:2577	FKBP7	protein_c	chr2:178463664-178
ENSG0000(	629	14.24299	chr2:2577	RNU7-104P	smallRNA	chr2:178831371-178
ENSG0000(	629	14.24299	chr2:2577	ENSG00000279205	TEC	chr2:176822030-176
ENSG0000(	629	14.24299	chr2:2577	RNA5SP112	Pseudoger	chr2:177138699-177
ENSG0000(	629	14.24299	chr2:2577	RPS2P18	Pseudoger	chr2:173297629-173
ENSG0000(	629	14.24299	chr2:2577	RPS15AP14	Pseudoger	chr2:175184215-175
ENSG0000(	629	14.24299	chr2:2577	ENSG00000236449	lncRNA	chr2:174547141-174
ENSG0000(	629	14.24299	chr2:2577	RNU6-629P	smallRNA	chr2:178038902-178
ENSG0000(	629	14.24299	chr2:2577	NUDCP2	Pseudoger	chr2:178454716-178
ENSG0000(	629	14.24299	chr2:2577	DNAJC19P5	Pseudoger	chr2:177229191-177
ENSG0000(	629	14.24299	chr2:2577	ENSG00000227098	lncRNA	chr2:176830839-176
ENSG0000(	629	14.24299	chr2:2577	LINC01960	lncRNA	chr2:174025280-174
ENSG0000(	629	14.24299	chr2:2577	ENSG00000270956	lncRNA	chr2:178541125-178
ENSG0000(	629	14.24299	chr2:2577	ENSG00000236664	lncRNA	chr2:177953111-178
ENSG0000(	629	14.24299	chr2:2577	ENSG00000235047	lncRNA	chr2:175897336-175
ENSG0000(	629	14.24299	chr2:2577	RAPGEF4-AS1	lncRNA	chr2:172677141-172
ENSG0000(	629	14.24299	chr2:2577	ENSG00000271825	lncRNA	chr2:177300600-177
ENSG0000(	629	14.24299	chr2:2577	OSBPL6	protein_c	chr2:178194481-178
ENSG0000(	629	14.24299	chr2:2577	MAP3K20-AS1	lncRNA	chr2:173166446-173
ENSG0000(	629	14.24299	chr2:2577	ENSG00000230104	Pseudoger	chr2:172674212-172
ENSG0000(	629	14.24299	chr2:2577	PPIAP67	Pseudoger	chr2:176338637-176
ENSG0000(	629	14.24299	chr2:2577	RPSAP25	Pseudoger	chr2:176242111-176
ENSG0000(	629	14.24299	chr2:2577	RAPGEF4	protein_c	chr2:172735274-173
ENSG0000(	629	14.24299	chr2:2577	RNU6-1290P	smallRNA	chr2:175029769-175
ENSG0000(	629	14.24299	chr2:2577	ENSG00000273258	lncRNA	chr2:174011856-174
ENSG0000(	629	14.24299	chr2:2577	ENSG00000229066	lncRNA	chr2:175257250-175
ENSG0000(	629	14.24299	chr2:2577	RPL5P7	Pseudoger	chr2:173871653-173
ENSG0000(	629	14.24299	chr2:2577	ENSG00000237617	Pseudoger	chr2:173705948-173
ENSG0000(	629	14.24299	chr2:2577	HNRNPA1P39	Pseudoger	chr2:174310015-174
ENSG0000(	629	14.24299	chr2:2577	AGPS	protein_c	chr2:177392746-177
ENSG0000(	629	14.24299	chr2:2577	HOXD-AS2	lncRNA	chr2:176121611-176
ENSG0000(	629	14.24299	chr2:2577	H3P7	Pseudoger	chr2:177344443-177
ENSG0000(	629	14.24299	chr2:2577	PDK1-AS1	lncRNA	chr2:172480840-172
ENSG0000(	629	14.24299	chr2:2577	CBY1P1	Pseudoger	chr2:173477996-173
ENSG0000(	629	14.24299	chr2:2577	PDE11A-AS1	lncRNA	chr2:177653419-177
ENSG0000(	629	14.24299	chr2:2577	ENSG00000229337	lncRNA	chr2:177111036-177
ENSG0000(	629	14.24299	chr2:2577	PPIAP66	Pseudoger	chr2:173485865-173
ENSG0000(	629	14.24299	chr2:2577	ENSG00000271996	lncRNA	chr2:177306373-177
ENSG0000(	629	14.24299	chr2:2577	TTN-AS1	lncRNA	chr2:178521183-178
ENSG0000(	629	14.24299	chr2:2577	ENSG00000229434	lncRNA	chr2:176506245-176
ENSG0000(	629	14.24299	chr2:2577	ENSG00000236231	lncRNA	chr2:176524879-176
ENSG0000(	629	14.24299	chr2:2577	ENSG00000236501	lncRNA	chr2:176989418-177
ENSG0000(	629	14.24299	chr2:2577	snoU13	smallRNA	chr2:177345858-177
ENSG0000(	629	14.24299	chr2:2577	AC007435.1	smallRNA	chr2:175034517-175
ENSG0000(	629	14.24299	chr2:2577	JPT1P1	Pseudoger	chr2:173431349-173
ENSG0000(	629	14.24299	chr2:2577	AC019046.1	smallRNA	chr2:173061732-173
ENSG0000(	629	14.24299	chr2:2577	MAP3K20	protein_c	chr2:173075435-173

ENSG0000(	629	14.24299	chr2:2577	Y_RNA		smallRNA	chr2:172558151-172
ENSG0000(	629	14.24299	chr2:2577	ENSG00000287149		lncRNA	chr2:178828349-179
ENSG0000(	629	14.24299	chr2:2577	RNU6ATAC14P		smallRNA	chr2:176664676-176
ENSG0000(	629	14.24299	chr2:2577	Y_RNA		smallRNA	chr2:174977803-174
ENSG0000(	629	14.24299	chr2:2577	CYCTP		Pseudoger	chr2:178092503-178
ENSG0000(	629	14.24299	chr2:2577	ATP5MC3		protein_c	chr2:175176258-175
ENSG0000(	629	14.24299	chr2:2577	RPA3P1		Pseudoger	chr2:174501952-174
ENSG0000(	629	14.24299	chr2:2577	LINC01305		lncRNA	chr2:174326027-174
ENSG0000(	629	14.24299	chr2:2577	ENSG00000236391		Pseudoger	chr2:173338219-173
ENSG0000(	629	14.24299	chr2:2577	MIR933		smallRNA	chr2:175167633-175
ENSG0000(	629	14.24299	chr7:2516	PIGCP2		Pseudoger	chr7:107808734-107
ENSG0000(	629	14.24299	chr2:2577	AC068706.2		smallRNA	chr2:176382232-176
ENSG0000(	629	14.24299	chr2:2577	RNU6-763P		smallRNA	chr2:175022214-175
ENSG0000(	629	14.24299	chr2:2577	HAGLROS		lncRNA	chr2:176177717-176
ENSG0000(	629	14.24299	chr2:2577	GPR155-DT		lncRNA	chr2:174487380-174
ENSG0000(	629	14.24299	chr2:2577	KRT8P40		Pseudoger	chr2:177197985-177
ENSG0000(	629	14.24299	chr2:2577	ENSG00000270574		lncRNA	chr2:178578790-178
ENSG0000(	629	14.24299	chr2:2577	ENSG00000279598		TEC	chr2:178554561-178
ENSG0000(	629	14.24299	chr2:2577	ENSG00000279160		TEC	chr2:176189919-176
ENSG0000(	629	14.24299	chr2:2577	TTC30B		protein_c	chr2:177548998-177
ENSG0000(	629	14.24299	chr2:2577	ENSG00000229750		lncRNA	chr2:175167904-175
ENSG0000(	629	14.24299	chr2:2577	SDHDP5		Pseudoger	chr2:178017993-178
ENSG0000(	629	14.24299	chr2:2577	ENSG00000280414		TEC	chr2:174334438-174
ENSG0000(	629	14.24299	chr2:2577	ENSG00000271011		lncRNA	chr2:178577103-178
ENSG0000(	629	14.24299	chr2:2577	PJVK		protein_c	chr2:178451346-178
ENSG0000(	629	14.24299	chr2:2577	STUB1P1		Pseudoger	chr2:177177695-177
ENSG0000(	629	14.24299	chr2:2577	MIR3128		smallRNA	chr2:177255945-177
ENSG0000(	629	14.24299	chr2:2577	ENSG00000237798		lncRNA	chr2:174575227-174
ENSG0000(	629	14.24299	chr2:2577	PDE11A		protein_c	chr2:177623244-178
ENSG0000(	629	14.24299	chr2:2577	CHRNA1		protein_c	chr2:174747592-174
ENSG0000(	629	14.24299	chr2:2577	LRRC2P1		Pseudoger	chr2:174299324-174
ENSG0000(	629	14.24299	chr2:2577	H3P6		Pseudoger	chr2:174719908-174
ENSG0000(	629	14.24299	chr2:2577	HOXD8		protein_c	chr2:176129694-176
ENSG0000(	629	14.24299	chr2:2577	ENSG00000270799		Pseudoger	chr2:174171891-174
ENSG0000(	629	14.24299	chr2:2577	MIR4444-1		smallRNA	chr2:177212726-177
ENSG0000(	629	14.24299	chr2:2577	LNPK		protein_c	chr2:175923882-176
ENSG0000(	629	14.24299	chr2:2577	ENSG00000213963		lncRNA	chr2:177271125-177
ENSG0000(	629	14.24299	chr2:2577	ENSG00000289296		lncRNA	chr2:173965488-173
ENSG0000(	629	14.24299	chr2:2577	HOXD11	NCGv7;AC	protein_c	chr2:176104216-176
ENSG0000(	629	14.24299	chr2:2577	AC068706.1		smallRNA	chr2:176383427-176
ENSG0000(	629	14.24299	chr2:2577	HOXD12		protein_c	chr2:176099795-176
ENSG0000(	629	14.24299	chr2:2577	CDCA7		protein_c	chr2:173354820-173
ENSG0000(	629	14.24299	chr2:2577	EVX2		protein_c	chr2:176077472-176
ENSG0000(	629	14.24299	chr2:2577	API5P2		Pseudoger	chr2:177997273-177
ENSG0000(	629	14.24299	chr2:2577	RPSAP24		Pseudoger	chr2:174043008-174
ENSG0000(	629	14.24299	chr2:2577	Y_RNA		smallRNA	chr2:174264878-174
ENSG0000(	629	14.24299	chr2:2577	TTC30A		protein_c	chr2:177612999-177
ENSG0000(	629	14.24299	chr2:2577	HOXD10		protein_c	chr2:176108790-176
ENSG0000(	629	14.24299	chr2:2577	Y_RNA		smallRNA	chr2:177194983-177
ENSG0000(	629	14.24299	chr2:2577	RPL21P31		Pseudoger	chr2:175033778-175
ENSG0000(	629	14.24299	chr2:2577	RPS6P2		Pseudoger	chr2:179001756-179

ENSG0000(	629	14.24299	chr2:2577	RNU6-5P		smallRNA	chr2:174557966-174
ENSG0000(	629	14.24299	chr2:2577	HOXD4		protein_c	chr2:176151550-176
ENSG0000(	629	14.24299	chr2:2577	HOXD9	AC	protein_c	chr2:176122719-176
ENSG0000(	629	14.24299	chr2:2577	MIR10B		smallRNA	chr2:176150303-176
ENSG0000(	629	14.24299	chr2:2577	RN7SL65P		smallRNA	chr2:174240142-174
ENSG0000(	629	14.24299	chr2:2577	CHN1	AC	protein_c	chr2:174798809-175
ENSG0000(	629	14.24299	chr2:2577	ENSG00000230552		lncRNA	chr2:176723614-176
ENSG0000(	629	14.24299	chr2:2577	ENSG00000271141		lncRNA	chr2:178616581-178
ENSG0000(	629	14.24299	chr2:2577	ENSG00000279884		TEC	chr2:174545385-174
ENSG0000(	629	14.24299	chr2:2577	ENSG00000272729		lncRNA	chr2:176164164-176
ENSG0000(	629	14.24299	chr2:2577	ENSG00000222043		lncRNA	chr2:177264359-177
ENSG0000(	629	14.24299	chr2:2577	SCRN3		protein_c	chr2:174395730-174
ENSG0000(	629	14.24299	chr2:2577	HNRNPA3	NCGv7	protein_c	chr2:177212694-177
ENSG0000(	629	14.24299	chr2:2577	LINC01117		lncRNA	chr2:176495255-176
ENSG0000(	629	14.24299	chr2:2577	NFE2L2	NCGv7;AC	protein_c	chr2:177218667-177
ENSG0000(	629	14.24299	chr2:2577	snoU13		smallRNA	chr2:172691299-172
ENSG0000(	629	14.24299	chr2:2577	ENSG00000227241		Pseudoger	chr2:178105637-178
ENSG0000(	629	14.24299	chr2:2577	MTX2		protein_c	chr2:176269395-176
ENSG0000(	629	14.24299	chr2:2577	HOXD3		protein_c	chr2:176136612-176
ENSG0000(	629	14.24299	chr2:2577	PLEKHA3		protein_c	chr2:178480457-178
ENSG0000(	629	14.24299	chr2:2577	HAGLR		lncRNA	chr2:176164051-176
ENSG0000(	629	14.24299	chr2:2577	CHROMR		lncRNA	chr2:178413635-178
ENSG0000(	629	14.24299	chr2:2577	EXTL2P1		Pseudoger	chr2:175842887-175
ENSG0000(	629	14.24299	chr2:2577	ENSG00000237016		Pseudoger	chr2:173575110-173
ENSG0000(	629	14.24299	chr2:2577	GPR155		protein_c	chr2:174431571-174
ENSG0000(	629	14.24299	chr2:2577	LINC01116		lncRNA	chr2:176611437-176
ENSG0000(	629	14.24299	chr2:2577	AC096649.4		smallRNA	chr2:175330373-175
ENSG0000(	629	14.24299	chr2:2577	ENSG00000271151		lncRNA	chr2:173968351-173
ENSG0000(	629	14.24299	chr2:2577	FUCA1P1		Pseudoger	chr2:176804987-176
ENSG0000(	629	14.24299	chr2:2577	ENSG00000270277		lncRNA	chr2:178548884-178
ENSG0000(	629	14.24299	chr2:2577	ENSG00000289349		lncRNA	chr2:175594179-175
ENSG0000(	629	14.24299	chr2:2577	CIR1		protein_c	chr2:174348022-174
ENSG0000(	629	14.24299	chr2:2577	ENSG00000267784		lncRNA	chr2:178723457-178
ENSG0000(	629	14.24299	chr2:2577	WIPF1		protein_c	chr2:174559572-174
ENSG0000(	629	14.24299	chr2:2577	CCDC141		protein_c	chr2:178829757-179
ENSG0000(	629	14.24299	chr2:2577	ATF2		protein_c	chr2:175072250-175
ENSG0000(	629	14.24299	chr2:2577	OLA1		protein_c	chr2:174072447-174
ENSG0000(	629	14.24299	chr2:2577	HOXD13	NCGv7;AC	protein_c	chr2:176092721-176
ENSG0000(	629	14.24299	chr2:2577	ENSG00000223777		Pseudoger	chr2:176809426-176
ENSG0000(	623	14.10713	chr5:4231	C5orf46		protein_c	chr5:147880726-147
ENSG0000(	623	14.10713	chr5:4231	ENSG00000253297		lncRNA	chr5:148644687-148
ENSG0000(	623	14.10713	chr5:4231	MARCOL		protein_c	chr5:148221650-148
ENSG0000(	623	14.10713	chr5:4231	ENSG00000253406		lncRNA	chr5:149216523-149
ENSG0000(	623	14.10713	chr5:4231	ENSG00000249518		Pseudoger	chr5:147851644-147
ENSG0000(	623	14.10713	chr5:4231	SPINK13		protein_c	chr5:148268180-148
ENSG0000(	623	14.10713	chr5:4231	RNU6-732P		smallRNA	chr5:149057554-149
ENSG0000(	623	14.10713	chr5:4231	SPINK1		protein_c	chr5:147824572-147
ENSG0000(	623	14.10713	chr2:2577	RNU4-48P		smallRNA	chr2:127472234-127
ENSG0000(	623	14.10713	chr5:4231	HTR4		protein_c	chr5:148451032-148
ENSG0000(	623	14.10713	chr5:4231	SCGB3A2		protein_c	chr5:147870682-147
ENSG0000(	623	14.10713	chr5:4231	ENSG00000248647		lncRNA	chr5:149163955-149

ENSG0000(	623	14.10713	chr5:4231	ENSG00000251330		lncRNA	chr5:148430159-148
ENSG0000(	623	14.10713	chr5:4231	ENSG00000288774		lncRNA	chr5:147874114-147
ENSG0000(	623	14.10713	chr5:4231	JAKMIP2-AS1		lncRNA	chr5:147559994-147
ENSG0000(	623	14.10713	chr5:4231	ABLIM3		protein_c	chr5:149141483-149
ENSG0000(	623	14.10713	chr5:4231	HMGN1P16		Pseudoger	chr5:148221360-148
ENSG0000(	623	14.10713	chr5:4231	FBXO38-DT		lncRNA	chr5:148088125-148
ENSG0000(	623	14.10713	chr5:4231	SPINK14		protein_c	chr5:148168546-148
ENSG0000(	623	14.10713	chr5:4231	ENSG00000272411		lncRNA	chr5:148970340-148
ENSG0000(	623	14.10713	chr5:4231	SPINK7		protein_c	chr5:148312419-148
ENSG0000(	623	14.10713	chr5:4231	ENSG00000248362		lncRNA	chr5:147886086-147
ENSG0000(	623	14.10713	chr5:4231	SH3TC2-DT		lncRNA	chr5:149063239-149
ENSG0000(	623	14.10713	chr5:4231	ADRB2		protein_c	chr5:148826611-148
ENSG0000(	623	14.10713	chr5:4231	FBXO38		protein_c	chr5:148383935-148
ENSG0000(	623	14.10713	chr5:4231	ENSG00000251320		lncRNA	chr5:147887112-147
ENSG0000(	623	14.10713	chr5:4231	SPINK5		protein_c	chr5:148025683-148
ENSG0000(	623	14.10713	chr5:4231	PGBD4P3		Pseudoger	chr5:148307298-148
ENSG0000(	623	14.10713	chr5:4231	ENSG00000287630		lncRNA	chr5:147725551-147
ENSG0000(	623	14.10713	chr5:4231	MIR584		smallRNA	chr5:149062313-149
ENSG0000(	623	14.10713	chr5:4231	SPINK6		protein_c	chr5:148202794-148
ENSG0000(	623	14.10713	chr5:4231	ENSG00000289430		lncRNA	chr5:148859611-148
ENSG0000(	623	14.10713	chr5:4231	EEF1GP2		Pseudoger	chr5:147922179-147
ENSG0000(	623	14.10713	chr5:4231	RN7SKP145		smallRNA	chr5:149116178-149
ENSG0000(	623	14.10713	chr5:4231	SH3TC2		protein_c	chr5:148923639-149
ENSG0000(	623	14.10713	chr5:4231	ENSG00000283653		Pseudoger	chr5:148362777-148
ENSG0000(	623	14.10713	chr5:4231	SPINK9		protein_c	chr5:148321203-148
ENSG0000(	623	14.10713	chr5:4231	JAKMIP2	NCGv7	protein_c	chr5:147585438-147
ENSG0000(	622	14.08449	chr7:1316	ENSG00000223718		Pseudoger	chr7:135660039-135
ENSG0000(	622	14.08449	chr2:8187	RPL26P15		Pseudoger	chr2:46003942-4600
ENSG0000(	620	14.0392	chr2:8187	FAM228B		protein_c	chr2:24076526-2416
ENSG0000(	620	14.0392	chr2:8187	ENSG00000271936		lncRNA	chr2:24825610-2482
ENSG0000(	620	14.0392	chr2:8187	HADHB		protein_c	chr2:26243170-2629
ENSG0000(	620	14.0392	chr2:8187	ASXL2	NCGv7	protein_c	chr2:25733753-2587
ENSG0000(	620	14.0392	chr2:8187	ENSG00000225378		lncRNA	chr2:26671254-2667
ENSG0000(	620	14.0392	chr2:8187	NDUFB4P4		Pseudoger	chr2:25886964-2588
ENSG0000(	620	14.0392	chr2:8187	DTNB-AS1		lncRNA	chr2:25421117-2542
ENSG0000(	620	14.0392	chr2:8187	MIR1301		smallRNA	chr2:25328640-2532
ENSG0000(	620	14.0392	chr2:8187	PGAM1P6		Pseudoger	chr2:23872268-2387
ENSG0000(	620	14.0392	chr2:8187	HADHA		protein_c	chr2:26190635-2624
ENSG0000(	620	14.0392	chr2:8187	ENSG00000286707		lncRNA	chr2:26298570-2630
ENSG0000(	620	14.0392	chr2:8187	ENSG00000289567		lncRNA	chr2:25362232-2536
ENSG0000(	620	14.0392	chr2:8187	ARNILA		lncRNA	chr2:25369136-2537
ENSG0000(	620	14.0392	chr2:8187	ITSN2		protein_c	chr2:24202864-2436
ENSG0000(	620	14.0392	chr2:8187	DNAJC27		protein_c	chr2:24943636-2497
ENSG0000(	620	14.0392	chr2:8187	LINC01381		lncRNA	chr2:25204313-2520
ENSG0000(	620	14.0392	chr2:8187	RNA5SP88		Pseudoger	chr2:24564630-2456
ENSG0000(	620	14.0392	chr2:8187	ENSG00000223530		lncRNA	chr2:23330664-2333
ENSG0000(	620	14.0392	chr2:8187	TP53I3		protein_c	chr2:24077433-2408
ENSG0000(	620	14.0392	chr2:8187	ENSG00000276087		protein_c	chr2:24124366-2419
ENSG0000(	620	14.0392	chr2:8187	PTGES3P2		Pseudoger	chr2:25822469-2582
ENSG0000(	620	14.0392	chr2:8187	GAREM2		protein_c	chr2:26173088-2618
ENSG0000(	620	14.0392	chr2:8187	MFSD2B		protein_c	chr2:24010081-2406

ENSG0000(	620	14.0392	chr2:8187	PTRHD1		protein_c	chr2:24789728-2479
ENSG0000(	620	14.0392	chr2:8187	ENSG00000223634		lncRNA	chr2:23357516-2336
ENSG0000(	620	14.0392	chr2:8187	ENSG00000232642		lncRNA	chr2:24165884-2417
ENSG0000(	620	14.0392	chr2:8187	DRC1		protein_c	chr2:26401920-2645
ENSG0000(	620	14.0392	chr2:8187	RNU6-942P		smallRNA	chr2:26042675-2604
ENSG0000(	620	14.0392	chr2:8187	RNU6-370P		smallRNA	chr2:24045835-2404
ENSG0000(	620	14.0392	chr2:8187	ENSG00000223754		lncRNA	chr2:24199839-2420
ENSG0000(	620	14.0392	chr2:8187	ADCY3	NCGv7	protein_c	chr2:24819169-2492
ENSG0000(	620	14.0392	chr2:8187	OTOF		protein_c	chr2:26457203-2655
ENSG0000(	620	14.0392	chr2:8187	RN7SL856P		smallRNA	chr2:25058032-2505
ENSG0000(	620	14.0392	chr2:8187	SCARNA21		smallRNA	chr2:24273614-2427
ENSG0000(	620	14.0392	chr2:8187	KCNK3		protein_c	chr2:26692722-2673
ENSG0000(	620	14.0392	chr2:8187	ADGRF3		protein_c	chr2:26308173-2634
ENSG0000(	620	14.0392	chr2:8187	CIB4		protein_c	chr2:26581205-2664
ENSG0000(	620	14.0392	chr2:8187	FAM166C		protein_c	chr2:26562585-2657
ENSG0000(	620	14.0392	chr2:8187	RPS2P15		Pseudoger	chr2:26101317-2610
ENSG0000(	620	14.0392	chr2:8187	HMGN2P20		Pseudoger	chr2:24330402-2433
ENSG0000(	620	14.0392	chr2:8187	DNAJC27-AS1		lncRNA	chr2:24971390-2503
ENSG0000(	620	14.0392	chr2:8187	TPM3P7		Pseudoger	chr2:25809925-2581
ENSG0000(	620	14.0392	chr2:8187	RAB10		protein_c	chr2:26034084-2613
ENSG0000(	620	14.0392	chr2:8187	RPS13P4		Pseudoger	chr2:23900693-2390
ENSG0000(	620	14.0392	chr2:8187	Y_RNA		smallRNA	chr2:25697076-2569
ENSG0000(	620	14.0392	chr2:8187	DTNB		protein_c	chr2:25377198-2567
ENSG0000(	620	14.0392	chr2:8187	POMC		protein_c	chr2:25160853-2516
ENSG0000(	620	14.0392	chr2:8187	WDCP	AC	protein_c	chr2:24029347-2404
ENSG0000(	620	14.0392	chr2:8187	ATAD2B		protein_c	chr2:23748664-2392
ENSG0000(	620	14.0392	chr2:8187	ENSG00000242628		lncRNA	chr2:24210650-2422
ENSG0000(	620	14.0392	chr2:8187	EMP2P1		Pseudoger	chr2:26140263-2614
ENSG0000(	620	14.0392	chr2:8187	ENSG00000227133		Pseudoger	chr2:26160023-2616
ENSG0000(	620	14.0392	chr2:8187	UBXN2A		protein_c	chr2:23927285-2400
ENSG0000(	620	14.0392	chr2:8187	RNU6-936P		smallRNA	chr2:24676309-2467
ENSG0000(	620	14.0392	chr2:8187	ENSG00000233714		lncRNA	chr2:23347654-2335
ENSG0000(	620	14.0392	chr2:8187	PPIL1P1		Pseudoger	chr2:26162056-2616
ENSG0000(	620	14.0392	chr2:8187	FKBP1B	DriverDBv	protein_c	chr2:24049701-2406
ENSG0000(	620	14.0392	chr2:8187	AC010150.2		smallRNA	chr2:25722192-2572
ENSG0000(	620	14.0392	chr2:8187	ENSG00000286645		lncRNA	chr2:25000147-2501
ENSG0000(	620	14.0392	chr2:8187	DNMT3A	NCGv7;AC	protein_c	chr2:25227855-2534
ENSG0000(	620	14.0392	chr2:8187	KLHL29		protein_c	chr2:23385179-2370
ENSG0000(	620	14.0392	chr2:8187	UQCRHP2		Pseudoger	chr2:25994794-2599
ENSG0000(	620	14.0392	chr2:8187	SUCLA2P3		Pseudoger	chr2:25079901-2508
ENSG0000(	620	14.0392	chr2:8187	LINC02923		lncRNA	chr2:23375229-2338
ENSG0000(	620	14.0392	chr2:8187	RPS13P5		Pseudoger	chr2:24968958-2496
ENSG0000(	620	14.0392	chr2:8187	SNORD14		smallRNA	chr2:24969388-2496
ENSG0000(	620	14.0392	chr2:8187	CENPO		protein_c	chr2:24793136-2482
ENSG0000(	620	14.0392	chr2:8187	TRMT112P6		Pseudoger	chr2:26028208-2602
ENSG0000(	620	14.0392	chr2:8187	SF3B6		protein_c	chr2:24067586-2407
ENSG0000(	620	14.0392	chr2:8187	KIF3C		protein_c	chr2:25926598-2598
ENSG0000(	620	14.0392	chr2:8187	RPL36AP13		Pseudoger	chr2:24334512-2433
ENSG0000(	620	14.0392	chr2:8187	SDHCP3		Pseudoger	chr2:23943846-2394
ENSG0000(	620	14.0392	chr2:8187	SELENOI		protein_c	chr2:26308547-2639
ENSG0000(	620	14.0392	chr2:8187	Y_RNA		smallRNA	chr2:25008845-2500

ENSG0000(	620	14.0392	chr2:8187	PFN4		protein_c	chr2:24114809-2412
ENSG0000(	620	14.0392	chr2:8187	ENSG00000218682		Pseudoger	chr2:25856461-2585
ENSG0000(	620	14.0392	chr2:8187	SMARCE1P6		Pseudoger	chr2:26149204-2615
ENSG0000(	620	14.0392	chr2:8187	FAM228A		protein_c	chr2:24175053-2420
ENSG0000(	620	14.0392	chr2:8187	EFR3B		protein_c	chr2:25042076-2515
ENSG0000(	620	14.0392	chr2:8187	RN7SL610P		smallRNA	chr2:23996797-2399
ENSG0000(	620	14.0392	chr2:8187	ENSG00000279526		TEC	chr2:23616499-2361
ENSG0000(	620	14.0392	chr2:8187	RPL37P11		Pseudoger	chr2:26678219-2667
ENSG0000(	620	14.0392	chr2:8187	NCOA1	IntOGen-I	protein_c	chr2:24491254-2477
ENSG0000(	620	14.0392	chr2:8187	ENSG00000283031		lncRNA	chr2:23667208-2368
ENSG0000(	620	14.0392	chr2:8187	ENSG00000286829		lncRNA	chr2:24402995-2441
ENSG0000(	620	14.0392	chr2:8187	ENSG00000224361		lncRNA	chr2:23507043-2352
ENSG0000(	617	13.97127	chr2:8187	ATP6V1E2	DriverDBv	protein_c	chr2:46490750-4654
ENSG0000(	617	13.97127	chr2:8187	ENSG00000229160		lncRNA	chr2:38132637-3813
ENSG0000(	617	13.97127	chr2:8187	ENSG00000287313		lncRNA	chr2:38408719-3841
ENSG0000(	617	13.97127	chr2:8187	RNU6-958P		smallRNA	chr2:43408307-4340
ENSG0000(	617	13.97127	chr2:8187	LINC01819		lncRNA	chr2:42972255-4304
ENSG0000(	617	13.97127	chr2:8187	ENSG00000285925		lncRNA	chr2:37339957-3734
ENSG0000(	617	13.97127	chr2:8187	ENSG00000287316		lncRNA	chr2:37466781-3752
ENSG0000(	617	13.97127	chr2:8187	ENSG00000238165		Pseudoger	chr2:50829442-5082
ENSG0000(	617	13.97127	chr2:8187	LINC01118		lncRNA	chr2:46698940-4682
ENSG0000(	617	13.97127	chr2:8187	HSPE1P13		Pseudoger	chr2:39098149-3909
ENSG0000(	617	13.97127	chr2:8187	VDAC1P13		Pseudoger	chr2:42463139-4246
ENSG0000(	617	13.97127	chr2:8187	KCNG3		protein_c	chr2:42442017-4249
ENSG0000(	617	13.97127	chr2:8187	SOCS5		protein_c	chr2:46698952-4678
ENSG0000(	617	13.97127	chr2:8187	HEATR5B		protein_c	chr2:36968383-3708
ENSG0000(	617	13.97127	chr2:8187	ENSG00000232518		lncRNA	chr2:38668202-3867
ENSG0000(	617	13.97127	chr2:8187	ENSG00000231336		lncRNA	chr2:46166789-4616
ENSG0000(	617	13.97127	chr2:8187	snoZ247		smallRNA	chr2:41734377-4173
ENSG0000(	617	13.97127	chr2:8187	AC009234.2		smallRNA	chr2:50696172-5069
ENSG0000(	617	13.97127	chr2:8187	GGCTP3		Pseudoger	chr2:52474073-5247
ENSG0000(	617	13.97127	chr2:8187	RPL7P12		Pseudoger	chr2:38231568-3823
ENSG0000(	617	13.97127	chr2:8187	VN1R18P		Pseudoger	chr2:47989625-4799
ENSG0000(	617	13.97127	chr2:8187	CHORDC1P1		Pseudoger	chr2:42826322-4282
ENSG0000(	617	13.97127	chr2:8187	ENSG00000272054		lncRNA	chr2:37208875-3721
ENSG0000(	617	13.97127	chr2:8187	ENSG00000289065		lncRNA	chr2:54115268-5411
ENSG0000(	617	13.97127	chr2:8187	CYP1B1-AS1		lncRNA	chr2:38073447-3823
ENSG0000(	617	13.97127	chr2:8187	SCARNA16		smallRNA	chr2:53470447-5347
ENSG0000(	617	13.97127	chr2:8187	SIX3		protein_c	chr2:44941702-4494
ENSG0000(	617	13.97127	chr2:8187	ENSG00000280276		TEC	chr2:38121935-3812
ENSG0000(	617	13.97127	chr2:8187	RNU6-851P		smallRNA	chr2:38884560-3888
ENSG0000(	617	13.97127	chr2:8187	ENSG00000269210		lncRNA	chr2:38959287-3896
ENSG0000(	617	13.97127	chr2:8187	STON1		protein_c	chr2:48529383-4859
ENSG0000(	617	13.97127	chr2:8187	ENSG00000289082		lncRNA	chr2:42795326-4279
ENSG0000(	617	13.97127	chr2:8187	KRTCAP2P1		Pseudoger	chr2:44996413-4499
ENSG0000(	617	13.97127	chr2:8187	ENSG00000223897		Pseudoger	chr2:53486144-5348
ENSG0000(	617	13.97127	chr2:8187	KNOP1P3		Pseudoger	chr2:51511058-5151
ENSG0000(	617	13.97127	chr2:8187	RPL7P13		Pseudoger	chr2:49878623-4987
ENSG0000(	617	13.97127	chr2:8187	PRKCE	AC	protein_c	chr2:45651345-4618
ENSG0000(	617	13.97127	chr2:8187	ENSG00000290100		lncRNA	chr2:37617328-3766
ENSG0000(	617	13.97127	chr2:8187	AC007682.2		smallRNA	chr2:50943292-5094

ENSG0000(	617	13.97127	chr2:8187	TTC39DP		Pseudoger	chr2:38763534-3876
ENSG0000(	617	13.97127	chr2:8187	ENSG00000228925		lncRNA	chr2:46899275-4690
ENSG0000(	617	13.97127	chr2:8187	LINC01126		lncRNA	chr2:43227210-4322
ENSG0000(	617	13.97127	chr2:8187	LHCGR		protein_c	chr2:48686774-4875
ENSG0000(	617	13.97127	chr2:8187	ENSG00000231156		lncRNA	chr2:45013214-4501
ENSG0000(	617	13.97127	chr2:8187	GPATCH11		protein_c	chr2:37084518-3709
ENSG0000(	617	13.97127	chr2:8187	SPTBN1		protein_c	chr2:54456317-5467
ENSG0000(	617	13.97127	chr2:8187	ENSG00000224058		Pseudoger	chr2:47731402-4773
ENSG0000(	617	13.97127	chr2:8187	ASB3		protein_c	chr2:53532672-5386
ENSG0000(	617	13.97127	chr2:8187	GEMIN6		protein_c	chr2:38751534-3878
ENSG0000(	617	13.97127	chr2:8187	ENSG00000235586		lncRNA	chr2:38601598-3860
ENSG0000(	617	13.97127	chr2:8187	PPIAP62		Pseudoger	chr2:47939738-4794
ENSG0000(	617	13.97127	chr2:8187	ASS1P2		Pseudoger	chr2:38810432-3881
ENSG0000(	617	13.97127	chr2:8187	CEBPZ		protein_c	chr2:37201612-3723
ENSG0000(	617	13.97127	chr2:8187	ENSG00000286519		lncRNA	chr2:45173722-4517
ENSG0000(	617	13.97127	chr2:8187	AC067957.1		smallRNA	chr2:44782046-4478
ENSG0000(	617	13.97127	chr2:8187	LINC02583		lncRNA	chr2:46429190-4644
ENSG0000(	617	13.97127	chr2:8187	ENSG00000226398		lncRNA	chr2:42015625-4202
ENSG0000(	617	13.97127	chr2:8187	PRKD3		protein_c	chr2:37250502-3732
ENSG0000(	617	13.97127	chr2:8187	NPLP1		Pseudoger	chr2:38769265-3877
ENSG0000(	617	13.97127	chr2:8187	QPCT		protein_c	chr2:37342827-3737
ENSG0000(	617	13.97127	chr2:8187	CCDC12P1		Pseudoger	chr2:51926882-5192
ENSG0000(	617	13.97127	chr2:8187	LINC01833		lncRNA	chr2:44921077-4493
ENSG0000(	617	13.97127	chr2:8187	FTOP1		Pseudoger	chr2:42797225-4279
ENSG0000(	617	13.97127	chr2:8187	LINC01913		lncRNA	chr2:41860155-4189
ENSG0000(	617	13.97127	chr2:8187	PRKD3-DT		lncRNA	chr2:37325340-3732
ENSG0000(	617	13.97127	chr2:8187	ENSG00000226548		lncRNA	chr2:46852020-4685
ENSG0000(	617	13.97127	chr2:8187	THUMPD2		protein_c	chr2:39736060-3977
ENSG0000(	617	13.97127	chr2:8187	ARHGEF33		protein_c	chr2:38889875-3897
ENSG0000(	617	13.97127	chr2:8187	AC010739.1		smallRNA	chr2:41596386-4159
ENSG0000(	617	13.97127	chr2:8187	RPLP0P6		Pseudoger	chr2:38481851-3848
ENSG0000(	617	13.97127	chr2:8187	ENSG00000232604		lncRNA	chr2:52864235-5286
ENSG0000(	617	13.97127	chr2:8187	SIX2	NCGv7	protein_c	chr2:45005182-4500
ENSG0000(	617	13.97127	chr2:8187	ENSG00000286412		lncRNA	chr2:51011777-5101
ENSG0000(	617	13.97127	chr2:8187	GTF2A1L		protein_c	chr2:48617798-4873
ENSG0000(	617	13.97127	chr2:8187	ENSG00000229695		Pseudoger	chr2:43680465-4368
ENSG0000(	617	13.97127	chr2:8187	ENSG00000279254		TEC	chr2:46668870-4667
ENSG0000(	617	13.97127	chr2:8187	ENSG00000233845		lncRNA	chr2:47035279-4704
ENSG0000(	617	13.97127	chr2:8187	RNU7-172P		smallRNA	chr2:54166944-5416
ENSG0000(	617	13.97127	chr2:8187	ENSG00000288886		lncRNA	chr2:42792299-4279
ENSG0000(	617	13.97127	chr2:8187	HMGB1P31		Pseudoger	chr2:54051334-5405
ENSG0000(	617	13.97127	chr2:8187	ENSG00000227292		lncRNA	chr2:38203363-3823
ENSG0000(	617	13.97127	chr2:8187	ENSG00000226523		Pseudoger	chr2:42680088-4268
ENSG0000(	617	13.97127	chr2:8187	snoU13		smallRNA	chr2:53839725-5383
ENSG0000(	617	13.97127	chr2:8187	MCFD2		protein_c	chr2:46901870-4694
ENSG0000(	617	13.97127	chr2:8187	HNRNPA1P57		Pseudoger	chr2:41143780-4115
ENSG0000(	617	13.97127	chr2:8187	RNU6-1185P		smallRNA	chr2:39393522-3939
ENSG0000(	617	13.97127	chr2:8187	NRXN1		protein_c	chr2:49918503-5122
ENSG0000(	617	13.97127	chr2:8187	SLC8A1		protein_c	chr2:40097270-4061
ENSG0000(	617	13.97127	chr2:8187	Y_RNA		smallRNA	chr2:42637961-4263
ENSG0000(	617	13.97127	chr2:8187	EPAS1	NCGv7	protein_c	chr2:46293667-4638

ENSG0000(	617	13.97127	chr2:8187	PIGF		protein_c	chr2:46580937-4661
ENSG0000(	617	13.97127	chr2:8187	ENSG00000287387		lncRNA	chr2:43219849-4322
ENSG0000(	617	13.97127	chr2:8187	RNU6-688P		smallRNA	chr2:47781379-4778
ENSG0000(	617	13.97127	chr2:8187	NDUFAF7		protein_c	chr2:37231631-3725
ENSG0000(	617	13.97127	chr2:8187	ENSG00000289013		lncRNA	chr2:40394673-4039
ENSG0000(	617	13.97127	chr2:8187	RNU4-49P		smallRNA	chr2:48340687-4834
ENSG0000(	617	13.97127	chr2:8187	THADA		protein_c	chr2:43230851-4359
ENSG0000(	617	13.97127	chr2:8187	CYP1B1		protein_c	chr2:38066973-3810
ENSG0000(	617	13.97127	chr2:8187	ENSG00000272814		lncRNA	chr2:46956615-4695
ENSG0000(	617	13.97127	chr2:8187	ENSG00000219391		Pseudoger	chr2:44065894-4406
ENSG0000(	617	13.97127	chr2:8187	PPM1B		protein_c	chr2:44167969-4424
ENSG0000(	617	13.97127	chr2:8187	MSH6	NCGv7;AC	protein_c	chr2:47695530-4781
ENSG0000(	617	13.97127	chr2:8187	MAP4K3	NCGv7	protein_c	chr2:39249266-3943
ENSG0000(	617	13.97127	chr2:8187	ENSG00000285898		lncRNA	chr2:40591285-4067
ENSG0000(	617	13.97127	chr2:8187	ENSG00000287344		lncRNA	chr2:51977787-5202
ENSG0000(	617	13.97127	chr2:8187	MIR4431		smallRNA	chr2:52702522-5270
ENSG0000(	617	13.97127	chr2:8187	ENSG00000285542		protein_c	chr2:44168851-4432
ENSG0000(	617	13.97127	chr2:8187	RNU6-939P		smallRNA	chr2:37331770-3733
ENSG0000(	617	13.97127	chr2:8187	ENSG00000236213		lncRNA	chr2:37562486-3764
ENSG0000(	617	13.97127	chr2:8187	MIR559		smallRNA	chr2:47377675-4737
ENSG0000(	617	13.97127	chr2:8187	ENSG00000226087		lncRNA	chr2:47225781-4724
ENSG0000(	617	13.97127	chr2:8187	ENSG00000233230		lncRNA	chr2:47905678-4790
ENSG0000(	617	13.97127	chr2:8187	MTCO1P42		Pseudoger	chr2:50588690-5058
ENSG0000(	617	13.97127	chr2:8187	DYNC2LI1		protein_c	chr2:43774039-4381
ENSG0000(	617	13.97127	chr2:8187	RMDN2		protein_c	chr2:37923187-3806
ENSG0000(	617	13.97127	chr2:8187	ENSG00000233978		lncRNA	chr2:43041193-4304
ENSG0000(	617	13.97127	chr2:8187	SRSF7		protein_c	chr2:38743599-3875
ENSG0000(	617	13.97127	chr2:8187	TMEM178A		protein_c	chr2:39664982-3971
ENSG0000(	617	13.97127	chr2:8187	CEBPZOS		protein_c	chr2:37196488-3721
ENSG0000(	617	13.97127	chr2:8187	SOS1	NCGv7	protein_c	chr2:38962206-3912
ENSG0000(	617	13.97127	chr2:8187	RN7SKP119		smallRNA	chr2:47359505-4735
ENSG0000(	617	13.97127	chr2:8187	AC007402.1		smallRNA	chr2:51435122-5143
ENSG0000(	617	13.97127	chr2:8187	MORN2		protein_c	chr2:38875976-3892
ENSG0000(	617	13.97127	chr2:8187	GAPDHP25		Pseudoger	chr2:38285410-3828
ENSG0000(	617	13.97127	chr2:8187	ENSG00000289003		lncRNA	chr2:39480783-3948
ENSG0000(	617	13.97127	chr2:8187	RNU6-1048P		smallRNA	chr2:43892690-4389
ENSG0000(	617	13.97127	chr2:8187	COX7A2L		protein_c	chr2:42333546-4242
ENSG0000(	617	13.97127	chr2:8187	ENSG00000288992		lncRNA	chr2:40450663-4045
ENSG0000(	617	13.97127	chr2:8187	ENSG00000230979		Pseudoger	chr2:47690716-4769
ENSG0000(	617	13.97127	chr2:8187	ENSG00000288994		lncRNA	chr2:38342476-3834
ENSG0000(	617	13.97127	chr2:8187	LINC02580		lncRNA	chr2:43092530-4321
ENSG0000(	617	13.97127	chr2:8187	SOS1-IT1		lncRNA	chr2:38992279-3899
ENSG0000(	617	13.97127	chr2:8187	ENSG00000271443		Pseudoger	chr2:38535258-3853
ENSG0000(	617	13.97127	chr2:8187	Y_RNA		smallRNA	chr2:43620878-4362
ENSG0000(	617	13.97127	chr2:8187	SLC3A1		protein_c	chr2:44275458-4432
ENSG0000(	617	13.97127	chr2:8187	RNU6-1116P		smallRNA	chr2:37435510-3743
ENSG0000(	617	13.97127	chr2:8187	ENSG00000215263		Pseudoger	chr2:42532766-4253
ENSG0000(	617	13.97127	chr2:8187	ENSG00000285548		lncRNA	chr2:50324643-5034
ENSG0000(	617	13.97127	chr2:8187	STON1-GTF2A1L		protein_c	chr2:48529925-4877
ENSG0000(	617	13.97127	chr2:8187	ENSG00000253515		lncRNA	chr2:46429229-4650
ENSG0000(	617	13.97127	chr2:8187	ENSG00000225284		lncRNA	chr2:38861720-3886

ENSG0000(	617	13.97127	chr2:8187	RNU6-242P		smallRNA	chr2:43091388-4309
ENSG0000(	617	13.97127	chr2:8187	BCYRN1		smallRNA	chr2:47335315-4733
ENSG0000(	617	13.97127	chr2:8187	ENSG00000287867		lncRNA	chr2:51441959-5145
ENSG0000(	617	13.97127	chr2:8187	ENSG00000289272		lncRNA	chr2:44228188-4422
ENSG0000(	617	13.97127	chr2:8187	RPS12P4		Pseudoger	chr2:41850203-4185
ENSG0000(	617	13.97127	chr2:8187	RNU6-566P		smallRNA	chr2:44154789-4415
ENSG0000(	617	13.97127	chr2:8187	ZFP36L2	NCGv7	protein_c	chr2:43222402-4322
ENSG0000(	617	13.97127	chr2:8187	PDSS1P2		Pseudoger	chr2:44166266-4416
ENSG0000(	617	13.97127	chr2:8187	ZNF863P		Pseudoger	chr2:52071355-5207
ENSG0000(	617	13.97127	chr2:8187	C1GALT1C1L		protein_c	chr2:43675151-4367
ENSG0000(	617	13.97127	chr2:8187	MSH2	NCGv7;AC	protein_c	chr2:47403067-4766
ENSG0000(	617	13.97127	chr2:8187	FSHR	NCGv7	protein_c	chr2:48962157-4915
ENSG0000(	617	13.97127	chr2:8187	ABCG5		protein_c	chr2:43812472-4383
ENSG0000(	617	13.97127	chr2:8187	ENSG00000282890		lncRNA	chr2:48809340-4941
ENSG0000(	617	13.97127	chr2:8187	ENSG00000273035		lncRNA	chr2:39323328-3932
ENSG0000(	617	13.97127	chr2:8187	ENSG00000289545		lncRNA	chr2:37489453-3760
ENSG0000(	617	13.97127	chr2:8187	ENSG00000286796		lncRNA	chr2:43128819-4313
ENSG0000(	617	13.97127	chr2:8187	FTH1P6		Pseudoger	chr2:52629743-5263
ENSG0000(	617	13.97127	chr2:8187	LINC01883		lncRNA	chr2:38431294-3843
ENSG0000(	617	13.97127	chr2:8187	EML4	NCGv7;AC	protein_c	chr2:42169353-4233
ENSG0000(	617	13.97127	chr2:8187	ENSG00000231054		lncRNA	chr2:45168583-4516
ENSG0000(	617	13.97127	chr2:8187	RNU6-137P		smallRNA	chr2:42712740-4271
ENSG0000(	617	13.97127	chr2:8187	FBXO11	NCGv7;AC	protein_c	chr2:47789316-4790
ENSG0000(	617	13.97127	chr2:8187	CALM2		protein_c	chr2:47160084-4717
ENSG0000(	617	13.97127	chr2:8187	ERLEC1		protein_c	chr2:53787009-5383
ENSG0000(	617	13.97127	chr2:8187	FOXN2		protein_c	chr2:48314637-4837
ENSG0000(	617	13.97127	chr2:8187	PSME4		protein_c	chr2:53864069-5397
ENSG0000(	617	13.97127	chr2:8187	PLEKHH2		protein_c	chr2:43637260-4376
ENSG0000(	617	13.97127	chr2:8187	SRBD1		protein_c	chr2:45388680-4561
ENSG0000(	617	13.97127	chr2:8187	snoU13		smallRNA	chr2:44239568-4423
ENSG0000(	617	13.97127	chr2:8187	PREPL		protein_c	chr2:44316281-4436
ENSG0000(	617	13.97127	chr2:8187	SNORA67		smallRNA	chr2:39283657-3928
ENSG0000(	617	13.97127	chr2:8187	ENSG00000234936		lncRNA	chr2:43229573-4323
ENSG0000(	617	13.97127	chr2:8187	SIX3-AS1		lncRNA	chr2:44940153-4494
ENSG0000(	617	13.97127	chr2:8187	TTC7A		protein_c	chr2:46915869-4707
ENSG0000(	617	13.97127	chr2:8187	Y_RNA		smallRNA	chr2:39128826-3912
ENSG0000(	617	13.97127	chr2:8187	ENSG00000232668		Pseudoger	chr2:52883243-5288
ENSG0000(	617	13.97127	chr2:8187	RNU6-846P		smallRNA	chr2:36959362-3695
ENSG0000(	617	13.97127	chr2:8187	CDC42EP3		protein_c	chr2:37641882-3773
ENSG0000(	617	13.97127	chr2:8187	RPL12P19		Pseudoger	chr2:44270621-4427
ENSG0000(	617	13.97127	chr2:8187	ENSG00000230773		lncRNA	chr2:47924181-4831
ENSG0000(	617	13.97127	chr2:8187	EPCAM-DT		lncRNA	chr2:47192405-4734
ENSG0000(	617	13.97127	chr2:8187	Y_RNA		smallRNA	chr2:52297995-5229
ENSG0000(	617	13.97127	chr2:8187	ELOBP3		Pseudoger	chr2:48780602-4878
ENSG0000(	617	13.97127	chr2:8187	LINC00211		lncRNA	chr2:37820498-3787
ENSG0000(	617	13.97127	chr2:8187	ENSG00000225187		lncRNA	chr2:47067822-4707
ENSG0000(	617	13.97127	chr2:8187	ENSG00000236572		lncRNA	chr2:37744333-3774
ENSG0000(	617	13.97127	chr2:8187	LINC01820		lncRNA	chr2:46392291-4639
ENSG0000(	617	13.97127	chr2:8187	MSH2-OT1		lncRNA	chr2:47527008-4753
ENSG0000(	617	13.97127	chr2:8187	ENSG00000236837		lncRNA	chr2:52494688-5250
ENSG0000(	617	13.97127	chr2:8187	CRYGGP		Pseudoger	chr2:51775258-5177

ENSG0000(	617	13.97127	chr2:8187	DHX57	protein_c	chr2:38797729-3887
ENSG0000(	617	13.97127	chr2:8187	RPS27AP7	Pseudoger	chr2:47883455-4788
ENSG0000(	617	13.97127	chr2:8187	RN7SKP66	smallRNA	chr2:43772120-4377
ENSG0000(	617	13.97127	chr2:8187	PPP1R21-DT	lncRNA	chr2:48440043-4844
ENSG0000(	617	13.97127	chr2:8187	RHOQ	protein_c	chr2:46541806-4658
ENSG0000(	617	13.97127	chr2:8187	ENSG00000225156	lncRNA	chr2:44954664-4496
ENSG0000(	617	13.97127	chr2:8187	ENSG00000288707	lncRNA	chr2:43995985-4399
ENSG0000(	617	13.97127	chr2:8187	AC007560.2	smallRNA	chr2:50798964-5079
ENSG0000(	617	13.97127	chr2:8187	ENSG00000232696	lncRNA	chr2:46078015-4607
ENSG0000(	617	13.97127	chr2:8187	EML4-AS1	lncRNA	chr2:42143238-4217
ENSG0000(	617	13.97127	chr2:8187	RPL31P16	Pseudoger	chr2:37194382-3719
ENSG0000(	617	13.97127	chr2:8187	ENSG00000283058	lncRNA	chr2:50620963-5063
ENSG0000(	617	13.97127	chr2:8187	RNU6-198P	smallRNA	chr2:39082589-3908
ENSG0000(	617	13.97127	chr2:8187	EIF2AK2	protein_c	chr2:37099210-3715
ENSG0000(	617	13.97127	chr2:8187	LINC01794	lncRNA	chr2:40746481-4076
ENSG0000(	617	13.97127	chr2:8187	RN7SL96P	smallRNA	chr2:38936880-3893
ENSG0000(	617	13.97127	chr2:8187	ENSG00000287468	lncRNA	chr2:40620667-4063
ENSG0000(	617	13.97127	chr2:8187	MAP4K3-DT	lncRNA	chr2:39436530-3966
ENSG0000(	617	13.97127	chr2:8187	ARL14EPP1	Pseudoger	chr2:37148530-3714
ENSG0000(	617	13.97127	chr2:8187	HAAO	protein_c	chr2:42767089-4279
ENSG0000(	617	13.97127	chr2:8187	OXER1	protein_c	chr2:42762499-4276
ENSG0000(	617	13.97127	chr2:8187	PKDCC	protein_c	chr2:42048021-4205
ENSG0000(	617	13.97127	chr2:8187	TMEM247	protein_c	chr2:46479565-4648
ENSG0000(	617	13.97127	chr2:8187	SNORA75	smallRNA	chr2:49888868-4988
ENSG0000(	617	13.97127	chr2:8187	RNU4-63P	smallRNA	chr2:41871271-4187
ENSG0000(	617	13.97127	chr2:8187	STPG4	protein_c	chr2:47045538-4715
ENSG0000(	617	13.97127	chr2:8187	ACYP2	protein_c	chr2:53970838-5430
ENSG0000(	617	13.97127	chr2:8187	AC007682.3	smallRNA	chr2:50961245-5096
ENSG0000(	617	13.97127	chr2:8187	ENSG00000278957	TEC	chr2:44927914-4492
ENSG0000(	617	13.97127	chr2:8187	ENSG00000273006	lncRNA	chr2:38193348-3819
ENSG0000(	617	13.97127	chr2:8187	ENSG00000287255	lncRNA	chr2:40511921-4054
ENSG0000(	617	13.97127	chr2:8187	RPL36AP15	Pseudoger	chr2:47797826-4779
ENSG0000(	617	13.97127	chr2:8187	LINC01914	lncRNA	chr2:41931599-4193
ENSG0000(	617	13.97127	chr2:8187	LINC01119	lncRNA	chr2:46816697-4685
ENSG0000(	617	13.97127	chr2:8187	ENSG00000241114	Pseudoger	chr2:54079974-5408
ENSG0000(	617	13.97127	chr2:8187	RPL36AP14	Pseudoger	chr2:46256860-4625
ENSG0000(	617	13.97127	chr2:8187	RPS13P3	Pseudoger	chr2:42469817-4247
ENSG0000(	617	13.97127	chr2:8187	ENSG00000272156	lncRNA	chr2:54082554-5408
ENSG0000(	617	13.97127	chr2:8187	LDHAP3	Pseudoger	chr2:41819747-4182
ENSG0000(	617	13.97127	chr2:8187	RMDN2-AS1	lncRNA	chr2:37949911-3806
ENSG0000(	617	13.97127	chr2:8187	CDKL4	protein_c	chr2:39168045-3924
ENSG0000(	617	13.97127	chr2:8187	CAMKMT	protein_c	chr2:44361947-4477
ENSG0000(	617	13.97127	chr2:8187	LINC02613	lncRNA	chr2:38406527-3851
ENSG0000(	617	13.97127	chr2:8187	ENSG00000287145	lncRNA	chr2:41716133-4173
ENSG0000(	617	13.97127	chr2:8187	CRTC1P1	Pseudoger	chr2:52570648-5257
ENSG0000(	617	13.97127	chr2:8187	RNU6-282P	smallRNA	chr2:48501922-4850
ENSG0000(	617	13.97127	chr2:8187	RN7SKP224	smallRNA	chr2:48217575-4821
ENSG0000(	617	13.97127	chr2:8187	SULT6B1	protein_c	chr2:37167820-3719
ENSG0000(	617	13.97127	chr2:8187	ENSG00000282998	lncRNA	chr2:49202126-4945
ENSG0000(	617	13.97127	chr2:8187	MIR3682	smallRNA	chr2:53849122-5384
ENSG0000(	617	13.97127	chr2:8187	PPM1B-DT	lncRNA	chr2:44167625-4416

ENSG0000(	617	13.97127	chr2:8187	LINC01121		lncRNA	chr2:45164816-4532
ENSG0000(	617	13.97127	chr2:8187	LINC02898		lncRNA	chr2:41935368-4195
ENSG0000(	617	13.97127	chr2:8187	LRPPRC		protein_c	chr2:43886224-4399
ENSG0000(	617	13.97127	chr2:8187	ENSG00000228033		lncRNA	chr2:52722671-5296
ENSG0000(	617	13.97127	chr2:8187	MTA3		protein_c	chr2:42494569-4275
ENSG0000(	617	13.97127	chr2:8187	CRIPT		protein_c	chr2:46616416-4663
ENSG0000(	617	13.97127	chr2:8187	Y_RNA		smallRNA	chr2:41857271-4185
ENSG0000(	617	13.97127	chr2:8187	RHOQ-AS1		lncRNA	chr2:46568256-4658
ENSG0000(	617	13.97127	chr2:8187	RPL21P30		Pseudoger	chr2:54029552-5403
ENSG0000(	617	13.97127	chr2:8187	LINC01867		lncRNA	chr2:52370602-5239
ENSG0000(	617	13.97127	chr2:8187	ENSG00000231918		lncRNA	chr2:51032601-5240
ENSG0000(	617	13.97127	chr2:8187	PRKCE-AS1		lncRNA	chr2:45674701-4567
ENSG0000(	617	13.97127	chr2:8187	SNORD75		smallRNA	chr2:42440377-4244
ENSG0000(	617	13.97127	chr2:8187	ENSG00000286728		lncRNA	chr2:45169616-4521
ENSG0000(	617	13.97127	chr2:8187	ENSG00000225402		Pseudoger	chr2:37816915-3781
ENSG0000(	617	13.97127	chr2:8187	PPP1R21		protein_c	chr2:48440598-4851
ENSG0000(	617	13.97127	chr2:8187	NME2P2		Pseudoger	chr2:47705468-4770
ENSG0000(	617	13.97127	chr2:8187	RN7SL817P		smallRNA	chr2:46448226-4644
ENSG0000(	617	13.97127	chr2:8187	ENSG00000230840		Pseudoger	chr2:51925692-5192
ENSG0000(	617	13.97127	chr2:8187	HNRNPLL		protein_c	chr2:38561969-3860
ENSG0000(	617	13.97127	chr2:8187	GPR75		protein_c	chr2:53852912-5385
ENSG0000(	617	13.97127	chr2:8187	CTBP2P5		Pseudoger	chr2:48915267-4891
ENSG0000(	617	13.97127	chr2:8187	ENSG00000284608		lncRNA	chr2:46429195-4648
ENSG0000(	617	13.97127	chr2:8187	SLC8A1-AS1		lncRNA	chr2:39786453-4025
ENSG0000(	617	13.97127	chr2:8187	ATL2		protein_c	chr2:38293954-3837
ENSG0000(	617	13.97127	chr2:8187	C2orf73		protein_c	chr2:54330034-5438
ENSG0000(	617	13.97127	chr2:8187	CHAC2		protein_c	chr2:53767804-5377
ENSG0000(	617	13.97127	chr2:8187	ENSG00000273269		protein_c	chr2:47065941-4717
ENSG0000(	617	13.97127	chr2:8187	RN7SL414P		smallRNA	chr2:45569201-4556
ENSG0000(	617	13.97127	chr2:8187	ABCG8	NCGv7	protein_c	chr2:43831942-4388
ENSG0000(	617	13.97127	chr2:8187	ENSG00000282828		lncRNA	chr2:49563388-4959
ENSG0000(	617	13.97127	chr2:8187	ENSG00000235653		Pseudoger	chr2:39929110-3992
ENSG0000(	617	13.97127	chr2:8187	TPT1P11		Pseudoger	chr2:48632856-4863
ENSG0000(	617	13.97127	chr2:8187	TSPYL6		protein_c	chr2:54253178-5425
ENSG0000(	617	13.97127	chr2:8187	RNU6-951P		smallRNA	chr2:38147415-3814
ENSG0000(	617	13.97127	chr2:8187	GALM		protein_c	chr2:38666081-3874
ENSG0000(	617	13.97127	chr2:8187	RNU6-439P		smallRNA	chr2:49233794-4923
ENSG0000(	617	13.97127	chr2:8187	KCNK12		protein_c	chr2:47509290-4757
ENSG0000(	617	13.97127	chr2:8187	EPCAM	AC	protein_c	chr2:47345158-4738
ENSG0000(	617	13.97127	chr2:8187	ENSG00000279956		protein_c	chr2:48632291-4875
ENSG0000(	616	13.94862	chr4:1865	UGT2A3P7		Pseudoger	chr4:69517667-6951
ENSG0000(	616	13.94862	chr4:1865	NPFFR2		protein_c	chr4:72031902-7214
ENSG0000(	616	13.94862	chr4:1865	RNU6-459P		smallRNA	chr4:70848136-7084
ENSG0000(	616	13.94862	chr4:1865	ENSG00000250828		Pseudoger	chr4:69437357-6944
ENSG0000(	616	13.94862	chr4:1865	ENSG00000251177		Pseudoger	chr4:69572391-6958
ENSG0000(	616	13.94862	chr4:1865	GNRHR		protein_c	chr4:67737118-6775
ENSG0000(	616	13.94862	chr4:1865	ENSG00000248567		lncRNA	chr4:71821305-7182
ENSG0000(	616	13.94862	chr4:1865	HTN1		protein_c	chr4:70050438-7005
ENSG0000(	616	13.94862	chr4:1865	YTHDC1		protein_c	chr4:68310387-6835
ENSG0000(	616	13.94862	chr4:1865	TMPRSS11I	NCGv7	protein_c	chr4:67820876-6788
ENSG0000(	616	13.94862	chr4:1865	SULT1D1P		Pseudoger	chr4:69791872-6981

ENSG0000(	616	13.94862	chr4:1865	UGT2B4		protein_c	chr4:69480165-6952
ENSG0000(	616	13.94862	chr4:1865	MT2P1		Pseudoger	chr4:68376323-6837
ENSG0000(	616	13.94862	chr4:1865	STATH		protein_c	chr4:69995966-7000
ENSG0000(	616	13.94862	chr4:1865	ENSG00000249472		Pseudoger	chr4:68614419-6861
ENSG0000(	616	13.94862	chr4:1865	UGT2B7		protein_c	chr4:69051363-6911
ENSG0000(	616	13.94862	chr4:1865	UGT2B11		protein_c	chr4:69199951-6921
ENSG0000(	616	13.94862	chr4:1865	UGT2A2	NCGv7	protein_c	chr4:69588417-6963
ENSG0000(	616	13.94862	chr4:1865	ENSG00000249763		Pseudoger	chr4:69242628-6925
ENSG0000(	616	13.94862	chr4:1865	ENSG00000289942		lncRNA	chr4:68376551-6837
ENSG0000(	616	13.94862	chr4:1865	SLC4A4		protein_c	chr4:71062667-7157
ENSG0000(	616	13.94862	chr4:1865	ENSG00000250696		lncRNA	chr4:69182100-6921
ENSG0000(	616	13.94862	chr4:1865	ENSG00000249735		Pseudoger	chr4:68985009-6898
ENSG0000(	616	13.94862	chr4:1865	UGT2B25P		Pseudoger	chr4:69389492-6940
ENSG0000(	616	13.94862	chr4:1865	ENSG00000250376		Pseudoger	chr4:68784618-6878
ENSG0000(	616	13.94862	chr4:1865	DCK	NCGv7	protein_c	chr4:70992538-7103
ENSG0000(	616	13.94862	chr4:1865	CSN1S1		protein_c	chr4:69931068-6994
ENSG0000(	616	13.94862	chr4:1865	STAP1		protein_c	chr4:67558727-6760
ENSG0000(	616	13.94862	chr4:1865	ENSG00000270257		Pseudoger	chr4:67638177-6763
ENSG0000(	616	13.94862	chr4:1865	RNU6-891P		smallRNA	chr4:70852130-7085
ENSG0000(	616	13.94862	chr4:1865	ENSG00000286848		lncRNA	chr4:70637745-7068
ENSG0000(	616	13.94862	chr4:1865	ENSG00000250642		Pseudoger	chr4:68282461-6828
ENSG0000(	616	13.94862	chr4:1865	ENSG00000249890		Pseudoger	chr4:69021656-6902
ENSG0000(	616	13.94862	chr4:1865	UGT2B26P		Pseudoger	chr4:69027831-6904
ENSG0000(	616	13.94862	chr4:1865	ENSG00000248763		Pseudoger	chr4:69066395-6906
ENSG0000(	616	13.94862	chr4:1865	ENSG00000251529		Pseudoger	chr4:68877626-6888
ENSG0000(	616	13.94862	chr4:1865	ST3GAL1P1		Pseudoger	chr4:67716375-6771
ENSG0000(	616	13.94862	chr4:1865	FTLP10		Pseudoger	chr4:68182292-6821
ENSG0000(	616	13.94862	chr4:1865	ENSG00000251236		Pseudoger	chr4:68813995-6881
ENSG0000(	616	13.94862	chr4:1865	ENSG00000251489		Pseudoger	chr4:69888877-6988
ENSG0000(	616	13.94862	chr4:1865	ENSG00000198277		Pseudoger	chr4:68914928-6892
ENSG0000(	616	13.94862	chr4:1865	ENSG00000290400		lncRNA	chr4:67991812-6799
ENSG0000(	616	13.94862	chr4:1865	ENSG00000249170		Pseudoger	chr4:71300258-7130
ENSG0000(	616	13.94862	chr4:1865	SYT14P1		Pseudoger	chr4:68061822-6806
ENSG0000(	616	13.94862	chr4:1865	UBA6-DT		lncRNA	chr4:67701209-6808
ENSG0000(	616	13.94862	chr4:1865	ENSG00000290407		lncRNA	chr4:68184129-6818
ENSG0000(	616	13.94862	chr4:1865	ENSG00000250030		Pseudoger	chr4:67446267-6744
ENSG0000(	616	13.94862	chr4:1865	RNA5SP163		Pseudoger	chr4:71759518-7175
ENSG0000(	616	13.94862	chr4:1865	CSN1S2AP		Pseudoger	chr4:70067386-7008
ENSG0000(	616	13.94862	chr4:1865	ENSG00000251424		Pseudoger	chr4:68834213-6883
ENSG0000(	616	13.94862	chr4:1865	HTN3		protein_c	chr4:70028455-7003
ENSG0000(	616	13.94862	chr4:1865	AMTN		protein_c	chr4:70518569-7053
ENSG0000(	616	13.94862	chr4:1865	ENSG00000251427		Pseudoger	chr4:69144734-6914
ENSG0000(	616	13.94862	chr4:1865	UTP3		protein_c	chr4:70688532-7069
ENSG0000(	616	13.94862	chr4:1865	TMPRSS11F		protein_c	chr4:68053198-6812
ENSG0000(	616	13.94862	chr4:1865	SULT1B1	NCGv7	protein_c	chr4:69721167-6978
ENSG0000(	616	13.94862	chr4:1865	ENSG00000248613		Pseudoger	chr4:68900651-6890
ENSG0000(	616	13.94862	chr4:1865	ENSG00000251498		Pseudoger	chr4:68972999-6897
ENSG0000(	616	13.94862	chr4:1865	snoU13		smallRNA	chr4:70812246-7081
ENSG0000(	616	13.94862	chr4:1865	PRR27		protein_c	chr4:70133616-7017
ENSG0000(	616	13.94862	chr4:1865	UGT2B29P		Pseudoger	chr4:68509441-6851
ENSG0000(	616	13.94862	chr4:1865	TMPRSS11GP		Pseudoger	chr4:67991684-6801

ENSG0000(	616	13.94862	chr4:186	JCHAIN		protein_c	chr4:70655541-7068
ENSG0000(	616	13.94862	chr4:186	ENSG00000251101		Pseudoger	chr4:68615393-6861
ENSG0000(	616	13.94862	chr4:186	ENSG00000248635		Pseudoger	chr4:68704600-6871
ENSG0000(	616	13.94862	chr4:186	ENAM		protein_c	chr4:70628744-7064
ENSG0000(	616	13.94862	chr4:186	UGT2B17	DriverDB	protein_c	chr4:68537173-6857
ENSG0000(	616	13.94862	chr4:186	ENSG00000249531		Pseudoger	chr4:68300325-6830
ENSG0000(	616	13.94862	chr4:186	RNU1-63P		smallRNA	chr4:67429591-6742
ENSG0000(	616	13.94862	chr4:186	ENSG00000272986		lncRNA	chr4:70703747-7070
ENSG0000(	616	13.94862	chr4:186	FDCSP		protein_c	chr4:70226124-7023
ENSG0000(	616	13.94862	chr4:186	ENSG00000284695		protein_c	chr4:69810780-6984
ENSG0000(	616	13.94862	chr4:186	APOOP4		Pseudoger	chr4:68304343-6830
ENSG0000(	616	13.94862	chr4:186	ENSG00000249985		Pseudoger	chr4:68907918-6890
ENSG0000(	616	13.94862	chr4:186	ENSG00000250100		Pseudoger	chr4:69131351-6913
ENSG0000(	616	13.94862	chr4:186	POLR2MP1		Pseudoger	chr4:68038544-6803
ENSG0000(	616	13.94862	chr4:186	ODAM	NCGv7	protein_c	chr4:70195725-7020
ENSG0000(	616	13.94862	chr4:186	ENSG00000270292		Pseudoger	chr4:67725183-6772
ENSG0000(	616	13.94862	chr4:186	CSN2		protein_c	chr4:69955256-6996
ENSG0000(	616	13.94862	chr4:186	SULT1E1		protein_c	chr4:69841212-6986
ENSG0000(	616	13.94862	chr4:186	ENSG00000270228		Pseudoger	chr4:67718996-6772
ENSG0000(	616	13.94862	chr4:186	MTND2P41		Pseudoger	chr4:68049706-6805
ENSG0000(	616	13.94862	chr4:186	MOB1B	NCGv7	protein_c	chr4:70902326-7102
ENSG0000(	616	13.94862	chr4:186	ENSG00000291282		lncRNA	chr4:68188403-6821
ENSG0000(	616	13.94862	chr4:186	TMPRSS11BNL		Pseudoger	chr4:68184081-6821
ENSG0000(	616	13.94862	chr4:186	ENSG00000251691		Pseudoger	chr4:69306469-6930
ENSG0000(	616	13.94862	chr4:186	AC108078.1		smallRNA	chr4:69479331-6947
ENSG0000(	616	13.94862	chr4:186	TMPRSS11E		protein_c	chr4:68447463-6849
ENSG0000(	616	13.94862	chr4:186	MUC7		protein_c	chr4:70430492-7048
ENSG0000(	616	13.94862	chr4:186	GRSF1	NCGv7	protein_c	chr4:70815783-7083
ENSG0000(	616	13.94862	chr4:186	AMBN		protein_c	chr4:70592256-7060
ENSG0000(	616	13.94862	chr4:186	ENSG00000250277		Pseudoger	chr4:68996935-6899
ENSG0000(	616	13.94862	chr4:186	RNU6-95P		smallRNA	chr4:68003895-6800
ENSG0000(	616	13.94862	chr4:186	CSN3		protein_c	chr4:70238382-7025
ENSG0000(	616	13.94862	chr4:186	LDHAL6EP		Pseudoger	chr4:71434405-7143
ENSG0000(	616	13.94862	chr4:186	ENSG00000272626		lncRNA	chr4:68901008-6890
ENSG0000(	616	13.94862	chr4:186	UGT2A3	NCGv7	protein_c	chr4:68928463-6895
ENSG0000(	616	13.94862	chr4:186	ENSG00000249686		Pseudoger	chr4:69450014-6945
ENSG0000(	616	13.94862	chr4:186	TMPRSS11CP		Pseudoger	chr4:67766480-6777
ENSG0000(	616	13.94862	chr4:186	ENSG00000250075		lncRNA	chr4:67417305-6746
ENSG0000(	616	13.94862	chr4:186	TMPRSS11A	NCGv7	protein_c	chr4:67909395-6796
ENSG0000(	616	13.94862	chr4:186	ENSG00000279464		TEC	chr4:67607856-6761
ENSG0000(	616	13.94862	chr4:186	CENPC		protein_c	chr4:67468762-6754
ENSG0000(	616	13.94862	chr4:186	TMPRSS11B		protein_c	chr4:68226653-6824
ENSG0000(	616	13.94862	chr4:186	UBA6	NCGv7	protein_c	chr4:67612652-6770
ENSG0000(	616	13.94862	chr4:186	RNU6-520P		smallRNA	chr4:70701755-7070
ENSG0000(	616	13.94862	chr4:186	ENSG00000268209		Pseudoger	chr4:69387580-6938
ENSG0000(	616	13.94862	chr4:186	RNU6-784P		smallRNA	chr4:70703018-7070
ENSG0000(	616	13.94862	chr4:186	ENSG00000251284		Pseudoger	chr4:69125274-6912
ENSG0000(	616	13.94862	chr4:186	ENSG00000248824		Pseudoger	chr4:69406931-6940
ENSG0000(	616	13.94862	chr4:186	UGT2A1	NCGv7	protein_c	chr4:69588417-6965
ENSG0000(	616	13.94862	chr4:186	UGT2B24P		Pseudoger	chr4:69408828-6942
ENSG0000(	616	13.94862	chr4:186	ENSG00000196472		Pseudoger	chr4:69181660-6918

ENSG0000(	616	13.94862	chr4:1865	ENSG00000251074		Pseudoger	chr4:68626847-6862
ENSG0000(	616	13.94862	chr4:1865	GC		protein_c	chr4:71741696-7180
ENSG0000(	616	13.94862	chr4:1865	OPRPN		protein_c	chr4:70397931-7041
ENSG0000(	616	13.94862	chr4:1865	ENSG00000248547		Pseudoger	chr4:68883885-6888
ENSG0000(	616	13.94862	chr4:1865	UGT2B27P		Pseudoger	chr4:69004862-6902
ENSG0000(	616	13.94862	chr4:1865	UGT2B15		protein_c	chr4:68646597-6867
ENSG0000(	616	13.94862	chr4:1865	SMR3B	AC	protein_c	chr4:70370093-7039
ENSG0000(	616	13.94862	chr4:1865	ENSG00000268803		Pseudoger	chr4:69215908-6921
ENSG0000(	616	13.94862	chr4:1865	ENSG00000289019		lncRNA	chr4:70899216-7090
ENSG0000(	616	13.94862	chr4:1865	UGT2B28		protein_c	chr4:69280475-6929
ENSG0000(	616	13.94862	chr4:1865	SNORA62		smallRNA	chr4:67747236-6774
ENSG0000(	616	13.94862	chr4:1865	UGT2B10	NCGv7	protein_c	chr4:68815994-6883
ENSG0000(	616	13.94862	chr4:1865	RUFY3		protein_c	chr4:70704204-7080
ENSG0000(	616	13.94862	chr4:1865	CABS1		protein_c	chr4:70334981-7033
ENSG0000(	616	13.94862	chr4:1865	ENSG00000250612		Pseudoger	chr4:69346609-6935
ENSG0000(	616	13.94862	chr4:1865	ENSG00000248639		Pseudoger	chr4:68293127-6829
ENSG0000(	616	13.94862	chr4:1865	SMR3A		protein_c	chr4:70360760-7036
ENSG0000(	615	13.92598	chr5:4231	GLRXP3		Pseudoger	chr5:161751448-161
ENSG0000(	614	13.90333	chr5:4231	snoU13		smallRNA	chr5:150543590-150
ENSG0000(	614	13.90333	chr3:3227	RNA5SP132		Pseudoger	chr3:51694465-5169
ENSG0000(	613	13.88069	chr3:3227	AC099332.1		smallRNA	chr3:39417480-3941
ENSG0000(	603	13.65425	chr5:4231	RNA5SP198		Pseudoger	chr5:151875566-151
ENSG0000(	603	13.65425	chr5:4231	ENSG00000288081		lncRNA	chr5:151595264-151
ENSG0000(	603	13.65425	chr5:4231	ENSG00000275765		lncRNA	chr5:151769783-151
ENSG0000(	603	13.65425	chr5:4231	NIPAL4-DT		lncRNA	chr5:157363382-157
ENSG0000(	603	13.65425	chr5:4231	LARP1	NCGv7	protein_c	chr5:154682986-154
ENSG0000(	603	13.65425	chr5:4231	ENSG00000271494		Pseudoger	chr5:151380341-151
ENSG0000(	603	13.65425	chr5:4231	GRIA1		protein_c	chr5:153489615-153
ENSG0000(	603	13.65425	chr5:4231	LINC01470		lncRNA	chr5:152618965-153
ENSG0000(	603	13.65425	chr5:4231	IL17B		protein_c	chr5:149371324-149
ENSG0000(	603	13.65425	chr5:4231	GARIN3		protein_c	chr5:157161846-157
ENSG0000(	603	13.65425	chr5:4231	CNOT8		protein_c	chr5:154857553-154
ENSG0000(	603	13.65425	chr5:4231	RNA5SP199		Pseudoger	chr5:155845437-155
ENSG0000(	603	13.65425	chr5:4231	RPL29P14		Pseudoger	chr5:149545383-149
ENSG0000(	603	13.65425	chr5:4231	ENSG00000248696		Pseudoger	chr5:150014785-150
ENSG0000(	603	13.65425	chr5:4231	HNRNPA3P7		Pseudoger	chr5:154337471-154
ENSG0000(	603	13.65425	chr5:4231	GM2A		protein_c	chr5:151212150-151
ENSG0000(	603	13.65425	chr5:4231	ENSG00000253673		lncRNA	chr5:158175396-158
ENSG0000(	603	13.65425	chr5:4231	RN7SKP232		smallRNA	chr5:151704289-151
ENSG0000(	603	13.65425	chr5:4231	AC010609.1		smallRNA	chr5:157235185-157
ENSG0000(	603	13.65425	chr5:4231	CSF1R	NCGv7;AC	protein_c	chr5:150053291-150
ENSG0000(	603	13.65425	chr5:4231	ENSG00000271477		Pseudoger	chr5:155397291-155
ENSG0000(	603	13.65425	chr5:4231	ENSG00000253370		Pseudoger	chr5:156458398-156
ENSG0000(	603	13.65425	chr5:4231	ENSG00000286331		lncRNA	chr5:150427904-150
ENSG0000(	603	13.65425	chr5:4231	Y_RNA		smallRNA	chr5:149940030-149
ENSG0000(	603	13.65425	chr5:4231	ENSG00000241187		Pseudoger	chr5:154729231-154
ENSG0000(	603	13.65425	chr5:4231	ENSG00000270442		Pseudoger	chr5:155491336-155
ENSG0000(	603	13.65425	chr5:4231	RNU6-588P		smallRNA	chr5:149606637-149
ENSG0000(	603	13.65425	chr5:4231	ENSG00000270978		Pseudoger	chr5:151848886-151
ENSG0000(	603	13.65425	chr5:4231	CD74	NCGv7;AC	protein_c	chr5:150401637-150
ENSG0000(	603	13.65425	chr5:4231	ENSG00000244331		Pseudoger	chr5:157931258-157

ENSG0000(	603	13.65425	chr5:4231	ENSG00000253134		lncRNA	chr5:158275711-158
ENSG0000(	603	13.65425	chr5:4231	ENSG00000253792		lncRNA	chr5:158173601-158
ENSG0000(	603	13.65425	chr5:4231	ENSG00000289970		lncRNA	chr5:151081195-151
ENSG0000(	603	13.65425	chr5:4231	ENSG00000275871		lncRNA	chr5:149425771-149
ENSG0000(	603	13.65425	chr5:4231	SGCD		protein_c	chr5:155870344-156
ENSG0000(	603	13.65425	chr5:4231	APOOP1		Pseudoger	chr5:156924119-156
ENSG0000(	603	13.65425	chr5:4231	CARMN		lncRNA	chr5:149406689-149
ENSG0000(	603	13.65425	chr5:4231	ENSG00000290991		lncRNA	chr5:151366299-151
ENSG0000(	603	13.65425	chr5:4231	CCDC69		protein_c	chr5:151181052-151
ENSG0000(	603	13.65425	chr5:4231	LINC02202		lncRNA	chr5:159100483-159
ENSG0000(	603	13.65425	chr5:4231	ENSG00000254246		lncRNA	chr5:157104788-157
ENSG0000(	603	13.65425	chr5:4231	ENSG00000253865		lncRNA	chr5:149372174-149
ENSG0000(	603	13.65425	chr5:4231	MIR378A		smallRNA	chr5:149732825-149
ENSG0000(	603	13.65425	chr5:4231	ENSG00000253261		Pseudoger	chr5:154993598-154
ENSG0000(	603	13.65425	chr5:4231	NIPAL4		protein_c	chr5:157460213-157
ENSG0000(	603	13.65425	chr5:4231	FNDC9		protein_c	chr5:157341598-157
ENSG0000(	603	13.65425	chr5:4231	ENSG00000283413		lncRNA	chr5:159484130-159
ENSG0000(	603	13.65425	chr5:4231	AC021078.1		smallRNA	chr5:149604276-149
ENSG0000(	603	13.65425	chr5:4231	MYOZ3-AS1		lncRNA	chr5:150670658-150
ENSG0000(	603	13.65425	chr5:4231	NMUR2	NCGv7	protein_c	chr5:152391546-152
ENSG0000(	603	13.65425	chr5:4231	ATOX1-AS1		lncRNA	chr5:151753992-151
ENSG0000(	603	13.65425	chr5:4231	ENSG00000254336		Pseudoger	chr5:157604707-157
ENSG0000(	603	13.65425	chr5:4231	NDST1-AS1		lncRNA	chr5:150475531-150
ENSG0000(	603	13.65425	chr5:4231	ADRA1B	NCGv7	protein_c	chr5:159865080-159
ENSG0000(	603	13.65425	chr5:4231	RPL7P1		Pseudoger	chr5:150094302-150
ENSG0000(	603	13.65425	chr5:4231	ENSG00000285736		lncRNA	chr5:149324220-149
ENSG0000(	603	13.65425	chr5:4231	CSNK1A1	NCGv7	protein_c	chr5:149492982-149
ENSG0000(	603	13.65425	chr5:4231	HMGXB3		protein_c	chr5:150000046-150
ENSG0000(	603	13.65425	chr5:4231	SNORA68		smallRNA	chr5:159230194-159
ENSG0000(	603	13.65425	chr5:4231	PDGFRB	NCGv7;AC	protein_c	chr5:150113839-150
ENSG0000(	603	13.65425	chr5:4231	ENSG00000253256		lncRNA	chr5:159209921-159
ENSG0000(	603	13.65425	chr5:4231	CDX1		protein_c	chr5:150166778-150
ENSG0000(	603	13.65425	chr5:4231	ENSG00000253897		Pseudoger	chr5:151366433-151
ENSG0000(	603	13.65425	chr5:4231	DCTN4		protein_c	chr5:150708440-150
ENSG0000(	603	13.65425	chr5:4231	RNU6-260P		smallRNA	chr5:157809098-157
ENSG0000(	603	13.65425	chr5:4231	RN7SL868P		smallRNA	chr5:149722070-149
ENSG0000(	603	13.65425	chr5:4231	FAXDC2	DriverDBv	protein_c	chr5:154818492-154
ENSG0000(	603	13.65425	chr5:4231	ENSG00000254135		lncRNA	chr5:158485190-158
ENSG0000(	603	13.65425	chr5:4231	AC008410.1		smallRNA	chr5:155024714-155
ENSG0000(	603	13.65425	chr5:4231	HAND1		protein_c	chr5:154474972-154
ENSG0000(	603	13.65425	chr5:4231	Metazoa_SRP		smallRNA	chr5:154718385-154
ENSG0000(	603	13.65425	chr5:4231	RNA5SP197		Pseudoger	chr5:151477459-151
ENSG0000(	603	13.65425	chr5:4231	HAVCR1	NCGv7	protein_c	chr5:157026742-157
ENSG0000(	603	13.65425	chr5:4231	ITK	NCGv7;AC	protein_c	chr5:157142933-157
ENSG0000(	603	13.65425	chr5:4231	MIR1303		smallRNA	chr5:154685776-154
ENSG0000(	603	13.65425	chr5:4231	THG1L		protein_c	chr5:157731420-157
ENSG0000(	603	13.65425	chr5:4231	ENSG00000287695		lncRNA	chr5:154346042-154
ENSG0000(	603	13.65425	chr5:4231	IL12B		protein_c	chr5:159314780-159
ENSG0000(	603	13.65425	chr5:4231	TTC1		protein_c	chr5:160009113-160
ENSG0000(	603	13.65425	chr5:4231	ENSG00000290601		lncRNA	chr5:157682985-157
ENSG0000(	603	13.65425	chr5:4231	snoU13		smallRNA	chr5:150029349-150

ENSG0000(	603	13.65425	chr5:4231	ATOX1	DriverDB\	protein_c	chr5:151742316-151
ENSG0000(	603	13.65425	chr5:4231	FAM114A2		protein_c	chr5:153990148-154
ENSG0000(	603	13.65425	chr5:4231	ENSG00000286749		lncRNA	chr5:152374998-152
ENSG0000(	603	13.65425	chr5:4231	CYFIP2		protein_c	chr5:157266079-157
ENSG0000(	603	13.65425	chr5:4231	ENSG00000254350		lncRNA	chr5:158424585-158
ENSG0000(	603	13.65425	chr5:4231	LINC02227		lncRNA	chr5:158320683-158
ENSG0000(	603	13.65425	chr5:4231	U3		smallRNA	chr5:149695749-149
ENSG0000(	603	13.65425	chr5:4231	AC008625.1		smallRNA	chr5:154340032-154
ENSG0000(	603	13.65425	chr5:4231	AC008703.2		smallRNA	chr5:159525938-159
ENSG0000(	603	13.65425	chr5:4231	PDE6A		protein_c	chr5:149857953-149
ENSG0000(	603	13.65425	chr5:4231	RN7SL439P		smallRNA	chr5:154703517-154
ENSG0000(	603	13.65425	chr5:4231	SPARC	AC	protein_c	chr5:151661096-151
ENSG0000(	603	13.65425	chr5:4231	UBLCP1		protein_c	chr5:159263290-159
ENSG0000(	603	13.65425	chr5:4231	AC034205.1		smallRNA	chr5:151368444-151
ENSG0000(	603	13.65425	chr5:4231	ENSG00000277866		Pseudoger	chr5:150978419-150
ENSG0000(	603	13.65425	chr5:4231	RN7SL655P		smallRNA	chr5:154349428-154
ENSG0000(	603	13.65425	chr5:4231	IRGM		protein_c	chr5:150846521-150
ENSG0000(	603	13.65425	chr5:4231	MFAP3		protein_c	chr5:154038959-154
ENSG0000(	603	13.65425	chr5:4231	MFFP2		Pseudoger	chr5:149932014-149
ENSG0000(	603	13.65425	chr5:4231	ENSG00000223908		Pseudoger	chr5:160023740-160
ENSG0000(	603	13.65425	chr5:4231	RNU6-556P		smallRNA	chr5:156814130-156
ENSG0000(	603	13.65425	chr5:4231	TIGD6		protein_c	chr5:149993118-150
ENSG0000(	603	13.65425	chr5:4231	GRPEL2		protein_c	chr5:149345430-149
ENSG0000(	603	13.65425	chr5:4231	ENSG00000253811		lncRNA	chr5:158983006-158
ENSG0000(	603	13.65425	chr5:4231	Y_RNA		smallRNA	chr5:150098406-150
ENSG0000(	603	13.65425	chr5:4231	MARK2P11		Pseudoger	chr5:157984658-157
ENSG0000(	603	13.65425	chr5:4231	ENSG00000288764		lncRNA	chr5:159262272-159
ENSG0000(	603	13.65425	chr5:4231	LINC01933		lncRNA	chr5:151949571-152
ENSG0000(	603	13.65425	chr5:4231	ARSI		protein_c	chr5:150296343-150
ENSG0000(	603	13.65425	chr5:4231	ENSG00000230551		lncRNA	chr5:149494314-149
ENSG0000(	603	13.65425	chr5:4231	SLC36A3		protein_c	chr5:151276358-151
ENSG0000(	603	13.65425	chr5:4231	CLMAT3		lncRNA	chr5:151676945-151
ENSG0000(	603	13.65425	chr5:4231	ENSG00000274235		Pseudoger	chr5:150950109-150
ENSG0000(	603	13.65425	chr5:4231	SLC36A2		protein_c	chr5:151314972-151
ENSG0000(	603	13.65425	chr5:4231	EBF1	NCGv7;AC	protein_c	chr5:158695916-159
ENSG0000(	603	13.65425	chr5:4231	Y_RNA		smallRNA	chr5:160008445-160
ENSG0000(	603	13.65425	chr5:4231	RNU6-390P		smallRNA	chr5:157579497-157
ENSG0000(	603	13.65425	chr5:4231	HAVCR2		protein_c	chr5:157085422-157
ENSG0000(	603	13.65425	chr5:4231	ENSG00000253422		Pseudoger	chr5:158078099-158
ENSG0000(	603	13.65425	chr5:4231	ENSG00000254047		Pseudoger	chr5:158188977-158
ENSG0000(	603	13.65425	chr5:4231	ENSG00000254298		lncRNA	chr5:151158106-151
ENSG0000(	603	13.65425	chr5:4231	RPL36AP20		Pseudoger	chr5:152495718-152
ENSG0000(	603	13.65425	chr5:4231	PPP1R2B		protein_c	chr5:156850295-156
ENSG0000(	603	13.65425	chr5:4231	MYOZ3	NCGv7	protein_c	chr5:150660882-150
ENSG0000(	603	13.65425	chr5:4231	RPS14		protein_c	chr5:150442635-150
ENSG0000(	603	13.65425	chr5:4231	SAP30L		protein_c	chr5:154445997-154
ENSG0000(	603	13.65425	chr5:4231	GALNT10	AC	protein_c	chr5:154190730-154
ENSG0000(	603	13.65425	chr5:4231	ENSG00000253449		lncRNA	chr5:158225352-158
ENSG0000(	603	13.65425	chr5:4231	ADAM19		protein_c	chr5:157395534-157
ENSG0000(	603	13.65425	chr5:4231	RN7SL177P		smallRNA	chr5:153755382-153
ENSG0000(	603	13.65425	chr5:4231	ENSG00000285868		protein_c	chr5:157341596-157

ENSG0000(	603	13.65425	chr5:4231	SOX30		protein_c	chr5:157625679-157
ENSG0000(	603	13.65425	chr5:4231	ENSG00000248544		lncRNA	chr5:157375741-157
ENSG0000(	603	13.65425	chr5:4231	RPS20P4		Pseudoger	chr5:150021567-150
ENSG0000(	603	13.65425	chr5:4231	ENSG00000253424		lncRNA	chr5:158256137-158
ENSG0000(	603	13.65425	chr5:4231	GPX3		protein_c	chr5:151020591-151
ENSG0000(	603	13.65425	chr5:4231	MIR1294		smallRNA	chr5:154347106-154
ENSG0000(	603	13.65425	chr5:4231	ENSG00000254293		lncRNA	chr5:154483917-154
ENSG0000(	603	13.65425	chr5:4231	MIR143		smallRNA	chr5:149428918-149
ENSG0000(	603	13.65425	chr5:4231	SLC6A7		protein_c	chr5:150190062-150
ENSG0000(	603	13.65425	chr5:4231	CLINT1		protein_c	chr5:157785743-157
ENSG0000(	603	13.65425	chr5:4231	C5orf52		protein_c	chr5:157671533-157
ENSG0000(	603	13.65425	chr5:4231	RPLP1P6		Pseudoger	chr5:151765859-151
ENSG0000(	603	13.65425	chr5:4231	RNU2-48P		smallRNA	chr5:157976766-157
ENSG0000(	603	13.65425	chr5:4231	RNF145		protein_c	chr5:159157409-159
ENSG0000(	603	13.65425	chr5:4231	TIMD4		protein_c	chr5:156919292-156
ENSG0000(	603	13.65425	chr5:4231	ENSG00000260581		lncRNA	chr5:151652275-151
ENSG0000(	603	13.65425	chr5:4231	AC010295.1		smallRNA	chr5:154093304-154
ENSG0000(	603	13.65425	chr5:4231	ENSG00000253653		lncRNA	chr5:157260122-157
ENSG0000(	603	13.65425	chr5:4231	ENSG00000253852		lncRNA	chr5:150608428-150
ENSG0000(	603	13.65425	chr5:4231	SLC36A1		protein_c	chr5:151437046-151
ENSG0000(	603	13.65425	chr5:4231	ENSG00000253456		lncRNA	chr5:158985806-158
ENSG0000(	603	13.65425	chr5:4231	ARHGEF37		protein_c	chr5:149551947-149
ENSG0000(	603	13.65425	chr5:4231	LINC01861		lncRNA	chr5:153887428-153
ENSG0000(	603	13.65425	chr5:4231	RPL6P32		Pseudoger	chr5:155111184-155
ENSG0000(	603	13.65425	chr5:4231	PDGFRL2P		Pseudoger	chr5:151173981-151
ENSG0000(	603	13.65425	chr5:4231	ENSG00000287963		lncRNA	chr5:155087430-155
ENSG0000(	603	13.65425	chr5:4231	ZNF300P1		lncRNA	chr5:150930456-150
ENSG0000(	603	13.65425	chr5:4231	AFAP1L1		protein_c	chr5:149271859-149
ENSG0000(	603	13.65425	chr5:4231	TCOF1		protein_c	chr5:150357629-150
ENSG0000(	603	13.65425	chr5:4231	CAMK2A		protein_c	chr5:150219491-150
ENSG0000(	603	13.65425	chr5:4231	ENSG00000253886		Pseudoger	chr5:154493576-154
ENSG0000(	603	13.65425	chr5:4231	ENSG00000213414		Pseudoger	chr5:157986146-157
ENSG0000(	603	13.65425	chr5:4231	LINC01845		lncRNA	chr5:159448556-159
ENSG0000(	603	13.65425	chr5:4231	FAT2	NCGv7	protein_c	chr5:151504092-151
ENSG0000(	603	13.65425	chr5:4231	ENSG00000253155		Pseudoger	chr5:157422439-157
ENSG0000(	603	13.65425	chr5:4231	ENSG00000249738		lncRNA	chr5:159310745-159
ENSG0000(	603	13.65425	chr5:4231	ZNF300		protein_c	chr5:150894392-150
ENSG0000(	603	13.65425	chr5:4231	LINC01847		lncRNA	chr5:159698586-159
ENSG0000(	603	13.65425	chr5:4231	G3BP1	NCGv7	protein_c	chr5:151771045-151
ENSG0000(	603	13.65425	chr5:4231	KIF4B		protein_c	chr5:155013755-155
ENSG0000(	603	13.65425	chr5:4231	PPARGC1B		protein_c	chr5:149730298-149
ENSG0000(	603	13.65425	chr5:4231	SLC26A2		protein_c	chr5:149960758-149
ENSG0000(	603	13.65425	chr5:4231	LSM11		protein_c	chr5:157743712-157
ENSG0000(	603	13.65425	chr5:4231	MED7		protein_c	chr5:157137424-157
ENSG0000(	603	13.65425	chr5:4231	TNIP1		protein_c	chr5:151029945-151
ENSG0000(	603	13.65425	chr5:4231	GLRA1		protein_c	chr5:151822513-151
ENSG0000(	603	13.65425	chr5:4231	CIR1P1		Pseudoger	chr5:154559308-154
ENSG0000(	603	13.65425	chr5:4231	PCYOX1L		protein_c	chr5:149358037-149
ENSG0000(	603	13.65425	chr5:4231	SMIM3		protein_c	chr5:150778757-150
ENSG0000(	603	13.65425	chr5:4231	ENSG00000271795		lncRNA	chr5:151509453-151
ENSG0000(	603	13.65425	chr5:4231	SAP30L-AS1		lncRNA	chr5:154325568-154

ENSG0000(	603	13.65425	chr5:4231	MRPL22		protein_c	chr5:154941073-154
ENSG0000(	603	13.65425	chr5:4231	GEMIN5		protein_c	chr5:154887411-154
ENSG0000(	603	13.65425	chr5:4231	ENSG00000279204		TEC	chr5:159106380-159
ENSG0000(	603	13.65425	chr5:4231	ENSG00000261382		lncRNA	chr5:153901459-153
ENSG0000(	603	13.65425	chr5:4231	NDST1		protein_c	chr5:150485818-150
ENSG0000(	603	13.65425	chr5:4231	ENSG00000254163		lncRNA	chr5:156704058-156
ENSG0000(	603	13.65425	chr5:4231	ENSG00000253630		Pseudoger	chr5:157747504-157
ENSG0000(	603	13.65425	chr5:4231	ANXA6		protein_c	chr5:151100706-151
ENSG0000(	603	13.65425	chr5:4231	ENSG00000253472		Pseudoger	chr5:151378003-151
ENSG0000(	603	13.65425	chr5:4231	ZNF300P1		Pseudoger	chr5:150930763-150
ENSG0000(	603	13.65425	chr5:4231	RNU4ATAC2P		smallRNA	chr5:159318042-159
ENSG0000(	603	13.65425	chr5:4231	ENSG00000272112		lncRNA	chr5:151724831-151
ENSG0000(	603	13.65425	chr5:4231	ENSG00000253798		Pseudoger	chr5:157689934-157
ENSG0000(	603	13.65425	chr5:4231	ENSG00000272085		lncRNA	chr5:158464465-158
ENSG0000(	603	13.65425	chr5:4231	ENSG00000286657		lncRNA	chr5:150621007-150
ENSG0000(	603	13.65425	chr5:4231	ENSG00000253980		lncRNA	chr5:157199242-157
ENSG0000(	603	13.65425	chr5:4231	SYNPO		protein_c	chr5:150601080-150
ENSG0000(	603	13.65425	chr5:4231	GAPDHP40		Pseudoger	chr5:159950493-159
ENSG0000(	603	13.65425	chr5:4231	ENSG00000286690		lncRNA	chr5:156019228-156
ENSG0000(	603	13.65425	chr5:4231	ENSG00000287323		lncRNA	chr5:149694237-149
ENSG0000(	603	13.65425	chr5:4231	LINC01932		lncRNA	chr5:159227715-159
ENSG0000(	603	13.65425	chr5:4231	AC026407.1		protein_c	chr5:157732995-157
ENSG0000(	603	13.65425	chr5:4231	ATP6V1G1P5		Pseudoger	chr5:151319422-151
ENSG0000(	603	13.65425	chr5:4231	RBM22		protein_c	chr5:150690792-150
ENSG0000(	603	13.65425	chr5:4231	ENSG00000253519		lncRNA	chr5:157565964-157
ENSG0000(	603	13.65425	chr5:4231	GRPEL2-AS1		lncRNA	chr5:149348116-149
ENSG0000(	603	13.65425	chr5:4231	MIR3141		smallRNA	chr5:154596012-154
ENSG0000(	602	13.63161	chr2:8187	RNU6-577P		smallRNA	chr2:36867398-3686
ENSG0000(	602	13.63161	chr2:8187	FEZ2		protein_c	chr2:36531805-3664
ENSG0000(	602	13.63161	chr2:8187	CRIM1-DT		lncRNA	chr2:36354744-3635
ENSG0000(	602	13.63161	chr2:8187	ENSG00000273090		lncRNA	chr2:36513255-3651
ENSG0000(	602	13.63161	chr2:8187	VIT	NCGv7	protein_c	chr2:36696690-3681
ENSG0000(	602	13.63161	chr2:8187	RACK1P2		Pseudoger	chr2:36656322-3665
ENSG0000(	602	13.63161	chr2:8187	STRN	IntOGen-I	protein_c	chr2:36837698-3696
ENSG0000(	602	13.63161	chr2:8187	ENSG00000279519		TEC	chr2:36839922-3684
ENSG0000(	602	13.63161	chr2:8187	CRIM1		protein_c	chr2:36355778-3655
ENSG0000(	602	13.63161	chr2:8187	RPL21P36		Pseudoger	chr2:36299388-3629
ENSG0000(	599	13.56368	chr2:2577	RNY4P7		smallRNA	chr2:127798903-127
ENSG0000(	599	13.56368	chr2:2577	TUBA3E		protein_c	chr2:130191745-130
ENSG0000(	599	13.56368	chr2:2577	LIMS2		protein_c	chr2:127638381-127
ENSG0000(	599	13.56368	chr2:2577	snosnR60_Z15		smallRNA	chr2:125128832-125
ENSG0000(	599	13.56368	chr2:2577	MZT2B		protein_c	chr2:130181737-130
ENSG0000(	599	13.56368	chr2:2577	ENSG00000286026		lncRNA	chr2:129990456-130
ENSG0000(	599	13.56368	chr2:2577	RAB6C-AS1		lncRNA	chr2:129966592-129
ENSG0000(	599	13.56368	chr2:2577	Y_RNA		smallRNA	chr2:128067800-128
ENSG0000(	599	13.56368	chr2:2577	MTND3P10		Pseudoger	chr2:120212935-120
ENSG0000(	599	13.56368	chr2:2577	RPL17P15		Pseudoger	chr2:119698623-119
ENSG0000(	599	13.56368	chr2:2577	ENSG00000290113		lncRNA	chr2:126220632-126
ENSG0000(	599	13.56368	chr2:2577	MTATP6P7		Pseudoger	chr2:130276507-130
ENSG0000(	599	13.56368	chr2:2577	CCDC74B		protein_c	chr2:130139287-130
ENSG0000(	599	13.56368	chr2:2577	ENSG00000279874		TEC	chr2:123850522-123

ENSG0000(	599	13.56368	chr2:2577	RPS26P19		Pseudoger	chr2:127846736-127
ENSG0000(	599	13.56368	chr2:2577	CNTNAP5-DT		lncRNA	chr2:124011984-124
ENSG0000(	599	13.56368	chr2:2577	RNU6-1147P		smallRNA	chr2:127316873-127
ENSG0000(	599	13.56368	chr2:2577	ENSG00000284619		Pseudoger	chr2:129988158-129
ENSG0000(	599	13.56368	chr2:2577	ARHGAP42P2		Pseudoger	chr2:130006200-130
ENSG0000(	599	13.56368	chr2:2577	Y_RNA		smallRNA	chr2:127829747-127
ENSG0000(	599	13.56368	chr2:2577	MTCO1P43		Pseudoger	chr2:117026537-117
ENSG0000(	599	13.56368	chr2:2577	PSMD14P1		Pseudoger	chr2:123762171-123
ENSG0000(	599	13.56368	chr2:2577	ENSG00000291278		lncRNA	chr2:130034788-130
ENSG0000(	599	13.56368	chr2:2577	GLI2	AC	protein_c	chr2:120735623-120
ENSG0000(	599	13.56368	chr2:2577	ENSG00000284171		Pseudoger	chr2:130278444-130
ENSG0000(	599	13.56368	chr2:2577	SCTR-AS1		lncRNA	chr2:119476428-119
ENSG0000(	599	13.56368	chr2:2577	ENSG00000286058		lncRNA	chr2:130026845-130
ENSG0000(	599	13.56368	chr2:2577	CLASP1	NCGv7	protein_c	chr2:121337776-121
ENSG0000(	599	13.56368	chr2:2577	Y_RNA		smallRNA	chr2:121040610-121
ENSG0000(	599	13.56368	chr2:2577	ENSG00000290590		lncRNA	chr2:117860333-117
ENSG0000(	599	13.56368	chr2:2577	RPS17P7		Pseudoger	chr2:121309995-121
ENSG0000(	599	13.56368	chr2:2577	MTCO3P43		Pseudoger	chr2:120211727-120
ENSG0000(	599	13.56368	chr2:2577	ENSG00000290591		lncRNA	chr2:130012065-130
ENSG0000(	599	13.56368	chr2:2577	RPL12P15		Pseudoger	chr2:121658475-121
ENSG0000(	599	13.56368	chr2:2577	TEKT4P3		Pseudoger	chr2:130313934-130
ENSG0000(	599	13.56368	chr2:2577	SRMP3		Pseudoger	chr2:128139792-128
ENSG0000(	599	13.56368	chr2:2577	ENSG00000270815		Pseudoger	chr2:124778454-124
ENSG0000(	599	13.56368	chr2:2577	LINC01826		lncRNA	chr2:123066151-123
ENSG0000(	599	13.56368	chr2:2577	ISCA1P6		Pseudoger	chr2:128518788-128
ENSG0000(	599	13.56368	chr2:2577	ENSG00000279721		TEC	chr2:121688203-121
ENSG0000(	599	13.56368	chr2:2577	ENSG00000227632		Pseudoger	chr2:130202312-130
ENSG0000(	599	13.56368	chr2:2577	ENSG00000228471		Pseudoger	chr2:130082092-130
ENSG0000(	599	13.56368	chr2:2577	Y_RNA		smallRNA	chr2:120651273-120
ENSG0000(	599	13.56368	chr2:2577	MED15P9		lncRNA	chr2:130129621-130
ENSG0000(	599	13.56368	chr2:2577	RNU6-259P		smallRNA	chr2:124989219-124
ENSG0000(	599	13.56368	chr2:2577	UGGT1		protein_c	chr2:128091200-128
ENSG0000(	599	13.56368	chr2:2577	MTND4P26		Pseudoger	chr2:120213631-120
ENSG0000(	599	13.56368	chr2:2577	HS6ST1		protein_c	chr2:128236716-128
ENSG0000(	599	13.56368	chr2:2577	FLJ14816		protein_c	chr2:120464335-120
ENSG0000(	599	13.56368	chr2:2577	ENSG00000224967		Pseudoger	chr2:117934025-117
ENSG0000(	599	13.56368	chr2:2577	CYP27C1		protein_c	chr2:127183832-127
ENSG0000(	599	13.56368	chr2:2577	ENSG00000259094		lncRNA	chr2:118949306-118
ENSG0000(	599	13.56368	chr2:2577	NPM1P32		Pseudoger	chr2:121708512-121
ENSG0000(	599	13.56368	chr2:2577	ENSG00000286873		lncRNA	chr2:127888829-127
ENSG0000(	599	13.56368	chr2:2577	ENSG00000224869		Pseudoger	chr2:116726453-116
ENSG0000(	599	13.56368	chr2:2577	RHOQP3		Pseudoger	chr2:130212870-130
ENSG0000(	599	13.56368	chr2:2577	NOC2LP1		Pseudoger	chr2:130229379-130
ENSG0000(	599	13.56368	chr2:2577	MTND5P29		Pseudoger	chr2:130280299-130
ENSG0000(	599	13.56368	chr2:2577	LINC01956		lncRNA	chr2:118766965-118
ENSG0000(	599	13.56368	chr2:2577	ENSG00000224789		lncRNA	chr2:120174885-120
ENSG0000(	599	13.56368	chr2:2577	ENSG00000286957		lncRNA	chr2:129877896-129
ENSG0000(	599	13.56368	chr2:2577	ENSG00000236255		lncRNA	chr2:117833937-117
ENSG0000(	599	13.56368	chr2:2577	ENSG00000286971		lncRNA	chr2:127024866-127
ENSG0000(	599	13.56368	chr2:2577	PLAC9P1		lncRNA	chr2:129893985-129
ENSG0000(	599	13.56368	chr2:2577	ENSG00000235066		lncRNA	chr2:117995397-118

ENSG0000(	599	13.56368	chr2:2577	MTCO1P7		Pseudoger	chr2:130273908-130
ENSG0000(	599	13.56368	chr2:2577	BIN1		protein_c	chr2:127048027-127
ENSG0000(	599	13.56368	chr2:2577	ENSG00000235128		Pseudoger	chr2:126679622-126
ENSG0000(	599	13.56368	chr2:2577	MTND2P22		Pseudoger	chr2:130272479-130
ENSG0000(	599	13.56368	chr2:2577	ENSG00000236145		lncRNA	chr2:121178327-121
ENSG0000(	599	13.56368	chr2:2577	C2orf76		protein_c	chr2:119302225-119
ENSG0000(	599	13.56368	chr2:2577	RNU4ATAC		smallRNA	chr2:121530881-121
ENSG0000(	599	13.56368	chr2:2577	LINC01101		TEC	chr2:120464335-120
ENSG0000(	599	13.56368	chr2:2577	Y_RNA		smallRNA	chr2:121603073-121
ENSG0000(	599	13.56368	chr2:2577	TMEM37		protein_c	chr2:119429901-119
ENSG0000(	599	13.56368	chr2:2577	GYPC	NCGv7	protein_c	chr2:126656133-126
ENSG0000(	599	13.56368	chr2:2577	AC092646.3		smallRNA	chr2:123682107-123
ENSG0000(	599	13.56368	chr2:2577	MAP3K2-DT		lncRNA	chr2:127388173-127
ENSG0000(	599	13.56368	chr2:2577	DYNLT3P2		Pseudoger	chr2:128199901-128
ENSG0000(	599	13.56368	chr2:2577	MTCO3P7		Pseudoger	chr2:130277184-130
ENSG0000(	599	13.56368	chr2:2577	ENSG00000229774		lncRNA	chr2:120686635-120
ENSG0000(	599	13.56368	chr2:2577	RN7SL206P		smallRNA	chr2:128122081-128
ENSG0000(	599	13.56368	chr2:2577	KLF2P1		Pseudoger	chr2:130036958-130
ENSG0000(	599	13.56368	chr2:2577	RN7SL468P		smallRNA	chr2:119169822-119
ENSG0000(	599	13.56368	chr2:2577	SLC6A14P3		Pseudoger	chr2:126311466-126
ENSG0000(	599	13.56368	chr2:2577	RN7SL111P		smallRNA	chr2:118016382-118
ENSG0000(	599	13.56368	chr2:2577	MED15P5		Pseudoger	chr2:130251568-130
ENSG0000(	599	13.56368	chr2:2577	RAB6C		protein_c	chr2:129979666-129
ENSG0000(	599	13.56368	chr2:2577	RPL22P7		Pseudoger	chr2:129939848-129
ENSG0000(	599	13.56368	chr2:2577	PTPN4		protein_c	chr2:119759922-119
ENSG0000(	599	13.56368	chr2:2577	DDX18	NCGv7	protein_c	chr2:117814691-117
ENSG0000(	599	13.56368	chr2:2577	ENSG00000232140		lncRNA	chr2:120552481-120
ENSG0000(	599	13.56368	chr2:2577	Y_RNA		smallRNA	chr2:128350829-128
ENSG0000(	599	13.56368	chr2:2577	STEAP3-AS1		lncRNA	chr2:119244422-119
ENSG0000(	599	13.56368	chr2:2577	ENSG00000234398		Pseudoger	chr2:130245971-130
ENSG0000(	599	13.56368	chr2:2577	RAB6C-AS1		Pseudoger	chr2:129968258-129
ENSG0000(	599	13.56368	chr2:2577	ENSG00000234455		lncRNA	chr2:121081437-121
ENSG0000(	599	13.56368	chr2:2577	RNA5SP102		Pseudoger	chr2:124932915-124
ENSG0000(	599	13.56368	chr2:2577	MTND5P22		Pseudoger	chr2:124680722-124
ENSG0000(	599	13.56368	chr2:2577	ENSG00000250207		Pseudoger	chr2:129992602-129
ENSG0000(	599	13.56368	chr2:2577	ENSG00000289374		lncRNA	chr2:129443088-129
ENSG0000(	599	13.56368	chr2:2577	RN7SKP102		smallRNA	chr2:123869256-123
ENSG0000(	599	13.56368	chr2:2577	RNU6-675P		smallRNA	chr2:126702839-126
ENSG0000(	599	13.56368	chr2:2577	ENSG00000235871		Pseudoger	chr2:116818086-116
ENSG0000(	599	13.56368	chr2:2577	ENSG00000232101		Pseudoger	chr2:128151154-128
ENSG0000(	599	13.56368	chr2:2577	POTEF-AS1		lncRNA	chr2:130107684-130
ENSG0000(	599	13.56368	chr2:2577	ENSG00000272895		lncRNA	chr2:117998745-117
ENSG0000(	599	13.56368	chr2:2577	PROC		protein_c	chr2:127418427-127
ENSG0000(	599	13.56368	chr2:2577	LINC01889		lncRNA	chr2:125710969-125
ENSG0000(	599	13.56368	chr2:2577	MTND6P8		Pseudoger	chr2:130282085-130
ENSG0000(	599	13.56368	chr2:2577	snoU13		smallRNA	chr2:129427422-129
ENSG0000(	599	13.56368	chr2:2577	RNU7-182P		smallRNA	chr2:127017421-127
ENSG0000(	599	13.56368	chr2:2577	RNU6-1049P		smallRNA	chr2:130109204-130
ENSG0000(	599	13.56368	chr2:2577	SMPD4		protein_c	chr2:130151392-130
ENSG0000(	599	13.56368	chr2:2577	MTND4LP14		Pseudoger	chr2:120213344-120
ENSG0000(	599	13.56368	chr2:2577	MTND4P27		Pseudoger	chr2:130278757-130

ENSG0000(	599	13.56368	chr2:2577	ENSG00000272789		lncRNA	chr2:127625997-127
ENSG0000(	599	13.56368	chr2:2577	ENSG00000235840		lncRNA	chr2:120319007-120
ENSG0000(	599	13.56368	chr2:2577	TFCP2L1		protein_c	chr2:121216587-121
ENSG0000(	599	13.56368	chr2:2577	EPB41L5		protein_c	chr2:120013077-120
ENSG0000(	599	13.56368	chr2:2577	STEAP3		protein_c	chr2:119223831-119
ENSG0000(	599	13.56368	chr2:2577	ENSG00000235774		lncRNA	chr2:126308494-126
ENSG0000(	599	13.56368	chr2:2577	ELOAP1		Pseudoger	chr2:123695302-123
ENSG0000(	599	13.56368	chr2:2577	CCDC115		protein_c	chr2:130337933-130
ENSG0000(	599	13.56368	chr2:2577	MTCO2P7		Pseudoger	chr2:130275592-130
ENSG0000(	599	13.56368	chr2:2577	SFT2D3		protein_c	chr2:127701497-127
ENSG0000(	599	13.56368	chr2:2577	MED15P9		Pseudoger	chr2:130135978-130
ENSG0000(	599	13.56368	chr2:2577	WDR33		protein_c	chr2:127701027-127
ENSG0000(	599	13.56368	chr2:2577	MTND5P28		Pseudoger	chr2:120215181-120
ENSG0000(	599	13.56368	chr2:2577	RALB	TAG	protein_c	chr2:120240064-120
ENSG0000(	599	13.56368	chr2:2577	SAP130		protein_c	chr2:127941217-128
ENSG0000(	599	13.56368	chr2:2577	ENSG00000238207		lncRNA	chr2:117754139-117
ENSG0000(	599	13.56368	chr2:2577	RPL21P34		Pseudoger	chr2:128217588-128
ENSG0000(	599	13.56368	chr2:2577	RNU7-190P		smallRNA	chr2:117139715-117
ENSG0000(	599	13.56368	chr2:2577	LINC01823		lncRNA	chr2:121779133-121
ENSG0000(	599	13.56368	chr2:2577	EN1	NCGv7	protein_c	chr2:118842171-118
ENSG0000(	599	13.56368	chr2:2577	ENSG00000288524		lncRNA	chr2:126640708-126
ENSG0000(	599	13.56368	chr2:2577	MTND1P28		Pseudoger	chr2:117023918-117
ENSG0000(	599	13.56368	chr2:2577	CFAP221		protein_c	chr2:119544432-119
ENSG0000(	599	13.56368	chr2:2577	INHBB		protein_c	chr2:120346136-120
ENSG0000(	599	13.56368	chr2:2577	ERCC3	NCGv7;AC	protein_c	chr2:127257290-127
ENSG0000(	599	13.56368	chr2:2577	IWS1	NCGv7	protein_c	chr2:127436207-127
ENSG0000(	599	13.56368	chr2:2577	AMMECR1L		protein_c	chr2:127861630-127
ENSG0000(	599	13.56368	chr2:2577	MIR4783		smallRNA	chr2:127423537-127
ENSG0000(	599	13.56368	chr2:2577	RNA5SP103		Pseudoger	chr2:128445081-128
ENSG0000(	599	13.56368	chr2:2577	POLR2D	NCGv7	protein_c	chr2:127843553-127
ENSG0000(	599	13.56368	chr2:2577	ENSG00000272667		lncRNA	chr2:127886556-127
ENSG0000(	599	13.56368	chr2:2577	GPR17		protein_c	chr2:127645864-127
ENSG0000(	599	13.56368	chr2:2577	ENSG00000238277		lncRNA	chr2:129063846-129
ENSG0000(	599	13.56368	chr2:2577	ENSG00000224410		Pseudoger	chr2:125823108-125
ENSG0000(	599	13.56368	chr2:2577	snoU13		smallRNA	chr2:121705967-121
ENSG0000(	599	13.56368	chr2:2577	snoU13		smallRNA	chr2:119917318-119
ENSG0000(	599	13.56368	chr2:2577	ENSG00000224352		Pseudoger	chr2:130330142-130
ENSG0000(	599	13.56368	chr2:2577	ENSG00000289091		lncRNA	chr2:120255848-120
ENSG0000(	599	13.56368	chr2:2577	TMEM177		protein_c	chr2:119679167-119
ENSG0000(	599	13.56368	chr2:2577	C1QL2	NCGv7	protein_c	chr2:119156243-119
ENSG0000(	599	13.56368	chr2:2577	PLAC9P1		Pseudoger	chr2:129923558-129
ENSG0000(	599	13.56368	chr2:2577	TSN		protein_c	chr2:121737103-121
ENSG0000(	599	13.56368	chr2:2577	AC016764.1		smallRNA	chr2:121035073-121
ENSG0000(	599	13.56368	chr2:2577	ENSG00000270798		Pseudoger	chr2:121743479-121
ENSG0000(	599	13.56368	chr2:2577	RPL14P6		Pseudoger	chr2:128203361-128
ENSG0000(	599	13.56368	chr2:2577	DBI	NCGv7	protein_c	chr2:119366924-119
ENSG0000(	599	13.56368	chr2:2577	ENSG00000286370		lncRNA	chr2:117536387-117
ENSG0000(	599	13.56368	chr2:2577	NIFK		protein_c	chr2:121726945-121
ENSG0000(	599	13.56368	chr2:2577	MYO7B		protein_c	chr2:127535683-127
ENSG0000(	599	13.56368	chr2:2577	ENSG00000226708		lncRNA	chr2:123443266-123
ENSG0000(	599	13.56368	chr2:2577	ENSG00000260163		lncRNA	chr2:127025211-127

ENSG0000(	599	13.56368	chr2:2577	ENSG00000286384	lncRNA	chr2:124695242-124
ENSG0000(	599	13.56368	chr2:2577	ENSG00000286400	lncRNA	chr2:127141976-127
ENSG0000(	599	13.56368	chr2:2577	ENSG00000231731	lncRNA	chr2:127455394-127
ENSG0000(	599	13.56368	chr2:2577	MTND1P29	Pseudoger	chr2:130270440-130
ENSG0000(	599	13.56368	chr2:2577	ENSG00000287854	lncRNA	chr2:123421486-123
ENSG0000(	599	13.56368	chr2:2577	POTEF	protein_c	chr2:130073535-130
ENSG0000(	599	13.56368	chr2:2577	MTND2P21	Pseudoger	chr2:117025077-117
ENSG0000(	599	13.56368	chr2:2577	ENSG00000286206	lncRNA	chr2:125665331-125
ENSG0000(	599	13.56368	chr2:2577	ENSG00000287871	lncRNA	chr2:122503366-122
ENSG0000(	599	13.56368	chr2:2577	ENSG00000279227	TEC	chr2:118014174-118
ENSG0000(	599	13.56368	chr2:2577	PPIAP65	Pseudoger	chr2:129496297-129
ENSG0000(	599	13.56368	chr2:2577	ENSG00000260634	lncRNA	chr2:127023537-127
ENSG0000(	599	13.56368	chr2:2577	MAP3K2	protein_c	chr2:127298668-127
ENSG0000(	599	13.56368	chr2:2577	ENSG00000271709	lncRNA	chr2:120866378-120
ENSG0000(	599	13.56368	chr2:2577	RPL27P7	Pseudoger	chr2:119993199-119
ENSG0000(	599	13.56368	chr2:2577	THORLNC	lncRNA	chr2:118132128-118
ENSG0000(	599	13.56368	chr2:2577	HTR5BP	Pseudoger	chr2:117859427-117
ENSG0000(	599	13.56368	chr2:2577	INSIG2	protein_c	chr2:118088452-118
ENSG0000(	599	13.56368	chr2:2577	MTCYBP8	Pseudoger	chr2:130282679-130
ENSG0000(	599	13.56368	chr2:2577	CCDC93	protein_c	chr2:117915478-118
ENSG0000(	599	13.56368	chr2:2577	RPL19P4	Pseudoger	chr2:130293822-130
ENSG0000(	599	13.56368	chr2:2577	ENSG00000237614	lncRNA	chr2:120542905-120
ENSG0000(	599	13.56368	chr2:2577	CLASP1-AS1	lncRNA	chr2:121530422-121
ENSG0000(	599	13.56368	chr2:2577	RNU6-395P	smallRNA	chr2:127845236-127
ENSG0000(	599	13.56368	chr2:2577	CYP4F27P	Pseudoger	chr2:130048426-130
ENSG0000(	599	13.56368	chr2:2577	TEX51	protein_c	chr2:126898864-126
ENSG0000(	599	13.56368	chr2:2577	ENSG00000271667	Pseudoger	chr2:116687218-116
ENSG0000(	599	13.56368	chr2:2577	LINC01941	lncRNA	chr2:126109761-126
ENSG0000(	599	13.56368	chr2:2577	LINC01856	lncRNA	chr2:129923177-129
ENSG0000(	599	13.56368	chr2:2577	MTND3P15	Pseudoger	chr2:130278036-130
ENSG0000(	599	13.56368	chr2:2577	CNTNAP5	protein_c	chr2:124025287-124
ENSG0000(	599	13.56368	chr2:2577	ENSG00000237532	lncRNA	chr2:128580491-128
ENSG0000(	599	13.56368	chr2:2577	ENSG00000289982	lncRNA	chr2:128090621-128
ENSG0000(	599	13.56368	chr2:2577	WBP11P2	Pseudoger	chr2:127247258-127
ENSG0000(	599	13.56368	chr2:2577	AC018804.1	smallRNA	chr2:130183443-130
ENSG0000(	599	13.56368	chr2:2577	SCTR	protein_c	chr2:119439843-119
ENSG0000(	599	13.56368	chr2:2577	NIFKP9	Pseudoger	chr2:127170011-127
ENSG0000(	599	13.56368	chr2:2577	ENSG00000286481	lncRNA	chr2:121902501-122
ENSG0000(	599	13.56368	chr2:2577	LINC02572	lncRNA	chr2:129825126-129
ENSG0000(	599	13.56368	chr2:2577	ZFP91P1	Pseudoger	chr2:127840606-127
ENSG0000(	599	13.56368	chr2:2577	ENSG00000217289	Pseudoger	chr2:129992889-129
ENSG0000(	599	13.56368	chr2:2577	ENSG00000237856	lncRNA	chr2:123065321-123
ENSG0000(	599	13.56368	chr2:2577	ENSG00000287742	lncRNA	chr2:127406731-127
ENSG0000(	599	13.56368	chr2:2577	ENSG00000286515	lncRNA	chr2:126154583-126
ENSG0000(	599	13.56368	chr2:2577	ENSG00000286145	lncRNA	chr2:127294212-127
ENSG0000(	599	13.56368	chr2:2577	NIFK-AS1	lncRNA	chr2:121649320-121
ENSG0000(	599	13.56368	chr2:2577	FAR2P1	Pseudoger	chr2:130028309-130
ENSG0000(	599	13.56368	chr2:2577	MARCO	protein_c	chr2:118942194-118
ENSG0000(	599	13.56368	chr2:2577	ENSG00000227291	Pseudoger	chr2:117180892-117
ENSG0000(	599	13.56368	chr2:2577	ENSG00000234044	Pseudoger	chr2:128067194-128
ENSG0000(	599	13.56368	chr2:2577	ENSG00000204399	Pseudoger	chr2:127931327-127

ENSG0000(	599	13.56368	chr2:2577	AC013275.1		smallRNA	chr2:119570900-119
ENSG0000(	599	13.56368	chr2:2577	TMEM185B		protein_c	chr2:120217479-120
ENSG0000(	599	13.56368	chr2:2577	LINC01854		lncRNA	chr2:129242173-129
ENSG0000(	599	13.56368	chr2:2577	Y_RNA		smallRNA	chr2:120192245-120
ENSG0000(	599	13.56368	chr2:2577	MTATP6P26		Pseudoger	chr2:120211054-120
ENSG0000(	599	13.56368	chr2:2577	MTCYBP39		Pseudoger	chr2:116751158-116
ENSG0000(	597	13.51839	chr5:4231	CKMT2		protein_c	chr5:81233320-8126
ENSG0000(	597	13.51839	chr5:4231	ST13P12		Pseudoger	chr5:82968888-8297
ENSG0000(	597	13.51839	chr5:4231	ATG10-IT1		lncRNA	chr5:81991995-8199
ENSG0000(	597	13.51839	chr5:4231	ENSG00000287938		lncRNA	chr5:81817531-8184
ENSG0000(	597	13.51839	chr5:4231	ENSG00000248112		lncRNA	chr5:82919376-8292
ENSG0000(	597	13.51839	chr5:4231	XRCC4		protein_c	chr5:83077498-8335
ENSG0000(	597	13.51839	chr5:4231	ENSG00000248393		lncRNA	chr5:82545862-8254
ENSG0000(	597	13.51839	chr5:4231	ENSG00000248105		Pseudoger	chr5:82824884-8282
ENSG0000(	597	13.51839	chr5:4231	ATG10-AS1		lncRNA	chr5:82073055-8207
ENSG0000(	597	13.51839	chr5:4231	ENSG00000248794		Pseudoger	chr5:81242330-8124
ENSG0000(	597	13.51839	chr5:4231	ACOT12		protein_c	chr5:81329996-8139
ENSG0000(	597	13.51839	chr5:4231	SSBP2	DriverDB\	protein_c	chr5:81412804-8175
ENSG0000(	597	13.51839	chr5:4231	TMEM167A		protein_c	chr5:83052846-8307
ENSG0000(	597	13.51839	chr5:4231	ENSG00000249100		lncRNA	chr5:82765404-8276
ENSG0000(	597	13.51839	chr5:4231	ATG10		protein_c	chr5:81972023-8227
ENSG0000(	597	13.51839	chr5:4231	ENSG00000249664		lncRNA	chr5:83012285-8301
ENSG0000(	597	13.51839	chr5:4231	AC114969.1		smallRNA	chr5:81978250-8197
ENSG0000(	597	13.51839	chr5:4231	ZCCHC9		protein_c	chr5:81301587-8131
ENSG0000(	597	13.51839	chr5:4231	SEM1P1		Pseudoger	chr5:81892490-8189
ENSG0000(	597	13.51839	chr5:4231	RPL5P16		Pseudoger	chr5:82777797-8277
ENSG0000(	597	13.51839	chr5:4231	ENSG00000286721		lncRNA	chr5:81408517-8141
ENSG0000(	597	13.51839	chr5:4231	RPS23		protein_c	chr5:82273320-8227
ENSG0000(	597	13.51839	chr5:4231	PPIAP11		Pseudoger	chr5:82009602-8201
ENSG0000(	597	13.51839	chr5:4231	ENSG00000248870		lncRNA	chr5:82586776-8258
ENSG0000(	597	13.51839	chr5:4231	ENSG00000249857		lncRNA	chr5:82940458-8294
ENSG0000(	597	13.51839	chr5:4231	ATP6AP1L		Pseudoger	chr5:82279462-8238
ENSG0000(	597	13.51839	chr5:4231	ENSG00000249483		lncRNA	chr5:81851601-8185
ENSG0000(	597	13.51839	chr5:4231	LINC01338		lncRNA	chr5:82807475-8286
ENSG0000(	597	13.51839	chr5:4231	ENSG00000271862		lncRNA	chr5:83049376-8305
ENSG0000(	597	13.51839	chr5:4231	ENSG00000251374		Pseudoger	chr5:82265157-8226
ENSG0000(	597	13.51839	chr5:4231	SCARNA18		smallRNA	chr5:83064204-8306
ENSG0000(	594	13.45046	chr2:8187	RPL30P3		Pseudoger	chr2:9081395-90817
ENSG0000(	594	13.45046	chr2:8187	LINC00276		lncRNA	chr2:13710531-1440
ENSG0000(	594	13.45046	chr2:8187	ENSG00000228496		lncRNA	chr2:11681434-1168
ENSG0000(	594	13.45046	chr2:8187	snoU13		smallRNA	chr2:15903669-1590
ENSG0000(	594	13.45046	chr2:8187	NT5C1B		protein_c	chr2:18562871-1858
ENSG0000(	594	13.45046	chr2:8187	ENSG00000261012		lncRNA	chr2:20999313-2100
ENSG0000(	594	13.45046	chr2:8187	TDRD15		protein_c	chr2:21123968-2114
ENSG0000(	594	13.45046	chr2:8187	SLC7A15P		Pseudoger	chr2:20386386-2039
ENSG0000(	594	13.45046	chr2:8187	NTSR2		protein_c	chr2:11658178-1167
ENSG0000(	594	13.45046	chr2:8187	ENSG00000260476		lncRNA	chr2:10021578-1002
ENSG0000(	594	13.45046	chr2:8187	OSR1		protein_c	chr2:19351485-1935
ENSG0000(	594	13.45046	chr2:8187	RPS26P18		Pseudoger	chr2:15397435-1539
ENSG0000(	594	13.45046	chr2:8187	ENSG00000231083		lncRNA	chr2:8600892-86229
ENSG0000(	594	13.45046	chr2:8187	GDF7		protein_c	chr2:20667144-2067

ENSG0000(	594	13.45046	chr2:8187	PIK3CDP1		Pseudoger	chr2:6495877-64967
ENSG0000(	594	13.45046	chr2:8187	snoU13		smallRNA	chr2:9532242-95323
ENSG0000(	594	13.45046	chr2:8187	ENSG00000228505		lncRNA	chr2:14616428-1463
ENSG0000(	594	13.45046	chr2:8187	RNA5SP86		Pseudoger	chr2:20401627-2040
ENSG0000(	594	13.45046	chr2:8187	ENSG00000223691		Pseudoger	chr2:22188336-2219
ENSG0000(	594	13.45046	chr2:8187	PDIA6		protein_c	chr2:10783391-1083
ENSG0000(	594	13.45046	chr2:8187	SNORD18		smallRNA	chr2:12030303-1203
ENSG0000(	594	13.45046	chr2:8187	ATP6V1C2		protein_c	chr2:10721100-1078
ENSG0000(	594	13.45046	chr2:8187	ENSG00000287119		lncRNA	chr2:7671330-76757
ENSG0000(	594	13.45046	chr2:8187	ENSG00000242136		lncRNA	chr2:9103440-91051
ENSG0000(	594	13.45046	chr2:8187	ENSG00000260331		lncRNA	chr2:18547386-1854
ENSG0000(	594	13.45046	chr2:8187	ENSG00000223536		lncRNA	chr2:16728121-1676
ENSG0000(	594	13.45046	chr2:8187	E2F6		protein_c	chr2:11444375-1146
ENSG0000(	594	13.45046	chr2:8187	CYRIA		protein_c	chr2:16549459-1666
ENSG0000(	594	13.45046	chr2:8187	ENSG00000228538		lncRNA	chr2:21638068-2164
ENSG0000(	594	13.45046	chr2:8187	RHOB	NCGv7	protein_c	chr2:20447074-2044
ENSG0000(	594	13.45046	chr2:8187	ENSG00000287956		lncRNA	chr2:21094101-2109
ENSG0000(	594	13.45046	chr2:8187	LINC01814		lncRNA	chr2:8461703-85929
ENSG0000(	594	13.45046	chr2:8187	ENSG00000287849		lncRNA	chr2:18492216-1851
ENSG0000(	594	13.45046	chr2:8187	ENSG00000236989		lncRNA	chr2:16085222-1610
ENSG0000(	594	13.45046	chr2:8187	ENSG00000243491		lncRNA	chr2:9757496-97703
ENSG0000(	594	13.45046	chr2:8187	PSMC1P10		Pseudoger	chr2:17385091-1738
ENSG0000(	594	13.45046	chr2:8187	RNU6-1288P		smallRNA	chr2:14274622-1427
ENSG0000(	594	13.45046	chr2:8187	RN7SKP27		smallRNA	chr2:22861552-2286
ENSG0000(	594	13.45046	chr2:8187	LINC02850		lncRNA	chr2:20859771-2086
ENSG0000(	594	13.45046	chr2:8187	ENSG00000228999		lncRNA	chr2:21932662-2253
ENSG0000(	594	13.45046	chr2:8187	ENSG00000287881		lncRNA	chr2:18403967-1850
ENSG0000(	594	13.45046	chr2:8187	ENSG00000231204		lncRNA	chr2:21317660-2156
ENSG0000(	594	13.45046	chr2:8187	GREB1		protein_c	chr2:11482341-1164
ENSG0000(	594	13.45046	chr2:8187	ENSG00000285354		lncRNA	chr2:16294445-1630
ENSG0000(	594	13.45046	chr2:8187	RN7SL104P		smallRNA	chr2:15950690-1595
ENSG0000(	594	13.45046	chr2:8187	EIF1P7		Pseudoger	chr2:9271292-92716
ENSG0000(	594	13.45046	chr2:8187	ENSG00000213774		Pseudoger	chr2:7324748-73250
ENSG0000(	594	13.45046	chr2:8187	IAH1		protein_c	chr2:9473658-94965
ENSG0000(	594	13.45046	chr2:8187	ENSG00000260077		lncRNA	chr2:10039092-1004
ENSG0000(	594	13.45046	chr2:8187	CMPK2		protein_c	chr2:6840570-68666
ENSG0000(	594	13.45046	chr2:8187	LPIN1		protein_c	chr2:11677595-1182
ENSG0000(	594	13.45046	chr2:8187	KCNS3		protein_c	chr2:17877847-1836
ENSG0000(	594	13.45046	chr2:8187	MYCN	NCGv7;AC	protein_c	chr2:15940550-1594
ENSG0000(	594	13.45046	chr2:8187	RSAD2		protein_c	chr2:6865557-68982
ENSG0000(	594	13.45046	chr2:8187	ROCK2		protein_c	chr2:11179759-1134
ENSG0000(	594	13.45046	chr2:8187	GRHL1		protein_c	chr2:9951693-10002
ENSG0000(	594	13.45046	chr2:8187	RNU2-13P		smallRNA	chr2:11561194-1156
ENSG0000(	594	13.45046	chr2:8187	SNORA40		smallRNA	chr2:16199203-1619
ENSG0000(	594	13.45046	chr2:8187	KIDINS220		protein_c	chr2:8721081-88376
ENSG0000(	594	13.45046	chr2:8187	LINC00299		lncRNA	chr2:7988683-84882
ENSG0000(	594	13.45046	chr2:8187	RPLP1P5		Pseudoger	chr2:15869939-1587
ENSG0000(	594	13.45046	chr2:8187	ENSG00000228950		lncRNA	chr2:20451042-2045
ENSG0000(	594	13.45046	chr2:8187	SNORA40		smallRNA	chr2:18040606-1804
ENSG0000(	594	13.45046	chr2:8187	RN7SKP168		smallRNA	chr2:17122841-1712
ENSG0000(	594	13.45046	chr2:8187	ENSG00000237633		lncRNA	chr2:16523176-1655

ENSG0000(	594	13.45046	chr2:8187	MBOAT2	NCGv7	protein_c	chr2:8852690-90037
ENSG0000(	594	13.45046	chr2:8187	ENSG00000270488		Pseudoger	chr2:10874291-1087
ENSG0000(	594	13.45046	chr2:8187	ENSG00000223360		lncRNA	chr2:12598987-1260
ENSG0000(	594	13.45046	chr2:8187	Y_RNA		smallRNA	chr2:20480844-2048
ENSG0000(	594	13.45046	chr2:8187	AIDAP1		Pseudoger	chr2:11308025-1130
ENSG0000(	594	13.45046	chr2:8187	LINC01866		lncRNA	chr2:16970034-1697
ENSG0000(	594	13.45046	chr2:8187	RNU6ATAC37P		smallRNA	chr2:7576841-75769
ENSG0000(	594	13.45046	chr2:8187	ENSG00000261104		lncRNA	chr2:9106593-91098
ENSG0000(	594	13.45046	chr2:8187	SNORA26		smallRNA	chr2:10090205-1009
ENSG0000(	594	13.45046	chr2:8187	ENSG00000231266		lncRNA	chr2:16227027-1622
ENSG0000(	594	13.45046	chr2:8187	ENSG00000287284		lncRNA	chr2:18939697-1894
ENSG0000(	594	13.45046	chr2:8187	ENSG00000261117		lncRNA	chr2:12715415-1271
ENSG0000(	594	13.45046	chr2:8187	ENSG00000287291		lncRNA	chr2:14705668-1499
ENSG0000(	594	13.45046	chr2:8187	KLF11		protein_c	chr2:10042849-1005
ENSG0000(	594	13.45046	chr2:8187	YWHAQ	AC	protein_c	chr2:9583967-96309
ENSG0000(	594	13.45046	chr2:8187	ENSG00000287305		lncRNA	chr2:9939088-99508
ENSG0000(	594	13.45046	chr2:8187	RN7SKP112		smallRNA	chr2:7141227-71415
ENSG0000(	594	13.45046	chr2:8187	ENSG00000228876		lncRNA	chr2:16224047-1633
ENSG0000(	594	13.45046	chr2:8187	ENSG00000276411		lncRNA	chr2:11673964-1167
ENSG0000(	594	13.45046	chr2:8187	ZFYVE9P2		Pseudoger	chr2:17284292-1728
ENSG0000(	594	13.45046	chr2:8187	ENSG00000236162		lncRNA	chr2:10717773-1072
ENSG0000(	594	13.45046	chr2:8187	MIR7515HG		lncRNA	chr2:6615389-66511
ENSG0000(	594	13.45046	chr2:8187	LINC01376		lncRNA	chr2:18986451-1934
ENSG0000(	594	13.45046	chr2:8187	RAD51AP2		protein_c	chr2:17510579-1751
ENSG0000(	594	13.45046	chr2:8187	RRM2		protein_c	chr2:10120698-1021
ENSG0000(	594	13.45046	chr2:8187	GACAT3		lncRNA	chr2:16013928-1608
ENSG0000(	594	13.45046	chr2:8187	ENSG00000244260		lncRNA	chr2:9671875-97084
ENSG0000(	594	13.45046	chr2:8187	GRASLND		lncRNA	chr2:6911754-69187
ENSG0000(	594	13.45046	chr2:8187	LINC00298		lncRNA	chr2:7922425-82781
ENSG0000(	594	13.45046	chr2:8187	RNF144A		protein_c	chr2:6917412-70682
ENSG0000(	594	13.45046	chr2:8187	ENSG00000226041		lncRNA	chr2:16202430-1620
ENSG0000(	594	13.45046	chr2:8187	LINC01954		lncRNA	chr2:10844224-1088
ENSG0000(	594	13.45046	chr2:8187	ENSG00000227718		lncRNA	chr2:13723048-1375
ENSG0000(	594	13.45046	chr2:8187	ENSG00000271947		lncRNA	chr2:6905724-69063
ENSG0000(	594	13.45046	chr2:8187	RPL6P4		Pseudoger	chr2:11102142-1110
ENSG0000(	594	13.45046	chr2:8187	MIR548S		smallRNA	chr2:11767444-1176
ENSG0000(	594	13.45046	chr2:8187	ENSG00000229370		lncRNA	chr2:13537673-1360
ENSG0000(	594	13.45046	chr2:8187	HMGB1P25		Pseudoger	chr2:9018293-90188
ENSG0000(	594	13.45046	chr2:8187	ENSG00000229740		lncRNA	chr2:8139335-81449
ENSG0000(	594	13.45046	chr2:8187	ENSG00000234022		lncRNA	chr2:15564170-1557
ENSG0000(	594	13.45046	chr2:8187	ASAP2	NCGv7	protein_c	chr2:9206765-94056
ENSG0000(	594	13.45046	chr2:8187	APOB	NCGv7	protein_c	chr2:21001429-2104
ENSG0000(	594	13.45046	chr2:8187	ADAM17		protein_c	chr2:9488486-95567
ENSG0000(	594	13.45046	chr2:8187	RNA5SP84		Pseudoger	chr2:11517397-1151
ENSG0000(	594	13.45046	chr2:8187	ENSG00000229727		lncRNA	chr2:7383227-74505
ENSG0000(	594	13.45046	chr2:8187	MIR4262		smallRNA	chr2:11836933-1183
ENSG0000(	594	13.45046	chr2:8187	NRIR		lncRNA	chr2:6819463-68404
ENSG0000(	594	13.45046	chr2:8187	LINC02973		lncRNA	chr2:7260871-72615
ENSG0000(	594	13.45046	chr2:8187	ENSG00000271855		lncRNA	chr2:9555899-95567
ENSG0000(	594	13.45046	chr2:8187	ENSG00000145063		lncRNA	chr2:11105317-1113
ENSG0000(	594	13.45046	chr2:8187	SNORA80B		smallRNA	chr2:10446714-1044

ENSG0000(	594	13.45046	chr2:8187	ENSG00000230790		lncRNA	chr2:11740997-1174
ENSG0000(	594	13.45046	chr2:8187	ENSG00000271315		Pseudoger	chr2:16149251-1614
ENSG0000(	594	13.45046	chr2:8187	ENSG00000272275		lncRNA	chr2:10767875-1077
ENSG0000(	594	13.45046	chr2:8187	LINC00487		lncRNA	chr2:6728177-67703
ENSG0000(	594	13.45046	chr2:8187	CYS1	NCGv7	protein_c	chr2:10056473-1008
ENSG0000(	594	13.45046	chr2:8187	ENSG00000280390		TEC	chr2:21023496-2102
ENSG0000(	594	13.45046	chr2:8187	ENSG00000188525		lncRNA	chr2:10001757-1000
ENSG0000(	594	13.45046	chr2:8187	ODC1-DT		lncRNA	chr2:10448654-1045
ENSG0000(	594	13.45046	chr2:8187	GEN1		protein_c	chr2:17753858-1778
ENSG0000(	594	13.45046	chr2:8187	ENSG00000234275		lncRNA	chr2:6258348-63417
ENSG0000(	594	13.45046	chr2:8187	SNRPEP5		Pseudoger	chr2:8603082-86033
ENSG0000(	594	13.45046	chr2:8187	MSGN1		protein_c	chr2:17816460-1781
ENSG0000(	594	13.45046	chr2:8187	ENSG00000225649		lncRNA	chr2:12780593-1300
ENSG0000(	594	13.45046	chr2:8187	LINC01830		lncRNA	chr2:22377594-2248
ENSG0000(	594	13.45046	chr2:8187	ENSG00000234189		Pseudoger	chr2:22317053-2231
ENSG0000(	594	13.45046	chr2:8187	TRIB2	AC	protein_c	chr2:12716910-1274
ENSG0000(	594	13.45046	chr2:8187	ENSG00000289856		lncRNA	chr2:12189604-1219
ENSG0000(	594	13.45046	chr2:8187	SNORA2		smallRNA	chr2:10155072-1015
ENSG0000(	594	13.45046	chr2:8187	LINC01247		lncRNA	chr2:6366010-63754
ENSG0000(	594	13.45046	chr2:8187	ENSG00000240980		lncRNA	chr2:9143166-91461
ENSG0000(	594	13.45046	chr2:8187	ENSG00000290092		lncRNA	chr2:7876670-78917
ENSG0000(	594	13.45046	chr2:8187	LINC01822		lncRNA	chr2:21687430-2171
ENSG0000(	594	13.45046	chr2:8187	ENSG00000233502		lncRNA	chr2:10083781-1008
ENSG0000(	594	13.45046	chr2:8187	PPIAP60		Pseudoger	chr2:11351627-1135
ENSG0000(	594	13.45046	chr2:8187	ENSG00000290023		lncRNA	chr2:9004095-90046
ENSG0000(	594	13.45046	chr2:8187	ENSG00000270100		lncRNA	chr2:20678254-2067
ENSG0000(	594	13.45046	chr2:8187	ENSG00000290030		lncRNA	chr2:12276707-1227
ENSG0000(	594	13.45046	chr2:8187	ENSG00000240960		Pseudoger	chr2:9746704-97468
ENSG0000(	594	13.45046	chr2:8187	ENSG00000227047		lncRNA	chr2:20499571-2050
ENSG0000(	594	13.45046	chr2:8187	RPS25P3		Pseudoger	chr2:20606280-2060
ENSG0000(	594	13.45046	chr2:8187	RNA5SP85		smallRNA	chr2:11561661-1156
ENSG0000(	594	13.45046	chr2:8187	AC011994.1		smallRNA	chr2:11607404-1160
ENSG0000(	594	13.45046	chr2:8187	RNU5E-7P		smallRNA	chr2:15864935-1586
ENSG0000(	594	13.45046	chr2:8187	ENSG00000244310		lncRNA	chr2:9110457-91169
ENSG0000(	594	13.45046	chr2:8187	ENSG00000290050		lncRNA	chr2:7748569-77492
ENSG0000(	594	13.45046	chr2:8187	RNU4-73P		smallRNA	chr2:9740643-97407
ENSG0000(	594	13.45046	chr2:8187	LINC01884		lncRNA	chr2:22508129-2254
ENSG0000(	594	13.45046	chr2:8187	NBAS		protein_c	chr2:15166916-1556
ENSG0000(	594	13.45046	chr2:8187	DDX1		protein_c	chr2:15591178-1563
ENSG0000(	594	13.45046	chr2:8187	LINC01824		lncRNA	chr2:6495651-65118
ENSG0000(	594	13.45046	chr2:8187	ENSG00000226506		lncRNA	chr2:7886767-78997
ENSG0000(	594	13.45046	chr2:8187	ENSG00000286427		lncRNA	chr2:12702764-1271
ENSG0000(	594	13.45046	chr2:8187	LINC03037		lncRNA	chr2:11357515-1136
ENSG0000(	594	13.45046	chr2:8187	ENSG00000229405		Pseudoger	chr2:7736438-77370
ENSG0000(	594	13.45046	chr2:8187	ENSG00000271787		lncRNA	chr2:10054421-1005
ENSG0000(	594	13.45046	chr2:8187	ENSG00000240687		lncRNA	chr2:9638707-97129
ENSG0000(	594	13.45046	chr2:8187	NDUFAF2P1		Pseudoger	chr2:20529467-2052
ENSG0000(	594	13.45046	chr2:8187	ENSG00000271629		Pseudoger	chr2:21198518-2119
ENSG0000(	594	13.45046	chr2:8187	ENSG00000203643		lncRNA	chr2:11721619-1172
ENSG0000(	594	13.45046	chr2:8187	MYCNOS		lncRNA	chr2:15918350-1594
ENSG0000(	594	13.45046	chr2:8187	ENSG00000226764		lncRNA	chr2:15997049-1606

ENSG0000(	594	13.45046	chr2:8187	ENSG00000290108		lncRNA	chr2:20446571-2044
ENSG0000(	594	13.45046	chr2:8187	ENSG00000279663		TEC	chr2:16541690-1654
ENSG0000(	594	13.45046	chr2:8187	RDH14		protein_c	chr2:18554723-1856
ENSG0000(	594	13.45046	chr2:8187	MIR4261		smallRNA	chr2:10192614-1019
ENSG0000(	594	13.45046	chr2:8187	ENSG00000231403		lncRNA	chr2:11388023-1139
ENSG0000(	594	13.45046	chr2:8187	ENSG00000237326		lncRNA	chr2:15801747-1581
ENSG0000(	594	13.45046	chr2:8187	ENSG00000234818		lncRNA	chr2:10589166-1060
ENSG0000(	594	13.45046	chr2:8187	ENSG00000224626		lncRNA	chr2:18784807-1878
ENSG0000(	594	13.45046	chr2:8187	snoU13		smallRNA	chr2:8978895-89789
ENSG0000(	594	13.45046	chr2:8187	LINC00570		lncRNA	chr2:11372612-1140
ENSG0000(	594	13.45046	chr2:8187	MIR3681HG		lncRNA	chr2:11833926-1270
ENSG0000(	594	13.45046	chr2:8187	ENSG00000224194		lncRNA	chr2:15668684-1568
ENSG0000(	594	13.45046	chr2:8187	ENSG00000289033		lncRNA	chr2:9996244-10009
ENSG0000(	594	13.45046	chr2:8187	RNU7-176P		smallRNA	chr2:10815485-1081
ENSG0000(	594	13.45046	chr2:8187	MIR4429		smallRNA	chr2:11540605-1154
ENSG0000(	594	13.45046	chr2:8187	RNU6-1081P		smallRNA	chr2:11233988-1123
ENSG0000(	594	13.45046	chr2:8187	RN7SL66P		smallRNA	chr2:10280631-1028
ENSG0000(	594	13.45046	chr2:8187	ENSG00000224400		lncRNA	chr2:16354256-1643
ENSG0000(	594	13.45046	chr2:8187	RN7SL832P		lncRNA	chr2:10690344-1069
ENSG0000(	594	13.45046	chr2:8187	ENSG00000286851		lncRNA	chr2:6649706-66508
ENSG0000(	594	13.45046	chr2:8187	RNU6-843P		smallRNA	chr2:12411398-1241
ENSG0000(	594	13.45046	chr2:8187	RNU7-138P		smallRNA	chr2:10744186-1074
ENSG0000(	594	13.45046	chr2:8187	RNU6-1215P		smallRNA	chr2:18583367-1858
ENSG0000(	594	13.45046	chr2:8187	ENSG00000269976		lncRNA	chr2:20586248-2058
ENSG0000(	594	13.45046	chr2:8187	ENSG00000289191		lncRNA	chr2:8281464-82825
ENSG0000(	594	13.45046	chr2:8187	ENSG00000232451		lncRNA	chr2:23018125-2319
ENSG0000(	594	13.45046	chr2:8187	ENSG00000285569		lncRNA	chr2:11405508-1142
ENSG0000(	594	13.45046	chr2:8187	ENSG00000232444		lncRNA	chr2:16316324-1631
ENSG0000(	594	13.45046	chr2:8187	ENSG00000232979		Pseudoger	chr2:7735645-77357
ENSG0000(	594	13.45046	chr2:8187	ENSG00000230515		lncRNA	chr2:7671604-76722
ENSG0000(	594	13.45046	chr2:8187	MYCNUT		lncRNA	chr2:15920399-1593
ENSG0000(	594	13.45046	chr2:8187	ENSG00000285872		lncRNA	chr2:10287704-1030
ENSG0000(	594	13.45046	chr2:8187	ENSG00000285876		lncRNA	chr2:13000953-1333
ENSG0000(	594	13.45046	chr2:8187	ENSG00000223884		lncRNA	chr2:7045329-70779
ENSG0000(	594	13.45046	chr2:8187	NT5C1B-RDH14		protein_c	chr2:18555545-1858
ENSG0000(	594	13.45046	chr2:8187	ENSG00000232056		lncRNA	chr2:10847577-1085
ENSG0000(	594	13.45046	chr2:8187	LINC01871		lncRNA	chr2:7725682-77322
ENSG0000(	594	13.45046	chr2:8187	NUTF2P8		Pseudoger	chr2:21362292-2136
ENSG0000(	594	13.45046	chr2:8187	ENSG00000235537		lncRNA	chr2:21607465-2163
ENSG0000(	594	13.45046	chr2:8187	ID2	AC	protein_c	chr2:8678845-86844
ENSG0000(	594	13.45046	chr2:8187	TAF1B		protein_c	chr2:9843443-99344
ENSG0000(	594	13.45046	chr2:8187	HPCAL1		protein_c	chr2:10302889-1042
ENSG0000(	594	13.45046	chr2:8187	ODC1	NCGv7	protein_c	chr2:10439968-1044
ENSG0000(	594	13.45046	chr2:8187	ENSG00000233005		lncRNA	chr2:21221169-2197
ENSG0000(	594	13.45046	chr2:8187	NOL10		protein_c	chr2:10562347-1068
ENSG0000(	594	13.45046	chr2:8187	ENSG00000224604		Pseudoger	chr2:16853820-1685
ENSG0000(	594	13.45046	chr2:8187	ENSG00000269973		lncRNA	chr2:9936360-99395
ENSG0000(	594	13.45046	chr2:8187	ID2-AS1		lncRNA	chr2:8666636-86818
ENSG0000(	594	13.45046	chr2:8187	CDK8P1		Pseudoger	chr2:11665369-1166
ENSG0000(	594	13.45046	chr2:8187	LDAH		protein_c	chr2:20684014-2082
ENSG0000(	594	13.45046	chr2:8187	HS1BP3	NCGv7	protein_c	chr2:20560448-2065

ENSG0000(	594	13.45046	chr2:8187	ITGB1BP1	protein_c	chr2:9403475-94235
ENSG0000(	594	13.45046	chr2:8187	C2orf50	protein_c	chr2:11133128-1115
ENSG0000(	594	13.45046	chr2:8187	MIR3681	smallRNA	chr2:12199130-1219
ENSG0000(	594	13.45046	chr2:8187	HS1BP3-IT1	lncRNA	chr2:20590775-2059
ENSG0000(	594	13.45046	chr2:8187	CPSF3	protein_c	chr2:9423651-94731
ENSG0000(	594	13.45046	chr2:8187	ENSG00000235127	lncRNA	chr2:14886647-1490
ENSG0000(	594	13.45046	chr2:8187	RNA5SP87	Pseudoger	chr2:22338886-2233
ENSG0000(	594	13.45046	chr2:8187	LINC01804	lncRNA	chr2:15690782-1574
ENSG0000(	594	13.45046	chr2:8187	MIR3125	smallRNA	chr2:12737367-1273
ENSG0000(	594	13.45046	chr2:8187	ENSG00000239300	lncRNA	chr2:9501466-95124
ENSG0000(	594	13.45046	chr2:8187	SNORA51	smallRNA	chr2:10296047-1029
ENSG0000(	594	13.45046	chr2:8187	RN7SL117P	smallRNA	chr2:21922993-2192
ENSG0000(	594	13.45046	chr2:8187	KCNF1	protein_c	chr2:10911934-1091
ENSG0000(	594	13.45046	chr2:8187	VSNL1	protein_c	chr2:17539126-1765
ENSG0000(	594	13.45046	chr2:8187	SMC6	protein_c	chr2:17663812-1780
ENSG0000(	594	13.45046	chr2:8187	Y_RNA	smallRNA	chr2:9615267-96153
ENSG0000(	594	13.45046	chr2:8187	LRATD1	protein_c	chr2:14632700-1465
ENSG0000(	594	13.45046	chr2:8187	ENSG00000285591	lncRNA	chr2:8488420-85050
ENSG0000(	594	13.45046	chr2:8187	ENSG00000286857	lncRNA	chr2:7937282-79534
ENSG0000(	594	13.45046	chr2:8187	ENSG00000289364	lncRNA	chr2:16452112-1647
ENSG0000(	594	13.45046	chr2:8187	SLC66A3	protein_c	chr2:11155198-1117
ENSG0000(	594	13.45046	chr2:8187	MIR4757	smallRNA	chr2:19348429-1934
ENSG0000(	593	13.42781	chr18:236	MIR187	smallRNA	chr18:35904818-359
ENSG0000(	589	13.33724	chr4:1865	ENSG00000248399	lncRNA	chr4:2463797-24641
ENSG0000(	587	13.29195	chr2:8187	ENSG00000270422	Pseudoger	chr2:31651381-3165
ENSG0000(	587	13.29195	chr2:8187	EMILIN1	protein_c	chr2:27078615-2708
ENSG0000(	587	13.29195	chr2:8187	SLC5A6	protein_c	chr2:27199587-2721
ENSG0000(	587	13.29195	chr2:8187	IFT172	protein_c	chr2:27444377-2748
ENSG0000(	587	13.29195	chr2:8187	ENSG00000270640	lncRNA	chr2:28396815-2839
ENSG0000(	587	13.29195	chr2:8187	TRIM54	protein_c	chr2:27282429-2730
ENSG0000(	587	13.29195	chr2:8187	PREB	protein_c	chr2:27130756-2713
ENSG0000(	587	13.29195	chr2:8187	SNRPGP7	Pseudoger	chr2:28460256-2846
ENSG0000(	587	13.29195	chr2:8187	TTC27	protein_c	chr2:32628032-3282
ENSG0000(	587	13.29195	chr2:8187	ENSG00000197644	lncRNA	chr2:29899597-2990
ENSG0000(	587	13.29195	chr2:8187	BIRC6-AS2	lncRNA	chr2:32557273-3257
ENSG0000(	587	13.29195	chr2:8187	EHD3	protein_c	chr2:31234152-3126
ENSG0000(	587	13.29195	chr2:8187	FAM133EP	Pseudoger	chr2:28015777-2801
ENSG0000(	587	13.29195	chr2:8187	MYG1P1	Pseudoger	chr2:27896116-2789
ENSG0000(	587	13.29195	chr2:8187	MRPL33	protein_c	chr2:27771717-2798
ENSG0000(	587	13.29195	chr2:8187	PRR30	protein_c	chr2:27136848-2713
ENSG0000(	587	13.29195	chr2:8187	Y_RNA	smallRNA	chr2:28972414-2897
ENSG0000(	587	13.29195	chr2:8187	SPAST	protein_c	chr2:32063556-3215
ENSG0000(	587	13.29195	chr2:8187	ATRAID	protein_c	chr2:27212041-2721
ENSG0000(	587	13.29195	chr2:8187	KHK	protein_c	chr2:27086747-2710
ENSG0000(	587	13.29195	chr2:8187	CGREF1	protein_c	chr2:27098889-2711
ENSG0000(	587	13.29195	chr2:8187	RNU6-986P	smallRNA	chr2:27475494-2747
ENSG0000(	587	13.29195	chr2:8187	GPN1	protein_c	chr2:27628247-2765
ENSG0000(	587	13.29195	chr2:8187	HNRNPA1P61	Pseudoger	chr2:33636502-3363
ENSG0000(	587	13.29195	chr2:8187	RN7SL516P	smallRNA	chr2:29681029-2968
ENSG0000(	587	13.29195	chr2:8187	TOGARAM2	protein_c	chr2:28956611-2905
ENSG0000(	587	13.29195	chr2:8187	LTBP1	protein_c	chr2:32946953-3339

ENSG0000(	587	13.29195	chr2:8187	AC097506.1		smallRNA	chr2:33927384-3392
ENSG0000(	587	13.29195	chr2:8187	ENSG00000270210		lncRNA	chr2:28425945-2842
ENSG0000(	587	13.29195	chr2:8187	ZNF512		protein_c	chr2:27582969-2762
ENSG0000(	587	13.29195	chr2:8187	SMIM7P1		Pseudoger	chr2:35219377-3521
ENSG0000(	587	13.29195	chr2:8187	FTH1P3		Pseudoger	chr2:27392784-2739
ENSG0000(	587	13.29195	chr2:8187	ENSG00000279544		TEC	chr2:32563328-3256
ENSG0000(	587	13.29195	chr2:8187	AC105398.1		smallRNA	chr2:29081270-2908
ENSG0000(	587	13.29195	chr2:8187	MIR4765		smallRNA	chr2:32635255-3263
ENSG0000(	587	13.29195	chr2:8187	SNX17		protein_c	chr2:27370496-2737
ENSG0000(	587	13.29195	chr2:8187	PPM1G		protein_c	chr2:27381195-2740
ENSG0000(	587	13.29195	chr2:8187	DDX50P1		Pseudoger	chr2:32201600-3220
ENSG0000(	587	13.29195	chr2:8187	CLIP4		protein_c	chr2:29097705-2919
ENSG0000(	587	13.29195	chr2:8187	PCARE		protein_c	chr2:29060976-2907
ENSG0000(	587	13.29195	chr2:8187	SNORD92		smallRNA	chr2:28913664-2891
ENSG0000(	587	13.29195	chr2:8187	H2ACP2		Pseudoger	chr2:33056333-3306
ENSG0000(	587	13.29195	chr2:8187	ENSG00000226994		lncRNA	chr2:34799850-3518
ENSG0000(	587	13.29195	chr2:8187	ABHD1	DriverDB\	protein_c	chr2:27123789-2713
ENSG0000(	587	13.29195	chr2:8187	RNA5SP92		Pseudoger	chr2:33332898-3333
ENSG0000(	587	13.29195	chr2:8187	BIRC6	NCGv7	protein_c	chr2:32357023-3261
ENSG0000(	587	13.29195	chr2:8187	ENSG00000272027		lncRNA	chr2:34692290-3470
ENSG0000(	587	13.29195	chr2:8187	XDH	NCGv7	protein_c	chr2:31334321-3141
ENSG0000(	587	13.29195	chr2:8187	ENSG00000289727		lncRNA	chr2:32233386-3226
ENSG0000(	587	13.29195	chr2:8187	ENSG00000272056		lncRNA	chr2:27053618-2705
ENSG0000(	587	13.29195	chr2:8187	CAD	AC	protein_c	chr2:27217369-2724
ENSG0000(	587	13.29195	chr2:8187	LINC00486		lncRNA	chr2:32825359-3292
ENSG0000(	587	13.29195	chr2:8187	ENSG00000285577		lncRNA	chr2:33274465-3328
ENSG0000(	587	13.29195	chr2:8187	MAPRE3	NCGv7	protein_c	chr2:26970637-2702
ENSG0000(	587	13.29195	chr2:8187	MIR4263		smallRNA	chr2:27996367-2799
ENSG0000(	587	13.29195	chr2:8187	ENSG00000230730		lncRNA	chr2:28633282-2866
ENSG0000(	587	13.29195	chr2:8187	GTF3C2		protein_c	chr2:27325849-2735
ENSG0000(	587	13.29195	chr2:8187	ENSG00000230286		lncRNA	chr2:26950308-2700
ENSG0000(	587	13.29195	chr2:8187	LBH		protein_c	chr2:30231534-3032
ENSG0000(	587	13.29195	chr2:8187	ENSG00000213620		Pseudoger	chr2:31290762-3129
ENSG0000(	587	13.29195	chr2:8187	MIR548AD		smallRNA	chr2:35471405-3547
ENSG0000(	587	13.29195	chr2:8187	RBKS		protein_c	chr2:27781379-2789
ENSG0000(	587	13.29195	chr2:8187	SLC30A6	NCGv7	protein_c	chr2:32165841-3222
ENSG0000(	587	13.29195	chr2:8187	AL121652.3		smallRNA	chr2:31823018-3182
ENSG0000(	587	13.29195	chr2:8187	RASGRP3		protein_c	chr2:33436324-3356
ENSG0000(	587	13.29195	chr2:8187	EIF2B4		protein_c	chr2:27364352-2737
ENSG0000(	587	13.29195	chr2:8187	NRBP1		protein_c	chr2:27427790-2744
ENSG0000(	587	13.29195	chr2:8187	FNDC4		protein_c	chr2:27491883-2749
ENSG0000(	587	13.29195	chr2:8187	SNORD53		smallRNA	chr2:28927067-2892
ENSG0000(	587	13.29195	chr2:8187	AGBL5	NCGv7	protein_c	chr2:27042364-2707
ENSG0000(	587	13.29195	chr2:8187	GCKR		protein_c	chr2:27496839-2752
ENSG0000(	587	13.29195	chr2:8187	ENSG00000230737		lncRNA	chr2:29890371-2989
ENSG0000(	587	13.29195	chr2:8187	ENSG00000288535		lncRNA	chr2:35263711-3528
ENSG0000(	587	13.29195	chr2:8187	TRMT61B		protein_c	chr2:28849821-2887
ENSG0000(	587	13.29195	chr2:8187	ENSG00000233862		lncRNA	chr2:30051066-3014
ENSG0000(	587	13.29195	chr2:8187	CDKN2AIPNLP2		Pseudoger	chr2:26827169-2682
ENSG0000(	587	13.29195	chr2:8187	NLRC4		protein_c	chr2:32224453-3226
ENSG0000(	587	13.29195	chr2:8187	SNORA36		smallRNA	chr2:27642043-2764

ENSG0000(	587	13.29195	chr2:8187	GTF3C2-AS2		lncRNA	chr2:27356246-2736
ENSG0000(	587	13.29195	chr2:8187	ENSG00000235267		Pseudoger	chr2:27455156-2745
ENSG0000(	587	13.29195	chr2:8187	CCDC121		protein_c	chr2:27625638-2762
ENSG0000(	587	13.29195	chr2:8187	RPL23AP34		Pseudoger	chr2:28308161-2830
ENSG0000(	587	13.29195	chr2:8187	GALNT14		protein_c	chr2:30910467-3115
ENSG0000(	587	13.29195	chr2:8187	ENSG00000225943		Pseudoger	chr2:30077093-3007
ENSG0000(	587	13.29195	chr2:8187	AC011748.1		smallRNA	chr2:34403664-3440
ENSG0000(	587	13.29195	chr2:8187	DPY30		protein_c	chr2:31867809-3203
ENSG0000(	587	13.29195	chr2:8187	MYADML		Pseudoger	chr2:33722721-3372
ENSG0000(	587	13.29195	chr2:8187	DPYSL5		protein_c	chr2:26847747-2695
ENSG0000(	587	13.29195	chr2:8187	LCLAT1		protein_c	chr2:30447226-3064
ENSG0000(	587	13.29195	chr2:8187	MEMO1		protein_c	chr2:31865060-3201
ENSG0000(	587	13.29195	chr2:8187	CAPN13		protein_c	chr2:30722771-3082
ENSG0000(	587	13.29195	chr2:8187	KRTCAP3		protein_c	chr2:27442366-2744
ENSG0000(	587	13.29195	chr2:8187	AL121652.2		smallRNA	chr2:31810195-3181
ENSG0000(	587	13.29195	chr2:8187	ENSG00000286415		lncRNA	chr2:33599442-3365
ENSG0000(	587	13.29195	chr2:8187	ENSG00000288553		lncRNA	chr2:29841187-2995
ENSG0000(	587	13.29195	chr2:8187	RN7SL602P		smallRNA	chr2:34809253-3480
ENSG0000(	587	13.29195	chr2:8187	WDR43		protein_c	chr2:28894667-2894
ENSG0000(	587	13.29195	chr2:8187	SPDYA		protein_c	chr2:28782517-2885
ENSG0000(	587	13.29195	chr2:8187	PLB1	NCGv7	protein_c	chr2:28457145-2864
ENSG0000(	587	13.29195	chr2:8187	PPP1CB-DT		lncRNA	chr2:28707511-2875
ENSG0000(	587	13.29195	chr2:8187	SLC4A1AP		protein_c	chr2:27663426-2769
ENSG0000(	587	13.29195	chr2:8187	ENSG00000272148		lncRNA	chr2:27062428-2706
ENSG0000(	587	13.29195	chr2:8187	ENSG00000273233		lncRNA	chr2:28810281-2881
ENSG0000(	587	13.29195	chr2:8187	ZNF513		protein_c	chr2:27377235-2738
ENSG0000(	587	13.29195	chr2:8187	UCN		protein_c	chr2:27307400-2730
ENSG0000(	587	13.29195	chr2:8187	ENSG00000273165		lncRNA	chr2:31852976-3185
ENSG0000(	587	13.29195	chr2:8187	DNAJC5G		protein_c	chr2:27275433-2728
ENSG0000(	587	13.29195	chr2:8187	RNA5SP91		Pseudoger	chr2:33285769-3328
ENSG0000(	587	13.29195	chr2:8187	LINC01460		lncRNA	chr2:27705786-2771
ENSG0000(	587	13.29195	chr2:8187	TCF23		protein_c	chr2:27149004-2715
ENSG0000(	587	13.29195	chr2:8187	BIRC6-AS1		lncRNA	chr2:32377631-3237
ENSG0000(	587	13.29195	chr2:8187	SRD5A2		protein_c	chr2:31522480-3158
ENSG0000(	587	13.29195	chr2:8187	MAPRE3-AS1		lncRNA	chr2:26984776-2701
ENSG0000(	587	13.29195	chr2:8187	H3P5		Pseudoger	chr2:30209995-3021
ENSG0000(	587	13.29195	chr2:8187	PPP1CB		protein_c	chr2:28751640-2880
ENSG0000(	587	13.29195	chr2:8187	ENSG00000276334		lncRNA	chr2:32521927-3252
ENSG0000(	587	13.29195	chr2:8187	YIPF4		protein_c	chr2:32277904-3231
ENSG0000(	587	13.29195	chr2:8187	FAM98A		protein_c	chr2:33532744-3359
ENSG0000(	587	13.29195	chr2:8187	YPEL5		protein_c	chr2:30146941-3016
ENSG0000(	587	13.29195	chr2:8187	Y_RNA		smallRNA	chr2:28927243-2892
ENSG0000(	587	13.29195	chr2:8187	RNA5SP89		Pseudoger	chr2:28683976-2868
ENSG0000(	587	13.29195	chr2:8187	ENSG00000232153		lncRNA	chr2:34732287-3482
ENSG0000(	587	13.29195	chr2:8187	SLC30A6-DT		lncRNA	chr2:32165041-3216
ENSG0000(	587	13.29195	chr2:8187	ENSG00000278908		TEC	chr2:30677762-3067
ENSG0000(	587	13.29195	chr2:8187	ENSG00000228563		lncRNA	chr2:31526942-3156
ENSG0000(	587	13.29195	chr2:8187	RNU6-647P		smallRNA	chr2:32214456-3221
ENSG0000(	587	13.29195	chr2:8187	TMEM214		protein_c	chr2:27032910-2704
ENSG0000(	587	13.29195	chr2:8187	ENSG00000288937		lncRNA	chr2:32039839-3204
ENSG0000(	587	13.29195	chr2:8187	ENSG00000259080		lncRNA	chr2:27583046-2763

ENSG0000(	587	13.29195	chr2:8187	SNORA64		smallRNA	chr2:30187434-3018
ENSG0000(	587	13.29195	chr2:8187	ENSG00000271228		lncRNA	chr2:32013061-3201
ENSG0000(	587	13.29195	chr2:8187	SUPT7L	NCGv7	protein_c	chr2:27650809-2766
ENSG0000(	587	13.29195	chr2:8187	ENSG00000229224		lncRNA	chr2:29088649-2909
ENSG0000(	587	13.29195	chr2:8187	CAPN14		protein_c	chr2:31173056-3123
ENSG0000(	587	13.29195	chr2:8187	ENSG00000237320		lncRNA	chr2:34998273-3500
ENSG0000(	587	13.29195	chr2:8187	FOSL2-AS1		lncRNA	chr2:28384409-2839
ENSG0000(	587	13.29195	chr2:8187	ALK	NCGv7;AC	protein_c	chr2:29192774-2992
ENSG0000(	587	13.29195	chr2:8187	C2orf16		protein_c	chr2:27537386-2758
ENSG0000(	587	13.29195	chr2:8187	ENSG00000234579		lncRNA	chr2:30986939-3099
ENSG0000(	587	13.29195	chr2:8187	ENSG00000285754		lncRNA	chr2:34134371-3422
ENSG0000(	587	13.29195	chr2:8187	MRPL50P1		Pseudoger	chr2:35724759-3572
ENSG0000(	587	13.29195	chr2:8187	AL121652.1		smallRNA	chr2:31919862-3191
ENSG0000(	587	13.29195	chr2:8187	AC073255.1		smallRNA	chr2:30432353-3043
ENSG0000(	587	13.29195	chr2:8187	AC016907.1		smallRNA	chr2:30066124-3006
ENSG0000(	587	13.29195	chr2:8187	KRT18P52		Pseudoger	chr2:31822591-3182
ENSG0000(	587	13.29195	chr2:8187	AC106899.1		smallRNA	chr2:29503907-2950
ENSG0000(	587	13.29195	chr2:8187	AK2P2		Pseudoger	chr2:31823413-3182
ENSG0000(	587	13.29195	chr2:8187	ENSG00000229013		Pseudoger	chr2:35471716-3547
ENSG0000(	587	13.29195	chr2:8187	ENSG00000287658		lncRNA	chr2:30343222-3034
ENSG0000(	587	13.29195	chr2:8187	AGBL5-IT1		lncRNA	chr2:27061038-2706
ENSG0000(	587	13.29195	chr2:8187	ENSG00000272754		lncRNA	chr2:32321638-3232
ENSG0000(	587	13.29195	chr2:8187	RNU6-1117P		smallRNA	chr2:35471605-3547
ENSG0000(	587	13.29195	chr2:8187	LINC00486		lncRNA	chr2:32927085-3294
ENSG0000(	587	13.29195	chr2:8187	AC074091.1		smallRNA	chr2:27581889-2758
ENSG0000(	587	13.29195	chr2:8187	AC009305.2		smallRNA	chr2:31035553-3103
ENSG0000(	587	13.29195	chr2:8187	OST4		protein_c	chr2:27070472-2707
ENSG0000(	587	13.29195	chr2:8187	BABAM2	NCGv7	protein_c	chr2:27889941-2833
ENSG0000(	587	13.29195	chr2:8187	GTF3C2-AS1		lncRNA	chr2:27335520-2734
ENSG0000(	587	13.29195	chr2:8187	Y_RNA		smallRNA	chr2:32945339-3294
ENSG0000(	587	13.29195	chr2:8187	AC069303.1		smallRNA	chr2:33786038-3378
ENSG0000(	587	13.29195	chr2:8187	LINC01946		lncRNA	chr2:31793823-3180
ENSG0000(	587	13.29195	chr2:8187	CENPA		protein_c	chr2:26764289-2680
ENSG0000(	587	13.29195	chr2:8187	ENSG00000227938		lncRNA	chr2:28448167-2845
ENSG0000(	587	13.29195	chr2:8187	ENSG00000289326		lncRNA	chr2:27752831-2775
ENSG0000(	587	13.29195	chr2:8187	SLC30A3		protein_c	chr2:27253684-2727
ENSG0000(	587	13.29195	chr2:8187	SLC25A5P2		Pseudoger	chr2:33839782-3384
ENSG0000(	587	13.29195	chr2:8187	ENSG00000286963		lncRNA	chr2:29319554-2935
ENSG0000(	587	13.29195	chr2:8187	FOSL2		protein_c	chr2:28392448-2841
ENSG0000(	587	13.29195	chr2:8187	BIRC6-AS2		Pseudoger	chr2:32557703-3255
ENSG0000(	587	13.29195	chr2:8187	ENSG00000223522		lncRNA	chr2:28307063-2831
ENSG0000(	587	13.29195	chr2:8187	LINC01936		lncRNA	chr2:30346623-3036
ENSG0000(	587	13.29195	chr2:8187	AL121655.1		smallRNA	chr2:32088304-3208
ENSG0000(	587	13.29195	chr2:8187	ENSG00000230118		Pseudoger	chr2:30746444-3074
ENSG0000(	587	13.29195	chr2:8187	ENSG00000285984		lncRNA	chr2:30887626-3089
ENSG0000(	587	13.29195	chr2:8187	SNORD53_SNORD92		smallRNA	chr2:28927983-2892
ENSG0000(	587	13.29195	chr2:8187	MIR558		smallRNA	chr2:32532153-3253
ENSG0000(	587	13.29195	chr2:8187	MPV17		protein_c	chr2:27309492-2732
ENSG0000(	587	13.29195	chr2:8187	ENSG00000276517		lncRNA	chr2:32526504-3252
ENSG0000(	587	13.29195	chr2:8187	RNA5SP90		Pseudoger	chr2:31228312-3122
ENSG0000(	587	13.29195	chr2:8187	LINC01320		lncRNA	chr2:33706886-3473

ENSG0000(	587	13.29195	chr2:8187	AGBL5-AS1	lncRNA	chr2:27049683-2705
ENSG0000(	587	13.29195	chr2:8187	SLC35F6	protein_c	chr2:26764284-2678
ENSG0000(	587	13.29195	chr2:8187	ATP6V0E1P3	Pseudoger	chr2:33602041-3360
ENSG0000(	587	13.29195	chr2:8187	LINC01318	lncRNA	chr2:34067226-3406
ENSG0000(	587	13.29195	chr2:8187	ENSG00000274159	lncRNA	chr2:32548675-3254
ENSG0000(	587	13.29195	chr2:8187	ENSG00000236099	Pseudoger	chr2:27158563-2715
ENSG0000(	587	13.29195	chr2:8187	SNORD112	smallRNA	chr2:32991259-3299
ENSG0000(	587	13.29195	chr2:8187	ENSG00000280154	TEC	chr2:30408170-3040
ENSG0000(	580	13.13344	chr2:2577	NEK2P4	Pseudoger	chr2:131177618-131
ENSG0000(	580	13.13344	chr2:2577	ENSG00000286833	lncRNA	chr2:132347468-132
ENSG0000(	580	13.13344	chr2:2577	MZT2A	protein_c	chr2:131464900-131
ENSG0000(	580	13.13344	chr2:2577	GPR148	protein_c	chr2:130729070-130
ENSG0000(	580	13.13344	chr2:2577	NCKAP5-IT1	lncRNA	chr2:133431666-133
ENSG0000(	580	13.13344	chr2:2577	MTND5P23	Pseudoger	chr2:131370306-131
ENSG0000(	580	13.13344	chr2:2577	POTEE	protein_c	chr2:131209536-131
ENSG0000(	580	13.13344	chr2:2577	CFC1	protein_c	chr2:130592165-130
ENSG0000(	580	13.13344	chr2:2577	MED15P4	Pseudoger	chr2:131535187-131
ENSG0000(	580	13.13344	chr2:2577	MIR3679	smallRNA	chr2:134127125-134
ENSG0000(	580	13.13344	chr2:2577	MTND2P18	Pseudoger	chr2:131384461-131
ENSG0000(	580	13.13344	chr2:2577	ENSG00000230065	lncRNA	chr2:132660526-132
ENSG0000(	580	13.13344	chr2:2577	ENSG00000232760	Pseudoger	chr2:131833783-131
ENSG0000(	580	13.13344	chr2:2577	ENSG00000273073	lncRNA	chr2:131461821-131
ENSG0000(	580	13.13344	chr2:2577	CCDC74A	protein_c	chr2:131527675-131
ENSG0000(	580	13.13344	chr2:2577	RNU6-473P	smallRNA	chr2:130497743-130
ENSG0000(	580	13.13344	chr2:2577	NBEAP2	Pseudoger	chr2:131722417-131
ENSG0000(	580	13.13344	chr2:2577	RNU6-617P	smallRNA	chr2:131602790-131
ENSG0000(	580	13.13344	chr2:2577	ENSG00000276460	Pseudoger	chr2:131398439-131
ENSG0000(	580	13.13344	chr2:2577	YBX1P7	Pseudoger	chr2:132488554-132
ENSG0000(	580	13.13344	chr2:2577	RNA5SP104	Pseudoger	chr2:134231195-134
ENSG0000(	580	13.13344	chr2:2577	ENSG00000274353	Pseudoger	chr2:131821987-131
ENSG0000(	580	13.13344	chr2:2577	ENSG00000287414	lncRNA	chr2:132010195-132
ENSG0000(	580	13.13344	chr2:2577	LINC01945	lncRNA	chr2:131983937-131
ENSG0000(	580	13.13344	chr2:2577	KLF2P4	Pseudoger	chr2:131299220-131
ENSG0000(	580	13.13344	chr2:2577	RN7SKP154	smallRNA	chr2:133391109-133
ENSG0000(	580	13.13344	chr2:2577	POTEJ	protein_c	chr2:130611440-130
ENSG0000(	580	13.13344	chr2:2577	RNU6-848P	smallRNA	chr2:130622838-130
ENSG0000(	580	13.13344	chr2:2577	MTATP6P4	Pseudoger	chr2:131380916-131
ENSG0000(	580	13.13344	chr2:2577	NF1P8	Pseudoger	chr2:131189834-131
ENSG0000(	580	13.13344	chr2:2577	NOC2LP2	Pseudoger	chr2:131442644-131
ENSG0000(	580	13.13344	chr2:2577	ZNF285CP	Pseudoger	chr2:132309309-132
ENSG0000(	580	13.13344	chr2:2577	AC104405.1	smallRNA	chr2:132523985-132
ENSG0000(	580	13.13344	chr2:2577	MIR663B	smallRNA	chr2:132256966-132
ENSG0000(	580	13.13344	chr2:2577	KLF2P3	Pseudoger	chr2:130692243-130
ENSG0000(	580	13.13344	chr2:2577	ENSG00000223430	lncRNA	chr2:133554315-133
ENSG0000(	580	13.13344	chr2:2577	ENSG00000284659	Pseudoger	chr2:131419665-131
ENSG0000(	580	13.13344	chr2:2577	ENSG00000288031	Pseudoger	chr2:131692392-131
ENSG0000(	580	13.13344	chr2:2577	ANKRD30BI NCGv7	protein_c	chr2:132147591-132
ENSG0000(	580	13.13344	chr2:2577	LINC01087	lncRNA	chr2:131637025-131
ENSG0000(	580	13.13344	chr2:2577	SSBP3P2	Pseudoger	chr2:131352214-131
ENSG0000(	580	13.13344	chr2:2577	RNU6-127P	smallRNA	chr2:131229607-131
ENSG0000(	580	13.13344	chr2:2577	PLEKHB2	protein_c	chr2:131104847-131

ENSG0000(	580	13.13344	chr2:2577	MTND3P18		Pseudoger	chr2:131379743-131
ENSG0000(	580	13.13344	chr2:2577	TOMM40P4		Pseudoger	chr2:131723253-131
ENSG0000(	580	13.13344	chr2:2577	IMP4	NCGv7	protein_c	chr2:130342877-130
ENSG0000(	580	13.13344	chr2:2577	ENSG00000235615		lncRNA	chr2:131964731-131
ENSG0000(	580	13.13344	chr2:2577	MTCO1P18		Pseudoger	chr2:131382643-131
ENSG0000(	580	13.13344	chr2:2577	CDRT15P3		Pseudoger	chr2:131795136-131
ENSG0000(	580	13.13344	chr2:2577	ENSG00000232408		lncRNA	chr2:130583549-130
ENSG0000(	580	13.13344	chr2:2577	CDRT15P4		Pseudoger	chr2:131766187-131
ENSG0000(	580	13.13344	chr2:2577	LINC01120		lncRNA	chr2:131402778-131
ENSG0000(	580	13.13344	chr2:2577	C2orf27A		lncRNA	chr2:131647990-131
ENSG0000(	580	13.13344	chr2:2577	GNAQP1		Pseudoger	chr2:131423801-131
ENSG0000(	580	13.13344	chr2:2577	AC103564.9		Pseudoger	chr2:131776567-131
ENSG0000(	580	13.13344	chr2:2577	CYP4F30P		Pseudoger	chr2:130681229-130
ENSG0000(	580	13.13344	chr2:2577	MTND1P26		Pseudoger	chr2:131385700-131
ENSG0000(	580	13.13344	chr2:2577	ENSG00000225448		Pseudoger	chr2:131181153-131
ENSG0000(	580	13.13344	chr2:2577	CYCSP8		Pseudoger	chr2:130776646-130
ENSG0000(	580	13.13344	chr2:2577	FAM168B		protein_c	chr2:131047876-131
ENSG0000(	580	13.13344	chr2:2577	LYPD1		protein_c	chr2:132643286-132
ENSG0000(	580	13.13344	chr2:2577	AC011755.1		protein_c	chr2:133117004-133
ENSG0000(	580	13.13344	chr2:2577	RNA5-8SP5		Pseudoger	chr2:132253154-132
ENSG0000(	580	13.13344	chr2:2577	MIR4784		smallRNA	chr2:131491160-131
ENSG0000(	580	13.13344	chr2:2577	TUBA3D		protein_c	chr2:131476119-131
ENSG0000(	580	13.13344	chr2:2577	ARHGAP42P1		Pseudoger	chr2:131328176-131
ENSG0000(	580	13.13344	chr2:2577	ENSG00000227745		Pseudoger	chr2:132132856-132
ENSG0000(	580	13.13344	chr2:2577	ENSG00000286068		lncRNA	chr2:132685224-132
ENSG0000(	580	13.13344	chr2:2577	ARHGEF4-AS1		lncRNA	chr2:130830978-130
ENSG0000(	580	13.13344	chr2:2577	MTCO3P18		Pseudoger	chr2:131380153-131
ENSG0000(	580	13.13344	chr2:2577	MTCYBP10		Pseudoger	chr2:131369062-131
ENSG0000(	580	13.13344	chr2:2577	CFC1B		protein_c	chr2:130521197-130
ENSG0000(	580	13.13344	chr2:2577	ENSG00000283303		lncRNA	chr2:131363364-131
ENSG0000(	580	13.13344	chr2:2577	MED15P3		Pseudoger	chr2:131395217-131
ENSG0000(	580	13.13344	chr2:2577	SMPD4BP		Pseudoger	chr2:131492813-131
ENSG0000(	580	13.13344	chr2:2577	ENSG00000284003		Pseudoger	chr2:131373647-131
ENSG0000(	580	13.13344	chr2:2577	ENSG00000244337		lncRNA	chr2:131958277-131
ENSG0000(	580	13.13344	chr2:2577	ARHGEF4		protein_c	chr2:130836914-131
ENSG0000(	580	13.13344	chr2:2577	MTND6P10		Pseudoger	chr2:131369785-131
ENSG0000(	580	13.13344	chr2:2577	MTND4P21		Pseudoger	chr2:131372294-131
ENSG0000(	580	13.13344	chr2:2577	POTEKP		Pseudoger	chr2:131591752-131
ENSG0000(	580	13.13344	chr2:2577	ENSG00000279512		TEC	chr2:133117004-133
ENSG0000(	580	13.13344	chr2:2577	ENSG00000286208		lncRNA	chr2:131555007-131
ENSG0000(	580	13.13344	chr2:2577	RN7SL701P		smallRNA	chr2:131874631-131
ENSG0000(	580	13.13344	chr2:2577	FAR2P3		Pseudoger	chr2:130690133-130
ENSG0000(	580	13.13344	chr2:2577	NCKAP5-AS2		lncRNA	chr2:133264968-133
ENSG0000(	580	13.13344	chr2:2577	RAB6D		protein_c	chr2:131360492-131
ENSG0000(	580	13.13344	chr2:2577	PTPN18		protein_c	chr2:130356045-130
ENSG0000(	580	13.13344	chr2:2577	FAM201B		Pseudoger	chr2:132352722-132
ENSG0000(	580	13.13344	chr2:2577	ENSG00000284706		Pseudoger	chr2:131335502-131
ENSG0000(	580	13.13344	chr2:2577	MED15P8		Pseudoger	chr2:131201142-131
ENSG0000(	580	13.13344	chr2:2577	RHOQP2		Pseudoger	chr2:131460999-131
ENSG0000(	580	13.13344	chr2:2577	GRAMD4P8		Pseudoger	chr2:131646704-131
ENSG0000(	580	13.13344	chr2:2577	PRSS40B		Pseudoger	chr2:130539839-130

ENSG0000(	580	13.13344	chr2:2577	ENSG00000290700	lncRNA	chr2:130693282-130
ENSG0000(	580	13.13344	chr2:2577	CYP4F30P	lncRNA	chr2:130680050-130
ENSG0000(	580	13.13344	chr2:2577	RN7SKP93	smallRNA	chr2:133596091-133
ENSG0000(	580	13.13344	chr2:2577	RN7SKP103	smallRNA	chr2:132523617-132
ENSG0000(	580	13.13344	chr2:2577	ENSG00000285819	lncRNA	chr2:131191641-131
ENSG0000(	580	13.13344	chr2:2577	ENSG00000290654	lncRNA	chr2:131279290-131
ENSG0000(	580	13.13344	chr2:2577	CDRT15P3	lncRNA	chr2:131794961-131
ENSG0000(	580	13.13344	chr2:2577	ENSG00000290616	lncRNA	chr2:131733656-131
ENSG0000(	580	13.13344	chr2:2577	PRSS40A	lncRNA	chr2:130570829-130
ENSG0000(	580	13.13344	chr2:2577	PRSS40B	lncRNA	chr2:130539095-130
ENSG0000(	580	13.13344	chr2:2577	FAR2P4	Pseudoger	chr2:131296843-131
ENSG0000(	580	13.13344	chr2:2577	ENSG00000228721	Pseudoger	chr2:132911096-132
ENSG0000(	580	13.13344	chr2:2577	ENSG00000290599	lncRNA	chr2:130429827-130
ENSG0000(	580	13.13344	chr2:2577	ENSG00000290596	lncRNA	chr2:130416755-130
ENSG0000(	580	13.13344	chr2:2577	ENSG00000290594	lncRNA	chr2:131758031-131
ENSG0000(	580	13.13344	chr2:2577	ENSG00000229203	lncRNA	chr2:131829801-131
ENSG0000(	580	13.13344	chr2:2577	GPR39	protein_c	chr2:132416805-132
ENSG0000(	580	13.13344	chr2:2577	ENSG00000271179	Pseudoger	chr2:132080290-132
ENSG0000(	580	13.13344	chr2:2577	ENSG00000284604	lncRNA	chr2:131303037-131
ENSG0000(	580	13.13344	chr2:2577	SMIM39	protein_c	chr2:131035092-131
ENSG0000(	580	13.13344	chr2:2577	PRSS40A	Pseudoger	chr2:130571659-130
ENSG0000(	580	13.13344	chr2:2577	ENSG00000226886	Pseudoger	chr2:132037787-132
ENSG0000(	580	13.13344	chr2:2577	MGAT5	protein_c	chr2:134119983-134
ENSG0000(	580	13.13344	chr2:2577	SCARNA4	smallRNA	chr2:130929762-130
ENSG0000(	580	13.13344	chr2:2577	RNU6-579P	smallRNA	chr2:133333829-133
ENSG0000(	580	13.13344	chr2:2577	POTEI	protein_c	chr2:130459455-130
ENSG0000(	580	13.13344	chr2:2577	CDC27P1	Pseudoger	chr2:132262328-132
ENSG0000(	580	13.13344	chr2:2577	AC140481.3	Pseudoger	chr2:130656999-130
ENSG0000(	580	13.13344	chr2:2577	RNU6-175P	smallRNA	chr2:132406752-132
ENSG0000(	580	13.13344	chr2:2577	ENSG00000289534	lncRNA	chr2:134201818-134
ENSG0000(	580	13.13344	chr2:2577	AC013269.1	Pseudoger	chr2:130463386-130
ENSG0000(	580	13.13344	chr2:2577	NCKAP5-AS1	lncRNA	chr2:132915557-132
ENSG0000(	580	13.13344	chr2:2577	ENSG00000230803	lncRNA	chr2:132285795-132
ENSG0000(	580	13.13344	chr2:2577	FAR2P2	Pseudoger	chr2:130419071-130
ENSG0000(	580	13.13344	chr2:2577	RNU6-1132P	smallRNA	chr2:132152243-132
ENSG0000(	580	13.13344	chr2:2577	MTCO2P18	Pseudoger	chr2:131381840-131
ENSG0000(	580	13.13344	chr2:2577	ENSG00000290902	lncRNA	chr2:132304806-132
ENSG0000(	580	13.13344	chr2:2577	ENSG00000225594	Pseudoger	chr2:132087289-132
ENSG0000(	580	13.13344	chr2:2577	AMER3	protein_c	chr2:130755540-130
ENSG0000(	580	13.13344	chr2:2577	ENSG00000282944	lncRNA	chr2:130681228-130
ENSG0000(	580	13.13344	chr2:2577	ENSG00000229797	lncRNA	chr2:130742959-130
ENSG0000(	580	13.13344	chr2:2577	KLF2P2	Pseudoger	chr2:130427731-130
ENSG0000(	580	13.13344	chr2:2577	NCKAP5	protein_c	chr2:132671788-133
ENSG0000(	580	13.13344	chr2:2577	ENSG00000225819	lncRNA	chr2:130515997-130
ENSG0000(	580	13.13344	chr2:2577	ENSG00000225341	lncRNA	chr2:130530275-130
ENSG0000(	580	13.13344	chr2:2577	ENSG00000272769	lncRNA	chr2:132345616-132
ENSG0000(	580	13.13344	chr2:2577	CYP4F62P	Pseudoger	chr2:130439285-130
ENSG0000(	580	13.13344	chr2:2577	ENSG00000286697	lncRNA	chr2:134028608-134
ENSG0000(	579	13.1108	chrX:1773	AC003658.1	smallRNA	chrX:14764975-1476
ENSG0000(	579	13.1108	chr22:220	IGLVI-42	Pseudoger	chr22:22397032-223
ENSG0000(	570	12.907	chr7:2516	ENSG00000225457	lncRNA	chr7:113100663-113

ENSG0000(	570	12.907	chr7:251(	IFRD1		protein_c	chr7:112422887-112
ENSG0000(	570	12.907	chr7:251(	MIPEPP1		Pseudoger	chr7:112735166-112
ENSG0000(	570	12.907	chr7:251(	SNORD112		smallRNA	chr7:104971287-104
ENSG0000(	570	12.907	chr7:251(	ENSG00000230192		Pseudoger	chr7:109660094-109
ENSG0000(	570	12.907	chr7:251(	ENSG00000228540		lncRNA	chr7:110724159-110
ENSG0000(	570	12.907	chr7:251(	MTCYBP24		Pseudoger	chr7:112374324-112
ENSG0000(	570	12.907	chr7:251(	CFTR		protein_c	chr7:117287120-117
ENSG0000(	570	12.907	chr7:251(	snoZ185		smallRNA	chr7:116433214-116
ENSG0000(	570	12.907	chr7:251(	ENSG00000234273		lncRNA	chr7:109521981-109
ENSG0000(	570	12.907	chr7:251(	ENSG00000243621		Pseudoger	chr7:111394875-111
ENSG0000(	570	12.907	chr7:251(	ENSG00000225647		lncRNA	chr7:108598352-108
ENSG0000(	570	12.907	chr7:251(	TMEM168		protein_c	chr7:112762377-112
ENSG0000(	570	12.907	chr7:251(	LINC01004		lncRNA	chr7:104950315-105
ENSG0000(	570	12.907	chr7:251(	ENSG00000278894		TEC	chr7:113451072-113
ENSG0000(	570	12.907	chr7:251(	EFCAB10		protein_c	chr7:105565120-105
ENSG0000(	570	12.907	chr7:251(	ENSG00000228368		lncRNA	chr7:116965846-116
ENSG0000(	570	12.907	chr7:251(	LHFPL3-AS2		lncRNA	chr7:104894628-104
ENSG0000(	570	12.907	chr7:251(	ATXN7L1	DriverDBv	protein_c	chr7:105604772-105
ENSG0000(	570	12.907	chr7:251(	GTF3AP6		Pseudoger	chr7:118880103-118
ENSG0000(	570	12.907	chr7:251(	DOCK4-AS1		lncRNA	chr7:111808516-111
ENSG0000(	570	12.907	chr7:251(	LINC02577		lncRNA	chr7:106774955-106
ENSG0000(	570	12.907	chr7:251(	EIF4BP6		Pseudoger	chr7:104667749-104
ENSG0000(	570	12.907	chr7:251(	LHFPL3	DriverDBv	protein_c	chr7:104328603-104
ENSG0000(	570	12.907	chr7:251(	RPL36P13		Pseudoger	chr7:114297114-114
ENSG0000(	570	12.907	chr7:251(	RPL7AP42		Pseudoger	chr7:111971222-111
ENSG0000(	570	12.907	chr7:251(	LINC01393		lncRNA	chr7:115030564-115
ENSG0000(	570	12.907	chr7:251(	AC005161.1		smallRNA	chr7:110928318-110
ENSG0000(	570	12.907	chr7:251(	ENSG00000228341		Pseudoger	chr7:107450083-107
ENSG0000(	570	12.907	chr7:251(	RNA5SP239		Pseudoger	chr7:116944286-116
ENSG0000(	570	12.907	chr7:251(	ENSG00000234826		lncRNA	chr7:117998858-118
ENSG0000(	570	12.907	chr7:251(	ENSG00000270425		Pseudoger	chr7:108720608-108
ENSG0000(	570	12.907	chr7:251(	AC006926.1		smallRNA	chr7:118462499-118
ENSG0000(	570	12.907	chr7:251(	LINC02476		lncRNA	chr7:119495024-119
ENSG0000(	570	12.907	chr7:251(	CCDC71L		protein_c	chr7:106654360-106
ENSG0000(	570	12.907	chr7:251(	ENSG00000287592		lncRNA	chr7:113486407-113
ENSG0000(	570	12.907	chr7:251(	POLR2DP2		Pseudoger	chr7:115503367-115
ENSG0000(	570	12.907	chr7:251(	RNA5SP237		Pseudoger	chr7:111953653-111
ENSG0000(	570	12.907	chr7:251(	MTND6P24		Pseudoger	chr7:112373733-112
ENSG0000(	570	12.907	chr7:251(	ENSG00000278424		Pseudoger	chr7:110215891-110
ENSG0000(	570	12.907	chr7:251(	ZNF277-AS1		lncRNA	chr7:112328189-112
ENSG0000(	570	12.907	chr7:251(	RNA5SP236		Pseudoger	chr7:106781600-106
ENSG0000(	570	12.907	chr7:251(	ENSG00000229603		lncRNA	chr7:108909453-108
ENSG0000(	570	12.907	chr7:251(	HRAT17		lncRNA	chr7:112953282-112
ENSG0000(	570	12.907	chr7:251(	ST7-AS2		lncRNA	chr7:117072072-117
ENSG0000(	570	12.907	chr7:251(	ENSG00000226624		Pseudoger	chr7:105819492-105
ENSG0000(	570	12.907	chr7:251(	KMT2E	NCGv7	protein_c	chr7:104940943-105
ENSG0000(	570	12.907	chr7:251(	ENSG00000227532		Pseudoger	chr7:117187548-117
ENSG0000(	570	12.907	chr7:251(	CFTR-AS1		lncRNA	chr7:117560733-117
ENSG0000(	570	12.907	chr7:251(	ST7		protein_c	chr7:116953238-117
ENSG0000(	570	12.907	chr7:251(	ENSG00000279288		TEC	chr7:113075124-113
ENSG0000(	570	12.907	chr7:251(	ENSG00000286390		lncRNA	chr7:117332761-117

ENSG0000(	570	12.907	chr7:251(	ST7-AS1		lncRNA	chr7:116952446-116
ENSG0000(	570	12.907	chr7:251(	ENSG00000226965		lncRNA	chr7:110108031-110
ENSG0000(	570	12.907	chr7:251(	ENSG00000287011		lncRNA	chr7:111757051-111
ENSG0000(	570	12.907	chr7:251(	RAC1P6		Pseudoger	chr7:115136475-115
ENSG0000(	570	12.907	chr7:251(	MTND4P6		Pseudoger	chr7:117263917-117
ENSG0000(	570	12.907	chr7:251(	ENSG00000243797		lncRNA	chr7:106372251-106
ENSG0000(	570	12.907	chr7:251(	PPP1R3A	NCGv7	protein_c	chr7:113876777-114
ENSG0000(	570	12.907	chr7:251(	ASZ1	NCGv7	protein_c	chr7:117363222-117
ENSG0000(	570	12.907	chr7:251(	PRKAR2B	NCGv7	protein_c	chr7:107044705-107
ENSG0000(	570	12.907	chr7:251(	SLC26A4-AS1		lncRNA	chr7:107650260-107
ENSG0000(	570	12.907	chr7:251(	ENSG00000224136		Pseudoger	chr7:117882859-117
ENSG0000(	570	12.907	chr7:251(	ENSG00000235427		lncRNA	chr7:116542718-116
ENSG0000(	570	12.907	chr7:251(	LINC01392		lncRNA	chr7:115061537-115
ENSG0000(	570	12.907	chr7:251(	ENSG00000234001		Pseudoger	chr7:117586207-117
ENSG0000(	570	12.907	chr7:251(	ENSG00000224595		lncRNA	chr7:114414244-114
ENSG0000(	570	12.907	chr7:251(	ENSG00000279086		TEC	chr7:116209234-116
ENSG0000(	570	12.907	chr7:251(	BANF1P5		Pseudoger	chr7:107642765-107
ENSG0000(	570	12.907	chr7:251(	ENSG00000280439		TEC	chr7:106035798-106
ENSG0000(	570	12.907	chr7:251(	RWDD4P1		Pseudoger	chr7:105301522-105
ENSG0000(	570	12.907	chr7:251(	LHFPL3-AS1		lncRNA	chr7:104738597-104
ENSG0000(	570	12.907	chr7:251(	ENSG00000286013		lncRNA	chr7:106624072-106
ENSG0000(	570	12.907	chr7:251(	DNAJB9	NCGv7	protein_c	chr7:108569867-108
ENSG0000(	570	12.907	chr7:251(	ENSG00000287186		lncRNA	chr7:110590082-110
ENSG0000(	570	12.907	chr7:251(	ENSG00000235945		Pseudoger	chr7:116873454-116
ENSG0000(	570	12.907	chr7:251(	ENSG00000227948		Pseudoger	chr7:111411319-111
ENSG0000(	570	12.907	chr7:251(	FOXP2	NCGv7	protein_c	chr7:114086327-114
ENSG0000(	570	12.907	chr7:251(	ENSG00000279043		TEC	chr7:108900105-108
ENSG0000(	570	12.907	chr7:251(	ENSG00000223646		lncRNA	chr7:112616440-112
ENSG0000(	570	12.907	chr7:251(	CDHR3	NCGv7	protein_c	chr7:105876796-106
ENSG0000(	570	12.907	chr7:251(	Y_RNA		smallRNA	chr7:116909877-116
ENSG0000(	570	12.907	chr7:251(	GPR85		protein_c	chr7:113078331-113
ENSG0000(	570	12.907	chr7:251(	BMT2		protein_c	chr7:112819147-112
ENSG0000(	570	12.907	chr7:251(	LSM8		protein_c	chr7:118184144-118
ENSG0000(	570	12.907	chr7:251(	COG5		protein_c	chr7:107201372-107
ENSG0000(	570	12.907	chr7:251(	DOCK4	NCGv7	protein_c	chr7:111726110-112
ENSG0000(	570	12.907	chr7:251(	ENSG00000223886		Pseudoger	chr7:105530209-105
ENSG0000(	570	12.907	chr7:251(	SYPL1		protein_c	chr7:106090505-106
ENSG0000(	570	12.907	chr7:251(	ENSG00000286076		lncRNA	chr7:106425278-106
ENSG0000(	570	12.907	chr7:251(	BUB3P1		Pseudoger	chr7:108994031-108
ENSG0000(	570	12.907	chr7:251(	AC004492.1		smallRNA	chr7:107294885-107
ENSG0000(	570	12.907	chr7:251(	EIF3IP1		Pseudoger	chr7:109959218-109
ENSG0000(	570	12.907	chr7:251(	snoU13		smallRNA	chr7:111109268-111
ENSG0000(	570	12.907	chr7:251(	LRRN3	NCGv7	protein_c	chr7:111091006-111
ENSG0000(	570	12.907	chr7:251(	ENSG00000273055		lncRNA	chr7:107942116-107
ENSG0000(	570	12.907	chr7:251(	ENSG00000230520		Pseudoger	chr7:118950386-118
ENSG0000(	570	12.907	chr7:251(	AC091320.2		smallRNA	chr7:119484325-119
ENSG0000(	570	12.907	chr7:251(	ENSG00000272918		lncRNA	chr7:105102838-105
ENSG0000(	570	12.907	chr7:251(	snoU109		smallRNA	chr7:107603363-107
ENSG0000(	570	12.907	chr7:251(	ENSG00000288914		protein_c	chr7:105040858-105
ENSG0000(	570	12.907	chr7:251(	ENSG00000272854		lncRNA	chr7:107579557-107
ENSG0000(	570	12.907	chr7:251(	MTCYBP6		Pseudoger	chr7:117264393-117

ENSG0000(	570	12.907	chr7:251(	ENSG00000288635		protein_c	chr7:116954391-117
ENSG0000(	570	12.907	chr7:251(	PNPLA8	NCGv7	protein_c	chr7:108470417-108
ENSG0000(	570	12.907	chr7:251(	RINT1	AC	protein_c	chr7:105532169-105
ENSG0000(	570	12.907	chr7:251(	TES	DriverDBv	protein_c	chr7:116210506-116
ENSG0000(	570	12.907	chr7:251(	MDFIC	NCGv7	protein_c	chr7:114922094-115
ENSG0000(	570	12.907	chr7:251(	WBP1LP2		Pseudoger	chr7:107628553-107
ENSG0000(	570	12.907	chr7:251(	ENSG00000272604		lncRNA	chr7:105571083-105
ENSG0000(	570	12.907	chr7:251(	ENSG00000289360		protein_c	chr7:105040848-105
ENSG0000(	570	12.907	chr7:251(	ENSG00000282859		lncRNA	chr7:114560961-114
ENSG0000(	570	12.907	chr7:251(	ENSG00000288640		protein_c	chr7:112450460-112
ENSG0000(	570	12.907	chr7:251(	ENSG00000288634		protein_c	chr7:112450487-112
ENSG0000(	570	12.907	chr7:251(	MET	NCGv7;AC	protein_c	chr7:116672196-116
ENSG0000(	570	12.907	chr7:251(	ENSG00000243243		lncRNA	chr7:116237929-116
ENSG0000(	570	12.907	chr7:251(	CAV2		protein_c	chr7:116287380-116
ENSG0000(	570	12.907	chr7:251(	TFEC		protein_c	chr7:115935148-116
ENSG0000(	570	12.907	chr7:251(	CBLL1	AC	protein_c	chr7:107743073-107
ENSG0000(	570	12.907	chr7:251(	CAPZA2		protein_c	chr7:116811070-116
ENSG0000(	570	12.907	chr7:251(	DUS4L		protein_c	chr7:107563484-107
ENSG0000(	570	12.907	chr7:251(	HBP1		protein_c	chr7:107168961-107
ENSG0000(	570	12.907	chr7:251(	PIK3CG	NCGv7	protein_c	chr7:106865278-106
ENSG0000(	570	12.907	chr7:251(	NAMPT	NCGv7	protein_c	chr7:106248298-106
ENSG0000(	570	12.907	chr7:251(	RPL36P12		Pseudoger	chr7:105244652-105
ENSG0000(	570	12.907	chr7:251(	YBX1P2		Pseudoger	chr7:105582258-105
ENSG0000(	570	12.907	chr7:251(	ENSG00000267052		lncRNA	chr7:106569876-106
ENSG0000(	570	12.907	chr7:251(	TPM3P1		Pseudoger	chr7:116972165-116
ENSG0000(	570	12.907	chr7:251(	PRKAR2B-AS1		lncRNA	chr7:107066591-107
ENSG0000(	570	12.907	chr7:251(	ENSG00000273320		lncRNA	chr7:106285200-106
ENSG0000(	570	12.907	chr7:251(	DUS4L-BCAP29		protein_c	chr7:107563971-107
ENSG0000(	570	12.907	chr7:251(	GPR22		protein_c	chr7:107470018-107
ENSG0000(	570	12.907	chr7:251(	Y_RNA		smallRNA	chr7:115833273-115
ENSG0000(	570	12.907	chr7:251(	U3		smallRNA	chr7:107999792-107
ENSG0000(	570	12.907	chr7:251(	KMT2E-AS1		lncRNA	chr7:105013277-105
ENSG0000(	570	12.907	chr7:251(	ENSG00000230785		Pseudoger	chr7:117262918-117
ENSG0000(	570	12.907	chr7:251(	DLD		protein_c	chr7:107891162-107
ENSG0000(	570	12.907	chr7:251(	RPL7P32		Pseudoger	chr7:108510233-108
ENSG0000(	570	12.907	chr7:251(	CTTNBP2		protein_c	chr7:117710651-117
ENSG0000(	570	12.907	chr7:251(	BCAP29		protein_c	chr7:107580246-107
ENSG0000(	570	12.907	chr7:251(	ENSG00000271482		Pseudoger	chr7:105204600-105
ENSG0000(	570	12.907	chr7:251(	RNU6-392P		smallRNA	chr7:106208167-106
ENSG0000(	570	12.907	chr7:251(	ENSG00000290116		lncRNA	chr7:106448382-106
ENSG0000(	570	12.907	chr7:251(	NPM1P14		Pseudoger	chr7:112520488-112
ENSG0000(	570	12.907	chr7:251(	ENSG00000271368		Pseudoger	chr7:109763574-109
ENSG0000(	570	12.907	chr7:251(	MTND5P8		Pseudoger	chr7:112372647-112
ENSG0000(	570	12.907	chr7:251(	CBLL1-AS1		lncRNA	chr7:107739999-107
ENSG0000(	570	12.907	chr7:251(	SLC26A3	NCGv7	protein_c	chr7:107765467-107
ENSG0000(	570	12.907	chr7:251(	RNU6-1322P		smallRNA	chr7:105332790-105
ENSG0000(	570	12.907	chr7:251(	AC007567.1		smallRNA	chr7:108358307-108
ENSG0000(	570	12.907	chr7:251(	ENSG00000242072		lncRNA	chr7:115647461-115
ENSG0000(	570	12.907	chr7:251(	ENSG00000242154		lncRNA	chr7:105304277-105
ENSG0000(	570	12.907	chr7:251(	DCAF13P1		Pseudoger	chr7:106125371-106
ENSG0000(	570	12.907	chr7:251(	AC020606.1		smallRNA	chr7:114629855-114

ENSG0000(	570	12.907	chr7:251(	ENSG00000270997		Pseudoger	chr7:113415689-113
ENSG0000(	570	12.907	chr7:251(	IMMP2L	NCGv7	protein_c	chr7:110662644-111
ENSG0000(	570	12.907	chr7:251(	RNA5SP238		Pseudoger	chr7:114613787-114
ENSG0000(	570	12.907	chr7:251(	SNORA25		smallRNA	chr7:115581315-115
ENSG0000(	570	12.907	chr7:251(	ENSG00000240973		lncRNA	chr7:115679345-115
ENSG0000(	570	12.907	chr7:251(	RN7SKP187		smallRNA	chr7:112288623-112
ENSG0000(	570	12.907	chr7:251(	SLC26A4		protein_c	chr7:107660828-107
ENSG0000(	570	12.907	chr7:251(	LAMB1	NCGv7	protein_c	chr7:107923799-108
ENSG0000(	570	12.907	chr7:251(	NRCAM		protein_c	chr7:108147623-108
ENSG0000(	570	12.907	chr7:251(	LAMB4	DriverDBv	protein_c	chr7:108023548-108
ENSG0000(	570	12.907	chr7:251(	PUS7		protein_c	chr7:105439661-105
ENSG0000(	570	12.907	chr7:251(	THAP5	NCGv7	protein_c	chr7:108554543-108
ENSG0000(	570	12.907	chr7:251(	MIR3666		smallRNA	chr7:114653345-114
ENSG0000(	570	12.907	chr7:251(	ENSG00000289630		lncRNA	chr7:113118666-113
ENSG0000(	570	12.907	chr7:251(	Y_RNA		smallRNA	chr7:115207894-115
ENSG0000(	570	12.907	chr7:251(	LINC02903		lncRNA	chr7:108883975-108
ENSG0000(	570	12.907	chr7:251(	ENSG00000272072		lncRNA	chr7:107192559-107
ENSG0000(	570	12.907	chr7:251(	ENSG00000270764		Pseudoger	chr7:105189190-105
ENSG0000(	570	12.907	chr7:251(	ENSG00000180019		Pseudoger	chr7:112446086-112
ENSG0000(	570	12.907	chr7:251(	ENSG00000083622		lncRNA	chr7:117604791-117
ENSG0000(	570	12.907	chr7:251(	ENSG00000230941		lncRNA	chr7:109322320-109
ENSG0000(	570	12.907	chr7:251(	U1		smallRNA	chr7:120005976-120
ENSG0000(	570	12.907	chr7:251(	LSMEM1		protein_c	chr7:112480853-112
ENSG0000(	570	12.907	chr7:251(	CAV1	AC	protein_c	chr7:116524994-116
ENSG0000(	570	12.907	chr7:251(	SRPK2		protein_c	chr7:105110704-105
ENSG0000(	570	12.907	chr7:251(	WNT2	NCGv7;AC	protein_c	chr7:117275451-117
ENSG0000(	570	12.907	chr7:251(	ENSG00000237870		lncRNA	chr7:116275606-116
ENSG0000(	570	12.907	chr7:251(	ENSG00000287829		lncRNA	chr7:117091678-117
ENSG0000(	570	12.907	chr7:251(	ENSG00000287827		lncRNA	chr7:118511942-118
ENSG0000(	570	12.907	chr7:251(	ENSG00000237974		Pseudoger	chr7:117487737-117
ENSG0000(	570	12.907	chr7:251(	ENSG00000237606		Pseudoger	chr7:104826336-104
ENSG0000(	570	12.907	chr7:251(	ENSG00000270516		Pseudoger	chr7:119178177-119
ENSG0000(	570	12.907	chr7:251(	ENSG00000237813		lncRNA	chr7:116238260-116
ENSG0000(	570	12.907	chr7:251(	RPL3P8		Pseudoger	chr7:109998434-109
ENSG0000(	570	12.907	chr7:251(	RN7SL8P		smallRNA	chr7:104911917-104
ENSG0000(	570	12.907	chr7:251(	AC002066.2		smallRNA	chr7:116345855-116
ENSG0000(	570	12.907	chr7:251(	SMIM30		protein_c	chr7:113116718-113
ENSG0000(	570	12.907	chr7:251(	ST7-OT4		lncRNA	chr7:116953899-117
ENSG0000(	570	12.907	chr7:251(	ENSG00000243345		lncRNA	chr7:115789729-115
ENSG0000(	570	12.907	chr7:251(	AC005048.1		smallRNA	chr7:108036846-108
ENSG0000(	570	12.907	chr7:251(	ZNF277		protein_c	chr7:112206695-112
ENSG0000(	570	12.907	chr7:251(	ANKRD49P4		Pseudoger	chr7:117439982-117
ENSG0000(	570	12.907	chr7:251(	LARP1BP2		Pseudoger	chr7:106315225-106
ENSG0000(	570	12.907	chr7:251(	ANKRD7		protein_c	chr7:118214669-118
ENSG0000(	570	12.907	chr7:251(	COMETT		lncRNA	chr7:116563594-116
ENSG0000(	566	12.81643	chr9:8075	RNA5SP293		Pseudoger	chr9:107918866-107
ENSG0000(	557	12.61263	chr4:7212	ENSG00000250622		Pseudoger	chr4:156972631-156
ENSG0000(	552	12.49941	chr4:1865	ENSG00000249320		Pseudoger	chr4:25472517-2547
ENSG0000(	552	12.49941	chr2:8187	SOX11	NCGv7	protein_c	chr2:5692384-57013
ENSG0000(	552	12.49941	chr4:1865	ENSG00000251410		lncRNA	chr4:27964517-2798
ENSG0000(	552	12.49941	chr4:1865	ENSG00000205830		lncRNA	chr4:27207505-2721

ENSG0000(	552	12.49941	chr4:1865	RN7SL101P		smallRNA	chr4:28711198-2871
ENSG0000(	552	12.49941	chr4:1865	ENSG00000251373		Pseudoger	chr4:29407100-2940
ENSG0000(	552	12.49941	chr4:1865	MTND3P5		Pseudoger	chr4:25720099-2572
ENSG0000(	552	12.49941	chr2:8187	ENSG00000232835		lncRNA	chr2:5549780-55560
ENSG0000(	552	12.49941	chr4:1865	SEL1L3		protein_c	chr4:25747433-2586
ENSG0000(	552	12.49941	chr4:1865	AC108218.1		smallRNA	chr4:25353970-2535
ENSG0000(	552	12.49941	chr4:1865	ENSG00000286596		lncRNA	chr4:30718805-3072
ENSG0000(	552	12.49941	chr4:1865	RPS3AP17		Pseudoger	chr4:29962644-2996
ENSG0000(	552	12.49941	chr4:1865	SMIM20		protein_c	chr4:25861830-2592
ENSG0000(	552	12.49941	chr4:1865	ENSG00000228154		Pseudoger	chr4:25622777-2562
ENSG0000(	552	12.49941	chr4:1865	ENSG00000251325		lncRNA	chr4:27262506-2726
ENSG0000(	552	12.49941	chr4:1865	ENSG00000248545		lncRNA	chr4:25529177-2561
ENSG0000(	552	12.49941	chr4:1865	ENSG00000287389		lncRNA	chr4:27917753-2794
ENSG0000(	552	12.49941	chr4:1865	STIM2	NCGv7	protein_c	chr4:26857601-2702
ENSG0000(	552	12.49941	chr4:1865	ENSG00000250541		lncRNA	chr4:25770266-2577
ENSG0000(	552	12.49941	chr4:1865	PCDH7		protein_c	chr4:30720369-3114
ENSG0000(	552	12.49941	chr4:1865	MTCYBP43		Pseudoger	chr4:30884443-3088
ENSG0000(	552	12.49941	chr4:1865	ENSG00000288940		lncRNA	chr4:30717369-3071
ENSG0000(	552	12.49941	chr2:8187	SILC1		lncRNA	chr2:5932543-60035
ENSG0000(	552	12.49941	chr4:1865	TBC1D19		protein_c	chr4:26576437-2675
ENSG0000(	552	12.49941	chr4:1865	LINC02364		lncRNA	chr4:28996498-2901
ENSG0000(	552	12.49941	chr4:1865	ENSG00000248608		Pseudoger	chr4:25504997-2550
ENSG0000(	552	12.49941	chr4:1865	STIM2-AS1		lncRNA	chr4:26859806-2686
ENSG0000(	552	12.49941	chr4:1865	PIMREGP4		Pseudoger	chr4:26873561-2687
ENSG0000(	552	12.49941	chr4:1865	RPS29P11		Pseudoger	chr4:25678850-2567
ENSG0000(	552	12.49941	chr4:1865	AC093807.1		smallRNA	chr4:26637390-2663
ENSG0000(	552	12.49941	chr4:1865	SLIRPP2		Pseudoger	chr4:25686202-2568
ENSG0000(	552	12.49941	chr4:1865	EEF1A1P21		Pseudoger	chr4:29748757-2975
ENSG0000(	552	12.49941	chr2:8187	LINC01810		lncRNA	chr2:5810641-58126
ENSG0000(	552	12.49941	chr4:1865	ENSG00000249564		Pseudoger	chr4:29907659-2990
ENSG0000(	552	12.49941	chr4:1865	ENSG00000248281		Pseudoger	chr4:30006951-3000
ENSG0000(	552	12.49941	chr4:1865	LINC02357		lncRNA	chr4:26070754-2610
ENSG0000(	552	12.49941	chr4:1865	ENSG00000249678		lncRNA	chr4:30776257-3079
ENSG0000(	552	12.49941	chr4:1865	LINC02261		lncRNA	chr4:27217479-2728
ENSG0000(	552	12.49941	chr4:1865	LINC02472		lncRNA	chr4:29214253-2929
ENSG0000(	552	12.49941	chr4:1865	ENSG00000249228		lncRNA	chr4:29046591-2904
ENSG0000(	552	12.49941	chr4:1865	SNORD74		smallRNA	chr4:26702309-2670
ENSG0000(	552	12.49941	chr4:1865	MTCO3P44		Pseudoger	chr4:25720516-2572
ENSG0000(	552	12.49941	chr4:1865	ENSG00000251434		lncRNA	chr4:31350284-3135
ENSG0000(	552	12.49941	chr4:1865	ENSG00000286141		lncRNA	chr4:28580991-2860
ENSG0000(	552	12.49941	chr4:1865	ENSG00000250038		lncRNA	chr4:28343862-2840
ENSG0000(	552	12.49941	chr4:1865	RNU7-126P		smallRNA	chr4:25598564-2559
ENSG0000(	552	12.49941	chr4:1865	ZCCHC4		protein_c	chr4:25312774-2537
ENSG0000(	552	12.49941	chr4:1865	ENSG00000250064		lncRNA	chr4:28435449-2860
ENSG0000(	552	12.49941	chr4:1865	ANAPC4		protein_c	chr4:25377263-2541
ENSG0000(	552	12.49941	chr4:1865	ENSG00000286321		lncRNA	chr4:28225969-2828
ENSG0000(	552	12.49941	chr4:1865	Y_RNA		smallRNA	chr4:27223319-2722
ENSG0000(	552	12.49941	chr4:1865	RBPJ	NCGv7	protein_c	chr4:26163455-2643
ENSG0000(	552	12.49941	chr4:1865	MTND4P9		Pseudoger	chr4:25718082-2571
ENSG0000(	552	12.49941	chr4:1865	SLC34A2	NCGv7;AC	protein_c	chr4:25648011-2567
ENSG0000(	552	12.49941	chr2:8187	ENSG00000242540		lncRNA	chr2:5696220-57196

ENSG0000(	552	12.49941	chr4:1865	ENSG00000276507	Pseudoger	chr4:29010619-2901
ENSG0000(	552	12.49941	chr2:8187	ENSG00000230090	lncRNA	chr2:5618327-56911
ENSG0000(	552	12.49941	chr4:1865	ENSG00000251009	lncRNA	chr4:25864881-2586
ENSG0000(	552	12.49941	chr4:1865	ENSG00000251080	lncRNA	chr4:27133996-2714
ENSG0000(	552	12.49941	chr4:1865	ENSG00000248452	Pseudoger	chr4:26111865-2611
ENSG0000(	552	12.49941	chr2:8187	ENSG00000236106	lncRNA	chr2:5726253-57303
ENSG0000(	552	12.49941	chr4:1865	RNU6-1221P	smallRNA	chr4:29510635-2951
ENSG0000(	552	12.49941	chr4:1865	MESTP3	Pseudoger	chr4:28823244-2882
ENSG0000(	552	12.49941	chr4:1865	CCKAR	protein_c	chr4:26481396-2649
ENSG0000(	552	12.49941	chr4:1865	MTND4LP22	Pseudoger	chr4:25719755-2572
ENSG0000(	552	12.49941	chr4:1865	ENSG00000251113	Pseudoger	chr4:29465701-2946
ENSG0000(	552	12.49941	chr4:1865	AC109351.1	smallRNA	chr4:29750196-2975
ENSG0000(	552	12.49941	chr4:1865	IGBP1P5	Pseudoger	chr4:27585145-2758
ENSG0000(	552	12.49941	chr4:1865	LINC02497	lncRNA	chr4:31171013-3121
ENSG0000(	552	12.49941	chr2:8187	LINC01248	lncRNA	chr2:5602505-56914
ENSG0000(	552	12.49941	chr4:1865	ENSG00000248176	lncRNA	chr4:29118304-2921
ENSG0000(	546	12.36355	chr17:289	ENSG00000274630	lncRNA	chr17:41867581-418
ENSG0000(	543	12.29562	chr3:6192	AC026166.1	smallRNA	chr3:12071038-1207
ENSG0000(	541	12.25033	chr2:2577	MIR4435-2	smallRNA	chr2:111321012-111
ENSG0000(	541	12.25033	chr2:2577	RPL39P16	Pseudoger	chr2:108878308-108
ENSG0000(	541	12.25033	chr4:1865	LINC02501	lncRNA	chr4:31506422-3155
ENSG0000(	541	12.25033	chr2:2577	LINC01789	lncRNA	chr2:107254691-107
ENSG0000(	541	12.25033	chr2:2577	LINC02936	lncRNA	chr2:113677702-113
ENSG0000(	541	12.25033	chr2:2577	AC110769.1	smallRNA	chr2:113990010-113
ENSG0000(	541	12.25033	chr2:2577	CDK8P2	Pseudoger	chr2:112932625-112
ENSG0000(	541	12.25033	chr2:2577	ENSG00000231747	Pseudoger	chr2:112621809-112
ENSG0000(	541	12.25033	chr2:2577	ENSG00000231583	Pseudoger	chr2:110500100-110
ENSG0000(	541	12.25033	chr2:2577	SRSF3P6	Pseudoger	chr2:109732731-109
ENSG0000(	541	12.25033	chr2:2577	ILRUNP1	Pseudoger	chr2:106344566-106
ENSG0000(	541	12.25033	chr2:2577	ENSG00000227294	lncRNA	chr2:106834464-106
ENSG0000(	541	12.25033	chr2:2577	ENSG00000261760	Pseudoger	chr2:110245756-110
ENSG0000(	541	12.25033	chr4:1865	ENSG00000249766	Pseudoger	chr4:33400538-3340
ENSG0000(	541	12.25033	chr2:2577	CAPZBP1	Pseudoger	chr2:103623630-103
ENSG0000(	541	12.25033	chr2:2577	ENSG00000235721	Pseudoger	chr2:110007675-110
ENSG0000(	541	12.25033	chr2:2577	ENSG00000230958	Pseudoger	chr2:111865798-111
ENSG0000(	541	12.25033	chr2:2577	SRSF3P4	Pseudoger	chr2:106253231-106
ENSG0000(	541	12.25033	chr2:2577	ANAPC1P6	Pseudoger	chr2:106369724-106
ENSG0000(	541	12.25033	chr2:2577	ENSG00000228968	lncRNA	chr2:104125268-104
ENSG0000(	541	12.25033	chr2:2577	SULT1C2	protein_c	chr2:108288639-108
ENSG0000(	541	12.25033	chr2:2577	ENSG00000235522	lncRNA	chr2:105846534-105
ENSG0000(	541	12.25033	chr2:2577	ENSG00000227574	lncRNA	chr2:110449242-110
ENSG0000(	541	12.25033	chr4:1865	ENSG00000251438	lncRNA	chr4:36311190-3639
ENSG0000(	541	12.25033	chr2:2577	ENSG00000233339	lncRNA	chr2:106179542-106
ENSG0000(	541	12.25033	chr2:2577	WASF1P1	Pseudoger	chr2:108276812-108
ENSG0000(	541	12.25033	chr2:2577	SH3RF3-AS1	lncRNA	chr2:109127327-109
ENSG0000(	541	12.25033	chr2:2577	ENSG00000226905	Pseudoger	chr2:106963193-106
ENSG0000(	541	12.25033	chr2:2577	IL1R1-AS1	lncRNA	chr2:102172621-102
ENSG0000(	541	12.25033	chr2:2577	RPS14P4	Pseudoger	chr2:111295629-111
ENSG0000(	541	12.25033	chr2:2577	MRPS9-AS1	lncRNA	chr2:105092368-105
ENSG0000(	541	12.25033	chr2:2577	ENSG00000236555	Pseudoger	chr2:112346011-112
ENSG0000(	541	12.25033	chr2:2577	RN7SL297P	smallRNA	chr2:111930175-111

ENSG0000(	541	12.25033	chr2:2577	MIR4265		smallRNA	chr2:109141490-109
ENSG0000(	541	12.25033	chr2:2577	LINC01123		lncRNA	chr2:109987063-109
ENSG0000(	541	12.25033	chr2:2577	ENSG00000235881		lncRNA	chr2:110709575-110
ENSG0000(	541	12.25033	chr2:2577	ENSG00000233494		Pseudoger	chr2:107902997-107
ENSG0000(	541	12.25033	chr2:2577	ENSG00000233479		lncRNA	chr2:113669166-113
ENSG0000(	541	12.25033	chr2:2577	ENSG00000226991		Pseudoger	chr2:110358790-110
ENSG0000(	541	12.25033	chr2:2577	ACOXL-AS1		lncRNA	chr2:111098345-111
ENSG0000(	541	12.25033	chr2:2577	MIR4436B1		smallRNA	chr2:110086433-110
ENSG0000(	541	12.25033	chr2:2577	SOCAR		lncRNA	chr2:111491272-111
ENSG0000(	541	12.25033	chr2:2577	ENSG00000227157		lncRNA	chr2:104406471-104
ENSG0000(	541	12.25033	chr4:1865	AC079772.1		smallRNA	chr4:33536744-3353
ENSG0000(	541	12.25033	chr4:1865	ENSG00000248417		lncRNA	chr4:33433510-3343
ENSG0000(	541	12.25033	chr2:2577	RPL23AP7		Pseudoger	chr2:113610502-113
ENSG0000(	541	12.25033	chr2:2577	ACTR1AP1		Pseudoger	chr2:110184054-110
ENSG0000(	541	12.25033	chr2:2577	ENSG00000229118		lncRNA	chr2:111265283-111
ENSG0000(	541	12.25033	chr4:1865	LINC02616		lncRNA	chr4:37001772-3702
ENSG0000(	541	12.25033	chr2:2577	ENSG00000227270		Pseudoger	chr2:107903964-107
ENSG0000(	541	12.25033	chr2:2577	ACTR3-AS1		lncRNA	chr2:113831049-113
ENSG0000(	541	12.25033	chr2:2577	ENSG00000227623		Pseudoger	chr2:103275904-103
ENSG0000(	541	12.25033	chr4:1865	ENSG00000251588		Pseudoger	chr4:36506043-3650
ENSG0000(	541	12.25033	chr2:2577	MIR4782		smallRNA	chr2:113721290-113
ENSG0000(	541	12.25033	chr2:2577	ENSG00000228251		lncRNA	chr2:112590796-112
ENSG0000(	541	12.25033	chr2:2577	LIMS1-AS1		lncRNA	chr2:108676795-108
ENSG0000(	541	12.25033	chr2:2577	AC013402.1		smallRNA	chr2:104568817-104
ENSG0000(	541	12.25033	chr2:2577	SDR42E1P5		Pseudoger	chr2:102411243-102
ENSG0000(	541	12.25033	chr2:2577	RPL37P12		Pseudoger	chr2:109552536-109
ENSG0000(	541	12.25033	chr2:2577	AC079753.1		smallRNA	chr2:112844311-112
ENSG0000(	541	12.25033	chr2:2577	LINC01102		lncRNA	chr2:104430130-104
ENSG0000(	541	12.25033	chr2:2577	ENSG00000231626		lncRNA	chr2:104580821-104
ENSG0000(	541	12.25033	chr2:2577	POU3F3	NCGv7	protein_c	chr2:104853287-104
ENSG0000(	541	12.25033	chr2:2577	SULT1C3		protein_c	chr2:108239968-108
ENSG0000(	541	12.25033	chr2:2577	RPL10P5		Pseudoger	chr2:108712262-108
ENSG0000(	541	12.25033	chr2:2577	MIR4266		smallRNA	chr2:109313571-109
ENSG0000(	541	12.25033	chr2:2577	ENSG00000228528		lncRNA	chr2:104702650-104
ENSG0000(	541	12.25033	chr2:2577	SETD6P1		Pseudoger	chr2:108226015-108
ENSG0000(	541	12.25033	chr2:2577	MIR4771-1		smallRNA	chr2:111771061-111
ENSG0000(	541	12.25033	chr2:2577	SNRPGP9		Pseudoger	chr2:109251422-109
ENSG0000(	541	12.25033	chr2:2577	RPL22P12		Pseudoger	chr2:110633947-110
ENSG0000(	541	12.25033	chr2:2577	SULT1C2P1		Pseudoger	chr2:108322353-108
ENSG0000(	541	12.25033	chr2:2577	CENPNP2		Pseudoger	chr2:111742586-111
ENSG0000(	541	12.25033	chr2:2577	DPP10-AS2		lncRNA	chr2:114833830-114
ENSG0000(	541	12.25033	chr2:2577	IGKV1OR2-108		protein_c	chr2:113406396-113
ENSG0000(	541	12.25033	chr2:2577	ENSG00000229209		lncRNA	chr2:103109759-103
ENSG0000(	541	12.25033	chr2:2577	MIR1302-3		smallRNA	chr2:113582959-113
ENSG0000(	541	12.25033	chr2:2577	LINC01885		lncRNA	chr2:107382715-107
ENSG0000(	541	12.25033	chr2:2577	GCC2-AS1		lncRNA	chr2:108507515-108
ENSG0000(	541	12.25033	chr2:2577	SLC30A6P1		Pseudoger	chr2:111952144-111
ENSG0000(	541	12.25033	chr2:2577	ENSG00000227842		Pseudoger	chr2:111717508-111
ENSG0000(	541	12.25033	chr2:2577	ENSG00000227680		lncRNA	chr2:102987323-102
ENSG0000(	541	12.25033	chr2:2577	PPP1R2P5		Pseudoger	chr2:106940880-106
ENSG0000(	541	12.25033	chr2:2577	PAFAH1B1P2		Pseudoger	chr2:111383752-111

ENSG0000(	541	12.25033	chr2:2577	PLGLA		Pseudoger	chr2:106382171-106
ENSG0000(	541	12.25033	chr2:2577	RNU6-744P		smallRNA	chr2:113681111-113
ENSG0000(	541	12.25033	chr2:2577	RPS21P2		Pseudoger	chr2:106288803-106
ENSG0000(	541	12.25033	chr4:1865	ENSG00000248936		lncRNA	chr4:37588087-3758
ENSG0000(	541	12.25033	chr4:1865	RNU7-131P		smallRNA	chr4:33784935-3378
ENSG0000(	541	12.25033	chr2:2577	CRLF3P1		Pseudoger	chr2:103494241-103
ENSG0000(	541	12.25033	chr2:2577	ENSG00000221849		Pseudoger	chr2:103527620-103
ENSG0000(	541	12.25033	chr2:2577	RPL22P10		Pseudoger	chr2:106247369-106
ENSG0000(	541	12.25033	chr2:2577	RNU6-1180P		smallRNA	chr2:113086805-113
ENSG0000(	541	12.25033	chr2:2577	HMGB3P11		Pseudoger	chr2:104771282-104
ENSG0000(	541	12.25033	chr4:1865	LINC02484		lncRNA	chr4:34120090-3433
ENSG0000(	541	12.25033	chr2:2577	ENSG00000231536		lncRNA	chr2:110610916-110
ENSG0000(	541	12.25033	chr2:2577	BMS1P19		Pseudoger	chr2:109667128-109
ENSG0000(	541	12.25033	chr2:2577	AC016745.1		protein_c	chr2:113447415-113
ENSG0000(	541	12.25033	chr4:1865	ENSG00000249452		Pseudoger	chr4:33238239-3323
ENSG0000(	541	12.25033	chr2:2577	MIR4267		smallRNA	chr2:110069961-110
ENSG0000(	541	12.25033	chr2:2577	ENSG00000227992		Pseudoger	chr2:111203964-111
ENSG0000(	541	12.25033	chr4:1865	LINC02506		lncRNA	chr4:31997376-3222
ENSG0000(	541	12.25033	chr2:2577	ENSG00000269707		lncRNA	chr2:104853285-104
ENSG0000(	541	12.25033	chr2:2577	ENSG00000243389		lncRNA	chr2:112589040-112
ENSG0000(	541	12.25033	chr2:2577	ENSG00000230499		lncRNA	chr2:111195963-111
ENSG0000(	541	12.25033	chr2:2577	LINC01935		lncRNA	chr2:102967408-102
ENSG0000(	541	12.25033	chr2:2577	ENSG00000234389		lncRNA	chr2:102438713-102
ENSG0000(	541	12.25033	chr2:2577	ENSG00000230650		Pseudoger	chr2:110360608-110
ENSG0000(	541	12.25033	chr2:2577	RGPD4-AS1		lncRNA	chr2:107823063-107
ENSG0000(	541	12.25033	chr2:2577	ENSG00000235242		lncRNA	chr2:114122274-114
ENSG0000(	541	12.25033	chr2:2577	EEF1E1P1		Pseudoger	chr2:111887914-111
ENSG0000(	541	12.25033	chr2:2577	NDUFB4P6		Pseudoger	chr2:112286057-112
ENSG0000(	541	12.25033	chr2:2577	ACTP1		Pseudoger	chr2:108099110-108
ENSG0000(	541	12.25033	chr2:2577	PGM5P4		Pseudoger	chr2:113541937-113
ENSG0000(	541	12.25033	chr4:1865	ENSG00000247193		lncRNA	chr4:36244116-3627
ENSG0000(	541	12.25033	chr2:2577	RPL5P9		Pseudoger	chr2:110967834-110
ENSG0000(	541	12.25033	chr2:2577	AC013268.1		smallRNA	chr2:110126732-110
ENSG0000(	541	12.25033	chr2:2577	ENSG00000232001		lncRNA	chr2:106521563-106
ENSG0000(	541	12.25033	chr2:2577	RTRAFP1		Pseudoger	chr2:111940302-111
ENSG0000(	541	12.25033	chr2:2577	SNORA72		smallRNA	chr2:104733234-104
ENSG0000(	541	12.25033	chr2:2577	ENSG00000230690		lncRNA	chr2:104659337-104
ENSG0000(	541	12.25033	chr2:2577	snoU13		smallRNA	chr2:113848133-113
ENSG0000(	541	12.25033	chr2:2577	ENSG00000230696		lncRNA	chr2:109594693-109
ENSG0000(	541	12.25033	chr2:2577	ENSG00000237666		Pseudoger	chr2:106470799-106
ENSG0000(	541	12.25033	chr2:2577	ENSG00000225588		lncRNA	chr2:107362282-107
ENSG0000(	541	12.25033	chr4:1865	Y_RNA		smallRNA	chr4:37699895-3770
ENSG0000(	541	12.25033	chr4:1865	ENSG00000248466		Pseudoger	chr4:36065060-3606
ENSG0000(	541	12.25033	chr4:1865	SEC63P2		Pseudoger	chr4:35487812-3548
ENSG0000(	541	12.25033	chr4:1865	DTHD1	NCGv7	protein_c	chr4:36281616-3634
ENSG0000(	541	12.25033	chr4:1865	LINC02353		lncRNA	chr4:32351038-3235
ENSG0000(	541	12.25033	chr2:2577	ENSG00000257207		protein_c	chr2:110402934-110
ENSG0000(	541	12.25033	chr4:1865	LINC02505		lncRNA	chr4:36496128-3664
ENSG0000(	541	12.25033	chr2:2577	snoU13		smallRNA	chr2:112271271-112
ENSG0000(	541	12.25033	chr4:1865	MIR4801		smallRNA	chr4:37241910-3724
ENSG0000(	541	12.25033	chr2:2577	RNU2-41P		smallRNA	chr2:114420125-114

ENSG0000(	541	12.25033	chr4:1865	snoU13	smallRNA	chr4:34966241-3496
ENSG0000(	541	12.25033	chr4:1865	ENSG00000250723	lncRNA	chr4:33850591-3398
ENSG0000(	541	12.25033	chr2:2577	DPP10-AS1	lncRNA	chr2:115126622-115
ENSG0000(	541	12.25033	chr2:2577	AC010884.2	smallRNA	chr2:104992902-104
ENSG0000(	541	12.25033	chr2:2577	LINC02966	lncRNA	chr2:113325009-113
ENSG0000(	541	12.25033	chr2:2577	CD8B2	protein_c	chr2:106487364-106
ENSG0000(	541	12.25033	chr2:2577	HMGN2P23	Pseudoger	chr2:112981166-112
ENSG0000(	541	12.25033	chr4:1865	AC093786.1	smallRNA	chr4:34386754-3438
ENSG0000(	541	12.25033	chr2:2577	RP11-65I12.1	lncRNA	chr2:113240730-113
ENSG0000(	541	12.25033	chr2:2577	SULT1C2P2	Pseudoger	chr2:108317725-108
ENSG0000(	541	12.25033	chr2:2577	MIR4436B2	smallRNA	chr2:110284853-110
ENSG0000(	541	12.25033	chr4:1865	ENSG00000247810	lncRNA	chr4:37073681-3713
ENSG0000(	541	12.25033	chr2:2577	LINC01594	lncRNA	chr2:108167125-108
ENSG0000(	541	12.25033	chr2:2577	RPL22P8	Pseudoger	chr2:107914948-107
ENSG0000(	541	12.25033	chr2:2577	SLC20A1-DT	lncRNA	chr2:112641832-112
ENSG0000(	541	12.25033	chr2:2577	ENSG00000236109	lncRNA	chr2:104583138-104
ENSG0000(	541	12.25033	chr2:2577	LINC01103	lncRNA	chr2:104488458-104
ENSG0000(	541	12.25033	chr2:2577	DPP10-AS3	lncRNA	chr2:114828432-114
ENSG0000(	541	12.25033	chr2:2577	ZBTB45P1	Pseudoger	chr2:109986939-109
ENSG0000(	541	12.25033	chr2:2577	Y_RNA	smallRNA	chr2:112579484-112
ENSG0000(	541	12.25033	chr2:2577	GPAA1P2	Pseudoger	chr2:110386377-110
ENSG0000(	541	12.25033	chr4:1865	ENSG00000250597	lncRNA	chr4:34657606-3466
ENSG0000(	541	12.25033	chr2:2577	ENSG00000230603	Pseudoger	chr2:109665209-109
ENSG0000(	541	12.25033	chr2:2577	snoU13	smallRNA	chr2:114281084-114
ENSG0000(	541	12.25033	chr2:2577	RPL23AP27	Pseudoger	chr2:104678223-104
ENSG0000(	541	12.25033	chr2:2577	LINC01191	lncRNA	chr2:113970719-114
ENSG0000(	541	12.25033	chr2:2577	GPAA1P1	Pseudoger	chr2:109984058-109
ENSG0000(	541	12.25033	chr2:2577	ST6GAL2-IT1	lncRNA	chr2:106822923-106
ENSG0000(	541	12.25033	chr2:2577	AHCYP3	Pseudoger	chr2:104395002-104
ENSG0000(	541	12.25033	chr2:2577	PGM5P4-AS1	lncRNA	chr2:113526836-113
ENSG0000(	541	12.25033	chr2:2577	ENSG00000229730	Pseudoger	chr2:110012265-110
ENSG0000(	541	12.25033	chr2:2577	RPL27AP4	Pseudoger	chr2:106304755-106
ENSG0000(	541	12.25033	chr2:2577	RPSAP23	Pseudoger	chr2:115468056-115
ENSG0000(	541	12.25033	chr2:2577	FAM183DP	Pseudoger	chr2:102249857-102
ENSG0000(	541	12.25033	chr2:2577	LINC01918	lncRNA	chr2:105144113-105
ENSG0000(	541	12.25033	chr2:2577	FAM138B	lncRNA	chr2:113577382-113
ENSG0000(	541	12.25033	chr2:2577	SOWAHC	protein_c	chr2:109614364-109
ENSG0000(	541	12.25033	chr2:2577	SEPHS1P7	Pseudoger	chr2:114260050-114
ENSG0000(	541	12.25033	chr2:2577	ENSG00000238273	lncRNA	chr2:105363038-105
ENSG0000(	541	12.25033	chr4:1865	ENSG00000239983	Pseudoger	chr4:33968174-3396
ENSG0000(	541	12.25033	chr4:1865	ENSG00000250954	lncRNA	chr4:33775498-3403
ENSG0000(	541	12.25033	chr2:2577	ENSG00000236525	lncRNA	chr2:102433957-102
ENSG0000(	541	12.25033	chr2:2577	ENSG00000243179	lncRNA	chr2:113979909-113
ENSG0000(	541	12.25033	chr2:2577	RPL22P11	Pseudoger	chr2:109737139-109
ENSG0000(	541	12.25033	chr2:2577	EEF1A1P12	Pseudoger	chr2:106697331-106
ENSG0000(	541	12.25033	chr2:2577	ENSG00000229682	Pseudoger	chr2:106368209-106
ENSG0000(	541	12.25033	chr2:2577	ENSG00000231505	lncRNA	chr2:106521903-106
ENSG0000(	541	12.25033	chr2:2577	MIR4772	smallRNA	chr2:102432289-102
ENSG0000(	541	12.25033	chr2:2577	ENSG00000236141	lncRNA	chr2:103856970-103
ENSG0000(	541	12.25033	chr2:2577	ENSG00000233648	Pseudoger	chr2:108694750-108
ENSG0000(	541	12.25033	chr4:1865	MAPRE1P2	Pseudoger	chr4:33010948-3301

ENSG0000(	541	12.25033	chr2:2577	PANTR1	lncRNA	chr2:104764932-104
ENSG0000(	541	12.25033	chr2:2577	ENSG00000232597	lncRNA	chr2:103855829-103
ENSG0000(	541	12.25033	chr2:2577	LINC01965	lncRNA	chr2:103874236-104
ENSG0000(	541	12.25033	chr2:2577	POLR2DP1	Pseudoger	chr2:112995517-112
ENSG0000(	541	12.25033	chr4:1865	RNU6-573P	smallRNA	chr4:35495898-3549
ENSG0000(	541	12.25033	chr2:2577	ENSG00000225744	lncRNA	chr2:111607841-111
ENSG0000(	541	12.25033	chr2:2577	AC012360.1	protein_c	chr2:105251302-105
ENSG0000(	541	12.25033	chr2:2577	LINC01831	lncRNA	chr2:104412145-104
ENSG0000(	541	12.25033	chr2:2577	LINC01114	lncRNA	chr2:104746638-104
ENSG0000(	541	12.25033	chr2:2577	ENSG00000234174	lncRNA	chr2:113171535-113
ENSG0000(	541	12.25033	chr2:2577	ENSG00000234162	lncRNA	chr2:105874509-105
ENSG0000(	541	12.25033	chr2:2577	LINC01961	lncRNA	chr2:113512222-113
ENSG0000(	541	12.25033	chr2:2577	ENSG00000244063	lncRNA	chr2:113829390-113
ENSG0000(	541	12.25033	chr2:2577	AC140479.1	smallRNA	chr2:110301312-110
ENSG0000(	541	12.25033	chr2:2577	LINC01593	lncRNA	chr2:108049200-108
ENSG0000(	541	12.25033	chr2:2577	DDX11L2	Pseudoger	chr2:113599036-113
ENSG0000(	541	12.25033	chr2:2577	LINC01886	lncRNA	chr2:107529292-107
ENSG0000(	541	12.25033	chr2:2577	LIMS4	protein_c	chr2:110445883-110
ENSG0000(	541	12.25033	chr2:2577	ENSG00000235319	lncRNA	chr2:105324210-105
ENSG0000(	541	12.25033	chr2:2577	MRPS9-AS2	lncRNA	chr2:104936239-105
ENSG0000(	541	12.25033	chr2:2577	SRSF3P5	Pseudoger	chr2:107920763-107
ENSG0000(	541	12.25033	chr2:2577	U3	smallRNA	chr2:114005441-114
ENSG0000(	541	12.25033	chr2:2577	RPL34P8	Pseudoger	chr2:111675026-111
ENSG0000(	541	12.25033	chr2:2577	LINC01159	lncRNA	chr2:104865407-104
ENSG0000(	541	12.25033	chr2:2577	LIMS3	protein_c	chr2:109898428-109
ENSG0000(	541	12.25033	chr2:2577	SULT1C4	protein_c	chr2:108377911-108
ENSG0000(	541	12.25033	chr2:2577	RGPD4	protein_c	chr2:107826892-107
ENSG0000(	541	12.25033	chr2:2577	U3	smallRNA	chr2:104532773-104
ENSG0000(	541	12.25033	chr2:2577	ZBTB45P2	Pseudoger	chr2:110383112-110
ENSG0000(	541	12.25033	chr2:2577	AC108868.1	protein_c	chr2:106483272-106
ENSG0000(	541	12.25033	chr2:2577	GACAT1	lncRNA	chr2:107754112-107
ENSG0000(	541	12.25033	chr4:1865	NWD2	protein_c	chr4:37244743-3744
ENSG0000(	541	12.25033	chr2:2577	ENSG00000287793	lncRNA	chr2:104423039-104
ENSG0000(	541	12.25033	chr4:1865	ENSG00000288321	lncRNA	chr4:33467398-3369
ENSG0000(	541	12.25033	chr2:2577	MALLP1	Pseudoger	chr2:110279606-110
ENSG0000(	541	12.25033	chr4:1865	ENSG00000288073	lncRNA	chr4:37454698-3747
ENSG0000(	541	12.25033	chr2:2577	ENSG00000282304	lncRNA	chr2:110245591-110
ENSG0000(	541	12.25033	chr2:2577	ENSG00000282033	lncRNA	chr2:109758799-109
ENSG0000(	541	12.25033	chr4:1865	ENSG00000287968	lncRNA	chr4:33150326-3320
ENSG0000(	541	12.25033	chr2:2577	ENSG00000280878	lncRNA	chr2:111468810-111
ENSG0000(	541	12.25033	chr2:2577	ENSG00000287937	lncRNA	chr2:112817076-112
ENSG0000(	541	12.25033	chr2:2577	TMEM182	protein_c	chr2:102736905-103
ENSG0000(	541	12.25033	chr2:2577	LINC01796	lncRNA	chr2:102873183-102
ENSG0000(	541	12.25033	chr2:2577	SEPTIN10	protein_c	chr2:109542799-109
ENSG0000(	541	12.25033	chr2:2577	PSD4	protein_c	chr2:113157325-113
ENSG0000(	541	12.25033	chr2:2577	ENSG00000286769	lncRNA	chr2:107698737-107
ENSG0000(	541	12.25033	chr2:2577	ENSG00000286776	lncRNA	chr2:115079354-115
ENSG0000(	541	12.25033	chr2:2577	ENSG00000272563	lncRNA	chr2:113432600-113
ENSG0000(	541	12.25033	chr2:2577	LINC01106	lncRNA	chr2:110375138-110
ENSG0000(	541	12.25033	chr2:2577	MTLN	protein_c	chr2:110211529-110
ENSG0000(	541	12.25033	chr2:2577	ENSG00000279904	TEC	chr2:104706612-104

ENSG0000(	541	12.25033	chr2:2577	DDX11L17		Pseudoger	chr2:113599784-113
ENSG0000(	541	12.25033	chr2:2577	ACRP1		Pseudoger	chr2:113667478-113
ENSG0000(	541	12.25033	chr2:2577	DPP10		protein_c	chr2:114442299-115
ENSG0000(	541	12.25033	chr4:1865	ENSG00000286784		lncRNA	chr4:32583855-3274
ENSG0000(	541	12.25033	chr2:2577	LIMS1		protein_c	chr2:108533671-108
ENSG0000(	541	12.25033	chr2:2577	BUB1		protein_c	chr2:110637528-110
ENSG0000(	541	12.25033	chr2:2577	RGPD8		protein_c	chr2:112368369-112
ENSG0000(	541	12.25033	chr2:2577	ENSG00000286545		lncRNA	chr2:112239621-112
ENSG0000(	541	12.25033	chr2:2577	DBF4P2		Pseudoger	chr2:111558413-111
ENSG0000(	541	12.25033	chr2:2577	POLR1B		protein_c	chr2:112541915-112
ENSG0000(	541	12.25033	chr2:2577	PAX8	AC	protein_c	chr2:113215997-113
ENSG0000(	541	12.25033	chr2:2577	ENSG00000289035		lncRNA	chr2:113370392-113
ENSG0000(	541	12.25033	chr2:2577	CHCHD5		protein_c	chr2:112584240-112
ENSG0000(	541	12.25033	chr2:2577	IL37		protein_c	chr2:112911165-112
ENSG0000(	541	12.25033	chr2:2577	IL1B	NCGv7	protein_c	chr2:112829751-112
ENSG0000(	541	12.25033	chr2:2577	FOXD4L1	IntOGen-I	protein_c	chr2:113498665-113
ENSG0000(	541	12.25033	chr2:2577	ENSG00000286290		lncRNA	chr2:103362082-103
ENSG0000(	541	12.25033	chr2:2577	SLC9A4		protein_c	chr2:102473226-102
ENSG0000(	541	12.25033	chr2:2577	XIAPP3		Pseudoger	chr2:112853328-112
ENSG0000(	541	12.25033	chr2:2577	VINAC1P		Pseudoger	chr2:112439312-112
ENSG0000(	541	12.25033	chr2:2577	ENSG00000283684		lncRNA	chr2:109937438-109
ENSG0000(	541	12.25033	chr2:2577	ENSG00000283283		Pseudoger	chr2:110022447-110
ENSG0000(	541	12.25033	chr2:2577	ENSG00000290733		lncRNA	chr2:110402914-110
ENSG0000(	541	12.25033	chr2:2577	ENSG00000290732		lncRNA	chr2:110231106-110
ENSG0000(	541	12.25033	chr2:2577	ENSG00000286513		lncRNA	chr2:114114679-114
ENSG0000(	541	12.25033	chr2:2577	ENSG00000290731		lncRNA	chr2:109933161-109
ENSG0000(	541	12.25033	chr2:2577	ENSG00000288565		lncRNA	chr2:104656135-104
ENSG0000(	541	12.25033	chr2:2577	ENSG00000283167		Pseudoger	chr2:110321402-110
ENSG0000(	541	12.25033	chr2:2577	CKAP2L		protein_c	chr2:112736349-112
ENSG0000(	541	12.25033	chr2:2577	ENSG00000278962		TEC	chr2:112188364-112
ENSG0000(	541	12.25033	chr2:2577	ST6GAL2	NCGv7	protein_c	chr2:106801600-106
ENSG0000(	541	12.25033	chr2:2577	NPHP1		protein_c	chr2:110122311-110
ENSG0000(	541	12.25033	chr2:2577	IL18RAP	NCGv7	protein_c	chr2:102418689-102
ENSG0000(	541	12.25033	chr2:2577	IL1RL1	NCGv7	protein_c	chr2:102311502-102
ENSG0000(	541	12.25033	chr2:2577	IL1RL2		protein_c	chr2:102187006-102
ENSG0000(	541	12.25033	chr2:2577	IL1R1		protein_c	chr2:102064544-102
ENSG0000(	541	12.25033	chr2:2577	ACTR3		protein_c	chr2:113890063-113
ENSG0000(	541	12.25033	chr2:2577	SLC35F5		protein_c	chr2:113705011-113
ENSG0000(	541	12.25033	chr2:2577	IL1A	NCGv7	protein_c	chr2:112773925-112
ENSG0000(	541	12.25033	chr2:2577	TTL	AC	protein_c	chr2:112482156-112
ENSG0000(	541	12.25033	chr2:2577	ENSG00000280228		TEC	chr2:112840328-112
ENSG0000(	541	12.25033	chr2:2577	C2orf49-DT		lncRNA	chr2:105333935-105
ENSG0000(	541	12.25033	chr2:2577	ENSG00000287451		lncRNA	chr2:115835129-115
ENSG0000(	541	12.25033	chr2:2577	ENSG00000287135		lncRNA	chr2:104928803-104
ENSG0000(	541	12.25033	chr2:2577	ENSG00000287141		lncRNA	chr2:112061084-112
ENSG0000(	541	12.25033	chr2:2577	ENSG00000186148		Pseudoger	chr2:109947672-109
ENSG0000(	541	12.25033	chr2:2577	ENSG00000287165		lncRNA	chr2:113582354-113
ENSG0000(	541	12.25033	chr2:2577	CCDC138		protein_c	chr2:108786757-108
ENSG0000(	541	12.25033	chr4:1865	ENSG00000287416		lncRNA	chr4:36902242-3691
ENSG0000(	541	12.25033	chr2:2577	MIR4435-2HG		lncRNA	chr2:111006015-111
ENSG0000(	541	12.25033	chr2:2577	WASH2P		lncRNA	chr2:113583593-113

ENSG0000(	541	12.25033	chr2:2577	WASH2P		Pseudoger	chr2:113588550-113
ENSG0000(	541	12.25033	chr2:2577	ENSG00000291125		lncRNA	chr2:106382110-106
ENSG0000(	541	12.25033	chr4:1865	ENSG00000287320		lncRNA	chr4:34140598-3418
ENSG0000(	541	12.25033	chr2:2577	IL18R1		protein_c	chr2:102311529-102
ENSG0000(	541	12.25033	chr2:2577	SLC9A2		protein_c	chr2:102619553-102
ENSG0000(	541	12.25033	chr2:2577	MALL		protein_c	chr2:110083870-110
ENSG0000(	541	12.25033	chr2:2577	FHL2	AC	protein_c	chr2:105357712-105
ENSG0000(	541	12.25033	chr2:2577	NT5DC4		protein_c	chr2:112721020-112
ENSG0000(	541	12.25033	chr2:2577	ENSG00000270190		lncRNA	chr2:111266868-111
ENSG0000(	541	12.25033	chr2:2577	RABL2A		protein_c	chr2:113627229-113
ENSG0000(	541	12.25033	chr2:2577	SLC20A1		protein_c	chr2:112645939-112
ENSG0000(	541	12.25033	chr2:2577	FBLN7		protein_c	chr2:112138385-112
ENSG0000(	541	12.25033	chr2:2577	SNRPA1P1		Pseudoger	chr2:113657908-113
ENSG0000(	541	12.25033	chr2:2577	ZC3H8		protein_c	chr2:112211529-112
ENSG0000(	541	12.25033	chr2:2577	ENSG00000286904		lncRNA	chr2:112039378-112
ENSG0000(	541	12.25033	chr2:2577	ECRG4		protein_c	chr2:106063246-106
ENSG0000(	541	12.25033	chr2:2577	WASH9P		Pseudoger	chr2:113588555-113
ENSG0000(	541	12.25033	chr2:2577	ENSG00000273595		Pseudoger	chr2:106207394-106
ENSG0000(	541	12.25033	chr2:2577	RGPD5		protein_c	chr2:109792758-109
ENSG0000(	541	12.25033	chr2:2577	ENSG00000273471		lncRNA	chr2:110386411-110
ENSG0000(	541	12.25033	chr2:2577	ENSG00000279957		TEC	chr2:113904086-113
ENSG0000(	541	12.25033	chr2:2577	ENSG00000290119		lncRNA	chr2:105790035-105
ENSG0000(	541	12.25033	chr2:2577	LINC02946		lncRNA	chr2:105600703-105
ENSG0000(	541	12.25033	chr2:2577	NCK2		protein_c	chr2:105744912-105
ENSG0000(	541	12.25033	chr2:2577	ENSG00000272861		lncRNA	chr2:105249404-105
ENSG0000(	541	12.25033	chr2:2577	SLC5A7		protein_c	chr2:107986523-108
ENSG0000(	541	12.25033	chr2:2577	ZC3H6		protein_c	chr2:112275597-112
ENSG0000(	541	12.25033	chr2:2577	UXS1		protein_c	chr2:106093308-106
ENSG0000(	541	12.25033	chr2:2577	ENSG00000284337		protein_c	chr2:109898432-109
ENSG0000(	541	12.25033	chr2:2577	ENSG00000287308		lncRNA	chr2:105601146-105
ENSG0000(	541	12.25033	chr2:2577	ENSG00000286218		lncRNA	chr2:106933739-106
ENSG0000(	541	12.25033	chr2:2577	MRPS9		protein_c	chr2:105038069-105
ENSG0000(	541	12.25033	chr2:2577	IL1F10		protein_c	chr2:113067970-113
ENSG0000(	541	12.25033	chr2:2577	IL36B		protein_c	chr2:113022089-113
ENSG0000(	541	12.25033	chr2:2577	ACOXL	NCGv7	protein_c	chr2:110732539-111
ENSG0000(	541	12.25033	chr2:2577	GMCL1P2		Pseudoger	chr2:108367116-108
ENSG0000(	541	12.25033	chr2:2577	BCL2L11	NCGv7	protein_c	chr2:111119378-111
ENSG0000(	541	12.25033	chr2:2577	SMIM12P1		Pseudoger	chr2:108423612-108
ENSG0000(	541	12.25033	chr2:2577	IL36RN		protein_c	chr2:113058638-113
ENSG0000(	541	12.25033	chr2:2577	IL36A	NCGv7	protein_c	chr2:113005459-113
ENSG0000(	541	12.25033	chr4:1865	ARAP2		protein_c	chr4:35948221-3624
ENSG0000(	541	12.25033	chr2:2577	ANAPC1		protein_c	chr2:111611639-111
ENSG0000(	541	12.25033	chr2:2577	TMEM87B		protein_c	chr2:112055269-112
ENSG0000(	541	12.25033	chr2:2577	MFSD9		protein_c	chr2:102714630-102
ENSG0000(	541	12.25033	chr2:2577	IL1RN		protein_c	chr2:113099315-113
ENSG0000(	541	12.25033	chr2:2577	EDAR	NCGv7	protein_c	chr2:108894471-108
ENSG0000(	541	12.25033	chr2:2577	IL36G		protein_c	chr2:112973203-112
ENSG0000(	541	12.25033	chr2:2577	TGFBRAP1		protein_c	chr2:105264391-105
ENSG0000(	541	12.25033	chr2:2577	RGPD3	NCGv7	protein_c	chr2:106391290-106
ENSG0000(	541	12.25033	chr2:2577	CBWD2		protein_c	chr2:113437691-113
ENSG0000(	541	12.25033	chr2:2577	MERTK	TAG;AC	protein_c	chr2:111898607-112

ENSG0000(	541	12.25033	chr2:2577	RGPD6		protein_c	chr2:110513802-110
ENSG0000(	541	12.25033	chr2:2577	ENSG00000279267		TEC	chr2:113605867-113
ENSG0000(	541	12.25033	chr2:2577	GCC2	NCGv7	protein_c	chr2:108449107-108
ENSG0000(	541	12.25033	chr2:2577	RANBP2	NCGv7	protein_c	chr2:108719482-108
ENSG0000(	541	12.25033	chr2:2577	ENSG00000289498		lncRNA	chr2:104860752-104
ENSG0000(	541	12.25033	chr2:2577	PAX8-AS1		lncRNA	chr2:113211421-113
ENSG0000(	541	12.25033	chr2:2577	ENSG00000270019		lncRNA	chr2:113888203-113
ENSG0000(	541	12.25033	chr2:2577	GPR45		protein_c	chr2:105241743-105
ENSG0000(	541	12.25033	chr2:2577	ENSG00000285016		lncRNA	chr2:111429324-111
ENSG0000(	541	12.25033	chr2:2577	C2orf49		protein_c	chr2:105337532-105
ENSG0000(	541	12.25033	chr4:1865	ENSG00000275250		Pseudoger	chr4:34714270-3471
ENSG0000(	541	12.25033	chr4:1865	C4orf19		protein_c	chr4:37453925-3762
ENSG0000(	541	12.25033	chr4:1865	ENSG00000286212		lncRNA	chr4:32005076-3202
ENSG0000(	541	12.25033	chr2:2577	ENSG00000271590		lncRNA	chr2:111210995-111
ENSG0000(	541	12.25033	chr2:2577	ENSG00000289202		lncRNA	chr2:110678205-110
ENSG0000(	541	12.25033	chr4:1865	RELL1		protein_c	chr4:37590800-3768
ENSG0000(	541	12.25033	chr2:2577	ENSG00000184115		Pseudoger	chr2:110397406-110
ENSG0000(	541	12.25033	chr2:2577	SULT1C2P1		lncRNA	chr2:108322238-108
ENSG0000(	540	12.22769	chr7:2516	CYCSP19		Pseudoger	chr7:121398452-121
ENSG0000(	540	12.22769	chr7:2516	IQUB	DriverDBv	protein_c	chr7:123452193-123
ENSG0000(	540	12.22769	chr7:2516	ENSG00000289578		lncRNA	chr7:121450948-121
ENSG0000(	540	12.22769	chr7:2516	WNT16		protein_c	chr7:121325367-121
ENSG0000(	540	12.22769	chr7:2516	LYPLA1P1		Pseudoger	chr7:123230120-123
ENSG0000(	540	12.22769	chr7:2516	TSPAN12	DriverDBv	protein_c	chr7:120787320-120
ENSG0000(	540	12.22769	chr7:2516	FEZF1	DriverDBv	protein_c	chr7:122301303-122
ENSG0000(	540	12.22769	chr7:2516	RNU6-296P		smallRNA	chr7:123457988-123
ENSG0000(	540	12.22769	chr7:2516	ENSG00000213302		Pseudoger	chr7:122234531-122
ENSG0000(	540	12.22769	chr7:2516	FEZF1-AS1		lncRNA	chr7:122303658-122
ENSG0000(	540	12.22769	chr7:2516	ENSG00000227371		Pseudoger	chr7:121419072-121
ENSG0000(	540	12.22769	chr7:2516	ENSG00000226636		Pseudoger	chr7:122159300-122
ENSG0000(	540	12.22769	chr7:2516	CPED1		protein_c	chr7:120988697-121
ENSG0000(	540	12.22769	chr7:2516	SLC13A1	NCGv7	protein_c	chr7:123113531-123
ENSG0000(	540	12.22769	chr7:2516	TAS2R16		protein_c	chr7:122994704-122
ENSG0000(	540	12.22769	chr7:2516	ENSG00000234418		lncRNA	chr7:122144405-122
ENSG0000(	540	12.22769	chr7:2516	ENSG00000225795		Pseudoger	chr7:122676580-122
ENSG0000(	540	12.22769	chr7:2516	ENSG00000234985		Pseudoger	chr7:121440834-121
ENSG0000(	540	12.22769	chr7:2516	RNU6-517P		smallRNA	chr7:121194948-121
ENSG0000(	540	12.22769	chr7:2516	PTPRZ1		protein_c	chr7:121873089-122
ENSG0000(	540	12.22769	chr7:2516	ENSG00000226680		lncRNA	chr7:123069249-123
ENSG0000(	540	12.22769	chr7:2516	RN7SKP277		smallRNA	chr7:121736443-121
ENSG0000(	540	12.22769	chr7:2516	FAM3C		protein_c	chr7:121348878-121
ENSG0000(	540	12.22769	chr7:2516	HMGN1P18		Pseudoger	chr7:121050927-121
ENSG0000(	540	12.22769	chr7:2516	ENSG00000233969		lncRNA	chr7:120166443-120
ENSG0000(	540	12.22769	chr7:2516	ENSG00000231295		lncRNA	chr7:120746738-120
ENSG0000(	540	12.22769	chr7:2516	RNU6-581P		smallRNA	chr7:120672871-120
ENSG0000(	540	12.22769	chr7:2516	ENSG00000240499		lncRNA	chr7:122328469-122
ENSG0000(	540	12.22769	chr7:2516	RNF133		protein_c	chr7:122697735-122
ENSG0000(	540	12.22769	chr7:2516	AC004875.1		smallRNA	chr7:121574669-121
ENSG0000(	540	12.22769	chr7:2516	RNF148		protein_c	chr7:122701668-122
ENSG0000(	540	12.22769	chr7:2516	AASS		protein_c	chr7:122064583-122
ENSG0000(	540	12.22769	chr7:2516	ENSG00000287554		lncRNA	chr7:121304657-121

ENSG0000(	540	12.22769	chr7:251(	ENSG00000232524		lncRNA	chr7:123456629-123
ENSG0000(	540	12.22769	chr7:251(	PNPT1P2		Pseudoger	chr7:121842368-121
ENSG0000(	540	12.22769	chr7:251(	RNA5SP240		Pseudoger	chr7:120981426-120
ENSG0000(	540	12.22769	chr7:251(	RPS26P31		Pseudoger	chr7:122681315-122
ENSG0000(	540	12.22769	chr7:251(	CADPS2		protein_c	chr7:122318411-122
ENSG0000(	540	12.22769	chr7:251(	ENSG00000227743		lncRNA	chr7:121643334-121
ENSG0000(	540	12.22769	chr7:251(	ENSG00000233417		lncRNA	chr7:120141016-120
ENSG0000(	540	12.22769	chr7:251(	RNA5SP241		Pseudoger	chr7:121083700-121
ENSG0000(	540	12.22769	chr7:251(	ENSG00000227573		Pseudoger	chr7:122849746-122
ENSG0000(	540	12.22769	chr7:251(	ING3		protein_c	chr7:120950763-120
ENSG0000(	540	12.22769	chr7:251(	KCND2		protein_c	chr7:120273175-120
ENSG0000(	540	12.22769	chr7:251(	RNU7-154P		smallRNA	chr7:122081720-122
ENSG0000(	538	12.1824	chr1:146!	SLAMF6P1		Pseudoger	chr1:162445549-162
ENSG0000(	538	12.1824	chr1:146!	DDR2	NCGv7	protein_c	chr1:162631373-162
ENSG0000(	538	12.1824	chr1:146!	SH2D1B		protein_c	chr1:162395268-162
ENSG0000(	538	12.1824	chr1:146!	AL390119.1		smallRNA	chr1:164983902-164
ENSG0000(	538	12.1824	chr1:146!	RNU6-171P		smallRNA	chr1:164639565-164
ENSG0000(	538	12.1824	chr1:146!	LRRC52		protein_c	chr1:165544000-165
ENSG0000(	538	12.1824	chr1:146!	RGS5		lncRNA	chr1:163244505-163
ENSG0000(	538	12.1824	chr1:146!	ENSG00000236206		lncRNA	chr1:165598356-165
ENSG0000(	538	12.1824	chr1:146!	RGS5-AS1		lncRNA	chr1:163161675-163
ENSG0000(	538	12.1824	chr1:146!	LMX1A-AS2		lncRNA	chr1:165210627-165
ENSG0000(	538	12.1824	chr1:146!	ENSG00000236364		lncRNA	chr1:165889725-165
ENSG0000(	538	12.1824	chr1:146!	RNU6-755P		smallRNA	chr1:164980035-164
ENSG0000(	538	12.1824	chr1:146!	ENSG00000229588		lncRNA	chr1:166165852-166
ENSG0000(	538	12.1824	chr1:146!	TMCO1-AS1		lncRNA	chr1:165768929-165
ENSG0000(	538	12.1824	chr1:146!	CCDC190		protein_c	chr1:162824458-162
ENSG0000(	538	12.1824	chr1:146!	LRRC52-AS1		lncRNA	chr1:165476833-165
ENSG0000(	538	12.1824	chr1:146!	HMGB3P6		Pseudoger	chr1:164356767-164
ENSG0000(	538	12.1824	chr1:146!	RNA5SP64		Pseudoger	chr1:166042244-166
ENSG0000(	538	12.1824	chr1:146!	ENSG00000273365		lncRNA	chr1:165706556-165
ENSG0000(	538	12.1824	chr1:146!	UAP1	NCGv7	protein_c	chr1:162561722-162
ENSG0000(	538	12.1824	chr1:146!	RGS4		protein_c	chr1:163068775-163
ENSG0000(	538	12.1824	chr1:146!	SNORD112		smallRNA	chr1:165072473-165
ENSG0000(	538	12.1824	chr1:146!	PBX1	NCGv7;AC	protein_c	chr1:164555584-164
ENSG0000(	538	12.1824	chr1:146!	ENSG00000225272		Pseudoger	chr1:165676310-165
ENSG0000(	538	12.1824	chr1:146!	ENSG00000269887		lncRNA	chr1:164680085-164
ENSG0000(	538	12.1824	chr1:146!	U3		smallRNA	chr1:163923670-163
ENSG0000(	538	12.1824	chr1:146!	RNA5SP63		Pseudoger	chr1:163509484-163
ENSG0000(	538	12.1824	chr1:146!	RPL35AP7		Pseudoger	chr1:164921318-164
ENSG0000(	538	12.1824	chr1:146!	ENSG00000225122		Pseudoger	chr1:163422405-163
ENSG0000(	538	12.1824	chr1:146!	HSD17B7		protein_c	chr1:162790702-162
ENSG0000(	538	12.1824	chr1:146!	ENSG00000289713		Pseudoger	chr1:162441192-162
ENSG0000(	538	12.1824	chr1:146!	RNU5F-6P		smallRNA	chr1:164351273-164
ENSG0000(	538	12.1824	chr1:146!	Y_RNA		smallRNA	chr1:165662585-165
ENSG0000(	538	12.1824	chr1:146!	ENSG00000230175		Pseudoger	chr1:165671256-165
ENSG0000(	538	12.1824	chr1:146!	RGS5		protein_c	chr1:163111121-163
ENSG0000(	538	12.1824	chr1:146!	ENSG00000237783		Pseudoger	chr1:165581613-165
ENSG0000(	538	12.1824	chr1:146!	PBX1-AS1		lncRNA	chr1:164769116-164
ENSG0000(	538	12.1824	chr1:146!	ENSG00000228289		Pseudoger	chr1:163769339-163
ENSG0000(	538	12.1824	chr1:146!	ENSG00000230659		Pseudoger	chr1:165819353-165

ENSG0000(	538	12.1824	chr1:146{	LMX1A-AS1		lncRNA	chr1:165215951-165
ENSG0000(	538	12.1824	chr1:146{	SNORD112		smallRNA	chr1:163385865-163
ENSG0000(	538	12.1824	chr1:146{	Y_RNA		smallRNA	chr1:164854231-164
ENSG0000(	538	12.1824	chr1:146{	LMX1A	NCGv7	protein_c	chr1:165201867-165
ENSG0000(	538	12.1824	chr1:146{	ENSG00000230739		Pseudoger	chr1:162824795-162
ENSG0000(	538	12.1824	chr1:146{	MIR921		smallRNA	chr1:166154743-166
ENSG0000(	538	12.1824	chr1:146{	PRELID1P7		Pseudoger	chr1:165497724-165
ENSG0000(	538	12.1824	chr1:146{	ENSG00000215838		Pseudoger	chr1:165698750-165
ENSG0000(	538	12.1824	chr1:146{	ENSG00000215835		Pseudoger	chr1:166275629-166
ENSG0000(	538	12.1824	chr1:146{	ENSG00000271527		Pseudoger	chr1:165941235-165
ENSG0000(	538	12.1824	chr1:146{	UAP1-DT		lncRNA	chr1:162560227-162
ENSG0000(	538	12.1824	chr1:146{	ENSG00000289408		lncRNA	chr1:164900169-164
ENSG0000(	538	12.1824	chr1:146{	FAM78B	NCGv7	protein_c	chr1:166057426-166
ENSG0000(	538	12.1824	chr1:146{	ENSG00000230898		lncRNA	chr1:166147782-166
ENSG0000(	538	12.1824	chr1:146{	UQCRBP2		Pseudoger	chr1:162541332-162
ENSG0000(	538	12.1824	chr1:146{	ENSG00000271917		lncRNA	chr1:164828436-164
ENSG0000(	538	12.1824	chr1:146{	ALDH9A1		protein_c	chr1:165662216-165
ENSG0000(	538	12.1824	chr1:146{	UHMK1		protein_c	chr1:162497251-162
ENSG0000(	538	12.1824	chr1:146{	RN7SL861P		smallRNA	chr1:162777730-162
ENSG0000(	538	12.1824	chr1:146{	RNA5SP62		Pseudoger	chr1:163468496-163
ENSG0000(	538	12.1824	chr1:146{	UCK2		protein_c	chr1:165827614-165
ENSG0000(	538	12.1824	chr1:146{	FAM78B-AS1		lncRNA	chr1:166081183-166
ENSG0000(	538	12.1824	chr1:146{	ENSG00000237756		lncRNA	chr1:163259850-163
ENSG0000(	538	12.1824	chr1:146{	NMNAT1P2		Pseudoger	chr1:164343005-164
ENSG0000(	538	12.1824	chr1:146{	ENSG00000272574		lncRNA	chr1:162593103-162
ENSG0000(	538	12.1824	chr1:146{	TMCO1		protein_c	chr1:165724293-165
ENSG0000(	538	12.1824	chr1:146{	NUF2	DriverDB\	protein_c	chr1:163266576-163
ENSG0000(	538	12.1824	chr1:146{	RXRG		protein_c	chr1:165400922-165
ENSG0000(	538	12.1824	chr1:146{	RPS3AP10		Pseudoger	chr1:166022215-166
ENSG0000(	538	12.1824	chr1:146{	ENSG00000227667		Pseudoger	chr1:162979551-162
ENSG0000(	538	12.1824	chr1:146{	MGST3		protein_c	chr1:165631213-165
ENSG0000(	538	12.1824	chr1:146{	ENSG00000225755		Pseudoger	chr1:163237214-163
ENSG0000(	535	12.11447	chr4:186{	ENSG00000286161		lncRNA	chr4:52712713-5272
ENSG0000(	535	12.11447	chr4:186{	PGM2		protein_c	chr4:37826660-3786
ENSG0000(	535	12.11447	chr4:186{	ATP8A1		protein_c	chr4:42408373-4265
ENSG0000(	535	12.11447	chr4:186{	RNU6-868P		smallRNA	chr4:48109353-4810
ENSG0000(	535	12.11447	chr4:186{	GLDCP1		Pseudoger	chr4:56593004-5659
ENSG0000(	535	12.11447	chr4:186{	POLR2B		protein_c	chr4:56977722-5703
ENSG0000(	535	12.11447	chr4:186{	ENSG00000248977		Pseudoger	chr4:40142198-4014
ENSG0000(	535	12.11447	chr4:186{	ENSG00000285454		lncRNA	chr4:42151028-4226
ENSG0000(	535	12.11447	chr4:186{	NDUFB4P12		Pseudoger	chr4:43898962-4389
ENSG0000(	535	12.11447	chr4:186{	ARL4AP2		Pseudoger	chr4:40786110-4078
ENSG0000(	535	12.11447	chr4:186{	RNU6-158P		smallRNA	chr4:48932755-4893
ENSG0000(	535	12.11447	chr4:186{	AC110298.1		smallRNA	chr4:54103580-5410
ENSG0000(	535	12.11447	chr4:186{	ENSG00000260918		lncRNA	chr4:47431960-4743
ENSG0000(	535	12.11447	chr4:186{	ENSG00000260878		lncRNA	chr4:46243548-4624
ENSG0000(	535	12.11447	chr4:186{	AC118282.1		smallRNA	chr4:49198207-4919
ENSG0000(	535	12.11447	chr4:186{	PRDX4P1		Pseudoger	chr4:44944015-4494
ENSG0000(	535	12.11447	chr4:186{	ENSG00000260120		lncRNA	chr4:52680609-5269
ENSG0000(	535	12.11447	chr4:186{	ENSG00000286294		lncRNA	chr4:47481001-4748
ENSG0000(	535	12.11447	chr4:186{	ENSG00000286097		lncRNA	chr4:59150924-5918

ENSG0000(	535	12.11447	chr4:186!	TBC1D1	AC	protein_c	chr4:37891084-3813
ENSG0000(	535	12.11447	chr4:186!	ENSG00000286349		lncRNA	chr4:39614465-3961
ENSG0000(	535	12.11447	chr4:186!	AC095061.1		smallRNA	chr4:60663949-6066
ENSG0000(	535	12.11447	chr4:186!	ENSG00000248583		Pseudoger	chr4:49486926-4948
ENSG0000(	535	12.11447	chr4:186!	ENSG00000287182		lncRNA	chr4:40743627-4075
ENSG0000(	535	12.11447	chr4:186!	ENSG00000248744		lncRNA	chr4:45009540-4505
ENSG0000(	535	12.11447	chr4:186!	EFL1P2		Pseudoger	chr4:65142703-6514
ENSG0000(	535	12.11447	chr4:186!	ENSG00000287174		lncRNA	chr4:59903404-5992
ENSG0000(	535	12.11447	chr4:186!	AC098869.1		smallRNA	chr4:40463716-4046
ENSG0000(	535	12.11447	chr4:186!	Y_RNA		smallRNA	chr4:39710085-3971
ENSG0000(	535	12.11447	chr4:186!	AC119751.3		smallRNA	chr4:49598423-4959
ENSG0000(	535	12.11447	chr4:186!	ENSG00000232471		Pseudoger	chr4:49550032-4955
ENSG0000(	535	12.11447	chr4:186!	RPL7AP31		Pseudoger	chr4:56356135-5635
ENSG0000(	535	12.11447	chr4:186!	RNU6-191P		smallRNA	chr4:64397694-6439
ENSG0000(	535	12.11447	chr4:186!	ENSG00000287262		lncRNA	chr4:39539395-3954
ENSG0000(	535	12.11447	chr4:186!	RNA5SP159		Pseudoger	chr4:39936753-3993
ENSG0000(	535	12.11447	chr4:186!	ENSG00000287060		lncRNA	chr4:55157877-5516
ENSG0000(	535	12.11447	chr4:186!	COMMD8		protein_c	chr4:47450787-4746
ENSG0000(	535	12.11447	chr4:186!	RNU6-699P		smallRNA	chr4:66897262-6689
ENSG0000(	535	12.11447	chr4:186!	LINC02835		lncRNA	chr4:65225867-6524
ENSG0000(	535	12.11447	chr4:186!	ATP8A1-DT		lncRNA	chr4:42657496-4265
ENSG0000(	535	12.11447	chr4:186!	ENSG00000242262		Pseudoger	chr4:47706372-4770
ENSG0000(	535	12.11447	chr4:186!	HOPX		protein_c	chr4:56647988-5668
ENSG0000(	535	12.11447	chr4:186!	TMEM156		protein_c	chr4:38966744-3903
ENSG0000(	535	12.11447	chr4:186!	PDS5A	NCGv7	protein_c	chr4:39822863-3997
ENSG0000(	535	12.11447	chr4:186!	PPAT		protein_c	chr4:56393362-5643
ENSG0000(	535	12.11447	chr4:186!	ENSG00000286093		lncRNA	chr4:56291601-5629
ENSG0000(	535	12.11447	chr4:186!	RPL22P13		Pseudoger	chr4:54221126-5422
ENSG0000(	535	12.11447	chr4:186!	RNU6-197P		smallRNA	chr4:56288485-5628
ENSG0000(	535	12.11447	chr4:186!	ENSG00000248847		Pseudoger	chr4:62489495-6249
ENSG0000(	535	12.11447	chr4:186!	ENSG00000286089		lncRNA	chr4:40187170-4019
ENSG0000(	535	12.11447	chr4:186!	ENSG00000242197		Pseudoger	chr4:40491733-4049
ENSG0000(	535	12.11447	chr4:186!	NFXL1		protein_c	chr4:47847233-4791
ENSG0000(	535	12.11447	chr4:186!	AC119751.1		smallRNA	chr4:49595610-4959
ENSG0000(	535	12.11447	chr4:186!	COX7B2		protein_c	chr4:46734827-4690
ENSG0000(	535	12.11447	chr4:186!	ENSG00000286891		lncRNA	chr4:43763134-4397
ENSG0000(	535	12.11447	chr4:186!	SRIP1		Pseudoger	chr4:58103147-5810
ENSG0000(	535	12.11447	chr4:186!	USP46-DT		lncRNA	chr4:52659406-5266
ENSG0000(	535	12.11447	chr4:186!	ENSG00000213851		Pseudoger	chr4:43410041-4341
ENSG0000(	535	12.11447	chr4:186!	ENSG00000285998		lncRNA	chr4:57841721-5785
ENSG0000(	535	12.11447	chr4:186!	RN7SKP82		smallRNA	chr4:42892396-4289
ENSG0000(	535	12.11447	chr4:186!	LIAS		protein_c	chr4:39459004-3948
ENSG0000(	535	12.11447	chr4:186!	AC119751.2		smallRNA	chr4:49598615-4959
ENSG0000(	535	12.11447	chr4:186!	ADGRL3-AS1		lncRNA	chr4:62071752-6216
ENSG0000(	535	12.11447	chr4:186!	GRXCR1		protein_c	chr4:42892713-4303
ENSG0000(	535	12.11447	chr4:186!	LINC02928		lncRNA	chr4:55373637-5538
ENSG0000(	535	12.11447	chr4:186!	RNU6-412P		smallRNA	chr4:46531237-4653
ENSG0000(	535	12.11447	chr4:186!	ENSG00000290817		lncRNA	chr4:56410642-5642
ENSG0000(	535	12.11447	chr4:186!	SNORA26		smallRNA	chr4:52748137-5274
ENSG0000(	535	12.11447	chr4:186!	SRD5A3		protein_c	chr4:55346213-5537
ENSG0000(	535	12.11447	chr4:186!	SPINK2		protein_c	chr4:56809860-5682

ENSG0000(	535	12.11447	chr4:1865	RHOH	NCGv7	protein_c	chr4:40191011-4024
ENSG0000(	535	12.11447	chr4:1865	SNORA26		smallRNA	chr4:52713249-5271
ENSG0000(	535	12.11447	chr4:1865	ENSG00000249892		lncRNA	chr4:59767816-5979
ENSG0000(	535	12.11447	chr4:1865	ENSG00000286599		lncRNA	chr4:55889743-5590
ENSG0000(	535	12.11447	chr4:1865	LIMCH1	NCGv7	protein_c	chr4:41359607-4170
ENSG0000(	535	12.11447	chr4:1865	RNU6-410P		smallRNA	chr4:55031965-5503
ENSG0000(	535	12.11447	chr4:1865	RASL11B		protein_c	chr4:52862317-5286
ENSG0000(	535	12.11447	chr4:1865	ENSG00000273369		lncRNA	chr4:44693946-4469
ENSG0000(	535	12.11447	chr4:1865	RNU7-11P		smallRNA	chr4:39621012-3962
ENSG0000(	535	12.11447	chr4:1865	ENSG00000260296		lncRNA	chr4:40166675-4016
ENSG0000(	535	12.11447	chr4:1865	PAICS		protein_c	chr4:56435741-5646
ENSG0000(	535	12.11447	chr4:1865	MTND3P22		Pseudoger	chr4:49246601-4924
ENSG0000(	535	12.11447	chr4:1865	ENSG00000248939		lncRNA	chr4:43133867-4323
ENSG0000(	535	12.11447	chr4:1865	KDR	NCGv7;AC	protein_c	chr4:55078481-5512
ENSG0000(	535	12.11447	chr4:1865	ENSG00000286664		lncRNA	chr4:64836361-6488
ENSG0000(	535	12.11447	chr4:1865	ENSG00000242431		Pseudoger	chr4:47490967-4749
ENSG0000(	535	12.11447	chr4:1865	RNU6-276P		smallRNA	chr4:55798636-5579
ENSG0000(	535	12.11447	chr4:1865	DPP3P1		Pseudoger	chr4:64430909-6443
ENSG0000(	535	12.11447	chr4:1865	UBE2K		protein_c	chr4:39698109-3978
ENSG0000(	535	12.11447	chr4:1865	snoU13		smallRNA	chr4:54123100-5412
ENSG0000(	535	12.11447	chr4:1865	N4BP2	NCGv7	protein_c	chr4:40056850-4015
ENSG0000(	535	12.11447	chr4:1865	Y_RNA		smallRNA	chr4:39441665-3944
ENSG0000(	535	12.11447	chr4:1865	ENSG00000250657		lncRNA	chr4:43340875-4334
ENSG0000(	535	12.11447	chr4:1865	MRPS33P2		Pseudoger	chr4:38006784-3800
ENSG0000(	535	12.11447	chr4:1865	ZBTB12BP		Pseudoger	chr4:39770081-3977
ENSG0000(	535	12.11447	chr4:1865	GSX2		protein_c	chr4:54099523-5410
ENSG0000(	535	12.11447	chr4:1865	LINC01259		lncRNA	chr4:38509729-3851
ENSG0000(	535	12.11447	chr4:1865	ENSG00000250646		lncRNA	chr4:55053060-5509
ENSG0000(	535	12.11447	chr4:1865	EXOC5P1		Pseudoger	chr4:62816826-6281
ENSG0000(	535	12.11447	chr4:1865	ENSG00000288944		lncRNA	chr4:56701545-5670
ENSG0000(	535	12.11447	chr4:1865	RNU6-931P		smallRNA	chr4:45480030-4548
ENSG0000(	535	12.11447	chr4:1865	ENSG00000249685		lncRNA	chr4:39133913-3913
ENSG0000(	535	12.11447	chr4:1865	RNA5SP161		Pseudoger	chr4:56097390-5609
ENSG0000(	535	12.11447	chr4:1865	snoU13		smallRNA	chr4:57492269-5749
ENSG0000(	535	12.11447	chr4:1865	ENSG00000250568		Pseudoger	chr4:39973128-3997
ENSG0000(	535	12.11447	chr4:1865	THEGL		protein_c	chr4:56530606-5660
ENSG0000(	535	12.11447	chr4:1865	RNU7-74P		smallRNA	chr4:40377452-4037
ENSG0000(	535	12.11447	chr4:1865	SRD5A3-AS1		lncRNA	chr4:55363971-5539
ENSG0000(	535	12.11447	chr4:1865	Y_RNA		smallRNA	chr4:55412636-5541
ENSG0000(	535	12.11447	chr4:1865	ENSG00000249706		lncRNA	chr4:53899871-5391
ENSG0000(	535	12.11447	chr4:1865	snoU13		smallRNA	chr4:52510013-5251
ENSG0000(	535	12.11447	chr4:1865	RNU6-652P		smallRNA	chr4:55885595-5588
ENSG0000(	535	12.11447	chr4:1865	ENSG00000249727		lncRNA	chr4:54836041-5484
ENSG0000(	535	12.11447	chr4:1865	ENSG00000250753		Pseudoger	chr4:49579833-4958
ENSG0000(	535	12.11447	chr4:1865	ENSG00000250769		Pseudoger	chr4:49507764-4950
ENSG0000(	535	12.11447	chr4:1865	ENSG00000251105		Pseudoger	chr4:54076625-5407
ENSG0000(	535	12.11447	chr4:1865	ENSG00000250775		lncRNA	chr4:63465511-6352
ENSG0000(	535	12.11447	chr4:1865	ANKRD20A17P		Pseudoger	chr4:49502145-4950
ENSG0000(	535	12.11447	chr4:1865	ENSG00000251055		lncRNA	chr4:63128311-6314
ENSG0000(	535	12.11447	chr4:1865	ENSG00000251049		lncRNA	chr4:57595940-5760
ENSG0000(	535	12.11447	chr4:1865	LINC02480		lncRNA	chr4:52044805-5204

ENSG0000(	535	12.11447	chr4:186⁵	snoU13		smallRNA	chr4:47305934-4730
ENSG0000(	535	12.11447	chr4:186⁵	snoU13		smallRNA	chr4:40868869-4086
ENSG0000(	535	12.11447	chr4:186⁵	LNX1-AS1		lncRNA	chr4:53496400-5354
ENSG0000(	535	12.11447	chr4:186⁵	ENSG00000250906		lncRNA	chr4:40812779-4082
ENSG0000(	535	12.11447	chr4:186⁵	ENSG00000250893		lncRNA	chr4:40426119-4042
ENSG0000(	535	12.11447	chr4:186⁵	NSUN7		protein_c	chr4:40749925-4081
ENSG0000(	535	12.11447	chr4:186⁵	ENSG00000271958		lncRNA	chr4:38618265-3861
ENSG0000(	535	12.11447	chr4:186⁵	snoU13		smallRNA	chr4:57106243-5710
ENSG0000(	535	12.11447	chr4:186⁵	ENSG00000250863		lncRNA	chr4:43972921-4402
ENSG0000(	535	12.11447	chr4:186⁵	AC118282.4		smallRNA	chr4:49209374-4920
ENSG0000(	535	12.11447	chr4:186⁵	ENSG00000288567		Pseudoger	chr4:62454245-6245
ENSG0000(	535	12.11447	chr4:186⁵	EPHA5-AS1		lncRNA	chr4:65669961-6569
ENSG0000(	535	12.11447	chr4:186⁵	AC110810.1		smallRNA	chr4:62595377-6259
ENSG0000(	535	12.11447	chr4:186⁵	snoU13		smallRNA	chr4:56994075-5699
ENSG0000(	535	12.11447	chr4:186⁵	Y_RNA		smallRNA	chr4:55501595-5550
ENSG0000(	535	12.11447	chr4:186⁵	ENSG00000288659		lncRNA	chr4:62133766-6222
ENSG0000(	535	12.11447	chr4:186⁵	EXOC1L		protein_c	chr4:55819790-5583
ENSG0000(	535	12.11447	chr4:186⁵	ENSG00000250815		Pseudoger	chr4:53927499-5392
ENSG0000(	535	12.11447	chr4:186⁵	ENSG00000250812		Pseudoger	chr4:55219344-5521
ENSG0000(	535	12.11447	chr4:186⁵	ENSG00000288695		protein_c	chr4:55346228-5538
ENSG0000(	535	12.11447	chr4:186⁵	ENSG00000250781		lncRNA	chr4:42281830-4239
ENSG0000(	535	12.11447	chr4:186⁵	ENSG00000249729		Pseudoger	chr4:44840380-4484
ENSG0000(	535	12.11447	chr4:186⁵	DUTP7		Pseudoger	chr4:51865050-5186
ENSG0000(	535	12.11447	chr4:186⁵	PHOX2B-AS1		lncRNA	chr4:41748293-4182
ENSG0000(	535	12.11447	chr4:186⁵	LRRC34P2		Pseudoger	chr4:55313011-5531
ENSG0000(	535	12.11447	chr4:186⁵	ENSG00000250249		Pseudoger	chr4:62291562-6229
ENSG0000(	535	12.11447	chr4:186⁵	EXOC1		protein_c	chr4:55853648-5590
ENSG0000(	535	12.11447	chr4:186⁵	ENSG00000250192		lncRNA	chr4:57154577-5715
ENSG0000(	535	12.11447	chr4:186⁵	MIR548AG1		smallRNA	chr4:60922619-6092
ENSG0000(	535	12.11447	chr4:186⁵	MTND5P13		Pseudoger	chr4:64609454-6461
ENSG0000(	535	12.11447	chr4:186⁵	MORF4L2P1		Pseudoger	chr4:54086926-5408
ENSG0000(	535	12.11447	chr4:186⁵	LINC02232		lncRNA	chr4:64885649-6502
ENSG0000(	535	12.11447	chr4:186⁵	ENSG00000249828		Pseudoger	chr4:49203171-4920
ENSG0000(	535	12.11447	chr4:186⁵	RN7SL357P		smallRNA	chr4:56805834-5680
ENSG0000(	535	12.11447	chr4:186⁵	ENSG00000289643		lncRNA	chr4:42285818-4229
ENSG0000(	535	12.11447	chr4:186⁵	RN7SL492P		smallRNA	chr4:56794350-5679
ENSG0000(	535	12.11447	chr4:186⁵	ENSG00000249831		lncRNA	chr4:57720035-5772
ENSG0000(	535	12.11447	chr4:186⁵	ENSG00000250078		lncRNA	chr4:58562160-5856
ENSG0000(	535	12.11447	chr4:186⁵	LINC02271		lncRNA	chr4:61143656-6115
ENSG0000(	535	12.11447	chr4:186⁵	RPS7P7		Pseudoger	chr4:42471810-4247
ENSG0000(	535	12.11447	chr4:186⁵	ELOCP33		Pseudoger	chr4:39932454-3993
ENSG0000(	535	12.11447	chr4:186⁵	SNX18P23		Pseudoger	chr4:49233289-4923
ENSG0000(	535	12.11447	chr4:186⁵	ENSG00000249863		Pseudoger	chr4:37868292-3786
ENSG0000(	535	12.11447	chr4:186⁵	ENSG00000289761		protein_c	chr4:41143022-4114
ENSG0000(	535	12.11447	chr4:186⁵	RAC1P2		Pseudoger	chr4:46723830-4672
ENSG0000(	535	12.11447	chr4:186⁵	AC084010.1		smallRNA	chr4:42445990-4244
ENSG0000(	535	12.11447	chr4:186⁵	ENSG00000249887		Pseudoger	chr4:41924180-4192
ENSG0000(	535	12.11447	chr4:186⁵	REST		protein_c	chr4:56907876-5696
ENSG0000(	535	12.11447	chr4:186⁵	NOA1		protein_c	chr4:56963350-5697
ENSG0000(	535	12.11447	chr4:186⁵	KCTD8	NCGv7	protein_c	chr4:44173903-4444
ENSG0000(	535	12.11447	chr4:186⁵	Y_RNA		smallRNA	chr4:59833175-5983

ENSG0000(	535	12.11447	chr4:186	PTTG2	AC	protein_c	chr4:37960398-3796
ENSG0000(	535	12.11447	chr4:186	RN7SL193P		smallRNA	chr4:43863274-4386
ENSG0000(	535	12.11447	chr4:186	LINC02494		lncRNA	chr4:58524515-5853
ENSG0000(	535	12.11447	chr4:186	LINC02260		lncRNA	chr4:54603211-5460
ENSG0000(	535	12.11447	chr4:186	ENSG00000289308		lncRNA	chr4:61749388-6176
ENSG0000(	535	12.11447	chr4:186	HMGB1P28		Pseudoger	chr4:41842154-4184
ENSG0000(	535	12.11447	chr4:186	Y_RNA		smallRNA	chr4:61906313-6190
ENSG0000(	535	12.11447	chr4:186	AC119751.5		smallRNA	chr4:49583025-4958
ENSG0000(	535	12.11447	chr4:186	UBE2CP3		Pseudoger	chr4:57072683-5707
ENSG0000(	535	12.11447	chr4:186	ENSG00000289393		lncRNA	chr4:56466900-5646
ENSG0000(	535	12.11447	chr4:186	ZAR1		protein_c	chr4:48490252-4849
ENSG0000(	535	12.11447	chr4:186	DCAF4L1		protein_c	chr4:41981756-4198
ENSG0000(	535	12.11447	chr4:186	ENSG00000250375		lncRNA	chr4:59047020-5907
ENSG0000(	535	12.11447	chr4:186	Y_RNA		smallRNA	chr4:48153434-4815
ENSG0000(	535	12.11447	chr4:186	RN7SL691P		smallRNA	chr4:43598542-4359
ENSG0000(	535	12.11447	chr4:186	RNA5SP160		Pseudoger	chr4:40990154-4099
ENSG0000(	535	12.11447	chr4:186	ENSG00000250338		lncRNA	chr4:40265472-4026
ENSG0000(	535	12.11447	chr4:186	ENSG00000250333		lncRNA	chr4:57605694-5765
ENSG0000(	535	12.11447	chr4:186	MRPL22P1		Pseudoger	chr4:56320719-5632
ENSG0000(	535	12.11447	chr4:186	ENSG00000249771		lncRNA	chr4:41883060-4193
ENSG0000(	535	12.11447	chr4:186	SNORA51		smallRNA	chr4:40082983-4008
ENSG0000(	535	12.11447	chr4:186	ENSG00000239532		Pseudoger	chr4:37821361-3782
ENSG0000(	535	12.11447	chr4:186	SCFD2		protein_c	chr4:52872982-5336
ENSG0000(	535	12.11447	chr4:186	RN7SL822P		smallRNA	chr4:55215626-5521
ENSG0000(	535	12.11447	chr4:186	LINC01618		lncRNA	chr4:52712394-5286
ENSG0000(	535	12.11447	chr4:186	RNU6-1325P		smallRNA	chr4:59834063-5983
ENSG0000(	535	12.11447	chr4:186	RPL12P20		Pseudoger	chr4:41389115-4138
ENSG0000(	535	12.11447	chr4:186	SHISA3		protein_c	chr4:42397488-4240
ENSG0000(	535	12.11447	chr4:186	FCF1P8		Pseudoger	chr4:55351812-5535
ENSG0000(	535	12.11447	chr4:186	ENSG00000249019		Pseudoger	chr4:39713842-3971
ENSG0000(	535	12.11447	chr4:186	ENSG00000251630		Pseudoger	chr4:49561285-4956
ENSG0000(	535	12.11447	chr4:186	COMMD5P1		Pseudoger	chr4:53575713-5357
ENSG0000(	535	12.11447	chr4:186	RNU6-1252P		smallRNA	chr4:52494849-5249
ENSG0000(	535	12.11447	chr4:186	TLR10		protein_c	chr4:38772238-3878
ENSG0000(	535	12.11447	chr4:186	TLR1		protein_c	chr4:38790677-3885
ENSG0000(	535	12.11447	chr4:186	ENSG00000249207		lncRNA	chr4:39066974-3918
ENSG0000(	535	12.11447	chr4:186	TLR6	NCGv7	protein_c	chr4:38822897-3885
ENSG0000(	535	12.11447	chr4:186	TMEM165	NCGv7	protein_c	chr4:55395957-5545
ENSG0000(	535	12.11447	chr4:186	Y_RNA		smallRNA	chr4:41303237-4130
ENSG0000(	535	12.11447	chr4:186	ATP1B1P1		Pseudoger	chr4:42029209-4203
ENSG0000(	535	12.11447	chr4:186	ENSG00000272862		lncRNA	chr4:41988741-4198
ENSG0000(	535	12.11447	chr4:186	ENSG00000249216		lncRNA	chr4:41688858-4169
ENSG0000(	535	12.11447	chr4:186	CHRNA9		protein_c	chr4:40335333-4035
ENSG0000(	535	12.11447	chr4:186	LINC02265		lncRNA	chr4:40316484-4033
ENSG0000(	535	12.11447	chr4:186	RNU6-310P		smallRNA	chr4:53265461-5326
ENSG0000(	535	12.11447	chr4:186	UGDH		protein_c	chr4:39498755-3952
ENSG0000(	535	12.11447	chr4:186	KLHL5	NCGv7	protein_c	chr4:39045039-3912
ENSG0000(	535	12.11447	chr4:186	KLF3		protein_c	chr4:38664197-3870
ENSG0000(	535	12.11447	chr4:186	SNX18P24		Pseudoger	chr4:49561479-4956
ENSG0000(	535	12.11447	chr4:186	ENSG00000251527		lncRNA	chr4:65858761-6586
ENSG0000(	535	12.11447	chr4:186	SRP72		protein_c	chr4:56467617-5650

ENSG0000(	535	12.11447	chr4:186{	LNX1		protein_c	chr4:53459301-5370
ENSG0000(	535	12.11447	chr4:186{	CEP135		protein_c	chr4:55948871-5603
ENSG0000(	535	12.11447	chr4:186{	ENSG00000251517		lncRNA	chr4:42706107-4270
ENSG0000(	535	12.11447	chr4:186{	ENSG00000287659		lncRNA	chr4:38276178-3827
ENSG0000(	535	12.11447	chr4:186{	ENSG00000251501		Pseudoger	chr4:43586328-4358
ENSG0000(	535	12.11447	chr4:186{	Y_RNA		smallRNA	chr4:52786537-5278
ENSG0000(	535	12.11447	chr4:186{	LINC02278		lncRNA	chr4:38564003-3857
ENSG0000(	535	12.11447	chr4:186{	LINC01258		lncRNA	chr4:38420662-3852
ENSG0000(	535	12.11447	chr4:186{	ENSG00000251642		lncRNA	chr4:38286994-3828
ENSG0000(	535	12.11447	chr4:186{	ENSG00000287369		lncRNA	chr4:57110547-5711
ENSG0000(	535	12.11447	chr4:186{	ENSG00000287382		lncRNA	chr4:55938153-5594
ENSG0000(	535	12.11447	chr4:186{	MTND3P24		Pseudoger	chr4:64610980-6461
ENSG0000(	535	12.11447	chr4:186{	ENSG00000287383		lncRNA	chr4:66169778-6632
ENSG0000(	535	12.11447	chr4:186{	KRT18P25		Pseudoger	chr4:40020240-4002
ENSG0000(	535	12.11447	chr4:186{	LINC02496		lncRNA	chr4:60750575-6079
ENSG0000(	535	12.11447	chr4:186{	RN7SKP215		smallRNA	chr4:47811464-4781
ENSG0000(	535	12.11447	chr4:186{	Y_RNA		smallRNA	chr4:59533786-5953
ENSG0000(	535	12.11447	chr4:186{	AC104066.1		smallRNA	chr4:52751147-5275
ENSG0000(	535	12.11447	chr4:186{	RNA5SP162		smallRNA	chr4:56331177-5633
ENSG0000(	535	12.11447	chr4:186{	ENSG00000249079		Pseudoger	chr4:49212251-4921
ENSG0000(	535	12.11447	chr4:186{	Y_RNA		smallRNA	chr4:56818218-5681
ENSG0000(	535	12.11447	chr4:186{	RNU6-836P		smallRNA	chr4:41084607-4108
ENSG0000(	535	12.11447	chr4:186{	OR5M14P		Pseudoger	chr4:41722538-4172
ENSG0000(	535	12.11447	chr4:186{	ENSG00000249105		Pseudoger	chr4:58117758-5811
ENSG0000(	535	12.11447	chr4:186{	ENSG00000249111		lncRNA	chr4:59152834-5917
ENSG0000(	535	12.11447	chr4:186{	ENSG00000249122		lncRNA	chr4:41750345-4175
ENSG0000(	535	12.11447	chr4:186{	MIR574		smallRNA	chr4:38868032-3886
ENSG0000(	535	12.11447	chr4:186{	IFITM3P1		Pseudoger	chr4:66094142-6609
ENSG0000(	535	12.11447	chr4:186{	MIR4802		smallRNA	chr4:40502040-4050
ENSG0000(	535	12.11447	chr4:186{	ENSG00000272969		lncRNA	chr4:55547112-5554
ENSG0000(	535	12.11447	chr4:186{	RNU5E-3P		smallRNA	chr4:48574453-4857
ENSG0000(	535	12.11447	chr4:186{	ENSG00000287534		lncRNA	chr4:54065239-5407
ENSG0000(	535	12.11447	chr4:186{	RNU6-998P		smallRNA	chr4:57002692-5700
ENSG0000(	535	12.11447	chr4:186{	ENSG00000272936		lncRNA	chr4:44704405-4470
ENSG0000(	535	12.11447	chr4:186{	ENSG00000249330		lncRNA	chr4:46390255-4651
ENSG0000(	535	12.11447	chr4:186{	SNX18P25		Pseudoger	chr4:49588772-4958
ENSG0000(	535	12.11447	chr4:186{	CRACD	NCGv7	protein_c	chr4:56049098-5633
ENSG0000(	535	12.11447	chr4:186{	NMU		protein_c	chr4:55595229-5563
ENSG0000(	535	12.11447	chr4:186{	ENSG00000251334		Pseudoger	chr4:48936582-4893
ENSG0000(	535	12.11447	chr4:186{	PSME2P4		Pseudoger	chr4:37995494-3799
ENSG0000(	535	12.11447	chr4:186{	RNU6-746P		smallRNA	chr4:55294490-5529
ENSG0000(	535	12.11447	chr4:186{	RNU1-49P		smallRNA	chr4:41771945-4177
ENSG0000(	535	12.11447	chr4:186{	ENSG00000251286		Pseudoger	chr4:52440494-5244
ENSG0000(	535	12.11447	chr4:186{	AC111194.1		smallRNA	chr4:55010833-5501
ENSG0000(	535	12.11447	chr4:186{	RPL17P20		Pseudoger	chr4:56710060-5671
ENSG0000(	535	12.11447	chr4:186{	LINC02429		lncRNA	chr4:58984215-5904
ENSG0000(	535	12.11447	chr4:186{	ENSG00000251264		lncRNA	chr4:54440502-5444
ENSG0000(	535	12.11447	chr4:186{	LINC02358		lncRNA	chr4:54845568-5485
ENSG0000(	535	12.11447	chr4:186{	RNU6-32P		smallRNA	chr4:39297605-3929
ENSG0000(	535	12.11447	chr4:186{	TXK		protein_c	chr4:48066393-4813
ENSG0000(	535	12.11447	chr4:186{	ENSG00000272304		lncRNA	chr4:66003281-6601

ENSG0000(	535	12.11447	chr4:186	MIR4449		smallRNA	chr4:52712682-5271
ENSG0000(	535	12.11447	chr4:186	MIR5591		smallRNA	chr4:39411910-3941
ENSG0000(	535	12.11447	chr4:186	ENSG00000237961		Pseudoger	chr4:49238032-4924
ENSG0000(	535	12.11447	chr4:186	ENSG00000205794		Pseudoger	chr4:40042917-4005
ENSG0000(	535	12.11447	chr4:186	UCHL1-DT		lncRNA	chr4:41220074-4125
ENSG0000(	535	12.11447	chr4:186	ENSG00000205682		lncRNA	chr4:61420246-6142
ENSG0000(	535	12.11447	chr4:186	RNU6-1112P		smallRNA	chr4:40077884-4007
ENSG0000(	535	12.11447	chr4:186	TECRL		protein_c	chr4:64275257-6440
ENSG0000(	535	12.11447	chr4:186	ENSG00000251159		Pseudoger	chr4:44533615-4453
ENSG0000(	535	12.11447	chr4:186	YIPF7		protein_c	chr4:44622088-4467
ENSG0000(	535	12.11447	chr4:186	ENSG00000287999		lncRNA	chr4:52252820-5255
ENSG0000(	535	12.11447	chr4:186	FRYL		protein_c	chr4:48497357-4878
ENSG0000(	535	12.11447	chr4:186	RPL17P19		Pseudoger	chr4:61211652-6121
ENSG0000(	535	12.11447	chr4:186	LINC02475		lncRNA	chr4:44016700-4402
ENSG0000(	535	12.11447	chr4:186	ENSG00000287762		lncRNA	chr4:41608312-4161
ENSG0000(	535	12.11447	chr4:186	PHOX2B	NCGv7;AC	protein_c	chr4:41744082-4174
ENSG0000(	535	12.11447	chr4:186	ENSG00000249341		lncRNA	chr4:53659208-5373
ENSG0000(	535	12.11447	chr4:186	CHIC2	AC	protein_c	chr4:54009789-5406
ENSG0000(	535	12.11447	chr4:186	UGDH-AS1		lncRNA	chr4:39527720-3959
ENSG0000(	535	12.11447	chr4:186	ENSG00000249351		Pseudoger	chr4:65216616-6521
ENSG0000(	535	12.11447	chr4:186	USP46		protein_c	chr4:52590960-5265
ENSG0000(	535	12.11447	chr4:186	DCUN1D4		protein_c	chr4:51843000-5191
ENSG0000(	535	12.11447	chr4:186	CWH43	NCGv7	protein_c	chr4:48986275-4906
ENSG0000(	535	12.11447	chr4:186	OCIAD1		protein_c	chr4:48805212-4886
ENSG0000(	535	12.11447	chr4:186	SLAIN2		protein_c	chr4:48341529-4842
ENSG0000(	535	12.11447	chr4:186	GABRA4		protein_c	chr4:46918900-4699
ENSG0000(	535	12.11447	chr4:186	TMEM33		protein_c	chr4:41935129-4196
ENSG0000(	535	12.11447	chr4:186	ENSG00000251459		lncRNA	chr4:57490808-5749
ENSG0000(	535	12.11447	chr4:186	ENSG00000272576		lncRNA	chr4:51918772-5191
ENSG0000(	535	12.11447	chr4:186	RNU6-1195P		smallRNA	chr4:41113942-4111
ENSG0000(	535	12.11447	chr4:186	Y_RNA		smallRNA	chr4:40826655-4082
ENSG0000(	535	12.11447	chr4:186	MTCO3P27		Pseudoger	chr4:64606418-6460
ENSG0000(	535	12.11447	chr4:186	ENSG00000272650		lncRNA	chr4:53997415-5399
ENSG0000(	535	12.11447	chr4:186	LINC02619		lncRNA	chr4:58939288-5898
ENSG0000(	535	12.11447	chr4:186	ENSG00000249392		lncRNA	chr4:59551142-5963
ENSG0000(	535	12.11447	chr4:186	AC131951.1		smallRNA	chr4:44448005-4444
ENSG0000(	535	12.11447	chr4:186	ENSG00000249413		lncRNA	chr4:65998846-6615
ENSG0000(	535	12.11447	chr4:186	RPL21P47		Pseudoger	chr4:62248294-6224
ENSG0000(	535	12.11447	chr4:186	RNU6-838P		smallRNA	chr4:48106081-4810
ENSG0000(	535	12.11447	chr4:186	MTCYBP16		Pseudoger	chr4:64607703-6460
ENSG0000(	535	12.11447	chr4:186	AC098680.1		smallRNA	chr4:38238613-3823
ENSG0000(	535	12.11447	chr4:186	MTND6P16		Pseudoger	chr4:64608905-6460
ENSG0000(	535	12.11447	chr4:186	ENSG00000280285		TEC	chr4:56662469-5666
ENSG0000(	535	12.11447	chr4:186	ENSG00000269848		Pseudoger	chr4:49524030-4952
ENSG0000(	535	12.11447	chr4:186	ENSG00000248317		lncRNA	chr4:54943626-5495
ENSG0000(	535	12.11447	chr4:186	AC021106.1		Pseudoger	chr4:37960435-3796
ENSG0000(	535	12.11447	chr4:186	GABRA2	NCGv7	protein_c	chr4:46243548-4647
ENSG0000(	535	12.11447	chr4:186	SLC30A9		protein_c	chr4:41990502-4209
ENSG0000(	535	12.11447	chr4:186	APBB2		protein_c	chr4:40810027-4121
ENSG0000(	535	12.11447	chr4:186	GUF1		protein_c	chr4:44678420-4470
ENSG0000(	535	12.11447	chr4:186	PABPC1P1		Pseudoger	chr4:39973444-3997

ENSG0000(	535	12.11447	chr4:186	RBM47		protein_c	chr4:40423267-4063
ENSG0000(	535	12.11447	chr4:186	SMIM14		protein_c	chr4:39546336-3963
ENSG0000(	535	12.11447	chr4:186	RPL9		protein_c	chr4:39452587-3945
ENSG0000(	535	12.11447	chr4:186	FIP1L1	AC	protein_c	chr4:53377569-5346
ENSG0000(	535	12.11447	chr4:186	EPHA5	NCGv7	protein_c	chr4:65319563-6567
ENSG0000(	535	12.11447	chr4:186	CORIN		protein_c	chr4:47593999-4783
ENSG0000(	535	12.11447	chr4:186	CCNL2P1		Pseudoger	chr4:42561457-4256
ENSG0000(	535	12.11447	chr4:186	ATP10D	NCGv7	protein_c	chr4:47485275-4759
ENSG0000(	535	12.11447	chr4:186	RFC1	NCGv7	protein_c	chr4:39287456-3936
ENSG0000(	535	12.11447	chr4:186	CNGA1		protein_c	chr4:47935977-4801
ENSG0000(	535	12.11447	chr4:186	LNX1-AS2		lncRNA	chr4:53592956-5360
ENSG0000(	535	12.11447	chr4:186	LINC02380		lncRNA	chr4:57424495-5747
ENSG0000(	535	12.11447	chr4:186	OCIAD2		protein_c	chr4:48885019-4890
ENSG0000(	535	12.11447	chr4:186	SLC10A4		protein_c	chr4:48483343-4848
ENSG0000(	535	12.11447	chr4:186	MTND4LP31		Pseudoger	chr4:64610616-6461
ENSG0000(	535	12.11447	chr4:186	ENSG00000248447		Pseudoger	chr4:65034634-6503
ENSG0000(	535	12.11447	chr4:186	OCIAD1-AS1		lncRNA	chr4:48852008-4886
ENSG0000(	535	12.11447	chr4:186	ENSG00000248254		lncRNA	chr4:47556731-4756
ENSG0000(	535	12.11447	chr4:186	FAM114A1		protein_c	chr4:38867677-3894
ENSG0000(	535	12.11447	chr4:186	LRRC66	NCGv7	protein_c	chr4:51993652-5202
ENSG0000(	535	12.11447	chr4:186	THAP12P9		Pseudoger	chr4:45323253-4532
ENSG0000(	535	12.11447	chr4:186	ENSG00000224097		Pseudoger	chr4:39480255-3948
ENSG0000(	535	12.11447	chr4:186	MTCO3P28		Pseudoger	chr4:64611374-6461
ENSG0000(	535	12.11447	chr4:186	LARP1BP1		Pseudoger	chr4:63350114-6335
ENSG0000(	535	12.11447	chr4:186	ENSG00000275959		Pseudoger	chr4:44994426-4499
ENSG0000(	535	12.11447	chr4:186	ENSG00000269506		lncRNA	chr4:54059597-5406
ENSG0000(	535	12.11447	chr4:186	RN7SL558P		smallRNA	chr4:39761000-3976
ENSG0000(	535	12.11447	chr4:186	RNU2-40P		smallRNA	chr4:65807240-6580
ENSG0000(	535	12.11447	chr4:186	KLF3-AS1		lncRNA	chr4:38602438-3866
ENSG0000(	535	12.11447	chr4:186	AC110611.1		smallRNA	chr4:55929982-5593
ENSG0000(	535	12.11447	chr4:186	RNU6-887P		smallRNA	chr4:39399149-3939
ENSG0000(	535	12.11447	chr4:186	AC119751.4		smallRNA	chr4:49599428-4959
ENSG0000(	535	12.11447	chr4:186	RPL38P3		Pseudoger	chr4:54976159-5497
ENSG0000(	535	12.11447	chr4:186	ENSG00000283156		Pseudoger	chr4:56410516-5643
ENSG0000(	535	12.11447	chr4:186	ENSG00000283043		Pseudoger	chr4:60038269-6003
ENSG0000(	535	12.11447	chr4:186	RN7SKP30		smallRNA	chr4:55540502-5554
ENSG0000(	535	12.11447	chr4:186	ENSG00000282917		lncRNA	chr4:47831330-4790
ENSG0000(	535	12.11447	chr4:186	RNA5SP158		Pseudoger	chr4:38758791-3875
ENSG0000(	535	12.11447	chr4:186	ENSG00000282904		lncRNA	chr4:47463590-4747
ENSG0000(	535	12.11447	chr4:186	IGFBP7-AS1		lncRNA	chr4:57109762-5720
ENSG0000(	535	12.11447	chr4:186	SMIM14-DT		lncRNA	chr4:39639107-3966
ENSG0000(	535	12.11447	chr4:186	ENSG00000248375		Pseudoger	chr4:52720081-5272
ENSG0000(	535	12.11447	chr4:186	ENSG00000282278		protein_c	chr4:53377839-5429
ENSG0000(	535	12.11447	chr4:186	ENSG00000277096		Pseudoger	chr4:43388157-4338
ENSG0000(	535	12.11447	chr4:186	ARL9		protein_c	chr4:56505209-5652
ENSG0000(	535	12.11447	chr4:186	AC118282.3		smallRNA	chr4:49197203-4919
ENSG0000(	535	12.11447	chr4:186	ENSG00000226439		Pseudoger	chr4:61775449-6177
ENSG0000(	535	12.11447	chr4:186	ENSG00000248479		lncRNA	chr4:65702202-6570
ENSG0000(	535	12.11447	chr4:186	HMGN1P11		Pseudoger	chr4:62510469-6251
ENSG0000(	535	12.11447	chr4:186	RN7SKP199		smallRNA	chr4:45995119-4599
ENSG0000(	535	12.11447	chr4:186	IGFBP7		protein_c	chr4:57030773-5711

ENSG0000(	535	12.11447	chr4:1865	LINC00682		lncRNA	chr4:41872741-4188
ENSG0000(	535	12.11447	chr4:1865	TEC	NCGv7;AC	protein_c	chr4:48135783-4826
ENSG0000(	535	12.11447	chr4:1865	ERVMER34-1		protein_c	chr4:52722618-5275
ENSG0000(	535	12.11447	chr4:1865	LINC02383		lncRNA	chr4:43457527-4349
ENSG0000(	535	12.11447	chr4:1865	KLB		protein_c	chr4:39406930-3945
ENSG0000(	535	12.11447	chr4:1865	ENSG00000244669		Pseudoger	chr4:64767130-6476
ENSG0000(	535	12.11447	chr4:1865	PDGFRA	NCGv7;AC	protein_c	chr4:54229280-5429
ENSG0000(	535	12.11447	chr4:1865	ADGRL3	NCGv7	protein_c	chr4:61200326-6207
ENSG0000(	535	12.11447	chr4:1865	WDR19		protein_c	chr4:39182504-3928
ENSG0000(	535	12.11447	chr4:1865	ENSG00000248115		lncRNA	chr4:52945649-5295
ENSG0000(	535	12.11447	chr4:1865	MTCO3P39		Pseudoger	chr4:49246021-4924
ENSG0000(	535	12.11447	chr4:1865	CLOCK		protein_c	chr4:55427903-5554
ENSG0000(	535	12.11447	chr4:1865	ENSG00000270147		lncRNA	chr4:56396312-5639
ENSG0000(	535	12.11447	chr4:1865	RPS23P3		Pseudoger	chr4:66431092-6643
ENSG0000(	535	12.11447	chr4:1865	ENSG00000269921		lncRNA	chr4:56387625-5638
ENSG0000(	535	12.11447	chr4:1865	ENSG00000280043		TEC	chr4:49229573-4923
ENSG0000(	535	12.11447	chr4:1865	ENSG00000280015		TEC	chr4:38390754-3839
ENSG0000(	535	12.11447	chr4:1865	DANCR		lncRNA	chr4:52712325-5272
ENSG0000(	535	12.11447	chr4:1865	UCHL1	AC	protein_c	chr4:41256413-4126
ENSG0000(	535	12.11447	chr4:1865	ENSG00000227040		Pseudoger	chr4:56760919-5676
ENSG0000(	535	12.11447	chr4:1865	MIR1269A		smallRNA	chr4:66276824-6627
ENSG0000(	535	12.11447	chr4:1865	RPS15AP17		Pseudoger	chr4:62105660-6210
ENSG0000(	535	12.11447	chr4:1865	RPS26P24		Pseudoger	chr4:57352999-5735
ENSG0000(	535	12.11447	chr4:1865	ENSG00000243929		Pseudoger	chr4:51978079-5197
ENSG0000(	535	12.11447	chr4:1865	TPI1P4		Pseudoger	chr4:49016682-4901
ENSG0000(	535	12.11447	chr4:1865	ENSG00000248532		Pseudoger	chr4:49523648-4952
ENSG0000(	535	12.11447	chr4:1865	MTCO3P42		Pseudoger	chr4:49548918-4954
ENSG0000(	535	12.11447	chr4:1865	RN7SL424P		smallRNA	chr4:55063735-5506
ENSG0000(	535	12.11447	chr4:1865	ENSG00000248518		lncRNA	chr4:54376002-5437
ENSG0000(	535	12.11447	chr4:1865	RPL6P10		Pseudoger	chr4:65573459-6557
ENSG0000(	535	12.11447	chr4:1865	GNPDA2	DriverDB\	protein_c	chr4:44682200-4472
ENSG0000(	535	12.11447	chr4:1865	GABRG1	NCGv7	protein_c	chr4:46035769-4612
ENSG0000(	535	12.11447	chr4:1865	AC107068.1		smallRNA	chr4:47976216-4797
ENSG0000(	535	12.11447	chr4:1865	AC118282.2		smallRNA	chr4:49198015-4919
ENSG0000(	535	12.11447	chr4:1865	ENSG00000279386		TEC	chr4:37866561-3786
ENSG0000(	535	12.11447	chr4:1865	SPATA18	NCGv7	protein_c	chr4:52051304-5209
ENSG0000(	535	12.11447	chr4:1865	SGCB		protein_c	chr4:52020706-5203
ENSG0000(	535	12.11447	chr4:1865	RPL21P44		Pseudoger	chr4:53986587-5398
ENSG0000(	535	12.11447	chr4:1865	ENSG00000269949		lncRNA	chr4:56960927-5696
ENSG0000(	535	12.11447	chr4:1865	ENSG00000259959		lncRNA	chr4:47840122-4784
ENSG0000(	535	12.11447	chr4:1865	LINC02283		lncRNA	chr4:54332892-5435
ENSG0000(	535	12.11447	chr4:1865	GABRB1		protein_c	chr4:46993723-4742
ENSG0000(	535	12.11447	chr4:1865	NIPAL1		protein_c	chr4:47914142-4804
ENSG0000(	535	12.11447	chr4:1865	ENSG00000248237		Pseudoger	chr4:60784115-6078
ENSG0000(	535	12.11447	chr4:1865	BEND4		protein_c	chr4:42110853-4215
ENSG0000(	535	12.11447	chr4:1865	KIT	NCGv7;AC	protein_c	chr4:54657267-5474
ENSG0000(	535	12.11447	chr4:1865	PDCL2		protein_c	chr4:55556519-5559
ENSG0000(	535	12.11447	chr4:1865	ENSG00000224560		Pseudoger	chr4:45414337-4541
ENSG0000(	535	12.11447	chr4:1865	AASDH		protein_c	chr4:56338287-5638
ENSG0000(	535	12.11447	chr4:1865	ENSG00000268967		Pseudoger	chr4:49548564-4954
ENSG0000(	535	12.11447	chr4:1865	LINC02513		lncRNA	chr4:38366914-3838

ENSG0000(	525	11.88803	chr1:2449	FMO9P		lncRNA	chr1:166603916-166
ENSG0000(	525	11.88803	chr1:2449	FMO9P		Pseudoger	chr1:166612470-166
ENSG0000(	525	11.88803	chr1:2449	ENSG00000213068		Pseudoger	chr1:167162423-167
ENSG0000(	525	11.88803	chr1:2449	ENSG00000273160		lncRNA	chr1:167457742-167
ENSG0000(	525	11.88803	chr1:2449	ENSG00000225325		lncRNA	chr1:166387727-166
ENSG0000(	525	11.88803	chr1:2449	RNA5SP65		Pseudoger	chr1:167005959-167
ENSG0000(	525	11.88803	chr1:2449	FMO10P		Pseudoger	chr1:166665885-166
ENSG0000(	525	11.88803	chr1:2449	TADA1	DriverDBv	protein_c	chr1:166856510-166
ENSG0000(	525	11.88803	chr1:2449	LINC01675		lncRNA	chr1:166474879-166
ENSG0000(	525	11.88803	chr1:2449	POU2F1-DT		lncRNA	chr1:167219822-167
ENSG0000(	525	11.88803	chr1:2449	ENSG00000287218		lncRNA	chr1:167455195-167
ENSG0000(	525	11.88803	chr1:2449	FMO8P		Pseudoger	chr1:166566178-166
ENSG0000(	525	11.88803	chr1:2449	POGK	DriverDBv	protein_c	chr1:166839447-166
ENSG0000(	525	11.88803	chr1:2449	GPA33		protein_c	chr1:167052836-167
ENSG0000(	525	11.88803	chr1:2449	ENSG00000227907		lncRNA	chr1:167052551-167
ENSG0000(	525	11.88803	chr1:2449	FMO7P		Pseudoger	chr1:166474745-166
ENSG0000(	525	11.88803	chr1:2449	POU2F1		protein_c	chr1:167220876-167
ENSG0000(	525	11.88803	chr1:2449	MAEL		protein_c	chr1:166975582-167
ENSG0000(	525	11.88803	chr1:2449	ILDR2		protein_c	chr1:166895711-166
ENSG0000(	525	11.88803	chr1:2449	ENSG00000272033		lncRNA	chr1:167379108-167
ENSG0000(	525	11.88803	chr1:2449	snoU13		smallRNA	chr1:167041435-167
ENSG0000(	525	11.88803	chr1:2449	DUTP6		Pseudoger	chr1:166868748-166
ENSG0000(	525	11.88803	chr1:2449	FMO11P		Pseudoger	chr1:166763334-166
ENSG0000(	525	11.88803	chr1:2449	RPL4P2		Pseudoger	chr1:166747379-166
ENSG0000(	525	11.88803	chr1:2449	CD247		protein_c	chr1:167425027-167
ENSG0000(	525	11.88803	chr1:2449	STYXL2		protein_c	chr1:167094075-167
ENSG0000(	525	11.88803	chr1:2449	CNN2P10		Pseudoger	chr1:166796266-166
ENSG0000(	525	11.88803	chr1:2449	LINC01363		lncRNA	chr1:167175363-167
ENSG0000(	525	11.88803	chr1:2449	ENSG00000233411		lncRNA	chr1:167457383-167
ENSG0000(	519	11.75217	chr6:9221	MIR3934		smallRNA	chr6:33698128-3369
ENSG0000(	518	11.72952	chr22:220	IGLV3-32		protein_c	chr22:22594528-225
ENSG0000(	517	11.70688	chr10:717	RN7SKP39		smallRNA	chr10:28442575-284
ENSG0000(	516	11.68424	chr6:1979	ENSG00000270987		Pseudoger	chr6:100889603-100
ENSG0000(	515	11.66159	chr10:717	ENSG00000236556		lncRNA	chr10:59282147-592
ENSG0000(	514	11.63895	chr3:3227	AC116038.2		smallRNA	chr3:38800296-3880
ENSG0000(	514	11.63895	chr17:289	PPIAP54		Pseudoger	chr17:40367952-403
ENSG0000(	514	11.63895	chr3:3227	MIR191		smallRNA	chr3:49020618-4902
ENSG0000(	512	11.59366	chr19:511	ENSG00000269681		Pseudoger	chr19:51155288-511
ENSG0000(	508	11.50308	chr22:220	IGLV2-34		Pseudoger	chr22:22580014-225
ENSG0000(	507	11.48044	chr22:220	RPS15AP38		Pseudoger	chr22:36421273-364
ENSG0000(	505	11.43515	chr11:144	ENSG00000254829		lncRNA	chr11:78015715-780
ENSG0000(	500	11.32193	chr3:3227	KRT8P18		Pseudoger	chr3:35215705-3521
ENSG0000(	500	11.32193	chr3:3227	UBP1		protein_c	chr3:33388336-3344
ENSG0000(	500	11.32193	chr3:3227	ITGA9-AS1		lncRNA	chr3:37693655-3786
ENSG0000(	500	11.32193	chr3:3227	DCLK3		protein_c	chr3:36712421-3676
ENSG0000(	500	11.32193	chr3:3227	NDUFAF4P3		Pseudoger	chr3:37789921-3779
ENSG0000(	500	11.32193	chr3:3227	snoU13		smallRNA	chr3:37196741-3719
ENSG0000(	500	11.32193	chr3:3227	VILL	NCGv7	protein_c	chr3:37988059-3800
ENSG0000(	500	11.32193	chr3:3227	MIR128-2		smallRNA	chr3:35744476-3574
ENSG0000(	500	11.32193	chr3:3227	LINC01811		lncRNA	chr3:33956972-3467
ENSG0000(	500	11.32193	chr3:3227	CTDSPL		protein_c	chr3:37861880-3798

ENSG0000(	500	11.32193	chr3:3227	STAC		protein_c	chr3:36380503-3654
ENSG0000(	500	11.32193	chr3:3227	ENSG00000271653		lncRNA	chr3:37216779-3721
ENSG0000(	500	11.32193	chr3:3227	ENSG00000213849		Pseudoger	chr3:32507955-3250
ENSG0000(	500	11.32193	chr3:3227	SUGT1P2		Pseudoger	chr3:32752910-3275
ENSG0000(	500	11.32193	chr3:3227	ENSG00000230119		Pseudoger	chr3:33190233-3319
ENSG0000(	500	11.32193	chr3:3227	RNU6ATAC4P		smallRNA	chr3:36968191-3696
ENSG0000(	500	11.32193	chr3:3227	HSPD1P6		Pseudoger	chr3:36767117-3678
ENSG0000(	500	11.32193	chr3:3227	PPP2R2DP1		Pseudoger	chr3:38051759-3805
ENSG0000(	500	11.32193	chr3:3227	DYNC1LI1		protein_c	chr3:32525974-3257
ENSG0000(	500	11.32193	chr3:3227	ENSG00000236452		lncRNA	chr3:34203244-3426
ENSG0000(	500	11.32193	chr3:3227	ITGA9		protein_c	chr3:37452115-3782
ENSG0000(	500	11.32193	chr3:3227	PDCD6IP-DT		lncRNA	chr3:33793644-3379
ENSG0000(	500	11.32193	chr3:3227	TRIM71		protein_c	chr3:32817997-3289
ENSG0000(	500	11.32193	chr3:3227	GOLGA4		protein_c	chr3:37243191-3736
ENSG0000(	500	11.32193	chr3:3227	ENSG00000290046		lncRNA	chr3:37176387-3720
ENSG0000(	500	11.32193	chr3:3227	ENSG00000237982		Pseudoger	chr3:38270283-3827
ENSG0000(	500	11.32193	chr3:3227	PLCD1	NCGv7	protein_c	chr3:38007496-3802
ENSG0000(	500	11.32193	chr3:3227	ENSG00000271993		lncRNA	chr3:37182107-3718
ENSG0000(	500	11.32193	chr3:3227	CMTM6		protein_c	chr3:32481312-3250
ENSG0000(	500	11.32193	chr3:3227	ENSG00000287641		lncRNA	chr3:34692820-3469
ENSG0000(	500	11.32193	chr3:3227	ENSG00000228112		Pseudoger	chr3:33727250-3372
ENSG0000(	500	11.32193	chr3:3227	ENSG00000290076		lncRNA	chr3:37862435-3786
ENSG0000(	500	11.32193	chr3:3227	LINC02033		lncRNA	chr3:36819276-3682
ENSG0000(	500	11.32193	chr3:3227	APRG1		lncRNA	chr3:37381062-3744
ENSG0000(	500	11.32193	chr3:3227	MYD88	NCGv7;AC	protein_c	chr3:38138478-3814
ENSG0000(	500	11.32193	chr3:3227	DLEC1	NCGv7	protein_c	chr3:38039205-3812
ENSG0000(	500	11.32193	chr3:3227	FBXL2		protein_c	chr3:33277025-3340
ENSG0000(	500	11.32193	chr3:3227	RNU6-1301P		smallRNA	chr3:37140327-3714
ENSG0000(	500	11.32193	chr3:3227	Y_RNA		smallRNA	chr3:37093133-3709
ENSG0000(	500	11.32193	chr3:3227	TMPPE		protein_c	chr3:33090421-3309
ENSG0000(	500	11.32193	chr3:3227	CNOT10-AS1		lncRNA	chr3:32730635-3273
ENSG0000(	500	11.32193	chr3:3227	RFC3P1		Pseudoger	chr3:36169767-3617
ENSG0000(	500	11.32193	chr3:3227	PRADC1P1		Pseudoger	chr3:36976316-3697
ENSG0000(	500	11.32193	chr3:3227	ENSG00000289460		lncRNA	chr3:33275247-3327
ENSG0000(	500	11.32193	chr3:3227	CMTM7		protein_c	chr3:32391698-3248
ENSG0000(	500	11.32193	chr3:3227	ENSG00000272334		lncRNA	chr3:36973117-3697
ENSG0000(	500	11.32193	chr3:3227	OXSR1		protein_c	chr3:38165089-3825
ENSG0000(	500	11.32193	chr3:3227	SLC22A13		protein_c	chr3:38265812-3827
ENSG0000(	500	11.32193	chr3:3227	TRANK1		protein_c	chr3:36826819-3694
ENSG0000(	500	11.32193	chr3:3227	CNOT10		protein_c	chr3:32685145-3277
ENSG0000(	500	11.32193	chr3:3227	SUMO2P10		Pseudoger	chr3:33077204-3307
ENSG0000(	500	11.32193	chr3:3227	ACAA1		protein_c	chr3:38103129-3813
ENSG0000(	500	11.32193	chr3:3227	CLASP2	NCGv7	protein_c	chr3:33496245-3371
ENSG0000(	500	11.32193	chr3:3227	RPL21P135		Pseudoger	chr3:37748110-3774
ENSG0000(	500	11.32193	chr3:3227	ENSG00000288972		lncRNA	chr3:37817408-3781
ENSG0000(	500	11.32193	chr3:3227	RPL30P4		Pseudoger	chr3:32635222-3263
ENSG0000(	500	11.32193	chr3:3227	SDAD1P3		Pseudoger	chr3:33771963-3377
ENSG0000(	500	11.32193	chr3:3227	ENSG00000287981		lncRNA	chr3:34152979-3415
ENSG0000(	500	11.32193	chr3:3227	ENSG00000226955		Pseudoger	chr3:32592972-3259
ENSG0000(	500	11.32193	chr3:3227	Y_RNA		smallRNA	chr3:38014568-3801
ENSG0000(	500	11.32193	chr3:3227	RNU6-243P		smallRNA	chr3:35256840-3525

ENSG0000(	500	11.32193	chr3:3227	RNU6-235P		smallRNA	chr3:38303773-3830
ENSG0000(	500	11.32193	chr3:3227	FECHP1		Pseudoger	chr3:34872641-3487
ENSG0000(	500	11.32193	chr3:3227	ARPP21		protein_c	chr3:35638945-3579
ENSG0000(	500	11.32193	chr3:3227	DLEC1P1		Pseudoger	chr3:38325237-3832
ENSG0000(	500	11.32193	chr3:3227	SLC22A14		protein_c	chr3:38282294-3831
ENSG0000(	500	11.32193	chr3:3227	UBE2FP1		Pseudoger	chr3:37143512-3714
ENSG0000(	500	11.32193	chr3:3227	RNA5SP129		Pseudoger	chr3:37341649-3734
ENSG0000(	500	11.32193	chr3:3227	GLB1		protein_c	chr3:32996609-3309
ENSG0000(	500	11.32193	chr3:3227	PDCD6IP		protein_c	chr3:33798571-3386
ENSG0000(	500	11.32193	chr3:3227	ENSG00000281100		TEC	chr3:36823151-3682
ENSG0000(	500	11.32193	chr3:3227	GOLGA4-AS1		lncRNA	chr3:37241789-3724
ENSG0000(	500	11.32193	chr3:3227	TCEA1P2		Pseudoger	chr3:37275693-3727
ENSG0000(	500	11.32193	chr3:3227	LRRFIP2		protein_c	chr3:37052626-3718
ENSG0000(	500	11.32193	chr3:3227	ENSG00000227498		lncRNA	chr3:34524818-3454
ENSG0000(	500	11.32193	chr3:3227	RN7SL296P		smallRNA	chr3:33103008-3310
ENSG0000(	500	11.32193	chr3:3227	RPL36AP17		Pseudoger	chr3:35871866-3587
ENSG0000(	500	11.32193	chr3:3227	SUSD5		protein_c	chr3:33150043-3321
ENSG0000(	500	11.32193	chr3:3227	ENSG00000234073		Pseudoger	chr3:36880184-3688
ENSG0000(	500	11.32193	chr3:3227	ARPP21-AS1		lncRNA	chr3:35650197-3565
ENSG0000(	500	11.32193	chr3:3227	EPM2AIP1		protein_c	chr3:36985043-3699
ENSG0000(	500	11.32193	chr3:3227	RNA5SP128		Pseudoger	chr3:33491983-3349
ENSG0000(	500	11.32193	chr3:3227	CCR4	NCGv7	protein_c	chr3:32951644-3295
ENSG0000(	500	11.32193	chr3:3227	MLH1	NCGv7;AC	protein_c	chr3:36993350-3705
ENSG0000(	500	11.32193	chr3:3227	AC104306.2		smallRNA	chr3:32515726-3251
ENSG0000(	500	11.32193	chr3:3227	UBE2D3P2		Pseudoger	chr3:37134658-3713
ENSG0000(	500	11.32193	chr3:3227	NBPF21P		Pseudoger	chr3:36616006-3663
ENSG0000(	500	11.32193	chr3:3227	RN7SKP227		smallRNA	chr3:36685040-3668
ENSG0000(	500	11.32193	chr3:3227	MIR26A1		smallRNA	chr3:37969404-3796
ENSG0000(	500	11.32193	chr3:3227	ENSG00000289420		lncRNA	chr3:35421084-3542
ENSG0000(	500	11.32193	chr3:3227	ENSG00000272149		lncRNA	chr3:33144104-3314
ENSG0000(	500	11.32193	chr3:3227	Y_RNA		smallRNA	chr3:38125292-3812
ENSG0000(	500	11.32193	chr3:3227	CRTAP		protein_c	chr3:33114014-3314
ENSG0000(	500	11.32193	chr3:3227	ENSG00000231449		Pseudoger	chr3:37196204-3719
ENSG0000(	500	11.32193	chr3:3227	COX6CP10		Pseudoger	chr3:33554636-3355
ENSG0000(	500	11.32193	chr3:3227	RPL23AP43		Pseudoger	chr3:32785646-3278
ENSG0000(	500	11.32193	chr3:3227	IGBP1P3		Pseudoger	chr3:32620903-3262
ENSG0000(	500	11.32193	chr3:3227	RNU7-110P		smallRNA	chr3:33414150-3341
ENSG0000(	500	11.32193	chr3:3227	SEC13P1		Pseudoger	chr3:33033863-3303
ENSG0000(	500	11.32193	chr3:3227	RNU7-73P		smallRNA	chr3:37573151-3757
ENSG0000(	500	11.32193	chr3:3227	RPL29P11		Pseudoger	chr3:37016523-3701
ENSG0000(	495	11.20871	chr11:144	ENSG00000254814		lncRNA	chr11:75800877-758
ENSG0000(	495	11.20871	chr11:144	AP002789.1		smallRNA	chr11:77595111-775
ENSG0000(	495	11.20871	chr11:144	OR2AT1P		Pseudoger	chr11:75131138-751
ENSG0000(	495	11.20871	chr11:144	RPS3		protein_c	chr11:75399515-754
ENSG0000(	495	11.20871	chr11:144	GDPD5		protein_c	chr11:75434640-755
ENSG0000(	495	11.20871	chr11:144	ENSG00000254810		lncRNA	chr11:76653597-766
ENSG0000(	495	11.20871	chr11:144	MIR326		smallRNA	chr11:75335092-753
ENSG0000(	495	11.20871	chr11:144	AQP11		protein_c	chr11:77589391-776
ENSG0000(	495	11.20871	chr11:144	MYO7A		protein_c	chr11:77128246-772
ENSG0000(	495	11.20871	chr11:144	ENSG00000261578		lncRNA	chr11:76800364-768
ENSG0000(	495	11.20871	chr11:144	DGAT2-DT		lncRNA	chr11:75758455-757

ENSG0000(	495	11.20871	chr11:144	GVQW3		protein_c	chr11:76381303-764
ENSG0000(	495	11.20871	chr11:144	RNA5SP344		Pseudoger	chr11:75934936-759
ENSG0000(	495	11.20871	chr11:144	ENSG00000268635		lncRNA	chr11:77473371-774
ENSG0000(	495	11.20871	chr11:144	Y_RNA		smallRNA	chr11:76404140-764
ENSG0000(	495	11.20871	chr11:144	ENSG00000254915		Pseudoger	chr11:75942129-759
ENSG0000(	495	11.20871	chr11:144	MOGAT2		protein_c	chr11:75717838-757
ENSG0000(	495	11.20871	chr10:173	AC012047.1		smallRNA	chr10:76127251-761
ENSG0000(	495	11.20871	chr11:144	ENSG00000254460		lncRNA	chr11:75506937-755
ENSG0000(	495	11.20871	chr11:144	GDPD4		protein_c	chr11:77216558-773
ENSG0000(	495	11.20871	chr11:144	SNORD15B		smallRNA	chr11:75404421-754
ENSG0000(	495	11.20871	chr11:144	ARRB1		protein_c	chr11:75260122-753
ENSG0000(	495	11.20871	chr11:144	ENSG00000236304		lncRNA	chr11:76656984-766
ENSG0000(	495	11.20871	chr11:144	SERPINH1		protein_c	chr11:75562056-755
ENSG0000(	495	11.20871	chr11:144	THAP12	NCGv7	protein_c	chr11:76349898-763
ENSG0000(	495	11.20871	chr11:144	ENSG00000255280		Pseudoger	chr11:75642600-756
ENSG0000(	495	11.20871	chr11:144	LRRC32	NCGv7	protein_c	chr11:76657524-766
ENSG0000(	495	11.20871	chr11:144	EMSY	AC	protein_c	chr11:76444923-765
ENSG0000(	495	11.20871	chr11:144	OMP	DriverDBv	protein_c	chr11:77102840-771
ENSG0000(	495	11.20871	chr11:144	KLHL35		protein_c	chr11:75422394-754
ENSG0000(	495	11.20871	chr11:144	ENSG00000291249		lncRNA	chr11:76703274-767
ENSG0000(	495	11.20871	chr11:144	ENSG00000254755		Pseudoger	chr11:76591023-765
ENSG0000(	495	11.20871	chr11:144	ENSG00000254988		lncRNA	chr11:76955417-769
ENSG0000(	495	11.20871	chr11:144	ACER3		protein_c	chr11:76860859-770
ENSG0000(	495	11.20871	chr11:144	AP002498.1		smallRNA	chr11:76878232-768
ENSG0000(	495	11.20871	chr11:144	TOMM20P1		Pseudoger	chr11:77313606-773
ENSG0000(	495	11.20871	chr11:144	PAK1	DriverDBv	protein_c	chr11:77322017-774
ENSG0000(	495	11.20871	chr11:144	ENSG00000279117		TEC	chr11:75260129-752
ENSG0000(	495	11.20871	chr11:144	TPBGL-AS1		lncRNA	chr11:75206048-752
ENSG0000(	495	11.20871	chr11:144	SLCO2B1		protein_c	chr11:75100563-752
ENSG0000(	495	11.20871	chr11:144	ENSG00000254632		lncRNA	chr11:76759916-767
ENSG0000(	495	11.20871	chr11:144	ENSG00000254826		lncRNA	chr11:75775904-757
ENSG0000(	495	11.20871	chr11:144	ENSG00000255434		lncRNA	chr11:75596144-755
ENSG0000(	495	11.20871	chr11:144	PPP1R1AP1		Pseudoger	chr11:75911204-759
ENSG0000(	495	11.20871	chr11:144	ENSG00000255326		lncRNA	chr11:75583196-755
ENSG0000(	495	11.20871	chr11:144	RSF1-IT2		lncRNA	chr11:77717712-777
ENSG0000(	495	11.20871	chr11:144	LINC02761		lncRNA	chr11:76210956-762
ENSG0000(	495	11.20871	chr11:144	RN7SL786P		smallRNA	chr11:75742129-757
ENSG0000(	495	11.20871	chr11:144	UVRAG-DT		lncRNA	chr11:75803431-758
ENSG0000(	495	11.20871	chr11:144	MAP6		protein_c	chr11:75586918-756
ENSG0000(	495	11.20871	chr11:144	ENSG00000255479		lncRNA	chr11:76625462-766
ENSG0000(	495	11.20871	chr11:144	CLNS1A		protein_c	chr11:77514936-776
ENSG0000(	495	11.20871	chr11:144	ENSG00000255081		lncRNA	chr11:75914201-759
ENSG0000(	495	11.20871	chr11:144	EMSY-DT		lncRNA	chr11:76435559-764
ENSG0000(	495	11.20871	chr11:144	SNORD15A		smallRNA	chr11:75400391-754
ENSG0000(	495	11.20871	chr11:144	ENSG00000255060		lncRNA	chr11:36425447-364
ENSG0000(	495	11.20871	chr11:144	TSKU		protein_c	chr11:76782251-767
ENSG0000(	495	11.20871	chr11:144	ENSG00000254975		lncRNA	chr11:76675079-767
ENSG0000(	495	11.20871	chr11:144	ZDHHC20P3		Pseudoger	chr11:75228322-752
ENSG0000(	495	11.20871	chr11:144	ENSG00000254630		lncRNA	chr11:75635883-756
ENSG0000(	495	11.20871	chr11:144	UVRAG		protein_c	chr11:75815210-761
ENSG0000(	495	11.20871	chr11:144	WNT11		protein_c	chr11:76186325-762

ENSG0000(	495	11.20871	chr11:14⁄	ENSG00000254933		lncRNA	chr11:76190725-761
ENSG0000(	495	11.20871	chr11:14⁄	ENSG00000255421		lncRNA	chr11:76137315-761
ENSG0000(	495	11.20871	chr11:14⁄	LINC02757		lncRNA	chr11:76607853-766
ENSG0000(	495	11.20871	chr11:14⁄	TPBGL		protein_c	chr11:75240774-752
ENSG0000(	495	11.20871	chr11:14⁄	Y_RNA		smallRNA	chr11:77691650-776
ENSG0000(	495	11.20871	chr11:14⁄	Y_RNA		smallRNA	chr11:75837544-758
ENSG0000(	495	11.20871	chr11:14⁄	DGAT2		protein_c	chr11:75759512-758
ENSG0000(	495	11.20871	chr11:14⁄	TSKU-AS1		lncRNA	chr11:76782581-767
ENSG0000(	495	11.20871	chr11:14⁄	ENSG00000290785		lncRNA	chr11:76712396-767
ENSG0000(	495	11.20871	chr11:14⁄	RNU7-59P		smallRNA	chr11:77566934-775
ENSG0000(	495	11.20871	chr11:14⁄	ENSG00000254963		lncRNA	chr11:75264289-752
ENSG0000(	495	11.20871	chr11:14⁄	ENSG00000254429		lncRNA	chr11:75260127-752
ENSG0000(	495	11.20871	chr11:14⁄	Y_RNA		smallRNA	chr11:75835215-758
ENSG0000(	495	11.20871	chr11:14⁄	CAPN5	DriverDB\	protein_c	chr11:77066961-771
ENSG0000(	495	11.20871	chr11:14⁄	GUCY2EP		Pseudoger	chr11:76694041-767
ENSG0000(	495	11.20871	chr11:14⁄	B3GNT6		protein_c	chr11:77034398-770
ENSG0000(	489	11.07285	chr16:23¢	ENSG00000289491		protein_c	chr16:30610211-306
ENSG0000(	485	10.98228	chr11:14⁄	KCNK4-TEX40		lncRNA	chr11:64291722-643
ENSG0000(	485	10.98228	chr11:14⁄	RPEP6		Pseudoger	chr11:72131282-721
ENSG0000(	485	10.98228	chr11:14⁄	ENSG00000228286		Pseudoger	chr11:72249856-722
ENSG0000(	485	10.98228	chr11:14⁄	RN7SL596P		smallRNA	chr11:63797788-637
ENSG0000(	485	10.98228	chr11:14⁄	ENSG00000257058		lncRNA	chr11:62545999-625
ENSG0000(	485	10.98228	chr11:14⁄	ENSG00000255118		lncRNA	chr11:62336911-623
ENSG0000(	485	10.98228	chr11:14⁄	DEFB131B		protein_c	chr11:71878453-718
ENSG0000(	485	10.98228	chr11:14⁄	LRRN4CL		protein_c	chr11:62686406-626
ENSG0000(	485	10.98228	chr11:14⁄	ENSG00000255119		lncRNA	chr11:67605521-676
ENSG0000(	485	10.98228	chr11:14⁄	FAU		protein_c	chr11:65120630-651
ENSG0000(	485	10.98228	chr11:14⁄	OVOL1-AS1		lncRNA	chr11:65789051-657
ENSG0000(	485	10.98228	chr11:14⁄	ENSG00000257086		lncRNA	chr11:64246939-642
ENSG0000(	485	10.98228	chr11:14⁄	ENSG00000255126		lncRNA	chr11:62391516-623
ENSG0000(	485	10.98228	chr11:14⁄	UNC93B6		Pseudoger	chr11:71603260-716
ENSG0000(	485	10.98228	chr11:14⁄	ART2P		Pseudoger	chr11:72519986-725
ENSG0000(	485	10.98228	chr11:14⁄	ENSG00000251143		lncRNA	chr11:72014291-720
ENSG0000(	485	10.98228	chr11:14⁄	TM7SF2		protein_c	chr11:65111845-651
ENSG0000(	485	10.98228	chr11:14⁄	CTTN	DriverDB\	protein_c	chr11:70398404-704
ENSG0000(	485	10.98228	chr11:14⁄	VPS51	NCGv7	protein_c	chr11:65089324-651
ENSG0000(	485	10.98228	chr11:14⁄	ENSG00000255446		lncRNA	chr11:62421845-624
ENSG0000(	485	10.98228	chr11:14⁄	RNU6-46P		smallRNA	chr11:67895631-678
ENSG0000(	485	10.98228	chr11:14⁄	SNORA57		smallRNA	chr11:62665422-626
ENSG0000(	485	10.98228	chr11:14⁄	PDCL2P2		Pseudoger	chr11:65160194-651
ENSG0000(	485	10.98228	chr11:14⁄	AP001266.1		Pseudoger	chr11:65777621-657
ENSG0000(	485	10.98228	chr11:14⁄	MTA2		protein_c	chr11:62593214-626
ENSG0000(	485	10.98228	chr11:14⁄	SNHG1		lncRNA	chr11:62851978-628
ENSG0000(	485	10.98228	chr11:14⁄	SLC22A8	NCGv7	protein_c	chr11:62989154-630
ENSG0000(	485	10.98228	chr11:14⁄	ENSG00000279459		TEC	chr11:70603304-706
ENSG0000(	485	10.98228	chr11:14⁄	ENSG00000255038		lncRNA	chr11:66067277-660
ENSG0000(	485	10.98228	chr11:14⁄	P4HA3		protein_c	chr11:74235801-743
ENSG0000(	485	10.98228	chr11:14⁄	LAMTOR1	DriverDB\	protein_c	chr11:72085895-721
ENSG0000(	485	10.98228	chr11:14⁄	RPS6KB2		protein_c	chr11:67428460-674
ENSG0000(	485	10.98228	chr11:14⁄	CCDC85B		protein_c	chr11:65890673-658
ENSG0000(	485	10.98228	chr11:14⁄	FOSL1	TAG;AC	protein_c	chr11:65892049-659

ENSG0000(	485	10.98228	chr11:14	P2RY2		protein_c	chr11:73218281-732
ENSG0000(	485	10.98228	chr11:14	RAB6A		protein_c	chr11:73675638-737
ENSG0000(	485	10.98228	chr11:14	ENSG00000255031		lncRNA	chr11:68050740-680
ENSG0000(	485	10.98228	chr11:14	MRPL48		protein_c	chr11:73787872-738
ENSG0000(	485	10.98228	chr11:14	Y_RNA		smallRNA	chr11:64296037-642
ENSG0000(	485	10.98228	chr11:14	PAAF1	NCGv7	protein_c	chr11:73876699-739
ENSG0000(	485	10.98228	chr11:14	C11orf68		protein_c	chr11:65916810-659
ENSG0000(	485	10.98228	chr11:14	UCP2		protein_c	chr11:73974672-739
ENSG0000(	485	10.98228	chr11:14	UCP3		protein_c	chr11:74000277-740
ENSG0000(	485	10.98228	chr11:14	DRAP1		protein_c	chr11:65919274-659
ENSG0000(	485	10.98228	chr11:14	CABP4		protein_c	chr11:67452406-674
ENSG0000(	485	10.98228	chr11:14	KCNE3		protein_c	chr11:74454841-744
ENSG0000(	485	10.98228	chr11:14	LIPT2		protein_c	chr11:74490519-744
ENSG0000(	485	10.98228	chr11:14	GPR152		protein_c	chr11:67451301-674
ENSG0000(	485	10.98228	chr11:14	TSGA10IP		protein_c	chr11:65945480-659
ENSG0000(	485	10.98228	chr11:14	CLCF1		protein_c	chr11:67364168-673
ENSG0000(	485	10.98228	chr11:14	INTS4	NCGv7;AC	protein_c	chr11:77874418-779
ENSG0000(	485	10.98228	chr11:14	ROM1		protein_c	chr11:62611722-626
ENSG0000(	485	10.98228	chr11:14	SNORA7		smallRNA	chr11:74252414-742
ENSG0000(	485	10.98228	chr11:14	EML3		protein_c	chr11:62602218-626
ENSG0000(	485	10.98228	chr11:14	PLAAT3		protein_c	chr11:63573195-636
ENSG0000(	485	10.98228	chr11:14	MIR4690		smallRNA	chr11:65636310-656
ENSG0000(	485	10.98228	chr11:14	RN7SL119P		smallRNA	chr11:62816830-628
ENSG0000(	485	10.98228	chr11:14	RNU6-672P		smallRNA	chr11:72869544-728
ENSG0000(	485	10.98228	chr11:14	FAM89B		protein_c	chr11:65572349-655
ENSG0000(	485	10.98228	chr11:14	ENSG00000255115		Pseudoger	chr11:77914990-779
ENSG0000(	485	10.98228	chr11:14	MIR548AL		smallRNA	chr11:74399237-743
ENSG0000(	485	10.98228	chr11:14	LINC02952		lncRNA	chr11:69438365-694
ENSG0000(	485	10.98228	chr11:14	ENSG00000287934		lncRNA	chr11:67252336-672
ENSG0000(	485	10.98228	chr11:14	CDC42EP2		protein_c	chr11:65314866-653
ENSG0000(	485	10.98228	chr11:14	ENSG00000287917		lncRNA	chr11:65305345-653
ENSG0000(	485	10.98228	chr11:14	MRPL49		protein_c	chr11:65122183-651
ENSG0000(	485	10.98228	chr11:14	PLCB3		protein_c	chr11:64251530-642
ENSG0000(	485	10.98228	chr11:14	FERMT3		protein_c	chr11:64205926-642
ENSG0000(	485	10.98228	chr11:14	NUDT22		protein_c	chr11:64225941-642
ENSG0000(	485	10.98228	chr11:14	INCENP		protein_c	chr11:62123998-621
ENSG0000(	485	10.98228	chr11:14	TRPT1		protein_c	chr11:64223799-642
ENSG0000(	485	10.98228	chr11:14	ENSG00000287851		lncRNA	chr11:67316539-673
ENSG0000(	485	10.98228	chr11:14	SLC22A9		protein_c	chr11:63369785-634
ENSG0000(	485	10.98228	chr11:14	RNU6-292P		smallRNA	chr11:72047873-720
ENSG0000(	485	10.98228	chr11:14	COX8A		protein_c	chr11:63974620-639
ENSG0000(	485	10.98228	chr11:14	GPHA2		protein_c	chr11:64934471-649
ENSG0000(	485	10.98228	chr11:14	ENSG00000287821		lncRNA	chr11:65260996-652
ENSG0000(	485	10.98228	chr11:14	LTO1		protein_c	chr11:69653076-696
ENSG0000(	485	10.98228	chr11:14	GSTP1		protein_c	chr11:67583742-675
ENSG0000(	485	10.98228	chr11:14	ENSG00000255084		lncRNA	chr11:78533176-785
ENSG0000(	485	10.98228	chr11:14	LINC02728		lncRNA	chr11:78423982-784
ENSG0000(	485	10.98228	chr11:14	B3GAT3		protein_c	chr11:62615296-626
ENSG0000(	485	10.98228	chr11:14	FAM86C1P		Pseudoger	chr11:71787537-717
ENSG0000(	485	10.98228	chr11:14	ENSG00000255143		lncRNA	chr11:70129297-701
ENSG0000(	485	10.98228	chr11:14	STX5-DT		lncRNA	chr11:62832234-628

ENSG0000(	485	10.98228	chr11:144	ARHGEF17-AS1	lncRNA	chr11:73307235-733
ENSG0000(	485	10.98228	chr11:144	ENSG00000255320	lncRNA	chr11:66244717-662
ENSG0000(	485	10.98228	chr11:144	CCDC87	protein_c	chr11:66590176-665
ENSG0000(	485	10.98228	chr11:144	DNAJB6P5	Pseudoger	chr11:69737298-697
ENSG0000(	485	10.98228	chr11:144	ALG1L8P	Pseudoger	chr11:67785273-677
ENSG0000(	485	10.98228	chr11:144	ENSG00000256041	Pseudoger	chr11:63032503-630
ENSG0000(	485	10.98228	chr11:144	Y_RNA	smallRNA	chr11:72766004-727
ENSG0000(	485	10.98228	chr11:144	ENSG00000227615	Pseudoger	chr11:74745716-747
ENSG0000(	485	10.98228	chr11:144	LINC02701	lncRNA	chr11:68870664-688
ENSG0000(	485	10.98228	chr11:144	ENSG00000256403	lncRNA	chr11:72410716-724
ENSG0000(	485	10.98228	chr11:144	ENSG00000256349	protein_c	chr11:66509079-665
ENSG0000(	485	10.98228	chr11:144	ENSG00000289194	lncRNA	chr11:62153730-621
ENSG0000(	485	10.98228	chr11:144	OR8R1P	Pseudoger	chr11:73248779-732
ENSG0000(	485	10.98228	chr11:144	ENSG00000255478	lncRNA	chr11:65367438-653
ENSG0000(	485	10.98228	chr11:144	RNU7-23P	smallRNA	chr11:66919762-669
ENSG0000(	485	10.98228	chr11:144	ENSG00000289231	lncRNA	chr11:65353024-653
ENSG0000(	485	10.98228	chr11:144	AP003064.2	smallRNA	chr11:62372656-623
ENSG0000(	485	10.98228	chr11:144	ENSG00000256448	lncRNA	chr11:73405297-734
ENSG0000(	485	10.98228	chr11:144	ENSG00000289560	lncRNA	chr11:66311859-663
ENSG0000(	485	10.98228	chr11:144	ENSG00000289074	lncRNA	chr11:70269189-702
ENSG0000(	485	10.98228	chr11:144	LINC02953	lncRNA	chr11:69425678-694
ENSG0000(	485	10.98228	chr11:144	MRGPRF-AS1	lncRNA	chr11:69012283-690
ENSG0000(	485	10.98228	chr11:144	ENSG00000255508	protein_c	chr11:62559603-625
ENSG0000(	485	10.98228	chr11:144	snoU13	smallRNA	chr11:67220908-672
ENSG0000(	485	10.98228	chr11:144	AP001992.1	smallRNA	chr11:74945981-749
ENSG0000(	485	10.98228	chr11:144	ENSG00000203520	lncRNA	chr11:63616308-636
ENSG0000(	485	10.98228	chr11:144	RPS6KB2-AS1	lncRNA	chr11:67431367-674
ENSG0000(	485	10.98228	chr11:144	RN7SKP239	smallRNA	chr11:67362414-673
ENSG0000(	485	10.98228	chr11:144	ENSG00000289058	lncRNA	chr11:64687682-646
ENSG0000(	485	10.98228	chr11:144	ARAP1-AS1	lncRNA	chr11:72685075-726
ENSG0000(	485	10.98228	chr11:144	ENSG00000289562	lncRNA	chr11:62574174-625
ENSG0000(	485	10.98228	chr11:144	H2AZP4	Pseudoger	chr11:70278921-702
ENSG0000(	485	10.98228	chr11:144	SNORA43	smallRNA	chr11:66432763-664
ENSG0000(	485	10.98228	chr11:144	ENSG00000256481	lncRNA	chr11:64081690-640
ENSG0000(	485	10.98228	chr11:144	ENSG00000256034	lncRNA	chr11:73760563-737
ENSG0000(	485	10.98228	chr11:144	RPL37P2	Pseudoger	chr11:67682772-676
ENSG0000(	485	10.98228	chr11:144	ENSG00000256341	lncRNA	chr11:64118272-641
ENSG0000(	485	10.98228	chr11:144	RN7SL12P	smallRNA	chr11:66712721-667
ENSG0000(	485	10.98228	chr11:144	ENSG00000256148	Pseudoger	chr11:73510658-735
ENSG0000(	485	10.98228	chr11:144	ENSG00000256189	Pseudoger	chr11:73991283-739
ENSG0000(	485	10.98228	chr11:144	RBM14 AC	protein_c	chr11:66616626-666
ENSG0000(	485	10.98228	chr11:144	ENSG00000256181	Pseudoger	chr11:63265836-632
ENSG0000(	485	10.98228	chr11:144	RNU6-1306P	smallRNA	chr11:63882587-638
ENSG0000(	485	10.98228	chr11:144	RPS3AP41	Pseudoger	chr11:71669520-716
ENSG0000(	485	10.98228	chr11:144	ENSG00000255449	lncRNA	chr11:77866412-778
ENSG0000(	485	10.98228	chr11:144	ENSG00000255440	lncRNA	chr11:74398825-744
ENSG0000(	485	10.98228	chr11:144	LINC01537	lncRNA	chr11:72570660-725
ENSG0000(	485	10.98228	chr11:144	CYCSP27	Pseudoger	chr11:74482250-744
ENSG0000(	485	10.98228	chr11:144	RN7SL309P	smallRNA	chr11:65695535-656
ENSG0000(	485	10.98228	chr11:144	ENSG00000256098	Pseudoger	chr11:74142151-741
ENSG0000(	485	10.98228	chr11:144	ENSG00000256116	lncRNA	chr11:64229214-642

ENSG0000(	485	10.98228	chr11:14∠	KCNK4	protein_c	chr11:64291302-643
ENSG0000(	485	10.98228	chr11:14∠	ENSG00000256100	protein_c	chr11:63974620-639
ENSG0000(	485	10.98228	chr11:14∠	ENSG00000255432	protein_c	chr11:62649694-626
ENSG0000(	485	10.98228	chr11:14∠	LINC02736	lncRNA	chr11:65487241-654
ENSG0000(	485	10.98228	chr11:14∠	ENSG00000255395	lncRNA	chr11:75099172-751
ENSG0000(	485	10.98228	chr11:14∠	COA4	protein_c	chr11:73872667-738
ENSG0000(	485	10.98228	chr11:14∠	ENSG00000289259	lncRNA	chr11:65471472-654
ENSG0000(	485	10.98228	chr11:14∠	B4GAT1-DT	lncRNA	chr11:66347950-663
ENSG0000(	485	10.98228	chr11:14∠	ENSG00000255404	lncRNA	chr11:65795946-657
ENSG0000(	485	10.98228	chr11:14∠	ATP5MGP1	Pseudoger	chr11:63834667-638
ENSG0000(	485	10.98228	chr11:14∠	RNU1-84P	smallRNA	chr11:66393449-663
ENSG0000(	485	10.98228	chr11:14∠	LINC02724	lncRNA	chr11:64449074-644
ENSG0000(	485	10.98228	chr11:14∠	RSF1-IT1	lncRNA	chr11:77738680-777
ENSG0000(	485	10.98228	chr11:14∠	ENSG00000289486	lncRNA	chr11:64184892-641
ENSG0000(	485	10.98228	chr11:14∠	MIR3164	smallRNA	chr11:69083176-690
ENSG0000(	485	10.98228	chr11:14∠	SHANK2-AS1	lncRNA	chr11:70626441-706
ENSG0000(	485	10.98228	chr11:14∠	ENSG00000255415	Pseudoger	chr11:71629045-716
ENSG0000(	485	10.98228	chr11:14∠	FTH1P16	Pseudoger	chr11:77734475-777
ENSG0000(	485	10.98228	chr11:14∠	ENSG00000289339	lncRNA	chr11:65575330-655
ENSG0000(	485	10.98228	chr11:14∠	ENSG00000289343	lncRNA	chr11:67508772-675
ENSG0000(	485	10.98228	chr11:14∠	ENSG00000255928	lncRNA	chr11:73722349-737
ENSG0000(	485	10.98228	chr11:14∠	ENSG00000256514	protein_c	chr11:67351572-673
ENSG0000(	485	10.98228	chr11:14∠	TALAM1	lncRNA	chr11:65499312-655
ENSG0000(	485	10.98228	chr11:14∠	AP003064.1	smallRNA	chr11:62463635-624
ENSG0000(	485	10.98228	chr11:14∠	KRTAP5-10	protein_c	chr11:71565563-715
ENSG0000(	485	10.98228	chr11:14∠	KRTAP5-11NCGv7	protein_c	chr11:71581855-716
ENSG0000(	485	10.98228	chr11:14∠	ENSG00000255191	lncRNA	chr11:69985876-700
ENSG0000(	485	10.98228	chr11:14∠	TMEM151A	protein_c	chr11:66291894-662
ENSG0000(	485	10.98228	chr11:14∠	XNDC1N-ZNF705EP-AI	protein_c	chr11:71804997-719
ENSG0000(	485	10.98228	chr11:14∠	PGAM1P8	Pseudoger	chr11:65174117-651
ENSG0000(	485	10.98228	chr11:14∠	CCND2P1	Pseudoger	chr11:63243085-632
ENSG0000(	485	10.98228	chr11:14∠	LINC02747	lncRNA	chr11:69475567-694
ENSG0000(	485	10.98228	chr11:14∠	ENSG00000256824	lncRNA	chr11:64035970-641
ENSG0000(	485	10.98228	chr11:14∠	SF3B2 NCGv7	protein_c	chr11:66050729-660
ENSG0000(	485	10.98228	chr11:14∠	NPM1P35	Pseudoger	chr11:62330946-623
ENSG0000(	485	10.98228	chr11:14∠	MIR4692	smallRNA	chr11:72783530-727
ENSG0000(	485	10.98228	chr11:14∠	ENSG00000256789	lncRNA	chr11:63637677-636
ENSG0000(	485	10.98228	chr11:14∠	ENSG00000255230	Pseudoger	chr11:67965873-679
ENSG0000(	485	10.98228	chr11:14∠	ENSG00000256739	lncRNA	chr11:72351347-723
ENSG0000(	485	10.98228	chr11:14∠	ENSG00000179038	Pseudoger	chr11:67195569-671
ENSG0000(	485	10.98228	chr11:14∠	OR7E1P	Pseudoger	chr11:67974286-679
ENSG0000(	485	10.98228	chr11:14∠	MIR139	smallRNA	chr11:72615063-726
ENSG0000(	485	10.98228	chr11:14∠	ENSG00000256928	lncRNA	chr11:73395559-733
ENSG0000(	485	10.98228	chr11:14∠	EVA1CP4	Pseudoger	chr11:67749043-677
ENSG0000(	485	10.98228	chr11:14∠	DEFB130C	Pseudoger	chr11:71856277-718
ENSG0000(	485	10.98228	chr11:14∠	ENSG00000257002	lncRNA	chr11:62909546-629
ENSG0000(	485	10.98228	chr11:14∠	ZNF75CP	Pseudoger	chr11:78384059-783
ENSG0000(	485	10.98228	chr11:14∠	ENSG00000255173	lncRNA	chr11:65117157-651
ENSG0000(	485	10.98228	chr11:14∠	PPP1R14B-AS1	lncRNA	chr11:64245838-642
ENSG0000(	485	10.98228	chr11:14∠	ENSG00000288538	lncRNA	chr11:78223815-782
ENSG0000(	485	10.98228	chr11:14∠	ARPC3P4	Pseudoger	chr11:73921665-739

ENSG0000(	485	10.98228	chr11:14<	ENSG00000255557		lncRNA	chr11:65745729-657
ENSG0000(	485	10.98228	chr11:14<	ENSG00000204971		lncRNA	chr11:72163322-722
ENSG0000(	485	10.98228	chr11:14<	UQCC3		protein_c	chr11:62670273-626
ENSG0000(	485	10.98228	chr11:14<	ENSG00000279093		TEC	chr11:65561484-655
ENSG0000(	485	10.98228	chr11:14<	SPDYC		protein_c	chr11:65170233-651
ENSG0000(	485	10.98228	chr11:14<	ENSG00000255741		lncRNA	chr11:68941503-689
ENSG0000(	485	10.98228	chr11:14<	ENSG00000256723		Pseudoger	chr11:73994972-739
ENSG0000(	485	10.98228	chr11:14<	CHKA-DT		lncRNA	chr11:68121624-681
ENSG0000(	485	10.98228	chr11:14<	OR7E4P		Pseudoger	chr11:71620020-716
ENSG0000(	485	10.98228	chr11:14<	ENSG00000255847		lncRNA	chr11:73963657-739
ENSG0000(	485	10.98228	chr11:14<	MIR4489		smallRNA	chr11:65649192-656
ENSG0000(	485	10.98228	chr11:14<	ENSG00000288853		lncRNA	chr11:78314796-783
ENSG0000(	485	10.98228	chr11:14<	IUR1		lncRNA	chr11:73157173-731
ENSG0000(	485	10.98228	chr11:14<	ENSG00000250659		lncRNA	chr11:62537312-625
ENSG0000(	485	10.98228	chr11:14<	ENSG00000227726		lncRNA	chr11:70477277-704
ENSG0000(	485	10.98228	chr11:14<	ENSG00000255306		lncRNA	chr11:68024809-680
ENSG0000(	485	10.98228	chr11:14<	RPL29P22		Pseudoger	chr11:63115880-631
ENSG0000(	485	10.98228	chr11:14<	ENSG00000255843		lncRNA	chr11:72302139-723
ENSG0000(	485	10.98228	chr11:14<	ENPP7P8		Pseudoger	chr11:71722052-717
ENSG0000(	485	10.98228	chr11:14<	FOLR1P1		Pseudoger	chr11:72158822-721
ENSG0000(	485	10.98228	chr11:14<	ENSG00000279353		TEC	chr11:74698231-746
ENSG0000(	485	10.98228	chr11:14<	GANAB		protein_c	chr11:62624826-626
ENSG0000(	485	10.98228	chr11:14<	ENSG00000255860		Pseudoger	chr11:72172455-721
ENSG0000(	485	10.98228	chr11:14<	DPP3-DT		lncRNA	chr11:66473490-664
ENSG0000(	485	10.98228	chr11:14<	ENSG00000288852		lncRNA	chr11:64183353-641
ENSG0000(	485	10.98228	chr11:14<	snoU13		smallRNA	chr11:72104074-721
ENSG0000(	485	10.98228	chr11:14<	MIX23P5		Pseudoger	chr11:73850469-738
ENSG0000(	485	10.98228	chr11:14<	ENSG00000255296		Pseudoger	chr11:71938671-719
ENSG0000(	485	10.98228	chr11:14<	AAMDC	DriverDB\	protein_c	chr11:77821109-779
ENSG0000(	485	10.98228	chr11:14<	ENSG00000227834		Pseudoger	chr11:67651576-676
ENSG0000(	485	10.98228	chr11:14<	ENSG00000255786		Pseudoger	chr11:73452020-734
ENSG0000(	485	10.98228	chr11:14<	KCTD14		protein_c	chr11:78015715-780
ENSG0000(	485	10.98228	chr11:14<	MIR4696		smallRNA	chr11:74720268-747
ENSG0000(	485	10.98228	chr11:14<	PDE2A-AS2		lncRNA	chr11:72584572-725
ENSG0000(	485	10.98228	chr11:14<	THRSP		protein_c	chr11:78063861-780
ENSG0000(	485	10.98228	chr11:14<	ENSG00000250105		lncRNA	chr11:66558866-665
ENSG0000(	485	10.98228	chr11:14<	snoU13		smallRNA	chr11:66053144-660
ENSG0000(	485	10.98228	chr11:14<	NDUFC2	DriverDB\	protein_c	chr11:78068297-780
ENSG0000(	485	10.98228	chr11:14<	PDE2A-AS1		lncRNA	chr11:72643237-726
ENSG0000(	485	10.98228	chr11:14<	ENSG00000256568		lncRNA	chr11:73157946-731
ENSG0000(	485	10.98228	chr11:14<	snoU13		smallRNA	chr11:66152621-661
ENSG0000(	485	10.98228	chr11:14<	ENSG00000255539		lncRNA	chr11:70324871-703
ENSG0000(	485	10.98228	chr11:14<	ALG8	DriverDB\	protein_c	chr11:78095244-781
ENSG0000(	485	10.98228	chr11:14<	AC004924.1		smallRNA	chr11:67933214-679
ENSG0000(	485	10.98228	chr11:14<	POLD4		protein_c	chr11:67350772-673
ENSG0000(	485	10.98228	chr11:14<	Y_RNA		smallRNA	chr11:73664515-736
ENSG0000(	485	10.98228	chr11:14<	PGM2L1		protein_c	chr11:74330316-743
ENSG0000(	485	10.98228	chr11:14<	CSKMT		protein_c	chr11:62665309-626
ENSG0000(	485	10.98228	chr11:14<	HNRNPUL2		protein_c	chr11:62712630-627
ENSG0000(	485	10.98228	chr11:14<	KRT8P26		Pseudoger	chr11:65726939-657
ENSG0000(	485	10.98228	chr11:14<	ZNF705EP		Pseudoger	chr11:71816512-718

ENSG0000(	485	10.98228	chr11:14	STARD10	NCGv7	protein_c	chr11:72754729-727
ENSG0000(	485	10.98228	chr11:14	PPME1		protein_c	chr11:74171267-742
ENSG0000(	485	10.98228	chr11:14	ENSG00000261276		lncRNA	chr11:69004394-690
ENSG0000(	485	10.98228	chr11:14	ENSG00000286688		lncRNA	chr11:66514306-665
ENSG0000(	485	10.98228	chr11:14	FOLR2		protein_c	chr11:72216601-722
ENSG0000(	485	10.98228	chr11:14	U3		smallRNA	chr11:66995479-669
ENSG0000(	485	10.98228	chr11:14	SCYL1		protein_c	chr11:65525077-655
ENSG0000(	485	10.98228	chr11:14	ENSG00000286708		lncRNA	chr11:70886090-708
ENSG0000(	485	10.98228	chr11:14	ENSG00000261347		lncRNA	chr11:69467598-694
ENSG0000(	485	10.98228	chr11:14	ENSG00000286756		lncRNA	chr11:65487884-654
ENSG0000(	485	10.98228	chr11:14	OR2AT2P		Pseudoger	chr11:75071148-750
ENSG0000(	485	10.98228	chr11:14	ENSG00000234751		Pseudoger	chr11:72940498-729
ENSG0000(	485	10.98228	chr11:14	ENSG00000286816		lncRNA	chr11:64486136-644
ENSG0000(	485	10.98228	chr11:14	ENSG00000274251		lncRNA	chr11:67353629-673
ENSG0000(	485	10.98228	chr11:14	INPPL1	NCGv7;AC	protein_c	chr11:72223701-722
ENSG0000(	485	10.98228	chr11:14	C11orf24		protein_c	chr11:68261338-682
ENSG0000(	485	10.98228	chr11:14	ENSG00000276109		Pseudoger	chr11:72551049-725
ENSG0000(	485	10.98228	chr11:14	NARS2		protein_c	chr11:78435620-785
ENSG0000(	485	10.98228	chr11:14	ENSG00000286369		lncRNA	chr11:68272100-682
ENSG0000(	485	10.98228	chr11:14	RP11-211G23.2		lncRNA	chr11:69371463-693
ENSG0000(	485	10.98228	chr11:14	ENSG00000274664		Pseudoger	chr11:72562044-725
ENSG0000(	485	10.98228	chr11:14	RNF121	DriverDB\	protein_c	chr11:71929018-719
ENSG0000(	485	10.98228	chr11:14	ENSG00000215841		lncRNA	chr11:73214851-732
ENSG0000(	485	10.98228	chr11:14	NDUFC2-KCTD14		protein_c	chr11:78016971-780
ENSG0000(	485	10.98228	chr11:14	ENSG00000260895		lncRNA	chr11:69103493-691
ENSG0000(	485	10.98228	chr11:14	ENSG00000286459		lncRNA	chr11:66043298-660
ENSG0000(	485	10.98228	chr11:14	ENSG00000261070		lncRNA	chr11:69147228-691
ENSG0000(	485	10.98228	chr11:14	RNA5SP342		Pseudoger	chr11:71845196-718
ENSG0000(	485	10.98228	chr11:14	TEX54		protein_c	chr11:62832319-628
ENSG0000(	485	10.98228	chr11:14	ENSG00000283257		Pseudoger	chr11:67538899-675
ENSG0000(	485	10.98228	chr11:14	RN7SKP297		smallRNA	chr11:74685224-746
ENSG0000(	485	10.98228	chr11:14	AP003498.2		smallRNA	chr11:71706479-717
ENSG0000(	485	10.98228	chr11:14	ENSG00000230835		Pseudoger	chr11:64881535-648
ENSG0000(	485	10.98228	chr11:14	ENSG00000286555		lncRNA	chr11:72689650-726
ENSG0000(	485	10.98228	chr11:14	PHOX2A		protein_c	chr11:72239077-722
ENSG0000(	485	10.98228	chr11:14	ENPP7P7		Pseudoger	chr11:67812557-678
ENSG0000(	485	10.98228	chr11:14	HNRNPUL2-BSCL2		protein_c	chr11:62690275-627
ENSG0000(	485	10.98228	chr11:14	IL18BP		protein_c	chr11:71998613-720
ENSG0000(	485	10.98228	chr11:14	AP5B1		protein_c	chr11:65773898-657
ENSG0000(	485	10.98228	chr11:14	MIR194-2HG		lncRNA	chr11:64889560-648
ENSG0000(	485	10.98228	chr11:14	ENSG00000254452		lncRNA	chr11:66276779-662
ENSG0000(	485	10.98228	chr11:14	RCC2P6		Pseudoger	chr11:62371146-623
ENSG0000(	485	10.98228	chr11:14	HIGD1AP10		Pseudoger	chr11:65145691-651
ENSG0000(	485	10.98228	chr11:14	ENSG00000254458		lncRNA	chr11:66312853-663
ENSG0000(	485	10.98228	chr11:14	ENSG00000254459		lncRNA	chr11:77829654-778
ENSG0000(	485	10.98228	chr11:14	ENSG00000254461		lncRNA	chr11:66259567-662
ENSG0000(	485	10.98228	chr11:14	XNDC1N		protein_c	chr11:71865504-719
ENSG0000(	485	10.98228	chr11:14	SNORD43		smallRNA	chr11:74716687-747
ENSG0000(	485	10.98228	chr11:14	ENSG00000280269		TEC	chr11:74204869-742
ENSG0000(	485	10.98228	chr11:14	FIBP		protein_c	chr11:65883740-658
ENSG0000(	485	10.98228	chr11:14	CARNS1	NCGv7	protein_c	chr11:67414968-674

ENSG0000(	485	10.98228	chr11:14⁄	PPP1CA		protein_c	chr11:67398181-674
ENSG0000(	485	10.98228	chr11:14⁄	OR7E145P		Pseudoger	chr11:67722483-677
ENSG0000(	485	10.98228	chr11:14⁄	CTSW		protein_c	chr11:65879809-658
ENSG0000(	485	10.98228	chr11:14⁄	RAD9A		protein_c	chr11:67317871-673
ENSG0000(	485	10.98228	chr11:14⁄	EFEMP2		protein_c	chr11:65866441-658
ENSG0000(	485	10.98228	chr11:14⁄	TMEM134		protein_c	chr11:67461710-674
ENSG0000(	485	10.98228	chr11:14⁄	ENSG00000254447		lncRNA	chr11:67735600-677
ENSG0000(	485	10.98228	chr11:14⁄	ENSG00000254420		lncRNA	chr11:78324758-784
ENSG0000(	485	10.98228	chr11:14⁄	RNU6-126P		smallRNA	chr11:78133420-781
ENSG0000(	485	10.98228	chr11:14⁄	NPM1P50		Pseudoger	chr11:75079265-750
ENSG0000(	485	10.98228	chr11:14⁄	CDC42BPG	NCGv7	protein_c	chr11:64823052-648
ENSG0000(	485	10.98228	chr11:14⁄	ENSG00000286943		lncRNA	chr11:74931724-749
ENSG0000(	485	10.98228	chr11:14⁄	ENSG00000286948		lncRNA	chr11:71563389-715
ENSG0000(	485	10.98228	chr11:14⁄	ARL2		protein_c	chr11:65014160-650
ENSG0000(	485	10.98228	chr11:14⁄	ENSG00000261625		lncRNA	chr11:69000765-690
ENSG0000(	485	10.98228	chr11:14⁄	C1QBPP2		Pseudoger	chr11:66761575-667
ENSG0000(	485	10.98228	chr11:14⁄	PTPRCAP		protein_c	chr11:67435510-674
ENSG0000(	485	10.98228	chr11:14⁄	ENSG00000213365		Pseudoger	chr11:72280151-722
ENSG0000(	485	10.98228	chr11:14⁄	OR2AT4		protein_c	chr11:75081753-750
ENSG0000(	485	10.98228	chr11:14⁄	LINC02584		lncRNA	chr11:70072434-700
ENSG0000(	485	10.98228	chr11:14⁄	IFITM9P		Pseudoger	chr11:69303412-693
ENSG0000(	485	10.98228	chr11:14⁄	USP35	DriverDB\	protein_c	chr11:78188812-782
ENSG0000(	485	10.98228	chr11:14⁄	SPCS2		protein_c	chr11:74949261-749
ENSG0000(	485	10.98228	chr11:14⁄	P2RY6		protein_c	chr11:73264498-733
ENSG0000(	485	10.98228	chr11:14⁄	SHANK2-AS3		lncRNA	chr11:70862790-708
ENSG0000(	485	10.98228	chr11:14⁄	RNU6-118P		smallRNA	chr11:62815966-628
ENSG0000(	485	10.98228	chr11:14⁄	ENSG00000254404		lncRNA	chr11:62213427-622
ENSG0000(	485	10.98228	chr11:14⁄	HNRNPA1P40		Pseudoger	chr11:74354443-743
ENSG0000(	485	10.98228	chr11:14⁄	NUMA1	NCGv7;AC	protein_c	chr11:72002864-720
ENSG0000(	485	10.98228	chr11:14⁄	SCGB1D2		protein_c	chr11:62242239-622
ENSG0000(	485	10.98228	chr11:14⁄	SART1		protein_c	chr11:65961728-659
ENSG0000(	485	10.98228	chr11:14⁄	MIR1237		smallRNA	chr11:64368602-643
ENSG0000(	485	10.98228	chr11:14⁄	ENSG00000232500		lncRNA	chr11:64500846-645
ENSG0000(	485	10.98228	chr11:14⁄	ENSG00000275484		lncRNA	chr11:67374416-673
ENSG0000(	485	10.98228	chr11:14⁄	BSCL2		protein_c	chr11:62689289-627
ENSG0000(	485	10.98228	chr11:14⁄	TESMIN	DriverDB\	protein_c	chr11:68707440-687
ENSG0000(	485	10.98228	chr11:14⁄	ALDH3B2		protein_c	chr11:67662155-676
ENSG0000(	485	10.98228	chr11:14⁄	POLR2G		protein_c	chr11:62761565-627
ENSG0000(	485	10.98228	chr11:14⁄	SLC3A2	AC	protein_c	chr11:62856004-628
ENSG0000(	485	10.98228	chr11:14⁄	ACY3		protein_c	chr11:67642555-676
ENSG0000(	485	10.98228	chr11:14⁄	IGHMBP2	DriverDB\	protein_c	chr11:68903863-689
ENSG0000(	485	10.98228	chr11:14⁄	MIR548K		smallRNA	chr11:70283955-702
ENSG0000(	485	10.98228	chr11:14⁄	PLAAT5		protein_c	chr11:63461404-634
ENSG0000(	485	10.98228	chr11:14⁄	ZNRD2-DT		lncRNA	chr11:65568482-655
ENSG0000(	485	10.98228	chr11:14⁄	SPINDOC		protein_c	chr11:63813456-638
ENSG0000(	485	10.98228	chr11:14⁄	ATG16L2		protein_c	chr11:72814406-728
ENSG0000(	485	10.98228	chr11:14⁄	C2CD3	NCGv7	protein_c	chr11:74012718-741
ENSG0000(	485	10.98228	chr11:14⁄	ENSG00000285656		lncRNA	chr11:62116470-621
ENSG0000(	485	10.98228	chr11:14⁄	FADD	DriverDB\	protein_c	chr11:70203296-702
ENSG0000(	485	10.98228	chr11:14⁄	LTBP3		protein_c	chr11:65538559-655
ENSG0000(	485	10.98228	chr11:14⁄	LRTOMT	DriverDB\	protein_c	chr11:72080331-721

ENSG0000(	485	10.98228	chr11:14∠	MACROD1	AC	protein_c	chr11:63998558-641
ENSG0000(	485	10.98228	chr11:14∠	SAC3D1		protein_c	chr11:65040901-650
ENSG0000(	485	10.98228	chr11:14∠	CDK2AP2		protein_c	chr11:67506497-675
ENSG0000(	485	10.98228	chr11:14∠	LINC01488		lncRNA	chr11:69481662-694
ENSG0000(	485	10.98228	chr11:14∠	ENSG00000285388		Pseudoger	chr11:62936427-629
ENSG0000(	485	10.98228	chr11:14∠	AP003498.1		smallRNA	chr11:71662547-716
ENSG0000(	485	10.98228	chr11:14∠	MEN1	NCGv7;AC	protein_c	chr11:64803510-648
ENSG0000(	485	10.98228	chr11:14∠	OTUB1	DriverDB\	protein_c	chr11:63985853-640
ENSG0000(	485	10.98228	chr11:14∠	RCOR2		protein_c	chr11:63911230-639
ENSG0000(	485	10.98228	chr11:14∠	CABP2		protein_c	chr11:67518912-675
ENSG0000(	485	10.98228	chr11:14∠	NDUFV1		protein_c	chr11:67605653-676
ENSG0000(	485	10.98228	chr11:14∠	DPF2		protein_c	chr11:65333843-653
ENSG0000(	485	10.98228	chr11:14∠	WDR74	NCGv7	protein_c	chr11:62832342-628
ENSG0000(	485	10.98228	chr11:14∠	NUDT8		protein_c	chr11:67627938-676
ENSG0000(	485	10.98228	chr11:14∠	TBX10		protein_c	chr11:67631303-676
ENSG0000(	485	10.98228	chr11:14∠	RELA-DT		lncRNA	chr11:65662988-656
ENSG0000(	485	10.98228	chr11:14∠	OR7E11P		Pseudoger	chr11:67735608-677
ENSG0000(	485	10.98228	chr11:14∠	PLAAT2		protein_c	chr11:63552770-635
ENSG0000(	485	10.98228	chr11:14∠	PLAAT4		protein_c	chr11:63536808-635
ENSG0000(	485	10.98228	chr11:14∠	RTN3		protein_c	chr11:63681446-637
ENSG0000(	485	10.98228	chr11:14∠	LGALS12		protein_c	chr11:63506052-635
ENSG0000(	485	10.98228	chr11:14∠	NAALADL1		protein_c	chr11:65044818-650
ENSG0000(	485	10.98228	chr11:14∠	BATF2		protein_c	chr11:64987945-649
ENSG0000(	485	10.98228	chr11:14∠	SCGB2A1		protein_c	chr11:62208673-622
ENSG0000(	485	10.98228	chr11:14∠	FLRT1		protein_c	chr11:64035931-641
ENSG0000(	485	10.98228	chr11:14∠	SMIM38		protein_c	chr11:69155478-691
ENSG0000(	485	10.98228	chr11:14∠	RNU2-2P		smallRNA	chr11:62841619-628
ENSG0000(	485	10.98228	chr11:14∠	AP000807.2		smallRNA	chr11:68505572-685
ENSG0000(	485	10.98228	chr11:14∠	CHRM1		protein_c	chr11:62908679-629
ENSG0000(	485	10.98228	chr11:14∠	TMEM223		protein_c	chr11:62771629-627
ENSG0000(	485	10.98228	chr11:14∠	ENSG00000231492		lncRNA	chr11:64420311-644
ENSG0000(	485	10.98228	chr11:14∠	ENSG00000284625		Pseudoger	chr11:71639551-716
ENSG0000(	485	10.98228	chr11:14∠	RNU2-23P		smallRNA	chr11:65147584-651
ENSG0000(	485	10.98228	chr11:14∠	PRDX5		protein_c	chr11:64318121-643
ENSG0000(	485	10.98228	chr11:14∠	SCGB1D1		protein_c	chr11:62190216-621
ENSG0000(	485	10.98228	chr11:14∠	FRMD8	NCGv7	protein_c	chr11:65386621-654
ENSG0000(	485	10.98228	chr11:14∠	RNF169		protein_c	chr11:74748849-748
ENSG0000(	485	10.98228	chr11:14∠	ENSG00000286264		protein_c	chr11:64241095-642
ENSG0000(	485	10.98228	chr11:14∠	XRRA1		protein_c	chr11:74807739-749
ENSG0000(	485	10.98228	chr11:14∠	FCHSD2		protein_c	chr11:72836745-731
ENSG0000(	485	10.98228	chr11:14∠	ENSG00000260808		lncRNA	chr11:68612899-686
ENSG0000(	485	10.98228	chr11:14∠	RNU6-1238P		smallRNA	chr11:67395210-673
ENSG0000(	485	10.98228	chr11:14∠	AHNAK	NCGv7	protein_c	chr11:62433542-625
ENSG0000(	485	10.98228	chr11:14∠	ENSG00000284722		lncRNA	chr11:75069243-750
ENSG0000(	485	10.98228	chr11:14∠	LINC02723		lncRNA	chr11:64394342-643
ENSG0000(	485	10.98228	chr11:14∠	SLC22A11	NCGv7	protein_c	chr11:64555690-645
ENSG0000(	485	10.98228	chr11:14∠	PPFIA1	DriverDB\	protein_c	chr11:70270690-703
ENSG0000(	485	10.98228	chr11:14∠	SF1	NCGv7	protein_c	chr11:64764606-647
ENSG0000(	485	10.98228	chr11:14∠	MAP4K2		protein_c	chr11:64784918-648
ENSG0000(	485	10.98228	chr11:14∠	MAJIN		protein_c	chr11:64937517-649
ENSG0000(	485	10.98228	chr11:14∠	CCDC88B		protein_c	chr11:64340204-643

ENSG0000(	485	10.98228	chr11:14⁄	ENSG00000285693		lncRNA	chr11:72793624-728
ENSG0000(	485	10.98228	chr11:14⁄	ENSG00000275598		Pseudoger	chr11:63469376-634
ENSG0000(	485	10.98228	chr11:14⁄	DOC2GP		Pseudoger	chr11:67612651-676
ENSG0000(	485	10.98228	chr11:14⁄	LINC02956		lncRNA	chr11:69414307-694
ENSG0000(	485	10.98228	chr11:14⁄	ANO1	DriverDB\	protein_c	chr11:69985907-701
ENSG0000(	485	10.98228	chr11:14⁄	TOMT		protein_c	chr11:72105924-721
ENSG0000(	485	10.98228	chr11:14⁄	ENSG00000260401		lncRNA	chr11:73238975-732
ENSG0000(	485	10.98228	chr11:14⁄	ENSG00000285816		protein_c	chr11:65261928-653
ENSG0000(	485	10.98228	chr11:14⁄	ENSG00000285864		lncRNA	chr11:72261731-722
ENSG0000(	485	10.98228	chr11:14⁄	HMGN2P38		Pseudoger	chr11:73580253-735
ENSG0000(	485	10.98228	chr11:14⁄	ENSG00000285933		lncRNA	chr11:71745331-717
ENSG0000(	485	10.98228	chr11:14⁄	STIP1		protein_c	chr11:64185272-642
ENSG0000(	485	10.98228	chr11:14⁄	ENSG00000219529		Pseudoger	chr11:77813319-778
ENSG0000(	485	10.98228	chr11:14⁄	CATSPERZ		protein_c	chr11:64300358-643
ENSG0000(	485	10.98228	chr11:14⁄	RNU6-311P		smallRNA	chr11:78579255-785
ENSG0000(	485	10.98228	chr11:14⁄	AP000807.1		smallRNA	chr11:68506083-685
ENSG0000(	485	10.98228	chr11:14⁄	AP002490.1		smallRNA	chr11:72078330-720
ENSG0000(	485	10.98228	chr11:14⁄	EHD1		protein_c	chr11:64851642-648
ENSG0000(	485	10.98228	chr11:14⁄	ANAPC15	DriverDB\	protein_c	chr11:72106378-721
ENSG0000(	485	10.98228	chr11:14⁄	FOLR1		protein_c	chr11:72189558-721
ENSG0000(	485	10.98228	chr11:14⁄	CCND1	NCGv7;AC	protein_c	chr11:69641156-696
ENSG0000(	485	10.98228	chr11:14⁄	CPT1A	DriverDB\	protein_c	chr11:68754620-688
ENSG0000(	485	10.98228	chr11:14⁄	NRXN2	NCGv7	protein_c	chr11:64606174-647
ENSG0000(	485	10.98228	chr11:14⁄	PPP6R3		protein_c	chr11:68460731-686
ENSG0000(	485	10.98228	chr11:14⁄	KMT5B		protein_c	chr11:68154863-682
ENSG0000(	485	10.98228	chr11:14⁄	UNC93B1		protein_c	chr11:67991100-680
ENSG0000(	485	10.98228	chr11:14⁄	BBS1		protein_c	chr11:66510606-665
ENSG0000(	485	10.98228	chr11:14⁄	ARHGEF17		protein_c	chr11:73308276-733
ENSG0000(	485	10.98228	chr11:14⁄	ATG2A	NCGv7	protein_c	chr11:64894546-649
ENSG0000(	485	10.98228	chr11:14⁄	SNX15		protein_c	chr11:65027439-650
ENSG0000(	485	10.98228	chr11:14⁄	PELI3	NCGv7	protein_c	chr11:66466327-664
ENSG0000(	485	10.98228	chr11:14⁄	DNAJC4		protein_c	chr11:64230278-642
ENSG0000(	485	10.98228	chr11:14⁄	MALAT1	AC	lncRNA	chr11:65497688-655
ENSG0000(	485	10.98228	chr11:14⁄	MRPL11		protein_c	chr11:66435075-664
ENSG0000(	485	10.98228	chr11:14⁄	NPAS4		protein_c	chr11:66421004-664
ENSG0000(	485	10.98228	chr11:14⁄	ENSG00000278879		TEC	chr11:74830574-748
ENSG0000(	485	10.98228	chr11:14⁄	FOLR3		protein_c	chr11:72114869-721
ENSG0000(	485	10.98228	chr11:14⁄	SNRPCP14		Pseudoger	chr11:71690453-716
ENSG0000(	485	10.98228	chr11:14⁄	SLC29A2	NCGv7	protein_c	chr11:66362521-663
ENSG0000(	485	10.98228	chr11:14⁄	ENSG00000280089		TEC	chr11:70705605-707
ENSG0000(	485	10.98228	chr11:14⁄	CHKA	TAG	protein_c	chr11:68052859-681
ENSG0000(	485	10.98228	chr11:14⁄	TCIRG1	IntOGen-I	protein_c	chr11:68039025-680
ENSG0000(	485	10.98228	chr11:14⁄	NDUFS8		protein_c	chr11:68030617-680
ENSG0000(	485	10.98228	chr11:14⁄	AIP		protein_c	chr11:67468174-674
ENSG0000(	485	10.98228	chr11:14⁄	PITPNM1		protein_c	chr11:67491768-675
ENSG0000(	485	10.98228	chr11:14⁄	NAA40		protein_c	chr11:63938959-639
ENSG0000(	485	10.98228	chr11:14⁄	CCS		protein_c	chr11:66593153-666
ENSG0000(	485	10.98228	chr11:14⁄	SCGB2A2		protein_c	chr11:62270158-622
ENSG0000(	485	10.98228	chr11:14⁄	CTSF		protein_c	chr11:66563464-665
ENSG0000(	485	10.98228	chr11:14⁄	OR7E128P		Pseudoger	chr11:71893410-718
ENSG0000(	485	10.98228	chr11:14⁄	TUBAP7		Pseudoger	chr11:63046785-630

ENSG0000C	485	10.98228	chr11:144	ENSG00000236935		lncRNA	chr11:64325050-643
ENSG0000C	485	10.98228	chr11:144	ENSG00000254756		lncRNA	chr11:66334494-663
ENSG0000C	485	10.98228	chr11:144	ZDHHC24		protein_c	chr11:66520637-665
ENSG0000C	485	10.98228	chr11:144	KLC2-AS2		lncRNA	chr11:66267635-662
ENSG0000C	485	10.98228	chr11:144	EEF1G		protein_c	chr11:62559596-625
ENSG0000C	485	10.98228	chr11:144	ZNHIT2		protein_c	chr11:65116403-651
ENSG0000C	485	10.98228	chr11:144	ENSG00000254792		Pseudoger	chr11:67840942-678
ENSG0000C	485	10.98228	chr11:144	LIPT2-AS1		lncRNA	chr11:74493366-744
ENSG0000C	485	10.98228	chr11:144	FAM86C2P		Pseudoger	chr11:67793196-678
ENSG0000C	485	10.98228	chr11:144	ENSG00000254484		lncRNA	chr11:70319928-703
ENSG0000C	485	10.98228	chr11:144	CST6		protein_c	chr11:66012008-660
ENSG0000C	485	10.98228	chr11:144	FTLP6		Pseudoger	chr11:66771246-667
ENSG0000C	485	10.98228	chr11:144	RNU6-45P		smallRNA	chr11:63970470-639
ENSG0000C	485	10.98228	chr11:144	Y_RNA		smallRNA	chr11:70705167-707
ENSG0000C	485	10.98228	chr11:144	DPP3		protein_c	chr11:66480013-665
ENSG0000C	485	10.98228	chr11:144	PACS1		protein_c	chr11:66070272-662
ENSG0000C	485	10.98228	chr11:144	ENSG00000237363		Pseudoger	chr11:64531044-645
ENSG0000C	485	10.98228	chr11:144	GAL3ST3		protein_c	chr11:66040765-660
ENSG0000C	485	10.98228	chr11:144	CATSPER1		protein_c	chr11:66016752-660
ENSG0000C	485	10.98228	chr11:144	KRTAP5-9		protein_c	chr11:71548420-715
ENSG0000C	485	10.98228	chr11:144	ALG1L9P		Pseudoger	chr11:71800541-718
ENSG0000C	485	10.98228	chr11:144	BANF1	TAG	protein_c	chr11:66002228-660
ENSG0000C	485	10.98228	chr11:144	EIF1AD		protein_c	chr11:65996545-660
ENSG0000C	485	10.98228	chr11:144	AP002495.1		smallRNA	chr11:71802585-718
ENSG0000C	485	10.98228	chr11:144	ART2BP		Pseudoger	chr11:72478221-724
ENSG0000C	485	10.98228	chr11:144	NRXN2-AS1		lncRNA	chr11:64646399-646
ENSG0000C	485	10.98228	chr11:144	ENSG00000287725		protein_c	chr11:69072915-691
ENSG0000C	485	10.98228	chr11:144	AP000560.1		smallRNA	chr11:74753474-747
ENSG0000C	485	10.98228	chr11:144	TBC1D10C		protein_c	chr11:67403915-674
ENSG0000C	485	10.98228	chr11:144	KLC2		protein_c	chr11:66257294-662
ENSG0000C	485	10.98228	chr11:144	ENSG00000254974		Pseudoger	chr11:74485580-744
ENSG0000C	485	10.98228	chr11:144	ENSG00000254850		Pseudoger	chr11:67935558-679
ENSG0000C	485	10.98228	chr11:144	CD248		protein_c	chr11:66314494-663
ENSG0000C	485	10.98228	chr11:144	KLC2-AS1		lncRNA	chr11:66264777-662
ENSG0000C	485	10.98228	chr11:144	B4GAT1		protein_c	chr11:66345374-663
ENSG0000C	485	10.98228	chr11:144	NDUFA3P2		Pseudoger	chr11:68488609-684
ENSG0000C	485	10.98228	chr11:144	ENSG00000254867		Pseudoger	chr11:65983679-659
ENSG0000C	485	10.98228	chr11:144	BRMS1		protein_c	chr11:66337333-663
ENSG0000C	485	10.98228	chr11:144	RIN1		protein_c	chr11:66330241-663
ENSG0000C	485	10.98228	chr11:144	ENSG00000254883		Pseudoger	chr11:67744322-677
ENSG0000C	485	10.98228	chr11:144	ANO1-AS1		lncRNA	chr11:70187788-701
ENSG0000C	485	10.98228	chr11:144	TUT1		protein_c	chr11:62575045-625
ENSG0000C	485	10.98228	chr11:144	ENSG00000278952		TEC	chr11:65118310-651
ENSG0000C	485	10.98228	chr11:144	YIF1A		protein_c	chr11:66284580-662
ENSG0000C	485	10.98228	chr11:144	CNIH2		protein_c	chr11:66278175-662
ENSG0000C	485	10.98228	chr11:144	SCGB1A1		protein_c	chr11:62405103-624
ENSG0000C	485	10.98228	chr11:144	ENSG00000254924		Pseudoger	chr11:71506061-715
ENSG0000C	485	10.98228	chr11:144	ENSG00000254928		lncRNA	chr11:74455348-744
ENSG0000C	485	10.98228	chr11:144	ENSG00000254964		lncRNA	chr11:62606161-626
ENSG0000C	485	10.98228	chr11:144	RAB1B		protein_c	chr11:66268590-662
ENSG0000C	485	10.98228	chr11:144	ENSG00000254972		lncRNA	chr11:71701268-717

ENSG0000(	485	10.98228	chr11:14<	BRD9P1		Pseudoger	chr11:66389609-663
ENSG0000(	485	10.98228	chr11:14<	RBM4		protein_c	chr11:66638667-666
ENSG0000(	485	10.98228	chr11:14<	ENSG00000254721		lncRNA	chr11:70206291-702
ENSG0000(	485	10.98228	chr11:14<	GRK2		protein_c	chr11:67266473-672
ENSG0000(	485	10.98228	chr11:14<	FGF19	NCGv7	protein_c	chr11:69698238-697
ENSG0000(	485	10.98228	chr11:14<	RNASEH2C		protein_c	chr11:65714005-657
ENSG0000(	485	10.98228	chr11:14<	ANKRD13D		protein_c	chr11:67289300-673
ENSG0000(	485	10.98228	chr11:14<	MRGPRF		protein_c	chr11:69004395-690
ENSG0000(	485	10.98228	chr11:14<	MRGPRD		protein_c	chr11:68980021-689
ENSG0000(	485	10.98228	chr11:14<	CAPN1-AS1		lncRNA	chr11:65177606-651
ENSG0000(	485	10.98228	chr11:14<	KAT5		protein_c	chr11:65711996-657
ENSG0000(	485	10.98228	chr11:14<	TPCN2	DriverDB\	protein_c	chr11:69048932-691
ENSG0000(	485	10.98228	chr11:14<	RELA	NCGv7	protein_c	chr11:65653599-656
ENSG0000(	485	10.98228	chr11:14<	ACTE1P		Pseudoger	chr11:71382601-714
ENSG0000(	485	10.98228	chr11:14<	LRP5		protein_c	chr11:68312591-684
ENSG0000(	485	10.98228	chr11:14<	TRMT112		protein_c	chr11:64316460-643
ENSG0000(	485	10.98228	chr11:14<	KDM2A	NCGv7;AC	protein_c	chr11:67119263-672
ENSG0000(	485	10.98228	chr11:14<	ESRRA	IntOGen-I	protein_c	chr11:64305497-643
ENSG0000(	485	10.98228	chr11:14<	RHOD		protein_c	chr11:67056847-670
ENSG0000(	485	10.98228	chr11:14<	SYT12	DriverDB\	protein_c	chr11:67006778-670
ENSG0000(	485	10.98228	chr11:14<	C11orf86		protein_c	chr11:66975277-669
ENSG0000(	485	10.98228	chr11:14<	GPR137		protein_c	chr11:64270062-642
ENSG0000(	485	10.98228	chr11:14<	ENSG00000287412		lncRNA	chr11:63495484-635
ENSG0000(	485	10.98228	chr11:14<	CDCA5		protein_c	chr11:65066300-650
ENSG0000(	485	10.98228	chr11:14<	RBM4B		protein_c	chr11:66664998-666
ENSG0000(	485	10.98228	chr11:14<	RNU7-105P		smallRNA	chr11:72621766-726
ENSG0000(	485	10.98228	chr11:14<	ENSG00000254495		lncRNA	chr11:70358198-703
ENSG0000(	485	10.98228	chr11:14<	RANP3		Pseudoger	chr11:74652636-746
ENSG0000(	485	10.98228	chr11:14<	CORO1B		protein_c	chr11:67435510-674
ENSG0000(	485	10.98228	chr11:14<	MUS81		protein_c	chr11:65857126-658
ENSG0000(	485	10.98228	chr11:14<	CFL1		protein_c	chr11:65823022-658
ENSG0000(	485	10.98228	chr11:14<	ENSG00000254501		lncRNA	chr11:65110714-651
ENSG0000(	485	10.98228	chr11:14<	ENSG00000254510		lncRNA	chr11:66409158-664
ENSG0000(	485	10.98228	chr11:14<	ENSG00000254563		lncRNA	chr11:78749250-787
ENSG0000(	485	10.98228	chr11:14<	OR7E126P		Pseudoger	chr11:71903194-719
ENSG0000(	485	10.98228	chr11:14<	ENSG00000254605		lncRNA	chr11:70014858-700
ENSG0000(	485	10.98228	chr11:14<	SHANK2-AS2		lncRNA	chr11:70646165-706
ENSG0000(	485	10.98228	chr11:14<	ENSG00000254596		Pseudoger	chr11:66454234-664
ENSG0000(	485	10.98228	chr11:14<	SNX32		protein_c	chr11:65833834-658
ENSG0000(	485	10.98228	chr11:14<	ENSG00000254604		lncRNA	chr11:70282367-703
ENSG0000(	485	10.98228	chr11:14<	OVOL1		protein_c	chr11:65787063-657
ENSG0000(	485	10.98228	chr11:14<	SSH3	NCGv7	protein_c	chr11:67303478-673
ENSG0000(	485	10.98228	chr11:14<	NADSYN1	DriverDB\	protein_c	chr11:71453109-715
ENSG0000(	485	10.98228	chr11:14<	DHCR7	DriverDB\	protein_c	chr11:71428193-714
ENSG0000(	485	10.98228	chr11:14<	ENSG00000287425		lncRNA	chr11:73983449-739
ENSG0000(	485	10.98228	chr11:14<	MAP3K11		protein_c	chr11:65597756-656
ENSG0000(	485	10.98228	chr11:14<	RPS6KA4		protein_c	chr11:64359148-643
ENSG0000(	485	10.98228	chr11:14<	MIR3664		smallRNA	chr11:70872270-708
ENSG0000(	485	10.98228	chr11:14<	LRFN4		protein_c	chr11:66856647-668
ENSG0000(	485	10.98228	chr11:14<	NEU3		protein_c	chr11:74988279-750
ENSG0000(	485	10.98228	chr11:14<	RCE1		protein_c	chr11:66842835-668

ENSG0000(	485	10.98228	chr11:14⁄	CLPB	DriverDB\	protein_c	chr11:72285495-724
ENSG0000(	485	10.98228	chr11:14⁄	C11orf80		protein_c	chr11:66744451-668
ENSG0000(	485	10.98228	chr11:14⁄	ENSG00000173727		Pseudoger	chr11:65455269-654
ENSG0000(	485	10.98228	chr11:14⁄	SHANK2	NCGv7	protein_c	chr11:70467854-712
ENSG0000(	485	10.98228	chr11:14⁄	MIR3165		smallRNA	chr11:72072228-720
ENSG0000(	485	10.98228	chr11:14⁄	C11orf98		protein_c	chr11:62662817-626
ENSG0000(	485	10.98228	chr11:14⁄	KCNK7		protein_c	chr11:65592836-655
ENSG0000(	485	10.98228	chr11:14⁄	ENSG00000254675		lncRNA	chr11:78022933-780
ENSG0000(	485	10.98228	chr11:14⁄	TIGD3	NCGv7	protein_c	chr11:65354751-653
ENSG0000(	485	10.98228	chr11:14⁄	MIR192		smallRNA	chr11:64891137-648
ENSG0000(	485	10.98228	chr11:14⁄	ENSG00000254682		lncRNA	chr11:71448562-714
ENSG0000(	485	10.98228	chr11:14⁄	ENSG00000254691		lncRNA	chr11:77850604-778
ENSG0000(	485	10.98228	chr11:14⁄	LINC02754		lncRNA	chr11:67886477-679
ENSG0000(	485	10.98228	chr11:14⁄	COPS8P3		Pseudoger	chr11:78581675-785
ENSG0000(	485	10.98228	chr11:14⁄	SPTBN2	NCGv7	protein_c	chr11:66682497-667
ENSG0000(	485	10.98228	chr11:14⁄	PC	DriverDB\	protein_c	chr11:66848417-669
ENSG0000(	485	10.98228	chr11:14⁄	EEF1DP8		Pseudoger	chr11:62169293-621
ENSG0000(	485	10.98228	chr11:14⁄	ENSG00000254649		lncRNA	chr11:78388061-783
ENSG0000(	485	10.98228	chr11:14⁄	ASRGL1		protein_c	chr11:62337448-623
ENSG0000(	485	10.98228	chr11:14⁄	ZFPL1		protein_c	chr11:65084210-650
ENSG0000(	485	10.98228	chr11:14⁄	SYVN1		protein_c	chr11:65121780-651
ENSG0000(	485	10.98228	chr11:14⁄	SLC25A45	NCGv7	protein_c	chr11:65375192-653
ENSG0000(	485	10.98228	chr11:14⁄	EHBP1L1		protein_c	chr11:65576046-655
ENSG0000(	485	10.98228	chr11:14⁄	STX5		protein_c	chr11:62806860-628
ENSG0000(	485	10.98228	chr11:14⁄	NXF1	NCGv7	protein_c	chr11:62792123-628
ENSG0000(	485	10.98228	chr11:14⁄	PPP1R14B		protein_c	chr11:64244479-642
ENSG0000(	485	10.98228	chr11:14⁄	ZNRD2		protein_c	chr11:65570460-655
ENSG0000(	485	10.98228	chr11:14⁄	ARL2-SNX15		protein_c	chr11:65014182-650
ENSG0000(	485	10.98228	chr11:14⁄	TAF6L		protein_c	chr11:62771357-627
ENSG0000(	485	10.98228	chr11:14⁄	FKBP2		protein_c	chr11:64241003-642
ENSG0000(	485	10.98228	chr11:14⁄	TTC9C		protein_c	chr11:62728069-627
ENSG0000(	485	10.98228	chr11:14⁄	VEGFB		protein_c	chr11:64234584-642
ENSG0000(	485	10.98228	chr11:14⁄	ENSG00000254631		lncRNA	chr11:74397549-744
ENSG0000(	485	10.98228	chr11:14⁄	LBHD1		protein_c	chr11:62662817-626
ENSG0000(	485	10.98228	chr11:14⁄	UBXN1		protein_c	chr11:62676498-626
ENSG0000(	485	10.98228	chr11:14⁄	GNG3	NCGv7	protein_c	chr11:62707676-627
ENSG0000(	485	10.98228	chr11:14⁄	SIPA1		protein_c	chr11:65638101-656
ENSG0000(	485	10.98228	chr11:14⁄	ENSG00000255672		lncRNA	chr11:72354516-723
ENSG0000(	485	10.98228	chr11:14⁄	KRTAP5-7		protein_c	chr11:71527267-715
ENSG0000(	485	10.98228	chr11:14⁄	ENSG00000290995		lncRNA	chr11:67934563-679
ENSG0000(	485	10.98228	chr11:14⁄	FAM86C2P		lncRNA	chr11:67791648-678
ENSG0000(	485	10.98228	chr11:14⁄	RASGRP2	NCGv7	protein_c	chr11:64726911-647
ENSG0000(	485	10.98228	chr11:14⁄	DEFB108B		protein_c	chr11:71833200-718
ENSG0000(	485	10.98228	chr11:14⁄	PPP2R5B		protein_c	chr11:64917553-649
ENSG0000(	485	10.98228	chr11:14⁄	PYGM		protein_c	chr11:64746389-647
ENSG0000(	485	10.98228	chr11:14⁄	RPS12P20		Pseudoger	chr11:72708186-727
ENSG0000(	485	10.98228	chr11:14⁄	GAL		protein_c	chr11:68683779-686
ENSG0000(	485	10.98228	chr11:14⁄	RN7SL59P		smallRNA	chr11:67541735-675
ENSG0000(	485	10.98228	chr11:14⁄	ZBTB3		protein_c	chr11:62748319-627
ENSG0000(	485	10.98228	chr11:14⁄	ENSG00000267811		lncRNA	chr11:62771120-627
ENSG0000(	485	10.98228	chr11:14⁄	RN7SKP243		smallRNA	chr11:73832382-738

ENSG0000(	485	10.98228	chr11:144	EEF1A1P18		Pseudoger	chr11:65025390-650
ENSG0000(	485	10.98228	chr11:144	POLA2		protein_c	chr11:65261920-653
ENSG0000(	485	10.98228	chr11:144	TMEM262		protein_c	chr11:65084979-650
ENSG0000(	485	10.98228	chr11:144	SLC22A20P		Pseudoger	chr11:65213840-652
ENSG0000(	485	10.98228	chr11:144	AP002958.1		smallRNA	chr11:78973721-789
ENSG0000(	485	10.98228	chr11:144	TMEM179B		protein_c	chr11:62787402-627
ENSG0000(	485	10.98228	chr11:144	ENSG00000291247		lncRNA	chr11:71722282-717
ENSG0000(	485	10.98228	chr11:144	RNU6-216P		smallRNA	chr11:74968189-749
ENSG0000(	485	10.98228	chr11:144	MARK2	NCGv7	protein_c	chr11:63838928-639
ENSG0000(	485	10.98228	chr11:144	PLEKHB1		protein_c	chr11:73646178-736
ENSG0000(	485	10.98228	chr11:144	IMMP1LP1		Pseudoger	chr11:63632233-636
ENSG0000(	485	10.98228	chr11:144	KRTAP5-14P		Pseudoger	chr11:71579728-715
ENSG0000(	485	10.98228	chr11:144	CAPN1		protein_c	chr11:65180566-652
ENSG0000(	485	10.98228	chr11:144	AP001362.1		protein_c	chr11:65591194-655
ENSG0000(	485	10.98228	chr11:144	ENSG00000241782		Pseudoger	chr11:77868722-778
ENSG0000(	485	10.98228	chr11:144	SNRPGP19		Pseudoger	chr11:65514403-655
ENSG0000(	485	10.98228	chr11:144	KCTD21-AS1		lncRNA	chr11:78139756-781
ENSG0000(	485	10.98228	chr11:144	LINC02753		lncRNA	chr11:70056230-700
ENSG0000(	485	10.98228	chr11:144	RPS3AP40		Pseudoger	chr11:67925651-679
ENSG0000(	485	10.98228	chr11:144	AP002761.1		smallRNA	chr11:73357135-733
ENSG0000(	485	10.98228	chr11:144	AP000719.1		smallRNA	chr11:71905107-719
ENSG0000(	485	10.98228	chr11:144	AP003096.1		smallRNA	chr11:68619499-686
ENSG0000(	485	10.98228	chr11:144	ENSG00000289908		lncRNA	chr11:68038252-680
ENSG0000(	485	10.98228	chr11:144	ENSG00000248903		Pseudoger	chr11:71568680-715
ENSG0000(	485	10.98228	chr11:144	OR7E87P		Pseudoger	chr11:71593454-715
ENSG0000(	485	10.98228	chr11:144	SLC22A24		protein_c	chr11:63079940-631
ENSG0000(	485	10.98228	chr11:144	ALG1L9P		lncRNA	chr11:71673885-718
ENSG0000(	485	10.98228	chr11:144	RN7SL114P		smallRNA	chr11:65049777-650
ENSG0000(	485	10.98228	chr11:144	MRPL21	NCGv7	protein_c	chr11:68891276-689
ENSG0000(	485	10.98228	chr11:144	ENSG00000291174		lncRNA	chr11:65455257-654
ENSG0000(	485	10.98228	chr11:144	SCGB1D4		protein_c	chr11:62296281-622
ENSG0000(	485	10.98228	chr11:144	RSF1	AC	protein_c	chr11:77660009-778
ENSG0000(	485	10.98228	chr11:144	ENSG00000291186		lncRNA	chr11:71787480-718
ENSG0000(	485	10.98228	chr11:144	MIR4691		smallRNA	chr11:68033897-680
ENSG0000(	485	10.98228	chr11:144	PCNX3		protein_c	chr11:65615776-656
ENSG0000(	485	10.98228	chr11:144	LRRC51		protein_c	chr11:72080337-720
ENSG0000(	485	10.98228	chr11:144	FGF3	TAG;AC	protein_c	chr11:69809968-698
ENSG0000(	485	10.98228	chr11:144	ALDH3B1		protein_c	chr11:68008578-680
ENSG0000(	485	10.98228	chr11:144	RBM14-RBM4		protein_c	chr11:66616626-666
ENSG0000(	485	10.98228	chr11:144	ENSG00000289883		lncRNA	chr11:65476515-654
ENSG0000(	485	10.98228	chr11:144	ENSG00000271100		Pseudoger	chr11:63698596-636
ENSG0000(	485	10.98228	chr11:144	CTTN-DT		lncRNA	chr11:70372246-703
ENSG0000(	485	10.98228	chr11:144	NDUFV1-DT		lncRNA	chr11:67602880-676
ENSG0000(	485	10.98228	chr11:144	P4HA3-AS1		lncRNA	chr11:74311362-743
ENSG0000(	485	10.98228	chr11:144	RN7SL239P		smallRNA	chr11:74845910-748
ENSG0000(	485	10.98228	chr11:144	BAD		protein_c	chr11:64269830-642
ENSG0000(	485	10.98228	chr11:144	NEAT1	AC	lncRNA	chr11:65422774-654
ENSG0000(	485	10.98228	chr11:144	RPL36AP38		Pseudoger	chr11:74738478-747
ENSG0000(	485	10.98228	chr11:144	RN7SL259P		smallRNA	chr11:62935984-629
ENSG0000(	485	10.98228	chr11:144	ZFTA		protein_c	chr11:63759892-637
ENSG0000(	485	10.98228	chr11:144	ENSG00000290026		lncRNA	chr11:65574399-655

ENSG0000(	485	10.98228	chr11:144	SF1-DT		lncRNA	chr11:64778954-647
ENSG0000(	485	10.98228	chr11:144	ARAP1	DriverDB\	protein_c	chr11:72685069-727
ENSG0000(	485	10.98228	chr11:144	ATL3		protein_c	chr11:63624087-636
ENSG0000(	485	10.98228	chr11:144	ENSG00000270323		Pseudoger	chr11:73640479-736
ENSG0000(	485	10.98228	chr11:144	GAB2	DriverDB\	protein_c	chr11:78215293-784
ENSG0000(	485	10.98228	chr11:144	DNAJB13		protein_c	chr11:73951026-739
ENSG0000(	485	10.98228	chr11:144	PDE2A		protein_c	chr11:72576141-726
ENSG0000(	485	10.98228	chr11:144	ENSG00000269463		lncRNA	chr11:62807682-628
ENSG0000(	485	10.98228	chr11:144	POLD3		protein_c	chr11:74493851-746
ENSG0000(	485	10.98228	chr11:144	CHRDL2		protein_c	chr11:74696429-747
ENSG0000(	485	10.98228	chr11:144	ENSG00000245156		lncRNA	chr11:66269832-662
ENSG0000(	485	10.98228	chr11:144	FAM168A		protein_c	chr11:73400487-735
ENSG0000(	485	10.98228	chr11:144	ENSG00000290736		lncRNA	chr11:71856277-718
ENSG0000(	485	10.98228	chr11:144	RELT		protein_c	chr11:73376399-733
ENSG0000(	485	10.98228	chr11:144	ENSG00000290016		lncRNA	chr11:72530155-725
ENSG0000(	485	10.98228	chr11:144	RNA5SP343		smallRNA	chr11:74198748-741
ENSG0000(	485	10.98228	chr11:144	ARAP1-AS2		lncRNA	chr11:72700474-727
ENSG0000(	485	10.98228	chr11:144	UNC93B5		Pseudoger	chr11:67711702-677
ENSG0000(	485	10.98228	chr11:144	RNU4-39P		smallRNA	chr11:66614964-666
ENSG0000(	485	10.98228	chr11:144	ENSG00000290775		lncRNA	chr11:71711740-717
ENSG0000(	485	10.98228	chr11:144	ENSG00000290057		lncRNA	chr11:65421448-654
ENSG0000(	485	10.98228	chr11:144	KRTAP5-8		protein_c	chr11:71538025-715
ENSG0000(	485	10.98228	chr11:144	MIR3163		smallRNA	chr11:66934434-669
ENSG0000(	485	10.98228	chr11:144	SLC22A6		protein_c	chr11:62936385-629
ENSG0000(	485	10.98228	chr11:144	RP11-869B15.1		lncRNA	chr11:64784921-647
ENSG0000(	485	10.98228	chr11:144	SLC22A10		protein_c	chr11:63268022-633
ENSG0000(	485	10.98228	chr11:144	ENSG00000290061		lncRNA	chr11:65492897-654
ENSG0000(	485	10.98228	chr11:144	SLC22A25		protein_c	chr11:63158437-632
ENSG0000(	485	10.98228	chr11:144	AP003385.1		smallRNA	chr11:67734521-677
ENSG0000(	485	10.98228	chr11:144	ENSG00000269176		lncRNA	chr11:62786023-627
ENSG0000(	485	10.98228	chr11:144	ACTN3		protein_c	chr11:66546395-665
ENSG0000(	485	10.98228	chr11:144	INTS5	AC	protein_c	chr11:62646848-626
ENSG0000(	485	10.98228	chr11:144	SLC22A12		protein_c	chr11:64590641-646
ENSG0000(	485	10.98228	chr11:144	ENSG00000270117		lncRNA	chr11:65498008-654
ENSG0000(	485	10.98228	chr11:144	RPL31P46		Pseudoger	chr11:74876286-749
ENSG0000(	485	10.98228	chr11:144	FGF4	TAG;AC	protein_c	chr11:69771022-697
ENSG0000(	485	10.98228	chr11:144	KCTD21		protein_c	chr11:78171249-781
ENSG0000(	482	10.91434	chr9:8075	CORO2A		protein_c	chr9:98120975-9819
ENSG0000(	482	10.91434	chr9:8075	TRMO		protein_c	chr9:97904489-9792
ENSG0000(	482	10.91434	chr9:8075	ANP32B		protein_c	chr9:97983341-9801
ENSG0000(	482	10.91434	chr9:8075	TRIM14		protein_c	chr9:98069275-9811
ENSG0000(	482	10.91434	chr9:8075	ENSG00000228957		Pseudoger	chr9:97386240-9739
ENSG0000(	482	10.91434	chr9:8075	ENSG00000260677		lncRNA	chr9:97743208-9774
ENSG0000(	482	10.91434	chr9:8075	NCBP1	NCGv7	protein_c	chr9:97633668-9767
ENSG0000(	482	10.91434	chr9:8075	XPA	NCGv7;AC	protein_c	chr9:97674909-9769
ENSG0000(	482	10.91434	chr9:8075	AL445531.1		smallRNA	chr9:97700234-9770
ENSG0000(	482	10.91434	chr9:8075	RNU6-918P		smallRNA	chr9:98032230-9803
ENSG0000(	482	10.91434	chr9:8075	HEMGN		protein_c	chr9:97926791-9794
ENSG0000(	482	10.91434	chr9:8075	ENSG00000287070		lncRNA	chr9:97921590-9792
ENSG0000(	482	10.91434	chr9:8075	TSTD2		protein_c	chr9:97600080-9763
ENSG0000(	482	10.91434	chr9:8075	Y_RNA		smallRNA	chr9:97915690-9791

ENSG0000(	482	10.91434	chr9:8075	PTCSC2		lncRNA	chr9:97699625-9785
ENSG0000(	482	10.91434	chr9:8075	TMOD1		protein_c	chr9:97501180-9760
ENSG0000(	482	10.91434	chr9:8075	ENSG00000231521		lncRNA	chr9:97634515-9763
ENSG0000(	482	10.91434	chr9:8075	NANS		protein_c	chr9:98056732-9808
ENSG0000(	482	10.91434	chr3:6192	ENSG00000272263		lncRNA	chr3:12832219-1283
ENSG0000(	482	10.91434	chr9:8075	ENSG00000236896		lncRNA	chr9:97986551-9798
ENSG0000(	482	10.91434	chr9:8075	TBC1D2		protein_c	chr9:98199011-9825
ENSG0000(	482	10.91434	chr9:8075	FOXE1	NCGv7	protein_c	chr9:97853226-9785
ENSG0000(	482	10.91434	chr9:8075	ENSG00000224001		Pseudoger	chr9:98234709-9823
ENSG0000(	482	10.91434	chr9:8075	ENSG00000228174		lncRNA	chr9:97512706-9751
ENSG0000(	482	10.91434	chr9:8075	KRT18P13		Pseudoger	chr9:97698922-9770
ENSG0000(	482	10.91434	chr9:8075	TDRD7		protein_c	chr9:97412096-9749
ENSG0000(	482	10.91434	chr9:8075	ENSG00000286375		lncRNA	chr9:97403709-9741
ENSG0000(	479	10.84641	chr16:239	ACA64		smallRNA	chr16:12297197-122
ENSG0000(	478	10.82377	chr10:717	RNA5SP313		smallRNA	chr10:46807458-468
ENSG0000(	478	10.82377	chr10:717	RNA5SP311		smallRNA	chr10:46807457-468
ENSG0000(	476	10.77848	chr16:239	ENSG00000275263		lncRNA	chr16:30956872-309
ENSG0000(	473	10.71055	chr3:3227	RPSA		protein_c	chr3:39406716-3942
ENSG0000(	473	10.71055	chr3:3227	NFU1P1		Pseudoger	chr3:39643638-3964
ENSG0000(	473	10.71055	chr3:3227	SNORA64		smallRNA	chr3:40238608-4023
ENSG0000(	473	10.71055	chr3:3227	ENSG00000234287		Pseudoger	chr3:40761545-4076
ENSG0000(	473	10.71055	chr3:3227	ENSG00000233096		lncRNA	chr3:40970541-4097
ENSG0000(	473	10.71055	chr3:3227	EXOG		protein_c	chr3:38496127-3854
ENSG0000(	473	10.71055	chr3:3227	HMGN2P24		Pseudoger	chr3:40699066-4069
ENSG0000(	473	10.71055	chr3:3227	XYLB		protein_c	chr3:38346760-3842
ENSG0000(	473	10.71055	chr3:3227	ENSG00000287958		lncRNA	chr3:39232531-3926
ENSG0000(	473	10.71055	chr3:3227	HNRNPA1P21		Pseudoger	chr3:39334979-3933
ENSG0000(	473	10.71055	chr3:3227	ENSG00000231873		lncRNA	chr3:40719859-4086
ENSG0000(	473	10.71055	chr3:3227	ENSG00000287995		lncRNA	chr3:39213069-3921
ENSG0000(	473	10.71055	chr3:3227	DSTNP4		Pseudoger	chr3:39214199-3921
ENSG0000(	473	10.71055	chr3:3227	ENSG00000226302		lncRNA	chr3:40603125-4060
ENSG0000(	473	10.71055	chr3:3227	MRPS31P1		Pseudoger	chr3:41189041-4118
ENSG0000(	473	10.71055	chr3:3227	ZNF620	NCGv7	protein_c	chr3:40477131-4051
ENSG0000(	473	10.71055	chr3:3227	ZNF619		protein_c	chr3:40477113-4049
ENSG0000(	473	10.71055	chr3:3227	EIF1B-AS1		lncRNA	chr3:40061110-4030
ENSG0000(	473	10.71055	chr3:3227	RNU6-1227P		smallRNA	chr3:39025987-3902
ENSG0000(	473	10.71055	chr3:3227	SNORA6		smallRNA	chr3:39408389-3940
ENSG0000(	473	10.71055	chr3:3227	RNU4-56P		smallRNA	chr3:39970125-3997
ENSG0000(	473	10.71055	chr3:3227	ENSG00000270367		Pseudoger	chr3:39285985-3928
ENSG0000(	473	10.71055	chr3:3227	SNORA62		smallRNA	chr3:39411054-3941
ENSG0000(	473	10.71055	chr3:3227	MYRIP		protein_c	chr3:39808914-4026
ENSG0000(	473	10.71055	chr3:3227	SLC25A38		protein_c	chr3:39383370-3939
ENSG0000(	473	10.71055	chr7:2516	MIR4284		smallRNA	chr7:73711317-7371
ENSG0000(	473	10.71055	chr3:3227	CSRNP1		protein_c	chr3:39141855-3915
ENSG0000(	473	10.71055	chr3:3227	SCN11A	NCGv7	protein_c	chr3:38845764-3905
ENSG0000(	473	10.71055	chr3:3227	EEF1A1P24		Pseudoger	chr3:39358545-3935
ENSG0000(	473	10.71055	chr3:3227	SCN10A	NCGv7	protein_c	chr3:38696802-3881
ENSG0000(	473	10.71055	chr3:3227	RPL14		protein_c	chr3:40457292-4046
ENSG0000(	473	10.71055	chr3:3227	ACVR2B	NCGv7	protein_c	chr3:38453890-3849
ENSG0000(	473	10.71055	chr3:3227	ENSG00000285885		lncRNA	chr3:39494837-3950
ENSG0000(	473	10.71055	chr3:3227	XIRP1		protein_c	chr3:39183210-3919

ENSG0000(	473	10.71055	chr3:3227	WDR48		protein_c	chr3:39052013-3909
ENSG0000(	473	10.71055	chr3:3227	GORASP1	NCGv7	protein_c	chr3:39095222-3910
ENSG0000(	473	10.71055	chr3:3227	CX3CR1	NCGv7	protein_c	chr3:39263495-3928
ENSG0000(	473	10.71055	chr3:3227	ENSG00000287780		lncRNA	chr3:39292556-3938
ENSG0000(	473	10.71055	chr3:3227	MOBP		protein_c	chr3:39467198-3952
ENSG0000(	473	10.71055	chr3:3227	ENSG00000289279		lncRNA	chr3:39519506-3952
ENSG0000(	473	10.71055	chr3:3227	EIF1B		protein_c	chr3:40309707-4031
ENSG0000(	473	10.71055	chr3:3227	ENSG00000289169		lncRNA	chr3:40623893-4062
ENSG0000(	473	10.71055	chr3:3227	ZNF621		protein_c	chr3:40524878-4057
ENSG0000(	473	10.71055	chr3:3227	DDTP1		Pseudoger	chr3:38515448-3851
ENSG0000(	473	10.71055	chr3:3227	ENSG00000287620		lncRNA	chr3:39177761-3917
ENSG0000(	473	10.71055	chr3:3227	RPL18AP7		Pseudoger	chr3:38526802-3852
ENSG0000(	473	10.71055	chr3:3227	RN7SL411P		smallRNA	chr3:40209005-4020
ENSG0000(	473	10.71055	chr3:3227	ENSG00000287415		lncRNA	chr3:40869548-4087
ENSG0000(	473	10.71055	chr3:3227	RNU5B-2P		smallRNA	chr3:40498891-4049
ENSG0000(	473	10.71055	chr3:3227	ENSG00000231243		lncRNA	chr3:38823902-3882
ENSG0000(	473	10.71055	chr3:3227	ENSG00000284669		lncRNA	chr3:39148281-3917
ENSG0000(	473	10.71055	chr3:3227	PGAM1P3		Pseudoger	chr3:40322715-4032
ENSG0000(	473	10.71055	chr3:3227	ENSG00000233919		lncRNA	chr3:41162287-4116
ENSG0000(	473	10.71055	chr3:3227	ENTPD3-AS1		lncRNA	chr3:40313802-4045
ENSG0000(	473	10.71055	chr3:3227	ENSG00000286781		lncRNA	chr3:39425342-3944
ENSG0000(	473	10.71055	chr3:3227	ENSG00000283849		lncRNA	chr3:39152906-3915
ENSG0000(	473	10.71055	chr3:3227	CCR8		protein_c	chr3:39329709-3933
ENSG0000(	473	10.71055	chr3:3227	ACVR2B-AS1		lncRNA	chr3:38451027-3845
ENSG0000(	473	10.71055	chr3:3227	ENTPD3		protein_c	chr3:40387184-4042
ENSG0000(	473	10.71055	chr3:3227	TTC21A		protein_c	chr3:39107680-3913
ENSG0000(	473	10.71055	chr3:3227	Y_RNA		smallRNA	chr3:38505480-3850
ENSG0000(	473	10.71055	chr3:3227	SCN5A	NCGv7	protein_c	chr3:38548057-3864
ENSG0000(	473	10.71055	chr3:3227	EEF1GP3		Pseudoger	chr3:40596207-4059
ENSG0000(	473	10.71055	chr3:3227	ENSG00000238267		Pseudoger	chr3:39338129-3933
ENSG0000(	473	10.71055	chr3:3227	AC116038.1		smallRNA	chr3:38907314-3890
ENSG0000(	473	10.71055	chr3:3227	RPL5P10		Pseudoger	chr3:40586535-4058
ENSG0000(	469	10.61997	chr12:201	LSM3P2		Pseudoger	chr12:94590821-945
ENSG0000(	468	10.59733	chr10:717	MIR5100		smallRNA	chr10:42997563-429
ENSG0000(	466	10.55204	chr18:236	ENSG00000266968		lncRNA	chr18:45646153-456
ENSG0000(	466	10.55204	chr18:236	RN7SKP26		smallRNA	chr18:45989653-459
ENSG0000(	466	10.55204	chr18:236	ENSG00000267558		Pseudoger	chr18:45788530-457
ENSG0000(	466	10.55204	chr18:236	EPG5	NCGv7	protein_c	chr18:45800581-459
ENSG0000(	466	10.55204	chr18:236	RP11-116018.3		lncRNA	chr18:45669367-457
ENSG0000(	466	10.55204	chr18:236	ENSG00000267293		Pseudoger	chr18:46028306-460
ENSG0000(	466	10.55204	chr18:236	ENSG00000287943		lncRNA	chr18:45571406-456
ENSG0000(	466	10.55204	chr18:236	SLC14A1		protein_c	chr18:45687025-457
ENSG0000(	466	10.55204	chr18:236	SIGLEC15		protein_c	chr18:45825675-458
ENSG0000(	465	10.5294	chr10:717	MIR605		smallRNA	chr10:51299573-512
ENSG0000(	465	10.5294	chr10:717	ENSG00000272892		lncRNA	chr10:67849525-678
ENSG0000(	465	10.5294	chr10:717	LRRTM3		protein_c	chr10:66926036-671
ENSG0000(	465	10.5294	chr10:717	SGMS1		protein_c	chr10:50305586-506
ENSG0000(	465	10.5294	chr10:717	ENSG00000277056		Pseudoger	chr10:66837457-668
ENSG0000(	465	10.5294	chr10:717	ENSG00000289270		lncRNA	chr10:51531638-515
ENSG0000(	465	10.5294	chr10:717	PRKG1-AS1		lncRNA	chr10:52230398-523
ENSG0000(	465	10.5294	chr10:717	WASHC2A		protein_c	chr10:50067888-501

ENSG0000(	465	10.5294	chr10:717	FAM25C		protein_c	chr10:47995322-479
ENSG0000(	465	10.5294	chr10:717	ERCC6	NCGv7	protein_c	chr10:49454168-495
ENSG0000(	465	10.5294	chr10:717	ENSG00000251413		lncRNA	chr10:48624004-486
ENSG0000(	465	10.5294	chr10:717	ANXA2P3		Pseudoger	chr10:64825572-648
ENSG0000(	465	10.5294	chr10:717	ENSG00000236958		lncRNA	chr10:54864382-548
ENSG0000(	465	10.5294	chr10:717	LINC02625		lncRNA	chr10:61781745-618
ENSG0000(	465	10.5294	chr10:717	LINC01515		lncRNA	chr10:65570338-658
ENSG0000(	465	10.5294	chr10:717	ENSG00000223800		lncRNA	chr10:55468327-554
ENSG0000(	465	10.5294	chr10:717	ENSG00000228048		lncRNA	chr10:55506219-555
ENSG0000(	465	10.5294	chr10:717	AC022538.1		smallRNA	chr10:65710060-657
ENSG0000(	465	10.5294	chr10:717	TMEM26-AS1		lncRNA	chr10:61452639-614
ENSG0000(	465	10.5294	chr10:717	RN7SL527P		smallRNA	chr10:47692444-476
ENSG0000(	465	10.5294	chr10:717	RP11-13E1.5		lncRNA	chr10:48155325-481
ENSG0000(	465	10.5294	chr10:717	ENSG00000232115		Pseudoger	chr10:58106423-581
ENSG0000(	465	10.5294	chr10:717	TATDN1P1		Pseudoger	chr10:63222155-632
ENSG0000(	465	10.5294	chr10:717	ENSG00000233665		lncRNA	chr10:48976554-489
ENSG0000(	465	10.5294	chr10:717	PTPN20CP		Pseudoger	chr10:48064308-481
ENSG0000(	465	10.5294	chr10:717	AC013737.1		smallRNA	chr10:54347963-543
ENSG0000(	465	10.5294	chr10:717	ASAH2C		protein_c	chr10:50187134-502
ENSG0000(	465	10.5294	chr10:717	NEK4P3		Pseudoger	chr10:65054460-650
ENSG0000(	465	10.5294	chr10:717	SNORA74		smallRNA	chr10:49954214-499
ENSG0000(	465	10.5294	chr10:717	ENSG00000289325		protein_c	chr10:66926308-669
ENSG0000(	465	10.5294	chr10:717	RNU2-72P		smallRNA	chr10:60912765-609
ENSG0000(	465	10.5294	chr10:717	RTKN2		protein_c	chr10:62183035-622
ENSG0000(	465	10.5294	chr10:717	ENSG00000241577		lncRNA	chr10:48883955-489
ENSG0000(	465	10.5294	chr10:717	CHAT		protein_c	chr10:49609095-496
ENSG0000(	465	10.5294	chr10:717	AC022537.1		smallRNA	chr10:51265325-512
ENSG0000(	465	10.5294	chr10:717	PCDH15	NCGv7	protein_c	chr10:53802771-556
ENSG0000(	465	10.5294	chr10:717	RPL21P92		Pseudoger	chr10:67793770-677
ENSG0000(	465	10.5294	chr10:717	LINC02929		lncRNA	chr10:62520448-626
ENSG0000(	465	10.5294	chr10:717	PRKG1	NCGv7	protein_c	chr10:50990888-522
ENSG0000(	465	10.5294	chr10:717	MIX23P2		Pseudoger	chr10:50962993-509
ENSG0000(	465	10.5294	chr10:717	AGAP12P		lncRNA	chr10:48010132-480
ENSG0000(	465	10.5294	chr10:717	AC067751.1		smallRNA	chr10:62658185-626
ENSG0000(	465	10.5294	chr10:717	ENSG00000224301		lncRNA	chr10:63123929-631
ENSG0000(	465	10.5294	chr10:717	GAPDHP21		Pseudoger	chr10:55667341-556
ENSG0000(	465	10.5294	chr10:717	ENSG00000236800		lncRNA	chr10:48664199-486
ENSG0000(	465	10.5294	chr10:717	ARID5B	NCGv7	protein_c	chr10:61901684-620
ENSG0000(	465	10.5294	chr10:717	JMJD1C-AS1		lncRNA	chr10:63465229-634
ENSG0000(	465	10.5294	chr10:717	ENSG00000236744		lncRNA	chr10:55247755-555
ENSG0000(	465	10.5294	chr10:717	Y_RNA		smallRNA	chr10:60081741-600
ENSG0000(	465	10.5294	chr10:717	DNAJC19P1		Pseudoger	chr10:67833763-678
ENSG0000(	465	10.5294	chr10:717	UBE2D1	NCGv7	protein_c	chr10:58334979-583
ENSG0000(	465	10.5294	chr10:717	ENSG00000290899		lncRNA	chr10:48105197-481
ENSG0000(	465	10.5294	chr10:717	RHOBTB1		protein_c	chr10:60869438-610
ENSG0000(	465	10.5294	chr10:717	VSTM4		protein_c	chr10:49014236-491
ENSG0000(	465	10.5294	chr10:717	THAP12P3		Pseudoger	chr10:52411135-524
ENSG0000(	465	10.5294	chr10:717	ADO		protein_c	chr10:62804720-628
ENSG0000(	465	10.5294	chr10:717	ENSG00000279822		TEC	chr10:48443836-484
ENSG0000(	465	10.5294	chr10:717	RN7SL220P		smallRNA	chr10:68007720-680
ENSG0000(	465	10.5294	chr10:717	MIR548F1		smallRNA	chr10:54607874-546

ENSG0000(	465	10.5294	chr10:717	ENSG00000261076		lncRNA	chr10:58325614-583
ENSG0000(	465	10.5294	chr10:717	RNA5SP312		smallRNA	chr10:48040462-480
ENSG0000(	465	10.5294	chr10:717	ENSG00000226426		lncRNA	chr10:64036345-640
ENSG0000(	465	10.5294	chr10:717	snoU13		smallRNA	chr10:55567739-555
ENSG0000(	465	10.5294	chr10:717	ENSG00000286401		lncRNA	chr10:50325697-503
ENSG0000(	465	10.5294	chr10:717	ENSG00000238280		lncRNA	chr10:62682652-628
ENSG0000(	465	10.5294	chr10:717	C10orf53		protein_c	chr10:49679651-497
ENSG0000(	465	10.5294	chr10:717	MIR4294		smallRNA	chr10:48985512-489
ENSG0000(	465	10.5294	chr10:717	SHQ1P1		Pseudoger	chr10:50629532-506
ENSG0000(	465	10.5294	chr10:717	MAPK6P6		Pseudoger	chr10:49771841-497
ENSG0000(	465	10.5294	chr10:717	LINC00843		lncRNA	chr10:49972763-499
ENSG0000(	465	10.5294	chr10:717	BMS1P2		Pseudoger	chr10:48046490-480
ENSG0000(	465	10.5294	chr10:717	RPL35AP24		Pseudoger	chr10:49909613-499
ENSG0000(	465	10.5294	chr10:717	FAM35DP		Pseudoger	chr10:47689707-477
ENSG0000(	465	10.5294	chr10:717	MIR3924		smallRNA	chr10:57304479-573
ENSG0000(	465	10.5294	chr10:717	ENSG00000288052		lncRNA	chr10:61493843-614
ENSG0000(	465	10.5294	chr10:717	PARG		protein_c	chr10:49818279-499
ENSG0000(	465	10.5294	chr10:717	RPS3AP38		Pseudoger	chr10:67960702-679
ENSG0000(	465	10.5294	chr10:717	ENSG00000288011		lncRNA	chr10:62339553-623
ENSG0000(	465	10.5294	chr10:717	RHEBP2		Pseudoger	chr10:47706203-477
ENSG0000(	465	10.5294	chr10:717	OGDHL		protein_c	chr10:49734641-497
ENSG0000(	465	10.5294	chr10:717	RPL7AP51		Pseudoger	chr10:67334123-673
ENSG0000(	465	10.5294	chr10:717	AGAP12P		Pseudoger	chr10:48009873-480
ENSG0000(	465	10.5294	chr10:717	TMEM273		protein_c	chr10:49154725-491
ENSG0000(	465	10.5294	chr10:717	BEND3P1		Pseudoger	chr10:50655967-506
ENSG0000(	465	10.5294	chr10:717	TRAF6P1		Pseudoger	chr10:59136625-591
ENSG0000(	465	10.5294	chr10:717	BMS1P7		Pseudoger	chr10:48050282-480
ENSG0000(	465	10.5294	chr10:717	TPT1P10		Pseudoger	chr10:58212541-582
ENSG0000(	465	10.5294	chr10:717	FAM21EP		lncRNA	chr10:50021182-500
ENSG0000(	465	10.5294	chr10:717	ASAH2B		protein_c	chr10:50739318-508
ENSG0000(	465	10.5294	chr10:717	SNORD2		smallRNA	chr10:56595963-565
ENSG0000(	465	10.5294	chr10:717	AGAP6		protein_c	chr10:49982190-500
ENSG0000(	465	10.5294	chr10:717	RN7SL591P		smallRNA	chr10:62350009-623
ENSG0000(	465	10.5294	chr10:717	ENSG00000289989		lncRNA	chr10:60944378-609
ENSG0000(	465	10.5294	chr10:717	SNRPEP8		Pseudoger	chr10:53038285-530
ENSG0000(	465	10.5294	chr10:717	SLC18A3		protein_c	chr10:49610310-496
ENSG0000(	465	10.5294	chr10:717	ENSG00000289834		lncRNA	chr10:61900107-619
ENSG0000(	465	10.5294	chr10:717	RPL17P35		Pseudoger	chr10:64620374-646
ENSG0000(	465	10.5294	chr10:717	ENSG00000233805		Pseudoger	chr10:53766400-537
ENSG0000(	465	10.5294	chr10:717	RNU7-107P		smallRNA	chr10:50590652-505
ENSG0000(	465	10.5294	chr10:717	Y_RNA		smallRNA	chr10:52852607-528
ENSG0000(	465	10.5294	chr10:717	SLC9A3P3		Pseudoger	chr10:50025135-500
ENSG0000(	465	10.5294	chr10:717	RN7SL453P		smallRNA	chr10:47692444-476
ENSG0000(	465	10.5294	chr10:717	RNA5SP317		Pseudoger	chr10:49979665-499
ENSG0000(	465	10.5294	chr10:717	LINC00845		lncRNA	chr10:61001088-611
ENSG0000(	465	10.5294	chr10:717	RNA5SP316		smallRNA	chr10:49979664-499
ENSG0000(	465	10.5294	chr10:717	EGR2	NCGv7	protein_c	chr10:62811996-628
ENSG0000(	465	10.5294	chr10:717	HMGB1P50		Pseudoger	chr10:49551308-495
ENSG0000(	465	10.5294	chr10:717	ENSG00000226168		Pseudoger	chr10:50695592-506
ENSG0000(	465	10.5294	chr10:717	IPMK		protein_c	chr10:58191517-582
ENSG0000(	465	10.5294	chr10:717	DRGX		protein_c	chr10:49364066-493

ENSG0000(	465	10.5294	chr10:717	DKK1		protein_c	chr10:52314281-523
ENSG0000(	465	10.5294	chr10:717	TFAM		protein_c	chr10:58385345-583
ENSG0000(	465	10.5294	chr10:717	CCDC6	NCGv7;AC	protein_c	chr10:59788747-599
ENSG0000(	465	10.5294	chr10:717	ZNF365		protein_c	chr10:62374192-624
ENSG0000(	465	10.5294	chr10:717	ANK3	NCGv7	protein_c	chr10:60026298-607
ENSG0000(	465	10.5294	chr10:717	CTSLP4		Pseudoger	chr10:50705380-507
ENSG0000(	465	10.5294	chr10:717	DNAJC12		protein_c	chr10:67796669-678
ENSG0000(	465	10.5294	chr10:717	CISD1		protein_c	chr10:58269162-582
ENSG0000(	465	10.5294	chr10:717	MRLN		protein_c	chr10:59736692-597
ENSG0000(	465	10.5294	chr10:717	ARL4AP1		Pseudoger	chr10:60684505-606
ENSG0000(	465	10.5294	chr10:717	BICC1		protein_c	chr10:58512872-588
ENSG0000(	465	10.5294	chr10:717	ENSG00000223841		Pseudoger	chr10:57955295-579
ENSG0000(	465	10.5294	chr10:717	ENSG00000272592		lncRNA	chr10:61684892-616
ENSG0000(	465	10.5294	chr10:717	MRPL35P2		Pseudoger	chr10:63634317-636
ENSG0000(	465	10.5294	chr10:717	MAPK8	TAG	protein_c	chr10:48306639-484
ENSG0000(	465	10.5294	chr10:717	SGMS1-AS1		lncRNA	chr10:50623466-506
ENSG0000(	465	10.5294	chr10:717	ZWINT	NCGv7	protein_c	chr10:56357227-563
ENSG0000(	465	10.5294	chr10:717	AC068062.1		smallRNA	chr10:51639743-516
ENSG0000(	465	10.5294	chr10:717	CSTF2T		protein_c	chr10:51695486-516
ENSG0000(	465	10.5294	chr10:717	snoU13		smallRNA	chr10:58238657-582
ENSG0000(	465	10.5294	chr10:717	ENSG00000226296		lncRNA	chr10:53291072-533
ENSG0000(	465	10.5294	chr10:717	ENSG00000287969		lncRNA	chr10:59357007-593
ENSG0000(	465	10.5294	chr10:717	LINC00844		lncRNA	chr10:58999482-590
ENSG0000(	465	10.5294	chr10:717	ANK3-DT		lncRNA	chr10:60734342-607
ENSG0000(	465	10.5294	chr10:717	C10orf71		protein_c	chr10:49299170-493
ENSG0000(	465	10.5294	chr10:717	ENSG00000286373		lncRNA	chr10:63630672-636
ENSG0000(	465	10.5294	chr10:717	RP11-144G6.10		lncRNA	chr10:47589059-475
ENSG0000(	465	10.5294	chr10:717	ENSG00000289158		lncRNA	chr10:57101444-572
ENSG0000(	465	10.5294	chr10:717	ENSG00000233724		Pseudoger	chr10:65248032-652
ENSG0000(	465	10.5294	chr10:717	snoU13		smallRNA	chr10:66615237-666
ENSG0000(	465	10.5294	chr10:717	ENSG00000231132		lncRNA	chr10:51244894-512
ENSG0000(	465	10.5294	chr10:717	LNCAROD		lncRNA	chr10:52450874-527
ENSG0000(	465	10.5294	chr10:717	LINC02675		lncRNA	chr10:47588681-476
ENSG0000(	465	10.5294	chr10:717	ENSG00000231906		Pseudoger	chr10:48546472-485
ENSG0000(	465	10.5294	chr10:717	ENSG00000289143		lncRNA	chr10:48010137-481
ENSG0000(	465	10.5294	chr10:717	ENSG00000234173		lncRNA	chr10:54486230-546
ENSG0000(	465	10.5294	chr10:717	AC067742.1		smallRNA	chr10:61926936-619
ENSG0000(	465	10.5294	chr10:717	LINC02621		lncRNA	chr10:62289521-623
ENSG0000(	465	10.5294	chr10:717	JMJD1C	NCGv7	protein_c	chr10:63167221-635
ENSG0000(	465	10.5294	chr10:717	ENSG00000287016		lncRNA	chr10:56361479-563
ENSG0000(	465	10.5294	chr10:717	RPL13AP19		Pseudoger	chr10:48745545-487
ENSG0000(	465	10.5294	chr10:717	NUTM2HP		Pseudoger	chr10:50676743-506
ENSG0000(	465	10.5294	chr10:717	ENSG00000285958		lncRNA	chr10:52946431-529
ENSG0000(	465	10.5294	chr10:717	MBL2		protein_c	chr10:52765380-527
ENSG0000(	465	10.5294	chr10:717	DBF4P1		Pseudoger	chr10:64168959-641
ENSG0000(	465	10.5294	chr10:717	MRPL50P4		Pseudoger	chr10:59551404-595
ENSG0000(	465	10.5294	chr10:717	RMRPP4		smallRNA	chr10:50585550-505
ENSG0000(	465	10.5294	chr10:717	RNU6-1250P		smallRNA	chr10:67808502-678
ENSG0000(	465	10.5294	chr10:717	ENSG00000232682		lncRNA	chr10:60050668-600
ENSG0000(	465	10.5294	chr10:717	ENSG00000228527		lncRNA	chr10:58304553-583
ENSG0000(	465	10.5294	chr10:717	WDFY4		protein_c	chr10:48684873-489

ENSG0000(	465	10.5294	chr10:717	ARHGAP22		protein_c	chr10:48446036-486
ENSG0000(	465	10.5294	chr10:717	RNU6-687P		smallRNA	chr10:54452423-544
ENSG0000(	465	10.5294	chr10:717	ENSG00000282121		lncRNA	chr10:60139912-601
ENSG0000(	465	10.5294	chr10:717	MIR1296		smallRNA	chr10:63372957-633
ENSG0000(	465	10.5294	chr10:717	C10orf71-AS1		lncRNA	chr10:49296112-492
ENSG0000(	465	10.5294	chr10:717	ENSG00000270541		Pseudoger	chr10:55746551-557
ENSG0000(	465	10.5294	chr10:717	AL671972.1		smallRNA	chr10:62116215-621
ENSG0000(	465	10.5294	chr10:717	CYP2C61P		Pseudoger	chr10:64762883-647
ENSG0000(	465	10.5294	chr10:717	ENSG00000235356		lncRNA	chr10:65271319-653
ENSG0000(	465	10.5294	chr10:717	ENSG00000273360		lncRNA	chr10:66079243-661
ENSG0000(	465	10.5294	chr10:717	REEP3		protein_c	chr10:63521401-636
ENSG0000(	465	10.5294	chr10:717	RNU6-543P		smallRNA	chr10:63110139-631
ENSG0000(	465	10.5294	chr10:717	ENSG00000235279		lncRNA	chr10:51302566-513
ENSG0000(	465	10.5294	chr10:717	ENSG00000270421		Pseudoger	chr10:63358745-633
ENSG0000(	465	10.5294	chr10:717	FRMPD2	NCGv7	protein_c	chr10:48153088-482
ENSG0000(	465	10.5294	chr10:717	ENSG00000289487		lncRNA	chr10:62814433-629
ENSG0000(	465	10.5294	chr10:717	ENSG00000287221		lncRNA	chr10:50736078-507
ENSG0000(	465	10.5294	chr10:717	ENSG00000228566		lncRNA	chr10:63664664-646
ENSG0000(	465	10.5294	chr10:717	ENSG00000224412		Pseudoger	chr10:63489461-634
ENSG0000(	465	10.5294	chr10:717	ENSG00000270352		Pseudoger	chr10:63658074-636
ENSG0000(	465	10.5294	chr10:717	ENSG00000285803		lncRNA	chr10:49981420-499
ENSG0000(	465	10.5294	chr10:717	RPS6P14		Pseudoger	chr10:48292699-482
ENSG0000(	465	10.5294	chr10:717	MYL6P3		Pseudoger	chr10:65169438-651
ENSG0000(	465	10.5294	chr10:717	RN7SKP196		smallRNA	chr10:58869184-588
ENSG0000(	465	10.5294	chr10:717	PRELID1P3		Pseudoger	chr10:63427297-634
ENSG0000(	465	10.5294	chr10:717	ENSG00000235810		Pseudoger	chr10:57513157-575
ENSG0000(	465	10.5294	chr10:717	LINC02671		lncRNA	chr10:64901136-650
ENSG0000(	465	10.5294	chr10:717	FAM170B		protein_c	chr10:49131154-491
ENSG0000(	465	10.5294	chr10:717	ENSG00000229711		lncRNA	chr10:50822692-508
ENSG0000(	465	10.5294	chr10:717	ENSG00000285837		protein_c	chr10:62374192-626
ENSG0000(	465	10.5294	chr10:717	RNA5SP315		Pseudoger	chr10:48040462-480
ENSG0000(	465	10.5294	chr10:717	ENSG00000285786		lncRNA	chr10:48246525-482
ENSG0000(	465	10.5294	chr10:717	ENSG00000261368		lncRNA	chr10:50964270-509
ENSG0000(	465	10.5294	chr10:717	MRPS35P3		Pseudoger	chr10:57982285-579
ENSG0000(	465	10.5294	chr10:717	RNA5SP318		Pseudoger	chr10:53458197-534
ENSG0000(	465	10.5294	chr10:717	LINC01553		lncRNA	chr10:59955430-599
ENSG0000(	465	10.5294	chr10:717	RPL21P89		Pseudoger	chr10:49815096-498
ENSG0000(	465	10.5294	chr10:717	FAM21EP		Pseudoger	chr10:50048202-500
ENSG0000(	465	10.5294	chr10:717	RPL12P8		Pseudoger	chr10:67874332-678
ENSG0000(	465	10.5294	chr10:717	snoR442		smallRNA	chr10:66494699-664
ENSG0000(	465	10.5294	chr10:717	ENSG00000235939		lncRNA	chr10:49419277-494
ENSG0000(	465	10.5294	chr10:717	ALDH7A1P4		Pseudoger	chr10:62741208-627
ENSG0000(	465	10.5294	chr10:717	LRRC18	NCGv7	protein_c	chr10:48909480-489
ENSG0000(	465	10.5294	chr10:717	SLC9A3P1		Pseudoger	chr10:50150603-501
ENSG0000(	465	10.5294	chr10:717	PHYHIPL		protein_c	chr10:59176643-592
ENSG0000(	465	10.5294	chr10:717	SLC16A9		protein_c	chr10:59650764-597
ENSG0000(	465	10.5294	chr10:717	ENSG00000282906		lncRNA	chr10:65123667-651
ENSG0000(	465	10.5294	chr10:717	ENSG00000289527		lncRNA	chr10:52033793-520
ENSG0000(	465	10.5294	chr10:717	ENSG00000223502		lncRNA	chr10:51062579-510
ENSG0000(	465	10.5294	chr10:717	ENSG00000228403		lncRNA	chr10:48878022-488
ENSG0000(	465	10.5294	chr10:717	TMEM26		protein_c	chr10:61406642-614

ENSG0000(	465	10.5294	chr10:717	DYNC1I2P1		Pseudoger	chr10:50264978-502
ENSG0000(	465	10.5294	chr10:717	FAM170B-AS1		lncRNA	chr10:49121839-491
ENSG0000(	465	10.5294	chr10:717	CTNNA3	NCGv7	protein_c	chr10:65912457-677
ENSG0000(	465	10.5294	chr10:717	A1CF	NCGv7	protein_c	chr10:50799409-508
ENSG0000(	465	10.5294	chr10:717	SIRT1	AC	protein_c	chr10:67884656-679
ENSG0000(	465	10.5294	chr10:717	NRBF2		protein_c	chr10:63133247-631
ENSG0000(	465	10.5294	chr10:717	FAM13C		protein_c	chr10:59246130-593
ENSG0000(	465	10.5294	chr10:717	RPL31P44		Pseudoger	chr10:52389112-523
ENSG0000(	465	10.5294	chr10:717	RN7SL394P		smallRNA	chr10:67825310-678
ENSG0000(	465	10.5294	chr10:717	RPL7AP50		Pseudoger	chr10:63902451-639
ENSG0000(	465	10.5294	chr10:717	NEFMP1		Pseudoger	chr10:54599678-546
ENSG0000(	465	10.5294	chr10:717	AKR1B10P1		Pseudoger	chr10:67750284-677
ENSG0000(	465	10.5294	chr10:717	ENSG00000289444		lncRNA	chr10:48036681-481
ENSG0000(	465	10.5294	chr10:717	RPLP1P10		Pseudoger	chr10:59088701-590
ENSG0000(	465	10.5294	chr10:717	ENSG00000279863		TEC	chr10:50334538-503
ENSG0000(	465	10.5294	chr10:717	ENSG00000286993		lncRNA	chr10:48835541-488
ENSG0000(	465	10.5294	chr10:717	ENSG00000226576		lncRNA	chr10:48984564-490
ENSG0000(	465	10.5294	chr10:717	ENSG00000225299		lncRNA	chr10:67052609-670
ENSG0000(	465	10.5294	chr10:717	FAM133CP		Pseudoger	chr10:58715554-587
ENSG0000(	465	10.5294	chr10:717	LINC02672		lncRNA	chr10:52972612-530
ENSG0000(	465	10.5294	chr10:717	CABCOCO1		protein_c	chr10:61662929-617
ENSG0000(	465	10.5294	chr10:717	PGGT1BP1		Pseudoger	chr10:50726947-507
ENSG0000(	465	10.5294	chr10:717	ARHGAP22-IT1		lncRNA	chr10:48510525-485
ENSG0000(	465	10.5294	chr10:717	ASAH2		protein_c	chr10:50182778-502
ENSG0000(	465	10.5294	chr10:717	ENSG00000225303		lncRNA	chr10:50472814-504
ENSG0000(	465	10.5294	chr10:717	POU5F1P5		Pseudoger	chr10:68010205-680
ENSG0000(	465	10.5294	chr10:717	RSU1P3		Pseudoger	chr10:51319826-513
ENSG0000(	465	10.5294	chr10:717	ENSG00000235140		lncRNA	chr10:59578467-596
ENSG0000(	465	10.5294	chr10:717	HERC4		protein_c	chr10:67921899-680
ENSG0000(	465	10.5294	chr10:717	CDK1	AC	protein_c	chr10:60778331-607
ENSG0000(	465	10.5294	chr10:717	AL356741.1		smallRNA	chr10:67970273-679
ENSG0000(	464	10.50675	chr5:4231	AC025470.1		smallRNA	chr5:57481820-5748
ENSG0000(	464	10.50675	chr10:173	SNORA11		smallRNA	chr10:73126080-731
ENSG0000(	463	10.48411	chr7:2516	FKBP6		protein_c	chr7:73328161-7335
ENSG0000(	463	10.48411	chr7:2516	BAZ1B		protein_c	chr7:73440406-7352
ENSG0000(	463	10.48411	chr7:2516	ENSG00000285886		lncRNA	chr7:72954797-7295
ENSG0000(	463	10.48411	chr7:2516	ENSG00000235581		Pseudoger	chr7:71942259-7194
ENSG0000(	463	10.48411	chr7:2516	SPDYE10		protein_c	chr7:73104008-7315
ENSG0000(	463	10.48411	chr7:2516	MIR3914-1		smallRNA	chr7:71307672-7130
ENSG0000(	463	10.48411	chr7:2516	MLXIPL	NCGv7	protein_c	chr7:73593194-7362
ENSG0000(	463	10.48411	chr7:2516	ENSG00000289042		lncRNA	chr7:72919205-7292
ENSG0000(	463	10.48411	chr7:2516	ENSG00000286466		lncRNA	chr7:69145185-6917
ENSG0000(	463	10.48411	chr7:2516	GTF2IP4		Pseudoger	chr7:73154938-7320
ENSG0000(	463	10.48411	chr7:2516	AC005488.1		protein_c	chr7:72988949-7299
ENSG0000(	463	10.48411	chr7:2516	RNA5SP232		Pseudoger	chr7:71913738-7191
ENSG0000(	463	10.48411	chr7:2516	SBDSP1		lncRNA	chr7:72829425-7283
ENSG0000(	463	10.48411	chr7:2516	PMS2P7		Pseudoger	chr7:73005541-7302
ENSG0000(	463	10.48411	chr7:2516	SPDYE7P		Pseudoger	chr7:72863903-7287
ENSG0000(	463	10.48411	chr7:2516	TMEM270		protein_c	chr7:73861159-7386
ENSG0000(	463	10.48411	chr7:2516	CLDN4		protein_c	chr7:73799542-7383
ENSG0000(	463	10.48411	chr7:2516	METTL27	DriverDBv	protein_c	chr7:73834590-7384

ENSG0000(	463	10.48411	chr7:251(	RN7SL371P		smallRNA	chr7:69536049-6953
ENSG0000(	463	10.48411	chr7:251(	TRIM50		protein_c	chr7:73312536-7332
ENSG0000(	463	10.48411	chr7:251(	LIMK1	NCGv7	protein_c	chr7:74082933-7412
ENSG0000(	463	10.48411	chr7:251(	MTCO1P25		Pseudoger	chr7:69331835-6933
ENSG0000(	463	10.48411	chr7:251(	POM121		protein_c	chr7:72879349-7295
ENSG0000(	463	10.48411	chr7:251(	ELN	NCGv7;AC	protein_c	chr7:74027789-7406
ENSG0000(	463	10.48411	chr7:251(	POM121B		Pseudoger	chr7:73293497-7330
ENSG0000(	463	10.48411	chr7:251(	RFC2		protein_c	chr7:74231499-7425
ENSG0000(	463	10.48411	chr7:251(	ENSG00000205584		Pseudoger	chr7:72969814-7297
ENSG0000(	463	10.48411	chr7:251(	TRIM74	DriverDB\	protein_c	chr7:72959485-7296
ENSG0000(	463	10.48411	chr7:251(	ABHD11	DriverDB\	protein_c	chr7:73736094-7373
ENSG0000(	463	10.48411	chr7:251(	STX1A		protein_c	chr7:73699206-7371
ENSG0000(	463	10.48411	chr7:251(	TYW1B	NCGv7	protein_c	chr7:72558744-7282
ENSG0000(	463	10.48411	chr7:251(	NSUN5P2		Pseudoger	chr7:72948485-7295
ENSG0000(	463	10.48411	chr7:251(	MIR4650-2		smallRNA	chr7:72697903-7269
ENSG0000(	463	10.48411	chr7:251(	CLDN3	DriverDB\	protein_c	chr7:73768997-7377
ENSG0000(	463	10.48411	chr7:251(	RNU6-229P		smallRNA	chr7:69401202-6940
ENSG0000(	463	10.48411	chr7:251(	ENSG00000261467		lncRNA	chr7:73985992-7398
ENSG0000(	463	10.48411	chr7:251(	EIF4H		protein_c	chr7:74174231-7419
ENSG0000(	463	10.48411	chr7:251(	Y_RNA		smallRNA	chr7:73011144-7301
ENSG0000(	463	10.48411	chr7:251(	ABCF2P2		Pseudoger	chr7:72103181-7210
ENSG0000(	463	10.48411	chr7:251(	CALN1		protein_c	chr7:71779491-7244
ENSG0000(	463	10.48411	chr7:251(	ENSG00000270555		Pseudoger	chr7:72768798-7276
ENSG0000(	463	10.48411	chr7:251(	AC091738.2		smallRNA	chr7:72673068-7267
ENSG0000(	463	10.48411	chr7:251(	RNU6-1080P		smallRNA	chr7:73339094-7333
ENSG0000(	463	10.48411	chr7:251(	AC004878.5		Pseudoger	chr7:73076167-7308
ENSG0000(	463	10.48411	chr7:251(	BCL7B		protein_c	chr7:73536356-7355
ENSG0000(	463	10.48411	chr7:251(	TBL2	NCGv7	protein_c	chr7:73567537-7357
ENSG0000(	463	10.48411	chr7:251(	RNA5SP231		Pseudoger	chr7:68723911-6872
ENSG0000(	463	10.48411	chr7:251(	MTCO2P25		Pseudoger	chr7:69330698-6933
ENSG0000(	463	10.48411	chr7:251(	VPS37D	DriverDB\	protein_c	chr7:73667831-7367
ENSG0000(	463	10.48411	chr7:251(	DNAJC30		protein_c	chr7:73680918-7368
ENSG0000(	463	10.48411	chr7:251(	NSUN5		protein_c	chr7:73302516-7330
ENSG0000(	463	10.48411	chr7:251(	ENSG00000236978		Pseudoger	chr7:70837738-7083
ENSG0000(	463	10.48411	chr7:251(	ENSG00000233689		lncRNA	chr7:69026433-6904
ENSG0000(	463	10.48411	chr7:251(	SBDSP1		Pseudoger	chr7:72829656-7283
ENSG0000(	463	10.48411	chr7:251(	Y_RNA		smallRNA	chr7:73095357-7309
ENSG0000(	463	10.48411	chr7:251(	GTF2IRD2P1		Pseudoger	chr7:73243271-7328
ENSG0000(	463	10.48411	chr7:251(	RN7SKP75		smallRNA	chr7:71685452-7168
ENSG0000(	463	10.48411	chr7:251(	PHB1P5		Pseudoger	chr7:73187969-7318
ENSG0000(	463	10.48411	chr7:251(	RNU6-1070P		smallRNA	chr7:74258526-7425
ENSG0000(	463	10.48411	chr7:251(	STAG3L3		Pseudoger	chr7:72998027-7300
ENSG0000(	463	10.48411	chr7:251(	PMS2P6		Pseudoger	chr7:73093657-7310
ENSG0000(	463	10.48411	chr7:251(	RN7SL265P		smallRNA	chr7:73732571-7373
ENSG0000(	463	10.48411	chr7:251(	ENSG00000272843		lncRNA	chr7:72924418-7292
ENSG0000(	463	10.48411	chr7:251(	Y_RNA		smallRNA	chr7:69507510-6950
ENSG0000(	463	10.48411	chr7:251(	RPL7AP77		Pseudoger	chr7:73314107-7331
ENSG0000(	463	10.48411	chr7:251(	RN7SL377P		smallRNA	chr7:72822898-7282
ENSG0000(	463	10.48411	chr7:251(	BUD23	NCGv7	protein_c	chr7:73683025-7370
ENSG0000(	463	10.48411	chr7:251(	Y_RNA		smallRNA	chr7:73067230-7306
ENSG0000(	463	10.48411	chr7:251(	ENSG00000290998		lncRNA	chr7:72862757-7286

ENSG0000(	463	10.48411	chr7:2516	ENSG00000225718		lncRNA	chr7:69186821-6943
ENSG0000(	463	10.48411	chr7:2516	ABHD11-AS1		Pseudoger	chr7:73734994-7373
ENSG0000(	463	10.48411	chr7:2516	ENSG00000290832		lncRNA	chr7:72969696-7300
ENSG0000(	463	10.48411	chr7:2516	ENSG00000290839		lncRNA	chr7:73242751-7327
ENSG0000(	463	10.48411	chr7:2516	Y_RNA		smallRNA	chr7:73403788-7340
ENSG0000(	463	10.48411	chr7:2516	RNU6-1198P		smallRNA	chr7:73507208-7350
ENSG0000(	463	10.48411	chr7:2516	AUTS2		protein_c	chr7:69598296-7079
ENSG0000(	463	10.48411	chr7:2516	LAT2		protein_c	chr7:74199652-7422
ENSG0000(	463	10.48411	chr7:2516	NCF1B		lncRNA	chr7:73220624-7323
ENSG0000(	463	10.48411	chr7:2516	MIR590		smallRNA	chr7:74191198-7419
ENSG0000(	463	10.48411	chr7:2516	ENSG00000236839		lncRNA	chr7:69187833-6918
ENSG0000(	463	10.48411	chr7:2516	ENSG00000274080		lncRNA	chr7:73609262-7361
ENSG0000(	463	10.48411	chr7:2516	NSUN5P2		lncRNA	chr7:72947581-7295
ENSG0000(	463	10.48411	chr7:2516	AC069280.1		smallRNA	chr7:69190141-6919
ENSG0000(	463	10.48411	chr7:2516	AC079398.1		smallRNA	chr7:71310459-7131
ENSG0000(	463	10.48411	chr7:2516	ELN-AS1		lncRNA	chr7:74058905-7406
ENSG0000(	463	10.48411	chr7:2516	FZD9		protein_c	chr7:73433778-7343
ENSG0000(	463	10.48411	chr7:2516	Y_RNA		smallRNA	chr7:72983361-7298
ENSG0000(	463	10.48411	chr7:2516	NCF1B		Pseudoger	chr7:73220646-7323
ENSG0000(	463	10.48411	chr7:2516	GALNT17		protein_c	chr7:71132144-7171
ENSG0000(	463	10.48411	chr7:2516	RNU6-832P		smallRNA	chr7:69125270-6912
ENSG0000(	463	10.48411	chr7:2516	SPDYE9		protein_c	chr7:73075971-7308
ENSG0000(	463	10.48411	chr7:2516	RN7SL625P		smallRNA	chr7:72841439-7284
ENSG0000(	463	10.48411	chr7:2516	ENSG00000270694		Pseudoger	chr7:72722885-7272
ENSG0000(	463	10.48411	chr7:2516	CT66		lncRNA	chr7:69594793-6959
ENSG0000(	461	10.43882	chr13:339	ENSG00000277831		lncRNA	chr13:44369365-443
ENSG0000(	460	10.41618	chr16:239	ENSG00000261369		lncRNA	chr16:47299447-473
ENSG0000(	460	10.41618	chr16:239	N4BP1	NCGv7	protein_c	chr16:48538726-486
ENSG0000(	460	10.41618	chr16:239	ENSG00000261267		lncRNA	chr16:48559661-485
ENSG0000(	460	10.41618	chr16:239	ENSG00000234337		Pseudoger	chr16:52655307-526
ENSG0000(	460	10.41618	chr16:239	ENSG00000260012		Pseudoger	chr16:47262741-472
ENSG0000(	460	10.41618	chr16:239	ZNF423		protein_c	chr16:49487524-498
ENSG0000(	460	10.41618	chr16:239	CBLN1		protein_c	chr16:49277917-492
ENSG0000(	460	10.41618	chr16:239	LONP2		protein_c	chr16:48244300-483
ENSG0000(	460	10.41618	chr16:239	ENSG00000260042		lncRNA	chr16:50856614-508
ENSG0000(	460	10.41618	chr16:239	ENSG00000280344		TEC	chr16:51176066-511
ENSG0000(	460	10.41618	chr10:717	CTSLP7		Pseudoger	chr10:47581211-475
ENSG0000(	460	10.41618	chr16:239	ENSG00000260605		lncRNA	chr16:51035118-510
ENSG0000(	460	10.41618	chr16:239	ENSG00000279779		TEC	chr16:52081568-520
ENSG0000(	460	10.41618	chr16:239	ENSG00000260616		lncRNA	chr16:50783859-508
ENSG0000(	460	10.41618	chr16:239	ENSG00000260620		lncRNA	chr16:51054728-510
ENSG0000(	460	10.41618	chr16:239	ENSG00000280189		TEC	chr16:49297143-492
ENSG0000(	460	10.41618	chr16:239	ENSG00000261017		Pseudoger	chr16:48059671-480
ENSG0000(	460	10.41618	chr16:239	LINC02179		lncRNA	chr16:49466106-494
ENSG0000(	460	10.41618	chr16:239	ENSG00000260381		lncRNA	chr16:50100339-501
ENSG0000(	460	10.41618	chr16:239	UNGP1		Pseudoger	chr16:51277852-512
ENSG0000(	460	10.41618	chr16:239	RPS2P44		Pseudoger	chr16:48577524-485
ENSG0000(	460	10.41618	chr16:239	ENSG00000260573		lncRNA	chr16:50407184-505
ENSG0000(	460	10.41618	chr16:239	TOX3	NCGv7	protein_c	chr16:52436417-525
ENSG0000(	460	10.41618	chr16:239	CYLD-AS1		lncRNA	chr16:50727417-507
ENSG0000(	460	10.41618	chr16:239	SALL1	NCGv7	protein_c	chr16:51135982-511

ENSG0000(	460	10.41618	chr16:23(	ENSG00000280376		TEC	chr16:46673443-466
ENSG0000(	460	10.41618	chr16:23(	ENSG00000261047		Pseudoger	chr16:51244054-512
ENSG0000(	460	10.41618	chr16:23(	ENSG00000261393		lncRNA	chr16:50368580-503
ENSG0000(	460	10.41618	chr16:23(	ENSG00000261421		lncRNA	chr16:49159508-491
ENSG0000(	460	10.41618	chr16:23(	RNA5SP424		smallRNA	chr16:47450347-474
ENSG0000(	460	10.41618	chr16:23(	ENSG00000261512		lncRNA	chr16:46622861-466
ENSG0000(	460	10.41618	chr16:23(	LINC02127		lncRNA	chr16:51017544-510
ENSG0000(	460	10.41618	chr16:23(	DNAJA2		protein_c	chr16:46955362-469
ENSG0000(	460	10.41618	chr16:23(	hsa-mir-3181		smallRNA	chr16:50742305-507
ENSG0000(	460	10.41618	chr16:23(	VPS35		protein_c	chr16:46656132-466
ENSG0000(	460	10.41618	chr16:23(	ENSG00000260850		lncRNA	chr16:51354456-515
ENSG0000(	460	10.41618	chr10:717	RP11-463P17.3		lncRNA	chr10:47380761-473
ENSG0000(	460	10.41618	chr16:23(	ENSG00000260029		lncRNA	chr16:50551824-505
ENSG0000(	460	10.41618	chr16:23(	RPL10P14		Pseudoger	chr16:50151280-501
ENSG0000(	460	10.41618	chr10:717	RBP3		protein_c	chr10:47348362-473
ENSG0000(	460	10.41618	chr16:23(	SOD1P2		Pseudoger	chr16:51107571-511
ENSG0000(	460	10.41618	chr16:23(	NOD2		protein_c	chr16:50693588-507
ENSG0000(	460	10.41618	chr16:23(	CKBP1		Pseudoger	chr16:46842632-468
ENSG0000(	460	10.41618	chr10:717	PTPN20A		protein_c	chr10:46911395-470
ENSG0000(	460	10.41618	chr16:23(	RNA5SP426		Pseudoger	chr16:50869455-508
ENSG0000(	460	10.41618	chr16:23(	ENSG00000260393		lncRNA	chr16:52607762-526
ENSG0000(	460	10.41618	chr16:23(	snoU13		smallRNA	chr16:50352189-503
ENSG0000(	460	10.41618	chr16:23(	ENSG00000261538		lncRNA	chr16:48164952-481
ENSG0000(	460	10.41618	chr16:23(	ENSG00000285800		lncRNA	chr16:52583694-525
ENSG0000(	460	10.41618	chr16:23(	ENSG00000280067		TEC	chr16:48343755-483
ENSG0000(	460	10.41618	chr16:23(	SNORD112		smallRNA	chr16:50498161-504
ENSG0000(	460	10.41618	chr16:23(	SNORA70		smallRNA	chr16:50151642-501
ENSG0000(	460	10.41618	chr16:23(	ENSG00000260688		Pseudoger	chr16:48470953-484
ENSG0000(	460	10.41618	chr16:23(	ENSG00000287469		lncRNA	chr16:49072543-492
ENSG0000(	460	10.41618	chr10:717	ANXA8		protein_c	chr10:47460161-474
ENSG0000(	460	10.41618	chr16:23(	ENSG00000272545		lncRNA	chr16:48640473-486
ENSG0000(	460	10.41618	chr16:23(	ENSG00000260184		Pseudoger	chr16:48849919-488
ENSG0000(	460	10.41618	chr16:23(	ENSG00000290429		lncRNA	chr16:46469341-465
ENSG0000(	460	10.41618	chr16:23(	ENSG00000260975		lncRNA	chr16:52198012-522
ENSG0000(	460	10.41618	chr16:23(	ENSG00000260033		Pseudoger	chr16:48600176-486
ENSG0000(	460	10.41618	chr16:23(	ENSG00000260963		lncRNA	chr16:52607006-526
ENSG0000(	460	10.41618	chr10:717	GDF2		protein_c	chr10:47322453-473
ENSG0000(	460	10.41618	chr16:23(	SHCBP1		protein_c	chr16:46578591-466
ENSG0000(	460	10.41618	chr16:23(	KLF8P1		Pseudoger	chr16:48718083-487
ENSG0000(	460	10.41618	chr16:23(	ENSG00000260251		Pseudoger	chr16:46573763-465
ENSG0000(	460	10.41618	chr16:23(	RN7SL54P		smallRNA	chr16:48414778-484
ENSG0000(	460	10.41618	chr16:23(	SIAH1		protein_c	chr16:48356364-484
ENSG0000(	460	10.41618	chr16:23(	SNX20		protein_c	chr16:50666300-506
ENSG0000(	460	10.41618	chr16:23(	NETO2	NCGv7	protein_c	chr16:47077703-471
ENSG0000(	460	10.41618	chr16:23(	ENSG00000260909		Pseudoger	chr16:46649126-466
ENSG0000(	460	10.41618	chr16:23(	ENSG00000260249		lncRNA	chr16:50664903-506
ENSG0000(	460	10.41618	chr16:23(	ENSG00000279249		lncRNA	chr16:49282021-493
ENSG0000(	460	10.41618	chr16:23(	CASC22		lncRNA	chr16:52258564-522
ENSG0000(	460	10.41618	chr16:23(	PHKB		protein_c	chr16:47461123-477
ENSG0000(	460	10.41618	chr16:23(	ENSG00000279842		TEC	chr16:49900019-499
ENSG0000(	460	10.41618	chr16:23(	RNA5SP425		Pseudoger	chr16:47505432-475

ENSG0000(	460	10.41618	chr16:239	ENSG00000261815		lncRNA	chr16:49170552-491
ENSG0000(	460	10.41618	chr16:239	ENSG00000261131		lncRNA	chr16:46660696-466
ENSG0000(	460	10.41618	chr16:239	ENSG00000260478		lncRNA	chr16:52238081-522
ENSG0000(	460	10.41618	chr16:239	ENSG00000262950		lncRNA	chr16:49442910-494
ENSG0000(	460	10.41618	chr16:239	ADAM3B		Pseudoger	chr16:49517762-495
ENSG0000(	460	10.41618	chr16:239	LINC02134		lncRNA	chr16:47965620-479
ENSG0000(	460	10.41618	chr16:239	ENSG00000261238		lncRNA	chr16:51149239-511
ENSG0000(	460	10.41618	chr16:239	ENSG00000261835		Pseudoger	chr16:50046496-500
ENSG0000(	460	10.41618	chr16:239	ENSG00000259866		Pseudoger	chr16:47561307-475
ENSG0000(	460	10.41618	chr16:239	MOCS1P1		Pseudoger	chr16:48496990-484
ENSG0000(	460	10.41618	chr16:239	RAB43P1		Pseudoger	chr16:46626404-466
ENSG0000(	460	10.41618	chr16:239	ANKRD26P1		Pseudoger	chr16:46518305-465
ENSG0000(	460	10.41618	chr16:239	DNAJA2-DT		lncRNA	chr16:46973789-469
ENSG0000(	460	10.41618	chr16:239	LINC02128		lncRNA	chr16:50840017-509
ENSG0000(	460	10.41618	chr16:239	LINC02178		lncRNA	chr16:50390916-503
ENSG0000(	460	10.41618	chr16:239	ENSG00000263110		lncRNA	chr16:49454229-494
ENSG0000(	460	10.41618	chr16:239	TENT4B	NCGv7	protein_c	chr16:50152911-502
ENSG0000(	460	10.41618	chr16:239	ENSG00000260086		lncRNA	chr16:48623415-487
ENSG0000(	460	10.41618	chr16:239	ENSG00000285367		lncRNA	chr16:51150770-514
ENSG0000(	460	10.41618	chr10:717	GDF10		protein_c	chr10:47300196-473
ENSG0000(	460	10.41618	chr16:239	ENSG00000259912		lncRNA	chr16:48620319-486
ENSG0000(	460	10.41618	chr16:239	MTND4LP25		Pseudoger	chr16:49066567-490
ENSG0000(	460	10.41618	chr10:717	RP11-463P17.1		lncRNA	chr10:47407164-474
ENSG0000(	460	10.41618	chr10:717	FAM25G		protein_c	chr10:47487218-474
ENSG0000(	460	10.41618	chr10:717	RP11-301J7.8		lncRNA	chr10:47477147-474
ENSG0000(	460	10.41618	chr16:239	AC007333.1		smallRNA	chr16:52255451-522
ENSG0000(	460	10.41618	chr16:239	AC007611.1		smallRNA	chr16:48651331-486
ENSG0000(	460	10.41618	chr16:239	LINC02133		lncRNA	chr16:47724568-479
ENSG0000(	460	10.41618	chr16:239	ENSG00000287256		lncRNA	chr16:51197769-512
ENSG0000(	460	10.41618	chr16:239	ENSG00000260782		Pseudoger	chr16:46789898-467
ENSG0000(	460	10.41618	chr16:239	ENSG00000261356		Pseudoger	chr16:46571537-465
ENSG0000(	460	10.41618	chr16:239	ENSG00000280131		TEC	chr16:49711273-497
ENSG0000(	460	10.41618	chr16:239	ENSG00000270120		lncRNA	chr16:50712844-507
ENSG0000(	460	10.41618	chr16:239	ABCC11		protein_c	chr16:48165773-482
ENSG0000(	460	10.41618	chr10:717	FRMPD2P1		Pseudoger	chr10:46870857-468
ENSG0000(	460	10.41618	chr16:239	NDUFA5P11		Pseudoger	chr16:47598654-475
ENSG0000(	460	10.41618	chr16:239	ENSG00000261802		lncRNA	chr16:48447904-484
ENSG0000(	460	10.41618	chr16:239	ACTG1P16		Pseudoger	chr16:50058150-500
ENSG0000(	460	10.41618	chr10:717	AL591684.1		protein_c	chr10:47460162-474
ENSG0000(	460	10.41618	chr16:239	MRPS21P7		Pseudoger	chr16:49708006-497
ENSG0000(	460	10.41618	chr16:239	ENSG00000260052		lncRNA	chr16:48637143-486
ENSG0000(	460	10.41618	chr16:239	CASC16		lncRNA	chr16:52552084-526
ENSG0000(	460	10.41618	chr16:239	RNU6-257P		smallRNA	chr16:48731704-487
ENSG0000(	460	10.41618	chr16:239	LINC02168		lncRNA	chr16:50806668-508
ENSG0000(	460	10.41618	chr16:239	ENSG00000288026		lncRNA	chr16:48050588-480
ENSG0000(	460	10.41618	chr16:239	ENSG00000259821		lncRNA	chr16:47004513-470
ENSG0000(	460	10.41618	chr16:239	AC007339.1		smallRNA	chr16:49679220-496
ENSG0000(	460	10.41618	chr16:239	ENSG00000262038		lncRNA	chr16:47529190-475
ENSG0000(	460	10.41618	chr16:239	ENSG00000261470		lncRNA	chr16:52078622-520
ENSG0000(	460	10.41618	chr16:239	LINC02192		lncRNA	chr16:47849307-478
ENSG0000(	460	10.41618	chr16:239	ITFG1-AS1		lncRNA	chr16:47144323-471

ENSG0000(	460	10.41618	chr16:239	ENSG00000287718		lncRNA	chr16:51089466-510
ENSG0000(	460	10.41618	chr16:239	ENSG00000260744		lncRNA	chr16:47196311-472
ENSG0000(	460	10.41618	chr10:717	DUSP8P4		Pseudoger	chr10:47564256-475
ENSG0000(	460	10.41618	chr16:239	LINC00919		lncRNA	chr16:52083064-520
ENSG0000(	460	10.41618	chr16:239	ENSG00000261725		Pseudoger	chr16:46850999-468
ENSG0000(	460	10.41618	chr16:239	ENSG00000205414		lncRNA	chr16:50606076-506
ENSG0000(	460	10.41618	chr16:239	LINC02911		lncRNA	chr16:52005487-520
ENSG0000(	460	10.41618	chr16:239	CNEP1R1		protein_c	chr16:50024410-500
ENSG0000(	460	10.41618	chr16:239	HEATR3-AS1		lncRNA	chr16:50046429-500
ENSG0000(	460	10.41618	chr10:717	FRMPD2P2		Pseudoger	chr10:46870857-468
ENSG0000(	460	10.41618	chr16:239	ENSG00000260497		Pseudoger	chr16:47760780-477
ENSG0000(	460	10.41618	chr16:239	ENSG00000261751		lncRNA	chr16:49920730-499
ENSG0000(	460	10.41618	chr16:239	LINC02180		lncRNA	chr16:52085210-520
ENSG0000(	460	10.41618	chr16:239	EIF4BP5		Pseudoger	chr16:47565090-475
ENSG0000(	460	10.41618	chr16:239	ENSG00000278909		TEC	chr16:50555355-505
ENSG0000(	460	10.41618	chr16:239	ADCY7		protein_c	chr16:50246137-503
ENSG0000(	460	10.41618	chr16:239	LINC01571		lncRNA	chr16:51755325-517
ENSG0000(	460	10.41618	chr16:239	MRPS21P8		Pseudoger	chr16:49764454-497
ENSG0000(	460	10.41618	chr16:239	ENSG00000259983		Pseudoger	chr16:47013957-470
ENSG0000(	460	10.41618	chr16:239	HNRNPA1L3		protein_c	chr16:51553436-516
ENSG0000(	460	10.41618	chr16:239	ENSG00000275155		lncRNA	chr16:49847018-498
ENSG0000(	460	10.41618	chr16:239	ITFG1		protein_c	chr16:47154387-474
ENSG0000(	460	10.41618	chr16:239	ENSG00000261261		lncRNA	chr16:52622115-526
ENSG0000(	460	10.41618	chr16:239	ENSG00000287444		lncRNA	chr16:50120064-501
ENSG0000(	460	10.41618	chr16:239	RNU6-845P		smallRNA	chr16:46556089-465
ENSG0000(	460	10.41618	chr16:239	snoU13		smallRNA	chr16:48658381-486
ENSG0000(	460	10.41618	chr16:239	snoU13		smallRNA	chr16:48492398-484
ENSG0000(	460	10.41618	chr16:239	MYLK3		protein_c	chr16:46702282-467
ENSG0000(	460	10.41618	chr10:717	RP11-301J7.9		Pseudoger	chr10:47484456-474
ENSG0000(	460	10.41618	chr16:239	NCOA5LP		Pseudoger	chr16:49954985-499
ENSG0000(	460	10.41618	chr16:239	ENSG00000289119		lncRNA	chr16:48610310-486
ENSG0000(	460	10.41618	chr16:239	HEATR3		protein_c	chr16:50065967-501
ENSG0000(	460	10.41618	chr16:239	snoU13		smallRNA	chr16:47120919-471
ENSG0000(	460	10.41618	chr10:717	PTPN20B		protein_c	chr10:46911395-470
ENSG0000(	460	10.41618	chr16:239	BRD7	NCGv7	protein_c	chr16:50313487-503
ENSG0000(	460	10.41618	chr10:717	AL731561.2		smallRNA	chr10:47351739-473
ENSG0000(	460	10.41618	chr10:717	ENSG00000289299		lncRNA	chr10:47207444-472
ENSG0000(	460	10.41618	chr16:239	RPL34P29		Pseudoger	chr16:49935028-499
ENSG0000(	460	10.41618	chr16:239	RPL23AP72		Pseudoger	chr16:47150132-471
ENSG0000(	460	10.41618	chr16:239	GPT2		protein_c	chr16:46884362-469
ENSG0000(	460	10.41618	chr16:239	C16orf78		protein_c	chr16:49373804-493
ENSG0000(	460	10.41618	chr16:239	Y_RNA		smallRNA	chr16:47313027-473
ENSG0000(	460	10.41618	chr16:239	ORC6		protein_c	chr16:46689643-466
ENSG0000(	460	10.41618	chr16:239	ABCC12		protein_c	chr16:48080882-481
ENSG0000(	460	10.41618	chr16:239	CYLD	NCGv7;AC	protein_c	chr16:50742050-508
ENSG0000(	460	10.41618	chr16:239	NKD1		protein_c	chr16:50548396-506
ENSG0000(	460	10.41618	chr16:239	ENSG00000279356		TEC	chr16:50072862-500
ENSG0000(	460	10.41618	chr16:239	RNY4P3		smallRNA	chr16:50098264-500
ENSG0000(	460	10.41618	chr16:239	UBA52P8		Pseudoger	chr16:48334471-483
ENSG0000(	460	10.41618	chr16:239	AC027348.1		smallRNA	chr16:49816685-498
ENSG0000(	460	10.41618	chr16:239	C16orf87		protein_c	chr16:46796603-468

ENSG0000(	460	10.41618	chr16:239	AC023818.1		smallRNA	chr16:48421127-484
ENSG0000(	460	10.41618	chr16:239	ENSG00000279509		TEC	chr16:47218026-472
ENSG0000(	460	10.41618	chr16:239	ENSG00000289907		lncRNA	chr16:52296987-523
ENSG0000(	460	10.41618	chr10:717	ZNF488		protein_c	chr10:47365495-473
ENSG0000(	460	10.41618	chr16:239	RN7SKP142		smallRNA	chr16:51671236-516
ENSG0000(	460	10.41618	chr16:239	ENSG00000279427		TEC	chr16:52436772-524
ENSG0000(	457	10.34825	chr17:289	RNU7-134P		smallRNA	chr17:57685529-576
ENSG0000(	456	10.3256	chr11:144	RPL34P22		Pseudoger	chr11:44629357-446
ENSG0000(	453	10.25767	chr1:3733	ENSG00000236948		lncRNA	chr1:5561709-56682
ENSG0000(	453	10.25767	chr1:3733	ENSG00000284616		lncRNA	chr1:5301928-53073
ENSG0000(	453	10.25767	chr1:3733	ESPN		protein_c	chr1:6424776-64613
ENSG0000(	453	10.25767	chr1:3733	ENSG00000271746		lncRNA	chr1:6393555-63943
ENSG0000(	453	10.25767	chr1:3733	TNFRSF25		protein_c	chr1:6460786-64661
ENSG0000(	453	10.25767	chr1:3733	RNU6-731P		smallRNA	chr1:6540854-65409
ENSG0000(	453	10.25767	chr1:3733	ENSG00000227950		Pseudoger	chr1:6834333-68346
ENSG0000(	453	10.25767	chr1:3733	THAP3		protein_c	chr1:6624868-66355
ENSG0000(	453	10.25767	chr1:3733	ENSG00000283356		Pseudoger	chr1:5554747-55548
ENSG0000(	453	10.25767	chr1:3733	CAMTA1-DT		lncRNA	chr1:6783892-67848
ENSG0000(	453	10.25767	chr1:3733	snoU13		smallRNA	chr1:6752719-67528
ENSG0000(	453	10.25767	chr1:3733	NPHP4		protein_c	chr1:5862811-59924
ENSG0000(	453	10.25767	chr1:3733	ENSG00000289893		lncRNA	chr1:5733358-57714
ENSG0000(	453	10.25767	chr1:3733	RNF207-AS1		lncRNA	chr1:6204840-62057
ENSG0000(	453	10.25767	chr1:3733	ICMT-DT		lncRNA	chr1:6234692-62394
ENSG0000(	453	10.25767	chr1:3733	MIR4689		smallRNA	chr1:5862672-58627
ENSG0000(	453	10.25767	chr1:3733	CAMTA1-IT1		lncRNA	chr1:7368942-73702
ENSG0000(	453	10.25767	chr1:3733	ENSG00000284744		lncRNA	chr1:6767954-67700
ENSG0000(	453	10.25767	chr1:3733	RNU1-8P		smallRNA	chr1:7219360-72195
ENSG0000(	453	10.25767	chr1:3733	HES2		protein_c	chr1:6412418-64246
ENSG0000(	453	10.25767	chr1:3733	ENSG00000284692		lncRNA	chr1:5478736-54930
ENSG0000(	453	10.25767	chr1:3733	CHD5	NCGv7	protein_c	chr1:6101787-61803
ENSG0000(	453	10.25767	chr1:3733	ENSG00000284666		lncRNA	chr1:5480787-54820
ENSG0000(	453	10.25767	chr1:3733	RPL22	NCGv7;AC	protein_c	chr1:6185020-62093
ENSG0000(	453	10.25767	chr1:3733	ENSG00000237365		lncRNA	chr1:7008376-70142
ENSG0000(	453	10.25767	chr1:3733	RNF207	NCGv7	protein_c	chr1:6205475-62212
ENSG0000(	453	10.25767	chr1:3733	GPR153		protein_c	chr1:6247353-62610
ENSG0000(	453	10.25767	chr1:3733	ZBTB48		protein_c	chr1:6579994-65892
ENSG0000(	453	10.25767	chr1:3733	ENSG00000231868		lncRNA	chr1:6443034-64470
ENSG0000(	453	10.25767	chr1:3733	LINC01672		lncRNA	chr1:6724637-67300
ENSG0000(	453	10.25767	chr1:3733	MIR4252		smallRNA	chr1:6429834-64298
ENSG0000(	453	10.25767	chr1:3733	ENSG00000260972		lncRNA	chr1:5492978-54946
ENSG0000(	453	10.25767	chr1:3733	LINC02782		lncRNA	chr1:5086459-50908
ENSG0000(	453	10.25767	chr1:3733	ACOT7		protein_c	chr1:6264269-63937
ENSG0000(	453	10.25767	chr1:3733	AL356261.1		smallRNA	chr1:5893839-58939
ENSG0000(	453	10.25767	chr1:3733	NOL9	NCGv7	protein_c	chr1:6521347-65545
ENSG0000(	453	10.25767	chr1:3733	KLHL21		protein_c	chr1:6590724-66146
ENSG0000(	453	10.25767	chr1:3733	ENSG00000285629		protein_c	chr1:6159430-61977
ENSG0000(	453	10.25767	chr1:3733	ICMT		protein_c	chr1:6221193-62359
ENSG0000(	453	10.25767	chr1:3733	DNAJC11		protein_c	chr1:6634168-67019
ENSG0000(	453	10.25767	chr1:3733	HES3		protein_c	chr1:6244179-62455
ENSG0000(	453	10.25767	chr1:3733	AL356693.1		smallRNA	chr1:5815871-58159
ENSG0000(	453	10.25767	chr1:3733	KCNAB2		protein_c	chr1:5990927-61011

ENSG0000(	453	10.25767	chr1:373:	MIR4417		smallRNA	chr1:5564071-55641
ENSG0000(	453	10.25767	chr1:373:	PHF13		protein_c	chr1:6613731-66240
ENSG0000(	453	10.25767	chr1:373:	PLEKHG5		protein_c	chr1:6467122-65200
ENSG0000(	453	10.25767	chr1:373:	ENSG00000229519		Pseudoger	chr1:6547905-65486
ENSG0000(	453	10.25767	chr1:373:	TAS1R1		protein_c	chr1:6555307-65797
ENSG0000(	452	10.23503	chr17:289	RPL9P28		Pseudoger	chr17:48691116-486
ENSG0000(	451	10.21238	chr10:717	RN7SL248P		smallRNA	chr10:46648947-466
ENSG0000(	451	10.21238	chr10:717	SHLD2P1		Pseudoger	chr10:46610473-466
ENSG0000(	451	10.21238	chr10:717	FAM25B		protein_c	chr10:46369093-463
ENSG0000(	451	10.21238	chr10:717	BMS1P6		Pseudoger	chr10:46788398-467
ENSG0000(	451	10.21238	chr10:717	CTSLP2		lncRNA	chr10:46753605-467
ENSG0000(	451	10.21238	chr10:717	ANTXRLP1		Pseudoger	chr10:46233885-462
ENSG0000(	451	10.21238	chr10:717	HNRNPA1P33		Pseudoger	chr10:46415852-464
ENSG0000(	451	10.21238	chr10:717	ENSG00000273760		lncRNA	chr10:46411164-464
ENSG0000(	451	10.21238	chr10:717	ANXA8L2		protein_c	chr10:46375676-463
ENSG0000(	451	10.21238	chr10:717	CTSLP2		Pseudoger	chr10:46758089-467
ENSG0000(	451	10.21238	chr10:717	FAM245B		lncRNA	chr10:46721002-467
ENSG0000(	451	10.21238	chr10:717	ANXA8L1	NCGv7	protein_c	chr10:46375718-463
ENSG0000(	451	10.21238	chr10:717	CTGLF8P		Pseudoger	chr10:46816515-468
ENSG0000(	451	10.21238	chr10:717	GLUD1P6		Pseudoger	chr10:46770737-467
ENSG0000(	451	10.21238	chr10:717	FAM25BP		Pseudoger	chr10:46369113-463
ENSG0000(	451	10.21238	chr10:717	RHEBP1		Pseudoger	chr10:46634910-466
ENSG0000(	451	10.21238	chr10:717	AGAP14P		Pseudoger	chr10:46337224-463
ENSG0000(	451	10.21238	chr10:717	GLUD1P7		Pseudoger	chr10:46770736-467
ENSG0000(	451	10.21238	chr10:717	DUSP8P2		Pseudoger	chr10:46774421-467
ENSG0000(	451	10.21238	chr10:717	GPRIN2	NCGv7	protein_c	chr10:46541735-465
ENSG0000(	451	10.21238	chr10:717	NPY4R2		protein_c	chr10:46461098-464
ENSG0000(	451	10.21238	chr10:717	LINC02637		lncRNA	chr10:46632386-466
ENSG0000(	451	10.21238	chr10:717	ANTXRL		protein_c	chr10:46286345-463
ENSG0000(	451	10.21238	chr10:717	SYT15B		protein_c	chr10:46578216-465
ENSG0000(	451	10.21238	chr10:717	ENSG00000290780		lncRNA	chr10:46370347-463
ENSG0000(	451	10.21238	chr10:717	DUSP8P3		Pseudoger	chr10:46774420-467
ENSG0000(	451	10.21238	chr10:717	SYT15-AS1		lncRNA	chr10:46580707-465
ENSG0000(	451	10.21238	chr10:717	AHCYP1		Pseudoger	chr10:46223143-462
ENSG0000(	451	10.21238	chr10:717	ENSG00000229227		lncRNA	chr10:46597917-466
ENSG0000(	451	10.21238	chr10:717	BMS1P1		Pseudoger	chr10:46788389-468
ENSG0000(	451	10.21238	chr10:717	SYT15		protein_c	chr10:46578216-465
ENSG0000(	451	10.21238	chr10:717	ENSG00000279458		lncRNA	chr10:46370759-463
ENSG0000(	451	10.21238	chr10:717	RP11-292F22.7		lncRNA	chr10:46284848-462
ENSG0000(	451	10.21238	chr10:717	NPY4R		protein_c	chr10:46461098-464
ENSG0000(	451	10.21238	chr10:717	AL603965.1		protein_c	chr10:46375702-463
ENSG0000(	451	10.21238	chr10:717	ENSG00000290913		lncRNA	chr10:46625928-466
ENSG0000(	451	10.21238	chr10:717	RP11-292F22.5		lncRNA	chr10:46199277-462
ENSG0000(	446	10.09916	chr18:236	MLECP1		Pseudoger	chr18:44522366-445
ENSG0000(	446	10.09916	chr18:236	LINC00907		lncRNA	chr18:42159283-426
ENSG0000(	446	10.09916	chr18:236	RPL17P45		Pseudoger	chr18:40143926-401
ENSG0000(	446	10.09916	chr18:236	SETBP1	NCGv7;AC	protein_c	chr18:44680173-450
ENSG0000(	446	10.09916	chr18:236	LINC01477		lncRNA	chr18:40066286-400
ENSG0000(	446	10.09916	chr18:236	FHOD3		protein_c	chr18:36297713-367
ENSG0000(	446	10.09916	chr18:236	CELF4	NCGv7	protein_c	chr18:37243040-375
ENSG0000(	446	10.09916	chr18:236	ENSG00000279034		TEC	chr18:40062903-400

ENSG0000(	446	10.09916	chr18:236	KRT8P5		Pseudoger	chr18:44320800-443
ENSG0000(	446	10.09916	chr18:236	LINC01901		lncRNA	chr18:39841174-399
ENSG0000(	446	10.09916	chr18:236	PIK3C3	NCGv7	protein_c	chr18:41955234-420
ENSG0000(	446	10.09916	chr18:236	SYT4	NCGv7	protein_c	chr18:43267892-432
ENSG0000(	446	10.09916	chr18:236	ENSG00000287007		lncRNA	chr18:42105729-421
ENSG0000(	446	10.09916	chr18:236	RNA5SP455		Pseudoger	chr18:44071583-440
ENSG0000(	446	10.09916	chr18:236	MIR5583-1		smallRNA	chr18:39676721-396
ENSG0000(	446	10.09916	chr18:236	ENSG00000276174		lncRNA	chr18:42052705-420
ENSG0000(	446	10.09916	chr18:236	SLC14A2		protein_c	chr18:45212995-456
ENSG0000(	446	10.09916	chr18:236	ENSG00000285940		lncRNA	chr18:37565281-377
ENSG0000(	446	10.09916	chr18:236	RIT2	NCGv7	protein_c	chr18:42743227-431
ENSG0000(	446	10.09916	chr18:236	SNORD112		smallRNA	chr18:36642613-366
ENSG0000(	446	10.09916	chr18:236	ENSG00000278986		TEC	chr18:35972151-359
ENSG0000(	446	10.09916	chr18:236	RNU7-145P		smallRNA	chr18:40472821-404
ENSG0000(	446	10.09916	chr18:236	LINC01902		lncRNA	chr18:39841678-398
ENSG0000(	446	10.09916	chr18:236	ENSG00000287209		lncRNA	chr18:43115728-431
ENSG0000(	446	10.09916	chr18:236	ENSG00000267627		lncRNA	chr18:35951803-359
ENSG0000(	446	10.09916	chr18:236	RPL12P40		Pseudoger	chr18:37672689-376
ENSG0000(	446	10.09916	chr18:236	MIR924HG		lncRNA	chr18:39206917-398
ENSG0000(	446	10.09916	chr18:236	ENSG00000278933		TEC	chr18:43499173-434
ENSG0000(	446	10.09916	chr18:236	ENSG00000285790		lncRNA	chr18:45491581-455
ENSG0000(	446	10.09916	chr18:236	ENSG00000267456		Pseudoger	chr18:36278735-362
ENSG0000(	446	10.09916	chr18:236	ENSG00000268566		lncRNA	chr18:38655209-386
ENSG0000(	446	10.09916	chr18:236	MIR3929		smallRNA	chr18:35934088-359
ENSG0000(	446	10.09916	chr18:236	SETBP1-DT		lncRNA	chr18:44676927-446
ENSG0000(	446	10.09916	chr18:236	ENSG00000289833		lncRNA	chr18:39970468-399
ENSG0000(	446	10.09916	chr18:236	ENSG00000283458		lncRNA	chr18:39314224-393
ENSG0000(	446	10.09916	chr18:236	ENSG00000267651		lncRNA	chr18:37234119-372
ENSG0000(	446	10.09916	chr18:236	NPM1P1		Pseudoger	chr18:41789162-417
ENSG0000(	446	10.09916	chr18:236	LINC01478		lncRNA	chr18:44280768-445
ENSG0000(	446	10.09916	chr18:236	ENSG00000267039		lncRNA	chr18:36901946-369
ENSG0000(	446	10.09916	chr18:236	RNU6-1242P		smallRNA	chr18:39678520-396
ENSG0000(	446	10.09916	chr18:236	ENSG00000267354		lncRNA	chr18:45507202-455
ENSG0000(	446	10.09916	chr18:236	ENSG00000267202		lncRNA	chr18:37273706-372
ENSG0000(	446	10.09916	chr18:236	ENSG00000286559		lncRNA	chr18:39655645-396
ENSG0000(	446	10.09916	chr18:236	ENSG00000280365		TEC	chr18:44732575-447
ENSG0000(	446	10.09916	chr18:236	ENSG00000285550		lncRNA	chr18:44214971-443
ENSG0000(	446	10.09916	chr18:236	MOCOS		protein_c	chr18:36187497-362
ENSG0000(	446	10.09916	chr18:236	MIR4318		smallRNA	chr18:37657135-376
ENSG0000(	446	10.09916	chr18:236	ENSG00000267716		Pseudoger	chr18:44039471-440
ENSG0000(	446	10.09916	chr18:236	ENSG00000274849		lncRNA	chr18:36189824-361
ENSG0000(	446	10.09916	chr18:236	ENSG00000267707		lncRNA	chr18:37243776-372
ENSG0000(	446	10.09916	chr18:236	ENSG00000288917		lncRNA	chr18:36128046-361
ENSG0000(	446	10.09916	chr18:236	ENSG00000279678		TEC	chr18:37305295-373
ENSG0000(	446	10.09916	chr18:236	ENSG00000279543		TEC	chr18:37473330-374
ENSG0000(	446	10.09916	chr18:236	RNU6-706P		smallRNA	chr18:39034007-390
ENSG0000(	446	10.09916	chr18:236	ENSG00000279319		TEC	chr18:44986109-449
ENSG0000(	446	10.09916	chr18:236	SLC14A2-AS1		lncRNA	chr18:45423764-454
ENSG0000(	446	10.09916	chr18:236	RNA5SP454		Pseudoger	chr18:42270589-422
ENSG0000(	446	10.09916	chr18:236	ENSG00000286976		lncRNA	chr18:42649520-426
ENSG0000(	446	10.09916	chr18:236	ENSG00000267101		lncRNA	chr18:45104361-451

ENSG0000(	446	10.09916	chr18:23(	ENSG00000266988		lncRNA	chr18:45483343-455
ENSG0000(	446	10.09916	chr18:23(	ENSG00000286890		lncRNA	chr18:36417055-364
ENSG0000(	446	10.09916	chr18:23(	ENSG00000286716		lncRNA	chr18:37960220-379
ENSG0000(	446	10.09916	chr18:23(	RNU6-443P		smallRNA	chr18:43832159-438
ENSG0000(	446	10.09916	chr18:23(	ENSG00000286844		lncRNA	chr18:40245879-402
ENSG0000(	446	10.09916	chr18:23(	KC6		lncRNA	chr18:41465783-416
ENSG0000(	446	10.09916	chr18:23(	ENSG00000267404		Pseudoger	chr18:36273574-362
ENSG0000(	446	10.09916	chr18:23(	KIAA1328		protein_c	chr18:36829106-372
ENSG0000(	446	10.09916	chr18:23(	ENSG00000267088		Pseudoger	chr18:42089312-420
ENSG0000(	446	10.09916	chr18:23(	MIR4319		smallRNA	chr18:44970082-449
ENSG0000(	446	10.09916	chr18:23(	SLC39A6		protein_c	chr18:36108531-361
ENSG0000(	446	10.09916	chr18:23(	TPGS2		protein_c	chr18:36777647-368
ENSG0000(	446	10.09916	chr18:23(	COSMOC		lncRNA	chr18:36179996-361
ENSG0000(	446	10.09916	chr18:23(	ENSG00000225253		Pseudoger	chr18:41819053-418
ENSG0000(	446	10.09916	chr18:23(	ELP2		protein_c	chr18:36129444-361
ENSG0000(	446	10.09916	chr18:23(	C18orf21		protein_c	chr18:35972625-359
ENSG0000(	446	10.09916	chr18:23(	RPRD1A		protein_c	chr18:35984387-360
ENSG0000(	446	10.09916	chr18:23(	RPL7AP66		Pseudoger	chr18:39334872-393
ENSG0000(	446	10.09916	chr18:23(	ENSG00000286328		lncRNA	chr18:41341319-415
ENSG0000(	446	10.09916	chr18:23(	RN7SKP182		smallRNA	chr18:39016047-390
ENSG0000(	444	10.05388	chr21:932	AP001137.1		smallRNA	chr21:21397748-213
ENSG0000(	442	10.00859	chr10:717	ENSG00000285712		lncRNA	chr10:43325833-433
ENSG0000(	442	10.00859	chr10:717	LINC00840		lncRNA	chr10:43778946-439
ENSG0000(	442	10.00859	chr10:717	TMEM72		protein_c	chr10:44911316-449
ENSG0000(	442	10.00859	chr10:717	RNU6-1207P		smallRNA	chr10:45188790-451
ENSG0000(	442	10.00859	chr10:717	RPS19P7		Pseudoger	chr10:45073146-450
ENSG0000(	442	10.00859	chr10:717	ENSG00000237389		lncRNA	chr10:43630882-436
ENSG0000(	442	10.00859	chr10:717	DEPP1		protein_c	chr10:44970981-449
ENSG0000(	442	10.00859	chr10:717	ENSG00000289092		lncRNA	chr10:46007580-460
ENSG0000(	442	10.00859	chr10:717	ENSG00000275312		Pseudoger	chr10:45180400-451
ENSG0000(	442	10.00859	chr10:717	ENSG00000227683		lncRNA	chr10:45164228-451
ENSG0000(	442	10.00859	chr10:717	ZNF22		protein_c	chr10:45000923-450
ENSG0000(	442	10.00859	chr10:717	ENSG00000290460		lncRNA	chr10:45718026-457
ENSG0000(	442	10.00859	chr10:717	ZNF22-AS1		lncRNA	chr10:44997698-450
ENSG0000(	442	10.00859	chr10:717	TIMM23		protein_c	chr10:45972488-460
ENSG0000(	442	10.00859	chr10:717	LINC02633		lncRNA	chr10:43323665-433
ENSG0000(	442	10.00859	chr10:717	ENSG00000228426		lncRNA	chr10:43665668-436
ENSG0000(	442	10.00859	chr10:717	MIR3156-1		smallRNA	chr10:45164014-451
ENSG0000(	442	10.00859	chr10:717	CXCL12		protein_c	chr10:44370165-443
ENSG0000(	442	10.00859	chr10:717	ZNF32-AS1		lncRNA	chr10:43643872-436
ENSG0000(	442	10.00859	chr10:717	RSU1P2		Pseudoger	chr10:45106610-451
ENSG0000(	442	10.00859	chr10:717	ENSG00000270767		Pseudoger	chr10:44488335-444
ENSG0000(	442	10.00859	chr10:717	RASSF4		protein_c	chr10:44959407-449
ENSG0000(	442	10.00859	chr10:717	RP11-592B15.6		Pseudoger	chr10:45940522-459
ENSG0000(	442	10.00859	chr10:717	AGAP10P		Pseudoger	chr10:45678692-457
ENSG0000(	442	10.00859	chr10:717	ENSG00000287852		lncRNA	chr10:43583498-435
ENSG0000(	442	10.00859	chr10:717	ZNF239		protein_c	chr10:43556344-435
ENSG0000(	442	10.00859	chr10:717	LINC02659		lncRNA	chr10:43900874-439
ENSG0000(	442	10.00859	chr10:717	RET	NCGv7;AC	protein_c	chr10:43077064-431
ENSG0000(	442	10.00859	chr10:717	ENSG00000270434		Pseudoger	chr10:45812648-458
ENSG0000(	442	10.00859	chr10:717	UQCRHP3		Pseudoger	chr10:43732334-437

ENSG0000(	442	10.00859	chr10:717	ENSG00000237590		lncRNA	chr10:44259602-442
ENSG0000(	442	10.00859	chr10:717	U3		smallRNA	chr10:43417250-434
ENSG0000(	442	10.00859	chr10:717	CAP1P2		Pseudoger	chr10:43604843-436
ENSG0000(	442	10.00859	chr10:717	FAM25E		Pseudoger	chr10:45815486-458
ENSG0000(	442	10.00859	chr10:717	BMS1		protein_c	chr10:42782795-428
ENSG0000(	442	10.00859	chr10:717	MSMB		protein_c	chr10:46033306-460
ENSG0000(	442	10.00859	chr10:717	FAM21FP		Pseudoger	chr10:45706431-457
ENSG0000(	442	10.00859	chr10:717	ENSG00000287901		lncRNA	chr10:44296805-443
ENSG0000(	442	10.00859	chr10:717	SNORA74		smallRNA	chr10:45984609-459
ENSG0000(	442	10.00859	chr10:717	AL512640.1		smallRNA	chr10:44034540-440
ENSG0000(	442	10.00859	chr10:717	RPL35AP23		Pseudoger	chr10:45940521-459
ENSG0000(	442	10.00859	chr10:717	AGAP7P		Pseudoger	chr10:46109620-461
ENSG0000(	442	10.00859	chr10:717	FXYD4		protein_c	chr10:43371636-433
ENSG0000(	442	10.00859	chr10:717	RPL21P88		Pseudoger	chr10:43630947-436
ENSG0000(	442	10.00859	chr10:717	OR13A1		protein_c	chr10:45302298-453
ENSG0000(	442	10.00859	chr10:717	CSGALNACT2-DT		lncRNA	chr10:43136824-431
ENSG0000(	442	10.00859	chr10:717	HNRNPA3P1		Pseudoger	chr10:43789249-437
ENSG0000(	442	10.00859	chr10:717	ENSG00000231964		lncRNA	chr10:45444570-454
ENSG0000(	442	10.00859	chr10:717	ENSG00000277757		lncRNA	chr10:45016080-451
ENSG0000(	442	10.00859	chr10:717	RNA5SP310		Pseudoger	chr10:45856307-458
ENSG0000(	442	10.00859	chr10:717	ANKRD54P1		Pseudoger	chr10:45151445-451
ENSG0000(	442	10.00859	chr10:717	RNU6ATAC11P		smallRNA	chr10:43341786-433
ENSG0000(	442	10.00859	chr10:717	ZFAND4	AC	protein_c	chr10:45615500-456
ENSG0000(	442	10.00859	chr10:717	NCOA4	NCGv7;AC	protein_c	chr10:46005087-460
ENSG0000(	442	10.00859	chr10:717	FAM25D		lncRNA	chr10:45815428-458
ENSG0000(	442	10.00859	chr10:717	RP11-481A12.2		Pseudoger	chr10:46060347-460
ENSG0000(	442	10.00859	chr10:717	RP11-175I17.2		Pseudoger	chr10:45854093-459
ENSG0000(	442	10.00859	chr10:717	ENSG00000278531		Pseudoger	chr10:45187292-451
ENSG0000(	442	10.00859	chr10:717	FAM25E		lncRNA	chr10:45815428-458
ENSG0000(	442	10.00859	chr10:717	RNU6-885P		smallRNA	chr10:42832447-428
ENSG0000(	442	10.00859	chr10:717	AGAP4		protein_c	chr10:45825594-458
ENSG0000(	442	10.00859	chr10:717	DUXAP4		Pseudoger	chr10:45055656-450
ENSG0000(	442	10.00859	chr10:717	snoU13		smallRNA	chr10:42810227-428
ENSG0000(	442	10.00859	chr10:717	LINC01264		lncRNA	chr10:42979017-429
ENSG0000(	442	10.00859	chr10:717	ENSG00000273363		lncRNA	chr10:44958917-449
ENSG0000(	442	10.00859	chr10:717	EIF2AP4		Pseudoger	chr10:44806443-448
ENSG0000(	442	10.00859	chr10:717	CSGALNACT2		protein_c	chr10:43138445-431
ENSG0000(	442	10.00859	chr10:717	ENSG00000223462		lncRNA	chr10:44937508-449
ENSG0000(	442	10.00859	chr10:717	HNRNPF		protein_c	chr10:43385617-434
ENSG0000(	442	10.00859	chr10:717	ALOX5		protein_c	chr10:45374176-454
ENSG0000(	442	10.00859	chr10:717	ZNF32		protein_c	chr10:43643860-436
ENSG0000(	442	10.00859	chr10:717	RP11-592B15.8		Pseudoger	chr10:45812646-458
ENSG0000(	442	10.00859	chr10:717	RPL23AP61		Pseudoger	chr10:46063249-460
ENSG0000(	442	10.00859	chr10:717	ENSG00000227029		lncRNA	chr10:43912444-439
ENSG0000(	442	10.00859	chr10:717	RNU7-193P		smallRNA	chr10:43458065-434
ENSG0000(	442	10.00859	chr10:717	ENSG00000229116		lncRNA	chr10:44282489-442
ENSG0000(	442	10.00859	chr10:717	ENSG00000234580		lncRNA	chr10:44591208-446
ENSG0000(	442	10.00859	chr10:717	RPL9P21		Pseudoger	chr10:44414649-444
ENSG0000(	442	10.00859	chr10:717	ENSG00000234504		lncRNA	chr10:44899614-449
ENSG0000(	442	10.00859	chr10:717	ZNF485		protein_c	chr10:43606419-436
ENSG0000(	442	10.00859	chr10:717	ZNF32-AS2		lncRNA	chr10:43645942-436

ENSG0000(	442	10.00859	chr10:717	ENSG00000236114	Pseudoger	chr10:43523256-435
ENSG0000(	442	10.00859	chr10:717	LINC00841	lncRNA	chr10:43909244-439
ENSG0000(	442	10.00859	chr10:717	RASGEF1A NCGv7	protein_c	chr10:43194535-432
ENSG0000(	442	10.00859	chr10:717	LINC02916	lncRNA	chr10:43420738-434
ENSG0000(	442	10.00859	chr10:717	ENSG00000289636	lncRNA	chr10:46092767-460
ENSG0000(	442	10.00859	chr10:717	ZNF32-AS3	lncRNA	chr10:43628817-436
ENSG0000(	442	10.00859	chr10:717	LINC02623	lncRNA	chr10:42871521-428
ENSG0000(	442	10.00859	chr10:717	ZNF487	protein_c	chr10:43436841-434
ENSG0000(	442	10.00859	chr10:717	LINC02658	lncRNA	chr10:43777562-437
ENSG0000(	442	10.00859	chr10:717	CUBNP2	Pseudoger	chr10:45232026-452
ENSG0000(	442	10.00859	chr10:717	ENSG00000243349	lncRNA	chr10:45147394-451
ENSG0000(	442	10.00859	chr10:717	CEP164P1	Pseudoger	chr10:45002222-450
ENSG0000(	442	10.00859	chr10:717	LINC02881	lncRNA	chr10:44292569-442
ENSG0000(	442	10.00859	chr10:717	ENSG00000228702	Pseudoger	chr10:45725694-457
ENSG0000(	442	10.00859	chr10:717	ENSG00000287173	lncRNA	chr10:44509708-445
ENSG0000(	442	10.00859	chr10:717	MARCHF8	protein_c	chr10:45454585-455
ENSG0000(	442	10.00859	chr10:717	SPRING1P1	Pseudoger	chr10:43728217-437
ENSG0000(	442	10.00859	chr10:717	CUBNP3	Pseudoger	chr10:45127318-451
ENSG0000(	442	10.00859	chr10:717	OR6D1P	Pseudoger	chr10:45255640-452
ENSG0000(	442	10.00859	chr10:717	ANKRD30BP3	Pseudoger	chr10:45156775-451
ENSG0000(	442	10.00859	chr10:717	ENSG00000290529	lncRNA	chr10:45099487-451
ENSG0000(	442	10.00859	chr10:717	TMEM72-AS1	lncRNA	chr10:44793119-449
ENSG0000(	442	10.00859	chr10:717	WASHC2C	protein_c	chr10:45727200-457
ENSG0000(	442	10.00859	chr10:717	ELOCP30	Pseudoger	chr10:43741437-437
ENSG0000(	440	9.963302	chr2:8187	RNU6-649P	smallRNA	chr2:4945277-49453
ENSG0000(	440	9.963302	chr2:8187	LINC01249	lncRNA	chr2:4628216-46564
ENSG0000(	440	9.963302	chr2:8187	ENSG00000228585	Pseudoger	chr2:5313740-53141
ENSG0000(	440	9.963302	chr2:8187	NPM1P48	Pseudoger	chr2:4514204-45150
ENSG0000(	440	9.963302	chr2:8187	ENSG00000289300	lncRNA	chr2:5071437-50734
ENSG0000(	440	9.963302	chr2:8187	SNORA31	smallRNA	chr2:4827001-48270
ENSG0000(	437	9.89537	chr17:289	KRTAP4-17P	Pseudoger	chr17:41186944-411
ENSG0000(	436	9.872726	chr3:6192	ENSG00000285654	lncRNA	chr3:16252123-1625
ENSG0000(	434	9.827438	chr10:717	Y_RNA	smallRNA	chr10:32056746-320
ENSG0000(	430	9.736863	chr2:8889	HIGD2AP1	Pseudoger	chr2:231437101-231
ENSG0000(	429	9.714219	chr6:1979	AL356739.1	smallRNA	chr6:145348758-145
ENSG0000(	429	9.714219	chr14:208	RNASE3	protein_c	chr14:20891385-208
ENSG0000(	425	9.623644	chr10:717	RPS4XP11	Pseudoger	chr10:32102522-321
ENSG0000(	425	9.623644	chr10:717	TMEM161BP1	Pseudoger	chr10:37337415-373
ENSG0000(	425	9.623644	chr10:717	SLC9B1P3	Pseudoger	chr10:38640971-386
ENSG0000(	425	9.623644	chr10:717	KSR1P1	Pseudoger	chr10:42149310-421
ENSG0000(	425	9.623644	chr10:717	ENSG00000291065	lncRNA	chr10:42254072-423
ENSG0000(	425	9.623644	chr10:717	ENSG00000275675	Pseudoger	chr10:38068449-380
ENSG0000(	425	9.623644	chr10:717	ENSG00000271650	Pseudoger	chr10:42209990-422
ENSG0000(	425	9.623644	chr10:717	LINC02630	lncRNA	chr10:35896874-358
ENSG0000(	425	9.623644	chr7:2516	ENSG00000241324	lncRNA	chr7:124337380-124
ENSG0000(	425	9.623644	chr10:717	ENSG00000226113	Pseudoger	chr10:38247922-383
ENSG0000(	425	9.623644	chr7:2516	EEF1GP1	Pseudoger	chr7:125033453-125
ENSG0000(	425	9.623644	chr7:2516	ENSG00000205898	Pseudoger	chr7:125159974-125
ENSG0000(	425	9.623644	chr10:717	ENSG00000274167	Pseudoger	chr10:42250985-422
ENSG0000(	425	9.623644	chr7:2516	ENSG00000241345	lncRNA	chr7:123994622-124
ENSG0000(	425	9.623644	chr7:2516	ENSG00000242593	lncRNA	chr7:124032126-124

ENSG0000(	425	9.623644	chr10:717	RNU6-794P		smallRNA	chr10:35231331-352
ENSG0000(	425	9.623644	chr10:717	ENSG00000233832		Pseudoger	chr10:38783004-387
ENSG0000(	425	9.623644	chr10:717	EIF3LP2		Pseudoger	chr10:42566677-425
ENSG0000(	425	9.623644	chr10:717	RNU7-77P		smallRNA	chr10:35135443-351
ENSG0000(	425	9.623644	chr10:717	HMGB1P7		Pseudoger	chr10:31913143-319
ENSG0000(	425	9.623644	chr10:717	ZNF37BP		Pseudoger	chr10:42513510-425
ENSG0000(	425	9.623644	chr10:717	ZNF33B		protein_c	chr10:42574185-426
ENSG0000(	425	9.623644	chr10:717	LINC01518		lncRNA	chr10:42644445-426
ENSG0000(	425	9.623644	chr10:717	ZNF25		protein_c	chr10:37949573-379
ENSG0000(	425	9.623644	chr10:717	EIF3LP3		Pseudoger	chr10:38080056-380
ENSG0000(	425	9.623644	chr10:717	CCDC7		protein_c	chr10:32446140-328
ENSG0000(	425	9.623644	chr10:717	VN1R54P		Pseudoger	chr10:42660728-426
ENSG0000(	425	9.623644	chr10:717	MKNK2P1		Pseudoger	chr10:36944111-369
ENSG0000(	425	9.623644	chr10:717	RNU6-193P		smallRNA	chr10:34942969-349
ENSG0000(	425	9.623644	chr10:717	ENSG00000259869		lncRNA	chr10:42751178-427
ENSG0000(	425	9.623644	chr10:717	ENSG00000230534		lncRNA	chr10:35098006-351
ENSG0000(	425	9.623644	chr10:717	FZD8		protein_c	chr10:35638247-356
ENSG0000(	425	9.623644	chr10:717	TACC1P1		Pseudoger	chr10:37523011-375
ENSG0000(	425	9.623644	chr10:717	ENSG00000277133		Pseudoger	chr10:38617255-386
ENSG0000(	425	9.623644	chr10:717	Y_RNA		smallRNA	chr10:34201980-342
ENSG0000(	425	9.623644	chr7:251(	ENSG00000234071		Pseudoger	chr7:125344969-125
ENSG0000(	425	9.623644	chr10:717	ENSG00000287945		lncRNA	chr10:35443411-354
ENSG0000(	425	9.623644	chr10:717	EPC1-AS1		lncRNA	chr10:32347397-323
ENSG0000(	425	9.623644	chr7:251(	ENSG00000197462		lncRNA	chr7:125917871-125
ENSG0000(	425	9.623644	chr7:251(	LINC02830		lncRNA	chr7:125151326-125
ENSG0000(	425	9.623644	chr7:251(	POT1-AS1		lncRNA	chr7:124929873-125
ENSG0000(	425	9.623644	chr10:717	ENSG00000215146		Pseudoger	chr10:42336101-423
ENSG0000(	425	9.623644	chr10:717	ABCD1P2		Pseudoger	chr10:38601418-386
ENSG0000(	425	9.623644	chr10:717	RPS12P16		Pseudoger	chr10:34675635-346
ENSG0000(	425	9.623644	chr10:717	AL031601.1		smallRNA	chr10:42234903-422
ENSG0000(	425	9.623644	chr10:717	ENSG00000272387		Pseudoger	chr10:42583241-425
ENSG0000(	425	9.623644	chr10:717	CHEK2P5		Pseudoger	chr10:38713195-387
ENSG0000(	425	9.623644	chr10:717	MTND4LP11		Pseudoger	chr10:33023368-330
ENSG0000(	425	9.623644	chr10:717	ENSG00000227253		lncRNA	chr10:32266289-322
ENSG0000(	425	9.623644	chr10:717	Y_RNA		smallRNA	chr10:37994863-379
ENSG0000(	425	9.623644	chr10:717	CCNYL4		Pseudoger	chr10:38175990-382
ENSG0000(	425	9.623644	chr7:251(	ENSG00000279419		TEC	chr7:124742312-124
ENSG0000(	425	9.623644	chr7:251(	TMEM229A		protein_c	chr7:124030921-124
ENSG0000(	425	9.623644	chr10:717	ENSG00000272319		Pseudoger	chr10:42579724-425
ENSG0000(	425	9.623644	chr10:717	CCNY		protein_c	chr10:35247025-355
ENSG0000(	425	9.623644	chr10:717	CUL2		protein_c	chr10:35008504-351
ENSG0000(	425	9.623644	chr10:717	ACTR3BP5		Pseudoger	chr10:38696424-386
ENSG0000(	425	9.623644	chr10:717	IGKV1OR10-1		Pseudoger	chr10:42185339-421
ENSG0000(	425	9.623644	chr10:717	ENSG00000233825		lncRNA	chr10:32346499-323
ENSG0000(	425	9.623644	chr7:251(	SPAM1	NCGv7	protein_c	chr7:123925237-123
ENSG0000(	425	9.623644	chr7:251(	PPIAP93		Pseudoger	chr7:125345825-125
ENSG0000(	425	9.623644	chr10:717	ENSG00000286409		lncRNA	chr10:32413499-324
ENSG0000(	425	9.623644	chr10:717	GJD4		protein_c	chr10:35605341-356
ENSG0000(	425	9.623644	chr10:717	RPL37P18		Pseudoger	chr10:34663859-346
ENSG0000(	425	9.623644	chr10:717	ELOBP4		Pseudoger	chr10:34488583-344
ENSG0000(	425	9.623644	chr10:717	RNU6-811P		smallRNA	chr10:37135237-371

ENSG0000(	425	9.623644	chr7:251(	ENSG00000225583		Pseudoger	chr7:123932132-123
ENSG0000(	425	9.623644	chr10:717	EPC1	NCGv7	protein_c	chr10:32267751-323
ENSG0000(	425	9.623644	chr10:717	ENSG00000290801		lncRNA	chr10:37309185-373
ENSG0000(	425	9.623644	chr10:717	ENSG00000285884		lncRNA	chr10:42638252-426
ENSG0000(	425	9.623644	chr10:717	ENSG00000273012		lncRNA	chr10:33341655-333
ENSG0000(	425	9.623644	chr10:717	NRP1	NCGv7	protein_c	chr10:33177492-333
ENSG0000(	425	9.623644	chr10:717	PABPC1P8		Pseudoger	chr10:42279734-422
ENSG0000(	425	9.623644	chr10:717	AL121748.1		smallRNA	chr10:33098535-330
ENSG0000(	425	9.623644	chr10:717	AL135791.1		smallRNA	chr10:37831889-378
ENSG0000(	425	9.623644	chr10:717	SS18L2P1		Pseudoger	chr10:34875210-348
ENSG0000(	425	9.623644	chr7:251(	ENSG00000243574		lncRNA	chr7:124274671-124
ENSG0000(	425	9.623644	chr10:717	HSD17B7P2		Pseudoger	chr10:38356455-383
ENSG0000(	425	9.623644	chr10:717	ENSG00000236514		lncRNA	chr10:37857740-378
ENSG0000(	425	9.623644	chr10:717	RN7SL825P		smallRNA	chr10:31992090-319
ENSG0000(	425	9.623644	chr10:717	RNU6-1170P		smallRNA	chr10:42605158-426
ENSG0000(	425	9.623644	chr7:251(	ENSG00000219445		lncRNA	chr7:125229579-125
ENSG0000(	425	9.623644	chr10:717	ENSG00000273312		lncRNA	chr10:35604485-356
ENSG0000(	425	9.623644	chr10:717	ENSG00000229878		Pseudoger	chr10:33035469-330
ENSG0000(	425	9.623644	chr10:717	ENSG00000272983		lncRNA	chr10:38137337-381
ENSG0000(	425	9.623644	chr7:251(	POT1	NCGv7;AC	protein_c	chr7:124822386-124
ENSG0000(	425	9.623644	chr10:717	MTND5P17		Pseudoger	chr10:36432882-364
ENSG0000(	425	9.623644	chr10:717	ARL6IP1P2		Pseudoger	chr10:36995023-369
ENSG0000(	425	9.623644	chr10:717	MTND4P18		Pseudoger	chr10:36434710-364
ENSG0000(	425	9.623644	chr10:717	LINC00993		Pseudoger	chr10:37248118-373
ENSG0000(	425	9.623644	chr10:717	AK3P5		Pseudoger	chr10:32944481-329
ENSG0000(	425	9.623644	chr10:717	LINC00839		lncRNA	chr10:42475480-424
ENSG0000(	425	9.623644	chr10:717	ENSG00000287278		lncRNA	chr10:33384891-333
ENSG0000(	425	9.623644	chr10:717	PABPC1P12		Pseudoger	chr10:38635908-386
ENSG0000(	425	9.623644	chr10:717	ENSG00000269952		lncRNA	chr10:35210416-352
ENSG0000(	425	9.623644	chr10:717	CCNYL2		Pseudoger	chr10:42409727-424
ENSG0000(	425	9.623644	chr10:717	ENSG00000290458		lncRNA	chr10:42242721-422
ENSG0000(	425	9.623644	chr10:717	FXYD6P2		Pseudoger	chr10:38091764-380
ENSG0000(	425	9.623644	chr10:717	VN1R53P		Pseudoger	chr10:37348616-373
ENSG0000(	425	9.623644	chr10:717	RPL7AP53		Pseudoger	chr10:33057615-330
ENSG0000(	425	9.623644	chr10:717	ENSG00000290535		lncRNA	chr10:38403189-384
ENSG0000(	425	9.623644	chr10:717	MIR3611		smallRNA	chr10:35079598-350
ENSG0000(	425	9.623644	chr10:717	ENSG00000226842		lncRNA	chr10:32281686-322
ENSG0000(	425	9.623644	chr10:717	CREM		protein_c	chr10:35126791-352
ENSG0000(	425	9.623644	chr10:717	CCNYL2		lncRNA	chr10:42408168-424
ENSG0000(	425	9.623644	chr10:717	LINC02635		lncRNA	chr10:34969881-349
ENSG0000(	425	9.623644	chr10:717	HSD17B7P2		lncRNA	chr10:38356380-383
ENSG0000(	425	9.623644	chr10:717	RNU6-795P		smallRNA	chr10:38043873-380
ENSG0000(	425	9.623644	chr10:717	AL117337.1		smallRNA	chr10:37954922-379
ENSG0000(	425	9.623644	chr10:717	RSU1P1		Pseudoger	chr10:42725857-427
ENSG0000(	425	9.623644	chr10:717	ENSG00000288893		lncRNA	chr10:35643844-356
ENSG0000(	425	9.623644	chr10:717	RPS24P13		Pseudoger	chr10:32231849-322
ENSG0000(	425	9.623644	chr10:717	LINC02634		lncRNA	chr10:35219894-352
ENSG0000(	425	9.623644	chr10:717	SEPTIN7P9		Pseudoger	chr10:38391983-383
ENSG0000(	425	9.623644	chr10:717	ENSG00000237002		lncRNA	chr10:36438616-364
ENSG0000(	425	9.623644	chr10:717	ATP6V1G1P4		Pseudoger	chr10:35158306-351
ENSG0000(	425	9.623644	chr10:717	RNU7-22P		smallRNA	chr10:32173684-321

ENSG0000(	425	9.623644	chr10:717	ENSG00000234918		lncRNA	chr10:37240887-372
ENSG0000(	425	9.623644	chr10:717	CCNY-AS1		lncRNA	chr10:35314552-353
ENSG0000(	425	9.623644	chr10:717	ENSG00000225965		Pseudoger	chr10:38161570-381
ENSG0000(	425	9.623644	chr10:717	LINC02632		lncRNA	chr10:42696012-427
ENSG0000(	425	9.623644	chr10:717	IATPR		lncRNA	chr10:33073486-331
ENSG0000(	425	9.623644	chr10:717	ENSG00000234811		Pseudoger	chr10:32111644-321
ENSG0000(	425	9.623644	chr10:717	CUBNP1		Pseudoger	chr10:42694371-427
ENSG0000(	425	9.623644	chr10:717	PRDX2P2		Pseudoger	chr10:34965069-349
ENSG0000(	425	9.623644	chr7:251(	GPR37		protein_c	chr7:124743885-124
ENSG0000(	425	9.623644	chr10:717	PPIAP31		Pseudoger	chr10:32188323-321
ENSG0000(	425	9.623644	chr10:717	LINC00999		protein_c	chr10:38428146-384
ENSG0000(	425	9.623644	chr10:717	Y_RNA		smallRNA	chr10:36626015-366
ENSG0000(	425	9.623644	chr10:717	KIF5B	IntOGen-I	protein_c	chr10:32009015-320
ENSG0000(	425	9.623644	chr10:717	Y_RNA		smallRNA	chr10:37303000-373
ENSG0000(	425	9.623644	chr7:251(	C7orf77		lncRNA	chr7:124777292-124
ENSG0000(	425	9.623644	chr10:717	RNU6-847P		smallRNA	chr10:34926775-349
ENSG0000(	425	9.623644	chr7:251(	RNU6-102P		smallRNA	chr7:124647719-124
ENSG0000(	425	9.623644	chr10:717	ENSG00000234941		Pseudoger	chr10:38250442-382
ENSG0000(	425	9.623644	chr10:717	ZNF33BP1		Pseudoger	chr10:37794276-377
ENSG0000(	425	9.623644	chr10:717	ENSG00000287528		lncRNA	chr10:35641097-356
ENSG0000(	425	9.623644	chr10:717	ENSG00000290887		lncRNA	chr10:38164648-382
ENSG0000(	425	9.623644	chr10:717	ZNF248		protein_c	chr10:37776526-378
ENSG0000(	425	9.623644	chr10:717	LINC02629		lncRNA	chr10:33909238-339
ENSG0000(	425	9.623644	chr10:717	ITGB1-DT		lncRNA	chr10:32958437-330
ENSG0000(	425	9.623644	chr10:717	ZNF33CP		Pseudoger	chr10:37894637-378
ENSG0000(	425	9.623644	chr10:717	DUXAP3		Pseudoger	chr10:42747544-427
ENSG0000(	425	9.623644	chr10:717	TLK2P2		Pseudoger	chr10:37818606-378
ENSG0000(	425	9.623644	chr10:717	LINC00838		lncRNA	chr10:33759713-337
ENSG0000(	425	9.623644	chr10:717	FXYD6P1		Pseudoger	chr10:42555440-425
ENSG0000(	425	9.623644	chr7:251(	ENSG00000213291		Pseudoger	chr7:125300504-125
ENSG0000(	425	9.623644	chr7:251(	ENSG00000213296		Pseudoger	chr7:124480524-124
ENSG0000(	425	9.623644	chr10:717	ENSG00000235113		Pseudoger	chr10:32109861-321
ENSG0000(	425	9.623644	chr10:717	MTND1P18		Pseudoger	chr10:37600796-376
ENSG0000(	425	9.623644	chr10:717	ITGB1		protein_c	chr10:32887273-330
ENSG0000(	425	9.623644	chr10:717	CICP9		Pseudoger	chr10:38452987-384
ENSG0000(	425	9.623644	chr10:717	ENSG00000276805		Pseudoger	chr10:38407631-384
ENSG0000(	425	9.623644	chr10:717	ANKRD30A		protein_c	chr10:37125598-372
ENSG0000(	425	9.623644	chr10:717	NAMPTP1		Pseudoger	chr10:36521721-365
ENSG0000(	425	9.623644	chr10:717	ENSG00000260137		lncRNA	chr10:37791580-377
ENSG0000(	425	9.623644	chr10:717	PARD3	NCGv7	protein_c	chr10:34109560-348
ENSG0000(	425	9.623644	chr10:717	RNU6-1167P		smallRNA	chr10:35250959-352
ENSG0000(	425	9.623644	chr10:717	ENSG00000226578		lncRNA	chr10:37775371-377
ENSG0000(	425	9.623644	chr10:717	ENSG00000277479		Pseudoger	chr10:42578500-425
ENSG0000(	425	9.623644	chr10:717	ENSG00000203496		lncRNA	chr10:38453181-384
ENSG0000(	425	9.623644	chr10:717	AL022345.9		Pseudoger	chr10:42583767-425
ENSG0000(	425	9.623644	chr10:717	RPL7P37		Pseudoger	chr10:35697300-356
ENSG0000(	425	9.623644	chr10:717	ZNF33A	NCGv7	protein_c	chr10:38010650-380
ENSG0000(	425	9.623644	chr7:251(	SSU72P8		protein_c	chr7:124476371-124
ENSG0000(	425	9.623644	chr10:717	RN7SL314P		smallRNA	chr10:37297398-372
ENSG0000(	425	9.623644	chr10:717	ENSG00000227475		lncRNA	chr10:36089086-360
ENSG0000(	425	9.623644	chr10:717	ENSG00000279239		Pseudoger	chr10:42470082-424

ENSG0000(	425	9.623644	chr10:717	ZNF25-DT		lncRNA	chr10:37976825-379
ENSG0000(	425	9.623644	chr10:717	SEPTIN7P9		lncRNA	chr10:38383023-384
ENSG0000(	425	9.623644	chr10:717	RPL34P19		Pseudoger	chr10:31983885-319
ENSG0000(	425	9.623644	chr10:717	ENSG00000288987		lncRNA	chr10:34018420-340
ENSG0000(	425	9.623644	chr10:717	PLD5P1		protein_c	chr10:38094368-383
ENSG0000(	425	9.623644	chr10:717	ZNF37CP		Pseudoger	chr10:37883594-378
ENSG0000(	425	9.623644	chr10:717	RN7SL847P		smallRNA	chr10:33034657-330
ENSG0000(	425	9.623644	chr10:717	RPL23P11		Pseudoger	chr10:33864313-338
ENSG0000(	425	9.623644	chr10:717	ENSG00000238176		lncRNA	chr10:36940109-369
ENSG0000(	425	9.623644	chr10:717	RNU6-1118P		smallRNA	chr10:38423625-384
ENSG0000(	425	9.623644	chr10:717	C1DP1		Pseudoger	chr10:32511336-325
ENSG0000(	425	9.623644	chr10:717	ENSG00000203565		lncRNA	chr10:33684755-337
ENSG0000(	425	9.623644	chr10:717	ENSG00000238258		lncRNA	chr10:33211277-332
ENSG0000(	425	9.623644	chr10:717	PARD3-DT		lncRNA	chr10:34815767-348
ENSG0000(	425	9.623644	chr10:717	RN7SL398P		smallRNA	chr10:33288306-332
ENSG0000(	425	9.623644	chr10:717	MIR4683		smallRNA	chr10:35641172-356
ENSG0000(	425	9.623644	chr10:717	ZNF37A		protein_c	chr10:38094334-381
ENSG0000(	425	9.623644	chr10:717	RNU6-1244P		smallRNA	chr10:32394558-323
ENSG0000(	425	9.623644	chr10:717	ENSG00000227679		Pseudoger	chr10:38762095-387
ENSG0000(	425	9.623644	chr10:717	PCAT5		lncRNA	chr10:35778302-358
ENSG0000(	425	9.623644	chr10:717	AL391839.1		smallRNA	chr10:32346864-323
ENSG0000(	425	9.623644	chr10:717	LINC02628		lncRNA	chr10:33578931-336
ENSG0000(	422	9.555712	chr1:373	ENSG00000270282		Pseudoger	chr1:8512653-85130
ENSG0000(	420	9.510424	chr17:204	ENSG00000287553		lncRNA	chr17:2040953-2041
ENSG0000(	419	9.48778	chrX:904	RN7SL266P		smallRNA	chrX:112855874-112
ENSG0000(	417	9.442493	chr16:239	RN7SL493P		smallRNA	chr16:10221777-102
ENSG0000(	416	9.419849	chr7:251	HYAL4		protein_c	chr7:123828983-123
ENSG0000(	416	9.419849	chr7:251	NDUFA5	NCGv7	protein_c	chr7:123536997-123
ENSG0000(	416	9.419849	chr7:251	ASB15	NCGv7	protein_c	chr7:123567010-123
ENSG0000(	416	9.419849	chr7:251	WASL-DT		lncRNA	chr7:123749068-123
ENSG0000(	416	9.419849	chr7:251	ASB15-AS1		lncRNA	chr7:123584859-123
ENSG0000(	416	9.419849	chr7:251	LMOD2		protein_c	chr7:123655866-123
ENSG0000(	416	9.419849	chr7:251	RNU6-11P		smallRNA	chr7:123790605-123
ENSG0000(	416	9.419849	chr7:251	HYAL6P		Pseudoger	chr7:123814139-123
ENSG0000(	416	9.419849	chr7:251	ENSG00000289345		lncRNA	chr7:123580881-123
ENSG0000(	416	9.419849	chr7:251	WASL		protein_c	chr7:123681943-123
ENSG0000(	415	9.397205	chr7:137	TAS2R5	DriverDB	protein_c	chr7:141790217-141
ENSG0000(	415	9.397205	chr7:137	TAS2R4	DriverDB	protein_c	chr7:141776674-141
ENSG0000(	415	9.397205	chr7:137	TAS2R39		protein_c	chr7:143183419-143
ENSG0000(	415	9.397205	chr7:137	TRPV5	NCGv7	protein_c	chr7:142908101-142
ENSG0000(	415	9.397205	chr7:137	MTCO1P55		Pseudoger	chr7:141801315-141
ENSG0000(	415	9.397205	chr7:137	AC073647.1		smallRNA	chr7:141859818-141
ENSG0000(	415	9.397205	chr7:137	RN7SL535P		smallRNA	chr7:143290615-143
ENSG0000(	415	9.397205	chr7:137	ENSG00000270512		Pseudoger	chr7:140282465-140
ENSG0000(	415	9.397205	chr7:137	RPS3AP28		Pseudoger	chr7:138490581-138
ENSG0000(	415	9.397205	chr7:137	ENSG00000228360		Pseudoger	chr7:139227537-139
ENSG0000(	415	9.397205	chr7:137	TAS2R3		protein_c	chr7:141764097-141
ENSG0000(	415	9.397205	chr7:137	TAS2R60	NCGv7	protein_c	chr7:143443453-143
ENSG0000(	415	9.397205	chr7:137	RNU6-206P		smallRNA	chr7:139315291-139
ENSG0000(	415	9.397205	chr7:137	TRBVB		Pseudoger	chr7:142711384-142
ENSG0000(	415	9.397205	chr7:137	TAS2R38		protein_c	chr7:141972631-141

ENSG00000	415	9.397205	chr7:1375	AC006452.1		smallRNA	chr7:140580287-140
ENSG00000	415	9.397205	chr7:1375	FAM131B-AS2		lncRNA	chr7:143379683-143
ENSG00000	415	9.397205	chr7:1375	OR6V1		protein_c	chr7:143052341-143
ENSG00000	415	9.397205	chr7:1375	PRSS3P1		Pseudoger	chr7:142760415-142
ENSG00000	415	9.397205	chr7:1375	ENSG00000241881		lncRNA	chr7:142285750-142
ENSG00000	415	9.397205	chr7:1375	ENSG00000231931		Pseudoger	chr7:138060056-138
ENSG00000	415	9.397205	chr7:1375	ENSG00000261629		lncRNA	chr7:141429711-141
ENSG00000	415	9.397205	chr7:1375	ENSG00000290761		lncRNA	chr7:143062330-143
ENSG00000	415	9.397205	chr7:1375	TRBV5-1		protein_c	chr7:142320677-142
ENSG00000	415	9.397205	chr7:1375	TRBV19		protein_c	chr7:142618849-142
ENSG00000	415	9.397205	chr7:1375	TRBV20-1		protein_c	chr7:142626649-142
ENSG00000	415	9.397205	chr7:1375	OR9N1P		Pseudoger	chr7:141911463-141
ENSG00000	415	9.397205	chr7:1375	TAS2R41		protein_c	chr7:143477873-143
ENSG00000	415	9.397205	chr7:1375	TRBV23-1		protein_c	chr7:142645961-142
ENSG00000	415	9.397205	chr7:1375	TRBV24-1		protein_c	chr7:142656701-142
ENSG00000	415	9.397205	chr7:1375	TRBV27		protein_c	chr7:142715346-142
ENSG00000	415	9.397205	chr7:1375	TRBV28		protein_c	chr7:142720660-142
ENSG00000	415	9.397205	chr7:1375	TRBJ2-1		protein_c	chr7:142796365-142
ENSG00000	415	9.397205	chr7:1375	TRBJ2-2		protein_c	chr7:142796560-142
ENSG00000	415	9.397205	chr7:1375	TRBJ2-2P		protein_c	chr7:142796697-142
ENSG00000	415	9.397205	chr7:1375	TRBJ2-3		protein_c	chr7:142796847-142
ENSG00000	415	9.397205	chr7:1375	TRBJ2-4		protein_c	chr7:142796998-142
ENSG00000	415	9.397205	chr7:1375	TRBJ2-5		protein_c	chr7:142797119-142
ENSG00000	415	9.397205	chr7:1375	TRBJ2-6		protein_c	chr7:142797239-142
ENSG00000	415	9.397205	chr7:1375	TRBV4-2		protein_c	chr7:142345421-142
ENSG00000	415	9.397205	chr7:1375	TRBV4-1		protein_c	chr7:142313184-142
ENSG00000	415	9.397205	chr7:1375	TRBV3-1		protein_c	chr7:142308542-142
ENSG00000	415	9.397205	chr7:1375	TRBV7-1		protein_c	chr7:142332182-142
ENSG00000	415	9.397205	chr7:1375	ENSG00000231923		Pseudoger	chr7:138046654-138
ENSG00000	415	9.397205	chr7:1375	TRBVA		Pseudoger	chr7:142681415-142
ENSG00000	415	9.397205	chr7:1375	RNA5SP248		Pseudoger	chr7:140386781-140
ENSG00000	415	9.397205	chr7:1375	ENSG00000289438		lncRNA	chr7:137846936-137
ENSG00000	415	9.397205	chr7:1375	NDUFB10P2		Pseudoger	chr7:141351977-141
ENSG00000	415	9.397205	chr7:2516	PPIAP82		Pseudoger	chr7:98454119-9845
ENSG00000	415	9.397205	chr7:1375	OR9A3P		Pseudoger	chr7:141862860-141
ENSG00000	415	9.397205	chr7:1375	TMEM178B		protein_c	chr7:141074064-141
ENSG00000	415	9.397205	chr7:1375	KLRG2	DriverDBv	protein_c	chr7:139452690-139
ENSG00000	415	9.397205	chr7:1375	U6		smallRNA	chr7:140884072-140
ENSG00000	415	9.397205	chr7:1375	OR9A2		protein_c	chr7:143026200-143
ENSG00000	415	9.397205	chr7:1375	MTND2P5		Pseudoger	chr7:141802482-141
ENSG00000	415	9.397205	chr7:1375	TMEM139		protein_c	chr7:143279957-143
ENSG00000	415	9.397205	chr7:1375	Y_RNA		smallRNA	chr7:138175045-138
ENSG00000	415	9.397205	chr7:1375	TRBV6-1		protein_c	chr7:142328297-142
ENSG00000	415	9.397205	chr7:1375	KDM7A-DT		lncRNA	chr7:140177184-140
ENSG00000	415	9.397205	chr7:1375	ENSG00000244701		lncRNA	chr7:141652381-141
ENSG00000	415	9.397205	chr7:1375	OR9A1P		protein_c	chr7:141887148-141
ENSG00000	415	9.397205	chr7:1375	RNU6-797P		smallRNA	chr7:140209563-140
ENSG00000	415	9.397205	chr7:1375	Y_RNA		smallRNA	chr7:140609847-140
ENSG00000	415	9.397205	chr7:1375	ZC3HAV1L		protein_c	chr7:139025706-139
ENSG00000	415	9.397205	chr7:1375	SVOPL		protein_c	chr7:138594285-138
ENSG00000	415	9.397205	chr7:1375	LLCFC1		protein_c	chr7:142939343-142

ENSG0000C	415	9.397205	chr7:1375	RNA5SP247		Pseudoger	chr7:140370441-140
ENSG0000C	415	9.397205	chr7:1375	MKRN1	NCGv7	protein_c	chr7:140453033-140
ENSG0000C	415	9.397205	chr7:1375	ENSG00000290099		lncRNA	chr7:142797704-142
ENSG0000C	415	9.397205	chr7:1375	PRSS3P3		Pseudoger	chr7:142287251-142
ENSG0000C	415	9.397205	chr7:1375	ADCK2		protein_c	chr7:140672945-140
ENSG0000C	415	9.397205	chr7:1375	UBN2		protein_c	chr7:139230356-139
ENSG0000C	415	9.397205	chr7:1375	ENSG00000234639		Pseudoger	chr7:138022458-138
ENSG0000C	415	9.397205	chr7:1375	ENSG00000234658		Pseudoger	chr7:138645671-138
ENSG0000C	415	9.397205	chr7:1375	ENSG00000204990		lncRNA	chr7:141414383-141
ENSG0000C	415	9.397205	chr7:1375	BRAF	NCGv7;AC	protein_c	chr7:140719327-140
ENSG0000C	415	9.397205	chr7:1375	TRBV21-1		Pseudoger	chr7:142636924-142
ENSG0000C	415	9.397205	chr7:1375	EPHA1	NCGv7	protein_c	chr7:143390289-143
ENSG0000C	415	9.397205	chr7:1375	AC024082.1		smallRNA	chr7:138068542-138
ENSG0000C	415	9.397205	chr7:1375	snoU13		smallRNA	chr7:138482480-138
ENSG0000C	415	9.397205	chr7:1375	RNU1-58P		smallRNA	chr7:140241870-140
ENSG0000C	415	9.397205	chr7:1375	WEE2	DriverDBv	protein_c	chr7:141708353-141
ENSG0000C	415	9.397205	chr7:1375	TMEM213		protein_c	chr7:138797952-138
ENSG0000C	415	9.397205	chr7:1375	RN7SL771P		smallRNA	chr7:140645844-140
ENSG0000C	415	9.397205	chr7:1375	TRPV6	NCGv7	protein_c	chr7:142871208-142
ENSG0000C	415	9.397205	chr7:1375	MIR4468		smallRNA	chr7:138123758-138
ENSG0000C	415	9.397205	chr7:1375	DENND2A		protein_c	chr7:140518420-140
ENSG0000C	415	9.397205	chr7:1375	snoU13		smallRNA	chr7:138121088-138
ENSG0000C	415	9.397205	chr7:1375	CREB3L2-AS1		lncRNA	chr7:137953348-137
ENSG0000C	415	9.397205	chr7:1375	TRBV30		protein_c	chr7:142812586-142
ENSG0000C	415	9.397205	chr7:1375	PARP12		protein_c	chr7:140023749-140
ENSG0000C	415	9.397205	chr7:1375	RAB19		protein_c	chr7:140404058-140
ENSG0000C	415	9.397205	chr7:1375	TBXAS1		protein_c	chr7:139777051-140
ENSG0000C	415	9.397205	chr7:1375	ENSG00000239254		Pseudoger	chr7:139172516-139
ENSG0000C	415	9.397205	chr7:1375	TRBV2		protein_c	chr7:142300924-142
ENSG0000C	415	9.397205	chr7:1375	ENSG00000271611		Pseudoger	chr7:140934867-140
ENSG0000C	415	9.397205	chr7:1375	Y_RNA		smallRNA	chr7:140094697-140
ENSG0000C	415	9.397205	chr7:1375	OR9A4		protein_c	chr7:141916399-141
ENSG0000C	415	9.397205	chr7:1375	MYL6P4		Pseudoger	chr7:141811805-141
ENSG0000C	415	9.397205	chr7:1375	Y_RNA		smallRNA	chr7:138415480-138
ENSG0000C	415	9.397205	chr7:1375	AGK-DT		lncRNA	chr7:141500079-141
ENSG0000C	415	9.397205	chr7:1375	ENSG00000286831		lncRNA	chr7:142725729-142
ENSG0000C	415	9.397205	chr7:1375	TRBC2		protein_c	chr7:142801041-142
ENSG0000C	415	9.397205	chr7:1375	SNORA51		smallRNA	chr7:138187998-138
ENSG0000C	415	9.397205	chr7:1375	PPP1R2P6		Pseudoger	chr7:140292752-140
ENSG0000C	415	9.397205	chr7:1375	IMPDH1P3		Pseudoger	chr7:138440690-138
ENSG0000C	415	9.397205	chr7:1375	DENND11		protein_c	chr7:141656728-141
ENSG0000C	415	9.397205	chr7:1375	PRSS37		protein_c	chr7:141836300-141
ENSG0000C	415	9.397205	chr7:1375	RN7SL481P		smallRNA	chr7:143298516-143
ENSG0000C	415	9.397205	chr7:1375	ENSG00000279223		TEC	chr7:143224042-143
ENSG0000C	415	9.397205	chr7:1375	ENSG00000270157		lncRNA	chr7:141662922-141
ENSG0000C	415	9.397205	chr7:1375	ENSG00000289938		lncRNA	chr7:142813393-142
ENSG0000C	415	9.397205	chr7:1375	ENSG00000271537		Pseudoger	chr7:139198557-139
ENSG0000C	415	9.397205	chr7:1375	AKR1D1	NCGv7	protein_c	chr7:138002324-138
ENSG0000C	415	9.397205	chr7:1375	RN7SKP223		smallRNA	chr7:138091254-138
ENSG0000C	415	9.397205	chr7:1375	ENSG00000224970		lncRNA	chr7:142875836-142
ENSG0000C	415	9.397205	chr7:1375	TRIM24	NCGv7	protein_c	chr7:138460259-138

ENSG0000(	415	9.397205	chr7:1375	RNU4-74P		smallRNA	chr7:141052249-141
ENSG0000(	415	9.397205	chr7:1375	ENSG00000243099		Pseudoger	chr7:138688980-138
ENSG0000(	415	9.397205	chr7:1375	KIAA1549	AC	protein_c	chr7:138831381-138
ENSG0000(	415	9.397205	chr7:1375	OR9P1P		Pseudoger	chr7:143047213-143
ENSG0000(	415	9.397205	chr7:1375	FAM131B-AS1		lncRNA	chr7:143363899-143
ENSG0000(	415	9.397205	chr7:1375	OR2R1P		Pseudoger	chr7:143488462-143
ENSG0000(	415	9.397205	chr7:1375	FAM131B		protein_c	chr7:143353400-143
ENSG0000(	415	9.397205	chr7:1375	MOXD2P		Pseudoger	chr7:142240740-142
ENSG0000(	415	9.397205	chr7:1375	CREB3L2	NCGv7;AC	protein_c	chr7:137874979-138
ENSG0000(	415	9.397205	chr7:1375	SLC37A3		protein_c	chr7:140293693-140
ENSG0000(	415	9.397205	chr7:1375	PRSS1	NCGv7	protein_c	chr7:142749468-142
ENSG0000(	415	9.397205	chr7:1375	TRBJ2-7		protein_c	chr7:142797456-142
ENSG0000(	415	9.397205	chr7:1375	LUC7L2	NCGv7	protein_c	chr7:139340359-139
ENSG0000(	415	9.397205	chr7:1375	NDUFB2	NCGv7	protein_c	chr7:140690777-140
ENSG0000(	415	9.397205	chr7:1375	FMC1-LUC7L2		protein_c	chr7:139341360-139
ENSG0000(	415	9.397205	chr7:1375	MTND1P3		Pseudoger	chr7:141803701-141
ENSG0000(	415	9.397205	chr7:1375	MTRNR2L6		protein_c	chr7:142666272-142
ENSG0000(	415	9.397205	chr7:1375	ENSG00000273391		lncRNA	chr7:139359032-139
ENSG0000(	415	9.397205	chr7:1375	ENSG00000290605		lncRNA	chr7:142240774-142
ENSG0000(	415	9.397205	chr7:1375	TRBV26		Pseudoger	chr7:142695699-142
ENSG0000(	415	9.397205	chr7:1375	CLEC5A		protein_c	chr7:141927357-141
ENSG0000(	415	9.397205	chr7:1375	snoU13		smallRNA	chr7:138176185-138
ENSG0000(	415	9.397205	chr7:1375	PRSS58		protein_c	chr7:142252143-142
ENSG0000(	415	9.397205	chr7:1375	RCC2P3		Pseudoger	chr7:138122202-138
ENSG0000(	415	9.397205	chr7:1375	HINT1P1		Pseudoger	chr7:143312684-143
ENSG0000(	415	9.397205	chr7:1375	ENSG00000285841		lncRNA	chr7:141392155-141
ENSG0000(	415	9.397205	chr7:1375	CLCN1		protein_c	chr7:143316111-143
ENSG0000(	415	9.397205	chr7:1375	ENSG00000261778		Pseudoger	chr7:141173043-141
ENSG0000(	415	9.397205	chr7:1375	SNORA40		smallRNA	chr7:138625060-138
ENSG0000(	415	9.397205	chr7:1375	TRBV25-1		protein_c	chr7:142670740-142
ENSG0000(	415	9.397205	chr7:1375	ENSG00000286569		lncRNA	chr7:142899365-142
ENSG0000(	415	9.397205	chr7:1375	RNU6-911P		smallRNA	chr7:139448740-139
ENSG0000(	415	9.397205	chr7:1375	MGAM	NCGv7	protein_c	chr7:141907813-142
ENSG0000(	415	9.397205	chr7:1375	CASP2		protein_c	chr7:143288215-143
ENSG0000(	415	9.397205	chr7:1375	ENSG00000253183		Pseudoger	chr7:139502453-139
ENSG0000(	415	9.397205	chr7:1375	ENSG00000225559		lncRNA	chr7:138163440-138
ENSG0000(	415	9.397205	chr7:1375	ENSG00000289788		lncRNA	chr7:140767530-140
ENSG0000(	415	9.397205	chr7:1375	ENSG00000268170		lncRNA	chr7:143220468-143
ENSG0000(	415	9.397205	chr7:1375	GSTK1		protein_c	chr7:143244093-143
ENSG0000(	415	9.397205	chr7:1375	RPL6P19		Pseudoger	chr7:137721985-137
ENSG0000(	415	9.397205	chr7:1375	ENSG00000103200		Pseudoger	chr7:140435316-140
ENSG0000(	415	9.397205	chr7:1375	FMC1		protein_c	chr7:139339457-139
ENSG0000(	415	9.397205	chr7:1375	RNU1-82P		smallRNA	chr7:141727984-141
ENSG0000(	415	9.397205	chr7:1375	ENSG00000290669		lncRNA	chr7:141863104-141
ENSG0000(	415	9.397205	chr7:1375	PTMAP10		Pseudoger	chr7:138404195-138
ENSG0000(	415	9.397205	chr7:1375	TRBV29-1		protein_c	chr7:142740206-142
ENSG0000(	415	9.397205	chr7:1375	ENSG00000213238		Pseudoger	chr7:138298088-138
ENSG0000(	415	9.397205	chr7:1375	MRPS33	NCGv7	protein_c	chr7:141002610-141
ENSG0000(	415	9.397205	chr7:1375	KEL	NCGv7	protein_c	chr7:142941114-142
ENSG0000(	415	9.397205	chr7:1375	TRBV22-1		Pseudoger	chr7:142641746-142
ENSG0000(	415	9.397205	chr7:1375	PRSS59P		Pseudoger	chr7:142265833-142

ENSG0000(	415	9.397205	chr7:1375	TRBD1		protein_c	chr7:142786213-142
ENSG0000(	415	9.397205	chr7:1375	OR6W1P		Pseudoger	chr7:143062330-143
ENSG0000(	415	9.397205	chr7:1375	NDUFB2-AS1		lncRNA	chr7:140695336-140
ENSG0000(	415	9.397205	chr7:1375	KDM7A		protein_c	chr7:140084746-140
ENSG0000(	415	9.397205	chr7:1375	ENSG00000229677		Pseudoger	chr7:139049456-139
ENSG0000(	415	9.397205	chr7:1375	ENSG00000288882		lncRNA	chr7:142716831-142
ENSG0000(	415	9.397205	chr7:1375	MGAM2		protein_c	chr7:142111718-142
ENSG0000(	415	9.397205	chr7:1375	SSBP1	NCGv7	protein_c	chr7:141738334-141
ENSG0000(	415	9.397205	chr7:1375	MZT1P2		Pseudoger	chr7:139228221-139
ENSG0000(	415	9.397205	chr7:1375	TAS2R62P		Pseudoger	chr7:143437034-143
ENSG0000(	415	9.397205	chr7:1375	WEE2-AS1		lncRNA	chr7:141704003-141
ENSG0000(	415	9.397205	chr7:1375	CLEC2L		protein_c	chr7:139523685-139
ENSG0000(	415	9.397205	chr7:1375	PGBD4P1		Pseudoger	chr7:142722358-142
ENSG0000(	415	9.397205	chr7:1375	TAS2R40		protein_c	chr7:143222037-143
ENSG0000(	415	9.397205	chr7:1375	CCT4P1		Pseudoger	chr7:140997952-140
ENSG0000(	415	9.397205	chr7:1375	PIP		protein_c	chr7:143132077-143
ENSG0000(	415	9.397205	chr7:1375	UQCRFS1P2		Pseudoger	chr7:138701607-138
ENSG0000(	415	9.397205	chr7:1375	TTC26	DriverDBv	protein_c	chr7:139133744-139
ENSG0000(	415	9.397205	chr7:1375	ZC3HAV1		protein_c	chr7:139043515-139
ENSG0000(	415	9.397205	chr7:1375	ATP6V0A4		protein_c	chr7:138706294-138
ENSG0000(	415	9.397205	chr7:1375	ZYX		protein_c	chr7:143381295-143
ENSG0000(	415	9.397205	chr7:1375	TMEM139-AS1		lncRNA	chr7:143255264-143
ENSG0000(	415	9.397205	chr7:1375	AGK		protein_c	chr7:141551278-141
ENSG0000(	415	9.397205	chr7:1375	ENSG00000290670		lncRNA	chr7:141911217-141
ENSG0000(	415	9.397205	chr7:1375	EPHB6	NCGv7	protein_c	chr7:142855061-142
ENSG0000(	415	9.397205	chr7:1375	ENSG00000261797		lncRNA	chr7:141512698-141
ENSG0000(	415	9.397205	chr7:1375	ERHP1		Pseudoger	chr7:139534240-139
ENSG0000(	415	9.397205	chr7:1375	TAS2R6P		Pseudoger	chr7:141787815-141
ENSG0000(	415	9.397205	chr7:1375	ENSG00000285904		lncRNA	chr7:140640909-140
ENSG0000(	415	9.397205	chr7:1375	snoU13		smallRNA	chr7:140375563-140
ENSG0000(	415	9.397205	chr7:1375	RNU6-1272P		smallRNA	chr7:138876359-138
ENSG0000(	415	9.397205	chr7:1375	TRBV1		Pseudoger	chr7:142299177-142
ENSG0000(	411	9.306629	chr12:201	RPSAP12		Pseudoger	chr12:68552995-685
ENSG0000(	409	9.261342	chr1:3733	LINC02781		lncRNA	chr1:4973381-49815
ENSG0000(	409	9.261342	chr1:3733	TP73-AS1		Pseudoger	chr1:3735511-37473
ENSG0000(	409	9.261342	chr1:3733	ENSG00000227169		lncRNA	chr1:4551735-45521
ENSG0000(	409	9.261342	chr1:3733	LINC02780		lncRNA	chr1:3976133-40127
ENSG0000(	409	9.261342	chr1:3733	AJAP1	NCGv7	protein_c	chr1:4654609-47925
ENSG0000(	409	9.261342	chr1:3733	ENSG00000284694		lncRNA	chr1:4479131-44843
ENSG0000(	409	9.261342	chr1:3733	DFFB		protein_c	chr1:3857267-38854
ENSG0000(	409	9.261342	chr1:3733	ENSG00000284703		lncRNA	chr1:4012921-40195
ENSG0000(	409	9.261342	chr1:3733	CEP104		protein_c	chr1:3812086-38573
ENSG0000(	409	9.261342	chr1:3733	LINC01345		lncRNA	chr1:3944547-39490
ENSG0000(	409	9.261342	chr1:3733	LRRC47	NCGv7	protein_c	chr1:3778559-37964
ENSG0000(	409	9.261342	chr1:3733	RN7SL574P		smallRNA	chr1:3782815-37831
ENSG0000(	409	9.261342	chr1:3733	ENSG00000284739		lncRNA	chr1:4963954-49732
ENSG0000(	409	9.261342	chr1:3733	ENSG00000272153		lncRNA	chr1:3785008-37855
ENSG0000(	409	9.261342	chr1:3733	ENSG00000287586		lncRNA	chr1:4730211-47349
ENSG0000(	409	9.261342	chr1:3733	LINC01346		lncRNA	chr1:3940486-39552
ENSG0000(	409	9.261342	chr1:3733	EEF1DP6		Pseudoger	chr1:4175528-41758
ENSG0000(	409	9.261342	chr1:3733	SMIM1		protein_c	chr1:3772749-37759

ENSG00000	409	9.261342	chr1:3733	LINC01134		lncRNA	chr1:3900347-39172
ENSG00000	409	9.261342	chr1:3733	LINC01777		lncRNA	chr1:4412027-44246
ENSG00000	409	9.261342	chr1:3733	AL691523.1		smallRNA	chr1:3884064-38841
ENSG00000	409	9.261342	chr1:3733	LINC01646		lncRNA	chr1:4571481-45940
ENSG00000	409	9.261342	chr1:3733	CCDC27		protein_c	chr1:3746460-37716
ENSG00000	409	9.261342	chr1:3733	C1orf174		protein_c	chr1:3889125-39002
ENSG00000	407	9.216054	chr2:8187	ENSG00000229550		lncRNA	chr2:4136644-41407
ENSG00000	407	9.216054	chr2:8187	LINC01304		lncRNA	chr2:3957652-39741
ENSG00000	407	9.216054	chr2:8187	AC068292.1		smallRNA	chr2:4228428-42285
ENSG00000	407	9.216054	chr2:8187	TRAPPC12		protein_c	chr2:3379675-34850
ENSG00000	407	9.216054	chr2:8187	ENSG00000242282		lncRNA	chr2:3531813-35368
ENSG00000	407	9.216054	chr2:8187	ENSG00000227364		lncRNA	chr2:2729908-27309
ENSG00000	407	9.216054	chr2:8187	ENSG00000224661		lncRNA	chr2:3702585-37041
ENSG00000	407	9.216054	chr2:8187	GAPDHP48		Pseudogen	chr2:3688021-36889
ENSG00000	407	9.216054	chr2:8187	ENSG00000237370		lncRNA	chr2:3603397-36042
ENSG00000	407	9.216054	chr2:8187	RPS7		protein_c	chr2:3575260-35809
ENSG00000	407	9.216054	chr2:8187	ENSG00000289608		lncRNA	chr2:2682109-26938
ENSG00000	407	9.216054	chr2:8187	SNORA73		smallRNA	chr2:3580570-35807
ENSG00000	407	9.216054	chr2:8187	RNASEH1		protein_c	chr2:3541430-35583
ENSG00000	407	9.216054	chr2:8187	RN7SL531P		smallRNA	chr2:4029681-40299
ENSG00000	407	9.216054	chr2:8187	ENSG00000287126		lncRNA	chr2:3568505-35751
ENSG00000	407	9.216054	chr2:8187	ENSG00000288786		lncRNA	chr2:2846699-28470
ENSG00000	407	9.216054	chr2:8187	ENSG00000236760		lncRNA	chr2:3131581-31457
ENSG00000	407	9.216054	chr2:8187	ENSG00000289136		lncRNA	chr2:3650922-36520
ENSG00000	407	9.216054	chr2:8187	EIPR1		protein_c	chr2:3188925-33778
ENSG00000	407	9.216054	chr2:8187	ENSG00000271868		lncRNA	chr2:3496956-34974
ENSG00000	407	9.216054	chr2:8187	ENSG00000237720		lncRNA	chr2:2707368-28405
ENSG00000	407	9.216054	chr2:8187	TMSB4XP2		Pseudogen	chr2:3617548-36177
ENSG00000	407	9.216054	chr2:8187	ENSG00000228391		lncRNA	chr2:2870558-28712
ENSG00000	407	9.216054	chr2:8187	ENSG00000287583		lncRNA	chr2:2697490-27018
ENSG00000	407	9.216054	chr2:8187	ENSG00000286905		protein_c	chr2:3532008-35583
ENSG00000	407	9.216054	chr2:8187	ENSG00000226649		lncRNA	chr2:3156756-31577
ENSG00000	407	9.216054	chr2:8187	snoU13		smallRNA	chr2:3075672-30757
ENSG00000	407	9.216054	chr2:8187	COLEC11		protein_c	chr2:3594832-36446
ENSG00000	407	9.216054	chr2:8187	DCDC2C		protein_c	chr2:3703575-38480
ENSG00000	407	9.216054	chr2:8187	AC074264.1		smallRNA	chr2:3017219-30173
ENSG00000	407	9.216054	chr2:8187	LINC01250		lncRNA	chr2:2895048-31260
ENSG00000	407	9.216054	chr2:8187	TRAPPC12-AS1		lncRNA	chr2:3481242-34824
ENSG00000	407	9.216054	chr2:8187	ADI1		protein_c	chr2:3497366-35195
ENSG00000	407	9.216054	chr2:8187	ENSG00000234929		lncRNA	chr2:2638178-26964
ENSG00000	407	9.216054	chr2:8187	RNASEH1-DT		lncRNA	chr2:3558419-35648
ENSG00000	407	9.216054	chr2:8187	ENSG00000290112		lncRNA	chr2:2613103-26133
ENSG00000	407	9.216054	chr2:8187	ALLC	NCGv7	protein_c	chr2:3658200-37026
ENSG00000	407	9.216054	chr2:8187	EIPR1-IT1		lncRNA	chr2:3298341-33014
ENSG00000	407	9.216054	chr2:8187	ENSG00000235078		lncRNA	chr2:3519275-35231
ENSG00000	401	9.080191	chr17:289	Y_RNA		smallRNA	chr17:38231043-382
ENSG00000	401	9.080191	chr17:289	ENSG00000273576		lncRNA	chr17:39566915-395
ENSG00000	401	9.080191	chr17:289	RARA-AS1		lncRNA	chr17:40340867-403
ENSG00000	401	9.080191	chr17:289	ENSG00000263466		lncRNA	chr17:38903704-389
ENSG00000	401	9.080191	chr17:289	RPL23AP75		Pseudogen	chr17:40439467-404
ENSG00000	401	9.080191	chr17:289	ENSG00000275173		lncRNA	chr17:37798894-378

ENSG0000(	401	9.080191	chr17:289	LINC00672		protein_c	chr17:38925168-389
ENSG0000(	401	9.080191	chr17:289	RARA	NCGv7;AC	protein_c	chr17:40309180-403
ENSG0000(	401	9.080191	chr17:289	ENSG00000266588		lncRNA	chr17:39003248-390
ENSG0000(	401	9.080191	chr17:289	PPP1R1B		protein_c	chr17:39626740-396
ENSG0000(	401	9.080191	chr17:289	LINC02079		lncRNA	chr17:39026715-390
ENSG0000(	401	9.080191	chr17:289	ENSG00000265799		lncRNA	chr17:40012226-400
ENSG0000(	401	9.080191	chr17:289	NPEPPSP1		Pseudoger	chr17:38195872-382
ENSG0000(	401	9.080191	chr17:289	RNU6-233P		smallRNA	chr17:39546104-395
ENSG0000(	401	9.080191	chr17:289	TCAP	DriverDB\	protein_c	chr17:39665349-396
ENSG0000(	401	9.080191	chr17:289	GPR179		protein_c	chr17:38324571-383
ENSG0000(	401	9.080191	chr17:289	ENSG00000266048		Pseudoger	chr17:39033926-390
ENSG0000(	401	9.080191	chr17:289	ORMDL3	DriverDB\	protein_c	chr17:39921041-399
ENSG0000(	401	9.080191	chr17:289	MIR4734		smallRNA	chr17:38702262-387
ENSG0000(	401	9.080191	chr17:289	SNORD124		smallRNA	chr17:40027542-400
ENSG0000(	401	9.080191	chr17:289	GSDMB	DriverDB\	protein_c	chr17:39904595-399
ENSG0000(	401	9.080191	chr17:289	AATF		protein_c	chr17:36948925-370
ENSG0000(	401	9.080191	chr17:289	MSL1	DriverDB\	protein_c	chr17:40121971-401
ENSG0000(	401	9.080191	chr17:289	ENSG00000264968		lncRNA	chr17:39927742-399
ENSG0000(	401	9.080191	chr17:289	WIPF2	NCGv7	protein_c	chr17:40219304-402
ENSG0000(	401	9.080191	chr17:289	STARD3	DriverDB\	protein_c	chr17:39637090-396
ENSG0000(	401	9.080191	chr17:289	ENSG00000279806		TEC	chr17:40473554-404
ENSG0000(	401	9.080191	chr17:289	ENSG00000277426		Pseudoger	chr17:39023394-390
ENSG0000(	401	9.080191	chr17:289	SNORA21		smallRNA	chr17:38852863-388
ENSG0000(	401	9.080191	chr17:289	RNU6-866P		smallRNA	chr17:38755279-387
ENSG0000(	401	9.080191	chr17:289	RNY4P8		smallRNA	chr17:40243223-402
ENSG0000(	401	9.080191	chr17:289	STAC2		protein_c	chr17:39210541-392
ENSG0000(	401	9.080191	chr17:289	ENSG00000275665		lncRNA	chr17:38715328-387
ENSG0000(	401	9.080191	chr17:289	ENSG00000261499		Pseudoger	chr17:38195880-382
ENSG0000(	401	9.080191	chr17:289	RP11-115K3.1		lncRNA	chr17:37745215-377
ENSG0000(	401	9.080191	chr17:289	CDK12	NCGv7;AC	protein_c	chr17:39461486-395
ENSG0000(	401	9.080191	chr17:289	ENSG00000277579		lncRNA	chr17:37034668-370
ENSG0000(	401	9.080191	chr17:289	IKZF3	NCGv7	protein_c	chr17:39757718-398
ENSG0000(	401	9.080191	chr17:289	ENSG00000276810		Pseudoger	chr17:37162616-371
ENSG0000(	401	9.080191	chr17:289	ENSG00000273965		lncRNA	chr17:37407936-374
ENSG0000(	401	9.080191	chr17:289	RPL23	AC	protein_c	chr17:38847860-388
ENSG0000(	401	9.080191	chr17:289	PSMB3		protein_c	chr17:38752741-387
ENSG0000(	401	9.080191	chr17:289	ENSG00000265784		lncRNA	chr17:38918801-389
ENSG0000(	401	9.080191	chr17:289	PIP4K2B		protein_c	chr17:38765691-388
ENSG0000(	401	9.080191	chr17:289	TBC1D3L		protein_c	chr17:37978155-379
ENSG0000(	401	9.080191	chr17:289	AC068669.1		smallRNA	chr17:40150136-401
ENSG0000(	401	9.080191	chr17:289	DUSP14		protein_c	chr17:37489891-375
ENSG0000(	401	9.080191	chr17:289	CDC6	AC	protein_c	chr17:40287879-403
ENSG0000(	401	9.080191	chr17:289	ENSG00000274308		Pseudoger	chr17:37140611-371
ENSG0000(	401	9.080191	chr17:289	LASP1	AC	protein_c	chr17:38869859-389
ENSG0000(	401	9.080191	chr17:289	ENSG00000287644		lncRNA	chr17:40317698-403
ENSG0000(	401	9.080191	chr17:289	LRRC37A11P		lncRNA	chr17:39029906-390
ENSG0000(	401	9.080191	chr17:289	ENSG00000286443		lncRNA	chr17:39086976-390
ENSG0000(	401	9.080191	chr17:289	PLXDC1	NCGv7	protein_c	chr17:39063313-391
ENSG0000(	401	9.080191	chr17:289	MED1	NCGv7	protein_c	chr17:39404285-394
ENSG0000(	401	9.080191	chr17:289	ENSG00000275532		lncRNA	chr17:38703472-387
ENSG0000(	401	9.080191	chr17:289	PGAP3	DriverDB\	protein_c	chr17:39671122-396

ENSG0000C	401	9.080191	chr17:289	KRT8P34		Pseudoger	chr17:39835037-398
ENSG0000C	401	9.080191	chr17:289	RNU6-981P		smallRNA	chr17:39482486-394
ENSG0000C	401	9.080191	chr17:289	RNA5SP441		Pseudoger	chr17:40374089-403
ENSG0000C	401	9.080191	chr17:289	ENSG00000278638		lncRNA	chr17:37045228-370
ENSG0000C	401	9.080191	chr17:289	ZPBP2	NCGv7	protein_c	chr17:39868202-398
ENSG0000C	401	9.080191	chr17:289	Y_RNA		smallRNA	chr17:40391232-403
ENSG0000C	401	9.080191	chr17:289	MED24	DriverDBv	protein_c	chr17:40019097-400
ENSG0000C	401	9.080191	chr17:289	RNA5SP440		Pseudoger	chr17:38731802-387
ENSG0000C	401	9.080191	chr17:289	TBC1D3	AC	protein_c	chr17:37978155-382
ENSG0000C	401	9.080191	chr17:289	CWC25		protein_c	chr17:38800441-388
ENSG0000C	401	9.080191	chr17:289	LRRC37A11P		Pseudoger	chr17:39027277-390
ENSG0000C	401	9.080191	chr17:289	ENSG00000276170		lncRNA	chr17:38450394-384
ENSG0000C	401	9.080191	chr17:289	ENSG00000214546		lncRNA	chr17:39619613-396
ENSG0000C	401	9.080191	chr17:289	SOCS7		protein_c	chr17:38351844-384
ENSG0000C	401	9.080191	chr17:289	MIR4728		smallRNA	chr17:39726495-397
ENSG0000C	401	9.080191	chr17:289	SNORA21		smallRNA	chr17:38851524-388
ENSG0000C	401	9.080191	chr17:289	IGFBP4		protein_c	chr17:40443450-404
ENSG0000C	401	9.080191	chr17:289	YWHAEP7		Pseudoger	chr17:37854303-378
ENSG0000C	401	9.080191	chr17:289	NEUROD2		protein_c	chr17:39603536-396
ENSG0000C	401	9.080191	chr17:289	ENSG00000278918		TEC	chr17:40348049-403
ENSG0000C	401	9.080191	chr17:289	RNA5SP440		smallRNA	chr17:38731802-387
ENSG0000C	401	9.080191	chr17:289	GJD3		protein_c	chr17:40360652-403
ENSG0000C	401	9.080191	chr17:289	HMGB1P24		Pseudoger	chr17:37143607-371
ENSG0000C	401	9.080191	chr17:289	CACNB1	DriverDBv	protein_c	chr17:39173453-391
ENSG0000C	401	9.080191	chr17:289	RN7SL102P		smallRNA	chr17:38621200-386
ENSG0000C	401	9.080191	chr17:289	C17orf78		protein_c	chr17:37375985-373
ENSG0000C	401	9.080191	chr17:289	ENSG00000289110		lncRNA	chr17:37488455-374
ENSG0000C	401	9.080191	chr17:289	ENSG00000265460		Pseudoger	chr17:39238466-392
ENSG0000C	401	9.080191	chr17:289	RPL19	DriverDBv	protein_c	chr17:39200283-392
ENSG0000C	401	9.080191	chr17:289	FBXO47		protein_c	chr17:38936432-389
ENSG0000C	401	9.080191	chr17:289	TBC1D3F		protein_c	chr17:37924426-379
ENSG0000C	401	9.080191	chr17:289	NR1D1	DriverDBv	protein_c	chr17:40092793-401
ENSG0000C	401	9.080191	chr17:289	MIR2909		smallRNA	chr17:37033745-370
ENSG0000C	401	9.080191	chr17:289	NPEPPSP1		Pseudoger	chr17:38195740-382
ENSG0000C	401	9.080191	chr17:289	FBXL20	DriverDBv	protein_c	chr17:39252663-394
ENSG0000C	401	9.080191	chr17:289	SYNRG	NCGv7	protein_c	chr17:37514807-376
ENSG0000C	401	9.080191	chr17:289	CSF3	AC	protein_c	chr17:40015361-400
ENSG0000C	401	9.080191	chr17:289	ENSG00000277911		lncRNA	chr17:36980167-369
ENSG0000C	401	9.080191	chr17:289	RP11-1407O15.3		Pseudoger	chr17:37974083-379
ENSG0000C	401	9.080191	chr17:289	TADA2A		protein_c	chr17:37406886-374
ENSG0000C	401	9.080191	chr17:289	CASC3	NCGv7	protein_c	chr17:40140318-401
ENSG0000C	401	9.080191	chr17:289	ENSG00000275839		Pseudoger	chr17:37454171-374
ENSG0000C	401	9.080191	chr17:289	EPOP		protein_c	chr17:38671703-386
ENSG0000C	401	9.080191	chr17:289	ENSG00000277589		lncRNA	chr17:36998598-370
ENSG0000C	401	9.080191	chr17:289	LHX1	AC	protein_c	chr17:36936785-369
ENSG0000C	401	9.080191	chr17:289	ENSG00000279199		TEC	chr17:40113215-401
ENSG0000C	401	9.080191	chr17:289	TBC1D3JP		Pseudoger	chr17:37978585-379
ENSG0000C	401	9.080191	chr17:289	RN7SL458P		smallRNA	chr17:38409799-384
ENSG0000C	401	9.080191	chr17:289	MIR4726		smallRNA	chr17:38719691-387
ENSG0000C	401	9.080191	chr17:289	CISD3		protein_c	chr17:38730341-387
ENSG0000C	401	9.080191	chr17:289	ENSG00000277688		lncRNA	chr17:37609739-376

ENSG0000(	401	9.080191	chr17:289	ENSG00000274996		lncRNA	chr17:38601049-386
ENSG0000(	401	9.080191	chr17:289	LRRC3C	DriverDBv	protein_c	chr17:39927732-399
ENSG0000(	401	9.080191	chr17:289	AC091178.1		smallRNA	chr17:39081065-390
ENSG0000(	401	9.080191	chr17:289	ENSG00000244086		Pseudoger	chr17:38855314-388
ENSG0000(	401	9.080191	chr17:289	YWHAEP7		lncRNA	chr17:37828305-378
ENSG0000(	401	9.080191	chr17:289	ENSG00000265428		Pseudoger	chr17:38996323-389
ENSG0000(	401	9.080191	chr17:289	ENSG00000266101		lncRNA	chr17:39173290-391
ENSG0000(	401	9.080191	chr17:289	AC005288.1		smallRNA	chr17:39297405-392
ENSG0000(	401	9.080191	chr17:289	NPEPPSP1		lncRNA	chr17:38195703-382
ENSG0000(	401	9.080191	chr17:289	ENSG00000266469		lncRNA	chr17:39401793-394
ENSG0000(	401	9.080191	chr17:289	RNU6-489P		smallRNA	chr17:37907957-379
ENSG0000(	401	9.080191	chr17:289	PSMD3	DriverDBv	protein_c	chr17:39980807-399
ENSG0000(	401	9.080191	chr17:289	MIR4727		smallRNA	chr17:38825838-388
ENSG0000(	401	9.080191	chr17:289	ARL5C		protein_c	chr17:39156894-391
ENSG0000(	401	9.080191	chr17:289	ENSG00000266088		lncRNA	chr17:40516892-405
ENSG0000(	401	9.080191	chr17:289	TBC1D3K		protein_c	chr17:37924415-379
ENSG0000(	401	9.080191	chr17:289	ENSG00000277182		lncRNA	chr17:38749360-387
ENSG0000(	401	9.080191	chr17:289	RAPGEFL1	DriverDBv	protein_c	chr17:40177010-401
ENSG0000(	401	9.080191	chr17:289	GSDMA	NCGv7	protein_c	chr17:39953263-399
ENSG0000(	401	9.080191	chr17:289	PNMT		protein_c	chr17:39667981-396
ENSG0000(	401	9.080191	chr17:289	ENSG00000279119		TEC	chr17:38727833-387
ENSG0000(	401	9.080191	chr17:289	MRPL45		protein_c	chr17:38297023-383
ENSG0000(	401	9.080191	chr17:289	HNF1B	NCGv7	protein_c	chr17:37686431-377
ENSG0000(	401	9.080191	chr17:289	TNS4	NCGv7;AC	protein_c	chr17:40475828-405
ENSG0000(	401	9.080191	chr17:289	ENSG00000276707		lncRNA	chr17:36940049-369
ENSG0000(	401	9.080191	chr17:289	ENSG00000277969		lncRNA	chr17:38702449-387
ENSG0000(	401	9.080191	chr17:289	C17orf98		protein_c	chr17:38835086-388
ENSG0000(	401	9.080191	chr17:289	DDX52		protein_c	chr17:37609739-376
ENSG0000(	401	9.080191	chr17:289	TOP2A	IntOGen-I	protein_c	chr17:40388525-404
ENSG0000(	401	9.080191	chr17:289	PCGF2		protein_c	chr17:38733898-387
ENSG0000(	401	9.080191	chr17:289	ARHGAP23		protein_c	chr17:38419280-385
ENSG0000(	401	9.080191	chr17:289	ENSG00000226117		Pseudoger	chr17:39839369-398
ENSG0000(	401	9.080191	chr17:289	ENSG00000274244		lncRNA	chr17:37649133-376
ENSG0000(	401	9.080191	chr17:289	ENSG00000277501		lncRNA	chr17:37642947-376
ENSG0000(	401	9.080191	chr17:289	GRB7	DriverDBv	protein_c	chr17:39737927-397
ENSG0000(	401	9.080191	chr17:289	ENSG00000276054		lncRNA	chr17:37386886-373
ENSG0000(	401	9.080191	chr17:289	THRA	DriverDBv	protein_c	chr17:40058290-400
ENSG0000(	401	9.080191	chr17:289	ERBB2	NCGv7;AC	protein_c	chr17:39687914-397
ENSG0000(	401	9.080191	chr17:289	SRCIN1		protein_c	chr17:38530031-386
ENSG0000(	401	9.080191	chr17:289	GJD3-AS1		lncRNA	chr17:40360655-403
ENSG0000(	401	9.080191	chr17:289	ENSG00000278346		Pseudoger	chr17:40380666-403
ENSG0000(	401	9.080191	chr17:289	RDM1P5		Pseudoger	chr17:39057019-391
ENSG0000(	401	9.080191	chr17:289	ACACA		protein_c	chr17:37084992-374
ENSG0000(	401	9.080191	chr17:289	MLLT6	AC	protein_c	chr17:38705273-387
ENSG0000(	401	9.080191	chr17:289	ENSG00000280177		TEC	chr17:39165073-391
ENSG0000(	401	9.080191	chr17:289	MIEN1	DriverDBv	protein_c	chr17:39728496-397
ENSG0000(	400	9.057547	chr16:133	PARN		protein_c	chr16:14435700-146
ENSG0000(	400	9.057547	chr16:133	ENSG00000270734		Pseudoger	chr16:14862380-148
ENSG0000(	400	9.057547	chr16:133	NPIPP1		Pseudoger	chr16:15104330-151
ENSG0000(	400	9.057547	chr16:133	TVP23CP2		Pseudoger	chr16:14200357-142
ENSG0000(	400	9.057547	chr16:133	ABCC6P2		lncRNA	chr16:14822432-148

ENSG0000(	400	9.057547	chr16:13[illegible]	PKD1P3		Pseudoger	chr16:14911551-149
ENSG0000(	400	9.057547	chr16:13[illegible]	hsa-mir-3180-3		smallRNA	chr16:14911220-149
ENSG0000(	400	9.057547	chr16:13[illegible]	ENSG00000275910		lncRNA	chr16:15015828-150
ENSG0000(	400	9.057547	chr16:13[illegible]	MIR193BHG		lncRNA	chr16:14301389-143
ENSG0000(	400	9.057547	chr16:13[illegible]	ENSG00000262732		lncRNA	chr16:13930677-139
ENSG0000(	400	9.057547	chr16:13[illegible]	Y_RNA		smallRNA	chr16:15150697-151
ENSG0000(	400	9.057547	chr16:13[illegible]	ENSG00000261695		lncRNA	chr16:14734685-147
ENSG0000(	400	9.057547	chr16:13[illegible]	ENSG00000262529		lncRNA	chr16:14191820-142
ENSG0000(	400	9.057547	chr16:13[illegible]	ENSG00000276564		lncRNA	chr16:14150833-141
ENSG0000(	400	9.057547	chr16:13[illegible]	ENSG00000260735		Pseudoger	chr16:15094411-151
ENSG0000(	400	9.057547	chr16:13[illegible]	ENSG00000258354		lncRNA	chr16:14909887-149
ENSG0000(	400	9.057547	chr16:13[illegible]	TMF1P1		Pseudoger	chr16:13458326-134
ENSG0000(	400	9.057547	chr16:13[illegible]	ENSG00000290394		lncRNA	chr16:15132720-151
ENSG0000(	400	9.057547	chr16:13[illegible]	RPS26P52		Pseudoger	chr16:13922332-139
ENSG0000(	400	9.057547	chr16:13[illegible]	Y_RNA		smallRNA	chr16:14275505-142
ENSG0000(	400	9.057547	chr16:13[illegible]	PLA2G10		protein_c	chr16:14672548-146
ENSG0000(	400	9.057547	chr16:13[illegible]	LINC02185		lncRNA	chr16:14001198-140
ENSG0000(	400	9.057547	chr16:13[illegible]	AC092291.1		Pseudoger	chr16:14454204-144
ENSG0000(	400	9.057547	chr16:13[illegible]	ENSG00000290391		lncRNA	chr16:14920336-149
ENSG0000(	400	9.057547	chr16:13[illegible]	ENSG00000261523		lncRNA	chr16:14695567-147
ENSG0000(	400	9.057547	chr16:13[illegible]	MIR193B		smallRNA	chr16:14303967-143
ENSG0000(	400	9.057547	chr16:13[illegible]	ENSG00000257769		lncRNA	chr16:15608474-156
ENSG0000(	400	9.057547	chr16:13[illegible]	NPIPA1		protein_c	chr16:14922802-149
ENSG0000(	400	9.057547	chr16:13[illegible]	PKD1P6		Pseudoger	chr16:15125242-151
ENSG0000(	400	9.057547	chr16:13[illegible]	MIR3670-1		smallRNA	chr16:14907717-149
ENSG0000(	400	9.057547	chr16:13[illegible]	NOMO1		protein_c	chr16:14833721-148
ENSG0000(	400	9.057547	chr16:13[illegible]	ENSG00000257264		lncRNA	chr16:14901499-149
ENSG0000(	400	9.057547	chr16:13[illegible]	ENSG00000263234		lncRNA	chr16:13953155-139
ENSG0000(	400	9.057547	chr16:13[illegible]	ENSG00000262267		lncRNA	chr16:13728654-137
ENSG0000(	400	9.057547	chr16:13[illegible]	Y_RNA		smallRNA	chr16:14915457-149
ENSG0000(	400	9.057547	chr16:13[illegible]	ENSG00000273804		Pseudoger	chr16:14953012-149
ENSG0000(	400	9.057547	chr16:13[illegible]	ENSG00000290396		lncRNA	chr16:15104312-151
ENSG0000(	400	9.057547	chr16:13[illegible]	NPIPA3		protein_c	chr16:14703411-147
ENSG0000(	400	9.057547	chr16:13[illegible]	PDXDC1	NCGv7	protein_c	chr16:14974591-151
ENSG0000(	400	9.057547	chr16:13[illegible]	NPIPA2		protein_c	chr16:14742533-147
ENSG0000(	400	9.057547	chr16:13[illegible]	ENSG00000261819		Pseudoger	chr16:14988259-149
ENSG0000(	400	9.057547	chr16:13[illegible]	ENSG00000263257		lncRNA	chr16:14407668-144
ENSG0000(	400	9.057547	chr16:13[illegible]	ENSG00000257391		lncRNA	chr16:15154903-151
ENSG0000(	400	9.057547	chr16:13[illegible]	BFAR		protein_c	chr16:14632931-146
ENSG0000(	400	9.057547	chr16:13[illegible]	MRTFB	AC	protein_c	chr16:14071319-142
ENSG0000(	400	9.057547	chr16:13[illegible]	ENSG00000255037		Pseudoger	chr16:14766402-147
ENSG0000(	400	9.057547	chr16:13[illegible]	LINC02186		lncRNA	chr16:14018880-140
ENSG0000(	400	9.057547	chr16:13[illegible]	BMERB1		protein_c	chr16:15434475-156
ENSG0000(	400	9.057547	chr16:13[illegible]	NPIPA5		protein_c	chr16:15363628-153
ENSG0000(	400	9.057547	chr16:13[illegible]	ENSG00000254609		Pseudoger	chr16:14727282-147
ENSG0000(	400	9.057547	chr16:13[illegible]	ENSG00000261130		protein_c	chr16:15395754-155
ENSG0000(	400	9.057547	chr16:13[illegible]	hsa-mir-3180-4		smallRNA	chr16:15154850-151
ENSG0000(	400	9.057547	chr16:13[illegible]	AC092291.2		protein_c	chr16:14470985-144
ENSG0000(	400	9.057547	chr16:13[illegible]	MIR365A		smallRNA	chr16:14309285-143
ENSG0000(	400	9.057547	chr16:13[illegible]	RN7SL274P		smallRNA	chr16:14601827-146
ENSG0000(	400	9.057547	chr16:13[illegible]	ENSG00000277770		Pseudoger	chr16:15358978-153

ENSG0000(	400	9.057547	chr16:13:	LINC02130		lncRNA	chr16:14363109-143
ENSG0000(	400	9.057547	chr16:13:	AC126763.1		smallRNA	chr16:15158457-151
ENSG0000(	400	9.057547	chr16:13:	ERCC4	NCGv7;AC	protein_c	chr16:13920138-139
ENSG0000(	400	9.057547	chr16:13:	MPV17L	DriverDBv	protein_c	chr16:15395754-154
ENSG0000(	400	9.057547	chr16:13:	ENSG00000291273		lncRNA	chr16:15135304-151
ENSG0000(	400	9.057547	chr16:13:	ENSG00000291272		lncRNA	chr16:15126659-151
ENSG0000(	400	9.057547	chr16:13:	MIR484		smallRNA	chr16:15643294-156
ENSG0000(	400	9.057547	chr16:13:	ABCC6P2		Pseudoger	chr16:14820792-148
ENSG0000(	400	9.057547	chr16:13:	PKD1P6-NPIPP1		lncRNA	chr16:15104723-151
ENSG0000(	400	9.057547	chr16:13:	NTAN1		protein_c	chr16:15037854-150
ENSG0000(	400	9.057547	chr16:13:	RP11-680G24.5		lncRNA	chr16:15018106-150
ENSG0000(	400	9.057547	chr16:13:	RRN3		protein_c	chr16:15060033-150
ENSG0000(	400	9.057547	chr16:13:	MIR1972-1		smallRNA	chr16:15010321-150
ENSG0000(	400	9.057547	chr16:13:	RNU7-125P		smallRNA	chr16:14643788-146
ENSG0000(	400	9.057547	chr16:13:	MARF1		protein_c	chr16:15594387-156
ENSG0000(	399	9.034903	chr4:721:	ENSG00000240669		Pseudoger	chr4:152613875-152
ENSG0000(	398	9.012259	chr17:28:	ZNHIT3		protein_c	chr17:36486629-364
ENSG0000(	398	9.012259	chr17:28:	SLFN11		protein_c	chr17:35350305-353
ENSG0000(	398	9.012259	chr17:28:	RDM1		protein_c	chr17:35918066-359
ENSG0000(	398	9.012259	chr17:28:	ENSG00000265697		lncRNA	chr17:33534110-335
ENSG0000(	398	9.012259	chr17:28:	RNU6-840P		smallRNA	chr17:35093909-350
ENSG0000(	398	9.012259	chr17:28:	ENSG00000267102		Pseudoger	chr17:35409602-354
ENSG0000(	398	9.012259	chr17:28:	ENSG00000242660		Pseudoger	chr17:35566517-355
ENSG0000(	398	9.012259	chr17:28:	PIGW		protein_c	chr17:36534987-365
ENSG0000(	398	9.012259	chr17:28:	RFFL		protein_c	chr17:35005990-350
ENSG0000(	398	9.012259	chr17:28:	CCL3-AS1		lncRNA	chr17:36072866-360
ENSG0000(	398	9.012259	chr17:28:	ENSG00000267592		Pseudoger	chr17:35596904-355
ENSG0000(	398	9.012259	chr17:28:	SLFN12L		protein_c	chr17:35464249-355
ENSG0000(	398	9.012259	chr17:28:	ENSG00000267711		lncRNA	chr17:35400878-354
ENSG0000(	398	9.012259	chr17:28:	snoU13		smallRNA	chr17:35054388-350
ENSG0000(	398	9.012259	chr17:28:	ENSG00000267648		Pseudoger	chr17:35406948-354
ENSG0000(	398	9.012259	chr17:28:	ENSG00000265115		lncRNA	chr17:33627027-336
ENSG0000(	398	9.012259	chr17:28:	RNA5SP439		Pseudoger	chr17:36491199-364
ENSG0000(	398	9.012259	chr17:28:	ENSG00000278395		Pseudoger	chr17:36241701-362
ENSG0000(	398	9.012259	chr17:28:	CCL18		protein_c	chr17:36064272-360
ENSG0000(	398	9.012259	chr17:28:	ENSG00000285559		lncRNA	chr17:34937306-349
ENSG0000(	398	9.012259	chr17:28:	ENSG00000267312		Pseudoger	chr17:35459481-354
ENSG0000(	398	9.012259	chr17:28:	MMP28		protein_c	chr17:35756249-357
ENSG0000(	398	9.012259	chr17:28:	SPACA3		protein_c	chr17:32970376-329
ENSG0000(	398	9.012259	chr17:28:	ENSG00000286030		Pseudoger	chr17:35522279-355
ENSG0000(	398	9.012259	chr17:28:	ENSG00000243423		Pseudoger	chr17:35076737-350
ENSG0000(	398	9.012259	chr17:28:	ENSG00000264458		lncRNA	chr17:32627739-326
ENSG0000(	398	9.012259	chr17:28:	TBC1D3H		protein_c	chr17:36377531-363
ENSG0000(	398	9.012259	chr17:28:	ENSG00000267457		lncRNA	chr17:35073831-350
ENSG0000(	398	9.012259	chr17:28:	ENSG00000278690		lncRNA	chr17:36012504-360
ENSG0000(	398	9.012259	chr17:28:	ENSG00000280349		TEC	chr17:33099969-331
ENSG0000(	398	9.012259	chr17:28:	RNU6-1192P		smallRNA	chr17:36404826-364
ENSG0000(	398	9.012259	chr17:28:	ENSG00000283417		lncRNA	chr17:33791838-338
ENSG0000(	398	9.012259	chr17:28:	ENSG00000267782		lncRNA	chr17:35164243-351
ENSG0000(	398	9.012259	chr17:28:	RNA5SP438		Pseudoger	chr17:34120056-341
ENSG0000(	398	9.012259	chr17:28:	CCL14		protein_c	chr17:35983288-359

ENSG0000(	398	9.012259	chr17:289	ENSG00000276508		lncRNA	chr17:36684754-366
ENSG0000(	398	9.012259	chr17:289	MYO19		protein_c	chr17:36495636-365
ENSG0000(	398	9.012259	chr17:289	SNHG30		lncRNA	chr17:35568050-355
ENSG0000(	398	9.012259	chr17:289	TBC1D3E		protein_c	chr17:36165740-361
ENSG0000(	398	9.012259	chr17:289	ENSG00000271268		Pseudoger	chr17:35861093-358
ENSG0000(	398	9.012259	chr17:289	ENSG00000267625		lncRNA	chr17:35561539-355
ENSG0000(	398	9.012259	chr17:289	CCL16		protein_c	chr17:35976493-359
ENSG0000(	398	9.012259	chr17:289	RPL17P42		Pseudoger	chr17:35719888-357
ENSG0000(	398	9.012259	chr17:289	ENSG00000274756		Pseudoger	chr17:36574462-365
ENSG0000(	398	9.012259	chr17:289	LIG3		protein_c	chr17:34980512-350
ENSG0000(	398	9.012259	chr17:289	CCL23		protein_c	chr17:36013056-360
ENSG0000(	398	9.012259	chr17:289	AC005549.2		smallRNA	chr17:34183084-341
ENSG0000(	398	9.012259	chr17:289	UFM1P2		Pseudoger	chr17:35377416-353
ENSG0000(	398	9.012259	chr17:289	CCT6B	NCGv7	protein_c	chr17:34927859-349
ENSG0000(	398	9.012259	chr17:289	ENSG00000275944		lncRNA	chr17:36001419-360
ENSG0000(	398	9.012259	chr17:289	SLFN5		protein_c	chr17:35243071-352
ENSG0000(	398	9.012259	chr17:289	ENSG00000270894		lncRNA	chr17:35818399-358
ENSG0000(	398	9.012259	chr17:289	ENSG00000270871		lncRNA	chr17:35816717-358
ENSG0000(	398	9.012259	chr17:289	AC005549.1		smallRNA	chr17:34174245-341
ENSG0000(	398	9.012259	chr17:289	ENSG00000267271		Pseudoger	chr17:35344231-353
ENSG0000(	398	9.012259	chr17:289	RN7SL301P		smallRNA	chr17:36110150-361
ENSG0000(	398	9.012259	chr17:289	ENSG00000263485		lncRNA	chr17:34080882-340
ENSG0000(	398	9.012259	chr17:289	ENSG00000267074		lncRNA	chr17:35499690-355
ENSG0000(	398	9.012259	chr17:289	ENSG00000289485		lncRNA	chr17:36116854-361
ENSG0000(	398	9.012259	chr17:289	HEATR9		protein_c	chr17:35854946-358
ENSG0000(	398	9.012259	chr17:289	ENSG00000289211		lncRNA	chr17:35242433-352
ENSG0000(	398	9.012259	chr17:289	FNDC8		protein_c	chr17:35121615-351
ENSG0000(	398	9.012259	chr17:289	ENSG00000264174		lncRNA	chr17:34725509-347
ENSG0000(	398	9.012259	chr17:289	ENSG00000270977		Pseudoger	chr17:35893707-359
ENSG0000(	398	9.012259	chr17:289	TAF5LP1		Pseudoger	chr17:35498218-354
ENSG0000(	398	9.012259	chr17:289	ENSG00000264791		lncRNA	chr17:34142947-341
ENSG0000(	398	9.012259	chr17:289	ENSG00000274767		lncRNA	chr17:36183235-361
ENSG0000(	398	9.012259	chr17:289	ENSG00000278860		TEC	chr17:34614409-346
ENSG0000(	398	9.012259	chr17:289	ENSG00000236377		lncRNA	chr17:32876726-329
ENSG0000(	398	9.012259	chr17:289	ENSG00000225582		lncRNA	chr17:34479272-344
ENSG0000(	398	9.012259	chr17:289	GAS2L2		protein_c	chr17:35744511-357
ENSG0000(	398	9.012259	chr17:289	ENSG00000265689		lncRNA	chr17:33688803-336
ENSG0000(	398	9.012259	chr17:289	ENSG00000267618		protein_c	chr17:35011349-351
ENSG0000(	398	9.012259	chr17:289	AC131056.5		lncRNA	chr17:36225246-362
ENSG0000(	398	9.012259	chr17:289	H2BN1		protein_c	chr17:32895433-329
ENSG0000(	398	9.012259	chr17:289	ENSG00000267364		lncRNA	chr17:35313442-353
ENSG0000(	398	9.012259	chr17:289	CCL4L1		protein_c	chr17:36210908-362
ENSG0000(	398	9.012259	chr17:289	GGNBP2		protein_c	chr17:36544912-365
ENSG0000(	398	9.012259	chr17:289	ENSG00000265125		lncRNA	chr17:33565764-336
ENSG0000(	398	9.012259	chr17:289	TBC1D3C	AC	protein_c	chr17:36377648-363
ENSG0000(	398	9.012259	chr17:289	ENSG00000276755		Pseudoger	chr17:36234447-362
ENSG0000(	398	9.012259	chr17:289	NLE1		protein_c	chr17:35128730-351
ENSG0000(	398	9.012259	chr17:289	ENSG00000265356		lncRNA	chr17:33935437-341
ENSG0000(	398	9.012259	chr17:289	CCL13		protein_c	chr17:34356480-343
ENSG0000(	398	9.012259	chr17:289	ENSG00000263435		lncRNA	chr17:33976531-339
ENSG0000(	398	9.012259	chr17:289	TBC1D3B	NCGv7	protein_c	chr17:36165683-361

ENSG0000(	398	9.012259	chr17:289	PEX12		protein_c	chr17:35574795-355
ENSG0000(	398	9.012259	chr17:289	TMEM132E		protein_c	chr17:34579487-346
ENSG0000(	398	9.012259	chr17:289	RAD51D		protein_c	chr17:35092221-351
ENSG0000(	398	9.012259	chr17:289	CCL15		protein_c	chr17:35996440-360
ENSG0000(	398	9.012259	chr17:289	CCL15-CCL14		protein_c	chr17:35983656-360
ENSG0000(	398	9.012259	chr17:289	ENSG00000273687		lncRNA	chr17:35018660-350
ENSG0000(	398	9.012259	chr17:289	SLFN12		protein_c	chr17:35410922-354
ENSG0000(	398	9.012259	chr17:289	CCL5		protein_c	chr17:35871491-358
ENSG0000(	398	9.012259	chr17:289	ENSG00000265775		lncRNA	chr17:34118347-341
ENSG0000(	398	9.012259	chr17:289	RNA5SP439		smallRNA	chr17:36491199-364
ENSG0000(	398	9.012259	chr17:289	ENSG00000282738		Pseudoger	chr17:36161607-361
ENSG0000(	398	9.012259	chr17:289	CCL3		protein_c	chr17:36088256-360
ENSG0000(	398	9.012259	chr17:289	TMEM98		protein_c	chr17:32927910-329
ENSG0000(	398	9.012259	chr17:289	CTB-91J4.3		Pseudoger	chr17:36378007-363
ENSG0000(	398	9.012259	chr17:289	ENSG00000267744		Pseudoger	chr17:35190961-351
ENSG0000(	398	9.012259	chr17:289	ENSG00000267554		Pseudoger	chr17:35470069-354
ENSG0000(	398	9.012259	chr17:289	ENSG00000270240		lncRNA	chr17:35868967-358
ENSG0000(	398	9.012259	chr17:289	ENSG00000291030		lncRNA	chr17:35963488-359
ENSG0000(	398	9.012259	chr17:289	CCL1		protein_c	chr17:34360328-343
ENSG0000(	398	9.012259	chr17:289	Y_RNA		smallRNA	chr17:32830219-328
ENSG0000(	398	9.012259	chr17:289	SNORD7		smallRNA	chr17:35573657-355
ENSG0000(	398	9.012259	chr17:289	ENSG00000265614		Pseudoger	chr17:34331862-343
ENSG0000(	398	9.012259	chr17:289	ENSG00000264598		lncRNA	chr17:33052107-330
ENSG0000(	398	9.012259	chr17:289	ENSG00000264622		lncRNA	chr17:34837871-348
ENSG0000(	398	9.012259	chr17:289	LYZL6		protein_c	chr17:35934518-359
ENSG0000(	398	9.012259	chr17:289	TOMM20P2		Pseudoger	chr17:35514766-355
ENSG0000(	398	9.012259	chr17:289	ENSG00000267547		lncRNA	chr17:35403837-354
ENSG0000(	398	9.012259	chr17:289	ENSG00000270829		lncRNA	chr17:35808489-358
ENSG0000(	398	9.012259	chr17:289	ENSG00000264990		lncRNA	chr17:34176538-341
ENSG0000(	398	9.012259	chr17:289	ENSG00000267359		lncRNA	chr17:35553205-355
ENSG0000(	398	9.012259	chr17:289	ENSG00000264643		lncRNA	chr17:33680577-336
ENSG0000(	398	9.012259	chr17:289	SLFN13	NCGv7	protein_c	chr17:35435096-354
ENSG0000(	398	9.012259	chr17:289	RN7SKP274		smallRNA	chr17:35889558-358
ENSG0000(	398	9.012259	chr17:289	DHRS11		protein_c	chr17:36591879-366
ENSG0000(	398	9.012259	chr17:289	CCL3L3		protein_c	chr17:36194869-361
ENSG0000(	398	9.012259	chr17:289	LHX1-DT		lncRNA	chr17:36861674-369
ENSG0000(	398	9.012259	chr17:289	TLK2P1		Pseudoger	chr17:34036681-340
ENSG0000(	398	9.012259	chr17:289	E2F3P1		Pseudoger	chr17:35490009-354
ENSG0000(	398	9.012259	chr17:289	ENSG00000267745		lncRNA	chr17:35406684-354
ENSG0000(	398	9.012259	chr17:289	TBC1D3G		protein_c	chr17:36377348-364
ENSG0000(	398	9.012259	chr17:289	ENSG00000267349		lncRNA	chr17:35477994-354
ENSG0000(	398	9.012259	chr17:289	CCL11		protein_c	chr17:34285742-342
ENSG0000(	398	9.012259	chr17:289	ENSG00000273736		Pseudoger	chr17:35963512-359
ENSG0000(	398	9.012259	chr17:289	TMEM132E-DT		lncRNA	chr17:34574123-345
ENSG0000(	398	9.012259	chr17:289	ENSG00000289011		lncRNA	chr17:36475918-364
ENSG0000(	398	9.012259	chr17:289	ENSG00000275720		lncRNA	chr17:36634069-366
ENSG0000(	398	9.012259	chr17:289	UNC45B		protein_c	chr17:35147817-351
ENSG0000(	398	9.012259	chr17:289	SLFN14		protein_c	chr17:35543985-355
ENSG0000(	398	9.012259	chr17:289	ENSG00000266535		lncRNA	chr17:33111858-331
ENSG0000(	398	9.012259	chr17:289	CCL7		protein_c	chr17:34270221-342
ENSG0000(	398	9.012259	chr17:289	ENSG00000279674		TEC	chr17:34000789-340

ENSG0000(	398	9.012259	chr17:289	C17orf50		protein_c	chr17:35760887-357
ENSG0000(	398	9.012259	chr17:289	ENSG00000275431		lncRNA	chr17:35983656-359
ENSG0000(	398	9.012259	chr17:289	AA06		lncRNA	chr17:33529787-335
ENSG0000(	398	9.012259	chr17:289	CCL4		protein_c	chr17:36103827-361
ENSG0000(	398	9.012259	chr17:289	ENSG00000283381		Pseudoger	chr17:33692229-336
ENSG0000(	398	9.012259	chr17:289	CCL8		protein_c	chr17:34319435-343
ENSG0000(	398	9.012259	chr17:289	ASIC2		protein_c	chr17:33013087-341
ENSG0000(	398	9.012259	chr17:289	ENSG00000275613		lncRNA	chr17:36722443-367
ENSG0000(	398	9.012259	chr17:289	AC024610.1		smallRNA	chr17:33874318-338
ENSG0000(	398	9.012259	chr17:289	TAF15	NCGv7;AC	protein_c	chr17:35809482-358
ENSG0000(	398	9.012259	chr17:289	LINC01989		lncRNA	chr17:34169372-341
ENSG0000(	398	9.012259	chr17:289	CCL2		protein_c	chr17:34255274-342
ENSG0000(	398	9.012259	chr17:289	LRRC37A9P		Pseudoger	chr17:35912635-359
ENSG0000(	398	9.012259	chr17:289	ENSG00000267035		lncRNA	chr17:35540039-355
ENSG0000(	398	9.012259	chr17:289	ENSG00000276241		lncRNA	chr17:36116177-361
ENSG0000(	398	9.012259	chr17:289	ENSG00000271392		lncRNA	chr17:35757199-357
ENSG0000(	398	9.012259	chr17:289	ENSG00000266981		lncRNA	chr17:35188522-351
ENSG0000(	398	9.012259	chr17:289	MRM1		protein_c	chr17:36601583-366
ENSG0000(	398	9.012259	chr17:289	SLC35G3		protein_c	chr17:35192520-351
ENSG0000(	398	9.012259	chr17:289	RASL10B		protein_c	chr17:35731639-357
ENSG0000(	398	9.012259	chr17:289	ENSG00000279668		lncRNA	chr17:33827699-340
ENSG0000(	398	9.012259	chr17:289	ZNF830	NCGv7	protein_c	chr17:34961540-349
ENSG0000(	398	9.012259	chr17:289	ENSG00000263571		lncRNA	chr17:34155566-341
ENSG0000(	398	9.012259	chr17:289	Vault		smallRNA	chr17:35097560-350
ENSG0000(	398	9.012259	chr17:289	ENSG00000265337		lncRNA	chr17:32529008-325
ENSG0000(	398	9.012259	chr17:289	ENSG00000266947		lncRNA	chr17:35231450-352
ENSG0000(	398	9.012259	chr17:289	AP2B1		protein_c	chr17:35578046-357
ENSG0000(	397	8.989615	chr1:2449	ENSG00000279401		TEC	chr1:185518651-185
ENSG0000(	397	8.989615	chr1:2449	Y_RNA		smallRNA	chr1:180519016-180
ENSG0000(	397	8.989615	chr1:2449	RNU6-152P		smallRNA	chr1:182327068-182
ENSG0000(	397	8.989615	chr1:2449	KIAA1614-AS1		lncRNA	chr1:180949699-180
ENSG0000(	397	8.989615	chr1:2449	ENSG00000276563		Pseudoger	chr1:178017127-178
ENSG0000(	397	8.989615	chr1:2449	ENSG00000227579		lncRNA	chr1:177392667-177
ENSG0000(	397	8.989615	chr1:2449	ENSG00000288574		lncRNA	chr1:181808927-181
ENSG0000(	397	8.989615	chr1:2449	VDAC1P4		Pseudoger	chr1:180434800-180
ENSG0000(	397	8.989615	chr1:2449	Y_RNA		smallRNA	chr1:184171714-184
ENSG0000(	397	8.989615	chr1:2449	RALGPS2-AS1		lncRNA	chr1:178724306-178
ENSG0000(	397	8.989615	chr1:2449	APOBEC4		protein_c	chr1:183646275-183
ENSG0000(	397	8.989615	chr1:2449	AL596220.1		protein_c	chr1:186394991-186
ENSG0000(	397	8.989615	chr1:2449	ENSG00000286966		lncRNA	chr1:183613537-183
ENSG0000(	397	8.989615	chr1:2449	RNU6-1240P		smallRNA	chr1:186311825-186
ENSG0000(	397	8.989615	chr1:2449	AL358354.1		smallRNA	chr1:180545832-180
ENSG0000(	397	8.989615	chr1:2449	DHX9-AS1		lncRNA	chr1:182837185-182
ENSG0000(	397	8.989615	chr1:2449	TOR1AIP2		protein_c	chr1:179839967-179
ENSG0000(	397	8.989615	chr1:2449	MEF2AP1		Pseudoger	chr1:179447578-179
ENSG0000(	397	8.989615	chr1:2449	ENSG00000270711		Pseudoger	chr1:180970837-180
ENSG0000(	397	8.989615	chr1:2449	TPR	NCGv7;AC	protein_c	chr1:186311652-186
ENSG0000(	397	8.989615	chr1:2449	TEX35		protein_c	chr1:178513109-178
ENSG0000(	397	8.989615	chr1:2449	ENSG00000273004		lncRNA	chr1:185317779-185
ENSG0000(	397	8.989615	chr1:2449	ODR4		protein_c	chr1:186375838-186
ENSG0000(	397	8.989615	chr1:2449	LINC01350		lncRNA	chr1:185558371-185

ENSG0000(	397	8.989615	chr1:2449	ENSG00000287452		lncRNA	chr1:181962889-181
ENSG0000(	397	8.989615	chr1:2449	SETP10		Pseudoger	chr1:179183734-179
ENSG0000(	397	8.989615	chr1:2449	HNRNPA1P54		Pseudoger	chr1:179447602-179
ENSG0000(	397	8.989615	chr1:2449	RN7SL374P		smallRNA	chr1:179364313-179
ENSG0000(	397	8.989615	chr1:2449	AL513344.1		smallRNA	chr1:182756919-182
ENSG0000(	397	8.989615	chr1:2449	ENSG00000225711		Pseudoger	chr1:179220938-179
ENSG0000(	397	8.989615	chr1:2449	ENSG00000289432		lncRNA	chr1:179017289-179
ENSG0000(	397	8.989615	chr1:2449	COLGALT2		protein_c	chr1:183929854-184
ENSG0000(	397	8.989615	chr1:2449	RNA5SP70		Pseudoger	chr1:181771566-181
ENSG0000(	397	8.989615	chr1:2449	EIF4A1P11		Pseudoger	chr1:179201705-179
ENSG0000(	397	8.989615	chr1:2449	RNA5SP71		smallRNA	chr1:182944365-182
ENSG0000(	397	8.989615	chr1:2449	LINC02818		lncRNA	chr1:179829609-179
ENSG0000(	397	8.989615	chr1:2449	TOR1AIP1		protein_c	chr1:179882042-179
ENSG0000(	397	8.989615	chr1:2449	TRMT1L		protein_c	chr1:185118101-185
ENSG0000(	397	8.989615	chr1:2449	RNU5F-2P		smallRNA	chr1:179576268-179
ENSG0000(	397	8.989615	chr1:2449	ENSG00000285847		lncRNA	chr1:184607599-184
ENSG0000(	397	8.989615	chr1:2449	PRG4	NCGv7	protein_c	chr1:186296279-186
ENSG0000(	397	8.989615	chr1:2449	ENSG00000289573		lncRNA	chr1:182149703-182
ENSG0000(	397	8.989615	chr1:2449	SMG7	NCGv7	protein_c	chr1:183472216-183
ENSG0000(	397	8.989615	chr1:2449	ACBD6		protein_c	chr1:180269653-180
ENSG0000(	397	8.989615	chr1:2449	NCF2	NCGv7	protein_c	chr1:183554461-183
ENSG0000(	397	8.989615	chr1:2449	PDC		protein_c	chr1:186443566-186
ENSG0000(	397	8.989615	chr1:2449	MIR4424		smallRNA	chr1:178677749-178
ENSG0000(	397	8.989615	chr1:2449	ENSG00000225359		lncRNA	chr1:181190471-181
ENSG0000(	397	8.989615	chr1:2449	CACNA1E	NCGv7	protein_c	chr1:181317690-181
ENSG0000(	397	8.989615	chr1:2449	SNORA67		smallRNA	chr1:179201487-179
ENSG0000(	397	8.989615	chr1:2449	ENSG00000228664		Pseudoger	chr1:182328497-182
ENSG0000(	397	8.989615	chr1:2449	C1orf21-DT		lncRNA	chr1:184385753-184
ENSG0000(	397	8.989615	chr1:2449	ENSG00000273384		lncRNA	chr1:178651706-178
ENSG0000(	397	8.989615	chr1:2449	OCLM		protein_c	chr1:186400572-186
ENSG0000(	397	8.989615	chr1:2449	ENSG00000227141		Pseudoger	chr1:179586705-179
ENSG0000(	397	8.989615	chr1:2449	LINC01633		lncRNA	chr1:184999710-185
ENSG0000(	397	8.989615	chr1:2449	ENSG00000226570		Pseudoger	chr1:182955390-182
ENSG0000(	397	8.989615	chr1:2449	RPL5P5		Pseudoger	chr1:185226808-185
ENSG0000(	397	8.989615	chr1:2449	C1orf220		lncRNA	chr1:178542752-178
ENSG0000(	397	8.989615	chr1:2449	ENSG00000213058		Pseudoger	chr1:178411616-178
ENSG0000(	397	8.989615	chr1:2449	RASAL2		protein_c	chr1:178094104-178
ENSG0000(	397	8.989615	chr1:2449	ENSG00000289099		lncRNA	chr1:183605128-183
ENSG0000(	397	8.989615	chr1:2449	ENSG00000231791		lncRNA	chr1:184329071-184
ENSG0000(	397	8.989615	chr1:2449	RN7SKP229		smallRNA	chr1:181839473-181
ENSG0000(	397	8.989615	chr1:2449	IVNS1ABP		protein_c	chr1:185296388-185
ENSG0000(	397	8.989615	chr1:2449	ENSG00000286372		lncRNA	chr1:183372870-183
ENSG0000(	397	8.989615	chr1:2449	SNORA67		smallRNA	chr1:179196473-179
ENSG0000(	397	8.989615	chr1:2449	TOR3A		protein_c	chr1:179082070-179
ENSG0000(	397	8.989615	chr1:2449	ENSG00000243062		lncRNA	chr1:179730191-179
ENSG0000(	397	8.989615	chr1:2449	TSEN15		protein_c	chr1:184051651-184
ENSG0000(	397	8.989615	chr1:2449	ENSG00000289589		lncRNA	chr1:181086644-181
ENSG0000(	397	8.989615	chr1:2449	ENSG00000261831		lncRNA	chr1:179926641-179
ENSG0000(	397	8.989615	chr1:2449	RALGPS2		protein_c	chr1:178725165-178
ENSG0000(	397	8.989615	chr1:2449	ENSG00000261817		lncRNA	chr1:180117140-180
ENSG0000(	397	8.989615	chr1:2449	ENSG00000223450		Pseudoger	chr1:180000438-180

ENSG0000(	397	8.989615	chr1:2449	ENSG00000289581		lncRNA	chr1:183874511-183
ENSG0000(	397	8.989615	chr1:2449	ANGPTL1		protein_c	chr1:178849535-178
ENSG0000(	397	8.989615	chr1:2449	FAM20B		protein_c	chr1:179025804-179
ENSG0000(	397	8.989615	chr1:2449	RNU7-13P		smallRNA	chr1:184821428-184
ENSG0000(	397	8.989615	chr1:2449	ENSG00000285910		lncRNA	chr1:178511563-178
ENSG0000(	397	8.989615	chr1:2449	NPHS2		protein_c	chr1:179550539-179
ENSG0000(	397	8.989615	chr1:2449	LINC00272		lncRNA	chr1:182407621-182
ENSG0000(	397	8.989615	chr1:2449	TEDDM1		protein_c	chr1:182398117-182
ENSG0000(	397	8.989615	chr1:2449	U6		smallRNA	chr1:180758722-180
ENSG0000(	397	8.989615	chr1:2449	QSOX1		protein_c	chr1:180154869-180
ENSG0000(	397	8.989615	chr1:2449	EDEM3		protein_c	chr1:184690237-184
ENSG0000(	397	8.989615	chr1:2449	C1orf21		protein_c	chr1:184387029-184
ENSG0000(	397	8.989615	chr1:2449	MCRIP2P2		Pseudoger	chr1:185435839-185
ENSG0000(	397	8.989615	chr1:2449	ENSG00000273198		lncRNA	chr1:186521773-186
ENSG0000(	397	8.989615	chr1:2449	SWT1		protein_c	chr1:185157080-185
ENSG0000(	397	8.989615	chr1:2449	ENSG00000224810		lncRNA	chr1:182062677-182
ENSG0000(	397	8.989615	chr1:2449	CRYZL2P-SEC16B		lncRNA	chr1:177928788-178
ENSG0000(	397	8.989615	chr1:2449	SMG7-AS1		lncRNA	chr1:183460874-183
ENSG0000(	397	8.989615	chr1:2449	LINC01688		lncRNA	chr1:182712862-182
ENSG0000(	397	8.989615	chr1:2449	RNASEL		protein_c	chr1:182573634-182
ENSG0000(	397	8.989615	chr1:2449	ENSG00000261060		lncRNA	chr1:179590372-179
ENSG0000(	397	8.989615	chr1:2449	RGS8		protein_c	chr1:182641816-182
ENSG0000(	397	8.989615	chr1:2449	MIR3121		smallRNA	chr1:180438314-180
ENSG0000(	397	8.989615	chr1:2449	STX6		protein_c	chr1:180972712-181
ENSG0000(	397	8.989615	chr1:2449	GLUL		protein_c	chr1:182378098-182
ENSG0000(	397	8.989615	chr1:2449	AL137800.1		smallRNA	chr1:183510675-183
ENSG0000(	397	8.989615	chr1:2449	ENSG00000234041		Pseudoger	chr1:179137764-179
ENSG0000(	397	8.989615	chr1:2449	ENSG00000290066		lncRNA	chr1:184629542-184
ENSG0000(	397	8.989615	chr1:2449	LINC01699		lncRNA	chr1:181236388-181
ENSG0000(	397	8.989615	chr1:2449	KRT18P28		Pseudoger	chr1:182959074-182
ENSG0000(	397	8.989615	chr1:2449	CLEC20A		protein_c	chr1:178479240-178
ENSG0000(	397	8.989615	chr1:2449	ABL2	NCGv7;AC	protein_c	chr1:179099330-179
ENSG0000(	397	8.989615	chr1:2449	ENSG00000287929		lncRNA	chr1:183252263-183
ENSG0000(	397	8.989615	chr1:2449	RN7SL654P		smallRNA	chr1:184335658-184
ENSG0000(	397	8.989615	chr1:2449	LINC01344		lncRNA	chr1:182096338-182
ENSG0000(	397	8.989615	chr1:2449	XPR1		protein_c	chr1:180632022-180
ENSG0000(	397	8.989615	chr1:2449	ENSG00000229407		lncRNA	chr1:179816184-179
ENSG0000(	397	8.989615	chr1:2449	ENSG00000286378		lncRNA	chr1:184664282-184
ENSG0000(	397	8.989615	chr1:2449	SHCBP1L		protein_c	chr1:182899865-182
ENSG0000(	397	8.989615	chr1:2449	RGS16		protein_c	chr1:182598623-182
ENSG0000(	397	8.989615	chr1:2449	NMNAT2		protein_c	chr1:183248237-183
ENSG0000(	397	8.989615	chr1:2449	ENSG00000288562		lncRNA	chr1:186624700-186
ENSG0000(	397	8.989615	chr1:2449	Y_RNA		smallRNA	chr1:185257911-185
ENSG0000(	397	8.989615	chr1:2449	ENSG00000227554		lncRNA	chr1:183754418-183
ENSG0000(	397	8.989615	chr1:2449	KIAA1614		protein_c	chr1:180912897-180
ENSG0000(	397	8.989615	chr1:2449	LAMC1	NCGv7	protein_c	chr1:183023420-183
ENSG0000(	397	8.989615	chr1:2449	RN7SL230P		smallRNA	chr1:179900262-179
ENSG0000(	397	8.989615	chr1:2449	LAMC1-AS1		lncRNA	chr1:183138402-183
ENSG0000(	397	8.989615	chr1:2449	ENSG00000260360		lncRNA	chr1:179953184-179
ENSG0000(	397	8.989615	chr1:2449	FTH1P25		Pseudoger	chr1:185071567-185
ENSG0000(	397	8.989615	chr1:2449	ENSG00000233196		Pseudoger	chr1:186580515-186

ENSG0000(	397	8.989615	chr1:244(	PACERR		lncRNA	chr1:186680601-186
ENSG0000(	397	8.989615	chr1:244(	PDC-AS1		lncRNA	chr1:186423481-186
ENSG0000(	397	8.989615	chr1:244(	SNORA63		smallRNA	chr1:178753654-178
ENSG0000(	397	8.989615	chr1:244(	GS1-279B7.1		Pseudoger	chr1:185321157-185
ENSG0000(	397	8.989615	chr1:244(	ENSG00000261729		lncRNA	chr1:185646463-185
ENSG0000(	397	8.989615	chr1:244(	NIBAN1		protein_c	chr1:184790724-184
ENSG0000(	397	8.989615	chr1:244(	CEP350		protein_c	chr1:179954674-180
ENSG0000(	397	8.989615	chr1:244(	Y_RNA		smallRNA	chr1:185634073-185
ENSG0000(	397	8.989615	chr1:244(	AL122019.1		smallRNA	chr1:177552870-177
ENSG0000(	397	8.989615	chr1:244(	CRYZL2P		Pseudoger	chr1:178006136-178
ENSG0000(	397	8.989615	chr1:244(	ENSG00000230470		lncRNA	chr1:184408337-184
ENSG0000(	397	8.989615	chr1:244(	ENSG00000270443		Pseudoger	chr1:182433893-182
ENSG0000(	397	8.989615	chr1:244(	AL590085.1		smallRNA	chr1:180827895-180
ENSG0000(	397	8.989615	chr1:244(	ENSG00000232750		Pseudoger	chr1:179035309-179
ENSG0000(	397	8.989615	chr1:244(	NPL		protein_c	chr1:182789293-182
ENSG0000(	397	8.989615	chr1:244(	RNU7-183P		smallRNA	chr1:185434244-185
ENSG0000(	397	8.989615	chr1:244(	RPSAP16		Pseudoger	chr1:179968686-179
ENSG0000(	397	8.989615	chr1:244(	DHX9	NCGv7	protein_c	chr1:182839347-182
ENSG0000(	397	8.989615	chr1:244(	ENSG00000272906		lncRNA	chr1:179881607-179
ENSG0000(	397	8.989615	chr1:244(	MR1		protein_c	chr1:181033374-181
ENSG0000(	397	8.989615	chr1:244(	FAM163A		protein_c	chr1:179743291-179
ENSG0000(	397	8.989615	chr1:244(	ENSG00000271269		Pseudoger	chr1:182733792-182
ENSG0000(	397	8.989615	chr1:244(	ARPC5		protein_c	chr1:183620846-183
ENSG0000(	397	8.989615	chr1:244(	RASAL2-AS1		lncRNA	chr1:178090677-178
ENSG0000(	397	8.989615	chr1:244(	SEC16B	DriverDB\	protein_c	chr1:177923956-177
ENSG0000(	397	8.989615	chr1:244(	RPS3AP8		Pseudoger	chr1:183266602-183
ENSG0000(	397	8.989615	chr1:244(	HMGN1P4		Pseudoger	chr1:182942115-182
ENSG0000(	397	8.989615	chr1:244(	LINC01741		lncRNA	chr1:177700524-177
ENSG0000(	397	8.989615	chr1:244(	SNORD112		smallRNA	chr1:184677934-184
ENSG0000(	397	8.989615	chr1:244(	AL450304.1		smallRNA	chr1:182959485-182
ENSG0000(	397	8.989615	chr1:244(	LINC01686		lncRNA	chr1:182615254-182
ENSG0000(	397	8.989615	chr1:244(	EIF1P3		Pseudoger	chr1:182336001-182
ENSG0000(	397	8.989615	chr1:244(	ENSG00000286655		lncRNA	chr1:184080657-184
ENSG0000(	397	8.989615	chr1:244(	PTGS2		protein_c	chr1:186671791-186
ENSG0000(	397	8.989615	chr1:244(	IER5		protein_c	chr1:181088700-181
ENSG0000(	397	8.989615	chr1:244(	ENSG00000224691		lncRNA	chr1:186176814-186
ENSG0000(	397	8.989615	chr1:244(	ENSG00000238061		Pseudoger	chr1:185280844-185
ENSG0000(	397	8.989615	chr1:244(	ENSG00000232309		lncRNA	chr1:182127297-182
ENSG0000(	397	8.989615	chr1:244(	Y_RNA		smallRNA	chr1:185251313-185
ENSG0000(	397	8.989615	chr1:244(	RNA5SP72		Pseudoger	chr1:185014951-185
ENSG0000(	397	8.989615	chr1:244(	ENSG00000228191		Pseudoger	chr1:179271116-179
ENSG0000(	397	8.989615	chr1:244(	YPEL5P1		Pseudoger	chr1:182182730-182
ENSG0000(	397	8.989615	chr1:244(	COX5BP8		Pseudoger	chr1:179255733-179
ENSG0000(	397	8.989615	chr1:244(	ENSG00000233583		Pseudoger	chr1:185262286-185
ENSG0000(	397	8.989615	chr1:244(	RNA5SP69		Pseudoger	chr1:178560913-178
ENSG0000(	397	8.989615	chr1:244(	Y_RNA		smallRNA	chr1:185630428-185
ENSG0000(	397	8.989615	chr1:244(	ENSG00000261250		lncRNA	chr1:179543201-179
ENSG0000(	397	8.989615	chr1:244(	ENSG00000243155		lncRNA	chr1:180944042-180
ENSG0000(	397	8.989615	chr1:244(	Y_RNA		smallRNA	chr1:185266535-185
ENSG0000(	397	8.989615	chr1:244(	Y_RNA		smallRNA	chr1:184315658-184
ENSG0000(	397	8.989615	chr1:244(	PTPN2P1		Pseudoger	chr1:178746683-178

ENSG0000(	397	8.989615	chr1:2449	RNU6-41P		smallRNA	chr1:182982212-182
ENSG0000(	397	8.989615	chr1:2449	HMCN1	NCGv7	protein_c	chr1:185734391-186
ENSG0000(	397	8.989615	chr1:2449	RPL22P24		Pseudoger	chr1:185171335-185
ENSG0000(	397	8.989615	chr1:2449	RGL1	NCGv7	protein_c	chr1:183636085-183
ENSG0000(	397	8.989615	chr1:2449	ENSG00000232036		Pseudoger	chr1:184566511-184
ENSG0000(	397	8.989615	chr1:2449	LINC01732		lncRNA	chr1:181174484-181
ENSG0000(	397	8.989615	chr1:2449	RNF2		protein_c	chr1:185045526-185
ENSG0000(	397	8.989615	chr1:2449	LHX4		protein_c	chr1:180230264-180
ENSG0000(	397	8.989615	chr1:2449	RGSL1		protein_c	chr1:182409192-182
ENSG0000(	397	8.989615	chr1:2449	TEDDM2P		Pseudoger	chr1:182441577-182
ENSG0000(	397	8.989615	chr1:2449	ENSG00000288078		lncRNA	chr1:186224472-186
ENSG0000(	397	8.989615	chr1:2449	ENSG00000270994		Pseudoger	chr1:183709305-183
ENSG0000(	397	8.989615	chr1:2449	ENSG00000228238		Pseudoger	chr1:186578279-186
ENSG0000(	397	8.989615	chr1:2449	AXDND1		protein_c	chr1:179365720-179
ENSG0000(	397	8.989615	chr1:2449	SOAT1	NCGv7	protein_c	chr1:179293714-179
ENSG0000(	397	8.989615	chr1:2449	TDRD5	NCGv7	protein_c	chr1:179591613-179
ENSG0000(	397	8.989615	chr1:2449	LINC02816		lncRNA	chr1:180906651-180
ENSG0000(	397	8.989615	chr1:2449	ZNF648		protein_c	chr1:182054570-182
ENSG0000(	397	8.989615	chr1:2449	OVAAL		lncRNA	chr1:180509380-180
ENSG0000(	397	8.989615	chr1:2449	ENSG00000289732		Pseudoger	chr1:183587174-183
ENSG0000(	397	8.989615	chr1:2449	ENSG00000279838		TEC	chr1:185292384-185
ENSG0000(	397	8.989615	chr1:2449	LINC01645		lncRNA	chr1:177351560-177
ENSG0000(	397	8.989615	chr1:2449	ENSG00000225982		lncRNA	chr1:182086551-182
ENSG0000(	397	8.989615	chr1:2449	LAMC2	TAG	protein_c	chr1:183186238-183
ENSG0000(	397	8.989615	chr1:2449	AL359853.1		smallRNA	chr1:179710250-179
ENSG0000(	397	8.989615	chr1:2449	ENSG00000251520		Pseudoger	chr1:180964511-180
ENSG0000(	397	8.989615	chr1:2449	ENSG00000270575		Pseudoger	chr1:178194342-178
ENSG0000(	395	8.944328	chr4:7212	AC093817.1		smallRNA	chr4:157588148-157
ENSG0000(	395	8.944328	chr4:7212	RN7SL446P		smallRNA	chr4:152264149-152
ENSG0000(	395	8.944328	chr4:7212	GRIA2	NCGv7	protein_c	chr4:157204182-157
ENSG0000(	395	8.944328	chr4:7212	NPY2R		protein_c	chr4:155208636-155
ENSG0000(	395	8.944328	chr4:7212	ENSG00000250398		Pseudoger	chr4:155383107-155
ENSG0000(	395	8.944328	chr4:7212	FAM192BP		Pseudoger	chr4:152927446-152
ENSG0000(	395	8.944328	chr4:7212	SFRP2		protein_c	chr4:153780591-153
ENSG0000(	395	8.944328	chr4:7212	ENSG00000278981		lncRNA	chr4:154235980-154
ENSG0000(	395	8.944328	chr4:7212	LINC02273		lncRNA	chr4:152090459-152
ENSG0000(	395	8.944328	chr4:7212	MTND5P9		Pseudoger	chr4:155453049-155
ENSG0000(	395	8.944328	chr4:7212	AC079340.1		smallRNA	chr4:152228468-152
ENSG0000(	395	8.944328	chr4:7212	RNU2-44P		smallRNA	chr4:154790291-154
ENSG0000(	395	8.944328	chr4:7212	MTND2P33		Pseudoger	chr4:155461633-155
ENSG0000(	395	8.944328	chr4:7212	ENSG00000270883		lncRNA	chr4:152317902-152
ENSG0000(	395	8.944328	chr4:7212	MTND6P17		Pseudoger	chr4:155452526-155
ENSG0000(	395	8.944328	chr4:7212	ENSG00000249200		Pseudoger	chr4:154373886-154
ENSG0000(	395	8.944328	chr4:7212	LINC02272		lncRNA	chr4:156634494-156
ENSG0000(	395	8.944328	chr4:7212	MTND1P22		Pseudoger	chr4:155462873-155
ENSG0000(	395	8.944328	chr4:7212	AC079298.1		smallRNA	chr4:154218909-154
ENSG0000(	395	8.944328	chr4:7212	ENSG00000290441		lncRNA	chr4:153640168-153
ENSG0000(	395	8.944328	chr4:7212	ENSG00000248571		lncRNA	chr4:152666368-152
ENSG0000(	395	8.944328	chr4:7212	LINC02433		lncRNA	chr4:157572490-157
ENSG0000(	395	8.944328	chr4:7212	TLR2	NCGv7	protein_c	chr4:153684070-153
ENSG0000(	395	8.944328	chr4:7212	ENSG00000287730		lncRNA	chr4:155442650-155

ENSG0000(	395	8.944328	chr4:7212	ENSG00000251244		lncRNA	chr4:155496110-155
ENSG0000(	395	8.944328	chr4:7212	AC108211.1		smallRNA	chr4:156296561-156
ENSG0000(	395	8.944328	chr4:7212	ENSG00000250706		lncRNA	chr4:152146385-152
ENSG0000(	395	8.944328	chr4:7212	DCHS2		protein_c	chr4:154231742-154
ENSG0000(	395	8.944328	chr4:7212	ENSG00000280005		lncRNA	chr4:154261735-154
ENSG0000(	395	8.944328	chr4:7212	ENSG00000249041		lncRNA	chr4:154754756-154
ENSG0000(	395	8.944328	chr4:7212	ANXA2P1		Pseudoger	chr4:153307792-153
ENSG0000(	395	8.944328	chr4:7212	RNU6-1285P		smallRNA	chr4:154543933-154
ENSG0000(	395	8.944328	chr4:7212	ENSG00000249022		lncRNA	chr4:153034211-153
ENSG0000(	395	8.944328	chr4:7212	NPY2R-AS1		lncRNA	chr4:155206529-155
ENSG0000(	395	8.944328	chr4:7212	TMEM154		protein_c	chr4:152618628-152
ENSG0000(	395	8.944328	chr4:7212	FGG		protein_c	chr4:154604134-154
ENSG0000(	395	8.944328	chr4:7212	TRIM2		protein_c	chr4:153152163-153
ENSG0000(	395	8.944328	chr4:7212	Y_RNA		smallRNA	chr4:153266909-153
ENSG0000(	395	8.944328	chr4:7212	ENSG00000279703		TEC	chr4:157818101-157
ENSG0000(	395	8.944328	chr4:7212	Y_RNA		smallRNA	chr4:157768013-157
ENSG0000(	395	8.944328	chr4:7212	ENSG00000287642		lncRNA	chr4:152934340-152
ENSG0000(	395	8.944328	chr4:7212	RBM46		protein_c	chr4:154781213-154
ENSG0000(	395	8.944328	chr4:7212	ENSG00000249309		lncRNA	chr4:153720327-153
ENSG0000(	395	8.944328	chr4:7212	MTND4P8		Pseudoger	chr4:155455050-155
ENSG0000(	395	8.944328	chr4:7212	RPS3AP18		Pseudoger	chr4:152551277-152
ENSG0000(	395	8.944328	chr4:7212	GLRB		protein_c	chr4:157076125-157
ENSG0000(	395	8.944328	chr4:7212	ENSG00000250771		Pseudoger	chr4:153678259-153
ENSG0000(	395	8.944328	chr4:7212	FBXW7	NCGv7;AC	protein_c	chr4:152320544-152
ENSG0000(	395	8.944328	chr4:7212	ENSG00000251511		lncRNA	chr4:156585979-156
ENSG0000(	395	8.944328	chr4:7212	ARFIP1		protein_c	chr4:152779937-152
ENSG0000(	395	8.944328	chr4:7212	YWHAEP4		Pseudoger	chr4:155381042-155
ENSG0000(	395	8.944328	chr4:7212	AC020703.1		smallRNA	chr4:153873367-153
ENSG0000(	395	8.944328	chr4:7212	LRAT		protein_c	chr4:154626945-154
ENSG0000(	395	8.944328	chr4:7212	TMEM131L		protein_c	chr4:153466346-153
ENSG0000(	395	8.944328	chr4:7212	MND1		protein_c	chr4:153344649-153
ENSG0000(	395	8.944328	chr4:7212	RNU2-66P		smallRNA	chr4:154787118-154
ENSG0000(	395	8.944328	chr4:7212	MIR3140		smallRNA	chr4:152489327-152
ENSG0000(	395	8.944328	chr4:7212	ENSG00000286720		lncRNA	chr4:151988526-152
ENSG0000(	395	8.944328	chr4:7212	MTCO3P9		Pseudoger	chr4:155457180-155
ENSG0000(	395	8.944328	chr4:7212	MTATP6P9		Pseudoger	chr4:155457961-155
ENSG0000(	395	8.944328	chr4:7212	MAP9-AS1		lncRNA	chr4:155173716-155
ENSG0000(	395	8.944328	chr4:7212	RPPH1-3P		smallRNA	chr4:156986372-156
ENSG0000(	395	8.944328	chr4:7212	ENSG00000249275		lncRNA	chr4:157637687-157
ENSG0000(	395	8.944328	chr4:7212	ENSG00000280241		lncRNA	chr4:153948718-154
ENSG0000(	395	8.944328	chr4:7212	ENSG00000249924		lncRNA	chr4:155286162-155
ENSG0000(	395	8.944328	chr4:7212	FGA	NCGv7	protein_c	chr4:154583128-154
ENSG0000(	395	8.944328	chr4:7212	MIR4453HG		lncRNA	chr4:152536264-152
ENSG0000(	395	8.944328	chr4:7212	ENSG00000248912		lncRNA	chr4:156686664-156
ENSG0000(	395	8.944328	chr4:7212	FGB	NCGv7	protein_c	chr4:154563011-154
ENSG0000(	395	8.944328	chr4:7212	PLRG1	NCGv7	protein_c	chr4:154535005-154
ENSG0000(	395	8.944328	chr4:7212	ENSG00000249479		lncRNA	chr4:156642580-156
ENSG0000(	395	8.944328	chr4:7212	RNF175		protein_c	chr4:153710160-153
ENSG0000(	395	8.944328	chr4:7212	PDGFC	NCGv7	protein_c	chr4:156760454-156
ENSG0000(	395	8.944328	chr4:7212	ENSG00000249692		Pseudoger	chr4:154770568-154
ENSG0000(	395	8.944328	chr4:7212	ENSG00000270302		lncRNA	chr4:152308283-152

ENSG0000(	395	8.944328	chr4:7212	ENSG00000249819		Pseudoger	chr4:157204462-157
ENSG0000(	395	8.944328	chr4:7212	MTCO1P9		Pseudoger	chr4:155459704-155
ENSG0000(	395	8.944328	chr4:7212	CTSO		protein_c	chr4:155921580-155
ENSG0000(	395	8.944328	chr4:7212	ENSG00000249627		Pseudoger	chr4:157815010-157
ENSG0000(	395	8.944328	chr4:7212	MTCO2P9		Pseudoger	chr4:155458870-155
ENSG0000(	395	8.944328	chr4:7212	ENSG00000280284		TEC	chr4:156876435-156
ENSG0000(	395	8.944328	chr4:7212	ENSG00000289320		lncRNA	chr4:155980938-156
ENSG0000(	395	8.944328	chr4:7212	ENSG00000287216		lncRNA	chr4:153633692-153
ENSG0000(	395	8.944328	chr4:7212	NSA2P6		Pseudoger	chr4:152796063-152
ENSG0000(	395	8.944328	chr4:7212	RNU6-1196P		smallRNA	chr4:153265662-153
ENSG0000(	395	8.944328	chr4:7212	AC099339.1		smallRNA	chr4:152900966-152
ENSG0000(	395	8.944328	chr4:7212	MTCYBP17		Pseudoger	chr4:155451617-155
ENSG0000(	395	8.944328	chr4:7212	ENSG00000278880		TEC	chr4:156886645-156
ENSG0000(	395	8.944328	chr4:7212	RN7SL419P		smallRNA	chr4:153381727-153
ENSG0000(	395	8.944328	chr4:7212	FTH1P21		Pseudoger	chr4:156006478-156
ENSG0000(	395	8.944328	chr4:7212	ENSG00000286133		lncRNA	chr4:157568731-157
ENSG0000(	395	8.944328	chr4:7212	Y_RNA		smallRNA	chr4:157064557-157
ENSG0000(	395	8.944328	chr4:7212	TDO2		protein_c	chr4:155854738-155
ENSG0000(	395	8.944328	chr4:7212	ENSG00000260244		lncRNA	chr4:155734448-155
ENSG0000(	395	8.944328	chr4:7212	ENSG00000248611		Pseudoger	chr4:154555208-154
ENSG0000(	395	8.944328	chr4:7212	GUCY1A1		protein_c	chr4:155666726-155
ENSG0000(	395	8.944328	chr4:7212	ENSG00000287770		Pseudoger	chr4:154605952-154
ENSG0000(	395	8.944328	chr4:7212	AC013477.1		smallRNA	chr4:153360533-153
ENSG0000(	395	8.944328	chr4:7212	ENSG00000287504		lncRNA	chr4:152113857-152
ENSG0000(	395	8.944328	chr4:7212	ENSG00000248629		lncRNA	chr4:156841359-156
ENSG0000(	395	8.944328	chr4:7212	ENSG00000288637		protein_c	chr4:153152435-153
ENSG0000(	395	8.944328	chr4:7212	AC079233.1		smallRNA	chr4:157360115-157
ENSG0000(	395	8.944328	chr4:7212	RNU6-582P		smallRNA	chr4:157201562-157
ENSG0000(	395	8.944328	chr4:7212	ASIC5		protein_c	chr4:155829729-155
ENSG0000(	395	8.944328	chr4:7212	ENSG00000287555		lncRNA	chr4:152448275-152
ENSG0000(	395	8.944328	chr4:7212	ENSG00000250609		Pseudoger	chr4:154523487-154
ENSG0000(	395	8.944328	chr4:7212	MTND3P3		Pseudoger	chr4:155456638-155
ENSG0000(	395	8.944328	chr4:7212	ENSG00000271606		Pseudoger	chr4:155385352-155
ENSG0000(	395	8.944328	chr4:7212	GUCY1B1		protein_c	chr4:155758992-155
ENSG0000(	395	8.944328	chr4:7212	FHDC1		protein_c	chr4:152936323-152
ENSG0000(	395	8.944328	chr4:7212	ENSG00000251377		lncRNA	chr4:152206987-152
ENSG0000(	395	8.944328	chr4:7212	TIGD4	DriverDB\	protein_c	chr4:152769354-152
ENSG0000(	395	8.944328	chr4:7212	NDUFB2P1		Pseudoger	chr4:154724586-154
ENSG0000(	395	8.944328	chr4:7212	FBXW7-AS1		lncRNA	chr4:152337655-152
ENSG0000(	395	8.944328	chr4:7212	MAP9		protein_c	chr4:155342658-155
ENSG0000(	395	8.944328	chr4:7212	WDR45P1		Pseudoger	chr4:153658572-153
ENSG0000(	394	8.921684	chr4:1154	snoU13		smallRNA	chr4:183329298-183
ENSG0000(	392	8.876396	chr17:289	ENSG00000263781		Pseudoger	chr17:29021325-290
ENSG0000(	390	8.831108	chr16:239	AC136932.1		smallRNA	chr16:34159185-341
ENSG0000(	390	8.831108	chr16:239	ENSG00000290419		lncRNA	chr16:33936430-339
ENSG0000(	390	8.831108	chr16:239	ENSG00000260522		Pseudoger	chr16:35722268-357
ENSG0000(	390	8.831108	chr16:239	ENSG00000262561		Pseudoger	chr16:34120470-341
ENSG0000(	390	8.831108	chr16:239	ENSG00000261445		lncRNA	chr16:35493754-354
ENSG0000(	390	8.831108	chr16:239	ENSG00000261566		lncRNA	chr16:35022851-350
ENSG0000(	390	8.831108	chr16:239	NAMPTP3		Pseudoger	chr16:35085896-350
ENSG0000(	390	8.831108	chr16:239	RNA5SP409		Pseudoger	chr16:35735625-357

ENSG0000(	390	8.831108	chr16:239	ENSG00000260525	Pseudoger	chr16:33875039-338
ENSG0000(	390	8.831108	chr16:239	DUX4L45	Pseudoger	chr16:34135736-341
ENSG0000(	390	8.831108	chr16:239	RNA5SP406	Pseudoger	chr16:35734393-357
ENSG0000(	390	8.831108	chr16:239	ENSG00000287448	lncRNA	chr16:35499494-355
ENSG0000(	390	8.831108	chr16:239	DUX4L46	Pseudoger	chr16:34141256-341
ENSG0000(	390	8.831108	chr16:239	BMS1P8	Pseudoger	chr16:33687330-336
ENSG0000(	390	8.831108	chr16:239	CHEK2P6	Pseudoger	chr16:33563030-335
ENSG0000(	390	8.831108	chr16:239	ENSG00000260857	lncRNA	chr16:35504370-355
ENSG0000(	390	8.831108	chr16:239	ENSG00000260308	Pseudoger	chr16:33771035-337
ENSG0000(	390	8.831108	chr16:239	ENSG00000261398	lncRNA	chr16:35195779-351
ENSG0000(	390	8.831108	chr16:239	ENSG00000259990	Pseudoger	chr16:34013394-340
ENSG0000(	390	8.831108	chr16:239	VN1R69P	Pseudoger	chr16:35134514-351
ENSG0000(	390	8.831108	chr16:239	IGHV3OR16-17	protein_c	chr16:33844784-338
ENSG0000(	390	8.831108	chr16:239	ENSG00000262885	Pseudoger	chr16:35023339-350
ENSG0000(	390	8.831108	chr16:239	ENSG00000261466	lncRNA	chr16:33548297-335
ENSG0000(	390	8.831108	chr16:239	KIF18BP1	Pseudoger	chr16:35636687-356
ENSG0000(	390	8.831108	chr16:239	C1QL1P1	Pseudoger	chr16:35574834-355
ENSG0000(	390	8.831108	chr16:239	ZNF971P	Pseudoger	chr16:35446898-354
ENSG0000(	390	8.831108	chr16:239	ENSG00000291264	lncRNA	chr16:33688222-337
ENSG0000(	390	8.831108	chr16:239	UBE2MP1	Pseudoger	chr16:35169692-351
ENSG0000(	390	8.831108	chr16:239	AGGF1P4	Pseudoger	chr16:35389203-353
ENSG0000(	390	8.831108	chr16:239	FRG2JP	Pseudoger	chr16:35245916-352
ENSG0000(	390	8.831108	chr16:239	ENSG00000280180	TEC	chr16:35148636-351
ENSG0000(	390	8.831108	chr16:239	AC116553.1	smallRNA	chr16:35901973-359
ENSG0000(	390	8.831108	chr16:239	C2orf69P1	Pseudoger	chr16:35552909-355
ENSG0000(	390	8.831108	chr16:239	RNA5SP420	Pseudoger	chr16:35754046-357
ENSG0000(	390	8.831108	chr16:239	RNA5SP419	Pseudoger	chr16:35753796-357
ENSG0000(	390	8.831108	chr16:239	FRG2DP	Pseudoger	chr16:35478617-354
ENSG0000(	390	8.831108	chr16:239	RARRES2P8	Pseudoger	chr16:35543279-355
ENSG0000(	390	8.831108	chr16:239	ENSG00000290420	lncRNA	chr16:33562649-335
ENSG0000(	390	8.831108	chr16:239	C2orf69P2	Pseudoger	chr16:35492324-354
ENSG0000(	390	8.831108	chr16:239	ENSG00000260312	lncRNA	chr16:33857935-338
ENSG0000(	390	8.831108	chr16:239	RNA5SP408	Pseudoger	chr16:35735372-357
ENSG0000(	390	8.831108	chr16:239	RNA5SP416	Pseudoger	chr16:35751574-357
ENSG0000(	390	8.831108	chr16:239	LINC00273	lncRNA	chr16:34158585-341
ENSG0000(	390	8.831108	chr16:239	TP53TG3HP	Pseudoger	chr16:35506592-355
ENSG0000(	390	8.831108	chr16:239	RNA5SP412	Pseudoger	chr16:35746772-357
ENSG0000(	390	8.831108	chr16:239	ENSG00000290414	lncRNA	chr16:33687025-336
ENSG0000(	390	8.831108	chr16:239	CCNYL3	Pseudoger	chr16:35040164-350
ENSG0000(	390	8.831108	chr16:239	ENSG00000261782	Pseudoger	chr16:35879036-358
ENSG0000(	390	8.831108	chr16:239	ENSG00000260073	lncRNA	chr16:35192687-351
ENSG0000(	390	8.831108	chr16:239	RNA5SP423	Pseudoger	chr16:35755177-357
ENSG0000(	390	8.831108	chr16:239	DUX4L47	Pseudoger	chr16:34142456-341
ENSG0000(	390	8.831108	chr16:239	AC007353.1	smallRNA	chr16:35276492-352
ENSG0000(	390	8.831108	chr16:239	RP11-244B22.14	Pseudoger	chr16:35140898-351
ENSG0000(	390	8.831108	chr16:239	ENSG00000261800	lncRNA	chr16:35143722-351
ENSG0000(	390	8.831108	chr16:239	ENSG00000284209	protein_c	chr16:35030719-350
ENSG0000(	390	8.831108	chr16:239	CTD-2144E22.7	Pseudoger	chr16:34979375-349
ENSG0000(	390	8.831108	chr16:239	RNA5SP417	Pseudoger	chr16:35751828-357
ENSG0000(	390	8.831108	chr16:239	ENSG00000279165	TEC	chr16:34941977-349
ENSG0000(	390	8.831108	chr16:239	RNA5SP413	Pseudoger	chr16:35747032-357

ENSG0000(	390	8.831108	chr16:239	RP11-244B22.12	Pseudoger	chr16:35143985-351
ENSG0000(	390	8.831108	chr16:239	RNA5-8SP2	Pseudoger	chr16:34162959-341
ENSG0000(	390	8.831108	chr16:239	C2orf69P4	Pseudoger	chr16:35352482-353
ENSG0000(	390	8.831108	chr16:239	IGHV3OR16-13	protein_c	chr16:33827214-338
ENSG0000(	390	8.831108	chr16:239	RNA5SP415	Pseudoger	chr16:35750061-357
ENSG0000(	390	8.831108	chr16:239	C2orf69P3	Pseudoger	chr16:35425364-354
ENSG0000(	390	8.831108	chr16:239	IGHV3OR16-16	Pseudoger	chr16:33949976-339
ENSG0000(	390	8.831108	chr16:239	RNA5SP407	Pseudoger	chr16:35734639-357
ENSG0000(	390	8.831108	chr16:239	ENPP7P13	Pseudoger	chr16:33769421-337
ENSG0000(	390	8.831108	chr16:239	ENSG00000261197	Pseudoger	chr16:34051140-340
ENSG0000(	390	8.831108	chr16:239	ENSG00000271691	Pseudoger	chr16:33851785-338
ENSG0000(	390	8.831108	chr16:239	ENSG00000283065	Pseudoger	chr16:34015260-340
ENSG0000(	390	8.831108	chr16:239	AGGF1P9	Pseudoger	chr16:35347385-353
ENSG0000(	390	8.831108	chr16:239	ENSG00000262090	Pseudoger	chr16:33495943-334
ENSG0000(	390	8.831108	chr16:239	RARRES2P6	Pseudoger	chr16:35255192-352
ENSG0000(	390	8.831108	chr16:239	RNA5SP421	Pseudoger	chr16:35754555-357
ENSG0000(	390	8.831108	chr16:239	ENSG00000287163	lncRNA	chr16:35450898-354
ENSG0000(	390	8.831108	chr16:239	ENSG00000287353	Pseudoger	chr16:34962970-349
ENSG0000(	390	8.831108	chr16:239	RARRES2P7	Pseudoger	chr16:35414279-354
ENSG0000(	390	8.831108	chr16:239	VPS35P1	Pseudoger	chr16:35090501-351
ENSG0000(	390	8.831108	chr16:239	ENSG00000259882	Pseudoger	chr16:33577502-336
ENSG0000(	390	8.831108	chr16:239	TP53TG3GP	Pseudoger	chr16:35207903-352
ENSG0000(	390	8.831108	chr16:239	AGGF1P7	Pseudoger	chr16:35547917-355
ENSG0000(	390	8.831108	chr16:239	AC023824.1	smallRNA	chr16:35208506-352
ENSG0000(	390	8.831108	chr16:239	FGFR3P5	Pseudoger	chr16:35232412-352
ENSG0000(	390	8.831108	chr16:239	RNA5SP410	Pseudoger	chr16:35745989-357
ENSG0000(	390	8.831108	chr16:239	IGHV3OR16-12	protein_c	chr16:33802764-338
ENSG0000(	390	8.831108	chr16:239	ENSG00000179755	lncRNA	chr16:35021749-350
ENSG0000(	390	8.831108	chr16:239	AGGF1P8	Pseudoger	chr16:35258630-352
ENSG0000(	390	8.831108	chr16:239	AGGF1P5	Pseudoger	chr16:35420291-354
ENSG0000(	390	8.831108	chr16:239	ARHGAP23P1	Pseudoger	chr16:33907419-339
ENSG0000(	390	8.831108	chr16:239	CLUHP11	Pseudoger	chr16:35068211-350
ENSG0000(	390	8.831108	chr16:239	IGHV3OR16-11	Pseudoger	chr16:33858896-338
ENSG0000(	390	8.831108	chr16:239	ENSG00000288300	lncRNA	chr16:35022001-350
ENSG0000(	390	8.831108	chr16:239	RNA5SP418	Pseudoger	chr16:35752826-357
ENSG0000(	390	8.831108	chr16:239	RARRES2P5	Pseudoger	chr16:35221583-352
ENSG0000(	390	8.831108	chr16:239	RNA5SP414	Pseudoger	chr16:35748520-357
ENSG0000(	390	8.831108	chr16:239	PCMTD1P2	Pseudoger	chr16:34139189-341
ENSG0000(	390	8.831108	chr16:239	ENSG00000279666	TEC	chr16:35903117-359
ENSG0000(	390	8.831108	chr16:239	AC136932.2	smallRNA	chr16:34143482-341
ENSG0000(	390	8.831108	chr16:239	ENSG00000261350	lncRNA	chr16:35491174-354
ENSG0000(	390	8.831108	chr16:239	ENSG00000260958	lncRNA	chr16:35207884-352
ENSG0000(	390	8.831108	chr16:239	ENSG00000290413	lncRNA	chr16:35477414-354
ENSG0000(	390	8.831108	chr16:239	ENSG00000286968	Pseudoger	chr16:34962970-349
ENSG0000(	390	8.831108	chr16:239	ENSG00000198555	Pseudoger	chr16:33976039-339
ENSG0000(	390	8.831108	chr16:239	RP11-244B22.13	Pseudoger	chr16:35149457-351
ENSG0000(	390	8.831108	chr16:239	ENSG00000260611	lncRNA	chr16:35785569-357
ENSG0000(	390	8.831108	chr16:239	ENSG00000205452	lncRNA	chr16:33964547-339
ENSG0000(	390	8.831108	chr16:239	LINC02184	lncRNA	chr16:34978744-349
ENSG0000(	390	8.831108	chr16:239	PPP1R1AP2	Pseudoger	chr16:35912120-359
ENSG0000(	390	8.831108	chr16:239	ENSG00000282924	Pseudoger	chr16:33723356-337

ENSG0000(	390	8.831108	chr16:239	ENSG00000270924		Pseudoger	chr16:33848476-338
ENSG0000(	390	8.831108	chr16:239	ENSG00000270401		Pseudoger	chr16:33965655-339
ENSG0000(	390	8.831108	chr16:239	RNA5SP422		Pseudoger	chr16:35754923-357
ENSG0000(	390	8.831108	chr6:9221	RNU6-464P		smallRNA	chr6:53153795-5315
ENSG0000(	390	8.831108	chr16:239	ENSG00000261153		Pseudoger	chr16:33808778-338
ENSG0000(	390	8.831108	chr16:239	FRG2HP		Pseudoger	chr16:35335277-353
ENSG0000(	390	8.831108	chr16:239	RARRES2P10		Pseudoger	chr16:35485766-354
ENSG0000(	390	8.831108	chr16:239	BCAP31P1		Pseudoger	chr16:33988406-339
ENSG0000(	390	8.831108	chr16:239	ENSG00000282946		Pseudoger	chr16:35528367-355
ENSG0000(	390	8.831108	chr16:239	VN1R70P		Pseudoger	chr16:35836886-358
ENSG0000(	390	8.831108	chr16:239	ENSG00000259791		lncRNA	chr16:35640029-356
ENSG0000(	390	8.831108	chr16:239	LINC01566		lncRNA	chr16:35363412-353
ENSG0000(	390	8.831108	chr16:239	ENSG00000261607		Pseudoger	chr16:33948278-339
ENSG0000(	390	8.831108	chr16:239	SLC25A1P4		Pseudoger	chr16:35173316-351
ENSG0000(	390	8.831108	chr16:239	ENSG00000261200		lncRNA	chr16:33541842-335
ENSG0000(	390	8.831108	chr16:239	LINC02167		lncRNA	chr16:35743268-357
ENSG0000(	390	8.831108	chr16:239	ENSG00000278681		Pseudoger	chr16:35435303-354
ENSG0000(	390	8.831108	chr11:103	ENSG00000271022		Pseudoger	chr11:121426645-12
ENSG0000(	390	8.831108	chr16:239	RARRES2P9		Pseudoger	chr16:35344091-353
ENSG0000(	390	8.831108	chr16:239	VN1R68P		Pseudoger	chr16:35118238-351
ENSG0000(	390	8.831108	chr16:239	AGGF1P6		Pseudoger	chr16:35489237-354
ENSG0000(	390	8.831108	chr16:239	AC135776.1		Pseudoger	chr16:34997735-349
ENSG0000(	390	8.831108	chr16:239	ENSG00000260626		lncRNA	chr16:33533816-335
ENSG0000(	390	8.831108	chr16:239	HMGN2P41		Pseudoger	chr16:35802869-358
ENSG0000(	390	8.831108	chr16:239	FRG2IP		Pseudoger	chr16:35405747-354
ENSG0000(	390	8.831108	chr16:239	IGHV3OR16-7		Pseudoger	chr16:33938337-339
ENSG0000(	390	8.831108	chr16:239	ENSG00000260590		Pseudoger	chr16:35193335-351
ENSG0000(	390	8.831108	chr16:239	FRG2GP		Pseudoger	chr16:35384144-353
ENSG0000(	390	8.831108	chr16:239	RNA5SP411		Pseudoger	chr16:35746523-357
ENSG0000(	389	8.808464	chr10:173	AC016821.1		smallRNA	chr10:69363555-693
ENSG0000(	389	8.808464	chr12:249	ENSG00000279334		TEC	chr12:124561869-12
ENSG0000(	387	8.763177	chr17:289	ENSG00000279431		TEC	chr17:30551193-305
ENSG0000(	386	8.740533	chr10:173	RPS3AP37		Pseudoger	chr10:68674356-686
ENSG0000(	386	8.740533	chr10:173	LINC02640		lncRNA	chr10:68233251-682
ENSG0000(	386	8.740533	chr10:173	CCAR1	NCGv7	protein_c	chr10:68721012-687
ENSG0000(	386	8.740533	chr10:173	RN7SKP202		smallRNA	chr10:68158079-681
ENSG0000(	386	8.740533	chr10:173	STOX1		protein_c	chr10:68827531-688
ENSG0000(	386	8.740533	chr10:173	SRGN		protein_c	chr10:69088103-691
ENSG0000(	386	8.740533	chr10:173	ENSG00000285871		lncRNA	chr10:68896920-689
ENSG0000(	386	8.740533	chr10:173	RN7SL373P		smallRNA	chr10:68966016-689
ENSG0000(	386	8.740533	chr10:173	SNORD98		smallRNA	chr10:68755172-687
ENSG0000(	386	8.740533	chr10:173	RPS12P17		Pseudoger	chr10:69161530-691
ENSG0000(	386	8.740533	chr10:173	RPL26P29		Pseudoger	chr10:68424560-684
ENSG0000(	386	8.740533	chr10:173	VPS26A		protein_c	chr10:69123512-691
ENSG0000(	386	8.740533	chr10:173	Y_RNA		smallRNA	chr10:68503152-685
ENSG0000(	386	8.740533	chr10:173	TET1	NCGv7	protein_c	chr10:68560337-686
ENSG0000(	386	8.740533	chr10:173	ACTBP14		Pseudoger	chr10:69022778-690
ENSG0000(	386	8.740533	chr10:173	TMEM14DP		Pseudoger	chr10:68544489-685
ENSG0000(	386	8.740533	chr10:173	ATOH7		protein_c	chr10:68230595-682
ENSG0000(	386	8.740533	chr10:173	RUFY2		protein_c	chr10:68341107-684
ENSG0000(	386	8.740533	chr1:2449	PLA2G4A		protein_c	chr1:186828949-186

ENSG0000(	386	8.740533	chr10:173	PBLD		protein_c	chr10:68282660-683
ENSG0000(	386	8.740533	chr10:173	SLC25A16		protein_c	chr10:68477998-685
ENSG0000(	386	8.740533	chr10:173	MIR1254-1		smallRNA	chr10:68759318-687
ENSG0000(	386	8.740533	chr10:173	SUPV3L1		protein_c	chr10:69180234-692
ENSG0000(	386	8.740533	chr10:173	RNU6-697P		smallRNA	chr10:68846805-688
ENSG0000(	386	8.740533	chr10:173	ENSG00000270494		Pseudoger	chr10:68717311-687
ENSG0000(	386	8.740533	chr10:173	HNRNPH3	NCGv7	protein_c	chr10:68331174-683
ENSG0000(	386	8.740533	chr10:173	snoU13		smallRNA	chr10:68712958-687
ENSG0000(	386	8.740533	chr10:173	RPL26P27		Pseudoger	chr10:68499532-684
ENSG0000(	386	8.740533	chr10:173	DNA2		protein_c	chr10:68414064-684
ENSG0000(	386	8.740533	chr10:173	RNU6-571P		smallRNA	chr10:68911653-689
ENSG0000(	386	8.740533	chr10:173	MYPN		protein_c	chr10:68087897-682
ENSG0000(	386	8.740533	chr10:173	DDX50	NCGv7	protein_c	chr10:68901286-689
ENSG0000(	386	8.740533	chr10:173	ENSG00000229261		lncRNA	chr10:69215333-692
ENSG0000(	386	8.740533	chr10:173	KRT19P4		Pseudoger	chr10:68260557-682
ENSG0000(	386	8.740533	chr10:173	RNA5SP319		Pseudoger	chr10:68461407-684
ENSG0000(	386	8.740533	chr10:173	KIFBP		protein_c	chr10:68988803-690
ENSG0000(	386	8.740533	chr10:173	MED28P1		Pseudoger	chr10:68991137-689
ENSG0000(	386	8.740533	chr10:173	Y_RNA		smallRNA	chr10:68735720-687
ENSG0000(	386	8.740533	chr10:173	ENSG00000260400		lncRNA	chr10:68698500-687
ENSG0000(	386	8.740533	chr10:173	DDX21		protein_c	chr10:68956135-689
ENSG0000(	386	8.740533	chr10:173	COX20P1		Pseudoger	chr10:68632371-686
ENSG0000(	386	8.740533	chr1:2449	ENSG00000271558		Pseudoger	chr1:187506838-187
ENSG0000(	384	8.695245	chr7:2516	RN7SL35P		smallRNA	chr7:80318253-8031
ENSG0000(	383	8.672601	chr16:239	MIR4517		smallRNA	chr16:28958583-289
ENSG0000(	382	8.649957	chr7:2516	CDK14	NCGv7;AC	protein_c	chr7:90466424-9121
ENSG0000(	382	8.649957	chr7:2516	GPC2	DriverDB\	protein_c	chr7:100169606-100
ENSG0000(	382	8.649957	chr1:2449	LRRN2		protein_c	chr1:204617170-204
ENSG0000(	382	8.649957	chr7:2516	VGF		protein_c	chr7:101162509-101
ENSG0000(	382	8.649957	chr1:2449	AL357932.1		smallRNA	chr1:195126681-195
ENSG0000(	382	8.649957	chr7:2516	STEAP1		protein_c	chr7:90154456-9016
ENSG0000(	382	8.649957	chr1:2449	KCNT2	NCGv7	protein_c	chr1:196225779-196
ENSG0000(	382	8.649957	chr1:2449	TUBA5P		Pseudoger	chr1:202852991-202
ENSG0000(	382	8.649957	chr7:2516	ZNF394	DriverDB\	protein_c	chr7:99473877-9950
ENSG0000(	382	8.649957	chr7:2516	SEM1	DriverDB\	protein_c	chr7:96481626-9670
ENSG0000(	382	8.649957	chr7:2516	CYP3A137P		Pseudoger	chr7:99820018-9982
ENSG0000(	382	8.649957	chr7:2516	PMS2P1		Pseudoger	chr7:100328836-100
ENSG0000(	382	8.649957	chr7:2516	PPP1R35		protein_c	chr7:100435282-100
ENSG0000(	382	8.649957	chr7:2516	OR7E38P		Pseudoger	chr7:97966090-9796
ENSG0000(	382	8.649957	chr7:2516	BRI3	DriverDB\	protein_c	chr7:98252379-9831
ENSG0000(	382	8.649957	chr7:2516	SEMA3E	NCGv7	protein_c	chr7:83363238-8364
ENSG0000(	382	8.649957	chr7:2516	ENSG00000274272		lncRNA	chr7:100572232-100
ENSG0000(	382	8.649957	chr1:2449	KIF21B	NCGv7	protein_c	chr1:200969390-201
ENSG0000(	382	8.649957	chr7:2516	LAMTOR4		protein_c	chr7:100148912-100
ENSG0000(	382	8.649957	chr1:2449	NR5A2		protein_c	chr1:200027614-200
ENSG0000(	382	8.649957	chr7:2516	GIGYF1		protein_c	chr7:100679507-100
ENSG0000(	382	8.649957	chr1:2449	CYCSP4		Pseudoger	chr1:202369526-202
ENSG0000(	382	8.649957	chr1:2449	ENSG00000227417		Pseudoger	chr1:203805621-203
ENSG0000(	382	8.649957	chr7:2516	snoU13		smallRNA	chr7:87624157-8762
ENSG0000(	382	8.649957	chr7:2516	STAG3L5P		Pseudoger	chr7:100338197-100
ENSG0000(	382	8.649957	chr7:2516	GNG11		protein_c	chr7:93921735-9392

ENSG0000(	382	8.649957	chr7:251(	IFT22		protein_c	chr7:101310914-101
ENSG0000(	382	8.649957	chr7:251(	PVRIG2P		Pseudoger	chr7:100352360-100
ENSG0000(	382	8.649957	chr7:251(	ENSG00000289886		lncRNA	chr7:100569732-100
ENSG0000(	382	8.649957	chr7:251(	HEPACAM2	NCGv7	protein_c	chr7:93188534-9322
ENSG0000(	382	8.649957	chr7:251(	AKAP9	NCGv7;AC	protein_c	chr7:91940840-9211
ENSG0000(	382	8.649957	chr7:251(	COL26A1		protein_c	chr7:101362875-101
ENSG0000(	382	8.649957	chr7:251(	RPL7P60		Pseudoger	chr7:100139629-100
ENSG0000(	382	8.649957	chr7:251(	ADAM22	NCGv7	protein_c	chr7:87934143-8820
ENSG0000(	382	8.649957	chr1:2449	CFHR3		protein_c	chr1:196774813-196
ENSG0000(	382	8.649957	chr1:2449	GPR37L1		protein_c	chr1:202122886-202
ENSG0000(	382	8.649957	chr1:2449	AL161793.1		smallRNA	chr1:204653102-204
ENSG0000(	382	8.649957	chr7:251(	DTX2P1		Pseudoger	chr7:76978617-7700
ENSG0000(	382	8.649957	chr7:251(	ENSG00000214243		Pseudoger	chr7:76650401-7665
ENSG0000(	382	8.649957	chr7:251(	PEG10		protein_c	chr7:94656325-9466
ENSG0000(	382	8.649957	chr7:251(	TRIP6	AC	protein_c	chr7:100867387-100
ENSG0000(	382	8.649957	chr7:251(	ENSG00000278388		Pseudoger	chr7:93914987-9391
ENSG0000(	382	8.649957	chr7:251(	ENSG00000288889		lncRNA	chr7:98616014-9861
ENSG0000(	382	8.649957	chr1:2449	ENSG00000224818		lncRNA	chr1:201464383-201
ENSG0000(	382	8.649957	chr7:251(	HINT1P2		Pseudoger	chr7:95018163-9501
ENSG0000(	382	8.649957	chr1:2449	ZC3H11A	NCGv7	protein_c	chr1:203795623-203
ENSG0000(	382	8.649957	chr7:251(	LINC01007		lncRNA	chr7:101562779-101
ENSG0000(	382	8.649957	chr7:251(	SPDYE3		protein_c	chr7:100307702-100
ENSG0000(	382	8.649957	chr1:2449	ENSG00000285986		Pseudoger	chr1:196850283-196
ENSG0000(	382	8.649957	chr7:251(	MBLAC1		protein_c	chr7:100126785-100
ENSG0000(	382	8.649957	chr7:251(	AZGP1P1		Pseudoger	chr7:99980762-9998
ENSG0000(	382	8.649957	chr7:251(	ENSG00000233683		Pseudoger	chr7:101388868-101
ENSG0000(	382	8.649957	chr7:251(	ENSG00000289691		lncRNA	chr7:100397383-100
ENSG0000(	382	8.649957	chr7:251(	TRAPPC14		protein_c	chr7:100154420-100
ENSG0000(	382	8.649957	chr7:251(	ENSG00000273299		lncRNA	chr7:90403434-9051
ENSG0000(	382	8.649957	chr1:2449	MIR4735		smallRNA	chr1:196582413-196
ENSG0000(	382	8.649957	chr7:251(	DPY19L2P4		Pseudoger	chr7:90119539-9012
ENSG0000(	382	8.649957	chr1:2449	ENSG00000282221		lncRNA	chr1:201399633-201
ENSG0000(	382	8.649957	chr7:251(	ENSG00000230617		Pseudoger	chr7:83424880-8342
ENSG0000(	382	8.649957	chr7:251(	ABCB1	NCGv7	protein_c	chr7:87503017-8771
ENSG0000(	382	8.649957	chr7:251(	ENSG00000289760		protein_c	chr7:100478099-100
ENSG0000(	382	8.649957	chr1:2449	RPL35AP5		Pseudoger	chr1:203835585-203
ENSG0000(	382	8.649957	chr1:2449	ENSG00000235449		Pseudoger	chr1:202767229-202
ENSG0000(	382	8.649957	chr7:251(	PPP1R35-AS1		lncRNA	chr7:100436204-100
ENSG0000(	382	8.649957	chr7:251(	ENSG00000235450		lncRNA	chr7:91380778-9155
ENSG0000(	382	8.649957	chr1:2449	ENSG00000230623		lncRNA	chr1:200333193-200
ENSG0000(	382	8.649957	chr7:251(	ATP5PBP2		Pseudoger	chr7:94738652-9473
ENSG0000(	382	8.649957	chr7:251(	KRIT1		protein_c	chr7:92197498-9224
ENSG0000(	382	8.649957	chr7:251(	NDUFAF4P2		Pseudoger	chr7:93844789-9384
ENSG0000(	382	8.649957	chr1:2449	Y_RNA		smallRNA	chr1:202914880-202
ENSG0000(	382	8.649957	chr1:2449	TMCC2-AS1		lncRNA	chr1:205233821-205
ENSG0000(	382	8.649957	chr1:2449	CCNQP1		Pseudoger	chr1:200213678-200
ENSG0000(	382	8.649957	chr1:2449	ENSG00000225172		lncRNA	chr1:198973379-198
ENSG0000(	382	8.649957	chr7:251(	PILRB		protein_c	chr7:100352176-100
ENSG0000(	382	8.649957	chr7:251(	ENSG00000233420		lncRNA	chr7:88420167-8875
ENSG0000(	382	8.649957	chr1:2449	ENSG00000288934		lncRNA	chr1:204411365-204
ENSG0000(	382	8.649957	chr1:2449	SNRPGP10		Pseudoger	chr1:205351247-205

ENSG0000(	382	8.649957	chr7:251(	ENSG00000234459		lncRNA	chr7:90266034-9027
ENSG0000(	382	8.649957	chr7:251(	PCOLCE-AS1		lncRNA	chr7:100589402-100
ENSG0000(	382	8.649957	chr7:251(	AZGP1P1		lncRNA	chr7:99980741-9998
ENSG0000(	382	8.649957	chr7:251(	ENSG00000286305		lncRNA	chr7:98989867-9899
ENSG0000(	382	8.649957	chr7:251(	ENSG00000289690		protein_c	chr7:100397577-100
ENSG0000(	382	8.649957	chr7:251(	OR2AE1		protein_c	chr7:99876062-9987
ENSG0000(	382	8.649957	chr1:244(	ENSG00000213045		Pseudoger	chr1:200329161-200
ENSG0000(	382	8.649957	chr1:244(	SNORD112		smallRNA	chr1:204904747-204
ENSG0000(	382	8.649957	chr7:251(	ENSG00000286411		lncRNA	chr7:91030149-9103
ENSG0000(	382	8.649957	chr7:251(	COL1A2	NCGv7	protein_c	chr7:94394895-9443
ENSG0000(	382	8.649957	chr1:244(	ENSG00000234132		lncRNA	chr1:201031136-201
ENSG0000(	382	8.649957	chr7:251(	SLC12A9		protein_c	chr7:100826820-100
ENSG0000(	382	8.649957	chr7:251(	AZGP1P2		Pseudoger	chr7:101287482-101
ENSG0000(	382	8.649957	chr1:244(	ATP2B4		protein_c	chr1:203626832-203
ENSG0000(	382	8.649957	chr7:251(	FIS1		protein_c	chr7:101239458-101
ENSG0000(	382	8.649957	chr7:251(	CPSF4		protein_c	chr7:99438922-9945
ENSG0000(	382	8.649957	chr7:251(	Y_RNA		smallRNA	chr7:76523605-7652
ENSG0000(	382	8.649957	chr7:251(	SRRT	NCGv7	protein_c	chr7:100875103-100
ENSG0000(	382	8.649957	chr1:244(	LMOD1		protein_c	chr1:201896456-201
ENSG0000(	382	8.649957	chr7:251(	LMTK2	DriverDB\	protein_c	chr7:98106862-9820
ENSG0000(	382	8.649957	chr7:251(	ACHE	DriverDB\	protein_c	chr7:100889994-100
ENSG0000(	382	8.649957	chr1:244(	ENSG00000240219		lncRNA	chr1:204626775-204
ENSG0000(	382	8.649957	chr7:251(	ELAPOR2		protein_c	chr7:86876906-8705
ENSG0000(	382	8.649957	chr1:244(	ELF3	NCGv7	protein_c	chr1:202007945-202
ENSG0000(	382	8.649957	chr7:251(	RPS3AP26		Pseudoger	chr7:98385801-9838
ENSG0000(	382	8.649957	chr1:244(	ERLNC1		lncRNA	chr1:204141404-204
ENSG0000(	382	8.649957	chr1:244(	ENSG00000213041		Pseudoger	chr1:205202191-205
ENSG0000(	382	8.649957	chr1:244(	KISS1		protein_c	chr1:204190341-204
ENSG0000(	382	8.649957	chr1:244(	ACTG1P25		Pseudoger	chr1:202861754-202
ENSG0000(	382	8.649957	chr1:244(	CRIP1P3		Pseudoger	chr1:202096759-202
ENSG0000(	382	8.649957	chr7:251(	KPNA2P2		Pseudoger	chr7:88564793-8856
ENSG0000(	382	8.649957	chr7:251(	ENSG00000286921		lncRNA	chr7:99974976-9997
ENSG0000(	382	8.649957	chr7:251(	SEMA3D		protein_c	chr7:84995553-8518
ENSG0000(	382	8.649957	chr1:244(	ENSG00000286572		lncRNA	chr1:204064533-204
ENSG0000(	382	8.649957	chr1:244(	ENSG00000261065		lncRNA	chr1:204131062-204
ENSG0000(	382	8.649957	chr7:251(	LINC03017		lncRNA	chr7:84532476-8458
ENSG0000(	382	8.649957	chr7:251(	RNU7-188P		smallRNA	chr7:96377857-9637
ENSG0000(	382	8.649957	chr1:244(	EEF1A1P32		Pseudoger	chr1:197688760-197
ENSG0000(	382	8.649957	chr7:251(	CASD1		protein_c	chr7:94509219-9455
ENSG0000(	382	8.649957	chr6:197(	RPL7P28		Pseudoger	chr6:109327175-109
ENSG0000(	382	8.649957	chr1:244(	RPL34P6		Pseudoger	chr1:200863808-200
ENSG0000(	382	8.649957	chr1:244(	ENSG00000234775		lncRNA	chr1:203115468-203
ENSG0000(	382	8.649957	chr1:244(	ZNF281		protein_c	chr1:200404940-200
ENSG0000(	382	8.649957	chr1:244(	ENSG00000273844		Pseudoger	chr1:196970495-196
ENSG0000(	382	8.649957	chr7:251(	GRPEL2P3		Pseudoger	chr7:94782886-9478
ENSG0000(	382	8.649957	chr7:251(	ENSG00000261462		lncRNA	chr7:87109539-8711
ENSG0000(	382	8.649957	chr7:251(	TRIM4		protein_c	chr7:99876958-9991
ENSG0000(	382	8.649957	chr7:251(	ENSG00000235077		lncRNA	chr7:100130964-100
ENSG0000(	382	8.649957	chr7:251(	ENSG00000284627		Pseudoger	chr7:97928082-9792
ENSG0000(	382	8.649957	chr1:244(	ENSG00000286383		lncRNA	chr1:203996532-204
ENSG0000(	382	8.649957	chr1:244(	ENSG00000229652		Pseudoger	chr1:203353365-203

ENSG0000(	382	8.649957	chr7:251(	HNRNPA1P8		Pseudoger	chr7:84983556-8498
ENSG0000(	382	8.649957	chr1:244(	LAX1		protein_c	chr1:203765177-203
ENSG0000(	382	8.649957	chr1:244(	MYOG		protein_c	chr1:203083129-203
ENSG0000(	382	8.649957	chr7:251(	ENSG00000278683		Pseudoger	chr7:101273322-101
ENSG0000(	382	8.649957	chr7:251(	TRIM56		protein_c	chr7:101085481-101
ENSG0000(	382	8.649957	chr7:251(	ENSG00000289836		lncRNA	chr7:90244783-9024
ENSG0000(	382	8.649957	chr1:244(	FMOD		protein_c	chr1:203340628-203
ENSG0000(	382	8.649957	chr7:251(	POMZP3		protein_c	chr7:76609986-7662
ENSG0000(	382	8.649957	chr7:251(	LINC02932		lncRNA	chr7:91311368-9151
ENSG0000(	382	8.649957	chr1:244(	CAMSAP2		protein_c	chr1:200738893-200
ENSG0000(	382	8.649957	chr7:251(	RBM48		protein_c	chr7:92528773-9254
ENSG0000(	382	8.649957	chr7:251(	ATP5MF-PTCD1		protein_c	chr7:99419749-9946
ENSG0000(	382	8.649957	chr1:244(	ENSG00000224671		lncRNA	chr1:203144694-203
ENSG0000(	382	8.649957	chr7:251(	CYP51A1	DriverDB\	protein_c	chr7:92084987-9213
ENSG0000(	382	8.649957	chr1:244(	LINC01222		lncRNA	chr1:199006040-199
ENSG0000(	382	8.649957	chr1:244(	ENSG00000227048		Pseudoger	chr1:201428975-201
ENSG0000(	382	8.649957	chr7:251(	AC092022.1		smallRNA	chr7:84665924-8466
ENSG0000(	382	8.649957	chr7:251(	PEX1		protein_c	chr7:92487020-9252
ENSG0000(	382	8.649957	chr7:251(	ANKIB1	DriverDB\	protein_c	chr7:92245974-9240
ENSG0000(	382	8.649957	chr7:251(	DYNLL1P7		Pseudoger	chr7:85381118-8538
ENSG0000(	382	8.649957	chr1:244(	ENSG00000224901		lncRNA	chr1:197757319-197
ENSG0000(	382	8.649957	chr7:251(	ENSG00000278819		Pseudoger	chr7:92540268-9255
ENSG0000(	382	8.649957	chr7:251(	EPHB4	NCGv7	protein_c	chr7:100802565-100
ENSG0000(	382	8.649957	chr7:251(	MTERF1		protein_c	chr7:91692008-9188
ENSG0000(	382	8.649957	chr7:251(	MEPCE		protein_c	chr7:100428322-100
ENSG0000(	382	8.649957	chr7:251(	MUC12-AS1		lncRNA	chr7:101014319-101
ENSG0000(	382	8.649957	chr1:244(	RABIF		protein_c	chr1:202878282-202
ENSG0000(	382	8.649957	chr7:251(	AC005071.1		smallRNA	chr7:100220027-100
ENSG0000(	382	8.649957	chr7:251(	LRCH4		protein_c	chr7:100574011-100
ENSG0000(	382	8.649957	chr7:251(	ZAN	NCGv7	protein_c	chr7:100733595-100
ENSG0000(	382	8.649957	chr7:251(	ZNF655		protein_c	chr7:99558406-9957
ENSG0000(	382	8.649957	chr7:251(	AZGP1	NCGv7	protein_c	chr7:99966720-9997
ENSG0000(	382	8.649957	chr7:251(	SGCE		protein_c	chr7:94524204-9465
ENSG0000(	382	8.649957	chr1:244(	ELF3-AS1		lncRNA	chr1:201995696-202
ENSG0000(	382	8.649957	chr7:251(	STEAP4		protein_c	chr7:88270892-8830
ENSG0000(	382	8.649957	chr7:251(	RN7SKP54		smallRNA	chr7:101058299-101
ENSG0000(	382	8.649957	chr7:251(	CASTOR3		lncRNA	chr7:100200653-100
ENSG0000(	382	8.649957	chr7:251(	ENSG00000286938		lncRNA	chr7:100482221-100
ENSG0000(	382	8.649957	chr1:244(	snoU13		smallRNA	chr1:202197628-202
ENSG0000(	382	8.649957	chr7:251(	ENSG00000223969		lncRNA	chr7:90590619-9059
ENSG0000(	382	8.649957	chr1:244(	RNU6-570P		smallRNA	chr1:200054061-200
ENSG0000(	382	8.649957	chr1:244(	ENSG00000235121		lncRNA	chr1:201723294-201
ENSG0000(	382	8.649957	chr1:244(	GPR25		protein_c	chr1:200872981-200
ENSG0000(	382	8.649957	chr7:251(	ENSG00000286923		lncRNA	chr7:99997690-1000
ENSG0000(	382	8.649957	chr1:244(	ENSG00000230260		lncRNA	chr1:197437976-197
ENSG0000(	382	8.649957	chr1:244(	DDX59		protein_c	chr1:200623896-200
ENSG0000(	382	8.649957	chr1:244(	TNNT2		protein_c	chr1:201359008-201
ENSG0000(	382	8.649957	chr7:251(	UPK3BP1		Pseudoger	chr7:77004662-7700
ENSG0000(	382	8.649957	chr7:251(	FAM133B		protein_c	chr7:92560758-9259
ENSG0000(	382	8.649957	chr7:251(	TFPI2-DT		lncRNA	chr7:93890913-9389
ENSG0000(	382	8.649957	chr1:244(	RBBP5		protein_c	chr1:205086142-205

ENSG0000(	382	8.649957	chr7:251(	RNU4-16P		smallRNA	chr7:95098227-9509
ENSG0000(	382	8.649957	chr1:244(	KLHL12		protein_c	chr1:202891116-202
ENSG0000(	382	8.649957	chr7:251(	RN7SL549P		smallRNA	chr7:100906299-100
ENSG0000(	382	8.649957	chr7:251(	STAG3L5P		lncRNA	chr7:100336079-100
ENSG0000(	382	8.649957	chr7:251(	ENSG00000250778		Pseudoger	chr7:76521611-7652
ENSG0000(	382	8.649957	chr1:244(	BTG2-DT		lncRNA	chr1:203298758-203
ENSG0000(	382	8.649957	chr1:244(	IGFN1	NCGv7	protein_c	chr1:201190824-201
ENSG0000(	382	8.649957	chr7:251(	TEX47		protein_c	chr7:88794106-8879
ENSG0000(	382	8.649957	chr1:244(	CFH		protein_c	chr1:196651754-196
ENSG0000(	382	8.649957	chr7:251(	RAD23BP2		Pseudoger	chr7:83663419-8366
ENSG0000(	382	8.649957	chr1:244(	KDM5B	AC	protein_c	chr1:202724495-202
ENSG0000(	382	8.649957	chr1:244(	RNU6-704P		smallRNA	chr1:200933345-200
ENSG0000(	382	8.649957	chr1:244(	RNA5SP75		Pseudoger	chr1:204707320-204
ENSG0000(	382	8.649957	chr7:251(	GNGT1		protein_c	chr7:93591573-9391
ENSG0000(	382	8.649957	chr7:251(	ENSG00000273407		lncRNA	chr7:99766543-9976
ENSG0000(	382	8.649957	chr7:251(	ENSG00000284292		protein_c	chr7:99325879-9939
ENSG0000(	382	8.649957	chr1:244(	ENSG00000286541		lncRNA	chr1:199040501-199
ENSG0000(	382	8.649957	chr1:244(	ENSG00000261573		lncRNA	chr1:198657553-198
ENSG0000(	382	8.649957	chr7:251(	CYP3A4		protein_c	chr7:99756960-9978
ENSG0000(	382	8.649957	chr7:251(	TVP23CP1		Pseudoger	chr7:90631906-9063
ENSG0000(	382	8.649957	chr1:244(	ENSG00000286600		lncRNA	chr1:201359018-201
ENSG0000(	382	8.649957	chr1:244(	ENSG00000229220		Pseudoger	chr1:200147531-200
ENSG0000(	382	8.649957	chr7:251(	ENSG00000233942		lncRNA	chr7:95471835-9547
ENSG0000(	382	8.649957	chr7:251(	PVRIG	NCGv7	protein_c	chr7:100218241-100
ENSG0000(	382	8.649957	chr1:244(	ENSG00000288644		protein_c	chr1:203802094-203
ENSG0000(	382	8.649957	chr1:244(	TMEM9		protein_c	chr1:201134772-201
ENSG0000(	382	8.649957	chr1:244(	DENND1B		protein_c	chr1:197504748-197
ENSG0000(	382	8.649957	chr7:251(	ENSG00000231255		lncRNA	chr7:86775081-8677
ENSG0000(	382	8.649957	chr7:251(	ENSG00000286742		lncRNA	chr7:92647906-9266
ENSG0000(	382	8.649957	chr1:244(	ENSG00000289697		protein_c	chr1:196651852-196
ENSG0000(	382	8.649957	chr1:244(	CFHR1		protein_c	chr1:196819731-196
ENSG0000(	382	8.649957	chr1:244(	SHISA4	NCGv7	protein_c	chr1:201888680-201
ENSG0000(	382	8.649957	chr7:251(	MUC17	NCGv7	protein_c	chr7:101020072-101
ENSG0000(	382	8.649957	chr7:251(	PILRA		protein_c	chr7:100367530-100
ENSG0000(	382	8.649957	chr7:251(	CYP3A52P		Pseudoger	chr7:99872168-9987
ENSG0000(	382	8.649957	chr1:244(	SNORA70		smallRNA	chr1:202527310-202
ENSG0000(	382	8.649957	chr1:244(	ENSG00000229191		lncRNA	chr1:201023949-201
ENSG0000(	382	8.649957	chr7:251(	snoU13		smallRNA	chr7:90607004-9060
ENSG0000(	382	8.649957	chr7:251(	CYP3A7		protein_c	chr7:99705036-9973
ENSG0000(	382	8.649957	chr7:251(	ENSG00000223665		lncRNA	chr7:91638847-9164
ENSG0000(	382	8.649957	chr1:244(	ENSG00000288925		lncRNA	chr1:203290128-203
ENSG0000(	382	8.649957	chr1:244(	INAVA		protein_c	chr1:200891048-200
ENSG0000(	382	8.649957	chr1:244(	ENSG00000229657		Pseudoger	chr1:204946608-204
ENSG0000(	382	8.649957	chr7:251(	ENSG00000288749		lncRNA	chr7:101302767-101
ENSG0000(	382	8.649957	chr7:251(	LINC00972		lncRNA	chr7:85421122-8548
ENSG0000(	382	8.649957	chr7:251(	ZCWPW1		protein_c	chr7:100400826-100
ENSG0000(	382	8.649957	chr7:251(	RPSAP46		Pseudoger	chr7:101203614-101
ENSG0000(	382	8.649957	chr7:251(	DPY19L2P4		lncRNA	chr7:90119358-9012
ENSG0000(	382	8.649957	chr1:244(	LINC01724		lncRNA	chr1:196044883-196
ENSG0000(	382	8.649957	chr7:251(	MIR3609		smallRNA	chr7:98881650-9888
ENSG0000(	382	8.649957	chr7:251(	TMEM130		protein_c	chr7:98846488-9887

ENSG00000	382	8.649957	chr1:2449	MROH3P		Pseudoger	chr1:200917460-200
ENSG00000	382	8.649957	chr7:2516	TRRAP	NCGv7;AC	protein_c	chr7:98877933-9905
ENSG00000	382	8.649957	chr1:2449	ENSG00000219133		Pseudoger	chr1:204346776-204
ENSG00000	382	8.649957	chr1:2449	KLHDC8A		protein_c	chr1:205336061-205
ENSG00000	382	8.649957	chr7:2516	ENSG00000284523		lncRNA	chr7:99252452-9932
ENSG00000	382	8.649957	chr7:2516	MIR5692A1		smallRNA	chr7:97963658-9796
ENSG00000	382	8.649957	chr7:2516	BHLHA15		protein_c	chr7:98211439-9821
ENSG00000	382	8.649957	chr7:2516	ENSG00000288976		lncRNA	chr7:100148341-100
ENSG00000	382	8.649957	chr7:2516	ENSG00000235243		Pseudoger	chr7:84847877-8484
ENSG00000	382	8.649957	chr1:2449	MGAT4FP		Pseudoger	chr1:202986557-202
ENSG00000	382	8.649957	chr1:2449	KIF14	AC	protein_c	chr1:200551497-200
ENSG00000	382	8.649957	chr1:2449	ENSG00000223881		lncRNA	chr1:198597724-198
ENSG00000	382	8.649957	chr1:2449	LINC01353		lncRNA	chr1:203273221-203
ENSG00000	382	8.649957	chr7:2516	COPS6		protein_c	chr7:100088969-100
ENSG00000	382	8.649957	chr7:2516	SAP25		protein_c	chr7:100572228-100
ENSG00000	382	8.649957	chr7:2516	TAC1	AC	protein_c	chr7:97732084-9774
ENSG00000	382	8.649957	chr7:2516	SPACDR		protein_c	chr7:100456620-100
ENSG00000	382	8.649957	chr7:2516	AC004745.1		smallRNA	chr7:97291514-9729
ENSG00000	382	8.649957	chr7:2516	ENSG00000241357		lncRNA	chr7:100435257-100
ENSG00000	382	8.649957	chr7:2516	ENSG00000230305		Pseudoger	chr7:76524515-7653
ENSG00000	382	8.649957	chr7:2516	AC006322.1		smallRNA	chr7:83919602-8391
ENSG00000	382	8.649957	chr1:2449	ENSG00000273093		lncRNA	chr1:200315435-200
ENSG00000	382	8.649957	chr7:2516	ENSG00000243107		lncRNA	chr7:92200014-9220
ENSG00000	382	8.649957	chr7:2516	Y_RNA		smallRNA	chr7:99936610-9993
ENSG00000	382	8.649957	chr7:2516	GRM3	NCGv7	protein_c	chr7:86643909-8686
ENSG00000	382	8.649957	chr1:2449	LINC00628		lncRNA	chr1:204368431-204
ENSG00000	382	8.649957	chr1:2449	ENSG00000236390		lncRNA	chr1:201673105-201
ENSG00000	382	8.649957	chr7:2516	HMGN2P11		Pseudoger	chr7:84876554-8487
ENSG00000	382	8.649957	chr7:2516	ENSG00000244055		lncRNA	chr7:92457564-9249
ENSG00000	382	8.649957	chr1:2449	PPP1R15B-AS1		lncRNA	chr1:204377850-204
ENSG00000	382	8.649957	chr7:2516	ENSG00000231183		lncRNA	chr7:76902480-7691
ENSG00000	382	8.649957	chr7:2516	ENSG00000270453		Pseudoger	chr7:92442077-9244
ENSG00000	382	8.649957	chr7:2516	ENSG00000225703		lncRNA	chr7:76972679-7697
ENSG00000	382	8.649957	chr7:2516	EIF4A1P13		Pseudoger	chr7:88448788-8844
ENSG00000	382	8.649957	chr7:2516	RNU6-532P		smallRNA	chr7:96330638-9633
ENSG00000	382	8.649957	chr7:2516	PON1	NCGv7	protein_c	chr7:95297676-9532
ENSG00000	382	8.649957	chr1:2449	SCARNA20		smallRNA	chr1:204727991-204
ENSG00000	382	8.649957	chr1:2449	MGAT4EP		Pseudoger	chr1:202820266-202
ENSG00000	382	8.649957	chr7:2516	CROT	AC	protein_c	chr7:87345664-8739
ENSG00000	382	8.649957	chr7:2516	MYH16		Pseudoger	chr7:99238829-9931
ENSG00000	382	8.649957	chr7:2516	ENSG00000228335		Pseudoger	chr7:99442890-9944
ENSG00000	382	8.649957	chr7:2516	ENSG00000242798		lncRNA	chr7:100115214-100
ENSG00000	382	8.649957	chr7:2516	ENSG00000272647		protein_c	chr7:99558695-9960
ENSG00000	382	8.649957	chr7:2516	MUC12	NCGv7	protein_c	chr7:100972000-101
ENSG00000	382	8.649957	chr7:2516	ENSG00000232097		Pseudoger	chr7:97938439-9793
ENSG00000	382	8.649957	chr7:2516	AC004980.1		smallRNA	chr7:76697502-7669
ENSG00000	382	8.649957	chr7:2516	ENSG00000226744		Pseudoger	chr7:97870167-9787
ENSG00000	382	8.649957	chr7:2516	ENSG00000236453		lncRNA	chr7:94022833-9406
ENSG00000	382	8.649957	chr7:2516	MIR5692C2		smallRNA	chr7:97964405-9796
ENSG00000	382	8.649957	chr7:2516	RNF14P3		Pseudoger	chr7:98998538-9899
ENSG00000	382	8.649957	chr7:2516	RNU6-364P		smallRNA	chr7:96341140-9634

ENSG0000(	382	8.649957	chr7:251(	CYP51A1-AS1		lncRNA	chr7:92134604-9218
ENSG0000(	382	8.649957	chr1:244(	ENSG00000236439		Pseudoger	chr1:202471864-202
ENSG0000(	382	8.649957	chr7:251(	ENSG00000285666		lncRNA	chr7:76818377-7690
ENSG0000(	382	8.649957	chr7:251(	AC005020.1		smallRNA	chr7:99586915-9958
ENSG0000(	382	8.649957	chr1:244(	KRT8P29		Pseudoger	chr1:203872574-203
ENSG0000(	382	8.649957	chr7:251(	RPL7P30		Pseudoger	chr7:84528122-8452
ENSG0000(	382	8.649957	chr7:251(	ENSG00000290101		lncRNA	chr7:95396497-9539
ENSG0000(	382	8.649957	chr7:251(	ABCB4		protein_c	chr7:87401696-8748
ENSG0000(	382	8.649957	chr1:244(	CFHR2	NCGv7	protein_c	chr1:196943738-196
ENSG0000(	382	8.649957	chr7:251(	AC006988.1		smallRNA	chr7:88640521-8864
ENSG0000(	382	8.649957	chr7:251(	ENSG00000227979		Pseudoger	chr7:88749470-8874
ENSG0000(	382	8.649957	chr1:244(	LARP7P1		Pseudoger	chr1:203400266-203
ENSG0000(	382	8.649957	chr1:244(	HNRNPA1P59		Pseudoger	chr1:202911812-202
ENSG0000(	382	8.649957	chr7:251(	SNORA67		smallRNA	chr7:88449092-8844
ENSG0000(	382	8.649957	chr1:244(	AC096645.1		smallRNA	chr1:203999245-203
ENSG0000(	382	8.649957	chr7:251(	FDPSP7		Pseudoger	chr7:76968197-7696
ENSG0000(	382	8.649957	chr7:251(	STAG3	NCGv7	protein_c	chr7:100177563-100
ENSG0000(	382	8.649957	chr7:251(	TECPR1		protein_c	chr7:98214624-9825
ENSG0000(	382	8.649957	chr7:251(	RNU6-1328P		smallRNA	chr7:94495299-9449
ENSG0000(	382	8.649957	chr7:251(	ENSG00000237551		Pseudoger	chr7:96283357-9628
ENSG0000(	382	8.649957	chr7:251(	STEAP2		protein_c	chr7:90167590-9023
ENSG0000(	382	8.649957	chr1:244(	ENSG00000225620		lncRNA	chr1:202632428-202
ENSG0000(	382	8.649957	chr7:251(	ENSG00000227863		lncRNA	chr7:89443946-8949
ENSG0000(	382	8.649957	chr1:244(	snoU13		smallRNA	chr1:202231664-202
ENSG0000(	382	8.649957	chr7:251(	ENSG00000289027		protein_c	chr7:92112176-9224
ENSG0000(	382	8.649957	chr1:244(	ENSG00000285718		lncRNA	chr1:194785517-194
ENSG0000(	382	8.649957	chr7:251(	ENSG00000285725		lncRNA	chr7:97966377-9797
ENSG0000(	382	8.649957	chr7:251(	TMEM225B		protein_c	chr7:99598267-9961
ENSG0000(	382	8.649957	chr1:244(	SLC25A39P1		Pseudoger	chr1:202796030-202
ENSG0000(	382	8.649957	chr7:251(	SAMD9		protein_c	chr7:93099513-9311
ENSG0000(	382	8.649957	chr7:251(	CASTOR3		Pseudoger	chr7:100222597-100
ENSG0000(	382	8.649957	chr7:251(	GATAD1	NCGv7	protein_c	chr7:92447482-9246
ENSG0000(	382	8.649957	chr1:244(	ASPM	NCGv7	protein_c	chr1:197084121-197
ENSG0000(	382	8.649957	chr1:244(	ENSG00000231984		Pseudoger	chr1:199752491-199
ENSG0000(	382	8.649957	chr7:251(	PPP1R9A-AS1		lncRNA	chr7:95035731-9521
ENSG0000(	382	8.649957	chr7:251(	ENSG00000237640		lncRNA	chr7:99929392-9994
ENSG0000(	382	8.649957	chr7:251(	ZNF789	DriverDBv	protein_c	chr7:99472890-9950
ENSG0000(	382	8.649957	chr7:251(	AC079781.1		smallRNA	chr7:97905972-9790
ENSG0000(	382	8.649957	chr7:251(	UFSP1	DriverDBv	protein_c	chr7:100888721-100
ENSG0000(	382	8.649957	chr7:251(	NAT16		protein_c	chr7:101170496-101
ENSG0000(	382	8.649957	chr1:244(	ENSG00000231691		lncRNA	chr1:204276901-204
ENSG0000(	382	8.649957	chr1:244(	ENSG00000249007		lncRNA	chr1:202011370-202
ENSG0000(	382	8.649957	chr7:251(	HNRNPA1P9		Pseudoger	chr7:87521461-8752
ENSG0000(	382	8.649957	chr7:251(	RNU6-1104P		smallRNA	chr7:101269938-101
ENSG0000(	382	8.649957	chr7:251(	VPS50	DriverDBv	protein_c	chr7:93232340-9336
ENSG0000(	382	8.649957	chr1:244(	PEBP1P3		Pseudoger	chr1:198679139-198
ENSG0000(	382	8.649957	chr7:251(	Y_RNA		smallRNA	chr7:100330777-100
ENSG0000(	382	8.649957	chr1:244(	SNORA77		smallRNA	chr1:203729581-203
ENSG0000(	382	8.649957	chr7:251(	ENSG00000232032		Pseudoger	chr7:97908256-9790
ENSG0000(	382	8.649957	chr1:244(	ENSG00000231547		Pseudoger	chr1:202999738-203
ENSG0000(	382	8.649957	chr7:251(	RNU6-274P		smallRNA	chr7:89754620-8975

ENSG0000(	382	8.649957	chr7:251(	PPP1R9A	NCGv7	protein_c	chr7:94907202-9529
ENSG0000(	382	8.649957	chr1:2449	ENSG00000260021		lncRNA	chr1:202810238-202
ENSG0000(	382	8.649957	chr7:251(	SNORA40		smallRNA	chr7:99952033-9995
ENSG0000(	382	8.649957	chr7:251(	DLX6		protein_c	chr7:97005553-9701
ENSG0000(	382	8.649957	chr7:251(	FZD1	NCGv7	protein_c	chr7:91264433-9127
ENSG0000(	382	8.649957	chr7:251(	DMTF1	DriverDB\	protein_c	chr7:87152409-8719
ENSG0000(	382	8.649957	chr7:251(	OCM2		protein_c	chr7:97984687-9799
ENSG0000(	382	8.649957	chr7:251(	AC092849.1		smallRNA	chr7:100483758-100
ENSG0000(	382	8.649957	chr7:251(	SLC12A9-AS1		lncRNA	chr7:100837314-100
ENSG0000(	382	8.649957	chr7:251(	CLDN12		protein_c	chr7:90383721-9051
ENSG0000(	382	8.649957	chr7:251(	BAIAP2L1	DriverDB\	protein_c	chr7:98291650-9840
ENSG0000(	382	8.649957	chr7:251(	DYNC1I1	NCGv7	protein_c	chr7:95772506-9611
ENSG0000(	382	8.649957	chr1:2449	PPP1R15B		protein_c	chr1:204396492-204
ENSG0000(	382	8.649957	chr1:2449	ENSG00000232626		Pseudoger	chr1:202438396-202
ENSG0000(	382	8.649957	chr7:251(	TMEM243	NCGv7	protein_c	chr7:87196160-8722
ENSG0000(	382	8.649957	chr7:251(	ZSCAN25		protein_c	chr7:99616946-9963
ENSG0000(	382	8.649957	chr1:2449	ZBTB41		protein_c	chr1:197153682-197
ENSG0000(	382	8.649957	chr7:251(	ENSG00000232019		lncRNA	chr7:84939349-8494
ENSG0000(	382	8.649957	chr1:2449	ENSG00000287364		lncRNA	chr1:195747024-195
ENSG0000(	382	8.649957	chr7:251(	MIR106B		smallRNA	chr7:100093993-100
ENSG0000(	382	8.649957	chr1:2449	Y_RNA		smallRNA	chr1:197685640-197
ENSG0000(	382	8.649957	chr7:251(	CNPY4		protein_c	chr7:100119634-100
ENSG0000(	382	8.649957	chr7:251(	ERVW-1		protein_c	chr7:92468380-9247
ENSG0000(	382	8.649957	chr1:2449	ENSG00000232296		Pseudoger	chr1:202028606-202
ENSG0000(	382	8.649957	chr1:2449	ENSG00000290127		lncRNA	chr1:197201486-197
ENSG0000(	382	8.649957	chr7:251(	LRRD1		protein_c	chr7:92141643-9217
ENSG0000(	382	8.649957	chr1:2449	ENSG00000228167		Pseudoger	chr1:194718795-194
ENSG0000(	382	8.649957	chr1:2449	TMEM81		protein_c	chr1:205083129-205
ENSG0000(	382	8.649957	chr1:2449	AL450244.1		smallRNA	chr1:199615188-199
ENSG0000(	382	8.649957	chr1:2449	ENSG00000290125		lncRNA	chr1:199932233-199
ENSG0000(	382	8.649957	chr7:251(	ENSG00000287631		lncRNA	chr7:100388809-100
ENSG0000(	382	8.649957	chr1:2449	GOLT1A	DriverDB\	protein_c	chr1:204198163-204
ENSG0000(	382	8.649957	chr7:251(	MIR489		smallRNA	chr7:93483936-9348
ENSG0000(	382	8.649957	chr1:2449	ENSG00000236779		Pseudoger	chr1:204528845-204
ENSG0000(	382	8.649957	chr7:251(	NIPA2P1		Pseudoger	chr7:91320128-9132
ENSG0000(	382	8.649957	chr1:2449	SNRPE		protein_c	chr1:203861599-203
ENSG0000(	382	8.649957	chr1:2449	AL583832.1		smallRNA	chr1:205062252-205
ENSG0000(	382	8.649957	chr7:251(	MIR4658		smallRNA	chr7:100156605-100
ENSG0000(	382	8.649957	chr7:251(	EMSLR		lncRNA	chr7:101308270-101
ENSG0000(	382	8.649957	chr1:2449	TMCC2		protein_c	chr1:205227946-205
ENSG0000(	382	8.649957	chr7:251(	ENSG00000285398		TEC	chr7:83372652-8337
ENSG0000(	382	8.649957	chr1:2449	ENSG00000228153		lncRNA	chr1:204663872-204
ENSG0000(	382	8.649957	chr1:2449	DDX59-AS1		lncRNA	chr1:200669507-200
ENSG0000(	382	8.649957	chr7:251(	ATP5MF		protein_c	chr7:99448475-9946
ENSG0000(	382	8.649957	chr7:251(	ENSG00000272950		lncRNA	chr7:98322853-9832
ENSG0000(	382	8.649957	chr7:251(	MIR93		smallRNA	chr7:100093768-100
ENSG0000(	382	8.649957	chr1:2449	ENSG00000223774		lncRNA	chr1:201893842-201
ENSG0000(	382	8.649957	chr7:251(	RN7SL478P		smallRNA	chr7:97998325-9799
ENSG0000(	382	8.649957	chr7:251(	ENSG00000279067		TEC	chr7:96118647-9611
ENSG0000(	382	8.649957	chr1:2449	EEF1A1P44		Pseudoger	chr1:199387141-199
ENSG0000(	382	8.649957	chr7:251(	MIR25		smallRNA	chr7:100093560-100

ENSG0000(	382	8.649957	chr1:244(	PHLDA3		protein_c	chr1:201464278-201
ENSG0000(	382	8.649957	chr7:251(	CYP3A51P		Pseudoger	chr7:99685145-9970
ENSG0000(	382	8.649957	chr7:251(	Y_RNA		smallRNA	chr7:90251923-9025
ENSG0000(	382	8.649957	chr7:251(	FAM237B		protein_c	chr7:90316503-9032
ENSG0000(	382	8.649957	chr1:244(	OPTC		protein_c	chr1:203494153-203
ENSG0000(	382	8.649957	chr1:244(	IPO9		protein_c	chr1:201829149-201
ENSG0000(	382	8.649957	chr7:251(	ENSG00000236938		lncRNA	chr7:94071759-9407
ENSG0000(	382	8.649957	chr1:244(	ENSG00000240710		lncRNA	chr1:204603035-204
ENSG0000(	382	8.649957	chr7:251(	ARPC1A	DriverDBv	protein_c	chr7:99325898-9936
ENSG0000(	382	8.649957	chr7:251(	TP53TG1		lncRNA	chr7:87322943-8734
ENSG0000(	382	8.649957	chr1:244(	RPL23AP16		Pseudoger	chr1:199371877-199
ENSG0000(	382	8.649957	chr7:251(	LINC03009		lncRNA	chr7:76549360-7662
ENSG0000(	382	8.649957	chr7:251(	OR7E7P		Pseudoger	chr7:97946987-9794
ENSG0000(	382	8.649957	chr7:251(	TMBIM7P		Pseudoger	chr7:92412550-9244
ENSG0000(	382	8.649957	chr7:251(	ENSG00000225898		Pseudoger	chr7:93777839-9377
ENSG0000(	382	8.649957	chr7:251(	PDK4		protein_c	chr7:95583499-9559
ENSG0000(	382	8.649957	chr1:244(	LHX9		protein_c	chr1:197911902-197
ENSG0000(	382	8.649957	chr7:251(	BET1-AS1		lncRNA	chr7:93969442-9401
ENSG0000(	382	8.649957	chr1:244(	ENSG00000237011		lncRNA	chr1:193684246-193
ENSG0000(	382	8.649957	chr7:251(	ENSG00000284707		lncRNA	chr7:97851688-9797
ENSG0000(	382	8.649957	chr1:244(	PRELP		protein_c	chr1:203475806-203
ENSG0000(	382	8.649957	chr7:251(	ASNS	DriverDBv	protein_c	chr7:97851677-9787
ENSG0000(	382	8.649957	chr7:251(	LNCPRESS1		lncRNA	chr7:101299558-101
ENSG0000(	382	8.649957	chr7:251(	RPS3AP25		Pseudoger	chr7:94695027-9469
ENSG0000(	382	8.649957	chr1:244(	MIR181A1		smallRNA	chr1:198859044-198
ENSG0000(	382	8.649957	chr7:251(	ZNF804B	NCGv7	protein_c	chr7:88759700-8933
ENSG0000(	382	8.649957	chr1:244(	LGR6		protein_c	chr1:202193799-202
ENSG0000(	382	8.649957	chr7:251(	snoU13		smallRNA	chr7:99413978-9941
ENSG0000(	382	8.649957	chr1:244(	RPL23AP22		Pseudoger	chr1:193756815-193
ENSG0000(	382	8.649957	chr7:251(	SMURF1	DriverDBv	protein_c	chr7:99027440-9914
ENSG0000(	382	8.649957	chr7:251(	ENSG00000228113		lncRNA	chr7:88219359-8830
ENSG0000(	382	8.649957	chr7:251(	ENSG00000287672		lncRNA	chr7:91880791-9188
ENSG0000(	382	8.649957	chr1:244(	CFHR5		protein_c	chr1:196975010-197
ENSG0000(	382	8.649957	chr1:244(	ENSG00000291234		lncRNA	chr1:202851828-202
ENSG0000(	382	8.649957	chr1:244(	RNU6-487P		smallRNA	chr1:203318996-203
ENSG0000(	382	8.649957	chr1:244(	ENSG00000271580		Pseudoger	chr1:205091163-205
ENSG0000(	382	8.649957	chr1:244(	MIR5191		smallRNA	chr1:201719508-201
ENSG0000(	382	8.649957	chr7:251(	RNU6-393P		smallRNA	chr7:98794718-9879
ENSG0000(	382	8.649957	chr1:244(	NPM1P40		Pseudoger	chr1:203255743-203
ENSG0000(	382	8.649957	chr1:244(	MIR181B1		smallRNA	chr1:198858873-198
ENSG0000(	382	8.649957	chr1:244(	CHIT1		protein_c	chr1:203212827-203
ENSG0000(	382	8.649957	chr7:251(	MIR1285-1		smallRNA	chr7:92204015-9220
ENSG0000(	382	8.649957	chr7:251(	AP1S2P1		Pseudoger	chr7:97437518-9743
ENSG0000(	382	8.649957	chr1:244(	RNU6-609P		smallRNA	chr1:200014689-200
ENSG0000(	382	8.649957	chr1:244(	RNU6-983P		smallRNA	chr1:194488103-194
ENSG0000(	382	8.649957	chr7:251(	AC004969.1		smallRNA	chr7:90171341-9017
ENSG0000(	382	8.649957	chr7:251(	ENSG00000285090		lncRNA	chr7:94278680-9439
ENSG0000(	382	8.649957	chr7:251(	MIR653		smallRNA	chr7:93482760-9348
ENSG0000(	382	8.649957	chr7:251(	DGAT2L7P		Pseudoger	chr7:101201809-101
ENSG0000(	382	8.649957	chr1:244(	CBX1P3		Pseudoger	chr1:203954640-203
ENSG0000(	382	8.649957	chr7:251(	MIR591		smallRNA	chr7:96219662-9621

ENSG0000(	382	8.649957	chr1:244(	MDM4	NCGv7;AC	protein_c	chr1:204516379-204
ENSG0000(	382	8.649957	chr7:251(	ENSG00000225807		lncRNA	chr7:100509416-100
ENSG0000(	382	8.649957	chr7:251(	ENSG00000280325		TEC	chr7:84939335-8494
ENSG0000(	382	8.649957	chr1:244(	RNU6-778P		smallRNA	chr1:199888159-199
ENSG0000(	382	8.649957	chr7:251(	RN7SL161P		smallRNA	chr7:100462829-100
ENSG0000(	382	8.649957	chr1:244(	DSTYK		protein_c	chr1:205142505-205
ENSG0000(	382	8.649957	chr7:251(	KPNA7		protein_c	chr7:99173572-9925
ENSG0000(	382	8.649957	chr1:244(	PIK3C2B	NCGv7	protein_c	chr1:204422628-204
ENSG0000(	382	8.649957	chr7:251(	ENSG00000237160		Pseudoger	chr7:95348718-9534
ENSG0000(	382	8.649957	chr1:244(	MYBPH		protein_c	chr1:203167811-203
ENSG0000(	382	8.649957	chr7:251(	ENSG00000270812		Pseudoger	chr7:88455309-8845
ENSG0000(	382	8.649957	chr7:251(	ASB4		protein_c	chr7:95478444-9554
ENSG0000(	382	8.649957	chr7:251(	SRI-AS1		lncRNA	chr7:88216660-8821
ENSG0000(	382	8.649957	chr1:244(	CFHR4		protein_c	chr1:196888014-196
ENSG0000(	382	8.649957	chr1:244(	CHI3L1		protein_c	chr1:203178931-203
ENSG0000(	382	8.649957	chr1:244(	ASCL5		protein_c	chr1:201113943-201
ENSG0000(	382	8.649957	chr1:244(	NAV1	NCGv7	protein_c	chr1:201539127-201
ENSG0000(	382	8.649957	chr7:251(	RN7SL750P		smallRNA	chr7:100821029-100
ENSG0000(	382	8.649957	chr7:251(	AC069294.1		smallRNA	chr7:99713037-9971
ENSG0000(	382	8.649957	chr1:244(	TIMM17A	NCGv7	protein_c	chr1:201955503-201
ENSG0000(	382	8.649957	chr1:244(	PCAT6		lncRNA	chr1:202810850-202
ENSG0000(	382	8.649957	chr7:251(	RN7SKP129		smallRNA	chr7:94801514-9480
ENSG0000(	382	8.649957	chr7:251(	DMTF1-AS1		lncRNA	chr7:87151419-8715
ENSG0000(	382	8.649957	chr7:251(	STAG3L5P-PVRIG2P-F		lncRNA	chr7:100336104-100
ENSG0000(	382	8.649957	chr7:251(	RN7SL252P		smallRNA	chr7:96940070-9694
ENSG0000(	382	8.649957	chr1:244(	CRB1	NCGv7	protein_c	chr1:197268204-197
ENSG0000(	382	8.649957	chr7:251(	SOCS5P1		Pseudoger	chr7:86216785-8621
ENSG0000(	382	8.649957	chr7:251(	RNA5SP235		Pseudoger	chr7:83017898-8301
ENSG0000(	382	8.649957	chr1:244(	ENSG00000228530		Pseudoger	chr1:199876978-199
ENSG0000(	382	8.649957	chr1:244(	MIR181A1HG		lncRNA	chr1:198777861-198
ENSG0000(	382	8.649957	chr7:251(	RNU6-956P		smallRNA	chr7:94712409-9471
ENSG0000(	382	8.649957	chr1:244(	ENSG00000237848		Pseudoger	chr1:204394541-204
ENSG0000(	382	8.649957	chr7:251(	PTTG1IP2		protein_c	chr7:90469639-9051
ENSG0000(	382	8.649957	chr7:251(	AGFG2	DriverDB\	protein_c	chr7:100539203-100
ENSG0000(	382	8.649957	chr1:244(	ENSG00000290909		lncRNA	chr1:202987277-202
ENSG0000(	382	8.649957	chr7:251(	PTP4A1P3		Pseudoger	chr7:91107374-9110
ENSG0000(	382	8.649957	chr1:244(	HSPE1P6		Pseudoger	chr1:203903723-203
ENSG0000(	382	8.649957	chr1:244(	SEPTIN14P12		Pseudoger	chr1:197138748-197
ENSG0000(	382	8.649957	chr1:244(	ENSG00000226640		lncRNA	chr1:193678894-193
ENSG0000(	382	8.649957	chr7:251(	FBXO24	DriverDB\	protein_c	chr7:100583982-100
ENSG0000(	382	8.649957	chr1:244(	ENSG00000237861		Pseudoger	chr1:197222222-197
ENSG0000(	382	8.649957	chr7:251(	PCOLCE		protein_c	chr7:100602363-100
ENSG0000(	382	8.649957	chr1:244(	PTPRC	NCGv7;AC	protein_c	chr1:198638457-198
ENSG0000(	382	8.649957	chr7:251(	MOSPD3		protein_c	chr7:100612102-100
ENSG0000(	382	8.649957	chr7:251(	ENSG00000223402		Pseudoger	chr7:98478551-9847
ENSG0000(	382	8.649957	chr7:251(	TFR2	DriverDB\	protein_c	chr7:100620416-100
ENSG0000(	382	8.649957	chr7:251(	PON3		protein_c	chr7:95359872-9539
ENSG0000(	382	8.649957	chr7:251(	ENSG00000235713		Pseudoger	chr7:99992397-9999
ENSG0000(	382	8.649957	chr1:244(	REN		protein_c	chr1:204154819-204
ENSG0000(	382	8.649957	chr7:251(	AC125387.1		smallRNA	chr7:101682015-101
ENSG0000(	382	8.649957	chr1:244(	CACNA1S		protein_c	chr1:201039512-201

ENSG0000(	382	8.649957	chr7:251(	RN7SL416P		smallRNA	chr7:100530364-100
ENSG0000(	382	8.649957	chr7:251(	ENSG00000235639		Pseudoger	chr7:88512029-8851
ENSG0000(	382	8.649957	chr1:244(	LINC00862		lncRNA	chr1:200253419-200
ENSG0000(	382	8.649957	chr7:251(	TAF6	DriverDB\	protein_c	chr7:100106876-100
ENSG0000(	382	8.649957	chr1:244(	BTG2	NCGv7	protein_c	chr1:203305491-203
ENSG0000(	382	8.649957	chr7:251(	BET1	DriverDB\	protein_c	chr7:93962762-9400
ENSG0000(	382	8.649957	chr7:251(	CCZ1P1		Pseudoger	chr7:97969005-9797
ENSG0000(	382	8.649957	chr7:251(	EPO		protein_c	chr7:100720468-100
ENSG0000(	382	8.649957	chr1:244(	ETNK2		protein_c	chr1:204131062-204
ENSG0000(	382	8.649957	chr7:251(	CDK6	NCGv7;AC	protein_c	chr7:92604921-9283
ENSG0000(	382	8.649957	chr7:251(	ENSG00000273138		lncRNA	chr7:95416108-9541
ENSG0000(	382	8.649957	chr7:251(	ENSG00000225498		lncRNA	chr7:90312496-9032
ENSG0000(	382	8.649957	chr7:251(	CYP3A43		protein_c	chr7:99828013-9986
ENSG0000(	382	8.649957	chr7:251(	ENSG00000275834		Pseudoger	chr7:96306810-9630
ENSG0000(	382	8.649957	chr7:251(	ZSCAN21		protein_c	chr7:100049774-100
ENSG0000(	382	8.649957	chr7:251(	MOGAT3	DriverDB\	protein_c	chr7:101195007-101
ENSG0000(	382	8.649957	chr7:251(	TFPI2		protein_c	chr7:93885396-9389
ENSG0000(	382	8.649957	chr7:251(	AP1S1	NCGv7	protein_c	chr7:101154456-101
ENSG0000(	382	8.649957	chr7:251(	SNRPCP9		Pseudoger	chr7:97885868-9788
ENSG0000(	382	8.649957	chr1:244(	ADIPOR1		protein_c	chr1:202940826-202
ENSG0000(	382	8.649957	chr7:251(	ENSG00000279525		TEC	chr7:95542145-9554
ENSG0000(	382	8.649957	chr7:251(	MIR4652		smallRNA	chr7:93716928-9371
ENSG0000(	382	8.649957	chr1:244(	CYB5R1		protein_c	chr1:202961873-202
ENSG0000(	382	8.649957	chr7:251(	SERPINE1		protein_c	chr7:101127104-101
ENSG0000(	382	8.649957	chr1:244(	SOX13		protein_c	chr1:204073115-204
ENSG0000(	382	8.649957	chr7:251(	ENSG00000291178		lncRNA	chr7:100300020-100
ENSG0000(	382	8.649957	chr1:244(	ENSG00000235811		Pseudoger	chr1:202039851-202
ENSG0000(	382	8.649957	chr7:251(	ZNF3	DriverDB\	protein_c	chr7:100064033-100
ENSG0000(	382	8.649957	chr7:251(	Y_RNA		smallRNA	chr7:87218611-8721
ENSG0000(	382	8.649957	chr7:251(	RPL7AP40		Pseudoger	chr7:97200708-9720
ENSG0000(	382	8.649957	chr7:251(	ARPC1B	NCGv7	protein_c	chr7:99374249-9939
ENSG0000(	382	8.649957	chr1:244(	PPP1R12B		protein_c	chr1:202348699-202
ENSG0000(	382	8.649957	chr1:244(	RNA5SP74		Pseudoger	chr1:204562413-204
ENSG0000(	382	8.649957	chr7:251(	CALCR	NCGv7	protein_c	chr7:93424486-9357
ENSG0000(	382	8.649957	chr1:244(	CSRP1-AS1		lncRNA	chr1:201507241-201
ENSG0000(	382	8.649957	chr7:251(	POP7		protein_c	chr7:100706121-100
ENSG0000(	382	8.649957	chr7:251(	SRI		protein_c	chr7:88205115-8822
ENSG0000(	382	8.649957	chr1:244(	ZBED6		protein_c	chr1:203795623-203
ENSG0000(	382	8.649957	chr1:244(	FAM204BP		Pseudoger	chr1:197746751-197
ENSG0000(	382	8.649957	chr7:251(	SEMA3A	NCGv7	protein_c	chr7:83955777-8449
ENSG0000(	382	8.649957	chr1:244(	U6		smallRNA	chr1:202410108-202
ENSG0000(	382	8.649957	chr1:244(	EEF1A1P14		Pseudoger	chr1:194188967-194
ENSG0000(	382	8.649957	chr1:244(	ENSG00000231714		lncRNA	chr1:194350943-194
ENSG0000(	382	8.649957	chr1:244(	U3		smallRNA	chr1:193731858-193
ENSG0000(	382	8.649957	chr7:251(	GRM3-AS1		lncRNA	chr7:86782357-8680
ENSG0000(	382	8.649957	chr1:244(	LINC02789		lncRNA	chr1:199148598-199
ENSG0000(	382	8.649957	chr7:251(	ENSG00000235503		lncRNA	chr7:83355154-8336
ENSG0000(	382	8.649957	chr7:251(	SLC25A13	DriverDB\	protein_c	chr7:96120220-9632
ENSG0000(	382	8.649957	chr7:251(	ENSG00000285953		protein_c	chr7:92144723-9224
ENSG0000(	382	8.649957	chr7:251(	RN7SL7P		smallRNA	chr7:92971004-9297
ENSG0000(	382	8.649957	chr1:244(	LINC01221		lncRNA	chr1:199016133-199

ENSG0000(	382	8.649957	chr7:251(	HMGB3P21		Pseudoger	chr7:97135015-9713
ENSG0000(	382	8.649957	chr7:251(	CYP3A7-CYP3A51P		protein_c	chr7:99684957-9973
ENSG0000(	382	8.649957	chr7:251(	RN7SKP104		smallRNA	chr7:97598933-9759
ENSG0000(	382	8.649957	chr1:244(	ENSG00000287989		lncRNA	chr1:198450666-198
ENSG0000(	382	8.649957	chr7:251(	ENSG00000285964		lncRNA	chr7:94311138-9434
ENSG0000(	382	8.649957	chr1:244(	TMEM183A		protein_c	chr1:203007374-203
ENSG0000(	382	8.649957	chr7:251(	snoU13		smallRNA	chr7:98884990-9888
ENSG0000(	382	8.649957	chr7:251(	SLC25A40		protein_c	chr7:87833568-8787
ENSG0000(	382	8.649957	chr7:251(	IRS3P		Pseudoger	chr7:100570131-100
ENSG0000(	382	8.649957	chr1:244(	PKP1		protein_c	chr1:201283452-201
ENSG0000(	382	8.649957	chr7:251(	ENSG00000228751		Pseudoger	chr7:95350164-9535
ENSG0000(	382	8.649957	chr7:251(	PON2		protein_c	chr7:95404862-9543
ENSG0000(	382	8.649957	chr7:251(	ZKSCAN1	DriverDB\	protein_c	chr7:100015572-100
ENSG0000(	382	8.649957	chr1:244(	ADORA1		protein_c	chr1:203090654-203
ENSG0000(	382	8.649957	chr7:251(	DLX6-AS1		lncRNA	chr7:96955141-9701
ENSG0000(	382	8.649957	chr7:251(	DLX5	AC	protein_c	chr7:97020396-9702
ENSG0000(	382	8.649957	chr1:244(	ENSG00000226862		lncRNA	chr1:202604268-202
ENSG0000(	382	8.649957	chr1:244(	ENSG00000235582		Pseudoger	chr1:197735636-197
ENSG0000(	382	8.649957	chr7:251(	SLC66A2P1		Pseudoger	chr7:88610241-8861
ENSG0000(	382	8.649957	chr7:251(	ENSG00000280440		TEC	chr7:90345873-9034
ENSG0000(	382	8.649957	chr7:251(	CYP3A5	DriverDB\	protein_c	chr7:99648194-9967
ENSG0000(	382	8.649957	chr7:251(	ENSG00000260445		Pseudoger	chr7:99869841-9986
ENSG0000(	382	8.649957	chr7:251(	PTCD1		protein_c	chr7:99416739-9946
ENSG0000(	382	8.649957	chr7:251(	BUD31	DriverDB\	protein_c	chr7:99408641-9941
ENSG0000(	382	8.649957	chr7:251(	ARF1P1		Pseudoger	chr7:94833904-9483
ENSG0000(	382	8.649957	chr7:251(	SAMD9L	NCGv7	protein_c	chr7:93130056-9314
ENSG0000(	382	8.649957	chr7:251(	PDAP1	NCGv7	protein_c	chr7:99392048-9940
ENSG0000(	382	8.649957	chr7:251(	NPTX2		protein_c	chr7:98617285-9862
ENSG0000(	382	8.649957	chr7:251(	PDK4-AS1		lncRNA	chr7:95545191-9561
ENSG0000(	382	8.649957	chr7:251(	GNB2		protein_c	chr7:100673567-100
ENSG0000(	382	8.649957	chr7:251(	ENSG00000231153		Pseudoger	chr7:95018407-9501
ENSG0000(	382	8.649957	chr1:244(	C1orf53		protein_c	chr1:197902630-197
ENSG0000(	382	8.649957	chr1:244(	RPL10P4		Pseudoger	chr1:201978642-201
ENSG0000(	382	8.649957	chr7:251(	MCM7	NCGv7	protein_c	chr7:100092728-100
ENSG0000(	382	8.649957	chr7:251(	Y_RNA		smallRNA	chr7:92202243-9220
ENSG0000(	382	8.649957	chr1:244(	RPS10P7		Pseudoger	chr1:201518703-201
ENSG0000(	382	8.649957	chr7:251(	CDK6-AS1		lncRNA	chr7:92836367-9291
ENSG0000(	382	8.649957	chr1:244(	CSRP1		protein_c	chr1:201483530-201
ENSG0000(	382	8.649957	chr7:251(	NYAP1	DriverDB\	protein_c	chr7:100483927-100
ENSG0000(	382	8.649957	chr1:244(	CNTN2	AC	protein_c	chr1:205042937-205
ENSG0000(	382	8.649957	chr1:244(	AC105941.1		smallRNA	chr1:199197080-199
ENSG0000(	382	8.649957	chr1:244(	NSA2P1		Pseudoger	chr1:203656969-203
ENSG0000(	382	8.649957	chr1:244(	SYT2	DriverDB\	protein_c	chr1:202590596-202
ENSG0000(	382	8.649957	chr1:244(	ATP6V1G3		protein_c	chr1:198523222-198
ENSG0000(	382	8.649957	chr1:244(	IPO9-AS1		lncRNA	chr1:201688259-201
ENSG0000(	382	8.649957	chr1:244(	MRPS21P3		Pseudoger	chr1:197363817-197
ENSG0000(	382	8.649957	chr7:251(	SPDYE16		protein_c	chr7:76531313-7654
ENSG0000(	382	8.649957	chr7:251(	RUNDC3B	DriverDB\	protein_c	chr7:87627548-8783
ENSG0000(	382	8.649957	chr7:251(	GAL3ST4		protein_c	chr7:100159244-100
ENSG0000(	382	8.649957	chr1:244(	PTPN7		protein_c	chr1:202147013-202
ENSG0000(	382	8.649957	chr1:244(	ENSG00000227747		Pseudoger	chr1:198949016-198

ENSG0000(	382	8.649957	chr7:251(	AC084368.1		smallRNA	chr7:96306760-9630
ENSG0000(	382	8.649957	chr1:2449	PLEKHA6		protein_c	chr1:204218853-204
ENSG0000(	382	8.649957	chr7:251(	FAM200A		protein_c	chr7:99546300-9955
ENSG0000(	382	8.649957	chr7:251(	ENSG00000278959		TEC	chr7:93954044-9395
ENSG0000(	382	8.649957	chr7:251(	ENSG00000231859		Pseudoger	chr7:97906429-9790
ENSG0000(	382	8.649957	chr1:2449	LINC00303		lncRNA	chr1:204032447-204
ENSG0000(	382	8.649957	chr1:2449	PTPRVP		Pseudoger	chr1:202168051-202
ENSG0000(	382	8.649957	chr1:2449	NUAK2	AC	protein_c	chr1:205302063-205
ENSG0000(	382	8.649957	chr1:2449	ENSG00000282849		lncRNA	chr1:200478020-200
ENSG0000(	382	8.649957	chr1:2449	NFASC		protein_c	chr1:204828651-205
ENSG0000(	382	8.649957	chr1:2449	ENSG00000287197		lncRNA	chr1:204822664-204
ENSG0000(	382	8.649957	chr7:251(	AP4M1		protein_c	chr7:100101549-100
ENSG0000(	382	8.649957	chr7:251(	DBF4	DriverDB\	protein_c	chr7:87876216-8790
ENSG0000(	382	8.649957	chr7:251(	TSC22D4		protein_c	chr7:100463359-100
ENSG0000(	382	8.649957	chr1:2449	TNNI1		protein_c	chr1:201403768-201
ENSG0000(	382	8.649957	chr7:251(	ZKSCAN5	DriverDB\	protein_c	chr7:99504662-9953
ENSG0000(	382	8.649957	chr1:2449	RNU6-501P		smallRNA	chr1:201733406-201
ENSG0000(	382	8.649957	chr7:251(	ACTL6B	NCGv7	protein_c	chr7:100643097-100
ENSG0000(	382	8.649957	chr7:251(	ENSG00000227785		Pseudoger	chr7:85636013-8563
ENSG0000(	382	8.649957	chr1:2449	ENSG00000236108		Pseudoger	chr1:205134646-205
ENSG0000(	382	8.649957	chr7:251(	SDHAF3		protein_c	chr7:97117698-9718
ENSG0000(	382	8.649957	chr7:251(	EEF1A1P28		Pseudoger	chr7:88639014-8864
ENSG0000(	382	8.649957	chr1:2449	F13B		protein_c	chr1:197038741-197
ENSG0000(	382	8.649957	chr1:2449	MIR1231		smallRNA	chr1:201808611-201
ENSG0000(	382	8.649957	chr7:251(	MARK2P10		Pseudoger	chr7:96858182-9685
ENSG0000(	382	8.649957	chr1:2449	ENSG00000229821		lncRNA	chr1:201222113-201
ENSG0000(	382	8.649957	chr7:251(	ENSG00000237729		Pseudoger	chr7:93669826-9367
ENSG0000(	382	8.649957	chr1:2449	RNPEP		protein_c	chr1:201982372-202
ENSG0000(	382	8.649957	chr7:251(	GJC3		protein_c	chr7:99923266-9992
ENSG0000(	382	8.649957	chr7:251(	RNU6-10P		smallRNA	chr7:92701708-9270
ENSG0000(	382	8.649957	chr7:251(	HSPA8P16		Pseudoger	chr7:85027828-8502
ENSG0000(	382	8.649957	chr7:251(	ENSG00000284840		Pseudoger	chr7:99032894-9903
ENSG0000(	382	8.649957	chr1:2449	RNU6-716P		smallRNA	chr1:200008505-200
ENSG0000(	382	8.649957	chr7:251(	LINC03009		Pseudoger	chr7:76549618-7655
ENSG0000(	382	8.649957	chr1:2449	ARL8A		protein_c	chr1:202133404-202
ENSG0000(	382	8.649957	chr1:2449	LAD1		protein_c	chr1:201380833-201
ENSG0000(	382	8.649957	chr7:251(	STEAP2-AS1		lncRNA	chr7:89882353-9021
ENSG0000(	382	8.649957	chr1:2449	NEK7		protein_c	chr1:198156994-198
ENSG0000(	382	8.649957	chr7:251(	ENSG00000238358		lncRNA	chr7:90119299-9012
ENSG0000(	382	8.649957	chr1:2449	AC096633.1		smallRNA	chr1:200144834-200
ENSG0000(	382	8.649957	chr7:251(	MYL10		protein_c	chr7:101613330-101
ENSG0000(	382	8.649957	chr7:251(	ENSG00000285772		protein_c	chr7:92112159-9217
ENSG0000(	382	8.649957	chr7:251(	snoU13		smallRNA	chr7:95276586-9527
ENSG0000(	382	8.649957	chr7:251(	CFAP69	DriverDB\	protein_c	chr7:90245174-9031
ENSG0000(	382	8.649957	chr7:251(	PLOD3	DriverDB\	protein_c	chr7:101205977-101
ENSG0000(	382	8.649957	chr7:251(	RPS29P15		Pseudoger	chr7:100928370-100
ENSG0000(	382	8.649957	chr7:251(	CLDN15	DriverDB\	protein_c	chr7:101232092-101
ENSG0000(	382	8.649957	chr1:2449	PRR13P1		Pseudoger	chr1:198197779-198
ENSG0000(	382	8.649957	chr7:251(	RPS3AP29		Pseudoger	chr7:97898347-9789
ENSG0000(	382	8.649957	chr1:2449	PPFIA4	DriverDB\	protein_c	chr1:203026491-203
ENSG0000(	382	8.649957	chr1:2449	ENSG00000229747		Pseudoger	chr1:199908921-199

ENSG0000C	382	8.649957	chr7:251€	RN7SL13P		smallRNA	chr7:98023729-9802
ENSG0000C	382	8.649957	chr1:2449	SNORA70		smallRNA	chr1:201978461-201
ENSG0000C	382	8.649957	chr7:251€	ZNHIT1		protein_c	chr7:101218165-101
ENSG0000C	382	8.649957	chr7:251€	AC004458.1		smallRNA	chr7:96468331-9646
ENSG0000C	382	8.649957	chr1:2449	ENSG00000225522		Pseudoger	chr1:204183006-204
ENSG0000C	382	8.649957	chr7:251€	ENSG00000224448		Pseudoger	chr7:99638242-9963
ENSG0000C	382	8.649957	chr7:251€	ENSG00000287932		lncRNA	chr7:92638198-9264
ENSG0000C	382	8.649957	chr1:2449	UBE2T		protein_c	chr1:202331544-202
ENSG0000C	382	8.649957	chr7:251€	GTPBP10	DriverDB\	protein_c	chr7:90335223-9039
ENSG0000C	382	8.649957	chr7:251€	MIR4653		smallRNA	chr7:101159473-101
ENSG0000C	382	8.649957	chr1:2449	LEMD1-AS1		lncRNA	chr1:205373252-205
ENSG0000C	381	8.627313	chr2:2577	ITGAV	NCGv7	protein_c	chr2:186590010-186
ENSG0000C	381	8.627313	chr2:2577	ENSG00000287129		lncRNA	chr2:185950469-185
ENSG0000C	381	8.627313	chr2:2577	UBE2E3		protein_c	chr2:180967248-181
ENSG0000C	381	8.627313	chr2:2577	KRT8P10		Pseudoger	chr2:183071040-183
ENSG0000C	381	8.627313	chr2:2577	ENSG00000272800		lncRNA	chr2:183178806-183
ENSG0000C	381	8.627313	chr2:2577	ZC3H15		protein_c	chr2:186486253-186
ENSG0000C	381	8.627313	chr2:2577	ENSG00000286980		lncRNA	chr2:184204372-184
ENSG0000C	381	8.627313	chr2:2577	U8		smallRNA	chr2:185661774-185
ENSG0000C	381	8.627313	chr2:2577	ENSG00000225258		lncRNA	chr2:180571712-180
ENSG0000C	381	8.627313	chr2:2577	ENSG00000226681		lncRNA	chr2:181422154-181
ENSG0000C	381	8.627313	chr2:2577	CERKL		protein_c	chr2:181535041-181
ENSG0000C	381	8.627313	chr2:2577	MED28P3		Pseudoger	chr2:186364598-186
ENSG0000C	381	8.627313	chr2:2577	RN7SL267P		smallRNA	chr2:182314284-182
ENSG0000C	381	8.627313	chr2:2577	NCKAP1		protein_c	chr2:182909115-183
ENSG0000C	381	8.627313	chr2:2577	ENSG00000225570		lncRNA	chr2:181690380-181
ENSG0000C	381	8.627313	chr2:2577	FSIP2-AS2		lncRNA	chr2:185719874-185
ENSG0000C	381	8.627313	chr2:2577	ELF2P4		Pseudoger	chr2:185547135-185
ENSG0000C	381	8.627313	chr2:2577	AC064837.1		smallRNA	chr2:182088429-182
ENSG0000C	381	8.627313	chr2:2577	ITPRID2		protein_c	chr2:181891730-181
ENSG0000C	381	8.627313	chr2:2577	ENSG00000287621		lncRNA	chr2:183253652-183
ENSG0000C	381	8.627313	chr2:2577	RPL23AP35		Pseudoger	chr2:186261419-186
ENSG0000C	381	8.627313	chr2:2577	LINC01934		lncRNA	chr2:181086076-181
ENSG0000C	381	8.627313	chr2:2577	KRT18P29		Pseudoger	chr2:181961212-181
ENSG0000C	381	8.627313	chr2:2577	MIR548AE1		smallRNA	chr2:184378975-184
ENSG0000C	381	8.627313	chr2:2577	FAM171B	NCGv7	protein_c	chr2:186694060-186
ENSG0000C	381	8.627313	chr2:2577	ITPRID2-DT		lncRNA	chr2:181887851-181
ENSG0000C	381	8.627313	chr2:2577	ENSG00000234172		lncRNA	chr2:183904529-183
ENSG0000C	381	8.627313	chr2:2577	ENSG00000270289		Pseudoger	chr2:180473137-180
ENSG0000C	381	8.627313	chr2:2577	ENSG00000234595		lncRNA	chr2:181683113-181
ENSG0000C	381	8.627313	chr2:2577	Y_RNA		smallRNA	chr2:183022859-183
ENSG0000C	381	8.627313	chr2:2577	ENSG00000289389		lncRNA	chr2:181305836-181
ENSG0000C	381	8.627313	chr2:2577	ENSG00000227227		lncRNA	chr2:186641339-186
ENSG0000C	381	8.627313	chr2:2577	CACYBPP2		Pseudoger	chr2:183607442-183
ENSG0000C	381	8.627313	chr2:2577	RNU6ATAC19P		smallRNA	chr2:181738606-181
ENSG0000C	381	8.627313	chr2:2577	ENSG00000286879		lncRNA	chr2:183897950-183
ENSG0000C	381	8.627313	chr2:2577	RNA5SP113		Pseudoger	chr2:182048822-182
ENSG0000C	381	8.627313	chr2:2577	ENSG00000279559		TEC	chr2:180801227-180
ENSG0000C	381	8.627313	chr2:2577	AC009478.2		smallRNA	chr2:180617118-180
ENSG0000C	381	8.627313	chr2:2577	FSIP2-AS1		lncRNA	chr2:185652374-185
ENSG0000C	381	8.627313	chr2:2577	DNAJC10		protein_c	chr2:182716255-182

ENSG0000(	381	8.627313	chr2:2577	RPL31P15		Pseudoger	chr2:182788098-182
ENSG0000(	381	8.627313	chr2:2577	RPL27AP3		Pseudoger	chr2:180938830-180
ENSG0000(	381	8.627313	chr2:2577	ITGA4	NCGv7	protein_c	chr2:181457202-181
ENSG0000(	381	8.627313	chr2:2577	UBE2E3-DT		lncRNA	chr2:180979427-180
ENSG0000(	381	8.627313	chr2:2577	RPL23AP33		Pseudoger	chr2:184902111-184
ENSG0000(	381	8.627313	chr2:2577	ENSG00000286152		lncRNA	chr2:184049467-184
ENSG0000(	381	8.627313	chr2:2577	FSIP2	NCGv7	protein_c	chr2:185738804-185
ENSG0000(	381	8.627313	chr2:2577	ZNF804A	NCGv7	protein_c	chr2:184598529-184
ENSG0000(	381	8.627313	chr2:2577	LIN28AP1		Pseudoger	chr2:183260642-183
ENSG0000(	381	8.627313	chr2:2577	DUSP19		protein_c	chr2:183078559-183
ENSG0000(	381	8.627313	chr2:2577	RPL21P32		Pseudoger	chr2:185956587-185
ENSG0000(	381	8.627313	chr2:2577	MIR4437		smallRNA	chr2:181305593-181
ENSG0000(	381	8.627313	chr2:2577	PPP1R1C		protein_c	chr2:181954241-182
ENSG0000(	381	8.627313	chr2:2577	ENSG00000226410		Pseudoger	chr2:186162949-186
ENSG0000(	381	8.627313	chr2:2577	LINC01473		lncRNA	chr2:186032884-186
ENSG0000(	381	8.627313	chr2:2577	NUP35		protein_c	chr2:183117513-183
ENSG0000(	381	8.627313	chr2:2577	SNORA77		smallRNA	chr2:183064235-183
ENSG0000(	381	8.627313	chr2:2577	snoU13		smallRNA	chr2:183949333-183
ENSG0000(	381	8.627313	chr2:2577	DPRXP1		Pseudoger	chr2:186488624-186
ENSG0000(	381	8.627313	chr2:2577	PDE1A		protein_c	chr2:182139968-182
ENSG0000(	381	8.627313	chr2:2577	FRZB		protein_c	chr2:182833275-182
ENSG0000(	381	8.627313	chr2:2577	ENSG00000259915		lncRNA	chr2:186354570-186
ENSG0000(	381	8.627313	chr2:2577	ZSWIM2		protein_c	chr2:186827475-186
ENSG0000(	381	8.627313	chr2:2577	RNU6-1122P		smallRNA	chr2:182873841-182
ENSG0000(	381	8.627313	chr2:2577	ENSG00000238171		lncRNA	chr2:181076051-181
ENSG0000(	381	8.627313	chr2:2577	ENSG00000283839		lncRNA	chr2:184593577-184
ENSG0000(	381	8.627313	chr2:2577	NEUROD1		protein_c	chr2:181668295-181
ENSG0000(	381	8.627313	chr2:2577	ENSG00000286807		lncRNA	chr2:183997372-184
ENSG0000(	381	8.627313	chr2:2577	ENSG00000287536		lncRNA	chr2:182278684-182
ENSG0000(	381	8.627313	chr2:2577	ENSG00000224643		lncRNA	chr2:183083405-183
ENSG0000(	381	8.627313	chr2:2577	Y_RNA		smallRNA	chr2:182828124-182
ENSG0000(	381	8.627313	chr2:2577	SAP18P2		Pseudoger	chr2:181694442-181
ENSG0000(	381	8.627313	chr2:2577	SCHLAP1		lncRNA	chr2:180692104-180
ENSG0000(	381	8.627313	chr2:2577	ENSG00000286797		lncRNA	chr2:185425063-185
ENSG0000(	381	8.627313	chr2:2577	FTH1P20		Pseudoger	chr2:180872867-180
ENSG0000(	380	8.60467	chr1:1021	ENSG00000228037		lncRNA	chr1:2581560-25845
ENSG0000(	380	8.60467	chr1:1021	ENSG00000272420		lncRNA	chr1:2363061-23636
ENSG0000(	380	8.60467	chr1:1021	PRDM16	NCGv7;AC	protein_c	chr1:3069168-34386
ENSG0000(	380	8.60467	chr1:1021	ENSG00000287828		lncRNA	chr1:3313052-33231
ENSG0000(	380	8.60467	chr1:1021	FAAP20		protein_c	chr1:2184461-22127
ENSG0000(	380	8.60467	chr1:1021	ENSG00000226286		lncRNA	chr1:3132927-31337
ENSG0000(	380	8.60467	chr1:1021	ENSG00000225931		TEC	chr1:2566410-25698
ENSG0000(	380	8.60467	chr1:1021	ENSG00000283259		lncRNA	chr1:2773603-27764
ENSG0000(	380	8.60467	chr1:1021	TP73-AS3		lncRNA	chr1:3658938-36687
ENSG0000(	380	8.60467	chr1:1021	TP73	NCGv7;AC	protein_c	chr1:3652516-37362
ENSG0000(	380	8.60467	chr1:1021	MMEL1	NCGv7	protein_c	chr1:2590639-26330
ENSG0000(	380	8.60467	chr1:1021	ENSG00000229393		lncRNA	chr1:2493437-24944
ENSG0000(	380	8.60467	chr1:1021	ENSG00000231630		lncRNA	chr1:2814432-28149
ENSG0000(	380	8.60467	chr1:1021	ACTRT2		protein_c	chr1:3021467-30229
ENSG0000(	380	8.60467	chr1:1021	ENSG00000272449		lncRNA	chr1:2530064-25474
ENSG0000(	380	8.60467	chr1:1021	HES5		protein_c	chr1:2528745-25302

ENSG0000(	380	8.60467	chr1:1021	ENSG00000271806		lncRNA	chr1:2141084-21452
ENSG0000(	380	8.60467	chr1:1021	ENSG00000284745		lncRNA	chr1:2960658-29687
ENSG0000(	380	8.60467	chr1:1021	ENSG00000286518		lncRNA	chr1:3367815-33739
ENSG0000(	380	8.60467	chr1:1021	ENSG00000279839		TEC	chr1:3205988-32086
ENSG0000(	380	8.60467	chr1:1021	MMEL1-AS1		lncRNA	chr1:2632568-26366
ENSG0000(	380	8.60467	chr1:1021	MEGF6		protein_c	chr1:3487951-36115
ENSG0000(	380	8.60467	chr1:1021	TNFRSF14-AS1		lncRNA	chr1:2549920-25570
ENSG0000(	380	8.60467	chr1:1021	PRDM16-DT		lncRNA	chr1:3059611-30690
ENSG0000(	380	8.60467	chr1:1021	ENSG00000234396		lncRNA	chr1:2212523-22207
ENSG0000(	380	8.60467	chr1:1021	ENSG00000272235		lncRNA	chr1:3306636-33100
ENSG0000(	380	8.60467	chr1:1021	ENSG00000289610		lncRNA	chr1:2585610-25860
ENSG0000(	380	8.60467	chr1:1021	ENSG00000233234		lncRNA	chr1:2811850-28126
ENSG0000(	380	8.60467	chr1:1021	RER1		protein_c	chr1:2391775-24054
ENSG0000(	380	8.60467	chr1:1021	SKI	NCGv7;AC	protein_c	chr1:2227388-23102
ENSG0000(	380	8.60467	chr1:1021	ARHGEF16		protein_c	chr1:3454665-34811
ENSG0000(	380	8.60467	chr1:1021	PEX10		protein_c	chr1:2403964-24137
ENSG0000(	380	8.60467	chr1:1021	ENSG00000224387		lncRNA	chr1:2492300-24932
ENSG0000(	380	8.60467	chr1:1021	ENSG00000287356		lncRNA	chr1:2315040-23230
ENSG0000(	380	8.60467	chr1:1021	PLCH2	NCGv7	protein_c	chr1:2425980-25055
ENSG0000(	380	8.60467	chr1:1021	PRKCZ-AS1		lncRNA	chr1:2181794-21843
ENSG0000(	380	8.60467	chr1:1021	TPRG1L	NCGv7	protein_c	chr1:3625015-36301
ENSG0000(	380	8.60467	chr1:1021	PANK4		protein_c	chr1:2508537-25265
ENSG0000(	380	8.60467	chr1:1021	ENSG00000272088		lncRNA	chr1:3487246-34876
ENSG0000(	380	8.60467	chr1:1021	TNFRSF14	NCGv7;AC	protein_c	chr1:2555639-25653
ENSG0000(	380	8.60467	chr1:1021	PRXL2B		protein_c	chr1:2586491-25914
ENSG0000(	380	8.60467	chr1:1021	ENSG00000287396		lncRNA	chr1:2814056-28177
ENSG0000(	380	8.60467	chr1:1021	ENSG00000238260		lncRNA	chr1:3622857-36247
ENSG0000(	380	8.60467	chr1:1021	MIR551A		smallRNA	chr1:3560695-35607
ENSG0000(	380	8.60467	chr1:1021	ENSG00000285945		lncRNA	chr1:2768091-27847
ENSG0000(	380	8.60467	chr6:1979	ENSG00000218793		Pseudoger	chr6:87441165-8744
ENSG0000(	380	8.60467	chr1:1021	MORN1	NCGv7	protein_c	chr1:2321253-23917
ENSG0000(	380	8.60467	chr1:1021	TP73-AS2		lncRNA	chr1:3712200-37142
ENSG0000(	380	8.60467	chr1:1021	ENSG00000269896		Pseudoger	chr1:2350414-23528
ENSG0000(	380	8.60467	chr1:1021	WRAP73	NCGv7	protein_c	chr1:3630767-36527
ENSG0000(	379	8.582026	chr9:3581	AL133410.1		smallRNA	chr9:35811476-3581
ENSG0000(	379	8.582026	chr17:289	RNU6-1267P		smallRNA	chr17:30288071-302
ENSG0000(	377	8.536738	chr10:173	ENSG00000227186		lncRNA	chr10:75023475-750
ENSG0000(	377	8.536738	chr10:173	PPIAP13		Pseudoger	chr10:75089248-750
ENSG0000(	377	8.536738	chr10:173	DUSP29		protein_c	chr10:75037472-750
ENSG0000(	377	8.536738	chr10:173	ENSG00000285810		lncRNA	chr10:75041876-750
ENSG0000(	377	8.536738	chr10:173	ENSG00000234149		lncRNA	chr10:75003055-750
ENSG0000(	374	8.468806	chr1:3733	ENO1-AS1		lncRNA	chr1:8878835-88798
ENSG0000(	374	8.468806	chr1:3733	PIK3CD	NCGv7	protein_c	chr1:9629889-97291
ENSG0000(	374	8.468806	chr1:3733	CAMTA1-AS2		lncRNA	chr1:7382487-73897
ENSG0000(	374	8.468806	chr1:3733	ENSG00000236266		lncRNA	chr1:7810242-78273
ENSG0000(	374	8.468806	chr1:3733	MTOR-AS1		lncRNA	chr1:11143898-1114
ENSG0000(	374	8.468806	chr1:3733	RN7SKP269		smallRNA	chr1:9947318-99476
ENSG0000(	374	8.468806	chr1:3733	SNORA77		smallRNA	chr1:8511795-85119
ENSG0000(	374	8.468806	chr1:3733	MIR34AHG		lncRNA	chr1:9148011-91989
ENSG0000(	374	8.468806	chr1:3733	ENSG00000232663		Pseudoger	chr1:8909742-89099
ENSG0000(	374	8.468806	chr1:3733	SLC2A5		protein_c	chr1:9035106-90884

ENSG0000(	374	8.468806	chr1:373:	MIR1273D		smallRNA	chr1:10227718-1022
ENSG0000(	374	8.468806	chr1:373:	Y_RNA		smallRNA	chr1:7982881-79829
ENSG0000(	374	8.468806	chr1:373:	CLSTN1		protein_c	chr1:9728926-98239
ENSG0000(	374	8.468806	chr1:373:	CA6		protein_c	chr1:8945867-89750
ENSG0000(	374	8.468806	chr1:373:	UBE2V2P3		Pseudoger	chr1:11278616-1127
ENSG0000(	374	8.468806	chr1:373:	ENSG00000233268		Pseudoger	chr1:9660828-96626
ENSG0000(	374	8.468806	chr1:373:	ENSG00000203469		lncRNA	chr1:10458555-1045
ENSG0000(	374	8.468806	chr1:373:	ENSG00000269925		lncRNA	chr1:7776383-77767
ENSG0000(	374	8.468806	chr1:373:	AL590639.1		Pseudoger	chr1:10018027-1001
ENSG0000(	374	8.468806	chr1:373:	C1orf127	NCGv7	protein_c	chr1:10946471-1098
ENSG0000(	374	8.468806	chr1:373:	CENPS		protein_c	chr1:10430433-1044
ENSG0000(	374	8.468806	chr1:373:	LINC01714		lncRNA	chr1:8201518-82152
ENSG0000(	374	8.468806	chr1:373:	TMEM274P		Pseudoger	chr1:9950572-99607
ENSG0000(	374	8.468806	chr1:373:	MIR5697		smallRNA	chr1:9967381-99674
ENSG0000(	374	8.468806	chr1:373:	ENSG00000290109		lncRNA	chr1:8970490-89712
ENSG0000(	374	8.468806	chr1:373:	MTOR	NCGv7;AC	protein_c	chr1:11106535-1126
ENSG0000(	374	8.468806	chr1:373:	ENSG00000284747		lncRNA	chr1:7991134-80053
ENSG0000(	374	8.468806	chr1:373:	MTCYBP45		Pseudoger	chr1:11425257-1142
ENSG0000(	374	8.468806	chr1:373:	ENSG00000224340		Pseudoger	chr1:10054445-1005
ENSG0000(	374	8.468806	chr1:373:	ERRFI1-DT		lncRNA	chr1:8026738-81227
ENSG0000(	374	8.468806	chr1:373:	ENSG00000272078		lncRNA	chr1:10639241-1065
ENSG0000(	374	8.468806	chr1:373:	RN7SL614P		smallRNA	chr1:10616836-1061
ENSG0000(	374	8.468806	chr1:373:	ENSG00000226545		Pseudoger	chr1:8798475-87995
ENSG0000(	374	8.468806	chr1:373:	ENSG00000270171		lncRNA	chr1:7693124-76948
ENSG0000(	374	8.468806	chr1:373:	ENSG00000228423		lncRNA	chr1:8805860-88070
ENSG0000(	374	8.468806	chr1:373:	ENSG00000287727		lncRNA	chr1:10612222-1061
ENSG0000(	374	8.468806	chr1:373:	ENSG00000241326		lncRNA	chr1:9983141-99845
ENSG0000(	374	8.468806	chr1:373:	ENSG00000285701		lncRNA	chr1:9900614-99080
ENSG0000(	374	8.468806	chr1:373:	PGAM1P11		Pseudoger	chr1:10058671-1005
ENSG0000(	374	8.468806	chr1:373:	ENSG00000233645		lncRNA	chr1:8218190-82205
ENSG0000(	374	8.468806	chr1:373:	ENSG00000231181		Pseudoger	chr1:9576427-95769
ENSG0000(	374	8.468806	chr1:373:	AL713997.1		smallRNA	chr1:10998176-1099
ENSG0000(	374	8.468806	chr1:373:	MZT1P1		Pseudoger	chr1:9780822-97812
ENSG0000(	374	8.468806	chr1:373:	ENSG00000223989		lncRNA	chr1:9848318-98501
ENSG0000(	374	8.468806	chr1:373:	SLC25A33		protein_c	chr1:9539465-95851
ENSG0000(	374	8.468806	chr1:373:	SLC45A1		protein_c	chr1:8318114-83441
ENSG0000(	374	8.468806	chr1:373:	RNU6-37P		smallRNA	chr1:10298966-1029
ENSG0000(	374	8.468806	chr1:373:	ENSG00000285833		lncRNA	chr1:11500803-1150
ENSG0000(	374	8.468806	chr1:373:	RERE-AS1		lncRNA	chr1:8424645-84350
ENSG0000(	374	8.468806	chr1:373:	RPL23AP19		Pseudoger	chr1:8831007-88313
ENSG0000(	374	8.468806	chr1:373:	RERE	AC	protein_c	chr1:8352397-88489
ENSG0000(	374	8.468806	chr1:373:	PIK3CD-AS2		lncRNA	chr1:9672405-96875
ENSG0000(	374	8.468806	chr1:373:	RNU6-304P		smallRNA	chr1:8883427-88835
ENSG0000(	374	8.468806	chr1:373:	RN7SL731P		smallRNA	chr1:10306465-1030
ENSG0000(	374	8.468806	chr1:373:	DFFA		protein_c	chr1:10456522-1047
ENSG0000(	374	8.468806	chr1:373:	H6PD		protein_c	chr1:9234774-92713
ENSG0000(	374	8.468806	chr1:373:	SRM		protein_c	chr1:11054584-1106
ENSG0000(	374	8.468806	chr1:373:	PARK7	TAG;AC	protein_c	chr1:7954291-79855
ENSG0000(	374	8.468806	chr1:373:	ENSG00000288816		lncRNA	chr1:8191692-81949
ENSG0000(	374	8.468806	chr1:373:	ERRFI1	NCGv7	protein_c	chr1:8004404-80263
ENSG0000(	374	8.468806	chr1:373:	ENSG00000289914		lncRNA	chr1:9162322-91629

ENSG0000(	374	8.468806	chr1:3733	PGD		protein_c	chr1:10398592-1042
ENSG0000(	374	8.468806	chr1:3733	RN7SL729P		smallRNA	chr1:8275124-82753
ENSG0000(	374	8.468806	chr1:3733	UTS2		protein_c	chr1:7843083-78535
ENSG0000(	374	8.468806	chr1:3733	VAMP3		protein_c	chr1:7771296-77814
ENSG0000(	374	8.468806	chr1:3733	SLC2A7		protein_c	chr1:9002973-90264
ENSG0000(	374	8.468806	chr1:3733	Y_RNA		smallRNA	chr1:8328067-83281
ENSG0000(	374	8.468806	chr1:3733	ENSG00000271989		lncRNA	chr1:10429881-1043
ENSG0000(	374	8.468806	chr1:3733	PER3	NCGv7	protein_c	chr1:7784291-78451
ENSG0000(	374	8.468806	chr1:3733	PEX14		protein_c	chr1:10472288-1063
ENSG0000(	374	8.468806	chr1:3733	RNU6-828P		smallRNA	chr1:10163268-1016
ENSG0000(	374	8.468806	chr1:3733	ENSG00000226849		lncRNA	chr1:11068471-1107
ENSG0000(	374	8.468806	chr1:3733	RNU6-991P		smallRNA	chr1:8292157-82922
ENSG0000(	374	8.468806	chr1:3733	RNA5SP40		Pseudoger	chr1:9437669-94377
ENSG0000(	374	8.468806	chr1:3733	SCARNA16		smallRNA	chr1:9082696-90828
ENSG0000(	374	8.468806	chr1:3733	SPSB1		protein_c	chr1:9292894-93695
ENSG0000(	374	8.468806	chr1:3733	ENSG00000284642		lncRNA	chr1:10395416-1039
ENSG0000(	374	8.468806	chr1:3733	snoU13		smallRNA	chr1:11169507-1116
ENSG0000(	374	8.468806	chr1:3733	ENSG00000284646		lncRNA	chr1:11311734-1131
ENSG0000(	374	8.468806	chr1:3733	Z98044.1		smallRNA	chr1:9278391-92785
ENSG0000(	374	8.468806	chr1:3733	CAMTA1	NCGv7;AC	protein_c	chr1:6785454-77697
ENSG0000(	374	8.468806	chr1:3733	MASP2		protein_c	chr1:11022009-1104
ENSG0000(	374	8.468806	chr1:3733	ENSG00000284652		lncRNA	chr1:9421098-94228
ENSG0000(	374	8.468806	chr1:3733	GPR157		protein_c	chr1:9100305-91291
ENSG0000(	374	8.468806	chr1:3733	TNFRSF9		protein_c	chr1:7915871-79431
ENSG0000(	374	8.468806	chr1:3733	KIF1B	NCGv7	protein_c	chr1:10210570-1038
ENSG0000(	374	8.468806	chr1:3733	EXOSC10		protein_c	chr1:11066618-1109
ENSG0000(	374	8.468806	chr1:3733	ENSG00000229305		Pseudoger	chr1:8189824-81927
ENSG0000(	374	8.468806	chr1:3733	ENSG00000235263		lncRNA	chr1:9501092-95034
ENSG0000(	374	8.468806	chr1:3733	RBP7		protein_c	chr1:9997206-10016
ENSG0000(	374	8.468806	chr1:3733	ANGPTL7		protein_c	chr1:11189355-1119
ENSG0000(	374	8.468806	chr1:3733	RNU1-7P		smallRNA	chr1:8206434-82065
ENSG0000(	374	8.468806	chr1:3733	RPL7AP18		Pseudoger	chr1:8057245-80584
ENSG0000(	374	8.468806	chr1:3733	CASZ1	NCGv7	protein_c	chr1:10636604-1079
ENSG0000(	374	8.468806	chr1:3733	UBE4B		protein_c	chr1:10032832-1018
ENSG0000(	374	8.468806	chr1:3733	DISP3	NCGv7	protein_c	chr1:11479155-1153
ENSG0000(	374	8.468806	chr1:3733	LZIC		protein_c	chr1:9922113-99434
ENSG0000(	374	8.468806	chr1:3733	ENO1		protein_c	chr1:8861000-88791
ENSG0000(	374	8.468806	chr1:3733	ENSG00000284735		lncRNA	chr1:10381906-1038
ENSG0000(	374	8.468806	chr1:3733	LINC02606		lncRNA	chr1:9425094-94405
ENSG0000(	374	8.468806	chr1:3733	Y_RNA		smallRNA	chr1:8796574-87966
ENSG0000(	374	8.468806	chr1:3733	ENSG00000284716		lncRNA	chr1:7998187-79999
ENSG0000(	374	8.468806	chr1:3733	ENSG00000270035		lncRNA	chr1:7698303-76988
ENSG0000(	374	8.468806	chr1:3733	HMGN2P17		Pseudoger	chr1:8893409-88941
ENSG0000(	374	8.468806	chr1:3733	TMEM201	NCGv7	protein_c	chr1:9588911-96148
ENSG0000(	374	8.468806	chr1:3733	CTNNBIP1		protein_c	chr1:9848276-99103
ENSG0000(	374	8.468806	chr1:3733	RPL39P6		Pseudoger	chr1:11232963-1123
ENSG0000(	374	8.468806	chr1:3733	NMNAT1		protein_c	chr1:9943428-99855
ENSG0000(	374	8.468806	chr1:3733	MIR34A		smallRNA	chr1:9151668-91517
ENSG0000(	374	8.468806	chr1:3733	RN7SL451P		smallRNA	chr1:8979578-89798
ENSG0000(	374	8.468806	chr1:3733	RN7SL721P		smallRNA	chr1:10390002-1039
ENSG0000(	374	8.468806	chr1:3733	CORT		protein_c	chr1:10450031-1045

ENSG0000(	374	8.468806	chr1:373	ENSG00000280113		TEC	chr1:9826289-98282
ENSG0000(	374	8.468806	chr1:373	PIK3CD-AS1		lncRNA	chr1:9652610-96545
ENSG0000(	374	8.468806	chr1:373	CFL1P6		Pseudoger	chr1:10990978-1099
ENSG0000(	374	8.468806	chr1:373	LNCTAM34A		lncRNA	chr1:9182004-91962
ENSG0000(	374	8.468806	chr1:373	RPL7P7		Pseudoger	chr1:8786211-87869
ENSG0000(	374	8.468806	chr1:373	ENSG00000228150		lncRNA	chr1:9942923-99499
ENSG0000(	374	8.468806	chr1:373	HSPE1P24		Pseudoger	chr1:10895761-1089
ENSG0000(	374	8.468806	chr1:373	ENSG00000270330		Pseudoger	chr1:7942372-79427
ENSG0000(	374	8.468806	chr1:373	ENSG00000269978		lncRNA	chr1:7700704-77009
ENSG0000(	374	8.468806	chr1:373	CENPS-CORT		protein_c	chr1:10430102-1045
ENSG0000(	374	8.468806	chr1:373	UBIAD1		protein_c	chr1:11273198-1129
ENSG0000(	374	8.468806	chr1:373	RPL7P11		Pseudoger	chr1:8750430-87510
ENSG0000(	374	8.468806	chr1:373	Y_RNA		smallRNA	chr1:10999862-1099
ENSG0000(	374	8.468806	chr1:373	TARDBP		protein_c	chr1:11012344-1103
ENSG0000(	374	8.468806	chr1:373	ENSG00000232208		Pseudoger	chr1:8907393-89077
ENSG0000(	374	8.468806	chr1:373	CAMTA1-AS1		lncRNA	chr1:7441096-74415
ENSG0000(	374	8.468806	chr1:373	RNU6-537P		smallRNA	chr1:11152350-1115
ENSG0000(	374	8.468806	chr1:373	RNU6-291P		smallRNA	chr1:11226254-1122
ENSG0000(	374	8.468806	chr1:373	EXOSC10-AS1		lncRNA	chr1:11099430-1110
ENSG0000(	373	8.446162	chr16:239	ENSG00000275857		lncRNA	chr16:29804430-298
ENSG0000(	373	8.446162	chr16:239	ENSG00000261588		Pseudoger	chr16:30587662-305
ENSG0000(	373	8.446162	chr1:2449	ENSG00000223344		lncRNA	chr1:191858707-191
ENSG0000(	373	8.446162	chr16:239	MIR4518		smallRNA	chr16:30503919-305
ENSG0000(	373	8.446162	chr16:239	BOLA2		protein_c	chr16:29453590-294
ENSG0000(	373	8.446162	chr16:239	CORO1A		protein_c	chr16:30182827-301
ENSG0000(	373	8.446162	chr1:2449	BRINP3	NCGv7	protein_c	chr1:190097658-190
ENSG0000(	373	8.446162	chr1:2449	ENSG00000230987		Pseudoger	chr1:189989570-189
ENSG0000(	373	8.446162	chr16:239	MVP-DT		lncRNA	chr16:29808636-298
ENSG0000(	373	8.446162	chr16:239	NPIPB13		protein_c	chr16:30222897-302
ENSG0000(	373	8.446162	chr16:239	TBX6		protein_c	chr16:30085793-300
ENSG0000(	373	8.446162	chr16:239	PPP4C	NCGv7	protein_c	chr16:30075978-300
ENSG0000(	373	8.446162	chr1:2449	GAPDHP75		Pseudoger	chr1:189132350-189
ENSG0000(	373	8.446162	chr1:2449	ZNF101P2		Pseudoger	chr1:192993449-192
ENSG0000(	373	8.446162	chr1:2449	B3GALT2		protein_c	chr1:193178730-193
ENSG0000(	373	8.446162	chr1:2449	RO60	NCGv7	protein_c	chr1:193059454-193
ENSG0000(	373	8.446162	chr16:239	SPN	NCGv7	protein_c	chr16:29662979-296
ENSG0000(	373	8.446162	chr16:239	ENSG00000261346		lncRNA	chr16:30477180-304
ENSG0000(	373	8.446162	chr1:2449	ENSG00000289995		lncRNA	chr1:192609359-192
ENSG0000(	373	8.446162	chr1:2449	UCHL5	NCGv7	protein_c	chr1:193012250-193
ENSG0000(	373	8.446162	chr1:2449	ENSG00000261182		lncRNA	chr1:188218400-188
ENSG0000(	373	8.446162	chr16:239	CORO1A-AS1		lncRNA	chr16:30183505-301
ENSG0000(	373	8.446162	chr1:2449	ENSG00000238054		lncRNA	chr1:188869474-188
ENSG0000(	373	8.446162	chr1:2449	MIR1278		smallRNA	chr1:193136503-193
ENSG0000(	373	8.446162	chr16:239	ZNF785		protein_c	chr16:30573740-305
ENSG0000(	373	8.446162	chr16:239	ENSG00000260413		Pseudoger	chr16:29312350-293
ENSG0000(	373	8.446162	chr16:239	SLX1B		protein_c	chr16:29454533-294
ENSG0000(	373	8.446162	chr16:239	ENSG00000258150		Pseudoger	chr16:29465222-294
ENSG0000(	373	8.446162	chr16:239	ZNF768		protein_c	chr16:30524004-305
ENSG0000(	373	8.446162	chr1:2449	ENSG00000226723		Pseudoger	chr1:192246708-192
ENSG0000(	373	8.446162	chr16:239	BOLA2B		protein_c	chr16:30192932-301
ENSG0000(	373	8.446162	chr16:239	ENSG00000274653		lncRNA	chr16:30359825-303

ENSG0000(	373	8.446162	chr1:2449	GLRX2		protein_c	chr1:193090866-193
ENSG0000(	373	8.446162	chr16:239	ENSG00000289181		lncRNA	chr16:29709156-297
ENSG0000(	373	8.446162	chr16:239	ZNF747		protein_c	chr16:30530367-305
ENSG0000(	373	8.446162	chr16:239	ZNF764		protein_c	chr16:30553764-305
ENSG0000(	373	8.446162	chr16:239	MIR3680-2		smallRNA	chr16:29599179-295
ENSG0000(	373	8.446162	chr16:239	ENSG00000290692		lncRNA	chr16:30204316-302
ENSG0000(	373	8.446162	chr1:2449	FDPSP1		Pseudoger	chr1:187563061-187
ENSG0000(	373	8.446162	chr1:2449	ENSG00000226814		Pseudoger	chr1:192800571-192
ENSG0000(	373	8.446162	chr16:239	Y_RNA		smallRNA	chr16:30453997-304
ENSG0000(	373	8.446162	chr1:2449	RGS18	NCGv7	protein_c	chr1:192158462-192
ENSG0000(	373	8.446162	chr16:239	BOLA2-SMG1P6		protein_c	chr16:29443230-294
ENSG0000(	373	8.446162	chr1:2449	ENSG00000236792		Pseudoger	chr1:192247505-192
ENSG0000(	373	8.446162	chr1:2449	ENSG00000236025		Pseudoger	chr1:190781787-190
ENSG0000(	373	8.446162	chr16:239	HIRIP3		protein_c	chr16:29992330-299
ENSG0000(	373	8.446162	chr16:239	ENSG00000258130		Pseudoger	chr16:30204571-302
ENSG0000(	373	8.446162	chr16:239	MVP		protein_c	chr16:29820394-298
ENSG0000(	373	8.446162	chr16:239	ENSG00000278078		Pseudoger	chr16:29380242-293
ENSG0000(	373	8.446162	chr16:239	KCTD13-DT		lncRNA	chr16:29926836-299
ENSG0000(	373	8.446162	chr16:239	SEPHS2		protein_c	chr16:30443631-304
ENSG0000(	373	8.446162	chr16:239	ENSG00000290678		lncRNA	chr16:29464967-294
ENSG0000(	373	8.446162	chr16:239	DOC2A	DriverDBv	protein_c	chr16:30005514-300
ENSG0000(	373	8.446162	chr1:2449	RN7SKP126		smallRNA	chr1:192875686-192
ENSG0000(	373	8.446162	chr16:239	YPEL3-DT		lncRNA	chr16:30096430-301
ENSG0000(	373	8.446162	chr1:2449	ENSG00000241505		lncRNA	chr1:190480379-190
ENSG0000(	373	8.446162	chr16:239	MYL11		protein_c	chr16:30370934-303
ENSG0000(	373	8.446162	chr16:239	RN7SKP127		smallRNA	chr16:29731051-297
ENSG0000(	373	8.446162	chr1:2449	RNA5SP73		Pseudoger	chr1:189666149-189
ENSG0000(	373	8.446162	chr1:2449	RGS1		protein_c	chr1:192575763-192
ENSG0000(	373	8.446162	chr1:2449	snoU109		smallRNA	chr1:193057281-193
ENSG0000(	373	8.446162	chr16:239	SNX29P2		Pseudoger	chr16:29355787-293
ENSG0000(	373	8.446162	chr16:239	SULT1A3		protein_c	chr16:30199228-302
ENSG0000(	373	8.446162	chr16:239	NPIPB12		protein_c	chr16:29484690-295
ENSG0000(	373	8.446162	chr16:239	SMG1P6		Pseudoger	chr16:29425800-294
ENSG0000(	373	8.446162	chr16:239	SMG1P5		lncRNA	chr16:30285018-303
ENSG0000(	373	8.446162	chr16:239	YPEL3		protein_c	chr16:30092314-300
ENSG0000(	373	8.446162	chr16:239	ENSG00000278887		Pseudoger	chr16:30205418-302
ENSG0000(	373	8.446162	chr16:239	ENSG00000261367		lncRNA	chr16:30107675-301
ENSG0000(	373	8.446162	chr1:2449	ENSG00000288950		lncRNA	chr1:193482540-193
ENSG0000(	373	8.446162	chr16:239	ENSG00000279228		Pseudoger	chr16:29466069-294
ENSG0000(	373	8.446162	chr16:239	TAOK2		protein_c	chr16:29973868-299
ENSG0000(	373	8.446162	chr16:239	snoU13		smallRNA	chr16:29437497-294
ENSG0000(	373	8.446162	chr16:239	ENSG00000280893		protein_c	chr16:29812261-298
ENSG0000(	373	8.446162	chr16:239	ENSG00000261680		Pseudoger	chr16:30624285-306
ENSG0000(	373	8.446162	chr16:239	ENSG00000198106		lncRNA	chr16:29291220-293
ENSG0000(	373	8.446162	chr1:2449	CDC73	NCGv7;AC	protein_c	chr1:193121983-193
ENSG0000(	373	8.446162	chr16:239	MAPK3		protein_c	chr16:30114105-301
ENSG0000(	373	8.446162	chr16:239	ALDOA	NCGv7	protein_c	chr16:30064164-300
ENSG0000(	373	8.446162	chr16:239	RNU7-61P		smallRNA	chr16:30483135-304
ENSG0000(	373	8.446162	chr16:239	GDPD3		protein_c	chr16:30104810-301
ENSG0000(	373	8.446162	chr1:2449	ENSG00000274702		Pseudoger	chr1:187632166-187
ENSG0000(	373	8.446162	chr16:239	ENSG00000259952		lncRNA	chr16:29806496-298

ENSG0000(	373	8.446162	chr16:239	TBC1D10B		protein_c	chr16:30357102-303
ENSG0000(	373	8.446162	chr16:239	ENSG00000279583		TEC	chr16:29595130-295
ENSG0000(	373	8.446162	chr1:2449	ENSG00000236069		lncRNA	chr1:192517190-192
ENSG0000(	373	8.446162	chr16:239	SLC7A5P1		Pseudoger	chr16:29613103-296
ENSG0000(	373	8.446162	chr16:239	ENSG00000260335		Pseudoger	chr16:29449191-294
ENSG0000(	373	8.446162	chr16:239	ENSG00000260719		lncRNA	chr16:29745247-297
ENSG0000(	373	8.446162	chr16:239	TLCD3B	NCGv7	protein_c	chr16:30024427-300
ENSG0000(	373	8.446162	chr16:239	CD2BP2	NCGv7	protein_c	chr16:30350773-303
ENSG0000(	373	8.446162	chr16:239	ENSG00000260167		lncRNA	chr16:30585907-306
ENSG0000(	373	8.446162	chr16:239	ENSG00000280607		lncRNA	chr16:29808679-298
ENSG0000(	373	8.446162	chr16:239	ZNF688		protein_c	chr16:30569672-305
ENSG0000(	373	8.446162	chr16:239	SMG1P2		Pseudoger	chr16:29527568-295
ENSG0000(	373	8.446162	chr1:2449	LINC01035		lncRNA	chr1:188905688-189
ENSG0000(	373	8.446162	chr1:2449	RGS2		protein_c	chr1:192809039-192
ENSG0000(	373	8.446162	chr16:239	ENSG00000290679		lncRNA	chr16:29467161-294
ENSG0000(	373	8.446162	chr16:239	ENSG00000278713		lncRNA	chr16:29862760-298
ENSG0000(	373	8.446162	chr16:239	ENSG00000274904		lncRNA	chr16:30064306-300
ENSG0000(	373	8.446162	chr1:2449	RGS13		protein_c	chr1:192636138-192
ENSG0000(	373	8.446162	chr1:2449	ENSG00000285894		lncRNA	chr1:188013642-188
ENSG0000(	373	8.446162	chr1:2449	ENSG00000224278		Pseudoger	chr1:188242139-188
ENSG0000(	373	8.446162	chr1:2449	LINC01701		lncRNA	chr1:189775465-189
ENSG0000(	373	8.446162	chr16:239	DCTPP1		protein_c	chr16:30423615-304
ENSG0000(	373	8.446162	chr16:239	ZNF771		protein_c	chr16:30407414-304
ENSG0000(	373	8.446162	chr1:2449	RN7SKP156		smallRNA	chr1:188155839-188
ENSG0000(	373	8.446162	chr16:239	QPRT	NCGv7	protein_c	chr16:29663279-296
ENSG0000(	373	8.446162	chr16:239	NPIPB11		protein_c	chr16:29381310-294
ENSG0000(	373	8.446162	chr1:2449	ENSG00000238108		Pseudoger	chr1:190797524-190
ENSG0000(	373	8.446162	chr16:239	Y_RNA		smallRNA	chr16:30471587-304
ENSG0000(	373	8.446162	chr6:9221	ERHP2		Pseudoger	chr6:54016479-5401
ENSG0000(	373	8.446162	chr16:239	snoU13		smallRNA	chr16:30279248-302
ENSG0000(	373	8.446162	chr16:239	ENSG00000257691		Pseudoger	chr16:29475064-294
ENSG0000(	373	8.446162	chr1:2449	LINC01720		lncRNA	chr1:190624890-190
ENSG0000(	373	8.446162	chr16:239	C16orf54		protein_c	chr16:29742463-297
ENSG0000(	373	8.446162	chr16:239	ENSG00000260678		Pseudoger	chr16:30615256-306
ENSG0000(	373	8.446162	chr1:2449	SLC4A1APP2		Pseudoger	chr1:187706561-187
ENSG0000(	373	8.446162	chr1:2449	LINC01680		lncRNA	chr1:191221159-191
ENSG0000(	373	8.446162	chr16:239	ENSG00000285043		protein_c	chr16:30053123-300
ENSG0000(	373	8.446162	chr16:239	CD2BP2-DT		lncRNA	chr16:30354665-303
ENSG0000(	373	8.446162	chr16:239	ZNF747-DT		lncRNA	chr16:30534752-305
ENSG0000(	373	8.446162	chr16:239	CDIPT		protein_c	chr16:29858357-298
ENSG0000(	373	8.446162	chr16:239	ENSG00000239791		lncRNA	chr16:30572039-305
ENSG0000(	373	8.446162	chr1:2449	ENSG00000286285		lncRNA	chr1:193457422-193
ENSG0000(	373	8.446162	chr16:239	ITGAL		protein_c	chr16:30472658-305
ENSG0000(	373	8.446162	chr16:239	ZNF689		protein_c	chr16:30602558-306
ENSG0000(	373	8.446162	chr16:239	MAZ	NCGv7	protein_c	chr16:29806106-298
ENSG0000(	373	8.446162	chr16:239	ENSG00000275371		lncRNA	chr16:30110895-301
ENSG0000(	373	8.446162	chr1:2449	ENSG00000237283		lncRNA	chr1:188705623-188
ENSG0000(	373	8.446162	chr1:2449	ENSG00000150732		Pseudoger	chr1:188067298-188
ENSG0000(	373	8.446162	chr1:2449	ENSG00000285638		lncRNA	chr1:190878145-191
ENSG0000(	373	8.446162	chr1:2449	ENSG00000227240		lncRNA	chr1:193473224-194
ENSG0000(	373	8.446162	chr16:239	SLX1A-SULT1A3		lncRNA	chr16:30193892-302

ENSG0000(	373	8.446162	chr16:239	PAGR1		protein_c	chr16:29816152-298
ENSG0000(	373	8.446162	chr16:239	ENSG00000288632		lncRNA	chr16:29442917-296
ENSG0000(	373	8.446162	chr16:239	CA5AP1		Pseudoger	chr16:29618785-296
ENSG0000(	373	8.446162	chr1:2449	ENSG00000285280		lncRNA	chr1:192167786-192
ENSG0000(	373	8.446162	chr1:2449	RPS3AP9		Pseudoger	chr1:188694320-188
ENSG0000(	373	8.446162	chr1:2449	ENSG00000271187		Pseudoger	chr1:191179521-191
ENSG0000(	373	8.446162	chr16:239	ENSG00000261459		protein_c	chr16:30525923-305
ENSG0000(	373	8.446162	chr16:239	ZG16	NCGv7	protein_c	chr16:29778256-297
ENSG0000(	373	8.446162	chr16:239	ENSG00000260487		lncRNA	chr16:30480588-304
ENSG0000(	373	8.446162	chr1:2449	HNRNPA1P46		Pseudoger	chr1:191146025-191
ENSG0000(	373	8.446162	chr16:239	ENSG00000279789		TEC	chr16:29913144-299
ENSG0000(	373	8.446162	chr16:239	ENSG00000291047		lncRNA	chr16:30270065-302
ENSG0000(	373	8.446162	chr16:239	PRRT2		protein_c	chr16:29811382-298
ENSG0000(	373	8.446162	chr1:2449	LINC02770		lncRNA	chr1:191823432-192
ENSG0000(	373	8.446162	chr16:239	ENSG00000290693		lncRNA	chr16:30206510-302
ENSG0000(	373	8.446162	chr16:239	ENSG00000257506		Pseudoger	chr16:29478879-294
ENSG0000(	373	8.446162	chr16:239	SNORA42		smallRNA	chr16:30419625-304
ENSG0000(	373	8.446162	chr1:2449	ENSG00000287472		lncRNA	chr1:189868381-189
ENSG0000(	373	8.446162	chr1:2449	ENSG00000238270		lncRNA	chr1:189868001-189
ENSG0000(	373	8.446162	chr1:2449	ENSG00000225006		lncRNA	chr1:188508538-188
ENSG0000(	373	8.446162	chr16:239	ENSG00000260514		Pseudoger	chr16:29624517-296
ENSG0000(	373	8.446162	chr1:2449	RPS27AP5		Pseudoger	chr1:192716183-192
ENSG0000(	373	8.446162	chr16:239	ASPHD1	NCGv7	protein_c	chr16:29900375-299
ENSG0000(	373	8.446162	chr16:239	SEZ6L2		protein_c	chr16:29871159-298
ENSG0000(	373	8.446162	chr16:239	ENSG00000273724		Pseudoger	chr16:30336400-303
ENSG0000(	373	8.446162	chr16:239	ENSG00000261332		lncRNA	chr16:30498766-304
ENSG0000(	373	8.446162	chr16:239	ENSG00000260494		Pseudoger	chr16:30537202-305
ENSG0000(	373	8.446162	chr16:239	INO80E		protein_c	chr16:29995715-300
ENSG0000(	373	8.446162	chr1:2449	ENSG00000261642		lncRNA	chr1:191151510-191
ENSG0000(	373	8.446162	chr1:2449	AL136987.1		smallRNA	chr1:192491128-192
ENSG0000(	373	8.446162	chr1:2449	CLPTM1LP1		Pseudoger	chr1:189035961-189
ENSG0000(	373	8.446162	chr16:239	ENSG00000261444		Pseudoger	chr16:30214409-302
ENSG0000(	373	8.446162	chr1:2449	BRINP3-DT		lncRNA	chr1:190478551-190
ENSG0000(	373	8.446162	chr16:239	snoU13		smallRNA	chr16:29539262-295
ENSG0000(	373	8.446162	chr16:239	ENSG00000260113		Pseudoger	chr16:30626976-306
ENSG0000(	373	8.446162	chr1:2449	RGS21		protein_c	chr1:192316992-192
ENSG0000(	373	8.446162	chr16:239	SMG1P5		Pseudoger	chr16:30267553-303
ENSG0000(	373	8.446162	chr16:239	C16orf92	DriverDBv	protein_c	chr16:30023198-300
ENSG0000(	373	8.446162	chr16:239	ENSG00000280137		TEC	chr16:30379763-303
ENSG0000(	373	8.446162	chr16:239	CDIPTOSP		Pseudoger	chr16:29863593-298
ENSG0000(	373	8.446162	chr16:239	SLX1B-SULT1A4		lncRNA	chr16:29455105-294
ENSG0000(	373	8.446162	chr16:239	SEPTIN1		protein_c	chr16:30378135-303
ENSG0000(	373	8.446162	chr1:2449	ENSG00000223847		Pseudoger	chr1:187714243-187
ENSG0000(	373	8.446162	chr16:239	ENSG00000281348		protein_c	chr16:29817239-298
ENSG0000(	373	8.446162	chr1:2449	LINC01036		lncRNA	chr1:187070700-187
ENSG0000(	373	8.446162	chr16:239	TMEM219		protein_c	chr16:29940885-299
ENSG0000(	373	8.446162	chr16:239	ZNF48		protein_c	chr16:30378106-304
ENSG0000(	373	8.446162	chr1:2449	ENSG00000235083		Pseudoger	chr1:188671353-188
ENSG0000(	373	8.446162	chr16:239	ENSG00000261203		Pseudoger	chr16:30218220-302
ENSG0000(	373	8.446162	chr1:2449	ENSG00000225811		lncRNA	chr1:190264898-190
ENSG0000(	373	8.446162	chr16:239	SULT1A4		protein_c	chr16:29459913-294

ENSG0000(	373	8.446162	chr1:2449	LINC01031		lncRNA	chr1:193304745-193
ENSG0000(	373	8.446162	chr16:239	ENSG00000290680		lncRNA	chr16:29350746-293
ENSG0000(	373	8.446162	chr16:239	ENSG00000278922		TEC	chr16:30526918-305
ENSG0000(	373	8.446162	chr16:239	KIF22		protein_c	chr16:29790727-298
ENSG0000(	373	8.446162	chr16:239	KCTD13		protein_c	chr16:29905012-299
ENSG0000(	373	8.446162	chr16:239	SLX1A		protein_c	chr16:30193875-301
ENSG0000(	372	8.423519	chr7:2516	DDX43P3		Pseudoger	chr7:81610884-8161
ENSG0000(	372	8.423519	chr7:2516	EIF4EP4		Pseudoger	chr7:81463036-8146
ENSG0000(	372	8.423519	chr7:2516	ENSG00000222024		Pseudoger	chr7:79353410-7935
ENSG0000(	372	8.423519	chr7:2516	SPDYE13		protein_c	chr7:75280790-7528
ENSG0000(	372	8.423519	chr10:173	ENSG00000230609		Pseudoger	chr10:72560264-725
ENSG0000(	372	8.423519	chr10:173	ENSG00000232342		lncRNA	chr10:74506081-745
ENSG0000(	372	8.423519	chr7:2516	UPK3B		protein_c	chr7:76510525-7651
ENSG0000(	372	8.423519	chr17:289	ENSG00000286194		lncRNA	chr17:30968785-309
ENSG0000(	372	8.423519	chr10:173	PCBD1		protein_c	chr10:70882280-708
ENSG0000(	372	8.423519	chr17:289	ENSG00000231421		lncRNA	chr17:30573471-305
ENSG0000(	372	8.423519	chr7:2516	GTF2IP1		lncRNA	chr7:75185385-7523
ENSG0000(	372	8.423519	chr7:2516	SPDYE15		protein_c	chr7:75335343-7534
ENSG0000(	372	8.423519	chr7:2516	ENSG00000280388		TEC	chr7:76043977-7604
ENSG0000(	372	8.423519	chr10:173	ENSG00000280166		TEC	chr10:72730557-727
ENSG0000(	372	8.423519	chr7:2516	PMS2P9		Pseudoger	chr7:77039944-7704
ENSG0000(	372	8.423519	chr7:2516	ENSG00000233491		Pseudoger	chr7:81489204-8169
ENSG0000(	372	8.423519	chr17:289	ENSG00000230113		lncRNA	chr17:30956280-309
ENSG0000(	372	8.423519	chr10:173	ENSG00000233144		lncRNA	chr10:73381433-733
ENSG0000(	372	8.423519	chr17:289	ANKRD13B		protein_c	chr17:29589769-296
ENSG0000(	372	8.423519	chr7:2516	RPL13AP17		Pseudoger	chr7:78347142-7835
ENSG0000(	372	8.423519	chr10:173	C10orf55		lncRNA	chr10:73909969-739
ENSG0000(	372	8.423519	chr7:2516	ENSG00000259628		lncRNA	chr7:77043721-7719
ENSG0000(	372	8.423519	chr7:2516	Y_RNA		smallRNA	chr7:77895880-7789
ENSG0000(	372	8.423519	chr17:289	MIR423		smallRNA	chr17:30117079-301
ENSG0000(	372	8.423519	chr7:2516	ENSG00000230882		Pseudoger	chr7:76071469-7607
ENSG0000(	372	8.423519	chr7:2516	SNRPBP1		Pseudoger	chr7:80377554-8037
ENSG0000(	372	8.423519	chr10:173	PSAP		protein_c	chr10:71816298-718
ENSG0000(	372	8.423519	chr17:289	SSH2		protein_c	chr17:29625938-299
ENSG0000(	372	8.423519	chr10:173	MTND2P15		Pseudoger	chr10:69594041-695
ENSG0000(	372	8.423519	chr7:2516	ENSG00000285892		lncRNA	chr7:78392626-7844
ENSG0000(	372	8.423519	chr10:173	NDST2		protein_c	chr10:73801911-738
ENSG0000(	372	8.423519	chr10:173	ENSG00000230526		lncRNA	chr10:71878356-718
ENSG0000(	372	8.423519	chr17:289	RNU6-920P		smallRNA	chr17:29641611-296
ENSG0000(	372	8.423519	chr10:173	ENSG00000231748		lncRNA	chr10:69265342-692
ENSG0000(	372	8.423519	chr10:173	ENSG00000280238		TEC	chr10:73791218-737
ENSG0000(	372	8.423519	chr10:173	TMEM256P1		Pseudoger	chr10:69523311-695
ENSG0000(	372	8.423519	chr17:289	RNU6ATAC7P		smallRNA	chr17:31563768-315
ENSG0000(	372	8.423519	chr17:289	ENSG00000280245		TEC	chr17:32504567-325
ENSG0000(	372	8.423519	chr7:2516	GNAI1	NCGv7	protein_c	chr7:79768028-8022
ENSG0000(	372	8.423519	chr7:2516	ENSG00000290834		lncRNA	chr7:75391955-7539
ENSG0000(	372	8.423519	chr7:2516	GATSL2		protein_c	chr7:74964817-7502
ENSG0000(	372	8.423519	chr7:2516	CACNA2D1-AS1		lncRNA	chr7:82009177-8202
ENSG0000(	372	8.423519	chr17:289	ENSG00000290974		lncRNA	chr17:32088160-320
ENSG0000(	372	8.423519	chr7:2516	STAG3L2		Pseudoger	chr7:74882705-7489
ENSG0000(	372	8.423519	chr17:289	ENSG00000290928		lncRNA	chr17:30709299-307

ENSG0000(	372	8.423519	chr7:251(	RSBN1L	NCGv7	protein_c	chr7:77696459-7778
ENSG0000(	372	8.423519	chr7:251(	HGF	NCGv7	protein_c	chr7:81699010-8177
ENSG0000(	372	8.423519	chr7:251(	PMS2P3		lncRNA	chr7:75507747-7552
ENSG0000(	372	8.423519	chr10:17:	LINC02636		lncRNA	chr10:69994276-700
ENSG0000(	372	8.423519	chr10:17:	RPS26P40		Pseudoger	chr10:70794230-707
ENSG0000(	372	8.423519	chr7:251(	NCF1C		Pseudoger	chr7:75156639-7517
ENSG0000(	372	8.423519	chr17:28(	ENSG00000275185		lncRNA	chr17:30899110-308
ENSG0000(	372	8.423519	chr17:28(	NSRP1	NCGv7	protein_c	chr17:30115521-301
ENSG0000(	372	8.423519	chr10:17:	CDH23-AS1		lncRNA	chr10:71508153-715
ENSG0000(	372	8.423519	chr17:28(	RNU6-711P		smallRNA	chr17:29424762-294
ENSG0000(	372	8.423519	chr10:17:	AIFM2		protein_c	chr10:70098223-701
ENSG0000(	372	8.423519	chr7:251(	PTPN12	NCGv7	protein_c	chr7:77537295-7764
ENSG0000(	372	8.423519	chr7:251(	POR		protein_c	chr7:75899200-7598
ENSG0000(	372	8.423519	chr7:251(	FGL2		protein_c	chr7:77193369-7719
ENSG0000(	372	8.423519	chr10:17:	ENSG00000282915		lncRNA	chr10:72467749-724
ENSG0000(	372	8.423519	chr7:251(	STYXL1		protein_c	chr7:75996338-7604
ENSG0000(	372	8.423519	chr7:251(	CCDC146		protein_c	chr7:77122434-7732
ENSG0000(	372	8.423519	chr7:251(	ENSG00000280958		TEC	chr7:78170195-7817
ENSG0000(	372	8.423519	chr17:28(	RNA5SP437		Pseudoger	chr17:31963805-319
ENSG0000(	372	8.423519	chr7:251(	GTF2IP1		Pseudoger	chr7:75187242-7521
ENSG0000(	372	8.423519	chr10:17:	TBATA		protein_c	chr10:70771238-707
ENSG0000(	372	8.423519	chr10:17:	ENSG00000285300		lncRNA	chr10:70927055-710
ENSG0000(	372	8.423519	chr10:17:	GLUD1P3		lncRNA	chr10:73730562-737
ENSG0000(	372	8.423519	chr7:251(	ENSG00000223514		Pseudoger	chr7:81335106-8133
ENSG0000(	372	8.423519	chr7:251(	ENSG00000290951		lncRNA	chr7:75359202-7539
ENSG0000(	372	8.423519	chr10:17:	CEP57L1P1		Pseudoger	chr10:70389426-703
ENSG0000(	372	8.423519	chr7:251(	AC004878.3		Pseudoger	chr7:75315964-7532
ENSG0000(	372	8.423519	chr7:251(	AC005159.1		smallRNA	chr7:82290077-8229
ENSG0000(	372	8.423519	chr17:28(	RHOT1		protein_c	chr17:32142454-322
ENSG0000(	372	8.423519	chr10:17:	RPS25P9		Pseudoger	chr10:70198988-701
ENSG0000(	372	8.423519	chr17:28(	ENSG00000290975		lncRNA	chr17:32106332-321
ENSG0000(	372	8.423519	chr17:28(	EVI2A	NCGv7;AC	protein_c	chr17:31316410-313
ENSG0000(	372	8.423519	chr10:17:	LINC02622		lncRNA	chr10:70918766-709
ENSG0000(	372	8.423519	chr10:17:	VCL		protein_c	chr10:73995193-741
ENSG0000(	372	8.423519	chr17:28(	OMG		protein_c	chr17:31272013-312
ENSG0000(	372	8.423519	chr7:251(	SPDYE14		protein_c	chr7:75308718-7531
ENSG0000(	372	8.423519	chr10:17:	USP54		protein_c	chr10:73497538-736
ENSG0000(	372	8.423519	chr10:17:	ENSG00000280401		TEC	chr10:70434829-704
ENSG0000(	372	8.423519	chr10:17:	RMRPP1		smallRNA	chr10:73774322-737
ENSG0000(	372	8.423519	chr7:251(	ENSG00000281008		TEC	chr7:78134079-7813
ENSG0000(	372	8.423519	chr10:17:	MSS51		protein_c	chr10:73423579-734
ENSG0000(	372	8.423519	chr7:251(	NSUN5P1		Pseudoger	chr7:75410368-7541
ENSG0000(	372	8.423519	chr7:251(	PHB1P6		Pseudoger	chr7:75203926-7520
ENSG0000(	372	8.423519	chr7:251(	TMEM60		protein_c	chr7:77793728-7779
ENSG0000(	372	8.423519	chr7:251(	CD36	NCGv7	protein_c	chr7:80369575-8067
ENSG0000(	372	8.423519	chr10:17:	RPL5P26		Pseudoger	chr10:69778962-697
ENSG0000(	372	8.423519	chr7:251(	PMS2P3		Pseudoger	chr7:75510931-7551
ENSG0000(	372	8.423519	chr17:28(	ENSG00000290858		lncRNA	chr17:31041088-310
ENSG0000(	372	8.423519	chr17:28(	ENSG00000267482		Pseudoger	chr17:30059054-300
ENSG0000(	372	8.423519	chr10:17:	ANXA7		protein_c	chr10:73375101-734
ENSG0000(	372	8.423519	chr10:17:	AL359832.1		smallRNA	chr10:71215800-712

ENSG0000(	372	8.423519	chr17:289	ABHD15	NCGv7	protein_c	chr17:29560547-295
ENSG0000(	372	8.423519	chr7:2516	GTF2I-AS1		lncRNA	chr7:74688864-7472
ENSG0000(	372	8.423519	chr10:173	ATP5MC1P7		Pseudoger	chr10:69432972-694
ENSG0000(	372	8.423519	chr10:173	PPP3CB-AS1		lncRNA	chr10:73495525-735
ENSG0000(	372	8.423519	chr10:173	ANAPC16		protein_c	chr10:72216000-722
ENSG0000(	372	8.423519	chr17:289	TP53I13		protein_c	chr17:29566052-295
ENSG0000(	372	8.423519	chr7:2516	DDX3ILA1		lncRNA	chr7:77990384-7799
ENSG0000(	372	8.423519	chr17:289	KRT17P3		Pseudoger	chr17:30567700-305
ENSG0000(	372	8.423519	chr17:289	RNU4-34P		smallRNA	chr17:29388560-293
ENSG0000(	372	8.423519	chr17:289	CORO6	DriverDBv	protein_c	chr17:29614756-296
ENSG0000(	372	8.423519	chr17:289	ENSG00000259928		lncRNA	chr17:30564057-305
ENSG0000(	372	8.423519	chr7:2516	RABGEF1P3		Pseudoger	chr7:76108434-7613
ENSG0000(	372	8.423519	chr17:289	RAB11FIP4	DriverDBv	protein_c	chr17:31391675-315
ENSG0000(	372	8.423519	chr10:173	AC025426.1		smallRNA	chr10:69812695-698
ENSG0000(	372	8.423519	chr7:2516	RPL10P11		Pseudoger	chr7:80096600-8009
ENSG0000(	372	8.423519	chr7:2516	ENSG00000219039		Pseudoger	chr7:75835663-7583
ENSG0000(	372	8.423519	chr7:2516	ENSG00000291121		lncRNA	chr7:77083681-7712
ENSG0000(	372	8.423519	chr10:173	SLC29A3		protein_c	chr10:71319259-713
ENSG0000(	372	8.423519	chr10:173	RPL26P6		Pseudoger	chr10:73422259-734
ENSG0000(	372	8.423519	chr17:289	ENSG00000277511		lncRNA	chr17:32127570-321
ENSG0000(	372	8.423519	chr10:173	FAM149B1		protein_c	chr10:73168119-732
ENSG0000(	372	8.423519	chr10:173	SNX19P4		Pseudoger	chr10:72483245-724
ENSG0000(	372	8.423519	chr7:2516	MAGI2	NCGv7	protein_c	chr7:78017055-7945
ENSG0000(	372	8.423519	chr7:2516	ENSG00000281120		lncRNA	chr7:79139829-7917
ENSG0000(	372	8.423519	chr10:173	ADAMTS14		protein_c	chr10:70672506-707
ENSG0000(	372	8.423519	chr10:173	ENSG00000229990		lncRNA	chr10:73841833-738
ENSG0000(	372	8.423519	chr10:173	NUDT13		protein_c	chr10:73110375-731
ENSG0000(	372	8.423519	chr17:289	ENSG00000276250		lncRNA	chr17:30803654-308
ENSG0000(	372	8.423519	chr10:173	CAMK2G-AS1		lncRNA	chr10:73813518-738
ENSG0000(	372	8.423519	chr10:173	SGPL1		protein_c	chr10:70815905-708
ENSG0000(	372	8.423519	chr10:173	OIT3		protein_c	chr10:72893584-729
ENSG0000(	372	8.423519	chr10:173	PLA2G12B		protein_c	chr10:72934762-729
ENSG0000(	372	8.423519	chr10:173	SYNPO2L		protein_c	chr10:73644881-736
ENSG0000(	372	8.423519	chr7:2516	ENSG00000277675		lncRNA	chr7:75225433-7523
ENSG0000(	372	8.423519	chr10:173	MTATP6P23		Pseudoger	chr10:69589880-695
ENSG0000(	372	8.423519	chr10:173	ASCC1		protein_c	chr10:72096032-722
ENSG0000(	372	8.423519	chr10:173	HMGN2P34		Pseudoger	chr10:72636497-726
ENSG0000(	372	8.423519	chr10:173	YY1P1		Pseudoger	chr10:70485336-704
ENSG0000(	372	8.423519	chr10:173	DDIT4		protein_c	chr10:72273920-722
ENSG0000(	372	8.423519	chr7:2516	ENSG00000275121		Pseudoger	chr7:75237293-7523
ENSG0000(	372	8.423519	chr10:173	ENSG00000232646		Pseudoger	chr10:73065126-730
ENSG0000(	372	8.423519	chr17:289	ENSG00000291063		lncRNA	chr17:31008497-310
ENSG0000(	372	8.423519	chr7:2516	ENSG00000229436		Pseudoger	chr7:80662331-8066
ENSG0000(	372	8.423519	chr7:2516	ENSG00000224134		Pseudoger	chr7:81431731-8143
ENSG0000(	372	8.423519	chr7:2516	ENSG00000232667		lncRNA	chr7:80312574-8039
ENSG0000(	372	8.423519	chr7:2516	HIP1	NCGv7;AC	protein_c	chr7:75533298-7573
ENSG0000(	372	8.423519	chr7:2516	Y_RNA		smallRNA	chr7:75381638-7538
ENSG0000(	372	8.423519	chr7:2516	RCC1L		protein_c	chr7:75027122-7507
ENSG0000(	372	8.423519	chr17:289	PSMD11		protein_c	chr17:32444379-324
ENSG0000(	372	8.423519	chr10:173	ENSG00000290737		lncRNA	chr10:73715843-737
ENSG0000(	372	8.423519	chr17:289	ABHD15-AS1		lncRNA	chr17:29560547-297

ENSG0000(	372	8.423519	chr10:17:	ENSG00000272748		lncRNA	chr10:74821610-748
ENSG0000(	372	8.423519	chr17:28(	ENSG00000264050		Pseudoger	chr17:29197071-291
ENSG0000(	372	8.423519	chr17:28(	NF1	NCGv7;AC	protein_c	chr17:31094927-313
ENSG0000(	372	8.423519	chr7:251(	RHBDD2		protein_c	chr7:75842602-7588
ENSG0000(	372	8.423519	chr10:17:	Y_RNA		smallRNA	chr10:72220752-722
ENSG0000(	372	8.423519	chr17:28(	C17orf75		protein_c	chr17:32324565-323
ENSG0000(	372	8.423519	chr10:17:	ENSG00000270808		Pseudoger	chr10:73740538-737
ENSG0000(	372	8.423519	chr17:28(	ENSG00000279781		TEC	chr17:32518322-325
ENSG0000(	372	8.423519	chr17:28(	ENSG00000264083		lncRNA	chr17:32430967-324
ENSG0000(	372	8.423519	chr17:28(	Y_RNA		smallRNA	chr17:30344503-303
ENSG0000(	372	8.423519	chr17:28(	UTP6		protein_c	chr17:31860904-319
ENSG0000(	372	8.423519	chr10:17:	ENSG00000272630		lncRNA	chr10:73098044-731
ENSG0000(	372	8.423519	chr10:17:	ENSG00000272627		lncRNA	chr10:72766560-727
ENSG0000(	372	8.423519	chr17:28(	MIR4733HG		lncRNA	chr17:31090787-310
ENSG0000(	372	8.423519	chr10:17:	ENSG00000279088		TEC	chr10:73742962-737
ENSG0000(	372	8.423519	chr17:28(	ENSG00000264007		lncRNA	chr17:29621617-296
ENSG0000(	372	8.423519	chr7:251(	Y_RNA		smallRNA	chr7:75353885-7535
ENSG0000(	372	8.423519	chr10:17:	TIMM9P1		Pseudoger	chr10:74344550-743
ENSG0000(	372	8.423519	chr17:28(	ENSG00000266599		lncRNA	chr17:32518953-325
ENSG0000(	372	8.423519	chr10:17:	DNAJC9-AS1		lncRNA	chr10:73247360-732
ENSG0000(	372	8.423519	chr17:28(	MIR193A		smallRNA	chr17:31559996-315
ENSG0000(	372	8.423519	chr17:28(	ENSG00000290404		lncRNA	chr17:30557112-305
ENSG0000(	372	8.423519	chr10:17:	ENSG00000272916		protein_c	chr10:73796514-738
ENSG0000(	372	8.423519	chr17:28(	ENSG00000290395		lncRNA	chr17:30600796-306
ENSG0000(	372	8.423519	chr17:28(	ENSG00000263860		lncRNA	chr17:30550493-305
ENSG0000(	372	8.423519	chr10:17:	Y_RNA		smallRNA	chr10:73346936-733
ENSG0000(	372	8.423519	chr10:17:	RPS26P41		Pseudoger	chr10:73537803-735
ENSG0000(	372	8.423519	chr17:28(	RN7SL316P		smallRNA	chr17:30702508-307
ENSG0000(	372	8.423519	chr7:251(	GTF2IRD2B		protein_c	chr7:75092573-7514
ENSG0000(	372	8.423519	chr10:17:	ENSG00000272791		lncRNA	chr10:73630556-736
ENSG0000(	372	8.423519	chr7:251(	AC006014.1		protein_c	chr7:75370632-7537
ENSG0000(	372	8.423519	chr17:28(	AC116407.1		smallRNA	chr17:32135729-321
ENSG0000(	372	8.423519	chr10:17:	RNU6-883P		smallRNA	chr10:73529051-735
ENSG0000(	372	8.423519	chr17:28(	ENSG00000263990		lncRNA	chr17:31873926-318
ENSG0000(	372	8.423519	chr7:251(	PMS2P11		Pseudoger	chr7:77011551-7701
ENSG0000(	372	8.423519	chr17:28(	ENSG00000264125		lncRNA	chr17:30204318-302
ENSG0000(	372	8.423519	chr17:28(	GOSR1		protein_c	chr17:30477362-305
ENSG0000(	372	8.423519	chr7:251(	RN7SL642P		smallRNA	chr7:75580643-7558
ENSG0000(	372	8.423519	chr10:17:	ENSG00000226163		lncRNA	chr10:72501746-725
ENSG0000(	372	8.423519	chr17:28(	ENSG00000264300		Pseudoger	chr17:32003110-320
ENSG0000(	372	8.423519	chr10:17:	PPP3CB		protein_c	chr10:73436433-734
ENSG0000(	372	8.423519	chr10:17:	MICU1		protein_c	chr10:72367340-726
ENSG0000(	372	8.423519	chr10:17:	SPOCK2		protein_c	chr10:72059034-720
ENSG0000(	372	8.423519	chr10:17:	VSIR		protein_c	chr10:71747556-717
ENSG0000(	372	8.423519	chr10:17:	CDH23	NCGv7	protein_c	chr10:71396920-718
ENSG0000(	372	8.423519	chr10:17:	UNC5B		protein_c	chr10:71212570-713
ENSG0000(	372	8.423519	chr17:28(	ENSG00000266490		lncRNA	chr17:30792372-307
ENSG0000(	372	8.423519	chr10:17:	PALD1		protein_c	chr10:70478767-706
ENSG0000(	372	8.423519	chr17:28(	MIR4724		smallRNA	chr17:31534883-315
ENSG0000(	372	8.423519	chr7:251(	ENSG00000287815		lncRNA	chr7:74280747-7428
ENSG0000(	372	8.423519	chr7:251(	RNU6-849P		smallRNA	chr7:79912104-7991

ENSG0000(	372	8.423519	chr17:289	ATAD5		protein_c	chr17:30831966-308
ENSG0000(	372	8.423519	chr7:2516	MAGI2-AS1		lncRNA	chr7:78939850-7894
ENSG0000(	372	8.423519	chr7:2516	SNORA15		smallRNA	chr7:65760052-6576
ENSG0000(	372	8.423519	chr17:289	ENSG00000264290		lncRNA	chr17:29569580-295
ENSG0000(	372	8.423519	chr7:2516	GCNT1P5		Pseudoger	chr7:77461458-7746
ENSG0000(	372	8.423519	chr10:173	Metazoa_SRP		smallRNA	chr10:72582803-725
ENSG0000(	372	8.423519	chr17:289	CRYBA1		protein_c	chr17:29246859-292
ENSG0000(	372	8.423519	chr17:289	CPD		protein_c	chr17:30378927-304
ENSG0000(	372	8.423519	chr17:289	BLMH		protein_c	chr17:30248203-302
ENSG0000(	372	8.423519	chr17:289	SLC6A4		protein_c	chr17:30194319-302
ENSG0000(	372	8.423519	chr17:289	ENSG00000264148		Pseudoger	chr17:31008154-310
ENSG0000(	372	8.423519	chr17:289	ENSG00000264164		Pseudoger	chr17:32083179-320
ENSG0000(	372	8.423519	chr17:289	RNY4P13		smallRNA	chr17:30059052-300
ENSG0000(	372	8.423519	chr17:289	LRRC37B		protein_c	chr17:32007383-320
ENSG0000(	372	8.423519	chr10:173	ENSG00000272599		lncRNA	chr10:73124573-731
ENSG0000(	372	8.423519	chr10:173	SNORA36		smallRNA	chr10:72180858-721
ENSG0000(	372	8.423519	chr17:289	ENSG00000279762		lncRNA	chr17:32423971-324
ENSG0000(	372	8.423519	chr10:173	ZSWIM8-AS1		lncRNA	chr10:73796514-738
ENSG0000(	372	8.423519	chr10:173	UNC5B-AS1		lncRNA	chr10:71217220-712
ENSG0000(	372	8.423519	chr17:289	ENSG00000290082		lncRNA	chr17:29567823-295
ENSG0000(	372	8.423519	chr17:289	GIT1		protein_c	chr17:29573475-295
ENSG0000(	372	8.423519	chr17:289	NUFIP2		protein_c	chr17:29255839-292
ENSG0000(	372	8.423519	chr17:289	OOSP1P2		Pseudoger	chr17:32343528-323
ENSG0000(	372	8.423519	chr17:289	MIR4523		smallRNA	chr17:29390662-293
ENSG0000(	372	8.423519	chr10:173	FAM32CP		Pseudoger	chr10:74744386-747
ENSG0000(	372	8.423519	chr10:173	MTCO2P23		Pseudoger	chr10:69590583-695
ENSG0000(	372	8.423519	chr17:289	SH3GL1P1		Pseudoger	chr17:32039974-320
ENSG0000(	372	8.423519	chr10:173	MTCO1P23		Pseudoger	chr10:69591406-695
ENSG0000(	372	8.423519	chr17:289	ENSG00000266775		lncRNA	chr17:30557732-305
ENSG0000(	372	8.423519	chr7:2516	ENSG00000228711		Pseudoger	chr7:82657035-8265
ENSG0000(	372	8.423519	chr7:2516	ZP3		protein_c	chr7:76397518-7644
ENSG0000(	372	8.423519	chr10:173	LINC02651		lncRNA	chr10:69684899-696
ENSG0000(	372	8.423519	chr17:289	SNORD63		smallRNA	chr17:30246757-302
ENSG0000(	372	8.423519	chr17:289	RNU6-298P		smallRNA	chr17:30861843-308
ENSG0000(	372	8.423519	chr17:289	MIR365BHG		lncRNA	chr17:31571142-315
ENSG0000(	372	8.423519	chr10:173	RNA5SP320		Pseudoger	chr10:73706490-737
ENSG0000(	372	8.423519	chr10:173	CHCHD1		protein_c	chr10:73782047-737
ENSG0000(	372	8.423519	chr7:2516	ENSG00000242073		Pseudoger	chr7:75474707-7548
ENSG0000(	372	8.423519	chr10:173	AGAP5		protein_c	chr10:73674287-736
ENSG0000(	372	8.423519	chr17:289	TBC1D29P	NCGv7	Pseudoger	chr17:30553502-305
ENSG0000(	372	8.423519	chr7:2516	GTF2IRD1		protein_c	chr7:74453790-7460
ENSG0000(	372	8.423519	chr10:173	LRRC20		protein_c	chr10:70298970-703
ENSG0000(	372	8.423519	chr17:289	COPRS		protein_c	chr17:31851871-318
ENSG0000(	372	8.423519	chr7:2516	RNU6-530P		smallRNA	chr7:79343266-7934
ENSG0000(	372	8.423519	chr7:2516	ENSG00000287519		lncRNA	chr7:77487317-7749
ENSG0000(	372	8.423519	chr17:289	ENSG00000278867		TEC	chr17:32051030-320
ENSG0000(	372	8.423519	chr7:2516	PCLO	DriverDBv	protein_c	chr7:82754012-8316
ENSG0000(	372	8.423519	chr17:289	ENSG00000280020		TEC	chr17:32178946-321
ENSG0000(	372	8.423519	chr17:289	ENSG00000273609		Pseudoger	chr17:30617680-306
ENSG0000(	372	8.423519	chr17:289	TAOK1		protein_c	chr17:29390363-295
ENSG0000(	372	8.423519	chr7:2516	RPL7L1P3		Pseudoger	chr7:75922755-7592

ENSG0000(	372	8.423519	chr10:173	CAMK2G		protein_c	chr10:73812501-738
ENSG0000(	372	8.423519	chr7:2516	ENSG00000228829		Pseudoger	chr7:76173733-7617
ENSG0000(	372	8.423519	chr7:2516	AC004851.1		smallRNA	chr7:74571347-7457
ENSG0000(	372	8.423519	chr7:2516	ENSG00000279996		TEC	chr7:75625089-7562
ENSG0000(	372	8.423519	chr10:173	DNAJB12		protein_c	chr10:72332830-723
ENSG0000(	372	8.423519	chr10:173	EIF4EBP2		protein_c	chr10:70404145-704
ENSG0000(	372	8.423519	chr10:173	DUSP8P5		Pseudoger	chr10:73731824-737
ENSG0000(	372	8.423519	chr10:173	NPFFR1		protein_c	chr10:70247329-702
ENSG0000(	372	8.423519	chr7:2516	ENSG00000273341		lncRNA	chr7:77416673-7742
ENSG0000(	372	8.423519	chr17:289	TEFM		protein_c	chr17:30897336-309
ENSG0000(	372	8.423519	chr7:2516	RNU6-337P		smallRNA	chr7:79030240-7903
ENSG0000(	372	8.423519	chr7:2516	PHB1P15		Pseudoger	chr7:74741457-7474
ENSG0000(	372	8.423519	chr10:173	MIR4676		smallRNA	chr10:72721029-727
ENSG0000(	372	8.423519	chr10:173	ENSG00000287306		lncRNA	chr10:69510166-695
ENSG0000(	372	8.423519	chr10:173	ENSG00000272988		lncRNA	chr10:72053294-720
ENSG0000(	372	8.423519	chr10:173	ENSG00000268584		lncRNA	chr10:73625996-736
ENSG0000(	372	8.423519	chr10:173	ENSG00000225761		lncRNA	chr10:74005137-740
ENSG0000(	372	8.423519	chr17:289	ENSG00000263567		lncRNA	chr17:31762440-317
ENSG0000(	372	8.423519	chr7:2516	RN7SL212P		smallRNA	chr7:76288791-7628
ENSG0000(	372	8.423519	chr7:2516	RNU6-863P		smallRNA	chr7:76087444-7608
ENSG0000(	372	8.423519	chr17:289	ENSG00000263603		lncRNA	chr17:30729469-307
ENSG0000(	372	8.423519	chr17:289	EVI2B		protein_c	chr17:31303770-313
ENSG0000(	372	8.423519	chr17:289	MIR632		smallRNA	chr17:32350109-323
ENSG0000(	372	8.423519	chr17:289	ENSG00000266718		lncRNA	chr17:32495536-325
ENSG0000(	372	8.423519	chr7:2516	ENSG00000287488		lncRNA	chr7:79657947-7967
ENSG0000(	372	8.423519	chr17:289	ENSG00000287506		lncRNA	chr17:32258615-322
ENSG0000(	372	8.423519	chr17:289	ENSG00000263657		lncRNA	chr17:29761103-297
ENSG0000(	372	8.423519	chr17:289	ENSG00000263674		lncRNA	chr17:32280387-322
ENSG0000(	372	8.423519	chr7:2516	AC005077.9		Pseudoger	chr7:76108434-7610
ENSG0000(	372	8.423519	chr17:289	ENSG00000263709		lncRNA	chr17:29140483-291
ENSG0000(	372	8.423519	chr17:289	ENSG00000263717		lncRNA	chr17:32512869-325
ENSG0000(	372	8.423519	chr17:289	AK4P1		Pseudoger	chr17:31345519-313
ENSG0000(	372	8.423519	chr17:289	ENSG00000263531		lncRNA	chr17:30863921-308
ENSG0000(	372	8.423519	chr7:2516	ENSG00000279005		TEC	chr7:74633510-7463
ENSG0000(	372	8.423519	chr7:2516	ENSG00000273069		lncRNA	chr7:74606913-7460
ENSG0000(	372	8.423519	chr7:2516	CCL26	NCGv7	protein_c	chr7:75769533-7578
ENSG0000(	372	8.423519	chr10:173	ENSG00000236154		lncRNA	chr10:69572906-695
ENSG0000(	372	8.423519	chr17:289	ENSG00000278977		TEC	chr17:31533171-315
ENSG0000(	372	8.423519	chr7:2516	GTF2I	NCGv7	protein_c	chr7:74650231-7476
ENSG0000(	372	8.423519	chr7:2516	ENSG00000263081		lncRNA	chr7:75232928-7523
ENSG0000(	372	8.423519	chr7:2516	PHTF2		protein_c	chr7:77798773-7795
ENSG0000(	372	8.423519	chr7:2516	GSAP	DriverDBv	protein_c	chr7:77310751-7741
ENSG0000(	372	8.423519	chr17:289	ENSG00000263369		Pseudoger	chr17:32106330-321
ENSG0000(	372	8.423519	chr17:289	ENSG00000263370		lncRNA	chr17:29639627-296
ENSG0000(	372	8.423519	chr7:2516	AC006145.1		smallRNA	chr7:81920203-8192
ENSG0000(	372	8.423519	chr17:289	ENSG00000263477		lncRNA	chr17:29863402-298
ENSG0000(	372	8.423519	chr7:2516	NCF1		protein_c	chr7:74774011-7478
ENSG0000(	372	8.423519	chr17:289	ENSG00000290450		lncRNA	chr17:30631755-306
ENSG0000(	372	8.423519	chr10:173	FUT11		protein_c	chr10:73772276-737
ENSG0000(	372	8.423519	chr7:2516	ENSG00000225726		Pseudoger	chr7:77071751-7707
ENSG0000(	372	8.423519	chr10:173	RNU6-833P		smallRNA	chr10:73279062-732

ENSG00000	372	8.423519	chr17:289	CRLF3		protein_c	chr17:30769388-308
ENSG00000	372	8.423519	chr17:289	ENSG00000266448		Pseudogen	chr17:31709568-317
ENSG00000	372	8.423519	chr7:2516	ENSG00000289346		protein_c	chr7:74796150-7489
ENSG00000	372	8.423519	chr7:2516	Y_RNA		smallRNA	chr7:74895816-7489
ENSG00000	372	8.423519	chr10:173	SAR1A		protein_c	chr10:70147289-701
ENSG00000	372	8.423519	chr17:289	snoU13		smallRNA	chr17:29883006-298
ENSG00000	372	8.423519	chr17:289	LRRC37BP1		Pseudogen	chr17:30629680-306
ENSG00000	372	8.423519	chr7:2516	ENSG00000279326		TEC	chr7:80972516-8097
ENSG00000	372	8.423519	chr17:289	RNU6-1034P		smallRNA	chr17:29445300-294
ENSG00000	372	8.423519	chr17:289	WDR45BP1		Pseudogen	chr17:32111562-321
ENSG00000	372	8.423519	chr10:173	ENSG00000289362		lncRNA	chr10:71551031-715
ENSG00000	372	8.423519	chr7:2516	RNA5SP234		Pseudogen	chr7:79654109-7965
ENSG00000	372	8.423519	chr7:2516	Y_RNA		smallRNA	chr7:75325959-7532
ENSG00000	372	8.423519	chr17:289	RPL35AP35		Pseudogen	chr17:29340482-293
ENSG00000	372	8.423519	chr7:2516	GTF2IRD2		protein_c	chr7:74796144-7485
ENSG00000	372	8.423519	chr10:173	MRPS16		protein_c	chr10:73248843-732
ENSG00000	372	8.423519	chr7:2516	Y_RNA		smallRNA	chr7:75513025-7551
ENSG00000	372	8.423519	chr17:289	ENSG00000279337		TEC	chr17:30724982-307
ENSG00000	372	8.423519	chr17:289	Y_RNA		smallRNA	chr17:32463374-324
ENSG00000	372	8.423519	chr10:173	ENSG00000289193		lncRNA	chr10:69808768-698
ENSG00000	372	8.423519	chr7:2516	DTX2P1-UPK3BP1-PMS		lncRNA	chr7:76959835-7704
ENSG00000	372	8.423519	chr10:173	Y_RNA		smallRNA	chr10:74551392-745
ENSG00000	372	8.423519	chr7:2516	snoU13		smallRNA	chr7:76112798-7611
ENSG00000	372	8.423519	chr10:173	PRF1	NCGv7;AC	protein_c	chr10:70597348-706
ENSG00000	372	8.423519	chr7:2516	ENSG00000289996		lncRNA	chr7:81186339-8121
ENSG00000	372	8.423519	chr10:173	PPA1		protein_c	chr10:70202835-702
ENSG00000	372	8.423519	chr7:2516	ENSG00000250614		lncRNA	chr7:76474587-7647
ENSG00000	372	8.423519	chr17:289	ENSG00000265334		lncRNA	chr17:30834325-308
ENSG00000	372	8.423519	chr10:173	snoU13		smallRNA	chr10:71325328-713
ENSG00000	372	8.423519	chr10:173	NDUFA8P1		Pseudogen	chr10:74641410-746
ENSG00000	372	8.423519	chr17:289	ENSG00000265394		lncRNA	chr17:30090366-301
ENSG00000	372	8.423519	chr10:173	RPL15P14		Pseudogen	chr10:72189941-721
ENSG00000	372	8.423519	chr7:2516	ENSG00000289059		lncRNA	chr7:76318253-7631
ENSG00000	372	8.423519	chr10:173	snoU13		smallRNA	chr10:73301781-733
ENSG00000	372	8.423519	chr7:2516	ENSG00000226671		Pseudogen	chr7:81191398-8119
ENSG00000	372	8.423519	chr10:173	RNU6-805P		smallRNA	chr10:72391964-723
ENSG00000	372	8.423519	chr17:289	ENSG00000265443		lncRNA	chr17:30726305-307
ENSG00000	372	8.423519	chr17:289	MIR4733		smallRNA	chr17:31094350-310
ENSG00000	372	8.423519	chr17:289	RNF135		protein_c	chr17:30970984-309
ENSG00000	372	8.423519	chr17:289	TMIGD1		protein_c	chr17:30316333-303
ENSG00000	372	8.423519	chr10:173	MACROH2A2		protein_c	chr10:70052544-701
ENSG00000	372	8.423519	chr17:289	ENSG00000265625		lncRNA	chr17:29644796-296
ENSG00000	372	8.423519	chr17:289	RPL9P30		Pseudogen	chr17:29855759-298
ENSG00000	372	8.423519	chr10:173	ENSG00000289607		lncRNA	chr10:71340814-713
ENSG00000	372	8.423519	chr17:289	ENSG00000266120		lncRNA	chr17:30238741-302
ENSG00000	372	8.423519	chr17:289	ENSG00000266111		lncRNA	chr17:29352069-294
ENSG00000	372	8.423519	chr17:289	GPR160P2		Pseudogen	chr17:31759795-317
ENSG00000	372	8.423519	chr10:173	ENSG00000279406		TEC	chr10:71364243-713
ENSG00000	372	8.423519	chr10:173	Y_RNA		smallRNA	chr10:73082494-730
ENSG00000	372	8.423519	chr17:289	RPL21P123		Pseudogen	chr17:29716279-297
ENSG00000	372	8.423519	chr17:289	MIR4725		smallRNA	chr17:31575269-315

ENSG0000(	372	8.423519	chr10:173	TSPAN15		protein_c	chr10:69451465-695
ENSG0000(	372	8.423519	chr10:173	ENSG00000289738		lncRNA	chr10:70815989-709
ENSG0000(	372	8.423519	chr10:173	ENSG00000289739		lncRNA	chr10:70929925-709
ENSG0000(	372	8.423519	chr7:2516	CACNA2D1	NCGv7	protein_c	chr7:81946444-8244
ENSG0000(	372	8.423519	chr17:289	RNU6-990P		smallRNA	chr17:30360463-303
ENSG0000(	372	8.423519	chr10:173	AL138925.1		smallRNA	chr10:69859214-698
ENSG0000(	372	8.423519	chr7:2516	GTF2IP7		Pseudoger	chr7:76099440-7610
ENSG0000(	372	8.423519	chr7:2516	MAGI2-AS2		lncRNA	chr7:79008988-7901
ENSG0000(	372	8.423519	chr10:173	ENSG00000289592		lncRNA	chr10:71888499-718
ENSG0000(	372	8.423519	chr7:2516	SNORA14A		smallRNA	chr7:75943782-7594
ENSG0000(	372	8.423519	chr7:2516	DTX2		protein_c	chr7:76461676-7650
ENSG0000(	372	8.423519	chr17:289	ENSG00000265798		Pseudoger	chr17:31038575-310
ENSG0000(	372	8.423519	chr17:289	RNU6-1134P		smallRNA	chr17:31713753-317
ENSG0000(	372	8.423519	chr7:2516	Y_RNA		smallRNA	chr7:75297990-7529
ENSG0000(	372	8.423519	chr10:173	MTND1P20		Pseudoger	chr10:69595487-695
ENSG0000(	372	8.423519	chr7:2516	Y_RNA		smallRNA	chr7:77041647-7704
ENSG0000(	372	8.423519	chr7:2516	Y_RNA		smallRNA	chr7:77013583-7701
ENSG0000(	372	8.423519	chr10:173	Y_RNA		smallRNA	chr10:72654165-726
ENSG0000(	372	8.423519	chr10:173	ENSG00000289506		lncRNA	chr10:72272288-722
ENSG0000(	372	8.423519	chr17:289	RN7SL79P		smallRNA	chr17:31465543-314
ENSG0000(	372	8.423519	chr17:289	ENSG00000265713		Pseudoger	chr17:29775747-297
ENSG0000(	372	8.423519	chr17:289	ENSG00000265739		lncRNA	chr17:30122429-301
ENSG0000(	372	8.423519	chr17:289	LINC02978		lncRNA	chr17:30964935-309
ENSG0000(	372	8.423519	chr10:173	ENSG00000279502		TEC	chr10:72756398-727
ENSG0000(	372	8.423519	chr17:289	ADAP2		protein_c	chr17:30906344-309
ENSG0000(	372	8.423519	chr17:289	ENSG00000265791		lncRNA	chr17:30781493-307
ENSG0000(	372	8.423519	chr17:289	ENSG00000265794		lncRNA	chr17:32324431-323
ENSG0000(	372	8.423519	chr17:289	RN7SL138P		smallRNA	chr17:30959565-309
ENSG0000(	372	8.423519	chr17:289	UBL5P2		Pseudoger	chr17:32227890-322
ENSG0000(	372	8.423519	chr10:173	EIF4A2P2		Pseudoger	chr10:73199826-732
ENSG0000(	372	8.423519	chr7:2516	AC006014.10		Pseudoger	chr7:75337138-7534
ENSG0000(	372	8.423519	chr7:2516	NUP35P2		Pseudoger	chr7:79543911-7954
ENSG0000(	372	8.423519	chr7:2516	SEMA3C	NCGv7	protein_c	chr7:80742538-8092
ENSG0000(	372	8.423519	chr17:289	ENSG00000264647		lncRNA	chr17:29591703-295
ENSG0000(	372	8.423519	chr7:2516	SRRM3		protein_c	chr7:76201896-7628
ENSG0000(	372	8.423519	chr7:2516	STAG3L1		Pseudoger	chr7:75361374-7536
ENSG0000(	372	8.423519	chr17:289	AC104996.1		smallRNA	chr17:30012033-300
ENSG0000(	372	8.423519	chr7:2516	AC138783.11		Pseudoger	chr7:75281243-7528
ENSG0000(	372	8.423519	chr7:2516	SPDYE18		protein_c	chr7:77050391-7706
ENSG0000(	372	8.423519	chr10:173	ENSG00000238215		Pseudoger	chr10:72643011-726
ENSG0000(	372	8.423519	chr10:173	MYOZ1		protein_c	chr10:73631612-736
ENSG0000(	372	8.423519	chr17:289	ENSG00000266385		lncRNA	chr17:32411217-324
ENSG0000(	372	8.423519	chr17:289	DPRXP4		Pseudoger	chr17:30975387-309
ENSG0000(	372	8.423519	chr17:289	TWF1P1		Pseudoger	chr17:29203426-292
ENSG0000(	372	8.423519	chr10:173	ENSG00000279689		TEC	chr10:73769264-737
ENSG0000(	372	8.423519	chr10:173	ENSG00000272140		lncRNA	chr10:73703735-737
ENSG0000(	372	8.423519	chr17:289	ENSG00000266379		Pseudoger	chr17:32088161-320
ENSG0000(	372	8.423519	chr7:2516	CCL24		protein_c	chr7:75810825-7582
ENSG0000(	372	8.423519	chr7:2516	HSPB1	AC	protein_c	chr7:76302673-7630
ENSG0000(	372	8.423519	chr7:2516	ENSG00000237896		lncRNA	chr7:81175508-8119
ENSG0000(	372	8.423519	chr17:289	SUZ12P1		Pseudoger	chr17:30731886-307

ENSG0000(	372	8.423519	chr7:251(	CLIP2	NCGv7	protein_c	chr7:74289407-7440
ENSG0000(	372	8.423519	chr17:28(	ENSG00000264373		Pseudoger	chr17:32434939-324
ENSG0000(	372	8.423519	chr17:28(	MYO1D		protein_c	chr17:32492522-328
ENSG0000(	372	8.423519	chr10:173	ENSG00000237768		lncRNA	chr10:73071295-730
ENSG0000(	372	8.423519	chr17:28(	CDK5R1		protein_c	chr17:32486993-324
ENSG0000(	372	8.423519	chr7:251(	ENSG00000226230		Pseudoger	chr7:78486530-7848
ENSG0000(	372	8.423519	chr10:173	AP3M1		protein_c	chr10:74120255-741
ENSG0000(	372	8.423519	chr17:28(	EFCAB5		protein_c	chr17:29929200-301
ENSG0000(	372	8.423519	chr7:251(	POM121C		protein_c	chr7:75416786-7548
ENSG0000(	372	8.423519	chr10:173	SEC24C		protein_c	chr10:73744372-737
ENSG0000(	372	8.423519	chr17:28(	ENSG00000264435		Pseudoger	chr17:29972514-299
ENSG0000(	372	8.423519	chr17:28(	ENSG00000264456		lncRNA	chr17:30971652-309
ENSG0000(	372	8.423519	chr10:173	MRPL35P3		Pseudoger	chr10:74527584-745
ENSG0000(	372	8.423519	chr17:28(	ENSG00000264497		Pseudoger	chr17:32024469-320
ENSG0000(	372	8.423519	chr10:173	TACR2		protein_c	chr10:69403903-694
ENSG0000(	372	8.423519	chr10:173	KAT6B	NCGv7;AC	protein_c	chr10:74824927-750
ENSG0000(	372	8.423519	chr10:173	ENSG00000288559		lncRNA	chr10:73247342-732
ENSG0000(	372	8.423519	chr17:28(	ARGFXP2		Pseudoger	chr17:32150433-321
ENSG0000(	372	8.423519	chr10:173	MCU		protein_c	chr10:72692143-728
ENSG0000(	372	8.423519	chr17:28(	ENSG00000265046		lncRNA	chr17:31830731-318
ENSG0000(	372	8.423519	chr17:28(	ENSG00000266340		lncRNA	chr17:30978610-309
ENSG0000(	372	8.423519	chr7:251(	TMEM120A		protein_c	chr7:75986831-7599
ENSG0000(	372	8.423519	chr17:28(	ENSG00000265118		protein_c	chr17:31305213-313
ENSG0000(	372	8.423519	chr10:173	ADK		protein_c	chr10:74151202-747
ENSG0000(	372	8.423519	chr17:28(	ENSG00000265139		lncRNA	chr17:32328441-323
ENSG0000(	372	8.423519	chr10:173	CFAP70	NCGv7	protein_c	chr10:73253762-733
ENSG0000(	372	8.423519	chr7:251(	RN7SL869P		smallRNA	chr7:80245926-8024
ENSG0000(	372	8.423519	chr17:28(	ENSG00000264808		lncRNA	chr17:29333910-293
ENSG0000(	372	8.423519	chr17:28(	ENSG00000265222		lncRNA	chr17:32509954-325
ENSG0000(	372	8.423519	chr10:173	SYNPO2L-AS1		lncRNA	chr10:73653980-736
ENSG0000(	372	8.423519	chr7:251(	SPDYE12		protein_c	chr7:74904289-7491
ENSG0000(	372	8.423519	chr10:173	AL513185.1		smallRNA	chr10:72576724-725
ENSG0000(	372	8.423519	chr10:173	ENSG00000288823		lncRNA	chr10:73780624-737
ENSG0000(	372	8.423519	chr17:28(	ENSG00000265289		lncRNA	chr17:30059339-300
ENSG0000(	372	8.423519	chr10:173	BMS1P4		lncRNA	chr10:73699151-737
ENSG0000(	372	8.423519	chr10:173	AL353731.1		smallRNA	chr10:73428667-734
ENSG0000(	372	8.423519	chr7:251(	MIR4651		smallRNA	chr7:75915197-7591
ENSG0000(	372	8.423519	chr7:251(	snoU13		smallRNA	chr7:77423683-7742
ENSG0000(	372	8.423519	chr17:28(	ENSG00000266371		lncRNA	chr17:31133182-311
ENSG0000(	372	8.423519	chr17:28(	SUZ12	NCGv7;AC	protein_c	chr17:31937007-320
ENSG0000(	372	8.423519	chr7:251(	TRIM73	DriverDB\	protein_c	chr7:75395063-7541
ENSG0000(	372	8.423519	chr7:251(	ENSG00000250990		lncRNA	chr7:77246340-7725
ENSG0000(	372	8.423519	chr17:28(	RN7SL45P		smallRNA	chr17:31518432-315
ENSG0000(	372	8.423519	chr10:173	NODAL		protein_c	chr10:70431936-704
ENSG0000(	372	8.423519	chr7:251(	AC004878.7		lncRNA	chr7:74974865-7497
ENSG0000(	372	8.423519	chr10:173	DNAJC9-AS1		lncRNA	chr10:73252791-732
ENSG0000(	372	8.423519	chr7:251(	RNA5SP233		Pseudoger	chr7:74487428-7448
ENSG0000(	372	8.423519	chr10:173	GLUD1P3		Pseudoger	chr10:73731176-737
ENSG0000(	372	8.423519	chr10:173	TYSND1	NCGv7	protein_c	chr10:70137981-701
ENSG0000(	372	8.423519	chr17:28(	SH3GL1P2		Pseudoger	chr17:30624413-306
ENSG0000(	372	8.423519	chr17:28(	ALOX12P1		Pseudoger	chr17:30529688-305

ENSG0000(	372	8.423519	chr10:17:	HK1		protein_c	chr10:69269984-694
ENSG0000(	372	8.423519	chr10:17:	HKDC1		protein_c	chr10:69220332-692
ENSG0000(	372	8.423519	chr10:17:	RNU7-38P		smallRNA	chr10:71844735-718
ENSG0000(	372	8.423519	chr17:28!	ENSG00000266865		Pseudoger	chr17:31008754-310
ENSG0000(	372	8.423519	chr17:28!	RHBDL3		protein_c	chr17:32265832-323
ENSG0000(	372	8.423519	chr10:17:	Y_RNA		smallRNA	chr10:73192223-731
ENSG0000(	372	8.423519	chr17:28!	ENSG00000214708		lncRNA	chr17:32141226-321
ENSG0000(	372	8.423519	chr10:17:	PLAU		protein_c	chr10:73909177-739
ENSG0000(	372	8.423519	chr7:251(	FAM185BP		Pseudoger	chr7:77083886-7712
ENSG0000(	372	8.423519	chr7:251(	SPDYE17		protein_c	chr7:77022306-7703
ENSG0000(	372	8.423519	chr10:17:	P4HA1		protein_c	chr10:73007217-730
ENSG0000(	372	8.423519	chr10:17:	NEUROG3		protein_c	chr10:69571698-695
ENSG0000(	372	8.423519	chr17:28!	ENSG00000214719		lncRNA	chr17:30576464-306
ENSG0000(	372	8.423519	chr17:28!	ENSG00000278546		lncRNA	chr17:31560132-315
ENSG0000(	372	8.423519	chr10:17:	CALM2P2		Pseudoger	chr10:70163685-701
ENSG0000(	372	8.423519	chr7:251(	FDPSP2		Pseudoger	chr7:76470162-7647
ENSG0000(	372	8.423519	chr7:251(	GNAT3		protein_c	chr7:80458635-8051
ENSG0000(	372	8.423519	chr10:17:	RPL17P50		Pseudoger	chr10:73005833-730
ENSG0000(	372	8.423519	chr7:251(	ENSG00000290730		lncRNA	chr7:76174309-7617
ENSG0000(	372	8.423519	chr7:251(	ENSG00000290729		lncRNA	chr7:76090431-7610
ENSG0000(	372	8.423519	chr10:17:	C10orf105		protein_c	chr10:71711701-717
ENSG0000(	372	8.423519	chr7:251(	ENSG00000213549		Pseudoger	chr7:76113026-7611
ENSG0000(	372	8.423519	chr17:28!	ENSG00000266987		lncRNA	chr17:30144062-301
ENSG0000(	372	8.423519	chr10:17:	DNAJC9		protein_c	chr10:73183362-732
ENSG0000(	372	8.423519	chr17:28!	ENSG00000278668		lncRNA	chr17:32410159-324
ENSG0000(	372	8.423519	chr10:17:	COL13A1		protein_c	chr10:69801880-699
ENSG0000(	372	8.423519	chr7:251(	ENSG00000234223		lncRNA	chr7:80246409-8031
ENSG0000(	372	8.423519	chr10:17:	NPM1P24		Pseudoger	chr10:72917641-729
ENSG0000(	372	8.423519	chr7:251(	MAGI2-AS3		lncRNA	chr7:79452877-7947
ENSG0000(	372	8.423519	chr17:28!	SMURF2P1		Pseudoger	chr17:30600865-306
ENSG0000(	372	8.423519	chr7:251(	ENSG00000146722		Pseudoger	chr7:75393365-7539
ENSG0000(	372	8.423519	chr7:251(	MDH2		protein_c	chr7:76048051-7606
ENSG0000(	372	8.423519	chr7:251(	MTHFD2P5		Pseudoger	chr7:82589848-8259
ENSG0000(	372	8.423519	chr17:28!	SNORA70		smallRNA	chr17:29777619-297
ENSG0000(	372	8.423519	chr10:17:	RAB5CP1		Pseudoger	chr10:74423435-744
ENSG0000(	372	8.423519	chr10:17:	BMS1P4-AGAP5		lncRNA	chr10:73674295-737
ENSG0000(	372	8.423519	chr7:251(	RPL7AP43		Pseudoger	chr7:77115399-7711
ENSG0000(	372	8.423519	chr7:251(	NSUN5P1		lncRNA	chr7:75410322-7541
ENSG0000(	372	8.423519	chr7:251(	ENSG00000229110		Pseudoger	chr7:79124739-7912
ENSG0000(	372	8.423519	chr7:251(	APTR		lncRNA	chr7:77657659-7769
ENSG0000(	372	8.423519	chr7:251(	CASTOR2	DriverDB\	protein_c	chr7:74964776-7503
ENSG0000(	372	8.423519	chr17:28!	ZNF207	NCGv7	protein_c	chr17:32350132-323
ENSG0000(	372	8.423519	chr10:17:	BMS1P4		Pseudoger	chr10:73717115-737
ENSG0000(	372	8.423519	chr10:17:	ECD		protein_c	chr10:73130155-731
ENSG0000(	372	8.423519	chr10:17:	CHST3		protein_c	chr10:71964395-720
ENSG0000(	372	8.423519	chr17:28!	ENSG00000242439		Pseudoger	chr17:30830901-308
ENSG0000(	372	8.423519	chr7:251(	YWHAG	AC	protein_c	chr7:76326799-7635
ENSG0000(	372	8.423519	chr10:17:	RPS15AP28		Pseudoger	chr10:69300432-693
ENSG0000(	372	8.423519	chr7:251(	PPIAP81		Pseudoger	chr7:76361768-7636
ENSG0000(	372	8.423519	chr7:251(	SPDYE5		protein_c	chr7:75492320-7550
ENSG0000(	372	8.423519	chr7:251(	ENSG00000286855		lncRNA	chr7:79335780-7935

ENSG0000(	372	8.423519	chr17:28(	ENSG00000280069		TEC	chr17:30738182-307
ENSG0000(	372	8.423519	chr17:28(	ENSG00000274341		lncRNA	chr17:32408013-324
ENSG0000(	372	8.423519	chr17:28(	ENSG00000280033		TEC	chr17:32159311-321
ENSG0000(	372	8.423519	chr7:251(	SSC4D		protein_c	chr7:76389334-7640
ENSG0000(	372	8.423519	chr17:28(	ENSG00000266877		lncRNA	chr17:31583162-316
ENSG0000(	372	8.423519	chr10:17(	ZSWIM8		protein_c	chr10:73785606-738
ENSG0000(	372	8.423519	chr10:17(	FAM241B		protein_c	chr10:69630247-696
ENSG0000(	372	8.423519	chr10:17(	POLR3DP1		Pseudoger	chr10:74654956-746
ENSG0000(	372	8.423519	chr17:28(	ENSG00000266876		Pseudoger	chr17:30068632-300
ENSG0000(	372	8.423519	chr10:17(	RPSAP6		Pseudoger	chr10:74371535-743
ENSG0000(	372	8.423519	chr17:28(	MIR365B		smallRNA	chr17:31575411-315
ENSG0000(	372	8.423519	chr17:28(	hsa-mir-423		lncRNA	chr17:30117079-301
ENSG0000(	372	8.423519	chr7:251(	PMS2P5		Pseudoger	chr7:74894116-7489
ENSG0000(	369	8.355587	chr16:23(	MIR4519		smallRNA	chr16:30875266-308
ENSG0000(	368	8.332943	chr16:23(	COX6A2		protein_c	chr16:31427731-314
ENSG0000(	368	8.332943	chr16:23(	ENSG00000277543		lncRNA	chr16:31428180-314
ENSG0000(	367	8.310299	chr12:20(	ENSG00000188646		Pseudoger	chr12:74663774-746
ENSG0000(	367	8.310299	chr6:197(	Y_RNA		smallRNA	chr6:119057880-119
ENSG0000(	367	8.310299	chr6:197(	ASF1A		protein_c	chr6:118894152-118
ENSG0000(	367	8.310299	chr6:197(	ENSG00000287100		lncRNA	chr6:119349886-119
ENSG0000(	367	8.310299	chr6:197(	MCM9		protein_c	chr6:118813442-118
ENSG0000(	367	8.310299	chr6:197(	FAM184A		protein_c	chr6:118959763-119
ENSG0000(	367	8.310299	chr6:197(	MIR548B		smallRNA	chr6:119069047-119
ENSG0000(	367	8.310299	chr6:197(	SELENOKP3		Pseudoger	chr6:118757518-118
ENSG0000(	367	8.310299	chr6:197(	ENSG00000253194		lncRNA	chr6:118934770-119
ENSG0000(	367	8.310299	chr6:197(	MAN1A1		protein_c	chr6:119177205-119
ENSG0000(	367	8.310299	chr6:197(	RNU6-194P		smallRNA	chr6:119327281-119
ENSG0000(	367	8.310299	chr6:197(	ENSG00000220139		Pseudoger	chr6:119159297-119
ENSG0000(	367	8.310299	chr6:197(	ENSG00000216316		Pseudoger	chr6:119269133-119
ENSG0000(	366	8.287655	chr3:619(	ENSG00000238138		Pseudoger	chr3:13460294-1346
ENSG0000(	366	8.287655	chr17:28(	KRTAP4-4		protein_c	chr17:41159649-411
ENSG0000(	365	8.265012	chr2:360(	ENSG00000216721		Pseudoger	chr2:215804731-215
ENSG0000(	365	8.265012	chr2:360(	ENSG00000237525		lncRNA	chr2:215533133-215
ENSG0000(	365	8.265012	chr2:360(	BARD1	NCGv7;AC	protein_c	chr2:214725646-214
ENSG0000(	365	8.265012	chr2:360(	ABCA12		protein_c	chr2:214931542-215
ENSG0000(	365	8.265012	chr2:360(	ATIC	AC	protein_c	chr2:215311956-215
ENSG0000(	365	8.265012	chr2:360(	LINC02862		lncRNA	chr2:215274992-215
ENSG0000(	365	8.265012	chr2:360(	ENSG00000228618		lncRNA	chr2:215476667-215
ENSG0000(	365	8.265012	chr2:360(	PECR		protein_c	chr2:215996329-216
ENSG0000(	365	8.265012	chr2:360(	POLHP1		Pseudoger	chr2:216156342-216
ENSG0000(	365	8.265012	chr2:360(	FN1	IntOGen-I	protein_c	chr2:215360440-215
ENSG0000(	365	8.265012	chr2:360(	FN1-DT		lncRNA	chr2:215436253-215
ENSG0000(	365	8.265012	chr2:360(	RPL10P6		Pseudoger	chr2:214847128-214
ENSG0000(	365	8.265012	chr2:360(	ENSAP3		Pseudoger	chr2:214697727-214
ENSG0000(	365	8.265012	chr2:360(	ENSG00000234938		lncRNA	chr2:215530447-215
ENSG0000(	365	8.265012	chr2:360(	ENSG00000225166		lncRNA	chr2:215453688-215
ENSG0000(	365	8.265012	chr2:360(	ENSG00000286836		lncRNA	chr2:215178791-215
ENSG0000(	365	8.265012	chr2:360(	SNORA70		smallRNA	chr2:214846947-214
ENSG0000(	365	8.265012	chr2:360(	ENSG00000288791		lncRNA	chr2:215758930-215
ENSG0000(	365	8.265012	chr2:360(	ENSG00000226276		lncRNA	chr2:215939308-215
ENSG0000(	365	8.265012	chr2:360(	snoU13		smallRNA	chr2:215302766-215

ENSG0000(	365	8.265012	chr2:360{	VWC2L-IT1		lncRNA	chr2:214510196-214
ENSG0000(	365	8.265012	chr2:360{	ENSG00000227824		lncRNA	chr2:215869923-215
ENSG0000(	365	8.265012	chr2:360{	AC122136.1		smallRNA	chr2:215878833-215
ENSG0000(	365	8.265012	chr2:360{	ENSG00000227769		lncRNA	chr2:215004782-215
ENSG0000(	365	8.265012	chr2:360{	TMEM169		protein_c	chr2:216081866-216
ENSG0000(	365	8.265012	chr2:360{	LINC01614		lncRNA	chr2:215718043-215
ENSG0000(	365	8.265012	chr2:360{	ENSG00000227981		lncRNA	chr2:215579407-215
ENSG0000(	365	8.265012	chr2:360{	SNHG31		lncRNA	chr2:214810181-214
ENSG0000(	365	8.265012	chr2:360{	MREG		protein_c	chr2:215942584-216
ENSG0000(	365	8.265012	chr2:360{	LINC00607		lncRNA	chr2:215611563-215
ENSG0000(	364	8.242368	chr22:22(	IGLV3-16		protein_c	chr22:22747383-227
ENSG0000(	363	8.219724	chr16:23(	ENSG00000279589		TEC	chr16:53310797-533
ENSG0000(	363	8.219724	chr16:23(	AC079412.1		smallRNA	chr16:54549303-545
ENSG0000(	363	8.219724	chr16:23(	Y_RNA		smallRNA	chr16:53250830-532
ENSG0000(	363	8.219724	chr16:23(	ENSG00000261997		lncRNA	chr16:55538200-555
ENSG0000(	363	8.219724	chr16:23(	MPHOSPH10P1		Pseudoger	chr16:53364982-533
ENSG0000(	363	8.219724	chr16:23(	CES1P2		Pseudoger	chr16:55728115-557
ENSG0000(	363	8.219724	chr16:23(	ENSG00000259283		lncRNA	chr16:55259969-553
ENSG0000(	363	8.219724	chr16:23(	snoU13		smallRNA	chr16:53334562-533
ENSG0000(	363	8.219724	chr16:23(	ENSG00000278928		TEC	chr16:55704119-557
ENSG0000(	363	8.219724	chr16:23(	CES5A		protein_c	chr16:55846154-559
ENSG0000(	363	8.219724	chr16:23(	ENSG00000286357		lncRNA	chr16:54847316-548
ENSG0000(	363	8.219724	chr16:23(	ENSG00000280392		TEC	chr16:53991012-539
ENSG0000(	363	8.219724	chr16:23(	ENSG00000283689		lncRNA	chr16:54285604-542
ENSG0000(	363	8.219724	chr16:23(	CES1P1		lncRNA	chr16:55760516-557
ENSG0000(	363	8.219724	chr16:23(	CES1		protein_c	chr16:55802851-558
ENSG0000(	363	8.219724	chr16:23(	RN7SL841P		smallRNA	chr16:55291381-552
ENSG0000(	363	8.219724	chr16:23(	RNU6-1153P		smallRNA	chr16:53443228-534
ENSG0000(	363	8.219724	chr16:23(	ENSG00000262714		lncRNA	chr16:53386944-533
ENSG0000(	363	8.219724	chr16:23(	IRX6	NCGv7	protein_c	chr16:55324203-553
ENSG0000(	363	8.219724	chr16:23(	ENSG00000279030		TEC	chr16:55486618-554
ENSG0000(	363	8.219724	chr16:23(	ENSG00000260194		lncRNA	chr16:53998313-539
ENSG0000(	363	8.219724	chr16:23(	ENSG00000288801		lncRNA	chr16:53518402-535
ENSG0000(	363	8.219724	chr16:23(	CES1P1		Pseudoger	chr16:55760642-557
ENSG0000(	363	8.219724	chr16:23(	MTND5P34		Pseudoger	chr16:55007982-550
ENSG0000(	363	8.219724	chr16:23(	ENSG00000279344		TEC	chr16:53478957-534
ENSG0000(	363	8.219724	chr16:23(	CAPNS2		protein_c	chr16:55566684-555
ENSG0000(	363	8.219724	chr16:23(	ENSG00000277639		protein_c	chr16:53035690-530
ENSG0000(	363	8.219724	chr16:23(	ENSG00000260939		Pseudoger	chr16:53010395-530
ENSG0000(	363	8.219724	chr16:23(	ENSG00000287909		lncRNA	chr16:53544339-535
ENSG0000(	363	8.219724	chr16:23(	ENSG00000260258		Pseudoger	chr16:52942139-529
ENSG0000(	363	8.219724	chr16:23(	ENSG00000279741		TEC	chr16:53389886-533
ENSG0000(	363	8.219724	chr16:23(	IRX5		protein_c	chr16:54930865-549
ENSG0000(	363	8.219724	chr16:23(	FTO	NCGv7	protein_c	chr16:53701692-541
ENSG0000(	363	8.219724	chr16:23(	RBL2		protein_c	chr16:53433977-534
ENSG0000(	363	8.219724	chr16:23(	ENSG00000279722		TEC	chr16:53487607-534
ENSG0000(	363	8.219724	chr16:23(	RPGRIP1L	NCGv7	protein_c	chr16:53598153-537
ENSG0000(	363	8.219724	chr16:23(	ENSG00000275191		lncRNA	chr16:53628256-536
ENSG0000(	363	8.219724	chr16:23(	CHD9	NCGv7	protein_c	chr16:53054991-533
ENSG0000(	363	8.219724	chr16:23(	ENSG00000261049		lncRNA	chr16:54033997-540
ENSG0000(	363	8.219724	chr16:23(	ENSG00000286845		lncRNA	chr16:54490281-544

ENSG0000(	363	8.219724	chr16:239	ENSG00000289307		lncRNA	chr16:54929495-549
ENSG0000(	363	8.219724	chr16:239	SLC6A2	NCGv7	protein_c	chr16:55655988-557
ENSG0000(	363	8.219724	chr16:239	LINC02183		lncRNA	chr16:54542808-545
ENSG0000(	363	8.219724	chr16:239	LPCAT2		protein_c	chr16:55509072-555
ENSG0000(	363	8.219724	chr16:239	MMP2		protein_c	chr16:55389700-555
ENSG0000(	363	8.219724	chr16:239	ENSG00000263207		lncRNA	chr16:55332355-553
ENSG0000(	363	8.219724	chr16:239	IRX3		protein_c	chr16:54283304-542
ENSG0000(	363	8.219724	chr16:239	ENSG00000280454		Pseudoger	chr16:54114697-541
ENSG0000(	363	8.219724	chr16:239	ENSG00000261056		Pseudoger	chr16:53298224-532
ENSG0000(	363	8.219724	chr16:239	MPHOSPH10P1		lncRNA	chr16:53365391-533
ENSG0000(	363	8.219724	chr16:239	ENSG00000261630		lncRNA	chr16:53981229-539
ENSG0000(	363	8.219724	chr16:239	RNA5SP427		Pseudoger	chr16:53337453-533
ENSG0000(	363	8.219724	chr16:239	ENSG00000283258		lncRNA	chr16:54628963-546
ENSG0000(	363	8.219724	chr16:239	PHB1P21		Pseudoger	chr16:52934813-529
ENSG0000(	363	8.219724	chr16:239	ENSG00000274508		lncRNA	chr16:55425574-554
ENSG0000(	363	8.219724	chr16:239	CRNDE		lncRNA	chr16:54845189-549
ENSG0000(	363	8.219724	chr16:239	ENSG00000259759		lncRNA	chr16:54239693-542
ENSG0000(	363	8.219724	chr16:239	ENSG00000259926		lncRNA	chr16:53448748-534
ENSG0000(	363	8.219724	chr16:239	ENSG00000259725		lncRNA	chr16:54937786-549
ENSG0000(	363	8.219724	chr16:239	ENSG00000259962		Pseudoger	chr16:53362019-533
ENSG0000(	363	8.219724	chr16:239	ENSG00000261804		lncRNA	chr16:53373479-533
ENSG0000(	363	8.219724	chr16:239	LINC02140		lncRNA	chr16:54365996-543
ENSG0000(	363	8.219724	chr16:239	ENSG00000277559		lncRNA	chr16:54290965-542
ENSG0000(	363	8.219724	chr16:239	ENSG00000280227		TEC	chr16:53227146-532
ENSG0000(	363	8.219724	chr16:239	ENSG00000261291		lncRNA	chr16:53168522-531
ENSG0000(	363	8.219724	chr16:239	ENSG00000259711		lncRNA	chr16:54934913-549
ENSG0000(	363	8.219724	chr16:239	AKTIP	AC	protein_c	chr16:53491040-535
ENSG0000(	363	8.219724	chr16:239	LINC02169		lncRNA	chr16:54245544-542
ENSG0000(	363	8.219724	chr16:239	MMP2-AS1		lncRNA	chr16:55426797-554
ENSG0000(	363	8.219724	chr16:239	ENSG00000283423		lncRNA	chr16:55321575-553
ENSG0000(	363	8.219724	chr16:239	RPL31P56		Pseudoger	chr16:55352721-553
ENSG0000(	363	8.219724	chr16:239	ENSG00000285775		lncRNA	chr16:55045700-550
ENSG0000(	363	8.219724	chr16:239	ENSG00000287885		lncRNA	chr16:54370265-543
ENSG0000(	363	8.219724	chr16:239	ENSG00000280027		TEC	chr16:53391089-533
ENSG0000(	361	8.174436	chr5:4231	AC008706.1		smallRNA	chr5:160129210-160
ENSG0000(	361	8.174436	chr5:4231	PWWP2A		protein_c	chr5:160061801-160
ENSG0000(	359	8.129148	chr2:8187	ENSG00000236665		lncRNA	chr2:1158310-11604
ENSG0000(	359	8.129148	chr2:8187	ENSG00000284600		lncRNA	chr2:1795525-18115
ENSG0000(	359	8.129148	chr2:8187	ENSG00000289941		lncRNA	chr2:2332434-23332
ENSG0000(	359	8.129148	chr2:8187	ENSG00000283766		lncRNA	chr2:1552445-15547
ENSG0000(	359	8.129148	chr2:8187	LINC01939		lncRNA	chr2:899462-905539
ENSG0000(	359	8.129148	chr2:8187	MYT1L	NCGv7	protein_c	chr2:1789113-23316
ENSG0000(	359	8.129148	chr2:8187	ENSG00000235403		lncRNA	chr2:1059133-10683
ENSG0000(	359	8.129148	chr2:8187	PXDN	NCGv7	protein_c	chr2:1631887-17448
ENSG0000(	359	8.129148	chr2:8187	ENSG00000231482		lncRNA	chr2:1366234-15807
ENSG0000(	359	8.129148	chr2:8187	ENSG00000232057		lncRNA	chr2:1824412-18286
ENSG0000(	359	8.129148	chr2:8187	SNTG2-AS1		lncRNA	chr2:949257-951502
ENSG0000(	359	8.129148	chr2:8187	MYT1L-AS1		lncRNA	chr2:2319232-23271
ENSG0000(	359	8.129148	chr2:8187	SNTG2		protein_c	chr2:950849-136761
ENSG0000(	359	8.129148	chr2:8187	ENSG00000233553		lncRNA	chr2:1341044-13465
ENSG0000(	359	8.129148	chr2:8187	TPO	NCGv7	protein_c	chr2:1374066-15437

ENSG0000(	359	8.129148	chr2:8187	ENSG00000203635		lncRNA	chr2:1620510-16254
ENSG0000(	359	8.129148	chr2:8187	ENSG00000234796		lncRNA	chr2:910926-921124
ENSG0000(	359	8.129148	chr2:8187	ENSG00000228613		lncRNA	chr2:1546665-16201
ENSG0000(	358	8.106504	chr16:239	ENSG00000289029		lncRNA	chr16:29288799-292
ENSG0000(	358	8.106504	chr16:239	ENSG00000277999		lncRNA	chr16:29272220-292
ENSG0000(	358	8.106504	chr16:239	ENSG00000260953		lncRNA	chr16:29262273-292
ENSG0000(	357	8.083861	chr2:2329	CALCRL		protein_c	chr2:187341964-187
ENSG0000(	357	8.083861	chr2:2329	RNU6-989P		smallRNA	chr2:187270698-187
ENSG0000(	357	8.083861	chr2:2329	GAPDHP59		Pseudoger	chr2:187415552-187
ENSG0000(	357	8.083861	chr2:2329	IMPDH1P7		Pseudoger	chr2:187001876-187
ENSG0000(	357	8.083861	chr2:2329	RN7SKP42		smallRNA	chr2:187122562-187
ENSG0000(	356	8.061217	chr14:106	IGHVII-26-2		Pseudoger	chr14:106314651-10
ENSG0000(	353	7.993285	chr6:9221	H4C11	NCGv7	protein_c	chr6:27824092-2782
ENSG0000(	350	7.925353	chr16:239	VN1R3		Pseudoger	chr16:31807926-318
ENSG0000(	350	7.925353	chr16:239	ENSG00000259934		Pseudoger	chr16:32845964-328
ENSG0000(	350	7.925353	chr16:239	ZNF843		protein_c	chr16:31432593-314
ENSG0000(	350	7.925353	chr16:239	ENSG00000260267		lncRNA	chr16:31456711-314
ENSG0000(	350	7.925353	chr16:239	STX4		protein_c	chr16:31032889-310
ENSG0000(	350	7.925353	chr16:239	PYCARD		protein_c	chr16:31201486-312
ENSG0000(	350	7.925353	chr16:239	ENSG00000259800		Pseudoger	chr16:33130235-331
ENSG0000(	350	7.925353	chr16:239	TRIM72		protein_c	chr16:31214091-312
ENSG0000(	350	7.925353	chr16:239	ENSG00000259874		Pseudoger	chr16:31870887-318
ENSG0000(	350	7.925353	chr16:239	BCKDK		protein_c	chr16:31106107-311
ENSG0000(	350	7.925353	chr16:239	PRSS8		protein_c	chr16:31131433-311
ENSG0000(	350	7.925353	chr16:239	ENSG00000259950		Pseudoger	chr16:31894542-318
ENSG0000(	350	7.925353	chr16:239	ENSG00000260827		lncRNA	chr16:33313853-333
ENSG0000(	350	7.925353	chr16:239	ENSG00000291267		lncRNA	chr16:33043609-330
ENSG0000(	350	7.925353	chr16:239	MIR762		smallRNA	chr16:30893903-308
ENSG0000(	350	7.925353	chr16:239	SNORA30		smallRNA	chr16:30710537-307
ENSG0000(	350	7.925353	chr16:239	ENSG00000290986		lncRNA	chr16:33098808-331
ENSG0000(	350	7.925353	chr16:239	FRG2KP		lncRNA	chr16:31563185-315
ENSG0000(	350	7.925353	chr16:239	FBXL19-AS1		lncRNA	chr16:30919319-309
ENSG0000(	350	7.925353	chr16:239	ZNF267		protein_c	chr16:31873807-319
ENSG0000(	350	7.925353	chr16:239	ENSG00000291268		lncRNA	chr16:33094204-330
ENSG0000(	350	7.925353	chr16:239	FAM153DP		Pseudoger	chr16:32602575-326
ENSG0000(	350	7.925353	chr16:239	ENSG00000261569		lncRNA	chr16:32649193-326
ENSG0000(	350	7.925353	chr16:239	ENSG00000291269		lncRNA	chr16:33111682-331
ENSG0000(	350	7.925353	chr16:239	ENSG00000279196		TEC	chr16:30984630-309
ENSG0000(	350	7.925353	chr16:239	ENSG00000291271		lncRNA	chr16:31981870-319
ENSG0000(	350	7.925353	chr16:239	RBM22P13		Pseudoger	chr16:31863633-318
ENSG0000(	350	7.925353	chr16:239	KAT8		protein_c	chr16:31114489-311
ENSG0000(	350	7.925353	chr16:239	RNF40	NCGv7	protein_c	chr16:30761745-307
ENSG0000(	350	7.925353	chr16:239	ENSG00000261263		Pseudoger	chr16:32650195-326
ENSG0000(	350	7.925353	chr16:239	SLC6A10P		Pseudoger	chr16:32878706-328
ENSG0000(	350	7.925353	chr16:239	ENSG00000260845		lncRNA	chr16:32735909-327
ENSG0000(	350	7.925353	chr16:239	ENSG00000262766		lncRNA	chr16:31118078-311
ENSG0000(	350	7.925353	chr16:239	PRR14	IntOGen-I	protein_c	chr16:30650717-306
ENSG0000(	350	7.925353	chr16:239	ITGAD		protein_c	chr16:31393335-314
ENSG0000(	350	7.925353	chr16:239	SRCAP	NCGv7	protein_c	chr16:30698209-307
ENSG0000(	350	7.925353	chr16:239	PRSS36		protein_c	chr16:31138926-311
ENSG0000(	350	7.925353	chr16:239	ACTR3BP3		Pseudoger	chr16:32379071-323

ENSG0000(	350	7.925353	chr16:239	ENSG00000259822	Pseudoger	chr16:32278866-322
ENSG0000(	350	7.925353	chr16:239	TP53TG3D	protein_c	chr16:32253286-322
ENSG0000(	350	7.925353	chr16:239	TP53TG3C	protein_c	chr16:33304306-333
ENSG0000(	350	7.925353	chr16:239	ABCD1P3	Pseudoger	chr16:32475051-324
ENSG0000(	350	7.925353	chr16:239	ENSG00000290927	lncRNA	chr16:31700558-317
ENSG0000(	350	7.925353	chr16:239	ENSG00000260847	lncRNA	chr16:32188333-321
ENSG0000(	350	7.925353	chr16:239	ENSG00000261259	Pseudoger	chr16:32951993-330
ENSG0000(	350	7.925353	chr16:239	ENSG00000260311	lncRNA	chr16:32663925-326
ENSG0000(	350	7.925353	chr16:239	ENSG00000259810	lncRNA	chr16:31788202-317
ENSG0000(	350	7.925353	chr16:239	CLUHP3	Pseudoger	chr16:31703168-317
ENSG0000(	350	7.925353	chr16:239	ENSG00000214614	lncRNA	chr16:32882586-328
ENSG0000(	350	7.925353	chr16:239	IGHV1OR16-2	Pseudoger	chr16:32978461-329
ENSG0000(	350	7.925353	chr16:239	ENSG00000274678	lncRNA	chr16:30821338-308
ENSG0000(	350	7.925353	chr16:239	PABPC1P13	Pseudoger	chr16:32439069-324
ENSG0000(	350	7.925353	chr16:239	ENSG00000260304	lncRNA	chr16:31182511-311
ENSG0000(	350	7.925353	chr16:239	ENSG00000261245	Pseudoger	chr16:31361278-313
ENSG0000(	350	7.925353	chr16:239	RNU6-1043P	smallRNA	chr16:30701337-307
ENSG0000(	350	7.925353	chr16:239	ENSG00000263343	Pseudoger	chr16:31165648-311
ENSG0000(	350	7.925353	chr16:239	IGHV1OR16-4	Pseudoger	chr16:33002333-330
ENSG0000(	350	7.925353	chr16:239	ENSG00000261289	Pseudoger	chr16:31952160-319
ENSG0000(	350	7.925353	chr16:239	KRBOX5P1	Pseudoger	chr16:31601593-316
ENSG0000(	350	7.925353	chr16:239	ZNF629	protein_c	chr16:30778456-307
ENSG0000(	350	7.925353	chr16:239	ORAI3	protein_c	chr16:30949068-309
ENSG0000(	350	7.925353	chr16:239	ENSG00000290953	lncRNA	chr16:31994821-319
ENSG0000(	350	7.925353	chr16:239	ENSG00000261111	Pseudoger	chr16:32635673-326
ENSG0000(	350	7.925353	chr16:239	VN1R66P	Pseudoger	chr16:31648307-316
ENSG0000(	350	7.925353	chr16:239	ENSG00000260082	Pseudoger	chr16:30821068-308
ENSG0000(	350	7.925353	chr16:239	MIR762HG	lncRNA	chr16:30875222-308
ENSG0000(	350	7.925353	chr16:239	IGHV3OR16-10	protein_c	chr16:32995048-329
ENSG0000(	350	7.925353	chr16:239	TP53TG3B	protein_c	chr16:33360841-333
ENSG0000(	350	7.925353	chr16:239	ITGAM	protein_c	chr16:31259967-313
ENSG0000(	350	7.925353	chr16:239	ENSG00000291274	lncRNA	chr16:32741314-327
ENSG0000(	350	7.925353	chr16:239	ABHD17AP7	Pseudoger	chr16:32726615-327
ENSG0000(	350	7.925353	chr16:239	ENSG00000223931	Pseudoger	chr16:32051825-320
ENSG0000(	350	7.925353	chr16:239	ENSG00000260158	lncRNA	chr16:32809556-328
ENSG0000(	350	7.925353	chr16:239	AC135050.1	smallRNA	chr16:31109230-311
ENSG0000(	350	7.925353	chr16:239	RP11-1437A8.6	lncRNA	chr16:33316511-333
ENSG0000(	350	7.925353	chr16:239	ENSG00000288983	lncRNA	chr16:30634507-306
ENSG0000(	350	7.925353	chr16:239	ENSG00000261474	lncRNA	chr16:31449535-314
ENSG0000(	350	7.925353	chr16:239	CTF1	protein_c	chr16:30896614-309
ENSG0000(	350	7.925353	chr16:239	ENSG00000260740	lncRNA	chr16:31487370-314
ENSG0000(	350	7.925353	chr16:239	LINC02190	lncRNA	chr16:31542748-315
ENSG0000(	350	7.925353	chr16:239	IGHV3OR16-9	protein_c	chr16:32066065-320
ENSG0000(	350	7.925353	chr16:239	ENSG00000261127	Pseudoger	chr16:32289547-323
ENSG0000(	350	7.925353	chr16:239	ENSG00000261124	lncRNA	chr16:31065495-310
ENSG0000(	350	7.925353	chr16:239	ENSG00000261487	lncRNA	chr16:30948386-309
ENSG0000(	350	7.925353	chr16:239	HERC2P4	Pseudoger	chr16:32152111-321
ENSG0000(	350	7.925353	chr16:239	ENSG00000260866	Pseudoger	chr16:32388135-324
ENSG0000(	350	7.925353	chr16:239	ENSG00000290984	lncRNA	chr16:32774929-327
ENSG0000(	350	7.925353	chr16:239	PYDC1	protein_c	chr16:31215962-312
ENSG0000(	350	7.925353	chr16:239	ENSG00000261457	Pseudoger	chr16:31802947-318

ENSG0000(	350	7.925353	chr16:239	ENSG00000291275		lncRNA	chr16:32760943-327
ENSG0000(	350	7.925353	chr16:239	ENSG00000261108		Pseudoger	chr16:32739627-327
ENSG0000(	350	7.925353	chr16:239	ENSG00000279780		TEC	chr16:32786394-327
ENSG0000(	350	7.925353	chr16:239	ENSG00000260141		lncRNA	chr16:33046529-330
ENSG0000(	350	7.925353	chr16:239	ENSG00000280132		TEC	chr16:31470204-314
ENSG0000(	350	7.925353	chr16:239	PYCARD-AS1		lncRNA	chr16:31201885-312
ENSG0000(	350	7.925353	chr16:239	BCAP31P2		Pseudoger	chr16:32869985-328
ENSG0000(	350	7.925353	chr16:239	IGHV3OR16-6		Pseudoger	chr16:32915074-329
ENSG0000(	350	7.925353	chr16:239	SLC6A10P		lncRNA	chr16:32877469-328
ENSG0000(	350	7.925353	chr16:239	PRSS53		protein_c	chr16:31083437-310
ENSG0000(	350	7.925353	chr16:239	ENSG00000290845		lncRNA	chr16:32169984-321
ENSG0000(	350	7.925353	chr16:239	IGHV1OR16-3		Pseudoger	chr16:32059084-320
ENSG0000(	350	7.925353	chr16:239	VN1R67P		Pseudoger	chr16:31801585-318
ENSG0000(	350	7.925353	chr16:239	IGHV2OR16-5		protein_c	chr16:32847713-328
ENSG0000(	350	7.925353	chr16:239	ENSG00000261385		lncRNA	chr16:31131433-311
ENSG0000(	350	7.925353	chr16:239	FBRS		protein_c	chr16:30658431-306
ENSG0000(	350	7.925353	chr16:239	ENSG00000255439		protein_c	chr16:31083439-310
ENSG0000(	350	7.925353	chr16:239	ENSG00000261391		Pseudoger	chr16:32715931-327
ENSG0000(	350	7.925353	chr16:239	AHSP		protein_c	chr16:31527900-315
ENSG0000(	350	7.925353	chr16:239	ENSG00000261541		lncRNA	chr16:32454612-324
ENSG0000(	350	7.925353	chr16:239	ENSG00000260757		lncRNA	chr16:31402565-314
ENSG0000(	350	7.925353	chr16:239	ENSG00000260218		Pseudoger	chr16:31963803-319
ENSG0000(	350	7.925353	chr16:239	ENSG00000291276		lncRNA	chr16:32103245-321
ENSG0000(	350	7.925353	chr16:239	ENSG00000260060		lncRNA	chr16:31196131-311
ENSG0000(	350	7.925353	chr16:239	ZNF668	NCGv7	protein_c	chr16:31060843-310
ENSG0000(	350	7.925353	chr16:239	ABHD17AP9		Pseudoger	chr16:33140850-331
ENSG0000(	350	7.925353	chr16:239	ZNF646		protein_c	chr16:31074422-310
ENSG0000(	350	7.925353	chr16:239	VKORC1		protein_c	chr16:31090842-310
ENSG0000(	350	7.925353	chr16:239	ENSG00000279795		TEC	chr16:32787962-327
ENSG0000(	350	7.925353	chr16:239	ENSG00000262721		lncRNA	chr16:30875766-308
ENSG0000(	350	7.925353	chr16:239	PHKG2		protein_c	chr16:30748293-307
ENSG0000(	350	7.925353	chr16:239	ENSG00000289930		lncRNA	chr16:31351767-313
ENSG0000(	350	7.925353	chr16:239	IGHV3OR16-15		Pseudoger	chr16:32903442-329
ENSG0000(	350	7.925353	chr16:239	ENSG00000260921		lncRNA	chr16:32995537-329
ENSG0000(	350	7.925353	chr16:239	SETD1A		protein_c	chr16:30957754-309
ENSG0000(	350	7.925353	chr16:239	RBM22P12		Pseudoger	chr16:31680847-316
ENSG0000(	350	7.925353	chr16:239	ENSG00000276867		lncRNA	chr16:31704728-317
ENSG0000(	350	7.925353	chr16:239	ENSG00000261731		lncRNA	chr16:31709113-317
ENSG0000(	350	7.925353	chr16:239	ENSG00000280160		TEC	chr16:31093727-310
ENSG0000(	350	7.925353	chr16:239	IGHV1OR16-1		Pseudoger	chr16:32034853-320
ENSG0000(	350	7.925353	chr16:239	RNU7-199P		smallRNA	chr16:31267437-312
ENSG0000(	350	7.925353	chr16:239	ENSG00000260568		lncRNA	chr16:31699676-317
ENSG0000(	350	7.925353	chr16:239	ENSG00000282927		Pseudoger	chr16:32650674-326
ENSG0000(	350	7.925353	chr16:239	CFAP119		protein_c	chr16:30757423-307
ENSG0000(	350	7.925353	chr16:239	ENSG00000260575		lncRNA	chr16:32250620-322
ENSG0000(	350	7.925353	chr16:239	ENSG00000260900		Pseudoger	chr16:33059936-330
ENSG0000(	350	7.925353	chr16:239	ENSG00000260419		lncRNA	chr16:33301641-333
ENSG0000(	350	7.925353	chr16:239	TGFB1I1		protein_c	chr16:31471585-314
ENSG0000(	350	7.925353	chr16:239	ENSG00000260662		Pseudoger	chr16:32460266-324
ENSG0000(	350	7.925353	chr16:239	HSD3B7		protein_c	chr16:30985207-309
ENSG0000(	350	7.925353	chr16:239	ENSG00000282973		Pseudoger	chr16:33345182-333

ENSG0000(	350	7.925353	chr16:239	CHEK2P7		Pseudoger	chr16:32356981-323
ENSG0000(	350	7.925353	chr16:239	TP53TG3E		protein_c	chr16:33304306-333
ENSG0000(	350	7.925353	chr16:239	ENSG00000260628		Pseudoger	chr16:31975803-320
ENSG0000(	350	7.925353	chr16:239	ENSG00000278950		TEC	chr16:32781883-327
ENSG0000(	350	7.925353	chr16:239	TMEM265		protein_c	chr16:30740642-307
ENSG0000(	350	7.925353	chr16:239	FRG2KP		Pseudoger	chr16:31563082-315
ENSG0000(	350	7.925353	chr16:239	ENSG00000278133		lncRNA	chr16:31122235-311
ENSG0000(	350	7.925353	chr16:239	TP53TG3F		protein_c	chr16:32673528-326
ENSG0000(	350	7.925353	chr16:239	ENSG00000261009		lncRNA	chr16:33370473-333
ENSG0000(	350	7.925353	chr16:239	ARMC5		protein_c	chr16:31458080-314
ENSG0000(	350	7.925353	chr16:239	RUSF1		protein_c	chr16:31489471-315
ENSG0000(	350	7.925353	chr16:239	ENSG00000261682		lncRNA	chr16:33129316-331
ENSG0000(	350	7.925353	chr16:239	ENSG00000261648		lncRNA	chr16:31546698-315
ENSG0000(	350	7.925353	chr16:239	ENSG00000260911		lncRNA	chr16:31043150-310
ENSG0000(	350	7.925353	chr16:239	ENSG00000282034		protein_c	chr16:30704067-307
ENSG0000(	350	7.925353	chr16:239	BCL7C		protein_c	chr16:30833626-308
ENSG0000(	350	7.925353	chr16:239	ABHD17AP8		Pseudoger	chr16:32199902-322
ENSG0000(	350	7.925353	chr16:239	ENSG00000260414		Pseudoger	chr16:33154352-331
ENSG0000(	350	7.925353	chr16:239	NDUFA3P6		Pseudoger	chr16:31174573-311
ENSG0000(	350	7.925353	chr16:239	ENSG00000289889		lncRNA	chr16:31516802-315
ENSG0000(	350	7.925353	chr16:239	RUSF1-DT		lncRNA	chr16:31508464-315
ENSG0000(	350	7.925353	chr16:239	ENSG00000260584		Pseudoger	chr16:32116700-321
ENSG0000(	350	7.925353	chr16:239	YBX3P1		Pseudoger	chr16:31568386-315
ENSG0000(	350	7.925353	chr16:239	ENSG00000260899		lncRNA	chr16:30740667-307
ENSG0000(	350	7.925353	chr16:239	SLC5A2		protein_c	chr16:31483002-314
ENSG0000(	350	7.925353	chr16:239	FBXL19		protein_c	chr16:30923055-309
ENSG0000(	350	7.925353	chr16:239	TP53TG3		protein_c	chr16:32673528-326
ENSG0000(	350	7.925353	chr16:239	ENSG00000260649		Pseudoger	chr16:32189262-321
ENSG0000(	350	7.925353	chr16:239	ENSG00000262187		Pseudoger	chr16:32618733-326
ENSG0000(	350	7.925353	chr16:239	ENSG00000260974		lncRNA	chr16:32675037-326
ENSG0000(	350	7.925353	chr16:239	ENSG00000278885		TEC	chr16:31566360-315
ENSG0000(	350	7.925353	chr16:239	RNU6-416P		smallRNA	chr16:30675300-306
ENSG0000(	350	7.925353	chr16:239	ENSG00000260610		Pseudoger	chr16:32931768-329
ENSG0000(	350	7.925353	chr16:239	HERC2P8		Pseudoger	chr16:33095029-331
ENSG0000(	350	7.925353	chr16:239	ENSG00000277304		Pseudoger	chr16:32916974-329
ENSG0000(	350	7.925353	chr16:239	FUS	NCGv7;AC	protein_c	chr16:31180138-311
ENSG0000(	350	7.925353	chr16:239	ITGAX		protein_c	chr16:31355134-313
ENSG0000(	350	7.925353	chr16:239	ENSG00000283110		Pseudoger	chr16:33386105-333
ENSG0000(	350	7.925353	chr16:239	ENSG00000232748		lncRNA	chr16:31056460-310
ENSG0000(	350	7.925353	chr16:239	ENSG00000286473		lncRNA	chr16:32126432-321
ENSG0000(	350	7.925353	chr16:239	ENSG00000289734		lncRNA	chr16:32600299-326
ENSG0000(	350	7.925353	chr16:239	ENSG00000260344		Pseudoger	chr16:32213380-322
ENSG0000(	350	7.925353	chr16:239	ENSG00000197476		Pseudoger	chr16:31962063-319
ENSG0000(	350	7.925353	chr16:239	STX1B		protein_c	chr16:30989256-310
ENSG0000(	350	7.925353	chr16:239	ENSG00000261727		Pseudoger	chr16:32008438-320
ENSG0000(	350	7.925353	chr16:239	VN1R65P		Pseudoger	chr16:31554105-315
ENSG0000(	350	7.925353	chr16:239	CTF2P		Pseudoger	chr16:30904348-309
ENSG0000(	350	7.925353	chr16:239	IGHV3OR16-8		protein_c	chr16:33009175-330
ENSG0000(	350	7.925353	chr16:239	ENSG00000280211		TEC	chr16:30773532-307
ENSG0000(	350	7.925353	chr16:239	ENSG00000260402		lncRNA	chr16:32262827-322
ENSG0000(	350	7.925353	chr16:239	HERC2P5		Pseudoger	chr16:32742353-327

ENSG0000(	350	7.925353	chr16:239	ENSG00000261719		Pseudoger	chr16:32811029-328
ENSG0000(	350	7.925353	chr16:239	VN1R64P		Pseudoger	chr16:31546805-315
ENSG0000(	350	7.925353	chr16:239	KRBOX5		protein_c	chr16:31713229-317
ENSG0000(	350	7.925353	chr16:239	ENSG00000279997		TEC	chr16:32185624-321
ENSG0000(	350	7.925353	chr16:239	ENSG00000261840		lncRNA	chr16:30697704-306
ENSG0000(	344	7.78949	chr5:4231	HMGB1P35		Pseudoger	chr5:77146568-7714
ENSG0000(	344	7.78949	chr6:9221	OR2B8P		protein_c	chr6:28053228-2805
ENSG0000(	341	7.721559	chr4:7212	NPY5R		protein_c	chr4:163343892-163
ENSG0000(	341	7.721559	chr4:7212	RN7SKP105		smallRNA	chr4:163828800-163
ENSG0000(	341	7.721559	chr4:7212	RPL35AP12		Pseudoger	chr4:163382179-163
ENSG0000(	341	7.721559	chr4:7212	ENSG00000249617		Pseudoger	chr4:165045969-165
ENSG0000(	341	7.721559	chr4:7212	ENSG00000270960		Pseudoger	chr4:164933086-164
ENSG0000(	341	7.721559	chr4:7212	TRIM75		protein_c	chr4:165053866-165
ENSG0000(	341	7.721559	chr4:7212	ENSG00000250547		Pseudoger	chr4:163265575-163
ENSG0000(	341	7.721559	chr4:7212	Y_RNA		smallRNA	chr4:159064297-159
ENSG0000(	341	7.721559	chr4:7212	KLHL2		protein_c	chr4:165207561-165
ENSG0000(	341	7.721559	chr4:7212	ENSG00000240674		Pseudoger	chr4:165012366-165
ENSG0000(	341	7.721559	chr4:7212	ENSG00000250712		Pseudoger	chr4:165010459-165
ENSG0000(	341	7.721559	chr4:7212	TOMM22P4		Pseudoger	chr4:162521682-162
ENSG0000(	341	7.721559	chr4:7212	ENSG00000279994		TEC	chr4:157945817-157
ENSG0000(	341	7.721559	chr4:7212	FABP5P12		Pseudoger	chr4:159026139-159
ENSG0000(	341	7.721559	chr4:7212	MIR578		smallRNA	chr4:165386242-165
ENSG0000(	341	7.721559	chr4:7212	ENSG00000250746		Pseudoger	chr4:164188311-164
ENSG0000(	341	7.721559	chr4:7212	ENSG00000236941		Pseudoger	chr4:164948079-164
ENSG0000(	341	7.721559	chr4:7212	ANP32C	AC	Pseudoger	chr4:164197007-164
ENSG0000(	341	7.721559	chr4:7212	RPS14P7		Pseudoger	chr4:160818422-160
ENSG0000(	341	7.721559	chr4:7212	AIDAP2		Pseudoger	chr4:158270378-158
ENSG0000(	341	7.721559	chr4:7212	ENSG00000289999		lncRNA	chr4:158841813-158
ENSG0000(	341	7.721559	chr4:7212	RAPGEF2		protein_c	chr4:159103013-159
ENSG0000(	341	7.721559	chr4:7212	ENSG00000248387		Pseudoger	chr4:158469586-158
ENSG0000(	341	7.721559	chr4:7212	RNU4-87P		smallRNA	chr4:165252580-165
ENSG0000(	341	7.721559	chr4:7212	U3		smallRNA	chr4:158700691-158
ENSG0000(	341	7.721559	chr4:7212	ENSG00000248431		lncRNA	chr4:162705359-162
ENSG0000(	341	7.721559	chr4:7212	ENSG00000248552		Pseudoger	chr4:159375099-159
ENSG0000(	341	7.721559	chr4:7212	AC106860.1		smallRNA	chr4:161003877-161
ENSG0000(	341	7.721559	chr4:7212	ENSG00000244146		Pseudoger	chr4:164920618-164
ENSG0000(	341	7.721559	chr4:7212	ENSG00000286558		lncRNA	chr4:158262995-158
ENSG0000(	341	7.721559	chr4:7212	APELA		protein_c	chr4:164877178-164
ENSG0000(	341	7.721559	chr4:7212	LINC02477		lncRNA	chr4:160539254-160
ENSG0000(	341	7.721559	chr4:7212	FNIP2		protein_c	chr4:158769026-158
ENSG0000(	341	7.721559	chr4:7212	ENSG00000249419		lncRNA	chr4:162022733-162
ENSG0000(	341	7.721559	chr4:7212	GK3P		protein_c	chr4:165277807-165
ENSG0000(	341	7.721559	chr4:7212	NACA3P		Pseudoger	chr4:164943290-164
ENSG0000(	341	7.721559	chr4:7212	AC093700.1		smallRNA	chr4:159000146-159
ENSG0000(	341	7.721559	chr4:7212	MSMO1		protein_c	chr4:165327667-165
ENSG0000(	341	7.721559	chr4:7212	PSME2P3		Pseudoger	chr4:159007119-159
ENSG0000(	341	7.721559	chr4:7212	TKTL2	NCGv7	protein_c	chr4:163471095-163
ENSG0000(	341	7.721559	chr4:7212	ENSG00000287072		lncRNA	chr4:162806370-162
ENSG0000(	341	7.721559	chr4:7212	MIR3688-2		smallRNA	chr4:159128802-159
ENSG0000(	341	7.721559	chr4:7212	GASK1B-AS1		lncRNA	chr4:158170752-158
ENSG0000(	341	7.721559	chr4:7212	ENSG00000250604		lncRNA	chr4:158199105-158

ENSG0000(	341	7.721559	chr4:7212	ENSG00000276623		Pseudoger	chr4:165016887-165
ENSG0000(	341	7.721559	chr4:7212	TRIM60P14		Pseudoger	chr4:164915565-164
ENSG0000(	341	7.721559	chr4:7212	ENSG00000285563		lncRNA	chr4:164920894-164
ENSG0000(	341	7.721559	chr4:7212	ENSG00000250887		lncRNA	chr4:165007086-165
ENSG0000(	341	7.721559	chr4:7212	LINC02233		lncRNA	chr4:159665833-159
ENSG0000(	341	7.721559	chr4:7212	ENSG00000248229		Pseudoger	chr4:164987265-164
ENSG0000(	341	7.721559	chr4:7212	NAF1		protein_c	chr4:163110073-163
ENSG0000(	341	7.721559	chr4:7212	U3		smallRNA	chr4:159506895-159
ENSG0000(	341	7.721559	chr4:7212	RNU6-128P		smallRNA	chr4:158794590-158
ENSG0000(	341	7.721559	chr4:7212	FAM218BP		Pseudoger	chr4:164929494-164
ENSG0000(	341	7.721559	chr4:7212	SMIM31		protein_c	chr4:164754064-164
ENSG0000(	341	7.721559	chr4:7212	AC095064.1		smallRNA	chr4:159123633-159
ENSG0000(	341	7.721559	chr4:7212	ENSG00000250997		Pseudoger	chr4:160521829-160
ENSG0000(	341	7.721559	chr4:7212	RXFP1		protein_c	chr4:158315311-158
ENSG0000(	341	7.721559	chr4:7212	RNU6-668P		smallRNA	chr4:164787290-164
ENSG0000(	341	7.721559	chr4:7212	ETFDH		protein_c	chr4:158671968-158
ENSG0000(	341	7.721559	chr4:7212	ENSG00000249568		lncRNA	chr4:161378953-161
ENSG0000(	341	7.721559	chr4:7212	ENSG00000287226		lncRNA	chr4:157929071-158
ENSG0000(	341	7.721559	chr4:7212	YWHAQP4		Pseudoger	chr4:163932526-163
ENSG0000(	341	7.721559	chr4:7212	TRIM60		protein_c	chr4:165016458-165
ENSG0000(	341	7.721559	chr4:7212	NPY1R		protein_c	chr4:163323962-163
ENSG0000(	341	7.721559	chr4:7212	RNU6-284P		smallRNA	chr4:164620732-164
ENSG0000(	341	7.721559	chr4:7212	MARCHF1		protein_c	chr4:163524298-164
ENSG0000(	341	7.721559	chr4:7212	PPID		protein_c	chr4:158709127-158
ENSG0000(	341	7.721559	chr4:7212	FSTL5	NCGv7	protein_c	chr4:161383897-162
ENSG0000(	341	7.721559	chr4:7212	ENSG00000279139		TEC	chr4:158109500-158
ENSG0000(	341	7.721559	chr4:7212	AC093788.1		smallRNA	chr4:163523292-163
ENSG0000(	341	7.721559	chr4:7212	NUDT19P5		Pseudoger	chr4:158182825-158
ENSG0000(	341	7.721559	chr4:7212	TRIM61		protein_c	chr4:164954446-164
ENSG0000(	341	7.721559	chr4:7212	ENSG00000248645		Pseudoger	chr4:164938322-164
ENSG0000(	341	7.721559	chr4:7212	TMEM192		protein_c	chr4:165070608-165
ENSG0000(	341	7.721559	chr4:7212	ENSG00000286862		lncRNA	chr4:157879902-157
ENSG0000(	341	7.721559	chr4:7212	ENSG00000250027		lncRNA	chr4:163108785-163
ENSG0000(	341	7.721559	chr4:7212	TMA16		protein_c	chr4:163494442-163
ENSG0000(	341	7.721559	chr4:7212	ENSG00000250430		Pseudoger	chr4:165026261-165
ENSG0000(	341	7.721559	chr4:7212	MTHFD2P4		Pseudoger	chr4:162322188-162
ENSG0000(	341	7.721559	chr4:7212	ENSG00000251162		Pseudoger	chr4:164597719-164
ENSG0000(	341	7.721559	chr4:7212	AC105316.1		smallRNA	chr4:159295714-159
ENSG0000(	341	7.721559	chr4:7212	ENSG00000273449		lncRNA	chr4:163529771-163
ENSG0000(	341	7.721559	chr4:7212	ENSG00000251123		Pseudoger	chr4:162984795-162
ENSG0000(	341	7.721559	chr4:7212	FAM218A		lncRNA	chr4:164956948-164
ENSG0000(	341	7.721559	chr4:7212	ENSG00000249901		lncRNA	chr4:158490766-158
ENSG0000(	341	7.721559	chr4:7212	CHORDC1P3		Pseudoger	chr4:164725310-164
ENSG0000(	341	7.721559	chr4:7212	ENSG00000250180		lncRNA	chr4:159398533-159
ENSG0000(	341	7.721559	chr4:7212	TMEM144		protein_c	chr4:158201604-158
ENSG0000(	341	7.721559	chr4:7212	ENSG00000287822		lncRNA	chr4:158178168-158
ENSG0000(	341	7.721559	chr4:7212	RNU4-79P		smallRNA	chr4:158395394-158
ENSG0000(	341	7.721559	chr4:7212	RP11-219C20.3		lncRNA	chr4:165070608-165
ENSG0000(	341	7.721559	chr4:7212	C4orf46		protein_c	chr4:158666675-158
ENSG0000(	341	7.721559	chr4:7212	C4orf45		protein_c	chr4:158893134-159
ENSG0000(	341	7.721559	chr4:7212	ENSG00000280140		TEC	chr4:158111361-158

ENSG0000(	341	7.721559	chr4:7212	GASK1B	protein_c	chr4:158124474-158
ENSG0000(	341	7.721559	chr4:7212	RPL6P11	Pseudoger	chr4:157968412-157
ENSG0000(	339	7.676271	chr7:2516	ENSG00000223948	Pseudoger	chr7:67769629-6777
ENSG0000(	339	7.676271	chr7:2516	Y_RNA	smallRNA	chr7:67297653-6729
ENSG0000(	339	7.676271	chr7:2516	MTCO1P57	Pseudoger	chr7:68097737-6809
ENSG0000(	339	7.676271	chr7:2516	ENSG00000233423	lncRNA	chr7:67691058-6769
ENSG0000(	339	7.676271	chr7:2516	MTND4P3	Pseudoger	chr7:68266319-6826
ENSG0000(	339	7.676271	chr7:2516	ENSG00000228019	Pseudoger	chr7:67307605-6730
ENSG0000(	339	7.676271	chr7:2516	AC073089.1	smallRNA	chr7:67139071-6713
ENSG0000(	339	7.676271	chr7:2516	MTATP6P21	Pseudoger	chr7:67627831-6762
ENSG0000(	339	7.676271	chr7:2516	ENSG00000225209	lncRNA	chr7:68020235-6803
ENSG0000(	339	7.676271	chr7:2516	MIR4650-1	smallRNA	chr7:67114322-6711
ENSG0000(	339	7.676271	chr7:2516	PMS2P4	Pseudoger	chr7:67295608-6729
ENSG0000(	339	7.676271	chr7:2516	AC006480.1	smallRNA	chr7:67356680-6735
ENSG0000(	339	7.676271	chr7:2516	ENSG00000236531	lncRNA	chr7:68091223-6811
ENSG0000(	339	7.676271	chr7:2516	ENSG00000273448	lncRNA	chr7:67333047-6733
ENSG0000(	339	7.676271	chr7:2516	ENSG00000237754	Pseudoger	chr7:68241637-6824
ENSG0000(	339	7.676271	chr7:2516	STAG3L4	Pseudoger	chr7:67305987-6730
ENSG0000(	339	7.676271	chr7:2516	MTCO3P41	Pseudoger	chr7:67628022-6762
ENSG0000(	339	7.676271	chr7:2516	STAG3L4	lncRNA	chr7:67302621-6736
ENSG0000(	339	7.676271	chr7:2516	PMS2P4	lncRNA	chr7:67139961-6730
ENSG0000(	339	7.676271	chr7:2516	ENSG00000233383	Pseudoger	chr7:67089257-6708
ENSG0000(	339	7.676271	chr7:2516	ENSG00000228429	Pseudoger	chr7:68640798-6864
ENSG0000(	339	7.676271	chr7:2516	ENSG00000226829	lncRNA	chr7:68149548-6831
ENSG0000(	337	7.630983	chr11:144	AP000781.1	smallRNA	chr11:57359722-573
ENSG0000(	335	7.585695	chr20:266	IDH3B-DT	lncRNA	chr20:2664352-2665
ENSG0000(	333	7.540408	chr2:2329	ENSG00000273240	lncRNA	chr2:189763859-189
ENSG0000(	333	7.540408	chr2:2329	ENSG00000284052	lncRNA	chr2:190607660-190
ENSG0000(	333	7.540408	chr2:2329	ENSG00000280083	TEC	chr2:191154118-191
ENSG0000(	333	7.540408	chr2:2329	CALCRL-AS1	lncRNA	chr2:187003220-187
ENSG0000(	333	7.540408	chr2:2329	DNAJB1P1	Pseudoger	chr2:191881182-191
ENSG0000(	333	7.540408	chr2:2329	LINC01090	lncRNA	chr2:187712816-188
ENSG0000(	333	7.540408	chr2:2329	ENSG00000288866	lncRNA	chr2:189439780-189
ENSG0000(	333	7.540408	chr2:2329	MIR1245A	smallRNA	chr2:188978092-188
ENSG0000(	333	7.540408	chr2:2329	ENSG00000288064	lncRNA	chr2:191045458-191
ENSG0000(	333	7.540408	chr2:2329	HNRNPCP2	Pseudoger	chr2:189923336-189
ENSG0000(	333	7.540408	chr2:2329	NEMP2	protein_c	chr2:190504338-190
ENSG0000(	333	7.540408	chr2:2329	ENSG00000228073	lncRNA	chr2:189095063-189
ENSG0000(	333	7.540408	chr2:2329	MIR3129	smallRNA	chr2:189133036-189
ENSG0000(	333	7.540408	chr2:2329	GULP1	protein_c	chr2:188291669-188
ENSG0000(	333	7.540408	chr22:220	D87007.1	smallRNA	chr22:22734575-227
ENSG0000(	333	7.540408	chr2:2329	ENSG00000223523	lncRNA	chr2:188598791-188
ENSG0000(	333	7.540408	chr2:2329	ENSG00000213115	Pseudoger	chr2:188227189-188
ENSG0000(	333	7.540408	chr2:2329	RNF11P1	Pseudoger	chr2:189930277-189
ENSG0000(	333	7.540408	chr2:2329	ASDURF	protein_c	chr2:189661452-189
ENSG0000(	333	7.540408	chr2:2329	ENSG00000288582	lncRNA	chr2:191733428-191
ENSG0000(	333	7.540408	chr2:2329	TFPI	protein_c	chr2:187464230-187
ENSG0000(	333	7.540408	chr2:2329	AC118063.1	smallRNA	chr2:189311671-189
ENSG0000(	333	7.540408	chr2:2329	ENSG00000230686	lncRNA	chr2:191017954-191
ENSG0000(	333	7.540408	chr2:2329	ENSG00000276828	Pseudoger	chr2:189625443-189
ENSG0000(	333	7.540408	chr2:2329	ENSG00000290089	lncRNA	chr2:190457656-190

ENSG0000(	333	7.540408	chr2:2329	MYO1B-AS1		lncRNA	chr2:191229165-191
ENSG0000(	333	7.540408	chr2:2329	NEMP2-DT		lncRNA	chr2:190534855-190
ENSG0000(	333	7.540408	chr2:2329	COL3A1	NCGv7	protein_c	chr2:188974373-189
ENSG0000(	333	7.540408	chr2:2329	C2orf88		protein_c	chr2:189879609-190
ENSG0000(	333	7.540408	chr2:2329	PMS1	NCGv7;AC	protein_c	chr2:189784085-189
ENSG0000(	333	7.540408	chr2:2329	STAT4-AS1		lncRNA	chr2:191021526-191
ENSG0000(	333	7.540408	chr2:2329	AC008122.1		smallRNA	chr2:189884764-189
ENSG0000(	333	7.540408	chr2:2329	CAVIN2		protein_c	chr2:191834310-191
ENSG0000(	333	7.540408	chr2:2329	OSGEPL1-AS1		lncRNA	chr2:189762704-189
ENSG0000(	333	7.540408	chr2:2329	MYO1B	NCGv7	protein_c	chr2:191245185-191
ENSG0000(	333	7.540408	chr2:2329	ANKAR		protein_c	chr2:189674290-189
ENSG0000(	333	7.540408	chr2:2329	OSGEPL1		protein_c	chr2:189746660-189
ENSG0000(	333	7.540408	chr2:2329	ORMDL1		protein_c	chr2:189770267-189
ENSG0000(	333	7.540408	chr2:2329	INPP1		protein_c	chr2:190343570-190
ENSG0000(	333	7.540408	chr2:2329	HMGB1P27		Pseudoger	chr2:191174233-191
ENSG0000(	333	7.540408	chr2:2329	MFSD6		protein_c	chr2:190408355-190
ENSG0000(	333	7.540408	chr2:2329	RNA5SP114		Pseudoger	chr2:188276732-188
ENSG0000(	333	7.540408	chr2:2329	ENSG00000288900		lncRNA	chr2:191677213-191
ENSG0000(	333	7.540408	chr2:2329	COL5A2		protein_c	chr2:189031898-189
ENSG0000(	333	7.540408	chr2:2329	ENSG00000235852		lncRNA	chr2:190880797-190
ENSG0000(	333	7.540408	chr2:2329	KDM3AP1		Pseudoger	chr2:189486480-189
ENSG0000(	333	7.540408	chr2:2329	ENSG00000287418		lncRNA	chr2:189537384-189
ENSG0000(	333	7.540408	chr2:2329	MSTN		protein_c	chr2:190055700-190
ENSG0000(	333	7.540408	chr2:2329	ENSG00000289205		lncRNA	chr2:188033794-188
ENSG0000(	333	7.540408	chr2:2329	ENSG00000286165		protein_c	chr2:189661519-189
ENSG0000(	333	7.540408	chr2:2329	ST13P2		Pseudoger	chr2:187825341-187
ENSG0000(	333	7.540408	chr2:2329	MIR561		smallRNA	chr2:188297492-188
ENSG0000(	333	7.540408	chr2:2329	NABP1	NCGv7	protein_c	chr2:191678068-191
ENSG0000(	333	7.540408	chr2:2329	KRT18P19		Pseudoger	chr2:189311263-189
ENSG0000(	333	7.540408	chr2:2329	TERF1P6		Pseudoger	chr2:189917254-189
ENSG0000(	333	7.540408	chr2:2329	WDR75	NCGv7	protein_c	chr2:189441446-189
ENSG0000(	333	7.540408	chr2:2329	ASNSD1		protein_c	chr2:189661385-189
ENSG0000(	333	7.540408	chr2:2329	Y_RNA		smallRNA	chr2:189753464-189
ENSG0000(	333	7.540408	chr2:2329	STAT1		protein_c	chr2:190908460-191
ENSG0000(	333	7.540408	chr2:2329	DIRC1		lncRNA	chr2:188734155-188
ENSG0000(	333	7.540408	chr2:2329	ENSG00000272979		lncRNA	chr2:190454092-190
ENSG0000(	333	7.540408	chr22:22(	IGLV2-18		protein_c	chr22:22734607-227
ENSG0000(	333	7.540408	chr2:2329	ENSG00000228509		lncRNA	chr2:190672297-190
ENSG0000(	333	7.540408	chr2:2329	RNU6-959P		smallRNA	chr2:191121950-191
ENSG0000(	333	7.540408	chr2:2329	SLC40A1		protein_c	chr2:189560590-189
ENSG0000(	333	7.540408	chr2:2329	NAB1		protein_c	chr2:190646746-190
ENSG0000(	333	7.540408	chr2:2329	ENSG00000225884		lncRNA	chr2:191792797-191
ENSG0000(	333	7.540408	chr2:2329	RAB1AP1		Pseudoger	chr2:190992639-190
ENSG0000(	333	7.540408	chr2:2329	RN7SKP179		smallRNA	chr2:190553264-190
ENSG0000(	333	7.540408	chr2:2329	AC068718.2		smallRNA	chr2:187742587-187
ENSG0000(	333	7.540408	chr2:2329	GLS		protein_c	chr2:190880821-190
ENSG0000(	333	7.540408	chr2:2329	RNU6-1045P		smallRNA	chr2:191370938-191
ENSG0000(	333	7.540408	chr2:2329	HIBCH		protein_c	chr2:190189735-190
ENSG0000(	333	7.540408	chr2:2329	CAVIN2-AS1		lncRNA	chr2:191846534-192
ENSG0000(	333	7.540408	chr2:2329	STAT4	NCGv7	protein_c	chr2:191029576-191
ENSG0000(	331	7.49512	chr6:1979	ENSG00000218857		Pseudoger	chr6:131184325-131

ENSG0000(	331	7.49512	chr6:1979	TAAR5		protein_c	chr6:132588592-132
ENSG0000(	331	7.49512	chr6:1979	EPB41L2		protein_c	chr6:130839347-131
ENSG0000(	331	7.49512	chr6:1979	TAAR4P		Pseudoger	chr6:132594398-132
ENSG0000(	331	7.49512	chr6:1979	CCNG1P1		Pseudoger	chr6:132698783-132
ENSG0000(	331	7.49512	chr6:1979	RPL23AP46		Pseudoger	chr6:132997551-132
ENSG0000(	331	7.49512	chr6:1979	HMGB1P13		Pseudoger	chr6:132868218-132
ENSG0000(	331	7.49512	chr6:1979	RPL21P66		Pseudoger	chr6:132518830-132
ENSG0000(	331	7.49512	chr6:1979	RNU4-18P		smallRNA	chr6:131642818-131
ENSG0000(	331	7.49512	chr6:1979	MIR548H5		smallRNA	chr6:131792172-131
ENSG0000(	331	7.49512	chr6:1979	ENSG00000234484		lncRNA	chr6:132752675-132
ENSG0000(	331	7.49512	chr6:1979	RN7SKP245		smallRNA	chr6:131820334-131
ENSG0000(	331	7.49512	chr6:1979	ENSG00000216917		Pseudoger	chr6:131780721-131
ENSG0000(	331	7.49512	chr6:1979	CTAGE9		protein_c	chr6:131708441-131
ENSG0000(	331	7.49512	chr6:1979	ENPP1		protein_c	chr6:131808016-131
ENSG0000(	331	7.49512	chr6:1979	ENSG00000272428		lncRNA	chr6:133540784-133
ENSG0000(	331	7.49512	chr6:1979	TAAR3P		Pseudoger	chr6:132608252-132
ENSG0000(	331	7.49512	chr6:1979	SNORD101		smallRNA	chr6:132815307-132
ENSG0000(	331	7.49512	chr6:1979	ENSG00000286438		lncRNA	chr6:133061240-133
ENSG0000(	331	7.49512	chr6:1979	ENSG00000280155		TEC	chr6:132130252-132
ENSG0000(	331	7.49512	chr6:1979	HSPE1P21		Pseudoger	chr6:133510386-133
ENSG0000(	331	7.49512	chr6:1979	HLFP1		Pseudoger	chr6:132674885-132
ENSG0000(	331	7.49512	chr6:1979	ENPP3		protein_c	chr6:131628442-131
ENSG0000(	331	7.49512	chr6:1979	SLC18B1		protein_c	chr6:132769370-132
ENSG0000(	331	7.49512	chr6:1979	SNORA33		smallRNA	chr6:132817219-132
ENSG0000(	331	7.49512	chr6:1979	CCN2	AC	protein_c	chr6:131948176-131
ENSG0000(	331	7.49512	chr6:1979	EYA4	NCGv7	protein_c	chr6:133240514-133
ENSG0000(	331	7.49512	chr6:1979	ARG1		protein_c	chr6:131470832-131
ENSG0000(	331	7.49512	chr6:1979	RPS12		protein_c	chr6:132814569-132
ENSG0000(	331	7.49512	chr6:1979	TAAR1		protein_c	chr6:132643312-132
ENSG0000(	331	7.49512	chr6:1979	VNN2		protein_c	chr6:132743870-132
ENSG0000(	331	7.49512	chr6:1979	VNN1		protein_c	chr6:132680849-132
ENSG0000(	331	7.49512	chr6:1979	TAAR7P		Pseudoger	chr6:132559024-132
ENSG0000(	331	7.49512	chr6:1979	MED23	NCGv7	protein_c	chr6:131573966-131
ENSG0000(	331	7.49512	chr6:1979	LINC01013		lncRNA	chr6:131901848-132
ENSG0000(	331	7.49512	chr6:1979	ENSG00000286663		lncRNA	chr6:132599972-132
ENSG0000(	331	7.49512	chr6:1979	ENSG00000223542		lncRNA	chr6:133435077-133
ENSG0000(	331	7.49512	chr6:1979	AKAP7		protein_c	chr6:131135467-131
ENSG0000(	331	7.49512	chr6:1979	TAAR8		protein_c	chr6:132552672-132
ENSG0000(	331	7.49512	chr6:1979	TAAR6		protein_c	chr6:132570322-132
ENSG0000(	331	7.49512	chr6:1979	ENSG00000290067		lncRNA	chr6:131217724-131
ENSG0000(	331	7.49512	chr6:1979	TAAR2		protein_c	chr6:132617022-132
ENSG0000(	331	7.49512	chr6:1979	ENSG00000234567		lncRNA	chr6:133452857-133
ENSG0000(	331	7.49512	chr6:1979	STX7		protein_c	chr6:132445867-132
ENSG0000(	331	7.49512	chr6:1979	MOXD1		protein_c	chr6:132296055-132
ENSG0000(	331	7.49512	chr6:1979	LINC00326		lncRNA	chr6:132954257-133
ENSG0000(	331	7.49512	chr6:1979	ENSG00000289262		lncRNA	chr6:131294417-131
ENSG0000(	331	7.49512	chr6:1979	MIR548AJ1		smallRNA	chr6:132115192-132
ENSG0000(	331	7.49512	chr6:1979	RPL21P67		Pseudoger	chr6:131469059-131
ENSG0000(	331	7.49512	chr6:1979	ENSG00000229923		lncRNA	chr6:130697312-130
ENSG0000(	331	7.49512	chr6:1979	VNN3P		Pseudoger	chr6:132722784-132
ENSG0000(	331	7.49512	chr6:1979	ENSG00000288977		lncRNA	chr6:131125625-131

ENSG0000(	331	7.49512	chr6:1979	TAAR9		protein_c	chr6:132538277-132
ENSG0000(	331	7.49512	chr6:1979	ENSG00000237115		Pseudoger	chr6:131825981-131
ENSG0000(	331	7.49512	chr6:1979	TAAR3P		lncRNA	chr6:132608225-132
ENSG0000(	331	7.49512	chr6:1979	MTCYBP4		Pseudoger	chr6:133150568-133
ENSG0000(	331	7.49512	chr6:1979	SELENOKP2		Pseudoger	chr6:131819803-131
ENSG0000(	331	7.49512	chr6:1979	RBM11P1		Pseudoger	chr6:132764660-132
ENSG0000(	331	7.49512	chr6:1979	SNORD100		smallRNA	chr6:132816802-132
ENSG0000(	331	7.49512	chr6:1979	SMLR1		protein_c	chr6:130827406-130
ENSG0000(	331	7.49512	chr6:1979	ENSG00000279960		TEC	chr6:132891924-132
ENSG0000(	331	7.49512	chr6:1979	OR2A4		protein_c	chr6:131699644-131
ENSG0000(	331	7.49512	chr6:1979	EEF1A1P36		Pseudoger	chr6:132271982-132
ENSG0000(	329	7.449832	chr16:239	ENSG00000279106		TEC	chr16:29204633-292
ENSG0000(	329	7.449832	chr16:239	ENSG00000284685		lncRNA	chr16:29139379-293
ENSG0000(	329	7.449832	chr2:3608	MIR26B		smallRNA	chr2:218402646-218
ENSG0000(	329	7.449832	chr16:239	ENSG00000260517		lncRNA	chr16:29139661-292
ENSG0000(	329	7.449832	chr16:239	ENSG00000259807		lncRNA	chr16:29215385-292
ENSG0000(	329	7.449832	chr16:239	ENSG00000289080		lncRNA	chr16:29145438-291
ENSG0000(	329	7.449832	chr16:239	ENSG00000273582		lncRNA	chr16:29225594-292
ENSG0000(	325	7.359257	chr5:4231	TLX3	NCGv7;AC	protein_c	chr5:171309248-171
ENSG0000(	325	7.359257	chr5:4231	RN7SL339P		smallRNA	chr5:171359117-171
ENSG0000(	325	7.359257	chr5:4231	RNU6-164P		smallRNA	chr5:162477891-162
ENSG0000(	325	7.359257	chr5:4231	RNU6-477P		smallRNA	chr5:169552449-169
ENSG0000(	325	7.359257	chr5:4231	AC008674.1		smallRNA	chr5:173730078-173
ENSG0000(	325	7.359257	chr5:4231	DUSP1		protein_c	chr5:172768096-172
ENSG0000(	325	7.359257	chr5:4231	ENSG00000253331		lncRNA	chr5:164119098-164
ENSG0000(	325	7.359257	chr5:4231	RARS1		protein_c	chr5:168486451-168
ENSG0000(	325	7.359257	chr5:4231	KRT18P41		Pseudoger	chr5:170140664-170
ENSG0000(	325	7.359257	chr5:4231	ENSG00000279739		TEC	chr5:168128856-168
ENSG0000(	325	7.359257	chr5:4231	ENSG00000213386		Pseudoger	chr5:172762521-172
ENSG0000(	325	7.359257	chr5:4231	WWC1		protein_c	chr5:168291645-168
ENSG0000(	325	7.359257	chr5:4231	RPL26L1-AS1		lncRNA	chr5:172954786-172
ENSG0000(	325	7.359257	chr5:4231	RANBP17	AC	protein_c	chr5:170861870-171
ENSG0000(	325	7.359257	chr5:4231	GABRP		protein_c	chr5:170763350-170
ENSG0000(	325	7.359257	chr5:4231	ERGIC1	AC	protein_c	chr5:172834251-172
ENSG0000(	325	7.359257	chr5:4231	INSYN2B	NCGv7	protein_c	chr5:169861303-169
ENSG0000(	325	7.359257	chr5:4231	ENSG00000224012		Pseudoger	chr5:163709210-163
ENSG0000(	325	7.359257	chr5:4231	ENSG00000213393		Pseudoger	chr5:171345343-171
ENSG0000(	325	7.359257	chr5:4231	RPL10P9		Pseudoger	chr5:168616352-168
ENSG0000(	325	7.359257	chr5:4231	ENSG00000253713		lncRNA	chr5:167116318-167
ENSG0000(	325	7.359257	chr5:4231	ENSG00000253925		lncRNA	chr5:168085329-168
ENSG0000(	325	7.359257	chr5:4231	BNIP1		protein_c	chr5:173144442-173
ENSG0000(	325	7.359257	chr5:4231	RN7SL623P		smallRNA	chr5:170866835-170
ENSG0000(	325	7.359257	chr7:2516	KCTD7	NCGv7	protein_c	chr7:66628881-6664
ENSG0000(	325	7.359257	chr5:4231	LINC01366		lncRNA	chr5:170307722-170
ENSG0000(	325	7.359257	chr5:4231	SLIT3-AS2		lncRNA	chr5:168706567-168
ENSG0000(	325	7.359257	chr5:4231	ENSG00000275038		lncRNA	chr5:171305980-171
ENSG0000(	325	7.359257	chr5:4231	ATP10B	NCGv7	protein_c	chr5:160563120-160
ENSG0000(	325	7.359257	chr7:2516	RABGEF1	NCGv7	protein_c	chr7:66682164-6681
ENSG0000(	325	7.359257	chr5:4231	ENSG00000288737		lncRNA	chr5:172807437-172
ENSG0000(	325	7.359257	chr5:4231	LINC02143		lncRNA	chr5:164448161-164
ENSG0000(	325	7.359257	chr5:4231	ENSG00000254391		lncRNA	chr5:160685351-160

ENSG0000(	325	7.359257	chr5:4231	ENSG00000254192		lncRNA	chr5:168654513-168
ENSG0000(	325	7.359257	chr5:4231	Y_RNA		smallRNA	chr5:172779982-172
ENSG0000(	325	7.359257	chr5:4231	LINC03000		lncRNA	chr5:164296696-165
ENSG0000(	325	7.359257	chr7:251(	SBDS	NCGv7;AC	protein_(	chr7:66987680-6699
ENSG0000(	325	7.359257	chr5:4231	ENSG00000280318		TEC	chr5:172609094-172
ENSG0000(	325	7.359257	chr5:4231	TENM2-AS1		lncRNA	chr5:168229583-168
ENSG0000(	325	7.359257	chr5:4231	AC122720.1		smallRNA	chr5:166159433-166
ENSG0000(	325	7.359257	chr7:251(	ENSG00000271064		Pseudoger	chr7:66748838-6674
ENSG0000(	325	7.359257	chr5:4231	CPEB4	NCGv7	protein_(	chr5:173888349-173
ENSG0000(	325	7.359257	chr5:4231	ENSG00000289264		lncRNA	chr5:167569078-167
ENSG0000(	325	7.359257	chr5:4231	ENSG00000253628		lncRNA	chr5:172816592-172
ENSG0000(	325	7.359257	chr7:251(	TMEM248		protein_(	chr7:66921225-6695
ENSG0000(	325	7.359257	chr7:251(	RNU6-96P		smallRNA	chr7:66395191-6639
ENSG0000(	325	7.359257	chr5:4231	ENSG00000253512		Pseudoger	chr5:165782805-165
ENSG0000(	325	7.359257	chr5:4231	ENSG00000250114		Pseudoger	chr5:163416525-163
ENSG0000(	325	7.359257	chr5:4231	MIR3142		smallRNA	chr5:160474402-160
ENSG0000(	325	7.359257	chr5:4231	AC011407.1		smallRNA	chr5:172439663-172
ENSG0000(	325	7.359257	chr5:4231	STC2		protein_(	chr5:173314723-173
ENSG0000(	325	7.359257	chr5:4231	ZBED8		protein_(	chr5:160393148-160
ENSG0000(	325	7.359257	chr5:4231	KLF3P1		Pseudoger	chr5:172254418-172
ENSG0000(	325	7.359257	chr5:4231	ENSG00000273569		Pseudoger	chr5:163855140-163
ENSG0000(	325	7.359257	chr5:4231	ATP6V0E1	NCGv7	protein_(	chr5:172983771-173
ENSG0000(	325	7.359257	chr5:4231	AC011363.1		smallRNA	chr5:160664587-160
ENSG0000(	325	7.359257	chr5:4231	SPDL1		protein_(	chr5:169583636-169
ENSG0000(	325	7.359257	chr5:4231	KCNIP1-OT1		lncRNA	chr5:170389493-170
ENSG0000(	325	7.359257	chr5:4231	SLIT3-AS1		lncRNA	chr5:169013227-169
ENSG0000(	325	7.359257	chr5:4231	ENSG00000253693		lncRNA	chr5:165349030-165
ENSG0000(	325	7.359257	chr5:4231	ENSG00000253141		lncRNA	chr5:173463484-173
ENSG0000(	325	7.359257	chr5:4231	ENSG00000254187		lncRNA	chr5:167653228-167
ENSG0000(	325	7.359257	chr5:4231	CREBRF		protein_(	chr5:173056352-173
ENSG0000(	325	7.359257	chr7:251(	ENSG00000272831		lncRNA	chr7:66739829-6674
ENSG0000(	325	7.359257	chr5:4231	RPSAP71		Pseudoger	chr5:171365595-171
ENSG0000(	325	7.359257	chr5:4231	ENSG00000254186		lncRNA	chr5:162424042-163
ENSG0000(	325	7.359257	chr5:4231	ENSG00000254164		lncRNA	chr5:173574938-173
ENSG0000(	325	7.359257	chr5:4231	ENSG00000253687		lncRNA	chr5:160613873-160
ENSG0000(	325	7.359257	chr5:4231	RNU6-168P		smallRNA	chr5:163796065-163
ENSG0000(	325	7.359257	chr5:4231	RPL12P22		Pseudoger	chr5:174039751-174
ENSG0000(	325	7.359257	chr5:4231	LINC01484		lncRNA	chr5:173707614-173
ENSG0000(	325	7.359257	chr5:4231	LINC01863		lncRNA	chr5:173642519-173
ENSG0000(	325	7.359257	chr7:251(	RN7SL43P		smallRNA	chr7:66980120-6698
ENSG0000(	325	7.359257	chr5:4231	Y_RNA		smallRNA	chr5:173254190-173
ENSG0000(	325	7.359257	chr5:4231	FOXI1		protein_(	chr5:170105897-170
ENSG0000(	325	7.359257	chr5:4231	AC022120.1		smallRNA	chr5:161230050-161
ENSG0000(	325	7.359257	chr5:4231	ENSG00000253110		lncRNA	chr5:171773652-171
ENSG0000(	325	7.359257	chr5:4231	ENSG00000253357		lncRNA	chr5:167721363-167
ENSG0000(	325	7.359257	chr5:4231	ENSG00000253683		Pseudoger	chr5:172656522-172
ENSG0000(	325	7.359257	chr5:4231	SNORA74B		smallRNA	chr5:173020728-173
ENSG0000(	325	7.359257	chr5:4231	KCNIP1		protein_(	chr5:170353487-170
ENSG0000(	325	7.359257	chr5:4231	LINC01202		lncRNA	chr5:161907111-162
ENSG0000(	325	7.359257	chr7:251(	RPL35P5		Pseudoger	chr7:66606738-6660
ENSG0000(	325	7.359257	chr5:4231	FBLL1		protein_(	chr5:168529305-168

ENSG0000(	325	7.359257	chr5:4231	RPL7P20		Pseudoger	chr5:166028567-166
ENSG0000(	325	7.359257	chr5:4231	RNU6-500P		smallRNA	chr5:173362134-173
ENSG0000(	325	7.359257	chr5:4231	UBTD2		protein_c	chr5:172209646-172
ENSG0000(	325	7.359257	chr5:4231	ENSG00000253966		Pseudoger	chr5:170896929-170
ENSG0000(	325	7.359257	chr5:4231	LINC01947		lncRNA	chr5:166905222-166
ENSG0000(	325	7.359257	chr5:4231	ARL2BPP5		Pseudoger	chr5:162761833-162
ENSG0000(	325	7.359257	chr5:4231	HMMR-AS1		lncRNA	chr5:163483065-163
ENSG0000(	325	7.359257	chr5:4231	SLIT3	NCGv7	protein_c	chr5:168661733-169
ENSG0000(	325	7.359257	chr5:4231	ENSG00000285914		lncRNA	chr5:171724575-171
ENSG0000(	325	7.359257	chr5:4231	RN7SKP60		smallRNA	chr5:165609441-165
ENSG0000(	325	7.359257	chr5:4231	CCNG1		protein_c	chr5:163437569-163
ENSG0000(	325	7.359257	chr7:251(	ENSG00000244657		Pseudoger	chr7:66485095-6648
ENSG0000(	325	7.359257	chr5:4231	ENSG00000289702		lncRNA	chr5:169856650-169
ENSG0000(	325	7.359257	chr5:4231	ENSG00000253946		Pseudoger	chr5:165905126-165
ENSG0000(	325	7.359257	chr7:251(	GTF2IRD1P1		lncRNA	chr7:66809993-6684
ENSG0000(	325	7.359257	chr5:4231	ENSG00000288799		lncRNA	chr5:172007100-172
ENSG0000(	325	7.359257	chr7:251(	ENSG00000226824		lncRNA	chr7:66654513-6668
ENSG0000(	325	7.359257	chr5:4231	GABRG2		protein_c	chr5:162000057-162
ENSG0000(	325	7.359257	chr5:4231	ENSG00000253947		lncRNA	chr5:167965187-167
ENSG0000(	325	7.359257	chr7:251(	GTF2IRD1P1		Pseudoger	chr7:66815836-6683
ENSG0000(	325	7.359257	chr7:251(	ENSG00000177418		Pseudoger	chr7:66556889-6655
ENSG0000(	325	7.359257	chr5:4231	LINC01485		lncRNA	chr5:173778527-173
ENSG0000(	325	7.359257	chr5:4231	SH3PXD2B		protein_c	chr5:172325000-172
ENSG0000(	325	7.359257	chr7:251(	RABGEF1P2		Pseudoger	chr7:66427949-6645
ENSG0000(	325	7.359257	chr5:4231	RPL26L1		protein_c	chr5:172958729-172
ENSG0000(	325	7.359257	chr5:4231	SMIM23		protein_c	chr5:171782432-171
ENSG0000(	325	7.359257	chr5:4231	KCNMB1		protein_c	chr5:170374671-170
ENSG0000(	325	7.359257	chr5:4231	AC008671.1		smallRNA	chr5:172279202-172
ENSG0000(	325	7.359257	chr5:4231	ENSG00000253660		lncRNA	chr5:167937717-167
ENSG0000(	325	7.359257	chr7:251(	snoU13		smallRNA	chr7:66790354-6679
ENSG0000(	325	7.359257	chr5:4231	PANK3		protein_c	chr5:168548495-168
ENSG0000(	325	7.359257	chr5:4231	ENSG00000253968		lncRNA	chr5:173383941-173
ENSG0000(	325	7.359257	chr7:251(	ENSG00000236928		Pseudoger	chr7:66434634-6643
ENSG0000(	325	7.359257	chr5:4231	ENSG00000254365		lncRNA	chr5:167287320-167
ENSG0000(	325	7.359257	chr7:251(	LINC03011		lncRNA	chr7:66493607-6649
ENSG0000(	325	7.359257	chr5:4231	ENSG00000253538		lncRNA	chr5:164209631-164
ENSG0000(	325	7.359257	chr5:4231	ENSG00000254373		Pseudoger	chr5:160258253-160
ENSG0000(	325	7.359257	chr5:4231	ENSG00000285590		lncRNA	chr5:163597352-163
ENSG0000(	325	7.359257	chr5:4231	ENSG00000253269		lncRNA	chr5:169772966-169
ENSG0000(	325	7.359257	chr5:4231	CCNJL		protein_c	chr5:160249083-160
ENSG0000(	325	7.359257	chr7:251(	RNU6-313P		smallRNA	chr7:66344304-6634
ENSG0000(	325	7.359257	chr5:4231	ENSG00000253772		Pseudoger	chr5:160247517-160
ENSG0000(	325	7.359257	chr5:4231	ENSG00000253858		lncRNA	chr5:170483806-170
ENSG0000(	325	7.359257	chr5:4231	LINC02159		lncRNA	chr5:160931778-160
ENSG0000(	325	7.359257	chr5:4231	SNORA40		smallRNA	chr5:168033091-168
ENSG0000(	325	7.359257	chr5:4231	NPM1	NCGv7;AC	protein_c	chr5:171387116-171
ENSG0000(	325	7.359257	chr5:4231	ENSG00000254328		Pseudoger	chr5:172986680-172
ENSG0000(	325	7.359257	chr7:251(	ENSG00000275400		Pseudoger	chr7:66553805-6655
ENSG0000(	325	7.359257	chr5:4231	ENSG00000253527		lncRNA	chr5:167306273-167
ENSG0000(	325	7.359257	chr7:251(	SAPCD2P3		Pseudoger	chr7:66556216-6655
ENSG0000(	325	7.359257	chr5:4231	SLC2A3P1		Pseudoger	chr5:168552277-168

ENSG0000(	325	7.359257	chr5:4231	LSM1P2		Pseudoger	chr5:163887506-163
ENSG0000(	325	7.359257	chr5:4231	ENSG00000290019		lncRNA	chr5:172802858-172
ENSG0000(	325	7.359257	chr5:4231	RPL10P8		Pseudoger	chr5:171366299-171
ENSG0000(	325	7.359257	chr5:4231	ENSG00000288615		lncRNA	chr5:170306557-170
ENSG0000(	325	7.359257	chr5:4231	ENSG00000253768		lncRNA	chr5:173562478-173
ENSG0000(	325	7.359257	chr5:4231	ENSG00000289085		lncRNA	chr5:173911682-173
ENSG0000(	325	7.359257	chr5:4231	MIR103A1		smallRNA	chr5:168560892-168
ENSG0000(	325	7.359257	chr5:4231	LCP2		protein_c	chr5:170246233-170
ENSG0000(	325	7.359257	chr5:4231	DOCK2	NCGv7	protein_c	chr5:169637268-170
ENSG0000(	325	7.359257	chr7:2516	ENSG00000244510		Pseudoger	chr7:66480394-6649
ENSG0000(	325	7.359257	chr7:2516	GTF2IP23		Pseudoger	chr7:66880708-6688
ENSG0000(	325	7.359257	chr5:4231	MRPL57P6		Pseudoger	chr5:163039291-163
ENSG0000(	325	7.359257	chr7:2516	TYW1		protein_c	chr7:66995173-6723
ENSG0000(	325	7.359257	chr5:4231	MIR3912		smallRNA	chr5:171386656-171
ENSG0000(	325	7.359257	chr5:4231	MIR378E		smallRNA	chr5:170028488-170
ENSG0000(	325	7.359257	chr7:2516	ENSG00000234500		Pseudoger	chr7:66511556-6654
ENSG0000(	325	7.359257	chr5:4231	NEURL1B		protein_c	chr5:172641263-172
ENSG0000(	325	7.359257	chr5:4231	RN7SL295P		smallRNA	chr5:160291788-160
ENSG0000(	325	7.359257	chr7:2516	ENSG00000279785		TEC	chr7:66474556-6647
ENSG0000(	325	7.359257	chr5:4231	STK10		protein_c	chr5:172042079-172
ENSG0000(	325	7.359257	chr5:4231	ENSG00000253447		lncRNA	chr5:174081506-174
ENSG0000(	325	7.359257	chr5:4231	SNORA57		smallRNA	chr5:172252334-172
ENSG0000(	325	7.359257	chr5:4231	ENSG00000285549		lncRNA	chr5:165129307-165
ENSG0000(	325	7.359257	chr5:4231	ENSG00000253445		lncRNA	chr5:172690454-172
ENSG0000(	325	7.359257	chr5:4231	BOD1	NCGv7	protein_c	chr5:173607145-173
ENSG0000(	325	7.359257	chr5:4231	PTTG1	TAG;AC	protein_c	chr5:160421855-160
ENSG0000(	325	7.359257	chr5:4231	ENSG00000253244		lncRNA	chr5:174056060-174
ENSG0000(	325	7.359257	chr5:4231	FBXW11		protein_c	chr5:171861549-172
ENSG0000(	325	7.359257	chr5:4231	MIR585		smallRNA	chr5:169263601-169
ENSG0000(	325	7.359257	chr7:2516	ENSG00000291136		lncRNA	chr7:66526088-6659
ENSG0000(	325	7.359257	chr7:2516	SKP1P1		Pseudoger	chr7:66423405-6642
ENSG0000(	325	7.359257	chr5:4231	CDC42P5		Pseudoger	chr5:173085342-173
ENSG0000(	325	7.359257	chr7:2516	GTF2IP9		Pseudoger	chr7:66407288-6640
ENSG0000(	325	7.359257	chr5:4231	MIR5003		smallRNA	chr5:172662165-172
ENSG0000(	325	7.359257	chr5:4231	SLU7		protein_c	chr5:160401641-160
ENSG0000(	325	7.359257	chr5:4231	ENSG00000248965		lncRNA	chr5:168993000-168
ENSG0000(	325	7.359257	chr7:2516	RNU6-1254P		smallRNA	chr7:66891188-6689
ENSG0000(	325	7.359257	chr5:4231	RNA5SP200		Pseudoger	chr5:173292517-173
ENSG0000(	325	7.359257	chr5:4231	C5orf58		protein_c	chr5:170232447-170
ENSG0000(	325	7.359257	chr7:2516	snoU13		smallRNA	chr7:66434507-6643
ENSG0000(	325	7.359257	chr5:4231	MIR218-2		smallRNA	chr5:168768146-168
ENSG0000(	325	7.359257	chr7:2516	ENSG00000289015		lncRNA	chr7:66681258-6668
ENSG0000(	325	7.359257	chr5:4231	LINC01942		lncRNA	chr5:173689459-173
ENSG0000(	325	7.359257	chr5:4231	RPLP0P9		Pseudoger	chr5:166382305-166
ENSG0000(	325	7.359257	chr7:2516	ENSG00000284461		protein_c	chr7:66628958-6681
ENSG0000(	325	7.359257	chr5:4231	NUDCD2		protein_c	chr5:163446526-163
ENSG0000(	325	7.359257	chr5:4231	ENSG00000254171		lncRNA	chr5:166128498-166
ENSG0000(	325	7.359257	chr5:4231	SNORA70		smallRNA	chr5:171366118-171
ENSG0000(	325	7.359257	chr5:4231	ENSG00000253348		lncRNA	chr5:170747047-170
ENSG0000(	325	7.359257	chr5:4231	ENSG00000253295		lncRNA	chr5:172755457-172
ENSG0000(	325	7.359257	chr5:4231	AC093304.1		smallRNA	chr5:167870545-167

ENSG0000(	325	7.359257	chr5:4231	C1QTNF2		protein_c	chr5:160347754-160
ENSG0000(	325	7.359257	chr5:4231	ENSG00000270612		Pseudoger	chr5:160326693-160
ENSG0000(	325	7.359257	chr5:4231	ENSG00000253600		Pseudoger	chr5:164601002-164
ENSG0000(	325	7.359257	chr5:4231	ENSG00000288912		lncRNA	chr5:169420745-169
ENSG0000(	325	7.359257	chr5:4231	AC113342.1		smallRNA	chr5:172080905-172
ENSG0000(	325	7.359257	chr5:4231	ENSG00000289493		lncRNA	chr5:173243111-173
ENSG0000(	325	7.359257	chr7:251(	ENSG00000179342		Pseudoger	chr7:66505155-6650
ENSG0000(	325	7.359257	chr5:4231	RNU6-209P		smallRNA	chr5:164935162-164
ENSG0000(	325	7.359257	chr5:4231	USP12P1		Pseudoger	chr5:171251866-171
ENSG0000(	325	7.359257	chr5:4231	FGF18		protein_c	chr5:171419647-171
ENSG0000(	325	7.359257	chr5:4231	Y_RNA		smallRNA	chr5:173052452-173
ENSG0000(	325	7.359257	chr7:251(	ENSG00000179131		Pseudoger	chr7:66914581-6691
ENSG0000(	325	7.359257	chr7:251(	LINC00174		lncRNA	chr7:66376044-6649
ENSG0000(	325	7.359257	chr5:4231	GABRA1	NCGv7	protein_c	chr5:161847063-161
ENSG0000(	325	7.359257	chr5:4231	ENSG00000253469		lncRNA	chr5:167164934-167
ENSG0000(	325	7.359257	chr5:4231	ENSG00000287814		lncRNA	chr5:172479274-172
ENSG0000(	325	7.359257	chr5:4231	ENSG00000253172		lncRNA	chr5:173144162-173
ENSG0000(	325	7.359257	chr5:4231	ENSG00000270243		Pseudoger	chr5:172785262-172
ENSG0000(	325	7.359257	chr5:4231	LINC01938		lncRNA	chr5:165220577-165
ENSG0000(	325	7.359257	chr5:4231	snoU13		smallRNA	chr5:171283659-171
ENSG0000(	325	7.359257	chr5:4231	ENSG00000250274		lncRNA	chr5:170308701-170
ENSG0000(	325	7.359257	chr5:4231	TENM2	NCGv7	protein_c	chr5:166979029-168
ENSG0000(	325	7.359257	chr5:4231	ENSG00000287003		lncRNA	chr5:173757562-173
ENSG0000(	325	7.359257	chr5:4231	LINC01187		lncRNA	chr5:170191579-170
ENSG0000(	325	7.359257	chr5:4231	MIR3142HG		lncRNA	chr5:160438594-160
ENSG0000(	325	7.359257	chr5:4231	ENSG00000253785		Pseudoger	chr5:172975511-172
ENSG0000(	325	7.359257	chr5:4231	HMMR	TAG	protein_c	chr5:163460203-163
ENSG0000(	325	7.359257	chr7:251(	ENSG00000232546		Pseudoger	chr7:66848496-6685
ENSG0000(	325	7.359257	chr5:4231	NKX2-5		protein_c	chr5:173232109-173
ENSG0000(	325	7.359257	chr5:4231	GABRB2	NCGv7	protein_c	chr5:161288429-161
ENSG0000(	325	7.359257	chr5:4231	ENSG00000289170		lncRNA	chr5:172772187-172
ENSG0000(	325	7.359257	chr5:4231	C5orf47		protein_c	chr5:173973779-174
ENSG0000(	325	7.359257	chr5:4231	ENSG00000253736		lncRNA	chr5:172762980-172
ENSG0000(	325	7.359257	chr5:4231	FABP6		protein_c	chr5:160187367-160
ENSG0000(	325	7.359257	chr5:4231	GABRA6	NCGv7	protein_c	chr5:161547063-161
ENSG0000(	325	7.359257	chr5:4231	MAT2B		protein_c	chr5:163503114-163
ENSG0000(	325	7.359257	chr5:4231	FABP6-AS1		lncRNA	chr5:160195744-160
ENSG0000(	325	7.359257	chr7:251(	ENSG00000289177		lncRNA	chr7:66845003-6684
ENSG0000(	325	7.359257	chr7:251(	LINC02604		lncRNA	chr7:66902857-6690
ENSG0000(	325	7.359257	chr5:4231	KCNIP1-AS1		lncRNA	chr5:170639158-170
ENSG0000(	325	7.359257	chr5:4231	Y_RNA		smallRNA	chr5:168791498-168
ENSG0000(	325	7.359257	chr5:4231	ENSG00000254297		lncRNA	chr5:167296234-167
ENSG0000(	325	7.359257	chr5:4231	EFCAB9		protein_c	chr5:172194172-172
ENSG0000(	325	7.359257	chr5:4231	LINC02995		lncRNA	chr5:173578275-173
ENSG0000(	325	7.359257	chr5:4231	AC011410.1		smallRNA	chr5:171770530-171
ENSG0000(	325	7.359257	chr5:4231	RPL7AP33		Pseudoger	chr5:173225836-173
ENSG0000(	325	7.359257	chr5:4231	ENSG00000254295		lncRNA	chr5:172954907-172
ENSG0000(	325	7.359257	chr5:4231	ENSG00000279752		TEC	chr5:169012148-169
ENSG0000(	325	7.359257	chr5:4231	LINC01944		lncRNA	chr5:172543403-172
ENSG0000(	325	7.359257	chr5:4231	ENSG00000253403		lncRNA	chr5:161687347-161
ENSG0000(	324	7.336613	chr12:201	ENSG00000277223		lncRNA	chr12:71448405-714

ENSG0000(	324	7.336613	chr12:201	ENSG00000258140		lncRNA	chr12:69212108-692
ENSG0000(	324	7.336613	chr12:201	ENSG00000258053		lncRNA	chr12:71047402-711
ENSG0000(	324	7.336613	chr12:201	ENSG00000256075		Pseudoger	chr12:68473741-684
ENSG0000(	324	7.336613	chr12:201	CCT2	DriverDB\	protein_c	chr12:69585426-696
ENSG0000(	324	7.336613	chr12:201	FRS2	DriverDB\	protein_c	chr12:69470349-695
ENSG0000(	324	7.336613	chr12:201	ENSG00000258052		protein_c	chr12:69801669-698
ENSG0000(	324	7.336613	chr12:201	LGR5		protein_c	chr12:71439798-715
ENSG0000(	324	7.336613	chr12:201	TMEM19		protein_c	chr12:71686082-717
ENSG0000(	324	7.336613	chr12:201	ENSG00000257991		Pseudoger	chr12:71835034-718
ENSG0000(	324	7.336613	chr12:201	RN7SL804P		smallRNA	chr12:69400903-694
ENSG0000(	324	7.336613	chr12:201	ENSG00000279530		TEC	chr12:70321542-703
ENSG0000(	324	7.336613	chr12:201	RPL10P12		Pseudoger	chr12:68626870-686
ENSG0000(	324	7.336613	chr12:201	LYZ		protein_c	chr12:69348381-693
ENSG0000(	324	7.336613	chr1:1021	ENSG00000269737		Pseudoger	chr1:1671990-16743
ENSG0000(	324	7.336613	chr12:201	MYRFL		protein_c	chr12:69825227-699
ENSG0000(	324	7.336613	chr12:201	ENSG00000289173		lncRNA	chr12:68610197-686
ENSG0000(	324	7.336613	chr12:201	ENSG00000277247		lncRNA	chr12:70570969-705
ENSG0000(	324	7.336613	chr1:1021	ENSG00000272004		lncRNA	chr1:1659325-16626
ENSG0000(	324	7.336613	chr12:201	ENSG00000279551		TEC	chr12:69660034-696
ENSG0000(	324	7.336613	chr12:201	ENSG00000256273		lncRNA	chr12:68344664-683
ENSG0000(	324	7.336613	chr1:1021	MMP23A		Pseudoger	chr1:1699942-17017
ENSG0000(	324	7.336613	chr12:201	ENSG00000289283		lncRNA	chr12:70317612-703
ENSG0000(	324	7.336613	chr12:201	ENSG00000256325		lncRNA	chr12:68828118-688
ENSG0000(	324	7.336613	chr12:201	ENSG00000258168		lncRNA	chr12:70468080-705
ENSG0000(	324	7.336613	chr12:201	RAB21		protein_c	chr12:71754863-718
ENSG0000(	324	7.336613	chr12:201	PTPRR		protein_c	chr12:70638073-709
ENSG0000(	324	7.336613	chr12:201	ENSG00000289595		lncRNA	chr12:68804873-688
ENSG0000(	324	7.336613	chr12:201	ENSG00000288530		lncRNA	chr12:71582293-715
ENSG0000(	324	7.336613	chr12:201	ENSG00000258064		protein_c	chr12:71674204-716
ENSG0000(	324	7.336613	chr12:201	RNU7-4P		smallRNA	chr12:68911921-689
ENSG0000(	324	7.336613	chr1:1021	SLC35E2B		protein_c	chr1:1659529-16927
ENSG0000(	324	7.336613	chr12:201	RNU4-65P		smallRNA	chr12:70443784-704
ENSG0000(	324	7.336613	chr12:201	YEATS4	NCGv7;AC	protein_c	chr12:69359710-693
ENSG0000(	324	7.336613	chr12:201	ENSG00000256664		Pseudoger	chr12:68841946-688
ENSG0000(	324	7.336613	chr12:201	PTPRB	NCGv7	protein_c	chr12:70515870-706
ENSG0000(	324	7.336613	chr12:201	AC133749.1		smallRNA	chr12:68956406-689
ENSG0000(	324	7.336613	chr12:201	Y_RNA		smallRNA	chr12:70906917-709
ENSG0000(	324	7.336613	chr12:201	SNORA17		smallRNA	chr12:71639355-716
ENSG0000(	324	7.336613	chr12:201	ZFC3H1		protein_c	chr12:71609599-716
ENSG0000(	324	7.336613	chr12:201	ENSG00000257454		lncRNA	chr12:71034122-711
ENSG0000(	324	7.336613	chr12:201	ENSG00000256917		Pseudoger	chr12:68487687-684
ENSG0000(	324	7.336613	chr12:201	RAP1B		protein_c	chr12:68610855-686
ENSG0000(	324	7.336613	chr12:201	PRELID2P1		Pseudoger	chr12:68957377-689
ENSG0000(	324	7.336613	chr12:201	IL22	NCGv7	protein_c	chr12:68248242-682
ENSG0000(	324	7.336613	chr12:201	TSPAN8		protein_c	chr12:71125085-714
ENSG0000(	324	7.336613	chr12:201	BEST3		protein_c	chr12:69643360-696
ENSG0000(	324	7.336613	chr12:201	RAB3IP	DriverDB\	protein_c	chr12:69738860-698
ENSG0000(	324	7.336613	chr12:201	SLC35E3	DriverDB\	protein_c	chr12:68746125-687
ENSG0000(	324	7.336613	chr12:201	ENSG00000256678		Pseudoger	chr12:68805011-688
ENSG0000(	324	7.336613	chr12:201	TBC1D15	AC	protein_c	chr12:71839707-719
ENSG0000(	324	7.336613	chr12:201	LINC02384		lncRNA	chr12:68332888-684

ENSG0000(	324	7.336613	chr12:201	LINC02373		lncRNA	chr12:69449470-694
ENSG0000(	324	7.336613	chr12:201	SNORA70G		smallRNA	chr12:68627234-686
ENSG0000(	324	7.336613	chr12:201	KCNMB4		protein_c	chr12:70366290-704
ENSG0000(	324	7.336613	chr12:201	CPM	DriverDB\	protein_c	chr12:68842197-689
ENSG0000(	324	7.336613	chr12:201	MDM2	NCGv7;AC	protein_c	chr12:68808177-688
ENSG0000(	324	7.336613	chr12:201	MIR3913-2		smallRNA	chr12:69584722-695
ENSG0000(	324	7.336613	chr12:201	ENSG00000257410		lncRNA	chr12:71793855-717
ENSG0000(	324	7.336613	chr12:201	FAHD2P1		Pseudoger	chr12:70671918-706
ENSG0000(	324	7.336613	chr12:201	KRT8P39		Pseudoger	chr12:68705634-687
ENSG0000(	324	7.336613	chr12:201	RPS26P45		Pseudoger	chr12:69422656-694
ENSG0000(	324	7.336613	chr12:201	LINC02821		lncRNA	chr12:70180338-702
ENSG0000(	324	7.336613	chr12:201	C1GALT1P1		Pseudoger	chr12:69284397-692
ENSG0000(	324	7.336613	chr12:201	ENSG00000257515		lncRNA	chr12:71709171-717
ENSG0000(	324	7.336613	chr12:201	ENSG00000247131		lncRNA	chr12:69713633-697
ENSG0000(	324	7.336613	chr12:201	PRANCR		lncRNA	chr12:69901918-702
ENSG0000(	324	7.336613	chr12:201	ENSG00000257181		lncRNA	chr12:68841288-688
ENSG0000(	324	7.336613	chr12:201	MRS2P2		Pseudoger	chr12:71849228-718
ENSG0000(	324	7.336613	chr12:201	ENSG00000257241		lncRNA	chr12:69946543-699
ENSG0000(	324	7.336613	chr12:201	ATP5PDP4		Pseudoger	chr12:68642519-686
ENSG0000(	324	7.336613	chr12:201	LRRC10		protein_c	chr12:69608564-696
ENSG0000(	324	7.336613	chr12:201	AC139931.1		smallRNA	chr12:69117568-691
ENSG0000(	324	7.336613	chr12:201	ENSG00000257265		lncRNA	chr12:71007773-710
ENSG0000(	324	7.336613	chr12:201	ENSG00000287132		lncRNA	chr12:70239114-702
ENSG0000(	324	7.336613	chr12:201	THAP2		protein_c	chr12:71664301-716
ENSG0000(	324	7.336613	chr12:201	RPL7P42		Pseudoger	chr12:68684595-686
ENSG0000(	324	7.336613	chr12:201	ENSG00000273824		lncRNA	chr12:68426331-684
ENSG0000(	324	7.336613	chr12:201	CPSF6	DriverDB\	protein_c	chr12:69239569-692
ENSG0000(	324	7.336613	chr12:201	CNOT2	DriverDB\	protein_c	chr12:70243002-703
ENSG0000(	324	7.336613	chr12:201	NUP107	DriverDB\	protein_c	chr12:68686951-687
ENSG0000(	324	7.336613	chr12:201	MDM1	DriverDB\	protein_c	chr12:68272443-683
ENSG0000(	324	7.336613	chr12:201	NUP107-DT		lncRNA	chr12:68674371-686
ENSG0000(	324	7.336613	chr12:201	ENSG00000274979		lncRNA	chr12:69326574-693
ENSG0000(	324	7.336613	chr12:201	ENSG00000257764		lncRNA	chr12:69353493-693
ENSG0000(	324	7.336613	chr12:201	ENSG00000257289		Pseudoger	chr12:68913804-689
ENSG0000(	322	7.291325	chr6:1089	RPL32P16		Pseudoger	chr6:151099529-151
ENSG0000(	322	7.291325	chr16:231	FTLP14		Pseudoger	chr16:68822587-688
ENSG0000(	322	7.291325	chr2:3608	ENSG00000232409		Pseudoger	chr2:208516908-208
ENSG0000(	322	7.291325	chr16:231	ENSG00000261469		lncRNA	chr16:68236845-682
ENSG0000(	322	7.291325	chrX:1773	ENSG00000274923		Pseudoger	chrX:7896514-78967
ENSG0000(	321	7.268681	chr11:103	APOA4		protein_c	chr11:116820700-11
ENSG0000(	321	7.268681	chr11:103	ENSG00000280143		TEC	chr11:117204967-11
ENSG0000(	321	7.268681	chr11:103	PAFAH1B2	AC	protein_c	chr11:117144284-11
ENSG0000(	321	7.268681	chr11:103	SCARNA11		smallRNA	chr11:117263799-11
ENSG0000(	321	7.268681	chr7:1238	ENSG00000223436		lncRNA	chr7:132352334-132
ENSG0000(	321	7.268681	chr11:103	ENSG00000270403		lncRNA	chr11:117668483-11
ENSG0000(	321	7.268681	chr11:103	ENSG00000250699		lncRNA	chr11:117316362-11
ENSG0000(	321	7.268681	chr7:1238	ENSG00000229532		Pseudoger	chr7:132086266-132
ENSG0000(	321	7.268681	chr11:103	BACE1		protein_c	chr11:117285232-11
ENSG0000(	321	7.268681	chr7:1238	ENSG00000227197		lncRNA	chr7:132830693-132
ENSG0000(	321	7.268681	chr7:1238	ENSG00000225144		lncRNA	chr7:132264152-132
ENSG0000(	321	7.268681	chr11:103	APOA1		protein_c	chr11:116835751-11

ENSG0000(	321	7.268681	chr11:10:	ENSG00000285513		lncRNA	chr11:116820645-11
ENSG0000(	321	7.268681	chr11:10:	ENSG00000279586		TEC	chr11:117611101-11
ENSG0000(	321	7.268681	chr11:10:	PRR13P3		Pseudoger	chr11:117336256-11
ENSG0000(	321	7.268681	chr7:1238	ENSG00000283041		Pseudoger	chr7:133034607-133
ENSG0000(	321	7.268681	chr11:10:	SIDT2		protein_c	chr11:117178736-11
ENSG0000(	321	7.268681	chr11:10:	ENSG00000234268		Pseudoger	chr11:117035797-11
ENSG0000(	321	7.268681	chr11:10:	TAGLN		protein_c	chr11:117199370-11
ENSG0000(	321	7.268681	chr7:1238	ENSG00000236386		Pseudoger	chr7:131893822-131
ENSG0000(	321	7.268681	chr11:10:	ENSG00000254851		Pseudoger	chr11:117135528-11
ENSG0000(	321	7.268681	chr11:10:	ENSG00000225236		Pseudoger	chr11:117117794-11
ENSG0000(	321	7.268681	chr7:1238	ENSG00000236395		Pseudoger	chr7:131702269-131
ENSG0000(	321	7.268681	chr11:10:	ENSG00000276505		lncRNA	chr11:117297005-11
ENSG0000(	321	7.268681	chr11:10:	BACE1-AS		lncRNA	chr11:117288453-11
ENSG0000(	321	7.268681	chr7:1238	EEF1B2P6		Pseudoger	chr7:131661952-131
ENSG0000(	321	7.268681	chr11:10:	LINC02151		lncRNA	chr11:116496568-11
ENSG0000(	321	7.268681	chr11:10:	RNF214		protein_c	chr11:117232625-11
ENSG0000(	321	7.268681	chr7:1238	ST13P7		Pseudoger	chr7:133169416-133
ENSG0000(	321	7.268681	chr11:10:	snoU13		smallRNA	chr11:116320344-11
ENSG0000(	321	7.268681	chr11:10:	APOA1-AS		lncRNA	chr11:116836117-11
ENSG0000(	321	7.268681	chr7:1238	SNORD46		smallRNA	chr7:132753023-132
ENSG0000(	321	7.268681	chr7:1238	CAPZA1P4		Pseudoger	chr7:131892616-131
ENSG0000(	321	7.268681	chr11:10:	RNY4P6		smallRNA	chr11:117015897-11
ENSG0000(	321	7.268681	chr11:10:	APOA5		protein_c	chr11:116789367-11
ENSG0000(	321	7.268681	chr7:1238	snosnR60_Z15		smallRNA	chr7:131916235-131
ENSG0000(	321	7.268681	chr7:1238	NDUFB9P2		Pseudoger	chr7:131753746-131
ENSG0000(	321	7.268681	chr11:10:	SIK3	NCGv7	protein_c	chr11:116843402-11
ENSG0000(	321	7.268681	chr7:1238	U6		smallRNA	chr7:133082829-133
ENSG0000(	321	7.268681	chr11:10:	PCSK7	AC	protein_c	chr11:117204337-11
ENSG0000(	321	7.268681	chr11:10:	CEP164		protein_c	chr11:117314557-11
ENSG0000(	321	7.268681	chr11:10:	ENSG00000254678		Pseudoger	chr11:117143891-11
ENSG0000(	321	7.268681	chr11:10:	BUD13		protein_c	chr11:116748170-11
ENSG0000(	321	7.268681	chr7:1238	PLXNA4		protein_c	chr7:132123340-132
ENSG0000(	321	7.268681	chr7:1238	CHCHD3		protein_c	chr7:132784870-133
ENSG0000(	321	7.268681	chr7:1238	ENSG00000224865		lncRNA	chr7:131897289-131
ENSG0000(	321	7.268681	chr16:231	snoU13		smallRNA	chr16:68313239-683
ENSG0000(	321	7.268681	chr11:10:	SIK3-IT1		lncRNA	chr11:116886046-11
ENSG0000(	321	7.268681	chr11:10:	APOC3		protein_c	chr11:116829706-11
ENSG0000(	321	7.268681	chr7:1238	ENSG00000225881		lncRNA	chr7:132758970-132
ENSG0000(	321	7.268681	chr11:10:	ENSG00000278945		TEC	chr11:116609995-11
ENSG0000(	321	7.268681	chr11:10:	BUD13-DT		lncRNA	chr11:116773389-11
ENSG0000(	321	7.268681	chr7:1238	AC008085.1		smallRNA	chr7:131852680-131
ENSG0000(	321	7.268681	chr11:10:	ZPR1		protein_c	chr11:116773799-11
ENSG0000(	321	7.268681	chr11:10:	LINC02702		lncRNA	chr11:116639422-11
ENSG0000(	321	7.268681	chr7:1238	FLJ40288		lncRNA	chr7:132648794-132
ENSG0000(	321	7.268681	chr11:10:	ENSG00000236267		lncRNA	chr11:116813204-11
ENSG0000(	321	7.268681	chr7:1238	ENSG00000236238		Pseudoger	chr7:131665507-131
ENSG0000(	321	7.268681	chr11:10:	ENSG00000224077		lncRNA	chr11:117098987-11
ENSG0000(	321	7.268681	chr11:10:	AP000936.2		smallRNA	chr11:117015430-11
ENSG0000(	320	7.246037	chr12:62	RPL21P99		Pseudoger	chr12:29658376-296
ENSG0000(	320	7.246037	chr12:62	CCDC91		protein_c	chr12:28133249-285
ENSG0000(	320	7.246037	chr12:62	ENSG00000273989		lncRNA	chr12:28236227-282

ENSG0000(	320	7.246037	chr12:624	ENSG00000275278		lncRNA	chr12:29156448-291
ENSG0000(	320	7.246037	chr12:624	ENSG00000256747		lncRNA	chr12:27779821-277
ENSG0000(	320	7.246037	chr12:624	TMTC1	DriverDB\	protein_c	chr12:29500840-297
ENSG0000(	320	7.246037	chr12:624	REP15		protein_c	chr12:27696447-276
ENSG0000(	320	7.246037	chr12:624	ENSG00000273680		lncRNA	chr12:29332733-293
ENSG0000(	320	7.246037	chr12:624	ENSG00000244712		Pseudoger	chr12:28564678-285
ENSG0000(	320	7.246037	chr12:624	Y_RNA		smallRNA	chr12:27752874-277
ENSG0000(	320	7.246037	chr12:624	RN7SKP15		smallRNA	chr12:27806082-278
ENSG0000(	320	7.246037	chr12:624	ENSG00000256512		lncRNA	chr12:27798641-278
ENSG0000(	320	7.246037	chr12:624	ENSG00000257042		lncRNA	chr12:27958517-279
ENSG0000(	320	7.246037	chr12:624	ENSG00000256513		lncRNA	chr12:28821975-288
ENSG0000(	320	7.246037	chr12:624	FAR2	DriverDB\	protein_c	chr12:29149016-293
ENSG0000(	320	7.246037	chr12:624	ENSG00000286566		lncRNA	chr12:30086342-301
ENSG0000(	320	7.246037	chr12:624	ENSG00000257456		lncRNA	chr12:29519731-295
ENSG0000(	320	7.246037	chr12:624	ENSG00000256504		lncRNA	chr12:27704568-277
ENSG0000(	320	7.246037	chr12:624	RNU4-54P		smallRNA	chr12:28319335-283
ENSG0000(	320	7.246037	chr12:624	MANSC4		protein_c	chr12:27762427-277
ENSG0000(	320	7.246037	chr12:624	OVCH1	NCGv7	protein_c	chr12:29412474-294
ENSG0000(	320	7.246037	chr12:624	ENSG00000257258		lncRNA	chr12:29142969-291
ENSG0000(	320	7.246037	chr12:624	RNA5SP355		Pseudoger	chr12:28505394-285
ENSG0000(	320	7.246037	chr12:624	PTHLH		protein_c	chr12:27958084-279
ENSG0000(	320	7.246037	chr12:624	U3		smallRNA	chr12:29899903-299
ENSG0000(	320	7.246037	chr12:624	KLHL42		protein_c	chr12:27780048-278
ENSG0000(	320	7.246037	chr12:624	MRPS35	DriverDB\	protein_c	chr12:27710822-277
ENSG0000(	320	7.246037	chr12:624	ERGIC2	DriverDB\	protein_c	chr12:29337352-293
ENSG0000(	320	7.246037	chr12:624	ENSG00000257176		lncRNA	chr12:29277955-293
ENSG0000(	320	7.246037	chr12:624	ENSG00000278733		lncRNA	chr12:28185625-281
ENSG0000(	320	7.246037	chr12:624	ENSG00000275476		lncRNA	chr12:29277397-292
ENSG0000(	320	7.246037	chr12:624	HMGB1P49		Pseudoger	chr12:27702338-277
ENSG0000(	320	7.246037	chr12:624	AC012150.1		smallRNA	chr12:29120783-291
ENSG0000(	320	7.246037	chr12:624	ENSG00000274315		lncRNA	chr12:29331434-293
ENSG0000(	320	7.246037	chr12:624	ENSG00000276261		lncRNA	chr12:27700066-277
ENSG0000(	320	7.246037	chr12:624	ENSG00000255951		Pseudoger	chr12:28164958-281
ENSG0000(	320	7.246037	chr12:624	ENSG00000256557		Pseudoger	chr12:27608733-276
ENSG0000(	320	7.246037	chr12:624	ENSG00000278687		Pseudoger	chr12:28978584-289
ENSG0000(	320	7.246037	chr12:624	OVCH1-AS1		lncRNA	chr12:29389289-294
ENSG0000(	320	7.246037	chr12:624	MRPS35-DT		lncRNA	chr12:27696388-277
ENSG0000(	320	7.246037	chr12:624	ENSG00000247934		lncRNA	chr12:28163298-281
ENSG0000(	320	7.246037	chr12:624	ENSG00000256378		Pseudoger	chr12:27935523-279
ENSG0000(	319	7.223394	chr6:1979	ENSG00000288054		lncRNA	chr6:135882780-135
ENSG0000(	319	7.223394	chr6:1979	LINC01010		lncRNA	chr6:134343307-134
ENSG0000(	319	7.223394	chr6:1979	AL356137.1		smallRNA	chr6:140660623-140
ENSG0000(	319	7.223394	chr6:1979	ENSG00000289312		lncRNA	chr6:136290014-136
ENSG0000(	319	7.223394	chr6:1979	RN7SL408P		smallRNA	chr6:134133573-134
ENSG0000(	319	7.223394	chr6:1979	HECA		protein_c	chr6:139135080-139
ENSG0000(	319	7.223394	chr6:1979	OLIG3		protein_c	chr6:137492199-137
ENSG0000(	319	7.223394	chr6:1979	MYB-AS1		lncRNA	chr6:135195083-135
ENSG0000(	319	7.223394	chr6:1979	ENSG00000287393		lncRNA	chr6:137900585-137
ENSG0000(	319	7.223394	chr6:1979	AL360178.1		smallRNA	chr6:135914755-135
ENSG0000(	319	7.223394	chr6:1979	PERP		protein_c	chr6:138088505-138
ENSG0000(	319	7.223394	chr6:1979	LINC01625		lncRNA	chr6:139435636-139

ENSG0000(	319	7.223394	chr6:1979	ENSG00000283265		lncRNA	chr6:137693068-137
ENSG0000(	319	7.223394	chr6:1979	PDE7B	NCGv7	protein_c	chr6:135851701-136
ENSG0000(	319	7.223394	chr6:1979	LINC02865		lncRNA	chr6:137945366-137
ENSG0000(	319	7.223394	chr6:1979	CITED2	NCGv7	protein_c	chr6:139371807-139
ENSG0000(	319	7.223394	chr6:1979	ENSG00000224374		lncRNA	chr6:135259996-135
ENSG0000(	319	7.223394	chr6:1979	TXLNB		protein_c	chr6:139240061-139
ENSG0000(	319	7.223394	chr6:1979	ENSG00000275138		Pseudoger	chr6:141019788-141
ENSG0000(	319	7.223394	chr6:1979	HBS1L		protein_c	chr6:134960378-135
ENSG0000(	319	7.223394	chr6:1979	ENSG00000277973		Pseudoger	chr6:136206478-136
ENSG0000(	319	7.223394	chr6:1979	CCDC28A-AS1		lncRNA	chr6:138725211-138
ENSG0000(	319	7.223394	chr6:1979	PEX7		protein_c	chr6:136822564-136
ENSG0000(	319	7.223394	chr6:1979	MIR548A2		smallRNA	chr6:135239160-135
ENSG0000(	319	7.223394	chr6:1979	ECT2L	NCGv7;AC	protein_c	chr6:138795911-138
ENSG0000(	319	7.223394	chr6:1979	ATP5PBP6		Pseudoger	chr6:139614438-139
ENSG0000(	319	7.223394	chr6:1979	ENSG00000286887		lncRNA	chr6:134296301-134
ENSG0000(	319	7.223394	chr6:1979	NHSL1-AS1		lncRNA	chr6:138692548-138
ENSG0000(	319	7.223394	chr6:1979	SNORA27		smallRNA	chr6:136855698-136
ENSG0000(	319	7.223394	chr6:1979	LINC02539		lncRNA	chr6:137730170-137
ENSG0000(	319	7.223394	chr6:1979	ENSG00000287974		lncRNA	chr6:134345688-134
ENSG0000(	319	7.223394	chr17:289	ENSG00000279570		TEC	chr17:78691266-786
ENSG0000(	319	7.223394	chr6:1979	SLC2A12		protein_c	chr6:133987581-134
ENSG0000(	319	7.223394	chr6:1979	MAP3K5	NCGv7	protein_c	chr6:136557046-136
ENSG0000(	319	7.223394	chr6:1979	ENSG00000232876		lncRNA	chr6:135055033-135
ENSG0000(	319	7.223394	chr6:1979	ENSG00000236389		lncRNA	chr6:134706060-134
ENSG0000(	319	7.223394	chr6:1979	ENSG00000220600		Pseudoger	chr6:138878899-138
ENSG0000(	319	7.223394	chr6:1979	ENSG00000220660		Pseudoger	chr6:136364129-136
ENSG0000(	319	7.223394	chr6:1979	SMIM28		protein_c	chr6:138377905-138
ENSG0000(	319	7.223394	chr6:1979	AL512290.1		smallRNA	chr6:135977811-135
ENSG0000(	319	7.223394	chr6:1979	ENSG00000234084		lncRNA	chr6:135301568-135
ENSG0000(	319	7.223394	chr6:1979	MTFR2		protein_c	chr6:136231024-136
ENSG0000(	319	7.223394	chr6:1979	CCDC28A	NCGv7;AC	protein_c	chr6:138773769-138
ENSG0000(	319	7.223394	chr6:1979	MAP3K5-AS2		lncRNA	chr6:136784045-136
ENSG0000(	319	7.223394	chr6:1979	HMGB1P17		Pseudoger	chr6:135636086-135
ENSG0000(	319	7.223394	chr6:1979	RN7SKP106		smallRNA	chr6:141486141-141
ENSG0000(	319	7.223394	chr6:1979	ENSG00000259828		lncRNA	chr6:141447011-141
ENSG0000(	319	7.223394	chr6:1979	RNA5SP219		Pseudoger	chr6:136630243-136
ENSG0000(	319	7.223394	chr6:1979	RNA5SP220		Pseudoger	chr6:140158591-140
ENSG0000(	319	7.223394	chr6:1979	ENSG00000260418		lncRNA	chr6:136335714-136
ENSG0000(	319	7.223394	chr6:1979	RPS3AP24		Pseudoger	chr6:140761529-140
ENSG0000(	319	7.223394	chr6:1979	ENSG00000287094		lncRNA	chr6:135323399-135
ENSG0000(	319	7.223394	chr6:1979	PBOV1		protein_c	chr6:138215986-138
ENSG0000(	319	7.223394	chr6:1979	ENSG00000287413		lncRNA	chr6:134074123-134
ENSG0000(	319	7.223394	chr6:1979	LINC02941		lncRNA	chr6:139976352-140
ENSG0000(	319	7.223394	chr6:1979	ENSG00000288529		protein_c	chr6:133953304-133
ENSG0000(	319	7.223394	chr6:1979	MAP7-AS1		lncRNA	chr6:136550661-136
ENSG0000(	319	7.223394	chr6:1979	MIR3668		smallRNA	chr6:140205252-140
ENSG0000(	319	7.223394	chr6:1979	KRT8P42		Pseudoger	chr6:134296997-134
ENSG0000(	319	7.223394	chr6:1979	AHI1-DT		lncRNA	chr6:135497422-135
ENSG0000(	319	7.223394	chr6:1979	RPL35AP3		Pseudoger	chr6:136973930-136
ENSG0000(	319	7.223394	chr6:1979	ENSG00000225148		lncRNA	chr6:139856104-139
ENSG0000(	319	7.223394	chr6:1979	ENSG00000220412		Pseudoger	chr6:137705423-137

ENSG0000(	319	7.223394	chr6:197(	ENSG00000205695		Pseudoger	chr6:139659928-139
ENSG0000(	319	7.223394	chr6:197(	ENSG00000236378		lncRNA	chr6:135807148-135
ENSG0000(	319	7.223394	chr6:197(	ENSG00000229722		lncRNA	chr6:134606299-134
ENSG0000(	319	7.223394	chr6:197(	IL22RA2		protein_c	chr6:137143820-137
ENSG0000(	319	7.223394	chr6:197(	MIR4465		smallRNA	chr6:140683814-140
ENSG0000(	319	7.223394	chr6:197(	MARCKSL1P2		Pseudoger	chr6:138402585-138
ENSG0000(	319	7.223394	chr6:197(	FAM8A6P		Pseudoger	chr6:134603564-134
ENSG0000(	319	7.223394	chr6:197(	ENSG00000240056		lncRNA	chr6:134941392-134
ENSG0000(	319	7.223394	chr6:197(	ENSG00000237596		lncRNA	chr6:135991936-136
ENSG0000(	319	7.223394	chr6:197(	TNFAIP3	NCGv7;AC	protein_c	chr6:137867214-137
ENSG0000(	319	7.223394	chr6:197(	HMGA1P7		Pseudoger	chr6:134115235-134
ENSG0000(	319	7.223394	chr6:197(	LINC02524		lncRNA	chr6:135628787-135
ENSG0000(	319	7.223394	chr6:197(	WAKMAR2		lncRNA	chr6:137823673-137
ENSG0000(	319	7.223394	chr6:197(	MTCH1P1		Pseudoger	chr6:138650226-138
ENSG0000(	319	7.223394	chr6:197(	ENSG00000278744		Pseudoger	chr6:134009033-134
ENSG0000(	319	7.223394	chr6:197(	IL20RA		protein_c	chr6:136999971-137
ENSG0000(	319	7.223394	chr6:197(	HEBP2		protein_c	chr6:138403531-138
ENSG0000(	319	7.223394	chr6:197(	TARID		lncRNA	chr6:133502252-133
ENSG0000(	319	7.223394	chr6:197(	Y_RNA		smallRNA	chr6:136934765-136
ENSG0000(	319	7.223394	chr6:197(	FILNC1		lncRNA	chr6:139677639-139
ENSG0000(	319	7.223394	chr6:197(	snoU13		smallRNA	chr6:134206411-134
ENSG0000(	319	7.223394	chr6:197(	ENSG00000227723		lncRNA	chr6:134636489-134
ENSG0000(	319	7.223394	chr6:197(	CHCHD2P4		Pseudoger	chr6:134393142-134
ENSG0000(	319	7.223394	chr6:197(	MEMO1P2		Pseudoger	chr6:134897874-134
ENSG0000(	319	7.223394	chr6:197(	COX5BP2		Pseudoger	chr6:136034553-136
ENSG0000(	319	7.223394	chr6:197(	RPL7AP37		Pseudoger	chr6:136900233-136
ENSG0000(	319	7.223394	chr6:197(	ENSG00000288714		lncRNA	chr6:140148490-140
ENSG0000(	319	7.223394	chr6:197(	BTF3L4P3		Pseudoger	chr6:137543897-137
ENSG0000(	319	7.223394	chr6:197(	GAPDHP73		Pseudoger	chr6:135619165-135
ENSG0000(	319	7.223394	chr6:197(	ENSG00000218565		Pseudoger	chr6:139338018-139
ENSG0000(	319	7.223394	chr6:197(	CT69		lncRNA	chr6:134428239-134
ENSG0000(	319	7.223394	chr6:197(	RNU6-427P		smallRNA	chr6:138859027-138
ENSG0000(	319	7.223394	chr6:197(	MYB	NCGv7;AC	protein_c	chr6:135181308-135
ENSG0000(	319	7.223394	chr6:197(	LINC01312		lncRNA	chr6:133821147-133
ENSG0000(	319	7.223394	chr6:197(	ENSG00000272446		lncRNA	chr6:139159157-139
ENSG0000(	319	7.223394	chr6:197(	MAP3K5-AS1		lncRNA	chr6:136629066-136
ENSG0000(	319	7.223394	chr6:197(	ENSG00000286313		lncRNA	chr6:135854053-135
ENSG0000(	319	7.223394	chr6:197(	TCF21	NCGv7	protein_c	chr6:133889113-133
ENSG0000(	319	7.223394	chr6:197(	ENSG00000216519		Pseudoger	chr6:136317961-136
ENSG0000(	319	7.223394	chr6:197(	ENSG00000290029		lncRNA	chr6:134373662-134
ENSG0000(	319	7.223394	chr6:197(	ENSG00000216548		Pseudoger	chr6:140922457-140
ENSG0000(	319	7.223394	chr6:197(	RPS3AP23		Pseudoger	chr6:141635650-141
ENSG0000(	319	7.223394	chr6:197(	ENSG00000216613		Pseudoger	chr6:136419847-136
ENSG0000(	319	7.223394	chr6:197(	REPS1	DriverDB\	protein_c	chr6:138903493-138
ENSG0000(	319	7.223394	chr6:197(	NDUFS5P1		Pseudoger	chr6:136475862-136
ENSG0000(	319	7.223394	chr6:197(	AL357060.1		smallRNA	chr6:137716948-137
ENSG0000(	319	7.223394	chr6:197(	SGK1	NCGv7;AC	protein_c	chr6:134169248-134
ENSG0000(	319	7.223394	chr6:197(	AHI1	NCGv7;AC	protein_c	chr6:135283407-135
ENSG0000(	319	7.223394	chr6:197(	NHSL1		protein_c	chr6:138422043-138
ENSG0000(	319	7.223394	chr6:197(	ENSG00000287820		lncRNA	chr6:139938864-139
ENSG0000(	319	7.223394	chr6:197(	ALDH8A1		protein_c	chr6:134917393-134

ENSG0000(	319	7.223394	chr6:1979	MAP7	NCGv7	protein_c	chr6:136342281-136
ENSG0000(	319	7.223394	chr6:1979	ENSG00000218499		Pseudoger	chr6:138393373-138
ENSG0000(	319	7.223394	chr6:1979	Y_RNA		smallRNA	chr6:134283092-134
ENSG0000(	319	7.223394	chr6:1979	ENSG00000235399		lncRNA	chr6:136995170-136
ENSG0000(	319	7.223394	chr6:1979	ARFGEF3		protein_c	chr6:138161939-138
ENSG0000(	319	7.223394	chr6:1979	Y_RNA		smallRNA	chr6:137784374-137
ENSG0000(	319	7.223394	chr6:1979	ENSG00000274594		Pseudoger	chr6:138464099-138
ENSG0000(	319	7.223394	chr6:1979	ENSG00000231329		lncRNA	chr6:139144204-139
ENSG0000(	319	7.223394	chr6:1979	ACKR4P1		Pseudoger	chr6:138822747-138
ENSG0000(	319	7.223394	chr6:1979	TBPL1	DriverDB\	protein_c	chr6:133952170-133
ENSG0000(	319	7.223394	chr6:1979	SNORD112		smallRNA	chr6:137540400-137
ENSG0000(	319	7.223394	chr6:1979	Y_RNA		smallRNA	chr6:135474504-135
ENSG0000(	319	7.223394	chr6:1979	ENSG00000226571		lncRNA	chr6:139271362-139
ENSG0000(	319	7.223394	chr6:1979	NHEG1		lncRNA	chr6:136982165-136
ENSG0000(	319	7.223394	chr6:1979	IFNGR1	IntOGen-I	protein_c	chr6:137197483-137
ENSG0000(	319	7.223394	chr6:1979	BCLAF1	NCGv7	protein_c	chr6:136256627-136
ENSG0000(	319	7.223394	chr6:1979	RNA5SP218		Pseudoger	chr6:134257035-134
ENSG0000(	319	7.223394	chr6:1979	ENSG00000234147		lncRNA	chr6:140575812-140
ENSG0000(	319	7.223394	chr6:1979	7SK		smallRNA	chr6:136545192-136
ENSG0000(	319	7.223394	chr6:1979	RPS29P32		Pseudoger	chr6:134211450-134
ENSG0000(	319	7.223394	chr6:1979	LINC03004		lncRNA	chr6:137657998-137
ENSG0000(	319	7.223394	chr6:1979	MIR3145		smallRNA	chr6:138435213-138
ENSG0000(	319	7.223394	chr6:1979	SLC35D3		protein_c	chr6:136922301-136
ENSG0000(	319	7.223394	chr6:1979	ABRACL		protein_c	chr6:139028745-139
ENSG0000(	319	7.223394	chr6:1979	LINC02528		lncRNA	chr6:137943079-137
ENSG0000(	319	7.223394	chr6:1979	RPSAP42		Pseudoger	chr6:137995270-137
ENSG0000(	319	7.223394	chr6:1979	ENSG00000286452		lncRNA	chr6:141403240-141
ENSG0000(	319	7.223394	chr6:1979	FTH1P26		Pseudoger	chr6:133676729-133
ENSG0000(	319	7.223394	chr6:1979	LINC03002		lncRNA	chr6:134520163-134
ENSG0000(	317	7.178106	chr17:289	RN7SL606P		smallRNA	chr17:60696313-606
ENSG0000(	317	7.178106	chr16:239	MIR548H2		smallRNA	chr16:11306440-113
ENSG0000(	315	7.132818	chr6:1979	LAMA4	NCGv7	protein_c	chr6:112107931-112
ENSG0000(	315	7.132818	chr6:1979	KRT18P65		Pseudoger	chr6:112361437-112
ENSG0000(	315	7.132818	chr6:1979	FCF1P10		Pseudoger	chr6:113010937-113
ENSG0000(	315	7.132818	chr6:1979	ENSG00000270661		lncRNA	chr6:112217640-112
ENSG0000(	315	7.132818	chr6:1979	CCN6	NCGv7	protein_c	chr6:112054075-112
ENSG0000(	315	7.132818	chr6:1979	ENSG00000287253		lncRNA	chr6:117262243-117
ENSG0000(	315	7.132818	chr6:1979	KRT18P22		Pseudoger	chr6:116457323-116
ENSG0000(	315	7.132818	chr6:1979	CDK19		protein_c	chr6:110609978-110
ENSG0000(	315	7.132818	chr6:1979	LINC02541		lncRNA	chr6:113616927-113
ENSG0000(	315	7.132818	chr6:1979	RNU6-1163P		smallRNA	chr6:112971493-112
ENSG0000(	315	7.132818	chr6:1979	SLC16A10		protein_c	chr6:111087503-111
ENSG0000(	315	7.132818	chr6:1979	RNU6-1115P		smallRNA	chr6:110856417-110
ENSG0000(	315	7.132818	chr6:1979	ENSG00000289372		lncRNA	chr6:117658838-117
ENSG0000(	315	7.132818	chr6:1979	ENSG00000281613		protein_c	chr6:112236806-112
ENSG0000(	315	7.132818	chr6:1979	PTCHD3P3		Pseudoger	chr6:109288571-109
ENSG0000(	315	7.132818	chr6:1979	AL357514.1		smallRNA	chr6:112532182-112
ENSG0000(	315	7.132818	chr6:1979	RPS27AP11		Pseudoger	chr6:113581501-113
ENSG0000(	315	7.132818	chr6:1979	ENSG00000289925		lncRNA	chr6:113635183-113
ENSG0000(	315	7.132818	chr6:1979	ENSG00000287268		lncRNA	chr6:110341973-110
ENSG0000(	315	7.132818	chr6:1979	ENSG00000219758		Pseudoger	chr6:113839279-113

ENSG0000(	315	7.132818	chr6:1979	ENSG00000289376		lncRNA	chr6:115901795-116
ENSG0000(	315	7.132818	chr6:1979	RPSAP45		Pseudoger	chr6:112355841-112
ENSG0000(	315	7.132818	chr6:1979	NIP7P3		Pseudoger	chr6:116137058-116
ENSG0000(	315	7.132818	chr6:1979	CDC40		protein_c	chr6:110180141-110
ENSG0000(	315	7.132818	chr6:1979	RNU6-960P		smallRNA	chr6:111091213-111
ENSG0000(	315	7.132818	chr6:1979	PA2G4P5		Pseudoger	chr6:112616703-112
ENSG0000(	315	7.132818	chr6:1979	ENSG00000219619		Pseudoger	chr6:115358498-115
ENSG0000(	315	7.132818	chr6:1979	ZBTB24-DT		lncRNA	chr6:109483638-109
ENSG0000(	315	7.132818	chr6:1979	LINC02527		lncRNA	chr6:111900305-111
ENSG0000(	315	7.132818	chr6:1979	RAP1BP3		Pseudoger	chr6:117431591-117
ENSG0000(	315	7.132818	chr6:1979	AL357515.1		smallRNA	chr6:110969773-110
ENSG0000(	315	7.132818	chr6:1979	HDAC2-AS2		lncRNA	chr6:113969701-114
ENSG0000(	315	7.132818	chr6:1979	FCF1P5		Pseudoger	chr6:111353702-111
ENSG0000(	315	7.132818	chr6:1979	HS3ST5		protein_c	chr6:114055596-114
ENSG0000(	315	7.132818	chr6:1979	NEPNP		Pseudoger	chr6:117633706-117
ENSG0000(	315	7.132818	chr6:1979	Y_RNA		smallRNA	chr6:109305494-109
ENSG0000(	315	7.132818	chr6:1979	ENSG00000289847		lncRNA	chr6:110530684-110
ENSG0000(	315	7.132818	chr6:1979	ENSG00000219150		Pseudoger	chr6:110706362-110
ENSG0000(	315	7.132818	chr6:1979	ENSG00000220506		Pseudoger	chr6:111494991-111
ENSG0000(	315	7.132818	chr6:1979	NUS1		protein_c	chr6:117675469-117
ENSG0000(	315	7.132818	chr6:1979	ENSG00000271498		Pseudoger	chr6:112825939-112
ENSG0000(	315	7.132818	chr6:1979	GTF3C6		protein_c	chr6:110958706-110
ENSG0000(	315	7.132818	chr6:1979	ENSG00000282218		protein_c	chr6:117318211-117
ENSG0000(	315	7.132818	chr6:1979	FEM1AP3		Pseudoger	chr6:112365704-112
ENSG0000(	315	7.132818	chr6:1979	ENSG00000219329		Pseudoger	chr6:110923566-110
ENSG0000(	315	7.132818	chr6:1979	LINC02534		lncRNA	chr6:115633540-115
ENSG0000(	315	7.132818	chr6:1979	TSPYL1	NCGv7	protein_c	chr6:116267760-116
ENSG0000(	315	7.132818	chr6:1979	ENSG00000233908		Pseudoger	chr6:109288440-109
ENSG0000(	315	7.132818	chr6:1979	ENSG00000287097		lncRNA	chr6:114822994-115
ENSG0000(	315	7.132818	chr6:1979	ZUP1		protein_c	chr6:116635618-116
ENSG0000(	315	7.132818	chr6:1979	SNORA40		smallRNA	chr6:110848546-110
ENSG0000(	315	7.132818	chr6:1979	RNA5SP213		Pseudoger	chr6:114220681-114
ENSG0000(	315	7.132818	chr6:1979	ENSG00000219559		Pseudoger	chr6:110562175-110
ENSG0000(	315	7.132818	chr6:1979	BRD7P4		Pseudoger	chr6:111430260-111
ENSG0000(	315	7.132818	chr6:1979	DCBLD1		protein_c	chr6:117453817-117
ENSG0000(	315	7.132818	chr6:1979	AK9		protein_c	chr6:109492855-109
ENSG0000(	315	7.132818	chr6:1979	ENSG00000271208		lncRNA	chr6:112234165-112
ENSG0000(	315	7.132818	chr6:1979	GSTM2P1		Pseudoger	chr6:111046868-111
ENSG0000(	315	7.132818	chr6:1979	ENSG00000213150		Pseudoger	chr6:110645699-110
ENSG0000(	315	7.132818	chr6:1979	CNN2P9		Pseudoger	chr6:110858239-110
ENSG0000(	315	7.132818	chr6:1979	CEP57L1		protein_c	chr6:109095110-109
ENSG0000(	315	7.132818	chr6:1979	ENSG00000286914		lncRNA	chr6:112392363-112
ENSG0000(	315	7.132818	chr6:1979	RPF2		protein_c	chr6:110982015-111
ENSG0000(	315	7.132818	chr6:1979	DNAJA1P4		Pseudoger	chr6:114349483-114
ENSG0000(	315	7.132818	chr6:1979	FAM162B		protein_c	chr6:116752197-116
ENSG0000(	315	7.132818	chr6:1979	SLC22A16		protein_c	chr6:110424687-110
ENSG0000(	315	7.132818	chr6:1979	CALHM4		protein_c	chr6:116529013-116
ENSG0000(	315	7.132818	chr6:1979	ENSG00000226079		lncRNA	chr6:114523443-114
ENSG0000(	315	7.132818	chr6:1979	ENSG00000223811		lncRNA	chr6:113357003-113
ENSG0000(	315	7.132818	chr6:1979	RPS29P13		Pseudoger	chr6:117048670-117
ENSG0000(	315	7.132818	chr6:1979	CALHM6-AS1		lncRNA	chr6:116460739-116

ENSG0000(	315	7.132818	chr6:1979	ENSG00000286339		lncRNA	chr6:118565660-118
ENSG0000(	315	7.132818	chr6:1979	CALHM6	NCGv7	protein_c	chr6:116461370-116
ENSG0000(	315	7.132818	chr6:1979	ENSG00000237234		lncRNA	chr6:112154765-112
ENSG0000(	315	7.132818	chr6:1979	ENSG00000217041		Pseudoger	chr6:110700562-110
ENSG0000(	315	7.132818	chr6:1979	AL357519.1		smallRNA	chr6:113704833-113
ENSG0000(	315	7.132818	chr6:1979	ENSG00000287728		lncRNA	chr6:113129707-113
ENSG0000(	315	7.132818	chr6:1979	PPIL6		protein_c	chr6:109390215-109
ENSG0000(	315	7.132818	chr6:1979	AL136446.1		smallRNA	chr6:114277385-114
ENSG0000(	315	7.132818	chr6:1979	SOCS5P5		Pseudoger	chr6:113222166-113
ENSG0000(	315	7.132818	chr6:1979	METTL24		protein_c	chr6:110243940-110
ENSG0000(	315	7.132818	chr6:1979	GPR6		protein_c	chr6:109978256-109
ENSG0000(	315	7.132818	chr6:1979	RNA5SP214		Pseudoger	chr6:117060682-117
ENSG0000(	315	7.132818	chr6:1979	RNU6-1226P		smallRNA	chr6:112196440-112
ENSG0000(	315	7.132818	chr6:1979	snoU13		smallRNA	chr6:110963466-110
ENSG0000(	315	7.132818	chr6:1979	AL513123.1		smallRNA	chr6:113514987-113
ENSG0000(	315	7.132818	chr6:1979	AL132671.1		smallRNA	chr6:117445390-117
ENSG0000(	315	7.132818	chr6:1979	ENSG00000271789		lncRNA	chr6:111297126-111
ENSG0000(	315	7.132818	chr6:1979	LNCPOIR		lncRNA	chr6:114477350-114
ENSG0000(	315	7.132818	chr6:1979	ENSG00000271730		lncRNA	chr6:108998482-108
ENSG0000(	315	7.132818	chr6:1979	ENSG00000286616		lncRNA	chr6:111505307-111
ENSG0000(	315	7.132818	chr6:1979	U3		smallRNA	chr6:113781315-113
ENSG0000(	315	7.132818	chr6:1979	FAM229B		protein_c	chr6:112087591-112
ENSG0000(	315	7.132818	chr6:1979	RNU6-653P		smallRNA	chr6:109059509-109
ENSG0000(	315	7.132818	chr6:1979	RNU6-253P		smallRNA	chr6:117457734-117
ENSG0000(	315	7.132818	chr6:1979	DDO		protein_c	chr6:110391771-110
ENSG0000(	315	7.132818	chr6:1979	CCDC162P		Pseudoger	chr6:109165831-109
ENSG0000(	315	7.132818	chr6:1979	LINC02518		lncRNA	chr6:113428540-113
ENSG0000(	315	7.132818	chr6:1979	ENSG00000288916		lncRNA	chr6:113586188-113
ENSG0000(	315	7.132818	chr6:1979	RNU6-906P		smallRNA	chr6:111008270-111
ENSG0000(	315	7.132818	chr6:1979	SLC35F1	NCGv7	protein_c	chr6:117907264-118
ENSG0000(	315	7.132818	chr6:1979	ENSG00000232299		lncRNA	chr6:112476538-112
ENSG0000(	315	7.132818	chr6:1979	RN7SKP51		smallRNA	chr6:117301455-117
ENSG0000(	315	7.132818	chr6:1979	AL365214.1		smallRNA	chr6:112361848-112
ENSG0000(	315	7.132818	chr6:1979	ENSG00000216809		Pseudoger	chr6:118452469-118
ENSG0000(	315	7.132818	chr6:1979	CBX3P9		Pseudoger	chr6:116453014-116
ENSG0000(	315	7.132818	chr6:1979	PLN		protein_c	chr6:118548296-118
ENSG0000(	315	7.132818	chr6:1979	ENSG00000287933		lncRNA	chr6:116033901-116
ENSG0000(	315	7.132818	chr6:1979	RPSAP43		Pseudoger	chr6:114084168-114
ENSG0000(	315	7.132818	chr6:1979	ENSG00000230202		Pseudoger	chr6:117998975-117
ENSG0000(	315	7.132818	chr6:1979	RFX6	NCGv7	protein_c	chr6:116877212-116
ENSG0000(	315	7.132818	chr6:1979	ENSG00000272356		lncRNA	chr6:111309203-111
ENSG0000(	315	7.132818	chr6:1979	HDAC2	NCGv7	protein_c	chr6:113933028-114
ENSG0000(	315	7.132818	chr6:1979	TUBE1		protein_c	chr6:112070663-112
ENSG0000(	315	7.132818	chr6:1979	FYN	NCGv7;AC	protein_c	chr6:111660332-111
ENSG0000(	315	7.132818	chr6:1979	snoU13		smallRNA	chr6:113840814-113
ENSG0000(	315	7.132818	chr6:1979	NT5DC1		protein_c	chr6:116100851-116
ENSG0000(	315	7.132818	chr6:1979	TSPYL4	NCGv7	protein_c	chr6:116249964-116
ENSG0000(	315	7.132818	chr6:1979	RN7SL617P		smallRNA	chr6:110439999-110
ENSG0000(	315	7.132818	chr6:1979	ENSG00000270934		Pseudoger	chr6:110598093-110
ENSG0000(	315	7.132818	chr6:1979	AL109947.2		smallRNA	chr6:109504387-109
ENSG0000(	315	7.132818	chr6:1979	RNU6-957P		smallRNA	chr6:110722250-110

ENSG0000(	315	7.132818	chr6:1979	CALHM5		protein_c	chr6:116511639-116
ENSG0000(	315	7.132818	chr6:1979	ENSG00000288560		lncRNA	chr6:112988311-113
ENSG0000(	315	7.132818	chr6:1979	VGLL2		protein_c	chr6:117265558-117
ENSG0000(	315	7.132818	chr6:1979	ENSG00000255389		lncRNA	chr6:111599875-111
ENSG0000(	315	7.132818	chr6:1979	ENSG00000233558		Pseudoger	chr6:116258493-116
ENSG0000(	315	7.132818	chr6:1979	LINC02880		lncRNA	chr6:113904132-113
ENSG0000(	315	7.132818	chr6:1979	NUDT19P3		Pseudoger	chr6:114019621-114
ENSG0000(	315	7.132818	chr6:1979	ENSG00000286691		lncRNA	chr6:110020011-110
ENSG0000(	315	7.132818	chr6:1979	ENSG00000216663		Pseudoger	chr6:112325753-112
ENSG0000(	315	7.132818	chr6:1979	MROCKI		lncRNA	chr6:113868013-113
ENSG0000(	315	7.132818	chr6:1979	snoU13		smallRNA	chr6:109291255-109
ENSG0000(	315	7.132818	chr6:1979	ENSG00000226181		lncRNA	chr6:117451130-117
ENSG0000(	315	7.132818	chr6:1979	CD164		protein_c	chr6:109366514-109
ENSG0000(	315	7.132818	chr6:1979	SMPD2		protein_c	chr6:109440724-109
ENSG0000(	315	7.132818	chr6:1979	MICAL1		protein_c	chr6:109444062-109
ENSG0000(	315	7.132818	chr6:1979	ENSG00000231912		lncRNA	chr6:113791829-113
ENSG0000(	315	7.132818	chr6:1979	LAMA4-AS1		lncRNA	chr6:112236093-112
ENSG0000(	315	7.132818	chr6:1979	TPI1P3		Pseudoger	chr6:116038756-116
ENSG0000(	315	7.132818	chr6:1979	RFPL4B		protein_c	chr6:112347330-112
ENSG0000(	315	7.132818	chr6:1979	TRAF3IP2-AS1		lncRNA	chr6:111483459-111
ENSG0000(	315	7.132818	chr6:1979	MARCKS		protein_c	chr6:113857345-113
ENSG0000(	315	7.132818	chr6:1979	ENSG00000285446		protein_c	chr6:116399395-116
ENSG0000(	315	7.132818	chr6:1979	ENSG00000289198		lncRNA	chr6:113855537-113
ENSG0000(	315	7.132818	chr6:1979	MFSD4B		protein_c	chr6:111259327-111
ENSG0000(	315	7.132818	chr6:1979	ROS1	NCGv7;AC	protein_c	chr6:117287353-117
ENSG0000(	315	7.132818	chr6:1979	ENSG00000260273		lncRNA	chr6:109382795-109
ENSG0000(	315	7.132818	chr6:1979	ENSG00000236347		lncRNA	chr6:113531118-113
ENSG0000(	315	7.132818	chr6:1979	MFSD4B-DT		lncRNA	chr6:111227747-111
ENSG0000(	315	7.132818	chr6:1979	BRD7P3		Pseudoger	chr6:118501430-118
ENSG0000(	315	7.132818	chr6:1979	snoU13		smallRNA	chr6:111646327-111
ENSG0000(	315	7.132818	chr6:1979	Z84488.1		smallRNA	chr6:116457732-116
ENSG0000(	315	7.132818	chr6:1979	ENSG00000289256		lncRNA	chr6:111873588-111
ENSG0000(	315	7.132818	chr6:1979	REV3L	NCGv7	protein_c	chr6:111299028-111
ENSG0000(	315	7.132818	chr6:1979	KPNA5		protein_c	chr6:116681187-116
ENSG0000(	315	7.132818	chr6:1979	ENSG00000234117		lncRNA	chr6:116492297-116
ENSG0000(	315	7.132818	chr6:1979	AL357519.2		smallRNA	chr6:113602915-113
ENSG0000(	315	7.132818	chr6:1979	SSXP10		Pseudoger	chr6:118589070-118
ENSG0000(	315	7.132818	chr6:1979	RN7SKP18		smallRNA	chr6:117299364-117
ENSG0000(	315	7.132818	chr6:1979	FRK		protein_c	chr6:115931149-116
ENSG0000(	315	7.132818	chr6:1979	DSE	NCGv7	protein_c	chr6:116254173-116
ENSG0000(	315	7.132818	chr6:1979	ENSG00000271607		Pseudoger	chr6:110863860-110
ENSG0000(	315	7.132818	chr6:1979	RWDD1		protein_c	chr6:116571409-116
ENSG0000(	315	7.132818	chr6:1979	COL10A1		protein_c	chr6:116118909-116
ENSG0000(	315	7.132818	chr6:1979	AMD1		protein_c	chr6:110874770-110
ENSG0000(	315	7.132818	chr6:1979	RSPH4A		protein_c	chr6:116616479-116
ENSG0000(	315	7.132818	chr6:1979	CEP85L		protein_c	chr6:118460772-118
ENSG0000(	315	7.132818	chr6:1979	ENSG00000289961		lncRNA	chr6:113345749-113
ENSG0000(	315	7.132818	chr6:1979	snoU13		smallRNA	chr6:112476726-112
ENSG0000(	315	7.132818	chr6:1979	ENSG00000236326		lncRNA	chr6:116244187-116
ENSG0000(	315	7.132818	chr6:1979	GOPC	NCGv7;AC	protein_c	chr6:117560269-117
ENSG0000(	315	7.132818	chr6:1979	TRAPPC3L		protein_c	chr6:116494989-116

ENSG0000(	315	7.132818	chr6:1979	ENSG00000289304		lncRNA	chr6:116569604-116
ENSG0000(	315	7.132818	chr6:1979	RNU6-475P		smallRNA	chr6:114866873-114
ENSG0000(	315	7.132818	chr6:1979	GPRC6A		protein_c	chr6:116792085-116
ENSG0000(	315	7.132818	chr6:1979	WASF1		protein_c	chr6:110099819-110
ENSG0000(	315	7.132818	chr6:1979	TRAF3IP2		protein_c	chr6:111555381-111
ENSG0000(	315	7.132818	chr6:1979	SESN1		protein_c	chr6:108984309-109
ENSG0000(	315	7.132818	chr6:1979	ENSG00000260188		lncRNA	chr6:110477907-110
ENSG0000(	315	7.132818	chr6:1979	ZBTB24		protein_c	chr6:109460632-109
ENSG0000(	315	7.132818	chr6:1979	REV3L-IT1		lncRNA	chr6:111360641-111
ENSG0000(	315	7.132818	chr6:1979	AL109947.1		smallRNA	chr6:109434174-109
ENSG0000(	315	7.132818	chr6:1979	FIG4		protein_c	chr6:109690609-109
ENSG0000(	315	7.132818	chr6:1979	ARMC2-AS1		lncRNA	chr6:108922976-108
ENSG0000(	314	7.110174	chr12:201	OSBPL9P5		Pseudoger	chr12:66563524-665
ENSG0000(	314	7.110174	chr12:201	ENSG00000257779		Pseudoger	chr12:63002469-630
ENSG0000(	314	7.110174	chr12:201	ENSG00000255629		lncRNA	chr12:64146388-641
ENSG0000(	314	7.110174	chr12:201	PABPC1P4		Pseudoger	chr12:63822021-638
ENSG0000(	314	7.110174	chr5:5659	FAM193B		protein_c	chr5:177519789-177
ENSG0000(	314	7.110174	chr12:201	APOOP3		Pseudoger	chr12:65134688-651
ENSG0000(	314	7.110174	chr12:201	RASSF3-DT		lncRNA	chr12:64599078-646
ENSG0000(	314	7.110174	chr12:201	ENSG00000257146		Pseudoger	chr12:59719698-597
ENSG0000(	314	7.110174	chr12:201	LINC02389		lncRNA	chr12:64883394-649
ENSG0000(	314	7.110174	chr12:201	ENSG00000257921		protein_c	chr12:57772660-577
ENSG0000(	314	7.110174	chr12:201	OSBPL9P4		Pseudoger	chr12:66563967-665
ENSG0000(	314	7.110174	chr12:201	LINC02388		lncRNA	chr12:58565959-587
ENSG0000(	314	7.110174	chr12:201	ENSG00000279444		TEC	chr12:63151065-631
ENSG0000(	314	7.110174	chr12:201	ENSG00000257953		lncRNA	chr12:57837092-578
ENSG0000(	314	7.110174	chr12:201	ENSG00000258133		Pseudoger	chr12:58394596-583
ENSG0000(	314	7.110174	chr12:201	ENSG00000277945		lncRNA	chr12:65830750-658
ENSG0000(	314	7.110174	chr12:201	ENSG00000256314		lncRNA	chr12:64654060-646
ENSG0000(	314	7.110174	chr12:201	ATP6V1E1P3		Pseudoger	chr12:64338178-643
ENSG0000(	314	7.110174	chr5:5659	GMCL2		protein_c	chr5:178184505-178
ENSG0000(	314	7.110174	chr5:5659	ENSG00000280207		lncRNA	chr5:177967004-177
ENSG0000(	314	7.110174	chr12:201	SRGAP1		protein_c	chr12:63843761-641
ENSG0000(	314	7.110174	chr5:5659	DOK3		protein_c	chr5:177501904-177
ENSG0000(	314	7.110174	chr12:201	ENSG00000279310		TEC	chr12:67065018-670
ENSG0000(	314	7.110174	chr12:201	LINC02421		lncRNA	chr12:67709047-677
ENSG0000(	314	7.110174	chr12:201	ENSG00000279134		TEC	chr12:58094914-580
ENSG0000(	314	7.110174	chr12:201	RAB11AP2		Pseudoger	chr12:67131567-671
ENSG0000(	314	7.110174	chr12:201	ENSG00000256399		Pseudoger	chr12:63887379-638
ENSG0000(	314	7.110174	chr12:201	LINC02448		lncRNA	chr12:59523278-595
ENSG0000(	314	7.110174	chr12:201	ENSG00000257235		lncRNA	chr12:63004338-630
ENSG0000(	314	7.110174	chr5:5659	FGFR4	NCGv7;AC	protein_c	chr5:177086905-177
ENSG0000(	314	7.110174	chr12:201	ENSG00000256571		lncRNA	chr12:63878787-638
ENSG0000(	314	7.110174	chr5:5659	ENSG00000248293		Pseudoger	chr5:174926541-174
ENSG0000(	314	7.110174	chr12:201	ENSG00000279411		TEC	chr12:58103907-581
ENSG0000(	314	7.110174	chr5:5659	RNF44	DriverDB\	protein_c	chr5:176526712-176
ENSG0000(	314	7.110174	chr12:201	ENSG00000255583		Pseudoger	chr12:63684748-637
ENSG0000(	314	7.110174	chr12:201	HMGA2-AS1		lncRNA	chr12:65851340-658
ENSG0000(	314	7.110174	chr5:5659	PFN3		protein_c	chr5:177400109-177
ENSG0000(	314	7.110174	chr12:201	NTAN1P3		Pseudoger	chr12:67424272-674
ENSG0000(	314	7.110174	chr5:5659	SFXN1		protein_c	chr5:175477062-175

ENSG0000(	314	7.110174	chr12:201	ENSG00000257159		Pseudoger	chr12:57967058-579
ENSG0000(	314	7.110174	chr12:201	ENSG00000280320		TEC	chr12:64696191-646
ENSG0000(	314	7.110174	chr12:201	ENSG00000255566		Pseudoger	chr12:64442510-644
ENSG0000(	314	7.110174	chr12:201	GIHCG		lncRNA	chr12:57930115-579
ENSG0000(	314	7.110174	chr12:201	ENSG00000269903		lncRNA	chr12:57814494-578
ENSG0000(	314	7.110174	chr12:201	ENSG00000258117		lncRNA	chr12:63292625-633
ENSG0000(	314	7.110174	chr5:5659	CPLX2		protein_c	chr5:175796310-175
ENSG0000(	314	7.110174	chr5:5659	RN7SL684P		smallRNA	chr5:176547435-176
ENSG0000(	314	7.110174	chr5:5659	ENSG00000246596		Pseudoger	chr5:177626436-177
ENSG0000(	314	7.110174	chr5:5659	HK3	NCGv7	protein_c	chr5:176880869-176
ENSG0000(	314	7.110174	chr12:201	LRIG3-DT		lncRNA	chr12:58920639-590
ENSG0000(	314	7.110174	chr5:5659	ARL2BPP6		Pseudoger	chr5:175290731-175
ENSG0000(	314	7.110174	chr12:201	ENSG00000257865		Pseudoger	chr12:60418983-604
ENSG0000(	314	7.110174	chr12:201	ENSG00000256083		lncRNA	chr12:65934777-659
ENSG0000(	314	7.110174	chr12:201	ENSG00000255817		lncRNA	chr12:64108763-641
ENSG0000(	314	7.110174	chr12:201	RASSF3		protein_c	chr12:64507001-646
ENSG0000(	314	7.110174	chr12:201	ENSG00000277895		lncRNA	chr12:64451591-644
ENSG0000(	314	7.110174	chr5:5659	N4BP3		protein_c	chr5:178113532-178
ENSG0000(	314	7.110174	chr12:201	ENSG00000257308		Pseudoger	chr12:61231159-612
ENSG0000(	314	7.110174	chr12:201	ENSG00000279505		TEC	chr12:67753701-677
ENSG0000(	314	7.110174	chr12:201	ENSG00000257005		Pseudoger	chr12:63651627-636
ENSG0000(	314	7.110174	chr12:201	ENSG00000255886		lncRNA	chr12:64038562-640
ENSG0000(	314	7.110174	chr5:5659	NHP2		protein_c	chr5:178149463-178
ENSG0000(	314	7.110174	chr12:201	LINC02442		lncRNA	chr12:67571197-675
ENSG0000(	314	7.110174	chr12:201	RPL39P28		Pseudoger	chr12:68001394-680
ENSG0000(	314	7.110174	chr12:201	LINC02403		lncRNA	chr12:58087892-580
ENSG0000(	314	7.110174	chr12:201	ENSG00000257288		Pseudoger	chr12:59052980-590
ENSG0000(	314	7.110174	chr5:5659	RMND5B		protein_c	chr5:178130996-178
ENSG0000(	314	7.110174	chr12:201	HMGA2	NCGv7;AC	protein_c	chr12:65824460-659
ENSG0000(	314	7.110174	chr12:201	ENSG00000255866		lncRNA	chr12:65556925-655
ENSG0000(	314	7.110174	chr12:201	ENSG00000280181		TEC	chr12:64709458-647
ENSG0000(	314	7.110174	chr12:201	RBMS1P1		Pseudoger	chr12:66234079-662
ENSG0000(	314	7.110174	chr12:201	ENSG00000257668		Pseudoger	chr12:57983795-579
ENSG0000(	314	7.110174	chr5:5659	ENSG00000280068		TEC	chr5:175903874-175
ENSG0000(	314	7.110174	chr12:201	RXYLT1-AS1		lncRNA	chr12:63804739-638
ENSG0000(	314	7.110174	chr5:5659	ENSG00000247679		lncRNA	chr5:177611240-177
ENSG0000(	314	7.110174	chr12:201	ENSG00000257083		lncRNA	chr12:66950754-670
ENSG0000(	314	7.110174	chr12:201	ENSG00000257294		lncRNA	chr12:60092864-600
ENSG0000(	314	7.110174	chr12:201	ENSG00000228144		protein_c	chr12:66123917-661
ENSG0000(	314	7.110174	chr12:201	LINC02420		lncRNA	chr12:67440998-674
ENSG0000(	314	7.110174	chr12:201	ENSG00000256708		Pseudoger	chr12:68143318-681
ENSG0000(	314	7.110174	chr5:5659	PRR7-AS1		lncRNA	chr5:177438503-177
ENSG0000(	314	7.110174	chr5:5659	ENSG00000291283		lncRNA	chr5:177619059-177
ENSG0000(	314	7.110174	chr12:201	ENSG00000270039		lncRNA	chr12:57803838-578
ENSG0000(	314	7.110174	chr12:201	ENSG00000245651		lncRNA	chr12:57869834-578
ENSG0000(	314	7.110174	chr12:201	ENSG00000225195		lncRNA	chr12:64628344-646
ENSG0000(	314	7.110174	chr5:5659	HIGD2A		protein_c	chr5:176388751-176
ENSG0000(	314	7.110174	chr12:201	LINC02454		lncRNA	chr12:65589111-656
ENSG0000(	314	7.110174	chr12:201	LINC01479		lncRNA	chr12:67929235-679
ENSG0000(	314	7.110174	chr12:201	LINC02425		lncRNA	chr12:66027725-660
ENSG0000(	314	7.110174	chr12:201	ENSG00000256072		lncRNA	chr12:66251745-662

ENSG0000(	314	7.110174	chr5:565(	ENSG00000291113		lncRNA	chr5:177809407-177
ENSG0000(	314	7.110174	chr5:565(	ENSG00000280139		TEC	chr5:176439090-176
ENSG0000(	314	7.110174	chr12:201	MIRLET7IHG		lncRNA	chr12:62602752-626
ENSG0000(	314	7.110174	chr12:201	WIF1	NCGv7;AC	protein_c	chr12:65050626-651
ENSG0000(	314	7.110174	chr12:201	ENSG00000257880		lncRNA	chr12:62139999-621
ENSG0000(	314	7.110174	chr12:201	ENSG00000257568		lncRNA	chr12:62482349-624
ENSG0000(	314	7.110174	chr12:201	PDCL3P7		Pseudoger	chr12:66275940-662
ENSG0000(	314	7.110174	chr12:201	KRT8P19		Pseudoger	chr12:61879318-618
ENSG0000(	314	7.110174	chr12:201	ENSG00000256835		Pseudoger	chr12:67978107-679
ENSG0000(	314	7.110174	chr5:565(	ENSG00000279821		TEC	chr5:177476631-177
ENSG0000(	314	7.110174	chr12:201	ENSG00000256199		lncRNA	chr12:64507166-645
ENSG0000(	314	7.110174	chr12:201	ENSG00000255780		Pseudoger	chr12:64032412-640
ENSG0000(	314	7.110174	chr12:201	RPS6P22		Pseudoger	chr12:59020169-590
ENSG0000(	314	7.110174	chr12:201	HNRNPA1P69		Pseudoger	chr12:63271404-632
ENSG0000(	314	7.110174	chr12:201	OOEPP2		Pseudoger	chr12:64266413-642
ENSG0000(	314	7.110174	chr12:201	ENSG00000256915		lncRNA	chr12:65622273-656
ENSG0000(	314	7.110174	chr12:201	GRIP1		protein_c	chr12:66347431-670
ENSG0000(	314	7.110174	chr12:201	TMBIM4		protein_c	chr12:66135846-661
ENSG0000(	314	7.110174	chr12:201	RSL24D1P5		Pseudoger	chr12:63055275-630
ENSG0000(	314	7.110174	chr12:201	MRPL40P1		Pseudoger	chr12:67351436-673
ENSG0000(	314	7.110174	chr5:565(	PDLIM7		protein_c	chr5:177483394-177
ENSG0000(	314	7.110174	chr5:565(	LMAN2		protein_c	chr5:177315805-177
ENSG0000(	314	7.110174	chr12:201	IFNG-AS1		lncRNA	chr12:67989445-682
ENSG0000(	314	7.110174	chr12:201	GAPDHP44		Pseudoger	chr12:62755227-627
ENSG0000(	314	7.110174	chr12:201	RPL32P26		Pseudoger	chr12:62658823-626
ENSG0000(	314	7.110174	chr12:201	AC090673.1		smallRNA	chr12:65858013-658
ENSG0000(	314	7.110174	chr12:201	RNA5SP362		Pseudoger	chr12:66066221-660
ENSG0000(	314	7.110174	chr5:565(	PRMT1P1		Pseudoger	chr5:177265580-177
ENSG0000(	314	7.110174	chr5:565(	SUMO2P6		Pseudoger	chr5:174561776-174
ENSG0000(	314	7.110174	chr5:565(	ENSG00000290935		lncRNA	chr5:176143078-176
ENSG0000(	314	7.110174	chr12:201	RNU6-871P		smallRNA	chr12:59450673-594
ENSG0000(	314	7.110174	chr5:565(	FAF2		protein_c	chr5:176447628-176
ENSG0000(	314	7.110174	chr12:201	AC108721.2		smallRNA	chr12:59528059-595
ENSG0000(	314	7.110174	chr5:565(	HRH2		protein_c	chr5:175658030-175
ENSG0000(	314	7.110174	chr5:565(	DBN1		protein_c	chr5:177456608-177
ENSG0000(	314	7.110174	chr5:565(	ZNF346		protein_c	chr5:177022696-177
ENSG0000(	314	7.110174	chr5:565(	UNC5A		protein_c	chr5:176810519-176
ENSG0000(	314	7.110174	chr5:565(	PDLIM7-AS1		lncRNA	chr5:177494995-177
ENSG0000(	314	7.110174	chr12:201	ENSG00000235872		lncRNA	chr12:67658991-676
ENSG0000(	314	7.110174	chr5:565(	ENSG00000248469		lncRNA	chr5:176049678-176
ENSG0000(	314	7.110174	chr12:201	LINC02231		lncRNA	chr12:64920845-649
ENSG0000(	314	7.110174	chr12:201	RPL36AP41		Pseudoger	chr12:63871739-638
ENSG0000(	314	7.110174	chr12:201	RNU6-1009P		smallRNA	chr12:64204793-642
ENSG0000(	314	7.110174	chr12:201	RNU6-279P		smallRNA	chr12:59369051-593
ENSG0000(	314	7.110174	chr5:565(	ENSG00000248943		lncRNA	chr5:177801204-177
ENSG0000(	314	7.110174	chr12:201	ENSG00000287014		lncRNA	chr12:60150187-602
ENSG0000(	314	7.110174	chr5:565(	HIGD1AP3		Pseudoger	chr5:174632932-174
ENSG0000(	314	7.110174	chr5:565(	ENSG00000248484		lncRNA	chr5:176707356-176
ENSG0000(	314	7.110174	chr12:201	RN7SKP65		smallRNA	chr12:57977965-579
ENSG0000(	314	7.110174	chr12:201	RPL21P18		Pseudoger	chr12:66037235-660
ENSG0000(	314	7.110174	chr12:201	PCNPP3		Pseudoger	chr12:65645992-656

ENSG0000(	314	7.110174	chr12:201	SNORA19		smallRNA	chr12:60363822-603
ENSG0000(	314	7.110174	chr12:201	RNU6ATAC42P		smallRNA	chr12:64939023-649
ENSG0000(	314	7.110174	chr12:201	RNU5A-7P		smallRNA	chr12:64228560-642
ENSG0000(	314	7.110174	chr12:201	MIR548C		smallRNA	chr12:64622509-646
ENSG0000(	314	7.110174	chr12:201	AVPR1A		protein_c	chr12:63142759-631
ENSG0000(	314	7.110174	chr12:201	AC078889.1		smallRNA	chr12:66235556-662
ENSG0000(	314	7.110174	chr5:5659	LINC01411		lncRNA	chr5:174336295-174
ENSG0000(	314	7.110174	chr5:5659	Y_RNA		smallRNA	chr5:176556704-176
ENSG0000(	314	7.110174	chr12:201	RN7SKP166		smallRNA	chr12:66292683-662
ENSG0000(	314	7.110174	chr12:201	KICS2		protein_c	chr12:64186316-642
ENSG0000(	314	7.110174	chr12:201	HNRNPA1P70		Pseudoger	chr12:68035767-680
ENSG0000(	314	7.110174	chr12:201	ENSG00000287618		lncRNA	chr12:64099415-641
ENSG0000(	314	7.110174	chr12:201	LEMD3	NCGv7	protein_c	chr12:65169583-652
ENSG0000(	314	7.110174	chr12:201	MSRB3		protein_c	chr12:65278643-654
ENSG0000(	314	7.110174	chr5:5659	ENSG00000249186		lncRNA	chr5:177856262-177
ENSG0000(	314	7.110174	chr5:5659	B4GALT7		protein_c	chr5:177600132-177
ENSG0000(	314	7.110174	chr5:5659	ENSG00000287579		lncRNA	chr5:176158610-176
ENSG0000(	314	7.110174	chr5:5659	SUDS3P1		Pseudoger	chr5:177971316-177
ENSG0000(	314	7.110174	chr12:201	CYP27B1	DriverDBv	protein_c	chr12:57762334-577
ENSG0000(	314	7.110174	chr12:201	RPSAP52		Pseudoger	chr12:65758020-658
ENSG0000(	314	7.110174	chr5:5659	ENSG00000290968		lncRNA	chr5:177632075-177
ENSG0000(	314	7.110174	chr5:5659	ZNF346-IT1		lncRNA	chr5:177051714-177
ENSG0000(	314	7.110174	chr5:5659	BRCC3P1		Pseudoger	chr5:176308063-176
ENSG0000(	314	7.110174	chr12:201	PPM1H		protein_c	chr12:62643994-629
ENSG0000(	314	7.110174	chr5:5659	ENSG00000251670		lncRNA	chr5:174751304-174
ENSG0000(	314	7.110174	chr12:201	TBC1D30		protein_c	chr12:64759484-648
ENSG0000(	314	7.110174	chr12:201	MIR26A2		smallRNA	chr12:57824609-578
ENSG0000(	314	7.110174	chr12:201	CAND1		protein_c	chr12:67269358-673
ENSG0000(	314	7.110174	chr12:201	IL26		protein_c	chr12:68201349-682
ENSG0000(	314	7.110174	chr12:201	IFNG	AC	protein_c	chr12:68154768-681
ENSG0000(	314	7.110174	chr5:5659	MXD3		protein_c	chr5:177301461-177
ENSG0000(	314	7.110174	chr12:201	RPS3P6		Pseudoger	chr12:62021570-620
ENSG0000(	314	7.110174	chr12:201	RPL21P103		Pseudoger	chr12:58244378-582
ENSG0000(	314	7.110174	chr5:5659	GAPDHP71		Pseudoger	chr5:174513305-174
ENSG0000(	314	7.110174	chr12:201	SNORD112		smallRNA	chr12:62117887-621
ENSG0000(	314	7.110174	chr5:5659	GPRIN1	NCGv7	protein_c	chr5:176595802-176
ENSG0000(	314	7.110174	chr5:5659	PRELID1		protein_c	chr5:177303799-177
ENSG0000(	314	7.110174	chr5:5659	RAB24		protein_c	chr5:177301198-177
ENSG0000(	314	7.110174	chr5:5659	RGS14	DriverDBv	protein_c	chr5:177357924-177
ENSG0000(	314	7.110174	chr12:201	ENSG00000286402		lncRNA	chr12:61668302-617
ENSG0000(	314	7.110174	chr12:201	ENSG00000286351		lncRNA	chr12:59102144-591
ENSG0000(	314	7.110174	chr5:5659	ENSG00000286350		lncRNA	chr5:175471414-175
ENSG0000(	314	7.110174	chr12:201	ENSG00000286279		lncRNA	chr12:59322745-594
ENSG0000(	314	7.110174	chr5:5659	THOC3		protein_c	chr5:175917873-176
ENSG0000(	314	7.110174	chr12:201	HELB		protein_c	chr12:66302493-663
ENSG0000(	314	7.110174	chr12:201	DYRK2		protein_c	chr12:67648338-676
ENSG0000(	314	7.110174	chr5:5659	ENSG00000218227		Pseudoger	chr5:178055604-178
ENSG0000(	314	7.110174	chr12:201	RPL7P39		Pseudoger	chr12:64867956-648
ENSG0000(	314	7.110174	chr5:5659	SLC34A1		protein_c	chr5:177379235-177
ENSG0000(	314	7.110174	chr5:5659	F12		protein_c	chr5:177402133-177
ENSG0000(	314	7.110174	chr5:5659	PRR7		protein_c	chr5:177446445-177

ENSG0000(	314	7.110174	chr5:565(	NOP16		protein_c	chr5:176383946-176
ENSG0000(	314	7.110174	chr5:565(	TSPAN17		protein_c	chr5:176647387-176
ENSG0000(	314	7.110174	chr12:201	ENSG00000275180		lncRNA	chr12:62603366-626
ENSG0000(	314	7.110174	chr5:565(	AC106795.1		smallRNA	chr5:177908465-177
ENSG0000(	314	7.110174	chr5:565(	ENSG00000248596		lncRNA	chr5:176143085-176
ENSG0000(	314	7.110174	chr5:565(	AC010297.1		smallRNA	chr5:176451762-176
ENSG0000(	314	7.110174	chr12:201	RPL21P104		Pseudoger	chr12:61775923-617
ENSG0000(	314	7.110174	chr12:201	TAFA2		protein_c	chr12:61708273-622
ENSG0000(	314	7.110174	chr12:201	ENSG00000198671		lncRNA	chr12:65017468-650
ENSG0000(	314	7.110174	chr12:201	ENSG00000290896		lncRNA	chr12:63682523-637
ENSG0000(	314	7.110174	chr12:201	ENSG00000286563		lncRNA	chr12:67329014-673
ENSG0000(	314	7.110174	chr12:201	KRT18P60		Pseudoger	chr12:65418731-654
ENSG0000(	314	7.110174	chr12:201	MIRLET7I		smallRNA	chr12:62603686-626
ENSG0000(	314	7.110174	chr12:201	LINC01465		lncRNA	chr12:62601751-626
ENSG0000(	314	7.110174	chr12:201	SLC16A7		protein_c	chr12:59596029-597
ENSG0000(	314	7.110174	chr12:201	RXYLT1		protein_c	chr12:63779833-638
ENSG0000(	314	7.110174	chr5:565(	RN7SKP148		smallRNA	chr5:175628188-175
ENSG0000(	314	7.110174	chr12:201	MON2		protein_c	chr12:62466817-626
ENSG0000(	314	7.110174	chr12:201	METTL1	DriverDB\	protein_c	chr12:57768471-577
ENSG0000(	314	7.110174	chr12:201	ENSG00000273805		lncRNA	chr12:57894232-578
ENSG0000(	314	7.110174	chr5:565(	MIR4634		smallRNA	chr5:174751734-174
ENSG0000(	314	7.110174	chr5:565(	MSX2	NCGv7	protein_c	chr5:174724582-174
ENSG0000(	314	7.110174	chr12:201	Y_RNA		smallRNA	chr12:60341824-603
ENSG0000(	314	7.110174	chr5:565(	LINC01574		lncRNA	chr5:176743205-176
ENSG0000(	314	7.110174	chr12:201	Y_RNA		smallRNA	chr12:62935701-629
ENSG0000(	314	7.110174	chr5:565(	OR1X5P		Pseudoger	chr5:177836434-177
ENSG0000(	314	7.110174	chr12:201	ATP23	DriverDB\	protein_c	chr12:57906039-579
ENSG0000(	314	7.110174	chr12:201	EEF1AKMT3	DriverDB\	protein_c	chr12:57771492-577
ENSG0000(	314	7.110174	chr12:201	ENSG00000286682		lncRNA	chr12:64820179-648
ENSG0000(	314	7.110174	chr5:565(	KIAA1191		protein_c	chr5:176346062-176
ENSG0000(	314	7.110174	chr12:201	GNS		protein_c	chr12:64713445-647
ENSG0000(	314	7.110174	chr12:201	USP15		protein_c	chr12:62260338-624
ENSG0000(	314	7.110174	chr5:565(	NSG2		protein_c	chr5:174045706-174
ENSG0000(	314	7.110174	chr5:565(	ENSG00000170089		Pseudoger	chr5:177875595-177
ENSG0000(	314	7.110174	chr12:201	ENSG00000243024		Pseudoger	chr12:64222333-643
ENSG0000(	314	7.110174	chr5:565(	SIMC1		protein_c	chr5:176238367-176
ENSG0000(	314	7.110174	chr5:565(	ENSG00000286634		lncRNA	chr5:177260340-177
ENSG0000(	314	7.110174	chr5:565(	FAM153A	DriverDB\	protein_c	chr5:177707981-177
ENSG0000(	314	7.110174	chr12:201	TSFM	DriverDB\	protein_c	chr12:57782761-578
ENSG0000(	314	7.110174	chr12:201	AVIL	DriverDB\	protein_c	chr12:57797376-578
ENSG0000(	314	7.110174	chr12:201	ENSG00000237176		Pseudoger	chr12:59812102-598
ENSG0000(	314	7.110174	chr5:565(	ENSG00000249109		lncRNA	chr5:177782197-177
ENSG0000(	314	7.110174	chr12:201	ENSG00000288665		lncRNA	chr12:64497968-645
ENSG0000(	314	7.110174	chr5:565(	GRK6		protein_c	chr5:177403204-177
ENSG0000(	314	7.110174	chr12:201	AC108721.1		smallRNA	chr12:59528177-595
ENSG0000(	314	7.110174	chr12:201	ENSG00000258231		lncRNA	chr12:58544124-588
ENSG0000(	314	7.110174	chr12:201	LINC02408		lncRNA	chr12:67443105-675
ENSG0000(	314	7.110174	chr5:565(	ENSG00000249684		lncRNA	chr5:177950335-177
ENSG0000(	314	7.110174	chr5:565(	ENSG00000270989		Pseudoger	chr5:176203314-176
ENSG0000(	314	7.110174	chr12:201	XPOT		protein_c	chr12:64404392-644
ENSG0000(	314	7.110174	chr12:201	MSRB3-AS1		lncRNA	chr12:65466820-656

ENSG0000	314	7.110174	chr5:5659	RNU6-226P		smallRNA	chr5:175703898-175
ENSG0000	314	7.110174	chr5:5659	THOC3-AS1		lncRNA	chr5:175972606-175
ENSG0000	314	7.110174	chr5:5659	ENSG00000250820		lncRNA	chr5:175906939-175
ENSG0000	314	7.110174	chr12:201	snoU13		smallRNA	chr12:64760868-647
ENSG0000	314	7.110174	chr12:201	SNORD83		smallRNA	chr12:64575665-645
ENSG0000	314	7.110174	chr5:5659	MIR4281		smallRNA	chr5:176629439-176
ENSG0000	314	7.110174	chr5:5659	ENSG00000254011		lncRNA	chr5:174173101-174
ENSG0000	314	7.110174	chr12:201	ENSG00000288591		protein_c	chr12:64759496-649
ENSG0000	314	7.110174	chr12:201	snoU13		smallRNA	chr12:66345719-663
ENSG0000	314	7.110174	chr5:5659	ENSG00000250909		lncRNA	chr5:176347941-176
ENSG0000	314	7.110174	chr5:5659	NSD1	NCGv7;AC	protein_c	chr5:177131830-177
ENSG0000	314	7.110174	chr12:201	snoU13		smallRNA	chr12:62651175-626
ENSG0000	314	7.110174	chr12:201	snoU13		smallRNA	chr12:64355762-643
ENSG0000	314	7.110174	chr5:5659	FAM153CP		lncRNA	chr5:178006348-178
ENSG0000	314	7.110174	chr5:5659	ENSG00000250992		lncRNA	chr5:176036593-176
ENSG0000	314	7.110174	chr5:5659	LINC01951		lncRNA	chr5:174919082-174
ENSG0000	314	7.110174	chr12:201	KLF17P1		Pseudoger	chr12:62234390-622
ENSG0000	314	7.110174	chr5:5659	TMED9		protein_c	chr5:177592203-177
ENSG0000	314	7.110174	chr5:5659	snoU13		smallRNA	chr5:175450816-175
ENSG0000	314	7.110174	chr12:201	RNU4-20P		smallRNA	chr12:59586793-595
ENSG0000	314	7.110174	chr12:201	LRIG3	NCGv7;AC	protein_c	chr12:58872149-589
ENSG0000	314	7.110174	chr12:201	ENSG00000258214		lncRNA	chr12:59366218-594
ENSG0000	314	7.110174	chr12:201	TBK1	NCGv7	protein_c	chr12:64452090-645
ENSG0000	314	7.110174	chr5:5659	AC138965.1		smallRNA	chr5:175935209-175
ENSG0000	314	7.110174	chr5:5659	RN7SL562P		smallRNA	chr5:177340674-177
ENSG0000	314	7.110174	chr5:5659	FAM153B		lncRNA	chr5:176058717-176
ENSG0000	314	7.110174	chr5:5659	ENSG00000249849		lncRNA	chr5:177682294-177
ENSG0000	314	7.110174	chr5:5659	ENSG00000289726		lncRNA	chr5:178154048-178
ENSG0000	314	7.110174	chr5:5659	UIMC1		protein_c	chr5:176905005-177
ENSG0000	314	7.110174	chr5:5659	ENSG00000250101		lncRNA	chr5:177939622-177
ENSG0000	314	7.110174	chr5:5659	DDX41	NCGv7	protein_c	chr5:177511577-177
ENSG0000	314	7.110174	chr12:201	ENSG00000240027		Pseudoger	chr12:63760358-637
ENSG0000	314	7.110174	chr12:201	IRAK3		protein_c	chr12:66189195-662
ENSG0000	314	7.110174	chr12:201	AC084357.1		smallRNA	chr12:63830754-638
ENSG0000	314	7.110174	chr5:5659	ENSG00000250256		lncRNA	chr5:174618164-174
ENSG0000	314	7.110174	chr12:201	ENSG00000250280		lncRNA	chr12:65281657-652
ENSG0000	314	7.110174	chr5:5659	ENSG00000239393		Pseudoger	chr5:177346073-177
ENSG0000	314	7.110174	chr12:201	RNU6-399P		smallRNA	chr12:62545582-625
ENSG0000	314	7.110174	chr12:201	ENSG00000276853		lncRNA	chr12:65171262-651
ENSG0000	314	7.110174	chr12:201	LLPH-DT		lncRNA	chr12:66130751-661
ENSG0000	314	7.110174	chr12:201	ENSG00000249753		lncRNA	chr12:63623788-637
ENSG0000	314	7.110174	chr12:201	ENSG00000258184		Pseudoger	chr12:60215994-602
ENSG0000	314	7.110174	chr12:201	ENSG00000289319		lncRNA	chr12:65168736-651
ENSG0000	314	7.110174	chr5:5659	ENSG00000240729		Pseudoger	chr5:177264766-177
ENSG0000	314	7.110174	chr12:201	RNU6-595P		smallRNA	chr12:62334091-623
ENSG0000	314	7.110174	chr5:5659	ENSG00000249722		lncRNA	chr5:174329612-174
ENSG0000	314	7.110174	chr12:201	LDHAL6CP		Pseudoger	chr12:63003553-630
ENSG0000	314	7.110174	chr5:5659	DRD1		protein_c	chr5:175440036-175
ENSG0000	314	7.110174	chr5:5659	MIR1271		smallRNA	chr5:176367946-176
ENSG0000	314	7.110174	chr12:201	RNU6-1083P		smallRNA	chr12:57806154-578
ENSG0000	314	7.110174	chr5:5659	ENSG00000251446		lncRNA	chr5:176726942-176

ENSG0000(	314	7.110174	chr12:201	DPY19L2		protein_c	chr12:63558913-636
ENSG0000(	314	7.110174	chr5:5659	ENSG00000251144		lncRNA	chr5:174820027-174
ENSG0000(	314	7.110174	chr5:5659	PROP1		protein_c	chr5:177992235-177
ENSG0000(	314	7.110174	chr5:5659	ENSG00000251211		Pseudoger	chr5:178165702-178
ENSG0000(	314	7.110174	chr5:5659	ARL10		protein_c	chr5:176365487-176
ENSG0000(	314	7.110174	chr5:5659	CLTB		protein_c	chr5:176392501-176
ENSG0000(	314	7.110174	chr12:201	RNU6-166P		smallRNA	chr12:65095749-650
ENSG0000(	314	7.110174	chr5:5659	ENSG00000249403		lncRNA	chr5:174986295-174
ENSG0000(	314	7.110174	chr12:201	AC091517.1		smallRNA	chr12:60026771-600
ENSG0000(	314	7.110174	chr5:5659	ENSG00000251414		lncRNA	chr5:176354206-176
ENSG0000(	314	7.110174	chr12:201	GGTA2P		Pseudoger	chr12:67265842-672
ENSG0000(	314	7.110174	chr5:5659	FAM193B-DT		lncRNA	chr5:177554824-177
ENSG0000(	314	7.110174	chr5:5659	EIF4E1B		protein_c	chr5:176630618-176
ENSG0000(	314	7.110174	chr5:5659	SNCB		protein_c	chr5:176620082-176
ENSG0000(	314	7.110174	chr5:5659	CDHR2	NCGv7	protein_c	chr5:176542511-176
ENSG0000(	314	7.110174	chr12:201	RNU1-83P		smallRNA	chr12:62850738-628
ENSG0000(	314	7.110174	chr5:5659	OR1X1P		Pseudoger	chr5:176006145-176
ENSG0000(	314	7.110174	chr5:5659	NIFKP2		Pseudoger	chr5:174923090-174
ENSG0000(	314	7.110174	chr12:201	C12orf56		protein_c	chr12:64264762-643
ENSG0000(	314	7.110174	chr12:201	AC025165.1		smallRNA	chr12:57774249-577
ENSG0000(	314	7.110174	chr5:5659	ENSG00000251458		lncRNA	chr5:176124210-176
ENSG0000(	314	7.110174	chr5:5659	ENSG00000283235		lncRNA	chr5:176173345-176
ENSG0000(	314	7.110174	chr12:201	CTDSP2	DriverDBv	protein_c	chr12:57819927-578
ENSG0000(	314	7.110174	chr5:5659	ENSG00000270839		Pseudoger	chr5:175483595-175
ENSG0000(	314	7.110174	chr12:201	DUX4L52		Pseudoger	chr12:61600067-616
ENSG0000(	314	7.110174	chr12:201	LLPH		protein_c	chr12:66116555-661
ENSG0000(	314	7.110174	chr5:5659	ENSG00000283559		Pseudoger	chr5:176145025-176
ENSG0000(	314	7.110174	chr12:201	RPL14P1		Pseudoger	chr12:62965325-629
ENSG0000(	313	7.08753	chr2:3608	RPL12P17		Pseudoger	chr2:208057248-208
ENSG0000(	313	7.08753	chr12:201	AC131213.1		smallRNA	chr12:72790451-727
ENSG0000(	313	7.08753	chr2:3608	ENSG00000271074		Pseudoger	chr2:211104934-211
ENSG0000(	313	7.08753	chr2:3608	ENSG00000224342		lncRNA	chr2:207173634-207
ENSG0000(	313	7.08753	chr2:3608	ENSG00000289422		lncRNA	chr2:208064701-208
ENSG0000(	313	7.08753	chr2:3608	ENSG00000240440		lncRNA	chr2:207235299-207
ENSG0000(	313	7.08753	chr2:3608	ENSG00000272519		lncRNA	chr2:213266995-213
ENSG0000(	313	7.08753	chr2:3608	ENSG00000287662		lncRNA	chr2:208885755-208
ENSG0000(	313	7.08753	chr2:3608	CRYGB		protein_c	chr2:208142573-208
ENSG0000(	313	7.08753	chr2:3608	ENSG00000279317		lncRNA	chr2:210171499-210
ENSG0000(	313	7.08753	chr2:3608	AC093865.1		smallRNA	chr2:212763570-212
ENSG0000(	313	7.08753	chr12:201	ENSG00000257587		lncRNA	chr12:73906940-739
ENSG0000(	313	7.08753	chr2:3608	MIR2355		smallRNA	chr2:207109987-207
ENSG0000(	313	7.08753	chr2:3608	RNA5SP117		Pseudoger	chr2:208920547-208
ENSG0000(	313	7.08753	chr2:3608	MEAF6P1		Pseudoger	chr2:209178804-209
ENSG0000(	313	7.08753	chr2:3608	ENSG00000286568		lncRNA	chr2:207217532-207
ENSG0000(	313	7.08753	chr12:201	AC090015.1		smallRNA	chr12:74017775-740
ENSG0000(	313	7.08753	chr2:3608	SPAG16-DT		lncRNA	chr2:213276552-213
ENSG0000(	313	7.08753	chr2:3608	RPSAP27		Pseudoger	chr2:208208135-208
ENSG0000(	313	7.08753	chr2:3608	ENSG00000272851		lncRNA	chr2:207753872-207
ENSG0000(	313	7.08753	chr12:201	AC131012.1		smallRNA	chr12:75724453-757
ENSG0000(	313	7.08753	chr2:3608	MIR4438		smallRNA	chr2:213758067-213
ENSG0000(	313	7.08753	chr12:201	ENSG00000257777		Pseudoger	chr12:75600047-756

ENSG0000C	313	7.08753	chr2:3608	CRYGFP		Pseudoger	chr2:209145241-209
ENSG0000C	313	7.08753	chr12:201	CAPS2-AS1		lncRNA	chr12:75234740-752
ENSG0000C	313	7.08753	chr12:201	TRHDE	NCGv7	protein_c	chr12:72087266-726
ENSG0000C	313	7.08753	chr2:3608	ENSG00000224529		Pseudoger	chr2:208135394-208
ENSG0000C	313	7.08753	chr2:3608	RPE	NCGv7	protein_c	chr2:210002565-210
ENSG0000C	313	7.08753	chr12:201	LINC02445		lncRNA	chr12:73758657-738
ENSG0000C	313	7.08753	chr2:3608	PKP4P1		Pseudoger	chr2:209178966-209
ENSG0000C	313	7.08753	chr12:201	CHCHD3P2		Pseudoger	chr12:72610678-726
ENSG0000C	313	7.08753	chr2:3608	MYL1		protein_c	chr2:210290150-210
ENSG0000C	313	7.08753	chr2:3608	VWC2L		protein_c	chr2:214411054-214
ENSG0000C	313	7.08753	chr2:3608	MTND2P23		Pseudoger	chr2:211777206-211
ENSG0000C	313	7.08753	chr2:3608	MYOSLID		lncRNA	chr2:207166120-207
ENSG0000C	313	7.08753	chr2:3608	CRYGC		protein_c	chr2:208128137-208
ENSG0000C	313	7.08753	chr2:3608	FZD5		protein_c	chr2:207762598-207
ENSG0000C	313	7.08753	chr2:3608	CCNYL1		protein_c	chr2:207711540-207
ENSG0000C	313	7.08753	chr2:3608	Y_RNA		smallRNA	chr2:210265963-210
ENSG0000C	313	7.08753	chr2:3608	ENSG00000272807		lncRNA	chr2:210028417-210
ENSG0000C	313	7.08753	chr2:3608	CRYGA		protein_c	chr2:208160740-208
ENSG0000C	313	7.08753	chr12:201	ENSG00000257823		Pseudoger	chr12:75305452-753
ENSG0000C	313	7.08753	chr2:3608	ENSG00000225916		lncRNA	chr2:207226949-207
ENSG0000C	313	7.08753	chr12:201	ENSG00000257682		lncRNA	chr12:73203815-732
ENSG0000C	313	7.08753	chr2:3608	ENSG00000197585		lncRNA	chr2:214241676-214
ENSG0000C	313	7.08753	chr2:3608	ENSG00000286249		lncRNA	chr2:209353977-209
ENSG0000C	313	7.08753	chr12:201	ENSG00000286259		lncRNA	chr12:75649195-756
ENSG0000C	313	7.08753	chr12:201	U8		smallRNA	chr12:73764279-737
ENSG0000C	313	7.08753	chr2:3608	RNA5SP116		Pseudoger	chr2:208054161-208
ENSG0000C	313	7.08753	chr2:3608	RPL9P14		Pseudoger	chr2:208031383-208
ENSG0000C	313	7.08753	chr2:3608	LANCL1		protein_c	chr2:210427131-210
ENSG0000C	313	7.08753	chr2:3608	C2orf80		protein_c	chr2:208165343-208
ENSG0000C	313	7.08753	chr12:201	ENSG00000257386		lncRNA	chr12:74538145-745
ENSG0000C	313	7.08753	chr12:201	RNU6-1012P		smallRNA	chr12:73679188-736
ENSG0000C	313	7.08753	chr12:201	VENTXP3		Pseudoger	chr12:74292324-742
ENSG0000C	313	7.08753	chr2:3608	KLF7		protein_c	chr2:207074137-207
ENSG0000C	313	7.08753	chr2:3608	CREB1	NCGv7;AC	protein_c	chr2:207529737-207
ENSG0000C	313	7.08753	chr2:3608	AC016697.3		smallRNA	chr2:208233444-208
ENSG0000C	313	7.08753	chr2:3608	CRYGD		protein_c	chr2:208121607-208
ENSG0000C	313	7.08753	chr2:3608	MIR4776-2		smallRNA	chr2:212926257-212
ENSG0000C	313	7.08753	chr2:3608	MYL6BP1		Pseudoger	chr2:208269343-208
ENSG0000C	313	7.08753	chr2:3608	ARPC1BP1		Pseudoger	chr2:208360631-208
ENSG0000C	313	7.08753	chr12:201	ENSG00000257183		lncRNA	chr12:74248637-742
ENSG0000C	313	7.08753	chr12:201	ENSG00000257323		Pseudoger	chr12:74656561-746
ENSG0000C	313	7.08753	chr2:3608	ACADL		protein_c	chr2:210187126-210
ENSG0000C	313	7.08753	chr2:3608	LANCL1-AS1		lncRNA	chr2:210324759-210
ENSG0000C	313	7.08753	chr2:3608	ENSG00000270659		lncRNA	chr2:213151972-213
ENSG0000C	313	7.08753	chr2:3608	PIKFYVE		protein_c	chr2:208266255-208
ENSG0000C	313	7.08753	chr12:201	H3P35		Pseudoger	chr12:72431722-724
ENSG0000C	313	7.08753	chr12:201	TRHDE-AS1		lncRNA	chr12:72249964-722
ENSG0000C	313	7.08753	chr2:3608	RNU6-360P		smallRNA	chr2:208019638-208
ENSG0000C	313	7.08753	chr2:3608	HSPA8P6		Pseudoger	chr2:209072597-209
ENSG0000C	313	7.08753	chr2:3608	MIR4775		smallRNA	chr2:207754807-207
ENSG0000C	313	7.08753	chr12:201	SNORA70		smallRNA	chr12:75689152-756

ENSG0000(	313	7.08753	chr2:360{	LINC01953		lncRNA	chr2:213155806-213
ENSG0000(	313	7.08753	chr2:360{	ERBB4	NCGv7;AC	protein_c	chr2:211375717-212
ENSG0000(	313	7.08753	chr2:360{	PLEKHM3	NCGv7	protein_c	chr2:207821288-208
ENSG0000(	313	7.08753	chr2:360{	RNA5SP119		Pseudoger	chr2:211535653-211
ENSG0000(	313	7.08753	chr2:360{	ENSG00000273118		lncRNA	chr2:212581357-213
ENSG0000(	313	7.08753	chr2:360{	snoU13		smallRNA	chr2:208074061-208
ENSG0000(	313	7.08753	chr2:360{	MAP2	IntOGen-I	protein_c	chr2:209424047-209
ENSG0000(	313	7.08753	chr12:201	LINC02882		lncRNA	chr12:73820315-744
ENSG0000(	313	7.08753	chr2:360{	AC006994.3		smallRNA	chr2:210171296-210
ENSG0000(	313	7.08753	chr2:360{	AC012362.1		smallRNA	chr2:208369561-208
ENSG0000(	313	7.08753	chr2:360{	MTCO1P46		Pseudoger	chr2:211778273-211
ENSG0000(	313	7.08753	chr2:360{	LINC01878		lncRNA	chr2:212795300-212
ENSG0000(	313	7.08753	chr2:360{	ENSG00000289113		lncRNA	chr2:209299674-209
ENSG0000(	313	7.08753	chr12:201	GLIPR1-AS1		lncRNA	chr12:75483454-754
ENSG0000(	313	7.08753	chr2:360{	Y_RNA		smallRNA	chr2:207687960-207
ENSG0000(	313	7.08753	chr2:360{	RNU6-664P		smallRNA	chr2:207680533-207
ENSG0000(	313	7.08753	chr2:360{	ENSG00000289002		lncRNA	chr2:213188417-213
ENSG0000(	313	7.08753	chr2:360{	LINC01802		lncRNA	chr2:207260445-207
ENSG0000(	313	7.08753	chr2:360{	CRYGEP		Pseudoger	chr2:208108233-208
ENSG0000(	313	7.08753	chr2:360{	KANSL1L-AS1		lncRNA	chr2:210030572-210
ENSG0000(	313	7.08753	chr12:201	ENSG00000273987		lncRNA	chr12:75333798-753
ENSG0000(	313	7.08753	chr12:201	CAPS2		protein_c	chr12:75275979-753
ENSG0000(	313	7.08753	chr12:201	KRR1		protein_c	chr12:75490863-755
ENSG0000(	313	7.08753	chr2:360{	ENSG00000225111		lncRNA	chr2:207868578-207
ENSG0000(	313	7.08753	chr12:201	GLIPR1L2	NCGv7	protein_c	chr12:75391089-754
ENSG0000(	313	7.08753	chr2:360{	RPL5P8		Pseudoger	chr2:214280638-214
ENSG0000(	313	7.08753	chr12:201	ENSG00000257113		Pseudoger	chr12:74274952-742
ENSG0000(	313	7.08753	chr2:360{	H3P9		Pseudoger	chr2:208466238-208
ENSG0000(	313	7.08753	chr12:201	ENSG00000257434		lncRNA	chr12:75020969-750
ENSG0000(	313	7.08753	chr2:360{	KLF7-IT1		lncRNA	chr2:207120884-207
ENSG0000(	313	7.08753	chr2:360{	ENSG00000234902		lncRNA	chr2:207186717-207
ENSG0000(	313	7.08753	chr2:360{	ENSG00000288732		lncRNA	chr2:210548375-210
ENSG0000(	313	7.08753	chr2:360{	ENSG00000286965		lncRNA	chr2:212932894-212
ENSG0000(	313	7.08753	chr12:201	AC136188.1		smallRNA	chr12:73899870-738
ENSG0000(	313	7.08753	chr12:201	GLIPR1L1		protein_c	chr12:75334670-753
ENSG0000(	313	7.08753	chr2:360{	SNAI1P1		Pseudoger	chr2:209808804-209
ENSG0000(	313	7.08753	chr2:360{	TPT1P2		Pseudoger	chr2:208214724-208
ENSG0000(	313	7.08753	chr2:360{	PPP1R14BP2		Pseudoger	chr2:207681590-207
ENSG0000(	313	7.08753	chr12:201	LINC02394		lncRNA	chr12:74039024-740
ENSG0000(	313	7.08753	chr2:360{	CPS1	NCGv7	protein_c	chr2:210477682-210
ENSG0000(	313	7.08753	chr2:360{	UNC80	NCGv7	protein_c	chr2:209771832-209
ENSG0000(	313	7.08753	chr12:201	ENSG00000243164		Pseudoger	chr12:73648666-736
ENSG0000(	313	7.08753	chr2:360{	ENSG00000223725		lncRNA	chr2:207239864-207
ENSG0000(	313	7.08753	chr2:360{	LINC01857		lncRNA	chr2:207662375-207
ENSG0000(	313	7.08753	chr2:360{	RPS29P9		Pseudoger	chr2:207654781-207
ENSG0000(	313	7.08753	chr12:201	GLIPR1		protein_c	chr12:75480753-755
ENSG0000(	313	7.08753	chr2:360{	IDH1	NCGv7;AC	protein_c	chr2:208236229-208
ENSG0000(	313	7.08753	chr2:360{	PTH2R		protein_c	chr2:208359714-208
ENSG0000(	313	7.08753	chr12:201	CCNG2P1		Pseudoger	chr12:75257635-752
ENSG0000(	313	7.08753	chr12:201	ENSG00000257998		Pseudoger	chr12:74728038-747
ENSG0000(	313	7.08753	chr12:201	TPH2		protein_c	chr12:71938845-721

ENSG0000(	313	7.08753	chr2:360(	IKZF2	NCGv7	protein_c	chr2:212999691-213
ENSG0000(	313	7.08753	chr2:360(	RNA5SP118		Pseudoger	chr2:209721913-209
ENSG0000(	313	7.08753	chr2:360(	PCED1CP		Pseudoger	chr2:212832161-212
ENSG0000(	313	7.08753	chr12:201	ENSG00000258115		lncRNA	chr12:72046149-720
ENSG0000(	313	7.08753	chr2:360(	SPAG16		protein_c	chr2:213284389-214
ENSG0000(	313	7.08753	chr12:201	ENSG00000258294		lncRNA	chr12:73115957-731
ENSG0000(	313	7.08753	chr2:360(	CPO		protein_c	chr2:206939518-206
ENSG0000(	313	7.08753	chr12:201	LINC02444		lncRNA	chr12:73159190-732
ENSG0000(	313	7.08753	chr2:360(	KANSL1L		protein_c	chr2:210021421-210
ENSG0000(	313	7.08753	chr2:360(	CPS1-IT1		lncRNA	chr2:210617571-210
ENSG0000(	313	7.08753	chr2:360(	ENSG00000223373		lncRNA	chr2:212911003-212
ENSG0000(	313	7.08753	chr12:201	ENSG00000258088		lncRNA	chr12:75694010-756
ENSG0000(	313	7.08753	chr2:360(	RPL6P6		Pseudoger	chr2:210088226-210
ENSG0000(	313	7.08753	chr12:201	KCNC2		protein_c	chr12:75040077-752
ENSG0000(	313	7.08753	chr12:201	ENSG00000258235		lncRNA	chr12:72727923-727
ENSG0000(	313	7.08753	chr2:360(	MIR548F2		smallRNA	chr2:212426263-212
ENSG0000(	313	7.08753	chr12:201	ATXN7L3B		protein_c	chr12:74537835-745
ENSG0000(	313	7.08753	chr2:360(	MIR1302-4		smallRNA	chr2:207269275-207
ENSG0000(	313	7.08753	chr2:360(	RPS27P10		Pseudoger	chr2:211298941-211
ENSG0000(	313	7.08753	chr12:201	ENSG00000258320		Pseudoger	chr12:74170445-741
ENSG0000(	313	7.08753	chr2:360(	METTL21A		protein_c	chr2:207580631-207
ENSG0000(	313	7.08753	chr2:360(	ENSG00000231896		lncRNA	chr2:208469850-208
ENSG0000(	313	7.08753	chr2:360(	ENSG00000290929		lncRNA	chr2:208108154-208
ENSG0000(	313	7.08753	chr12:201	RPL10P13		Pseudoger	chr12:75688794-756
ENSG0000(	313	7.08753	chr2:360(	IDH1-AS1		lncRNA	chr2:208255214-208
ENSG0000(	312	7.064887	chr7:131(	CNOT4		protein_c	chr7:135361795-135
ENSG0000(	312	7.064887	chr7:131(	ENSG00000224375		lncRNA	chr7:134684144-134
ENSG0000(	312	7.064887	chr7:131(	RPS3AP27		Pseudoger	chr7:133732493-133
ENSG0000(	312	7.064887	chr7:131(	Y_RNA		smallRNA	chr7:136129980-136
ENSG0000(	312	7.064887	chr7:131(	EXOC4		protein_c	chr7:133253073-134
ENSG0000(	312	7.064887	chr7:131(	SNORD81		smallRNA	chr7:137287687-137
ENSG0000(	312	7.064887	chr7:131(	AC009784.1		smallRNA	chr7:136244446-136
ENSG0000(	312	7.064887	chr7:131(	ENSG00000234352		lncRNA	chr7:136685559-137
ENSG0000(	312	7.064887	chr7:131(	AKR1B15		protein_c	chr7:134549110-134
ENSG0000(	312	7.064887	chr7:131(	COX5BP3		Pseudoger	chr7:133727368-133
ENSG0000(	312	7.064887	chr7:131(	FAM180A		protein_c	chr7:135728348-135
ENSG0000(	312	7.064887	chr7:131(	ENSG00000229177		lncRNA	chr7:134346071-134
ENSG0000(	312	7.064887	chr7:131(	SLC23A4P		Pseudoger	chr7:135270017-135
ENSG0000(	312	7.064887	chr7:131(	ENSG00000289175		lncRNA	chr7:134953657-134
ENSG0000(	312	7.064887	chr7:131(	ENSG00000230649		lncRNA	chr7:136025717-136
ENSG0000(	312	7.064887	chr7:131(	ENSG00000272941		lncRNA	chr7:135168403-135
ENSG0000(	312	7.064887	chr7:131(	RNU6-223P		smallRNA	chr7:135960703-135
ENSG0000(	312	7.064887	chr7:131(	AC083875.1		smallRNA	chr7:133262640-133
ENSG0000(	312	7.064887	chr7:131(	MIR490		smallRNA	chr7:136903167-136
ENSG0000(	312	7.064887	chr7:131(	AKR1B10	NCGv7	protein_c	chr7:134527567-134
ENSG0000(	312	7.064887	chr7:131(	WDR91		protein_c	chr7:135183839-135
ENSG0000(	312	7.064887	chr7:131(	MTPN		protein_c	chr7:135926760-135
ENSG0000(	312	7.064887	chr7:131(	PTN	NCGv7	protein_c	chr7:137227341-137
ENSG0000(	312	7.064887	chr7:131(	STMP1		protein_c	chr7:135662496-135
ENSG0000(	312	7.064887	chr7:131(	ENSG00000290805		lncRNA	chr7:135246113-135
ENSG0000(	312	7.064887	chr7:131(	CHRM2		protein_c	chr7:136868652-137

ENSG0000(	312	7.064887	chr7:131(	ENSG00000224469		Pseudoger	chr7:137164043-137
ENSG0000(	312	7.064887	chr7:131(	ENSG00000231794		lncRNA	chr7:135198401-135
ENSG0000(	312	7.064887	chr14:20(	TRAJ42		protein_c	chr14:22496887-224
ENSG0000(	312	7.064887	chr7:131(	NUP205		protein_c	chr7:135557917-135
ENSG0000(	312	7.064887	chr7:131(	LRGUK	DriverDB\	protein_c	chr7:134127299-134
ENSG0000(	312	7.064887	chr7:131(	TRPC6P8		Pseudoger	chr7:136262558-136
ENSG0000(	312	7.064887	chr7:131(	SLC35B4		protein_c	chr7:134289332-134
ENSG0000(	312	7.064887	chr7:131(	RNF14P4		Pseudoger	chr7:135128444-135
ENSG0000(	312	7.064887	chr7:131(	DGKI	NCGv7	protein_c	chr7:137381037-137
ENSG0000(	312	7.064887	chr7:131(	AC083862.1		protein_c	chr7:135148045-135
ENSG0000(	312	7.064887	chr7:131(	ENSG00000232053		lncRNA	chr7:136092913-136
ENSG0000(	312	7.064887	chr7:131(	STRA8		protein_c	chr7:135231979-135
ENSG0000(	312	7.064887	chr7:131(	ENSG00000279483		Pseudoger	chr7:137513859-137
ENSG0000(	312	7.064887	chr7:131(	AC091736.1		smallRNA	chr7:135798006-135
ENSG0000(	312	7.064887	chr7:131(	snoU13		smallRNA	chr7:136841093-136
ENSG0000(	312	7.064887	chr7:131(	ENSG00000224746		lncRNA	chr7:135774521-135
ENSG0000(	312	7.064887	chr7:131(	ENSG00000286458		lncRNA	chr7:134816543-134
ENSG0000(	312	7.064887	chr7:131(	ENSG00000231098		lncRNA	chr7:134284500-134
ENSG0000(	312	7.064887	chr7:131(	RNU6-1154P		smallRNA	chr7:135665599-135
ENSG0000(	312	7.064887	chr7:131(	ENSG00000289600		lncRNA	chr7:135980929-135
ENSG0000(	312	7.064887	chr7:131(	ENSG00000273297		lncRNA	chr7:134368737-134
ENSG0000(	312	7.064887	chr7:131(	ZP3P2		Pseudoger	chr7:136485656-136
ENSG0000(	312	7.064887	chr7:131(	AGBL3	DriverDB\	protein_c	chr7:134986508-135
ENSG0000(	312	7.064887	chr7:131(	BPGM		protein_c	chr7:134646811-134
ENSG0000(	312	7.064887	chr7:131(	ENSG00000271414		Pseudoger	chr7:135650972-135
ENSG0000(	312	7.064887	chr7:131(	ENSG00000235429		Pseudoger	chr7:133315009-133
ENSG0000(	312	7.064887	chr7:131(	ENSG00000231114		lncRNA	chr7:137318592-137
ENSG0000(	312	7.064887	chr7:131(	TUBB3P2		Pseudoger	chr7:134734898-134
ENSG0000(	312	7.064887	chr7:131(	SDHDP2		Pseudoger	chr7:135444461-135
ENSG0000(	312	7.064887	chr7:131(	AKR1B1		protein_c	chr7:134442356-134
ENSG0000(	312	7.064887	chr7:131(	ENSG00000228031		lncRNA	chr7:137344930-137
ENSG0000(	312	7.064887	chr7:131(	CYREN		protein_c	chr7:135092363-135
ENSG0000(	312	7.064887	chr7:131(	CALD1	NCGv7	protein_c	chr7:134744252-134
ENSG0000(	312	7.064887	chr7:131(	TMEM140	AC	protein_c	chr7:135148072-135
ENSG0000(	312	7.064887	chr7:131(	KRT8P51		Pseudoger	chr7:136938280-136
ENSG0000(	312	7.064887	chr7:131(	ENSG00000273219		lncRNA	chr7:135704537-135
ENSG0000(	312	7.064887	chr7:131(	PSMC1P3		Pseudoger	chr7:136713871-136
ENSG0000(	312	7.064887	chr7:131(	SLC13A4	DriverDB\	protein_c	chr7:135681231-135
ENSG0000(	312	7.064887	chr7:131(	ENSG00000287733		lncRNA	chr7:135170816-135
ENSG0000(	311	7.042243	chr5:4231	ZBED3		protein_c	chr5:77072072-7708
ENSG0000(	311	7.042243	chr5:4231	RPS2P24		Pseudoger	chr5:77545397-7754
ENSG0000(	311	7.042243	chr5:4231	ENSG00000284762		protein_c	chr5:77086732-7742
ENSG0000(	311	7.042243	chr5:4231	snoU13		smallRNA	chr5:76784096-7678
ENSG0000(	311	7.042243	chr5:4231	SCAMP1		protein_c	chr5:78360611-7848
ENSG0000(	311	7.042243	chr5:4231	SCAMP1-AS1		lncRNA	chr5:78342333-7836
ENSG0000(	311	7.042243	chr5:4231	HMGB1P21		Pseudoger	chr5:78644265-7864
ENSG0000(	311	7.042243	chr5:4231	SNORA47		smallRNA	chr5:77080434-7708
ENSG0000(	311	7.042243	chr5:4231	OTP		protein_c	chr5:77628712-7763
ENSG0000(	311	7.042243	chr5:4231	ENSG00000285000		protein_c	chr5:77030902-7715
ENSG0000(	311	7.042243	chr5:4231	ENSG00000250615		lncRNA	chr5:77073881-7707
ENSG0000(	311	7.042243	chr5:4231	TBCA		protein_c	chr5:77691166-7786

ENSG0000(	311	7.042243	chr5:4231	ACTBP2		Pseudoger	chr5:77784881-7778
ENSG0000(	311	7.042243	chr5:4231	AP3B1		protein_c	chr5:78000522-7829
ENSG0000(	311	7.042243	chr5:4231	ENSG00000287162		lncRNA	chr5:77958198-7795
ENSG0000(	311	7.042243	chr5:4231	RNU6ATAC36P		smallRNA	chr5:76948791-7694
ENSG0000(	311	7.042243	chr5:4231	AC024568.1		smallRNA	chr5:78056624-7805
ENSG0000(	311	7.042243	chr5:4231	RNU6-183P		smallRNA	chr5:78314916-7831
ENSG0000(	311	7.042243	chr5:4231	WDR41		protein_c	chr5:77425970-7762
ENSG0000(	311	7.042243	chr5:4231	ENSG00000289924		lncRNA	chr5:76818312-7681
ENSG0000(	311	7.042243	chr5:4231	ENSG00000254310		Pseudoger	chr5:78753596-7875
ENSG0000(	311	7.042243	chr5:4231	F2RL1		protein_c	chr5:76818933-7683
ENSG0000(	311	7.042243	chr5:4231	ZBED3-AS1		lncRNA	chr5:77086688-7716
ENSG0000(	311	7.042243	chr5:4231	ENSG00000254170		Pseudoger	chr5:78708734-7871
ENSG0000(	311	7.042243	chr5:4231	RPL7P23		Pseudoger	chr5:77582376-7758
ENSG0000(	311	7.042243	chr5:4231	Y_RNA		smallRNA	chr5:78317036-7831
ENSG0000(	311	7.042243	chr5:4231	ENSG00000251605		Pseudoger	chr5:78307503-7830
ENSG0000(	311	7.042243	chr5:4231	RN7SL208P		smallRNA	chr5:76836902-7683
ENSG0000(	311	7.042243	chr5:4231	AGGF1	NCGv7	protein_c	chr5:77029251-7706
ENSG0000(	311	7.042243	chr5:4231	S100Z		protein_c	chr5:76850001-7692
ENSG0000(	311	7.042243	chr5:4231	ALDH7A1P1		Pseudoger	chr5:77290268-7729
ENSG0000(	311	7.042243	chr5:4231	AC008581.1		smallRNA	chr5:77137967-7713
ENSG0000(	311	7.042243	chr5:4231	LHFPL2		protein_c	chr5:78485215-7877
ENSG0000(	311	7.042243	chr5:4231	PDE8B		protein_c	chr5:77210449-7742
ENSG0000(	311	7.042243	chr5:4231	ENSG00000253558		Pseudoger	chr5:78041888-7804
ENSG0000(	311	7.042243	chr5:4231	ENSG00000253572		lncRNA	chr5:77942757-7794
ENSG0000(	311	7.042243	chr5:4231	ATP6V1G1P6		Pseudoger	chr5:78222184-7822
ENSG0000(	311	7.042243	chr5:4231	CRHBP		protein_c	chr5:76953045-7698
ENSG0000(	307	6.951667	chr6:9221	H2BC9	NCGv7	protein_c	chr6:26251614-2625
ENSG0000(	306	6.929023	chr22:22(	IGLV1-47		protein_c	chr22:22357739-223
ENSG0000(	305	6.906379	chr7:251(	MTCO1P10		Pseudoger	chr7:57187892-5718
ENSG0000(	305	6.906379	chr7:251(	ENSG00000287580		lncRNA	chr7:64801958-6480
ENSG0000(	305	6.906379	chr7:251(	ENSG00000270749		Pseudoger	chr7:57629285-5763
ENSG0000(	305	6.906379	chr7:251(	ZNF92		protein_c	chr7:65373799-6540
ENSG0000(	305	6.906379	chr7:251(	SUMO2P3		Pseudoger	chr7:55732144-5573
ENSG0000(	305	6.906379	chr7:251(	ENSG00000234185		lncRNA	chr7:66119603-6616
ENSG0000(	305	6.906379	chr7:251(	RN7SL816P		smallRNA	chr7:56965248-5696
ENSG0000(	305	6.906379	chr7:251(	ENSG00000231317		Pseudoger	chr7:55656768-5567
ENSG0000(	305	6.906379	chr7:251(	MTCO2P8		Pseudoger	chr7:64107829-6410
ENSG0000(	305	6.906379	chr7:251(	PSPH	DriverDB\	protein_c	chr7:56011051-5605
ENSG0000(	305	6.906379	chr7:251(	VN1R25P		Pseudoger	chr7:56559221-5655
ENSG0000(	305	6.906379	chr7:251(	SEPTIN7P5		Pseudoger	chr7:63495799-6350
ENSG0000(	305	6.906379	chr7:251(	GUSB	NCGv7	protein_c	chr7:65960684-6598
ENSG0000(	305	6.906379	chr7:251(	LINC03006		Pseudoger	chr7:65770473-6577
ENSG0000(	305	6.906379	chr7:251(	ENSG00000279072		lncRNA	chr7:56809214-5684
ENSG0000(	305	6.906379	chr7:251(	GTF2IP14		Pseudoger	chr7:65084103-6510
ENSG0000(	305	6.906379	chr7:251(	ENSG00000233918		Pseudoger	chr7:62275360-6227
ENSG0000(	305	6.906379	chr7:251(	SNORA63		smallRNA	chr7:65327396-6532
ENSG0000(	305	6.906379	chr7:251(	ENSG00000237210		Pseudoger	chr7:55244247-5524
ENSG0000(	305	6.906379	chr7:251(	ENSG00000237236		Pseudoger	chr7:57822201-5782
ENSG0000(	305	6.906379	chr7:251(	ENSG00000287699		lncRNA	chr7:64294516-6430
ENSG0000(	305	6.906379	chr7:251(	ENSG00000286477		lncRNA	chr7:56659149-5667
ENSG0000(	305	6.906379	chr7:251(	ENSG00000237268		Pseudoger	chr7:56421857-5643

ENSG0000(	305	6.906379	chr7:251(	ENSG00000231232		Pseudoger	chr7:57822465-5782
ENSG0000(	305	6.906379	chr7:251(	ENSG00000286456		lncRNA	chr7:64574673-6461
ENSG0000(	305	6.906379	chr7:251(	ENSG00000233960		lncRNA	chr7:52165235-5219
ENSG0000(	305	6.906379	chr7:251(	MTATP6P10		Pseudoger	chr7:57190461-5719
ENSG0000(	305	6.906379	chr7:251(	MTND2P6		Pseudoger	chr7:57186412-5718
ENSG0000(	305	6.906379	chr7:251(	VKORC1L1		protein_c	chr7:65873074-6595
ENSG0000(	305	6.906379	chr7:251(	EGFR	NCGv7;AC	protein_c	chr7:55019017-5521
ENSG0000(	305	6.906379	chr7:251(	ENSG00000286436		lncRNA	chr7:57140112-5715
ENSG0000(	305	6.906379	chr7:251(	GUSBP12		Pseudoger	chr7:57200825-5720
ENSG0000(	305	6.906379	chr7:251(	ZNF680P1		Pseudoger	chr7:64469185-6447
ENSG0000(	305	6.906379	chr7:251(	ENSG00000286404		lncRNA	chr7:54721724-5473
ENSG0000(	305	6.906379	chr7:251(	MTND3P2		Pseudoger	chr7:64105654-6410
ENSG0000(	305	6.906379	chr7:251(	ENSG00000224155		Pseudoger	chr7:56304678-5630
ENSG0000(	305	6.906379	chr7:251(	ENSG00000223889		Pseudoger	chr7:63393749-6339
ENSG0000(	305	6.906379	chr7:251(	ENSG00000249319		protein_c	chr7:66087761-6615
ENSG0000(	305	6.906379	chr7:251(	ENSG00000287588		lncRNA	chr7:63925832-6393
ENSG0000(	305	6.906379	chr7:251(	ENSG00000234085		Pseudoger	chr7:57654822-5765
ENSG0000(	305	6.906379	chr7:251(	CCT6A	DriverDB\	protein_c	chr7:56051685-5606
ENSG0000(	305	6.906379	chr7:251(	ENSG00000236907		Pseudoger	chr7:57817601-5781
ENSG0000(	305	6.906379	chr7:251(	SEPTIN14P24		Pseudoger	chr7:56360362-5636
ENSG0000(	305	6.906379	chr7:251(	MTND2P4		Pseudoger	chr7:64110547-6411
ENSG0000(	305	6.906379	chr7:251(	ENSG00000234105		lncRNA	chr7:53514992-5351
ENSG0000(	305	6.906379	chr7:251(	NIPSNAP2	DriverDB\	protein_c	chr7:55951793-5600
ENSG0000(	305	6.906379	chr7:251(	AC092634.1		smallRNA	chr7:63901068-6390
ENSG0000(	305	6.906379	chr7:251(	ENSG00000234089		lncRNA	chr7:57209865-5722
ENSG0000(	305	6.906379	chr7:251(	RPL6P20		Pseudoger	chr7:64141538-6414
ENSG0000(	305	6.906379	chr7:251(	ENSG00000229301		lncRNA	chr7:63354457-6335
ENSG0000(	305	6.906379	chr7:251(	ENSG00000274299		Pseudoger	chr7:57270839-5727
ENSG0000(	305	6.906379	chr7:251(	ENSG00000233962		Pseudoger	chr7:57652736-5765
ENSG0000(	305	6.906379	chr7:251(	RNU6-417P		smallRNA	chr7:63011463-6301
ENSG0000(	305	6.906379	chr7:251(	RNU6-389P		smallRNA	chr7:55685977-5568
ENSG0000(	305	6.906379	chr7:251(	ARAFP1		Pseudoger	chr7:63937905-6393
ENSG0000(	305	6.906379	chr7:251(	SLC29A4P1		Pseudoger	chr7:57014285-5702
ENSG0000(	305	6.906379	chr7:251(	ENSG00000290193		lncRNA	chr7:56482723-5649
ENSG0000(	305	6.906379	chr7:251(	ENSG00000237026		Pseudoger	chr7:65235790-6523
ENSG0000(	305	6.906379	chr7:251(	ENSG00000233977		lncRNA	chr7:55592074-5559
ENSG0000(	305	6.906379	chr7:251(	ENSG00000230271		Pseudoger	chr7:57772584-5777
ENSG0000(	305	6.906379	chr7:251(	SNORA22		smallRNA	chr7:65065999-6506
ENSG0000(	305	6.906379	chr7:251(	MTCYBP29		Pseudoger	chr7:57173357-5717
ENSG0000(	305	6.906379	chr7:251(	ENSG00000287517		lncRNA	chr7:57242681-5724
ENSG0000(	305	6.906379	chr7:251(	SLC25A1P2		Pseudoger	chr7:63398046-6339
ENSG0000(	305	6.906379	chr7:251(	ENSG00000278577		Pseudoger	chr7:63993904-6399
ENSG0000(	305	6.906379	chr7:251(	ENSG00000286908		lncRNA	chr7:52493152-5250
ENSG0000(	305	6.906379	chr7:251(	ERV3-1		protein_c	chr7:64990356-6500
ENSG0000(	305	6.906379	chr7:251(	GABPAP		Pseudoger	chr7:63893286-6389
ENSG0000(	305	6.906379	chr7:251(	ENSG00000235738		Pseudoger	chr7:55797946-5579
ENSG0000(	305	6.906379	chr7:251(	VN1R35P		Pseudoger	chr7:63963431-6396
ENSG0000(	305	6.906379	chr7:251(	RSL24D1P3		Pseudoger	chr7:65335174-6533
ENSG0000(	305	6.906379	chr7:251(	SNORA63		smallRNA	chr7:65326719-6532
ENSG0000(	305	6.906379	chr7:251(	HNRNPCP7		Pseudoger	chr7:64500825-6450
ENSG0000(	305	6.906379	chr7:251(	ENSG00000225488		lncRNA	chr7:56477588-5648

ENSG0000(	305	6.906379	chr7:251(	PHKG1P2	Pseudoger	chr7:63509303-6351
ENSG0000(	305	6.906379	chr7:251(	ENSG00000228627	lncRNA	chr7:53559214-5357
ENSG0000(	305	6.906379	chr7:251(	RNU2-29P	smallRNA	chr7:53776136-5377
ENSG0000(	305	6.906379	chr7:251(	ENSG00000275833	Pseudoger	chr7:65764535-6576
ENSG0000(	305	6.906379	chr7:251(	LINC03006	lncRNA	chr7:65647010-6577
ENSG0000(	305	6.906379	chr7:251(	ENSG00000291184	lncRNA	chr7:56426859-5644
ENSG0000(	305	6.906379	chr7:251(	EEF1DP4	Pseudoger	chr7:64862999-6486
ENSG0000(	305	6.906379	chr7:251(	SNORA73	smallRNA	chr7:54865818-5486
ENSG0000(	305	6.906379	chr7:251(	ENSG00000213642	Pseudoger	chr7:64569428-6457
ENSG0000(	305	6.906379	chr7:251(	VN1R37P	Pseudoger	chr7:64149685-6415
ENSG0000(	305	6.906379	chr7:251(	MTCO3P10	Pseudoger	chr7:57167880-5716
ENSG0000(	305	6.906379	chr7:251(	SAPCD2P1	Pseudoger	chr7:64181710-6418
ENSG0000(	305	6.906379	chr7:251(	SEPTIN14P1	Pseudoger	chr7:63117501-6315
ENSG0000(	305	6.906379	chr7:251(	SLC25A1P3	Pseudoger	chr7:63931141-6393
ENSG0000(	305	6.906379	chr7:251(	ENSG00000213650	Pseudoger	chr7:56567906-5656
ENSG0000(	305	6.906379	chr7:251(	ENSG00000273720	Pseudoger	chr7:55743073-5574
ENSG0000(	305	6.906379	chr7:251(	ENSG00000225451	Pseudoger	chr7:63876341-6387
ENSG0000(	305	6.906379	chr7:251(	ENSG00000286987	lncRNA	chr7:51773836-5178
ENSG0000(	305	6.906379	chr7:251(	SLC29A4P2	Pseudoger	chr7:63556598-6356
ENSG0000(	305	6.906379	chr7:251(	CICP8	Pseudoger	chr7:56362458-5636
ENSG0000(	305	6.906379	chr7:251(	MTCO3P4	Pseudoger	chr7:57191141-5719
ENSG0000(	305	6.906379	chr7:251(	VN1R38P	Pseudoger	chr7:64180006-6418
ENSG0000(	305	6.906379	chr7:251(	ENSG00000287019	lncRNA	chr7:56615084-5661
ENSG0000(	305	6.906379	chr7:251(	ENSG00000277206	Pseudoger	chr7:64768156-6476
ENSG0000(	305	6.906379	chr7:251(	IFITM3P4	Pseudoger	chr7:56163145-5616
ENSG0000(	305	6.906379	chr7:251(	ENSG00000213067	Pseudoger	chr7:56597379-5659
ENSG0000(	305	6.906379	chr7:251(	MTND4P4	Pseudoger	chr7:57169406-5717
ENSG0000(	305	6.906379	chr7:251(	ENSG00000287039	lncRNA	chr7:51849086-5186
ENSG0000(	305	6.906379	chr7:251(	ENSG00000235349	Pseudoger	chr7:64566814-6456
ENSG0000(	305	6.906379	chr7:251(	SAPCD2P2	Pseudoger	chr7:57424503-5742
ENSG0000(	305	6.906379	chr7:251(	ENSG00000235421	Pseudoger	chr7:65525629-6555
ENSG0000(	305	6.906379	chr7:251(	HAUS6P3	Pseudoger	chr7:53862233-5386
ENSG0000(	305	6.906379	chr7:251(	ENSG00000290553	lncRNA	chr7:65647864-6571
ENSG0000(	305	6.906379	chr7:251(	CICP12	Pseudoger	chr7:55798034-5579
ENSG0000(	305	6.906379	chr7:251(	ENSG00000235095	Pseudoger	chr7:57147986-5715
ENSG0000(	305	6.906379	chr7:251(	ENSG00000268181	lncRNA	chr7:63396341-6339
ENSG0000(	305	6.906379	chr7:251(	ENSG00000228897	Pseudoger	chr7:51386363-5138
ENSG0000(	305	6.906379	chr7:251(	RN7SKP218	smallRNA	chr7:53490148-5349
ENSG0000(	305	6.906379	chr7:251(	EGFR-AS1	lncRNA	chr7:55179750-5518
ENSG0000(	305	6.906379	chr7:251(	ENSG00000225244	Pseudoger	chr7:56940341-5694
ENSG0000(	305	6.906379	chr7:251(	ENSG00000228735	lncRNA	chr7:54576052-5457
ENSG0000(	305	6.906379	chr7:251(	ENSG00000290564	lncRNA	chr7:64120431-6415
ENSG0000(	305	6.906379	chr7:251(	ENSG00000290558	lncRNA	chr7:63291552-6330
ENSG0000(	305	6.906379	chr7:251(	MTND1P2	Pseudoger	chr7:64111752-6411
ENSG0000(	305	6.906379	chr7:251(	CDC42P2	Pseudoger	chr7:55638274-5563
ENSG0000(	305	6.906379	chr7:251(	SLC25A5P3	Pseudoger	chr7:54419444-5442
ENSG0000(	305	6.906379	chr7:251(	INTS4P1	lncRNA	chr7:65141032-6523
ENSG0000(	305	6.906379	chr7:251(	ENSG00000236529	lncRNA	chr7:65840055-6584
ENSG0000(	305	6.906379	chr7:251(	ENSG00000277544	Pseudoger	chr7:65290429-6529
ENSG0000(	305	6.906379	chr7:251(	ZNF90P3	Pseudoger	chr7:63087070-6308
ENSG0000(	305	6.906379	chr7:251(	ENSG00000284534	lncRNA	chr7:63959844-6398

ENSG0000(	305	6.906379	chr7:251(	ENSG00000284474		Pseudoger	chr7:57167039-5716
ENSG0000(	305	6.906379	chr7:251(	INTS4P2		Pseudoger	chr7:65647823-6571
ENSG0000(	305	6.906379	chr7:251(	ZNF736		protein_c	chr7:64307459-6435
ENSG0000(	305	6.906379	chr7:251(	ZNF734P		Pseudoger	chr7:63449819-6345
ENSG0000(	305	6.906379	chr7:251(	MTCO2P10		Pseudoger	chr7:57189578-5719
ENSG0000(	305	6.906379	chr7:251(	POM121L12		protein_c	chr7:53035633-5303
ENSG0000(	305	6.906379	chr7:251(	ENSG00000284221		Pseudoger	chr7:57192405-5719
ENSG0000(	305	6.906379	chr7:251(	ENSG00000234387		lncRNA	chr7:63394277-6339
ENSG0000(	305	6.906379	chr7:251(	ENSG00000261275		lncRNA	chr7:56493124-5649
ENSG0000(	305	6.906379	chr7:251(	ZNF679		protein_c	chr7:64228474-6426
ENSG0000(	305	6.906379	chr7:251(	ENSG00000228303		Pseudoger	chr7:56638943-5665
ENSG0000(	305	6.906379	chr7:251(	ENSG00000231394		lncRNA	chr7:55593777-5559
ENSG0000(	305	6.906379	chr7:251(	ENSG00000234338		Pseudoger	chr7:64835280-6483
ENSG0000(	305	6.906379	chr7:251(	snoU2_19		smallRNA	chr7:63175940-6317
ENSG0000(	305	6.906379	chr7:251(	ENSG00000284098		Pseudoger	chr7:57190300-5719
ENSG0000(	305	6.906379	chr7:251(	ENSG00000236574		Pseudoger	chr7:63843314-6384
ENSG0000(	305	6.906379	chr7:251(	ENSG00000225018		Pseudoger	chr7:55876738-5587
ENSG0000(	305	6.906379	chr7:251(	SAPCD2P4		Pseudoger	chr7:63113635-6311
ENSG0000(	305	6.906379	chr7:251(	SGO1P2		Pseudoger	chr7:52891837-5289
ENSG0000(	305	6.906379	chr7:251(	ZNF727	DriverDBv	protein_c	chr7:64045434-6408
ENSG0000(	305	6.906379	chr7:251(	ENSG00000236638		Pseudoger	chr7:63263065-6326
ENSG0000(	305	6.906379	chr7:251(	LINC01445		lncRNA	chr7:54330670-5445
ENSG0000(	305	6.906379	chr7:251(	ZNF680	IntOGen-I	protein_c	chr7:64519878-6456
ENSG0000(	305	6.906379	chr7:251(	VSTM2A		protein_c	chr7:54542325-5457
ENSG0000(	305	6.906379	chr7:251(	ENSG00000284558		Pseudoger	chr7:57191997-5719
ENSG0000(	305	6.906379	chr7:251(	NMD3P2		Pseudoger	chr7:56502284-5650
ENSG0000(	305	6.906379	chr7:251(	ENSG00000243981		Pseudoger	chr7:57628479-5764
ENSG0000(	305	6.906379	chr7:251(	LINC01005		lncRNA	chr7:64024409-6403
ENSG0000(	305	6.906379	chr7:251(	ENSG00000234716		Pseudoger	chr7:56577848-5657
ENSG0000(	305	6.906379	chr7:251(	RNU1-14P		smallRNA	chr7:53366058-5336
ENSG0000(	305	6.906379	chr7:251(	SEC61G-DT		lncRNA	chr7:54759313-5481
ENSG0000(	305	6.906379	chr7:251(	ENSG00000287317		lncRNA	chr7:64651655-6465
ENSG0000(	305	6.906379	chr7:251(	MTCO1P8		Pseudoger	chr7:64108625-6411
ENSG0000(	305	6.906379	chr7:251(	ENSG00000275875		Pseudoger	chr7:55741525-5574
ENSG0000(	305	6.906379	chr7:251(	MTCYBP5		Pseudoger	chr7:57196638-5719
ENSG0000(	305	6.906379	chr7:251(	ENSG00000230132		Pseudoger	chr7:63480598-6348
ENSG0000(	305	6.906379	chr7:251(	ENSG00000231484		Pseudoger	chr7:57828331-5783
ENSG0000(	305	6.906379	chr7:251(	ENSG00000236261		Pseudoger	chr7:57652875-5765
ENSG0000(	305	6.906379	chr7:251(	BNIP3P42		Pseudoger	chr7:64551498-6455
ENSG0000(	305	6.906379	chr7:251(	VN1R31P		Pseudoger	chr7:63367427-6336
ENSG0000(	305	6.906379	chr7:251(	RRBP1P1		Pseudoger	chr7:53002420-5300
ENSG0000(	305	6.906379	chr7:251(	ENSG00000236299		lncRNA	chr7:63888200-6390
ENSG0000(	305	6.906379	chr7:251(	ENSG00000229064		Pseudoger	chr7:65247608-6524
ENSG0000(	305	6.906379	chr7:251(	MTND5P6		Pseudoger	chr7:57170974-5717
ENSG0000(	305	6.906379	chr7:251(	ZNF138		protein_c	chr7:64794388-6483
ENSG0000(	305	6.906379	chr7:251(	ENSG00000284572		Pseudoger	chr7:57196056-5719
ENSG0000(	305	6.906379	chr7:251(	CCT6P1		Pseudoger	chr7:65751142-6576
ENSG0000(	305	6.906379	chr7:251(	CCT6P3		Pseudoger	chr7:65064999-6507
ENSG0000(	305	6.906379	chr7:251(	SNORA15		smallRNA	chr7:56060470-5606
ENSG0000(	305	6.906379	chr7:251(	ENSG00000290090		lncRNA	chr7:66114830-6611
ENSG0000(	305	6.906379	chr7:251(	ENSG00000286397		lncRNA	chr7:64035644-6403

ENSG0000(	305	6.906379	chr7:251(	VOPP1-DT		lncRNA	chr7:55573171-5558
ENSG0000(	305	6.906379	chr7:251(	ENSG00000224484		Pseudoger	chr7:57776972-5777
ENSG0000(	305	6.906379	chr7:251(	ENSG00000271696		Pseudoger	chr7:57835309-5783
ENSG0000(	305	6.906379	chr7:251(	ENSG00000232906		Pseudoger	chr7:65355934-6535
ENSG0000(	305	6.906379	chr7:251(	ENSG00000289108		lncRNA	chr7:65219563-6525
ENSG0000(	305	6.906379	chr7:251(	ENSG00000227305		lncRNA	chr7:63393745-6342
ENSG0000(	305	6.906379	chr7:251(	ARAFP3		Pseudoger	chr7:63342711-6334
ENSG0000(	305	6.906379	chr7:251(	ENSG00000232817		lncRNA	chr7:63388808-6339
ENSG0000(	305	6.906379	chr7:251(	ZNF117		protein_c	chr7:64971772-6500
ENSG0000(	305	6.906379	chr7:251(	ENSG00000282381		lncRNA	chr7:65269359-6531
ENSG0000(	305	6.906379	chr7:251(	ENSG00000291021		lncRNA	chr7:56875385-5688
ENSG0000(	305	6.906379	chr7:251(	GUSBP6		Pseudoger	chr7:64100305-6412
ENSG0000(	305	6.906379	chr7:251(	RNU6-1335P		smallRNA	chr7:56340231-5634
ENSG0000(	305	6.906379	chr7:251(	HAUS6P1		Pseudoger	chr7:53187388-5318
ENSG0000(	305	6.906379	chr7:251(	ENSG00000271047		Pseudoger	chr7:56603410-5660
ENSG0000(	305	6.906379	chr7:251(	CICP28		Pseudoger	chr7:56805336-5680
ENSG0000(	305	6.906379	chr7:251(	VN1R42P		Pseudoger	chr7:64933273-6493
ENSG0000(	305	6.906379	chr7:251(	ZNF716	DriverDB\	protein_c	chr7:57450177-5747
ENSG0000(	305	6.906379	chr7:251(	CICP17		Pseudoger	chr7:51382284-5138
ENSG0000(	305	6.906379	chr7:251(	BNIP3P11		Pseudoger	chr7:64678954-6468
ENSG0000(	305	6.906379	chr7:251(	ENSG00000226767		Pseudoger	chr7:65508773-6550
ENSG0000(	305	6.906379	chr7:251(	ENSG00000229762		Pseudoger	chr7:55810779-5581
ENSG0000(	305	6.906379	chr7:251(	MIR3147		smallRNA	chr7:57405025-5740
ENSG0000(	305	6.906379	chr7:251(	ZNF107		protein_c	chr7:64666099-6471
ENSG0000(	305	6.906379	chr7:251(	VN1R36P		Pseudoger	chr7:63965834-6396
ENSG0000(	305	6.906379	chr7:251(	ENSG00000203462		Pseudoger	chr7:56322804-5632
ENSG0000(	305	6.906379	chr7:251(	TNRC18P3		Pseudoger	chr7:56991888-5700
ENSG0000(	305	6.906379	chr7:251(	ENSG00000270957		Pseudoger	chr7:57485166-5748
ENSG0000(	305	6.906379	chr7:251(	VN1R32P		Pseudoger	chr7:63377329-6337
ENSG0000(	305	6.906379	chr7:251(	CCNJP1		Pseudoger	chr7:56228634-5622
ENSG0000(	305	6.906379	chr7:251(	ENSG00000230796		Pseudoger	chr7:57650381-5765
ENSG0000(	305	6.906379	chr7:251(	ENSG00000276650		Pseudoger	chr7:64425623-6442
ENSG0000(	305	6.906379	chr7:251(	MTND1P4		Pseudoger	chr7:57185788-5718
ENSG0000(	305	6.906379	chr7:251(	RNU6-1052P		smallRNA	chr7:56402070-5640
ENSG0000(	305	6.906379	chr7:251(	AC092685.1		smallRNA	chr7:65263414-6526
ENSG0000(	305	6.906379	chr7:251(	snoU13		smallRNA	chr7:56100659-5610
ENSG0000(	305	6.906379	chr7:251(	SUMF2	DriverDB\	protein_c	chr7:56064002-5608
ENSG0000(	305	6.906379	chr7:251(	ENSG00000233028		Pseudoger	chr7:56175634-5617
ENSG0000(	305	6.906379	chr7:251(	MIR4283-2		smallRNA	chr7:63621090-6362
ENSG0000(	305	6.906379	chr7:251(	ENSG00000282879		Pseudoger	chr7:56736396-5673
ENSG0000(	305	6.906379	chr7:251(	RNU6-912P		smallRNA	chr7:65814672-6581
ENSG0000(	305	6.906379	chr7:251(	ENSG00000230386		Pseudoger	chr7:65617082-6561
ENSG0000(	305	6.906379	chr7:251(	MTATP6P8		Pseudoger	chr7:57167200-5716
ENSG0000(	305	6.906379	chr7:251(	ENSG00000288798		lncRNA	chr7:51327615-5135
ENSG0000(	305	6.906379	chr7:251(	VN1R33P		Pseudoger	chr7:63401385-6340
ENSG0000(	305	6.906379	chr7:251(	ENSG00000223740		Pseudoger	chr7:56525428-5652
ENSG0000(	305	6.906379	chr7:251(	LINC02848		lncRNA	chr7:63900313-6392
ENSG0000(	305	6.906379	chr7:251(	ENSG00000226587		lncRNA	chr7:63326674-6335
ENSG0000(	305	6.906379	chr7:251(	AC104057.1		protein_c	chr7:65647167-6564
ENSG0000(	305	6.906379	chr7:251(	ENSG00000227397		Pseudoger	chr7:63789909-6379
ENSG0000(	305	6.906379	chr7:251(	SEPTIN7P15		Pseudoger	chr7:57067950-5707

ENSG0000(	305	6.906379	chr7:251(	ENSG00000232944		Pseudoger	chr7:56408699-5640
ENSG0000(	305	6.906379	chr7:251(	ENSG00000250618		Pseudoger	chr7:57817996-5781
ENSG0000(	305	6.906379	chr7:251(	TRIM60P17		Pseudoger	chr7:64085560-6408
ENSG0000(	305	6.906379	chr7:251(	RN7SL64P		smallRNA	chr7:55892726-5589
ENSG0000(	305	6.906379	chr7:251(	SEPTIN7P4		Pseudoger	chr7:63241590-6324
ENSG0000(	305	6.906379	chr7:251(	RAC1P9		Pseudoger	chr7:53779783-5378
ENSG0000(	305	6.906379	chr7:251(	GTF2IP5		Pseudoger	chr7:65773620-6580
ENSG0000(	305	6.906379	chr7:251(	ENSG00000229881		lncRNA	chr7:64140495-6415
ENSG0000(	305	6.906379	chr7:251(	ENSG00000229886		Pseudoger	chr7:66025126-6603
ENSG0000(	305	6.906379	chr7:251(	ENSG00000227113		Pseudoger	chr7:65075023-6507
ENSG0000(	305	6.906379	chr7:251(	TNRC18P2		Pseudoger	chr7:63567743-6358
ENSG0000(	305	6.906379	chr7:251(	ENSG00000244550		lncRNA	chr7:63233115-6324
ENSG0000(	305	6.906379	chr7:251(	AC073136.1		smallRNA	chr7:56269783-5626
ENSG0000(	305	6.906379	chr7:251(	CALM1P2		Pseudoger	chr7:55259809-5526
ENSG0000(	305	6.906379	chr7:251(	ENSG00000227080		Pseudoger	chr7:51388430-5138
ENSG0000(	305	6.906379	chr7:251(	ENSG00000232418		Pseudoger	chr7:53787167-5378
ENSG0000(	305	6.906379	chr7:251(	TRIM60P16		Pseudoger	chr7:56631739-5663
ENSG0000(	305	6.906379	chr7:251(	ZNF273		protein_c	chr7:64870172-6493
ENSG0000(	305	6.906379	chr7:251(	ENSG00000189316		lncRNA	chr7:64888527-6489
ENSG0000(	305	6.906379	chr7:251(	ENSG00000285544		lncRNA	chr7:64045429-6411
ENSG0000(	305	6.906379	chr7:251(	ZNF735		protein_c	chr7:64207203-6422
ENSG0000(	305	6.906379	chr7:251(	MTND5P7		Pseudoger	chr7:57194257-5719
ENSG0000(	305	6.906379	chr7:251(	MTATP6P18		Pseudoger	chr7:64106822-6410
ENSG0000(	305	6.906379	chr7:251(	ENSG00000277174		Pseudoger	chr7:56685036-5668
ENSG0000(	305	6.906379	chr7:251(	RNF138P2		Pseudoger	chr7:53316149-5331
ENSG0000(	305	6.906379	chr7:251(	ENSG00000232165		Pseudoger	chr7:63345840-6334
ENSG0000(	305	6.906379	chr7:251(	ENSG00000232161		Pseudoger	chr7:57770966-5777
ENSG0000(	305	6.906379	chr7:251(	ENSG00000227015		Pseudoger	chr7:57770619-5777
ENSG0000(	305	6.906379	chr7:251(	PHKG1		protein_c	chr7:56080283-5609
ENSG0000(	305	6.906379	chr7:251(	RNU6-1229P		smallRNA	chr7:65023204-6502
ENSG0000(	305	6.906379	chr7:251(	AC069285.1		smallRNA	chr7:63068087-6306
ENSG0000(	305	6.906379	chr7:251(	CCT6P3		lncRNA	chr7:65038354-6507
ENSG0000(	305	6.906379	chr7:251(	ENSG00000239985		Pseudoger	chr7:65038372-6503
ENSG0000(	305	6.906379	chr7:251(	LANCL2	DriverDB\	protein_c	chr7:55365337-5543
ENSG0000(	305	6.906379	chr7:251(	SEPTIN14		protein_c	chr7:55793540-5586
ENSG0000(	305	6.906379	chr7:251(	VOPP1	DriverDB\	protein_c	chr7:55436056-5557
ENSG0000(	305	6.906379	chr7:251(	YWHAEP1		Pseudoger	chr7:64433830-6443
ENSG0000(	305	6.906379	chr7:251(	ENSG00000285741		lncRNA	chr7:51471717-5172
ENSG0000(	305	6.906379	chr7:251(	CICP24		Pseudoger	chr7:63768257-6377
ENSG0000(	305	6.906379	chr7:251(	ENSG00000249773		protein_c	chr7:55887277-5595
ENSG0000(	305	6.906379	chr7:251(	ENSG00000224370		Pseudoger	chr7:56380976-5638
ENSG0000(	305	6.906379	chr7:251(	ARAFP2		Pseudoger	chr7:63404842-6340
ENSG0000(	305	6.906379	chr7:251(	INTS4P1		Pseudoger	chr7:65141241-6521
ENSG0000(	305	6.906379	chr7:251(	ENSG00000224365		Pseudoger	chr7:66004017-6600
ENSG0000(	305	6.906379	chr7:251(	VN1R28P		Pseudoger	chr7:57422830-5742
ENSG0000(	305	6.906379	chr7:251(	SEPHS1P1		Pseudoger	chr7:64852397-6485
ENSG0000(	305	6.906379	chr7:251(	ENSG00000223559		Pseudoger	chr7:56288230-5629
ENSG0000(	305	6.906379	chr7:251(	FKBP9P1		lncRNA	chr7:55681074-5568
ENSG0000(	305	6.906379	chr7:251(	ENSG00000230600		lncRNA	chr7:63380466-6338
ENSG0000(	305	6.906379	chr7:251(	RPL31P35		Pseudoger	chr7:54656358-5465
ENSG0000(	305	6.906379	chr7:251(	ENSG00000227148		lncRNA	chr7:63044827-6305

ENSG0000(	305	6.906379	chr7:251(	MRPS17	DriverDB\	protein_c	chr7:55951877-5595
ENSG0000(	305	6.906379	chr7:251(	ENSG00000285670		lncRNA	chr7:56214979-5622
ENSG0000(	305	6.906379	chr7:251(	VSTM2A-OT1		lncRNA	chr7:54556970-5457
ENSG0000(	305	6.906379	chr7:251(	MTND4LP2		Pseudoger	chr7:64105292-6410
ENSG0000(	305	6.906379	chr7:251(	RNU6-973P		smallRNA	chr7:65859660-6585
ENSG0000(	305	6.906379	chr7:251(	SEC61G	NCGv7	protein_c	chr7:54752250-5475
ENSG0000(	305	6.906379	chr7:251(	ENSG00000278205		Pseudoger	chr7:56462536-5646
ENSG0000(	305	6.906379	chr7:251(	TUBBP6		Pseudoger	chr7:55645620-5564
ENSG0000(	305	6.906379	chr7:251(	RN7SL855P		smallRNA	chr7:63611445-6361
ENSG0000(	305	6.906379	chr7:251(	ENSG00000176232		Pseudoger	chr7:63980385-6398
ENSG0000(	305	6.906379	chr7:251(	ASL	NCGv7	protein_c	chr7:66075800-6609
ENSG0000(	305	6.906379	chr7:251(	LINC01446		lncRNA	chr7:53655508-5381
ENSG0000(	305	6.906379	chr7:251(	SNORA31		smallRNA	chr7:52269437-5226
ENSG0000(	305	6.906379	chr7:251(	ENSG00000224172		Pseudoger	chr7:64735933-6473
ENSG0000(	305	6.906379	chr7:251(	ENSG00000237572		Pseudoger	chr7:63209219-6321
ENSG0000(	305	6.906379	chr7:251(	MTCO3P8		Pseudoger	chr7:64106065-6410
ENSG0000(	305	6.906379	chr7:251(	ENSG00000224653		Pseudoger	chr7:57819819-5782
ENSG0000(	305	6.906379	chr7:251(	ENSG00000205596		Pseudoger	chr7:66070904-6607
ENSG0000(	305	6.906379	chr7:251(	RNU6-1125P		smallRNA	chr7:54621025-5462
ENSG0000(	305	6.906379	chr7:251(	MTND4P2		Pseudoger	chr7:64104093-6410
ENSG0000(	305	6.906379	chr7:251(	ZNF722		protein_c	chr7:63998849-6401
ENSG0000(	305	6.906379	chr7:251(	MTND6P29		Pseudoger	chr7:57172772-5717
ENSG0000(	305	6.906379	chr7:251(	ENSG00000238124		lncRNA	chr7:65463071-6546
ENSG0000(	305	6.906379	chr7:251(	NMD3P1		Pseudoger	chr7:63908966-6391
ENSG0000(	305	6.906379	chr7:251(	LINC02854		lncRNA	chr7:53926676-5394
ENSG0000(	305	6.906379	chr7:251(	PHKG1P4		Pseudoger	chr7:57060590-5706
ENSG0000(	305	6.906379	chr7:251(	ZNF479	NCGv7	protein_c	chr7:57119614-5713
ENSG0000(	305	6.906379	chr7:251(	ENSG00000288525		Pseudoger	chr7:54676217-5467
ENSG0000(	305	6.906379	chr7:251(	NCOR1P3		Pseudoger	chr7:57599794-5761
ENSG0000(	305	6.906379	chr7:251(	VN1R40P		Pseudoger	chr7:64443802-6444
ENSG0000(	305	6.906379	chr7:251(	ENSG00000233288		lncRNA	chr7:56528253-5653
ENSG0000(	305	6.906379	chr7:251(	SNORA22		smallRNA	chr7:56055365-5605
ENSG0000(	305	6.906379	chr7:251(	ZNF733P		Pseudoger	chr7:63291518-6330
ENSG0000(	305	6.906379	chr7:251(	SNORA22		smallRNA	chr7:65755526-6575
ENSG0000(	305	6.906379	chr7:251(	MIR4283-1		smallRNA	chr7:56955785-5695
ENSG0000(	305	6.906379	chr7:251(	ROBO2P1		Pseudoger	chr7:51600286-5160
ENSG0000(	305	6.906379	chr7:251(	FKBP9P1		Pseudoger	chr7:55682652-5571
ENSG0000(	305	6.906379	chr7:251(	ENSG00000280920		lncRNA	chr7:54201224-5420
ENSG0000(	305	6.906379	chr7:251(	ENSG00000283431		Pseudoger	chr7:57163168-5716
ENSG0000(	305	6.906379	chr7:251(	ENSG00000233437		Pseudoger	chr7:56876500-5687
ENSG0000(	305	6.906379	chr7:251(	ENSG00000227923		Pseudoger	chr7:63354033-6335
ENSG0000(	305	6.906379	chr7:251(	ENSG00000287869		lncRNA	chr7:64867427-6487
ENSG0000(	305	6.906379	chr7:251(	VN1R34P		Pseudoger	chr7:63934449-6393
ENSG0000(	305	6.906379	chr7:251(	ENSG00000287953		lncRNA	chr7:54185071-5425
ENSG0000(	305	6.906379	chr7:251(	PSPHP1		Pseudoger	chr7:55764797-5577
ENSG0000(	305	6.906379	chr7:251(	CHCHD2	DriverDB\	protein_c	chr7:56101573-5610
ENSG0000(	305	6.906379	chr7:251(	ENSG00000287985		lncRNA	chr7:64369241-6437
ENSG0000(	305	6.906379	chr7:251(	ENSG00000224669		Pseudoger	chr7:64582733-6458
ENSG0000(	305	6.906379	chr7:251(	RNU6-1126P		smallRNA	chr7:55789368-5578
ENSG0000(	305	6.906379	chr7:251(	ENSG00000233454		Pseudoger	chr7:63057453-6306
ENSG0000(	305	6.906379	chr7:251(	ENSG00000227910		Pseudoger	chr7:63924787-6392

ENSG0000(	305	6.906379	chr7:251(	CICP11		Pseudoger	chr7:55736779-5573
ENSG0000(	305	6.906379	chr7:251(	SNORA15		smallRNA	chr7:65070538-6507
ENSG0000(	305	6.906379	chr7:251(	ENSG00000230000		lncRNA	chr7:63348861-6335
ENSG0000(	305	6.906379	chr7:251(	CRCP		protein_c	chr7:66114604-6615
ENSG0000(	305	6.906379	chr7:251(	MTDHP1		Pseudoger	chr7:64942143-6494
ENSG0000(	305	6.906379	chr7:251(	ENSG00000237639		Pseudoger	chr7:57650521-5765
ENSG0000(	305	6.906379	chr7:251(	ENSG00000226411		Pseudoger	chr7:63699390-6370
ENSG0000(	305	6.906379	chr7:251(	ENSG00000230936		Pseudoger	chr7:55342316-5534
ENSG0000(	305	6.906379	chr7:251(	PHKG1P1		Pseudoger	chr7:63233035-6323
ENSG0000(	305	6.906379	chr7:251(	ENSG00000218586		Pseudoger	chr7:54933699-5493
ENSG0000(	305	6.906379	chr7:251(	ENSG00000291124		lncRNA	chr7:65751037-6576
ENSG0000(	305	6.906379	chr7:251(	snoU13		smallRNA	chr7:54359892-5435
ENSG0000(	305	6.906379	chr7:251(	TRIM60P18		Pseudoger	chr7:64355078-6435
ENSG0000(	305	6.906379	chr7:251(	ENSG00000227545		Pseudoger	chr7:63349154-6335
ENSG0000(	305	6.906379	chr7:251(	RNU7-157P		smallRNA	chr7:57227001-5722
ENSG0000(	305	6.906379	chr7:251(	ENSG00000283230		Pseudoger	chr7:57168725-5716
ENSG0000(	305	6.906379	chr7:251(	ENSG00000250923		Pseudoger	chr7:57773183-5777
ENSG0000(	305	6.906379	chr7:251(	snoU13		smallRNA	chr7:52323798-5232
ENSG0000(	305	6.906379	chr7:251(	NUPR2		protein_c	chr7:56114681-5611
ENSG0000(	305	6.906379	chr7:251(	ELDR		lncRNA	chr7:55235965-5525
ENSG0000(	305	6.906379	chr7:251(	AC092634.2		smallRNA	chr7:63926836-6392
ENSG0000(	305	6.906379	chr7:251(	RBM22P3		Pseudoger	chr7:56490682-5649
ENSG0000(	305	6.906379	chr7:251(	BSNDP4		Pseudoger	chr7:57638725-5763
ENSG0000(	305	6.906379	chr7:251(	AC068533.1		smallRNA	chr7:66056234-6605
ENSG0000(	305	6.906379	chr7:251(	ENSG00000286342		lncRNA	chr7:64947571-6495
ENSG0000(	305	6.906379	chr7:251(	MTND4P5		Pseudoger	chr7:57192695-5719
ENSG0000(	305	6.906379	chr7:251(	ENSG00000280225		TEC	chr7:57402181-5740
ENSG0000(	305	6.906379	chr7:251(	GUSBP10		Pseudoger	chr7:57177409-5718
ENSG0000(	305	6.906379	chr7:251(	ENSG00000223836		Pseudoger	chr7:57820032-5782
ENSG0000(	305	6.906379	chr7:251(	ENSG00000227499		Pseudoger	chr7:55877912-5587
ENSG0000(	305	6.906379	chr7:251(	RN7SL292P		smallRNA	chr7:51716658-5171
ENSG0000(	305	6.906379	chr7:251(	ZNF713	DriverDB\	protein_c	chr7:55887456-5594
ENSG0000(	305	6.906379	chr7:251(	ENSG00000226401		Pseudoger	chr7:63632608-6363
ENSG0000(	305	6.906379	chr7:251(	ENSG00000229403		lncRNA	chr7:51614251-5163
ENSG0000(	305	6.906379	chr7:251(	ENSG00000276475		Pseudoger	chr7:64590599-6459
ENSG0000(	305	6.906379	chr7:251(	MTND4LP32		Pseudoger	chr7:57169143-5716
ENSG0000(	302	6.838448	chr3:619:	RN7SL4P		smallRNA	chr3:15738515-1573
ENSG0000(	301	6.815804	chr1:1021	ENSG00000274481		Pseudoger	chr1:1583510-15839
ENSG0000(	301	6.815804	chr1:1021	ENSG00000290854		lncRNA	chr1:1724838-17459
ENSG0000(	301	6.815804	chr1:1021	GNB1-DT		lncRNA	chr1:1891223-18927
ENSG0000(	301	6.815804	chr2:360{	MIR4268		smallRNA	chr2:219906502-219
ENSG0000(	301	6.815804	chr1:1021	ENSG00000227775		Pseudoger	chr1:1724512-17372
ENSG0000(	301	6.815804	chr1:1021	NADK	NCGv7	protein_c	chr1:1751232-17804
ENSG0000(	301	6.815804	chr1:1021	ENSG00000233542		lncRNA	chr1:2013213-20156
ENSG0000(	301	6.815804	chr1:1021	MMP23B		protein_c	chr1:1632163-16352
ENSG0000(	301	6.815804	chr1:1021	CALML6		protein_c	chr1:1915108-19172
ENSG0000(	301	6.815804	chr1:1021	ENSG00000286989		lncRNA	chr1:1600314-16027
ENSG0000(	301	6.815804	chr1:1021	MIB2		protein_c	chr1:1615454-16306
ENSG0000(	301	6.815804	chr1:1021	TMEM240		protein_c	chr1:1534778-15406
ENSG0000(	301	6.815804	chr1:1021	PRKCZ	NCGv7	protein_c	chr1:2050411-21853
ENSG0000(	301	6.815804	chr1:1021	TMEM52		protein_c	chr1:1917590-19192

ENSG0000(	301	6.815804	chr1:1021	PRKCZ-DT		lncRNA	chr1:2049203-20500
ENSG0000(	301	6.815804	chr1:1021	GNB1	NCGv7	protein_c	chr1:1785285-18922
ENSG0000(	301	6.815804	chr1:1021	SSU72		protein_c	chr1:1541673-15748
ENSG0000(	301	6.815804	chr1:1021	ENSG00000268575		lncRNA	chr1:1702736-17376
ENSG0000(	301	6.815804	chr1:1021	ENSG00000272106		lncRNA	chr1:1613758-16157
ENSG0000(	301	6.815804	chr1:1021	SLC35E2A		Pseudoger	chr1:1734690-17395
ENSG0000(	301	6.815804	chr1:1021	FNDC10		protein_c	chr1:1598012-16001
ENSG0000(	301	6.815804	chr1:1021	GABRD		protein_c	chr1:2019329-20307
ENSG0000(	301	6.815804	chr1:1021	CFAP74	NCGv7	protein_c	chr1:1921951-20038
ENSG0000(	301	6.815804	chr1:1021	CDK11A	NCGv7	protein_c	chr1:1702379-17243
ENSG0000(	301	6.815804	chr1:1021	ENSG00000215014		lncRNA	chr1:1574102-15770
ENSG0000(	300	6.79316	chr3:3227	ENSG00000229863		Pseudoger	chr3:57134142-5713
ENSG0000(	300	6.79316	chr9:1311	RNU6-881P		smallRNA	chr9:131126990-131
ENSG0000(	298	6.747872	chr5:4231	THBS4-AS1		lncRNA	chr5:80052374-8008
ENSG0000(	298	6.747872	chr5:4231	RASGRF2-AS1		lncRNA	chr5:80947694-8096
ENSG0000(	298	6.747872	chr5:4231	DHFR		protein_c	chr5:80626226-8065
ENSG0000(	298	6.747872	chr5:4231	RPL7P24		Pseudoger	chr5:80500332-8050
ENSG0000(	298	6.747872	chr5:4231	RBMX2P5		Pseudoger	chr5:80331573-8033
ENSG0000(	298	6.747872	chr5:4231	DBIP2		Pseudoger	chr5:80603383-8060
ENSG0000(	298	6.747872	chr5:4231	ENSG00000248967		Pseudoger	chr5:80746339-8074
ENSG0000(	298	6.747872	chr5:4231	KRT18P45		Pseudoger	chr5:80288449-8028
ENSG0000(	298	6.747872	chr5:4231	ENSG00000249829		Pseudoger	chr5:79191549-7919
ENSG0000(	298	6.747872	chr5:4231	LINC01455		lncRNA	chr5:79936579-7996
ENSG0000(	298	6.747872	chr5:4231	RNY3P1		smallRNA	chr5:79170234-7917
ENSG0000(	298	6.747872	chr5:4231	CKMT2-AS1		lncRNA	chr5:81201341-8130
ENSG0000(	298	6.747872	chr5:4231	snoU13		smallRNA	chr5:80239487-8023
ENSG0000(	298	6.747872	chr5:4231	SPZ1		protein_c	chr5:80319625-8032
ENSG0000(	298	6.747872	chr5:4231	FAM151B-DT		lncRNA	chr5:80411231-8048
ENSG0000(	298	6.747872	chr5:4231	RBX1P2		Pseudoger	chr5:80019609-8001
ENSG0000(	298	6.747872	chr5:4231	ENSG00000251675		lncRNA	chr5:80128361-8014
ENSG0000(	298	6.747872	chr5:4231	ENSG00000238254		Pseudoger	chr5:79849552-7984
ENSG0000(	298	6.747872	chr5:4231	ENSG00000249655		lncRNA	chr5:80630313-8063
ENSG0000(	298	6.747872	chr5:4231	ANKRD34B		protein_c	chr5:80556755-8057
ENSG0000(	298	6.747872	chr5:4231	ENSG00000288846		lncRNA	chr5:79543786-7954
ENSG0000(	298	6.747872	chr5:4231	ENSG00000251001		Pseudoger	chr5:80997183-8099
ENSG0000(	298	6.747872	chr5:4231	RPL7AP32		Pseudoger	chr5:79529302-7953
ENSG0000(	298	6.747872	chr5:4231	ENSG00000288741		lncRNA	chr5:80256200-8025
ENSG0000(	298	6.747872	chr5:4231	ENSG00000244076		Pseudoger	chr5:80315671-8031
ENSG0000(	298	6.747872	chr5:4231	HOMER1		protein_c	chr5:79372636-7951
ENSG0000(	298	6.747872	chr5:4231	JMY		protein_c	chr5:79236131-7932
ENSG0000(	298	6.747872	chr5:4231	FAM151B		protein_c	chr5:80487969-8054
ENSG0000(	298	6.747872	chr5:4231	SERINC5		protein_c	chr5:80111651-8025
ENSG0000(	298	6.747872	chr5:4231	ENSG00000289317		lncRNA	chr5:80407323-8040
ENSG0000(	298	6.747872	chr5:4231	ENSG00000244630		Pseudoger	chr5:80855507-8085
ENSG0000(	298	6.747872	chr5:4231	ARSB		protein_c	chr5:78777209-7898
ENSG0000(	298	6.747872	chr5:4231	TENT2		protein_c	chr5:79612120-7968
ENSG0000(	298	6.747872	chr5:4231	RNU6-211P		smallRNA	chr5:80365726-8036
ENSG0000(	298	6.747872	chr5:4231	ENSG00000250258		lncRNA	chr5:79774348-7981
ENSG0000(	298	6.747872	chr5:4231	TRMT112P2		Pseudoger	chr5:80033171-8003
ENSG0000(	298	6.747872	chr5:4231	Y_RNA		smallRNA	chr5:79534853-7953
ENSG0000(	298	6.747872	chr5:4231	ENSG00000249772		lncRNA	chr5:81113385-8111

ENSG0000(	298	6.747872	chr5:4231	MTX3		protein_c	chr5:79976716-7999
ENSG0000(	298	6.747872	chr5:4231	ENSG00000184188		Pseudoger	chr5:80299678-8030
ENSG0000(	298	6.747872	chr5:4231	ENSG00000250330		Pseudoger	chr5:79229904-7923
ENSG0000(	298	6.747872	chr5:4231	LINC01337		lncRNA	chr5:80608623-8062
ENSG0000(	298	6.747872	chr5:4231	SNORA18		smallRNA	chr5:79220942-7922
ENSG0000(	298	6.747872	chr5:4231	RASGRF2	NCGv7	protein_c	chr5:80960363-8123
ENSG0000(	298	6.747872	chr5:4231	MSH3	NCGv7	protein_c	chr5:80654652-8087
ENSG0000(	298	6.747872	chr5:4231	THBS4		protein_c	chr5:79991311-8008
ENSG0000(	298	6.747872	chr5:4231	CMYA5		protein_c	chr5:79689836-7980
ENSG0000(	298	6.747872	chr5:4231	SNORA31		smallRNA	chr5:80306235-8030
ENSG0000(	298	6.747872	chr5:4231	ZFYVE16		protein_c	chr5:80408013-8048
ENSG0000(	298	6.747872	chr5:4231	BHMT2	NCGv7	protein_c	chr5:79069767-7909
ENSG0000(	298	6.747872	chr5:4231	BHMT		protein_c	chr5:79111809-7913
ENSG0000(	298	6.747872	chr5:4231	DMGDH		protein_c	chr5:78997564-7923
ENSG0000(	298	6.747872	chr5:4231	RPS3AP20		Pseudoger	chr5:79284132-7928
ENSG0000(	298	6.747872	chr5:4231	ENSG00000213755		Pseudoger	chr5:79510434-7951
ENSG0000(	298	6.747872	chr5:4231	ENSG00000248569		Pseudoger	chr5:80351021-8035
ENSG0000(	298	6.747872	chr5:4231	ENSG00000286818		lncRNA	chr5:79840797-7984
ENSG0000(	298	6.747872	chr5:4231	HNRNPA1P12		Pseudoger	chr5:80359080-8036
ENSG0000(	298	6.747872	chr5:4231	ENSG00000214890		Pseudoger	chr5:79000112-7900
ENSG0000(	298	6.747872	chr5:4231	RPS27AP9		Pseudoger	chr5:80498534-8049
ENSG0000(	295	6.679941	chr7:251(	TPST1	DriverDB\	protein_c	chr7:66205317-6642
ENSG0000(	294	6.657297	chr6:197(	ENSG00000217776		Pseudoger	chr6:87070156-8707
ENSG0000(	294	6.657297	chr6:197(	ENSG00000218561		Pseudoger	chr6:86729708-8673
ENSG0000(	294	6.657297	chr6:197(	ENSG00000260574		lncRNA	chr6:81969453-8196
ENSG0000(	294	6.657297	chr6:197(	RNU6-130P		smallRNA	chr6:82210338-8221
ENSG0000(	294	6.657297	chr6:197(	ENSG00000218418		Pseudoger	chr6:80064286-8007
ENSG0000(	294	6.657297	chr6:197(	CEP162		protein_c	chr6:84124241-8422
ENSG0000(	294	6.657297	chr6:197(	ENSG00000218313		Pseudoger	chr6:87173368-8717
ENSG0000(	294	6.657297	chr6:197(	ENSG00000272129		lncRNA	chr6:80355424-8035
ENSG0000(	294	6.657297	chr6:197(	SYNCRIP	NCGv7	protein_c	chr6:85607779-8564
ENSG0000(	294	6.657297	chr6:197(	ENSG00000260645		lncRNA	chr6:80466958-8046
ENSG0000(	294	6.657297	chr6:197(	Y_RNA		smallRNA	chr6:85777639-8577
ENSG0000(	294	6.657297	chr6:197(	AK4P5		Pseudoger	chr6:80077729-8007
ENSG0000(	294	6.657297	chr6:197(	TPBG		protein_c	chr6:82363206-8236
ENSG0000(	294	6.657297	chr6:197(	ENSG00000217769		Pseudoger	chr6:86897118-8689
ENSG0000(	294	6.657297	chr6:197(	ENSG00000287294		lncRNA	chr6:84769010-8477
ENSG0000(	294	6.657297	chr6:197(	ENSG00000288071		lncRNA	chr6:80046715-8008
ENSG0000(	294	6.657297	chr6:197(	ENSG00000288021		lncRNA	chr6:85949690-8599
ENSG0000(	294	6.657297	chr6:197(	ENSG00000288009		lncRNA	chr6:89130887-8914
ENSG0000(	294	6.657297	chr6:197(	SMARCE1P2		Pseudoger	chr6:84429584-8443
ENSG0000(	294	6.657297	chr6:197(	ENSG00000217334		Pseudoger	chr6:85731371-8573
ENSG0000(	294	6.657297	chr6:197(	GAPDHP63		Pseudoger	chr6:79953005-7995
ENSG0000(	294	6.657297	chr6:197(	ENSG00000213204		protein_c	chr6:87408012-8751
ENSG0000(	294	6.657297	chr6:197(	ENSG00000272008		lncRNA	chr6:87151159-8715
ENSG0000(	294	6.657297	chr6:197(	ST13P16		Pseudoger	chr6:87444395-8744
ENSG0000(	294	6.657297	chr6:197(	ENSG00000219702		Pseudoger	chr6:81764211-8176
ENSG0000(	294	6.657297	chr6:197(	ENSG00000280232		TEC	chr6:85498441-8549
ENSG0000(	294	6.657297	chr6:197(	ME1		protein_c	chr6:83210402-8343
ENSG0000(	294	6.657297	chr6:197(	ENSG00000219604		Pseudoger	chr6:84689292-8468
ENSG0000(	294	6.657297	chr6:197(	ENSG00000279616		TEC	chr6:87356831-8735

ENSG0000(	294	6.657297	chr6:197(	snoU13		smallRNA	chr6:87720915-8772
ENSG0000(	294	6.657297	chr6:197(	RPL17P25		Pseudoger	chr6:80374015-8037
ENSG0000(	294	6.657297	chr6:197(	ENSG00000219500		Pseudoger	chr6:86432244-8643
ENSG0000(	294	6.657297	chr6:197(	RPSAP72		Pseudoger	chr6:80470071-8047
ENSG0000(	294	6.657297	chr6:197(	ENSG00000219190		Pseudoger	chr6:86768522-8676
ENSG0000(	294	6.657297	chr6:197(	PNRC1-DT		lncRNA	chr6:89080164-8908
ENSG0000(	294	6.657297	chr6:197(	RN7SL183P		smallRNA	chr6:87845650-8784
ENSG0000(	294	6.657297	chr6:197(	LINC02857		lncRNA	chr6:84019204-8402
ENSG0000(	294	6.657297	chr6:197(	ENSG00000218766		Pseudoger	chr6:85868953-8586
ENSG0000(	294	6.657297	chr6:197(	AL138919.1		smallRNA	chr6:87847545-8784
ENSG0000(	294	6.657297	chr6:197(	LINC02542		lncRNA	chr6:81844602-8216
ENSG0000(	294	6.657297	chr6:197(	TBX18	NCGv7	protein_c	chr6:84687351-8476
ENSG0000(	294	6.657297	chr6:197(	ENSG00000237874		lncRNA	chr6:83728055-8373
ENSG0000(	294	6.657297	chr6:197(	TENT5A		protein_c	chr6:81491439-8175
ENSG0000(	294	6.657297	chr6:197(	ZNF292	NCGv7	protein_c	chr6:87151803-8726
ENSG0000(	294	6.657297	chr6:197(	MTHFD2P2		Pseudoger	chr6:87000045-8700
ENSG0000(	294	6.657297	chr6:197(	PRSS35		protein_c	chr6:83512534-8352
ENSG0000(	294	6.657297	chr6:197(	SMIM8		protein_c	chr6:87322583-8739
ENSG0000(	294	6.657297	chr6:197(	NT5E		protein_c	chr6:85449584-8549
ENSG0000(	294	6.657297	chr6:197(	MRAP2		protein_c	chr6:84033772-8409
ENSG0000(	294	6.657297	chr6:197(	Y_RNA		smallRNA	chr6:87826173-8782
ENSG0000(	294	6.657297	chr6:197(	RN7SKP209		smallRNA	chr6:87083233-8708
ENSG0000(	294	6.657297	chr6:197(	ENSG00000234426		lncRNA	chr6:88177804-8844
ENSG0000(	294	6.657297	chr6:197(	AKIRIN2	AC	protein_c	chr6:87674860-8770
ENSG0000(	294	6.657297	chr6:197(	ENSG00000233835		lncRNA	chr6:79871873-7987
ENSG0000(	294	6.657297	chr6:197(	IBTK		protein_c	chr6:82169986-8224
ENSG0000(	294	6.657297	chr6:197(	ORC3		protein_c	chr6:87590067-8766
ENSG0000(	294	6.657297	chr6:197(	RPL7P29		Pseudoger	chr6:86970466-8697
ENSG0000(	294	6.657297	chr6:197(	TPT1P6		Pseudoger	chr6:85427453-8542
ENSG0000(	294	6.657297	chr6:197(	ENSG00000287705		lncRNA	chr6:83852312-8385
ENSG0000(	294	6.657297	chr6:197(	RCN1P1		Pseudoger	chr6:87121693-8712
ENSG0000(	294	6.657297	chr6:197(	ENSG00000279022		TEC	chr6:80440730-8044
ENSG0000(	294	6.657297	chr6:197(	TBX18-AS1		lncRNA	chr6:84687712-8470
ENSG0000(	294	6.657297	chr6:197(	RN7SL336P		smallRNA	chr6:89090946-8909
ENSG0000(	294	6.657297	chr6:197(	ENSG00000233967		lncRNA	chr6:80441295-8046
ENSG0000(	294	6.657297	chr6:197(	CGA		protein_c	chr6:87085498-8709
ENSG0000(	294	6.657297	chr6:197(	LINC01526		lncRNA	chr6:81813286-8181
ENSG0000(	294	6.657297	chr6:197(	LINC02535		lncRNA	chr6:85387219-8539
ENSG0000(	294	6.657297	chr6:197(	RNU4-72P		smallRNA	chr6:85751581-8575
ENSG0000(	294	6.657297	chr6:197(	ENSG00000226089		lncRNA	chr6:81527102-8153
ENSG0000(	294	6.657297	chr6:197(	RNGTT		protein_c	chr6:88609897-8896
ENSG0000(	294	6.657297	chr6:197(	RAB1AP2		Pseudoger	chr6:86170447-8617
ENSG0000(	294	6.657297	chr6:197(	GJB7		protein_c	chr6:87282980-8732
ENSG0000(	294	6.657297	chr6:197(	ENSG00000286875		lncRNA	chr6:82353861-8236
ENSG0000(	294	6.657297	chr6:197(	PNRC1	NCGv7	protein_c	chr6:89080751-8908
ENSG0000(	294	6.657297	chr6:197(	PM20D2		protein_c	chr6:89146055-8916
ENSG0000(	294	6.657297	chr6:197(	RNU6-444P		smallRNA	chr6:87488445-8748
ENSG0000(	294	6.657297	chr6:197(	RARS2		protein_c	chr6:87513459-8759
ENSG0000(	294	6.657297	chr6:197(	ENSG00000217060		Pseudoger	chr6:85286076-8529
ENSG0000(	294	6.657297	chr6:197(	RNU2-61P		smallRNA	chr6:89063500-8906
ENSG0000(	294	6.657297	chr6:197(	HSPD1P10		Pseudoger	chr6:87298772-8730

ENSG0000(	294	6.657297	chr6:1979	LINC01611		lncRNA	chr6:84421028-8455
ENSG0000(	294	6.657297	chr6:1979	SLC35A1		protein_c	chr6:87470623-8751
ENSG0000(	294	6.657297	chr6:1979	ENSG00000287816		lncRNA	chr6:79947810-7998
ENSG0000(	294	6.657297	chr6:1979	ENSG00000216902		Pseudoger	chr6:85257328-8525
ENSG0000(	294	6.657297	chr6:1979	CFAP206		protein_c	chr6:87407972-8746
ENSG0000(	294	6.657297	chr6:1979	SNX14		protein_c	chr6:85504776-8559
ENSG0000(	294	6.657297	chr6:1979	SNORA70		smallRNA	chr6:81764024-8176
ENSG0000(	294	6.657297	chr6:1979	ENSG00000216523		Pseudoger	chr6:84966397-8496
ENSG0000(	294	6.657297	chr6:1979	DUTP5		Pseudoger	chr6:85426657-8542
ENSG0000(	294	6.657297	chr6:1979	ENSG00000216352		Pseudoger	chr6:80555841-8055
ENSG0000(	294	6.657297	chr6:1979	ENSG00000286278		lncRNA	chr6:88047056-8804
ENSG0000(	294	6.657297	chr6:1979	ENSG00000216324		Pseudoger	chr6:87425795-8742
ENSG0000(	294	6.657297	chr6:1979	HTR1E	NCGv7	protein_c	chr6:86937528-8701
ENSG0000(	294	6.657297	chr6:1979	ENSG00000286871		lncRNA	chr6:88378280-8838
ENSG0000(	294	6.657297	chr6:1979	TTK	NCGv7	protein_c	chr6:80003887-8004
ENSG0000(	294	6.657297	chr6:1979	CYB5R4		protein_c	chr6:83859656-8396
ENSG0000(	294	6.657297	chr6:1979	ENSG00000289931		lncRNA	chr6:88963824-8896
ENSG0000(	294	6.657297	chr6:1979	ENSG00000228679		lncRNA	chr6:83983728-8400
ENSG0000(	294	6.657297	chr6:1979	PKMP3		Pseudoger	chr6:85659892-8566
ENSG0000(	294	6.657297	chr6:1979	RNA5SP210		Pseudoger	chr6:81622200-8162
ENSG0000(	294	6.657297	chr6:1979	ENSG00000279565		TEC	chr6:88172261-8817
ENSG0000(	294	6.657297	chr6:1979	ENSG00000181705		Pseudoger	chr6:80499196-8049
ENSG0000(	294	6.657297	chr6:1979	SPACA1		protein_c	chr6:88047841-8806
ENSG0000(	294	6.657297	chr6:1979	NDUFA5P9		Pseudoger	chr6:86214237-8621
ENSG0000(	294	6.657297	chr6:1979	ENSG00000232031		lncRNA	chr6:81724722-8173
ENSG0000(	294	6.657297	chr6:1979	ENSG00000280277		TEC	chr6:88964527-8896
ENSG0000(	294	6.657297	chr6:1979	ENSG00000220918		Pseudoger	chr6:79854684-7985
ENSG0000(	294	6.657297	chr6:1979	SNORA73		smallRNA	chr6:88714242-8871
ENSG0000(	294	6.657297	chr6:1979	RNU4-12P		smallRNA	chr6:85993021-8599
ENSG0000(	294	6.657297	chr6:1979	ENSG00000227215		lncRNA	chr6:82932601-8293
ENSG0000(	294	6.657297	chr6:1979	ENSG00000220960		Pseudoger	chr6:89059628-8906
ENSG0000(	294	6.657297	chr6:1979	AL391417.1		smallRNA	chr6:86473487-8647
ENSG0000(	294	6.657297	chr6:1979	RPL35AP18		Pseudoger	chr6:79964215-7996
ENSG0000(	294	6.657297	chr6:1979	CNR1		protein_c	chr6:88139864-8816
ENSG0000(	294	6.657297	chr6:1979	ENSG00000271114		Pseudoger	chr6:87891424-8789
ENSG0000(	294	6.657297	chr6:1979	RWDD2A		protein_c	chr6:83193357-8319
ENSG0000(	294	6.657297	chr6:1979	UBE3D		protein_c	chr6:82892390-8306
ENSG0000(	294	6.657297	chr6:1979	ELOVL4		protein_c	chr6:79914814-7994
ENSG0000(	294	6.657297	chr6:1979	AL391416.1		smallRNA	chr6:83367706-8336
ENSG0000(	294	6.657297	chr6:1979	ENSG00000285586		lncRNA	chr6:79868402-7987
ENSG0000(	294	6.657297	chr6:1979	SMIM11P1		Pseudoger	chr6:85735744-8573
ENSG0000(	294	6.657297	chr6:1979	SRSF12		protein_c	chr6:89095959-8911
ENSG0000(	294	6.657297	chr6:1979	DOP1A		protein_c	chr6:83067666-8317
ENSG0000(	294	6.657297	chr6:1979	AL132875.1		smallRNA	chr6:79826793-7982
ENSG0000(	294	6.657297	chr6:1979	BCKDHB	AC	protein_c	chr6:80106647-8034
ENSG0000(	294	6.657297	chr6:1979	KRT18P64		Pseudoger	chr6:85287789-8528
ENSG0000(	294	6.657297	chr6:1979	ENSG00000277797		lncRNA	chr6:81551686-8155
ENSG0000(	294	6.657297	chr6:1979	ENSG00000220537		Pseudoger	chr6:82263996-8226
ENSG0000(	294	6.657297	chr6:1979	PGM3		protein_c	chr6:83147324-8319
ENSG0000(	294	6.657297	chr6:1979	LAP3P1		Pseudoger	chr6:82924829-8292
ENSG0000(	294	6.657297	chr6:1979	C6orf163		protein_c	chr6:87344813-8736

ENSG0000(	294	6.657297	chr6:1979	SNAP91		protein_c	chr6:83552880-8370
ENSG0000(	294	6.657297	chr6:1979	ACTBP8		Pseudoger	chr6:88275882-8827
ENSG0000(	294	6.657297	chr6:1979	RN7SL643P		smallRNA	chr6:86435852-8643
ENSG0000(	294	6.657297	chr6:1979	ENSG00000220131		Pseudoger	chr6:88265158-8826
ENSG0000(	294	6.657297	chr6:1979	ENSG00000271793		protein_c	chr6:85504776-8567
ENSG0000(	294	6.657297	chr6:1979	RPL7P27		Pseudoger	chr6:86086415-8608
ENSG0000(	294	6.657297	chr6:1979	RIPPLY2		protein_c	chr6:83853360-8385
ENSG0000(	294	6.657297	chr6:1979	SNHG5		lncRNA	chr6:85650491-8567
ENSG0000(	294	6.657297	chr6:1979	ENSG00000220130		Pseudoger	chr6:89001548-8900
ENSG0000(	294	6.657297	chr6:1979	ENSG00000220240		Pseudoger	chr6:86897547-8689
ENSG0000(	294	6.657297	chr6:1979	ENSG00000220291		Pseudoger	chr6:85155667-8516
ENSG0000(	294	6.657297	chr6:1979	ENSG00000219951		Pseudoger	chr6:83392416-8339
ENSG0000(	293	6.634653	chr10:817	MIR4482-1		smallRNA	chr10:104268322-10
ENSG0000(	289	6.544078	chr6:1979	ENSG00000285598		lncRNA	chr6:145296545-145
ENSG0000(	289	6.544078	chr6:1979	PHACTR2-AS1		lncRNA	chr6:143554325-143
ENSG0000(	289	6.544078	chr6:1979	ENSG00000233138		lncRNA	chr6:142748443-142
ENSG0000(	289	6.544078	chr6:1979	ENSG00000237851		lncRNA	chr6:142788123-142
ENSG0000(	289	6.544078	chr6:1979	FUCA2	DriverDBv	protein_c	chr6:143494812-143
ENSG0000(	289	6.544078	chr6:1979	MRPL42P3		Pseudoger	chr6:144136450-144
ENSG0000(	289	6.544078	chr6:1979	ENSG00000217231		Pseudoger	chr6:144036618-144
ENSG0000(	289	6.544078	chr6:1979	ENSG00000225752		lncRNA	chr6:143094034-143
ENSG0000(	289	6.544078	chr6:1979	ENSG00000270655		Pseudoger	chr6:143386581-143
ENSG0000(	289	6.544078	chr6:1979	ENSG00000219409		Pseudoger	chr6:144397959-144
ENSG0000(	289	6.544078	chr6:1979	ENSG00000280148		protein_c	chr6:143857318-143
ENSG0000(	289	6.544078	chr6:1979	ENSG00000217195		Pseudoger	chr6:144706733-144
ENSG0000(	289	6.544078	chr6:1979	AIG1		protein_c	chr6:143060496-143
ENSG0000(	289	6.544078	chr6:1979	HIVEP2		protein_c	chr6:142751469-142
ENSG0000(	289	6.544078	chr6:1979	HIVEP2-DT		lncRNA	chr6:142946406-142
ENSG0000(	289	6.544078	chr6:1979	ENSG00000227192		lncRNA	chr6:143039425-143
ENSG0000(	289	6.544078	chr6:1979	LINC02919		lncRNA	chr6:142251847-142
ENSG0000(	289	6.544078	chr6:1979	ENSG00000270983		Pseudoger	chr6:142062717-142
ENSG0000(	289	6.544078	chr6:1979	TPT1P4		Pseudoger	chr6:144200447-144
ENSG0000(	289	6.544078	chr6:1979	ENSG00000216475		Pseudoger	chr6:144257034-144
ENSG0000(	289	6.544078	chr6:1979	ENSG00000270890		Pseudoger	chr6:143858062-143
ENSG0000(	289	6.544078	chr6:1979	GJE1		protein_c	chr6:142132925-142
ENSG0000(	289	6.544078	chr6:1979	ENSG00000217612		Pseudoger	chr6:145489630-145
ENSG0000(	289	6.544078	chr6:1979	LINC01277		lncRNA	chr6:142966293-143
ENSG0000(	289	6.544078	chr6:1979	ENSG00000217495		Pseudoger	chr6:143298770-143
ENSG0000(	289	6.544078	chr6:1979	ENSG00000257065		protein_c	chr6:143807092-143
ENSG0000(	289	6.544078	chr6:1979	NMBR-AS1		lncRNA	chr6:142088233-142
ENSG0000(	289	6.544078	chr6:1979	ENSG00000288836		lncRNA	chr6:144139977-144
ENSG0000(	289	6.544078	chr6:1979	SF3B5		protein_c	chr6:144094884-144
ENSG0000(	289	6.544078	chr6:1979	NMBR	NCGv7	protein_c	chr6:142058330-142
ENSG0000(	289	6.544078	chr6:1979	ENSG00000287084		lncRNA	chr6:142671972-142
ENSG0000(	289	6.544078	chr6:1979	TUBB8P2		Pseudoger	chr6:143436216-143
ENSG0000(	289	6.544078	chr6:1979	STX11		protein_c	chr6:144150487-144
ENSG0000(	289	6.544078	chr6:1979	UTRN		protein_c	chr6:144285335-144
ENSG0000(	289	6.544078	chr6:1979	RNA5SP221		Pseudoger	chr6:143449809-143
ENSG0000(	289	6.544078	chr6:1979	ENSG00000289850		lncRNA	chr6:144922315-144
ENSG0000(	289	6.544078	chr6:1979	PHACTR2	DriverDBv	protein_c	chr6:143536845-143
ENSG0000(	289	6.544078	chr6:1979	AL360007.1		smallRNA	chr6:142262643-142

ENSG0000(	289	6.544078	chr6:1979	ADGRG6	NCGv7	protein_c	chr6:142301854-142
ENSG0000(	289	6.544078	chr6:1979	PLAGL1		protein_c	chr6:143940300-144
ENSG0000(	289	6.544078	chr6:1979	LTV1	DriverDBv	protein_c	chr6:143843338-143
ENSG0000(	289	6.544078	chr6:1979	RNU1-33P		smallRNA	chr6:145463238-145
ENSG0000(	289	6.544078	chr6:1979	ENSG00000220739		Pseudoger	chr6:144708106-144
ENSG0000(	289	6.544078	chr6:1979	ENSG00000225311		lncRNA	chr6:144311699-144
ENSG0000(	289	6.544078	chr6:1979	VTA1		protein_c	chr6:142147162-142
ENSG0000(	289	6.544078	chr12:624	TUBB4BP1		Pseudoger	chr12:25440875-254
ENSG0000(	289	6.544078	chr6:1979	ZC2HC1B		protein_c	chr6:143864436-143
ENSG0000(	289	6.544078	chr6:1979	ADAT2		protein_c	chr6:143422832-143
ENSG0000(	289	6.544078	chr6:1979	PEX3	DriverDBv	protein_c	chr6:143450805-143
ENSG0000(	289	6.544078	chr6:1979	HYMAI		lncRNA	chr6:144004916-144
ENSG0000(	289	6.544078	chr6:1979	ENSG00000217648		Pseudoger	chr6:143342246-143
ENSG0000(	289	6.544078	chr6:1979	ENSG00000278206		lncRNA	chr6:143484979-143
ENSG0000(	289	6.544078	chr6:1979	ENSG00000236366		lncRNA	chr6:142526455-142
ENSG0000(	289	6.544078	chr6:1979	ENSG00000216642		Pseudoger	chr6:143327275-143
ENSG0000(	288	6.521434	chr22:220	ENSG00000232515		Pseudoger	chr22:22347807-223
ENSG0000(	288	6.521434	chr11:144	OR7E5P		Pseudoger	chr11:55979398-559
ENSG0000(	287	6.49879	chr17:289	ZNF652-AS1		lncRNA	chr17:49361150-493
ENSG0000(	287	6.49879	chr17:289	ENSG00000275025		lncRNA	chr17:50098460-500
ENSG0000(	287	6.49879	chr17:289	snoU13		smallRNA	chr17:50244896-502
ENSG0000(	287	6.49879	chr17:289	GNGT2		protein_c	chr17:49202791-492
ENSG0000(	287	6.49879	chr17:289	ENSG00000248702		Pseudoger	chr17:50797366-507
ENSG0000(	287	6.49879	chr17:289	ENSG00000272763		lncRNA	chr17:48635923-486
ENSG0000(	287	6.49879	chr17:289	ENSG00000290053		lncRNA	chr17:50793281-507
ENSG0000(	287	6.49879	chr17:289	ENSG00000250838		lncRNA	chr17:48995266-489
ENSG0000(	287	6.49879	chr17:289	XYLT2	DriverDBv	protein_c	chr17:50346126-503
ENSG0000(	287	6.49879	chr17:289	TMEM92-AS1		lncRNA	chr17:50281577-502
ENSG0000(	287	6.49879	chr17:289	ENSG00000251461		lncRNA	chr17:49004731-490
ENSG0000(	287	6.49879	chr17:289	ENSG00000244559		Pseudoger	chr17:48690481-486
ENSG0000(	287	6.49879	chr17:289	PHB1		protein_c	chr17:49404049-494
ENSG0000(	287	6.49879	chr17:289	Y_RNA		smallRNA	chr17:50696644-506
ENSG0000(	287	6.49879	chr17:289	ENSG00000230532		lncRNA	chr17:48931791-489
ENSG0000(	287	6.49879	chr17:289	LUC7L3		protein_c	chr17:50719565-507
ENSG0000(	287	6.49879	chr17:289	ABCC3	NCGv7	protein_c	chr17:50634777-506
ENSG0000(	287	6.49879	chr17:289	LRRC59	DriverDBv	protein_c	chr17:50375059-503
ENSG0000(	287	6.49879	chr17:289	MRPL27	DriverDBv	protein_c	chr17:50367857-503
ENSG0000(	287	6.49879	chr17:289	ENSG00000240995		Pseudoger	chr17:49148339-491
ENSG0000(	287	6.49879	chr17:289	TMEM92		protein_c	chr17:50271406-502
ENSG0000(	287	6.49879	chr17:289	EME1	DriverDBv	protein_c	chr17:50373220-503
ENSG0000(	287	6.49879	chr17:289	ENSG00000251550		Pseudoger	chr17:49037122-490
ENSG0000(	287	6.49879	chr17:289	ENSG00000250948		lncRNA	chr17:49375380-493
ENSG0000(	287	6.49879	chr17:289	ANKRD40CL		protein_c	chr17:50761029-507
ENSG0000(	287	6.49879	chr17:289	ENSG00000204584		lncRNA	chr17:49845910-498
ENSG0000(	287	6.49879	chr17:289	NGFR-AS1		lncRNA	chr17:49505657-495
ENSG0000(	287	6.49879	chr17:289	ENSG00000285829		lncRNA	chr17:50781306-507
ENSG0000(	287	6.49879	chr17:289	EIF4EP2		Pseudoger	chr17:49424269-494
ENSG0000(	287	6.49879	chr17:289	HOXB2		protein_c	chr17:48540894-485
ENSG0000(	287	6.49879	chr17:289	PHOSPHO1		protein_c	chr17:49223366-492
ENSG0000(	287	6.49879	chr17:289	ENSG00000249176		Pseudoger	chr17:49939122-499
ENSG0000(	287	6.49879	chr17:289	ENSG00000250976		lncRNA	chr17:50563203-505

ENSG0000(	287	6.49879	chr17:28(	ENSG00000254039		lncRNA	chr17:49914403-499
ENSG0000(	287	6.49879	chr17:28(	PRAC2		protein_c	chr17:48723185-487
ENSG0000(	287	6.49879	chr17:28(	EPN3	DriverDB\	protein_c	chr17:50532682-505
ENSG0000(	287	6.49879	chr17:28(	PDK2	NCGv7	protein_c	chr17:50094737-501
ENSG0000(	287	6.49879	chr17:28(	ACSF2		protein_c	chr17:50426158-504
ENSG0000(	287	6.49879	chr17:28(	B4GALNT2	DriverDB\	protein_c	chr17:49132460-491
ENSG0000(	287	6.49879	chr17:28(	ENSG00000270781		Pseudoger	chr17:48899131-488
ENSG0000(	287	6.49879	chr17:28(	ENSG00000248172		lncRNA	chr17:49996889-500
ENSG0000(	287	6.49879	chr17:28(	SGCA		protein_c	chr17:50164214-501
ENSG0000(	287	6.49879	chr17:28(	COL1A1	NCGv7;AC	protein_c	chr17:50184101-502
ENSG0000(	287	6.49879	chr17:28(	PPP1R9B	NCGv7	protein_c	chr17:50133737-501
ENSG0000(	287	6.49879	chr17:28(	LINC02086		lncRNA	chr17:48646923-487
ENSG0000(	287	6.49879	chr17:28(	snoU13		smallRNA	chr17:48956650-489
ENSG0000(	287	6.49879	chr17:28(	HOXB6		protein_c	chr17:48595751-486
ENSG0000(	287	6.49879	chr17:28(	NXPH3		protein_c	chr17:49575871-495
ENSG0000(	287	6.49879	chr17:28(	ANKRD40	DriverDB\	protein_c	chr17:50693198-507
ENSG0000(	287	6.49879	chr17:28(	ENSG00000250282		lncRNA	chr17:50100704-501
ENSG0000(	287	6.49879	chr17:28(	TAC4		protein_c	chr17:49838300-498
ENSG0000(	287	6.49879	chr17:28(	HOXB-AS2		lncRNA	chr17:48557262-485
ENSG0000(	287	6.49879	chr17:28(	SAMD14		protein_c	chr17:50110040-501
ENSG0000(	287	6.49879	chr17:28(	ENSG00000249451		lncRNA	chr17:50503412-505
ENSG0000(	287	6.49879	chr17:28(	Y_RNA		smallRNA	chr17:48479097-484
ENSG0000(	287	6.49879	chr17:28(	ENSG00000250286		lncRNA	chr17:50525520-505
ENSG0000(	287	6.49879	chr17:28(	HOXB-AS3		lncRNA	chr17:48549630-486
ENSG0000(	287	6.49879	chr17:28(	ENSG00000289021		lncRNA	chr17:48944890-489
ENSG0000(	287	6.49879	chr17:28(	SUMO2P17		Pseudoger	chr17:48874860-489
ENSG0000(	287	6.49879	chr17:28(	ENSG00000250310		lncRNA	chr17:49544788-495
ENSG0000(	287	6.49879	chr17:28(	HOXB7		protein_c	chr17:48607232-486
ENSG0000(	287	6.49879	chr17:28(	ENSG00000251239		lncRNA	chr17:50627032-506
ENSG0000(	287	6.49879	chr17:28(	ENSG00000250186		lncRNA	chr17:49404081-494
ENSG0000(	287	6.49879	chr17:28(	ENSG00000249406		lncRNA	chr17:50199876-502
ENSG0000(	287	6.49879	chr17:28(	ENSG00000276851		lncRNA	chr17:50094065-500
ENSG0000(	287	6.49879	chr17:28(	ENSG00000250751		lncRNA	chr17:49708334-497
ENSG0000(	287	6.49879	chr17:28(	DLX4	DriverDB\	protein_c	chr17:49968970-499
ENSG0000(	287	6.49879	chr17:28(	ABI3	NCGv7	protein_c	chr17:49210411-492
ENSG0000(	287	6.49879	chr17:28(	SUMO2P7		Pseudoger	chr17:50235845-502
ENSG0000(	287	6.49879	chr17:28(	ENSG00000279792		TEC	chr17:50362967-503
ENSG0000(	287	6.49879	chr17:28(	LINC02075		lncRNA	chr17:49457861-494
ENSG0000(	287	6.49879	chr17:28(	AC105030.1		smallRNA	chr17:49049540-490
ENSG0000(	287	6.49879	chr17:28(	Y_RNA		smallRNA	chr17:49416744-494
ENSG0000(	287	6.49879	chr17:28(	RNU1-42P		smallRNA	chr17:48949361-489
ENSG0000(	287	6.49879	chr17:28(	FLJ40194		lncRNA	chr17:49230853-493
ENSG0000(	287	6.49879	chr17:28(	CACNA1G-AS1		lncRNA	chr17:50556207-505
ENSG0000(	287	6.49879	chr17:28(	ZNF652	DriverDB\	protein_c	chr17:49289206-493
ENSG0000(	287	6.49879	chr17:28(	RN7SL125P		smallRNA	chr17:48765556-487
ENSG0000(	287	6.49879	chr17:28(	Metazoa_SRP		smallRNA	chr17:49109762-491
ENSG0000(	287	6.49879	chr17:28(	ENSG00000264451		lncRNA	chr17:48460370-484
ENSG0000(	287	6.49879	chr17:28(	ENSG00000253102		lncRNA	chr17:50396438-503
ENSG0000(	287	6.49879	chr17:28(	HOXB4		protein_c	chr17:48575507-485
ENSG0000(	287	6.49879	chr17:28(	Y_RNA		smallRNA	chr17:50386145-503
ENSG0000(	287	6.49879	chr17:28(	MRPS21P9		Pseudoger	chr17:50450179-504

ENSG0000(	287	6.49879	chr17:289	CACNA1G		protein_c	chr17:50560715-506
ENSG0000(	287	6.49879	chr17:289	SPATA20	DriverDB\	protein_c	chr17:50543058-505
ENSG0000(	287	6.49879	chr17:289	RNU6-826P		smallRNA	chr17:49036352-490
ENSG0000(	287	6.49879	chr17:289	HOXB-AS1		lncRNA	chr17:48543551-485
ENSG0000(	287	6.49879	chr17:289	ENSG00000257178		lncRNA	chr17:48579630-485
ENSG0000(	287	6.49879	chr17:289	SPOP	NCGv7	protein_c	chr17:49598884-496
ENSG0000(	287	6.49879	chr17:289	SLC35B1	DriverDB\	protein_c	chr17:49700934-497
ENSG0000(	287	6.49879	chr17:289	ENSG00000262039		lncRNA	chr17:49370740-494
ENSG0000(	287	6.49879	chr17:289	FAM117A	NCGv7	protein_c	chr17:49710332-497
ENSG0000(	287	6.49879	chr17:289	KAT7	NCGv7	protein_c	chr17:49788648-498
ENSG0000(	287	6.49879	chr17:289	CHAD		protein_c	chr17:50464496-504
ENSG0000(	287	6.49879	chr17:289	MIR196A1		smallRNA	chr17:48632490-486
ENSG0000(	287	6.49879	chr17:289	ENSG00000248895		Pseudoger	chr17:48881122-488
ENSG0000(	287	6.49879	chr17:289	ENSG00000236472		lncRNA	chr17:50135586-501
ENSG0000(	287	6.49879	chr17:289	ENSG00000261959		lncRNA	chr17:50163523-501
ENSG0000(	287	6.49879	chr17:289	PICART1		lncRNA	chr17:50050349-500
ENSG0000(	287	6.49879	chr17:289	MIR3185		smallRNA	chr17:48724408-487
ENSG0000(	287	6.49879	chr17:289	ENSG00000186244		Pseudoger	chr17:49400429-494
ENSG0000(	287	6.49879	chr17:289	RNU6-1313P		smallRNA	chr17:49966075-499
ENSG0000(	287	6.49879	chr17:289	SNORA68		smallRNA	chr17:48880840-488
ENSG0000(	287	6.49879	chr17:289	B4GALNT2P1		Pseudoger	chr17:49085511-490
ENSG0000(	287	6.49879	chr17:289	ENSG00000275897		lncRNA	chr17:50475819-504
ENSG0000(	287	6.49879	chr17:289	MYCBPAP		protein_c	chr17:50508384-505
ENSG0000(	287	6.49879	chr17:289	HOXB9		protein_c	chr17:48621156-486
ENSG0000(	287	6.49879	chr17:289	HOXB1		protein_c	chr17:48528526-485
ENSG0000(	287	6.49879	chr17:289	RP11-708H21.4		lncRNA	chr17:49171895-491
ENSG0000(	287	6.49879	chr17:289	ENSG00000262967		lncRNA	chr17:50693448-506
ENSG0000(	287	6.49879	chr17:289	HOXB3		protein_c	chr17:48548870-486
ENSG0000(	287	6.49879	chr17:289	RNU7-135P		smallRNA	chr17:49325840-493
ENSG0000(	287	6.49879	chr17:289	ENSG00000263176		lncRNA	chr17:50212933-502
ENSG0000(	287	6.49879	chr17:289	ENSG00000278963		TEC	chr17:50722074-507
ENSG0000(	287	6.49879	chr17:289	ENSG00000262837		lncRNA	chr17:49834239-498
ENSG0000(	287	6.49879	chr17:289	HOXB5		protein_c	chr17:48591257-485
ENSG0000(	287	6.49879	chr17:289	HOXB8		protein_c	chr17:48611377-486
ENSG0000(	287	6.49879	chr17:289	PRAC1		protein_c	chr17:48721719-487
ENSG0000(	287	6.49879	chr17:289	HOXB13		protein_c	chr17:48724763-487
ENSG0000(	287	6.49879	chr17:289	ATP5MC1		protein_c	chr17:48892765-488
ENSG0000(	287	6.49879	chr17:289	UBE2Z	DriverDB\	protein_c	chr17:48908407-489
ENSG0000(	287	6.49879	chr17:289	SNF8	DriverDB\	protein_c	chr17:48929316-489
ENSG0000(	287	6.49879	chr17:289	IGF2BP1	AC	protein_c	chr17:48997385-490
ENSG0000(	287	6.49879	chr17:289	GIP		protein_c	chr17:48958554-489
ENSG0000(	287	6.49879	chr17:289	TTLL6		protein_c	chr17:48762234-488
ENSG0000(	287	6.49879	chr17:289	ENSG00000253730		lncRNA	chr17:50158333-501
ENSG0000(	287	6.49879	chr17:289	COX6B1P2		Pseudoger	chr17:48713730-487
ENSG0000(	287	6.49879	chr17:289	H1-9P		Pseudoger	chr17:50171428-501
ENSG0000(	287	6.49879	chr17:289	AC006487.1		smallRNA	chr17:49561002-495
ENSG0000(	287	6.49879	chr17:289	HOXB-AS4		lncRNA	chr17:48628675-486
ENSG0000(	287	6.49879	chr17:289	ENSG00000242407		lncRNA	chr17:48733091-487
ENSG0000(	287	6.49879	chr17:289	ITGA3	AC	protein_c	chr17:50055968-500
ENSG0000(	287	6.49879	chr17:289	CALCOCO2	DriverDB\	protein_c	chr17:48831018-488
ENSG0000(	287	6.49879	chr17:289	NGFR		protein_c	chr17:49495293-495

ENSG0000(	287	6.49879	chr17:289	ENSG00000248954		lncRNA	chr17:49887598-499
ENSG0000(	287	6.49879	chr17:289	RSAD1		protein_c	chr17:50478860-504
ENSG0000(	287	6.49879	chr17:289	ENSG00000279036		TEC	chr17:49494223-494
ENSG0000(	287	6.49879	chr17:289	SRP14P3		Pseudoger	chr17:49795343-497
ENSG0000(	287	6.49879	chr17:289	DLX3	DriverDB\	protein_c	chr17:49990005-499
ENSG0000(	285	6.453502	chr16:239	LINC01567		lncRNA	chr16:24661422-246
ENSG0000(	285	6.453502	chr3:6192	TPRXL		Pseudoger	chr3:13937273-1408
ENSG0000(	285	6.453502	chr3:6192	LINC00620		lncRNA	chr3:13650696-1374
ENSG0000(	285	6.453502	chr3:6192	COL6A4P1		Pseudoger	chr3:15151833-1518
ENSG0000(	285	6.453502	chr3:6192	ENSG00000235240		Pseudoger	chr3:11488300-1148
ENSG0000(	285	6.453502	chr3:6192	ENSG00000286962		lncRNA	chr3:10914196-1097
ENSG0000(	285	6.453502	chr3:6192	ENSG00000268279		protein_c	chr3:14135217-1414
ENSG0000(	285	6.453502	chr3:6192	ENSG00000226621		Pseudoger	chr3:11290406-1129
ENSG0000(	285	6.453502	chr3:6192	ENSG00000290072		lncRNA	chr3:12664310-1266
ENSG0000(	285	6.453502	chr3:6192	IQSEC1		protein_c	chr3:12897043-1328
ENSG0000(	285	6.453502	chr3:6192	ENSG00000270409		Pseudoger	chr3:15732252-1573
ENSG0000(	285	6.453502	chr16:239	ENSG00000279276		TEC	chr16:23962951-239
ENSG0000(	285	6.453502	chr3:6192	AC093611.1		smallRNA	chr3:13738060-1373
ENSG0000(	285	6.453502	chr3:6192	ENSG00000285906		lncRNA	chr3:11193439-1122
ENSG0000(	285	6.453502	chr16:239	RBBP6	NCGv7	protein_c	chr16:24537693-245
ENSG0000(	285	6.453502	chr16:239	AC008938.1		smallRNA	chr16:24438988-244
ENSG0000(	285	6.453502	chr3:6192	METTL6		protein_c	chr3:15381275-1544
ENSG0000(	285	6.453502	chr3:6192	COLQ		protein_c	chr3:15450133-1552
ENSG0000(	285	6.453502	chr3:6192	ANKRD28		protein_c	chr3:15667236-1585
ENSG0000(	285	6.453502	chr3:6192	LINC00606		lncRNA	chr3:10759350-1076
ENSG0000(	285	6.453502	chr3:6192	FGD5P1		lncRNA	chr3:13933056-1393
ENSG0000(	285	6.453502	chr3:6192	MIR3134		smallRNA	chr3:15697298-1569
ENSG0000(	285	6.453502	chr3:6192	ENSG00000288952		lncRNA	chr3:12178694-1223
ENSG0000(	285	6.453502	chr3:6192	ENSG00000288969		lncRNA	chr3:11183856-1118
ENSG0000(	285	6.453502	chr3:6192	AC093611.2		smallRNA	chr3:13717918-1371
ENSG0000(	285	6.453502	chr3:6192	SNORA7A		smallRNA	chr3:12840312-1284
ENSG0000(	285	6.453502	chr3:6192	IMPDH1P8		Pseudoger	chr3:15878047-1587
ENSG0000(	285	6.453502	chr3:6192	HDAC11		protein_c	chr3:13479724-1350
ENSG0000(	285	6.453502	chr3:6192	ENSG00000272529		lncRNA	chr3:16345126-1634
ENSG0000(	285	6.453502	chr3:6192	COL6A4P1		lncRNA	chr3:15165275-1520
ENSG0000(	285	6.453502	chr3:6192	ENSG00000223398		Pseudoger	chr3:13460424-1346
ENSG0000(	285	6.453502	chr3:6192	SYN2	NCGv7	protein_c	chr3:12004388-1219
ENSG0000(	285	6.453502	chr3:6192	TIMP4		protein_c	chr3:12153068-1215
ENSG0000(	285	6.453502	chr3:6192	ENSG00000287059		lncRNA	chr3:14038647-1404
ENSG0000(	285	6.453502	chr3:6192	ENSG00000272498		lncRNA	chr3:16339308-1633
ENSG0000(	285	6.453502	chr16:239	ENSG00000279756		TEC	chr16:23753689-237
ENSG0000(	285	6.453502	chr3:6192	ENSG00000271716		lncRNA	chr3:11607184-1161
ENSG0000(	285	6.453502	chr3:6192	HRH1		protein_c	chr3:11137093-1126
ENSG0000(	285	6.453502	chr3:6192	ACTG1P12		Pseudoger	chr3:12070195-1207
ENSG0000(	285	6.453502	chr3:6192	SLC6A1		protein_c	chr3:10992186-1103
ENSG0000(	285	6.453502	chr3:6192	ENSG00000272483		lncRNA	chr3:11611602-1161
ENSG0000(	285	6.453502	chr3:6192	RHBDF1P1		Pseudoger	chr3:14572852-1457
ENSG0000(	285	6.453502	chr3:6192	snoU13		smallRNA	chr3:15316620-1531
ENSG0000(	285	6.453502	chr3:6192	Y_RNA		smallRNA	chr3:15237000-1523
ENSG0000(	285	6.453502	chr3:6192	MTCO1P5		Pseudoger	chr3:12165299-1216
ENSG0000(	285	6.453502	chr3:6192	ENSG00000290787		lncRNA	chr3:15732827-1573

ENSG0000(	285	6.453502	chr3:619:	ENSG00000287964		lncRNA	chr3:11060973-1109
ENSG0000(	285	6.453502	chr3:619:	ENSG00000287377		lncRNA	chr3:16361348-1637
ENSG0000(	285	6.453502	chr3:619:	ENSG00000269391		lncRNA	chr3:16524800-1653
ENSG0000(	285	6.453502	chr3:619:	ENSG00000271964		lncRNA	chr3:16314439-1631
ENSG0000(	285	6.453502	chr16:23!	ENSG00000260072		lncRNA	chr16:24472798-244
ENSG0000(	285	6.453502	chr16:23!	ENSG00000279202		TEC	chr16:24098239-240
ENSG0000(	285	6.453502	chr16:23!	AC008731.1		Pseudoger	chr16:24919393-249
ENSG0000(	285	6.453502	chr3:619:	LSM3		protein_c	chr3:14178817-1420
ENSG0000(	285	6.453502	chr3:619:	ENSG00000289809		protein_c	chr3:12825768-1287
ENSG0000(	285	6.453502	chr3:619:	MKRN2	DriverDBv	protein_c	chr3:12557057-1258
ENSG0000(	285	6.453502	chr3:619:	ENSG00000250939		Pseudoger	chr3:12850659-1285
ENSG0000(	285	6.453502	chr3:619:	GSTM5P1		Pseudoger	chr3:12257801-1225
ENSG0000(	285	6.453502	chr16:23!	ERN2	NCGv7	protein_c	chr16:23690310-237
ENSG0000(	285	6.453502	chr3:619:	ENSG00000290932		lncRNA	chr3:15151843-1515
ENSG0000(	285	6.453502	chr3:619:	FGD5P1		Pseudoger	chr3:13934255-1393
ENSG0000(	285	6.453502	chr3:619:	ENSG00000251038		Pseudoger	chr3:11909634-1191
ENSG0000(	285	6.453502	chr3:619:	HMGN2P7		Pseudoger	chr3:15375373-1537
ENSG0000(	285	6.453502	chr3:619:	CDYLP1		Pseudoger	chr3:16692745-1669
ENSG0000(	285	6.453502	chr3:619:	VGLL4		protein_c	chr3:11556069-1177
ENSG0000(	285	6.453502	chr3:619:	RN7SL110P		smallRNA	chr3:15506625-1550
ENSG0000(	285	6.453502	chr3:619:	CRIP1P1		Pseudoger	chr3:12673699-1267
ENSG0000(	285	6.453502	chr3:619:	RPL32		protein_c	chr3:12834485-1284
ENSG0000(	285	6.453502	chr3:619:	RNU6-454P		smallRNA	chr3:15339749-1533
ENSG0000(	285	6.453502	chr3:619:	TAMM41	NCGv7	protein_c	chr3:11790442-1184
ENSG0000(	285	6.453502	chr3:619:	XPC-AS1		lncRNA	chr3:14144637-1416
ENSG0000(	285	6.453502	chr3:619:	ENSG00000290044		lncRNA	chr3:14144198-1414
ENSG0000(	285	6.453502	chr3:619:	TMEM43		protein_c	chr3:14125015-1414
ENSG0000(	285	6.453502	chr16:23!	PRKCB	NCGv7	protein_c	chr16:23835983-242
ENSG0000(	285	6.453502	chr3:619:	ENSG00000288077		lncRNA	chr3:15501439-1550
ENSG0000(	285	6.453502	chr3:619:	CHCHD4P4		Pseudoger	chr3:11234256-1123
ENSG0000(	285	6.453502	chr3:619:	RNU6-1024P		smallRNA	chr3:15433110-1543
ENSG0000(	285	6.453502	chr16:23!	ENSG00000261583		lncRNA	chr16:23711990-237
ENSG0000(	285	6.453502	chr3:619:	NR2C2		protein_c	chr3:14947583-1505
ENSG0000(	285	6.453502	chr3:619:	MARK2P14		Pseudoger	chr3:11906527-1191
ENSG0000(	285	6.453502	chr16:23!	ENSG00000260714		Pseudoger	chr16:25015978-250
ENSG0000(	285	6.453502	chr3:619:	KRT18P17		Pseudoger	chr3:12787393-1278
ENSG0000(	285	6.453502	chr3:619:	MKRN2OS	DriverDBv	protein_c	chr3:12514934-1256
ENSG0000(	285	6.453502	chr3:619:	SH3BP5-AS1		lncRNA	chr3:15254184-1526
ENSG0000(	285	6.453502	chr3:619:	CYCSP12		Pseudoger	chr3:11878830-1187
ENSG0000(	285	6.453502	chr3:619:	ENSG00000285969		lncRNA	chr3:14791534-1479
ENSG0000(	285	6.453502	chr3:619:	CAND2		protein_c	chr3:12796472-1283
ENSG0000(	285	6.453502	chr3:619:	ENSG00000229271		lncRNA	chr3:16647666-1669
ENSG0000(	285	6.453502	chr3:619:	VN1R20P		Pseudoger	chr3:13926813-1392
ENSG0000(	285	6.453502	chr3:619:	ENSG00000283392		lncRNA	chr3:14602035-1464
ENSG0000(	285	6.453502	chr3:619:	ENSG00000279465		TEC	chr3:15731877-1573
ENSG0000(	285	6.453502	chr3:619:	ENSG00000255021		lncRNA	chr3:14272373-1430
ENSG0000(	285	6.453502	chr3:619:	FANCD2P2		Pseudoger	chr3:11859674-1189
ENSG0000(	285	6.453502	chr3:619:	RNA5SP124		Pseudoger	chr3:14394648-1439
ENSG0000(	285	6.453502	chr3:619:	ENSG00000288050		lncRNA	chr3:16444540-1644
ENSG0000(	285	6.453502	chr16:23!	SLC5A11		protein_c	chr16:24845841-249
ENSG0000(	285	6.453502	chr3:619:	LINC02922		lncRNA	chr3:14648194-1464

ENSG0000(	285	6.453502	chr3:619?	RPS3AP53		Pseudoger	chr3:15141380-1514
ENSG0000(	285	6.453502	chr16:23?	ENSG00000289171		lncRNA	chr16:24550351-245
ENSG0000(	285	6.453502	chr3:619?	OXNAD1	NCGv7	protein_c	chr3:16265160-1635
ENSG0000(	285	6.453502	chr3:619?	ENSG00000287042		lncRNA	chr3:15860112-1613
ENSG0000(	285	6.453502	chr16:23?	ARHGAP17		protein_c	chr16:24919389-250
ENSG0000(	285	6.453502	chr3:619?	AC010139.1		smallRNA	chr3:16619521-1661
ENSG0000(	285	6.453502	chr3:619?	SLC6A11	NCGv7	protein_c	chr3:10816201-1094
ENSG0000(	285	6.453502	chr3:619?	CAPN7		protein_c	chr3:15206152-1525
ENSG0000(	285	6.453502	chr3:619?	HACL1		protein_c	chr3:15560699-1560
ENSG0000(	285	6.453502	chr3:619?	EAF1-AS1		lncRNA	chr3:15436171-1545
ENSG0000(	285	6.453502	chr3:619?	PPARG	NCGv7;AC	protein_c	chr3:12287368-1243
ENSG0000(	285	6.453502	chr3:619?	NUP210	DriverDB\	protein_c	chr3:13316235-1342
ENSG0000(	285	6.453502	chr3:619?	RNU6-905P		smallRNA	chr3:14530982-1453
ENSG0000(	285	6.453502	chr3:619?	DPH3	NCGv7	protein_c	chr3:16257061-1626
ENSG0000(	285	6.453502	chr3:619?	HDAC11-AS1		lncRNA	chr3:13476982-1348
ENSG0000(	285	6.453502	chr3:619?	MIR563		smallRNA	chr3:15873771-1587
ENSG0000(	285	6.453502	chr3:619?	BTD		protein_c	chr3:15601341-1572
ENSG0000(	285	6.453502	chr3:619?	MIR4270		smallRNA	chr3:15496239-1549
ENSG0000(	285	6.453502	chr3:619?	AC090954.1		smallRNA	chr3:15134383-1513
ENSG0000(	285	6.453502	chr3:619?	RFTN1	NCGv7	protein_c	chr3:16313574-1651
ENSG0000(	285	6.453502	chr3:619?	C3orf20		protein_c	chr3:14675141-1477
ENSG0000(	285	6.453502	chr3:619?	RBSN		protein_c	chr3:15070073-1509
ENSG0000(	285	6.453502	chr3:619?	NUP210P2		Pseudoger	chr3:11900011-1190
ENSG0000(	285	6.453502	chr3:619?	LINC02022		lncRNA	chr3:12877522-1288
ENSG0000(	285	6.453502	chr3:619?	ENSG00000228874		Pseudoger	chr3:15131848-1513
ENSG0000(	285	6.453502	chr3:619?	LINC02011		lncRNA	chr3:14799331-1481
ENSG0000(	285	6.453502	chr3:619?	ENSG00000286689		lncRNA	chr3:15970003-1597
ENSG0000(	285	6.453502	chr16:23?	CHP2		protein_c	chr16:23755026-237
ENSG0000(	285	6.453502	chr3:619?	RNU6-404P		smallRNA	chr3:12501623-1250
ENSG0000(	285	6.453502	chr3:619?	RNU6-377P		smallRNA	chr3:12514706-1251
ENSG0000(	285	6.453502	chr3:619?	ENSG00000270739		Pseudoger	chr3:16275060-1627
ENSG0000(	285	6.453502	chr3:619?	LINC01267		lncRNA	chr3:14348451-1435
ENSG0000(	285	6.453502	chr3:619?	RAF1	NCGv7;AC	protein_c	chr3:12582101-1266
ENSG0000(	285	6.453502	chr3:619?	ATG7	NCGv7	protein_c	chr3:11272309-1155
ENSG0000(	285	6.453502	chr3:619?	SLC6A6		protein_c	chr3:14402576-1448
ENSG0000(	285	6.453502	chr3:619?	DAZL		protein_c	chr3:16586792-1667
ENSG0000(	285	6.453502	chr3:619?	GALNT15		protein_c	chr3:16174680-1623
ENSG0000(	285	6.453502	chr3:619?	ENSG00000225026		Pseudoger	chr3:12328003-1232
ENSG0000(	285	6.453502	chr3:619?	RNA5SP123		Pseudoger	chr3:12511095-1251
ENSG0000(	285	6.453502	chr16:23?	CACNG3		protein_c	chr16:24256335-243
ENSG0000(	285	6.453502	chr3:619?	SH3BP5		protein_c	chr3:15254353-1534
ENSG0000(	285	6.453502	chr3:619?	ENSG00000230599		lncRNA	chr3:10767464-1077
ENSG0000(	285	6.453502	chr16:23?	TNRC6A	NCGv7	protein_c	chr16:24610209-248
ENSG0000(	285	6.453502	chr3:619?	RN7SL147P		smallRNA	chr3:11952790-1195
ENSG0000(	285	6.453502	chr16:23?	LINC02194		lncRNA	chr16:24236104-242
ENSG0000(	285	6.453502	chr3:619?	AC137674.2		smallRNA	chr3:30138960-3013
ENSG0000(	285	6.453502	chr16:23?	RNU7-24P		smallRNA	chr16:24400056-244
ENSG0000(	285	6.453502	chr3:619?	TSEN2	DriverDB\	protein_c	chr3:12484421-1254
ENSG0000(	285	6.453502	chr3:619?	ENSG00000231362		Pseudoger	chr3:13052967-1305
ENSG0000(	285	6.453502	chr16:23?	SNORA1		smallRNA	chr16:24333415-243
ENSG0000(	285	6.453502	chr16:23?	ENSG00000278912		TEC	chr16:23696665-236

ENSG0000(	285	6.453502	chr3:619	GRIP2		protein_c	chr3:14489107-1455
ENSG0000(	285	6.453502	chr16:23	ENSG00000261669		lncRNA	chr16:24803451-248
ENSG0000(	285	6.453502	chr3:619	TMEM40	NCGv7	protein_c	chr3:12733528-1276
ENSG0000(	285	6.453502	chr3:619	LINC00690		lncRNA	chr3:16524771-1654
ENSG0000(	285	6.453502	chr3:619	FBLN2	NCGv7	protein_c	chr3:13549125-1363
ENSG0000(	285	6.453502	chr16:23	ENSG00000279554		TEC	chr16:24208105-242
ENSG0000(	285	6.453502	chr3:619	VN1R21P		Pseudoger	chr3:14089107-1408
ENSG0000(	285	6.453502	chr3:619	FGD5-AS1		lncRNA	chr3:14920347-1494
ENSG0000(	285	6.453502	chr3:619	EAF1		protein_c	chr3:15427598-1545
ENSG0000(	285	6.453502	chr3:619	WNT7A		protein_c	chr3:13816258-1388
ENSG0000(	285	6.453502	chr3:619	XPC	NCGv7;AC	protein_c	chr3:14145147-1417
ENSG0000(	285	6.453502	chr3:619	ENSG00000270531		Pseudoger	chr3:11529488-1152
ENSG0000(	285	6.453502	chr3:619	ENSG00000230172		lncRNA	chr3:14764952-1476
ENSG0000(	285	6.453502	chr3:619	CHCHD4		protein_c	chr3:14112077-1412
ENSG0000(	285	6.453502	chr3:619	MRPS25		protein_c	chr3:15009611-1506
ENSG0000(	285	6.453502	chr3:619	CCDC174		protein_c	chr3:14651762-1467
ENSG0000(	285	6.453502	chr3:619	FGD5	IntOGen-I	protein_c	chr3:14810853-1493
ENSG0000(	285	6.453502	chr3:619	snoU13		smallRNA	chr3:12632734-1263
ENSG0000(	285	6.453502	chr3:619	SLC6A1-AS1		lncRNA	chr3:11006098-1101
ENSG0000(	285	6.453502	chr3:619	ENSG00000289750		lncRNA	chr3:15048548-1509
ENSG0000(	284	6.430858	chr3:322	MIR1226		smallRNA	chr3:47849555-4784
ENSG0000(	283	6.408214	chr2:360	RUFY4		protein_c	chr2:218034960-218
ENSG0000(	283	6.408214	chr2:360	PSMB3P2		Pseudoger	chr2:216610342-216
ENSG0000(	283	6.408214	chr2:360	STK16		protein_c	chr2:219245455-219
ENSG0000(	283	6.408214	chr2:360	ABCB6		protein_c	chr2:219209772-219
ENSG0000(	283	6.408214	chr2:360	CNPPD1		protein_c	chr2:219171897-219
ENSG0000(	283	6.408214	chr2:360	ENSG00000279348		TEC	chr2:216211404-216
ENSG0000(	283	6.408214	chr2:360	ENSG00000280323		TEC	chr2:219427477-219
ENSG0000(	283	6.408214	chr2:360	RETREG2		protein_c	chr2:219176225-219
ENSG0000(	283	6.408214	chr2:360	ENSG00000231597		lncRNA	chr2:216854389-216
ENSG0000(	283	6.408214	chr2:360	TUBA4B		protein_c	chr2:219253243-219
ENSG0000(	283	6.408214	chr2:360	DIRC3-AS1		lncRNA	chr2:216694404-217
ENSG0000(	283	6.408214	chr2:360	RESP18	NCGv7	protein_c	chr2:219327407-219
ENSG0000(	283	6.408214	chr2:360	RPL23AP31		Pseudoger	chr2:218841599-218
ENSG0000(	283	6.408214	chr2:360	RN7SKP43		smallRNA	chr2:217251556-217
ENSG0000(	283	6.408214	chr2:360	ENSG00000273361		lncRNA	chr2:218398743-218
ENSG0000(	283	6.408214	chr2:360	CYP27A1		protein_c	chr2:218781749-218
ENSG0000(	283	6.408214	chr2:360	snoU13		smallRNA	chr2:218184979-218
ENSG0000(	283	6.408214	chr2:360	SPEGNB		protein_c	chr2:219496131-219
ENSG0000(	283	6.408214	chr2:360	MIR3132		smallRNA	chr2:219549073-219
ENSG0000(	283	6.408214	chr2:360	CXCR1		protein_c	chr2:218162841-218
ENSG0000(	283	6.408214	chr2:360	SMARCAL1-AS1		lncRNA	chr2:216385288-216
ENSG0000(	283	6.408214	chr2:360	RPL37A		protein_c	chr2:216498825-216
ENSG0000(	283	6.408214	chr2:360	CRYBA2		protein_c	chr2:218990189-218
ENSG0000(	283	6.408214	chr2:360	CXCR2P1		Pseudoger	chr2:218060339-218
ENSG0000(	283	6.408214	chr2:360	LINC01803		lncRNA	chr2:219903900-219
ENSG0000(	283	6.408214	chr2:360	CDK5R2-AS1		lncRNA	chr2:218944629-218
ENSG0000(	283	6.408214	chr2:360	CXCR2	NCGv7	protein_c	chr2:218125289-218
ENSG0000(	283	6.408214	chr2:360	ENSG00000230432		lncRNA	chr2:219299002-219
ENSG0000(	283	6.408214	chr2:360	RN7SL764P		smallRNA	chr2:219102434-219
ENSG0000(	283	6.408214	chr2:360	IHH		protein_c	chr2:219054424-219

ENSG0000(	283	6.408214	chr2:360{	CDK5R2	AC	protein_c	chr2:218959666-218
ENSG0000(	283	6.408214	chr2:360{	GLB1L		protein_c	chr2:219236598-219
ENSG0000(	283	6.408214	chr2:360{	IGFBP2		protein_c	chr2:216632828-216
ENSG0000(	283	6.408214	chr2:360{	LINC02832		lncRNA	chr2:219685381-219
ENSG0000(	283	6.408214	chr2:360{	RPL37A-DT		lncRNA	chr2:216479030-216
ENSG0000(	283	6.408214	chr2:360{	SLC4A3		protein_c	chr2:219627394-219
ENSG0000(	283	6.408214	chr2:360{	TNS1		protein_c	chr2:217799588-218
ENSG0000(	283	6.408214	chr2:360{	STK11IP	NCGv7	protein_c	chr2:219597857-219
ENSG0000(	283	6.408214	chr2:360{	XRCC5	NCGv7	protein_c	chr2:216107464-216
ENSG0000(	283	6.408214	chr2:360{	ANKZF1		protein_c	chr2:219229783-219
ENSG0000(	283	6.408214	chr2:360{	CFAP65		protein_c	chr2:219002846-219
ENSG0000(	283	6.408214	chr2:360{	GMPPA		protein_c	chr2:219498865-219
ENSG0000(	283	6.408214	chr2:360{	TMBIM1		protein_c	chr2:218274197-218
ENSG0000(	283	6.408214	chr2:360{	ENSG00000224414		Pseudoger	chr2:217693895-217
ENSG0000(	283	6.408214	chr2:360{	FEV	NCGv7;AC	protein_c	chr2:218981087-218
ENSG0000(	283	6.408214	chr2:360{	RPL19P5		Pseudoger	chr2:218354075-218
ENSG0000(	283	6.408214	chr2:360{	MARCHF4		protein_c	chr2:216257865-216
ENSG0000(	283	6.408214	chr2:360{	GPBAR1		protein_c	chr2:218259496-218
ENSG0000(	283	6.408214	chr2:360{	ARPC2		protein_c	chr2:218217141-218
ENSG0000(	283	6.408214	chr2:360{	CTDSP1		protein_c	chr2:218398256-218
ENSG0000(	283	6.408214	chr2:360{	LINC01280		lncRNA	chr2:216590426-216
ENSG0000(	283	6.408214	chr2:360{	AC068946.1		smallRNA	chr2:219224023-219
ENSG0000(	283	6.408214	chr2:360{	ASIC4-AS1		lncRNA	chr2:219482073-219
ENSG0000(	283	6.408214	chr2:360{	snoU13		smallRNA	chr2:218328580-218
ENSG0000(	283	6.408214	chr2:360{	ENSG00000232789		Pseudoger	chr2:219358870-219
ENSG0000(	283	6.408214	chr2:360{	CNOT9	NCGv7	protein_c	chr2:218568580-218
ENSG0000(	283	6.408214	chr2:360{	RNF25		protein_c	chr2:218663892-218
ENSG0000(	283	6.408214	chr2:360{	WNT6		protein_c	chr2:218859805-218
ENSG0000(	283	6.408214	chr2:360{	IGFBP5		protein_c	chr2:216672105-216
ENSG0000(	283	6.408214	chr2:360{	ENSG00000288819		lncRNA	chr2:218368113-218
ENSG0000(	283	6.408214	chr2:360{	DIRC3		lncRNA	chr2:217284019-217
ENSG0000(	283	6.408214	chr2:360{	STK36		protein_c	chr2:218672069-218
ENSG0000(	283	6.408214	chr2:360{	PRKAG3		protein_c	chr2:218822308-218
ENSG0000(	283	6.408214	chr2:360{	ZNF142		protein_c	chr2:218633329-218
ENSG0000(	283	6.408214	chr2:360{	ENSG00000288898		lncRNA	chr2:218909158-218
ENSG0000(	283	6.408214	chr2:360{	ENSG00000288902		lncRNA	chr2:219947834-220
ENSG0000(	283	6.408214	chr2:360{	PLCD4		protein_c	chr2:218607855-218
ENSG0000(	283	6.408214	chr2:360{	ENSG00000228973		lncRNA	chr2:219625059-219
ENSG0000(	283	6.408214	chr2:360{	ENSG00000280537		protein_c	chr2:219075329-219
ENSG0000(	283	6.408214	chr2:360{	ENSG00000284820		protein_c	chr2:219209772-219
ENSG0000(	283	6.408214	chr2:360{	TMEM198		protein_c	chr2:219543663-219
ENSG0000(	283	6.408214	chr2:360{	ENSG00000268896		lncRNA	chr2:219547211-219
ENSG0000(	283	6.408214	chr2:360{	ENSG00000237479		lncRNA	chr2:216866332-216
ENSG0000(	283	6.408214	chr2:360{	ENSG00000286154		lncRNA	chr2:218799729-218
ENSG0000(	283	6.408214	chr2:360{	ENSG00000224090		lncRNA	chr2:219002215-219
ENSG0000(	283	6.408214	chr2:360{	CHPF		protein_c	chr2:219538954-219
ENSG0000(	283	6.408214	chr2:360{	ENSG00000286143		protein_c	chr2:219491867-219
ENSG0000(	283	6.408214	chr2:360{	TNP1		protein_c	chr2:216859458-216
ENSG0000(	283	6.408214	chr2:360{	ENSG00000234638		lncRNA	chr2:219425071-219
ENSG0000(	283	6.408214	chr2:360{	RPL23P4		Pseudoger	chr2:219697799-219
ENSG0000(	283	6.408214	chr2:360{	LINC00608		lncRNA	chr2:218975393-218

ENSG0000(	283	6.408214	chr2:360{	RPL7L1P9		Pseudoger	chr2:217760547-217
ENSG0000(	283	6.408214	chr2:360{	ENSG00000233581		lncRNA	chr2:216303325-216
ENSG0000(	283	6.408214	chr2:360{	LINC01963		lncRNA	chr2:216217045-216
ENSG0000(	283	6.408214	chr2:360{	ENSG00000261338		lncRNA	chr2:218255319-218
ENSG0000(	283	6.408214	chr2:360{	HMGB1P9		Pseudoger	chr2:218200434-218
ENSG0000(	283	6.408214	chr2:360{	ASIC4		protein_c	chr2:219514170-219
ENSG0000(	283	6.408214	chr2:360{	MIR3131		smallRNA	chr2:219058688-219
ENSG0000(	283	6.408214	chr2:360{	TTLL4		protein_c	chr2:218710835-218
ENSG0000(	283	6.408214	chr2:360{	FABP5P14		Pseudoger	chr2:216805737-216
ENSG0000(	283	6.408214	chr2:360{	DNAJB2	NCGv7	protein_c	chr2:219279342-219
ENSG0000(	283	6.408214	chr2:360{	ENSG00000231092		lncRNA	chr2:216259595-216
ENSG0000(	283	6.408214	chr2:360{	INHA		protein_c	chr2:219569162-219
ENSG0000(	283	6.408214	chr2:360{	ENSG00000236295		lncRNA	chr2:216870441-216
ENSG0000(	283	6.408214	chr2:360{	MIR375		smallRNA	chr2:219001640-219
ENSG0000(	283	6.408214	chr2:360{	ENSG00000267919		lncRNA	chr2:219645090-219
ENSG0000(	283	6.408214	chr2:360{	OBSL1		protein_c	chr2:219550728-219
ENSG0000(	283	6.408214	chr2:360{	NHEJ1		protein_c	chr2:219069355-219
ENSG0000(	283	6.408214	chr2:360{	RPL31P14		Pseudoger	chr2:216785774-216
ENSG0000(	283	6.408214	chr2:360{	AC010887.1		smallRNA	chr2:217176498-217
ENSG0000(	283	6.408214	chr2:360{	ENSG00000290060		lncRNA	chr2:216395085-216
ENSG0000(	283	6.408214	chr2:360{	DNPEP		protein_c	chr2:219372043-219
ENSG0000(	283	6.408214	chr2:360{	ENSG00000290093		lncRNA	chr2:219177068-219
ENSG0000(	283	6.408214	chr2:360{	CATIP	NCGv7	protein_c	chr2:218356857-218
ENSG0000(	283	6.408214	chr2:360{	ZFAND2B		protein_c	chr2:219195237-219
ENSG0000(	283	6.408214	chr2:360{	RNU6-642P		smallRNA	chr2:218916184-218
ENSG0000(	283	6.408214	chr2:360{	SLC11A1		protein_c	chr2:218382029-218
ENSG0000(	283	6.408214	chr2:360{	USP37		protein_c	chr2:218450251-218
ENSG0000(	283	6.408214	chr2:360{	ATG9A		protein_c	chr2:219219380-219
ENSG0000(	283	6.408214	chr2:360{	ENSG00000241520		lncRNA	chr2:216483032-216
ENSG0000(	283	6.408214	chr2:360{	RNA5SP120		Pseudoger	chr2:216793489-216
ENSG0000(	283	6.408214	chr2:360{	RNU6-136P		smallRNA	chr2:218589214-218
ENSG0000(	283	6.408214	chr2:360{	LINC01921		lncRNA	chr2:216859948-216
ENSG0000(	283	6.408214	chr2:360{	U3		smallRNA	chr2:218619801-218
ENSG0000(	283	6.408214	chr2:360{	CATIP-AS2		lncRNA	chr2:218326889-218
ENSG0000(	283	6.408214	chr2:360{	RN7SKP38		smallRNA	chr2:218500729-218
ENSG0000(	283	6.408214	chr2:360{	ENSG00000225794		lncRNA	chr2:216594359-216
ENSG0000(	283	6.408214	chr2:360{	PTPRN		protein_c	chr2:219289623-219
ENSG0000(	283	6.408214	chr2:360{	PNKD	NCGv7	protein_c	chr2:218269651-218
ENSG0000(	283	6.408214	chr2:360{	DNAJB6P3		Pseudoger	chr2:219684030-219
ENSG0000(	283	6.408214	chr2:360{	AAMP		protein_c	chr2:218264129-218
ENSG0000(	283	6.408214	chr2:360{	ENSG00000287529		lncRNA	chr2:219198640-219
ENSG0000(	283	6.408214	chr2:360{	VIL1		protein_c	chr2:218419121-218
ENSG0000(	283	6.408214	chr2:360{	ENSG00000268603		lncRNA	chr2:219497611-219
ENSG0000(	283	6.408214	chr2:360{	DNPEP-AS1		lncRNA	chr2:219388496-219
ENSG0000(	283	6.408214	chr2:360{	TNS1-AS1		lncRNA	chr2:217978707-217
ENSG0000(	283	6.408214	chr2:360{	TUBA4A		protein_c	chr2:219249710-219
ENSG0000(	283	6.408214	chr2:360{	DES		protein_c	chr2:219418377-219
ENSG0000(	283	6.408214	chr2:360{	ENSG00000286606		lncRNA	chr2:219065308-219
ENSG0000(	283	6.408214	chr2:360{	BCS1L		protein_c	chr2:218658764-218
ENSG0000(	283	6.408214	chr2:360{	LINC01494		lncRNA	chr2:218900811-218
ENSG0000(	283	6.408214	chr2:360{	MIR153-1		smallRNA	chr2:219294111-219

ENSG0000(	283	6.408214	chr2:360{	ENSG00000287498		lncRNA	chr2:217061067-217
ENSG0000(	283	6.408214	chr2:360{	ENSG00000272555		lncRNA	chr2:218818690-218
ENSG0000(	283	6.408214	chr2:360{	ENSG00000286199		lncRNA	chr2:219904983-219
ENSG0000(	283	6.408214	chr2:360{	ENSG00000291236		lncRNA	chr2:218059155-218
ENSG0000(	283	6.408214	chr2:360{	SLC23A3		protein_c	chr2:219161465-219
ENSG0000(	283	6.408214	chr2:360{	KRT8P30		Pseudoger	chr2:218953401-218
ENSG0000(	283	6.408214	chr2:360{	SMARCAL1		protein_c	chr2:216412383-216
ENSG0000(	283	6.408214	chr2:360{	ENSG00000236478		Pseudoger	chr2:216174896-216
ENSG0000(	283	6.408214	chr2:360{	SPEG	NCGv7	protein_c	chr2:219434843-219
ENSG0000(	283	6.408214	chr2:360{	RN7SKP213		smallRNA	chr2:219590687-219
ENSG0000(	283	6.408214	chr2:360{	WNT10A	AC	protein_c	chr2:218880852-218
ENSG0000(	283	6.408214	chr2:360{	Y_RNA		smallRNA	chr2:216351403-216
ENSG0000(	283	6.408214	chr2:360{	ENSG00000225911		Pseudoger	chr2:220048822-220
ENSG0000(	283	6.408214	chr2:360{	CATIP-AS1		lncRNA	chr2:218366661-218
ENSG0000(	283	6.408214	chr2:360{	ENSG00000269068		lncRNA	chr2:219559083-219
ENSG0000(	281	6.362927	chr11:14	ENSG00000284715		Pseudoger	chr11:104975356-10
ENSG0000(	281	6.362927	chr11:14	ENSG00000287846		Pseudoger	chr11:106106398-10
ENSG0000(	281	6.362927	chr11:14	CASP1P2		Pseudoger	chr11:105063345-10
ENSG0000(	281	6.362927	chr11:14	CASP5		protein_c	chr11:104994235-10
ENSG0000(	281	6.362927	chr11:14	CASP1		protein_c	chr11:105025397-10
ENSG0000(	281	6.362927	chr11:14	OR2AL1P		Pseudoger	chr11:105194440-10
ENSG0000(	281	6.362927	chr11:14	GRIA4		protein_c	chr11:105609535-10
ENSG0000(	281	6.362927	chr11:14	ENSG00000255548		lncRNA	chr11:103945548-10
ENSG0000(	281	6.362927	chr12:201	AC013283.1		smallRNA	chr12:98605874-986
ENSG0000(	281	6.362927	chr11:14	ENSG00000289232		lncRNA	chr11:106579494-10
ENSG0000(	281	6.362927	chr11:14	MIR4693		smallRNA	chr11:103849906-10
ENSG0000(	281	6.362927	chr11:14	ENSG00000270449		Pseudoger	chr11:104682383-10
ENSG0000(	281	6.362927	chr11:14	CARD18		protein_c	chr11:105137714-10
ENSG0000(	281	6.362927	chr11:14	ENSG00000255065		Pseudoger	chr11:106310045-10
ENSG0000(	281	6.362927	chr11:14	CASP4		protein_c	chr11:104942866-10
ENSG0000(	281	6.362927	chr11:14	GUCY1A2	NCGv7	protein_c	chr11:106674019-10
ENSG0000(	281	6.362927	chr11:14	ENSG00000254811		Pseudoger	chr11:106264315-10
ENSG0000(	281	6.362927	chr11:14	AP001282.1		smallRNA	chr11:106801349-10
ENSG0000(	281	6.362927	chr11:14	MSANTD4		protein_c	chr11:105995623-10
ENSG0000(	281	6.362927	chr11:14	ENSG00000286345		lncRNA	chr11:106241974-10
ENSG0000(	281	6.362927	chr11:14	ENSG00000254569		Pseudoger	chr11:104873264-10
ENSG0000(	281	6.362927	chr11:14	CARD17		Pseudoger	chr11:105092486-10
ENSG0000(	281	6.362927	chr11:14	KBTBD3		protein_c	chr11:106051098-10
ENSG0000(	281	6.362927	chr11:14	ASS1P13		Pseudoger	chr11:107176286-10
ENSG0000(	281	6.362927	chr11:14	LINC02552		lncRNA	chr11:104445868-10
ENSG0000(	281	6.362927	chr11:14	LINC02719		lncRNA	chr11:106112459-10
ENSG0000(	281	6.362927	chr11:14	RNA5SP348		smallRNA	chr11:104252579-10
ENSG0000(	281	6.362927	chr11:14	HSPD1P13		Pseudoger	chr11:105706299-10
ENSG0000(	281	6.362927	chr11:14	ENSG00000274584		Pseudoger	chr11:106824025-10
ENSG0000(	281	6.362927	chr11:14	ENSG00000288255		Pseudoger	chr11:105055567-10
ENSG0000(	281	6.362927	chr11:14	ENSG00000290797		lncRNA	chr11:105092469-10
ENSG0000(	281	6.362927	chr11:14	RNU4-55P		smallRNA	chr11:105824634-10
ENSG0000(	281	6.362927	chr11:14	CASP4LP		Pseudoger	chr11:104903453-10
ENSG0000(	281	6.362927	chr17:28	RNU6-362P		smallRNA	chr17:75031097-750
ENSG0000(	281	6.362927	chr11:14	CASP12		protein_c	chr11:104885718-10
ENSG0000(	281	6.362927	chr11:14	CARD16		protein_c	chr11:105041326-10

ENSG0000	281	6.362927	chr11:14	ENSG00000255336		Pseudoge	chr11:105246880-10
ENSG0000	281	6.362927	chr11:14	ENSG00000290498		lncRNA	chr11:106250019-10
ENSG0000	281	6.362927	chr11:14	DDI1		protein_	chr11:104036640-10
ENSG0000	281	6.362927	chr11:14	AP000673.1		smallRNA	chr11:105933954-10
ENSG0000	281	6.362927	chr11:14	ENSG00000287802		lncRNA	chr11:106465985-10
ENSG0000	281	6.362927	chr6:197	MTHFD2P3		Pseudoge	chr6:107985811-107
ENSG0000	281	6.362927	chr11:14	CASP4LP		lncRNA	chr11:104901549-10
ENSG0000	281	6.362927	chr11:14	PDGFD		protein_	chr11:103907189-10
ENSG0000	281	6.362927	chr11:14	AASDHPPT		protein_	chr11:106075501-10
ENSG0000	281	6.362927	chr11:14	CASP1P1		Pseudoge	chr11:105122661-10
ENSG0000	281	6.362927	chr11:14	ENSG00000289383		lncRNA	chr11:105181864-10
ENSG0000	281	6.362927	chr11:14	ENSG00000213252		Pseudoge	chr11:106826392-10
ENSG0000	281	6.362927	chr11:14	ENSG00000254433		lncRNA	chr11:106085990-10
ENSG0000	281	6.362927	chr11:14	ENSG00000285813		lncRNA	chr11:105995185-10
ENSG0000	281	6.362927	chr11:14	RNU6-277P		smallRNA	chr11:105974826-10
ENSG0000	278	6.294995	chr20:29	MIR3616		smallRNA	chr20:47166967-471
ENSG0000	276	6.249707	chr22:22	IGLV1-51		protein_	chr22:22322472-223
ENSG0000	276	6.249707	chr22:22	IGLV5-52		protein_	chr22:22318727-223
ENSG0000	276	6.249707	chr5:432	Y_RNA		smallRNA	chr5:10337277-1033
ENSG0000	276	6.249707	chr22:22	IGLV3-30		Pseudoge	chr22:22618572-226
ENSG0000	276	6.249707	chr22:22	LL22NC03-63E9.3		lncRNA	chr22:22559343-225
ENSG0000	276	6.249707	chr22:22	ASH2LP1		Pseudoge	chr22:22420326-224
ENSG0000	276	6.249707	chr5:432	ATPSCKMT		protein_	chr5:10225507-1024
ENSG0000	276	6.249707	chr5:432	ROPN1L		protein_	chr5:10441524-1047
ENSG0000	276	6.249707	chr5:432	MARCHF6		protein_	chr5:10353695-1044
ENSG0000	276	6.249707	chr22:22	IGLVVII-41-1		Pseudoge	chr22:22398849-223
ENSG0000	276	6.249707	chr5:432	ENSG00000259802		lncRNA	chr5:10323547-1035
ENSG0000	276	6.249707	chr22:22	IGLV3-29		Pseudoge	chr22:22661299-226
ENSG0000	276	6.249707	chr5:432	CMBL	DriverDB	protein_	chr5:10275875-1030
ENSG0000	276	6.249707	chr22:22	ASH2LP3		Pseudoge	chr22:22339573-223
ENSG0000	276	6.249707	chr22:22	GGTLC2		protein_	chr22:22644614-226
ENSG0000	276	6.249707	chr22:22	ASH2LP2		Pseudoge	chr22:22366210-223
ENSG0000	276	6.249707	chr22:22	BCRP4		Pseudoge	chr22:22630065-226
ENSG0000	276	6.249707	chr5:432	ENSG00000248968		lncRNA	chr5:10248325-1024
ENSG0000	276	6.249707	chr22:22	snoU13		smallRNA	chr22:22517780-225
ENSG0000	276	6.249707	chr22:22	IGLVI-38		Pseudoge	chr22:22425821-224
[truncated: 2,624,399 more chars]
