# Supplementary material for: MUC20 regulated by extrachromosomal circular DNA attenuates proteasome inhibitor resistance of multiple myeloma by modulating cuproptosis
Source: J Exp Clin Cancer Res. 2024 Mar 5;43:68. doi: 10.1186/s13046-024-02972-6 (PMC10913264; doi:10.1186/s13046-024-02972-6)
Supplement: Supplementary file 2 — Additional file 2: Supplementary Table S1. Demographic characteristics of HDs and patients with MM. Supplementary Table S2. Demographic characteristics of patients with NDMM and RRMM. Supplementary Table S8. DEED-amplified encoding genes both in PI-resistant KAS-6/1 and U266 cells. [file 13046_2024_2972_MOESM2_ESM.zip › Supplementary Table S5.pdf]

GeneID	bp_evid	ences_Nor	eccDNA_ID	ene_symbo	rDriverGe	ne_bioty	GeneLocus
ENSG0000(	3464	86.63786	chr11:76(	RN7SKP243		smallRNA	chr11:73832382-738
ENSG0000(	3318	82.98626	chr11:76(	AP002761.1		smallRNA	chr11:73357135-733
ENSG0000(	3307	82.71114	chr11:76(	RNU6-216P		smallRNA	chr11:74968189-749
ENSG0000(	3294	82.386	chr11:76(	ENSG00000254826		lncRNA	chr11:75775904-757
ENSG0000(	3294	82.386	chr11:76(	ENSG00000255786		Pseudoger	chr11:73452020-734
ENSG0000(	3294	82.386	chr11:76(	NEU3		protein_c	chr11:74988279-750
ENSG0000(	3294	82.386	chr11:76(	ENSG00000279117		TEC	chr11:75260129-752
ENSG0000(	3294	82.386	chr11:76(	SNORA7		smallRNA	chr11:74252414-742
ENSG0000(	3294	82.386	chr11:76(	ENSG00000254755		Pseudoger	chr11:76591023-765
ENSG0000(	3294	82.386	chr11:76(	GUCY2EP		Pseudoger	chr11:76694041-767
ENSG0000(	3294	82.386	chr11:76(	ENSG00000286943		lncRNA	chr11:74931724-749
ENSG0000(	3294	82.386	chr11:76(	ENSG00000279353		TEC	chr11:74698231-746
ENSG0000(	3294	82.386	chr11:76(	DNAJB13		protein_c	chr11:73951026-739
ENSG0000(	3294	82.386	chr11:76(	ENSG00000254810		lncRNA	chr11:76653597-766
ENSG0000(	3294	82.386	chr11:76(	ENSG00000254814		lncRNA	chr11:75800877-758
ENSG0000(	3294	82.386	chr11:76(	ENSG00000254829		lncRNA	chr11:78015715-780
ENSG0000(	3294	82.386	chr11:76(	ZDHHC20P3		Pseudoger	chr11:75228322-752
ENSG0000(	3294	82.386	chr11:76(	LIPT2-AS1		lncRNA	chr11:74493366-744
ENSG0000(	3294	82.386	chr11:76(	AP002789.1		smallRNA	chr11:77595111-775
ENSG0000(	3294	82.386	chr11:76(	DGAT2		protein_c	chr11:75759512-758
ENSG0000(	3294	82.386	chr11:76(	RPL36AP38		Pseudoger	chr11:74738478-747
ENSG0000(	3294	82.386	chr11:76(	HNRNPA1P40		Pseudoger	chr11:74354443-743
ENSG0000(	3294	82.386	chr11:76(	RPL31P46		Pseudoger	chr11:74876286-749
ENSG0000(	3294	82.386	chr11:76(	ENSG00000268635		lncRNA	chr11:77473371-774
ENSG0000(	3294	82.386	chr11:76(	UVRAG-DT		lncRNA	chr11:75803431-758
ENSG0000(	3294	82.386	chr11:76(	ENSG00000290785		lncRNA	chr11:76712396-767
ENSG0000(	3294	82.386	chr11:76(	ENSG00000241782		Pseudoger	chr11:77868722-778
ENSG0000(	3294	82.386	chr11:76(	TOMM20P1		Pseudoger	chr11:77313606-773
ENSG0000(	3294	82.386	chr11:76(	ENSG00000255479		lncRNA	chr11:76625462-766
ENSG0000(	3294	82.386	chr11:76(	ENSG00000255847		lncRNA	chr11:73963657-739
ENSG0000(	3294	82.386	chr11:76(	COPS8P3		Pseudoger	chr11:78581675-785
ENSG0000(	3294	82.386	chr11:76(	ENSG00000254691		lncRNA	chr11:77850604-778
ENSG0000(	3294	82.386	chr11:76(	ENSG00000236304		lncRNA	chr11:76656984-766
ENSG0000(	3294	82.386	chr11:76(	AAMDC	DriverDBv	protein_c	chr11:77821109-779
ENSG0000(	3294	82.386	chr11:76(	RANP3		Pseudoger	chr11:74652636-746
ENSG0000(	3294	82.386	chr11:76(	SNORD15B		smallRNA	chr11:75404421-754
ENSG0000(	3294	82.386	chr11:76(	ENSG00000256448		lncRNA	chr11:73405297-734
ENSG0000(	3294	82.386	chr11:76(	ENSG00000287425		lncRNA	chr11:73983449-739
ENSG0000(	3294	82.386	chr11:76(	OMP	DriverDBv	protein_c	chr11:77102840-771
ENSG0000(	3294	82.386	chr11:76(	OR8R1P		Pseudoger	chr11:73248779-732
ENSG0000(	3294	82.386	chr11:76(	ENSG00000278879		TEC	chr11:74830574-748
ENSG0000(	3294	82.386	chr11:76(	MAP6		protein_c	chr11:75586918-756
ENSG0000(	3294	82.386	chr11:76(	ENSG00000254630		lncRNA	chr11:75635883-756
ENSG0000(	3294	82.386	chr11:76(	ENSG00000254631		lncRNA	chr11:74397549-744
ENSG0000(	3294	82.386	chr11:76(	ENSG00000254632		lncRNA	chr11:76759916-767
ENSG0000(	3294	82.386	chr11:76(	ENSG00000254649		lncRNA	chr11:78388061-783
ENSG0000(	3294	82.386	chr11:76(	ENSG00000255928		lncRNA	chr11:73722349-737
ENSG0000(	3294	82.386	chr11:76(	OR2AT4		protein_c	chr11:75081753-750
ENSG0000(	3294	82.386	chr11:76(	ENSG00000256189		Pseudoger	chr11:73991283-739
ENSG0000(	3294	82.386	chr11:76(	ENSG00000256148		Pseudoger	chr11:73510658-735

ENSG0000(	3294	82.386	chr11:76(	ENSG00000256098		Pseudoger	chr11:74142151-741
ENSG0000(	3294	82.386	chr11:76(	ENSG00000254675		lncRNA	chr11:78022933-780
ENSG0000(	3294	82.386	chr11:76(	KCTD14		protein_c	chr11:78015715-780
ENSG0000(	3294	82.386	chr11:76(	THRSP		protein_c	chr11:78063861-780
ENSG0000(	3294	82.386	chr11:76(	NDUFC2	DriverDBv	protein_c	chr11:78068297-780
ENSG0000(	3294	82.386	chr11:76(	ENSG00000256034		lncRNA	chr11:73760563-737
ENSG0000(	3294	82.386	chr11:76(	ENSG00000255084		lncRNA	chr11:78533176-785
ENSG0000(	3294	82.386	chr11:76(	P2RY6		protein_c	chr11:73264498-733
ENSG0000(	3294	82.386	chr11:76(	KCTD21		protein_c	chr11:78171249-781
ENSG0000(	3294	82.386	chr11:76(	GAB2	DriverDBv	protein_c	chr11:78215293-784
ENSG0000(	3294	82.386	chr11:76(	ENSG00000255449		lncRNA	chr11:77866412-778
ENSG0000(	3294	82.386	chr11:76(	OR2AT1P		Pseudoger	chr11:75131138-751
ENSG0000(	3294	82.386	chr11:76(	ENSG00000280269		TEC	chr11:74204869-742
ENSG0000(	3294	82.386	chr11:76(	RSF1-IT2		lncRNA	chr11:77717712-777
ENSG0000(	3294	82.386	chr11:76(	ENSG00000254988		lncRNA	chr11:76955417-769
ENSG0000(	3294	82.386	chr11:76(	DGAT2-DT		lncRNA	chr11:75758455-757
ENSG0000(	3294	82.386	chr11:76(	GVQW3		protein_c	chr11:76381303-764
ENSG0000(	3294	82.386	chr11:76(	GDPD4		protein_c	chr11:77216558-773
ENSG0000(	3294	82.386	chr11:76(	AQP11		protein_c	chr11:77589391-776
ENSG0000(	3294	82.386	chr11:76(	ALG8	DriverDBv	protein_c	chr11:78095244-781
ENSG0000(	3294	82.386	chr11:76(	SNORD15A		smallRNA	chr11:75400391-754
ENSG0000(	3294	82.386	chr11:76(	HMGN2P38		Pseudoger	chr11:73580253-735
ENSG0000(	3294	82.386	chr11:76(	LIPT2		protein_c	chr11:74490519-744
ENSG0000(	3294	82.386	chr11:76(	EMSY	AC	protein_c	chr11:76444923-765
ENSG0000(	3294	82.386	chr11:76(	GDPD5		protein_c	chr11:75434640-755
ENSG0000(	3294	82.386	chr11:76(	KCNE3		protein_c	chr11:74454841-744
ENSG0000(	3294	82.386	chr11:76(	PPP1R1AP1		Pseudoger	chr11:75911204-759
ENSG0000(	3294	82.386	chr11:76(	PLEKHB1		protein_c	chr11:73646178-736
ENSG0000(	3294	82.386	chr11:76(	PPME1		protein_c	chr11:74171267-742
ENSG0000(	3294	82.386	chr11:76(	UCP3		protein_c	chr11:74000277-740
ENSG0000(	3294	82.386	chr11:76(	TPBGL-AS1		lncRNA	chr11:75206048-752
ENSG0000(	3294	82.386	chr11:76(	EMSY-DT		lncRNA	chr11:76435559-764
ENSG0000(	3294	82.386	chr11:76(	UCP2		protein_c	chr11:73974672-739
ENSG0000(	3294	82.386	chr11:76(	ENSG00000255081		lncRNA	chr11:75914201-759
ENSG0000(	3294	82.386	chr11:76(	P2RY2		protein_c	chr11:73218281-732
ENSG0000(	3294	82.386	chr11:76(	ENSG00000255115		Pseudoger	chr11:77914990-779
ENSG0000(	3294	82.386	chr11:76(	PAAF1	NCGv7	protein_c	chr11:73876699-739
ENSG0000(	3294	82.386	chr11:76(	RAB6A		protein_c	chr11:73675638-737
ENSG0000(	3294	82.386	chr11:76(	MRPL48		protein_c	chr11:73787872-738
ENSG0000(	3294	82.386	chr11:76(	P4HA3-AS1		lncRNA	chr11:74311362-743
ENSG0000(	3294	82.386	chr11:76(	ENSG00000270323		Pseudoger	chr11:73640479-736
ENSG0000(	3294	82.386	chr11:76(	RN7SL239P		smallRNA	chr11:74845910-748
ENSG0000(	3294	82.386	chr11:76(	LINC02757		lncRNA	chr11:76607853-766
ENSG0000(	3294	82.386	chr11:76(	CYCSP27		Pseudoger	chr11:74482250-744
ENSG0000(	3294	82.386	chr11:76(	ENSG00000255440		lncRNA	chr11:74398825-744
ENSG0000(	3294	82.386	chr11:76(	ENSG00000255434		lncRNA	chr11:75596144-755
ENSG0000(	3294	82.386	chr11:76(	AP002498.1		smallRNA	chr11:76878232-768
ENSG0000(	3294	82.386	chr11:76(	ENSG00000255421		lncRNA	chr11:76137315-761
ENSG0000(	3294	82.386	chr11:76(	ENSG00000254915		Pseudoger	chr11:75942129-759
ENSG0000(	3294	82.386	chr11:76(	RSF1-IT1		lncRNA	chr11:77738680-777
ENSG0000(	3294	82.386	chr11:76(	RNA5SP343		smallRNA	chr11:74198748-741

ENSG0000(	3294	82.386	chr11:76(	ENSG00000255395		lncRNA	chr11:75099172-751
ENSG0000(	3294	82.386	chr11:76(	TSKU		protein_c	chr11:76782251-767
ENSG0000(	3294	82.386	chr11:76(	ENSG00000284722		lncRNA	chr11:75069243-750
ENSG0000(	3294	82.386	chr11:76(	COA4		protein_c	chr11:73872667-738
ENSG0000(	3294	82.386	chr11:76(	LINC02761		lncRNA	chr11:76210956-762
ENSG0000(	3294	82.386	chr11:76(	ENSG00000255280		Pseudoger	chr11:75642600-756
ENSG0000(	3294	82.386	chr11:76(	SNORD43		smallRNA	chr11:74716687-747
ENSG0000(	3294	82.386	chr11:76(	ENSG00000254928		lncRNA	chr11:74455348-744
ENSG0000(	3294	82.386	chr11:76(	ENSG00000254933		lncRNA	chr11:76190725-761
ENSG0000(	3294	82.386	chr11:76(	ENSG00000255326		lncRNA	chr11:75583196-755
ENSG0000(	3294	82.386	chr11:76(	RN7SKP297		smallRNA	chr11:74685224-746
ENSG0000(	3294	82.386	chr11:76(	ENSG00000254963		lncRNA	chr11:75264289-752
ENSG0000(	3294	82.386	chr11:76(	ENSG00000219529		Pseudoger	chr11:77813319-778
ENSG0000(	3294	82.386	chr11:76(	ARHGEF17		protein_c	chr11:73308276-733
ENSG0000(	3294	82.386	chr11:76(	ENSG00000254974		Pseudoger	chr11:74485580-744
ENSG0000(	3294	82.386	chr11:76(	ENSG00000254975		lncRNA	chr11:76675079-767
ENSG0000(	3294	82.386	chr11:76(	KCTD21-AS1		lncRNA	chr11:78139756-781
ENSG0000(	3294	82.386	chr11:76(	ENSG00000227615		Pseudoger	chr11:74745716-747
ENSG0000(	3294	82.386	chr11:76(	P4HA3		protein_c	chr11:74235801-743
ENSG0000(	3294	82.386	chr11:76(	RNU7-59P		smallRNA	chr11:77566934-775
ENSG0000(	3294	82.386	chr11:76(	TSKU-AS1		lncRNA	chr11:76782581-767
ENSG0000(	3294	82.386	chr11:76(	RNU6-126P		smallRNA	chr11:78133420-781
ENSG0000(	3294	82.386	chr11:76(	Y_RNA		smallRNA	chr11:76404140-764
ENSG0000(	3294	82.386	chr11:76(	ENSG00000256928		lncRNA	chr11:73395559-733
ENSG0000(	3294	82.386	chr11:76(	ARPC3P4		Pseudoger	chr11:73921665-739
ENSG0000(	3294	82.386	chr11:76(	ENSG00000288538		lncRNA	chr11:78223815-782
ENSG0000(	3294	82.386	chr11:76(	IUR1		lncRNA	chr11:73157173-731
ENSG0000(	3294	82.386	chr11:76(	CHRDL2		protein_c	chr11:74696429-747
ENSG0000(	3294	82.386	chr11:76(	B3GNT6		protein_c	chr11:77034398-770
ENSG0000(	3294	82.386	chr11:76(	RSF1	AC	protein_c	chr11:77660009-778
ENSG0000(	3294	82.386	chr11:76(	ENSG00000256723		Pseudoger	chr11:73994972-739
ENSG0000(	3294	82.386	chr11:76(	ARHGEF17-AS1		lncRNA	chr11:73307235-733
ENSG0000(	3294	82.386	chr11:76(	ENSG00000254429		lncRNA	chr11:75260127-752
ENSG0000(	3294	82.386	chr11:76(	SPCS2		protein_c	chr11:74949261-749
ENSG0000(	3294	82.386	chr11:76(	POLD3		protein_c	chr11:74493851-746
ENSG0000(	3294	82.386	chr11:76(	MIX23P5		Pseudoger	chr11:73850469-738
ENSG0000(	3294	82.386	chr11:76(	LINC02728		lncRNA	chr11:78423982-784
ENSG0000(	3294	82.386	chr11:76(	NDUFC2-KCTD14		protein_c	chr11:78016971-780
ENSG0000(	3294	82.386	chr11:76(	ENSG00000256568		lncRNA	chr11:73157946-731
ENSG0000(	3294	82.386	chr11:76(	MIR4696		smallRNA	chr11:74720268-747
ENSG0000(	3294	82.386	chr11:76(	ENSG00000254420		lncRNA	chr11:78324758-784
ENSG0000(	3294	82.386	chr11:76(	PGM2L1		protein_c	chr11:74330316-743
ENSG0000(	3294	82.386	chr11:76(	WNT11		protein_c	chr11:76186325-762
ENSG0000(	3294	82.386	chr11:76(	Y_RNA		smallRNA	chr11:77691650-776
ENSG0000(	3294	82.386	chr11:76(	ENSG00000254460		lncRNA	chr11:75506937-755
ENSG0000(	3294	82.386	chr11:76(	THAP12	NCGv7	protein_c	chr11:76349898-763
ENSG0000(	3294	82.386	chr11:76(	SLCO2B1		protein_c	chr11:75100563-752
ENSG0000(	3294	82.386	chr11:76(	LRRC32	NCGv7	protein_c	chr11:76657524-766
ENSG0000(	3294	82.386	chr11:76(	ARRB1		protein_c	chr11:75260122-753
ENSG0000(	3294	82.386	chr11:76(	MYO7A		protein_c	chr11:77128246-772
ENSG0000(	3294	82.386	chr11:76(	USP35	DriverDB\	protein_c	chr11:78188812-782

ENSG0000(	3294	82.386	chr11:76(	NARS2		protein_c	chr11:78435620-785
ENSG0000(	3294	82.386	chr11:76(	NPM1P50		Pseudoger	chr11:75079265-750
ENSG0000(	3294	82.386	chr11:76(	FAM168A		protein_c	chr11:73400487-735
ENSG0000(	3294	82.386	chr11:76(	Y_RNA		smallRNA	chr11:73664515-736
ENSG0000(	3294	82.386	chr11:76(	MIR326		smallRNA	chr11:75335092-753
ENSG0000(	3294	82.386	chr11:76(	FTH1P16		Pseudoger	chr11:77734475-777
ENSG0000(	3294	82.386	chr11:76(	AP001992.1		smallRNA	chr11:74945981-749
ENSG0000(	3294	82.386	chr11:76(	ZNF75CP		Pseudoger	chr11:78384059-783
ENSG0000(	3294	82.386	chr11:76(	ACER3		protein_c	chr11:76860859-770
ENSG0000(	3294	82.386	chr11:76(	OR2AT2P		Pseudoger	chr11:75071148-750
ENSG0000(	3294	82.386	chr11:76(	RELT		protein_c	chr11:73376399-733
ENSG0000(	3294	82.386	chr11:76(	Y_RNA		smallRNA	chr11:75837544-758
ENSG0000(	3294	82.386	chr11:76(	UVRAG		protein_c	chr11:75815210-761
ENSG0000(	3294	82.386	chr11:76(	ENSG00000215841		lncRNA	chr11:73214851-732
ENSG0000(	3294	82.386	chr11:76(	ENSG00000254459		lncRNA	chr11:77829654-778
ENSG0000(	3294	82.386	chr11:76(	MOGAT2		protein_c	chr11:75717838-757
ENSG0000(	3294	82.386	chr11:76(	RPS3		protein_c	chr11:75399515-754
ENSG0000(	3294	82.386	chr11:76(	RN7SL786P		smallRNA	chr11:75742129-757
ENSG0000(	3294	82.386	chr11:76(	XRRA1		protein_c	chr11:74807739-749
ENSG0000(	3294	82.386	chr11:76(	PAK1	DriverDB\	protein_c	chr11:77322017-774
ENSG0000(	3294	82.386	chr11:76(	INTS4	NCGv7;AC	protein_c	chr11:77874418-779
ENSG0000(	3294	82.386	chr11:76(	MIR548AL		smallRNA	chr11:74399237-743
ENSG0000(	3294	82.386	chr11:76(	RNF169		protein_c	chr11:74748849-748
ENSG0000(	3294	82.386	chr11:76(	ENSG00000260401		lncRNA	chr11:73238975-732
ENSG0000(	3294	82.386	chr11:76(	RNA5SP344		Pseudoger	chr11:75934936-759
ENSG0000(	3294	82.386	chr11:76(	CAPN5	DriverDB\	protein_c	chr11:77066961-771
ENSG0000(	3294	82.386	chr11:76(	CLNS1A		protein_c	chr11:77514936-776
ENSG0000(	3294	82.386	chr11:76(	SERPINH1		protein_c	chr11:75562056-755
ENSG0000(	3294	82.386	chr11:76(	AP000560.1		smallRNA	chr11:74753474-747
ENSG0000(	3294	82.386	chr11:76(	ENSG00000261578		lncRNA	chr11:76800364-768
ENSG0000(	3294	82.386	chr11:76(	TPBGL		protein_c	chr11:75240774-752
ENSG0000(	3294	82.386	chr11:76(	ENSG00000288853		lncRNA	chr11:78314796-783
ENSG0000(	3294	82.386	chr11:76(	Y_RNA		smallRNA	chr11:75835215-758
ENSG0000(	3294	82.386	chr11:76(	ENSG00000291249		lncRNA	chr11:76703274-767
ENSG0000(	3294	82.386	chr11:76(	KLHL35		protein_c	chr11:75422394-754
ENSG0000(	3294	82.386	chr11:76(	C2CD3	NCGv7	protein_c	chr11:74012718-741
ENSG0000(	3294	82.386	chr11:76(	RNU6-311P		smallRNA	chr11:78579255-785
ENSG0000(	2802	70.08062	chr11:76(	ENSG00000255345		lncRNA	chr11:79092848-790
ENSG0000(	2802	70.08062	chr11:76(	TENM4		protein_c	chr11:78652829-794
ENSG0000(	2802	70.08062	chr11:76(	ENSG00000254885		lncRNA	chr11:79191558-791
ENSG0000(	2802	70.08062	chr11:76(	AP002958.1		smallRNA	chr11:78973721-789
ENSG0000(	2802	70.08062	chr11:76(	MIR708		smallRNA	chr11:79402022-794
ENSG0000(	2802	70.08062	chr11:76(	ENSG00000255209		lncRNA	chr11:79604967-796
ENSG0000(	2802	70.08062	chr11:76(	ENSG00000254563		lncRNA	chr11:78749250-787
ENSG0000(	2802	70.08062	chr11:76(	AP000478.1		smallRNA	chr11:79692379-796
ENSG0000(	2802	70.08062	chr11:76(	MIR5579		smallRNA	chr11:79422169-794
ENSG0000(	2794	69.88054	chr11:76(	ENSG00000285568		lncRNA	chr11:81015849-810
ENSG0000(	2794	69.88054	chr11:76(	ENSG00000279900		TEC	chr11:83083687-830
ENSG0000(	2794	69.88054	chr11:76(	RBMXP3		Pseudoger	chr11:82805354-828
ENSG0000(	2794	69.88054	chr11:76(	RNU6-544P		smallRNA	chr11:80527960-805
ENSG0000(	2794	69.88054	chr11:76(	BCAS2P1		Pseudoger	chr11:83132056-831

ENSG0000(	2794	69.88054	chr11:76(	RAB30		protein_c	chr11:82973133-830
ENSG0000(	2794	69.88054	chr11:76(	SNORA70E		smallRNA	chr11:83041464-830
ENSG0000(	2794	69.88054	chr11:76(	PRCP		protein_c	chr11:82822936-829
ENSG0000(	2794	69.88054	chr11:76(	ENSG00000270701		Pseudoger	chr11:81842435-818
ENSG0000(	2794	69.88054	chr11:76(	LINC02720		lncRNA	chr11:80751200-807
ENSG0000(	2794	69.88054	chr11:76(	SNORD112		smallRNA	chr11:82572009-825
ENSG0000(	2794	69.88054	chr11:76(	ENSG00000255246		lncRNA	chr11:82603529-826
ENSG0000(	2794	69.88054	chr11:76(	RPS28P7		Pseudoger	chr11:82689559-826
ENSG0000(	2794	69.88054	chr11:76(	C1DP5		Pseudoger	chr11:83111060-831
ENSG0000(	2794	69.88054	chr11:76(	ANKRD42-DT		lncRNA	chr11:83184491-831
ENSG0000(	2794	69.88054	chr11:76(	CCDC90B-AS1		lncRNA	chr11:83286120-834
ENSG0000(	2794	69.88054	chr11:76(	CYCSP28		Pseudoger	chr11:83328926-833
ENSG0000(	2794	69.88054	chr11:76(	RAB30-DT		lncRNA	chr11:83072052-831
ENSG0000(	2794	69.88054	chr11:76(	COX5BP4		Pseudoger	chr11:83106580-831
ENSG0000(	2794	69.88054	chr11:76(	MIR4300HG		lncRNA	chr11:81821272-827
ENSG0000(	2794	69.88054	chr11:76(	PCF11-AS1		lncRNA	chr11:83185521-831
ENSG0000(	2794	69.88054	chr11:76(	DLG2-AS2		lncRNA	chr11:83643602-837
ENSG0000(	2794	69.88054	chr11:76(	MTND6P25		Pseudoger	chr11:81555910-815
ENSG0000(	2794	69.88054	chr11:76(	ARL6IP1P3		Pseudoger	chr11:80653429-806
ENSG0000(	2794	69.88054	chr11:76(	PCF11	NCGv7	protein_c	chr11:83156988-831
ENSG0000(	2794	69.88054	chr11:76(	DDIAS		protein_c	chr11:82899975-829
ENSG0000(	2794	69.88054	chr11:76(	MIR4300		smallRNA	chr11:81890741-818
ENSG0000(	2794	69.88054	chr11:76(	FAM181B		protein_c	chr11:82729940-827
ENSG0000(	2794	69.88054	chr11:76(	LINC02734		lncRNA	chr11:82781502-828
ENSG0000(	2794	69.88054	chr11:76(	RPL7AP54		Pseudoger	chr11:82957423-829
ENSG0000(	2794	69.88054	chr11:76(	ENSG00000254471		Pseudoger	chr11:79987513-799
ENSG0000(	2794	69.88054	chr11:76(	ENSG00000254511		lncRNA	chr11:81970994-819
ENSG0000(	2794	69.88054	chr11:76(	ENSG00000254629		Pseudoger	chr11:83789977-837
ENSG0000(	2794	69.88054	chr11:76(	MTND4LP18		Pseudoger	chr11:81552226-815
ENSG0000(	2794	69.88054	chr11:76(	ENSG00000254551		lncRNA	chr11:83209431-832
ENSG0000(	2794	69.88054	chr11:76(	ENSG00000254698		lncRNA	chr11:82963681-830
ENSG0000(	2794	69.88054	chr11:76(	LDHAL6DP		Pseudoger	chr11:83814571-838
ENSG0000(	2794	69.88054	chr11:76(	snoU13		smallRNA	chr11:82843885-828
ENSG0000(	2794	69.88054	chr11:76(	RPL32P24		Pseudoger	chr11:83213005-832
ENSG0000(	2794	69.88054	chr11:76(	CKS1BP4		Pseudoger	chr11:83209399-832
ENSG0000(	2794	69.88054	chr11:76(	ENSG00000287912		lncRNA	chr11:81175201-814
ENSG0000(	2794	69.88054	chr11:76(	ENSG00000254676		lncRNA	chr11:83180144-831
ENSG0000(	2794	69.88054	chr11:76(	ANKRD42		protein_c	chr11:83193712-832
ENSG0000(	2794	69.88054	chr11:76(	CCDC90B		protein_c	chr11:83259081-832
ENSG0000(	2794	69.88054	chr11:76(	ENSG00000254434		lncRNA	chr11:80321620-803
ENSG0000(	2794	69.88054	chr11:76(	ENSG00000254437		Pseudoger	chr11:80957317-809
ENSG0000(	2794	69.88054	chr11:76(	LINC02951		lncRNA	chr11:83072402-830
ENSG0000(	2794	69.88054	chr11:76(	ENSG00000254522		Pseudoger	chr11:83040806-830
ENSG0000(	2794	69.88054	chr11:76(	EIF2S2P6		Pseudoger	chr11:82835509-828
ENSG0000(	2794	69.88054	chr11:76(	ENSG00000279295		TEC	chr11:79966805-799
ENSG0000(	2660	66.52907	chr4:909:	MIR4798		smallRNA	chr4:7310450-73105
ENSG0000(	2610	65.27853	chr11:109	ATP5MG		protein_c	chr11:118401346-11
ENSG0000(	2610	65.27853	chr11:109	RPL5P30		Pseudoger	chr11:118560690-11
ENSG0000(	2610	65.27853	chr11:109	Metazoa_SRP		smallRNA	chr11:118067237-11
ENSG0000(	2610	65.27853	chr11:109	RNU6-1157P		smallRNA	chr11:118593988-11
ENSG0000(	2610	65.27853	chr11:109	JAML		protein_c	chr11:118193725-11

ENSG0000(	2610	65.27853	chr11:109	TTC36		protein_c	chr11:118527472-11
ENSG0000(	2610	65.27853	chr11:109	ARCN1		protein_c	chr11:118572390-11
ENSG0000(	2610	65.27853	chr11:109	ENSG00000255384		lncRNA	chr11:118433121-11
ENSG0000(	2610	65.27853	chr11:109	ENSG00000280032		TEC	chr11:118264593-11
ENSG0000(	2610	65.27853	chr11:109	CD3D		protein_c	chr11:118339075-11
ENSG0000(	2610	65.27853	chr11:109	SMIM35		protein_c	chr11:118003634-11
ENSG0000(	2610	65.27853	chr11:109	ENSG00000254992		Pseudoger	chr11:118322789-11
ENSG0000(	2610	65.27853	chr11:109	ENSG00000285827		protein_c	chr11:118401602-11
ENSG0000(	2610	65.27853	chr11:109	KMT2A	NCGv7;AC	protein_c	chr11:118436456-11
ENSG0000(	2610	65.27853	chr11:109	IL10RA		protein_c	chr11:117986370-11
ENSG0000(	2610	65.27853	chr11:109	TMEM25		protein_c	chr11:118531041-11
ENSG0000(	2610	65.27853	chr11:109	ENSG00000254873		lncRNA	chr11:118397095-11
ENSG0000(	2610	65.27853	chr11:109	TMPRSS4	NCGv7	protein_c	chr11:118077012-11
ENSG0000(	2610	65.27853	chr11:109	CD3G	NCGv7	protein_c	chr11:118344344-11
ENSG0000(	2610	65.27853	chr11:109	TTC36-AS1		lncRNA	chr11:118510273-11
ENSG0000(	2610	65.27853	chr11:109	MPZL2		protein_c	chr11:118253416-11
ENSG0000(	2610	65.27853	chr11:109	SCN2B		protein_c	chr11:118162806-11
ENSG0000(	2610	65.27853	chr11:109	RN7SL86P		smallRNA	chr11:118430408-11
ENSG0000(	2610	65.27853	chr11:109	IFT46		protein_c	chr11:118544528-11
ENSG0000(	2610	65.27853	chr11:109	SCN4B		protein_c	chr11:118133377-11
ENSG0000(	2610	65.27853	chr11:109	UBE4A		protein_c	chr11:118359600-11
ENSG0000(	2610	65.27853	chr11:109	CD3E		protein_c	chr11:118304730-11
ENSG0000(	2610	65.27853	chr11:109	ENSG00000269944		lncRNA	chr11:118415977-11
ENSG0000(	2610	65.27853	chr11:109	HSPE1P18		Pseudoger	chr11:118208932-11
ENSG0000(	2610	65.27853	chr11:109	MPZL3		protein_c	chr11:118226690-11
ENSG0000(	2596	64.92837	chr11:109	ENSG00000257012		Pseudoger	chr11:95040411-950
ENSG0000(	2596	64.92837	chr11:109	ENSG00000274486		Pseudoger	chr11:95030549-950
ENSG0000(	2596	64.92837	chr11:109	SRSF8BP		Pseudoger	chr11:95037482-950
ENSG0000(	2550	63.77787	chr11:109	ENSG00000254626		lncRNA	chr11:112787304-11
ENSG0000(	2550	63.77787	chr11:109	Y_RNA		smallRNA	chr11:118836010-11
ENSG0000(	2550	63.77787	chr11:109	SETP16		Pseudoger	chr11:118833846-11
ENSG0000(	2550	63.77787	chr11:109	NNMT		protein_c	chr11:114257787-11
ENSG0000(	2550	63.77787	chr11:109	ENSG00000271390		lncRNA	chr11:111089870-11
ENSG0000(	2550	63.77787	chr11:109	USP28	NCGv7	protein_c	chr11:113797874-11
ENSG0000(	2550	63.77787	chr11:109	ENSG00000254478		Pseudoger	chr11:118821895-11
ENSG0000(	2550	63.77787	chr11:109	HSPB2		protein_c	chr11:111912734-11
ENSG0000(	2550	63.77787	chr11:109	RNU6-44P		smallRNA	chr11:112352556-11
ENSG0000(	2550	63.77787	chr11:109	PAFAH1B2	AC	protein_c	chr11:117144284-11
ENSG0000(	2550	63.77787	chr11:109	ENSG00000276505		lncRNA	chr11:117297005-11
ENSG0000(	2550	63.77787	chr11:109	CRYAB	AC	protein_c	chr11:111908564-11
ENSG0000(	2550	63.77787	chr11:109	ENSG00000278376		lncRNA	chr11:118791202-11
ENSG0000(	2550	63.77787	chr11:109	ENSG00000235286		Pseudoger	chr11:111670956-11
ENSG0000(	2550	63.77787	chr11:109	ALG9-IT1		lncRNA	chr11:111817214-11
ENSG0000(	2550	63.77787	chr11:109	ENSG00000278945		TEC	chr11:116609995-11
ENSG0000(	2550	63.77787	chr11:109	HSPB2-C11orf52		protein_c	chr11:111912736-11
ENSG0000(	2550	63.77787	chr11:109	LINC02762		lncRNA	chr11:112270748-11
ENSG0000(	2550	63.77787	chr11:109	SIK3-IT1		lncRNA	chr11:116886046-11
ENSG0000(	2550	63.77787	chr11:109	AP000908.1		smallRNA	chr11:114086811-11
ENSG0000(	2550	63.77787	chr11:109	RNF214		protein_c	chr11:117232625-11
ENSG0000(	2550	63.77787	chr11:109	BACE1-AS		lncRNA	chr11:117288453-11
ENSG0000(	2550	63.77787	chr11:109	FXYD6-AS1		lncRNA	chr11:117833719-11

ENSG0000(	2550	63.77787	chr11:10(	ENSG00000234268		Pseudoger	chr11:117035797-11
ENSG0000(	2550	63.77787	chr11:10(	PRR13P3		Pseudoger	chr11:117336256-11
ENSG0000(	2550	63.77787	chr11:10(	ENSG00000254678		Pseudoger	chr11:117143891-11
ENSG0000(	2550	63.77787	chr11:10(	ZBTB16	NCGv7;AC	protein_c	chr11:114059041-11
ENSG0000(	2550	63.77787	chr11:10(	POU2AF1	NCGv7;AC	protein_c	chr11:111352255-11
ENSG0000(	2550	63.77787	chr11:10(	TREH	AC	protein_c	chr11:118657316-11
ENSG0000(	2550	63.77787	chr11:10(	TFAMP2		Pseudoger	chr11:109907004-10
ENSG0000(	2550	63.77787	chr11:10(	RNU2-60P		smallRNA	chr11:111383092-11
ENSG0000(	2550	63.77787	chr11:10(	RNU6-1107P		smallRNA	chr11:113859346-11
ENSG0000(	2550	63.77787	chr11:10(	ENSG00000290078		lncRNA	chr11:110328408-11
ENSG0000(	2550	63.77787	chr11:10(	ENSG00000254844		lncRNA	chr11:117818443-11
ENSG0000(	2550	63.77787	chr11:10(	DDX6	NCGv7;AC	protein_c	chr11:118747763-11
ENSG0000(	2550	63.77787	chr11:10(	ENSG00000289465		lncRNA	chr11:109803905-11
ENSG0000(	2550	63.77787	chr11:10(	APOA1		protein_c	chr11:116835751-11
ENSG0000(	2550	63.77787	chr11:10(	ENSG00000254990		lncRNA	chr11:111768668-11
ENSG0000(	2550	63.77787	chr11:10(	ENSG00000254851		Pseudoger	chr11:117135528-11
ENSG0000(	2550	63.77787	chr11:10(	SCARNA11		smallRNA	chr11:117263799-11
ENSG0000(	2550	63.77787	chr11:10(	ENSG00000279771		TEC	chr11:115659168-11
ENSG0000(	2550	63.77787	chr11:10(	CXCR5		protein_c	chr11:118883892-11
ENSG0000(	2550	63.77787	chr11:10(	ACA59		smallRNA	chr11:115128218-11
ENSG0000(	2550	63.77787	chr11:10(	PCSK7	AC	protein_c	chr11:117204337-11
ENSG0000(	2550	63.77787	chr11:10(	SIK3	NCGv7	protein_c	chr11:116843402-11
ENSG0000(	2550	63.77787	chr11:10(	ENSG00000279818		TEC	chr11:115731942-11
ENSG0000(	2550	63.77787	chr11:10(	ENSG00000279586		TEC	chr11:117611101-11
ENSG0000(	2550	63.77787	chr11:10(	LINC02732		lncRNA	chr11:110355130-11
ENSG0000(	2550	63.77787	chr11:10(	CEP164		protein_c	chr11:117314557-11
ENSG0000(	2550	63.77787	chr11:10(	ENSG00000225236		Pseudoger	chr11:117117794-11
ENSG0000(	2550	63.77787	chr11:10(	ENSG00000254638		lncRNA	chr11:112165197-11
ENSG0000(	2550	63.77787	chr11:10(	ZPR1		protein_c	chr11:116773799-11
ENSG0000(	2550	63.77787	chr11:10(	ENSG00000254980		lncRNA	chr11:111514043-11
ENSG0000(	2550	63.77787	chr11:10(	ANKK1		protein_c	chr11:113387779-11
ENSG0000(	2550	63.77787	chr11:10(	LINC02715		lncRNA	chr11:109741625-10
ENSG0000(	2550	63.77787	chr11:10(	ENSG00000250699		lncRNA	chr11:117316362-11
ENSG0000(	2550	63.77787	chr11:10(	SIK2		protein_c	chr11:111602449-11
ENSG0000(	2550	63.77787	chr11:10(	CLDN25		protein_c	chr11:113779796-11
ENSG0000(	2550	63.77787	chr11:10(	LINC02763		lncRNA	chr11:112393118-11
ENSG0000(	2550	63.77787	chr11:10(	KCTD9P4		Pseudoger	chr11:112180773-11
ENSG0000(	2550	63.77787	chr11:10(	RPL23AP62		Pseudoger	chr11:112461468-11
ENSG0000(	2550	63.77787	chr11:10(	COLCA2		protein_c	chr11:111298546-11
ENSG0000(	2550	63.77787	chr11:10(	APOA5		protein_c	chr11:116789367-11
ENSG0000(	2550	63.77787	chr11:10(	ST13P10		Pseudoger	chr11:112267290-11
ENSG0000(	2550	63.77787	chr11:10(	GNG5P3		Pseudoger	chr11:111864254-11
ENSG0000(	2550	63.77787	chr11:10(	APOA4		protein_c	chr11:116820700-11
ENSG0000(	2550	63.77787	chr11:10(	APOC3		protein_c	chr11:116829706-11
ENSG0000(	2550	63.77787	chr11:10(	LINC02550		lncRNA	chr11:111091932-11
ENSG0000(	2550	63.77787	chr11:10(	ENSG00000270204		Pseudoger	chr11:114517864-11
ENSG0000(	2550	63.77787	chr11:10(	ENSG00000255093		Pseudoger	chr11:111448450-11
ENSG0000(	2550	63.77787	chr11:10(	RNU6-376P		smallRNA	chr11:118702342-11
ENSG0000(	2550	63.77787	chr11:10(	DIXDC1		protein_c	chr11:111927144-11
ENSG0000(	2550	63.77787	chr11:10(	ENSG00000287245		lncRNA	chr11:109946581-10
ENSG0000(	2550	63.77787	chr11:10(	POU2AF2		protein_c	chr11:111245725-11

ENSG0000(	2550	63.77787	chr11:10(	NXPE1		protein_c	chr11:114518934-11
ENSG0000(	2550	63.77787	chr11:10(	COLCA1		lncRNA	chr11:111290787-11
ENSG0000(	2550	63.77787	chr11:10(	snoU13		smallRNA	chr11:114527129-11
ENSG0000(	2550	63.77787	chr11:10(	snoU13		smallRNA	chr11:116320344-11
ENSG0000(	2550	63.77787	chr11:10(	ENSG00000256281		lncRNA	chr11:115532322-11
ENSG0000(	2550	63.77787	chr11:10(	CADM1-AS1		lncRNA	chr11:115396756-11
ENSG0000(	2550	63.77787	chr11:10(	APOA1-AS		lncRNA	chr11:116836117-11
ENSG0000(	2550	63.77787	chr11:10(	PIH1D2		protein_c	chr11:112063218-11
ENSG0000(	2550	63.77787	chr11:10(	RNU6-893P		smallRNA	chr11:112032499-11
ENSG0000(	2550	63.77787	chr11:10(	ENSG00000256452		lncRNA	chr11:113818077-11
ENSG0000(	2550	63.77787	chr11:10(	TAGLN		protein_c	chr11:117199370-11
ENSG0000(	2550	63.77787	chr11:10(	SIDT2		protein_c	chr11:117178736-11
ENSG0000(	2550	63.77787	chr11:10(	ENSG00000287556		lncRNA	chr11:111013452-11
ENSG0000(	2550	63.77787	chr11:10(	BCO2		protein_c	chr11:112175510-11
ENSG0000(	2550	63.77787	chr11:10(	ENSG00000256533		Pseudoger	chr11:114453064-11
ENSG0000(	2550	63.77787	chr11:10(	LINC02702		lncRNA	chr11:116639422-11
ENSG0000(	2550	63.77787	chr11:10(	HTR3B		protein_c	chr11:113904796-11
ENSG0000(	2550	63.77787	chr11:10(	DLAT		protein_c	chr11:112025408-11
ENSG0000(	2550	63.77787	chr11:10(	NKAPD1		protein_c	chr11:112074086-11
ENSG0000(	2550	63.77787	chr11:10(	ENSG00000255663		protein_c	chr11:114400682-11
ENSG0000(	2550	63.77787	chr11:10(	RPL12P46		Pseudoger	chr11:115578049-11
ENSG0000(	2550	63.77787	chr11:10(	ENSG00000255689		lncRNA	chr11:115582283-11
ENSG0000(	2550	63.77787	chr11:10(	ENSG00000255710		Pseudoger	chr11:113820856-11
ENSG0000(	2550	63.77787	chr11:10(	PLET1		protein_c	chr11:112248153-11
ENSG0000(	2550	63.77787	chr11:10(	ENSG00000287028		lncRNA	chr11:111097155-11
ENSG0000(	2550	63.77787	chr11:10(	MIR34C		smallRNA	chr11:111513439-11
ENSG0000(	2550	63.77787	chr11:10(	NCAM1-AS1		lncRNA	chr11:113265137-11
ENSG0000(	2550	63.77787	chr11:10(	RNU7-187P		smallRNA	chr11:112977353-11
ENSG0000(	2550	63.77787	chr11:10(	ENSG00000271025		Pseudoger	chr11:112036627-11
ENSG0000(	2550	63.77787	chr11:10(	ENSG00000255870		Pseudoger	chr11:113789242-11
ENSG0000(	2550	63.77787	chr11:10(	RNA5SP351		Pseudoger	chr11:111928400-11
ENSG0000(	2550	63.77787	chr11:10(	TIMM8B		protein_c	chr11:112084800-11
ENSG0000(	2550	63.77787	chr11:10(	NXPE2P1		Pseudoger	chr11:114512706-11
ENSG0000(	2550	63.77787	chr11:10(	PPIHP1		Pseudoger	chr11:112029858-11
ENSG0000(	2550	63.77787	chr11:10(	MTRF1LP1		Pseudoger	chr11:113711720-11
ENSG0000(	2550	63.77787	chr11:10(	ATF4P4		Pseudoger	chr11:113789231-11
ENSG0000(	2550	63.77787	chr11:10(	ENSG00000256195		lncRNA	chr11:114343052-11
ENSG0000(	2550	63.77787	chr11:10(	PTS		protein_c	chr11:112226367-11
ENSG0000(	2550	63.77787	chr11:10(	TEX12		protein_c	chr11:112167372-11
ENSG0000(	2550	63.77787	chr11:10(	IL18		protein_c	chr11:112143253-11
ENSG0000(	2550	63.77787	chr11:10(	ENSG00000287238		lncRNA	chr11:118721104-11
ENSG0000(	2550	63.77787	chr11:10(	C11orf52		protein_c	chr11:111918032-11
ENSG0000(	2550	63.77787	chr11:10(	DRD2		protein_c	chr11:113409605-11
ENSG0000(	2550	63.77787	chr11:10(	NCAM1		protein_c	chr11:112961247-11
ENSG0000(	2550	63.77787	chr11:10(	ENSG00000224077		lncRNA	chr11:117098987-11
ENSG0000(	2550	63.77787	chr11:10(	BTG4		protein_c	chr11:111467526-11
ENSG0000(	2550	63.77787	chr11:10(	RDX		protein_c	chr11:109864295-11
ENSG0000(	2550	63.77787	chr11:10(	PPP2R1B		protein_c	chr11:111726908-11
ENSG0000(	2550	63.77787	chr11:10(	FDX1	NCGv7	protein_c	chr11:110429948-11
ENSG0000(	2550	63.77787	chr11:10(	C11orf1		protein_c	chr11:111878935-11
ENSG0000(	2550	63.77787	chr11:10(	FXYD6		protein_c	chr11:117836976-11

ENSG0000(	2550	63.77787	chr11:109	ARHGAP20		protein_c	chr11:110577042-11
ENSG0000(	2550	63.77787	chr11:109	FXYD2		protein_c	chr11:117800844-11
ENSG0000(	2550	63.77787	chr11:109	TMPRSS13	NCGv7	protein_c	chr11:117900641-11
ENSG0000(	2550	63.77787	chr11:109	RBM7	NCGv7	protein_c	chr11:114400030-11
ENSG0000(	2550	63.77787	chr11:109	TTC12		protein_c	chr11:113314579-11
ENSG0000(	2550	63.77787	chr11:109	ENSG00000260254		lncRNA	chr11:115638563-11
ENSG0000(	2550	63.77787	chr11:109	AP000936.2		smallRNA	chr11:117015430-11
ENSG0000(	2550	63.77787	chr11:109	AP002884.1		smallRNA	chr11:112247830-11
ENSG0000(	2550	63.77787	chr11:109	REXO2		protein_c	chr11:114439435-11
ENSG0000(	2550	63.77787	chr11:109	MIR4492		smallRNA	chr11:118910708-11
ENSG0000(	2550	63.77787	chr11:109	MIR4491		smallRNA	chr11:111347757-11
ENSG0000(	2550	63.77787	chr11:109	ENSG00000236267		lncRNA	chr11:116813204-11
ENSG0000(	2550	63.77787	chr11:109	LINC02151		lncRNA	chr11:116496568-11
ENSG0000(	2550	63.77787	chr11:109	BUD13-DT		lncRNA	chr11:116773389-11
ENSG0000(	2550	63.77787	chr11:109	RNA5SP350		Pseudoger	chr11:111040098-11
ENSG0000(	2550	63.77787	chr11:109	ENSG00000288097		lncRNA	chr11:111320379-11
ENSG0000(	2550	63.77787	chr11:109	BUD13		protein_c	chr11:116748170-11
ENSG0000(	2550	63.77787	chr11:109	NXPE4		protein_c	chr11:114570591-11
ENSG0000(	2550	63.77787	chr11:109	ZC3H12C		protein_c	chr11:110093392-11
ENSG0000(	2550	63.77787	chr11:109	ALG9		protein_c	chr11:111782195-11
ENSG0000(	2550	63.77787	chr11:109	ZW10		protein_c	chr11:113733187-11
ENSG0000(	2550	63.77787	chr11:109	ENSG00000256603		lncRNA	chr11:113770393-11
ENSG0000(	2550	63.77787	chr11:109	ENSG00000256674		Pseudoger	chr11:114583253-11
ENSG0000(	2550	63.77787	chr11:109	LINC02698		lncRNA	chr11:115659658-11
ENSG0000(	2550	63.77787	chr11:109	RN7SKP273		smallRNA	chr11:111683111-11
ENSG0000(	2550	63.77787	chr11:109	ENSG00000256757		lncRNA	chr11:113405321-11
ENSG0000(	2550	63.77787	chr11:109	ENSG00000287897		lncRNA	chr11:115332529-11
ENSG0000(	2550	63.77787	chr11:109	ENSG00000256947		lncRNA	chr11:114210616-11
ENSG0000(	2550	63.77787	chr11:109	ENSG00000256972		lncRNA	chr11:115333577-11
ENSG0000(	2550	63.77787	chr11:109	LINC02703		lncRNA	chr11:115920248-11
ENSG0000(	2550	63.77787	chr11:109	ENSG00000257070		Pseudoger	chr11:113769660-11
ENSG0000(	2550	63.77787	chr11:109	ENSG00000257087		Pseudoger	chr11:115538869-11
ENSG0000(	2550	63.77787	chr11:109	LRRC37A13P		Pseudoger	chr11:113791466-11
ENSG0000(	2550	63.77787	chr11:109	MIR4301		smallRNA	chr11:113450023-11
ENSG0000(	2550	63.77787	chr11:109	ENSG00000258529		protein_c	chr11:111786286-11
ENSG0000(	2550	63.77787	chr11:109	ENSG00000288020		lncRNA	chr11:115734848-11
ENSG0000(	2550	63.77787	chr11:109	ENSG00000288070		lncRNA	chr11:112822806-11
ENSG0000(	2550	63.77787	chr11:109	ENSG00000287006		lncRNA	chr11:112698772-11
ENSG0000(	2550	63.77787	chr11:109	TMPRSS5		protein_c	chr11:113687547-11
ENSG0000(	2550	63.77787	chr11:109	snosnR66		smallRNA	chr11:112602354-11
ENSG0000(	2550	63.77787	chr11:109	ENSG00000285769		lncRNA	chr11:112637324-11
ENSG0000(	2550	63.77787	chr11:109	PHLDB1		protein_c	chr11:118606440-11
ENSG0000(	2550	63.77787	chr11:109	HOATZ		protein_c	chr11:111514778-11
ENSG0000(	2550	63.77787	chr11:109	ENSG00000286463		lncRNA	chr11:114159791-11
ENSG0000(	2550	63.77787	chr11:109	ENSG00000255422		lncRNA	chr11:118700415-11
ENSG0000(	2550	63.77787	chr11:109	FDXACB1		protein_c	chr11:111874056-11
ENSG0000(	2550	63.77787	chr11:109	MIR34B		smallRNA	chr11:111512938-11
ENSG0000(	2550	63.77787	chr11:109	BACE1		protein_c	chr11:117285232-11
ENSG0000(	2550	63.77787	chr11:109	ENSG00000270423		Pseudoger	chr11:109985243-10
ENSG0000(	2550	63.77787	chr11:109	LAYN		protein_c	chr11:111540280-11
ENSG0000(	2550	63.77787	chr11:109	SDHD	NCGv7;AC	protein_c	chr11:112086824-11

ENSG0000(	2550	63.77787	chr11:10(	ENSG00000268472		lncRNA	chr11:112260265-11
ENSG0000(	2550	63.77787	chr11:10(	NXPE2		protein_c	chr11:114678527-11
ENSG0000(	2550	63.77787	chr11:10(	ENSG00000255210		Pseudoger	chr11:110049528-11
ENSG0000(	2550	63.77787	chr11:10(	ENSG00000270202		Pseudoger	chr11:111107060-11
ENSG0000(	2550	63.77787	chr11:10(	ENSG00000289230		lncRNA	chr11:114057969-11
ENSG0000(	2550	63.77787	chr11:10(	ENSG00000255428		lncRNA	chr11:111414242-11
ENSG0000(	2550	63.77787	chr11:10(	RPSAP50		Pseudoger	chr11:109982192-10
ENSG0000(	2550	63.77787	chr11:10(	RNY4P6		smallRNA	chr11:117015897-11
ENSG0000(	2550	63.77787	chr11:10(	MIR34BHG		lncRNA	chr11:111510600-11
ENSG0000(	2550	63.77787	chr11:10(	MRPS36P4		Pseudoger	chr11:112208601-11
ENSG0000(	2550	63.77787	chr11:10(	ENSG00000247416		lncRNA	chr11:112959279-11
ENSG0000(	2550	63.77787	chr11:10(	TREHP1		Pseudoger	chr11:118688033-11
ENSG0000(	2550	63.77787	chr11:10(	CADM1		protein_c	chr11:115169218-11
ENSG0000(	2550	63.77787	chr11:10(	ENSG00000270403		lncRNA	chr11:117668483-11
ENSG0000(	2550	63.77787	chr11:10(	LINC00900		lncRNA	chr11:115753889-11
ENSG0000(	2550	63.77787	chr11:10(	RPS6P16		Pseudoger	chr11:112235371-11
ENSG0000(	2550	63.77787	chr11:10(	ENSG00000255580		lncRNA	chr11:115363629-11
ENSG0000(	2550	63.77787	chr11:10(	HTR3A		protein_c	chr11:113975075-11
ENSG0000(	2550	63.77787	chr11:10(	ENSG00000255292		protein_c	chr11:112086903-11
ENSG0000(	2550	63.77787	chr11:10(	ENSG00000245869		lncRNA	chr11:118885841-11
ENSG0000(	2550	63.77787	chr11:10(	snoU13		smallRNA	chr11:115627082-11
ENSG0000(	2550	63.77787	chr11:10(	ENSG00000270179		lncRNA	chr11:113368478-11
ENSG0000(	2550	63.77787	chr11:10(	RPS17P15		Pseudoger	chr11:111105547-11
ENSG0000(	2550	63.77787	chr11:10(	C11orf71		protein_c	chr11:114391443-11
ENSG0000(	2550	63.77787	chr11:10(	ENSG00000280143		TEC	chr11:117204967-11
ENSG0000(	2550	63.77787	chr11:10(	TTC12-DT		lncRNA	chr11:113278250-11
ENSG0000(	2550	63.77787	chr11:10(	RPL37AP8		Pseudoger	chr11:111889199-11
ENSG0000(	2550	63.77787	chr11:10(	ENSG00000255149		Pseudoger	chr11:110876994-11
ENSG0000(	2550	63.77787	chr11:10(	ENSG00000255286		Pseudoger	chr11:111945639-11
ENSG0000(	2550	63.77787	chr11:10(	DSCAML1		protein_c	chr11:117427772-11
ENSG0000(	2550	63.77787	chr11:10(	ENSG00000239600		Pseudoger	chr11:115951105-11
ENSG0000(	2550	63.77787	chr11:10(	ENSG00000285513		lncRNA	chr11:116820645-11
ENSG0000(	2550	63.77787	chr11:10(	ENSG00000255334		lncRNA	chr11:112015307-11
ENSG0000(	2550	63.77787	chr11:10(	FXYD6-FXYD2		protein_c	chr11:117820163-11
ENSG0000(	2550	63.77787	chr11:10(	ENSG00000255176		lncRNA	chr11:118636620-11
ENSG0000(	2550	63.77787	chr11:10(	ENSG00000244259		Pseudoger	chr11:115950196-11
ENSG0000(	2550	63.77787	chr11:10(	HNRNPA1P60		Pseudoger	chr11:110788026-11
ENSG0000(	2550	63.77787	chr11:10(	RPS12P21		Pseudoger	chr11:112218326-11
ENSG0000(	2550	63.77787	chr11:10(	RPS29P19		Pseudoger	chr11:113751116-11
ENSG0000(	2550	63.77787	chr11:10(	ENSG00000255599		lncRNA	chr11:115628065-11
ENSG0000(	2550	63.77787	chr11:10(	LINC02764		lncRNA	chr11:112534220-11
ENSG0000(	2546	63.67783	chr11:10(	RN7SL529P		smallRNA	chr11:118994334-11
ENSG0000(	2545	63.65281	chr11:10(	MIR1261		smallRNA	chr11:90869121-908
ENSG0000(	2545	63.65281	chr11:10(	ENSG00000288018		lncRNA	chr11:88337839-884
ENSG0000(	2545	63.65281	chr11:10(	FZD4-DT		lncRNA	chr11:86955616-870
ENSG0000(	2545	63.65281	chr11:10(	ENSG00000213287		Pseudoger	chr11:87258851-872
ENSG0000(	2545	63.65281	chr11:10(	ENSG00000250946		Pseudoger	chr11:89820082-898
ENSG0000(	2545	63.65281	chr11:10(	TRIM64EP		Pseudoger	chr11:90057605-900
ENSG0000(	2545	63.65281	chr11:10(	HNRNPCP6		Pseudoger	chr11:85020785-850
ENSG0000(	2545	63.65281	chr11:10(	CTSC		protein_c	chr11:88265069-883
ENSG0000(	2545	63.65281	chr11:10(	GRM5	NCGv7	protein_c	chr11:88504576-890

ENSG0000(	2545	63.65281	chr11:109	ENSG00000280307		TEC	chr11:87879473-878
ENSG0000(	2545	63.65281	chr11:109	PICALM	NCGv7;AC	protein_c	chr11:85957175-860
ENSG0000(	2545	63.65281	chr11:109	ENSG00000280124		TEC	chr11:91184934-911
ENSG0000(	2545	63.65281	chr11:109	RNU6-16P		smallRNA	chr11:88612805-886
ENSG0000(	2545	63.65281	chr11:109	EED	NCGv7	protein_c	chr11:86244753-862
ENSG0000(	2545	63.65281	chr11:109	ENSG00000288809		lncRNA	chr11:86244353-862
ENSG0000(	2545	63.65281	chr11:109	ENSG00000280093		TEC	chr11:91382909-913
ENSG0000(	2545	63.65281	chr11:109	ENSG00000280201		TEC	chr11:89576913-895
ENSG0000(	2545	63.65281	chr11:109	RNU6-560P		smallRNA	chr11:86153227-861
ENSG0000(	2545	63.65281	chr11:109	TRIM53AP		Pseudoger	chr11:89993536-899
ENSG0000(	2545	63.65281	chr11:109	TYR		protein_c	chr11:89177875-892
ENSG0000(	2545	63.65281	chr11:109	TRIM49		protein_c	chr11:89797655-898
ENSG0000(	2545	63.65281	chr11:109	RNU6-1063P		smallRNA	chr11:87648489-876
ENSG0000(	2545	63.65281	chr11:109	NAALAD2		protein_c	chr11:90131515-901
ENSG0000(	2545	63.65281	chr11:109	ENSG00000280339		TEC	chr11:86908990-869
ENSG0000(	2545	63.65281	chr11:109	ENSG00000280085		TEC	chr11:90972316-909
ENSG0000(	2545	63.65281	chr11:109	ENSG00000280367		TEC	chr11:90223153-902
ENSG0000(	2545	63.65281	chr11:109	ENSG00000280379		TEC	chr11:93171134-931
ENSG0000(	2545	63.65281	chr11:109	ENSG00000280385		TEC	chr11:90193614-901
ENSG0000(	2545	63.65281	chr11:109	ENSG00000280430		TEC	chr11:90731110-907
ENSG0000(	2545	63.65281	chr11:109	CCDC89		protein_c	chr11:85683848-856
ENSG0000(	2545	63.65281	chr11:109	CHORDC1		protein_c	chr11:90200429-902
ENSG0000(	2545	63.65281	chr11:109	NOX4	NCGv7	protein_c	chr11:89324353-894
ENSG0000(	2545	63.65281	chr11:109	LINC02746		lncRNA	chr11:92913227-929
ENSG0000(	2545	63.65281	chr11:109	ENSG00000286665		lncRNA	chr11:90110638-901
ENSG0000(	2545	63.65281	chr11:109	ENSG00000279701		TEC	chr11:87490144-874
ENSG0000(	2545	63.65281	chr11:109	TRIM64B		protein_c	chr11:89870438-898
ENSG0000(	2545	63.65281	chr11:109	ENSG00000278989		TEC	chr11:86434924-864
ENSG0000(	2545	63.65281	chr11:109	ENSG00000279697		TEC	chr11:89545128-895
ENSG0000(	2545	63.65281	chr11:109	ENSG00000279684		TEC	chr11:93286629-932
ENSG0000(	2545	63.65281	chr11:109	ENSG00000240174		Pseudoger	chr11:84639993-846
ENSG0000(	2545	63.65281	chr11:109	GAPDHP70		Pseudoger	chr11:88408179-884
ENSG0000(	2545	63.65281	chr11:109	RN7SL225P		smallRNA	chr11:86324014-863
ENSG0000(	2545	63.65281	chr11:109	ENSG00000279603		TEC	chr11:89300709-893
ENSG0000(	2545	63.65281	chr11:109	ENSG00000279056		TEC	chr11:89556860-895
ENSG0000(	2545	63.65281	chr11:109	ENSG00000279163		TEC	chr11:89704164-897
ENSG0000(	2545	63.65281	chr11:109	SNORD56		smallRNA	chr11:90118391-901
ENSG0000(	2545	63.65281	chr11:109	ENSG00000279209		TEC	chr11:91013787-910
ENSG0000(	2545	63.65281	chr11:109	ENSG00000279454		TEC	chr11:93587703-935
ENSG0000(	2545	63.65281	chr11:109	ENSG00000279248		TEC	chr11:89533645-895
ENSG0000(	2545	63.65281	chr11:109	AP004242.1		smallRNA	chr11:93407825-934
ENSG0000(	2545	63.65281	chr11:109	ENSG00000279438		TEC	chr11:91093875-910
ENSG0000(	2545	63.65281	chr11:109	ENSG00000279269		TEC	chr11:93206990-932
ENSG0000(	2545	63.65281	chr11:109	ENSG00000279297		TEC	chr11:89297717-892
ENSG0000(	2545	63.65281	chr11:109	ENSG00000279299		TEC	chr11:90125658-901
ENSG0000(	2545	63.65281	chr11:109	ENSG00000279304		TEC	chr11:93133855-931
ENSG0000(	2545	63.65281	chr11:109	ENSG00000279341		TEC	chr11:87718667-877
ENSG0000(	2545	63.65281	chr11:109	RN7SL223P		smallRNA	chr11:93556913-935
ENSG0000(	2545	63.65281	chr11:109	ENSG00000278980		TEC	chr11:91208838-912
ENSG0000(	2545	63.65281	chr11:109	ENSG00000279733		TEC	chr11:88165575-881
ENSG0000(	2545	63.65281	chr11:109	ENSG00000279742		TEC	chr11:85852557-858

ENSG0000(	2545	63.65281	chr11:109	TRIM51EP		Pseudoger	chr11:89981441-899
ENSG0000(	2545	63.65281	chr11:109	OR7E13P		Pseudoger	chr11:86832540-868
ENSG0000(	2545	63.65281	chr11:109	SLC36A4		protein_c	chr11:93144174-931
ENSG0000(	2545	63.65281	chr11:109	RPS3AP42		Pseudoger	chr11:92498152-924
ENSG0000(	2545	63.65281	chr11:109	NDUFB11P1		Pseudoger	chr11:92336032-923
ENSG0000(	2545	63.65281	chr11:109	snoU13		smallRNA	chr11:92729330-927
ENSG0000(	2545	63.65281	chr11:109	CBX3P7		Pseudoger	chr11:89293745-892
ENSG0000(	2545	63.65281	chr11:109	PGAM1P9		Pseudoger	chr11:92366496-923
ENSG0000(	2545	63.65281	chr11:109	TMEM126A		protein_c	chr11:85647967-856
ENSG0000(	2545	63.65281	chr11:109	TMEM126B		protein_c	chr11:85628573-856
ENSG0000(	2545	63.65281	chr11:109	FZD4		protein_c	chr11:86945679-869
ENSG0000(	2545	63.65281	chr11:109	RPL26P31		Pseudoger	chr11:93052395-930
ENSG0000(	2545	63.65281	chr11:109	ENSG00000279836		TEC	chr11:87121058-871
ENSG0000(	2545	63.65281	chr11:109	RPL7AP57		Pseudoger	chr11:92161106-921
ENSG0000(	2545	63.65281	chr11:109	ENSG00000278837		Pseudoger	chr11:89954593-899
ENSG0000(	2545	63.65281	chr11:109	snoU13		smallRNA	chr11:86094192-860
ENSG0000(	2545	63.65281	chr11:109	ENSG00000278859		TEC	chr11:91992833-919
ENSG0000(	2545	63.65281	chr11:109	ENSG00000278892		TEC	chr11:93071529-930
ENSG0000(	2545	63.65281	chr11:109	ENSG00000278929		TEC	chr11:87565994-875
ENSG0000(	2545	63.65281	chr11:109	ENSG00000278953		TEC	chr11:93592187-935
ENSG0000(	2545	63.65281	chr11:109	ENSG00000279793		TEC	chr11:87816999-878
ENSG0000(	2545	63.65281	chr11:109	ENSG00000279045		TEC	chr11:89548130-895
ENSG0000(	2545	63.65281	chr11:109	SRP14P2		Pseudoger	chr11:93535468-935
ENSG0000(	2545	63.65281	chr11:109	TRIM64		protein_c	chr11:89966037-899
ENSG0000(	2545	63.65281	chr11:109	ENSG00000254705		lncRNA	chr11:92400191-924
ENSG0000(	2545	63.65281	chr11:109	HNRNPCP8		Pseudoger	chr11:87024583-870
ENSG0000(	2545	63.65281	chr11:109	TRIM51BP		Pseudoger	chr11:89854953-898
ENSG0000(	2545	63.65281	chr11:109	ENSG00000255250		lncRNA	chr11:86727355-867
ENSG0000(	2545	63.65281	chr11:109	RAB38		protein_c	chr11:88113251-881
ENSG0000(	2545	63.65281	chr11:109	ENSG00000254733		lncRNA	chr11:86431590-866
ENSG0000(	2545	63.65281	chr11:109	ENSG00000254731		lncRNA	chr11:86703099-867
ENSG0000(	2545	63.65281	chr11:109	MIR4490		smallRNA	chr11:90555774-905
ENSG0000(	2545	63.65281	chr11:109	PTP4A1P6		Pseudoger	chr11:86432098-864
ENSG0000(	2545	63.65281	chr11:109	HNRNPA1P72		Pseudoger	chr11:84545131-845
ENSG0000(	2545	63.65281	chr11:109	ENSG00000255285		Pseudoger	chr11:89789412-897
ENSG0000(	2545	63.65281	chr11:109	SNRPGP16		Pseudoger	chr11:92937441-929
ENSG0000(	2545	63.65281	chr11:109	H3P34		Pseudoger	chr11:89498748-894
ENSG0000(	2545	63.65281	chr11:109	TRIM64DP		Pseudoger	chr11:89776981-897
ENSG0000(	2545	63.65281	chr11:109	ENSG00000255305		Pseudoger	chr11:89978540-899
ENSG0000(	2545	63.65281	chr11:109	CCDC83		protein_c	chr11:85855101-859
ENSG0000(	2545	63.65281	chr11:109	TRIM49C	IntOGen-I	protein_c	chr11:90031106-900
ENSG0000(	2545	63.65281	chr11:109	LINC02695		lncRNA	chr11:86192787-861
ENSG0000(	2545	63.65281	chr11:109	AP002364.1		smallRNA	chr11:90324776-903
ENSG0000(	2545	63.65281	chr11:109	ENSG00000254684		Pseudoger	chr11:85336075-853
ENSG0000(	2545	63.65281	chr11:109	OSBPL9P2		Pseudoger	chr11:91114724-911
ENSG0000(	2545	63.65281	chr11:109	ENSG00000254655		Pseudoger	chr11:89960422-899
ENSG0000(	2545	63.65281	chr11:109	MTCYBP41		Pseudoger	chr11:87815035-878
ENSG0000(	2545	63.65281	chr11:109	ENSG00000254617		Pseudoger	chr11:89887366-898
ENSG0000(	2545	63.65281	chr11:109	TUBB4BP4		Pseudoger	chr11:91949731-919
ENSG0000(	2545	63.65281	chr11:109	DLG2	NCGv7	protein_c	chr11:83455012-856
ENSG0000(	2545	63.65281	chr11:109	ENSG00000204456		Pseudoger	chr11:89766440-897

ENSG0000(	2545	63.65281	chr11:109	TRIM49D2		protein_c	chr11:89924064-899
ENSG0000(	2545	63.65281	chr11:109	XIAPP2		Pseudoger	chr11:87094734-870
ENSG0000(	2545	63.65281	chr11:109	ENSG00000254897		Pseudoger	chr11:85452282-854
ENSG0000(	2545	63.65281	chr11:109	FOLH1B		Pseudoger	chr11:89639237-896
ENSG0000(	2545	63.65281	chr11:109	FOLH1B		lncRNA	chr11:89659297-896
ENSG0000(	2545	63.65281	chr11:109	LINC02748		lncRNA	chr11:91157994-912
ENSG0000(	2545	63.65281	chr11:109	ENSG00000254916		Pseudoger	chr11:90051837-900
ENSG0000(	2545	63.65281	chr11:109	ENSG00000254888		Pseudoger	chr11:90074839-900
ENSG0000(	2545	63.65281	chr11:109	ENSG00000255184		Pseudoger	chr11:90017611-900
ENSG0000(	2545	63.65281	chr11:109	SETP17		Pseudoger	chr11:86294760-862
ENSG0000(	2545	63.65281	chr11:109	ENSG00000270510		Pseudoger	chr11:87323709-873
ENSG0000(	2545	63.65281	chr11:109	ENSG00000254874		lncRNA	chr11:92965797-929
ENSG0000(	2545	63.65281	chr11:109	LINC02711		lncRNA	chr11:87718354-877
ENSG0000(	2545	63.65281	chr11:109	ENSG00000255233		lncRNA	chr11:93240133-932
ENSG0000(	2545	63.65281	chr11:109	TRIM49D1		protein_c	chr11:89911111-899
ENSG0000(	2545	63.65281	chr11:109	ENSG00000255235		Pseudoger	chr11:89883927-898
ENSG0000(	2545	63.65281	chr11:109	ENSG00000255241		lncRNA	chr11:88098591-881
ENSG0000(	2545	63.65281	chr11:109	ENSG00000254818		Pseudoger	chr11:89944035-899
ENSG0000(	2545	63.65281	chr11:109	TUBAP2		Pseudoger	chr11:90282560-902
ENSG0000(	2545	63.65281	chr11:109	ENSG00000254803		Pseudoger	chr11:90049945-900
ENSG0000(	2545	63.65281	chr11:109	ENSG00000254794		Pseudoger	chr11:87917948-879
ENSG0000(	2545	63.65281	chr11:109	CCDC81	NCGv7	protein_c	chr11:86374736-864
ENSG0000(	2545	63.65281	chr11:109	ENSG00000254787		lncRNA	chr11:84720826-848
ENSG0000(	2545	63.65281	chr11:109	ENSG00000254785		Pseudoger	chr11:89895799-899
ENSG0000(	2545	63.65281	chr11:109	ENSG00000254783		Pseudoger	chr11:86283927-862
ENSG0000(	2545	63.65281	chr11:109	HIKESHI	NCGv7	protein_c	chr11:86302211-863
ENSG0000(	2545	63.65281	chr11:109	OR7E2P		Pseudoger	chr11:86857059-868
ENSG0000(	2545	63.65281	chr11:109	SYTL2	NCGv7	protein_c	chr11:85694224-858
ENSG0000(	2545	63.65281	chr11:109	PSMA2P1		Pseudoger	chr11:87329407-873
ENSG0000(	2545	63.65281	chr11:109	GRM5-AS1		lncRNA	chr11:88504576-885
ENSG0000(	2545	63.65281	chr11:109	ENSG00000255429		lncRNA	chr11:89546637-895
ENSG0000(	2545	63.65281	chr11:109	Y_RNA		smallRNA	chr11:88099995-881
ENSG0000(	2545	63.65281	chr11:109	ENSG00000255516		lncRNA	chr11:88050106-880
ENSG0000(	2545	63.65281	chr11:109	ENSG00000255445		Pseudoger	chr11:93152075-931
ENSG0000(	2545	63.65281	chr11:109	OSBPL9P3		Pseudoger	chr11:91115961-911
ENSG0000(	2545	63.65281	chr11:109	ENSG00000269895		lncRNA	chr11:86833068-868
ENSG0000(	2545	63.65281	chr11:109	FAT3	NCGv7	protein_c	chr11:92224818-928
ENSG0000(	2545	63.65281	chr11:109	DEUP1		protein_c	chr11:93329971-934
ENSG0000(	2545	63.65281	chr11:109	ENSG00000255515		Pseudoger	chr11:93609760-936
ENSG0000(	2545	63.65281	chr11:109	ENSG00000255506		lncRNA	chr11:92748732-927
ENSG0000(	2545	63.65281	chr11:109	SMCO4		protein_c	chr11:93478472-935
ENSG0000(	2545	63.65281	chr11:109	ENSG00000255102		lncRNA	chr11:88061774-880
ENSG0000(	2545	63.65281	chr11:109	ENSG00000255540		Pseudoger	chr11:89731017-897
ENSG0000(	2545	63.65281	chr11:109	TRIM53BP		Pseudoger	chr11:89841997-898
ENSG0000(	2545	63.65281	chr11:109	RNU6-1135P		smallRNA	chr11:87606783-876
ENSG0000(	2545	63.65281	chr11:109	PRSS23-AS1		lncRNA	chr11:86892214-869
ENSG0000(	2545	63.65281	chr11:109	ENSG00000290774		lncRNA	chr11:92915884-929
ENSG0000(	2545	63.65281	chr11:109	DISC1FP1		lncRNA	chr11:90251204-909
ENSG0000(	2545	63.65281	chr11:109	RNU6-1292P		smallRNA	chr11:85509938-855
ENSG0000(	2545	63.65281	chr11:109	ME3		protein_c	chr11:86441108-866
ENSG0000(	2545	63.65281	chr11:109	ENSG00000255162		Pseudoger	chr11:89785945-897

ENSG0000(	2545	63.65281	chr11:10(	EEF1A1P49		Pseudoger	chr11:92914603-929
ENSG0000(	2545	63.65281	chr11:10(	TMEM135		protein_c	chr11:87037844-873
ENSG0000(	2545	63.65281	chr11:10(	ENSG00000255486		Pseudoger	chr11:89723482-897
ENSG0000(	2545	63.65281	chr11:10(	LINC02756		lncRNA	chr11:91794319-918
ENSG0000(	2545	63.65281	chr11:10(	AP003305.1		smallRNA	chr11:84229560-842
ENSG0000(	2545	63.65281	chr11:10(	UBTFL2		Pseudoger	chr11:89752785-897
ENSG0000(	2545	63.65281	chr11:10(	UBTFL1		protein_c	chr11:90085950-900
ENSG0000(	2545	63.65281	chr11:10(	CREBZF	NCGv7	protein_c	chr11:85657742-856
ENSG0000(	2545	63.65281	chr11:10(	PRSS23		protein_c	chr11:86791059-869
ENSG0000(	2545	63.65281	chr11:10(	ENSG00000254558		Pseudoger	chr11:89863848-898
ENSG0000(	2545	63.65281	chr11:10(	ENSG00000254971		lncRNA	chr11:89753982-897
ENSG0000(	2545	63.65281	chr11:10(	TRIM77		protein_c	chr11:89710299-897
ENSG0000(	2545	63.65281	chr11:10(	MIR3166		smallRNA	chr11:88176502-881
ENSG0000(	2545	63.65281	chr11:10(	FNTAP1		Pseudoger	chr11:86195132-861
ENSG0000(	2545	63.65281	chr11:10(	AP001482.1		smallRNA	chr11:89112710-891
ENSG0000(	2545	63.65281	chr11:10(	ENSG00000233737		Pseudoger	chr11:93221486-932
ENSG0000(	2545	63.65281	chr11:10(	ENSG00000255005		lncRNA	chr11:85916502-859
ENSG0000(	2545	63.65281	chr11:10(	ENSG00000255360		Pseudoger	chr11:89955146-899
ENSG0000(	2545	63.65281	chr11:10(	MTNR1B		protein_c	chr11:92969651-929
ENSG0000(	2545	63.65281	chr11:10(	ENSG00000255555		lncRNA	chr11:84936689-849
ENSG0000(	2545	63.65281	chr11:10(	ENSG00000255011		Pseudoger	chr11:90071136-900
ENSG0000(	2545	63.65281	chr11:10(	MOB4P2		Pseudoger	chr11:86821143-868
ENSG0000(	2545	63.65281	chr11:10(	SLC25A1P1		Pseudoger	chr11:85934737-859
ENSG0000(	2545	63.65281	chr11:10(	ENSG00000254436		Pseudoger	chr11:90098421-900
ENSG0000(	2545	63.65281	chr11:10(	ENSG00000255022		Pseudoger	chr11:87816224-878
ENSG0000(	2545	63.65281	chr11:10(	ENSG00000255170		Pseudoger	chr11:89764884-897
ENSG0000(	2545	63.65281	chr11:10(	ENSG00000290492		lncRNA	chr11:89777155-897
ENSG0000(	2545	63.65281	chr11:10(	UBTFL10		Pseudoger	chr11:89700764-897
ENSG0000(	2545	63.65281	chr11:10(	ENSG00000255391		Pseudoger	chr11:87480736-874
ENSG0000(	2545	63.65281	chr11:10(	ENSG00000255396		Pseudoger	chr11:84997226-849
ENSG0000(	2545	63.65281	chr11:10(	MTND1P35		Pseudoger	chr11:89908540-899
ENSG0000(	2537	63.45273	chr11:10(	MIR3656		smallRNA	chr11:119018944-11
ENSG0000(	2519	63.00253	chr11:10(	AP005718.1		smallRNA	chr11:108469455-10
ENSG0000(	2514	62.87748	chr11:10(	ENSG00000255893		lncRNA	chr11:94472908-944
ENSG0000(	2514	62.87748	chr11:10(	KDM4F		protein_c	chr11:95049422-950
ENSG0000(	2514	62.87748	chr11:10(	MMP10		protein_c	chr11:102770502-10
ENSG0000(	2514	62.87748	chr11:10(	RNU6-262P		smallRNA	chr11:119289562-11
ENSG0000(	2514	62.87748	chr11:10(	PIWIL4		protein_c	chr11:94543840-946
ENSG0000(	2514	62.87748	chr11:10(	ENSG00000255548		lncRNA	chr11:103945548-10
ENSG0000(	2514	62.87748	chr11:10(	KDM4D		protein_c	chr11:94973709-949
ENSG0000(	2514	62.87748	chr11:10(	BCL9L	NCGv7;AC	protein_c	chr11:118893875-11
ENSG0000(	2514	62.87748	chr11:10(	CENATAC		protein_c	chr11:118998138-11
ENSG0000(	2514	62.87748	chr11:10(	ENSG00000286345		lncRNA	chr11:106241974-10
ENSG0000(	2514	62.87748	chr11:10(	RPL23AP64		Pseudoger	chr11:119003012-11
ENSG0000(	2514	62.87748	chr11:10(	RPA2P3		Pseudoger	chr11:100336856-10
ENSG0000(	2514	62.87748	chr11:10(	ENSG00000255528		lncRNA	chr11:109002465-10
ENSG0000(	2514	62.87748	chr11:10(	C11orf87		protein_c	chr11:109422190-10
ENSG0000(	2514	62.87748	chr11:10(	MTMR12P1		Pseudoger	chr11:101208905-10
ENSG0000(	2514	62.87748	chr11:10(	LINC02713		lncRNA	chr11:97878475-979
ENSG0000(	2514	62.87748	chr11:10(	CARD18		protein_c	chr11:105137714-10
ENSG0000(	2514	62.87748	chr11:10(	SNORA40		smallRNA	chr11:93735111-937

ENSG0000(	2514	62.87748	chr11:10(	ENSG00000285921		lncRNA	chr11:96092374-961
ENSG0000(	2514	62.87748	chr11:10(	ENSG00000285878		lncRNA	chr11:103252217-10
ENSG0000(	2514	62.87748	chr11:10(	RPS2P39		Pseudoger	chr11:108690289-10
ENSG0000(	2514	62.87748	chr11:10(	GRIA4		protein_c	chr11:105609535-10
ENSG0000(	2514	62.87748	chr11:10(	ENSG00000240652		Pseudoger	chr11:107908420-10
ENSG0000(	2514	62.87748	chr11:10(	SNORD5		smallRNA	chr11:93733228-937
ENSG0000(	2514	62.87748	chr11:10(	JRKL-AS1		lncRNA	chr11:96447132-965
ENSG0000(	2514	62.87748	chr11:10(	LINC02700		lncRNA	chr11:94638045-946
ENSG0000(	2514	62.87748	chr11:10(	ENSG00000255653		Pseudoger	chr11:95011858-950
ENSG0000(	2514	62.87748	chr11:10(	snoU13		smallRNA	chr11:102058305-10
ENSG0000(	2514	62.87748	chr11:10(	GUCY1A2	NCGv7	protein_c	chr11:106674019-10
ENSG0000(	2514	62.87748	chr11:10(	H2AX		protein_c	chr11:119093874-11
ENSG0000(	2514	62.87748	chr11:10(	KRT8P7		Pseudoger	chr11:119602875-11
ENSG0000(	2514	62.87748	chr11:10(	TMEM123		protein_c	chr11:102396332-10
ENSG0000(	2514	62.87748	chr11:10(	RN7SL688P		smallRNA	chr11:118931211-11
ENSG0000(	2514	62.87748	chr11:10(	CWF19L2		protein_c	chr11:107326360-10
ENSG0000(	2514	62.87748	chr11:10(	ENSG00000239861		Pseudoger	chr11:103045237-10
ENSG0000(	2514	62.87748	chr11:10(	DYNC2H1		protein_c	chr11:103109410-10
ENSG0000(	2514	62.87748	chr11:10(	ANGPTL5		protein_c	chr11:101890674-10
ENSG0000(	2514	62.87748	chr11:10(	ENSG00000255605		lncRNA	chr11:95698086-957
ENSG0000(	2514	62.87748	chr11:10(	PIWIL4-AS1		lncRNA	chr11:94545330-947
ENSG0000(	2514	62.87748	chr11:10(	TRAPPC4		protein_c	chr11:119018763-11
ENSG0000(	2514	62.87748	chr11:10(	ENSG00000255995		Pseudoger	chr11:93991346-939
ENSG0000(	2514	62.87748	chr11:10(	ENSG00000288528		lncRNA	chr11:102316173-10
ENSG0000(	2514	62.87748	chr11:10(	FAM76B		protein_c	chr11:95768953-957
ENSG0000(	2514	62.87748	chr11:10(	SNORD39		smallRNA	chr11:109263494-10
ENSG0000(	2514	62.87748	chr11:10(	ALKBH8		protein_c	chr11:107502727-10
ENSG0000(	2514	62.87748	chr11:10(	CASP5		protein_c	chr11:104994235-10
ENSG0000(	2514	62.87748	chr11:10(	CASP1		protein_c	chr11:105025397-10
ENSG0000(	2514	62.87748	chr11:10(	RNU6-1123P		smallRNA	chr11:119656311-11
ENSG0000(	2514	62.87748	chr11:10(	MMP13	NCGv7	protein_c	chr11:102942995-10
ENSG0000(	2514	62.87748	chr11:10(	SLC37A4		protein_c	chr11:119023751-11
ENSG0000(	2514	62.87748	chr11:10(	RNA5SP347		Pseudoger	chr11:97657464-976
ENSG0000(	2514	62.87748	chr11:10(	YAP1	AC	protein_c	chr11:102110447-10
ENSG0000(	2514	62.87748	chr11:10(	DCUN1D5		protein_c	chr11:103050686-10
ENSG0000(	2514	62.87748	chr11:10(	CFAP300		protein_c	chr11:102047437-10
ENSG0000(	2514	62.87748	chr11:10(	MMP27		protein_c	chr11:102691487-10
ENSG0000(	2514	62.87748	chr11:10(	MMP20		protein_c	chr11:102576832-10
ENSG0000(	2514	62.87748	chr11:10(	MMP7	DriverDBv	protein_c	chr11:102520508-10
ENSG0000(	2514	62.87748	chr11:10(	ENSG00000288255		Pseudoger	chr11:105055567-10
ENSG0000(	2514	62.87748	chr11:10(	RNA5SP345		Pseudoger	chr11:95840017-958
ENSG0000(	2514	62.87748	chr11:10(	MCAM		protein_c	chr11:119308529-11
ENSG0000(	2514	62.87748	chr11:10(	Y_RNA		smallRNA	chr11:118970498-11
ENSG0000(	2514	62.87748	chr11:10(	ENSG00000261098		lncRNA	chr11:107312132-10
ENSG0000(	2514	62.87748	chr11:10(	RNU4-55P		smallRNA	chr11:105824634-10
ENSG0000(	2514	62.87748	chr11:10(	AP000673.1		smallRNA	chr11:105933954-10
ENSG0000(	2514	62.87748	chr11:10(	Y_RNA		smallRNA	chr11:93719603-937
ENSG0000(	2514	62.87748	chr11:10(	MMP12	AC	protein_c	chr11:102862736-10
ENSG0000(	2514	62.87748	chr11:10(	ENSG00000288833		lncRNA	chr11:102346213-10
ENSG0000(	2514	62.87748	chr11:10(	THY1-AS1		lncRNA	chr11:119417951-11
ENSG0000(	2514	62.87748	chr11:10(	MIR3920		smallRNA	chr11:101519820-10

ENSG0000(	2514	62.87748	chr11:10(	AP000765.1		smallRNA	chr11:94416710-944
ENSG0000(	2514	62.87748	chr11:10(	AP001282.1		smallRNA	chr11:106801349-10
ENSG0000(	2514	62.87748	chr11:10(	ENSG00000288789		lncRNA	chr11:94650324-946
ENSG0000(	2514	62.87748	chr11:10(	ENSG00000260966		lncRNA	chr11:103050687-10
ENSG0000(	2514	62.87748	chr11:10(	SNORA18		smallRNA	chr11:93733466-937
ENSG0000(	2514	62.87748	chr11:10(	ACAT1		protein_c	chr11:108116695-10
ENSG0000(	2514	62.87748	chr11:10(	MIR4693		smallRNA	chr11:103849906-10
ENSG0000(	2514	62.87748	chr11:10(	AP003730.1		smallRNA	chr11:97912797-979
ENSG0000(	2514	62.87748	chr11:10(	ENSG00000260008		lncRNA	chr11:102107886-10
ENSG0000(	2514	62.87748	chr11:10(	TRPC6	NCGv7	protein_c	chr11:101451564-10
ENSG0000(	2514	62.87748	chr11:10(	ENSG00000288047		lncRNA	chr11:119892700-11
ENSG0000(	2514	62.87748	chr11:10(	ENSG00000256035		Pseudoger	chr11:102806948-10
ENSG0000(	2514	62.87748	chr11:10(	MMP3		protein_c	chr11:102835801-10
ENSG0000(	2514	62.87748	chr11:10(	ENSG00000287545		lncRNA	chr11:119832966-11
ENSG0000(	2514	62.87748	chr11:10(	CASP4		protein_c	chr11:104942866-10
ENSG0000(	2514	62.87748	chr11:10(	snoU13		smallRNA	chr11:100934274-10
ENSG0000(	2514	62.87748	chr11:10(	ENSG00000256469		lncRNA	chr11:94874052-949
ENSG0000(	2514	62.87748	chr11:10(	snoU13		smallRNA	chr11:93797213-937
ENSG0000(	2514	62.87748	chr11:10(	LINC02552		lncRNA	chr11:104445868-10
ENSG0000(	2514	62.87748	chr11:10(	ENSG00000285842		lncRNA	chr11:95571040-957
ENSG0000(	2514	62.87748	chr11:10(	CNTN5		protein_c	chr11:99020949-100
ENSG0000(	2514	62.87748	chr11:10(	MIR1260B		smallRNA	chr11:96341438-963
ENSG0000(	2514	62.87748	chr11:10(	MMP1		protein_c	chr11:102789401-10
ENSG0000(	2514	62.87748	chr11:10(	CWC15		protein_c	chr11:94962620-949
ENSG0000(	2514	62.87748	chr11:10(	RNU7-159P		smallRNA	chr11:102903892-10
ENSG0000(	2514	62.87748	chr11:10(	FUT4		protein_c	chr11:94543921-945
ENSG0000(	2514	62.87748	chr11:10(	HMBS		protein_c	chr11:119084866-11
ENSG0000(	2514	62.87748	chr11:10(	ENSG00000256254		Pseudoger	chr11:102766801-10
ENSG0000(	2514	62.87748	chr11:10(	AP003558.1		smallRNA	chr11:99424938-994
ENSG0000(	2514	62.87748	chr11:10(	HYOU1		protein_c	chr11:119044188-11
ENSG0000(	2514	62.87748	chr11:10(	AASDHPPT		protein_c	chr11:106075501-10
ENSG0000(	2514	62.87748	chr11:10(	AC015600.1		smallRNA	chr11:100794441-10
ENSG0000(	2514	62.87748	chr11:10(	ENSG00000287846		Pseudoger	chr11:106106398-10
ENSG0000(	2514	62.87748	chr11:10(	ENSG00000288012		lncRNA	chr11:108008678-10
ENSG0000(	2514	62.87748	chr11:10(	RN7SL195P		smallRNA	chr11:93800483-938
ENSG0000(	2514	62.87748	chr11:10(	PGR	NCGv7	protein_c	chr11:101029624-10
ENSG0000(	2514	62.87748	chr11:10(	C11orf97		protein_c	chr11:94512461-945
ENSG0000(	2514	62.87748	chr11:10(	ENSG00000256916		lncRNA	chr11:102606916-10
ENSG0000(	2514	62.87748	chr11:10(	HPRT1P3		Pseudoger	chr11:93998643-939
ENSG0000(	2514	62.87748	chr11:10(	ARPC3P3		Pseudoger	chr11:94188449-941
ENSG0000(	2514	62.87748	chr11:10(	SNORA8		smallRNA	chr11:93732361-937
ENSG0000(	2514	62.87748	chr11:10(	ATM	NCGv7;AC	protein_c	chr11:108223044-10
ENSG0000(	2514	62.87748	chr11:10(	LINC02737		lncRNA	chr11:96508425-965
ENSG0000(	2514	62.87748	chr11:10(	ENSG00000287802		lncRNA	chr11:106465985-10
ENSG0000(	2514	62.87748	chr11:10(	SESN3		protein_c	chr11:95165513-952
ENSG0000(	2514	62.87748	chr11:10(	ENDOD1		protein_c	chr11:95089846-951
ENSG0000(	2514	62.87748	chr11:10(	CCDC82		protein_c	chr11:96349241-963
ENSG0000(	2514	62.87748	chr11:10(	NPAT		protein_c	chr11:108157215-10
ENSG0000(	2514	62.87748	chr11:10(	MTMR2		protein_c	chr11:95821766-959
ENSG0000(	2514	62.87748	chr11:10(	RN7SL222P		smallRNA	chr11:100641978-10
ENSG0000(	2514	62.87748	chr11:10(	PHB1P16		Pseudoger	chr11:94055023-940

ENSG0000(	2514	62.87748	chr11:109	MAML2	NCGv7;AC	protein_c	chr11:95976598-963
ENSG0000(	2514	62.87748	chr11:109	EXPH5	NCGv7	protein_c	chr11:108505435-10
ENSG0000(	2514	62.87748	chr11:109	ENSG00000254730		Pseudoger	chr11:108727741-10
ENSG0000(	2514	62.87748	chr11:109	C2CD2L		protein_c	chr11:119102198-11
ENSG0000(	2514	62.87748	chr11:109	PDZD3		protein_c	chr11:119185457-11
ENSG0000(	2514	62.87748	chr11:109	ABCG4		protein_c	chr11:119149052-11
ENSG0000(	2514	62.87748	chr11:109	HINFP	NCGv7	protein_c	chr11:119121580-11
ENSG0000(	2514	62.87748	chr11:109	ENSG00000254702		Pseudoger	chr11:107736009-10
ENSG0000(	2514	62.87748	chr11:109	DPAGT1		protein_c	chr11:119096025-11
ENSG0000(	2514	62.87748	chr11:109	ENSG00000254599		lncRNA	chr11:98676391-986
ENSG0000(	2514	62.87748	chr11:109	ENSG00000254590		Pseudoger	chr11:119402792-11
ENSG0000(	2514	62.87748	chr11:109	ENSG00000254587		lncRNA	chr11:96590317-969
ENSG0000(	2514	62.87748	chr11:109	LINC02719		lncRNA	chr11:106112459-10
ENSG0000(	2514	62.87748	chr11:109	ENSG00000254569		Pseudoger	chr11:104873264-10
ENSG0000(	2514	62.87748	chr11:109	MTND2P26		Pseudoger	chr11:103406213-10
ENSG0000(	2514	62.87748	chr11:109	ENSG00000254555		Pseudoger	chr11:97908253-979
ENSG0000(	2514	62.87748	chr11:109	PLS1P1		Pseudoger	chr11:101765935-10
ENSG0000(	2514	62.87748	chr11:109	GPR83		protein_c	chr11:94377316-944
ENSG0000(	2514	62.87748	chr11:109	VPS11-DT		lncRNA	chr11:119067374-11
ENSG0000(	2514	62.87748	chr11:109	CASP1P2		Pseudoger	chr11:105063345-10
ENSG0000(	2514	62.87748	chr11:109	ENSG00000271751		lncRNA	chr11:119065263-11
ENSG0000(	2514	62.87748	chr11:109	ENSG00000254892		Pseudoger	chr11:119403963-11
ENSG0000(	2514	62.87748	chr11:109	ENSG00000254890		Pseudoger	chr11:109486968-10
ENSG0000(	2514	62.87748	chr11:109	ENSG00000271600		Pseudoger	chr11:102308557-10
ENSG0000(	2514	62.87748	chr11:109	VPS11		protein_c	chr11:119067818-11
ENSG0000(	2514	62.87748	chr11:109	NLRX1		protein_c	chr11:119166568-11
ENSG0000(	2514	62.87748	chr11:109	NECTIN1-DT		lncRNA	chr11:119729583-11
ENSG0000(	2514	62.87748	chr11:109	ENSG00000254830		lncRNA	chr11:98938912-989
ENSG0000(	2514	62.87748	chr11:109	ENSG00000254758		Pseudoger	chr11:107642887-10
ENSG0000(	2514	62.87748	chr11:109	ENSG00000254824		Pseudoger	chr11:103409580-10
ENSG0000(	2514	62.87748	chr11:109	ENSG00000279696		TEC	chr11:93726654-937
ENSG0000(	2514	62.87748	chr11:109	ENSG00000254811		Pseudoger	chr11:106264315-10
ENSG0000(	2514	62.87748	chr11:109	RNF26	NCGv7	protein_c	chr11:119334527-11
ENSG0000(	2514	62.87748	chr11:109	SMARCE1P1		Pseudoger	chr11:107403404-10
ENSG0000(	2514	62.87748	chr11:109	OR2AL1P		Pseudoger	chr11:105194440-10
ENSG0000(	2514	62.87748	chr11:109	ENSG00000254506		Pseudoger	chr11:101584295-10
ENSG0000(	2514	62.87748	chr11:109	DDI1		protein_c	chr11:104036640-10
ENSG0000(	2514	62.87748	chr11:109	PDGFD		protein_c	chr11:103907189-10
ENSG0000(	2514	62.87748	chr11:109	ENSG00000276521		Pseudoger	chr11:102963460-10
ENSG0000(	2514	62.87748	chr11:109	ST13P11		Pseudoger	chr11:94913047-949
ENSG0000(	2514	62.87748	chr11:109	ANKRD49		protein_c	chr11:94493979-944
ENSG0000(	2514	62.87748	chr11:109	snoU13		smallRNA	chr11:102056963-10
ENSG0000(	2514	62.87748	chr11:109	ARHGAP42		protein_c	chr11:100687288-10
ENSG0000(	2514	62.87748	chr11:109	CEP295		protein_c	chr11:93661682-937
ENSG0000(	2514	62.87748	chr11:109	TAF1D		protein_c	chr11:93729948-937
ENSG0000(	2514	62.87748	chr11:109	AMOTL1	NCGv7	protein_c	chr11:94706431-948
ENSG0000(	2514	62.87748	chr11:109	MMP8		protein_c	chr11:102711796-10
ENSG0000(	2514	62.87748	chr11:109	CEP57		protein_c	chr11:95789965-958
ENSG0000(	2514	62.87748	chr11:109	CUL5		protein_c	chr11:108008898-10
ENSG0000(	2514	62.87748	chr11:109	ENSG00000225678		Pseudoger	chr11:102751070-10
ENSG0000(	2514	62.87748	chr11:109	C11orf65		protein_c	chr11:108308519-10

ENSG0000(	2514	62.87748	chr11:10(	RNA5SP348	smallRNA	chr11:104252579-10
ENSG0000(	2514	62.87748	chr11:10(	RNU6-277P	smallRNA	chr11:105974826-10
ENSG0000(	2514	62.87748	chr11:10(	ENSG00000277459	lncRNA	chr11:102109827-10
ENSG0000(	2514	62.87748	chr11:10(	RPS25	protein_c	chr11:119015712-11
ENSG0000(	2514	62.87748	chr11:10(	MSANTD4	protein_c	chr11:105995623-10
ENSG0000(	2514	62.87748	chr11:10(	ENSG00000273600	Pseudoger	chr11:95756959-957
ENSG0000(	2514	62.87748	chr11:10(	ENSG00000250390	lncRNA	chr11:95482406-954
ENSG0000(	2514	62.87748	chr11:10(	ENSG00000254482	Pseudoger	chr11:109637468-10
ENSG0000(	2514	62.87748	chr11:10(	ENSG00000250519	lncRNA	chr11:94185439-942
ENSG0000(	2514	62.87748	chr11:10(	SLN	protein_c	chr11:107707378-10
ENSG0000(	2514	62.87748	chr11:10(	MIR1304	smallRNA	chr11:93733674-937
ENSG0000(	2514	62.87748	chr11:10(	ENSG00000254433	lncRNA	chr11:106085990-10
ENSG0000(	2514	62.87748	chr11:10(	ENSG00000254428	lncRNA	chr11:119003742-11
ENSG0000(	2514	62.87748	chr11:10(	MIR548L	smallRNA	chr11:94466495-944
ENSG0000(	2514	62.87748	chr11:10(	ENSG00000254422	lncRNA	chr11:102229851-10
ENSG0000(	2514	62.87748	chr11:10(	ENSG00000254406	Pseudoger	chr11:119821304-11
ENSG0000(	2514	62.87748	chr11:10(	ENSG00000277984	Pseudoger	chr11:95057282-950
ENSG0000(	2514	62.87748	chr11:10(	ENSG00000213252	Pseudoger	chr11:106826392-10
ENSG0000(	2514	62.87748	chr11:10(	MTND1P36	Pseudoger	chr11:103407441-10
ENSG0000(	2514	62.87748	chr11:10(	ENSG00000274584	Pseudoger	chr11:106824025-10
ENSG0000(	2514	62.87748	chr11:10(	MTCO3P15	Pseudoger	chr11:103402013-10
ENSG0000(	2514	62.87748	chr11:10(	ELMOD1	protein_c	chr11:107591091-10
ENSG0000(	2514	62.87748	chr11:10(	AP001925.1	protein_c	chr11:108259528-10
ENSG0000(	2514	62.87748	chr11:10(	SLC35F2	protein_c	chr11:107790991-10
ENSG0000(	2514	62.87748	chr11:10(	ENSG00000243777	Pseudoger	chr11:102295060-10
ENSG0000(	2514	62.87748	chr11:10(	DUXAP5	Pseudoger	chr11:119444991-11
ENSG0000(	2514	62.87748	chr11:10(	ENSG00000255376	Pseudoger	chr11:96642671-966
ENSG0000(	2514	62.87748	chr11:10(	ENSG00000284057	protein_c	chr11:93741664-938
ENSG0000(	2514	62.87748	chr11:10(	HEPHL1	protein_c	chr11:94021354-941
ENSG0000(	2514	62.87748	chr11:10(	ASS1P13	Pseudoger	chr11:107176286-10
ENSG0000(	2514	62.87748	chr11:10(	MTCO1P15	Pseudoger	chr11:103404309-10
ENSG0000(	2514	62.87748	chr11:10(	TMEM123-DT	lncRNA	chr11:102452919-10
ENSG0000(	2514	62.87748	chr11:10(	ENSG00000255336	Pseudoger	chr11:105246880-10
ENSG0000(	2514	62.87748	chr11:10(	USP2-AS1	lncRNA	chr11:119356467-11
ENSG0000(	2514	62.87748	chr11:10(	ENSG00000245385	lncRNA	chr11:119336249-11
ENSG0000(	2514	62.87748	chr11:10(	LNCRNA-IUR	lncRNA	chr11:95150539-952
ENSG0000(	2514	62.87748	chr11:10(	ACA64	smallRNA	chr11:119323293-11
ENSG0000(	2514	62.87748	chr11:10(	WTAPP1	Pseudoger	chr11:102746968-10
ENSG0000(	2514	62.87748	chr11:10(	NECTIN1-AS1	lncRNA	chr11:119709920-11
ENSG0000(	2514	62.87748	chr11:10(	KBTBD3	protein_c	chr11:106051098-10
ENSG0000(	2514	62.87748	chr11:10(	ENSG00000284715	Pseudoger	chr11:104975356-10
ENSG0000(	2514	62.87748	chr11:10(	CYCSP29	Pseudoger	chr11:108822333-10
ENSG0000(	2514	62.87748	chr11:10(	ENSG00000255467	lncRNA	chr11:108105074-10
ENSG0000(	2514	62.87748	chr11:10(	ENSG00000285813	lncRNA	chr11:105995185-10
ENSG0000(	2514	62.87748	chr11:10(	ENSG00000255483	Pseudoger	chr11:107600379-10
ENSG0000(	2514	62.87748	chr11:10(	ENSG00000255482	lncRNA	chr11:102467255-10
ENSG0000(	2514	62.87748	chr11:10(	ENSG00000285696	lncRNA	chr11:108142458-10
ENSG0000(	2514	62.87748	chr11:10(	BOLA3P1	Pseudoger	chr11:102880492-10
ENSG0000(	2514	62.87748	chr11:10(	IZUMO1R	protein_c	chr11:94304580-943
ENSG0000(	2514	62.87748	chr11:10(	JRKL	protein_c	chr11:96389989-965
ENSG0000(	2514	62.87748	chr11:10(	ENSG00000255380	Pseudoger	chr11:98130239-981

ENSG0000(	2514	62.87748	chr11:10(	RPL32P25		Pseudoger	chr11:95963219-959
ENSG0000(	2514	62.87748	chr11:10(	THY1		protein_c	chr11:119415476-11
ENSG0000(	2514	62.87748	chr11:10(	CASP1P1		Pseudoger	chr11:105122661-10
ENSG0000(	2514	62.87748	chr11:10(	MTCO2P15		Pseudoger	chr11:103403512-10
ENSG0000(	2514	62.87748	chr11:10(	HSPD1P13		Pseudoger	chr11:105706299-10
ENSG0000(	2514	62.87748	chr11:10(	C11orf54		protein_c	chr11:93741591-937
ENSG0000(	2514	62.87748	chr11:10(	ENSG00000282834		Pseudoger	chr11:95203727-952
ENSG0000(	2514	62.87748	chr11:10(	ENSG00000270449		Pseudoger	chr11:104682383-10
ENSG0000(	2514	62.87748	chr11:10(	PGR-AS1		lncRNA	chr11:101129077-10
ENSG0000(	2514	62.87748	chr11:10(	CEP126		protein_c	chr11:101915010-10
ENSG0000(	2514	62.87748	chr11:10(	LINC02553		lncRNA	chr11:97222644-972
ENSG0000(	2514	62.87748	chr11:10(	ENSG00000255028		lncRNA	chr11:109355085-10
ENSG0000(	2514	62.87748	chr11:10(	ENSG00000254987		lncRNA	chr11:103675994-10
ENSG0000(	2514	62.87748	chr11:10(	PANX1		protein_c	chr11:94128841-941
ENSG0000(	2514	62.87748	chr11:10(	ENSG00000254939		Pseudoger	chr11:98565074-985
ENSG0000(	2514	62.87748	chr11:10(	MTATP6P15		Pseudoger	chr11:103402588-10
ENSG0000(	2514	62.87748	chr11:10(	VSTM5		protein_c	chr11:93818232-938
ENSG0000(	2514	62.87748	chr11:10(	ENSG00000255114		lncRNA	chr11:119044188-11
ENSG0000(	2514	62.87748	chr11:10(	BIRC2	AC	protein_c	chr11:102347211-10
ENSG0000(	2514	62.87748	chr11:10(	SCARNA9		lncRNA	chr11:93721513-937
ENSG0000(	2514	62.87748	chr11:10(	UPK2		protein_c	chr11:118925164-11
ENSG0000(	2514	62.87748	chr11:10(	CBL	NCGv7;AC	protein_c	chr11:119206298-11
ENSG0000(	2514	62.87748	chr11:10(	NECTIN1		protein_c	chr11:119623408-11
ENSG0000(	2514	62.87748	chr11:10(	ENSG00000254909		Pseudoger	chr11:119005727-11
ENSG0000(	2514	62.87748	chr11:10(	PPIAP43		Pseudoger	chr11:100666459-10
ENSG0000(	2514	62.87748	chr11:10(	ENSG00000270969		Pseudoger	chr11:107585901-10
ENSG0000(	2514	62.87748	chr11:10(	RAB39A		protein_c	chr11:107928448-10
ENSG0000(	2514	62.87748	chr11:10(	DDX10	NCGv7;AC	protein_c	chr11:108665058-10
ENSG0000(	2514	62.87748	chr11:10(	ARHGAP42-AS1		lncRNA	chr11:100684162-10
ENSG0000(	2514	62.87748	chr11:10(	ENSG00000281655		lncRNA	chr11:102641078-10
ENSG0000(	2514	62.87748	chr11:10(	CARD17		Pseudoger	chr11:105092486-10
ENSG0000(	2514	62.87748	chr11:10(	RNU6-952P		smallRNA	chr11:102313722-10
ENSG0000(	2514	62.87748	chr11:10(	ENSG00000270578		Pseudoger	chr11:95145437-951
ENSG0000(	2514	62.87748	chr11:10(	POGLUT3		protein_c	chr11:108472112-10
ENSG0000(	2514	62.87748	chr11:10(	MED28P5		Pseudoger	chr11:97000274-970
ENSG0000(	2514	62.87748	chr11:10(	CENATAC-DT		lncRNA	chr11:118994824-11
ENSG0000(	2514	62.87748	chr11:10(	BUD13P1		Pseudoger	chr11:95143637-951
ENSG0000(	2514	62.87748	chr11:10(	CCDC153		protein_c	chr11:119190250-11
ENSG0000(	2514	62.87748	chr11:10(	ENSG00000270753		Pseudoger	chr11:97086160-970
ENSG0000(	2514	62.87748	chr11:10(	ENSG00000280167		TEC	chr11:94559018-945
ENSG0000(	2514	62.87748	chr11:10(	ENSG00000270868		Pseudoger	chr11:102306112-10
ENSG0000(	2514	62.87748	chr11:10(	FOXR1	NCGv7	protein_c	chr11:118971712-11
ENSG0000(	2514	62.87748	chr11:10(	ENSG00000255065		Pseudoger	chr11:106310045-10
ENSG0000(	2514	62.87748	chr11:10(	SRSF8		protein_c	chr11:95066919-950
ENSG0000(	2514	62.87748	chr11:10(	BIRC3	NCGv7;AC	protein_c	chr11:102317450-10
ENSG0000(	2514	62.87748	chr11:10(	ENSG00000290797		lncRNA	chr11:105092469-10
ENSG0000(	2514	62.87748	chr11:10(	CARD16		protein_c	chr11:105041326-10
ENSG0000(	2514	62.87748	chr11:10(	CASP12		protein_c	chr11:104885718-10
ENSG0000(	2514	62.87748	chr11:10(	RN7SKP115		smallRNA	chr11:100839630-10
ENSG0000(	2514	62.87748	chr11:10(	ENSG00000233536		lncRNA	chr11:94638038-946
ENSG0000(	2514	62.87748	chr11:10(	SNORA25		smallRNA	chr11:93730513-937

ENSG0000(	2514	62.87748	chr11:109	ENSG00000290498		lncRNA	chr11:106250019-10
ENSG0000(	2514	62.87748	chr11:109	MRE11	AC	protein_c	chr11:94415570-944
ENSG0000(	2514	62.87748	chr11:109	ENSG00000289124		lncRNA	chr11:119101493-11
ENSG0000(	2514	62.87748	chr11:109	ENSG00000203334		lncRNA	chr11:108957718-10
ENSG0000(	2514	62.87748	chr11:109	ENSG00000289232		lncRNA	chr11:106579494-10
ENSG0000(	2514	62.87748	chr11:109	SNORD6		smallRNA	chr11:93731502-937
ENSG0000(	2514	62.87748	chr11:109	RN7SKP53		smallRNA	chr11:99120176-991
ENSG0000(	2514	62.87748	chr11:109	USP2		protein_c	chr11:119355215-11
ENSG0000(	2514	62.87748	chr11:109	RNA5SP349		Pseudoger	chr11:109120878-10
ENSG0000(	2514	62.87748	chr11:109	Y_RNA		smallRNA	chr11:108229503-10
ENSG0000(	2514	62.87748	chr11:109	MED17		protein_c	chr11:93784227-938
ENSG0000(	2514	62.87748	chr11:109	C1QTNF5		protein_c	chr11:119338942-11
ENSG0000(	2514	62.87748	chr11:109	CASP4LP		Pseudoger	chr11:104903453-10
ENSG0000(	2514	62.87748	chr11:109	SNORA32		smallRNA	chr11:93730979-937
ENSG0000(	2514	62.87748	chr11:109	CASP4LP		lncRNA	chr11:104901549-10
ENSG0000(	2514	62.87748	chr11:109	SNORA1		smallRNA	chr11:93732004-937
ENSG0000(	2514	62.87748	chr11:109	Y_RNA		smallRNA	chr11:108084913-10
ENSG0000(	2514	62.87748	chr11:109	ENSG00000289383		lncRNA	chr11:105181864-10
ENSG0000(	2514	62.87748	chr11:109	MFRP		protein_c	chr11:119338942-11
ENSG0000(	2514	62.87748	chr11:109	KDM4E		protein_c	chr11:95025258-950
ENSG0000(	2514	62.87748	chr11:109	Y_RNA		smallRNA	chr11:95473272-954
ENSG0000(	2514	62.87748	chr11:109	Y_RNA		smallRNA	chr11:93827622-938
ENSG0000(	2514	62.87748	chr11:109	ENSG00000290773		lncRNA	chr11:102752123-10
ENSG0000(	2514	62.87748	chr11:109	ENSG00000289553		lncRNA	chr11:119584444-11
ENSG0000(	2514	62.87748	chr11:109	RNU6-654P		smallRNA	chr11:109005517-10
ENSG0000(	2514	62.87748	chr11:109	RNA5SP346		Pseudoger	chr11:96474572-964
ENSG0000(	2376	59.42597	chr11:446	ARHGEF12	NCGv7;AC	protein_c	chr11:120336413-12
ENSG0000(	2376	59.42597	chr11:446	POU2F3		protein_c	chr11:120236640-12
ENSG0000(	2376	59.42597	chr11:446	ELOCP22		Pseudoger	chr11:120619934-12
ENSG0000(	2376	59.42597	chr11:446	ENSG00000255216		lncRNA	chr11:120008044-12
ENSG0000(	2376	59.42597	chr11:446	ENSG00000286992		lncRNA	chr11:119975745-12
ENSG0000(	2376	59.42597	chr11:446	ENSG00000286731		lncRNA	chr11:120026753-12
ENSG0000(	2376	59.42597	chr11:446	OAF		protein_c	chr11:120211032-12
ENSG0000(	2376	59.42597	chr11:446	TLCD5		protein_c	chr11:120325296-12
ENSG0000(	2376	59.42597	chr11:446	LINC02744		lncRNA	chr11:119987952-11
ENSG0000(	2376	59.42597	chr11:446	ENSG00000176984		lncRNA	chr11:120168977-12
ENSG0000(	2376	59.42597	chr11:446	ENSG00000259541		lncRNA	chr11:120249759-12
ENSG0000(	2376	59.42597	chr11:446	TRIM29		protein_c	chr11:120111286-12
ENSG0000(	1973	49.34656	chr6:105(	H3C3	NCGv7	protein_c	chr6:26045384-2604
ENSG0000(	1687	42.19344	chr11:76(	ENSG00000290061		lncRNA	chr11:65492897-654
ENSG0000(	1607	40.19256	chr6:105(	RPL36P10		Pseudoger	chr6:46438310-4643
ENSG0000(	1591	39.79239	chr6:105(	ENSG00000262048		Pseudoger	chr6:79406240-7940
ENSG0000(	1590	39.76738	chr6:105(	MIR4647		smallRNA	chr6:44254206-4425
ENSG0000(	1590	39.76738	chr6:105(	AL109615.1		smallRNA	chr6:44013001-4401
ENSG0000(	1590	39.76738	chr6:105(	C6orf223		lncRNA	chr6:44000580-4400
ENSG0000(	1590	39.76738	chr6:105(	ENSG00000283573		lncRNA	chr6:43803193-4384
ENSG0000(	1590	39.76738	chr6:105(	MRPL14	DriverDB\	protein_c	chr6:44113451-4412
ENSG0000(	1590	39.76738	chr6:105(	RCAN2	NCGv7	protein_c	chr6:46220736-4649
ENSG0000(	1590	39.76738	chr6:105(	TMEM63B		protein_c	chr6:44126914-4415
ENSG0000(	1590	39.76738	chr6:105(	AARS2	DriverDB\	protein_c	chr6:44298731-4431
ENSG0000(	1590	39.76738	chr6:105(	ENSG00000272442		protein_c	chr6:44273194-4437

ENSG0000(	1590	39.76738	chr6:105(	ENSG00000272114		lncRNA	chr6:43770429-4377
ENSG0000(	1590	39.76738	chr6:105(	SLC29A1	DriverDBv	protein_c	chr6:44219553-4423
ENSG0000(	1590	39.76738	chr6:105(	LINC01512		lncRNA	chr6:43891045-4393
ENSG0000(	1590	39.76738	chr6:105(	AL355353.1		smallRNA	chr6:47462851-4746
ENSG0000(	1590	39.76738	chr6:105(	ENSG00000287562		lncRNA	chr6:44090787-4409
ENSG0000(	1590	39.76738	chr6:105(	RUNX2	TAG	protein_c	chr6:45328157-4566
ENSG0000(	1590	39.76738	chr6:105(	OPN5		protein_c	chr6:47781982-4783
ENSG0000(	1590	39.76738	chr6:105(	ENSG00000183239		Pseudoger	chr6:44089242-4408
ENSG0000(	1590	39.76738	chr6:105(	ENSG00000223469		lncRNA	chr6:43851757-4385
ENSG0000(	1590	39.76738	chr6:105(	CAPN11		protein_c	chr6:44158811-4418
ENSG0000(	1590	39.76738	chr6:105(	ADGRF5		protein_c	chr6:46852522-4695
ENSG0000(	1590	39.76738	chr6:105(	ENSG00000236961		lncRNA	chr6:43722786-4373
ENSG0000(	1590	39.76738	chr6:105(	SCIRT		lncRNA	chr6:43931572-4407
ENSG0000(	1590	39.76738	chr6:105(	ENSG00000276156		Pseudoger	chr6:45097496-4509
ENSG0000(	1590	39.76738	chr6:105(	ENSG00000219384		Pseudoger	chr6:45158870-4515
ENSG0000(	1590	39.76738	chr6:105(	ENSG00000226594		lncRNA	chr6:47729827-4774
ENSG0000(	1590	39.76738	chr6:105(	ADGRF5-AS1		lncRNA	chr6:46903471-4690
ENSG0000(	1590	39.76738	chr6:105(	PLA2G7		protein_c	chr6:46704201-4673
ENSG0000(	1590	39.76738	chr6:105(	TNFRSF21		protein_c	chr6:47231532-4730
ENSG0000(	1590	39.76738	chr6:105(	ENSG00000231881		lncRNA	chr6:44058792-4408
ENSG0000(	1590	39.76738	chr6:105(	RN7SKP116		smallRNA	chr6:47715283-4771
ENSG0000(	1590	39.76738	chr6:105(	RNU6-515P		smallRNA	chr6:45646104-4564
ENSG0000(	1590	39.76738	chr6:105(	TMEM151B		protein_c	chr6:44270450-4430
ENSG0000(	1590	39.76738	chr6:105(	RNU6-754P		smallRNA	chr6:46018745-4601
ENSG0000(	1590	39.76738	chr6:105(	ACTG1P9		Pseudoger	chr6:46204729-4620
ENSG0000(	1590	39.76738	chr6:105(	ENSG00000231769		lncRNA	chr6:46096004-4612
ENSG0000(	1590	39.76738	chr6:105(	TCTE1		protein_c	chr6:44278734-4429
ENSG0000(	1590	39.76738	chr6:105(	ENSG00000236164		lncRNA	chr6:46758296-4676
ENSG0000(	1590	39.76738	chr6:105(	NFKBIE	NCGv7	protein_c	chr6:44258166-4426
ENSG0000(	1590	39.76738	chr6:105(	ENSG00000237530		lncRNA	chr6:44513262-4452
ENSG0000(	1590	39.76738	chr6:105(	CYP39A1		protein_c	chr6:46549580-4665
ENSG0000(	1590	39.76738	chr6:105(	SPATS1		protein_c	chr6:44342650-4438
ENSG0000(	1590	39.76738	chr6:105(	RBM22P4		Pseudoger	chr6:45098021-4509
ENSG0000(	1590	39.76738	chr6:105(	ADGRF2	NCGv7	Pseudoger	chr6:47656487-4769
ENSG0000(	1590	39.76738	chr6:105(	TDRD6		protein_c	chr6:46687875-4670
ENSG0000(	1590	39.76738	chr6:105(	ENPP4		protein_c	chr6:46129989-4614
ENSG0000(	1590	39.76738	chr6:105(	CD2AP		protein_c	chr6:47477789-4762
ENSG0000(	1590	39.76738	chr6:105(	ENSG00000289609		lncRNA	chr6:43990798-4399
ENSG0000(	1590	39.76738	chr6:105(	LINC02537		lncRNA	chr6:43844878-4385
ENSG0000(	1590	39.76738	chr6:105(	TDRD6-AS1		lncRNA	chr6:46670444-4668
ENSG0000(	1590	39.76738	chr6:105(	B3GNTL1P2		Pseudoger	chr6:47368943-4736
ENSG0000(	1590	39.76738	chr6:105(	ADGRF4		protein_c	chr6:47685864-4772
ENSG0000(	1590	39.76738	chr6:105(	HSP90AB1	NCGv7	protein_c	chr6:44246166-4425
ENSG0000(	1590	39.76738	chr6:105(	ENSG00000216813		Pseudoger	chr6:47562622-4756
ENSG0000(	1590	39.76738	chr6:105(	MEP1A	NCGv7	protein_c	chr6:46793389-4683
ENSG0000(	1590	39.76738	chr6:105(	ANKRD66		protein_c	chr6:46746933-4675
ENSG0000(	1590	39.76738	chr6:105(	SUPT3H		protein_c	chr6:44809317-4537
ENSG0000(	1590	39.76738	chr6:105(	RCAN2-DT		lncRNA	chr6:46492052-4660
ENSG0000(	1590	39.76738	chr6:105(	ENSG00000287485		lncRNA	chr6:47374561-4739
ENSG0000(	1590	39.76738	chr6:105(	RNU1-105P		smallRNA	chr6:47823390-4782
ENSG0000(	1590	39.76738	chr6:105(	MIR586		smallRNA	chr6:45197674-4519

ENSG0000(	1590	39.76738	chr6:105(	Y_RNA		smallRNA	chr6:47489067-4748
ENSG0000(	1590	39.76738	chr6:105(	SLC35B2		protein_c	chr6:44254096-4425
ENSG0000(	1590	39.76738	chr6:105(	ADGRF1	AC	protein_c	chr6:46997708-4704
ENSG0000(	1590	39.76738	chr6:105(	MYMX		protein_c	chr6:44216926-4421
ENSG0000(	1590	39.76738	chr6:105(	NUDT19P4		Pseudoger	chr6:44898711-4489
ENSG0000(	1590	39.76738	chr6:105(	CDC5L	NCGv7;AC	protein_c	chr6:44387706-4445
ENSG0000(	1590	39.76738	chr6:105(	CD2AP-DT		lncRNA	chr6:47477208-4747
ENSG0000(	1590	39.76738	chr6:105(	ENPP5		protein_c	chr6:46159185-4617
ENSG0000(	1590	39.76738	chr6:105(	RUNX2-AS1		lncRNA	chr6:45573346-4557
ENSG0000(	1590	39.76738	chr6:105(	ENSG00000279076		TEC	chr6:44551577-4455
ENSG0000(	1590	39.76738	chr6:105(	CLIC5	NCGv7	protein_c	chr6:45880827-4608
ENSG0000(	1590	39.76738	chr6:105(	VEGFA	DriverDBv	protein_c	chr6:43770184-4378
ENSG0000(	1590	39.76738	chr6:105(	ENSG00000271857		lncRNA	chr6:45421079-4542
ENSG0000(	1590	39.76738	chr6:105(	MIR4642		smallRNA	chr6:44435641-4443
ENSG0000(	1590	39.76738	chr6:105(	SLC25A27		protein_c	chr6:46652915-4667
ENSG0000(	1590	39.76738	chr6:105(	ENSG00000216616		Pseudoger	chr6:47752828-4775
ENSG0000(	1590	39.76738	chr6:105(	ENSG00000286417		lncRNA	chr6:44727921-4483
ENSG0000(	1587	39.69234	chr6:105(	ENSG00000288614		protein_c	chr6:53041266-5304
ENSG0000(	1555	38.89199	chr6:105(	ENSG00000272541		lncRNA	chr6:57855891-5785
ENSG0000(	1552	38.81696	chr6:105(	ENSG00000261745		lncRNA	chr6:53125644-5312
ENSG0000(	1540	38.51683	chr6:105(	AC002485.1		smallRNA	chr6:66359425-6635
ENSG0000(	1533	38.34175	chr6:105(	HMGN3-AS1		lncRNA	chr6:79233699-7923
ENSG0000(	1533	38.34175	chr6:105(	HMGN3		protein_c	chr6:79201245-7923
ENSG0000(	1533	38.34175	chr6:105(	ENSG00000286340		lncRNA	chr6:79077841-7914
ENSG0000(	1533	38.34175	chr6:105(	SNORD112		smallRNA	chr6:77937201-7793
ENSG0000(	1533	38.34175	chr6:105(	ENSG00000279659		TEC	chr6:79573877-7957
ENSG0000(	1533	38.34175	chr6:105(	ENSG00000287811		lncRNA	chr6:79537185-7953
ENSG0000(	1533	38.34175	chr6:105(	ENSG00000231533		lncRNA	chr6:79420172-7952
ENSG0000(	1533	38.34175	chr6:105(	ENSG00000229495		lncRNA	chr6:78809715-7881
ENSG0000(	1533	38.34175	chr6:105(	PHIP	NCGv7	protein_c	chr6:78934419-7907
ENSG0000(	1533	38.34175	chr6:105(	IRAK1BP1		protein_c	chr6:78867551-7894
ENSG0000(	1533	38.34175	chr6:105(	SH3BGRL2		protein_c	chr6:79631329-7970
ENSG0000(	1533	38.34175	chr6:105(	Y_RNA		smallRNA	chr6:79301963-7930
ENSG0000(	1533	38.34175	chr6:105(	ENSG00000261970		Pseudoger	chr6:79552794-7955
ENSG0000(	1533	38.34175	chr6:105(	AL132875.1		smallRNA	chr6:79826793-7982
ENSG0000(	1533	38.34175	chr6:105(	LINC01621		lncRNA	chr6:79803574-7983
ENSG0000(	1533	38.34175	chr6:105(	ENSG00000230309		lncRNA	chr6:78604467-7860
ENSG0000(	1533	38.34175	chr6:105(	ENSG00000217512		Pseudoger	chr6:79067692-7906
ENSG0000(	1533	38.34175	chr6:105(	ENSG00000276064		Pseudoger	chr6:77343557-7734
ENSG0000(	1533	38.34175	chr6:105(	ENSG00000271945		lncRNA	chr6:76774966-7709
ENSG0000(	1533	38.34175	chr6:105(	ENSG00000285586		lncRNA	chr6:79868402-7987
ENSG0000(	1533	38.34175	chr6:105(	ENSG00000217786		Pseudoger	chr6:79326568-7932
ENSG0000(	1533	38.34175	chr6:105(	RPS6P7		Pseudoger	chr6:77496119-7749
ENSG0000(	1533	38.34175	chr6:105(	AL356776.1		smallRNA	chr6:78961496-7896
ENSG0000(	1533	38.34175	chr6:105(	ENSG00000233835		lncRNA	chr6:79871873-7987
ENSG0000(	1533	38.34175	chr6:105(	DBIP1		Pseudoger	chr6:79436908-7943
ENSG0000(	1533	38.34175	chr6:105(	LCA5		protein_c	chr6:79484991-7953
ENSG0000(	1533	38.34175	chr6:105(	ENSG00000220918		Pseudoger	chr6:79854684-7985
ENSG0000(	1533	38.34175	chr6:105(	ENSG00000272137		lncRNA	chr6:79561132-7956
ENSG0000(	1533	38.34175	chr6:105(	GAPDHP63		Pseudoger	chr6:79953005-7995
ENSG0000(	1533	38.34175	chr6:105(	HTR1B		protein_c	chr6:77460924-7746

ENSG0000(	1533	38.34175	chr6:105(	ELOVL4		protein_c	chr6:79914814-7994
ENSG0000(	1533	38.34175	chr6:105(	ENSG00000218029		Pseudoger	chr6:79354443-7935
ENSG0000(	1533	38.34175	chr6:105(	ENSG00000220154		Pseudoger	chr6:79278105-7927
ENSG0000(	1533	38.34175	chr6:105(	LCAL1		lncRNA	chr6:79307669-7931
ENSG0000(	1533	38.34175	chr6:105(	MEI4		protein_c	chr6:77650274-7792
ENSG0000(	1533	38.34175	chr6:105(	RPL35AP18		Pseudoger	chr6:79964215-7996
ENSG0000(	1526	38.16668	chr6:105(	SNORD66		smallRNA	chr6:51464690-5146
ENSG0000(	1526	38.16668	chr6:105(	MIR206		smallRNA	chr6:52144349-5214
ENSG0000(	1526	38.16668	chr6:105(	DEFB113		protein_c	chr6:49968677-4996
ENSG0000(	1526	38.16668	chr6:105(	ENSG00000270306		Pseudoger	chr6:51385282-5138
ENSG0000(	1526	38.16668	chr6:105(	RNU7-65P		smallRNA	chr6:49344800-4934
ENSG0000(	1526	38.16668	chr6:105(	ENSG00000233470		lncRNA	chr6:50514035-5052
ENSG0000(	1526	38.16668	chr6:105(	DEFB114		protein_c	chr6:49960249-4996
ENSG0000(	1526	38.16668	chr6:105(	DEFB133		Pseudoger	chr6:49946101-4994
ENSG0000(	1526	38.16668	chr6:105(	ENSG00000287137		lncRNA	chr6:48754649-4879
ENSG0000(	1526	38.16668	chr6:105(	ENSG00000290804		lncRNA	chr6:49946021-4995
ENSG0000(	1526	38.16668	chr6:105(	MMUT		protein_c	chr6:49430360-4946
ENSG0000(	1526	38.16668	chr6:105(	DEFB110		protein_c	chr6:50009138-5002
ENSG0000(	1526	38.16668	chr6:105(	ENSG00000214641		Pseudoger	chr6:51537155-5153
ENSG0000(	1526	38.16668	chr6:105(	ENSG00000271162		Pseudoger	chr6:48952070-4895
ENSG0000(	1526	38.16668	chr6:105(	CRISP1		protein_c	chr6:49834257-4987
ENSG0000(	1526	38.16668	chr6:105(	ENSG00000289276		lncRNA	chr6:52364084-5236
ENSG0000(	1526	38.16668	chr6:105(	CENPQ		protein_c	chr6:49463370-4949
ENSG0000(	1526	38.16668	chr6:105(	C6orf141		protein_c	chr6:49550666-4956
ENSG0000(	1526	38.16668	chr6:105(	HNRNPA3P4		Pseudoger	chr6:48149203-4815
ENSG0000(	1526	38.16668	chr6:105(	TFAP2B		protein_c	chr6:50818723-5084
ENSG0000(	1526	38.16668	chr6:105(	TFAP2D	NCGv7	protein_c	chr6:50713526-5077
ENSG0000(	1526	38.16668	chr6:105(	ENSG00000286811		lncRNA	chr6:48069213-4811
ENSG0000(	1526	38.16668	chr6:105(	SLC25A20P1		Pseudoger	chr6:52246460-5224
ENSG0000(	1526	38.16668	chr6:105(	ENSG00000218337		Pseudoger	chr6:49077712-4907
ENSG0000(	1526	38.16668	chr6:105(	RN7SL580P		smallRNA	chr6:51975660-5197
ENSG0000(	1526	38.16668	chr6:105(	AL391538.1		smallRNA	chr6:48849923-4885
ENSG0000(	1526	38.16668	chr6:105(	FTH1P5		Pseudoger	chr6:50912712-5091
ENSG0000(	1526	38.16668	chr6:105(	PTCHD4	NCGv7	protein_c	chr6:47856673-4811
ENSG0000(	1526	38.16668	chr6:105(	ENSG00000274867		Pseudoger	chr6:49115537-4911
ENSG0000(	1526	38.16668	chr6:105(	CRISP3		protein_c	chr6:49727376-4974
ENSG0000(	1526	38.16668	chr6:105(	GLYATL3		protein_c	chr6:49499923-4952
ENSG0000(	1526	38.16668	chr6:105(	ENSG00000232702		Pseudoger	chr6:51410081-5141
ENSG0000(	1526	38.16668	chr6:105(	ENSG00000286405		lncRNA	chr6:50411844-5041
ENSG0000(	1526	38.16668	chr6:105(	ENSG00000230472		lncRNA	chr6:50587607-5063
ENSG0000(	1526	38.16668	chr6:105(	CRISP2		protein_c	chr6:49692358-4971
ENSG0000(	1526	38.16668	chr6:105(	DEFB112		protein_c	chr6:50042099-5004
ENSG0000(	1526	38.16668	chr6:105(	RHAG		protein_c	chr6:49605175-4963
ENSG0000(	1526	38.16668	chr6:105(	PKHD1	NCGv7	protein_c	chr6:51615299-5208
ENSG0000(	1526	38.16668	chr6:105(	EEF1A1P42		Pseudoger	chr6:49358185-4936
ENSG0000(	1526	38.16668	chr6:105(	ENSG00000217631		Pseudoger	chr6:49273600-4927
ENSG0000(	1526	38.16668	chr6:105(	MCM3		protein_c	chr6:52264014-5228
ENSG0000(	1526	38.16668	chr6:105(	ENSG00000226733		lncRNA	chr6:50093389-5018
ENSG0000(	1526	38.16668	chr6:105(	MIR133B		smallRNA	chr6:52148923-5214
ENSG0000(	1526	38.16668	chr6:105(	AL590391.1		smallRNA	chr6:52061615-5206
ENSG0000(	1526	38.16668	chr6:105(	ENSG00000216913		Pseudoger	chr6:50897615-5089

ENSG0000(	1526	38.16668	chr6:105(	LINCMD1		lncRNA	chr6:52146814-5215
ENSG0000(	1526	38.16668	chr6:105(	IL17F		protein_c	chr6:52236681-5224
ENSG0000(	1526	38.16668	chr6:105(	IL17A		protein_c	chr6:52186375-5219
ENSG0000(	1526	38.16668	chr6:105(	ENSG00000235122		lncRNA	chr6:49714325-4982
ENSG0000(	1526	38.16668	chr6:105(	ENSG00000228689		lncRNA	chr6:51599723-5162
ENSG0000(	1526	38.16668	chr6:105(	PAQR8		protein_c	chr6:52361421-5240
ENSG0000(	1526	38.16668	chr6:105(	RBMXP1		Pseudoger	chr6:48213604-4821
ENSG0000(	1526	38.16668	chr6:105(	RPS17P5		Pseudoger	chr6:50857255-5085
ENSG0000(	1526	38.16668	chr6:105(	ENSG00000226707		lncRNA	chr6:49823712-4982
ENSG0000(	1526	38.16668	chr6:105(	ENSG00000279127		TEC	chr6:49721931-4972
ENSG0000(	1526	38.16668	chr6:105(	ENSG00000278736		Pseudoger	chr6:48701491-4870
ENSG0000(	1526	38.16668	chr6:105(	CYP2AC1P		Pseudoger	chr6:49565924-4958
ENSG0000(	1526	38.16668	chr6:105(	PGK2		protein_c	chr6:49785660-4978
ENSG0000(	1523	38.09165	chr6:105(	MIR4463		smallRNA	chr6:75428407-7542
ENSG0000(	1516	37.91657	chr6:105(	FBXO9	NCGv7	protein_c	chr6:53051991-5310
ENSG0000(	1516	37.91657	chr6:105(	RN7SL244P		smallRNA	chr6:53090961-5309
ENSG0000(	1516	37.91657	chr6:105(	ENSG00000261116		lncRNA	chr6:54943167-5494
ENSG0000(	1516	37.91657	chr6:105(	snoU13		smallRNA	chr6:73393913-7339
ENSG0000(	1516	37.91657	chr6:105(	EEF1A1-AS1		lncRNA	chr6:73523618-7357
ENSG0000(	1516	37.91657	chr6:105(	ENSG00000271761		lncRNA	chr6:57902609-5790
ENSG0000(	1516	37.91657	chr6:105(	ENSG00000232120		lncRNA	chr6:64377795-6441
ENSG0000(	1516	37.91657	chr6:105(	SDCBP2P1		Pseudoger	chr6:73322837-7332
ENSG0000(	1516	37.91657	chr6:105(	RN7SK		smallRNA	chr6:52995620-5299
ENSG0000(	1516	37.91657	chr6:105(	ENSG00000236740		lncRNA	chr6:53930022-5399
ENSG0000(	1516	37.91657	chr6:105(	RNU7-48P		smallRNA	chr6:70513294-7051
ENSG0000(	1516	37.91657	chr6:105(	RPSAP44		Pseudoger	chr6:54624605-5462
ENSG0000(	1516	37.91657	chr6:105(	NANOGP3		Pseudoger	chr6:53418452-5341
ENSG0000(	1516	37.91657	chr6:105(	BMP5	NCGv7	protein_c	chr6:55753653-5587
ENSG0000(	1516	37.91657	chr6:105(	PTP4A1	AC	protein_c	chr6:63521746-6358
ENSG0000(	1516	37.91657	chr6:105(	KHDC1-AS1		lncRNA	chr6:73263212-7330
ENSG0000(	1516	37.91657	chr6:105(	CILK1	NCGv7	protein_c	chr6:53001279-5306
ENSG0000(	1516	37.91657	chr6:105(	ENSG00000271367		lncRNA	chr6:53350158-5335
ENSG0000(	1516	37.91657	chr6:105(	H3P27		Pseudoger	chr6:75586122-7558
ENSG0000(	1516	37.91657	chr6:105(	AL354933.1		smallRNA	chr6:71585089-7158
ENSG0000(	1516	37.91657	chr6:105(	CD109-AS1		lncRNA	chr6:73693903-7369
ENSG0000(	1516	37.91657	chr6:105(	LINC02540		lncRNA	chr6:76521640-7659
ENSG0000(	1516	37.91657	chr6:105(	KCNQ5-DT		lncRNA	chr6:72614386-7262
ENSG0000(	1516	37.91657	chr6:105(	ENSG00000285838		lncRNA	chr6:68040290-6809
ENSG0000(	1516	37.91657	chr6:105(	KHDRBS2	NCGv7	protein_c	chr6:61679961-6228
ENSG0000(	1516	37.91657	chr6:105(	ENSG00000266680		lncRNA	chr6:63571005-6357
ENSG0000(	1516	37.91657	chr6:105(	EIF3EP1		Pseudoger	chr6:73291962-7329
ENSG0000(	1516	37.91657	chr6:105(	MYO6		protein_c	chr6:75749201-7591
ENSG0000(	1516	37.91657	chr6:105(	ADH5P4		Pseudoger	chr6:65836930-6583
ENSG0000(	1516	37.91657	chr6:105(	RAB23	TAG;AC	protein_c	chr6:57186992-5722
ENSG0000(	1516	37.91657	chr6:105(	AL606923.1		smallRNA	chr6:68493439-6849
ENSG0000(	1516	37.91657	chr6:105(	BAG2		protein_c	chr6:57172326-5718
ENSG0000(	1516	37.91657	chr6:105(	KRASP1		Pseudoger	chr6:54770583-5477
ENSG0000(	1516	37.91657	chr6:105(	NPM1P37		Pseudoger	chr6:69705287-6970
ENSG0000(	1516	37.91657	chr6:105(	RCC2P7		Pseudoger	chr6:56431950-5643
ENSG0000(	1516	37.91657	chr6:105(	PGAM1P10		Pseudoger	chr6:73055097-7305
ENSG0000(	1516	37.91657	chr6:105(	FTH1P15		Pseudoger	chr6:57004520-5700

ENSG0000(	1516	37.91657	chr6:105(	ZNF451	NCGv7	protein_c	chr6:57086844-5717
ENSG0000(	1516	37.91657	chr6:105(	ENSG00000220725		Pseudoger	chr6:55680642-5568
ENSG0000(	1516	37.91657	chr6:105(	ENSG00000232295		lncRNA	chr6:71221457-7132
ENSG0000(	1516	37.91657	chr6:105(	ENSG00000288712		protein_c	chr6:68635890-6863
ENSG0000(	1516	37.91657	chr6:105(	ENSG00000266579		lncRNA	chr6:60386769-6039
ENSG0000(	1516	37.91657	chr6:105(	SOD1P1		Pseudoger	chr6:53196720-5319
ENSG0000(	1516	37.91657	chr6:105(	NUFIP1P1		Pseudoger	chr6:66093431-6609
ENSG0000(	1516	37.91657	chr6:105(	ENSG00000285401		Pseudoger	chr6:75744275-7574
ENSG0000(	1516	37.91657	chr6:105(	RNU6-411P		smallRNA	chr6:71182612-7118
ENSG0000(	1516	37.91657	chr6:105(	AL590874.1		smallRNA	chr6:67149509-6714
ENSG0000(	1516	37.91657	chr6:105(	ENSG00000218713		Pseudoger	chr6:53206598-5320
ENSG0000(	1516	37.91657	chr6:105(	ENSG00000291036		lncRNA	chr6:52939726-5297
ENSG0000(	1516	37.91657	chr6:105(	MIR5685		smallRNA	chr6:53276993-5327
ENSG0000(	1516	37.91657	chr6:105(	ENSG00000243501		protein_c	chr6:73209746-7326
ENSG0000(	1516	37.91657	chr6:105(	RBPMS2P1		Pseudoger	chr6:73157517-7315
ENSG0000(	1516	37.91657	chr6:105(	ENSG00000218617		Pseudoger	chr6:63392222-6339
ENSG0000(	1516	37.91657	chr6:105(	ENSG00000272243		lncRNA	chr6:74530248-7473
ENSG0000(	1516	37.91657	chr6:105(	RPL37P15		Pseudoger	chr6:70098390-7009
ENSG0000(	1516	37.91657	chr6:105(	RNU4-66P		smallRNA	chr6:71652474-7165
ENSG0000(	1516	37.91657	chr6:105(	ENSG00000272316		lncRNA	chr6:57908560-5791
ENSG0000(	1516	37.91657	chr6:105(	ENSG00000231683		lncRNA	chr6:53561289-5361
ENSG0000(	1516	37.91657	chr6:105(	ENSG00000218520		Pseudoger	chr6:63229087-6323
ENSG0000(	1516	37.91657	chr6:105(	COL12A1	NCGv7	protein_c	chr6:75084326-7520
ENSG0000(	1516	37.91657	chr6:105(	ENSG00000243828		Pseudoger	chr6:64728837-6473
ENSG0000(	1516	37.91657	chr6:105(	U6		smallRNA	chr6:76557653-7655
ENSG0000(	1516	37.91657	chr6:105(	ENSG00000287679		lncRNA	chr6:61870097-6189
ENSG0000(	1516	37.91657	chr6:105(	ENSG00000290002		lncRNA	chr6:73388408-7338
ENSG0000(	1516	37.91657	chr6:105(	ENSG00000217477		Pseudoger	chr6:63193072-6319
ENSG0000(	1516	37.91657	chr6:105(	GSTA1		protein_c	chr6:52791371-5280
ENSG0000(	1516	37.91657	chr6:105(	ENSG00000218483		Pseudoger	chr6:74610449-7461
ENSG0000(	1516	37.91657	chr6:105(	ENSG00000287598		lncRNA	chr6:60789805-6094
ENSG0000(	1516	37.91657	chr6:105(	AL035467.1		smallRNA	chr6:71538884-7153
ENSG0000(	1516	37.91657	chr6:105(	ENSG00000218732		Pseudoger	chr6:72316599-7231
ENSG0000(	1516	37.91657	chr6:105(	KLHL31		protein_c	chr6:53647916-5366
ENSG0000(	1516	37.91657	chr6:105(	ENSG00000288646		protein_c	chr6:53065602-5306
ENSG0000(	1516	37.91657	chr6:105(	ENSG00000271967		lncRNA	chr6:70596438-7059
ENSG0000(	1516	37.91657	chr6:105(	Metazoa_SRP		smallRNA	chr6:73487592-7348
ENSG0000(	1516	37.91657	chr6:105(	ENSG00000287380		lncRNA	chr6:71238743-7124
ENSG0000(	1516	37.91657	chr6:105(	MIR548U		smallRNA	chr6:57390132-5739
ENSG0000(	1516	37.91657	chr6:105(	SLC25A6P6		Pseudoger	chr6:70663502-7066
ENSG0000(	1516	37.91657	chr6:105(	ENSG00000218834		Pseudoger	chr6:68598204-6859
ENSG0000(	1516	37.91657	chr6:105(	ENSG00000291006		lncRNA	chr6:52664366-5266
ENSG0000(	1516	37.91657	chr6:105(	COL21A1	NCGv7	protein_c	chr6:56056590-5639
ENSG0000(	1516	37.91657	chr6:105(	ENSG00000218813		Pseudoger	chr6:63797189-6379
ENSG0000(	1516	37.91657	chr6:105(	MIR4282		smallRNA	chr6:72967687-7296
ENSG0000(	1516	37.91657	chr6:105(	ENSG00000289286		lncRNA	chr6:73570461-7357
ENSG0000(	1516	37.91657	chr6:105(	FAM135A-AS1		lncRNA	chr6:70412828-7041
ENSG0000(	1516	37.91657	chr6:105(	ENSG00000287557		lncRNA	chr6:67888977-6799
ENSG0000(	1516	37.91657	chr6:105(	AC019205.2		smallRNA	chr6:73432984-7343
ENSG0000(	1516	37.91657	chr6:105(	AL591034.1		smallRNA	chr6:53456447-5345
ENSG0000(	1516	37.91657	chr6:105(	GSTA9P		Pseudoger	chr6:52939906-5295

ENSG0000(	1516	37.91657	chr6:105(	RPL39P3		Pseudoger	chr6:73373108-7337
ENSG0000(	1516	37.91657	chr6:105(	ENSG00000238156		lncRNA	chr6:75454944-7545
ENSG0000(	1516	37.91657	chr6:105(	GSTA5		protein_c	chr6:52831655-5284
ENSG0000(	1516	37.91657	chr6:105(	RNA5SP209		Pseudoger	chr6:75865239-7586
ENSG0000(	1516	37.91657	chr6:105(	ENSG00000285976		protein_c	chr6:63572472-6358
ENSG0000(	1516	37.91657	chr6:105(	KHDC1		protein_c	chr6:73241314-7331
ENSG0000(	1516	37.91657	chr6:105(	TMEM30A	NCGv7	protein_c	chr6:75252924-7528
ENSG0000(	1516	37.91657	chr6:105(	ENSG00000230597		lncRNA	chr6:71328942-7132
ENSG0000(	1516	37.91657	chr6:105(	ENSG00000262803		Pseudoger	chr6:70693839-7069
ENSG0000(	1516	37.91657	chr6:105(	ENSG00000223504		lncRNA	chr6:68055351-6806
ENSG0000(	1516	37.91657	chr6:105(	IMPG1		protein_c	chr6:75921114-7607
ENSG0000(	1516	37.91657	chr6:105(	GSTA12P		Pseudoger	chr6:52765280-5277
ENSG0000(	1516	37.91657	chr6:105(	ENSG00000262651		Pseudoger	chr6:63612761-6361
ENSG0000(	1516	37.91657	chr6:105(	ENSG00000262566		Pseudoger	chr6:62611153-6261
ENSG0000(	1516	37.91657	chr6:105(	TXNP7		Pseudoger	chr6:74004520-7400
ENSG0000(	1516	37.91657	chr6:105(	SENP6		protein_c	chr6:75601509-7571
ENSG0000(	1516	37.91657	chr6:105(	RNU7-66P		smallRNA	chr6:66728843-6672
ENSG0000(	1516	37.91657	chr6:105(	Y_RNA		smallRNA	chr6:75928513-7592
ENSG0000(	1516	37.91657	chr6:105(	COL9A1		protein_c	chr6:70216040-7030
ENSG0000(	1516	37.91657	chr6:105(	ENSG00000270382		Pseudoger	chr6:52657373-5265
ENSG0000(	1516	37.91657	chr6:105(	ENSG00000219575		Pseudoger	chr6:71550958-7155
ENSG0000(	1516	37.91657	chr6:105(	LINC00680		lncRNA	chr6:57946074-5796
ENSG0000(	1516	37.91657	chr6:105(	NPM1P36		Pseudoger	chr6:55939790-5594
ENSG0000(	1516	37.91657	chr6:105(	ENSG00000286564		lncRNA	chr6:68223667-6824
ENSG0000(	1516	37.91657	chr6:105(	HMGB1P20		Pseudoger	chr6:53235621-5323
ENSG0000(	1516	37.91657	chr6:105(	ADGRB3-DT		lncRNA	chr6:68627879-6863
ENSG0000(	1516	37.91657	chr6:105(	COX7A2		protein_c	chr6:75237675-7525
ENSG0000(	1516	37.91657	chr6:105(	ENSG00000270509		Pseudoger	chr6:67933485-6793
ENSG0000(	1516	37.91657	chr6:105(	ENSG00000270521		Pseudoger	chr6:66710242-6671
ENSG0000(	1516	37.91657	chr6:105(	RP11-452D24.1		Pseudoger	chr6:61060454-6106
ENSG0000(	1516	37.91657	chr6:105(	ENSG00000227602		lncRNA	chr6:56331788-5633
ENSG0000(	1516	37.91657	chr6:105(	RNU6-975P		smallRNA	chr6:73464073-7346
ENSG0000(	1516	37.91657	chr6:105(	FAM136FP		Pseudoger	chr6:74282266-7428
ENSG0000(	1516	37.91657	chr6:105(	SDHAF4		protein_c	chr6:70566917-7058
ENSG0000(	1516	37.91657	chr6:105(	snoU13		smallRNA	chr6:75865964-7586
ENSG0000(	1516	37.91657	chr6:105(	ENSG00000269966		lncRNA	chr6:71295173-7141
ENSG0000(	1516	37.91657	chr6:105(	EYS	NCGv7	protein_c	chr6:63719980-6570
ENSG0000(	1516	37.91657	chr6:105(	ENSG00000217067		Pseudoger	chr6:62547427-6254
ENSG0000(	1516	37.91657	chr6:105(	ELOVL5		protein_c	chr6:53267398-5334
ENSG0000(	1516	37.91657	chr6:105(	SCAT8		lncRNA	chr6:63805797-6382
ENSG0000(	1516	37.91657	chr6:105(	ENSG00000279289		TEC	chr6:71386852-7139
ENSG0000(	1516	37.91657	chr6:105(	ENSG00000224583		lncRNA	chr6:74642384-7465
ENSG0000(	1516	37.91657	chr6:105(	DPPA5		protein_c	chr6:73353063-7335
ENSG0000(	1516	37.91657	chr6:105(	AC019205.1		smallRNA	chr6:73263008-7326
ENSG0000(	1516	37.91657	chr6:105(	KHDC3L	NCGv7	protein_c	chr6:73362658-7336
ENSG0000(	1516	37.91657	chr6:105(	RNU6-248P		smallRNA	chr6:76092834-7609
ENSG0000(	1516	37.91657	chr6:105(	ENSG00000286723		lncRNA	chr6:57880071-5788
ENSG0000(	1516	37.91657	chr6:105(	OOEP		protein_c	chr6:73368555-7339
ENSG0000(	1516	37.91657	chr6:105(	ENSG00000286680		lncRNA	chr6:67884041-6798
ENSG0000(	1516	37.91657	chr6:105(	RPSAP41		Pseudoger	chr6:73290403-7329
ENSG0000(	1516	37.91657	chr6:105(	GSTA10P		Pseudoger	chr6:52873014-5288

ENSG0000(	1516	37.91657	chr6:105(	GFRAL	NCGv7	protein_c	chr6:55327469-5540
ENSG0000(	1516	37.91657	chr6:105(	GUSBP4		Pseudoger	chr6:57919784-5793
ENSG0000(	1516	37.91657	chr6:105(	ENSG00000290597		lncRNA	chr6:60353963-6054
ENSG0000(	1516	37.91657	chr6:105(	GCNT1P4		Pseudoger	chr6:63857441-6385
ENSG0000(	1516	37.91657	chr6:105(	DHFRP5		Pseudoger	chr6:62460940-6246
ENSG0000(	1516	37.91657	chr6:105(	BECN1P2		Pseudoger	chr6:71075564-7107
ENSG0000(	1516	37.91657	chr6:105(	GCLC		protein_c	chr6:53497341-5361
ENSG0000(	1516	37.91657	chr6:105(	ENSG00000216775		Pseudoger	chr6:52665274-5266
ENSG0000(	1516	37.91657	chr6:105(	ENSG00000214558		protein_c	chr6:65301476-6530
ENSG0000(	1516	37.91657	chr6:105(	DHFRP6		Pseudoger	chr6:56276529-5627
ENSG0000(	1516	37.91657	chr6:105(	LINC01564		lncRNA	chr6:53616471-5370
ENSG0000(	1516	37.91657	chr6:105(	ENSG00000216687		Pseudoger	chr6:58071720-5807
ENSG0000(	1516	37.91657	chr6:105(	ENSG00000271111		Pseudoger	chr6:67456346-6745
ENSG0000(	1516	37.91657	chr6:105(	ENSG00000227706		lncRNA	chr6:67878316-6788
ENSG0000(	1516	37.91657	chr6:105(	RNU6-261P		smallRNA	chr6:76446988-7644
ENSG0000(	1516	37.91657	chr6:105(	GSTA8P		Pseudoger	chr6:52687930-5270
ENSG0000(	1516	37.91657	chr6:105(	RNU1-136P		smallRNA	chr6:53219261-5321
ENSG0000(	1516	37.91657	chr6:105(	ENSG00000271218		lncRNA	chr6:53918974-5392
ENSG0000(	1516	37.91657	chr6:105(	SREK1IP1P2		Pseudoger	chr6:52688661-5268
ENSG0000(	1516	37.91657	chr6:105(	B3GAT2		protein_c	chr6:70856679-7095
ENSG0000(	1516	37.91657	chr6:105(	SLC25A51P1		Pseudoger	chr6:65788417-6578
ENSG0000(	1516	37.91657	chr6:105(	SMAP1		protein_c	chr6:70667776-7086
ENSG0000(	1516	37.91657	chr6:105(	AL590558.1		smallRNA	chr6:62983084-6298
ENSG0000(	1516	37.91657	chr6:105(	RNU1-34P		smallRNA	chr6:75473738-7547
ENSG0000(	1516	37.91657	chr6:105(	ENSG00000271338		Pseudoger	chr6:53381519-5338
ENSG0000(	1516	37.91657	chr6:105(	MTO1		protein_c	chr6:73461578-7350
ENSG0000(	1516	37.91657	chr6:105(	ADGRB3	NCGv7	protein_c	chr6:68635282-6939
ENSG0000(	1516	37.91657	chr6:105(	ENSG00000285963		lncRNA	chr6:71532070-7153
ENSG0000(	1516	37.91657	chr6:105(	ENSG00000220030		Pseudoger	chr6:61240897-6124
ENSG0000(	1516	37.91657	chr6:105(	ENSG00000287300		lncRNA	chr6:70345919-7035
ENSG0000(	1516	37.91657	chr6:105(	RPL17P26		Pseudoger	chr6:56871191-5687
ENSG0000(	1516	37.91657	chr6:105(	RBBP4P4		Pseudoger	chr6:58119741-5812
ENSG0000(	1516	37.91657	chr6:105(	EEF1A1	NCGv7;AC	protein_c	chr6:73489308-7352
ENSG0000(	1516	37.91657	chr6:105(	CD109		protein_c	chr6:73695785-7382
ENSG0000(	1516	37.91657	chr6:105(	RPL26P20		Pseudoger	chr6:75499705-7550
ENSG0000(	1516	37.91657	chr6:105(	HMGB1P39		Pseudoger	chr6:75319101-7531
ENSG0000(	1516	37.91657	chr6:105(	EFHC1		protein_c	chr6:52362123-5252
ENSG0000(	1516	37.91657	chr6:105(	TMEM14A		protein_c	chr6:52671113-5268
ENSG0000(	1516	37.91657	chr6:105(	MIR30A		smallRNA	chr6:71403551-7140
ENSG0000(	1516	37.91657	chr6:105(	ENSG00000219736		Pseudoger	chr6:74849077-7485
ENSG0000(	1516	37.91657	chr6:105(	GSTA11P		Pseudoger	chr6:52847910-5287
ENSG0000(	1516	37.91657	chr6:105(	DST-AS1		lncRNA	chr6:56843928-5686
ENSG0000(	1516	37.91657	chr6:105(	LINC00472		lncRNA	chr6:71343427-7142
ENSG0000(	1516	37.91657	chr6:105(	Y_RNA		smallRNA	chr6:67625356-6762
ENSG0000(	1516	37.91657	chr6:105(	OSTCP6		Pseudoger	chr6:56975606-5697
ENSG0000(	1516	37.91657	chr6:105(	RPL31P28		Pseudoger	chr6:53354715-5335
ENSG0000(	1516	37.91657	chr6:105(	BEND6		protein_c	chr6:56955107-5702
ENSG0000(	1516	37.91657	chr6:105(	GAPDHP42		Pseudoger	chr6:69745871-6974
ENSG0000(	1516	37.91657	chr6:105(	DST	NCGv7	protein_c	chr6:56457987-5695
ENSG0000(	1516	37.91657	chr6:105(	AL137008.1		smallRNA	chr6:56432379-5643
ENSG0000(	1516	37.91657	chr6:105(	SPTLC1P3		Pseudoger	chr6:63227485-6322

ENSG0000(	1516	37.91657	chr6:105(	KCNQ5	NCGv7	protein_c	chr6:72621792-7319
ENSG0000(	1516	37.91657	chr6:105(	GAPDHP15		Pseudoger	chr6:57967687-5796
ENSG0000(	1516	37.91657	chr6:105(	GSTA6P		Pseudoger	chr6:52805613-5281
ENSG0000(	1516	37.91657	chr6:105(	AL121931.1		smallRNA	chr6:62726835-6272
ENSG0000(	1516	37.91657	chr6:105(	LINC00680		Pseudoger	chr6:57959029-5796
ENSG0000(	1516	37.91657	chr6:105(	RN7SK		smallRNA	chr6:52995621-5299
ENSG0000(	1516	37.91657	chr6:105(	RPL31P33		Pseudoger	chr6:53368670-5336
ENSG0000(	1516	37.91657	chr6:105(	MLIP-AS1		lncRNA	chr6:53978549-5407
ENSG0000(	1516	37.91657	chr6:105(	LMBRD1		protein_c	chr6:69672757-6986
ENSG0000(	1516	37.91657	chr6:105(	KHDC1P1		Pseudoger	chr6:73209083-7321
ENSG0000(	1516	37.91657	chr6:105(	RNU6-1016P		smallRNA	chr6:75644084-7564
ENSG0000(	1516	37.91657	chr6:105(	KNOP1P4		Pseudoger	chr6:72986313-7298
ENSG0000(	1516	37.91657	chr6:105(	ENSG00000287939		lncRNA	chr6:71028582-7105
ENSG0000(	1516	37.91657	chr6:105(	PRIM2		protein_c	chr6:57314805-6054
ENSG0000(	1516	37.91657	chr6:105(	GSTA7P		Pseudoger	chr6:52739590-5274
ENSG0000(	1516	37.91657	chr6:105(	KHDRBS2-OT1		lncRNA	chr6:61630233-6168
ENSG0000(	1516	37.91657	chr6:105(	MIR30C2		smallRNA	chr6:71376960-7137
ENSG0000(	1516	37.91657	chr6:105(	AL590684.1		smallRNA	chr6:74719746-7471
ENSG0000(	1516	37.91657	chr6:105(	ENSG00000280511		lncRNA	chr6:76561328-7656
ENSG0000(	1516	37.91657	chr6:105(	MLIP		protein_c	chr6:53929982-5426
ENSG0000(	1516	37.91657	chr6:105(	LINC01610		lncRNA	chr6:70394880-7040
ENSG0000(	1516	37.91657	chr6:105(	ENSG00000218048		Pseudoger	chr6:63440766-6344
ENSG0000(	1516	37.91657	chr6:105(	TRAM2		protein_c	chr6:52497408-5257
ENSG0000(	1516	37.91657	chr6:105(	HMGCLL1		protein_c	chr6:55434373-5557
ENSG0000(	1516	37.91657	chr6:105(	ENSG00000253809		lncRNA	chr6:70222758-7024
ENSG0000(	1516	37.91657	chr6:105(	KIAA1586		protein_c	chr6:57046532-5705
ENSG0000(	1516	37.91657	chr6:105(	RNU6-1338P		smallRNA	chr6:75593055-7559
ENSG0000(	1516	37.91657	chr6:105(	PRIM2BP		Pseudoger	chr6:60400251-6054
ENSG0000(	1516	37.91657	chr6:105(	RNU6-155P		smallRNA	chr6:75768059-7576
ENSG0000(	1516	37.91657	chr6:105(	OGFRL1		protein_c	chr6:71288811-7130
ENSG0000(	1516	37.91657	chr6:105(	TINAG		protein_c	chr6:54307859-5439
ENSG0000(	1516	37.91657	chr6:105(	SLC17A5	NCGv7	protein_c	chr6:73593379-7365
ENSG0000(	1516	37.91657	chr6:105(	HCRTR2		protein_c	chr6:55106460-5528
ENSG0000(	1516	37.91657	chr6:105(	KRT19P1		Pseudoger	chr6:71584721-7158
ENSG0000(	1516	37.91657	chr6:105(	LRRC1		protein_c	chr6:53794497-5392
ENSG0000(	1516	37.91657	chr6:105(	GCM1		protein_c	chr6:53126961-5314
ENSG0000(	1516	37.91657	chr6:105(	ENSG00000283352		lncRNA	chr6:57919912-5796
ENSG0000(	1516	37.91657	chr6:105(	UBE2V1P15		Pseudoger	chr6:75465123-7546
ENSG0000(	1516	37.91657	chr6:105(	ENSG00000225096		lncRNA	chr6:57961438-5843
ENSG0000(	1516	37.91657	chr6:105(	FAM83B	NCGv7	protein_c	chr6:54846771-5494
ENSG0000(	1516	37.91657	chr6:105(	ENSG00000289611		lncRNA	chr6:68840383-6884
ENSG0000(	1516	37.91657	chr6:105(	ENSG00000218274		Pseudoger	chr6:63395007-6339
ENSG0000(	1516	37.91657	chr6:105(	Y_RNA		smallRNA	chr6:52979158-5297
ENSG0000(	1516	37.91657	chr6:105(	AL589736.1		smallRNA	chr6:62683178-6268
ENSG0000(	1516	37.91657	chr6:105(	RPL7AP34		Pseudoger	chr6:63548708-6354
ENSG0000(	1516	37.91657	chr6:105(	RPS6P8		Pseudoger	chr6:73391038-7339
ENSG0000(	1516	37.91657	chr6:105(	TMEM30A-DT		lncRNA	chr6:75284992-7531
ENSG0000(	1516	37.91657	chr6:105(	GAPDHP41		Pseudoger	chr6:60719222-6072
ENSG0000(	1516	37.91657	chr6:105(	TRAM2-AS1		lncRNA	chr6:52576787-5264
ENSG0000(	1516	37.91657	chr6:105(	PHF3	NCGv7	protein_c	chr6:63635802-6377
ENSG0000(	1516	37.91657	chr6:105(	RPL10P10		Pseudoger	chr6:54602487-5460

ENSG0000(	1516	37.91657	chr6:105(	GSTA3		protein_c	chr6:52896639-5290
ENSG0000(	1516	37.91657	chr6:105(	AL356131.1		smallRNA	chr6:61180831-6118
ENSG0000(	1516	37.91657	chr6:105(	PAICSP3		Pseudoger	chr6:73327524-7332
ENSG0000(	1516	37.91657	chr6:105(	RNU6-626P		smallRNA	chr6:56945730-5694
ENSG0000(	1516	37.91657	chr6:105(	ENSG00000232389		Pseudoger	chr6:70608234-7060
ENSG0000(	1516	37.91657	chr6:105(	ENSG00000279790		TEC	chr6:73971346-7397
ENSG0000(	1516	37.91657	chr6:105(	RPS16P5		Pseudoger	chr6:53336943-5333
ENSG0000(	1516	37.91657	chr6:105(	MLIP-IT1		lncRNA	chr6:53998890-5400
ENSG0000(	1516	37.91657	chr6:105(	ZNF451-AS1		lncRNA	chr6:57114894-5717
ENSG0000(	1516	37.91657	chr6:105(	POM121L14P		Pseudoger	chr6:57937458-5793
ENSG0000(	1516	37.91657	chr6:105(	LGSN	NCGv7	protein_c	chr6:63275951-6331
ENSG0000(	1516	37.91657	chr6:105(	FILIP1		protein_c	chr6:75291859-7549
ENSG0000(	1516	37.91657	chr6:105(	ENSG00000223786		lncRNA	chr6:74069451-7469
ENSG0000(	1516	37.91657	chr6:105(	ENSG00000249379		lncRNA	chr6:53503109-5350
ENSG0000(	1516	37.91657	chr6:105(	CGAS		protein_c	chr6:73413515-7345
ENSG0000(	1516	37.91657	chr6:105(	TINAG-AS1		lncRNA	chr6:54365335-5436
ENSG0000(	1516	37.91657	chr6:105(	ENSG00000274844		Pseudoger	chr6:60826185-6082
ENSG0000(	1516	37.91657	chr6:105(	FKBP1C		protein_c	chr6:63211446-6321
ENSG0000(	1516	37.91657	chr6:105(	OOEP-AS1		lncRNA	chr6:73369704-7338
ENSG0000(	1516	37.91657	chr6:105(	ERHP2		Pseudoger	chr6:54016479-5401
ENSG0000(	1516	37.91657	chr6:105(	RNU6-464P		smallRNA	chr6:53153795-5315
ENSG0000(	1516	37.91657	chr6:105(	RN7SKP256		smallRNA	chr6:53415294-5341
ENSG0000(	1516	37.91657	chr6:105(	RPL9P18		Pseudoger	chr6:63615827-6361
ENSG0000(	1516	37.91657	chr6:105(	ENSG00000275046		Pseudoger	chr6:58386799-5838
ENSG0000(	1516	37.91657	chr6:105(	RPA3P2		Pseudoger	chr6:53378503-5337
ENSG0000(	1516	37.91657	chr6:105(	FAM135A		protein_c	chr6:70412941-7056
ENSG0000(	1516	37.91657	chr6:105(	COL19A1		protein_c	chr6:69866556-7021
ENSG0000(	1516	37.91657	chr6:105(	ENSG00000217488		Pseudoger	chr6:75610958-7561
ENSG0000(	1516	37.91657	chr6:105(	CLNS1AP1		Pseudoger	chr6:54485169-5448
ENSG0000(	1516	37.91657	chr6:105(	U3		smallRNA	chr6:53147808-5314
ENSG0000(	1516	37.91657	chr6:105(	ENSG00000288014		Pseudoger	chr6:52970650-5297
ENSG0000(	1516	37.91657	chr6:105(	RBBP4P3		Pseudoger	chr6:60873360-6087
ENSG0000(	1516	37.91657	chr6:105(	RNA5SP208		Pseudoger	chr6:67467231-6746
ENSG0000(	1516	37.91657	chr6:105(	RNU6-280P		smallRNA	chr6:67546651-6754
ENSG0000(	1516	37.91657	chr6:105(	RNU6-1023P		smallRNA	chr6:54786387-5478
ENSG0000(	1516	37.91657	chr6:105(	U3		smallRNA	chr6:75398861-7539
ENSG0000(	1516	37.91657	chr6:105(	AL358133.1		smallRNA	chr6:69621373-6962
ENSG0000(	1516	37.91657	chr6:105(	ENSG00000224984		lncRNA	chr6:54840118-5484
ENSG0000(	1516	37.91657	chr6:105(	AL109612.1		smallRNA	chr6:65467004-6546
ENSG0000(	1516	37.91657	chr6:105(	KHDC1L		protein_c	chr6:73223544-7322
ENSG0000(	1516	37.91657	chr6:105(	LINC01626		lncRNA	chr6:71450834-7145
ENSG0000(	1516	37.91657	chr6:105(	ENSG00000223967		Pseudoger	chr6:73526744-7352
ENSG0000(	1516	37.91657	chr6:105(	RPS27P15		Pseudoger	chr6:73618346-7361
ENSG0000(	1516	37.91657	chr6:105(	RN7SKP163		smallRNA	chr6:75654970-7565
ENSG0000(	1516	37.91657	chr6:105(	LINC02549		lncRNA	chr6:68226972-6832
ENSG0000(	1516	37.91657	chr6:105(	AL158051.1		smallRNA	chr6:69214730-6921
ENSG0000(	1516	37.91657	chr6:105(	DDX43		protein_c	chr6:73394828-7341
ENSG0000(	1516	37.91657	chr6:105(	ENSG00000237174		lncRNA	chr6:75357214-7539
ENSG0000(	1516	37.91657	chr6:105(	HNRNPDP2		Pseudoger	chr6:64631205-6463
ENSG0000(	1516	37.91657	chr6:105(	GSTA4	NCGv7	protein_c	chr6:52977948-5299
ENSG0000(	1516	37.91657	chr6:105(	U3		smallRNA	chr6:71126894-7112

ENSG0000(	1516	37.91657	chr6:105(	KCNQ5-AS1		lncRNA	chr6:73130646-7314
ENSG0000(	1516	37.91657	chr6:105(	ENSG00000288088		lncRNA	chr6:68332019-6857
ENSG0000(	1516	37.91657	chr6:105(	MRPL30P1		Pseudoger	chr6:57029521-5702
ENSG0000(	1516	37.91657	chr6:105(	ENSG00000227885		lncRNA	chr6:53739266-5379
ENSG0000(	1516	37.91657	chr6:105(	ENSG00000287745		lncRNA	chr6:54190206-5419
ENSG0000(	1516	37.91657	chr6:105(	RIMS1	NCGv7	protein_c	chr6:71886550-7240
ENSG0000(	1516	37.91657	chr6:105(	AL603910.1		smallRNA	chr6:73477058-7347
ENSG0000(	1516	37.91657	chr6:105(	NDUFAB1P1		Pseudoger	chr6:70734503-7073
ENSG0000(	1516	37.91657	chr6:105(	EEF1B2P5		Pseudoger	chr6:63480134-6348
ENSG0000(	1516	37.91657	chr6:105(	AL445256.1		smallRNA	chr6:72226707-7222
ENSG0000(	1516	37.91657	chr6:105(	ENSG00000275773		lncRNA	chr6:60723148-6072
ENSG0000(	1516	37.91657	chr6:105(	AL109922.1		smallRNA	chr6:64974393-6497
ENSG0000(	1516	37.91657	chr6:105(	ENSG00000217483		Pseudoger	chr6:72522641-7252
ENSG0000(	1516	37.91657	chr6:105(	ENSG00000289911		lncRNA	chr6:63378892-6356
ENSG0000(	1516	37.91657	chr6:105(	ENSG00000276127		Pseudoger	chr6:71251758-7125
ENSG0000(	1516	37.91657	chr6:105(	SNORD65		smallRNA	chr6:67210408-6721
ENSG0000(	1516	37.91657	chr6:105(	ENSG00000181514		Pseudoger	chr6:72598451-7259
ENSG0000(	1516	37.91657	chr6:105(	ENSG00000231762		Pseudoger	chr6:76660486-7666
ENSG0000(	1516	37.91657	chr6:105(	GSTA2		protein_c	chr6:52750087-5276
ENSG0000(	1516	37.91657	chr6:105(	LYPLA1P3		Pseudoger	chr6:71165076-7116
ENSG0000(	1505	37.64145	chr6:105(	SNORA70		smallRNA	chr6:81764024-8176
ENSG0000(	1475	36.89112	chr6:105(	ENSG00000277797		lncRNA	chr6:81551686-8155
ENSG0000(	1475	36.89112	chr6:105(	ENSG00000287816		lncRNA	chr6:79947810-7998
ENSG0000(	1475	36.89112	chr6:105(	RNU6-130P		smallRNA	chr6:82210338-8221
ENSG0000(	1475	36.89112	chr6:105(	ENSG00000218418		Pseudoger	chr6:80064286-8007
ENSG0000(	1475	36.89112	chr6:105(	ENSG00000226089		lncRNA	chr6:81527102-8153
ENSG0000(	1475	36.89112	chr6:105(	TPBG		protein_c	chr6:82363206-8236
ENSG0000(	1475	36.89112	chr6:105(	ENSG00000216352		Pseudoger	chr6:80555841-8055
ENSG0000(	1475	36.89112	chr6:105(	ENSG00000220537		Pseudoger	chr6:82263996-8226
ENSG0000(	1475	36.89112	chr6:105(	ENSG00000260645		lncRNA	chr6:80466958-8046
ENSG0000(	1475	36.89112	chr6:105(	IBTK		protein_c	chr6:82169986-8224
ENSG0000(	1475	36.89112	chr6:105(	ENSG00000286875		lncRNA	chr6:82353861-8236
ENSG0000(	1475	36.89112	chr6:105(	ENSG00000288071		lncRNA	chr6:80046715-8008
ENSG0000(	1475	36.89112	chr6:105(	ENSG00000181705		Pseudoger	chr6:80499196-8049
ENSG0000(	1475	36.89112	chr6:105(	BCKDHB	AC	protein_c	chr6:80106647-8034
ENSG0000(	1475	36.89112	chr6:105(	ENSG00000279022		TEC	chr6:80440730-8044
ENSG0000(	1475	36.89112	chr6:105(	ENSG00000232031		lncRNA	chr6:81724722-8173
ENSG0000(	1475	36.89112	chr6:105(	RPSAP72		Pseudoger	chr6:80470071-8047
ENSG0000(	1475	36.89112	chr6:105(	RPL17P25		Pseudoger	chr6:80374015-8037
ENSG0000(	1475	36.89112	chr6:105(	LINC02542		lncRNA	chr6:81844602-8216
ENSG0000(	1475	36.89112	chr6:105(	AK4P5		Pseudoger	chr6:80077729-8007
ENSG0000(	1475	36.89112	chr6:105(	ENSG00000260574		lncRNA	chr6:81969453-8196
ENSG0000(	1475	36.89112	chr6:105(	TTK	NCGv7	protein_c	chr6:80003887-8004
ENSG0000(	1475	36.89112	chr6:105(	ENSG00000219702		Pseudoger	chr6:81764211-8176
ENSG0000(	1475	36.89112	chr6:105(	ENSG00000272129		lncRNA	chr6:80355424-8035
ENSG0000(	1475	36.89112	chr6:105(	RNA5SP210		Pseudoger	chr6:81622200-8162
ENSG0000(	1475	36.89112	chr6:105(	TENT5A		protein_c	chr6:81491439-8175
ENSG0000(	1475	36.89112	chr6:105(	LINC01526		lncRNA	chr6:81813286-8181
ENSG0000(	1475	36.89112	chr1:1005	ENSG00000270066		lncRNA	chr1:109100193-109
ENSG0000(	1475	36.89112	chr6:105(	ENSG00000233967		lncRNA	chr6:80441295-8046
ENSG0000(	1356	33.91482	chr6:105(	KLC4-AS1		lncRNA	chr6:43074331-4307

ENSG0000(	1347	33.68972	chr16:291	COX6CP16		Pseudoger	chr16:85278820-852
ENSG0000(	1343	33.58968	chr6:105(	RNU1-88P		smallRNA	chr6:36639545-3663
ENSG0000(	1337	33.43961	chr4:909:	ENSG00000290803		lncRNA	chr4:6200733-62399
ENSG0000(	1337	33.43961	chr4:909:	RN7SKP36		smallRNA	chr4:7112088-71123
ENSG0000(	1337	33.43961	chr4:909:	SORCS2	NCGv7	protein_c	chr4:7192538-77428
ENSG0000(	1337	33.43961	chr4:909:	MRFAP1L1		protein_c	chr4:6707701-67098
ENSG0000(	1337	33.43961	chr4:909:	Y_RNA		smallRNA	chr4:5426885-54269
ENSG0000(	1337	33.43961	chr4:909:	PPP2R2C		protein_c	chr4:6320578-65636
ENSG0000(	1337	33.43961	chr4:909:	CRMP1		protein_c	chr4:5748084-58930
ENSG0000(	1337	33.43961	chr4:909:	ENSG00000251408		lncRNA	chr4:6202328-62066
ENSG0000(	1337	33.43961	chr4:909:	GRPEL1		protein_c	chr4:7058895-70680
ENSG0000(	1337	33.43961	chr4:909:	EVC		protein_c	chr4:5711201-58143
ENSG0000(	1337	33.43961	chr4:909:	MIR4274		smallRNA	chr4:7460028-74601
ENSG0000(	1337	33.43961	chr4:909:	JAKMIP1		protein_c	chr4:6026199-62005
ENSG0000(	1337	33.43961	chr4:909:	ENSG00000287104		lncRNA	chr4:6985913-69870
ENSG0000(	1337	33.43961	chr4:909:	AC093323.1		protein_c	chr4:6692055-66924
ENSG0000(	1337	33.43961	chr4:909:	MIR378D1		smallRNA	chr4:5923275-59233
ENSG0000(	1337	33.43961	chr4:909:	ENSG00000286176		lncRNA	chr4:6292369-63086
ENSG0000(	1337	33.43961	chr4:909:	ENSG00000279859		TEC	chr4:6691995-66929
ENSG0000(	1337	33.43961	chr4:909:	MAN2B2		protein_c	chr4:6575189-66233
ENSG0000(	1337	33.43961	chr4:909:	TBC1D14		protein_c	chr4:6909242-70331
ENSG0000(	1337	33.43961	chr4:909:	JAKMIP1-DT		Pseudoger	chr4:6227614-62298
ENSG0000(	1337	33.43961	chr4:909:	ENSG00000289414		lncRNA	chr4:5890366-58915
ENSG0000(	1337	33.43961	chr4:909:	RN7SKP275		smallRNA	chr4:5458581-54588
ENSG0000(	1337	33.43961	chr4:909:	ENSG00000187904		lncRNA	chr4:6995341-69989
ENSG0000(	1337	33.43961	chr4:909:	BLOC1S4		protein_c	chr4:6716174-67176
ENSG0000(	1337	33.43961	chr4:909:	ENSG00000287331		lncRNA	chr4:6763508-67676
ENSG0000(	1337	33.43961	chr4:909:	PSAPL1		protein_c	chr4:7430285-74349
ENSG0000(	1337	33.43961	chr4:909:	LINC02482		lncRNA	chr4:6648695-66739
ENSG0000(	1337	33.43961	chr4:909:	STK32B		protein_c	chr4:5051480-55009
ENSG0000(	1337	33.43961	chr4:909:	WFS1		protein_c	chr4:6269849-63032
ENSG0000(	1337	33.43961	chr4:909:	AC097382.1		smallRNA	chr4:6997526-69976
ENSG0000(	1337	33.43961	chr4:909:	LINC01587		lncRNA	chr4:5524569-55278
ENSG0000(	1337	33.43961	chr4:909:	MRFAP1		protein_c	chr4:6640091-66427
ENSG0000(	1337	33.43961	chr4:909:	S100P		protein_c	chr4:6693878-66971
ENSG0000(	1337	33.43961	chr4:909:	ENSG00000170846		protein_c	chr4:6663396-66767
ENSG0000(	1337	33.43961	chr4:909:	RP11-1406H17.1		lncRNA	chr4:6239441-62399
ENSG0000(	1337	33.43961	chr4:909:	KIAA0232	DriverDBv	protein_c	chr4:6781375-68841
ENSG0000(	1337	33.43961	chr4:909:	ENSG00000282742		TEC	chr4:6697861-66988
ENSG0000(	1337	33.43961	chr4:909:	LINC02481		lncRNA	chr4:6687448-66905
ENSG0000(	1337	33.43961	chr4:909:	ENSG00000284847		Pseudoger	chr4:6658690-66589
ENSG0000(	1337	33.43961	chr4:909:	ENSG00000288588		lncRNA	chr4:6245563-62616
ENSG0000(	1337	33.43961	chr4:909:	C4orf50		protein_c	chr4:5897373-62005
ENSG0000(	1337	33.43961	chr4:909:	TADA2B	DriverDBv	protein_c	chr4:7041899-70579
ENSG0000(	1337	33.43961	chr4:909:	CCDC96		protein_c	chr4:7040849-70430
ENSG0000(	1337	33.43961	chr4:909:	ENSG00000284684		protein_c	chr4:6064977-60701
ENSG0000(	1337	33.43961	chr4:909:	AC092463.1		smallRNA	chr4:6953289-69533
ENSG0000(	1337	33.43961	chr4:909:	ENSG00000287786		lncRNA	chr4:6178186-61849
ENSG0000(	1337	33.43961	chr4:909:	EVC2	NCGv7	protein_c	chr4:5542772-57095
ENSG0000(	1337	33.43961	chr4:909:	LINC02447		lncRNA	chr4:7093776-71033
ENSG0000(	1337	33.43961	chr4:909:	ENSG00000245748		lncRNA	chr4:7030554-70462

ENSG0000(	1337	33.43961	chr4:909:	RN7SKP292		smallRNA	chr4:6997012-69973
ENSG0000(	1303	32.58924	chr4:909:	ENSG00000280310		TEC	chr4:4820405-48214
ENSG0000(	1303	32.58924	chr4:909:	LINC01396		lncRNA	chr4:4844188-48508
ENSG0000(	1303	32.58924	chr4:909:	CYTL1		protein_c	chr4:5014586-50194
ENSG0000(	1303	32.58924	chr4:909:	STX18-AS1		lncRNA	chr4:4542131-47873
ENSG0000(	1303	32.58924	chr4:909:	LDHAP1		Pseudoger	chr4:4894182-48951
ENSG0000(	1303	32.58924	chr4:909:	MSX1		protein_c	chr4:4859665-48639
ENSG0000(	1303	32.58924	chr4:909:	RPS7P15		Pseudoger	chr4:4507078-45077
ENSG0000(	1303	32.58924	chr4:909:	RN7SKP113		smallRNA	chr4:4920770-49210
ENSG0000(	1300	32.51421	chr6:105(	BDH2P1		Pseudoger	chr6:99174744-9917
ENSG0000(	1297	32.43918	chr1:116(	RN7SL326P		smallRNA	chr1:40804846-4080
ENSG0000(	1282	32.06401	chr1:152:	ENSG00000231349		Pseudoger	chr1:86404176-8640
ENSG0000(	1277	31.93896	chr1:152:	AL109843.1		smallRNA	chr1:67239440-6723
ENSG0000(	1252	31.31368	chr7:330(	RPL23AP95		Pseudoger	chr7:103152007-103
ENSG0000(	1236	30.91351	chr6:105(	ENSG00000219023		Pseudoger	chr6:35555873-3555
ENSG0000(	1233	30.83848	chr1:813	Y_RNA		smallRNA	chr1:95125511-9512
ENSG0000(	1210	30.26322	chr7:330(	snoU13		smallRNA	chr7:130319279-130
ENSG0000(	1208	30.2132	chr6:105(	RN7SL273P		smallRNA	chr6:37361185-3736
ENSG0000(	1201	30.03813	chr11:76(	ENSG00000254596		Pseudoger	chr11:66454234-664
ENSG0000(	1198	29.96309	chr1:152:	ARL5AP3		Pseudoger	chr1:68049360-6804
ENSG0000(	1189	29.73799	chr6:105(	RNU6-761P		smallRNA	chr6:42018408-4201
ENSG0000(	1179	29.48789	chr6:105(	TBC1D22B		protein_c	chr6:37257772-3733
ENSG0000(	1179	29.48789	chr6:105(	XPO5		protein_c	chr6:43522334-4357
ENSG0000(	1179	29.48789	chr6:105(	RRP36	DriverDB\	protein_c	chr6:43021623-4303
ENSG0000(	1179	29.48789	chr6:105(	RN7SL285P		smallRNA	chr6:37832922-3783
ENSG0000(	1179	29.48789	chr6:105(	ENSG00000290049		lncRNA	chr6:42980542-4298
ENSG0000(	1179	29.48789	chr6:105(	TRERF1	NCGv7	protein_c	chr6:42224931-4245
ENSG0000(	1179	29.48789	chr6:105(	TMEM217		protein_c	chr6:37212180-3725
ENSG0000(	1179	29.48789	chr6:105(	ENSG00000286672		lncRNA	chr6:37301210-3730
ENSG0000(	1179	29.48789	chr6:105(	ABCC10		protein_c	chr6:43427366-4345
ENSG0000(	1179	29.48789	chr6:105(	TREML3P		Pseudoger	chr6:41209634-4121
ENSG0000(	1179	29.48789	chr6:105(	ENSG00000219470		Pseudoger	chr6:43538822-4353
ENSG0000(	1179	29.48789	chr6:105(	SNORD45		smallRNA	chr6:38207274-3820
ENSG0000(	1179	29.48789	chr6:105(	POLR1C	DriverDB\	protein_c	chr6:43509702-4356
ENSG0000(	1179	29.48789	chr6:105(	KIF6		protein_c	chr6:39329990-3972
ENSG0000(	1179	29.48789	chr6:105(	ENSG00000124593		protein_c	chr6:41780349-4179
ENSG0000(	1179	29.48789	chr6:105(	OARD1	DriverDB\	protein_c	chr6:41033627-4109
ENSG0000(	1179	29.48789	chr6:105(	DLK2		protein_c	chr6:43450352-4345
ENSG0000(	1179	29.48789	chr6:105(	UNC5CL		protein_c	chr6:41026895-4103
ENSG0000(	1179	29.48789	chr6:105(	ZNF318		protein_c	chr6:43307134-4336
ENSG0000(	1179	29.48789	chr6:105(	KCNK5		protein_c	chr6:39188971-3922
ENSG0000(	1179	29.48789	chr6:105(	RN7SL465P		smallRNA	chr6:38744086-3874
ENSG0000(	1179	29.48789	chr6:105(	MOCS1		protein_c	chr6:39899578-3993
ENSG0000(	1179	29.48789	chr6:105(	MED20	DriverDB\	protein_c	chr6:41905354-4192
ENSG0000(	1179	29.48789	chr6:105(	TBCC	DriverDB\	protein_c	chr6:42744498-4274
ENSG0000(	1179	29.48789	chr6:105(	PEX6		protein_c	chr6:42963865-4297
ENSG0000(	1179	29.48789	chr6:105(	GTPBP2	DriverDB\	protein_c	chr6:43605316-4362
ENSG0000(	1179	29.48789	chr6:105(	MAD2L1BP	DriverDB\	protein_c	chr6:43629540-4364
ENSG0000(	1179	29.48789	chr6:105(	ENSG00000279284		TEC	chr6:41381392-4138
ENSG0000(	1179	29.48789	chr6:105(	ENSG00000278745		Pseudoger	chr6:41252696-4125
ENSG0000(	1179	29.48789	chr6:105(	UBR2	DriverDB\	protein_c	chr6:42564029-4269

ENSG0000(	1179	29.48789	chr6:105(	RPS2P28		Pseudoger	chr6:43363479-4336
ENSG0000(	1179	29.48789	chr6:105(	TDRG1		lncRNA	chr6:40334775-4038
ENSG0000(	1179	29.48789	chr6:105(	LRRC73		protein_c	chr6:43506968-4351
ENSG0000(	1179	29.48789	chr6:105(	FOXP4-AS1		lncRNA	chr6:41452889-4154
ENSG0000(	1179	29.48789	chr6:105(	RPL24P4		Pseudoger	chr6:42956345-4295
ENSG0000(	1179	29.48789	chr6:105(	ENSG00000227920		lncRNA	chr6:37545145-3755
ENSG0000(	1179	29.48789	chr6:105(	PTCRA		protein_c	chr6:42915989-4292
ENSG0000(	1179	29.48789	chr6:105(	LINC00951		lncRNA	chr6:40344346-4038
ENSG0000(	1179	29.48789	chr6:105(	RNA5SP207		Pseudoger	chr6:41239520-4123
ENSG0000(	1179	29.48789	chr6:105(	ENSG00000272223		lncRNA	chr6:43033897-4303
ENSG0000(	1179	29.48789	chr6:105(	C6orf132		protein_c	chr6:42092233-4214
ENSG0000(	1179	29.48789	chr6:105(	RPL32P15		Pseudoger	chr6:41308166-4130
ENSG0000(	1179	29.48789	chr6:105(	RNU1-54P		smallRNA	chr6:39620345-3962
ENSG0000(	1179	29.48789	chr6:105(	CUL7	NCGv7;AC	protein_c	chr6:43037617-4305
ENSG0000(	1179	29.48789	chr6:105(	TREML4		protein_c	chr6:41228339-4123
ENSG0000(	1179	29.48789	chr6:105(	ENSG00000281969		TEC	chr6:39818751-3982
ENSG0000(	1179	29.48789	chr6:105(	TOMM6		protein_c	chr6:41787662-4178
ENSG0000(	1179	29.48789	chr6:105(	U3		smallRNA	chr6:42412200-4241
ENSG0000(	1179	29.48789	chr6:105(	ZFAND3-DT		lncRNA	chr6:37815777-3781
ENSG0000(	1179	29.48789	chr6:105(	USP49	DriverDB\	protein_c	chr6:41789896-4189
ENSG0000(	1179	29.48789	chr6:105(	RNU6-890P		smallRNA	chr6:42664162-4266
ENSG0000(	1179	29.48789	chr6:105(	ENSG00000290034		lncRNA	chr6:41154450-4117
ENSG0000(	1179	29.48789	chr6:105(	TREML5P		Pseudoger	chr6:41247369-4124
ENSG0000(	1179	29.48789	chr6:105(	ADCY10P1		Pseudoger	chr6:41101022-4113
ENSG0000(	1179	29.48789	chr6:105(	TREML1		protein_c	chr6:41149337-4115
ENSG0000(	1179	29.48789	chr6:105(	ENSG00000275550		Pseudoger	chr6:37295584-3729
ENSG0000(	1179	29.48789	chr6:105(	TMEM217B		protein_c	chr6:37212181-3725
ENSG0000(	1179	29.48789	chr6:105(	KLHDC3	DriverDB\	protein_c	chr6:43014103-4302
ENSG0000(	1179	29.48789	chr6:105(	MRPS10		protein_c	chr6:42206807-4221
ENSG0000(	1179	29.48789	chr6:105(	APOBEC2	NCGv7	protein_c	chr6:41053202-4106
ENSG0000(	1179	29.48789	chr6:105(	ZFAND3		protein_c	chr6:37819727-3815
ENSG0000(	1179	29.48789	chr6:105(	PTK7	DriverDB\	protein_c	chr6:43076307-4316
ENSG0000(	1179	29.48789	chr6:105(	ENSG00000280371		TEC	chr6:41405819-4140
ENSG0000(	1179	29.48789	chr6:105(	TSPO2		protein_c	chr6:41042467-4104
ENSG0000(	1179	29.48789	chr6:105(	SNORD112		smallRNA	chr6:37183597-3718
ENSG0000(	1179	29.48789	chr6:105(	DAAM2-AS1		lncRNA	chr6:39881804-3990
ENSG0000(	1179	29.48789	chr6:105(	ENSG00000180211		Pseudoger	chr6:39958414-3995
ENSG0000(	1179	29.48789	chr6:105(	SNORA8		smallRNA	chr6:41832854-4183
ENSG0000(	1179	29.48789	chr6:105(	ENSG00000245261		lncRNA	chr6:43213801-4322
ENSG0000(	1179	29.48789	chr6:105(	TREML2		protein_c	chr6:41189749-4120
ENSG0000(	1179	29.48789	chr6:105(	PPP2R5D		protein_c	chr6:42984553-4301
ENSG0000(	1179	29.48789	chr6:105(	BICRAL	DriverDB\	protein_c	chr6:42746958-4286
ENSG0000(	1179	29.48789	chr6:105(	PRPH2		protein_c	chr6:42696598-4272
ENSG0000(	1179	29.48789	chr6:105(	ENSG00000271754		lncRNA	chr6:43519180-4351
ENSG0000(	1179	29.48789	chr6:105(	GUCA1B		protein_c	chr6:42183284-4219
ENSG0000(	1179	29.48789	chr6:105(	RNU1-87P		smallRNA	chr6:37915573-3791
ENSG0000(	1179	29.48789	chr6:105(	TUBBP9		Pseudoger	chr6:39934595-4000
ENSG0000(	1179	29.48789	chr6:105(	POLH-AS1		lncRNA	chr6:43588230-4359
ENSG0000(	1179	29.48789	chr6:105(	ENSG00000289216		lncRNA	chr6:42191657-4219
ENSG0000(	1179	29.48789	chr6:105(	BYSL		protein_c	chr6:41921499-4193
ENSG0000(	1179	29.48789	chr6:105(	CCND3	NCGv7;AC	protein_c	chr6:41934934-4205

ENSG0000(	1179	29.48789	chr6:105(	RNU6-643P		smallRNA	chr6:41302466-4130
ENSG0000(	1179	29.48789	chr6:105(	RNU6-250P		smallRNA	chr6:40407853-4040
ENSG0000(	1179	29.48789	chr6:105(	PRICKLE4		protein_c	chr6:41780782-4178
ENSG0000(	1179	29.48789	chr6:105(	ENSG00000220614		Pseudoger	chr6:43328134-4332
ENSG0000(	1179	29.48789	chr6:105(	PI16		protein_c	chr6:36948263-3696
ENSG0000(	1179	29.48789	chr6:105(	C6orf226		protein_c	chr6:42890265-4289
ENSG0000(	1179	29.48789	chr6:105(	SAYSD1		protein_c	chr6:39104063-3911
ENSG0000(	1179	29.48789	chr6:105(	SRF		protein_c	chr6:43171269-4318
ENSG0000(	1179	29.48789	chr6:105(	ENSG00000220076		Pseudoger	chr6:38928031-3892
ENSG0000(	1179	29.48789	chr6:105(	SNORA8		smallRNA	chr6:38822307-3882
ENSG0000(	1179	29.48789	chr6:105(	GUCA1A		protein_c	chr6:42173364-4218
ENSG0000(	1179	29.48789	chr6:105(	BTBD9		protein_c	chr6:38168451-3864
ENSG0000(	1179	29.48789	chr6:105(	MDFI		protein_c	chr6:41636882-4165
ENSG0000(	1179	29.48789	chr6:105(	ENSG00000290147		protein_c	chr6:42155406-4218
ENSG0000(	1179	29.48789	chr6:105(	GNMT		protein_c	chr6:42960754-4296
ENSG0000(	1179	29.48789	chr6:105(	TFEB	NCGv7;AC	protein_c	chr6:41683978-4173
ENSG0000(	1179	29.48789	chr6:105(	DNAH8	NCGv7	protein_c	chr6:38715311-3903
ENSG0000(	1179	29.48789	chr6:105(	TREM1		protein_c	chr6:41267926-4128
ENSG0000(	1179	29.48789	chr6:105(	MEA1		protein_c	chr6:43011143-4301
ENSG0000(	1179	29.48789	chr6:105(	ENSG00000290563		lncRNA	chr6:41208713-4121
ENSG0000(	1179	29.48789	chr6:105(	SCARNA15		smallRNA	chr6:43544144-4354
ENSG0000(	1179	29.48789	chr6:105(	GLO1	AC	protein_c	chr6:38675925-3870
ENSG0000(	1179	29.48789	chr6:105(	KCNK17		protein_c	chr6:39299001-3931
ENSG0000(	1179	29.48789	chr6:105(	CUL9	NCGv7	protein_c	chr6:43182184-4322
ENSG0000(	1179	29.48789	chr6:105(	ENSG00000220556		Pseudoger	chr6:39039603-3903
ENSG0000(	1179	29.48789	chr6:105(	RN7SL403P		smallRNA	chr6:43036198-4303
ENSG0000(	1179	29.48789	chr6:105(	ATP6V0CP3		Pseudoger	chr6:42727234-4272
ENSG0000(	1179	29.48789	chr6:105(	RNF8		protein_c	chr6:37353979-3739
ENSG0000(	1179	29.48789	chr6:105(	MDGA1		protein_c	chr6:37630679-3769
ENSG0000(	1179	29.48789	chr6:105(	POLH	DriverDB\	protein_c	chr6:43576185-4362
ENSG0000(	1179	29.48789	chr6:105(	DNPH1	DriverDB\	protein_c	chr6:43225629-4322
ENSG0000(	1179	29.48789	chr6:105(	ENSG00000274256		Pseudoger	chr6:41250851-4125
ENSG0000(	1179	29.48789	chr6:105(	ENSG00000229559		Pseudoger	chr6:37543553-3754
ENSG0000(	1179	29.48789	chr6:105(	ENSG00000279942		TEC	chr6:37567716-3757
ENSG0000(	1179	29.48789	chr6:105(	LRFN2	NCGv7	protein_c	chr6:40391591-4058
ENSG0000(	1179	29.48789	chr6:105(	GLP1R		protein_c	chr6:39048781-3909
ENSG0000(	1179	29.48789	chr6:105(	ADCY10P1		lncRNA	chr6:41101034-4114
ENSG0000(	1179	29.48789	chr6:105(	MRPL2		protein_c	chr6:43054029-4305
ENSG0000(	1179	29.48789	chr6:105(	RSPH9	DriverDB\	protein_c	chr6:43645036-4367
ENSG0000(	1179	29.48789	chr6:105(	TAF8		protein_c	chr6:42050513-4208
ENSG0000(	1179	29.48789	chr6:105(	KLC4	DriverDB\	protein_c	chr6:43040777-4307
ENSG0000(	1179	29.48789	chr6:105(	RPL12P47		Pseudoger	chr6:43310231-4331
ENSG0000(	1179	29.48789	chr6:105(	TREM2		protein_c	chr6:41158506-4116
ENSG0000(	1179	29.48789	chr6:105(	DNAH8-AS1		lncRNA	chr6:38923029-3895
ENSG0000(	1179	29.48789	chr6:105(	KCNK16		protein_c	chr6:39314698-3932
ENSG0000(	1179	29.48789	chr6:105(	PPIL1	NCGv7	protein_c	chr6:36854827-3687
ENSG0000(	1179	29.48789	chr6:105(	FOXP4	DriverDB\	protein_c	chr6:41546381-4160
ENSG0000(	1179	29.48789	chr6:105(	ENSG00000288564		protein_c	chr6:43051066-4305
ENSG0000(	1179	29.48789	chr6:105(	ENSG00000287266		lncRNA	chr6:43403815-4340
ENSG0000(	1179	29.48789	chr6:105(	ENSG00000218986		Pseudoger	chr6:39353747-3935
ENSG0000(	1179	29.48789	chr6:105(	ENSG00000269387		lncRNA	chr6:41764292-4176

ENSG0000(	1179	29.48789	chr6:105(	NFYA	DriverDB\	protein_c	chr6:41072974-4110
ENSG0000(	1179	29.48789	chr6:105(	CNPY3	DriverDB\	protein_c	chr6:42929480-4293
ENSG0000(	1179	29.48789	chr6:105(	MRPS18A	DriverDB\	protein_c	chr6:43671202-4368
ENSG0000(	1179	29.48789	chr6:105(	ENSG00000226454		lncRNA	chr6:40505507-4052
ENSG0000(	1179	29.48789	chr6:105(	ENSG00000227131		lncRNA	chr6:40271566-4027
ENSG0000(	1179	29.48789	chr6:105(	snoU13		smallRNA	chr6:37251204-3725
ENSG0000(	1179	29.48789	chr6:105(	MIR4641		smallRNA	chr6:41598723-4159
ENSG0000(	1179	29.48789	chr6:105(	Y_RNA		smallRNA	chr6:41917416-4191
ENSG0000(	1179	29.48789	chr6:105(	ENSG00000287055		lncRNA	chr6:43370026-4342
ENSG0000(	1179	29.48789	chr6:105(	snoU13		smallRNA	chr6:42505500-4250
ENSG0000(	1179	29.48789	chr6:105(	ENSG00000218809		Pseudoger	chr6:41269875-4127
ENSG0000(	1179	29.48789	chr6:105(	ENSG00000268745		lncRNA	chr6:41791410-4179
ENSG0000(	1179	29.48789	chr6:105(	TJAP1	DriverDB\	protein_c	chr6:43477523-4350
ENSG0000(	1179	29.48789	chr6:105(	ENSG00000227516		lncRNA	chr6:41868622-4186
ENSG0000(	1179	29.48789	chr6:105(	RPL12P2		Pseudoger	chr6:37091314-3709
ENSG0000(	1179	29.48789	chr6:105(	ENSG00000223946		lncRNA	chr6:42030053-4203
ENSG0000(	1179	29.48789	chr6:105(	YIPF3	DriverDB\	protein_c	chr6:43511832-4351
ENSG0000(	1179	29.48789	chr6:105(	PIM1	NCGv7;AC	protein_c	chr6:37170152-3717
ENSG0000(	1179	29.48789	chr6:105(	GUCA1ANB		protein_c	chr6:42155406-4216
ENSG0000(	1179	29.48789	chr6:105(	SLC22A7		protein_c	chr6:43295694-4330
ENSG0000(	1179	29.48789	chr6:105(	MTCH1		protein_c	chr6:36965807-3698
ENSG0000(	1179	29.48789	chr6:105(	CMTR1	NCGv7	protein_c	chr6:37433219-3748
ENSG0000(	1179	29.48789	chr6:105(	ENSG00000288721		protein_c	chr6:41793314-4192
ENSG0000(	1179	29.48789	chr6:105(	MIR4462		smallRNA	chr6:37555365-3755
ENSG0000(	1179	29.48789	chr6:105(	ENSG00000226558		Pseudoger	chr6:43364220-4336
ENSG0000(	1179	29.48789	chr6:105(	DAAM2		protein_c	chr6:39792298-3990
ENSG0000(	1179	29.48789	chr6:105(	FRS3		protein_c	chr6:41770176-4178
ENSG0000(	1179	29.48789	chr6:105(	ENSG00000287825		lncRNA	chr6:42927686-4292
ENSG0000(	1179	29.48789	chr6:105(	COX6A1P2		Pseudoger	chr6:37044860-3704
ENSG0000(	1179	29.48789	chr6:105(	ENSG00000288010		lncRNA	chr6:42893761-4290
ENSG0000(	1179	29.48789	chr6:105(	DNAH8-DT		lncRNA	chr6:38714051-3871
ENSG0000(	1179	29.48789	chr6:105(	CRIP3		protein_c	chr6:43299710-4330
ENSG0000(	1179	29.48789	chr6:105(	LINC01276		lncRNA	chr6:41499033-4151
ENSG0000(	1179	29.48789	chr6:105(	ENSG00000218107		Pseudoger	chr6:43705949-4370
ENSG0000(	1179	29.48789	chr6:105(	NPM1P51		Pseudoger	chr6:41666906-4166
ENSG0000(	1179	29.48789	chr6:105(	ENSG00000236075		lncRNA	chr6:40501631-4050
ENSG0000(	1179	29.48789	chr6:105(	ENSG00000219273		Pseudoger	chr6:38762905-3876
ENSG0000(	1179	29.48789	chr6:105(	E2F4P1		Pseudoger	chr6:39553811-3955
ENSG0000(	1179	29.48789	chr6:105(	ENSG00000287891		lncRNA	chr6:36841430-3684
ENSG0000(	1179	29.48789	chr6:105(	LINC02520		lncRNA	chr6:37507348-3753
ENSG0000(	1179	29.48789	chr6:105(	ENSG00000232598		lncRNA	chr6:36940071-3694
ENSG0000(	1179	29.48789	chr6:105(	ENSG00000218521		Pseudoger	chr6:38002832-3800
ENSG0000(	1179	29.48789	chr6:105(	FGD2		protein_c	chr6:37005646-3702
ENSG0000(	1179	29.48789	chr6:105(	ENSG00000237947		lncRNA	chr6:40713411-4071
ENSG0000(	1179	29.48789	chr6:105(	Y_RNA		smallRNA	chr6:38565950-3856
ENSG0000(	1179	29.48789	chr6:105(	ENSG00000287678		lncRNA	chr6:41080624-4110
ENSG0000(	1179	29.48789	chr6:105(	AL136967.1		smallRNA	chr6:41355449-4135
ENSG0000(	1179	29.48789	chr6:105(	TTBK1		protein_c	chr6:43243481-4328
ENSG0000(	1179	29.48789	chr6:105(	ANKRD18EP		Pseudoger	chr6:39110321-3911
ENSG0000(	1179	29.48789	chr6:105(	NCR2		protein_c	chr6:41335608-4135
ENSG0000(	1179	29.48789	chr6:105(	PGC		protein_c	chr6:41736711-4175

ENSG0000(	1179	29.48789	chr6:105(	BTBD9-AS1		lncRNA	chr6:38481692-3848
ENSG0000(	1179	29.48789	chr6:105(	TFGP1		Pseudoger	chr6:38587323-3858
ENSG0000(	1179	29.48789	chr6:105(	RPL7L1	DriverDB\	protein_c	chr6:42879616-4288
ENSG0000(	1179	29.48789	chr6:105(	ENSG00000231102		lncRNA	chr6:41720396-4173
ENSG0000(	1179	29.48789	chr6:105(	RNU6-1113P		smallRNA	chr6:43474186-4347
ENSG0000(	1179	29.48789	chr6:105(	RPL36AP5		Pseudoger	chr6:42499710-4250
ENSG0000(	1179	29.48789	chr6:105(	LINC02976		lncRNA	chr6:42940364-4294
ENSG0000(	1179	29.48789	chr6:105(	C6orf89		protein_c	chr6:36871870-3692
ENSG0000(	1179	29.48789	chr6:105(	AL031905.1		smallRNA	chr6:38359148-3835
ENSG0000(	1179	29.48789	chr6:105(	CCDC167		protein_c	chr6:37482938-3749
ENSG0000(	1177	29.43786	chr11:76(	RN7SL23P		smallRNA	chr11:61444130-614
ENSG0000(	1160	29.01268	chr6:105(	ETV7		protein_c	chr6:36354091-3638
ENSG0000(	1160	29.01268	chr6:105(	Z85986.1		smallRNA	chr6:36510517-3651
ENSG0000(	1160	29.01268	chr6:105(	BRPF3	NCGv7	protein_c	chr6:36196744-3623
ENSG0000(	1160	29.01268	chr6:105(	RAB44		protein_c	chr6:36697826-3673
ENSG0000(	1160	29.01268	chr6:105(	RN7SL748P		smallRNA	chr6:36522191-3652
ENSG0000(	1160	29.01268	chr6:105(	DINOL		lncRNA	chr6:36677609-3667
ENSG0000(	1160	29.01268	chr6:105(	BNIP5		protein_c	chr6:36315761-3633
ENSG0000(	1160	29.01268	chr6:105(	CDKN1A	NCGv7;AC	protein_c	chr6:36676460-3668
ENSG0000(	1160	29.01268	chr6:105(	CPNE5	NCGv7	protein_c	chr6:36740775-3683
ENSG0000(	1160	29.01268	chr6:105(	ENSG00000285888		lncRNA	chr6:36768485-3677
ENSG0000(	1160	29.01268	chr6:105(	PXT1		protein_c	chr6:36390551-3644
ENSG0000(	1160	29.01268	chr6:105(	ETV7-AS1		lncRNA	chr6:36386831-3639
ENSG0000(	1160	29.01268	chr6:105(	PNPLA1		protein_c	chr6:36243203-3631
ENSG0000(	1160	29.01268	chr6:105(	PANDAR		lncRNA	chr6:36673621-3667
ENSG0000(	1160	29.01268	chr6:105(	BRPF3-AS1		lncRNA	chr6:36146698-3619
ENSG0000(	1160	29.01268	chr6:105(	RN7SL502P		smallRNA	chr6:36450915-3645
ENSG0000(	1160	29.01268	chr6:105(	STK38		protein_c	chr6:36493892-3654
ENSG0000(	1160	29.01268	chr6:105(	ENSG00000220349		Pseudoger	chr6:36737050-3673
ENSG0000(	1160	29.01268	chr6:105(	MIR3925		smallRNA	chr6:36622436-3662
ENSG0000(	1160	29.01268	chr6:105(	LAP3P2		Pseudoger	chr6:36673817-3667
ENSG0000(	1160	29.01268	chr6:105(	SRSF3	NCGv7;AC	protein_c	chr6:36594353-3660
ENSG0000(	1160	29.01268	chr6:105(	KCTD20		protein_c	chr6:36442767-3649
ENSG0000(	1160	29.01268	chr6:105(	Z95152.1		smallRNA	chr6:36140498-3614
ENSG0000(	1160	29.01268	chr6:105(	Y_RNA		smallRNA	chr6:36672838-3667
ENSG0000(	1150	28.76257	chr6:105(	SLC26A8		protein_c	chr6:35943516-3602
ENSG0000(	1150	28.76257	chr6:105(	ENSG00000288747		lncRNA	chr6:35764401-3576
ENSG0000(	1150	28.76257	chr6:105(	ENSG00000287458		lncRNA	chr6:35650997-3565
ENSG0000(	1150	28.76257	chr6:105(	SRPK1		protein_c	chr6:35832966-3592
ENSG0000(	1150	28.76257	chr6:105(	AL138721.1		smallRNA	chr6:35133454-3513
ENSG0000(	1150	28.76257	chr6:105(	TULP1		protein_c	chr6:35497874-3551
ENSG0000(	1150	28.76257	chr6:105(	CLPSL2		protein_c	chr6:35776594-3577
ENSG0000(	1150	28.76257	chr6:105(	ENSG00000232909		lncRNA	chr6:35733867-3573
ENSG0000(	1150	28.76257	chr6:105(	DEF6		protein_c	chr6:35297818-3532
ENSG0000(	1150	28.76257	chr6:105(	CLPSL1		protein_c	chr6:35781019-3579
ENSG0000(	1150	28.76257	chr6:105(	snoU13		smallRNA	chr6:34682798-3468
ENSG0000(	1150	28.76257	chr6:105(	ARMC12		protein_c	chr6:35737032-3574
ENSG0000(	1150	28.76257	chr6:105(	ENSG00000220734		Pseudoger	chr6:35765908-3576
ENSG0000(	1150	28.76257	chr6:105(	ENSG00000286550		lncRNA	chr6:35041116-3505
ENSG0000(	1150	28.76257	chr6:105(	MIR5690		smallRNA	chr6:35664717-3566
ENSG0000(	1150	28.76257	chr6:105(	MAPK13		protein_c	chr6:36127809-3614

ENSG0000(	1150	28.76257	chr6:105(	Y_RNA		smallRNA	chr6:34821445-3482
ENSG0000(	1150	28.76257	chr6:105(	ENSG00000186328		Pseudoger	chr6:34715613-3471
ENSG0000(	1150	28.76257	chr6:105(	LHFPL5		protein_c	chr6:35797206-3584
ENSG0000(	1150	28.76257	chr6:105(	IFITM3P3		Pseudoger	chr6:34576258-3457
ENSG0000(	1150	28.76257	chr6:105(	ENSG00000220643		Pseudoger	chr6:34686602-3468
ENSG0000(	1150	28.76257	chr6:105(	AL157823.1		smallRNA	chr6:35701003-3570
ENSG0000(	1150	28.76257	chr6:105(	ILRUN-AS1		lncRNA	chr6:34696317-3469
ENSG0000(	1150	28.76257	chr6:105(	SCUBE3	NCGv7	protein_c	chr6:35213956-3525
ENSG0000(	1150	28.76257	chr6:105(	ENSG00000272374		lncRNA	chr6:35220370-3522
ENSG0000(	1150	28.76257	chr6:105(	ENSG00000228559		lncRNA	chr6:35539838-3554
ENSG0000(	1150	28.76257	chr6:105(	ENSG00000217130		Pseudoger	chr6:34744176-3474
ENSG0000(	1150	28.76257	chr6:105(	ENSG00000217004		Pseudoger	chr6:35279177-3527
ENSG0000(	1150	28.76257	chr6:105(	snoU13		smallRNA	chr6:34692780-3469
ENSG0000(	1150	28.76257	chr6:105(	ILRUN		protein_c	chr6:34587288-3469
ENSG0000(	1150	28.76257	chr6:105(	FKBP5		protein_c	chr6:35573585-3572
ENSG0000(	1150	28.76257	chr6:105(	MKRN6P		Pseudoger	chr6:35443044-3544
ENSG0000(	1150	28.76257	chr6:105(	TAF11	NCGv7	protein_c	chr6:34877462-3488
ENSG0000(	1150	28.76257	chr6:105(	FANCE	NCGv7;AC	protein_c	chr6:35452338-3546
ENSG0000(	1150	28.76257	chr6:105(	HSPE1P11		Pseudoger	chr6:35023522-3502
ENSG0000(	1150	28.76257	chr6:105(	ENSG00000289456		lncRNA	chr6:35563135-3556
ENSG0000(	1150	28.76257	chr6:105(	MAPK14		protein_c	chr6:36027782-3611
ENSG0000(	1150	28.76257	chr6:105(	PPARD	NCGv7	protein_c	chr6:35342558-3542
ENSG0000(	1150	28.76257	chr6:105(	ZNF76		protein_c	chr6:35258909-3529
ENSG0000(	1150	28.76257	chr6:105(	TEAD3		protein_c	chr6:35473597-3549
ENSG0000(	1150	28.76257	chr6:105(	DPRXP2		Pseudoger	chr6:35989515-3599
ENSG0000(	1150	28.76257	chr6:105(	ENSG00000273870		Pseudoger	chr6:35070871-3507
ENSG0000(	1150	28.76257	chr6:105(	ANKS1A		protein_c	chr6:34889255-3509
ENSG0000(	1150	28.76257	chr6:105(	SNORA40		smallRNA	chr6:35651818-3565
ENSG0000(	1150	28.76257	chr6:105(	RPL10A		protein_c	chr6:35468401-3547
ENSG0000(	1150	28.76257	chr6:105(	UHRF1BP1		protein_c	chr6:34792083-3487
ENSG0000(	1150	28.76257	chr6:105(	RPL36P9		Pseudoger	chr6:35607628-3560
ENSG0000(	1150	28.76257	chr6:105(	ENSG00000237719		Pseudoger	chr6:36091991-3609
ENSG0000(	1150	28.76257	chr6:105(	TCP11	NCGv7	protein_c	chr6:35118071-3514
ENSG0000(	1150	28.76257	chr6:105(	SNRPC		protein_c	chr6:34757505-3477
ENSG0000(	1150	28.76257	chr6:105(	RN7SL200P		smallRNA	chr6:34685357-3468
ENSG0000(	1150	28.76257	chr6:105(	CLPS		protein_c	chr6:35794982-3579
ENSG0000(	1150	28.76257	chr6:105(	RPL7P25		Pseudoger	chr6:34616538-3461
ENSG0000(	1148	28.71255	chr1:116(	ENSG00000235002		Pseudoger	chr1:42412398-4241
ENSG0000(	1139	28.48745	chr1:152:	ENSG00000230546		Pseudoger	chr1:58084419-5808
ENSG0000(	1132	28.31237	chr11:76(	MIR129-2		smallRNA	chr11:43581394-435
ENSG0000(	1118	27.96222	chr11:76(	TBC1D10C		protein_c	chr11:67403915-674
ENSG0000(	1118	27.96222	chr11:76(	ENSG00000285933		lncRNA	chr11:71745331-717
ENSG0000(	1118	27.96222	chr11:76(	CLCF1		protein_c	chr11:67364168-673
ENSG0000(	1118	27.96222	chr11:76(	DPP3		protein_c	chr11:66480013-665
ENSG0000(	1118	27.96222	chr11:76(	POLD4		protein_c	chr11:67350772-673
ENSG0000(	1118	27.96222	chr11:76(	ENSG00000248903		Pseudoger	chr11:71568680-715
ENSG0000(	1118	27.96222	chr11:76(	SART1		protein_c	chr11:65961728-659
ENSG0000(	1118	27.96222	chr11:76(	RPS6KB2		protein_c	chr11:67428460-674
ENSG0000(	1118	27.96222	chr11:76(	RNU1-84P		smallRNA	chr11:66393449-663
ENSG0000(	1118	27.96222	chr11:76(	FTLP6		Pseudoger	chr11:66771246-667
ENSG0000(	1118	27.96222	chr11:76(	ENSG00000254924		Pseudoger	chr11:71506061-715

ENSG0000(	1118	27.96222	chr11:76(	XNDC1N-ZNF705EP-AI		protein_c	chr11:71804997-719
ENSG0000(	1118	27.96222	chr11:76(	GAL		protein_c	chr11:68683779-686
ENSG0000(	1118	27.96222	chr11:76(	KRTAP5-9		protein_c	chr11:71548420-715
ENSG0000(	1118	27.96222	chr11:76(	RNU6-46P		smallRNA	chr11:67895631-678
ENSG0000(	1118	27.96222	chr11:76(	FAM86C1P		Pseudoger	chr11:71787537-717
ENSG0000(	1118	27.96222	chr11:76(	EIF1AD		protein_c	chr11:65996545-660
ENSG0000(	1118	27.96222	chr11:76(	ENSG00000287934		lncRNA	chr11:67252336-672
ENSG0000(	1118	27.96222	chr11:76(	LINC02753		lncRNA	chr11:70056230-700
ENSG0000(	1118	27.96222	chr11:76(	RBM14-RBM4		protein_c	chr11:66616626-666
ENSG0000(	1118	27.96222	chr11:76(	TSGA10IP		protein_c	chr11:65945480-659
ENSG0000(	1118	27.96222	chr11:76(	ENSG00000284625		Pseudoger	chr11:71639551-716
ENSG0000(	1118	27.96222	chr11:76(	INPPL1	NCGv7;AC	protein_c	chr11:72223701-722
ENSG0000(	1118	27.96222	chr11:76(	ENSG00000227726		lncRNA	chr11:70477277-704
ENSG0000(	1118	27.96222	chr11:76(	ENSG00000255038		lncRNA	chr11:66067277-660
ENSG0000(	1118	27.96222	chr11:76(	FOSL1	TAG;AC	protein_c	chr11:65892049-659
ENSG0000(	1118	27.96222	chr11:76(	ZNF705EP		Pseudoger	chr11:71816512-718
ENSG0000(	1118	27.96222	chr11:76(	LINC02953		lncRNA	chr11:69425678-694
ENSG0000(	1118	27.96222	chr11:76(	C11orf68		protein_c	chr11:65916810-659
ENSG0000(	1118	27.96222	chr11:76(	FOLR2		protein_c	chr11:72216601-722
ENSG0000(	1118	27.96222	chr11:76(	ENSG00000255031		lncRNA	chr11:68050740-680
ENSG0000(	1118	27.96222	chr11:76(	CABP4		protein_c	chr11:67452406-674
ENSG0000(	1118	27.96222	chr11:76(	ENSG00000254972		lncRNA	chr11:71701268-717
ENSG0000(	1118	27.96222	chr11:76(	U3		smallRNA	chr11:66995479-669
ENSG0000(	1118	27.96222	chr11:76(	MIR4691		smallRNA	chr11:68033897-680
ENSG0000(	1118	27.96222	chr11:76(	CCDC85B		protein_c	chr11:65890673-658
ENSG0000(	1118	27.96222	chr11:76(	ALG1L9P		lncRNA	chr11:71673885-718
ENSG0000(	1118	27.96222	chr11:76(	ACTN3		protein_c	chr11:66546395-665
ENSG0000(	1118	27.96222	chr11:76(	ENSG00000280089		TEC	chr11:70705605-707
ENSG0000(	1118	27.96222	chr11:76(	UNC93B5		Pseudoger	chr11:67711702-677
ENSG0000(	1118	27.96222	chr11:76(	RPS6KB2-AS1		lncRNA	chr11:67431367-674
ENSG0000(	1118	27.96222	chr11:76(	ALG1L9P		Pseudoger	chr11:71800541-718
ENSG0000(	1118	27.96222	chr11:76(	GPR152		protein_c	chr11:67451301-674
ENSG0000(	1118	27.96222	chr11:76(	NUMA1	NCGv7;AC	protein_c	chr11:72002864-720
ENSG0000(	1118	27.96222	chr11:76(	AP000807.2		smallRNA	chr11:68505572-685
ENSG0000(	1118	27.96222	chr11:76(	SNORA43		smallRNA	chr11:66432763-664
ENSG0000(	1118	27.96222	chr11:76(	ENSG00000287725		protein_c	chr11:69072915-691
ENSG0000(	1118	27.96222	chr11:76(	SMIM38		protein_c	chr11:69155478-691
ENSG0000(	1118	27.96222	chr11:76(	ENSG00000289908		lncRNA	chr11:68038252-680
ENSG0000(	1118	27.96222	chr11:76(	XNDC1N		protein_c	chr11:71865504-719
ENSG0000(	1118	27.96222	chr11:76(	ENSG00000254461		lncRNA	chr11:66259567-662
ENSG0000(	1118	27.96222	chr11:76(	ENSG00000254458		lncRNA	chr11:66312853-663
ENSG0000(	1118	27.96222	chr11:76(	ENSG00000254452		lncRNA	chr11:66276779-662
ENSG0000(	1118	27.96222	chr11:76(	ENSG00000254447		lncRNA	chr11:67735600-677
ENSG0000(	1118	27.96222	chr11:76(	ALDH3B1		protein_c	chr11:68008578-680
ENSG0000(	1118	27.96222	chr11:76(	AP000807.1		smallRNA	chr11:68506083-685
ENSG0000(	1118	27.96222	chr11:76(	CCDC87		protein_c	chr11:66590176-665
ENSG0000(	1118	27.96222	chr11:76(	MRPL21	NCGv7	protein_c	chr11:68891276-689
ENSG0000(	1118	27.96222	chr11:76(	KRTAP5-7		protein_c	chr11:71527267-715
ENSG0000(	1118	27.96222	chr11:76(	TOMT		protein_c	chr11:72105924-721
ENSG0000(	1118	27.96222	chr11:76(	LAMTOR1	DriverDB\	protein_c	chr11:72085895-721
ENSG0000(	1118	27.96222	chr11:76(	AP002490.1		smallRNA	chr11:72078330-720

ENSG0000(	1118	27.96222	chr11:76(	ENSG00000204971		lncRNA	chr11:72163322-722
ENSG0000(	1118	27.96222	chr11:76(	LRTOMT	DriverDBv	protein_c	chr11:72080331-721
ENSG0000(	1118	27.96222	chr11:76(	ENSG00000254792		Pseudoger	chr11:67840942-678
ENSG0000(	1118	27.96222	chr11:76(	KLC2-AS2		lncRNA	chr11:66267635-662
ENSG0000(	1118	27.96222	chr11:76(	ENSG00000254756		lncRNA	chr11:66334494-663
ENSG0000(	1118	27.96222	chr11:76(	ENSG00000254484		lncRNA	chr11:70319928-703
ENSG0000(	1118	27.96222	chr11:76(	RNU6-1175P		smallRNA	chr11:70075363-700
ENSG0000(	1118	27.96222	chr11:76(	FGF19	NCGv7	protein_c	chr11:69698238-697
ENSG0000(	1118	27.96222	chr11:76(	TPCN2	DriverDBv	protein_c	chr11:69048932-691
ENSG0000(	1118	27.96222	chr11:76(	LRP5		protein_c	chr11:68312591-684
ENSG0000(	1118	27.96222	chr11:76(	ENSG00000254682		lncRNA	chr11:71448562-714
ENSG0000(	1118	27.96222	chr11:76(	ENSG00000254605		lncRNA	chr11:70014858-700
ENSG0000(	1118	27.96222	chr11:76(	SHANK2	NCGv7	protein_c	chr11:70467854-712
ENSG0000(	1118	27.96222	chr11:76(	ENSG00000254604		lncRNA	chr11:70282367-703
ENSG0000(	1118	27.96222	chr11:76(	MIR3164		smallRNA	chr11:69083176-690
ENSG0000(	1118	27.96222	chr11:76(	ENSG00000254721		lncRNA	chr11:70206291-702
ENSG0000(	1118	27.96222	chr11:76(	ENSG00000254495		lncRNA	chr11:70358198-703
ENSG0000(	1118	27.96222	chr11:76(	SF3B2	NCGv7	protein_c	chr11:66050729-660
ENSG0000(	1118	27.96222	chr11:76(	RPS3AP40		Pseudoger	chr11:67925651-679
ENSG0000(	1118	27.96222	chr11:76(	BRD9P1		Pseudoger	chr11:66389609-663
ENSG0000(	1118	27.96222	chr11:76(	OR7E126P		Pseudoger	chr11:71903194-719
ENSG0000(	1118	27.96222	chr11:76(	RPEP6		Pseudoger	chr11:72131282-721
ENSG0000(	1118	27.96222	chr11:76(	RN7SL59P		smallRNA	chr11:67541735-675
ENSG0000(	1118	27.96222	chr11:76(	RNU6-292P		smallRNA	chr11:72047873-720
ENSG0000(	1118	27.96222	chr11:76(	ENSG00000254510		lncRNA	chr11:66409158-664
ENSG0000(	1118	27.96222	chr11:76(	LINC02584		lncRNA	chr11:70072434-700
ENSG0000(	1118	27.96222	chr11:76(	ENPP7P7		Pseudoger	chr11:67812557-678
ENSG0000(	1118	27.96222	chr11:76(	DEFB108B		protein_c	chr11:71833200-718
ENSG0000(	1118	27.96222	chr11:76(	FOLR1P1		Pseudoger	chr11:72158822-721
ENSG0000(	1118	27.96222	chr11:76(	GSTP1		protein_c	chr11:67583742-675
ENSG0000(	1118	27.96222	chr11:76(	ENSG00000254850		Pseudoger	chr11:67935558-679
ENSG0000(	1118	27.96222	chr11:76(	CTTN-DT		lncRNA	chr11:70372246-703
ENSG0000(	1118	27.96222	chr11:76(	ENSG00000289343		lncRNA	chr11:67508772-675
ENSG0000(	1118	27.96222	chr11:76(	ENSG00000256349		protein_c	chr11:66509079-665
ENSG0000(	1118	27.96222	chr11:76(	KLC2-AS1		lncRNA	chr11:66264777-662
ENSG0000(	1118	27.96222	chr11:76(	ENSG00000291186		lncRNA	chr11:71787480-718
ENSG0000(	1118	27.96222	chr11:76(	NDUFA3P2		Pseudoger	chr11:68488609-684
ENSG0000(	1118	27.96222	chr11:76(	TMEM151A		protein_c	chr11:66291894-662
ENSG0000(	1118	27.96222	chr11:76(	ENSG00000254867		Pseudoger	chr11:65983679-659
ENSG0000(	1118	27.96222	chr11:76(	ENSG00000287851		lncRNA	chr11:67316539-673
ENSG0000(	1118	27.96222	chr11:76(	RNA5SP342		Pseudoger	chr11:71845196-718
ENSG0000(	1118	27.96222	chr11:76(	ENSG00000179038		Pseudoger	chr11:67195569-671
ENSG0000(	1118	27.96222	chr11:76(	ENSG00000254883		Pseudoger	chr11:67744322-677
ENSG0000(	1118	27.96222	chr11:76(	OR7E87P		Pseudoger	chr11:71593454-715
ENSG0000(	1118	27.96222	chr11:76(	LRRC51		protein_c	chr11:72080337-720
ENSG0000(	1118	27.96222	chr11:76(	ANO1-AS1		lncRNA	chr11:70187788-701
ENSG0000(	1118	27.96222	chr11:76(	FAM86C2P		Pseudoger	chr11:67793196-678
ENSG0000(	1118	27.96222	chr11:76(	NDUFV1-DT		lncRNA	chr11:67602880-676
ENSG0000(	1118	27.96222	chr11:76(	RNF121	DriverDBv	protein_c	chr11:71929018-719
ENSG0000(	1118	27.96222	chr11:76(	RNU6-1238P		smallRNA	chr11:67395210-673
ENSG0000(	1118	27.96222	chr11:76(	DEFB131B		protein_c	chr11:71878453-718

ENSG0000(	1118	27.96222	chr11:76(	ENSG00000290995		lncRNA	chr11:67934563-679
ENSG0000(	1118	27.96222	chr11:76(	ENSG00000289074		lncRNA	chr11:70269189-702
ENSG0000(	1118	27.96222	chr11:76(	ENSG00000256514		protein_c	chr11:67351572-673
ENSG0000(	1118	27.96222	chr11:76(	MRGPRF-AS1		lncRNA	chr11:69012283-690
ENSG0000(	1118	27.96222	chr11:76(	LINC01488		lncRNA	chr11:69481662-694
ENSG0000(	1118	27.96222	chr11:76(	CTTN	DriverDB\	protein_c	chr11:70398404-704
ENSG0000(	1118	27.96222	chr11:76(	LTO1		protein_c	chr11:69653076-696
ENSG0000(	1118	27.96222	chr11:76(	MIR3163		smallRNA	chr11:66934434-669
ENSG0000(	1118	27.96222	chr11:76(	AP003385.1		smallRNA	chr11:67734521-677
ENSG0000(	1118	27.96222	chr11:76(	DNAJB6P5		Pseudoger	chr11:69737298-697
ENSG0000(	1118	27.96222	chr11:76(	OR7E11P		Pseudoger	chr11:67735608-677
ENSG0000(	1118	27.96222	chr11:76(	ENSG00000274251		lncRNA	chr11:67353629-673
ENSG0000(	1118	27.96222	chr11:76(	FAM86C2P		lncRNA	chr11:67791648-678
ENSG0000(	1118	27.96222	chr11:76(	ENSG00000283257		Pseudoger	chr11:67538899-675
ENSG0000(	1118	27.96222	chr11:76(	SNRPCP14		Pseudoger	chr11:71690453-716
ENSG0000(	1118	27.96222	chr11:76(	ENSG00000245156		lncRNA	chr11:66269832-662
ENSG0000(	1118	27.96222	chr11:76(	RN7SKP239		smallRNA	chr11:67362414-673
ENSG0000(	1118	27.96222	chr11:76(	IL18BP		protein_c	chr11:71998613-720
ENSG0000(	1118	27.96222	chr11:76(	CST6		protein_c	chr11:66012008-660
ENSG0000(	1118	27.96222	chr11:76(	BANF1	TAG	protein_c	chr11:66002228-660
ENSG0000(	1118	27.96222	chr11:76(	DRAP1		protein_c	chr11:65919274-659
ENSG0000(	1118	27.96222	chr11:76(	CATSPER1		protein_c	chr11:66016752-660
ENSG0000(	1118	27.96222	chr11:76(	FGF3	TAG;AC	protein_c	chr11:69809968-698
ENSG0000(	1118	27.96222	chr11:76(	OR7E145P		Pseudoger	chr11:67722483-677
ENSG0000(	1118	27.96222	chr11:76(	LINC02701		lncRNA	chr11:68870664-688
ENSG0000(	1118	27.96222	chr11:76(	Y_RNA		smallRNA	chr11:70705167-707
ENSG0000(	1118	27.96222	chr11:76(	OR7E4P		Pseudoger	chr11:71620020-716
ENSG0000(	1118	27.96222	chr11:76(	C11orf86		protein_c	chr11:66975277-669
ENSG0000(	1118	27.96222	chr11:76(	ANO1	DriverDB\	protein_c	chr11:69985907-701
ENSG0000(	1118	27.96222	chr11:76(	PPFIA1	DriverDB\	protein_c	chr11:70270690-703
ENSG0000(	1118	27.96222	chr11:76(	SYT12	DriverDB\	protein_c	chr11:67006778-670
ENSG0000(	1118	27.96222	chr11:76(	ALG1L8P		Pseudoger	chr11:67785273-677
ENSG0000(	1118	27.96222	chr11:76(	RHOD		protein_c	chr11:67056847-670
ENSG0000(	1118	27.96222	chr11:76(	ENSG00000260808		lncRNA	chr11:68612899-686
ENSG0000(	1118	27.96222	chr11:76(	KDM2A	NCGv7;AC	protein_c	chr11:67119263-672
ENSG0000(	1118	27.96222	chr11:76(	ENSG00000279459		TEC	chr11:70603304-706
ENSG0000(	1118	27.96222	chr11:76(	AP002495.1		smallRNA	chr11:71802585-718
ENSG0000(	1118	27.96222	chr11:76(	ENSG00000255539		lncRNA	chr11:70324871-703
ENSG0000(	1118	27.96222	chr11:76(	RNU4-39P		smallRNA	chr11:66614964-666
ENSG0000(	1118	27.96222	chr11:76(	C11orf24		protein_c	chr11:68261338-682
ENSG0000(	1118	27.96222	chr11:76(	GRK2		protein_c	chr11:67266473-672
ENSG0000(	1118	27.96222	chr11:76(	ENSG00000255296		Pseudoger	chr11:71938671-719
ENSG0000(	1118	27.96222	chr11:76(	ENSG00000255306		lncRNA	chr11:68024809-680
ENSG0000(	1118	27.96222	chr11:76(	DOC2GP		Pseudoger	chr11:67612651-676
ENSG0000(	1118	27.96222	chr11:76(	DPP3-DT		lncRNA	chr11:66473490-664
ENSG0000(	1118	27.96222	chr11:76(	ENPP7P8		Pseudoger	chr11:71722052-717
ENSG0000(	1118	27.96222	chr11:76(	PC	DriverDB\	protein_c	chr11:66848417-669
ENSG0000(	1118	27.96222	chr11:76(	OR7E1P		Pseudoger	chr11:67974286-679
ENSG0000(	1118	27.96222	chr11:76(	ENSG00000255741		lncRNA	chr11:68941503-689
ENSG0000(	1118	27.96222	chr11:76(	LRFN4		protein_c	chr11:66856647-668
ENSG0000(	1118	27.96222	chr11:76(	PELI3	NCGv7	protein_c	chr11:66466327-664

ENSG0000(	1118	27.96222	chr11:76(	ENSG00000286459		lncRNA	chr11:66043298-660
ENSG0000(	1118	27.96222	chr11:76(	BBS1		protein_c	chr11:66510606-665
ENSG0000(	1118	27.96222	chr11:76(	KRTAP5-11	NCGv7	protein_c	chr11:71581855-716
ENSG0000(	1118	27.96222	chr11:76(	KRTAP5-14P		Pseudoger	chr11:71579728-715
ENSG0000(	1118	27.96222	chr11:76(	C1QBPP2		Pseudoger	chr11:66761575-667
ENSG0000(	1118	27.96222	chr11:76(	PTPRCAP		protein_c	chr11:67435510-674
ENSG0000(	1118	27.96222	chr11:76(	LINC02952		lncRNA	chr11:69438365-694
ENSG0000(	1118	27.96222	chr11:76(	ENSG00000251143		lncRNA	chr11:72014291-720
ENSG0000(	1118	27.96222	chr11:76(	ZDHHC24		protein_c	chr11:66520637-665
ENSG0000(	1118	27.96222	chr11:76(	ENSG00000255230		Pseudoger	chr11:67965873-679
ENSG0000(	1118	27.96222	chr11:76(	CTSF		protein_c	chr11:66563464-665
ENSG0000(	1118	27.96222	chr11:76(	CCS		protein_c	chr11:66593153-666
ENSG0000(	1118	27.96222	chr11:76(	CHKA-DT		lncRNA	chr11:68121624-681
ENSG0000(	1118	27.96222	chr11:76(	RBM4		protein_c	chr11:66638667-666
ENSG0000(	1118	27.96222	chr11:76(	snoU13		smallRNA	chr11:67220908-672
ENSG0000(	1118	27.96222	chr11:76(	RBM4B		protein_c	chr11:66664998-666
ENSG0000(	1118	27.96222	chr11:76(	SPTBN2	NCGv7	protein_c	chr11:66682497-667
ENSG0000(	1118	27.96222	chr11:76(	C11orf80		protein_c	chr11:66744451-668
ENSG0000(	1118	27.96222	chr11:76(	AP003498.1		smallRNA	chr11:71662547-716
ENSG0000(	1118	27.96222	chr11:76(	IFITM9P		Pseudoger	chr11:69303412-693
ENSG0000(	1118	27.96222	chr11:76(	RCE1		protein_c	chr11:66842835-668
ENSG0000(	1118	27.96222	chr11:76(	UNC93B6		Pseudoger	chr11:71603260-716
ENSG0000(	1118	27.96222	chr11:76(	ENSG00000255320		lncRNA	chr11:66244717-662
ENSG0000(	1118	27.96222	chr11:76(	AC004924.1		smallRNA	chr11:67933214-679
ENSG0000(	1118	27.96222	chr11:76(	H2AZP4		Pseudoger	chr11:70278921-702
ENSG0000(	1118	27.96222	chr11:76(	MRGPRD		protein_c	chr11:68980021-689
ENSG0000(	1118	27.96222	chr11:76(	TMEM134		protein_c	chr11:67461710-674
ENSG0000(	1118	27.96222	chr11:76(	ENSG00000290775		lncRNA	chr11:71711740-717
ENSG0000(	1118	27.96222	chr11:76(	B4GAT1-DT		lncRNA	chr11:66347950-663
ENSG0000(	1118	27.96222	chr11:76(	EFEMP2		protein_c	chr11:65866441-658
ENSG0000(	1118	27.96222	chr11:76(	RAD9A		protein_c	chr11:67317871-673
ENSG0000(	1118	27.96222	chr11:76(	MIR3664		smallRNA	chr11:70872270-708
ENSG0000(	1118	27.96222	chr11:76(	ENSG00000261625		lncRNA	chr11:69000765-690
ENSG0000(	1118	27.96222	chr11:76(	SHANK2-AS2		lncRNA	chr11:70646165-706
ENSG0000(	1118	27.96222	chr11:76(	ENSG00000255415		Pseudoger	chr11:71629045-716
ENSG0000(	1118	27.96222	chr11:76(	MIR3165		smallRNA	chr11:72072228-720
ENSG0000(	1118	27.96222	chr11:76(	IGHMBP2	DriverDB\	protein_c	chr11:68903863-689
ENSG0000(	1118	27.96222	chr11:76(	ACY3		protein_c	chr11:67642555-676
ENSG0000(	1118	27.96222	chr11:76(	ALDH3B2		protein_c	chr11:67662155-676
ENSG0000(	1118	27.96222	chr11:76(	TESMIN	DriverDB\	protein_c	chr11:68707440-687
ENSG0000(	1118	27.96222	chr11:76(	OR7E128P		Pseudoger	chr11:71893410-718
ENSG0000(	1118	27.96222	chr11:76(	CTSW		protein_c	chr11:65879809-658
ENSG0000(	1118	27.96222	chr11:76(	RBM14	AC	protein_c	chr11:66616626-666
ENSG0000(	1118	27.96222	chr11:76(	PPP1CA		protein_c	chr11:67398181-674
ENSG0000(	1118	27.96222	chr11:76(	ENSG00000290736		lncRNA	chr11:71856277-718
ENSG0000(	1118	27.96222	chr11:76(	CARNS1	NCGv7	protein_c	chr11:67414968-674
ENSG0000(	1118	27.96222	chr11:76(	FIBP		protein_c	chr11:65883740-658
ENSG0000(	1118	27.96222	chr11:76(	ENSG00000286948		lncRNA	chr11:71563389-715
ENSG0000(	1118	27.96222	chr11:76(	ENSG00000289560		lncRNA	chr11:66311859-663
ENSG0000(	1118	27.96222	chr11:76(	RNU7-23P		smallRNA	chr11:66919762-669
ENSG0000(	1118	27.96222	chr11:76(	RPS3AP41		Pseudoger	chr11:71669520-716

ENSG0000(	1118	27.96222	chr11:76(	CORO1B		protein_c	chr11:67435510-674
ENSG0000(	1118	27.96222	chr11:76(	ENSG00000261276		lncRNA	chr11:69004394-690
ENSG0000(	1118	27.96222	chr11:76(	AP003498.2		smallRNA	chr11:71706479-717
ENSG0000(	1118	27.96222	chr11:76(	MRGPRF		protein_c	chr11:69004395-690
ENSG0000(	1118	27.96222	chr11:76(	RP11-211G23.2		lncRNA	chr11:69371463-693
ENSG0000(	1118	27.96222	chr11:76(	ENSG00000260895		lncRNA	chr11:69103493-691
ENSG0000(	1118	27.96222	chr11:76(	ANKRD13D		protein_c	chr11:67289300-673
ENSG0000(	1118	27.96222	chr11:76(	SHANK2-AS1		lncRNA	chr11:70626441-706
ENSG0000(	1118	27.96222	chr11:76(	ACTE1P		Pseudoger	chr11:71382601-714
ENSG0000(	1118	27.96222	chr11:76(	ENSG00000261070		lncRNA	chr11:69147228-691
ENSG0000(	1118	27.96222	chr11:76(	ENSG00000286688		lncRNA	chr11:66514306-665
ENSG0000(	1118	27.96222	chr11:76(	FGF4	TAG;AC	protein_c	chr11:69771022-697
ENSG0000(	1118	27.96222	chr11:76(	ENSG00000286708		lncRNA	chr11:70886090-708
ENSG0000(	1118	27.96222	chr11:76(	MUS81		protein_c	chr11:65857126-658
ENSG0000(	1118	27.96222	chr11:76(	DHCR7	DriverDB	protein_c	chr11:71428193-714
ENSG0000(	1118	27.96222	chr11:76(	RPL37P2		Pseudoger	chr11:67682772-676
ENSG0000(	1118	27.96222	chr11:76(	RN7SL12P		smallRNA	chr11:66712721-667
ENSG0000(	1118	27.96222	chr11:76(	NADSYN1	DriverDB	protein_c	chr11:71453109-715
ENSG0000(	1118	27.96222	chr11:76(	SSH3	NCGv7	protein_c	chr11:67303478-673
ENSG0000(	1118	27.96222	chr11:76(	ENSG00000250105		lncRNA	chr11:66558866-665
ENSG0000(	1118	27.96222	chr11:76(	SHANK2-AS3		lncRNA	chr11:70862790-708
ENSG0000(	1118	27.96222	chr11:76(	SNX32		protein_c	chr11:65833834-658
ENSG0000(	1118	27.96222	chr11:76(	CFL1		protein_c	chr11:65823022-658
ENSG0000(	1118	27.96222	chr11:76(	ENSG00000261347		lncRNA	chr11:69467598-694
ENSG0000(	1118	27.96222	chr11:76(	MRPL11		protein_c	chr11:66435075-664
ENSG0000(	1118	27.96222	chr11:76(	ENSG00000227834		Pseudoger	chr11:67651576-676
ENSG0000(	1118	27.96222	chr11:76(	LINC02754		lncRNA	chr11:67886477-679
ENSG0000(	1118	27.96222	chr11:76(	CD248		protein_c	chr11:66314494-663
ENSG0000(	1118	27.96222	chr11:76(	PACS1		protein_c	chr11:66070272-662
ENSG0000(	1118	27.96222	chr11:76(	ENSG00000286369		lncRNA	chr11:68272100-682
ENSG0000(	1118	27.96222	chr11:76(	CPT1A	DriverDB	protein_c	chr11:68754620-688
ENSG0000(	1118	27.96222	chr11:76(	TBX10		protein_c	chr11:67631303-676
ENSG0000(	1118	27.96222	chr11:76(	CCND1	NCGv7;AC	protein_c	chr11:69641156-696
ENSG0000(	1118	27.96222	chr11:76(	DEFB130C		Pseudoger	chr11:71856277-718
ENSG0000(	1118	27.96222	chr11:76(	LINC02956		lncRNA	chr11:69414307-694
ENSG0000(	1118	27.96222	chr11:76(	KLC2		protein_c	chr11:66257294-662
ENSG0000(	1118	27.96222	chr11:76(	FOLR1		protein_c	chr11:72189558-721
ENSG0000(	1118	27.96222	chr11:76(	ANAPC15	DriverDB	protein_c	chr11:72106378-721
ENSG0000(	1118	27.96222	chr11:76(	EVA1CP4		Pseudoger	chr11:67749043-677
ENSG0000(	1118	27.96222	chr11:76(	FOLR3		protein_c	chr11:72114869-721
ENSG0000(	1118	27.96222	chr11:76(	snoU13		smallRNA	chr11:72104074-721
ENSG0000(	1118	27.96222	chr11:76(	snoU13		smallRNA	chr11:66152621-661
ENSG0000(	1118	27.96222	chr11:76(	NUDT8		protein_c	chr11:67627938-676
ENSG0000(	1118	27.96222	chr11:76(	ENSG00000255143		lncRNA	chr11:70129297-701
ENSG0000(	1118	27.96222	chr11:76(	YIF1A		protein_c	chr11:66284580-662
ENSG0000(	1118	27.96222	chr11:76(	CNIH2		protein_c	chr11:66278175-662
ENSG0000(	1118	27.96222	chr11:76(	CDK2AP2		protein_c	chr11:67506497-675
ENSG0000(	1118	27.96222	chr11:76(	CHKA	TAG	protein_c	chr11:68052859-681
ENSG0000(	1118	27.96222	chr11:76(	AIP		protein_c	chr11:67468174-674
ENSG0000(	1118	27.96222	chr11:76(	TCIRG1	IntOGen-I	protein_c	chr11:68039025-680
ENSG0000(	1118	27.96222	chr11:76(	KRTAP5-10		protein_c	chr11:71565563-715

ENSG0000(	1118	27.96222	chr11:76(	NDUFS8		protein_c	chr11:68030617-680
ENSG0000(	1118	27.96222	chr11:76(	CABP2		protein_c	chr11:67518912-675
ENSG0000(	1118	27.96222	chr11:76(	AP003096.1		smallRNA	chr11:68619499-686
ENSG0000(	1118	27.96222	chr11:76(	KRTAP5-8		protein_c	chr11:71538025-715
ENSG0000(	1118	27.96222	chr11:76(	MIR548K		smallRNA	chr11:70283955-702
ENSG0000(	1118	27.96222	chr11:76(	NDUFV1		protein_c	chr11:67605653-676
ENSG0000(	1118	27.96222	chr11:76(	SLC29A2	NCGv7	protein_c	chr11:66362521-663
ENSG0000(	1118	27.96222	chr11:76(	AP000719.1		smallRNA	chr11:71905107-719
ENSG0000(	1118	27.96222	chr11:76(	ENSG00000275484		lncRNA	chr11:67374416-673
ENSG0000(	1118	27.96222	chr11:76(	B4GAT1		protein_c	chr11:66345374-663
ENSG0000(	1118	27.96222	chr11:76(	PITPNM1		protein_c	chr11:67491768-675
ENSG0000(	1118	27.96222	chr11:76(	LINC02747		lncRNA	chr11:69475567-694
ENSG0000(	1118	27.96222	chr11:76(	snoU13		smallRNA	chr11:66053144-660
ENSG0000(	1118	27.96222	chr11:76(	UNC93B1		protein_c	chr11:67991100-680
ENSG0000(	1118	27.96222	chr11:76(	ENSG00000291247		lncRNA	chr11:71722282-717
ENSG0000(	1118	27.96222	chr11:76(	KMT5B		protein_c	chr11:68154863-682
ENSG0000(	1118	27.96222	chr11:76(	RAB1B		protein_c	chr11:66268590-662
ENSG0000(	1118	27.96222	chr11:76(	BRMS1		protein_c	chr11:66337333-663
ENSG0000(	1118	27.96222	chr11:76(	GAL3ST3		protein_c	chr11:66040765-660
ENSG0000(	1118	27.96222	chr11:76(	RIN1		protein_c	chr11:66330241-663
ENSG0000(	1118	27.96222	chr11:76(	PPP6R3		protein_c	chr11:68460731-686
ENSG0000(	1118	27.96222	chr11:76(	NPAS4		protein_c	chr11:66421004-664
ENSG0000(	1118	27.96222	chr11:76(	ENSG00000255860		Pseudoger	chr11:72172455-721
ENSG0000(	1118	27.96222	chr11:76(	ENSG00000255119		lncRNA	chr11:67605521-676
ENSG0000(	1118	27.96222	chr11:76(	FADD	DriverDB\	protein_c	chr11:70203296-702
ENSG0000(	1118	27.96222	chr11:76(	ENSG00000255191		lncRNA	chr11:69985876-700
ENSG0000(	1115	27.88719	chr1:1522	ENSG00000272691		lncRNA	chr1:85578500-8557
ENSG0000(	1107	27.6871	chr6:105(	PNRC1-DT		lncRNA	chr6:89080164-8908
ENSG0000(	1107	27.6871	chr6:2135	RPL23AP46		Pseudoger	chr6:132997551-132
ENSG0000(	1106	27.66209	chr1:116(	ENSG00000227527		lncRNA	chr1:42335386-4233
ENSG0000(	1106	27.66209	chr1:116(	RNU6-369P		smallRNA	chr1:44390722-4439
ENSG0000(	1106	27.66209	chr1:116(	FOXJ3		protein_c	chr1:42176539-4233
ENSG0000(	1106	27.66209	chr1:116(	SLC2A1		protein_c	chr1:42925353-4295
ENSG0000(	1106	27.66209	chr1:116(	ENSG00000288955		lncRNA	chr1:42924460-4292
ENSG0000(	1106	27.66209	chr1:116(	GUCA2A		protein_c	chr1:42162690-4216
ENSG0000(	1106	27.66209	chr1:116(	ENSG00000284989		protein_c	chr1:43650149-4393
ENSG0000(	1106	27.66209	chr1:116(	TMSB4XP1		Pseudoger	chr1:42500205-4250
ENSG0000(	1106	27.66209	chr1:116(	ENSG00000271329		Pseudoger	chr1:44187943-4418
ENSG0000(	1106	27.66209	chr1:116(	KDM4A		protein_c	chr1:43650149-4370
ENSG0000(	1106	27.66209	chr1:116(	ENSG00000287587		lncRNA	chr1:42036143-4205
ENSG0000(	1106	27.66209	chr1:116(	EBNA1BP2		protein_c	chr1:43164175-4327
ENSG0000(	1106	27.66209	chr1:116(	ENSG00000227994		Pseudoger	chr1:44172506-4417
ENSG0000(	1106	27.66209	chr1:116(	P3H1		protein_c	chr1:42746335-4276
ENSG0000(	1106	27.66209	chr1:116(	CDC20	NCGv7	protein_c	chr1:43358981-4336
ENSG0000(	1106	27.66209	chr1:116(	CCDC30		protein_c	chr1:42463221-4265
ENSG0000(	1106	27.66209	chr1:116(	RNU6-1058P		smallRNA	chr1:43716467-4371
ENSG0000(	1106	27.66209	chr1:116(	Y_RNA		smallRNA	chr1:44153385-4415
ENSG0000(	1106	27.66209	chr1:116(	ELOVL1		protein_c	chr1:43363398-4336
ENSG0000(	1106	27.66209	chr1:116(	RP11-7O11.3		lncRNA	chr1:43944370-4394
ENSG0000(	1106	27.66209	chr1:116(	ENSG00000234917		lncRNA	chr1:42678735-4268
ENSG0000(	1106	27.66209	chr1:116(	MIR5584		smallRNA	chr1:44545493-4454

ENSG0000(	1106	27.66209	chr1:116(	C1orf50-AS1		lncRNA	chr1:42775813-4277
ENSG0000(	1106	27.66209	chr1:116(	DPH2		protein_c	chr1:43970000-4397
ENSG0000(	1106	27.66209	chr1:116(	ATP6V0CP4		Pseudoger	chr1:42952202-4295
ENSG0000(	1106	27.66209	chr1:116(	CFAP57		protein_c	chr1:43172330-4325
ENSG0000(	1106	27.66209	chr1:116(	RNU6-536P		smallRNA	chr1:42569033-4256
ENSG0000(	1106	27.66209	chr1:116(	ENSG00000288772		lncRNA	chr1:43368180-4336
ENSG0000(	1106	27.66209	chr1:116(	HNRNPFP1		Pseudoger	chr1:42040597-4204
ENSG0000(	1106	27.66209	chr1:116(	GUCA2B		protein_c	chr1:42153410-4215
ENSG0000(	1106	27.66209	chr1:116(	ENSG00000283973		lncRNA	chr1:42959065-4296
ENSG0000(	1106	27.66209	chr1:116(	ERI3-IT1		lncRNA	chr1:44243408-4424
ENSG0000(	1106	27.66209	chr1:116(	SHMT1P1		Pseudoger	chr1:43850300-4385
ENSG0000(	1106	27.66209	chr1:116(	OOSP1P1		Pseudoger	chr1:44155028-4415
ENSG0000(	1106	27.66209	chr1:116(	SLC2A1-DT		lncRNA	chr1:42959049-4299
ENSG0000(	1106	27.66209	chr1:116(	PPIH		protein_c	chr1:42658335-4267
ENSG0000(	1106	27.66209	chr1:116(	ENSG00000233674		Pseudoger	chr1:43743471-4374
ENSG0000(	1106	27.66209	chr1:116(	ARTN		protein_c	chr1:43933320-4393
ENSG0000(	1106	27.66209	chr1:116(	ZMYND12		protein_c	chr1:42430329-4245
ENSG0000(	1106	27.66209	chr1:116(	KLF17		protein_c	chr1:44118821-4413
ENSG0000(	1106	27.66209	chr1:116(	RPS3AP11		Pseudoger	chr1:42491739-4249
ENSG0000(	1106	27.66209	chr1:116(	ENSG00000227163		Pseudoger	chr1:44087958-4408
ENSG0000(	1106	27.66209	chr1:116(	ENSG00000283580		protein_c	chr1:42767292-4279
ENSG0000(	1106	27.66209	chr1:116(	AL390776.1		smallRNA	chr1:44333105-4433
ENSG0000(	1106	27.66209	chr1:116(	RN7SL479P		smallRNA	chr1:44117100-4411
ENSG0000(	1106	27.66209	chr1:116(	MPL	NCGv7;AC	protein_c	chr1:43337818-4335
ENSG0000(	1106	27.66209	chr1:116(	ENSG00000230615		lncRNA	chr1:44030414-4411
ENSG0000(	1106	27.66209	chr1:116(	KDM4A-AS1		lncRNA	chr1:43685123-4370
ENSG0000(	1106	27.66209	chr1:116(	CLDN19		protein_c	chr1:42733093-4274
ENSG0000(	1106	27.66209	chr1:116(	TIE1		protein_c	chr1:43300982-4332
ENSG0000(	1106	27.66209	chr1:116(	YBX1	AC	protein_c	chr1:42682418-4270
ENSG0000(	1106	27.66209	chr1:116(	KRT8P47		Pseudoger	chr1:44103306-4410
ENSG0000(	1106	27.66209	chr1:116(	KLF18		protein_c	chr1:44137821-4414
ENSG0000(	1106	27.66209	chr1:116(	RNA5SP46		Pseudoger	chr1:43196417-4319
ENSG0000(	1106	27.66209	chr1:116(	SZT2		protein_c	chr1:43389882-4345
ENSG0000(	1106	27.66209	chr1:116(	FAM183A		protein_c	chr1:43145153-4315
ENSG0000(	1106	27.66209	chr1:116(	PTPRF		protein_c	chr1:43525187-4362
ENSG0000(	1106	27.66209	chr1:116(	HYI-AS1		lncRNA	chr1:43453927-4345
ENSG0000(	1106	27.66209	chr1:116(	ENSG00000277513		Pseudoger	chr1:43104086-4310
ENSG0000(	1106	27.66209	chr1:116(	RNU6-880P		smallRNA	chr1:42991438-4299
ENSG0000(	1106	27.66209	chr1:116(	ENSG00000285728		lncRNA	chr1:42658687-4268
ENSG0000(	1106	27.66209	chr1:116(	PPCS		protein_c	chr1:42456117-4247
ENSG0000(	1106	27.66209	chr1:116(	ST3GAL3-AS1		lncRNA	chr1:43709392-4372
ENSG0000(	1106	27.66209	chr1:116(	RNU6-870P		smallRNA	chr1:43023549-4302
ENSG0000(	1106	27.66209	chr1:116(	SZT2-AS1		lncRNA	chr1:43447776-4344
ENSG0000(	1106	27.66209	chr1:116(	HYI		protein_c	chr1:43450989-4345
ENSG0000(	1106	27.66209	chr1:116(	CDC20-DT		lncRNA	chr1:43354684-4335
ENSG0000(	1106	27.66209	chr1:116(	MED8-AS1		lncRNA	chr1:43385113-4338
ENSG0000(	1106	27.66209	chr1:116(	ZNF691-DT		lncRNA	chr1:42832522-4284
ENSG0000(	1106	27.66209	chr1:116(	ENSG00000236180		Pseudoger	chr1:42570970-4257
ENSG0000(	1106	27.66209	chr1:116(	ENSG00000285649		lncRNA	chr1:43968351-4397
ENSG0000(	1106	27.66209	chr1:116(	TMEM125		protein_c	chr1:43269983-4327
ENSG0000(	1106	27.66209	chr1:116(	SLC6A9	NCGv7	protein_c	chr1:43991500-4403

ENSG0000(	1106	27.66209	chr1:116(	ST3GAL3		protein_c	chr1:43705824-4393
ENSG0000(	1106	27.66209	chr1:116(	TMEM53		protein_c	chr1:44635238-4467
ENSG0000(	1106	27.66209	chr1:116(	ENSG00000233514		Pseudoger	chr1:44122153-4412
ENSG0000(	1106	27.66209	chr1:116(	ENSG00000226804		Pseudoger	chr1:44150594-4415
ENSG0000(	1106	27.66209	chr1:116(	TMEM269		protein_c	chr1:42784991-4281
ENSG0000(	1106	27.66209	chr1:116(	MED8		protein_c	chr1:43383917-4338
ENSG0000(	1106	27.66209	chr1:116(	RNF220		protein_c	chr1:44405194-4465
ENSG0000(	1106	27.66209	chr1:116(	ZNF691		protein_c	chr1:42846573-4285
ENSG0000(	1106	27.66209	chr1:116(	ENSG00000233708		Pseudoger	chr1:42886597-4288
ENSG0000(	1106	27.66209	chr1:116(	ATP6V1E1P1		Pseudoger	chr1:42903232-4290
ENSG0000(	1106	27.66209	chr1:116(	B4GALT2		protein_c	chr1:43978943-4399
ENSG0000(	1106	27.66209	chr1:116(	C1orf210		protein_c	chr1:43281877-4328
ENSG0000(	1106	27.66209	chr1:116(	IPO13		protein_c	chr1:43946950-4396
ENSG0000(	1106	27.66209	chr1:116(	RIMKLA	DriverDB\	protein_c	chr1:42380792-4242
ENSG0000(	1106	27.66209	chr1:116(	SVBP		protein_c	chr1:42807052-4281
ENSG0000(	1106	27.66209	chr1:116(	ATP6V0B	NCGv7	protein_c	chr1:43974487-4397
ENSG0000(	1106	27.66209	chr1:116(	AL451006.1		smallRNA	chr1:41759141-4175
ENSG0000(	1106	27.66209	chr1:116(	ENSG00000228776		Pseudoger	chr1:42140635-4214
ENSG0000(	1106	27.66209	chr1:116(	ENSG00000287113		lncRNA	chr1:43348288-4334
ENSG0000(	1106	27.66209	chr1:116(	ERMAP		protein_c	chr1:42817122-4284
ENSG0000(	1106	27.66209	chr1:116(	CCDC24		protein_c	chr1:43991359-4399
ENSG0000(	1106	27.66209	chr1:116(	ERI3		protein_c	chr1:44221070-4435
ENSG0000(	1106	27.66209	chr1:116(	C1orf50		protein_c	chr1:42767245-4277
ENSG0000(	1106	27.66209	chr1:116(	MKRN8P		Pseudoger	chr1:42891094-4289
ENSG0000(	1106	27.66209	chr1:116(	DMAP1		protein_c	chr1:44213455-4422
ENSG0000(	1105	27.63708	chr6:105(	RN7SKP209		smallRNA	chr6:87083233-8708
ENSG0000(	1095	27.38697	chr1:152:	ENSG00000272672		lncRNA	chr1:89939601-8994
ENSG0000(	1095	27.38697	chr1:152:	RPAP2		protein_c	chr1:92299059-9240
ENSG0000(	1095	27.38697	chr1:152:	ZNF644		protein_c	chr1:90915298-9102
ENSG0000(	1095	27.38697	chr1:152:	ENSG00000229067		Pseudoger	chr1:91600171-9160
ENSG0000(	1095	27.38697	chr1:152:	PKN2-AS1		lncRNA	chr1:87620803-8868
ENSG0000(	1095	27.38697	chr1:152:	RBMXL1		protein_c	chr1:88979456-8899
ENSG0000(	1095	27.38697	chr1:152:	EPHX4		protein_c	chr1:92029985-9206
ENSG0000(	1095	27.38697	chr1:152:	GBP2		protein_c	chr1:89106132-8915
ENSG0000(	1095	27.38697	chr1:152:	ZNF326		protein_c	chr1:89995110-9003
ENSG0000(	1095	27.38697	chr1:152:	GBP4		protein_c	chr1:89181144-8919
ENSG0000(	1095	27.38697	chr1:152:	LINC02801		lncRNA	chr1:87212669-8726
ENSG0000(	1095	27.38697	chr1:152:	GBP7		protein_c	chr1:89131742-8917
ENSG0000(	1095	27.38697	chr1:152:	HFM1		protein_c	chr1:91260766-9140
ENSG0000(	1095	27.38697	chr1:152:	RN7SKP272		smallRNA	chr1:89987713-8998
ENSG0000(	1095	27.38697	chr1:152:	GFI1	TAG;AC	protein_c	chr1:92473043-9248
ENSG0000(	1095	27.38697	chr1:152:	RNU6-695P		smallRNA	chr1:90253456-9025
ENSG0000(	1095	27.38697	chr1:152:	ENSG00000289483		lncRNA	chr1:92028938-9202
ENSG0000(	1095	27.38697	chr1:152:	ENSG00000279778		TEC	chr1:87805286-8780
ENSG0000(	1095	27.38697	chr1:152:	LRRC8D-DT		lncRNA	chr1:89820174-8982
ENSG0000(	1095	27.38697	chr1:152:	AL451010.1		smallRNA	chr1:92229256-9222
ENSG0000(	1095	27.38697	chr1:152:	ODF2L		protein_c	chr1:86346824-8639
ENSG0000(	1095	27.38697	chr1:152:	ENSG00000223787		Pseudoger	chr1:92580476-9258
ENSG0000(	1095	27.38697	chr1:152:	ENSG00000235308		Pseudoger	chr1:88923370-8892
ENSG0000(	1095	27.38697	chr1:152:	CLCA4-AS1		lncRNA	chr1:86569024-8670
ENSG0000(	1095	27.38697	chr1:152:	ENSG00000225505		Pseudoger	chr1:92732000-9273

ENSG0000(	1095	27.38697	chr1:152	LINC02787		lncRNA	chr1:90510910-9053
ENSG0000(	1095	27.38697	chr1:152	AL139139.1		smallRNA	chr1:87151298-8715
ENSG0000(	1095	27.38697	chr1:152	ENSG00000226773		Pseudoger	chr1:92203148-9220
ENSG0000(	1095	27.38697	chr1:152	GBP3		protein_c	chr1:89006679-8902
ENSG0000(	1095	27.38697	chr1:152	RP4-604K5.2		lncRNA	chr1:86943685-8694
ENSG0000(	1095	27.38697	chr1:152	RNA5SP52		Pseudoger	chr1:87453240-8745
ENSG0000(	1095	27.38697	chr1:152	GBP1		protein_c	chr1:89051882-8906
ENSG0000(	1095	27.38697	chr1:152	ENSG00000289712		Pseudoger	chr1:89236843-8923
ENSG0000(	1095	27.38697	chr1:152	LINC01364		lncRNA	chr1:87353521-8737
ENSG0000(	1095	27.38697	chr1:152	ENSG00000225568		Pseudoger	chr1:87045875-8704
ENSG0000(	1095	27.38697	chr1:152	PKN2		protein_c	chr1:88684222-8883
ENSG0000(	1095	27.38697	chr1:152	RNU4-59P		smallRNA	chr1:92700819-9270
ENSG0000(	1095	27.38697	chr1:152	ENSG00000289582		lncRNA	chr1:89127160-8912
ENSG0000(	1095	27.38697	chr1:152	LRRC8D		protein_c	chr1:89821014-8993
ENSG0000(	1095	27.38697	chr1:152	LRRC8C		protein_c	chr1:89633072-8976
ENSG0000(	1095	27.38697	chr1:152	Y_RNA		smallRNA	chr1:91261625-9126
ENSG0000(	1095	27.38697	chr1:152	ENSG00000288629		protein_c	chr1:89579592-8957
ENSG0000(	1095	27.38697	chr1:152	LINC01763		lncRNA	chr1:90851122-9085
ENSG0000(	1095	27.38697	chr1:152	U3		smallRNA	chr1:90657750-9065
ENSG0000(	1095	27.38697	chr1:152	ENSG00000273487		lncRNA	chr1:92189237-9219
ENSG0000(	1095	27.38697	chr1:152	CLCA2		protein_c	chr1:86424171-8645
ENSG0000(	1095	27.38697	chr1:152	GBP1P1		Pseudoger	chr1:89410319-8942
ENSG0000(	1095	27.38697	chr1:152	FEN1P1		Pseudoger	chr1:91328369-9132
ENSG0000(	1095	27.38697	chr1:152	RPL5P6		Pseudoger	chr1:91023919-9102
ENSG0000(	1095	27.38697	chr1:152	AL356270.1		smallRNA	chr1:86606270-8660
ENSG0000(	1095	27.38697	chr1:152	KYAT3		protein_c	chr1:88935773-8899
ENSG0000(	1095	27.38697	chr1:152	BARHL2		protein_c	chr1:90711539-9071
ENSG0000(	1095	27.38697	chr1:152	LMO4	AC	protein_c	chr1:87328880-8734
ENSG0000(	1095	27.38697	chr1:152	ENSG00000235251		Pseudoger	chr1:87044935-8704
ENSG0000(	1095	27.38697	chr1:152	ENSG00000237568		lncRNA	chr1:89260582-8926
ENSG0000(	1095	27.38697	chr1:152	GLMN		protein_c	chr1:92246402-9229
ENSG0000(	1095	27.38697	chr1:152	CAPNS1P1		Pseudoger	chr1:89394033-8939
ENSG0000(	1095	27.38697	chr1:152	GEMIN8P4		Pseudoger	chr1:89993593-8999
ENSG0000(	1095	27.38697	chr1:152	RNU6-125P		smallRNA	chr1:88816779-8881
ENSG0000(	1095	27.38697	chr1:152	PTGES3P1		Pseudoger	chr1:89104285-8910
ENSG0000(	1095	27.38697	chr1:152	BRDT	NCGv7	protein_c	chr1:91949343-9201
ENSG0000(	1095	27.38697	chr1:152	GTF2B	NCGv7	protein_c	chr1:88852633-8889
ENSG0000(	1095	27.38697	chr1:152	snoU13		smallRNA	chr1:89768212-8976
ENSG0000(	1095	27.38697	chr1:152	H3P3		Pseudoger	chr1:92749175-9274
ENSG0000(	1095	27.38697	chr1:152	SELENOF		protein_c	chr1:86862445-8691
ENSG0000(	1095	27.38697	chr1:152	ENSG00000230735		lncRNA	chr1:89629725-8967
ENSG0000(	1095	27.38697	chr1:152	RN7SL653P		smallRNA	chr1:91829776-9183
ENSG0000(	1095	27.38697	chr1:152	RN7SL235P		smallRNA	chr1:91939269-9193
ENSG0000(	1095	27.38697	chr1:152	LRRC8C-DT		lncRNA	chr1:89581291-8963
ENSG0000(	1095	27.38697	chr1:152	SETSIP		protein_c	chr1:92074533-9207
ENSG0000(	1095	27.38697	chr1:152	RPL36AP10		Pseudoger	chr1:88577880-8857
ENSG0000(	1095	27.38697	chr1:152	ENSG00000272094		lncRNA	chr1:90860550-9086
ENSG0000(	1095	27.38697	chr1:152	LINC02788		lncRNA	chr1:90835660-9084
ENSG0000(	1095	27.38697	chr1:152	ENSG00000287372		lncRNA	chr1:90388193-9042
ENSG0000(	1095	27.38697	chr1:152	ENSG00000270507		Pseudoger	chr1:88313153-8831
ENSG0000(	1095	27.38697	chr1:152	ENSG00000230053		Pseudoger	chr1:88498309-8849

ENSG0000(	1095	27.38697	chr1:1522	RN7SL583P		smallRNA	chr1:88477831-8847
ENSG0000(	1095	27.38697	chr1:1522	GBP1P1		lncRNA	chr1:89407679-8942
ENSG0000(	1095	27.38697	chr1:1522	HSP90B3P		Pseudoger	chr1:91642516-9164
ENSG0000(	1095	27.38697	chr1:1522	BTBD8		protein_c	chr1:92080305-9218
ENSG0000(	1095	27.38697	chr1:1522	CDCA4P2		Pseudoger	chr1:86552625-8655
ENSG0000(	1095	27.38697	chr1:1522	ENSG00000286758		lncRNA	chr1:88462936-8846
ENSG0000(	1095	27.38697	chr1:1522	ACTBP12		Pseudoger	chr1:92229018-9222
ENSG0000(	1095	27.38697	chr1:1522	LINC01140		lncRNA	chr1:87129765-8716
ENSG0000(	1095	27.38697	chr1:1522	LPCAT2BP		Pseudoger	chr1:92066306-9206
ENSG0000(	1095	27.38697	chr1:1522	CCNJP2		Pseudoger	chr1:92755794-9275
ENSG0000(	1095	27.38697	chr1:1522	GBP6		protein_c	chr1:89364059-8938
ENSG0000(	1095	27.38697	chr1:1522	ENSG00000233235		Pseudoger	chr1:89324522-8933
ENSG0000(	1095	27.38697	chr1:1522	ENSG00000226394		Pseudoger	chr1:89661212-8966
ENSG0000(	1095	27.38697	chr1:1522	RN7SL824P		smallRNA	chr1:92402391-9240
ENSG0000(	1095	27.38697	chr1:1522	ENSG00000286802		lncRNA	chr1:89128432-8914
ENSG0000(	1095	27.38697	chr1:1522	GBP5		protein_c	chr1:89256189-8927
ENSG0000(	1095	27.38697	chr1:1522	SH3GLB1	NCGv7	protein_c	chr1:86704570-8674
ENSG0000(	1095	27.38697	chr1:1522	ENSG00000230721		Pseudoger	chr1:86784913-8678
ENSG0000(	1095	27.38697	chr1:1522	C1orf146		protein_c	chr1:92217915-9224
ENSG0000(	1095	27.38697	chr1:1522	ENSG00000287015		lncRNA	chr1:90045998-9004
ENSG0000(	1095	27.38697	chr1:1522	PHKA1P1		Pseudoger	chr1:90892992-9089
ENSG0000(	1095	27.38697	chr1:1522	ENSG00000267734		lncRNA	chr1:86932199-8693
ENSG0000(	1095	27.38697	chr1:1522	LRRC8B		protein_c	chr1:89524829-8959
ENSG0000(	1095	27.38697	chr1:1522	ENSG00000267561		protein_c	chr1:86993009-8716
ENSG0000(	1095	27.38697	chr1:1522	PRKAR1AP1		Pseudoger	chr1:92125301-9212
ENSG0000(	1095	27.38697	chr1:1522	WDR82P2		Pseudoger	chr1:91534666-9153
ENSG0000(	1095	27.38697	chr1:1522	ENSG00000286548		lncRNA	chr1:89427533-8952
ENSG0000(	1095	27.38697	chr1:1522	CLCA3P		lncRNA	chr1:86634273-8665
ENSG0000(	1095	27.38697	chr1:1522	CLCA4		protein_c	chr1:86547078-8658
ENSG0000(	1095	27.38697	chr1:1522	ENSG00000287406		lncRNA	chr1:90242088-9028
ENSG0000(	1095	27.38697	chr1:1522	HMGB3P9		Pseudoger	chr1:92647048-9264
ENSG0000(	1095	27.38697	chr1:1522	ENSG00000284846		lncRNA	chr1:86821558-8683
ENSG0000(	1095	27.38697	chr1:1522	ENSG00000231613		lncRNA	chr1:89788914-8979
ENSG0000(	1095	27.38697	chr1:1522	TGFBR3	NCGv7	protein_c	chr1:91680343-9190
ENSG0000(	1095	27.38697	chr1:1522	Y_RNA		smallRNA	chr1:89020246-8902
ENSG0000(	1095	27.38697	chr1:1522	CDC7		protein_c	chr1:91500851-9152
ENSG0000(	1095	27.38697	chr1:1522	HS2ST1	NCGv7	protein_c	chr1:86914635-8710
ENSG0000(	1095	27.38697	chr1:1522	CLCA1		protein_c	chr1:86468368-8650
ENSG0000(	1095	27.38697	chr1:1522	LINC02609		lncRNA	chr1:90769086-9085
ENSG0000(	1095	27.38697	chr1:1522	ENSG00000238081		Pseudoger	chr1:89289676-8929
ENSG0000(	1095	27.38697	chr1:1522	GAPDHP46		Pseudoger	chr1:92114803-9211
ENSG0000(	1095	27.38697	chr1:1522	EVI5	AC	protein_c	chr1:92508696-9279
ENSG0000(	1095	27.38697	chr1:1522	ENSG00000271949		protein_c	chr1:89633140-8993
ENSG0000(	1095	27.38697	chr1:1522	ENSG00000284734		lncRNA	chr1:89198714-8920
ENSG0000(	1095	27.38697	chr1:1522	ELOCP19		Pseudoger	chr1:88829102-8882
ENSG0000(	1095	27.38697	chr1:1522	CLCA3P		Pseudoger	chr1:86634276-8665
ENSG0000(	1095	27.38697	chr1:1522	ENSG00000287076		lncRNA	chr1:90719576-9072
ENSG0000(	1095	27.38697	chr1:1522	ENSG00000284637		Pseudoger	chr1:89203280-8920
ENSG0000(	1083	27.08684	chr1:1005	ST13P21		Pseudoger	chr1:108502358-108
ENSG0000(	1083	27.08684	chr1:1005	SLC25A24P2		Pseudoger	chr1:108383736-108
ENSG0000(	1083	27.08684	chr1:1005	ENSG00000283354		Pseudoger	chr1:108495475-108

ENSG0000(	1083	27.08684	chr1:1005	NBPF6		protein_c	chr1:108450282-108
ENSG0000(	1083	27.08684	chr1:1005	ENSG00000285923		lncRNA	chr1:108661533-108
ENSG0000(	1083	27.08684	chr1:1005	ENSG00000224698		lncRNA	chr1:108420689-108
ENSG0000(	1083	27.08684	chr1:1005	ENSG00000290552		lncRNA	chr1:108375838-108
ENSG0000(	1083	27.08684	chr1:1005	NBPF5P		Pseudoger	chr1:108376119-108
ENSG0000(	1083	27.08684	chr1:1005	FAM102B		protein_c	chr1:108560089-108
ENSG0000(	1083	27.08684	chr1:1005	ENSG00000290126		lncRNA	chr1:108690627-108
ENSG0000(	1083	27.08684	chr1:1005	HENMT1		protein_c	chr1:108648290-108
ENSG0000(	1083	27.08684	chr1:1005	ENSG00000226483		Pseudoger	chr1:108508574-108
ENSG0000(	1083	27.08684	chr1:1005	PRPF38B	NCGv7	protein_c	chr1:108692310-108
ENSG0000(	1081	27.03681	chr1:1522	RN7SL290P		smallRNA	chr1:51995740-5199
ENSG0000(	1075	26.88675	chr6:1050	ENSG00000220130		Pseudoger	chr6:89001548-8900
ENSG0000(	1074	26.86174	chr1:1522	SNORA58		smallRNA	chr1:53771018-5377
ENSG0000(	1070	26.76169	chr1:1005	ENSG00000260879		lncRNA	chr1:108199926-108
ENSG0000(	1070	26.76169	chr1:1005	ENSG00000271277		Pseudoger	chr1:101882516-101
ENSG0000(	1070	26.76169	chr1:1005	ENSG00000271578		Pseudoger	chr1:101190520-101
ENSG0000(	1070	26.76169	chr1:1005	ENSG00000289612		lncRNA	chr1:107140235-107
ENSG0000(	1070	26.76169	chr1:1005	ENSG00000233359		lncRNA	chr1:102199739-102
ENSG0000(	1070	26.76169	chr1:1005	PPIAP7		Pseudoger	chr1:101270875-101
ENSG0000(	1070	26.76169	chr1:1005	ENSG00000290547		lncRNA	chr1:108272943-108
ENSG0000(	1070	26.76169	chr1:1005	ENSG00000289355		lncRNA	chr1:101150560-101
ENSG0000(	1070	26.76169	chr1:1005	FTLP17		Pseudoger	chr1:104153306-104
ENSG0000(	1070	26.76169	chr1:1005	DPH5-DT		lncRNA	chr1:101025844-101
ENSG0000(	1070	26.76169	chr1:1005	LINC01661		lncRNA	chr1:106818224-106
ENSG0000(	1070	26.76169	chr1:1005	SCARNA16		smallRNA	chr1:101133153-101
ENSG0000(	1070	26.76169	chr1:1005	DPH5		protein_c	chr1:100989623-101
ENSG0000(	1070	26.76169	chr1:1005	RPL36AP12		Pseudoger	chr1:100651947-100
ENSG0000(	1070	26.76169	chr1:1005	ENSG00000234441		Pseudoger	chr1:103668071-103
ENSG0000(	1070	26.76169	chr1:1005	AC114491.1		smallRNA	chr1:107448469-107
ENSG0000(	1070	26.76169	chr1:1005	RNU6-352P		smallRNA	chr1:101859851-101
ENSG0000(	1070	26.76169	chr1:1005	LINC02785		lncRNA	chr1:108040263-108
ENSG0000(	1070	26.76169	chr1:1005	ENSG00000232952		Pseudoger	chr1:105891739-105
ENSG0000(	1070	26.76169	chr1:1005	RNU6-965P		smallRNA	chr1:101728642-101
ENSG0000(	1070	26.76169	chr1:1005	SLC25A24		protein_c	chr1:108134043-108
ENSG0000(	1070	26.76169	chr1:1005	ENSG00000285525		lncRNA	chr1:100628230-100
ENSG0000(	1070	26.76169	chr1:1005	RPL7AP17		Pseudoger	chr1:100586649-100
ENSG0000(	1070	26.76169	chr1:1005	AMY2B	NCGv7	protein_c	chr1:103553815-103
ENSG0000(	1070	26.76169	chr1:1005	CDK4P1		Pseudoger	chr1:105433994-105
ENSG0000(	1070	26.76169	chr1:1005	NBPF4		protein_c	chr1:108222464-108
ENSG0000(	1070	26.76169	chr1:1005	OLFM3	NCGv7	protein_c	chr1:101802560-101
ENSG0000(	1070	26.76169	chr1:1005	S1PR1	NCGv7	protein_c	chr1:101236865-101
ENSG0000(	1070	26.76169	chr1:1005	RP11-347K2.1		lncRNA	chr1:103414879-103
ENSG0000(	1070	26.76169	chr1:1005	ENSG00000280186		TEC	chr1:108200413-108
ENSG0000(	1070	26.76169	chr1:1005	RNPC3-DT		lncRNA	chr1:103415980-103
ENSG0000(	1070	26.76169	chr1:1005	AL390036.1		smallRNA	chr1:108018653-108
ENSG0000(	1070	26.76169	chr1:1005	HNRNPA1P68		Pseudoger	chr1:100941017-100
ENSG0000(	1070	26.76169	chr1:1005	MTATP6P14		Pseudoger	chr1:106802755-106
ENSG0000(	1070	26.76169	chr1:1005	ENSG00000289192		lncRNA	chr1:101964793-101
ENSG0000(	1070	26.76169	chr1:1005	AMY1C		protein_c	chr1:103745323-103
ENSG0000(	1070	26.76169	chr1:1005	RPSAP19		Pseudoger	chr1:101786340-101
ENSG0000(	1070	26.76169	chr1:1005	LINC01677		lncRNA	chr1:105927620-106

ENSG0000(	1070	26.76169	chr1:100{	VAV3	TAG;AC	protein_c	chr1:107571161-107
ENSG0000(	1070	26.76169	chr1:100{	ENSG00000225191		Pseudoger	chr1:103926567-103
ENSG0000(	1070	26.76169	chr1:100{	ENSG00000230864		lncRNA	chr1:102763322-102
ENSG0000(	1070	26.76169	chr1:100{	ENSG00000285981		lncRNA	chr1:104998406-105
ENSG0000(	1070	26.76169	chr1:100{	RN7SKP285		smallRNA	chr1:103523562-103
ENSG0000(	1070	26.76169	chr1:100{	LINC01349		lncRNA	chr1:100627049-100
ENSG0000(	1070	26.76169	chr1:100{	ENSG00000270342		Pseudoger	chr1:106544342-106
ENSG0000(	1070	26.76169	chr1:100{	ENSG00000237480		lncRNA	chr1:105956694-106
ENSG0000(	1070	26.76169	chr1:100{	snoU13		smallRNA	chr1:101228664-101
ENSG0000(	1070	26.76169	chr1:100{	AMY1B		protein_c	chr1:103687415-103
ENSG0000(	1070	26.76169	chr1:100{	VAV3-AS1		lncRNA	chr1:107964443-107
ENSG0000(	1070	26.76169	chr1:100{	ACTG1P4		Pseudoger	chr1:103569553-103
ENSG0000(	1070	26.76169	chr1:100{	ENSG00000235795		lncRNA	chr1:100995473-100
ENSG0000(	1070	26.76169	chr1:100{	LINC01307		lncRNA	chr1:101323337-101
ENSG0000(	1070	26.76169	chr1:100{	MTCO1P14		Pseudoger	chr1:106804474-106
ENSG0000(	1070	26.76169	chr1:100{	AMYP1		Pseudoger	chr1:103713723-103
ENSG0000(	1070	26.76169	chr1:100{	LINC01676		lncRNA	chr1:105587575-105
ENSG0000(	1070	26.76169	chr1:100{	SOD2P1		Pseudoger	chr1:103100143-103
ENSG0000(	1070	26.76169	chr1:100{	ENSG00000230932		Pseudoger	chr1:106080801-106
ENSG0000(	1070	26.76169	chr1:100{	ENSG00000273204		lncRNA	chr1:100894928-100
ENSG0000(	1070	26.76169	chr1:100{	S1PR1-DT		lncRNA	chr1:101234555-101
ENSG0000(	1070	26.76169	chr1:100{	PRMT6		protein_c	chr1:107056674-107
ENSG0000(	1070	26.76169	chr1:100{	ENSG00000230759		lncRNA	chr1:103414879-103
ENSG0000(	1070	26.76169	chr1:100{	NTNG1		protein_c	chr1:107140007-107
ENSG0000(	1070	26.76169	chr1:100{	ENSG00000270976		Pseudoger	chr1:106780223-106
ENSG0000(	1070	26.76169	chr1:100{	RP11-347K2.2		lncRNA	chr1:103418079-103
ENSG0000(	1070	26.76169	chr1:100{	ENSG00000237897		Pseudoger	chr1:105890693-105
ENSG0000(	1070	26.76169	chr1:100{	AL591042.1		smallRNA	chr1:107776174-107
ENSG0000(	1070	26.76169	chr1:100{	RNPC3		protein_c	chr1:103525691-103
ENSG0000(	1070	26.76169	chr1:100{	ENSG00000215869		Pseudoger	chr1:104072983-104
ENSG0000(	1070	26.76169	chr1:100{	ENSG00000228399		Pseudoger	chr1:101256274-101
ENSG0000(	1070	26.76169	chr1:100{	SEPTIN2P1		Pseudoger	chr1:105698039-105
ENSG0000(	1070	26.76169	chr1:100{	DNAJA1P5		Pseudoger	chr1:101893105-101
ENSG0000(	1070	26.76169	chr1:100{	SLC30A7		protein_c	chr1:100896076-100
ENSG0000(	1070	26.76169	chr1:100{	EXTL2		protein_c	chr1:100872372-100
ENSG0000(	1070	26.76169	chr1:100{	LINC01709		lncRNA	chr1:101639509-101
ENSG0000(	1070	26.76169	chr1:100{	VCAM1		protein_c	chr1:100719742-100
ENSG0000(	1070	26.76169	chr1:100{	AC093157.1		protein_c	chr1:100990205-100
ENSG0000(	1070	26.76169	chr1:100{	AMY2A		protein_c	chr1:103617427-103
ENSG0000(	1070	26.76169	chr1:100{	NDUFA4P1		Pseudoger	chr1:107505203-107
ENSG0000(	1070	26.76169	chr1:100{	COL11A1	NCGv7	protein_c	chr1:102876467-103
ENSG0000(	1070	26.76169	chr1:100{	ENSG00000238122		lncRNA	chr1:108261196-108
ENSG0000(	1070	26.76169	chr1:100{	SLC25A24P1		Pseudoger	chr1:108273139-108
ENSG0000(	1070	26.76169	chr1:100{	AMY1A		protein_c	chr1:103655760-103
ENSG0000(	1067	26.68666	chr1:813{	BRI3P1		Pseudoger	chr1:100213293-100
ENSG0000(	1066	26.66165	chr1:152{	ANKRD13C		protein_c	chr1:70258999-7035
ENSG0000(	1066	26.66165	chr1:152{	ZRANB2-DT		lncRNA	chr1:71081324-7148
ENSG0000(	1066	26.66165	chr1:152{	HHLA3-AS1		lncRNA	chr1:70359562-7036
ENSG0000(	1066	26.66165	chr1:152{	FPGT		protein_c	chr1:74198238-7423
ENSG0000(	1066	26.66165	chr1:152{	ENSG00000229440		Pseudoger	chr1:68381441-6838
ENSG0000(	1066	26.66165	chr1:152{	HHLA3		lncRNA	chr1:70354786-7038

ENSG0000(	1066	26.66165	chr1:1522	RN7SKP19		smallRNA	chr1:73191604-7319
ENSG0000(	1066	26.66165	chr1:1522	ZRANB2-AS1		lncRNA	chr1:71048855-7106
ENSG0000(	1066	26.66165	chr1:1522	RN7SL242P		smallRNA	chr1:70180146-7018
ENSG0000(	1066	26.66165	chr1:1522	RNA5SP50		Pseudoger	chr1:73749517-7374
ENSG0000(	1066	26.66165	chr1:1522	PTGER3		protein_c	chr1:70852353-7104
ENSG0000(	1066	26.66165	chr1:1522	RN7SL538P		smallRNA	chr1:69879592-6987
ENSG0000(	1066	26.66165	chr1:1522	ENSG00000271992		lncRNA	chr1:70445071-7044
ENSG0000(	1066	26.66165	chr1:1522	GADD45A		protein_c	chr1:67685201-6768
ENSG0000(	1066	26.66165	chr1:1522	AL157407.1		smallRNA	chr1:68058177-6805
ENSG0000(	1066	26.66165	chr1:1522	WLS	NCGv7	protein_c	chr1:68098473-6823
ENSG0000(	1066	26.66165	chr1:1522	LRRIQ3	NCGv7	protein_c	chr1:74026015-7419
ENSG0000(	1066	26.66165	chr1:1522	LRRC7-AS1		lncRNA	chr1:70013982-7003
ENSG0000(	1066	26.66165	chr1:1522	ENSG00000286863		lncRNA	chr1:72283170-7275
ENSG0000(	1066	26.66165	chr1:1522	RN7SL392P		smallRNA	chr1:67656833-6765
ENSG0000(	1066	26.66165	chr1:1522	LINC01788		lncRNA	chr1:70706441-7078
ENSG0000(	1066	26.66165	chr1:1522	ENSG00000285041		lncRNA	chr1:68633701-6864
ENSG0000(	1066	26.66165	chr1:1522	GNG12-AS1		lncRNA	chr1:67832293-6820
ENSG0000(	1066	26.66165	chr1:1522	ENSG00000280317		TEC	chr1:72979014-7297
ENSG0000(	1066	26.66165	chr1:1522	ENSG00000271618		Pseudoger	chr1:71738173-7173
ENSG0000(	1066	26.66165	chr1:1522	LRRC7	NCGv7	protein_c	chr1:69567922-7015
ENSG0000(	1066	26.66165	chr1:1522	HNRNPCP9		Pseudoger	chr1:67660155-6766
ENSG0000(	1066	26.66165	chr1:1522	PIN1P1		Pseudoger	chr1:69919322-6992
ENSG0000(	1066	26.66165	chr1:1522	GNG12		protein_c	chr1:67701475-6783
ENSG0000(	1066	26.66165	chr1:1522	ENSG00000231985		lncRNA	chr1:71570956-7157
ENSG0000(	1066	26.66165	chr1:1522	CASP3P1		Pseudoger	chr1:70660657-7066
ENSG0000(	1066	26.66165	chr1:1522	RNU4ATAC8P		smallRNA	chr1:73883713-7388
ENSG0000(	1066	26.66165	chr1:1522	CHORDC1P5		Pseudoger	chr1:70530526-7053
ENSG0000(	1066	26.66165	chr1:1522	LINC02238		lncRNA	chr1:73635216-7371
ENSG0000(	1066	26.66165	chr1:1522	ENSG00000228988		lncRNA	chr1:70218589-7022
ENSG0000(	1066	26.66165	chr1:1522	ENSG00000269933		lncRNA	chr1:71005854-7100
ENSG0000(	1066	26.66165	chr1:1522	ENSG00000235055		Pseudoger	chr1:68043330-6804
ENSG0000(	1066	26.66165	chr1:1522	KRT8P21		Pseudoger	chr1:73104792-7310
ENSG0000(	1066	26.66165	chr1:1522	ENSG00000285407		lncRNA	chr1:68679202-6894
ENSG0000(	1066	26.66165	chr1:1522	ZRANB2		protein_c	chr1:71063291-7108
ENSG0000(	1066	26.66165	chr1:1522	NEGR1-IT1		lncRNA	chr1:71794232-7183
ENSG0000(	1066	26.66165	chr1:1522	LINC02796		lncRNA	chr1:72765031-7279
ENSG0000(	1066	26.66165	chr1:1522	ENSG00000233589		lncRNA	chr1:68479129-6848
ENSG0000(	1066	26.66165	chr1:1522	LINC01707		lncRNA	chr1:69055838-6922
ENSG0000(	1066	26.66165	chr1:1522	SRSF11		protein_c	chr1:70205682-7025
ENSG0000(	1066	26.66165	chr1:1522	ENSG00000226208		lncRNA	chr1:70715933-7072
ENSG0000(	1066	26.66165	chr1:1522	ENSG00000235782		lncRNA	chr1:70947379-7095
ENSG0000(	1066	26.66165	chr1:1522	TXNP2		Pseudoger	chr1:68514375-6851
ENSG0000(	1066	26.66165	chr1:1522	ENSG00000226324		Pseudoger	chr1:71367054-7136
ENSG0000(	1066	26.66165	chr1:1522	DIRAS3		protein_c	chr1:68045886-6805
ENSG0000(	1066	26.66165	chr1:1522	ENSG00000285473		lncRNA	chr1:68974010-6902
ENSG0000(	1066	26.66165	chr1:1522	LINC02797		lncRNA	chr1:72793104-7285
ENSG0000(	1066	26.66165	chr1:1522	LINC01758		lncRNA	chr1:69433255-6943
ENSG0000(	1066	26.66165	chr1:1522	LINC01360		lncRNA	chr1:73305609-7335
ENSG0000(	1066	26.66165	chr1:1522	RPL31P12		Pseudoger	chr1:72301472-7230
ENSG0000(	1066	26.66165	chr1:1522	LRRC40		protein_c	chr1:70144805-7020
ENSG0000(	1066	26.66165	chr1:1522	GDI2P2		Pseudoger	chr1:72274552-7227

ENSG0000(	1066	26.66165	chr1:1522	RNU6-1246P		smallRNA	chr1:72717663-7271
ENSG0000(	1066	26.66165	chr1:1522	CTBP2P8		Pseudoger	chr1:68161761-6816
ENSG0000(	1066	26.66165	chr1:1522	DEPDC1	DriverDB\	protein_c	chr1:68474152-6849
ENSG0000(	1066	26.66165	chr1:1522	ENSG00000287453		lncRNA	chr1:69551848-6956
ENSG0000(	1066	26.66165	chr1:1522	RPS7P4		Pseudoger	chr1:68242474-6824
ENSG0000(	1066	26.66165	chr1:1522	CTH	DriverDB\	protein_c	chr1:70411218-7043
ENSG0000(	1066	26.66165	chr1:1522	AL360297.1		smallRNA	chr1:71141975-7114
ENSG0000(	1066	26.66165	chr1:1522	RNU7-80P		smallRNA	chr1:67772593-6777
ENSG0000(	1066	26.66165	chr1:1522	SGO1P1		Pseudoger	chr1:69606855-6960
ENSG0000(	1066	26.66165	chr1:1522	ELOCP18		Pseudoger	chr1:68375327-6837
ENSG0000(	1066	26.66165	chr1:1522	MIR1262		smallRNA	chr1:68183518-6818
ENSG0000(	1066	26.66165	chr1:1522	MIR186		smallRNA	chr1:71067631-7106
ENSG0000(	1066	26.66165	chr1:1522	ENSG00000225087		lncRNA	chr1:72636547-7289
ENSG0000(	1066	26.66165	chr1:1522	ENSG00000285778		lncRNA	chr1:73787370-7391
ENSG0000(	1066	26.66165	chr1:1522	ENSG00000287283		lncRNA	chr1:69706950-6971
ENSG0000(	1066	26.66165	chr1:1522	COX6B1P7		Pseudoger	chr1:68282388-6828
ENSG0000(	1066	26.66165	chr1:1522	ENSG00000237324		lncRNA	chr1:74341579-7437
ENSG0000(	1066	26.66165	chr1:1522	RPE65		protein_c	chr1:68428822-6844
ENSG0000(	1066	26.66165	chr1:1522	LINC02791		lncRNA	chr1:69215835-6924
ENSG0000(	1066	26.66165	chr1:1522	DEPDC1-AS1		lncRNA	chr1:68496676-6853
ENSG0000(	1066	26.66165	chr1:1522	NEGR1		protein_c	chr1:71395943-7228
ENSG0000(	1064	26.61163	chr6:105(	ENSG00000275716		Pseudoger	chr6:100093311-100
ENSG0000(	1064	26.61163	chr6:105(	ARMC2-AS1		lncRNA	chr6:108922976-108
ENSG0000(	1064	26.61163	chr6:105(	ENSG00000287294		lncRNA	chr6:84769010-8477
ENSG0000(	1064	26.61163	chr6:105(	KRT18P50		Pseudoger	chr6:95991107-9599
ENSG0000(	1064	26.61163	chr6:105(	ENSG00000230248		lncRNA	chr6:108275642-108
ENSG0000(	1064	26.61163	chr6:105(	ENSG00000270987		Pseudoger	chr6:100889603-100
ENSG0000(	1064	26.61163	chr6:105(	ENSG00000286511		lncRNA	chr6:108798929-108
ENSG0000(	1064	26.61163	chr6:105(	ENSG00000219150		Pseudoger	chr6:110706362-110
ENSG0000(	1064	26.61163	chr6:105(	ENSG00000287268		lncRNA	chr6:110341973-110
ENSG0000(	1064	26.61163	chr6:105(	RTN4IP1		protein_c	chr6:106570771-106
ENSG0000(	1064	26.61163	chr6:105(	OSTM1-AS1		lncRNA	chr6:108123457-108
ENSG0000(	1064	26.61163	chr6:105(	ENSG00000219755		Pseudoger	chr6:99575712-9957
ENSG0000(	1064	26.61163	chr6:105(	LINC02532		lncRNA	chr6:106705328-106
ENSG0000(	1064	26.61163	chr6:105(	RNU6-957P		smallRNA	chr6:110722250-110
ENSG0000(	1064	26.61163	chr6:105(	ENSG00000219757		Pseudoger	chr6:102453367-102
ENSG0000(	1064	26.61163	chr6:105(	PIMREGP3		Pseudoger	chr6:89814709-8981
ENSG0000(	1064	26.61163	chr6:105(	ENSG00000288084		lncRNA	chr6:103866023-103
ENSG0000(	1064	26.61163	chr6:105(	RN7SL415P		smallRNA	chr6:91739609-9173
ENSG0000(	1064	26.61163	chr6:105(	ENSG00000216324		Pseudoger	chr6:87425795-8742
ENSG0000(	1064	26.61163	chr6:105(	MTRES1		protein_c	chr6:107028199-107
ENSG0000(	1064	26.61163	chr6:105(	PNISR-AS1		lncRNA	chr6:99424911-9943
ENSG0000(	1064	26.61163	chr6:105(	ENSG00000288085		lncRNA	chr6:93720163-9374
ENSG0000(	1064	26.61163	chr6:105(	QRSL1		protein_c	chr6:106629578-106
ENSG0000(	1064	26.61163	chr6:105(	ENSG00000260273		lncRNA	chr6:109382795-109
ENSG0000(	1064	26.61163	chr6:105(	ENSG00000260188		lncRNA	chr6:110477907-110
ENSG0000(	1064	26.61163	chr6:105(	ENSG00000260271		lncRNA	chr6:90295507-9036
ENSG0000(	1064	26.61163	chr6:105(	Y_RNA		smallRNA	chr6:109305494-109
ENSG0000(	1064	26.61163	chr6:105(	RNU2-61P		smallRNA	chr6:89063500-8906
ENSG0000(	1064	26.61163	chr6:105(	ENSG00000276620		Pseudoger	chr6:107192300-107
ENSG0000(	1064	26.61163	chr6:105(	ENSG00000231628		lncRNA	chr6:105279016-105

ENSG0000(	1064	26.61163	chr6:105(	KLHL32		protein_c	chr6:96924620-9714
ENSG0000(	1064	26.61163	chr6:105(	ENSG00000235099		lncRNA	chr6:93070387-9310
ENSG0000(	1064	26.61163	chr6:105(	ENSG00000217769		Pseudoger	chr6:86897118-8689
ENSG0000(	1064	26.61163	chr6:105(	U3		smallRNA	chr6:92119522-9211
ENSG0000(	1064	26.61163	chr6:105(	HTR1E	NCGv7	protein_c	chr6:86937528-8701
ENSG0000(	1064	26.61163	chr6:105(	ENSG00000237027		lncRNA	chr6:89560875-8956
ENSG0000(	1064	26.61163	chr6:105(	ENSG00000270934		Pseudoger	chr6:110598093-110
ENSG0000(	1064	26.61163	chr6:105(	RN7SL617P		smallRNA	chr6:110439999-110
ENSG0000(	1064	26.61163	chr6:105(	CDC40		protein_c	chr6:110180141-110
ENSG0000(	1064	26.61163	chr6:105(	RPL35P3		Pseudoger	chr6:105302453-105
ENSG0000(	1064	26.61163	chr6:105(	ENSG00000217776		Pseudoger	chr6:87070156-8707
ENSG0000(	1064	26.61163	chr6:105(	ENSG00000289433		lncRNA	chr6:107595351-107
ENSG0000(	1064	26.61163	chr6:105(	ENSG00000288101		lncRNA	chr6:92631066-9263
ENSG0000(	1064	26.61163	chr6:105(	ENSG00000286278		lncRNA	chr6:88047056-8804
ENSG0000(	1064	26.61163	chr6:105(	ENSG00000219867		Pseudoger	chr6:91815843-9181
ENSG0000(	1064	26.61163	chr6:105(	ENSG00000289020		lncRNA	chr6:106702878-106
ENSG0000(	1064	26.61163	chr6:105(	ENSG00000260000		lncRNA	chr6:100881450-100
ENSG0000(	1064	26.61163	chr6:105(	TYMSP1		Pseudoger	chr6:96653274-9665
ENSG0000(	1064	26.61163	chr6:105(	PTCHD3P3		Pseudoger	chr6:109288571-109
ENSG0000(	1064	26.61163	chr6:105(	RN7SL183P		smallRNA	chr6:87845650-8784
ENSG0000(	1064	26.61163	chr6:105(	MCHR2	NCGv7	protein_c	chr6:99918519-9999
ENSG0000(	1064	26.61163	chr6:105(	PRDM1	NCGv7;AC	protein_c	chr6:105993463-106
ENSG0000(	1064	26.61163	chr6:105(	CYCSP17		Pseudoger	chr6:95504182-9550
ENSG0000(	1064	26.61163	chr6:105(	SIM1		protein_c	chr6:100385009-100
ENSG0000(	1064	26.61163	chr6:105(	FIG4		protein_c	chr6:109690609-109
ENSG0000(	1064	26.61163	chr6:105(	ZBTB24		protein_c	chr6:109460632-109
ENSG0000(	1064	26.61163	chr6:105(	ENSG00000213150		Pseudoger	chr6:110645699-110
ENSG0000(	1064	26.61163	chr6:105(	SNX3		protein_c	chr6:108211222-108
ENSG0000(	1064	26.61163	chr6:105(	NR2E1		protein_c	chr6:108166022-108
ENSG0000(	1064	26.61163	chr6:105(	SOBP		protein_c	chr6:107490106-107
ENSG0000(	1064	26.61163	chr6:105(	PRDX2P4		Pseudoger	chr6:100136064-100
ENSG0000(	1064	26.61163	chr6:105(	CRYBG1		protein_c	chr6:106360717-106
ENSG0000(	1064	26.61163	chr6:105(	ENSG00000219240		Pseudoger	chr6:89886156-8988
ENSG0000(	1064	26.61163	chr6:105(	MIR587		smallRNA	chr6:106784125-106
ENSG0000(	1064	26.61163	chr6:105(	WASF1		protein_c	chr6:110099819-110
ENSG0000(	1064	26.61163	chr6:105(	BVES		protein_c	chr6:105096822-105
ENSG0000(	1064	26.61163	chr6:105(	ASCC3	NCGv7	protein_c	chr6:100508194-100
ENSG0000(	1064	26.61163	chr6:105(	Y_RNA		smallRNA	chr6:85777639-8577
ENSG0000(	1064	26.61163	chr6:105(	LINC01611		lncRNA	chr6:84421028-8455
ENSG0000(	1064	26.61163	chr6:105(	RPL7AP35		Pseudoger	chr6:105298149-105
ENSG0000(	1064	26.61163	chr6:105(	PRDM13	AC	protein_c	chr6:99606774-9961
ENSG0000(	1064	26.61163	chr6:105(	CCNC	NCGv7	protein_c	chr6:99542387-9956
ENSG0000(	1064	26.61163	chr6:105(	FBXL4		protein_c	chr6:98868535-9894
ENSG0000(	1064	26.61163	chr6:105(	POPDC3		protein_c	chr6:105157900-105
ENSG0000(	1064	26.61163	chr6:105(	GPR63		protein_c	chr6:96794125-9683
ENSG0000(	1064	26.61163	chr6:105(	FHL5		protein_c	chr6:96562548-9661
ENSG0000(	1064	26.61163	chr6:105(	PNISR		protein_c	chr6:99397629-9942
ENSG0000(	1064	26.61163	chr6:105(	Y_RNA		smallRNA	chr6:89841329-8984
ENSG0000(	1064	26.61163	chr6:105(	ENSG00000286691		lncRNA	chr6:110020011-110
ENSG0000(	1064	26.61163	chr6:105(	ENSG00000219190		Pseudoger	chr6:86768522-8676
ENSG0000(	1064	26.61163	chr6:105(	ENSG00000216523		Pseudoger	chr6:84966397-8496

ENSG0000(	1064	26.61163	chr6:105(	ENSG00000287044		lncRNA	chr6:108441880-108
ENSG0000(	1064	26.61163	chr6:105(	SRSF12		protein_c	chr6:89095959-8911
ENSG0000(	1064	26.61163	chr6:105(	CNN2P9		Pseudoger	chr6:110858239-110
ENSG0000(	1064	26.61163	chr6:105(	ENSG00000228679		lncRNA	chr6:83983728-8400
ENSG0000(	1064	26.61163	chr6:105(	ENSG00000227535		lncRNA	chr6:104831129-104
ENSG0000(	1064	26.61163	chr6:105(	HSPD1P10		Pseudoger	chr6:87298772-8730
ENSG0000(	1064	26.61163	chr6:105(	AL109947.1		smallRNA	chr6:109434174-109
ENSG0000(	1064	26.61163	chr6:105(	TSTD3		protein_c	chr6:99520976-9958
ENSG0000(	1064	26.61163	chr6:105(	FUT9		protein_c	chr6:96015974-9621
ENSG0000(	1064	26.61163	chr6:105(	RPL21P65		Pseudoger	chr6:106642463-106
ENSG0000(	1064	26.61163	chr6:105(	MANEA		protein_c	chr6:95577485-9560
ENSG0000(	1064	26.61163	chr6:105(	ENSG00000217041		Pseudoger	chr6:110700562-110
ENSG0000(	1064	26.61163	chr6:105(	RNU6-653P		smallRNA	chr6:109059509-109
ENSG0000(	1064	26.61163	chr6:105(	ENSG00000217060		Pseudoger	chr6:85286076-8529
ENSG0000(	1064	26.61163	chr6:105(	ENSG00000217120		Pseudoger	chr6:103002514-103
ENSG0000(	1064	26.61163	chr6:105(	TBX18	NCGv7	protein_c	chr6:84687351-8476
ENSG0000(	1064	26.61163	chr6:105(	RN7SL11P		smallRNA	chr6:89478141-8947
ENSG0000(	1064	26.61163	chr6:105(	ENSG00000286871		lncRNA	chr6:88378280-8838
ENSG0000(	1064	26.61163	chr6:105(	MTHFD2P2		Pseudoger	chr6:87000045-8700
ENSG0000(	1064	26.61163	chr6:105(	MANEA-DT		lncRNA	chr6:95575183-9557
ENSG0000(	1064	26.61163	chr6:105(	Y_RNA		smallRNA	chr6:87826173-8782
ENSG0000(	1064	26.61163	chr6:105(	RAB1AP2		Pseudoger	chr6:86170447-8617
ENSG0000(	1064	26.61163	chr6:105(	MTATP6P25		Pseudoger	chr6:91726810-9172
ENSG0000(	1064	26.61163	chr6:105(	ENSG00000216902		Pseudoger	chr6:85257328-8525
ENSG0000(	1064	26.61163	chr6:105(	ENSG00000217331		Pseudoger	chr6:96672129-9667
ENSG0000(	1064	26.61163	chr6:105(	MTCYBP36		Pseudoger	chr6:94446740-9444
ENSG0000(	1064	26.61163	chr6:105(	ENSG00000217334		Pseudoger	chr6:85731371-8573
ENSG0000(	1064	26.61163	chr6:105(	MIR4643		smallRNA	chr6:91521660-9152
ENSG0000(	1064	26.61163	chr6:105(	RN7SL509P		smallRNA	chr6:96914701-9691
ENSG0000(	1064	26.61163	chr6:105(	ZBTB24-DT		lncRNA	chr6:109483638-109
ENSG0000(	1064	26.61163	chr6:105(	R3HDM2P2		Pseudoger	chr6:104017633-104
ENSG0000(	1064	26.61163	chr6:105(	NPM1P38		Pseudoger	chr6:100050372-100
ENSG0000(	1064	26.61163	chr6:105(	ATF1P1		Pseudoger	chr6:92887251-9288
ENSG0000(	1064	26.61163	chr6:105(	COQ3		protein_c	chr6:99369401-9939
ENSG0000(	1064	26.61163	chr6:105(	BACH2	NCGv7	protein_c	chr6:89926528-9029
ENSG0000(	1064	26.61163	chr6:105(	EEF1GP6		Pseudoger	chr6:96750824-9675
ENSG0000(	1064	26.61163	chr6:105(	ENSG00000279498		TEC	chr6:108359084-108
ENSG0000(	1064	26.61163	chr6:105(	ENSG00000271042		Pseudoger	chr6:99082449-9908
ENSG0000(	1064	26.61163	chr6:105(	RNU6-1115P		smallRNA	chr6:110856417-110
ENSG0000(	1064	26.61163	chr6:105(	COPS5P1		Pseudoger	chr6:93091976-9309
ENSG0000(	1064	26.61163	chr6:105(	UFL1		protein_c	chr6:96521595-9655
ENSG0000(	1064	26.61163	chr6:105(	ENSG00000187472		Pseudoger	chr6:98780257-9878
ENSG0000(	1064	26.61163	chr6:105(	CCDC162P		Pseudoger	chr6:109165831-109
ENSG0000(	1064	26.61163	chr6:105(	RNU4-72P		smallRNA	chr6:85751581-8575
ENSG0000(	1064	26.61163	chr6:105(	ZNF292	NCGv7	protein_c	chr6:87151803-8726
ENSG0000(	1064	26.61163	chr6:105(	DDO		protein_c	chr6:110391771-110
ENSG0000(	1064	26.61163	chr6:105(	SUMO2P8		Pseudoger	chr6:108618000-108
ENSG0000(	1064	26.61163	chr6:105(	RN7SKP110		smallRNA	chr6:90001222-9000
ENSG0000(	1064	26.61163	chr6:105(	ENSG00000236920		lncRNA	chr6:98210020-9821
ENSG0000(	1064	26.61163	chr6:105(	Y_RNA		smallRNA	chr6:99642237-9964
ENSG0000(	1064	26.61163	chr6:105(	ENSG00000213204		protein_c	chr6:87408012-8751

ENSG0000(	1064	26.61163	chr6:105(	ENSG00000261038	lncRNA	chr6:92387841-9238
ENSG0000(	1064	26.61163	chr6:105(	ENSG00000219549	Pseudoger	chr6:95630115-9563
ENSG0000(	1064	26.61163	chr6:105(	KRT18P64	Pseudoger	chr6:85287789-8528
ENSG0000(	1064	26.61163	chr6:105(	snoU13	smallRNA	chr6:108243455-108
ENSG0000(	1064	26.61163	chr6:105(	ENSG00000279565	TEC	chr6:88172261-8817
ENSG0000(	1064	26.61163	chr6:105(	ENSG00000219559	Pseudoger	chr6:110562175-110
ENSG0000(	1064	26.61163	chr6:105(	ENSG00000279616	TEC	chr6:87356831-8735
ENSG0000(	1064	26.61163	chr6:105(	ZPR1P1	Pseudoger	chr6:108782126-108
ENSG0000(	1064	26.61163	chr6:105(	ENSG00000219604	Pseudoger	chr6:84689292-8468
ENSG0000(	1064	26.61163	chr6:105(	ENSG00000270484	Pseudoger	chr6:94442030-9444
ENSG0000(	1064	26.61163	chr6:105(	U3	smallRNA	chr6:92790453-9279
ENSG0000(	1064	26.61163	chr6:105(	ENSG00000286562	lncRNA	chr6:108261288-108
ENSG0000(	1064	26.61163	chr6:105(	ENSG00000289501	lncRNA	chr6:98832905-9883
ENSG0000(	1064	26.61163	chr6:105(	ENSG00000233511	Pseudoger	chr6:108252214-108
ENSG0000(	1064	26.61163	chr6:105(	ATG5	protein_c	chr6:106045423-106
ENSG0000(	1064	26.61163	chr6:105(	AL359709.1	smallRNA	chr6:105181149-105
ENSG0000(	1064	26.61163	chr6:105(	LINC00222	lncRNA	chr6:108751654-108
ENSG0000(	1064	26.61163	chr6:105(	LINC02857	lncRNA	chr6:84019204-8402
ENSG0000(	1064	26.61163	chr6:105(	MDN1	protein_c	chr6:89642498-8981
ENSG0000(	1064	26.61163	chr6:105(	ENSG00000289178	lncRNA	chr6:94546175-9473
ENSG0000(	1064	26.61163	chr6:105(	RN7SL336P	smallRNA	chr6:89090946-8909
ENSG0000(	1064	26.61163	chr6:105(	AK9	protein_c	chr6:109492855-109
ENSG0000(	1064	26.61163	chr6:105(	RIPPLY2	protein_c	chr6:83853360-8385
ENSG0000(	1064	26.61163	chr6:105(	SNHG5	lncRNA	chr6:85650491-8567
ENSG0000(	1064	26.61163	chr6:105(	SNORA73	smallRNA	chr6:88714242-8871
ENSG0000(	1064	26.61163	chr6:105(	C6orf163	protein_c	chr6:87344813-8736
ENSG0000(	1064	26.61163	chr6:105(	DUTP5	Pseudoger	chr6:85426657-8542
ENSG0000(	1064	26.61163	chr6:105(	LIN28B AC	protein_c	chr6:104936616-105
ENSG0000(	1064	26.61163	chr6:105(	ENSG00000224605	lncRNA	chr6:91390313-9139
ENSG0000(	1064	26.61163	chr6:105(	PGM3	protein_c	chr6:83147324-8319
ENSG0000(	1064	26.61163	chr6:105(	CDK19	protein_c	chr6:110609978-110
ENSG0000(	1064	26.61163	chr6:105(	ATP5MFP2	Pseudoger	chr6:108907615-108
ENSG0000(	1064	26.61163	chr6:105(	ENSG00000279398	TEC	chr6:108178871-108
ENSG0000(	1064	26.61163	chr6:105(	RWDD2A	protein_c	chr6:83193357-8319
ENSG0000(	1064	26.61163	chr6:105(	ENSG00000279403	TEC	chr6:94397664-9439
ENSG0000(	1064	26.61163	chr6:105(	LIN28B-AS1	lncRNA	chr6:104864464-104
ENSG0000(	1064	26.61163	chr6:105(	snoU13	smallRNA	chr6:100957160-100
ENSG0000(	1064	26.61163	chr6:105(	RPS7P8	Pseudoger	chr6:96648489-9664
ENSG0000(	1064	26.61163	chr6:105(	BVES-AS1	lncRNA	chr6:105136308-105
ENSG0000(	1064	26.61163	chr6:105(	GABRR2	protein_c	chr6:89254464-8931
ENSG0000(	1064	26.61163	chr6:105(	RNGTT	protein_c	chr6:88609897-8896
ENSG0000(	1064	26.61163	chr6:105(	SMARCE1P2	Pseudoger	chr6:84429584-8443
ENSG0000(	1064	26.61163	chr6:105(	ENSG00000219500	Pseudoger	chr6:86432244-8643
ENSG0000(	1064	26.61163	chr6:105(	ENSG00000216378	Pseudoger	chr6:100177209-100
ENSG0000(	1064	26.61163	chr6:105(	SMIM8	protein_c	chr6:87322583-8739
ENSG0000(	1064	26.61163	chr6:105(	ENSG00000219951	Pseudoger	chr6:83392416-8339
ENSG0000(	1064	26.61163	chr6:105(	ENSG00000271114	Pseudoger	chr6:87891424-8789
ENSG0000(	1064	26.61163	chr6:105(	ENSG00000288021	lncRNA	chr6:85949690-8599
ENSG0000(	1064	26.61163	chr6:105(	AL589947.1	smallRNA	chr6:92812041-9281
ENSG0000(	1064	26.61163	chr6:105(	ENSG00000282408	lncRNA	chr6:106451496-106
ENSG0000(	1064	26.61163	chr6:105(	SNAP91	protein_c	chr6:83552880-8370

ENSG0000(	1064	26.61163	chr6:105(	MICAL1		protein_c	chr6:109444062-109
ENSG0000(	1064	26.61163	chr6:105(	ENSG00000233941		lncRNA	chr6:106358566-106
ENSG0000(	1064	26.61163	chr6:105(	ENSG00000271793		protein_c	chr6:85504776-8567
ENSG0000(	1064	26.61163	chr6:105(	CYB5R4		protein_c	chr6:83859656-8396
ENSG0000(	1064	26.61163	chr6:105(	RNU4-70P		smallRNA	chr6:96649453-9664
ENSG0000(	1064	26.61163	chr6:105(	PRSS35		protein_c	chr6:83512534-8352
ENSG0000(	1064	26.61163	chr6:105(	MMS22L	NCGv7;AC	protein_c	chr6:97142161-9728
ENSG0000(	1064	26.61163	chr6:105(	RNU6-527P		smallRNA	chr6:106607716-106
ENSG0000(	1064	26.61163	chr6:105(	RNU6-437P		smallRNA	chr6:107930088-107
ENSG0000(	1064	26.61163	chr6:105(	ENSG00000271860		lncRNA	chr6:97283303-9840
ENSG0000(	1064	26.61163	chr6:105(	ME1		protein_c	chr6:83210402-8343
ENSG0000(	1064	26.61163	chr6:105(	AL109947.2		smallRNA	chr6:109504387-109
ENSG0000(	1064	26.61163	chr6:105(	SLC22A16		protein_c	chr6:110424687-110
ENSG0000(	1064	26.61163	chr6:105(	RPL23AP50		Pseudoger	chr6:107931800-107
ENSG0000(	1064	26.61163	chr6:105(	PDSS2	NCGv7	protein_c	chr6:107152562-107
ENSG0000(	1064	26.61163	chr6:105(	FAXC		protein_c	chr6:99271168-9935
ENSG0000(	1064	26.61163	chr6:105(	ENSG00000283010		lncRNA	chr6:98829967-9883
ENSG0000(	1064	26.61163	chr6:105(	GRIK2	NCGv7	protein_c	chr6:100962701-102
ENSG0000(	1064	26.61163	chr6:105(	CASC6		lncRNA	chr6:91557292-9169
ENSG0000(	1064	26.61163	chr6:105(	SLC35A1		protein_c	chr6:87470623-8751
ENSG0000(	1064	26.61163	chr6:105(	GJB7		protein_c	chr6:87282980-8732
ENSG0000(	1064	26.61163	chr6:105(	GABRR1		protein_c	chr6:89177504-8923
ENSG0000(	1064	26.61163	chr6:105(	ENSG00000289847		lncRNA	chr6:110530684-110
ENSG0000(	1064	26.61163	chr6:105(	SNORA73		smallRNA	chr6:107985659-107
ENSG0000(	1064	26.61163	chr6:105(	PNRC1	NCGv7	protein_c	chr6:89080751-8908
ENSG0000(	1064	26.61163	chr6:105(	PM20D2		protein_c	chr6:89146055-8916
ENSG0000(	1064	26.61163	chr6:105(	SMPD2		protein_c	chr6:109440724-109
ENSG0000(	1064	26.61163	chr6:105(	LINC02836		lncRNA	chr6:105612667-105
ENSG0000(	1064	26.61163	chr6:105(	Y_RNA		smallRNA	chr6:108060818-108
ENSG0000(	1064	26.61163	chr6:105(	CNR1		protein_c	chr6:88139864-8816
ENSG0000(	1064	26.61163	chr6:105(	ENSG00000224384		lncRNA	chr6:96785137-9679
ENSG0000(	1064	26.61163	chr6:105(	RNU6-117P		smallRNA	chr6:106738948-106
ENSG0000(	1064	26.61163	chr6:105(	MCHR2-AS1		lncRNA	chr6:99993934-1001
ENSG0000(	1064	26.61163	chr6:105(	SNORA33		smallRNA	chr6:103583135-103
ENSG0000(	1064	26.61163	chr6:105(	BEND3		protein_c	chr6:107065182-107
ENSG0000(	1064	26.61163	chr6:105(	ENSG00000234426		lncRNA	chr6:88177804-8844
ENSG0000(	1064	26.61163	chr6:105(	RN7SKP211		smallRNA	chr6:105904373-105
ENSG0000(	1064	26.61163	chr6:105(	ENSG00000218173		Pseudoger	chr6:104687241-104
ENSG0000(	1064	26.61163	chr6:105(	CASP8AP2		protein_c	chr6:89829894-8987
ENSG0000(	1064	26.61163	chr6:105(	snoU13		smallRNA	chr6:109291255-109
ENSG0000(	1064	26.61163	chr6:105(	ENSG00000271607		Pseudoger	chr6:110863860-110
ENSG0000(	1064	26.61163	chr6:105(	ENSG00000271608		Pseudoger	chr6:108551018-108
ENSG0000(	1064	26.61163	chr6:105(	UBE3D		protein_c	chr6:82892390-8306
ENSG0000(	1064	26.61163	chr6:105(	SPACA1		protein_c	chr6:88047841-8806
ENSG0000(	1064	26.61163	chr6:105(	ARMC2		protein_c	chr6:108848416-108
ENSG0000(	1064	26.61163	chr6:105(	Y_RNA		smallRNA	chr6:106454828-106
ENSG0000(	1064	26.61163	chr6:105(	ENSG00000220695		Pseudoger	chr6:100530276-100
ENSG0000(	1064	26.61163	chr6:105(	ENSG00000233908		Pseudoger	chr6:109288440-109
ENSG0000(	1064	26.61163	chr6:105(	Y_RNA		smallRNA	chr6:89898283-8989
ENSG0000(	1064	26.61163	chr6:105(	RNU6-770P		smallRNA	chr6:108392073-108
ENSG0000(	1064	26.61163	chr6:105(	AL359709.2		smallRNA	chr6:105196853-105

ENSG0000(	1064	26.61163	chr6:105(	RN7SL797P		smallRNA	chr6:96282567-9628
ENSG0000(	1064	26.61163	chr6:105(	DOP1A		protein_c	chr6:83067666-8317
ENSG0000(	1064	26.61163	chr6:105(	RNU6-897P		smallRNA	chr6:104766822-104
ENSG0000(	1064	26.61163	chr6:105(	LYRM2		protein_c	chr6:89568144-8963
ENSG0000(	1064	26.61163	chr6:105(	AFG1L		protein_c	chr6:108294991-108
ENSG0000(	1064	26.61163	chr6:105(	ENSG00000271730		lncRNA	chr6:108998482-108
ENSG0000(	1064	26.61163	chr6:105(	FOXO3	NCGv7	protein_c	chr6:108559835-108
ENSG0000(	1064	26.61163	chr6:105(	RARS2		protein_c	chr6:87513459-8759
ENSG0000(	1064	26.61163	chr6:105(	ENSG00000287789		lncRNA	chr6:90073051-9007
ENSG0000(	1064	26.61163	chr6:105(	MDN1-AS1		lncRNA	chr6:89673469-8967
ENSG0000(	1064	26.61163	chr6:105(	LINC02535		lncRNA	chr6:85387219-8539
ENSG0000(	1064	26.61163	chr6:105(	MIR548AI		smallRNA	chr6:99124609-9912
ENSG0000(	1064	26.61163	chr6:105(	LINC02526		lncRNA	chr6:106695535-106
ENSG0000(	1064	26.61163	chr6:105(	ENSG00000287616		lncRNA	chr6:104487095-104
ENSG0000(	1064	26.61163	chr6:105(	AL359987.1		smallRNA	chr6:92644741-9264
ENSG0000(	1064	26.61163	chr6:105(	RNU6-1106P		smallRNA	chr6:105045562-105
ENSG0000(	1064	26.61163	chr6:105(	ST13P16		Pseudoger	chr6:87444395-8744
ENSG0000(	1064	26.61163	chr6:105(	ENSG00000234206		lncRNA	chr6:107509803-107
ENSG0000(	1064	26.61163	chr6:105(	ENSG00000220960		Pseudoger	chr6:89059628-8906
ENSG0000(	1064	26.61163	chr6:105(	ENSG00000269919		lncRNA	chr6:106100140-106
ENSG0000(	1064	26.61163	chr6:105(	ENSG00000289931		lncRNA	chr6:88963824-8896
ENSG0000(	1064	26.61163	chr6:105(	AL391417.1		smallRNA	chr6:86473487-8647
ENSG0000(	1064	26.61163	chr6:105(	ENSG00000287705		lncRNA	chr6:83852312-8385
ENSG0000(	1064	26.61163	chr6:105(	RPL7P28		Pseudoger	chr6:109327175-109
ENSG0000(	1064	26.61163	chr6:105(	ENSG00000237874		lncRNA	chr6:83728055-8373
ENSG0000(	1064	26.61163	chr6:105(	SCML4		protein_c	chr6:107702154-107
ENSG0000(	1064	26.61163	chr6:105(	RNU4-12P		smallRNA	chr6:85993021-8599
ENSG0000(	1064	26.61163	chr6:105(	ENSG00000218561		Pseudoger	chr6:86729708-8673
ENSG0000(	1064	26.61163	chr6:105(	ENSG00000218536		Pseudoger	chr6:102078872-102
ENSG0000(	1064	26.61163	chr6:105(	USP45		protein_c	chr6:99432325-9952
ENSG0000(	1064	26.61163	chr6:105(	ENSG00000289988		lncRNA	chr6:90376124-9037
ENSG0000(	1064	26.61163	chr6:105(	NDUFAF4		protein_c	chr6:96889315-9689
ENSG0000(	1064	26.61163	chr6:105(	ENSG00000290011		lncRNA	chr6:104941624-104
ENSG0000(	1064	26.61163	chr6:105(	ACTG1P18		Pseudoger	chr6:101430411-101
ENSG0000(	1064	26.61163	chr6:105(	ENSG00000231559		Pseudoger	chr6:108817680-108
ENSG0000(	1064	26.61163	chr6:105(	MTHFD2P3		Pseudoger	chr6:107985811-107
ENSG0000(	1064	26.61163	chr6:105(	ENSG00000287683		lncRNA	chr6:93416951-9367
ENSG0000(	1064	26.61163	chr6:105(	CFAP206		protein_c	chr6:87407972-8746
ENSG0000(	1064	26.61163	chr6:105(	ENSG00000272476		lncRNA	chr6:107957413-107
ENSG0000(	1064	26.61163	chr6:105(	ENSG00000218730		Pseudoger	chr6:90116700-9011
ENSG0000(	1064	26.61163	chr6:105(	LINC02531		lncRNA	chr6:92723003-9272
ENSG0000(	1064	26.61163	chr6:105(	RPL36AP24		Pseudoger	chr6:108318079-108
ENSG0000(	1064	26.61163	chr6:105(	ENSG00000238079		Pseudoger	chr6:108372772-108
ENSG0000(	1064	26.61163	chr6:105(	ENSG00000289081		lncRNA	chr6:93419821-9342
ENSG0000(	1064	26.61163	chr6:105(	ENSG00000287499		lncRNA	chr6:102350271-102
ENSG0000(	1064	26.61163	chr6:105(	AL138919.1		smallRNA	chr6:87847545-8784
ENSG0000(	1064	26.61163	chr6:105(	RN7SL47P		smallRNA	chr6:106283415-106
ENSG0000(	1064	26.61163	chr6:105(	PREP		protein_c	chr6:105273218-105
ENSG0000(	1064	26.61163	chr6:105(	HACE1		protein_c	chr6:104728094-104
ENSG0000(	1064	26.61163	chr6:105(	GPR6		protein_c	chr6:109978256-109
ENSG0000(	1064	26.61163	chr6:105(	MIR2113		smallRNA	chr6:98024531-9802

ENSG0000(	1064	26.61163	chr6:105(	NDUFA5P9		Pseudoger	chr6:86214237-8621
ENSG0000(	1064	26.61163	chr6:105(	SIM1-AS1		lncRNA	chr6:100427118-100
ENSG0000(	1064	26.61163	chr6:105(	RNU6-1144P		smallRNA	chr6:108292766-108
ENSG0000(	1064	26.61163	chr6:105(	ENSG00000272008		lncRNA	chr6:87151159-8715
ENSG0000(	1064	26.61163	chr6:105(	RPL22P14		Pseudoger	chr6:89876544-8987
ENSG0000(	1064	26.61163	chr6:105(	NACAP7		Pseudoger	chr6:89422099-8942
ENSG0000(	1064	26.61163	chr6:105(	ENSG00000218872		Pseudoger	chr6:108856400-108
ENSG0000(	1064	26.61163	chr6:105(	CEP57L1		protein_c	chr6:109095110-109
ENSG0000(	1064	26.61163	chr6:105(	RN7SL643P		smallRNA	chr6:86435852-8643
ENSG0000(	1064	26.61163	chr6:105(	ENSG00000231143		lncRNA	chr6:94163920-9419
ENSG0000(	1064	26.61163	chr6:105(	ENSG00000287578		lncRNA	chr6:95917143-9601
ENSG0000(	1064	26.61163	chr6:105(	ENSG00000284999		lncRNA	chr6:105679378-105
ENSG0000(	1064	26.61163	chr6:105(	ENSG00000218793		Pseudoger	chr6:87441165-8744
ENSG0000(	1064	26.61163	chr6:105(	ENSG00000229600		lncRNA	chr6:93886900-9388
ENSG0000(	1064	26.61163	chr6:105(	ENSG00000218766		Pseudoger	chr6:85868953-8586
ENSG0000(	1064	26.61163	chr6:105(	ENSG00000224987		Pseudoger	chr6:107985089-107
ENSG0000(	1064	26.61163	chr6:105(	ENSG00000226455		lncRNA	chr6:89950116-8995
ENSG0000(	1064	26.61163	chr6:105(	ENSG00000218313		Pseudoger	chr6:87173368-8717
ENSG0000(	1064	26.61163	chr6:105(	DNAJC19P6		Pseudoger	chr6:89797598-8979
ENSG0000(	1064	26.61163	chr6:105(	RNA5SP212		Pseudoger	chr6:108252401-108
ENSG0000(	1064	26.61163	chr6:105(	CD164		protein_c	chr6:109366514-109
ENSG0000(	1064	26.61163	chr6:105(	ENSG00000280451		TEC	chr6:93811825-9381
ENSG0000(	1064	26.61163	chr6:105(	EIF4EBP2P3		Pseudoger	chr6:98179499-9817
ENSG0000(	1064	26.61163	chr6:105(	RRAGD		protein_c	chr6:89364616-8941
ENSG0000(	1064	26.61163	chr6:105(	SNORA40		smallRNA	chr6:110848546-110
ENSG0000(	1064	26.61163	chr6:105(	OSTM1		protein_c	chr6:108029245-108
ENSG0000(	1064	26.61163	chr6:105(	LAP3P1		Pseudoger	chr6:82924829-8292
ENSG0000(	1064	26.61163	chr6:105(	AL391416.1		smallRNA	chr6:83367706-8336
ENSG0000(	1064	26.61163	chr6:105(	ENSG00000227215		lncRNA	chr6:82932601-8293
ENSG0000(	1064	26.61163	chr6:105(	ANKRD6		protein_c	chr6:89433152-8963
ENSG0000(	1064	26.61163	chr6:105(	RPS24P12		Pseudoger	chr6:107229759-107
ENSG0000(	1064	26.61163	chr6:105(	CEP162		protein_c	chr6:84124241-8422
ENSG0000(	1064	26.61163	chr6:105(	SEC63		protein_c	chr6:107867756-107
ENSG0000(	1064	26.61163	chr6:105(	SESN1		protein_c	chr6:108984309-109
ENSG0000(	1064	26.61163	chr6:105(	SYNCRIP	NCGv7	protein_c	chr6:85607779-8564
ENSG0000(	1064	26.61163	chr6:105(	ENSG00000226207		lncRNA	chr6:97710953-9771
ENSG0000(	1064	26.61163	chr6:105(	SNX14		protein_c	chr6:85504776-8559
ENSG0000(	1064	26.61163	chr6:105(	ENSG00000280232		TEC	chr6:85498441-8549
ENSG0000(	1064	26.61163	chr6:105(	NT5E		protein_c	chr6:85449584-8549
ENSG0000(	1064	26.61163	chr6:105(	MRAP2		protein_c	chr6:84033772-8409
ENSG0000(	1064	26.61163	chr6:105(	ENSG00000280277		TEC	chr6:88964527-8896
ENSG0000(	1064	26.61163	chr6:105(	EPHA7	NCGv7	protein_c	chr6:93240020-9341
ENSG0000(	1064	26.61163	chr6:105(	NPM1P10		Pseudoger	chr6:104025540-104
ENSG0000(	1064	26.61163	chr6:105(	SNORA18		smallRNA	chr6:93879106-9387
ENSG0000(	1064	26.61163	chr6:105(	TUBB3P1		Pseudoger	chr6:89301783-8930
ENSG0000(	1064	26.61163	chr6:105(	ENSG00000227072		Pseudoger	chr6:108387512-108
ENSG0000(	1064	26.61163	chr6:105(	snoU13		smallRNA	chr6:89823295-8982
ENSG0000(	1064	26.61163	chr6:105(	ACTBP8		Pseudoger	chr6:88275882-8827
ENSG0000(	1064	26.61163	chr6:105(	PPIL6		protein_c	chr6:109390215-109
ENSG0000(	1064	26.61163	chr6:105(	MIR4464		smallRNA	chr6:90312742-9031
ENSG0000(	1064	26.61163	chr6:105(	ENSG00000220291		Pseudoger	chr6:85155667-8516

ENSG0000(	1064	26.61163	chr6:105(	ENSG00000271099		Pseudoger	chr6:104860449-104
ENSG0000(	1064	26.61163	chr6:105(	MTCO1P56		Pseudoger	chr6:91727282-9172
ENSG0000(	1064	26.61163	chr6:105(	ENSG00000286084		lncRNA	chr6:105403207-105
ENSG0000(	1064	26.61163	chr6:105(	ENSG00000220370		Pseudoger	chr6:94344737-9434
ENSG0000(	1064	26.61163	chr6:105(	RCN1P1		Pseudoger	chr6:87121693-8712
ENSG0000(	1064	26.61163	chr6:105(	ENSG00000288009		lncRNA	chr6:89130887-8914
ENSG0000(	1064	26.61163	chr6:105(	MTND4LP19		Pseudoger	chr6:91727159-9172
ENSG0000(	1064	26.61163	chr6:105(	ENSG00000219088		Pseudoger	chr6:105666326-105
ENSG0000(	1064	26.61163	chr6:105(	TPT1P6		Pseudoger	chr6:85427453-8542
ENSG0000(	1064	26.61163	chr6:105(	RPL7P29		Pseudoger	chr6:86970466-8697
ENSG0000(	1064	26.61163	chr6:105(	METTL24		protein_c	chr6:110243940-110
ENSG0000(	1064	26.61163	chr6:105(	ENSG00000220131		Pseudoger	chr6:88265158-8826
ENSG0000(	1064	26.61163	chr6:105(	UBE2J1		protein_c	chr6:89326625-8935
ENSG0000(	1064	26.61163	chr6:105(	ENSG00000280135		TEC	chr6:107697299-107
ENSG0000(	1064	26.61163	chr6:105(	RNU6-1299P		smallRNA	chr6:107133071-107
ENSG0000(	1064	26.61163	chr6:105(	snoU13		smallRNA	chr6:87720915-8772
ENSG0000(	1064	26.61163	chr6:105(	SMIM11P1		Pseudoger	chr6:85735744-8573
ENSG0000(	1064	26.61163	chr6:105(	ENSG00000285961		lncRNA	chr6:92002610-9218
ENSG0000(	1064	26.61163	chr6:105(	UFL1-AS1		lncRNA	chr6:96199840-9652
ENSG0000(	1064	26.61163	chr6:105(	ORC3		protein_c	chr6:87590067-8766
ENSG0000(	1064	26.61163	chr6:105(	GJA10		protein_c	chr6:89894469-8992
ENSG0000(	1064	26.61163	chr6:105(	PKMP3		Pseudoger	chr6:85659892-8566
ENSG0000(	1064	26.61163	chr6:105(	CGA		protein_c	chr6:87085498-8709
ENSG0000(	1064	26.61163	chr6:105(	MAP3K7	AC	protein_c	chr6:90513573-9058
ENSG0000(	1064	26.61163	chr6:105(	AL391559.1		smallRNA	chr6:90446772-9044
ENSG0000(	1064	26.61163	chr6:105(	RPL3P7		Pseudoger	chr6:108004357-108
ENSG0000(	1064	26.61163	chr6:105(	Y_RNA		smallRNA	chr6:106420706-106
ENSG0000(	1064	26.61163	chr6:105(	RPL5P19		Pseudoger	chr6:92013842-9201
ENSG0000(	1064	26.61163	chr6:105(	RPL7P27		Pseudoger	chr6:86086415-8608
ENSG0000(	1064	26.61163	chr6:105(	RNU6-444P		smallRNA	chr6:87488445-8748
ENSG0000(	1064	26.61163	chr6:105(	RNA5SP211		Pseudoger	chr6:106449381-106
ENSG0000(	1064	26.61163	chr6:105(	TBX18-AS1		lncRNA	chr6:84687712-8470
ENSG0000(	1064	26.61163	chr6:105(	POU3F2		protein_c	chr6:98834574-9883
ENSG0000(	1064	26.61163	chr6:105(	ENSG00000287397		lncRNA	chr6:101452917-101
ENSG0000(	1064	26.61163	chr6:105(	ENSG00000220240		Pseudoger	chr6:86897547-8689
ENSG0000(	1064	26.61163	chr6:105(	AKIRIN2	AC	protein_c	chr6:87674860-8770
ENSG0000(	1064	26.61163	chr6:105(	RNU6-344P		smallRNA	chr6:106304176-106
ENSG0000(	1062	26.56161	chr7:330(	ENSG00000237160		Pseudoger	chr7:95348718-9534
ENSG0000(	1054	26.36152	chr1:1522	TCEANC2		protein_c	chr1:54053584-5411
ENSG0000(	1054	26.36152	chr1:1522	ENSG00000279049		Pseudoger	chr1:54099968-5410
ENSG0000(	1054	26.36152	chr1:1522	MIR4422		smallRNA	chr1:55225641-5522
ENSG0000(	1054	26.36152	chr1:1522	PIGPP2		Pseudoger	chr1:62189131-6218
ENSG0000(	1054	26.36152	chr1:1522	LINC01748		lncRNA	chr1:60515716-6064
ENSG0000(	1054	26.36152	chr1:1522	PRKAA2		protein_c	chr1:56645314-5671
ENSG0000(	1054	26.36152	chr1:1522	C1orf141		protein_c	chr1:67092165-6723
ENSG0000(	1054	26.36152	chr1:1522	LINC02777		lncRNA	chr1:58882868-5893
ENSG0000(	1054	26.36152	chr1:1522	USP24		protein_c	chr1:55066359-5521
ENSG0000(	1054	26.36152	chr1:1522	AK4		protein_c	chr1:65147549-6523
ENSG0000(	1054	26.36152	chr1:1522	EFCAB7		protein_c	chr1:63523372-6357
ENSG0000(	1054	26.36152	chr1:1522	ACOT11		protein_c	chr1:54542257-5463
ENSG0000(	1054	26.36152	chr1:1522	JAK1	NCGv7;AC	protein_c	chr1:64833223-6506

ENSG0000(	1054	26.36152	chr1:1522	SLC35D1		protein_c	chr1:66999350-6705
ENSG0000(	1054	26.36152	chr1:1522	RAVER2		protein_c	chr1:64745075-6483
ENSG0000(	1054	26.36152	chr1:1522	LEPR	NCGv7	protein_c	chr1:65420652-6564
ENSG0000(	1054	26.36152	chr1:1522	PLPP3		protein_c	chr1:56494761-5664
ENSG0000(	1054	26.36152	chr1:1522	AL353898.2		Pseudoger	chr1:54033143-5403
ENSG0000(	1054	26.36152	chr1:1522	SLC1A7		protein_c	chr1:53087179-5314
ENSG0000(	1054	26.36152	chr1:1522	PDE4B-AS1		lncRNA	chr1:66042500-6605
ENSG0000(	1054	26.36152	chr1:1522	SSBP3		protein_c	chr1:54225433-5441
ENSG0000(	1054	26.36152	chr1:1522	AC119674.1		smallRNA	chr1:56377333-5637
ENSG0000(	1054	26.36152	chr1:1522	CDCP2		protein_c	chr1:54132687-5415
ENSG0000(	1054	26.36152	chr1:1522	BSND		protein_c	chr1:54998933-5501
ENSG0000(	1054	26.36152	chr1:1522	ENSG00000233216		Pseudoger	chr1:58228682-5822
ENSG0000(	1054	26.36152	chr1:1522	HNRNPA1P63		Pseudoger	chr1:54536796-5453
ENSG0000(	1054	26.36152	chr1:1522	CZIB		protein_c	chr1:53214099-5322
ENSG0000(	1054	26.36152	chr1:1522	AC099796.1		smallRNA	chr1:54504668-5450
ENSG0000(	1054	26.36152	chr1:1522	HNRNPA3P12		Pseudoger	chr1:53974969-5397
ENSG0000(	1054	26.36152	chr1:1522	RP4-758J24.5		lncRNA	chr1:54026683-5402
ENSG0000(	1054	26.36152	chr1:1522	INSL5		protein_c	chr1:66797740-6680
ENSG0000(	1054	26.36152	chr1:1522	MIR4794		smallRNA	chr1:64579847-6457
ENSG0000(	1054	26.36152	chr1:1522	AL049745.1		smallRNA	chr1:53828792-5382
ENSG0000(	1054	26.36152	chr1:1522	RNU7-123P		smallRNA	chr1:63536711-6353
ENSG0000(	1054	26.36152	chr1:1522	AL138847.1		protein_c	chr1:62607766-6260
ENSG0000(	1054	26.36152	chr1:1522	FAM151A		protein_c	chr1:54609181-5462
ENSG0000(	1054	26.36152	chr1:1522	MIR3671		smallRNA	chr1:65057755-6505
ENSG0000(	1054	26.36152	chr1:1522	ENSG00000272226		lncRNA	chr1:58812808-5881
ENSG0000(	1054	26.36152	chr1:1522	C1orf87		protein_c	chr1:59987269-6007
ENSG0000(	1054	26.36152	chr1:1522	NFIA		protein_c	chr1:60865259-6146
ENSG0000(	1054	26.36152	chr1:1522	LRP8-DT		lncRNA	chr1:53328233-5333
ENSG0000(	1054	26.36152	chr1:1522	ENSG00000290013		lncRNA	chr1:58784270-5878
ENSG0000(	1054	26.36152	chr1:1522	MRPL37		protein_c	chr1:54184041-5422
ENSG0000(	1054	26.36152	chr1:1522	RPS15AP7		Pseudoger	chr1:62190522-6219
ENSG0000(	1054	26.36152	chr1:1522	YIPF1		protein_c	chr1:53851719-5388
ENSG0000(	1054	26.36152	chr1:1522	LRRC42		protein_c	chr1:53946085-5396
ENSG0000(	1054	26.36152	chr1:1522	TM2D1		protein_c	chr1:61681046-6172
ENSG0000(	1054	26.36152	chr1:1522	RPS26P15		Pseudoger	chr1:58056133-5805
ENSG0000(	1054	26.36152	chr1:1522	CZIB-DT		lncRNA	chr1:53220663-5322
ENSG0000(	1054	26.36152	chr1:1522	AC096534.1		smallRNA	chr1:61083455-6108
ENSG0000(	1054	26.36152	chr1:1522	OMA1	NCGv7	protein_c	chr1:58415384-5854
ENSG0000(	1054	26.36152	chr1:1522	MYSM1		protein_c	chr1:58643440-5870
ENSG0000(	1054	26.36152	chr1:1522	COX6CP13		Pseudoger	chr1:65298755-6529
ENSG0000(	1054	26.36152	chr1:1522	RN7SL475P		smallRNA	chr1:59974759-5997
ENSG0000(	1054	26.36152	chr1:1522	LDLRAD1	NCGv7	protein_c	chr1:54007298-5401
ENSG0000(	1054	26.36152	chr1:1522	KANK4		protein_c	chr1:62236165-6231
ENSG0000(	1054	26.36152	chr1:1522	ENSG00000275678		lncRNA	chr1:67121605-6712
ENSG0000(	1054	26.36152	chr1:1522	ENSG00000272506		lncRNA	chr1:65003470-6500
ENSG0000(	1054	26.36152	chr1:1522	USP1		protein_c	chr1:62436297-6245
ENSG0000(	1054	26.36152	chr1:1522	ENSG00000227935		lncRNA	chr1:57386576-5738
ENSG0000(	1054	26.36152	chr1:1522	ANGPTL3		protein_c	chr1:62597520-6260
ENSG0000(	1054	26.36152	chr1:1522	IL23R		protein_c	chr1:67138907-6725
ENSG0000(	1054	26.36152	chr1:1522	TMEM59		protein_c	chr1:54026681-5405
ENSG0000(	1054	26.36152	chr1:1522	DOCK7		protein_c	chr1:62454298-6268

ENSG0000(	1054	26.36152	chr1:1522	MAGOH-DT		lncRNA	chr1:53238550-5324
ENSG0000(	1054	26.36152	chr1:1522	AC099791.1		smallRNA	chr1:61629031-6162
ENSG0000(	1054	26.36152	chr1:1522	PATJ	NCGv7	protein_c	chr1:61742477-6217
ENSG0000(	1054	26.36152	chr1:1522	C8B		protein_c	chr1:56929207-5697
ENSG0000(	1054	26.36152	chr1:1522	DLEU2L		lncRNA	chr1:63547082-6355
ENSG0000(	1054	26.36152	chr1:1522	NDC1		protein_c	chr1:53765478-5383
ENSG0000(	1054	26.36152	chr1:1522	MAGOH		protein_c	chr1:53226900-5323
ENSG0000(	1054	26.36152	chr1:1522	PATJ-DT		lncRNA	chr1:61741998-6174
ENSG0000(	1054	26.36152	chr1:1522	DAB1-AS1		lncRNA	chr1:57860532-5788
ENSG0000(	1054	26.36152	chr1:1522	RP5-1024G6.8		lncRNA	chr1:53242364-5324
ENSG0000(	1054	26.36152	chr1:1522	GOT2P1		Pseudoger	chr1:55367466-5536
ENSG0000(	1054	26.36152	chr1:1522	SGIP1		protein_c	chr1:66533267-6675
ENSG0000(	1054	26.36152	chr1:1522	ENSG00000277397		Pseudoger	chr1:53180921-5318
ENSG0000(	1054	26.36152	chr1:1522	SERBP1		protein_c	chr1:67407810-6743
ENSG0000(	1054	26.36152	chr1:1522	ITGB3BP		protein_c	chr1:63440770-6359
ENSG0000(	1054	26.36152	chr1:1522	LINC01359		lncRNA	chr1:64972225-6500
ENSG0000(	1054	26.36152	chr1:1522	ENSG00000290094		lncRNA	chr1:65310302-6531
ENSG0000(	1054	26.36152	chr1:1522	SNORA31		smallRNA	chr1:67102645-6710
ENSG0000(	1054	26.36152	chr1:1522	RN7SL713P		smallRNA	chr1:58565629-5856
ENSG0000(	1054	26.36152	chr1:1522	DHCR24		protein_c	chr1:54849627-5488
ENSG0000(	1054	26.36152	chr1:1522	MROH7-TTC4		protein_c	chr1:54641786-5474
ENSG0000(	1054	26.36152	chr1:1522	ENSG00000232993		lncRNA	chr1:53069938-5308
ENSG0000(	1054	26.36152	chr1:1522	ENSG00000233271		lncRNA	chr1:54980950-5499
ENSG0000(	1054	26.36152	chr1:1522	ENSG00000226938		lncRNA	chr1:53348488-5334
ENSG0000(	1054	26.36152	chr1:1522	AL136985.1		smallRNA	chr1:58764849-5876
ENSG0000(	1054	26.36152	chr1:1522	ENSG00000270209		Pseudoger	chr1:58552913-5855
ENSG0000(	1054	26.36152	chr1:1522	ATG4C		protein_c	chr1:62784132-6286
ENSG0000(	1054	26.36152	chr1:1522	MIR4781		smallRNA	chr1:54054079-5405
ENSG0000(	1054	26.36152	chr1:1522	ENSG00000226883		lncRNA	chr1:59754747-5978
ENSG0000(	1054	26.36152	chr1:1522	ENSG00000213703		Pseudoger	chr1:62641122-6264
ENSG0000(	1054	26.36152	chr1:1522	ENSG00000225475		Pseudoger	chr1:56619409-5661
ENSG0000(	1054	26.36152	chr1:1522	RPSAP65		Pseudoger	chr1:63262113-6326
ENSG0000(	1054	26.36152	chr1:1522	Y_RNA		smallRNA	chr1:63338263-6333
ENSG0000(	1054	26.36152	chr1:1522	LINC01755		lncRNA	chr1:55868254-5595
ENSG0000(	1054	26.36152	chr1:1522	DMRTB1		protein_c	chr1:53459399-5346
ENSG0000(	1054	26.36152	chr1:1522	RNU6-387P		smallRNA	chr1:67417214-6741
ENSG0000(	1054	26.36152	chr1:1522	ENSG00000270457		lncRNA	chr1:59289303-5928
ENSG0000(	1054	26.36152	chr1:1522	ENSG00000203605		lncRNA	chr1:63139250-6316
ENSG0000(	1054	26.36152	chr1:1522	ENSG00000228734		lncRNA	chr1:63249920-6325
ENSG0000(	1054	26.36152	chr1:1522	SNORD112		smallRNA	chr1:54525386-5452
ENSG0000(	1054	26.36152	chr1:1522	MTCO2P34		Pseudoger	chr1:55372710-5537
ENSG0000(	1054	26.36152	chr1:1522	TMEM61		protein_c	chr1:54980628-5499
ENSG0000(	1054	26.36152	chr1:1522	DYNLT5		protein_c	chr1:66752459-6677
ENSG0000(	1054	26.36152	chr1:1522	DNAI4		protein_c	chr1:66812885-6692
ENSG0000(	1054	26.36152	chr1:1522	PCSK9		protein_c	chr1:55039447-5506
ENSG0000(	1054	26.36152	chr1:1522	RNU4-88P		smallRNA	chr1:66094461-6609
ENSG0000(	1054	26.36152	chr1:1522	ENSG00000233877		Pseudoger	chr1:64941979-6494
ENSG0000(	1054	26.36152	chr1:1522	ENSG00000270549		Pseudoger	chr1:62530636-6253
ENSG0000(	1054	26.36152	chr1:1522	ENSG00000234784		Pseudoger	chr1:64918443-6491
ENSG0000(	1054	26.36152	chr1:1522	MIR4711		smallRNA	chr1:59733227-5973
ENSG0000(	1054	26.36152	chr1:1522	LINC01135		lncRNA	chr1:58784384-5890

ENSG0000(	1054	26.36152	chr1:1522	DHCR24-DT		lncRNA	chr1:54887563-5488
ENSG0000(	1054	26.36152	chr1:1522	RN7SL488P		smallRNA	chr1:63529617-6352
ENSG0000(	1054	26.36152	chr1:1522	ENSG00000234578		lncRNA	chr1:53267935-5326
ENSG0000(	1054	26.36152	chr1:1522	ENSG00000225632		lncRNA	chr1:54285404-5428
ENSG0000(	1054	26.36152	chr1:1522	CFL1P3		Pseudoger	chr1:63843196-6384
ENSG0000(	1054	26.36152	chr1:1522	DNAJB6P4		Pseudoger	chr1:67278052-6727
ENSG0000(	1054	26.36152	chr1:1522	PIGQP1		Pseudoger	chr1:55938714-5593
ENSG0000(	1054	26.36152	chr1:1522	CYP2J2		protein_c	chr1:59893308-5992
ENSG0000(	1054	26.36152	chr1:1522	C8A		protein_c	chr1:56854768-5691
ENSG0000(	1054	26.36152	chr1:1522	PARS2		protein_c	chr1:54756898-5476
ENSG0000(	1054	26.36152	chr1:1522	CPT2		protein_c	chr1:53196792-5321
ENSG0000(	1054	26.36152	chr1:1522	LRP8		protein_c	chr1:53242364-5332
ENSG0000(	1054	26.36152	chr1:1522	ENSG00000234318		lncRNA	chr1:62896009-6290
ENSG0000(	1054	26.36152	chr1:1522	MIR3116-1		smallRNA	chr1:62078786-6207
ENSG0000(	1054	26.36152	chr1:1522	CYB5RL		protein_c	chr1:54169651-5420
ENSG0000(	1054	26.36152	chr1:1522	TTC22		protein_c	chr1:54779712-5480
ENSG0000(	1054	26.36152	chr1:1522	Y_RNA		smallRNA	chr1:55484871-5548
ENSG0000(	1054	26.36152	chr1:1522	ENSG00000228838		lncRNA	chr1:53288024-5328
ENSG0000(	1054	26.36152	chr1:1522	PHB1P3		Pseudoger	chr1:58999676-5900
ENSG0000(	1054	26.36152	chr1:1522	ENSG00000260971		lncRNA	chr1:56154545-5647
ENSG0000(	1054	26.36152	chr1:1522	ENSG00000278967		TEC	chr1:62607766-6260
ENSG0000(	1054	26.36152	chr1:1522	ENSG00000232762		lncRNA	chr1:53304536-5330
ENSG0000(	1054	26.36152	chr1:1522	HIGD1AP11		Pseudoger	chr1:53073110-5307
ENSG0000(	1054	26.36152	chr1:1522	ENSG00000290536		lncRNA	chr1:58047889-5804
ENSG0000(	1054	26.36152	chr1:1522	ENSG00000234810		lncRNA	chr1:55329288-5607
ENSG0000(	1054	26.36152	chr1:1522	ENSG00000232245		lncRNA	chr1:54416256-5442
ENSG0000(	1054	26.36152	chr1:1522	MRPS21P1		Pseudoger	chr1:65092392-6509
ENSG0000(	1054	26.36152	chr1:1522	RNU6-586P		smallRNA	chr1:67196140-6719
ENSG0000(	1054	26.36152	chr1:1522	MIR3117		smallRNA	chr1:66628440-6662
ENSG0000(	1054	26.36152	chr1:1522	ENSG00000256407		protein_c	chr1:54132686-5420
ENSG0000(	1054	26.36152	chr1:1522	LEPROT		protein_c	chr1:65420587-6543
ENSG0000(	1054	26.36152	chr1:1522	RNA5SP49		Pseudoger	chr1:63186336-6318
ENSG0000(	1054	26.36152	chr1:1522	CACHD1	NCGv7	protein_c	chr1:64470129-6469
ENSG0000(	1054	26.36152	chr1:1522	ENSG00000227485		lncRNA	chr1:63024207-6302
ENSG0000(	1054	26.36152	chr1:1522	RNU6-830P		smallRNA	chr1:55398514-5539
ENSG0000(	1054	26.36152	chr1:1522	HOOK1		protein_c	chr1:59814786-5987
ENSG0000(	1054	26.36152	chr1:1522	RNU4ATAC4P		smallRNA	chr1:67267601-6726
ENSG0000(	1054	26.36152	chr1:1522	LEXM		protein_c	chr1:54806063-5484
ENSG0000(	1054	26.36152	chr1:1522	DNAJC6		protein_c	chr1:65248219-6541
ENSG0000(	1054	26.36152	chr1:1522	FGGY		protein_c	chr1:59296638-5981
ENSG0000(	1054	26.36152	chr1:1522	ENSG00000235038		lncRNA	chr1:58060139-5808
ENSG0000(	1054	26.36152	chr1:1522	AL137855.1		smallRNA	chr1:57757084-5775
ENSG0000(	1054	26.36152	chr1:1522	ENSG00000225183		Pseudoger	chr1:54089856-5409
ENSG0000(	1054	26.36152	chr1:1522	ENSG00000287224		lncRNA	chr1:61588049-6158
ENSG0000(	1054	26.36152	chr1:1522	snoU13		smallRNA	chr1:65571549-6557
ENSG0000(	1054	26.36152	chr1:1522	RNU7-95P		smallRNA	chr1:53688749-5368
ENSG0000(	1054	26.36152	chr1:1522	ENSG00000235804		Pseudoger	chr1:65077413-6507
ENSG0000(	1054	26.36152	chr1:1522	FOXD3-AS1		lncRNA	chr1:63320878-6332
ENSG0000(	1054	26.36152	chr1:1522	GLIS1		protein_c	chr1:53506237-5373
ENSG0000(	1054	26.36152	chr1:1522	ENSG00000286918		lncRNA	chr1:58546168-5856
ENSG0000(	1054	26.36152	chr1:1522	RN7SL854P		smallRNA	chr1:65761060-6576

ENSG0000(	1054	26.36152	chr1:152:	ENSG00000230728		lncRNA	chr1:54621477-5462
ENSG0000(	1054	26.36152	chr1:152:	ENSG00000224570		Pseudoger	chr1:65576129-6557
ENSG0000(	1054	26.36152	chr1:152:	Y_RNA		smallRNA	chr1:58722279-5872
ENSG0000(	1054	26.36152	chr1:152:	FYB2	NCGv7	protein_c	chr1:56718789-5681
ENSG0000(	1054	26.36152	chr1:152:	FGGY-DT		lncRNA	chr1:59131932-5929
ENSG0000(	1054	26.36152	chr1:152:	LINC02784		lncRNA	chr1:54516412-5451
ENSG0000(	1054	26.36152	chr1:152:	FOXD3		protein_c	chr1:63322567-6332
ENSG0000(	1054	26.36152	chr1:152:	RPS29P7		Pseudoger	chr1:65154480-6515
ENSG0000(	1054	26.36152	chr1:152:	L1TD1		protein_c	chr1:62194849-6221
ENSG0000(	1054	26.36152	chr1:152:	ENSG00000286455		lncRNA	chr1:63011197-6301
ENSG0000(	1054	26.36152	chr1:152:	ENSG00000286429		lncRNA	chr1:63487957-6350
ENSG0000(	1054	26.36152	chr1:152:	RNU6-1031P		smallRNA	chr1:67541127-6754
ENSG0000(	1054	26.36152	chr1:152:	RNU7-62P		smallRNA	chr1:64384398-6438
ENSG0000(	1054	26.36152	chr1:152:	ENSG00000223920		Pseudoger	chr1:61654194-6165
ENSG0000(	1054	26.36152	chr1:152:	NFIA-AS1		lncRNA	chr1:61248945-6125
ENSG0000(	1054	26.36152	chr1:152:	IL12RB2		protein_c	chr1:67307364-6739
ENSG0000(	1054	26.36152	chr1:152:	ENSG00000237453		lncRNA	chr1:54792885-5479
ENSG0000(	1054	26.36152	chr1:152:	LINC00466		lncRNA	chr1:63159083-6331
ENSG0000(	1054	26.36152	chr1:152:	AL353898.3		Pseudoger	chr1:54099968-5410
ENSG0000(	1054	26.36152	chr1:152:	MIER1		protein_c	chr1:66924895-6698
ENSG0000(	1054	26.36152	chr1:152:	LINC01767		lncRNA	chr1:56414918-5641
ENSG0000(	1054	26.36152	chr1:152:	ENSG00000287724		lncRNA	chr1:54874672-5488
ENSG0000(	1054	26.36152	chr1:152:	DAB1	NCGv7	protein_c	chr1:56994778-5854
ENSG0000(	1054	26.36152	chr1:152:	ENSG00000237852		lncRNA	chr1:65486406-6549
ENSG0000(	1054	26.36152	chr1:152:	ROR1-AS1		lncRNA	chr1:64094379-6417
ENSG0000(	1054	26.36152	chr1:152:	AL353898.1		Pseudoger	chr1:54015654-5401
ENSG0000(	1054	26.36152	chr1:152:	NFIA-AS2		lncRNA	chr1:60912675-6105
ENSG0000(	1054	26.36152	chr1:152:	GYG1P3		Pseudoger	chr1:55222379-5522
ENSG0000(	1054	26.36152	chr1:152:	ALG6		protein_c	chr1:63367575-6343
ENSG0000(	1054	26.36152	chr1:152:	MIR4422HG		lncRNA	chr1:55217645-5532
ENSG0000(	1054	26.36152	chr1:152:	RNU6-371P		smallRNA	chr1:62298149-6229
ENSG0000(	1054	26.36152	chr1:152:	ENSG00000231080		lncRNA	chr1:66826942-6682
ENSG0000(	1054	26.36152	chr1:152:	ENSG00000238139		Pseudoger	chr1:67561073-6756
ENSG0000(	1054	26.36152	chr1:152:	ENSG00000287582		lncRNA	chr1:54514417-5451
ENSG0000(	1054	26.36152	chr1:152:	LINC02778		lncRNA	chr1:60114875-6014
ENSG0000(	1054	26.36152	chr1:152:	Y_RNA		smallRNA	chr1:64066640-6406
ENSG0000(	1054	26.36152	chr1:152:	ENSG00000241042		lncRNA	chr1:59054397-5905
ENSG0000(	1054	26.36152	chr1:152:	ENSG00000185839		Pseudoger	chr1:58630841-5863
ENSG0000(	1054	26.36152	chr1:152:	LINC01739		lncRNA	chr1:62975751-6302
ENSG0000(	1054	26.36152	chr1:152:	RPL23AP85		Pseudoger	chr1:56585612-5658
ENSG0000(	1054	26.36152	chr1:152:	ENSG00000284601		lncRNA	chr1:54974900-5498
ENSG0000(	1054	26.36152	chr1:152:	ENSG00000229687		Pseudoger	chr1:53841547-5384
ENSG0000(	1054	26.36152	chr1:152:	RNU6-1176P		smallRNA	chr1:65022968-6502
ENSG0000(	1054	26.36152	chr1:152:	ENSG00000231740		lncRNA	chr1:58838448-5885
ENSG0000(	1054	26.36152	chr1:152:	ENSG00000289394		lncRNA	chr1:66925327-6692
ENSG0000(	1054	26.36152	chr1:152:	RN7SL130P		smallRNA	chr1:63655743-6365
ENSG0000(	1054	26.36152	chr1:152:	ENSG00000283445		lncRNA	chr1:58715609-5877
ENSG0000(	1054	26.36152	chr1:152:	RNU6-1177P		smallRNA	chr1:61852499-6185
ENSG0000(	1054	26.36152	chr1:152:	PGBD4P8		Pseudoger	chr1:60097415-6009
ENSG0000(	1054	26.36152	chr1:152:	ENSG00000284686		protein_c	chr1:56173433-5652
ENSG0000(	1054	26.36152	chr1:152:	ENSG00000248458		lncRNA	chr1:66665864-6667

ENSG0000(	1054	26.36152	chr1:1522	ENSG00000229294		lncRNA	chr1:65279456-6530
ENSG0000(	1054	26.36152	chr1:1522	ENSG00000280425		lncRNA	chr1:54137746-5414
ENSG0000(	1054	26.36152	chr1:1522	ENSG00000280378		TEC	chr1:54033126-5403
ENSG0000(	1054	26.36152	chr1:1522	JUN	NCGv7;AC	protein_c	chr1:58776845-5878
ENSG0000(	1054	26.36152	chr1:1522	ENSG00000177452		Pseudoger	chr1:63788721-6378
ENSG0000(	1054	26.36152	chr1:1522	UBE2U		protein_c	chr1:64203623-6426
ENSG0000(	1054	26.36152	chr1:1522	AL357673.1		protein_c	chr1:54169660-5417
ENSG0000(	1054	26.36152	chr1:1522	AL354978.1		smallRNA	chr1:66730967-6673
ENSG0000(	1054	26.36152	chr1:1522	TTC4	NCGv7	protein_c	chr1:54715861-5474
ENSG0000(	1054	26.36152	chr1:1522	DOCK7-DT		lncRNA	chr1:62688482-6271
ENSG0000(	1054	26.36152	chr1:1522	LINC01753		lncRNA	chr1:55915603-5594
ENSG0000(	1054	26.36152	chr1:1522	TACSTD2	AC	protein_c	chr1:58575433-5857
ENSG0000(	1054	26.36152	chr1:1522	ROR1		protein_c	chr1:63774017-6418
ENSG0000(	1054	26.36152	chr1:1522	SLC2A3P2		Pseudoger	chr1:64984608-6498
ENSG0000(	1054	26.36152	chr1:1522	LINC02812		lncRNA	chr1:53366656-5336
ENSG0000(	1054	26.36152	chr1:1522	ENSG00000285954		lncRNA	chr1:53344031-5336
ENSG0000(	1054	26.36152	chr1:1522	DIO1		protein_c	chr1:53891239-5391
ENSG0000(	1054	26.36152	chr1:1522	PDE4B		protein_c	chr1:65792514-6637
ENSG0000(	1054	26.36152	chr1:1522	RNU6-414P		smallRNA	chr1:61816419-6181
ENSG0000(	1054	26.36152	chr1:1522	ENSG00000235612		lncRNA	chr1:56145721-5615
ENSG0000(	1054	26.36152	chr1:1522	MROH7		protein_c	chr1:54641754-5471
ENSG0000(	1054	26.36152	chr1:1522	ENSG00000242396		lncRNA	chr1:54886812-5497
ENSG0000(	1054	26.36152	chr1:1522	ENSG00000284808		lncRNA	chr1:61481087-6153
ENSG0000(	1054	26.36152	chr1:1522	LINC01702		lncRNA	chr1:67522299-6753
ENSG0000(	1054	26.36152	chr1:1522	ENSG00000235215		lncRNA	chr1:59055999-5907
ENSG0000(	1054	26.36152	chr1:1522	ENSG00000229913		lncRNA	chr1:56823679-5682
ENSG0000(	1054	26.36152	chr1:1522	HNRNPA1P6		Pseudoger	chr1:58048175-5804
ENSG0000(	1054	26.36152	chr1:1522	RPSAP20		Pseudoger	chr1:56207567-5620
ENSG0000(	1054	26.36152	chr1:1522	RN7SL180P		smallRNA	chr1:62072448-6207
ENSG0000(	1054	26.36152	chr1:1522	ENSG00000235563		lncRNA	chr1:53114576-5311
ENSG0000(	1054	26.36152	chr1:1522	ENSG00000285079		lncRNA	chr1:65703962-6571
ENSG0000(	1054	26.36152	chr1:1522	ENSG00000284928		lncRNA	chr1:64186791-6419
ENSG0000(	1054	26.36152	chr1:1522	HSPB11		protein_c	chr1:53916574-5394
ENSG0000(	1054	26.36152	chr1:1522	ENSG00000229225		lncRNA	chr1:63078081-6307
ENSG0000(	1054	26.36152	chr1:1522	RNU6-809P		smallRNA	chr1:64028894-6402
ENSG0000(	1054	26.36152	chr1:1522	ENSG00000237163		Pseudoger	chr1:62905180-6290
ENSG0000(	1054	26.36152	chr1:1522	ENSG00000288527		Pseudoger	chr1:53441268-5344
ENSG0000(	1054	26.36152	chr1:1522	PGM1		protein_c	chr1:63593411-6366
ENSG0000(	1054	26.36152	chr1:1522	AL161740.1		smallRNA	chr1:56966280-5696
ENSG0000(	1054	26.36152	chr1:1522	LINC01358		lncRNA	chr1:58933643-5924
ENSG0000(	1054	26.36152	chr1:1522	SSBP3-AS1		lncRNA	chr1:54236440-5423
ENSG0000(	1054	26.36152	chr1:1522	RP4-535B20.1		lncRNA	chr1:65066627-6506
ENSG0000(	1054	26.36152	chr1:1522	RPL21P23		Pseudoger	chr1:56538452-5653
ENSG0000(	1054	26.36152	chr1:1522	ENSG00000288804		lncRNA	chr1:65067808-6506
ENSG0000(	1054	26.36152	chr1:1522	RPS20P5		Pseudoger	chr1:57605847-5760
ENSG0000(	1054	26.36152	chr1:1522	MIR101-1		smallRNA	chr1:65058434-6505
ENSG0000(	1054	26.36152	chr1:1522	ENSG00000236341		lncRNA	chr1:56963886-5699
ENSG0000(	1054	26.36152	chr1:1522	Y_RNA		smallRNA	chr1:62211557-6221
ENSG0000(	1054	26.36152	chr1:1522	RN7SKP291		smallRNA	chr1:55376526-5537
ENSG0000(	1054	26.36152	chr1:1522	ENSG00000236674		Pseudoger	chr1:63359823-6336
ENSG0000(	1054	26.36152	chr1:1522	RNU2-15P		smallRNA	chr1:65415816-6541

ENSG0000(	1054	26.36152	chr1:1522	ENSG00000237173		Pseudoger	chr1:54524824-5452
ENSG0000(	1054	26.36152	chr1:1522	SLC25A3P1		Pseudoger	chr1:53413149-5344
ENSG0000(	1054	26.36152	chr1:1522	LAMTOR5P1		Pseudoger	chr1:62038842-6203
ENSG0000(	1054	26.36152	chr1:1522	ENSG00000237227		Pseudoger	chr1:62208136-6220
ENSG0000(	1054	26.36152	chr1:1522	ENSG00000231252		lncRNA	chr1:60659631-6086
ENSG0000(	1054	26.36152	chr1:1522	ENSG00000236723		lncRNA	chr1:53209783-5321
ENSG0000(	1051	26.28649	chr1:8137	MIR553		smallRNA	chr1:100281241-100
ENSG0000(	1051	26.28649	chr1:8137	AC104457.1		smallRNA	chr1:100378682-100
ENSG0000(	1051	26.28649	chr1:8137	DPYD-AS2		lncRNA	chr1:97796921-9779
ENSG0000(	1051	26.28649	chr1:8137	RN7SL692P		smallRNA	chr1:92974829-9297
ENSG0000(	1051	26.28649	chr1:8137	RTCA-AS1		lncRNA	chr1:100251528-100
ENSG0000(	1051	26.28649	chr1:8137	ENSG00000233482		lncRNA	chr1:94145111-9414
ENSG0000(	1051	26.28649	chr1:8137	CCDC18-AS1		lncRNA	chr1:93262186-9334
ENSG0000(	1051	26.28649	chr1:8137	RPL36AP11		Pseudoger	chr1:93190740-9319
ENSG0000(	1051	26.28649	chr1:8137	ARHGAP29-AS1		lncRNA	chr1:94247819-9441
ENSG0000(	1051	26.28649	chr1:8137	SLC44A3	NCGv7	protein_c	chr1:94820342-9489
ENSG0000(	1051	26.28649	chr1:8137	LINC01650		lncRNA	chr1:95351251-9535
ENSG0000(	1051	26.28649	chr1:8137	ALG14-AS1		lncRNA	chr1:95061596-9506
ENSG0000(	1051	26.28649	chr1:8137	NFU1P2		Pseudoger	chr1:98077000-9807
ENSG0000(	1051	26.28649	chr1:8137	FRRS1		protein_c	chr1:99703970-9976
ENSG0000(	1051	26.28649	chr1:8137	MFSD14A		protein_c	chr1:100038095-100
ENSG0000(	1051	26.28649	chr1:8137	NDUFS5P2		Pseudoger	chr1:96584422-9658
ENSG0000(	1051	26.28649	chr1:8137	ENSG00000225923		Pseudoger	chr1:96390652-9639
ENSG0000(	1051	26.28649	chr1:8137	AL356479.1		smallRNA	chr1:95504976-9550
ENSG0000(	1051	26.28649	chr1:8137	CNN3-DT		lncRNA	chr1:94927361-9496
ENSG0000(	1051	26.28649	chr1:8137	ENSG00000229567		Pseudoger	chr1:93278961-9327
ENSG0000(	1051	26.28649	chr1:8137	SEC63P1		Pseudoger	chr1:97545701-9754
ENSG0000(	1051	26.28649	chr1:8137	BCAS2P2		Pseudoger	chr1:100393033-100
ENSG0000(	1051	26.28649	chr1:8137	ENSG00000223675		lncRNA	chr1:94585556-9459
ENSG0000(	1051	26.28649	chr1:8137	ENSG00000227034		Pseudoger	chr1:99008218-9900
ENSG0000(	1051	26.28649	chr1:8137	SNORA51		smallRNA	chr1:92846059-9284
ENSG0000(	1051	26.28649	chr1:8137	ENSG00000229052		Pseudoger	chr1:92930696-9293
ENSG0000(	1051	26.28649	chr1:8137	ENSG00000229635		Pseudoger	chr1:93384487-9338
ENSG0000(	1051	26.28649	chr1:8137	DPYD	NCGv7	protein_c	chr1:97077743-9799
ENSG0000(	1051	26.28649	chr1:8137	RN7SKP270		smallRNA	chr1:96695856-9669
ENSG0000(	1051	26.28649	chr1:8137	MTND3P21		Pseudoger	chr1:93924386-9392
ENSG0000(	1051	26.28649	chr1:8137	HMGB3P10		Pseudoger	chr1:99698242-9969
ENSG0000(	1051	26.28649	chr1:8137	ENSG00000288810		lncRNA	chr1:98047173-9804
ENSG0000(	1051	26.28649	chr1:8137	ENSG00000260464		lncRNA	chr1:93847174-9384
ENSG0000(	1051	26.28649	chr1:8137	ENSG00000228852		lncRNA	chr1:95243167-9527
ENSG0000(	1051	26.28649	chr1:8137	ENSG00000230718		Pseudoger	chr1:97774669-9777
ENSG0000(	1051	26.28649	chr1:8137	DIPK1A		protein_c	chr1:92832737-9296
ENSG0000(	1051	26.28649	chr1:8137	MTCO1P21		Pseudoger	chr1:93927714-9392
ENSG0000(	1051	26.28649	chr1:8137	RNU6-750P		smallRNA	chr1:99978939-9997
ENSG0000(	1051	26.28649	chr1:8137	ENSG00000226952		Pseudoger	chr1:100099239-100
ENSG0000(	1051	26.28649	chr1:8137	ENSG00000283761		protein_c	chr1:99970011-1000
ENSG0000(	1051	26.28649	chr1:8137	ENSG00000286692		lncRNA	chr1:94417743-9441
ENSG0000(	1051	26.28649	chr1:8137	SASS6		protein_c	chr1:100083563-100
ENSG0000(	1051	26.28649	chr1:8137	MTND4P11		Pseudoger	chr1:93922574-9392
ENSG0000(	1051	26.28649	chr1:8137	MTF2		protein_c	chr1:93079235-9313
ENSG0000(	1051	26.28649	chr1:8137	ENSG00000231992		lncRNA	chr1:95120147-9513

ENSG0000(	1051	26.28649	chr1:8137	CCDC18		protein_c	chr1:93179919-9327
ENSG0000(	1051	26.28649	chr1:8137	SNX7	NCGv7	protein_c	chr1:98661701-9876
ENSG0000(	1051	26.28649	chr1:8137	RNU6-1318P		smallRNA	chr1:100000637-100
ENSG0000(	1051	26.28649	chr1:8137	Y_RNA		smallRNA	chr1:99791662-9979
ENSG0000(	1051	26.28649	chr1:8137	LINC01761		lncRNA	chr1:95474737-9547
ENSG0000(	1051	26.28649	chr1:8137	MTCO2P21		Pseudoger	chr1:93926615-9392
ENSG0000(	1051	26.28649	chr1:8137	LINC01776		lncRNA	chr1:98210747-9827
ENSG0000(	1051	26.28649	chr1:8137	AC092812.1		smallRNA	chr1:95886540-9588
ENSG0000(	1051	26.28649	chr1:8137	ALG14		protein_c	chr1:94974405-9507
ENSG0000(	1051	26.28649	chr1:8137	ENSG00000285530		lncRNA	chr1:100220488-100
ENSG0000(	1051	26.28649	chr1:8137	ENSG00000231996		Pseudoger	chr1:99842610-9984
ENSG0000(	1051	26.28649	chr1:8137	ENSG00000241073		lncRNA	chr1:100057990-100
ENSG0000(	1051	26.28649	chr1:8137	MTATP6P13		Pseudoger	chr1:93925406-9392
ENSG0000(	1051	26.28649	chr1:8137	DPYD-IT1		lncRNA	chr1:97394154-9742
ENSG0000(	1051	26.28649	chr1:8137	SNORA66		smallRNA	chr1:92838018-9283
ENSG0000(	1051	26.28649	chr1:8137	AL592205.1		smallRNA	chr1:96902699-9690
ENSG0000(	1051	26.28649	chr1:8137	ENSG00000289544		lncRNA	chr1:92961858-9296
ENSG0000(	1051	26.28649	chr1:8137	ENSG00000233983		Pseudoger	chr1:99464378-9946
ENSG0000(	1051	26.28649	chr1:8137	ENSG00000288736		lncRNA	chr1:94541937-9455
ENSG0000(	1051	26.28649	chr1:8137	AL160056.1		smallRNA	chr1:98373385-9837
ENSG0000(	1051	26.28649	chr1:8137	Y_RNA		smallRNA	chr1:93027410-9302
ENSG0000(	1051	26.28649	chr1:8137	RPL26P9		Pseudoger	chr1:97585862-9758
ENSG0000(	1051	26.28649	chr1:8137	RWDD3		protein_c	chr1:95234210-9524
ENSG0000(	1051	26.28649	chr1:8137	MIR760		smallRNA	chr1:93846832-9384
ENSG0000(	1051	26.28649	chr1:8137	LRRC39		protein_c	chr1:100148448-100
ENSG0000(	1051	26.28649	chr1:8137	TRMT13		protein_c	chr1:100133150-100
ENSG0000(	1051	26.28649	chr1:8137	ENSG00000270911		Pseudoger	chr1:97855575-9785
ENSG0000(	1051	26.28649	chr1:8137	ENSG00000230287		Pseudoger	chr1:100249090-100
ENSG0000(	1051	26.28649	chr1:8137	GPR88		protein_c	chr1:100538139-100
ENSG0000(	1051	26.28649	chr1:8137	ENSG00000228084		lncRNA	chr1:99968382-9996
ENSG0000(	1051	26.28649	chr1:8137	TLCD4-RWDD3		protein_c	chr1:95117923-9524
ENSG0000(	1051	26.28649	chr1:8137	SNORD21		smallRNA	chr1:92837289-9283
ENSG0000(	1051	26.28649	chr1:8137	RNU4-75P		smallRNA	chr1:99784740-9978
ENSG0000(	1051	26.28649	chr1:8137	RWDD3-DT		lncRNA	chr1:95161676-9523
ENSG0000(	1051	26.28649	chr1:8137	GCLM		protein_c	chr1:93885199-9390
ENSG0000(	1051	26.28649	chr1:8137	ENSG00000271252		lncRNA	chr1:95743096-9575
ENSG0000(	1051	26.28649	chr1:8137	RN7SL831P		smallRNA	chr1:96583209-9658
ENSG0000(	1051	26.28649	chr1:8137	LINC01787		lncRNA	chr1:96254069-9637
ENSG0000(	1051	26.28649	chr1:8137	AGL		protein_c	chr1:99850361-9992
ENSG0000(	1051	26.28649	chr1:8137	ENSG00000285922		lncRNA	chr1:98052077-9805
ENSG0000(	1051	26.28649	chr1:8137	LINC01760		lncRNA	chr1:95310928-9531
ENSG0000(	1051	26.28649	chr1:8137	EEF1A1P11		Pseudoger	chr1:96446930-9644
ENSG0000(	1051	26.28649	chr1:8137	RPL23AP90		Pseudoger	chr1:100196816-100
ENSG0000(	1051	26.28649	chr1:8137	BCAR3-AS1		lncRNA	chr1:93591966-9361
ENSG0000(	1051	26.28649	chr1:8137	RPL5	NCGv7;AC	protein_c	chr1:92832013-9284
ENSG0000(	1051	26.28649	chr1:8137	DNTTIP2		protein_c	chr1:93866284-9387
ENSG0000(	1051	26.28649	chr1:8137	SLC44A3-AS1		Pseudoger	chr1:94613814-9485
ENSG0000(	1051	26.28649	chr1:8137	PALMD		protein_c	chr1:99646113-9969
ENSG0000(	1051	26.28649	chr1:8137	ENSG00000280040		TEC	chr1:98660388-9866
ENSG0000(	1051	26.28649	chr1:8137	ENSG00000228086		lncRNA	chr1:100462399-100
ENSG0000(	1051	26.28649	chr1:8137	RN7SL440P		smallRNA	chr1:94150738-9415

ENSG0000(	1051	26.28649	chr1:8137	MTCO3P21		Pseudoger	chr1:93924743-9392
ENSG0000(	1051	26.28649	chr1:8137	ENSG00000223906		lncRNA	chr1:100344477-100
ENSG0000(	1051	26.28649	chr1:8137	LINC01708		lncRNA	chr1:99472332-9960
ENSG0000(	1051	26.28649	chr1:8137	RNU6-970P		smallRNA	chr1:92969604-9296
ENSG0000(	1051	26.28649	chr1:8137	ENSG00000233129		Pseudoger	chr1:93934479-9393
ENSG0000(	1051	26.28649	chr1:8137	ENSG00000259946		lncRNA	chr1:97967005-9796
ENSG0000(	1051	26.28649	chr1:8137	ENSG00000287797		lncRNA	chr1:92978265-9298
ENSG0000(	1051	26.28649	chr1:8137	GAPDHP29		Pseudoger	chr1:94302038-9430
ENSG0000(	1051	26.28649	chr1:8137	RNU1-130P		smallRNA	chr1:96225901-9622
ENSG0000(	1051	26.28649	chr1:8137	PLPPR4		protein_c	chr1:99264292-9930
ENSG0000(	1051	26.28649	chr1:8137	CHCHD2P5		Pseudoger	chr1:93921268-9392
ENSG0000(	1051	26.28649	chr1:8137	UBE2WP1		Pseudoger	chr1:96418594-9641
ENSG0000(	1051	26.28649	chr1:8137	ENSG00000250890		Pseudoger	chr1:93926032-9392
ENSG0000(	1051	26.28649	chr1:8137	SLC35A3		protein_c	chr1:99969351-1000
ENSG0000(	1051	26.28649	chr1:8137	PLPPR5-AS1		lncRNA	chr1:99004276-9924
ENSG0000(	1051	26.28649	chr1:8137	AL451051.1		smallRNA	chr1:99829465-9982
ENSG0000(	1051	26.28649	chr1:8137	snoU13		smallRNA	chr1:94151418-9415
ENSG0000(	1051	26.28649	chr1:8137	LINC02607		lncRNA	chr1:95510059-9578
ENSG0000(	1051	26.28649	chr1:8137	RNU6-210P		smallRNA	chr1:93010257-9301
ENSG0000(	1051	26.28649	chr1:8137	KATNBL1P2		Pseudoger	chr1:94650544-9465
ENSG0000(	1051	26.28649	chr1:8137	CDC14A		protein_c	chr1:100345001-100
ENSG0000(	1051	26.28649	chr1:8137	AL592205.2		smallRNA	chr1:96935545-9693
ENSG0000(	1051	26.28649	chr1:8137	MIR137HG		lncRNA	chr1:97933474-9804
ENSG0000(	1051	26.28649	chr1:8137	Y_RNA		smallRNA	chr1:93385711-9338
ENSG0000(	1051	26.28649	chr1:8137	ENSG00000232918		Pseudoger	chr1:94406395-9440
ENSG0000(	1051	26.28649	chr1:8137	ENSG00000236098		lncRNA	chr1:94318479-9432
ENSG0000(	1051	26.28649	chr1:8137	ENSG00000237954		lncRNA	chr1:95356229-9538
ENSG0000(	1051	26.28649	chr1:8137	ENSG00000215871		Pseudoger	chr1:100331804-100
ENSG0000(	1051	26.28649	chr1:8137	RN7SKP123		smallRNA	chr1:93026252-9302
ENSG0000(	1051	26.28649	chr1:8137	PTBP2		protein_c	chr1:96721665-9682
ENSG0000(	1051	26.28649	chr1:8137	PLPPR5		protein_c	chr1:98890245-9922
ENSG0000(	1051	26.28649	chr1:8137	ENSG00000287919		lncRNA	chr1:95282233-9528
ENSG0000(	1051	26.28649	chr1:8137	AC093577.1		smallRNA	chr1:92982237-9298
ENSG0000(	1051	26.28649	chr1:8137	SNORA66		smallRNA	chr1:92840719-9284
ENSG0000(	1051	26.28649	chr1:8137	RTCA	NCGv7	protein_c	chr1:100266216-100
ENSG0000(	1051	26.28649	chr1:8137	DBT		protein_c	chr1:100186919-100
ENSG0000(	1051	26.28649	chr1:8137	ABCA4		protein_c	chr1:93992834-9412
ENSG0000(	1051	26.28649	chr1:8137	ENSG00000288826		lncRNA	chr1:100036632-100
ENSG0000(	1051	26.28649	chr1:8137	ENSG00000225297		Pseudoger	chr1:93199755-9319
ENSG0000(	1051	26.28649	chr1:8137	RPL7P9		Pseudoger	chr1:96678874-9667
ENSG0000(	1051	26.28649	chr1:8137	MIR378G		smallRNA	chr1:94745860-9474
ENSG0000(	1051	26.28649	chr1:8137	TMED5		protein_c	chr1:93149742-9318
ENSG0000(	1051	26.28649	chr1:8137	ARHGAP29		protein_c	chr1:94148988-9427
ENSG0000(	1051	26.28649	chr1:8137	DR1		protein_c	chr1:93345907-9336
ENSG0000(	1051	26.28649	chr1:8137	CNN3		protein_c	chr1:94896949-9492
ENSG0000(	1051	26.28649	chr1:8137	TLCD4		protein_c	chr1:95117355-9519
ENSG0000(	1051	26.28649	chr1:8137	BCAR3		protein_c	chr1:93561741-9384
ENSG0000(	1051	26.28649	chr1:8137	FNBP1L	NCGv7	protein_c	chr1:93448118-9355
ENSG0000(	1051	26.28649	chr1:8137	LINC02790		lncRNA	chr1:95937901-9602
ENSG0000(	1051	26.28649	chr1:8137	RNA5SP53		Pseudoger	chr1:93488333-9348
ENSG0000(	1051	26.28649	chr1:8137	ABCD3		protein_c	chr1:94418389-9451

ENSG0000C	1051	26.28649	chr1:8137	DPYD-AS1		lncRNA	chr1:97095923-9732
ENSG0000C	1051	26.28649	chr1:8137	F3		protein_c	chr1:94529173-9454
ENSG0000C	1048	26.21145	chr1:1522	EPS15	NCGv7;AC	protein_c	chr1:51354263-5151
ENSG0000C	1048	26.21145	chr1:1522	RNU6-1026P		smallRNA	chr1:50582404-5058
ENSG0000C	1048	26.21145	chr1:1522	ENSG00000232027		Pseudoger	chr1:51372270-5137
ENSG0000C	1048	26.21145	chr1:1522	FAF1		protein_c	chr1:50437028-5096
ENSG0000C	1048	26.21145	chr1:1522	Y_RNA		smallRNA	chr1:51107222-5110
ENSG0000C	1048	26.21145	chr1:1522	ENSG00000284700		lncRNA	chr1:50423609-5042
ENSG0000C	1048	26.21145	chr1:1522	TXNDC12		protein_c	chr1:52020131-5205
ENSG0000C	1048	26.21145	chr1:1522	CFL1P2		Pseudoger	chr1:51157788-5115
ENSG0000C	1048	26.21145	chr1:1522	Y_RNA		smallRNA	chr1:50499758-5049
ENSG0000C	1048	26.21145	chr1:1522	ENSG00000223390		lncRNA	chr1:52033391-5204
ENSG0000C	1048	26.21145	chr1:1522	ENSG00000272175		lncRNA	chr1:51801028-5180
ENSG0000C	1048	26.21145	chr1:1522	CALR4P		Pseudoger	chr1:51561866-5159
ENSG0000C	1048	26.21145	chr1:1522	OSBPL9		protein_c	chr1:51577179-5179
ENSG0000C	1048	26.21145	chr1:1522	ENSG00000290102		lncRNA	chr1:50967883-5096
ENSG0000C	1048	26.21145	chr1:1522	RNA5SP48		Pseudoger	chr1:51973410-5197
ENSG0000C	1048	26.21145	chr1:1522	AL589663.1		smallRNA	chr1:51900920-5190
ENSG0000C	1048	26.21145	chr1:1522	TTC39A-AS1		lncRNA	chr1:51329654-5133
ENSG0000C	1048	26.21145	chr1:1522	CDKN2C	NCGv7;AC	protein_c	chr1:50960745-5097
ENSG0000C	1048	26.21145	chr1:1522	TTC39A		protein_c	chr1:51287258-5134
ENSG0000C	1048	26.21145	chr1:1522	GAPDHP51		Pseudoger	chr1:51707138-5170
ENSG0000C	1048	26.21145	chr1:1522	SLC25A6P3		Pseudoger	chr1:51709062-5170
ENSG0000C	1048	26.21145	chr1:1522	MIR4421		smallRNA	chr1:51059837-5105
ENSG0000C	1048	26.21145	chr1:1522	MIR761		smallRNA	chr1:51836341-5183
ENSG0000C	1048	26.21145	chr1:1522	Y_RNA		smallRNA	chr1:51865633-5186
ENSG0000C	1048	26.21145	chr1:1522	EPS15-AS1		lncRNA	chr1:51518288-5156
ENSG0000C	1048	26.21145	chr1:1522	RP5-850015.4		lncRNA	chr1:50437028-5043
ENSG0000C	1048	26.21145	chr1:1522	ENSG00000285839		protein_c	chr1:52020153-5203
ENSG0000C	1048	26.21145	chr1:1522	KTI12		protein_c	chr1:52032103-5203
ENSG0000C	1048	26.21145	chr1:1522	RNF11		protein_c	chr1:51236273-5127
ENSG0000C	1048	26.21145	chr1:1522	AL162430.1		smallRNA	chr1:51190883-5119
ENSG0000C	1048	26.21145	chr1:1522	PHB1P12		Pseudoger	chr1:50780340-5078
ENSG0000C	1048	26.21145	chr1:1522	SNORA26		smallRNA	chr1:51724775-5172
ENSG0000C	1048	26.21145	chr1:1522	AL162430.2		smallRNA	chr1:51188463-5118
ENSG0000C	1048	26.21145	chr1:1522	TSEN15P2		Pseudoger	chr1:51859778-5186
ENSG0000C	1048	26.21145	chr1:1522	RNU6-1281P		smallRNA	chr1:51538625-5153
ENSG0000C	1048	26.21145	chr1:1522	RNU6-1253P		smallRNA	chr1:50750296-5075
ENSG0000C	1048	26.21145	chr1:1522	snoU13		smallRNA	chr1:51231253-5123
ENSG0000C	1048	26.21145	chr1:1522	RAB3B		protein_c	chr1:51907956-5199
ENSG0000C	1048	26.21145	chr1:1522	ENSG00000229032		Pseudoger	chr1:51980473-5198
ENSG0000C	1048	26.21145	chr1:1522	TXNDC12-AS1		lncRNA	chr1:52050918-5205
ENSG0000C	1048	26.21145	chr1:1522	ENSG00000233406		Pseudoger	chr1:51250603-5125
ENSG0000C	1048	26.21145	chr1:1522	NRDC		protein_c	chr1:51789191-5187
ENSG0000C	1048	26.21145	chr1:1522	RNU6-877P		smallRNA	chr1:51382308-5138
ENSG0000C	1048	26.21145	chr1:1522	BTF3L4		protein_c	chr1:52056199-5209
ENSG0000C	1048	26.21145	chr1:1522	ENSG00000236434		lncRNA	chr1:51264916-5126
ENSG0000C	1048	26.21145	chr1:1522	ENSG00000238140		lncRNA	chr1:51461721-5146
ENSG0000C	1048	26.21145	chr1:1522	LINC01562		lncRNA	chr1:51195095-5123
ENSG0000C	1048	26.21145	chr1:1522	FAF1-AS1		lncRNA	chr1:50461469-5047
ENSG0000C	1048	26.21145	chr1:1522	ENSG00000266993		lncRNA	chr1:51793934-5179

ENSG0000(	1048	26.21145	chr1:1522	MRPS6P2		Pseudoger	chr1:50846468-5084
ENSG0000(	1048	26.21145	chr1:1522	C1orf185	NCGv7	protein_c	chr1:51102221-5114
ENSG0000(	1044	26.11141	chr7:330(	MIR93		smallRNA	chr7:100093768-100
ENSG0000(	1044	26.11141	chr7:330(	MIR25		smallRNA	chr7:100093560-100
ENSG0000(	1044	26.11141	chr7:330(	RPS29P15		Pseudoger	chr7:100928370-100
ENSG0000(	1041	26.03638	chr1:1522	ENSG00000234953		lncRNA	chr1:81513880-8155
ENSG0000(	1041	26.03638	chr1:1522	TNNI3K		protein_c	chr1:74235387-7454
ENSG0000(	1041	26.03638	chr1:1522	ENSG00000273338		lncRNA	chr1:78004346-7800
ENSG0000(	1041	26.03638	chr1:1522	ENSG00000213560		Pseudoger	chr1:78091499-7809
ENSG0000(	1041	26.03638	chr1:1522	FUBP1	NCGv7;AC	protein_c	chr1:77944055-7797
ENSG0000(	1041	26.03638	chr1:1522	ENSG00000229486		Pseudoger	chr1:84015865-8401
ENSG0000(	1041	26.03638	chr1:1522	TXN2P1		Pseudoger	chr1:84085741-8408
ENSG0000(	1041	26.03638	chr1:1522	ENSG00000280099		TEC	chr1:85152487-8515
ENSG0000(	1041	26.03638	chr1:1522	LINC01361		lncRNA	chr1:82970820-8298
ENSG0000(	1041	26.03638	chr1:1522	HSPE1P25		Pseudoger	chr1:77853355-7785
ENSG0000(	1041	26.03638	chr1:1522	ENSG00000282057		lncRNA	chr1:85482281-8557
ENSG0000(	1041	26.03638	chr1:1522	ADGRL2	NCGv7	protein_c	chr1:81306147-8199
ENSG0000(	1041	26.03638	chr1:1522	ENSG00000229505		Pseudoger	chr1:86029854-8603
ENSG0000(	1041	26.03638	chr1:1522	ENSG00000226084		Pseudoger	chr1:77129114-7712
ENSG0000(	1041	26.03638	chr1:1522	CRYZ		protein_c	chr1:74705482-7473
ENSG0000(	1041	26.03638	chr1:1522	NEXN		protein_c	chr1:77888513-7794
ENSG0000(	1041	26.03638	chr1:1522	SNORA2		smallRNA	chr1:84277321-8427
ENSG0000(	1041	26.03638	chr1:1522	ENSG00000289881		lncRNA	chr1:84614068-8462
ENSG0000(	1041	26.03638	chr1:1522	RPL7P10		Pseudoger	chr1:81098267-8109
ENSG0000(	1041	26.03638	chr1:1522	ENSG00000213579		Pseudoger	chr1:75582099-7558
ENSG0000(	1041	26.03638	chr1:1522	ENSG00000282898		lncRNA	chr1:79323769-7932
ENSG0000(	1041	26.03638	chr1:1522	LINC01555		lncRNA	chr1:84628230-8463
ENSG0000(	1041	26.03638	chr1:1522	AC096951.1		smallRNA	chr1:77007744-7700
ENSG0000(	1041	26.03638	chr1:1522	ENSG00000288822		lncRNA	chr1:79488644-7949
ENSG0000(	1041	26.03638	chr1:1522	RNA5SP23		Pseudoger	chr1:78375164-7837
ENSG0000(	1041	26.03638	chr1:1522	AC104169.1		smallRNA	chr1:84793068-8479
ENSG0000(	1041	26.03638	chr1:1522	MIGA1		protein_c	chr1:77779624-7787
ENSG0000(	1041	26.03638	chr1:1522	ENSG00000228187		Pseudoger	chr1:77194825-7719
ENSG0000(	1041	26.03638	chr1:1522	DNAJB4		protein_c	chr1:77979175-7801
ENSG0000(	1041	26.03638	chr1:1522	HNRNPA1P64		Pseudoger	chr1:80451083-8045
ENSG0000(	1041	26.03638	chr1:1522	ERICH3-AS1		lncRNA	chr1:74577430-7462
ENSG0000(	1041	26.03638	chr1:1522	RPF1		protein_c	chr1:84479259-8449
ENSG0000(	1041	26.03638	chr1:1522	ENSG00000227960		lncRNA	chr1:81505099-8150
ENSG0000(	1041	26.03638	chr1:1522	C1orf52		protein_c	chr1:85249953-8525
ENSG0000(	1041	26.03638	chr1:1522	ENSG00000235400		Pseudoger	chr1:78749073-7875
ENSG0000(	1041	26.03638	chr1:1522	RNU6-1102P		smallRNA	chr1:78088988-7808
ENSG0000(	1041	26.03638	chr1:1522	DNAI3		protein_c	chr1:84999147-8513
ENSG0000(	1041	26.03638	chr1:1522	BCL10-AS1		lncRNA	chr1:85276388-8544
ENSG0000(	1041	26.03638	chr1:1522	RNU6-161P		smallRNA	chr1:76753135-7675
ENSG0000(	1041	26.03638	chr1:1522	MTND2P30		Pseudoger	chr1:81080790-8108
ENSG0000(	1041	26.03638	chr1:1522	ACADM		protein_c	chr1:75724431-7578
ENSG0000(	1041	26.03638	chr1:1522	LINC01781		lncRNA	chr1:80535755-8064
ENSG0000(	1041	26.03638	chr1:1522	ENSG00000272855		lncRNA	chr1:76636877-7663
ENSG0000(	1041	26.03638	chr1:1522	ST6GALNAC5		protein_c	chr1:76867480-7706
ENSG0000(	1041	26.03638	chr1:1522	RNA5SP51		Pseudoger	chr1:85883680-8588
ENSG0000(	1041	26.03638	chr1:1522	ENSG00000272864		lncRNA	chr1:74698769-7469

ENSG0000(	1041	26.03638	chr1:1522	AC095030.1		smallRNA	chr1:77391422-7739
ENSG0000(	1041	26.03638	chr1:1522	PTGFR	NCGv7	protein_c	chr1:78303884-7854
ENSG0000(	1041	26.03638	chr1:1522	RN7SKP247		smallRNA	chr1:81251789-8125
ENSG0000(	1041	26.03638	chr1:1522	LRRC53		protein_c	chr1:74469376-7451
ENSG0000(	1041	26.03638	chr1:1522	ZNHIT6	AC	protein_c	chr1:85649417-8570
ENSG0000(	1041	26.03638	chr1:1522	DLSTP1		Pseudoger	chr1:75743423-7574
ENSG0000(	1041	26.03638	chr1:1522	SSX2IP		protein_c	chr1:84643706-8469
ENSG0000(	1041	26.03638	chr1:1522	ERICH3		protein_c	chr1:74568117-7467
ENSG0000(	1041	26.03638	chr1:1522	ENSG00000234108		Pseudoger	chr1:80495903-8049
ENSG0000(	1041	26.03638	chr1:1522	ADGRL4		protein_c	chr1:78889764-7928
ENSG0000(	1041	26.03638	chr1:1522	TYW3		protein_c	chr1:74733152-7476
ENSG0000(	1041	26.03638	chr1:1522	HMGB1P18		Pseudoger	chr1:80283352-8028
ENSG0000(	1041	26.03638	chr1:1522	LHX8		protein_c	chr1:75128434-7516
ENSG0000(	1041	26.03638	chr1:1522	SNORD81		smallRNA	chr1:85592280-8559
ENSG0000(	1041	26.03638	chr1:1522	AC104837.1		smallRNA	chr1:78687060-7868
ENSG0000(	1041	26.03638	chr1:1522	ENSG00000273264		lncRNA	chr1:85467295-8546
ENSG0000(	1041	26.03638	chr1:1522	CTBS		protein_c	chr1:84549611-8457
ENSG0000(	1041	26.03638	chr1:1522	SPATA1		protein_c	chr1:84506300-8456
ENSG0000(	1041	26.03638	chr1:1522	ENSG00000213561		Pseudoger	chr1:78043383-7804
ENSG0000(	1041	26.03638	chr1:1522	CCN1		protein_c	chr1:85580761-8558
ENSG0000(	1041	26.03638	chr1:1522	MCOLN2		protein_c	chr1:84925583-8499
ENSG0000(	1041	26.03638	chr1:1522	DDAH1		protein_c	chr1:85318481-8557
ENSG0000(	1041	26.03638	chr1:1522	ENSG00000224493		Pseudoger	chr1:75521562-7552
ENSG0000(	1041	26.03638	chr1:1522	ENSG00000230863		Pseudoger	chr1:75641178-7572
ENSG0000(	1041	26.03638	chr1:1522	HNRNPA3P14		Pseudoger	chr1:81426456-8142
ENSG0000(	1041	26.03638	chr1:1522	RNFT1P2		Pseudoger	chr1:78170481-7817
ENSG0000(	1041	26.03638	chr1:1522	AC104458.1		smallRNA	chr1:76718140-7671
ENSG0000(	1041	26.03638	chr1:1522	ENSG00000219201		Pseudoger	chr1:77810861-7781
ENSG0000(	1041	26.03638	chr1:1522	LINC01362		lncRNA	chr1:82903183-8316
ENSG0000(	1041	26.03638	chr1:1522	LINC02567		lncRNA	chr1:76758124-7677
ENSG0000(	1041	26.03638	chr1:1522	ENSG00000260322		lncRNA	chr1:80114943-8011
ENSG0000(	1041	26.03638	chr1:1522	AL035706.1		smallRNA	chr1:83793877-8379
ENSG0000(	1041	26.03638	chr1:1522	RN7SL370P		smallRNA	chr1:77645324-7764
ENSG0000(	1041	26.03638	chr1:1522	ASB17		protein_c	chr1:75918873-7593
ENSG0000(	1041	26.03638	chr1:1522	ENSG00000235089		Pseudoger	chr1:81208568-8120
ENSG0000(	1041	26.03638	chr1:1522	AK5		protein_c	chr1:77282019-7755
ENSG0000(	1041	26.03638	chr1:1522	NEDD8P1		Pseudoger	chr1:84244334-8424
ENSG0000(	1041	26.03638	chr1:1522	PSAT1P3		Pseudoger	chr1:79054945-7905
ENSG0000(	1041	26.03638	chr1:1522	ENSG00000235011		lncRNA	chr1:79323769-7932
ENSG0000(	1041	26.03638	chr1:1522	ADH5P2		Pseudoger	chr1:79521080-7952
ENSG0000(	1041	26.03638	chr1:1522	ACTG1P21		Pseudoger	chr1:77773865-7777
ENSG0000(	1041	26.03638	chr1:1522	SAMD13		protein_c	chr1:84298366-8438
ENSG0000(	1041	26.03638	chr1:1522	ENSG00000227556		Pseudoger	chr1:78317157-7831
ENSG0000(	1041	26.03638	chr1:1522	ENSG00000287870		lncRNA	chr1:77248633-7725
ENSG0000(	1041	26.03638	chr1:1522	ENSG00000235756		Pseudoger	chr1:80092103-8009
ENSG0000(	1041	26.03638	chr1:1522	ENSG00000237076		lncRNA	chr1:83766417-8380
ENSG0000(	1041	26.03638	chr1:1522	ENSG00000233290		lncRNA	chr1:82212413-8284
ENSG0000(	1041	26.03638	chr1:1522	ENSG00000277670		Pseudoger	chr1:80124004-8012
ENSG0000(	1041	26.03638	chr1:1522	ARID3BP1		Pseudoger	chr1:81501794-8150
ENSG0000(	1041	26.03638	chr1:1522	MCOLN3		protein_c	chr1:85018082-8504
ENSG0000(	1041	26.03638	chr1:1522	ENSG00000224326		lncRNA	chr1:80534978-8058

ENSG00000	1041	26.03638	chr1:1522	ENSG00000238015		Pseudoger	chr1:78666272-7866
ENSG00000	1041	26.03638	chr1:1522	AL606519.1		smallRNA	chr1:80329379-8032
ENSG00000	1041	26.03638	chr1:1522	MIR4423		smallRNA	chr1:85133794-8513
ENSG00000	1041	26.03638	chr1:1522	SNORD45A		smallRNA	chr1:75787889-7578
ENSG00000	1041	26.03638	chr1:1522	LPAR3	NCGv7	protein_c	chr1:84811602-8489
ENSG00000	1041	26.03638	chr1:1522	ENSG00000227062		Pseudoger	chr1:80464301-8046
ENSG00000	1041	26.03638	chr1:1522	AC093430.1		smallRNA	chr1:79135133-7913
ENSG00000	1041	26.03638	chr1:1522	NEXN-AS1		lncRNA	chr1:77881348-7788
ENSG00000	1041	26.03638	chr1:1522	TPI1P1		Pseudoger	chr1:76699789-7670
ENSG00000	1041	26.03638	chr1:1522	LINC01725		lncRNA	chr1:83575776-8386
ENSG00000	1041	26.03638	chr1:1522	TTLL7-IT1		lncRNA	chr1:83979118-8398
ENSG00000	1041	26.03638	chr1:1522	SNORD45C		smallRNA	chr1:75787072-7578
ENSG00000	1041	26.03638	chr1:1522	ENSG00000233099		lncRNA	chr1:77346046-7734
ENSG00000	1041	26.03638	chr1:1522	ST13P20		Pseudoger	chr1:81721693-8172
ENSG00000	1041	26.03638	chr1:1522	AL445464.1		smallRNA	chr1:75842632-7584
ENSG00000	1041	26.03638	chr1:1522	ENSG00000223905		Pseudoger	chr1:76353583-7635
ENSG00000	1041	26.03638	chr1:1522	ENSG00000224149		lncRNA	chr1:75129974-7513
ENSG00000	1041	26.03638	chr1:1522	Y_RNA		smallRNA	chr1:85264296-8526
ENSG00000	1041	26.03638	chr1:1522	COL24A1		protein_c	chr1:85729233-8615
ENSG00000	1041	26.03638	chr1:1522	GNG5		protein_c	chr1:84498323-8450
ENSG00000	1041	26.03638	chr1:1522	AL590113.1		smallRNA	chr1:85284610-8528
ENSG00000	1041	26.03638	chr1:1522	SYDE2		protein_c	chr1:85156889-8520
ENSG00000	1041	26.03638	chr1:1522	ENSG00000234683		Pseudoger	chr1:81596157-8159
ENSG00000	1041	26.03638	chr1:1522	UOX		Pseudoger	chr1:84363706-8439
ENSG00000	1041	26.03638	chr1:1522	RNU6-503P		smallRNA	chr1:75538015-7553
ENSG00000	1041	26.03638	chr1:1522	LINC01712		lncRNA	chr1:83445967-8348
ENSG00000	1041	26.03638	chr1:1522	IFI44L	NCGv7	protein_c	chr1:78619902-7864
ENSG00000	1041	26.03638	chr1:1522	SNORD45B		smallRNA	chr1:75789477-7578
ENSG00000	1041	26.03638	chr1:1522	Y_RNA		smallRNA	chr1:85435175-8543
ENSG00000	1041	26.03638	chr1:1522	RNA5SP22		Pseudoger	chr1:78094807-7809
ENSG00000	1041	26.03638	chr1:1522	ENSG00000233894		lncRNA	chr1:74468195-7446
ENSG00000	1041	26.03638	chr1:1522	ENSG00000229943		lncRNA	chr1:74963314-7496
ENSG00000	1041	26.03638	chr1:1522	MED28P8		Pseudoger	chr1:81557121-8155
ENSG00000	1041	26.03638	chr1:1522	RNU7-8P		smallRNA	chr1:77420325-7742
ENSG00000	1041	26.03638	chr1:1522	ENSG00000236676		lncRNA	chr1:81585941-8162
ENSG00000	1041	26.03638	chr1:1522	ZZZ3		protein_c	chr1:77562416-7768
ENSG00000	1041	26.03638	chr1:1522	LINC02792		lncRNA	chr1:79325008-7934
ENSG00000	1041	26.03638	chr1:1522	ENSG00000285409		lncRNA	chr1:79967733-8005
ENSG00000	1041	26.03638	chr1:1522	ENSG00000285374		lncRNA	chr1:84607099-8461
ENSG00000	1041	26.03638	chr1:1522	ENSG00000285361		lncRNA	chr1:84477039-8447
ENSG00000	1041	26.03638	chr1:1522	ENSG00000285325		lncRNA	chr1:84785427-8478
ENSG00000	1041	26.03638	chr1:1522	LINC02795		lncRNA	chr1:86288704-8632
ENSG00000	1041	26.03638	chr1:1522	ENSG00000285201		lncRNA	chr1:84038529-8406
ENSG00000	1041	26.03638	chr1:1522	GIPC2		protein_c	chr1:77979542-7813
ENSG00000	1041	26.03638	chr1:1522	MSH4		protein_c	chr1:75796882-7591
ENSG00000	1041	26.03638	chr1:1522	IFI44		protein_c	chr1:78649796-7866
ENSG00000	1041	26.03638	chr1:1522	RNA5SP20		Pseudoger	chr1:77614869-7761
ENSG00000	1041	26.03638	chr1:1522	ENSG00000285179		lncRNA	chr1:81209834-8122
ENSG00000	1041	26.03638	chr1:1522	SLC44A5	NCGv7	protein_c	chr1:75202129-7561
ENSG00000	1041	26.03638	chr1:1522	ENSG00000261213		lncRNA	chr1:75122518-7512
ENSG00000	1041	26.03638	chr1:1522	ENSG00000284882		lncRNA	chr1:84574114-8458

ENSG0000(	1041	26.03638	chr1:1522	DNASE2B		protein_c	chr1:84398484-8441
ENSG0000(	1041	26.03638	chr1:1522	ST6GALNA(	NCGv7	protein_c	chr1:76074746-7663
ENSG0000(	1041	26.03638	chr1:1522	ENSG00000287647		lncRNA	chr1:77431314-7743
ENSG0000(	1041	26.03638	chr1:1522	PRKACB-DT		lncRNA	chr1:84076331-8407
ENSG0000(	1041	26.03638	chr1:1522	PIGK		protein_c	chr1:77088989-7721
ENSG0000(	1041	26.03638	chr1:1522	RNU6-622P		smallRNA	chr1:75183045-7518
ENSG0000(	1041	26.03638	chr1:1522	ENSG00000288543		lncRNA	chr1:77067920-7707
ENSG0000(	1041	26.03638	chr1:1522	ENSG00000225598		lncRNA	chr1:80373364-8037
ENSG0000(	1041	26.03638	chr1:1522	ENSG00000225605		lncRNA	chr1:75926454-7601
ENSG0000(	1041	26.03638	chr1:1522	ENSG00000289212		lncRNA	chr1:77219520-7722
ENSG0000(	1041	26.03638	chr1:1522	FPGT-TNNI3K		protein_c	chr1:74198235-7454
ENSG0000(	1041	26.03638	chr1:1522	ENSG00000230285		lncRNA	chr1:85599131-8560
ENSG0000(	1041	26.03638	chr1:1522	TTLL7		protein_c	chr1:83865024-8399
ENSG0000(	1041	26.03638	chr1:1522	ENSG00000224127		lncRNA	chr1:75127830-7513
ENSG0000(	1041	26.03638	chr1:1522	USP33		protein_c	chr1:77695987-7775
ENSG0000(	1041	26.03638	chr1:1522	MGC27382		lncRNA	chr1:78229599-7836
ENSG0000(	1041	26.03638	chr1:1522	ENSG00000249237		Pseudoger	chr1:84344678-8434
ENSG0000(	1041	26.03638	chr1:1522	RABGGTB		protein_c	chr1:75786197-7579
ENSG0000(	1041	26.03638	chr1:1522	PRKACB		protein_c	chr1:84078062-8423
ENSG0000(	1041	26.03638	chr1:1522	ENSG00000232622		Pseudoger	chr1:84636158-8463
ENSG0000(	1041	26.03638	chr1:1522	ENSG00000230027		lncRNA	chr1:76041691-7606
ENSG0000(	1041	26.03638	chr1:1522	ENSG00000285928		lncRNA	chr1:78022565-7802
ENSG0000(	1041	26.03638	chr1:1522	BCL10	NCGv7;AC	protein_c	chr1:85265776-8527
ENSG0000(	1041	26.03638	chr1:1522	NSRP1P1		Pseudoger	chr1:77847110-7784
ENSG0000(	1041	26.03638	chr1:1522	ENSG00000285851		lncRNA	chr1:84498350-8455
ENSG0000(	1041	26.03638	chr1:1522	RNA5SP21		Pseudoger	chr1:77779904-7777
ENSG0000(	1041	26.03638	chr1:1522	ENSG00000285782		lncRNA	chr1:83397555-8342
ENSG0000(	1036	25.91132	chr7:330(	BANF1P5		Pseudoger	chr7:107642765-107
ENSG0000(	1034	25.8613	chr1:1522	ORC1		protein_c	chr1:52372829-5240
ENSG0000(	1034	25.8613	chr1:1522	H3P2		Pseudoger	chr1:52943536-5294
ENSG0000(	1034	25.8613	chr1:1522	DNAJC19P7		Pseudoger	chr1:52252062-5225
ENSG0000(	1034	25.8613	chr1:1522	PDCL3P6		Pseudoger	chr1:52179848-5218
ENSG0000(	1034	25.8613	chr1:1522	MIR1273G		smallRNA	chr1:52940314-5294
ENSG0000(	1034	25.8613	chr1:1522	ENSG00000236004		Pseudoger	chr1:52189916-5219
ENSG0000(	1034	25.8613	chr1:1522	ENSG00000272100		lncRNA	chr1:52353487-5235
ENSG0000(	1034	25.8613	chr1:1522	ENSG00000223429		Pseudoger	chr1:52162186-5216
ENSG0000(	1034	25.8613	chr1:1522	PLA2G12AP1		Pseudoger	chr1:52368677-5236
ENSG0000(	1034	25.8613	chr1:1522	TUBBP10		Pseudoger	chr1:52994726-5299
ENSG0000(	1034	25.8613	chr1:1522	SHISAL2A		protein_c	chr1:52633168-5266
ENSG0000(	1034	25.8613	chr1:1522	ENSG00000272371		lncRNA	chr1:52554818-5255
ENSG0000(	1034	25.8613	chr1:1522	COA7		protein_c	chr1:52684449-5269
ENSG0000(	1034	25.8613	chr1:1522	ZYG11B		protein_c	chr1:52726453-5282
ENSG0000(	1034	25.8613	chr1:1522	ENSG00000242391		lncRNA	chr1:52881216-5288
ENSG0000(	1034	25.8613	chr1:1522	NDUFS5P3		Pseudoger	chr1:52709122-5270
ENSG0000(	1034	25.8613	chr1:1522	RN7SL788P		smallRNA	chr1:52150105-5215
ENSG0000(	1034	25.8613	chr1:1522	ECHDC2		protein_c	chr1:52895910-5292
ENSG0000(	1034	25.8613	chr1:1522	ZFYVE9		protein_c	chr1:52142089-5234
ENSG0000(	1034	25.8613	chr1:1522	RN7SL62P		smallRNA	chr1:52714399-5271
ENSG0000(	1034	25.8613	chr1:1522	MIR5095		smallRNA	chr1:52934930-5293
ENSG0000(	1034	25.8613	chr1:1522	ZYG11A		protein_c	chr1:52842511-5289
ENSG0000(	1034	25.8613	chr1:1522	CC2D1B		protein_c	chr1:52345723-5236

ENSG0000(	1034	25.8613	chr1:1522	snoU13		smallRNA	chr1:52411442-5241
ENSG0000(	1034	25.8613	chr1:1522	ENSG00000287078		lncRNA	chr1:52365443-5236
ENSG0000(	1034	25.8613	chr1:1522	RNU6-969P		smallRNA	chr1:52805108-5280
ENSG0000(	1034	25.8613	chr1:1522	TUT4		protein_c	chr1:52408282-5255
ENSG0000(	1034	25.8613	chr1:1522	PRPF38A		protein_c	chr1:52404602-5242
ENSG0000(	1034	25.8613	chr1:1522	ENSG00000230953		Pseudoger	chr1:52920422-5292
ENSG0000(	1034	25.8613	chr1:1522	RRAS2P1		Pseudoger	chr1:52993201-5299
ENSG0000(	1034	25.8613	chr1:1522	ANAPC10P1		Pseudoger	chr1:52253621-5225
ENSG0000(	1034	25.8613	chr1:1522	MIR1273F		smallRNA	chr1:52928674-5292
ENSG0000(	1034	25.8613	chr1:1522	EEF1GP7		Pseudoger	chr1:52573114-5257
ENSG0000(	1034	25.8613	chr1:1522	ENSG00000231866		Pseudoger	chr1:52925249-5292
ENSG0000(	1034	25.8613	chr1:1522	ENSG00000228407		Pseudoger	chr1:52160261-5216
ENSG0000(	1034	25.8613	chr1:1522	RPS13P2		Pseudoger	chr1:52772194-5277
ENSG0000(	1034	25.8613	chr1:1522	GPX7		protein_c	chr1:52602371-5260
ENSG0000(	1034	25.8613	chr1:1522	SCP2		protein_c	chr1:52927276-5305
ENSG0000(	1034	25.8613	chr1:1522	RNU2-30P		smallRNA	chr1:52754322-5275
ENSG0000(	1034	25.8613	chr1:1522	PODN		protein_c	chr1:53062052-5308
ENSG0000(	1032	25.81128	chr7:330(	EIF4BP6		Pseudoger	chr7:104667749-104
ENSG0000(	1032	25.81128	chr7:330(	ENSG00000237606		Pseudoger	chr7:104826336-104
ENSG0000(	1032	25.81128	chr7:330(	LHFPL3-AS1		lncRNA	chr7:104738597-104
ENSG0000(	1032	25.81128	chr7:330(	LHFPL3-AS2		lncRNA	chr7:104894628-104
ENSG0000(	1032	25.81128	chr7:330(	RN7SL8P		smallRNA	chr7:104911917-104
ENSG0000(	1020	25.51115	chr7:330(	LRWD1	NCGv7	protein_c	chr7:102464956-102
ENSG0000(	1020	25.51115	chr7:330(	MIR5480		smallRNA	chr7:102405742-102
ENSG0000(	1020	25.51115	chr7:330(	ENSG00000272604		lncRNA	chr7:105571083-105
ENSG0000(	1020	25.51115	chr7:330(	KMT2E-AS1		lncRNA	chr7:105013277-105
ENSG0000(	1020	25.51115	chr7:330(	RPL36P12		Pseudoger	chr7:105244652-105
ENSG0000(	1020	25.51115	chr7:330(	FBXL13		protein_c	chr7:102812838-103
ENSG0000(	1020	25.51115	chr7:330(	ENSG00000278586		Pseudoger	chr7:102264706-102
ENSG0000(	1020	25.51115	chr7:330(	SPDYE2B		protein_c	chr7:102650319-102
ENSG0000(	1020	25.51115	chr7:330(	AC005088.1		smallRNA	chr7:102238321-102
ENSG0000(	1020	25.51115	chr7:330(	MIR5090		smallRNA	chr7:102465742-102
ENSG0000(	1020	25.51115	chr7:330(	ENSG00000239486		Pseudoger	chr7:102380465-102
ENSG0000(	1020	25.51115	chr7:330(	ENSG00000270764		Pseudoger	chr7:105189190-105
ENSG0000(	1020	25.51115	chr7:330(	ENSG00000224415		Pseudoger	chr7:103141349-103
ENSG0000(	1020	25.51115	chr7:330(	Y_RNA		smallRNA	chr7:102336869-102
ENSG0000(	1020	25.51115	chr7:330(	ENSG00000286076		lncRNA	chr7:106425278-106
ENSG0000(	1020	25.51115	chr7:330(	SYPL1		protein_c	chr7:106090505-106
ENSG0000(	1020	25.51115	chr7:330(	DCAF13P1		Pseudoger	chr7:106125371-106
ENSG0000(	1020	25.51115	chr7:330(	RELN	NCGv7	protein_c	chr7:103471381-103
ENSG0000(	1020	25.51115	chr7:330(	RNA5SP236		Pseudoger	chr7:106781600-106
ENSG0000(	1020	25.51115	chr7:330(	RINT1	AC	protein_c	chr7:105532169-105
ENSG0000(	1020	25.51115	chr7:330(	SRPK2		protein_c	chr7:105110704-105
ENSG0000(	1020	25.51115	chr7:330(	ENSG00000286013		lncRNA	chr7:106624072-106
ENSG0000(	1020	25.51115	chr7:330(	AC093668.3		smallRNA	chr7:102480080-102
ENSG0000(	1020	25.51115	chr7:330(	ORAI2		protein_c	chr7:102433106-102
ENSG0000(	1020	25.51115	chr7:330(	ALKBH4		protein_c	chr7:102456238-102
ENSG0000(	1020	25.51115	chr7:330(	SH2B2		protein_c	chr7:102285091-102
ENSG0000(	1020	25.51115	chr7:330(	ENSG00000289613		lncRNA	chr7:103315169-103
ENSG0000(	1020	25.51115	chr7:330(	SNORA48		smallRNA	chr7:102194076-102
ENSG0000(	1020	25.51115	chr7:330(	EFCAB10		protein_c	chr7:105565120-105

ENSG0000(	1020	25.51115	chr7:330(	ENSG00000239480		lncRNA	chr7:102426818-102
ENSG0000(	1020	25.51115	chr7:330(	ENSG00000271482		Pseudoger	chr7:105204600-105
ENSG0000(	1020	25.51115	chr7:330(	ENSG00000279724		Pseudoger	chr7:102188599-102
ENSG0000(	1020	25.51115	chr7:330(	ENSG00000242154		lncRNA	chr7:105304277-105
ENSG0000(	1020	25.51115	chr7:330(	ENSG00000279482		TEC	chr7:103161947-103
ENSG0000(	1020	25.51115	chr7:330(	LINC02577		lncRNA	chr7:106774955-106
ENSG0000(	1020	25.51115	chr7:330(	ENSG00000290830		lncRNA	chr7:102364162-102
ENSG0000(	1020	25.51115	chr7:330(	ENSG00000223886		Pseudoger	chr7:105530209-105
ENSG0000(	1020	25.51115	chr7:330(	UPK3BL1		protein_c	chr7:102637025-102
ENSG0000(	1020	25.51115	chr7:330(	DPY19L2P2		Pseudoger	chr7:103175343-103
ENSG0000(	1020	25.51115	chr7:330(	ENSG00000279168		lncRNA	chr7:102579104-102
ENSG0000(	1020	25.51115	chr7:330(	ENSG00000267052		lncRNA	chr7:106569876-106
ENSG0000(	1020	25.51115	chr7:330(	YBX1P2		Pseudoger	chr7:105582258-105
ENSG0000(	1020	25.51115	chr7:330(	KMT2E	NCGv7	protein_c	chr7:104940943-105
ENSG0000(	1020	25.51115	chr7:330(	RASA4DP		Pseudoger	chr7:102681836-102
ENSG0000(	1020	25.51115	chr7:330(	ENSG00000286830		lncRNA	chr7:102699228-102
ENSG0000(	1020	25.51115	chr7:330(	NFE4		lncRNA	chr7:102973483-102
ENSG0000(	1020	25.51115	chr7:330(	MIR4285		smallRNA	chr7:102293103-102
ENSG0000(	1020	25.51115	chr7:330(	ARMC10		protein_c	chr7:103074881-103
ENSG0000(	1020	25.51115	chr7:330(	S100A11P1		Pseudoger	chr7:103262000-103
ENSG0000(	1020	25.51115	chr7:330(	POLR2J		protein_c	chr7:102473128-102
ENSG0000(	1020	25.51115	chr7:330(	ENSG00000236226		lncRNA	chr7:103030104-103
ENSG0000(	1020	25.51115	chr7:330(	ENSG00000290116		lncRNA	chr7:106448382-106
ENSG0000(	1020	25.51115	chr7:330(	RASA4B		protein_c	chr7:102479976-102
ENSG0000(	1020	25.51115	chr7:330(	POLR2J2		protein_c	chr7:102665368-102
ENSG0000(	1020	25.51115	chr7:330(	RWDD4P1		Pseudoger	chr7:105301522-105
ENSG0000(	1020	25.51115	chr7:330(	ENSG00000261535		lncRNA	chr7:102153355-102
ENSG0000(	1020	25.51115	chr7:330(	ORC5	DriverDB\	protein_c	chr7:104126341-104
ENSG0000(	1020	25.51115	chr7:330(	ENSG00000259294		lncRNA	chr7:101822247-101
ENSG0000(	1020	25.51115	chr7:330(	RN7SKP86		smallRNA	chr7:103484208-103
ENSG0000(	1020	25.51115	chr7:330(	PSMC2		protein_c	chr7:103328570-103
ENSG0000(	1020	25.51115	chr7:330(	DPY19L2P2		lncRNA	chr7:103175133-103
ENSG0000(	1020	25.51115	chr7:330(	NAPEPLD		protein_c	chr7:103099776-103
ENSG0000(	1020	25.51115	chr7:330(	ENSG00000272918		lncRNA	chr7:105102838-105
ENSG0000(	1020	25.51115	chr7:330(	ENSG00000267645		protein_c	chr7:102637049-102
ENSG0000(	1020	25.51115	chr7:330(	AC073127.1		smallRNA	chr7:103014256-103
ENSG0000(	1020	25.51115	chr7:330(	ENSG00000288914		protein_c	chr7:105040858-105
ENSG0000(	1020	25.51115	chr7:330(	LHFPL3	DriverDB\	protein_c	chr7:104328603-104
ENSG0000(	1020	25.51115	chr7:330(	NAMPT	NCGv7	protein_c	chr7:106248298-106
ENSG0000(	1020	25.51115	chr7:330(	PMS2P12		Pseudoger	chr7:102337316-102
ENSG0000(	1020	25.51115	chr7:330(	SLC26A5		protein_c	chr7:103352730-103
ENSG0000(	1020	25.51115	chr7:330(	ENSG00000226624		Pseudoger	chr7:105819492-105
ENSG0000(	1020	25.51115	chr7:330(	CDHR3	NCGv7	protein_c	chr7:105876796-106
ENSG0000(	1020	25.51115	chr7:330(	PRKRIP1		protein_c	chr7:102363872-102
ENSG0000(	1020	25.51115	chr7:330(	ENSG00000273320		lncRNA	chr7:106285200-106
ENSG0000(	1020	25.51115	chr7:330(	SLC26A5-AS1		lncRNA	chr7:103445207-103
ENSG0000(	1020	25.51115	chr7:330(	LINC01004		lncRNA	chr7:104950315-105
ENSG0000(	1020	25.51115	chr7:330(	SPDYE6		protein_c	chr7:102345746-102
ENSG0000(	1020	25.51115	chr7:330(	AC093668.1		smallRNA	chr7:102507203-102
ENSG0000(	1020	25.51115	chr7:330(	RNU6-1322P		smallRNA	chr7:105332790-105
ENSG0000(	1020	25.51115	chr7:330(	ENSG00000270249		protein_c	chr7:102541501-102

ENSG0000(	1020	25.51115	chr7:330(	AC005086.4		Pseudoger	chr7:102188599-102
ENSG0000(	1020	25.51115	chr7:330(	DNAJC2		protein_c	chr7:103312289-103
ENSG0000(	1020	25.51115	chr7:330(	PMPCB		protein_c	chr7:103297435-103
ENSG0000(	1020	25.51115	chr7:330(	Y_RNA		smallRNA	chr7:103433461-103
ENSG0000(	1020	25.51115	chr7:330(	LARP1BP2		Pseudoger	chr7:106315225-106
ENSG0000(	1020	25.51115	chr7:330(	RASA4	DriverDB\	protein_c	chr7:102579646-102
ENSG0000(	1020	25.51115	chr7:330(	RN7SKP198		smallRNA	chr7:102857450-102
ENSG0000(	1020	25.51115	chr7:330(	RPL7AP39		Pseudoger	chr7:102755146-102
ENSG0000(	1020	25.51115	chr7:330(	FAM185A		protein_c	chr7:102748971-102
ENSG0000(	1020	25.51115	chr7:330(	ENSG00000272219		lncRNA	chr7:101960116-101
ENSG0000(	1020	25.51115	chr7:330(	Y_RNA		smallRNA	chr7:103434994-103
ENSG0000(	1020	25.51115	chr7:330(	ATXN7L1	DriverDB\	protein_c	chr7:105604772-105
ENSG0000(	1020	25.51115	chr7:330(	LRRC17	NCGv7	protein_c	chr7:102913000-102
ENSG0000(	1020	25.51115	chr7:330(	CCDC71L		protein_c	chr7:106654360-106
ENSG0000(	1020	25.51115	chr7:330(	SNORD112		smallRNA	chr7:104971287-104
ENSG0000(	1020	25.51115	chr7:330(	ENSG00000280439		TEC	chr7:106035798-106
ENSG0000(	1020	25.51115	chr7:330(	UPK3BL2		protein_c	chr7:102537919-102
ENSG0000(	1020	25.51115	chr7:330(	RPS29P16		Pseudoger	chr7:103348601-103
ENSG0000(	1020	25.51115	chr7:330(	CUX1	NCGv7;AC	protein_c	chr7:101815904-102
ENSG0000(	1020	25.51115	chr7:330(	AC005086.1		Pseudoger	chr7:102161120-102
ENSG0000(	1020	25.51115	chr7:330(	ENSG00000170409		Pseudoger	chr7:102327256-102
ENSG0000(	1020	25.51115	chr7:330(	POLR2J3		protein_c	chr7:102562133-102
ENSG0000(	1020	25.51115	chr7:330(	AC105052.1		smallRNA	chr7:102606366-102
ENSG0000(	1020	25.51115	chr7:330(	ENSG00000280404		Pseudoger	chr7:102161120-102
ENSG0000(	1020	25.51115	chr7:330(	CRYZP1		Pseudoger	chr7:103088664-103
ENSG0000(	1020	25.51115	chr7:330(	PUS7		protein_c	chr7:105439661-105
ENSG0000(	1020	25.51115	chr7:330(	RNU6-392P		smallRNA	chr7:106208167-106
ENSG0000(	1020	25.51115	chr7:330(	ENSG00000289956		lncRNA	chr7:103152899-103
ENSG0000(	1020	25.51115	chr7:330(	ENSG00000280004		Pseudoger	chr7:102186819-102
ENSG0000(	1020	25.51115	chr7:330(	ENSG00000239969		Pseudoger	chr7:102375808-102
ENSG0000(	1020	25.51115	chr7:330(	SPDYE2		protein_c	chr7:102551226-102
ENSG0000(	1020	25.51115	chr7:330(	RNU6-1136P		smallRNA	chr7:102834605-102
ENSG0000(	1020	25.51115	chr7:330(	ENSG00000243797		lncRNA	chr7:106372251-106
ENSG0000(	1020	25.51115	chr7:330(	AC093668.2		Pseudoger	chr7:102479732-102
ENSG0000(	1020	25.51115	chr7:330(	AC005086.2		Pseudoger	chr7:102186819-102
ENSG0000(	1020	25.51115	chr7:330(	ENSG00000205236		protein_c	chr7:102582523-102
ENSG0000(	1020	25.51115	chr7:330(	ENSG00000289360		protein_c	chr7:105040848-105
ENSG0000(	1020	25.51115	chr7:330(	MIR4467		smallRNA	chr7:102471469-102
ENSG0000(	1020	25.51115	chr7:330(	AC005086.3		Pseudoger	chr7:102162296-102
ENSG0000(	1020	25.51115	chr7:330(	PIK3CG	NCGv7	protein_c	chr7:106865278-106
ENSG0000(	1020	25.51115	chr7:330(	POLR2J3		protein_c	chr7:102537918-102
ENSG0000(	1012	25.31106	chr19:13:	COX6CP7		Pseudoger	chr19:49502432-495
ENSG0000(	1009	25.23603	chr6:213!	MFSD4B		protein_c	chr6:111259327-111
ENSG0000(	1009	25.23603	chr6:213!	ENSG00000220326		Pseudoger	chr6:121641009-121
ENSG0000(	1009	25.23603	chr6:213!	MIR548B		smallRNA	chr6:119069047-119
ENSG0000(	1009	25.23603	chr6:213!	ENSG00000227678		lncRNA	chr6:130133410-130
ENSG0000(	1009	25.23603	chr6:213!	ENSG00000234117		lncRNA	chr6:116492297-116
ENSG0000(	1009	25.23603	chr6:213!	HMGB3P18		Pseudoger	chr6:121858179-121
ENSG0000(	1009	25.23603	chr6:213!	ENSG00000289961		lncRNA	chr6:113345749-113
ENSG0000(	1009	25.23603	chr6:213!	ENSG00000220139		Pseudoger	chr6:119159297-119
ENSG0000(	1009	25.23603	chr6:213!	ENSG00000230202		Pseudoger	chr6:117998975-117

ENSG0000(	1009	25.23603	chr6:213	RNA5SP214		Pseudoger	chr6:117060682-117
ENSG0000(	1009	25.23603	chr6:213	RPL21P66		Pseudoger	chr6:132518830-132
ENSG0000(	1009	25.23603	chr6:213	ENSG00000220110		Pseudoger	chr6:128639188-128
ENSG0000(	1009	25.23603	chr6:213	LNCPOIR		lncRNA	chr6:114477350-114
ENSG0000(	1009	25.23603	chr6:213	ENSG00000220522		Pseudoger	chr6:127416535-127
ENSG0000(	1009	25.23603	chr6:213	NEPNP		Pseudoger	chr6:117633706-117
ENSG0000(	1009	25.23603	chr6:213	ENSG00000279114		TEC	chr6:122471923-122
ENSG0000(	1009	25.23603	chr6:213	TAAR2		protein_c	chr6:132617022-132
ENSG0000(	1009	25.23603	chr6:213	TAAR6		protein_c	chr6:132570322-132
ENSG0000(	1009	25.23603	chr6:213	TAAR8		protein_c	chr6:132552672-132
ENSG0000(	1009	25.23603	chr6:213	TAAR1		protein_c	chr6:132643312-132
ENSG0000(	1009	25.23603	chr6:213	SLC18B1		protein_c	chr6:132769370-132
ENSG0000(	1009	25.23603	chr6:213	ENSG00000270661		lncRNA	chr6:112217640-112
ENSG0000(	1009	25.23603	chr6:213	RAP1BP3		Pseudoger	chr6:117431591-117
ENSG0000(	1009	25.23603	chr6:213	TRDN		protein_c	chr6:123216339-123
ENSG0000(	1009	25.23603	chr6:213	HEY2-AS1		lncRNA	chr6:125577545-125
ENSG0000(	1009	25.23603	chr6:213	RPSAP43		Pseudoger	chr6:114084168-114
ENSG0000(	1009	25.23603	chr6:213	ENSG00000219699		Pseudoger	chr6:127659690-127
ENSG0000(	1009	25.23603	chr6:213	AL136446.1		smallRNA	chr6:114277385-114
ENSG0000(	1009	25.23603	chr6:213	CALHM6-AS1		lncRNA	chr6:116460739-116
ENSG0000(	1009	25.23603	chr6:213	ENSG00000219666		Pseudoger	chr6:129783769-129
ENSG0000(	1009	25.23603	chr6:213	BMPR1AP1		Pseudoger	chr6:129157523-129
ENSG0000(	1009	25.23603	chr6:213	RN7SL564P		smallRNA	chr6:122745274-122
ENSG0000(	1009	25.23603	chr6:213	ENSG00000286540		lncRNA	chr6:120462511-120
ENSG0000(	1009	25.23603	chr6:213	RN7SKP56		smallRNA	chr6:125875168-125
ENSG0000(	1009	25.23603	chr6:213	ARHGAP18		protein_c	chr6:129576132-129
ENSG0000(	1009	25.23603	chr6:213	PTPRK-AS1		lncRNA	chr6:128027886-128
ENSG0000(	1009	25.23603	chr6:213	ENSG00000289198		lncRNA	chr6:113855537-113
ENSG0000(	1009	25.23603	chr6:213	CTAGE9		protein_c	chr6:131708441-131
ENSG0000(	1009	25.23603	chr6:213	ENSG00000286215		Pseudoger	chr6:126304223-127
ENSG0000(	1009	25.23603	chr6:213	ENSG00000287818		lncRNA	chr6:122711887-122
ENSG0000(	1009	25.23603	chr6:213	ENSG00000224733		lncRNA	chr6:128500527-128
ENSG0000(	1009	25.23603	chr6:213	SNORA33		smallRNA	chr6:132817219-132
ENSG0000(	1009	25.23603	chr6:213	ENSG00000286299		lncRNA	chr6:125268087-125
ENSG0000(	1009	25.23603	chr6:213	ENSG00000219784		Pseudoger	chr6:121478273-121
ENSG0000(	1009	25.23603	chr6:213	RPL21P67		Pseudoger	chr6:131469059-131
ENSG0000(	1009	25.23603	chr6:213	RPSAP45		Pseudoger	chr6:112355841-112
ENSG0000(	1009	25.23603	chr6:213	RNU4-35P		smallRNA	chr6:121453981-121
ENSG0000(	1009	25.23603	chr6:213	ENSG00000286339		lncRNA	chr6:118565660-118
ENSG0000(	1009	25.23603	chr6:213	ENSG00000219758		Pseudoger	chr6:113839279-113
ENSG0000(	1009	25.23603	chr6:213	ENSG00000289925		lncRNA	chr6:113635183-113
ENSG0000(	1009	25.23603	chr6:213	TBC1D32	NCGv7	protein_c	chr6:121079494-121
ENSG0000(	1009	25.23603	chr6:213	CLVS2		protein_c	chr6:122996235-123
ENSG0000(	1009	25.23603	chr6:213	RNF217	NCGv7	protein_c	chr6:124962437-125
ENSG0000(	1009	25.23603	chr6:213	RSPO3	NCGv7	protein_c	chr6:127118671-127
ENSG0000(	1009	25.23603	chr6:213	ENSG00000233558		Pseudoger	chr6:116258493-116
ENSG0000(	1009	25.23603	chr6:213	TRAF3IP2		protein_c	chr6:111555381-111
ENSG0000(	1009	25.23603	chr6:213	ENSG00000288977		lncRNA	chr6:131125625-131
ENSG0000(	1009	25.23603	chr6:213	PLN		protein_c	chr6:118548296-118
ENSG0000(	1009	25.23603	chr6:213	L3MBTL3		protein_c	chr6:130013699-130
ENSG0000(	1009	25.23603	chr6:213	ENSG00000237234		lncRNA	chr6:112154765-112

ENSG0000(	1009	25.23603	chr6:213!	ENSG00000272472		lncRNA	chr6:122643388-122
ENSG0000(	1009	25.23603	chr6:213!	ENSG00000216316		Pseudoger	chr6:119269133-119
ENSG0000(	1009	25.23603	chr6:213!	NCOA7		protein_c	chr6:125781161-125
ENSG0000(	1009	25.23603	chr6:213!	ENSG00000290064		lncRNA	chr6:121619478-121
ENSG0000(	1009	25.23603	chr6:213!	ENSG00000285446		protein_c	chr6:116399395-116
ENSG0000(	1009	25.23603	chr6:213!	RNA5SP217		Pseudoger	chr6:127317883-127
ENSG0000(	1009	25.23603	chr6:213!	SOGA3		protein_c	chr6:127472806-127
ENSG0000(	1009	25.23603	chr6:213!	EPB41L2		protein_c	chr6:130839347-131
ENSG0000(	1009	25.23603	chr6:213!	ENSG00000290067		lncRNA	chr6:131217724-131
ENSG0000(	1009	25.23603	chr6:213!	MOXD1		protein_c	chr6:132296055-132
ENSG0000(	1009	25.23603	chr6:213!	KRT18P65		Pseudoger	chr6:112361437-112
ENSG0000(	1009	25.23603	chr6:213!	STX7		protein_c	chr6:132445867-132
ENSG0000(	1009	25.23603	chr6:213!	ENSG00000288560		lncRNA	chr6:112988311-113
ENSG0000(	1009	25.23603	chr6:213!	Y_RNA		smallRNA	chr6:128761717-128
ENSG0000(	1009	25.23603	chr6:213!	RPL5P18		Pseudoger	chr6:127362513-127
ENSG0000(	1009	25.23603	chr6:213!	ENSG00000271789		lncRNA	chr6:111297126-111
ENSG0000(	1009	25.23603	chr6:213!	HINT3		protein_c	chr6:125956770-125
ENSG0000(	1009	25.23603	chr6:213!	ENSG00000289262		lncRNA	chr6:131294417-131
ENSG0000(	1009	25.23603	chr6:213!	TPD52L1		protein_c	chr6:125119049-125
ENSG0000(	1009	25.23603	chr6:213!	HDDC2		protein_c	chr6:125219962-125
ENSG0000(	1009	25.23603	chr6:213!	ENSG00000285652		lncRNA	chr6:122975198-122
ENSG0000(	1009	25.23603	chr6:213!	ENSG00000287992		lncRNA	chr6:120840733-120
ENSG0000(	1009	25.23603	chr6:213!	Z97352.1		smallRNA	chr6:129747737-129
ENSG0000(	1009	25.23603	chr6:213!	ENSG00000226149		lncRNA	chr6:129439485-129
ENSG0000(	1009	25.23603	chr6:213!	FAM162B		protein_c	chr6:116752197-116
ENSG0000(	1009	25.23603	chr6:213!	RNA5SP216		Pseudoger	chr6:125979812-125
ENSG0000(	1009	25.23603	chr6:213!	VNN2		protein_c	chr6:132743870-132
ENSG0000(	1009	25.23603	chr6:213!	LINC02527		lncRNA	chr6:111900305-111
ENSG0000(	1009	25.23603	chr6:213!	TAAR9		protein_c	chr6:132538277-132
ENSG0000(	1009	25.23603	chr6:213!	VNN1		protein_c	chr6:132680849-132
ENSG0000(	1009	25.23603	chr6:213!	MED23	NCGv7	protein_c	chr6:131573966-131
ENSG0000(	1009	25.23603	chr6:213!	ENSG00000237115		Pseudoger	chr6:131825981-131
ENSG0000(	1009	25.23603	chr6:213!	RNU6-475P		smallRNA	chr6:114866873-114
ENSG0000(	1009	25.23603	chr6:213!	SNORD101		smallRNA	chr6:132815307-132
ENSG0000(	1009	25.23603	chr6:213!	ENSG00000260212		Pseudoger	chr6:127659424-127
ENSG0000(	1009	25.23603	chr6:213!	AMD1		protein_c	chr6:110874770-110
ENSG0000(	1009	25.23603	chr6:213!	COL10A1		protein_c	chr6:116118909-116
ENSG0000(	1009	25.23603	chr6:213!	RN7SKP51		smallRNA	chr6:117301455-117
ENSG0000(	1009	25.23603	chr6:213!	AL354936.1		smallRNA	chr6:123740434-123
ENSG0000(	1009	25.23603	chr6:213!	MESTP1		Pseudoger	chr6:128928995-128
ENSG0000(	1009	25.23603	chr6:213!	AL357519.1		smallRNA	chr6:113704833-113
ENSG0000(	1009	25.23603	chr6:213!	TRDN-AS1		lncRNA	chr6:123389421-123
ENSG0000(	1009	25.23603	chr6:213!	RN7SKP18		smallRNA	chr6:117299364-117
ENSG0000(	1009	25.23603	chr6:213!	ENSG00000272356		lncRNA	chr6:111309203-111
ENSG0000(	1009	25.23603	chr6:213!	TRMT11		protein_c	chr6:125986479-126
ENSG0000(	1009	25.23603	chr6:213!	ENSG00000289304		lncRNA	chr6:116569604-116
ENSG0000(	1009	25.23603	chr6:213!	ENSG00000226079		lncRNA	chr6:114523443-114
ENSG0000(	1009	25.23603	chr6:213!	SLC16A10		protein_c	chr6:111087503-111
ENSG0000(	1009	25.23603	chr6:213!	FEM1AP3		Pseudoger	chr6:112365704-112
ENSG0000(	1009	25.23603	chr6:213!	ENSG00000237321		lncRNA	chr6:123823240-123
ENSG0000(	1009	25.23603	chr6:213!	ENSG00000285691		lncRNA	chr6:123519697-123

ENSG0000(	1009	25.23603	chr6:213[illegible]	RPS27AP11		Pseudoger	chr6:113581501-113
ENSG0000(	1009	25.23603	chr6:213[illegible]	SMPDL3A		protein_c	chr6:122789049-122
ENSG0000(	1009	25.23603	chr6:213[illegible]	FRK		protein_c	chr6:115931149-116
ENSG0000(	1009	25.23603	chr6:213[illegible]	ENSG00000285941		lncRNA	chr6:123589711-123
ENSG0000(	1009	25.23603	chr6:213[illegible]	ENSG00000220506		Pseudoger	chr6:111494991-111
ENSG0000(	1009	25.23603	chr6:213[illegible]	ENSG00000279453		TEC	chr6:122436789-122
ENSG0000(	1009	25.23603	chr6:213[illegible]	ENSG00000287933		lncRNA	chr6:116033901-116
ENSG0000(	1009	25.23603	chr6:213[illegible]	TAAR4P		Pseudoger	chr6:132594398-132
ENSG0000(	1009	25.23603	chr6:213[illegible]	HSF2		protein_c	chr6:122399551-122
ENSG0000(	1009	25.23603	chr6:213[illegible]	DNAJA1P4		Pseudoger	chr6:114349483-114
ENSG0000(	1009	25.23603	chr6:213[illegible]	ENSG00000220447		Pseudoger	chr6:121381472-121
ENSG0000(	1009	25.23603	chr6:213[illegible]	LINC02534		lncRNA	chr6:115633540-115
ENSG0000(	1009	25.23603	chr6:213[illegible]	CCN6	NCGv7	protein_c	chr6:112054075-112
ENSG0000(	1009	25.23603	chr6:213[illegible]	NCOA7-AS1		lncRNA	chr6:125797856-125
ENSG0000(	1009	25.23603	chr6:213[illegible]	FCF1P5		Pseudoger	chr6:111353702-111
ENSG0000(	1009	25.23603	chr6:213[illegible]	KRT18P22		Pseudoger	chr6:116457323-116
ENSG0000(	1009	25.23603	chr6:213[illegible]	ENSG00000271208		lncRNA	chr6:112234165-112
ENSG0000(	1009	25.23603	chr6:213[illegible]	RN7SKP245		smallRNA	chr6:131820334-131
ENSG0000(	1009	25.23603	chr6:213[illegible]	SLC25A5P7		Pseudoger	chr6:121653795-121
ENSG0000(	1009	25.23603	chr6:213[illegible]	RFX6	NCGv7	protein_c	chr6:116877212-116
ENSG0000(	1009	25.23603	chr6:213[illegible]	EEF1DP5		Pseudoger	chr6:128580113-128
ENSG0000(	1009	25.23603	chr6:213[illegible]	HS3ST5		protein_c	chr6:114055596-114
ENSG0000(	1009	25.23603	chr6:213[illegible]	ENSG00000218187		Pseudoger	chr6:127654860-127
ENSG0000(	1009	25.23603	chr6:213[illegible]	LAMA4	NCGv7	protein_c	chr6:112107931-112
ENSG0000(	1009	25.23603	chr6:213[illegible]	HLFP1		Pseudoger	chr6:132674885-132
ENSG0000(	1009	25.23603	chr6:213[illegible]	C6orf58		protein_c	chr6:127519455-127
ENSG0000(	1009	25.23603	chr6:213[illegible]	ENSG00000287731		lncRNA	chr6:126719560-126
ENSG0000(	1009	25.23603	chr6:213[illegible]	ENSG00000226181		lncRNA	chr6:117451130-117
ENSG0000(	1009	25.23603	chr6:213[illegible]	Y_RNA		smallRNA	chr6:130573996-130
ENSG0000(	1009	25.23603	chr6:213[illegible]	Y_RNA		smallRNA	chr6:128584390-128
ENSG0000(	1009	25.23603	chr6:213[illegible]	ENSG00000220694		Pseudoger	chr6:127435636-127
ENSG0000(	1009	25.23603	chr6:213[illegible]	ENSG00000229923		lncRNA	chr6:130697312-130
ENSG0000(	1009	25.23603	chr6:213[illegible]	RNU6-960P		smallRNA	chr6:111091213-111
ENSG0000(	1009	25.23603	chr6:213[illegible]	SERINC1		protein_c	chr6:122443351-122
ENSG0000(	1009	25.23603	chr6:213[illegible]	LINC01013		lncRNA	chr6:131901848-132
ENSG0000(	1009	25.23603	chr6:213[illegible]	THEMIS		protein_c	chr6:127708072-127
ENSG0000(	1009	25.23603	chr6:213[illegible]	YAP1P3		Pseudoger	chr6:126627484-126
ENSG0000(	1009	25.23603	chr6:213[illegible]	MAN1A1		protein_c	chr6:119177205-119
ENSG0000(	1009	25.23603	chr6:213[illegible]	FAM184A		protein_c	chr6:118959763-119
ENSG0000(	1009	25.23603	chr6:213[illegible]	MCM9		protein_c	chr6:118813442-118
ENSG0000(	1009	25.23603	chr6:213[illegible]	ENSG00000271498		Pseudoger	chr6:112825939-112
ENSG0000(	1009	25.23603	chr6:213[illegible]	ASF1A		protein_c	chr6:118894152-118
ENSG0000(	1009	25.23603	chr6:213[illegible]	PPP1R14BP5		Pseudoger	chr6:126257921-126
ENSG0000(	1009	25.23603	chr6:213[illegible]	RBM11P1		Pseudoger	chr6:132764660-132
ENSG0000(	1009	25.23603	chr6:213[illegible]	HMGB1P13		Pseudoger	chr6:132868218-132
ENSG0000(	1009	25.23603	chr6:213[illegible]	YWHAZP4		Pseudoger	chr6:127355756-127
ENSG0000(	1009	25.23603	chr6:213[illegible]	CEP85L		protein_c	chr6:118460772-118
ENSG0000(	1009	25.23603	chr6:213[illegible]	ENSG00000289256		lncRNA	chr6:111873588-111
ENSG0000(	1009	25.23603	chr6:213[illegible]	RSPH4A		protein_c	chr6:116616479-116
ENSG0000(	1009	25.23603	chr6:213[illegible]	ENSG00000272714		Pseudoger	chr6:121389324-121
ENSG0000(	1009	25.23603	chr6:213[illegible]	RWDD1		protein_c	chr6:116571409-116

ENSG0000(	1009	25.23603	chr6:2135	DSE	NCGv7	protein_c	chr6:116254173-116
ENSG0000(	1009	25.23603	chr6:2135	PA2G4P5		Pseudoger	chr6:112616703-112
ENSG0000(	1009	25.23603	chr6:2135	RPS12		protein_c	chr6:132814569-132
ENSG0000(	1009	25.23603	chr6:2135	ENSG00000219619		Pseudoger	chr6:115358498-115
ENSG0000(	1009	25.23603	chr6:2135	FYN	NCGv7;AC	protein_c	chr6:111660332-111
ENSG0000(	1009	25.23603	chr6:2135	RPF2		protein_c	chr6:110982015-111
ENSG0000(	1009	25.23603	chr6:2135	KIAA0408		protein_c	chr6:127438406-127
ENSG0000(	1009	25.23603	chr6:2135	RNU6-1163P		smallRNA	chr6:112971493-112
ENSG0000(	1009	25.23603	chr6:2135	B3GALNT2P1		Pseudoger	chr6:129800908-129
ENSG0000(	1009	25.23603	chr6:2135	RPL23AP48		Pseudoger	chr6:121679972-121
ENSG0000(	1009	25.23603	chr6:2135	PRELID1P1		Pseudoger	chr6:126643488-126
ENSG0000(	1009	25.23603	chr6:2135	CCN2	AC	protein_c	chr6:131948176-131
ENSG0000(	1009	25.23603	chr6:2135	ARG1		protein_c	chr6:131470832-131
ENSG0000(	1009	25.23603	chr6:2135	RNF146		protein_c	chr6:127266726-127
ENSG0000(	1009	25.23603	chr6:2135	ENSG00000223811		lncRNA	chr6:113357003-113
ENSG0000(	1009	25.23603	chr6:2135	TAAR3P		Pseudoger	chr6:132608252-132
ENSG0000(	1009	25.23603	chr6:2135	snoU13		smallRNA	chr6:110963466-110
ENSG0000(	1009	25.23603	chr6:2135	AKAP7		protein_c	chr6:131135467-131
ENSG0000(	1009	25.23603	chr6:2135	ENSG00000287253		lncRNA	chr6:117262243-117
ENSG0000(	1009	25.23603	chr6:2135	ENSG00000281613		protein_c	chr6:112236806-112
ENSG0000(	1009	25.23603	chr6:2135	ENSG00000287258		lncRNA	chr6:122562172-122
ENSG0000(	1009	25.23603	chr6:2135	MIR548H5		smallRNA	chr6:131792172-131
ENSG0000(	1009	25.23603	chr6:2135	ENSG00000216917		Pseudoger	chr6:131780721-131
ENSG0000(	1009	25.23603	chr6:2135	RNU6-1226P		smallRNA	chr6:112196440-112
ENSG0000(	1009	25.23603	chr6:2135	LINC02536		lncRNA	chr6:127664554-127
ENSG0000(	1009	25.23603	chr6:2135	NT5DC1		protein_c	chr6:116100851-116
ENSG0000(	1009	25.23603	chr6:2135	ECHDC1		protein_c	chr6:127288712-127
ENSG0000(	1009	25.23603	chr6:2135	VNN3P		Pseudoger	chr6:132722784-132
ENSG0000(	1009	25.23603	chr6:2135	LINC02880		lncRNA	chr6:113904132-113
ENSG0000(	1009	25.23603	chr6:2135	AL357515.1		smallRNA	chr6:110969773-110
ENSG0000(	1009	25.23603	chr6:2135	RPL5P21		Pseudoger	chr6:129756298-129
ENSG0000(	1009	25.23603	chr6:2135	ENSG00000287100		lncRNA	chr6:119349886-119
ENSG0000(	1009	25.23603	chr6:2135	MARCKS		protein_c	chr6:113857345-113
ENSG0000(	1009	25.23603	chr6:2135	RNU6-214P		smallRNA	chr6:120526294-120
ENSG0000(	1009	25.23603	chr6:2135	SOCS5P5		Pseudoger	chr6:113222166-113
ENSG0000(	1009	25.23603	chr6:2135	FABP7		protein_c	chr6:122779716-122
ENSG0000(	1009	25.23603	chr6:2135	CALHM4		protein_c	chr6:116529013-116
ENSG0000(	1009	25.23603	chr6:2135	DCBLD1		protein_c	chr6:117453817-117
ENSG0000(	1009	25.23603	chr6:2135	ENSG00000233351		lncRNA	chr6:129479615-129
ENSG0000(	1009	25.23603	chr6:2135	ENSG00000287097		lncRNA	chr6:114822994-115
ENSG0000(	1009	25.23603	chr6:2135	SAMD3		protein_c	chr6:130144315-130
ENSG0000(	1009	25.23603	chr6:2135	TMEM200A		protein_c	chr6:130366017-130
ENSG0000(	1009	25.23603	chr6:2135	LINC02523		lncRNA	chr6:125674353-125
ENSG0000(	1009	25.23603	chr6:2135	ENSG00000282218		protein_c	chr6:117318211-117
ENSG0000(	1009	25.23603	chr6:2135	PTPRK	NCGv7	protein_c	chr6:127968785-128
ENSG0000(	1009	25.23603	chr6:2135	snoU13		smallRNA	chr6:128613201-128
ENSG0000(	1009	25.23603	chr6:2135	GJA1		protein_c	chr6:121435595-121
ENSG0000(	1009	25.23603	chr6:2135	MROCKI		lncRNA	chr6:113868013-113
ENSG0000(	1009	25.23603	chr6:2135	BRD7P4		Pseudoger	chr6:111430260-111
ENSG0000(	1009	25.23603	chr6:2135	SSXP10		Pseudoger	chr6:118589070-118
ENSG0000(	1009	25.23603	chr6:2135	REV3L	NCGv7	protein_c	chr6:111299028-111

ENSG0000(	1009	25.23603	chr6:2135	AL132671.1		smallRNA	chr6:117445390-117
ENSG0000(	1009	25.23603	chr6:2135	ENSG00000226409		lncRNA	chr6:125370034-125
ENSG0000(	1009	25.23603	chr6:2135	TSPYL1	NCGv7	protein_c	chr6:116267760-116
ENSG0000(	1009	25.23603	chr6:2135	RNU6-1286P		smallRNA	chr6:121354354-121
ENSG0000(	1009	25.23603	chr6:2135	VGLL2		protein_c	chr6:117265558-117
ENSG0000(	1009	25.23603	chr6:2135	ENSG00000275339		Pseudoger	chr6:122454358-122
ENSG0000(	1009	25.23603	chr6:2135	ENSG00000234484		lncRNA	chr6:132752675-132
ENSG0000(	1009	25.23603	chr6:2135	CCNG1P1		Pseudoger	chr6:132698783-132
ENSG0000(	1009	25.23603	chr6:2135	RNU1-18P		smallRNA	chr6:122211648-122
ENSG0000(	1009	25.23603	chr6:2135	RPS29P13		Pseudoger	chr6:117048670-117
ENSG0000(	1009	25.23603	chr6:2135	HEY2		protein_c	chr6:125747664-125
ENSG0000(	1009	25.23603	chr6:2135	PKIB		protein_c	chr6:122471931-122
ENSG0000(	1009	25.23603	chr6:2135	TAAR5		protein_c	chr6:132588592-132
ENSG0000(	1009	25.23603	chr6:2135	TRAPPC3L		protein_c	chr6:116494989-116
ENSG0000(	1009	25.23603	chr6:2135	TRAF3IP2-AS1		lncRNA	chr6:111483459-111
ENSG0000(	1009	25.23603	chr6:2135	ENSG00000289372		lncRNA	chr6:117658838-117
ENSG0000(	1009	25.23603	chr6:2135	RNU6-906P		smallRNA	chr6:111008270-111
ENSG0000(	1009	25.23603	chr6:2135	AL365214.1		smallRNA	chr6:112361848-112
ENSG0000(	1009	25.23603	chr6:2135	ENSG00000217139		Pseudoger	chr6:121682925-121
ENSG0000(	1009	25.23603	chr6:2135	LINC02518		lncRNA	chr6:113428540-113
ENSG0000(	1009	25.23603	chr6:2135	GPRC6A		protein_c	chr6:116792085-116
ENSG0000(	1009	25.23603	chr6:2135	KPNA5		protein_c	chr6:116681187-116
ENSG0000(	1009	25.23603	chr6:2135	GSTM2P1		Pseudoger	chr6:111046868-111
ENSG0000(	1009	25.23603	chr6:2135	ENSG00000218857		Pseudoger	chr6:131184325-131
ENSG0000(	1009	25.23603	chr6:2135	LAMA4-AS1		lncRNA	chr6:112236093-112
ENSG0000(	1009	25.23603	chr6:2135	AL513123.1		smallRNA	chr6:113514987-113
ENSG0000(	1009	25.23603	chr6:2135	ENSG00000280155		TEC	chr6:132130252-132
ENSG0000(	1009	25.23603	chr6:2135	ENSG00000289376		lncRNA	chr6:115901795-116
ENSG0000(	1009	25.23603	chr6:2135	ENSG00000236326		lncRNA	chr6:116244187-116
ENSG0000(	1009	25.23603	chr6:2135	Y_RNA		smallRNA	chr6:121378797-121
ENSG0000(	1009	25.23603	chr6:2135	ENSG00000236347		lncRNA	chr6:113531118-113
ENSG0000(	1009	25.23603	chr6:2135	SMLR1		protein_c	chr6:130827406-130
ENSG0000(	1009	25.23603	chr6:2135	AL357519.2		smallRNA	chr6:113602915-113
ENSG0000(	1009	25.23603	chr6:2135	CALHM5		protein_c	chr6:116511639-116
ENSG0000(	1009	25.23603	chr6:2135	EEF1A1P36		Pseudoger	chr6:132271982-132
ENSG0000(	1009	25.23603	chr6:2135	CBX3P9		Pseudoger	chr6:116453014-116
ENSG0000(	1009	25.23603	chr6:2135	SLC35F1	NCGv7	protein_c	chr6:117907264-118
ENSG0000(	1009	25.23603	chr6:2135	snoU13		smallRNA	chr6:113840814-113
ENSG0000(	1009	25.23603	chr6:2135	LINC02541		lncRNA	chr6:113616927-113
ENSG0000(	1009	25.23603	chr6:2135	REV3L-IT1		lncRNA	chr6:111360641-111
ENSG0000(	1009	25.23603	chr6:2135	RNU6-253P		smallRNA	chr6:117457734-117
ENSG0000(	1009	25.23603	chr6:2135	LAMA2	NCGv7	protein_c	chr6:128883138-129
ENSG0000(	1009	25.23603	chr6:2135	HDAC2	NCGv7	protein_c	chr6:113933028-114
ENSG0000(	1009	25.23603	chr6:2135	RNA5SP215		Pseudoger	chr6:120686621-120
ENSG0000(	1009	25.23603	chr6:2135	RNU2-8P		smallRNA	chr6:121580332-121
ENSG0000(	1009	25.23603	chr6:2135	AL357514.1		smallRNA	chr6:112532182-112
ENSG0000(	1009	25.23603	chr6:2135	RNU6-200P		smallRNA	chr6:126590287-126
ENSG0000(	1009	25.23603	chr6:2135	ENSG00000231912		lncRNA	chr6:113791829-113
ENSG0000(	1009	25.23603	chr6:2135	MFSD4B-DT		lncRNA	chr6:111227747-111
ENSG0000(	1009	25.23603	chr6:2135	AL137251.1		smallRNA	chr6:130434265-130
ENSG0000(	1009	25.23603	chr6:2135	ENSG00000232299		lncRNA	chr6:112476538-112

ENSG0000(	1009	25.23603	chr6:213	RNU4-18P		smallRNA	chr6:131642818-131
ENSG0000(	1009	25.23603	chr6:213	RNU6-861P		smallRNA	chr6:129436875-129
ENSG0000(	1009	25.23603	chr6:213	Y_RNA		smallRNA	chr6:119057880-119
ENSG0000(	1009	25.23603	chr6:213	ENSG00000288916		lncRNA	chr6:113586188-113
ENSG0000(	1009	25.23603	chr6:213	snoU13		smallRNA	chr6:111646327-111
ENSG0000(	1009	25.23603	chr6:213	BRD7P3		Pseudoger	chr6:118501430-118
ENSG0000(	1009	25.23603	chr6:213	TAAR3P		lncRNA	chr6:132608225-132
ENSG0000(	1009	25.23603	chr6:213	ATP5MGP2		Pseudoger	chr6:122859678-122
ENSG0000(	1009	25.23603	chr6:213	ENSG00000286616		lncRNA	chr6:111505307-111
ENSG0000(	1009	25.23603	chr6:213	TUBE1		protein_c	chr6:112070663-112
ENSG0000(	1009	25.23603	chr6:213	MIR548AJ1		smallRNA	chr6:132115192-132
ENSG0000(	1009	25.23603	chr6:213	RFPL4B		protein_c	chr6:112347330-112
ENSG0000(	1009	25.23603	chr6:213	NUS1		protein_c	chr6:117675469-117
ENSG0000(	1009	25.23603	chr6:213	ENSG00000289190		lncRNA	chr6:128520728-128
ENSG0000(	1009	25.23603	chr6:213	ZUP1		protein_c	chr6:116635618-116
ENSG0000(	1009	25.23603	chr6:213	ENSG00000219284		Pseudoger	chr6:127632958-127
ENSG0000(	1009	25.23603	chr6:213	SNORD100		smallRNA	chr6:132816802-132
ENSG0000(	1009	25.23603	chr6:213	MIR3144		smallRNA	chr6:120015179-120
ENSG0000(	1009	25.23603	chr6:213	ENSG00000279960		TEC	chr6:132891924-132
ENSG0000(	1009	25.23603	chr6:213	ENSG00000216663		Pseudoger	chr6:112325753-112
ENSG0000(	1009	25.23603	chr6:213	ROS1	NCGv7;AC	protein_c	chr6:117287353-117
ENSG0000(	1009	25.23603	chr6:213	NKAIN2		protein_c	chr6:123803865-124
ENSG0000(	1009	25.23603	chr6:213	ENSG00000287728		lncRNA	chr6:113129707-113
ENSG0000(	1009	25.23603	chr6:213	RNA5SP213		Pseudoger	chr6:114220681-114
ENSG0000(	1009	25.23603	chr6:213	ENSG00000219302		Pseudoger	chr6:129819637-129
ENSG0000(	1009	25.23603	chr6:213	ENSG00000255330		protein_c	chr6:127438406-127
ENSG0000(	1009	25.23603	chr6:213	ENSG00000286663		lncRNA	chr6:132599972-132
ENSG0000(	1009	25.23603	chr6:213	Z84488.1		smallRNA	chr6:116457732-116
ENSG0000(	1009	25.23603	chr6:213	COX6A1P3		Pseudoger	chr6:120781220-120
ENSG0000(	1009	25.23603	chr6:213	ENSG00000289871		lncRNA	chr6:121857813-121
ENSG0000(	1009	25.23603	chr6:213	NUDT19P3		Pseudoger	chr6:114019621-114
ENSG0000(	1009	25.23603	chr6:213	ENSG00000255389		lncRNA	chr6:111599875-111
ENSG0000(	1009	25.23603	chr6:213	NIP7P3		Pseudoger	chr6:116137058-116
ENSG0000(	1009	25.23603	chr6:213	RNF217-AS	AC	lncRNA	chr6:124644434-124
ENSG0000(	1009	25.23603	chr6:213	ENSG00000219329		Pseudoger	chr6:110923566-110
ENSG0000(	1009	25.23603	chr6:213	ENPP3		protein_c	chr6:131628442-131
ENSG0000(	1009	25.23603	chr6:213	MIR588		smallRNA	chr6:126484631-126
ENSG0000(	1009	25.23603	chr6:213	ENSG00000216809		Pseudoger	chr6:118452469-118
ENSG0000(	1009	25.23603	chr6:213	ENSG00000286914		lncRNA	chr6:112392363-112
ENSG0000(	1009	25.23603	chr6:213	GTF3C6		protein_c	chr6:110958706-110
ENSG0000(	1009	25.23603	chr6:213	OR2A4		protein_c	chr6:131699644-131
ENSG0000(	1009	25.23603	chr6:213	RPS4XP9		Pseudoger	chr6:126683036-126
ENSG0000(	1009	25.23603	chr6:213	SELENOKP3		Pseudoger	chr6:118757518-118
ENSG0000(	1009	25.23603	chr6:213	RNU6-194P		smallRNA	chr6:119327281-119
ENSG0000(	1009	25.23603	chr6:213	TMEM244		protein_c	chr6:129831244-129
ENSG0000(	1009	25.23603	chr6:213	TPI1P3		Pseudoger	chr6:116038756-116
ENSG0000(	1009	25.23603	chr6:213	RNU4-76P		smallRNA	chr6:121542486-121
ENSG0000(	1009	25.23603	chr6:213	ENSG00000253194		lncRNA	chr6:118934770-119
ENSG0000(	1009	25.23603	chr6:213	FAM229B		protein_c	chr6:112087591-112
ENSG0000(	1009	25.23603	chr6:213	FCF1P10		Pseudoger	chr6:113010937-113
ENSG0000(	1009	25.23603	chr6:213	HDAC2-AS2		lncRNA	chr6:113969701-114

ENSG0000(	1009	25.23603	chr6:213E	U3		smallRNA	chr6:113781315-113
ENSG0000(	1009	25.23603	chr6:213E	SELENOKP2		Pseudoger	chr6:131819803-131
ENSG0000(	1009	25.23603	chr6:213E	TAAR7P		Pseudoger	chr6:132559024-132
ENSG0000(	1009	25.23603	chr6:213E	CALHM6	NCGv7	protein_c	chr6:116461370-116
ENSG0000(	1009	25.23603	chr6:213E	snoU13		smallRNA	chr6:112476726-112
ENSG0000(	1009	25.23603	chr6:213E	ENPP1		protein_c	chr6:131808016-131
ENSG0000(	1009	25.23603	chr6:213E	MRPS17P5		Pseudoger	chr6:127909833-127
ENSG0000(	1009	25.23603	chr6:213E	CENPW	AC	protein_c	chr6:126340115-126
ENSG0000(	1009	25.23603	chr6:213E	TSPYL4	NCGv7	protein_c	chr6:116249964-116
ENSG0000(	1009	25.23603	chr6:213E	GOPC	NCGv7;AC	protein_c	chr6:117560269-117
ENSG0000(	1009	25.23603	chr6:213E	ENSG00000277408		Pseudoger	chr6:122531123-122
ENSG0000(	1007	25.18601	chr1:116(	RNU5E-6P		smallRNA	chr1:44819883-4481
ENSG0000(	1006	25.16099	chr1:100E	RNU7-122P		smallRNA	chr1:109207794-109
ENSG0000(	1005	25.13598	chr7:330(	IFRD1		protein_c	chr7:112422887-112
ENSG0000(	1005	25.13598	chr7:330(	ENSG00000234273		lncRNA	chr7:109521981-109
ENSG0000(	1005	25.13598	chr7:330(	ZNF277-AS1		lncRNA	chr7:112328189-112
ENSG0000(	1005	25.13598	chr7:330(	ENSG00000289630		lncRNA	chr7:113118666-113
ENSG0000(	1005	25.13598	chr7:330(	CBLL1	AC	protein_c	chr7:107743073-107
ENSG0000(	1005	25.13598	chr7:330(	THAP5	NCGv7	protein_c	chr7:108554543-108
ENSG0000(	1005	25.13598	chr7:330(	MIR3666		smallRNA	chr7:114653345-114
ENSG0000(	1005	25.13598	chr7:330(	LAMB4	DriverDB\	protein_c	chr7:108023548-108
ENSG0000(	1005	25.13598	chr7:330(	ENSG00000243621		Pseudoger	chr7:111394875-111
ENSG0000(	1005	25.13598	chr7:330(	NRCAM		protein_c	chr7:108147623-108
ENSG0000(	1005	25.13598	chr7:330(	TMEM168		protein_c	chr7:112762377-112
ENSG0000(	1005	25.13598	chr7:330(	ENSG00000224595		lncRNA	chr7:114414244-114
ENSG0000(	1005	25.13598	chr7:330(	LAMB1	NCGv7	protein_c	chr7:107923799-108
ENSG0000(	1005	25.13598	chr7:330(	DLD		protein_c	chr7:107891162-107
ENSG0000(	1005	25.13598	chr7:330(	MTND6P24		Pseudoger	chr7:112373733-112
ENSG0000(	1005	25.13598	chr7:330(	SLC26A4		protein_c	chr7:107660828-107
ENSG0000(	1005	25.13598	chr7:330(	SLC26A3	NCGv7	protein_c	chr7:107765467-107
ENSG0000(	1005	25.13598	chr7:330(	RPL36P13		Pseudoger	chr7:114297114-114
ENSG0000(	1005	25.13598	chr7:330(	ENSG00000225457		lncRNA	chr7:113100663-113
ENSG0000(	1005	25.13598	chr7:330(	LSMEM1		protein_c	chr7:112480853-112
ENSG0000(	1005	25.13598	chr7:330(	PRKAR2B	NCGv7	protein_c	chr7:107044705-107
ENSG0000(	1005	25.13598	chr7:330(	ENSG00000228341		Pseudoger	chr7:107450083-107
ENSG0000(	1005	25.13598	chr7:330(	ENSG00000272072		lncRNA	chr7:107192559-107
ENSG0000(	1005	25.13598	chr7:330(	MTND5P8		Pseudoger	chr7:112372647-112
ENSG0000(	1005	25.13598	chr7:330(	EIF3IP1		Pseudoger	chr7:109959218-109
ENSG0000(	1005	25.13598	chr7:330(	LINC02903		lncRNA	chr7:108883975-108
ENSG0000(	1005	25.13598	chr7:330(	ENSG00000278424		Pseudoger	chr7:110215891-110
ENSG0000(	1005	25.13598	chr7:330(	ENSG00000279043		TEC	chr7:108900105-108
ENSG0000(	1005	25.13598	chr7:330(	RNA5SP237		Pseudoger	chr7:111953653-111
ENSG0000(	1005	25.13598	chr7:330(	AC020606.1		smallRNA	chr7:114629855-114
ENSG0000(	1005	25.13598	chr7:330(	ENSG00000279288		TEC	chr7:113075124-113
ENSG0000(	1005	25.13598	chr7:330(	ENSG00000227948		Pseudoger	chr7:111411319-111
ENSG0000(	1005	25.13598	chr7:330(	ENSG00000270425		Pseudoger	chr7:108720608-108
ENSG0000(	1005	25.13598	chr7:330(	AC005048.1		smallRNA	chr7:108036846-108
ENSG0000(	1005	25.13598	chr7:330(	RPL7P32		Pseudoger	chr7:108510233-108
ENSG0000(	1005	25.13598	chr7:330(	ENSG00000228540		lncRNA	chr7:110724159-110
ENSG0000(	1005	25.13598	chr7:330(	PRKAR2B-AS1		lncRNA	chr7:107066591-107
ENSG0000(	1005	25.13598	chr7:330(	RAC1P6		Pseudoger	chr7:115136475-115

ENSG0000(	1005	25.13598	chr7:330(	ENSG00000226965		lncRNA	chr7:110108031-110
ENSG0000(	1005	25.13598	chr7:330(	NPM1P14		Pseudoger	chr7:112520488-112
ENSG0000(	1005	25.13598	chr7:330(	U3		smallRNA	chr7:107999792-107
ENSG0000(	1005	25.13598	chr7:330(	HBP1		protein_c	chr7:107168961-107
ENSG0000(	1005	25.13598	chr7:330(	DUS4L		protein_c	chr7:107563484-107
ENSG0000(	1005	25.13598	chr7:330(	SMIM30		protein_c	chr7:113116718-113
ENSG0000(	1005	25.13598	chr7:330(	PPP1R3A	NCGv7	protein_c	chr7:113876777-114
ENSG0000(	1005	25.13598	chr7:330(	snoU109		smallRNA	chr7:107603363-107
ENSG0000(	1005	25.13598	chr7:330(	RNA5SP238		Pseudoger	chr7:114613787-114
ENSG0000(	1005	25.13598	chr7:330(	ENSG00000288640		protein_c	chr7:112450460-112
ENSG0000(	1005	25.13598	chr7:330(	ENSG00000278894		TEC	chr7:113451072-113
ENSG0000(	1005	25.13598	chr7:330(	MTCYBP24		Pseudoger	chr7:112374324-112
ENSG0000(	1005	25.13598	chr7:330(	Y_RNA		smallRNA	chr7:115207894-115
ENSG0000(	1005	25.13598	chr7:330(	RPL3P8		Pseudoger	chr7:109998434-109
ENSG0000(	1005	25.13598	chr7:330(	ENSG00000223646		lncRNA	chr7:112616440-112
ENSG0000(	1005	25.13598	chr7:330(	WBP1LP2		Pseudoger	chr7:107628553-107
ENSG0000(	1005	25.13598	chr7:330(	ENSG00000287186		lncRNA	chr7:110590082-110
ENSG0000(	1005	25.13598	chr7:330(	RN7SKP187		smallRNA	chr7:112288623-112
ENSG0000(	1005	25.13598	chr7:330(	BUB3P1		Pseudoger	chr7:108994031-108
ENSG0000(	1005	25.13598	chr7:330(	ENSG00000288634		protein_c	chr7:112450487-112
ENSG0000(	1005	25.13598	chr7:330(	snoU13		smallRNA	chr7:111109268-111
ENSG0000(	1005	25.13598	chr7:330(	ENSG00000282859		lncRNA	chr7:114560961-114
ENSG0000(	1005	25.13598	chr7:330(	SLC26A4-AS1		lncRNA	chr7:107650260-107
ENSG0000(	1005	25.13598	chr7:330(	ENSG00000271368		Pseudoger	chr7:109763574-109
ENSG0000(	1005	25.13598	chr7:330(	ENSG00000230192		Pseudoger	chr7:109660094-109
ENSG0000(	1005	25.13598	chr7:330(	GPR85		protein_c	chr7:113078331-113
ENSG0000(	1005	25.13598	chr7:330(	ENSG00000225647		lncRNA	chr7:108598352-108
ENSG0000(	1005	25.13598	chr7:330(	LRRN3	NCGv7	protein_c	chr7:111091006-111
ENSG0000(	1005	25.13598	chr7:330(	BMT2		protein_c	chr7:112819147-112
ENSG0000(	1005	25.13598	chr7:330(	COG5		protein_c	chr7:107201372-107
ENSG0000(	1005	25.13598	chr7:330(	AC004492.1		smallRNA	chr7:107294885-107
ENSG0000(	1005	25.13598	chr7:330(	ENSG00000287011		lncRNA	chr7:111757051-111
ENSG0000(	1005	25.13598	chr7:330(	CBLL1-AS1		lncRNA	chr7:107739999-107
ENSG0000(	1005	25.13598	chr7:330(	IMMP2L	NCGv7	protein_c	chr7:110662644-111
ENSG0000(	1005	25.13598	chr7:330(	DNAJB9	NCGv7	protein_c	chr7:108569867-108
ENSG0000(	1005	25.13598	chr7:330(	DOCK4	NCGv7	protein_c	chr7:111726110-112
ENSG0000(	1005	25.13598	chr7:330(	ENSG00000272854		lncRNA	chr7:107579557-107
ENSG0000(	1005	25.13598	chr7:330(	ENSG00000180019		Pseudoger	chr7:112446086-112
ENSG0000(	1005	25.13598	chr7:330(	MIPEPP1		Pseudoger	chr7:112735166-112
ENSG0000(	1005	25.13598	chr7:330(	DOCK4-AS1		lncRNA	chr7:111808516-111
ENSG0000(	1005	25.13598	chr7:330(	PNPLA8	NCGv7	protein_c	chr7:108470417-108
ENSG0000(	1005	25.13598	chr7:330(	BCAP29		protein_c	chr7:107580246-107
ENSG0000(	1005	25.13598	chr7:330(	FOXP2	NCGv7	protein_c	chr7:114086327-114
ENSG0000(	1005	25.13598	chr7:330(	ENSG00000229603		lncRNA	chr7:108909453-108
ENSG0000(	1005	25.13598	chr7:330(	ENSG00000273055		lncRNA	chr7:107942116-107
ENSG0000(	1005	25.13598	chr7:330(	RPL7AP42		Pseudoger	chr7:111971222-111
ENSG0000(	1005	25.13598	chr7:330(	LINC01392		lncRNA	chr7:115061537-115
ENSG0000(	1005	25.13598	chr7:330(	ZNF277		protein_c	chr7:112206695-112
ENSG0000(	1005	25.13598	chr7:330(	ENSG00000230941		lncRNA	chr7:109322320-109
ENSG0000(	1005	25.13598	chr7:330(	PIGCP2		Pseudoger	chr7:107808734-107
ENSG0000(	1005	25.13598	chr7:330(	AC005161.1		smallRNA	chr7:110928318-110

ENSG0000(	1005	25.13598	chr7:330(	LINC01393		lncRNA	chr7:115030564-115
ENSG0000(	1005	25.13598	chr7:330(	ENSG00000287592		lncRNA	chr7:113486407-113
ENSG0000(	1005	25.13598	chr7:330(	HRAT17		lncRNA	chr7:112953282-112
ENSG0000(	1005	25.13598	chr7:330(	GPR22		protein_c	chr7:107470018-107
ENSG0000(	1005	25.13598	chr7:330(	AC007567.1		smallRNA	chr7:108358307-108
ENSG0000(	1005	25.13598	chr7:330(	ENSG00000270997		Pseudoger	chr7:113415689-113
ENSG0000(	1005	25.13598	chr7:330(	DUS4L-BCAP29		protein_c	chr7:107563971-107
ENSG0000(	1005	25.13598	chr7:330(	MDFIC	NCGv7	protein_c	chr7:114922094-115
ENSG0000(	987	24.68579	chr1:1005	ENSG00000228076		Pseudoger	chr1:108766841-108
ENSG0000(	986	24.66078	chr7:330(	ENSG00000278683		Pseudoger	chr7:101273322-101
ENSG0000(	986	24.66078	chr7:330(	EPO		protein_c	chr7:100720468-100
ENSG0000(	986	24.66078	chr7:330(	ENSG00000233683		Pseudoger	chr7:101388868-101
ENSG0000(	986	24.66078	chr7:330(	SLC12A9		protein_c	chr7:100826820-100
ENSG0000(	986	24.66078	chr7:330(	LINC01007		lncRNA	chr7:101562779-101
ENSG0000(	986	24.66078	chr7:330(	RNU6-1104P		smallRNA	chr7:101269938-101
ENSG0000(	986	24.66078	chr7:330(	ZAN	NCGv7	protein_c	chr7:100733595-100
ENSG0000(	986	24.66078	chr7:330(	IFT22		protein_c	chr7:101310914-101
ENSG0000(	986	24.66078	chr7:330(	EPHB4	NCGv7	protein_c	chr7:100802565-100
ENSG0000(	986	24.66078	chr7:330(	MUC17	NCGv7	protein_c	chr7:101020072-101
ENSG0000(	986	24.66078	chr7:330(	COL26A1		protein_c	chr7:101362875-101
ENSG0000(	986	24.66078	chr7:330(	TRIM56		protein_c	chr7:101085481-101
ENSG0000(	986	24.66078	chr7:330(	DGAT2L7P		Pseudoger	chr7:101201809-101
ENSG0000(	986	24.66078	chr7:330(	RN7SL549P		smallRNA	chr7:100906299-100
ENSG0000(	986	24.66078	chr7:330(	RPSAP46		Pseudoger	chr7:101203614-101
ENSG0000(	986	24.66078	chr7:330(	MUC12	NCGv7	protein_c	chr7:100972000-101
ENSG0000(	986	24.66078	chr7:330(	AC125387.1		smallRNA	chr7:101682015-101
ENSG0000(	986	24.66078	chr7:330(	MIR4653		smallRNA	chr7:101159473-101
ENSG0000(	986	24.66078	chr7:330(	SRRT	NCGv7	protein_c	chr7:100875103-100
ENSG0000(	986	24.66078	chr7:330(	TRIP6	AC	protein_c	chr7:100867387-100
ENSG0000(	986	24.66078	chr7:330(	LNCPRESS1		lncRNA	chr7:101299558-101
ENSG0000(	986	24.66078	chr7:330(	ACHE	DriverDB\	protein_c	chr7:100889994-100
ENSG0000(	986	24.66078	chr7:330(	MUC12-AS1		lncRNA	chr7:101014319-101
ENSG0000(	986	24.66078	chr7:330(	VGF		protein_c	chr7:101162509-101
ENSG0000(	986	24.66078	chr7:330(	AP1S1	NCGv7	protein_c	chr7:101154456-101
ENSG0000(	986	24.66078	chr7:330(	SLC12A9-AS1		lncRNA	chr7:100837314-100
ENSG0000(	986	24.66078	chr7:330(	ZNHIT1		protein_c	chr7:101218165-101
ENSG0000(	986	24.66078	chr7:330(	ENSG00000288749		lncRNA	chr7:101302767-101
ENSG0000(	986	24.66078	chr7:330(	MYL10		protein_c	chr7:101613330-101
ENSG0000(	986	24.66078	chr7:330(	AZGP1P2		Pseudoger	chr7:101287482-101
ENSG0000(	986	24.66078	chr7:330(	RN7SKP54		smallRNA	chr7:101058299-101
ENSG0000(	986	24.66078	chr7:330(	PLOD3	DriverDB\	protein_c	chr7:101205977-101
ENSG0000(	986	24.66078	chr7:330(	FIS1		protein_c	chr7:101239458-101
ENSG0000(	986	24.66078	chr7:330(	RN7SL750P		smallRNA	chr7:100821029-100
ENSG0000(	986	24.66078	chr7:330(	UFSP1	DriverDB\	protein_c	chr7:100888721-100
ENSG0000(	986	24.66078	chr7:330(	MOGAT3	DriverDB\	protein_c	chr7:101195007-101
ENSG0000(	986	24.66078	chr7:330(	EMSLR		lncRNA	chr7:101308270-101
ENSG0000(	986	24.66078	chr7:330(	SERPINE1		protein_c	chr7:101127104-101
ENSG0000(	986	24.66078	chr7:330(	CLDN15	DriverDB\	protein_c	chr7:101232092-101
ENSG0000(	986	24.66078	chr7:330(	NAT16		protein_c	chr7:101170496-101
ENSG0000(	981	24.53572	chr7:330(	CAPZA1P4		Pseudoger	chr7:131892616-131
ENSG0000(	977	24.43568	chr7:330(	FDPSP7		Pseudoger	chr7:76968197-7696

ENSG0000(	977	24.43568	chr7:330(	LINC03009		lncRNA	chr7:76549360-7662
ENSG0000(	977	24.43568	chr7:330(	POMZP3		protein_c	chr7:76609986-7662
ENSG0000(	977	24.43568	chr7:330(	ENSG00000214243		Pseudoger	chr7:76650401-7665
ENSG0000(	977	24.43568	chr7:330(	ENSG00000231183		lncRNA	chr7:76902480-7691
ENSG0000(	977	24.43568	chr7:330(	SPDYE16		protein_c	chr7:76531313-7654
ENSG0000(	977	24.43568	chr7:330(	Y_RNA		smallRNA	chr7:76523605-7652
ENSG0000(	977	24.43568	chr7:330(	ENSG00000225703		lncRNA	chr7:76972679-7697
ENSG0000(	977	24.43568	chr7:330(	AC004980.1		smallRNA	chr7:76697502-7669
ENSG0000(	977	24.43568	chr7:330(	LINC03009		Pseudoger	chr7:76549618-7655
ENSG0000(	977	24.43568	chr7:330(	UPK3B		protein_c	chr7:76510525-7651
ENSG0000(	977	24.43568	chr7:330(	ENSG00000230305		Pseudoger	chr7:76524515-7653
ENSG0000(	977	24.43568	chr7:330(	ENSG00000250778		Pseudoger	chr7:76521611-7652
ENSG0000(	977	24.43568	chr7:330(	ENSG00000285666		lncRNA	chr7:76818377-7690
ENSG0000(	976	24.41067	chr11:76(	MIR4489		smallRNA	chr11:65649192-656
ENSG0000(	969	24.23559	chr11:76(	OR4D6		protein_c	chr11:59456961-594
ENSG0000(	969	24.23559	chr11:76(	OR5AN2P		Pseudoger	chr11:59309681-593
ENSG0000(	969	24.23559	chr11:76(	FADS3		protein_c	chr11:61873519-618
ENSG0000(	969	24.23559	chr11:76(	GLYATL1B		protein_c	chr11:59086307-590
ENSG0000(	969	24.23559	chr11:76(	GLYATL1		protein_c	chr11:58905398-590
ENSG0000(	969	24.23559	chr11:76(	GLYATL1P1		Pseudoger	chr11:58978787-589
ENSG0000(	969	24.23559	chr11:76(	RN7SL435P		smallRNA	chr11:59291053-592
ENSG0000(	969	24.23559	chr11:76(	MS4A19P		Pseudoger	chr11:60577856-606
ENSG0000(	969	24.23559	chr11:76(	OR4D10		protein_c	chr11:59473315-594
ENSG0000(	969	24.23559	chr11:76(	RNU6-933P		smallRNA	chr11:60985061-609
ENSG0000(	969	24.23559	chr11:76(	ENSG00000254477		lncRNA	chr11:59752578-597
ENSG0000(	969	24.23559	chr11:76(	OR5A2		protein_c	chr11:59416969-594
ENSG0000(	969	24.23559	chr11:76(	EEF1DP8		Pseudoger	chr11:62169293-621
ENSG0000(	969	24.23559	chr11:76(	ENSG00000244176		Pseudoger	chr11:62011751-620
ENSG0000(	969	24.23559	chr11:76(	RNU6-779P		smallRNA	chr11:59498992-594
ENSG0000(	969	24.23559	chr11:76(	TMA16P1		Pseudoger	chr11:58796237-587
ENSG0000(	969	24.23559	chr11:76(	TMEM216	NCGv7	protein_c	chr11:61392393-613
ENSG0000(	969	24.23559	chr11:76(	OR5A1		protein_c	chr11:59436469-594
ENSG0000(	969	24.23559	chr11:76(	ENSG00000255240		lncRNA	chr11:58933643-590
ENSG0000(	969	24.23559	chr11:76(	OR10V1		protein_c	chr11:59712916-597
ENSG0000(	969	24.23559	chr11:76(	NPM1P35		Pseudoger	chr11:62330946-623
ENSG0000(	969	24.23559	chr11:76(	ENSG00000289194		lncRNA	chr11:62153730-621
ENSG0000(	969	24.23559	chr11:76(	GLYATL2		protein_c	chr11:58834065-589
ENSG0000(	969	24.23559	chr11:76(	MS4A1		protein_c	chr11:60455846-604
ENSG0000(	969	24.23559	chr11:76(	ENSG00000279491		TEC	chr11:61967729-619
ENSG0000(	969	24.23559	chr11:76(	ENSG00000255126		lncRNA	chr11:62391516-623
ENSG0000(	969	24.23559	chr11:76(	ENSG00000255139		lncRNA	chr11:59565923-596
ENSG0000(	969	24.23559	chr11:76(	LINC02739		lncRNA	chr11:59560234-595
ENSG0000(	969	24.23559	chr11:76(	ENSG00000254877		Pseudoger	chr11:58967596-589
ENSG0000(	969	24.23559	chr11:76(	CCDC86		protein_c	chr11:60842113-608
ENSG0000(	969	24.23559	chr11:76(	PRPF19		protein_c	chr11:60890547-609
ENSG0000(	969	24.23559	chr11:76(	MS4A5		protein_c	chr11:60429572-604
ENSG0000(	969	24.23559	chr11:76(	TMEM109		protein_c	chr11:60914158-609
ENSG0000(	969	24.23559	chr11:76(	MS4A14		protein_c	chr11:60378530-604
ENSG0000(	969	24.23559	chr11:76(	MS4A7		protein_c	chr11:60378485-603
ENSG0000(	969	24.23559	chr11:76(	ENSG00000254926		Pseudoger	chr11:58748999-587
ENSG0000(	969	24.23559	chr11:76(	MS4A6E	NCGv7	protein_c	chr11:60327255-603

ENSG0000(	969	24.23559	chr11:76(	ENSG00000250230		lncRNA	chr11:61588442-616
ENSG0000(	969	24.23559	chr11:76(	LINC02705		lncRNA	chr11:60159687-601
ENSG0000(	969	24.23559	chr11:76(	RNU6-1243P		smallRNA	chr11:61937894-619
ENSG0000(	969	24.23559	chr11:76(	SLC15A3		protein_c	chr11:60937060-609
ENSG0000(	969	24.23559	chr11:76(	CD5		protein_c	chr11:61102489-611
ENSG0000(	969	24.23559	chr11:76(	MRPL16		protein_c	chr11:59806140-598
ENSG0000(	969	24.23559	chr11:76(	OR5BB1P		Pseudoger	chr11:59391354-593
ENSG0000(	969	24.23559	chr11:76(	SDHAF2	NCGv7	protein_c	chr11:61430042-614
ENSG0000(	969	24.23559	chr11:76(	STX3		protein_c	chr11:59713456-598
ENSG0000(	969	24.23559	chr11:76(	DDB1		protein_c	chr11:61299451-613
ENSG0000(	969	24.23559	chr11:76(	VPS37C		protein_c	chr11:61130257-611
ENSG0000(	969	24.23559	chr11:76(	PATL1		protein_c	chr11:59636716-596
ENSG0000(	969	24.23559	chr11:76(	FABP5P7		Pseudoger	chr11:59781318-597
ENSG0000(	969	24.23559	chr11:76(	VWCE		protein_c	chr11:61258286-612
ENSG0000(	969	24.23559	chr11:76(	RAB3IL1		protein_c	chr11:61897301-619
ENSG0000(	969	24.23559	chr11:76(	BEST1		protein_c	chr11:61950063-619
ENSG0000(	969	24.23559	chr11:76(	FTH1	NCGv7	protein_c	chr11:61959718-619
ENSG0000(	969	24.23559	chr11:76(	ENSG00000255118		lncRNA	chr11:62336911-623
ENSG0000(	969	24.23559	chr11:76(	RN7SL42P		smallRNA	chr11:59214407-592
ENSG0000(	969	24.23559	chr11:76(	SCGB2A2		protein_c	chr11:62270158-622
ENSG0000(	969	24.23559	chr11:76(	SRD5A3P1		Pseudoger	chr11:59898185-598
ENSG0000(	969	24.23559	chr11:76(	CD6		protein_c	chr11:60971680-610
ENSG0000(	969	24.23559	chr11:76(	PATL1-DT		lncRNA	chr11:59669312-596
ENSG0000(	969	24.23559	chr11:76(	RCC2P6		Pseudoger	chr11:62371146-623
ENSG0000(	969	24.23559	chr11:76(	RNU7-58P		smallRNA	chr11:59558800-595
ENSG0000(	969	24.23559	chr11:76(	MS4A13		protein_c	chr11:60515392-605
ENSG0000(	969	24.23559	chr11:76(	OR4D8P		Pseudoger	chr11:59491652-594
ENSG0000(	969	24.23559	chr11:76(	ENSG00000255959		lncRNA	chr11:60835996-608
ENSG0000(	969	24.23559	chr11:76(	ENSG00000255947		lncRNA	chr11:61654665-616
ENSG0000(	969	24.23559	chr11:76(	ENSG00000289635		lncRNA	chr11:59105304-591
ENSG0000(	969	24.23559	chr11:76(	ENSG00000255931		lncRNA	chr11:61496440-615
ENSG0000(	969	24.23559	chr11:76(	ENSG00000287264		lncRNA	chr11:59545602-595
ENSG0000(	969	24.23559	chr11:76(	ENSG00000255845		lncRNA	chr11:60813932-608
ENSG0000(	969	24.23559	chr11:76(	PGA4		protein_c	chr11:61222347-612
ENSG0000(	969	24.23559	chr11:76(	TCN1		protein_c	chr11:59852800-598
ENSG0000(	969	24.23559	chr11:76(	TMEM258		protein_c	chr11:61768501-617
ENSG0000(	969	24.23559	chr11:76(	FADS2		protein_c	chr11:61792980-618
ENSG0000(	969	24.23559	chr11:76(	CBLIF		protein_c	chr11:59829273-598
ENSG0000(	969	24.23559	chr11:76(	DAGLA		protein_c	chr11:61680391-617
ENSG0000(	969	24.23559	chr11:76(	ENSG00000279549		TEC	chr11:61143380-611
ENSG0000(	969	24.23559	chr11:76(	MIR4488		smallRNA	chr11:61508596-615
ENSG0000(	969	24.23559	chr11:76(	OR4D11		protein_c	chr11:59503576-595
ENSG0000(	969	24.23559	chr11:76(	ENSG00000279632		TEC	chr11:61426448-614
ENSG0000(	969	24.23559	chr11:76(	FAM111A	NCGv7	protein_c	chr11:59142748-591
ENSG0000(	969	24.23559	chr11:76(	INCENP		protein_c	chr11:62123998-621
ENSG0000(	969	24.23559	chr11:76(	LRRC10B		protein_c	chr11:61508749-615
ENSG0000(	969	24.23559	chr11:76(	ENSG00000289621		lncRNA	chr11:58770377-587
ENSG0000(	969	24.23559	chr11:76(	CPSF7		protein_c	chr11:61402641-614
ENSG0000(	969	24.23559	chr11:76(	MS4A3		protein_c	chr11:60056587-600
ENSG0000(	969	24.23559	chr11:76(	OOSP2	NCGv7	protein_c	chr11:60040409-600
ENSG0000(	969	24.23559	chr11:76(	ZP1		protein_c	chr11:60867542-608

ENSG0000(	969	24.23559	chr11:76(	ENSG00000256443		lncRNA	chr11:61539516-615
ENSG0000(	969	24.23559	chr11:76(	FADS1		protein_c	chr11:61799627-618
ENSG0000(	969	24.23559	chr11:76(	MIR1908		smallRNA	chr11:61815161-618
ENSG0000(	969	24.23559	chr11:76(	OR5AN1		protein_c	chr11:59358895-593
ENSG0000(	969	24.23559	chr11:76(	TMEM138		protein_c	chr11:61361964-613
ENSG0000(	969	24.23559	chr11:76(	ENSG00000279246		TEC	chr11:61352244-613
ENSG0000(	969	24.23559	chr11:76(	ENSG00000256220		lncRNA	chr11:61227168-612
ENSG0000(	969	24.23559	chr11:76(	ENSG00000256196		lncRNA	chr11:60916339-609
ENSG0000(	969	24.23559	chr11:76(	ENSG00000290884		lncRNA	chr11:58878302-588
ENSG0000(	969	24.23559	chr11:76(	TKFC		protein_c	chr11:61333220-613
ENSG0000(	969	24.23559	chr11:76(	AP003064.1		smallRNA	chr11:62463635-624
ENSG0000(	969	24.23559	chr11:76(	TMEM132A		protein_c	chr11:60924460-609
ENSG0000(	969	24.23559	chr11:76(	WARS1P1		Pseudoger	chr11:59255360-592
ENSG0000(	969	24.23559	chr11:76(	SCGB1D4		protein_c	chr11:62296281-622
ENSG0000(	969	24.23559	chr11:76(	ENSG00000256813		lncRNA	chr11:60841806-608
ENSG0000(	969	24.23559	chr11:76(	MS4A18		protein_c	chr11:60729304-607
ENSG0000(	969	24.23559	chr11:76(	OR10Y1P		Pseudoger	chr11:59728519-597
ENSG0000(	969	24.23559	chr11:76(	TMEM109-DT		lncRNA	chr11:60913166-609
ENSG0000(	969	24.23559	chr11:76(	SCGB1D1		protein_c	chr11:62190216-621
ENSG0000(	969	24.23559	chr11:76(	ENSG00000254404		lncRNA	chr11:62213427-622
ENSG0000(	969	24.23559	chr11:76(	FEN1	NCGv7	protein_c	chr11:61792911-617
ENSG0000(	969	24.23559	chr11:76(	OOSP4B		protein_c	chr11:59978020-600
ENSG0000(	969	24.23559	chr11:76(	LINC02733		lncRNA	chr11:62049863-620
ENSG0000(	969	24.23559	chr11:76(	ENSG00000254424		Pseudoger	chr11:62072882-620
ENSG0000(	969	24.23559	chr11:76(	OR5BR1P		Pseudoger	chr11:59332757-593
ENSG0000(	969	24.23559	chr11:76(	ENSG00000255381		Pseudoger	chr11:59142121-591
ENSG0000(	969	24.23559	chr11:76(	MS4A4A		protein_c	chr11:60185657-603
ENSG0000(	969	24.23559	chr11:76(	ENSG00000279878		TEC	chr11:61525708-615
ENSG0000(	969	24.23559	chr11:76(	PRPF19-DT		lncRNA	chr11:60906789-609
ENSG0000(	969	24.23559	chr11:76(	ENSG00000255331		Pseudoger	chr11:60077350-600
ENSG0000(	969	24.23559	chr11:76(	GLYATL1P4		Pseudoger	chr11:59042737-590
ENSG0000(	969	24.23559	chr11:76(	RN7SKP192		smallRNA	chr11:59706057-597
ENSG0000(	969	24.23559	chr11:76(	MIR611		smallRNA	chr11:61792495-617
ENSG0000(	969	24.23559	chr11:76(	SYT7		protein_c	chr11:61513714-615
ENSG0000(	969	24.23559	chr11:76(	FAM111B	NCGv7	protein_c	chr11:59107185-591
ENSG0000(	969	24.23559	chr11:76(	OR10V2P		Pseudoger	chr11:59748811-597
ENSG0000(	969	24.23559	chr11:76(	ENSG00000255523		lncRNA	chr11:58917015-589
ENSG0000(	969	24.23559	chr11:76(	FAM111A-DT		lncRNA	chr11:59130133-591
ENSG0000(	969	24.23559	chr11:76(	MPEG1	NCGv7	protein_c	chr11:59208510-592
ENSG0000(	969	24.23559	chr11:76(	SCGB1A1		protein_c	chr11:62405103-624
ENSG0000(	969	24.23559	chr11:76(	ENSG00000256591		protein_c	chr11:61429220-614
ENSG0000(	969	24.23559	chr11:76(	ENSG00000214797		lncRNA	chr11:59268876-592
ENSG0000(	969	24.23559	chr11:76(	ENSG00000214788		lncRNA	chr11:59942879-599
ENSG0000(	969	24.23559	chr11:76(	MS4A4E		protein_c	chr11:60200270-602
ENSG0000(	969	24.23559	chr11:76(	ENSG00000288788		lncRNA	chr11:59101577-591
ENSG0000(	969	24.23559	chr11:76(	PGA5		protein_c	chr11:61241175-612
ENSG0000(	969	24.23559	chr11:76(	AP003064.2		smallRNA	chr11:62372656-623
ENSG0000(	969	24.23559	chr11:76(	LINC02954		lncRNA	chr11:61055392-610
ENSG0000(	969	24.23559	chr11:76(	ENSG00000255446		lncRNA	chr11:62421845-624
ENSG0000(	969	24.23559	chr11:76(	ENSG00000285656		lncRNA	chr11:62116470-621
ENSG0000(	969	24.23559	chr11:76(	MS4A2		protein_c	chr11:60088261-600

ENSG0000(	969	24.23559	chr11:76(	MS4A6A		protein_c	chr11:60172015-601
ENSG0000(	969	24.23559	chr11:76(	MIR3162		smallRNA	chr11:59595077-595
ENSG0000(	969	24.23559	chr11:76(	OR4D7P		Pseudoger	chr11:59531754-595
ENSG0000(	969	24.23559	chr11:76(	MYRF-AS1		lncRNA	chr11:61746493-617
ENSG0000(	969	24.23559	chr11:76(	LINC00301		Pseudoger	chr11:60615729-606
ENSG0000(	969	24.23559	chr11:76(	PGA3		protein_c	chr11:61203307-612
ENSG0000(	969	24.23559	chr11:76(	GLYATL1P2		Pseudoger	chr11:58884947-588
ENSG0000(	969	24.23559	chr11:76(	ASRGL1		protein_c	chr11:62337448-623
ENSG0000(	969	24.23559	chr11:76(	OOSP1		protein_c	chr11:59938432-599
ENSG0000(	969	24.23559	chr11:76(	MS4A8		protein_c	chr11:60699585-607
ENSG0000(	969	24.23559	chr11:76(	MS4A12		protein_c	chr11:60492778-605
ENSG0000(	969	24.23559	chr11:76(	DTX4	NCGv7	protein_c	chr11:59171430-592
ENSG0000(	969	24.23559	chr11:76(	MYRF		protein_c	chr11:61752636-617
ENSG0000(	969	24.23559	chr11:76(	MS4A15		protein_c	chr11:60756867-607
ENSG0000(	969	24.23559	chr11:76(	OOSP3		protein_c	chr11:59878782-598
ENSG0000(	969	24.23559	chr11:76(	ENSG00000254704		Pseudoger	chr11:59287339-592
ENSG0000(	969	24.23559	chr11:76(	SLC25A47P1		Pseudoger	chr11:59263389-592
ENSG0000(	969	24.23559	chr11:76(	PPP1R32		protein_c	chr11:61481120-614
ENSG0000(	969	24.23559	chr11:76(	CYB561A3		protein_c	chr11:61348754-613
ENSG0000(	969	24.23559	chr11:76(	RPLP0P2		Pseudoger	chr11:61615036-616
ENSG0000(	969	24.23559	chr11:76(	OR10V3P		Pseudoger	chr11:59741325-597
ENSG0000(	969	24.23559	chr11:76(	MS4A10		protein_c	chr11:60785333-608
ENSG0000(	969	24.23559	chr11:76(	OSBP		protein_c	chr11:59574398-596
ENSG0000(	969	24.23559	chr11:76(	PTGDR2	NCGv7	protein_c	chr11:60850933-608
ENSG0000(	969	24.23559	chr11:76(	ENSG00000254786		Pseudoger	chr11:58995439-589
ENSG0000(	969	24.23559	chr11:76(	SCGB2A1		protein_c	chr11:62208673-622
ENSG0000(	969	24.23559	chr11:76(	SCGB1D2		protein_c	chr11:62242239-622
ENSG0000(	969	24.23559	chr11:76(	OR4D9		protein_c	chr11:59511368-595
ENSG0000(	969	24.23559	chr11:76(	ENSG00000289268		lncRNA	chr11:61797424-617
ENSG0000(	969	24.23559	chr11:76(	OOSP4A		protein_c	chr11:59964033-599
ENSG0000(	959	23.98548	chr7:330(	ENSG00000275834		Pseudoger	chr7:96306810-9630
ENSG0000(	957	23.93546	chr11:76(	Y_RNA		smallRNA	chr11:64296037-642
ENSG0000(	956	23.91045	chr7:330(	Y_RNA		smallRNA	chr7:116909877-116
ENSG0000(	955	23.88544	chr11:76(	ENSG00000254487		Pseudoger	chr11:49817778-498
ENSG0000(	955	23.88544	chr11:76(	ENSG00000265973		Pseudoger	chr11:49843488-498
ENSG0000(	955	23.88544	chr11:76(	TRIM51FP		Pseudoger	chr11:49833140-498
ENSG0000(	955	23.88544	chr11:76(	ENSG00000255268		Pseudoger	chr11:49824839-498
ENSG0000(	955	23.88544	chr17:454	ENSG00000273982		lncRNA	chr17:61354763-613
ENSG0000(	954	23.86043	chr7:330(	CYP3A137P		Pseudoger	chr7:99820018-9982
ENSG0000(	953	23.83542	chr1:1005	ENSG00000288803		lncRNA	chr1:110680508-110
ENSG0000(	953	23.83542	chr1:1005	GSTM1	DriverDB\	protein_c	chr1:109687814-109
ENSG0000(	953	23.83542	chr1:1005	GSTM4		protein_c	chr1:109656099-109
ENSG0000(	953	23.83542	chr1:1005	ENSG00000244716		Pseudoger	chr1:108992282-108
ENSG0000(	953	23.83542	chr1:1005	GNAT2		protein_c	chr1:109603091-109
ENSG0000(	953	23.83542	chr1:1005	AMIGO1		protein_c	chr1:109504178-109
ENSG0000(	953	23.83542	chr1:1005	SLC6A17-AS1		lncRNA	chr1:110165948-110
ENSG0000(	953	23.83542	chr1:1005	ENSG00000254942		lncRNA	chr1:109539906-109
ENSG0000(	953	23.83542	chr1:1005	ENSG00000290117		lncRNA	chr1:109546610-109
ENSG0000(	953	23.83542	chr1:1005	ENSG00000235526		lncRNA	chr1:110177643-110
ENSG0000(	953	23.83542	chr1:1005	CYMP-AS1		lncRNA	chr1:110487680-110
ENSG0000(	953	23.83542	chr1:1005	AMPD2		protein_c	chr1:109616104-109

ENSG0000(	953	23.83542	chr1:1005	GSTM2		protein_c	chr1:109668022-109
ENSG0000(	953	23.83542	chr1:1005	ENSG00000235005		lncRNA	chr1:109884176-109
ENSG0000(	953	23.83542	chr1:1005	SLC16A4-AS1		lncRNA	chr1:110370154-110
ENSG0000(	953	23.83542	chr1:1005	LAMTOR5	AC	protein_c	chr1:110401249-110
ENSG0000(	953	23.83542	chr1:1005	ENSG00000282887		lncRNA	chr1:110472543-110
ENSG0000(	953	23.83542	chr1:1005	ENSG00000251484		Pseudoger	chr1:109103535-109
ENSG0000(	953	23.83542	chr1:1005	RPL7P8		Pseudoger	chr1:109651370-109
ENSG0000(	953	23.83542	chr1:1005	LINC01768		lncRNA	chr1:109828355-109
ENSG0000(	953	23.83542	chr1:1005	GPSM2		protein_c	chr1:108875350-108
ENSG0000(	953	23.83542	chr1:1005	CLCC1		protein_c	chr1:108881885-108
ENSG0000(	953	23.83542	chr1:1005	STXBP3		protein_c	chr1:108746674-108
ENSG0000(	953	23.83542	chr1:1005	ENSG00000260246		lncRNA	chr1:109693117-109
ENSG0000(	953	23.83542	chr1:1005	LINC01397		lncRNA	chr1:110082651-110
ENSG0000(	953	23.83542	chr1:1005	PROK1		protein_c	chr1:110451149-110
ENSG0000(	953	23.83542	chr1:1005	CELSR2		protein_c	chr1:109249539-109
ENSG0000(	953	23.83542	chr1:1005	SNORA25		smallRNA	chr1:110272484-110
ENSG0000(	953	23.83542	chr1:1005	TMEM167B		protein_c	chr1:109090764-109
ENSG0000(	953	23.83542	chr1:1005	ENSG00000228665		Pseudoger	chr1:109030067-109
ENSG0000(	953	23.83542	chr1:1005	GSTM5	NCGv7	protein_c	chr1:109711780-109
ENSG0000(	953	23.83542	chr1:1005	ENSG00000241720		lncRNA	chr1:109725820-109
ENSG0000(	953	23.83542	chr1:1005	CYB561D1		protein_c	chr1:109494052-109
ENSG0000(	953	23.83542	chr1:1005	ENSG00000282852		Pseudoger	chr1:110256754-110
ENSG0000(	953	23.83542	chr1:1005	ATXN7L2		protein_c	chr1:109483479-109
ENSG0000(	953	23.83542	chr1:1005	CFAP276		protein_c	chr1:109105951-109
ENSG0000(	953	23.83542	chr1:1005	CSF1	AC	protein_c	chr1:109910242-109
ENSG0000(	953	23.83542	chr1:1005	SORT1		protein_c	chr1:109309568-109
ENSG0000(	953	23.83542	chr1:1005	PSRC1		protein_c	chr1:109279556-109
ENSG0000(	953	23.83542	chr1:1005	AKNAD1		protein_c	chr1:108815898-108
ENSG0000(	953	23.83542	chr1:1005	GSTM3		protein_c	chr1:109733932-109
ENSG0000(	953	23.83542	chr1:1005	AL365361.1		smallRNA	chr1:110652942-110
ENSG0000(	953	23.83542	chr1:1005	ENSG00000283999		lncRNA	chr1:110473756-110
ENSG0000(	953	23.83542	chr1:1005	ENSG00000228703		lncRNA	chr1:109628417-109
ENSG0000(	953	23.83542	chr1:1005	KCNC4		protein_c	chr1:110210314-110
ENSG0000(	953	23.83542	chr1:1005	KCNA10		protein_c	chr1:110517217-110
ENSG0000(	953	23.83542	chr1:1005	EPS8L3		protein_c	chr1:109750080-109
ENSG0000(	953	23.83542	chr1:1005	SPATA42		lncRNA	chr1:108857217-108
ENSG0000(	953	23.83542	chr1:1005	ENSG00000237349		Pseudoger	chr1:108986963-108
ENSG0000(	953	23.83542	chr1:1005	LAMTOR5-AS1		lncRNA	chr1:110347116-110
ENSG0000(	953	23.83542	chr1:1005	Y_RNA		smallRNA	chr1:110764408-110
ENSG0000(	953	23.83542	chr1:1005	KCNA3		protein_c	chr1:110653560-110
ENSG0000(	953	23.83542	chr1:1005	RBM15-AS1		lncRNA	chr1:110286375-110
ENSG0000(	953	23.83542	chr1:1005	RNU6V		smallRNA	chr1:109591534-109
ENSG0000(	953	23.83542	chr1:1005	TAF13		protein_c	chr1:109062496-109
ENSG0000(	953	23.83542	chr1:1005	KCNA2		protein_c	chr1:110519837-110
ENSG0000(	953	23.83542	chr1:1005	RBM15	NCGv7;AC	protein_c	chr1:110338506-110
ENSG0000(	953	23.83542	chr1:1005	ALX3		protein_c	chr1:110059870-110
ENSG0000(	953	23.83542	chr1:1005	NDUFA5P10		Pseudoger	chr1:109810642-109
ENSG0000(	953	23.83542	chr1:1005	CYMP		Pseudoger	chr1:110480752-110
ENSG0000(	953	23.83542	chr1:1005	ENSG00000270380		lncRNA	chr1:110456505-110
ENSG0000(	953	23.83542	chr1:1005	FNDC7		protein_c	chr1:108712908-108
ENSG0000(	953	23.83542	chr1:1005	ENSG00000232971		lncRNA	chr1:108734256-108

ENSG0000(	953	23.83542	chr1:1005	STRIP1		protein_c	chr1:110031577-110
ENSG0000(	953	23.83542	chr1:1005	GPR61		protein_c	chr1:109539872-109
ENSG0000(	953	23.83542	chr1:1005	ELAPOR1		protein_c	chr1:109113679-109
ENSG0000(	953	23.83542	chr1:1005	ENSG00000258634		lncRNA	chr1:110058340-110
ENSG0000(	953	23.83542	chr1:1005	TMEM167B-DT		lncRNA	chr1:109087971-109
ENSG0000(	953	23.83542	chr1:1005	KCNC4-DT		lncRNA	chr1:110208834-110
ENSG0000(	953	23.83542	chr1:1005	AHCYL1	AC	protein_c	chr1:109984765-110
ENSG0000(	953	23.83542	chr1:1005	PSMA5		protein_c	chr1:109399042-109
ENSG0000(	953	23.83542	chr1:1005	UBL4B		protein_c	chr1:110112443-110
ENSG0000(	953	23.83542	chr1:1005	SLC16A4	NCGv7	protein_c	chr1:110362851-110
ENSG0000(	953	23.83542	chr1:1005	WDR47		protein_c	chr1:108970214-109
ENSG0000(	953	23.83542	chr1:1005	MYBPHL		protein_c	chr1:109292365-109
ENSG0000(	953	23.83542	chr1:1005	ENSG00000225113		lncRNA	chr1:109596225-109
ENSG0000(	953	23.83542	chr1:1005	MIR197		smallRNA	chr1:109598893-109
ENSG0000(	953	23.83542	chr1:1005	ENSG00000261055		lncRNA	chr1:109895973-109
ENSG0000(	953	23.83542	chr1:1005	SLC6A17		protein_c	chr1:110150494-110
ENSG0000(	953	23.83542	chr1:1005	SARS1		protein_c	chr1:109213918-109
ENSG0000(	953	23.83542	chr1:1005	RANP5		Pseudoger	chr1:109046828-109
ENSG0000(	953	23.83542	chr1:1005	SYPL2		protein_c	chr1:109466546-109
ENSG0000(	953	23.83542	chr1:1005	GNAI3		protein_c	chr1:109548615-109
ENSG0000(	951	23.78539	chr7:330(	TUBB3P2		Pseudoger	chr7:134734898-134
ENSG0000(	951	23.78539	chr7:330(	ENSG00000259920		lncRNA	chr7:130481491-130
ENSG0000(	951	23.78539	chr7:330(	ENSG00000225144		lncRNA	chr7:132264152-132
ENSG0000(	951	23.78539	chr7:330(	CHCHD3		protein_c	chr7:132784870-133
ENSG0000(	951	23.78539	chr7:330(	NDUFB9P2		Pseudoger	chr7:131753746-131
ENSG0000(	951	23.78539	chr7:330(	ENSG00000271204		lncRNA	chr7:130930209-130
ENSG0000(	951	23.78539	chr7:330(	LRGUK	DriverDB\	protein_c	chr7:134127299-134
ENSG0000(	951	23.78539	chr7:330(	LINC-PINT		lncRNA	chr7:130791264-131
ENSG0000(	951	23.78539	chr7:330(	PODXL		protein_c	chr7:131500262-131
ENSG0000(	951	23.78539	chr7:330(	ENSG00000273319		lncRNA	chr7:130936464-130
ENSG0000(	951	23.78539	chr7:330(	EXOC4		protein_c	chr7:133253073-134
ENSG0000(	951	23.78539	chr7:330(	PLXNA4		protein_c	chr7:132123340-132
ENSG0000(	951	23.78539	chr7:330(	ENSG00000225881		lncRNA	chr7:132758970-132
ENSG0000(	951	23.78539	chr7:330(	MKLN1		protein_c	chr7:131110096-131
ENSG0000(	951	23.78539	chr7:330(	ENSG00000273489		lncRNA	chr7:131493964-131
ENSG0000(	951	23.78539	chr7:330(	BPGM		protein_c	chr7:134646811-134
ENSG0000(	951	23.78539	chr7:330(	AC083875.1		smallRNA	chr7:133262640-133
ENSG0000(	951	23.78539	chr7:330(	ENSG00000270953		lncRNA	chr7:130507660-130
ENSG0000(	951	23.78539	chr7:330(	LINC00513		lncRNA	chr7:130853720-130
ENSG0000(	951	23.78539	chr7:330(	SLC35B4		protein_c	chr7:134289332-134
ENSG0000(	951	23.78539	chr7:330(	ENSG00000270823		lncRNA	chr7:130495794-130
ENSG0000(	951	23.78539	chr7:330(	AKR1B10	NCGv7	protein_c	chr7:134527567-134
ENSG0000(	951	23.78539	chr7:330(	CEP41		protein_c	chr7:130393771-130
ENSG0000(	951	23.78539	chr7:330(	ENSG00000224545		Pseudoger	chr7:131520137-131
ENSG0000(	951	23.78539	chr7:330(	RNA5SP246		Pseudoger	chr7:130602795-130
ENSG0000(	951	23.78539	chr7:330(	ENSG00000283041		Pseudoger	chr7:133034607-133
ENSG0000(	951	23.78539	chr7:330(	MEST	DriverDB\	protein_c	chr7:130486171-130
ENSG0000(	951	23.78539	chr7:330(	MKLN1-AS		lncRNA	chr7:131309469-131
ENSG0000(	951	23.78539	chr7:330(	MIR335		smallRNA	chr7:130496111-130
ENSG0000(	951	23.78539	chr7:330(	ENSG00000229532		Pseudoger	chr7:132086266-132
ENSG0000(	951	23.78539	chr7:330(	ENSG00000233287		Pseudoger	chr7:131242590-131

ENSG0000(	951	23.78539	chr7:330(	AKR1B1		protein_c	chr7:134442356-134
ENSG0000(	951	23.78539	chr7:330(	H4P1		Pseudoger	chr7:130823205-130
ENSG0000(	951	23.78539	chr7:330(	ENSG00000229858		Pseudoger	chr7:130822868-130
ENSG0000(	951	23.78539	chr7:330(	RNU6-1010P		smallRNA	chr7:131054886-131
ENSG0000(	951	23.78539	chr7:330(	ENSG00000273297		lncRNA	chr7:134368737-134
ENSG0000(	951	23.78539	chr7:330(	ENSG00000236238		Pseudoger	chr7:131665507-131
ENSG0000(	951	23.78539	chr7:330(	ENSG00000232716		Pseudoger	chr7:130840204-130
ENSG0000(	951	23.78539	chr7:330(	COX5BP3		Pseudoger	chr7:133727368-133
ENSG0000(	951	23.78539	chr7:330(	ENSG00000223436		lncRNA	chr7:132352334-132
ENSG0000(	951	23.78539	chr7:330(	ST13P7		Pseudoger	chr7:133169416-133
ENSG0000(	951	23.78539	chr7:330(	ENSG00000235429		Pseudoger	chr7:133315009-133
ENSG0000(	951	23.78539	chr7:330(	U6		smallRNA	chr7:133082829-133
ENSG0000(	951	23.78539	chr7:330(	ENSG00000231098		lncRNA	chr7:134284500-134
ENSG0000(	951	23.78539	chr7:330(	SNORD46		smallRNA	chr7:132753023-132
ENSG0000(	951	23.78539	chr7:330(	ENSG00000229177		lncRNA	chr7:134346071-134
ENSG0000(	951	23.78539	chr7:330(	ENSG00000224375		lncRNA	chr7:134684144-134
ENSG0000(	951	23.78539	chr7:330(	ENSG00000236395		Pseudoger	chr7:131702269-131
ENSG0000(	951	23.78539	chr7:330(	ENSG00000226045		Pseudoger	chr7:130645225-130
ENSG0000(	951	23.78539	chr7:330(	CPA1		protein_c	chr7:130380339-130
ENSG0000(	951	23.78539	chr7:330(	ENSG00000227197		lncRNA	chr7:132830693-132
ENSG0000(	951	23.78539	chr7:330(	EEF1B2P6		Pseudoger	chr7:131661952-131
ENSG0000(	951	23.78539	chr7:330(	ENSG00000224865		lncRNA	chr7:131897289-131
ENSG0000(	951	23.78539	chr7:330(	RPS3AP27		Pseudoger	chr7:133732493-133
ENSG0000(	951	23.78539	chr7:330(	KLF14		protein_c	chr7:130730697-130
ENSG0000(	951	23.78539	chr7:330(	ENSG00000271522		lncRNA	chr7:130790208-130
ENSG0000(	951	23.78539	chr7:330(	AC008085.1		smallRNA	chr7:131852680-131
ENSG0000(	951	23.78539	chr7:330(	ENSG00000287547		lncRNA	chr7:130668852-130
ENSG0000(	951	23.78539	chr7:330(	MESTIT1		lncRNA	chr7:130486042-130
ENSG0000(	951	23.78539	chr7:330(	snosnR60_Z15		smallRNA	chr7:131916235-131
ENSG0000(	951	23.78539	chr7:330(	FLJ40288		lncRNA	chr7:132648794-132
ENSG0000(	951	23.78539	chr7:330(	AKR1B15		protein_c	chr7:134549110-134
ENSG0000(	951	23.78539	chr7:330(	TSGA13		protein_c	chr7:130668643-130
ENSG0000(	951	23.78539	chr7:330(	ENSG00000236386		Pseudoger	chr7:131893822-131
ENSG0000(	948	23.71036	chr7:330(	ASZ1	NCGv7	protein_c	chr7:117363222-117
ENSG0000(	948	23.71036	chr7:330(	SNORA25		smallRNA	chr7:115581315-115
ENSG0000(	948	23.71036	chr7:330(	ENSG00000241345		lncRNA	chr7:123994622-124
ENSG0000(	948	23.71036	chr7:330(	LYPLA1P1		Pseudoger	chr7:123230120-123
ENSG0000(	948	23.71036	chr7:330(	WNT2	NCGv7;AC	protein_c	chr7:117275451-117
ENSG0000(	948	23.71036	chr7:330(	ENSG00000243243		lncRNA	chr7:116237929-116
ENSG0000(	948	23.71036	chr7:330(	ENSG00000230520		Pseudoger	chr7:118950386-118
ENSG0000(	948	23.71036	chr7:330(	CFTR		protein_c	chr7:117287120-117
ENSG0000(	948	23.71036	chr7:330(	ST7-AS1		lncRNA	chr7:116952446-116
ENSG0000(	948	23.71036	chr7:330(	ASB15	NCGv7	protein_c	chr7:123567010-123
ENSG0000(	948	23.71036	chr7:330(	ENSG00000226636		Pseudoger	chr7:122159300-122
ENSG0000(	948	23.71036	chr7:330(	ENSG00000287827		lncRNA	chr7:118511942-118
ENSG0000(	948	23.71036	chr7:330(	KCND2		protein_c	chr7:120273175-120
ENSG0000(	948	23.71036	chr7:330(	ENSG00000287829		lncRNA	chr7:117091678-117
ENSG0000(	948	23.71036	chr7:330(	ENSG00000083622		lncRNA	chr7:117604791-117
ENSG0000(	948	23.71036	chr7:330(	ENSG00000240499		lncRNA	chr7:122328469-122
ENSG0000(	948	23.71036	chr7:330(	SLC13A1	NCGv7	protein_c	chr7:123113531-123
ENSG0000(	948	23.71036	chr7:330(	ST7-OT4		lncRNA	chr7:116953899-117

ENSG0000(	948	23.71036	chr7:330(	TES	DriverDB\	protein_c	chr7:116210506-116
ENSG0000(	948	23.71036	chr7:330(	ENSG00000225795		Pseudoger	chr7:122676580-122
ENSG0000(	948	23.71036	chr7:330(	RNF133		protein_c	chr7:122697735-122
ENSG0000(	948	23.71036	chr7:330(	ENSG00000288635		protein_c	chr7:116954391-117
ENSG0000(	948	23.71036	chr7:330(	Y_RNA		smallRNA	chr7:115833273-115
ENSG0000(	948	23.71036	chr7:330(	LINC02476		lncRNA	chr7:119495024-119
ENSG0000(	948	23.71036	chr7:330(	ENSG00000224136		Pseudoger	chr7:117882859-117
ENSG0000(	948	23.71036	chr7:330(	ST7		protein_c	chr7:116953238-117
ENSG0000(	948	23.71036	chr7:330(	MET	NCGv7;AC	protein_c	chr7:116672196-116
ENSG0000(	948	23.71036	chr7:330(	RNA5SP241		Pseudoger	chr7:121083700-121
ENSG0000(	948	23.71036	chr7:330(	AC091320.2		smallRNA	chr7:119484325-119
ENSG0000(	948	23.71036	chr7:330(	RN7SKP277		smallRNA	chr7:121736443-121
ENSG0000(	948	23.71036	chr7:330(	AC002066.2		smallRNA	chr7:116345855-116
ENSG0000(	948	23.71036	chr7:330(	RNU7-154P		smallRNA	chr7:122081720-122
ENSG0000(	948	23.71036	chr7:330(	POLR2DP2		Pseudoger	chr7:115503367-115
ENSG0000(	948	23.71036	chr7:330(	FEZF1-AS1		lncRNA	chr7:122303658-122
ENSG0000(	948	23.71036	chr7:330(	ST7-AS2		lncRNA	chr7:117072072-117
ENSG0000(	948	23.71036	chr7:330(	snoZ185		smallRNA	chr7:116433214-116
ENSG0000(	948	23.71036	chr7:330(	AC006926.1		smallRNA	chr7:118462499-118
ENSG0000(	948	23.71036	chr7:330(	ENSG00000232524		lncRNA	chr7:123456629-123
ENSG0000(	948	23.71036	chr7:330(	ASB15-AS1		lncRNA	chr7:123584859-123
ENSG0000(	948	23.71036	chr7:330(	ENSG00000226680		lncRNA	chr7:123069249-123
ENSG0000(	948	23.71036	chr7:330(	WASL-DT		lncRNA	chr7:123749068-123
ENSG0000(	948	23.71036	chr7:330(	ENSG00000286390		lncRNA	chr7:117332761-117
ENSG0000(	948	23.71036	chr7:330(	ENSG00000234001		Pseudoger	chr7:117586207-117
ENSG0000(	948	23.71036	chr7:330(	GTF3AP6		Pseudoger	chr7:118880103-118
ENSG0000(	948	23.71036	chr7:330(	ENSG00000225583		Pseudoger	chr7:123932132-123
ENSG0000(	948	23.71036	chr7:330(	TMEM229A		protein_c	chr7:124030921-124
ENSG0000(	948	23.71036	chr7:330(	MTND4P6		Pseudoger	chr7:117263917-117
ENSG0000(	948	23.71036	chr7:330(	ING3		protein_c	chr7:120950763-120
ENSG0000(	948	23.71036	chr7:330(	ENSG00000243345		lncRNA	chr7:115789729-115
ENSG0000(	948	23.71036	chr7:330(	ENSG00000234985		Pseudoger	chr7:121440834-121
ENSG0000(	948	23.71036	chr7:330(	RPS26P31		Pseudoger	chr7:122681315-122
ENSG0000(	948	23.71036	chr7:330(	ENSG00000237974		Pseudoger	chr7:117487737-117
ENSG0000(	948	23.71036	chr7:330(	ENSG00000227532		Pseudoger	chr7:117187548-117
ENSG0000(	948	23.71036	chr7:330(	CAV1	AC	protein_c	chr7:116524994-116
ENSG0000(	948	23.71036	chr7:330(	HMGN1P18		Pseudoger	chr7:121050927-121
ENSG0000(	948	23.71036	chr7:330(	CAV2		protein_c	chr7:116287380-116
ENSG0000(	948	23.71036	chr7:330(	ENSG00000237813		lncRNA	chr7:116238260-116
ENSG0000(	948	23.71036	chr7:330(	ENSG00000270516		Pseudoger	chr7:119178177-119
ENSG0000(	948	23.71036	chr7:330(	RNU6-581P		smallRNA	chr7:120672871-120
ENSG0000(	948	23.71036	chr7:330(	ENSG00000240973		lncRNA	chr7:115679345-115
ENSG0000(	948	23.71036	chr7:330(	ENSG00000233969		lncRNA	chr7:120166443-120
ENSG0000(	948	23.71036	chr7:330(	TFEC		protein_c	chr7:115935148-116
ENSG0000(	948	23.71036	chr7:330(	ENSG00000230785		Pseudoger	chr7:117262918-117
ENSG0000(	948	23.71036	chr7:330(	SPAM1	NCGv7	protein_c	chr7:123925237-123
ENSG0000(	948	23.71036	chr7:330(	ENSG00000233417		lncRNA	chr7:120141016-120
ENSG0000(	948	23.71036	chr7:330(	PNPT1P2		Pseudoger	chr7:121842368-121
ENSG0000(	948	23.71036	chr7:330(	CAPZA2		protein_c	chr7:116811070-116
ENSG0000(	948	23.71036	chr7:330(	CYCSP19		Pseudoger	chr7:121398452-121
ENSG0000(	948	23.71036	chr7:330(	TAS2R16		protein_c	chr7:122994704-122

ENSG0000(	948	23.71036	chr7:330(	WASL		protein_c	chr7:123681943-123
ENSG0000(	948	23.71036	chr7:330(	ENSG00000234418		lncRNA	chr7:122144405-122
ENSG0000(	948	23.71036	chr7:330(	ENSG00000279086		TEC	chr7:116209234-116
ENSG0000(	948	23.71036	chr7:330(	WNT16		protein_c	chr7:121325367-121
ENSG0000(	948	23.71036	chr7:330(	RNA5SP239		Pseudoger	chr7:116944286-116
ENSG0000(	948	23.71036	chr7:330(	ENSG00000213302		Pseudoger	chr7:122234531-122
ENSG0000(	948	23.71036	chr7:330(	CTTNBP2		protein_c	chr7:117710651-117
ENSG0000(	948	23.71036	chr7:330(	ENSG00000289345		lncRNA	chr7:123580881-123
ENSG0000(	948	23.71036	chr7:330(	CPED1		protein_c	chr7:120988697-121
ENSG0000(	948	23.71036	chr7:330(	RNU6-517P		smallRNA	chr7:121194948-121
ENSG0000(	948	23.71036	chr7:330(	ENSG00000231295		lncRNA	chr7:120746738-120
ENSG0000(	948	23.71036	chr7:330(	ENSG00000227573		Pseudoger	chr7:122849746-122
ENSG0000(	948	23.71036	chr7:330(	RNU6-11P		smallRNA	chr7:123790605-123
ENSG0000(	948	23.71036	chr7:330(	PTPRZ1		protein_c	chr7:121873089-122
ENSG0000(	948	23.71036	chr7:330(	HYAL4		protein_c	chr7:123828983-123
ENSG0000(	948	23.71036	chr6:105(	OR2B7P		Pseudoger	chr6:28046434-2804
ENSG0000(	948	23.71036	chr7:330(	ANKRD49P4		Pseudoger	chr7:117439982-117
ENSG0000(	948	23.71036	chr7:330(	CADPS2		protein_c	chr7:122318411-122
ENSG0000(	948	23.71036	chr7:330(	RNF148		protein_c	chr7:122701668-122
ENSG0000(	948	23.71036	chr7:330(	ENSG00000242072		lncRNA	chr7:115647461-115
ENSG0000(	948	23.71036	chr7:330(	LMOD2		protein_c	chr7:123655866-123
ENSG0000(	948	23.71036	chr7:330(	ENSG00000227743		lncRNA	chr7:121643334-121
ENSG0000(	948	23.71036	chr7:330(	TPM3P1		Pseudoger	chr7:116972165-116
ENSG0000(	948	23.71036	chr7:330(	ENSG00000228368		lncRNA	chr7:116965846-116
ENSG0000(	948	23.71036	chr7:330(	COMETT		lncRNA	chr7:116563594-116
ENSG0000(	948	23.71036	chr7:330(	AASS		protein_c	chr7:122064583-122
ENSG0000(	948	23.71036	chr7:330(	AC004875.1		smallRNA	chr7:121574669-121
ENSG0000(	948	23.71036	chr7:330(	TSPAN12	DriverDB\	protein_c	chr7:120787320-120
ENSG0000(	948	23.71036	chr7:330(	MTCYBP6		Pseudoger	chr7:117264393-117
ENSG0000(	948	23.71036	chr7:330(	HYAL6P		Pseudoger	chr7:123814139-123
ENSG0000(	948	23.71036	chr7:330(	U1		smallRNA	chr7:120005976-120
ENSG0000(	948	23.71036	chr7:330(	LSM8		protein_c	chr7:118184144-118
ENSG0000(	948	23.71036	chr7:330(	ANKRD7		protein_c	chr7:118214669-118
ENSG0000(	948	23.71036	chr7:330(	NDUFA5	NCGv7	protein_c	chr7:123536997-123
ENSG0000(	948	23.71036	chr7:330(	ENSG00000287554		lncRNA	chr7:121304657-121
ENSG0000(	948	23.71036	chr7:330(	IQUB	DriverDB\	protein_c	chr7:123452193-123
ENSG0000(	948	23.71036	chr7:330(	CFTR-AS1		lncRNA	chr7:117560733-117
ENSG0000(	948	23.71036	chr7:330(	RNU6-296P		smallRNA	chr7:123457988-123
ENSG0000(	948	23.71036	chr7:330(	ENSG00000237870		lncRNA	chr7:116275606-116
ENSG0000(	948	23.71036	chr7:330(	ENSG00000235427		lncRNA	chr7:116542718-116
ENSG0000(	948	23.71036	chr7:330(	FAM3C		protein_c	chr7:121348878-121
ENSG0000(	948	23.71036	chr7:330(	ENSG00000234826		lncRNA	chr7:117998858-118
ENSG0000(	948	23.71036	chr7:330(	ENSG00000235945		Pseudoger	chr7:116873454-116
ENSG0000(	948	23.71036	chr7:330(	ENSG00000227371		Pseudoger	chr7:121419072-121
ENSG0000(	948	23.71036	chr7:330(	ENSG00000289578		lncRNA	chr7:121450948-121
ENSG0000(	948	23.71036	chr7:330(	FEZF1	DriverDB\	protein_c	chr7:122301303-122
ENSG0000(	948	23.71036	chr7:330(	RNA5SP240		Pseudoger	chr7:120981426-120
ENSG0000(	947	23.68535	chr22:231	ENSG00000259421		Pseudoger	chr22:39070414-390
ENSG0000(	944	23.61032	chr1:116(	RNU1-1		smallRNA	chr1:16514122-1651
ENSG0000(	941	23.53528	chr7:330(	RNU6-863P		smallRNA	chr7:76087444-7608
ENSG0000(	941	23.53528	chr7:330(	DTX2P1-UPK3BP1-PMS		lncRNA	chr7:76959835-7704

ENSG0000(	941	23.53528	chr7:330(	RN7SL869P		smallRNA	chr7:80245926-8024
ENSG0000(	941	23.53528	chr7:330(	CNPY4		protein_c	chr7:100119634-100
ENSG0000(	941	23.53528	chr7:330(	CASTOR3		lncRNA	chr7:100200653-100
ENSG0000(	941	23.53528	chr7:330(	ENSG00000229110		Pseudoger	chr7:79124739-7912
ENSG0000(	941	23.53528	chr7:330(	MAGI2-AS1		lncRNA	chr7:78939850-7894
ENSG0000(	941	23.53528	chr7:330(	MAGI2-AS2		lncRNA	chr7:79008988-7901
ENSG0000(	941	23.53528	chr7:330(	ENSG00000226744		Pseudoger	chr7:97870167-9787
ENSG0000(	941	23.53528	chr7:330(	ENSG00000288889		lncRNA	chr7:98616014-9861
ENSG0000(	941	23.53528	chr7:330(	ENSG00000286742		lncRNA	chr7:92647906-9266
ENSG0000(	941	23.53528	chr7:330(	Y_RNA		smallRNA	chr7:77895880-7789
ENSG0000(	941	23.53528	chr7:330(	RN7SKP129		smallRNA	chr7:94801514-9480
ENSG0000(	941	23.53528	chr7:330(	ENSG00000286921		lncRNA	chr7:99974976-9997
ENSG0000(	941	23.53528	chr7:330(	RHBDD2		protein_c	chr7:75842602-7588
ENSG0000(	941	23.53528	chr7:330(	ZNF804B	NCGv7	protein_c	chr7:88759700-8933
ENSG0000(	941	23.53528	chr7:330(	ENSG00000261462		lncRNA	chr7:87109539-8711
ENSG0000(	941	23.53528	chr7:330(	DPY19L2P4		lncRNA	chr7:90119358-9012
ENSG0000(	941	23.53528	chr7:330(	ENSG00000289886		lncRNA	chr7:100569732-100
ENSG0000(	941	23.53528	chr7:330(	MIR489		smallRNA	chr7:93483936-9348
ENSG0000(	941	23.53528	chr7:330(	PON3		protein_c	chr7:95359872-9539
ENSG0000(	941	23.53528	chr7:330(	ENSG00000273407		lncRNA	chr7:99766543-9976
ENSG0000(	941	23.53528	chr7:330(	ZSCAN25		protein_c	chr7:99616946-9963
ENSG0000(	941	23.53528	chr7:330(	DTX2		protein_c	chr7:76461676-7650
ENSG0000(	941	23.53528	chr7:330(	SRI-AS1		lncRNA	chr7:88216660-8821
ENSG0000(	941	23.53528	chr7:330(	ENSG00000228335		Pseudoger	chr7:99442890-9944
ENSG0000(	941	23.53528	chr7:330(	Y_RNA		smallRNA	chr7:99936610-9993
ENSG0000(	941	23.53528	chr7:330(	RN7SL478P		smallRNA	chr7:97998325-9799
ENSG0000(	941	23.53528	chr7:330(	BHLHA15		protein_c	chr7:98211439-9821
ENSG0000(	941	23.53528	chr7:330(	ENSG00000286938		lncRNA	chr7:100482221-100
ENSG0000(	941	23.53528	chr7:330(	ENSG00000223514		Pseudoger	chr7:81335106-8133
ENSG0000(	941	23.53528	chr7:330(	ENSG00000286923		lncRNA	chr7:99997690-1000
ENSG0000(	941	23.53528	chr7:330(	ENSG00000243107		lncRNA	chr7:92200014-9220
ENSG0000(	941	23.53528	chr7:330(	AC004745.1		smallRNA	chr7:97291514-9729
ENSG0000(	941	23.53528	chr7:330(	snoU13		smallRNA	chr7:98884990-9888
ENSG0000(	941	23.53528	chr7:330(	AC092849.1		smallRNA	chr7:100483758-100
ENSG0000(	941	23.53528	chr7:330(	CASTOR3		Pseudoger	chr7:100222597-100
ENSG0000(	941	23.53528	chr7:330(	snoU13		smallRNA	chr7:77423683-7742
ENSG0000(	941	23.53528	chr7:330(	AC006145.1		smallRNA	chr7:81920203-8192
ENSG0000(	941	23.53528	chr7:330(	ENSG00000225898		Pseudoger	chr7:93777839-9377
ENSG0000(	941	23.53528	chr7:330(	ENSG00000285090		lncRNA	chr7:94278680-9439
ENSG0000(	941	23.53528	chr7:330(	ENSG00000286855		lncRNA	chr7:79335780-7935
ENSG0000(	941	23.53528	chr7:330(	LINC02932		lncRNA	chr7:91311368-9151
ENSG0000(	941	23.53528	chr7:330(	SEMA3D		protein_c	chr7:84995553-8518
ENSG0000(	941	23.53528	chr7:330(	ENSG00000278959		TEC	chr7:93954044-9395
ENSG0000(	941	23.53528	chr7:330(	GRPEL2P3		Pseudoger	chr7:94782886-9478
ENSG0000(	941	23.53528	chr7:330(	APTR		lncRNA	chr7:77657659-7769
ENSG0000(	941	23.53528	chr7:330(	ENSG00000260445		Pseudoger	chr7:99869841-9986
ENSG0000(	941	23.53528	chr7:330(	HSPA8P16		Pseudoger	chr7:85027828-8502
ENSG0000(	941	23.53528	chr7:330(	RN7SL13P		smallRNA	chr7:98023729-9802
ENSG0000(	941	23.53528	chr7:330(	RN7SL252P		smallRNA	chr7:96940070-9694
ENSG0000(	941	23.53528	chr7:330(	ENSG00000278388		Pseudoger	chr7:93914987-9391
ENSG0000(	941	23.53528	chr7:330(	ENSG00000237896		lncRNA	chr7:81175508-8119

ENSG0000(	941	23.53528	chr7:330(	AC004458.1		smallRNA	chr7:96468331-9646
ENSG0000(	941	23.53528	chr7:330(	RNU6-337P		smallRNA	chr7:79030240-7903
ENSG0000(	941	23.53528	chr7:330(	SPDYE3		protein_c	chr7:100307702-100
ENSG0000(	941	23.53528	chr7:330(	ENSG00000223402		Pseudoger	chr7:98478551-9847
ENSG0000(	941	23.53528	chr7:330(	snoU13		smallRNA	chr7:95276586-9527
ENSG0000(	941	23.53528	chr7:330(	LAMTOR4		protein_c	chr7:100148912-100
ENSG0000(	941	23.53528	chr7:330(	GAL3ST4		protein_c	chr7:100159244-100
ENSG0000(	941	23.53528	chr7:330(	TP53TG1		lncRNA	chr7:87322943-8734
ENSG0000(	941	23.53528	chr7:330(	HEPACAM2	NCGv7	protein_c	chr7:93188534-9322
ENSG0000(	941	23.53528	chr7:330(	ENSG00000287519		lncRNA	chr7:77487317-7749
ENSG0000(	941	23.53528	chr7:330(	HMGB3P21		Pseudoger	chr7:97135015-9713
ENSG0000(	941	23.53528	chr7:330(	PVRIG	NCGv7	protein_c	chr7:100218241-100
ENSG0000(	941	23.53528	chr7:330(	FDPSP2		Pseudoger	chr7:76470162-7647
ENSG0000(	941	23.53528	chr7:330(	ENSG00000290729		lncRNA	chr7:76090431-7610
ENSG0000(	941	23.53528	chr7:330(	ZCWPW1		protein_c	chr7:100400826-100
ENSG0000(	941	23.53528	chr7:330(	ZP3		protein_c	chr7:76397518-7644
ENSG0000(	941	23.53528	chr7:330(	ENSG00000287488		lncRNA	chr7:79657947-7967
ENSG0000(	941	23.53528	chr7:330(	ENSG00000291121		lncRNA	chr7:77083681-7712
ENSG0000(	941	23.53528	chr7:330(	PON1	NCGv7	protein_c	chr7:95297676-9532
ENSG0000(	941	23.53528	chr7:330(	ABCB4		protein_c	chr7:87401696-8748
ENSG0000(	941	23.53528	chr7:330(	STAG3L5P		lncRNA	chr7:100336079-100
ENSG0000(	941	23.53528	chr7:330(	ENSG00000280325		TEC	chr7:84939335-8494
ENSG0000(	941	23.53528	chr7:330(	CROT	AC	protein_c	chr7:87345664-8739
ENSG0000(	941	23.53528	chr7:330(	EIF4EP4		Pseudoger	chr7:81463036-8146
ENSG0000(	941	23.53528	chr7:330(	ENSG00000223665		lncRNA	chr7:91638847-9164
ENSG0000(	941	23.53528	chr7:330(	OR7E7P		Pseudoger	chr7:97946987-9794
ENSG0000(	941	23.53528	chr7:330(	SAMD9L	NCGv7	protein_c	chr7:93130056-9314
ENSG0000(	941	23.53528	chr7:330(	MIR5692A1		smallRNA	chr7:97963658-9796
ENSG0000(	941	23.53528	chr7:330(	YWHAG	AC	protein_c	chr7:76326799-7635
ENSG0000(	941	23.53528	chr7:330(	ENSG00000238358		lncRNA	chr7:90119299-9012
ENSG0000(	941	23.53528	chr7:330(	ENSG00000290730		lncRNA	chr7:76174309-7617
ENSG0000(	941	23.53528	chr7:330(	FAM133B		protein_c	chr7:92560758-9259
ENSG0000(	941	23.53528	chr7:330(	ENSG00000289690		protein_c	chr7:100397577-100
ENSG0000(	941	23.53528	chr7:330(	SRI		protein_c	chr7:88205115-8822
ENSG0000(	941	23.53528	chr7:330(	BAIAP2L1	DriverDB\	protein_c	chr7:98291650-9840
ENSG0000(	941	23.53528	chr7:330(	ENSG00000226671		Pseudoger	chr7:81191398-8119
ENSG0000(	941	23.53528	chr7:330(	TEX47		protein_c	chr7:88794106-8879
ENSG0000(	941	23.53528	chr7:330(	COL1A2	NCGv7	protein_c	chr7:94394895-9443
ENSG0000(	941	23.53528	chr7:330(	SNORA40		smallRNA	chr7:99952033-9995
ENSG0000(	941	23.53528	chr7:330(	RN7SL212P		smallRNA	chr7:76288791-7628
ENSG0000(	941	23.53528	chr7:330(	OCM2		protein_c	chr7:97984687-9799
ENSG0000(	941	23.53528	chr7:330(	TMEM243	NCGv7	protein_c	chr7:87196160-8722
ENSG0000(	941	23.53528	chr7:330(	CCDC146		protein_c	chr7:77122434-7732
ENSG0000(	941	23.53528	chr7:330(	TAC1	AC	protein_c	chr7:97732084-9774
ENSG0000(	941	23.53528	chr7:330(	MIR3609		smallRNA	chr7:98881650-9888
ENSG0000(	941	23.53528	chr7:330(	TMEM60		protein_c	chr7:77793728-7779
ENSG0000(	941	23.53528	chr7:330(	CD36	NCGv7	protein_c	chr7:80369575-8067
ENSG0000(	941	23.53528	chr7:330(	ENSG00000229436		Pseudoger	chr7:80662331-8066
ENSG0000(	941	23.53528	chr7:330(	CYP3A51P		Pseudoger	chr7:99685145-9970
ENSG0000(	941	23.53528	chr7:330(	ELAPOR2		protein_c	chr7:86876906-8705
ENSG0000(	941	23.53528	chr7:330(	STEAP1		protein_c	chr7:90154456-9016

ENSG0000(	941	23.53528	chr7:330(	ZNF655		protein_c	chr7:99558406-9957
ENSG0000(	941	23.53528	chr7:330(	RPL13AP17		Pseudoger	chr7:78347142-7835
ENSG0000(	941	23.53528	chr7:330(	CYP3A7-CYP3A51P		protein_c	chr7:99684957-9973
ENSG0000(	941	23.53528	chr7:330(	CCL26	NCGv7	protein_c	chr7:75769533-7578
ENSG0000(	941	23.53528	chr7:330(	HMGN2P11		Pseudoger	chr7:84876554-8487
ENSG0000(	941	23.53528	chr7:330(	RNU6-530P		smallRNA	chr7:79343266-7934
ENSG0000(	941	23.53528	chr7:330(	PON2		protein_c	chr7:95404862-9543
ENSG0000(	941	23.53528	chr7:330(	LINC00972		lncRNA	chr7:85421122-8548
ENSG0000(	941	23.53528	chr7:330(	GNB2		protein_c	chr7:100673567-100
ENSG0000(	941	23.53528	chr7:330(	RPL7AP40		Pseudoger	chr7:97200708-9720
ENSG0000(	941	23.53528	chr7:330(	AC006322.1		smallRNA	chr7:83919602-8391
ENSG0000(	941	23.53528	chr7:330(	TMEM120A		protein_c	chr7:75986831-7599
ENSG0000(	941	23.53528	chr7:330(	Y_RNA		smallRNA	chr7:92202243-9220
ENSG0000(	941	23.53528	chr7:330(	snoU13		smallRNA	chr7:76112798-7611
ENSG0000(	941	23.53528	chr7:330(	ENSG00000225807		lncRNA	chr7:100509416-100
ENSG0000(	941	23.53528	chr7:330(	TFPI2		protein_c	chr7:93885396-9389
ENSG0000(	941	23.53528	chr7:330(	BRI3	DriverDB\	protein_c	chr7:98252379-9831
ENSG0000(	941	23.53528	chr7:330(	ENSG00000225498		lncRNA	chr7:90312496-9032
ENSG0000(	941	23.53528	chr7:330(	DMTF1	DriverDB\	protein_c	chr7:87152409-8719
ENSG0000(	941	23.53528	chr7:330(	PHTF2		protein_c	chr7:77798773-7795
ENSG0000(	941	23.53528	chr7:330(	RUNDC3B	DriverDB\	protein_c	chr7:87627548-8783
ENSG0000(	941	23.53528	chr7:330(	MBLAC1		protein_c	chr7:100126785-100
ENSG0000(	941	23.53528	chr7:330(	ENSG00000224448		Pseudoger	chr7:99638242-9963
ENSG0000(	941	23.53528	chr7:330(	ENSG00000281120		lncRNA	chr7:79139829-7917
ENSG0000(	941	23.53528	chr7:330(	CFAP69	DriverDB\	protein_c	chr7:90245174-9031
ENSG0000(	941	23.53528	chr7:330(	RNU6-532P		smallRNA	chr7:96330638-9633
ENSG0000(	941	23.53528	chr7:330(	ADAM22	NCGv7	protein_c	chr7:87934143-8820
ENSG0000(	941	23.53528	chr7:330(	ENSG00000281008		TEC	chr7:78134079-7813
ENSG0000(	941	23.53528	chr7:330(	TMBIM7P		Pseudoger	chr7:92412550-9244
ENSG0000(	941	23.53528	chr7:330(	snoU13		smallRNA	chr7:90607004-9060
ENSG0000(	941	23.53528	chr7:330(	SNORA14A		smallRNA	chr7:75943782-7594
ENSG0000(	941	23.53528	chr7:330(	GTPBP10	DriverDB\	protein_c	chr7:90335223-9039
ENSG0000(	941	23.53528	chr7:330(	RN7SL7P		smallRNA	chr7:92971004-9297
ENSG0000(	941	23.53528	chr7:330(	RNU7-188P		smallRNA	chr7:96377857-9637
ENSG0000(	941	23.53528	chr7:330(	RNU6-364P		smallRNA	chr7:96341140-9634
ENSG0000(	941	23.53528	chr7:330(	ENSG00000284707		lncRNA	chr7:97851688-9797
ENSG0000(	941	23.53528	chr7:330(	CDK6	NCGv7;AC	protein_c	chr7:92604921-9283
ENSG0000(	941	23.53528	chr7:330(	ENSG00000250990		lncRNA	chr7:77246340-7725
ENSG0000(	941	23.53528	chr7:330(	GTF2IP7		Pseudoger	chr7:76099440-7610
ENSG0000(	941	23.53528	chr7:330(	ENSG00000280958		TEC	chr7:78170195-7817
ENSG0000(	941	23.53528	chr7:330(	DLX6		protein_c	chr7:97005553-9701
ENSG0000(	941	23.53528	chr7:330(	AZGP1P1		Pseudoger	chr7:99980762-9998
ENSG0000(	941	23.53528	chr7:330(	TRRAP	NCGv7;AC	protein_c	chr7:98877933-9905
ENSG0000(	941	23.53528	chr7:330(	MIR4658		smallRNA	chr7:100156605-100
ENSG0000(	941	23.53528	chr7:330(	RNA5SP234		Pseudoger	chr7:79654109-7965
ENSG0000(	941	23.53528	chr7:330(	TFPI2-DT		lncRNA	chr7:93890913-9389
ENSG0000(	941	23.53528	chr7:330(	ENSG00000235077		lncRNA	chr7:100130964-100
ENSG0000(	941	23.53528	chr7:330(	RNF14P3		Pseudoger	chr7:98998538-9899
ENSG0000(	941	23.53528	chr7:330(	LMTK2	DriverDB\	protein_c	chr7:98106862-9820
ENSG0000(	941	23.53528	chr7:330(	ENSG00000287631		lncRNA	chr7:100388809-100
ENSG0000(	941	23.53528	chr7:330(	snoU13		smallRNA	chr7:87624157-8762

ENSG0000(	941	23.53528	chr7:330(	GJC3		protein_c	chr7:99923266-9992
ENSG0000(	941	23.53528	chr7:330(	ASB4		protein_c	chr7:95478444-9554
ENSG0000(	941	23.53528	chr7:330(	MIR5692C2		smallRNA	chr7:97964405-9796
ENSG0000(	941	23.53528	chr7:330(	ENSG00000279525		TEC	chr7:95542145-9554
ENSG0000(	941	23.53528	chr7:330(	FAM237B		protein_c	chr7:90316503-9032
ENSG0000(	941	23.53528	chr7:330(	ENSG00000228113		lncRNA	chr7:88219359-8830
ENSG0000(	941	23.53528	chr7:330(	Y_RNA		smallRNA	chr7:90251923-9025
ENSG0000(	941	23.53528	chr7:330(	ENSG00000232019		lncRNA	chr7:84939349-8494
ENSG0000(	941	23.53528	chr7:330(	GRM3-AS1		lncRNA	chr7:86782357-8680
ENSG0000(	941	23.53528	chr7:330(	ENSG00000274272		lncRNA	chr7:100572232-100
ENSG0000(	941	23.53528	chr7:330(	ENSG00000228829		Pseudoger	chr7:76173733-7617
ENSG0000(	941	23.53528	chr7:330(	SDHAF3		protein_c	chr7:97117698-9718
ENSG0000(	941	23.53528	chr7:330(	ENSG00000233420		lncRNA	chr7:88420167-8875
ENSG0000(	941	23.53528	chr7:330(	snoU13		smallRNA	chr7:99413978-9941
ENSG0000(	941	23.53528	chr7:330(	ENSG00000236453		lncRNA	chr7:94022833-9406
ENSG0000(	941	23.53528	chr7:330(	POP7		protein_c	chr7:100706121-100
ENSG0000(	941	23.53528	chr7:330(	OR2AE1		protein_c	chr7:99876062-9987
ENSG0000(	941	23.53528	chr7:330(	ENSG00000228751		Pseudoger	chr7:95350164-9535
ENSG0000(	941	23.53528	chr7:330(	ZKSCAN5	DriverDBv	protein_c	chr7:99504662-9953
ENSG0000(	941	23.53528	chr7:330(	SRRM3		protein_c	chr7:76201896-7628
ENSG0000(	941	23.53528	chr7:330(	ENSG00000234459		lncRNA	chr7:90266034-9027
ENSG0000(	941	23.53528	chr7:330(	RNU4-16P		smallRNA	chr7:95098227-9509
ENSG0000(	941	23.53528	chr7:330(	ENSG00000280388		TEC	chr7:76043977-7604
ENSG0000(	941	23.53528	chr7:330(	ENSG00000219039		Pseudoger	chr7:75835663-7583
ENSG0000(	941	23.53528	chr7:330(	ENSG00000289760		protein_c	chr7:100478099-100
ENSG0000(	941	23.53528	chr7:330(	ENSG00000228711		Pseudoger	chr7:82657035-8265
ENSG0000(	941	23.53528	chr7:330(	ENSG00000284840		Pseudoger	chr7:99032894-9903
ENSG0000(	941	23.53528	chr7:330(	HNRNPA1P8		Pseudoger	chr7:84983556-8498
ENSG0000(	941	23.53528	chr7:330(	TMEM130		protein_c	chr7:98846488-9887
ENSG0000(	941	23.53528	chr7:330(	ATP5MF-PTCD1		protein_c	chr7:99419749-9946
ENSG0000(	941	23.53528	chr7:330(	CACNA2D1	NCGv7	protein_c	chr7:81946444-8244
ENSG0000(	941	23.53528	chr7:330(	NIPA2P1		Pseudoger	chr7:91320128-9132
ENSG0000(	941	23.53528	chr7:330(	RNU6-274P		smallRNA	chr7:89754620-8975
ENSG0000(	941	23.53528	chr7:330(	CYP51A1-AS1		lncRNA	chr7:92134604-9218
ENSG0000(	941	23.53528	chr7:330(	MCM7	NCGv7	protein_c	chr7:100092728-100
ENSG0000(	941	23.53528	chr7:330(	ENSG00000287672		lncRNA	chr7:91880791-9188
ENSG0000(	941	23.53528	chr7:330(	AC004969.1		smallRNA	chr7:90171341-9017
ENSG0000(	941	23.53528	chr7:330(	DBF4	DriverDBv	protein_c	chr7:87876216-8790
ENSG0000(	941	23.53528	chr7:330(	ENSG00000230882		Pseudoger	chr7:76071469-7607
ENSG0000(	941	23.53528	chr7:330(	PTTG1IP2		protein_c	chr7:90469639-9051
ENSG0000(	941	23.53528	chr7:330(	ZSCAN21		protein_c	chr7:100049774-100
ENSG0000(	941	23.53528	chr7:330(	RPL7L1P3		Pseudoger	chr7:75922755-7592
ENSG0000(	941	23.53528	chr7:330(	SEMA3E	NCGv7	protein_c	chr7:83363238-8364
ENSG0000(	941	23.53528	chr7:330(	PMS2P1		Pseudoger	chr7:100328836-100
ENSG0000(	941	23.53528	chr7:330(	RPL10P11		Pseudoger	chr7:80096600-8009
ENSG0000(	941	23.53528	chr7:330(	CCZ1P1		Pseudoger	chr7:97969005-9797
ENSG0000(	941	23.53528	chr7:330(	ENSG00000225726		Pseudoger	chr7:77071751-7707
ENSG0000(	941	23.53528	chr7:330(	MAGI2-AS3		lncRNA	chr7:79452877-7947
ENSG0000(	941	23.53528	chr7:330(	RNU6-849P		smallRNA	chr7:79912104-7991
ENSG0000(	941	23.53528	chr7:330(	RNU6-1328P		smallRNA	chr7:94495299-9449
ENSG0000(	941	23.53528	chr7:330(	DPY19L2P4		Pseudoger	chr7:90119539-9012

ENSG0000(	941	23.53528	chr7:330(	ENSG00000289027		protein_c	chr7:92112176-9224
ENSG0000(	941	23.53528	chr7:330(	ENSG00000280440		TEC	chr7:90345873-9034
ENSG0000(	941	23.53528	chr7:330(	AC069294.1		smallRNA	chr7:99713037-9971
ENSG0000(	941	23.53528	chr7:330(	Y_RNA		smallRNA	chr7:87218611-8721
ENSG0000(	941	23.53528	chr7:330(	AC084368.1		smallRNA	chr7:96306760-9630
ENSG0000(	941	23.53528	chr7:330(	ENSG00000250614		lncRNA	chr7:76474587-7647
ENSG0000(	941	23.53528	chr7:330(	ENSG00000289836		lncRNA	chr7:90244783-9024
ENSG0000(	941	23.53528	chr7:330(	MTHFD2P5		Pseudoger	chr7:82589848-8259
ENSG0000(	941	23.53528	chr7:330(	ENSG00000235450		lncRNA	chr7:91380778-9155
ENSG0000(	941	23.53528	chr7:330(	DMTF1-AS1		lncRNA	chr7:87151419-8715
ENSG0000(	941	23.53528	chr7:330(	ZNF3	DriverDB\	protein_c	chr7:100064033-100
ENSG0000(	941	23.53528	chr7:330(	RNU6-956P		smallRNA	chr7:94712409-9471
ENSG0000(	941	23.53528	chr7:330(	BET1	DriverDB\	protein_c	chr7:93962762-9400
ENSG0000(	941	23.53528	chr7:330(	AC005159.1		smallRNA	chr7:82290077-8229
ENSG0000(	941	23.53528	chr7:330(	GPC2	DriverDB\	protein_c	chr7:100169606-100
ENSG0000(	941	23.53528	chr7:330(	CPSF4		protein_c	chr7:99438922-9945
ENSG0000(	941	23.53528	chr7:330(	CYP3A52P		Pseudoger	chr7:99872168-9987
ENSG0000(	941	23.53528	chr7:330(	ENSG00000286305		lncRNA	chr7:98989867-9899
ENSG0000(	941	23.53528	chr7:330(	ARPC1B	NCGv7	protein_c	chr7:99374249-9939
ENSG0000(	941	23.53528	chr7:330(	Y_RNA		smallRNA	chr7:100330777-100
ENSG0000(	941	23.53528	chr7:330(	ENSG00000285953		protein_c	chr7:92144723-9224
ENSG0000(	941	23.53528	chr7:330(	SNRPCP9		Pseudoger	chr7:97885868-9788
ENSG0000(	941	23.53528	chr7:330(	ENSG00000284627		Pseudoger	chr7:97928082-9792
ENSG0000(	941	23.53528	chr7:330(	ENSG00000287932		lncRNA	chr7:92638198-9264
ENSG0000(	941	23.53528	chr7:330(	CYP3A43		protein_c	chr7:99828013-9986
ENSG0000(	941	23.53528	chr7:330(	GSAP	DriverDB\	protein_c	chr7:77310751-7741
ENSG0000(	941	23.53528	chr7:330(	OR7E38P		Pseudoger	chr7:97966090-9796
ENSG0000(	941	23.53528	chr7:330(	PCOLCE-AS1		lncRNA	chr7:100589402-100
ENSG0000(	941	23.53528	chr7:330(	ENSG00000285964		lncRNA	chr7:94311138-9434
ENSG0000(	941	23.53528	chr7:330(	ENSG00000270812		Pseudoger	chr7:88455309-8845
ENSG0000(	941	23.53528	chr7:330(	AC079781.1		smallRNA	chr7:97905972-9790
ENSG0000(	941	23.53528	chr7:330(	ENSG00000234223		lncRNA	chr7:80246409-8031
ENSG0000(	941	23.53528	chr7:330(	RPL7P60		Pseudoger	chr7:100139629-100
ENSG0000(	941	23.53528	chr7:330(	ERVW-1		protein_c	chr7:92468380-9247
ENSG0000(	941	23.53528	chr7:330(	ARF1P1		Pseudoger	chr7:94833904-9483
ENSG0000(	941	23.53528	chr7:330(	ZKSCAN1	DriverDB\	protein_c	chr7:100015572-100
ENSG0000(	941	23.53528	chr7:330(	ENSG00000235639		Pseudoger	chr7:88512029-8851
ENSG0000(	941	23.53528	chr7:330(	FAM185BP		Pseudoger	chr7:77083886-7712
ENSG0000(	941	23.53528	chr7:330(	DYNC1I1	NCGv7	protein_c	chr7:95772506-9611
ENSG0000(	941	23.53528	chr7:330(	ATP5PBP2		Pseudoger	chr7:94738652-9473
ENSG0000(	941	23.53528	chr7:330(	AZGP1P1		lncRNA	chr7:99980741-9998
ENSG0000(	941	23.53528	chr7:330(	RNU6-10P		smallRNA	chr7:92701708-9270
ENSG0000(	941	23.53528	chr7:330(	MIR106B		smallRNA	chr7:100093993-100
ENSG0000(	941	23.53528	chr7:330(	PPP1R9A	NCGv7	protein_c	chr7:94907202-9529
ENSG0000(	941	23.53528	chr7:330(	CYP3A5	DriverDB\	protein_c	chr7:99648194-9967
ENSG0000(	941	23.53528	chr7:330(	PTCD1		protein_c	chr7:99416739-9946
ENSG0000(	941	23.53528	chr7:330(	ENSG00000213549		Pseudoger	chr7:76113026-7611
ENSG0000(	941	23.53528	chr7:330(	BUD31	DriverDB\	protein_c	chr7:99408641-9941
ENSG0000(	941	23.53528	chr7:330(	PILRA		protein_c	chr7:100367530-100
ENSG0000(	941	23.53528	chr7:330(	ENSG00000236938		lncRNA	chr7:94071759-9407
ENSG0000(	941	23.53528	chr7:330(	PDAP1	NCGv7	protein_c	chr7:99392048-9940

ENSG0000(	941	23.53528	chr7:330(	AKAP9	NCGv7;AC	protein_c	chr7:91940840-9211
ENSG0000(	941	23.53528	chr7:330(	Y_RNA		smallRNA	chr7:77013583-7701
ENSG0000(	941	23.53528	chr7:330(	GNG11		protein_c	chr7:93921735-9392
ENSG0000(	941	23.53528	chr7:330(	ENSG00000231153		Pseudoger	chr7:95018407-9501
ENSG0000(	941	23.53528	chr7:330(	SEM1	DriverDB\	protein_c	chr7:96481626-9670
ENSG0000(	941	23.53528	chr7:330(	HGF	NCGv7	protein_c	chr7:81699010-8177
ENSG0000(	941	23.53528	chr7:330(	GNGT1		protein_c	chr7:93591573-9391
ENSG0000(	941	23.53528	chr7:330(	PTPN12	NCGv7	protein_c	chr7:77537295-7764
ENSG0000(	941	23.53528	chr7:330(	MIR1285-1		smallRNA	chr7:92204015-9220
ENSG0000(	941	23.53528	chr7:330(	POR		protein_c	chr7:75899200-7598
ENSG0000(	941	23.53528	chr7:330(	FGL2		protein_c	chr7:77193369-7719
ENSG0000(	941	23.53528	chr7:330(	ENSG00000237640		lncRNA	chr7:99929392-9994
ENSG0000(	941	23.53528	chr7:330(	STYXL1		protein_c	chr7:75996338-7604
ENSG0000(	941	23.53528	chr7:330(	STEAP4		protein_c	chr7:88270892-8830
ENSG0000(	941	23.53528	chr7:330(	GNAI1	NCGv7	protein_c	chr7:79768028-8022
ENSG0000(	941	23.53528	chr7:330(	STEAP2-AS1		lncRNA	chr7:89882353-9021
ENSG0000(	941	23.53528	chr7:330(	PEX1		protein_c	chr7:92487020-9252
ENSG0000(	941	23.53528	chr7:330(	MTERF1		protein_c	chr7:91692008-9188
ENSG0000(	941	23.53528	chr7:330(	SGCE		protein_c	chr7:94524204-9465
ENSG0000(	941	23.53528	chr7:330(	RN7SKP104		smallRNA	chr7:97598933-9759
ENSG0000(	941	23.53528	chr7:330(	RBM48		protein_c	chr7:92528773-9254
ENSG0000(	941	23.53528	chr7:330(	CASD1		protein_c	chr7:94509219-9455
ENSG0000(	941	23.53528	chr7:330(	ACTL6B	NCGv7	protein_c	chr7:100643097-100
ENSG0000(	941	23.53528	chr7:330(	AGFG2	DriverDB\	protein_c	chr7:100539203-100
ENSG0000(	941	23.53528	chr7:330(	TAF6	DriverDB\	protein_c	chr7:100106876-100
ENSG0000(	941	23.53528	chr7:330(	AC092022.1		smallRNA	chr7:84665924-8466
ENSG0000(	941	23.53528	chr7:330(	DDX43P3		Pseudoger	chr7:81610884-8161
ENSG0000(	941	23.53528	chr7:330(	BET1-AS1		lncRNA	chr7:93969442-9401
ENSG0000(	941	23.53528	chr7:330(	ANKIB1	DriverDB\	protein_c	chr7:92245974-9240
ENSG0000(	941	23.53528	chr7:330(	PDK4-AS1		lncRNA	chr7:95545191-9561
ENSG0000(	941	23.53528	chr7:330(	ATP5MF		protein_c	chr7:99448475-9946
ENSG0000(	941	23.53528	chr7:330(	MIR4651		smallRNA	chr7:75915197-7591
ENSG0000(	941	23.53528	chr7:330(	PCOLCE		protein_c	chr7:100602363-100
ENSG0000(	941	23.53528	chr7:330(	SLC25A13	DriverDB\	protein_c	chr7:96120220-9632
ENSG0000(	941	23.53528	chr7:330(	ASNS	DriverDB\	protein_c	chr7:97851677-9787
ENSG0000(	941	23.53528	chr7:330(	SAP25		protein_c	chr7:100572228-100
ENSG0000(	941	23.53528	chr7:330(	RPS3AP25		Pseudoger	chr7:94695027-9469
ENSG0000(	941	23.53528	chr7:330(	ENSG00000244055		lncRNA	chr7:92457564-9249
ENSG0000(	941	23.53528	chr7:330(	TECPR1		protein_c	chr7:98214624-9825
ENSG0000(	941	23.53528	chr7:330(	RAD23BP2		Pseudoger	chr7:83663419-8366
ENSG0000(	941	23.53528	chr7:330(	PTP4A1P3		Pseudoger	chr7:91107374-9110
ENSG0000(	941	23.53528	chr7:330(	TFR2	DriverDB\	protein_c	chr7:100620416-100
ENSG0000(	941	23.53528	chr7:330(	PDK4		protein_c	chr7:95583499-9559
ENSG0000(	941	23.53528	chr7:330(	ENSG00000237551		Pseudoger	chr7:96283357-9628
ENSG0000(	941	23.53528	chr7:330(	PMS2P11		Pseudoger	chr7:77011551-7701
ENSG0000(	941	23.53528	chr7:330(	PPIAP81		Pseudoger	chr7:76361768-7636
ENSG0000(	941	23.53528	chr7:330(	ENSG00000241357		lncRNA	chr7:100435257-100
ENSG0000(	941	23.53528	chr7:330(	VPS50	DriverDB\	protein_c	chr7:93232340-9336
ENSG0000(	941	23.53528	chr7:330(	Y_RNA		smallRNA	chr7:77041647-7704
ENSG0000(	941	23.53528	chr7:330(	ENSG00000291178		lncRNA	chr7:100300020-100
ENSG0000(	941	23.53528	chr7:330(	ENSG00000272950		lncRNA	chr7:98322853-9832

ENSG0000(	941	23.53528	chr7:330(	SAMD9		protein_c	chr7:93099513-9311
ENSG0000(	941	23.53528	chr7:330(	ENSG00000223969		lncRNA	chr7:90590619-9059
ENSG0000(	941	23.53528	chr7:330(	ENSG00000227863		lncRNA	chr7:89443946-8949
ENSG0000(	941	23.53528	chr7:330(	GCNT1P5		Pseudoger	chr7:77461458-7746
ENSG0000(	941	23.53528	chr7:330(	ENSG00000288976		lncRNA	chr7:100148341-100
ENSG0000(	941	23.53528	chr7:330(	CALCR	NCGv7	protein_c	chr7:93424486-9357
ENSG0000(	941	23.53528	chr7:330(	AC005077.9		Pseudoger	chr7:76108434-7610
ENSG0000(	941	23.53528	chr7:330(	STEAP2		protein_c	chr7:90167590-9023
ENSG0000(	941	23.53528	chr7:330(	FBXO24	DriverDBv	protein_c	chr7:100583982-100
ENSG0000(	941	23.53528	chr7:330(	PILRB		protein_c	chr7:100352176-100
ENSG0000(	941	23.53528	chr7:330(	ZNF789	DriverDBv	protein_c	chr7:99472890-9950
ENSG0000(	941	23.53528	chr7:330(	DDX3ILA1		lncRNA	chr7:77990384-7799
ENSG0000(	941	23.53528	chr7:330(	CLDN12		protein_c	chr7:90383721-9051
ENSG0000(	941	23.53528	chr7:330(	KRIT1		protein_c	chr7:92197498-9224
ENSG0000(	941	23.53528	chr7:330(	MIR591		smallRNA	chr7:96219662-9621
ENSG0000(	941	23.53528	chr7:330(	FZD1	NCGv7	protein_c	chr7:91264433-9127
ENSG0000(	941	23.53528	chr7:330(	CYP51A1	DriverDBv	protein_c	chr7:92084987-9213
ENSG0000(	941	23.53528	chr7:330(	GATAD1	NCGv7	protein_c	chr7:92447482-9246
ENSG0000(	941	23.53528	chr7:330(	ENSG00000289691		lncRNA	chr7:100397383-100
ENSG0000(	941	23.53528	chr7:330(	SPACDR		protein_c	chr7:100456620-100
ENSG0000(	941	23.53528	chr7:330(	HINT1P2		Pseudoger	chr7:95018163-9501
ENSG0000(	941	23.53528	chr7:330(	ARPC1A	DriverDBv	protein_c	chr7:99325898-9936
ENSG0000(	941	23.53528	chr7:330(	AC005071.1		smallRNA	chr7:100220027-100
ENSG0000(	941	23.53528	chr7:330(	ENSG00000231859		Pseudoger	chr7:97906429-9790
ENSG0000(	941	23.53528	chr7:330(	MIR653		smallRNA	chr7:93482760-9348
ENSG0000(	941	23.53528	chr7:330(	ENSG00000232667		lncRNA	chr7:80312574-8039
ENSG0000(	941	23.53528	chr7:330(	ENSG00000226230		Pseudoger	chr7:78486530-7848
ENSG0000(	941	23.53528	chr7:330(	ENSG00000224134		Pseudoger	chr7:81431731-8143
ENSG0000(	941	23.53528	chr7:330(	FAM200A		protein_c	chr7:99546300-9955
ENSG0000(	941	23.53528	chr7:330(	KPNA7		protein_c	chr7:99173572-9925
ENSG0000(	941	23.53528	chr7:330(	ENSG00000273138		lncRNA	chr7:95416108-9541
ENSG0000(	941	23.53528	chr7:330(	ENSG00000235503		lncRNA	chr7:83355154-8336
ENSG0000(	941	23.53528	chr7:330(	SOCS5P1		Pseudoger	chr7:86216785-8621
ENSG0000(	941	23.53528	chr7:330(	RNU6-393P		smallRNA	chr7:98794718-9879
ENSG0000(	941	23.53528	chr7:330(	ENSG00000235243		Pseudoger	chr7:84847877-8484
ENSG0000(	941	23.53528	chr7:330(	ENSG00000290101		lncRNA	chr7:95396497-9539
ENSG0000(	941	23.53528	chr7:330(	PPIAP82		Pseudoger	chr7:98454119-9845
ENSG0000(	941	23.53528	chr7:330(	UPK3BP1		Pseudoger	chr7:77004662-7700
ENSG0000(	941	23.53528	chr7:330(	MEPCE		protein_c	chr7:100428322-100
ENSG0000(	941	23.53528	chr7:330(	ENSG00000289059		lncRNA	chr7:76318253-7631
ENSG0000(	941	23.53528	chr7:330(	TRIM4		protein_c	chr7:99876958-9991
ENSG0000(	941	23.53528	chr7:330(	PPP1R35-AS1		lncRNA	chr7:100436204-100
ENSG0000(	941	23.53528	chr7:330(	STAG3	NCGv7	protein_c	chr7:100177563-100
ENSG0000(	941	23.53528	chr7:330(	RN7SL35P		smallRNA	chr7:80318253-8031
ENSG0000(	941	23.53528	chr7:330(	GIGYF1		protein_c	chr7:100679507-100
ENSG0000(	941	23.53528	chr7:330(	TRAPPC14		protein_c	chr7:100154420-100
ENSG0000(	941	23.53528	chr7:330(	ENSG00000273299		lncRNA	chr7:90403434-9051
ENSG0000(	941	23.53528	chr7:330(	TVP23CP1		Pseudoger	chr7:90631906-9063
ENSG0000(	941	23.53528	chr7:330(	GRM3	NCGv7	protein_c	chr7:86643909-8686
ENSG0000(	941	23.53528	chr7:330(	ENSG00000285725		lncRNA	chr7:97966377-9797
ENSG0000(	941	23.53528	chr7:330(	ENSG00000232032		Pseudoger	chr7:97908256-9790

ENSG0000(	941	23.53528	chr7:330(	RPL7AP43		Pseudoger	chr7:77115399-7711
ENSG0000(	941	23.53528	chr7:330(	RNA5SP235		Pseudoger	chr7:83017898-8301
ENSG0000(	941	23.53528	chr7:330(	DTX2P1		Pseudoger	chr7:76978617-7700
ENSG0000(	941	23.53528	chr7:330(	COPS6		protein_c	chr7:100088969-100
ENSG0000(	941	23.53528	chr7:330(	SNORA67		smallRNA	chr7:88449092-8844
ENSG0000(	941	23.53528	chr7:330(	ENSG00000233491		Pseudoger	chr7:81489204-8169
ENSG0000(	941	23.53528	chr7:330(	STAG3L5P		Pseudoger	chr7:100338197-100
ENSG0000(	941	23.53528	chr7:330(	AP1S2P1		Pseudoger	chr7:97437518-9743
ENSG0000(	941	23.53528	chr7:330(	PVRIG2P		Pseudoger	chr7:100352360-100
ENSG0000(	941	23.53528	chr7:330(	ENSG00000289996		lncRNA	chr7:81186339-8121
ENSG0000(	941	23.53528	chr7:330(	ENSG00000273341		lncRNA	chr7:77416673-7742
ENSG0000(	941	23.53528	chr7:330(	RSBN1L	NCGv7	protein_c	chr7:77696459-7778
ENSG0000(	941	23.53528	chr7:330(	NDUFAF4P2		Pseudoger	chr7:93844789-9384
ENSG0000(	941	23.53528	chr7:330(	MIR4652		smallRNA	chr7:93716928-9371
ENSG0000(	941	23.53528	chr7:330(	ZNF394	DriverDB\	protein_c	chr7:99473877-9950
ENSG0000(	941	23.53528	chr7:330(	CYP3A7		protein_c	chr7:99705036-9973
ENSG0000(	941	23.53528	chr7:330(	ENSG00000230617		Pseudoger	chr7:83424880-8342
ENSG0000(	941	23.53528	chr7:330(	CYP3A4		protein_c	chr7:99756960-9978
ENSG0000(	941	23.53528	chr7:330(	AZGP1	NCGv7	protein_c	chr7:99966720-9997
ENSG0000(	941	23.53528	chr7:330(	PMS2P9		Pseudoger	chr7:77039944-7704
ENSG0000(	941	23.53528	chr7:330(	LINC03017		lncRNA	chr7:84532476-8458
ENSG0000(	941	23.53528	chr7:330(	RPL7P30		Pseudoger	chr7:84528122-8452
ENSG0000(	941	23.53528	chr7:330(	PPP1R35		protein_c	chr7:100435282-100
ENSG0000(	941	23.53528	chr7:330(	SEMA3A	NCGv7	protein_c	chr7:83955777-8449
ENSG0000(	941	23.53528	chr7:330(	DLX5	AC	protein_c	chr7:97020396-9702
ENSG0000(	941	23.53528	chr7:330(	SEMA3C	NCGv7	protein_c	chr7:80742538-8092
ENSG0000(	941	23.53528	chr7:330(	DYNLL1P7		Pseudoger	chr7:85381118-8538
ENSG0000(	941	23.53528	chr7:330(	ENSG00000227979		Pseudoger	chr7:88749470-8874
ENSG0000(	941	23.53528	chr7:330(	ENSG00000284523		lncRNA	chr7:99252452-9932
ENSG0000(	941	23.53528	chr7:330(	SNRPBP1		Pseudoger	chr7:80377554-8037
ENSG0000(	941	23.53528	chr7:330(	AC005020.1		smallRNA	chr7:99586915-9958
ENSG0000(	941	23.53528	chr7:330(	TMEM225B		protein_c	chr7:99598267-9961
ENSG0000(	941	23.53528	chr7:330(	MAGI2	NCGv7	protein_c	chr7:78017055-7945
ENSG0000(	941	23.53528	chr7:330(	ENSG00000231255		lncRNA	chr7:86775081-8677
ENSG0000(	941	23.53528	chr7:330(	ENSG00000242798		lncRNA	chr7:100115214-100
ENSG0000(	941	23.53528	chr7:330(	ENSG00000272647		protein_c	chr7:99558695-9960
ENSG0000(	941	23.53528	chr7:330(	ENSG00000259628		lncRNA	chr7:77043721-7719
ENSG0000(	941	23.53528	chr7:330(	ENSG00000270453		Pseudoger	chr7:92442077-9244
ENSG0000(	941	23.53528	chr7:330(	CDK14	NCGv7;AC	protein_c	chr7:90466424-9121
ENSG0000(	941	23.53528	chr7:330(	NYAP1	DriverDB\	protein_c	chr7:100483927-100
ENSG0000(	941	23.53528	chr7:330(	ENSG00000285892		lncRNA	chr7:78392626-7844
ENSG0000(	941	23.53528	chr7:330(	MDH2		protein_c	chr7:76048051-7606
ENSG0000(	941	23.53528	chr7:330(	ENSG00000235713		Pseudoger	chr7:99992397-9999
ENSG0000(	941	23.53528	chr7:330(	ENSG00000284292		protein_c	chr7:99325879-9939
ENSG0000(	941	23.53528	chr7:330(	KPNA2P2		Pseudoger	chr7:88564793-8856
ENSG0000(	941	23.53528	chr7:330(	ENSG00000278819		Pseudoger	chr7:92540268-9255
ENSG0000(	941	23.53528	chr7:330(	IRS3P		Pseudoger	chr7:100570131-100
ENSG0000(	941	23.53528	chr7:330(	GNAT3		protein_c	chr7:80458635-8051
ENSG0000(	941	23.53528	chr7:330(	AP4M1		protein_c	chr7:100101549-100
ENSG0000(	941	23.53528	chr7:330(	ENSG00000237729		Pseudoger	chr7:93669826-9367
ENSG0000(	941	23.53528	chr7:330(	CACNA2D1-AS1		lncRNA	chr7:82009177-8202

ENSG0000(	941	23.53528	chr7:330(	LRRD1		protein_c	chr7:92141643-9217
ENSG0000(	941	23.53528	chr7:330(	EEF1A1P28		Pseudoger	chr7:88639014-8864
ENSG0000(	941	23.53528	chr7:330(	SSC4D		protein_c	chr7:76389334-7640
ENSG0000(	941	23.53528	chr7:330(	MARK2P10		Pseudoger	chr7:96858182-9685
ENSG0000(	941	23.53528	chr7:330(	STAG3L5P-PVRIG2P-F		lncRNA	chr7:100336104-100
ENSG0000(	941	23.53528	chr7:330(	ABCB1	NCGv7	protein_c	chr7:87503017-8771
ENSG0000(	941	23.53528	chr7:330(	MYH16		Pseudoger	chr7:99238829-9931
ENSG0000(	941	23.53528	chr7:330(	CCL24		protein_c	chr7:75810825-7582
ENSG0000(	941	23.53528	chr7:330(	ENSG00000279326		TEC	chr7:80972516-8097
ENSG0000(	941	23.53528	chr7:330(	HSPB1	AC	protein_c	chr7:76302673-7630
ENSG0000(	941	23.53528	chr7:330(	ENSG00000286411		lncRNA	chr7:91030149-9103
ENSG0000(	941	23.53528	chr7:330(	AC006988.1		smallRNA	chr7:88640521-8864
ENSG0000(	941	23.53528	chr7:330(	NPTX2		protein_c	chr7:98617285-9862
ENSG0000(	941	23.53528	chr7:330(	ENSG00000232097		Pseudoger	chr7:97938439-9793
ENSG0000(	941	23.53528	chr7:330(	RN7SL416P		smallRNA	chr7:100530364-100
ENSG0000(	941	23.53528	chr7:330(	ENSG00000233942		lncRNA	chr7:95471835-9547
ENSG0000(	941	23.53528	chr7:330(	PCLO	DriverDB\	protein_c	chr7:82754012-8316
ENSG0000(	941	23.53528	chr7:330(	MOSPD3		protein_c	chr7:100612102-100
ENSG0000(	941	23.53528	chr7:330(	TSC22D4		protein_c	chr7:100463359-100
ENSG0000(	941	23.53528	chr7:330(	ENSG00000222024		Pseudoger	chr7:79353410-7935
ENSG0000(	941	23.53528	chr7:330(	SPDYE17		protein_c	chr7:77022306-7703
ENSG0000(	941	23.53528	chr7:330(	ENSG00000285772		protein_c	chr7:92112159-9217
ENSG0000(	941	23.53528	chr7:330(	EIF4A1P13		Pseudoger	chr7:88448788-8844
ENSG0000(	941	23.53528	chr7:330(	CDK6-AS1		lncRNA	chr7:92836367-9291
ENSG0000(	941	23.53528	chr7:330(	ENSG00000227785		Pseudoger	chr7:85636013-8563
ENSG0000(	941	23.53528	chr7:330(	SLC25A40		protein_c	chr7:87833568-8787
ENSG0000(	941	23.53528	chr7:330(	PEG10		protein_c	chr7:94656325-9466
ENSG0000(	941	23.53528	chr7:330(	NUP35P2		Pseudoger	chr7:79543911-7954
ENSG0000(	941	23.53528	chr7:330(	SLC66A2P1		Pseudoger	chr7:88610241-8861
ENSG0000(	941	23.53528	chr7:330(	RN7SL161P		smallRNA	chr7:100462829-100
ENSG0000(	941	23.53528	chr7:330(	LRCH4		protein_c	chr7:100574011-100
ENSG0000(	941	23.53528	chr7:330(	SMURF1	DriverDB\	protein_c	chr7:99027440-9914
ENSG0000(	941	23.53528	chr7:330(	SPDYE18		protein_c	chr7:77050391-7706
ENSG0000(	941	23.53528	chr7:330(	DLX6-AS1		lncRNA	chr7:96955141-9701
ENSG0000(	941	23.53528	chr7:330(	HNRNPA1P9		Pseudoger	chr7:87521461-8752
ENSG0000(	941	23.53528	chr7:330(	RPS3AP26		Pseudoger	chr7:98385801-9838
ENSG0000(	941	23.53528	chr11:76(	ENSG00000271543		Pseudoger	chr11:47796169-477
ENSG0000(	941	23.53528	chr7:330(	RPS3AP29		Pseudoger	chr7:97898347-9789
ENSG0000(	941	23.53528	chr7:330(	ENSG00000279067		TEC	chr7:96118647-9611
ENSG0000(	941	23.53528	chr7:330(	RABGEF1P3		Pseudoger	chr7:76108434-7613
ENSG0000(	941	23.53528	chr7:330(	PPP1R9A-AS1		lncRNA	chr7:95035731-9521
ENSG0000(	941	23.53528	chr7:330(	ENSG00000285398		TEC	chr7:83372652-8337
ENSG0000(	939	23.48526	chr1:116(	ENSG00000229447		Pseudoger	chr1:31263245-3126
ENSG0000(	936	23.41023	chr6:105(	ENSG00000288879		lncRNA	chr6:34236075-3423
ENSG0000(	936	23.41023	chr6:105(	KRT18P9		Pseudoger	chr6:34189780-3419
ENSG0000(	936	23.41023	chr6:105(	PACSIN1	NCGv7	protein_c	chr6:34466061-3453
ENSG0000(	936	23.41023	chr6:105(	RPL35P2		Pseudoger	chr6:34263311-3426
ENSG0000(	936	23.41023	chr6:105(	RP11-513I15.6		lncRNA	chr6:34279679-3428
ENSG0000(	936	23.41023	chr6:105(	BX255972.1		smallRNA	chr6:34470790-3447
ENSG0000(	936	23.41023	chr6:105(	NUDT3		protein_c	chr6:34279679-3439
ENSG0000(	936	23.41023	chr6:105(	CYCSP55		Pseudoger	chr6:34219439-3422

ENSG0000(	936	23.41023	chr6:105(	HMGA1	NCGv7;AC	protein_c	chr6:34236873-3424
ENSG0000(	936	23.41023	chr6:105(	RPS10-NUDT3		protein_c	chr6:34284887-3442
ENSG0000(	936	23.41023	chr6:105(	SPDEF		protein_c	chr6:34537802-3455
ENSG0000(	936	23.41023	chr6:105(	AL590403.1		smallRNA	chr6:34069157-3406
ENSG0000(	936	23.41023	chr6:105(	SMIM29		protein_c	chr6:34246381-3424
ENSG0000(	936	23.41023	chr6:105(	RPS10		protein_c	chr6:34417454-3442
ENSG0000(	935	23.38522	chr1:116(	ENSG00000224066		lncRNA	chr1:32204769-3220
ENSG0000(	935	23.38522	chr1:116(	ENSG00000291132		lncRNA	chr1:32231656-3224
ENSG0000(	935	23.38522	chr1:116(	RNF19B		protein_c	chr1:32936445-3296
ENSG0000(	935	23.38522	chr1:116(	ZBTB8A		protein_c	chr1:32539427-3260
ENSG0000(	935	23.38522	chr1:116(	LCK	NCGv7;AC	protein_c	chr1:32251244-3228
ENSG0000(	935	23.38522	chr1:116(	YARS1		protein_c	chr1:32775237-3281
ENSG0000(	935	23.38522	chr1:116(	ENSG00000287691		lncRNA	chr1:32925454-3295
ENSG0000(	935	23.38522	chr1:116(	FNDC5		protein_c	chr1:32862268-3287
ENSG0000(	935	23.38522	chr1:116(	BSDC1		protein_c	chr1:32364633-3239
ENSG0000(	935	23.38522	chr1:116(	Y_RNA		smallRNA	chr1:32286452-3228
ENSG0000(	935	23.38522	chr1:116(	EIF3I	AC	protein_c	chr1:32221077-3224
ENSG0000(	935	23.38522	chr1:116(	ZBTB8B		protein_c	chr1:32465072-3249
ENSG0000(	935	23.38522	chr1:116(	S100PBP		protein_c	chr1:32816767-3285
ENSG0000(	935	23.38522	chr1:116(	MARCKSL1		protein_c	chr1:32333839-3233
ENSG0000(	935	23.38522	chr1:116(	Y_RNA		smallRNA	chr1:32639951-3264
ENSG0000(	935	23.38522	chr1:116(	TXLNA		protein_c	chr1:32179675-3219
ENSG0000(	935	23.38522	chr1:116(	DCDC2B		protein_c	chr1:32209089-3221
ENSG0000(	935	23.38522	chr1:116(	SYNC		protein_c	chr1:32679906-3270
ENSG0000(	935	23.38522	chr1:116(	RBBP4		protein_c	chr1:32651142-3268
ENSG0000(	935	23.38522	chr1:116(	ENSG00000270850		Pseudoger	chr1:32421979-3242
ENSG0000(	935	23.38522	chr1:116(	ENSG00000290045		lncRNA	chr1:32240526-3224
ENSG0000(	935	23.38522	chr1:116(	MTMR9LP		Pseudoger	chr1:32231847-3224
ENSG0000(	935	23.38522	chr1:116(	HPCA		protein_c	chr1:32885994-3289
ENSG0000(	935	23.38522	chr1:116(	TMEM54		protein_c	chr1:32894594-3290
ENSG0000(	935	23.38522	chr1:116(	GAPDHP20		Pseudoger	chr1:32402109-3240
ENSG0000(	935	23.38522	chr1:116(	KIAA1522		protein_c	chr1:32741830-3277
ENSG0000(	935	23.38522	chr1:116(	ENSG00000254553		protein_c	chr1:32465057-3260
ENSG0000(	935	23.38522	chr1:116(	LRRC37A12P		Pseudoger	chr1:32423214-3242
ENSG0000(	935	23.38522	chr1:116(	ENSG00000224409		lncRNA	chr1:32717734-3272
ENSG0000(	935	23.38522	chr1:116(	ENSG00000233775		lncRNA	chr1:32349194-3235
ENSG0000(	935	23.38522	chr1:116(	TSSK3		protein_c	chr1:32351521-3236
ENSG0000(	935	23.38522	chr1:116(	FAM167B		protein_c	chr1:32247222-3224
ENSG0000(	935	23.38522	chr1:116(	ZBTB8OS		protein_c	chr1:32600172-3265
ENSG0000(	935	23.38522	chr1:116(	CCDC28B		protein_c	chr1:32200595-3220
ENSG0000(	935	23.38522	chr1:116(	IQCC		protein_c	chr1:32205671-3220
ENSG0000(	935	23.38522	chr1:116(	ENSG00000250135		lncRNA	chr1:32170733-3217
ENSG0000(	935	23.38522	chr1:116(	AL031602.1		smallRNA	chr1:32926294-3292
ENSG0000(	935	23.38522	chr1:116(	FAM229A		protein_c	chr1:32361270-3236
ENSG0000(	935	23.38522	chr1:116(	TMEM234		protein_c	chr1:32214472-3222
ENSG0000(	935	23.38522	chr1:116(	RN7SL122P		smallRNA	chr1:32457835-3245
ENSG0000(	935	23.38522	chr1:116(	HDAC1	NCGv7;AC	protein_c	chr1:32292083-3233
ENSG0000(	934	23.36021	chr1:8137	snoU13		smallRNA	chr1:112371004-112
ENSG0000(	934	23.36021	chr1:8137	ENSG00000229283		lncRNA	chr1:111317600-111
ENSG0000(	934	23.36021	chr1:8137	ENSG00000232240		Pseudoger	chr1:111323833-111
ENSG0000(	934	23.36021	chr1:8137	DRAM2		protein_c	chr1:111117163-111

ENSG0000(	934	23.36021	chr1:8137	Y_RNA		smallRNA	chr1:111446798-111
ENSG0000(	934	23.36021	chr1:8137	TXNP3		Pseudoger	chr1:112363281-112
ENSG0000(	934	23.36021	chr1:8137	KCND3-IT1		lncRNA	chr1:111853762-111
ENSG0000(	934	23.36021	chr1:8137	OVGP1		protein_c	chr1:111414319-111
ENSG0000(	934	23.36021	chr1:8137	TMIGD3		protein_c	chr1:111483348-111
ENSG0000(	934	23.36021	chr1:8137	C1orf162		protein_c	chr1:111473792-111
ENSG0000(	934	23.36021	chr1:8137	ENSG00000273221		lncRNA	chr1:111184415-111
ENSG0000(	934	23.36021	chr1:8137	ENSG00000261654		lncRNA	chr1:110936369-110
ENSG0000(	934	23.36021	chr1:8137	NRBF2P3		Pseudoger	chr1:110848077-110
ENSG0000(	934	23.36021	chr1:8137	CCNT2P1		Pseudoger	chr1:111007700-111
ENSG0000(	934	23.36021	chr1:8137	HIGD1AP12		Pseudoger	chr1:111380291-111
ENSG0000(	934	23.36021	chr1:8137	ENSG00000260948		lncRNA	chr1:111431046-111
ENSG0000(	934	23.36021	chr1:8137	RNA5SP54		Pseudoger	chr1:111041834-111
ENSG0000(	934	23.36021	chr1:8137	DENND2D		protein_c	chr1:111185969-111
ENSG0000(	934	23.36021	chr1:8137	ENSG00000284830		lncRNA	chr1:111745299-111
ENSG0000(	934	23.36021	chr1:8137	ST7L		protein_c	chr1:112523514-112
ENSG0000(	934	23.36021	chr1:8137	CEPT1		protein_c	chr1:111139479-111
ENSG0000(	934	23.36021	chr1:8137	KRT18P57		Pseudoger	chr1:111648291-111
ENSG0000(	934	23.36021	chr1:8137	MRPL53P1		Pseudoger	chr1:112625906-112
ENSG0000(	934	23.36021	chr1:8137	CHIAP2		lncRNA	chr1:111280059-111
ENSG0000(	934	23.36021	chr1:8137	snoU13		smallRNA	chr1:112195502-112
ENSG0000(	934	23.36021	chr1:8137	KCND3-AS1		lncRNA	chr1:111909336-111
ENSG0000(	934	23.36021	chr1:8137	LRIF1		protein_c	chr1:110947190-110
ENSG0000(	934	23.36021	chr1:8137	UBE2FP3		Pseudoger	chr1:111437514-111
ENSG0000(	934	23.36021	chr1:8137	ENSG00000243960		lncRNA	chr1:111438638-111
ENSG0000(	934	23.36021	chr1:8137	CHIAP2		Pseudoger	chr1:111280060-111
ENSG0000(	934	23.36021	chr1:8137	CHIAP3		Pseudoger	chr1:111353275-111
ENSG0000(	934	23.36021	chr1:8137	PGBP		Pseudoger	chr1:111382860-111
ENSG0000(	934	23.36021	chr1:8137	LINC01750		lncRNA	chr1:111989770-111
ENSG0000(	934	23.36021	chr1:8137	RAP1A	AC	protein_c	chr1:111542218-111
ENSG0000(	934	23.36021	chr1:8137	ATP5PB		protein_c	chr1:111448864-111
ENSG0000(	934	23.36021	chr1:8137	ENSG00000273483		lncRNA	chr1:112517799-112
ENSG0000(	934	23.36021	chr1:8137	WDR77		protein_c	chr1:111439890-111
ENSG0000(	934	23.36021	chr1:8137	RNU6-792P		smallRNA	chr1:111490317-111
ENSG0000(	934	23.36021	chr1:8137	ENSG00000232811		lncRNA	chr1:110943467-110
ENSG0000(	934	23.36021	chr1:8137	PGBP		lncRNA	chr1:111384519-111
ENSG0000(	934	23.36021	chr1:8137	LINC01160		lncRNA	chr1:111599655-111
ENSG0000(	934	23.36021	chr1:8137	ADORA3		protein_c	chr1:111499429-111
ENSG0000(	934	23.36021	chr1:8137	CHI3L2		protein_c	chr1:111200771-111
ENSG0000(	934	23.36021	chr1:8137	CHIAP1		Pseudoger	chr1:111250254-111
ENSG0000(	934	23.36021	chr1:8137	ENSG00000272982		lncRNA	chr1:111181374-111
ENSG0000(	934	23.36021	chr1:8137	INKA2		protein_c	chr1:111680630-111
ENSG0000(	934	23.36021	chr1:8137	INKA2-AS1		lncRNA	chr1:111739579-111
ENSG0000(	934	23.36021	chr1:8137	CHIA		protein_c	chr1:111290851-111
ENSG0000(	934	23.36021	chr1:8137	WNT2B		protein_c	chr1:112466541-112
ENSG0000(	934	23.36021	chr1:8137	CTTNBP2NL		protein_c	chr1:112396214-112
ENSG0000(	934	23.36021	chr1:8137	KCND3		protein_c	chr1:111770662-111
ENSG0000(	934	23.36021	chr1:8137	CD53		protein_c	chr1:110871188-110
ENSG0000(	934	23.36021	chr1:8137	ENSG00000273010		lncRNA	chr1:110963302-110
ENSG0000(	934	23.36021	chr1:8137	PIFO		protein_c	chr1:111346600-111
ENSG0000(	934	23.36021	chr1:8137	OR11I1P		Pseudoger	chr1:110853939-110

ENSG0000(	934	23.36021	chr1:8137	RNU6-151P		smallRNA	chr1:111650431-111
ENSG0000(	934	23.36021	chr1:8137	LINC02884		lncRNA	chr1:112176836-112
ENSG0000(	934	23.36021	chr1:8137	DDX20	NCGv7	protein_c	chr1:111754832-111
ENSG0000(	933	23.3352	chr11:76(	KCNK7		protein_c	chr11:65592836-655
ENSG0000(	933	23.3352	chr11:76(	ENSG00000256824		lncRNA	chr11:64035970-641
ENSG0000(	933	23.3352	chr11:76(	ROM1		protein_c	chr11:62611722-626
ENSG0000(	933	23.3352	chr11:76(	AC024475.1		smallRNA	chr11:46113122-461
ENSG0000(	933	23.3352	chr11:76(	OR4A47		protein_c	chr11:48488793-484
ENSG0000(	933	23.3352	chr11:76(	MIR5582		smallRNA	chr11:46753125-467
ENSG0000(	933	23.3352	chr11:76(	SLC22A6		protein_c	chr11:62936385-629
ENSG0000(	933	23.3352	chr11:76(	ART2BP		Pseudoger	chr11:72478221-724
ENSG0000(	933	23.3352	chr11:76(	PCNX3		protein_c	chr11:65615776-656
ENSG0000(	933	23.3352	chr11:76(	ENSG00000288434		Pseudoger	chr11:49048271-490
ENSG0000(	933	23.3352	chr11:76(	ENSG00000256897		lncRNA	chr11:47220218-472
ENSG0000(	933	23.3352	chr11:76(	OR4C4P		Pseudoger	chr11:48345351-483
ENSG0000(	933	23.3352	chr11:76(	MTA2		protein_c	chr11:62593214-626
ENSG0000(	933	23.3352	chr11:76(	ENSG00000257086		lncRNA	chr11:64246939-642
ENSG0000(	933	23.3352	chr11:76(	OR9I1		protein_c	chr11:58116742-581
ENSG0000(	933	23.3352	chr11:76(	OR1S2		protein_c	chr11:58203204-582
ENSG0000(	933	23.3352	chr11:76(	KAT5		protein_c	chr11:65711996-657
ENSG0000(	933	23.3352	chr11:76(	OR5AR1		protein_c	chr11:56663686-566
ENSG0000(	933	23.3352	chr11:76(	SLC22A20P		Pseudoger	chr11:65213840-652
ENSG0000(	933	23.3352	chr11:76(	CCND2P1		Pseudoger	chr11:63243085-632
ENSG0000(	933	23.3352	chr11:76(	AC090559.1		smallRNA	chr11:47485822-474
ENSG0000(	933	23.3352	chr11:76(	RNU6-45P		smallRNA	chr11:63970470-639
ENSG0000(	933	23.3352	chr11:76(	OR5M12P		Pseudoger	chr11:56628932-566
ENSG0000(	933	23.3352	chr11:76(	OR5B17	NCGv7	protein_c	chr11:58358124-583
ENSG0000(	933	23.3352	chr11:76(	PPP1R14B-AS1		lncRNA	chr11:64245838-642
ENSG0000(	933	23.3352	chr11:76(	AP002517.1		smallRNA	chr11:56637987-566
ENSG0000(	933	23.3352	chr11:76(	ENSG00000288534		protein_c	chr11:57712582-578
ENSG0000(	933	23.3352	chr11:76(	OR6Q1		protein_c	chr11:58030953-580
ENSG0000(	933	23.3352	chr11:76(	CLP1		protein_c	chr11:57648188-576
ENSG0000(	933	23.3352	chr11:76(	ENSG00000278952		TEC	chr11:65118310-651
ENSG0000(	933	23.3352	chr11:76(	OR5T3		protein_c	chr11:56252200-562
ENSG0000(	933	23.3352	chr11:76(	ENSG00000288852		lncRNA	chr11:64183353-641
ENSG0000(	933	23.3352	chr11:76(	RN7SL652P		smallRNA	chr11:47557536-475
ENSG0000(	933	23.3352	chr11:76(	HNRNPUL2-BSCL2		protein_c	chr11:62690275-627
ENSG0000(	933	23.3352	chr11:76(	FOLH1	NCGv7	protein_c	chr11:49145092-492
ENSG0000(	933	23.3352	chr11:76(	ENSG00000237363		Pseudoger	chr11:64531044-645
ENSG0000(	933	23.3352	chr11:76(	GANAB		protein_c	chr11:62624826-626
ENSG0000(	933	23.3352	chr11:76(	EML3		protein_c	chr11:62602218-626
ENSG0000(	933	23.3352	chr11:76(	OR5B2		protein_c	chr11:58421238-584
ENSG0000(	933	23.3352	chr11:76(	LINC02736		lncRNA	chr11:65487241-654
ENSG0000(	933	23.3352	chr11:76(	EHBP1L1		protein_c	chr11:65576046-655
ENSG0000(	933	23.3352	chr11:76(	ENSG00000231492		lncRNA	chr11:64420311-644
ENSG0000(	933	23.3352	chr11:76(	SLC22A12		protein_c	chr11:64590641-646
ENSG0000(	933	23.3352	chr11:76(	AC110283.1		smallRNA	chr11:54855185-548
ENSG0000(	933	23.3352	chr11:76(	OR9G4		protein_c	chr11:56741223-567
ENSG0000(	933	23.3352	chr11:76(	ENSG00000287984		lncRNA	chr11:45008266-450
ENSG0000(	933	23.3352	chr11:76(	OR4C16		protein_c	chr11:55572128-555
ENSG0000(	933	23.3352	chr11:76(	ENSG00000257002		lncRNA	chr11:62909546-629

ENSG0000(	933	23.3352	chr11:76(	LINC02716		lncRNA	chr11:45771416-457
ENSG0000(	933	23.3352	chr11:76(	B3GAT3		protein_c	chr11:62615296-626
ENSG0000(	933	23.3352	chr11:76(	ENSG00000256789		lncRNA	chr11:63637677-636
ENSG0000(	933	23.3352	chr11:76(	OR5G5P		Pseudoger	chr11:56801856-568
ENSG0000(	933	23.3352	chr11:76(	OR10W1		protein_c	chr11:58266792-582
ENSG0000(	933	23.3352	chr11:76(	OVOL1		protein_c	chr11:65787063-657
ENSG0000(	933	23.3352	chr11:76(	AP000781.1		smallRNA	chr11:57359722-573
ENSG0000(	933	23.3352	chr11:76(	ENSG00000289562		lncRNA	chr11:62574174-625
ENSG0000(	933	23.3352	chr11:76(	PHKG1P3		Pseudoger	chr11:50246554-502
ENSG0000(	933	23.3352	chr11:76(	OR8I2		protein_c	chr11:56093308-560
ENSG0000(	933	23.3352	chr11:76(	SLC43A1		protein_c	chr11:57484534-575
ENSG0000(	933	23.3352	chr11:76(	MIR4688		smallRNA	chr11:46376402-463
ENSG0000(	933	23.3352	chr11:76(	SSRP1		protein_c	chr11:57325986-573
ENSG0000(	933	23.3352	chr11:76(	OR5F1		protein_c	chr11:55993681-559
ENSG0000(	933	23.3352	chr11:76(	SERPING1	NCGv7	protein_c	chr11:57597387-576
ENSG0000(	933	23.3352	chr11:76(	GLYAT	NCGv7	protein_c	chr11:58640426-587
ENSG0000(	933	23.3352	chr11:76(	MIR4690		smallRNA	chr11:65636310-656
ENSG0000(	933	23.3352	chr11:76(	TMEM230P2		Pseudoger	chr11:56690465-566
ENSG0000(	933	23.3352	chr11:76(	OR5L2		protein_c	chr11:55827219-558
ENSG0000(	933	23.3352	chr11:76(	ENSG00000279093		TEC	chr11:65561484-655
ENSG0000(	933	23.3352	chr11:76(	ART2P		Pseudoger	chr11:72519986-725
ENSG0000(	933	23.3352	chr11:76(	TNKS1BP1		protein_c	chr11:57299638-573
ENSG0000(	933	23.3352	chr11:76(	RNASEH2C		protein_c	chr11:65714005-657
ENSG0000(	933	23.3352	chr11:76(	DGKZ		protein_c	chr11:46332905-463
ENSG0000(	933	23.3352	chr11:76(	CTNND1	NCGv7	protein_c	chr11:57753243-578
ENSG0000(	933	23.3352	chr11:76(	GRM5P1		Pseudoger	chr11:49560818-498
ENSG0000(	933	23.3352	chr11:76(	OR4C11		protein_c	chr11:55602360-556
ENSG0000(	933	23.3352	chr11:76(	PTPRJ		protein_c	chr11:47980425-481
ENSG0000(	933	23.3352	chr11:76(	OR4X2		protein_c	chr11:48245104-482
ENSG0000(	933	23.3352	chr11:76(	ENSG00000291174		lncRNA	chr11:65455257-654
ENSG0000(	933	23.3352	chr11:76(	GPR137		protein_c	chr11:64270062-642
ENSG0000(	933	23.3352	chr11:76(	ENSG00000205044		Pseudoger	chr11:49010179-490
ENSG0000(	933	23.3352	chr11:76(	ENSG00000287821		lncRNA	chr11:65260996-652
ENSG0000(	933	23.3352	chr11:76(	ENSG00000257058		lncRNA	chr11:62545999-625
ENSG0000(	933	23.3352	chr11:76(	C11orf98		protein_c	chr11:62662817-626
ENSG0000(	933	23.3352	chr11:76(	OR4C50P		Pseudoger	chr11:54591479-545
ENSG0000(	933	23.3352	chr11:76(	OR5B21		protein_c	chr11:58506807-585
ENSG0000(	933	23.3352	chr11:76(	ENSG00000291248		lncRNA	chr11:49015484-490
ENSG0000(	933	23.3352	chr11:76(	CELF1	NCGv7	protein_c	chr11:47465933-475
ENSG0000(	933	23.3352	chr11:76(	RNU2-23P		smallRNA	chr11:65147584-651
ENSG0000(	933	23.3352	chr11:76(	ARFGAP2		protein_c	chr11:47164299-471
ENSG0000(	933	23.3352	chr11:76(	CSTPP1		protein_c	chr11:46936689-471
ENSG0000(	933	23.3352	chr11:76(	MIR4692		smallRNA	chr11:72783530-727
ENSG0000(	933	23.3352	chr11:76(	OR5B3		protein_c	chr11:58402464-584
ENSG0000(	933	23.3352	chr11:76(	AC090559.2		smallRNA	chr11:47386082-473
ENSG0000(	933	23.3352	chr11:76(	ZNRD2		protein_c	chr11:65570460-655
ENSG0000(	933	23.3352	chr11:76(	MIR3160-2		smallRNA	chr11:46451805-464
ENSG0000(	933	23.3352	chr11:76(	OR8U1		protein_c	chr11:56375624-563
ENSG0000(	933	23.3352	chr11:76(	Y_RNA		smallRNA	chr11:47726894-477
ENSG0000(	933	23.3352	chr11:76(	OR5D16		protein_c	chr11:55838752-558
ENSG0000(	933	23.3352	chr11:76(	PPP1R14B		protein_c	chr11:64244479-642

ENSG0000(	933	23.3352	chr11:76(	ENSG00000197254		Pseudoger	chr11:58685086-586
ENSG0000(	933	23.3352	chr11:76(	BAD		protein_c	chr11:64269830-642
ENSG0000(	933	23.3352	chr11:76(	ENSG00000256739		lncRNA	chr11:72351347-723
ENSG0000(	933	23.3352	chr11:76(	C1QTNF4		protein_c	chr11:47589667-475
ENSG0000(	933	23.3352	chr11:76(	RN7SL596P		smallRNA	chr11:63797788-637
ENSG0000(	933	23.3352	chr11:76(	TRMT112		protein_c	chr11:64316460-643
ENSG0000(	933	23.3352	chr11:76(	SCYL1		protein_c	chr11:65525077-655
ENSG0000(	933	23.3352	chr11:76(	VN2R9P		Pseudoger	chr11:58040708-580
ENSG0000(	933	23.3352	chr11:76(	ENSG00000232500		lncRNA	chr11:64500846-645
ENSG0000(	933	23.3352	chr11:76(	MADD-AS1		lncRNA	chr11:47270657-472
ENSG0000(	933	23.3352	chr11:76(	TRIM51CP		Pseudoger	chr11:48945694-489
ENSG0000(	933	23.3352	chr11:76(	MARK2	NCGv7	protein_c	chr11:63838928-639
ENSG0000(	933	23.3352	chr11:76(	ENSG00000234751		Pseudoger	chr11:72940498-729
ENSG0000(	933	23.3352	chr11:76(	CDCA5		protein_c	chr11:65066300-650
ENSG0000(	933	23.3352	chr11:76(	RN7SL119P		smallRNA	chr11:62816830-628
ENSG0000(	933	23.3352	chr11:76(	OR5L1		protein_c	chr11:55811367-558
ENSG0000(	933	23.3352	chr11:76(	ENSG00000288177		lncRNA	chr11:49852096-498
ENSG0000(	933	23.3352	chr11:76(	C11orf94		protein_c	chr11:45906513-459
ENSG0000(	933	23.3352	chr11:76(	SLC22A8	NCGv7	protein_c	chr11:62989154-630
ENSG0000(	933	23.3352	chr11:76(	ENSG00000289058		lncRNA	chr11:64687682-646
ENSG0000(	933	23.3352	chr11:76(	CDC42BPG	NCGv7	protein_c	chr11:64823052-648
ENSG0000(	933	23.3352	chr11:76(	NRXN2-AS1		lncRNA	chr11:64646399-646
ENSG0000(	933	23.3352	chr11:76(	RELA	NCGv7	protein_c	chr11:65653599-656
ENSG0000(	933	23.3352	chr11:76(	OR4C13		protein_c	chr11:49952391-499
ENSG0000(	933	23.3352	chr11:76(	VEGFB		protein_c	chr11:64234584-642
ENSG0000(	933	23.3352	chr11:76(	STX5-DT		lncRNA	chr11:62832234-628
ENSG0000(	933	23.3352	chr11:76(	ZNRD2-DT		lncRNA	chr11:65568482-655
ENSG0000(	933	23.3352	chr11:76(	ENSG00000250659		lncRNA	chr11:62537312-625
ENSG0000(	933	23.3352	chr11:76(	TUT1		protein_c	chr11:62575045-625
ENSG0000(	933	23.3352	chr11:76(	LINC01537		lncRNA	chr11:72570660-725
ENSG0000(	933	23.3352	chr11:76(	ENSG00000236935		lncRNA	chr11:64325050-643
ENSG0000(	933	23.3352	chr11:76(	ENSG00000287917		lncRNA	chr11:65305345-653
ENSG0000(	933	23.3352	chr11:76(	OR5AP2		protein_c	chr11:56641489-566
ENSG0000(	933	23.3352	chr11:76(	OR2AH1P		Pseudoger	chr11:56669017-566
ENSG0000(	933	23.3352	chr11:76(	MIR4487		smallRNA	chr11:47400970-474
ENSG0000(	933	23.3352	chr11:76(	ESRRA	IntOGen-I	protein_c	chr11:64305497-643
ENSG0000(	933	23.3352	chr11:76(	SLC22A24		protein_c	chr11:63079940-631
ENSG0000(	933	23.3352	chr11:76(	SEPTIN7P11		lncRNA	chr11:50258255-502
ENSG0000(	933	23.3352	chr11:76(	RN7SL114P		smallRNA	chr11:65049777-650
ENSG0000(	933	23.3352	chr11:76(	AP000445.3		smallRNA	chr11:58665801-586
ENSG0000(	933	23.3352	chr11:76(	OR5B12		protein_c	chr11:58438994-584
ENSG0000(	933	23.3352	chr11:76(	FCHSD2		protein_c	chr11:72836745-731
ENSG0000(	933	23.3352	chr11:76(	MIR3161		smallRNA	chr11:48096782-480
ENSG0000(	933	23.3352	chr11:76(	OR8J1		protein_c	chr11:56360215-563
ENSG0000(	933	23.3352	chr11:76(	PDE2A-AS2		lncRNA	chr11:72584572-725
ENSG0000(	933	23.3352	chr11:76(	MAP3K11		protein_c	chr11:65597756-656
ENSG0000(	933	23.3352	chr11:76(	FKBP2		protein_c	chr11:64241003-642
ENSG0000(	933	23.3352	chr11:76(	KCNK4-TEX40		lncRNA	chr11:64291722-643
ENSG0000(	933	23.3352	chr11:76(	RNU6-118P		smallRNA	chr11:62815966-628
ENSG0000(	933	23.3352	chr11:76(	ENSG00000255266		Pseudoger	chr11:57870322-578
ENSG0000(	933	23.3352	chr11:76(	OR10AG1		protein_c	chr11:55965755-559

ENSG0000(	933	23.3352	chr11:76(	OR5B15P		Pseudoger	chr11:58387583-583
ENSG0000(	933	23.3352	chr11:76(	ENSG00000289259		lncRNA	chr11:65471472-654
ENSG0000(	933	23.3352	chr11:76(	ENSG00000289883		lncRNA	chr11:65476515-654
ENSG0000(	933	23.3352	chr11:76(	OR4A19P		Pseudoger	chr11:49920312-499
ENSG0000(	933	23.3352	chr11:76(	OR8K4P		Pseudoger	chr11:56142516-561
ENSG0000(	933	23.3352	chr11:76(	NUP160		protein_c	chr11:47778087-478
ENSG0000(	933	23.3352	chr11:76(	ENSG00000254953		Pseudoger	chr11:56736316-567
ENSG0000(	933	23.3352	chr11:76(	OR4S2		protein_c	chr11:55648327-556
ENSG0000(	933	23.3352	chr11:76(	ENSG00000254964		lncRNA	chr11:62606161-626
ENSG0000(	933	23.3352	chr11:76(	RPL5P29		Pseudoger	chr11:56357328-563
ENSG0000(	933	23.3352	chr11:76(	OR8J3		protein_c	chr11:56134721-561
ENSG0000(	933	23.3352	chr11:76(	ENSG00000244313		Pseudoger	chr11:46428653-464
ENSG0000(	933	23.3352	chr11:76(	ENSG00000285864		lncRNA	chr11:72261731-722
ENSG0000(	933	23.3352	chr11:76(	ENSG00000254979		protein_c	chr11:57387365-574
ENSG0000(	933	23.3352	chr11:76(	OR5I1		protein_c	chr11:55935456-559
ENSG0000(	933	23.3352	chr11:76(	ENSG00000285816		protein_c	chr11:65261928-653
ENSG0000(	933	23.3352	chr11:76(	ENSG00000285895		lncRNA	chr11:58491773-584
ENSG0000(	933	23.3352	chr11:76(	OR4A5		protein_c	chr11:54706831-547
ENSG0000(	933	23.3352	chr11:76(	OR5J2		protein_c	chr11:56176618-561
ENSG0000(	933	23.3352	chr11:76(	TRIM77BP		Pseudoger	chr11:49117010-491
ENSG0000(	933	23.3352	chr11:76(	ENSG00000255001		Pseudoger	chr11:50290749-502
ENSG0000(	933	23.3352	chr11:76(	LINC02696		lncRNA	chr11:45355371-453
ENSG0000(	933	23.3352	chr11:76(	YPEL4		protein_c	chr11:57645087-576
ENSG0000(	933	23.3352	chr11:76(	ENSG00000256481		lncRNA	chr11:64081690-640
ENSG0000(	933	23.3352	chr11:76(	LINC02489		lncRNA	chr11:46256264-462
ENSG0000(	933	23.3352	chr11:76(	OR5M3		protein_c	chr11:56469274-564
ENSG0000(	933	23.3352	chr11:76(	NR1H3		protein_c	chr11:47248300-472
ENSG0000(	933	23.3352	chr11:76(	OR5M1		protein_c	chr11:56609236-566
ENSG0000(	933	23.3352	chr11:76(	SIPA1		protein_c	chr11:65638101-656
ENSG0000(	933	23.3352	chr11:76(	ENSG00000271350		Pseudoger	chr11:47041027-470
ENSG0000(	933	23.3352	chr11:76(	ENSG00000276109		Pseudoger	chr11:72551049-725
ENSG0000(	933	23.3352	chr11:76(	OR4C9P		Pseudoger	chr11:48464053-484
ENSG0000(	933	23.3352	chr11:76(	OR5BQ1P		Pseudoger	chr11:57029408-570
ENSG0000(	933	23.3352	chr11:76(	ARL2		protein_c	chr11:65014160-650
ENSG0000(	933	23.3352	chr11:76(	ENSG00000254501		lncRNA	chr11:65110714-651
ENSG0000(	933	23.3352	chr11:76(	OR5G1P		Pseudoger	chr11:56775261-567
ENSG0000(	933	23.3352	chr11:76(	ENSG00000254497		lncRNA	chr11:45253884-452
ENSG0000(	933	23.3352	chr11:76(	CBX3P8		Pseudoger	chr11:49406190-494
ENSG0000(	933	23.3352	chr11:76(	CNTF		protein_c	chr11:58622665-586
ENSG0000(	933	23.3352	chr11:76(	OR5M7P		Pseudoger	chr11:56500300-565
ENSG0000(	933	23.3352	chr11:76(	OR5AK3P		protein_c	chr11:56971050-569
ENSG0000(	933	23.3352	chr11:76(	OR5AK2		protein_c	chr11:56988914-569
ENSG0000(	933	23.3352	chr11:76(	ENSG00000285658		lncRNA	chr11:46572948-465
ENSG0000(	933	23.3352	chr11:76(	FBLIM1P2		Pseudoger	chr11:46142716-461
ENSG0000(	933	23.3352	chr11:76(	OR4A49P		Pseudoger	chr11:49915013-499
ENSG0000(	933	23.3352	chr11:76(	AP5B1		protein_c	chr11:65773898-657
ENSG0000(	933	23.3352	chr11:76(	ENSG00000285693		lncRNA	chr11:72793624-728
ENSG0000(	933	23.3352	chr11:76(	LRRC55	NCGv7	protein_c	chr11:57181747-571
ENSG0000(	933	23.3352	chr11:76(	FRMD8	NCGv7	protein_c	chr11:65386621-654
ENSG0000(	933	23.3352	chr11:76(	HIGD1AP10		Pseudoger	chr11:65145691-651
ENSG0000(	933	23.3352	chr11:76(	PRDX5		protein_c	chr11:64318121-643

ENSG0000(	933	23.3352	chr11:76(	OR5W1P		Pseudoger	chr11:55903341-559
ENSG0000(	933	23.3352	chr11:76(	PTPRJ-AS1		lncRNA	chr11:48014406-480
ENSG0000(	933	23.3352	chr11:76(	MALAT1	AC	lncRNA	chr11:65497688-655
ENSG0000(	933	23.3352	chr11:76(	OR4A3P		Pseudoger	chr11:54662579-546
ENSG0000(	933	23.3352	chr11:76(	FLRT1		protein_c	chr11:64035931-641
ENSG0000(	933	23.3352	chr11:76(	OR4A9P		Pseudoger	chr11:55388529-553
ENSG0000(	933	23.3352	chr11:76(	RNA5SP341		Pseudoger	chr11:57450183-574
ENSG0000(	933	23.3352	chr11:76(	OTUB1	DriverDB\	protein_c	chr11:63985853-640
ENSG0000(	933	23.3352	chr11:76(	RCOR2		protein_c	chr11:63911230-639
ENSG0000(	933	23.3352	chr11:76(	OR8L1P		Pseudoger	chr11:56381635-563
ENSG0000(	933	23.3352	chr11:76(	ENSG00000254920		Pseudoger	chr11:48893256-488
ENSG0000(	933	23.3352	chr11:76(	YPEL5P2		Pseudoger	chr11:47841324-478
ENSG0000(	933	23.3352	chr11:76(	OR5D2P		Pseudoger	chr11:55714799-557
ENSG0000(	933	23.3352	chr11:76(	SELENOH		protein_c	chr11:57741491-577
ENSG0000(	933	23.3352	chr11:76(	OR5AQ1P		Pseudoger	chr11:56054377-560
ENSG0000(	933	23.3352	chr11:76(	OR4B1		protein_c	chr11:48216810-482
ENSG0000(	933	23.3352	chr11:76(	SPINDOC		protein_c	chr11:63813456-638
ENSG0000(	933	23.3352	chr11:76(	TMEM179B		protein_c	chr11:62787402-627
ENSG0000(	933	23.3352	chr11:76(	SLC3A2	AC	protein_c	chr11:62856004-628
ENSG0000(	933	23.3352	chr11:76(	OR5G3		protein_c	chr11:56819573-568
ENSG0000(	933	23.3352	chr11:76(	ENSG00000255111		Pseudoger	chr11:49892262-498
ENSG0000(	933	23.3352	chr11:76(	OR4A48P		Pseudoger	chr11:48492026-484
ENSG0000(	933	23.3352	chr11:76(	ENSG00000290411		lncRNA	chr11:55850347-558
ENSG0000(	933	23.3352	chr11:76(	OR10Q1		protein_c	chr11:58227882-582
ENSG0000(	933	23.3352	chr11:76(	CREB3L1	NCGv7;AC	protein_c	chr11:46277662-463
ENSG0000(	933	23.3352	chr11:76(	ZBTB3		protein_c	chr11:62748319-627
ENSG0000(	933	23.3352	chr11:76(	TSPAN18	NCGv7	protein_c	chr11:44726465-449
ENSG0000(	933	23.3352	chr11:76(	PLAAT5		protein_c	chr11:63461404-634
ENSG0000(	933	23.3352	chr11:76(	OR5M5P		Pseudoger	chr11:56526568-565
ENSG0000(	933	23.3352	chr11:76(	ENSG00000290412		lncRNA	chr11:50307681-503
ENSG0000(	933	23.3352	chr11:76(	ENSG00000289231		lncRNA	chr11:65353024-653
ENSG0000(	933	23.3352	chr11:76(	ATG16L2		protein_c	chr11:72814406-728
ENSG0000(	933	23.3352	chr11:76(	ATL3		protein_c	chr11:63624087-636
ENSG0000(	933	23.3352	chr11:76(	ENSG00000290417		lncRNA	chr11:55295406-552
ENSG0000(	933	23.3352	chr11:76(	OVOL1-AS1		lncRNA	chr11:65789051-657
ENSG0000(	933	23.3352	chr11:76(	Y_RNA		smallRNA	chr11:47614898-476
ENSG0000(	933	23.3352	chr11:76(	HARBI1		protein_c	chr11:46602861-466
ENSG0000(	933	23.3352	chr11:76(	OR9L1P		Pseudoger	chr11:58077348-580
ENSG0000(	933	23.3352	chr11:76(	ENSG00000283338		Pseudoger	chr11:47323983-473
ENSG0000(	933	23.3352	chr11:76(	OR8K2P		Pseudoger	chr11:56335142-563
ENSG0000(	933	23.3352	chr11:76(	OR4C46		protein_c	chr11:54603068-546
ENSG0000(	933	23.3352	chr11:76(	OR4C12		protein_c	chr11:49981473-499
ENSG0000(	933	23.3352	chr11:76(	MDK		protein_c	chr11:46380756-463
ENSG0000(	933	23.3352	chr11:76(	ENSG00000286264		protein_c	chr11:64241095-642
ENSG0000(	933	23.3352	chr11:76(	FADS2B		Pseudoger	chr11:56890613-569
ENSG0000(	933	23.3352	chr11:76(	OR8J2		protein_c	chr11:56208985-562
ENSG0000(	933	23.3352	chr11:76(	AMBRA1		protein_c	chr11:46396414-465
ENSG0000(	933	23.3352	chr11:76(	ENSG00000255110		Pseudoger	chr11:55279949-552
ENSG0000(	933	23.3352	chr11:76(	ENSG00000290405		lncRNA	chr11:55506635-555
ENSG0000(	933	23.3352	chr11:76(	OR5B10P		Pseudoger	chr11:58348989-583
ENSG0000(	933	23.3352	chr11:76(	PPIAP42		Pseudoger	chr11:57718044-577

ENSG0000(	933	23.3352	chr11:76(	OR4C10P		Pseudoger	chr11:48432217-484
ENSG0000(	933	23.3352	chr11:76(	OR5D15P		Pseudoger	chr11:55786964-557
ENSG0000(	933	23.3352	chr11:76(	ENSG00000254412		Pseudoger	chr11:49098163-491
ENSG0000(	933	23.3352	chr11:76(	TSPAN18-AS1		lncRNA	chr11:44719392-447
ENSG0000(	933	23.3352	chr11:76(	ENSG00000254411		Pseudoger	chr11:56495760-564
ENSG0000(	933	23.3352	chr11:76(	OR4A1P		Pseudoger	chr11:49898267-498
ENSG0000(	933	23.3352	chr11:76(	SLC22A10		protein_c	chr11:63268022-633
ENSG0000(	933	23.3352	chr11:76(	ENSG00000255041		lncRNA	chr11:45387215-455
ENSG0000(	933	23.3352	chr11:76(	SEPTIN7P11		Pseudoger	chr11:50284949-502
ENSG0000(	933	23.3352	chr11:76(	OR4A44P		Pseudoger	chr11:48627546-486
ENSG0000(	933	23.3352	chr11:76(	PDCL2P2		Pseudoger	chr11:65160194-651
ENSG0000(	933	23.3352	chr11:76(	Y_RNA		smallRNA	chr11:72766004-727
ENSG0000(	933	23.3352	chr11:76(	STARD10	NCGv7	protein_c	chr11:72754729-727
ENSG0000(	933	23.3352	chr11:76(	BSCL2		protein_c	chr11:62689289-627
ENSG0000(	933	23.3352	chr11:76(	ZFP91-CNTF		protein_c	chr11:58579172-586
ENSG0000(	933	23.3352	chr11:76(	CHRM4		protein_c	chr11:46383789-463
ENSG0000(	933	23.3352	chr11:76(	OR4X7P		Pseudoger	chr11:55411526-554
ENSG0000(	933	23.3352	chr11:76(	OR4A6P		Pseudoger	chr11:54724846-547
ENSG0000(	933	23.3352	chr11:76(	INTS5	AC	protein_c	chr11:62646848-626
ENSG0000(	933	23.3352	chr11:76(	ENSG00000271100		Pseudoger	chr11:63698596-636
ENSG0000(	933	23.3352	chr11:76(	MIR130A		smallRNA	chr11:57641198-576
ENSG0000(	933	23.3352	chr11:76(	POLR2G		protein_c	chr11:62761565-627
ENSG0000(	933	23.3352	chr11:76(	LINC02704		lncRNA	chr11:44694863-446
ENSG0000(	933	23.3352	chr11:76(	OR8V1P		Pseudoger	chr11:56188379-561
ENSG0000(	933	23.3352	chr11:76(	OR5AK1P		Pseudoger	chr11:57018100-570
ENSG0000(	933	23.3352	chr11:76(	GRM5P1		lncRNA	chr11:49558546-498
ENSG0000(	933	23.3352	chr11:76(	OR5AZ1P		Pseudoger	chr11:57917297-579
ENSG0000(	933	23.3352	chr11:76(	ENSG00000255091		lncRNA	chr11:45651529-456
ENSG0000(	933	23.3352	chr11:76(	ENSG00000290402		lncRNA	chr11:49366071-493
ENSG0000(	933	23.3352	chr11:76(	OR5BP1P		Pseudoger	chr11:57055587-570
ENSG0000(	933	23.3352	chr11:76(	ENSG00000254853		Pseudoger	chr11:58072378-580
ENSG0000(	933	23.3352	chr11:76(	OR5BN2P		Pseudoger	chr11:56116639-561
ENSG0000(	933	23.3352	chr11:76(	STX5		protein_c	chr11:62806860-628
ENSG0000(	933	23.3352	chr11:76(	OR4A15		protein_c	chr11:55367974-553
ENSG0000(	933	23.3352	chr11:76(	OR4A16		protein_c	chr11:55343201-553
ENSG0000(	933	23.3352	chr11:76(	MIR194-2HG		lncRNA	chr11:64889560-648
ENSG0000(	933	23.3352	chr11:76(	TRIM49B		protein_c	chr11:49027501-490
ENSG0000(	933	23.3352	chr11:76(	ENSG00000243802		Pseudoger	chr11:47191181-471
ENSG0000(	933	23.3352	chr11:76(	OR4A8		protein_c	chr11:54682876-546
ENSG0000(	933	23.3352	chr11:76(	OR4A18P		Pseudoger	chr11:49917391-499
ENSG0000(	933	23.3352	chr11:76(	FNBP4		protein_c	chr11:47716494-477
ENSG0000(	933	23.3352	chr11:76(	RPS6KA4		protein_c	chr11:64359148-643
ENSG0000(	933	23.3352	chr11:76(	ZFPL1		protein_c	chr11:65084210-650
ENSG0000(	933	23.3352	chr11:76(	SYVN1		protein_c	chr11:65121780-651
ENSG0000(	933	23.3352	chr11:76(	OR10Q2P		Pseudoger	chr11:58291826-582
ENSG0000(	933	23.3352	chr11:76(	RNU7-105P		smallRNA	chr11:72621766-726
ENSG0000(	933	23.3352	chr11:76(	SLC25A45	NCGv7	protein_c	chr11:65375192-653
ENSG0000(	933	23.3352	chr11:76(	NXF1	NCGv7	protein_c	chr11:62792123-628
ENSG0000(	933	23.3352	chr11:76(	OR4A21P		Pseudoger	chr11:55491205-554
ENSG0000(	933	23.3352	chr11:76(	TAF6L		protein_c	chr11:62771357-627
ENSG0000(	933	23.3352	chr11:76(	OR4R3P		Pseudoger	chr11:49923049-499

ENSG0000(	933	23.3352	chr11:76(	TTC9C		protein_c	chr11:62728069-627
ENSG0000(	933	23.3352	chr11:76(	LBHD1		protein_c	chr11:62662817-626
ENSG0000(	933	23.3352	chr11:76(	UBXN1		protein_c	chr11:62676498-626
ENSG0000(	933	23.3352	chr11:76(	GNG3	NCGv7	protein_c	chr11:62707676-627
ENSG0000(	933	23.3352	chr11:76(	KBTBD4	NCGv7	protein_c	chr11:47572197-475
ENSG0000(	933	23.3352	chr11:76(	TYRL		Pseudoger	chr11:49405091-494
ENSG0000(	933	23.3352	chr11:76(	CAPN1-AS1		lncRNA	chr11:65177606-651
ENSG0000(	933	23.3352	chr11:76(	CLPB	DriverDB\	protein_c	chr11:72285495-724
ENSG0000(	933	23.3352	chr11:76(	ENSG00000290016		lncRNA	chr11:72530155-725
ENSG0000(	933	23.3352	chr11:76(	OR5M6P		Pseudoger	chr11:56512235-565
ENSG0000(	933	23.3352	chr11:76(	FAM8A2P		Pseudoger	chr11:56331347-563
ENSG0000(	933	23.3352	chr11:76(	OR4A12P		Pseudoger	chr11:55325756-553
ENSG0000(	933	23.3352	chr11:76(	OR4A13P		Pseudoger	chr11:55466771-554
ENSG0000(	933	23.3352	chr11:76(	OR4C15	NCGv7	protein_c	chr11:55554307-555
ENSG0000(	933	23.3352	chr11:76(	OR5BN1P		Pseudoger	chr11:56132666-561
ENSG0000(	933	23.3352	chr11:76(	LINC02685		lncRNA	chr11:44973770-449
ENSG0000(	933	23.3352	chr11:76(	ENSG00000254662		lncRNA	chr11:57325603-573
ENSG0000(	933	23.3352	chr11:76(	OR4A11P		Pseudoger	chr11:55318560-553
ENSG0000(	933	23.3352	chr11:76(	ENSG00000254664		lncRNA	chr11:45215815-452
ENSG0000(	933	23.3352	chr11:76(	OR4A42P		Pseudoger	chr11:48610063-486
ENSG0000(	933	23.3352	chr11:76(	ENSG00000289990		lncRNA	chr11:46242492-462
ENSG0000(	933	23.3352	chr11:76(	PEX16	NCGv7	protein_c	chr11:45909663-459
ENSG0000(	933	23.3352	chr11:76(	CHST1	NCGv7	protein_c	chr11:45647689-456
ENSG0000(	933	23.3352	chr11:76(	OR5T1		protein_c	chr11:56274154-562
ENSG0000(	933	23.3352	chr11:76(	OR5T2		protein_c	chr11:56231282-562
ENSG0000(	933	23.3352	chr11:76(	OR8K5	NCGv7	protein_c	chr11:56159394-561
ENSG0000(	933	23.3352	chr11:76(	CRY2		protein_c	chr11:45847118-458
ENSG0000(	933	23.3352	chr11:76(	MTCH2		protein_c	chr11:47617315-476
ENSG0000(	933	23.3352	chr11:76(	MAPK8IP1	NCGv7	protein_c	chr11:45885651-459
ENSG0000(	933	23.3352	chr11:76(	OR8H3		protein_c	chr11:56122373-561
ENSG0000(	933	23.3352	chr11:76(	OR8H2		protein_c	chr11:56103687-561
ENSG0000(	933	23.3352	chr11:76(	OR8H1		protein_c	chr11:56288462-562
ENSG0000(	933	23.3352	chr11:76(	OR5J1P		Pseudoger	chr11:56071111-560
ENSG0000(	933	23.3352	chr11:76(	OR5AS1		protein_c	chr11:56027654-560
ENSG0000(	933	23.3352	chr11:76(	OR9I3P		Pseudoger	chr11:58108720-581
ENSG0000(	933	23.3352	chr11:76(	ENSG00000254653		lncRNA	chr11:46116578-461
ENSG0000(	933	23.3352	chr11:76(	OR5AO1P		Pseudoger	chr11:57045091-570
ENSG0000(	933	23.3352	chr11:76(	SLC35C1		protein_c	chr11:45804072-458
ENSG0000(	933	23.3352	chr11:76(	OR5D17P		Pseudoger	chr11:55754977-557
ENSG0000(	933	23.3352	chr11:76(	OR4C6		protein_c	chr11:55662201-556
ENSG0000(	933	23.3352	chr11:76(	OR5BL1P		Pseudoger	chr11:58171098-581
ENSG0000(	933	23.3352	chr11:76(	LINC02724		lncRNA	chr11:64449074-644
ENSG0000(	933	23.3352	chr11:76(	SPI1	NCGv7;AC	protein_c	chr11:47354860-473
ENSG0000(	933	23.3352	chr11:76(	OR4P4		protein_c	chr11:55635113-556
ENSG0000(	933	23.3352	chr11:76(	ENSG00000254651		lncRNA	chr11:45399448-454
ENSG0000(	933	23.3352	chr11:76(	ENSG00000254639		lncRNA	chr11:46238382-462
ENSG0000(	933	23.3352	chr11:76(	ATG13	NCGv7	protein_c	chr11:46617527-466
ENSG0000(	933	23.3352	chr11:76(	ENSG00000254728		Pseudoger	chr11:48881723-489
ENSG0000(	933	23.3352	chr11:76(	ENSG00000290026		lncRNA	chr11:65574399-655
ENSG0000(	933	23.3352	chr11:76(	OR10AK1P		Pseudoger	chr11:55957216-559
ENSG0000(	933	23.3352	chr11:76(	SNX15		protein_c	chr11:65027439-650

ENSG0000(	933	23.3352	chr11:76(	LPXN		protein_c	chr11:58526871-585
ENSG0000(	933	23.3352	chr11:76(	OR5AP1P		Pseudoger	chr11:56633164-566
ENSG0000(	933	23.3352	chr11:76(	TP53I11		protein_c	chr11:44885903-449
ENSG0000(	933	23.3352	chr11:76(	ENSG00000290057		lncRNA	chr11:65421448-654
ENSG0000(	933	23.3352	chr11:76(	ATG2A	NCGv7	protein_c	chr11:64894546-649
ENSG0000(	933	23.3352	chr11:76(	EHD1		protein_c	chr11:64851642-648
ENSG0000(	933	23.3352	chr11:76(	ENSG00000280010		TEC	chr11:58627435-586
ENSG0000(	933	23.3352	chr11:76(	ENSG00000275598		Pseudoger	chr11:63469376-634
ENSG0000(	933	23.3352	chr11:76(	ENSG00000254800		Pseudoger	chr11:49882999-498
ENSG0000(	933	23.3352	chr11:76(	ENSG00000254519		lncRNA	chr11:45722279-457
ENSG0000(	933	23.3352	chr11:76(	ENSG00000254801		Pseudoger	chr11:49070752-490
ENSG0000(	933	23.3352	chr11:76(	ENSG00000254804		lncRNA	chr11:55684141-556
ENSG0000(	933	23.3352	chr11:76(	ENSG00000285388		Pseudoger	chr11:62936427-629
ENSG0000(	933	23.3352	chr11:76(	TRIM51	NCGv7	protein_c	chr11:55883297-558
ENSG0000(	933	23.3352	chr11:76(	RNU6-1306P		smallRNA	chr11:63882587-638
ENSG0000(	933	23.3352	chr11:76(	AHNAK	NCGv7	protein_c	chr11:62433542-625
ENSG0000(	933	23.3352	chr11:76(	LINC02750		lncRNA	chr11:50298579-503
ENSG0000(	933	23.3352	chr11:76(	OR9G2P		Pseudoger	chr11:56751023-567
ENSG0000(	933	23.3352	chr11:76(	NRXN2	NCGv7	protein_c	chr11:64606174-647
ENSG0000(	933	23.3352	chr11:76(	OR9G3P		Pseudoger	chr11:56740179-567
ENSG0000(	933	23.3352	chr11:76(	ENSG00000275725		Pseudoger	chr11:44656771-446
ENSG0000(	933	23.3352	chr11:76(	ENSG00000254828		Pseudoger	chr11:55302290-553
ENSG0000(	933	23.3352	chr11:76(	RELA-DT		lncRNA	chr11:65662988-656
ENSG0000(	933	23.3352	chr11:76(	OR4A40P		Pseudoger	chr11:48512273-485
ENSG0000(	933	23.3352	chr11:76(	OR5M10		protein_c	chr11:56576736-565
ENSG0000(	933	23.3352	chr11:76(	ENSG00000254517		Pseudoger	chr11:48959928-489
ENSG0000(	933	23.3352	chr11:76(	ENSG00000254840		Pseudoger	chr11:50315414-503
ENSG0000(	933	23.3352	chr11:76(	OR4V1P		Pseudoger	chr11:55673536-556
ENSG0000(	933	23.3352	chr11:76(	ENSG00000254514		lncRNA	chr11:45582525-455
ENSG0000(	933	23.3352	chr11:76(	ZNF408		protein_c	chr11:46701030-467
ENSG0000(	933	23.3352	chr11:76(	OR8I4P		Pseudoger	chr11:56097244-560
ENSG0000(	933	23.3352	chr11:76(	MIR139		smallRNA	chr11:72615063-726
ENSG0000(	933	23.3352	chr11:76(	ENSG00000254780		Pseudoger	chr11:47905323-479
ENSG0000(	933	23.3352	chr11:76(	ENSG00000254732		protein_c	chr11:57741779-578
ENSG0000(	933	23.3352	chr11:76(	OR5AL1		protein_c	chr11:56412696-564
ENSG0000(	933	23.3352	chr11:76(	ENSG00000254746		lncRNA	chr11:45486867-455
ENSG0000(	933	23.3352	chr11:76(	MIR130AHG		lncRNA	chr11:57638024-576
ENSG0000(	933	23.3352	chr11:76(	CYCSP26		Pseudoger	chr11:58005386-580
ENSG0000(	933	23.3352	chr11:76(	ARL2-SNX15		protein_c	chr11:65014182-650
ENSG0000(	933	23.3352	chr11:76(	EIF4A2P3		Pseudoger	chr11:58242043-582
ENSG0000(	933	23.3352	chr11:76(	OR4C1P		Pseudoger	chr11:55509729-555
ENSG0000(	933	23.3352	chr11:76(	ARHGAP1		protein_c	chr11:46677080-467
ENSG0000(	933	23.3352	chr11:76(	OR5F2P		Pseudoger	chr11:56015017-560
ENSG0000(	933	23.3352	chr11:76(	TRIM51G		protein_c	chr11:48975498-489
ENSG0000(	933	23.3352	chr11:76(	KCNK4		protein_c	chr11:64291302-643
ENSG0000(	933	23.3352	chr11:76(	OR5BD1P		Pseudoger	chr11:57945598-579
ENSG0000(	933	23.3352	chr11:76(	P2RX3		protein_c	chr11:57338352-573
ENSG0000(	933	23.3352	chr11:76(	RPS12P20		Pseudoger	chr11:72708186-727
ENSG0000(	933	23.3352	chr11:76(	UBTFL9		Pseudoger	chr11:49133653-491
ENSG0000(	933	23.3352	chr11:76(	OR5M2P		Pseudoger	chr11:56479492-564
ENSG0000(	933	23.3352	chr11:76(	SNORD67		smallRNA	chr11:46762389-467

ENSG0000(	933	23.3352	chr11:76(	CKAP5		protein_c	chr11:46743048-468
ENSG0000(	933	23.3352	chr11:76(	LINC02723		lncRNA	chr11:64394342-643
ENSG0000(	933	23.3352	chr11:76(	TRIM53CP		Pseudoger	chr11:48985868-489
ENSG0000(	933	23.3352	chr11:76(	OR4A4P		Pseudoger	chr11:54659666-546
ENSG0000(	933	23.3352	chr11:76(	Y_RNA		smallRNA	chr11:57510559-575
ENSG0000(	933	23.3352	chr11:76(	OR5M8		protein_c	chr11:56490435-564
ENSG0000(	933	23.3352	chr11:76(	OR4C2P		Pseudoger	chr11:48420210-484
ENSG0000(	933	23.3352	chr11:76(	ENSG00000254547		Pseudoger	chr11:55867569-558
ENSG0000(	933	23.3352	chr11:76(	ENSG00000284732		protein_c	chr11:56459221-564
ENSG0000(	933	23.3352	chr11:76(	EEF1G		protein_c	chr11:62559596-625
ENSG0000(	933	23.3352	chr11:76(	DNAJC4		protein_c	chr11:64230278-642
ENSG0000(	933	23.3352	chr11:76(	OR5M13P		Pseudoger	chr11:56597426-565
ENSG0000(	933	23.3352	chr11:76(	RN7SL259P		smallRNA	chr11:62935984-629
ENSG0000(	933	23.3352	chr11:76(	TUBAP7		Pseudoger	chr11:63046785-630
ENSG0000(	933	23.3352	chr11:76(	OR4A43P		Pseudoger	chr11:48526064-485
ENSG0000(	933	23.3352	chr11:76(	PHOX2A		protein_c	chr11:72239077-722
ENSG0000(	933	23.3352	chr11:76(	COX8A		protein_c	chr11:63974620-639
ENSG0000(	933	23.3352	chr11:76(	ENSG00000228286		Pseudoger	chr11:72249856-722
ENSG0000(	933	23.3352	chr11:76(	TALAM1		lncRNA	chr11:65499312-655
ENSG0000(	933	23.3352	chr11:76(	ENSG00000230835		Pseudoger	chr11:64881535-648
ENSG0000(	933	23.3352	chr11:76(	DPF2		protein_c	chr11:65333843-653
ENSG0000(	933	23.3352	chr11:76(	MEN1	NCGv7;AC	protein_c	chr11:64803510-648
ENSG0000(	933	23.3352	chr11:76(	ENSG00000255508		protein_c	chr11:62559603-625
ENSG0000(	933	23.3352	chr11:76(	OR4B2P		Pseudoger	chr11:48227429-482
ENSG0000(	933	23.3352	chr11:76(	ENSG00000255519		Pseudoger	chr11:45514187-455
ENSG0000(	933	23.3352	chr11:76(	ENSG00000255520		lncRNA	chr11:47123104-471
ENSG0000(	933	23.3352	chr11:76(	ENSG00000255527		Pseudoger	chr11:48907103-489
ENSG0000(	933	23.3352	chr11:76(	ENSG00000213365		Pseudoger	chr11:72280151-722
ENSG0000(	933	23.3352	chr11:76(	ENSG00000255532		Pseudoger	chr11:49305714-493
ENSG0000(	933	23.3352	chr11:76(	ENSG00000255543		Pseudoger	chr11:55267408-552
ENSG0000(	933	23.3352	chr11:76(	ENSG00000255499		Pseudoger	chr11:55865709-558
ENSG0000(	933	23.3352	chr11:76(	ENSG00000255550		Pseudoger	chr11:49854080-498
ENSG0000(	933	23.3352	chr11:76(	ENSG00000255551		Pseudoger	chr11:48880111-488
ENSG0000(	933	23.3352	chr11:76(	ENSG00000255557		lncRNA	chr11:65745729-657
ENSG0000(	933	23.3352	chr11:76(	OR4R2P		Pseudoger	chr11:54657119-546
ENSG0000(	933	23.3352	chr11:76(	OR4C14P		Pseudoger	chr11:55537002-555
ENSG0000(	933	23.3352	chr11:76(	LARGE2		protein_c	chr11:45921621-459
ENSG0000(	933	23.3352	chr11:76(	ENSG00000231880		lncRNA	chr11:47577725-475
ENSG0000(	933	23.3352	chr11:76(	ENSG00000267811		lncRNA	chr11:62771120-627
ENSG0000(	933	23.3352	chr11:76(	RNU5E-10P		smallRNA	chr11:47576471-475
ENSG0000(	933	23.3352	chr11:76(	ENSG00000290827		lncRNA	chr11:57861948-578
ENSG0000(	933	23.3352	chr11:76(	ENSG00000255672		lncRNA	chr11:72354516-723
ENSG0000(	933	23.3352	chr11:76(	PACSIN3		protein_c	chr11:47177522-471
ENSG0000(	933	23.3352	chr11:76(	SLC39A13		protein_c	chr11:47407132-474
ENSG0000(	933	23.3352	chr11:76(	SNHG1		lncRNA	chr11:62851978-628
ENSG0000(	933	23.3352	chr11:76(	ENSG00000255500		Pseudoger	chr11:50268161-502
ENSG0000(	933	23.3352	chr11:76(	ENSG00000255498		lncRNA	chr11:45905941-459
ENSG0000(	933	23.3352	chr11:76(	TMEM223		protein_c	chr11:62771629-627
ENSG0000(	933	23.3352	chr11:76(	OR5AL2P		Pseudoger	chr11:56393729-563
ENSG0000(	933	23.3352	chr11:76(	ENSG00000255442		Pseudoger	chr11:50170156-501
ENSG0000(	933	23.3352	chr11:76(	ENSG00000255447		lncRNA	chr11:45813219-458

ENSG0000(	933	23.3352	chr11:76(	ENSG00000290749		lncRNA	chr11:56802076-568
ENSG0000(	933	23.3352	chr11:76(	ENSG00000255146		lncRNA	chr11:58044110-580
ENSG0000(	933	23.3352	chr11:76(	ENSG00000290752		lncRNA	chr11:56667545-566
ENSG0000(	933	23.3352	chr11:76(	ENSG00000255452		Pseudoger	chr11:49092404-490
ENSG0000(	933	23.3352	chr11:76(	MACROD1	AC	protein_c	chr11:63998558-641
ENSG0000(	933	23.3352	chr11:76(	WDR74	NCGv7	protein_c	chr11:62832342-628
ENSG0000(	933	23.3352	chr11:76(	LGALS12		protein_c	chr11:63506052-635
ENSG0000(	933	23.3352	chr11:76(	RTN3		protein_c	chr11:63681446-637
ENSG0000(	933	23.3352	chr11:76(	PLAAT4		protein_c	chr11:63536808-635
ENSG0000(	933	23.3352	chr11:76(	PLAAT2		protein_c	chr11:63552770-635
ENSG0000(	933	23.3352	chr11:76(	ENSG00000290753		lncRNA	chr11:56476287-564
ENSG0000(	933	23.3352	chr11:76(	OR8I1P		Pseudoger	chr11:56296541-562
ENSG0000(	933	23.3352	chr11:76(	ENSG00000269570		lncRNA	chr11:58611119-586
ENSG0000(	933	23.3352	chr11:76(	OR4A10P		Pseudoger	chr11:55430478-554
ENSG0000(	933	23.3352	chr11:76(	ENSG00000289486		lncRNA	chr11:64184892-641
ENSG0000(	933	23.3352	chr11:76(	ENSG00000255478		lncRNA	chr11:65367438-653
ENSG0000(	933	23.3352	chr11:76(	RN7SKP259		smallRNA	chr11:57451690-574
ENSG0000(	933	23.3352	chr11:76(	ENSG00000269463		lncRNA	chr11:62807682-628
ENSG0000(	933	23.3352	chr11:76(	ENSG00000286983		lncRNA	chr11:50213420-502
ENSG0000(	933	23.3352	chr11:76(	PYGM		protein_c	chr11:64746389-647
ENSG0000(	933	23.3352	chr11:76(	OR5G4P		Pseudoger	chr11:56790206-567
ENSG0000(	933	23.3352	chr11:76(	RP11-869B15.1		lncRNA	chr11:64784921-647
ENSG0000(	933	23.3352	chr11:76(	OR4A45P		Pseudoger	chr11:48579436-485
ENSG0000(	933	23.3352	chr11:76(	ENSG00000269176		lncRNA	chr11:62786023-627
ENSG0000(	933	23.3352	chr11:76(	ZNHIT2		protein_c	chr11:65116403-651
ENSG0000(	933	23.3352	chr11:76(	OR5D13		protein_c	chr11:55773438-557
ENSG0000(	933	23.3352	chr11:76(	SF1-DT		lncRNA	chr11:64778954-647
ENSG0000(	933	23.3352	chr11:76(	AP001362.1		protein_c	chr11:65591194-655
ENSG0000(	933	23.3352	chr11:76(	PSMC3	NCGv7	protein_c	chr11:47418769-474
ENSG0000(	933	23.3352	chr11:76(	RAPSN		protein_c	chr11:47437764-474
ENSG0000(	933	23.3352	chr11:76(	LRP4		protein_c	chr11:46856717-469
ENSG0000(	933	23.3352	chr11:76(	TRIM48	NCGv7	protein_c	chr11:55262155-552
ENSG0000(	933	23.3352	chr11:76(	ENSG00000256181		Pseudoger	chr11:63265836-632
ENSG0000(	933	23.3352	chr11:76(	PHF21A	NCGv7	protein_c	chr11:45929319-461
ENSG0000(	933	23.3352	chr11:76(	ENSG00000287412		lncRNA	chr11:63495484-635
ENSG0000(	933	23.3352	chr11:76(	RNU6-1302P		smallRNA	chr11:47440006-474
ENSG0000(	933	23.3352	chr11:76(	SLC22A25		protein_c	chr11:63158437-632
ENSG0000(	933	23.3352	chr11:76(	OR1S1		protein_c	chr11:58212720-582
ENSG0000(	933	23.3352	chr11:76(	ATP5MGP1		Pseudoger	chr11:63834667-638
ENSG0000(	933	23.3352	chr11:76(	SNORD67		smallRNA	chr11:46758766-467
ENSG0000(	933	23.3352	chr11:76(	RN7SL772P		smallRNA	chr11:47205845-472
ENSG0000(	933	23.3352	chr11:76(	ENSG00000256341		lncRNA	chr11:64118272-641
ENSG0000(	933	23.3352	chr11:76(	FAM180B		protein_c	chr11:47586678-475
ENSG0000(	933	23.3352	chr11:76(	UQCC3		protein_c	chr11:62670273-626
ENSG0000(	933	23.3352	chr11:76(	OR5M9		protein_c	chr11:56462469-564
ENSG0000(	933	23.3352	chr11:76(	OR8K1	NCGv7	protein_c	chr11:56346039-563
ENSG0000(	933	23.3352	chr11:76(	TRIM64C		protein_c	chr11:49053714-490
ENSG0000(	933	23.3352	chr11:76(	MYBPC3		protein_c	chr11:47331406-473
ENSG0000(	933	23.3352	chr11:76(	ENSG00000256403		lncRNA	chr11:72410716-724
ENSG0000(	933	23.3352	chr11:76(	ENSG00000290992		lncRNA	chr11:49009959-490
ENSG0000(	933	23.3352	chr11:76(	VPS51	NCGv7	protein_c	chr11:65089324-651

ENSG0000(	933	23.3352	chr11:76(	TM7SF2		protein_c	chr11:65111845-651
ENSG0000(	933	23.3352	chr11:76(	FAU		protein_c	chr11:65120630-651
ENSG0000(	933	23.3352	chr11:76(	ENSG00000287538		lncRNA	chr11:50184250-502
ENSG0000(	933	23.3352	chr11:76(	CDC42EP2		protein_c	chr11:65314866-653
ENSG0000(	933	23.3352	chr11:76(	MRPL49		protein_c	chr11:65122183-651
ENSG0000(	933	23.3352	chr11:76(	PLCB3		protein_c	chr11:64251530-642
ENSG0000(	933	23.3352	chr11:76(	FERMT3		protein_c	chr11:64205926-642
ENSG0000(	933	23.3352	chr11:76(	NUDT22		protein_c	chr11:64225941-642
ENSG0000(	933	23.3352	chr11:76(	TRPT1		protein_c	chr11:64223799-642
ENSG0000(	933	23.3352	chr11:76(	SLC22A9		protein_c	chr11:63369785-634
ENSG0000(	933	23.3352	chr11:76(	GPHA2		protein_c	chr11:64934471-649
ENSG0000(	933	23.3352	chr11:76(	RNU2-2P		smallRNA	chr11:62841619-628
ENSG0000(	933	23.3352	chr11:76(	OR8K3		protein_c	chr11:56315144-563
ENSG0000(	933	23.3352	chr11:76(	LRRN4CL		protein_c	chr11:62686406-626
ENSG0000(	933	23.3352	chr11:76(	snoU13		smallRNA	chr11:47727546-477
ENSG0000(	933	23.3352	chr11:76(	DDB2	NCGv7;AC	protein_c	chr11:47214465-472
ENSG0000(	933	23.3352	chr11:76(	ACP2		protein_c	chr11:47239302-472
ENSG0000(	933	23.3352	chr11:76(	AGBL2	NCGv7	protein_c	chr11:47659591-477
ENSG0000(	933	23.3352	chr11:76(	PDE2A-AS1		lncRNA	chr11:72643237-726
ENSG0000(	933	23.3352	chr11:76(	NDUFS3		protein_c	chr11:47565336-475
ENSG0000(	933	23.3352	chr11:76(	PLAAT3		protein_c	chr11:63573195-636
ENSG0000(	933	23.3352	chr11:76(	RNU6-899P		smallRNA	chr11:58026706-580
ENSG0000(	933	23.3352	chr11:76(	OR4C5	NCGv7	protein_c	chr11:48365485-483
ENSG0000(	933	23.3352	chr11:76(	OR4C3		protein_c	chr11:48324920-483
ENSG0000(	933	23.3352	chr11:76(	SLC43A3		protein_c	chr11:57406954-574
ENSG0000(	933	23.3352	chr11:76(	TIMM10		protein_c	chr11:57528464-575
ENSG0000(	933	23.3352	chr11:76(	ENSG00000255843		lncRNA	chr11:72302139-723
ENSG0000(	933	23.3352	chr11:76(	APLNR	NCGv7	protein_c	chr11:57233577-572
ENSG0000(	933	23.3352	chr11:76(	TIGD3	NCGv7	protein_c	chr11:65354751-653
ENSG0000(	933	23.3352	chr11:76(	OR4S1		protein_c	chr11:48306223-483
ENSG0000(	933	23.3352	chr11:76(	ENSG00000173727		Pseudoger	chr11:65455269-654
ENSG0000(	933	23.3352	chr11:76(	snoU13		smallRNA	chr11:57328859-573
ENSG0000(	933	23.3352	chr11:76(	SMTNL1		protein_c	chr11:57537595-575
ENSG0000(	933	23.3352	chr11:76(	ARAP1-AS1		lncRNA	chr11:72685075-726
ENSG0000(	933	23.3352	chr11:76(	ENSG00000256041		Pseudoger	chr11:63032503-630
ENSG0000(	933	23.3352	chr11:76(	OR4X1		protein_c	chr11:48263861-482
ENSG0000(	933	23.3352	chr11:76(	SPDYC		protein_c	chr11:65170233-651
ENSG0000(	933	23.3352	chr11:76(	SNORA57		smallRNA	chr11:62665422-626
ENSG0000(	933	23.3352	chr11:76(	ENSG00000256100		protein_c	chr11:63974620-639
ENSG0000(	933	23.3352	chr11:76(	ENSG00000256116		lncRNA	chr11:64229214-642
ENSG0000(	933	23.3352	chr11:76(	FAM89B		protein_c	chr11:65572349-655
ENSG0000(	933	23.3352	chr11:76(	OR7E5P		Pseudoger	chr11:55979398-559
ENSG0000(	933	23.3352	chr11:76(	TRIM51HP		Pseudoger	chr11:55291883-552
ENSG0000(	933	23.3352	chr11:76(	ENSG00000214883		Pseudoger	chr11:50409042-504
ENSG0000(	933	23.3352	chr11:76(	ENSG00000290745		lncRNA	chr11:56082801-560
ENSG0000(	933	23.3352	chr11:76(	PPP2R5B		protein_c	chr11:64917553-649
ENSG0000(	933	23.3352	chr11:76(	ENSG00000290744		lncRNA	chr11:56049785-560
ENSG0000(	933	23.3352	chr11:76(	MAP4K2		protein_c	chr11:64784918-648
ENSG0000(	933	23.3352	chr11:76(	MAJIN		protein_c	chr11:64937517-649
ENSG0000(	933	23.3352	chr11:76(	ENSG00000286450		lncRNA	chr11:46920880-469
ENSG0000(	933	23.3352	chr11:76(	ENSG00000255214		Pseudoger	chr11:49871826-498

ENSG0000(	933	23.3352	chr11:76(	OR4R1P		Pseudoger	chr11:48486363-484
ENSG0000(	933	23.3352	chr11:76(	CCDC88B		protein_c	chr11:64340204-643
ENSG0000(	933	23.3352	chr11:76(	OR5J7P		Pseudoger	chr11:56165500-561
ENSG0000(	933	23.3352	chr11:76(	OR5BC1P		Pseudoger	chr11:58317637-583
ENSG0000(	933	23.3352	chr11:76(	OR9I2P		Pseudoger	chr11:58144495-581
ENSG0000(	933	23.3352	chr11:76(	SNRPGP19		Pseudoger	chr11:65514403-655
ENSG0000(	933	23.3352	chr11:76(	OR9Q1		protein_c	chr11:58023881-581
ENSG0000(	933	23.3352	chr11:76(	OR5M11		protein_c	chr11:56542340-565
ENSG0000(	933	23.3352	chr11:76(	ENSG00000203520		lncRNA	chr11:63616308-636
ENSG0000(	933	23.3352	chr11:76(	OR9Q2		protein_c	chr11:58189070-581
ENSG0000(	933	23.3352	chr11:76(	LINC02690		lncRNA	chr11:45749454-457
ENSG0000(	933	23.3352	chr11:76(	OR5B1P		Pseudoger	chr11:58365836-583
ENSG0000(	933	23.3352	chr11:76(	ARAP1	DriverDBv	protein_c	chr11:72685069-727
ENSG0000(	933	23.3352	chr11:76(	ENSG00000286555		lncRNA	chr11:72689650-726
ENSG0000(	933	23.3352	chr11:76(	PDE2A		protein_c	chr11:72576141-726
ENSG0000(	933	23.3352	chr11:76(	PRG2		protein_c	chr11:57386780-573
ENSG0000(	933	23.3352	chr11:76(	ZFP91	NCGv7	protein_c	chr11:58579063-586
ENSG0000(	933	23.3352	chr11:76(	F2		protein_c	chr11:46719196-467
ENSG0000(	933	23.3352	chr11:76(	NAA40		protein_c	chr11:63938959-639
ENSG0000(	933	23.3352	chr11:76(	OR5D3P		protein_c	chr11:55723776-557
ENSG0000(	933	23.3352	chr11:76(	RTN4RL2		protein_c	chr11:57460528-574
ENSG0000(	933	23.3352	chr11:76(	LINC02735		lncRNA	chr11:56848478-568
ENSG0000(	933	23.3352	chr11:76(	RPS4XP13		Pseudoger	chr11:57576292-575
ENSG0000(	933	23.3352	chr11:76(	TMEM262		protein_c	chr11:65084979-650
ENSG0000(	933	23.3352	chr11:76(	OR9G1	NCGv7	protein_c	chr11:56699095-567
ENSG0000(	933	23.3352	chr11:76(	LINC02687		lncRNA	chr11:45371397-453
ENSG0000(	933	23.3352	chr11:76(	PRG3		protein_c	chr11:57376769-573
ENSG0000(	933	23.3352	chr11:76(	ENSG00000286418		lncRNA	chr11:45397590-454
ENSG0000(	933	23.3352	chr11:76(	NEAT1	AC	lncRNA	chr11:65422774-654
ENSG0000(	933	23.3352	chr11:76(	UBE2L6		protein_c	chr11:57551656-575
ENSG0000(	933	23.3352	chr11:76(	MADD		protein_c	chr11:47269161-473
ENSG0000(	933	23.3352	chr11:76(	EEF1A1P18		Pseudoger	chr11:65025390-650
ENSG0000(	933	23.3352	chr11:76(	OR5AK4P		Pseudoger	chr11:57037534-570
ENSG0000(	933	23.3352	chr11:76(	OR5D14		protein_c	chr11:55795556-557
ENSG0000(	933	23.3352	chr11:76(	OR5D18		protein_c	chr11:55819630-558
ENSG0000(	933	23.3352	chr11:76(	OR9M1P		Pseudoger	chr11:55855592-558
ENSG0000(	933	23.3352	chr11:76(	ENSG00000226268		Pseudoger	chr11:49433480-494
ENSG0000(	933	23.3352	chr11:76(	LTBP3		protein_c	chr11:65538559-655
ENSG0000(	933	23.3352	chr11:76(	OR5AM1P		Pseudoger	chr11:56619779-566
ENSG0000(	933	23.3352	chr11:76(	ENSG00000255173		lncRNA	chr11:65117157-651
ENSG0000(	933	23.3352	chr11:76(	TEX54		protein_c	chr11:62832319-628
ENSG0000(	933	23.3352	chr11:76(	SYT13		protein_c	chr11:45240302-452
ENSG0000(	933	23.3352	chr11:76(	NAALADL1		protein_c	chr11:65044818-650
ENSG0000(	933	23.3352	chr11:76(	SAC3D1		protein_c	chr11:65040901-650
ENSG0000(	933	23.3352	chr11:76(	PRDM11		protein_c	chr11:45095806-452
ENSG0000(	933	23.3352	chr11:76(	PTPMT1		protein_c	chr11:47565430-475
ENSG0000(	933	23.3352	chr11:76(	TRIM51DP		Pseudoger	chr11:49874945-498
ENSG0000(	933	23.3352	chr11:76(	OR4A17P		Pseudoger	chr11:55444435-554
ENSG0000(	933	23.3352	chr11:76(	ENSG00000255197		lncRNA	chr11:47381509-474
ENSG0000(	933	23.3352	chr11:76(	GTF2IP11		Pseudoger	chr11:50100892-501
ENSG0000(	933	23.3352	chr11:76(	PGAM1P8		Pseudoger	chr11:65174117-651

ENSG0000(	933	23.3352	chr11:76(	ENSG00000255204		Pseudoger	chr11:55863932-558
ENSG0000(	933	23.3352	chr11:76(	OR5M4P		Pseudoger	chr11:56448668-564
ENSG0000(	933	23.3352	chr11:76(	BATF2		protein_c	chr11:64987945-649
ENSG0000(	933	23.3352	chr11:76(	SLC22A11	NCGv7	protein_c	chr11:64555690-645
ENSG0000(	933	23.3352	chr11:76(	SF1	NCGv7	protein_c	chr11:64764606-647
ENSG0000(	933	23.3352	chr11:76(	TMX2		protein_c	chr11:57712593-577
ENSG0000(	933	23.3352	chr11:76(	MED19		protein_c	chr11:57703710-577
ENSG0000(	933	23.3352	chr11:76(	ZDHHC5		protein_c	chr11:57667747-577
ENSG0000(	933	23.3352	chr11:76(	IMMP1LP1		Pseudoger	chr11:63632233-636
ENSG0000(	933	23.3352	chr11:76(	ARAP1-AS2		lncRNA	chr11:72700474-727
ENSG0000(	933	23.3352	chr11:76(	LINC02710		lncRNA	chr11:46213150-462
ENSG0000(	933	23.3352	chr11:76(	ENSG00000270072		lncRNA	chr11:47513605-475
ENSG0000(	933	23.3352	chr11:76(	CATSPERZ		protein_c	chr11:64300358-643
ENSG0000(	933	23.3352	chr11:76(	ENSG00000270060		lncRNA	chr11:47168281-471
ENSG0000(	933	23.3352	chr11:76(	ENSG00000255338		Pseudoger	chr11:49110756-491
ENSG0000(	933	23.3352	chr11:76(	Y_RNA		smallRNA	chr11:47820203-478
ENSG0000(	933	23.3352	chr11:76(	OR4A7P		Pseudoger	chr11:54692449-546
ENSG0000(	933	23.3352	chr11:76(	ENSG00000286704		Pseudoger	chr11:56750330-567
ENSG0000(	933	23.3352	chr11:76(	ENSG00000286756		lncRNA	chr11:65487884-654
ENSG0000(	933	23.3352	chr11:76(	ZFTA		protein_c	chr11:63759892-637
ENSG0000(	933	23.3352	chr11:76(	UBTFL7		Pseudoger	chr11:49081907-490
ENSG0000(	933	23.3352	chr11:76(	MIR192		smallRNA	chr11:64891137-648
ENSG0000(	933	23.3352	chr11:76(	ENSG00000286816		lncRNA	chr11:64486136-644
ENSG0000(	933	23.3352	chr11:76(	STIP1		protein_c	chr11:64185272-642
ENSG0000(	933	23.3352	chr11:76(	HNRNPUL2		protein_c	chr11:62712630-627
ENSG0000(	933	23.3352	chr11:76(	CSKMT		protein_c	chr11:62665309-626
ENSG0000(	933	23.3352	chr11:76(	OR4A2P		Pseudoger	chr11:54667297-546
ENSG0000(	933	23.3352	chr11:76(	OR5BE1P		Pseudoger	chr11:56082801-560
ENSG0000(	933	23.3352	chr11:76(	ENSG00000255404		lncRNA	chr11:65795946-657
ENSG0000(	933	23.3352	chr11:76(	OR10AF1P		Pseudoger	chr11:55948120-559
ENSG0000(	933	23.3352	chr11:76(	MIR1237		smallRNA	chr11:64368602-643
ENSG0000(	933	23.3352	chr11:76(	RN7SL309P		smallRNA	chr11:65695535-656
ENSG0000(	933	23.3352	chr11:76(	RASGRP2	NCGv7	protein_c	chr11:64726911-647
ENSG0000(	933	23.3352	chr11:76(	CHRM1		protein_c	chr11:62908679-629
ENSG0000(	933	23.3352	chr11:76(	BTBD18		protein_c	chr11:57743514-577
ENSG0000(	933	23.3352	chr11:76(	OR4P1P		Pseudoger	chr11:55683239-556
ENSG0000(	933	23.3352	chr11:76(	ENSG00000255426		lncRNA	chr11:45733994-457
ENSG0000(	933	23.3352	chr11:76(	NAA50P1		Pseudoger	chr11:56205125-562
ENSG0000(	933	23.3352	chr11:76(	RNU6-672P		smallRNA	chr11:72869544-728
ENSG0000(	933	23.3352	chr11:76(	OR5B19P		Pseudoger	chr11:58343737-583
ENSG0000(	933	23.3352	chr11:76(	ENSG00000255432		protein_c	chr11:62649694-626
ENSG0000(	933	23.3352	chr11:76(	RNA5SP340		Pseudoger	chr11:47804296-478
ENSG0000(	933	23.3352	chr11:76(	ENSG00000254660		Pseudoger	chr11:56509573-565
ENSG0000(	933	23.3352	chr11:76(	RPL29P22		Pseudoger	chr11:63115880-631
ENSG0000(	933	23.3352	chr11:76(	OR4C7P		Pseudoger	chr11:54635036-546
ENSG0000(	933	23.3352	chr11:76(	ENSG00000255301		lncRNA	chr11:57476493-574
ENSG0000(	933	23.3352	chr11:76(	OR4A41P		Pseudoger	chr11:48589760-485
ENSG0000(	933	23.3352	chr11:76(	OR5BA1P		Pseudoger	chr11:57866298-578
ENSG0000(	933	23.3352	chr11:76(	OR4A46P		Pseudoger	chr11:48496348-484
ENSG0000(	933	23.3352	chr11:76(	ENSG00000274664		Pseudoger	chr11:72562044-725
ENSG0000(	933	23.3352	chr11:76(	OR5W2		protein_c	chr11:55913650-559

ENSG0000(	933	23.3352	chr11:76(	OR4A50P		Pseudoger	chr11:55475943-554
ENSG0000(	933	23.3352	chr11:76(	POLA2		protein_c	chr11:65261920-653
ENSG0000(	933	23.3352	chr11:76(	CAPN1		protein_c	chr11:65180566-652
ENSG0000(	933	23.3352	chr11:76(	KRT8P26		Pseudoger	chr11:65726939-657
ENSG0000(	933	23.3352	chr11:76(	OR8U3		protein_c	chr11:56417258-564
ENSG0000(	933	23.3352	chr11:76(	ENSG00000255314		lncRNA	chr11:46123031-461
ENSG0000(	933	23.3352	chr11:76(	ENSG00000255283		Pseudoger	chr11:55279447-552
ENSG0000(	933	23.3352	chr11:76(	AP001266.1		Pseudoger	chr11:65777621-657
ENSG0000(	933	23.3352	chr11:76(	ENSG00000289339		lncRNA	chr11:65575330-655
ENSG0000(	933	23.3352	chr11:76(	ENSG00000270117		lncRNA	chr11:65498008-654
ENSG0000(	933	23.3352	chr11:76(	LRP4-AS1		lncRNA	chr11:46846410-468
ENSG0000(	933	23.3352	chr11:76(	ENSG00000255333		Pseudoger	chr11:58354004-583
ENSG0000(	933	23.3352	chr11:76(	ENSG00000255299		lncRNA	chr11:58497888-585
ENSG0000(	930	23.26016	chr1:116(	TLR12P		Pseudoger	chr1:33466249-3346
ENSG0000(	930	23.26016	chr1:116(	ENSG00000236274		Pseudoger	chr1:35509742-3551
ENSG0000(	930	23.26016	chr1:116(	EVA1B		protein_c	chr1:36322030-3632
ENSG0000(	930	23.26016	chr1:116(	ADPRS		protein_c	chr1:36088892-3609
ENSG0000(	930	23.26016	chr1:116(	MRPS15		protein_c	chr1:36455718-3646
ENSG0000(	930	23.26016	chr1:116(	HMGB4		protein_c	chr1:33860475-3386
ENSG0000(	930	23.26016	chr1:116(	MAP7D1		protein_c	chr1:36155579-3618
ENSG0000(	930	23.26016	chr1:116(	C1orf94		protein_c	chr1:34166883-3421
ENSG0000(	930	23.26016	chr1:116(	ZMYM6		protein_c	chr1:34986165-3503
ENSG0000(	930	23.26016	chr1:116(	KIAA0319L		protein_c	chr1:35393883-3555
ENSG0000(	930	23.26016	chr1:116(	CSF3R	NCGv7;AC	protein_c	chr1:36466043-3648
ENSG0000(	930	23.26016	chr1:116(	SNORA62		smallRNA	chr1:35310274-3531
ENSG0000(	930	23.26016	chr1:116(	SMIM12		protein_c	chr1:34712737-3485
ENSG0000(	930	23.26016	chr1:116(	C1orf216		protein_c	chr1:35713877-3571
ENSG0000(	930	23.26016	chr1:116(	EFCAB14P1		Pseudoger	chr1:35122022-3512
ENSG0000(	930	23.26016	chr1:116(	OSCP1		protein_c	chr1:36415827-3645
ENSG0000(	930	23.26016	chr1:116(	ENSG00000279179		TEC	chr1:33162851-3316
ENSG0000(	930	23.26016	chr1:116(	TFAP2E		protein_c	chr1:35573314-3559
ENSG0000(	930	23.26016	chr1:116(	Y_RNA		smallRNA	chr1:35195969-3519
ENSG0000(	930	23.26016	chr1:116(	AC115286.1		smallRNA	chr1:34175866-3417
ENSG0000(	930	23.26016	chr1:116(	AL138837.1		smallRNA	chr1:33442025-3344
ENSG0000(	930	23.26016	chr1:116(	ENSG00000234481		lncRNA	chr1:36769812-3677
ENSG0000(	930	23.26016	chr1:116(	RN7SL136P		smallRNA	chr1:35264222-3526
ENSG0000(	930	23.26016	chr1:116(	SNORD112		smallRNA	chr1:34943756-3494
ENSG0000(	930	23.26016	chr1:116(	ZMYM1		protein_c	chr1:35032172-3511
ENSG0000(	930	23.26016	chr1:116(	ENSG00000271741		protein_c	chr1:34981533-3503
ENSG0000(	930	23.26016	chr1:116(	ENSG00000230163		lncRNA	chr1:34850694-3485
ENSG0000(	930	23.26016	chr1:116(	ENSG00000236065		lncRNA	chr1:32987075-3303
ENSG0000(	930	23.26016	chr1:116(	ENSG00000286899		lncRNA	chr1:35908980-3591
ENSG0000(	930	23.26016	chr1:116(	ENSG00000232862		Pseudoger	chr1:36080066-3608
ENSG0000(	930	23.26016	chr1:116(	ENSG00000225313		lncRNA	chr1:33307348-3334
ENSG0000(	930	23.26016	chr1:116(	RPL5P4		Pseudoger	chr1:35350722-3535
ENSG0000(	930	23.26016	chr1:116(	A3GALT2		protein_c	chr1:33306766-3332
ENSG0000(	930	23.26016	chr1:116(	ZNF362	NCGv7	protein_c	chr1:33256492-3330
ENSG0000(	930	23.26016	chr1:116(	ENSG00000271554		lncRNA	chr1:35992109-3601
ENSG0000(	930	23.26016	chr1:116(	SNORA63		smallRNA	chr1:36418450-3641
ENSG0000(	930	23.26016	chr1:116(	GJB3		protein_c	chr1:34781214-3478
ENSG0000(	930	23.26016	chr1:116(	UBE2V2P4		Pseudoger	chr1:36241898-3624

ENSG0000(	930	23.26016	chr1:116(	FKSG48		protein_c	chr1:32973667-3297
ENSG0000(	930	23.26016	chr1:116(	AGO3		protein_c	chr1:35930718-3607
ENSG0000(	930	23.26016	chr1:116(	PSMB2		protein_c	chr1:35599541-3564
ENSG0000(	930	23.26016	chr1:116(	RN7SKP16		smallRNA	chr1:33336566-3333
ENSG0000(	930	23.26016	chr1:116(	GJB5		protein_c	chr1:34755047-3475
ENSG0000(	930	23.26016	chr1:116(	PHC2-AS1		lncRNA	chr1:33350352-3336
ENSG0000(	930	23.26016	chr1:116(	CFAP97P1		Pseudoger	chr1:35873270-3587
ENSG0000(	930	23.26016	chr1:116(	FTLP18		Pseudoger	chr1:36630335-3663
ENSG0000(	930	23.26016	chr1:116(	PHC2	NCGv7	protein_c	chr1:33323623-3343
ENSG0000(	930	23.26016	chr1:116(	AK2		protein_c	chr1:33007986-3308
ENSG0000(	930	23.26016	chr1:116(	ZMYM4-AS1		lncRNA	chr1:35358822-3536
ENSG0000(	930	23.26016	chr1:116(	ENSG00000287703		lncRNA	chr1:34640157-3468
ENSG0000(	930	23.26016	chr1:116(	ENSG00000270241		Pseudoger	chr1:34276859-3427
ENSG0000(	930	23.26016	chr1:116(	ENSG00000232335		lncRNA	chr1:35739389-3574
ENSG0000(	930	23.26016	chr1:116(	RN7SL503P		smallRNA	chr1:35292200-3529
ENSG0000(	930	23.26016	chr1:116(	ENSG00000270115		lncRNA	chr1:33261212-3326
ENSG0000(	930	23.26016	chr1:116(	ZMYM4		protein_c	chr1:35268709-3542
ENSG0000(	930	23.26016	chr1:116(	ENSG00000286379		lncRNA	chr1:36329630-3633
ENSG0000(	930	23.26016	chr1:116(	NCDN		protein_c	chr1:35557473-3556
ENSG0000(	930	23.26016	chr1:116(	CSMD2-AS1		lncRNA	chr1:33868953-3389
ENSG0000(	930	23.26016	chr1:116(	GPR199P		Pseudoger	chr1:34975699-3497
ENSG0000(	930	23.26016	chr1:116(	RN7SL281P		smallRNA	chr1:35706025-3570
ENSG0000(	930	23.26016	chr1:116(	ENSG00000239670		Pseudoger	chr1:32986952-3298
ENSG0000(	930	23.26016	chr1:116(	TFAP2E-AS1		lncRNA	chr1:35569807-3557
ENSG0000(	930	23.26016	chr1:116(	MIR552		smallRNA	chr1:34669599-3466
ENSG0000(	930	23.26016	chr1:116(	RNU4-27P		smallRNA	chr1:36402721-3640
ENSG0000(	930	23.26016	chr1:116(	SH3D21		protein_c	chr1:36306368-3632
ENSG0000(	930	23.26016	chr1:116(	RN7SL131P		smallRNA	chr1:36191915-3619
ENSG0000(	930	23.26016	chr1:116(	GJA4		protein_c	chr1:34792999-3479
ENSG0000(	930	23.26016	chr1:116(	RPL12P45		Pseudoger	chr1:35053468-3505
ENSG0000(	930	23.26016	chr1:116(	AGO1		protein_c	chr1:35869808-3593
ENSG0000(	930	23.26016	chr1:116(	HSPD1P14		Pseudoger	chr1:33838523-3384
ENSG0000(	930	23.26016	chr1:116(	ENSG00000217644		Pseudoger	chr1:32979947-3298
ENSG0000(	930	23.26016	chr1:116(	TMEM35B		protein_c	chr1:34981380-3498
ENSG0000(	930	23.26016	chr1:116(	COL8A2		protein_c	chr1:36095239-3612
ENSG0000(	930	23.26016	chr1:116(	CLSPN		protein_c	chr1:35720218-3576
ENSG0000(	930	23.26016	chr1:116(	AL121988.1		smallRNA	chr1:34778561-3477
ENSG0000(	930	23.26016	chr1:116(	ENSG00000284640		lncRNA	chr1:35141515-3514
ENSG0000(	930	23.26016	chr1:116(	TEKT2		protein_c	chr1:36084094-3608
ENSG0000(	930	23.26016	chr1:116(	MIR3605		smallRNA	chr1:33332393-3333
ENSG0000(	930	23.26016	chr1:116(	THRAP3	NCGv7;AC	protein_c	chr1:36224432-3630
ENSG0000(	930	23.26016	chr1:116(	ENSG00000235907		Pseudoger	chr1:33512008-3351
ENSG0000(	930	23.26016	chr1:116(	TRIM62		protein_c	chr1:33145399-3318
ENSG0000(	930	23.26016	chr1:116(	LSM10		protein_c	chr1:36391238-3639
ENSG0000(	930	23.26016	chr1:116(	DLGAP3		protein_c	chr1:34865436-3492
ENSG0000(	930	23.26016	chr1:116(	AZIN2		protein_c	chr1:33081104-3312
ENSG0000(	930	23.26016	chr1:116(	SFPQ	NCGv7;AC	protein_c	chr1:35176378-3519
ENSG0000(	930	23.26016	chr1:116(	CSMD2	NCGv7	protein_c	chr1:33513998-3416
ENSG0000(	930	23.26016	chr1:116(	ZSCAN20		protein_c	chr1:33472645-3350
ENSG0000(	930	23.26016	chr1:116(	RNA5SP42		Pseudoger	chr1:34112949-3411
ENSG0000(	930	23.26016	chr1:116(	AL513327.1		Pseudoger	chr1:33299374-3330

ENSG0000(	930	23.26016	chr1:116(	STK40		protein_c	chr1:36339624-3638
ENSG0000(	930	23.26016	chr1:116(	GJB4		protein_c	chr1:34759740-3476
ENSG0000(	930	23.26016	chr1:116(	ENSG00000278966		TEC	chr1:32973553-3297
ENSG0000(	930	23.26016	chr1:116(	ENSG00000284721		lncRNA	chr1:33194788-3320
ENSG0000(	930	23.26016	chr1:116(	TRAPPC3		protein_c	chr1:36136570-3615
ENSG0000(	930	23.26016	chr1:116(	ENSG00000271914		lncRNA	chr1:35929720-3593
ENSG0000(	930	23.26016	chr1:116(	ENSG00000284720		lncRNA	chr1:36768122-3676
ENSG0000(	930	23.26016	chr1:116(	AGO4	NCGv7	protein_c	chr1:35808016-3585
ENSG0000(	930	23.26016	chr1:116(	ENSG00000284705		lncRNA	chr1:36703953-3671
ENSG0000(	930	23.26016	chr1:116(	RNY5P1		smallRNA	chr1:35427816-3542
ENSG0000(	930	23.26016	chr1:116(	ENSG00000278997		TEC	chr1:33141871-3314
ENSG0000(	930	23.26016	chr1:116(	ENSG00000255811		lncRNA	chr1:34761426-3478
ENSG0000(	930	23.26016	chr1:116(	ENSG00000284773		protein_c	chr1:34974356-3498
ENSG0000(	929	23.23515	chr7:330(	ENSG00000290951		lncRNA	chr7:75359202-7539
ENSG0000(	929	23.23515	chr7:330(	ENSG00000275121		Pseudoger	chr7:75237293-7523
ENSG0000(	929	23.23515	chr7:330(	ENSG00000277675		lncRNA	chr7:75225433-7523
ENSG0000(	929	23.23515	chr7:330(	ENSG00000290834		lncRNA	chr7:75391955-7539
ENSG0000(	929	23.23515	chr7:330(	Y_RNA		smallRNA	chr7:75353885-7535
ENSG0000(	929	23.23515	chr7:330(	Y_RNA		smallRNA	chr7:75381638-7538
ENSG0000(	929	23.23515	chr7:330(	AC006014.1		protein_c	chr7:75370632-7537
ENSG0000(	929	23.23515	chr7:330(	GTF2IP1		lncRNA	chr7:75185385-7523
ENSG0000(	929	23.23515	chr7:330(	GTF2IP1		Pseudoger	chr7:75187242-7521
ENSG0000(	929	23.23515	chr7:330(	SPDYE5		protein_c	chr7:75492320-7550
ENSG0000(	929	23.23515	chr7:330(	ENSG00000263081		lncRNA	chr7:75232928-7523
ENSG0000(	929	23.23515	chr7:330(	TRIM73	DriverDB\	protein_c	chr7:75395063-7541
ENSG0000(	929	23.23515	chr7:330(	PMS2P3		lncRNA	chr7:75507747-7552
ENSG0000(	929	23.23515	chr7:330(	NCF1C		Pseudoger	chr7:75156639-7517
ENSG0000(	929	23.23515	chr7:330(	GTF2IRD2B		protein_c	chr7:75092573-7514
ENSG0000(	929	23.23515	chr7:330(	Y_RNA		smallRNA	chr7:75297990-7529
ENSG0000(	929	23.23515	chr7:330(	PMS2P3		Pseudoger	chr7:75510931-7551
ENSG0000(	929	23.23515	chr7:330(	RCC1L		protein_c	chr7:75027122-7507
ENSG0000(	929	23.23515	chr7:330(	SPDYE15		protein_c	chr7:75335343-7534
ENSG0000(	929	23.23515	chr7:330(	POM121C		protein_c	chr7:75416786-7548
ENSG0000(	929	23.23515	chr7:330(	RN7SL642P		smallRNA	chr7:75580643-7558
ENSG0000(	929	23.23515	chr7:330(	STAG3L1		Pseudoger	chr7:75361374-7536
ENSG0000(	929	23.23515	chr7:330(	AC138783.11		Pseudoger	chr7:75281243-7528
ENSG0000(	929	23.23515	chr7:330(	Y_RNA		smallRNA	chr7:75325959-7532
ENSG0000(	929	23.23515	chr7:330(	Y_RNA		smallRNA	chr7:75513025-7551
ENSG0000(	929	23.23515	chr7:330(	AC006014.10		Pseudoger	chr7:75337138-7534
ENSG0000(	929	23.23515	chr7:330(	ENSG00000279996		TEC	chr7:75625089-7562
ENSG0000(	929	23.23515	chr7:330(	ENSG00000242073		Pseudoger	chr7:75474707-7548
ENSG0000(	929	23.23515	chr7:330(	HIP1	NCGv7;AC	protein_c	chr7:75533298-7573
ENSG0000(	929	23.23515	chr7:330(	AC004878.3		Pseudoger	chr7:75315964-7532
ENSG0000(	929	23.23515	chr7:330(	NSUN5P1		Pseudoger	chr7:75410368-7541
ENSG0000(	929	23.23515	chr7:330(	ENSG00000146722		Pseudoger	chr7:75393365-7539
ENSG0000(	929	23.23515	chr7:330(	SPDYE14		protein_c	chr7:75308718-7531
ENSG0000(	929	23.23515	chr7:330(	NSUN5P1		lncRNA	chr7:75410322-7541
ENSG0000(	929	23.23515	chr7:330(	PHB1P6		Pseudoger	chr7:75203926-7520
ENSG0000(	929	23.23515	chr7:330(	SPDYE13		protein_c	chr7:75280790-7528
ENSG0000(	925	23.13511	chr1:116(	PEF1		protein_c	chr1:31629866-3164
ENSG0000(	925	23.13511	chr1:116(	ENSG00000254545		lncRNA	chr1:31789130-3179

ENSG0000(	925	23.13511	chr1:116(	RP11-439L8.3	lncRNA	chr1:31506240-3150
ENSG0000(	925	23.13511	chr1:116(	EEF1A1P46	Pseudoger	chr1:31487589-3148
ENSG0000(	925	23.13511	chr1:116(	TINAGL1	protein_c	chr1:31576485-3158
ENSG0000(	925	23.13511	chr1:116(	SELENOWP1	Pseudoger	chr1:31094987-3109
ENSG0000(	925	23.13511	chr1:116(	SNORD103B	smallRNA	chr1:30949117-3094
ENSG0000(	925	23.13511	chr1:116(	MATN1-AS1	lncRNA	chr1:30718504-3072
ENSG0000(	925	23.13511	chr1:116(	SDC3	protein_c	chr1:30869466-3090
ENSG0000(	925	23.13511	chr1:116(	SNORD103A	smallRNA	chr1:30935688-3093
ENSG0000(	925	23.13511	chr1:116(	ENSG00000228176	lncRNA	chr1:29708851-2970
ENSG0000(	925	23.13511	chr1:116(	ENSG00000203620	lncRNA	chr1:31842019-3185
ENSG0000(	925	23.13511	chr1:116(	MATN1	protein_c	chr1:30711277-3072
ENSG0000(	925	23.13511	chr1:116(	ENSG00000236335	lncRNA	chr1:30409560-3041
ENSG0000(	925	23.13511	chr1:116(	LAPTM5	protein_c	chr1:30732469-3075
ENSG0000(	925	23.13511	chr1:116(	SERINC2	protein_c	chr1:31409565-3143
ENSG0000(	925	23.13511	chr1:116(	RNU6-40P	smallRNA	chr1:31497577-3149
ENSG0000(	925	23.13511	chr1:116(	PEF1-AS1	lncRNA	chr1:31644049-3166
ENSG0000(	925	23.13511	chr1:116(	ENSG00000232768	Pseudoger	chr1:31050872-3105
ENSG0000(	925	23.13511	chr1:116(	ENSG00000229044	lncRNA	chr1:31333067-3134
ENSG0000(	925	23.13511	chr1:116(	SNORD85	smallRNA	chr1:30968164-3096
ENSG0000(	925	23.13511	chr1:116(	ENSG00000231251	Pseudoger	chr1:30226523-3022
ENSG0000(	925	23.13511	chr1:116(	ENSG00000235143	lncRNA	chr1:30858158-3086
ENSG0000(	925	23.13511	chr1:116(	ENSG00000237329	Pseudoger	chr1:31036734-3103
ENSG0000(	925	23.13511	chr1:116(	ENSG00000231949	lncRNA	chr1:30415825-3042
ENSG0000(	925	23.13511	chr1:116(	PTP4A2 AC	protein_c	chr1:31906421-3194
ENSG0000(	925	23.13511	chr1:116(	ENSG00000233372	lncRNA	chr1:30140263-3014
ENSG0000(	925	23.13511	chr1:116(	AL136115.1	protein_c	chr1:31913573-3191
ENSG0000(	925	23.13511	chr1:116(	LINC01648	lncRNA	chr1:30013952-3003
ENSG0000(	925	23.13511	chr1:116(	ENSG00000228634	lncRNA	chr1:31933020-3193
ENSG0000(	925	23.13511	chr1:116(	ENSG00000229607	lncRNA	chr1:30810378-3081
ENSG0000(	925	23.13511	chr1:116(	ENSG00000229167	lncRNA	chr1:31571585-3157
ENSG0000(	925	23.13511	chr1:116(	ENSG00000203325	lncRNA	chr1:32052291-3207
ENSG0000(	925	23.13511	chr1:116(	LINC01778	lncRNA	chr1:30824217-3083
ENSG0000(	925	23.13511	chr1:116(	ENSG00000264078	lncRNA	chr1:31644694-3164
ENSG0000(	925	23.13511	chr1:116(	AL645944.1	smallRNA	chr1:29884902-2988
ENSG0000(	925	23.13511	chr1:116(	FABP3	protein_c	chr1:31365253-3137
ENSG0000(	925	23.13511	chr1:116(	KHDRBS1	protein_c	chr1:32013868-3206
ENSG0000(	925	23.13511	chr1:116(	NKAIN1	protein_c	chr1:31179745-3123
ENSG0000(	925	23.13511	chr1:116(	COL16A1	protein_c	chr1:31652263-3170
ENSG0000(	925	23.13511	chr1:116(	ENSG00000225142	Pseudoger	chr1:31108188-3110
ENSG0000(	925	23.13511	chr1:116(	RN7SKP91	smallRNA	chr1:30843823-3084
ENSG0000(	925	23.13511	chr1:116(	AC114494.1	protein_c	chr1:31429345-3142
ENSG0000(	925	23.13511	chr1:116(	SPOCD1	protein_c	chr1:31790422-3181
ENSG0000(	925	23.13511	chr1:116(	KPNA6	protein_c	chr1:32108056-3217
ENSG0000(	925	23.13511	chr1:116(	ENSG00000269967	lncRNA	chr1:31851913-3192
ENSG0000(	925	23.13511	chr1:116(	ENSG00000287510	lncRNA	chr1:30731693-3073
ENSG0000(	925	23.13511	chr1:116(	ENSG00000289710	protein_c	chr1:30576655-3057
ENSG0000(	925	23.13511	chr1:116(	MIR4420	smallRNA	chr1:30739156-3073
ENSG0000(	925	23.13511	chr1:116(	ENSG00000270927	Pseudoger	chr1:29904865-2990
ENSG0000(	925	23.13511	chr1:116(	PUM1	protein_c	chr1:30931506-3106
ENSG0000(	925	23.13511	chr1:116(	TMEM39B	protein_c	chr1:32072031-3210
ENSG0000(	925	23.13511	chr1:116(	ZCCHC17	protein_c	chr1:31296982-3136

ENSG0000(	925	23.13511	chr1:116(	LINC01226		lncRNA	chr1:31506226-3158
ENSG0000(	925	23.13511	chr1:116(	MIR5585		smallRNA	chr1:32086949-3208
ENSG0000(	925	23.13511	chr1:116(	MIR4254		smallRNA	chr1:31758660-3175
ENSG0000(	925	23.13511	chr1:116(	HCRTR1		protein_c	chr1:31617686-3163
ENSG0000(	925	23.13511	chr1:116(	ENSG00000288678		protein_c	chr1:31919563-3191
ENSG0000(	925	23.13511	chr1:116(	SNRNP40		protein_c	chr1:31259568-3129
ENSG0000(	925	23.13511	chr1:116(	ADGRB2		protein_c	chr1:31727117-3176
ENSG0000(	925	23.13511	chr1:116(	ENSG00000284702		lncRNA	chr1:31972189-3198
ENSG0000(	925	23.13511	chr1:116(	ENSG00000284676		lncRNA	chr1:29755175-2979
ENSG0000(	918	22.96003	chr11:76(	CD82		protein_c	chr11:44564427-446
ENSG0000(	918	22.96003	chr11:76(	ENSG00000255451		lncRNA	chr11:44468464-444
ENSG0000(	918	22.96003	chr11:76(	RPL7AP79		Pseudoger	chr11:44539778-445
ENSG0000(	918	22.96003	chr11:76(	ENSG00000255092		lncRNA	chr11:44606170-446
ENSG0000(	918	22.96003	chr11:76(	ENSG00000254693		lncRNA	chr11:44604508-446
ENSG0000(	918	22.96003	chr11:76(	RPL34P22		Pseudoger	chr11:44629357-446
ENSG0000(	914	22.85999	chr7:330(	AC004851.1		smallRNA	chr7:74571347-7457
ENSG0000(	914	22.85999	chr7:330(	NCF1		protein_c	chr7:74774011-7478
ENSG0000(	914	22.85999	chr7:330(	CASTOR2	DriverDB\	protein_c	chr7:74964776-7503
ENSG0000(	914	22.85999	chr7:330(	ENSG00000273069		lncRNA	chr7:74606913-7460
ENSG0000(	914	22.85999	chr7:330(	RNU6-1070P		smallRNA	chr7:74258526-7425
ENSG0000(	914	22.85999	chr7:330(	GTF2I-AS1		lncRNA	chr7:74688864-7472
ENSG0000(	914	22.85999	chr7:330(	ENSG00000279005		TEC	chr7:74633510-7463
ENSG0000(	914	22.85999	chr7:330(	LIMK1	NCGv7	protein_c	chr7:74082933-7412
ENSG0000(	914	22.85999	chr7:330(	RFC2		protein_c	chr7:74231499-7425
ENSG0000(	914	22.85999	chr7:330(	GTF2IRD1		protein_c	chr7:74453790-7460
ENSG0000(	914	22.85999	chr7:330(	ELN	NCGv7;AC	protein_c	chr7:74027789-7406
ENSG0000(	914	22.85999	chr1:1234	Y_RNA		smallRNA	chr1:156484098-156
ENSG0000(	914	22.85999	chr7:330(	EIF4H		protein_c	chr7:74174231-7419
ENSG0000(	914	22.85999	chr7:330(	Y_RNA		smallRNA	chr7:74895816-7489
ENSG0000(	914	22.85999	chr7:330(	SPDYE12		protein_c	chr7:74904289-7491
ENSG0000(	914	22.85999	chr7:330(	PMS2P5		Pseudoger	chr7:74894116-7489
ENSG0000(	914	22.85999	chr7:330(	CLIP2	NCGv7	protein_c	chr7:74289407-7440
ENSG0000(	914	22.85999	chr7:330(	AC004878.7		lncRNA	chr7:74974865-7497
ENSG0000(	914	22.85999	chr7:330(	MIR590		smallRNA	chr7:74191198-7419
ENSG0000(	914	22.85999	chr7:330(	LAT2		protein_c	chr7:74199652-7422
ENSG0000(	914	22.85999	chr7:330(	STAG3L2		Pseudoger	chr7:74882705-7489
ENSG0000(	914	22.85999	chr7:330(	GTF2I	NCGv7	protein_c	chr7:74650231-7476
ENSG0000(	914	22.85999	chr7:330(	ENSG00000287815		lncRNA	chr7:74280747-7428
ENSG0000(	914	22.85999	chr7:330(	ENSG00000289346		protein_c	chr7:74796150-7489
ENSG0000(	914	22.85999	chr7:330(	GATSL2		protein_c	chr7:74964817-7502
ENSG0000(	914	22.85999	chr7:330(	GTF2IRD2		protein_c	chr7:74796144-7485
ENSG0000(	914	22.85999	chr7:330(	RNA5SP233		Pseudoger	chr7:74487428-7448
ENSG0000(	914	22.85999	chr7:330(	PHB1P15		Pseudoger	chr7:74741457-7474
ENSG0000(	914	22.85999	chr7:330(	ELN-AS1		lncRNA	chr7:74058905-7406
ENSG0000(	912	22.80997	chr1:8137	AL157904.1		smallRNA	chr1:116905150-116
ENSG0000(	908	22.70992	chr5:169(	ENSG00000213755		Pseudoger	chr5:79510434-7951
ENSG0000(	903	22.58487	chr7:330(	ENSG00000224138		lncRNA	chr7:127350128-127
ENSG0000(	903	22.58487	chr7:330(	ATP6V1FNB		protein_c	chr7:128866330-128
ENSG0000(	903	22.58487	chr7:330(	PRELID3BP10		Pseudoger	chr7:127295620-127
ENSG0000(	903	22.58487	chr7:330(	ENSG00000243230		lncRNA	chr7:129209775-129
ENSG0000(	903	22.58487	chr7:330(	ENSG00000224163		Pseudoger	chr7:128912732-128

ENSG0000(	903	22.58487	chr7:330(	ENSG00000272915		lncRNA	chr7:128264526-128
ENSG0000(	903	22.58487	chr7:330(	ENSG00000243302		Pseudoger	chr7:128651185-128
ENSG0000(	903	22.58487	chr7:330(	RNU6-102P		smallRNA	chr7:124647719-124
ENSG0000(	903	22.58487	chr7:330(	TMEM209		protein_c	chr7:130164713-130
ENSG0000(	903	22.58487	chr7:330(	MIR96		smallRNA	chr7:129774692-129
ENSG0000(	903	22.58487	chr7:330(	PPIAP93		Pseudoger	chr7:125345825-125
ENSG0000(	903	22.58487	chr7:330(	FLNC-AS1		lncRNA	chr7:128850162-128
ENSG0000(	903	22.58487	chr7:330(	ENSG00000287702		lncRNA	chr7:126495312-126
ENSG0000(	903	22.58487	chr7:330(	PRRT4		protein_c	chr7:128350325-128
ENSG0000(	903	22.58487	chr7:330(	RNU6-177P		smallRNA	chr7:128627172-128
ENSG0000(	903	22.58487	chr7:330(	EFCAB3P1		Pseudoger	chr7:128466563-128
ENSG0000(	903	22.58487	chr7:330(	ENSG00000242593		lncRNA	chr7:124032126-124
ENSG0000(	903	22.58487	chr7:330(	ENSG00000242588		lncRNA	chr7:128531707-128
ENSG0000(	903	22.58487	chr7:330(	ENSG00000290319		lncRNA	chr7:129780410-129
ENSG0000(	903	22.58487	chr7:330(	ENSG00000197462		lncRNA	chr7:125917871-125
ENSG0000(	903	22.58487	chr7:330(	LINC02830		lncRNA	chr7:125151326-125
ENSG0000(	903	22.58487	chr7:330(	CDC26P1		Pseudoger	chr7:129410113-129
ENSG0000(	903	22.58487	chr7:330(	POT1-AS1		lncRNA	chr7:124929873-125
ENSG0000(	903	22.58487	chr7:330(	ENSG00000243574		lncRNA	chr7:124274671-124
ENSG0000(	903	22.58487	chr7:330(	HILPDA		protein_c	chr7:128455849-128
ENSG0000(	903	22.58487	chr7:330(	ENSG00000243679		Pseudoger	chr7:128653969-128
ENSG0000(	903	22.58487	chr7:330(	Y_RNA		smallRNA	chr7:129954693-129
ENSG0000(	903	22.58487	chr7:330(	LINC03008		lncRNA	chr7:130141707-130
ENSG0000(	903	22.58487	chr7:330(	RNA5SP243		Pseudoger	chr7:128697710-128
ENSG0000(	903	22.58487	chr7:330(	ENSG00000280347		TEC	chr7:127359785-127
ENSG0000(	903	22.58487	chr7:330(	ENSG00000275106		TEC	chr7:128952527-128
ENSG0000(	903	22.58487	chr7:330(	ENSG00000280828		Pseudoger	chr7:128533652-128
ENSG0000(	903	22.58487	chr7:330(	CICP14		Pseudoger	chr7:128655962-128
ENSG0000(	903	22.58487	chr7:330(	ENSG00000229413		Pseudoger	chr7:128653690-128
ENSG0000(	903	22.58487	chr7:330(	GCC1		protein_c	chr7:127580628-127
ENSG0000(	903	22.58487	chr7:330(	GRM8-AS1		lncRNA	chr7:127215127-127
ENSG0000(	903	22.58487	chr7:330(	GRM8	NCGv7	protein_c	chr7:126438598-127
ENSG0000(	903	22.58487	chr7:330(	ODCP		Pseudoger	chr7:129028889-129
ENSG0000(	903	22.58487	chr7:330(	RNU1-72P		smallRNA	chr7:129484504-129
ENSG0000(	903	22.58487	chr7:330(	ENSG00000273329		lncRNA	chr7:129604548-129
ENSG0000(	903	22.58487	chr7:330(	Y_RNA		smallRNA	chr7:129961989-129
ENSG0000(	903	22.58487	chr7:330(	RN7SL306P		smallRNA	chr7:128970734-128
ENSG0000(	903	22.58487	chr7:330(	ENSG00000273270		lncRNA	chr7:128524016-128
ENSG0000(	903	22.58487	chr7:330(	RN7SL81P		smallRNA	chr7:128761337-128
ENSG0000(	903	22.58487	chr7:330(	NRF1		protein_c	chr7:129611720-129
ENSG0000(	903	22.58487	chr7:330(	KCP	DriverDBv	protein_c	chr7:128862042-128
ENSG0000(	903	22.58487	chr7:330(	ENSG00000273184		lncRNA	chr7:128455840-128
ENSG0000(	903	22.58487	chr7:330(	ENSG00000224981		Pseudoger	chr7:126868767-126
ENSG0000(	903	22.58487	chr7:330(	RNA5SP244		Pseudoger	chr7:129756266-129
ENSG0000(	903	22.58487	chr7:330(	IMPDH1		protein_c	chr7:128392277-128
ENSG0000(	903	22.58487	chr7:330(	RBM28		protein_c	chr7:128297685-128
ENSG0000(	903	22.58487	chr7:330(	PAX4	AC	protein_c	chr7:127610292-127
ENSG0000(	903	22.58487	chr7:330(	UBE2H-DT		lncRNA	chr7:129953234-130
ENSG0000(	903	22.58487	chr7:330(	FSCN3		protein_c	chr7:127591409-127
ENSG0000(	903	22.58487	chr7:330(	GARIN1B		protein_c	chr7:128709061-128
ENSG0000(	903	22.58487	chr7:330(	ENSG00000242261		Pseudoger	chr7:128306649-128

ENSG0000(	903	22.58487	chr7:330(	SND1	NCGv7	protein_c	chr7:127652194-128
ENSG0000(	903	22.58487	chr7:330(	ENSG00000242162		Pseudoger	chr7:129066021-129
ENSG0000(	903	22.58487	chr7:330(	ENSG00000242078		lncRNA	chr7:129783370-129
ENSG0000(	903	22.58487	chr7:330(	POT1	NCGv7;AC	protein_c	chr7:124822386-124
ENSG0000(	903	22.58487	chr7:330(	ENSG00000230626		Pseudoger	chr7:129126518-129
ENSG0000(	903	22.58487	chr7:330(	STRIP2		protein_c	chr7:129434432-129
ENSG0000(	903	22.58487	chr7:330(	MIR129-1		smallRNA	chr7:128207871-128
ENSG0000(	903	22.58487	chr7:330(	AHCYL2	NCGv7	protein_c	chr7:129225030-129
ENSG0000(	903	22.58487	chr7:330(	FLNC		protein_c	chr7:128830406-128
ENSG0000(	903	22.58487	chr7:330(	TSPAN33		protein_c	chr7:129144884-129
ENSG0000(	903	22.58487	chr7:330(	LRRC4		protein_c	chr7:128027071-128
ENSG0000(	903	22.58487	chr7:330(	CALU	DriverDB\	protein_c	chr7:128739292-128
ENSG0000(	903	22.58487	chr7:330(	CCDC136		protein_c	chr7:128790757-128
ENSG0000(	903	22.58487	chr7:330(	SMO	NCGv7;AC	protein_c	chr7:129188633-129
ENSG0000(	903	22.58487	chr7:330(	IRF5	NCGv7	protein_c	chr7:128937457-128
ENSG0000(	903	22.58487	chr7:330(	KLHDC10		protein_c	chr7:130070534-130
ENSG0000(	903	22.58487	chr7:330(	OPN1SW		protein_c	chr7:128772485-128
ENSG0000(	903	22.58487	chr7:330(	RNU7-54P		smallRNA	chr7:128443449-128
ENSG0000(	903	22.58487	chr7:330(	IMP3P2		Pseudoger	chr7:128693450-128
ENSG0000(	903	22.58487	chr7:330(	MIR592		smallRNA	chr7:127058088-127
ENSG0000(	903	22.58487	chr7:330(	MIR183		smallRNA	chr7:129774905-129
ENSG0000(	903	22.58487	chr7:330(	ENSG00000286722		lncRNA	chr7:129366827-129
ENSG0000(	903	22.58487	chr7:330(	TNPO3		protein_c	chr7:128954180-129
ENSG0000(	903	22.58487	chr7:330(	ENSG00000230715		Pseudoger	chr7:128652841-128
ENSG0000(	903	22.58487	chr7:330(	snoU13		smallRNA	chr7:129061531-129
ENSG0000(	903	22.58487	chr7:330(	ENSG00000270992		Pseudoger	chr7:130255902-130
ENSG0000(	903	22.58487	chr7:330(	RNU7-27P		smallRNA	chr7:128344081-128
ENSG0000(	903	22.58487	chr7:330(	ENSG00000289434		lncRNA	chr7:128221388-128
ENSG0000(	903	22.58487	chr7:330(	MIR593		smallRNA	chr7:128081861-128
ENSG0000(	903	22.58487	chr7:330(	RNA5SP242		Pseudoger	chr7:128697439-128
ENSG0000(	903	22.58487	chr7:330(	ENSG00000230820		Pseudoger	chr7:126980967-126
ENSG0000(	903	22.58487	chr7:330(	snoU13		smallRNA	chr7:130344415-130
ENSG0000(	903	22.58487	chr7:330(	CPA4		protein_c	chr7:130293134-130
ENSG0000(	903	22.58487	chr7:330(	ENSG00000219445		lncRNA	chr7:125229579-125
ENSG0000(	903	22.58487	chr7:330(	CPA2		protein_c	chr7:130266863-130
ENSG0000(	903	22.58487	chr7:330(	ENSG00000286380		lncRNA	chr7:129763060-129
ENSG0000(	903	22.58487	chr7:330(	RNA5SP245		Pseudoger	chr7:130027277-130
ENSG0000(	903	22.58487	chr7:330(	ENSG00000287568		lncRNA	chr7:126533665-126
ENSG0000(	903	22.58487	chr7:330(	ENSG00000241921		lncRNA	chr7:126378970-126
ENSG0000(	903	22.58487	chr7:330(	ENSG00000241573		Pseudoger	chr7:129096027-129
ENSG0000(	903	22.58487	chr7:330(	ENSG00000241493		Pseudoger	chr7:128669087-128
ENSG0000(	903	22.58487	chr7:330(	MIR182		smallRNA	chr7:129770383-129
ENSG0000(	903	22.58487	chr7:330(	ENSG00000241324		lncRNA	chr7:124337380-124
ENSG0000(	903	22.58487	chr7:330(	SSU72P8		protein_c	chr7:124476371-124
ENSG0000(	903	22.58487	chr7:330(	ENSG00000234071		Pseudoger	chr7:125344969-125
ENSG0000(	903	22.58487	chr7:330(	Y_RNA		smallRNA	chr7:129164849-129
ENSG0000(	903	22.58487	chr7:330(	ENSG00000241102		Pseudoger	chr7:129095301-129
ENSG0000(	903	22.58487	chr7:330(	ENSG00000271553		lncRNA	chr7:128667043-128
ENSG0000(	903	22.58487	chr7:330(	TPI1P2		Pseudoger	chr7:129055223-129
ENSG0000(	903	22.58487	chr7:330(	SND1-DT		lncRNA	chr7:127644685-127
ENSG0000(	903	22.58487	chr7:330(	ZC3HC1	NCGv7	protein_c	chr7:130018287-130

ENSG0000(	903	22.58487	chr7:330(	HILPDA-AS1	lncRNA	chr7:128455937-128
ENSG0000(	903	22.58487	chr7:330(	ZNF800	protein_c	chr7:127346790-127
ENSG0000(	903	22.58487	chr7:330(	UBE2H	protein_c	chr7:129830732-129
ENSG0000(	903	22.58487	chr7:330(	ENSG00000240579	Pseudoger	chr7:127857852-127
ENSG0000(	903	22.58487	chr7:330(	ENSG00000240571	lncRNA	chr7:130173718-130
ENSG0000(	903	22.58487	chr7:330(	EEF1GP1	Pseudoger	chr7:125033453-125
ENSG0000(	903	22.58487	chr7:330(	ENSG00000271344	lncRNA	chr7:128690451-128
ENSG0000(	903	22.58487	chr7:330(	CYCSP20	Pseudoger	chr7:129117513-129
ENSG0000(	903	22.58487	chr7:330(	SMKR1	protein_c	chr7:129502531-129
ENSG0000(	903	22.58487	chr7:330(	METTL2B	protein_c	chr7:128476729-128
ENSG0000(	903	22.58487	chr7:330(	ENSG00000227249	Pseudoger	chr7:126511135-126
ENSG0000(	903	22.58487	chr7:330(	SNRPGP3	Pseudoger	chr7:129477875-129
ENSG0000(	903	22.58487	chr7:330(	CPA5	protein_c	chr7:130344816-130
ENSG0000(	903	22.58487	chr7:330(	ATP6V1F	protein_c	chr7:128862856-128
ENSG0000(	903	22.58487	chr7:330(	ENSG00000288881	lncRNA	chr7:129642758-129
ENSG0000(	903	22.58487	chr7:330(	SND1-IT1	lncRNA	chr7:127997597-128
ENSG0000(	903	22.58487	chr7:330(	AC073320.1	smallRNA	chr7:129985647-129
ENSG0000(	903	22.58487	chr7:330(	ENSG00000279419	TEC	chr7:124742312-124
ENSG0000(	903	22.58487	chr7:330(	ENSG00000213280	Pseudoger	chr7:128570241-128
ENSG0000(	903	22.58487	chr7:330(	ARF5	protein_c	chr7:127588386-127
ENSG0000(	903	22.58487	chr7:330(	ENSG00000213296	Pseudoger	chr7:124480524-124
ENSG0000(	903	22.58487	chr7:330(	AC003968.1	smallRNA	chr7:126040229-126
ENSG0000(	903	22.58487	chr7:330(	ENSG00000279265	TEC	chr7:127349833-127
ENSG0000(	903	22.58487	chr7:330(	LEP	protein_c	chr7:128241278-128
ENSG0000(	903	22.58487	chr7:330(	ENSG00000213291	Pseudoger	chr7:125300504-125
ENSG0000(	903	22.58487	chr7:330(	AC018635.1	smallRNA	chr7:128290004-128
ENSG0000(	903	22.58487	chr7:330(	RNU7-16P	smallRNA	chr7:129405635-129
ENSG0000(	903	22.58487	chr7:330(	SSMEM1	protein_c	chr7:130206344-130
ENSG0000(	903	22.58487	chr7:330(	AC025594.1	smallRNA	chr7:128906772-128
ENSG0000(	903	22.58487	chr7:330(	C7orf77	lncRNA	chr7:124777292-124
ENSG0000(	903	22.58487	chr7:330(	GPR37	protein_c	chr7:124743885-124
ENSG0000(	903	22.58487	chr7:330(	ENSG00000228700	Pseudoger	chr7:128433422-128
ENSG0000(	903	22.58487	chr7:330(	GARIN1A	protein_c	chr7:128671693-128
ENSG0000(	903	22.58487	chr7:330(	LINC03012	lncRNA	chr7:127476883-127
ENSG0000(	903	22.58487	chr7:330(	ENSG00000205898	Pseudoger	chr7:125159974-125
ENSG0000(	896	22.40979	chr1:1522	ENSG00000232514	Pseudoger	chr1:48497263-4849
ENSG0000(	896	22.40979	chr1:1522	ENSG00000286597	lncRNA	chr1:48926021-4893
ENSG0000(	896	22.40979	chr1:1522	ENSG00000279324	TEC	chr1:49994318-4999
ENSG0000(	896	22.40979	chr1:1522	ENSG00000279214	TEC	chr1:48262230-4826
ENSG0000(	896	22.40979	chr1:1522	ENSG00000279096	TEC	chr1:48102068-4810
ENSG0000(	896	22.40979	chr1:1522	AL645730.1	smallRNA	chr1:49982215-4998
ENSG0000(	896	22.40979	chr1:1522	ENSG00000234080	Pseudoger	chr1:50326131-5032
ENSG0000(	896	22.40979	chr1:1522	ENSG00000291246	lncRNA	chr1:48164710-4818
ENSG0000(	896	22.40979	chr1:1522	PPP1R8P1	Pseudoger	chr1:48325080-4832
ENSG0000(	896	22.40979	chr1:1522	RNU4-61P	smallRNA	chr1:48447936-4844
ENSG0000(	896	22.40979	chr1:1522	ENSG00000272491	lncRNA	chr1:48227888-4822
ENSG0000(	896	22.40979	chr1:1522	AGBL4	protein_c	chr1:48532854-5002
ENSG0000(	896	22.40979	chr1:1522	RNU6-723P	smallRNA	chr1:48344209-4834
ENSG0000(	896	22.40979	chr1:1522	SLC5A9	protein_c	chr1:48222685-4824
ENSG0000(	896	22.40979	chr1:1522	AL109659.1	protein_c	chr1:48103634-4810
ENSG0000(	896	22.40979	chr1:1522	ENSG00000287661	lncRNA	chr1:48552991-4855

ENSG0000(	896	22.40979	chr1:1522	AGBL4-AS1		lncRNA	chr1:49257411-4926
ENSG0000(	896	22.40979	chr1:1522	LINC02794		lncRNA	chr1:48050659-4809
ENSG0000(	896	22.40979	chr1:1522	ZNF859P		Pseudoger	chr1:49841821-4984
ENSG0000(	896	22.40979	chr1:1522	SKINT1L		Pseudoger	chr1:48161799-4817
ENSG0000(	896	22.40979	chr1:1522	ELAVL4	NCGv7	protein_c	chr1:50024029-5020
ENSG0000(	896	22.40979	chr1:1522	AGBL4-IT1		lncRNA	chr1:49374201-4947
ENSG0000(	896	22.40979	chr1:1522	ENSG00000231413		lncRNA	chr1:48078787-4808
ENSG0000(	896	22.40979	chr1:1522	ENSG00000290466		lncRNA	chr1:48096092-4816
ENSG0000(	896	22.40979	chr1:1522	AL645730.2		protein_c	chr1:49994318-4999
ENSG0000(	896	22.40979	chr1:1522	ENSG00000235105		Pseudoger	chr1:48435967-4843
ENSG0000(	896	22.40979	chr1:1522	DMRTA2		protein_c	chr1:50417550-5042
ENSG0000(	896	22.40979	chr1:1522	ENSG00000284645		lncRNA	chr1:50252569-5025
ENSG0000(	896	22.40979	chr1:1522	CYP46A4P		Pseudoger	chr1:48089368-4808
ENSG0000(	896	22.40979	chr1:1522	ENSG00000230828		Pseudoger	chr1:50114937-5011
ENSG0000(	896	22.40979	chr1:1522	LINC02808		lncRNA	chr1:50229662-5032
ENSG0000(	896	22.40979	chr1:1522	snoU13		smallRNA	chr1:49151392-4915
ENSG0000(	896	22.40979	chr1:1522	ENSG00000233407		lncRNA	chr1:50206084-5022
ENSG0000(	896	22.40979	chr1:1522	HMGB1P45		Pseudoger	chr1:50398825-5039
ENSG0000(	896	22.40979	chr1:1522	BEND5		protein_c	chr1:48727519-4877
ENSG0000(	896	22.40979	chr1:1522	ENSG00000237478		Pseudoger	chr1:49691262-4969
ENSG0000(	896	22.40979	chr1:1522	ENSG00000229846		lncRNA	chr1:49025595-4918
ENSG0000(	896	22.40979	chr1:1522	FCF1P6		Pseudoger	chr1:50405430-5040
ENSG0000(	896	22.40979	chr1:1522	ELAVL4-AS1		lncRNA	chr1:50174306-5017
ENSG0000(	896	22.40979	chr1:1522	ENSG00000223720		lncRNA	chr1:48172972-4820
ENSG0000(	896	22.40979	chr1:1522	SPATA6	NCGv7	protein_c	chr1:48295373-4847
ENSG0000(	896	22.40979	chr1:1522	MTND2P29		Pseudoger	chr1:50017092-5001
ENSG0000(	893	22.33476	chr7:330(	ENSG00000287733		lncRNA	chr7:135170816-135
ENSG0000(	893	22.33476	chr7:330(	MTPN		protein_c	chr7:135926760-135
ENSG0000(	893	22.33476	chr7:330(	WDR91		protein_c	chr7:135183839-135
ENSG0000(	893	22.33476	chr7:330(	Y_RNA		smallRNA	chr7:136129980-136
ENSG0000(	893	22.33476	chr7:330(	ENSG00000225559		lncRNA	chr7:138163440-138
ENSG0000(	893	22.33476	chr7:330(	AC083862.1		protein_c	chr7:135148045-135
ENSG0000(	893	22.33476	chr7:330(	CREB3L2	NCGv7;AC	protein_c	chr7:137874979-138
ENSG0000(	893	22.33476	chr7:330(	PTN	NCGv7	protein_c	chr7:137227341-137
ENSG0000(	893	22.33476	chr7:330(	ATP6V0A4		protein_c	chr7:138706294-138
ENSG0000(	893	22.33476	chr7:330(	TMEM213		protein_c	chr7:138797952-138
ENSG0000(	893	22.33476	chr7:330(	ENSG00000213238		Pseudoger	chr7:138298088-138
ENSG0000(	893	22.33476	chr7:330(	ENSG00000243099		Pseudoger	chr7:138688980-138
ENSG0000(	893	22.33476	chr7:330(	RNU6-911P		smallRNA	chr7:139448740-139
ENSG0000(	893	22.33476	chr7:330(	CNOT4		protein_c	chr7:135361795-135
ENSG0000(	893	22.33476	chr7:330(	PSMC1P3		Pseudoger	chr7:136713871-136
ENSG0000(	893	22.33476	chr7:330(	STMP1		protein_c	chr7:135662496-135
ENSG0000(	893	22.33476	chr7:330(	PTMAP10		Pseudoger	chr7:138404195-138
ENSG0000(	893	22.33476	chr7:330(	AKR1D1	NCGv7	protein_c	chr7:138002324-138
ENSG0000(	893	22.33476	chr7:330(	CREB3L2-AS1		lncRNA	chr7:137953348-137
ENSG0000(	893	22.33476	chr7:330(	CALD1	NCGv7	protein_c	chr7:134744252-134
ENSG0000(	893	22.33476	chr7:330(	ZP3P2		Pseudoger	chr7:136485656-136
ENSG0000(	893	22.33476	chr7:330(	CYREN		protein_c	chr7:135092363-135
ENSG0000(	893	22.33476	chr7:330(	TRIM24	NCGv7	protein_c	chr7:138460259-138
ENSG0000(	893	22.33476	chr7:330(	KIAA1549	AC	protein_c	chr7:138831381-138
ENSG0000(	893	22.33476	chr7:330(	RN7SKP223		smallRNA	chr7:138091254-138

ENSG0000(	893	22.33476	chr7:330(	ENSG00000272941		lncRNA	chr7:135168403-135
ENSG0000(	893	22.33476	chr7:330(	KRT8P51		Pseudoger	chr7:136938280-136
ENSG0000(	893	22.33476	chr7:330(	ENSG00000231114		lncRNA	chr7:137318592-137
ENSG0000(	893	22.33476	chr7:330(	AC091736.1		smallRNA	chr7:135798006-135
ENSG0000(	893	22.33476	chr7:330(	NUP205		protein_c	chr7:135557917-135
ENSG0000(	893	22.33476	chr7:330(	ENSG00000223718		Pseudoger	chr7:135660039-135
ENSG0000(	893	22.33476	chr7:330(	ENSG00000289600		lncRNA	chr7:135980929-135
ENSG0000(	893	22.33476	chr7:330(	UBN2		protein_c	chr7:139230356-139
ENSG0000(	893	22.33476	chr7:330(	ENSG00000231794		lncRNA	chr7:135198401-135
ENSG0000(	893	22.33476	chr7:330(	SVOPL		protein_c	chr7:138594285-138
ENSG0000(	893	22.33476	chr7:330(	DGKI	NCGv7	protein_c	chr7:137381037-137
ENSG0000(	893	22.33476	chr7:330(	UQCRFS1P2		Pseudoger	chr7:138701607-138
ENSG0000(	893	22.33476	chr7:330(	SLC23A4P		Pseudoger	chr7:135270017-135
ENSG0000(	893	22.33476	chr7:330(	ENSG00000239254		Pseudoger	chr7:139172516-139
ENSG0000(	893	22.33476	chr7:330(	RPS3AP28		Pseudoger	chr7:138490581-138
ENSG0000(	893	22.33476	chr7:330(	MIR490		smallRNA	chr7:136903167-136
ENSG0000(	893	22.33476	chr7:330(	ENSG00000230649		lncRNA	chr7:136025717-136
ENSG0000(	893	22.33476	chr7:330(	snoU13		smallRNA	chr7:138121088-138
ENSG0000(	893	22.33476	chr7:330(	snoU13		smallRNA	chr7:138482480-138
ENSG0000(	893	22.33476	chr7:330(	KLRG2	DriverDB\	protein_c	chr7:139452690-139
ENSG0000(	893	22.33476	chr7:330(	AGBL3	DriverDB\	protein_c	chr7:134986508-135
ENSG0000(	893	22.33476	chr7:330(	STRA8		protein_c	chr7:135231979-135
ENSG0000(	893	22.33476	chr7:330(	ZC3HAV1L		protein_c	chr7:139025706-139
ENSG0000(	893	22.33476	chr7:330(	TMEM140	AC	protein_c	chr7:135148072-135
ENSG0000(	893	22.33476	chr7:330(	LUC7L2	NCGv7	protein_c	chr7:139340359-139
ENSG0000(	893	22.33476	chr7:330(	snoU13		smallRNA	chr7:138176185-138
ENSG0000(	893	22.33476	chr7:330(	ENSG00000289438		lncRNA	chr7:137846936-137
ENSG0000(	893	22.33476	chr7:330(	ENSG00000224469		Pseudoger	chr7:137164043-137
ENSG0000(	893	22.33476	chr7:330(	AC024082.1		smallRNA	chr7:138068542-138
ENSG0000(	893	22.33476	chr7:330(	ENSG00000234658		Pseudoger	chr7:138645671-138
ENSG0000(	893	22.33476	chr7:330(	ENSG00000289175		lncRNA	chr7:134953657-134
ENSG0000(	893	22.33476	chr7:330(	ENSG00000228360		Pseudoger	chr7:139227537-139
ENSG0000(	893	22.33476	chr7:330(	ENSG00000232053		lncRNA	chr7:136092913-136
ENSG0000(	893	22.33476	chr7:330(	AC009784.1		smallRNA	chr7:136244446-136
ENSG0000(	893	22.33476	chr7:330(	IMPDH1P3		Pseudoger	chr7:138440690-138
ENSG0000(	893	22.33476	chr7:330(	RCC2P3		Pseudoger	chr7:138122202-138
ENSG0000(	893	22.33476	chr7:330(	SNORA40		smallRNA	chr7:138625060-138
ENSG0000(	893	22.33476	chr7:330(	Y_RNA		smallRNA	chr7:138415480-138
ENSG0000(	893	22.33476	chr7:330(	SDHDP2		Pseudoger	chr7:135444461-135
ENSG0000(	893	22.33476	chr7:330(	TTC26	DriverDB\	protein_c	chr7:139133744-139
ENSG0000(	893	22.33476	chr7:330(	ENSG00000224746		lncRNA	chr7:135774521-135
ENSG0000(	893	22.33476	chr7:330(	SNORD81		smallRNA	chr7:137287687-137
ENSG0000(	893	22.33476	chr7:330(	ENSG00000271537		Pseudoger	chr7:139198557-139
ENSG0000(	893	22.33476	chr7:330(	RPL6P19		Pseudoger	chr7:137721985-137
ENSG0000(	893	22.33476	chr7:330(	ENSG00000234639		Pseudoger	chr7:138022458-138
ENSG0000(	893	22.33476	chr7:330(	FMC1-LUC7L2		protein_c	chr7:139341360-139
ENSG0000(	893	22.33476	chr7:330(	ENSG00000279483		Pseudoger	chr7:137513859-137
ENSG0000(	893	22.33476	chr7:330(	ENSG00000286458		lncRNA	chr7:134816543-134
ENSG0000(	893	22.33476	chr7:330(	ENSG00000271414		Pseudoger	chr7:135650972-135
ENSG0000(	893	22.33476	chr7:330(	ENSG00000234352		lncRNA	chr7:136685559-137
ENSG0000(	893	22.33476	chr7:330(	MZT1P2		Pseudoger	chr7:139228221-139

ENSG0000(	893	22.33476	chr7:330(	ENSG00000231931		Pseudoger	chr7:138060056-138
ENSG0000(	893	22.33476	chr7:330(	ENSG00000231923		Pseudoger	chr7:138046654-138
ENSG0000(	893	22.33476	chr7:330(	snoU13		smallRNA	chr7:136841093-136
ENSG0000(	893	22.33476	chr7:330(	RNU6-206P		smallRNA	chr7:139315291-139
ENSG0000(	893	22.33476	chr7:330(	SNORA51		smallRNA	chr7:138187998-138
ENSG0000(	893	22.33476	chr7:330(	ZC3HAV1		protein_c	chr7:139043515-139
ENSG0000(	893	22.33476	chr7:330(	FAM180A		protein_c	chr7:135728348-135
ENSG0000(	893	22.33476	chr7:330(	SLC13A4	DriverDBv	protein_c	chr7:135681231-135
ENSG0000(	893	22.33476	chr7:330(	FMC1		protein_c	chr7:139339457-139
ENSG0000(	893	22.33476	chr7:330(	ENSG00000273219		lncRNA	chr7:135704537-135
ENSG0000(	893	22.33476	chr7:330(	ENSG00000290805		lncRNA	chr7:135246113-135
ENSG0000(	893	22.33476	chr7:330(	ERHP1		Pseudoger	chr7:139534240-139
ENSG0000(	893	22.33476	chr7:330(	ENSG00000228031		lncRNA	chr7:137344930-137
ENSG0000(	893	22.33476	chr7:330(	CLEC2L		protein_c	chr7:139523685-139
ENSG0000(	893	22.33476	chr7:330(	RNF14P4		Pseudoger	chr7:135128444-135
ENSG0000(	893	22.33476	chr7:330(	RNU6-1272P		smallRNA	chr7:138876359-138
ENSG0000(	893	22.33476	chr7:330(	TRPC6P8		Pseudoger	chr7:136262558-136
ENSG0000(	893	22.33476	chr7:330(	MIR4468		smallRNA	chr7:138123758-138
ENSG0000(	893	22.33476	chr7:330(	ENSG00000273391		lncRNA	chr7:139359032-139
ENSG0000(	893	22.33476	chr7:330(	ENSG00000229677		Pseudoger	chr7:139049456-139
ENSG0000(	893	22.33476	chr7:330(	ENSG00000253183		Pseudoger	chr7:139502453-139
ENSG0000(	893	22.33476	chr7:330(	CHRM2		protein_c	chr7:136868652-137
ENSG0000(	893	22.33476	chr7:330(	Y_RNA		smallRNA	chr7:138175045-138
ENSG0000(	893	22.33476	chr7:330(	RNU6-223P		smallRNA	chr7:135960703-135
ENSG0000(	893	22.33476	chr7:330(	RNU6-1154P		smallRNA	chr7:135665599-135
ENSG0000(	889	22.23472	chr1:1166	SNORD55		smallRNA	chr1:44775864-4477
ENSG0000(	887	22.18469	chr11:76(	KRT18P14		Pseudoger	chr11:35860249-358
ENSG0000(	887	22.18469	chr11:76(	ENSG00000283217		lncRNA	chr11:43556436-436
ENSG0000(	887	22.18469	chr11:76(	ENSG00000244953		lncRNA	chr11:43943787-439
ENSG0000(	887	22.18469	chr11:76(	ENSG00000254919		lncRNA	chr11:35915051-359
ENSG0000(	887	22.18469	chr11:76(	ENSG00000240975		Pseudoger	chr11:39161453-391
ENSG0000(	887	22.18469	chr11:76(	RN7SKP158		smallRNA	chr11:28261194-282
ENSG0000(	887	22.18469	chr11:76(	ENSG00000254566		lncRNA	chr11:36321158-363
ENSG0000(	887	22.18469	chr11:76(	ENSG00000254914		Pseudoger	chr11:42939775-429
ENSG0000(	887	22.18469	chr11:76(	Y_RNA		smallRNA	chr11:33004250-330
ENSG0000(	887	22.18469	chr11:76(	ENSG00000254907		lncRNA	chr11:43328748-433
ENSG0000(	887	22.18469	chr11:76(	MIR3973		smallRNA	chr11:36010098-360
ENSG0000(	887	22.18469	chr11:76(	THEM7P		Pseudoger	chr11:32112049-323
ENSG0000(	887	22.18469	chr11:76(	LINC00678		lncRNA	chr11:27617626-276
ENSG0000(	887	22.18469	chr11:76(	MIR610		smallRNA	chr11:28056815-280
ENSG0000(	887	22.18469	chr11:76(	ENSG00000271369		Pseudoger	chr11:34709600-347
ENSG0000(	887	22.18469	chr11:76(	SLC1A2-AS1		lncRNA	chr11:35281813-352
ENSG0000(	887	22.18469	chr11:76(	CCDC73		protein_c	chr11:32602721-327
ENSG0000(	887	22.18469	chr11:76(	CYCSP25		Pseudoger	chr11:31280672-312
ENSG0000(	887	22.18469	chr11:76(	ENSG00000255375		lncRNA	chr11:32132657-321
ENSG0000(	887	22.18469	chr11:76(	Y_RNA		smallRNA	chr11:33171381-331
ENSG0000(	887	22.18469	chr11:76(	Y_RNA		smallRNA	chr11:40351564-403
ENSG0000(	887	22.18469	chr11:76(	CIR1P3		Pseudoger	chr11:34430880-344
ENSG0000(	887	22.18469	chr11:76(	ENSG00000246250		lncRNA	chr11:43827517-438
ENSG0000(	887	22.18469	chr11:76(	AL137224.1		smallRNA	chr11:34484436-344
ENSG0000(	887	22.18469	chr11:76(	LINC02741		lncRNA	chr11:41518895-417

ENSG0000(	887	22.18469	chr11:76(	BDNF-AS		lncRNA	chr11:27506830-276
ENSG0000(	887	22.18469	chr11:76(	ALKBH3-AS1		lncRNA	chr11:43909289-439
ENSG0000(	887	22.18469	chr11:76(	LINC01616		lncRNA	chr11:29980113-299
ENSG0000(	887	22.18469	chr11:76(	Y_RNA		smallRNA	chr11:34189174-341
ENSG0000(	887	22.18469	chr11:76(	ENSG00000238149		Pseudoger	chr11:28287235-282
ENSG0000(	887	22.18469	chr11:76(	ENSG00000283341		lncRNA	chr11:43578889-438
ENSG0000(	887	22.18469	chr11:76(	ENSG00000215380		Pseudoger	chr11:35374265-353
ENSG0000(	887	22.18469	chr11:76(	RPL12P31		Pseudoger	chr11:35997226-359
ENSG0000(	887	22.18469	chr11:76(	WT1	NCGv7;AC	protein_c	chr11:32387775-324
ENSG0000(	887	22.18469	chr11:76(	LINC02758		lncRNA	chr11:28679183-286
ENSG0000(	887	22.18469	chr11:76(	AL122015.1		smallRNA	chr11:33354465-333
ENSG0000(	887	22.18469	chr11:76(	RPL29P23		Pseudoger	chr11:33190062-331
ENSG0000(	887	22.18469	chr11:76(	DNAAF11P1		Pseudoger	chr11:38211389-382
ENSG0000(	887	22.18469	chr11:76(	EIF4A2P5		Pseudoger	chr11:32085111-320
ENSG0000(	887	22.18469	chr11:76(	ENSG00000244535		Pseudoger	chr11:32758268-327
ENSG0000(	887	22.18469	chr11:76(	IFTAP		protein_c	chr11:36594369-366
ENSG0000(	887	22.18469	chr11:76(	RAG1	NCGv7	protein_c	chr11:36510372-365
ENSG0000(	887	22.18469	chr11:76(	TRIM44		protein_c	chr11:35662775-358
ENSG0000(	887	22.18469	chr11:76(	ENSG00000254734		lncRNA	chr11:29713909-298
ENSG0000(	887	22.18469	chr11:76(	AC110056.1		smallRNA	chr11:29276689-292
ENSG0000(	887	22.18469	chr11:76(	ENSG00000184566		lncRNA	chr11:33880643-338
ENSG0000(	887	22.18469	chr11:76(	ENSG00000255252		lncRNA	chr11:32097143-321
ENSG0000(	887	22.18469	chr11:76(	ENSG00000283483		Pseudoger	chr11:43733761-437
ENSG0000(	887	22.18469	chr11:76(	ENSG00000283393		Pseudoger	chr11:43718676-437
ENSG0000(	887	22.18469	chr11:76(	ENSG00000254822		lncRNA	chr11:41876634-418
ENSG0000(	887	22.18469	chr11:76(	ENSG00000283375		lncRNA	chr11:43855913-438
ENSG0000(	887	22.18469	chr11:76(	EEF1A1P47		Pseudoger	chr11:29275655-292
ENSG0000(	887	22.18469	chr11:76(	CD44-AS1		lncRNA	chr11:35210343-352
ENSG0000(	887	22.18469	chr11:76(	RCN1		protein_c	chr11:32091074-321
ENSG0000(	887	22.18469	chr11:76(	ENSG00000241255		Pseudoger	chr11:33237008-332
ENSG0000(	887	22.18469	chr11:76(	MIR670HG		lncRNA	chr11:43569306-435
ENSG0000(	887	22.18469	chr11:76(	CAPRIN1		protein_c	chr11:34051731-341
ENSG0000(	887	22.18469	chr11:76(	ENSG00000254409		lncRNA	chr11:43921059-440
ENSG0000(	887	22.18469	chr11:76(	PRRG4		protein_c	chr11:32829927-328
ENSG0000(	887	22.18469	chr11:76(	ENSG00000254836		lncRNA	chr11:32052843-320
ENSG0000(	887	22.18469	chr11:76(	LGR4-AS1		lncRNA	chr11:27471729-274
ENSG0000(	887	22.18469	chr11:76(	ENSG00000279675		TEC	chr11:40107244-401
ENSG0000(	887	22.18469	chr11:76(	ENSG00000255477		lncRNA	chr11:38498995-385
ENSG0000(	887	22.18469	chr11:76(	ELF5	NCGv7	protein_c	chr11:34478791-345
ENSG0000(	887	22.18469	chr11:76(	Y_RNA		smallRNA	chr11:33196547-331
ENSG0000(	887	22.18469	chr11:76(	ENSG00000255480		lncRNA	chr11:30425552-304
ENSG0000(	887	22.18469	chr11:76(	RPL7AP58		Pseudoger	chr11:29725764-297
ENSG0000(	887	22.18469	chr11:76(	ENSG00000255347		Pseudoger	chr11:40083710-400
ENSG0000(	887	22.18469	chr11:76(	CSTF3-DT		lncRNA	chr11:33161657-331
ENSG0000(	887	22.18469	chr11:76(	LINC02721		lncRNA	chr11:33813873-338
ENSG0000(	887	22.18469	chr11:76(	ENSG00000255165		lncRNA	chr11:44071462-440
ENSG0000(	887	22.18469	chr11:76(	ENSG00000255161		Pseudoger	chr11:33787054-337
ENSG0000(	887	22.18469	chr11:76(	DCDC1		protein_c	chr11:30830369-313
ENSG0000(	887	22.18469	chr11:76(	CBX3P1		Pseudoger	chr11:27806443-278
ENSG0000(	887	22.18469	chr11:76(	ENSG00000255272		lncRNA	chr11:33776188-337
ENSG0000(	887	22.18469	chr11:76(	HNRNPA3P9		Pseudoger	chr11:32591793-325

ENSG0000(	887	22.18469	chr11:76(	QSER1		protein_c	chr11:32892811-329
ENSG0000(	887	22.18469	chr11:76(	ENSG00000255132		lncRNA	chr11:41394595-414
ENSG0000(	887	22.18469	chr11:76(	CSTF3		protein_c	chr11:33077188-331
ENSG0000(	887	22.18469	chr11:76(	LINC00294		lncRNA	chr11:33076149-330
ENSG0000(	887	22.18469	chr11:76(	AC108456.1		smallRNA	chr11:31435470-314
ENSG0000(	887	22.18469	chr11:76(	ENSG00000255279		lncRNA	chr11:41855920-418
ENSG0000(	887	22.18469	chr11:76(	LINC02546		lncRNA	chr11:29594326-296
ENSG0000(	887	22.18469	chr11:76(	ENSG00000255281		Pseudoger	chr11:29391525-293
ENSG0000(	887	22.18469	chr11:76(	ENSG00000271028		Pseudoger	chr11:43063637-430
ENSG0000(	887	22.18469	chr11:76(	ENSG00000255288		Pseudoger	chr11:30167163-301
ENSG0000(	887	22.18469	chr11:76(	ENSG00000289526		lncRNA	chr11:35014075-350
ENSG0000(	887	22.18469	chr11:76(	LINC02722		lncRNA	chr11:33804768-338
ENSG0000(	887	22.18469	chr11:76(	LINC01499		lncRNA	chr11:41714534-418
ENSG0000(	887	22.18469	chr11:76(	LINC02759		lncRNA	chr11:38618264-386
ENSG0000(	887	22.18469	chr11:76(	ENSG00000270491		Pseudoger	chr11:34742906-347
ENSG0000(	887	22.18469	chr11:76(	ENSG00000255227		lncRNA	chr11:29445487-294
ENSG0000(	887	22.18469	chr11:76(	HNRNPKP3		Pseudoger	chr11:43120762-432
ENSG0000(	887	22.18469	chr11:76(	BDNF		protein_c	chr11:27654893-277
ENSG0000(	887	22.18469	chr11:76(	ENSG00000255256		lncRNA	chr11:35972428-359
ENSG0000(	887	22.18469	chr11:76(	ENSG00000270588		Pseudoger	chr11:39873782-398
ENSG0000(	887	22.18469	chr11:76(	ENSG00000255207		Pseudoger	chr11:33450646-334
ENSG0000(	887	22.18469	chr11:76(	RNU6-365P		smallRNA	chr11:41122907-411
ENSG0000(	887	22.18469	chr11:76(	U3		smallRNA	chr11:32081764-320
ENSG0000(	887	22.18469	chr11:76(	ENSG00000280321		TEC	chr11:36196248-361
ENSG0000(	887	22.18469	chr11:76(	ENSG00000280331		TEC	chr11:36510361-365
ENSG0000(	887	22.18469	chr11:76(	ENSG00000255202		lncRNA	chr11:33665220-336
ENSG0000(	887	22.18469	chr11:76(	DNAJC24		protein_c	chr11:31369840-314
ENSG0000(	887	22.18469	chr11:76(	ENSG00000255271		lncRNA	chr11:34533014-345
ENSG0000(	887	22.18469	chr11:76(	ENSG00000255186		lncRNA	chr11:36386521-363
ENSG0000(	887	22.18469	chr11:76(	LINC02740		lncRNA	chr11:42183292-422
ENSG0000(	887	22.18469	chr11:76(	FSHB		protein_c	chr11:30231014-302
ENSG0000(	887	22.18469	chr11:76(	ENSG00000255094		lncRNA	chr11:27978669-280
ENSG0000(	887	22.18469	chr11:76(	TTC17		protein_c	chr11:43358920-434
ENSG0000(	887	22.18469	chr11:76(	AC090692.1		smallRNA	chr11:35860654-358
ENSG0000(	887	22.18469	chr11:76(	C11orf91		protein_c	chr11:33698261-337
ENSG0000(	887	22.18469	chr11:76(	AC104387.1		smallRNA	chr11:40759522-407
ENSG0000(	887	22.18469	chr11:76(	RNU6-99P		smallRNA	chr11:39261107-392
ENSG0000(	887	22.18469	chr12:12(	ENSG00000257905		Pseudoger	chr12:47593208-475
ENSG0000(	887	22.18469	chr11:76(	C11orf96		protein_c	chr11:43942637-439
ENSG0000(	887	22.18469	chr11:76(	ENSG00000255340		lncRNA	chr11:43378882-433
ENSG0000(	887	22.18469	chr11:76(	LINC02742		lncRNA	chr11:28702607-290
ENSG0000(	887	22.18469	chr11:76(	ENSG00000240036		Pseudoger	chr11:27581680-275
ENSG0000(	887	22.18469	chr11:76(	AL133376.1		smallRNA	chr11:31173924-311
ENSG0000(	887	22.18469	chr11:76(	ENSG00000255029		lncRNA	chr11:29482807-295
ENSG0000(	887	22.18469	chr11:76(	ENSG00000255496		lncRNA	chr11:27696312-278
ENSG0000(	887	22.18469	chr11:76(	ACCSL		protein_c	chr11:44047981-440
ENSG0000(	887	22.18469	chr11:76(	ATP5MGP8		Pseudoger	chr11:28144907-281
ENSG0000(	887	22.18469	chr11:76(	MPPED2	NCGv7	protein_c	chr11:30384493-305
ENSG0000(	887	22.18469	chr11:76(	LGR4		protein_c	chr11:27365961-274
ENSG0000(	887	22.18469	chr11:76(	ALX4	NCGv7	protein_c	chr11:44260440-443
ENSG0000(	887	22.18469	chr11:76(	ENSG00000220204		Pseudoger	chr11:34404663-344

ENSG0000(	887	22.18469	chr11:76(	FJX1		protein_c	chr11:35618460-356
ENSG0000(	887	22.18469	chr11:76(	AC090720.1		smallRNA	chr11:40614102-406
ENSG0000(	887	22.18469	chr11:76(	LINC02745		lncRNA	chr11:41993381-421
ENSG0000(	887	22.18469	chr11:76(	HSP90AA2P		Pseudoger	chr11:27888838-278
ENSG0000(	887	22.18469	chr11:76(	ENSG00000287373		lncRNA	chr11:30773913-308
ENSG0000(	887	22.18469	chr11:76(	ENSG00000255060		lncRNA	chr11:36425447-364
ENSG0000(	887	22.18469	chr11:76(	ENSG00000279004		TEC	chr11:38013510-380
ENSG0000(	887	22.18469	chr11:76(	TCP11L1		protein_c	chr11:33039417-331
ENSG0000(	887	22.18469	chr11:76(	PAUPAR		lncRNA	chr11:31812307-320
ENSG0000(	887	22.18469	chr11:76(	LDLRAD3		protein_c	chr11:35943981-362
ENSG0000(	887	22.18469	chr11:76(	ENSG00000286626		lncRNA	chr11:34049786-340
ENSG0000(	887	22.18469	chr11:76(	HNRNPRP2		Pseudoger	chr11:29668886-296
ENSG0000(	887	22.18469	chr11:76(	SNORA31		smallRNA	chr11:37702125-377
ENSG0000(	887	22.18469	chr11:76(	ENSG00000251194		lncRNA	chr11:35212550-352
ENSG0000(	887	22.18469	chr11:76(	ENSG00000228061		lncRNA	chr11:31618124-317
ENSG0000(	887	22.18469	chr11:76(	MIR1343		smallRNA	chr11:34941837-349
ENSG0000(	887	22.18469	chr11:76(	MMADHCP2		Pseudoger	chr11:34335118-343
ENSG0000(	887	22.18469	chr11:76(	LINC02707		lncRNA	chr11:34570876-345
ENSG0000(	887	22.18469	chr11:76(	ARL14EP		protein_c	chr11:30323104-303
ENSG0000(	887	22.18469	chr11:76(	MIR670		smallRNA	chr11:43559656-435
ENSG0000(	887	22.18469	chr11:76(	ACCS		protein_c	chr11:44065925-440
ENSG0000(	887	22.18469	chr11:76(	ENSG00000254668		Pseudoger	chr11:41797543-417
ENSG0000(	887	22.18469	chr11:76(	ENSG00000254669		lncRNA	chr11:35579430-355
ENSG0000(	887	22.18469	chr11:76(	WEE2P1		Pseudoger	chr11:35967010-359
ENSG0000(	887	22.18469	chr11:76(	ARL14EP-DT		lncRNA	chr11:30044053-303
ENSG0000(	887	22.18469	chr11:76(	ELP4		protein_c	chr11:31509755-317
ENSG0000(	887	22.18469	chr11:76(	PAMR1		protein_c	chr11:35431823-355
ENSG0000(	887	22.18469	chr11:76(	LINC02755		lncRNA	chr11:29335878-299
ENSG0000(	887	22.18469	chr11:76(	METTL15		protein_c	chr11:28108248-285
ENSG0000(	887	22.18469	chr11:76(	CCDC34		protein_c	chr11:27330827-273
ENSG0000(	887	22.18469	chr11:76(	ENSG00000285283		protein_c	chr11:31812391-321
ENSG0000(	887	22.18469	chr11:76(	ENSG00000254526		lncRNA	chr11:29159956-292
ENSG0000(	887	22.18469	chr11:76(	EIF3M		protein_c	chr11:32583798-326
ENSG0000(	887	22.18469	chr11:76(	WT1-AS		lncRNA	chr11:32435518-325
ENSG0000(	887	22.18469	chr11:76(	API5	NCGv7	protein_c	chr11:43311963-433
ENSG0000(	887	22.18469	chr11:76(	PIGCP1		Pseudoger	chr11:33075566-330
ENSG0000(	887	22.18469	chr11:76(	ALKBH3		protein_c	chr11:43880811-439
ENSG0000(	887	22.18469	chr11:76(	RPL7AP56		Pseudoger	chr11:37725666-377
ENSG0000(	887	22.18469	chr11:76(	ENSG00000255563		Pseudoger	chr11:41660030-416
ENSG0000(	887	22.18469	chr11:76(	EXT2	NCGv7;AC	protein_c	chr11:44095648-442
ENSG0000(	887	22.18469	chr11:76(	LINC02760		lncRNA	chr11:37938601-379
ENSG0000(	887	22.18469	chr11:76(	CD59		protein_c	chr11:33703010-337
ENSG0000(	887	22.18469	chr11:76(	ENSG00000255542		lncRNA	chr11:35419057-354
ENSG0000(	887	22.18469	chr11:76(	ENSG00000242729		Pseudoger	chr11:41538434-415
ENSG0000(	887	22.18469	chr11:76(	AC021749.1		smallRNA	chr11:39861506-398
ENSG0000(	887	22.18469	chr11:76(	PHB1P2		Pseudoger	chr11:43733759-437
ENSG0000(	887	22.18469	chr11:76(	FBXO3-DT		lncRNA	chr11:33774699-337
ENSG0000(	887	22.18469	chr11:76(	PAX6	NCGv7	protein_c	chr11:31784779-318
ENSG0000(	887	22.18469	chr11:76(	ENSG00000254686		lncRNA	chr11:35656694-356
ENSG0000(	887	22.18469	chr11:76(	RN7SKP287		smallRNA	chr11:43435132-434
ENSG0000(	887	22.18469	chr11:76(	AL162614.1		smallRNA	chr11:31205759-312

ENSG0000(	887	22.18469	chr11:76(	OR2BH1P		Pseudoger	chr11:28986635-289
ENSG0000(	887	22.18469	chr11:76(	CAT		protein_c	chr11:34438934-344
ENSG0000(	887	22.18469	chr11:76(	DEPDC7		protein_c	chr11:33015876-330
ENSG0000(	887	22.18469	chr11:76(	ABTB2		protein_c	chr11:34150987-343
ENSG0000(	887	22.18469	chr11:76(	LINC01493		lncRNA	chr11:38646451-386
ENSG0000(	887	22.18469	chr11:76(	KIAA1549L		protein_c	chr11:33376108-336
ENSG0000(	887	22.18469	chr11:76(	ENSG00000254577		lncRNA	chr11:43390283-433
ENSG0000(	887	22.18469	chr11:76(	HSD17B12		protein_c	chr11:43680680-438
ENSG0000(	887	22.18469	chr11:76(	ENSG00000254579		Pseudoger	chr11:40444371-405
ENSG0000(	887	22.18469	chr11:76(	APIP		protein_c	chr11:34853094-349
ENSG0000(	887	22.18469	chr11:76(	IMMP1L		protein_c	chr11:31432401-315
ENSG0000(	887	22.18469	chr11:76(	RPL23AP63		Pseudoger	chr11:43718676-437
ENSG0000(	887	22.18469	chr11:76(	LINC03031		lncRNA	chr11:32035979-320
ENSG0000(	887	22.18469	chr11:76(	LRRC4C	NCGv7	protein_c	chr11:40114203-414
ENSG0000(	887	22.18469	chr11:76(	HIPK3		protein_c	chr11:33256672-333
ENSG0000(	887	22.18469	chr11:76(	LIN7C		protein_c	chr11:27494418-275
ENSG0000(	887	22.18469	chr11:76(	CTBP2P6		Pseudoger	chr11:43522036-435
ENSG0000(	887	22.18469	chr11:76(	KCNA4	NCGv7	protein_c	chr11:30009730-300
ENSG0000(	887	22.18469	chr11:76(	FBXO3		protein_c	chr11:33740939-337
ENSG0000(	887	22.18469	chr11:76(	RN7SL240P		smallRNA	chr11:29721399-297
ENSG0000(	887	22.18469	chr11:76(	RAG2	NCGv7	protein_c	chr11:36575574-365
ENSG0000(	887	22.18469	chr11:76(	PDHX		protein_c	chr11:34915829-350
ENSG0000(	887	22.18469	chr11:76(	TRAF6	NCGv7;AC	protein_c	chr11:36483769-365
ENSG0000(	887	22.18469	chr11:76(	ENSG00000254606		lncRNA	chr11:28516832-285
ENSG0000(	887	22.18469	chr11:76(	ENSG00000254619		lncRNA	chr11:33810145-338
ENSG0000(	887	22.18469	chr11:76(	SLC1A2		protein_c	chr11:35251205-354
ENSG0000(	887	22.18469	chr11:76(	ENSG00000254537		Pseudoger	chr11:33403216-334
ENSG0000(	887	22.18469	chr11:76(	COMMD9		protein_c	chr11:36269284-362
ENSG0000(	887	22.18469	chr11:76(	ENSG00000254627		lncRNA	chr11:32064912-320
ENSG0000(	887	22.18469	chr11:76(	ENSG00000284969		protein_c	chr11:33698261-337
ENSG0000(	887	22.18469	chr11:76(	KIF18A		protein_c	chr11:28020619-281
ENSG0000(	887	22.18469	chr11:76(	SEC14L1P1		Pseudoger	chr11:43897456-438
ENSG0000(	887	22.18469	chr11:76(	ENSG00000254498		Pseudoger	chr11:36696317-366
ENSG0000(	887	22.18469	chr11:76(	ENSG00000254725		Pseudoger	chr11:43065686-430
ENSG0000(	887	22.18469	chr11:76(	PRR5L		protein_c	chr11:36296288-364
ENSG0000(	887	22.18469	chr11:76(	CD44		protein_c	chr11:35138882-352
ENSG0000(	887	22.18469	chr11:76(	ENSG00000285705		lncRNA	chr11:35824192-358
ENSG0000(	887	22.18469	chr11:76(	ENSG00000255525		lncRNA	chr11:31305685-313
ENSG0000(	887	22.18469	chr11:76(	EHF		protein_c	chr11:34621093-346
ENSG0000(	887	22.18469	chr11:76(	CD44-DT		lncRNA	chr11:35132655-351
ENSG0000(	887	22.18469	chr11:76(	Y_RNA		smallRNA	chr11:43331261-433
ENSG0000(	887	22.18469	chr11:76(	PPIAP41		Pseudoger	chr11:43466392-434
ENSG0000(	887	22.18469	chr11:76(	ENSG00000285740		lncRNA	chr11:35894987-358
ENSG0000(	887	22.18469	chr11:76(	AC027806.1		smallRNA	chr11:39730809-397
ENSG0000(	887	22.18469	chr11:76(	MPPED2-AS1		lncRNA	chr11:30584112-306
ENSG0000(	887	22.18469	chr11:76(	ENSG00000285751		lncRNA	chr11:39024631-391
ENSG0000(	887	22.18469	chr11:76(	LMO2	NCGv7;AC	protein_c	chr11:33858576-338
ENSG0000(	887	22.18469	chr11:76(	ENSG00000242527		Pseudoger	chr11:27483850-274
ENSG0000(	887	22.18469	chr11:76(	RPL12P30		Pseudoger	chr11:30368148-303
ENSG0000(	887	22.18469	chr11:76(	NAT10	NCGv7	protein_c	chr11:34105617-341
ENSG0000(	887	22.18469	chr11:76(	RNA5SP339		Pseudoger	chr11:27521605-275

ENSG0000(	879	21.98461	chr1:116(	SNORD38B		smallRNA	chr1:44778390-4477
ENSG0000(	871	21.78452	chr1:152(	AL591415.1		smallRNA	chr1:47621093-4762
ENSG0000(	871	21.78452	chr1:152(	TRABD2B		protein_c	chr1:47760528-4799
ENSG0000(	871	21.78452	chr1:152(	LINC01738		lncRNA	chr1:47688463-4770
ENSG0000(	871	21.78452	chr1:152(	FLJ00388		protein_c	chr1:47761307-4776
ENSG0000(	871	21.78452	chr1:152(	ENSG00000225028		lncRNA	chr1:47818066-4782
ENSG0000(	871	21.78452	chr1:152(	ENSG00000223814		lncRNA	chr1:47761132-4776
ENSG0000(	867	21.68448	chr1:116(	ENSG00000281133		Pseudoger	chr1:45580892-4558
ENSG0000(	864	21.60944	chr11:13(	ENSG00000254989		lncRNA	chr11:134671426-13
ENSG0000(	859	21.48439	chr1:116(	POMGNT1		protein_c	chr1:46188683-4622
ENSG0000(	859	21.48439	chr1:116(	ENSG00000280836		Pseudoger	chr1:45581219-4558
ENSG0000(	859	21.48439	chr1:116(	TSPAN1	AC	protein_c	chr1:46175073-4618
ENSG0000(	859	21.48439	chr1:116(	FOXO6		protein_c	chr1:41361922-4138
ENSG0000(	859	21.48439	chr1:116(	PLK3		protein_c	chr1:44800377-4480
ENSG0000(	859	21.48439	chr1:116(	ENSG00000233114		Pseudoger	chr1:46104950-4610
ENSG0000(	859	21.48439	chr1:116(	RIMS3		protein_c	chr1:40620680-4066
ENSG0000(	859	21.48439	chr1:116(	RNA5SP45		Pseudoger	chr1:41466937-4146
ENSG0000(	859	21.48439	chr1:116(	NFYC		protein_c	chr1:40691648-4077
ENSG0000(	859	21.48439	chr1:116(	DYNLT4		protein_c	chr1:44805893-4480
ENSG0000(	859	21.48439	chr1:116(	ENSG00000284895		protein_c	chr1:41585306-4162
ENSG0000(	859	21.48439	chr1:116(	P3R3URF		protein_c	chr1:46175486-4617
ENSG0000(	859	21.48439	chr1:116(	ENSG00000228940		Pseudoger	chr1:40938104-4093
ENSG0000(	859	21.48439	chr1:116(	ARMH1		protein_c	chr1:44674692-4472
ENSG0000(	859	21.48439	chr1:116(	ENSG00000290041		lncRNA	chr1:44807524-4480
ENSG0000(	859	21.48439	chr1:116(	MAST2		protein_c	chr1:45786987-4603
ENSG0000(	859	21.48439	chr1:116(	UROD		protein_c	chr1:45010950-4501
ENSG0000(	859	21.48439	chr1:116(	ENSG00000226957		lncRNA	chr1:46046818-4604
ENSG0000(	859	21.48439	chr1:116(	ENSG00000287743		lncRNA	chr1:40659848-4066
ENSG0000(	859	21.48439	chr1:116(	TESK2		protein_c	chr1:45343883-4549
ENSG0000(	859	21.48439	chr1:116(	RPS8		protein_c	chr1:44775251-4477
ENSG0000(	859	21.48439	chr1:116(	PPIAP35		Pseudoger	chr1:44988234-4498
ENSG0000(	859	21.48439	chr1:116(	SLFNL1-AS1		lncRNA	chr1:41014590-4104
ENSG0000(	859	21.48439	chr1:116(	ENSG00000291157		lncRNA	chr1:41302911-4130
ENSG0000(	859	21.48439	chr1:116(	ENSG00000286668		lncRNA	chr1:40939294-4094
ENSG0000(	859	21.48439	chr1:116(	PIK3R3	NCGv7	protein_c	chr1:46040140-4613
ENSG0000(	859	21.48439	chr1:116(	RNU5D-1		smallRNA	chr1:44731055-4473
ENSG0000(	859	21.48439	chr1:116(	PRDX1		protein_c	chr1:45510914-4554
ENSG0000(	859	21.48439	chr1:116(	AKR1A1		protein_c	chr1:45550543-4557
ENSG0000(	859	21.48439	chr1:116(	SLFNL1		protein_c	chr1:41015589-4102
ENSG0000(	859	21.48439	chr1:116(	ENSG00000281825		Pseudoger	chr1:45605657-4560
ENSG0000(	859	21.48439	chr1:116(	PTCH2	AC	protein_c	chr1:44819844-4484
ENSG0000(	859	21.48439	chr1:116(	SCMH1-DT		lncRNA	chr1:41242373-4128
ENSG0000(	859	21.48439	chr1:116(	CTPS1		protein_c	chr1:40979300-4101
ENSG0000(	859	21.48439	chr1:116(	ENSG00000286640		Pseudoger	chr1:41302938-4130
ENSG0000(	859	21.48439	chr1:116(	LINC01144		lncRNA	chr1:45303910-4530
ENSG0000(	859	21.48439	chr1:116(	AL592294.1		smallRNA	chr1:45232218-4523
ENSG0000(	859	21.48439	chr1:116(	ENSG00000230638		Pseudoger	chr1:41542069-4154
ENSG0000(	859	21.48439	chr1:116(	EIF2B3		protein_c	chr1:44850522-4498
ENSG0000(	859	21.48439	chr1:116(	KCNQ4	DriverDB\	protein_c	chr1:40783787-4084
ENSG0000(	859	21.48439	chr1:116(	CCNB1IP1P1		Pseudoger	chr1:44958557-4495
ENSG0000(	859	21.48439	chr1:116(	ENSG00000227857		lncRNA	chr1:46134531-4613

ENSG0000(	859	21.48439	chr1:116(	LURAP1		protein_c	chr1:46203334-4622
ENSG0000(	859	21.48439	chr1:116(	ENSG00000281112		Pseudoger	chr1:45592722-4559
ENSG0000(	859	21.48439	chr1:116(	ZSWIM5		protein_c	chr1:45016399-4530
ENSG0000(	859	21.48439	chr1:116(	BEST4		protein_c	chr1:44783585-4478
ENSG0000(	859	21.48439	chr1:116(	MIR30E		smallRNA	chr1:40754355-4075
ENSG0000(	859	21.48439	chr1:116(	ENSG00000287400		lncRNA	chr1:41241772-4133
ENSG0000(	859	21.48439	chr1:116(	UBE2V1P8		Pseudoger	chr1:40942251-4094
ENSG0000(	859	21.48439	chr1:116(	KIF2C		protein_c	chr1:44739818-4476
ENSG0000(	859	21.48439	chr1:116(	CCDC163		protein_c	chr1:45493866-4550
ENSG0000(	859	21.48439	chr1:116(	HMGB1P48		Pseudoger	chr1:45530927-4553
ENSG0000(	859	21.48439	chr1:116(	RNA5SP47		Pseudoger	chr1:44932323-4493
ENSG0000(	859	21.48439	chr1:116(	NFYC-AS1		lncRNA	chr1:40690380-4069
ENSG0000(	859	21.48439	chr1:116(	PPIAP36		Pseudoger	chr1:45415020-4541
ENSG0000(	859	21.48439	chr1:116(	RPL36AP9		Pseudoger	chr1:41264550-4126
ENSG0000(	859	21.48439	chr1:116(	AL359473.1		smallRNA	chr1:45033969-4503
ENSG0000(	859	21.48439	chr1:116(	TMA16P2		Pseudoger	chr1:45846994-4584
ENSG0000(	859	21.48439	chr1:116(	ENSG00000230896		lncRNA	chr1:45694684-4569
ENSG0000(	859	21.48439	chr1:116(	MUTYH	NCGv7;AC	protein_c	chr1:45329163-4534
ENSG0000(	859	21.48439	chr1:116(	SNORD46		smallRNA	chr1:44776490-4477
ENSG0000(	859	21.48439	chr1:116(	RPS15AP11		Pseudoger	chr1:44780331-4478
ENSG0000(	859	21.48439	chr1:116(	RPL23AP17		Pseudoger	chr1:41098638-4109
ENSG0000(	859	21.48439	chr1:116(	FOXO6-AS1		lncRNA	chr1:41375004-4137
ENSG0000(	859	21.48439	chr1:116(	EDN2		protein_c	chr1:41478775-4148
ENSG0000(	859	21.48439	chr1:116(	HIVEP3		protein_c	chr1:41506365-4203
ENSG0000(	859	21.48439	chr1:116(	ENSG00000226499		Pseudoger	chr1:44843921-4484
ENSG0000(	859	21.48439	chr1:116(	NASP		protein_c	chr1:45583846-4561
ENSG0000(	859	21.48439	chr1:116(	RPS15AP10		Pseudoger	chr1:45645816-4564
ENSG0000(	859	21.48439	chr1:116(	MMACHC	NCGv7	protein_c	chr1:45500300-4551
ENSG0000(	859	21.48439	chr1:116(	MIR30C1		smallRNA	chr1:40757284-4075
ENSG0000(	859	21.48439	chr1:116(	snoU13		smallRNA	chr1:45358652-4535
ENSG0000(	859	21.48439	chr1:116(	TOE1		protein_c	chr1:45340052-4534
ENSG0000(	859	21.48439	chr1:116(	AL136380.1		smallRNA	chr1:44913068-4491
ENSG0000(	859	21.48439	chr1:116(	MRPS17P1		Pseudoger	chr1:44988705-4499
ENSG0000(	859	21.48439	chr1:116(	ENSG00000230881		lncRNA	chr1:41535443-4153
ENSG0000(	859	21.48439	chr1:116(	HECTD3		protein_c	chr1:45002540-4501
ENSG0000(	859	21.48439	chr1:116(	P3R3URF-PIK3R3		protein_c	chr1:46043661-4617
ENSG0000(	859	21.48439	chr1:116(	ENSG00000229528		lncRNA	chr1:40863914-4087
ENSG0000(	859	21.48439	chr1:116(	CITED4		protein_c	chr1:40861054-4086
ENSG0000(	859	21.48439	chr1:116(	HPDL		protein_c	chr1:45326895-4532
ENSG0000(	859	21.48439	chr1:116(	IPP		protein_c	chr1:45694324-4575
ENSG0000(	859	21.48439	chr1:116(	SCMH1		protein_c	chr1:41027202-4124
ENSG0000(	859	21.48439	chr1:116(	ENSG00000289407		lncRNA	chr1:45583238-4558
ENSG0000(	859	21.48439	chr1:116(	ENSG00000237899		lncRNA	chr1:40669089-4068
ENSG0000(	859	21.48439	chr1:116(	RPL7AP16		Pseudoger	chr1:45651039-4565
ENSG0000(	859	21.48439	chr1:116(	RPL6P1		Pseudoger	chr1:45781277-4578
ENSG0000(	859	21.48439	chr1:116(	OSTCP5		Pseudoger	chr1:45069977-4507
ENSG0000(	859	21.48439	chr1:116(	BTBD19		protein_c	chr1:44808523-4481
ENSG0000(	859	21.48439	chr1:116(	TMEM69		protein_c	chr1:45688181-4569
ENSG0000(	859	21.48439	chr1:116(	RNU5F-1		smallRNA	chr1:44721786-4472
ENSG0000(	859	21.48439	chr1:116(	ENSG00000225721		lncRNA	chr1:44759037-4477
ENSG0000(	859	21.48439	chr1:116(	CCDC17		protein_c	chr1:45620044-4562

ENSG0000(	859	21.48439	chr1:116(	SNORD38A		smallRNA	chr1:44777843-4477
ENSG0000(	859	21.48439	chr1:116(	GPBP1L1		protein_c	chr1:45627304-4568
ENSG0000(	859	21.48439	chr1:116(	ENSG00000288208		protein_c	chr1:45329262-4549
ENSG0000(	852	21.30931	chr14:987	MIR2392		smallRNA	chr14:100814491-10
ENSG0000(	846	21.15925	chr7:330(	Y_RNA		smallRNA	chr7:140609847-140
ENSG0000(	846	21.15925	chr7:330(	SSBP1	NCGv7	protein_c	chr7:141738334-141
ENSG0000(	846	21.15925	chr7:330(	ENSG00000290670		lncRNA	chr7:141911217-141
ENSG0000(	846	21.15925	chr7:330(	TRBV2		protein_c	chr7:142300924-142
ENSG0000(	846	21.15925	chr7:330(	ENSG00000270512		Pseudoger	chr7:140282465-140
ENSG0000(	846	21.15925	chr7:330(	ENSG00000290669		lncRNA	chr7:141863104-141
ENSG0000(	846	21.15925	chr7:330(	ENSG00000261797		lncRNA	chr7:141512698-141
ENSG0000(	846	21.15925	chr7:330(	TRBVB		Pseudoger	chr7:142711384-142
ENSG0000(	846	21.15925	chr7:330(	snoU13		smallRNA	chr7:140375563-140
ENSG0000(	846	21.15925	chr7:330(	ENSG00000285841		lncRNA	chr7:141392155-141
ENSG0000(	846	21.15925	chr7:330(	RAB19		protein_c	chr7:140404058-140
ENSG0000(	846	21.15925	chr7:330(	ENSG00000286831		lncRNA	chr7:142725729-142
ENSG0000(	846	21.15925	chr7:330(	ENSG00000204990		lncRNA	chr7:141414383-141
ENSG0000(	846	21.15925	chr7:330(	ENSG00000288882		lncRNA	chr7:142716831-142
ENSG0000(	846	21.15925	chr7:330(	TAS2R6P		Pseudoger	chr7:141787815-141
ENSG0000(	846	21.15925	chr7:330(	TRBV21-1		Pseudoger	chr7:142636924-142
ENSG0000(	846	21.15925	chr7:330(	AC073647.1		smallRNA	chr7:141859818-141
ENSG0000(	846	21.15925	chr7:330(	PARP12		protein_c	chr7:140023749-140
ENSG0000(	846	21.15925	chr7:330(	TBXAS1		protein_c	chr7:139777051-140
ENSG0000(	846	21.15925	chr7:330(	RNU1-82P		smallRNA	chr7:141727984-141
ENSG0000(	846	21.15925	chr7:330(	RNU6-797P		smallRNA	chr7:140209563-140
ENSG0000(	846	21.15925	chr7:330(	MKRN1	NCGv7	protein_c	chr7:140453033-140
ENSG0000(	846	21.15925	chr7:330(	WEE2	DriverDBv	protein_c	chr7:141708353-141
ENSG0000(	846	21.15925	chr7:330(	ENSG00000261778		Pseudoger	chr7:141173043-141
ENSG0000(	846	21.15925	chr7:330(	MTRNR2L6		protein_c	chr7:142666272-142
ENSG0000(	846	21.15925	chr7:330(	WEE2-AS1		lncRNA	chr7:141704003-141
ENSG0000(	846	21.15925	chr7:330(	ENSG00000285904		lncRNA	chr7:140640909-140
ENSG0000(	846	21.15925	chr7:330(	MTCO1P55		Pseudoger	chr7:141801315-141
ENSG0000(	846	21.15925	chr7:330(	CLEC5A		protein_c	chr7:141927357-141
ENSG0000(	846	21.15925	chr7:330(	PRSS58		protein_c	chr7:142252143-142
ENSG0000(	846	21.15925	chr7:330(	RNU4-74P		smallRNA	chr7:141052249-141
ENSG0000(	846	21.15925	chr7:330(	BRAF	NCGv7;AC	protein_c	chr7:140719327-140
ENSG0000(	846	21.15925	chr7:330(	DENND2A		protein_c	chr7:140518420-140
ENSG0000(	846	21.15925	chr7:330(	SLC37A3		protein_c	chr7:140293693-140
ENSG0000(	846	21.15925	chr7:330(	OR9A4		protein_c	chr7:141916399-141
ENSG0000(	846	21.15925	chr7:330(	KDM7A		protein_c	chr7:140084746-140
ENSG0000(	846	21.15925	chr7:330(	ENSG00000290605		lncRNA	chr7:142240774-142
ENSG0000(	846	21.15925	chr7:330(	MYL6P4		Pseudoger	chr7:141811805-141
ENSG0000(	846	21.15925	chr7:330(	TRBV29-1		protein_c	chr7:142740206-142
ENSG0000(	846	21.15925	chr7:330(	RNA5SP247		Pseudoger	chr7:140370441-140
ENSG0000(	846	21.15925	chr7:330(	TRBV6-1		protein_c	chr7:142328297-142
ENSG0000(	846	21.15925	chr7:330(	TRBV25-1		protein_c	chr7:142670740-142
ENSG0000(	846	21.15925	chr7:330(	OR9A1P		protein_c	chr7:141887148-141
ENSG0000(	846	21.15925	chr7:330(	TRBV22-1		Pseudoger	chr7:142641746-142
ENSG0000(	846	21.15925	chr7:330(	ENSG00000103200		Pseudoger	chr7:140435316-140
ENSG0000(	846	21.15925	chr7:330(	MGAM	NCGv7	protein_c	chr7:141907813-142
ENSG0000(	846	21.15925	chr7:330(	TRBV28		protein_c	chr7:142720660-142

ENSG0000(	846	21.15925	chr7:330(	CCT4P1		Pseudoger	chr7:140997952-140
ENSG0000(	846	21.15925	chr7:330(	TRBV27		protein_c	chr7:142715346-142
ENSG0000(	846	21.15925	chr7:330(	TRBV7-1		protein_c	chr7:142332182-142
ENSG0000(	846	21.15925	chr7:330(	Y_RNA		smallRNA	chr7:140094697-140
ENSG0000(	846	21.15925	chr7:330(	TRBV24-1		protein_c	chr7:142656701-142
ENSG0000(	846	21.15925	chr7:330(	TRBV23-1		protein_c	chr7:142645961-142
ENSG0000(	846	21.15925	chr7:330(	MGAM2		protein_c	chr7:142111718-142
ENSG0000(	846	21.15925	chr7:330(	TRBV1		Pseudoger	chr7:142299177-142
ENSG0000(	846	21.15925	chr7:330(	PRSS59P		Pseudoger	chr7:142265833-142
ENSG0000(	846	21.15925	chr7:330(	TRBV20-1		protein_c	chr7:142626649-142
ENSG0000(	846	21.15925	chr7:330(	TRBV19		protein_c	chr7:142618849-142
ENSG0000(	846	21.15925	chr7:330(	TRBV4-2		protein_c	chr7:142345421-142
ENSG0000(	846	21.15925	chr7:330(	NDUFB2-AS1		lncRNA	chr7:140695336-140
ENSG0000(	846	21.15925	chr7:330(	PRSS37		protein_c	chr7:141836300-141
ENSG0000(	846	21.15925	chr7:330(	TRBV4-1		protein_c	chr7:142313184-142
ENSG0000(	846	21.15925	chr7:330(	NDUFB2	NCGv7	protein_c	chr7:140690777-140
ENSG0000(	846	21.15925	chr7:330(	TAS2R3		protein_c	chr7:141764097-141
ENSG0000(	846	21.15925	chr7:330(	TAS2R5	DriverDBv	protein_c	chr7:141790217-141
ENSG0000(	846	21.15925	chr7:330(	MTND2P5		Pseudoger	chr7:141802482-141
ENSG0000(	846	21.15925	chr7:330(	TAS2R4	DriverDBv	protein_c	chr7:141776674-141
ENSG0000(	846	21.15925	chr7:330(	MRPS33	NCGv7	protein_c	chr7:141002610-141
ENSG0000(	846	21.15925	chr7:330(	TRBV5-1		protein_c	chr7:142320677-142
ENSG0000(	846	21.15925	chr7:330(	RNU1-58P		smallRNA	chr7:140241870-140
ENSG0000(	846	21.15925	chr7:330(	TRBD1		protein_c	chr7:142786213-142
ENSG0000(	846	21.15925	chr7:330(	MOXD2P		Pseudoger	chr7:142240740-142
ENSG0000(	846	21.15925	chr7:330(	MTND1P3		Pseudoger	chr7:141803701-141
ENSG0000(	846	21.15925	chr7:330(	ENSG00000270157		lncRNA	chr7:141662922-141
ENSG0000(	846	21.15925	chr7:330(	RNA5SP248		Pseudoger	chr7:140386781-140
ENSG0000(	846	21.15925	chr7:330(	DENND11		protein_c	chr7:141656728-141
ENSG0000(	846	21.15925	chr7:330(	ENSG00000241881		lncRNA	chr7:142285750-142
ENSG0000(	846	21.15925	chr7:330(	PRSS3P1		Pseudoger	chr7:142760415-142
ENSG0000(	846	21.15925	chr7:330(	ENSG00000261629		lncRNA	chr7:141429711-141
ENSG0000(	846	21.15925	chr7:330(	AGK		protein_c	chr7:141551278-141
ENSG0000(	846	21.15925	chr7:330(	TAS2R38		protein_c	chr7:141972631-141
ENSG0000(	846	21.15925	chr7:330(	U6		smallRNA	chr7:140884072-140
ENSG0000(	846	21.15925	chr7:330(	NDUFB10P2		Pseudoger	chr7:141351977-141
ENSG0000(	846	21.15925	chr7:330(	PPP1R2P6		Pseudoger	chr7:140292752-140
ENSG0000(	846	21.15925	chr7:330(	TMEM178B		protein_c	chr7:141074064-141
ENSG0000(	846	21.15925	chr7:330(	KDM7A-DT		lncRNA	chr7:140177184-140
ENSG0000(	846	21.15925	chr7:330(	AGK-DT		lncRNA	chr7:141500079-141
ENSG0000(	846	21.15925	chr7:330(	PGBD4P1		Pseudoger	chr7:142722358-142
ENSG0000(	846	21.15925	chr7:330(	PRSS1	NCGv7	protein_c	chr7:142749468-142
ENSG0000(	846	21.15925	chr7:330(	PRSS3P3		Pseudoger	chr7:142287251-142
ENSG0000(	846	21.15925	chr7:330(	RN7SL771P		smallRNA	chr7:140645844-140
ENSG0000(	846	21.15925	chr7:330(	OR9A3P		Pseudoger	chr7:141862860-141
ENSG0000(	846	21.15925	chr7:330(	ENSG00000289788		lncRNA	chr7:140767530-140
ENSG0000(	846	21.15925	chr7:330(	TRBV26		Pseudoger	chr7:142695699-142
ENSG0000(	846	21.15925	chr7:330(	TRBVA		Pseudoger	chr7:142681415-142
ENSG0000(	846	21.15925	chr7:330(	TRBV3-1		protein_c	chr7:142308542-142
ENSG0000(	846	21.15925	chr7:330(	OR9N1P		Pseudoger	chr7:141911463-141
ENSG0000(	846	21.15925	chr7:330(	ENSG00000271611		Pseudoger	chr7:140934867-140

ENSG0000(	846	21.15925	chr7:330(	ENSG00000244701		lncRNA	chr7:141652381-141
ENSG0000(	846	21.15925	chr7:330(	ADCK2		protein_c	chr7:140672945-140
ENSG0000(	846	21.15925	chr7:330(	AC006452.1		smallRNA	chr7:140580287-140
ENSG0000(	840	21.00918	chr6:105(	ENSG00000270666		Pseudoger	chr6:27515039-2751
ENSG0000(	839	20.98417	chr1:8137	LINC02798		lncRNA	chr1:121396754-121
ENSG0000(	839	20.98417	chr1:8137	ENSG00000272583		lncRNA	chr1:121518365-121
ENSG0000(	839	20.98417	chr1:8137	EMBP1		Pseudoger	chr1:121519345-121
ENSG0000(	839	20.98417	chr1:8137	SRGAP2-AS1		lncRNA	chr1:121360156-121
ENSG0000(	839	20.98417	chr1:8137	LINC01691		lncRNA	chr1:121573946-121
ENSG0000(	839	20.98417	chr1:8137	RP11-343N15.5		lncRNA	chr1:121391395-121
ENSG0000(	839	20.98417	chr1:8137	ENSG00000228826		lncRNA	chr1:121494329-121
ENSG0000(	839	20.98417	chr1:8137	EMBP1		lncRNA	chr1:121519103-121
ENSG0000(	839	20.98417	chr1:8137	SRGAP2C		Pseudoger	chr1:121365263-121
ENSG0000(	839	20.98417	chr1:8137	MTIF2P1		Pseudoger	chr1:121502344-121
ENSG0000(	839	20.98417	chr1:8137	RNVU1-5		smallRNA	chr1:120942599-120
ENSG0000(	834	20.85911	chr7:330(	AC004889.1		Pseudoger	chr7:144183455-144
ENSG0000(	834	20.85911	chr7:330(	TCAF1	NCGv7	protein_c	chr7:143851375-143
ENSG0000(	834	20.85911	chr7:330(	ARHGEF35		protein_c	chr7:144186083-144
ENSG0000(	834	20.85911	chr7:330(	EI24P4		Pseudoger	chr7:145005058-145
ENSG0000(	834	20.85911	chr7:330(	TPK1	DriverDB\	protein_c	chr7:144451941-144
ENSG0000(	834	20.85911	chr7:330(	KEL	NCGv7	protein_c	chr7:142941114-142
ENSG0000(	834	20.85911	chr7:330(	OR2F1		protein_c	chr7:143954844-143
ENSG0000(	834	20.85911	chr7:330(	CNTNAP2-AS1		lncRNA	chr7:147080934-147
ENSG0000(	834	20.85911	chr7:330(	FAM131B-AS2		lncRNA	chr7:143379683-143
ENSG0000(	834	20.85911	chr7:330(	MIR548F4		smallRNA	chr7:147378017-147
ENSG0000(	834	20.85911	chr7:330(	OR2R1P		Pseudoger	chr7:143488462-143
ENSG0000(	834	20.85911	chr7:330(	RN7SKP174		smallRNA	chr7:144849755-144
ENSG0000(	834	20.85911	chr7:330(	GSTK1		protein_c	chr7:143244093-143
ENSG0000(	834	20.85911	chr7:330(	TAS2R39		protein_c	chr7:143183419-143
ENSG0000(	834	20.85911	chr7:330(	ENSG00000268170		lncRNA	chr7:143220468-143
ENSG0000(	834	20.85911	chr7:330(	CTAGE4	DriverDB\	protein_c	chr7:144183466-144
ENSG0000(	834	20.85911	chr7:330(	RNU6-1184P		smallRNA	chr7:147831770-147
ENSG0000(	834	20.85911	chr7:330(	OR2A20P		Pseudoger	chr7:144250671-144
ENSG0000(	834	20.85911	chr7:330(	HINT1P1		Pseudoger	chr7:143312684-143
ENSG0000(	834	20.85911	chr7:330(	RNU6ATAC40P		smallRNA	chr7:144451681-144
ENSG0000(	834	20.85911	chr7:330(	TRBV30		protein_c	chr7:142812586-142
ENSG0000(	834	20.85911	chr7:330(	OR6V1		protein_c	chr7:143052341-143
ENSG0000(	834	20.85911	chr7:330(	CNTNAP2	NCGv7	protein_c	chr7:146116002-148
ENSG0000(	834	20.85911	chr7:330(	TRBJ2-3		protein_c	chr7:142796847-142
ENSG0000(	834	20.85911	chr7:330(	ENSG00000232145		Pseudoger	chr7:143796295-143
ENSG0000(	834	20.85911	chr7:330(	FAM131B-AS1		lncRNA	chr7:143363899-143
ENSG0000(	834	20.85911	chr7:330(	TCAF1P1		Pseudoger	chr7:143598039-143
ENSG0000(	834	20.85911	chr7:330(	TRBJ2-1		protein_c	chr7:142796365-142
ENSG0000(	834	20.85911	chr7:330(	TRBJ2-2		protein_c	chr7:142796560-142
ENSG0000(	834	20.85911	chr7:330(	TRBJ2-2P		protein_c	chr7:142796697-142
ENSG0000(	834	20.85911	chr7:330(	TRBJ2-4		protein_c	chr7:142796998-142
ENSG0000(	834	20.85911	chr7:330(	ENSG00000290602		lncRNA	chr7:143810373-143
ENSG0000(	834	20.85911	chr7:330(	TRBJ2-5		protein_c	chr7:142797119-142
ENSG0000(	834	20.85911	chr7:330(	TRBJ2-6		protein_c	chr7:142797239-142
ENSG0000(	834	20.85911	chr7:330(	TRBJ2-7		protein_c	chr7:142797456-142
ENSG0000(	834	20.85911	chr7:330(	TRBC2		protein_c	chr7:142801041-142

ENSG0000(	834	20.85911	chr7:330(	PPIAP83		Pseudoger	chr7:144434826-144
ENSG0000(	834	20.85911	chr7:330(	OR2A3P		Pseudoger	chr7:144157226-144
ENSG0000(	834	20.85911	chr7:330(	PAICSP5		Pseudoger	chr7:143545186-143
ENSG0000(	834	20.85911	chr7:330(	OR2A42	NCGv7	protein_c	chr7:144228244-144
ENSG0000(	834	20.85911	chr7:330(	ENSG00000226592		Pseudoger	chr7:145269514-145
ENSG0000(	834	20.85911	chr7:330(	OR6B1		protein_c	chr7:144000320-144
ENSG0000(	834	20.85911	chr7:330(	ENSG00000229977		Pseudoger	chr7:143532749-143
ENSG0000(	834	20.85911	chr7:330(	SLC16A1P1		Pseudoger	chr7:143985142-143
ENSG0000(	834	20.85911	chr7:330(	RN7SL456P		smallRNA	chr7:147940004-147
ENSG0000(	834	20.85911	chr7:330(	OR2A5		protein_c	chr7:144048948-144
ENSG0000(	834	20.85911	chr7:330(	TAS2R41		protein_c	chr7:143477873-143
ENSG0000(	834	20.85911	chr4:909:	AC093628.1		smallRNA	chr4:104490876-104
ENSG0000(	834	20.85911	chr7:330(	OR2A12		protein_c	chr7:144086278-144
ENSG0000(	834	20.85911	chr7:330(	OR2F2	NCGv7	protein_c	chr7:143935233-143
ENSG0000(	834	20.85911	chr7:330(	ENSG00000230190		lncRNA	chr7:147671711-147
ENSG0000(	834	20.85911	chr7:330(	OR2A25		protein_c	chr7:144069811-144
ENSG0000(	834	20.85911	chr7:330(	TAS2R62P		Pseudoger	chr7:143437034-143
ENSG0000(	834	20.85911	chr7:330(	TAS2R40		protein_c	chr7:143222037-143
ENSG0000(	834	20.85911	chr7:330(	OR2A14		protein_c	chr7:144123176-144
ENSG0000(	834	20.85911	chr7:330(	ARHGEF34P		Pseudoger	chr7:144272445-144
ENSG0000(	834	20.85911	chr7:330(	DUTP3		Pseudoger	chr7:147146342-147
ENSG0000(	834	20.85911	chr7:330(	PAICSP6		Pseudoger	chr7:143782667-143
ENSG0000(	834	20.85911	chr7:330(	ENSG00000253882		Pseudoger	chr7:143833245-143
ENSG0000(	834	20.85911	chr7:330(	ENSG00000290815		lncRNA	chr7:144153486-144
ENSG0000(	834	20.85911	chr7:330(	TCAF2C		protein_c	chr7:143639230-143
ENSG0000(	834	20.85911	chr7:330(	OR2A1-AS1		lncRNA	chr7:144300395-144
ENSG0000(	834	20.85911	chr7:330(	ENSG00000214035		Pseudoger	chr7:145009961-145
ENSG0000(	834	20.85911	chr7:330(	ENSG00000290816		lncRNA	chr7:144250045-144
ENSG0000(	834	20.85911	chr7:330(	ENSG00000290818		lncRNA	chr7:144294480-144
ENSG0000(	834	20.85911	chr7:330(	OR9A2		protein_c	chr7:143026200-143
ENSG0000(	834	20.85911	chr7:330(	ENSG00000290761		lncRNA	chr7:143062330-143
ENSG0000(	834	20.85911	chr7:330(	OR6W1P		Pseudoger	chr7:143062330-143
ENSG0000(	834	20.85911	chr7:330(	ENSG00000280144		TEC	chr7:146050062-146
ENSG0000(	834	20.85911	chr7:330(	TMEM139		protein_c	chr7:143279957-143
ENSG0000(	834	20.85911	chr7:330(	LLCFC1		protein_c	chr7:142939343-142
ENSG0000(	834	20.85911	chr7:330(	TRPV6	NCGv7	protein_c	chr7:142871208-142
ENSG0000(	834	20.85911	chr7:330(	OR10AC1		protein_c	chr7:143509256-143
ENSG0000(	834	20.85911	chr7:330(	OR2Q1P		Pseudoger	chr7:143980905-143
ENSG0000(	834	20.85911	chr7:330(	ENSG00000283537		Pseudoger	chr7:143623762-143
ENSG0000(	834	20.85911	chr7:330(	RPL7P59		Pseudoger	chr7:145039999-145
ENSG0000(	834	20.85911	chr7:330(	RN7SL207P		smallRNA	chr7:146096087-146
ENSG0000(	834	20.85911	chr7:330(	OR2A13P		Pseudoger	chr7:144142009-144
ENSG0000(	834	20.85911	chr7:330(	ARHGEF35-AS1		lncRNA	chr7:144194858-144
ENSG0000(	834	20.85911	chr7:330(	RNU6-267P		smallRNA	chr7:143754628-143
ENSG0000(	834	20.85911	chr7:330(	NOBOX		protein_c	chr7:144397240-144
ENSG0000(	834	20.85911	chr7:330(	EPHA1-AS1		lncRNA	chr7:143407784-143
ENSG0000(	834	20.85911	chr7:330(	CASP2		protein_c	chr7:143288215-143
ENSG0000(	834	20.85911	chr7:330(	EPHB6	NCGv7	protein_c	chr7:142855061-142
ENSG0000(	834	20.85911	chr7:330(	ENSG00000290786		lncRNA	chr7:143620943-143
ENSG0000(	834	20.85911	chr7:330(	OR2A7	DriverDB\	protein_c	chr7:144257663-144
ENSG0000(	834	20.85911	chr7:330(	ENSG00000284644		lncRNA	chr7:144251264-144

ENSG0000(	834	20.85911	chr7:330(	EEF1A1P10		Pseudoger	chr7:144647186-144
ENSG0000(	834	20.85911	chr7:330(	AC084872.1		smallRNA	chr7:146896597-146
ENSG0000(	834	20.85911	chr7:330(	ENSG00000224970		lncRNA	chr7:142875836-142
ENSG0000(	834	20.85911	chr7:330(	TAS2R60	NCGv7	protein_c	chr7:143443453-143
ENSG0000(	834	20.85911	chr7:330(	ENSG00000290099		lncRNA	chr7:142797704-142
ENSG0000(	834	20.85911	chr7:330(	OR2A41P		Pseudoger	chr7:144077721-144
ENSG0000(	834	20.85911	chr7:330(	ZYX		protein_c	chr7:143381295-143
ENSG0000(	834	20.85911	chr7:330(	PIP		protein_c	chr7:143132077-143
ENSG0000(	834	20.85911	chr7:330(	TRPV5	NCGv7	protein_c	chr7:142908101-142
ENSG0000(	834	20.85911	chr7:330(	RANP2		Pseudoger	chr7:147167344-147
ENSG0000(	834	20.85911	chr7:330(	OR2A2	NCGv7	protein_c	chr7:144109583-144
ENSG0000(	834	20.85911	chr7:330(	ENSG00000230746		lncRNA	chr7:145615942-145
ENSG0000(	834	20.85911	chr7:330(	OR2A01P		Pseudoger	chr7:144176740-144
ENSG0000(	834	20.85911	chr7:330(	RNU6-162P		smallRNA	chr7:143574746-143
ENSG0000(	834	20.85911	chr7:330(	RN7SL535P		smallRNA	chr7:143290615-143
ENSG0000(	834	20.85911	chr7:330(	OR2A9P		Pseudoger	chr7:144299373-144
ENSG0000(	834	20.85911	chr7:330(	ENSG00000230556		Pseudoger	chr7:143578907-143
ENSG0000(	834	20.85911	chr7:330(	CTAGE6		protein_c	chr7:143755089-143
ENSG0000(	834	20.85911	chr7:330(	RN7SL481P		smallRNA	chr7:143298516-143
ENSG0000(	834	20.85911	chr7:330(	ENSG00000286569		lncRNA	chr7:142899365-142
ENSG0000(	834	20.85911	chr7:330(	CTAGE15		protein_c	chr7:143571801-143
ENSG0000(	834	20.85911	chr7:330(	ARHGEF5	TAG;AC	protein_c	chr7:144355288-144
ENSG0000(	834	20.85911	chr7:330(	OR2A15P		Pseudoger	chr7:144118461-144
ENSG0000(	834	20.85911	chr7:330(	CTAGE8		protein_c	chr7:144266674-144
ENSG0000(	834	20.85911	chr7:330(	CLCN1		protein_c	chr7:143316111-143
ENSG0000(	834	20.85911	chr7:330(	DPY19L4P2		Pseudoger	chr7:145583197-145
ENSG0000(	834	20.85911	chr7:330(	TMEM139-AS1		lncRNA	chr7:143255264-143
ENSG0000(	834	20.85911	chr7:330(	ENSG00000291149		lncRNA	chr7:143721660-143
ENSG0000(	834	20.85911	chr7:330(	RNA5SP249		Pseudoger	chr7:147849884-147
ENSG0000(	834	20.85911	chr7:330(	ENSG00000279223		TEC	chr7:143224042-143
ENSG0000(	834	20.85911	chr7:330(	FAM131B		protein_c	chr7:143353400-143
ENSG0000(	834	20.85911	chr7:330(	OR9P1P		Pseudoger	chr7:143047213-143
ENSG0000(	834	20.85911	chr7:330(	ENSG00000289938		lncRNA	chr7:142813393-142
ENSG0000(	834	20.85911	chr7:330(	EPHA1	NCGv7	protein_c	chr7:143390289-143
ENSG0000(	834	20.85911	chr7:330(	ENSG00000270634		Pseudoger	chr7:147704294-147
ENSG0000(	834	20.85911	chr7:330(	OR2A1		protein_c	chr7:144312419-144
ENSG0000(	834	20.85911	chr7:330(	CAPZA1P5		Pseudoger	chr7:143979121-143
ENSG0000(	834	20.85911	chr7:330(	TCAF2P1		Pseudoger	chr7:143800732-143
ENSG0000(	829	20.73406	chr1:3732	MIR557		smallRNA	chr1:168375524-168
ENSG0000(	828	20.70905	chr2:4707	PPIAP66		Pseudoger	chr2:173485865-173
ENSG0000(	826	20.65903	chr7:330(	VN1R42P		Pseudoger	chr7:64933273-6493
ENSG0000(	822	20.55898	chr12:685	RPL29P25		Pseudoger	chr12:110841538-11
ENSG0000(	819	20.48395	chr1:8137	RNVU1-15		smallRNA	chr1:144412575-144
ENSG0000(	819	20.48395	chr1:8137	ENSG00000278431		lncRNA	chr1:145961387-145
ENSG0000(	819	20.48395	chr1:8137	GPR89B		protein_c	chr1:147928393-147
ENSG0000(	819	20.48395	chr1:8137	KMT2CP3		Pseudoger	chr1:143461220-143
ENSG0000(	819	20.48395	chr1:8137	Y_RNA		smallRNA	chr1:147420199-147
ENSG0000(	819	20.48395	chr1:8137	HYDIN2		Pseudoger	chr1:146875321-146
ENSG0000(	819	20.48395	chr1:8137	ENSG00000280778		protein_c	chr1:145927257-145
ENSG0000(	819	20.48395	chr1:8137	ENSG00000224481		lncRNA	chr1:148295895-148
ENSG0000(	819	20.48395	chr1:8137	ENSG00000277702		Pseudoger	chr1:143419624-143

ENSG0000(	819	20.48395	chr1:8137	ENSG00000275129		Pseudoger	chr1:144965024-144
ENSG0000(	819	20.48395	chr1:8137	ENSG00000287190		lncRNA	chr1:146050440-146
ENSG0000(	819	20.48395	chr1:8137	ENSG00000227700		Pseudoger	chr1:148246169-148
ENSG0000(	819	20.48395	chr1:8137	Y_RNA		smallRNA	chr1:148330271-148
ENSG0000(	819	20.48395	chr1:8137	RNVU1-1		smallRNA	chr1:148362370-148
ENSG0000(	819	20.48395	chr1:8137	ENSG00000228626		Pseudoger	chr1:148288001-148
ENSG0000(	819	20.48395	chr1:8137	ENSG00000273059		lncRNA	chr1:148011799-148
ENSG0000(	819	20.48395	chr1:8137	RNU1-135P		smallRNA	chr1:148385829-148
ENSG0000(	819	20.48395	chr1:8137	RNU1-120P		smallRNA	chr1:148263476-148
ENSG0000(	819	20.48395	chr1:8137	RP4-565E6.1		lncRNA	chr1:148162787-148
ENSG0000(	819	20.48395	chr1:8137	RNU1-92P		smallRNA	chr1:143720510-143
ENSG0000(	819	20.48395	chr1:8137	ENSG00000287374		lncRNA	chr1:145475606-145
ENSG0000(	819	20.48395	chr1:8137	H3-7		protein_c	chr1:143894544-143
ENSG0000(	819	20.48395	chr1:8137	PFN1P4		Pseudoger	chr1:148129497-148
ENSG0000(	819	20.48395	chr1:8137	LINC02804		lncRNA	chr1:148013203-148
ENSG0000(	819	20.48395	chr1:8137	NBPF17P		Pseudoger	chr1:143595216-143
ENSG0000(	819	20.48395	chr1:8137	RPL7AP15		Pseudoger	chr1:147223554-147
ENSG0000(	819	20.48395	chr1:8137	SSBL4P		Pseudoger	chr1:147082338-147
ENSG0000(	819	20.48395	chr1:8137	ENSG00000270962		Pseudoger	chr1:143784376-143
ENSG0000(	819	20.48395	chr1:8137	NBPF13P		Pseudoger	chr1:147099482-147
ENSG0000(	819	20.48395	chr1:8137	ENSG00000244619		lncRNA	chr1:145892846-145
ENSG0000(	819	20.48395	chr1:8137	LINC00624		lncRNA	chr1:147258885-147
ENSG0000(	819	20.48395	chr1:8137	RNA5SP58		smallRNA	chr1:148193716-148
ENSG0000(	819	20.48395	chr1:8137	PDIA3P1		Pseudoger	chr1:147172744-147
ENSG0000(	819	20.48395	chr1:8137	GJA8		protein_c	chr1:147902795-147
ENSG0000(	819	20.48395	chr1:8137	CHD1L	TAG;AC	protein_c	chr1:147242654-147
ENSG0000(	819	20.48395	chr1:8137	ENSG00000274415		lncRNA	chr1:147757185-147
ENSG0000(	819	20.48395	chr1:8137	PEX11B		protein_c	chr1:145911349-145
ENSG0000(	819	20.48395	chr1:8137	FMO5	DriverDBv	protein_c	chr1:147175351-147
ENSG0000(	819	20.48395	chr1:8137	DRD5P2		Pseudoger	chr1:143449275-143
ENSG0000(	819	20.48395	chr1:8137	PFN1P8		Pseudoger	chr1:146957117-146
ENSG0000(	819	20.48395	chr1:8137	RP11-495P10.7		lncRNA	chr1:148295792-148
ENSG0000(	819	20.48395	chr1:8137	PIAS3		protein_c	chr1:145848521-145
ENSG0000(	819	20.48395	chr1:8137	PRKAB2	NCGv7	protein_c	chr1:147155106-147
ENSG0000(	819	20.48395	chr1:8137	ENSG00000288626		protein_c	chr1:147611590-147
ENSG0000(	819	20.48395	chr1:8137	RP11-439A17.7		lncRNA	chr1:143972630-143
ENSG0000(	819	20.48395	chr1:8137	FAM72B		protein_c	chr1:143955289-143
ENSG0000(	819	20.48395	chr1:8137	ENSG00000237188		lncRNA	chr1:147172755-147
ENSG0000(	819	20.48395	chr1:8137	PPIAL4E		protein_c	chr1:144372874-144
ENSG0000(	819	20.48395	chr1:8137	BCL9	NCGv7;AC	protein_c	chr1:147541501-147
ENSG0000(	819	20.48395	chr1:8137	NOTCH2NLR		protein_c	chr1:146155128-146
ENSG0000(	819	20.48395	chr1:8137	RNVU1-10		smallRNA	chr1:148362370-148
ENSG0000(	819	20.48395	chr1:8137	ENSG00000276509		lncRNA	chr1:146235805-146
ENSG0000(	819	20.48395	chr1:8137	RNU1-13P		smallRNA	chr1:148388490-148
ENSG0000(	819	20.48395	chr1:8137	RP11-289H16.1		lncRNA	chr1:144917169-144
ENSG0000(	819	20.48395	chr1:8137	CD160		protein_c	chr1:145719470-145
ENSG0000(	819	20.48395	chr1:8137	RNVU1-17		smallRNA	chr1:143699456-143
ENSG0000(	819	20.48395	chr1:8137	RPL22P6		Pseudoger	chr1:143929994-143
ENSG0000(	819	20.48395	chr1:8137	GNRHR2		Pseudoger	chr1:145919012-145
ENSG0000(	819	20.48395	chr1:8137	NOTCH2NLB		protein_c	chr1:146149342-146
ENSG0000(	819	20.48395	chr1:8137	ANKRD34A		protein_c	chr1:145959440-145

ENSG0000(	819	20.48395	chr1:8137	GPR89A	protein_c	chr1:145607987-145
ENSG0000(	819	20.48395	chr1:8137	ENSG00000230186	lncRNA	chr1:143905487-143
ENSG0000(	819	20.48395	chr1:8137	RNVU1-7	smallRNA	chr1:145465617-145
ENSG0000(	819	20.48395	chr1:8137	BX842679.1	protein_c	chr1:148159688-148
ENSG0000(	819	20.48395	chr1:8137	HJV	protein_c	chr1:146017467-146
ENSG0000(	819	20.48395	chr1:8137	RP11-337C18.9	lncRNA	chr1:147175602-147
ENSG0000(	819	20.48395	chr1:8137	RNVU1-8	smallRNA	chr1:147084616-147
ENSG0000(	819	20.48395	chr1:8137	ENSG00000224335	Pseudoger	chr1:148234273-148
ENSG0000(	819	20.48395	chr1:8137	KMT2CP1	Pseudoger	chr1:143461247-143
ENSG0000(	819	20.48395	chr1:8137	XXyac-YX155B6.2	Pseudoger	chr1:148080598-148
ENSG0000(	819	20.48395	chr1:8137	ENSG00000286185	protein_c	chr1:146069621-146
ENSG0000(	819	20.48395	chr1:8137	POLR3C	protein_c	chr1:145824052-145
ENSG0000(	819	20.48395	chr1:8137	ENSG00000213226	Pseudoger	chr1:147319110-147
ENSG0000(	819	20.48395	chr1:8137	LINC02806	lncRNA	chr1:148295180-148
ENSG0000(	819	20.48395	chr1:8137	NOTCH2NLC	protein_c	chr1:146148864-146
ENSG0000(	819	20.48395	chr1:8137	ENSG00000271644	Pseudoger	chr1:144965024-144
ENSG0000(	819	20.48395	chr1:8137	ENSG00000225603	Pseudoger	chr1:147050817-147
ENSG0000(	819	20.48395	chr1:8137	LIX1L	protein_c	chr1:145933422-145
ENSG0000(	819	20.48395	chr1:8137	FAM91A3P	Pseudoger	chr1:143766540-143
ENSG0000(	819	20.48395	chr1:8137	RP11-403I13.7	lncRNA	chr1:143790010-143
ENSG0000(	819	20.48395	chr1:8137	OR13Z2P	Pseudoger	chr1:147445579-147
ENSG0000(	819	20.48395	chr1:8137	RN7SL261P	smallRNA	chr1:147689256-147
ENSG0000(	819	20.48395	chr1:8137	RP11-666A1.5	lncRNA	chr1:144418122-144
ENSG0000(	819	20.48395	chr1:8137	CCT8P1	Pseudoger	chr1:147203276-147
ENSG0000(	819	20.48395	chr1:8137	POLR3GL	protein_c	chr1:145964689-145
ENSG0000(	819	20.48395	chr1:8137	CR812485.1	smallRNA	chr1:143828767-143
ENSG0000(	819	20.48395	chr1:8137	RP6-137J22.3	Pseudoger	chr1:145269604-145
ENSG0000(	819	20.48395	chr1:8137	PFN1P12	Pseudoger	chr1:143619994-143
ENSG0000(	819	20.48395	chr1:8137	ACP6	protein_c	chr1:147629652-147
ENSG0000(	819	20.48395	chr1:8137	RNVU1-2	smallRNA	chr1:148385829-148
ENSG0000(	819	20.48395	chr1:8137	NKAIN1P1	Pseudoger	chr1:143487771-143
ENSG0000(	819	20.48395	chr1:8137	PDZK1	protein_c	chr1:145670851-145
ENSG0000(	819	20.48395	chr1:8137	XXyac-YX155B6.6	lncRNA	chr1:148162787-148
ENSG0000(	819	20.48395	chr1:8137	ABHD17AP2	Pseudoger	chr1:148146395-148
ENSG0000(	819	20.48395	chr1:8137	ENSG00000237503	Pseudoger	chr1:143846097-143
ENSG0000(	819	20.48395	chr1:8137	ANKRD35	protein_c	chr1:145866559-145
ENSG0000(	819	20.48395	chr1:8137	RP11-495P10.8	lncRNA	chr1:148290890-148
ENSG0000(	819	20.48395	chr1:8137	RPL22P5	Pseudoger	chr1:143929994-143
ENSG0000(	819	20.48395	chr1:8137	HIST2H3DP1	Pseudoger	chr1:143905555-143
ENSG0000(	819	20.48395	chr1:8137	ENSG00000272824	lncRNA	chr1:148358245-148
ENSG0000(	819	20.48395	chr1:8137	GPR89C	protein_c	chr1:147953335-147
ENSG0000(	819	20.48395	chr1:8137	RP11-458D21.6	lncRNA	chr1:146237251-146
ENSG0000(	819	20.48395	chr1:8137	ENSG00000229002	Pseudoger	chr1:144472534-144
ENSG0000(	819	20.48395	chr1:8137	RNU1-143P	smallRNA	chr1:143791542-143
ENSG0000(	819	20.48395	chr1:8137	FCGR1BP	Pseudoger	chr1:143876113-143
ENSG0000(	819	20.48395	chr1:8137	NBPF10	protein_c	chr1:146075000-146
ENSG0000(	819	20.48395	chr1:8137	OR13Z3P	Pseudoger	chr1:147482238-147
ENSG0000(	819	20.48395	chr1:8137	ENSG00000271439	Pseudoger	chr1:144401068-144
ENSG0000(	819	20.48395	chr1:8137	NUDT17	protein_c	chr1:145845629-145
ENSG0000(	819	20.48395	chr1:8137	RNA5SP57	smallRNA	chr1:148193716-148
ENSG0000(	819	20.48395	chr1:8137	AL109844.1	smallRNA	chr1:143828767-143

ENSG0000(	819	20.48395	chr1:8137	LINC01719		lncRNA	chr1:146052565-146
ENSG0000(	819	20.48395	chr1:8137	RNVU1-12		smallRNA	chr1:148402715-148
ENSG0000(	819	20.48395	chr1:8137	LIX1L-AS1		lncRNA	chr1:145926589-145
ENSG0000(	819	20.48395	chr1:8137	ENSG00000234225		lncRNA	chr1:147001931-147
ENSG0000(	819	20.48395	chr1:8137	AL732363.1		smallRNA	chr1:143541645-143
ENSG0000(	819	20.48395	chr1:8137	LINC02805		lncRNA	chr1:148156139-148
ENSG0000(	819	20.48395	chr1:8137	RNU1-129P		smallRNA	chr1:148014417-148
ENSG0000(	819	20.48395	chr1:8137	RP11-439A17.9		lncRNA	chr1:143877730-143
ENSG0000(	819	20.48395	chr1:8137	ENSG00000223495		Pseudoger	chr1:143498784-143
ENSG0000(	819	20.48395	chr1:8137	ENSG00000227193		lncRNA	chr1:143905552-143
ENSG0000(	819	20.48395	chr1:8137	LINC01731		lncRNA	chr1:148271884-148
ENSG0000(	819	20.48395	chr1:8137	PFN1P6		Pseudoger	chr1:144442605-144
ENSG0000(	819	20.48395	chr1:8137	PFN1P5		Pseudoger	chr1:148129480-148
ENSG0000(	819	20.48395	chr1:8137	RNVU1-16		smallRNA	chr1:145281115-145
ENSG0000(	819	20.48395	chr1:8137	ENSG00000227242		Pseudoger	chr1:147019656-147
ENSG0000(	819	20.48395	chr1:8137	RNVU1-6		smallRNA	chr1:146052080-146
ENSG0000(	819	20.48395	chr1:8137	ENSG00000289318		lncRNA	chr1:143972669-143
ENSG0000(	819	20.48395	chr1:8137	TXNIP	NCGv7	protein_c	chr1:145992434-145
ENSG0000(	819	20.48395	chr1:8137	H2BP1		lncRNA	chr1:143894527-143
ENSG0000(	819	20.48395	chr1:8137	GJA5		protein_c	chr1:147756199-147
ENSG0000(	819	20.48395	chr1:8137	ENSG00000232721		lncRNA	chr1:143735983-143
ENSG0000(	819	20.48395	chr1:8137	ENSG00000223728		Pseudoger	chr1:147840962-147
ENSG0000(	819	20.48395	chr1:8137	ENSG00000223779		Pseudoger	chr1:143745249-143
ENSG0000(	819	20.48395	chr1:8137	ENSG00000289565		protein_c	chr1:145917713-145
ENSG0000(	819	20.48395	chr1:8137	H2BP1		Pseudoger	chr1:143904287-143
ENSG0000(	819	20.48395	chr1:8137	ENSG00000234190		lncRNA	chr1:147777590-147
ENSG0000(	819	20.48395	chr1:8137	ENSG00000227139		lncRNA	chr1:147697794-147
ENSG0000(	819	20.48395	chr1:8137	RNA5SP59		Pseudoger	chr1:143439604-143
ENSG0000(	819	20.48395	chr1:8137	RNVU1-3		smallRNA	chr1:148402715-148
ENSG0000(	819	20.48395	chr1:8137	FAM72C		protein_c	chr1:143944179-143
ENSG0000(	819	20.48395	chr1:8137	ENSG00000203825		Pseudoger	chr1:143541768-143
ENSG0000(	819	20.48395	chr1:8137	ABHD17AP1		Pseudoger	chr1:148146394-148
ENSG0000(	819	20.48395	chr1:8137	LINC02799		lncRNA	chr1:143499186-143
ENSG0000(	819	20.48395	chr1:8137	NBPF24		protein_c	chr1:148102151-148
ENSG0000(	819	20.48395	chr1:8137	ENSG00000290705		lncRNA	chr1:147993862-148
ENSG0000(	819	20.48395	chr1:8137	RP11-403I13.9		lncRNA	chr1:143811359-143
ENSG0000(	819	20.48395	chr1:8137	ENSG00000290735		lncRNA	chr1:143875171-143
ENSG0000(	819	20.48395	chr1:8137	NOTCH2NLA		protein_c	chr1:146146202-146
ENSG0000(	819	20.48395	chr1:8137	FCGR1CP		Pseudoger	chr1:143874793-143
ENSG0000(	819	20.48395	chr1:8137	WI2-925H4.1		lncRNA	chr1:145601945-145
ENSG0000(	819	20.48395	chr1:8137	ENSG00000264145		Pseudoger	chr1:143449570-143
ENSG0000(	819	20.48395	chr1:8137	RNU1-122P		smallRNA	chr1:148334612-148
ENSG0000(	819	20.48395	chr1:8137	ENSG00000235988		Pseudoger	chr1:148317683-148
ENSG0000(	819	20.48395	chr1:8137	RNF115		protein_c	chr1:145738867-145
ENSG0000(	819	20.48395	chr1:8137	RNVU1-18		smallRNA	chr1:143729407-143
ENSG0000(	819	20.48395	chr1:8137	PDZK1P1		Pseudoger	chr1:147994301-148
ENSG0000(	819	20.48395	chr1:8137	FAM72D	NCGv7	protein_c	chr1:143955287-143
ENSG0000(	819	20.48395	chr1:8137	RNU1-114P		smallRNA	chr1:143652050-143
ENSG0000(	819	20.48395	chr1:8137	ITGA10		protein_c	chr1:145891207-145
ENSG0000(	819	20.48395	chr1:8137	ENSG00000223612		Pseudoger	chr1:145233001-145
ENSG0000(	819	20.48395	chr1:8137	RNVU1-11		smallRNA	chr1:148388490-148

ENSG0000(	819	20.48395	chr1:8137	RP11-439A17.10		lncRNA	chr1:143874925-143
ENSG0000(	819	20.48395	chr1:8137	RNVU1-9		smallRNA	chr1:148038753-148
ENSG0000(	819	20.48395	chr1:8137	LSP1P5		lncRNA	chr1:143401427-143
ENSG0000(	819	20.48395	chr1:8137	RBM8A	NCGv7	protein_c	chr1:145921555-145
ENSG0000(	819	20.48395	chr1:8137	RNU1-137P		smallRNA	chr1:145431527-145
ENSG0000(	819	20.48395	chr1:8137	RP11-666A1.3		Pseudoger	chr1:144418677-144
ENSG0000(	819	20.48395	chr1:8137	RP11-14N7.2		lncRNA	chr1:143401429-143
ENSG0000(	819	20.48395	chr1:8137	ENSG00000289419		lncRNA	chr1:147608331-147
ENSG0000(	819	20.48395	chr1:8137	OR13Z1P		Pseudoger	chr1:147419053-147
ENSG0000(	819	20.48395	chr1:8137	RNVU1-8		smallRNA	chr1:147079746-147
ENSG0000(	806	20.15881	chr7:330(	METTL27	DriverDB\	protein_c	chr7:73834590-7384
ENSG0000(	806	20.15881	chr7:330(	STAG3L3		Pseudoger	chr7:72998027-7300
ENSG0000(	806	20.15881	chr7:330(	FZD9		protein_c	chr7:73433778-7343
ENSG0000(	806	20.15881	chr7:330(	CLDN3	DriverDB\	protein_c	chr7:73768997-7377
ENSG0000(	806	20.15881	chr7:330(	STX1A		protein_c	chr7:73699206-7371
ENSG0000(	806	20.15881	chr7:330(	BUD23	NCGv7	protein_c	chr7:73683025-7370
ENSG0000(	806	20.15881	chr7:330(	DNAJC30		protein_c	chr7:73680918-7368
ENSG0000(	806	20.15881	chr7:330(	AC004878.5		Pseudoger	chr7:73076167-7308
ENSG0000(	806	20.15881	chr7:330(	PMS2P7		Pseudoger	chr7:73005541-7302
ENSG0000(	806	20.15881	chr7:330(	ABHD11	DriverDB\	protein_c	chr7:73736094-7373
ENSG0000(	806	20.15881	chr7:330(	VPS37D	DriverDB\	protein_c	chr7:73667831-7367
ENSG0000(	806	20.15881	chr7:330(	SPDYE7P		Pseudoger	chr7:72863903-7287
ENSG0000(	806	20.15881	chr7:330(	GTF2IP4		Pseudoger	chr7:73154938-7320
ENSG0000(	806	20.15881	chr7:330(	ENSG00000290998		lncRNA	chr7:72862757-7286
ENSG0000(	806	20.15881	chr7:330(	RNU6-1198P		smallRNA	chr7:73507208-7350
ENSG0000(	806	20.15881	chr7:330(	Y_RNA		smallRNA	chr7:73067230-7306
ENSG0000(	806	20.15881	chr7:330(	NSUN5P2		Pseudoger	chr7:72948485-7295
ENSG0000(	806	20.15881	chr7:330(	PMS2P6		Pseudoger	chr7:73093657-7310
ENSG0000(	806	20.15881	chr7:330(	MLXIPL	NCGv7	protein_c	chr7:73593194-7362
ENSG0000(	806	20.15881	chr7:330(	MIR4650-2		smallRNA	chr7:72697903-7269
ENSG0000(	806	20.15881	chr7:330(	RPL7AP77		Pseudoger	chr7:73314107-7331
ENSG0000(	806	20.15881	chr7:330(	TRIM50		protein_c	chr7:73312536-7332
ENSG0000(	806	20.15881	chr7:330(	BAZ1B		protein_c	chr7:73440406-7352
ENSG0000(	806	20.15881	chr7:330(	ENSG00000285886		lncRNA	chr7:72954797-7295
ENSG0000(	806	20.15881	chr7:330(	TBL2	NCGv7	protein_c	chr7:73567537-7357
ENSG0000(	806	20.15881	chr7:330(	POM121B		Pseudoger	chr7:73293497-7330
ENSG0000(	806	20.15881	chr7:330(	BCL7B		protein_c	chr7:73536356-7355
ENSG0000(	806	20.15881	chr7:330(	AC091738.2		smallRNA	chr7:72673068-7267
ENSG0000(	806	20.15881	chr7:330(	RN7SL265P		smallRNA	chr7:73732571-7373
ENSG0000(	806	20.15881	chr7:330(	TMEM270		protein_c	chr7:73861159-7386
ENSG0000(	806	20.15881	chr7:330(	TYW1B	NCGv7	protein_c	chr7:72558744-7282
ENSG0000(	806	20.15881	chr7:330(	FKBP6		protein_c	chr7:73328161-7335
ENSG0000(	806	20.15881	chr7:330(	ABHD11-AS1		Pseudoger	chr7:73734994-7373
ENSG0000(	806	20.15881	chr7:330(	RNU6-1080P		smallRNA	chr7:73339094-7333
ENSG0000(	806	20.15881	chr7:330(	Y_RNA		smallRNA	chr7:72983361-7298
ENSG0000(	806	20.15881	chr7:330(	AC005488.1		protein_c	chr7:72988949-7299
ENSG0000(	806	20.15881	chr7:330(	RN7SL377P		smallRNA	chr7:72822898-7282
ENSG0000(	806	20.15881	chr7:330(	TRIM74	DriverDB\	protein_c	chr7:72959485-7296
ENSG0000(	806	20.15881	chr7:330(	ENSG00000270694		Pseudoger	chr7:72722885-7272
ENSG0000(	806	20.15881	chr7:330(	ENSG00000261467		lncRNA	chr7:73985992-7398
ENSG0000(	806	20.15881	chr7:330(	ENSG00000290832		lncRNA	chr7:72969696-7300

ENSG0000(	806	20.15881	chr7:330(	CLDN4		protein_c	chr7:73799542-7383
ENSG0000(	806	20.15881	chr7:330(	SBDSP1		lncRNA	chr7:72829425-7283
ENSG0000(	806	20.15881	chr7:330(	SPDYE9		protein_c	chr7:73075971-7308
ENSG0000(	806	20.15881	chr7:330(	SBDSP1		Pseudoger	chr7:72829656-7283
ENSG0000(	806	20.15881	chr7:330(	ENSG00000274080		lncRNA	chr7:73609262-7361
ENSG0000(	806	20.15881	chr7:330(	PHB1P5		Pseudoger	chr7:73187969-7318
ENSG0000(	806	20.15881	chr7:330(	GTF2IRD2P1		Pseudoger	chr7:73243271-7328
ENSG0000(	806	20.15881	chr7:330(	ENSG00000270555		Pseudoger	chr7:72768798-7276
ENSG0000(	806	20.15881	chr7:330(	NCF1B		Pseudoger	chr7:73220646-7323
ENSG0000(	806	20.15881	chr7:330(	Y_RNA		smallRNA	chr7:73011144-7301
ENSG0000(	806	20.15881	chr7:330(	MIR4284		smallRNA	chr7:73711317-7371
ENSG0000(	806	20.15881	chr7:330(	POM121		protein_c	chr7:72879349-7295
ENSG0000(	806	20.15881	chr7:330(	SPDYE10		protein_c	chr7:73104008-7315
ENSG0000(	806	20.15881	chr7:330(	NCF1B		lncRNA	chr7:73220624-7323
ENSG0000(	806	20.15881	chr7:330(	Y_RNA		smallRNA	chr7:73095357-7309
ENSG0000(	806	20.15881	chr7:330(	NSUN5		protein_c	chr7:73302516-7330
ENSG0000(	806	20.15881	chr7:330(	Y_RNA		smallRNA	chr7:73403788-7340
ENSG0000(	806	20.15881	chr7:330(	ENSG00000289042		lncRNA	chr7:72919205-7292
ENSG0000(	806	20.15881	chr7:330(	ENSG00000272843		lncRNA	chr7:72924418-7292
ENSG0000(	806	20.15881	chr7:330(	ENSG00000205584		Pseudoger	chr7:72969814-7297
ENSG0000(	806	20.15881	chr7:330(	NSUN5P2		lncRNA	chr7:72947581-7295
ENSG0000(	806	20.15881	chr7:330(	ENSG00000290839		lncRNA	chr7:73242751-7327
ENSG0000(	806	20.15881	chr7:330(	RN7SL625P		smallRNA	chr7:72841439-7284
ENSG0000(	797	19.93371	chr1:1522	NENFP1		Pseudoger	chr1:46665910-4666
ENSG0000(	796	19.9087	chr7:330(	POM121L12		protein_c	chr7:53035633-5303
ENSG0000(	796	19.9087	chr7:330(	ENSG00000233960		lncRNA	chr7:52165235-5219
ENSG0000(	796	19.9087	chr7:330(	IFITM3P4		Pseudoger	chr7:56163145-5616
ENSG0000(	796	19.9087	chr7:330(	NUPR2		protein_c	chr7:56114681-5611
ENSG0000(	796	19.9087	chr7:330(	MIR4283-2		smallRNA	chr7:63621090-6362
ENSG0000(	796	19.9087	chr7:330(	ZNF107		protein_c	chr7:64666099-6471
ENSG0000(	796	19.9087	chr7:330(	ENSG00000290090		lncRNA	chr7:66114830-6611
ENSG0000(	796	19.9087	chr7:330(	MTND1P2		Pseudoger	chr7:64111752-6411
ENSG0000(	796	19.9087	chr7:330(	MTND2P4		Pseudoger	chr7:64110547-6411
ENSG0000(	796	19.9087	chr7:330(	ENSG00000230132		Pseudoger	chr7:63480598-6348
ENSG0000(	796	19.9087	chr7:330(	ENSG00000238124		lncRNA	chr7:65463071-6546
ENSG0000(	796	19.9087	chr7:330(	ENSG00000233977		lncRNA	chr7:55592074-5559
ENSG0000(	796	19.9087	chr7:330(	ENSG00000227080		Pseudoger	chr7:51388430-5138
ENSG0000(	796	19.9087	chr7:330(	ZNF733P		Pseudoger	chr7:63291518-6330
ENSG0000(	796	19.9087	chr7:330(	ENSG00000287039		lncRNA	chr7:51849086-5186
ENSG0000(	796	19.9087	chr7:330(	LINC02854		lncRNA	chr7:53926676-5394
ENSG0000(	796	19.9087	chr7:330(	ENSG00000233962		Pseudoger	chr7:57652736-5765
ENSG0000(	796	19.9087	chr7:330(	ENSG00000230936		Pseudoger	chr7:55342316-5534
ENSG0000(	796	19.9087	chr7:330(	ENSG00000271696		Pseudoger	chr7:57835309-5783
ENSG0000(	796	19.9087	chr7:330(	ZNF479	NCGv7	protein_c	chr7:57119614-5713
ENSG0000(	796	19.9087	chr7:330(	GTF2IP5		Pseudoger	chr7:65773620-6580
ENSG0000(	796	19.9087	chr7:330(	ENSG00000233918		Pseudoger	chr7:62275360-6227
ENSG0000(	796	19.9087	chr7:330(	ENSG00000290553		lncRNA	chr7:65647864-6571
ENSG0000(	796	19.9087	chr7:330(	RN7SL292P		smallRNA	chr7:51716658-5171
ENSG0000(	796	19.9087	chr7:330(	ENSG00000235095		Pseudoger	chr7:57147986-5715
ENSG0000(	796	19.9087	chr7:330(	NCOR1P3		Pseudoger	chr7:57599794-5761
ENSG0000(	796	19.9087	chr7:330(	LINC03006		Pseudoger	chr7:65770473-6577

ENSG0000(	796	19.9087	chr7:330(	ENSG00000203462	Pseudoger	chr7:56322804-5632
ENSG0000(	796	19.9087	chr7:330(	ENSG00000232161	Pseudoger	chr7:57770966-5777
ENSG0000(	796	19.9087	chr7:330(	INTS4P1	lncRNA	chr7:65141032-6523
ENSG0000(	796	19.9087	chr7:330(	ENSG00000223889	Pseudoger	chr7:63393749-6339
ENSG0000(	796	19.9087	chr7:330(	ENSG00000232165	Pseudoger	chr7:63345840-6334
ENSG0000(	796	19.9087	chr7:330(	SNORA22	smallRNA	chr7:65065999-6506
ENSG0000(	796	19.9087	chr7:330(	TNRC18P2	Pseudoger	chr7:63567743-6358
ENSG0000(	796	19.9087	chr7:330(	MTND3P2	Pseudoger	chr7:64105654-6410
ENSG0000(	796	19.9087	chr7:330(	ENSG00000290558	lncRNA	chr7:63291552-6330
ENSG0000(	796	19.9087	chr7:330(	ENSG00000290564	lncRNA	chr7:64120431-6415
ENSG0000(	796	19.9087	chr7:330(	GUSBP6	Pseudoger	chr7:64100305-6412
ENSG0000(	796	19.9087	chr7:330(	ENSG00000233454	Pseudoger	chr7:63057453-6306
ENSG0000(	796	19.9087	chr7:330(	ENSG00000285544	lncRNA	chr7:64045429-6411
ENSG0000(	796	19.9087	chr7:330(	TNRC18P3	Pseudoger	chr7:56991888-5700
ENSG0000(	796	19.9087	chr7:330(	VN1R38P	Pseudoger	chr7:64180006-6418
ENSG0000(	796	19.9087	chr7:330(	ENSG00000287317	lncRNA	chr7:64651655-6465
ENSG0000(	796	19.9087	chr7:330(	ENSG00000189316	lncRNA	chr7:64888527-6489
ENSG0000(	796	19.9087	chr7:330(	ENSG00000223559	Pseudoger	chr7:56288230-5629
ENSG0000(	796	19.9087	chr7:330(	ENSG00000229881	lncRNA	chr7:64140495-6415
ENSG0000(	796	19.9087	chr7:330(	ENSG00000227015	Pseudoger	chr7:57770619-5777
ENSG0000(	796	19.9087	chr7:330(	HAUS6P1	Pseudoger	chr7:53187388-5318
ENSG0000(	796	19.9087	chr7:330(	ENSG00000287699	lncRNA	chr7:64294516-6430
ENSG0000(	796	19.9087	chr7:330(	ENSG00000224484	Pseudoger	chr7:57776972-5777
ENSG0000(	796	19.9087	chr7:330(	RNU6-1335P	smallRNA	chr7:56340231-5634
ENSG0000(	796	19.9087	chr7:330(	MIR4283-1	smallRNA	chr7:56955785-5695
ENSG0000(	796	19.9087	chr7:330(	RNU6-1126P	smallRNA	chr7:55789368-5578
ENSG0000(	796	19.9087	chr7:330(	ENSG00000230000	lncRNA	chr7:63348861-6335
ENSG0000(	796	19.9087	chr7:330(	MTCYBP29	Pseudoger	chr7:57173357-5717
ENSG0000(	796	19.9087	chr7:330(	ZNF90P3	Pseudoger	chr7:63087070-6308
ENSG0000(	796	19.9087	chr7:330(	PHKG1P1	Pseudoger	chr7:63233035-6323
ENSG0000(	796	19.9087	chr7:330(	AC104057.1	protein_c	chr7:65647167-6564
ENSG0000(	796	19.9087	chr7:330(	CRCP	protein_c	chr7:66114604-6615
ENSG0000(	796	19.9087	chr7:330(	ENSG00000285741	lncRNA	chr7:51471717-5172
ENSG0000(	796	19.9087	chr7:330(	LINC02848	lncRNA	chr7:63900313-6392
ENSG0000(	796	19.9087	chr7:330(	GABPAP	Pseudoger	chr7:63893286-6389
ENSG0000(	796	19.9087	chr7:330(	ENSG00000235738	Pseudoger	chr7:55797946-5579
ENSG0000(	796	19.9087	chr7:330(	RNU6-1125P	smallRNA	chr7:54621025-5462
ENSG0000(	796	19.9087	chr7:330(	ENSG00000226587	lncRNA	chr7:63326674-6335
ENSG0000(	796	19.9087	chr7:330(	ENSG00000290193	lncRNA	chr7:56482723-5649
ENSG0000(	796	19.9087	chr7:330(	MTATP6P18	Pseudoger	chr7:64106822-6410
ENSG0000(	796	19.9087	chr7:330(	TRIM60P16	Pseudoger	chr7:56631739-5663
ENSG0000(	796	19.9087	chr7:330(	ENSG00000285670	lncRNA	chr7:56214979-5622
ENSG0000(	796	19.9087	chr7:330(	ENSG00000229886	Pseudoger	chr7:66025126-6603
ENSG0000(	796	19.9087	chr7:330(	VN1R40P	Pseudoger	chr7:64443802-6444
ENSG0000(	796	19.9087	chr7:330(	BNIP3P11	Pseudoger	chr7:64678954-6468
ENSG0000(	796	19.9087	chr7:330(	ZNF735	protein_c	chr7:64207203-6422
ENSG0000(	796	19.9087	chr7:330(	SLC25A1P2	Pseudoger	chr7:63398046-6339
ENSG0000(	796	19.9087	chr7:330(	ENSG00000227305	lncRNA	chr7:63393745-6342
ENSG0000(	796	19.9087	chr7:330(	ENSG00000227397	Pseudoger	chr7:63789909-6379
ENSG0000(	796	19.9087	chr7:330(	CCT6P3	Pseudoger	chr7:65064999-6507
ENSG0000(	796	19.9087	chr7:330(	ARAFP3	Pseudoger	chr7:63342711-6334

ENSG0000(	796	19.9087	chr7:330(	MTND4P5		Pseudoger	chr7:57192695-5719
ENSG0000(	796	19.9087	chr7:330(	HAUS6P3		Pseudoger	chr7:53862233-5386
ENSG0000(	796	19.9087	chr7:330(	SLC29A4P1		Pseudoger	chr7:57014285-5702
ENSG0000(	796	19.9087	chr7:330(	RNU6-973P		smallRNA	chr7:65859660-6585
ENSG0000(	796	19.9087	chr7:330(	snoU13		smallRNA	chr7:52323798-5232
ENSG0000(	796	19.9087	chr7:330(	PSPH	DriverDB\	protein_c	chr7:56011051-5605
ENSG0000(	796	19.9087	chr7:330(	ENSG00000239985		Pseudoger	chr7:65038372-6503
ENSG0000(	796	19.9087	chr7:330(	ENSG00000230600		lncRNA	chr7:63380466-6338
ENSG0000(	796	19.9087	chr7:330(	ENSG00000230271		Pseudoger	chr7:57772584-5777
ENSG0000(	796	19.9087	chr7:330(	SEPTIN14		protein_c	chr7:55793540-5586
ENSG0000(	796	19.9087	chr7:330(	VOPP1	DriverDB\	protein_c	chr7:55436056-5557
ENSG0000(	796	19.9087	chr7:330(	SEC61G	NCGv7	protein_c	chr7:54752250-5475
ENSG0000(	796	19.9087	chr7:330(	LANCL2	DriverDB\	protein_c	chr7:55365337-5543
ENSG0000(	796	19.9087	chr7:330(	ZNF736		protein_c	chr7:64307459-6435
ENSG0000(	796	19.9087	chr7:330(	CICP17		Pseudoger	chr7:51382284-5138
ENSG0000(	796	19.9087	chr7:330(	RNU6-912P		smallRNA	chr7:65814672-6581
ENSG0000(	796	19.9087	chr7:330(	CALM1P2		Pseudoger	chr7:55259809-5526
ENSG0000(	796	19.9087	chr7:330(	RAC1P9		Pseudoger	chr7:53779783-5378
ENSG0000(	796	19.9087	chr7:330(	ENSG00000224653		Pseudoger	chr7:57819819-5782
ENSG0000(	796	19.9087	chr7:330(	RNU7-157P		smallRNA	chr7:57227001-5722
ENSG0000(	796	19.9087	chr7:330(	MRPS17	DriverDB\	protein_c	chr7:55951877-5595
ENSG0000(	796	19.9087	chr7:330(	VN1R36P		Pseudoger	chr7:63965834-6396
ENSG0000(	796	19.9087	chr7:330(	CCT6A	DriverDB\	protein_c	chr7:56051685-5606
ENSG0000(	796	19.9087	chr7:330(	MTCYBP5		Pseudoger	chr7:57196638-5719
ENSG0000(	796	19.9087	chr7:330(	SAPCD2P2		Pseudoger	chr7:57424503-5742
ENSG0000(	796	19.9087	chr7:330(	EGFR	NCGv7;AC	protein_c	chr7:55019017-5521
ENSG0000(	796	19.9087	chr7:330(	RNU6-1229P		smallRNA	chr7:65023204-6502
ENSG0000(	796	19.9087	chr7:330(	ENSG00000230796		Pseudoger	chr7:57650381-5765
ENSG0000(	796	19.9087	chr7:330(	MTDHP1		Pseudoger	chr7:64942143-6494
ENSG0000(	796	19.9087	chr7:330(	ENSG00000270957		Pseudoger	chr7:57485166-5748
ENSG0000(	796	19.9087	chr7:330(	ENSG00000286908		lncRNA	chr7:52493152-5250
ENSG0000(	796	19.9087	chr7:330(	ENSG00000286987		lncRNA	chr7:51773836-5178
ENSG0000(	796	19.9087	chr7:330(	ENSG00000287019		lncRNA	chr7:56615084-5661
ENSG0000(	796	19.9087	chr7:330(	ENSG00000270749		Pseudoger	chr7:57629285-5763
ENSG0000(	796	19.9087	chr7:330(	ENSG00000235421		Pseudoger	chr7:65525629-6555
ENSG0000(	796	19.9087	chr7:330(	MTND1P4		Pseudoger	chr7:57185788-5718
ENSG0000(	796	19.9087	chr7:330(	CDC42P2		Pseudoger	chr7:55638274-5563
ENSG0000(	796	19.9087	chr7:330(	ENSG00000271047		Pseudoger	chr7:56603410-5660
ENSG0000(	796	19.9087	chr7:330(	SUMF2	DriverDB\	protein_c	chr7:56064002-5608
ENSG0000(	796	19.9087	chr7:330(	NIPSNAP2	DriverDB\	protein_c	chr7:55951793-5600
ENSG0000(	796	19.9087	chr7:330(	AC092634.2		smallRNA	chr7:63926836-6392
ENSG0000(	796	19.9087	chr7:330(	ENSG00000234716		Pseudoger	chr7:56577848-5657
ENSG0000(	796	19.9087	chr7:330(	RN7SKP218		smallRNA	chr7:53490148-5349
ENSG0000(	796	19.9087	chr7:330(	SEC61G-DT		lncRNA	chr7:54759313-5481
ENSG0000(	796	19.9087	chr7:330(	RNF138P2		Pseudoger	chr7:53316149-5331
ENSG0000(	796	19.9087	chr7:330(	ENSG00000286658		lncRNA	chr7:50641725-5064
ENSG0000(	796	19.9087	chr7:330(	MTCO1P8		Pseudoger	chr7:64108625-6411
ENSG0000(	796	19.9087	chr7:330(	VKORC1L1		protein_c	chr7:65873074-6595
ENSG0000(	796	19.9087	chr7:330(	ENSG00000224370		Pseudoger	chr7:56380976-5638
ENSG0000(	796	19.9087	chr7:330(	SLC29A4P2		Pseudoger	chr7:63556598-6356
ENSG0000(	796	19.9087	chr7:330(	snoU13		smallRNA	chr7:56100659-5610

ENSG0000(	796	19.9087	chr7:330(	SNORA15		smallRNA	chr7:65760052-6576
ENSG0000(	796	19.9087	chr7:330(	SNORA4		smallRNA	chr7:50935350-5093
ENSG0000(	796	19.9087	chr7:330(	VN1R32P		Pseudoger	chr7:63377329-6337
ENSG0000(	796	19.9087	chr7:330(	VN1R33P		Pseudoger	chr7:63401385-6340
ENSG0000(	796	19.9087	chr7:330(	ENSG00000227545		Pseudoger	chr7:63349154-6335
ENSG0000(	796	19.9087	chr7:330(	ENSG00000227148		lncRNA	chr7:63044827-6305
ENSG0000(	796	19.9087	chr7:330(	CICP24		Pseudoger	chr7:63768257-6377
ENSG0000(	796	19.9087	chr7:330(	GUSBP10		Pseudoger	chr7:57177409-5718
ENSG0000(	796	19.9087	chr7:330(	ZNF138		protein_c	chr7:64794388-6483
ENSG0000(	796	19.9087	chr7:330(	PSPHP1		Pseudoger	chr7:55764797-5577
ENSG0000(	796	19.9087	chr7:330(	ENSG00000234185		lncRNA	chr7:66119603-6616
ENSG0000(	796	19.9087	chr7:330(	ENSG00000286342		lncRNA	chr7:64947571-6495
ENSG0000(	796	19.9087	chr7:330(	ENSG00000234105		lncRNA	chr7:53514992-5351
ENSG0000(	796	19.9087	chr7:330(	ASL	NCGv7	protein_c	chr7:66075800-6609
ENSG0000(	796	19.9087	chr7:330(	ZNF722		protein_c	chr7:63998849-6401
ENSG0000(	796	19.9087	chr7:330(	ENSG00000234089		lncRNA	chr7:57209865-5722
ENSG0000(	796	19.9087	chr7:330(	ENSG00000223836		Pseudoger	chr7:57820032-5782
ENSG0000(	796	19.9087	chr7:330(	ZNF727	DriverDB\	protein_c	chr7:64045434-6408
ENSG0000(	796	19.9087	chr7:330(	ZNF679		protein_c	chr7:64228474-6426
ENSG0000(	796	19.9087	chr7:330(	ENSG00000234085		Pseudoger	chr7:57654822-5765
ENSG0000(	796	19.9087	chr7:330(	ENSG00000287580		lncRNA	chr7:64801958-6480
ENSG0000(	796	19.9087	chr7:330(	ENSG00000287588		lncRNA	chr7:63925832-6393
ENSG0000(	796	19.9087	chr7:330(	ENSG00000227113		Pseudoger	chr7:65075023-6507
ENSG0000(	796	19.9087	chr7:330(	ENSG00000223740		Pseudoger	chr7:56525428-5652
ENSG0000(	796	19.9087	chr7:330(	MTCO2P8		Pseudoger	chr7:64107829-6410
ENSG0000(	796	19.9087	chr7:330(	MTND5P7		Pseudoger	chr7:57194257-5719
ENSG0000(	796	19.9087	chr7:330(	TRIM60P17		Pseudoger	chr7:64085560-6408
ENSG0000(	796	19.9087	chr7:330(	ENSG00000234387		lncRNA	chr7:63394277-6339
ENSG0000(	796	19.9087	chr7:330(	NMD3P1		Pseudoger	chr7:63908966-6391
ENSG0000(	796	19.9087	chr7:330(	RNU6-417P		smallRNA	chr7:63011463-6301
ENSG0000(	796	19.9087	chr7:330(	ENSG00000224669		Pseudoger	chr7:64582733-6458
ENSG0000(	796	19.9087	chr7:330(	RNU2-29P		smallRNA	chr7:53776136-5377
ENSG0000(	796	19.9087	chr7:330(	ENSG00000234338		Pseudoger	chr7:64835280-6483
ENSG0000(	796	19.9087	chr7:330(	SEPTIN7P4		Pseudoger	chr7:63241590-6324
ENSG0000(	796	19.9087	chr7:330(	SAPCD2P4		Pseudoger	chr7:63113635-6311
ENSG0000(	796	19.9087	chr7:330(	ARAFP2		Pseudoger	chr7:63404842-6340
ENSG0000(	796	19.9087	chr7:330(	ENSG00000286477		lncRNA	chr7:56659149-5667
ENSG0000(	796	19.9087	chr7:330(	SGO1P2		Pseudoger	chr7:52891837-5289
ENSG0000(	796	19.9087	chr7:330(	ENSG00000224365		Pseudoger	chr7:66004017-6600
ENSG0000(	796	19.9087	chr7:330(	ENSG00000286456		lncRNA	chr7:64574673-6461
ENSG0000(	796	19.9087	chr7:330(	ENSG00000287517		lncRNA	chr7:57242681-5724
ENSG0000(	796	19.9087	chr7:330(	ENSG00000286436		lncRNA	chr7:57140112-5715
ENSG0000(	796	19.9087	chr7:330(	ENSG00000286404		lncRNA	chr7:54721724-5473
ENSG0000(	796	19.9087	chr7:330(	ZNF92		protein_c	chr7:65373799-6540
ENSG0000(	796	19.9087	chr7:330(	ENSG00000286397		lncRNA	chr7:64035644-6403
ENSG0000(	796	19.9087	chr7:330(	VN1R28P		Pseudoger	chr7:57422830-5742
ENSG0000(	796	19.9087	chr7:330(	AC092685.1		smallRNA	chr7:65263414-6526
ENSG0000(	796	19.9087	chr7:330(	ENSG00000230386		Pseudoger	chr7:65617082-6561
ENSG0000(	796	19.9087	chr7:330(	ENSG00000235349		Pseudoger	chr7:64566814-6456
ENSG0000(	796	19.9087	chr7:330(	snoU13		smallRNA	chr7:54359892-5435
ENSG0000(	796	19.9087	chr7:330(	VN1R25P		Pseudoger	chr7:56559221-5655

ENSG0000(	796	19.9087	chr7:330(	PHKG1P2	Pseudoger	chr7:63509303-6351
ENSG0000(	796	19.9087	chr7:330(	SLC25A5P3	Pseudoger	chr7:54419444-5442
ENSG0000(	796	19.9087	chr7:330(	EGFR-AS1	lncRNA	chr7:55179750-5518
ENSG0000(	796	19.9087	chr7:330(	RPL31P35	Pseudoger	chr7:54656358-5465
ENSG0000(	796	19.9087	chr7:330(	ENSG00000232944	Pseudoger	chr7:56408699-5640
ENSG0000(	796	19.9087	chr7:330(	ENSG00000249319	protein_(	chr7:66087761-6615
ENSG0000(	796	19.9087	chr7:330(	ENSG00000213650	Pseudoger	chr7:56567906-5656
ENSG0000(	796	19.9087	chr7:330(	ENSG00000226767	Pseudoger	chr7:65508773-6550
ENSG0000(	796	19.9087	chr7:330(	SLC25A1P3	Pseudoger	chr7:63931141-6393
ENSG0000(	796	19.9087	chr7:330(	MTCO3P10	Pseudoger	chr7:57167880-5716
ENSG0000(	796	19.9087	chr7:330(	SAPCD2P1	Pseudoger	chr7:64181710-6418
ENSG0000(	796	19.9087	chr7:330(	ENSG00000291021	lncRNA	chr7:56875385-5688
ENSG0000(	796	19.9087	chr7:330(	ENSG00000288525	Pseudoger	chr7:54676217-5467
ENSG0000(	796	19.9087	chr7:330(	VN1R37P	Pseudoger	chr7:64149685-6415
ENSG0000(	796	19.9087	chr7:330(	ENSG00000237026	Pseudoger	chr7:65235790-6523
ENSG0000(	796	19.9087	chr7:330(	SNORA63	smallRNA	chr7:65327396-6532
ENSG0000(	796	19.9087	chr7:330(	AC069285.1	smallRNA	chr7:63068087-6306
ENSG0000(	796	19.9087	chr7:330(	RRBP1P1	Pseudoger	chr7:53002420-5300
ENSG0000(	796	19.9087	chr7:330(	ENSG00000213642	Pseudoger	chr7:64569428-6457
ENSG0000(	796	19.9087	chr7:330(	RPL6P20	Pseudoger	chr7:64141538-6414
ENSG0000(	796	19.9087	chr7:330(	snoU2_19	smallRNA	chr7:63175940-6317
ENSG0000(	796	19.9087	chr7:330(	ENSG00000232906	Pseudoger	chr7:65355934-6535
ENSG0000(	796	19.9087	chr7:330(	ENSG00000229064	Pseudoger	chr7:65247608-6524
ENSG0000(	796	19.9087	chr7:330(	EEF1DP4	Pseudoger	chr7:64862999-6486
ENSG0000(	796	19.9087	chr7:330(	ENSG00000276650	Pseudoger	chr7:64425623-6442
ENSG0000(	796	19.9087	chr7:330(	ENSG00000225451	Pseudoger	chr7:63876341-6387
ENSG0000(	796	19.9087	chr7:330(	ENSG00000224155	Pseudoger	chr7:56304678-5630
ENSG0000(	796	19.9087	chr7:330(	VN1R34P	Pseudoger	chr7:63934449-6393
ENSG0000(	796	19.9087	chr7:330(	SNORA15	smallRNA	chr7:56060470-5606
ENSG0000(	796	19.9087	chr7:330(	ARAFP1	Pseudoger	chr7:63937905-6393
ENSG0000(	796	19.9087	chr7:330(	ENSG00000224172	Pseudoger	chr7:64735933-6473
ENSG0000(	796	19.9087	chr7:330(	ENSG00000280225	TEC	chr7:57402181-5740
ENSG0000(	796	19.9087	chr7:330(	PHKG1	protein_(	chr7:56080283-5609
ENSG0000(	796	19.9087	chr7:330(	ENSG00000228204	lncRNA	chr7:50866747-5102
ENSG0000(	796	19.9087	chr7:330(	ENSG00000237268	Pseudoger	chr7:56421857-5643
ENSG0000(	796	19.9087	chr7:330(	ENSG00000228735	lncRNA	chr7:54576052-5457
ENSG0000(	796	19.9087	chr7:330(	FKBP9P1	Pseudoger	chr7:55682652-5571
ENSG0000(	796	19.9087	chr7:330(	RNU6-389P	smallRNA	chr7:55685977-5568
ENSG0000(	796	19.9087	chr7:330(	ENSG00000228303	Pseudoger	chr7:56638943-5665
ENSG0000(	796	19.9087	chr7:330(	ENSG00000176232	Pseudoger	chr7:63980385-6398
ENSG0000(	796	19.9087	chr7:330(	ENSG00000237236	Pseudoger	chr7:57822201-5782
ENSG0000(	796	19.9087	chr7:330(	SEPTIN14P24	Pseudoger	chr7:56360362-5636
ENSG0000(	796	19.9087	chr7:330(	ENSG00000237210	Pseudoger	chr7:55244247-5524
ENSG0000(	796	19.9087	chr7:330(	LINC03006	lncRNA	chr7:65647010-6577
ENSG0000(	796	19.9087	chr7:330(	ENSG00000233028	Pseudoger	chr7:56175634-5617
ENSG0000(	796	19.9087	chr7:330(	VOPP1-DT	lncRNA	chr7:55573171-5558
ENSG0000(	796	19.9087	chr7:330(	ENSG00000231317	Pseudoger	chr7:55656768-5567
ENSG0000(	796	19.9087	chr7:330(	ENSG00000232418	Pseudoger	chr7:53787167-5378
ENSG0000(	796	19.9087	chr7:330(	AC073136.1	smallRNA	chr7:56269783-5626
ENSG0000(	796	19.9087	chr7:330(	ENSG00000261275	lncRNA	chr7:56493124-5649
ENSG0000(	796	19.9087	chr7:330(	CCT6P3	lncRNA	chr7:65038354-6507

ENSG0000(	796	19.9087	chr7:330(	FKBP9P1		lncRNA	chr7:55681074-5568
ENSG0000(	796	19.9087	chr7:330(	ENSG00000291124		lncRNA	chr7:65751037-6576
ENSG0000(	796	19.9087	chr7:330(	CICP12		Pseudoger	chr7:55798034-5579
ENSG0000(	796	19.9087	chr7:330(	CCT6P1		Pseudoger	chr7:65751142-6576
ENSG0000(	796	19.9087	chr7:330(	ENSG00000236638		Pseudoger	chr7:63263065-6326
ENSG0000(	796	19.9087	chr7:330(	ENSG00000232817		lncRNA	chr7:63388808-6339
ENSG0000(	796	19.9087	chr7:330(	RNU1-14P		smallRNA	chr7:53366058-5336
ENSG0000(	796	19.9087	chr7:330(	MTCO1P10		Pseudoger	chr7:57187892-5718
ENSG0000(	796	19.9087	chr7:330(	ENSG00000279072		lncRNA	chr7:56809214-5684
ENSG0000(	796	19.9087	chr7:330(	ENSG00000275875		Pseudoger	chr7:55741525-5574
ENSG0000(	796	19.9087	chr7:330(	ENSG00000275833		Pseudoger	chr7:65764535-6576
ENSG0000(	796	19.9087	chr7:330(	ENSG00000228897		Pseudoger	chr7:51386363-5138
ENSG0000(	796	19.9087	chr7:330(	MTND5P6		Pseudoger	chr7:57170974-5717
ENSG0000(	796	19.9087	chr7:330(	ERV3-1		protein_c	chr7:64990356-6500
ENSG0000(	796	19.9087	chr7:330(	ENSG00000236299		lncRNA	chr7:63888200-6390
ENSG0000(	796	19.9087	chr7:330(	ENSG00000231394		lncRNA	chr7:55593777-5559
ENSG0000(	796	19.9087	chr7:330(	VN1R31P		Pseudoger	chr7:63367427-6336
ENSG0000(	796	19.9087	chr7:330(	MTCO3P4		Pseudoger	chr7:57191141-5719
ENSG0000(	796	19.9087	chr7:330(	YWHAEP1		Pseudoger	chr7:64433830-6443
ENSG0000(	796	19.9087	chr7:330(	RPL7L1P2		Pseudoger	chr7:51259429-5126
ENSG0000(	796	19.9087	chr7:330(	MTND4P4		Pseudoger	chr7:57169406-5717
ENSG0000(	796	19.9087	chr7:330(	ENSG00000226411		Pseudoger	chr7:63699390-6370
ENSG0000(	796	19.9087	chr7:330(	MIR3147		smallRNA	chr7:57405025-5740
ENSG0000(	796	19.9087	chr7:330(	ENSG00000225244		Pseudoger	chr7:56940341-5694
ENSG0000(	796	19.9087	chr7:330(	ENSG00000236261		Pseudoger	chr7:57652875-5765
ENSG0000(	796	19.9087	chr7:330(	ENSG00000236574		Pseudoger	chr7:63843314-6384
ENSG0000(	796	19.9087	chr7:330(	ENSG00000276475		Pseudoger	chr7:64590599-6459
ENSG0000(	796	19.9087	chr7:330(	ENSG00000250618		Pseudoger	chr7:57817996-5781
ENSG0000(	796	19.9087	chr7:330(	AC092634.1		smallRNA	chr7:63901068-6390
ENSG0000(	796	19.9087	chr7:330(	VSTM2A		protein_c	chr7:54542325-5457
ENSG0000(	796	19.9087	chr7:330(	ENSG00000213067		Pseudoger	chr7:56597379-5659
ENSG0000(	796	19.9087	chr7:330(	SNORA63		smallRNA	chr7:65326719-6532
ENSG0000(	796	19.9087	chr7:330(	SNORA73		smallRNA	chr7:54865818-5486
ENSG0000(	796	19.9087	chr7:330(	ENSG00000236907		Pseudoger	chr7:57817601-5781
ENSG0000(	796	19.9087	chr7:330(	ZNF680	IntOGen-I	protein_c	chr7:64519878-6456
ENSG0000(	796	19.9087	chr7:330(	RBM22P3		Pseudoger	chr7:56490682-5649
ENSG0000(	796	19.9087	chr7:330(	LINC01445		lncRNA	chr7:54330670-5445
ENSG0000(	796	19.9087	chr7:330(	RNU6-1052P		smallRNA	chr7:56402070-5640
ENSG0000(	796	19.9087	chr7:330(	ENSG00000218586		Pseudoger	chr7:54933699-5493
ENSG0000(	796	19.9087	chr7:330(	CICP8		Pseudoger	chr7:56362458-5636
ENSG0000(	796	19.9087	chr7:330(	BNIP3P42		Pseudoger	chr7:64551498-6455
ENSG0000(	796	19.9087	chr7:330(	ENSG00000249773		protein_c	chr7:55887277-5595
ENSG0000(	796	19.9087	chr7:330(	ENSG00000278577		Pseudoger	chr7:63993904-6399
ENSG0000(	796	19.9087	chr7:330(	RN7SL816P		smallRNA	chr7:56965248-5696
ENSG0000(	796	19.9087	chr7:330(	SEPTIN14P1		Pseudoger	chr7:63117501-6315
ENSG0000(	796	19.9087	chr7:330(	NMD3P2		Pseudoger	chr7:56502284-5650
ENSG0000(	796	19.9087	chr7:330(	MTND4P2		Pseudoger	chr7:64104093-6410
ENSG0000(	796	19.9087	chr7:330(	SEPTIN7P5		Pseudoger	chr7:63495799-6350
ENSG0000(	796	19.9087	chr7:330(	INTS4P1		Pseudoger	chr7:65141241-6521
ENSG0000(	796	19.9087	chr7:330(	ENSG00000237572		Pseudoger	chr7:63209219-6321
ENSG0000(	796	19.9087	chr7:330(	ENSG00000284534		lncRNA	chr7:63959844-6398

ENSG0000(	796	19.9087	chr7:330(	CICP28		Pseudoger	chr7:56805336-5680
ENSG0000(	796	19.9087	chr7:330(	ENSG00000284474		Pseudoger	chr7:57167039-5716
ENSG0000(	796	19.9087	chr7:330(	COBL	DriverDBv	protein_c	chr7:51016212-5131
ENSG0000(	796	19.9087	chr7:330(	ENSG00000277206		Pseudoger	chr7:64768156-6476
ENSG0000(	796	19.9087	chr7:330(	ENSG00000243981		Pseudoger	chr7:57628479-5764
ENSG0000(	796	19.9087	chr7:330(	MTCO2P10		Pseudoger	chr7:57189578-5719
ENSG0000(	796	19.9087	chr7:330(	ENSG00000237639		Pseudoger	chr7:57650521-5765
ENSG0000(	796	19.9087	chr7:330(	CHCHD2	DriverDBv	protein_c	chr7:56101573-5610
ENSG0000(	796	19.9087	chr7:330(	ENSG00000284221		Pseudoger	chr7:57192405-5719
ENSG0000(	796	19.9087	chr7:330(	ENSG00000277174		Pseudoger	chr7:56685036-5668
ENSG0000(	796	19.9087	chr7:330(	ENSG00000284098		Pseudoger	chr7:57190300-5719
ENSG0000(	796	19.9087	chr7:330(	MTATP6P10		Pseudoger	chr7:57190461-5719
ENSG0000(	796	19.9087	chr7:330(	ENSG00000268181		lncRNA	chr7:63396341-6339
ENSG0000(	796	19.9087	chr7:330(	ENSG00000225488		lncRNA	chr7:56477588-5648
ENSG0000(	796	19.9087	chr7:330(	MTATP6P8		Pseudoger	chr7:57167200-5716
ENSG0000(	796	19.9087	chr7:330(	RN7SL64P		smallRNA	chr7:55892726-5589
ENSG0000(	796	19.9087	chr7:330(	SNORA15		smallRNA	chr7:65070538-6507
ENSG0000(	796	19.9087	chr7:330(	BSNDP4		Pseudoger	chr7:57638725-5763
ENSG0000(	796	19.9087	chr7:330(	MTND4LP32		Pseudoger	chr7:57169143-5716
ENSG0000(	796	19.9087	chr7:330(	RN7SL855P		smallRNA	chr7:63611445-6361
ENSG0000(	796	19.9087	chr7:330(	ENSG00000284558		Pseudoger	chr7:57191997-5719
ENSG0000(	796	19.9087	chr7:330(	ENSG00000284572		Pseudoger	chr7:57196056-5719
ENSG0000(	796	19.9087	chr7:330(	ZNF716	DriverDBv	protein_c	chr7:57450177-5747
ENSG0000(	796	19.9087	chr7:330(	ENSG00000231484		Pseudoger	chr7:57828331-5783
ENSG0000(	796	19.9087	chr7:330(	ENSG00000233437		Pseudoger	chr7:56876500-5687
ENSG0000(	796	19.9087	chr7:330(	CICP11		Pseudoger	chr7:55736779-5573
ENSG0000(	796	19.9087	chr7:330(	ENSG00000228627		lncRNA	chr7:53559214-5357
ENSG0000(	796	19.9087	chr7:330(	GTF2IP14		Pseudoger	chr7:65084103-6510
ENSG0000(	796	19.9087	chr7:330(	SNORA22		smallRNA	chr7:65755526-6575
ENSG0000(	796	19.9087	chr7:330(	ENSG00000277544		Pseudoger	chr7:65290429-6529
ENSG0000(	796	19.9087	chr7:330(	HNRNPCP7		Pseudoger	chr7:64500825-6450
ENSG0000(	796	19.9087	chr7:330(	SEPHS1P1		Pseudoger	chr7:64852397-6485
ENSG0000(	796	19.9087	chr7:330(	ZNF734P		Pseudoger	chr7:63449819-6345
ENSG0000(	796	19.9087	chr7:330(	ENSG00000227910		Pseudoger	chr7:63924787-6392
ENSG0000(	796	19.9087	chr7:330(	VN1R35P		Pseudoger	chr7:63963431-6396
ENSG0000(	796	19.9087	chr7:330(	ZNF273		protein_c	chr7:64870172-6493
ENSG0000(	796	19.9087	chr7:330(	ENSG00000289108		lncRNA	chr7:65219563-6525
ENSG0000(	796	19.9087	chr7:330(	INTS4P2		Pseudoger	chr7:65647823-6571
ENSG0000(	796	19.9087	chr7:330(	ENSG00000229762		Pseudoger	chr7:55810779-5581
ENSG0000(	796	19.9087	chr7:330(	TUBBP6		Pseudoger	chr7:55645620-5564
ENSG0000(	796	19.9087	chr7:330(	ZNF117		protein_c	chr7:64971772-6500
ENSG0000(	796	19.9087	chr7:330(	SUMO2P3		Pseudoger	chr7:55732144-5573
ENSG0000(	796	19.9087	chr7:330(	ENSG00000227923		Pseudoger	chr7:63354033-6335
ENSG0000(	796	19.9087	chr7:330(	MTCO3P8		Pseudoger	chr7:64106065-6410
ENSG0000(	796	19.9087	chr7:330(	GUSBP12		Pseudoger	chr7:57200825-5720
ENSG0000(	796	19.9087	chr7:330(	ENSG00000283431		Pseudoger	chr7:57163168-5716
ENSG0000(	796	19.9087	chr7:330(	ENSG00000288798		lncRNA	chr7:51327615-5135
ENSG0000(	796	19.9087	chr7:330(	ENSG00000287953		lncRNA	chr7:54185071-5425
ENSG0000(	796	19.9087	chr7:330(	ENSG00000282381		lncRNA	chr7:65269359-6531
ENSG0000(	796	19.9087	chr7:330(	ENSG00000231232		Pseudoger	chr7:57822465-5782
ENSG0000(	796	19.9087	chr7:330(	ENSG00000205596		Pseudoger	chr7:66070904-6607

ENSG0000(	796	19.9087	chr7:330(	ENSG00000229403		lncRNA	chr7:51614251-5163
ENSG0000(	796	19.9087	chr7:330(	ENSG00000228085		Pseudoger	chr7:50843578-5084
ENSG0000(	796	19.9087	chr7:330(	GUSB	NCGv7	protein_c	chr7:65960684-6598
ENSG0000(	796	19.9087	chr7:330(	ENSG00000274299		Pseudoger	chr7:57270839-5727
ENSG0000(	796	19.9087	chr7:330(	ENSG00000236046		lncRNA	chr7:50839363-5084
ENSG0000(	796	19.9087	chr7:330(	ENSG00000287985		lncRNA	chr7:64369241-6437
ENSG0000(	796	19.9087	chr7:330(	ZNF713	DriverDB\	protein_c	chr7:55887456-5594
ENSG0000(	796	19.9087	chr7:330(	MTND4LP2		Pseudoger	chr7:64105292-6410
ENSG0000(	796	19.9087	chr7:330(	ENSG00000280920		lncRNA	chr7:54201224-5420
ENSG0000(	796	19.9087	chr7:330(	ELDR		lncRNA	chr7:55235965-5525
ENSG0000(	796	19.9087	chr7:330(	ENSG00000229301		lncRNA	chr7:63354457-6335
ENSG0000(	796	19.9087	chr7:330(	SNORA31		smallRNA	chr7:52269437-5226
ENSG0000(	796	19.9087	chr7:330(	MTND2P6		Pseudoger	chr7:57186412-5718
ENSG0000(	796	19.9087	chr7:330(	SEPTIN7P15		Pseudoger	chr7:57067950-5707
ENSG0000(	796	19.9087	chr7:330(	AC068533.1		smallRNA	chr7:66056234-6605
ENSG0000(	796	19.9087	chr7:330(	ENSG00000278205		Pseudoger	chr7:56462536-5646
ENSG0000(	796	19.9087	chr7:330(	SNORA22		smallRNA	chr7:56055365-5605
ENSG0000(	796	19.9087	chr7:330(	ENSG00000236529		lncRNA	chr7:65840055-6584
ENSG0000(	796	19.9087	chr7:330(	ENSG00000244550		lncRNA	chr7:63233115-6324
ENSG0000(	796	19.9087	chr7:330(	ENSG00000291184		lncRNA	chr7:56426859-5644
ENSG0000(	796	19.9087	chr7:330(	ROBO2P1		Pseudoger	chr7:51600286-5160
ENSG0000(	796	19.9087	chr7:330(	ZNF680P1		Pseudoger	chr7:64469185-6447
ENSG0000(	796	19.9087	chr7:330(	TRIM60P18		Pseudoger	chr7:64355078-6435
ENSG0000(	796	19.9087	chr7:330(	ENSG00000226401		Pseudoger	chr7:63632608-6363
ENSG0000(	796	19.9087	chr7:330(	ENSG00000233288		lncRNA	chr7:56528253-5653
ENSG0000(	796	19.9087	chr7:330(	ENSG00000287869		lncRNA	chr7:64867427-6487
ENSG0000(	796	19.9087	chr7:330(	ENSG00000273720		Pseudoger	chr7:55743073-5574
ENSG0000(	796	19.9087	chr7:330(	VSTM2A-OT1		lncRNA	chr7:54556970-5457
ENSG0000(	796	19.9087	chr7:330(	RSL24D1P3		Pseudoger	chr7:65335174-6533
ENSG0000(	796	19.9087	chr7:330(	MTND6P29		Pseudoger	chr7:57172772-5717
ENSG0000(	796	19.9087	chr7:330(	CCNJP1		Pseudoger	chr7:56228634-5622
ENSG0000(	796	19.9087	chr7:330(	ENSG00000283230		Pseudoger	chr7:57168725-5716
ENSG0000(	796	19.9087	chr7:330(	ENSG00000250923		Pseudoger	chr7:57773183-5777
ENSG0000(	796	19.9087	chr7:330(	ENSG00000225018		Pseudoger	chr7:55876738-5587
ENSG0000(	796	19.9087	chr7:330(	LINC01005		lncRNA	chr7:64024409-6403
ENSG0000(	796	19.9087	chr7:330(	LINC01446		lncRNA	chr7:53655508-5381
ENSG0000(	796	19.9087	chr7:330(	ENSG00000227499		Pseudoger	chr7:55877912-5587
ENSG0000(	796	19.9087	chr7:330(	ENSG00000282879		Pseudoger	chr7:56736396-5673
ENSG0000(	796	19.9087	chr7:330(	PHKG1P4		Pseudoger	chr7:57060590-5706
ENSG0000(	795	19.88369	chr7:330(	AC079398.1		smallRNA	chr7:71310459-7131
ENSG0000(	786	19.65859	chr7:330(	GTF2IP9		Pseudoger	chr7:66407288-6640
ENSG0000(	786	19.65859	chr7:330(	SAPCD2P3		Pseudoger	chr7:66556216-6655
ENSG0000(	786	19.65859	chr7:330(	TPST1	DriverDB\	protein_c	chr7:66205317-6642
ENSG0000(	786	19.65859	chr7:330(	ENSG00000232546		Pseudoger	chr7:66848496-6685
ENSG0000(	786	19.65859	chr7:330(	RNA5SP232		Pseudoger	chr7:71913738-7191
ENSG0000(	786	19.65859	chr7:330(	ABCF2P2		Pseudoger	chr7:72103181-7210
ENSG0000(	786	19.65859	chr7:330(	RPL35P5		Pseudoger	chr7:66606738-6660
ENSG0000(	786	19.65859	chr7:330(	GTF2IRD1P1		lncRNA	chr7:66809993-6684
ENSG0000(	786	19.65859	chr7:330(	CALN1		protein_c	chr7:71779491-7244
ENSG0000(	786	19.65859	chr7:330(	PMS2P4		lncRNA	chr7:67139961-6730
ENSG0000(	786	19.65859	chr7:330(	SBDS	NCGv7;AC	protein_c	chr7:66987680-6699

ENSG0000(	786	19.65859	chr7:330(	GTF2IP23		Pseudoger	chr7:66880708-6688
ENSG0000(	786	19.65859	chr7:330(	ENSG00000179131		Pseudoger	chr7:66914581-6691
ENSG0000(	786	19.65859	chr7:330(	ENSG00000179342		Pseudoger	chr7:66505155-6650
ENSG0000(	786	19.65859	chr7:330(	LINC00174		lncRNA	chr7:66376044-6649
ENSG0000(	786	19.65859	chr7:330(	GALNT17		protein_c	chr7:71132144-7171
ENSG0000(	786	19.65859	chr7:330(	ENSG00000228019		Pseudoger	chr7:67307605-6730
ENSG0000(	786	19.65859	chr7:330(	RABGEF1P2		Pseudoger	chr7:66427949-6645
ENSG0000(	786	19.65859	chr7:330(	ENSG00000244657		Pseudoger	chr7:66485095-6648
ENSG0000(	786	19.65859	chr7:330(	ENSG00000273448		lncRNA	chr7:67333047-6733
ENSG0000(	786	19.65859	chr7:330(	ENSG00000244510		Pseudoger	chr7:66480394-6649
ENSG0000(	786	19.65859	chr7:330(	STAG3L4		Pseudoger	chr7:67305987-6730
ENSG0000(	786	19.65859	chr7:330(	TMEM248		protein_c	chr7:66921225-6695
ENSG0000(	786	19.65859	chr7:330(	LINC02604		lncRNA	chr7:66902857-6690
ENSG0000(	786	19.65859	chr7:330(	ENSG00000284461		protein_c	chr7:66628958-6681
ENSG0000(	786	19.65859	chr7:330(	ENSG00000233689		lncRNA	chr7:69026433-6904
ENSG0000(	786	19.65859	chr7:330(	MTND4P3		Pseudoger	chr7:68266319-6826
ENSG0000(	786	19.65859	chr7:330(	ENSG00000233383		Pseudoger	chr7:67089257-6708
ENSG0000(	786	19.65859	chr7:330(	KCTD7	NCGv7	protein_c	chr7:66628881-6664
ENSG0000(	786	19.65859	chr7:330(	ENSG00000226824		lncRNA	chr7:66654513-6668
ENSG0000(	786	19.65859	chr7:330(	ENSG00000226829		lncRNA	chr7:68149548-6831
ENSG0000(	786	19.65859	chr7:330(	ENSG00000233423		lncRNA	chr7:67691058-6769
ENSG0000(	786	19.65859	chr7:330(	ENSG00000177418		Pseudoger	chr7:66556889-6655
ENSG0000(	786	19.65859	chr7:330(	CT66		lncRNA	chr7:69594793-6959
ENSG0000(	786	19.65859	chr7:330(	STAG3L4		lncRNA	chr7:67302621-6736
ENSG0000(	786	19.65859	chr7:330(	RNU6-1254P		smallRNA	chr7:66891188-6689
ENSG0000(	786	19.65859	chr7:330(	ENSG00000271064		Pseudoger	chr7:66748838-6674
ENSG0000(	786	19.65859	chr7:330(	AC073089.1		smallRNA	chr7:67139071-6713
ENSG0000(	786	19.65859	chr7:330(	ENSG00000228429		Pseudoger	chr7:68640798-6864
ENSG0000(	786	19.65859	chr7:330(	ENSG00000291136		lncRNA	chr7:66526088-6659
ENSG0000(	786	19.65859	chr7:330(	ENSG00000275400		Pseudoger	chr7:66553805-6655
ENSG0000(	786	19.65859	chr7:330(	MTCO2P25		Pseudoger	chr7:69330698-6933
ENSG0000(	786	19.65859	chr7:330(	AUTS2		protein_c	chr7:69598296-7079
ENSG0000(	786	19.65859	chr7:330(	ENSG00000225718		lncRNA	chr7:69186821-6943
ENSG0000(	786	19.65859	chr7:330(	RN7SL43P		smallRNA	chr7:66980120-6698
ENSG0000(	786	19.65859	chr7:330(	ENSG00000225209		lncRNA	chr7:68020235-6803
ENSG0000(	786	19.65859	chr7:330(	ENSG00000234500		Pseudoger	chr7:66511556-6654
ENSG0000(	786	19.65859	chr7:330(	Y_RNA		smallRNA	chr7:67297653-6729
ENSG0000(	786	19.65859	chr7:330(	RNU6-313P		smallRNA	chr7:66344304-6634
ENSG0000(	786	19.65859	chr7:330(	MTCO1P25		Pseudoger	chr7:69331835-6933
ENSG0000(	786	19.65859	chr7:330(	ENSG00000279785		TEC	chr7:66474556-6647
ENSG0000(	786	19.65859	chr7:330(	snoU13		smallRNA	chr7:66434507-6643
ENSG0000(	786	19.65859	chr7:330(	GTF2IRD1P1		Pseudoger	chr7:66815836-6683
ENSG0000(	786	19.65859	chr7:330(	ENSG00000286466		lncRNA	chr7:69145185-6917
ENSG0000(	786	19.65859	chr7:330(	ENSG00000272831		lncRNA	chr7:66739829-6674
ENSG0000(	786	19.65859	chr7:330(	RNU6-229P		smallRNA	chr7:69401202-6940
ENSG0000(	786	19.65859	chr7:330(	MTCO1P57		Pseudoger	chr7:68097737-6809
ENSG0000(	786	19.65859	chr7:330(	snoU13		smallRNA	chr7:66790354-6679
ENSG0000(	786	19.65859	chr7:330(	RN7SKP75		smallRNA	chr7:71685452-7168
ENSG0000(	786	19.65859	chr7:330(	Y_RNA		smallRNA	chr7:69507510-6950
ENSG0000(	786	19.65859	chr7:330(	ENSG00000289177		lncRNA	chr7:66845003-6684
ENSG0000(	786	19.65859	chr7:330(	ENSG00000236531		lncRNA	chr7:68091223-6811

ENSG0000(	786	19.65859	chr7:330(	AC006480.1		smallRNA	chr7:67356680-6735
ENSG0000(	786	19.65859	chr7:330(	RNA5SP231		Pseudoger	chr7:68723911-6872
ENSG0000(	786	19.65859	chr7:330(	RN7SL371P		smallRNA	chr7:69536049-6953
ENSG0000(	786	19.65859	chr7:330(	SKP1P1		Pseudoger	chr7:66423405-6642
ENSG0000(	786	19.65859	chr7:330(	ENSG00000236928		Pseudoger	chr7:66434634-6643
ENSG0000(	786	19.65859	chr7:330(	RNU6-96P		smallRNA	chr7:66395191-6639
ENSG0000(	786	19.65859	chr7:330(	MIR4650-1		smallRNA	chr7:67114322-6711
ENSG0000(	786	19.65859	chr7:330(	TYW1		protein_c	chr7:66995173-6723
ENSG0000(	786	19.65859	chr7:330(	MIR3914-1		smallRNA	chr7:71307672-7130
ENSG0000(	786	19.65859	chr7:330(	RABGEF1	NCGv7	protein_c	chr7:66682164-6681
ENSG0000(	786	19.65859	chr7:330(	ENSG00000236978		Pseudoger	chr7:70837738-7083
ENSG0000(	786	19.65859	chr7:330(	AC069280.1		smallRNA	chr7:69190141-6919
ENSG0000(	786	19.65859	chr7:330(	MTCO3P41		Pseudoger	chr7:67628022-6762
ENSG0000(	786	19.65859	chr7:330(	ENSG00000223948		Pseudoger	chr7:67769629-6777
ENSG0000(	786	19.65859	chr7:330(	ENSG00000289015		lncRNA	chr7:66681258-6668
ENSG0000(	786	19.65859	chr7:330(	ENSG00000237754		Pseudoger	chr7:68241637-6824
ENSG0000(	786	19.65859	chr7:330(	ENSG00000235581		Pseudoger	chr7:71942259-7194
ENSG0000(	786	19.65859	chr7:330(	RNU6-832P		smallRNA	chr7:69125270-6912
ENSG0000(	786	19.65859	chr7:330(	MTATP6P21		Pseudoger	chr7:67627831-6762
ENSG0000(	786	19.65859	chr7:330(	PMS2P4		Pseudoger	chr7:67295608-6729
ENSG0000(	786	19.65859	chr7:330(	ENSG00000236839		lncRNA	chr7:69187833-6918
ENSG0000(	786	19.65859	chr7:330(	LINC03011		lncRNA	chr7:66493607-6649
ENSG0000(	782	19.55855	chr1:8137	CAPZA1		protein_c	chr1:112619805-112
ENSG0000(	782	19.55855	chr1:8137	MRPL57P1		Pseudoger	chr1:114279011-114
ENSG0000(	782	19.55855	chr1:8137	U3		smallRNA	chr1:116278606-116
ENSG0000(	782	19.55855	chr1:8137	RN7SL432P		smallRNA	chr1:114697629-114
ENSG0000(	782	19.55855	chr1:8137	ATP1A1-AS1		lncRNA	chr1:116378437-116
ENSG0000(	782	19.55855	chr1:8137	LINC02868		lncRNA	chr1:116694112-116
ENSG0000(	782	19.55855	chr1:8137	RN7SL420P		smallRNA	chr1:115606471-115
ENSG0000(	782	19.55855	chr1:8137	HSD3B2		protein_c	chr1:119414931-119
ENSG0000(	782	19.55855	chr1:8137	GAPDHP27		Pseudoger	chr1:119558755-119
ENSG0000(	782	19.55855	chr1:8137	ENSG00000232895		lncRNA	chr1:114206427-114
ENSG0000(	782	19.55855	chr1:8137	AKR7A2P1		Pseudoger	chr1:112923423-112
ENSG0000(	782	19.55855	chr1:8137	CNOT7P2		Pseudoger	chr1:115564601-115
ENSG0000(	782	19.55855	chr1:8137	ENSG00000272715		lncRNA	chr1:116909149-116
ENSG0000(	782	19.55855	chr1:8137	HSD3BP1		Pseudoger	chr1:119467221-119
ENSG0000(	782	19.55855	chr1:8137	PPM1J-DT		lncRNA	chr1:112715672-112
ENSG0000(	782	19.55855	chr1:8137	TENT5C	NCGv7;AC	protein_c	chr1:117606048-117
ENSG0000(	782	19.55855	chr1:8137	CSDE1	NCGv7	protein_c	chr1:114716913-114
ENSG0000(	782	19.55855	chr1:8137	GAPDHP58		Pseudoger	chr1:119495836-119
ENSG0000(	782	19.55855	chr1:8137	PPM1J	NCGv7	protein_c	chr1:112709994-112
ENSG0000(	782	19.55855	chr1:8137	ENSG00000226973		Pseudoger	chr1:115471941-115
ENSG0000(	782	19.55855	chr1:8137	TBX15		protein_c	chr1:118883046-118
ENSG0000(	782	19.55855	chr1:8137	SLC16A1		protein_c	chr1:112911847-112
ENSG0000(	782	19.55855	chr1:8137	ENSG00000226984		Pseudoger	chr1:114459934-114
ENSG0000(	782	19.55855	chr1:8137	AL512638.1		smallRNA	chr1:115485651-115
ENSG0000(	782	19.55855	chr1:8137	GAPDHP33		Pseudoger	chr1:119596167-119
ENSG0000(	782	19.55855	chr1:8137	RHOC	AC	protein_c	chr1:112701127-112
ENSG0000(	782	19.55855	chr1:8137	ENSG00000231128		lncRNA	chr1:113812379-113
ENSG0000(	782	19.55855	chr1:8137	MOV10	NCGv7	protein_c	chr1:112673141-112
ENSG0000(	782	19.55855	chr1:8137	MAB21L3		protein_c	chr1:116111399-116

ENSG0000(	782	19.55855	chr1:8137	ENSG00000271143		Pseudoger	chr1:115916497-115
ENSG0000(	782	19.55855	chr1:8137	TENT5C-DT		lncRNA	chr1:117596832-117
ENSG0000(	782	19.55855	chr1:8137	HNRNPA1P43		Pseudoger	chr1:115856910-115
ENSG0000(	782	19.55855	chr1:8137	HSD3BP3		Pseudoger	chr1:119538509-119
ENSG0000(	782	19.55855	chr1:8137	ENSG00000224950		lncRNA	chr1:116493016-116
ENSG0000(	782	19.55855	chr1:8137	ENSG00000236887		Pseudoger	chr1:113198825-113
ENSG0000(	782	19.55855	chr1:8137	AMPD1		protein_c	chr1:114673090-114
ENSG0000(	782	19.55855	chr1:8137	ENSG00000227712		lncRNA	chr1:119230313-119
ENSG0000(	782	19.55855	chr1:8137	BCAS2		protein_c	chr1:114567557-114
ENSG0000(	782	19.55855	chr1:8137	OLFML3		protein_c	chr1:113979391-114
ENSG0000(	782	19.55855	chr1:8137	VPS25P1		Pseudoger	chr1:117549415-117
ENSG0000(	782	19.55855	chr1:8137	PHTF1		protein_c	chr1:113696831-113
ENSG0000(	782	19.55855	chr1:8137	VANGL1	NCGv7	protein_c	chr1:115641970-115
ENSG0000(	782	19.55855	chr1:8137	WARS2-AS1		lncRNA	chr1:119140391-119
ENSG0000(	782	19.55855	chr1:8137	CD58	NCGv7	protein_c	chr1:116514534-116
ENSG0000(	782	19.55855	chr1:8137	GDAP2		protein_c	chr1:117863485-117
ENSG0000(	782	19.55855	chr1:8137	CD2		protein_c	chr1:116754430-116
ENSG0000(	782	19.55855	chr1:8137	HSD3BP4		Pseudoger	chr1:119564066-119
ENSG0000(	782	19.55855	chr1:8137	HSD3B1		protein_c	chr1:119507198-119
ENSG0000(	782	19.55855	chr1:8137	TTF2		protein_c	chr1:117060326-117
ENSG0000(	782	19.55855	chr1:8137	WARS2		protein_c	chr1:119031216-119
ENSG0000(	782	19.55855	chr1:8137	HAO2		protein_c	chr1:119368779-119
ENSG0000(	782	19.55855	chr1:8137	ENSG00000279513		TEC	chr1:117493515-117
ENSG0000(	782	19.55855	chr1:8137	AL355794.1		smallRNA	chr1:116592106-116
ENSG0000(	782	19.55855	chr1:8137	ENSG00000273406		lncRNA	chr1:120076616-120
ENSG0000(	782	19.55855	chr1:8137	WARS2-IT1		lncRNA	chr1:119047405-119
ENSG0000(	782	19.55855	chr1:8137	ELOCP20		Pseudoger	chr1:115556826-115
ENSG0000(	782	19.55855	chr1:8137	HSD3BP2		Pseudoger	chr1:119439001-119
ENSG0000(	782	19.55855	chr1:8137	AL136376.1		protein_c	chr1:116373268-116
ENSG0000(	782	19.55855	chr1:8137	MTND5P20		Pseudoger	chr1:113576757-113
ENSG0000(	782	19.55855	chr1:8137	FTH1P22		Pseudoger	chr1:116775104-116
ENSG0000(	782	19.55855	chr1:8137	LINC01357		lncRNA	chr1:112849821-112
ENSG0000(	782	19.55855	chr1:8137	NAP1L4P1		Pseudoger	chr1:116532936-116
ENSG0000(	782	19.55855	chr1:8137	HIPK1		protein_c	chr1:113929324-113
ENSG0000(	782	19.55855	chr1:8137	GAPDHP23		Pseudoger	chr1:119462029-119
ENSG0000(	782	19.55855	chr1:8137	RP11-277L2.6		Pseudoger	chr1:120861539-120
ENSG0000(	782	19.55855	chr1:8137	ENSG00000225075		lncRNA	chr1:112693688-112
ENSG0000(	782	19.55855	chr1:8137	NGF-AS1		lncRNA	chr1:115283034-115
ENSG0000(	782	19.55855	chr1:8137	NBPF7P		Pseudoger	chr1:119834870-119
ENSG0000(	782	19.55855	chr1:8137	LINC01356		lncRNA	chr1:112820170-112
ENSG0000(	782	19.55855	chr1:8137	PNRC2P1		Pseudoger	chr1:117778087-117
ENSG0000(	782	19.55855	chr1:8137	LINC01649		lncRNA	chr1:115904855-115
ENSG0000(	782	19.55855	chr1:8137	LRIG2	NCGv7	protein_c	chr1:113073198-113
ENSG0000(	782	19.55855	chr1:8137	ENSG00000286276		lncRNA	chr1:116493350-116
ENSG0000(	782	19.55855	chr1:8137	ENSG00000274468		Pseudoger	chr1:115479428-115
ENSG0000(	782	19.55855	chr1:8137	LRIG2-DT		lncRNA	chr1:113011687-113
ENSG0000(	782	19.55855	chr1:8137	GAPDHP64		Pseudoger	chr1:116713833-116
ENSG0000(	782	19.55855	chr1:8137	RLIMP2		Pseudoger	chr1:113125321-113
ENSG0000(	782	19.55855	chr1:8137	MAGI3		protein_c	chr1:113390515-113
ENSG0000(	782	19.55855	chr1:8137	RSBN1	NCGv7	protein_c	chr1:113761832-113
ENSG0000(	782	19.55855	chr1:8137	DCLRE1B		protein_c	chr1:113904619-113

ENSG0000(	782	19.55855	chr1:8137	ENSG00000226172		lncRNA	chr1:119000344-119
ENSG0000(	782	19.55855	chr1:8137	HSD3BP5		Pseudoger	chr1:119601340-119
ENSG0000(	782	19.55855	chr1:8137	CASQ2		protein_c	chr1:115700021-115
ENSG0000(	782	19.55855	chr1:8137	RNVU1-19		smallRNA	chr1:120850819-120
ENSG0000(	782	19.55855	chr1:8137	NHLH2		protein_c	chr1:115836377-115
ENSG0000(	782	19.55855	chr1:8137	RPL6P2		Pseudoger	chr1:119219314-119
ENSG0000(	782	19.55855	chr1:8137	Y_RNA		smallRNA	chr1:116452536-116
ENSG0000(	782	19.55855	chr1:8137	ENSG00000271810		protein_c	chr1:112702614-112
ENSG0000(	782	19.55855	chr1:8137	AP4B1-AS1		lncRNA	chr1:113856635-113
ENSG0000(	782	19.55855	chr1:8137	TRIM33	NCGv7;AC	protein_c	chr1:114392790-114
ENSG0000(	782	19.55855	chr1:8137	RNVU1-13		smallRNA	chr1:120850818-120
ENSG0000(	782	19.55855	chr1:8137	PSMC1P12		Pseudoger	chr1:118614333-118
ENSG0000(	782	19.55855	chr1:8137	ENSG00000237993		lncRNA	chr1:116013813-116
ENSG0000(	782	19.55855	chr1:8137	LINC00622		lncRNA	chr1:119597702-119
ENSG0000(	782	19.55855	chr1:8137	CD101-AS1		lncRNA	chr1:117025482-117
ENSG0000(	782	19.55855	chr1:8137	ENSG00000229911		Pseudoger	chr1:118712999-118
ENSG0000(	782	19.55855	chr1:8137	NUTF2P4		Pseudoger	chr1:112748095-112
ENSG0000(	782	19.55855	chr1:8137	ENSG00000285698		lncRNA	chr1:115270767-115
ENSG0000(	782	19.55855	chr1:8137	RNU6-817P		smallRNA	chr1:116413766-116
ENSG0000(	782	19.55855	chr1:8137	MIR942		smallRNA	chr1:117094643-117
ENSG0000(	782	19.55855	chr1:8137	ENSG00000228040		Pseudoger	chr1:112890767-112
ENSG0000(	782	19.55855	chr1:8137	NEFHP1		Pseudoger	chr1:116739981-116
ENSG0000(	782	19.55855	chr1:8137	RP3-328E19.4		Pseudoger	chr1:120844645-120
ENSG0000(	782	19.55855	chr1:8137	HIPK1-AS1		lncRNA	chr1:113924000-113
ENSG0000(	782	19.55855	chr1:8137	ENSG00000271419		Pseudoger	chr1:114353986-114
ENSG0000(	782	19.55855	chr1:8137	RNA5SP55		Pseudoger	chr1:116962347-116
ENSG0000(	782	19.55855	chr1:8137	ENSG00000271427		lncRNA	chr1:117364899-117
ENSG0000(	782	19.55855	chr1:8137	LINC01765		lncRNA	chr1:115099580-115
ENSG0000(	782	19.55855	chr1:8137	PFN1P9		Pseudoger	chr1:119853316-119
ENSG0000(	782	19.55855	chr1:8137	TAFA3		protein_c	chr1:112718905-112
ENSG0000(	782	19.55855	chr1:8137	ENSG00000282048		lncRNA	chr1:114032377-114
ENSG0000(	782	19.55855	chr1:8137	DENND2C		protein_c	chr1:114582848-114
ENSG0000(	782	19.55855	chr1:8137	ENSG00000230381		lncRNA	chr1:116429049-116
ENSG0000(	782	19.55855	chr1:8137	ZNF697	NCGv7	protein_c	chr1:119619377-119
ENSG0000(	782	19.55855	chr1:8137	IGSF3		protein_c	chr1:116574399-116
ENSG0000(	782	19.55855	chr1:8137	LINC01762		lncRNA	chr1:116423724-116
ENSG0000(	782	19.55855	chr1:8137	ENSG00000233839		Pseudoger	chr1:113168994-113
ENSG0000(	782	19.55855	chr1:8137	MIR320B1		smallRNA	chr1:116671749-116
ENSG0000(	782	19.55855	chr1:8137	ENSG00000287980		lncRNA	chr1:118185349-118
ENSG0000(	782	19.55855	chr1:8137	Y_RNA		smallRNA	chr1:114727720-114
ENSG0000(	782	19.55855	chr1:8137	MIR548AC		smallRNA	chr1:116560024-116
ENSG0000(	782	19.55855	chr1:8137	RNU7-70P		smallRNA	chr1:112634719-112
ENSG0000(	782	19.55855	chr1:8137	SYCP1		protein_c	chr1:114854863-114
ENSG0000(	782	19.55855	chr1:8137	RBMX2P3		Pseudoger	chr1:119084998-119
ENSG0000(	782	19.55855	chr1:8137	NR1H5P		Pseudoger	chr1:114837227-114
ENSG0000(	782	19.55855	chr1:8137	SIKE1		protein_c	chr1:114769479-114
ENSG0000(	782	19.55855	chr1:8137	GAPDHP74		Pseudoger	chr1:119434166-119
ENSG0000(	782	19.55855	chr1:8137	SNORA40		smallRNA	chr1:117688621-117
ENSG0000(	782	19.55855	chr1:8137	ENSG00000287103		lncRNA	chr1:115356664-115
ENSG0000(	782	19.55855	chr1:8137	RP3-328E19.5		Pseudoger	chr1:120861538-120
ENSG0000(	782	19.55855	chr1:8137	TSPAN2		protein_c	chr1:115048011-115

ENSG0000(	782	19.55855	chr1:8137	CD101		protein_c	chr1:117001750-117
ENSG0000(	782	19.55855	chr1:8137	VTCN1		protein_c	chr1:117143587-117
ENSG0000(	782	19.55855	chr1:8137	NGF		protein_c	chr1:115285904-115
ENSG0000(	782	19.55855	chr1:8137	PKMP1		Pseudoger	chr1:114535995-114
ENSG0000(	782	19.55855	chr1:8137	RP11-763B22.10		Pseudoger	chr1:120861539-120
ENSG0000(	782	19.55855	chr1:8137	REG4		protein_c	chr1:119794017-119
ENSG0000(	782	19.55855	chr1:8137	Y_RNA		smallRNA	chr1:114490724-114
ENSG0000(	782	19.55855	chr1:8137	SLC16A1-AS1		lncRNA	chr1:112956415-113
ENSG0000(	782	19.55855	chr1:8137	snoU13		smallRNA	chr1:119259041-119
ENSG0000(	782	19.55855	chr1:8137	RP6-42F4.1		lncRNA	chr1:120150758-120
ENSG0000(	782	19.55855	chr1:8137	MAN1A2	NCGv7	protein_c	chr1:117367449-117
ENSG0000(	782	19.55855	chr1:8137	BCL2L15		protein_c	chr1:113876816-113
ENSG0000(	782	19.55855	chr1:8137	SPAG17	NCGv7	protein_c	chr1:117953590-118
ENSG0000(	782	19.55855	chr1:8137	ENSG00000270780		Pseudoger	chr1:114001433-114
ENSG0000(	782	19.55855	chr1:8137	WDR3		protein_c	chr1:117929720-117
ENSG0000(	782	19.55855	chr1:8137	AP4B1		protein_c	chr1:113894194-113
ENSG0000(	782	19.55855	chr1:8137	RNU6-465P		smallRNA	chr1:120126974-120
ENSG0000(	782	19.55855	chr1:8137	EIF2S2P5		Pseudoger	chr1:114468315-114
ENSG0000(	782	19.55855	chr1:8137	VDAC2P3		Pseudoger	chr1:117640812-117
ENSG0000(	782	19.55855	chr1:8137	TRIM45		protein_c	chr1:117111060-117
ENSG0000(	782	19.55855	chr1:8137	ENSG00000270631		Pseudoger	chr1:115577229-115
ENSG0000(	782	19.55855	chr1:8137	LINC01779		lncRNA	chr1:116164209-116
ENSG0000(	782	19.55855	chr1:8137	LINC01525		lncRNA	chr1:117272182-117
ENSG0000(	782	19.55855	chr1:8137	RNU1-75P		smallRNA	chr1:119331397-119
ENSG0000(	782	19.55855	chr1:8137	HAO2-IT1		lncRNA	chr1:119368946-119
ENSG0000(	782	19.55855	chr1:8137	SNORA42		smallRNA	chr1:115621872-115
ENSG0000(	782	19.55855	chr1:8137	NOTCH2P1		Pseudoger	chr1:119886304-119
ENSG0000(	782	19.55855	chr1:8137	ENSG00000287217		lncRNA	chr1:116289237-116
ENSG0000(	782	19.55855	chr1:8137	ENSG00000232450		Pseudoger	chr1:113698884-113
ENSG0000(	782	19.55855	chr1:8137	SLC22A15		protein_c	chr1:115976513-116
ENSG0000(	782	19.55855	chr1:8137	ATP1A1	NCGv7;AC	protein_c	chr1:116372668-116
ENSG0000(	782	19.55855	chr1:8137	SYT6		protein_c	chr1:114089291-114
ENSG0000(	782	19.55855	chr1:8137	NOTCH2	NCGv7;AC	protein_c	chr1:119911553-120
ENSG0000(	782	19.55855	chr1:8137	HMGCS2		protein_c	chr1:119748002-119
ENSG0000(	782	19.55855	chr1:8137	PTPN22	NCGv7	protein_c	chr1:113813811-113
ENSG0000(	782	19.55855	chr1:8137	ENSG00000232499		Pseudoger	chr1:113449700-113
ENSG0000(	782	19.55855	chr1:8137	PTGFRN	NCGv7	protein_c	chr1:116909916-116
ENSG0000(	782	19.55855	chr1:8137	ADAM30		protein_c	chr1:119893533-119
ENSG0000(	782	19.55855	chr1:8137	TSHB		protein_c	chr1:115029826-115
ENSG0000(	782	19.55855	chr1:8137	NRAS	NCGv7;AC	protein_c	chr1:114704469-114
ENSG0000(	782	19.55855	chr1:8137	snoU13		smallRNA	chr1:112652588-112
ENSG0000(	782	19.55855	chr1:8137	GAPDHP32		Pseudoger	chr1:119533749-119
ENSG0000(	782	19.55855	chr1:8137	LINC01780		lncRNA	chr1:119327399-119
ENSG0000(	782	19.55855	chr1:8137	ENSG00000270719		Pseudoger	chr1:117700029-117
ENSG0000(	782	19.55855	chr1:8137	ENSG00000287807		lncRNA	chr1:112978610-112
ENSG0000(	782	19.55855	chr1:8137	RNA5SP56		Pseudoger	chr1:118264372-118
ENSG0000(	782	19.55855	chr1:8137	PHGDH	NCGv7	protein_c	chr1:119648411-119
ENSG0000(	782	19.55855	chr1:8137	RP5-1042I8.7		lncRNA	chr1:119909255-119
ENSG0000(	782	19.55855	chr1:8137	RPS3AP12		Pseudoger	chr1:119126539-119
ENSG0000(	782	19.55855	chr1:8137	RPS15AP9		Pseudoger	chr1:117138229-117
ENSG0000(	782	19.55855	chr1:8137	ENSG00000239216		lncRNA	chr1:119000618-119

ENSG0000(	777	19.43349	chr1:1522	ATP6V0E1P4		Pseudoger	chr1:47550196-4755
ENSG0000(	777	19.43349	chr1:1522	ENSG00000225779		Pseudoger	chr1:46370586-4637
ENSG0000(	777	19.43349	chr1:1522	TUBAP9		Pseudoger	chr1:47074778-4707
ENSG0000(	777	19.43349	chr1:1522	TEX38		protein_c	chr1:46668855-4667
ENSG0000(	777	19.43349	chr1:1522	LINC01398		lncRNA	chr1:46446600-4645
ENSG0000(	777	19.43349	chr1:1522	ENSG00000226252		lncRNA	chr1:47225797-4723
ENSG0000(	777	19.43349	chr1:1522	TMEM275		protein_c	chr1:46532166-4654
ENSG0000(	777	19.43349	chr1:1522	CYP4A26P		Pseudoger	chr1:46967679-4696
ENSG0000(	777	19.43349	chr1:1522	RPL21P24		Pseudoger	chr1:47497894-4749
ENSG0000(	777	19.43349	chr1:1522	CYP4A43P		Pseudoger	chr1:46994382-4699
ENSG0000(	777	19.43349	chr1:1522	CYP4A11	NCGv7	protein_c	chr1:46929177-4694
ENSG0000(	777	19.43349	chr1:1522	CYP4Z2P		Pseudoger	chr1:46843178-4690
ENSG0000(	777	19.43349	chr1:1522	CYP4Z2P		lncRNA	chr1:46843095-4690
ENSG0000(	777	19.43349	chr1:1522	CYP4B1	NCGv7	protein_c	chr1:46757838-4681
ENSG0000(	777	19.43349	chr1:1522	FAAHP1		Pseudoger	chr1:46432129-4644
ENSG0000(	777	19.43349	chr1:1522	ENSG00000271355		Pseudoger	chr1:47483698-4748
ENSG0000(	777	19.43349	chr1:1522	CYP4Z1	NCGv7	protein_c	chr1:47067231-4711
ENSG0000(	777	19.43349	chr1:1522	LRRC41		protein_c	chr1:46261196-4630
ENSG0000(	777	19.43349	chr1:1522	FAAH		protein_c	chr1:46394317-4641
ENSG0000(	777	19.43349	chr1:1522	FOXD2-AS1		lncRNA	chr1:47432133-4743
ENSG0000(	777	19.43349	chr1:1522	TAL1	NCGv7;AC	protein_c	chr1:47216290-4723
ENSG0000(	777	19.43349	chr1:1522	MKNK1-AS1		lncRNA	chr1:46538611-4657
ENSG0000(	777	19.43349	chr1:1522	PDZK1IP1	AC	protein_c	chr1:47183582-4719
ENSG0000(	777	19.43349	chr1:1522	CYP4X1		protein_c	chr1:47023669-4705
ENSG0000(	777	19.43349	chr1:1522	CYP4A22		protein_c	chr1:47137435-4714
ENSG0000(	777	19.43349	chr1:1522	EFCAB14		protein_c	chr1:46674659-4671
ENSG0000(	777	19.43349	chr1:1522	NSUN4		protein_c	chr1:46340789-4636
ENSG0000(	777	19.43349	chr1:1522	FOXD2		protein_c	chr1:47438044-4744
ENSG0000(	777	19.43349	chr1:1522	FOXE3		protein_c	chr1:47416285-4741
ENSG0000(	777	19.43349	chr1:1522	ENSG00000236476		Pseudoger	chr1:46742329-4674
ENSG0000(	777	19.43349	chr1:1522	ATPAF1		protein_c	chr1:46632737-4667
ENSG0000(	777	19.43349	chr1:1522	STIL	NCGv7;AC	protein_c	chr1:47250139-4731
ENSG0000(	777	19.43349	chr1:1522	DMBX1		protein_c	chr1:46489836-4651
ENSG0000(	777	19.43349	chr1:1522	LINC01389		lncRNA	chr1:47380928-4740
ENSG0000(	777	19.43349	chr1:1522	MKNK1	NCGv7	protein_c	chr1:46557407-4661
ENSG0000(	777	19.43349	chr1:1522	KNCN		protein_c	chr1:46545641-4655
ENSG0000(	777	19.43349	chr1:1522	EFCAB14-AS1		lncRNA	chr1:46674036-4669
ENSG0000(	777	19.43349	chr1:1522	MOB3C		protein_c	chr1:46607719-4661
ENSG0000(	777	19.43349	chr1:1522	CMPK1		protein_c	chr1:47333790-4739
ENSG0000(	777	19.43349	chr1:1522	LINC00853		lncRNA	chr1:47179250-4718
ENSG0000(	777	19.43349	chr1:1522	CYP4A44P		Pseudoger	chr1:47002995-4700
ENSG0000(	777	19.43349	chr1:1522	AL356458.1		smallRNA	chr1:47502504-4750
ENSG0000(	777	19.43349	chr1:1522	CYP4A22-AS1		lncRNA	chr1:47096653-4717
ENSG0000(	777	19.43349	chr1:1522	UQCRH		protein_c	chr1:46303698-4631
ENSG0000(	777	19.43349	chr1:1522	CYP4A27P		Pseudoger	chr1:47000898-4700
ENSG0000(	777	19.43349	chr1:1522	TUBAP8		Pseudoger	chr1:46891639-4689
ENSG0000(	777	19.43349	chr1:1522	ENSG00000290081		lncRNA	chr1:47180175-4718
ENSG0000(	777	19.43349	chr1:1522	RAD54L		protein_c	chr1:46246461-4627
ENSG0000(	777	19.43349	chr1:1522	MTND1P34		Pseudoger	chr1:47164510-4716
ENSG0000(	777	19.43349	chr1:1522	ENSG00000291138		lncRNA	chr1:46433827-4648
ENSG0000(	772	19.30844	chr1:8137	RN7SL444P		smallRNA	chr1:151300667-151

ENSG0000(	769	19.2334	chr1:8137	ENSG00000291233		lncRNA	chr1:148889429-148
ENSG0000(	769	19.2334	chr1:8137	ENSG00000271546		Pseudoger	chr1:148795796-148
ENSG0000(	769	19.2334	chr1:8137	NBPF25P		Pseudoger	chr1:149058924-149
ENSG0000(	769	19.2334	chr1:8137	ENSG00000289642		lncRNA	chr1:149013782-149
ENSG0000(	769	19.2334	chr1:8137	ENSG00000254539		lncRNA	chr1:149048575-149
ENSG0000(	769	19.2334	chr1:8137	NUDT4P2		Pseudoger	chr1:148748952-148
ENSG0000(	769	19.2334	chr1:8137	RN7SKP88		smallRNA	chr1:148839482-148
ENSG0000(	769	19.2334	chr1:8137	ENSG00000254913		lncRNA	chr1:149006308-149
ENSG0000(	769	19.2334	chr1:8137	SEC22B2P		Pseudoger	chr1:148772639-148
ENSG0000(	769	19.2334	chr1:8137	ENSG00000290999		lncRNA	chr1:148962571-149
ENSG0000(	769	19.2334	chr1:8137	AL138796.1		smallRNA	chr1:149007076-149
ENSG0000(	769	19.2334	chr1:8137	NUDT4B		protein_c	chr1:148748773-148
ENSG0000(	769	19.2334	chr1:8137	ENSG00000272755		lncRNA	chr1:148865453-148
ENSG0000(	769	19.2334	chr1:8137	AL590452.1		protein_c	chr1:148893126-148
ENSG0000(	769	19.2334	chr1:8137	RNU6-1071P		smallRNA	chr1:148739378-148
ENSG0000(	769	19.2334	chr1:8137	ENSG00000255148		lncRNA	chr1:149018670-149
ENSG0000(	769	19.2334	chr1:8137	SEC22B4P		Pseudoger	chr1:148772639-148
ENSG0000(	769	19.2334	chr1:8137	NBPF9		protein_c	chr1:149054026-149
ENSG0000(	769	19.2334	chr1:8137	SEC22B		protein_c	chr1:148770173-148
ENSG0000(	769	19.2334	chr1:8137	PDE4DIPP2		Pseudoger	chr1:148808504-149
ENSG0000(	769	19.2334	chr1:8137	RNU2-38P		smallRNA	chr1:148939738-148
ENSG0000(	769	19.2334	chr1:8137	PDE4DIP	NCGv7;AC	protein_c	chr1:148808139-149
ENSG0000(	769	19.2334	chr1:8137	SEC22B3P		Pseudoger	chr1:148772639-148
ENSG0000(	763	19.08334	chr6:105(	DDX39B-AS1		lncRNA	chr6:31542304-3154
ENSG0000(	762	19.05833	chr1:8137	ENSG00000290790		lncRNA	chr1:149676889-149
ENSG0000(	762	19.05833	chr1:8137	GABPB2		protein_c	chr1:151070578-151
ENSG0000(	762	19.05833	chr1:8137	ENSG00000231073		lncRNA	chr1:150973123-150
ENSG0000(	762	19.05833	chr1:8137	H3C15		protein_c	chr1:149852608-149
ENSG0000(	762	19.05833	chr1:8137	RPL6P31		Pseudoger	chr1:150053864-150
ENSG0000(	762	19.05833	chr1:8137	GOLPH3L		protein_c	chr1:150646230-150
ENSG0000(	762	19.05833	chr1:8137	RNU6-1309P		smallRNA	chr1:150812591-150
ENSG0000(	762	19.05833	chr1:8137	RP11-277L2.3		lncRNA	chr1:149607448-149
ENSG0000(	762	19.05833	chr1:8137	HORMAD1	NCGv7	protein_c	chr1:150698060-150
ENSG0000(	762	19.05833	chr1:8137	H2AC21		protein_c	chr1:149887469-149
ENSG0000(	762	19.05833	chr1:8137	H2AC20	NCGv7	protein_c	chr1:149886918-149
ENSG0000(	762	19.05833	chr1:8137	H2AC19		protein_c	chr1:149851061-149
ENSG0000(	762	19.05833	chr1:8137	RP11-277L2.5		lncRNA	chr1:149618320-149
ENSG0000(	762	19.05833	chr1:8137	ENSG00000275557		lncRNA	chr1:149607765-149
ENSG0000(	762	19.05833	chr1:8137	ENSG00000226500		Pseudoger	chr1:149754301-149
ENSG0000(	762	19.05833	chr1:8137	ENSG00000288880		lncRNA	chr1:150629814-150
ENSG0000(	762	19.05833	chr1:8137	ENSG00000289288		lncRNA	chr1:151146793-151
ENSG0000(	762	19.05833	chr1:8137	PDE4DIPP4		lncRNA	chr1:149677473-149
ENSG0000(	762	19.05833	chr1:8137	ECM1		protein_c	chr1:150508062-150
ENSG0000(	762	19.05833	chr1:8137	PFN1P2		Pseudoger	chr1:149084616-149
ENSG0000(	762	19.05833	chr1:8137	RN7SL480P		smallRNA	chr1:150211632-150
ENSG0000(	762	19.05833	chr1:8137	PRUNE1		protein_c	chr1:151008420-151
ENSG0000(	762	19.05833	chr1:8137	RNU1-68P		smallRNA	chr1:149700151-149
ENSG0000(	762	19.05833	chr1:8137	RNU6-1042P		smallRNA	chr1:150701866-150
ENSG0000(	762	19.05833	chr1:8137	TARS2		protein_c	chr1:150487414-150
ENSG0000(	762	19.05833	chr1:8137	PPIAL4G	NCGv7	protein_c	chr1:148482548-148
ENSG0000(	762	19.05833	chr1:8137	RP11-277L2.4		lncRNA	chr1:149606196-149

ENSG0000(	762	19.05833	chr1:8137	RP11-353N4.4		lncRNA	chr1:149701425-149
ENSG0000(	762	19.05833	chr1:8137	LINC00869		Pseudoger	chr1:149655747-149
ENSG0000(	762	19.05833	chr1:8137	H4C15		protein_c	chr1:149854045-149
ENSG0000(	762	19.05833	chr1:8137	FALEC		lncRNA	chr1:150515757-150
ENSG0000(	762	19.05833	chr1:8137	CDC42SE1		protein_c	chr1:151050971-151
ENSG0000(	762	19.05833	chr1:8137	CYCSP51		Pseudoger	chr1:150903896-150
ENSG0000(	762	19.05833	chr1:8137	CA14		protein_c	chr1:150257251-150
ENSG0000(	762	19.05833	chr1:8137	C1orf54		protein_c	chr1:150268200-150
ENSG0000(	762	19.05833	chr1:8137	RPS27AP6		Pseudoger	chr1:150881236-150
ENSG0000(	762	19.05833	chr1:8137	BOLA1		protein_c	chr1:149887890-149
ENSG0000(	762	19.05833	chr1:8137	OTUD7B		protein_c	chr1:149937812-150
ENSG0000(	762	19.05833	chr1:8137	ENSG00000289041		lncRNA	chr1:150281114-150
ENSG0000(	762	19.05833	chr1:8137	H2BC21		protein_c	chr1:149884459-149
ENSG0000(	762	19.05833	chr1:8137	RN7SL600P		smallRNA	chr1:150568973-150
ENSG0000(	762	19.05833	chr1:8137	RP11-196G18.23		lncRNA	chr1:149831312-149
ENSG0000(	762	19.05833	chr1:8137	RN7SL473P		smallRNA	chr1:150566564-150
ENSG0000(	762	19.05833	chr1:8137	ADAMTSL4-AS2		lncRNA	chr1:150548562-150
ENSG0000(	762	19.05833	chr1:8137	H2BC19P		Pseudoger	chr1:149843041-149
ENSG0000(	762	19.05833	chr1:8137	SF3B4		protein_c	chr1:149923317-149
ENSG0000(	762	19.05833	chr1:8137	ENSG00000289457		lncRNA	chr1:150561466-150
ENSG0000(	762	19.05833	chr1:8137	AL356356.1		protein_c	chr1:150549421-150
ENSG0000(	762	19.05833	chr1:8137	ZNF687	NCGv7	protein_c	chr1:151281618-151
ENSG0000(	762	19.05833	chr1:8137	ENSG00000284964		Pseudoger	chr1:149607467-149
ENSG0000(	762	19.05833	chr1:8137	ENSG00000290791		lncRNA	chr1:149842875-149
ENSG0000(	762	19.05833	chr1:8137	RP6-206I17.3		lncRNA	chr1:148435062-148
ENSG0000(	762	19.05833	chr1:8137	SNORA40		smallRNA	chr1:150600539-150
ENSG0000(	762	19.05833	chr1:8137	Y_RNA		smallRNA	chr1:150882451-150
ENSG0000(	762	19.05833	chr1:8137	ENSG00000275296		Pseudoger	chr1:149754303-149
ENSG0000(	762	19.05833	chr1:8137	ENSG00000290792		lncRNA	chr1:149844498-149
ENSG0000(	762	19.05833	chr1:8137	SETDB1	NCGv7;AC	protein_c	chr1:150926263-150
ENSG0000(	762	19.05833	chr1:8137	BNIPL	DriverDB\	protein_c	chr1:151036321-151
ENSG0000(	762	19.05833	chr1:8137	HIST2H2AA3		protein_c	chr1:149841933-149
ENSG0000(	762	19.05833	chr1:8137	ENSG00000225871		Pseudoger	chr1:148435105-148
ENSG0000(	762	19.05833	chr1:8137	H2BC18		protein_c	chr1:149782689-149
ENSG0000(	762	19.05833	chr1:8137	H3C13		protein_c	chr1:149813225-149
ENSG0000(	762	19.05833	chr1:8137	ENSA		protein_c	chr1:150600851-150
ENSG0000(	762	19.05833	chr1:8137	RPRD2		protein_c	chr1:150363091-150
ENSG0000(	762	19.05833	chr1:8137	SEMA6C		protein_c	chr1:151131685-151
ENSG0000(	762	19.05833	chr1:8137	CTSS	NCGv7	protein_c	chr1:150730079-150
ENSG0000(	762	19.05833	chr1:8137	CTXND2		protein_c	chr1:150887136-150
ENSG0000(	762	19.05833	chr1:8137	RP6-206I17.4		Pseudoger	chr1:148435103-148
ENSG0000(	762	19.05833	chr1:8137	ARNT	NCGv7;AC	protein_c	chr1:150809713-150
ENSG0000(	762	19.05833	chr1:8137	CERS2		protein_c	chr1:150960583-150
ENSG0000(	762	19.05833	chr1:8137	TNFAIP8L2		protein_c	chr1:151156649-151
ENSG0000(	762	19.05833	chr1:8137	LYSMD1		protein_c	chr1:151159748-151
ENSG0000(	762	19.05833	chr1:8137	RP11-353N4.1		lncRNA	chr1:149621576-149
ENSG0000(	762	19.05833	chr1:8137	ENSG00000224645		lncRNA	chr1:151340648-151
ENSG0000(	762	19.05833	chr1:8137	ENSG00000259357		lncRNA	chr1:150965245-150
ENSG0000(	762	19.05833	chr1:8137	PDE4DIPP6		Pseudoger	chr1:148415258-148
ENSG0000(	762	19.05833	chr1:8137	SCNM1		protein_c	chr1:151156664-151
ENSG0000(	762	19.05833	chr1:8137	TMOD4		protein_c	chr1:151169986-151

ENSG0000(	762	19.05833	chr1:8137	ENSG00000261168		lncRNA	chr1:151130075-151
ENSG0000(	762	19.05833	chr1:8137	VPS72		protein_c	chr1:151176304-151
ENSG0000(	762	19.05833	chr1:8137	H4C14		protein_c	chr1:149832657-149
ENSG0000(	762	19.05833	chr1:8137	ENSG00000273481		lncRNA	chr1:151327949-151
ENSG0000(	762	19.05833	chr1:8137	PLEKHO1		protein_c	chr1:150149183-150
ENSG0000(	762	19.05833	chr1:8137	RNU2-17P		smallRNA	chr1:150236967-150
ENSG0000(	762	19.05833	chr1:8137	H3C14		protein_c	chr1:149840687-149
ENSG0000(	762	19.05833	chr1:8137	FCGR1A		protein_c	chr1:149782671-149
ENSG0000(	762	19.05833	chr1:8137	ZNF687-AS1		lncRNA	chr1:151279678-151
ENSG0000(	762	19.05833	chr1:8137	ENSG00000285184		lncRNA	chr1:150045660-150
ENSG0000(	762	19.05833	chr1:8137	ADAMTSL4		protein_c	chr1:150549369-150
ENSG0000(	762	19.05833	chr1:8137	MCL1	NCGv7;AC	protein_c	chr1:150560895-150
ENSG0000(	762	19.05833	chr1:8137	ADAMTSL4-AS1		lncRNA	chr1:150560202-150
ENSG0000(	762	19.05833	chr1:8137	CTSK		protein_c	chr1:150794880-150
ENSG0000(	762	19.05833	chr1:8137	MTMR11		protein_c	chr1:149928651-149
ENSG0000(	762	19.05833	chr1:8137	RNU6-884P		smallRNA	chr1:151022746-151
ENSG0000(	762	19.05833	chr1:8137	ENSG00000276110		lncRNA	chr1:150255095-150
ENSG0000(	762	19.05833	chr1:8137	ANXA9		protein_c	chr1:150982249-150
ENSG0000(	762	19.05833	chr1:8137	AL358813.3		smallRNA	chr1:149737309-149
ENSG0000(	762	19.05833	chr1:8137	AL358813.1		Pseudoger	chr1:149717832-149
ENSG0000(	762	19.05833	chr1:8137	LINC02988		lncRNA	chr1:150173049-150
ENSG0000(	762	19.05833	chr1:8137	PI4KB		protein_c	chr1:151291797-151
ENSG0000(	762	19.05833	chr1:8137	PIP5K1A		protein_c	chr1:151197949-151
ENSG0000(	762	19.05833	chr1:8137	ANP32E		protein_c	chr1:150218417-150
ENSG0000(	762	19.05833	chr1:8137	MINDY1		protein_c	chr1:150996549-151
ENSG0000(	762	19.05833	chr1:8137	snoU13		smallRNA	chr1:150261694-150
ENSG0000(	762	19.05833	chr1:8137	ENSG00000291232		lncRNA	chr1:148402516-148
ENSG0000(	762	19.05833	chr1:8137	MIR4257		smallRNA	chr1:150551929-150
ENSG0000(	762	19.05833	chr1:8137	RP11-289I10.3		Pseudoger	chr1:148511083-148
ENSG0000(	762	19.05833	chr1:8137	ENSG00000290074		lncRNA	chr1:150579917-150
ENSG0000(	762	19.05833	chr1:8137	RNVU1-20		smallRNA	chr1:149636766-149
ENSG0000(	762	19.05833	chr1:8137	PRPF3	NCGv7	protein_c	chr1:150321479-150
ENSG0000(	762	19.05833	chr1:8137	C1orf56		protein_c	chr1:151047751-151
ENSG0000(	762	19.05833	chr1:8137	AL358813.2		protein_c	chr1:149704411-149
ENSG0000(	762	19.05833	chr1:8137	PFN1P3		Pseudoger	chr1:149084616-149
ENSG0000(	762	19.05833	chr1:8137	RP11-353N4.3		Pseudoger	chr1:149693026-149
ENSG0000(	762	19.05833	chr1:8137	CIART		protein_c	chr1:150282543-150
ENSG0000(	762	19.05833	chr1:8137	MLLT11	DriverDB\	protein_c	chr1:151060397-151
ENSG0000(	762	19.05833	chr1:8137	UBE2D3P3		Pseudoger	chr1:150800473-150
ENSG0000(	762	19.05833	chr1:8137	RP6-206I17.2		lncRNA	chr1:148402453-148
ENSG0000(	762	19.05833	chr1:8137	PSMD4		protein_c	chr1:151254709-151
ENSG0000(	762	19.05833	chr1:8137	APH1A		protein_c	chr1:150265399-150
ENSG0000(	762	19.05833	chr1:8137	VPS45	NCGv7	protein_c	chr1:150067279-150
ENSG0000(	762	19.05833	chr1:8137	HIST2H2BC		Pseudoger	chr1:149850193-149
ENSG0000(	762	19.05833	chr1:8137	RNU1-59P		smallRNA	chr1:149162782-149
ENSG0000(	762	19.05833	chr1:8137	ENSG00000236713		Pseudoger	chr1:150780272-150
ENSG0000(	762	19.05833	chr1:8137	MRPS21	NCGv7	protein_c	chr1:150293861-150
ENSG0000(	762	19.05833	chr1:8137	ENSG00000233030		lncRNA	chr1:149785659-149
ENSG0000(	762	19.05833	chr1:8137	SV2A		protein_c	chr1:149903318-149
ENSG0000(	762	19.05833	chr1:8137	RPS29P29		Pseudoger	chr1:151111912-151
ENSG0000(	752	18.80822	chr1:8137	C2CD4D	DriverDB\	protein_c	chr1:151837818-151

ENSG0000(	752	18.80822	chr1:8137	OAZ3		protein_c	chr1:151762899-151
ENSG0000(	752	18.80822	chr1:8137	ENSG00000232536		lncRNA	chr1:151540516-151
ENSG0000(	752	18.80822	chr1:8137	SELENBP1		protein_c	chr1:151364304-151
ENSG0000(	752	18.80822	chr1:8137	TDRKH	DriverDB\	protein_c	chr1:151770107-151
ENSG0000(	752	18.80822	chr1:8137	ENSG00000227045		lncRNA	chr1:151701026-151
ENSG0000(	752	18.80822	chr1:8137	ENSG00000223861		Pseudoger	chr1:151557446-151
ENSG0000(	752	18.80822	chr1:8137	C2CD4D-AS1		lncRNA	chr1:151841877-151
ENSG0000(	752	18.80822	chr1:8137	ENSG00000249602		lncRNA	chr1:151763384-151
ENSG0000(	752	18.80822	chr1:8137	ENSG00000268288		lncRNA	chr1:151766486-151
ENSG0000(	752	18.80822	chr1:8137	Y_RNA		smallRNA	chr1:151841736-151
ENSG0000(	752	18.80822	chr1:8137	ENSG00000269489		lncRNA	chr1:151798054-151
ENSG0000(	752	18.80822	chr1:8137	PSMB4		protein_c	chr1:151399560-151
ENSG0000(	752	18.80822	chr1:8137	CELF3		protein_c	chr1:151700058-151
ENSG0000(	752	18.80822	chr1:8137	MIR554		smallRNA	chr1:151545796-151
ENSG0000(	752	18.80822	chr1:8137	LINGO4		protein_c	chr1:151800264-151
ENSG0000(	752	18.80822	chr1:8137	SNX27		protein_c	chr1:151612006-151
ENSG0000(	752	18.80822	chr1:8137	RFX5		protein_c	chr1:151340640-151
ENSG0000(	752	18.80822	chr1:8137	ENSG00000250734		lncRNA	chr1:151612038-151
ENSG0000(	752	18.80822	chr1:8137	SNORA44		smallRNA	chr1:151527831-151
ENSG0000(	752	18.80822	chr1:8137	RNU6-1062P		smallRNA	chr1:151629324-151
ENSG0000(	752	18.80822	chr1:8137	RFX5-AS1		lncRNA	chr1:151346938-151
ENSG0000(	752	18.80822	chr1:8137	ENSG00000285651		lncRNA	chr1:151885251-151
ENSG0000(	752	18.80822	chr1:8137	ENSG00000232937		lncRNA	chr1:151765709-151
ENSG0000(	752	18.80822	chr1:8137	RIIAD1		protein_c	chr1:151710433-151
ENSG0000(	752	18.80822	chr1:8137	TUFT1		protein_c	chr1:151540305-151
ENSG0000(	752	18.80822	chr1:8137	POGZ		protein_c	chr1:151402724-151
ENSG0000(	752	18.80822	chr1:8137	CGN	DriverDB\	protein_c	chr1:151510510-151
ENSG0000(	752	18.80822	chr1:8137	TDRKH-AS1		lncRNA	chr1:151790804-151
ENSG0000(	752	18.80822	chr1:8137	ENSG00000269621		lncRNA	chr1:151755541-151
ENSG0000(	752	18.80822	chr1:8137	RORC	DriverDB\	protein_c	chr1:151806071-151
ENSG0000(	752	18.80822	chr1:8137	RNY4P25		smallRNA	chr1:151439000-151
ENSG0000(	752	18.80822	chr1:8137	THEM5	DriverDB\	protein_c	chr1:151847101-151
ENSG0000(	752	18.80822	chr1:8137	AL589765.1		protein_c	chr1:151710433-151
ENSG0000(	752	18.80822	chr1:8137	ENSG00000236940		Pseudoger	chr1:151757659-151
ENSG0000(	752	18.80822	chr1:8137	RNU6-662P		smallRNA	chr1:151747597-151
ENSG0000(	752	18.80822	chr1:8137	MRPL9		protein_c	chr1:151759647-151
ENSG0000(	749	18.73319	chr7:330(	GRB10		protein_c	chr7:50590063-5079
ENSG0000(	749	18.73319	chr7:330(	ENSG00000233539		lncRNA	chr7:46673785-4675
ENSG0000(	749	18.73319	chr7:330(	ENSG00000229459		lncRNA	chr7:46261064-4629
ENSG0000(	749	18.73319	chr7:330(	RNU6-241P		smallRNA	chr7:45789585-4578
ENSG0000(	749	18.73319	chr7:330(	ENSG00000290107		lncRNA	chr7:45690599-4569
ENSG0000(	749	18.73319	chr7:330(	ABCA13	NCGv7	protein_c	chr7:48171458-4864
ENSG0000(	749	18.73319	chr7:330(	MRPL42P4		Pseudoger	chr7:47026128-4702
ENSG0000(	749	18.73319	chr7:330(	ENSG00000229628		lncRNA	chr7:45990905-4600
ENSG0000(	749	18.73319	chr7:330(	ENSG00000226838		Pseudoger	chr7:45816557-4582
ENSG0000(	749	18.73319	chr7:330(	LINC02902		lncRNA	chr7:47655244-4766
ENSG0000(	749	18.73319	chr7:330(	ENSG00000290114		lncRNA	chr7:50450421-5045
ENSG0000(	749	18.73319	chr7:330(	LINC02838		lncRNA	chr7:48708327-4871
ENSG0000(	749	18.73319	chr7:330(	UPP1	DriverDB\	protein_c	chr7:48088628-4810
ENSG0000(	749	18.73319	chr7:330(	EPS15P1		Pseudoger	chr7:46781373-4678
ENSG0000(	749	18.73319	chr7:330(	RNU6-326P		smallRNA	chr7:45843634-4584

ENSG0000(	749	18.73319	chr7:330(	ENSG00000226999		Pseudoger	chr7:45534523-4553
ENSG0000(	749	18.73319	chr7:330(	ENSG00000232072		lncRNA	chr7:46890625-4704
ENSG0000(	749	18.73319	chr7:330(	ENSG00000237760		lncRNA	chr7:46302120-4634
ENSG0000(	749	18.73319	chr7:330(	ENSG00000228005		lncRNA	chr7:50141540-5014
ENSG0000(	749	18.73319	chr7:330(	DDC-AS1		lncRNA	chr7:50531759-5054
ENSG0000(	749	18.73319	chr7:330(	CICP20		Pseudoger	chr7:45816216-4581
ENSG0000(	749	18.73319	chr7:330(	GNL2P1		Pseudoger	chr7:49942251-4994
ENSG0000(	749	18.73319	chr7:330(	SPATA48		protein_c	chr7:50095883-5015
ENSG0000(	749	18.73319	chr7:330(	FTLP15		Pseudoger	chr7:45997540-4599
ENSG0000(	749	18.73319	chr7:330(	ENSG00000285165		lncRNA	chr7:50388489-5040
ENSG0000(	749	18.73319	chr7:330(	CCDC201		protein_c	chr7:45859994-4587
ENSG0000(	749	18.73319	chr7:330(	ENSG00000237471		lncRNA	chr7:45940449-4598
ENSG0000(	749	18.73319	chr7:330(	LINC01447		lncRNA	chr7:47608465-4762
ENSG0000(	749	18.73319	chr7:330(	ENSG00000228173		lncRNA	chr7:48660125-4866
ENSG0000(	749	18.73319	chr7:330(	ENSG00000235620		lncRNA	chr7:50274790-5027
ENSG0000(	749	18.73319	chr7:330(	ZPBP	DriverDB	protein_c	chr7:49850421-5012
ENSG0000(	749	18.73319	chr7:330(	DDC		protein_c	chr7:50458436-5056
ENSG0000(	749	18.73319	chr7:330(	ENSG00000229192		lncRNA	chr7:47000620-4707
ENSG0000(	749	18.73319	chr7:330(	FIGNL1	NCGv7	protein_c	chr7:50444128-5054
ENSG0000(	749	18.73319	chr7:330(	LINC00525		lncRNA	chr7:47761476-4776
ENSG0000(	749	18.73319	chr7:330(	PKD1L1		protein_c	chr7:47740202-4794
ENSG0000(	749	18.73319	chr7:330(	SEPTIN7P2		Pseudoger	chr7:45736787-4576
ENSG0000(	749	18.73319	chr7:330(	ENSG00000223829		lncRNA	chr7:46969644-4702
ENSG0000(	749	18.73319	chr7:330(	ENSG00000225507		Pseudoger	chr7:47956793-4795
ENSG0000(	749	18.73319	chr7:330(	ENSG00000291208		lncRNA	chr7:45769060-4581
ENSG0000(	749	18.73319	chr7:330(	ENSG00000291207		lncRNA	chr7:45723780-4576
ENSG0000(	749	18.73319	chr7:330(	RNU6-1091P		smallRNA	chr7:50435380-5043
ENSG0000(	749	18.73319	chr7:330(	TTC4P1		Pseudoger	chr7:45999621-4600
ENSG0000(	749	18.73319	chr7:330(	ENSG00000279578		TEC	chr7:48847766-4885
ENSG0000(	749	18.73319	chr7:330(	ZNF619P1		Pseudoger	chr7:46144937-4614
ENSG0000(	749	18.73319	chr7:330(	TNS3		protein_c	chr7:47275154-4758
ENSG0000(	749	18.73319	chr7:330(	ENSG00000225705		Pseudoger	chr7:48846426-4885
ENSG0000(	749	18.73319	chr7:330(	ENSG00000279104		TEC	chr7:49760897-4976
ENSG0000(	749	18.73319	chr7:330(	ENSG00000230680		lncRNA	chr7:46477822-4648
ENSG0000(	749	18.73319	chr7:330(	ENSG00000286315		lncRNA	chr7:47252139-4725
ENSG0000(	749	18.73319	chr7:330(	HUS1		protein_c	chr7:47963288-4797
ENSG0000(	749	18.73319	chr7:330(	PKD1L1-AS1		lncRNA	chr7:47795291-4781
ENSG0000(	749	18.73319	chr7:330(	ENSG00000234686		lncRNA	chr7:49230137-4925
ENSG0000(	749	18.73319	chr7:330(	IGFBP3		protein_c	chr7:45912245-4592
ENSG0000(	749	18.73319	chr7:330(	C7orf57		protein_c	chr7:48035511-4806
ENSG0000(	749	18.73319	chr7:330(	DDX43P2		Pseudoger	chr7:49258493-4925
ENSG0000(	749	18.73319	chr7:330(	SRSF8CP		Pseudoger	chr7:47052793-4705
ENSG0000(	749	18.73319	chr7:330(	ENSG00000231681		lncRNA	chr7:50202001-5026
ENSG0000(	749	18.73319	chr7:330(	HMGN1P19		Pseudoger	chr7:46634614-4663
ENSG0000(	749	18.73319	chr7:330(	VWC2		protein_c	chr7:49773638-4992
ENSG0000(	749	18.73319	chr7:330(	ENSG00000251378		Pseudoger	chr7:45818582-4581
ENSG0000(	749	18.73319	chr7:330(	CDC14C		protein_c	chr7:48924547-4892
ENSG0000(	749	18.73319	chr7:330(	ENSG00000240355		lncRNA	chr7:46476457-4647
ENSG0000(	749	18.73319	chr7:330(	ENSG00000287521		lncRNA	chr7:50093279-5009
ENSG0000(	749	18.73319	chr7:330(	IGFBP1		protein_c	chr7:45888360-4589
ENSG0000(	749	18.73319	chr7:330(	ADCY1	NCGv7	protein_c	chr7:45574140-4572

ENSG0000(	749	18.73319	chr7:330(	ENSG00000286995		lncRNA	chr7:49524208-4958
ENSG0000(	749	18.73319	chr7:330(	RNU7-76P		smallRNA	chr7:45975377-4597
ENSG0000(	749	18.73319	chr7:330(	SUN3	DriverDB\	protein_c	chr7:47987148-4802
ENSG0000(	749	18.73319	chr7:330(	GDI2P1		Pseudoger	chr7:48902556-4890
ENSG0000(	747	18.68316	chr2:4707	HMGN2P20		Pseudoger	chr2:24330402-2433
ENSG0000(	743	18.58312	chr4:909?	ENPP7P11		Pseudoger	chr4:9677308-96779
ENSG0000(	737	18.43305	chr1:373?	RNU6-1310P		smallRNA	chr1:168263375-168
ENSG0000(	727	18.18295	chr15:40?	ENSG00000259346		Pseudoger	chr15:65301922-653
ENSG0000(	726	18.15793	chr7:330(	RNY4		smallRNA	chr7:148963315-148
ENSG0000(	726	18.15793	chr7:330(	RNY3		smallRNA	chr7:148983755-148
ENSG0000(	726	18.15793	chr7:330(	ENSG00000274133		Pseudoger	chr7:148445786-148
ENSG0000(	726	18.15793	chr7:330(	ENSG00000290600		lncRNA	chr7:149285281-149
ENSG0000(	726	18.15793	chr7:330(	AC005229.1		smallRNA	chr7:148730829-148
ENSG0000(	726	18.15793	chr7:330(	CUL1	NCGv7	protein_c	chr7:148697914-148
ENSG0000(	726	18.15793	chr7:330(	ZNF425		protein_c	chr7:149102784-149
ENSG0000(	726	18.15793	chr7:330(	ZNF783		protein_c	chr7:149262171-149
ENSG0000(	726	18.15793	chr7:330(	COX6B1P1		Pseudoger	chr7:149053961-149
ENSG0000(	726	18.15793	chr7:330(	ZNF398		protein_c	chr7:149126416-149
ENSG0000(	726	18.15793	chr7:330(	RNY5		smallRNA	chr7:148941488-148
ENSG0000(	726	18.15793	chr7:330(	ENSG00000283504		lncRNA	chr7:148473599-148
ENSG0000(	726	18.15793	chr7:330(	RNU6-650P		smallRNA	chr7:149033057-149
ENSG0000(	726	18.15793	chr7:330(	ENSG00000244560		Pseudoger	chr7:149287614-149
ENSG0000(	726	18.15793	chr7:330(	RN7SL521P		smallRNA	chr7:149125693-149
ENSG0000(	726	18.15793	chr7:330(	NPM1P12		Pseudoger	chr7:149334024-149
ENSG0000(	726	18.15793	chr7:330(	ENSG00000239719		Pseudoger	chr7:149191043-149
ENSG0000(	726	18.15793	chr7:330(	ENSG00000273314		lncRNA	chr7:148696467-148
ENSG0000(	726	18.15793	chr7:330(	PDIA4	NCGv7	protein_c	chr7:149003062-149
ENSG0000(	726	18.15793	chr7:330(	ENSG00000228151		lncRNA	chr7:149398204-149
ENSG0000(	726	18.15793	chr7:330(	C7orf33		protein_c	chr7:148590766-148
ENSG0000(	726	18.15793	chr7:330(	ENSG00000286180		lncRNA	chr7:148940265-148
ENSG0000(	726	18.15793	chr7:330(	ENSG00000261842		lncRNA	chr7:149422675-149
ENSG0000(	726	18.15793	chr7:330(	ENSG00000286171		lncRNA	chr7:148941483-148
ENSG0000(	726	18.15793	chr7:330(	ENSG00000270200		Pseudoger	chr7:148649547-148
ENSG0000(	726	18.15793	chr7:330(	U3		smallRNA	chr7:148389649-148
ENSG0000(	726	18.15793	chr7:330(	RN7SL72P		smallRNA	chr7:148438309-148
ENSG0000(	726	18.15793	chr7:330(	ZNF212		protein_c	chr7:149239651-149
ENSG0000(	726	18.15793	chr7:330(	ENSG00000271664		Pseudoger	chr7:149275619-149
ENSG0000(	726	18.15793	chr7:330(	RNY1		smallRNA	chr7:148987136-148
ENSG0000(	726	18.15793	chr7:330(	RPL32P17		Pseudoger	chr7:148580401-148
ENSG0000(	726	18.15793	chr7:330(	ZNF786		protein_c	chr7:149069641-149
ENSG0000(	726	18.15793	chr7:330(	EZH2	NCGv7;AC	protein_c	chr7:148807257-148
ENSG0000(	726	18.15793	chr7:330(	ZNF282		protein_c	chr7:149195546-149
ENSG0000(	726	18.15793	chr7:330(	RN7SL569P		smallRNA	chr7:148890214-148
ENSG0000(	726	18.15793	chr7:330(	ENSG00000213209		Pseudoger	chr7:148637179-148
ENSG0000(	726	18.15793	chr7:330(	GHET1		lncRNA	chr7:148987527-148
ENSG0000(	726	18.15793	chr7:330(	ENSG00000287636		lncRNA	chr7:148584818-148
ENSG0000(	726	18.15793	chr7:330(	ENSG00000283648		lncRNA	chr7:148543677-148
ENSG0000(	726	18.15793	chr7:330(	ENSG00000231397		Pseudoger	chr7:149321865-149
ENSG0000(	726	18.15793	chr7:330(	RNU7-20P		smallRNA	chr7:148821012-148
ENSG0000(	726	18.15793	chr7:330(	SNORD112		smallRNA	chr7:149286442-149
ENSG0000(	723	18.0829	chr12:68?	IFT81		protein_c	chr12:110124335-11

ENSG0000(	723	18.0829	chr12:68	ENSG00000277299		lncRNA	chr12:109948389-10
ENSG0000(	723	18.0829	chr12:68	MIR4497		smallRNA	chr12:109833348-10
ENSG0000(	723	18.0829	chr12:68	GLTP		protein_c	chr12:109850945-10
ENSG0000(	723	18.0829	chr12:68	HVCN1	NCGv7	protein_c	chr12:110627841-11
ENSG0000(	723	18.0829	chr12:68	FAM216A		protein_c	chr12:110468415-11
ENSG0000(	723	18.0829	chr12:68	PPTC7		protein_c	chr12:110533245-11
ENSG0000(	723	18.0829	chr12:68	ENSG00000279925		TEC	chr12:110744549-11
ENSG0000(	723	18.0829	chr12:68	RN7SL387P		smallRNA	chr12:110625647-11
ENSG0000(	723	18.0829	chr12:68	ENSG00000277595		lncRNA	chr12:110032245-11
ENSG0000(	723	18.0829	chr12:68	ENSG00000289311		lncRNA	chr12:110279086-11
ENSG0000(	723	18.0829	chr12:68	CCDC63	NCGv7	protein_c	chr12:110846769-11
ENSG0000(	723	18.0829	chr12:68	ENSG00000258210		lncRNA	chr12:110387463-11
ENSG0000(	723	18.0829	chr12:68	GIT2		protein_c	chr12:109929804-10
ENSG0000(	723	18.0829	chr12:68	ENSG00000279360		TEC	chr12:110056916-11
ENSG0000(	723	18.0829	chr12:68	HMGA1P3		Pseudoger	chr12:110429959-11
ENSG0000(	723	18.0829	chr12:68	ANAPC7		protein_c	chr12:110372900-11
ENSG0000(	723	18.0829	chr12:68	RAD9B		protein_c	chr12:110501655-11
ENSG0000(	723	18.0829	chr12:68	ENSG00000278993		TEC	chr12:110501614-11
ENSG0000(	723	18.0829	chr12:68	ANKRD13A	NCGv7	protein_c	chr12:109999186-11
ENSG0000(	723	18.0829	chr12:68	MYL2		protein_c	chr12:110910819-11
ENSG0000(	723	18.0829	chr12:68	VPS29		protein_c	chr12:110491083-11
ENSG0000(	723	18.0829	chr12:68	GPN3		protein_c	chr12:110452486-11
ENSG0000(	723	18.0829	chr12:68	ARPC3		protein_c	chr12:110434823-11
ENSG0000(	723	18.0829	chr12:68	ENSG00000256351		Pseudoger	chr12:109859767-10
ENSG0000(	723	18.0829	chr12:68	TCHP		protein_c	chr12:109900264-10
ENSG0000(	723	18.0829	chr12:68	ENSG00000280426		TEC	chr12:109997419-10
ENSG0000(	723	18.0829	chr12:68	C12orf76		protein_c	chr12:110027028-11
ENSG0000(	723	18.0829	chr12:68	LINC01404		lncRNA	chr12:110951683-11
ENSG0000(	723	18.0829	chr12:68	TCTN1	DriverDB	protein_c	chr12:110614027-11
ENSG0000(	723	18.0829	chr12:68	ATP2A2	NCGv7	protein_c	chr12:110280756-11
ENSG0000(	723	18.0829	chr12:68	ENSG00000286220		Pseudoger	chr12:110050991-11
ENSG0000(	723	18.0829	chr12:68	ENSG00000257268		lncRNA	chr12:110831779-11
ENSG0000(	723	18.0829	chr12:68	LINC01405		lncRNA	chr12:110934590-11
ENSG0000(	723	18.0829	chr12:68	RN7SL441P		smallRNA	chr12:109866216-10
ENSG0000(	723	18.0829	chr12:68	AC144522.1		smallRNA	chr12:110632604-11
ENSG0000(	723	18.0829	chr12:68	RPL31P49		Pseudoger	chr12:110460988-11
ENSG0000(	723	18.0829	chr12:68	SNORD50		smallRNA	chr12:110496352-11
ENSG0000(	723	18.0829	chr12:68	AC002978.1		smallRNA	chr12:111073968-11
ENSG0000(	723	18.0829	chr12:68	RN7SL769P		smallRNA	chr12:110360450-11
ENSG0000(	723	18.0829	chr12:68	PPP1CC		protein_c	chr12:110719680-11
ENSG0000(	723	18.0829	chr12:68	ENSG00000290863		lncRNA	chr12:110049360-11
ENSG0000(	723	18.0829	chr12:68	ENSG00000249094		lncRNA	chr12:109880676-10
ENSG0000(	721	18.03288	chr4:909	RN7SL671P		smallRNA	chr4:1869060-18693
ENSG0000(	714	17.8578	chr6:105(	MIR4640		smallRNA	chr6:30890883-3089
ENSG0000(	709	17.73275	chr6:105(	ENSG00000290047		lncRNA	chr6:30570638-3057
ENSG0000(	705	17.63271	chr4:909	AC097473.1		smallRNA	chr4:108789200-108
ENSG0000(	696	17.40761	chr6:105(	ENSG00000233183		lncRNA	chr6:33891327-3392
ENSG0000(	696	17.40761	chr6:105(	ETF1P1		Pseudoger	chr6:30031713-3003
ENSG0000(	696	17.40761	chr6:105(	AL669914.1		smallRNA	chr6:30088821-3008
ENSG0000(	696	17.40761	chr6:105(	ENSG00000290870		lncRNA	chr6:29849550-2988
ENSG0000(	696	17.40761	chr6:105(	TRIM15		protein_c	chr6:30163206-3017

ENSG0000(	696	17.40761	chr6:105(	GABBR1		protein_c	chr6:29555629-2963
ENSG0000(	696	17.40761	chr6:105(	MIR4646		smallRNA	chr6:31701029-3170
ENSG0000(	696	17.40761	chr6:105(	MIR5004		smallRNA	chr6:33438331-3343
ENSG0000(	696	17.40761	chr6:105(	TRIM10		protein_c	chr6:30151943-3016
ENSG0000(	696	17.40761	chr6:105(	WASF5P		Pseudoger	chr6:31287510-3128
ENSG0000(	696	17.40761	chr6:105(	OR12D1		protein_c	chr6:29414928-2941
ENSG0000(	696	17.40761	chr6:105(	HCG22		protein_c	chr6:31053450-3105
ENSG0000(	696	17.40761	chr6:105(	GTF2H4		protein_c	chr6:30908207-3091
ENSG0000(	696	17.40761	chr6:105(	MAS1L	NCGv7	protein_c	chr6:29486697-2948
ENSG0000(	696	17.40761	chr6:105(	RANP1		Pseudoger	chr6:30485940-3048
ENSG0000(	696	17.40761	chr6:105(	TNXA		Pseudoger	chr6:32008614-3201
ENSG0000(	696	17.40761	chr6:105(	ENSG00000288751		lncRNA	chr6:33207081-3320
ENSG0000(	696	17.40761	chr6:105(	HCG4		Pseudoger	chr6:29791753-2979
ENSG0000(	696	17.40761	chr6:105(	NAPGP2		Pseudoger	chr6:30961403-3096
ENSG0000(	696	17.40761	chr6:105(	STK19B		Pseudoger	chr6:32013270-3201
ENSG0000(	696	17.40761	chr6:105(	MIR3135B		smallRNA	chr6:32749912-3274
ENSG0000(	696	17.40761	chr6:105(	ENSG00000250264		protein_c	chr6:32813767-3283
ENSG0000(	696	17.40761	chr6:105(	LYPLA2P1		Pseudoger	chr6:33365548-3336
ENSG0000(	696	17.40761	chr6:105(	DHFRP2		Pseudoger	chr6:31360865-3136
ENSG0000(	696	17.40761	chr6:105(	HLA-V		Pseudoger	chr6:29792234-2979
ENSG0000(	696	17.40761	chr6:105(	UBD		protein_c	chr6:29555515-2955
ENSG0000(	696	17.40761	chr6:105(	ENSG00000244349		lncRNA	chr6:28986800-2898
ENSG0000(	696	17.40761	chr6:105(	EHMT2		protein_c	chr6:31879759-3189
ENSG0000(	696	17.40761	chr6:105(	TRIM39-RPP21		protein_c	chr6:30328907-3034
ENSG0000(	696	17.40761	chr6:105(	RPL7AP7		Pseudoger	chr6:29803195-2980
ENSG0000(	696	17.40761	chr6:105(	HCP5		lncRNA	chr6:31463170-3147
ENSG0000(	696	17.40761	chr6:105(	GNL1		protein_c	chr6:30541381-3055
ENSG0000(	696	17.40761	chr6:105(	LINC02571		lncRNA	chr6:31293908-3130
ENSG0000(	696	17.40761	chr6:105(	OR12D3		protein_c	chr6:29373423-2937
ENSG0000(	696	17.40761	chr6:105(	ZBTB12	DriverDB\	protein_c	chr6:31899613-3190
ENSG0000(	696	17.40761	chr6:105(	MCCD1P1		Pseudoger	chr6:29907783-2990
ENSG0000(	696	17.40761	chr6:105(	SLC39A7		protein_c	chr6:33200305-3320
ENSG0000(	696	17.40761	chr6:105(	PHF1		protein_c	chr6:33410399-3341
ENSG0000(	696	17.40761	chr6:105(	NELFE	NCGv7	protein_c	chr6:31952087-3195
ENSG0000(	696	17.40761	chr6:105(	CUTA		protein_c	chr6:33416442-3341
ENSG0000(	696	17.40761	chr6:105(	HLA-E		protein_c	chr6:30489509-3049
ENSG0000(	696	17.40761	chr6:105(	OR14J1		protein_c	chr6:29301701-2931
ENSG0000(	696	17.40761	chr6:105(	OR11A1		protein_c	chr6:29425504-2945
ENSG0000(	696	17.40761	chr6:105(	RNU6-603P		smallRNA	chr6:32352877-3235
ENSG0000(	696	17.40761	chr6:105(	PTMAP1		Pseudoger	chr6:30633632-3063
ENSG0000(	696	17.40761	chr6:105(	RNU1-61P		smallRNA	chr6:32549940-3255
ENSG0000(	696	17.40761	chr6:105(	HLA-P		Pseudoger	chr6:29800415-2980
ENSG0000(	696	17.40761	chr6:105(	TRIM39		protein_c	chr6:30326479-3034
ENSG0000(	696	17.40761	chr6:105(	TNF	NCGv7	protein_c	chr6:31575565-3157
ENSG0000(	696	17.40761	chr6:105(	ATP6V1G2	NCGv7	protein_c	chr6:31544444-3154
ENSG0000(	696	17.40761	chr6:105(	OR2H1		protein_c	chr6:29457155-2946
ENSG0000(	696	17.40761	chr6:105(	ENSG00000263756		lncRNA	chr6:32972065-3297
ENSG0000(	696	17.40761	chr6:105(	ENSG00000244255		protein_c	chr6:31927698-3195
ENSG0000(	696	17.40761	chr6:105(	HLA-DPA1		protein_c	chr6:33064569-3308
ENSG0000(	696	17.40761	chr6:105(	RPS18		protein_c	chr6:33272075-3327
ENSG0000(	696	17.40761	chr6:105(	AL662800.2		smallRNA	chr6:30648666-3064

ENSG0000(	696	17.40761	chr6:105(	TRIM40		protein_c	chr6:30136124-3014
ENSG0000(	696	17.40761	chr6:105(	RPL15P4		Pseudoger	chr6:31528114-3152
ENSG0000(	696	17.40761	chr6:105(	TNXB		protein_c	chr6:32041153-3211
ENSG0000(	696	17.40761	chr6:105(	AL662890.3		smallRNA	chr6:28795972-2879
ENSG0000(	696	17.40761	chr6:105(	HLA-DPA2		Pseudoger	chr6:33091753-3309
ENSG0000(	696	17.40761	chr6:105(	ENSG00000288805		lncRNA	chr6:30460825-3048
ENSG0000(	696	17.40761	chr6:105(	COL11A2P1		Pseudoger	chr6:33103794-3310
ENSG0000(	696	17.40761	chr6:105(	ENSG00000288813		lncRNA	chr6:31272240-3127
ENSG0000(	696	17.40761	chr6:105(	ZNRD1ASP		Pseudoger	chr6:29976042-3006
ENSG0000(	696	17.40761	chr6:105(	RPL3P2		Pseudoger	chr6:31280317-3128
ENSG0000(	696	17.40761	chr6:105(	MUCL3		protein_c	chr6:30934523-3095
ENSG0000(	696	17.40761	chr6:105(	STK19	NCGv7	protein_c	chr6:31971091-3198
ENSG0000(	696	17.40761	chr6:105(	RN7SL471P		smallRNA	chr6:28977475-2897
ENSG0000(	696	17.40761	chr6:105(	UBQLN1P1		Pseudoger	chr6:30363112-3036
ENSG0000(	696	17.40761	chr6:105(	KRT18P1		Pseudoger	chr6:28969130-2897
ENSG0000(	696	17.40761	chr6:105(	LY6G6E		Pseudoger	chr6:31711771-3171
ENSG0000(	696	17.40761	chr6:105(	CYP21A1P		Pseudoger	chr6:32005689-3200
ENSG0000(	696	17.40761	chr6:105(	KIFC1	IntOGen-I	protein_c	chr6:33391823-3340
ENSG0000(	696	17.40761	chr6:105(	OR2W1-AS1		lncRNA	chr6:29036021-2907
ENSG0000(	696	17.40761	chr6:105(	UBDP1		Pseudoger	chr6:29464596-2946
ENSG0000(	696	17.40761	chr6:105(	ENSG00000290788		lncRNA	chr6:32005636-3200
ENSG0000(	696	17.40761	chr6:105(	FKBPL		protein_c	chr6:32128707-3213
ENSG0000(	696	17.40761	chr6:105(	LINC02829		lncRNA	chr6:29497475-2951
ENSG0000(	696	17.40761	chr6:105(	PRRT1	NCGv7	protein_c	chr6:32148359-3215
ENSG0000(	696	17.40761	chr6:105(	AGPAT1		protein_c	chr6:32168212-3217
ENSG0000(	696	17.40761	chr6:105(	RNF5		protein_c	chr6:32178405-3218
ENSG0000(	696	17.40761	chr6:105(	HCG9		lncRNA	chr6:29975112-2997
ENSG0000(	696	17.40761	chr6:105(	AGER		protein_c	chr6:32180968-3218
ENSG0000(	696	17.40761	chr6:105(	ENSG00000277881		Pseudoger	chr6:29450210-2945
ENSG0000(	696	17.40761	chr6:105(	DXO		protein_c	chr6:31969810-3197
ENSG0000(	696	17.40761	chr6:105(	HLA-DQB2		protein_c	chr6:32756098-3276
ENSG0000(	696	17.40761	chr6:105(	HNRNPA1P2		Pseudoger	chr6:32325219-3232
ENSG0000(	696	17.40761	chr6:105(	RNU6-930P		smallRNA	chr6:28915645-2891
ENSG0000(	696	17.40761	chr6:105(	TRIM31		protein_c	chr6:30102897-3011
ENSG0000(	696	17.40761	chr6:105(	RNF39		protein_c	chr6:30070266-3007
ENSG0000(	696	17.40761	chr6:105(	LY6G6D		protein_c	chr6:31715348-3171
ENSG0000(	696	17.40761	chr6:105(	SKIV2L		protein_c	chr6:31959116-3196
ENSG0000(	696	17.40761	chr6:105(	OR12D2		protein_c	chr6:29395631-2939
ENSG0000(	696	17.40761	chr6:105(	RPL35AP4		Pseudoger	chr6:33389374-3338
ENSG0000(	696	17.40761	chr6:105(	LY6G6F-LY6G6D		protein_c	chr6:31706904-3171
ENSG0000(	696	17.40761	chr6:105(	RN7SL353P		smallRNA	chr6:30751038-3075
ENSG0000(	696	17.40761	chr6:105(	USP8P1		Pseudoger	chr6:31275572-3127
ENSG0000(	696	17.40761	chr6:105(	PPP1R11		protein_c	chr6:30066709-3007
ENSG0000(	696	17.40761	chr6:105(	HLA-J		Pseudoger	chr6:30006606-3000
ENSG0000(	696	17.40761	chr6:105(	OR2H2		protein_c	chr6:29585121-2959
ENSG0000(	696	17.40761	chr6:105(	TAP1	NCGv7	protein_c	chr6:32845209-3285
ENSG0000(	696	17.40761	chr6:105(	MOG	NCGv7	protein_c	chr6:29657002-2967
ENSG0000(	696	17.40761	chr6:105(	MIR3934		smallRNA	chr6:33698128-3369
ENSG0000(	696	17.40761	chr6:105(	ZFP57		protein_c	chr6:29672483-2968
ENSG0000(	696	17.40761	chr6:105(	LINC01623		lncRNA	chr6:28859625-2886
ENSG0000(	696	17.40761	chr6:105(	HLA-F	NCGv7	protein_c	chr6:29722775-2973

ENSG0000(	696	17.40761	chr6:105(	LINC00243		lncRNA	chr6:30798211-3083
ENSG0000(	696	17.40761	chr6:105(	ZNF70P1		Pseudoger	chr6:33215705-3321
ENSG0000(	696	17.40761	chr6:105(	HLA-G		protein_c	chr6:29826967-2983
ENSG0000(	696	17.40761	chr6:105(	SAPCD1		protein_c	chr6:31762656-3176
ENSG0000(	696	17.40761	chr6:105(	TRIM26BP		Pseudoger	chr6:30238301-3024
ENSG0000(	696	17.40761	chr6:105(	GPR53P		Pseudoger	chr6:29537704-2953
ENSG0000(	696	17.40761	chr6:105(	ENSG00000263020		protein_c	chr6:31666102-3167
ENSG0000(	696	17.40761	chr6:105(	OR2P1P		Pseudoger	chr6:29071824-2907
ENSG0000(	696	17.40761	chr6:105(	NEU1		protein_c	chr6:31857659-3186
ENSG0000(	696	17.40761	chr6:105(	SLC44A4		protein_c	chr6:31863192-3187
ENSG0000(	696	17.40761	chr6:105(	MICB	DriverDBv	protein_c	chr6:31494881-3151
ENSG0000(	696	17.40761	chr6:105(	POU5F1	NCGv7;AC	protein_c	chr6:31164337-3118
ENSG0000(	696	17.40761	chr6:105(	HTATSF1P1		Pseudoger	chr6:33237799-3323
ENSG0000(	696	17.40761	chr6:105(	HLA-DPA3		Pseudoger	chr6:33131216-3314
ENSG0000(	696	17.40761	chr6:105(	PSORS1C3		lncRNA	chr6:31173735-3117
ENSG0000(	696	17.40761	chr6:105(	GPSM3		protein_c	chr6:32190766-3219
ENSG0000(	696	17.40761	chr6:105(	PRR3		protein_c	chr6:30557280-3056
ENSG0000(	696	17.40761	chr6:105(	HCG14		lncRNA	chr6:28896530-2889
ENSG0000(	696	17.40761	chr6:105(	HLA-C		protein_c	chr6:31268749-3127
ENSG0000(	696	17.40761	chr6:105(	MIR1275		smallRNA	chr6:33999972-3400
ENSG0000(	696	17.40761	chr6:105(	EHMT2-AS1		lncRNA	chr6:31877808-3188
ENSG0000(	696	17.40761	chr6:105(	DDX39B		protein_c	chr6:31530219-3154
ENSG0000(	696	17.40761	chr6:105(	MICA		protein_c	chr6:31399784-3141
ENSG0000(	696	17.40761	chr6:105(	HCG20		lncRNA	chr6:30743790-3079
ENSG0000(	696	17.40761	chr6:105(	ENSG00000248993		protein_c	chr6:32937364-3295
ENSG0000(	696	17.40761	chr6:105(	HLA-DRB5	NCGv7	protein_c	chr6:32517353-3253
ENSG0000(	696	17.40761	chr6:105(	MCCD1		protein_c	chr6:31528962-3153
ENSG0000(	696	17.40761	chr6:105(	NFKBIL1		protein_c	chr6:31546870-3155
ENSG0000(	696	17.40761	chr6:105(	LINC01016		lncRNA	chr6:33867506-3389
ENSG0000(	696	17.40761	chr6:105(	TMPOP1		Pseudoger	chr6:30466452-3046
ENSG0000(	696	17.40761	chr6:105(	HLA-U		Pseudoger	chr6:29934101-2993
ENSG0000(	696	17.40761	chr6:105(	LST1		protein_c	chr6:31586124-3158
ENSG0000(	696	17.40761	chr6:105(	NCR3		protein_c	chr6:31588895-3159
ENSG0000(	696	17.40761	chr6:105(	ENSG00000288473		lncRNA	chr6:30908242-3092
ENSG0000(	696	17.40761	chr6:105(	C2		protein_c	chr6:31897785-3194
ENSG0000(	696	17.40761	chr6:105(	AIF1		protein_c	chr6:31615217-3161
ENSG0000(	696	17.40761	chr6:105(	HLA-DRB6		lncRNA	chr6:32552713-3256
ENSG0000(	696	17.40761	chr6:105(	PRRC2A		protein_c	chr6:31620715-3163
ENSG0000(	696	17.40761	chr6:105(	C4A		protein_c	chr6:31982057-3200
ENSG0000(	696	17.40761	chr6:105(	AL662890.1		smallRNA	chr6:28775916-2877
ENSG0000(	696	17.40761	chr6:105(	ZBED9-AS1		lncRNA	chr6:28587378-2859
ENSG0000(	696	17.40761	chr6:105(	HCG25		lncRNA	chr6:33249534-3325
ENSG0000(	696	17.40761	chr6:105(	HLA-S		Pseudoger	chr6:31382074-3138
ENSG0000(	696	17.40761	chr6:105(	C6orf136	NCGv7	protein_c	chr6:30647039-3065
ENSG0000(	696	17.40761	chr6:105(	RNA5SP206		Pseudoger	chr6:32078508-3207
ENSG0000(	696	17.40761	chr6:105(	DHX16	NCGv7	protein_c	chr6:30653119-3067
ENSG0000(	696	17.40761	chr6:105(	MRPS18B		protein_c	chr6:30617840-3062
ENSG0000(	696	17.40761	chr6:105(	MUC21		protein_c	chr6:30983718-3098
ENSG0000(	696	17.40761	chr6:105(	RPSAP2		Pseudoger	chr6:28732017-2873
ENSG0000(	696	17.40761	chr6:105(	RPL13AP		Pseudoger	chr6:29582508-2958
ENSG0000(	696	17.40761	chr6:105(	PPP1R10	AC	protein_c	chr6:30600413-3061

ENSG0000(	696	17.40761	chr6:105(	ZBTB22		protein_c	chr6:33314406-3331
ENSG0000(	696	17.40761	chr6:105(	RGL2		protein_c	chr6:33291654-3329
ENSG0000(	696	17.40761	chr6:105(	ABCF1	NCGv7	protein_c	chr6:30571393-3059
ENSG0000(	696	17.40761	chr6:105(	C6orf15		protein_c	chr6:31111223-3111
ENSG0000(	696	17.40761	chr6:105(	ENSG00000289047		lncRNA	chr6:32894299-3290
ENSG0000(	696	17.40761	chr6:105(	HCG4P8		Pseudoger	chr6:29827385-2982
ENSG0000(	696	17.40761	chr6:105(	PSORS1C1		protein_c	chr6:31114750-3114
ENSG0000(	696	17.40761	chr6:105(	CDSN		protein_c	chr6:31115087-3112
ENSG0000(	696	17.40761	chr6:105(	HLA-A	NCGv7	protein_c	chr6:29941260-2994
ENSG0000(	696	17.40761	chr6:105(	OR10C1		protein_c	chr6:29439306-2944
ENSG0000(	696	17.40761	chr6:105(	PSORS1C2		protein_c	chr6:31137534-3113
ENSG0000(	696	17.40761	chr6:105(	CCHCR1		protein_c	chr6:31142439-3115
ENSG0000(	696	17.40761	chr6:105(	MIR219-1		smallRNA	chr6:33207835-3320
ENSG0000(	696	17.40761	chr6:105(	TRIM31-AS1		lncRNA	chr6:30105240-3011
ENSG0000(	696	17.40761	chr6:105(	MIR1236		smallRNA	chr6:31956839-3195
ENSG0000(	696	17.40761	chr6:105(	HLA-DQB1		protein_c	chr6:32659467-3266
ENSG0000(	696	17.40761	chr6:105(	CLIC1		protein_c	chr6:31730581-3173
ENSG0000(	696	17.40761	chr6:105(	ATF6B		protein_c	chr6:32115264-3212
ENSG0000(	696	17.40761	chr6:105(	ENSG00000280128		TEC	chr6:30282349-3028
ENSG0000(	696	17.40761	chr6:105(	ENSG00000255899		Pseudoger	chr6:31224342-3122
ENSG0000(	696	17.40761	chr6:105(	BAG6		protein_c	chr6:31639028-3165
ENSG0000(	696	17.40761	chr6:105(	OR2U1P		Pseudoger	chr6:29262703-2926
ENSG0000(	696	17.40761	chr6:105(	VWA7	NCGv7	protein_c	chr6:31765590-3177
ENSG0000(	696	17.40761	chr6:105(	ENSG00000204422		lncRNA	chr6:31686962-3171
ENSG0000(	696	17.40761	chr6:105(	OR2B3		protein_c	chr6:29086208-2908
ENSG0000(	696	17.40761	chr6:105(	LY6G6C		protein_c	chr6:31718648-3172
ENSG0000(	696	17.40761	chr6:105(	HLA-DRB6		Pseudoger	chr6:32553046-3255
ENSG0000(	696	17.40761	chr6:105(	ENSG00000291111		lncRNA	chr6:33112440-3311
ENSG0000(	696	17.40761	chr6:105(	RPS17P1		Pseudoger	chr6:29489271-2948
ENSG0000(	696	17.40761	chr6:105(	ZBTB9		protein_c	chr6:33453970-3345
ENSG0000(	696	17.40761	chr6:105(	MPIG6B		protein_c	chr6:31718594-3172
ENSG0000(	696	17.40761	chr6:105(	Y_RNA		smallRNA	chr6:31402152-3140
ENSG0000(	696	17.40761	chr6:105(	MICD		Pseudoger	chr6:29970801-2997
ENSG0000(	696	17.40761	chr6:105(	SAR1AP1		Pseudoger	chr6:29076573-2907
ENSG0000(	696	17.40761	chr6:105(	MSH5	NCGv7	protein_c	chr6:31739677-3176
ENSG0000(	696	17.40761	chr6:105(	VPS52		protein_c	chr6:33250272-3327
ENSG0000(	696	17.40761	chr6:105(	VARS1		protein_c	chr6:31777518-3179
ENSG0000(	696	17.40761	chr6:105(	DDAH2		protein_c	chr6:31727038-3173
ENSG0000(	696	17.40761	chr6:105(	OR2J1		protein_c	chr6:29099490-2910
ENSG0000(	696	17.40761	chr6:105(	LSM2		protein_c	chr6:31797396-3180
ENSG0000(	696	17.40761	chr6:105(	TSBP1-AS1		lncRNA	chr6:32254640-3240
ENSG0000(	696	17.40761	chr6:105(	HSPA1L	NCGv7	protein_c	chr6:31809619-3181
ENSG0000(	696	17.40761	chr6:105(	HSPA1A	AC	protein_c	chr6:31815543-3181
ENSG0000(	696	17.40761	chr6:105(	Y_RNA		smallRNA	chr6:30932618-3093
ENSG0000(	696	17.40761	chr6:105(	MICE		Pseudoger	chr6:29741731-2974
ENSG0000(	696	17.40761	chr6:105(	OR2J3		protein_c	chr6:29108058-2911
ENSG0000(	696	17.40761	chr6:105(	ENSG00000273333		lncRNA	chr6:32184733-3218
ENSG0000(	696	17.40761	chr6:105(	HSPA1B		protein_c	chr6:31827738-3183
ENSG0000(	696	17.40761	chr6:105(	SNHG32		lncRNA	chr6:31834608-3183
ENSG0000(	696	17.40761	chr6:105(	OR2J2		protein_c	chr6:29170907-2917
ENSG0000(	696	17.40761	chr6:105(	C2-AS1		lncRNA	chr6:31934474-3194

ENSG0000(	696	17.40761	chr6:105(	OR2W1		protein_c	chr6:29044213-2904
ENSG0000(	696	17.40761	chr6:105(	LY6G6F		protein_c	chr6:31706866-3171
ENSG0000(	696	17.40761	chr6:105(	MYL12BP3		Pseudoger	chr6:33338978-3333
ENSG0000(	696	17.40761	chr6:105(	HLA-H		Pseudoger	chr6:29887752-2989
ENSG0000(	696	17.40761	chr6:105(	HLA-F-AS1		lncRNA	chr6:29726601-2974
ENSG0000(	696	17.40761	chr6:105(	HCP5B		lncRNA	chr6:29871895-2987
ENSG0000(	696	17.40761	chr6:105(	MICG		Pseudoger	chr6:29812565-2981
ENSG0000(	696	17.40761	chr6:105(	DDR1		protein_c	chr6:30876421-3090
ENSG0000(	696	17.40761	chr6:105(	MUC22		protein_c	chr6:31010474-3103
ENSG0000(	696	17.40761	chr6:105(	APOM		protein_c	chr6:31652416-3165
ENSG0000(	696	17.40761	chr6:105(	Y_RNA		smallRNA	chr6:29750371-2975
ENSG0000(	696	17.40761	chr6:105(	RNU6-850P		smallRNA	chr6:31756951-3175
ENSG0000(	696	17.40761	chr6:105(	C6orf47		protein_c	chr6:31658298-3166
ENSG0000(	696	17.40761	chr6:105(	GPANK1		protein_c	chr6:31661228-3166
ENSG0000(	696	17.40761	chr6:105(	ENSG00000225173		lncRNA	chr6:28837240-2883
ENSG0000(	696	17.40761	chr6:105(	CSNK2B	NCGv7	protein_c	chr6:31665227-3167
ENSG0000(	696	17.40761	chr6:105(	OR2J4P		Pseudoger	chr6:29181510-2918
ENSG0000(	696	17.40761	chr6:105(	ENSG00000225864		Pseudoger	chr6:29722981-2972
ENSG0000(	696	17.40761	chr6:105(	RPL13P		Pseudoger	chr6:28861416-2886
ENSG0000(	696	17.40761	chr6:105(	OR2G1P		Pseudoger	chr6:29229227-2923
ENSG0000(	696	17.40761	chr6:105(	ENSG00000255726		Pseudoger	chr6:31222913-3122
ENSG0000(	696	17.40761	chr6:105(	TRIM27	NCGv7;AC	protein_c	chr6:28903002-2892
ENSG0000(	696	17.40761	chr6:105(	SNORA48		smallRNA	chr6:30132805-3013
ENSG0000(	696	17.40761	chr6:105(	ENSG00000288587		lncRNA	chr6:31400702-3146
ENSG0000(	696	17.40761	chr6:105(	LINC01556		lncRNA	chr6:28943877-2894
ENSG0000(	696	17.40761	chr6:105(	SYNGAP1-AS1		lncRNA	chr6:33437363-3345
ENSG0000(	696	17.40761	chr6:105(	HLA-DQA2		protein_c	chr6:32741391-3274
ENSG0000(	696	17.40761	chr6:105(	HCG27		lncRNA	chr6:31197760-3120
ENSG0000(	696	17.40761	chr6:105(	LY6G5C	DriverDB	protein_c	chr6:31676684-3168
ENSG0000(	696	17.40761	chr6:105(	ABHD16A		protein_c	chr6:31686955-3170
ENSG0000(	696	17.40761	chr6:105(	HLA-L		lncRNA	chr6:30259548-3029
ENSG0000(	696	17.40761	chr6:105(	PBX2	AC	protein_c	chr6:32184733-3219
ENSG0000(	696	17.40761	chr6:105(	ENSG00000287279		lncRNA	chr6:28634861-2864
ENSG0000(	696	17.40761	chr6:105(	NOTCH4	NCGv7;AC	protein_c	chr6:32194843-3222
ENSG0000(	696	17.40761	chr6:105(	ENSG00000233902		Pseudoger	chr6:31462728-3146
ENSG0000(	696	17.40761	chr6:105(	ATP6V1G2-DDX39B		protein_c	chr6:31530219-3154
ENSG0000(	696	17.40761	chr6:105(	SYNGAP1		protein_c	chr6:33419661-3345
ENSG0000(	696	17.40761	chr6:105(	RPP21		protein_c	chr6:30345131-3034
ENSG0000(	696	17.40761	chr6:105(	EGFL8		protein_c	chr6:32164595-3216
ENSG0000(	696	17.40761	chr6:105(	HCG18		lncRNA	chr6:30286690-3032
ENSG0000(	696	17.40761	chr6:105(	ENSG00000271821		lncRNA	chr6:31200165-3120
ENSG0000(	696	17.40761	chr6:105(	WDR46		protein_c	chr6:33279108-3328
ENSG0000(	696	17.40761	chr6:105(	LINC01149		lncRNA	chr6:31441667-3144
ENSG0000(	696	17.40761	chr6:105(	MAS1LP1		Pseudoger	chr6:29475025-2947
ENSG0000(	696	17.40761	chr6:105(	SAPCD1-AS1		lncRNA	chr6:31764310-3176
ENSG0000(	696	17.40761	chr6:105(	RPL32P1		Pseudoger	chr6:33079451-3307
ENSG0000(	696	17.40761	chr6:105(	DDX39BP2		Pseudoger	chr6:29993209-2999
ENSG0000(	696	17.40761	chr6:105(	MDC1-AS1		lncRNA	chr6:30703067-3071
ENSG0000(	696	17.40761	chr6:105(	RN7SKP186		smallRNA	chr6:30864250-3086
ENSG0000(	696	17.40761	chr6:105(	ENSG00000289203		lncRNA	chr6:29485157-2949
ENSG0000(	696	17.40761	chr6:105(	LINC00336		lncRNA	chr6:33586104-3359

ENSG0000(	696	17.40761	chr6:105(	LINC03003		Pseudoger	chr6:29290711-2929
ENSG0000(	696	17.40761	chr6:105(	POLR2LP1		Pseudoger	chr6:31140727-3114
ENSG0000(	696	17.40761	chr6:105(	LINC00533		lncRNA	chr6:28648286-2864
ENSG0000(	696	17.40761	chr6:105(	C6orf47-AS1		lncRNA	chr6:31658329-3166
ENSG0000(	696	17.40761	chr6:105(	ENSG00000271440		Pseudoger	chr6:28633381-2863
ENSG0000(	696	17.40761	chr6:105(	NOP56P1		Pseudoger	chr6:28783633-2878
ENSG0000(	696	17.40761	chr6:105(	PPT2	DriverDBv	protein_c	chr6:32153441-3216
ENSG0000(	696	17.40761	chr6:105(	SNORD48		smallRNA	chr6:31835263-3183
ENSG0000(	696	17.40761	chr6:105(	ENSG00000289282		protein_c	chr6:31623874-3162
ENSG0000(	696	17.40761	chr6:105(	TRIM26		protein_c	chr6:30184455-3021
ENSG0000(	696	17.40761	chr6:105(	ENSG00000223837		lncRNA	chr6:32970232-3297
ENSG0000(	696	17.40761	chr6:105(	HCG24		lncRNA	chr6:33144783-3314
ENSG0000(	696	17.40761	chr6:105(	ENSG00000286301		lncRNA	chr6:30451139-3045
ENSG0000(	696	17.40761	chr6:105(	Y_RNA		smallRNA	chr6:30736304-3073
ENSG0000(	696	17.40761	chr6:105(	HLA-DOB		protein_c	chr6:32812763-3282
ENSG0000(	696	17.40761	chr6:105(	ENSG00000271581		Pseudoger	chr6:31356647-3135
ENSG0000(	696	17.40761	chr6:105(	ENSG00000289975		lncRNA	chr6:33247976-3324
ENSG0000(	696	17.40761	chr6:105(	ZDHHC20P1		Pseudoger	chr6:29708125-2970
ENSG0000(	696	17.40761	chr6:105(	GRM4		protein_c	chr6:34018643-3415
ENSG0000(	696	17.40761	chr6:105(	RPL7P4		Pseudoger	chr6:30696806-3069
ENSG0000(	696	17.40761	chr6:105(	RN7SL26P		smallRNA	chr6:33540694-3354
ENSG0000(	696	17.40761	chr6:105(	MCCD1P2		Pseudoger	chr6:29994437-2999
ENSG0000(	696	17.40761	chr6:105(	DDX39BP1		Pseudoger	chr6:29906543-2990
ENSG0000(	696	17.40761	chr6:105(	LINC02570		lncRNA	chr6:30838324-3084
ENSG0000(	696	17.40761	chr6:105(	ENSG00000272501		lncRNA	chr6:31195200-3119
ENSG0000(	696	17.40761	chr6:105(	HLA-DPB1		protein_c	chr6:33075990-3308
ENSG0000(	696	17.40761	chr6:105(	HLA-DMB		protein_c	chr6:32934629-3294
ENSG0000(	696	17.40761	chr6:105(	C4A-AS1		lncRNA	chr6:31999976-3200
ENSG0000(	696	17.40761	chr6:105(	ENSG00000285647		lncRNA	chr6:31367057-3137
ENSG0000(	696	17.40761	chr6:105(	ENSG00000272540		lncRNA	chr6:30723105-3072
ENSG0000(	696	17.40761	chr6:105(	LTA		protein_c	chr6:31572054-3157
ENSG0000(	696	17.40761	chr6:105(	ENSG00000290478		lncRNA	chr6:29212886-2921
ENSG0000(	696	17.40761	chr6:105(	ENSG00000290479		lncRNA	chr6:29137410-2914
ENSG0000(	696	17.40761	chr6:105(	LINC02569		lncRNA	chr6:30516266-3051
ENSG0000(	696	17.40761	chr6:105(	LINC03003		lncRNA	chr6:29223973-2929
ENSG0000(	696	17.40761	chr6:105(	HLA-DQB3		Pseudoger	chr6:32730758-3273
ENSG0000(	696	17.40761	chr6:105(	OR2U2P		Pseudoger	chr6:29268462-2926
ENSG0000(	696	17.40761	chr6:105(	MICC		Pseudoger	chr6:30414715-3041
ENSG0000(	696	17.40761	chr6:105(	RNY4P10		smallRNA	chr6:33199601-3319
ENSG0000(	696	17.40761	chr6:105(	ENSG00000272221		lncRNA	chr6:31394289-3139
ENSG0000(	696	17.40761	chr6:105(	DDX6P1		Pseudoger	chr6:29329626-2933
ENSG0000(	696	17.40761	chr6:105(	PAIP1P1		Pseudoger	chr6:30186798-3018
ENSG0000(	696	17.40761	chr6:105(	IP6K3		protein_c	chr6:33721662-3374
ENSG0000(	696	17.40761	chr6:105(	LEMD2	NCGv7	protein_c	chr6:33771202-3378
ENSG0000(	696	17.40761	chr6:105(	OR2I1P		protein_c	chr6:29550407-2955
ENSG0000(	696	17.40761	chr6:105(	ENSG00000272217		lncRNA	chr6:33246075-3324
ENSG0000(	696	17.40761	chr6:105(	ENSG00000272236		lncRNA	chr6:29751965-2975
ENSG0000(	696	17.40761	chr6:105(	ENSG00000232080		lncRNA	chr6:32718005-3271
ENSG0000(	696	17.40761	chr6:105(	BAK1		protein_c	chr6:33572547-3358
ENSG0000(	696	17.40761	chr6:105(	ZBED9		protein_c	chr6:28570535-2861
ENSG0000(	696	17.40761	chr6:105(	IER3-AS1		lncRNA	chr6:30742757-3074

ENSG0000(	696	17.40761	chr6:105(	AL121932.1		smallRNA	chr6:28648197-2864
ENSG0000(	696	17.40761	chr6:105(	ENSG00000285799		Pseudoger	chr6:29852363-2985
ENSG0000(	696	17.40761	chr6:105(	ENSG00000285761		lncRNA	chr6:29752573-2976
ENSG0000(	696	17.40761	chr6:105(	ENSG00000227766		Pseudoger	chr6:29942075-2994
ENSG0000(	696	17.40761	chr6:105(	SNORD117		smallRNA	chr6:31536374-3153
ENSG0000(	696	17.40761	chr6:105(	HCG21		lncRNA	chr6:30945979-3095
ENSG0000(	696	17.40761	chr6:105(	FLOT1		protein_c	chr6:30727709-3074
ENSG0000(	696	17.40761	chr6:105(	PPIAP9		Pseudoger	chr6:31519480-3152
ENSG0000(	696	17.40761	chr6:105(	ENSG00000286974		lncRNA	chr6:32108406-3211
ENSG0000(	696	17.40761	chr6:105(	ENSG00000270896		Pseudoger	chr6:29763258-2976
ENSG0000(	696	17.40761	chr6:105(	HLA-K		Pseudoger	chr6:29926459-2992
ENSG0000(	696	17.40761	chr6:105(	MICB-DT		lncRNA	chr6:31479973-3149
ENSG0000(	696	17.40761	chr6:105(	MTCO3P1		Pseudoger	chr6:32706124-3270
ENSG0000(	696	17.40761	chr6:105(	SMIM40		protein_c	chr6:33323628-3332
ENSG0000(	696	17.40761	chr6:105(	Y_RNA		smallRNA	chr6:31663288-3166
ENSG0000(	696	17.40761	chr6:105(	PPT2-EGFL8		protein_c	chr6:32153845-3217
ENSG0000(	696	17.40761	chr6:105(	RNU6-1133P		smallRNA	chr6:31083010-3108
ENSG0000(	696	17.40761	chr6:105(	SNORA38		smallRNA	chr6:31623079-3162
ENSG0000(	696	17.40761	chr6:105(	ENSG00000289559		lncRNA	chr6:32893197-3289
ENSG0000(	696	17.40761	chr6:105(	AL662800.1		smallRNA	chr6:30616229-3061
ENSG0000(	696	17.40761	chr6:105(	RPL23AP1		Pseudoger	chr6:29726669-2972
ENSG0000(	696	17.40761	chr6:105(	UQCC2		protein_c	chr6:33694293-3371
ENSG0000(	696	17.40761	chr6:105(	MSH5-SAPCD1		protein_c	chr6:31740020-3176
ENSG0000(	696	17.40761	chr6:105(	ENSG00000289375		lncRNA	chr6:31619847-3162
ENSG0000(	696	17.40761	chr6:105(	AL671883.1		smallRNA	chr6:31374318-3137
ENSG0000(	696	17.40761	chr6:105(	ENSG00000289406		lncRNA	chr6:31560244-3157
ENSG0000(	696	17.40761	chr6:105(	MLN		protein_c	chr6:33794673-3380
ENSG0000(	696	17.40761	chr6:105(	ITPR3	NCGv7	protein_c	chr6:33620365-3369
ENSG0000(	696	17.40761	chr6:105(	SUMO2P1		Pseudoger	chr6:29636060-2963
ENSG0000(	696	17.40761	chr6:105(	HLA-DRB1	NCGv7	protein_c	chr6:32577902-3258
ENSG0000(	696	17.40761	chr6:105(	TUBB		protein_c	chr6:30717435-3072
ENSG0000(	696	17.40761	chr6:105(	HCG17		lncRNA	chr6:30234039-3032
ENSG0000(	696	17.40761	chr6:105(	ENSG00000224486		Pseudoger	chr6:30359278-3035
ENSG0000(	696	17.40761	chr6:105(	TMEM183AP1		Pseudoger	chr6:29577459-2957
ENSG0000(	696	17.40761	chr6:105(	ENSG00000287089		lncRNA	chr6:33800142-3380
ENSG0000(	696	17.40761	chr6:105(	SNORD84		smallRNA	chr6:31541101-3154
ENSG0000(	696	17.40761	chr6:105(	HLA-W		Pseudoger	chr6:29956596-2995
ENSG0000(	696	17.40761	chr6:105(	HLA-Z		Pseudoger	chr6:32896416-3289
ENSG0000(	696	17.40761	chr6:105(	SFTA2		protein_c	chr6:30931353-3095
ENSG0000(	696	17.40761	chr6:105(	HLA-DRB9		Pseudoger	chr6:32459821-3247
ENSG0000(	696	17.40761	chr6:105(	TCF19		protein_c	chr6:31158547-3116
ENSG0000(	696	17.40761	chr6:105(	IER3		protein_c	chr6:30743199-3074
ENSG0000(	696	17.40761	chr6:105(	FGFR3P1		Pseudoger	chr6:31377419-3137
ENSG0000(	696	17.40761	chr6:105(	MDC1	NCGv7	protein_c	chr6:30699807-3071
ENSG0000(	696	17.40761	chr6:105(	LY6G5B		protein_c	chr6:31669976-3167
ENSG0000(	696	17.40761	chr6:105(	PSMB9		protein_c	chr6:32844136-3285
ENSG0000(	696	17.40761	chr6:105(	RN7SL175P		smallRNA	chr6:30906878-3090
ENSG0000(	696	17.40761	chr6:105(	HCG15		lncRNA	chr6:28986203-2898
ENSG0000(	696	17.40761	chr6:105(	HLA-N		Pseudoger	chr6:30351416-3035
ENSG0000(	696	17.40761	chr6:105(	ENSG00000289829		lncRNA	chr6:31826841-3182
ENSG0000(	696	17.40761	chr6:105(	RNU6-283P		smallRNA	chr6:31370134-3137

ENSG0000(	696	17.40761	chr6:105(	HLA-DQA1	NCGv7	protein_c	chr6:32628179-3264
ENSG0000(	696	17.40761	chr6:105(	ENSG00000230521		Pseudoger	chr6:29887294-2988
ENSG0000(	696	17.40761	chr6:105(	TAPBP		protein_c	chr6:33299694-3331
ENSG0000(	696	17.40761	chr6:105(	ENSG00000271362		Pseudoger	chr6:33883170-3388
ENSG0000(	696	17.40761	chr6:105(	Y_RNA		smallRNA	chr6:31496689-3149
ENSG0000(	696	17.40761	chr6:105(	OR2AD1P		Pseudoger	chr6:29026680-2902
ENSG0000(	696	17.40761	chr6:105(	HCG9P5		Pseudoger	chr6:29748289-2974
ENSG0000(	696	17.40761	chr6:105(	SNORD52		smallRNA	chr6:31837076-3183
ENSG0000(	696	17.40761	chr6:105(	OR2H4P		Pseudoger	chr6:29215236-2921
ENSG0000(	696	17.40761	chr6:105(	C4B		protein_c	chr6:32014795-3203
ENSG0000(	696	17.40761	chr6:105(	POLR1H		protein_c	chr6:30058899-3006
ENSG0000(	696	17.40761	chr6:105(	LINC01015		lncRNA	chr6:29528721-2953
ENSG0000(	696	17.40761	chr6:105(	ATAT1	DriverDB\	protein_c	chr6:30626842-3064
ENSG0000(	696	17.40761	chr6:105(	NRM		protein_c	chr6:30688047-3069
ENSG0000(	696	17.40761	chr6:105(	VARS2	NCGv7	protein_c	chr6:30914205-3092
ENSG0000(	696	17.40761	chr6:105(	SNORD32B		smallRNA	chr6:29582249-2958
ENSG0000(	696	17.40761	chr6:105(	HLA-DPB2		Pseudoger	chr6:33112516-3312
ENSG0000(	696	17.40761	chr6:105(	HLA-B	NCGv7	protein_c	chr6:31353872-3136
ENSG0000(	696	17.40761	chr6:105(	ENSG00000289637		lncRNA	chr6:31821149-3182
ENSG0000(	696	17.40761	chr6:105(	HCG4B		Pseudoger	chr6:29925983-2992
ENSG0000(	696	17.40761	chr6:105(	PPP1R18		protein_c	chr6:30676389-3068
ENSG0000(	696	17.40761	chr6:105(	CYP21A2		protein_c	chr6:32038327-3204
ENSG0000(	696	17.40761	chr6:105(	ZDHHC20P2		Pseudoger	chr6:31380411-3138
ENSG0000(	696	17.40761	chr6:105(	PPP1R2P1		Pseudoger	chr6:32879171-3287
ENSG0000(	696	17.40761	chr6:105(	Y_RNA		smallRNA	chr6:31778817-3177
ENSG0000(	696	17.40761	chr6:105(	UQCRHP1		Pseudoger	chr6:31611083-3161
ENSG0000(	696	17.40761	chr6:105(	OR2N1P		Pseudoger	chr6:29137880-2913
ENSG0000(	696	17.40761	chr6:105(	ENSG00000227206		lncRNA	chr6:29124210-2912
ENSG0000(	696	17.40761	chr6:105(	DAXX	NCGv7;AC	protein_c	chr6:33318558-3332
ENSG0000(	696	17.40761	chr6:105(	AL645941.1		smallRNA	chr6:32936916-3293
ENSG0000(	696	17.40761	chr6:105(	ENSG00000290574		lncRNA	chr6:30005971-3000
ENSG0000(	696	17.40761	chr6:105(	HLA-V		lncRNA	chr6:29790954-2979
ENSG0000(	696	17.40761	chr6:105(	ENSG00000285085		protein_c	chr6:32150495-3215
ENSG0000(	696	17.40761	chr6:105(	OR5V1		protein_c	chr6:29353749-2943
ENSG0000(	696	17.40761	chr6:105(	ENSG00000284954		lncRNA	chr6:32152802-3215
ENSG0000(	696	17.40761	chr6:105(	LTB	NCGv7	protein_c	chr6:31580525-3158
ENSG0000(	696	17.40761	chr6:105(	MIR877		smallRNA	chr6:30584332-3058
ENSG0000(	696	17.40761	chr6:105(	HLA-DOA		protein_c	chr6:33004182-3300
ENSG0000(	696	17.40761	chr6:105(	RPL12P1		Pseudoger	chr6:33400059-3340
ENSG0000(	696	17.40761	chr6:105(	OR2H5P		Pseudoger	chr6:29573909-2957
ENSG0000(	696	17.40761	chr6:105(	COL11A2		protein_c	chr6:33162681-3319
ENSG0000(	696	17.40761	chr6:105(	HLA-DRA		protein_c	chr6:32439878-3244
ENSG0000(	696	17.40761	chr6:105(	HLA-T		Pseudoger	chr6:29896654-2989
ENSG0000(	696	17.40761	chr6:105(	ENSG00000289100		lncRNA	chr6:33299479-3330
ENSG0000(	696	17.40761	chr6:105(	BTNL2		protein_c	chr6:32393339-3240
ENSG0000(	696	17.40761	chr6:105(	PFDN6		protein_c	chr6:33289302-3329
ENSG0000(	696	17.40761	chr6:105(	ENSG00000237669		Pseudoger	chr6:30006121-3000
ENSG0000(	696	17.40761	chr6:105(	ENSG00000285064		protein_c	chr6:33321386-3332
ENSG0000(	696	17.40761	chr6:105(	RXRB		protein_c	chr6:33193588-3320
ENSG0000(	696	17.40761	chr6:105(	HLA-DQB1-AS1		lncRNA	chr6:32659880-3266
ENSG0000(	696	17.40761	chr6:105(	HLA-DMA		protein_c	chr6:32948613-3296

ENSG0000(	696	17.40761	chr6:105(	PSMB8-AS1		lncRNA	chr6:32844078-3284
ENSG0000(	696	17.40761	chr6:105(	HSD17B8	DriverDBv	protein_c	chr6:33204655-3320
ENSG0000(	696	17.40761	chr6:105(	ENSG00000284656		lncRNA	chr6:29162475-2916
ENSG0000(	696	17.40761	chr6:105(	ENSG00000229836		Pseudoger	chr6:31307815-3130
ENSG0000(	696	17.40761	chr6:105(	BRD2		protein_c	chr6:32968594-3298
ENSG0000(	696	17.40761	chr6:105(	PSMB8		protein_c	chr6:32840717-3284
ENSG0000(	696	17.40761	chr6:105(	CFB		protein_c	chr6:31945650-3195
ENSG0000(	696	17.40761	chr6:105(	C4B-AS1		lncRNA	chr6:32032713-3203
ENSG0000(	696	17.40761	chr6:105(	ZNF311	DriverDBv	protein_c	chr6:28994785-2900
ENSG0000(	696	17.40761	chr6:105(	RING1	AC	protein_c	chr6:33208500-3321
ENSG0000(	696	17.40761	chr6:105(	IFITM4P		Pseudoger	chr6:29750729-2975
ENSG0000(	696	17.40761	chr6:105(	TAP2		protein_c	chr6:32821833-3283
ENSG0000(	696	17.40761	chr6:105(	DDR1-DT		lncRNA	chr6:30866982-3087
ENSG0000(	696	17.40761	chr6:105(	SUCLA2P1		Pseudoger	chr6:30468882-3047
ENSG0000(	696	17.40761	chr6:105(	ENSG00000256851		Pseudoger	chr6:31515979-3151
ENSG0000(	696	17.40761	chr6:105(	ENSG00000285565		lncRNA	chr6:31828834-3183
ENSG0000(	696	17.40761	chr6:105(	GGNBP1		Pseudoger	chr6:33540046-3358
ENSG0000(	696	17.40761	chr6:105(	B3GALT4		protein_c	chr6:33277123-3328
ENSG0000(	696	17.40761	chr6:105(	TSBP1		protein_c	chr6:32288526-3237
ENSG0000(	696	17.40761	chr6:105(	HLA-L		Pseudoger	chr6:30259625-3026
ENSG0000(	696	17.40761	chr6:105(	ENSG00000284829		lncRNA	chr6:32098176-3211
ENSG0000(	696	17.40761	chr6:105(	ZNF90P2		Pseudoger	chr6:28888832-2888
ENSG0000(	696	17.40761	chr6:105(	AL645922.1		protein_c	chr6:32006168-3200
ENSG0000(	695	17.3826	chr1:116(	ENSG00000290111		lncRNA	chr1:40394763-4039
ENSG0000(	692	17.30756	chr4:909:	RNA5SP159		Pseudoger	chr4:39936753-3993
ENSG0000(	692	17.30756	chr7:330(	ENSG00000284048		lncRNA	chr7:150379329-150
ENSG0000(	692	17.30756	chr7:330(	ENSG00000241449		lncRNA	chr7:150033653-150
ENSG0000(	692	17.30756	chr7:330(	ENSG00000273293		lncRNA	chr7:149881359-149
ENSG0000(	692	17.30756	chr7:330(	ENSG00000224016		Pseudoger	chr7:149891191-149
ENSG0000(	692	17.30756	chr7:330(	REPIN1		protein_c	chr7:150368189-150
ENSG0000(	692	17.30756	chr7:330(	ENSG00000279536		TEC	chr7:149881477-149
ENSG0000(	692	17.30756	chr7:330(	REPIN1-AS1		lncRNA	chr7:150363777-150
ENSG0000(	692	17.30756	chr7:330(	KRBA1		protein_c	chr7:149714781-149
ENSG0000(	692	17.30756	chr7:330(	ZNF467		protein_c	chr7:149764182-149
ENSG0000(	692	17.30756	chr7:330(	ENSG00000288997		lncRNA	chr7:149495204-149
ENSG0000(	692	17.30756	chr7:330(	ENSG00000275295		Pseudoger	chr7:45303620-4530
ENSG0000(	692	17.30756	chr7:330(	ENSG00000273419		lncRNA	chr7:149858400-149
ENSG0000(	692	17.30756	chr7:330(	ENSG00000260555		lncRNA	chr7:150000752-150
ENSG0000(	692	17.30756	chr7:330(	ZBED10P		Pseudoger	chr7:150322639-150
ENSG0000(	692	17.30756	chr7:330(	ATP6V0E2		protein_c	chr7:149872968-149
ENSG0000(	692	17.30756	chr7:330(	ENSG00000276538		Pseudoger	chr7:150047609-150
ENSG0000(	692	17.30756	chr7:330(	ZNF777		protein_c	chr7:149431363-149
ENSG0000(	692	17.30756	chr7:330(	ENSG00000261305		lncRNA	chr7:150337483-150
ENSG0000(	692	17.30756	chr7:330(	ENSG00000286912		lncRNA	chr7:150234194-150
ENSG0000(	692	17.30756	chr7:330(	ENSG00000280149		TEC	chr7:149851572-149
ENSG0000(	692	17.30756	chr7:330(	ZNF746		protein_c	chr7:149472696-149
ENSG0000(	692	17.30756	chr7:330(	SSPOP		Pseudoger	chr7:149776042-149
ENSG0000(	692	17.30756	chr7:330(	LRRC61		protein_c	chr7:150323263-150
ENSG0000(	692	17.30756	chr7:330(	LINC00996		lncRNA	chr7:150433654-150
ENSG0000(	692	17.30756	chr7:330(	ENSG00000286738		lncRNA	chr7:45268681-4538
ENSG0000(	692	17.30756	chr7:330(	ZNF767P		Pseudoger	chr7:149619924-149

ENSG0000(	692	17.30756	chr7:330(	ZNF775-AS1		lncRNA	chr7:150379854-150
ENSG0000(	692	17.30756	chr7:330(	RARRES2	NCGv7	protein_c	chr7:150338317-150
ENSG0000(	692	17.30756	chr7:330(	ACTR3C		protein_c	chr7:150243916-150
ENSG0000(	692	17.30756	chr7:330(	ELK1P1		Pseudoger	chr7:45391626-4539
ENSG0000(	692	17.30756	chr7:330(	ZNF862	DriverDB\	protein_c	chr7:149838375-149
ENSG0000(	692	17.30756	chr7:330(	ENSG00000284691		protein_c	chr7:150400702-150
ENSG0000(	692	17.30756	chr7:330(	ENSG00000225537		lncRNA	chr7:45460712-4554
ENSG0000(	692	17.30756	chr7:330(	ATP6V0E2-AS1		lncRNA	chr7:149867697-149
ENSG0000(	692	17.30756	chr7:330(	ZNF775		protein_c	chr7:150368790-150
ENSG0000(	692	17.30756	chr7:330(	ENSG00000273011		lncRNA	chr7:149890739-149
ENSG0000(	692	17.30756	chr7:330(	ZNF767P		lncRNA	chr7:149547154-149
ENSG0000(	691	17.28255	chr12:10(	RNA5SP373		Pseudoger	chr12:111339527-11
ENSG0000(	691	17.28255	chr12:10(	CUX2		protein_c	chr12:111034165-11
ENSG0000(	691	17.28255	chr12:10(	HSPA8P14		Pseudoger	chr12:111381867-11
ENSG0000(	691	17.28255	chr12:10(	LINC02356		lncRNA	chr12:111369261-11
ENSG0000(	691	17.28255	chr12:10(	AC002979.1		smallRNA	chr12:111302800-11
ENSG0000(	691	17.28255	chr12:10(	PHETA1		protein_c	chr12:111360651-11
ENSG0000(	688	17.20752	chr6:105(	ZNF204P		Pseudoger	chr6:27358252-2735
ENSG0000(	688	17.20752	chr6:105(	VN1R10P		Pseudoger	chr6:27324894-2732
ENSG0000(	688	17.20752	chr6:105(	ZNF184		protein_c	chr6:27450743-2747
ENSG0000(	688	17.20752	chr6:105(	ENSG00000286652		lncRNA	chr6:27473194-2749
ENSG0000(	688	17.20752	chr6:105(	ENSG00000278332		Pseudoger	chr6:27510501-2751
ENSG0000(	688	17.20752	chr6:105(	ENSG00000285849		lncRNA	chr6:27454568-2745
ENSG0000(	688	17.20752	chr6:105(	ENSG00000291112		lncRNA	chr6:27356451-2737
ENSG0000(	688	17.20752	chr6:105(	POM121L2		protein_c	chr6:27285903-2731
ENSG0000(	688	17.20752	chr6:105(	ZNF391	DriverDB\	protein_c	chr6:27374615-2740
ENSG0000(	688	17.20752	chr6:105(	HNRNPA1P1		Pseudoger	chr6:27523076-2752
ENSG0000(	688	17.20752	chr6:105(	MCFD2P1		Pseudoger	chr6:27407697-2740
ENSG0000(	688	17.20752	chr6:105(	CD83P1		Pseudoger	chr6:27560822-2756
ENSG0000(	688	17.20752	chr6:105(	ENSG00000271755		lncRNA	chr6:27404010-2740
ENSG0000(	688	17.20752	chr6:105(	ENSG00000285703		lncRNA	chr6:27491422-2751
ENSG0000(	688	17.20752	chr4:909(	Y_RNA		smallRNA	chr4:75662121-7566
ENSG0000(	685	17.13249	chr14:71(	ENSG00000259079		Pseudoger	chr14:71330342-713
ENSG0000(	676	16.90739	chr4:909(	AC092846.2		smallRNA	chr4:24429832-2442
ENSG0000(	673	16.83236	chr6:105(	RNU7-26P		smallRNA	chr6:27897504-2789
ENSG0000(	667	16.68229	chr4:909(	PPBPP1		Pseudoger	chr4:73847866-7384
ENSG0000(	661	16.53222	chr15:69(	AC009677.1		smallRNA	chr15:69730740-697
ENSG0000(	658	16.45719	chr4:909(	ENSG00000273179		lncRNA	chr4:1167778-11681
ENSG0000(	654	16.35715	chr4:909(	ENSG00000287389		lncRNA	chr4:27917753-2794
ENSG0000(	654	16.35715	chr4:909(	MIR573		smallRNA	chr4:24520192-2452
ENSG0000(	654	16.35715	chr4:909(	RN7SL315P		smallRNA	chr4:17595068-1759
ENSG0000(	654	16.35715	chr4:909(	PABPC1P1		Pseudoger	chr4:39973444-3997
ENSG0000(	654	16.35715	chr4:909(	RN7SL822P		smallRNA	chr4:55215626-5521
ENSG0000(	654	16.35715	chr4:909(	ENSG00000286596		lncRNA	chr4:30718805-3072
ENSG0000(	654	16.35715	chr4:909(	LINC02261		lncRNA	chr4:27217479-2728
ENSG0000(	654	16.35715	chr4:909(	ENSG00000249547		lncRNA	chr4:23234625-2328
ENSG0000(	654	16.35715	chr4:909(	ENSG00000249564		Pseudoger	chr4:29907659-2990
ENSG0000(	654	16.35715	chr4:909(	ENSG00000279386		TEC	chr4:37866561-3786
ENSG0000(	654	16.35715	chr4:909(	ENSG00000289643		lncRNA	chr4:42285818-4229
ENSG0000(	654	16.35715	chr4:909(	LINC01258		lncRNA	chr4:38420662-3852
ENSG0000(	654	16.35715	chr4:909(	ENSG00000249766		Pseudoger	chr4:33400538-3340

ENSG0000(	654	16.35715	chr4:909:	ENSG00000239532		Pseudoger	chr4:37821361-3782
ENSG0000(	654	16.35715	chr4:909:	ENSG00000249863		Pseudoger	chr4:37868292-3786
ENSG0000(	654	16.35715	chr4:909:	ZEB2P1		Pseudoger	chr4:16360686-1639
ENSG0000(	654	16.35715	chr4:909:	EEF1A1P21		Pseudoger	chr4:29748757-2975
ENSG0000(	654	16.35715	chr4:909:	RNU6-931P		smallRNA	chr4:45480030-4548
ENSG0000(	654	16.35715	chr4:909:	ENSG00000249828		Pseudoger	chr4:49203171-4920
ENSG0000(	654	16.35715	chr4:909:	LINC01259		lncRNA	chr4:38509729-3851
ENSG0000(	654	16.35715	chr4:909:	ENSG00000249706		lncRNA	chr4:53899871-5391
ENSG0000(	654	16.35715	chr4:909:	MRPS33P2		Pseudoger	chr4:38006784-3800
ENSG0000(	654	16.35715	chr4:909:	TBC1D1	AC	protein_c	chr4:37891084-3813
ENSG0000(	654	16.35715	chr4:909:	RPL12P20		Pseudoger	chr4:41389115-4138
ENSG0000(	654	16.35715	chr4:909:	RNU6-746P		smallRNA	chr4:55294490-5529
ENSG0000(	654	16.35715	chr4:909:	AC093807.1		smallRNA	chr4:26637390-2663
ENSG0000(	654	16.35715	chr4:909:	ENSG00000271172		lncRNA	chr4:25220403-2522
ENSG0000(	654	16.35715	chr4:909:	HMGB1P28		Pseudoger	chr4:41842154-4184
ENSG0000(	654	16.35715	chr4:909:	snoU13		smallRNA	chr4:17528937-1752
ENSG0000(	654	16.35715	chr4:909:	ENSG00000279379		TEC	chr4:19257951-1925
ENSG0000(	654	16.35715	chr4:909:	RN7SL193P		smallRNA	chr4:43863274-4386
ENSG0000(	654	16.35715	chr4:909:	CLRN2		protein_c	chr4:17515165-1752
ENSG0000(	654	16.35715	chr4:909:	ENSG00000249727		lncRNA	chr4:54836041-5484
ENSG0000(	654	16.35715	chr4:909:	ENSG00000249771		lncRNA	chr4:41883060-4193
ENSG0000(	654	16.35715	chr4:909:	ENSG00000249678		lncRNA	chr4:30776257-3079
ENSG0000(	654	16.35715	chr4:909:	ENSG00000249729		Pseudoger	chr4:44840380-4484
ENSG0000(	654	16.35715	chr4:909:	ANAPC4		protein_c	chr4:25377263-2541
ENSG0000(	654	16.35715	chr4:909:	ENSG00000249685		lncRNA	chr4:39133913-3913
ENSG0000(	654	16.35715	chr4:909:	AC093786.1		smallRNA	chr4:34386754-3438
ENSG0000(	654	16.35715	chr4:909:	PACRGL		protein_c	chr4:20696282-2075
ENSG0000(	654	16.35715	chr4:909:	ENSG00000244538		Pseudoger	chr4:22594196-2259
ENSG0000(	654	16.35715	chr4:909:	MED28-DT		lncRNA	chr4:17586267-1761
ENSG0000(	654	16.35715	chr4:909:	NACAP5		Pseudoger	chr4:17552117-1755
ENSG0000(	654	16.35715	chr4:909:	KLF3-AS1		lncRNA	chr4:38602438-3866
ENSG0000(	654	16.35715	chr4:909:	SPATA18	NCGv7	protein_c	chr4:52051304-5209
ENSG0000(	654	16.35715	chr4:909:	ENSG00000280043		TEC	chr4:49229573-4923
ENSG0000(	654	16.35715	chr4:909:	ENSG00000280015		TEC	chr4:38390754-3839
ENSG0000(	654	16.35715	chr4:909:	ENSG00000249207		lncRNA	chr4:39066974-3918
ENSG0000(	654	16.35715	chr4:909:	ATP1B1P1		Pseudoger	chr4:42029209-4203
ENSG0000(	654	16.35715	chr4:909:	ENSG00000287762		lncRNA	chr4:41608312-4161
ENSG0000(	654	16.35715	chr4:909:	ENSG00000249216		lncRNA	chr4:41688858-4169
ENSG0000(	654	16.35715	chr4:909:	ENSG00000249228		lncRNA	chr4:29046591-2904
ENSG0000(	654	16.35715	chr4:909:	ENSG00000249234		lncRNA	chr4:16178939-1618
ENSG0000(	654	16.35715	chr4:909:	GRXCR1		protein_c	chr4:42892713-4303
ENSG0000(	654	16.35715	chr4:909:	snoU13		smallRNA	chr4:22349450-2234
ENSG0000(	654	16.35715	chr4:909:	LINC02265		lncRNA	chr4:40316484-4033
ENSG0000(	654	16.35715	chr4:909:	RNA5SP160		Pseudoger	chr4:40990154-4099
ENSG0000(	654	16.35715	chr4:909:	PROM1	NCGv7	protein_c	chr4:15963076-1608
ENSG0000(	654	16.35715	chr4:909:	ENSG00000288073		lncRNA	chr4:37454698-3747
ENSG0000(	654	16.35715	chr4:909:	ATP5MGP3		Pseudoger	chr4:24658072-2465
ENSG0000(	654	16.35715	chr4:909:	PPARGC1A		protein_c	chr4:23755041-2390
ENSG0000(	654	16.35715	chr4:909:	SGCB		protein_c	chr4:52020706-5203
ENSG0000(	654	16.35715	chr4:909:	UGDH		protein_c	chr4:39498755-3952
ENSG0000(	654	16.35715	chr4:909:	ENSG00000289902		lncRNA	chr4:24321454-2432

ENSG0000(	654	16.35715	chr4:909:	KRT18P25		Pseudoger	chr4:40020240-4002
ENSG0000(	654	16.35715	chr4:909:	ENSG00000249079		Pseudoger	chr4:49212251-4921
ENSG0000(	654	16.35715	chr4:909:	ENSG00000260878		lncRNA	chr4:46243548-4624
ENSG0000(	654	16.35715	chr4:909:	OR5M14P		Pseudoger	chr4:41722538-4172
ENSG0000(	654	16.35715	chr4:909:	FAM184B		protein_c	chr4:17629306-1778
ENSG0000(	654	16.35715	chr4:909:	DHX15	NCGv7	protein_c	chr4:24517441-2458
ENSG0000(	654	16.35715	chr4:909:	SOD3		protein_c	chr4:24789912-2480
ENSG0000(	654	16.35715	chr4:909:	SEPSECS		protein_c	chr4:25120014-2516
ENSG0000(	654	16.35715	chr4:909:	NCAPG	NCGv7	protein_c	chr4:17810979-1784
ENSG0000(	654	16.35715	chr4:909:	RN7SKP82		smallRNA	chr4:42892396-4289
ENSG0000(	654	16.35715	chr4:909:	TBC1D19		protein_c	chr4:26576437-2675
ENSG0000(	654	16.35715	chr4:909:	STIM2	NCGv7	protein_c	chr4:26857601-2702
ENSG0000(	654	16.35715	chr4:909:	ENSG00000260918		lncRNA	chr4:47431960-4743
ENSG0000(	654	16.35715	chr4:909:	KLF3		protein_c	chr4:38664197-3870
ENSG0000(	654	16.35715	chr4:909:	ENSG00000249122		lncRNA	chr4:41750345-4175
ENSG0000(	654	16.35715	chr4:909:	KLHL5	NCGv7	protein_c	chr4:39045039-3912
ENSG0000(	654	16.35715	chr4:909:	SLIRPP2		Pseudoger	chr4:25686202-2568
ENSG0000(	654	16.35715	chr4:909:	ENSG00000289849		lncRNA	chr4:22237413-2231
ENSG0000(	654	16.35715	chr4:909:	AC098869.1		smallRNA	chr4:40463716-4046
ENSG0000(	654	16.35715	chr4:909:	NWD2		protein_c	chr4:37244743-3744
ENSG0000(	654	16.35715	chr4:909:	SEL1L3		protein_c	chr4:25747433-2586
ENSG0000(	654	16.35715	chr4:909:	CHRNA9		protein_c	chr4:40335333-4035
ENSG0000(	654	16.35715	chr4:909:	ENSG00000239983		Pseudoger	chr4:33968174-3396
ENSG0000(	654	16.35715	chr4:909:	Y_RNA		smallRNA	chr4:40826655-4082
ENSG0000(	654	16.35715	chr4:909:	ENSG00000224097		Pseudoger	chr4:39480255-3948
ENSG0000(	654	16.35715	chr4:909:	ENSG00000249441		lncRNA	chr4:19747179-1975
ENSG0000(	654	16.35715	chr4:909:	MIR218-1		smallRNA	chr4:20528275-2052
ENSG0000(	654	16.35715	chr4:909:	TLR6	NCGv7	protein_c	chr4:38822897-3885
ENSG0000(	654	16.35715	chr4:909:	AC079772.1		smallRNA	chr4:33536744-3353
ENSG0000(	654	16.35715	chr4:909:	TLR1		protein_c	chr4:38790677-3885
ENSG0000(	654	16.35715	chr4:909:	TLR10		protein_c	chr4:38772238-3878
ENSG0000(	654	16.35715	chr4:909:	ENSG00000249452		Pseudoger	chr4:33238239-3323
ENSG0000(	654	16.35715	chr4:909:	ENSG00000249453		lncRNA	chr4:23779590-2378
ENSG0000(	654	16.35715	chr4:909:	RNU6-412P		smallRNA	chr4:46531237-4653
ENSG0000(	654	16.35715	chr4:909:	COMMD5P1		Pseudoger	chr4:53575713-5357
ENSG0000(	654	16.35715	chr4:909:	RNU1-49P		smallRNA	chr4:41771945-4177
ENSG0000(	654	16.35715	chr4:909:	RNU6-1195P		smallRNA	chr4:41113942-4111
ENSG0000(	654	16.35715	chr4:909:	ENSG00000287416		lncRNA	chr4:36902242-3691
ENSG0000(	654	16.35715	chr4:909:	RASL11B		protein_c	chr4:52862317-5286
ENSG0000(	654	16.35715	chr4:909:	ENSG00000279918		TEC	chr4:19098199-1909
ENSG0000(	654	16.35715	chr4:909:	KDR	NCGv7;AC	protein_c	chr4:55078481-5512
ENSG0000(	654	16.35715	chr4:909:	LINC02473		lncRNA	chr4:24659856-2467
ENSG0000(	654	16.35715	chr4:909:	ENSG00000288321		lncRNA	chr4:33467398-3369
ENSG0000(	654	16.35715	chr4:909:	ENSG00000249320		Pseudoger	chr4:25472517-2547
ENSG0000(	654	16.35715	chr4:909:	AC119751.1		smallRNA	chr4:49595610-4959
ENSG0000(	654	16.35715	chr4:909:	ENSG00000249330		lncRNA	chr4:46390255-4651
ENSG0000(	654	16.35715	chr4:909:	SNX18P25		Pseudoger	chr4:49588772-4958
ENSG0000(	654	16.35715	chr4:909:	ENSG00000272995		lncRNA	chr4:20766808-2076
ENSG0000(	654	16.35715	chr4:909:	UBE2K		protein_c	chr4:39698109-3978
ENSG0000(	654	16.35715	chr4:909:	ENSG00000249341		lncRNA	chr4:53659208-5373
ENSG0000(	654	16.35715	chr4:909:	ELOCP33		Pseudoger	chr4:39932454-3993

ENSG0000(	654	16.35715	chr4:909:	UGDH-AS1		lncRNA	chr4:39527720-3959
ENSG0000(	654	16.35715	chr4:909:	RPS7P7		Pseudoger	chr4:42471810-4247
ENSG0000(	654	16.35715	chr4:909:	N4BP2	NCGv7	protein_c	chr4:40056850-4015
ENSG0000(	654	16.35715	chr4:909:	STIM2-AS1		lncRNA	chr4:26859806-2686
ENSG0000(	654	16.35715	chr4:909:	RNU6-1221P		smallRNA	chr4:29510635-2951
ENSG0000(	654	16.35715	chr4:909:	ENSG00000289761		protein_c	chr4:41143022-4114
ENSG0000(	654	16.35715	chr4:909:	MTND4P9		Pseudoger	chr4:25718082-2571
ENSG0000(	654	16.35715	chr4:909:	RNU6-887P		smallRNA	chr4:39399149-3939
ENSG0000(	654	16.35715	chr4:909:	AC107068.1		smallRNA	chr4:47976216-4797
ENSG0000(	654	16.35715	chr4:909:	BEND4		protein_c	chr4:42110853-4215
ENSG0000(	654	16.35715	chr4:909:	ENSG00000287060		lncRNA	chr4:55157877-5516
ENSG0000(	654	16.35715	chr4:909:	RNU7-74P		smallRNA	chr4:40377452-4037
ENSG0000(	654	16.35715	chr4:909:	LAP3		protein_c	chr4:17577198-1760
ENSG0000(	654	16.35715	chr4:909:	ENSG00000250863		lncRNA	chr4:43972921-4402
ENSG0000(	654	16.35715	chr4:909:	ENSG00000251373		Pseudoger	chr4:29407100-2940
ENSG0000(	654	16.35715	chr4:909:	PCDH7		protein_c	chr4:30720369-3114
ENSG0000(	654	16.35715	chr4:909:	ENSG00000250893		lncRNA	chr4:40426119-4042
ENSG0000(	654	16.35715	chr4:909:	ENSG00000287081		lncRNA	chr4:16598718-1660
ENSG0000(	654	16.35715	chr4:909:	NIPAL1		protein_c	chr4:47914142-4804
ENSG0000(	654	16.35715	chr4:909:	MTND3P5		Pseudoger	chr4:25720099-2572
ENSG0000(	654	16.35715	chr4:909:	LINC02475		lncRNA	chr4:44016700-4402
ENSG0000(	654	16.35715	chr4:909:	TAPT1		protein_c	chr4:16160505-1622
ENSG0000(	654	16.35715	chr4:909:	LDB2		protein_c	chr4:16501541-1689
ENSG0000(	654	16.35715	chr4:909:	ENSG00000250906		lncRNA	chr4:40812779-4082
ENSG0000(	654	16.35715	chr4:909:	ENSG00000251334		Pseudoger	chr4:48936582-4893
ENSG0000(	654	16.35715	chr4:909:	PSME2P4		Pseudoger	chr4:37995494-3799
ENSG0000(	654	16.35715	chr4:909:	RNU6-420P		smallRNA	chr4:22118983-2211
ENSG0000(	654	16.35715	chr4:909:	IGBP1P5		Pseudoger	chr4:27585145-2758
ENSG0000(	654	16.35715	chr4:909:	ZCCHC4		protein_c	chr4:25312774-2537
ENSG0000(	654	16.35715	chr4:909:	ENSG00000251325		lncRNA	chr4:27262506-2726
ENSG0000(	654	16.35715	chr4:909:	CCKAR		protein_c	chr4:26481396-2649
ENSG0000(	654	16.35715	chr4:909:	ENSG00000250657		lncRNA	chr4:43340875-4334
ENSG0000(	654	16.35715	chr4:909:	snoU13		smallRNA	chr4:52510013-5251
ENSG0000(	654	16.35715	chr4:909:	TPI1P4		Pseudoger	chr4:49016682-4901
ENSG0000(	654	16.35715	chr4:909:	GABRG1	NCGv7	protein_c	chr4:46035769-4612
ENSG0000(	654	16.35715	chr4:909:	GABRB1		protein_c	chr4:46993723-4742
ENSG0000(	654	16.35715	chr4:909:	ENSG00000243929		Pseudoger	chr4:51978079-5197
ENSG0000(	654	16.35715	chr4:909:	Y_RNA		smallRNA	chr4:41303237-4130
ENSG0000(	654	16.35715	chr4:909:	AC131951.1		smallRNA	chr4:44448005-4444
ENSG0000(	654	16.35715	chr4:909:	ENSG00000251410		lncRNA	chr4:27964517-2798
ENSG0000(	654	16.35715	chr4:909:	RBPJ	NCGv7	protein_c	chr4:26163455-2643
ENSG0000(	654	16.35715	chr4:909:	ENSG00000250723		lncRNA	chr4:33850591-3398
ENSG0000(	654	16.35715	chr4:909:	ENSG00000250753		Pseudoger	chr4:49579833-4958
ENSG0000(	654	16.35715	chr4:909:	ENSG00000250769		Pseudoger	chr4:49507764-4950
ENSG0000(	654	16.35715	chr4:909:	ENSG00000250781		lncRNA	chr4:42281830-4239
ENSG0000(	654	16.35715	chr4:909:	RNU6-836P		smallRNA	chr4:41084607-4108
ENSG0000(	654	16.35715	chr4:909:	ENSG00000250812		Pseudoger	chr4:55219344-5521
ENSG0000(	654	16.35715	chr4:909:	ENSG00000250815		Pseudoger	chr4:53927499-5392
ENSG0000(	654	16.35715	chr4:909:	LINC02493		lncRNA	chr4:17171757-1718
ENSG0000(	654	16.35715	chr4:909:	LINC02353		lncRNA	chr4:32351038-3235
ENSG0000(	654	16.35715	chr4:909:	LRRC66	NCGv7	protein_c	chr4:51993652-5202

ENSG0000(	654	16.35715	chr4:9093	LINC02501		lncRNA	chr4:31506422-3155
ENSG0000(	654	16.35715	chr4:9093	ENSG00000251113		Pseudoger	chr4:29465701-2946
ENSG0000(	654	16.35715	chr4:9093	ANKRD20A17P		Pseudoger	chr4:49502145-4950
ENSG0000(	654	16.35715	chr4:9093	ENSG00000287182		lncRNA	chr4:40743627-4075
ENSG0000(	654	16.35715	chr4:9093	MTND5P4		Pseudoger	chr4:17061878-1706
ENSG0000(	654	16.35715	chr4:9093	SNORD74		smallRNA	chr4:26702309-2670
ENSG0000(	654	16.35715	chr4:9093	snoU13		smallRNA	chr4:54123100-5412
ENSG0000(	654	16.35715	chr4:9093	ENSG00000251080		lncRNA	chr4:27133996-2714
ENSG0000(	654	16.35715	chr4:9093	ENSG00000232471		Pseudoger	chr4:49550032-4955
ENSG0000(	654	16.35715	chr4:9093	ENSG00000251105		Pseudoger	chr4:54076625-5407
ENSG0000(	654	16.35715	chr4:9093	LINC02497		lncRNA	chr4:31171013-3121
ENSG0000(	654	16.35715	chr4:9093	ENSG00000251048		lncRNA	chr4:18488062-1848
ENSG0000(	654	16.35715	chr4:9093	COMMD8		protein_c	chr4:47450787-4746
ENSG0000(	654	16.35715	chr4:9093	LINC02506		lncRNA	chr4:31997376-3222
ENSG0000(	654	16.35715	chr4:9093	Y_RNA		smallRNA	chr4:52786537-5278
ENSG0000(	654	16.35715	chr4:9093	RNU6-1252P		smallRNA	chr4:52494849-5249
ENSG0000(	654	16.35715	chr4:9093	ENSG00000251159		Pseudoger	chr4:44533615-4453
ENSG0000(	654	16.35715	chr4:9093	UCHL1-DT		lncRNA	chr4:41220074-4125
ENSG0000(	654	16.35715	chr4:9093	LIMCH1	NCGv7	protein_c	chr4:41359607-4170
ENSG0000(	654	16.35715	chr4:9093	Y_RNA		smallRNA	chr4:39441665-3944
ENSG0000(	654	16.35715	chr4:9093	RFPL4AP3		Pseudoger	chr4:23514940-2351
ENSG0000(	654	16.35715	chr4:9093	LINC02480		lncRNA	chr4:52044805-5204
ENSG0000(	654	16.35715	chr4:9093	RHOH	NCGv7	protein_c	chr4:40191011-4024
ENSG0000(	654	16.35715	chr4:9093	ENSG00000251286		Pseudoger	chr4:52440494-5244
ENSG0000(	654	16.35715	chr4:9093	TAPT1-AS1		lncRNA	chr4:16226685-1632
ENSG0000(	654	16.35715	chr4:9093	MESTP3		Pseudoger	chr4:28823244-2882
ENSG0000(	654	16.35715	chr4:9093	snoU13		smallRNA	chr4:34966241-3496
ENSG0000(	654	16.35715	chr4:9093	RN7SKP199		smallRNA	chr4:45995119-4599
ENSG0000(	654	16.35715	chr4:9093	LNX1-AS1		lncRNA	chr4:53496400-5354
ENSG0000(	654	16.35715	chr4:9093	AC092846.1		smallRNA	chr4:24421176-2442
ENSG0000(	654	16.35715	chr4:9093	ENSG00000250954		lncRNA	chr4:33775498-3403
ENSG0000(	654	16.35715	chr4:9093	ERVH-1		lncRNA	chr4:23723262-2373
ENSG0000(	654	16.35715	chr4:9093	ENSG00000277096		Pseudoger	chr4:43388157-4338
ENSG0000(	654	16.35715	chr4:9093	LINC02358		lncRNA	chr4:54845568-5485
ENSG0000(	654	16.35715	chr4:9093	CCDC149		protein_c	chr4:24803514-2498
ENSG0000(	654	16.35715	chr4:9093	ENSG00000251009		lncRNA	chr4:25864881-2586
ENSG0000(	654	16.35715	chr4:9093	ENSG00000205794		Pseudoger	chr4:40042917-4005
ENSG0000(	654	16.35715	chr4:9093	AC118282.1		smallRNA	chr4:49198207-4919
ENSG0000(	654	16.35715	chr4:9093	ENSG00000205830		lncRNA	chr4:27207505-2721
ENSG0000(	654	16.35715	chr4:9093	ENSG00000251264		lncRNA	chr4:54440502-5444
ENSG0000(	654	16.35715	chr4:9093	ENSG00000287320		lncRNA	chr4:34140598-3418
ENSG0000(	654	16.35715	chr4:9093	PGM2		protein_c	chr4:37826660-3786
ENSG0000(	654	16.35715	chr4:9093	ENSG00000251434		lncRNA	chr4:31350284-3135
ENSG0000(	654	16.35715	chr4:9093	ENSG00000250646		lncRNA	chr4:55053060-5509
ENSG0000(	654	16.35715	chr4:9093	GNPDA2	DriverDB\	protein_c	chr4:44682200-4472
ENSG0000(	654	16.35715	chr4:9093	AC119751.2		smallRNA	chr4:49598615-4959
ENSG0000(	654	16.35715	chr4:9093	AC104066.1		smallRNA	chr4:52751147-5275
ENSG0000(	654	16.35715	chr4:9093	SLC34A2	NCGv7;AC	protein_c	chr4:25648011-2567
ENSG0000(	654	16.35715	chr4:9093	RPS3AP17		Pseudoger	chr4:29962644-2996
ENSG0000(	654	16.35715	chr4:9093	ENSG00000250064		lncRNA	chr4:28435449-2860
ENSG0000(	654	16.35715	chr4:9093	MAPRE1P2		Pseudoger	chr4:33010948-3301

ENSG0000(	654	16.35715	chr4:909:	ENSG00000250092		lncRNA	chr4:21582096-2161
ENSG0000(	654	16.35715	chr4:909:	RNU6-350P		smallRNA	chr4:16008676-1600
ENSG0000(	654	16.35715	chr4:909:	RNU7-131P		smallRNA	chr4:33784935-3378
ENSG0000(	654	16.35715	chr4:909:	AC119751.3		smallRNA	chr4:49598423-4959
ENSG0000(	654	16.35715	chr4:909:	ENSG00000250039		lncRNA	chr4:21949015-2233
ENSG0000(	654	16.35715	chr4:909:	RNU5E-3P		smallRNA	chr4:48574453-4857
ENSG0000(	654	16.35715	chr4:909:	ENSG00000250137		lncRNA	chr4:23560923-2376
ENSG0000(	654	16.35715	chr4:909:	ENSG00000286888		lncRNA	chr4:16322954-1632
ENSG0000(	654	16.35715	chr4:909:	MORF4L2P1		Pseudoger	chr4:54086926-5408
ENSG0000(	654	16.35715	chr4:909:	LINC02364		lncRNA	chr4:28996498-2901
ENSG0000(	654	16.35715	chr4:909:	ENSG00000250243		lncRNA	chr4:21304468-2135
ENSG0000(	654	16.35715	chr4:909:	PTTG2	AC	protein_c	chr4:37960398-3796
ENSG0000(	654	16.35715	chr4:909:	ENSG00000286891		lncRNA	chr4:43763134-4397
ENSG0000(	654	16.35715	chr4:909:	ENSG00000249019		Pseudoger	chr4:39713842-3971
ENSG0000(	654	16.35715	chr4:909:	ENSG00000250038		lncRNA	chr4:28343862-2840
ENSG0000(	654	16.35715	chr4:909:	ENSG00000276507		Pseudoger	chr4:29010619-2901
ENSG0000(	654	16.35715	chr4:909:	GBA3		protein_c	chr4:22692914-2281
ENSG0000(	654	16.35715	chr4:909:	ENSG00000286784		lncRNA	chr4:32583855-3274
ENSG0000(	654	16.35715	chr4:909:	ENSG00000288940		lncRNA	chr4:30717369-3071
ENSG0000(	654	16.35715	chr4:909:	ENSG00000249887		Pseudoger	chr4:41924180-4192
ENSG0000(	654	16.35715	chr4:909:	ENSG00000224560		Pseudoger	chr4:45414337-4541
ENSG0000(	654	16.35715	chr4:909:	AC084010.1		smallRNA	chr4:42445990-4244
ENSG0000(	654	16.35715	chr4:909:	RN7SL101P		smallRNA	chr4:28711198-2871
ENSG0000(	654	16.35715	chr4:909:	LNX1		protein_c	chr4:53459301-5370
ENSG0000(	654	16.35715	chr4:909:	RAC1P2		Pseudoger	chr4:46723830-4672
ENSG0000(	654	16.35715	chr4:909:	RN7SL691P		smallRNA	chr4:43598542-4359
ENSG0000(	654	16.35715	chr4:909:	WDR19		protein_c	chr4:39182504-3928
ENSG0000(	654	16.35715	chr4:909:	FGFBP2		protein_c	chr4:15960245-1596
ENSG0000(	654	16.35715	chr4:909:	FGFBP1	NCGv7	protein_c	chr4:15935577-1593
ENSG0000(	654	16.35715	chr4:909:	Y_RNA		smallRNA	chr4:27223319-2722
ENSG0000(	654	16.35715	chr4:909:	RN7SKP215		smallRNA	chr4:47811464-4781
ENSG0000(	654	16.35715	chr4:909:	RN7SL424P		smallRNA	chr4:55063735-5506
ENSG0000(	654	16.35715	chr4:909:	ENSG00000249998		lncRNA	chr4:16973275-1707
ENSG0000(	654	16.35715	chr4:909:	SNX18P23		Pseudoger	chr4:49233289-4923
ENSG0000(	654	16.35715	chr4:909:	RNA5SP157		Pseudoger	chr4:19181814-1918
ENSG0000(	654	16.35715	chr4:909:	HPRT1P1		Pseudoger	chr4:15864938-1586
ENSG0000(	654	16.35715	chr4:909:	ENSG00000251642		lncRNA	chr4:38286994-3828
ENSG0000(	654	16.35715	chr4:909:	ENSG00000251638		Pseudoger	chr4:24768049-2476
ENSG0000(	654	16.35715	chr4:909:	COX7B2		protein_c	chr4:46734827-4690
ENSG0000(	654	16.35715	chr4:909:	RNA5SP158		Pseudoger	chr4:38758791-3875
ENSG0000(	654	16.35715	chr4:909:	ENSG00000251517		lncRNA	chr4:42706107-4270
ENSG0000(	654	16.35715	chr4:909:	ENSG00000251516		Pseudoger	chr4:22607501-2261
ENSG0000(	654	16.35715	chr4:909:	RNU7-126P		smallRNA	chr4:25598564-2559
ENSG0000(	654	16.35715	chr4:909:	ENSG00000250541		lncRNA	chr4:25770266-2577
ENSG0000(	654	16.35715	chr4:909:	DCAF16		protein_c	chr4:17800655-1781
ENSG0000(	654	16.35715	chr4:909:	ENSG00000251501		Pseudoger	chr4:43586328-4358
ENSG0000(	654	16.35715	chr4:909:	AC098680.1		smallRNA	chr4:38238613-3823
ENSG0000(	654	16.35715	chr4:909:	ENSG00000250568		Pseudoger	chr4:39973128-3997
ENSG0000(	654	16.35715	chr4:909:	CD38		protein_c	chr4:15778275-1585
ENSG0000(	654	16.35715	chr4:909:	MIR4802		smallRNA	chr4:40502040-4050
ENSG0000(	654	16.35715	chr4:909:	NFXL1		protein_c	chr4:47847233-4791

ENSG0000(	654	16.35715	chr4:909:	ENSG00000250597		lncRNA	chr4:34657606-3466
ENSG0000(	654	16.35715	chr4:909:	ENSG00000250611		Pseudoger	chr4:20037560-2003
ENSG0000(	654	16.35715	chr4:909:	ENSG00000251438		lncRNA	chr4:36311190-3639
ENSG0000(	654	16.35715	chr4:909:	TEC	NCGv7;AC	protein_c	chr4:48135783-4826
ENSG0000(	654	16.35715	chr4:909:	MTCYBP43		Pseudoger	chr4:30884443-3088
ENSG0000(	654	16.35715	chr4:909:	TXK		protein_c	chr4:48066393-4813
ENSG0000(	654	16.35715	chr4:909:	DUTP7		Pseudoger	chr4:51865050-5186
ENSG0000(	654	16.35715	chr4:909:	RELL1		protein_c	chr4:37590800-3768
ENSG0000(	654	16.35715	chr4:909:	PHOX2B-AS1		lncRNA	chr4:41748293-4182
ENSG0000(	654	16.35715	chr4:909:	LRRC34P2		Pseudoger	chr4:55313011-5531
ENSG0000(	654	16.35715	chr4:909:	LINC02278		lncRNA	chr4:38564003-3857
ENSG0000(	654	16.35715	chr4:909:	LINC01618		lncRNA	chr4:52712394-5286
ENSG0000(	654	16.35715	chr4:909:	ENSG00000251630		Pseudoger	chr4:49561285-4956
ENSG0000(	654	16.35715	chr4:909:	FRYL		protein_c	chr4:48497357-4878
ENSG0000(	654	16.35715	chr4:909:	KRT18P63		Pseudoger	chr4:17911674-1791
ENSG0000(	654	16.35715	chr4:909:	SMIM20		protein_c	chr4:25861830-2592
ENSG0000(	654	16.35715	chr4:909:	ENSG00000250338		lncRNA	chr4:40265472-4026
ENSG0000(	654	16.35715	chr4:909:	ENSG00000251588		Pseudoger	chr4:36506043-3650
ENSG0000(	654	16.35715	chr4:909:	DCAF4L1		protein_c	chr4:41981756-4198
ENSG0000(	654	16.35715	chr4:909:	AC021106.1		Pseudoger	chr4:37960435-3796
ENSG0000(	654	16.35715	chr4:909:	LINC02616		lncRNA	chr4:37001772-3702
ENSG0000(	654	16.35715	chr4:909:	KIT	NCGv7;AC	protein_c	chr4:54657267-5474
ENSG0000(	654	16.35715	chr4:909:	ZAR1		protein_c	chr4:48490252-4849
ENSG0000(	654	16.35715	chr4:909:	SEC63P2		Pseudoger	chr4:35487812-3548
ENSG0000(	654	16.35715	chr4:909:	LINC02260		lncRNA	chr4:54603211-5460
ENSG0000(	654	16.35715	chr4:909:	ENSG00000275959		Pseudoger	chr4:44994426-4499
ENSG0000(	654	16.35715	chr4:909:	SNX18P24		Pseudoger	chr4:49561479-4956
ENSG0000(	654	16.35715	chr4:909:	MTCO3P42		Pseudoger	chr4:49548918-4954
ENSG0000(	654	16.35715	chr4:909:	QDPR		protein_c	chr4:17460261-1751
ENSG0000(	654	16.35715	chr4:909:	ENSG00000248452		Pseudoger	chr4:26111865-2611
ENSG0000(	654	16.35715	chr4:909:	ENSG00000286089		lncRNA	chr4:40187170-4019
ENSG0000(	654	16.35715	chr4:909:	ENSG00000248375		Pseudoger	chr4:52720081-5272
ENSG0000(	654	16.35715	chr4:909:	ENSG00000248417		lncRNA	chr4:33433510-3343
ENSG0000(	654	16.35715	chr4:909:	ENSG00000271958		lncRNA	chr4:38618265-3861
ENSG0000(	654	16.35715	chr4:909:	LCORL	DriverDB\	protein_c	chr4:17841187-1802
ENSG0000(	654	16.35715	chr4:909:	HNRNPA1P65		Pseudoger	chr4:24771467-2477
ENSG0000(	654	16.35715	chr4:909:	PDS5A	NCGv7	protein_c	chr4:39822863-3997
ENSG0000(	654	16.35715	chr4:909:	ENSG00000248466		Pseudoger	chr4:36065060-3606
ENSG0000(	654	16.35715	chr4:909:	ENSG00000286046		lncRNA	chr4:18418662-1888
ENSG0000(	654	16.35715	chr4:909:	RFC1	NCGv7	protein_c	chr4:39287456-3936
ENSG0000(	654	16.35715	chr4:909:	SNORA26		smallRNA	chr4:52748137-5274
ENSG0000(	654	16.35715	chr4:909:	AC109351.1		smallRNA	chr4:29750196-2975
ENSG0000(	654	16.35715	chr4:909:	ENSG00000213851		Pseudoger	chr4:43410041-4341
ENSG0000(	654	16.35715	chr4:909:	LNX1-AS2		lncRNA	chr4:53592956-5360
ENSG0000(	654	16.35715	chr4:909:	ENSG00000248515		lncRNA	chr4:19455418-1993
ENSG0000(	654	16.35715	chr4:909:	SHISA3		protein_c	chr4:42397488-4240
ENSG0000(	654	16.35715	chr4:909:	ENSG00000228154		Pseudoger	chr4:25622777-2562
ENSG0000(	654	16.35715	chr4:909:	GABRA4		protein_c	chr4:46918900-4699
ENSG0000(	654	16.35715	chr4:909:	LIAS		protein_c	chr4:39459004-3948
ENSG0000(	654	16.35715	chr4:909:	LINC02438		lncRNA	chr4:19172335-1945
ENSG0000(	654	16.35715	chr4:909:	ATP8A1		protein_c	chr4:42408373-4265

ENSG0000(	654	16.35715	chr4:909:	ERVMER34-1		protein_c	chr4:52722618-5275
ENSG0000(	654	16.35715	chr4:909:	ENSG00000248254		lncRNA	chr4:47556731-4756
ENSG0000(	654	16.35715	chr4:909:	OCIAD1-AS1		lncRNA	chr4:48852008-4886
ENSG0000(	654	16.35715	chr4:909:	ENSG00000248281		Pseudoger	chr4:30006951-3000
ENSG0000(	654	16.35715	chr4:909:	CCNL2P1		Pseudoger	chr4:42561457-4256
ENSG0000(	654	16.35715	chr4:909:	ENSG00000248343		lncRNA	chr4:21697450-2171
ENSG0000(	654	16.35715	chr4:909:	TMEM156		protein_c	chr4:38966744-3903
ENSG0000(	654	16.35715	chr4:909:	ENSG00000248317		lncRNA	chr4:54943626-5495
ENSG0000(	654	16.35715	chr4:909:	THAP12P9		Pseudoger	chr4:45323253-4532
ENSG0000(	654	16.35715	chr4:909:	ENSG00000269848		Pseudoger	chr4:49524030-4952
ENSG0000(	654	16.35715	chr4:909:	LINC02472		lncRNA	chr4:29214253-2929
ENSG0000(	654	16.35715	chr4:909:	PIMREGP4		Pseudoger	chr4:26873561-2687
ENSG0000(	654	16.35715	chr4:909:	ENSG00000248518		lncRNA	chr4:54376002-5437
ENSG0000(	654	16.35715	chr4:909:	ENSG00000248532		Pseudoger	chr4:49523648-4952
ENSG0000(	654	16.35715	chr4:909:	ENSG00000272862		lncRNA	chr4:41988741-4198
ENSG0000(	654	16.35715	chr4:909:	SLIT2	NCGv7	protein_c	chr4:20251905-2062
ENSG0000(	654	16.35715	chr4:909:	MIR4449		smallRNA	chr4:52712682-5271
ENSG0000(	654	16.35715	chr4:909:	KCNIP4		protein_c	chr4:20728606-2194
ENSG0000(	654	16.35715	chr4:909:	ENSG00000286212		lncRNA	chr4:32005076-3202
ENSG0000(	654	16.35715	chr4:909:	LGI2		protein_c	chr4:24998847-2503
ENSG0000(	654	16.35715	chr4:909:	PDGFRA	NCGv7;AC	protein_c	chr4:54229280-5429
ENSG0000(	654	16.35715	chr4:909:	MIR574		smallRNA	chr4:38868032-3886
ENSG0000(	654	16.35715	chr4:909:	RNU6-158P		smallRNA	chr4:48932755-4893
ENSG0000(	654	16.35715	chr4:909:	ENSG00000248545		lncRNA	chr4:25529177-2561
ENSG0000(	654	16.35715	chr4:909:	ENSG00000248744		lncRNA	chr4:45009540-4505
ENSG0000(	654	16.35715	chr4:909:	Y_RNA		smallRNA	chr4:48153434-4815
ENSG0000(	654	16.35715	chr4:909:	PHOX2B	NCGv7;AC	protein_c	chr4:41744082-4174
ENSG0000(	654	16.35715	chr4:909:	ARL4AP2		Pseudoger	chr4:40786110-4078
ENSG0000(	654	16.35715	chr4:909:	DTHD1	NCGv7	protein_c	chr4:36281616-3634
ENSG0000(	654	16.35715	chr4:909:	TMEM33		protein_c	chr4:41935129-4196
ENSG0000(	654	16.35715	chr4:909:	AC119751.4		smallRNA	chr4:49599428-4959
ENSG0000(	654	16.35715	chr4:909:	MED28		protein_c	chr4:17614641-1763
ENSG0000(	654	16.35715	chr4:909:	RNU6-310P		smallRNA	chr4:53265461-5326
ENSG0000(	654	16.35715	chr4:909:	LINC02484		lncRNA	chr4:34120090-3433
ENSG0000(	654	16.35715	chr4:909:	AC118282.4		smallRNA	chr4:49209374-4920
ENSG0000(	654	16.35715	chr4:909:	PI4K2B		protein_c	chr4:25160663-2527
ENSG0000(	654	16.35715	chr4:909:	Y_RNA		smallRNA	chr4:39710085-3971
ENSG0000(	654	16.35715	chr4:909:	ENSG00000286161		lncRNA	chr4:52712713-5272
ENSG0000(	654	16.35715	chr4:909:	MTCO3P44		Pseudoger	chr4:25720516-2572
ENSG0000(	654	16.35715	chr4:909:	ENSG00000286141		lncRNA	chr4:28580991-2860
ENSG0000(	654	16.35715	chr4:909:	ENSG00000287999		lncRNA	chr4:52252820-5255
ENSG0000(	654	16.35715	chr4:909:	ENSG00000248608		Pseudoger	chr4:25504997-2550
ENSG0000(	654	16.35715	chr4:909:	ENSG00000248583		Pseudoger	chr4:49486926-4948
ENSG0000(	654	16.35715	chr4:909:	YIPF7		protein_c	chr4:44622088-4467
ENSG0000(	654	16.35715	chr4:909:	ENSG00000241612		Pseudoger	chr4:19813749-1981
ENSG0000(	654	16.35715	chr4:909:	RNU6-868P		smallRNA	chr4:48109353-4810
ENSG0000(	654	16.35715	chr4:909:	KCNIP4-IT1		lncRNA	chr4:21843341-2185
ENSG0000(	654	16.35715	chr4:909:	NDUFB4P12		Pseudoger	chr4:43898962-4389
ENSG0000(	654	16.35715	chr4:909:	SLIT2-IT1		lncRNA	chr4:20392154-2039
ENSG0000(	654	16.35715	chr4:909:	LINC02513		lncRNA	chr4:38366914-3838
ENSG0000(	654	16.35715	chr4:909:	ENSG00000282917		lncRNA	chr4:47831330-4790

ENSG0000(	654	16.35715	chr4:909:	RPL22P13		Pseudoger	chr4:54221126-5422
ENSG0000(	654	16.35715	chr4:909:	ENSG00000272576		lncRNA	chr4:51918772-5191
ENSG0000(	654	16.35715	chr4:909:	Y_RNA		smallRNA	chr4:37699895-3770
ENSG0000(	654	16.35715	chr4:909:	RNU6-1112P		smallRNA	chr4:40077884-4007
ENSG0000(	654	16.35715	chr4:909:	RPS29P11		Pseudoger	chr4:25678850-2567
ENSG0000(	654	16.35715	chr4:909:	LINC00682		lncRNA	chr4:41872741-4188
ENSG0000(	654	16.35715	chr4:909:	MTCO3P39		Pseudoger	chr4:49246021-4924
ENSG0000(	654	16.35715	chr4:909:	ENSG00000287659		lncRNA	chr4:38276178-3827
ENSG0000(	654	16.35715	chr4:909:	ENSG00000282904		lncRNA	chr4:47463590-4747
ENSG0000(	654	16.35715	chr4:909:	ENSG00000260296		lncRNA	chr4:40166675-4016
ENSG0000(	654	16.35715	chr4:909:	FAM114A1		protein_c	chr4:38867677-3894
ENSG0000(	654	16.35715	chr4:909:	KCTD8	NCGv7	protein_c	chr4:44173903-4444
ENSG0000(	654	16.35715	chr4:909:	ENSG00000247193		lncRNA	chr4:36244116-3627
ENSG0000(	654	16.35715	chr4:909:	ENSG00000242431		Pseudoger	chr4:47490967-4749
ENSG0000(	654	16.35715	chr4:909:	ENSG00000283219		Pseudoger	chr4:17557892-1755
ENSG0000(	654	16.35715	chr4:909:	CNGA1		protein_c	chr4:47935977-4801
ENSG0000(	654	16.35715	chr4:909:	ENSG00000272650		lncRNA	chr4:53997415-5399
ENSG0000(	654	16.35715	chr4:909:	RNU6-32P		smallRNA	chr4:39297605-3929
ENSG0000(	654	16.35715	chr4:909:	ENSG00000287262		lncRNA	chr4:39539395-3954
ENSG0000(	654	16.35715	chr4:909:	RNU6-573P		smallRNA	chr4:35495898-3549
ENSG0000(	654	16.35715	chr4:909:	ATP8A1-DT		lncRNA	chr4:42657496-4265
ENSG0000(	654	16.35715	chr4:909:	ENSG00000268967		Pseudoger	chr4:49548564-4954
ENSG0000(	654	16.35715	chr4:909:	AC111194.1		smallRNA	chr4:55010833-5501
ENSG0000(	654	16.35715	chr4:909:	RNU6-838P		smallRNA	chr4:48106081-4810
ENSG0000(	654	16.35715	chr4:909:	ENSG00000242768		Pseudoger	chr4:21656511-2165
ENSG0000(	654	16.35715	chr4:909:	ZBTB12BP		Pseudoger	chr4:39770081-3977
ENSG0000(	654	16.35715	chr4:909:	MIR4801		smallRNA	chr4:37241910-3724
ENSG0000(	654	16.35715	chr4:909:	RPL21P44		Pseudoger	chr4:53986587-5398
ENSG0000(	654	16.35715	chr4:909:	AC119751.5		smallRNA	chr4:49583025-4958
ENSG0000(	654	16.35715	chr4:909:	GSX2		protein_c	chr4:54099523-5410
ENSG0000(	654	16.35715	chr4:909:	DANCR		lncRNA	chr4:52712325-5272
ENSG0000(	654	16.35715	chr4:909:	ENSG00000285454		lncRNA	chr4:42151028-4226
ENSG0000(	654	16.35715	chr4:909:	RPS21P4		Pseudoger	chr4:16256308-1625
ENSG0000(	654	16.35715	chr4:909:	LINC02505		lncRNA	chr4:36496128-3664
ENSG0000(	654	16.35715	chr4:909:	SMIM14-DT		lncRNA	chr4:39639107-3966
ENSG0000(	654	16.35715	chr4:909:	SMIM14		protein_c	chr4:39546336-3963
ENSG0000(	654	16.35715	chr4:909:	ENSG00000259959		lncRNA	chr4:47840122-4784
ENSG0000(	654	16.35715	chr4:909:	RPL9		protein_c	chr4:39452587-3945
ENSG0000(	654	16.35715	chr4:909:	MTND4LP22		Pseudoger	chr4:25719755-2572
ENSG0000(	654	16.35715	chr4:909:	ENSG00000248138		lncRNA	chr4:16400430-1651
ENSG0000(	654	16.35715	chr4:909:	LINC02383		lncRNA	chr4:43457527-4349
ENSG0000(	654	16.35715	chr4:909:	AC110298.1		smallRNA	chr4:54103580-5410
ENSG0000(	654	16.35715	chr4:909:	RPL38P3		Pseudoger	chr4:54976159-5497
ENSG0000(	654	16.35715	chr4:909:	CDC42P6		Pseudoger	chr4:22727375-2272
ENSG0000(	654	16.35715	chr4:909:	RN7SL16P		smallRNA	chr4:24563195-2456
ENSG0000(	654	16.35715	chr4:909:	SEPSECS-AS1		lncRNA	chr4:25160641-2520
ENSG0000(	654	16.35715	chr4:909:	AC108218.1		smallRNA	chr4:25353970-2535
ENSG0000(	654	16.35715	chr4:909:	ENSG00000248176		lncRNA	chr4:29118304-2921
ENSG0000(	654	16.35715	chr4:909:	LINC02283		lncRNA	chr4:54332892-5435
ENSG0000(	654	16.35715	chr4:909:	ENSG00000289201		lncRNA	chr4:24457352-2447
ENSG0000(	654	16.35715	chr4:909:	ENSG00000248115		lncRNA	chr4:52945649-5295

ENSG0000C	654	16.35715	chr4:9093	ENSG00000242197		Pseudoger	chr4:40491733-4049
ENSG0000C	654	16.35715	chr4:9093	SLC30A9		protein_c	chr4:41990502-4209
ENSG0000C	654	16.35715	chr4:9093	RBM47		protein_c	chr4:40423267-4063
ENSG0000C	654	16.35715	chr4:9093	APBB2		protein_c	chr4:40810027-4121
ENSG0000C	654	16.35715	chr4:9093	NSUN7		protein_c	chr4:40749925-4081
ENSG0000C	654	16.35715	chr4:9093	ENSG00000242262		Pseudoger	chr4:47706372-4770
ENSG0000C	654	16.35715	chr4:9093	ENSG00000237961		Pseudoger	chr4:49238032-4924
ENSG0000C	654	16.35715	chr4:9093	SCFD2		protein_c	chr4:52872982-5336
ENSG0000C	654	16.35715	chr4:9093	ENSG00000287968		lncRNA	chr4:33150326-3320
ENSG0000C	654	16.35715	chr4:9093	ENSG00000273369		lncRNA	chr4:44693946-4469
ENSG0000C	654	16.35715	chr4:9093	ENSG00000247810		lncRNA	chr4:37073681-3713
ENSG0000C	654	16.35715	chr4:9093	AC118282.3		smallRNA	chr4:49197203-4919
ENSG0000C	654	16.35715	chr4:9093	ENSG00000282278		protein_c	chr4:53377839-5429
ENSG0000C	654	16.35715	chr4:9093	ENSG00000260120		lncRNA	chr4:52680609-5269
ENSG0000C	654	16.35715	chr4:9093	ENSG00000269506		lncRNA	chr4:54059597-5406
ENSG0000C	654	16.35715	chr4:9093	ENSG00000275250		Pseudoger	chr4:34714270-3471
ENSG0000C	654	16.35715	chr4:9093	LINC02357		lncRNA	chr4:26070754-2610
ENSG0000C	654	16.35715	chr4:9093	CORIN		protein_c	chr4:47593999-4783
ENSG0000C	654	16.35715	chr4:9093	RNU7-11P		smallRNA	chr4:39621012-3962
ENSG0000C	654	16.35715	chr4:9093	CWH43	NCGv7	protein_c	chr4:48986275-4906
ENSG0000C	654	16.35715	chr4:9093	SNORA26		smallRNA	chr4:52713249-5271
ENSG0000C	654	16.35715	chr4:9093	SNORA51		smallRNA	chr4:40082983-4008
ENSG0000C	654	16.35715	chr4:9093	SLC10A4		protein_c	chr4:48483343-4848
ENSG0000C	654	16.35715	chr4:9093	ENSG00000248977		Pseudoger	chr4:40142198-4014
ENSG0000C	654	16.35715	chr4:9093	ENSG00000286280		lncRNA	chr4:16114233-1611
ENSG0000C	654	16.35715	chr4:9093	PRDX4P1		Pseudoger	chr4:44944015-4494
ENSG0000C	654	16.35715	chr4:9093	GUF1		protein_c	chr4:44678420-4470
ENSG0000C	654	16.35715	chr4:9093	DCUN1D4		protein_c	chr4:51843000-5191
ENSG0000C	654	16.35715	chr4:9093	USP46		protein_c	chr4:52590960-5265
ENSG0000C	654	16.35715	chr4:9093	GABRA2	NCGv7	protein_c	chr4:46243548-4647
ENSG0000C	654	16.35715	chr4:9093	snoU13		smallRNA	chr4:40868869-4086
ENSG0000C	654	16.35715	chr4:9093	ENSG00000287534		lncRNA	chr4:54065239-5407
ENSG0000C	654	16.35715	chr4:9093	SNORA75		smallRNA	chr4:17320746-1732
ENSG0000C	654	16.35715	chr4:9093	ENSG00000286321		lncRNA	chr4:28225969-2828
ENSG0000C	654	16.35715	chr4:9093	ADGRA3		protein_c	chr4:22345071-2251
ENSG0000C	654	16.35715	chr4:9093	ENSG00000286349		lncRNA	chr4:39614465-3961
ENSG0000C	654	16.35715	chr4:9093	MTND3P22		Pseudoger	chr4:49246601-4924
ENSG0000C	654	16.35715	chr4:9093	OCIAD1		protein_c	chr4:48805212-4886
ENSG0000C	654	16.35715	chr4:9093	ENSG00000248939		lncRNA	chr4:43133867-4323
ENSG0000C	654	16.35715	chr4:9093	ENSG00000248851		Pseudoger	chr4:16307456-1630
ENSG0000C	654	16.35715	chr4:9093	RNU6-410P		smallRNA	chr4:55031965-5503
ENSG0000C	654	16.35715	chr4:9093	ATP10D	NCGv7	protein_c	chr4:47485275-4759
ENSG0000C	654	16.35715	chr4:9093	OCIAD2		protein_c	chr4:48885019-4890
ENSG0000C	654	16.35715	chr4:9093	ENSG00000286318		lncRNA	chr4:21520854-2152
ENSG0000C	654	16.35715	chr4:9093	KLB		protein_c	chr4:39406930-3945
ENSG0000C	654	16.35715	chr4:9093	C4orf19		protein_c	chr4:37453925-3762
ENSG0000C	654	16.35715	chr4:9093	USP46-DT		lncRNA	chr4:52659406-5266
ENSG0000C	654	16.35715	chr4:9093	UCHL1	AC	protein_c	chr4:41256413-4126
ENSG0000C	654	16.35715	chr4:9093	ENSG00000286294		lncRNA	chr4:47481001-4748
ENSG0000C	654	16.35715	chr4:9093	MIR5591		smallRNA	chr4:39411910-3941
ENSG0000C	654	16.35715	chr4:9093	ARAP2		protein_c	chr4:35948221-3624

ENSG0000(	654	16.35715	chr4:909:	ENSG00000272936		lncRNA	chr4:44704405-4470
ENSG0000(	654	16.35715	chr4:909:	AC118282.2		smallRNA	chr4:49198015-4919
ENSG0000(	654	16.35715	chr4:909:	ENSG00000248936		lncRNA	chr4:37588087-3758
ENSG0000(	654	16.35715	chr4:909:	ENSG00000248837		lncRNA	chr4:22989147-2319
ENSG0000(	654	16.35715	chr4:909:	RPS7P6		Pseudoger	chr4:17427696-1742
ENSG0000(	654	16.35715	chr4:909:	FIP1L1	AC	protein_c	chr4:53377569-5346
ENSG0000(	654	16.35715	chr4:909:	CHIC2	AC	protein_c	chr4:54009789-5406
ENSG0000(	654	16.35715	chr4:909:	snoU13		smallRNA	chr4:47305934-4730
ENSG0000(	654	16.35715	chr4:909:	RN7SL558P		smallRNA	chr4:39761000-3976
ENSG0000(	654	16.35715	chr4:909:	SLAIN2		protein_c	chr4:48341529-4842
ENSG0000(	650	16.2571	chr1:116(	ENSG00000235673		Pseudoger	chr1:37840986-3784
ENSG0000(	649	16.23209	chr4:909:	SNRPCP13		Pseudoger	chr4:9583942-95841
ENSG0000(	646	16.15706	chr4:909:	ENSG00000250670		lncRNA	chr4:104556960-104
ENSG0000(	646	16.15706	chr4:909:	FAM13A-AS1		lncRNA	chr4:88709298-8873
ENSG0000(	646	16.15706	chr4:909:	LAMTOR3		protein_c	chr4:99878336-9989
ENSG0000(	646	16.15706	chr4:909:	ENSG00000291282		lncRNA	chr4:68188403-6821
ENSG0000(	646	16.15706	chr4:909:	ENSG00000289308		lncRNA	chr4:61749388-6176
ENSG0000(	646	16.15706	chr4:909:	FTLP10		Pseudoger	chr4:68182292-6821
ENSG0000(	646	16.15706	chr4:909:	ENSG00000249257		Pseudoger	chr4:108773613-108
ENSG0000(	646	16.15706	chr4:909:	ENSG00000248926		Pseudoger	chr4:77958000-7795
ENSG0000(	646	16.15706	chr4:909:	ENSG00000289379		lncRNA	chr4:77820363-7782
ENSG0000(	646	16.15706	chr4:909:	HSP90AB3P		Pseudoger	chr4:87891843-8789
ENSG0000(	646	16.15706	chr4:909:	ADH7		protein_c	chr4:99412261-9943
ENSG0000(	646	16.15706	chr4:909:	LINC02499		lncRNA	chr4:73508803-7353
ENSG0000(	646	16.15706	chr4:909:	TMPRSS11CP		Pseudoger	chr4:67766480-6777
ENSG0000(	646	16.15706	chr4:909:	RNU6-1059P		smallRNA	chr4:95868667-9586
ENSG0000(	646	16.15706	chr4:909:	UBA6-DT		lncRNA	chr4:67701209-6808
ENSG0000(	646	16.15706	chr4:909:	ST3GAL1P1		Pseudoger	chr4:67716375-6771
ENSG0000(	646	16.15706	chr4:909:	ENSG00000250655		Pseudoger	chr4:96212279-9621
ENSG0000(	646	16.15706	chr4:909:	ENSG00000285330		protein_c	chr4:109713916-109
ENSG0000(	646	16.15706	chr4:909:	ENSG00000250677		Pseudoger	chr4:83237303-8323
ENSG0000(	646	16.15706	chr4:909:	KPNA2P1		Pseudoger	chr4:80079532-8008
ENSG0000(	646	16.15706	chr4:909:	CCSER1		protein_c	chr4:90127394-9160
ENSG0000(	646	16.15706	chr4:909:	ENSG00000250735		lncRNA	chr4:75401195-7542
ENSG0000(	646	16.15706	chr4:909:	ENSG00000250775		lncRNA	chr4:63465511-6352
ENSG0000(	646	16.15706	chr4:909:	ENSG00000242318		Pseudoger	chr4:98409290-9840
ENSG0000(	646	16.15706	chr4:909:	DPP3P1		Pseudoger	chr4:64430909-6443
ENSG0000(	646	16.15706	chr4:909:	ODAM	NCGv7	protein_c	chr4:70195725-7020
ENSG0000(	646	16.15706	chr4:909:	ENSG00000288019		lncRNA	chr4:74076533-7407
ENSG0000(	646	16.15706	chr4:909:	CFI		protein_c	chr4:109731008-109
ENSG0000(	646	16.15706	chr4:909:	SNORA62		smallRNA	chr4:67747236-6774
ENSG0000(	646	16.15706	chr4:909:	ENSG00000250403		Pseudoger	chr4:99990737-9999
ENSG0000(	646	16.15706	chr4:909:	ENSG00000249171		lncRNA	chr4:83668510-8373
ENSG0000(	646	16.15706	chr4:909:	ENSG00000250740		lncRNA	chr4:105927060-105
ENSG0000(	646	16.15706	chr4:909:	MANBA		protein_c	chr4:102630770-102
ENSG0000(	646	16.15706	chr4:909:	ENSG00000289942		lncRNA	chr4:68376551-6837
ENSG0000(	646	16.15706	chr4:909:	ENSG00000249262		Pseudoger	chr4:87410644-8741
ENSG0000(	646	16.15706	chr4:909:	SNORA3		smallRNA	chr4:73263960-7326
ENSG0000(	646	16.15706	chr4:909:	ENSG00000248242		lncRNA	chr4:104653874-104
ENSG0000(	646	16.15706	chr4:909:	SMR3A		protein_c	chr4:70360760-7036
ENSG0000(	646	16.15706	chr4:909:	AREG	NCGv7	protein_c	chr4:74445136-7445

ENSG0000(	646	16.15706	chr4:909:	NFKB1		protein_c	chr4:102501330-102
ENSG0000(	646	16.15706	chr4:909:	SEC24B-AS1		lncRNA	chr4:109347475-109
ENSG0000(	646	16.15706	chr4:909:	IBSP		protein_c	chr4:87799554-8781
ENSG0000(	646	16.15706	chr4:909:	ENSG00000250696		lncRNA	chr4:69182100-6921
ENSG0000(	646	16.15706	chr4:909:	PF4V1		protein_c	chr4:73853296-7385
ENSG0000(	646	16.15706	chr4:909:	PABPC1P7		Pseudoger	chr4:102896725-102
ENSG0000(	646	16.15706	chr4:909:	ENSG00000286490		lncRNA	chr4:82612184-8261
ENSG0000(	646	16.15706	chr4:909:	ENSG00000289530		lncRNA	chr4:73706323-7370
ENSG0000(	646	16.15706	chr4:909:	NUDT9		protein_c	chr4:87422573-8745
ENSG0000(	646	16.15706	chr4:909:	RNF14P2		Pseudoger	chr4:110264865-110
ENSG0000(	646	16.15706	chr4:909:	UBA6	NCGv7	protein_c	chr4:67612652-6770
ENSG0000(	646	16.15706	chr4:909:	RACK1P3		Pseudoger	chr4:92821986-9282
ENSG0000(	646	16.15706	chr4:909:	TECRP1		Pseudoger	chr4:86949669-8695
ENSG0000(	646	16.15706	chr4:909:	ENSG00000250572		lncRNA	chr4:87261931-8726
ENSG0000(	646	16.15706	chr4:909:	COL25A1	NCGv7	protein_c	chr4:108808725-109
ENSG0000(	646	16.15706	chr4:909:	UGT2B29P		Pseudoger	chr4:68509441-6851
ENSG0000(	646	16.15706	chr4:909:	ENSG00000250560		Pseudoger	chr4:75194867-7519
ENSG0000(	646	16.15706	chr4:909:	ADH1C		protein_c	chr4:99336497-9935
ENSG0000(	646	16.15706	chr4:909:	EXOC7P1		Pseudoger	chr4:108417705-108
ENSG0000(	646	16.15706	chr4:909:	ABT1P1		Pseudoger	chr4:99022311-9902
ENSG0000(	646	16.15706	chr4:909:	ACTR6P1		Pseudoger	chr4:106836498-106
ENSG0000(	646	16.15706	chr4:909:	HSD17B13		protein_c	chr4:87303789-8732
ENSG0000(	646	16.15706	chr4:909:	LINC02994		lncRNA	chr4:83796436-8429
ENSG0000(	646	16.15706	chr4:909:	ENSG00000250522		lncRNA	chr4:105540190-105
ENSG0000(	646	16.15706	chr4:909:	ENSG00000250532		lncRNA	chr4:74418917-7444
ENSG0000(	646	16.15706	chr4:909:	ELOVL6		protein_c	chr4:110045846-110
ENSG0000(	646	16.15706	chr4:909:	ENSG00000248161		lncRNA	chr4:102418602-102
ENSG0000(	646	16.15706	chr4:909:	ENSG00000248165		lncRNA	chr4:74993877-7503
ENSG0000(	646	16.15706	chr4:909:	UGT2B17	DriverDB\	protein_c	chr4:68537173-6857
ENSG0000(	646	16.15706	chr4:909:	ENSG00000287512		lncRNA	chr4:98961083-9896
ENSG0000(	646	16.15706	chr4:909:	TMEM150C		protein_c	chr4:82483170-8256
ENSG0000(	646	16.15706	chr4:909:	RPS15AP17		Pseudoger	chr4:62105660-6210
ENSG0000(	646	16.15706	chr4:909:	ENSG00000289532		lncRNA	chr4:98259390-9826
ENSG0000(	646	16.15706	chr4:909:	SHROOM3-AS1		lncRNA	chr4:76708853-7680
ENSG0000(	646	16.15706	chr4:909:	RN7SKP48		smallRNA	chr4:85100496-8510
ENSG0000(	646	16.15706	chr4:909:	RN7SL218P		smallRNA	chr4:74011578-7401
ENSG0000(	646	16.15706	chr4:909:	ENSG00000248113		Pseudoger	chr4:82580117-8258
ENSG0000(	646	16.15706	chr4:909:	RN7SL127P		smallRNA	chr4:78898855-7889
ENSG0000(	646	16.15706	chr4:909:	RNA5SP163		Pseudoger	chr4:71759518-7175
ENSG0000(	646	16.15706	chr4:909:	ENSG00000248200		Pseudoger	chr4:110146374-110
ENSG0000(	646	16.15706	chr4:909:	MICOS10P4		Pseudoger	chr4:78379197-7837
ENSG0000(	646	16.15706	chr4:909:	Y_RNA		smallRNA	chr4:88330176-8833
ENSG0000(	646	16.15706	chr4:909:	snoU13		smallRNA	chr4:87768485-8776
ENSG0000(	646	16.15706	chr4:909:	ENSG00000248196		Pseudoger	chr4:87166844-8716
ENSG0000(	646	16.15706	chr4:909:	IFITM3P1		Pseudoger	chr4:66094142-6609
ENSG0000(	646	16.15706	chr4:909:	ENSG00000286978		lncRNA	chr4:88358945-8836
ENSG0000(	646	16.15706	chr4:909:	RNU6-34P		smallRNA	chr4:96152297-9615
ENSG0000(	646	16.15706	chr4:909:	GAPDHP60		Pseudoger	chr4:87207092-8720
ENSG0000(	646	16.15706	chr4:909:	snoU13		smallRNA	chr4:70812246-7081
ENSG0000(	646	16.15706	chr4:909:	ENSG00000249235		Pseudoger	chr4:68861332-6886
ENSG0000(	646	16.15706	chr4:909:	APOOP4		Pseudoger	chr4:68304343-6830

ENSG0000(	646	16.15706	chr4:9093	SNCA-AS1		lncRNA	chr4:89836408-8984
ENSG0000(	646	16.15706	chr4:9093	VAMP9P		Pseudoger	chr4:82284971-8234
ENSG0000(	646	16.15706	chr4:9093	HERC3		protein_c	chr4:88523810-8870
ENSG0000(	646	16.15706	chr4:9093	ENSG00000250612		Pseudoger	chr4:69346609-6935
ENSG0000(	646	16.15706	chr4:9093	ENSG00000248128		Pseudoger	chr4:78003143-7800
ENSG0000(	646	16.15706	chr4:9093	ENSG00000250642		Pseudoger	chr4:68282461-6828
ENSG0000(	646	16.15706	chr4:9093	SULT1D1P		Pseudoger	chr4:69791872-6981
ENSG0000(	646	16.15706	chr4:9093	ENSG00000287037		lncRNA	chr4:73997933-7400
ENSG0000(	646	16.15706	chr4:9093	ENSG00000249170		Pseudoger	chr4:71300258-7130
ENSG0000(	646	16.15706	chr4:9093	RPL34	NCGv7	protein_c	chr4:108620569-108
ENSG0000(	646	16.15706	chr4:9093	PRKG2-AS1		lncRNA	chr4:81164922-8119
ENSG0000(	646	16.15706	chr4:9093	SDAD1		protein_c	chr4:75940950-7599
ENSG0000(	646	16.15706	chr4:9093	RNU6-33P		smallRNA	chr4:88684848-8868
ENSG0000(	646	16.15706	chr4:9093	ENSG00000244669		Pseudoger	chr4:64767130-6476
ENSG0000(	646	16.15706	chr4:9093	ENSG00000289443		lncRNA	chr4:77048964-7705
ENSG0000(	646	16.15706	chr4:9093	TIGD2		protein_c	chr4:89111533-8911
ENSG0000(	646	16.15706	chr4:9093	EFL1P2		Pseudoger	chr4:65142703-6514
ENSG0000(	646	16.15706	chr4:9093	ENSG00000251055		lncRNA	chr4:63128311-6314
ENSG0000(	646	16.15706	chr4:9093	WDFY3-AS2		lncRNA	chr4:84965534-8501
ENSG0000(	646	16.15706	chr4:9093	CXCL10		protein_c	chr4:76021118-7602
ENSG0000(	646	16.15706	chr4:9093	RNU6ATAC31P		smallRNA	chr4:88206427-8820
ENSG0000(	646	16.15706	chr4:9093	CXCL11		protein_c	chr4:76033682-7604
ENSG0000(	646	16.15706	chr4:9093	ENSG00000287181		lncRNA	chr4:88352222-8835
ENSG0000(	646	16.15706	chr4:9093	SNORD50		smallRNA	chr4:76402076-7640
ENSG0000(	646	16.15706	chr4:9093	RRH		protein_c	chr4:109827972-109
ENSG0000(	646	16.15706	chr4:9093	MRPL1		protein_c	chr4:77862830-7795
ENSG0000(	646	16.15706	chr4:9093	ENSG00000242727		Pseudoger	chr4:76891034-7689
ENSG0000(	646	16.15706	chr4:9093	FAM47E		protein_c	chr4:76214040-7628
ENSG0000(	646	16.15706	chr4:9093	RNU6-1000P		smallRNA	chr4:76356610-7635
ENSG0000(	646	16.15706	chr4:9093	THAP9-AS1		lncRNA	chr4:82893009-8290
ENSG0000(	646	16.15706	chr4:9093	HIGD1AP13		Pseudoger	chr4:78648954-7864
ENSG0000(	646	16.15706	chr4:9093	RPL5P12		Pseudoger	chr4:98025390-9802
ENSG0000(	646	16.15706	chr4:9093	ENSG00000289480		lncRNA	chr4:82348252-8234
ENSG0000(	646	16.15706	chr4:9093	RNU6-553P		smallRNA	chr4:105406997-105
ENSG0000(	646	16.15706	chr4:9093	ENSG00000251017		Pseudoger	chr4:74085995-7408
ENSG0000(	646	16.15706	chr4:9093	GAR1		protein_c	chr4:109815510-109
ENSG0000(	646	16.15706	chr4:9093	ENSG00000277695		lncRNA	chr4:89747802-8975
ENSG0000(	646	16.15706	chr4:9093	ENSG00000288888		lncRNA	chr4:77056585-7705
ENSG0000(	646	16.15706	chr4:9093	Y_RNA		smallRNA	chr4:78632273-7863
ENSG0000(	646	16.15706	chr4:9093	ENSG00000249051		Pseudoger	chr4:73777636-7377
ENSG0000(	646	16.15706	chr4:9093	ENSG00000251170		lncRNA	chr4:104230380-104
ENSG0000(	646	16.15706	chr4:9093	RNU6-145P		smallRNA	chr4:76532222-7653
ENSG0000(	646	16.15706	chr4:9093	ENSG00000287239		lncRNA	chr4:89995223-8999
ENSG0000(	646	16.15706	chr4:9093	snoU13		smallRNA	chr4:78640100-7864
ENSG0000(	646	16.15706	chr4:9093	ENSG00000287841		lncRNA	chr4:96841995-9685
ENSG0000(	646	16.15706	chr4:9093	RUFY3		protein_c	chr4:70704204-7080
ENSG0000(	646	16.15706	chr4:9093	RNU6-95P		smallRNA	chr4:68003895-6800
ENSG0000(	646	16.15706	chr4:9093	MTND5P5		Pseudoger	chr4:101972423-101
ENSG0000(	646	16.15706	chr4:9093	ENSG00000229717		lncRNA	chr4:75822966-7583
ENSG0000(	646	16.15706	chr4:9093	ENSG00000249036		lncRNA	chr4:77394491-7749
ENSG0000(	646	16.15706	chr4:9093	MTND3P24		Pseudoger	chr4:64610980-6461

ENSG0000(	646	16.15706	chr4:9093	ENSG00000249049		lncRNA	chr4:91319034-9132
ENSG0000(	646	16.15706	chr4:9093	RNU2-16P		smallRNA	chr4:75829454-7582
ENSG0000(	646	16.15706	chr4:9093	LIN54		protein_c	chr4:82909973-8301
ENSG0000(	646	16.15706	chr4:9093	TBCAP3		Pseudoger	chr4:98909537-9890
ENSG0000(	646	16.15706	chr4:9093	ENSG00000249001		lncRNA	chr4:87568035-8773
ENSG0000(	646	16.15706	chr4:9093	PCNAP1		Pseudoger	chr4:99160514-9916
ENSG0000(	646	16.15706	chr4:9093	ENSG00000251101		Pseudoger	chr4:68615393-6861
ENSG0000(	646	16.15706	chr4:9093	DAPP1		protein_c	chr4:99816827-9987
ENSG0000(	646	16.15706	chr4:9093	PARM1	AC	protein_c	chr4:74933095-7505
ENSG0000(	646	16.15706	chr4:9093	EXOC5P1		Pseudoger	chr4:62816826-6281
ENSG0000(	646	16.15706	chr4:9093	ENSG00000251095		lncRNA	chr4:89410960-8972
ENSG0000(	646	16.15706	chr4:9093	ENSG00000251081		lncRNA	chr4:107258700-107
ENSG0000(	646	16.15706	chr4:9093	ENSG00000289019		lncRNA	chr4:70899216-7090
ENSG0000(	646	16.15706	chr4:9093	ENSG00000251074		Pseudoger	chr4:68626847-6862
ENSG0000(	646	16.15706	chr4:9093	ENSG00000249072		Pseudoger	chr4:78008512-7800
ENSG0000(	646	16.15706	chr4:9093	ENSG00000290407		lncRNA	chr4:68184129-6818
ENSG0000(	646	16.15706	chr4:9093	ENSG00000248984		lncRNA	chr4:91108023-9111
ENSG0000(	646	16.15706	chr4:9093	CYP2U1-AS1		lncRNA	chr4:107863473-107
ENSG0000(	646	16.15706	chr4:9093	LINC02835		lncRNA	chr4:65225867-6524
ENSG0000(	646	16.15706	chr4:9093	TMPRSS11E		protein_c	chr4:68447463-6849
ENSG0000(	646	16.15706	chr4:9093	PPM1K-DT		lncRNA	chr4:88284507-8834
ENSG0000(	646	16.15706	chr4:9093	ENSG00000198277		Pseudoger	chr4:68914928-6892
ENSG0000(	646	16.15706	chr4:9093	HTN3		protein_c	chr4:70028455-7003
ENSG0000(	646	16.15706	chr4:9093	ENSG00000285458		protein_c	chr4:86886472-8693
ENSG0000(	646	16.15706	chr4:9093	SMARCAD1-DT		lncRNA	chr4:94117792-9420
ENSG0000(	646	16.15706	chr4:9093	SNORD75		smallRNA	chr4:77702746-7770
ENSG0000(	646	16.15706	chr4:9093	UBE2D3-AS1		lncRNA	chr4:102827611-102
ENSG0000(	646	16.15706	chr4:9093	TACR3		protein_c	chr4:103586031-103
ENSG0000(	646	16.15706	chr4:9093	COL25A1-DT		lncRNA	chr4:109303035-109
ENSG0000(	646	16.15706	chr4:9093	RNU6-499P		smallRNA	chr4:82174547-8217
ENSG0000(	646	16.15706	chr4:9093	ENSG00000250877		lncRNA	chr4:72323028-7233
ENSG0000(	646	16.15706	chr4:9093	RNU6-112P		smallRNA	chr4:88275205-8827
ENSG0000(	646	16.15706	chr4:9093	RPL30P6		Pseudoger	chr4:95644952-9564
ENSG0000(	646	16.15706	chr4:9093	ENSG00000224207		Pseudoger	chr4:102734358-102
ENSG0000(	646	16.15706	chr4:9093	SNORA75		smallRNA	chr4:79843102-7984
ENSG0000(	646	16.15706	chr4:9093	MAPK10		protein_c	chr4:85990007-8659
ENSG0000(	646	16.15706	chr4:9093	LARP1BP1		Pseudoger	chr4:63350114-6335
ENSG0000(	646	16.15706	chr4:9093	EPHA5-AS1		lncRNA	chr4:65669961-6569
ENSG0000(	646	16.15706	chr4:9093	UBE2D3		protein_c	chr4:102794383-102
ENSG0000(	646	16.15706	chr4:9093	MTND4LP31		Pseudoger	chr4:64610616-6461
ENSG0000(	646	16.15706	chr4:9093	ENSG00000250828		Pseudoger	chr4:69437357-6944
ENSG0000(	646	16.15706	chr4:9093	LNCPRESS2		lncRNA	chr4:92268767-9227
ENSG0000(	646	16.15706	chr4:9093	HNRNPA3P13		Pseudoger	chr4:82128535-8212
ENSG0000(	646	16.15706	chr4:9093	SNORD42		smallRNA	chr4:82402638-8240
ENSG0000(	646	16.15706	chr4:9093	PPBPP2		lncRNA	chr4:74054038-7405
ENSG0000(	646	16.15706	chr4:9093	HMGB3P15		Pseudoger	chr4:94195940-9419
ENSG0000(	646	16.15706	chr4:9093	CFAP299		protein_c	chr4:80335730-8096
ENSG0000(	646	16.15706	chr4:9093	H2AZ1-DT		lncRNA	chr4:99950006-1001
ENSG0000(	646	16.15706	chr4:9093	ENSG00000289515		lncRNA	chr4:76305887-7630
ENSG0000(	646	16.15706	chr4:9093	snoU13		smallRNA	chr4:102859393-102
ENSG0000(	646	16.15706	chr4:9093	CXCL8		protein_c	chr4:73740519-7374

ENSG0000(	646	16.15706	chr4:909:	CXXC4-AS1		lncRNA	chr4:104490849-104
ENSG0000(	646	16.15706	chr4:909:	ENSG00000224218		lncRNA	chr4:76758554-7680
ENSG0000(	646	16.15706	chr4:909:	RASSF6	NCGv7	protein_c	chr4:73571550-7362
ENSG0000(	646	16.15706	chr4:909:	MTND2P41		Pseudoger	chr4:68049706-6805
ENSG0000(	646	16.15706	chr4:909:	RNU1-63P		smallRNA	chr4:67429591-6742
ENSG0000(	646	16.15706	chr4:909:	ENSG00000289186		lncRNA	chr4:82566385-8257
ENSG0000(	646	16.15706	chr4:909:	UNC5C	NCGv7	protein_c	chr4:95162504-9554
ENSG0000(	646	16.15706	chr4:909:	ENSG00000289496		lncRNA	chr4:77076049-7707
ENSG0000(	646	16.15706	chr4:909:	KRT8P46		Pseudoger	chr4:102728746-102
ENSG0000(	646	16.15706	chr4:909:	ENSG00000290776		lncRNA	chr4:79587302-7958
ENSG0000(	646	16.15706	chr4:909:	ENSG00000290400		lncRNA	chr4:67991812-6799
ENSG0000(	646	16.15706	chr4:909:	HMGB1P44		Pseudoger	chr4:77963940-7796
ENSG0000(	646	16.15706	chr4:909:	RNU6-891P		smallRNA	chr4:70852130-7085
ENSG0000(	646	16.15706	chr4:909:	RPL6P14		Pseudoger	chr4:104886118-104
ENSG0000(	646	16.15706	chr4:909:	LRIT3		protein_c	chr4:109848107-109
ENSG0000(	646	16.15706	chr4:909:	ENSG00000288796		protein_c	chr4:73981074-7398
ENSG0000(	646	16.15706	chr4:909:	SDAD1-AS1		lncRNA	chr4:75980790-7600
ENSG0000(	646	16.15706	chr4:909:	ATP5F1EP1		Pseudoger	chr4:105532475-105
ENSG0000(	646	16.15706	chr4:909:	ENSG00000250920		lncRNA	chr4:103550586-103
ENSG0000(	646	16.15706	chr4:909:	UGT2B26P		Pseudoger	chr4:69027831-6904
ENSG0000(	646	16.15706	chr4:909:	TECRL		protein_c	chr4:64275257-6440
ENSG0000(	646	16.15706	chr4:909:	ENSG00000246090		lncRNA	chr4:99088805-9930
ENSG0000(	646	16.15706	chr4:909:	ENSG00000250908		lncRNA	chr4:93318623-9331
ENSG0000(	646	16.15706	chr4:909:	ENSG00000280056		TEC	chr4:92260367-9226
ENSG0000(	646	16.15706	chr4:909:	ZACNP1		Pseudoger	chr4:108415220-108
ENSG0000(	646	16.15706	chr4:909:	LINC00989		lncRNA	chr4:79491802-7962
ENSG0000(	646	16.15706	chr4:909:	ENSG00000250376		Pseudoger	chr4:68784618-6878
ENSG0000(	646	16.15706	chr4:909:	HSD17B11		protein_c	chr4:87336515-8739
ENSG0000(	646	16.15706	chr4:909:	ENSG00000287392		lncRNA	chr4:92183235-9226
ENSG0000(	646	16.15706	chr4:909:	ENSG00000248646		lncRNA	chr4:75361207-7543
ENSG0000(	646	16.15706	chr4:909:	RN7SL89P		smallRNA	chr4:105293658-105
ENSG0000(	646	16.15706	chr4:909:	ENSG00000279464		TEC	chr4:67607856-6761
ENSG0000(	646	16.15706	chr4:909:	AF213884.3		smallRNA	chr4:102565875-102
ENSG0000(	646	16.15706	chr4:909:	RNU6-784P		smallRNA	chr4:70703018-7070
ENSG0000(	646	16.15706	chr4:909:	PARM1-AS1		lncRNA	chr4:74955974-7497
ENSG0000(	646	16.15706	chr4:909:	ENSG00000196472		Pseudoger	chr4:69181660-6918
ENSG0000(	646	16.15706	chr4:909:	SETP20		Pseudoger	chr4:109553243-109
ENSG0000(	646	16.15706	chr4:909:	GK2		protein_c	chr4:79406361-7940
ENSG0000(	646	16.15706	chr4:909:	RNU6-35P		smallRNA	chr4:109992325-109
ENSG0000(	646	16.15706	chr4:909:	NAP1L5		protein_c	chr4:88695913-8869
ENSG0000(	646	16.15706	chr4:909:	TMPRSS11GP		Pseudoger	chr4:67991684-6801
ENSG0000(	646	16.15706	chr4:909:	ENSG00000239793		Pseudoger	chr4:78768499-7876
ENSG0000(	646	16.15706	chr4:909:	DDIT4L-AS1		lncRNA	chr4:100190033-100
ENSG0000(	646	16.15706	chr4:909:	OSTC		protein_c	chr4:108650585-108
ENSG0000(	646	16.15706	chr4:909:	ENSG00000286147		lncRNA	chr4:106525401-106
ENSG0000(	646	16.15706	chr4:909:	ENSG00000286150		lncRNA	chr4:100421655-100
ENSG0000(	646	16.15706	chr4:909:	UGT2B15		protein_c	chr4:68646597-6867
ENSG0000(	646	16.15706	chr4:909:	RPL21P47		Pseudoger	chr4:62248294-6224
ENSG0000(	646	16.15706	chr4:909:	YTHDC1		protein_c	chr4:68310387-6835
ENSG0000(	646	16.15706	chr4:909:	ENSG00000286291		lncRNA	chr4:103871890-103
ENSG0000(	646	16.15706	chr4:909:	ENSG00000249686		Pseudoger	chr4:69450014-6945

ENSG0000(	646	16.15706	chr4:9093	KRT19P3	Pseudoger	chr4:109879070-109
ENSG0000(	646	16.15706	chr4:9093	ENSG00000248676	lncRNA	chr4:99594799-9962
ENSG0000(	646	16.15706	chr4:9093	ENSG00000249351	Pseudoger	chr4:65216616-6521
ENSG0000(	646	16.15706	chr4:9093	TXNP6	Pseudoger	chr4:76958860-7695
ENSG0000(	646	16.15706	chr4:9093	RN7SL55P	smallRNA	chr4:109450775-109
ENSG0000(	646	16.15706	chr4:9093	GPRIN3	protein_c	chr4:89236383-8930
ENSG0000(	646	16.15706	chr4:9093	ENSG00000248847	Pseudoger	chr4:62489495-6249
ENSG0000(	646	16.15706	chr4:9093	ENSG00000248635	Pseudoger	chr4:68704600-6871
ENSG0000(	646	16.15706	chr4:9093	RNU2-40P	smallRNA	chr4:65807240-6580
ENSG0000(	646	16.15706	chr4:9093	ENSG00000287383	lncRNA	chr4:66169778-6632
ENSG0000(	646	16.15706	chr4:9093	PPBPP2	Pseudoger	chr4:74054041-7405
ENSG0000(	646	16.15706	chr4:9093	ENSG00000279913	TEC	chr4:99844138-9984
ENSG0000(	646	16.15706	chr4:9093	RPL6P10	Pseudoger	chr4:65573459-6557
ENSG0000(	646	16.15706	chr4:9093	RNU6-520P	smallRNA	chr4:70701755-7070
ENSG0000(	646	16.15706	chr4:9093	RNU6-733P	smallRNA	chr4:107867807-107
ENSG0000(	646	16.15706	chr4:9093	Y_RNA	smallRNA	chr4:61906313-6190
ENSG0000(	646	16.15706	chr4:9093	MIR576	smallRNA	chr4:109488698-109
ENSG0000(	646	16.15706	chr4:9093	ENSG00000248613	Pseudoger	chr4:68900651-6890
ENSG0000(	646	16.15706	chr4:9093	RNU6-774P	smallRNA	chr4:84233657-8423
ENSG0000(	646	16.15706	chr4:9093	ENSG00000248627	lncRNA	chr4:92833685-9283
ENSG0000(	646	16.15706	chr4:9093	NPFFR2	protein_c	chr4:72031902-7214
ENSG0000(	646	16.15706	chr4:9093	ENSG00000286136	lncRNA	chr4:108669949-108
ENSG0000(	646	16.15706	chr4:9093	ENSG00000248639	Pseudoger	chr4:68293127-6829
ENSG0000(	646	16.15706	chr4:9093	ENSG00000286124	lncRNA	chr4:101640946-101
ENSG0000(	646	16.15706	chr4:9093	HNRNPA1P56	Pseudoger	chr4:77987860-7798
ENSG0000(	646	16.15706	chr4:9093	ENSG00000249764	Pseudoger	chr4:98713804-9871
ENSG0000(	646	16.15706	chr4:9093	ADH6	protein_c	chr4:99202638-9921
ENSG0000(	646	16.15706	chr4:9093	ENSG00000249763	Pseudoger	chr4:69242628-6925
ENSG0000(	646	16.15706	chr4:9093	ENSG00000249755	Pseudoger	chr4:88527160-8852
ENSG0000(	646	16.15706	chr4:9093	HMGA1P2	Pseudoger	chr4:73098822-7309
ENSG0000(	646	16.15706	chr4:9093	ENSG00000286664	lncRNA	chr4:64836361-6488
ENSG0000(	646	16.15706	chr4:9093	ENSG00000249735	Pseudoger	chr4:68985009-6898
ENSG0000(	646	16.15706	chr4:9093	HPSE	protein_c	chr4:83292461-8333
ENSG0000(	646	16.15706	chr4:9093	COQ2	protein_c	chr4:83261536-8328
ENSG0000(	646	16.15706	chr4:9093	ENSG00000284695	protein_c	chr4:69810780-6984
ENSG0000(	646	16.15706	chr4:9093	BIN2P1	Pseudoger	chr4:82275071-8227
ENSG0000(	646	16.15706	chr4:9093	RN7SKP28	smallRNA	chr4:96348738-9634
ENSG0000(	646	16.15706	chr4:9093	ENSG00000248778	Pseudoger	chr4:105679050-105
ENSG0000(	646	16.15706	chr4:9093	MIR575	smallRNA	chr4:82753337-8275
ENSG0000(	646	16.15706	chr4:9093	ENSG00000287401	lncRNA	chr4:76240740-7631
ENSG0000(	646	16.15706	chr4:9093	TMSB4XP8	Pseudoger	chr4:90838903-9083
ENSG0000(	646	16.15706	chr4:9093	GNRHR	protein_c	chr4:67737118-6775
ENSG0000(	646	16.15706	chr4:9093	ENSG00000248749	lncRNA	chr4:84371393-8438
ENSG0000(	646	16.15706	chr4:9093	ENSG00000249472	Pseudoger	chr4:68614419-6861
ENSG0000(	646	16.15706	chr4:9093	ENSG00000248750	Pseudoger	chr4:92884663-9288
ENSG0000(	646	16.15706	chr4:9093	RBMXP4	Pseudoger	chr4:109346326-109
ENSG0000(	646	16.15706	chr4:9093	ENSG00000248763	Pseudoger	chr4:69066395-6906
ENSG0000(	646	16.15706	chr4:9093	RPSAP34	Pseudoger	chr4:108407843-108
ENSG0000(	646	16.15706	chr4:9093	ENSG00000284968	lncRNA	chr4:86924630-8693
ENSG0000(	646	16.15706	chr4:9093	ENSG00000286242	lncRNA	chr4:102814252-102
ENSG0000(	646	16.15706	chr4:9093	ENSG00000248831	Pseudoger	chr4:77350370-7735

ENSG0000C	646	16.15706	chr4:9093	TMPRSS11B		protein_c	chr4:68226653-6824
ENSG0000C	646	16.15706	chr4:9093	ENSG00000287552		lncRNA	chr4:94315486-9434
ENSG0000C	646	16.15706	chr4:9093	SLC4A4		protein_c	chr4:71062667-7157
ENSG0000C	646	16.15706	chr4:9093	RPL34-DT		lncRNA	chr4:108538190-108
ENSG0000C	646	16.15706	chr4:9093	PRR27		protein_c	chr4:70133616-7017
ENSG0000C	646	16.15706	chr4:9093	ENSG00000288563		lncRNA	chr4:89582507-8970
ENSG0000C	646	16.15706	chr4:9093	HIGD1AP14		Pseudoger	chr4:109673843-109
ENSG0000C	646	16.15706	chr4:9093	RN7SL728P		smallRNA	chr4:102348394-102
ENSG0000C	646	16.15706	chr4:9093	ENSG00000248824		Pseudoger	chr4:69406931-6940
ENSG0000C	646	16.15706	chr4:9093	PMPCAP1		Pseudoger	chr4:92182477-9218
ENSG0000C	646	16.15706	chr4:9093	ENSG00000288567		Pseudoger	chr4:62454245-6245
ENSG0000C	646	16.15706	chr4:9093	ENSG00000249413		lncRNA	chr4:65998846-6615
ENSG0000C	646	16.15706	chr4:9093	ENSG00000289034		lncRNA	chr4:88006143-8800
ENSG0000C	646	16.15706	chr4:9093	ENSG00000249531		Pseudoger	chr4:68300325-6830
ENSG0000C	646	16.15706	chr4:9093	Y_RNA		smallRNA	chr4:83636196-8363
ENSG0000C	646	16.15706	chr4:9093	ADH1A		protein_c	chr4:99276369-9929
ENSG0000C	646	16.15706	chr4:9093	PRDM8-AS1		lncRNA	chr4:80182637-8019
ENSG0000C	646	16.15706	chr4:9093	ENSG00000286618		lncRNA	chr4:87974385-8800
ENSG0000C	646	16.15706	chr4:9093	ADGRL3-AS1		lncRNA	chr4:62071752-6216
ENSG0000C	646	16.15706	chr4:9093	TMPRSS11A	NCGv7	protein_c	chr4:67909395-6796
ENSG0000C	646	16.15706	chr4:9093	OR7E94P		Pseudoger	chr4:79587757-7958
ENSG0000C	646	16.15706	chr4:9093	C4orf54		protein_c	chr4:99636529-9965
ENSG0000C	646	16.15706	chr4:9093	ENSG00000249635		lncRNA	chr4:106003317-106
ENSG0000C	646	16.15706	chr4:9093	RNU6-1298P		smallRNA	chr4:88226729-8822
ENSG0000C	646	16.15706	chr4:9093	MOB1B	NCGv7	protein_c	chr4:70902326-7102
ENSG0000C	646	16.15706	chr4:9093	LINC02503		lncRNA	chr4:103961616-104
ENSG0000C	646	16.15706	chr4:9093	ADH1B	NCGv7	protein_c	chr4:99304971-9935
ENSG0000C	646	16.15706	chr4:9093	LINC02173		lncRNA	chr4:106433489-106
ENSG0000C	646	16.15706	chr4:9093	ENSG00000249604		lncRNA	chr4:107936031-107
ENSG0000C	646	16.15706	chr4:9093	BMPR1B-DT		lncRNA	chr4:94743668-9475
ENSG0000C	646	16.15706	chr4:9093	RNU6-431P		smallRNA	chr4:108652150-108
ENSG0000C	646	16.15706	chr4:9093	AMTN		protein_c	chr4:70518569-7053
ENSG0000C	646	16.15706	chr4:9093	SULT1B1	NCGv7	protein_c	chr4:69721167-6978
ENSG0000C	646	16.15706	chr4:9093	UGT2A1	NCGv7	protein_c	chr4:69588417-6965
ENSG0000C	646	16.15706	chr4:9093	ENSG00000286189		lncRNA	chr4:84970180-8497
ENSG0000C	646	16.15706	chr4:9093	RNU1-36P		smallRNA	chr4:88000237-8800
ENSG0000C	646	16.15706	chr4:9093	AC093680.1		smallRNA	chr4:106415706-106
ENSG0000C	646	16.15706	chr4:9093	RPL35AP11		Pseudoger	chr4:94369833-9437
ENSG0000C	646	16.15706	chr4:9093	SERBP1P5		Pseudoger	chr4:78180866-7818
ENSG0000C	646	16.15706	chr4:9093	RPS23P3		Pseudoger	chr4:66431092-6643
ENSG0000C	646	16.15706	chr4:9093	ENSG00000248725		Pseudoger	chr4:85246157-8524
ENSG0000C	646	16.15706	chr4:9093	LINC02428		lncRNA	chr4:103255822-103
ENSG0000C	646	16.15706	chr4:9093	EPGN		protein_c	chr4:74308470-7431
ENSG0000C	646	16.15706	chr4:9093	SNX5P1		Pseudoger	chr4:76344550-7634
ENSG0000C	646	16.15706	chr4:9093	RPL30P5		Pseudoger	chr4:83502699-8350
ENSG0000C	646	16.15706	chr4:9093	RPSAP39		Pseudoger	chr4:80161129-8016
ENSG0000C	646	16.15706	chr4:9093	ENSG00000289241		lncRNA	chr4:74099403-7409
ENSG0000C	646	16.15706	chr4:9093	CRYZP2		Pseudoger	chr4:97916353-9791
ENSG0000C	646	16.15706	chr4:9093	ENSG00000250277		Pseudoger	chr4:68996935-6899
ENSG0000C	646	16.15706	chr4:9093	MTCO3P28		Pseudoger	chr4:64611374-6461
ENSG0000C	646	16.15706	chr4:9093	HMGN1P11		Pseudoger	chr4:62510469-6251

ENSG0000(	646	16.15706	chr4:909:	RPL6P13	Pseudoger	chr4:86870191-8687
ENSG0000(	646	16.15706	chr4:909:	ENSG00000250249	Pseudoger	chr4:62291562-6229
ENSG0000(	646	16.15706	chr4:909:	Y_RNA	smallRNA	chr4:82944738-8294
ENSG0000(	646	16.15706	chr4:909:	ENSG00000249278	Pseudoger	chr4:76509284-7650
ENSG0000(	646	16.15706	chr4:909:	LINC01216	lncRNA	chr4:100660279-100
ENSG0000(	646	16.15706	chr4:909:	ANKRD17-DT	lncRNA	chr4:73259209-7331
ENSG0000(	646	16.15706	chr4:909:	ENSG00000250214	Pseudoger	chr4:77864508-7786
ENSG0000(	646	16.15706	chr4:909:	RNU6-462P	smallRNA	chr4:101723876-101
ENSG0000(	646	16.15706	chr4:909:	ENSG00000250202	Pseudoger	chr4:86876338-8687
ENSG0000(	646	16.15706	chr4:909:	SULT1E1	protein_c	chr4:69841212-6986
ENSG0000(	646	16.15706	chr4:909:	RCC2P8	Pseudoger	chr4:108788745-108
ENSG0000(	646	16.15706	chr4:909:	ENSG00000287375	lncRNA	chr4:74881174-7488
ENSG0000(	646	16.15706	chr4:909:	MTND5P13	Pseudoger	chr4:64609454-6461
ENSG0000(	646	16.15706	chr4:909:	LEF1-AS1	lncRNA	chr4:108167525-108
ENSG0000(	646	16.15706	chr4:909:	LINC02232	lncRNA	chr4:64885649-6502
ENSG0000(	646	16.15706	chr4:909:	ENSG00000248373	lncRNA	chr4:104900125-105
ENSG0000(	646	16.15706	chr4:909:	ENSG00000288913	lncRNA	chr4:110197678-110
ENSG0000(	646	16.15706	chr4:909:	LINC00575	lncRNA	chr4:82610974-8262
ENSG0000(	646	16.15706	chr4:909:	RNU6-907P	smallRNA	chr4:89130852-8913
ENSG0000(	646	16.15706	chr4:909:	COX7A2P2	Pseudoger	chr4:96902801-9690
ENSG0000(	646	16.15706	chr4:909:	ENSG00000286848	lncRNA	chr4:70637745-7068
ENSG0000(	646	16.15706	chr4:909:	ENSG00000250100	Pseudoger	chr4:69131351-6913
ENSG0000(	646	16.15706	chr4:909:	ENSG00000248401	Pseudoger	chr4:83247179-8324
ENSG0000(	646	16.15706	chr4:909:	GIMD1	protein_c	chr4:106357392-106
ENSG0000(	646	16.15706	chr4:909:	UGT2A3P7	Pseudoger	chr4:69517667-6951
ENSG0000(	646	16.15706	chr4:909:	AMBN	protein_c	chr4:70592256-7060
ENSG0000(	646	16.15706	chr4:909:	PIGY-DT	lncRNA	chr4:88523826-8852
ENSG0000(	646	16.15706	chr4:909:	RNU6ATAC5P	smallRNA	chr4:73026748-7302
ENSG0000(	646	16.15706	chr4:909:	ARHGEF38	protein_c	chr4:105552620-105
ENSG0000(	646	16.15706	chr4:909:	HSPE1P23	Pseudoger	chr4:74917822-7491
ENSG0000(	646	16.15706	chr4:909:	RNU6-818P	smallRNA	chr4:88201703-8820
ENSG0000(	646	16.15706	chr4:909:	MTCYBP44	Pseudoger	chr4:81733385-8173
ENSG0000(	646	16.15706	chr4:909:	CXCL1P1	Pseudoger	chr4:73944011-7394
ENSG0000(	646	16.15706	chr4:909:	EEF1A1P9	Pseudoger	chr4:105484698-105
ENSG0000(	646	16.15706	chr4:909:	Y_RNA	smallRNA	chr4:98173784-9817
ENSG0000(	646	16.15706	chr4:909:	ENSG00000241981	Pseudoger	chr4:102662611-102
ENSG0000(	646	16.15706	chr4:909:	DYNLL1P6	Pseudoger	chr4:100041841-100
ENSG0000(	646	16.15706	chr4:909:	RN7SL681P	smallRNA	chr4:87386886-8738
ENSG0000(	646	16.15706	chr4:909:	IGBP1P4	Pseudoger	chr4:82401578-8240
ENSG0000(	646	16.15706	chr4:909:	RNU5A-2P	smallRNA	chr4:81334303-8133
ENSG0000(	646	16.15706	chr4:909:	ENSG00000250300	Pseudoger	chr4:99469598-9949
ENSG0000(	646	16.15706	chr4:909:	LRRC37A15P	Pseudoger	chr4:102727274-102
ENSG0000(	646	16.15706	chr4:909:	ENSG00000250315	Pseudoger	chr4:75101477-7510
ENSG0000(	646	16.15706	chr4:909:	MUC7	protein_c	chr4:70430492-7048
ENSG0000(	646	16.15706	chr4:909:	OPRPN	protein_c	chr4:70397931-7041
ENSG0000(	646	16.15706	chr4:909:	SMR3B AC	protein_c	chr4:70370093-7039
ENSG0000(	646	16.15706	chr4:909:	ZBED1P1	Pseudoger	chr4:110291644-110
ENSG0000(	646	16.15706	chr4:909:	CSN3	protein_c	chr4:70238382-7025
ENSG0000(	646	16.15706	chr4:909:	ENSG00000286035	lncRNA	chr4:83075957-8308
ENSG0000(	646	16.15706	chr4:909:	AFP	protein_c	chr4:73431138-7345
ENSG0000(	646	16.15706	chr4:909:	UGT2B7	protein_c	chr4:69051363-6911

ENSG0000(	646	16.15706	chr4:9093	CSN1S2AP		Pseudoger	chr4:70067386-7008
ENSG0000(	646	16.15706	chr4:9093	ENSG00000232327		Pseudoger	chr4:80386178-8038
ENSG0000(	646	16.15706	chr4:9093	STAP1		protein_c	chr4:67558727-6760
ENSG0000(	646	16.15706	chr4:9093	LINC02469		lncRNA	chr4:79663761-7969
ENSG0000(	646	16.15706	chr4:9093	ACTR3BP4		Pseudoger	chr4:102961956-102
ENSG0000(	646	16.15706	chr4:9093	NPM1P41		Pseudoger	chr4:82010665-8201
ENSG0000(	646	16.15706	chr4:9093	CDC42P4		Pseudoger	chr4:109555170-109
ENSG0000(	646	16.15706	chr4:9093	ENSG00000249960		Pseudoger	chr4:82571137-8257
ENSG0000(	646	16.15706	chr4:9093	UGT2B24P		Pseudoger	chr4:69408828-6942
ENSG0000(	646	16.15706	chr4:9093	ENSG00000249951		lncRNA	chr4:94675245-9470
ENSG0000(	646	16.15706	chr4:9093	NCOA4P2		Pseudoger	chr4:88508591-8851
ENSG0000(	646	16.15706	chr4:9093	ENSG00000279098		TEC	chr4:99942081-9994
ENSG0000(	646	16.15706	chr4:9093	RN7SL552P		smallRNA	chr4:84687728-8468
ENSG0000(	646	16.15706	chr4:9093	HNRNPA1P55		Pseudoger	chr4:73938604-7393
ENSG0000(	646	16.15706	chr4:9093	ATOH1		protein_c	chr4:93828753-9383
ENSG0000(	646	16.15706	chr4:9093	MTND6P16		Pseudoger	chr4:64608905-6460
ENSG0000(	646	16.15706	chr4:9093	ADH4		protein_c	chr4:99123657-9915
ENSG0000(	646	16.15706	chr4:9093	ADH5		protein_c	chr4:99070978-9908
ENSG0000(	646	16.15706	chr4:9093	KRT19P6		Pseudoger	chr4:91885046-9188
ENSG0000(	646	16.15706	chr4:9093	ENSG00000249970		Pseudoger	chr4:73543822-7354
ENSG0000(	646	16.15706	chr4:9093	ENSG00000249890		Pseudoger	chr4:69021656-6902
ENSG0000(	646	16.15706	chr4:9093	AC019131.1		smallRNA	chr4:98929923-9892
ENSG0000(	646	16.15706	chr4:9093	TMPRSS11F		protein_c	chr4:68053198-6812
ENSG0000(	646	16.15706	chr4:9093	ENSG00000248547		Pseudoger	chr4:68883885-6888
ENSG0000(	646	16.15706	chr4:9093	ARHGEF38-IT1		lncRNA	chr4:105561591-105
ENSG0000(	646	16.15706	chr4:9093	MIR1269A		smallRNA	chr4:66276824-6627
ENSG0000(	646	16.15706	chr4:9093	ENSG00000288659		lncRNA	chr4:62133766-6222
ENSG0000(	646	16.15706	chr4:9093	AC121157.1		smallRNA	chr4:100129945-100
ENSG0000(	646	16.15706	chr4:9093	ENSG00000248567		lncRNA	chr4:71821305-7182
ENSG0000(	646	16.15706	chr4:9093	RNU6-1187P		smallRNA	chr4:77150328-7715
ENSG0000(	646	16.15706	chr4:9093	FDCSP		protein_c	chr4:70226124-7023
ENSG0000(	646	16.15706	chr4:9093	AFF1	IntOGen-I	protein_c	chr4:86935002-8714
ENSG0000(	646	16.15706	chr4:9093	ENSG00000248511		lncRNA	chr4:92297251-9230
ENSG0000(	646	16.15706	chr4:9093	CHCHD2P7		Pseudoger	chr4:87785920-8778
ENSG0000(	646	16.15706	chr4:9093	CXCL2	AC	protein_c	chr4:74097040-7409
ENSG0000(	646	16.15706	chr4:9093	ENSG00000286074		lncRNA	chr4:76148561-7620
ENSG0000(	646	16.15706	chr4:9093	ENSG00000250075		lncRNA	chr4:67417305-6746
ENSG0000(	646	16.15706	chr4:9093	Y_RNA		smallRNA	chr4:87743952-8774
ENSG0000(	646	16.15706	chr4:9093	MCUB		protein_c	chr4:109560209-109
ENSG0000(	646	16.15706	chr4:9093	RNU6-205P		smallRNA	chr4:110278185-110
ENSG0000(	646	16.15706	chr4:9093	MAPK10-AS1		lncRNA	chr4:86117912-8621
ENSG0000(	646	16.15706	chr4:9093	ENSG00000248447		Pseudoger	chr4:65034634-6503
ENSG0000(	646	16.15706	chr4:9093	ENSG00000241853		Pseudoger	chr4:88163579-8816
ENSG0000(	646	16.15706	chr4:9093	ENSG00000279013		TEC	chr4:91603275-9160
ENSG0000(	646	16.15706	chr4:9093	ENSG00000250057		lncRNA	chr4:83233512-8324
ENSG0000(	646	16.15706	chr4:9093	COX5BP1		Pseudoger	chr4:81919995-8192
ENSG0000(	646	16.15706	chr4:9093	AFM	NCGv7	protein_c	chr4:73481745-7350
ENSG0000(	646	16.15706	chr4:9093	ENSG00000287632		lncRNA	chr4:78669690-7869
ENSG0000(	646	16.15706	chr4:9093	RPL36AP18		Pseudoger	chr4:76401251-7640
ENSG0000(	646	16.15706	chr4:9093	ENSG00000249976		Pseudoger	chr4:73337233-7333
ENSG0000(	646	16.15706	chr4:9093	ENSG00000250030		Pseudoger	chr4:67446267-6744

ENSG0000(	646	16.15706	chr4:909:	TMPRSS11BNL		Pseudoger	chr4:68184081-6821
ENSG0000(	646	16.15706	chr4:909:	LINC01088		lncRNA	chr4:78939485-7930
ENSG0000(	646	16.15706	chr4:909:	UGT2B10	NCGv7	protein_c	chr4:68815994-6883
ENSG0000(	646	16.15706	chr4:909:	ENSG00000250006		Pseudoger	chr4:77311397-7731
ENSG0000(	646	16.15706	chr4:909:	BTC		protein_c	chr4:74744759-7479
ENSG0000(	646	16.15706	chr4:909:	THAP6		protein_c	chr4:75513946-7555
ENSG0000(	646	16.15706	chr4:909:	ENSG00000248479		lncRNA	chr4:65702202-6570
ENSG0000(	646	16.15706	chr4:909:	ENSG00000249985		Pseudoger	chr4:68907918-6890
ENSG0000(	646	16.15706	chr4:909:	ENSG00000289586		lncRNA	chr4:76908814-7694
ENSG0000(	646	16.15706	chr4:909:	ODAPH		protein_c	chr4:75556048-7556
ENSG0000(	646	16.15706	chr4:909:	LINC02267		lncRNA	chr4:96310701-9681
ENSG0000(	646	16.15706	chr4:909:	SOWAHB		protein_c	chr4:76894152-7689
ENSG0000(	646	16.15706	chr4:909:	ENSG00000249052		lncRNA	chr4:91887886-9190
ENSG0000(	646	16.15706	chr4:909:	STPG2		protein_c	chr4:97184093-9814
ENSG0000(	646	16.15706	chr4:909:	ENSG00000269559		lncRNA	chr4:74156511-7415
ENSG0000(	646	16.15706	chr4:909:	SMARCAD1		protein_c	chr4:94207611-9429
ENSG0000(	646	16.15706	chr4:909:	DDX3P3		Pseudoger	chr4:103572089-103
ENSG0000(	646	16.15706	chr4:909:	HPGDS		protein_c	chr4:94298535-9434
ENSG0000(	646	16.15706	chr4:909:	RN7SKP96		smallRNA	chr4:86336318-8633
ENSG0000(	646	16.15706	chr4:909:	SNORA31		smallRNA	chr4:81928313-8192
ENSG0000(	646	16.15706	chr4:909:	PDLIM5	NCGv7	protein_c	chr4:94451857-9466
ENSG0000(	646	16.15706	chr4:909:	TMPRSS11I	NCGv7	protein_c	chr4:67820876-6788
ENSG0000(	646	16.15706	chr4:909:	PDHA2		protein_c	chr4:95840093-9584
ENSG0000(	646	16.15706	chr4:909:	UGT2B11		protein_c	chr4:69199951-6921
ENSG0000(	646	16.15706	chr4:909:	AC083829.1		smallRNA	chr4:88493933-8849
ENSG0000(	646	16.15706	chr4:909:	AC112719.1		smallRNA	chr4:76095408-7609
ENSG0000(	646	16.15706	chr4:909:	ENSG00000272986		lncRNA	chr4:70703747-7070
ENSG0000(	646	16.15706	chr4:909:	snoR442		smallRNA	chr4:82949168-8294
ENSG0000(	646	16.15706	chr4:909:	PAQR3		protein_c	chr4:78887127-7893
ENSG0000(	646	16.15706	chr4:909:	ANTXR2		protein_c	chr4:79901146-8012
ENSG0000(	646	16.15706	chr4:909:	HELQ		protein_c	chr4:83407343-8345
ENSG0000(	646	16.15706	chr4:909:	ENSG00000268803		Pseudoger	chr4:69215908-6921
ENSG0000(	646	16.15706	chr4:909:	MRPS18C		protein_c	chr4:83455932-8346
ENSG0000(	646	16.15706	chr4:909:	RNU6-459P		smallRNA	chr4:70848136-7084
ENSG0000(	646	16.15706	chr4:909:	AC084209.1		smallRNA	chr4:108577072-108
ENSG0000(	646	16.15706	chr4:909:	ABRAXAS1		protein_c	chr4:83459517-8352
ENSG0000(	646	16.15706	chr4:909:	ENSG00000251529		Pseudoger	chr4:68877626-6888
ENSG0000(	646	16.15706	chr4:909:	ENSG00000251572		Pseudoger	chr4:102461250-102
ENSG0000(	646	16.15706	chr4:909:	TACR3-AS1		lncRNA	chr4:103548745-103
ENSG0000(	646	16.15706	chr4:909:	RNU6-699P		smallRNA	chr4:66897262-6689
ENSG0000(	646	16.15706	chr4:909:	LINC01218		lncRNA	chr4:100812255-100
ENSG0000(	646	16.15706	chr4:909:	USO1		protein_c	chr4:75724577-7581
ENSG0000(	646	16.15706	chr4:909:	CNOT6L		protein_c	chr4:77713387-7781
ENSG0000(	646	16.15706	chr4:909:	CCNG2		protein_c	chr4:77157207-7743
ENSG0000(	646	16.15706	chr4:909:	ENSG00000272626		lncRNA	chr4:68901008-6890
ENSG0000(	646	16.15706	chr4:909:	HNRNPD-DT		lncRNA	chr4:82374142-8238
ENSG0000(	646	16.15706	chr4:909:	SCARB2		protein_c	chr4:76158737-7623
ENSG0000(	646	16.15706	chr4:909:	BTF3P13		Pseudoger	chr4:98740742-9874
ENSG0000(	646	16.15706	chr4:909:	FRAS1		protein_c	chr4:78057323-7854
ENSG0000(	646	16.15706	chr4:909:	ENSG00000251647		Pseudoger	chr4:83377363-8337
ENSG0000(	646	16.15706	chr4:909:	ENSG00000272777		lncRNA	chr4:99067256-9906

ENSG0000(	646	16.15706	chr4:909:	SEPTIN11		protein_c	chr4:76949751-7704
ENSG0000(	646	16.15706	chr4:909:	ENSG00000272856		lncRNA	chr4:87460807-8746
ENSG0000(	646	16.15706	chr4:909:	GAR1-DT		lncRNA	chr4:109815047-109
ENSG0000(	646	16.15706	chr4:909:	STPG2-AS1		lncRNA	chr4:97366681-9749
ENSG0000(	646	16.15706	chr4:909:	MIR1255A		smallRNA	chr4:101330302-101
ENSG0000(	646	16.15706	chr4:909:	AC097470.1		smallRNA	chr4:74326886-7432
ENSG0000(	646	16.15706	chr4:909:	MT2P1		Pseudoger	chr4:68376323-6837
ENSG0000(	646	16.15706	chr4:909:	TET2-AS1		lncRNA	chr4:105171354-105
ENSG0000(	646	16.15706	chr4:909:	AC104687.1		smallRNA	chr4:76853964-7685
ENSG0000(	646	16.15706	chr4:909:	AC098870.1		smallRNA	chr4:85910478-8591
ENSG0000(	646	16.15706	chr4:909:	RNU7-151P		smallRNA	chr4:102837047-102
ENSG0000(	646	16.15706	chr4:909:	DUTP8		Pseudoger	chr4:98048555-9804
ENSG0000(	646	16.15706	chr4:909:	ENSG00000251527		lncRNA	chr4:65858761-6586
ENSG0000(	646	16.15706	chr4:909:	ENSG00000251523		lncRNA	chr4:98496364-9850
ENSG0000(	646	16.15706	chr4:909:	PPBP		protein_c	chr4:73986439-7398
ENSG0000(	646	16.15706	chr4:909:	DSPP	NCGv7	protein_c	chr4:87608529-8761
ENSG0000(	646	16.15706	chr4:909:	PKD2	NCGv7	protein_c	chr4:88007635-8807
ENSG0000(	646	16.15706	chr4:909:	ENSG00000268209		Pseudoger	chr4:69387580-6938
ENSG0000(	646	16.15706	chr4:909:	WDFY3	NCGv7	protein_c	chr4:84668765-8496
ENSG0000(	646	16.15706	chr4:909:	COX18		protein_c	chr4:73052362-7306
ENSG0000(	646	16.15706	chr4:909:	ENSG00000251411		Pseudoger	chr4:86913266-8691
ENSG0000(	646	16.15706	chr4:909:	MEPE		protein_c	chr4:87821398-8784
ENSG0000(	646	16.15706	chr4:909:	DMP1		protein_c	chr4:87650280-8766
ENSG0000(	646	16.15706	chr4:909:	PTPN13	NCGv7	protein_c	chr4:86594315-8681
ENSG0000(	646	16.15706	chr4:909:	ABCG2	NCGv7	protein_c	chr4:88090150-8823
ENSG0000(	646	16.15706	chr4:909:	ALB	NCGv7	protein_c	chr4:73397114-7342
ENSG0000(	646	16.15706	chr4:909:	SPARCL1		protein_c	chr4:87473335-8753
ENSG0000(	646	16.15706	chr4:909:	PRDM8	AC	protein_c	chr4:80183879-8020
ENSG0000(	646	16.15706	chr4:909:	SPP1		protein_c	chr4:87975667-8798
ENSG0000(	646	16.15706	chr4:909:	ENSG00000251401		Pseudoger	chr4:90682996-9068
ENSG0000(	646	16.15706	chr4:909:	STBD1		protein_c	chr4:76306733-7631
ENSG0000(	646	16.15706	chr4:909:	NAAA	AC	protein_c	chr4:75913660-7594
ENSG0000(	646	16.15706	chr4:909:	ENSG00000251399		lncRNA	chr4:79596542-7959
ENSG0000(	646	16.15706	chr4:909:	C4orf36		protein_c	chr4:86876205-8689
ENSG0000(	646	16.15706	chr4:909:	CCNI	NCGv7	protein_c	chr4:77047155-7707
ENSG0000(	646	16.15706	chr4:909:	THAP9		protein_c	chr4:82900684-8291
ENSG0000(	646	16.15706	chr4:909:	PPM1K		protein_c	chr4:88257620-8828
ENSG0000(	646	16.15706	chr4:909:	CXCL3	AC	protein_c	chr4:74036589-7403
ENSG0000(	646	16.15706	chr4:909:	CDS1		protein_c	chr4:84583127-8465
ENSG0000(	646	16.15706	chr4:909:	NKX6-1		protein_c	chr4:84491985-8449
ENSG0000(	646	16.15706	chr4:909:	BANK1		protein_c	chr4:101411286-102
ENSG0000(	646	16.15706	chr4:909:	CXCL9		protein_c	chr4:76001275-7600
ENSG0000(	646	16.15706	chr4:909:	ENSG00000251498		Pseudoger	chr4:68972999-6897
ENSG0000(	646	16.15706	chr4:909:	ENSG00000251489		Pseudoger	chr4:69888877-6988
ENSG0000(	646	16.15706	chr4:909:	ENSG00000251473		Pseudoger	chr4:105102891-105
ENSG0000(	646	16.15706	chr4:909:	G3BP2	NCGv7	protein_c	chr4:75641849-7572
ENSG0000(	646	16.15706	chr4:909:	AC112249.1		smallRNA	chr4:76493247-7649
ENSG0000(	646	16.15706	chr4:909:	FLJ20021		lncRNA	chr4:101347752-101
ENSG0000(	646	16.15706	chr4:909:	ENSG00000251454		lncRNA	chr4:75341279-7535
ENSG0000(	646	16.15706	chr4:909:	ENSG00000273156		lncRNA	chr4:82344876-8234
ENSG0000(	646	16.15706	chr4:909:	MTND1P19		Pseudoger	chr4:92702345-9270

ENSG0000(	646	16.15706	chr4:909:	BMP2K		protein_c	chr4:78776342-7891
ENSG0000(	646	16.15706	chr4:909:	LINC01094		lncRNA	chr4:78638780-7868
ENSG0000(	646	16.15706	chr4:909:	BMP3		protein_c	chr4:81030708-8105
ENSG0000(	646	16.15706	chr4:909:	RPL7P17		Pseudoger	chr4:77082403-7708
ENSG0000(	646	16.15706	chr4:909:	NUP54	NCGv7	protein_c	chr4:76107562-7614
ENSG0000(	646	16.15706	chr4:909:	RNU4ATAC9P		smallRNA	chr4:72965178-7296
ENSG0000(	646	16.15706	chr4:909:	ENSG00000276542		lncRNA	chr4:89748283-8974
ENSG0000(	646	16.15706	chr4:909:	MIR5096		smallRNA	chr4:78820752-7882
ENSG0000(	646	16.15706	chr4:909:	NDUFS5P4		Pseudoger	chr4:98976800-9897
ENSG0000(	646	16.15706	chr4:909:	MTCO3P27		Pseudoger	chr4:64606418-6460
ENSG0000(	646	16.15706	chr4:909:	ENSG00000251427		Pseudoger	chr4:69144734-6914
ENSG0000(	646	16.15706	chr4:909:	ENSG00000251424		Pseudoger	chr4:68834213-6883
ENSG0000(	646	16.15706	chr4:909:	HNRNPDL	NCGv7	protein_c	chr4:82422565-8243
ENSG0000(	646	16.15706	chr4:909:	CENPC		protein_c	chr4:67468762-6754
ENSG0000(	646	16.15706	chr4:909:	UGT2B27P		Pseudoger	chr4:69004862-6902
ENSG0000(	646	16.15706	chr4:909:	ENSG00000251691		Pseudoger	chr4:69306469-6930
ENSG0000(	646	16.15706	chr4:909:	UNC5C-AS1		lncRNA	chr4:95549129-9555
ENSG0000(	646	16.15706	chr4:909:	LEF1	NCGv7;AC	protein_c	chr4:108047545-108
ENSG0000(	646	16.15706	chr4:909:	CASP6		protein_c	chr4:109688622-109
ENSG0000(	646	16.15706	chr4:909:	INTS12	NCGv7	protein_c	chr4:105682627-105
ENSG0000(	646	16.15706	chr4:909:	GSTCD		protein_c	chr4:105708778-105
ENSG0000(	646	16.15706	chr4:909:	CENPE		protein_c	chr4:103105349-103
ENSG0000(	646	16.15706	chr4:909:	PPA2		protein_c	chr4:105369077-105
ENSG0000(	646	16.15706	chr4:909:	ANXA3		protein_c	chr4:78551747-7861
ENSG0000(	646	16.15706	chr4:909:	SLC25A14P1		Pseudoger	chr4:83477524-8347
ENSG0000(	646	16.15706	chr4:909:	SHROOM3	NCGv7	protein_c	chr4:76435229-7678
ENSG0000(	646	16.15706	chr4:909:	CDKL2		protein_c	chr4:75576496-7563
ENSG0000(	646	16.15706	chr4:909:	PIMREGP2		Pseudoger	chr4:105526596-105
ENSG0000(	646	16.15706	chr4:909:	HADH		protein_c	chr4:107989714-108
ENSG0000(	646	16.15706	chr4:909:	ENSG00000227304		Pseudoger	chr4:82494786-8249
ENSG0000(	646	16.15706	chr4:909:	HTN1		protein_c	chr4:70050438-7005
ENSG0000(	646	16.15706	chr4:909:	UGT2B25P		Pseudoger	chr4:69389492-6940
ENSG0000(	646	16.15706	chr4:909:	SYT14P1		Pseudoger	chr4:68061822-6806
ENSG0000(	646	16.15706	chr4:909:	STATH		protein_c	chr4:69995966-7000
ENSG0000(	646	16.15706	chr4:909:	CSN1S1		protein_c	chr4:69931068-6994
ENSG0000(	646	16.15706	chr4:909:	ENSG00000271676		Pseudoger	chr4:77112495-7711
ENSG0000(	646	16.15706	chr4:909:	NAA11		protein_c	chr4:79225694-7932
ENSG0000(	646	16.15706	chr4:909:	CXCL13		protein_c	chr4:77511753-7761
ENSG0000(	646	16.15706	chr4:909:	ART3		protein_c	chr4:76011184-7611
ENSG0000(	646	16.15706	chr4:909:	HNRNPA1P67		Pseudoger	chr4:72807267-7280
ENSG0000(	646	16.15706	chr4:909:	ENSG00000270669		Pseudoger	chr4:77216416-7721
ENSG0000(	646	16.15706	chr4:909:	EPHA5	NCGv7	protein_c	chr4:65319563-6567
ENSG0000(	646	16.15706	chr4:909:	HSBP1P2		Pseudoger	chr4:110251871-110
ENSG0000(	646	16.15706	chr4:909:	POLR2MP1		Pseudoger	chr4:68038544-6803
ENSG0000(	646	16.15706	chr4:909:	RNU6-191P		smallRNA	chr4:64397694-6439
ENSG0000(	646	16.15706	chr4:909:	RNU6-469P		smallRNA	chr4:84886386-8488
ENSG0000(	646	16.15706	chr4:909:	PIGY	NCGv7	protein_c	chr4:88520998-8852
ENSG0000(	646	16.15706	chr4:909:	MIR3684		smallRNA	chr4:98997387-9899
ENSG0000(	646	16.15706	chr4:909:	UGT2A2	NCGv7	protein_c	chr4:69588417-6963
ENSG0000(	646	16.15706	chr4:909:	ENSG00000214559		lncRNA	chr4:98251688-9826
ENSG0000(	646	16.15706	chr4:909:	SNORA31		smallRNA	chr4:105105987-105

ENSG0000(	646	16.15706	chr4:909:	AC110810.1		smallRNA	chr4:62595377-6259
ENSG0000(	646	16.15706	chr4:909:	MTTP		protein_c	chr4:99564081-9962
ENSG0000(	646	16.15706	chr4:909:	RPL7AP26		Pseudoger	chr4:82490823-8249
ENSG0000(	646	16.15706	chr4:909:	ENSG00000270720		lncRNA	chr4:89119284-8911
ENSG0000(	646	16.15706	chr4:909:	ENSG00000214980		Pseudoger	chr4:84244003-8424
ENSG0000(	646	16.15706	chr4:909:	SLC39A8		protein_c	chr4:102251080-102
ENSG0000(	646	16.15706	chr4:909:	MIR548AH		smallRNA	chr4:76575551-7657
ENSG0000(	646	16.15706	chr4:909:	PPP3CA	NCGv7	protein_c	chr4:101023409-101
ENSG0000(	646	16.15706	chr4:909:	C4orf17		protein_c	chr4:99511012-9954
ENSG0000(	646	16.15706	chr4:909:	ENSG00000270842		Pseudoger	chr4:82775461-8277
ENSG0000(	646	16.15706	chr4:909:	SEC24B		protein_c	chr4:109433772-109
ENSG0000(	646	16.15706	chr4:909:	PAPSS1		protein_c	chr4:107590276-107
ENSG0000(	646	16.15706	chr4:909:	RPL3P13		Pseudoger	chr4:84544304-8454
ENSG0000(	646	16.15706	chr4:909:	EGF		protein_c	chr4:109912883-110
ENSG0000(	646	16.15706	chr4:909:	RN7SKP244		smallRNA	chr4:88583666-8858
ENSG0000(	646	16.15706	chr4:909:	ENSG00000270480		Pseudoger	chr4:82691737-8269
ENSG0000(	646	16.15706	chr4:909:	PPEF2		protein_c	chr4:75859864-7590
ENSG0000(	646	16.15706	chr4:909:	SNORD112		smallRNA	chr4:107117332-107
ENSG0000(	646	16.15706	chr4:909:	RN7SL275P		smallRNA	chr4:110117736-110
ENSG0000(	646	16.15706	chr4:909:	TRMT10A		protein_c	chr4:99546709-9956
ENSG0000(	646	16.15706	chr4:909:	CYP2U1	DriverDB	protein_c	chr4:107931549-107
ENSG0000(	646	16.15706	chr4:909:	DKK2		protein_c	chr4:106921802-107
ENSG0000(	646	16.15706	chr4:909:	ENSG00000272304		lncRNA	chr4:66003281-6601
ENSG0000(	646	16.15706	chr4:909:	GC		protein_c	chr4:71741696-7180
ENSG0000(	646	16.15706	chr4:909:	RNU7-149P		smallRNA	chr4:98966815-9896
ENSG0000(	646	16.15706	chr4:909:	GRSF1	NCGv7	protein_c	chr4:70815783-7083
ENSG0000(	646	16.15706	chr4:909:	CABS1		protein_c	chr4:70334981-7033
ENSG0000(	646	16.15706	chr4:909:	FAM47E-STBD1		protein_c	chr4:76251721-7631
ENSG0000(	646	16.15706	chr4:909:	ENAM		protein_c	chr4:70628744-7064
ENSG0000(	646	16.15706	chr4:909:	ADAMTS3	NCGv7	protein_c	chr4:72280969-7256
ENSG0000(	646	16.15706	chr4:909:	JCHAIN		protein_c	chr4:70655541-7068
ENSG0000(	646	16.15706	chr4:909:	Y_RNA		smallRNA	chr4:87412228-8741
ENSG0000(	646	16.15706	chr4:909:	ENOPH1		protein_c	chr4:82430590-8246
ENSG0000(	646	16.15706	chr4:909:	ANKRD17	NCGv7	protein_c	chr4:73073376-7325
ENSG0000(	646	16.15706	chr4:909:	UTP3		protein_c	chr4:70688532-7069
ENSG0000(	646	16.15706	chr4:909:	PLAC8	AC	protein_c	chr4:83090048-8313
ENSG0000(	646	16.15706	chr4:909:	ENSG00000263923		lncRNA	chr4:98928897-9899
ENSG0000(	646	16.15706	chr4:909:	UMLILO		lncRNA	chr4:73710302-7371
ENSG0000(	646	16.15706	chr4:909:	SCD5		protein_c	chr4:82629539-8279
ENSG0000(	646	16.15706	chr4:909:	SLC10A6		protein_c	chr4:86823468-8684
ENSG0000(	646	16.15706	chr4:909:	KLHL8	NCGv7	protein_c	chr4:87160103-8724
ENSG0000(	646	16.15706	chr4:909:	SNCA		protein_c	chr4:89700345-8983
ENSG0000(	646	16.15706	chr4:909:	PYURF		protein_c	chr4:88520998-8852
ENSG0000(	646	16.15706	chr4:909:	TBCK		protein_c	chr4:106041599-106
ENSG0000(	646	16.15706	chr4:909:	RNA5SP164		Pseudoger	chr4:93820171-9382
ENSG0000(	646	16.15706	chr4:909:	DCK	NCGv7	protein_c	chr4:70992538-7103
ENSG0000(	646	16.15706	chr4:909:	UGT2B4		protein_c	chr4:69480165-6952
ENSG0000(	646	16.15706	chr4:909:	RNU6-351P		smallRNA	chr4:104974662-104
ENSG0000(	646	16.15706	chr4:909:	ENSG00000270292		Pseudoger	chr4:67725183-6772
ENSG0000(	646	16.15706	chr4:909:	ENSG00000270257		Pseudoger	chr4:67638177-6763
ENSG0000(	646	16.15706	chr4:909:	RNU6-615P		smallRNA	chr4:83003412-8300

ENSG0000(	646	16.15706	chr4:909:	EREG		protein_c	chr4:74365145-7438
ENSG0000(	646	16.15706	chr4:909:	ENSG00000270244		Pseudoger	chr4:76886029-7688
ENSG0000(	646	16.15706	chr4:909:	CXCL6		protein_c	chr4:73836640-7384
ENSG0000(	646	16.15706	chr4:909:	AC034154.1		smallRNA	chr4:97486806-9748
ENSG0000(	646	16.15706	chr4:909:	RNU6-635P		smallRNA	chr4:103924540-103
ENSG0000(	646	16.15706	chr4:909:	RNU6-551P		smallRNA	chr4:107435118-107
ENSG0000(	646	16.15706	chr4:909:	MIR5705		smallRNA	chr4:87300495-8730
ENSG0000(	646	16.15706	chr4:909:	ENSG00000270228		Pseudoger	chr4:67718996-6772
ENSG0000(	646	16.15706	chr4:909:	AC108078.1		smallRNA	chr4:69479331-6947
ENSG0000(	646	16.15706	chr4:909:	RAC1P5		Pseudoger	chr4:107203349-107
ENSG0000(	646	16.15706	chr4:909:	DDIT4L		protein_c	chr4:100185870-100
ENSG0000(	646	16.15706	chr4:909:	CISD2		protein_c	chr4:102868974-102
ENSG0000(	646	16.15706	chr4:909:	PLA2G12A		protein_c	chr4:109709989-109
ENSG0000(	646	16.15706	chr4:909:	LDHAL6EP		Pseudoger	chr4:71434405-7143
ENSG0000(	646	16.15706	chr4:909:	CXCL5		protein_c	chr4:73995642-7399
ENSG0000(	646	16.15706	chr4:909:	RN7SKP248		smallRNA	chr4:90370123-9037
ENSG0000(	646	16.15706	chr4:909:	WDFY3-AS1		lncRNA	chr4:84796614-8481
ENSG0000(	646	16.15706	chr4:909:	FAM177A1P1		Pseudoger	chr4:98955982-9895
ENSG0000(	646	16.15706	chr4:909:	DNAJB14		protein_c	chr4:99896248-9994
ENSG0000(	646	16.15706	chr4:909:	H2AZ1		protein_c	chr4:99948086-9995
ENSG0000(	646	16.15706	chr4:909:	ENSG00000251259		lncRNA	chr4:105137280-105
ENSG0000(	646	16.15706	chr4:909:	EMCN		protein_c	chr4:100395341-100
ENSG0000(	646	16.15706	chr4:909:	SLC9B1		protein_c	chr4:102885048-103
ENSG0000(	646	16.15706	chr4:909:	GPAT3		protein_c	chr4:83535914-8360
ENSG0000(	646	16.15706	chr4:909:	FGF5	NCGv7;AC	protein_c	chr4:80266639-8033
ENSG0000(	646	16.15706	chr4:909:	SLC9B2		protein_c	chr4:103019868-103
ENSG0000(	646	16.15706	chr4:909:	METAP1		protein_c	chr4:98995659-9906
ENSG0000(	646	16.15706	chr4:909:	UGT2B28		protein_c	chr4:69280475-6929
ENSG0000(	646	16.15706	chr4:909:	MIR4451		smallRNA	chr4:85722468-8572
ENSG0000(	646	16.15706	chr4:909:	EIF4E	AC	protein_c	chr4:98879276-9892
ENSG0000(	646	16.15706	chr4:909:	ENSG00000251236		Pseudoger	chr4:68813995-6881
ENSG0000(	646	16.15706	chr4:909:	BDH2		protein_c	chr4:103077592-103
ENSG0000(	646	16.15706	chr4:909:	ENSG00000251177		Pseudoger	chr4:69572391-6958
ENSG0000(	646	16.15706	chr4:909:	SEC31A		protein_c	chr4:82818509-8290
ENSG0000(	646	16.15706	chr4:909:	BMPR1B		protein_c	chr4:94757955-9515
ENSG0000(	646	16.15706	chr4:909:	GSTCD-AS1		lncRNA	chr4:105746245-105
ENSG0000(	646	16.15706	chr4:909:	ENSG00000254044		lncRNA	chr4:97334635-9763
ENSG0000(	646	16.15706	chr4:909:	SGMS2		protein_c	chr4:107824563-107
ENSG0000(	646	16.15706	chr4:909:	RPL36P8		Pseudoger	chr4:76036750-7603
ENSG0000(	646	16.15706	chr4:909:	ENSG00000251288		Pseudoger	chr4:102751401-102
ENSG0000(	646	16.15706	chr4:909:	ENSG00000260651		lncRNA	chr4:102500841-102
ENSG0000(	646	16.15706	chr4:909:	ENSG00000251285		Pseudoger	chr4:88220569-8822
ENSG0000(	646	16.15706	chr4:909:	ARHGAP24		protein_c	chr4:85475150-8600
ENSG0000(	646	16.15706	chr4:909:	ENSG00000251284		Pseudoger	chr4:69125274-6912
ENSG0000(	646	16.15706	chr4:909:	AIMP1		protein_c	chr4:106315544-106
ENSG0000(	646	16.15706	chr4:909:	RAP1GDS1	NCGv7;AC	protein_c	chr4:98261384-9844
ENSG0000(	646	16.15706	chr4:909:	MIR4452		smallRNA	chr4:86542482-8654
ENSG0000(	646	16.15706	chr4:909:	HERC3		protein_c	chr4:88592434-8870
ENSG0000(	646	16.15706	chr4:909:	LINC02562		lncRNA	chr4:75081702-7508
ENSG0000(	646	16.15706	chr4:909:	ENSG00000253170		lncRNA	chr4:97120701-9713
ENSG0000(	646	16.15706	chr4:909:	AC004052.1		smallRNA	chr4:104278107-104

ENSG0000(	646	16.15706	chr4:909:	HERC6		protein_c	chr4:88378739-8844
ENSG0000(	646	16.15706	chr4:909:	ENSG00000251185		lncRNA	chr4:75269068-7536
ENSG0000(	646	16.15706	chr4:909:	MMRN1		protein_c	chr4:89879532-8995
ENSG0000(	646	16.15706	chr4:909:	UGT2A3	NCGv7	protein_c	chr4:68928463-6895
ENSG0000(	646	16.15706	chr4:909:	HERC5	DriverDB\	protein_c	chr4:88457119-8850
ENSG0000(	646	16.15706	chr4:909:	AC093897.1		smallRNA	chr4:78188336-7818
ENSG0000(	646	16.15706	chr4:909:	GRID2		protein_c	chr4:92303966-9381
ENSG0000(	646	16.15706	chr4:909:	ENSG00000226439		Pseudoger	chr4:61775449-6177
ENSG0000(	646	16.15706	chr4:909:	CCDC158		protein_c	chr4:76312997-7642
ENSG0000(	646	16.15706	chr4:909:	ENSG00000274154		Pseudoger	chr4:83369416-8336
ENSG0000(	646	16.15706	chr4:909:	RCHY1		protein_c	chr4:75479033-7551
ENSG0000(	646	16.15706	chr4:909:	CXCL1	TAG;AC	protein_c	chr4:73869393-7387
ENSG0000(	646	16.15706	chr4:909:	MTCYBP16		Pseudoger	chr4:64607703-6460
ENSG0000(	646	16.15706	chr4:909:	PCAT4		lncRNA	chr4:79827471-7987
ENSG0000(	646	16.15706	chr4:909:	MTHFD2L		protein_c	chr4:74114174-7430
ENSG0000(	646	16.15706	chr4:909:	ENSG00000276992		Pseudoger	chr4:104996900-104
ENSG0000(	646	16.15706	chr4:909:	CXXC4	NCGv7	protein_c	chr4:104468308-104
ENSG0000(	646	16.15706	chr4:909:	RASGEF1B		protein_c	chr4:81426393-8204
ENSG0000(	646	16.15706	chr4:909:	ENSG00000274238		lncRNA	chr4:89743792-8974
ENSG0000(	646	16.15706	chr4:909:	PF4		protein_c	chr4:73980811-7398
ENSG0000(	646	16.15706	chr4:909:	TET2	NCGv7;AC	protein_c	chr4:105145875-105
ENSG0000(	646	16.15706	chr4:909:	PRKG2		protein_c	chr4:81087370-8121
ENSG0000(	646	16.15706	chr4:909:	TSPAN5-DT		lncRNA	chr4:98658894-9866
ENSG0000(	646	16.15706	chr4:909:	FAM13A		protein_c	chr4:88725955-8911
ENSG0000(	646	16.15706	chr4:909:	BMP2K-DT		lncRNA	chr4:78773654-7877
ENSG0000(	646	16.15706	chr4:909:	COPS4		protein_c	chr4:83034447-8307
ENSG0000(	646	16.15706	chr4:909:	ENSG00000273447		lncRNA	chr4:109692004-109
ENSG0000(	646	16.15706	chr4:909:	LINC01217		lncRNA	chr4:100778582-100
ENSG0000(	646	16.15706	chr4:909:	TSPAN5		protein_c	chr4:98470367-9865
ENSG0000(	646	16.15706	chr4:909:	LINC02483		lncRNA	chr4:75354076-7536
ENSG0000(	646	16.15706	chr4:909:	ETNPPL		protein_c	chr4:108742048-108
ENSG0000(	646	16.15706	chr4:909:	ENSG00000251309		lncRNA	chr4:101976894-102
ENSG0000(	646	16.15706	chr4:909:	ENSG00000255723		lncRNA	chr4:87317170-8734
ENSG0000(	646	16.15706	chr4:909:	HNRNPD	NCGv7	protein_c	chr4:82352498-8237
ENSG0000(	646	16.15706	chr4:909:	CSN2		protein_c	chr4:69955256-6996
ENSG0000(	646	16.15706	chr4:909:	NPNT	NCGv7	protein_c	chr4:105894775-106
ENSG0000(	646	16.15706	chr4:909:	MIR4450		smallRNA	chr4:76573568-7657
ENSG0000(	642	16.05702	chr5:169(	ENSG00000271926		lncRNA	chr5:72953635-7295
ENSG0000(	638	15.95697	chr6:105(	TOB2P1		Pseudoger	chr6:28217643-2821
ENSG0000(	638	15.95697	chr6:105(	OR2B8P		protein_c	chr6:28053228-2805
ENSG0000(	638	15.95697	chr6:105(	ZSCAN16-AS1		lncRNA	chr6:28015122-2813
ENSG0000(	638	15.95697	chr6:105(	GPX6		protein_c	chr6:28503296-2852
ENSG0000(	638	15.95697	chr6:105(	RSL24D1P1		Pseudoger	chr6:27780619-2778
ENSG0000(	638	15.95697	chr6:105(	ENSG00000287674		lncRNA	chr6:27679971-2768
ENSG0000(	638	15.95697	chr6:105(	H4C10P		Pseudoger	chr6:27807075-2780
ENSG0000(	638	15.95697	chr6:105(	H4C11	NCGv7	protein_c	chr6:27824092-2782
ENSG0000(	638	15.95697	chr6:105(	H3C11	NCGv7	protein_c	chr6:27871845-2787
ENSG0000(	638	15.95697	chr6:105(	ENSG00000290051		lncRNA	chr6:28335995-2833
ENSG0000(	638	15.95697	chr6:105(	H2AC17	NCGv7	protein_c	chr6:27892699-2789
ENSG0000(	638	15.95697	chr6:105(	OR2E1P		Pseudoger	chr6:28455648-2845
ENSG0000(	638	15.95697	chr6:105(	ZNF165	NCGv7	protein_c	chr6:28080568-2808

ENSG0000(	638	15.95697	chr6:105(	ZSCAN12P1		Pseudoger	chr6:28092427-2809
ENSG0000(	638	15.95697	chr6:105(	NKAPL		protein_c	chr6:28259297-2826
ENSG0000(	638	15.95697	chr6:105(	ENSG00000287252		lncRNA	chr6:27757416-2776
ENSG0000(	638	15.95697	chr6:105(	H2BC13		protein_c	chr6:27807479-2780
ENSG0000(	638	15.95697	chr6:105(	SMIM15P2		Pseudoger	chr6:28319660-2831
ENSG0000(	638	15.95697	chr6:105(	ZKSCAN3	NCGv7	protein_c	chr6:28349947-2836
ENSG0000(	638	15.95697	chr6:105(	ENSG00000290891		lncRNA	chr6:28107748-2811
ENSG0000(	638	15.95697	chr6:105(	RNU2-45P		smallRNA	chr6:28410270-2841
ENSG0000(	638	15.95697	chr6:105(	OR1F12P		Pseudoger	chr6:28073316-2807
ENSG0000(	638	15.95697	chr6:105(	ZKSCAN8P2		Pseudoger	chr6:28188050-2818
ENSG0000(	638	15.95697	chr6:105(	OR2B6		protein_c	chr6:27957241-2795
ENSG0000(	638	15.95697	chr6:105(	ENSG00000272009		lncRNA	chr6:28078792-2808
ENSG0000(	638	15.95697	chr6:105(	TRNAI6		smallRNA	chr6:27631409-2763
ENSG0000(	638	15.95697	chr6:105(	RPLP2P1		Pseudoger	chr6:27965175-2796
ENSG0000(	638	15.95697	chr6:105(	ZKSCAN8P1		protein_c	chr6:28161769-2816
ENSG0000(	638	15.95697	chr6:105(	RPL8P1		Pseudoger	chr6:27652602-2765
ENSG0000(	638	15.95697	chr6:105(	ENSG00000291008		lncRNA	chr6:28091137-2809
ENSG0000(	638	15.95697	chr6:105(	H1-5	NCGv7	protein_c	chr6:27866792-2786
ENSG0000(	638	15.95697	chr6:105(	H2BC16P		Pseudoger	chr6:27864062-2786
ENSG0000(	638	15.95697	chr6:105(	OR2W4P		Pseudoger	chr6:27977150-2797
ENSG0000(	638	15.95697	chr6:105(	IQCB2P		Pseudoger	chr6:28010723-2801
ENSG0000(	638	15.95697	chr6:105(	H2AC13		protein_c	chr6:27808173-2780
ENSG0000(	638	15.95697	chr6:105(	ENSG00000280107		TEC	chr6:28170845-2817
ENSG0000(	638	15.95697	chr6:105(	ZNF602P		Pseudoger	chr6:28115628-2811
ENSG0000(	638	15.95697	chr6:105(	ZSCAN16		protein_c	chr6:28107689-2813
ENSG0000(	638	15.95697	chr6:105(	H3C10	NCGv7	protein_c	chr6:27810051-2781
ENSG0000(	638	15.95697	chr6:105(	LINC01012		lncRNA	chr6:27694026-2771
ENSG0000(	638	15.95697	chr6:105(	ZSCAN26		protein_c	chr6:28267058-2827
ENSG0000(	638	15.95697	chr6:105(	PGBD1		protein_c	chr6:28281572-2830
ENSG0000(	638	15.95697	chr6:105(	H3C12	NCGv7	protein_c	chr6:27890315-2789
ENSG0000(	638	15.95697	chr6:105(	ZSCAN9		protein_c	chr6:28224886-2823
ENSG0000(	638	15.95697	chr6:105(	H2AC14		protein_c	chr6:27814302-2781
ENSG0000(	638	15.95697	chr6:105(	GPR89P		Pseudoger	chr6:27737000-2773
ENSG0000(	638	15.95697	chr6:105(	H2AC15		protein_c	chr6:27837880-2783
ENSG0000(	638	15.95697	chr6:105(	ENSG00000289467		lncRNA	chr6:28335662-2833
ENSG0000(	638	15.95697	chr6:105(	ZNF603P		Pseudoger	chr6:28176188-2817
ENSG0000(	638	15.95697	chr6:105(	ZKSCAN4		protein_c	chr6:28241697-2825
ENSG0000(	638	15.95697	chr6:105(	ZSCAN23		protein_c	chr6:28431930-2844
ENSG0000(	638	15.95697	chr6:105(	ENSG00000287804		lncRNA	chr6:28476650-2848
ENSG0000(	638	15.95697	chr6:105(	OR2W6P		Pseudoger	chr6:27937465-2793
ENSG0000(	638	15.95697	chr6:105(	COX11P1		Pseudoger	chr6:28446973-2844
ENSG0000(	638	15.95697	chr6:105(	ZSCAN31	NCGv7	protein_c	chr6:28324693-2835
ENSG0000(	638	15.95697	chr6:105(	ENSG00000261839		lncRNA	chr6:28136849-2813
ENSG0000(	638	15.95697	chr6:105(	H2BC17	NCGv7	protein_c	chr6:27893425-2789
ENSG0000(	638	15.95697	chr6:105(	RNU6-471P		smallRNA	chr6:27596412-2759
ENSG0000(	638	15.95697	chr6:105(	H2AC16	NCGv7	protein_c	chr6:27865317-2786
ENSG0000(	638	15.95697	chr6:105(	ENSG00000273712		Pseudoger	chr6:28315613-2831
ENSG0000(	638	15.95697	chr6:105(	H2BC15		protein_c	chr6:27838545-2785
ENSG0000(	638	15.95697	chr6:105(	H2BC14		protein_c	chr6:27815022-2781
ENSG0000(	638	15.95697	chr6:105(	ENSG00000286819		lncRNA	chr6:28489579-2849
ENSG0000(	638	15.95697	chr6:105(	OR2B2		protein_c	chr6:27911185-2791

ENSG0000(	638	15.95697	chr6:105(	H4C12		protein_c	chr6:27831174-2783
ENSG0000(	638	15.95697	chr6:105(	GPX5	NCGv7	protein_c	chr6:28525881-2853
ENSG0000(	638	15.95697	chr6:105(	ZSCAN12		protein_c	chr6:28378955-2839
ENSG0000(	638	15.95697	chr6:105(	ENSG00000276302		protein_c	chr6:28267121-2828
ENSG0000(	638	15.95697	chr6:105(	U3		smallRNA	chr6:28015568-2801
ENSG0000(	638	15.95697	chr6:105(	H4C13		protein_c	chr6:27873148-2787
ENSG0000(	638	15.95697	chr6:105(	ZKSCAN8		protein_c	chr6:28141883-2815
ENSG0000(	638	15.95697	chr6:105(	OR2W2P		Pseudoger	chr6:28033947-2803
ENSG0000(	635	15.88194	chr4:909[illegible]	OR7E83P		Pseudoger	chr4:9512905-95138
ENSG0000(	635	15.88194	chr4:909[illegible]	Y_RNA		smallRNA	chr4:56818218-5681
ENSG0000(	635	15.88194	chr4:909[illegible]	USP17L30		protein_c	chr4:9363129-93647
ENSG0000(	635	15.88194	chr4:909[illegible]	ENSG00000250098		lncRNA	chr4:12947574-1294
ENSG0000(	635	15.88194	chr4:909[illegible]	snoU13		smallRNA	chr4:57106243-5710
ENSG0000(	635	15.88194	chr4:909[illegible]	ENSG00000251186		lncRNA	chr4:8453410-84549
ENSG0000(	635	15.88194	chr4:909[illegible]	ARL9		protein_c	chr4:56505209-5652
ENSG0000(	635	15.88194	chr4:909[illegible]	USP17L27		protein_c	chr4:9344148-93457
ENSG0000(	635	15.88194	chr4:909[illegible]	ADGRL3	NCGv7	protein_c	chr4:61200326-6207
ENSG0000(	635	15.88194	chr4:909[illegible]	RPL32P12		Pseudoger	chr4:13647697-1364
ENSG0000(	635	15.88194	chr4:909[illegible]	ENSG00000251412		lncRNA	chr4:14383123-1440
ENSG0000(	635	15.88194	chr4:909[illegible]	ENSG00000248262		lncRNA	chr4:11914667-1191
ENSG0000(	635	15.88194	chr4:909[illegible]	SNORA63		smallRNA	chr4:14690710-1469
ENSG0000(	635	15.88194	chr4:909[illegible]	ENSG00000250078		lncRNA	chr4:58562160-5856
ENSG0000(	635	15.88194	chr4:909[illegible]	DEFB131A		protein_c	chr4:9444414-94506
ENSG0000(	635	15.88194	chr4:909[illegible]	ENSG00000227040		Pseudoger	chr4:56760919-5676
ENSG0000(	635	15.88194	chr4:909[illegible]	ENSG00000250074		Pseudoger	chr4:10267895-1026
ENSG0000(	635	15.88194	chr4:909[illegible]	AFAP1		protein_c	chr4:7758714-79399
ENSG0000(	635	15.88194	chr4:909[illegible]	USP17L10		protein_c	chr4:9210657-92122
ENSG0000(	635	15.88194	chr4:909[illegible]	ENSG00000287164		lncRNA	chr4:7798527-78080
ENSG0000(	635	15.88194	chr4:909[illegible]	ENSG00000287174		lncRNA	chr4:59903404-5992
ENSG0000(	635	15.88194	chr4:909[illegible]	USP17L12		protein_c	chr4:9220152-92217
ENSG0000(	635	15.88194	chr4:909[illegible]	FAM86KP		Pseudoger	chr4:9153296-91654
ENSG0000(	635	15.88194	chr4:909[illegible]	LINC02360		lncRNA	chr4:11740948-1176
ENSG0000(	635	15.88194	chr4:909[illegible]	LINC02429		lncRNA	chr4:58984215-5904
ENSG0000(	635	15.88194	chr4:909[illegible]	ENSG00000214846		Pseudoger	chr4:15730962-1573
ENSG0000(	635	15.88194	chr4:909[illegible]	FCF1P8		Pseudoger	chr4:55351812-5535
ENSG0000(	635	15.88194	chr4:909[illegible]	CLNK		protein_c	chr4:10486395-1068
ENSG0000(	635	15.88194	chr4:909[illegible]	SLC2A9		protein_c	chr4:9771153-10054
ENSG0000(	635	15.88194	chr4:909[illegible]	CPZ		protein_c	chr4:8592660-86197
ENSG0000(	635	15.88194	chr4:909[illegible]	LINC01182		lncRNA	chr4:13654374-1400
ENSG0000(	635	15.88194	chr4:909[illegible]	LINC01097		lncRNA	chr4:13526319-1353
ENSG0000(	635	15.88194	chr4:909[illegible]	USP17L11		protein_c	chr4:9215405-92169
ENSG0000(	635	15.88194	chr4:909[illegible]	LINC01085		Pseudoger	chr4:14111968-1414
ENSG0000(	635	15.88194	chr4:909[illegible]	RNU6-998P		smallRNA	chr4:57002692-5700
ENSG0000(	635	15.88194	chr4:909[illegible]	RNU6-962P		smallRNA	chr4:13051342-1305
ENSG0000(	635	15.88194	chr4:909[illegible]	RN7SKP30		smallRNA	chr4:55540502-5554
ENSG0000(	635	15.88194	chr4:909[illegible]	ENSG00000250505		Pseudoger	chr4:10284961-1028
ENSG0000(	635	15.88194	chr4:909[illegible]	LINC02928		lncRNA	chr4:55373637-5538
ENSG0000(	635	15.88194	chr4:909[illegible]	NKX3-2		protein_c	chr4:13540830-1354
ENSG0000(	635	15.88194	chr4:909[illegible]	ENSG00000287117		lncRNA	chr4:9567474-96917
ENSG0000(	635	15.88194	chr4:909[illegible]	BST1		protein_c	chr4:15703065-1573
ENSG0000(	635	15.88194	chr4:909[illegible]	USP17L17		protein_c	chr4:9243879-92454

ENSG0000(	635	15.88194	chr4:909:	ENSG00000273267	lncRNA	chr4:8022665-80231
ENSG0000(	635	15.88194	chr4:909:	ENSG00000249105	Pseudoger	chr4:58117758-5811
ENSG0000(	635	15.88194	chr4:909:	OR7E85P	Pseudoger	chr4:9483718-94847
ENSG0000(	635	15.88194	chr4:909:	ENSG00000287154	lncRNA	chr4:10456745-1053
ENSG0000(	635	15.88194	chr4:909:	ENSG00000249111	lncRNA	chr4:59152834-5917
ENSG0000(	635	15.88194	chr4:909:	MIR572	smallRNA	chr4:11368827-1136
ENSG0000(	635	15.88194	chr4:909:	LINC02517	lncRNA	chr4:8320105-83271
ENSG0000(	635	15.88194	chr4:909:	EXOC1	protein_c	chr4:55853648-5590
ENSG0000(	635	15.88194	chr4:909:	RAF1P1	Pseudoger	chr4:10254926-1025
ENSG0000(	635	15.88194	chr4:909:	ENSG00000249148	Pseudoger	chr4:13631820-1363
ENSG0000(	635	15.88194	chr4:909:	ENSG00000286599	lncRNA	chr4:55889743-5590
ENSG0000(	635	15.88194	chr4:909:	MTND2P31	Pseudoger	chr4:14505865-1450
ENSG0000(	635	15.88194	chr4:909:	snoU13	smallRNA	chr4:57492269-5749
ENSG0000(	635	15.88194	chr4:909:	ENSG00000287382	lncRNA	chr4:55938153-5594
ENSG0000(	635	15.88194	chr4:909:	ENSG00000271544	Pseudoger	chr4:10238213-1023
ENSG0000(	635	15.88194	chr4:909:	RNU6-276P	smallRNA	chr4:55798636-5579
ENSG0000(	635	15.88194	chr4:909:	ENSG00000249392	lncRNA	chr4:59551142-5963
ENSG0000(	635	15.88194	chr4:909:	REST	protein_c	chr4:56907876-5696
ENSG0000(	635	15.88194	chr4:909:	SRP72	protein_c	chr4:56467617-5650
ENSG0000(	635	15.88194	chr4:909:	ENSG00000249930	Pseudoger	chr4:15492729-1549
ENSG0000(	635	15.88194	chr4:909:	ENSG00000290817	lncRNA	chr4:56410642-5642
ENSG0000(	635	15.88194	chr4:909:	CEP135	protein_c	chr4:55948871-5603
ENSG0000(	635	15.88194	chr4:909:	AC073648.1	smallRNA	chr4:9023333-90234
ENSG0000(	635	15.88194	chr4:909:	NOA1	protein_c	chr4:56963350-5697
ENSG0000(	635	15.88194	chr4:909:	USP17L20	protein_c	chr4:9258124-92597
ENSG0000(	635	15.88194	chr4:909:	SH3TC1	protein_c	chr4:8182072-82418
ENSG0000(	635	15.88194	chr4:909:	RNA5SP153	Pseudoger	chr4:9411178-94112
ENSG0000(	635	15.88194	chr4:909:	RNU6-578P	smallRNA	chr4:12312588-1231
ENSG0000(	635	15.88194	chr4:909:	USP17L29	protein_c	chr4:9353638-93552
ENSG0000(	635	15.88194	chr4:909:	ENSG00000228919	lncRNA	chr4:7939001-79402
ENSG0000(	635	15.88194	chr4:909:	USP17L18	protein_c	chr4:9248630-92502
ENSG0000(	635	15.88194	chr4:909:	ALG1L3P	Pseudoger	chr4:9703754-97108
ENSG0000(	635	15.88194	chr4:909:	ENSG00000287360	lncRNA	chr4:14359400-1445
ENSG0000(	635	15.88194	chr4:909:	ENSG00000286097	lncRNA	chr4:59150924-5918
ENSG0000(	635	15.88194	chr4:909:	AC095061.1	smallRNA	chr4:60663949-6066
ENSG0000(	635	15.88194	chr4:909:	RPL17P19	Pseudoger	chr4:61211652-6121
ENSG0000(	635	15.88194	chr4:909:	Y_RNA	smallRNA	chr4:59833175-5983
ENSG0000(	635	15.88194	chr4:909:	ENSG00000249347	Pseudoger	chr4:8990455-89908
ENSG0000(	635	15.88194	chr4:909:	OR7E111FP	Pseudoger	chr4:8981436-89823
ENSG0000(	635	15.88194	chr4:909:	AC098830.1	smallRNA	chr4:13658627-1365
ENSG0000(	635	15.88194	chr4:909:	EXOC1L	protein_c	chr4:55819790-5583
ENSG0000(	635	15.88194	chr4:909:	ENSG00000249334	lncRNA	chr4:10685003-1069
ENSG0000(	635	15.88194	chr4:909:	RPL17P20	Pseudoger	chr4:56710060-5671
ENSG0000(	635	15.88194	chr4:909:	ENSG00000249892	lncRNA	chr4:59767816-5979
ENSG0000(	635	15.88194	chr4:909:	LINC02619	lncRNA	chr4:58939288-5898
ENSG0000(	635	15.88194	chr4:909:	ENSG00000286093	lncRNA	chr4:56291601-5629
ENSG0000(	635	15.88194	chr4:909:	USP17L13	protein_c	chr4:9224896-92264
ENSG0000(	635	15.88194	chr4:909:	ENSG00000205959	lncRNA	chr4:8482270-85167
ENSG0000(	635	15.88194	chr4:909:	SRIP1	Pseudoger	chr4:58103147-5810
ENSG0000(	635	15.88194	chr4:909:	LINC02270	lncRNA	chr4:12223445-1225
ENSG0000(	635	15.88194	chr4:909:	GPR78	protein_c	chr4:8558725-86197

ENSG0000(	635	15.88194	chr4:909:	ENSG00000249219		lncRNA	chr4:9922814-99241
ENSG0000(	635	15.88194	chr4:909:	ECM1P2		Pseudoger	chr4:12639086-1264
ENSG0000(	635	15.88194	chr4:909:	ENSG00000288695		protein_c	chr4:55346228-5538
ENSG0000(	635	15.88194	chr4:909:	ENSG00000287369		lncRNA	chr4:57110547-5711
ENSG0000(	635	15.88194	chr4:909:	RNA5SP162		smallRNA	chr4:56331177-5633
ENSG0000(	635	15.88194	chr4:909:	ENSG00000249443		Pseudoger	chr4:9521910-95222
ENSG0000(	635	15.88194	chr4:909:	RPL7AP31		Pseudoger	chr4:56356135-5635
ENSG0000(	635	15.88194	chr4:909:	ENSG00000270147		lncRNA	chr4:56396312-5639
ENSG0000(	635	15.88194	chr4:909:	ENSG00000242034		Pseudoger	chr4:9553614-95539
ENSG0000(	635	15.88194	chr4:909:	Y_RNA		smallRNA	chr4:55501595-5550
ENSG0000(	635	15.88194	chr4:909:	CLOCK		protein_c	chr4:55427903-5554
ENSG0000(	635	15.88194	chr4:909:	snoU13		smallRNA	chr4:56994075-5699
ENSG0000(	635	15.88194	chr4:909:	POLR2B		protein_c	chr4:56977722-5703
ENSG0000(	635	15.88194	chr4:909:	ENSG00000251379		lncRNA	chr4:15358141-1542
ENSG0000(	635	15.88194	chr4:909:	TRMT44	NCGv7	protein_c	chr4:8436140-84935
ENSG0000(	635	15.88194	chr4:909:	ENSG00000219492		Pseudoger	chr4:9384017-93889
ENSG0000(	635	15.88194	chr4:909:	ENSG00000249988		lncRNA	chr4:14164455-1424
ENSG0000(	635	15.88194	chr4:909:	UNC93B8		Pseudoger	chr4:8959628-89639
ENSG0000(	635	15.88194	chr4:909:	C1QTNF7-AS1		lncRNA	chr4:15004942-1542
ENSG0000(	635	15.88194	chr4:909:	GLDCP1		Pseudoger	chr4:56593004-5659
ENSG0000(	635	15.88194	chr4:909:	ENSG00000248237		Pseudoger	chr4:60784115-6078
ENSG0000(	635	15.88194	chr4:909:	SRD5A3-AS1		lncRNA	chr4:55363971-5539
ENSG0000(	635	15.88194	chr4:909:	CPEB2-DT		lncRNA	chr4:14909961-1500
ENSG0000(	635	15.88194	chr4:909:	ENSG00000289865		lncRNA	chr4:10117089-1011
ENSG0000(	635	15.88194	chr4:909:	RNU6-1325P		smallRNA	chr4:59834063-5983
ENSG0000(	635	15.88194	chr4:909:	ENSG00000286297		lncRNA	chr4:13491185-1351
ENSG0000(	635	15.88194	chr4:909:	USP17L24		protein_c	chr4:9325165-93267
ENSG0000(	635	15.88194	chr4:909:	USP17L26		protein_c	chr4:9334658-93362
ENSG0000(	635	15.88194	chr4:909:	THEGL		protein_c	chr4:56530606-5660
ENSG0000(	635	15.88194	chr4:909:	RNU6-197P		smallRNA	chr4:56288485-5628
ENSG0000(	635	15.88194	chr4:909:	ENSG00000251049		lncRNA	chr4:57595940-5760
ENSG0000(	635	15.88194	chr4:909:	CC2D2A		protein_c	chr4:15469865-1560
ENSG0000(	635	15.88194	chr4:909:	ENSG00000285998		lncRNA	chr4:57841721-5785
ENSG0000(	635	15.88194	chr4:909:	ENSG00000250192		lncRNA	chr4:57154577-5715
ENSG0000(	635	15.88194	chr4:909:	ENSG00000250497		lncRNA	chr4:13777377-1378
ENSG0000(	635	15.88194	chr4:909:	ENSG00000251278		Pseudoger	chr4:10292195-1029
ENSG0000(	635	15.88194	chr4:909:	ENSG00000250393		Pseudoger	chr4:10143367-1014
ENSG0000(	635	15.88194	chr4:909:	CRACD	NCGv7	protein_c	chr4:56049098-5633
ENSG0000(	635	15.88194	chr4:909:	NMU		protein_c	chr4:55595229-5563
ENSG0000(	635	15.88194	chr4:909:	ENSG00000272969		lncRNA	chr4:55547112-5554
ENSG0000(	635	15.88194	chr4:909:	OR7E85BP		Pseudoger	chr4:8949630-89505
ENSG0000(	635	15.88194	chr4:909:	ENSG00000273133		lncRNA	chr4:15563698-1556
ENSG0000(	635	15.88194	chr4:909:	WDR1		protein_c	chr4:10068089-1011
ENSG0000(	635	15.88194	chr4:909:	RN7SKP170		smallRNA	chr4:15093767-1509
ENSG0000(	635	15.88194	chr4:909:	UBE2CP3		Pseudoger	chr4:57072683-5707
ENSG0000(	635	15.88194	chr4:909:	ENSG00000258507		lncRNA	chr4:8858715-88608
ENSG0000(	635	15.88194	chr4:909:	AC108519.1		smallRNA	chr4:9168229-91682
ENSG0000(	635	15.88194	chr4:909:	RPS3AP19		Pseudoger	chr4:9563171-95639
ENSG0000(	635	15.88194	chr4:909:	RNA5SP154		Pseudoger	chr4:9792751-97928
ENSG0000(	635	15.88194	chr4:909:	ENPP7P10		Pseudoger	chr4:9079023-91416
ENSG0000(	635	15.88194	chr4:909:	ENSG00000283043		Pseudoger	chr4:60038269-6003

ENSG0000(	635	15.88194	chr4:909(	ZNF518B		protein_c	chr4:10439880-1045
ENSG0000(	635	15.88194	chr4:909(	AC110611.1		smallRNA	chr4:55929982-5593
ENSG0000(	635	15.88194	chr4:909(	USP17L19		protein_c	chr4:9253378-92549
ENSG0000(	635	15.88194	chr4:909(	ENSG00000250375		lncRNA	chr4:59047020-5907
ENSG0000(	635	15.88194	chr4:909(	ENSG00000248419		Pseudoger	chr4:10478524-1047
ENSG0000(	635	15.88194	chr4:909(	LINC02271		lncRNA	chr4:61143656-6115
ENSG0000(	635	15.88194	chr4:909(	ENSG00000251338		Pseudoger	chr4:10294463-1029
ENSG0000(	635	15.88194	chr4:909(	USP17L22		protein_c	chr4:9267619-92692
ENSG0000(	635	15.88194	chr4:909(	AASDH		protein_c	chr4:56338287-5638
ENSG0000(	635	15.88194	chr4:909(	RNU6-652P		smallRNA	chr4:55885595-5588
ENSG0000(	635	15.88194	chr4:909(	MIR3138		smallRNA	chr4:10078611-1007
ENSG0000(	635	15.88194	chr4:909(	DEFB108F		Pseudoger	chr4:9399204-94035
ENSG0000(	635	15.88194	chr4:909(	ENSG00000250371		lncRNA	chr4:12859101-1286
ENSG0000(	635	15.88194	chr4:909(	BOD1L1		protein_c	chr4:13568738-1362
ENSG0000(	635	15.88194	chr4:909(	MIR548AG1		smallRNA	chr4:60922619-6092
ENSG0000(	635	15.88194	chr4:909(	ENSG00000251296		Pseudoger	chr4:10167159-1016
ENSG0000(	635	15.88194	chr4:909(	FAM200B		protein_c	chr4:15681662-1569
ENSG0000(	635	15.88194	chr4:909(	ENSG00000248425		lncRNA	chr4:14390439-1439
ENSG0000(	635	15.88194	chr4:909(	LINC01096		lncRNA	chr4:13546075-1354
ENSG0000(	635	15.88194	chr5:103(	AC093267.1		smallRNA	chr5:122776431-122
ENSG0000(	635	15.88194	chr4:909(	RN7SL357P		smallRNA	chr4:56805834-5680
ENSG0000(	635	15.88194	chr4:909(	LINC02494		lncRNA	chr4:58524515-5853
ENSG0000(	635	15.88194	chr4:909(	AC097493.1		smallRNA	chr4:9600328-96004
ENSG0000(	635	15.88194	chr4:909(	ACOX3		protein_c	chr4:8366282-84407
ENSG0000(	635	15.88194	chr4:909(	DEFB130D		Pseudoger	chr4:9422313-94297
ENSG0000(	635	15.88194	chr4:909(	ENSG00000251313		Pseudoger	chr4:9030286-90310
ENSG0000(	635	15.88194	chr4:909(	ENSG00000287972		lncRNA	chr4:9076949-90925
ENSG0000(	635	15.88194	chr4:909(	GMPSP1		Pseudoger	chr4:8174421-81749
ENSG0000(	635	15.88194	chr4:909(	PAICS		protein_c	chr4:56435741-5646
ENSG0000(	635	15.88194	chr4:909(	MIR548I2		smallRNA	chr4:9556168-95563
ENSG0000(	635	15.88194	chr4:909(	ENSG00000288944		lncRNA	chr4:56701545-5670
ENSG0000(	635	15.88194	chr4:909(	CPEB2	NCGv7	protein_c	chr4:15002481-1507
ENSG0000(	635	15.88194	chr4:909(	DRD5	NCGv7	protein_c	chr4:9781634-97840
ENSG0000(	635	15.88194	chr4:909(	PDCL2		protein_c	chr4:55556519-5559
ENSG0000(	635	15.88194	chr4:909(	ENSG00000284636		Pseudoger	chr4:9000841-90010
ENSG0000(	635	15.88194	chr4:909(	PPAT		protein_c	chr4:56393362-5643
ENSG0000(	635	15.88194	chr4:909(	RPS26P24		Pseudoger	chr4:57352999-5735
ENSG0000(	635	15.88194	chr4:909(	ENSG00000250915		lncRNA	chr4:8745391-87477
ENSG0000(	635	15.88194	chr4:909(	SPINK2		protein_c	chr4:56809860-5682
ENSG0000(	635	15.88194	chr4:909(	ENSG00000288951		lncRNA	chr4:13764787-1384
ENSG0000(	635	15.88194	chr4:909(	ENSG00000283156		Pseudoger	chr4:56410516-5643
ENSG0000(	635	15.88194	chr4:909(	C1QTNF7		protein_c	chr4:15339818-1544
ENSG0000(	635	15.88194	chr4:909(	HS3ST1		protein_c	chr4:11393150-1142
ENSG0000(	635	15.88194	chr4:909(	SLC2A9-AS1		lncRNA	chr4:10006482-1000
ENSG0000(	635	15.88194	chr4:909(	ENSG00000289393		lncRNA	chr4:56466900-5646
ENSG0000(	635	15.88194	chr4:909(	IGFBP7		protein_c	chr4:57030773-5711
ENSG0000(	635	15.88194	chr4:909(	ENSG00000248777		Pseudoger	chr4:10199823-1020
ENSG0000(	635	15.88194	chr4:909(	LINC02498		lncRNA	chr4:10737558-1074
ENSG0000(	635	15.88194	chr4:909(	OR7E35P		Pseudoger	chr4:9754898-97557
ENSG0000(	635	15.88194	chr4:909(	ENSG00000284648		Pseudoger	chr4:9532429-95326
ENSG0000(	635	15.88194	chr4:909(	SRD5A3		protein_c	chr4:55346213-5537

ENSG0000(	635	15.88194	chr4:909:	RN7SL492P		smallRNA	chr4:56794350-5679
ENSG0000(	635	15.88194	chr4:909:	USP17L23		protein_c	chr4:9272364-92729
ENSG0000(	635	15.88194	chr4:909:	RAB28		protein_c	chr4:13361354-1348
ENSG0000(	635	15.88194	chr4:909:	HTRA3		protein_c	chr4:8269754-83070
ENSG0000(	635	15.88194	chr4:909:	OR7E84P		Pseudoger	chr4:9468974-94699
ENSG0000(	635	15.88194	chr4:909:	SNRPCP16		Pseudoger	chr4:9051842-90520
ENSG0000(	635	15.88194	chr4:909:	MIR95		smallRNA	chr4:8005301-80053
ENSG0000(	635	15.88194	chr4:909:	ALG1L14P		Pseudoger	chr4:9166297-91702
ENSG0000(	635	15.88194	chr4:909:	ENSG00000250613		Pseudoger	chr4:10410996-1041
ENSG0000(	635	15.88194	chr4:909:	LINC02496		lncRNA	chr4:60750575-6079
ENSG0000(	635	15.88194	chr4:909:	MIR5091		smallRNA	chr4:13627865-1362
ENSG0000(	635	15.88194	chr4:909:	USP17L5		protein_c	chr4:9339403-93409
ENSG0000(	635	15.88194	chr4:909:	ENSG00000251679		lncRNA	chr4:14134936-1414
ENSG0000(	635	15.88194	chr4:909:	FAM86MP		Pseudoger	chr4:9692495-97029
ENSG0000(	635	15.88194	chr4:909:	USP17L15		protein_c	chr4:9234385-92360
ENSG0000(	635	15.88194	chr4:909:	AFAP1-AS1		lncRNA	chr4:7754077-77789
ENSG0000(	635	15.88194	chr4:909:	LINC00504		lncRNA	chr4:14470465-1488
ENSG0000(	635	15.88194	chr4:909:	ENSG00000287778		lncRNA	chr4:11426874-1146
ENSG0000(	635	15.88194	chr4:909:	LINC02380		lncRNA	chr4:57424495-5747
ENSG0000(	635	15.88194	chr4:909:	ENSG00000269921		lncRNA	chr4:56387625-5638
ENSG0000(	635	15.88194	chr4:909:	Y_RNA		smallRNA	chr4:59533786-5953
ENSG0000(	635	15.88194	chr4:909:	ENSG00000249631		lncRNA	chr4:11625651-1182
ENSG0000(	635	15.88194	chr4:909:	USP17L21		protein_c	chr4:9262872-92644
ENSG0000(	635	15.88194	chr4:909:	Y_RNA		smallRNA	chr4:55412636-5541
ENSG0000(	635	15.88194	chr4:909:	FAM90A26		protein_c	chr4:9170409-91767
ENSG0000(	635	15.88194	chr4:909:	RNA5SP156		Pseudoger	chr4:11414051-1141
ENSG0000(	635	15.88194	chr4:909:	AC097452.1		smallRNA	chr4:12013347-1201
ENSG0000(	635	15.88194	chr4:909:	FBXL5		protein_c	chr4:15604381-1568
ENSG0000(	635	15.88194	chr4:909:	RNA5SP155		Pseudoger	chr4:10115756-1011
ENSG0000(	635	15.88194	chr4:909:	USP17L16P		Pseudoger	chr4:9239130-92407
ENSG0000(	635	15.88194	chr4:909:	ENSG00000288606		lncRNA	chr4:15681756-1570
ENSG0000(	635	15.88194	chr4:909:	RNA5SP161		Pseudoger	chr4:56097390-5609
ENSG0000(	635	15.88194	chr4:909:	ENSG00000269949		lncRNA	chr4:56960927-5696
ENSG0000(	635	15.88194	chr4:909:	ENSG00000280285		TEC	chr4:56662469-5666
ENSG0000(	635	15.88194	chr4:909:	ENSG00000249831		lncRNA	chr4:57720035-5772
ENSG0000(	635	15.88194	chr4:909:	USP17L14P		Pseudoger	chr4:9229641-92312
ENSG0000(	635	15.88194	chr4:909:	TMEM165	NCGv7	protein_c	chr4:55395957-5545
ENSG0000(	635	15.88194	chr4:909:	USP17L9P		Pseudoger	chr4:9358383-93599
ENSG0000(	635	15.88194	chr4:909:	ENSG00000205682		lncRNA	chr4:61420246-6142
ENSG0000(	635	15.88194	chr4:909:	HOPX		protein_c	chr4:56647988-5668
ENSG0000(	635	15.88194	chr4:909:	USP17L6P		Pseudoger	chr4:9367874-93690
ENSG0000(	635	15.88194	chr4:909:	ENSG00000248188		Pseudoger	chr4:15743196-1574
ENSG0000(	635	15.88194	chr4:909:	USP17L28		protein_c	chr4:9348893-93504
ENSG0000(	635	15.88194	chr4:909:	ENSG00000271057		Pseudoger	chr4:9020857-90211
ENSG0000(	635	15.88194	chr4:909:	ENSG00000251460		lncRNA	chr4:8066528-80677
ENSG0000(	635	15.88194	chr4:909:	ENSG00000249780		Pseudoger	chr4:12640298-1264
ENSG0000(	635	15.88194	chr4:909:	RNA5SP152		Pseudoger	chr4:8393015-83931
ENSG0000(	635	15.88194	chr4:909:	MRPL22P1		Pseudoger	chr4:56320719-5632
ENSG0000(	635	15.88194	chr4:909:	ENSG00000290015		lncRNA	chr4:8159049-81601
ENSG0000(	635	15.88194	chr4:909:	ABLIM2	NCGv7	protein_c	chr4:7965310-81588
ENSG0000(	635	15.88194	chr4:909:	ENSG00000251615		lncRNA	chr4:8355090-83583

ENSG0000(	635	15.88194	chr4:909:	USP17L25		protein_c	chr4:9329911-93315
ENSG0000(	635	15.88194	chr4:909:	ENSG00000251459		lncRNA	chr4:57490808-5749
ENSG0000(	635	15.88194	chr4:909:	ENSG00000251152		lncRNA	chr4:11469250-1147
ENSG0000(	635	15.88194	chr4:909:	ENSG00000224569		Pseudoger	chr4:13977166-1397
ENSG0000(	635	15.88194	chr4:909:	ENSG00000250573		Pseudoger	chr4:10259445-1026
ENSG0000(	635	15.88194	chr4:909:	ENSG00000250333		lncRNA	chr4:57605694-5765
ENSG0000(	635	15.88194	chr4:909:	OR7E86P		Pseudoger	chr4:9459255-94602
ENSG0000(	635	15.88194	chr4:909:	UNC93B7		Pseudoger	chr4:9493736-94980
ENSG0000(	635	15.88194	chr4:909:	RNPS1P1		Pseudoger	chr4:11371975-1137
ENSG0000(	635	15.88194	chr4:909:	HSP90AB2P		Pseudoger	chr4:13333414-1333
ENSG0000(	635	15.88194	chr4:909:	IGFBP7-AS1		lncRNA	chr4:57109762-5720
ENSG0000(	635	15.88194	chr4:909:	EVA1CP1		Pseudoger	chr4:9733370-97423
ENSG0000(	635	15.88194	chr4:909:	HMX1		protein_c	chr4:8846076-88718
ENSG0000(	632	15.80691	chr1:245(	ENSG00000238224		lncRNA	chr1:245614773-245
ENSG0000(	630	15.75689	chr5:103(	RNU6-752P		smallRNA	chr5:126755316-126
ENSG0000(	629	15.73187	chr5:169(	SNORA50		smallRNA	chr5:69160806-6916
ENSG0000(	618	15.45675	chr5:169(	SNORA47		smallRNA	chr5:77080434-7708
ENSG0000(	615	15.38172	chr1:373:	snoU13		smallRNA	chr1:202231664-202
ENSG0000(	613	15.3317	chr5:169(	PMCHL2		lncRNA	chr5:71375786-7138
ENSG0000(	613	15.3317	chr5:169(	GUSBP16		Pseudoger	chr5:70751184-7079
ENSG0000(	613	15.3317	chr5:169(	OCLNP1		Pseudoger	chr5:71074225-7109
ENSG0000(	613	15.3317	chr5:169(	ENSG00000249588		lncRNA	chr5:68523878-6853
ENSG0000(	613	15.3317	chr5:169(	ENSG00000253333		Pseudoger	chr5:70495100-7050
ENSG0000(	613	15.3317	chr5:169(	RPL7P22		Pseudoger	chr5:72725419-7272
ENSG0000(	613	15.3317	chr5:169(	GUSBP14		Pseudoger	chr5:70219918-7025
ENSG0000(	613	15.3317	chr5:169(	RNU6-1232P		smallRNA	chr5:68159061-6815
ENSG0000(	613	15.3317	chr5:169(	GTF2H2B		Pseudoger	chr5:70415352-7044
ENSG0000(	613	15.3317	chr5:169(	CDH12P3		Pseudoger	chr5:70132679-7013
ENSG0000(	613	15.3317	chr5:169(	MRPS27		protein_c	chr5:72214953-7232
ENSG0000(	613	15.3317	chr5:169(	HMGN1P12		Pseudoger	chr5:71537652-7153
ENSG0000(	613	15.3317	chr5:169(	MIR4803		smallRNA	chr5:72169467-7216
ENSG0000(	613	15.3317	chr5:169(	LINC02219		lncRNA	chr5:68189876-6819
ENSG0000(	613	15.3317	chr5:169(	NAIP		protein_c	chr5:70968166-7102
ENSG0000(	613	15.3317	chr5:169(	NDUFB9P1		Pseudoger	chr5:69349936-6935
ENSG0000(	613	15.3317	chr5:169(	GUSBP17		Pseudoger	chr5:71220356-7125
ENSG0000(	613	15.3317	chr5:169(	ENSG00000251158		Pseudoger	chr5:69898867-6990
ENSG0000(	613	15.3317	chr5:169(	YBX1P5		Pseudoger	chr5:72417489-7241
ENSG0000(	613	15.3317	chr5:169(	AC145141.2		smallRNA	chr5:71387276-7138
ENSG0000(	613	15.3317	chr5:169(	LINC02198		lncRNA	chr5:68970692-6903
ENSG0000(	613	15.3317	chr5:169(	ENSG00000253536		Pseudoger	chr5:71475761-7147
ENSG0000(	613	15.3317	chr5:169(	PMCHL2		Pseudoger	chr5:71375830-7137
ENSG0000(	613	15.3317	chr5:169(	CHCHD2P2		Pseudoger	chr5:69333929-6933
ENSG0000(	613	15.3317	chr5:169(	H2BL1P		Pseudoger	chr5:72733833-7273
ENSG0000(	613	15.3317	chr5:169(	TNPO1	NCGv7	protein_c	chr5:72816312-7291
ENSG0000(	613	15.3317	chr5:169(	ENSG00000289810		lncRNA	chr5:71032670-7103
ENSG0000(	613	15.3317	chr5:169(	ENSG00000285204		lncRNA	chr5:70931244-7093
ENSG0000(	613	15.3317	chr5:169(	RP11-1198D22.2		Pseudoger	chr5:71233327-7123
ENSG0000(	613	15.3317	chr5:169(	RN7SL103P		smallRNA	chr5:69160036-6916
ENSG0000(	613	15.3317	chr5:169(	SMN2		protein_c	chr5:70049638-7007
ENSG0000(	613	15.3317	chr5:169(	SERF1B		protein_c	chr5:70025247-7004
ENSG0000(	613	15.3317	chr5:169(	ENSG00000248884		lncRNA	chr5:68430339-6843

ENSG0000(	613	15.3317	chr5:169(	NAIPP2		Pseudoger	chr5:70094659-7012
ENSG0000(	613	15.3317	chr5:169(	ZNF366		protein_c	chr5:72439903-7250
ENSG0000(	613	15.3317	chr5:169(	RP11-1198D22.3		Pseudoger	chr5:71215306-7121
ENSG0000(	613	15.3317	chr5:169(	LINC02197		lncRNA	chr5:71337182-7144
ENSG0000(	613	15.3317	chr5:169(	CHP1P1		Pseudoger	chr5:73020700-7302
ENSG0000(	613	15.3317	chr5:169(	ENSG00000285151		lncRNA	chr5:70055820-7005
ENSG0000(	613	15.3317	chr5:169(	GUSBP9		Pseudoger	chr5:71197646-7120
ENSG0000(	613	15.3317	chr5:169(	CCDC125		protein_c	chr5:69280175-6933
ENSG0000(	613	15.3317	chr5:169(	ENSG00000269983		lncRNA	chr5:70449636-7045
ENSG0000(	613	15.3317	chr5:169(	ENSG00000251613		lncRNA	chr5:72687112-7277
ENSG0000(	613	15.3317	chr5:169(	GTF2H2C		protein_c	chr5:69560191-6959
ENSG0000(	613	15.3317	chr5:169(	LINC02056		lncRNA	chr5:72574120-7266
ENSG0000(	613	15.3317	chr5:169(	NAIPP4		Pseudoger	chr5:71102898-7112
ENSG0000(	613	15.3317	chr5:169(	CARTPT		protein_c	chr5:71719275-7172
ENSG0000(	613	15.3317	chr5:169(	PTCD2		protein_c	chr5:72320367-7236
ENSG0000(	613	15.3317	chr5:169(	ENSG00000248359		lncRNA	chr5:68508223-6856
ENSG0000(	613	15.3317	chr5:169(	RPL35AP13		Pseudoger	chr5:72878868-7287
ENSG0000(	613	15.3317	chr5:169(	CTC-498J12.1		lncRNA	chr5:69038518-6904
ENSG0000(	613	15.3317	chr5:169(	snoU13		smallRNA	chr5:69175824-6917
ENSG0000(	613	15.3317	chr5:169(	EEF1B2P2		Pseudoger	chr5:68159218-6815
ENSG0000(	613	15.3317	chr5:169(	RP11-1415C14.1		Pseudoger	chr5:70214942-7021
ENSG0000(	613	15.3317	chr5:169(	OCLN		protein_c	chr5:69492292-6955
ENSG0000(	613	15.3317	chr5:169(	RP11-497H16.4		Pseudoger	chr5:70529371-7053
ENSG0000(	613	15.3317	chr5:169(	MRPS36		protein_c	chr5:69217760-6923
ENSG0000(	613	15.3317	chr5:169(	CCNB1	AC	protein_c	chr5:69167135-6917
ENSG0000(	613	15.3317	chr5:169(	GUSBP13		Pseudoger	chr5:69875271-6992
ENSG0000(	613	15.3317	chr5:169(	ENSG00000248664		lncRNA	chr5:69113109-6916
ENSG0000(	613	15.3317	chr5:169(	NAIPP1		Pseudoger	chr5:70473448-7047
ENSG0000(	613	15.3317	chr5:169(	RP11-1415C14.2		Pseudoger	chr5:70232992-7023
ENSG0000(	613	15.3317	chr5:169(	ENSG00000291221		lncRNA	chr5:70462244-7047
ENSG0000(	613	15.3317	chr5:169(	MIR4804		smallRNA	chr5:72878591-7287
ENSG0000(	613	15.3317	chr5:169(	TAF9		protein_c	chr5:69362026-6937
ENSG0000(	613	15.3317	chr5:169(	RPS27P14		Pseudoger	chr5:69469883-6947
ENSG0000(	613	15.3317	chr5:169(	RN7SL153P		smallRNA	chr5:72314399-7231
ENSG0000(	613	15.3317	chr5:169(	ENSG00000291220		lncRNA	chr5:70415396-7044
ENSG0000(	613	15.3317	chr5:169(	AK6		protein_c	chr5:69350984-6937
ENSG0000(	613	15.3317	chr5:169(	MARVELD2		protein_c	chr5:69415065-6944
ENSG0000(	613	15.3317	chr5:169(	RAD17	NCGv7	protein_c	chr5:69369293-6941
ENSG0000(	613	15.3317	chr5:169(	RNU6-724P		smallRNA	chr5:69530613-6953
ENSG0000(	613	15.3317	chr5:169(	U8		smallRNA	chr5:68873954-6887
ENSG0000(	613	15.3317	chr5:169(	NAIPP3		Pseudoger	chr5:69618313-6962
ENSG0000(	613	15.3317	chr5:169(	ENSG00000251467		Pseudoger	chr5:72996920-7299
ENSG0000(	613	15.3317	chr5:169(	CDH12P1		Pseudoger	chr5:70860285-7086
ENSG0000(	613	15.3317	chr5:169(	ENSG00000244061		Pseudoger	chr5:72381794-7238
ENSG0000(	613	15.3317	chr5:169(	ENSG00000280187		TEC	chr5:69186359-6918
ENSG0000(	613	15.3317	chr5:169(	RN7SL476P		smallRNA	chr5:69455575-6945
ENSG0000(	613	15.3317	chr5:169(	CENPH		protein_c	chr5:69189574-6921
ENSG0000(	613	15.3317	chr5:169(	ENSG00000248769		Pseudoger	chr5:69653248-6965
ENSG0000(	613	15.3317	chr5:169(	RP11-497H16.6		Pseudoger	chr5:70508079-7051
ENSG0000(	613	15.3317	chr5:169(	CDK7		protein_c	chr5:69234795-6927
ENSG0000(	613	15.3317	chr5:169(	TNPO1-DT		lncRNA	chr5:72794405-7281

ENSG0000(	613	15.3317	chr5:1696	VWA8P1		Pseudoger	chr5:68854910-6885
ENSG0000(	613	15.3317	chr5:1696	Y_RNA		smallRNA	chr5:72768702-7276
ENSG0000(	613	15.3317	chr5:1696	ENSG00000290560		lncRNA	chr5:69639459-6967
ENSG0000(	613	15.3317	chr5:1696	SUMO2P4		Pseudoger	chr5:69068925-6906
ENSG0000(	613	15.3317	chr5:1696	RN7SL9P		smallRNA	chr5:70074846-7007
ENSG0000(	613	15.3317	chr5:1696	ENSG00000254701		Pseudoger	chr5:70197255-7020
ENSG0000(	613	15.3317	chr5:1696	ENSG00000250066		lncRNA	chr5:68963246-6896
ENSG0000(	613	15.3317	chr5:1696	ENSG00000288349		Pseudoger	chr5:70775506-7077
ENSG0000(	613	15.3317	chr5:1696	CDH12P2		Pseudoger	chr5:69985204-6998
ENSG0000(	613	15.3317	chr5:1696	FCHO2-DT		lncRNA	chr5:72955206-7295
ENSG0000(	613	15.3317	chr5:1696	MCCC2		protein_c	chr5:71579531-7165
ENSG0000(	613	15.3317	chr5:1696	ENSG00000290556		lncRNA	chr5:69607099-6962
ENSG0000(	613	15.3317	chr5:1696	GUSBP15		Pseudoger	chr5:70516387-7055
ENSG0000(	613	15.3317	chr5:1696	ENSG00000249981		lncRNA	chr5:71445616-7144
ENSG0000(	613	15.3317	chr5:1696	PIK3R1	NCGv7;AC	protein_c	chr5:68215740-6830
ENSG0000(	613	15.3317	chr5:1696	SERF1A		protein_c	chr5:70900669-7091
ENSG0000(	613	15.3317	chr5:1696	GUSBP3		Pseudoger	chr5:69640266-6967
ENSG0000(	613	15.3317	chr5:1696	SMN1		protein_c	chr5:70925030-7095
ENSG0000(	613	15.3317	chr5:1696	ENSG00000253985		lncRNA	chr5:71372676-7137
ENSG0000(	613	15.3317	chr5:1696	MAP1B	DriverDBv	protein_c	chr5:72107234-7220
ENSG0000(	613	15.3317	chr5:1696	CDH12P4		Pseudoger	chr5:71132993-7113
ENSG0000(	613	15.3317	chr5:1696	ENSG00000249295		lncRNA	chr5:69477472-6950
ENSG0000(	613	15.3317	chr5:1696	snoU13		smallRNA	chr5:69539161-6953
ENSG0000(	613	15.3317	chr5:1696	RP11-497H16.2		Pseudoger	chr5:70487602-7049
ENSG0000(	613	15.3317	chr5:1696	ENSG00000249335		lncRNA	chr5:68792609-6904
ENSG0000(	613	15.3317	chr5:1696	BDP1		protein_c	chr5:71455651-7156
ENSG0000(	613	15.3317	chr5:1696	GTF2H2		protein_c	chr5:71035016-7106
ENSG0000(	613	15.3317	chr5:1696	SLC30A5		protein_c	chr5:69093991-6913
ENSG0000(	613	15.3317	chr5:1696	ENSG00000285804		lncRNA	chr5:72087782-7210
ENSG0000(	613	15.3317	chr5:1696	ENSG00000250138		Pseudoger	chr5:69631963-6963
ENSG0000(	613	15.3317	chr5:1696	RN7SL616P		smallRNA	chr5:69478993-6947
ENSG0000(	613	15.3317	chr5:1696	AC143336.1		smallRNA	chr5:71614984-7161
ENSG0000(	613	15.3317	chr5:1696	ENSG00000278824		Pseudoger	chr5:71754378-7175
ENSG0000(	613	15.3317	chr5:1696	snoU13		smallRNA	chr5:71088632-7108
ENSG0000(	613	15.3317	chr5:1696	CFL1P5		Pseudoger	chr5:69313371-6931
ENSG0000(	613	15.3317	chr5:1696	ENSG00000261269		lncRNA	chr5:72439903-7244
ENSG0000(	607	15.18163	chr4:9093	SCARNA22		smallRNA	chr4:1974636-19747
ENSG0000(	601	15.03157	chr4:9093	NAT8L	NCGv7	protein_c	chr4:2059327-20690
ENSG0000(	601	15.03157	chr4:9093	IDUA		protein_c	chr4:986997-100456
ENSG0000(	601	15.03157	chr4:9093	STX18-IT1		lncRNA	chr4:4476121-44817
ENSG0000(	601	15.03157	chr4:9093	ENSG00000253917		lncRNA	chr4:3915183-39554
ENSG0000(	601	15.03157	chr4:9093	HGFAC		protein_c	chr4:3441968-34494
ENSG0000(	601	15.03157	chr4:9093	DGKQ		protein_c	chr4:958887-986895
ENSG0000(	601	15.03157	chr4:9093	EVA1CP2		Pseudoger	chr4:3915187-39242
ENSG0000(	601	15.03157	chr4:9093	FGFRL1		protein_c	chr4:1009936-10268
ENSG0000(	601	15.03157	chr4:9093	TMED11P		Pseudoger	chr4:1115197-11537
ENSG0000(	601	15.03157	chr4:9093	COX6B1P5		Pseudoger	chr4:2234251-22344
ENSG0000(	601	15.03157	chr4:9093	TMEM175		protein_c	chr4:932387-958656
ENSG0000(	601	15.03157	chr4:9093	TACC3		protein_c	chr4:1712858-17451
ENSG0000(	601	15.03157	chr4:9093	ENSG00000248516		lncRNA	chr4:4321962-43341
ENSG0000(	601	15.03157	chr4:9093	NELFA		protein_c	chr4:1982717-20419

ENSG0000(	601	15.03157	chr4:909:	NSD2	NCGv7;AC	protein_c	chr4:1871393-19822
ENSG0000(	601	15.03157	chr4:909:	ENSG00000249077		Pseudoger	chr4:2324467-23250
ENSG0000(	601	15.03157	chr4:909:	FAM86EP		lncRNA	chr4:3941760-39554
ENSG0000(	601	15.03157	chr4:909:	FAM193A		protein_c	chr4:2536647-27325
ENSG0000(	601	15.03157	chr4:909:	SLC26A1		protein_c	chr4:979073-993440
ENSG0000(	601	15.03157	chr4:909:	OR7E162P		Pseudoger	chr4:3901566-39025
ENSG0000(	601	15.03157	chr4:909:	GRK4		protein_c	chr4:2963571-30407
ENSG0000(	601	15.03157	chr4:909:	LYAR		protein_c	chr4:4267701-42901
ENSG0000(	601	15.03157	chr4:909:	ENSG00000290180		protein_c	chr4:2512477-26253
ENSG0000(	601	15.03157	chr4:909:	ENSG00000248669		Pseudoger	chr4:4117925-41182
ENSG0000(	601	15.03157	chr4:909:	RGS12	NCGv7	protein_c	chr4:3293021-34399
ENSG0000(	601	15.03157	chr4:909:	MIR943		smallRNA	chr4:1986384-19864
ENSG0000(	601	15.03157	chr4:909:	ENSG00000244459		lncRNA	chr4:1574055-15805
ENSG0000(	601	15.03157	chr4:909:	HAUS3		protein_c	chr4:2227464-22421
ENSG0000(	601	15.03157	chr4:909:	MFSD10	NCGv7	protein_c	chr4:2930561-29348
ENSG0000(	601	15.03157	chr4:909:	CTBP1		protein_c	chr4:1211445-12503
ENSG0000(	601	15.03157	chr4:909:	ENSG00000249522		Pseudoger	chr4:3910283-39106
ENSG0000(	601	15.03157	chr4:909:	ADRA2C		protein_c	chr4:3766348-37685
ENSG0000(	601	15.03157	chr4:909:	ENSG00000249006		Pseudoger	chr4:2088718-20906
ENSG0000(	601	15.03157	chr4:909:	OTOP1	NCGv7	protein_c	chr4:4188726-42269
ENSG0000(	601	15.03157	chr4:909:	LRPAP1	DriverDB\	protein_c	chr4:3503612-35324
ENSG0000(	601	15.03157	chr4:909:	SPON2		protein_c	chr4:1166932-12089
ENSG0000(	601	15.03157	chr4:909:	ENSG00000248399		lncRNA	chr4:2463797-24641
ENSG0000(	601	15.03157	chr4:909:	ENSG00000248155		Pseudoger	chr4:2607870-26081
ENSG0000(	601	15.03157	chr4:909:	ENSG00000290263		protein_c	chr4:2078998-22422
ENSG0000(	601	15.03157	chr4:909:	SLBP	DriverDB\	protein_c	chr4:1692731-17123
ENSG0000(	601	15.03157	chr4:909:	RNF212	DriverDB\	protein_c	chr4:1056250-11135
ENSG0000(	601	15.03157	chr4:909:	DOK7		protein_c	chr4:3463306-35014
ENSG0000(	601	15.03157	chr4:909:	ENSG00000284727		Pseudoger	chr4:4107280-41074
ENSG0000(	601	15.03157	chr4:909:	CTBP1-AS		lncRNA	chr4:1210120-12185
ENSG0000(	601	15.03157	chr4:909:	UVSSA		protein_c	chr4:1345691-13959
ENSG0000(	601	15.03157	chr4:909:	AL132868.1		Pseudoger	chr4:1960041-19603
ENSG0000(	601	15.03157	chr4:909:	ENSG00000254094		lncRNA	chr4:1356581-13580
ENSG0000(	601	15.03157	chr4:909:	ZFYVE28		protein_c	chr4:2269582-24186
ENSG0000(	601	15.03157	chr4:909:	MIR4800		smallRNA	chr4:2250077-22501
ENSG0000(	601	15.03157	chr4:909:	FAM53A		protein_c	chr4:1617915-16843
ENSG0000(	601	15.03157	chr4:909:	SNORA48		smallRNA	chr4:1118884-11190
ENSG0000(	601	15.03157	chr4:909:	ENSG00000272783		lncRNA	chr4:1550284-15505
ENSG0000(	601	15.03157	chr4:909:	RPL7AP29		Pseudoger	chr4:3323977-33247
ENSG0000(	601	15.03157	chr4:909:	ENSG00000248840		Pseudoger	chr4:3312512-33130
ENSG0000(	601	15.03157	chr4:909:	MXD4		protein_c	chr4:2247432-22621
ENSG0000(	601	15.03157	chr4:909:	ENSG00000227189		lncRNA	chr4:1151372-11537
ENSG0000(	601	15.03157	chr4:909:	RPS3AP16		Pseudoger	chr4:4076711-40774
ENSG0000(	601	15.03157	chr4:909:	Y_RNA		smallRNA	chr4:2615124-26152
ENSG0000(	601	15.03157	chr4:909:	ENSG00000253399		lncRNA	chr4:1358479-13594
ENSG0000(	601	15.03157	chr4:909:	C4orf48		protein_c	chr4:2041995-20439
ENSG0000(	601	15.03157	chr4:909:	NOP14-AS1		lncRNA	chr4:2934882-29617
ENSG0000(	601	15.03157	chr4:909:	AL590235.1		protein_c	chr4:3509796-35099
ENSG0000(	601	15.03157	chr4:909:	OR7E99P		Pseudoger	chr4:4156522-41574
ENSG0000(	601	15.03157	chr4:909:	SH3BP2		protein_c	chr4:2793071-28410
ENSG0000(	601	15.03157	chr4:909:	Y_RNA		smallRNA	chr4:1683420-16835

ENSG0000(	601	15.03157	chr4:9093	RN7SL589P		smallRNA	chr4:2316199-23165
ENSG0000(	601	15.03157	chr4:9093	RP11-529E10.6		lncRNA	chr4:3503597-35044
ENSG0000(	601	15.03157	chr4:9093	RP11-572017.1		lncRNA	chr4:1712821-17136
ENSG0000(	601	15.03157	chr4:9093	ENPP7P9		Pseudoger	chr4:3967034-39676
ENSG0000(	601	15.03157	chr4:9093	FGFR3	NCGv7;AC	protein_c	chr4:1793293-18088
ENSG0000(	601	15.03157	chr4:9093	HTT-AS		lncRNA	chr4:3049094-30745
ENSG0000(	601	15.03157	chr4:9093	ENSG00000250623		lncRNA	chr4:2139673-21410
ENSG0000(	601	15.03157	chr4:9093	NKX1-1		protein_c	chr4:1402932-14064
ENSG0000(	601	15.03157	chr4:9093	LINC02171		lncRNA	chr4:3673593-36778
ENSG0000(	601	15.03157	chr4:9093	ENSG00000250681		lncRNA	chr4:3633029-36339
ENSG0000(	601	15.03157	chr4:9093	CFAP99		protein_c	chr4:2418974-24629
ENSG0000(	601	15.03157	chr4:9093	NOP14		protein_c	chr4:2937933-29634
ENSG0000(	601	15.03157	chr4:9093	FAM86EP		Pseudoger	chr4:3943274-39554
ENSG0000(	601	15.03157	chr4:9093	AC116562.1		smallRNA	chr4:4084334-40844
ENSG0000(	601	15.03157	chr4:9093	ENSG00000290888		lncRNA	chr4:4048211-41075
ENSG0000(	601	15.03157	chr4:9093	ENSG00000250940		Pseudoger	chr4:4086638-40868
ENSG0000(	601	15.03157	chr4:9093	POLN		protein_c	chr4:2071918-22421
ENSG0000(	601	15.03157	chr4:9093	OR7E43P		Pseudoger	chr4:4174319-41752
ENSG0000(	601	15.03157	chr4:9093	OR7E103P		Pseudoger	chr4:4126659-41275
ENSG0000(	601	15.03157	chr4:9093	MSANTD1		protein_c	chr4:3244369-32717
ENSG0000(	601	15.03157	chr4:9093	ENSG00000287099		lncRNA	chr4:2859983-28626
ENSG0000(	601	15.03157	chr4:9093	ENSG00000251229		Pseudoger	chr4:2505081-25062
ENSG0000(	601	15.03157	chr4:9093	LINC02600		lncRNA	chr4:3758748-37633
ENSG0000(	601	15.03157	chr4:9093	ALG1L7P		Pseudoger	chr4:3935447-39425
ENSG0000(	601	15.03157	chr4:9093	ENSG00000270090		lncRNA	chr4:3544555-35487
ENSG0000(	601	15.03157	chr4:9093	ENSG00000286900		lncRNA	chr4:3450638-34531
ENSG0000(	601	15.03157	chr4:9093	ADD1		protein_c	chr4:2843844-29300
ENSG0000(	601	15.03157	chr4:9093	NSG1		protein_c	chr4:4348140-44190
ENSG0000(	601	15.03157	chr4:9093	ENSG00000250259		lncRNA	chr4:1027678-10289
ENSG0000(	601	15.03157	chr4:9093	ENSG00000251652		lncRNA	chr4:1113639-11329
ENSG0000(	601	15.03157	chr4:9093	ENSG00000251639		Pseudoger	chr4:1100016-11015
ENSG0000(	601	15.03157	chr4:9093	MAEA		protein_c	chr4:1289887-13401
ENSG0000(	601	15.03157	chr4:9093	AC116562.2		smallRNA	chr4:4037084-40371
ENSG0000(	601	15.03157	chr4:9093	OR7E163P		Pseudoger	chr4:3889356-38902
ENSG0000(	601	15.03157	chr4:9093	LETM1	NCGv7	protein_c	chr4:1811479-18561
ENSG0000(	601	15.03157	chr4:9093	TMEM129	DriverDBv	protein_c	chr4:1715952-17213
ENSG0000(	601	15.03157	chr4:9093	CTBP1-DT		lncRNA	chr4:1249300-12882
ENSG0000(	601	15.03157	chr4:9093	LINC00955		lncRNA	chr4:3576869-35907
ENSG0000(	601	15.03157	chr4:9093	ENSG00000251148		lncRNA	chr4:2295584-23190
ENSG0000(	601	15.03157	chr4:9093	RNF4	NCGv7	protein_c	chr4:2462220-25158
ENSG0000(	601	15.03157	chr4:9093	ZBTB49		protein_c	chr4:4290251-43217
ENSG0000(	601	15.03157	chr4:9093	RNU6-204P		smallRNA	chr4:3044101-30442
ENSG0000(	601	15.03157	chr4:9093	TNIP2		protein_c	chr4:2741648-27563
ENSG0000(	601	15.03157	chr4:9093	STX18		protein_c	chr4:4415742-45423
ENSG0000(	601	15.03157	chr4:9093	TMEM128	NCGv7	protein_c	chr4:4235542-42482
ENSG0000(	601	15.03157	chr4:9093	ENSG00000289032		lncRNA	chr4:4248245-42487
ENSG0000(	601	15.03157	chr4:9093	UNC93B4		Pseudoger	chr4:4143458-41504
ENSG0000(	601	15.03157	chr4:9093	ENSG00000288589		lncRNA	chr4:3972360-39732
ENSG0000(	601	15.03157	chr4:9093	HTT		protein_c	chr4:3041363-32439
ENSG0000(	597	14.93152	chr1:3732	Y_RNA		smallRNA	chr1:173808489-173
ENSG0000(	591	14.78146	chr1:1234	RNU7-57P		smallRNA	chr1:154338743-154

ENSG0000(	587	14.68142	chr5:169(	MAST4-AS1		lncRNA	chr5:67001383-6700
ENSG0000(	587	14.68142	chr5:169(	MAST4-IT1		lncRNA	chr5:66662331-6666
ENSG0000(	587	14.68142	chr5:169(	ENSG00000250421		lncRNA	chr5:67699429-6790
ENSG0000(	587	14.68142	chr5:169(	ENSG00000250978		lncRNA	chr5:67463809-6747
ENSG0000(	587	14.68142	chr5:169(	BCL9P1		Pseudoger	chr5:67636382-6763
ENSG0000(	587	14.68142	chr5:169(	MAST4	NCGv7	protein_c	chr5:66596380-6716
ENSG0000(	587	14.68142	chr5:169(	ENSG00000273860		Pseudoger	chr5:67855735-6785
ENSG0000(	587	14.68142	chr5:169(	ENSG00000248803		Pseudoger	chr5:66622868-6662
ENSG0000(	587	14.68142	chr5:169(	ENSG00000286062		lncRNA	chr5:66350148-6644
ENSG0000(	587	14.68142	chr5:169(	LINC02997		lncRNA	chr5:67379378-6780
ENSG0000(	587	14.68142	chr5:169(	AC010376.1		smallRNA	chr5:67967445-6796
ENSG0000(	587	14.68142	chr5:169(	LINC02229		lncRNA	chr5:66507380-6651
ENSG0000(	587	14.68142	chr5:169(	ENSG00000249894		lncRNA	chr5:67800740-6789
ENSG0000(	587	14.68142	chr5:169(	CD180		protein_c	chr5:67179613-6719
ENSG0000(	587	14.68142	chr5:169(	ENSG00000250669		Pseudoger	chr5:67690033-6769
ENSG0000(	587	14.68142	chr5:169(	ENSG00000286647		lncRNA	chr5:67356663-6736
ENSG0000(	587	14.68142	chr5:169(	ENSG00000251391		lncRNA	chr5:66298394-6639
ENSG0000(	587	14.68142	chr5:169(	ENSG00000253801		Pseudoger	chr5:68087161-6808
ENSG0000(	587	14.68142	chr5:169(	TILRLS		lncRNA	chr5:67268022-6727
ENSG0000(	587	14.68142	chr5:169(	LINC02242		lncRNA	chr5:67632266-6764
ENSG0000(	587	14.68142	chr5:169(	ENSG00000249721		Pseudoger	chr5:67692812-6769
ENSG0000(	587	14.68142	chr5:169(	PPIAP78		Pseudoger	chr5:66572057-6657
ENSG0000(	587	14.68142	chr5:169(	ENSG00000239870		Pseudoger	chr5:67608654-6760
ENSG0000(	587	14.68142	chr5:169(	AC079467.1		smallRNA	chr5:67464522-6746
ENSG0000(	586	14.6564	chr1:373:	ENSG00000241666		lncRNA	chr1:167627385-167
ENSG0000(	586	14.6564	chr1:373:	POU2F1		protein_c	chr1:167220876-167
ENSG0000(	586	14.6564	chr1:373:	ENSG00000250762		Pseudoger	chr1:167819898-167
ENSG0000(	586	14.6564	chr1:373:	TBX19		protein_c	chr1:168280877-168
ENSG0000(	586	14.6564	chr1:373:	TADA1	DriverDB\	protein_c	chr1:166856510-166
ENSG0000(	586	14.6564	chr1:373:	GPA33		protein_c	chr1:167052836-167
ENSG0000(	586	14.6564	chr1:373:	CREG1	DriverDB\	protein_c	chr1:167529117-167
ENSG0000(	586	14.6564	chr1:373:	DCAF6		protein_c	chr1:167935783-168
ENSG0000(	586	14.6564	chr1:373:	MAEL		protein_c	chr1:166975582-167
ENSG0000(	586	14.6564	chr1:373:	MPC2	DriverDB\	protein_c	chr1:167916675-167
ENSG0000(	586	14.6564	chr1:373:	POGK	DriverDB\	protein_c	chr1:166839447-166
ENSG0000(	586	14.6564	chr1:373:	DUTP6		Pseudoger	chr1:166868748-166
ENSG0000(	586	14.6564	chr1:373:	ENSG00000287218		lncRNA	chr1:167455195-167
ENSG0000(	586	14.6564	chr1:373:	TIPRL		protein_c	chr1:168178962-168
ENSG0000(	586	14.6564	chr1:373:	GPR161		protein_c	chr1:168079542-168
ENSG0000(	586	14.6564	chr1:373:	RPL34P1		Pseudoger	chr1:168210616-168
ENSG0000(	586	14.6564	chr1:373:	ENSG00000273160		lncRNA	chr1:167457742-167
ENSG0000(	586	14.6564	chr1:373:	ILDR2		protein_c	chr1:166895711-166
ENSG0000(	586	14.6564	chr1:373:	SFT2D2		protein_c	chr1:168225938-168
ENSG0000(	586	14.6564	chr1:373:	ENSG00000213068		Pseudoger	chr1:167162423-167
ENSG0000(	586	14.6564	chr1:373:	MIR1255B2		smallRNA	chr1:167998660-167
ENSG0000(	586	14.6564	chr1:373:	ENSG00000231029		Pseudoger	chr1:167591392-167
ENSG0000(	586	14.6564	chr1:373:	snoU13		smallRNA	chr1:167041435-167
ENSG0000(	586	14.6564	chr1:373:	ANKRD36BP1		Pseudoger	chr1:168245565-168
ENSG0000(	586	14.6564	chr1:373:	LINC01363		lncRNA	chr1:167175363-167
ENSG0000(	586	14.6564	chr1:373:	FMO11P		Pseudoger	chr1:166763334-166
ENSG0000(	586	14.6564	chr1:373:	ENSG00000227907		lncRNA	chr1:167052551-167

ENSG0000(	586	14.6564	chr1:373:	MPZL1		protein_c	chr1:167721192-167
ENSG0000(	586	14.6564	chr1:373:	POU2F1-DT		lncRNA	chr1:167219822-167
ENSG0000(	586	14.6564	chr1:373:	GCSHP5		Pseudoger	chr1:168055901-168
ENSG0000(	586	14.6564	chr1:373:	ENSG00000272033		lncRNA	chr1:167379108-167
ENSG0000(	586	14.6564	chr1:373:	RNA5SP65		Pseudoger	chr1:167005959-167
ENSG0000(	586	14.6564	chr1:373:	ENSG00000233411		lncRNA	chr1:167457383-167
ENSG0000(	586	14.6564	chr1:373:	ADCY10	DriverDB\	protein_c	chr1:167809386-167
ENSG0000(	586	14.6564	chr1:373:	AKR1D1P1		Pseudoger	chr1:167519536-167
ENSG0000(	586	14.6564	chr1:373:	RCSD1		protein_c	chr1:167630093-167
ENSG0000(	586	14.6564	chr1:373:	ENSG00000232194		lncRNA	chr1:167820406-167
ENSG0000(	586	14.6564	chr1:373:	CD247		protein_c	chr1:167425027-167
ENSG0000(	586	14.6564	chr1:373:	ENSG00000237131		Pseudoger	chr1:168215405-168
ENSG0000(	586	14.6564	chr1:373:	STYXL2		protein_c	chr1:167094075-167
ENSG0000(	586	14.6564	chr1:373:	CNN2P10		Pseudoger	chr1:166796266-166
ENSG0000(	585	14.63139	chr5:169(	RPL27AP5		Pseudoger	chr5:74990189-7499
ENSG0000(	585	14.63139	chr5:169(	COQ10BP2		Pseudoger	chr5:83279921-8328
ENSG0000(	585	14.63139	chr5:169(	MIR4280		smallRNA	chr5:87114879-8711
ENSG0000(	585	14.63139	chr5:169(	RNU6-620P		smallRNA	chr5:83703448-8370
ENSG0000(	585	14.63139	chr5:169(	DDX18P4		Pseudoger	chr5:98679402-9868
ENSG0000(	585	14.63139	chr5:169(	ENSG00000247121		lncRNA	chr5:96814028-9693
ENSG0000(	585	14.63139	chr5:169(	MIR3607		smallRNA	chr5:86620506-8662
ENSG0000(	585	14.63139	chr5:169(	ENSG00000248870		lncRNA	chr5:82586776-8258
ENSG0000(	585	14.63139	chr5:169(	OR7H2P		Pseudoger	chr5:101816475-101
ENSG0000(	585	14.63139	chr5:169(	ENSG00000248881		lncRNA	chr5:75598482-7559
ENSG0000(	585	14.63139	chr5:169(	RN7SL802P		smallRNA	chr5:101581830-101
ENSG0000(	585	14.63139	chr5:169(	ENSG00000251193		Pseudoger	chr5:97737894-9773
ENSG0000(	585	14.63139	chr5:169(	ACTBP2		Pseudoger	chr5:77784881-7778
ENSG0000(	585	14.63139	chr5:169(	ENSG00000253321		lncRNA	chr5:90353037-9035
ENSG0000(	585	14.63139	chr5:169(	ENSG00000232578		Pseudoger	chr5:94592771-9459
ENSG0000(	585	14.63139	chr5:169(	ENSG00000251023		lncRNA	chr5:93860669-9386
ENSG0000(	585	14.63139	chr5:169(	CTBP2P4		Pseudoger	chr5:98576341-9857
ENSG0000(	585	14.63139	chr5:169(	HMGB1P35		Pseudoger	chr5:77146568-7714
ENSG0000(	585	14.63139	chr5:169(	RPS3AP20		Pseudoger	chr5:79284132-7928
ENSG0000(	585	14.63139	chr5:169(	CRLF3P2		Pseudoger	chr5:99948295-9994
ENSG0000(	585	14.63139	chr5:169(	GUSBP19		Pseudoger	chr5:100375700-100
ENSG0000(	585	14.63139	chr5:169(	ENSG00000243385		Pseudoger	chr5:83201229-8320
ENSG0000(	585	14.63139	chr5:169(	LINC01846		lncRNA	chr5:98085866-9816
ENSG0000(	585	14.63139	chr5:169(	SLF1		protein_c	chr5:94618669-9473
ENSG0000(	585	14.63139	chr5:169(	FUNDC2P1		Pseudoger	chr5:73508685-7350
ENSG0000(	585	14.63139	chr5:169(	HMGB1P21		Pseudoger	chr5:78644265-7864
ENSG0000(	585	14.63139	chr5:169(	PSME2P1		Pseudoger	chr5:98213402-9821
ENSG0000(	585	14.63139	chr5:169(	CHD1	NCGv7	protein_c	chr5:98853985-9892
ENSG0000(	585	14.63139	chr5:169(	ENSG00000289008		lncRNA	chr5:88685191-8868
ENSG0000(	585	14.63139	chr5:169(	PDCD5P2		Pseudoger	chr5:76280956-7628
ENSG0000(	585	14.63139	chr5:169(	RNA5SP186		Pseudoger	chr5:76091708-7609
ENSG0000(	585	14.63139	chr5:169(	MEF2C-AS1		lncRNA	chr5:88883328-8946
ENSG0000(	585	14.63139	chr5:169(	MTND5P12		Pseudoger	chr5:94567461-9456
ENSG0000(	585	14.63139	chr5:169(	CAST	NCGv7	protein_c	chr5:96525267-9677
ENSG0000(	585	14.63139	chr5:169(	ENSG00000290550		lncRNA	chr5:86282460-8629
ENSG0000(	585	14.63139	chr5:169(	GCNT4		protein_c	chr5:75025346-7505
ENSG0000(	585	14.63139	chr5:169(	ENSG00000251001		Pseudoger	chr5:80997183-8099

ENSG0000(	585	14.63139	chr5:169(	EDIL3		protein_c	chr5:83940554-8438
ENSG0000(	585	14.63139	chr5:169(	TMEM161B		protein_c	chr5:88189633-8826
ENSG0000(	585	14.63139	chr5:169(	ENSG00000225407		lncRNA	chr5:76691439-7671
ENSG0000(	585	14.63139	chr5:169(	AC120120.1		smallRNA	chr5:92405946-9240
ENSG0000(	585	14.63139	chr5:169(	LINC02230		lncRNA	chr5:73337906-7333
ENSG0000(	585	14.63139	chr5:169(	LIX1-AS1		lncRNA	chr5:97089075-9743
ENSG0000(	585	14.63139	chr5:169(	LINC02059		lncRNA	chr5:86746818-8674
ENSG0000(	585	14.63139	chr5:169(	FAM174A-DT		lncRNA	chr5:100428124-100
ENSG0000(	585	14.63139	chr5:169(	MTX3		protein_c	chr5:79976716-7999
ENSG0000(	585	14.63139	chr5:169(	PPIAP11		Pseudoger	chr5:82009602-8201
ENSG0000(	585	14.63139	chr5:169(	FOXD1		protein_c	chr5:73444827-7344
ENSG0000(	585	14.63139	chr5:169(	RPL5P17		Pseudoger	chr5:85464434-8546
ENSG0000(	585	14.63139	chr5:169(	ENSG00000248794		Pseudoger	chr5:81242330-8124
ENSG0000(	585	14.63139	chr5:169(	RHOBTB3		protein_c	chr5:95713522-9582
ENSG0000(	585	14.63139	chr5:169(	CETN3		protein_c	chr5:90392257-9040
ENSG0000(	585	14.63139	chr5:169(	RPL7P18		Pseudoger	chr5:94825961-9482
ENSG0000(	585	14.63139	chr5:169(	SLC25A5P9		Pseudoger	chr5:75752570-7575
ENSG0000(	585	14.63139	chr5:169(	ENSG00000248170		Pseudoger	chr5:85237681-8523
ENSG0000(	585	14.63139	chr5:169(	ENSG00000251093		lncRNA	chr5:91226475-9122
ENSG0000(	585	14.63139	chr5:169(	ENSG00000214890		Pseudoger	chr5:79000112-7900
ENSG0000(	585	14.63139	chr5:169(	ENSG00000287701		lncRNA	chr5:74899115-7490
ENSG0000(	585	14.63139	chr5:169(	LINC01332		lncRNA	chr5:74327995-7433
ENSG0000(	585	14.63139	chr5:169(	PCBP2P3		Pseudoger	chr5:91843687-9184
ENSG0000(	585	14.63139	chr5:169(	FAM81B		protein_c	chr5:95391366-9545
ENSG0000(	585	14.63139	chr5:169(	FOXD1-AS1		lncRNA	chr5:73446357-7344
ENSG0000(	585	14.63139	chr5:169(	ARSK		protein_c	chr5:95555101-9560
ENSG0000(	585	14.63139	chr5:169(	RNU6-448P		smallRNA	chr5:84196883-8419
ENSG0000(	585	14.63139	chr5:169(	LINC02161		lncRNA	chr5:89581209-8967
ENSG0000(	585	14.63139	chr5:169(	ENSG00000284762		protein_c	chr5:77086732-7742
ENSG0000(	585	14.63139	chr5:169(	ENSG00000250362		lncRNA	chr5:95861786-9587
ENSG0000(	585	14.63139	chr5:169(	ENSG00000250377		lncRNA	chr5:88691757-8869
ENSG0000(	585	14.63139	chr5:169(	ZBED3		protein_c	chr5:77072072-7708
ENSG0000(	585	14.63139	chr5:169(	ENSG00000288741		lncRNA	chr5:80256200-8025
ENSG0000(	585	14.63139	chr5:169(	LINC01554		lncRNA	chr5:95838245-9586
ENSG0000(	585	14.63139	chr5:169(	AP3B1		protein_c	chr5:78000522-7829
ENSG0000(	585	14.63139	chr5:169(	Y_RNA		smallRNA	chr5:100021804-100
ENSG0000(	585	14.63139	chr5:169(	HMGN2P4		Pseudoger	chr5:76242024-7624
ENSG0000(	585	14.63139	chr5:169(	ENSG00000285618		lncRNA	chr5:91132303-9114
ENSG0000(	585	14.63139	chr5:169(	ENSG00000248261		lncRNA	chr5:102141893-102
ENSG0000(	585	14.63139	chr5:169(	RPS2P24		Pseudoger	chr5:77545397-7754
ENSG0000(	585	14.63139	chr5:169(	SV2C-AS1		lncRNA	chr5:76081946-7608
ENSG0000(	585	14.63139	chr5:169(	ENSG00000242858		Pseudoger	chr5:83746388-8374
ENSG0000(	585	14.63139	chr5:169(	RNU6ATAC36P		smallRNA	chr5:76948791-7694
ENSG0000(	585	14.63139	chr5:169(	LYSMD3		protein_c	chr5:90515611-9052
ENSG0000(	585	14.63139	chr5:169(	ENSG00000251605		Pseudoger	chr5:78307503-7830
ENSG0000(	585	14.63139	chr5:169(	ENSG00000228367		Pseudoger	chr5:75920670-7592
ENSG0000(	585	14.63139	chr5:169(	ENSG00000250306		Pseudoger	chr5:88382948-8838
ENSG0000(	585	14.63139	chr5:169(	ENSG00000249017		lncRNA	chr5:102500541-102
ENSG0000(	585	14.63139	chr5:169(	BHMT2	NCGv7	protein_c	chr5:79069767-7909
ENSG0000(	585	14.63139	chr5:169(	DMGDH		protein_c	chr5:78997564-7923
ENSG0000(	585	14.63139	chr5:169(	ENSG00000249023		lncRNA	chr5:85429702-8543

ENSG0000(	585	14.63139	chr5:169(	ENSG00000253558		Pseudoger	chr5:78041888-7804
ENSG0000(	585	14.63139	chr5:169(	ENSG00000288740		lncRNA	chr5:89301289-8930
ENSG0000(	585	14.63139	chr5:169(	ENSG00000248112		lncRNA	chr5:82919376-8292
ENSG0000(	585	14.63139	chr5:169(	F2RL1		protein_c	chr5:76818933-7683
ENSG0000(	585	14.63139	chr5:169(	AC010595.1		smallRNA	chr5:85634412-8563
ENSG0000(	585	14.63139	chr5:169(	FAM151B-DT		lncRNA	chr5:80411231-8048
ENSG0000(	585	14.63139	chr5:169(	SUMO2P5		Pseudoger	chr5:75225424-7522
ENSG0000(	585	14.63139	chr5:169(	RNU4-90P		smallRNA	chr5:91270727-9127
ENSG0000(	585	14.63139	chr5:169(	ENSG00000250258		lncRNA	chr5:79774348-7981
ENSG0000(	585	14.63139	chr5:169(	ENSG00000253572		lncRNA	chr5:77942757-7794
ENSG0000(	585	14.63139	chr5:169(	RPS27AP9		Pseudoger	chr5:80498534-8049
ENSG0000(	585	14.63139	chr5:169(	ENSG00000251668		Pseudoger	chr5:76170930-7617
ENSG0000(	585	14.63139	chr5:169(	EDIL3-DT		lncRNA	chr5:84382424-8449
ENSG0000(	585	14.63139	chr5:169(	MBLAC2	DriverDB\	protein_c	chr5:90458209-9047
ENSG0000(	585	14.63139	chr5:169(	RBMX2P5		Pseudoger	chr5:80331573-8033
ENSG0000(	585	14.63139	chr5:169(	LINC01335		lncRNA	chr5:74258689-7432
ENSG0000(	585	14.63139	chr5:169(	ENSG00000248928		Pseudoger	chr5:99594880-9959
ENSG0000(	585	14.63139	chr5:169(	RNU4-11P		smallRNA	chr5:83803554-8380
ENSG0000(	585	14.63139	chr5:169(	ENSG00000285000		protein_c	chr5:77030902-7715
ENSG0000(	585	14.63139	chr5:169(	MTCYBP40		Pseudoger	chr5:97677547-9767
ENSG0000(	585	14.63139	chr5:169(	NR2F1-AS1		lncRNA	chr5:93360779-9358
ENSG0000(	585	14.63139	chr5:169(	RPL7AP32		Pseudoger	chr5:79529302-7953
ENSG0000(	585	14.63139	chr5:169(	PTP4A1P4		Pseudoger	chr5:86087526-8608
ENSG0000(	585	14.63139	chr5:169(	RGMB-AS1		lncRNA	chr5:98769618-9877
ENSG0000(	585	14.63139	chr5:169(	WDR41		protein_c	chr5:77425970-7762
ENSG0000(	585	14.63139	chr5:169(	ENSG00000250348		lncRNA	chr5:76285542-7631
ENSG0000(	585	14.63139	chr5:169(	RNU6-1119P		smallRNA	chr5:100153672-100
ENSG0000(	585	14.63139	chr5:169(	VCAN	NCGv7	protein_c	chr5:83471618-8358
ENSG0000(	585	14.63139	chr5:169(	ENSG00000251585		Pseudoger	chr5:86402465-8640
ENSG0000(	585	14.63139	chr5:169(	ENSG00000251599		lncRNA	chr5:73132008-7315
ENSG0000(	585	14.63139	chr5:169(	ZFYVE16		protein_c	chr5:80408013-8048
ENSG0000(	585	14.63139	chr5:169(	SV2C		protein_c	chr5:76083383-7635
ENSG0000(	585	14.63139	chr5:169(	POLD2P1		Pseudoger	chr5:93267429-9326
ENSG0000(	585	14.63139	chr5:169(	ENSG00000176183		Pseudoger	chr5:98338744-9833
ENSG0000(	585	14.63139	chr5:169(	MIR3660		smallRNA	chr5:90016621-9001
ENSG0000(	585	14.63139	chr5:169(	ENSG00000285190		lncRNA	chr5:87412342-8749
ENSG0000(	585	14.63139	chr5:169(	LDHBP3		Pseudoger	chr5:92840807-9284
ENSG0000(	585	14.63139	chr5:169(	LINC01340		lncRNA	chr5:97504663-9769
ENSG0000(	585	14.63139	chr5:169(	ENSG00000250330		Pseudoger	chr5:79229904-7923
ENSG0000(	585	14.63139	chr5:169(	Y_RNA		smallRNA	chr5:79534853-7953
ENSG0000(	585	14.63139	chr5:169(	POLK		protein_c	chr5:75511756-7560
ENSG0000(	585	14.63139	chr5:169(	ENSG00000227836		Pseudoger	chr5:96942299-9694
ENSG0000(	585	14.63139	chr5:169(	MIR548P		smallRNA	chr5:100816482-100
ENSG0000(	585	14.63139	chr5:169(	ENSG00000248967		Pseudoger	chr5:80746339-8074
ENSG0000(	585	14.63139	chr5:169(	AGGF1	NCGv7	protein_c	chr5:77029251-7706
ENSG0000(	585	14.63139	chr5:169(	LINC01385		lncRNA	chr5:73451498-7345
ENSG0000(	585	14.63139	chr5:169(	ENSG00000248758		lncRNA	chr5:97188090-9720
ENSG0000(	585	14.63139	chr5:169(	SPZ1		protein_c	chr5:80319625-8032
ENSG0000(	585	14.63139	chr5:169(	ATG10-AS1		lncRNA	chr5:82073055-8207
ENSG0000(	585	14.63139	chr5:169(	ST13P12		Pseudoger	chr5:82968888-8297
ENSG0000(	585	14.63139	chr5:169(	RN7SKP62		smallRNA	chr5:100733058-100

ENSG0000(	585	14.63139	chr5:169(	ENSG00000287938		lncRNA	chr5:81817531-8184
ENSG0000(	585	14.63139	chr5:169(	ENSG00000251361		lncRNA	chr5:93019663-9306
ENSG0000(	585	14.63139	chr5:169(	DBIP2		Pseudoger	chr5:80603383-8060
ENSG0000(	585	14.63139	chr5:169(	PPIAP79		Pseudoger	chr5:84999812-8500
ENSG0000(	585	14.63139	chr5:169(	LINC01455		lncRNA	chr5:79936579-7996
ENSG0000(	585	14.63139	chr5:169(	ATG10		protein_c	chr5:81972023-8227
ENSG0000(	585	14.63139	chr5:169(	LINC02058		lncRNA	chr5:92907180-9293
ENSG0000(	585	14.63139	chr5:169(	ENSG00000290580		lncRNA	chr5:99489559-9949
ENSG0000(	585	14.63139	chr5:169(	SNORA40		smallRNA	chr5:74882656-7488
ENSG0000(	585	14.63139	chr5:169(	POC5	NCGv7	protein_c	chr5:75674124-7571
ENSG0000(	585	14.63139	chr5:169(	RN7SKP295		smallRNA	chr5:83685866-8368
ENSG0000(	585	14.63139	chr5:169(	ANKRA2		protein_c	chr5:73552190-7356
ENSG0000(	585	14.63139	chr5:169(	ARRDC3-AS1		lncRNA	chr5:91380349-9161
ENSG0000(	585	14.63139	chr5:169(	ENSG00000244630		Pseudoger	chr5:80855507-8085
ENSG0000(	585	14.63139	chr5:169(	HSPD1P11		Pseudoger	chr5:95768999-9577
ENSG0000(	585	14.63139	chr5:169(	ENSG00000248393		lncRNA	chr5:82545862-8254
ENSG0000(	585	14.63139	chr5:169(	ENSG00000250874		lncRNA	chr5:85663232-8566
ENSG0000(	585	14.63139	chr5:169(	LINC01339		lncRNA	chr5:90153052-9029
ENSG0000(	585	14.63139	chr5:169(	LINC00492		lncRNA	chr5:102581368-102
ENSG0000(	585	14.63139	chr5:169(	RBX1P2		Pseudoger	chr5:80019609-8001
ENSG0000(	585	14.63139	chr5:169(	ENSG00000251374		Pseudoger	chr5:82265157-8226
ENSG0000(	585	14.63139	chr5:169(	ENSG00000288846		lncRNA	chr5:79543786-7954
ENSG0000(	585	14.63139	chr5:169(	FAM151B		protein_c	chr5:80487969-8054
ENSG0000(	585	14.63139	chr5:169(	TENT2		protein_c	chr5:79612120-7968
ENSG0000(	585	14.63139	chr5:169(	RNU1-73P		smallRNA	chr5:97175950-9717
ENSG0000(	585	14.63139	chr5:169(	JMY		protein_c	chr5:79236131-7932
ENSG0000(	585	14.63139	chr5:169(	HOMER1		protein_c	chr5:79372636-7951
ENSG0000(	585	14.63139	chr5:169(	RNA5SP187		Pseudoger	chr5:88274258-8827
ENSG0000(	585	14.63139	chr5:169(	ENSG00000248569		Pseudoger	chr5:80351021-8035
ENSG0000(	585	14.63139	chr5:169(	RAB5CP2		Pseudoger	chr5:91476382-9147
ENSG0000(	585	14.63139	chr5:169(	GPR150		protein_c	chr5:95620087-9562
ENSG0000(	585	14.63139	chr5:169(	ENSG00000247372		lncRNA	chr5:75320155-7533
ENSG0000(	585	14.63139	chr5:169(	CKMT2-AS1		lncRNA	chr5:81201341-8130
ENSG0000(	585	14.63139	chr5:169(	SEM1P1		Pseudoger	chr5:81892490-8189
ENSG0000(	585	14.63139	chr5:169(	GFM2		protein_c	chr5:74721206-7476
ENSG0000(	585	14.63139	chr5:169(	ENSG00000251054		lncRNA	chr5:97223371-9722
ENSG0000(	585	14.63139	chr5:169(	MTND6P3		Pseudoger	chr5:94568929-9456
ENSG0000(	585	14.63139	chr5:169(	ENSG00000247402		lncRNA	chr5:102605635-102
ENSG0000(	585	14.63139	chr5:169(	ENSG00000250955		lncRNA	chr5:95964999-9598
ENSG0000(	585	14.63139	chr5:169(	ATG10-IT1		lncRNA	chr5:81991995-8199
ENSG0000(	585	14.63139	chr5:169(	FAM169A		protein_c	chr5:74777574-7486
ENSG0000(	585	14.63139	chr5:169(	MEF2C-AS2		lncRNA	chr5:88676014-8877
ENSG0000(	585	14.63139	chr5:169(	F2RL2		protein_c	chr5:76615482-7662
ENSG0000(	585	14.63139	chr5:169(	NSA2		protein_c	chr5:74766991-7478
ENSG0000(	585	14.63139	chr5:169(	MEF2C	NCGv7;AC	protein_c	chr5:88717117-8890
ENSG0000(	585	14.63139	chr5:169(	GUSBP7		Pseudoger	chr5:100062275-100
ENSG0000(	585	14.63139	chr5:169(	ENSG00000254170		Pseudoger	chr5:78708734-7871
ENSG0000(	585	14.63139	chr5:169(	LINC01338		lncRNA	chr5:82807475-8286
ENSG0000(	585	14.63139	chr5:169(	CHD1-DT		lncRNA	chr5:98929163-9899
ENSG0000(	585	14.63139	chr5:169(	ENSG00000251314		lncRNA	chr5:95962001-9663
ENSG0000(	585	14.63139	chr5:169(	SCAMP1-AS1		lncRNA	chr5:78342333-7836

ENSG0000(	585	14.63139	chr5:169(	ENSG00000254310	Pseudoger	chr5:78753596-7875
ENSG0000(	585	14.63139	chr5:169(	LINC00461	lncRNA	chr5:88507546-8869
ENSG0000(	585	14.63139	chr5:169(	UTP15	protein_c	chr5:73565443-7358
ENSG0000(	585	14.63139	chr5:169(	CCNH	protein_c	chr5:87318416-8741
ENSG0000(	585	14.63139	chr5:169(	LINC01386	lncRNA	chr5:73454187-7347
ENSG0000(	585	14.63139	chr5:169(	MTCYBP35	Pseudoger	chr5:94569540-9457
ENSG0000(	585	14.63139	chr5:169(	LINC02122	lncRNA	chr5:74084068-7410
ENSG0000(	585	14.63139	chr5:169(	TTC37	protein_c	chr5:95461755-9555
ENSG0000(	585	14.63139	chr5:169(	ENSG00000251342	Pseudoger	chr5:76078666-7607
ENSG0000(	585	14.63139	chr5:169(	ENSG00000273957	Pseudoger	chr5:100388853-100
ENSG0000(	585	14.63139	chr5:169(	POU5F2	protein_c	chr5:93733220-9374
ENSG0000(	585	14.63139	chr5:169(	DHFR	protein_c	chr5:80626226-8065
ENSG0000(	585	14.63139	chr5:169(	LINC01337	lncRNA	chr5:80608623-8062
ENSG0000(	585	14.63139	chr5:169(	AC106732.1	smallRNA	chr5:74271682-7427
ENSG0000(	585	14.63139	chr5:169(	ENSG00000248588	lncRNA	chr5:92823935-9284
ENSG0000(	585	14.63139	chr5:169(	RNU6-308P	smallRNA	chr5:95521369-9552
ENSG0000(	585	14.63139	chr5:169(	ENSG00000288883	lncRNA	chr5:74294413-7430
ENSG0000(	585	14.63139	chr5:169(	SCAMP1	protein_c	chr5:78360611-7848
ENSG0000(	585	14.63139	chr5:169(	RNA5SP188	Pseudoger	chr5:102131007-102
ENSG0000(	585	14.63139	chr5:169(	ENSG00000248701	lncRNA	chr5:86797685-8680
ENSG0000(	585	14.63139	chr5:169(	AC026700.1	smallRNA	chr5:85528044-8552
ENSG0000(	585	14.63139	chr5:169(	ENSG00000287783	lncRNA	chr5:87767608-8777
ENSG0000(	585	14.63139	chr5:169(	LINC02144	lncRNA	chr5:87665345-8773
ENSG0000(	585	14.63139	chr5:169(	TMEM161B-DT	lncRNA	chr5:88268864-8843
ENSG0000(	585	14.63139	chr5:169(	SNRPCP2	Pseudoger	chr5:76376675-7637
ENSG0000(	585	14.63139	chr5:169(	CMYA5	protein_c	chr5:79689836-7980
ENSG0000(	585	14.63139	chr5:169(	SLCO6A1	protein_c	chr5:102371774-102
ENSG0000(	585	14.63139	chr5:169(	ERAP2	protein_c	chr5:96875986-9691
ENSG0000(	585	14.63139	chr5:169(	ENSG00000250615	lncRNA	chr5:77073881-7707
ENSG0000(	585	14.63139	chr5:169(	ERAP1	protein_c	chr5:96760810-9680
ENSG0000(	585	14.63139	chr5:169(	RNU6-680P	smallRNA	chr5:75709495-7570
ENSG0000(	585	14.63139	chr5:169(	XRCC4	protein_c	chr5:83077498-8335
ENSG0000(	585	14.63139	chr5:169(	GGCTP1	Pseudoger	chr5:95834424-9583
ENSG0000(	585	14.63139	chr5:169(	LUCAT1	lncRNA	chr5:91054834-9131
ENSG0000(	585	14.63139	chr5:169(	ENSG00000248734	lncRNA	chr5:96784777-9678
ENSG0000(	585	14.63139	chr5:169(	AC008394.1	protein_c	chr5:87216606-8723
ENSG0000(	585	14.63139	chr5:169(	RN7SL208P	smallRNA	chr5:76836902-7683
ENSG0000(	585	14.63139	chr5:169(	RASGRF2-AS1	lncRNA	chr5:80947694-8096
ENSG0000(	585	14.63139	chr5:169(	ENSG00000244076	Pseudoger	chr5:80315671-8031
ENSG0000(	585	14.63139	chr5:169(	ENSG00000248195	lncRNA	chr5:86335024-8633
ENSG0000(	585	14.63139	chr5:169(	ENSG00000250574	Pseudoger	chr5:76510440-7651
ENSG0000(	585	14.63139	chr5:169(	SERINC5	protein_c	chr5:80111651-8025
ENSG0000(	585	14.63139	chr5:169(	ENSG00000250555	lncRNA	chr5:88692651-8869
ENSG0000(	585	14.63139	chr5:169(	MIR583HG	lncRNA	chr5:96050115-9621
ENSG0000(	585	14.63139	chr5:169(	RNU6-658P	smallRNA	chr5:74725099-7472
ENSG0000(	585	14.63139	chr5:169(	ENSG00000259968	lncRNA	chr5:73952940-7395
ENSG0000(	585	14.63139	chr5:169(	F2R	protein_c	chr5:76716126-7673
ENSG0000(	585	14.63139	chr5:169(	BIN2P2	Pseudoger	chr5:75910565-7591
ENSG0000(	585	14.63139	chr5:169(	RBBP4P6	Pseudoger	chr5:85190629-8519
ENSG0000(	585	14.63139	chr5:169(	LINC02234	lncRNA	chr5:97840912-9792
ENSG0000(	585	14.63139	chr5:169(	ENSG00000287862	lncRNA	chr5:87863703-8814

ENSG0000(	585	14.63139	chr5:169(	Y_RNA		smallRNA	chr5:78317036-7831
ENSG0000(	585	14.63139	chr5:169(	ENSG00000250831		lncRNA	chr5:89466537-8947
ENSG0000(	585	14.63139	chr5:169(	MRPS35P2		Pseudoger	chr5:98402542-9840
ENSG0000(	585	14.63139	chr5:169(	ENSG00000248363		lncRNA	chr5:86353803-8636
ENSG0000(	585	14.63139	chr5:169(	ENSG00000255647		lncRNA	chr5:90410000-9041
ENSG0000(	585	14.63139	chr5:169(	YTHDF1P1		Pseudoger	chr5:97368776-9737
ENSG0000(	585	14.63139	chr5:169(	ENSG00000248236		Pseudoger	chr5:99594329-9959
ENSG0000(	585	14.63139	chr5:169(	ENSG00000250806		lncRNA	chr5:100654112-100
ENSG0000(	585	14.63139	chr5:169(	ZBED3-AS1		lncRNA	chr5:77086688-7716
ENSG0000(	585	14.63139	chr5:169(	AC109496.1		smallRNA	chr5:83973796-8397
ENSG0000(	585	14.63139	chr5:169(	FTH1P9		Pseudoger	chr5:83426676-8342
ENSG0000(	585	14.63139	chr5:169(	RPL7P23		Pseudoger	chr5:77582376-7758
ENSG0000(	585	14.63139	chr5:169(	ELL2		protein_c	chr5:95885098-9596
ENSG0000(	585	14.63139	chr5:169(	ATP6AP1L		Pseudoger	chr5:82279462-8238
ENSG0000(	585	14.63139	chr5:169(	RN7SL814P		smallRNA	chr5:74317986-7431
ENSG0000(	585	14.63139	chr5:169(	ALDH7A1P1		Pseudoger	chr5:77290268-7729
ENSG0000(	585	14.63139	chr5:169(	ENSG00000250747		Pseudoger	chr5:73152416-7315
ENSG0000(	585	14.63139	chr5:169(	NBPF22P		Pseudoger	chr5:86282766-8629
ENSG0000(	585	14.63139	chr5:169(	ADGRV1	NCGv7	protein_c	chr5:90529344-9116
ENSG0000(	585	14.63139	chr5:169(	ENSG00000248667		lncRNA	chr5:85420028-8542
ENSG0000(	585	14.63139	chr5:169(	ENSG00000251409		lncRNA	chr5:95835521-9585
ENSG0000(	585	14.63139	chr5:169(	TMEM174	DriverDB\	protein_c	chr5:73173193-7317
ENSG0000(	585	14.63139	chr5:169(	ENSG00000251066		Pseudoger	chr5:85549409-8554
ENSG0000(	585	14.63139	chr5:169(	H3P23		Pseudoger	chr5:88602780-8860
ENSG0000(	585	14.63139	chr5:169(	LINC00491		lncRNA	chr5:102604220-102
ENSG0000(	585	14.63139	chr5:169(	RNU6-402P		smallRNA	chr5:98889731-9888
ENSG0000(	585	14.63139	chr5:169(	LINC01331		lncRNA	chr5:74111690-7453
ENSG0000(	585	14.63139	chr5:169(	ENSG00000251419		Pseudoger	chr5:75831255-7583
ENSG0000(	585	14.63139	chr5:169(	RNU6-1330P		smallRNA	chr5:74779309-7477
ENSG0000(	585	14.63139	chr5:169(	ENSG00000248105		Pseudoger	chr5:82824884-8282
ENSG0000(	585	14.63139	chr5:169(	ARSB		protein_c	chr5:78777209-7898
ENSG0000(	585	14.63139	chr5:169(	MTCO2P24		Pseudoger	chr5:98409675-9841
ENSG0000(	585	14.63139	chr5:169(	SSBP2	DriverDB\	protein_c	chr5:81412804-8175
ENSG0000(	585	14.63139	chr5:169(	BHMT		protein_c	chr5:79111809-7913
ENSG0000(	585	14.63139	chr5:169(	TMEM171		protein_c	chr5:73120569-7313
ENSG0000(	585	14.63139	chr5:169(	LINC02113		lncRNA	chr5:99549432-9957
ENSG0000(	585	14.63139	chr5:169(	FCHO2		protein_c	chr5:72956041-7309
ENSG0000(	585	14.63139	chr5:169(	TMEM251P1		Pseudoger	chr5:91030990-9103
ENSG0000(	585	14.63139	chr5:169(	AC113407.1		smallRNA	chr5:100050365-100
ENSG0000(	585	14.63139	chr5:169(	ENSG00000249293		lncRNA	chr5:73778039-7378
ENSG0000(	585	14.63139	chr5:169(	ENSG00000289337		lncRNA	chr5:96165522-9617
ENSG0000(	585	14.63139	chr5:169(	RTRAFP2		Pseudoger	chr5:95440044-9544
ENSG0000(	585	14.63139	chr5:169(	ENSG00000249301		Pseudoger	chr5:75910283-7591
ENSG0000(	585	14.63139	chr5:169(	THBS4-AS1		lncRNA	chr5:80052374-8008
ENSG0000(	585	14.63139	chr5:169(	ANKRD31		protein_c	chr5:75068275-7523
ENSG0000(	585	14.63139	chr5:169(	FAM172A		protein_c	chr5:93617725-9411
ENSG0000(	585	14.63139	chr5:169(	IQGAP2	NCGv7	protein_c	chr5:76403285-7670
ENSG0000(	585	14.63139	chr5:169(	CRHBP		protein_c	chr5:76953045-7698
ENSG0000(	585	14.63139	chr5:169(	RASA1	NCGv7	protein_c	chr5:87267883-8739
ENSG0000(	585	14.63139	chr5:169(	ENSG00000249495		Pseudoger	chr5:101976296-101
ENSG0000(	585	14.63139	chr5:169(	ENSG00000271904		lncRNA	chr5:88433892-8849

ENSG0000(	585	14.63139	chr5:169(	MIR583		smallRNA	chr5:96079138-9607
ENSG0000(	585	14.63139	chr5:169(	AC116345.1		smallRNA	chr5:73154111-7315
ENSG0000(	585	14.63139	chr5:169(	ENSG00000249829		Pseudoger	chr5:79191549-7919
ENSG0000(	585	14.63139	chr5:169(	ENSG00000249483		lncRNA	chr5:81851601-8185
ENSG0000(	585	14.63139	chr5:169(	LIX1		protein_c	chr5:97091867-9714
ENSG0000(	585	14.63139	chr5:169(	GIN1		protein_c	chr5:103086000-103
ENSG0000(	585	14.63139	chr5:169(	PAM	DriverDBv	protein_c	chr5:102753981-103
ENSG0000(	585	14.63139	chr5:169(	ENSG00000289317		lncRNA	chr5:80407323-8040
ENSG0000(	585	14.63139	chr5:169(	MTCYBP22		Pseudoger	chr5:100045928-100
ENSG0000(	585	14.63139	chr5:169(	CCT7P2		Pseudoger	chr5:92889387-9289
ENSG0000(	585	14.63139	chr5:169(	LHFPL2		protein_c	chr5:78485215-7877
ENSG0000(	585	14.63139	chr5:169(	ENSG00000289639		lncRNA	chr5:74868003-7486
ENSG0000(	585	14.63139	chr5:169(	SNORA31		smallRNA	chr5:80306235-8030
ENSG0000(	585	14.63139	chr5:169(	AC008581.1		smallRNA	chr5:77137967-7713
ENSG0000(	585	14.63139	chr5:169(	ENSG00000249776		lncRNA	chr5:92410256-9266
ENSG0000(	585	14.63139	chr5:169(	ENSG00000249984		lncRNA	chr5:92675956-9268
ENSG0000(	585	14.63139	chr5:169(	SAP18P1		Pseudoger	chr5:76075531-7607
ENSG0000(	585	14.63139	chr5:169(	ENSG00000272040		lncRNA	chr5:75608817-7560
ENSG0000(	585	14.63139	chr5:169(	TRMT112P2		Pseudoger	chr5:80033171-8003
ENSG0000(	585	14.63139	chr5:169(	RPS2P25		Pseudoger	chr5:85762559-8576
ENSG0000(	585	14.63139	chr5:169(	ENSG00000249977		Pseudoger	chr5:97449358-9744
ENSG0000(	585	14.63139	chr5:169(	ENSG00000234292		lncRNA	chr5:91280097-9128
ENSG0000(	585	14.63139	chr5:169(	ENSG00000272021		lncRNA	chr5:95849309-9584
ENSG0000(	585	14.63139	chr5:169(	ENSG00000249787		lncRNA	chr5:100399047-100
ENSG0000(	585	14.63139	chr5:169(	MTCO2P22		Pseudoger	chr5:100052859-100
ENSG0000(	585	14.63139	chr5:169(	snoU13		smallRNA	chr5:76784096-7678
ENSG0000(	585	14.63139	chr5:169(	MCTP1-AS1		lncRNA	chr5:94979151-9498
ENSG0000(	585	14.63139	chr5:169(	RNU6-524P		smallRNA	chr5:96210121-9621
ENSG0000(	585	14.63139	chr5:169(	ENSG00000286953		lncRNA	chr5:97799404-9788
ENSG0000(	585	14.63139	chr5:169(	MTND6P22		Pseudoger	chr5:100046450-100
ENSG0000(	585	14.63139	chr5:169(	ZCCHC9		protein_c	chr5:81301587-8131
ENSG0000(	585	14.63139	chr5:169(	EEF1A1P20		Pseudoger	chr5:99996547-9999
ENSG0000(	585	14.63139	chr5:169(	CKMT2		protein_c	chr5:81233320-8126
ENSG0000(	585	14.63139	chr5:169(	ENSG00000249792		lncRNA	chr5:85212958-8521
ENSG0000(	585	14.63139	chr5:169(	LNPEP		protein_c	chr5:96935394-9703
ENSG0000(	585	14.63139	chr5:169(	RN7SKP68		smallRNA	chr5:102302504-102
ENSG0000(	585	14.63139	chr5:169(	HAPLN1	NCGv7	protein_c	chr5:83637805-8372
ENSG0000(	585	14.63139	chr5:169(	MTND4P35		Pseudoger	chr5:100048988-100
ENSG0000(	585	14.63139	chr5:169(	ENSG00000271862		lncRNA	chr5:83049376-8305
ENSG0000(	585	14.63139	chr5:169(	MTATP6P2		Pseudoger	chr5:100051920-100
ENSG0000(	585	14.63139	chr5:169(	ARRDC3		protein_c	chr5:91368631-9138
ENSG0000(	585	14.63139	chr5:169(	NPM1P27		Pseudoger	chr5:93682838-9368
ENSG0000(	585	14.63139	chr5:169(	ANKRD34B		protein_c	chr5:80556755-8057
ENSG0000(	585	14.63139	chr5:169(	ENSG00000286121		lncRNA	chr5:91642643-9179
ENSG0000(	585	14.63139	chr5:169(	HEXB		protein_c	chr5:74640023-7472
ENSG0000(	585	14.63139	chr5:169(	ENSG00000286338		lncRNA	chr5:102664610-102
ENSG0000(	585	14.63139	chr5:169(	ENSG00000249349		lncRNA	chr5:85112342-8511
ENSG0000(	585	14.63139	chr5:169(	ENSG00000249444		Pseudoger	chr5:99499070-9949
ENSG0000(	585	14.63139	chr5:169(	FAM169A-AS1		lncRNA	chr5:74865893-7486
ENSG0000(	585	14.63139	chr5:169(	ACOT12		protein_c	chr5:81329996-8139
ENSG0000(	585	14.63139	chr5:169(	MTND5P10		Pseudoger	chr5:100046977-100

ENSG0000(	585	14.63139	chr5:169(	ENSG00000260871		lncRNA	chr5:90388468-9038
ENSG0000(	585	14.63139	chr5:169(	SCARNA18		smallRNA	chr5:83064204-8306
ENSG0000(	585	14.63139	chr5:169(	KRT18P45		Pseudoger	chr5:80288449-8028
ENSG0000(	585	14.63139	chr5:169(	LINC02488		lncRNA	chr5:87662040-8770
ENSG0000(	585	14.63139	chr5:169(	RGMB		protein_c	chr5:98768650-9879
ENSG0000(	585	14.63139	chr5:169(	RNU7-196P		smallRNA	chr5:73828012-7382
ENSG0000(	585	14.63139	chr5:169(	ATP6V1G1P6		Pseudoger	chr5:78222184-7822
ENSG0000(	585	14.63139	chr5:169(	ENSG00000289274		lncRNA	chr5:93598593-9360
ENSG0000(	585	14.63139	chr5:169(	PDE8B		protein_c	chr5:77210449-7742
ENSG0000(	585	14.63139	chr5:169(	ENSG00000241059		lncRNA	chr5:91355380-9135
ENSG0000(	585	14.63139	chr5:169(	RN7SL629P		smallRNA	chr5:87294191-8729
ENSG0000(	585	14.63139	chr5:169(	ENSG00000287180		lncRNA	chr5:93621683-9367
ENSG0000(	585	14.63139	chr5:169(	CERT1		protein_c	chr5:75356345-7551
ENSG0000(	585	14.63139	chr5:169(	HMGCR		protein_c	chr5:75336329-7536
ENSG0000(	585	14.63139	chr5:169(	ENSG00000212930		lncRNA	chr5:91223419-9122
ENSG0000(	585	14.63139	chr5:169(	ENSG00000279232		lncRNA	chr5:98792861-9879
ENSG0000(	585	14.63139	chr5:169(	RAP1BL		Pseudoger	chr5:76173629-7617
ENSG0000(	585	14.63139	chr5:169(	SNORA18		smallRNA	chr5:79220942-7922
ENSG0000(	585	14.63139	chr5:169(	FAM174A	DriverDB\	protein_c	chr5:100535374-100
ENSG0000(	585	14.63139	chr5:169(	ENSG00000287090		lncRNA	chr5:73195314-7320
ENSG0000(	585	14.63139	chr5:169(	ARHGEF28		protein_c	chr5:73626158-7394
ENSG0000(	585	14.63139	chr5:169(	ENSG00000214942		lncRNA	chr5:89900664-8999
ENSG0000(	585	14.63139	chr5:169(	VCAN-AS1		lncRNA	chr5:83531352-8358
ENSG0000(	585	14.63139	chr5:169(	SLCO4C1		protein_c	chr5:102233986-102
ENSG0000(	585	14.63139	chr5:169(	ENSG00000241597		Pseudoger	chr5:98954394-9895
ENSG0000(	585	14.63139	chr5:169(	MTND4LP5		Pseudoger	chr5:100050359-100
ENSG0000(	585	14.63139	chr5:169(	TMEM167A		protein_c	chr5:83052846-8307
ENSG0000(	585	14.63139	chr5:169(	POLR3G		protein_c	chr5:90471748-9051
ENSG0000(	585	14.63139	chr5:169(	ENSG00000249842		lncRNA	chr5:85446974-8544
ENSG0000(	585	14.63139	chr5:169(	RASGRF2	NCGv7	protein_c	chr5:80960363-8123
ENSG0000(	585	14.63139	chr5:169(	MTCO3P22		Pseudoger	chr5:100051133-100
ENSG0000(	585	14.63139	chr5:169(	MSH3	NCGv7	protein_c	chr5:80654652-8087
ENSG0000(	585	14.63139	chr5:169(	SNORA70		smallRNA	chr5:88382772-8838
ENSG0000(	585	14.63139	chr5:169(	LINC01333		lncRNA	chr5:74321293-7434
ENSG0000(	585	14.63139	chr5:169(	ENSG00000287162		lncRNA	chr5:77958198-7795
ENSG0000(	585	14.63139	chr5:169(	RN7SKP34		smallRNA	chr5:87102603-8710
ENSG0000(	585	14.63139	chr5:169(	THBS4		protein_c	chr5:79991311-8008
ENSG0000(	585	14.63139	chr5:169(	BTF3		protein_c	chr5:73498408-7350
ENSG0000(	585	14.63139	chr5:169(	KIAA0825		protein_c	chr5:94150851-9461
ENSG0000(	585	14.63139	chr5:169(	ANKDD1B		protein_c	chr5:75611182-7568
ENSG0000(	585	14.63139	chr5:169(	ENSG00000249856		lncRNA	chr5:74917726-7502
ENSG0000(	585	14.63139	chr5:169(	ENSG00000249857		lncRNA	chr5:82940458-8294
ENSG0000(	585	14.63139	chr5:169(	MTND3P19		Pseudoger	chr5:100050719-100
ENSG0000(	585	14.63139	chr5:169(	MTCO1P24		Pseudoger	chr5:98410235-9841
ENSG0000(	585	14.63139	chr5:169(	SPATA9		protein_c	chr5:95652181-9569
ENSG0000(	585	14.63139	chr5:169(	COX7C	NCGv7	protein_c	chr5:86617928-8662
ENSG0000(	585	14.63139	chr5:169(	BTF3-DT		lncRNA	chr5:73497549-7349
ENSG0000(	585	14.63139	chr5:169(	ST8SIA4		protein_c	chr5:100806933-100
ENSG0000(	585	14.63139	chr5:169(	snoU13		smallRNA	chr5:80239487-8023
ENSG0000(	585	14.63139	chr5:169(	ENSG00000249169		lncRNA	chr5:92654848-9267
ENSG0000(	585	14.63139	chr5:169(	ENSG00000242477		Pseudoger	chr5:86884231-8688

ENSG0000(	585	14.63139	chr5:169(	RPL7P24		Pseudoger	chr5:80500332-8050
ENSG0000(	585	14.63139	chr5:169(	ENSG00000287447		lncRNA	chr5:93790505-9380
ENSG0000(	585	14.63139	chr5:169(	ENSG00000289462		lncRNA	chr5:88140403-8814
ENSG0000(	585	14.63139	chr5:169(	AC024568.1		smallRNA	chr5:78056624-7805
ENSG0000(	585	14.63139	chr5:169(	ENSG00000249153		lncRNA	chr5:85848502-8584
ENSG0000(	585	14.63139	chr5:169(	ENSG00000249157		Pseudoger	chr5:75006713-7500
ENSG0000(	585	14.63139	chr5:169(	RNU6-183P		smallRNA	chr5:78314916-7831
ENSG0000(	585	14.63139	chr5:169(	RPS3AP22		Pseudoger	chr5:88381957-8838
ENSG0000(	585	14.63139	chr5:169(	KRT8P32		Pseudoger	chr5:98392070-9839
ENSG0000(	585	14.63139	chr5:169(	TBCA		protein_c	chr5:77691166-7786
ENSG0000(	585	14.63139	chr5:169(	RNU6-804P		smallRNA	chr5:86663144-8666
ENSG0000(	585	14.63139	chr5:169(	HNRNPA1P12		Pseudoger	chr5:80359080-8036
ENSG0000(	585	14.63139	chr5:169(	ENSG00000286818		lncRNA	chr5:79840797-7984
ENSG0000(	585	14.63139	chr5:169(	LINC02060		lncRNA	chr5:88408982-8843
ENSG0000(	585	14.63139	chr5:169(	Y_RNA		smallRNA	chr5:98936638-9893
ENSG0000(	585	14.63139	chr5:169(	RNU6-606P		smallRNA	chr5:87299687-8729
ENSG0000(	585	14.63139	chr5:169(	RN7SL378P		smallRNA	chr5:82078427-8207
ENSG0000(	585	14.63139	chr5:169(	MTCO1P22		Pseudoger	chr5:100053686-100
ENSG0000(	585	14.63139	chr5:169(	AC099522.1		Pseudoger	chr5:73446256-7344
ENSG0000(	585	14.63139	chr5:169(	RIOK2	NCGv7	protein_c	chr5:97160867-9718
ENSG0000(	585	14.63139	chr5:169(	ENSG00000249149		Pseudoger	chr5:73382384-7341
ENSG0000(	585	14.63139	chr5:169(	ENSG00000289535		lncRNA	chr5:74948146-7497
ENSG0000(	585	14.63139	chr5:169(	S100Z		protein_c	chr5:76850001-7692
ENSG0000(	585	14.63139	chr5:169(	ENSG00000249135		Pseudoger	chr5:98203059-9820
ENSG0000(	585	14.63139	chr5:169(	OTP		protein_c	chr5:77628712-7763
ENSG0000(	585	14.63139	chr5:169(	ENSG00000249101		Pseudoger	chr5:98025965-9802
ENSG0000(	585	14.63139	chr5:169(	ENSG00000249664		lncRNA	chr5:83012285-8301
ENSG0000(	585	14.63139	chr5:169(	ENSG00000249655		lncRNA	chr5:80630313-8063
ENSG0000(	585	14.63139	chr5:169(	RNU7-175P		smallRNA	chr5:75538598-7553
ENSG0000(	585	14.63139	chr5:169(	ENSG00000250124		lncRNA	chr5:86380660-8638
ENSG0000(	585	14.63139	chr5:169(	MCTP1		protein_c	chr5:94703690-9528
ENSG0000(	585	14.63139	chr5:169(	RNY3P1		smallRNA	chr5:79170234-7917
ENSG0000(	585	14.63139	chr5:169(	ENSG00000206356		Pseudoger	chr5:99522311-9952
ENSG0000(	585	14.63139	chr5:169(	RFESD		protein_c	chr5:95646754-9568
ENSG0000(	585	14.63139	chr5:169(	MIR2277		smallRNA	chr5:93620696-9362
ENSG0000(	585	14.63139	chr5:169(	PCSK1		protein_c	chr5:96390333-9643
ENSG0000(	585	14.63139	chr5:169(	ENSG00000239517		Pseudoger	chr5:74931662-7493
ENSG0000(	585	14.63139	chr5:169(	ENSG00000286721		lncRNA	chr5:81408517-8141
ENSG0000(	585	14.63139	chr5:169(	ENSG00000286638		lncRNA	chr5:91280229-9128
ENSG0000(	585	14.63139	chr5:169(	ENC1	NCGv7	protein_c	chr5:74627406-7464
ENSG0000(	585	14.63139	chr5:169(	ENSG00000272406		lncRNA	chr5:93741640-9374
ENSG0000(	585	14.63139	chr5:169(	ENSG00000184188		Pseudoger	chr5:80299678-8030
ENSG0000(	585	14.63139	chr5:169(	AC026781.1		smallRNA	chr5:92717432-9271
ENSG0000(	585	14.63139	chr5:169(	ENSG00000250240		lncRNA	chr5:95701249-9573
ENSG0000(	585	14.63139	chr5:169(	ENSG00000240388		Pseudoger	chr5:91354478-9135
ENSG0000(	585	14.63139	chr5:169(	ENSG00000249100		lncRNA	chr5:82765404-8276
ENSG0000(	585	14.63139	chr5:169(	AC093311.1		smallRNA	chr5:94578769-9457
ENSG0000(	585	14.63139	chr5:169(	ENSG00000280009		TEC	chr5:86305233-8630
ENSG0000(	585	14.63139	chr5:169(	ENSG00000270133		lncRNA	chr5:94611906-9461
ENSG0000(	585	14.63139	chr5:169(	ENSG00000251675		lncRNA	chr5:80128361-8014
ENSG0000(	585	14.63139	chr5:169(	ZP3P1		Pseudoger	chr5:83875166-8387

ENSG0000(	585	14.63139	chr5:169(	RNU6-727P		smallRNA	chr5:87420341-8742
ENSG0000(	585	14.63139	chr5:169(	ENSG00000196390		Pseudoger	chr5:74929880-7493
ENSG0000(	585	14.63139	chr5:169(	ENSG00000289912		lncRNA	chr5:100903394-100
ENSG0000(	585	14.63139	chr5:169(	SETP22		Pseudoger	chr5:97056402-9705
ENSG0000(	585	14.63139	chr5:169(	ENSG00000250158		lncRNA	chr5:96247776-9627
ENSG0000(	585	14.63139	chr5:169(	ENSG00000286509		lncRNA	chr5:93088856-9309
ENSG0000(	585	14.63139	chr5:169(	ENSG00000249175		lncRNA	chr5:94788789-9479
ENSG0000(	585	14.63139	chr5:169(	GUSBP8		Pseudoger	chr5:99532628-9953
ENSG0000(	585	14.63139	chr5:169(	ENSG00000272109		lncRNA	chr5:96803688-9680
ENSG0000(	585	14.63139	chr5:169(	RPS23		protein_c	chr5:82273320-8227
ENSG0000(	585	14.63139	chr5:169(	ENSG00000249061		lncRNA	chr5:86967321-8713
ENSG0000(	585	14.63139	chr5:169(	ENSG00000250253		Pseudoger	chr5:85210060-8521
ENSG0000(	585	14.63139	chr5:169(	Y_RNA		smallRNA	chr5:73100073-7310
ENSG0000(	585	14.63139	chr5:169(	CSNK1A1P3		Pseudoger	chr5:98833382-9883
ENSG0000(	585	14.63139	chr5:169(	ENSG00000249772		lncRNA	chr5:81113385-8111
ENSG0000(	585	14.63139	chr5:169(	FABP5P5		Pseudoger	chr5:95973041-9597
ENSG0000(	585	14.63139	chr5:169(	ENSG00000249761		Pseudoger	chr5:97336858-9733
ENSG0000(	585	14.63139	chr5:169(	EIF3KP1		Pseudoger	chr5:103032376-103
ENSG0000(	585	14.63139	chr5:169(	ENSG00000249746		lncRNA	chr5:96213263-9621
ENSG0000(	585	14.63139	chr5:169(	AC022142.1		smallRNA	chr5:98639097-9863
ENSG0000(	585	14.63139	chr5:169(	ENSG00000249180		lncRNA	chr5:96741079-9674
ENSG0000(	585	14.63139	chr5:169(	GLRX		protein_c	chr5:95751319-9582
ENSG0000(	585	14.63139	chr5:169(	ENSG00000286828		lncRNA	chr5:97183827-9721
ENSG0000(	585	14.63139	chr5:169(	ENSG00000249713		lncRNA	chr5:76606608-7660
ENSG0000(	585	14.63139	chr5:169(	ENSG00000289184		lncRNA	chr5:88143177-8814
ENSG0000(	585	14.63139	chr5:169(	RPL5P16		Pseudoger	chr5:82777797-8277
ENSG0000(	585	14.63139	chr5:169(	ENSG00000184084		Pseudoger	chr5:73803296-7380
ENSG0000(	585	14.63139	chr5:169(	RNU6-211P		smallRNA	chr5:80365726-8036
ENSG0000(	585	14.63139	chr5:169(	ENSG00000278905		TEC	chr5:93600357-9360
ENSG0000(	585	14.63139	chr5:169(	AC020900.2		smallRNA	chr5:96694531-9669
ENSG0000(	585	14.63139	chr5:169(	AC020900.1		smallRNA	chr5:96590192-9659
ENSG0000(	585	14.63139	chr5:169(	ENSG00000238254		Pseudoger	chr5:79849552-7984
ENSG0000(	585	14.63139	chr5:169(	ENSG00000249743		lncRNA	chr5:73213930-7329
ENSG0000(	585	14.63139	chr5:169(	ENSG00000289924		lncRNA	chr5:76818312-7681
ENSG0000(	585	14.63139	chr5:169(	NR2F1		protein_c	chr5:93583222-9359
ENSG0000(	585	14.63139	chr5:169(	ENSG00000250071		Pseudoger	chr5:74798994-7480
ENSG0000(	585	14.63139	chr5:169(	ENSG00000242198		Pseudoger	chr5:75374463-7537
ENSG0000(	585	14.63139	chr5:169(	Y_RNA		smallRNA	chr5:96962656-9696
ENSG0000(	585	14.63139	chr5:169(	AC114969.1		smallRNA	chr5:81978250-8197
ENSG0000(	585	14.63139	chr5:169(	ENSG00000286577		lncRNA	chr5:94111720-9417
ENSG0000(	584	14.60638	chr1:116(	MIR3972		smallRNA	chr1:17277889-1727
ENSG0000(	583	14.58137	chr1:123(	MIR4258		smallRNA	chr1:154975693-154
ENSG0000(	579	14.48133	chr1:116(	ENSG00000290006		lncRNA	chr1:26692586-2669
ENSG0000(	579	14.48133	chr1:4061	RN7SL668P		smallRNA	chr1:234904186-234
ENSG0000(	578	14.45632	chr5:103(	RAB9BP1		Pseudoger	chr5:105099473-105
ENSG0000(	578	14.45632	chr1:116(	ENSG00000227311		Pseudoger	chr1:40333078-4033
ENSG0000(	578	14.45632	chr1:116(	ZC3H12A	NCGv7	protein_c	chr1:37474580-3748
ENSG0000(	578	14.45632	chr1:116(	snoU13		smallRNA	chr1:37750202-3775
ENSG0000(	578	14.45632	chr1:116(	CAP1		protein_c	chr1:40040233-4007
ENSG0000(	578	14.45632	chr1:116(	MEAF6		protein_c	chr1:37489993-3751
ENSG0000(	578	14.45632	chr5:103(	RN7SL255P		smallRNA	chr5:104260533-104

ENSG0000(	578	14.45632	chr1:116(	DNALI1		protein_c	chr1:37556919-3756
ENSG0000(	578	14.45632	chr1:116(	ZFP69		protein_c	chr1:40477290-4049
ENSG0000(	578	14.45632	chr1:116(	MIR3659HG		lncRNA	chr1:38047314-3811
ENSG0000(	578	14.45632	chr5:103(	ENSG00000253776		lncRNA	chr5:104773641-104
ENSG0000(	578	14.45632	chr1:116(	ZFP69B		protein_c	chr1:40450102-4046
ENSG0000(	578	14.45632	chr1:116(	ENSG00000227416		Pseudoger	chr1:37782457-3778
ENSG0000(	578	14.45632	chr1:116(	SNIP1		protein_c	chr1:37534449-3755
ENSG0000(	578	14.45632	chr1:116(	ENSG00000287987		lncRNA	chr1:38193619-3821
ENSG0000(	578	14.45632	chr1:116(	HEYL		protein_c	chr1:39623435-3963
ENSG0000(	578	14.45632	chr1:116(	ENSG00000279667		TEC	chr1:40473055-4047
ENSG0000(	578	14.45632	chr1:116(	ZC3H12A-DT		lncRNA	chr1:37350934-3747
ENSG0000(	578	14.45632	chr1:116(	RSPO1		protein_c	chr1:37611350-3763
ENSG0000(	578	14.45632	chr1:116(	LINC01343		lncRNA	chr1:38209034-3821
ENSG0000(	578	14.45632	chr1:116(	ENSG00000236505		Pseudoger	chr1:40563534-4056
ENSG0000(	578	14.45632	chr1:116(	RLF		protein_c	chr1:40161387-4024
ENSG0000(	578	14.45632	chr1:116(	ZNF684		protein_c	chr1:40531573-4054
ENSG0000(	578	14.45632	chr1:116(	ENSG00000286838		lncRNA	chr1:40559666-4058
ENSG0000(	578	14.45632	chr1:116(	CDCA8		protein_c	chr1:37692481-3770
ENSG0000(	578	14.45632	chr1:116(	ENSG00000286552		lncRNA	chr1:38149544-3816
ENSG0000(	578	14.45632	chr1:116(	ENSG00000289711		Pseudoger	chr1:39733327-3973
ENSG0000(	578	14.45632	chr1:116(	ENSG00000231296		Pseudoger	chr1:40262672-4026
ENSG0000(	578	14.45632	chr1:116(	MYCL-AS1		lncRNA	chr1:39897745-3989
ENSG0000(	578	14.45632	chr5:103(	LINC01950		lncRNA	chr5:106815197-107
ENSG0000(	578	14.45632	chr1:116(	RPS29P6		Pseudoger	chr1:37330852-3733
ENSG0000(	578	14.45632	chr1:116(	GNL2		protein_c	chr1:37566816-3759
ENSG0000(	578	14.45632	chr1:116(	MYCL	NCGv7;AC	protein_c	chr1:39895426-3990
ENSG0000(	578	14.45632	chr5:103(	ENSG00000253584		lncRNA	chr5:104917492-105
ENSG0000(	578	14.45632	chr1:116(	NT5C1A		protein_c	chr1:39651229-3967
ENSG0000(	578	14.45632	chr1:116(	MFSD2A		protein_c	chr1:39955112-3996
ENSG0000(	578	14.45632	chr1:116(	SNORA55		smallRNA	chr1:39567374-3956
ENSG0000(	578	14.45632	chr1:116(	RNU6-584P		smallRNA	chr1:37885023-3788
ENSG0000(	578	14.45632	chr1:116(	OXCT2	NCGv7	protein_c	chr1:39769523-3977
ENSG0000(	578	14.45632	chr1:116(	C1orf109		protein_c	chr1:37681570-3769
ENSG0000(	578	14.45632	chr1:116(	AL929472.1		protein_c	chr1:37826560-3782
ENSG0000(	578	14.45632	chr5:103(	NIHCOLE		lncRNA	chr5:104079847-104
ENSG0000(	578	14.45632	chr1:116(	EXO5		protein_c	chr1:40508741-4051
ENSG0000(	578	14.45632	chr1:116(	HPCAL4		protein_c	chr1:39678648-3969
ENSG0000(	578	14.45632	chr1:116(	TMCO2		protein_c	chr1:40245947-4025
ENSG0000(	578	14.45632	chr1:116(	BMP8B		protein_c	chr1:39757182-3978
ENSG0000(	578	14.45632	chr1:116(	MTF1		protein_c	chr1:37809574-3785
ENSG0000(	578	14.45632	chr1:116(	PABPC4-AS1		lncRNA	chr1:39565052-3957
ENSG0000(	578	14.45632	chr5:103(	ENSG00000251204		Pseudoger	chr5:106415576-106
ENSG0000(	578	14.45632	chr1:116(	ENSG00000227278		lncRNA	chr1:40514461-4051
ENSG0000(	578	14.45632	chr1:116(	BMP8A		protein_c	chr1:39491636-3952
ENSG0000(	578	14.45632	chr5:103(	RNA5SP189		Pseudoger	chr5:105922994-105
ENSG0000(	578	14.45632	chr1:116(	BMP8B-AS1		lncRNA	chr1:39779969-3978
ENSG0000(	578	14.45632	chr1:116(	LINC02811		lncRNA	chr1:39801414-3981
ENSG0000(	578	14.45632	chr1:116(	ENSG00000284677		lncRNA	chr1:40436199-4045
ENSG0000(	578	14.45632	chr1:116(	ACTN4P2		Pseudoger	chr1:37776670-3777
ENSG0000(	578	14.45632	chr1:116(	GTF2F2P2		Pseudoger	chr1:40593633-4059
ENSG0000(	578	14.45632	chr1:116(	Y_RNA		smallRNA	chr1:39944890-3994

ENSG0000(	578	14.45632	chr1:116(	ENSG00000229213		Pseudoger	chr1:39795843-3979
ENSG0000(	578	14.45632	chr1:116(	PPT1		protein_c	chr1:40072710-4009
ENSG0000(	578	14.45632	chr5:103(	RNU6-334P		smallRNA	chr5:104780288-104
ENSG0000(	578	14.45632	chr1:116(	ENSG00000223589		Pseudoger	chr1:38080572-3808
ENSG0000(	578	14.45632	chr1:116(	LINC02786		lncRNA	chr1:38129464-3814
ENSG0000(	578	14.45632	chr1:373	ENSG00000288925		lncRNA	chr1:203290128-203
ENSG0000(	578	14.45632	chr1:116(	COL9A2	NCGv7	protein_c	chr1:40300489-4031
ENSG0000(	578	14.45632	chr1:116(	PPIEL		Pseudoger	chr1:39531838-3955
ENSG0000(	578	14.45632	chr1:116(	ZMPSTE24-DT		lncRNA	chr1:40256333-4025
ENSG0000(	578	14.45632	chr1:116(	SNORA63		smallRNA	chr1:37884237-3788
ENSG0000(	578	14.45632	chr1:116(	ENSG00000260920		lncRNA	chr1:40464319-4046
ENSG0000(	578	14.45632	chr1:116(	RNU7-121P		smallRNA	chr1:39723566-3972
ENSG0000(	578	14.45632	chr1:116(	ENSG00000237749		Pseudoger	chr1:37556247-3755
ENSG0000(	578	14.45632	chr1:116(	INPP5B		protein_c	chr1:37860697-3794
ENSG0000(	578	14.45632	chr1:116(	C1orf122		protein_c	chr1:37806979-3780
ENSG0000(	578	14.45632	chr1:116(	MIR5581		smallRNA	chr1:37500935-3750
ENSG0000(	578	14.45632	chr1:116(	YRDC		protein_c	chr1:37802945-3780
ENSG0000(	578	14.45632	chr1:116(	Y_RNA		smallRNA	chr1:39881566-3988
ENSG0000(	578	14.45632	chr1:116(	ENSG00000261798		lncRNA	chr1:39788976-3979
ENSG0000(	578	14.45632	chr1:116(	ENSG00000284719		lncRNA	chr1:39799419-3980
ENSG0000(	578	14.45632	chr5:103(	PSMC1P5		Pseudoger	chr5:107195156-107
ENSG0000(	578	14.45632	chr1:116(	EXO5-DT		lncRNA	chr1:40493157-4050
ENSG0000(	578	14.45632	chr1:116(	UTP11		protein_c	chr1:38009258-3802
ENSG0000(	578	14.45632	chr1:116(	PABPC4	NCGv7	protein_c	chr1:39560709-3957
ENSG0000(	578	14.45632	chr1:116(	SF3A3		protein_c	chr1:37956975-3799
ENSG0000(	578	14.45632	chr1:116(	FHL3		protein_c	chr1:37996770-3800
ENSG0000(	578	14.45632	chr1:116(	ENSG00000238186		lncRNA	chr1:40515754-4051
ENSG0000(	578	14.45632	chr1:116(	ENSG00000228477		Pseudoger	chr1:39962680-3996
ENSG0000(	578	14.45632	chr5:103(	ENSG00000250145		Pseudoger	chr5:106543066-106
ENSG0000(	578	14.45632	chr5:103(	ENSG00000278958		TEC	chr5:104434772-104
ENSG0000(	578	14.45632	chr1:116(	ENSG00000225333		Pseudoger	chr1:39718028-3971
ENSG0000(	578	14.45632	chr1:116(	ENSG00000213172		Pseudoger	chr1:40364766-4036
ENSG0000(	578	14.45632	chr1:116(	TRIT1		protein_c	chr1:39838110-3988
ENSG0000(	578	14.45632	chr1:116(	OAZ1P1		Pseudoger	chr1:40132764-4013
ENSG0000(	578	14.45632	chr5:103(	ENSG00000251574		lncRNA	chr5:104383298-105
ENSG0000(	578	14.45632	chr1:116(	ENSG00000284748		lncRNA	chr1:37596126-3760
ENSG0000(	578	14.45632	chr5:103(	RNU1-140P		smallRNA	chr5:104098874-104
ENSG0000(	578	14.45632	chr1:116(	Y_RNA		smallRNA	chr1:37737955-3773
ENSG0000(	578	14.45632	chr1:116(	MIR3659		smallRNA	chr1:38089231-3808
ENSG0000(	578	14.45632	chr1:116(	PPIEL		lncRNA	chr1:39522280-3955
ENSG0000(	578	14.45632	chr1:116(	ENSG00000230955		lncRNA	chr1:37860697-3786
ENSG0000(	578	14.45632	chr1:116(	MANEAL		protein_c	chr1:37793847-3780
ENSG0000(	578	14.45632	chr1:116(	EPHA10	DriverDB	protein_c	chr1:37713880-3776
ENSG0000(	578	14.45632	chr1:116(	RNU6-1237P		smallRNA	chr1:40177843-4017
ENSG0000(	578	14.45632	chr5:103(	SNORA31		smallRNA	chr5:106546665-106
ENSG0000(	578	14.45632	chr1:116(	FTH1P1		Pseudoger	chr1:37544763-3754
ENSG0000(	578	14.45632	chr1:116(	RNU6-510P		smallRNA	chr1:37991462-3799
ENSG0000(	578	14.45632	chr1:116(	ZMPSTE24		protein_c	chr1:40258041-4029
ENSG0000(	578	14.45632	chr1:116(	PPIE		protein_c	chr1:39692182-3976
ENSG0000(	578	14.45632	chr1:116(	ENSG00000225903		lncRNA	chr1:39633416-3963
ENSG0000(	578	14.45632	chr1:116(	ENSG00000233728		lncRNA	chr1:37799720-3780

ENSG0000(	578	14.45632	chr1:116(	POU3F1	protein_c	chr1:38043829-3804
ENSG0000(	578	14.45632	chr1:116(	SMAP2	protein_c	chr1:40344850-4042
ENSG0000(	578	14.45632	chr5:103(	ENSG00000283462	lncRNA	chr5:103880129-103
ENSG0000(	578	14.45632	chr1:116(	OXCT2P1	Pseudoger	chr1:39514956-3951
ENSG0000(	576	14.4063	chr1:116(	ENSG00000270733	Pseudoger	chr1:26263041-2626
ENSG0000(	576	14.4063	chr2:744(	RN7SL297P	smallRNA	chr2:111930175-111
ENSG0000(	575	14.38128	chr2:470(	ENSG00000235586	lncRNA	chr2:38601598-3860
ENSG0000(	574	14.35627	chr1:373(	SIGLEC30P	Pseudoger	chr1:170115636-170
ENSG0000(	574	14.35627	chr1:373(	RN7SL333P	smallRNA	chr1:169859756-169
ENSG0000(	574	14.35627	chr1:373(	ENSG00000289425	lncRNA	chr1:174967328-174
ENSG0000(	574	14.35627	chr1:373(	ENSG00000226375	lncRNA	chr1:173174300-173
ENSG0000(	574	14.35627	chr1:373(	GORAB-AS1	lncRNA	chr1:170460453-170
ENSG0000(	574	14.35627	chr1:373(	ENSG00000289426	lncRNA	chr1:173637713-173
ENSG0000(	574	14.35627	chr1:373(	LINC01741	lncRNA	chr1:177700524-177
ENSG0000(	574	14.35627	chr1:373(	QRSL1P1	Pseudoger	chr1:168449672-168
ENSG0000(	574	14.35627	chr1:373(	RN7SKP160	smallRNA	chr1:173791548-173
ENSG0000(	574	14.35627	chr1:373(	ISCUP1	Pseudoger	chr1:170211010-170
ENSG0000(	574	14.35627	chr1:373(	SLC25A38P1	Pseudoger	chr1:172748560-172
ENSG0000(	574	14.35627	chr1:373(	ENSG00000234604	Pseudoger	chr1:169474060-169
ENSG0000(	574	14.35627	chr1:373(	ENSG00000227815	Pseudoger	chr1:176272483-176
ENSG0000(	574	14.35627	chr1:373(	GM2AP2	Pseudoger	chr1:171392229-171
ENSG0000(	574	14.35627	chr1:373(	ENSG00000227579	lncRNA	chr1:177392667-177
ENSG0000(	574	14.35627	chr1:373(	TEX50	protein_c	chr1:173635338-173
ENSG0000(	574	14.35627	chr1:373(	MIR488	smallRNA	chr1:177029363-177
ENSG0000(	574	14.35627	chr1:373(	ENSG00000285777	protein_c	chr1:173596060-173
ENSG0000(	574	14.35627	chr1:373(	ENSG00000230704	lncRNA	chr1:169762929-169
ENSG0000(	574	14.35627	chr1:373(	ENSG00000231424	lncRNA	chr1:170748573-171
ENSG0000(	574	14.35627	chr1:373(	ENSG00000287831	lncRNA	chr1:168898633-168
ENSG0000(	574	14.35627	chr1:373(	ENSG00000235575	lncRNA	chr1:169310665-169
ENSG0000(	574	14.35627	chr1:373(	RPS29P4	Pseudoger	chr1:175297080-175
ENSG0000(	574	14.35627	chr1:373(	ENSG00000230687	lncRNA	chr1:175203228-175
ENSG0000(	574	14.35627	chr1:373(	LINC01681	lncRNA	chr1:170173865-170
ENSG0000(	574	14.35627	chr1:373(	ENTR1P2	Pseudoger	chr1:175044626-175
ENSG0000(	574	14.35627	chr1:373(	RN7SL269P	smallRNA	chr1:169957944-169
ENSG0000(	574	14.35627	chr1:373(	ENSG00000236741	Pseudoger	chr1:171762074-171
ENSG0000(	574	14.35627	chr1:373(	BANF1P4	Pseudoger	chr1:174756850-174
ENSG0000(	574	14.35627	chr1:373(	ANKRD45	protein_c	chr1:173608336-173
ENSG0000(	574	14.35627	chr1:373(	ENSG00000238272	lncRNA	chr1:173555251-173
ENSG0000(	574	14.35627	chr1:373(	ENSG00000285622	lncRNA	chr1:168695874-169
ENSG0000(	574	14.35627	chr1:373(	ENSG00000227722	Pseudoger	chr1:168317497-168
ENSG0000(	574	14.35627	chr1:373(	ENSG00000224600	Pseudoger	chr1:171824610-171
ENSG0000(	574	14.35627	chr1:373(	BX284613.1	smallRNA	chr1:171041347-171
ENSG0000(	574	14.35627	chr1:373(	ENSG00000231020	Pseudoger	chr1:176305672-176
ENSG0000(	574	14.35627	chr1:373(	LINC02803	lncRNA	chr1:175904762-175
ENSG0000(	574	14.35627	chr1:373(	ENSG00000289466	TEC	chr1:169112164-169
ENSG0000(	574	14.35627	chr1:373(	FMO4	protein_c	chr1:171314183-171
ENSG0000(	574	14.35627	chr1:373(	KLHL20	protein_c	chr1:173714941-173
ENSG0000(	574	14.35627	chr1:373(	AL021398.1	smallRNA	chr1:177186360-177
ENSG0000(	574	14.35627	chr1:373(	snoU13	smallRNA	chr1:174200129-174
ENSG0000(	574	14.35627	chr1:373(	NDUFAF4P4	Pseudoger	chr1:174849667-174
ENSG0000(	574	14.35627	chr1:373(	DNM3OS	lncRNA	chr1:172138397-172

ENSG0000(	574	14.35627	chr1:3732	COP1-DT		lncRNA	chr1:176207646-176
ENSG0000(	574	14.35627	chr1:3732	MIR214		smallRNA	chr1:172138798-172
ENSG0000(	574	14.35627	chr1:3732	ENSG00000235869		Pseudoger	chr1:174922107-174
ENSG0000(	574	14.35627	chr1:3732	snoU13		smallRNA	chr1:171481907-171
ENSG0000(	574	14.35627	chr1:3732	RABGAP1L-DT		lncRNA	chr1:174110268-174
ENSG0000(	574	14.35627	chr1:3732	SLC9C2	NCGv7	protein_c	chr1:173500460-173
ENSG0000(	574	14.35627	chr1:3732	ENSG00000224228		lncRNA	chr1:172775905-173
ENSG0000(	574	14.35627	chr1:3732	RNU6-773P		smallRNA	chr1:171519816-171
ENSG0000(	574	14.35627	chr1:3732	PTP4A1P7		Pseudoger	chr1:176616273-176
ENSG0000(	574	14.35627	chr1:3732	RNU6-290P		smallRNA	chr1:171418644-171
ENSG0000(	574	14.35627	chr1:3732	ENSG00000286754		lncRNA	chr1:176829128-176
ENSG0000(	574	14.35627	chr1:3732	snoU13		smallRNA	chr1:173660097-173
ENSG0000(	574	14.35627	chr1:3732	SUMO1P2		Pseudoger	chr1:168898136-168
ENSG0000(	574	14.35627	chr1:3732	RNU6-157P		smallRNA	chr1:172366540-172
ENSG0000(	574	14.35627	chr1:3732	HMGB1P11		Pseudoger	chr1:171270954-171
ENSG0000(	574	14.35627	chr1:3732	RC3H1-IT1		lncRNA	chr1:174009267-174
ENSG0000(	574	14.35627	chr1:3732	SNORD78		smallRNA	chr1:173865622-173
ENSG0000(	574	14.35627	chr1:3732	ENSG00000224000		lncRNA	chr1:172906900-172
ENSG0000(	574	14.35627	chr1:3732	SRP14P4		Pseudoger	chr1:171345105-171
ENSG0000(	574	14.35627	chr1:3732	ENSG00000226552		Pseudoger	chr1:171083565-171
ENSG0000(	574	14.35627	chr1:3732	Y_RNA		smallRNA	chr1:171253906-171
ENSG0000(	574	14.35627	chr1:3732	PIGC	NCGv7	protein_c	chr1:172370189-172
ENSG0000(	574	14.35627	chr1:3732	RPL7AP19		Pseudoger	chr1:168542737-168
ENSG0000(	574	14.35627	chr1:3732	CYCSP53		Pseudoger	chr1:171444699-171
ENSG0000(	574	14.35627	chr1:3732	ENSG00000271459		Pseudoger	chr1:171755803-171
ENSG0000(	574	14.35627	chr1:3732	RPL4P3		Pseudoger	chr1:171683128-171
ENSG0000(	574	14.35627	chr1:3732	AL022400.1		smallRNA	chr1:174348265-174
ENSG0000(	574	14.35627	chr1:3732	TNR-IT1		lncRNA	chr1:175538775-175
ENSG0000(	574	14.35627	chr1:3732	RABGAP1L		protein_c	chr1:174159410-174
ENSG0000(	574	14.35627	chr1:3732	BRINP2		protein_c	chr1:177170958-177
ENSG0000(	574	14.35627	chr1:3732	FMO2		protein_c	chr1:171185249-171
ENSG0000(	574	14.35627	chr1:3732	ENSG00000237317		Pseudoger	chr1:174367105-174
ENSG0000(	574	14.35627	chr1:3732	MIR199A2		smallRNA	chr1:172144535-172
ENSG0000(	574	14.35627	chr1:3732	SUCO		protein_c	chr1:172532349-172
ENSG0000(	574	14.35627	chr1:3732	DNM3		protein_c	chr1:171817887-172
ENSG0000(	574	14.35627	chr1:3732	RABGAP1L-AS1		lncRNA	chr1:174934947-174
ENSG0000(	574	14.35627	chr1:3732	ASTN1	NCGv7	protein_c	chr1:176857302-177
ENSG0000(	574	14.35627	chr1:3732	ENSG00000287282		lncRNA	chr1:169059576-169
ENSG0000(	574	14.35627	chr1:3732	ENSG00000287336		lncRNA	chr1:172210711-172
ENSG0000(	574	14.35627	chr1:3732	MYOCOS		protein_c	chr1:171600621-171
ENSG0000(	574	14.35627	chr1:3732	RC3H1		protein_c	chr1:173931084-174
ENSG0000(	574	14.35627	chr1:3732	RNA5SP68		Pseudoger	chr1:173969318-173
ENSG0000(	574	14.35627	chr1:3732	ENSG00000237707		lncRNA	chr1:169104124-169
ENSG0000(	574	14.35627	chr1:3732	GOT2P2		Pseudoger	chr1:173141100-173
ENSG0000(	574	14.35627	chr1:3732	C1orf105		protein_c	chr1:172420685-172
ENSG0000(	574	14.35627	chr1:3732	MYOC		protein_c	chr1:171635417-171
ENSG0000(	574	14.35627	chr1:3732	RC3H1-DT		lncRNA	chr1:174022509-174
ENSG0000(	574	14.35627	chr1:3732	ENSG00000235736		lncRNA	chr1:168763365-168
ENSG0000(	574	14.35627	chr1:3732	AL122019.1		smallRNA	chr1:177552870-177
ENSG0000(	574	14.35627	chr1:3732	LINC01645		lncRNA	chr1:177351560-177
ENSG0000(	574	14.35627	chr1:3732	MORF4L1P7		Pseudoger	chr1:176367699-176

ENSG0000(	574	14.35627	chr1:3732	GAS5-AS1		lncRNA	chr1:173862473-173
ENSG0000(	574	14.35627	chr1:3732	KIAA0040		protein_c	chr1:175156986-175
ENSG0000(	574	14.35627	chr1:3732	ENSG00000235303		lncRNA	chr1:170598854-170
ENSG0000(	574	14.35627	chr1:3732	ENSG00000288139		Pseudoger	chr1:169915004-169
ENSG0000(	574	14.35627	chr1:3732	TNN	NCGv7	protein_c	chr1:175067833-175
ENSG0000(	574	14.35627	chr1:3732	MRPS14	NCGv7	protein_c	chr1:175010789-175
ENSG0000(	574	14.35627	chr1:3732	RN7SL425P		smallRNA	chr1:171492411-171
ENSG0000(	574	14.35627	chr1:3732	RPS29P5		Pseudoger	chr1:175921975-175
ENSG0000(	574	14.35627	chr1:3732	GAS5		lncRNA	chr1:173858559-173
ENSG0000(	574	14.35627	chr1:3732	AIMP1P2		Pseudoger	chr1:172885947-172
ENSG0000(	574	14.35627	chr1:3732	CENPL	DriverDB\	protein_c	chr1:173799550-173
ENSG0000(	574	14.35627	chr1:3732	LINC01657		lncRNA	chr1:175877343-175
ENSG0000(	574	14.35627	chr1:3732	SELL		protein_c	chr1:169690665-169
ENSG0000(	574	14.35627	chr1:3732	ENSG00000287697		lncRNA	chr1:174998353-174
ENSG0000(	574	14.35627	chr1:3732	TNFSF18		protein_c	chr1:173039202-173
ENSG0000(	574	14.35627	chr1:3732	RPL7AP21		Pseudoger	chr1:168578653-168
ENSG0000(	574	14.35627	chr1:3732	SEC16B	DriverDB\	protein_c	chr1:177923956-177
ENSG0000(	574	14.35627	chr1:3732	GORAB	DriverDB\	protein_c	chr1:170531819-170
ENSG0000(	574	14.35627	chr1:3732	MRPS10P1		Pseudoger	chr1:169990067-169
ENSG0000(	574	14.35627	chr1:3732	ENSG00000231615		Pseudoger	chr1:173362397-173
ENSG0000(	574	14.35627	chr1:3732	ZBTB37	DriverDB\	protein_c	chr1:173868082-173
ENSG0000(	574	14.35627	chr1:3732	F5	NCGv7	protein_c	chr1:169511951-169
ENSG0000(	574	14.35627	chr1:3732	ENSG00000237249		Pseudoger	chr1:174892417-174
ENSG0000(	574	14.35627	chr1:3732	LINC01142		lncRNA	chr1:170271395-170
ENSG0000(	574	14.35627	chr1:3732	ENSG00000271811		lncRNA	chr1:170667381-170
ENSG0000(	574	14.35627	chr1:3732	ENSG00000283255		lncRNA	chr1:168401483-168
ENSG0000(	574	14.35627	chr1:3732	ENSG00000232463		Pseudoger	chr1:176231200-176
ENSG0000(	574	14.35627	chr1:3732	KIFAP3		protein_c	chr1:169921326-170
ENSG0000(	574	14.35627	chr1:3732	FMO3		protein_c	chr1:171090901-171
ENSG0000(	574	14.35627	chr1:3732	ENSG00000225243		lncRNA	chr1:171199244-171
ENSG0000(	574	14.35627	chr1:3732	SELE		protein_c	chr1:169722640-169
ENSG0000(	574	14.35627	chr1:3732	RNU6-693P		smallRNA	chr1:172613428-172
ENSG0000(	574	14.35627	chr1:3732	SLC19A2	DriverDB\	protein_c	chr1:169463909-169
ENSG0000(	574	14.35627	chr1:3732	FMO1		protein_c	chr1:171248471-171
ENSG0000(	574	14.35627	chr1:3732	ENSG00000260990		lncRNA	chr1:175307218-175
ENSG0000(	574	14.35627	chr1:3732	PAPPA2		protein_c	chr1:176463171-176
ENSG0000(	574	14.35627	chr1:3732	ENSG00000279061		TEC	chr1:172752586-172
ENSG0000(	574	14.35627	chr1:3732	snoU13		smallRNA	chr1:173281077-173
ENSG0000(	574	14.35627	chr1:3732	CACYBP		protein_c	chr1:174999163-175
ENSG0000(	574	14.35627	chr1:3732	TNR	NCGv7	protein_c	chr1:175315194-175
ENSG0000(	574	14.35627	chr1:3732	PRRX1	NCGv7	protein_c	chr1:170662728-170
ENSG0000(	574	14.35627	chr1:3732	METTL18		protein_c	chr1:169792529-169
ENSG0000(	574	14.35627	chr1:3732	ENSG00000232959		lncRNA	chr1:170024077-170
ENSG0000(	574	14.35627	chr1:3732	RABGAP1L-IT1		lncRNA	chr1:174896958-174
ENSG0000(	574	14.35627	chr1:3732	SCARNA3		smallRNA	chr1:175968398-175
ENSG0000(	574	14.35627	chr1:3732	RNA5SP66		Pseudoger	chr1:169067264-169
ENSG0000(	574	14.35627	chr1:3732	RNU2-12P		smallRNA	chr1:176243862-176
ENSG0000(	574	14.35627	chr1:3732	ENSG00000232751		Pseudoger	chr1:173351689-173
ENSG0000(	574	14.35627	chr1:3732	ENSG00000225591		Pseudoger	chr1:173741674-173
ENSG0000(	574	14.35627	chr1:3732	SCARNA20		smallRNA	chr1:171768070-171
ENSG0000(	574	14.35627	chr1:3732	MIR1295A		smallRNA	chr1:171101728-171

ENSG0000(	574	14.35627	chr1:3732	LINC00626		lncRNA	chr1:168784012-168
ENSG0000(	574	14.35627	chr1:3732	ENSG00000225545		lncRNA	chr1:170587249-170
ENSG0000(	574	14.35627	chr1:3732	ENSG00000228697		lncRNA	chr1:168400829-168
ENSG0000(	574	14.35627	chr1:3732	NTMT2		protein_c	chr1:170145959-170
ENSG0000(	574	14.35627	chr1:3732	PRDX6-AS1		lncRNA	chr1:173417793-173
ENSG0000(	574	14.35627	chr1:3732	RPL30P1		Pseudoger	chr1:174090136-174
ENSG0000(	574	14.35627	chr1:3732	AL049798.1		smallRNA	chr1:168747796-168
ENSG0000(	574	14.35627	chr1:3732	ENSG00000228686		lncRNA	chr1:176017277-176
ENSG0000(	574	14.35627	chr1:3732	ENSG00000232261		Pseudoger	chr1:171751543-171
ENSG0000(	574	14.35627	chr1:3732	MROH9		protein_c	chr1:170935526-171
ENSG0000(	574	14.35627	chr1:3732	BLZF1	DriverDB\	protein_c	chr1:169367970-169
ENSG0000(	574	14.35627	chr1:3732	FMO6P		Pseudoger	chr1:171137740-171
ENSG0000(	574	14.35627	chr1:3732	COP1		protein_c	chr1:175944831-176
ENSG0000(	574	14.35627	chr1:3732	DPT		protein_c	chr1:168695468-168
ENSG0000(	574	14.35627	chr1:3732	PRRC2C	DriverDB\	protein_c	chr1:171485530-171
ENSG0000(	574	14.35627	chr1:3732	VAMP4	NCGv7	protein_c	chr1:171700160-171
ENSG0000(	574	14.35627	chr1:3732	FASLG		protein_c	chr1:172659103-172
ENSG0000(	574	14.35627	chr1:3732	XCL2	NCGv7	protein_c	chr1:168540768-168
ENSG0000(	574	14.35627	chr1:3732	XCL1	DriverDB\	protein_c	chr1:168576605-168
ENSG0000(	574	14.35627	chr1:3732	SNORD112		smallRNA	chr1:172348143-172
ENSG0000(	574	14.35627	chr1:3732	TNFSF4		protein_c	chr1:173183731-173
ENSG0000(	574	14.35627	chr1:3732	PRDX6		protein_c	chr1:173477330-173
ENSG0000(	574	14.35627	chr1:3732	DARS2		protein_c	chr1:173824653-173
ENSG0000(	574	14.35627	chr1:3732	METTL13		protein_c	chr1:171781660-171
ENSG0000(	574	14.35627	chr1:3732	SERPINC1		protein_c	chr1:173903800-173
ENSG0000(	574	14.35627	chr1:3732	RNU6-307P		smallRNA	chr1:174996524-174
ENSG0000(	574	14.35627	chr1:3732	NME7		protein_c	chr1:169132531-169
ENSG0000(	574	14.35627	chr1:3732	SELP	NCGv7	protein_c	chr1:169588849-169
ENSG0000(	574	14.35627	chr1:3732	ATP1B1		protein_c	chr1:169105697-169
ENSG0000(	574	14.35627	chr1:3732	RNA5SP67		Pseudoger	chr1:173921070-173
ENSG0000(	574	14.35627	chr1:3732	PFN1P1		Pseudoger	chr1:171670517-171
ENSG0000(	574	14.35627	chr11:76(	SNORA45		smallRNA	chr11:8685439-8685
ENSG0000(	574	14.35627	chr1:3732	ENSG00000213060		Pseudoger	chr1:171803517-171
ENSG0000(	574	14.35627	chr1:3732	Y_RNA		smallRNA	chr1:171814512-171
ENSG0000(	574	14.35627	chr1:3732	ENSG00000213062		lncRNA	chr1:169486076-169
ENSG0000(	574	14.35627	chr1:3732	RPL29P7		Pseudoger	chr1:168938467-168
ENSG0000(	574	14.35627	chr1:3732	GPR52		protein_c	chr1:174447964-174
ENSG0000(	574	14.35627	chr1:3732	CCDC181		protein_c	chr1:169394870-169
ENSG0000(	574	14.35627	chr1:3732	C1orf112	DriverDB\	protein_c	chr1:169662007-169
ENSG0000(	574	14.35627	chr1:3732	LINC00970		lncRNA	chr1:168903905-169
ENSG0000(	574	14.35627	chr1:3732	AL354732.1		smallRNA	chr1:170370213-170
ENSG0000(	574	14.35627	chr1:3732	SCYL3		protein_c	chr1:169849631-169
ENSG0000(	574	14.35627	chr1:3732	HAUS4P1		Pseudoger	chr1:170369223-170
ENSG0000(	574	14.35627	chr1:3732	Y_RNA		smallRNA	chr1:175022479-175
ENSG0000(	574	14.35627	chr1:3732	MIR3119-2		smallRNA	chr1:170151378-170
ENSG0000(	574	14.35627	chr1:3732	DNM3-IT1		lncRNA	chr1:171864187-171
ENSG0000(	564	14.10616	chr1:116(	AKIRIN1	DriverDB\	protein_c	chr1:38991276-3900
ENSG0000(	564	14.10616	chr1:116(	MACF1	NCGv7	protein_c	chr1:39081316-3948
ENSG0000(	564	14.10616	chr1:116(	ENSG00000274944		protein_c	chr1:38864501-3888
ENSG0000(	564	14.10616	chr1:116(	Y_RNA		smallRNA	chr1:38950825-3895
ENSG0000(	564	14.10616	chr1:116(	RNA5SP44		Pseudoger	chr1:39154164-3915

ENSG0000(	564	14.10616	chr1:116(	RRAGC	IntOGen-I	protein_c	chr1:38838198-3885
ENSG0000(	564	14.10616	chr1:116(	RRAGC-DT		lncRNA	chr1:38859912-3896
ENSG0000(	564	14.10616	chr1:116(	RHBDL2		protein_c	chr1:38885807-3894
ENSG0000(	564	14.10616	chr1:116(	ENSG00000275350		Pseudoger	chr1:38383838-3838
ENSG0000(	564	14.10616	chr1:116(	ENSG00000273637		lncRNA	chr1:38839333-3887
ENSG0000(	564	14.10616	chr1:116(	MYCBP		protein_c	chr1:38862493-3887
ENSG0000(	564	14.10616	chr1:116(	LINC01685		lncRNA	chr1:38474825-3851
ENSG0000(	564	14.10616	chr1:116(	ENSG00000226438		lncRNA	chr1:39249838-3925
ENSG0000(	564	14.10616	chr1:116(	EIF1P2		Pseudoger	chr1:38958275-3895
ENSG0000(	564	14.10616	chr1:116(	RNU6-753P		smallRNA	chr1:38396659-3839
ENSG0000(	564	14.10616	chr1:116(	HSPA5P1		Pseudoger	chr1:38708931-3871
ENSG0000(	564	14.10616	chr1:116(	ENSG00000284632		lncRNA	chr1:38754216-3881
ENSG0000(	564	14.10616	chr1:116(	HSPE1P8		Pseudoger	chr1:39304294-3930
ENSG0000(	564	14.10616	chr1:116(	NDUFS5		protein_c	chr1:39026318-3903
ENSG0000(	564	14.10616	chr1:116(	RNU6-605P		smallRNA	chr1:38926870-3892
ENSG0000(	564	14.10616	chr1:116(	GJA9		protein_c	chr1:38874069-3888
ENSG0000(	564	14.10616	chr1:116(	ENSG00000287422		lncRNA	chr1:39226670-3923
ENSG0000(	564	14.10616	chr1:116(	RNU6-608P		smallRNA	chr1:39120940-3912
ENSG0000(	563	14.08115	chr1:373:	ENSG00000236846		lncRNA	chr1:223144049-223
ENSG0000(	562	14.05614	chr6:213!	Y_RNA		smallRNA	chr6:134283092-134
ENSG0000(	562	14.05614	chr6:213!	snoU13		smallRNA	chr6:134206411-134
ENSG0000(	562	14.05614	chr6:213!	ENSG00000229722		lncRNA	chr6:134606299-134
ENSG0000(	562	14.05614	chr6:213!	ENSG00000286438		lncRNA	chr6:133061240-133
ENSG0000(	562	14.05614	chr6:213!	HBS1L		protein_c	chr6:134960378-135
ENSG0000(	562	14.05614	chr6:213!	ENSG00000278744		Pseudoger	chr6:134009033-134
ENSG0000(	562	14.05614	chr6:213!	ENSG00000234567		lncRNA	chr6:133452857-133
ENSG0000(	562	14.05614	chr6:213!	MIR548A2		smallRNA	chr6:135239160-135
ENSG0000(	562	14.05614	chr6:213!	ENSG00000227723		lncRNA	chr6:134636489-134
ENSG0000(	562	14.05614	chr6:213!	HMGA1P7		Pseudoger	chr6:134115235-134
ENSG0000(	562	14.05614	chr6:213!	ENSG00000287094		lncRNA	chr6:135323399-135
ENSG0000(	562	14.05614	chr6:213!	ENSG00000287974		lncRNA	chr6:134345688-134
ENSG0000(	562	14.05614	chr6:213!	RN7SL408P		smallRNA	chr6:134133573-134
ENSG0000(	562	14.05614	chr6:213!	CHCHD2P4		Pseudoger	chr6:134393142-134
ENSG0000(	562	14.05614	chr6:213!	ENSG00000272428		lncRNA	chr6:133540784-133
ENSG0000(	562	14.05614	chr6:213!	FAM8A6P		Pseudoger	chr6:134603564-134
ENSG0000(	562	14.05614	chr6:213!	LINC00326		lncRNA	chr6:132954257-133
ENSG0000(	562	14.05614	chr6:213!	HSPE1P21		Pseudoger	chr6:133510386-133
ENSG0000(	562	14.05614	chr6:213!	LINC01312		lncRNA	chr6:133821147-133
ENSG0000(	562	14.05614	chr6:213!	ENSG00000240056		lncRNA	chr6:134941392-134
ENSG0000(	562	14.05614	chr6:213!	RNA5SP218		Pseudoger	chr6:134257035-134
ENSG0000(	562	14.05614	chr6:213!	LINC01010		lncRNA	chr6:134343307-134
ENSG0000(	562	14.05614	chr6:213!	ENSG00000224374		lncRNA	chr6:135259996-135
ENSG0000(	562	14.05614	chr6:213!	TARID		lncRNA	chr6:133502252-133
ENSG0000(	562	14.05614	chr6:213!	EYA4	NCGv7	protein_c	chr6:133240514-133
ENSG0000(	562	14.05614	chr6:213!	MYB	NCGv7;AC	protein_c	chr6:135181308-135
ENSG0000(	562	14.05614	chr6:213!	ENSG00000287413		lncRNA	chr6:134074123-134
ENSG0000(	562	14.05614	chr6:213!	ENSG00000223542		lncRNA	chr6:133435077-133
ENSG0000(	562	14.05614	chr6:213!	ENSG00000234084		lncRNA	chr6:135301568-135
ENSG0000(	562	14.05614	chr6:213!	ALDH8A1		protein_c	chr6:134917393-134
ENSG0000(	562	14.05614	chr6:213!	MYB-AS1		lncRNA	chr6:135195083-135
ENSG0000(	562	14.05614	chr6:213!	ENSG00000286887		lncRNA	chr6:134296301-134

ENSG0000(	562	14.05614	chr6:213	KRT8P42		Pseudoger	chr6:134296997-134
ENSG0000(	562	14.05614	chr6:213	SGK1	NCGv7;AC	protein_c	chr6:134169248-134
ENSG0000(	562	14.05614	chr6:213	RPS29P32		Pseudoger	chr6:134211450-134
ENSG0000(	562	14.05614	chr6:213	ENSG00000232876		lncRNA	chr6:135055033-135
ENSG0000(	562	14.05614	chr6:213	TCF21	NCGv7	protein_c	chr6:133889113-133
ENSG0000(	562	14.05614	chr6:213	MTCYBP4		Pseudoger	chr6:133150568-133
ENSG0000(	562	14.05614	chr6:213	SLC2A12		protein_c	chr6:133987581-134
ENSG0000(	562	14.05614	chr6:213	FTH1P26		Pseudoger	chr6:133676729-133
ENSG0000(	562	14.05614	chr6:213	LINC03002		lncRNA	chr6:134520163-134
ENSG0000(	562	14.05614	chr6:213	TBPL1	DriverDB	protein_c	chr6:133952170-133
ENSG0000(	562	14.05614	chr6:213	ENSG00000288529		protein_c	chr6:133953304-133
ENSG0000(	562	14.05614	chr6:213	ENSG00000290029		lncRNA	chr6:134373662-134
ENSG0000(	562	14.05614	chr6:213	CT69		lncRNA	chr6:134428239-134
ENSG0000(	562	14.05614	chr6:213	ENSG00000236389		lncRNA	chr6:134706060-134
ENSG0000(	562	14.05614	chr6:213	MEMO1P2		Pseudoger	chr6:134897874-134
ENSG0000(	561	14.03113	chr12:10	ENSG00000274191		lncRNA	chr12:122634130-12
ENSG0000(	559	13.98111	chr5:169	NUDT12	DriverDB	protein_c	chr5:103548855-103
ENSG0000(	559	13.98111	chr5:169	ENSG00000248203		lncRNA	chr5:103246048-103
ENSG0000(	559	13.98111	chr5:169	LINC02115		lncRNA	chr5:103528434-103
ENSG0000(	559	13.98111	chr5:169	MACIR		protein_c	chr5:103258763-103
ENSG0000(	559	13.98111	chr5:169	PPIP5K2		protein_c	chr5:103120149-103
ENSG0000(	559	13.98111	chr5:169	PDZPH1P		Pseudoger	chr5:103430406-103
ENSG0000(	559	13.98111	chr5:169	ENSG00000250567		lncRNA	chr5:103408941-103
ENSG0000(	553	13.83104	chr5:103	ENSG00000243304		Pseudoger	chr5:115264669-115
ENSG0000(	553	13.83104	chr5:103	DCP2		protein_c	chr5:112976702-113
ENSG0000(	553	13.83104	chr5:103	CSNK1G3		protein_c	chr5:123512177-123
ENSG0000(	553	13.83104	chr5:103	LINC02147		lncRNA	chr5:117730515-118
ENSG0000(	553	13.83104	chr5:103	AC027320.1		smallRNA	chr5:119257340-119
ENSG0000(	553	13.83104	chr5:103	snoU13		smallRNA	chr5:123009542-123
ENSG0000(	553	13.83104	chr5:103	RNU6-718P		smallRNA	chr5:120337549-120
ENSG0000(	553	13.83104	chr5:103	snoU13		smallRNA	chr5:119468959-119
ENSG0000(	553	13.83104	chr5:103	LINCADL		lncRNA	chr5:115956571-115
ENSG0000(	553	13.83104	chr5:103	snoU13		smallRNA	chr5:119610202-119
ENSG0000(	553	13.83104	chr5:103	SRFBP1		protein_c	chr5:121961975-122
ENSG0000(	553	13.83104	chr5:103	PRELID3BP8		Pseudoger	chr5:120658077-120
ENSG0000(	553	13.83104	chr5:103	MIR5706		smallRNA	chr5:119154637-119
ENSG0000(	553	13.83104	chr5:103	RN7SL174P		smallRNA	chr5:119306344-119
ENSG0000(	553	13.83104	chr5:103	AC034236.2		smallRNA	chr5:116030018-116
ENSG0000(	553	13.83104	chr5:103	snoU13		smallRNA	chr5:119069297-119
ENSG0000(	553	13.83104	chr5:103	SEPTIN7P10		Pseudoger	chr5:119126782-119
ENSG0000(	553	13.83104	chr5:103	FABP5P6		Pseudoger	chr5:119555250-119
ENSG0000(	553	13.83104	chr5:103	ENSG00000288697		Pseudoger	chr5:128932070-128
ENSG0000(	553	13.83104	chr5:103	RNU7-34P		smallRNA	chr5:118758211-118
ENSG0000(	553	13.83104	chr5:103	ENSG00000248799		lncRNA	chr5:127651693-127
ENSG0000(	553	13.83104	chr5:103	PPIC-AS1		lncRNA	chr5:123036271-123
ENSG0000(	553	13.83104	chr5:103	ENSG00000250015		lncRNA	chr5:116302354-116
ENSG0000(	553	13.83104	chr5:103	ALDH7A1		protein_c	chr5:126531200-126
ENSG0000(	553	13.83104	chr5:103	PHAX	NCGv7	protein_c	chr5:126600925-126
ENSG0000(	553	13.83104	chr5:103	ENSG00000261036		lncRNA	chr5:120345907-120
ENSG0000(	553	13.83104	chr5:103	GRAMD2B		protein_c	chr5:126360132-126
ENSG0000(	553	13.83104	chr5:103	ENSG00000287390		lncRNA	chr5:130655383-130

ENSG0000(	553	13.83104	chr5:1036	FTMT		protein_c	chr5:121851882-121
ENSG0000(	553	13.83104	chr5:1036	SEMA6A	NCGv7	protein_c	chr5:116443555-116
ENSG0000(	553	13.83104	chr5:1036	CBX3P3		Pseudoger	chr5:112777630-112
ENSG0000(	553	13.83104	chr5:1036	AC004769.1		smallRNA	chr5:130277625-130
ENSG0000(	553	13.83104	chr5:1036	ENSG00000250080		Pseudoger	chr5:127170535-127
ENSG0000(	553	13.83104	chr5:1036	RNU4ATAC13P		smallRNA	chr5:113493835-113
ENSG0000(	553	13.83104	chr5:1036	MRPS5P3		Pseudoger	chr5:127143082-127
ENSG0000(	553	13.83104	chr5:1036	HMGN2P27		Pseudoger	chr5:116218026-116
ENSG0000(	553	13.83104	chr5:1036	RNU6-963P		smallRNA	chr5:126553302-126
ENSG0000(	553	13.83104	chr5:1036	TMED7-TI(	DriverDBv	protein_c	chr5:115578642-115
ENSG0000(	553	13.83104	chr5:1036	RPL35AP15		Pseudoger	chr5:117301104-117
ENSG0000(	553	13.83104	chr5:1036	SLC27A6		protein_c	chr5:128538013-129
ENSG0000(	553	13.83104	chr5:1036	RNU6ATAC10P		smallRNA	chr5:129974891-129
ENSG0000(	553	13.83104	chr5:1036	TICAM2		protein_c	chr5:115578496-115
ENSG0000(	553	13.83104	chr5:1036	TSSK1B		protein_c	chr5:113432553-113
ENSG0000(	553	13.83104	chr5:1036	ZNF474		protein_c	chr5:122129546-122
ENSG0000(	553	13.83104	chr5:1036	LOX	IntOGen-I	protein_c	chr5:122063195-122
ENSG0000(	553	13.83104	chr5:1036	PRRC1		protein_c	chr5:127517640-127
ENSG0000(	553	13.83104	chr5:1036	ISOC1		protein_c	chr5:129094749-129
ENSG0000(	553	13.83104	chr5:1036	ENSG00000248853		Pseudoger	chr5:121059244-121
ENSG0000(	553	13.83104	chr5:1036	ENSG00000249904		Pseudoger	chr5:125886285-125
ENSG0000(	553	13.83104	chr5:1036	ENSG00000249950		Pseudoger	chr5:125862746-125
ENSG0000(	553	13.83104	chr5:1036	LMNB1		protein_c	chr5:126776623-126
ENSG0000(	553	13.83104	chr5:1036	ENSG00000249916		lncRNA	chr5:122369762-122
ENSG0000(	553	13.83104	chr5:1036	ENSG00000248927		lncRNA	chr5:120781218-120
ENSG0000(	553	13.83104	chr5:1036	RP11-395P13.2		lncRNA	chr5:125368568-125
ENSG0000(	553	13.83104	chr5:1036	ENSG00000230929		Pseudoger	chr5:126628019-126
ENSG0000(	553	13.83104	chr5:1036	ENSG00000249944		Pseudoger	chr5:115489634-115
ENSG0000(	553	13.83104	chr5:1036	XBP1P1		Pseudoger	chr5:112885094-112
ENSG0000(	553	13.83104	chr5:1036	ENSG00000249791		lncRNA	chr5:115188563-115
ENSG0000(	553	13.83104	chr5:1036	ENSG00000286745		lncRNA	chr5:118581242-118
ENSG0000(	553	13.83104	chr5:1036	DMXL1	NCGv7	protein_c	chr5:119037772-119
ENSG0000(	553	13.83104	chr5:1036	LVRN		protein_c	chr5:115962454-116
ENSG0000(	553	13.83104	chr5:1036	ENSG00000251135		Pseudoger	chr5:121040090-121
ENSG0000(	553	13.83104	chr5:1036	ATG12		protein_c	chr5:115828200-115
ENSG0000(	553	13.83104	chr5:1036	MEGF10	DriverDBv	protein_c	chr5:127290796-127
ENSG0000(	553	13.83104	chr5:1036	ADAMTS19	NCGv7	protein_c	chr5:129460281-129
ENSG0000(	553	13.83104	chr5:1036	YTHDC2		protein_c	chr5:113513694-113
ENSG0000(	553	13.83104	chr5:1036	ENSG00000185641		Pseudoger	chr5:116051917-116
ENSG0000(	553	13.83104	chr5:1036	ENSG00000251132		lncRNA	chr5:115031273-115
ENSG0000(	553	13.83104	chr5:1036	PRDM6-AS1		lncRNA	chr5:123087248-123
ENSG0000(	553	13.83104	chr5:1036	ENSG00000279860		TEC	chr5:121363002-121
ENSG0000(	553	13.83104	chr5:1036	AC034236.1		smallRNA	chr5:116043171-116
ENSG0000(	553	13.83104	chr5:1036	RNU7-53P		smallRNA	chr5:130386621-130
ENSG0000(	553	13.83104	chr5:1036	HMGB3P17		Pseudoger	chr5:123468781-123
ENSG0000(	553	13.83104	chr5:1036	snoU13		smallRNA	chr5:122796125-122
ENSG0000(	553	13.83104	chr5:1036	ENSG00000272265		lncRNA	chr5:116078110-116
ENSG0000(	553	13.83104	chr5:1036	ENSG00000285809		lncRNA	chr5:117313085-117
ENSG0000(	553	13.83104	chr5:1036	BOLA3P3		Pseudoger	chr5:126663337-126
ENSG0000(	553	13.83104	chr5:1036	SEMA6A-AS2		lncRNA	chr5:116574482-116
ENSG0000(	553	13.83104	chr5:1036	SELENOTP2		Pseudoger	chr5:127176696-127

ENSG0000(	553	13.83104	chr5:103(	ENSG00000249418		Pseudoger	chr5:130598348-130
ENSG0000(	553	13.83104	chr5:103(	ADAMTS19-AS1		lncRNA	chr5:129424782-129
ENSG0000(	553	13.83104	chr5:103(	ENSG00000286274		lncRNA	chr5:129150677-129
ENSG0000(	553	13.83104	chr5:103(	ENSG00000249150		lncRNA	chr5:116819220-116
ENSG0000(	553	13.83104	chr5:103(	LINC02215		lncRNA	chr5:118596188-118
ENSG0000(	553	13.83104	chr5:103(	COMMD10		protein_c	chr5:116085016-116
ENSG0000(	553	13.83104	chr5:103(	RNU2-49P		smallRNA	chr5:115774319-115
ENSG0000(	553	13.83104	chr5:103(	RNA5SP191		Pseudoger	chr5:130114409-130
ENSG0000(	553	13.83104	chr5:103(	ARL2BPP4		Pseudoger	chr5:130529419-130
ENSG0000(	553	13.83104	chr5:103(	LINC02039		lncRNA	chr5:126179565-126
ENSG0000(	553	13.83104	chr5:103(	ENSG00000249261		lncRNA	chr5:124868972-124
ENSG0000(	553	13.83104	chr5:103(	TICAM2-AS1		lncRNA	chr5:115602057-115
ENSG0000(	553	13.83104	chr5:103(	PRR16		protein_c	chr5:120464300-120
ENSG0000(	553	13.83104	chr5:103(	RPSAP37		Pseudoger	chr5:125966777-125
ENSG0000(	553	13.83104	chr5:103(	ZNF608	NCGv7	protein_c	chr5:124636913-124
ENSG0000(	553	13.83104	chr5:103(	ENSG00000271918		lncRNA	chr5:116083807-116
ENSG0000(	553	13.83104	chr5:103(	ENSG00000213663		Pseudoger	chr5:119547131-119
ENSG0000(	553	13.83104	chr5:103(	ENSG00000213661		Pseudoger	chr5:121616247-121
ENSG0000(	553	13.83104	chr5:103(	RN7SL689P		smallRNA	chr5:123022489-123
ENSG0000(	553	13.83104	chr5:103(	ENSG00000213655		Pseudoger	chr5:123236079-123
ENSG0000(	553	13.83104	chr5:103(	FEM1C		protein_c	chr5:115520908-115
ENSG0000(	553	13.83104	chr5:103(	PPIC		protein_c	chr5:123023250-123
ENSG0000(	553	13.83104	chr5:103(	ZRSR2P1		Pseudoger	chr5:112891610-112
ENSG0000(	553	13.83104	chr5:103(	CEP120		protein_c	chr5:123344890-123
ENSG0000(	553	13.83104	chr5:103(	ENSG00000271797		lncRNA	chr5:115262505-115
ENSG0000(	553	13.83104	chr5:103(	ENSG00000272139		lncRNA	chr5:122311297-122
ENSG0000(	553	13.83104	chr5:103(	ENSG00000234259		Pseudoger	chr5:119680197-119
ENSG0000(	553	13.83104	chr5:103(	ENSG00000286111		lncRNA	chr5:125493261-125
ENSG0000(	553	13.83104	chr5:103(	TNFAIP8	NCGv7	protein_c	chr5:119268692-119
ENSG0000(	553	13.83104	chr5:103(	RNU6-701P		smallRNA	chr5:119138859-119
ENSG0000(	553	13.83104	chr5:103(	ENSG00000271766		lncRNA	chr5:127478295-127
ENSG0000(	553	13.83104	chr5:103(	ENSG00000249426		lncRNA	chr5:118760474-118
ENSG0000(	553	13.83104	chr5:103(	ENSG00000249112		lncRNA	chr5:124707827-124
ENSG0000(	553	13.83104	chr5:103(	ENSG00000279772		TEC	chr5:123508736-123
ENSG0000(	553	13.83104	chr5:103(	RNA5SP190		Pseudoger	chr5:119445716-119
ENSG0000(	553	13.83104	chr5:103(	ENSG00000286615		lncRNA	chr5:127231959-127
ENSG0000(	553	13.83104	chr5:103(	ACTBP4		Pseudoger	chr5:131659046-131
ENSG0000(	553	13.83104	chr5:103(	ENSG00000229855		lncRNA	chr5:121164687-121
ENSG0000(	553	13.83104	chr5:103(	ENSG00000289458		lncRNA	chr5:124497294-124
ENSG0000(	553	13.83104	chr5:103(	LAMTOR3P2		Pseudoger	chr5:119132651-119
ENSG0000(	553	13.83104	chr5:103(	RPS14P8		Pseudoger	chr5:116562562-116
ENSG0000(	553	13.83104	chr5:103(	ENSG00000290020		lncRNA	chr5:131210048-131
ENSG0000(	553	13.83104	chr5:103(	HMGN1P15		Pseudoger	chr5:115289036-115
ENSG0000(	553	13.83104	chr5:103(	ENSG00000248955		Pseudoger	chr5:131008583-131
ENSG0000(	553	13.83104	chr5:103(	ENSG00000279446		TEC	chr5:127073587-127
ENSG0000(	553	13.83104	chr5:103(	KRT18P16		Pseudoger	chr5:123636110-123
ENSG0000(	553	13.83104	chr5:103(	ENSG00000289497		lncRNA	chr5:115364197-115
ENSG0000(	553	13.83104	chr5:103(	Y_RNA		smallRNA	chr5:123510436-123
ENSG0000(	553	13.83104	chr5:103(	SLC12A2		protein_c	chr5:128083766-128
ENSG0000(	553	13.83104	chr5:103(	ENSG00000286839		lncRNA	chr5:113738106-113
ENSG0000(	553	13.83104	chr5:103(	SNX24		protein_c	chr5:122843439-123

ENSG0000(	553	13.83104	chr5:103(	SNCAIP		protein_c	chr5:122311354-122
ENSG0000(	553	13.83104	chr5:103(	CDO1		protein_c	chr5:115804733-115
ENSG0000(	553	13.83104	chr5:103(	REEP5	NCGv7	protein_c	chr5:112876385-112
ENSG0000(	553	13.83104	chr5:103(	MIR4633		smallRNA	chr5:129097688-129
ENSG0000(	553	13.83104	chr5:103(	HSPE1P10		Pseudoger	chr5:126737438-126
ENSG0000(	553	13.83104	chr5:103(	ENSG00000279370		TEC	chr5:131261321-131
ENSG0000(	553	13.83104	chr5:103(	RP11-395P13.3		lncRNA	chr5:125370165-125
ENSG0000(	553	13.83104	chr5:103(	RN7SKP117		smallRNA	chr5:125350868-125
ENSG0000(	553	13.83104	chr5:103(	ENSG00000249433		Pseudoger	chr5:115493557-115
ENSG0000(	553	13.83104	chr5:103(	ENSG00000249600		Pseudoger	chr5:121671373-121
ENSG0000(	553	13.83104	chr5:103(	MARCHF3		protein_c	chr5:126867714-127
ENSG0000(	553	13.83104	chr5:103(	DMXL1-DT		lncRNA	chr5:119006347-119
ENSG0000(	553	13.83104	chr5:103(	ENSG00000258864		protein_c	chr5:112827213-112
ENSG0000(	553	13.83104	chr5:103(	LINC02216		lncRNA	chr5:118575575-118
ENSG0000(	553	13.83104	chr5:103(	MINAR2		protein_c	chr5:129748094-129
ENSG0000(	553	13.83104	chr5:103(	ENSG00000249577		Pseudoger	chr5:127465822-127
ENSG0000(	553	13.83104	chr5:103(	FBN2	NCGv7	protein_c	chr5:128257909-128
ENSG0000(	553	13.83104	chr5:103(	LYRM7		protein_c	chr5:131170944-131
ENSG0000(	553	13.83104	chr5:103(	AC011416.1		smallRNA	chr5:127260936-127
ENSG0000(	553	13.83104	chr5:103(	RPL7L1P4		Pseudoger	chr5:117778826-117
ENSG0000(	553	13.83104	chr5:103(	RAPGEF6		protein_c	chr5:131423921-131
ENSG0000(	553	13.83104	chr5:103(	PRDM6	AC	protein_c	chr5:123089241-123
ENSG0000(	553	13.83104	chr5:103(	CCDC192		protein_c	chr5:127703391-127
ENSG0000(	553	13.83104	chr5:103(	CDC42SE2		protein_c	chr5:131245493-131
ENSG0000(	553	13.83104	chr5:103(	LINC01170		lncRNA	chr5:124059794-124
ENSG0000(	553	13.83104	chr5:103(	ZNF474-AS1		lncRNA	chr5:122154496-122
ENSG0000(	553	13.83104	chr5:103(	H3P24		Pseudoger	chr5:115770586-115
ENSG0000(	553	13.83104	chr5:103(	ENSG00000249621		lncRNA	chr5:122321291-122
ENSG0000(	553	13.83104	chr5:103(	CTNNA1P1		Pseudoger	chr5:115389643-115
ENSG0000(	553	13.83104	chr5:103(	ARGFXP1		Pseudoger	chr5:122675795-122
ENSG0000(	553	13.83104	chr5:103(	ENSG00000249021		lncRNA	chr5:115691462-115
ENSG0000(	553	13.83104	chr5:103(	ENSG00000242814		Pseudoger	chr5:124808828-124
ENSG0000(	553	13.83104	chr5:103(	ENSG00000250194		Pseudoger	chr5:125074665-125
ENSG0000(	553	13.83104	chr5:103(	ENSG00000279118		TEC	chr5:126496279-126
ENSG0000(	553	13.83104	chr5:103(	LINC01957		lncRNA	chr5:114576041-114
ENSG0000(	553	13.83104	chr5:103(	HINT1		protein_c	chr5:131155383-131
ENSG0000(	553	13.83104	chr5:103(	FAM170A	DriverDB\	protein_c	chr5:119629558-119
ENSG0000(	553	13.83104	chr5:103(	ENSG00000247311		lncRNA	chr5:122114598-122
ENSG0000(	553	13.83104	chr5:103(	DTWD2	NCGv7	protein_c	chr5:118836074-118
ENSG0000(	553	13.83104	chr5:103(	C5orf63		protein_c	chr5:127045235-127
ENSG0000(	553	13.83104	chr5:103(	RN7SKP89		smallRNA	chr5:114253513-114
ENSG0000(	553	13.83104	chr5:103(	RN7SL711P		smallRNA	chr5:123070782-123
ENSG0000(	553	13.83104	chr5:103(	ENSG00000250949		lncRNA	chr5:117031200-117
ENSG0000(	553	13.83104	chr5:103(	RNU6-644P		smallRNA	chr5:116188455-116
ENSG0000(	553	13.83104	chr5:103(	ENSG00000250650		Pseudoger	chr5:117019548-117
ENSG0000(	553	13.83104	chr5:103(	ARL14EPL		protein_c	chr5:116032324-116
ENSG0000(	553	13.83104	chr5:103(	ENSG00000246316		lncRNA	chr5:114475339-114
ENSG0000(	553	13.83104	chr5:103(	KCNN2	NCGv7	protein_c	chr5:114055926-114
ENSG0000(	553	13.83104	chr5:103(	CHSY3		protein_c	chr5:129904465-130
ENSG0000(	553	13.83104	chr5:103(	HSPA8P4		Pseudoger	chr5:130140031-130
ENSG0000(	553	13.83104	chr5:103(	ENSG00000287794		Pseudoger	chr5:122373512-122

ENSG0000(	553	13.83104	chr5:103(	PGGT1B	protein_(	chr5:115204012-115
ENSG0000(	553	13.83104	chr5:103(	ENSG00000250928	Pseudoger	chr5:119011471-119
ENSG0000(	553	13.83104	chr5:103(	ENSG00000251421	lncRNA	chr5:124395603-124
ENSG0000(	553	13.83104	chr5:103(	ENSG00000250678	Pseudoger	chr5:119010060-119
ENSG0000(	553	13.83104	chr5:103(	HMGB1P22	Pseudoger	chr5:125265528-125
ENSG0000(	553	13.83104	chr5:103(	ENSG00000289054	lncRNA	chr5:122326610-122
ENSG0000(	553	13.83104	chr5:103(	RNU6-482P	smallRNA	chr5:112778363-112
ENSG0000(	553	13.83104	chr5:103(	ENSG00000260686	lncRNA	chr5:122832356-122
ENSG0000(	553	13.83104	chr5:103(	POGLUT2P1	Pseudoger	chr5:128109486-128
ENSG0000(	553	13.83104	chr5:103(	ENSG00000232633	lncRNA	chr5:113323028-113
ENSG0000(	553	13.83104	chr5:103(	MGC32805	lncRNA	chr5:122436497-122
ENSG0000(	553	13.83104	chr5:103(	ENSG00000248709	lncRNA	chr5:115738978-115
ENSG0000(	553	13.83104	chr5:103(	ENSG00000288008	lncRNA	chr5:124993408-124
ENSG0000(	553	13.83104	chr5:103(	LINC00992	lncRNA	chr5:117415509-117
ENSG0000(	553	13.83104	chr5:103(	RNU6-373P	smallRNA	chr5:119007571-119
ENSG0000(	553	13.83104	chr5:103(	ENSG00000251293	lncRNA	chr5:120245448-120
ENSG0000(	553	13.83104	chr5:103(	LINC02201	lncRNA	chr5:122628952-122
ENSG0000(	553	13.83104	chr5:103(	HSD17B4	protein_(	chr5:119452465-119
ENSG0000(	553	13.83104	chr5:103(	TRIM36-IT1	lncRNA	chr5:115148764-115
ENSG0000(	553	13.83104	chr5:103(	ENSG00000248634	lncRNA	chr5:128663978-128
ENSG0000(	553	13.83104	chr5:103(	PTMAP2	Pseudoger	chr5:118973796-118
ENSG0000(	553	13.83104	chr5:103(	CTXN3	protein_(	chr5:127649082-127
ENSG0000(	553	13.83104	chr5:103(	SNX2	protein_(	chr5:122775079-122
ENSG0000(	553	13.83104	chr5:103(	CCT5P1	Pseudoger	chr5:115512077-115
ENSG0000(	553	13.83104	chr5:103(	ENSG00000248443	lncRNA	chr5:124829472-124
ENSG0000(	553	13.83104	chr5:103(	ENSG00000248600	lncRNA	chr5:124452277-124
ENSG0000(	553	13.83104	chr5:103(	KRT8P33	Pseudoger	chr5:123400922-123
ENSG0000(	553	13.83104	chr5:103(	LMNB1-DT	lncRNA	chr5:126751963-126
ENSG0000(	553	13.83104	chr5:103(	ENSG00000251477	Pseudoger	chr5:117719240-117
ENSG0000(	553	13.83104	chr5:103(	SRP19	protein_(	chr5:112861188-112
ENSG0000(	553	13.83104	chr5:103(	ENSG00000250438	lncRNA	chr5:124469591-124
ENSG0000(	553	13.83104	chr5:103(	LINC02148	lncRNA	chr5:118282575-118
ENSG0000(	553	13.83104	chr5:103(	SLC12A2-DT	lncRNA	chr5:127939152-128
ENSG0000(	553	13.83104	chr5:103(	RP11-395P13.6	lncRNA	chr5:125395379-125
ENSG0000(	553	13.83104	chr5:103(	ENSG00000250405	Pseudoger	chr5:130994253-130
ENSG0000(	553	13.83104	chr5:103(	ENSG00000251456	lncRNA	chr5:124734618-124
ENSG0000(	553	13.83104	chr5:103(	RPS17P2	Pseudoger	chr5:116716243-116
ENSG0000(	553	13.83104	chr5:103(	ENSG00000250602	lncRNA	chr5:126372477-126
ENSG0000(	553	13.83104	chr5:103(	RP11-395P13.4	lncRNA	chr5:125376834-125
ENSG0000(	553	13.83104	chr5:103(	ENSG00000250956	Pseudoger	chr5:127179756-127
ENSG0000(	553	13.83104	chr5:103(	ENSG00000250603	lncRNA	chr5:127838486-127
ENSG0000(	553	13.83104	chr5:103(	ENSG00000282925	lncRNA	chr5:127588746-127
ENSG0000(	553	13.83104	chr5:103(	ENSG00000288890	lncRNA	chr5:123619519-123
ENSG0000(	553	13.83104	chr5:103(	LINC02240	lncRNA	chr5:124808981-125
ENSG0000(	553	13.83104	chr5:103(	LINC02214	lncRNA	chr5:116742991-116
ENSG0000(	553	13.83104	chr5:103(	DDX43P1	Pseudoger	chr5:115974513-115
ENSG0000(	553	13.83104	chr5:103(	AC010235.1	smallRNA	chr5:126196835-126
ENSG0000(	553	13.83104	chr5:103(	ENSG00000251680	lncRNA	chr5:129500361-129
ENSG0000(	553	13.83104	chr5:103(	ENSG00000288766	lncRNA	chr5:123511177-123
ENSG0000(	553	13.83104	chr5:103(	TRIM36	protein_(	chr5:115124762-115
ENSG0000(	553	13.83104	chr5:103(	AK3P4	Pseudoger	chr5:115402380-115

ENSG00000	553	13.83104	chr5:1036	ENSG00000283897		lncRNA	chr5:127215159-127
ENSG00000	553	13.83104	chr5:1036	ENSG00000250242		lncRNA	chr5:115087892-115
ENSG00000	553	13.83104	chr5:1036	CCDC112		protein_c	chr5:115267190-115
ENSG00000	553	13.83104	chr5:1036	ENSG00000250847		Pseudoger	chr5:121195980-121
ENSG00000	553	13.83104	chr5:1036	HNRNPKP1		Pseudoger	chr5:127511464-127
ENSG00000	553	13.83104	chr5:1036	HMGB1P29		Pseudoger	chr5:124220579-124
ENSG00000	553	13.83104	chr5:1036	AP3S1		protein_c	chr5:115841592-115
ENSG00000	553	13.83104	chr5:1036	MIR4460		smallRNA	chr5:129397062-129
ENSG00000	553	13.83104	chr5:1036	RPL23AP44		Pseudoger	chr5:121575848-121
ENSG00000	553	13.83104	chr5:1036	Y_RNA		smallRNA	chr5:128371099-128
ENSG00000	553	13.83104	chr5:1036	TEX43		protein_c	chr5:126631705-126
ENSG00000	553	13.83104	chr5:1036	RNU4-69P		smallRNA	chr5:120710698-120
ENSG00000	553	13.83104	chr5:1036	LINC02208		lncRNA	chr5:118000253-118
ENSG00000	553	13.83104	chr5:1036	CUL1P1		Pseudoger	chr5:127784618-127
ENSG00000	553	13.83104	chr5:1036	TUBAP15		Pseudoger	chr5:119652523-119
ENSG00000	553	13.83104	chr5:1036	ENSG00000248752		lncRNA	chr5:125333369-126
ENSG00000	553	13.83104	chr5:1036	ENSG00000251214		lncRNA	chr5:124459912-124
ENSG00000	553	13.83104	chr5:1036	MCC	AC	protein_c	chr5:113022106-113
ENSG00000	553	13.83104	chr5:1036	TMED7		protein_c	chr5:115613210-115
ENSG00000	553	13.83104	chr5:1036	SEMA6A-AS1		lncRNA	chr5:116447547-116
ENSG00000	553	13.83104	chr5:1036	ENSG00000250803		protein_c	chr5:122129622-122
ENSG00000	553	13.83104	chr5:1036	ENSG00000244192		Pseudoger	chr5:130989897-130
ENSG00000	553	13.83104	chr5:1036	ENSG00000248296		lncRNA	chr5:124492775-124
ENSG00000	553	13.83104	chr5:1036	RP11-395P13.5		lncRNA	chr5:125382859-125
ENSG00000	553	13.83104	chr5:1036	AC008536.1		smallRNA	chr5:112905638-112
ENSG00000	552	13.80603	chr1:1166	SCARNA11		smallRNA	chr1:13696070-1369
ENSG00000	550	13.75601	chr1:1166	AL590683.2		smallRNA	chr1:24279767-2427
ENSG00000	550	13.75601	chr1:1234	AL589685.1		Pseudoger	chr1:156323511-156
ENSG00000	546	13.65597	chr3:8573	RNU2-64P		smallRNA	chr3:73110992-7311
ENSG00000	544	13.60595	chr1:1234	S100A7A	NCGv7	protein_c	chr1:153416520-153
ENSG00000	544	13.60595	chr1:1234	RUSC1-AS1		lncRNA	chr1:155316863-155
ENSG00000	544	13.60595	chr1:1234	ENSG00000271380		lncRNA	chr1:154961825-154
ENSG00000	544	13.60595	chr1:1234	S100A6		protein_c	chr1:153534599-153
ENSG00000	544	13.60595	chr1:1234	FLAD1		protein_c	chr1:154983338-154
ENSG00000	544	13.60595	chr1:1234	ENSG00000289935		lncRNA	chr1:153945551-153
ENSG00000	544	13.60595	chr1:1234	SPRR2G		protein_c	chr1:153149582-153
ENSG00000	544	13.60595	chr1:1234	SLC27A3		protein_c	chr1:153774354-153
ENSG00000	544	13.60595	chr1:1234	ENSG00000226716		lncRNA	chr1:152122534-152
ENSG00000	544	13.60595	chr1:1234	SNORD59		smallRNA	chr1:154288460-154
ENSG00000	544	13.60595	chr1:1234	S100A7	NCGv7;AC	protein_c	chr1:153457744-153
ENSG00000	544	13.60595	chr1:1234	SNAPIN		protein_c	chr1:153658703-153
ENSG00000	544	13.60595	chr1:1234	ENSG00000223503		Pseudoger	chr1:155590601-155
ENSG00000	544	13.60595	chr1:1234	PSMD8P1		Pseudoger	chr1:154414369-154
ENSG00000	544	13.60595	chr1:1234	LINC02962		lncRNA	chr1:152205858-152
ENSG00000	544	13.60595	chr1:1234	LCE1A		protein_c	chr1:152827473-152
ENSG00000	544	13.60595	chr1:1234	NUP210L	NCGv7	protein_c	chr1:153992685-154
ENSG00000	544	13.60595	chr1:1234	ZBTB7B	NCGv7	protein_c	chr1:155002630-155
ENSG00000	544	13.60595	chr1:1234	U3		smallRNA	chr1:153998041-153
ENSG00000	544	13.60595	chr1:1234	RN7SL372P		smallRNA	chr1:153704088-153
ENSG00000	544	13.60595	chr1:1234	RNU6-106P		smallRNA	chr1:155358712-155
ENSG00000	544	13.60595	chr1:1234	PGLYRP3		protein_c	chr1:153297116-153

ENSG0000(	544	13.60595	chr1:1234	SHC1		protein_c	chr1:154962298-154
ENSG0000(	544	13.60595	chr1:1234	HRNR	NCGv7	protein_c	chr1:152212076-152
ENSG0000(	544	13.60595	chr1:1234	DAP3P1		Pseudoger	chr1:155586644-155
ENSG0000(	544	13.60595	chr1:1234	TCHH	NCGv7	protein_c	chr1:152106317-152
ENSG0000(	544	13.60595	chr1:1234	THEM4		protein_c	chr1:151870866-151
ENSG0000(	544	13.60595	chr1:1234	POU5F1P4		Pseudoger	chr1:155433178-155
ENSG0000(	544	13.60595	chr1:1234	SLC39A1		protein_c	chr1:153959099-153
ENSG0000(	544	13.60595	chr1:1234	NBPF18P		Pseudoger	chr1:152018662-152
ENSG0000(	544	13.60595	chr1:1234	ENSG00000236263		lncRNA	chr1:155211151-155
ENSG0000(	544	13.60595	chr1:1234	RNU6-121P		smallRNA	chr1:154297650-154
ENSG0000(	544	13.60595	chr1:1234	UBAP2L		protein_c	chr1:154220179-154
ENSG0000(	544	13.60595	chr1:1234	ENSG00000285818		Pseudoger	chr1:152079557-152
ENSG0000(	544	13.60595	chr1:1234	RPS7P2		Pseudoger	chr1:154078866-154
ENSG0000(	544	13.60595	chr1:1234	ENSG00000231827		Pseudoger	chr1:153795173-153
ENSG0000(	544	13.60595	chr1:1234	MIR190B		smallRNA	chr1:154193665-154
ENSG0000(	544	13.60595	chr1:1234	SPRR2C		Pseudoger	chr1:153140491-153
ENSG0000(	544	13.60595	chr1:1234	LCE4A	NCGv7	protein_c	chr1:152708160-152
ENSG0000(	544	13.60595	chr1:1234	RPS27	NCGv7	protein_c	chr1:153990762-153
ENSG0000(	544	13.60595	chr1:1234	RPLP0P4		Pseudoger	chr1:153225080-153
ENSG0000(	544	13.60595	chr1:1234	ADAR		protein_c	chr1:154581695-154
ENSG0000(	544	13.60595	chr1:1234	JTB-DT		lncRNA	chr1:153977727-153
ENSG0000(	544	13.60595	chr1:1234	LCE2A		protein_c	chr1:152698345-152
ENSG0000(	544	13.60595	chr1:1234	DCST1-AS1		lncRNA	chr1:155045191-155
ENSG0000(	544	13.60595	chr1:1234	LCE2C		protein_c	chr1:152675279-152
ENSG0000(	544	13.60595	chr1:1234	SPRR1A		protein_c	chr1:152984081-152
ENSG0000(	544	13.60595	chr1:1234	CHTOP		protein_c	chr1:153633982-153
ENSG0000(	544	13.60595	chr1:1234	CRCT1		protein_c	chr1:152514482-152
ENSG0000(	544	13.60595	chr1:1234	TPM3	NCGv7;AC	protein_c	chr1:154155308-154
ENSG0000(	544	13.60595	chr1:1234	FLG2		protein_c	chr1:152348735-152
ENSG0000(	544	13.60595	chr1:1234	LCE3E		protein_c	chr1:152565654-152
ENSG0000(	544	13.60595	chr1:1234	ENSG00000287839		lncRNA	chr1:155626755-155
ENSG0000(	544	13.60595	chr1:1234	PRR9		protein_c	chr1:153217584-153
ENSG0000(	544	13.60595	chr1:1234	GEMIN2P1		Pseudoger	chr1:153717303-153
ENSG0000(	544	13.60595	chr1:1234	LELP1		protein_c	chr1:153203430-153
ENSG0000(	544	13.60595	chr1:1234	CRNN		protein_c	chr1:152409243-152
ENSG0000(	544	13.60595	chr1:1234	LCEP3		Pseudoger	chr1:152656332-152
ENSG0000(	544	13.60595	chr1:1234	CKS1B	AC	protein_c	chr1:154974653-154
ENSG0000(	544	13.60595	chr1:1234	ENSG00000233222		lncRNA	chr1:153750983-153
ENSG0000(	544	13.60595	chr1:1234	ENSG00000285641		protein_c	chr1:153975850-153
ENSG0000(	544	13.60595	chr1:1234	SPRR4		protein_c	chr1:152970648-152
ENSG0000(	544	13.60595	chr1:1234	S100A1	NCGv7	protein_c	chr1:153627926-153
ENSG0000(	544	13.60595	chr1:1234	ENSG00000271267		Pseudoger	chr1:155566050-155
ENSG0000(	544	13.60595	chr1:1234	LCE1E		protein_c	chr1:152786214-152
ENSG0000(	544	13.60595	chr1:1234	IL6R-AS1		lncRNA	chr1:154402328-154
ENSG0000(	544	13.60595	chr1:1234	SPRR5		protein_c	chr1:152947206-152
ENSG0000(	544	13.60595	chr1:1234	RNU6-160P		smallRNA	chr1:153331622-153
ENSG0000(	544	13.60595	chr1:1234	MIR555		smallRNA	chr1:155346350-155
ENSG0000(	544	13.60595	chr1:1234	Y_RNA		smallRNA	chr1:155120490-155
ENSG0000(	544	13.60595	chr1:1234	ATP8B2	NCGv7	protein_c	chr1:154325525-154
ENSG0000(	544	13.60595	chr1:1234	ENSG00000286581		lncRNA	chr1:151944890-151
ENSG0000(	544	13.60595	chr1:1234	ENSG00000232519		lncRNA	chr1:155609776-155

ENSG0000(	544	13.60595	chr1:123⁄	MTX1		protein_c	chr1:155208695-155
ENSG0000(	544	13.60595	chr1:123⁄	ADAM15		protein_c	chr1:155050566-155
ENSG0000(	544	13.60595	chr1:123⁄	KPRP		protein_c	chr1:152758025-152
ENSG0000(	544	13.60595	chr1:123⁄	JTB		protein_c	chr1:153974269-153
ENSG0000(	544	13.60595	chr1:123⁄	SNORA58		smallRNA	chr1:154259727-154
ENSG0000(	544	13.60595	chr1:123⁄	S100A8	NCGv7;AC	protein_c	chr1:153390032-153
ENSG0000(	544	13.60595	chr1:123⁄	LCE5A		protein_c	chr1:152510803-152
ENSG0000(	544	13.60595	chr1:123⁄	THBS3		protein_c	chr1:155195588-155
ENSG0000(	544	13.60595	chr1:123⁄	ASH1L-IT1		lncRNA	chr1:155396010-155
ENSG0000(	544	13.60595	chr1:123⁄	ENSG00000285779		protein_c	chr1:153959151-153
ENSG0000(	544	13.60595	chr1:123⁄	ENSG00000286391		lncRNA	chr1:154564855-154
ENSG0000(	544	13.60595	chr1:123⁄	ENSG00000236327		Pseudoger	chr1:153890595-153
ENSG0000(	544	13.60595	chr1:123⁄	LCE2D		protein_c	chr1:152663380-152
ENSG0000(	544	13.60595	chr1:123⁄	S100A3		protein_c	chr1:153547329-153
ENSG0000(	544	13.60595	chr1:123⁄	ENSG00000229021		lncRNA	chr1:151994531-152
ENSG0000(	544	13.60595	chr1:123⁄	AL606500.1		protein_c	chr1:154612591-154
ENSG0000(	544	13.60595	chr1:123⁄	S100A7P1		Pseudoger	chr1:153427020-153
ENSG0000(	544	13.60595	chr1:123⁄	LCE3A		protein_c	chr1:152622834-152
ENSG0000(	544	13.60595	chr1:123⁄	LCE1F		protein_c	chr1:152775140-152
ENSG0000(	544	13.60595	chr1:123⁄	RAB13		protein_c	chr1:153981617-153
ENSG0000(	544	13.60595	chr1:123⁄	LORICRIN		protein_c	chr1:153259687-153
ENSG0000(	544	13.60595	chr1:123⁄	ENSG00000291199		lncRNA	chr1:153793937-153
ENSG0000(	544	13.60595	chr1:123⁄	HMGN3P1		Pseudoger	chr1:152399577-152
ENSG0000(	544	13.60595	chr1:123⁄	LCE3B		protein_c	chr1:152613811-152
ENSG0000(	544	13.60595	chr1:123⁄	SPTLC1P4		Pseudoger	chr1:152077952-152
ENSG0000(	544	13.60595	chr1:123⁄	SPRR2E		protein_c	chr1:153093135-153
ENSG0000(	544	13.60595	chr1:123⁄	EFNA1		protein_c	chr1:155127876-155
ENSG0000(	544	13.60595	chr1:123⁄	AL713999.1		smallRNA	chr1:155236749-155
ENSG0000(	544	13.60595	chr1:123⁄	ENSG00000271853		lncRNA	chr1:153626332-153
ENSG0000(	544	13.60595	chr1:123⁄	PUDPP2		Pseudoger	chr1:152124016-152
ENSG0000(	544	13.60595	chr1:123⁄	PMVK		protein_c	chr1:154924740-154
ENSG0000(	544	13.60595	chr1:123⁄	THBS3-AS1		lncRNA	chr1:155194996-155
ENSG0000(	544	13.60595	chr1:123⁄	MIR5698		smallRNA	chr1:154104521-154
ENSG0000(	544	13.60595	chr1:123⁄	PBXIP1		protein_c	chr1:154944076-154
ENSG0000(	544	13.60595	chr1:123⁄	LCEP4		Pseudoger	chr1:152644393-152
ENSG0000(	544	13.60595	chr1:123⁄	PYGO2		protein_c	chr1:154957026-154
ENSG0000(	544	13.60595	chr1:123⁄	SLC50A1		protein_c	chr1:155135344-155
ENSG0000(	544	13.60595	chr1:123⁄	LENEP	DriverDB\	protein_c	chr1:154993586-154
ENSG0000(	544	13.60595	chr1:123⁄	HCN3		protein_c	chr1:155277463-155
ENSG0000(	544	13.60595	chr1:123⁄	DCST2	DriverDB\	protein_c	chr1:155018520-155
ENSG0000(	544	13.60595	chr1:123⁄	ENSG00000289062		lncRNA	chr1:152897800-152
ENSG0000(	544	13.60595	chr1:123⁄	DPM3		protein_c	chr1:155139891-155
ENSG0000(	544	13.60595	chr1:123⁄	DCST1	DriverDB\	protein_c	chr1:155033824-155
ENSG0000(	544	13.60595	chr1:123⁄	PKLR		protein_c	chr1:155289293-155
ENSG0000(	544	13.60595	chr1:123⁄	LCE1D	NCGv7	protein_c	chr1:152796721-152
ENSG0000(	544	13.60595	chr1:123⁄	INTS3	AC	protein_c	chr1:153728050-153
ENSG0000(	544	13.60595	chr1:123⁄	LCE7A		protein_c	chr1:152859996-152
ENSG0000(	544	13.60595	chr1:123⁄	ENSG00000234262		Pseudoger	chr1:153023962-153
ENSG0000(	544	13.60595	chr1:123⁄	RNU6-179P		smallRNA	chr1:154039916-154
ENSG0000(	544	13.60595	chr1:123⁄	RNU6-1297P		smallRNA	chr1:155419397-155
ENSG0000(	544	13.60595	chr1:123⁄	CFAP141		protein_c	chr1:154199085-154

ENSG0000(	544	13.60595	chr1:123<	MSTO1		protein_c	chr1:155563235-155
ENSG0000(	544	13.60595	chr1:123<	S100A4	AC	protein_c	chr1:153543613-153
ENSG0000(	544	13.60595	chr1:123<	RN7SL431P		smallRNA	chr1:154166247-154
ENSG0000(	544	13.60595	chr1:123<	S100A12		protein_c	chr1:153373711-153
ENSG0000(	544	13.60595	chr1:123<	S100A15A		Pseudoger	chr1:153396591-153
ENSG0000(	544	13.60595	chr1:123<	S100A9		protein_c	chr1:153357854-153
ENSG0000(	544	13.60595	chr1:123<	NPR1		protein_c	chr1:153678688-153
ENSG0000(	544	13.60595	chr1:123<	ENSG00000270361		lncRNA	chr1:154937370-154
ENSG0000(	544	13.60595	chr1:123<	RPTN		protein_c	chr1:152153595-152
ENSG0000(	544	13.60595	chr1:123<	LCEP2		Pseudoger	chr1:152737518-152
ENSG0000(	544	13.60595	chr1:123<	ENSG00000229699		lncRNA	chr1:153174518-153
ENSG0000(	544	13.60595	chr1:123<	SPRR2D		protein_c	chr1:153039732-153
ENSG0000(	544	13.60595	chr1:123<	LCE1B		protein_c	chr1:152811971-152
ENSG0000(	544	13.60595	chr1:123<	KRTCAP2		protein_c	chr1:155169408-155
ENSG0000(	544	13.60595	chr1:123<	SPRR2F		protein_c	chr1:153112121-153
ENSG0000(	544	13.60595	chr1:123<	ENSG00000273110		lncRNA	chr1:154480012-154
ENSG0000(	544	13.60595	chr1:123<	ENSG00000231416		Pseudoger	chr1:153995632-153
ENSG0000(	544	13.60595	chr1:123<	ASH1L-AS1		lncRNA	chr1:155562026-155
ENSG0000(	544	13.60595	chr1:123<	ENSG00000289103		lncRNA	chr1:155000799-155
ENSG0000(	544	13.60595	chr1:123<	ENSG00000251246		protein_c	chr1:155063748-155
ENSG0000(	544	13.60595	chr1:123<	Y_RNA		smallRNA	chr1:153726252-153
ENSG0000(	544	13.60595	chr1:123<	Y_RNA		smallRNA	chr1:153785720-153
ENSG0000(	544	13.60595	chr1:123<	S100A2		protein_c	chr1:153561108-153
ENSG0000(	544	13.60595	chr1:123<	HMGN2P18		Pseudoger	chr1:155148544-155
ENSG0000(	544	13.60595	chr1:123<	ENSG00000272030		lncRNA	chr1:153631438-153
ENSG0000(	544	13.60595	chr1:123<	SPRR2B	NCGv7	protein_c	chr1:153070226-153
ENSG0000(	544	13.60595	chr1:123<	LCE3C		protein_c	chr1:152600234-152
ENSG0000(	544	13.60595	chr1:123<	ENSG00000233875		lncRNA	chr1:154579065-154
ENSG0000(	544	13.60595	chr1:123<	TRIM46	DriverDB\	protein_c	chr1:155173787-155
ENSG0000(	544	13.60595	chr1:123<	snoU13		smallRNA	chr1:153754124-153
ENSG0000(	544	13.60595	chr1:123<	RNU7-150P		smallRNA	chr1:155143271-155
ENSG0000(	544	13.60595	chr1:123<	SPRR2A		protein_c	chr1:153056120-153
ENSG0000(	544	13.60595	chr1:123<	SNORA31		smallRNA	chr1:153012482-153
ENSG0000(	544	13.60595	chr1:123<	LCE6A		protein_c	chr1:152842856-152
ENSG0000(	544	13.60595	chr1:123<	ENSG00000273088		protein_c	chr1:155169409-155
ENSG0000(	544	13.60595	chr1:123<	ENSG00000238279		lncRNA	chr1:153533430-153
ENSG0000(	544	13.60595	chr1:123<	LCEP1		Pseudoger	chr1:152744299-152
ENSG0000(	544	13.60595	chr1:123<	SHE	NCGv7	protein_c	chr1:154469772-154
ENSG0000(	544	13.60595	chr1:123<	LAPTM4BP1		Pseudoger	chr1:153379821-153
ENSG0000(	544	13.60595	chr1:123<	S100A5		protein_c	chr1:153537147-153
ENSG0000(	544	13.60595	chr1:123<	LCE1C		protein_c	chr1:152804832-152
ENSG0000(	544	13.60595	chr1:123<	FLG	NCGv7	protein_c	chr1:152302165-152
ENSG0000(	544	13.60595	chr1:123<	RN7SL44P		smallRNA	chr1:153500467-153
ENSG0000(	544	13.60595	chr1:123<	YY1AP1	AC	protein_c	chr1:155659443-155
ENSG0000(	544	13.60595	chr1:123<	PGLYRP4		protein_c	chr1:153330120-153
ENSG0000(	544	13.60595	chr1:123<	TDRD10	NCGv7	protein_c	chr1:154502219-154
ENSG0000(	544	13.60595	chr1:123<	ENSG00000243613		lncRNA	chr1:153746851-153
ENSG0000(	544	13.60595	chr1:123<	SCAMP3		protein_c	chr1:155255979-155
ENSG0000(	544	13.60595	chr1:123<	MTX1P1		Pseudoger	chr1:155230975-155
ENSG0000(	544	13.60595	chr1:123<	GBAP1		lncRNA	chr1:155213821-155
ENSG0000(	544	13.60595	chr1:123<	HAX1	NCGv7;AC	protein_c	chr1:154272355-154

ENSG0000(	544	13.60595	chr1:1234	CREB3L4	NCGv7	protein_c	chr1:153967534-153
ENSG0000(	544	13.60595	chr1:1234	FLG-AS1		lncRNA	chr1:152168125-152
ENSG0000(	544	13.60595	chr1:1234	ASH1L	NCGv7	protein_c	chr1:155335268-155
ENSG0000(	544	13.60595	chr1:1234	ENSG00000284738		lncRNA	chr1:153923284-153
ENSG0000(	544	13.60595	chr1:1234	RPSAP17		Pseudoger	chr1:154378207-154
ENSG0000(	544	13.60595	chr1:1234	EFNA3		protein_c	chr1:155078837-155
ENSG0000(	544	13.60595	chr1:1234	S100A7L2		Pseudoger	chr1:153437058-153
ENSG0000(	544	13.60595	chr1:1234	GBAP1		Pseudoger	chr1:155214368-155
ENSG0000(	544	13.60595	chr1:1234	CLK2		protein_c	chr1:155262868-155
ENSG0000(	544	13.60595	chr1:1234	S100A13		protein_c	chr1:153618787-153
ENSG0000(	544	13.60595	chr1:1234	FAM189B	AC	protein_c	chr1:155247205-155
ENSG0000(	544	13.60595	chr1:1234	LINC01527		lncRNA	chr1:152930040-152
ENSG0000(	544	13.60595	chr1:1234	ENSG00000287064		lncRNA	chr1:154671593-154
ENSG0000(	544	13.60595	chr1:1234	ENSG00000285867		lncRNA	chr1:153586813-153
ENSG0000(	544	13.60595	chr1:1234	ENSG00000223599		Pseudoger	chr1:153852106-153
ENSG0000(	544	13.60595	chr1:1234	IL6R		protein_c	chr1:154405193-154
ENSG0000(	544	13.60595	chr1:1234	TCHHL1		protein_c	chr1:152084141-152
ENSG0000(	544	13.60595	chr1:1234	GBA		protein_c	chr1:155234452-155
ENSG0000(	544	13.60595	chr1:1234	S100A16		protein_c	chr1:153606886-153
ENSG0000(	544	13.60595	chr1:1234	KRT8P28		Pseudoger	chr1:151949523-151
ENSG0000(	544	13.60595	chr1:1234	S100A10		protein_c	chr1:151982915-151
ENSG0000(	544	13.60595	chr1:1234	ENSG00000237920		Pseudoger	chr1:154376966-154
ENSG0000(	544	13.60595	chr1:1234	RUSC1		protein_c	chr1:155320894-155
ENSG0000(	544	13.60595	chr1:1234	UBE2Q1		protein_c	chr1:154548577-154
ENSG0000(	544	13.60595	chr1:1234	CHRNB2	NCGv7	protein_c	chr1:154567778-154
ENSG0000(	544	13.60595	chr1:1234	SPRR1B		protein_c	chr1:153031203-153
ENSG0000(	544	13.60595	chr1:1234	CRTC2		protein_c	chr1:153947669-153
ENSG0000(	544	13.60595	chr1:1234	FDPS	AC	protein_c	chr1:155308748-155
ENSG0000(	544	13.60595	chr1:1234	RNU6-239P		smallRNA	chr1:154295503-154
ENSG0000(	544	13.60595	chr1:1234	AQP10		protein_c	chr1:154321090-154
ENSG0000(	544	13.60595	chr1:1234	EFNA4		protein_c	chr1:155063737-155
ENSG0000(	544	13.60595	chr1:1234	LCE2B		protein_c	chr1:152686123-152
ENSG0000(	544	13.60595	chr1:1234	snoU13		smallRNA	chr1:155415558-155
ENSG0000(	544	13.60595	chr1:1234	S100A11		protein_c	chr1:152032506-152
ENSG0000(	544	13.60595	chr1:1234	ENSG00000273026		lncRNA	chr1:153966516-153
ENSG0000(	544	13.60595	chr1:1234	C1orf43		protein_c	chr1:154206696-154
ENSG0000(	544	13.60595	chr1:1234	LCE3D		protein_c	chr1:152579381-152
ENSG0000(	544	13.60595	chr1:1234	SMCP		protein_c	chr1:152878322-152
ENSG0000(	544	13.60595	chr1:1234	C1orf68		protein_c	chr1:152719522-152
ENSG0000(	544	13.60595	chr1:1234	GATAD2B		protein_c	chr1:153789030-153
ENSG0000(	544	13.60595	chr1:1234	ENSG00000246203		Pseudoger	chr1:155614726-155
ENSG0000(	544	13.60595	chr1:1234	KCNN3	NCGv7	protein_c	chr1:154697455-154
ENSG0000(	544	13.60595	chr1:1234	AL590431.1		smallRNA	chr1:154254445-154
ENSG0000(	544	13.60595	chr1:1234	ENSG00000278694		Pseudoger	chr1:154312462-154
ENSG0000(	544	13.60595	chr1:1234	S100A14		protein_c	chr1:153614255-153
ENSG0000(	544	13.60595	chr1:1234	ENSG00000282386		lncRNA	chr1:153964361-153
ENSG0000(	544	13.60595	chr1:1234	ILF2		protein_c	chr1:153661788-153
ENSG0000(	544	13.60595	chr1:1234	MUC1	DriverDBv	protein_c	chr1:155185824-155
ENSG0000(	544	13.60595	chr1:1234	DENND4B	NCGv7	protein_c	chr1:153929501-153
ENSG0000(	544	13.60595	chr1:1234	SPRR3		protein_c	chr1:153001747-153
ENSG0000(	544	13.60595	chr1:1234	MIR92B		smallRNA	chr1:155195177-155

ENSG0000(	544	13.60595	chr1:1234	UBE2Q1-AS1		lncRNA	chr1:154553609-154
ENSG0000(	544	13.60595	chr1:1234	IVL		protein_c	chr1:152908546-152
ENSG0000(	540	13.5059	chr1:1234	VHLL		protein_c	chr1:156298624-156
ENSG0000(	537	13.43087	chr22:227	IGLV3-22		protein_c	chr22:22704265-227
ENSG0000(	536	13.40586	chr1:1166	RN7SL386P		smallRNA	chr1:21987481-2198
ENSG0000(	536	13.40586	chr1:1166	RNU6-1022P		smallRNA	chr1:21987816-2198
ENSG0000(	536	13.40586	chr1:1166	RN7SL421P		smallRNA	chr1:21978951-2197
ENSG0000(	533	13.33083	chr6:105(	H2AC9P		Pseudoger	chr6:26233122-2623
ENSG0000(	529	13.23078	chr1:1166	SELENON		protein_c	chr1:25800193-2581
ENSG0000(	529	13.23078	chr1:1166	RNU6-1171P		smallRNA	chr1:25340971-2534
ENSG0000(	529	13.23078	chr1:1166	PDIK1L		protein_c	chr1:26111165-2612
ENSG0000(	529	13.23078	chr1:1166	RUNX3-AS1		lncRNA	chr1:24961345-2496
ENSG0000(	529	13.23078	chr1:1166	ENSG00000225854		Pseudoger	chr1:26326688-2632
ENSG0000(	529	13.23078	chr1:1166	ENSG00000272478		lncRNA	chr1:25831913-2583
ENSG0000(	529	13.23078	chr1:1166	LINC02793		lncRNA	chr1:25041136-2504
ENSG0000(	529	13.23078	chr1:1166	STMN1	AC	protein_c	chr1:25884181-2590
ENSG0000(	529	13.23078	chr1:1166	AL033528.1		smallRNA	chr1:25911749-2591
ENSG0000(	529	13.23078	chr1:1166	RSRP1		protein_c	chr1:25242249-2533
ENSG0000(	529	13.23078	chr1:1166	HMGN2		protein_c	chr1:26472440-2647
ENSG0000(	529	13.23078	chr1:1166	ENSG00000231953		lncRNA	chr1:25208139-2520
ENSG0000(	529	13.23078	chr1:1166	CD52		protein_c	chr1:26317958-2632
ENSG0000(	529	13.23078	chr1:1166	C1orf232		protein_c	chr1:26164101-2616
ENSG0000(	529	13.23078	chr1:1234	SNORA26		smallRNA	chr1:156192063-156
ENSG0000(	529	13.23078	chr1:1166	AL031284.1		smallRNA	chr1:25406231-2540
ENSG0000(	529	13.23078	chr1:1166	SDHDP6		Pseudoger	chr1:25294164-2529
ENSG0000(	529	13.23078	chr1:1166	MAN1C1		protein_c	chr1:25616791-2578
ENSG0000(	529	13.23078	chr1:1166	MTFR1L	NCGv7	protein_c	chr1:25818640-2583
ENSG0000(	529	13.23078	chr1:1166	ENSG00000272432		lncRNA	chr1:25247837-2524
ENSG0000(	529	13.23078	chr1:1166	MIR4425		smallRNA	chr1:25023503-2502
ENSG0000(	529	13.23078	chr1:1166	SCARNA17		smallRNA	chr1:26006006-2600
ENSG0000(	529	13.23078	chr1:1166	ENSG00000236528		lncRNA	chr1:25859580-2586
ENSG0000(	529	13.23078	chr1:1166	RNU6-110P		smallRNA	chr1:25964197-2596
ENSG0000(	529	13.23078	chr1:1166	SYF2	NCGv7	protein_c	chr1:25222276-2523
ENSG0000(	529	13.23078	chr1:1166	ENSG00000233478		lncRNA	chr1:25644544-2565
ENSG0000(	529	13.23078	chr1:1166	RUNX3	AC	protein_c	chr1:24899511-2496
ENSG0000(	529	13.23078	chr1:1166	AUNIP		protein_c	chr1:25831913-2585
ENSG0000(	529	13.23078	chr1:1166	SCARNA18		smallRNA	chr1:26006216-2600
ENSG0000(	529	13.23078	chr1:1166	CEP85		protein_c	chr1:26234200-2627
ENSG0000(	529	13.23078	chr1:1166	RHCE		protein_c	chr1:25362249-2543
ENSG0000(	529	13.23078	chr1:1166	RN7SL490P		smallRNA	chr1:26348465-2634
ENSG0000(	529	13.23078	chr1:1166	RN7SL679P		smallRNA	chr1:26593246-2659
ENSG0000(	529	13.23078	chr1:1166	LIN28A	AC	protein_c	chr1:26410817-2642
ENSG0000(	529	13.23078	chr1:1166	MACO1		protein_c	chr1:25430858-2550
ENSG0000(	529	13.23078	chr1:1166	ENSG00000284657		lncRNA	chr1:25239494-2524
ENSG0000(	529	13.23078	chr1:1166	PAFAH2		protein_c	chr1:25959767-2599
ENSG0000(	529	13.23078	chr1:1166	EXTL1		protein_c	chr1:26019884-2603
ENSG0000(	529	13.23078	chr1:1166	ENSG00000261349		Pseudoger	chr1:25266102-2526
ENSG0000(	529	13.23078	chr1:1166	ENSG00000255054		protein_c	chr1:25811470-2582
ENSG0000(	529	13.23078	chr1:1166	CNKSR1	NCGv7	protein_c	chr1:26177484-2618
ENSG0000(	529	13.23078	chr1:1166	SLC30A2		protein_c	chr1:26037252-2604
ENSG0000(	529	13.23078	chr1:1166	TRIM63		protein_c	chr1:26051301-2606

ENSG0000(	529	13.23078	chr1:116(	CRYBG2		protein_c	chr1:26321698-2636
ENSG0000(	529	13.23078	chr1:116(	AL391650.1		smallRNA	chr1:26071578-2607
ENSG0000(	529	13.23078	chr1:116(	DPPA2P2		Pseudoger	chr1:26519354-2652
ENSG0000(	529	13.23078	chr1:116(	ENSG00000225643		lncRNA	chr1:25581478-2559
ENSG0000(	529	13.23078	chr1:116(	ZNF593		protein_c	chr1:26169908-2617
ENSG0000(	529	13.23078	chr1:116(	ENSG00000259984		Pseudoger	chr1:25336429-2533
ENSG0000(	529	13.23078	chr1:116(	ENSG00000228172		lncRNA	chr1:25816749-2582
ENSG0000(	529	13.23078	chr1:116(	MIR1976		smallRNA	chr1:26554542-2655
ENSG0000(	529	13.23078	chr1:116(	ENSG00000289452		lncRNA	chr1:26620707-2662
ENSG0000(	529	13.23078	chr1:116(	UBXN11		protein_c	chr1:26281328-2631
ENSG0000(	529	13.23078	chr1:116(	ZNF683		protein_c	chr1:26361634-2637
ENSG0000(	529	13.23078	chr1:116(	FAM110D		protein_c	chr1:26159079-2616
ENSG0000(	529	13.23078	chr1:116(	ENSG00000284602		lncRNA	chr1:25232586-2523
ENSG0000(	529	13.23078	chr1:116(	ENSG00000261025		lncRNA	chr1:24968423-2497
ENSG0000(	529	13.23078	chr1:116(	CATSPER4		protein_c	chr1:26190561-2620
ENSG0000(	529	13.23078	chr1:116(	RPS6KA1		protein_c	chr1:26529761-2657
ENSG0000(	529	13.23078	chr1:116(	LDLRAP1		protein_c	chr1:25543606-2556
ENSG0000(	529	13.23078	chr1:116(	ENSG00000278572		Pseudoger	chr1:26218581-2622
ENSG0000(	529	13.23078	chr1:116(	IFITM3P7		Pseudoger	chr1:25125053-2512
ENSG0000(	529	13.23078	chr1:116(	ENSG00000238084		Pseudoger	chr1:25398721-2539
ENSG0000(	529	13.23078	chr1:116(	DHDDS-AS1		lncRNA	chr1:26462756-2646
ENSG0000(	529	13.23078	chr1:116(	ENSG00000284309		lncRNA	chr1:26046665-2604
ENSG0000(	529	13.23078	chr1:116(	snoU13		smallRNA	chr1:25346274-2534
ENSG0000(	529	13.23078	chr1:116(	SNRPFP2		Pseudoger	chr1:25887360-2588
ENSG0000(	529	13.23078	chr1:116(	MIR3917		smallRNA	chr1:25906362-2590
ENSG0000(	529	13.23078	chr1:116(	ENSG00000223624		Pseudoger	chr1:25888970-2588
ENSG0000(	529	13.23078	chr1:116(	Y_RNA		smallRNA	chr1:26593940-2659
ENSG0000(	529	13.23078	chr1:116(	ENSG00000233755		lncRNA	chr1:25043707-2511
ENSG0000(	529	13.23078	chr1:116(	ZNF593OS		protein_c	chr1:26169516-2617
ENSG0000(	529	13.23078	chr1:116(	RNU6-1208P		smallRNA	chr1:24777873-2477
ENSG0000(	529	13.23078	chr1:116(	DHDDS		protein_c	chr1:26432282-2647
ENSG0000(	529	13.23078	chr1:116(	TMEM50A		protein_c	chr1:25338317-2536
ENSG0000(	529	13.23078	chr1:116(	Y_RNA		smallRNA	chr1:25877496-2587
ENSG0000(	529	13.23078	chr1:116(	RHD	DriverDBv	protein_c	chr1:25272393-2533
ENSG0000(	529	13.23078	chr1:116(	ENSG00000223583		Pseudoger	chr1:26454653-2645
ENSG0000(	529	13.23078	chr1:116(	PAQR7		protein_c	chr1:25861484-2587
ENSG0000(	529	13.23078	chr1:116(	ZPLD2P		Pseudoger	chr1:26209741-2622
ENSG0000(	529	13.23078	chr1:116(	SH3BGRL3		protein_c	chr1:26280086-2628
ENSG0000(	529	13.23078	chr1:3732	RNU6-755P		smallRNA	chr1:164980035-164
ENSG0000(	528	13.20577	chr1:1234	GLRX5P2		Pseudoger	chr1:161034834-161
ENSG0000(	524	13.10573	chr1:116(	SH2D5		protein_c	chr1:20719731-2073
ENSG0000(	524	13.10573	chr1:116(	MIR378F		smallRNA	chr1:23929070-2392
ENSG0000(	524	13.10573	chr1:116(	ENSG00000289014		lncRNA	chr1:23167098-2316
ENSG0000(	524	13.10573	chr1:116(	ID3	NCGv7	protein_c	chr1:23557926-2355
ENSG0000(	524	13.10573	chr1:116(	ENSG00000233069		lncRNA	chr1:20732880-2073
ENSG0000(	524	13.10573	chr1:116(	RPS15AP6		Pseudoger	chr1:21003550-2100
ENSG0000(	524	13.10573	chr1:116(	RNU7-200P		smallRNA	chr1:20841241-2084
ENSG0000(	524	13.10573	chr1:116(	MIR4418		smallRNA	chr1:22266239-2226
ENSG0000(	524	13.10573	chr1:116(	PNRC2		protein_c	chr1:23956839-2396
ENSG0000(	524	13.10573	chr1:116(	IFNLR1		protein_c	chr1:24154168-2418
ENSG0000(	524	13.10573	chr1:116(	LINC00339		lncRNA	chr1:22024558-2203

ENSG0000(	524	13.10573	chr1:116(	ENSG00000287192		lncRNA	chr1:21177054-2117
ENSG0000(	524	13.10573	chr1:116(	H3P1		Pseudoger	chr1:23949016-2394
ENSG0000(	524	13.10573	chr1:116(	MIR4253		smallRNA	chr1:22863159-2286
ENSG0000(	524	13.10573	chr1:116(	MIR4419A		smallRNA	chr1:23057858-2305
ENSG0000(	524	13.10573	chr1:116(	EPHA8		protein_c	chr1:22563489-2260
ENSG0000(	524	13.10573	chr1:116(	CDC42	NCGv7	protein_c	chr1:22052627-2210
ENSG0000(	524	13.10573	chr1:116(	USP48		protein_c	chr1:21678298-2178
ENSG0000(	524	13.10573	chr1:116(	NIPAL3		protein_c	chr1:24415802-2447
ENSG0000(	524	13.10573	chr1:116(	RN7SL186P		smallRNA	chr1:22010650-2201
ENSG0000(	524	13.10573	chr1:116(	ENSG00000232557		lncRNA	chr1:23907111-2390
ENSG0000(	524	13.10573	chr1:116(	EIF4G3		protein_c	chr1:20806292-2117
ENSG0000(	524	13.10573	chr1:116(	RCAN3AS		lncRNA	chr1:24496254-2453
ENSG0000(	524	13.10573	chr1:116(	ZBTB40-IT1		lncRNA	chr1:22517474-2251
ENSG0000(	524	13.10573	chr1:116(	ENSG00000232482		Pseudoger	chr1:23410832-2341
ENSG0000(	524	13.10573	chr1:116(	snoU13		smallRNA	chr1:23882255-2388
ENSG0000(	524	13.10573	chr1:116(	RN7SL857P		smallRNA	chr1:24529455-2452
ENSG0000(	524	13.10573	chr1:116(	ENSG00000223944		lncRNA	chr1:37154761-3732
ENSG0000(	524	13.10573	chr1:116(	LINC01355		lncRNA	chr1:23281307-2328
ENSG0000(	524	13.10573	chr1:116(	AL391357.1		Pseudoger	chr1:20650363-2065
ENSG0000(	524	13.10573	chr1:116(	ENSG00000225315		lncRNA	chr1:24040835-2408
ENSG0000(	524	13.10573	chr1:116(	RNA5SP43		Pseudoger	chr1:37264677-3726
ENSG0000(	524	13.10573	chr1:116(	BTBD6P1		Pseudoger	chr1:23901471-2390
ENSG0000(	524	13.10573	chr1:116(	ENSG00000236009		lncRNA	chr1:21415898-2141
ENSG0000(	524	13.10573	chr1:116(	GRHL3-AS1		lncRNA	chr1:24307556-2432
ENSG0000(	524	13.10573	chr1:116(	MIR4684		smallRNA	chr1:22719517-2271
ENSG0000(	524	13.10573	chr1:116(	RP1-224A6.9		lncRNA	chr1:22100613-2210
ENSG0000(	524	13.10573	chr1:116(	ENSG00000289835		lncRNA	chr1:23790609-2379
ENSG0000(	524	13.10573	chr1:116(	GALE		protein_c	chr1:23795599-2380
ENSG0000(	524	13.10573	chr1:116(	MIR3115		smallRNA	chr1:23044305-2304
ENSG0000(	524	13.10573	chr1:116(	ENSG00000236073		Pseudoger	chr1:21073639-2107
ENSG0000(	524	13.10573	chr1:116(	PPIAP34		Pseudoger	chr1:22322840-2232
ENSG0000(	524	13.10573	chr1:116(	EPHB2		protein_c	chr1:22710839-2292
ENSG0000(	524	13.10573	chr1:116(	ENSG00000288982		lncRNA	chr1:24476362-2447
ENSG0000(	524	13.10573	chr1:116(	HNRNPR		protein_c	chr1:23303771-2334
ENSG0000(	524	13.10573	chr1:116(	CDA		protein_c	chr1:20589086-2061
ENSG0000(	524	13.10573	chr1:116(	LDLRAD2		protein_c	chr1:21812265-2182
ENSG0000(	524	13.10573	chr1:116(	HSPE1P27		Pseudoger	chr1:21161475-2116
ENSG0000(	524	13.10573	chr1:116(	C1QB		protein_c	chr1:22652762-2266
ENSG0000(	524	13.10573	chr1:116(	PINK1-AS		lncRNA	chr1:20642657-2065
ENSG0000(	524	13.10573	chr1:116(	C1QA		protein_c	chr1:22635077-2263
ENSG0000(	524	13.10573	chr1:116(	ENSG00000236936		lncRNA	chr1:21266082-2126
ENSG0000(	524	13.10573	chr1:116(	ENSG00000235052		lncRNA	chr1:23549139-2355
ENSG0000(	524	13.10573	chr1:116(	ENSG00000289694		protein_c	chr1:22025142-2209
ENSG0000(	524	13.10573	chr1:116(	ENSG00000289692		protein_c	chr1:22636506-2264
ENSG0000(	524	13.10573	chr1:116(	STPG1		protein_c	chr1:24356999-2441
ENSG0000(	524	13.10573	chr1:116(	ENSG00000229010		Pseudoger	chr1:23140325-2314
ENSG0000(	524	13.10573	chr1:116(	CELA3A		protein_c	chr1:22001657-2201
ENSG0000(	524	13.10573	chr1:116(	RNU6-776P		smallRNA	chr1:22010985-2201
ENSG0000(	524	13.10573	chr1:116(	PINK1		protein_c	chr1:20633458-2065
ENSG0000(	524	13.10573	chr1:116(	ZNF436		protein_c	chr1:23359448-2336
ENSG0000(	524	13.10573	chr1:116(	ENSG00000227312		Pseudoger	chr1:24563627-2456

ENSG0000(	524	13.10573	chr1:116(	LUZP1	NCGv7	protein_c	chr1:23084030-2317
ENSG0000(	524	13.10573	chr1:116(	NBPF3		protein_c	chr1:21440128-2148
ENSG0000(	524	13.10573	chr1:116(	HSPG2	NCGv7	protein_c	chr1:21822244-2193
ENSG0000(	524	13.10573	chr1:116(	PITHD1		protein_c	chr1:23778418-2378
ENSG0000(	524	13.10573	chr1:116(	ENSG00000279625		TEC	chr1:22364630-2236
ENSG0000(	524	13.10573	chr1:116(	HP1BP3		protein_c	chr1:20740266-2078
ENSG0000(	524	13.10573	chr1:116(	C1QC		protein_c	chr1:22643014-2264
ENSG0000(	524	13.10573	chr1:116(	ENSG00000240553		lncRNA	chr1:23020147-2308
ENSG0000(	524	13.10573	chr1:116(	RN7SL768P		smallRNA	chr1:22003585-2200
ENSG0000(	524	13.10573	chr1:116(	CROCCP5		Pseudoger	chr1:21434318-2143
ENSG0000(	524	13.10573	chr1:116(	Y_RNA		smallRNA	chr1:23370254-2337
ENSG0000(	524	13.10573	chr1:116(	MPHOSPH6P1		Pseudoger	chr1:22068340-2206
ENSG0000(	524	13.10573	chr1:116(	RAP1GAP		protein_c	chr1:21596221-2166
ENSG0000(	524	13.10573	chr1:116(	RNU6-636P		smallRNA	chr1:37203610-3720
ENSG0000(	524	13.10573	chr1:116(	HS6ST1P1		Pseudoger	chr1:21428303-2142
ENSG0000(	524	13.10573	chr1:116(	KIF17		protein_c	chr1:20664014-2071
ENSG0000(	524	13.10573	chr1:116(	ASAP3		protein_c	chr1:23428563-2348
ENSG0000(	524	13.10573	chr1:116(	SRRM1		protein_c	chr1:24631716-2467
ENSG0000(	524	13.10573	chr1:116(	HMGCL		protein_c	chr1:23801885-2383
ENSG0000(	524	13.10573	chr1:116(	ECE1-AS1		lncRNA	chr1:21293290-2129
ENSG0000(	524	13.10573	chr1:116(	snoU13		smallRNA	chr1:24666983-2466
ENSG0000(	524	13.10573	chr1:116(	CNR2	AC	protein_c	chr1:23870515-2391
ENSG0000(	524	13.10573	chr1:116(	PPP1R11P1		Pseudoger	chr1:21397987-2139
ENSG0000(	524	13.10573	chr1:116(	ECE1		protein_c	chr1:21217247-2134
ENSG0000(	524	13.10573	chr1:116(	E2F2	NCGv7	protein_c	chr1:23506438-2353
ENSG0000(	524	13.10573	chr1:116(	ENSG00000271420		lncRNA	chr1:23378380-2337
ENSG0000(	524	13.10573	chr1:116(	ENSG00000235432		Pseudoger	chr1:20692734-2069
ENSG0000(	524	13.10573	chr1:116(	Y_RNA		smallRNA	chr1:24625411-2462
ENSG0000(	524	13.10573	chr1:116(	SRSF10		protein_c	chr1:23964347-2398
ENSG0000(	524	13.10573	chr1:116(	MDS2	AC	lncRNA	chr1:23581495-2364
ENSG0000(	524	13.10573	chr1:116(	MYOM3-AS1		lncRNA	chr1:24066774-2408
ENSG0000(	524	13.10573	chr1:116(	CELA3B		protein_c	chr1:21977022-2199
ENSG0000(	524	13.10573	chr1:116(	LINC01635		lncRNA	chr1:22023990-2202
ENSG0000(	524	13.10573	chr1:116(	CLIC4		protein_c	chr1:24745382-2484
ENSG0000(	524	13.10573	chr1:116(	RNU6-514P		smallRNA	chr1:23162704-2316
ENSG0000(	524	13.10573	chr1:116(	MYOM3	NCGv7	protein_c	chr1:24056035-2411
ENSG0000(	524	13.10573	chr1:116(	KDM1A	NCGv7	protein_c	chr1:23019443-2308
ENSG0000(	524	13.10573	chr1:116(	ENSG00000289715		protein_c	chr1:21547404-2155
ENSG0000(	524	13.10573	chr1:116(	RPL11		protein_c	chr1:23691742-2369
ENSG0000(	524	13.10573	chr1:116(	GRHL3		protein_c	chr1:24199558-2436
ENSG0000(	524	13.10573	chr1:116(	IL22RA1		protein_c	chr1:24119771-2414
ENSG0000(	524	13.10573	chr1:116(	ELOA-AS1		lncRNA	chr1:23706901-2377
ENSG0000(	524	13.10573	chr1:116(	AL031005.1		smallRNA	chr1:21176566-2117
ENSG0000(	524	13.10573	chr1:116(	PDE4DIPP10		Pseudoger	chr1:21411460-2141
ENSG0000(	524	13.10573	chr1:116(	FUCA1		protein_c	chr1:23845077-2386
ENSG0000(	524	13.10573	chr1:116(	MIR1256		smallRNA	chr1:20988314-2098
ENSG0000(	524	13.10573	chr1:116(	GRIK3	NCGv7	protein_c	chr1:36795527-3703
ENSG0000(	524	13.10573	chr1:116(	RPL36P5		Pseudoger	chr1:24007881-2400
ENSG0000(	524	13.10573	chr1:116(	ENSG00000285873		lncRNA	chr1:22142850-2215
ENSG0000(	524	13.10573	chr1:116(	CDC42-AS1		lncRNA	chr1:22028317-2205
ENSG0000(	524	13.10573	chr1:116(	RNU6-135P		smallRNA	chr1:23163953-2316

ENSG0000(	524	13.10573	chr1:116(	AL611946.1		smallRNA	chr1:22748817-2274
ENSG0000(	524	13.10573	chr1:116(	AL359815.1		smallRNA	chr1:21592411-2159
ENSG0000(	524	13.10573	chr1:116(	LACTBL1		protein_c	chr1:22953043-2297
ENSG0000(	524	13.10573	chr1:116(	TEX46		protein_c	chr1:23010834-2301
ENSG0000(	524	13.10573	chr1:116(	PFN1P10		Pseudoger	chr1:21459756-2146
ENSG0000(	524	13.10573	chr1:116(	LINC02800		lncRNA	chr1:24200240-2421
ENSG0000(	524	13.10573	chr1:116(	FAM43B		protein_c	chr1:20552573-2055
ENSG0000(	524	13.10573	chr1:116(	RPS4XP4		Pseudoger	chr1:20525989-2052
ENSG0000(	524	13.10573	chr1:116(	ZNF436-AS1		lncRNA	chr1:23368939-2337
ENSG0000(	524	13.10573	chr1:116(	ZBTB40		protein_c	chr1:22428838-2253
ENSG0000(	524	13.10573	chr1:116(	ENSG00000285959		lncRNA	chr1:21983606-2203
ENSG0000(	524	13.10573	chr1:116(	LINC02596		lncRNA	chr1:21586472-2159
ENSG0000(	524	13.10573	chr1:116(	EEF1A1P48		Pseudoger	chr1:23670294-2368
ENSG0000(	524	13.10573	chr1:116(	ENSG00000284726		lncRNA	chr1:23297797-2330
ENSG0000(	524	13.10573	chr1:116(	ENSG00000284650		lncRNA	chr1:37133489-3713
ENSG0000(	524	13.10573	chr1:116(	ENSG00000285802		lncRNA	chr1:23576436-2359
ENSG0000(	524	13.10573	chr1:116(	ENSG00000225952		lncRNA	chr1:22835713-2283
ENSG0000(	524	13.10573	chr1:116(	LYPLA2		protein_c	chr1:23791145-2379
ENSG0000(	524	13.10573	chr1:116(	ENSG00000285794		Pseudoger	chr1:22030527-2203
ENSG0000(	524	13.10573	chr1:116(	ENSG00000231978		Pseudoger	chr1:21768269-2176
ENSG0000(	524	13.10573	chr1:116(	ELOA		protein_c	chr1:23743448-2376
ENSG0000(	524	13.10573	chr1:116(	NCMAP-DT		lncRNA	chr1:24538802-2455
ENSG0000(	524	13.10573	chr1:116(	ENSG00000284699		lncRNA	chr1:24704894-2471
ENSG0000(	524	13.10573	chr1:116(	ENSG00000232037		Pseudoger	chr1:21908098-2190
ENSG0000(	524	13.10573	chr1:116(	DDOST		protein_c	chr1:20651767-2066
ENSG0000(	524	13.10573	chr1:116(	TCEA3		protein_c	chr1:23380909-2342
ENSG0000(	524	13.10573	chr1:116(	RCAN3		protein_c	chr1:24502351-2454
ENSG0000(	524	13.10573	chr1:116(	NCMAP		protein_c	chr1:24556087-2460
ENSG0000(	524	13.10573	chr1:116(	MIR4255		smallRNA	chr1:37161563-3716
ENSG0000(	524	13.10573	chr1:116(	ENSG00000283234		Pseudoger	chr1:21950679-2195
ENSG0000(	524	13.10573	chr1:116(	RN7SL532P		smallRNA	chr1:23736610-2373
ENSG0000(	524	13.10573	chr1:116(	RN7SL24P		smallRNA	chr1:23881794-2388
ENSG0000(	524	13.10573	chr1:116(	AL590683.1		smallRNA	chr1:24227917-2422
ENSG0000(	524	13.10573	chr1:116(	RP5-886K2.1		Pseudoger	chr1:23705801-2370
ENSG0000(	524	13.10573	chr1:116(	NBPF2P		Pseudoger	chr1:21424625-2142
ENSG0000(	524	13.10573	chr1:116(	HTR1D		protein_c	chr1:23191895-2321
ENSG0000(	524	13.10573	chr1:116(	CDC42-IT1		lncRNA	chr1:22059197-2206
ENSG0000(	524	13.10573	chr1:116(	WNT4		protein_c	chr1:22117313-2214
ENSG0000(	524	13.10573	chr1:116(	ALPL		protein_c	chr1:21509397-2157
ENSG0000(	524	13.10573	chr1:116(	ENSG00000215381		Pseudoger	chr1:23244765-2324
ENSG0000(	524	13.10573	chr1:116(	RP5-930J4.4		lncRNA	chr1:20742987-2074
ENSG0000(	523	13.08072	chr1:123<	ARHGEF2-AS2		lncRNA	chr1:155978799-155
ENSG0000(	523	13.08072	chr1:123<	ENSG00000272971		lncRNA	chr1:156614742-156
ENSG0000(	523	13.08072	chr1:123<	ARHGEF2	AC	protein_c	chr1:155946851-156
ENSG0000(	523	13.08072	chr1:123<	ACA64		smallRNA	chr1:161141208-161
ENSG0000(	523	13.08072	chr1:123<	ENSG00000228239		lncRNA	chr1:157232231-157
ENSG0000(	523	13.08072	chr1:123<	ATF6		protein_c	chr1:161766298-161
ENSG0000(	523	13.08072	chr1:123<	B4GALT3	NCGv7	protein_c	chr1:161171310-161
ENSG0000(	523	13.08072	chr1:123<	MRPS21P2		Pseudoger	chr1:157861197-157
ENSG0000(	523	13.08072	chr1:123<	GON4L		protein_c	chr1:155749659-155
ENSG0000(	523	13.08072	chr1:123<	CD84		protein_c	chr1:160541095-160

ENSG0000(	523	13.08072	chr1:123<	ENSG00000234937		Pseudoger	chr1:155845367-155
ENSG0000(	523	13.08072	chr1:123<	ENSG00000231700		Pseudoger	chr1:157709086-157
ENSG0000(	523	13.08072	chr1:123<	LAMTOR2		protein_c	chr1:156054782-156
ENSG0000(	523	13.08072	chr1:123<	ENSG00000215840		Pseudoger	chr1:161406068-161
ENSG0000(	523	13.08072	chr1:123<	ADAMTS4		protein_c	chr1:161184302-161
ENSG0000(	523	13.08072	chr1:123<	MEF2D	NCGv7	protein_c	chr1:156463727-156
ENSG0000(	523	13.08072	chr1:123<	ENSG00000224515		lncRNA	chr1:161556290-161
ENSG0000(	523	13.08072	chr1:123<	ENSG00000258465		protein_c	chr1:160216800-160
ENSG0000(	523	13.08072	chr1:123<	CFAP126		protein_c	chr1:161364733-161
ENSG0000(	523	13.08072	chr1:123<	AL121987.1		smallRNA	chr1:160205377-160
ENSG0000(	523	13.08072	chr1:123<	ENSG00000272668		lncRNA	chr1:159854870-159
ENSG0000(	523	13.08072	chr1:123<	DEDD	DriverDB\	protein_c	chr1:161120974-161
ENSG0000(	523	13.08072	chr1:123<	VSIG8		protein_c	chr1:159854316-159
ENSG0000(	523	13.08072	chr1:123<	SCARNA4		smallRNA	chr1:155925958-155
ENSG0000(	523	13.08072	chr1:123<	SLAMF9		protein_c	chr1:159951492-159
ENSG0000(	523	13.08072	chr1:123<	snoU13		smallRNA	chr1:156529330-156
ENSG0000(	523	13.08072	chr1:123<	LINC01704		lncRNA	chr1:158131983-158
ENSG0000(	523	13.08072	chr1:123<	ENSG00000285677		lncRNA	chr1:156001953-156
ENSG0000(	523	13.08072	chr1:123<	HMGN1P5		Pseudoger	chr1:158266753-158
ENSG0000(	523	13.08072	chr1:123<	ENSG00000270149		protein_c	chr1:160997957-161
ENSG0000(	523	13.08072	chr1:123<	SNORA42		smallRNA	chr1:155919909-155
ENSG0000(	523	13.08072	chr1:123<	ENSG00000289593		lncRNA	chr1:156504660-156
ENSG0000(	523	13.08072	chr1:123<	OR2AQ1P		Pseudoger	chr1:158796014-158
ENSG0000(	523	13.08072	chr1:123<	CYCSP52		Pseudoger	chr1:157128362-157
ENSG0000(	523	13.08072	chr1:123<	OR10J3		protein_c	chr1:159313720-159
ENSG0000(	523	13.08072	chr1:123<	NECTIN4-AS1		lncRNA	chr1:161084465-161
ENSG0000(	523	13.08072	chr1:123<	MPZ		protein_c	chr1:161304735-161
ENSG0000(	523	13.08072	chr1:123<	TOMM40L		protein_c	chr1:161225939-161
ENSG0000(	523	13.08072	chr1:123<	SEMA4A		protein_c	chr1:156147366-156
ENSG0000(	523	13.08072	chr1:123<	APOA2		protein_c	chr1:161222292-161
ENSG0000(	523	13.08072	chr1:123<	OR10J1		protein_c	chr1:159437845-159
ENSG0000(	523	13.08072	chr1:123<	FCER1G		protein_c	chr1:161215234-161
ENSG0000(	523	13.08072	chr1:123<	NDUFS2		protein_c	chr1:161197104-161
ENSG0000(	523	13.08072	chr1:123<	OR6K2		protein_c	chr1:158699678-158
ENSG0000(	523	13.08072	chr1:123<	OR10X1		protein_c	chr1:158578919-158
ENSG0000(	523	13.08072	chr1:123<	RIT1	NCGv7;AC	protein_c	chr1:155897808-155
ENSG0000(	523	13.08072	chr1:123<	SNORD64		smallRNA	chr1:159851906-159
ENSG0000(	523	13.08072	chr1:123<	MPTX1		Pseudoger	chr1:159276503-159
ENSG0000(	523	13.08072	chr1:123<	KCNJ9	NCGv7	protein_c	chr1:160081538-160
ENSG0000(	523	13.08072	chr1:123<	TSTD1	DriverDB\	protein_c	chr1:161037631-161
ENSG0000(	523	13.08072	chr1:123<	ENSG00000289273		lncRNA	chr1:161518705-161
ENSG0000(	523	13.08072	chr1:123<	ENSG00000287040		lncRNA	chr1:159900475-159
ENSG0000(	523	13.08072	chr1:123<	LY9		protein_c	chr1:160796074-160
ENSG0000(	523	13.08072	chr1:123<	ACKR1		protein_c	chr1:159203307-159
ENSG0000(	523	13.08072	chr1:123<	OLFML2B		protein_c	chr1:161983192-162
ENSG0000(	523	13.08072	chr1:123<	SLAMF6		protein_c	chr1:160485030-160
ENSG0000(	523	13.08072	chr1:123<	SLAMF8		protein_c	chr1:159826811-159
ENSG0000(	523	13.08072	chr1:123<	RPL31P11		Pseudoger	chr1:161683695-161
ENSG0000(	523	13.08072	chr1:123<	RNU6-481P		smallRNA	chr1:161401289-161
ENSG0000(	523	13.08072	chr1:123<	TTC24		protein_c	chr1:156579723-156
ENSG0000(	523	13.08072	chr1:123<	TAGLN2		protein_c	chr1:159918107-159

ENSG0000(	523	13.08072	chr1:1234	VANGL2		protein_c	chr1:160400564-160
ENSG0000(	523	13.08072	chr1:1234	NCSTN	NCGv7	protein_c	chr1:160343294-160
ENSG0000(	523	13.08072	chr1:1234	PEAR1	NCGv7	protein_c	chr1:156893698-156
ENSG0000(	523	13.08072	chr1:1234	COPA		protein_c	chr1:160288594-160
ENSG0000(	523	13.08072	chr1:1234	PEX19		protein_c	chr1:160276807-160
ENSG0000(	523	13.08072	chr1:1234	KIRREL1-IT1		lncRNA	chr1:158025550-158
ENSG0000(	523	13.08072	chr1:1234	OR10K1		protein_c	chr1:158461574-158
ENSG0000(	523	13.08072	chr1:1234	CADM3		protein_c	chr1:159171609-159
ENSG0000(	523	13.08072	chr1:1234	ENSG00000227741		lncRNA	chr1:160202199-160
ENSG0000(	523	13.08072	chr1:1234	CD1D	NCGv7	protein_c	chr1:158178030-158
ENSG0000(	523	13.08072	chr1:1234	ENSG00000289484		lncRNA	chr1:159501664-159
ENSG0000(	523	13.08072	chr1:1234	CD1A	NCGv7	protein_c	chr1:158254424-158
ENSG0000(	523	13.08072	chr1:1234	CD1C		protein_c	chr1:158289923-158
ENSG0000(	523	13.08072	chr1:1234	SETP9		Pseudoger	chr1:160670148-160
ENSG0000(	523	13.08072	chr1:1234	PEA15		protein_c	chr1:160205380-160
ENSG0000(	523	13.08072	chr1:1234	CD1B	NCGv7	protein_c	chr1:158327951-158
ENSG0000(	523	13.08072	chr1:1234	CD1E		protein_c	chr1:158353696-158
ENSG0000(	523	13.08072	chr1:1234	IGSF8		protein_c	chr1:160091340-160
ENSG0000(	523	13.08072	chr1:1234	SUMO1P3		Pseudoger	chr1:160317403-160
ENSG0000(	523	13.08072	chr1:1234	ENSG00000285570		lncRNA	chr1:156689676-156
ENSG0000(	523	13.08072	chr1:1234	FCRLB	DriverDB\	protein_c	chr1:161721544-161
ENSG0000(	523	13.08072	chr1:1234	FCGR3B		protein_c	chr1:161623196-161
ENSG0000(	523	13.08072	chr1:1234	LINC02819		lncRNA	chr1:159466321-159
ENSG0000(	523	13.08072	chr1:1234	OR6N2		protein_c	chr1:158774222-158
ENSG0000(	523	13.08072	chr1:1234	KRT8P45		Pseudoger	chr1:157073257-157
ENSG0000(	523	13.08072	chr1:1234	NIT1		protein_c	chr1:161118086-161
ENSG0000(	523	13.08072	chr1:1234	CFAP45	NCGv7	protein_c	chr1:159872364-159
ENSG0000(	523	13.08072	chr1:1234	CRPP1		Pseudoger	chr1:159704983-159
ENSG0000(	523	13.08072	chr1:1234	PPIAP37		Pseudoger	chr1:160848010-160
ENSG0000(	523	13.08072	chr1:1234	ENSG00000229808		Pseudoger	chr1:161890833-161
ENSG0000(	523	13.08072	chr1:1234	USF1		protein_c	chr1:161039251-161
ENSG0000(	523	13.08072	chr1:1234	F11R	DriverDB\	protein_c	chr1:160995211-161
ENSG0000(	523	13.08072	chr1:1234	HSPA7		Pseudoger	chr1:161606291-161
ENSG0000(	523	13.08072	chr1:1234	CD244		protein_c	chr1:160830160-160
ENSG0000(	523	13.08072	chr1:1234	ENSG00000176320		lncRNA	chr1:158197922-158
ENSG0000(	523	13.08072	chr1:1234	ENSG00000227673		lncRNA	chr1:155710098-155
ENSG0000(	523	13.08072	chr1:1234	ENSG00000232188		lncRNA	chr1:160931739-160
ENSG0000(	523	13.08072	chr1:1234	ENSG00000213080		Pseudoger	chr1:160894980-160
ENSG0000(	523	13.08072	chr1:1234	ENSG00000279430		TEC	chr1:159910094-159
ENSG0000(	523	13.08072	chr1:1234	RXFP4		protein_c	chr1:155941638-155
ENSG0000(	523	13.08072	chr1:1234	NAXE		protein_c	chr1:156591756-156
ENSG0000(	523	13.08072	chr1:1234	OR10J6P		Pseudoger	chr1:159598298-159
ENSG0000(	523	13.08072	chr1:1234	Y_RNA		smallRNA	chr1:160326104-160
ENSG0000(	523	13.08072	chr1:1234	HSPA6		protein_c	chr1:161524540-161
ENSG0000(	523	13.08072	chr1:1234	ENSG00000235226		Pseudoger	chr1:159759170-159
ENSG0000(	523	13.08072	chr1:1234	OR10J4		protein_c	chr1:159432204-159
ENSG0000(	523	13.08072	chr1:1234	DUSP23	DriverDB\	protein_c	chr1:159780932-159
ENSG0000(	523	13.08072	chr1:1234	PYHIN5P		Pseudoger	chr1:158878746-158
ENSG0000(	523	13.08072	chr1:1234	SNHG28		lncRNA	chr1:159834480-159
ENSG0000(	523	13.08072	chr1:1234	KLHDC9	DriverDB\	protein_c	chr1:161098361-161
ENSG0000(	523	13.08072	chr1:1234	ENSG00000284592		Pseudoger	chr1:157204779-157

ENSG0000(	523	13.08072	chr1:1234	PFDN2	DriverDB\	protein_c	chr1:161100556-161
ENSG0000(	523	13.08072	chr1:1234	RP11-122G18.8		lncRNA	chr1:161374762-161
ENSG0000(	523	13.08072	chr1:1234	PMF1-BGLAP		protein_c	chr1:156212982-156
ENSG0000(	523	13.08072	chr1:1234	ENSG00000233691		Pseudoger	chr1:160935537-160
ENSG0000(	523	13.08072	chr1:1234	ENSG00000237390		lncRNA	chr1:156388226-156
ENSG0000(	523	13.08072	chr1:1234	GLMP		protein_c	chr1:156290089-156
ENSG0000(	523	13.08072	chr1:1234	OR10R3P		Pseudoger	chr1:158491219-158
ENSG0000(	523	13.08072	chr1:1234	ENSG00000277882		Pseudoger	chr1:161411597-161
ENSG0000(	523	13.08072	chr1:1234	ENSG00000237409		Pseudoger	chr1:160020300-160
ENSG0000(	523	13.08072	chr1:1234	DUSP12	AC	protein_c	chr1:161749758-161
ENSG0000(	523	13.08072	chr1:1234	RPS23P10		Pseudoger	chr1:161536571-161
ENSG0000(	523	13.08072	chr1:1234	PCP4L1		protein_c	chr1:161258745-161
ENSG0000(	523	13.08072	chr1:1234	ENSG00000290592		lncRNA	chr1:157171116-157
ENSG0000(	523	13.08072	chr1:1234	ENSG00000228560		lncRNA	chr1:159346166-159
ENSG0000(	523	13.08072	chr1:1234	ENSG00000288835		lncRNA	chr1:156503083-156
ENSG0000(	523	13.08072	chr1:1234	ENSG00000273933		Pseudoger	chr1:159972548-159
ENSG0000(	523	13.08072	chr1:1234	CD5L		protein_c	chr1:157830911-157
ENSG0000(	523	13.08072	chr1:1234	NTRK1	NCGv7;AC	protein_c	chr1:156815640-156
ENSG0000(	523	13.08072	chr1:1234	OR6K1P		Pseudoger	chr1:158694539-158
ENSG0000(	523	13.08072	chr1:1234	ATF6-DT		lncRNA	chr1:161749452-161
ENSG0000(	523	13.08072	chr1:1234	ENSG00000198358		lncRNA	chr1:160932465-160
ENSG0000(	523	13.08072	chr1:1234	ENSG00000233712		Pseudoger	chr1:157636300-157
ENSG0000(	523	13.08072	chr1:1234	ENSG00000237588		lncRNA	chr1:156687695-156
ENSG0000(	523	13.08072	chr1:1234	FCGR2C		protein_c	chr1:161581339-161
ENSG0000(	523	13.08072	chr1:1234	ENSG00000223356		lncRNA	chr1:156712212-156
ENSG0000(	523	13.08072	chr1:1234	ENSG00000283317		lncRNA	chr1:161433444-161
ENSG0000(	523	13.08072	chr1:1234	AL591806.1		protein_c	chr1:161065865-161
ENSG0000(	523	13.08072	chr1:1234	OR10AA1P		Pseudoger	chr1:158808399-158
ENSG0000(	523	13.08072	chr1:1234	ENSG00000291226		lncRNA	chr1:157925974-157
ENSG0000(	523	13.08072	chr1:1234	KCNJ10		protein_c	chr1:159998651-160
ENSG0000(	523	13.08072	chr1:1234	OR6K6		protein_c	chr1:158754720-158
ENSG0000(	523	13.08072	chr1:1234	LINC01133		lncRNA	chr1:159958035-159
ENSG0000(	523	13.08072	chr1:1234	OR6K4P		Pseudoger	chr1:158724113-158
ENSG0000(	523	13.08072	chr1:1234	ENSG00000274562		Pseudoger	chr1:160776975-160
ENSG0000(	523	13.08072	chr1:1234	MSTO2P		Pseudoger	chr1:155745829-155
ENSG0000(	523	13.08072	chr1:1234	SLAMF7		protein_c	chr1:160739057-160
ENSG0000(	523	13.08072	chr1:1234	FCER1A	NCGv7	protein_c	chr1:159289714-159
ENSG0000(	523	13.08072	chr1:1234	ENSG00000260460		lncRNA	chr1:156509854-156
ENSG0000(	523	13.08072	chr1:1234	ATP1A2		protein_c	chr1:160115759-160
ENSG0000(	523	13.08072	chr1:1234	ENSG00000288670		lncRNA	chr1:161368022-161
ENSG0000(	523	13.08072	chr1:1234	ENSG00000236957		Pseudoger	chr1:157925065-157
ENSG0000(	523	13.08072	chr1:1234	ETV3L		protein_c	chr1:157092043-157
ENSG0000(	523	13.08072	chr1:1234	OR6K5P		Pseudoger	chr1:158742146-158
ENSG0000(	523	13.08072	chr1:1234	RNA5SP60		Pseudoger	chr1:159178473-159
ENSG0000(	523	13.08072	chr1:1234	ENSG00000236731		Pseudoger	chr1:157629939-157
ENSG0000(	523	13.08072	chr1:1234	MIR5187		smallRNA	chr1:161227186-161
ENSG0000(	523	13.08072	chr1:1234	ENSG00000256029		protein_c	chr1:159834474-159
ENSG0000(	523	13.08072	chr1:1234	Y_RNA		smallRNA	chr1:161699506-161
ENSG0000(	523	13.08072	chr1:1234	SMIM42		protein_c	chr1:158127287-158
ENSG0000(	523	13.08072	chr1:1234	AL355388.1		smallRNA	chr1:156077373-156
ENSG0000(	523	13.08072	chr1:1234	ENSG00000236656		lncRNA	chr1:158474454-158

ENSG0000(	523	13.08072	chr1:123<	ENSG00000288093		lncRNA	chr1:161399998-161
ENSG0000(	523	13.08072	chr1:123<	OR10T1P		Pseudoger	chr1:158445068-158
ENSG0000(	523	13.08072	chr1:123<	DCAF8-DT		lncRNA	chr1:160261731-160
ENSG0000(	523	13.08072	chr1:123<	HSP90AA3P		Pseudoger	chr1:158523672-158
ENSG0000(	523	13.08072	chr1:123<	RP11-85G21.2		lncRNA	chr1:157287703-157
ENSG0000(	523	13.08072	chr1:123<	OR10Z1		protein_c	chr1:158605268-158
ENSG0000(	523	13.08072	chr1:123<	OR10R2	NCGv7	protein_c	chr1:158472220-158
ENSG0000(	523	13.08072	chr1:123<	RAD1P2		Pseudoger	chr1:159081133-159
ENSG0000(	523	13.08072	chr1:123<	SMG5	NCGv7	protein_c	chr1:156249224-156
ENSG0000(	523	13.08072	chr1:123<	ENSG00000288775		lncRNA	chr1:159776325-159
ENSG0000(	523	13.08072	chr1:123<	ITLN1	NCGv7	protein_c	chr1:160876540-160
ENSG0000(	523	13.08072	chr1:123<	hsa-mir-4259		smallRNA	chr1:159899979-159
ENSG0000(	523	13.08072	chr1:123<	OR10J7P		Pseudoger	chr1:159351093-159
ENSG0000(	523	13.08072	chr1:123<	RRM2P2		Pseudoger	chr1:161378707-161
ENSG0000(	523	13.08072	chr1:123<	FCGR3A	NCGv7	protein_c	chr1:161541759-161
ENSG0000(	523	13.08072	chr1:123<	OR6K3		protein_c	chr1:158716327-158
ENSG0000(	523	13.08072	chr1:123<	ENSG00000276632		Pseudoger	chr1:159649151-159
ENSG0000(	523	13.08072	chr1:123<	ENSG00000283360		lncRNA	chr1:161403409-161
ENSG0000(	523	13.08072	chr1:123<	ENSG00000290105		lncRNA	chr1:159890207-159
ENSG0000(	523	13.08072	chr1:123<	ENSG00000289106		lncRNA	chr1:161364221-161
ENSG0000(	523	13.08072	chr1:123<	APCS		protein_c	chr1:159587826-159
ENSG0000(	523	13.08072	chr1:123<	HAPLN2		protein_c	chr1:156619331-156
ENSG0000(	523	13.08072	chr1:123<	RAB25		protein_c	chr1:156061160-156
ENSG0000(	523	13.08072	chr1:123<	ARHGEF11	NCGv7	protein_c	chr1:156934840-157
ENSG0000(	523	13.08072	chr1:123<	CRP	NCGv7	protein_c	chr1:159712289-159
ENSG0000(	523	13.08072	chr1:123<	BCAN		protein_c	chr1:156641390-156
ENSG0000(	523	13.08072	chr1:123<	FCRL1	NCGv7	protein_c	chr1:157794403-157
ENSG0000(	523	13.08072	chr1:123<	NES	NCGv7	protein_c	chr1:156668763-156
ENSG0000(	523	13.08072	chr1:123<	ATP1A4		protein_c	chr1:160151586-160
ENSG0000(	523	13.08072	chr1:123<	KHDC4		protein_c	chr1:155913045-155
ENSG0000(	523	13.08072	chr1:123<	ENSG00000290115		lncRNA	chr1:161165695-161
ENSG0000(	523	13.08072	chr1:123<	RHBG	NCGv7	protein_c	chr1:156369211-156
ENSG0000(	523	13.08072	chr1:123<	FCRL4	NCGv7	protein_c	chr1:157573747-157
ENSG0000(	523	13.08072	chr1:123<	DAP3		protein_c	chr1:155687960-155
ENSG0000(	523	13.08072	chr1:123<	OR10J8P		Pseudoger	chr1:159366161-159
ENSG0000(	523	13.08072	chr1:123<	FCRL2		protein_c	chr1:157745733-157
ENSG0000(	523	13.08072	chr1:123<	ENSG00000289121		lncRNA	chr1:161046027-161
ENSG0000(	523	13.08072	chr1:123<	RPS23P9		Pseudoger	chr1:161617992-161
ENSG0000(	523	13.08072	chr1:123<	SSR2		protein_c	chr1:156009048-156
ENSG0000(	523	13.08072	chr1:123<	TMEM79		protein_c	chr1:156282935-156
ENSG0000(	523	13.08072	chr1:123<	ARHGAP30		protein_c	chr1:161046946-161
ENSG0000(	523	13.08072	chr1:123<	CCT3		protein_c	chr1:156308968-156
ENSG0000(	523	13.08072	chr1:123<	CADM3-AS1		lncRNA	chr1:159194325-159
ENSG0000(	523	13.08072	chr1:123<	ENSG00000289141		lncRNA	chr1:161389547-161
ENSG0000(	523	13.08072	chr1:123<	TSACC		protein_c	chr1:156337314-156
ENSG0000(	523	13.08072	chr1:123<	ENSG00000273112		lncRNA	chr1:161513176-161
ENSG0000(	523	13.08072	chr1:123<	AL359753.1		smallRNA	chr1:159059249-159
ENSG0000(	523	13.08072	chr1:123<	NHLH1		protein_c	chr1:160367071-160
ENSG0000(	523	13.08072	chr1:123<	FCRLA		protein_c	chr1:161706972-161
ENSG0000(	523	13.08072	chr1:123<	ENSG00000228863		lncRNA	chr1:160670778-160
ENSG0000(	523	13.08072	chr1:123<	SPTA1	NCGv7	protein_c	chr1:158610704-158

ENSG0000(	523	13.08072	chr1:1234	DCAF8		protein_c	chr1:160215715-160
ENSG0000(	523	13.08072	chr1:1234	IGSF9	DriverDB\	protein_c	chr1:159927039-159
ENSG0000(	523	13.08072	chr1:1234	OR10J2P		Pseudoger	chr1:159279041-159
ENSG0000(	523	13.08072	chr1:1234	RP11-226L15.5		lncRNA	chr1:160024953-160
ENSG0000(	523	13.08072	chr1:1234	RN7SL612P		smallRNA	chr1:156985757-156
ENSG0000(	523	13.08072	chr1:1234	FCGR2B	NCGv7;AC	protein_c	chr1:161663143-161
ENSG0000(	523	13.08072	chr1:1234	RPSAP18		Pseudoger	chr1:160266340-160
ENSG0000(	523	13.08072	chr1:1234	AL590714.1		protein_c	chr1:161220370-161
ENSG0000(	523	13.08072	chr1:1234	INSRR		protein_c	chr1:156840063-156
ENSG0000(	523	13.08072	chr1:1234	OR10K2		protein_c	chr1:158418210-158
ENSG0000(	523	13.08072	chr1:1234	ARHGEF2-AS1		lncRNA	chr1:155991390-156
ENSG0000(	523	13.08072	chr1:1234	SMU1P1		Pseudoger	chr1:157059232-157
ENSG0000(	523	13.08072	chr1:1234	VDAC1P9		Pseudoger	chr1:157724180-157
ENSG0000(	523	13.08072	chr1:1234	AL138930.2		smallRNA	chr1:160545648-160
ENSG0000(	523	13.08072	chr1:1234	EI24P2		Pseudoger	chr1:158454198-158
ENSG0000(	523	13.08072	chr1:1234	SH2D2A		protein_c	chr1:156806243-156
ENSG0000(	523	13.08072	chr1:1234	AL365181.1		smallRNA	chr1:156587856-156
ENSG0000(	523	13.08072	chr1:1234	AL590560.1		protein_c	chr1:159910094-159
ENSG0000(	523	13.08072	chr1:1234	MNDA		protein_c	chr1:158831351-158
ENSG0000(	523	13.08072	chr1:1234	ENSG00000231100		Pseudoger	chr1:159557368-159
ENSG0000(	523	13.08072	chr1:1234	OR6Y1		protein_c	chr1:158544550-158
ENSG0000(	523	13.08072	chr1:1234	ENSG00000287624		lncRNA	chr1:156768105-156
ENSG0000(	523	13.08072	chr1:1234	ENSG00000224985		lncRNA	chr1:161153760-161
ENSG0000(	523	13.08072	chr1:1234	FCRL6		protein_c	chr1:159800511-159
ENSG0000(	523	13.08072	chr1:1234	OR6N1		protein_c	chr1:158747814-158
ENSG0000(	523	13.08072	chr1:1166	MIR3675		smallRNA	chr1:16858949-1685
ENSG0000(	523	13.08072	chr1:1234	AIM2		protein_c	chr1:159061599-159
ENSG0000(	523	13.08072	chr1:1234	ENSG00000275801		Pseudoger	chr1:160775954-160
ENSG0000(	523	13.08072	chr1:1234	OR10R1P		Pseudoger	chr1:158514785-158
ENSG0000(	523	13.08072	chr1:1234	ENSG00000283696		lncRNA	chr1:161399409-161
ENSG0000(	523	13.08072	chr1:1234	IFI16		protein_c	chr1:158999968-159
ENSG0000(	523	13.08072	chr1:1234	PYHIN1		protein_c	chr1:158930796-158
ENSG0000(	523	13.08072	chr1:1234	SYT11		protein_c	chr1:155859567-155
ENSG0000(	523	13.08072	chr1:1234	ENSG00000225279		lncRNA	chr1:160062461-160
ENSG0000(	523	13.08072	chr1:1234	ITLN2		protein_c	chr1:160945025-160
ENSG0000(	523	13.08072	chr1:1234	MEX3A	DriverDB\	protein_c	chr1:156072013-156
ENSG0000(	523	13.08072	chr1:1234	HDGF		protein_c	chr1:156742109-156
ENSG0000(	523	13.08072	chr1:1234	CD48		protein_c	chr1:160678746-160
ENSG0000(	523	13.08072	chr1:1234	ENSG00000229953		lncRNA	chr1:156646507-156
ENSG0000(	523	13.08072	chr1:1234	ENSG00000227217		lncRNA	chr1:157691762-157
ENSG0000(	523	13.08072	chr1:1234	OR10J9P		Pseudoger	chr1:159405423-159
ENSG0000(	523	13.08072	chr1:1234	IQGAP3	DriverDB\	protein_c	chr1:156525405-156
ENSG0000(	523	13.08072	chr1:1234	ETV3	NCGv7;AC	protein_c	chr1:157121191-157
ENSG0000(	523	13.08072	chr1:1234	PYDC5		protein_c	chr1:158999971-159
ENSG0000(	523	13.08072	chr1:1234	RN7SL466P		smallRNA	chr1:161735808-161
ENSG0000(	523	13.08072	chr1:1234	KIRREL1		protein_c	chr1:157993273-158
ENSG0000(	523	13.08072	chr1:1234	CRABP2		protein_c	chr1:156699606-156
ENSG0000(	523	13.08072	chr1:1234	FCRL3		protein_c	chr1:157674321-157
ENSG0000(	523	13.08072	chr1:1234	NR1I3	DriverDB\	protein_c	chr1:161229666-161
ENSG0000(	523	13.08072	chr1:1234	LRRC71		protein_c	chr1:156920632-156
ENSG0000(	523	13.08072	chr1:1234	GPATCH4	NCGv7	protein_c	chr1:156594301-156

ENSG0000(	523	13.08072	chr1:1234	OR10AE1P		Pseudoger	chr1:159581620-159
ENSG0000(	523	13.08072	chr1:1234	ENSG00000229961		Pseudoger	chr1:157182860-157
ENSG0000(	523	13.08072	chr1:1234	PIGM	DriverDBv	protein_c	chr1:160024953-160
ENSG0000(	523	13.08072	chr1:1234	MIR9-1HG		lncRNA	chr1:156404250-156
ENSG0000(	523	13.08072	chr1:1234	ENSG00000286151		Pseudoger	chr1:157400927-157
ENSG0000(	523	13.08072	chr1:1234	ENSG00000289768		protein_c	chr1:161544807-161
ENSG0000(	523	13.08072	chr1:1234	FCGR2A		protein_c	chr1:161505430-161
ENSG0000(	523	13.08072	chr1:1234	AL138930.1		smallRNA	chr1:160503376-160
ENSG0000(	523	13.08072	chr1:1234	SLAMF1	NCGv7	protein_c	chr1:160608106-160
ENSG0000(	523	13.08072	chr1:1234	FCRL5		protein_c	chr1:157513377-157
ENSG0000(	523	13.08072	chr1:1234	NECTIN4	DriverDBv	protein_c	chr1:161070998-161
ENSG0000(	523	13.08072	chr1:1234	BCAN-AS1		lncRNA	chr1:156637783-156
ENSG0000(	523	13.08072	chr1:1234	UFC1	DriverDBv	protein_c	chr1:161152776-161
ENSG0000(	523	13.08072	chr1:1234	PRCC	AC	protein_c	chr1:156750610-156
ENSG0000(	523	13.08072	chr1:1234	OR10J5		protein_c	chr1:159535078-159
ENSG0000(	523	13.08072	chr1:1234	ENSG00000234211		lncRNA	chr1:161671978-161
ENSG0000(	523	13.08072	chr1:1234	OR6P1		protein_c	chr1:158560606-158
ENSG0000(	523	13.08072	chr1:1234	METTL25B		protein_c	chr1:156728442-156
ENSG0000(	523	13.08072	chr1:1234	ISG20L2		protein_c	chr1:156721891-156
ENSG0000(	523	13.08072	chr1:1234	CASQ1		protein_c	chr1:160190575-160
ENSG0000(	523	13.08072	chr1:1234	BGLAP		protein_c	chr1:156242184-156
ENSG0000(	523	13.08072	chr1:1234	ENSG00000286073		Pseudoger	chr1:157287267-157
ENSG0000(	523	13.08072	chr1:1234	PPOX	DriverDBv	protein_c	chr1:161166056-161
ENSG0000(	523	13.08072	chr1:1234	OR10T2		protein_c	chr1:158398522-158
ENSG0000(	523	13.08072	chr1:1234	MRPL24		protein_c	chr1:156737303-156
ENSG0000(	523	13.08072	chr1:1234	ELL2P1		Pseudoger	chr1:158175850-158
ENSG0000(	523	13.08072	chr1:1234	ENSG00000272405		lncRNA	chr1:156641666-156
ENSG0000(	523	13.08072	chr1:1234	SLC25A44		protein_c	chr1:156193932-156
ENSG0000(	523	13.08072	chr1:1234	UBQLN4		protein_c	chr1:156035299-156
ENSG0000(	523	13.08072	chr1:1234	ENSG00000229914		Pseudoger	chr1:158195633-158
ENSG0000(	523	13.08072	chr1:1234	MIR765		smallRNA	chr1:156936131-156
ENSG0000(	523	13.08072	chr1:1234	SDHC	NCGv7;AC	protein_c	chr1:161314381-161
ENSG0000(	523	13.08072	chr1:1234	AL121985.1		Pseudoger	chr1:160697970-160
ENSG0000(	523	13.08072	chr1:1234	LMNA		protein_c	chr1:156082573-156
ENSG0000(	523	13.08072	chr1:1234	ENSG00000234425		lncRNA	chr1:160537073-160
ENSG0000(	523	13.08072	chr1:1234	RNU4-19P		smallRNA	chr1:155894281-155
ENSG0000(	523	13.08072	chr1:1234	LINC02772		lncRNA	chr1:157273760-157
ENSG0000(	523	13.08072	chr1:1234	MIR9-1		smallRNA	chr1:156420331-156
ENSG0000(	523	13.08072	chr1:1234	USP21	DriverDBv	protein_c	chr1:161159450-161
ENSG0000(	523	13.08072	chr1:1234	RNU4-42P		smallRNA	chr1:160392768-160
ENSG0000(	523	13.08072	chr1:1234	ENSG00000286005		Pseudoger	chr1:157437484-157
ENSG0000(	523	13.08072	chr1:1234	PMF1		protein_c	chr1:156212993-156
ENSG0000(	523	13.08072	chr1:1234	PAQR6		protein_c	chr1:156243320-156
ENSG0000(	522	13.05571	chr5:1036	ENSG00000250383		lncRNA	chr5:108818041-108
ENSG0000(	522	13.05571	chr5:1036	RNU6-47P		smallRNA	chr5:109014834-109
ENSG0000(	522	13.05571	chr5:1036	ENSG00000286882		lncRNA	chr5:109165289-109
ENSG0000(	522	13.05571	chr5:1036	RACK1P1		Pseudoger	chr5:108784098-108
ENSG0000(	522	13.05571	chr5:1036	ENSG00000248876		lncRNA	chr5:109470843-109
ENSG0000(	522	13.05571	chr5:1036	ENSG00000248867		Pseudoger	chr5:109448349-109
ENSG0000(	522	13.05571	chr5:1036	KRT18P42		Pseudoger	chr5:109588338-109
ENSG0000(	522	13.05571	chr5:1036	RN7SL782P		smallRNA	chr5:107734775-107

ENSG0000(	522	13.05571	chr5:103(	Y_RNA		smallRNA	chr5:108891437-108
ENSG0000(	522	13.05571	chr5:103(	PJA2		protein_c	chr5:109334713-109
ENSG0000(	522	13.05571	chr5:103(	MAN2A1-DT		lncRNA	chr5:109687802-109
ENSG0000(	522	13.05571	chr5:103(	GJA1P1		Pseudoger	chr5:109051315-109
ENSG0000(	522	13.05571	chr5:103(	ENSG00000289260		lncRNA	chr5:108727866-108
ENSG0000(	522	13.05571	chr5:103(	ENSG00000248440		lncRNA	chr5:109497877-109
ENSG0000(	522	13.05571	chr5:103(	ENSG00000249959		lncRNA	chr5:107699392-107
ENSG0000(	522	13.05571	chr5:103(	ENSG00000251367		lncRNA	chr5:109467353-109
ENSG0000(	522	13.05571	chr5:103(	PGAM5P1		Pseudoger	chr5:109884610-109
ENSG0000(	522	13.05571	chr5:103(	FER	TAG	protein_c	chr5:108747841-109
ENSG0000(	522	13.05571	chr5:103(	ENSG00000248827		Pseudoger	chr5:107724961-107
ENSG0000(	522	13.05571	chr5:103(	ENSG00000286503		lncRNA	chr5:108382156-108
ENSG0000(	522	13.05571	chr5:103(	ENSG00000249476		lncRNA	chr5:109237120-109
ENSG0000(	522	13.05571	chr5:103(	AC012603.1		smallRNA	chr5:109685580-109
ENSG0000(	522	13.05571	chr5:103(	MAN2A1	NCGv7	protein_c	chr5:109689927-109
ENSG0000(	522	13.05571	chr5:103(	RN7SKP230		smallRNA	chr5:109699500-109
ENSG0000(	522	13.05571	chr5:103(	FBXL17	NCGv7	protein_c	chr5:107859035-108
ENSG0000(	522	13.05571	chr5:103(	ENSG00000250441		Pseudoger	chr5:108894347-108
ENSG0000(	522	13.05571	chr5:103(	EFNA5		protein_c	chr5:107376894-107
ENSG0000(	522	13.05571	chr5:103(	LINC01848		lncRNA	chr5:109883182-109
ENSG0000(	522	13.05571	chr5:103(	ENSG00000249068		Pseudoger	chr5:109840128-109
ENSG0000(	522	13.05571	chr5:103(	LINC01023		lncRNA	chr5:108725707-108
ENSG0000(	522	13.05571	chr5:103(	ENSG00000244245		Pseudoger	chr5:108593609-108
ENSG0000(	522	13.05571	chr5:103(	RN7SKP122		smallRNA	chr5:107810629-107
ENSG0000(	519	12.98067	chr1:116(	RP11-242O24.5		lncRNA	chr1:29152489-2915
ENSG0000(	519	12.98067	chr1:116(	ZDHHC18		protein_c	chr1:26826688-2685
ENSG0000(	519	12.98067	chr1:116(	ENSG00000270103		lncRNA	chr1:28648600-2864
ENSG0000(	519	12.98067	chr1:116(	AL353354.1		protein_c	chr1:28200502-2820
ENSG0000(	519	12.98067	chr1:116(	Y_RNA		smallRNA	chr1:28881726-2888
ENSG0000(	519	12.98067	chr1:116(	ENSG00000289291		lncRNA	chr1:28736044-2873
ENSG0000(	519	12.98067	chr1:116(	STX12		protein_c	chr1:27773219-2782
ENSG0000(	519	12.98067	chr1:116(	DNAJC8		protein_c	chr1:28199456-2823
ENSG0000(	519	12.98067	chr1:116(	NPM1P39		Pseudoger	chr1:27206930-2720
ENSG0000(	519	12.98067	chr1:116(	SCARNA24		smallRNA	chr1:28689665-2868
ENSG0000(	519	12.98067	chr1:116(	RPL32P6		Pseudoger	chr1:26983628-2698
ENSG0000(	519	12.98067	chr1:116(	RNU7-29P		smallRNA	chr1:28144156-2814
ENSG0000(	519	12.98067	chr1:116(	PTAFR		protein_c	chr1:28147166-2819
ENSG0000(	519	12.98067	chr1:116(	ENSG00000287244		lncRNA	chr1:27724822-2772
ENSG0000(	519	12.98067	chr1:116(	PRDX3P2		Pseudoger	chr1:28526318-2852
ENSG0000(	519	12.98067	chr1:116(	ENSG00000235069		Pseudoger	chr1:26647447-2664
ENSG0000(	519	12.98067	chr1:116(	LINC02574		lncRNA	chr1:27660328-2766
ENSG0000(	519	12.98067	chr1:116(	SYTL1	NCGv7	protein_c	chr1:27342020-2735
ENSG0000(	519	12.98067	chr1:116(	SNORA73B		smallRNA	chr1:28508559-2850
ENSG0000(	519	12.98067	chr1:116(	ENSG00000233427		lncRNA	chr1:28870483-2887
ENSG0000(	519	12.98067	chr1:116(	SNHG12		lncRNA	chr1:28578538-2858
ENSG0000(	519	12.98067	chr1:116(	GPN2		protein_c	chr1:26876132-2689
ENSG0000(	519	12.98067	chr1:116(	MECR		protein_c	chr1:29192657-2923
ENSG0000(	519	12.98067	chr1:116(	RP1-212P9.2		lncRNA	chr1:28867575-2887
ENSG0000(	519	12.98067	chr1:116(	SRSF4		protein_c	chr1:29147743-2918
ENSG0000(	519	12.98067	chr1:116(	ENSG00000214812		Pseudoger	chr1:28120449-2812
ENSG0000(	519	12.98067	chr1:116(	OPRD1	NCGv7	protein_c	chr1:28812170-2887

ENSG0000(	519	12.98067	chr1:116(	ENSG00000229820		Pseudoger	chr1:28453541-2845
ENSG0000(	519	12.98067	chr1:116(	TRNAU1AP		protein_c	chr1:28553085-2857
ENSG0000(	519	12.98067	chr1:116(	GPR3		protein_c	chr1:27392622-2739
ENSG0000(	519	12.98067	chr1:116(	RN7SL165P		smallRNA	chr1:26814822-2681
ENSG0000(	519	12.98067	chr1:116(	EYA3		protein_c	chr1:27970344-2808
ENSG0000(	519	12.98067	chr1:116(	Y_RNA		smallRNA	chr1:28422555-2842
ENSG0000(	519	12.98067	chr1:116(	XKR8		protein_c	chr1:27959588-2796
ENSG0000(	519	12.98067	chr1:116(	AL109927.1		protein_c	chr1:27850574-2785
ENSG0000(	519	12.98067	chr19:115	ENSG00000213304		Pseudoger	chr19:11521968-115
ENSG0000(	519	12.98067	chr1:116(	ENSG00000241169		lncRNA	chr1:27457198-2745
ENSG0000(	519	12.98067	chr1:116(	CHMP1AP1		Pseudoger	chr1:27686810-2768
ENSG0000(	519	12.98067	chr1:116(	ENSG00000229247		Pseudoger	chr1:26640377-2664
ENSG0000(	519	12.98067	chr1:116(	NR0B2		protein_c	chr1:26911489-2691
ENSG0000(	519	12.98067	chr1:116(	AL353354.2		protein_c	chr1:28200559-2820
ENSG0000(	519	12.98067	chr1:116(	RCC1	NCGv7	protein_c	chr1:28505943-2853
ENSG0000(	519	12.98067	chr1:116(	snoU13		smallRNA	chr1:28517476-2851
ENSG0000(	519	12.98067	chr1:116(	ENSG00000225011		Pseudoger	chr1:29488193-2949
ENSG0000(	519	12.98067	chr1:116(	RNU6-1245P		smallRNA	chr1:27824538-2782
ENSG0000(	519	12.98067	chr1:116(	SPCS2P4		Pseudoger	chr1:28095742-2809
ENSG0000(	519	12.98067	chr1:116(	WDTC1-DT		lncRNA	chr1:27229106-2723
ENSG0000(	519	12.98067	chr1:116(	GPATCH3		protein_c	chr1:26890488-2690
ENSG0000(	519	12.98067	chr1:116(	ENSG00000243659		Pseudoger	chr1:27311240-2731
ENSG0000(	519	12.98067	chr1:116(	ENSG00000225886		lncRNA	chr1:27669468-2770
ENSG0000(	519	12.98067	chr1:116(	FAM76A		protein_c	chr1:27725961-2776
ENSG0000(	519	12.98067	chr1:116(	ENSG00000279443		TEC	chr1:28544460-2854
ENSG0000(	519	12.98067	chr1:116(	RN7SL501P		smallRNA	chr1:26763624-2676
ENSG0000(	519	12.98067	chr1:116(	FCN3		protein_c	chr1:27369110-2737
ENSG0000(	519	12.98067	chr1:116(	SNHG3		lncRNA	chr1:28505980-2851
ENSG0000(	519	12.98067	chr1:116(	ENSG00000237429		lncRNA	chr1:27525805-2753
ENSG0000(	519	12.98067	chr1:116(	RAB42	NCGv7	protein_c	chr1:28592200-2859
ENSG0000(	519	12.98067	chr1:116(	WASF2		protein_c	chr1:27404230-2749
ENSG0000(	519	12.98067	chr1:116(	ENSG00000237934		lncRNA	chr1:29223933-2922
ENSG0000(	519	12.98067	chr1:116(	RNU6-424P		smallRNA	chr1:27693731-2769
ENSG0000(	519	12.98067	chr1:116(	MAP3K6	NCGv7	protein_c	chr1:27355184-2736
ENSG0000(	519	12.98067	chr1:116(	ENSG00000290123		lncRNA	chr1:28234080-2823
ENSG0000(	519	12.98067	chr1:116(	TENT5B		protein_c	chr1:27005020-2701
ENSG0000(	519	12.98067	chr1:116(	Y_RNA		smallRNA	chr1:27255464-2725
ENSG0000(	519	12.98067	chr1:116(	ACTG1P20		Pseudoger	chr1:27325329-2732
ENSG0000(	519	12.98067	chr1:116(	RNU6-176P		smallRNA	chr1:28142737-2814
ENSG0000(	519	12.98067	chr1:116(	ENSG00000227050		lncRNA	chr1:27938875-2796
ENSG0000(	519	12.98067	chr1:116(	ENSG00000231207		Pseudoger	chr1:27389468-2739
ENSG0000(	519	12.98067	chr1:116(	ENSG00000228943		Pseudoger	chr1:28109739-2811
ENSG0000(	519	12.98067	chr1:116(	SNRPEP7		Pseudoger	chr1:27211265-2721
ENSG0000(	519	12.98067	chr1:116(	ENSG00000235912		Pseudoger	chr1:27649419-2764
ENSG0000(	519	12.98067	chr1:116(	ENSG00000269971		lncRNA	chr1:27773858-2777
ENSG0000(	519	12.98067	chr1:116(	RNU6-48P		smallRNA	chr1:27325219-2732
ENSG0000(	519	12.98067	chr1:116(	RPL12P13		Pseudoger	chr1:26980165-2698
ENSG0000(	519	12.98067	chr1:116(	TMEM200B		protein_c	chr1:29119429-2912
ENSG0000(	519	12.98067	chr1:116(	Y_RNA		smallRNA	chr1:28985710-2898
ENSG0000(	519	12.98067	chr1:116(	EPB41	NCGv7	protein_c	chr1:28887091-2912
ENSG0000(	519	12.98067	chr1:116(	ENSG00000289576		lncRNA	chr1:28116812-2812

ENSG0000(	519	12.98067	chr1:116(	ENSG00000225750		lncRNA	chr1:29144494-2914
ENSG0000(	519	12.98067	chr1:116(	ENSG00000270031		lncRNA	chr1:27819983-2782
ENSG0000(	519	12.98067	chr1:116(	RPEP3		Pseudoger	chr1:27739954-2774
ENSG0000(	519	12.98067	chr1:116(	AL645859.1		smallRNA	chr1:29253542-2925
ENSG0000(	519	12.98067	chr1:116(	IFI6		protein_c	chr1:27666064-2767
ENSG0000(	519	12.98067	chr1:116(	ENSG00000238231		Pseudoger	chr1:27990158-2799
ENSG0000(	519	12.98067	chr1:116(	PHACTR4		protein_c	chr1:28369582-2850
ENSG0000(	519	12.98067	chr1:116(	SNORD99		smallRNA	chr1:28578749-2857
ENSG0000(	519	12.98067	chr1:116(	ENSG00000260063		lncRNA	chr1:26692132-2669
ENSG0000(	519	12.98067	chr1:116(	LINC01756		lncRNA	chr1:29329620-2935
ENSG0000(	519	12.98067	chr1:116(	ENSG00000287810		Pseudoger	chr1:26817300-2681
ENSG0000(	519	12.98067	chr1:116(	ENSG00000270605		lncRNA	chr1:28239509-2824
ENSG0000(	519	12.98067	chr16:65(	ENSG00000261815		lncRNA	chr16:49170552-491
ENSG0000(	519	12.98067	chr1:116(	KDF1		protein_c	chr1:26949562-2696
ENSG0000(	519	12.98067	chr1:116(	snoU13		smallRNA	chr1:26642286-2664
ENSG0000(	519	12.98067	chr1:116(	SLC9A1		protein_c	chr1:27098809-2716
ENSG0000(	519	12.98067	chr1:116(	THEMIS2		protein_c	chr1:27872543-2788
ENSG0000(	519	12.98067	chr1:116(	MED18		protein_c	chr1:28329002-2833
ENSG0000(	519	12.98067	chr1:116(	ENSG00000226698		lncRNA	chr1:26876133-2687
ENSG0000(	519	12.98067	chr1:116(	NUDC		protein_c	chr1:26900238-2694
ENSG0000(	519	12.98067	chr1:116(	ENSG00000271398		lncRNA	chr1:28247144-2824
ENSG0000(	519	12.98067	chr1:116(	AL139151.1		smallRNA	chr1:29432481-2943
ENSG0000(	519	12.98067	chr1:116(	TAF12-DT		lncRNA	chr1:28643228-2864
ENSG0000(	519	12.98067	chr1:116(	FGR	TAG;AC	protein_c	chr1:27612064-2763
ENSG0000(	519	12.98067	chr1:116(	ATP5IF1		protein_c	chr1:28236109-2824
ENSG0000(	519	12.98067	chr1:116(	TMEM222		protein_c	chr1:27322145-2733
ENSG0000(	519	12.98067	chr1:116(	SMPDL3B		protein_c	chr1:27935000-2795
ENSG0000(	519	12.98067	chr1:116(	TRNP1		protein_c	chr1:26993692-2700
ENSG0000(	519	12.98067	chr1:116(	SCARNA1		smallRNA	chr1:27834401-2783
ENSG0000(	519	12.98067	chr1:116(	SESN2	NCGv7	protein_c	chr1:28259518-2828
ENSG0000(	519	12.98067	chr1:116(	SFN		protein_c	chr1:26863149-2686
ENSG0000(	519	12.98067	chr1:116(	RNU6-949P		smallRNA	chr1:27675603-2767
ENSG0000(	519	12.98067	chr1:116(	CD164L2		protein_c	chr1:27379176-2738
ENSG0000(	519	12.98067	chr1:116(	ENSG00000289554		lncRNA	chr1:26881109-2688
ENSG0000(	519	12.98067	chr1:116(	WDTC1	NCGv7	protein_c	chr1:27234632-2730
ENSG0000(	519	12.98067	chr1:116(	RPA2		protein_c	chr1:27891524-2791
ENSG0000(	519	12.98067	chr1:116(	PTPRU	NCGv7	protein_c	chr1:29236516-2932
ENSG0000(	519	12.98067	chr1:116(	ARID1A	NCGv7;AC	protein_c	chr1:26693236-2678
ENSG0000(	519	12.98067	chr1:116(	TAF12		protein_c	chr1:28587829-2864
ENSG0000(	519	12.98067	chr1:116(	AHDC1		protein_c	chr1:27534035-2760
ENSG0000(	519	12.98067	chr1:116(	CHCHD3P3		Pseudoger	chr1:27200834-2720
ENSG0000(	519	12.98067	chr1:116(	OSTCP2		Pseudoger	chr1:26985897-2698
ENSG0000(	519	12.98067	chr1:116(	RN7SL559P		smallRNA	chr1:28031886-2803
ENSG0000(	519	12.98067	chr1:116(	RNU6ATAC27P		smallRNA	chr1:28481362-2848
ENSG0000(	519	12.98067	chr1:116(	ENSG00000286433		lncRNA	chr1:27827812-2783
ENSG0000(	519	12.98067	chr1:116(	PIGV		protein_c	chr1:26787054-2680
ENSG0000(	519	12.98067	chr1:116(	ENSG00000229985		Pseudoger	chr1:27176751-2717
ENSG0000(	519	12.98067	chr1:116(	GMEB1		protein_c	chr1:28668778-2871
ENSG0000(	519	12.98067	chr1:116(	YTHDF2	NCGv7	protein_c	chr1:28736621-2876
ENSG0000(	519	12.98067	chr1:116(	AC092265.1		smallRNA	chr1:29644545-2964
ENSG0000(	519	12.98067	chr1:116(	ENSG00000231344		Pseudoger	chr1:27739091-2773

ENSG0000(	519	12.98067	chr1:116(	ENSG00000225616		Pseudoger	chr1:28982278-2898
ENSG0000(	519	12.98067	chr1:116(	PPP1R8		protein_c	chr1:27830782-2785
ENSG0000(	517	12.93065	chr17:374	RNU6-227P		smallRNA	chr17:76656320-766
ENSG0000(	515	12.88063	chr5:103(	SNORA13		smallRNA	chr5:112161485-112
ENSG0000(	515	12.88063	chr5:103(	EPB41L4A		protein_c	chr5:112142441-112
ENSG0000(	515	12.88063	chr5:103(	AC008967.1		smallRNA	chr5:111555895-111
ENSG0000(	515	12.88063	chr5:103(	EPB41L4A-DT		lncRNA	chr5:112419583-112
ENSG0000(	515	12.88063	chr5:103(	ENSG00000248350		Pseudoger	chr5:112363456-112
ENSG0000(	515	12.88063	chr5:103(	ENSG00000248268		lncRNA	chr5:111277517-111
ENSG0000(	515	12.88063	chr5:103(	RN7SKP57		smallRNA	chr5:111719769-111
ENSG0000(	515	12.88063	chr5:103(	BCLAF1P1		Pseudoger	chr5:110948029-110
ENSG0000(	515	12.88063	chr5:103(	ENSG00000250728		Pseudoger	chr5:111155244-111
ENSG0000(	515	12.88063	chr5:103(	ENSG00000248428		lncRNA	chr5:110970951-111
ENSG0000(	515	12.88063	chr5:103(	ENSG00000290027		lncRNA	chr5:112521511-112
ENSG0000(	515	12.88063	chr5:103(	HMGB3P16		Pseudoger	chr5:112452703-112
ENSG0000(	515	12.88063	chr5:103(	SNORA51		smallRNA	chr5:110684794-110
ENSG0000(	515	12.88063	chr5:103(	TSLP		protein_c	chr5:111070062-111
ENSG0000(	515	12.88063	chr5:103(	CAMK4		protein_c	chr5:111223653-111
ENSG0000(	515	12.88063	chr5:103(	ENSG00000253613		lncRNA	chr5:111076921-111
ENSG0000(	515	12.88063	chr5:103(	LINC02200		lncRNA	chr5:112628436-112
ENSG0000(	515	12.88063	chr1:116(	RNU4-28P		smallRNA	chr1:19510593-1951
ENSG0000(	515	12.88063	chr5:103(	ENSG00000288965		lncRNA	chr5:111238810-111
ENSG0000(	515	12.88063	chr5:103(	ENSG00000250882		lncRNA	chr5:112173570-112
ENSG0000(	515	12.88063	chr5:103(	RPS3AP21		Pseudoger	chr5:111192226-111
ENSG0000(	515	12.88063	chr5:103(	STARD4		protein_c	chr5:111496033-111
ENSG0000(	515	12.88063	chr5:103(	ENSG00000270779		Pseudoger	chr5:110908284-110
ENSG0000(	515	12.88063	chr5:103(	HMGN1P14		Pseudoger	chr5:111846050-111
ENSG0000(	515	12.88063	chr5:103(	ENSG00000251099		Pseudoger	chr5:111572236-111
ENSG0000(	515	12.88063	chr5:103(	ENSG00000249318		lncRNA	chr5:111265809-111
ENSG0000(	515	12.88063	chr5:103(	NREP		protein_c	chr5:111662621-111
ENSG0000(	515	12.88063	chr5:103(	TMEM232	DriverDB\	protein_c	chr5:110289233-110
ENSG0000(	515	12.88063	chr5:103(	EPB41L4A-AS1		lncRNA	chr5:112160526-112
ENSG0000(	515	12.88063	chr5:103(	NREP-AS1		lncRNA	chr5:111912508-112
ENSG0000(	515	12.88063	chr5:103(	SLC25A46		protein_c	chr5:110738136-110
ENSG0000(	515	12.88063	chr5:103(	ENSG00000251076		lncRNA	chr5:112228283-112
ENSG0000(	515	12.88063	chr5:103(	MIR548F3		smallRNA	chr5:110513829-110
ENSG0000(	515	12.88063	chr5:103(	ENSG00000251627		Pseudoger	chr5:110983563-110
ENSG0000(	515	12.88063	chr5:103(	WDR36		protein_c	chr5:111092321-111
ENSG0000(	515	12.88063	chr5:103(	APC	NCGv7;AC	protein_c	chr5:112707498-112
ENSG0000(	515	12.88063	chr5:103(	ENSG00000251014		Pseudoger	chr5:112546973-112
ENSG0000(	515	12.88063	chr5:103(	HMGN1P13		Pseudoger	chr5:111572102-111
ENSG0000(	515	12.88063	chr5:103(	STARD4-AS1		lncRNA	chr5:111510396-111
ENSG0000(	515	12.88063	chr5:103(	ENSG00000251187		lncRNA	chr5:112192020-112
ENSG0000(	513	12.83061	chr22:229	IGLJ4		protein_c	chr22:22910574-229
ENSG0000(	513	12.83061	chr22:229	IGLC4		Pseudoger	chr22:22910828-229
ENSG0000(	512	12.8056	chr17:709	ENSG00000266651		lncRNA	chr17:16440479-164
ENSG0000(	512	12.8056	chr8:127(	ENSG00000241385		Pseudoger	chr8:109899246-109
ENSG0000(	510	12.75557	chr1:116(	AKR7L		protein_c	chr1:19265982-1927
ENSG0000(	510	12.75557	chr1:116(	AKR7A2		protein_c	chr1:19303965-1931
ENSG0000(	510	12.75557	chr1:116(	MIR1290		smallRNA	chr1:18897071-1889
ENSG0000(	510	12.75557	chr1:116(	PDE4DIPP8		Pseudoger	chr1:16548651-1655

ENSG0000(	510	12.75557	chr1:116(	ENSG00000272084		lncRNA	chr1:19072110-1907
ENSG0000(	510	12.75557	chr1:116(	ACTL8		protein_c	chr1:17755333-1782
ENSG0000(	510	12.75557	chr1:116(	LINC01654		lncRNA	chr1:18065657-1807
ENSG0000(	510	12.75557	chr1:116(	RNF186-AS1		lncRNA	chr1:19814367-1981
ENSG0000(	510	12.75557	chr1:116(	ENSG00000284710		lncRNA	chr1:20272018-2027
ENSG0000(	510	12.75557	chr1:116(	AL137127.1		smallRNA	chr1:19083552-1908
ENSG0000(	510	12.75557	chr1:116(	ENSG00000227066		lncRNA	chr1:20154171-2016
ENSG0000(	510	12.75557	chr1:116(	IGSF21		protein_c	chr1:18107798-1837
ENSG0000(	510	12.75557	chr1:116(	NECAP2		protein_c	chr1:16440721-1646
ENSG0000(	510	12.75557	chr1:116(	ENSG00000270728		Pseudoger	chr1:19297080-1929
ENSG0000(	510	12.75557	chr1:116(	snoU13		smallRNA	chr1:19532170-1953
ENSG0000(	510	12.75557	chr1:116(	AL021920.2		protein_c	chr1:16733952-1673
ENSG0000(	510	12.75557	chr1:116(	IFFO2		protein_c	chr1:18904280-1895
ENSG0000(	510	12.75557	chr1:116(	ENSG00000224174		lncRNA	chr1:16520694-1652
ENSG0000(	510	12.75557	chr1:116(	MRTO4		protein_c	chr1:19251805-1926
ENSG0000(	510	12.75557	chr1:116(	ENSG00000284743		lncRNA	chr1:20478779-2048
ENSG0000(	510	12.75557	chr1:116(	AL355149.2		protein_c	chr1:16539066-1653
ENSG0000(	510	12.75557	chr1:116(	IGSF21-AS1		lncRNA	chr1:18166929-1817
ENSG0000(	510	12.75557	chr1:116(	RNU1-3		smallRNA	chr1:16666785-1666
ENSG0000(	510	12.75557	chr1:116(	AL137798.1		protein_c	chr1:16673003-1667
ENSG0000(	510	12.75557	chr1:116(	CROCCP2		Pseudoger	chr1:16618969-1665
ENSG0000(	510	12.75557	chr1:116(	ENSG00000279151		lncRNA	chr1:16701546-1670
ENSG0000(	510	12.75557	chr1:116(	PADI6		protein_c	chr1:17372196-1740
ENSG0000(	510	12.75557	chr1:116(	ENSG00000272426		lncRNA	chr1:16904339-1690
ENSG0000(	510	12.75557	chr1:116(	PLA2G2E		protein_c	chr1:19920009-1992
ENSG0000(	510	12.75557	chr1:116(	snoU13		smallRNA	chr1:17449763-1744
ENSG0000(	510	12.75557	chr1:116(	ENSG00000226396		Pseudoger	chr1:19608114-1960
ENSG0000(	510	12.75557	chr1:116(	PADI1		protein_c	chr1:17205128-1724
ENSG0000(	510	12.75557	chr1:116(	OTUD3		protein_c	chr1:19882395-1991
ENSG0000(	510	12.75557	chr1:116(	ENSG00000286898		lncRNA	chr1:16976302-1697
ENSG0000(	510	12.75557	chr1:116(	ENSG00000228549		lncRNA	chr1:16870945-1688
ENSG0000(	510	12.75557	chr1:116(	ESPNP		Pseudoger	chr1:16692280-1672
ENSG0000(	510	12.75557	chr1:116(	PADI3		protein_c	chr1:17249098-1728
ENSG0000(	510	12.75557	chr1:116(	PLA2G2D		protein_c	chr1:20111939-2011
ENSG0000(	510	12.75557	chr1:116(	ENSG00000284653		lncRNA	chr1:18015712-1804
ENSG0000(	510	12.75557	chr1:116(	VWA5B1		protein_c	chr1:20290875-2035
ENSG0000(	510	12.75557	chr1:116(	LINC01783		lncRNA	chr1:16533886-1653
ENSG0000(	510	12.75557	chr1:116(	RNF186		protein_c	chr1:19814029-1981
ENSG0000(	510	12.75557	chr1:116(	EIF1AXP1		Pseudoger	chr1:16685621-1668
ENSG0000(	510	12.75557	chr1:116(	ENSG00000261135		lncRNA	chr1:16514645-1651
ENSG0000(	510	12.75557	chr1:116(	PLA2G2F		protein_c	chr1:20139323-2015
ENSG0000(	510	12.75557	chr1:116(	RNU1-4		smallRNA	chr1:16740516-1674
ENSG0000(	510	12.75557	chr1:116(	ENSG00000226487		lncRNA	chr1:20412304-2041
ENSG0000(	510	12.75557	chr1:116(	ENSG00000290849		lncRNA	chr1:16687339-1669
ENSG0000(	510	12.75557	chr1:116(	HTR6		protein_c	chr1:19664875-1968
ENSG0000(	510	12.75557	chr1:116(	NBL1	NCGv7	protein_c	chr1:19596979-1965
ENSG0000(	510	12.75557	chr1:116(	LINC02783		lncRNA	chr1:17189783-1719
ENSG0000(	510	12.75557	chr1:116(	LINC02810		lncRNA	chr1:17717625-1774
ENSG0000(	510	12.75557	chr1:116(	ENSG00000238142		lncRNA	chr1:16887577-1688
ENSG0000(	510	12.75557	chr1:116(	RNU6-1099P		smallRNA	chr1:19305076-1930
ENSG0000(	510	12.75557	chr1:116(	LINC01772		lncRNA	chr1:16460948-1646

ENSG0000(	510	12.75557	chr1:116(	ENSG00000290851	lncRNA	chr1:16754910-1677
ENSG0000(	510	12.75557	chr1:116(	RN7SL85P	smallRNA	chr1:19319805-1932
ENSG0000(	510	12.75557	chr1:116(	ENSG00000286064	Pseudoger	chr1:19260521-1926
ENSG0000(	510	12.75557	chr1:116(	MICOS10	protein_c	chr1:19484403-1962
ENSG0000(	510	12.75557	chr1:116(	PLA2G2A	protein_c	chr1:19975431-1998
ENSG0000(	510	12.75557	chr1:116(	CROCCP3	lncRNA	chr1:16467436-1649
ENSG0000(	510	12.75557	chr1:116(	ENSG00000223643	lncRNA	chr1:16851257-1685
ENSG0000(	510	12.75557	chr1:116(	UBR4	protein_c	chr1:19074510-1921
ENSG0000(	510	12.75557	chr1:116(	AC004824.1	smallRNA	chr1:17413631-1741
ENSG0000(	510	12.75557	chr1:116(	ENSG00000282740	lncRNA	chr1:16739938-1675
ENSG0000(	510	12.75557	chr1:116(	MIR4695	smallRNA	chr1:18883202-1888
ENSG0000(	510	12.75557	chr1:116(	ENSG00000290850	lncRNA	chr1:16740280-1675
ENSG0000(	510	12.75557	chr1:116(	Y_RNA	smallRNA	chr1:17158197-1715
ENSG0000(	510	12.75557	chr1:116(	RN7SL304P	smallRNA	chr1:19970969-1997
ENSG0000(	510	12.75557	chr1:116(	ENSG00000285853	lncRNA	chr1:16515034-1652
ENSG0000(	510	12.75557	chr1:116(	ENSG00000288636	protein_c	chr1:17005068-1701
ENSG0000(	510	12.75557	chr1:116(	LINC01757	lncRNA	chr1:20243095-2024
ENSG0000(	510	12.75557	chr1:116(	ATP13A2	protein_c	chr1:16985958-1701
ENSG0000(	510	12.75557	chr1:116(	PADI4	protein_c	chr1:17308195-1736
ENSG0000(	510	12.75557	chr1:116(	RCC2-AS1	lncRNA	chr1:17406760-1740
ENSG0000(	510	12.75557	chr1:116(	CROCCP2	lncRNA	chr1:16618253-1664
ENSG0000(	510	12.75557	chr1:116(	ENSG00000226526	lncRNA	chr1:16978926-1700
ENSG0000(	510	12.75557	chr1:116(	ALDH4A1	protein_c	chr1:18871430-1890
ENSG0000(	510	12.75557	chr1:116(	AKR7A3	protein_c	chr1:19282573-1928
ENSG0000(	510	12.75557	chr1:116(	MST1L	Pseudoger	chr1:16757232-1676
ENSG0000(	510	12.75557	chr1:116(	RCC2	protein_c	chr1:17406760-1743
ENSG0000(	510	12.75557	chr1:116(	ENSG00000283773	Pseudoger	chr1:16642767-1664
ENSG0000(	510	12.75557	chr1:116(	ENSG00000280222	TEC	chr1:18109389-1811
ENSG0000(	510	12.75557	chr1:116(	MUL1	protein_c	chr1:20499448-2050
ENSG0000(	510	12.75557	chr1:116(	RN7SL277P	smallRNA	chr1:19424384-1942
ENSG0000(	510	12.75557	chr1:116(	KLHDC7A	protein_c	chr1:18480930-1848
ENSG0000(	510	12.75557	chr1:116(	ENSG00000282143	lncRNA	chr1:16656879-1666
ENSG0000(	510	12.75557	chr1:116(	TAS1R2	protein_c	chr1:18839599-1885
ENSG0000(	510	12.75557	chr1:116(	TMCO4	protein_c	chr1:19682240-1979
ENSG0000(	510	12.75557	chr1:116(	UBXN10	protein_c	chr1:20186096-2019
ENSG0000(	510	12.75557	chr1:116(	CAMK2N1	protein_c	chr1:20482391-2048
ENSG0000(	510	12.75557	chr1:116(	LINC01141	lncRNA	chr1:20360579-2043
ENSG0000(	510	12.75557	chr1:116(	AL355149.1	smallRNA	chr1:16548914-1654
ENSG0000(	510	12.75557	chr1:116(	CAPZB	protein_c	chr1:19338775-1948
ENSG0000(	510	12.75557	chr1:116(	PLA2G5	protein_c	chr1:20028179-2009
ENSG0000(	510	12.75557	chr1:116(	ARHGEF10INCGv7	protein_c	chr1:17539698-1769
ENSG0000(	510	12.75557	chr1:116(	EMC1	protein_c	chr1:19215660-1925
ENSG0000(	510	12.75557	chr1:116(	RP13-279N23.2	protein_c	chr1:18849273-1892
ENSG0000(	510	12.75557	chr1:116(	ENSG00000280114	Pseudoger	chr1:16681097-1668
ENSG0000(	510	12.75557	chr1:116(	AL021920.1	smallRNA	chr1:16681255-1668
ENSG0000(	510	12.75557	chr1:116(	PLA2G2C	protein_c	chr1:20161253-2018
ENSG0000(	510	12.75557	chr1:116(	ENSG00000284641	lncRNA	chr1:20476222-2047
ENSG0000(	510	12.75557	chr1:116(	SLC66A1	protein_c	chr1:19312326-1932
ENSG0000(	510	12.75557	chr1:116(	MICOS10-DT	lncRNA	chr1:19591802-1959
ENSG0000(	510	12.75557	chr1:116(	ENSG00000290096	lncRNA	chr1:17439186-1743
ENSG0000(	510	12.75557	chr1:116(	ENSG00000271732	lncRNA	chr1:16617391-1661

ENSG0000(	510	12.75557	chr1:1166	ENSG00000225478		Pseudoger	chr1:18595414-1859
ENSG0000(	510	12.75557	chr1:1166	PDE4DIPP9		Pseudoger	chr1:16855407-1685
ENSG0000(	510	12.75557	chr1:1166	AC004824.2		protein_c	chr1:17329012-1734
ENSG0000(	510	12.75557	chr1:1166	CROCCP4		Pseudoger	chr1:16750233-1675
ENSG0000(	510	12.75557	chr1:1166	ENSG00000235241		Pseudoger	chr1:16889095-1688
ENSG0000(	510	12.75557	chr1:1166	ENSG00000225387		lncRNA	chr1:18385829-1838
ENSG0000(	510	12.75557	chr1:1166	DYNLL1P3		Pseudoger	chr1:18513118-1851
ENSG0000(	510	12.75557	chr1:1166	CROCCP3		Pseudoger	chr1:16474396-1649
ENSG0000(	510	12.75557	chr1:1166	PAX7	AC	protein_c	chr1:18630846-1874
ENSG0000(	510	12.75557	chr1:1166	ENSG00000289402		lncRNA	chr1:20486358-2048
ENSG0000(	510	12.75557	chr1:1166	RNU1-2		smallRNA	chr1:16895980-1689
ENSG0000(	510	12.75557	chr1:1166	ENSG00000226664		lncRNA	chr1:20294211-2032
ENSG0000(	510	12.75557	chr1:1166	MFAP2		protein_c	chr1:16974502-1698
ENSG0000(	510	12.75557	chr1:1166	MICOS10-NBL1		protein_c	chr1:19597067-1965
ENSG0000(	510	12.75557	chr1:1166	ENSG00000290122		lncRNA	chr1:16905199-1690
ENSG0000(	510	12.75557	chr1:1166	PADI2	AC	protein_c	chr1:17066761-1711
ENSG0000(	510	12.75557	chr1:1166	NBPF1	NCGv7	protein_c	chr1:16562319-1661
ENSG0000(	510	12.75557	chr1:1166	EMC1-AS1		lncRNA	chr1:19210348-1924
ENSG0000(	510	12.75557	chr1:1166	MST1P2		Pseudoger	chr1:16645622-1665
ENSG0000(	510	12.75557	chr1:1166	ENSG00000282843		lncRNA	chr1:17193232-1720
ENSG0000(	510	12.75557	chr1:1166	SDHB	NCGv7;AC	protein_c	chr1:17018722-1705
ENSG0000(	508	12.70555	chr1:3732	ENSG00000236206		lncRNA	chr1:165598356-165
ENSG0000(	508	12.70555	chr1:3732	FMO9P		lncRNA	chr1:166603916-166
ENSG0000(	508	12.70555	chr1:3732	ENSG00000215838		Pseudoger	chr1:165698750-165
ENSG0000(	508	12.70555	chr1:3732	ENSG00000237756		lncRNA	chr1:163259850-163
ENSG0000(	508	12.70555	chr1:3732	TMCO1		protein_c	chr1:165724293-165
ENSG0000(	508	12.70555	chr1:3732	ENSG00000254706		protein_c	chr1:162365407-162
ENSG0000(	508	12.70555	chr1:3732	LMX1A-AS1		lncRNA	chr1:165215951-165
ENSG0000(	508	12.70555	chr1:3732	CCDC190		protein_c	chr1:162824458-162
ENSG0000(	508	12.70555	chr1:3732	UQCRBP2		Pseudoger	chr1:162541332-162
ENSG0000(	508	12.70555	chr1:3732	Y_RNA		smallRNA	chr1:165662585-165
ENSG0000(	508	12.70555	chr1:3732	UCK2		protein_c	chr1:165827614-165
ENSG0000(	508	12.70555	chr1:3732	RXRG		protein_c	chr1:165400922-165
ENSG0000(	508	12.70555	chr1:3732	MIR4654		smallRNA	chr1:162157107-162
ENSG0000(	508	12.70555	chr1:3732	UAP1-DT		lncRNA	chr1:162560227-162
ENSG0000(	508	12.70555	chr1:3732	FMO8P		Pseudoger	chr1:166566178-166
ENSG0000(	508	12.70555	chr1:3732	RNA5SP61		Pseudoger	chr1:162338643-162
ENSG0000(	508	12.70555	chr1:3732	ENSG00000273365		lncRNA	chr1:165706556-165
ENSG0000(	508	12.70555	chr1:3732	U3		smallRNA	chr1:163923670-163
ENSG0000(	508	12.70555	chr1:3732	RNU6-171P		smallRNA	chr1:164639565-164
ENSG0000(	508	12.70555	chr1:3732	RNA5SP62		Pseudoger	chr1:163468496-163
ENSG0000(	508	12.70555	chr1:3732	FMO7P		Pseudoger	chr1:166474745-166
ENSG0000(	508	12.70555	chr1:3732	ENSG00000230898		lncRNA	chr1:166147782-166
ENSG0000(	508	12.70555	chr1:3732	ENSG00000269887		lncRNA	chr1:164680085-164
ENSG0000(	508	12.70555	chr1:3732	ENSG00000229588		lncRNA	chr1:166165852-166
ENSG0000(	508	12.70555	chr1:3732	ENSG00000225325		lncRNA	chr1:166387727-166
ENSG0000(	508	12.70555	chr1:3732	RNA5SP63		Pseudoger	chr1:163509484-163
ENSG0000(	508	12.70555	chr1:3732	ENSG00000225272		Pseudoger	chr1:165676310-165
ENSG0000(	508	12.70555	chr1:3732	RGS5		lncRNA	chr1:163244505-163
ENSG0000(	508	12.70555	chr1:3732	ENSG00000237783		Pseudoger	chr1:165581613-165
ENSG0000(	508	12.70555	chr1:3732	RPL4P2		Pseudoger	chr1:166747379-166

ENSG0000(	508	12.70555	chr1:3732	ENSG00000225122		Pseudoger	chr1:163422405-163
ENSG0000(	508	12.70555	chr1:3732	SLAMF6P1		Pseudoger	chr1:162445549-162
ENSG0000(	508	12.70555	chr11:76(	PCNAP4		Pseudoger	chr11:19274540-192
ENSG0000(	508	12.70555	chr1:3732	RPS3AP10		Pseudoger	chr1:166022215-166
ENSG0000(	508	12.70555	chr1:3732	RNA5SP64		Pseudoger	chr1:166042244-166
ENSG0000(	508	12.70555	chr1:3732	PBX1	NCGv7;AC	protein_c	chr1:164555584-164
ENSG0000(	508	12.70555	chr1:3732	SPATA46		protein_c	chr1:162373203-162
ENSG0000(	508	12.70555	chr1:3732	RGS5-AS1		lncRNA	chr1:163161675-163
ENSG0000(	508	12.70555	chr1:3732	LINC01675		lncRNA	chr1:166474879-166
ENSG0000(	508	12.70555	chr1:3732	ENSG00000289713		Pseudoger	chr1:162441192-162
ENSG0000(	508	12.70555	chr1:3732	HSD17B7		protein_c	chr1:162790702-162
ENSG0000(	508	12.70555	chr1:3732	TMCO1-AS1		lncRNA	chr1:165768929-165
ENSG0000(	508	12.70555	chr1:3732	ENSG00000230659		Pseudoger	chr1:165819353-165
ENSG0000(	508	12.70555	chr1:3732	NUF2	DriverDB\	protein_c	chr1:163266576-163
ENSG0000(	508	12.70555	chr1:3732	FMO10P		Pseudoger	chr1:166665885-166
ENSG0000(	508	12.70555	chr1:3732	ENSG00000285636		lncRNA	chr1:162146709-162
ENSG0000(	508	12.70555	chr1:3732	RNU5F-6P		smallRNA	chr1:164351273-164
ENSG0000(	508	12.70555	chr1:3732	LRRC52-AS1		lncRNA	chr1:165476833-165
ENSG0000(	508	12.70555	chr1:3732	ENSG00000230175		Pseudoger	chr1:165671256-165
ENSG0000(	508	12.70555	chr1:3732	SH2D1B		protein_c	chr1:162395268-162
ENSG0000(	508	12.70555	chr1:3732	UHMK1		protein_c	chr1:162497251-162
ENSG0000(	508	12.70555	chr1:3732	FAM78B-AS1		lncRNA	chr1:166081183-166
ENSG0000(	508	12.70555	chr1:3732	LMX1A-AS2		lncRNA	chr1:165210627-165
ENSG0000(	508	12.70555	chr1:3732	ENSG00000272574		lncRNA	chr1:162593103-162
ENSG0000(	508	12.70555	chr1:3732	SNORD112		smallRNA	chr1:165072473-165
ENSG0000(	508	12.70555	chr1:3732	ENSG00000225755		Pseudoger	chr1:163237214-163
ENSG0000(	508	12.70555	chr1:3732	RGS5		protein_c	chr1:163111121-163
ENSG0000(	508	12.70555	chr1:3732	AL390119.1		smallRNA	chr1:164983902-164
ENSG0000(	508	12.70555	chr1:3732	ENSG00000289408		lncRNA	chr1:164900169-164
ENSG0000(	508	12.70555	chr1:3732	ENSG00000228289		Pseudoger	chr1:163769339-163
ENSG0000(	508	12.70555	chr1:3732	MGST3		protein_c	chr1:165631213-165
ENSG0000(	508	12.70555	chr1:3732	MIR921		smallRNA	chr1:166154743-166
ENSG0000(	508	12.70555	chr1:3732	PBX1-AS1		lncRNA	chr1:164769116-164
ENSG0000(	508	12.70555	chr1:3732	SNORD112		smallRNA	chr1:163385865-163
ENSG0000(	508	12.70555	chr1:3732	ENSG00000227667		Pseudoger	chr1:162979551-162
ENSG0000(	508	12.70555	chr1:3732	RN7SL861P		smallRNA	chr1:162777730-162
ENSG0000(	508	12.70555	chr1:3732	ENSG00000271527		Pseudoger	chr1:165941235-165
ENSG0000(	508	12.70555	chr1:3732	UAP1	NCGv7	protein_c	chr1:162561722-162
ENSG0000(	508	12.70555	chr1:3732	DDR2	NCGv7	protein_c	chr1:162631373-162
ENSG0000(	508	12.70555	chr1:3732	ENSG00000227818		lncRNA	chr1:162039016-162
ENSG0000(	508	12.70555	chr1:3732	MIR556		smallRNA	chr1:162342546-162
ENSG0000(	508	12.70555	chr1:3732	PRELID1P7		Pseudoger	chr1:165497724-165
ENSG0000(	508	12.70555	chr1:3732	RGS4		protein_c	chr1:163068775-163
ENSG0000(	508	12.70555	chr1:3732	ENSG00000230739		Pseudoger	chr1:162824795-162
ENSG0000(	508	12.70555	chr1:3732	ENSG00000271917		lncRNA	chr1:164828436-164
ENSG0000(	508	12.70555	chr1:3732	ALDH9A1		protein_c	chr1:165662216-165
ENSG0000(	508	12.70555	chr1:3732	C1orf226		protein_c	chr1:162378841-162
ENSG0000(	508	12.70555	chr1:3732	LRRC52		protein_c	chr1:165544000-165
ENSG0000(	508	12.70555	chr19:132	ENSG00000277744		lncRNA	chr19:41373971-413
ENSG0000(	508	12.70555	chr1:3732	LMX1A	NCGv7	protein_c	chr1:165201867-165
ENSG0000(	508	12.70555	chr1:3732	ENSG00000236364		lncRNA	chr1:165889725-165

ENSG0000C	508	12.70555	chr1:3732	FAM78B	NCGv7	protein_c	chr1:166057426-166
ENSG0000C	508	12.70555	chr1:3732	RPL35AP7		Pseudoger	chr1:164921318-164
ENSG0000C	508	12.70555	chr1:3732	ENSG00000215835		Pseudoger	chr1:166275629-166
ENSG0000C	508	12.70555	chr1:3732	NOS1AP		protein_c	chr1:162069691-162
ENSG0000C	508	12.70555	chr1:3732	ENSG00000227094		lncRNA	chr1:162316852-162
ENSG0000C	508	12.70555	chr1:3732	HMGB3P6		Pseudoger	chr1:164356767-164
ENSG0000C	508	12.70555	chr1:3732	FMO9P		Pseudoger	chr1:166612470-166
ENSG0000C	508	12.70555	chr1:3732	Y_RNA		smallRNA	chr1:164854231-164
ENSG0000C	508	12.70555	chr1:3732	NMNAT1P2		Pseudoger	chr1:164343005-164
ENSG0000C	504	12.60551	chr16:209	MIR3680-1		smallRNA	chr16:21506049-215
ENSG0000C	500	12.50546	chr12:109	ENSG00000255839		lncRNA	chr12:123707602-12
ENSG0000C	496	12.40542	chr1:3732	FAM20B		protein_c	chr1:179025804-179
ENSG0000C	496	12.40542	chr1:3732	FAM163A		protein_c	chr1:179743291-179
ENSG0000C	496	12.40542	chr1:3732	CRYZL2P		Pseudoger	chr1:178006136-178
ENSG0000C	496	12.40542	chr1:3732	SNORA67		smallRNA	chr1:179201487-179
ENSG0000C	496	12.40542	chr1:3732	ENSG00000232750		Pseudoger	chr1:179035309-179
ENSG0000C	496	12.40542	chr1:3732	CRYZL2P-SEC16B		lncRNA	chr1:177928788-178
ENSG0000C	496	12.40542	chr1:3732	TEX35		protein_c	chr1:178513109-178
ENSG0000C	496	12.40542	chr1:3732	RASAL2-AS1		lncRNA	chr1:178090677-178
ENSG0000C	496	12.40542	chr1:3732	SNORA63		smallRNA	chr1:178753654-178
ENSG0000C	496	12.40542	chr1:3732	RALGPS2-AS1		lncRNA	chr1:178724306-178
ENSG0000C	496	12.40542	chr1:3732	MEF2AP1		Pseudoger	chr1:179447578-179
ENSG0000C	496	12.40542	chr1:3732	ENSG00000227141		Pseudoger	chr1:179586705-179
ENSG0000C	496	12.40542	chr1:3732	AL359853.1		smallRNA	chr1:179710250-179
ENSG0000C	496	12.40542	chr1:3732	ENSG00000261060		lncRNA	chr1:179590372-179
ENSG0000C	496	12.40542	chr1:3732	ENSG00000225711		Pseudoger	chr1:179220938-179
ENSG0000C	496	12.40542	chr1:3732	RNU5F-2P		smallRNA	chr1:179576268-179
ENSG0000C	496	12.40542	chr1:3732	PTPN2P1		Pseudoger	chr1:178746683-178
ENSG0000C	496	12.40542	chr1:3732	RASAL2		protein_c	chr1:178094104-178
ENSG0000C	496	12.40542	chr1:3732	LINC02818		lncRNA	chr1:179829609-179
ENSG0000C	496	12.40542	chr1:3732	EIF4A1P11		Pseudoger	chr1:179201705-179
ENSG0000C	496	12.40542	chr1:3732	ENSG00000273384		lncRNA	chr1:178651706-178
ENSG0000C	496	12.40542	chr1:3732	ENSG00000229407		lncRNA	chr1:179816184-179
ENSG0000C	496	12.40542	chr1:3732	ENSG00000228191		Pseudoger	chr1:179271116-179
ENSG0000C	496	12.40542	chr1:3732	ENSG00000243062		lncRNA	chr1:179730191-179
ENSG0000C	496	12.40542	chr1:3732	SETP10		Pseudoger	chr1:179183734-179
ENSG0000C	496	12.40542	chr1:3732	RALGPS2		protein_c	chr1:178725165-178
ENSG0000C	496	12.40542	chr1:3732	TDRD5	NCGv7	protein_c	chr1:179591613-179
ENSG0000C	496	12.40542	chr1:3732	ENSG00000276563		Pseudoger	chr1:178017127-178
ENSG0000C	496	12.40542	chr1:3732	HNRNPA1P54		Pseudoger	chr1:179447602-179
ENSG0000C	496	12.40542	chr1:3732	ENSG00000270575		Pseudoger	chr1:178194342-178
ENSG0000C	496	12.40542	chr1:3732	ENSG00000261250		lncRNA	chr1:179543201-179
ENSG0000C	496	12.40542	chr1:3732	RN7SL374P		smallRNA	chr1:179364313-179
ENSG0000C	496	12.40542	chr1:3732	COX5BP8		Pseudoger	chr1:179255733-179
ENSG0000C	496	12.40542	chr1:3732	NPHS2		protein_c	chr1:179550539-179
ENSG0000C	496	12.40542	chr1:3732	ABL2	NCGv7;AC	protein_c	chr1:179099330-179
ENSG0000C	496	12.40542	chr1:3732	MIR4424		smallRNA	chr1:178677749-178
ENSG0000C	496	12.40542	chr1:3732	TOR3A		protein_c	chr1:179082070-179
ENSG0000C	496	12.40542	chr1:3732	C1orf220		lncRNA	chr1:178542752-178
ENSG0000C	496	12.40542	chr1:3732	RNA5SP69		Pseudoger	chr1:178560913-178
ENSG0000C	496	12.40542	chr1:3732	ENSG00000289432		lncRNA	chr1:179017289-179

ENSG0000(	496	12.40542	chr1:3732	ENSG00000285910		lncRNA	chr1:178511563-178
ENSG0000(	496	12.40542	chr1:3732	SNORA67		smallRNA	chr1:179196473-179
ENSG0000(	496	12.40542	chr1:3732	SOAT1	NCGv7	protein_c	chr1:179293714-179
ENSG0000(	496	12.40542	chrX:1657	OOEPP1		Pseudoger	chrX:39791580-3979
ENSG0000(	496	12.40542	chr1:3732	CLEC20A		protein_c	chr1:178479240-178
ENSG0000(	496	12.40542	chr1:3732	ENSG00000234041		Pseudoger	chr1:179137764-179
ENSG0000(	496	12.40542	chr1:3732	AXDND1		protein_c	chr1:179365720-179
ENSG0000(	496	12.40542	chr1:3732	ENSG00000213058		Pseudoger	chr1:178411616-178
ENSG0000(	496	12.40542	chr1:3732	ANGPTL1		protein_c	chr1:178849535-178
ENSG0000(	494	12.3554	chr20:473	AL031666.1		smallRNA	chr20:47314525-473
ENSG0000(	493	12.33039	chr13:204	MIR4499		smallRNA	chr13:20433778-204
ENSG0000(	491	12.28037	chr1:3732	RPL23AP16		Pseudoger	chr1:199371877-199
ENSG0000(	491	12.28037	chr1:3732	ENSG00000273093		lncRNA	chr1:200315435-200
ENSG0000(	491	12.28037	chr1:3732	TMEM9		protein_c	chr1:201134772-201
ENSG0000(	491	12.28037	chr1:3732	TMEM183A		protein_c	chr1:203007374-203
ENSG0000(	491	12.28037	chr1:3732	ENSG00000235121		lncRNA	chr1:201723294-201
ENSG0000(	491	12.28037	chr1:3732	NR5A2		protein_c	chr1:200027614-200
ENSG0000(	491	12.28037	chr1:3732	ELF3	NCGv7	protein_c	chr1:202007945-202
ENSG0000(	491	12.28037	chr1:3732	LMOD1		protein_c	chr1:201896456-201
ENSG0000(	491	12.28037	chr1:3732	RNU6-778P		smallRNA	chr1:199888159-199
ENSG0000(	491	12.28037	chr1:3732	ENSG00000223881		lncRNA	chr1:198597724-198
ENSG0000(	491	12.28037	chr1:3732	RPS10P7		Pseudoger	chr1:201518703-201
ENSG0000(	491	12.28037	chr1:3732	INAVA		protein_c	chr1:200891048-200
ENSG0000(	491	12.28037	chr1:3732	ENSG00000230623		lncRNA	chr1:200333193-200
ENSG0000(	491	12.28037	chr1:3732	ENSG00000286600		lncRNA	chr1:201359018-201
ENSG0000(	491	12.28037	chr1:3732	IGFN1	NCGv7	protein_c	chr1:201190824-201
ENSG0000(	491	12.28037	chr1:3732	snoU13		smallRNA	chr1:202197628-202
ENSG0000(	491	12.28037	chr1:3732	SEPTIN14P12		Pseudoger	chr1:197138748-197
ENSG0000(	491	12.28037	chr1:3732	ELF3-AS1		lncRNA	chr1:201995696-202
ENSG0000(	491	12.28037	chr1:3732	KIF21B	NCGv7	protein_c	chr1:200969390-201
ENSG0000(	491	12.28037	chr1:3732	RNU6-704P		smallRNA	chr1:200933345-200
ENSG0000(	491	12.28037	chr1:3732	LINC00862		lncRNA	chr1:200253419-200
ENSG0000(	491	12.28037	chr1:3732	ENSG00000232626		Pseudoger	chr1:202438396-202
ENSG0000(	491	12.28037	chr1:3732	LINC01222		lncRNA	chr1:199006040-199
ENSG0000(	491	12.28037	chr1:3732	ENSG00000231547		Pseudoger	chr1:202999738-203
ENSG0000(	491	12.28037	chr1:3732	EEF1A1P32		Pseudoger	chr1:197688760-197
ENSG0000(	491	12.28037	chr1:3732	ADORA1		protein_c	chr1:203090654-203
ENSG0000(	491	12.28037	chr1:3732	ENSG00000224818		lncRNA	chr1:201464383-201
ENSG0000(	491	12.28037	chr1:3732	PPP1R12B		protein_c	chr1:202348699-202
ENSG0000(	491	12.28037	chr1:3732	UBE2T		protein_c	chr1:202331544-202
ENSG0000(	491	12.28037	chr1:3732	ENSG00000282221		lncRNA	chr1:201399633-201
ENSG0000(	491	12.28037	chr1:3732	LAD1		protein_c	chr1:201380833-201
ENSG0000(	491	12.28037	chr1:3732	ENSG00000230260		lncRNA	chr1:197437976-197
ENSG0000(	491	12.28037	chr1:3732	TNNI1		protein_c	chr1:201403768-201
ENSG0000(	491	12.28037	chr1:3732	U6		smallRNA	chr1:202410108-202
ENSG0000(	491	12.28037	chr1:3732	CSRP1		protein_c	chr1:201483530-201
ENSG0000(	491	12.28037	chr1:3732	RNU6-609P		smallRNA	chr1:200014689-200
ENSG0000(	491	12.28037	chr1:3732	ASCL5		protein_c	chr1:201113943-201
ENSG0000(	491	12.28037	chr1:3732	ENSG00000224901		lncRNA	chr1:197757319-197
ENSG0000(	491	12.28037	chr1:3732	SLC25A39P1		Pseudoger	chr1:202796030-202
ENSG0000(	491	12.28037	chr1:3732	ENSG00000234775		lncRNA	chr1:203115468-203

ENSG0000(	491	12.28037	chr1:3732	MGAT4FP			Pseudoger	chr1:202986557-202
ENSG0000(	491	12.28037	chr1:3732	Y_RNA			smallRNA	chr1:202914880-202
ENSG0000(	491	12.28037	chr1:3732	ENSG00000260021			lncRNA	chr1:202810238-202
ENSG0000(	491	12.28037	chr1:3732	ENSG00000227747			Pseudoger	chr1:198949016-198
ENSG0000(	491	12.28037	chr1:3732	CCNQP1			Pseudoger	chr1:200213678-200
ENSG0000(	491	12.28037	chr1:3732	ENSG00000224671			lncRNA	chr1:203144694-203
ENSG0000(	491	12.28037	chr1:3732	ENSG00000286541			lncRNA	chr1:199040501-199
ENSG0000(	491	12.28037	chr1:3732	SNORA70			smallRNA	chr1:201978461-201
ENSG0000(	491	12.28037	chr1:3732	ADIPOR1			protein_c	chr1:202940826-202
ENSG0000(	491	12.28037	chr1:3732	CYB5R1			protein_c	chr1:202961873-202
ENSG0000(	491	12.28037	chr1:3732	BTG2	NCGv7		protein_c	chr1:203305491-203
ENSG0000(	491	12.28037	chr1:3732	MIR181A1			smallRNA	chr1:198859044-198
ENSG0000(	491	12.28037	chr1:3732	NEK7			protein_c	chr1:198156994-198
ENSG0000(	491	12.28037	chr1:3732	MRPS21P3			Pseudoger	chr1:197363817-197
ENSG0000(	491	12.28037	chr1:3732	CHI3L1			protein_c	chr1:203178931-203
ENSG0000(	491	12.28037	chr1:3732	HNRNPA1P59			Pseudoger	chr1:202911812-202
ENSG0000(	491	12.28037	chr1:3732	PRR13P1			Pseudoger	chr1:198197779-198
ENSG0000(	491	12.28037	chr1:3732	ZNF281			protein_c	chr1:200404940-200
ENSG0000(	491	12.28037	chr1:3732	FMOD			protein_c	chr1:203340628-203
ENSG0000(	491	12.28037	chr1:3732	LGR6			protein_c	chr1:202193799-202
ENSG0000(	491	12.28037	chr1:3732	CHIT1			protein_c	chr1:203212827-203
ENSG0000(	491	12.28037	chr1:3732	MYOG			protein_c	chr1:203083129-203
ENSG0000(	491	12.28037	chr1:3732	MROH3P			Pseudoger	chr1:200917460-200
ENSG0000(	491	12.28037	chr1:3732	ENSG00000235811			Pseudoger	chr1:202039851-202
ENSG0000(	491	12.28037	chr1:3732	BTG2-DT			lncRNA	chr1:203298758-203
ENSG0000(	491	12.28037	chr1:3732	MYBPH			protein_c	chr1:203167811-203
ENSG0000(	491	12.28037	chr1:3732	ENSG00000249007			lncRNA	chr1:202011370-202
ENSG0000(	491	12.28037	chr1:3732	PCAT6			lncRNA	chr1:202810850-202
ENSG0000(	491	12.28037	chr1:3732	MIR1231			smallRNA	chr1:201808611-201
ENSG0000(	491	12.28037	chr1:3732	KDM5B	AC		protein_c	chr1:202724495-202
ENSG0000(	491	12.28037	chr1:3732	ENSG00000229652			Pseudoger	chr1:203353365-203
ENSG0000(	491	12.28037	chr1:3732	AL450244.1			smallRNA	chr1:199615188-199
ENSG0000(	491	12.28037	chr1:3732	LHX9			protein_c	chr1:197911902-197
ENSG0000(	491	12.28037	chr1:3732	ACTG1P25			Pseudoger	chr1:202861754-202
ENSG0000(	491	12.28037	chr1:3732	CRIP1P3			Pseudoger	chr1:202096759-202
ENSG0000(	491	12.28037	chr1:3732	DDX59-AS1			lncRNA	chr1:200669507-200
ENSG0000(	491	12.28037	chr1:3732	MIR181A1HG			lncRNA	chr1:198777861-198
ENSG0000(	491	12.28037	chr1:3732	RPL34P6			Pseudoger	chr1:200863808-200
ENSG0000(	491	12.28037	chr1:3732	ENSG00000213045			Pseudoger	chr1:200329161-200
ENSG0000(	491	12.28037	chr1:3732	MIR181B1			smallRNA	chr1:198858873-198
ENSG0000(	491	12.28037	chr1:3732	ATP6V1G3			protein_c	chr1:198523222-198
ENSG0000(	491	12.28037	chr1:3732	RNU6-487P			smallRNA	chr1:203318996-203
ENSG0000(	491	12.28037	chr1:3732	NPM1P40			Pseudoger	chr1:203255743-203
ENSG0000(	491	12.28037	chr1:3732	RNU6-716P			smallRNA	chr1:200008505-200
ENSG0000(	491	12.28037	chr1:3732	AC096633.1			smallRNA	chr1:200144834-200
ENSG0000(	491	12.28037	chr1:3732	ZBTB41			protein_c	chr1:197153682-197
ENSG0000(	491	12.28037	chr1:3732	TIMM17A	NCGv7		protein_c	chr1:201955503-201
ENSG0000(	491	12.28037	chr1:3732	CRB1	NCGv7		protein_c	chr1:197268204-197
ENSG0000(	491	12.28037	chr1:3732	CFHR5			protein_c	chr1:196975010-197
ENSG0000(	491	12.28037	chr1:3732	SYT2		DriverDB\	protein_c	chr1:202590596-202
ENSG0000(	491	12.28037	chr1:3732	IPO9			protein_c	chr1:201829149-201

ENSG0000(	491	12.28037	chr1:3732	PEBP1P3		Pseudoger	chr1:198679139-198
ENSG0000(	491	12.28037	chr1:3732	PTPN7		protein_c	chr1:202147013-202
ENSG0000(	491	12.28037	chr1:3732	KLHL12		protein_c	chr1:202891116-202
ENSG0000(	491	12.28037	chr1:3732	ENSG00000223774		lncRNA	chr1:201893842-201
ENSG0000(	491	12.28037	chr1:3732	MIR5191		smallRNA	chr1:201719508-201
ENSG0000(	491	12.28037	chr1:3732	ENSG00000231984		Pseudoger	chr1:199752491-199
ENSG0000(	491	12.28037	chr1:3732	ENSG00000236439		Pseudoger	chr1:202471864-202
ENSG0000(	491	12.28037	chr1:3732	PKP1		protein_c	chr1:201283452-201
ENSG0000(	491	12.28037	chr1:3732	CACNA1S		protein_c	chr1:201039512-201
ENSG0000(	491	12.28037	chr1:3732	ENSG00000235582		Pseudoger	chr1:197735636-197
ENSG0000(	491	12.28037	chr1:3732	ENSG00000229191		lncRNA	chr1:201023949-201
ENSG0000(	491	12.28037	chr1:3732	ENSG00000237861		Pseudoger	chr1:197222222-197
ENSG0000(	491	12.28037	chr1:3732	ENSG00000290909		lncRNA	chr1:202987277-202
ENSG0000(	491	12.28037	chr1:3732	PTPRC	NCGv7;AC	protein_c	chr1:198638457-198
ENSG0000(	491	12.28037	chr1:3732	PPFIA4	DriverDBv	protein_c	chr1:203026491-203
ENSG0000(	491	12.28037	chr1:3732	TNNT2		protein_c	chr1:201359008-201
ENSG0000(	491	12.28037	chr1:3732	CYCSP4		Pseudoger	chr1:202369526-202
ENSG0000(	491	12.28037	chr1:3732	SNORA70		smallRNA	chr1:202527310-202
ENSG0000(	491	12.28037	chr1:3732	CSRP1-AS1		lncRNA	chr1:201507241-201
ENSG0000(	491	12.28037	chr1:3732	ENSG00000228530		Pseudoger	chr1:199876978-199
ENSG0000(	491	12.28037	chr1:3732	GPR37L1		protein_c	chr1:202122886-202
ENSG0000(	491	12.28037	chr1:3732	AC105941.1		smallRNA	chr1:199197080-199
ENSG0000(	491	12.28037	chr1:3732	GPR25		protein_c	chr1:200872981-200
ENSG0000(	491	12.28037	chr1:3732	LINC01221		lncRNA	chr1:199016133-199
ENSG0000(	491	12.28037	chr1:3732	ENSG00000273844		Pseudoger	chr1:196970495-196
ENSG0000(	491	12.28037	chr1:3732	ENSG00000235449		Pseudoger	chr1:202767229-202
ENSG0000(	491	12.28037	chr1:3732	ASPM	NCGv7	protein_c	chr1:197084121-197
ENSG0000(	491	12.28037	chr1:3732	DDX59		protein_c	chr1:200623896-200
ENSG0000(	491	12.28037	chr1:3732	IPO9-AS1		lncRNA	chr1:201688259-201
ENSG0000(	491	12.28037	chr1:3732	RNPEP		protein_c	chr1:201982372-202
ENSG0000(	491	12.28037	chr1:3732	ENSG00000236390		lncRNA	chr1:201673105-201
ENSG0000(	491	12.28037	chr1:3732	ENSG00000291234		lncRNA	chr1:202851828-202
ENSG0000(	491	12.28037	chr1:3732	PTPRVP		Pseudoger	chr1:202168051-202
ENSG0000(	491	12.28037	chr1:3732	ENSG00000225620		lncRNA	chr1:202632428-202
ENSG0000(	491	12.28037	chr1:3732	CFHR4		protein_c	chr1:196888014-196
ENSG0000(	491	12.28037	chr1:3732	F13B		protein_c	chr1:197038741-197
ENSG0000(	491	12.28037	chr1:3732	LINC02789		lncRNA	chr1:199148598-199
ENSG0000(	491	12.28037	chr1:3732	ENSG00000227048		Pseudoger	chr1:201428975-201
ENSG0000(	491	12.28037	chr1:3732	NAV1	NCGv7	protein_c	chr1:201539127-201
ENSG0000(	491	12.28037	chr1:3732	CAMSAP2		protein_c	chr1:200738893-200
ENSG0000(	491	12.28037	chr1:3732	ARL8A		protein_c	chr1:202133404-202
ENSG0000(	491	12.28037	chr1:3732	LINC01353		lncRNA	chr1:203273221-203
ENSG0000(	491	12.28037	chr1:3732	DENND1B		protein_c	chr1:197504748-197
ENSG0000(	491	12.28037	chr1:3732	RNU6-501P		smallRNA	chr1:201733406-201
ENSG0000(	491	12.28037	chr1:3732	ENSG00000229821		lncRNA	chr1:201222113-201
ENSG0000(	491	12.28037	chr1:3732	ENSG00000261573		lncRNA	chr1:198657553-198
ENSG0000(	491	12.28037	chr1:3732	MGAT4EP		Pseudoger	chr1:202820266-202
ENSG0000(	491	12.28037	chr1:3732	ENSG00000290125		lncRNA	chr1:199932233-199
ENSG0000(	491	12.28037	chr1:3732	PHLDA3		protein_c	chr1:201464278-201
ENSG0000(	491	12.28037	chr1:3732	ENSG00000226862		lncRNA	chr1:202604268-202
ENSG0000(	491	12.28037	chr1:3732	ENSG00000232296		Pseudoger	chr1:202028606-202

ENSG0000(	491	12.28037	chr1:3732	ENSG00000287989		lncRNA	chr1:198450666-198
ENSG0000(	491	12.28037	chr1:3732	ENSG00000290127		lncRNA	chr1:197201486-197
ENSG0000(	491	12.28037	chr1:3732	SHISA4	NCGv7	protein_c	chr1:201888680-201
ENSG0000(	491	12.28037	chr1:3732	RABIF		protein_c	chr1:202878282-202
ENSG0000(	491	12.28037	chr1:3732	C1orf53		protein_c	chr1:197902630-197
ENSG0000(	491	12.28037	chr1:3732	FAM204BP		Pseudoger	chr1:197746751-197
ENSG0000(	491	12.28037	chr1:3732	RPL10P4		Pseudoger	chr1:201978642-201
ENSG0000(	491	12.28037	chr1:3732	ENSG00000225172		lncRNA	chr1:198973379-198
ENSG0000(	491	12.28037	chr1:3732	CFHR2	NCGv7	protein_c	chr1:196943738-196
ENSG0000(	491	12.28037	chr1:3732	ENSG00000282849		lncRNA	chr1:200478020-200
ENSG0000(	491	12.28037	chr1:3732	Y_RNA		smallRNA	chr1:197685640-197
ENSG0000(	491	12.28037	chr1:3732	ENSG00000229747		Pseudoger	chr1:199908921-199
ENSG0000(	491	12.28037	chr1:3732	ENSG00000234132		lncRNA	chr1:201031136-201
ENSG0000(	491	12.28037	chr1:3732	KIF14	AC	protein_c	chr1:200551497-200
ENSG0000(	491	12.28037	chr1:3732	EEF1A1P44		Pseudoger	chr1:199387141-199
ENSG0000(	491	12.28037	chr1:3732	RNU6-570P		smallRNA	chr1:200054061-200
ENSG0000(	491	12.28037	chr1:3732	ENSG00000229220		Pseudoger	chr1:200147531-200
ENSG0000(	491	12.28037	chr1:3732	TUBA5P		Pseudoger	chr1:202852991-202
ENSG0000(	490	12.25536	chr6:3405	LINC02524		lncRNA	chr6:135628787-135
ENSG0000(	490	12.25536	chr6:3405	Y_RNA		smallRNA	chr6:136934765-136
ENSG0000(	490	12.25536	chr6:3405	ENSG00000217648		Pseudoger	chr6:143342246-143
ENSG0000(	490	12.25536	chr6:3405	UTRN		protein_c	chr6:144285335-144
ENSG0000(	490	12.25536	chr6:3405	WAKMAR2		lncRNA	chr6:137823673-137
ENSG0000(	490	12.25536	chr6:3405	ECT2L	NCGv7;AC	protein_c	chr6:138795911-138
ENSG0000(	490	12.25536	chr6:3405	AHI1-DT		lncRNA	chr6:135497422-135
ENSG0000(	490	12.25536	chr6:3405	AL360007.1		smallRNA	chr6:142262643-142
ENSG0000(	490	12.25536	chr6:3405	GJE1		protein_c	chr6:142132925-142
ENSG0000(	490	12.25536	chr6:3405	ENSG00000283265		lncRNA	chr6:137693068-137
ENSG0000(	490	12.25536	chr6:3405	HIVEP2-DT		lncRNA	chr6:142946406-142
ENSG0000(	490	12.25536	chr6:3405	AL357060.1		smallRNA	chr6:137716948-137
ENSG0000(	490	12.25536	chr6:3405	ENSG00000217195		Pseudoger	chr6:144706733-144
ENSG0000(	490	12.25536	chr6:3405	MAP7-AS1		lncRNA	chr6:136550661-136
ENSG0000(	490	12.25536	chr6:3405	ENSG00000237596		lncRNA	chr6:135991936-136
ENSG0000(	490	12.25536	chr6:3405	MIR3668		smallRNA	chr6:140205252-140
ENSG0000(	490	12.25536	chr6:3405	SNORA27		smallRNA	chr6:136855698-136
ENSG0000(	490	12.25536	chr6:3405	PDE7B	NCGv7	protein_c	chr6:135851701-136
ENSG0000(	490	12.25536	chr6:3405	OLIG3		protein_c	chr6:137492199-137
ENSG0000(	490	12.25536	chr6:3405	ENSG00000278206		lncRNA	chr6:143484979-143
ENSG0000(	490	12.25536	chr6:3405	ENSG00000287820		lncRNA	chr6:139938864-139
ENSG0000(	490	12.25536	chr6:3405	MTCH1P1		Pseudoger	chr6:138650226-138
ENSG0000(	490	12.25536	chr6:3405	MAP3K5-AS1		lncRNA	chr6:136629066-136
ENSG0000(	490	12.25536	chr6:3405	SF3B5		protein_c	chr6:144094884-144
ENSG0000(	490	12.25536	chr6:3405	ENSG00000236366		lncRNA	chr6:142526455-142
ENSG0000(	490	12.25536	chr6:3405	Y_RNA		smallRNA	chr6:137784374-137
ENSG0000(	490	12.25536	chr6:3405	ENSG00000260418		lncRNA	chr6:136335714-136
ENSG0000(	490	12.25536	chr6:3405	SNORD112		smallRNA	chr6:137540400-137
ENSG0000(	490	12.25536	chr6:3405	ENSG00000236378		lncRNA	chr6:135807148-135
ENSG0000(	490	12.25536	chr6:3405	NMBR-AS1		lncRNA	chr6:142088233-142
ENSG0000(	490	12.25536	chr6:3405	MIR4465		smallRNA	chr6:140683814-140
ENSG0000(	490	12.25536	chr6:3405	CCDC28A-AS1		lncRNA	chr6:138725211-138
ENSG0000(	490	12.25536	chr6:3405	RPS3AP24		Pseudoger	chr6:140761529-140

ENSG0000(	490	12.25536	chr6:340E	ENSG00000220739		Pseudoger	chr6:144708106-144
ENSG0000(	490	12.25536	chr6:340E	HYMAI		lncRNA	chr6:144004916-144
ENSG0000(	490	12.25536	chr6:340E	MAP3K5	NCGv7	protein_c	chr6:136557046-136
ENSG0000(	490	12.25536	chr6:340E	NHSL1		protein_c	chr6:138422043-138
ENSG0000(	490	12.25536	chr6:340E	CITED2	NCGv7	protein_c	chr6:139371807-139
ENSG0000(	490	12.25536	chr6:340E	ENSG00000227192		lncRNA	chr6:143039425-143
ENSG0000(	490	12.25536	chr6:340E	ENSG00000288714		lncRNA	chr6:140148490-140
ENSG0000(	490	12.25536	chr6:340E	ACKR4P1		Pseudoger	chr6:138822747-138
ENSG0000(	490	12.25536	chr6:340E	AHI1	NCGv7;AC	protein_c	chr6:135283407-135
ENSG0000(	490	12.25536	chr6:340E	FILNC1		lncRNA	chr6:139677639-139
ENSG0000(	490	12.25536	chr6:340E	NHEG1		lncRNA	chr6:136982165-136
ENSG0000(	490	12.25536	chr6:340E	ENSG00000217231		Pseudoger	chr6:144036618-144
ENSG0000(	490	12.25536	chr6:340E	TXLNB		protein_c	chr6:139240061-139
ENSG0000(	490	12.25536	chr6:340E	IFNGR1	IntOGen-I	protein_c	chr6:137197483-137
ENSG0000(	490	12.25536	chr6:340E	MIR3145		smallRNA	chr6:138435213-138
ENSG0000(	490	12.25536	chr6:340E	ENSG00000237851		lncRNA	chr6:142788123-142
ENSG0000(	490	12.25536	chr6:340E	LTV1	DriverDB\	protein_c	chr6:143843338-143
ENSG0000(	490	12.25536	chr6:340E	LINC01625		lncRNA	chr6:139435636-139
ENSG0000(	490	12.25536	chr6:340E	MRPL42P3		Pseudoger	chr6:144136450-144
ENSG0000(	490	12.25536	chr6:340E	MAP7	NCGv7	protein_c	chr6:136342281-136
ENSG0000(	490	12.25536	chr6:340E	ENSG00000275138		Pseudoger	chr6:141019788-141
ENSG0000(	490	12.25536	chr6:340E	ENSG00000288054		lncRNA	chr6:135882780-135
ENSG0000(	490	12.25536	chr6:340E	SMIM28		protein_c	chr6:138377905-138
ENSG0000(	490	12.25536	chr6:340E	RNA5SP221		Pseudoger	chr6:143449809-143
ENSG0000(	490	12.25536	chr6:340E	ENSG00000205695		Pseudoger	chr6:139659928-139
ENSG0000(	490	12.25536	chr6:340E	NMBR	NCGv7	protein_c	chr6:142058330-142
ENSG0000(	490	12.25536	chr6:340E	BCLAF1	NCGv7	protein_c	chr6:136256627-136
ENSG0000(	490	12.25536	chr6:340E	ENSG00000231329		lncRNA	chr6:139144204-139
ENSG0000(	490	12.25536	chr6:340E	LINC02919		lncRNA	chr6:142251847-142
ENSG0000(	490	12.25536	chr6:340E	ENSG00000225311		lncRNA	chr6:144311699-144
ENSG0000(	490	12.25536	chr6:340E	ENSG00000288836		lncRNA	chr6:144139977-144
ENSG0000(	490	12.25536	chr6:340E	ENSG00000289850		lncRNA	chr6:144922315-144
ENSG0000(	490	12.25536	chr6:340E	CCDC28A	NCGv7;AC	protein_c	chr6:138773769-138
ENSG0000(	490	12.25536	chr6:340E	ENSG00000225148		lncRNA	chr6:139856104-139
ENSG0000(	490	12.25536	chr6:340E	STX11		protein_c	chr6:144150487-144
ENSG0000(	490	12.25536	chr6:340E	REPS1	DriverDB\	protein_c	chr6:138903493-138
ENSG0000(	490	12.25536	chr6:340E	IL22RA2		protein_c	chr6:137143820-137
ENSG0000(	490	12.25536	chr6:340E	PBOV1		protein_c	chr6:138215986-138
ENSG0000(	490	12.25536	chr6:340E	IL20RA		protein_c	chr6:136999971-137
ENSG0000(	490	12.25536	chr6:340E	ENSG00000277973		Pseudoger	chr6:136206478-136
ENSG0000(	490	12.25536	chr6:340E	FUCA2	DriverDB\	protein_c	chr6:143494812-143
ENSG0000(	490	12.25536	chr6:340E	TUBB8P2		Pseudoger	chr6:143436216-143
ENSG0000(	490	12.25536	chr6:340E	ENSG00000218499		Pseudoger	chr6:138393373-138
ENSG0000(	490	12.25536	chr6:340E	ENSG00000274594		Pseudoger	chr6:138464099-138
ENSG0000(	490	12.25536	chr6:340E	LINC02941		lncRNA	chr6:139976352-140
ENSG0000(	490	12.25536	chr6:340E	VTA1		protein_c	chr6:142147162-142
ENSG0000(	490	12.25536	chr6:340E	7SK		smallRNA	chr6:136545192-136
ENSG0000(	490	12.25536	chr6:340E	ENSG00000218565		Pseudoger	chr6:139338018-139
ENSG0000(	490	12.25536	chr6:340E	ENSG00000216548		Pseudoger	chr6:140922457-140
ENSG0000(	490	12.25536	chr6:340E	ENSG00000220600		Pseudoger	chr6:138878899-138
ENSG0000(	490	12.25536	chr6:340E	ENSG00000220660		Pseudoger	chr6:136364129-136

ENSG0000C	490	12.25536	chr6:340E	RPS3AP23		Pseudoger	chr6:141635650-141
ENSG0000C	490	12.25536	chr6:340E	ENSG00000257065		protein_c	chr6:143807092-143
ENSG0000C	490	12.25536	chr6:340E	SLC35D3		protein_c	chr6:136922301-136
ENSG0000C	490	12.25536	chr6:340E	ZC2HC1B		protein_c	chr6:143864436-143
ENSG0000C	490	12.25536	chr6:340E	Y_RNA		smallRNA	chr6:135474504-135
ENSG0000C	490	12.25536	chr6:340E	HEBP2		protein_c	chr6:138403531-138
ENSG0000C	490	12.25536	chr6:340E	MAP3K5-AS2		lncRNA	chr6:136784045-136
ENSG0000C	490	12.25536	chr6:340E	PEX3	DriverDBv	protein_c	chr6:143450805-143
ENSG0000C	490	12.25536	chr6:340E	NDUFS5P1		Pseudoger	chr6:136475862-136
ENSG0000C	490	12.25536	chr6:340E	ENSG00000225752		lncRNA	chr6:143094034-143
ENSG0000C	490	12.25536	chr6:340E	ENSG00000259828		lncRNA	chr6:141447011-141
ENSG0000C	490	12.25536	chr6:340E	RNA5SP220		Pseudoger	chr6:140158591-140
ENSG0000C	490	12.25536	chr6:340E	ENSG00000216475		Pseudoger	chr6:144257034-144
ENSG0000C	490	12.25536	chr6:340E	PHACTR2-AS1		lncRNA	chr6:143554325-143
ENSG0000C	490	12.25536	chr6:340E	AL512290.1		smallRNA	chr6:135977811-135
ENSG0000C	490	12.25536	chr6:340E	PLAGL1		protein_c	chr6:143940300-144
ENSG0000C	490	12.25536	chr6:340E	ENSG00000220412		Pseudoger	chr6:137705423-137
ENSG0000C	490	12.25536	chr6:340E	ENSG00000226571		lncRNA	chr6:139271362-139
ENSG0000C	490	12.25536	chr6:340E	RPSAP42		Pseudoger	chr6:137995270-137
ENSG0000C	490	12.25536	chr6:340E	ENSG00000287393		lncRNA	chr6:137900585-137
ENSG0000C	490	12.25536	chr6:340E	ENSG00000216519		Pseudoger	chr6:136317961-136
ENSG0000C	490	12.25536	chr6:340E	ENSG00000286452		lncRNA	chr6:141403240-141
ENSG0000C	490	12.25536	chr6:340E	TNFAIP3	NCGv7;AC	protein_c	chr6:137867214-137
ENSG0000C	490	12.25536	chr6:340E	LINC02539		lncRNA	chr6:137730170-137
ENSG0000C	490	12.25536	chr6:340E	ENSG00000280148		protein_c	chr6:143857318-143
ENSG0000C	490	12.25536	chr6:340E	RNU6-427P		smallRNA	chr6:138859027-138
ENSG0000C	490	12.25536	chr6:340E	AIG1		protein_c	chr6:143060496-143
ENSG0000C	490	12.25536	chr6:340E	PEX7		protein_c	chr6:136822564-136
ENSG0000C	490	12.25536	chr6:340E	ENSG00000233138		lncRNA	chr6:142748443-142
ENSG0000C	490	12.25536	chr6:340E	PHACTR2	DriverDBv	protein_c	chr6:143536845-143
ENSG0000C	490	12.25536	chr6:340E	ENSG00000272446		lncRNA	chr6:139159157-139
ENSG0000C	490	12.25536	chr6:340E	ADAT2		protein_c	chr6:143422832-143
ENSG0000C	490	12.25536	chr6:340E	ENSG00000287084		lncRNA	chr6:142671972-142
ENSG0000C	490	12.25536	chr6:340E	ENSG00000289312		lncRNA	chr6:136290014-136
ENSG0000C	490	12.25536	chr6:340E	ADGRG6	NCGv7	protein_c	chr6:142301854-142
ENSG0000C	490	12.25536	chr6:340E	HECA		protein_c	chr6:139135080-139
ENSG0000C	490	12.25536	chr6:340E	ARFGEF3		protein_c	chr6:138161939-138
ENSG0000C	490	12.25536	chr6:340E	PERP		protein_c	chr6:138088505-138
ENSG0000C	490	12.25536	chr6:340E	ENSG00000286313		lncRNA	chr6:135854053-135
ENSG0000C	490	12.25536	chr6:340E	AL360178.1		smallRNA	chr6:135914755-135
ENSG0000C	490	12.25536	chr6:340E	ENSG00000234147		lncRNA	chr6:140575812-140
ENSG0000C	490	12.25536	chr6:340E	BTF3L4P3		Pseudoger	chr6:137543897-137
ENSG0000C	490	12.25536	chr6:340E	RPL7AP37		Pseudoger	chr6:136900233-136
ENSG0000C	490	12.25536	chr6:340E	ATP5PBP6		Pseudoger	chr6:139614438-139
ENSG0000C	490	12.25536	chr6:340E	ENSG00000270890		Pseudoger	chr6:143858062-143
ENSG0000C	490	12.25536	chr6:340E	LINC02528		lncRNA	chr6:137943079-137
ENSG0000C	490	12.25536	chr6:340E	COX5BP2		Pseudoger	chr6:136034553-136
ENSG0000C	490	12.25536	chr6:340E	ENSG00000235399		lncRNA	chr6:136995170-136
ENSG0000C	490	12.25536	chr6:340E	ENSG00000270983		Pseudoger	chr6:142062717-142
ENSG0000C	490	12.25536	chr6:340E	RPL35AP3		Pseudoger	chr6:136973930-136
ENSG0000C	490	12.25536	chr6:340E	NHSL1-AS1		lncRNA	chr6:138692548-138

ENSG0000(	490	12.25536	chr6:340!	HIVEP2		protein_c	chr6:142751469-142
ENSG0000(	490	12.25536	chr6:340!	LINC01277		lncRNA	chr6:142966293-143
ENSG0000(	490	12.25536	chr6:340!	ENSG00000270655		Pseudoger	chr6:143386581-143
ENSG0000(	490	12.25536	chr6:340!	ENSG00000219409		Pseudoger	chr6:144397959-144
ENSG0000(	490	12.25536	chr6:340!	HMGB1P17		Pseudoger	chr6:135636086-135
ENSG0000(	490	12.25536	chr6:340!	LINC02865		lncRNA	chr6:137945366-137
ENSG0000(	490	12.25536	chr6:340!	MTFR2		protein_c	chr6:136231024-136
ENSG0000(	490	12.25536	chr6:340!	ENSG00000216613		Pseudoger	chr6:136419847-136
ENSG0000(	490	12.25536	chr6:340!	TPT1P4		Pseudoger	chr6:144200447-144
ENSG0000(	490	12.25536	chr6:340!	ENSG00000217495		Pseudoger	chr6:143298770-143
ENSG0000(	490	12.25536	chr6:340!	RN7SKP106		smallRNA	chr6:141486141-141
ENSG0000(	490	12.25536	chr6:340!	ENSG00000216642		Pseudoger	chr6:143327275-143
ENSG0000(	490	12.25536	chr6:340!	LINC03004		lncRNA	chr6:137657998-137
ENSG0000(	490	12.25536	chr6:340!	RNA5SP219		Pseudoger	chr6:136630243-136
ENSG0000(	490	12.25536	chr6:340!	MARCKSL1P2		Pseudoger	chr6:138402585-138
ENSG0000(	490	12.25536	chr6:340!	ABRACL		protein_c	chr6:139028745-139
ENSG0000(	490	12.25536	chr6:340!	GAPDHP73		Pseudoger	chr6:135619165-135
ENSG0000(	490	12.25536	chr6:340!	AL356137.1		smallRNA	chr6:140660623-140
ENSG0000(	489	12.23034	chr1:116(	FBXO42		protein_c	chr1:16246840-1635
ENSG0000(	489	12.23034	chr1:116(	HSPB7		protein_c	chr1:16014028-1601
ENSG0000(	489	12.23034	chr1:116(	PLEKHM2		protein_c	chr1:15684320-1573
ENSG0000(	489	12.23034	chr1:116(	RSC1A1		protein_c	chr1:15659713-1566
ENSG0000(	489	12.23034	chr1:116(	EPHA2	NCGv7;AC	protein_c	chr1:16124337-1615
ENSG0000(	489	12.23034	chr1:116(	SCARNA21		smallRNA	chr1:15542165-1554
ENSG0000(	489	12.23034	chr1:116(	EFHD2-AS1		lncRNA	chr1:15402979-1540
ENSG0000(	489	12.23034	chr1:116(	SPEN	NCGv7	protein_c	chr1:15836095-1594
ENSG0000(	489	12.23034	chr1:116(	CELA2B		protein_c	chr1:15465909-1549
ENSG0000(	489	12.23034	chr1:116(	CASP9	NCGv7	protein_c	chr1:15490832-1552
ENSG0000(	489	12.23034	chr1:116(	RPL12P14		Pseudoger	chr1:15792796-1579
ENSG0000(	489	12.23034	chr1:116(	AGMAT		protein_c	chr1:15571699-1558
ENSG0000(	489	12.23034	chr1:116(	ZBTB2P1		Pseudoger	chr1:15226373-1522
ENSG0000(	489	12.23034	chr1:116(	ENSG00000275503		Pseudoger	chr1:15989140-1598
ENSG0000(	489	12.23034	chr1:116(	SLC25A34		protein_c	chr1:15736258-1574
ENSG0000(	489	12.23034	chr1:116(	TBC1D3P6		Pseudoger	chr1:15989871-1599
ENSG0000(	489	12.23034	chr1:116(	C1orf134		protein_c	chr1:16228873-1622
ENSG0000(	489	12.23034	chr1:116(	ENSG00000231353		Pseudoger	chr1:15988182-1598
ENSG0000(	489	12.23034	chr1:116(	FAM131C2P		Pseudoger	chr1:16035178-1604
ENSG0000(	489	12.23034	chr1:116(	CPLANE2		protein_c	chr1:16231692-1623
ENSG0000(	489	12.23034	chr1:116(	MFFP1		Pseudoger	chr1:15191828-1519
ENSG0000(	489	12.23034	chr1:116(	ENSG00000272510		lncRNA	chr1:15565611-1556
ENSG0000(	489	12.23034	chr1:116(	SZRD1		protein_c	chr1:16352575-1639
ENSG0000(	489	12.23034	chr1:116(	ENSG00000237301		lncRNA	chr1:15586136-1560
ENSG0000(	489	12.23034	chr1:116(	SLC25A34-AS1		lncRNA	chr1:15740048-1574
ENSG0000(	489	12.23034	chr1:116(	EFHD2	NCGv7	protein_c	chr1:15409888-1543
ENSG0000(	489	12.23034	chr1:116(	CD24P1		Pseudoger	chr1:15614643-1561
ENSG0000(	489	12.23034	chr1:116(	TMEM51-AS2		lncRNA	chr1:15164344-1517
ENSG0000(	489	12.23034	chr9:197!	RN7SL665P		smallRNA	chr9:130400266-130
ENSG0000(	489	12.23034	chr1:116(	ENSG00000271742		lncRNA	chr1:15682873-1568
ENSG0000(	489	12.23034	chr1:116(	ENSG00000233078		lncRNA	chr1:16006160-1600
ENSG0000(	489	12.23034	chr1:116(	ARHGEF19		protein_c	chr1:16197854-1621
ENSG0000(	489	12.23034	chr1:116(	FHAD1	DriverDB\	protein_c	chr1:15236521-1540

ENSG0000(	489	12.23034	chr1:116(	CLCNKA	protein_c	chr1:16018875-1603
ENSG0000(	489	12.23034	chr1:116(	MT1XP1	Pseudoger	chr1:16241213-1624
ENSG0000(	489	12.23034	chr1:116(	ANO7L1	Pseudoger	chr1:16216469-1622
ENSG0000(	489	12.23034	chr1:116(	RPL22P3	Pseudoger	chr1:16369150-1636
ENSG0000(	489	12.23034	chr1:116(	snoU13	smallRNA	chr1:15910897-1591
ENSG0000(	489	12.23034	chr1:116(	ENSG00000237938	lncRNA	chr1:15720312-1573
ENSG0000(	489	12.23034	chr1:116(	ARHGEF19-AS1	lncRNA	chr1:16197854-1619
ENSG0000(	489	12.23034	chr1:116(	ZBTB17	protein_c	chr1:15941869-1597
ENSG0000(	489	12.23034	chr1:116(	ENSG00000224621	lncRNA	chr1:16159266-1616
ENSG0000(	489	12.23034	chr1:116(	SPEN-AS1	lncRNA	chr1:15834474-1584
ENSG0000(	489	12.23034	chr1:116(	ENSG00000236045	lncRNA	chr1:15334166-1533
ENSG0000(	489	12.23034	chr1:116(	FHAD1-AS1	lncRNA	chr1:15326680-1534
ENSG0000(	489	12.23034	chr1:116(	DDI2	protein_c	chr1:15617458-1566
ENSG0000(	489	12.23034	chr1:116(	TMEM51	protein_c	chr1:15152532-1522
ENSG0000(	489	12.23034	chr1:116(	SPATA21	protein_c	chr1:16387117-1643
ENSG0000(	489	12.23034	chr1:116(	DNAJC16	protein_c	chr1:15526813-1559
ENSG0000(	489	12.23034	chr1:116(	ENSG00000291077	lncRNA	chr1:16215907-1621
ENSG0000(	489	12.23034	chr1:116(	TMEM82	protein_c	chr1:15742499-1574
ENSG0000(	489	12.23034	chr1:116(	ENSG00000270620	Pseudoger	chr1:15917698-1591
ENSG0000(	489	12.23034	chr1:116(	EPHA2-AS1	lncRNA	chr1:16155176-1615
ENSG0000(	489	12.23034	chr1:116(	CLCNKB	protein_c	chr1:16040252-1605
ENSG0000(	489	12.23034	chr1:116(	SRARP	protein_c	chr1:16004236-1600
ENSG0000(	489	12.23034	chr1:116(	ENSG00000234607	Pseudoger	chr1:15969632-1597
ENSG0000(	489	12.23034	chr1:116(	UQCRHL	protein_c	chr1:15807169-1580
ENSG0000(	489	12.23034	chr1:116(	AL121992.1	smallRNA	chr1:15684472-1568
ENSG0000(	489	12.23034	chr1:116(	FBLIM1	protein_c	chr1:15756607-1578
ENSG0000(	489	12.23034	chr1:116(	RNU7-179P	smallRNA	chr1:15608078-1560
ENSG0000(	489	12.23034	chr1:116(	CELA2A	protein_c	chr1:15456728-1547
ENSG0000(	489	12.23034	chr1:116(	FAM131C	protein_c	chr1:16057769-1607
ENSG0000(	489	12.23034	chr1:116(	CHCHD2P6	Pseudoger	chr1:15604597-1560
ENSG0000(	489	12.23034	chr1:116(	CTRC	protein_c	chr1:15438442-1544
ENSG0000(	489	12.23034	chr1:116(	TMEM51-AS1	lncRNA	chr1:15111815-1515
ENSG0000(	489	12.23034	chr1:116(	ENSG00000178715	Pseudoger	chr1:15828232-1582
ENSG0000(	489	12.23034	chr1:116(	ENSG00000288398	lncRNA	chr1:16228674-1623
ENSG0000(	487	12.18032	chr1:116(	RP13-221M14.2	lncRNA	chr1:13079329-1307
ENSG0000(	487	12.18032	chr1:116(	PRAMEF35P	Pseudoger	chr1:13104403-1310
ENSG0000(	487	12.18032	chr1:116(	PRAMEF7	protein_c	chr1:12916610-1292
ENSG0000(	487	12.18032	chr1:116(	PRAMEF2	protein_c	chr1:12857086-1286
ENSG0000(	487	12.18032	chr1:116(	PRAMEF29P	Pseudoger	chr1:12926162-1292
ENSG0000(	487	12.18032	chr1:116(	PRAMEF6	protein_c	chr1:12938472-1294
ENSG0000(	487	12.18032	chr1:116(	PRAMEF10	protein_c	chr1:12892896-1289
ENSG0000(	487	12.18032	chr1:116(	RNU6-1072P	smallRNA	chr1:12922554-1292
ENSG0000(	487	12.18032	chr1:116(	RP13-221M14.3	Pseudoger	chr1:13095174-1309
ENSG0000(	487	12.18032	chr1:116(	HNRNPCL2	protein_c	chr1:13115488-1311
ENSG0000(	487	12.18032	chr1:116(	PRAMEF4 NCGv7	protein_c	chr1:12879212-1288
ENSG0000(	487	12.18032	chr1:116(	PRAMEF34P	Pseudoger	chr1:13095179-1309
ENSG0000(	483	12.08028	chr1:373(	ENSG00000233583	Pseudoger	chr1:185262286-185
ENSG0000(	483	12.08028	chr1:373(	LINC01720	lncRNA	chr1:190624890-190
ENSG0000(	483	12.08028	chr1:373(	Y_RNA	smallRNA	chr1:184171714-184
ENSG0000(	483	12.08028	chr1:373(	ENSG00000231714	lncRNA	chr1:194350943-194
ENSG0000(	483	12.08028	chr1:373(	ENSG00000226570	Pseudoger	chr1:182955390-182

ENSG0000(	483	12.08028	chr1:3732	RNASEL		protein_c	chr1:182573634-182
ENSG0000(	483	12.08028	chr1:3732	ENSG00000285986		Pseudoger	chr1:196850283-196
ENSG0000(	483	12.08028	chr1:3732	ENSG00000224691		lncRNA	chr1:186176814-186
ENSG0000(	483	12.08028	chr1:3732	FDPSP1		Pseudoger	chr1:187563061-187
ENSG0000(	483	12.08028	chr1:3732	ENSG00000286966		lncRNA	chr1:183613537-183
ENSG0000(	483	12.08028	chr1:3732	MCRIP2P2		Pseudoger	chr1:185435839-185
ENSG0000(	483	12.08028	chr1:3732	RGS8		protein_c	chr1:182641816-182
ENSG0000(	483	12.08028	chr1:3732	PRG4	NCGv7	protein_c	chr1:186296279-186
ENSG0000(	483	12.08028	chr1:3732	ENSG00000243155		lncRNA	chr1:180944042-180
ENSG0000(	483	12.08028	chr1:3732	SMG7	NCGv7	protein_c	chr1:183472216-183
ENSG0000(	483	12.08028	chr1:3732	OCLM		protein_c	chr1:186400572-186
ENSG0000(	483	12.08028	chr1:3732	RNA5SP71		smallRNA	chr1:182944365-182
ENSG0000(	483	12.08028	chr1:3732	RPS3AP8		Pseudoger	chr1:183266602-183
ENSG0000(	483	12.08028	chr1:3732	C1orf21		protein_c	chr1:184387029-184
ENSG0000(	483	12.08028	chr1:3732	ODR4		protein_c	chr1:186375838-186
ENSG0000(	483	12.08028	chr1:3732	LHX4		protein_c	chr1:180230264-180
ENSG0000(	483	12.08028	chr1:3732	NPL		protein_c	chr1:182789293-182
ENSG0000(	483	12.08028	chr1:3732	SWT1		protein_c	chr1:185157080-185
ENSG0000(	483	12.08028	chr1:3732	PTGS2		protein_c	chr1:186671791-186
ENSG0000(	483	12.08028	chr1:3732	ENSG00000270711		Pseudoger	chr1:180970837-180
ENSG0000(	483	12.08028	chr1:3732	RGSL1		protein_c	chr1:182409192-182
ENSG0000(	483	12.08028	chr1:3732	NMNAT2		protein_c	chr1:183248237-183
ENSG0000(	483	12.08028	chr1:3732	RN7SKP156		smallRNA	chr1:188155839-188
ENSG0000(	483	12.08028	chr1:3732	SHCBP1L		protein_c	chr1:182899865-182
ENSG0000(	483	12.08028	chr1:3732	ENSG00000274702		Pseudoger	chr1:187632166-187
ENSG0000(	483	12.08028	chr1:3732	IVNS1ABP		protein_c	chr1:185296388-185
ENSG0000(	483	12.08028	chr1:3732	ENSG00000272906		lncRNA	chr1:179881607-179
ENSG0000(	483	12.08028	chr1:3732	AL137800.1		smallRNA	chr1:183510675-183
ENSG0000(	483	12.08028	chr1:3732	TOR1AIP2		protein_c	chr1:179839967-179
ENSG0000(	483	12.08028	chr1:3732	NIBAN1		protein_c	chr1:184790724-184
ENSG0000(	483	12.08028	chr1:3732	SLC4A1APP2		Pseudoger	chr1:187706561-187
ENSG0000(	483	12.08028	chr1:3732	C1orf21-DT		lncRNA	chr1:184385753-184
ENSG0000(	483	12.08028	chr1:3732	AL450304.1		smallRNA	chr1:182959485-182
ENSG0000(	483	12.08028	chr1:3732	RGS13		protein_c	chr1:192636138-192
ENSG0000(	483	12.08028	chr1:3732	DHX9	NCGv7	protein_c	chr1:182839347-182
ENSG0000(	483	12.08028	chr1:3732	ENSG00000287929		lncRNA	chr1:183252263-183
ENSG0000(	483	12.08028	chr1:3732	CDC73	NCGv7;AC	protein_c	chr1:193121983-193
ENSG0000(	483	12.08028	chr1:3732	BRINP3-DT		lncRNA	chr1:190478551-190
ENSG0000(	483	12.08028	chr1:3732	ENSG00000228167		Pseudoger	chr1:194718795-194
ENSG0000(	483	12.08028	chr1:3732	ENSG00000261831		lncRNA	chr1:179926641-179
ENSG0000(	483	12.08028	chr1:3732	LINC01688		lncRNA	chr1:182712862-182
ENSG0000(	483	12.08028	chr1:3732	CEP350		protein_c	chr1:179954674-180
ENSG0000(	483	12.08028	chr1:3732	TRMT1L		protein_c	chr1:185118101-185
ENSG0000(	483	12.08028	chr1:3732	KIAA1614		protein_c	chr1:180912897-180
ENSG0000(	483	12.08028	chr1:3732	RPL5P5		Pseudoger	chr1:185226808-185
ENSG0000(	483	12.08028	chr1:3732	GAPDHP75		Pseudoger	chr1:189132350-189
ENSG0000(	483	12.08028	chr1:3732	RNF2		protein_c	chr1:185045526-185
ENSG0000(	483	12.08028	chr1:3732	ENSG00000290066		lncRNA	chr1:184629542-184
ENSG0000(	483	12.08028	chr1:3732	COLGALT2		protein_c	chr1:183929854-184
ENSG0000(	483	12.08028	chr1:3732	ENSG00000228664		Pseudoger	chr1:182328497-182
ENSG0000(	483	12.08028	chr1:3732	STX6		protein_c	chr1:180972712-181

ENSG0000(	483	12.08028	chr1:3732	RNA5SP72		Pseudoger	chr1:185014951-185
ENSG0000(	483	12.08028	chr1:3732	VDAC1P4		Pseudoger	chr1:180434800-180
ENSG0000(	483	12.08028	chr1:3732	MIR1278		smallRNA	chr1:193136503-193
ENSG0000(	483	12.08028	chr1:3732	RGL1	NCGv7	protein_c	chr1:183636085-183
ENSG0000(	483	12.08028	chr1:3732	ARPC5		protein_c	chr1:183620846-183
ENSG0000(	483	12.08028	chr1:3732	HMCN1	NCGv7	protein_c	chr1:185734391-186
ENSG0000(	483	12.08028	chr1:3732	RNU6-152P		smallRNA	chr1:182327068-182
ENSG0000(	483	12.08028	chr1:3732	LINC01036		lncRNA	chr1:187070700-187
ENSG0000(	483	12.08028	chr1:3732	ENSG00000235083		Pseudoger	chr1:188671353-188
ENSG0000(	483	12.08028	chr1:3732	ENSG00000285718		lncRNA	chr1:194785517-194
ENSG0000(	483	12.08028	chr1:3732	ENSG00000288078		lncRNA	chr1:186224472-186
ENSG0000(	483	12.08028	chr1:3732	RPL23AP22		Pseudoger	chr1:193756815-193
ENSG0000(	483	12.08028	chr1:3732	RPS27AP5		Pseudoger	chr1:192716183-192
ENSG0000(	483	12.08028	chr1:3732	LINC01350		lncRNA	chr1:185558371-185
ENSG0000(	483	12.08028	chr1:3732	ENSG00000150732		Pseudoger	chr1:188067298-188
ENSG0000(	483	12.08028	chr1:3732	CFH		protein_c	chr1:196651754-196
ENSG0000(	483	12.08028	chr1:3732	LAMC2	TAG	protein_c	chr1:183186238-183
ENSG0000(	483	12.08028	chr1:3732	RN7SKP126		smallRNA	chr1:192875686-192
ENSG0000(	483	12.08028	chr1:3732	ENSG00000233196		Pseudoger	chr1:186580515-186
ENSG0000(	483	12.08028	chr1:3732	RGS18	NCGv7	protein_c	chr1:192158462-192
ENSG0000(	483	12.08028	chr1:3732	Y_RNA		smallRNA	chr1:185251313-185
ENSG0000(	483	12.08028	chr1:3732	APOBEC4		protein_c	chr1:183646275-183
ENSG0000(	483	12.08028	chr1:3732	ENSG00000271269		Pseudoger	chr1:182733792-182
ENSG0000(	483	12.08028	chr1:3732	ENSG00000230470		lncRNA	chr1:184408337-184
ENSG0000(	483	12.08028	chr1:3732	Y_RNA		smallRNA	chr1:180519016-180
ENSG0000(	483	12.08028	chr1:3732	ENSG00000285638		lncRNA	chr1:190878145-191
ENSG0000(	483	12.08028	chr1:3732	ENSG00000289573		lncRNA	chr1:182149703-182
ENSG0000(	483	12.08028	chr1:3732	GLRX2		protein_c	chr1:193090866-193
ENSG0000(	483	12.08028	chr1:3732	ENSG00000237011		lncRNA	chr1:193684246-193
ENSG0000(	483	12.08028	chr1:3732	OVAAL		lncRNA	chr1:180509380-180
ENSG0000(	483	12.08028	chr1:3732	ACBD6		protein_c	chr1:180269653-180
ENSG0000(	483	12.08028	chr1:3732	B3GALT2		protein_c	chr1:193178730-193
ENSG0000(	483	12.08028	chr1:3732	AL358354.1		smallRNA	chr1:180545832-180
ENSG0000(	483	12.08028	chr1:3732	ENSG00000231791		lncRNA	chr1:184329071-184
ENSG0000(	483	12.08028	chr1:3732	RN7SL230P		smallRNA	chr1:179900262-179
ENSG0000(	483	12.08028	chr1:3732	ENSG00000226814		Pseudoger	chr1:192800571-192
ENSG0000(	483	12.08028	chr1:3732	BRINP3	NCGv7	protein_c	chr1:190097658-190
ENSG0000(	483	12.08028	chr1:3732	ENSG00000288562		lncRNA	chr1:186624700-186
ENSG0000(	483	12.08028	chr1:3732	RNU6-1240P		smallRNA	chr1:186311825-186
ENSG0000(	483	12.08028	chr1:3732	ENSG00000260360		lncRNA	chr1:179953184-179
ENSG0000(	483	12.08028	chr1:3732	KCNT2	NCGv7	protein_c	chr1:196225779-196
ENSG0000(	483	12.08028	chr1:3732	ENSG00000289581		lncRNA	chr1:183874511-183
ENSG0000(	483	12.08028	chr1:3732	RNU7-13P		smallRNA	chr1:184821428-184
ENSG0000(	483	12.08028	chr1:3732	ENSG00000286655		lncRNA	chr1:184080657-184
ENSG0000(	483	12.08028	chr1:3732	RNU7-183P		smallRNA	chr1:185434244-185
ENSG0000(	483	12.08028	chr1:3732	LINC01732		lncRNA	chr1:181174484-181
ENSG0000(	483	12.08028	chr1:3732	LINC01699		lncRNA	chr1:181236388-181
ENSG0000(	483	12.08028	chr1:3732	ENSG00000289589		lncRNA	chr1:181086644-181
ENSG0000(	483	12.08028	chr1:3732	AL513344.1		smallRNA	chr1:182756919-182
ENSG0000(	483	12.08028	chr1:3732	RO60	NCGv7	protein_c	chr1:193059454-193
ENSG0000(	483	12.08028	chr1:3732	RGS2		protein_c	chr1:192809039-192

ENSG0000(	483	12.08028	chr1:3732	U3		smallRNA	chr1:193731858-193
ENSG0000(	483	12.08028	chr1:3732	ZNF648		protein_c	chr1:182054570-182
ENSG0000(	483	12.08028	chr1:3732	ENSG00000279401		TEC	chr1:185518651-185
ENSG0000(	483	12.08028	chr1:3732	ENSG00000271187		Pseudoger	chr1:191179521-191
ENSG0000(	483	12.08028	chr1:3732	PLA2G4A		protein_c	chr1:186828949-186
ENSG0000(	483	12.08028	chr1:3732	KIAA1614-AS1		lncRNA	chr1:180949699-180
ENSG0000(	483	12.08028	chr1:3732	PDC		protein_c	chr1:186443566-186
ENSG0000(	483	12.08028	chr1:3732	HNRNPA1P46		Pseudoger	chr1:191146025-191
ENSG0000(	483	12.08028	chr1:3732	CLPTM1LP1		Pseudoger	chr1:189035961-189
ENSG0000(	483	12.08028	chr1:3732	NCF2	NCGv7	protein_c	chr1:183554461-183
ENSG0000(	483	12.08028	chr1:3732	ENSG00000270994		Pseudoger	chr1:183709305-183
ENSG0000(	483	12.08028	chr1:3732	GLUL		protein_c	chr1:182378098-182
ENSG0000(	483	12.08028	chr1:3732	RGS21		protein_c	chr1:192316992-192
ENSG0000(	483	12.08028	chr1:3732	ENSG00000285280		lncRNA	chr1:192167786-192
ENSG0000(	483	12.08028	chr1:3732	TSEN15		protein_c	chr1:184051651-184
ENSG0000(	483	12.08028	chr1:3732	ENSG00000236792		Pseudoger	chr1:192247505-192
ENSG0000(	483	12.08028	chr1:3732	CFHR3		protein_c	chr1:196774813-196
ENSG0000(	483	12.08028	chr1:3732	RPSAP16		Pseudoger	chr1:179968686-179
ENSG0000(	483	12.08028	chr1:3732	ENSG00000286378		lncRNA	chr1:184664282-184
ENSG0000(	483	12.08028	chr1:3732	TEDDM2P		Pseudoger	chr1:182441577-182
ENSG0000(	483	12.08028	chr1:3732	ENSG00000288574		lncRNA	chr1:181808927-181
ENSG0000(	483	12.08028	chr1:3732	TEDDM1		protein_c	chr1:182398117-182
ENSG0000(	483	12.08028	chr1:3732	ENSG00000289995		lncRNA	chr1:192609359-192
ENSG0000(	483	12.08028	chr1:3732	AL357932.1		smallRNA	chr1:195126681-195
ENSG0000(	483	12.08028	chr1:3732	ENSG00000224810		lncRNA	chr1:182062677-182
ENSG0000(	483	12.08028	chr1:3732	UCHL5	NCGv7	protein_c	chr1:193012250-193
ENSG0000(	483	12.08028	chr1:3732	ENSG00000228687		Pseudoger	chr1:192796533-192
ENSG0000(	483	12.08028	chr1:3732	ENSG00000286372		lncRNA	chr1:183372870-183
ENSG0000(	483	12.08028	chr1:3732	Y_RNA		smallRNA	chr1:185266535-185
ENSG0000(	483	12.08028	chr1:3732	IER5		protein_c	chr1:181088700-181
ENSG0000(	483	12.08028	chr1:3732	TOR1AIP1		protein_c	chr1:179882042-179
ENSG0000(	483	12.08028	chr1:3732	ENSG00000237283		lncRNA	chr1:188705623-188
ENSG0000(	483	12.08028	chr1:3732	AL590085.1		smallRNA	chr1:180827895-180
ENSG0000(	483	12.08028	chr1:3732	LINC00272		lncRNA	chr1:182407621-182
ENSG0000(	483	12.08028	chr1:3732	RGS1		protein_c	chr1:192575763-192
ENSG0000(	483	12.08028	chr1:3732	snoU109		smallRNA	chr1:193057281-193
ENSG0000(	483	12.08028	chr1:3732	QSOX1		protein_c	chr1:180154869-180
ENSG0000(	483	12.08028	chr1:3732	ENSG00000225811		lncRNA	chr1:190264898-190
ENSG0000(	483	12.08028	chr1:3732	ENSG00000232309		lncRNA	chr1:182127297-182
ENSG0000(	483	12.08028	chr1:3732	LAMC1-AS1		lncRNA	chr1:183138402-183
ENSG0000(	483	12.08028	chr1:3732	LINC01686		lncRNA	chr1:182615254-182
ENSG0000(	483	12.08028	chr1:3732	ENSG00000261729		lncRNA	chr1:185646463-185
ENSG0000(	483	12.08028	chr1:3732	ENSG00000261642		lncRNA	chr1:191151510-191
ENSG0000(	483	12.08028	chr1:3732	ENSG00000286285		lncRNA	chr1:193457422-193
ENSG0000(	483	12.08028	chr1:3732	ENSG00000236069		lncRNA	chr1:192517190-192
ENSG0000(	483	12.08028	chr1:3732	SNORD112		smallRNA	chr1:184677934-184
ENSG0000(	483	12.08028	chr1:3732	EDEM3		protein_c	chr1:184690237-184
ENSG0000(	483	12.08028	chr1:3732	ENSG00000270443		Pseudoger	chr1:182433893-182
ENSG0000(	483	12.08028	chr1:3732	ENSG00000232036		Pseudoger	chr1:184566511-184
ENSG0000(	483	12.08028	chr1:3732	ENSG00000224278		Pseudoger	chr1:188242139-188
ENSG0000(	483	12.08028	chr1:3732	LINC01724		lncRNA	chr1:196044883-196

ENSG0000(	483	12.08028	chr1:3732	ENSG00000227554		lncRNA	chr1:183754418-183
ENSG0000(	483	12.08028	chr1:3732	Y_RNA		smallRNA	chr1:184315658-184
ENSG0000(	483	12.08028	chr1:3732	RN7SL654P		smallRNA	chr1:184335658-184
ENSG0000(	483	12.08028	chr1:3732	GS1-279B7.1		Pseudoger	chr1:185321157-185
ENSG0000(	483	12.08028	chr1:3732	RNA5SP73		Pseudoger	chr1:189666149-189
ENSG0000(	483	12.08028	chr1:3732	ENSG00000289732		Pseudoger	chr1:183587174-183
ENSG0000(	483	12.08028	chr1:3732	ENSG00000227240		lncRNA	chr1:193473224-194
ENSG0000(	483	12.08028	chr1:3732	RGS16		protein_c	chr1:182598623-182
ENSG0000(	483	12.08028	chr1:3732	XPR1		protein_c	chr1:180632022-180
ENSG0000(	483	12.08028	chr1:3732	ZNF101P2		Pseudoger	chr1:192993449-192
ENSG0000(	483	12.08028	chr1:3732	LINC01035		lncRNA	chr1:188905688-189
ENSG0000(	483	12.08028	chr1:3732	TPR	NCGv7;AC	protein_c	chr1:186311652-186
ENSG0000(	483	12.08028	chr1:3732	RNA5SP70		Pseudoger	chr1:181771566-181
ENSG0000(	483	12.08028	chr1:3732	ENSG00000251520		Pseudoger	chr1:180964511-180
ENSG0000(	483	12.08028	chr1:3732	LINC01680		lncRNA	chr1:191221159-191
ENSG0000(	483	12.08028	chr1:3732	ENSG00000273004		lncRNA	chr1:185317779-185
ENSG0000(	483	12.08028	chr1:3732	U6		smallRNA	chr1:180758722-180
ENSG0000(	483	12.08028	chr1:3732	LINC01344		lncRNA	chr1:182096338-182
ENSG0000(	483	12.08028	chr1:3732	LINC02770		lncRNA	chr1:191823432-192
ENSG0000(	483	12.08028	chr1:3732	RNU6-41P		smallRNA	chr1:182982212-182
ENSG0000(	483	12.08028	chr1:3732	ENSG00000223344		lncRNA	chr1:191858707-191
ENSG0000(	483	12.08028	chr1:3732	ENSG00000226640		lncRNA	chr1:193678894-193
ENSG0000(	483	12.08028	chr1:3732	RPL22P24		Pseudoger	chr1:185171335-185
ENSG0000(	483	12.08028	chr1:3732	YPEL5P1		Pseudoger	chr1:182182730-182
ENSG0000(	483	12.08028	chr1:3732	MR1		protein_c	chr1:181033374-181
ENSG0000(	483	12.08028	chr1:3732	ENSG00000271558		Pseudoger	chr1:187506838-187
ENSG0000(	483	12.08028	chr1:3732	ENSG00000238054		lncRNA	chr1:188869474-188
ENSG0000(	483	12.08028	chr1:3732	AL596220.1		protein_c	chr1:186394991-186
ENSG0000(	483	12.08028	chr1:3732	ENSG00000238108		Pseudoger	chr1:190797524-190
ENSG0000(	483	12.08028	chr1:3732	ENSG00000225359		lncRNA	chr1:181190471-181
ENSG0000(	483	12.08028	chr1:3732	ENSG00000223847		Pseudoger	chr1:187714243-187
ENSG0000(	483	12.08028	chr1:3732	RN7SKP229		smallRNA	chr1:181839473-181
ENSG0000(	483	12.08028	chr1:3732	RNU6-983P		smallRNA	chr1:194488103-194
ENSG0000(	483	12.08028	chr1:3732	ENSG00000287364		lncRNA	chr1:195747024-195
ENSG0000(	483	12.08028	chr1:3732	ENSG00000238061		Pseudoger	chr1:185280844-185
ENSG0000(	483	12.08028	chr1:3732	CACNA1E	NCGv7	protein_c	chr1:181317690-181
ENSG0000(	483	12.08028	chr1:3732	EEF1A1P14		Pseudoger	chr1:194188967-194
ENSG0000(	483	12.08028	chr1:3732	EIF1P3		Pseudoger	chr1:182336001-182
ENSG0000(	483	12.08028	chr1:3732	MIR4735		smallRNA	chr1:196582413-196
ENSG0000(	483	12.08028	chr1:3732	ENSG00000225006		lncRNA	chr1:188508538-188
ENSG0000(	483	12.08028	chr1:3732	ENSG00000225982		lncRNA	chr1:182086551-182
ENSG0000(	483	12.08028	chr1:3732	PACERR		lncRNA	chr1:186680601-186
ENSG0000(	483	12.08028	chr1:3732	ENSG00000289099		lncRNA	chr1:183605128-183
ENSG0000(	483	12.08028	chr1:3732	ENSG00000288950		lncRNA	chr1:193482540-193
ENSG0000(	483	12.08028	chr1:3732	ENSG00000289697		protein_c	chr1:196651852-196
ENSG0000(	483	12.08028	chr1:3732	Y_RNA		smallRNA	chr1:185630428-185
ENSG0000(	483	12.08028	chr1:3732	ENSG00000223450		Pseudoger	chr1:180000438-180
ENSG0000(	483	12.08028	chr1:3732	LINC02816		lncRNA	chr1:180906651-180
ENSG0000(	483	12.08028	chr1:3732	ENSG00000285847		lncRNA	chr1:184607599-184
ENSG0000(	483	12.08028	chr1:3732	HMGN1P4		Pseudoger	chr1:182942115-182
ENSG0000(	483	12.08028	chr1:3732	KRT18P28		Pseudoger	chr1:182959074-182

ENSG0000(	483	12.08028	chr1:3732	ENSG00000238270		lncRNA	chr1:189868001-189
ENSG0000(	483	12.08028	chr1:3732	DHX9-AS1		lncRNA	chr1:182837185-182
ENSG0000(	483	12.08028	chr1:3732	PDC-AS1		lncRNA	chr1:186423481-186
ENSG0000(	483	12.08028	chr1:3732	AL136987.1		smallRNA	chr1:192491128-192
ENSG0000(	483	12.08028	chr1:3732	LINC01031		lncRNA	chr1:193304745-193
ENSG0000(	483	12.08028	chr1:3732	ENSG00000241505		lncRNA	chr1:190480379-190
ENSG0000(	483	12.08028	chr1:3732	ENSG00000230987		Pseudoger	chr1:189989570-189
ENSG0000(	483	12.08028	chr1:3732	ENSG00000261817		lncRNA	chr1:180117140-180
ENSG0000(	483	12.08028	chr1:3732	RPS3AP9		Pseudoger	chr1:188694320-188
ENSG0000(	483	12.08028	chr1:3732	Y_RNA		smallRNA	chr1:185257911-185
ENSG0000(	483	12.08028	chr1:3732	LINC01633		lncRNA	chr1:184999710-185
ENSG0000(	483	12.08028	chr1:3732	ENSG00000226723		Pseudoger	chr1:192246708-192
ENSG0000(	483	12.08028	chr1:3732	Y_RNA		smallRNA	chr1:185634073-185
ENSG0000(	483	12.08028	chr1:3732	ENSG00000236025		Pseudoger	chr1:190781787-190
ENSG0000(	483	12.08028	chr1:3732	SMG7-AS1		lncRNA	chr1:183460874-183
ENSG0000(	483	12.08028	chr1:3732	CFHR1		protein_c	chr1:196819731-196
ENSG0000(	483	12.08028	chr1:3732	ENSG00000285894		lncRNA	chr1:188013642-188
ENSG0000(	483	12.08028	chr1:3732	LINC01701		lncRNA	chr1:189775465-189
ENSG0000(	483	12.08028	chr1:3732	ENSG00000287452		lncRNA	chr1:181962889-181
ENSG0000(	483	12.08028	chr1:3732	ENSG00000287472		lncRNA	chr1:189868381-189
ENSG0000(	483	12.08028	chr1:3732	ENSG00000228238		Pseudoger	chr1:186578279-186
ENSG0000(	483	12.08028	chr1:3732	MIR3121		smallRNA	chr1:180438314-180
ENSG0000(	483	12.08028	chr1:3732	FTH1P25		Pseudoger	chr1:185071567-185
ENSG0000(	483	12.08028	chr1:3732	ENSG00000261182		lncRNA	chr1:188218400-188
ENSG0000(	483	12.08028	chr1:3732	ENSG00000279838		TEC	chr1:185292384-185
ENSG0000(	483	12.08028	chr1:3732	ENSG00000273198		lncRNA	chr1:186521773-186
ENSG0000(	483	12.08028	chr1:3732	LAMC1	NCGv7	protein_c	chr1:183023420-183
ENSG0000(	479	11.98024	chr1:1166	PRAMEF12		protein_c	chr1:12773738-1277
ENSG0000(	479	11.98024	chr1:1166	ENSG00000289380		lncRNA	chr1:13757778-1375
ENSG0000(	479	11.98024	chr1:1166	ENSG00000288927		lncRNA	chr1:12618389-1261
ENSG0000(	479	11.98024	chr1:1166	XX-FW84067D5.2		Pseudoger	chr1:13305955-1330
ENSG0000(	479	11.98024	chr1:1166	RP11-219C24.10		lncRNA	chr1:13324039-1332
ENSG0000(	479	11.98024	chr1:1166	PRAMEF1		protein_c	chr1:12791397-1279
ENSG0000(	479	11.98024	chr1:1166	snoU13		smallRNA	chr1:12739736-1273
ENSG0000(	479	11.98024	chr1:1166	PRAMEF30P		Pseudoger	chr1:12838125-1284
ENSG0000(	479	11.98024	chr1:1166	PRAMEF8		protein_c	chr1:13281035-1328
ENSG0000(	479	11.98024	chr1:1166	PRDM2	NCGv7	protein_c	chr1:13700188-1382
ENSG0000(	479	11.98024	chr1:1166	RNU6-771P		smallRNA	chr1:13279125-1327
ENSG0000(	479	11.98024	chr1:1166	ENSG00000226166		Pseudoger	chr1:12692909-1269
ENSG0000(	479	11.98024	chr1:1166	PRAMEF13		protein_c	chr1:13341907-1334
ENSG0000(	479	11.98024	chr1:1166	DHRS3		protein_c	chr1:12567910-1261
ENSG0000(	479	11.98024	chr1:1166	PRAMEF19		protein_c	chr1:13368431-1337
ENSG0000(	479	11.98024	chr1:1166	RP11-248D7.2		Pseudoger	chr1:13245863-1324
ENSG0000(	479	11.98024	chr1:1166	PRAMEF14		protein_c	chr1:13341892-1334
ENSG0000(	479	11.98024	chr1:1166	TBCAP2		Pseudoger	chr1:14692129-1469
ENSG0000(	479	11.98024	chr1:1166	RP11-219C24.6		Pseudoger	chr1:13305955-1330
ENSG0000(	479	11.98024	chr1:1166	ENSG00000231606		lncRNA	chr1:14221887-1430
ENSG0000(	479	11.98024	chr1:1166	RNU6-1265P		smallRNA	chr1:14124233-1412
ENSG0000(	479	11.98024	chr1:1166	PRAMEF28P		Pseudoger	chr1:13342528-1334
ENSG0000(	479	11.98024	chr1:1166	AL359771.1		smallRNA	chr1:13623902-1362
ENSG0000(	479	11.98024	chr1:1166	PRAMEF26		protein_c	chr1:13148905-1315

ENSG0000(	479	11.98024	chr1:1166	LRRC38		protein_c	chr1:13474973-1351
ENSG0000(	479	11.98024	chr1:1166	PRAMEF32P		Pseudoger	chr1:13273539-1327
ENSG0000(	479	11.98024	chr1:1166	ENSG00000259961		lncRNA	chr1:13513220-1351
ENSG0000(	479	11.98024	chr1:1166	PDPN		protein_c	chr1:13583465-1361
ENSG0000(	479	11.98024	chr1:1166	KAZN-AS1		lncRNA	chr1:14338825-1441
ENSG0000(	479	11.98024	chr1:1166	PRAMEF21		protein_c	chr1:13410450-1342
ENSG0000(	479	11.98024	chr1:1166	PRAMEF11		protein_c	chr1:12824610-1283
ENSG0000(	479	11.98024	chr1:1166	PRAMEF9		protein_c	chr1:13315581-1332
ENSG0000(	479	11.98024	chr1:1166	PRAMEF16		protein_c	chr1:13389628-1339
ENSG0000(	479	11.98024	chr1:1166	PRAMEF18		protein_c	chr1:13369067-1337
ENSG0000(	479	11.98024	chr1:1166	AADACL4	NCGv7	protein_c	chr1:12644085-1266
ENSG0000(	479	11.98024	chr1:1166	HNRNPCL1	NCGv7	protein_c	chr1:12847377-1284
ENSG0000(	479	11.98024	chr1:1166	PRAMEF5		protein_c	chr1:13254198-1326
ENSG0000(	479	11.98024	chr1:1166	RNU6ATAC18P		smallRNA	chr1:12569972-1257
ENSG0000(	479	11.98024	chr1:1166	SNORA59A	AC	smallRNA	chr1:12507246-1250
ENSG0000(	479	11.98024	chr1:1166	PRAMEF17		protein_c	chr1:13389632-1339
ENSG0000(	479	11.98024	chr1:1166	CFAP107		protein_c	chr1:12746200-1276
ENSG0000(	479	11.98024	chr1:1166	KAZN		protein_c	chr1:13892792-1511
ENSG0000(	479	11.98024	chr1:1166	LINC02766		lncRNA	chr1:12525716-1253
ENSG0000(	479	11.98024	chr1:1166	LINC01784		lncRNA	chr1:12822686-1282
ENSG0000(	479	11.98024	chr1:1166	ENSG00000287756		lncRNA	chr1:14774469-1477
ENSG0000(	479	11.98024	chr1:1166	PRAMEF20		protein_c	chr1:13410450-1342
ENSG0000(	479	11.98024	chr1:1166	AADACL3		protein_c	chr1:12716110-1272
ENSG0000(	479	11.98024	chr1:1166	ENSG00000272482		lncRNA	chr1:12618900-1261
ENSG0000(	479	11.98024	chr1:1166	BRWD1P1		Pseudoger	chr1:13555001-1355
ENSG0000(	479	11.98024	chr1:1166	ENSG00000237445		lncRNA	chr1:13657311-1365
ENSG0000(	479	11.98024	chr1:1166	RNA5SP41		Pseudoger	chr1:13623184-1362
ENSG0000(	475	11.88019	chr2:4707	ACA59		smallRNA	chr2:63883249-6388
ENSG0000(	474	11.85518	chr3:8573	HMGN2P25		Pseudoger	chr3:141865012-141
ENSG0000(	470	11.75514	chr2:4707	RPL21P36		Pseudoger	chr2:36299388-3629
ENSG0000(	468	11.70511	chr12:109	DYNLL1P4		Pseudoger	chr12:113789542-11
ENSG0000(	464	11.60507	chr1:1166	RNU5E-1		smallRNA	chr1:11908152-1190
ENSG0000(	464	11.60507	chr1:1166	SNORA70		smallRNA	chr1:12221148-1222
ENSG0000(	464	11.60507	chr1:1166	ENSG00000285604		lncRNA	chr1:12088441-1209
ENSG0000(	464	11.60507	chr1:1166	ENSG00000287384		lncRNA	chr1:11979533-1198
ENSG0000(	464	11.60507	chr1:1166	SBF1P2		Pseudoger	chr1:11877770-1188
ENSG0000(	464	11.60507	chr1:1166	TNFRSF8	NCGv7	protein_c	chr1:12063303-1214
ENSG0000(	464	11.60507	chr1:1166	NPPB		protein_c	chr1:11857464-1185
ENSG0000(	464	11.60507	chr1:1166	C1orf167-AS1		lncRNA	chr1:11777077-1177
ENSG0000(	464	11.60507	chr1:1166	DRAXIN		protein_c	chr1:11691710-1172
ENSG0000(	464	11.60507	chr1:1166	TNFRSF1B	NCGv7;AC	protein_c	chr1:12166991-1220
ENSG0000(	464	11.60507	chr1:1166	ENSG00000285646		lncRNA	chr1:11907940-1191
ENSG0000(	464	11.60507	chr1:1166	CLCN6		protein_c	chr1:11806096-1184
ENSG0000(	464	11.60507	chr1:1166	VPS13D		protein_c	chr1:12230030-1251
ENSG0000(	464	11.60507	chr1:1166	MTHFR	NCGv7	protein_c	chr1:11785723-1180
ENSG0000(	464	11.60507	chr1:1166	RNU6-777P		smallRNA	chr1:12077881-1207
ENSG0000(	464	11.60507	chr1:1166	FBXO6		protein_c	chr1:11664200-1167
ENSG0000(	464	11.60507	chr1:1166	C1orf167		protein_c	chr1:11761787-1178
ENSG0000(	464	11.60507	chr1:1166	AGTRAP		protein_c	chr1:11736084-1175
ENSG0000(	464	11.60507	chr1:1166	MFN2		protein_c	chr1:11980181-1201
ENSG0000(	464	11.60507	chr1:1166	NPPA		protein_c	chr1:11845709-1184

ENSG0000(	464	11.60507	chr1:116(	RNU5E-4P	smallRNA	chr1:11909808-1190
ENSG0000(	464	11.60507	chr1:116(	KIAA2013	protein_c	chr1:11919591-1192
ENSG0000(	464	11.60507	chr1:116(	RN7SL649P	smallRNA	chr1:12036742-1203
ENSG0000(	464	11.60507	chr1:116(	PLOD1	protein_c	chr1:11934205-1197
ENSG0000(	464	11.60507	chr1:116(	MIR4632	smallRNA	chr1:12191713-1219
ENSG0000(	464	11.60507	chr1:116(	RPL10P17	Pseudoger	chr1:12220794-1222
ENSG0000(	464	11.60507	chr1:116(	MIIP	protein_c	chr1:12019466-1203
ENSG0000(	464	11.60507	chr1:116(	RPL23AP89	Pseudoger	chr1:12080293-1208
ENSG0000(	464	11.60507	chr1:116(	ENSG00000270914	Pseudoger	chr1:12017216-1201
ENSG0000(	464	11.60507	chr1:116(	Y_RNA	smallRNA	chr1:12024012-1202
ENSG0000(	462	11.55505	chr5:169(	AC008391.1	smallRNA	chr5:56457038-5645
ENSG0000(	461	11.53004	chr12:10(	RN7SL133P	smallRNA	chr12:123016180-12
ENSG0000(	461	11.53004	chr12:10(	LINC00507	lncRNA	chr12:127914707-12
ENSG0000(	461	11.53004	chr12:10(	ENSG00000257658	Pseudoger	chr12:112321759-11
ENSG0000(	461	11.53004	chr12:10(	RNU6-1188P	smallRNA	chr12:116082570-11
ENSG0000(	461	11.53004	chr12:10(	ENSG00000256695	lncRNA	chr12:120389502-12
ENSG0000(	461	11.53004	chr12:10(	LINC02393	lncRNA	chr12:127881616-12
ENSG0000(	461	11.53004	chr12:10(	LINC02463	lncRNA	chr12:115810359-11
ENSG0000(	461	11.53004	chr12:10(	SNORA9	smallRNA	chr12:123616708-12
ENSG0000(	461	11.53004	chr12:10(	TMEM132D-AS2	lncRNA	chr12:129208601-12
ENSG0000(	461	11.53004	chr12:10(	ENSG00000257997	lncRNA	chr12:114080381-11
ENSG0000(	461	11.53004	chr12:10(	SNRPGP18	Pseudoger	chr12:116169045-11
ENSG0000(	461	11.53004	chr12:10(	LINC02376	lncRNA	chr12:127274265-12
ENSG0000(	461	11.53004	chr12:10(	ENSG00000256827	Pseudoger	chr12:124005871-12
ENSG0000(	461	11.53004	chr12:10(	ENSG00000256249	lncRNA	chr12:122687125-12
ENSG0000(	461	11.53004	chr12:10(	AC002395.1	smallRNA	chr12:111487478-11
ENSG0000(	461	11.53004	chr12:10(	ENSG00000258034	lncRNA	chr12:115961187-11
ENSG0000(	461	11.53004	chr12:10(	RNU4-1	smallRNA	chr12:120293097-12
ENSG0000(	461	11.53004	chr12:10(	LINC02441	lncRNA	chr12:128023788-12
ENSG0000(	461	11.53004	chr12:10(	ENSG00000256286	lncRNA	chr12:126874804-12
ENSG0000(	461	11.53004	chr12:10(	ENSG00000256630	Pseudoger	chr12:128437778-12
ENSG0000(	461	11.53004	chr12:10(	LINC02457	lncRNA	chr12:116482073-11
ENSG0000(	461	11.53004	chr12:10(	LINC02985	lncRNA	chr12:122063306-12
ENSG0000(	461	11.53004	chr12:10(	LINC02419	lncRNA	chr12:130070325-13
ENSG0000(	461	11.53004	chr12:10(	ENSG00000257603	lncRNA	chr12:114077133-11
ENSG0000(	461	11.53004	chr12:10(	ENSG00000257624	Pseudoger	chr12:112000739-11
ENSG0000(	461	11.53004	chr12:10(	SDSL	protein_c	chr12:113422380-11
ENSG0000(	461	11.53004	chr12:10(	LINC02368	lncRNA	chr12:128116730-12
ENSG0000(	461	11.53004	chr12:10(	ENSG00000289831	lncRNA	chr12:122423304-12
ENSG0000(	461	11.53004	chr12:10(	ENSG00000257654	lncRNA	chr12:116698336-11
ENSG0000(	461	11.53004	chr12:10(	ENSG00000256884	lncRNA	chr12:119174065-11
ENSG0000(	461	11.53004	chr12:10(	ENSG00000256609	lncRNA	chr12:119182048-11
ENSG0000(	461	11.53004	chr12:10(	ENSG00000256861	protein_c	chr12:122207779-12
ENSG0000(	461	11.53004	chr12:10(	GATC	protein_c	chr12:120446444-12
ENSG0000(	461	11.53004	chr12:10(	MIR3657	smallRNA	chr12:112037599-11
ENSG0000(	461	11.53004	chr12:10(	LINC02825	lncRNA	chr12:126400792-12
ENSG0000(	461	11.53004	chr12:10(	RNU6-558P	smallRNA	chr12:116782105-11
ENSG0000(	461	11.53004	chr12:10(	AC069240.1	smallRNA	chr12:114550762-11
ENSG0000(	461	11.53004	chr12:10(	ENSG00000256732	lncRNA	chr12:126609704-12
ENSG0000(	461	11.53004	chr12:10(	ENSG00000289101	lncRNA	chr12:114445798-11
ENSG0000(	461	11.53004	chr12:10(	MIR3612	smallRNA	chr12:128294092-12

ENSG0000(	461	11.53004	chr12:10(	GLULP5	Pseudoger	chr12:114104542-11
ENSG0000(	461	11.53004	chr12:10(	ENSG00000256502	Pseudoger	chr12:127344258-12
ENSG0000(	461	11.53004	chr12:10(	TBX3-AS1	lncRNA	chr12:114682292-11
ENSG0000(	461	11.53004	chr12:10(	LINC02423	lncRNA	chr12:118758217-11
ENSG0000(	461	11.53004	chr12:10(	Y_RNA	smallRNA	chr12:120504571-12
ENSG0000(	461	11.53004	chr12:10(	AC002070.1	smallRNA	chr12:119580792-11
ENSG0000(	461	11.53004	chr12:10(	ENSG00000257883	lncRNA	chr12:116661582-11
ENSG0000(	461	11.53004	chr12:10(	RN7SKP216	smallRNA	chr12:114504876-11
ENSG0000(	461	11.53004	chr12:10(	ABCB9	protein_c	chr12:122920951-12
ENSG0000(	461	11.53004	chr12:10(	RILPL2	protein_c	chr12:123415039-12
ENSG0000(	461	11.53004	chr12:10(	DHX37	protein_c	chr12:124946825-12
ENSG0000(	461	11.53004	chr12:10(	UBC	protein_c	chr12:124911604-12
ENSG0000(	461	11.53004	chr12:10(	ENSG00000256811	lncRNA	chr12:121856259-12
ENSG0000(	461	11.53004	chr12:10(	MIR4419B	smallRNA	chr12:128244506-12
ENSG0000(	461	11.53004	chr12:10(	MAPKAPK5-AS1	lncRNA	chr12:111839758-11
ENSG0000(	461	11.53004	chr12:10(	LHX5-AS1	lncRNA	chr12:113471981-11
ENSG0000(	461	11.53004	chr12:10(	ENSG00000256596	lncRNA	chr12:124206228-12
ENSG0000(	461	11.53004	chr12:10(	SCARB1	protein_c	chr12:124776856-12
ENSG0000(	461	11.53004	chr12:10(	ENSG00000257726	lncRNA	chr12:115363012-11
ENSG0000(	461	11.53004	chr12:10(	ENSG00000257767	protein_c	chr12:111753890-11
ENSG0000(	461	11.53004	chr12:10(	ENSG00000257958	lncRNA	chr12:115077325-11
ENSG0000(	461	11.53004	chr12:10(	ENSG00000257781	lncRNA	chr12:115755262-11
ENSG0000(	461	11.53004	chr12:10(	RNF10	protein_c	chr12:120533480-12
ENSG0000(	461	11.53004	chr12:10(	ENSG00000256814	lncRNA	chr12:125150058-12
ENSG0000(	461	11.53004	chr12:10(	ENSG00000256496	Pseudoger	chr12:127317709-12
ENSG0000(	461	11.53004	chr12:10(	MIR3908	smallRNA	chr12:123536409-12
ENSG0000(	461	11.53004	chr12:10(	Y_RNA	smallRNA	chr12:112069104-11
ENSG0000(	461	11.53004	chr12:10(	MIR4472-2	smallRNA	chr12:116428252-11
ENSG0000(	461	11.53004	chr12:10(	ENSG00000288863	lncRNA	chr12:113361328-11
ENSG0000(	461	11.53004	chr12:10(	AC004812.1	smallRNA	chr12:120172039-12
ENSG0000(	461	11.53004	chr12:10(	KDM2B-DT	lncRNA	chr12:121580792-12
ENSG0000(	461	11.53004	chr12:10(	Y_RNA	smallRNA	chr12:113156765-11
ENSG0000(	461	11.53004	chr12:10(	ENSG00000257519	lncRNA	chr12:115263170-11
ENSG0000(	461	11.53004	chr12:10(	SLC25A3P2	Pseudoger	chr12:111992996-11
ENSG0000(	461	11.53004	chr12:10(	RITA1	protein_c	chr12:113185526-11
ENSG0000(	461	11.53004	chr12:10(	LINC02375	lncRNA	chr12:127324152-12
ENSG0000(	461	11.53004	chr12:10(	SNORA70	smallRNA	chr12:121107043-12
ENSG0000(	461	11.53004	chr12:10(	ENSG00000256364	lncRNA	chr12:120697124-12
ENSG0000(	461	11.53004	chr12:10(	ENSG00000257025	lncRNA	chr12:128399978-12
ENSG0000(	461	11.53004	chr12:10(	RHOF	protein_c	chr12:121777754-12
ENSG0000(	461	11.53004	chr12:10(	RNA5SP374	Pseudoger	chr12:118829681-11
ENSG0000(	461	11.53004	chr12:10(	HSPE1P20	Pseudoger	chr12:126912034-12
ENSG0000(	461	11.53004	chr12:10(	MIR4304	smallRNA	chr12:123010667-12
ENSG0000(	461	11.53004	chr12:10(	RN7SKP71	smallRNA	chr12:112267077-11
ENSG0000(	461	11.53004	chr12:10(	ENSG00000289401	lncRNA	chr12:120572901-12
ENSG0000(	461	11.53004	chr12:10(	Y_RNA	smallRNA	chr12:112149360-11
ENSG0000(	461	11.53004	chr12:10(	LINC00508	lncRNA	chr12:127933689-12
ENSG0000(	461	11.53004	chr12:10(	ENSG00000257407	lncRNA	chr12:115582061-11
ENSG0000(	461	11.53004	chr12:10(	ENSG00000259862	lncRNA	chr12:130249679-13
ENSG0000(	461	11.53004	chr12:10(	ENSG00000257452	lncRNA	chr12:112907628-11
ENSG0000(	461	11.53004	chr12:10(	ELOCP32	Pseudoger	chr12:117245551-11

ENSG0000(	461	11.53004	chr12:10(	ENSG00000289940		lncRNA	chr12:124742270-12
ENSG0000(	461	11.53004	chr12:10(	ENSG00000257035		Pseudoger	chr12:127624153-12
ENSG0000(	461	11.53004	chr12:10(	SNORA38		smallRNA	chr12:118888434-11
ENSG0000(	461	11.53004	chr12:10(	ENSG00000261650		lncRNA	chr12:130024493-13
ENSG0000(	461	11.53004	chr12:10(	SNORD56		smallRNA	chr12:115852648-11
ENSG0000(	461	11.53004	chr12:10(	ENSG00000223538		Pseudoger	chr12:121107220-12
ENSG0000(	461	11.53004	chr12:10(	ENSG00000257095		lncRNA	chr12:119031039-11
ENSG0000(	461	11.53004	chr12:10(	ENSG00000257279		lncRNA	chr12:116977442-11
ENSG0000(	461	11.53004	chr12:10(	MIR4700		smallRNA	chr12:120723193-12
ENSG0000(	461	11.53004	chr12:10(	SBNO1		protein_c	chr12:123289109-12
ENSG0000(	461	11.53004	chr12:10(	ENSG00000257286		lncRNA	chr12:113185624-11
ENSG0000(	461	11.53004	chr12:10(	HAUS8P1		Pseudoger	chr12:114097682-11
ENSG0000(	461	11.53004	chr12:10(	MORN3		protein_c	chr12:121648742-12
ENSG0000(	461	11.53004	chr12:10(	ENSG00000257359		lncRNA	chr12:113932569-11
ENSG0000(	461	11.53004	chr12:10(	SETD1B	NCGv7	protein_c	chr12:121804009-12
ENSG0000(	461	11.53004	chr12:10(	AC060226.1		smallRNA	chr12:116255883-11
ENSG0000(	461	11.53004	chr12:10(	ADAM1B		Pseudoger	chr12:111927018-11
ENSG0000(	461	11.53004	chr12:10(	VPS33A		protein_c	chr12:122229564-12
ENSG0000(	461	11.53004	chr12:10(	VPS37B		protein_c	chr12:122865330-12
ENSG0000(	461	11.53004	chr12:10(	ENSG00000290033		lncRNA	chr12:116817906-11
ENSG0000(	461	11.53004	chr12:10(	IL31		protein_c	chr12:122172029-12
ENSG0000(	461	11.53004	chr12:10(	ENSG00000256963		Pseudoger	chr12:121079842-12
ENSG0000(	461	11.53004	chr12:10(	Y_RNA		smallRNA	chr12:122768099-12
ENSG0000(	461	11.53004	chr12:10(	ENSG00000258435		lncRNA	chr12:121391962-12
ENSG0000(	461	11.53004	chr12:10(	PCNPP1		Pseudoger	chr12:111669852-11
ENSG0000(	461	11.53004	chr12:10(	ENSG00000257494		lncRNA	chr12:112256800-11
ENSG0000(	461	11.53004	chr12:10(	RNU4-2		smallRNA	chr12:120291763-12
ENSG0000(	461	11.53004	chr12:10(	ENSG00000257517		lncRNA	chr12:114768674-11
ENSG0000(	461	11.53004	chr12:10(	LINC02369		lncRNA	chr12:128086621-12
ENSG0000(	461	11.53004	chr12:10(	TMEM132B	NCGv7	protein_c	chr12:125186386-12
ENSG0000(	461	11.53004	chr12:10(	MIR5188		smallRNA	chr12:124915547-12
ENSG0000(	461	11.53004	chr12:10(	ENSG00000256569		lncRNA	chr12:120721507-12
ENSG0000(	461	11.53004	chr12:10(	NDUFA5P6		Pseudoger	chr12:126524248-12
ENSG0000(	461	11.53004	chr12:10(	ENSG00000258346		lncRNA	chr12:116368764-11
ENSG0000(	461	11.53004	chr12:10(	Y_RNA		smallRNA	chr12:113178423-11
ENSG0000(	461	11.53004	chr12:10(	RNA5SP375		Pseudoger	chr12:123282916-12
ENSG0000(	461	11.53004	chr12:10(	SLC15A4		protein_c	chr12:128793194-12
ENSG0000(	461	11.53004	chr12:10(	ENSG00000256298		lncRNA	chr12:130047132-13
ENSG0000(	461	11.53004	chr12:10(	MPHOSPH9		protein_c	chr12:123152320-12
ENSG0000(	461	11.53004	chr12:10(	Y_RNA		smallRNA	chr12:127619336-12
ENSG0000(	461	11.53004	chr12:10(	NLRP9P1		Pseudoger	chr12:129013336-12
ENSG0000(	461	11.53004	chr12:10(	RPL35AP30		Pseudoger	chr12:119959424-11
ENSG0000(	461	11.53004	chr12:10(	ATXN2-AS		lncRNA	chr12:111599498-11
ENSG0000(	461	11.53004	chr12:10(	DENR		protein_c	chr12:122752824-12
ENSG0000(	461	11.53004	chr12:10(	LINC02459		lncRNA	chr12:114238970-11
ENSG0000(	461	11.53004	chr12:10(	ATXN2		protein_c	chr12:111443485-11
ENSG0000(	461	11.53004	chr12:10(	IMMP1LP2		Pseudoger	chr12:113015607-11
ENSG0000(	461	11.53004	chr12:10(	ENSG00000256343		lncRNA	chr12:130651342-13
ENSG0000(	461	11.53004	chr12:10(	SRRM4		protein_c	chr12:118981541-11
ENSG0000(	461	11.53004	chr12:10(	ENSG00000258108		lncRNA	chr12:115569394-11
ENSG0000(	461	11.53004	chr12:10(	OSTF1P1		Pseudoger	chr12:114588194-11

ENSG0000(	461	11.53004	chr12:10(	ZCCHC8	NCGv7	protein_c	chr12:122471599-12
ENSG0000(	461	11.53004	chr12:10(	MAP1LC3B2		protein_c	chr12:116548105-11
ENSG0000(	461	11.53004	chr12:10(	ENSG00000258337		lncRNA	chr12:116174502-11
ENSG0000(	461	11.53004	chr12:10(	ENSG00000258249		lncRNA	chr12:116599270-11
ENSG0000(	461	11.53004	chr12:10(	ENSG00000258254		lncRNA	chr12:114621761-11
ENSG0000(	461	11.53004	chr12:10(	RN7SL534P		smallRNA	chr12:129779308-12
ENSG0000(	461	11.53004	chr12:10(	ENSG00000256950		protein_c	chr12:121888809-12
ENSG0000(	461	11.53004	chr12:10(	TESC-AS1		lncRNA	chr12:117099481-11
ENSG0000(	461	11.53004	chr12:10(	ENSG00000256311		lncRNA	chr12:119225834-11
ENSG0000(	461	11.53004	chr12:10(	ENSG00000258323		lncRNA	chr12:112063909-11
ENSG0000(	461	11.53004	chr12:10(	CLIP1-AS1		lncRNA	chr12:122395542-12
ENSG0000(	461	11.53004	chr12:10(	ENSG00000271184		Pseudoger	chr12:125900421-12
ENSG0000(	461	11.53004	chr12:10(	RPL21P1		Pseudoger	chr12:122364782-12
ENSG0000(	461	11.53004	chr12:10(	AC026366.1		smallRNA	chr12:118166799-11
ENSG0000(	461	11.53004	chr12:10(	PXN		protein_c	chr12:120210439-12
ENSG0000(	461	11.53004	chr12:10(	ENSG00000111780		protein_c	chr12:120438198-12
ENSG0000(	461	11.53004	chr12:10(	RPLP0		protein_c	chr12:120196699-12
ENSG0000(	461	11.53004	chr12:10(	SRSF9	NCGv7	protein_c	chr12:120461672-12
ENSG0000(	461	11.53004	chr12:10(	HECTD4		protein_c	chr12:112160188-11
ENSG0000(	461	11.53004	chr12:10(	GCN1		protein_c	chr12:120127202-12
ENSG0000(	461	11.53004	chr12:10(	OAS1		protein_c	chr12:112905856-11
ENSG0000(	461	11.53004	chr12:10(	ENSG00000280272		TEC	chr12:126141952-12
ENSG0000(	461	11.53004	chr12:10(	LHX5		protein_c	chr12:113462033-11
ENSG0000(	461	11.53004	chr12:10(	RNU6-927P		smallRNA	chr12:124901512-12
ENSG0000(	461	11.53004	chr12:10(	KDM2B	NCGv7	protein_c	chr12:121429096-12
ENSG0000(	461	11.53004	chr12:10(	SLC8B1		protein_c	chr12:113298759-11
ENSG0000(	461	11.53004	chr12:10(	SNRNP35	NCGv7	protein_c	chr12:123458088-12
ENSG0000(	461	11.53004	chr12:10(	ANAPC5		protein_c	chr12:121308245-12
ENSG0000(	461	11.53004	chr12:10(	ENSG00000287112		lncRNA	chr12:127484243-12
ENSG0000(	461	11.53004	chr12:10(	MTRFR		protein_c	chr12:123233385-12
ENSG0000(	461	11.53004	chr12:10(	P2RX7		protein_c	chr12:121132819-12
ENSG0000(	461	11.53004	chr12:10(	HIP1R		protein_c	chr12:122834453-12
ENSG0000(	461	11.53004	chr12:10(	MAPKAPK5	NCGv7	protein_c	chr12:111842228-11
ENSG0000(	461	11.53004	chr12:10(	RPL6		protein_c	chr12:112405189-11
ENSG0000(	461	11.53004	chr12:10(	ENSG00000279343		TEC	chr12:130350996-13
ENSG0000(	461	11.53004	chr12:10(	SIRT4	NCGv7	protein_c	chr12:120302316-12
ENSG0000(	461	11.53004	chr12:10(	RPH3A		protein_c	chr12:112570380-11
ENSG0000(	461	11.53004	chr12:10(	PEBP1		protein_c	chr12:118136124-11
ENSG0000(	461	11.53004	chr12:10(	TPCN1		protein_c	chr12:113221050-11
ENSG0000(	461	11.53004	chr12:10(	snoU13		smallRNA	chr12:120048430-12
ENSG0000(	461	11.53004	chr12:10(	ENSG00000280196		TEC	chr12:129839928-12
ENSG0000(	461	11.53004	chr12:10(	LINC02359		lncRNA	chr12:126094112-12
ENSG0000(	461	11.53004	chr12:10(	ENSG00000269938		lncRNA	chr12:123968023-12
ENSG0000(	461	11.53004	chr12:10(	ENSG00000279527		TEC	chr12:124529722-12
ENSG0000(	461	11.53004	chr12:10(	NOS1	NCGv7	protein_c	chr12:117208142-11
ENSG0000(	461	11.53004	chr12:10(	ENSG00000279500		TEC	chr12:128813186-12
ENSG0000(	461	11.53004	chr12:10(	ERP29		protein_c	chr12:112013348-11
ENSG0000(	461	11.53004	chr12:10(	SUDS3		protein_c	chr12:118376555-11
ENSG0000(	461	11.53004	chr12:10(	RPL31P52		Pseudoger	chr12:120450437-12
ENSG0000(	461	11.53004	chr12:10(	TBX5		protein_c	chr12:114353911-11
ENSG0000(	461	11.53004	chr12:10(	ENSG00000279478		TEC	chr12:130383364-13

ENSG0000(	461	11.53004	chr12:109	PRKAB1		protein_c	chr12:119667864-11
ENSG0000(	461	11.53004	chr12:109	ENSG00000279475		TEC	chr12:127825938-12
ENSG0000(	461	11.53004	chr12:109	IFITM3P5		Pseudoger	chr12:111581379-11
ENSG0000(	461	11.53004	chr12:109	RPS20P31		Pseudoger	chr12:120247460-12
ENSG0000(	461	11.53004	chr12:109	ENSG00000279462		TEC	chr12:125300936-12
ENSG0000(	461	11.53004	chr12:109	BRAP		protein_c	chr12:111642146-11
ENSG0000(	461	11.53004	chr12:109	RAB35		protein_c	chr12:120095099-12
ENSG0000(	461	11.53004	chr12:109	COX6A1		protein_c	chr12:120438090-12
ENSG0000(	461	11.53004	chr12:109	TESC	NCGv7	protein_c	chr12:117038923-11
ENSG0000(	461	11.53004	chr12:109	DYNLL1	NCGv7	protein_c	chr12:120469850-12
ENSG0000(	461	11.53004	chr12:109	CCDC62		protein_c	chr12:122774526-12
ENSG0000(	461	11.53004	chr12:109	ENSG00000274859		lncRNA	chr12:118066398-11
ENSG0000(	461	11.53004	chr12:109	ENSG00000280398		TEC	chr12:126130166-12
ENSG0000(	461	11.53004	chr12:109	RNU7-170P		smallRNA	chr12:121908103-12
ENSG0000(	461	11.53004	chr12:109	ENSG00000213144		Pseudoger	chr12:119194850-11
ENSG0000(	461	11.53004	chr12:109	ENSG00000286991		lncRNA	chr12:115878748-11
ENSG0000(	461	11.53004	chr12:109	ENSG00000280405		TEC	chr12:130144954-13
ENSG0000(	461	11.53004	chr12:109	RPL7AP60		Pseudoger	chr12:112301870-11
ENSG0000(	461	11.53004	chr12:109	ATP6V0A2		protein_c	chr12:123712353-12
ENSG0000(	461	11.53004	chr12:109	KNTC1		protein_c	chr12:122527246-12
ENSG0000(	461	11.53004	chr12:109	ENSG00000280415		TEC	chr12:125858862-12
ENSG0000(	461	11.53004	chr12:109	ENSG00000280444		TEC	chr12:124623391-12
ENSG0000(	461	11.53004	chr12:109	TMED2		protein_c	chr12:123584533-12
ENSG0000(	461	11.53004	chr12:109	MIR1178		smallRNA	chr12:119713634-11
ENSG0000(	461	11.53004	chr12:109	DNAH10OS		lncRNA	chr12:123925461-12
ENSG0000(	461	11.53004	chr12:109	ENSG00000275759		lncRNA	chr12:118428281-11
ENSG0000(	461	11.53004	chr12:109	RPL11P5		Pseudoger	chr12:120594015-12
ENSG0000(	461	11.53004	chr12:109	POP5		protein_c	chr12:120578764-12
ENSG0000(	461	11.53004	chr12:109	ENSG00000279001		TEC	chr12:121096027-12
ENSG0000(	461	11.53004	chr12:109	MIR1302-1		smallRNA	chr12:112695034-11
ENSG0000(	461	11.53004	chr12:109	THRIL		lncRNA	chr12:125025434-12
ENSG0000(	461	11.53004	chr12:109	ENSG00000286922		lncRNA	chr12:127486938-12
ENSG0000(	461	11.53004	chr12:109	ARF1P2		Pseudoger	chr12:120845110-12
ENSG0000(	461	11.53004	chr12:109	ENSG00000279071		TEC	chr12:124537844-12
ENSG0000(	461	11.53004	chr12:109	ENSG00000279334		TEC	chr12:124561869-12
ENSG0000(	461	11.53004	chr12:109	ENSG00000279180		TEC	chr12:128288159-12
ENSG0000(	461	11.53004	chr12:109	ENSG00000280300		TEC	chr12:123754246-12
ENSG0000(	461	11.53004	chr12:109	ENSG00000287622		lncRNA	chr12:127563908-12
ENSG0000(	461	11.53004	chr12:109	CLIP1	NCGv7	protein_c	chr12:122271432-12
ENSG0000(	461	11.53004	chr12:109	HCAR2		protein_c	chr12:122701293-12
ENSG0000(	461	11.53004	chr12:109	LINC02372		lncRNA	chr12:126869010-12
ENSG0000(	461	11.53004	chr12:109	ENSG00000280354		TEC	chr12:124542503-12
ENSG0000(	461	11.53004	chr12:109	ENSG00000279233		TEC	chr12:125138245-12
ENSG0000(	461	11.53004	chr12:109	ENSG00000275409		lncRNA	chr12:118430147-11
ENSG0000(	461	11.53004	chr12:109	ENSG00000279193		TEC	chr12:130121925-13
ENSG0000(	461	11.53004	chr12:109	DNAH10		protein_c	chr12:123762188-12
ENSG0000(	461	11.53004	chr12:109	ENSG00000279087		TEC	chr12:124637999-12
ENSG0000(	461	11.53004	chr12:109	ENSG00000279171		TEC	chr12:126677262-12
ENSG0000(	461	11.53004	chr12:109	ENSG00000279146		TEC	chr12:130465008-13
ENSG0000(	461	11.53004	chr12:109	ENSG00000280364		TEC	chr12:126106459-12
ENSG0000(	461	11.53004	chr12:109	ENSG00000279121		TEC	chr12:124555905-12

ENSG0000(	461	11.53004	chr12:109	ENSG00000275467		lncRNA	chr12:116801023-11
ENSG0000(	461	11.53004	chr12:109	ENSG00000248636		lncRNA	chr12:119361247-11
ENSG0000(	461	11.53004	chr12:109	TMEM120B		protein_c	chr12:121712752-12
ENSG0000(	461	11.53004	chr12:109	ENSG00000280381		TEC	chr12:123087256-12
ENSG0000(	461	11.53004	chr12:109	ENSG00000274874		lncRNA	chr12:123973215-12
ENSG0000(	461	11.53004	chr12:109	ENSG00000284934		protein_c	chr12:122207662-12
ENSG0000(	461	11.53004	chr12:109	IQCD		protein_c	chr12:113195441-11
ENSG0000(	461	11.53004	chr12:109	RPS2P5		Pseudoger	chr12:118246084-11
ENSG0000(	461	11.53004	chr12:109	RFC5		protein_c	chr12:118013588-11
ENSG0000(	461	11.53004	chr12:109	LINC00939		lncRNA	chr12:125958688-12
ENSG0000(	461	11.53004	chr12:109	B3GNT4		protein_c	chr12:122203681-12
ENSG0000(	461	11.53004	chr12:109	ENSG00000287229		lncRNA	chr12:113863402-11
ENSG0000(	461	11.53004	chr12:109	ENSG00000270482		lncRNA	chr12:118375350-11
ENSG0000(	461	11.53004	chr12:109	LINC01089		lncRNA	chr12:121795267-12
ENSG0000(	461	11.53004	chr12:109	ENSG00000270130		lncRNA	chr12:123960717-12
ENSG0000(	461	11.53004	chr12:109	PSMD9		protein_c	chr12:121888732-12
ENSG0000(	461	11.53004	chr12:109	ENSG00000279953		TEC	chr12:123649068-12
ENSG0000(	461	11.53004	chr12:109	NCOR2	NCGv7	protein_c	chr12:124324415-12
ENSG0000(	461	11.53004	chr12:109	ENSG00000270095		lncRNA	chr12:123971457-12
ENSG0000(	461	11.53004	chr12:109	COQ5		protein_c	chr12:120503279-12
ENSG0000(	461	11.53004	chr12:109	ENSG00000279952		TEC	chr12:127462507-12
ENSG0000(	461	11.53004	chr12:109	ENSG00000279931		TEC	chr12:124725524-12
ENSG0000(	461	11.53004	chr12:109	RNU6-1004P		smallRNA	chr12:121604902-12
ENSG0000(	461	11.53004	chr12:109	ENSG00000275265		lncRNA	chr12:122501187-12
ENSG0000(	461	11.53004	chr12:109	ENSG00000272849		lncRNA	chr12:121797511-12
ENSG0000(	461	11.53004	chr12:109	ENSG00000270061		lncRNA	chr12:123969990-12
ENSG0000(	461	11.53004	chr12:109	RPS15AP32		Pseudoger	chr12:113054062-11
ENSG0000(	461	11.53004	chr12:109	ENSG00000286394		lncRNA	chr12:129696599-12
ENSG0000(	461	11.53004	chr12:109	LINC02405		lncRNA	chr12:126915199-12
ENSG0000(	461	11.53004	chr12:109	LINC00943		lncRNA	chr12:126723412-12
ENSG0000(	461	11.53004	chr12:109	SNORA27		smallRNA	chr12:114737704-11
ENSG0000(	461	11.53004	chr12:109	FBXW8		protein_c	chr12:116910950-11
ENSG0000(	461	11.53004	chr12:109	CFAP73		protein_c	chr12:113149724-11
ENSG0000(	461	11.53004	chr12:109	ENSG00000214650		lncRNA	chr12:124513222-12
ENSG0000(	461	11.53004	chr12:109	RNU6-1088P		smallRNA	chr12:120313238-12
ENSG0000(	461	11.53004	chr12:109	ENSG00000280125		TEC	chr12:125808668-12
ENSG0000(	461	11.53004	chr12:109	CCDC60		protein_c	chr12:119334712-11
ENSG0000(	461	11.53004	chr12:109	ENSG00000280117		TEC	chr12:125160525-12
ENSG0000(	461	11.53004	chr12:109	ENSG00000242963		Pseudoger	chr12:130216807-13
ENSG0000(	461	11.53004	chr12:109	ENSG00000287339		lncRNA	chr12:127149955-12
ENSG0000(	461	11.53004	chr12:109	ENSG00000286493		lncRNA	chr12:120904702-12
ENSG0000(	461	11.53004	chr12:109	UNC119B		protein_c	chr12:120710458-12
ENSG0000(	461	11.53004	chr12:109	ENSG00000280138		TEC	chr12:122870059-12
ENSG0000(	461	11.53004	chr12:109	ENSG00000275389		lncRNA	chr12:124085761-12
ENSG0000(	461	11.53004	chr12:109	ENSG00000287365		lncRNA	chr12:127451346-12
ENSG0000(	461	11.53004	chr12:109	ENSG00000280097		TEC	chr12:124593181-12
ENSG0000(	461	11.53004	chr12:109	TMEM132D-AS1		lncRNA	chr12:129109629-12
ENSG0000(	461	11.53004	chr12:109	ENSG00000287311		lncRNA	chr12:128052122-12
ENSG0000(	461	11.53004	chr12:109	ENSG00000280051		TEC	chr12:129262345-12
ENSG0000(	461	11.53004	chr12:109	ENSG00000280024		TEC	chr12:127372243-12
ENSG0000(	461	11.53004	chr12:109	ENSG00000287242		lncRNA	chr12:124786783-12

ENSG0000(	461	11.53004	chr12:10(	ENSG00000287240		lncRNA	chr12:125159331-12
ENSG0000(	461	11.53004	chr12:10(	COPS5P2		Pseudoger	chr12:123441517-12
ENSG0000(	461	11.53004	chr12:10(	ADAM1A		Pseudoger	chr12:111899263-11
ENSG0000(	461	11.53004	chr12:10(	ENSG00000279905		TEC	chr12:130425004-13
ENSG0000(	461	11.53004	chr12:10(	RNU7-114P		smallRNA	chr12:113602791-11
ENSG0000(	461	11.53004	chr12:10(	MLEC		protein_c	chr12:120687149-12
ENSG0000(	461	11.53004	chr12:10(	GTF2H3		protein_c	chr12:123633739-12
ENSG0000(	461	11.53004	chr12:10(	ENSG00000287493		lncRNA	chr12:121887540-12
ENSG0000(	461	11.53004	chr12:10(	RP11-380L11.4		lncRNA	chr12:123925461-12
ENSG0000(	461	11.53004	chr12:10(	DDX54	NCGv7	protein_c	chr12:113157173-11
ENSG0000(	461	11.53004	chr12:10(	RP11-380L11.3		lncRNA	chr12:123935622-12
ENSG0000(	461	11.53004	chr12:10(	LINC01234		lncRNA	chr12:113583886-11
ENSG0000(	461	11.53004	chr12:10(	MED13L		protein_c	chr12:115957905-11
ENSG0000(	461	11.53004	chr12:10(	ENSG00000269997		lncRNA	chr12:123966077-12
ENSG0000(	461	11.53004	chr12:10(	RASAL1	NCGv7	protein_c	chr12:113098819-11
ENSG0000(	461	11.53004	chr12:10(	ENSG00000269980		lncRNA	chr12:123262060-12
ENSG0000(	461	11.53004	chr12:10(	EIF2B1		protein_c	chr12:123620406-12
ENSG0000(	461	11.53004	chr12:10(	SNORA9		smallRNA	chr12:122492113-12
ENSG0000(	461	11.53004	chr12:10(	DDX55	NCGv7	protein_c	chr12:123602077-12
ENSG0000(	461	11.53004	chr12:10(	snoU13		smallRNA	chr12:128398432-12
ENSG0000(	461	11.53004	chr12:10(	ENSG00000286354		lncRNA	chr12:116357578-11
ENSG0000(	461	11.53004	chr12:10(	HCAR1		protein_c	chr12:122726076-12
ENSG0000(	461	11.53004	chr12:10(	SPRING1		protein_c	chr12:116710171-11
ENSG0000(	461	11.53004	chr12:10(	LINC02826		lncRNA	chr12:125983702-12
ENSG0000(	461	11.53004	chr12:10(	FZD10		protein_c	chr12:130162459-13
ENSG0000(	461	11.53004	chr12:10(	HCAR3		protein_c	chr12:122714756-12
ENSG0000(	461	11.53004	chr12:10(	TBX5-AS1		lncRNA	chr12:114408131-11
ENSG0000(	461	11.53004	chr12:10(	RN7SKP197		smallRNA	chr12:119631090-11
ENSG0000(	461	11.53004	chr12:10(	NME2P1		Pseudoger	chr12:120282303-12
ENSG0000(	461	11.53004	chr12:10(	CAMKK2	NCGv7	protein_c	chr12:121237675-12
ENSG0000(	461	11.53004	chr12:10(	CIT		protein_c	chr12:119685791-11
ENSG0000(	461	11.53004	chr12:10(	BCL7A	NCGv7;AC	protein_c	chr12:122019422-12
ENSG0000(	461	11.53004	chr12:10(	RSRC2		protein_c	chr12:122503454-12
ENSG0000(	461	11.53004	chr12:10(	VSIG10		protein_c	chr12:118063593-11
ENSG0000(	461	11.53004	chr12:10(	KMT5A		protein_c	chr12:123384132-12
ENSG0000(	461	11.53004	chr12:10(	WSB2		protein_c	chr12:118032687-11
ENSG0000(	461	11.53004	chr12:10(	RBM19		protein_c	chr12:113816738-11
ENSG0000(	461	11.53004	chr12:10(	SH2B3	NCGv7;AC	protein_c	chr12:111405923-11
ENSG0000(	461	11.53004	chr12:10(	ENSG00000286386		lncRNA	chr12:126165730-12
ENSG0000(	461	11.53004	chr12:10(	LINC00173		lncRNA	chr12:116533422-11
ENSG0000(	461	11.53004	chr12:10(	ACAD10		protein_c	chr12:111686053-11
ENSG0000(	461	11.53004	chr12:10(	DIABLO		protein_c	chr12:122207663-12
ENSG0000(	461	11.53004	chr12:10(	ALDH2	AC	protein_c	chr12:111766887-11
ENSG0000(	461	11.53004	chr12:10(	PITPNM2	NCGv7	protein_c	chr12:122983480-12
ENSG0000(	461	11.53004	chr12:10(	NAA25		protein_c	chr12:112026689-11
ENSG0000(	461	11.53004	chr12:10(	OGFOD2		protein_c	chr12:122974580-12
ENSG0000(	461	11.53004	chr12:10(	CDK2AP1		protein_c	chr12:123250112-12
ENSG0000(	461	11.53004	chr12:10(	ENSG00000270048		lncRNA	chr12:123962555-12
ENSG0000(	461	11.53004	chr12:10(	OAS3		protein_c	chr12:112938051-11
ENSG0000(	461	11.53004	chr12:10(	ACADS		protein_c	chr12:120725774-12
ENSG0000(	461	11.53004	chr12:10(	OAS2		protein_c	chr12:112978395-11

ENSG0000(	461	11.53004	chr12:10(	ENSG00000278973		TEC	chr12:124622290-12
ENSG0000(	461	11.53004	chr12:10(	ENSG00000280120		TEC	chr12:123152324-12
ENSG0000(	461	11.53004	chr12:10(	RN7SL508P		smallRNA	chr12:118993857-11
ENSG0000(	461	11.53004	chr12:10(	ENSG00000274292		lncRNA	chr12:121800797-12
ENSG0000(	461	11.53004	chr12:10(	TMEM132D	NCGv7	protein_c	chr12:129071725-12
ENSG0000(	461	11.53004	chr12:10(	GLT1D1		protein_c	chr12:128853427-12
ENSG0000(	461	11.53004	chr12:10(	RILPL1		protein_c	chr12:123470054-12
ENSG0000(	461	11.53004	chr12:10(	ENSG00000276308		lncRNA	chr12:115318657-11
ENSG0000(	461	11.53004	chr12:10(	AC079949.1		smallRNA	chr12:127166071-12
ENSG0000(	461	11.53004	chr12:10(	ENSG00000278344		lncRNA	chr12:120500735-12
ENSG0000(	461	11.53004	chr12:10(	ENSG00000255853		Pseudoger	chr12:126510910-12
ENSG0000(	461	11.53004	chr12:10(	UBA52P7		Pseudoger	chr12:115592604-11
ENSG0000(	461	11.53004	chr12:10(	ENSG00000255923		Pseudoger	chr12:126864465-12
ENSG0000(	461	11.53004	chr12:10(	RPL36P15		Pseudoger	chr12:116908312-11
ENSG0000(	461	11.53004	chr12:10(	ENSG00000255856		lncRNA	chr12:122007434-12
ENSG0000(	461	11.53004	chr12:10(	PXN-AS1		lncRNA	chr12:120201274-12
ENSG0000(	461	11.53004	chr12:10(	ENSG00000276487		lncRNA	chr12:128187225-12
ENSG0000(	461	11.53004	chr12:10(	CFAP251		protein_c	chr12:121918592-12
ENSG0000(	461	11.53004	chr12:10(	ENSG00000255900		Pseudoger	chr12:126616344-12
ENSG0000(	461	11.53004	chr12:10(	ENSG00000277840		lncRNA	chr12:117002463-11
ENSG0000(	461	11.53004	chr12:10(	ENSG00000255838		Pseudoger	chr12:128944274-12
ENSG0000(	461	11.53004	chr12:10(	ENSG00000286016		lncRNA	chr12:127130005-12
ENSG0000(	461	11.53004	chr12:10(	ENSG00000276292		lncRNA	chr12:118037869-11
ENSG0000(	461	11.53004	chr12:10(	Y_RNA		smallRNA	chr12:114657949-11
ENSG0000(	461	11.53004	chr12:10(	RFLNA	NCGv7	protein_c	chr12:123973241-12
ENSG0000(	461	11.53004	chr12:10(	TBX3	NCGv7;AC	protein_c	chr12:114670255-11
ENSG0000(	461	11.53004	chr12:10(	ENSG00000255757		Pseudoger	chr12:127876999-12
ENSG0000(	461	11.53004	chr12:10(	ENSG00000276122		lncRNA	chr12:130138693-13
ENSG0000(	461	11.53004	chr12:10(	ENSG00000286233		lncRNA	chr12:118988010-11
ENSG0000(	461	11.53004	chr12:10(	RPS2P41		Pseudoger	chr12:111879338-11
ENSG0000(	461	11.53004	chr12:10(	ENSG00000256085		lncRNA	chr12:129521303-12
ENSG0000(	461	11.53004	chr12:10(	ENSG00000256071		lncRNA	chr12:117889454-11
ENSG0000(	461	11.53004	chr12:10(	ENSG00000276188		lncRNA	chr12:120709112-12
ENSG0000(	461	11.53004	chr12:10(	RPL17P37		Pseudoger	chr12:118737964-11
ENSG0000(	461	11.53004	chr12:10(	LINC02439		lncRNA	chr12:118782926-11
ENSG0000(	461	11.53004	chr12:10(	ENSG00000255830		Pseudoger	chr12:114548854-11
ENSG0000(	461	11.53004	chr12:10(	ENSG00000274029		lncRNA	chr12:121190868-12
ENSG0000(	461	11.53004	chr12:10(	PITPNM2-AS1		lncRNA	chr12:123081384-12
ENSG0000(	461	11.53004	chr12:10(	PIWIL1	AC	protein_c	chr12:130337887-13
ENSG0000(	461	11.53004	chr12:10(	NRAV		lncRNA	chr12:120488079-12
ENSG0000(	461	11.53004	chr12:10(	ENSG00000286092		lncRNA	chr12:123713408-12
ENSG0000(	461	11.53004	chr12:10(	ENSG00000287982		lncRNA	chr12:114733757-11
ENSG0000(	461	11.53004	chr12:10(	ENSG00000278084		lncRNA	chr12:121874193-12
ENSG0000(	461	11.53004	chr12:10(	ZNF664		protein_c	chr12:123971845-12
ENSG0000(	461	11.53004	chr12:10(	LINC02411		lncRNA	chr12:127631248-12
ENSG0000(	461	11.53004	chr12:10(	CABP1-DT		lncRNA	chr12:120628830-12
ENSG0000(	461	11.53004	chr12:10(	ENSG00000255965		Pseudoger	chr12:124383381-12
ENSG0000(	461	11.53004	chr12:10(	ENSG00000255972		Pseudoger	chr12:122563735-12
ENSG0000(	461	11.53004	chr12:10(	ENSG00000278112		lncRNA	chr12:123519390-12
ENSG0000(	461	11.53004	chr12:10(	MIR4498		smallRNA	chr12:120155434-12
ENSG0000(	461	11.53004	chr12:10(	LINC02824		lncRNA	chr12:126688341-12

ENSG0000(	461	11.53004	chr12:10(	ENSG00000256064		lncRNA	chr12:130419535-13
ENSG0000(	461	11.53004	chr12:10(	ENSG00000274227		lncRNA	chr12:112018804-11
ENSG0000(	461	11.53004	chr12:10(	ENSG00000256001		lncRNA	chr12:127142029-12
ENSG0000(	461	11.53004	chr12:10(	SDS		protein_c	chr12:113392445-11
ENSG0000(	461	11.53004	chr12:10(	PTPN11	NCGv7;AC	protein_c	chr12:112418351-11
ENSG0000(	461	11.53004	chr12:10(	HPD	NCGv7	protein_c	chr12:121839527-12
ENSG0000(	461	11.53004	chr12:10(	TAOK3		protein_c	chr12:118149801-11
ENSG0000(	461	11.53004	chr12:10(	ENSG00000277873		lncRNA	chr12:118024817-11
ENSG0000(	461	11.53004	chr12:10(	CLIC1P1		Pseudoger	chr12:120914400-12
ENSG0000(	461	11.53004	chr12:10(	ENSG00000276972		lncRNA	chr12:115332146-11
ENSG0000(	461	11.53004	chr12:10(	ENSG00000286067		lncRNA	chr12:120218070-12
ENSG0000(	461	11.53004	chr12:10(	TMEM132C	NCGv7	protein_c	chr12:128267170-12
ENSG0000(	461	11.53004	chr12:10(	BRI3BP		protein_c	chr12:124993645-12
ENSG0000(	461	11.53004	chr12:10(	AC107020.1		smallRNA	chr12:128669880-12
ENSG0000(	461	11.53004	chr12:10(	MIR620		smallRNA	chr12:116148560-11
ENSG0000(	461	11.53004	chr12:10(	ENSG00000256044		Pseudoger	chr12:122749481-12
ENSG0000(	461	11.53004	chr12:10(	FBXO21		protein_c	chr12:117141991-11
ENSG0000(	461	11.53004	chr12:10(	ENSG00000255944		lncRNA	chr12:126628172-12
ENSG0000(	461	11.53004	chr12:10(	HNF1A	NCGv7	protein_c	chr12:120978543-12
ENSG0000(	461	11.53004	chr12:10(	ENSG00000255945		lncRNA	chr12:127598168-12
ENSG0000(	461	11.53004	chr12:10(	MSI1	AC	protein_c	chr12:120341330-12
ENSG0000(	461	11.53004	chr12:10(	ENSG00000255946		lncRNA	chr12:120740470-12
ENSG0000(	461	11.53004	chr12:10(	ENSG00000278266		lncRNA	chr12:127147149-12
ENSG0000(	461	11.53004	chr12:10(	ENSG00000256028		lncRNA	chr12:122975320-12
ENSG0000(	461	11.53004	chr12:10(	ENSG00000288523		lncRNA	chr12:127726339-12
ENSG0000(	461	11.53004	chr12:10(	HNF1A-AS1		lncRNA	chr12:120941728-12
ENSG0000(	461	11.53004	chr12:10(	TMEM233		protein_c	chr12:119593774-11
ENSG0000(	461	11.53004	chr12:10(	ENSG00000286662		lncRNA	chr12:127758832-12
ENSG0000(	461	11.53004	chr12:10(	RNF34		protein_c	chr12:121400083-12
ENSG0000(	461	11.53004	chr12:10(	TMED2-DT		lncRNA	chr12:123575891-12
ENSG0000(	461	11.53004	chr12:10(	FZD10-AS1		lncRNA	chr12:130144315-13
ENSG0000(	461	11.53004	chr12:10(	ENSG00000255575		Pseudoger	chr12:120057035-12
ENSG0000(	461	11.53004	chr12:10(	U7		smallRNA	chr12:111564821-11
ENSG0000(	461	11.53004	chr12:10(	ENSG00000277283		lncRNA	chr12:120116907-12
ENSG0000(	461	11.53004	chr12:10(	ENSG00000274554		lncRNA	chr12:116948738-11
ENSG0000(	461	11.53004	chr12:10(	KSR2	AC	protein_c	chr12:117453012-11
ENSG0000(	461	11.53004	chr12:10(	Y_RNA		smallRNA	chr12:123348999-12
ENSG0000(	461	11.53004	chr12:10(	ENSG00000239374		Pseudoger	chr12:116755395-11
ENSG0000(	461	11.53004	chr12:10(	RPS27P25		Pseudoger	chr12:120369440-12
ENSG0000(	461	11.53004	chr12:10(	CABP1		protein_c	chr12:120640626-12
ENSG0000(	461	11.53004	chr12:10(	ENSG00000255595		lncRNA	chr12:126191151-12
ENSG0000(	461	11.53004	chr12:10(	SPPL3		protein_c	chr12:120762510-12
ENSG0000(	461	11.53004	chr12:10(	RNU1-104P		smallRNA	chr12:127321355-12
ENSG0000(	461	11.53004	chr12:10(	LINC02440		lncRNA	chr12:118773031-11
ENSG0000(	461	11.53004	chr12:10(	ENSG00000288623		protein_c	chr12:120534697-12
ENSG0000(	461	11.53004	chr12:10(	OASL		protein_c	chr12:121017763-12
ENSG0000(	461	11.53004	chr12:10(	TMEM116	NCGv7	protein_c	chr12:111931282-11
ENSG0000(	461	11.53004	chr12:10(	ENSG00000277566		lncRNA	chr12:113249466-11
ENSG0000(	461	11.53004	chr12:10(	LINC00934		lncRNA	chr12:119283825-11
ENSG0000(	461	11.53004	chr12:10(	LINC00944		lncRNA	chr12:126729787-12
ENSG0000(	461	11.53004	chr12:10(	ENSG00000278866		TEC	chr12:126675895-12

ENSG0000(	461	11.53004	chr12:109	ENSG00000278861		TEC	chr12:123655528-12
ENSG0000(	461	11.53004	chr12:109	RPL27P12		Pseudoger	chr12:123721246-12
ENSG0000(	461	11.53004	chr12:109	RPL12P33		Pseudoger	chr12:120916745-12
ENSG0000(	461	11.53004	chr12:109	ENSG00000256149		lncRNA	chr12:118849354-11
ENSG0000(	461	11.53004	chr12:109	ENSG00000274695		lncRNA	chr12:128826836-12
ENSG0000(	461	11.53004	chr12:109	ENSG00000277423		lncRNA	chr12:120703867-12
ENSG0000(	461	11.53004	chr12:109	CCDC92		protein_c	chr12:123918660-12
ENSG0000(	461	11.53004	chr12:109	RPL29P24		Pseudoger	chr12:120522664-12
ENSG0000(	461	11.53004	chr12:109	LINC02418		lncRNA	chr12:130032928-13
ENSG0000(	461	11.53004	chr12:109	ENSG00000283459		lncRNA	chr12:114894632-11
ENSG0000(	461	11.53004	chr12:109	RP11-216P16.2		lncRNA	chr12:121000486-12
ENSG0000(	461	11.53004	chr12:109	ENSG00000256137		lncRNA	chr12:129681427-12
ENSG0000(	461	11.53004	chr12:109	ARL6IP4		protein_c	chr12:122980060-12
ENSG0000(	461	11.53004	chr12:109	ENSG00000271579		lncRNA	chr12:115299588-11
ENSG0000(	461	11.53004	chr12:109	PLBD2		protein_c	chr12:113358566-11
ENSG0000(	461	11.53004	chr12:109	ENSG00000274427		lncRNA	chr12:123515275-12
ENSG0000(	461	11.53004	chr12:109	ENSG00000256152		lncRNA	chr12:122865335-12
ENSG0000(	461	11.53004	chr12:109	RN7SL865P		smallRNA	chr12:115717725-11
ENSG0000(	461	11.53004	chr12:109	SBNO1-AS1		lncRNA	chr12:123363868-12
ENSG0000(	461	11.53004	chr12:109	P2RX4		protein_c	chr12:121210065-12
ENSG0000(	461	11.53004	chr12:109	RNFT2	DriverDBv	protein_c	chr12:116738178-11
ENSG0000(	461	11.53004	chr12:109	TCTN2		protein_c	chr12:123671110-12
ENSG0000(	461	11.53004	chr12:109	HRK		protein_c	chr12:116856144-11
ENSG0000(	461	11.53004	chr12:109	ENSG00000275936		lncRNA	chr12:120224744-12
ENSG0000(	461	11.53004	chr12:109	C12orf43		protein_c	chr12:121000486-12
ENSG0000(	461	11.53004	chr12:109	ENSG00000255686		lncRNA	chr12:117453012-11
ENSG0000(	461	11.53004	chr12:109	ENSG00000251536		lncRNA	chr12:129852208-12
ENSG0000(	461	11.53004	chr12:109	AACS		protein_c	chr12:125065434-12
ENSG0000(	461	11.53004	chr12:109	ENSG00000255692		lncRNA	chr12:119699768-11
ENSG0000(	461	11.53004	chr12:109	LINC02460		lncRNA	chr12:118645315-11
ENSG0000(	461	11.53004	chr12:109	ORAI1		protein_c	chr12:121626509-12
ENSG0000(	461	11.53004	chr12:109	ENSG00000286791		lncRNA	chr12:127146535-12
ENSG0000(	461	11.53004	chr12:109	HSPB8	DriverDBv	protein_c	chr12:119171555-11
ENSG0000(	461	11.53004	chr12:109	TRIAP1		protein_c	chr12:120443964-12
ENSG0000(	461	11.53004	chr12:109	LRRC43		protein_c	chr12:122167738-12
ENSG0000(	461	11.53004	chr12:109	BICDL1		protein_c	chr12:119989236-12
ENSG0000(	461	11.53004	chr12:109	ENSG00000275898		lncRNA	chr12:116580974-11
ENSG0000(	461	11.53004	chr12:109	PLA2G1B		protein_c	chr12:120322115-12
ENSG0000(	461	11.53004	chr12:109	ENSG00000286248		lncRNA	chr12:121177919-12
ENSG0000(	461	11.53004	chr12:109	DTX1	NCGv7	protein_c	chr12:113056730-11
ENSG0000(	461	11.53004	chr12:109	ENSG00000286246		lncRNA	chr12:129622929-12
ENSG0000(	461	11.53004	chr12:109	ENSG00000286586		lncRNA	chr12:121687187-12
ENSG0000(	461	11.53004	chr12:109	LINC02347		lncRNA	chr12:126438837-12
ENSG0000(	461	11.53004	chr12:109	ENSG00000256093		Pseudoger	chr12:125056359-12
ENSG0000(	461	11.53004	chr12:109	RPL22P19		Pseudoger	chr12:124935455-12
ENSG0000(	461	11.53004	chr12:109	TRAFD1		protein_c	chr12:112125538-11
ENSG0000(	461	11.53004	chr12:109	OASL2P		Pseudoger	chr12:121053732-12
ENSG0000(	461	11.53004	chr12:109	FAM32EP		Pseudoger	chr12:126458462-12
ENSG0000(	459	11.48002	chr2:4707	RNA5SP96		Pseudoger	chr2:69181897-6918
ENSG0000(	456	11.40498	chr5:1696	MRPS30-DT		lncRNA	chr5:44742420-4480
ENSG0000(	456	11.40498	chr5:1696	LINC02224		lncRNA	chr5:44495099-4465

ENSG0000(	456	11.40498	chr5:169(	RPL29P12		Pseudoger	chr5:43666766-4366
ENSG0000(	456	11.40498	chr5:169(	RN7SL383P		smallRNA	chr5:44716190-4471
ENSG0000(	456	11.40498	chr5:169(	ENSG00000249276		lncRNA	chr5:43874367-4388
ENSG0000(	456	11.40498	chr1:3732	RNA5SP74		Pseudoger	chr1:204562413-204
ENSG0000(	456	11.40498	chr5:169(	AMD1P3		Pseudoger	chr5:43586918-4358
ENSG0000(	456	11.40498	chr5:169(	ENSG00000250017		Pseudoger	chr5:45574590-4557
ENSG0000(	456	11.40498	chr5:169(	HCN1	NCGv7	protein_c	chr5:45254948-4569
ENSG0000(	456	11.40498	chr5:169(	ENSG00000272335		lncRNA	chr5:44826076-4482
ENSG0000(	456	11.40498	chr5:169(	FGF10	AC	protein_c	chr5:44300247-4438
ENSG0000(	456	11.40498	chr5:169(	FGF10-AS1		lncRNA	chr5:44388732-4441
ENSG0000(	456	11.40498	chr5:169(	ENSG00000279557		TEC	chr5:43586367-4358
ENSG0000(	456	11.40498	chr5:169(	ENSG00000250122		lncRNA	chr5:45890216-4589
ENSG0000(	456	11.40498	chr5:169(	ENSG00000250422		Pseudoger	chr5:45557919-4555
ENSG0000(	456	11.40498	chr5:169(	RNU6-381P		smallRNA	chr5:44066624-4406
ENSG0000(	456	11.40498	chr5:169(	ENSG00000250418		lncRNA	chr5:44698431-4470
ENSG0000(	456	11.40498	chr5:169(	ENSG00000287540		lncRNA	chr5:45226286-4522
ENSG0000(	456	11.40498	chr5:169(	NNT-AS1		lncRNA	chr5:43571594-4360
ENSG0000(	456	11.40498	chr5:169(	NNT		protein_c	chr5:43602692-4370
ENSG0000(	456	11.40498	chr5:169(	ENSG00000248779		lncRNA	chr5:44752949-4476
ENSG0000(	456	11.40498	chr5:169(	MRPS30		protein_c	chr5:44808947-4482
ENSG0000(	456	11.40498	chr5:169(	ENSG00000248148		lncRNA	chr5:45035199-4509
ENSG0000(	455	11.37997	chrX:1657	MIR421		smallRNA	chrX:74218377-7421
ENSG0000(	455	11.37997	chrX:1657	MIR374B		smallRNA	chrX:74218547-7421
ENSG0000(	455	11.37997	chr1:3732	RNA5SP80		Pseudoger	chr1:231281414-231
ENSG0000(	444	11.10485	chr1:3732	ENSG00000270598		Pseudoger	chr1:226127178-226
ENSG0000(	439	10.9798	chr2:4707	snoU13		smallRNA	chr2:64908587-6490
ENSG0000(	431	10.77971	chr12:583	RPL7AP9		Pseudoger	chr12:76599671-766
ENSG0000(	430	10.7547	chr6:3405	ENSG00000289851		lncRNA	chr6:146914424-146
ENSG0000(	430	10.7547	chr2:4707	RNU6-649P		smallRNA	chr2:4945277-49453
ENSG0000(	424	10.60463	chr10:265	ELOBP4		Pseudoger	chr10:34488583-344
ENSG0000(	423	10.57962	chr5:169(	RNU6-913P		smallRNA	chr5:61664145-6166
ENSG0000(	423	10.57962	chr5:169(	ZSWIM6	NCGv7	protein_c	chr5:61332258-6154
ENSG0000(	423	10.57962	chr5:169(	SMIM15		protein_c	chr5:61157704-6116
ENSG0000(	423	10.57962	chr5:169(	ENSG00000248529		lncRNA	chr5:61658474-6169
ENSG0000(	423	10.57962	chr5:169(	ENSG00000248586		Pseudoger	chr5:57317989-5732
ENSG0000(	423	10.57962	chr5:169(	HTR1A		protein_c	chr5:63957874-6396
ENSG0000(	423	10.57962	chr5:169(	ENSG00000286853		lncRNA	chr5:58114598-5814
ENSG0000(	423	10.57962	chr5:169(	ENSG00000248935		lncRNA	chr5:60021249-6003
ENSG0000(	423	10.57962	chr5:169(	GAPT		protein_c	chr5:58491435-5849
ENSG0000(	423	10.57962	chr5:169(	ENSG00000248652		Pseudoger	chr5:57081745-5708
ENSG0000(	423	10.57962	chr5:169(	GNL3LP1		Pseudoger	chr5:60891935-6089
ENSG0000(	423	10.57962	chr5:169(	ENSG00000264281		Pseudoger	chr5:55944656-5594
ENSG0000(	423	10.57962	chr5:169(	ENSG00000271257		Pseudoger	chr5:58503295-5850
ENSG0000(	423	10.57962	chr5:169(	snoU13		smallRNA	chr5:56857148-5685
ENSG0000(	423	10.57962	chr5:169(	DEPDC1B		protein_c	chr5:60596912-6070
ENSG0000(	423	10.57962	chr5:169(	AC008836.1		smallRNA	chr5:61557283-6155
ENSG0000(	423	10.57962	chr5:169(	SETP21		Pseudoger	chr5:60459848-6046
ENSG0000(	423	10.57962	chr5:169(	IL6ST-DT		lncRNA	chr5:55995153-5600
ENSG0000(	423	10.57962	chr5:169(	RPL26P19		Pseudoger	chr5:56504635-5650
ENSG0000(	423	10.57962	chr5:169(	RPL17P22		Pseudoger	chr5:56136979-5613
ENSG0000(	423	10.57962	chr5:169(	ENSG00000248288		Pseudoger	chr5:65209921-6521

ENSG0000(	423	10.57962	chr5:169(	ENSG00000248475		lncRNA	chr5:58741581-5881
ENSG0000(	423	10.57962	chr5:169(	MAP3K1	NCGv7	protein_c	chr5:56815549-5689
ENSG0000(	423	10.57962	chr5:169(	RNU6-299P		smallRNA	chr5:56125671-5612
ENSG0000(	423	10.57962	chr5:169(	ENSG00000233943		Pseudoger	chr5:56975593-5697
ENSG0000(	423	10.57962	chr5:169(	PSMC1P4		Pseudoger	chr5:56275304-5627
ENSG0000(	423	10.57962	chr5:169(	RPL5P15		Pseudoger	chr5:58822503-5882
ENSG0000(	423	10.57962	chr5:169(	ENSG00000248285		lncRNA	chr5:63957893-6398
ENSG0000(	423	10.57962	chr5:169(	RNA5SP185		Pseudoger	chr5:56260333-5626
ENSG0000(	423	10.57962	chr5:169(	ENSG00000213896		Pseudoger	chr5:60429903-6043
ENSG0000(	423	10.57962	chr5:169(	snoU13		smallRNA	chr5:58070188-5807
ENSG0000(	423	10.57962	chr5:169(	SLC38A9		protein_c	chr5:55625845-5577
ENSG0000(	423	10.57962	chr5:169(	ENSG00000287179		lncRNA	chr5:63569419-6357
ENSG0000(	423	10.57962	chr5:169(	IL31RA		protein_c	chr5:55851357-5592
ENSG0000(	423	10.57962	chr5:169(	RPL3P6		Pseudoger	chr5:61390668-6139
ENSG0000(	423	10.57962	chr5:169(	LINC02101		lncRNA	chr5:58107631-5812
ENSG0000(	423	10.57962	chr5:169(	MIR582		smallRNA	chr5:59703606-5970
ENSG0000(	423	10.57962	chr5:169(	ELOVL7	DriverDB\	protein_c	chr5:60751791-6084
ENSG0000(	423	10.57962	chr5:169(	ENSG00000248185		Pseudoger	chr5:63301523-6330
ENSG0000(	423	10.57962	chr5:169(	NDUFAF2		protein_c	chr5:60945177-6115
ENSG0000(	423	10.57962	chr5:169(	ENSG00000248199		lncRNA	chr5:61878109-6192
ENSG0000(	423	10.57962	chr5:169(	SNORA76		smallRNA	chr5:65961183-6596
ENSG0000(	423	10.57962	chr5:169(	ENSG00000248733		lncRNA	chr5:58846885-5886
ENSG0000(	423	10.57962	chr5:169(	LINC01948		lncRNA	chr5:56451627-5648
ENSG0000(	423	10.57962	chr5:169(	SHLD3		protein_c	chr5:65625027-6563
ENSG0000(	423	10.57962	chr5:169(	ERCC8-AS1		lncRNA	chr5:60917490-6091
ENSG0000(	423	10.57962	chr5:169(	RNF180	DriverDB\	protein_c	chr5:64165843-6437
ENSG0000(	423	10.57962	chr5:169(	C5orf67		lncRNA	chr5:56511567-5660
ENSG0000(	423	10.57962	chr5:169(	ENSG00000247345		lncRNA	chr5:59039761-5906
ENSG0000(	423	10.57962	chr5:169(	LINC02065		lncRNA	chr5:66205120-6620
ENSG0000(	423	10.57962	chr5:169(	AC008780.1		smallRNA	chr5:57642895-5764
ENSG0000(	423	10.57962	chr5:169(	GPBP1		protein_c	chr5:57173948-5726
ENSG0000(	423	10.57962	chr5:169(	ENSG00000253744		lncRNA	chr5:66144156-6614
ENSG0000(	423	10.57962	chr5:169(	C5orf64		lncRNA	chr5:61610424-6179
ENSG0000(	423	10.57962	chr5:169(	HNRNPH1P3		Pseudoger	chr5:55838801-5584
ENSG0000(	423	10.57962	chr5:169(	PGAM1P1		Pseudoger	chr5:58161197-5816
ENSG0000(	423	10.57962	chr5:169(	RPL35AP14		Pseudoger	chr5:62575951-6257
ENSG0000(	423	10.57962	chr5:169(	ANKRD55		protein_c	chr5:56099680-5623
ENSG0000(	423	10.57962	chr5:169(	ENSG00000235635		lncRNA	chr5:56941307-5694
ENSG0000(	423	10.57962	chr5:169(	MRPL49P1		Pseudoger	chr5:64674853-6467
ENSG0000(	423	10.57962	chr5:169(	ENSG00000249878		Pseudoger	chr5:61831947-6183
ENSG0000(	423	10.57962	chr5:169(	DDX4		protein_c	chr5:55738017-5581
ENSG0000(	423	10.57962	chr5:169(	ENSG00000251368		Pseudoger	chr5:55750206-5575
ENSG0000(	423	10.57962	chr5:169(	C1GALT1P2		Pseudoger	chr5:56192530-5619
ENSG0000(	423	10.57962	chr5:169(	RNU6-540P		smallRNA	chr5:65604661-6560
ENSG0000(	423	10.57962	chr5:169(	ADAMTS6	NCGv7	protein_c	chr5:65148738-6548
ENSG0000(	423	10.57962	chr5:169(	ENSG00000262211		lncRNA	chr5:55936143-5594
ENSG0000(	423	10.57962	chr5:169(	ENSG00000285999		lncRNA	chr5:66085405-6609
ENSG0000(	423	10.57962	chr5:169(	ENSG00000289709		lncRNA	chr5:56766395-5677
ENSG0000(	423	10.57962	chr5:169(	NDUFB4P2		Pseudoger	chr5:59528861-5952
ENSG0000(	423	10.57962	chr5:169(	SHISAL2B		protein_c	chr5:64690442-6471
ENSG0000(	423	10.57962	chr5:169(	ERBIN	NCGv7	protein_c	chr5:65883128-6608

ENSG0000(	423	10.57962	chr5:169(	ENSG00000286314		lncRNA	chr5:65965373-6596
ENSG0000(	423	10.57962	chr5:169(	SREK1		protein_c	chr5:66139971-6618
ENSG0000(	423	10.57962	chr5:169(	CAB39P1		Pseudoger	chr5:60630514-6063
ENSG0000(	423	10.57962	chr5:169(	PLK2		protein_c	chr5:58453982-5846
ENSG0000(	423	10.57962	chr5:169(	RNA5SP184		Pseudoger	chr5:56146739-5614
ENSG0000(	423	10.57962	chr5:169(	ENSG00000225230		lncRNA	chr5:56900041-5691
ENSG0000(	423	10.57962	chr5:169(	RN7SKP157		smallRNA	chr5:62146665-6214
ENSG0000(	423	10.57962	chr5:169(	RMEL3		lncRNA	chr5:57395060-5753
ENSG0000(	423	10.57962	chr5:169(	LRRC70		protein_c	chr5:62578819-6258
ENSG0000(	423	10.57962	chr5:169(	ENSG00000205644		Pseudoger	chr5:65415353-6541
ENSG0000(	423	10.57962	chr5:169(	CENPK		protein_c	chr5:65517766-6556
ENSG0000(	423	10.57962	chr5:169(	ERCC8		protein_c	chr5:60866454-6094
ENSG0000(	423	10.57962	chr5:169(	PDE4D		protein_c	chr5:58969038-6052
ENSG0000(	423	10.57962	chr5:169(	ENSG00000249697		lncRNA	chr5:56313905-5632
ENSG0000(	423	10.57962	chr5:169(	RPEP1		Pseudoger	chr5:65251127-6525
ENSG0000(	423	10.57962	chr5:169(	ENSG00000234553		lncRNA	chr5:56536583-5653
ENSG0000(	423	10.57962	chr5:169(	KRT8P31		Pseudoger	chr5:60743563-6074
ENSG0000(	423	10.57962	chr5:169(	LINC02108		lncRNA	chr5:58541567-5855
ENSG0000(	423	10.57962	chr5:169(	ENSG00000237705		lncRNA	chr5:56842016-5686
ENSG0000(	423	10.57962	chr5:169(	ENSG00000289916		lncRNA	chr5:61902617-6191
ENSG0000(	423	10.57962	chr5:169(	RNU6-294P		smallRNA	chr5:64573569-6457
ENSG0000(	423	10.57962	chr5:169(	ENSG00000250081		lncRNA	chr5:65486444-6548
ENSG0000(	423	10.57962	chr5:169(	PART1		lncRNA	chr5:60487713-6054
ENSG0000(	423	10.57962	chr5:169(	TRAPPC13		protein_c	chr5:65625004-6566
ENSG0000(	423	10.57962	chr5:169(	TRIM23	NCGv7	protein_c	chr5:65589690-6562
ENSG0000(	423	10.57962	chr5:169(	ISCA1P1		Pseudoger	chr5:62776877-6277
ENSG0000(	423	10.57962	chr5:169(	HMGN1P17		Pseudoger	chr5:56381781-5638
ENSG0000(	423	10.57962	chr5:169(	PPWD1		protein_c	chr5:65563236-6558
ENSG0000(	423	10.57962	chr5:169(	SREK1IP1		protein_c	chr5:64718148-6476
ENSG0000(	423	10.57962	chr5:169(	CWC27		protein_c	chr5:64766368-6510
ENSG0000(	423	10.57962	chr5:169(	DIMT1		protein_c	chr5:62347284-6240
ENSG0000(	423	10.57962	chr5:169(	IPO11		protein_c	chr5:62403972-6262
ENSG0000(	423	10.57962	chr5:169(	SGTB		protein_c	chr5:65665928-6572
ENSG0000(	423	10.57962	chr5:169(	ENSG00000287709		lncRNA	chr5:57650632-5775
ENSG0000(	423	10.57962	chr5:169(	ENSG00000288936		lncRNA	chr5:61321498-6133
ENSG0000(	423	10.57962	chr5:169(	ENSG00000276945		Pseudoger	chr5:61145846-6114
ENSG0000(	423	10.57962	chr5:169(	CKS1BP3		Pseudoger	chr5:62512246-6251
ENSG0000(	423	10.57962	chr5:169(	NT5ELP		Pseudoger	chr5:64404512-6448
ENSG0000(	423	10.57962	chr5:169(	SMIM15-AS1		lncRNA	chr5:61162070-6123
ENSG0000(	423	10.57962	chr5:169(	NLN	NCGv7	protein_c	chr5:65722205-6587
ENSG0000(	423	10.57962	chr5:169(	ENSG00000251648		Pseudoger	chr5:65732973-6573
ENSG0000(	423	10.57962	chr5:169(	MIER3	NCGv7	protein_c	chr5:56919602-5697
ENSG0000(	423	10.57962	chr5:169(	ENSG00000251682		Pseudoger	chr5:61347126-6134
ENSG0000(	423	10.57962	chr5:169(	RNA5SP183		Pseudoger	chr5:55777469-5577
ENSG0000(	423	10.57962	chr5:169(	LINC02057		lncRNA	chr5:61201304-6130
ENSG0000(	423	10.57962	chr5:169(	RN7SL169P		smallRNA	chr5:64539504-6453
ENSG0000(	423	10.57962	chr5:169(	RNU6-806P		smallRNA	chr5:60304047-6030
ENSG0000(	423	10.57962	chr5:169(	ENSG00000271828		lncRNA	chr5:56927874-5692
ENSG0000(	423	10.57962	chr5:169(	ENSG00000250480		Pseudoger	chr5:57120796-5712
ENSG0000(	423	10.57962	chr5:169(	ENSG00000279985		TEC	chr5:61114432-6111
ENSG0000(	423	10.57962	chr5:169(	SALL4P1		Pseudoger	chr5:57422464-5742

ENSG0000(	423	10.57962	chr5:169(	ENSG00000249236		lncRNA	chr5:55978248-5611
ENSG0000(	423	10.57962	chr5:169(	ACTBL2		protein_c	chr5:57480018-5748
ENSG0000(	423	10.57962	chr5:169(	ENSG00000249198		lncRNA	chr5:57751192-5775
ENSG0000(	423	10.57962	chr5:169(	AC116606.1		smallRNA	chr5:57872939-5787
ENSG0000(	423	10.57962	chr5:169(	Y_RNA		smallRNA	chr5:57169108-5716
ENSG0000(	423	10.57962	chr5:169(	C5orf64-AS1		lncRNA	chr5:61732774-6173
ENSG0000(	423	10.57962	chr5:169(	RGS7BP		protein_c	chr5:64506015-6461
ENSG0000(	423	10.57962	chr5:169(	ENSG00000228650		lncRNA	chr5:56770799-5677
ENSG0000(	423	10.57962	chr5:169(	ENSG00000285997		lncRNA	chr5:56488486-5649
ENSG0000(	423	10.57962	chr5:169(	ENSG00000288643		protein_c	chr5:62306206-6253
ENSG0000(	423	10.57962	chr5:169(	ENSG00000250461		Pseudoger	chr5:61375112-6137
ENSG0000(	423	10.57962	chr5:169(	SETD9		protein_c	chr5:56909260-5692
ENSG0000(	423	10.57962	chr5:169(	ENSG00000276214		Pseudoger	chr5:59314110-5931
ENSG0000(	423	10.57962	chr5:169(	AC025470.1		smallRNA	chr5:57481820-5748
ENSG0000(	423	10.57962	chr5:169(	snoU13		smallRNA	chr5:66231129-6623
ENSG0000(	423	10.57962	chr5:169(	MIR548AE2		smallRNA	chr5:58530043-5853
ENSG0000(	423	10.57962	chr5:169(	RNU6-661P		smallRNA	chr5:62443176-6244
ENSG0000(	423	10.57962	chr5:169(	LINC02225		lncRNA	chr5:57890327-5789
ENSG0000(	423	10.57962	chr5:169(	RNU6ATAC2P		smallRNA	chr5:56297564-5629
ENSG0000(	423	10.57962	chr5:169(	snoU13		smallRNA	chr5:56000518-5600
ENSG0000(	423	10.57962	chr5:169(	ENSG00000249579		Pseudoger	chr5:63022919-6302
ENSG0000(	423	10.57962	chr5:169(	ERBIN-DT		lncRNA	chr5:65924629-6592
ENSG0000(	423	10.57962	chr5:169(	IL6ST	NCGv7;AC	protein_c	chr5:55935095-5599
ENSG0000(	423	10.57962	chr5:169(	RAB3C		protein_c	chr5:58582221-5885
ENSG0000(	423	10.57962	chr5:169(	ENSG00000287434		lncRNA	chr5:60699729-6070
ENSG0000(	423	10.57962	chr5:169(	ENSG00000241991		Pseudoger	chr5:62561859-6256
ENSG0000(	423	10.57962	chr5:169(	LNCBRM		lncRNA	chr5:57574027-5761
ENSG0000(	423	10.57962	chr5:169(	ENSG00000289152		lncRNA	chr5:57172842-5717
ENSG0000(	423	10.57962	chr5:169(	KIF2A		protein_c	chr5:62306162-6239
ENSG0000(	423	10.57962	chr5:169(	Y_RNA		smallRNA	chr5:65123369-6512
ENSG0000(	422	10.55461	chr5:169(	AC024569.1		smallRNA	chr5:53609587-5360
ENSG0000(	422	10.55461	chr5:169(	LINC02105		lncRNA	chr5:53776048-5381
ENSG0000(	422	10.55461	chr5:169(	AC025175.1		smallRNA	chr5:53782698-5378
ENSG0000(	422	10.55461	chr5:169(	NDUFS4		protein_c	chr5:53560633-5368
ENSG0000(	420	10.50459	chrX:1657	MTND2P24		Pseudoger	chrX:55179194-5517
ENSG0000(	420	10.50459	chrX:1657	DMRTC1B		protein_c	chrX:72776890-7284
ENSG0000(	420	10.50459	chrX:1657	ENSG00000229662		Pseudoger	chrX:48244894-4824
ENSG0000(	420	10.50459	chrX:1657	ETF1P3		Pseudoger	chrX:65794345-6579
ENSG0000(	420	10.50459	chrX:1657	SATL1		protein_c	chrX:85092284-8524
ENSG0000(	420	10.50459	chrX:1657	MIR502		smallRNA	chrX:50014598-5001
ENSG0000(	420	10.50459	chrX:1657	ENSG00000230100		lncRNA	chrX:48333675-4833
ENSG0000(	420	10.50459	chrX:1657	RN7SL264P		smallRNA	chrX:72223244-7222
ENSG0000(	420	10.50459	chrX:1657	ENSG00000230105		lncRNA	chrX:56618391-5662
ENSG0000(	420	10.50459	chrX:1657	ENSG00000286031		lncRNA	chrX:49279677-4928
ENSG0000(	420	10.50459	chrX:1657	RPL37P24		Pseudoger	chrX:54147372-5414
ENSG0000(	420	10.50459	chrX:1657	BUD31P2		Pseudoger	chrX:75201491-7520
ENSG0000(	420	10.50459	chrX:1657	EIF4A2P4		Pseudoger	chrX:52832704-5283
ENSG0000(	420	10.50459	chrX:1657	ENSG00000230187		Pseudoger	chrX:70672895-7067
ENSG0000(	420	10.50459	chrX:1657	ENSG00000225925		Pseudoger	chrX:68474710-6847
ENSG0000(	420	10.50459	chrX:1657	AF196972.2		Pseudoger	chrX:48412303-4841
ENSG0000(	420	10.50459	chrX:1657	ENSG00000234391		Pseudoger	chrX:48306769-4830

ENSG0000(	420	10.50459	chrX:1657	MIR325		smallRNA	chrX:77005404-7700
ENSG0000(	420	10.50459	chrX:1657	ENSG00000229601		Pseudoger	chrX:71413834-7141
ENSG0000(	420	10.50459	chrX:1657	SPRYD7P1		Pseudoger	chrX:77374212-7738
ENSG0000(	420	10.50459	chrX:1657	ENSG00000283743		lncRNA	chrX:47575128-4762
ENSG0000(	420	10.50459	chrX:1657	ENSG00000283446		Pseudoger	chrX:85143427-8515
ENSG0000(	420	10.50459	chrX:1657	SSXP4		Pseudoger	chrX:52598494-5260
ENSG0000(	420	10.50459	chrX:1657	ENSG00000229594		Pseudoger	chrX:55633031-5563
ENSG0000(	420	10.50459	chrX:1657	RP11-472D17.2		Pseudoger	chrX:52448586-5245
ENSG0000(	420	10.50459	chrX:1657	ENSG00000260118		lncRNA	chrX:68013470-6801
ENSG0000(	420	10.50459	chrX:1657	SSX2	NCGv7;AC	protein_c	chrX:52696896-5270
ENSG0000(	420	10.50459	chrX:1657	USP27X-DT		lncRNA	chrX:49876724-4987
ENSG0000(	420	10.50459	chrX:1657	TEX11		protein_c	chrX:70528940-7090
ENSG0000(	420	10.50459	chrX:1657	FAM236C		protein_c	chrX:72912615-7291
ENSG0000(	420	10.50459	chrX:1657	ENSG00000283599		protein_c	chrX:71667542-7167
ENSG0000(	420	10.50459	chrX:1657	MIRLET7F2		smallRNA	chrX:53557192-5355
ENSG0000(	420	10.50459	chrX:1657	RNU6-1056P		smallRNA	chrX:48724455-4872
ENSG0000(	420	10.50459	chrX:1657	MIR362		smallRNA	chrX:50008964-5000
ENSG0000(	420	10.50459	chrX:1657	ARR3		protein_c	chrX:70268305-7028
ENSG0000(	420	10.50459	chrX:1657	PDZD11		protein_c	chrX:70281118-7029
ENSG0000(	420	10.50459	chrX:1657	PJA1		protein_c	chrX:69160851-6916
ENSG0000(	420	10.50459	chrX:1657	MIR98		smallRNA	chrX:53556223-5355
ENSG0000(	420	10.50459	chrX:1657	SNORA11		smallRNA	chrX:54927305-5492
ENSG0000(	420	10.50459	chrX:1657	ENSG00000224799		lncRNA	chrX:56973510-5697
ENSG0000(	420	10.50459	chrX:1657	ENSG00000286077		lncRNA	chrX:70037840-7003
ENSG0000(	420	10.50459	chrX:1657	TPMTP4		Pseudoger	chrX:86128893-8612
ENSG0000(	420	10.50459	chrX:1657	SEPHS1P4		Pseudoger	chrX:73769248-7377
ENSG0000(	420	10.50459	chrX:1657	PHKA1-AS1		lncRNA	chrX:72688950-7271
ENSG0000(	420	10.50459	chrX:1657	ENSG00000289890		lncRNA	chrX:47555936-4756
ENSG0000(	420	10.50459	chrX:1657	ENSG00000231963		lncRNA	chrX:73080167-7308
ENSG0000(	420	10.50459	chrX:1657	FRMD8P1		Pseudoger	chrX:65550898-6555
ENSG0000(	420	10.50459	chrX:1657	POF1B	NCGv7	protein_c	chrX:85277396-8537
ENSG0000(	420	10.50459	chrX:1657	RBM22P9		Pseudoger	chrX:52509904-5251
ENSG0000(	420	10.50459	chrX:1657	RPSAP14		Pseudoger	chrX:74031462-7403
ENSG0000(	420	10.50459	chrX:1657	ENSG00000226010		Pseudoger	chrX:64144872-6414
ENSG0000(	420	10.50459	chrX:1657	XIST		lncRNA	chrX:73820649-7385
ENSG0000(	420	10.50459	chrX:1657	PFN5P		Pseudoger	chrX:64405473-6440
ENSG0000(	420	10.50459	chrX:1657	ENSG00000229968		Pseudoger	chrX:47776827-4777
ENSG0000(	420	10.50459	chrX:1657	RBM10	NCGv7	protein_c	chrX:47145221-4718
ENSG0000(	420	10.50459	chrX:1657	FAM120C		protein_c	chrX:54068324-5418
ENSG0000(	420	10.50459	chrX:1657	RPL23AP83		Pseudoger	chrX:56242937-5624
ENSG0000(	420	10.50459	chrX:1657	ENSG00000227058		Pseudoger	chrX:52545151-5254
ENSG0000(	420	10.50459	chrX:1657	SNORA4		smallRNA	chrX:82561201-8256
ENSG0000(	420	10.50459	chrX:1657	GPR173		protein_c	chrX:53048789-5308
ENSG0000(	420	10.50459	chrX:1657	TSPYL2	NCGv7	protein_c	chrX:53082367-5308
ENSG0000(	420	10.50459	chrX:1657	DGAT2L6	NCGv7	protein_c	chrX:70177483-7020
ENSG0000(	420	10.50459	chrX:1657	RNU6-245P		smallRNA	chrX:68539443-6853
ENSG0000(	420	10.50459	chrX:1657	RBMXP5		Pseudoger	chrX:65956289-6595
ENSG0000(	420	10.50459	chrX:1657	CYLC1	NCGv7	protein_c	chrX:83861126-8388
ENSG0000(	420	10.50459	chrX:1657	NUDT10	NCGv7	protein_c	chrX:51332231-5133
ENSG0000(	420	10.50459	chrX:1657	YBX1P8		Pseudoger	chrX:46684765-4668
ENSG0000(	420	10.50459	chrX:1657	PAGE2		protein_c	chrX:55089018-5509

ENSG0000(	420	10.50459	chrX:1657	ENSG00000285547		protein_c	chrX:72301691-7257
ENSG0000(	420	10.50459	chrX:1657	MIR676		smallRNA	chrX:70022857-7002
ENSG0000(	420	10.50459	chrX:1657	XAGE1C		protein_c	chrX:52512076-5251
ENSG0000(	420	10.50459	chrX:1657	GAGE12H		protein_c	chrX:49579949-4958
ENSG0000(	420	10.50459	chrX:1657	NDUFA5P7		Pseudoger	chrX:86066227-8606
ENSG0000(	420	10.50459	chrX:1657	ENSG00000232009		Pseudoger	chrX:47389961-4739
ENSG0000(	420	10.50459	chrX:1657	ENSG00000229885		Pseudoger	chrX:52801400-5280
ENSG0000(	420	10.50459	chrX:1657	AL158069.1		smallRNA	chrX:69504705-6950
ENSG0000(	420	10.50459	chrX:1657	RTL5		protein_c	chrX:72127110-7213
ENSG0000(	420	10.50459	chrX:1657	ENSG00000285171		protein_c	chrX:71103987-7111
ENSG0000(	420	10.50459	chrX:1657	ENSG00000229826		Pseudoger	chrX:52735901-5273
ENSG0000(	420	10.50459	chrX:1657	RN7SL581P		smallRNA	chrX:70222008-7022
ENSG0000(	420	10.50459	chrX:1657	ENSG00000234019		lncRNA	chrX:53093710-5309
ENSG0000(	420	10.50459	chrX:1657	5S_rRNA		smallRNA	chrX:69672479-6967
ENSG0000(	420	10.50459	chrX:1657	ENSG00000182776		lncRNA	chrX:52050860-5205
ENSG0000(	420	10.50459	chrX:1657	RP3-326L13.3		lncRNA	chrX:83511296-8351
ENSG0000(	420	10.50459	chrX:1657	MED12	NCGv7;AC	protein_c	chrX:71118543-7114
ENSG0000(	420	10.50459	chrX:1657	GAGE10		protein_c	chrX:49303646-4931
ENSG0000(	420	10.50459	chrX:1657	IGBP1-AS2		lncRNA	chrX:70148582-7014
ENSG0000(	420	10.50459	chrX:1657	ENSG00000225957		Pseudoger	chrX:52382053-5238
ENSG0000(	420	10.50459	chrX:1657	PYY3		Pseudoger	chrX:50156159-5015
ENSG0000(	420	10.50459	chrX:1657	GAGE12G		protein_c	chrX:49570400-4957
ENSG0000(	420	10.50459	chrX:1657	AC003001.1		smallRNA	chrX:85244095-8524
ENSG0000(	420	10.50459	chrX:1657	ENSG00000284618		lncRNA	chrX:81000150-8100
ENSG0000(	420	10.50459	chrX:1657	INE1		lncRNA	chrX:47204921-4720
ENSG0000(	420	10.50459	chrX:1657	AL353698.1		protein_c	chrX:56074324-5607
ENSG0000(	420	10.50459	chrX:1657	CCNYL5		Pseudoger	chrX:65821255-6582
ENSG0000(	420	10.50459	chrX:1657	ENSG00000284391		lncRNA	chrX:70427450-7043
ENSG0000(	420	10.50459	chrX:1657	RN7SL799P		smallRNA	chrX:64210690-6421
ENSG0000(	420	10.50459	chrX:1657	AMER1	NCGv7	protein_c	chrX:64185117-6420
ENSG0000(	420	10.50459	chrX:1657	YIPF6		protein_c	chrX:68498562-6853
ENSG0000(	420	10.50459	chrX:1657	RNU6-867P		smallRNA	chrX:76244968-7624
ENSG0000(	420	10.50459	chrX:1657	AF207550.1		Pseudoger	chrX:48939992-4894
ENSG0000(	420	10.50459	chrX:1657	PRAF2	DriverDB\	protein_c	chrX:49071161-4907
ENSG0000(	420	10.50459	chrX:1657	FAM104B		protein_c	chrX:55143102-5516
ENSG0000(	420	10.50459	chrX:1657	ENSG00000226110		Pseudoger	chrX:53768986-5376
ENSG0000(	420	10.50459	chrX:1657	PABPC1L2B		protein_c	chrX:73002939-7300
ENSG0000(	420	10.50459	chrX:1657	FNDC3CP		Pseudoger	chrX:78165696-7817
ENSG0000(	420	10.50459	chrX:1657	CENPVL1		protein_c	chrX:51710512-5171
ENSG0000(	420	10.50459	chrX:1657	BMI1P1		Pseudoger	chrX:67791955-6779
ENSG0000(	420	10.50459	chrX:1657	FXYD6P3		Pseudoger	chrX:73875068-7387
ENSG0000(	420	10.50459	chrX:1657	RN7SL641P		smallRNA	chrX:74453623-7445
ENSG0000(	420	10.50459	chrX:1657	CHMP5P1		Pseudoger	chrX:46725164-4672
ENSG0000(	420	10.50459	chrX:1657	UHRF2P1		Pseudoger	chrX:74105572-7410
ENSG0000(	420	10.50459	chrX:1657	MIR501		smallRNA	chrX:50009722-5000
ENSG0000(	420	10.50459	chrX:1657	INGX		Pseudoger	chrX:71491682-7149
ENSG0000(	420	10.50459	chrX:1657	MTCO1P52		Pseudoger	chrX:55178207-5517
ENSG0000(	420	10.50459	chrX:1657	FOXO4	NCGv7	protein_c	chrX:71095851-7110
ENSG0000(	420	10.50459	chrX:1657	TBX22	NCGv7	protein_c	chrX:80014753-8003
ENSG0000(	420	10.50459	chrX:1657	MATR3P1		Pseudoger	chrX:72660470-7266
ENSG0000(	420	10.50459	chrX:1657	POMPP1		Pseudoger	chrX:83559896-8356

ENSG0000(	420	10.50459	chrX:1657	USP27X		protein_c	chrX:49879484-4988
ENSG0000(	420	10.50459	chrX:1657	RNA5SP508		Pseudoger	chrX:76655009-7665
ENSG0000(	420	10.50459	chrX:1657	ACTR3P2		Pseudoger	chrX:68771322-6877
ENSG0000(	420	10.50459	chrX:1657	SAR1AP4		Pseudoger	chrX:75884546-7588
ENSG0000(	420	10.50459	chrX:1657	ENSG00000279155		TEC	chrX:48939992-4894
ENSG0000(	420	10.50459	chrX:1657	HMGN1P35		Pseudoger	chrX:69174124-6917
ENSG0000(	420	10.50459	chrX:1657	ENSG00000228427		lncRNA	chrX:71183382-7119
ENSG0000(	420	10.50459	chrX:1657	RP13-216E22.4		lncRNA	chrX:73948973-7394
ENSG0000(	420	10.50459	chrX:1657	LINC01545		lncRNA	chrX:46887417-4689
ENSG0000(	420	10.50459	chrX:1657	FAM236A		protein_c	chrX:72938163-7293
ENSG0000(	420	10.50459	chrX:1657	RBM22P6		Pseudoger	chrX:52485046-5248
ENSG0000(	420	10.50459	chrX:1657	ENSG00000250084		Pseudoger	chrX:52448587-5245
ENSG0000(	420	10.50459	chrX:1657	IPO7P1		Pseudoger	chrX:51921864-5192
ENSG0000(	420	10.50459	chrX:1657	DDX3P1		Pseudoger	chrX:74121012-7413
ENSG0000(	420	10.50459	chrX:1657	ENSG00000291017		lncRNA	chrX:71662991-7166
ENSG0000(	420	10.50459	chrX:1657	RAB11FIP1P1		Pseudoger	chrX:74202834-7420
ENSG0000(	420	10.50459	chrX:1657	ENSG00000228827		Pseudoger	chrX:51903338-5190
ENSG0000(	420	10.50459	chrX:1657	RNU7-37P		smallRNA	chrX:53130481-5313
ENSG0000(	420	10.50459	chrX:1657	SPACA5		protein_c	chrX:48004336-4800
ENSG0000(	420	10.50459	chrX:1657	SSX6P		Pseudoger	chrX:48109981-4811
ENSG0000(	420	10.50459	chrX:1657	SPACA5B		protein_c	chrX:48130626-4813
ENSG0000(	420	10.50459	chrX:1657	GAGE12E		protein_c	chrX:49551289-4955
ENSG0000(	420	10.50459	chrX:1657	MIR4769		smallRNA	chrX:47587429-4758
ENSG0000(	420	10.50459	chrX:1657	RNU6-722P		smallRNA	chrX:48959179-4895
ENSG0000(	420	10.50459	chrX:1657	GLOD5		protein_c	chrX:48761747-4877
ENSG0000(	420	10.50459	chrX:1657	PSMA1P1		Pseudoger	chrX:80709928-8071
ENSG0000(	420	10.50459	chrX:1657	ENSG00000233139		Pseudoger	chrX:48153868-4815
ENSG0000(	420	10.50459	chrX:1657	XAGE3		protein_c	chrX:52862525-5286
ENSG0000(	420	10.50459	chrX:1657	SYN1		protein_c	chrX:47571901-4761
ENSG0000(	420	10.50459	chrX:1657	RPL22P22		Pseudoger	chrX:82506434-8250
ENSG0000(	420	10.50459	chrX:1657	ENSG00000279750		lncRNA	chrX:52053176-5205
ENSG0000(	420	10.50459	chrX:1657	ENSG00000228343		lncRNA	chrX:48579774-4858
ENSG0000(	420	10.50459	chrX:1657	TENT5D	NCGv7	protein_c	chrX:80335504-8044
ENSG0000(	420	10.50459	chrX:1657	MRPL32P2		Pseudoger	chrX:53807776-5380
ENSG0000(	420	10.50459	chrX:1657	RPL21P134		Pseudoger	chrX:75384346-7538
ENSG0000(	420	10.50459	chrX:1657	ENSG00000233250		lncRNA	chrX:53432722-5343
ENSG0000(	420	10.50459	chrX:1657	PABPN1P1		Pseudoger	chrX:71420083-7142
ENSG0000(	420	10.50459	chrX:1657	ENSG00000229030		Pseudoger	chrX:71848775-7184
ENSG0000(	420	10.50459	chrX:1657	U3		smallRNA	chrX:54064845-5406
ENSG0000(	420	10.50459	chrX:1657	ENSG00000204620		lncRNA	chrX:48568014-4857
ENSG0000(	420	10.50459	chrX:1657	CNOT7P1		Pseudoger	chrX:69937166-6993
ENSG0000(	420	10.50459	chrX:1657	RNU6-1225P		smallRNA	chrX:68102068-6810
ENSG0000(	420	10.50459	chrX:1657	SSX9P		Pseudoger	chrX:48296816-4830
ENSG0000(	420	10.50459	chrX:1657	ENSG00000228354		Pseudoger	chrX:52655207-5265
ENSG0000(	420	10.50459	chrX:1657	ENSG00000279528		TEC	chrX:48580741-4858
ENSG0000(	420	10.50459	chrX:1657	CYSLTR1		protein_c	chrX:78271468-7832
ENSG0000(	420	10.50459	chrX:1657	RN7SL139P		smallRNA	chrX:49007559-4900
ENSG0000(	420	10.50459	chrX:1657	PGAM4		protein_c	chrX:77967949-7796
ENSG0000(	420	10.50459	chrX:1657	ENSG00000279437		TEC	chrX:83506023-8350
ENSG0000(	420	10.50459	chrX:1657	SSXP3		Pseudoger	chrX:48156367-4816
ENSG0000(	420	10.50459	chrX:1657	PHF8		protein_c	chrX:53936676-5404

ENSG0000(	420	10.50459	chrX:1657	MORF4L1P6		Pseudoger	chrX:73474563-7347
ENSG0000(	420	10.50459	chrX:1657	XAGE5		protein_c	chrX:52811287-5281
ENSG0000(	420	10.50459	chrX:1657	CLCN5		protein_c	chrX:49922596-5009
ENSG0000(	420	10.50459	chrX:1657	PGK1P1		Pseudoger	chrX:68070520-6807
ENSG0000(	420	10.50459	chrX:1657	ENSG00000276929		Pseudoger	chrX:55137930-5514
ENSG0000(	420	10.50459	chrX:1657	CCT4P2		Pseudoger	chrX:65270913-6527
ENSG0000(	420	10.50459	chrX:1657	JPX		lncRNA	chrX:73944182-7407
ENSG0000(	420	10.50459	chrX:1657	TPT1P15		Pseudoger	chrX:72159845-7216
ENSG0000(	420	10.50459	chrX:1657	RPS6P26		Pseudoger	chrX:74376125-7437
ENSG0000(	420	10.50459	chrX:1657	ENSG00000291285		lncRNA	chrX:48107992-4811
ENSG0000(	420	10.50459	chrX:1657	SNORA11C		smallRNA	chrX:47388649-4738
ENSG0000(	420	10.50459	chrX:1657	GJB1		protein_c	chrX:71212811-7122
ENSG0000(	420	10.50459	chrX:1657	KANTR		protein_c	chrX:53094142-5317
ENSG0000(	420	10.50459	chrX:1657	STIP1P3		Pseudoger	chrX:86084716-8608
ENSG0000(	420	10.50459	chrX:1657	TPMTP3		Pseudoger	chrX:51979223-5198
ENSG0000(	420	10.50459	chrX:1657	LAS1L		protein_c	chrX:65438549-6553
ENSG0000(	420	10.50459	chrX:1657	APEX2		protein_c	chrX:55000363-5500
ENSG0000(	420	10.50459	chrX:1657	ENSG00000169164		Pseudoger	chrX:55654709-5565
ENSG0000(	420	10.50459	chrX:1657	HK2P1		Pseudoger	chrX:80571871-8057
ENSG0000(	420	10.50459	chrX:1657	AR	NCGv7;AC	protein_c	chrX:67544021-6773
ENSG0000(	420	10.50459	chrX:1657	GAGE2E		protein_c	chrX:49331626-4933
ENSG0000(	420	10.50459	chrX:1657	GAGE1		protein_c	chrX:49599020-4960
ENSG0000(	420	10.50459	chrX:1657	RN7SL460P		smallRNA	chrX:77885377-7788
ENSG0000(	420	10.50459	chrX:1657	ENSG00000277289		Pseudoger	chrX:52531709-5253
ENSG0000(	420	10.50459	chrX:1657	ZNF630-AS1		lncRNA	chrX:48056310-4806
ENSG0000(	420	10.50459	chrX:1657	ENSG00000277516		Pseudoger	chrX:55520382-5552
ENSG0000(	420	10.50459	chrX:1657	Z97054.1		smallRNA	chrX:53486672-5348
ENSG0000(	420	10.50459	chrX:1657	ENSG00000277499		Pseudoger	chrX:63087071-6308
ENSG0000(	420	10.50459	chrX:1657	ENSG00000278160		Pseudoger	chrX:52481515-5248
ENSG0000(	420	10.50459	chrX:1657	ENSG00000276897		Pseudoger	chrX:52409772-5241
ENSG0000(	420	10.50459	chrX:1657	RP11-472D17.1		Pseudoger	chrX:52458937-5246
ENSG0000(	420	10.50459	chrX:1657	ENSG00000276892		Pseudoger	chrX:85851575-8585
ENSG0000(	420	10.50459	chrX:1657	ARL5AP5		Pseudoger	chrX:76029895-7603
ENSG0000(	420	10.50459	chrX:1657	FOXN3P2		Pseudoger	chrX:75888700-7588
ENSG0000(	420	10.50459	chrX:1657	ENSG00000232828		lncRNA	chrX:48698963-4873
ENSG0000(	420	10.50459	chrX:1657	YWHAZP8		Pseudoger	chrX:73274785-7327
ENSG0000(	420	10.50459	chrX:1657	S100A11P6		Pseudoger	chrX:48274904-4827
ENSG0000(	420	10.50459	chrX:1657	CHMP1B2P		Pseudoger	chrX:80228489-8033
ENSG0000(	420	10.50459	chrX:1657	HNRNPDP1		Pseudoger	chrX:64044305-6404
ENSG0000(	420	10.50459	chrX:1657	ENSG00000232765		Pseudoger	chrX:55279839-5528
ENSG0000(	420	10.50459	chrX:1657	RBM22P7		Pseudoger	chrX:52509902-5251
ENSG0000(	420	10.50459	chrX:1657	FAM236D		protein_c	chrX:72807425-7280
ENSG0000(	420	10.50459	chrX:1657	ENSG00000225397		Pseudoger	chrX:52722338-5272
ENSG0000(	420	10.50459	chrX:1657	ENSG00000278358		Pseudoger	chrX:52480836-5248
ENSG0000(	420	10.50459	chrX:1657	ATG4AP1		Pseudoger	chrX:82998699-8299
ENSG0000(	420	10.50459	chrX:1657	ENSG00000228771		Pseudoger	chrX:52583989-5258
ENSG0000(	420	10.50459	chrX:1657	RP11-204I15.1		Pseudoger	chrX:52448587-5245
ENSG0000(	420	10.50459	chrX:1657	MIR548I4		smallRNA	chrX:84225752-8422
ENSG0000(	420	10.50459	chrX:1657	ENSG00000276391		Pseudoger	chrX:85207187-8521
ENSG0000(	420	10.50459	chrX:1657	PABPC1L2B-AS1		lncRNA	chrX:72998388-7300
ENSG0000(	420	10.50459	chrX:1657	ENSG00000276474		Pseudoger	chrX:52326166-5232

ENSG0000(	420	10.50459	chrX:1657	ENSG00000278319		Pseudoger	chrX:55101637-5510
ENSG0000(	420	10.50459	chrX:1657	ENSG00000276689		Pseudoger	chrX:65786763-6578
ENSG0000(	420	10.50459	chrX:1657	SNORA11D		smallRNA	chrX:52190621-5219
ENSG0000(	420	10.50459	chrX:1657	ENSG00000278283		Pseudoger	chrX:55044749-5504
ENSG0000(	420	10.50459	chrX:1657	RNU6-504P		smallRNA	chrX:51870706-5187
ENSG0000(	420	10.50459	chrX:1657	LDHBP2		Pseudoger	chrX:76334841-7633
ENSG0000(	420	10.50459	chrX:1657	CENPVP2		Pseudoger	chrX:51682067-5168
ENSG0000(	420	10.50459	chrX:1657	ENSG00000226971		Pseudoger	chrX:48446893-4844
ENSG0000(	420	10.50459	chrX:1657	SHISA5P2		Pseudoger	chrX:74066083-7406
ENSG0000(	420	10.50459	chrX:1657	ENSG00000203402		lncRNA	chrX:47297852-4729
ENSG0000(	420	10.50459	chrX:1657	ACAA2P1		Pseudoger	chrX:48775644-4877
ENSG0000(	420	10.50459	chrX:1657	ENSG00000290520		lncRNA	chrX:55654721-5565
ENSG0000(	420	10.50459	chrX:1657	ENSG00000179028		lncRNA	chrX:52199840-5220
ENSG0000(	420	10.50459	chrX:1657	SLC38A5		protein_c	chrX:48458537-4847
ENSG0000(	420	10.50459	chrX:1657	BX276092.1		smallRNA	chrX:71759385-7175
ENSG0000(	420	10.50459	chrX:1657	IGBP1-AS1		lncRNA	chrX:70163842-7016
ENSG0000(	420	10.50459	chrX:1657	ENSG00000274398		Pseudoger	chrX:76250183-7625
ENSG0000(	420	10.50459	chrX:1657	ENSG00000233484		Pseudoger	chrX:84403898-8440
ENSG0000(	420	10.50459	chrX:1657	AKIRIN1P2		Pseudoger	chrX:68132868-6813
ENSG0000(	420	10.50459	chrX:1657	WAS	NCGv7;AC	protein_c	chrX:48676596-4869
ENSG0000(	420	10.50459	chrX:1657	P2RY10BP		Pseudoger	chrX:79084936-7908
ENSG0000(	420	10.50459	chrX:1657	RP11-262D11.1		Pseudoger	chrX:72132150-7213
ENSG0000(	420	10.50459	chrX:1657	MIR325HG		lncRNA	chrX:76656866-7701
ENSG0000(	420	10.50459	chrX:1657	NUTF2P7		Pseudoger	chrX:71016531-7101
ENSG0000(	420	10.50459	chrX:1657	VDAC1P2		Pseudoger	chrX:49632500-4963
ENSG0000(	420	10.50459	chrX:1657	TLE1P1		Pseudoger	chrX:65408084-6540
ENSG0000(	420	10.50459	chrX:1657	ENSG00000228160		lncRNA	chrX:69569635-6957
ENSG0000(	420	10.50459	chrX:1657	MIR223HG		lncRNA	chrX:66015414-6602
ENSG0000(	420	10.50459	chrX:1657	DGKK		protein_c	chrX:50365409-5047
ENSG0000(	420	10.50459	chrX:1657	C4orf46P2		Pseudoger	chrX:77910682-7796
ENSG0000(	420	10.50459	chrX:1657	GRPEL2P2		Pseudoger	chrX:64697878-6469
ENSG0000(	420	10.50459	chrX:1657	U3		smallRNA	chrX:69692956-6969
ENSG0000(	420	10.50459	chrX:1657	LINC00891		lncRNA	chrX:71697196-7170
ENSG0000(	420	10.50459	chrX:1657	H3P44		Pseudoger	chrX:50905438-5090
ENSG0000(	420	10.50459	chrX:1657	MAGED1	NCGv7	protein_c	chrX:51803007-5190
ENSG0000(	420	10.50459	chrX:1657	ENSG00000233710		Pseudoger	chrX:67533163-6753
ENSG0000(	420	10.50459	chrX:1657	ENSG00000290184		protein_c	chrX:49258343-4927
ENSG0000(	420	10.50459	chrX:1657	MTCYBP31		Pseudoger	chrX:70120353-7012
ENSG0000(	420	10.50459	chrX:1657	U3		smallRNA	chrX:72726123-7272
ENSG0000(	420	10.50459	chrX:1657	MYCLP2		Pseudoger	chrX:57933837-5793
ENSG0000(	420	10.50459	chrX:1657	Y_RNA		smallRNA	chrX:50171197-5017
ENSG0000(	420	10.50459	chrX:1657	BX119917.1		smallRNA	chrX:72152336-7215
ENSG0000(	420	10.50459	chrX:1657	AL357115.1		smallRNA	chrX:81240820-8124
ENSG0000(	420	10.50459	chrX:1657	ENSG00000283178		Pseudoger	chrX:71869763-7187
ENSG0000(	420	10.50459	chrX:1657	PGAM1P7		Pseudoger	chrX:46646594-4664
ENSG0000(	420	10.50459	chrX:1657	CENPVL2		protein_c	chrX:51681212-5168
ENSG0000(	420	10.50459	chrX:1657	UBE2DNL		Pseudoger	chrX:84934113-8493
ENSG0000(	420	10.50459	chrX:1657	KIF4CP		Pseudoger	chrX:79323446-7932
ENSG0000(	420	10.50459	chrX:1657	SNORA11		smallRNA	chrX:54814370-5481
ENSG0000(	420	10.50459	chrX:1657	EIF3JP1		Pseudoger	chrX:82497669-8249
ENSG0000(	420	10.50459	chrX:1657	SPIN4-AS1		lncRNA	chrX:63349646-6335

ENSG0000(	420	10.50459	chrX:1657	RNU6-1189P		smallRNA	chrX:47087506-4708
ENSG0000(	420	10.50459	chrX:1657	WBP11P3		Pseudoger	chrX:80560146-8056
ENSG0000(	420	10.50459	chrX:1657	USP51	NCGv7	protein_c	chrX:55484616-5548
ENSG0000(	420	10.50459	chrX:1657	SNORA11E		smallRNA	chrX:52063347-5206
ENSG0000(	420	10.50459	chrX:1657	ENSG00000225055		Pseudoger	chrX:48357125-4835
ENSG0000(	420	10.50459	chrX:1657	FAM156B		protein_c	chrX:52891306-5290
ENSG0000(	420	10.50459	chrX:1657	RTL3		protein_c	chrX:78656068-7865
ENSG0000(	420	10.50459	chrX:1657	Y_RNA		smallRNA	chrX:68361075-6836
ENSG0000(	420	10.50459	chrX:1657	PDK1P2		Pseudoger	chrX:81600072-8160
ENSG0000(	420	10.50459	chrX:1657	RPS7P14		Pseudoger	chrX:74409518-7441
ENSG0000(	420	10.50459	chrX:1657	SERBP1P1		Pseudoger	chrX:68783472-6878
ENSG0000(	420	10.50459	chrX:1657	NBDY		protein_c	chrX:56729241-5681
ENSG0000(	420	10.50459	chrX:1657	ENSG00000275387		Pseudoger	chrX:54842014-5484
ENSG0000(	420	10.50459	chrX:1657	PAGE3		protein_c	chrX:55258415-5526
ENSG0000(	420	10.50459	chrX:1657	ATP7A		protein_c	chrX:77910690-7805
ENSG0000(	420	10.50459	chrX:1657	HDX		protein_c	chrX:84317874-8450
ENSG0000(	420	10.50459	chrX:1657	BRWD3		protein_c	chrX:80669503-8080
ENSG0000(	420	10.50459	chrX:1657	SLC7A3		protein_c	chrX:70925579-7093
ENSG0000(	420	10.50459	chrX:1657	RNU1-56P		smallRNA	chrX:71020275-7102
ENSG0000(	420	10.50459	chrX:1657	SSX5		protein_c	chrX:48186220-4819
ENSG0000(	420	10.50459	chrX:1657	SPANXN5		protein_c	chrX:52796144-5279
ENSG0000(	420	10.50459	chrX:1657	ENSG00000204368		Pseudoger	chrX:48212791-4821
ENSG0000(	420	10.50459	chrX:1657	SSX3		protein_c	chrX:48346427-4835
ENSG0000(	420	10.50459	chrX:1657	FAAH2	NCGv7	protein_c	chrX:57286706-5748
ENSG0000(	420	10.50459	chrX:1657	XAGE1E		protein_c	chrX:52495667-5250
ENSG0000(	420	10.50459	chrX:1657	XAGE1D		protein_c	chrX:52495667-5250
ENSG0000(	420	10.50459	chrX:1657	XAGE1A		protein_c	chrX:52512077-5251
ENSG0000(	420	10.50459	chrX:1657	ENSG00000280116		Pseudoger	chrX:49155242-4915
ENSG0000(	420	10.50459	chrX:1657	XAGE1B		protein_c	chrX:52492086-5250
ENSG0000(	420	10.50459	chrX:1657	RNU6-493P		smallRNA	chrX:80900757-8090
ENSG0000(	420	10.50459	chrX:1657	snoU13		smallRNA	chrX:71440517-7144
ENSG0000(	420	10.50459	chrX:1657	RHOG2P		Pseudoger	chrX:71352418-7135
ENSG0000(	420	10.50459	chrX:1657	ENSG00000229151		lncRNA	chrX:51190598-5139
ENSG0000(	420	10.50459	chrX:1657	CAPZA1P3		Pseudoger	chrX:72727977-7272
ENSG0000(	420	10.50459	chrX:1657	SPIN3		protein_c	chrX:56818298-5699
ENSG0000(	420	10.50459	chrX:1657	WASHC3P1		Pseudoger	chrX:70857163-7085
ENSG0000(	420	10.50459	chrX:1657	ENSG00000226870		Pseudoger	chrX:71719142-7172
ENSG0000(	420	10.50459	chrX:1657	KPNA4P1		Pseudoger	chrX:64305047-6430
ENSG0000(	420	10.50459	chrX:1657	CXXC1P1		lncRNA	chrX:47707191-4775
ENSG0000(	420	10.50459	chrX:1657	ENSG00000226867		Pseudoger	chrX:52781452-5278
ENSG0000(	420	10.50459	chrX:1657	ENSG00000280375		TEC	chrX:74122134-7412
ENSG0000(	420	10.50459	chrX:1657	PRICKLE3	DriverDBv	protein_c	chrX:49174802-4918
ENSG0000(	420	10.50459	chrX:1657	ENSG00000290686		lncRNA	chrX:52622935-5263
ENSG0000(	420	10.50459	chrX:1657	SSX9P		lncRNA	chrX:48301550-4830
ENSG0000(	420	10.50459	chrX:1657	ENSG00000290713		lncRNA	chrX:71719925-7172
ENSG0000(	420	10.50459	chrX:1657	ENSG00000290714		lncRNA	chrX:71760764-7176
ENSG0000(	420	10.50459	chrX:1657	ENSG00000290734		lncRNA	chrX:48117084-4812
ENSG0000(	420	10.50459	chrX:1657	ENSG00000226854		lncRNA	chrX:75903105-7590
ENSG0000(	420	10.50459	chrX:1657	MTND2P25		Pseudoger	chrX:62843697-6284
ENSG0000(	420	10.50459	chrX:1657	SNORD112		smallRNA	chrX:55903587-5590
ENSG0000(	420	10.50459	chrX:1657	RNU6-995P		smallRNA	chrX:80936434-8093

ENSG0000(	420	10.50459	chrX:1657	ENSG00000290748		lncRNA	chrX:55281371-5528
ENSG0000(	420	10.50459	chrX:1657	BMP2KL		Pseudoger	chrX:74185929-7418
ENSG0000(	420	10.50459	chrX:1657	CHIC1	AC	protein_c	chrX:73563197-7368
ENSG0000(	420	10.50459	chrX:1657	NAP1L6P		Pseudoger	chrX:73126037-7312
ENSG0000(	420	10.50459	chrX:1657	ENSG00000226820		Pseudoger	chrX:66437345-6644
ENSG0000(	420	10.50459	chrX:1657	NHSL2		protein_c	chrX:71910845-7216
ENSG0000(	420	10.50459	chrX:1657	CXorf65		protein_c	chrX:71103889-7110
ENSG0000(	420	10.50459	chrX:1657	TERF1P7		Pseudoger	chrX:75326772-7532
ENSG0000(	420	10.50459	chrX:1657	AWAT1		protein_c	chrX:70234655-7024
ENSG0000(	420	10.50459	chrX:1657	RNA5SP503		Pseudoger	chrX:48206258-4820
ENSG0000(	420	10.50459	chrX:1657	MPV17L2P1		Pseudoger	chrX:53340986-5334
ENSG0000(	420	10.50459	chrX:1657	IQSEC2		protein_c	chrX:53225828-5332
ENSG0000(	420	10.50459	chrX:1657	ENSG00000286118		lncRNA	chrX:52925956-5292
ENSG0000(	420	10.50459	chrX:1657	GSPT2		protein_c	chrX:51743442-5174
ENSG0000(	420	10.50459	chrX:1657	ENSG00000226280		Pseudoger	chrX:67373573-6737
ENSG0000(	420	10.50459	chrX:1657	GAGE2A		protein_c	chrX:49589496-4959
ENSG0000(	420	10.50459	chrX:1657	Y_RNA		smallRNA	chrX:72284945-7228
ENSG0000(	420	10.50459	chrX:1657	SHC1P1		Pseudoger	chrX:64432401-6443
ENSG0000(	420	10.50459	chrX:1657	EDA2R		protein_c	chrX:66595637-6663
ENSG0000(	420	10.50459	chrX:1657	ARHGEF9		protein_c	chrX:63634967-6380
ENSG0000(	420	10.50459	chrX:1657	SH3BGRL		protein_c	chrX:81202102-8129
ENSG0000(	420	10.50459	chrX:1657	COX7B		protein_c	chrX:77899440-7790
ENSG0000(	420	10.50459	chrX:1657	ENSG00000270497		Pseudoger	chrX:53065053-5306
ENSG0000(	420	10.50459	chrX:1657	RLIM	NCGv7	protein_c	chrX:74582976-7461
ENSG0000(	420	10.50459	chrX:1657	CDX4	NCGv7	protein_c	chrX:73447053-7345
ENSG0000(	420	10.50459	chrX:1657	ABCB7		protein_c	chrX:75051048-7515
ENSG0000(	420	10.50459	chrX:1657	ENSG00000287215		lncRNA	chrX:52195836-5226
ENSG0000(	420	10.50459	chrX:1657	Y_RNA		smallRNA	chrX:55582733-5558
ENSG0000(	420	10.50459	chrX:1657	FOXR2	NCGv7	protein_c	chrX:55623400-5562
ENSG0000(	420	10.50459	chrX:1657	CYCSP43		Pseudoger	chrX:69485343-6948
ENSG0000(	420	10.50459	chrX:1657	RGN		protein_c	chrX:47078355-4709
ENSG0000(	420	10.50459	chrX:1657	UBA1		protein_c	chrX:47190861-4721
ENSG0000(	420	10.50459	chrX:1657	S100A11P9		Pseudoger	chrX:48336899-4833
ENSG0000(	420	10.50459	chrX:1657	GNL3L		protein_c	chrX:54530183-5462
ENSG0000(	420	10.50459	chrX:1657	ENSG00000286977		lncRNA	chrX:57222706-5722
ENSG0000(	420	10.50459	chrX:1657	TIPINP1		Pseudoger	chrX:53456273-5345
ENSG0000(	420	10.50459	chrX:1657	BMP15		protein_c	chrX:50910735-5091
ENSG0000(	420	10.50459	chrX:1657	ENSG00000224523		Pseudoger	chrX:81408938-8142
ENSG0000(	420	10.50459	chrX:1657	MIR500B		smallRNA	chrX:50010671-5001
ENSG0000(	420	10.50459	chrX:1657	CBX1P1		Pseudoger	chrX:63299247-6329
ENSG0000(	420	10.50459	chrX:1657	TSIX		lncRNA	chrX:73792205-7382
ENSG0000(	420	10.50459	chrX:1657	RPL7P54		Pseudoger	chrX:78763226-7876
ENSG0000(	420	10.50459	chrX:1657	LINC01278		lncRNA	chrX:63222993-6356
ENSG0000(	420	10.50459	chrX:1657	SLC9A7		protein_c	chrX:46599251-4675
ENSG0000(	420	10.50459	chrX:1657	snoU13		smallRNA	chrX:48561702-4856
ENSG0000(	420	10.50459	chrX:1657	ACA64		smallRNA	chrX:80857076-8085
ENSG0000(	420	10.50459	chrX:1657	ENSG00000235461		lncRNA	chrX:85210684-8522
ENSG0000(	420	10.50459	chrX:1657	RP11-472D17.3		Pseudoger	chrX:52421452-5242
ENSG0000(	420	10.50459	chrX:1657	OTUD6A		protein_c	chrX:70062457-7006
ENSG0000(	420	10.50459	chrX:1657	WDR13	DriverDBv	protein_c	chrX:48590042-4860
ENSG0000(	420	10.50459	chrX:1657	ENSG00000230926		Pseudoger	chrX:51082818-5109

ENSG0000(	420	10.50459	chrX:1657	HDAC6		protein_c	chrX:48801377-4882
ENSG0000(	420	10.50459	chrX:1657	TFE3	NCGv7;AC	protein_c	chrX:49028726-4904
ENSG0000(	420	10.50459	chrX:1657	TBC1D25		protein_c	chrX:48539714-4856
ENSG0000(	420	10.50459	chrX:1657	GPKOW	DriverDBv	protein_c	chrX:49113407-4912
ENSG0000(	420	10.50459	chrX:1657	GRIPAP1		protein_c	chrX:48973720-4900
ENSG0000(	420	10.50459	chrX:1657	FTSJ1	DriverDBv	protein_c	chrX:48476021-4848
ENSG0000(	420	10.50459	chrX:1657	VSIG4		protein_c	chrX:66021738-6604
ENSG0000(	420	10.50459	chrX:1657	NLGN3		protein_c	chrX:71144821-7117
ENSG0000(	420	10.50459	chrX:1657	XAGE2		protein_c	chrX:52369021-5237
ENSG0000(	420	10.50459	chrX:1657	RNA5SP507		Pseudoger	chrX:70253042-7025
ENSG0000(	420	10.50459	chrX:1657	ENSG00000287370		lncRNA	chrX:64205974-6423
ENSG0000(	420	10.50459	chrX:1657	NUDT11	NCGv7	protein_c	chrX:51490011-5149
ENSG0000(	420	10.50459	chrX:1657	ENSG00000196395		Pseudoger	chrX:52824269-5282
ENSG0000(	420	10.50459	chrX:1657	ENSG00000226310		lncRNA	chrX:57121572-5713
ENSG0000(	420	10.50459	chrX:1657	FGF16		protein_c	chrX:77454157-7745
ENSG0000(	420	10.50459	chrX:1657	OTUD5	DriverDBv	protein_c	chrX:48922024-4895
ENSG0000(	420	10.50459	chrX:1657	MDH1P1		Pseudoger	chrX:57766320-5776
ENSG0000(	420	10.50459	chrX:1657	RN7SL746P		smallRNA	chrX:71084489-7108
ENSG0000(	420	10.50459	chrX:1657	NUS1P1		Pseudoger	chrX:47512602-4751
ENSG0000(	420	10.50459	chrX:1657	RNU6-935P		smallRNA	chrX:50649641-5064
ENSG0000(	420	10.50459	chrX:1657	RPSAP62		Pseudoger	chrX:53322990-5332
ENSG0000(	420	10.50459	chrX:1657	PHKA1		protein_c	chrX:72578814-7271
ENSG0000(	420	10.50459	chrX:1657	CORO1CP1		Pseudoger	chrX:79369364-7937
ENSG0000(	420	10.50459	chrX:1657	snoU13		smallRNA	chrX:47279127-4727
ENSG0000(	420	10.50459	chrX:1657	TRO	NCGv7	protein_c	chrX:54920462-5493
ENSG0000(	420	10.50459	chrX:1657	DLG3-AS1		lncRNA	chrX:70452958-7045
ENSG0000(	420	10.50459	chrX:1657	MKRN5P		Pseudoger	chrX:74161062-7416
ENSG0000(	420	10.50459	chrX:1657	KRT8P27		Pseudoger	chrX:64623117-6462
ENSG0000(	420	10.50459	chrX:1657	ENSG00000289245		lncRNA	chrX:49155862-4915
ENSG0000(	420	10.50459	chrX:1657	ENSG00000230934		Pseudoger	chrX:71691932-7169
ENSG0000(	420	10.50459	chrX:1657	UPRT		protein_c	chrX:75156388-7530
ENSG0000(	420	10.50459	chrX:1657	RNU6-1044P		smallRNA	chrX:73397531-7339
ENSG0000(	420	10.50459	chrX:1657	RNU2-68P		smallRNA	chrX:72376979-7237
ENSG0000(	420	10.50459	chrX:1657	VN1R110P		Pseudoger	chrX:48636165-4863
ENSG0000(	420	10.50459	chrX:1657	GDPD2		protein_c	chrX:70423031-7043
ENSG0000(	420	10.50459	chrX:1657	NALF2		protein_c	chrX:69504326-6953
ENSG0000(	420	10.50459	chrX:1657	MTND4P31		Pseudoger	chrX:70124972-7012
ENSG0000(	420	10.50459	chrX:1657	AKAP4		protein_c	chrX:50190777-5020
ENSG0000(	420	10.50459	chrX:1657	KRT8P17		Pseudoger	chrX:57984686-5798
ENSG0000(	420	10.50459	chrX:1657	RN7SL790P		smallRNA	chrX:74390692-7439
ENSG0000(	420	10.50459	chrX:1657	HDAC8		protein_c	chrX:72329516-7257
ENSG0000(	420	10.50459	chrX:1657	ENSG00000271199		lncRNA	chrX:73958059-7396
ENSG0000(	420	10.50459	chrX:1657	PSMA5P1		Pseudoger	chrX:55599663-5560
ENSG0000(	420	10.50459	chrX:1657	SLC16A2		protein_c	chrX:74421493-7453
ENSG0000(	420	10.50459	chrX:1657	CXXC1P1		Pseudoger	chrX:47722331-4772
ENSG0000(	420	10.50459	chrX:1657	PPP1R11P2		Pseudoger	chrX:57229034-5722
ENSG0000(	420	10.50459	chrX:1657	ZCCHC13		protein_c	chrX:74304180-7430
ENSG0000(	420	10.50459	chrX:1657	RN7SL388P		smallRNA	chrX:72198712-7219
ENSG0000(	420	10.50459	chrX:1657	ZNF157		protein_c	chrX:47370578-4741
ENSG0000(	420	10.50459	chrX:1657	THAP12P1		Pseudoger	chrX:74396871-7439
ENSG0000(	420	10.50459	chrX:1657	ZNF182		protein_c	chrX:47974851-4800

ENSG0000(	420	10.50459	chrX:1657	MTND1P31		Pseudoger	chrX:62842542-6284
ENSG0000(	420	10.50459	chrX:1657	SHROOM4		protein_c	chrX:50586796-5081
ENSG0000(	420	10.50459	chrX:1657	RIBC1		protein_c	chrX:53422690-5343
ENSG0000(	420	10.50459	chrX:1657	SSX7		protein_c	chrX:52644061-5265
ENSG0000(	420	10.50459	chrX:1657	ENSG00000226515		Pseudoger	chrX:74342713-7434
ENSG0000(	420	10.50459	chrX:1657	ENSG00000224617		Pseudoger	chrX:71789386-7178
ENSG0000(	420	10.50459	chrX:1657	AL121865.1		smallRNA	chrX:50645118-5064
ENSG0000(	420	10.50459	chrX:1657	UBE2V1P7		Pseudoger	chrX:78554412-7855
ENSG0000(	420	10.50459	chrX:1657	ALAS2		protein_c	chrX:55009055-5503
ENSG0000(	420	10.50459	chrX:1657	MAGEH1		protein_c	chrX:55452127-5545
ENSG0000(	420	10.50459	chrX:1657	RBM22P10		Pseudoger	chrX:52485045-5248
ENSG0000(	420	10.50459	chrX:1657	ENSG00000227329		Pseudoger	chrX:53164391-5316
ENSG0000(	420	10.50459	chrX:1657	MSN	NCGv7;AC	protein_c	chrX:65588377-6574
ENSG0000(	420	10.50459	chrX:1657	PFKFB1		protein_c	chrX:54932961-5499
ENSG0000(	420	10.50459	chrX:1657	ERAS	AC	protein_c	chrX:48826513-4883
ENSG0000(	420	10.50459	chrX:1657	TSR2		protein_c	chrX:54440404-5444
ENSG0000(	420	10.50459	chrX:1657	EZHIP		protein_c	chrX:51406948-5140
ENSG0000(	420	10.50459	chrX:1657	SPIN2A		protein_c	chrX:57134530-5713
ENSG0000(	420	10.50459	chrX:1657	RPS23P8		Pseudoger	chrX:70962964-7096
ENSG0000(	420	10.50459	chrX:1657	NDUFB11		protein_c	chrX:47142071-4714
ENSG0000(	420	10.50459	chrX:1657	ZNF41		protein_c	chrX:47445178-4748
ENSG0000(	420	10.50459	chrX:1657	UBQLN2		protein_c	chrX:56563627-5656
ENSG0000(	420	10.50459	chrX:1657	SALL1P1		Pseudoger	chrX:49664844-4966
ENSG0000(	420	10.50459	chrX:1657	IL2RG	NCGv7	protein_c	chrX:71107404-7111
ENSG0000(	420	10.50459	chrX:1657	GCNA		protein_c	chrX:71578437-7161
ENSG0000(	420	10.50459	chrX:1657	CPSF1P2		Pseudoger	chrX:49911620-4991
ENSG0000(	420	10.50459	chrX:1657	ZNF711	NCGv7	protein_c	chrX:85243991-8527
ENSG0000(	420	10.50459	chrX:1657	MRPL32P1		Pseudoger	chrX:48583093-4858
ENSG0000(	420	10.50459	chrX:1657	ARHGEF9-IT1		lncRNA	chrX:63670196-6367
ENSG0000(	420	10.50459	chrX:1657	CTHRC1P1		Pseudoger	chrX:79177200-7917
ENSG0000(	420	10.50459	chrX:1657	ENSG00000224556		Pseudoger	chrX:52422069-5245
ENSG0000(	420	10.50459	chrX:1657	ENSG00000230781		Pseudoger	chrX:84973217-8497
ENSG0000(	420	10.50459	chrX:1657	ENSG00000235350		Pseudoger	chrX:48433201-4844
ENSG0000(	420	10.50459	chrX:1657	GEMIN8P3		Pseudoger	chrX:86304367-8630
ENSG0000(	420	10.50459	chrX:1657	CHM		protein_c	chrX:85861180-8604
ENSG0000(	420	10.50459	chrX:1657	WASF4P		Pseudoger	chrX:47803296-4780
ENSG0000(	420	10.50459	chrX:1657	STARD8		protein_c	chrX:68647666-6872
ENSG0000(	420	10.50459	chrX:1657	ITGB1BP2		protein_c	chrX:71301750-7130
ENSG0000(	420	10.50459	chrX:1657	SNX12		protein_c	chrX:71056332-7107
ENSG0000(	420	10.50459	chrX:1657	RAB41		protein_c	chrX:70282093-7028
ENSG0000(	420	10.50459	chrX:1657	CCDC120	NCGv7	protein_c	chrX:49053572-4906
ENSG0000(	420	10.50459	chrX:1657	ZMYM3	NCGv7	protein_c	chrX:71239624-7125
ENSG0000(	420	10.50459	chrX:1657	TAF1	NCGv7;AC	protein_c	chrX:71366222-7153
ENSG0000(	420	10.50459	chrX:1657	GPR174	NCGv7	protein_c	chrX:79144688-7917
ENSG0000(	420	10.50459	chrX:1657	NONO	IntOGen-I	protein_c	chrX:71254814-7130
ENSG0000(	420	10.50459	chrX:1657	ENSG00000235224		Pseudoger	chrX:53113018-5311
ENSG0000(	420	10.50459	chrX:1657	PABPC1P3		Pseudoger	chrX:74583088-7458
ENSG0000(	420	10.50459	chrX:1657	LPAR4	NCGv7	protein_c	chrX:78747709-7875
ENSG0000(	420	10.50459	chrX:1657	OGT		protein_c	chrX:71533087-7157
ENSG0000(	420	10.50459	chrX:1657	SSX8P		Pseudoger	chrX:52624998-5263
ENSG0000(	420	10.50459	chrX:1657	RNU4-52P		smallRNA	chrX:49082028-4908

ENSG0000(	420	10.50459	chrX:1657	KDM5C-IT1		lncRNA	chrX:53212408-5321
ENSG0000(	420	10.50459	chrX:1657	EBP	DriverDB\	protein_c	chrX:48521799-4852
ENSG0000(	420	10.50459	chrX:1657	AWAT2		protein_c	chrX:70040542-7004
ENSG0000(	420	10.50459	chrX:1657	RNU6-854P		smallRNA	chrX:77837289-7783
ENSG0000(	420	10.50459	chrX:1657	RNU4-81P		smallRNA	chrX:70450879-7045
ENSG0000(	420	10.50459	chrX:1657	RPL7P57		Pseudoger	chrX:47840963-4784
ENSG0000(	420	10.50459	chrX:1657	PAGE1		protein_c	chrX:49687447-4969
ENSG0000(	420	10.50459	chrX:1657	NICN2P		Pseudoger	chrX:47273366-4727
ENSG0000(	420	10.50459	chrX:1657	AL451105.1		Pseudoger	chrX:75899804-7589
ENSG0000(	420	10.50459	chrX:1657	GAGE12D		protein_c	chrX:49541733-4954
ENSG0000(	420	10.50459	chrX:1657	RBM22P11		Pseudoger	chrX:52526971-5252
ENSG0000(	420	10.50459	chrX:1657	ENSG00000237311		lncRNA	chrX:65925836-6600
ENSG0000(	420	10.50459	chrX:1657	EIF4BP9		Pseudoger	chrX:66074835-6607
ENSG0000(	420	10.50459	chrX:1657	ENSG00000288783		lncRNA	chrX:50161928-5016
ENSG0000(	420	10.50459	chrX:1657	RBM22P8		Pseudoger	chrX:52527284-5252
ENSG0000(	420	10.50459	chrX:1657	U3		smallRNA	chrX:70846081-7084
ENSG0000(	420	10.50459	chrX:1657	ASB12		protein_c	chrX:64224194-6423
ENSG0000(	420	10.50459	chrX:1657	AF196779.1		smallRNA	chrX:49203242-4920
ENSG0000(	420	10.50459	chrX:1657	S100A11P10		Pseudoger	chrX:52687363-5268
ENSG0000(	420	10.50459	chrX:1657	Y_RNA		smallRNA	chrX:71491066-7149
ENSG0000(	420	10.50459	chrX:1657	ENSG00000237265		Pseudoger	chrX:71663276-7166
ENSG0000(	420	10.50459	chrX:1657	MMADHCP1		Pseudoger	chrX:76222637-7622
ENSG0000(	420	10.50459	chrX:1657	RP11-472D17.6		Pseudoger	chrX:52409771-5241
ENSG0000(	420	10.50459	chrX:1657	SYP-AS1		lncRNA	chrX:49198966-4920
ENSG0000(	420	10.50459	chrX:1657	ENSG00000237345		Pseudoger	chrX:48279423-4828
ENSG0000(	420	10.50459	chrX:1657	ITPK1P1		Pseudoger	chrX:47156638-4715
ENSG0000(	420	10.50459	chrX:1657	RNU6-330P		smallRNA	chrX:74680053-7468
ENSG0000(	420	10.50459	chrX:1657	GAGE12F		protein_c	chrX:49551278-4956
ENSG0000(	420	10.50459	chrX:1657	EIF3MP1		Pseudoger	chrX:82875649-8287
ENSG0000(	420	10.50459	chrX:1657	AL590762.1		Pseudoger	chrX:71300730-7130
ENSG0000(	420	10.50459	chrX:1657	RRAGB		protein_c	chrX:55717749-5575
ENSG0000(	420	10.50459	chrX:1657	FCF1P9		Pseudoger	chrX:86481532-8648
ENSG0000(	420	10.50459	chrX:1657	UQCR10P1		Pseudoger	chrX:51923552-5192
ENSG0000(	420	10.50459	chrX:1657	ZXDB	IntOGen-I	protein_c	chrX:57592011-5759
ENSG0000(	420	10.50459	chrX:1657	UXT-AS1		lncRNA	chrX:47658833-4769
ENSG0000(	420	10.50459	chrX:1657	HNRNPA1P25		Pseudoger	chrX:74473017-7447
ENSG0000(	420	10.50459	chrX:1657	RPL7P53		Pseudoger	chrX:73535503-7353
ENSG0000(	420	10.50459	chrX:1657	DLG3	NCGv7	protein_c	chrX:70444835-7050
ENSG0000(	420	10.50459	chrX:1657	VDAC1P1		Pseudoger	chrX:80929500-8093
ENSG0000(	420	10.50459	chrX:1657	ENSG00000227493		Pseudoger	chrX:51030422-5103
ENSG0000(	420	10.50459	chrX:1657	ZCRB1P1		Pseudoger	chrX:71314912-7131
ENSG0000(	420	10.50459	chrX:1657	MAGEE1		protein_c	chrX:76427710-7643
ENSG0000(	420	10.50459	chrX:1657	OPHN1		protein_c	chrX:67949349-6843
ENSG0000(	420	10.50459	chrX:1657	ENSG00000288759		lncRNA	chrX:47129317-4713
ENSG0000(	420	10.50459	chrX:1657	RNA5SP504		Pseudoger	chrX:52665231-5266
ENSG0000(	420	10.50459	chrX:1657	LRRFIP2P1		Pseudoger	chrX:73237647-7323
ENSG0000(	420	10.50459	chrX:1657	Y_RNA		smallRNA	chrX:86019608-8601
ENSG0000(	420	10.50459	chrX:1657	ARAF	NCGv7;AC	protein_c	chrX:47561205-4757
ENSG0000(	420	10.50459	chrX:1657	BLOC1S2P1		Pseudoger	chrX:64726748-6472
ENSG0000(	420	10.50459	chrX:1657	RPL31P63		Pseudoger	chrX:68600099-6860
ENSG0000(	420	10.50459	chrX:1657	GAGE12B		protein_c	chrX:49529869-4952

ENSG0000(	420	10.50459	chrX:1657	CENPVL3		protein_c	chrX:51617020-5161
ENSG0000(	420	10.50459	chrX:1657	MIR374A		smallRNA	chrX:74287286-7428
ENSG0000(	420	10.50459	chrX:1657	BTF3P8		Pseudoger	chrX:63766875-6376
ENSG0000(	420	10.50459	chrX:1657	AL445523.1		smallRNA	chrX:65215237-6521
ENSG0000(	420	10.50459	chrX:1657	ENSG00000288661		protein_c	chrX:63754485-6375
ENSG0000(	420	10.50459	chrX:1657	SSBL2P		Pseudoger	chrX:62779250-6278
ENSG0000(	420	10.50459	chrX:1657	VTRNA3-1P		smallRNA	chrX:53462209-5346
ENSG0000(	420	10.50459	chrX:1657	MTFR1P1		Pseudoger	chrX:66360766-6636
ENSG0000(	420	10.50459	chrX:1657	ACTG1P10		Pseudoger	chrX:53142832-5314
ENSG0000(	420	10.50459	chrX:1657	MTRNR2L10		protein_c	chrX:55181391-5518
ENSG0000(	420	10.50459	chrX:1657	SETP4		Pseudoger	chrX:84755136-8475
ENSG0000(	420	10.50459	chrX:1657	MIR1468		smallRNA	chrX:63786002-6378
ENSG0000(	420	10.50459	chrX:1657	ENSG00000236571		Pseudoger	chrX:53337783-5333
ENSG0000(	420	10.50459	chrX:1657	ENSG00000236576		Pseudoger	chrX:51856968-5185
ENSG0000(	420	10.50459	chrX:1657	ENSG00000288053		protein_c	chrX:49071470-4907
ENSG0000(	420	10.50459	chrX:1657	ENSG00000237182		Pseudoger	chrX:71736454-7173
ENSG0000(	420	10.50459	chrX:1657	ENSG00000288059		lncRNA	chrX:81475532-8149
ENSG0000(	420	10.50459	chrX:1657	ENSG00000288739		lncRNA	chrX:55488883-5549
ENSG0000(	420	10.50459	chrX:1657	AL139396.1		smallRNA	chrX:53143034-5314
ENSG0000(	420	10.50459	chrX:1657	RNU6-562P		smallRNA	chrX:75202703-7520
ENSG0000(	420	10.50459	chrX:1657	MIR361		smallRNA	chrX:85903636-8590
ENSG0000(	420	10.50459	chrX:1657	ENSG00000227486		lncRNA	chrX:55908123-5620
ENSG0000(	420	10.50459	chrX:1657	ITM2A		protein_c	chrX:79360384-7936
ENSG0000(	420	10.50459	chrX:1657	P2RY10		protein_c	chrX:78945332-7896
ENSG0000(	420	10.50459	chrX:1657	FABP5P15		Pseudoger	chrX:77727867-7772
ENSG0000(	420	10.50459	chrX:1657	PCNPP4		Pseudoger	chrX:75537547-7553
ENSG0000(	420	10.50459	chrX:1657	SSX4	NCGv7;AC	protein_c	chrX:48383516-4839
ENSG0000(	420	10.50459	chrX:1657	ZXDA		protein_c	chrX:57905430-5791
ENSG0000(	420	10.50459	chrX:1657	AL590764.1		smallRNA	chrX:71141185-7114
ENSG0000(	420	10.50459	chrX:1657	HMGN5	AC	protein_c	chrX:81113699-8120
ENSG0000(	420	10.50459	chrX:1657	WDR45	NCGv7	protein_c	chrX:49074433-4910
ENSG0000(	420	10.50459	chrX:1657	SOCS5P4		Pseudoger	chrX:71043214-7104
ENSG0000(	420	10.50459	chrX:1657	PAGE2B		protein_c	chrX:55075030-5507
ENSG0000(	420	10.50459	chrX:1657	HEPH		protein_c	chrX:66162671-6626
ENSG0000(	420	10.50459	chrX:1657	Y_RNA		smallRNA	chrX:53324562-5332
ENSG0000(	420	10.50459	chrX:1657	IGBP1		protein_c	chrX:70133447-7016
ENSG0000(	420	10.50459	chrX:1657	ENSG00000223958		Pseudoger	chrX:52707602-5270
ENSG0000(	420	10.50459	chrX:1657	TRAPPC2LP1		Pseudoger	chrX:70361486-7036
ENSG0000(	420	10.50459	chrX:1657	HMGB1P15		Pseudoger	chrX:50931114-5093
ENSG0000(	420	10.50459	chrX:1657	SSXP1		Pseudoger	chrX:52606535-5261
ENSG0000(	420	10.50459	chrX:1657	RN7SL648P		smallRNA	chrX:74242610-7424
ENSG0000(	420	10.50459	chrX:1657	FAM226B		lncRNA	chrX:72777608-7277
ENSG0000(	420	10.50459	chrX:1657	MAP2K4P1		Pseudoger	chrX:73524275-7356
ENSG0000(	420	10.50459	chrX:1657	ENSG00000289038		lncRNA	chrX:68498309-6849
ENSG0000(	420	10.50459	chrX:1657	RP11-552J9.15		Pseudoger	chrX:52748497-5275
ENSG0000(	420	10.50459	chrX:1657	RPS26P11		Pseudoger	chrX:72044545-7204
ENSG0000(	420	10.50459	chrX:1657	MRPS18CP7		Pseudoger	chrX:53825887-5382
ENSG0000(	420	10.50459	chrX:1657	GOT2P6		Pseudoger	chrX:55961208-5596
ENSG0000(	420	10.50459	chrX:1657	KIF4A		protein_c	chrX:70290104-7042
ENSG0000(	420	10.50459	chrX:1657	APOOL		protein_c	chrX:85003877-8509
ENSG0000(	420	10.50459	chrX:1657	WNK3		protein_c	chrX:54192823-5435

ENSG0000(	420	10.50459	chrX:1657	snoU13		smallRNA	chrX:48081767-4808
ENSG0000(	420	10.50459	chrX:1657	DDX3P2		Pseudoger	chrX:74133004-7413
ENSG0000(	420	10.50459	chrX:1657	RP11-344N17.12		Pseudoger	chrX:48380150-4838
ENSG0000(	420	10.50459	chrX:1657	ENSG00000231593		Pseudoger	chrX:52561769-5256
ENSG0000(	420	10.50459	chrX:1657	LINC01560		lncRNA	chrX:47483571-4748
ENSG0000(	420	10.50459	chrX:1657	MAGED4		protein_c	chrX:52184876-5219
ENSG0000(	420	10.50459	chrX:1657	EFNB1		protein_c	chrX:68829021-6884
ENSG0000(	420	10.50459	chrX:1657	ENSG00000289132		lncRNA	chrX:80810091-8081
ENSG0000(	420	10.50459	chrX:1657	POU3F4		protein_c	chrX:83508290-8351
ENSG0000(	420	10.50459	chrX:1657	PKMP2		Pseudoger	chrX:66497748-6649
ENSG0000(	420	10.50459	chrX:1657	ENSG00000270012		lncRNA	chrX:49273054-4927
ENSG0000(	420	10.50459	chrX:1657	S100A11P7		Pseudoger	chrX:48228833-4822
ENSG0000(	420	10.50459	chrX:1657	ATP5MKP1		Pseudoger	chrX:74173890-7417
ENSG0000(	420	10.50459	chrX:1657	AP1M2P1		Pseudoger	chrX:65469087-6547
ENSG0000(	420	10.50459	chrX:1657	MTND1P30		Pseudoger	chrX:55180377-5518
ENSG0000(	420	10.50459	chrX:1657	ENSG00000237717		Pseudoger	chrX:71744828-7174
ENSG0000(	420	10.50459	chrX:1657	ENSG00000287757		lncRNA	chrX:48071223-4807
ENSG0000(	420	10.50459	chrX:1657	UQCRBP1		Pseudoger	chrX:56737242-5673
ENSG0000(	420	10.50459	chrX:1657	FAM156A		protein_c	chrX:52926402-5299
ENSG0000(	420	10.50459	chrX:1657	RN7SL785P		smallRNA	chrX:47280815-4728
ENSG0000(	420	10.50459	chrX:1657	ENSG00000288908		lncRNA	chrX:48958643-4895
ENSG0000(	420	10.50459	chrX:1657	ENSG00000287767		lncRNA	chrX:51325790-5133
ENSG0000(	420	10.50459	chrX:1657	SPIN2P1		Pseudoger	chrX:57068914-5706
ENSG0000(	420	10.50459	chrX:1657	SSX2B	AC	protein_c	chrX:52751132-5279
ENSG0000(	420	10.50459	chrX:1657	RPS4X		protein_c	chrX:72255679-7227
ENSG0000(	420	10.50459	chrX:1657	ENSG00000231489		Pseudoger	chrX:48423342-4842
ENSG0000(	420	10.50459	chrX:1657	RNU6-394P		smallRNA	chrX:66676258-6667
ENSG0000(	420	10.50459	chrX:1657	ATRX	NCGv7;AC	protein_c	chrX:77504880-7778
ENSG0000(	420	10.50459	chrX:1657	hsa-mir-4536-2		smallRNA	chrX:55451495-5545
ENSG0000(	420	10.50459	chrX:1657	GAGE12C		protein_c	chrX:49532177-4953
ENSG0000(	420	10.50459	chrX:1657	HSD17B10		protein_c	chrX:53431258-5343
ENSG0000(	420	10.50459	chrX:1657	SMC1A	NCGv7	protein_c	chrX:53374149-5342
ENSG0000(	420	10.50459	chrX:1657	S100A11P5		Pseudoger	chrX:48177731-4817
ENSG0000(	420	10.50459	chrX:1657	MAGIX		protein_c	chrX:49162987-4916
ENSG0000(	420	10.50459	chrX:1657	RNU6-29P		smallRNA	chrX:48776965-4877
ENSG0000(	420	10.50459	chrX:1657	SSX4B		protein_c	chrX:48402082-4841
ENSG0000(	420	10.50459	chrX:1657	ENSG00000236190		Pseudoger	chrX:86888586-8688
ENSG0000(	420	10.50459	chrX:1657	ENSG00000237971		Pseudoger	chrX:65185018-6518
ENSG0000(	420	10.50459	chrX:1657	PRXL2CP1		Pseudoger	chrX:65356890-6535
ENSG0000(	420	10.50459	chrX:1657	DMRTC1		protein_c	chrX:72872025-7294
ENSG0000(	420	10.50459	chrX:1657	ENSG00000237926		Pseudoger	chrX:51162864-5116
ENSG0000(	420	10.50459	chrX:1657	ZNF81		protein_c	chrX:47836902-4800
ENSG0000(	420	10.50459	chrX:1657	RNU6-434P		smallRNA	chrX:54343546-5434
ENSG0000(	420	10.50459	chrX:1657	RPS6KA6		protein_c	chrX:84058346-8420
ENSG0000(	420	10.50459	chrX:1657	S100A11P8		Pseudoger	chrX:52770809-5277
ENSG0000(	420	10.50459	chrX:1657	HUWE1	NCGv7	protein_c	chrX:53532096-5368
ENSG0000(	420	10.50459	chrX:1657	FAM236B		protein_c	chrX:72781865-7278
ENSG0000(	420	10.50459	chrX:1657	PORCN-DT		lncRNA	chrX:48506523-4850
ENSG0000(	420	10.50459	chrX:1657	AF222686.1		smallRNA	chrX:50006656-5000
ENSG0000(	420	10.50459	chrX:1657	TRAPPC13P1		Pseudoger	chrX:76655498-7665
ENSG0000(	420	10.50459	chrX:1657	SSXP5		Pseudoger	chrX:52672718-5267

ENSG0000(	420	10.50459	chrX:1657	YWHAZP7		Pseudoger	chrX:64612632-6461
ENSG0000(	420	10.50459	chrX:1657	TERF1P4		Pseudoger	chrX:83748825-8374
ENSG0000(	420	10.50459	chrX:1657	RNU6-421P		smallRNA	chrX:49945336-4994
ENSG0000(	420	10.50459	chrX:1657	GATA1	NCGv7;AC	protein_c	chrX:48786540-4879
ENSG0000(	420	10.50459	chrX:1657	RBM3	TAG;AC	protein_c	chrX:48574449-4858
ENSG0000(	420	10.50459	chrX:1657	TOMM20P4		Pseudoger	chrX:73223124-7322
ENSG0000(	420	10.50459	chrX:1657	MAGED2		protein_c	chrX:54807599-5481
ENSG0000(	420	10.50459	chrX:1657	RNU1-112P		smallRNA	chrX:72740706-7274
ENSG0000(	420	10.50459	chrX:1657	ENSG00000231875		Pseudoger	chrX:81363268-8136
ENSG0000(	420	10.50459	chrX:1657	MTHFD1P1		Pseudoger	chrX:57392646-5739
ENSG0000(	420	10.50459	chrX:1657	EEF1A1P29		Pseudoger	chrX:86160264-8616
ENSG0000(	420	10.50459	chrX:1657	MAGT1		protein_c	chrX:77825747-7789
ENSG0000(	420	10.50459	chrX:1657	RP11-472D17.4		Pseudoger	chrX:52382052-5238
ENSG0000(	420	10.50459	chrX:1657	ENSG00000234780		Pseudoger	chrX:48197221-4819
ENSG0000(	420	10.50459	chrX:1657	RPL7AP71		Pseudoger	chrX:54223324-5422
ENSG0000(	420	10.50459	chrX:1657	CXCR3		protein_c	chrX:71615916-7161
ENSG0000(	420	10.50459	chrX:1657	PGK1		protein_c	chrX:77910739-7812
ENSG0000(	420	10.50459	chrX:1657	LINC01496		lncRNA	chrX:51498490-5151
ENSG0000(	420	10.50459	chrX:1657	PAGE5		protein_c	chrX:55220346-5522
ENSG0000(	420	10.50459	chrX:1657	NPM1P49		Pseudoger	chrX:47438736-4743
ENSG0000(	420	10.50459	chrX:1657	ENSG00000224735		Pseudoger	chrX:53759026-5375
ENSG0000(	420	10.50459	chrX:1657	MIR223		smallRNA	chrX:66018870-6601
ENSG0000(	420	10.50459	chrX:1657	ENSG00000286268		protein_c	chrX:48521806-4854
ENSG0000(	420	10.50459	chrX:1657	AARSD1P1		Pseudoger	chrX:74069276-7407
ENSG0000(	420	10.50459	chrX:1657	PQBP1		protein_c	chrX:48890197-4890
ENSG0000(	420	10.50459	chrX:1657	SPIN2B		protein_c	chrX:57118551-5712
ENSG0000(	420	10.50459	chrX:1657	EDA		protein_c	chrX:69616067-7003
ENSG0000(	420	10.50459	chrX:1657	TTC3P1		Pseudoger	chrX:75740831-7574
ENSG0000(	420	10.50459	chrX:1657	SOCS6P1		Pseudoger	chrX:71527814-7153
ENSG0000(	420	10.50459	chrX:1657	CXorf49		protein_c	chrX:71714371-7171
ENSG0000(	420	10.50459	chrX:1657	CXorf49B		protein_c	chrX:71763349-7176
ENSG0000(	420	10.50459	chrX:1657	RNA5SP509		Pseudoger	chrX:77066709-7706
ENSG0000(	420	10.50459	chrX:1657	PCSK1N		protein_c	chrX:48831096-4883
ENSG0000(	420	10.50459	chrX:1657	RNU6-1078P		smallRNA	chrX:71965972-7196
ENSG0000(	420	10.50459	chrX:1657	ITIH6		protein_c	chrX:54748918-5479
ENSG0000(	420	10.50459	chrX:1657	DACH2	NCGv7	protein_c	chrX:86148451-8683
ENSG0000(	420	10.50459	chrX:1657	NAP1L2	NCGv7	protein_c	chrX:73212299-7321
ENSG0000(	420	10.50459	chrX:1657	MORF4L1P5		Pseudoger	chrX:65327988-6533
ENSG0000(	420	10.50459	chrX:1657	PORCN	NCGv7	protein_c	chrX:48508959-4852
ENSG0000(	420	10.50459	chrX:1657	PIN4		protein_c	chrX:72181353-7230
ENSG0000(	420	10.50459	chrX:1657	AL590763.1		smallRNA	chrX:71625753-7162
ENSG0000(	420	10.50459	chrX:1657	FGD1		protein_c	chrX:54445454-5449
ENSG0000(	420	10.50459	chrX:1657	MIR188		smallRNA	chrX:50003503-5000
ENSG0000(	420	10.50459	chrX:1657	SMSP1		Pseudoger	chrX:47718002-4771
ENSG0000(	420	10.50459	chrX:1657	PABPC1L2A		protein_c	chrX:73077276-7307
ENSG0000(	420	10.50459	chrX:1657	MIR545		smallRNA	chrX:74287104-7428
ENSG0000(	420	10.50459	chrX:1657	ENSG00000271533		lncRNA	chrX:74209976-7421
ENSG0000(	420	10.50459	chrX:1657	LINC01284		lncRNA	chrX:51095836-5122
ENSG0000(	420	10.50459	chrX:1657	CDK16	DriverDB\	protein_c	chrX:47217860-4722
ENSG0000(	420	10.50459	chrX:1657	P2RY4		protein_c	chrX:70258166-7026
ENSG0000(	420	10.50459	chrX:1657	RNU6-149P		smallRNA	chrX:47351843-4735

ENSG0000(	420	10.50459	chrX:1657	USP11		protein_c	chrX:47232866-4724
ENSG0000(	420	10.50459	chrX:1657	ZC4H2	NCGv7	protein_c	chrX:64915802-6503
ENSG0000(	420	10.50459	chrX:1657	MIR532		smallRNA	chrX:50003148-5000
ENSG0000(	420	10.50459	chrX:1657	RNU6-707P		smallRNA	chrX:48153980-4815
ENSG0000(	420	10.50459	chrX:1657	COPS8P1		Pseudoger	chrX:86798598-8679
ENSG0000(	420	10.50459	chrX:1657	SSXP9		Pseudoger	chrX:48322349-4832
ENSG0000(	420	10.50459	chrX:1657	ZFRP1		Pseudoger	chrX:62878811-6288
ENSG0000(	420	10.50459	chrX:1657	ENSG00000234792		Pseudoger	chrX:52612656-5261
ENSG0000(	420	10.50459	chrX:1657	NANOGP9		Pseudoger	chrX:65772741-6577
ENSG0000(	420	10.50459	chrX:1657	SFR1P2		Pseudoger	chrX:85849410-8585
ENSG0000(	420	10.50459	chrX:1657	PPATP2		Pseudoger	chrX:78699384-7869
ENSG0000(	420	10.50459	chrX:1657	SSX1	NCGv7;AC	protein_c	chrX:48255392-4826
ENSG0000(	420	10.50459	chrX:1657	ERCC6L		protein_c	chrX:72204657-7223
ENSG0000(	420	10.50459	chrX:1657	UXT		protein_c	chrX:47651796-4765
ENSG0000(	420	10.50459	chrX:1657	RP2		protein_c	chrX:46837043-4688
ENSG0000(	420	10.50459	chrX:1657	CFP		protein_c	chrX:47623172-4763
ENSG0000(	420	10.50459	chrX:1657	ELK1	TAG;AC	protein_c	chrX:47635521-4765
ENSG0000(	420	10.50459	chrX:1657	TIMM17B		protein_c	chrX:48893447-4889
ENSG0000(	420	10.50459	chrX:1657	ZNF630		protein_c	chrX:47983356-4807
ENSG0000(	420	10.50459	chrX:1657	ENSG00000271589		Pseudoger	chrX:74769639-7476
ENSG0000(	420	10.50459	chrX:1657	TIMP1		protein_c	chrX:47582408-4758
ENSG0000(	420	10.50459	chrX:1657	MAGED4B		protein_c	chrX:52061827-5206
ENSG0000(	420	10.50459	chrX:1657	MIR500A		smallRNA	chrX:50008431-5000
ENSG0000(	420	10.50459	chrX:1657	HNRNPH3P1		Pseudoger	chrX:80529037-8053
ENSG0000(	420	10.50459	chrX:1657	ENSG00000186678		Pseudoger	chrX:55172717-5517
ENSG0000(	420	10.50459	chrX:1657	ZC3H12B		protein_c	chrX:65034788-6550
ENSG0000(	420	10.50459	chrX:1657	MAGEE2		protein_c	chrX:75782987-7578
ENSG0000(	420	10.50459	chrX:1657	KDM5C	NCGv7;AC	protein_c	chrX:53176283-5322
ENSG0000(	420	10.50459	chrX:1657	SPIN4		protein_c	chrX:63347228-6335
ENSG0000(	420	10.50459	chrX:1657	RNA5SP505		Pseudoger	chrX:53909054-5390
ENSG0000(	420	10.50459	chrX:1657	MTMR8	NCGv7	protein_c	chrX:64268081-6439
ENSG0000(	420	10.50459	chrX:1657	ENSG00000230241		Pseudoger	chrX:48135658-4813
ENSG0000(	420	10.50459	chrX:1657	CITED1		protein_c	chrX:72301638-7230
ENSG0000(	420	10.50459	chrX:1657	ATXN7L3P1		Pseudoger	chrX:66055587-6605
ENSG0000(	420	10.50459	chrX:1657	NEXMIF		protein_c	chrX:74732856-7492
ENSG0000(	420	10.50459	chrX:1657	ENSG00000234448		Pseudoger	chrX:48371905-4837
ENSG0000(	420	10.50459	chrX:1657	RNU6-974P		smallRNA	chrX:82001220-8200
ENSG0000(	420	10.50459	chrX:1657	FOXP3		protein_c	chrX:49250438-4926
ENSG0000(	420	10.50459	chrX:1657	TAF9B		protein_c	chrX:78129748-7813
ENSG0000(	420	10.50459	chrX:1657	NLRP2B		protein_c	chrX:57677067-5768
ENSG0000(	420	10.50459	chrX:1657	PLP2	DriverDB\	protein_c	chrX:49171898-4917
ENSG0000(	420	10.50459	chrX:1657	SYP		protein_c	chrX:49187815-4920
ENSG0000(	420	10.50459	chrX:1657	PGAM4P1		Pseudoger	chrX:54671985-5467
ENSG0000(	420	10.50459	chrX:1657	CACNA1F		protein_c	chrX:49205063-4923
ENSG0000(	420	10.50459	chrX:1657	ZDHHC15		protein_c	chrX:75368427-7552
ENSG0000(	420	10.50459	chrX:1657	ENSG00000234442		Pseudoger	chrX:71761561-7176
ENSG0000(	420	10.50459	chrX:1657	RN7SL262P		smallRNA	chrX:49152651-4915
ENSG0000(	420	10.50459	chrX:1657	PPP1R3F		protein_c	chrX:49269793-4930
ENSG0000(	420	10.50459	chrX:1657	BRAFP1		Pseudoger	chrX:75582676-7558
ENSG0000(	420	10.50459	chrX:1657	CCDC22		protein_c	chrX:49235470-4925
ENSG0000(	420	10.50459	chrX:1657	MIR660		smallRNA	chrX:50013241-5001

ENSG0000(	420	10.50459	chrX:1657	HDGFL3P1		Pseudoger	chrX:55054945-5505
ENSG0000(	420	10.50459	chrX:1657	PIM2	DriverDBv	protein_c	chrX:48913182-4891
ENSG0000(	420	10.50459	chrX:1657	HSPB1P2		Pseudoger	chrX:49233956-4923
ENSG0000(	420	10.50459	chrX:1657	SLC35A2	DriverDBv	protein_c	chrX:48903180-4891
ENSG0000(	420	10.50459	chrX:1657	ENSG00000241207		Pseudoger	chrX:48276704-4827
ENSG0000(	420	10.50459	chrX:1657	ENSG00000226530		lncRNA	chrX:51395915-5146
ENSG0000(	420	10.50459	chrX:1657	FTX		lncRNA	chrX:73940435-7429
ENSG0000(	420	10.50459	chrX:1657	HMGN1P34		Pseudoger	chrX:78519593-7852
ENSG0000(	420	10.50459	chrX:1657	SUV39H1		protein_c	chrX:48695554-4870
ENSG0000(	420	10.50459	chrX:1657	PAGE4		protein_c	chrX:49829260-4983
ENSG0000(	420	10.50459	chrX:1657	KCND1		protein_c	chrX:48961378-4897
ENSG0000(	420	10.50459	chrX:1657	RNY4P23		smallRNA	chrX:70396279-7039
ENSG0000(	420	10.50459	chrX:1657	LINC00269		lncRNA	chrX:69179557-6920
ENSG0000(	420	10.50459	chrX:1657	KLF8	AC	protein_c	chrX:56232356-5629
ENSG0000(	420	10.50459	chrX:1657	RNU12-2P		smallRNA	chrX:47132671-4713
ENSG0000(	420	10.50459	chrX:1657	ENSG00000286181		lncRNA	chrX:49262866-4927
ENSG0000(	420	10.50459	chrX:1657	COX6CP12		Pseudoger	chrX:68645326-6864
ENSG0000(	420	10.50459	chrX:1657	PBDC1		protein_c	chrX:76173040-7617
ENSG0000(	418	10.45457	chr1:3732	MIR205HG		lncRNA	chr1:209428817-209
ENSG0000(	418	10.45457	chr1:3732	C1orf147		lncRNA	chr1:206491116-206
ENSG0000(	418	10.45457	chr1:3732	LINC01693		lncRNA	chr1:211639440-211
ENSG0000(	418	10.45457	chr1:3732	snoU13		smallRNA	chr1:207038694-207
ENSG0000(	418	10.45457	chr1:3732	NSL1		protein_c	chr1:212726153-212
ENSG0000(	418	10.45457	chr1:3732	NENF	DriverDBv	protein_c	chr1:212432920-212
ENSG0000(	418	10.45457	chr1:3732	RCOR3		protein_c	chr1:211258377-211
ENSG0000(	418	10.45457	chr1:3732	ENSG00000286383		lncRNA	chr1:203996532-204
ENSG0000(	418	10.45457	chr1:3732	LAMB3		protein_c	chr1:209614870-209
ENSG0000(	418	10.45457	chr1:3732	ENSG00000285719		lncRNA	chr1:207909992-207
ENSG0000(	418	10.45457	chr1:3732	ENSG00000287354		lncRNA	chr1:210362861-210
ENSG0000(	418	10.45457	chr1:3732	NEK2	NCGv7	protein_c	chr1:211658657-211
ENSG0000(	418	10.45457	chr1:3732	ENSG00000213041		Pseudoger	chr1:205202191-205
ENSG0000(	418	10.45457	chr1:3732	ENSG00000289071		lncRNA	chr1:207822903-207
ENSG0000(	418	10.45457	chr1:3732	ENSG00000223649		lncRNA	chr1:211492255-211
ENSG0000(	418	10.45457	chr1:3732	LINC01698		lncRNA	chr1:209367662-209
ENSG0000(	418	10.45457	chr1:3732	ENSG00000275392		lncRNA	chr1:207127010-207
ENSG0000(	418	10.45457	chr1:3732	ENSG00000229832		lncRNA	chr1:212357418-212
ENSG0000(	418	10.45457	chr1:3732	LEMD1-DT		lncRNA	chr1:205455929-205
ENSG0000(	418	10.45457	chr1:3732	RNA5SP75		Pseudoger	chr1:204707320-204
ENSG0000(	418	10.45457	chr1:3732	RNU6-423P		smallRNA	chr1:212692264-212
ENSG0000(	418	10.45457	chr1:3732	ENSG00000285521		lncRNA	chr1:205775559-205
ENSG0000(	418	10.45457	chr1:3732	RBBP5		protein_c	chr1:205086142-205
ENSG0000(	418	10.45457	chr1:3732	RP11-31207.2		lncRNA	chr1:206203345-206
ENSG0000(	418	10.45457	chr1:3732	ENSG00000240710		lncRNA	chr1:204603035-204
ENSG0000(	418	10.45457	chr1:3732	CR2		protein_c	chr1:207453024-207
ENSG0000(	418	10.45457	chr1:3732	C1orf74		protein_c	chr1:209779208-209
ENSG0000(	418	10.45457	chr1:3732	ENSG00000285239		lncRNA	chr1:207709024-207
ENSG0000(	418	10.45457	chr1:3732	ANGEL2		protein_c	chr1:212992182-213
ENSG0000(	418	10.45457	chr1:3732	RN7SL512P		smallRNA	chr1:212615708-212
ENSG0000(	418	10.45457	chr1:3732	ENSG00000279946		TEC	chr1:206541758-206
ENSG0000(	418	10.45457	chr1:3732	PPP1R15B-AS1		lncRNA	chr1:204377850-204
ENSG0000(	418	10.45457	chr1:3732	RNU6-418P		smallRNA	chr1:205595041-205

ENSG0000(	418	10.45457	chr1:3732	ENSG00000285417		Pseudoger	chr1:206035252-206
ENSG0000(	418	10.45457	chr1:3732	HSD11B1-AS1		lncRNA	chr1:209661356-209
ENSG0000(	418	10.45457	chr1:3732	CD55		protein_c	chr1:207321519-207
ENSG0000(	418	10.45457	chr1:3732	ATF3	AC	protein_c	chr1:212565334-212
ENSG0000(	418	10.45457	chr1:3732	FLVCR1		protein_c	chr1:212858275-212
ENSG0000(	418	10.45457	chr1:3732	GARIN4		protein_c	chr1:212624474-212
ENSG0000(	418	10.45457	chr1:3732	TRAF3IP3	NCGv7	protein_c	chr1:209756032-209
ENSG0000(	418	10.45457	chr1:3732	GOLT1A	DriverDBv	protein_c	chr1:204198163-204
ENSG0000(	418	10.45457	chr1:3732	TMEM81		protein_c	chr1:205083129-205
ENSG0000(	418	10.45457	chr1:3732	HSD11B1		protein_c	chr1:209686178-209
ENSG0000(	418	10.45457	chr1:3732	CD34		protein_c	chr1:207880972-207
ENSG0000(	418	10.45457	chr1:3732	AL592063.1		smallRNA	chr1:213609696-213
ENSG0000(	418	10.45457	chr1:3732	UTP25		protein_c	chr1:209827972-209
ENSG0000(	418	10.45457	chr1:3732	ENSG00000235862		lncRNA	chr1:212624284-212
ENSG0000(	418	10.45457	chr1:3732	NUCKS1		protein_c	chr1:205712822-205
ENSG0000(	418	10.45457	chr1:3732	PM20D1	NCGv7	protein_c	chr1:205828025-205
ENSG0000(	418	10.45457	chr1:3732	IRF6	NCGv7	protein_c	chr1:209785617-209
ENSG0000(	418	10.45457	chr1:3732	ENSG00000287445		lncRNA	chr1:212430269-212
ENSG0000(	418	10.45457	chr1:3732	KLHDC8A		protein_c	chr1:205336061-205
ENSG0000(	418	10.45457	chr1:3732	ENSG00000287432		lncRNA	chr1:206147163-206
ENSG0000(	418	10.45457	chr1:3732	CDK18		protein_c	chr1:205504596-205
ENSG0000(	418	10.45457	chr1:3732	RAB29		protein_c	chr1:205767986-205
ENSG0000(	418	10.45457	chr1:3732	ENSG00000226036		Pseudoger	chr1:212647296-212
ENSG0000(	418	10.45457	chr1:3732	SLC26A9	DriverDBv	protein_c	chr1:205913048-205
ENSG0000(	418	10.45457	chr1:3732	MFSD4A		protein_c	chr1:205568885-205
ENSG0000(	418	10.45457	chr1:3732	FAM72A	AC	protein_c	chr1:206186178-206
ENSG0000(	418	10.45457	chr1:3732	ENSG00000237848		Pseudoger	chr1:204394541-204
ENSG0000(	418	10.45457	chr1:3732	MIR4260		smallRNA	chr1:209623444-209
ENSG0000(	418	10.45457	chr1:3732	ENSG00000286198		lncRNA	chr1:208244966-208
ENSG0000(	418	10.45457	chr1:3732	LRRN2		protein_c	chr1:204617170-204
ENSG0000(	418	10.45457	chr1:3732	SLC30A1		protein_c	chr1:211571568-211
ENSG0000(	418	10.45457	chr1:3732	AC092017.1		smallRNA	chr1:211210989-211
ENSG0000(	418	10.45457	chr1:3732	ENSG00000286213		lncRNA	chr1:212504178-212
ENSG0000(	418	10.45457	chr1:3732	ENSG00000230063		lncRNA	chr1:212297448-212
ENSG0000(	418	10.45457	chr1:3732	KISS1		protein_c	chr1:204190341-204
ENSG0000(	418	10.45457	chr1:3732	ENSG00000219133		Pseudoger	chr1:204346776-204
ENSG0000(	418	10.45457	chr1:3732	ENSG00000288007		lncRNA	chr1:212559363-212
ENSG0000(	418	10.45457	chr1:3732	ENSG00000271580		Pseudoger	chr1:205091163-205
ENSG0000(	418	10.45457	chr1:3732	CBX1P3		Pseudoger	chr1:203954640-203
ENSG0000(	418	10.45457	chr1:3732	MDM4	NCGv7;AC	protein_c	chr1:204516379-204
ENSG0000(	418	10.45457	chr1:3732	KRT8P29		Pseudoger	chr1:203872574-203
ENSG0000(	418	10.45457	chr1:3732	ENSG00000287902		lncRNA	chr1:209531216-209
ENSG0000(	418	10.45457	chr1:3732	RD3		protein_c	chr1:211476522-211
ENSG0000(	418	10.45457	chr1:3732	LEMD1		protein_c	chr1:205381378-205
ENSG0000(	418	10.45457	chr1:3732	ENSG00000278684		Pseudoger	chr1:209001338-209
ENSG0000(	418	10.45457	chr1:3732	LINC01717		lncRNA	chr1:208728665-208
ENSG0000(	418	10.45457	chr1:3732	SERTAD4	DriverDBv	protein_c	chr1:210232796-210
ENSG0000(	418	10.45457	chr1:3732	RPL23AP18		Pseudoger	chr1:212309051-212
ENSG0000(	418	10.45457	chr1:3732	TRAF5	NCGv7	protein_c	chr1:211326615-211
ENSG0000(	418	10.45457	chr1:3732	LINC01735		lncRNA	chr1:208606564-208
ENSG0000(	418	10.45457	chr1:3732	ENSG00000271680		Pseudoger	chr1:206905928-206

ENSG0000(	418	10.45457	chr1:373;	C4BPAP2		Pseudoger	chr1:207225798-207
ENSG0000(	418	10.45457	chr1:373;	FLVCR1-DT		lncRNA	chr1:212852105-212
ENSG0000(	418	10.45457	chr1:373;	ENSG00000288738		lncRNA	chr1:211605839-211
ENSG0000(	418	10.45457	chr1:373;	ENSG00000236889		lncRNA	chr1:206175059-206
ENSG0000(	418	10.45457	chr1:373;	RPL31P13		Pseudoger	chr1:213428708-213
ENSG0000(	418	10.45457	chr1:373;	IKBKE	AC	protein_c	chr1:206470476-206
ENSG0000(	418	10.45457	chr1:373;	ENSG00000236905		Pseudoger	chr1:212824027-212
ENSG0000(	418	10.45457	chr1:373;	ENSG00000236911		lncRNA	chr1:207551925-207
ENSG0000(	418	10.45457	chr1:373;	Y_RNA		smallRNA	chr1:206747980-206
ENSG0000(	418	10.45457	chr1:373;	PLXNA2	NCGv7	protein_c	chr1:208022242-208
ENSG0000(	418	10.45457	chr1:373;	SNX25P1		Pseudoger	chr1:211417025-211
ENSG0000(	418	10.45457	chr1:373;	ENSG00000236779		Pseudoger	chr1:204528845-204
ENSG0000(	418	10.45457	chr1:373;	ENSG00000236942		Pseudoger	chr1:205625483-205
ENSG0000(	418	10.45457	chr1:373;	LINC01774		lncRNA	chr1:208972454-208
ENSG0000(	418	10.45457	chr1:373;	ENSG00000232537		lncRNA	chr1:209147220-209
ENSG0000(	418	10.45457	chr1:373;	ENSG00000224114		Pseudoger	chr1:206695837-206
ENSG0000(	418	10.45457	chr1:373;	ENSG00000225522		Pseudoger	chr1:204183006-204
ENSG0000(	418	10.45457	chr1:373;	RPL22P4		Pseudoger	chr1:206160886-206
ENSG0000(	418	10.45457	chr1:373;	FDPSP8		Pseudoger	chr1:211660189-211
ENSG0000(	418	10.45457	chr1:373;	RPL21P28		Pseudoger	chr1:212051524-212
ENSG0000(	418	10.45457	chr1:373;	RN7SL344P		smallRNA	chr1:211792113-211
ENSG0000(	418	10.45457	chr1:373;	SPATA45		protein_c	chr1:212830141-212
ENSG0000(	418	10.45457	chr1:373;	LINC02942		lncRNA	chr1:207240122-207
ENSG0000(	418	10.45457	chr1:373;	MIR135B		smallRNA	chr1:205448302-205
ENSG0000(	418	10.45457	chr1:373;	ENSG00000228792		lncRNA	chr1:211635865-211
ENSG0000(	418	10.45457	chr1:373;	C4BPB	DriverDBv	protein_c	chr1:207088860-207
ENSG0000(	418	10.45457	chr1:373;	NEK2-DT		lncRNA	chr1:211675749-211
ENSG0000(	418	10.45457	chr1:373;	ENSG00000261000		lncRNA	chr1:206503948-206
ENSG0000(	418	10.45457	chr1:373;	ENSG00000229983		lncRNA	chr1:212168207-212
ENSG0000(	418	10.45457	chr1:373;	CR1L		protein_c	chr1:207645113-207
ENSG0000(	418	10.45457	chr1:373;	LINC00467		lncRNA	chr1:211382736-211
ENSG0000(	418	10.45457	chr1:373;	AVPR1B-DT		lncRNA	chr1:206117782-206
ENSG0000(	418	10.45457	chr1:373;	HHAT	NCGv7	protein_c	chr1:210328252-210
ENSG0000(	418	10.45457	chr1:373;	ATP5MC2P1		Pseudoger	chr1:209267798-209
ENSG0000(	418	10.45457	chr1:373;	LINC02773		lncRNA	chr1:212653305-212
ENSG0000(	418	10.45457	chr1:373;	SNORA72		smallRNA	chr1:205731221-205
ENSG0000(	418	10.45457	chr1:373;	ENSG00000279333		TEC	chr1:210678315-210
ENSG0000(	418	10.45457	chr1:373;	CNTN2	AC	protein_c	chr1:205042937-205
ENSG0000(	418	10.45457	chr1:373;	ENSG00000225233		lncRNA	chr1:213492288-213
ENSG0000(	418	10.45457	chr1:373;	ADORA2BP1		Pseudoger	chr1:209744373-209
ENSG0000(	418	10.45457	chr1:373;	IL10		protein_c	chr1:206767602-206
ENSG0000(	418	10.45457	chr1:373;	ENSG00000236108		Pseudoger	chr1:205134646-205
ENSG0000(	418	10.45457	chr1:373;	CAMK1G		protein_c	chr1:209583714-209
ENSG0000(	418	10.45457	chr1:373;	RPS6KC1		protein_c	chr1:213051233-213
ENSG0000(	418	10.45457	chr1:373;	TMCC2		protein_c	chr1:205227946-205
ENSG0000(	418	10.45457	chr1:373;	SLC41A1		protein_c	chr1:205789094-205
ENSG0000(	418	10.45457	chr1:373;	DSTYK		protein_c	chr1:205142505-205
ENSG0000(	418	10.45457	chr1:373;	ARPC3P2		Pseudoger	chr1:211442274-211
ENSG0000(	418	10.45457	chr1:373;	PIK3C2B	NCGv7	protein_c	chr1:204422628-204
ENSG0000(	418	10.45457	chr1:373;	SCARNA20		smallRNA	chr1:204727991-204
ENSG0000(	418	10.45457	chr1:373;	KCNH1		protein_c	chr1:210676823-211

ENSG0000(	418	10.45457	chr1:3732	KCNH1-IT1		lncRNA	chr1:211132588-211
ENSG0000(	418	10.45457	chr1:3732	ENSG00000288934		lncRNA	chr1:204411365-204
ENSG0000(	418	10.45457	chr1:3732	SNORA16B		smallRNA	chr1:212352816-212
ENSG0000(	418	10.45457	chr1:3732	ENSG00000261314		lncRNA	chr1:211583015-211
ENSG0000(	418	10.45457	chr1:3732	C4BPA		protein_c	chr1:207104233-207
ENSG0000(	418	10.45457	chr1:3732	DYRK3-AS1		lncRNA	chr1:206634184-206
ENSG0000(	418	10.45457	chr1:3732	AL583832.1		smallRNA	chr1:205062252-205
ENSG0000(	418	10.45457	chr1:3732	PFKFB2	DriverDBv	protein_c	chr1:207034366-207
ENSG0000(	418	10.45457	chr1:3732	HSPE1P6		Pseudoger	chr1:203903723-203
ENSG0000(	418	10.45457	chr1:3732	ENSG00000224260		lncRNA	chr1:209528455-209
ENSG0000(	418	10.45457	chr1:3732	AVPR1B	DriverDBv	protein_c	chr1:206106935-206
ENSG0000(	418	10.45457	chr1:3732	ENSG00000226565		Pseudoger	chr1:207150205-207
ENSG0000(	418	10.45457	chr1:3732	CDCA4P4		Pseudoger	chr1:207762584-207
ENSG0000(	418	10.45457	chr1:3732	G0S2		protein_c	chr1:209675412-209
ENSG0000(	418	10.45457	chr1:3732	BATF3		protein_c	chr1:212686417-212
ENSG0000(	418	10.45457	chr1:3732	ENSG00000236317		Pseudoger	chr1:212855175-212
ENSG0000(	418	10.45457	chr1:3732	LPGAT1		protein_c	chr1:211743457-211
ENSG0000(	418	10.45457	chr1:3732	ENSG00000261065		lncRNA	chr1:204131062-204
ENSG0000(	418	10.45457	chr1:3732	IL19		protein_c	chr1:206770764-206
ENSG0000(	418	10.45457	chr1:3732	AC096645.1		smallRNA	chr1:203999245-203
ENSG0000(	418	10.45457	chr1:3732	RHEX		protein_c	chr1:206053172-206
ENSG0000(	418	10.45457	chr1:3732	ENSG00000289700		protein_c	chr1:209779629-209
ENSG0000(	418	10.45457	chr1:3732	RASSF5		protein_c	chr1:206507531-206
ENSG0000(	418	10.45457	chr1:3732	SYT14		protein_c	chr1:209900923-210
ENSG0000(	418	10.45457	chr1:3732	CD46		protein_c	chr1:207752037-207
ENSG0000(	418	10.45457	chr1:3732	IPO8P1		Pseudoger	chr1:210859177-210
ENSG0000(	418	10.45457	chr1:3732	SERTAD4-AS1		lncRNA	chr1:210231456-210
ENSG0000(	418	10.45457	chr1:3732	LINC02769		lncRNA	chr1:208626741-208
ENSG0000(	418	10.45457	chr1:3732	PLEKHA6		protein_c	chr1:204218853-204
ENSG0000(	418	10.45457	chr1:3732	AL691452.1		smallRNA	chr1:207515285-207
ENSG0000(	418	10.45457	chr1:3732	SNRPGP10		Pseudoger	chr1:205351247-205
ENSG0000(	418	10.45457	chr1:3732	BPNT2P1		Pseudoger	chr1:210462345-210
ENSG0000(	418	10.45457	chr1:3732	SNORA26		smallRNA	chr1:212025561-212
ENSG0000(	418	10.45457	chr1:3732	SNRPE		protein_c	chr1:203861599-203
ENSG0000(	418	10.45457	chr1:3732	LINC02608		lncRNA	chr1:212180141-212
ENSG0000(	418	10.45457	chr1:3732	ENSG00000240219		lncRNA	chr1:204626775-204
ENSG0000(	418	10.45457	chr1:3732	DTL	NCGv7	protein_c	chr1:212035553-212
ENSG0000(	418	10.45457	chr1:3732	LINC00628		lncRNA	chr1:204368431-204
ENSG0000(	418	10.45457	chr1:3732	Y_RNA		smallRNA	chr1:211803017-211
ENSG0000(	418	10.45457	chr1:3732	ENSG00000228153		lncRNA	chr1:204663872-204
ENSG0000(	418	10.45457	chr1:3732	ENSG00000233455		Pseudoger	chr1:210303684-210
ENSG0000(	418	10.45457	chr1:3732	CR1	NCGv7	protein_c	chr1:207496147-207
ENSG0000(	418	10.45457	chr1:3732	ETNK2		protein_c	chr1:204131062-204
ENSG0000(	418	10.45457	chr1:3732	SOX13		protein_c	chr1:204073115-204
ENSG0000(	418	10.45457	chr1:3732	REN		protein_c	chr1:204154819-204
ENSG0000(	418	10.45457	chr1:3732	RPS26P13		Pseudoger	chr1:208697369-208
ENSG0000(	418	10.45457	chr1:3732	ENSG00000287157		lncRNA	chr1:210386657-210
ENSG0000(	418	10.45457	chr1:3732	RP11-61J19.4		lncRNA	chr1:212557833-212
ENSG0000(	418	10.45457	chr1:3732	SNORD112		smallRNA	chr1:204904747-204
ENSG0000(	418	10.45457	chr1:3732	ENSG00000226843		Pseudoger	chr1:208255290-208
ENSG0000(	418	10.45457	chr1:3732	ERLNC1		lncRNA	chr1:204141404-204

ENSG0000(	418	10.45457	chr1:3732	PPP2R5A	NCGv7	protein_c	chr1:212285410-212
ENSG0000(	418	10.45457	chr1:3732	ENSG00000287046		lncRNA	chr1:209107503-209
ENSG0000(	418	10.45457	chr1:3732	ENSG00000243636		lncRNA	chr1:207179296-207
ENSG0000(	418	10.45457	chr1:3732	ENSG00000287033		lncRNA	chr1:211376804-211
ENSG0000(	418	10.45457	chr1:3732	PACC1		protein_c	chr1:212363928-212
ENSG0000(	418	10.45457	chr1:3732	LPGAT1-AS1		lncRNA	chr1:211829636-211
ENSG0000(	418	10.45457	chr1:3732	ENSG00000226868		lncRNA	chr1:211715928-211
ENSG0000(	418	10.45457	chr1:3732	LINC02771		lncRNA	chr1:212466699-212
ENSG0000(	418	10.45457	chr1:3732	TFDP1P1		Pseudoger	chr1:209232196-209
ENSG0000(	418	10.45457	chr1:3732	MIR29B2CHG		lncRNA	chr1:207801518-207
ENSG0000(	418	10.45457	chr1:3732	TATDN3		protein_c	chr1:212791828-212
ENSG0000(	418	10.45457	chr1:3732	C1orf116		protein_c	chr1:207018522-207
ENSG0000(	418	10.45457	chr1:3732	LINC01696		lncRNA	chr1:209325392-209
ENSG0000(	418	10.45457	chr1:3732	LINC01740		lncRNA	chr1:212545694-212
ENSG0000(	418	10.45457	chr1:3732	PRELID1P5		Pseudoger	chr1:211207239-211
ENSG0000(	418	10.45457	chr1:3732	AL356310.1		smallRNA	chr1:211653456-211
ENSG0000(	418	10.45457	chr1:3732	ENSG00000233626		Pseudoger	chr1:211936249-211
ENSG0000(	418	10.45457	chr1:3732	ENSG00000286619		lncRNA	chr1:205813322-205
ENSG0000(	418	10.45457	chr1:3732	ENSG00000282718		lncRNA	chr1:212916787-212
ENSG0000(	418	10.45457	chr1:3732	MIR3122		smallRNA	chr1:212077613-212
ENSG0000(	418	10.45457	chr1:3732	ENSG00000229509		Pseudoger	chr1:206333327-206
ENSG0000(	418	10.45457	chr1:3732	ENSG00000283044		lncRNA	chr1:207401691-207
ENSG0000(	418	10.45457	chr1:3732	CD46P1		Pseudoger	chr1:207645234-207
ENSG0000(	418	10.45457	chr1:3732	SLC45A3	IntOGen-I	protein_c	chr1:205657851-205
ENSG0000(	418	10.45457	chr1:3732	ENSG00000286572		lncRNA	chr1:204064533-204
ENSG0000(	418	10.45457	chr1:3732	ELK4	NCGv7;AC	protein_c	chr1:205597556-205
ENSG0000(	418	10.45457	chr1:3732	PPP1R15B		protein_c	chr1:204396492-204
ENSG0000(	418	10.45457	chr1:3732	YOD1		protein_c	chr1:207043849-207
ENSG0000(	418	10.45457	chr1:3732	ENSG00000228081		Pseudoger	chr1:209173014-209
ENSG0000(	418	10.45457	chr1:3732	RPL7AP20		Pseudoger	chr1:206528915-206
ENSG0000(	418	10.45457	chr1:3732	ENSG00000283952		protein_c	chr1:211082872-211
ENSG0000(	418	10.45457	chr1:3732	LINC02767		lncRNA	chr1:207959292-207
ENSG0000(	418	10.45457	chr1:3732	ENSG00000284376		lncRNA	chr1:211108445-211
ENSG0000(	418	10.45457	chr1:3732	LEMD1-AS1		lncRNA	chr1:205373252-205
ENSG0000(	418	10.45457	chr1:3732	BLACAT1		protein_c	chr1:205434885-205
ENSG0000(	418	10.45457	chr1:3732	ENSG00000234915		lncRNA	chr1:212299495-212
ENSG0000(	418	10.45457	chr1:3732	ENSG00000227687		lncRNA	chr1:205935128-205
ENSG0000(	418	10.45457	chr1:3732	ENSG00000226945		Pseudoger	chr1:206907619-206
ENSG0000(	418	10.45457	chr1:3732	RNU5A-8P		smallRNA	chr1:210374154-210
ENSG0000(	418	10.45457	chr1:3732	ENSG00000229657		Pseudoger	chr1:204946608-204
ENSG0000(	418	10.45457	chr1:3732	SRGAP2		protein_c	chr1:206342846-206
ENSG0000(	418	10.45457	chr1:3732	TMCC2-AS1		lncRNA	chr1:205233821-205
ENSG0000(	418	10.45457	chr1:3732	ST13P19		Pseudoger	chr1:210265636-210
ENSG0000(	418	10.45457	chr1:3732	ENSG00000235182		Pseudoger	chr1:212853280-212
ENSG0000(	418	10.45457	chr1:3732	NFASC		protein_c	chr1:204828651-205
ENSG0000(	418	10.45457	chr1:3732	NUAK2	AC	protein_c	chr1:205302063-205
ENSG0000(	418	10.45457	chr1:3732	ENSG00000231691		lncRNA	chr1:204276901-204
ENSG0000(	418	10.45457	chr1:3732	RNU2-19P		smallRNA	chr1:205566716-205
ENSG0000(	418	10.45457	chr1:3732	CDCA4P3		Pseudoger	chr1:207658454-207
ENSG0000(	418	10.45457	chr1:3732	EIF2D		protein_c	chr1:206571292-206
ENSG0000(	418	10.45457	chr1:3732	SNORD112		smallRNA	chr1:206681963-206

ENSG0000(	418	10.45457	chr1:3732	LINC00303		lncRNA	chr1:204032447-204
ENSG0000(	418	10.45457	chr1:3732	CTSE		protein_c	chr1:206009146-206
ENSG0000(	418	10.45457	chr1:3732	ENSG00000287220		lncRNA	chr1:208106102-208
ENSG0000(	418	10.45457	chr1:3732	RN7SKP98		smallRNA	chr1:212099521-212
ENSG0000(	418	10.45457	chr1:3732	ENSG00000234004		Pseudoger	chr1:211173488-211
ENSG0000(	418	10.45457	chr1:3732	VASH2		protein_c	chr1:212950520-212
ENSG0000(	418	10.45457	chr1:3732	INTS7	NCGv7;AC	protein_c	chr1:211940399-212
ENSG0000(	418	10.45457	chr1:3732	FCAMR		protein_c	chr1:206957965-206
ENSG0000(	418	10.45457	chr1:3732	SNORD60		smallRNA	chr1:206080238-206
ENSG0000(	418	10.45457	chr1:3732	PIGR	DriverDBv	protein_c	chr1:206928522-206
ENSG0000(	418	10.45457	chr1:3732	MAPKAPK2		protein_c	chr1:206684905-206
ENSG0000(	418	10.45457	chr1:3732	ENSG00000228255		lncRNA	chr1:213731416-213
ENSG0000(	418	10.45457	chr1:3732	FCMR		protein_c	chr1:206903317-206
ENSG0000(	418	10.45457	chr1:3732	C4BPAP1		Pseudoger	chr1:207165496-207
ENSG0000(	418	10.45457	chr1:3732	ENSG00000287343		lncRNA	chr1:209987333-209
ENSG0000(	418	10.45457	chr1:3732	ENSG00000287197		lncRNA	chr1:204822664-204
ENSG0000(	418	10.45457	chr1:3732	IL24		protein_c	chr1:206897443-206
ENSG0000(	418	10.45457	chr1:3732	DYRK3		protein_c	chr1:206635536-206
ENSG0000(	418	10.45457	chr1:3732	IL20		protein_c	chr1:206865623-206
ENSG0000(	418	10.45457	chr1:3732	AL161793.1		smallRNA	chr1:204653102-204
ENSG0000(	417	10.42956	chr16:209	ENSG00000283421		Pseudoger	chr16:21299903-213
ENSG0000(	415	10.37954	chrX:1657	AL596268.1		smallRNA	chrX:35056331-3505
ENSG0000(	412	10.3045	chr5:1696	RNU6-272P		smallRNA	chr5:53912020-5391
ENSG0000(	412	10.3045	chr5:1696	MIR581		smallRNA	chr5:53951504-5395
ENSG0000(	412	10.3045	chr5:1696	ITGA2-AS1		lncRNA	chr5:52930606-5299
ENSG0000(	412	10.3045	chr5:1696	MOCS2-DT		lncRNA	chr5:53109816-5312
ENSG0000(	412	10.3045	chr5:1696	GZMA		protein_c	chr5:55102646-5511
ENSG0000(	412	10.3045	chr5:1696	ENSG00000248898		lncRNA	chr5:52673186-5279
ENSG0000(	412	10.3045	chr5:1696	ITGA1		protein_c	chr5:52787916-5295
ENSG0000(	412	10.3045	chr5:1696	ENSG00000288035		lncRNA	chr5:51438792-5166
ENSG0000(	412	10.3045	chr5:1696	ENSG00000240052		Pseudoger	chr5:53413504-5341
ENSG0000(	412	10.3045	chr5:1696	ENSG00000213956		Pseudoger	chr5:50633124-5063
ENSG0000(	412	10.3045	chr5:1696	ENSG00000286048		lncRNA	chr5:53024924-5303
ENSG0000(	412	10.3045	chr5:1696	ENSG00000288957		lncRNA	chr5:54310711-5431
ENSG0000(	412	10.3045	chr5:1696	MIR449A		smallRNA	chr5:55170532-5517
ENSG0000(	412	10.3045	chr5:1696	PARP8		protein_c	chr5:50665899-5084
ENSG0000(	412	10.3045	chr5:1696	ENSG00000241809		Pseudoger	chr5:52903908-5290
ENSG0000(	412	10.3045	chr5:1696	ITGA2		protein_c	chr5:52989340-5309
ENSG0000(	412	10.3045	chr5:1696	MCIDAS		protein_c	chr5:55219564-5522
ENSG0000(	412	10.3045	chr5:1696	ENSG00000250360		lncRNA	chr5:50965687-5096
ENSG0000(	412	10.3045	chr5:1696	Y_RNA		smallRNA	chr5:55040847-5504
ENSG0000(	412	10.3045	chr5:1696	SNX18		protein_c	chr5:54517759-5454
ENSG0000(	412	10.3045	chr5:1696	snoU13		smallRNA	chr5:51321983-5132
ENSG0000(	412	10.3045	chr5:1696	MIR4459		smallRNA	chr5:54075518-5407
ENSG0000(	412	10.3045	chr5:1696	ASS1P9		Pseudoger	chr5:53859166-5386
ENSG0000(	412	10.3045	chr5:1696	MFSD4BP1		Pseudoger	chr5:52579555-5258
ENSG0000(	412	10.3045	chr5:1696	ENSG00000272416		lncRNA	chr5:53880293-5388
ENSG0000(	412	10.3045	chr5:1696	ENSG00000249843		Pseudoger	chr5:54878238-5487
ENSG0000(	412	10.3045	chr5:1696	RNF138P1		Pseudoger	chr5:55530156-5553
ENSG0000(	412	10.3045	chr5:1696	RNA5SP182		Pseudoger	chr5:51886688-5188
ENSG0000(	412	10.3045	chr5:1696	PLPP1		protein_c	chr5:55424854-5553

ENSG0000(	412	10.3045	chr5:169(	ENSG00000240627		Pseudoger	chr5:51275253-5127
ENSG0000(	412	10.3045	chr5:169(	EMB		protein_c	chr5:50396192-5044
ENSG0000(	412	10.3045	chr5:169(	ISL1		protein_c	chr5:51383448-5139
ENSG0000(	412	10.3045	chr5:169(	MIR5687		smallRNA	chr5:55508850-5550
ENSG0000(	412	10.3045	chr5:169(	LINC02106		lncRNA	chr5:50969217-5097
ENSG0000(	412	10.3045	chr5:169(	ENSG00000251601		lncRNA	chr5:50929484-5093
ENSG0000(	412	10.3045	chr5:169(	CCNO		protein_c	chr5:55231152-5523
ENSG0000(	412	10.3045	chr5:169(	RPL37P25		Pseudoger	chr5:54399439-5440
ENSG0000(	412	10.3045	chr5:169(	PELO		protein_c	chr5:52787916-5280
ENSG0000(	412	10.3045	chr5:169(	ENSG00000279883		TEC	chr5:55484403-5548
ENSG0000(	412	10.3045	chr5:169(	ENSG00000272123		lncRNA	chr5:53089016-5308
ENSG0000(	412	10.3045	chr5:169(	ESM1		protein_c	chr5:54977867-5502
ENSG0000(	412	10.3045	chr5:169(	MTREX	NCGv7	protein_c	chr5:55307989-5542
ENSG0000(	412	10.3045	chr5:169(	ISL1-DT		lncRNA	chr5:51372736-5138
ENSG0000(	412	10.3045	chr5:169(	DHX29		protein_c	chr5:55256055-5530
ENSG0000(	412	10.3045	chr5:169(	MIR449C		smallRNA	chr5:55172262-5517
ENSG0000(	412	10.3045	chr5:169(	ENSG00000248029		lncRNA	chr5:55063179-5506
ENSG0000(	412	10.3045	chr5:169(	ENSG00000287367		lncRNA	chr5:54663327-5473
ENSG0000(	412	10.3045	chr5:169(	CDC20B		protein_c	chr5:55112971-5517
ENSG0000(	412	10.3045	chr5:169(	GPX8		protein_c	chr5:55160167-5516
ENSG0000(	412	10.3045	chr5:169(	ENSG00000251376		lncRNA	chr5:55054428-5505
ENSG0000(	412	10.3045	chr5:169(	FST		protein_c	chr5:53480626-5348
ENSG0000(	412	10.3045	chr5:169(	ENSG00000249016		lncRNA	chr5:54956235-5495
ENSG0000(	412	10.3045	chr5:169(	KATNBL1P4		Pseudoger	chr5:51930808-5193
ENSG0000(	412	10.3045	chr5:169(	RN7SL801P		smallRNA	chr5:54048009-5404
ENSG0000(	412	10.3045	chr5:169(	ENSG00000248918		lncRNA	chr5:50969660-5097
ENSG0000(	412	10.3045	chr5:169(	ENSG00000285831		lncRNA	chr5:53297872-5332
ENSG0000(	412	10.3045	chr5:169(	MOCS2	DriverDB\	protein_c	chr5:53095679-5311
ENSG0000(	412	10.3045	chr5:169(	ENSG00000213940		Pseudoger	chr5:53206561-5320
ENSG0000(	412	10.3045	chr5:169(	GZMK		protein_c	chr5:55024256-5503
ENSG0000(	412	10.3045	chr5:169(	ENSG00000279638		TEC	chr5:54286194-5428
ENSG0000(	412	10.3045	chr5:169(	AC091866.1		smallRNA	chr5:51061469-5106
ENSG0000(	412	10.3045	chr5:169(	ENSG00000288539		lncRNA	chr5:52853183-5287
ENSG0000(	412	10.3045	chr5:169(	ENSG00000289056		lncRNA	chr5:50667071-5066
ENSG0000(	412	10.3045	chr5:169(	ENSG00000289060		lncRNA	chr5:54883635-5488
ENSG0000(	412	10.3045	chr5:169(	HSPB3		protein_c	chr5:54455699-5445
ENSG0000(	412	10.3045	chr5:169(	B3GNTL1P1		Pseudoger	chr5:52889666-5288
ENSG0000(	412	10.3045	chr5:169(	RNU6-480P		smallRNA	chr5:51055353-5105
ENSG0000(	412	10.3045	chr5:169(	HMGB1P47		Pseudoger	chr5:51409617-5141
ENSG0000(	412	10.3045	chr5:169(	ENSG00000271752		lncRNA	chr5:50662859-5066
ENSG0000(	412	10.3045	chr5:169(	LINC01033		lncRNA	chr5:54313564-5441
ENSG0000(	412	10.3045	chr5:169(	RNU6-1296P		smallRNA	chr5:51167541-5116
ENSG0000(	412	10.3045	chr5:169(	LINC02118		lncRNA	chr5:52008031-5208
ENSG0000(	412	10.3045	chr5:169(	CSPG4BP		Pseudoger	chr5:54808210-5487
ENSG0000(	412	10.3045	chr5:169(	ENSG00000251125		lncRNA	chr5:51451158-5146
ENSG0000(	412	10.3045	chr5:169(	ENSG00000287087		lncRNA	chr5:53897464-5389
ENSG0000(	412	10.3045	chr5:169(	ENSG00000271410		Pseudoger	chr5:55356308-5535
ENSG0000(	412	10.3045	chr5:169(	RPS17P11		Pseudoger	chr5:52282567-5228
ENSG0000(	412	10.3045	chr5:169(	MIR449B		smallRNA	chr5:55170646-5517
ENSG0000(	412	10.3045	chr5:169(	ARL15		protein_c	chr5:53883942-5431
ENSG0000(	412	10.3045	chr5:169(	GZMAP1		Pseudoger	chr5:55073015-5509

ENSG0000(	412	10.3045	chr5:169(	AC112198.2	smallRNA	chr5:54852846-5485
ENSG0000(	412	10.3045	chr5:169(	ENSG00000280159	TEC	chr5:54643557-5464
ENSG0000(	412	10.3045	chr5:169(	AK4P2	Pseudoger	chr5:55551885-5555
ENSG0000(	412	10.3045	chr5:169(	ENSG00000249405	lncRNA	chr5:50858760-5086
ENSG0000(	412	10.3045	chr5:169(	ENSG00000271455	Pseudoger	chr5:50603229-5060
ENSG0000(	412	10.3045	chr5:169(	ENSG00000278734	Pseudoger	chr5:51101172-5110
ENSG0000(	412	10.3045	chr5:169(	ENSG00000226810	Pseudoger	chr5:54857272-5485
ENSG0000(	412	10.3045	chr5:169(	ENSG00000279470	TEC	chr5:51963992-5196
ENSG0000(	412	10.3045	chr5:169(	ENSG00000248891	Pseudoger	chr5:51427996-5142
ENSG0000(	412	10.3045	chr5:169(	ENSG00000240535	lncRNA	chr5:55021299-5504
ENSG0000(	412	10.3045	chr5:169(	ENSG00000270830	Pseudoger	chr5:50903608-5090
ENSG0000(	412	10.3045	chr5:169(	CCNO-DT	lncRNA	chr5:55233934-5529
ENSG0000(	409	10.22947	chr17:33(	ENSG00000278860	TEC	chr17:34614409-346
ENSG0000(	402	10.05439	chr20:459	RPL7AP12	Pseudoger	chr20:4590993-4591
ENSG0000(	402	10.05439	chr1:3732	RPS15AP12	Pseudoger	chr1:220143964-220
ENSG0000(	401	10.02938	chrX:1657	PCNAP3	Pseudoger	chrX:46058751-4605
ENSG0000(	401	10.02938	chrX:1657	ENSG00000225384	Pseudoger	chrX:34717440-3471
ENSG0000(	401	10.02938	chrX:1657	YWHAQP8	Pseudoger	chrX:101191115-101
ENSG0000(	401	10.02938	chrX:1657	FMN2P1	Pseudoger	chrX:26496936-2650
ENSG0000(	401	10.02938	chrX:1657	Y_RNA	smallRNA	chrX:19985058-1998
ENSG0000(	401	10.02938	chrX:1657	RPL7P58	Pseudoger	chrX:26686026-2668
ENSG0000(	401	10.02938	chrX:1657	DNAJA1P3	Pseudoger	chrX:107351650-107
ENSG0000(	401	10.02938	chrX:1657	PHEX-AS1	lncRNA	chrX:22162733-2217
ENSG0000(	401	10.02938	chrX:1657	IMPDH1P2	Pseudoger	chrX:40359183-4036
ENSG0000(	401	10.02938	chrX:1657	VEZTP1	Pseudoger	chrX:46454097-4645
ENSG0000(	401	10.02938	chrX:1657	CTNNBL1P1	Pseudoger	chrX:46418450-4642
ENSG0000(	401	10.02938	chrX:1657	Z97356.1	smallRNA	chrX:105860689-105
ENSG0000(	401	10.02938	chrX:1657	MIR548F5	smallRNA	chrX:32641474-3264
ENSG0000(	401	10.02938	chrX:1657	ORC1P1	Pseudoger	chrX:27893348-2789
ENSG0000(	401	10.02938	chrX:1657	ENSG00000231772	lncRNA	chrX:42252459-4269
ENSG0000(	401	10.02938	chrX:1657	RPL19P20	Pseudoger	chrX:44649129-4464
ENSG0000(	401	10.02938	chrX:1657	TRMT2B-AS1	lncRNA	chrX:101043564-101
ENSG0000(	401	10.02938	chrX:1657	ENSG00000214071	Pseudoger	chrX:37077851-3707
ENSG0000(	401	10.02938	chrX:1657	ENSG00000229491	lncRNA	chrX:45183251-4533
ENSG0000(	401	10.02938	chrX:1657	RARRES2P3	Pseudoger	chrX:21421822-2142
ENSG0000(	401	10.02938	chrX:1657	Z73964.1	smallRNA	chrX:103810384-103
ENSG0000(	401	10.02938	chrX:1657	MDM4P1	Pseudoger	chrX:17933349-1793
ENSG0000(	401	10.02938	chrX:1657	NUDT19P2	Pseudoger	chrX:103007461-103
ENSG0000(	401	10.02938	chrX:1657	CLDN7P1	Pseudoger	chrX:40760104-4076
ENSG0000(	401	10.02938	chrX:1657	MIR548AJ2	smallRNA	chrX:38023895-3802
ENSG0000(	401	10.02938	chrX:1657	HNRNPDLP1	Pseudoger	chrX:95638375-9563
ENSG0000(	401	10.02938	chrX:1657	LINC01456	lncRNA	chrX:17970197-1810
ENSG0000(	401	10.02938	chrX:1657	ENSG00000232446	Pseudoger	chrX:26147774-2614
ENSG0000(	401	10.02938	chrX:1657	NAP1L4P2	Pseudoger	chrX:106466542-106
ENSG0000(	401	10.02938	chrX:1657	TDGF1P1	Pseudoger	chrX:38395369-3839
ENSG0000(	401	10.02938	chrX:1657	SIAH1P1	Pseudoger	chrX:35626142-3562
ENSG0000(	401	10.02938	chrX:1657	SRIP2	Pseudoger	chrX:89367435-8936
ENSG0000(	401	10.02938	chrX:1657	RPS29P28	Pseudoger	chrX:96195477-9619
ENSG0000(	401	10.02938	chrX:1657	HMGB1P16	Pseudoger	chrX:36211096-3621
ENSG0000(	401	10.02938	chrX:1657	RNU6-30P	smallRNA	chrX:101634006-101
ENSG0000(	401	10.02938	chrX:1657	ENSG00000224610	lncRNA	chrX:39138146-3929

ENSG0000(	401	10.02938	chrX:1657	TCP11X2	protein_c	chrX:102456862-102
ENSG0000(	401	10.02938	chrX:1657	H3P43	Pseudoger	chrX:28659065-2865
ENSG0000(	401	10.02938	chrX:1657	RPS15AP39	Pseudoger	chrX:41748222-4174
ENSG0000(	401	10.02938	chrX:1657	ENSG00000224054	Pseudoger	chrX:44466691-4446
ENSG0000(	401	10.02938	chrX:1657	RDXP2	Pseudoger	chrX:27517771-2751
ENSG0000(	401	10.02938	chrX:1657	RNU6-49P	smallRNA	chrX:37870109-3787
ENSG0000(	401	10.02938	chrX:1657	H3P45	Pseudoger	chrX:102615473-102
ENSG0000(	401	10.02938	chrX:1657	ENSG00000225012	lncRNA	chrX:89423738-8944
ENSG0000(	401	10.02938	chrX:1657	EIF1AX-AS1	lncRNA	chrX:20139968-2014
ENSG0000(	401	10.02938	chrX:1657	AL590285.1	smallRNA	chrX:36577396-3657
ENSG0000(	401	10.02938	chrX:1657	Y_RNA	smallRNA	chrX:40492840-4049
ENSG0000(	401	10.02938	chrX:1657	SERBP1P4	Pseudoger	chrX:91560436-9156
ENSG0000(	401	10.02938	chrX:1657	ENSG00000223753	Pseudoger	chrX:101683780-101
ENSG0000(	401	10.02938	chrX:1657	RPL26P36	Pseudoger	chrX:92676519-9267
ENSG0000(	401	10.02938	chrX:1657	CBX1P4	Pseudoger	chrX:17282637-1728
ENSG0000(	401	10.02938	chrX:1657	RPS2P55	Pseudoger	chrX:40934982-4093
ENSG0000(	401	10.02938	chrX:1657	RRM2P3	Pseudoger	chrX:44309314-4431
ENSG0000(	401	10.02938	chrX:1657	RPL12P49	Pseudoger	chrX:16803233-1680
ENSG0000(	401	10.02938	chrX:1657	CHP1P3	Pseudoger	chrX:17705339-1770
ENSG0000(	401	10.02938	chrX:1657	FDPSP5	Pseudoger	chrX:44476027-4447
ENSG0000(	401	10.02938	chrX:1657	RPSAP61	Pseudoger	chrX:44741087-4474
ENSG0000(	401	10.02938	chrX:1657	RPSAP8	Pseudoger	chrX:100155885-100
ENSG0000(	401	10.02938	chrX:1657	RPS11P7	Pseudoger	chrX:39865424-3986
ENSG0000(	401	10.02938	chrX:1657	RNU6-133P	smallRNA	chrX:21214637-2121
ENSG0000(	401	10.02938	chrX:1657	ENSG00000206062	lncRNA	chrX:91307781-9130
ENSG0000(	401	10.02938	chrX:1657	AL096700.1	smallRNA	chrX:18863564-1886
ENSG0000(	401	10.02938	chrX:1657	FRMPD3-AS1	lncRNA	chrX:107512983-107
ENSG0000(	401	10.02938	chrX:1657	SNORD77	smallRNA	chrX:45892912-4589
ENSG0000(	401	10.02938	chrX:1657	MIR222	smallRNA	chrX:45747015-4574
ENSG0000(	401	10.02938	chrX:1657	ARMCX3-AS1	lncRNA	chrX:101622983-101
ENSG0000(	401	10.02938	chrX:1657	RBM39P1	Pseudoger	chrX:44029332-4403
ENSG0000(	401	10.02938	chrX:1657	FTLP2	Pseudoger	chrX:30630298-3063
ENSG0000(	401	10.02938	chrX:1657	AL035422.1	smallRNA	chrX:101427393-101
ENSG0000(	401	10.02938	chrX:1657	ENSG00000226679	lncRNA	chrX:38221391-3822
ENSG0000(	401	10.02938	chrX:1657	SUPT20HL1	protein_c	chrX:24360639-2436
ENSG0000(	401	10.02938	chrX:1657	LINC01204	lncRNA	chrX:45505388-4563
ENSG0000(	401	10.02938	chrX:1657	H2BP7	Pseudoger	chrX:24632257-2463
ENSG0000(	401	10.02938	chrX:1657	ENSG00000223742	lncRNA	chrX:28571532-2858
ENSG0000(	401	10.02938	chrX:1657	PDCL2P1	Pseudoger	chrX:23198339-2319
ENSG0000(	401	10.02938	chrX:1657	RANBP1P1	Pseudoger	chrX:25645722-2564
ENSG0000(	401	10.02938	chrX:1657	MIR548M	smallRNA	chrX:95063141-9506
ENSG0000(	401	10.02938	chrX:1657	SHISA5P1	Pseudoger	chrX:41322601-4132
ENSG0000(	401	10.02938	chrX:1657	BAG1P1	Pseudoger	chrX:38479215-3847
ENSG0000(	401	10.02938	chrX:1657	CBX1P2	Pseudoger	chrX:17099782-1710
ENSG0000(	401	10.02938	chrX:1657	PPP4R3C	protein_c	chrX:27460207-2746
ENSG0000(	401	10.02938	chrX:1657	HAUS1P2	Pseudoger	chrX:18865771-1886
ENSG0000(	401	10.02938	chrX:1657	MTND6P32	Pseudoger	chrX:102806164-102
ENSG0000(	401	10.02938	chrX:1657	LINC00630	lncRNA	chrX:102769158-102
ENSG0000(	401	10.02938	chrX:1657	TMEM230P1	Pseudoger	chrX:107939263-107
ENSG0000(	401	10.02938	chrX:1657	AL035088.1	smallRNA	chrX:107428989-107
ENSG0000(	401	10.02938	chrX:1657	STAU2P1	Pseudoger	chrX:90039213-9003

ENSG0000(	401	10.02938	chrX:1657	SLC35C2P1	Pseudoger	chrX:16897651-1689
ENSG0000(	401	10.02938	chrX:1657	YY2	protein_c	chrX:21855987-2185
ENSG0000(	401	10.02938	chrX:1657	CAPZA1P1	Pseudoger	chrX:88096393-8809
ENSG0000(	401	10.02938	chrX:1657	GTF3C6P2	Pseudoger	chrX:107131659-107
ENSG0000(	401	10.02938	chrX:1657	ENSG00000231069	Pseudoger	chrX:36985523-3698
ENSG0000(	401	10.02938	chrX:1657	MIR221	smallRNA	chrX:45746157-4574
ENSG0000(	401	10.02938	chrX:1657	BEND7P1	Pseudoger	chrX:102705784-102
ENSG0000(	401	10.02938	chrX:1657	EIF4A1P10	Pseudoger	chrX:92113246-9211
ENSG0000(	401	10.02938	chrX:1657	RNU6-587P	smallRNA	chrX:101691580-101
ENSG0000(	401	10.02938	chrX:1657	TFDP1P2	Pseudoger	chrX:36962196-3696
ENSG0000(	401	10.02938	chrX:1657	SERPINA7P1	Pseudoger	chrX:106562774-106
ENSG0000(	401	10.02938	chrX:1657	SUPT20HL2	protein_c	chrX:24308210-2431
ENSG0000(	401	10.02938	chrX:1657	MTATP6P19	Pseudoger	chrX:102798396-102
ENSG0000(	401	10.02938	chrX:1657	H2BP9	Pseudoger	chrX:103904322-103
ENSG0000(	401	10.02938	chrX:1657	FTLP16	Pseudoger	chrX:38484752-3848
ENSG0000(	401	10.02938	chrX:1657	DCAF8L1	protein_c	chrX:27977992-2798
ENSG0000(	401	10.02938	chrX:1657	CTDSPL2P2	Pseudoger	chrX:106070481-106
ENSG0000(	401	10.02938	chrX:1657	GAPDHP1	Pseudoger	chrX:39787132-3978
ENSG0000(	401	10.02938	chrX:1657	TAB3-AS1	lncRNA	chrX:30834623-3083
ENSG0000(	401	10.02938	chrX:1657	ENSG00000228933	lncRNA	chrX:27174920-2739
ENSG0000(	401	10.02938	chrX:1657	KRT18P68	Pseudoger	chrX:45983365-4598
ENSG0000(	401	10.02938	chrX:1657	PIGFP3	Pseudoger	chrX:30017160-3001
ENSG0000(	401	10.02938	chrX:1657	SC4MOP	Pseudoger	chrX:37945884-3794
ENSG0000(	401	10.02938	chrX:1657	ENSG00000229731	Pseudoger	chrX:27811885-2781
ENSG0000(	401	10.02938	chrX:1657	RPP40P1	Pseudoger	chrX:25591353-2559
ENSG0000(	401	10.02938	chrX:1657	RNU4-6P	smallRNA	chrX:16875146-1687
ENSG0000(	401	10.02938	chrX:1657	MTND2P2	Pseudoger	chrX:102786813-102
ENSG0000(	401	10.02938	chrX:1657	Y_RNA	smallRNA	chrX:107901145-107
ENSG0000(	401	10.02938	chrX:1657	RNU6-1124P	smallRNA	chrX:42174279-4217
ENSG0000(	401	10.02938	chrX:1657	MED28P4	Pseudoger	chrX:45951762-4595
ENSG0000(	401	10.02938	chrX:1657	AC112778.1	smallRNA	chrX:25206370-2520
ENSG0000(	401	10.02938	chrX:1657	SNX3P1X	Pseudoger	chrX:92515323-9251
ENSG0000(	401	10.02938	chrX:1657	ENSG00000230478	Pseudoger	chrX:87425583-8742
ENSG0000(	401	10.02938	chrX:1657	ENSG00000228345	Pseudoger	chrX:27854323-2785
ENSG0000(	401	10.02938	chrX:1657	Y_RNA	smallRNA	chrX:18395994-1839
ENSG0000(	401	10.02938	chrX:1657	KRT18P49	Pseudoger	chrX:107428630-107
ENSG0000(	401	10.02938	chrX:1657	RNU6-630P	smallRNA	chrX:42139425-4213
ENSG0000(	401	10.02938	chrX:1657	HMGB1P32	Pseudoger	chrX:98422138-9842
ENSG0000(	401	10.02938	chrX:1657	METTL15P3	Pseudoger	chrX:22435598-2243
ENSG0000(	401	10.02938	chrX:1657	MTCO3P19	Pseudoger	chrX:102799563-102
ENSG0000(	401	10.02938	chrX:1657	ENSG00000231590	Pseudoger	chrX:102425343-102
ENSG0000(	401	10.02938	chrX:1657	MTCYBP32	Pseudoger	chrX:102806741-102
ENSG0000(	401	10.02938	chrX:1657	MTND4P32	Pseudoger	chrX:102799942-102
ENSG0000(	401	10.02938	chrX:1657	Y_RNA	smallRNA	chrX:21872707-2187
ENSG0000(	401	10.02938	chrX:1657	ENSG00000229090	Pseudoger	chrX:101720340-101
ENSG0000(	401	10.02938	chrX:1657	PPIAP89	Pseudoger	chrX:100800303-100
ENSG0000(	401	10.02938	chrX:1657	AL390966.1	smallRNA	chrX:95134279-9513
ENSG0000(	401	10.02938	chrX:1657	LINC02595	lncRNA	chrX:45847042-4585
ENSG0000(	401	10.02938	chrX:1657	KRT8P14	Pseudoger	chrX:45632292-4563
ENSG0000(	401	10.02938	chrX:1657	RNU6-207P	smallRNA	chrX:105676071-105
ENSG0000(	401	10.02938	chrX:1657	ZNF674-AS1	lncRNA	chrX:46545438-4654

ENSG0000(	401	10.02938	chrX:1657	B3GNT2P1		Pseudoger	chrX:99886740-9988
ENSG0000(	401	10.02938	chrX:1657	Y_RNA		smallRNA	chrX:96701507-9670
ENSG0000(	401	10.02938	chrX:1657	DPPA3P1		Pseudoger	chrX:103961811-103
ENSG0000(	401	10.02938	chrX:1657	CCNB1IP1P3		Pseudoger	chrX:94776739-9477
ENSG0000(	401	10.02938	chrX:1657	FAM136GP		Pseudoger	chrX:17769005-1776
ENSG0000(	401	10.02938	chrX:1657	ENSG00000231011		Pseudoger	chrX:22616744-2261
ENSG0000(	401	10.02938	chrX:1657	RNF19BPX		Pseudoger	chrX:90209796-9021
ENSG0000(	401	10.02938	chrX:1657	GEMIN7P1		Pseudoger	chrX:41472264-4147
ENSG0000(	401	10.02938	chrX:1657	ENSG00000226199		Pseudoger	chrX:27832175-2783
ENSG0000(	401	10.02938	chrX:1657	ENSG00000228780		Pseudoger	chrX:42150511-4215
ENSG0000(	401	10.02938	chrX:1657	RPL7P55		Pseudoger	chrX:93573834-9357
ENSG0000(	401	10.02938	chrX:1657	RNU6-1321P		smallRNA	chrX:41570923-4157
ENSG0000(	401	10.02938	chrX:1657	YWHAZP10		Pseudoger	chrX:41675760-4167
ENSG0000(	401	10.02938	chrX:1657	ENSG00000224692		Pseudoger	chrX:103258572-103
ENSG0000(	401	10.02938	chrX:1657	CALM1P1		Pseudoger	chrX:95499604-9550
ENSG0000(	401	10.02938	chrX:1657	LINC02601		lncRNA	chrX:41275739-4127
ENSG0000(	401	10.02938	chrX:1657	FTH1P19		Pseudoger	chrX:37492021-3749
ENSG0000(	401	10.02938	chrX:1657	ACTBP1		Pseudoger	chrX:46288016-4628
ENSG0000(	401	10.02938	chrX:1657	ATP5MC2P4		Pseudoger	chrX:42098754-4209
ENSG0000(	401	10.02938	chrX:1657	ENSG00000226241		Pseudoger	chrX:103087198-103
ENSG0000(	401	10.02938	chrX:1657	TCEAL3-AS1		lncRNA	chrX:103626076-103
ENSG0000(	401	10.02938	chrX:1657	EEF1A1P40		Pseudoger	chrX:107032248-107
ENSG0000(	401	10.02938	chrX:1657	Y_RNA		smallRNA	chrX:101288917-101
ENSG0000(	401	10.02938	chrX:1657	MAGEB1		protein_c	chrX:30243715-3025
ENSG0000(	401	10.02938	chrX:1657	NANOGP10		Pseudoger	chrX:43407665-4340
ENSG0000(	401	10.02938	chrX:1657	KRT18P11		Pseudoger	chrX:92459670-9246
ENSG0000(	401	10.02938	chrX:1657	AL121869.1		smallRNA	chrX:92460452-9246
ENSG0000(	401	10.02938	chrX:1657	MORF4L2-AS1		lncRNA	chrX:103687284-103
ENSG0000(	401	10.02938	chrX:1657	H2AL3		protein_c	chrX:37994272-3799
ENSG0000(	401	10.02938	chrX:1657	ENSG00000215310		Pseudoger	chrX:33410944-3341
ENSG0000(	401	10.02938	chrX:1657	NHS-AS1		lncRNA	chrX:17552349-1755
ENSG0000(	401	10.02938	chrX:1657	RNU6-591P		smallRNA	chrX:39081180-3908
ENSG0000(	401	10.02938	chrX:1657	DDX3X	NCGv7	protein_c	chrX:41333348-4136
ENSG0000(	401	10.02938	chrX:1657	SRSF6P1		Pseudoger	chrX:45913118-4591
ENSG0000(	401	10.02938	chrX:1657	EEF1A1P15		Pseudoger	chrX:98389512-9839
ENSG0000(	401	10.02938	chrX:1657	RNA5SP501		Pseudoger	chrX:32102065-3210
ENSG0000(	401	10.02938	chrX:1657	SLC25A53P1		Pseudoger	chrX:103928099-103
ENSG0000(	401	10.02938	chrX:1657	AC004673.1		smallRNA	chrX:23120832-2312
ENSG0000(	401	10.02938	chrX:1657	UBE2V1P9		Pseudoger	chrX:90289536-9028
ENSG0000(	401	10.02938	chrX:1657	RPS26P58		Pseudoger	chrX:24429573-2442
ENSG0000(	401	10.02938	chrX:1657	ENSG00000227511		Pseudoger	chrX:26170052-2617
ENSG0000(	401	10.02938	chrX:1657	ENSG00000229347		Pseudoger	chrX:35058927-3505
ENSG0000(	401	10.02938	chrX:1657	TBCAP1		Pseudoger	chrX:33041395-3304
ENSG0000(	401	10.02938	chrX:1657	ENSG00000228464		Pseudoger	chrX:26345236-2634
ENSG0000(	401	10.02938	chrX:1657	RNU6-332P		smallRNA	chrX:94003815-9400
ENSG0000(	401	10.02938	chrX:1657	USP12PX		Pseudoger	chrX:90112650-9011
ENSG0000(	401	10.02938	chrX:1657	TMSB15B-AS1		lncRNA	chrX:103845151-103
ENSG0000(	401	10.02938	chrX:1657	RN7SKP194		smallRNA	chrX:96410432-9641
ENSG0000(	401	10.02938	chrX:1657	MAGEB6B		protein_c	chrX:26160601-2616
ENSG0000(	401	10.02938	chrX:1657	AL591708.1		smallRNA	chrX:94893501-9489
ENSG0000(	401	10.02938	chrX:1657	ENSG00000226484		lncRNA	chrX:36365626-3644

ENSG0000(	401	10.02938	chrX:1657	ENSG00000227393		Pseudoger	chrX:29369001-2936
ENSG0000(	401	10.02938	chrX:1657	RNU6-555P		smallRNA	chrX:90597235-9059
ENSG0000(	401	10.02938	chrX:1657	MTND1P32		Pseudoger	chrX:102785667-102
ENSG0000(	401	10.02938	chrX:1657	NDUFB5P2		Pseudoger	chrX:96953630-9695
ENSG0000(	401	10.02938	chrX:1657	AL008708.1		smallRNA	chrX:103068865-103
ENSG0000(	401	10.02938	chrX:1657	SNRPEP9		Pseudoger	chrX:24513450-2451
ENSG0000(	401	10.02938	chrX:1657	PINCR		lncRNA	chrX:43176994-4322
ENSG0000(	401	10.02938	chrX:1657	PAICSP7		Pseudoger	chrX:94636663-9463
ENSG0000(	401	10.02938	chrX:1657	HIKESHIP1		Pseudoger	chrX:23351013-2335
ENSG0000(	401	10.02938	chrX:1657	AL161723.1		smallRNA	chrX:89790335-8979
ENSG0000(	401	10.02938	chrX:1657	RNA5SP511		Pseudoger	chrX:103850797-103
ENSG0000(	401	10.02938	chrX:1657	SKP2P1		Pseudoger	chrX:96620654-9662
ENSG0000(	401	10.02938	chrX:1657	XRCC6P5		Pseudoger	chrX:99719364-9972
ENSG0000(	401	10.02938	chrX:1657	ENSG00000223486		lncRNA	chrX:40009276-4001
ENSG0000(	401	10.02938	chrX:1657	TUBB4BP8		Pseudoger	chrX:94319038-9432
ENSG0000(	401	10.02938	chrX:1657	TMEM184CP1		Pseudoger	chrX:88061590-8806
ENSG0000(	401	10.02938	chrX:1657	RNU6-641P		smallRNA	chrX:36021939-3602
ENSG0000(	401	10.02938	chrX:1657	RNU2-26P		smallRNA	chrX:91798093-9179
ENSG0000(	401	10.02938	chrX:1657	GK-IT1		lncRNA	chrX:30671635-3067
ENSG0000(	401	10.02938	chrX:1657	C3orf49P1		Pseudoger	chrX:102006883-102
ENSG0000(	401	10.02938	chrX:1657	GTF3C6P1		Pseudoger	chrX:95584303-9558
ENSG0000(	401	10.02938	chrX:1657	PAFAH1B2P1		Pseudoger	chrX:25029545-2503
ENSG0000(	401	10.02938	chrX:1657	RPL18AP14		Pseudoger	chrX:104803915-104
ENSG0000(	401	10.02938	chrX:1657	TSPAN6		protein_c	chrX:100627108-100
ENSG0000(	401	10.02938	chrX:1657	EEF1B2P3		Pseudoger	chrX:24788392-2478
ENSG0000(	401	10.02938	chrX:1657	DCAF8L2		protein_c	chrX:27590344-2774
ENSG0000(	401	10.02938	chrX:1657	MORF4L2		protein_c	chrX:103675496-103
ENSG0000(	401	10.02938	chrX:1657	H2BW1		protein_c	chrX:104011147-104
ENSG0000(	401	10.02938	chrX:1657	snoU13		smallRNA	chrX:17357930-1735
ENSG0000(	401	10.02938	chrX:1657	RAB9B		protein_c	chrX:103822327-103
ENSG0000(	401	10.02938	chrX:1657	NRK	NCGv7	protein_c	chrX:105822539-105
ENSG0000(	401	10.02938	chrX:1657	FAM199X		protein_c	chrX:104166453-104
ENSG0000(	401	10.02938	chrX:1657	ESX1		protein_c	chrX:104250038-104
ENSG0000(	401	10.02938	chrX:1657	MAOA		protein_c	chrX:43654907-4374
ENSG0000(	401	10.02938	chrX:1657	RPL32P36		Pseudoger	chrX:40907987-4090
ENSG0000(	401	10.02938	chrX:1657	SNORD45		smallRNA	chrX:87146733-8714
ENSG0000(	401	10.02938	chrX:1657	snoU13		smallRNA	chrX:40285096-4028
ENSG0000(	401	10.02938	chrX:1657	FAM47B		protein_c	chrX:34942796-3494
ENSG0000(	401	10.02938	chrX:1657	IL1RAPL2		protein_c	chrX:104566199-105
ENSG0000(	401	10.02938	chrX:1657	snoU13		smallRNA	chrX:32847907-3284
ENSG0000(	401	10.02938	chrX:1657	DUSP21		protein_c	chrX:44844021-4484
ENSG0000(	401	10.02938	chrX:1657	MAGEB16		protein_c	chrX:35798342-3580
ENSG0000(	401	10.02938	chrX:1657	NDP		protein_c	chrX:43948776-4397
ENSG0000(	401	10.02938	chrX:1657	USP9X	NCGv7	protein_c	chrX:41085445-4123
ENSG0000(	401	10.02938	chrX:1657	RNU7-164P		smallRNA	chrX:40535502-4053
ENSG0000(	401	10.02938	chrX:1657	SERPINA7		protein_c	chrX:106032435-106
ENSG0000(	401	10.02938	chrX:1657	RNU7-56P		smallRNA	chrX:16767844-1676
ENSG0000(	401	10.02938	chrX:1657	PLP1		protein_c	chrX:103773718-103
ENSG0000(	401	10.02938	chrX:1657	MPC1L		protein_c	chrX:40623428-4062
ENSG0000(	401	10.02938	chrX:1657	MKRN4P		Pseudoger	chrX:40834485-4083
ENSG0000(	401	10.02938	chrX:1657	RDXP3		Pseudoger	chrX:27517884-2751

ENSG0000(	401	10.02938	chrX:1657	NXF4		Pseudoger	chrX:102561935-102
ENSG0000(	401	10.02938	chrX:1657	snoU13		smallRNA	chrX:23507211-2350
ENSG0000(	401	10.02938	chrX:1657	ENSG00000274022		Pseudoger	chrX:90208895-9020
ENSG0000(	401	10.02938	chrX:1657	ENSG00000273746		Pseudoger	chrX:102259043-102
ENSG0000(	401	10.02938	chrX:1657	ENSG00000273704		Pseudoger	chrX:34768327-3476
ENSG0000(	401	10.02938	chrX:1657	Y_RNA		smallRNA	chrX:41316488-4131
ENSG0000(	401	10.02938	chrX:1657	TASL		protein_c	chrX:30558809-3057
ENSG0000(	401	10.02938	chrX:1657	MAGEB4		protein_c	chrX:30242000-3024
ENSG0000(	401	10.02938	chrX:1657	BLOC1S6P1		Pseudoger	chrX:18838709-1883
ENSG0000(	401	10.02938	chrX:1657	TCEAL3		protein_c	chrX:103607451-103
ENSG0000(	401	10.02938	chrX:1657	ARMCX4		protein_c	chrX:101418287-101
ENSG0000(	401	10.02938	chrX:1657	RNU7-7P		smallRNA	chrX:38608215-3860
ENSG0000(	401	10.02938	chrX:1657	ACOT9		protein_c	chrX:23701055-2376
ENSG0000(	401	10.02938	chrX:1657	PRDX4		protein_c	chrX:23664262-2368
ENSG0000(	401	10.02938	chrX:1657	NYX		protein_c	chrX:41447343-4147
ENSG0000(	401	10.02938	chrX:1657	TRMT2B		protein_c	chrX:101009346-101
ENSG0000(	401	10.02938	chrX:1657	ARMCX5		protein_c	chrX:102599168-102
ENSG0000(	401	10.02938	chrX:1657	ENSG00000239407		lncRNA	chrX:102884414-102
ENSG0000(	401	10.02938	chrX:1657	SAT1		protein_c	chrX:23783173-2378
ENSG0000(	401	10.02938	chrX:1657	USP37P1		Pseudoger	chrX:93776911-9377
ENSG0000(	401	10.02938	chrX:1657	NANOGNBP3		Pseudoger	chrX:101337725-101
ENSG0000(	401	10.02938	chrX:1657	ENSG00000270794		Pseudoger	chrX:30209423-3021
ENSG0000(	401	10.02938	chrX:1657	DPRXP6		Pseudoger	chrX:40758483-4075
ENSG0000(	401	10.02938	chrX:1657	ENSG00000270745		Pseudoger	chrX:88982215-8898
ENSG0000(	401	10.02938	chrX:1657	EIF2S3		protein_c	chrX:24054946-2407
ENSG0000(	401	10.02938	chrX:1657	PRRG1	NCGv7	protein_c	chrX:37349309-3745
ENSG0000(	401	10.02938	chrX:1657	ENSG00000270583		Pseudoger	chrX:95720243-9572
ENSG0000(	401	10.02938	chrX:1657	MKI67P1		Pseudoger	chrX:46560928-4656
ENSG0000(	401	10.02938	chrX:1657	RPL34P36		Pseudoger	chrX:87806433-8780
ENSG0000(	401	10.02938	chrX:1657	TEX101P1		Pseudoger	chrX:99656997-9965
ENSG0000(	401	10.02938	chrX:1657	SMIM15P1		Pseudoger	chrX:43000174-4300
ENSG0000(	401	10.02938	chrX:1657	PDHA1		protein_c	chrX:19343893-1936
ENSG0000(	401	10.02938	chrX:1657	RAI2		protein_c	chrX:17800049-1786
ENSG0000(	401	10.02938	chrX:1657	MIR222HG		lncRNA	chrX:45745210-4577
ENSG0000(	401	10.02938	chrX:1657	FTHL17		protein_c	chrX:31071233-3107
ENSG0000(	401	10.02938	chrX:1657	MRPS22P1		Pseudoger	chrX:87807549-8780
ENSG0000(	401	10.02938	chrX:1657	HNRNPA1P26		Pseudoger	chrX:100855288-100
ENSG0000(	401	10.02938	chrX:1657	ENSG00000271693		Pseudoger	chrX:88369343-8836
ENSG0000(	401	10.02938	chrX:1657	ENSG00000271088		Pseudoger	chrX:35741600-3574
ENSG0000(	401	10.02938	chrX:1657	GLRA4		Pseudoger	chrX:103702210-103
ENSG0000(	401	10.02938	chrX:1657	HNRNPH2		protein_c	chrX:101408222-101
ENSG0000(	401	10.02938	chrX:1657	ARMCX1		protein_c	chrX:101550547-101
ENSG0000(	401	10.02938	chrX:1657	TMEM35A		protein_c	chrX:101078879-101
ENSG0000(	401	10.02938	chrX:1657	NXF5		Pseudoger	chrX:101832537-101
ENSG0000(	401	10.02938	chrX:1657	TIMM8A		protein_c	chrX:101345661-101
ENSG0000(	401	10.02938	chrX:1657	ENSG00000271519		Pseudoger	chrX:35864858-3586
ENSG0000(	401	10.02938	chrX:1657	ENSG00000271457		Pseudoger	chrX:87143854-8714
ENSG0000(	401	10.02938	chrX:1657	ENSG00000271322		Pseudoger	chrX:98348451-9834
ENSG0000(	401	10.02938	chrX:1657	CYTH1P1		Pseudoger	chrX:21939461-2194
ENSG0000(	401	10.02938	chrX:1657	BRDTP1		Pseudoger	chrX:96337236-9633
ENSG0000(	401	10.02938	chrX:1657	MAGEB5		protein_c	chrX:26216169-2621

ENSG0000(	401	10.02938	chrX:1657	ENSG00000271205	Pseudoger	chrX:87723089-8772
ENSG0000(	401	10.02938	chrX:1657	ENSG00000271157	Pseudoger	chrX:88827554-8882
ENSG0000(	401	10.02938	chrX:1657	ARMCX5-GPRASP2	lncRNA	chrX:102599512-102
ENSG0000(	401	10.02938	chrX:1657	ENSG00000271136	Pseudoger	chrX:34516920-3451
ENSG0000(	401	10.02938	chrX:1657	ENSG00000271094	Pseudoger	chrX:95453369-9545
ENSG0000(	401	10.02938	chrX:1657	ENSG00000275314	Pseudoger	chrX:36794500-3679
ENSG0000(	401	10.02938	chrX:1657	ENSG00000275320	Pseudoger	chrX:100815865-100
ENSG0000(	401	10.02938	chrX:1657	MID1IP1-AS1	lncRNA	chrX:38801568-3880
ENSG0000(	401	10.02938	chrX:1657	ENSG00000283737	TEC	chrX:42285130-4228
ENSG0000(	401	10.02938	chrX:1657	ENSG00000283631	lncRNA	chrX:20311838-2031
ENSG0000(	401	10.02938	chrX:1657	NDP-AS1	lncRNA	chrX:43949732-4397
ENSG0000(	401	10.02938	chrX:1657	NPM1P8	Pseudoger	chrX:32205902-3220
ENSG0000(	401	10.02938	chrX:1657	ENSG00000283546	lncRNA	chrX:21131496-2116
ENSG0000(	401	10.02938	chrX:1657	ENSG00000283400	lncRNA	chrX:20443326-2049
ENSG0000(	401	10.02938	chrX:1657	ENSG00000283380	lncRNA	chrX:20769718-2137
ENSG0000(	401	10.02938	chrX:1657	ENSG00000282914	lncRNA	chrX:87707579-8775
ENSG0000(	401	10.02938	chrX:1657	ENSG00000236393	lncRNA	chrX:40262917-4029
ENSG0000(	401	10.02938	chrX:1657	ENSG00000236413	Pseudoger	chrX:91422309-9142
ENSG0000(	401	10.02938	chrX:1657	Y_RNA	smallRNA	chrX:107202577-107
ENSG0000(	401	10.02938	chrX:1657	ENSG00000236487	Pseudoger	chrX:24681909-2468
ENSG0000(	401	10.02938	chrX:1657	ENSG00000281566	lncRNA	chrX:98450688-9889
ENSG0000(	401	10.02938	chrX:1657	ENSG00000281091	lncRNA	chrX:103404777-103
ENSG0000(	401	10.02938	chrX:1657	HMGA1P1	Pseudoger	chrX:26748029-2674
ENSG0000(	401	10.02938	chrX:1657	RNA5SP502	Pseudoger	chrX:41292798-4129
ENSG0000(	401	10.02938	chrX:1657	LINC01282	lncRNA	chrX:39367285-3939
ENSG0000(	401	10.02938	chrX:1657	LINC01186	lncRNA	chrX:46256759-4632
ENSG0000(	401	10.02938	chrX:1657	DIAPH2-AS1	lncRNA	chrX:97431286-9764
ENSG0000(	401	10.02938	chrX:1657	RPL6P29	Pseudoger	chrX:98251679-9825
ENSG0000(	401	10.02938	chrX:1657	PCYT1B-AS1	lncRNA	chrX:24650073-2465
ENSG0000(	401	10.02938	chrX:1657	ENSG00000284800	protein_c	chrX:102360395-102
ENSG0000(	401	10.02938	chrX:1657	ENSG00000235834	lncRNA	chrX:17528435-1758
ENSG0000(	401	10.02938	chrX:1657	ENSG00000286327	lncRNA	chrX:102659983-102
ENSG0000(	401	10.02938	chrX:1657	ENSG00000286306	lncRNA	chrX:46400686-4640
ENSG0000(	401	10.02938	chrX:1657	ARMCX5-GPRASP2	protein_c	chrX:102712495-102
ENSG0000(	401	10.02938	chrX:1657	H2BW3P	Pseudoger	chrX:104062542-104
ENSG0000(	401	10.02938	chrX:1657	ENSG00000286000	lncRNA	chrX:101640421-101
ENSG0000(	401	10.02938	chrX:1657	H2BP8	Pseudoger	chrX:101989481-101
ENSG0000(	401	10.02938	chrX:1657	H2AL1MP	Pseudoger	chrX:37700005-3770
ENSG0000(	401	10.02938	chrX:1657	ENSG00000285900	lncRNA	chrX:31411033-3141
ENSG0000(	401	10.02938	chrX:1657	ENSG00000285899	lncRNA	chrX:43279560-4343
ENSG0000(	401	10.02938	chrX:1657	ENSG00000236064	lncRNA	chrX:107857364-107
ENSG0000(	401	10.02938	chrX:1657	SRSF2P1	Pseudoger	chrX:34387722-3438
ENSG0000(	401	10.02938	chrX:1657	ENSG00000285709	lncRNA	chrX:39908309-3991
ENSG0000(	401	10.02938	chrX:1657	ENSG00000236160	Pseudoger	chrX:26657291-2665
ENSG0000(	401	10.02938	chrX:1657	TUSC2P2	Pseudoger	chrX:92770855-9277
ENSG0000(	401	10.02938	chrX:1657	HNRNPDLP3	Pseudoger	chrX:95610578-9561
ENSG0000(	401	10.02938	chrX:1657	GJA6P	Pseudoger	chrX:18535079-1853
ENSG0000(	401	10.02938	chrX:1657	DMD-AS3	lncRNA	chrX:32754940-3275
ENSG0000(	401	10.02938	chrX:1657	Y_RNA	smallRNA	chrX:16912215-1691
ENSG0000(	401	10.02938	chrX:1657	RAD21P1	Pseudoger	chrX:100801188-100
ENSG0000(	401	10.02938	chrX:1657	TATDN2P1	Pseudoger	chrX:44283916-4428

ENSG0000(	401	10.02938	chrX:1657	ENSG00000276298		Pseudoger	chrX:103909126-103
ENSG0000(	401	10.02938	chrX:1657	MTCO2P19		Pseudoger	chrX:102797531-102
ENSG0000(	401	10.02938	chrX:1657	FAM47C	NCGv7	protein_c	chrX:37008366-3701
ENSG0000(	401	10.02938	chrX:1657	TNIP2P1		Pseudoger	chrX:40831844-4083
ENSG0000(	401	10.02938	chrX:1657	EIF5P2		Pseudoger	chrX:19496378-1949
ENSG0000(	401	10.02938	chrX:1657	NUP62CL		protein_c	chrX:107123427-107
ENSG0000(	401	10.02938	chrX:1657	PRKCIP1		Pseudoger	chrX:101537485-101
ENSG0000(	401	10.02938	chrX:1657	NCKAP1P1		Pseudoger	chrX:97906644-9790
ENSG0000(	401	10.02938	chrX:1657	PLLPP1		Pseudoger	chrX:45381527-4538
ENSG0000(	401	10.02938	chrX:1657	PHKA2-AS1		lncRNA	chrX:18890296-1889
ENSG0000(	401	10.02938	chrX:1657	DLGAP5P2		Pseudoger	chrX:93042490-9304
ENSG0000(	401	10.02938	chrX:1657	ENSG00000275681		Pseudoger	chrX:21841794-2184
ENSG0000(	401	10.02938	chrX:1657	ENSG00000237875		Pseudoger	chrX:41790192-4179
ENSG0000(	401	10.02938	chrX:1657	CLIC4P3		Pseudoger	chrX:41076999-4107
ENSG0000(	401	10.02938	chrX:1657	ENSG00000237994		lncRNA	chrX:28942050-2894
ENSG0000(	401	10.02938	chrX:1657	ENSG00000238049		Pseudoger	chrX:103347462-103
ENSG0000(	401	10.02938	chrX:1657	RPL9P7		Pseudoger	chrX:23836742-2383
ENSG0000(	401	10.02938	chrX:1657	ENSG00000276467		Pseudoger	chrX:17897711-1790
ENSG0000(	401	10.02938	chrX:1657	PLCE1P1		Pseudoger	chrX:30290047-3029
ENSG0000(	401	10.02938	chrX:1657	RNU6-50P		smallRNA	chrX:46517766-4651
ENSG0000(	401	10.02938	chrX:1657	RPSAP15		Pseudoger	chrX:87703343-8770
ENSG0000(	401	10.02938	chrX:1657	CTDSPL2P1		Pseudoger	chrX:100149783-100
ENSG0000(	401	10.02938	chrX:1657	ENSG00000237019		lncRNA	chrX:22698457-2276
ENSG0000(	401	10.02938	chrX:1657	ENSG00000279894		TEC	chrX:103596608-103
ENSG0000(	401	10.02938	chrX:1657	ENSG00000237171		Pseudoger	chrX:26223903-2622
ENSG0000(	401	10.02938	chrX:1657	IMPDH1P4		Pseudoger	chrX:43278125-4327
ENSG0000(	401	10.02938	chrX:1657	ARMCX6		protein_c	chrX:101615118-101
ENSG0000(	401	10.02938	chrX:1657	PPEF1-AS1		lncRNA	chrX:18688643-1869
ENSG0000(	401	10.02938	chrX:1657	DMD	NCGv7	protein_c	chrX:31097677-3333
ENSG0000(	401	10.02938	chrX:1657	GPRASP1		protein_c	chrX:102651092-102
ENSG0000(	401	10.02938	chrX:1657	BHLHB9		protein_c	chrX:102720688-102
ENSG0000(	401	10.02938	chrX:1657	MTCO1P19		Pseudoger	chrX:102796251-102
ENSG0000(	401	10.02938	chrX:1657	GK		protein_c	chrX:30653359-3073
ENSG0000(	401	10.02938	chrX:1657	MAGEB3		protein_c	chrX:30230657-3023
ENSG0000(	401	10.02938	chrX:1657	NLRP3P1		Pseudoger	chrX:107974078-107
ENSG0000(	401	10.02938	chrX:1657	ENSG00000277876		Pseudoger	chrX:30821815-3082
ENSG0000(	401	10.02938	chrX:1657	NT5DC1P1		Pseudoger	chrX:93388075-9338
ENSG0000(	401	10.02938	chrX:1657	ENSG00000277673		Pseudoger	chrX:106566021-106
ENSG0000(	401	10.02938	chrX:1657	ENSG00000270050		lncRNA	chrX:102599657-102
ENSG0000(	401	10.02938	chrX:1657	MORC4	NCGv7	protein_c	chrX:106813871-107
ENSG0000(	401	10.02938	chrX:1657	H2AP		protein_c	chrX:37990779-3799
ENSG0000(	401	10.02938	chrX:1657	TMEM31		protein_c	chrX:103710909-103
ENSG0000(	401	10.02938	chrX:1657	NSA2P3		Pseudoger	chrX:100910712-100
ENSG0000(	401	10.02938	chrX:1657	CKS1BP6		Pseudoger	chrX:30617454-3061
ENSG0000(	401	10.02938	chrX:1657	GK4P		Pseudoger	chrX:101778969-101
ENSG0000(	401	10.02938	chrX:1657	MAGEB10		protein_c	chrX:27807990-2782
ENSG0000(	401	10.02938	chrX:1657	BEND2		protein_c	chrX:18162931-1822
ENSG0000(	401	10.02938	chrX:1657	RPS6KA3	NCGv7	protein_c	chrX:20149911-2026
ENSG0000(	401	10.02938	chrX:1657	METTL1P1		Pseudoger	chrX:25194381-2519
ENSG0000(	401	10.02938	chrX:1657	MAGEB18		protein_c	chrX:26138343-2614
ENSG0000(	401	10.02938	chrX:1657	MAGEB6		protein_c	chrX:26192440-2619

ENSG0000(	401	10.02938	chrX:1657	FOXN3P1		Pseudoger	chrX:102546734-102
ENSG0000(	401	10.02938	chrX:1657	CBLL2		protein_c	chrX:22272913-2227
ENSG0000(	401	10.02938	chrX:1657	RN7SKP183		smallRNA	chrX:20452108-2045
ENSG0000(	401	10.02938	chrX:1657	RNU6-894P		smallRNA	chrX:31348192-3134
ENSG0000(	401	10.02938	chrX:1657	RNU6-345P		smallRNA	chrX:101915589-101
ENSG0000(	401	10.02938	chrX:1657	PABPC5		protein_c	chrX:91434595-9143
ENSG0000(	401	10.02938	chrX:1657	CFAP47	NCGv7	protein_c	chrX:35919734-3638
ENSG0000(	401	10.02938	chrX:1657	FAM47DP		Pseudoger	chrX:37541077-3754
ENSG0000(	401	10.02938	chrX:1657	FAM133A	NCGv7	protein_c	chrX:93674013-9371
ENSG0000(	401	10.02938	chrX:1657	TMSB15B		protein_c	chrX:103918896-103
ENSG0000(	401	10.02938	chrX:1657	DYNLT3		protein_c	chrX:37836757-3784
ENSG0000(	401	10.02938	chrX:1657	GPRASP2		protein_c	chrX:102712445-102
ENSG0000(	401	10.02938	chrX:1657	RPL36A-HNRNPH2		protein_c	chrX:101391011-101
ENSG0000(	401	10.02938	chrX:1657	XKRX		protein_c	chrX:100913445-100
ENSG0000(	401	10.02938	chrX:1657	CSGALNACT2P2		Pseudoger	chrX:106327534-106
ENSG0000(	401	10.02938	chrX:1657	CNKSR2		protein_c	chrX:21372801-2165
ENSG0000(	401	10.02938	chrX:1657	ATP6AP2		protein_c	chrX:40579372-4060
ENSG0000(	401	10.02938	chrX:1657	KCTD9P2		Pseudoger	chrX:105405647-105
ENSG0000(	401	10.02938	chrX:1657	TGIF2LX		protein_c	chrX:89921908-8992
ENSG0000(	401	10.02938	chrX:1657	TCEAL8		protein_c	chrX:103252995-103
ENSG0000(	401	10.02938	chrX:1657	MAP3K15		protein_c	chrX:19360056-1951
ENSG0000(	401	10.02938	chrX:1657	TSPAN7		protein_c	chrX:38561542-3868
ENSG0000(	401	10.02938	chrX:1657	RPGR	NCGv7	protein_c	chrX:38269163-3832
ENSG0000(	401	10.02938	chrX:1657	CNEP1R1P1		Pseudoger	chrX:103651735-103
ENSG0000(	401	10.02938	chrX:1657	PWWP3B		protein_c	chrX:106168305-106
ENSG0000(	401	10.02938	chrX:1657	TSC22D3		protein_c	chrX:107713221-107
ENSG0000(	401	10.02938	chrX:1657	TAB3	NCGv7	protein_c	chrX:30827442-3097
ENSG0000(	401	10.02938	chrX:1657	MED14		protein_c	chrX:40648305-4073
ENSG0000(	401	10.02938	chrX:1657	TMSB15A		protein_c	chrX:102513682-102
ENSG0000(	401	10.02938	chrX:1657	CYBB		protein_c	chrX:37780018-3781
ENSG0000(	401	10.02938	chrX:1657	MID1IP1		protein_c	chrX:38801440-3880
ENSG0000(	401	10.02938	chrX:1657	CSGALNACT2P1		Pseudoger	chrX:100956593-100
ENSG0000(	401	10.02938	chrX:1657	ZCCHC18		protein_c	chrX:104112131-104
ENSG0000(	401	10.02938	chrX:1657	SNORA31		smallRNA	chrX:38308112-3830
ENSG0000(	401	10.02938	chrX:1657	ACA64		smallRNA	chrX:88148792-8814
ENSG0000(	401	10.02938	chrX:1657	SCARNA23		smallRNA	chrX:24744441-2474
ENSG0000(	401	10.02938	chrX:1657	RAB40A	NCGv7	protein_c	chrX:103499130-103
ENSG0000(	401	10.02938	chrX:1657	TCEAL1		protein_c	chrX:103628704-103
ENSG0000(	401	10.02938	chrX:1657	RNU6-202P		smallRNA	chrX:41919254-4191
ENSG0000(	401	10.02938	chrX:1657	TCP11X3P		Pseudoger	chrX:102173100-102
ENSG0000(	401	10.02938	chrX:1657	ZNF674		protein_c	chrX:46497725-4654
ENSG0000(	401	10.02938	chrX:1657	NR0B1		protein_c	chrX:30304206-3030
ENSG0000(	401	10.02938	chrX:1657	IL1RAPL1		protein_c	chrX:28587446-2995
ENSG0000(	401	10.02938	chrX:1657	GPR34		protein_c	chrX:41688973-4169
ENSG0000(	401	10.02938	chrX:1657	GPR82		protein_c	chrX:41724181-4173
ENSG0000(	401	10.02938	chrX:1657	REPS2		protein_c	chrX:16946658-1715
ENSG0000(	401	10.02938	chrX:1657	SYAP1		protein_c	chrX:16719612-1676
ENSG0000(	401	10.02938	chrX:1657	S100G		protein_c	chrX:16650158-1665
ENSG0000(	401	10.02938	chrX:1657	ENSG00000250349		protein_c	chrX:37349330-3868
ENSG0000(	401	10.02938	chrX:1657	NCBP2L		protein_c	chrX:107774899-107
ENSG0000(	401	10.02938	chrX:1657	RNU6-523P		smallRNA	chrX:44885505-4488

ENSG0000(	401	10.02938	chrX:1657	BEX3		protein_c	chrX:103376395-103
ENSG0000(	401	10.02938	chrX:1657	CXorf58		protein_c	chrX:23908006-2393
ENSG0000(	401	10.02938	chrX:1657	AL136137.1		smallRNA	chrX:44577354-4457
ENSG0000(	401	10.02938	chrX:1657	PTCHD1		protein_c	chrX:23334849-2340
ENSG0000(	401	10.02938	chrX:1657	PCDH19		protein_c	chrX:100291644-100
ENSG0000(	401	10.02938	chrX:1657	CLDN2		protein_c	chrX:106900164-106
ENSG0000(	401	10.02938	chrX:1657	ARL13A		protein_c	chrX:100969708-100
ENSG0000(	401	10.02938	chrX:1657	RNU1-142P		smallRNA	chrX:26790142-2679
ENSG0000(	401	10.02938	chrX:1657	FAM3C2P		Pseudoger	chrX:23075758-2307
ENSG0000(	401	10.02938	chrX:1657	KLHL15		protein_c	chrX:23983716-2402
ENSG0000(	401	10.02938	chrX:1657	H2AL1Q		protein_c	chrX:36719829-3672
ENSG0000(	401	10.02938	chrX:1657	Y_RNA		smallRNA	chrX:27252119-2725
ENSG0000(	401	10.02938	chrX:1657	AL109750.1		smallRNA	chrX:99385195-9938
ENSG0000(	401	10.02938	chrX:1657	RNU6-1087P		smallRNA	chrX:35594173-3559
ENSG0000(	401	10.02938	chrX:1657	SNORA25		smallRNA	chrX:100100084-100
ENSG0000(	401	10.02938	chrX:1657	ADGRG2		protein_c	chrX:18989307-1912
ENSG0000(	401	10.02938	chrX:1657	BCLAF3		protein_c	chrX:19912860-1999
ENSG0000(	401	10.02938	chrX:1657	EIF1AX	NCGv7	protein_c	chrX:20124525-2014
ENSG0000(	401	10.02938	chrX:1657	RNU6-589P		smallRNA	chrX:102678707-102
ENSG0000(	401	10.02938	chrX:1657	ZMAT1		protein_c	chrX:101882288-101
ENSG0000(	401	10.02938	chrX:1657	FRMPD3		protein_c	chrX:107449652-107
ENSG0000(	401	10.02938	chrX:1657	RADX		protein_c	chrX:106611930-106
ENSG0000(	401	10.02938	chrX:1657	BEX2		protein_c	chrX:103309346-103
ENSG0000(	401	10.02938	chrX:1657	MIR4768		smallRNA	chrX:17425881-1742
ENSG0000(	401	10.02938	chrX:1657	MIR548AM		smallRNA	chrX:16627012-1662
ENSG0000(	401	10.02938	chrX:1657	NAP1L3		protein_c	chrX:93670930-9367
ENSG0000(	401	10.02938	chrX:1657	AC004552.1		smallRNA	chrX:24348809-2434
ENSG0000(	401	10.02938	chrX:1657	RN7SL732P		smallRNA	chrX:39786526-3978
ENSG0000(	401	10.02938	chrX:1657	RN7SL15P		smallRNA	chrX:41345582-4134
ENSG0000(	401	10.02938	chrX:1657	MIR23C		smallRNA	chrX:20017088-2001
ENSG0000(	401	10.02938	chrX:1657	Z95400.1		smallRNA	chrX:87485879-8748
ENSG0000(	401	10.02938	chrX:1657	KLHL34		protein_c	chrX:21654690-2165
ENSG0000(	401	10.02938	chrX:1657	AC004655.1		smallRNA	chrX:24698122-2469
ENSG0000(	401	10.02938	chrX:1657	AC096509.1		smallRNA	chrX:24286545-2428
ENSG0000(	401	10.02938	chrX:1657	MIR4666B		smallRNA	chrX:29574278-2957
ENSG0000(	401	10.02938	chrX:1657	MIR1587		smallRNA	chrX:39837561-3983
ENSG0000(	401	10.02938	chrX:1657	MIR3937		smallRNA	chrX:39661216-3966
ENSG0000(	401	10.02938	chrX:1657	CXorf38		protein_c	chrX:40626921-4064
ENSG0000(	401	10.02938	chrX:1657	MIR3915		smallRNA	chrX:32583656-3258
ENSG0000(	401	10.02938	chrX:1657	FTH1P14		Pseudoger	chrX:34147036-3414
ENSG0000(	401	10.02938	chrX:1657	RPL36A		protein_c	chrX:101390824-101
ENSG0000(	401	10.02938	chrX:1657	RN7SL74P		smallRNA	chrX:97341046-9734
ENSG0000(	401	10.02938	chrX:1657	AC004656.1		smallRNA	chrX:24517240-2451
ENSG0000(	401	10.02938	chrX:1657	MIR548AN		smallRNA	chrX:106639814-106
ENSG0000(	401	10.02938	chrX:1657	RN7SL406P		smallRNA	chrX:41637474-4163
ENSG0000(	401	10.02938	chrX:1657	RNF128		protein_c	chrX:106693794-106
ENSG0000(	401	10.02938	chrX:1657	TBC1D8B		protein_c	chrX:106802673-106
ENSG0000(	401	10.02938	chrX:1657	TCEAL4		protein_c	chrX:103576231-103
ENSG0000(	401	10.02938	chrX:1657	BEX1		protein_c	chrX:103062651-103
ENSG0000(	401	10.02938	chrX:1657	ENSG00000269902		lncRNA	chrX:45764772-4576
ENSG0000(	401	10.02938	chrX:1657	SLC25A53		protein_c	chrX:104099214-104

ENSG0000(	401	10.02938	chrX:1657	NXF2B		protein_c	chrX:102360395-102
ENSG0000(	401	10.02938	chrX:1657	NXF2		protein_c	chrX:102247167-102
ENSG0000(	401	10.02938	chrX:1657	TEX13A		protein_c	chrX:105218929-105
ENSG0000(	401	10.02938	chrX:1657	TCP11X1		protein_c	chrX:102215298-102
ENSG0000(	401	10.02938	chrX:1657	AL928874.1		smallRNA	chrX:21563072-2156
ENSG0000(	401	10.02938	chrX:1657	RN7SL48P		smallRNA	chrX:18915247-1891
ENSG0000(	401	10.02938	chrX:1657	RN7SL91P		smallRNA	chrX:25060651-2506
ENSG0000(	401	10.02938	chrX:1657	AL049610.1		smallRNA	chrX:103618505-103
ENSG0000(	401	10.02938	chrX:1657	AC005926.1		smallRNA	chrX:30315085-3031
ENSG0000(	401	10.02938	chrX:1657	RN7SL436P		smallRNA	chrX:22054153-2205
ENSG0000(	401	10.02938	chrX:1657	AC092832.1		smallRNA	chrX:22636992-2263
ENSG0000(	401	10.02938	chrX:1657	FAM47A		protein_c	chrX:34129752-3413
ENSG0000(	401	10.02938	chrX:1657	AC078988.1		smallRNA	chrX:28376963-2837
ENSG0000(	401	10.02938	chrX:1657	PRPS1		protein_c	chrX:107628428-107
ENSG0000(	401	10.02938	chrX:1657	VENTXP1		Pseudoger	chrX:26557675-2688
ENSG0000(	401	10.02938	chrX:1657	RN7SL379P		smallRNA	chrX:96023360-9602
ENSG0000(	401	10.02938	chrX:1657	FTHL18P		Pseudoger	chrX:37043023-3704
ENSG0000(	401	10.02938	chrX:1657	GK-AS1		lncRNA	chrX:30699998-3072
ENSG0000(	401	10.02938	chrX:1657	SH3KBP1		protein_c	chrX:19533977-1988
ENSG0000(	401	10.02938	chrX:1657	TCEAL7		protein_c	chrX:103330229-103
ENSG0000(	401	10.02938	chrX:1657	TMEM47		protein_c	chrX:34627075-3465
ENSG0000(	401	10.02938	chrX:1657	LANCL3		protein_c	chrX:37571569-3768
ENSG0000(	401	10.02938	chrX:1657	SYTL5		protein_c	chrX:38006553-3812
ENSG0000(	401	10.02938	chrX:1657	CASK		protein_c	chrX:41514934-4192
ENSG0000(	401	10.02938	chrX:1657	KDM6A	NCGv7;AC	protein_c	chrX:44873188-4511
ENSG0000(	401	10.02938	chrX:1657	DIPK2B		protein_c	chrX:45148373-4520
ENSG0000(	401	10.02938	chrX:1657	CHST7		protein_c	chrX:46573765-4659
ENSG0000(	401	10.02938	chrX:1657	KRBOX4		protein_c	chrX:46447292-4649
ENSG0000(	401	10.02938	chrX:1657	CPXCR1		protein_c	chrX:88747225-8875
ENSG0000(	401	10.02938	chrX:1657	DIAPH2	NCGv7	protein_c	chrX:96684712-9760
ENSG0000(	401	10.02938	chrX:1657	NXF3		protein_c	chrX:103075810-103
ENSG0000(	401	10.02938	chrX:1657	RIPPLY1		protein_c	chrX:106900063-106
ENSG0000(	401	10.02938	chrX:1657	BCOR	NCGv7;AC	protein_c	chrX:40049815-4017
ENSG0000(	401	10.02938	chrX:1657	ENSG00000259977		lncRNA	chrX:37906147-3794
ENSG0000(	401	10.02938	chrX:1657	AC131011.1		smallRNA	chrX:23756172-2375
ENSG0000(	401	10.02938	chrX:1657	EFHC2		protein_c	chrX:44147872-4434
ENSG0000(	401	10.02938	chrX:1657	TCEAL9		protein_c	chrX:103356489-103
ENSG0000(	401	10.02938	chrX:1657	ENSG00000241607		Pseudoger	chrX:37441523-3744
ENSG0000(	401	10.02938	chrX:1657	ENSG00000261435		lncRNA	chrX:39837536-3984
ENSG0000(	401	10.02938	chrX:1657	ENSG00000241886		lncRNA	chrX:30698207-3072
ENSG0000(	401	10.02938	chrX:1657	RP11-524D16__A.3		lncRNA	chrX:100673330-100
ENSG0000(	401	10.02938	chrX:1657	TCEAL2		protein_c	chrX:102125679-102
ENSG0000(	401	10.02938	chrX:1657	ENSG00000261101		lncRNA	chrX:101627868-101
ENSG0000(	401	10.02938	chrX:1657	ARMCX2		protein_c	chrX:101655281-101
ENSG0000(	401	10.02938	chrX:1657	APOO		protein_c	chrX:23833353-2390
ENSG0000(	401	10.02938	chrX:1657	DDX53		protein_c	chrX:22999960-2300
ENSG0000(	401	10.02938	chrX:1657	GHc-362H12.3		lncRNA	chrX:103943056-103
ENSG0000(	401	10.02938	chrX:1657	ENSG00000242021		lncRNA	chrX:27042907-2717
ENSG0000(	401	10.02938	chrX:1657	RN7SL291P		smallRNA	chrX:44772775-4477
ENSG0000(	401	10.02938	chrX:1657	BEX5		protein_c	chrX:102153708-102
ENSG0000(	401	10.02938	chrX:1657	MAP7D2		protein_c	chrX:20006713-2011

ENSG0000(	401	10.02938	chrX:1657	RPL21P132		Pseudoger	chrX:101339917-101
ENSG0000(	401	10.02938	chrX:1657	RN7SL144P		smallRNA	chrX:41683171-4168
ENSG0000(	401	10.02938	chrX:1657	ENSG00000235806		lncRNA	chrX:38770331-3879
ENSG0000(	401	10.02938	chrX:1657	NHS		protein_c	chrX:17375200-1773
ENSG0000(	401	10.02938	chrX:1657	TEX13B		protein_c	chrX:107980864-107
ENSG0000(	401	10.02938	chrX:1657	RPA4		protein_c	chrX:96883908-9688
ENSG0000(	401	10.02938	chrX:1657	TCEAL6	NCGv7	protein_c	chrX:102140476-102
ENSG0000(	401	10.02938	chrX:1657	TCEAL5		protein_c	chrX:103273691-103
ENSG0000(	401	10.02938	chrX:1657	MYCLP1		Pseudoger	chrX:107272582-107
ENSG0000(	401	10.02938	chrX:1657	ENSG00000289479		lncRNA	chrX:92100276-9210
ENSG0000(	401	10.02938	chrX:1657	ENSG00000289472		lncRNA	chrX:24168659-2416
ENSG0000(	401	10.02938	chrX:1657	MTND6P13		Pseudoger	chrX:102008524-102
ENSG0000(	401	10.02938	chrX:1657	ENSG00000287098		lncRNA	chrX:40620701-4062
ENSG0000(	401	10.02938	chrX:1657	KPNB1P1		Pseudoger	chrX:97975878-9797
ENSG0000(	401	10.02938	chrX:1657	MTND5P26		Pseudoger	chrX:102803041-102
ENSG0000(	401	10.02938	chrX:1657	MAGEB2		protein_c	chrX:30215563-3022
ENSG0000(	401	10.02938	chrX:1657	CHTF8P1		Pseudoger	chrX:44633692-4463
ENSG0000(	401	10.02938	chrX:1657	ENSG00000287223		lncRNA	chrX:42723209-4275
ENSG0000(	401	10.02938	chrX:1657	ENSG00000233571		lncRNA	chrX:34206725-3441
ENSG0000(	401	10.02938	chrX:1657	RNA5SP500		Pseudoger	chrX:28982852-2898
ENSG0000(	401	10.02938	chrX:1657	PDK3		protein_c	chrX:24465244-2455
ENSG0000(	401	10.02938	chrX:1657	ENSG00000289215		lncRNA	chrX:32353833-3235
ENSG0000(	401	10.02938	chrX:1657	ENSG00000203262		Pseudoger	chrX:96070669-9607
ENSG0000(	401	10.02938	chrX:1657	ENSG00000233663		Pseudoger	chrX:35710911-3571
ENSG0000(	401	10.02938	chrX:1657	HNRNPA1P27		Pseudoger	chrX:100887636-100
ENSG0000(	401	10.02938	chrX:1657	VDAC1P3		Pseudoger	chrX:91982655-9198
ENSG0000(	401	10.02938	chrX:1657	ENSG00000289127		lncRNA	chrX:38870767-3901
ENSG0000(	401	10.02938	chrX:1657	snoU2-30		smallRNA	chrX:20136066-2013
ENSG0000(	401	10.02938	chrX:1657	ENSG00000233403		lncRNA	chrX:25878339-2589
ENSG0000(	401	10.02938	chrX:1657	VSIG1		protein_c	chrX:108044970-108
ENSG0000(	401	10.02938	chrX:1657	RNU6-266P		smallRNA	chrX:22527835-2252
ENSG0000(	401	10.02938	chrX:1657	OTC		protein_c	chrX:38352586-3842
ENSG0000(	401	10.02938	chrX:1657	H2BW2		protein_c	chrX:104039956-104
ENSG0000(	401	10.02938	chrX:1657	CSTF2		protein_c	chrX:100820391-100
ENSG0000(	401	10.02938	chrX:1657	TNMD		protein_c	chrX:100584936-100
ENSG0000(	401	10.02938	chrX:1657	LINC01281		lncRNA	chrX:39304956-3932
ENSG0000(	401	10.02938	chrX:1657	ENSG00000233103		lncRNA	chrX:42047883-4205
ENSG0000(	401	10.02938	chrX:1657	PHKA2	NCGv7	protein_c	chrX:18892298-1898
ENSG0000(	401	10.02938	chrX:1657	ENSG00000289923		lncRNA	chrX:107676136-107
ENSG0000(	401	10.02938	chrX:1657	CTPS2		protein_c	chrX:16587999-1671
ENSG0000(	401	10.02938	chrX:1657	XK		protein_c	chrX:37685791-3773
ENSG0000(	401	10.02938	chrX:1657	SCML1		protein_c	chrX:17737449-1775
ENSG0000(	401	10.02938	chrX:1657	ENSG00000289708		Pseudoger	chrX:36984358-3698
ENSG0000(	401	10.02938	chrX:1657	TMSB10P2		Pseudoger	chrX:18341216-1834
ENSG0000(	401	10.02938	chrX:1657	ENSG00000235196		Pseudoger	chrX:101911529-101
ENSG0000(	401	10.02938	chrX:1657	ENSG00000286724		lncRNA	chrX:18984291-1906
ENSG0000(	401	10.02938	chrX:1657	VKORC1P1		Pseudoger	chrX:27863893-2786
ENSG0000(	401	10.02938	chrX:1657	ENSG00000289575		lncRNA	chrX:87723928-8772
ENSG0000(	401	10.02938	chrX:1657	ENSG00000286794		lncRNA	chrX:102142563-102
ENSG0000(	401	10.02938	chrX:1657	ENSG00000289570		lncRNA	chrX:39991276-4001
ENSG0000(	401	10.02938	chrX:1657	ENSG00000286846		lncRNA	chrX:102945971-102

ENSG0000(	401	10.02938	chrX:1657	H2BFXP	Pseudoger	chrX:103975957-103
ENSG0000(	401	10.02938	chrX:1657	ENSG00000289122	lncRNA	chrX:40006365-4000
ENSG0000(	401	10.02938	chrX:1657	SNORA68	smallRNA	chrX:24133186-2413
ENSG0000(	401	10.02938	chrX:1657	FUNDC1	protein_c	chrX:44523639-4454
ENSG0000(	401	10.02938	chrX:1657	SMPX	protein_c	chrX:21705978-2175
ENSG0000(	401	10.02938	chrX:1657	SNORA9	smallRNA	chrX:100822620-100
ENSG0000(	401	10.02938	chrX:1657	ENSG00000288706	protein_c	chrX:23783278-2378
ENSG0000(	401	10.02938	chrX:1657	ENSG00000287700	lncRNA	chrX:33718958-3372
ENSG0000(	401	10.02938	chrX:1657	PPEF1	protein_c	chrX:18675909-1882
ENSG0000(	401	10.02938	chrX:1657	SNORD74	smallRNA	chrX:28924069-2892
ENSG0000(	401	10.02938	chrX:1657	Y_RNA	smallRNA	chrX:19376774-1937
ENSG0000(	401	10.02938	chrX:1657	ENSG00000234405	lncRNA	chrX:103486461-103
ENSG0000(	401	10.02938	chrX:1657	FTH1P27	Pseudoger	chrX:37505310-3750
ENSG0000(	401	10.02938	chrX:1657	TXLNG	protein_c	chrX:16786432-1684
ENSG0000(	401	10.02938	chrX:1657	ELF2P1	Pseudoger	chrX:103910305-103
ENSG0000(	401	10.02938	chrX:1657	ENSG00000288597	lncRNA	chrX:103707034-103
ENSG0000(	401	10.02938	chrX:1657	ENSG00000234050	lncRNA	chrX:102937770-102
ENSG0000(	401	10.02938	chrX:1657	ST13P18	Pseudoger	chrX:93287733-9328
ENSG0000(	401	10.02938	chrX:1657	ENSG00000287729	lncRNA	chrX:38779584-3880
ENSG0000(	401	10.02938	chrX:1657	MID2	protein_c	chrX:107825755-107
ENSG0000(	401	10.02938	chrX:1657	DNAAF6	protein_c	chrX:107206611-107
ENSG0000(	401	10.02938	chrX:1657	RNU6-934P	smallRNA	chrX:101325404-101
ENSG0000(	401	10.02938	chrX:1657	AP2B1P1	Pseudoger	chrX:93222220-9322
ENSG0000(	401	10.02938	chrX:1657	PABPC5-AS1	lncRNA	chrX:91414878-9143
ENSG0000(	401	10.02938	chrX:1657	LINC01283	lncRNA	chrX:39401252-3943
ENSG0000(	401	10.02938	chrX:1657	ZFX-AS1	lncRNA	chrX:24146225-2414
ENSG0000(	401	10.02938	chrX:1657	snoU2_19	smallRNA	chrX:20136306-2013
ENSG0000(	401	10.02938	chrX:1657	ENSG00000233928	lncRNA	chrX:33726337-3434
ENSG0000(	401	10.02938	chrX:1657	RPS15AP40	Pseudoger	chrX:36374793-3637
ENSG0000(	401	10.02938	chrX:1657	ENSG00000287549	lncRNA	chrX:41008427-4102
ENSG0000(	401	10.02938	chrX:1657	MAOB	protein_c	chrX:43766610-4388
ENSG0000(	401	10.02938	chrX:1657	ENSG00000289084	lncRNA	chrX:22259797-2329
ENSG0000(	401	10.02938	chrX:1657	ENSG00000289068	lncRNA	chrX:39653211-3967
ENSG0000(	401	10.02938	chrX:1657	MED14OS	lncRNA	chrX:40735400-4073
ENSG0000(	401	10.02938	chrX:1657	Y_RNA	smallRNA	chrX:94178005-9417
ENSG0000(	401	10.02938	chrX:1657	Y_RNA	smallRNA	chrX:106426568-106
ENSG0000(	401	10.02938	chrX:1657	ENSG00000234613	lncRNA	chrX:45913267-4591
ENSG0000(	401	10.02938	chrX:1657	SNORA16	smallRNA	chrX:17044380-1704
ENSG0000(	401	10.02938	chrX:1657	ENSG00000233785	lncRNA	chrX:23772992-2378
ENSG0000(	401	10.02938	chrX:1657	PPIAP90	Pseudoger	chrX:101443707-101
ENSG0000(	401	10.02938	chrX:1657	ENSG00000288832	lncRNA	chrX:21652655-2165
ENSG0000(	401	10.02938	chrX:1657	RBM41	protein_c	chrX:107061885-107
ENSG0000(	401	10.02938	chrX:1657	AMZ2P3	Pseudoger	chrX:27218131-2721
ENSG0000(	401	10.02938	chrX:1657	MOB1AP2	Pseudoger	chrX:37291168-3729
ENSG0000(	401	10.02938	chrX:1657	ENSG00000287619	lncRNA	chrX:103563917-103
ENSG0000(	401	10.02938	chrX:1657	ENSG00000288856	lncRNA	chrX:40171944-4017
ENSG0000(	401	10.02938	chrX:1657	NFYCP1	Pseudoger	chrX:35761499-3576
ENSG0000(	401	10.02938	chrX:1657	Y_RNA	smallRNA	chrX:23782210-2378
ENSG0000(	401	10.02938	chrX:1657	RNA5SP510	Pseudoger	chrX:95582064-9558
ENSG0000(	401	10.02938	chrX:1657	ENSG00000233887	Pseudoger	chrX:97847263-9784
ENSG0000(	401	10.02938	chrX:1657	RPSAP59	Pseudoger	chrX:103206010-103

ENSG0000(	401	10.02938	chrX:1657	ARMCX7P		Pseudoger	chrX:101597510-101
ENSG0000(	401	10.02938	chrX:1657	ENSG00000290743		lncRNA	chrX:102182315-102
ENSG0000(	401	10.02938	chrX:1657	ARMCX3	NCGv7	protein_c	chrX:101622797-101
ENSG0000(	401	10.02938	chrX:1657	ENSG00000232644		Pseudoger	chrX:26200042-2620
ENSG0000(	401	10.02938	chrX:1657	DRP2		protein_c	chrX:101219769-101
ENSG0000(	401	10.02938	chrX:1657	TAF7L		protein_c	chrX:101268253-101
ENSG0000(	401	10.02938	chrX:1657	ENSG00000235685		Pseudoger	chrX:40514895-4051
ENSG0000(	401	10.02938	chrX:1657	Y_RNA		smallRNA	chrX:103156120-103
ENSG0000(	401	10.02938	chrX:1657	GLA		protein_c	chrX:101393273-101
ENSG0000(	401	10.02938	chrX:1657	BEX4		protein_c	chrX:103215108-103
ENSG0000(	401	10.02938	chrX:1657	SRPX	NCGv7	protein_c	chrX:38149336-3822
ENSG0000(	401	10.02938	chrX:1657	MFFP3		Pseudoger	chrX:45730752-4573
ENSG0000(	401	10.02938	chrX:1657	SCML2		protein_c	chrX:18239313-1835
ENSG0000(	401	10.02938	chrX:1657	ENSG00000235510		Pseudoger	chrX:36957913-3695
ENSG0000(	401	10.02938	chrX:1657	ENSG00000290908		lncRNA	chrX:101598017-101
ENSG0000(	401	10.02938	chrX:1657	PCYT1B		protein_c	chrX:24558087-2467
ENSG0000(	401	10.02938	chrX:1657	PHEX		protein_c	chrX:22032325-2249
ENSG0000(	401	10.02938	chrX:1657	GAPDHP65		Pseudoger	chrX:46439709-4644
ENSG0000(	401	10.02938	chrX:1657	PHB1P10		Pseudoger	chrX:104647216-104
ENSG0000(	401	10.02938	chrX:1657	MBTPS2		protein_c	chrX:21839617-2188
ENSG0000(	401	10.02938	chrX:1657	PPP1R2C		protein_c	chrX:42777366-4277
ENSG0000(	401	10.02938	chrX:1657	CENPI		protein_c	chrX:101098188-101
ENSG0000(	401	10.02938	chrX:1657	SYTL4		protein_c	chrX:100671783-100
ENSG0000(	401	10.02938	chrX:1657	PTP4A1P5		Pseudoger	chrX:27427777-2742
ENSG0000(	401	10.02938	chrX:1657	KLHL4		protein_c	chrX:87517409-8767
ENSG0000(	401	10.02938	chrX:1657	ENSG00000290955		lncRNA	chrX:36840649-3698
ENSG0000(	401	10.02938	chrX:1657	SRPX2		protein_c	chrX:100644195-100
ENSG0000(	401	10.02938	chrX:1657	TAB3-AS2		lncRNA	chrX:30854321-3085
ENSG0000(	401	10.02938	chrX:1657	Y_RNA		smallRNA	chrX:44401603-4440
ENSG0000(	401	10.02938	chrX:1657	KAT7P1		Pseudoger	chrX:95973328-9597
ENSG0000(	401	10.02938	chrX:1657	RBBP7		protein_c	chrX:16839283-1687
ENSG0000(	401	10.02938	chrX:1657	BTK	NCGv7	protein_c	chrX:101349338-101
ENSG0000(	401	10.02938	chrX:1657	ENSG00000232834		Pseudoger	chrX:27847588-2784
ENSG0000(	401	10.02938	chrX:1657	PCDH11X	NCGv7	protein_c	chrX:91779261-9262
ENSG0000(	401	10.02938	chrX:1657	SMS		protein_c	chrX:21940709-2199
ENSG0000(	401	10.02938	chrX:1657	ENSG00000290798		lncRNA	chrX:101832112-101
ENSG0000(	401	10.02938	chrX:1657	RAB40AL		protein_c	chrX:102937272-102
ENSG0000(	401	10.02938	chrX:1657	CDKL5		protein_c	chrX:18425583-1865
ENSG0000(	401	10.02938	chrX:1657	ARX		protein_c	chrX:25003694-2501
ENSG0000(	401	10.02938	chrX:1657	ZFX	NCGv7	protein_c	chrX:24149173-2421
ENSG0000(	401	10.02938	chrX:1657	SDCBPP3		Pseudoger	chrX:40890647-4089
ENSG0000(	401	10.02938	chrX:1657	RS1	NCGv7	protein_c	chrX:18639688-1867
ENSG0000(	401	10.02938	chrX:1657	NXF4		lncRNA	chrX:102549965-102
ENSG0000(	401	10.02938	chrX:1657	ENSG00000286475		lncRNA	chrX:36678678-3686
ENSG0000(	401	10.02938	chrX:1657	RP11-40F8.2		lncRNA	chrX:22259797-2329
ENSG0000(	401	10.02938	chrX:1657	PSMD10	AC	protein_c	chrX:108084207-108
ENSG0000(	401	10.02938	chrX:1657	ATG4A		protein_c	chrX:108091668-108
ENSG0000(	401	10.02938	chrX:1657	CASK-AS1		lncRNA	chrX:41520036-4152
ENSG0000(	401	10.02938	chrX:1657	ENSG00000232576		Pseudoger	chrX:88908403-8890
ENSG0000(	401	10.02938	chrX:1657	ENSG00000286523		lncRNA	chrX:96127747-9637
ENSG0000(	401	10.02938	chrX:1657	POLA1		protein_c	chrX:24693873-2499

ENSG0000(	401	10.02938	chrX:1657	NOX1		protein_c	chrX:100843324-100
ENSG0000(	401	10.02938	chrX:1657	RPS7P13		Pseudoger	chrX:95679130-9567
ENSG0000(	401	10.02938	chrX:1657	UBTFL11		Pseudoger	chrX:38531923-3853
ENSG0000(	397	9.929339	chr1:3732	SEPTIN7P13		Pseudoger	chr1:223995895-224
ENSG0000(	397	9.929339	chr1:3732	RN7SL276P		smallRNA	chr1:222708237-222
ENSG0000(	397	9.929339	chr1:3732	MTARC1		protein_c	chr1:220786352-220
ENSG0000(	397	9.929339	chr1:3732	IARS2		protein_c	chr1:220094132-220
ENSG0000(	397	9.929339	chr1:3732	LYPLAL1		protein_c	chr1:219173869-219
ENSG0000(	397	9.929339	chr1:3732	MIR3620		smallRNA	chr1:228097263-228
ENSG0000(	397	9.929339	chr1:3732	RNA5SP76		Pseudoger	chr1:219761789-219
ENSG0000(	397	9.929339	chr1:3732	MORF4L1P1		Pseudoger	chr1:220253570-220
ENSG0000(	397	9.929339	chr1:3732	TAF1A		protein_c	chr1:222557902-222
ENSG0000(	397	9.929339	chr1:3732	ENSG00000288999		lncRNA	chr1:223846081-223
ENSG0000(	397	9.929339	chr1:3732	SMYD2		protein_c	chr1:214281102-214
ENSG0000(	397	9.929339	chr1:3732	SNORD116		smallRNA	chr1:215630026-215
ENSG0000(	397	9.929339	chr1:3732	AL592310.1		smallRNA	chr1:227645280-227
ENSG0000(	397	9.929339	chr1:3732	SUSD4	DriverDB\	protein_c	chr1:223220819-223
ENSG0000(	397	9.929339	chr1:3732	ENSG00000229016		Pseudoger	chr1:218301262-218
ENSG0000(	397	9.929339	chr1:3732	USH2A-AS2		lncRNA	chr1:216072465-216
ENSG0000(	397	9.929339	chr1:3732	ENSG00000223869		Pseudoger	chr1:214943123-214
ENSG0000(	397	9.929339	chr1:3732	LINC01705		lncRNA	chr1:222010825-222
ENSG0000(	397	9.929339	chr1:3732	AC096643.1		smallRNA	chr1:219663377-219
ENSG0000(	397	9.929339	chr1:3732	NUP133	NCGv7	protein_c	chr1:229440259-229
ENSG0000(	397	9.929339	chr1:3732	NXNP1		Pseudoger	chr1:218881600-218
ENSG0000(	397	9.929339	chr1:3732	ENSG00000226601		lncRNA	chr1:223181144-223
ENSG0000(	397	9.929339	chr1:3732	ENSG00000289142		lncRNA	chr1:220878225-220
ENSG0000(	397	9.929339	chr1:3732	AIDA		protein_c	chr1:222668013-222
ENSG0000(	397	9.929339	chr1:3732	RNA5S13		smallRNA	chr1:228637096-228
ENSG0000(	397	9.929339	chr1:3732	XRCC6P3		Pseudoger	chr1:220313945-220
ENSG0000(	397	9.929339	chr1:3732	ENSG00000230331		Pseudoger	chr1:229425020-229
ENSG0000(	397	9.929339	chr1:3732	PRSS38	NCGv7	protein_c	chr1:227815675-227
ENSG0000(	397	9.929339	chr1:3732	LINC01653		lncRNA	chr1:218043505-218
ENSG0000(	397	9.929339	chr1:3732	ENSG00000226643		lncRNA	chr1:222452738-222
ENSG0000(	397	9.929339	chr1:3732	MIR5008		smallRNA	chr1:227941590-227
ENSG0000(	397	9.929339	chr1:3732	GAPDHP24		Pseudoger	chr1:214870734-214
ENSG0000(	397	9.929339	chr1:3732	MIXL1		protein_c	chr1:226223618-226
ENSG0000(	397	9.929339	chr1:3732	DNAH14	NCGv7	protein_c	chr1:224896262-225
ENSG0000(	397	9.929339	chr1:3732	ENSG00000233706		lncRNA	chr1:226992140-226
ENSG0000(	397	9.929339	chr1:3732	COQ8A		protein_c	chr1:226940286-226
ENSG0000(	397	9.929339	chr1:3732	RN7SKP276		smallRNA	chr1:229410500-229
ENSG0000(	397	9.929339	chr1:3732	ENSG00000288674		protein_c	chr1:226870184-226
ENSG0000(	397	9.929339	chr1:3732	ENSG00000270104		lncRNA	chr1:228384114-228
ENSG0000(	397	9.929339	chr1:3732	ENSG00000270110		lncRNA	chr1:228295549-228
ENSG0000(	397	9.929339	chr1:3732	H3-4		protein_c	chr1:228424845-228
ENSG0000(	397	9.929339	chr1:3732	ITPKB-AS1		lncRNA	chr1:226668897-226
ENSG0000(	397	9.929339	chr1:3732	ENSG00000288644		protein_c	chr1:203802094-203
ENSG0000(	397	9.929339	chr1:3732	RNF187		protein_c	chr1:228487382-228
ENSG0000(	397	9.929339	chr1:3732	ENSG00000270287		Pseudoger	chr1:226411615-226
ENSG0000(	397	9.929339	chr1:3732	ENSG00000228625		lncRNA	chr1:227178333-227
ENSG0000(	397	9.929339	chr1:3732	ENSG00000223375		Pseudoger	chr1:218338541-218
ENSG0000(	397	9.929339	chr1:3732	RNA5SP19		Pseudoger	chr1:228555793-228

ENSG0000(	397	9.929339	chr1:3732	RNU6-1304P		smallRNA	chr1:225741275-225
ENSG0000(	397	9.929339	chr1:3732	SPATA17-AS1		lncRNA	chr1:217781198-217
ENSG0000(	397	9.929339	chr1:3732	SNX2P1		Pseudoger	chr1:220207618-220
ENSG0000(	397	9.929339	chr1:3732	ENSG00000232436		lncRNA	chr1:221508559-221
ENSG0000(	397	9.929339	chr1:3732	ENSG00000270708		Pseudoger	chr1:220220291-220
ENSG0000(	397	9.929339	chr1:3732	LEFTY3P		Pseudoger	chr1:225803148-225
ENSG0000(	397	9.929339	chr1:3732	JMJD4		protein_c	chr1:227730425-227
ENSG0000(	397	9.929339	chr1:3732	ENSG00000291068		lncRNA	chr1:223951394-223
ENSG0000(	397	9.929339	chr1:3732	ENSG00000228729		Pseudoger	chr1:227234269-227
ENSG0000(	397	9.929339	chr1:3732	ABHD17AP3		Pseudoger	chr1:214605470-214
ENSG0000(	397	9.929339	chr1:3732	ENSG00000230714		lncRNA	chr1:218031835-218
ENSG0000(	397	9.929339	chr1:3732	RNA5S15		smallRNA	chr1:228641568-228
ENSG0000(	397	9.929339	chr1:3732	MIR194-1		smallRNA	chr1:220118157-220
ENSG0000(	397	9.929339	chr1:3732	ENSG00000270094		lncRNA	chr1:228394290-228
ENSG0000(	397	9.929339	chr1:3732	LYPLAL1-AS1		lncRNA	chr1:219409039-219
ENSG0000(	397	9.929339	chr1:3732	CICP5		Pseudoger	chr1:223947605-223
ENSG0000(	397	9.929339	chr1:3732	RAB4A		protein_c	chr1:229271062-229
ENSG0000(	397	9.929339	chr1:3732	KCTD3		protein_c	chr1:215567304-215
ENSG0000(	397	9.929339	chr1:3732	HLX		protein_c	chr1:220879431-220
ENSG0000(	397	9.929339	chr1:3732	PROX1-AS1		lncRNA	chr1:213817751-213
ENSG0000(	397	9.929339	chr1:3732	EPRS1	NCGv7	protein_c	chr1:219968600-220
ENSG0000(	397	9.929339	chr1:3732	ITPKB-IT1		lncRNA	chr1:226656080-226
ENSG0000(	397	9.929339	chr1:3732	RN7SKP165		smallRNA	chr1:226445937-226
ENSG0000(	397	9.929339	chr1:3732	HHIPL2		protein_c	chr1:222522258-222
ENSG0000(	397	9.929339	chr1:3732	MIR4666A		smallRNA	chr1:228462074-228
ENSG0000(	397	9.929339	chr1:3732	MIR4742		smallRNA	chr1:224398227-224
ENSG0000(	397	9.929339	chr1:3732	ENSG00000288862		lncRNA	chr1:229407361-229
ENSG0000(	397	9.929339	chr1:3732	AL596330.1		smallRNA	chr1:224722467-224
ENSG0000(	397	9.929339	chr1:3732	ENSG00000232628		lncRNA	chr1:224208741-224
ENSG0000(	397	9.929339	chr1:3732	RNA5SP77		Pseudoger	chr1:227561181-227
ENSG0000(	397	9.929339	chr1:3732	RNA5S16		smallRNA	chr1:228643809-228
ENSG0000(	397	9.929339	chr1:3732	ENSG00000233920		lncRNA	chr1:229223457-229
ENSG0000(	397	9.929339	chr1:3732	LINC02474		lncRNA	chr1:221966341-221
ENSG0000(	397	9.929339	chr1:3732	RNU4-57P		smallRNA	chr1:223373822-223
ENSG0000(	397	9.929339	chr1:3732	ENSG00000269934		lncRNA	chr1:228238241-228
ENSG0000(	397	9.929339	chr1:3732	ENSG00000228470		lncRNA	chr1:214344172-214
ENSG0000(	397	9.929339	chr1:3732	ENSG00000226349		lncRNA	chr1:225710968-225
ENSG0000(	397	9.929339	chr1:3732	ENSG00000290989		lncRNA	chr1:223992743-224
ENSG0000(	397	9.929339	chr1:3732	ENSG00000228525		Pseudoger	chr1:226958069-226
ENSG0000(	397	9.929339	chr1:3732	TLR5	NCGv7	protein_c	chr1:223109404-223
ENSG0000(	397	9.929339	chr1:3732	DUSP10		protein_c	chr1:221701424-221
ENSG0000(	397	9.929339	chr1:3732	SNAP47-AS1		lncRNA	chr1:227743831-227
ENSG0000(	397	9.929339	chr1:3732	TP53BP2	NCGv7	protein_c	chr1:223779893-223
ENSG0000(	397	9.929339	chr1:3732	MIA3		protein_c	chr1:222618097-222
ENSG0000(	397	9.929339	chr1:3732	ENSG00000227585		Pseudoger	chr1:221549786-221
ENSG0000(	397	9.929339	chr1:3732	LYPLAL1-DT		lncRNA	chr1:218976672-219
ENSG0000(	397	9.929339	chr1:3732	CICP26		Pseudoger	chr1:227975390-227
ENSG0000(	397	9.929339	chr1:3732	LINC02869		lncRNA	chr1:218459265-218
ENSG0000(	397	9.929339	chr1:3732	CCSAP		protein_c	chr1:229321011-229
ENSG0000(	397	9.929339	chr1:3732	ENAH		protein_c	chr1:225486765-225
ENSG0000(	397	9.929339	chr1:3732	TRIM11		protein_c	chr1:228393673-228

ENSG0000(	397	9.929339	chr1:3732	H2BU2P		Pseudoger	chr1:228464213-228
ENSG0000(	397	9.929339	chr1:3732	OBSCN	NCGv7	protein_c	chr1:228208044-228
ENSG0000(	397	9.929339	chr1:3732	H2AW		protein_c	chr1:228434777-228
ENSG0000(	397	9.929339	chr1:3732	WNT3A		protein_c	chr1:228006998-228
ENSG0000(	397	9.929339	chr1:3732	DISP1	NCGv7	protein_c	chr1:222815022-223
ENSG0000(	397	9.929339	chr1:3732	ZNF678		protein_c	chr1:227563543-227
ENSG0000(	397	9.929339	chr1:3732	PHB1P11		Pseudoger	chr1:223856579-223
ENSG0000(	397	9.929339	chr1:3732	RNA5SP18		Pseudoger	chr1:228647912-228
ENSG0000(	397	9.929339	chr1:3732	ENSG00000289602		lncRNA	chr1:225653285-225
ENSG0000(	397	9.929339	chr1:3732	ENSG00000223570		Pseudoger	chr1:226188870-226
ENSG0000(	397	9.929339	chr1:3732	IBA57		protein_c	chr1:228165804-228
ENSG0000(	397	9.929339	chr1:3732	ENSG00000227496		lncRNA	chr1:225700264-225
ENSG0000(	397	9.929339	chr1:3732	RN7SL464P		smallRNA	chr1:220571743-220
ENSG0000(	397	9.929339	chr1:3732	PTPN14	NCGv7	protein_c	chr1:214348700-214
ENSG0000(	397	9.929339	chr1:3732	ENSG00000255835		protein_c	chr1:225886696-225
ENSG0000(	397	9.929339	chr1:3732	LEFTY1		protein_c	chr1:225886282-225
ENSG0000(	397	9.929339	chr1:3732	ENSG00000229742		Pseudoger	chr1:224297646-224
ENSG0000(	397	9.929339	chr1:3732	RNU6-1319P		smallRNA	chr1:223976146-223
ENSG0000(	397	9.929339	chr1:3732	ATP2B4		protein_c	chr1:203626832-203
ENSG0000(	397	9.929339	chr1:3732	QRSL1P2		Pseudoger	chr1:222261833-222
ENSG0000(	397	9.929339	chr1:3732	ENSG00000227625		Pseudoger	chr1:228134785-228
ENSG0000(	397	9.929339	chr1:3732	ZC3H11A	NCGv7	protein_c	chr1:203795623-203
ENSG0000(	397	9.929339	chr1:3732	BPNT1		protein_c	chr1:220057482-220
ENSG0000(	397	9.929339	chr1:3732	ENSG00000229242		lncRNA	chr1:215886582-215
ENSG0000(	397	9.929339	chr1:3732	TRIM17		protein_c	chr1:228407935-228
ENSG0000(	397	9.929339	chr1:3732	WDR26		protein_c	chr1:224385146-224
ENSG0000(	397	9.929339	chr1:3732	OBSCN-AS1		lncRNA	chr1:228203503-228
ENSG0000(	397	9.929339	chr1:3732	CDKN2AIPNLP1		Pseudoger	chr1:226493188-226
ENSG0000(	397	9.929339	chr1:3732	RAB4A-AS1		lncRNA	chr1:229256892-229
ENSG0000(	397	9.929339	chr1:3732	MRPL55		protein_c	chr1:228106679-228
ENSG0000(	397	9.929339	chr1:3732	CAPN2		protein_c	chr1:223701593-223
ENSG0000(	397	9.929339	chr1:3732	TMEM78		lncRNA	chr1:229249636-229
ENSG0000(	397	9.929339	chr1:3732	BROX		protein_c	chr1:222712553-222
ENSG0000(	397	9.929339	chr1:3732	C1orf115	DriverDB\	protein_c	chr1:220690363-220
ENSG0000(	397	9.929339	chr1:3732	SPATA17		protein_c	chr1:217631324-217
ENSG0000(	397	9.929339	chr1:3732	LINC01655		lncRNA	chr1:221819842-221
ENSG0000(	397	9.929339	chr1:3732	ENSG00000289880		lncRNA	chr1:222742640-222
ENSG0000(	397	9.929339	chr1:3732	CCDC185		protein_c	chr1:223393415-223
ENSG0000(	397	9.929339	chr1:3732	ENSG00000248322		lncRNA	chr1:225936411-225
ENSG0000(	397	9.929339	chr1:3732	HDAC1P2		Pseudoger	chr1:220625740-220
ENSG0000(	397	9.929339	chr1:3732	ENSG00000290037		lncRNA	chr1:229431366-229
ENSG0000(	397	9.929339	chr1:3732	ENSG00000229399		Pseudoger	chr1:222641414-222
ENSG0000(	397	9.929339	chr1:3732	CNIH3-AS1		lncRNA	chr1:224717504-224
ENSG0000(	397	9.929339	chr1:3732	ENSG00000289962		lncRNA	chr1:225467092-225
ENSG0000(	397	9.929339	chr1:3732	RNU6-1248P		smallRNA	chr1:223690051-223
ENSG0000(	397	9.929339	chr1:3732	USH2A	NCGv7	protein_c	chr1:215622891-216
ENSG0000(	397	9.929339	chr1:3732	ENSG00000227711		Pseudoger	chr1:227509028-227
ENSG0000(	397	9.929339	chr1:3732	U3		smallRNA	chr1:218541691-218
ENSG0000(	397	9.929339	chr1:3732	TGFB2-AS1		lncRNA	chr1:218344190-218
ENSG0000(	397	9.929339	chr1:3732	UBBP2		Pseudoger	chr1:217850403-217
ENSG0000(	397	9.929339	chr1:3732	ZBED6		protein_c	chr1:203795623-203

ENSG0000(	397	9.929339	chr1:3732	ACTA1		protein_c	chr1:229430365-229
ENSG0000(	397	9.929339	chr1:3732	GUK1		protein_c	chr1:228139962-228
ENSG0000(	397	9.929339	chr1:3732	ENSG00000226920		lncRNA	chr1:229440284-229
ENSG0000(	397	9.929339	chr1:3732	RPS3AP7		Pseudoger	chr1:226438564-226
ENSG0000(	397	9.929339	chr1:3732	EPHX1	DriverDB\	protein_c	chr1:225810124-225
ENSG0000(	397	9.929339	chr1:3732	WNT9A		protein_c	chr1:227918656-227
ENSG0000(	397	9.929339	chr1:3732	LBR		protein_c	chr1:225401502-225
ENSG0000(	397	9.929339	chr1:3732	PYCR2		protein_c	chr1:225919877-225
ENSG0000(	397	9.929339	chr1:3732	PSEN2		protein_c	chr1:226870184-226
ENSG0000(	397	9.929339	chr1:3732	PARP1		protein_c	chr1:226360210-226
ENSG0000(	397	9.929339	chr1:3732	AKR1B1P1		Pseudoger	chr1:224574434-224
ENSG0000(	397	9.929339	chr1:3732	C1orf35		protein_c	chr1:228100726-228
ENSG0000(	397	9.929339	chr1:3732	CNIH3		protein_c	chr1:224434660-224
ENSG0000(	397	9.929339	chr1:3732	CDC42BPA		protein_c	chr1:226989865-227
ENSG0000(	397	9.929339	chr1:3732	ITPKB	NCGv7	protein_c	chr1:226631690-226
ENSG0000(	397	9.929339	chr1:3732	ENSG00000260505		lncRNA	chr1:220401122-220
ENSG0000(	397	9.929339	chr1:3732	ENSG00000223842		lncRNA	chr1:219222248-219
ENSG0000(	397	9.929339	chr1:3732	CNIH4		protein_c	chr1:224356858-224
ENSG0000(	397	9.929339	chr1:3732	LEFTY2		protein_c	chr1:225936598-225
ENSG0000(	397	9.929339	chr1:3732	H3-3A	NCGv7;AC	protein_c	chr1:226061851-226
ENSG0000(	397	9.929339	chr1:3732	ARF1		protein_c	chr1:228082708-228
ENSG0000(	397	9.929339	chr1:3732	FBXO28	NCGv7	protein_c	chr1:224114111-224
ENSG0000(	397	9.929339	chr1:3732	DEGS1		protein_c	chr1:224175756-224
ENSG0000(	397	9.929339	chr1:3732	SDE2		protein_c	chr1:225982702-225
ENSG0000(	397	9.929339	chr1:3732	ENSG00000213036		Pseudoger	chr1:214482813-214
ENSG0000(	397	9.929339	chr1:3732	NVL		protein_c	chr1:224227334-224
ENSG0000(	397	9.929339	chr1:3732	SRP9		protein_c	chr1:225777813-225
ENSG0000(	397	9.929339	chr1:3732	SNAP47		protein_c	chr1:227728200-227
ENSG0000(	397	9.929339	chr1:3732	ENSG00000226927		lncRNA	chr1:220359731-220
ENSG0000(	397	9.929339	chr1:3732	SNRPEP10		Pseudoger	chr1:223644110-223
ENSG0000(	397	9.929339	chr1:3732	RNU6-180P		smallRNA	chr1:229383128-229
ENSG0000(	397	9.929339	chr1:3732	IBA57-DT		lncRNA	chr1:228164086-228
ENSG0000(	397	9.929339	chr1:3732	HLX-AS1		lncRNA	chr1:220832763-220
ENSG0000(	397	9.929339	chr1:3732	ENSG00000227417		Pseudoger	chr1:203805621-203
ENSG0000(	397	9.929339	chr1:3732	RPLP0P5		Pseudoger	chr1:220316667-220
ENSG0000(	397	9.929339	chr1:3732	CNIH3-AS2		lncRNA	chr1:224608130-224
ENSG0000(	397	9.929339	chr1:3732	AC092765.1		smallRNA	chr1:222013007-222
ENSG0000(	397	9.929339	chr1:3732	NUCKS1P1		Pseudoger	chr1:227410617-227
ENSG0000(	397	9.929339	chr1:3732	RPL35AP5		Pseudoger	chr1:203835585-203
ENSG0000(	397	9.929339	chr1:3732	ACBD3		protein_c	chr1:226144679-226
ENSG0000(	397	9.929339	chr1:3732	RN7SKP49		smallRNA	chr1:224107282-224
ENSG0000(	397	9.929339	chr1:3732	ENSG00000289348		lncRNA	chr1:226349171-226
ENSG0000(	397	9.929339	chr1:3732	ENSG00000289341		lncRNA	chr1:225999615-226
ENSG0000(	397	9.929339	chr1:3732	STUM		protein_c	chr1:226548764-226
ENSG0000(	397	9.929339	chr1:3732	ISCA1P2		Pseudoger	chr1:229042171-229
ENSG0000(	397	9.929339	chr1:3732	DUSP5P1		Pseudoger	chr1:228650241-228
ENSG0000(	397	9.929339	chr1:3732	VDAC1P10		Pseudoger	chr1:215376484-215
ENSG0000(	397	9.929339	chr1:3732	ENSG00000242861		lncRNA	chr1:225840883-225
ENSG0000(	397	9.929339	chr1:3732	BTNL10	DriverDB\	Pseudoger	chr1:228510425-228
ENSG0000(	397	9.929339	chr1:3732	ZNF847P		Pseudoger	chr1:227696892-227
ENSG0000(	397	9.929339	chr1:3732	NDUFA3P3		Pseudoger	chr1:225964179-225

ENSG0000(	397	9.929339	chr1:3732	ZC3H11B		protein_c	chr1:219608012-219
ENSG0000(	397	9.929339	chr1:3732	KRT18P12		Pseudoger	chr1:214532195-214
ENSG0000(	397	9.929339	chr1:3732	ENSG00000242757		Pseudoger	chr1:227430526-227
ENSG0000(	397	9.929339	chr1:3732	RRP15		protein_c	chr1:218285293-218
ENSG0000(	397	9.929339	chr1:3732	AURKAP1		Pseudoger	chr1:220266706-220
ENSG0000(	397	9.929339	chr1:3732	LIN9		protein_c	chr1:226231149-226
ENSG0000(	397	9.929339	chr1:3732	ENSG00000229930		Pseudoger	chr1:224030704-224
ENSG0000(	397	9.929339	chr1:3732	SNORA72		smallRNA	chr1:224179641-224
ENSG0000(	397	9.929339	chr1:3732	ENSG00000286421		lncRNA	chr1:221555550-221
ENSG0000(	397	9.929339	chr1:3732	RNA5S7		smallRNA	chr1:228623667-228
ENSG0000(	397	9.929339	chr1:3732	ESRRG	NCGv7	protein_c	chr1:216503246-217
ENSG0000(	397	9.929339	chr1:3732	ENSG00000287532		lncRNA	chr1:227123895-227
ENSG0000(	397	9.929339	chr1:3732	LINC02814		lncRNA	chr1:229087114-229
ENSG0000(	397	9.929339	chr1:3732	YBX1P9		Pseudoger	chr1:226318015-226
ENSG0000(	397	9.929339	chr1:3732	ENSG00000287525		lncRNA	chr1:227280449-227
ENSG0000(	397	9.929339	chr1:3732	ENSG00000279306		TEC	chr1:228486188-228
ENSG0000(	397	9.929339	chr1:3732	RNU6ATAC35P		lncRNA	chr1:220825620-220
ENSG0000(	397	9.929339	chr1:3732	FAM133FP		Pseudoger	chr1:227598424-227
ENSG0000(	397	9.929339	chr1:3732	TAF1A-AS1		lncRNA	chr1:222589825-222
ENSG0000(	397	9.929339	chr1:3732	RIMKLBP2		Pseudoger	chr1:219199914-219
ENSG0000(	397	9.929339	chr1:3732	SLC30A10		protein_c	chr1:219685427-219
ENSG0000(	397	9.929339	chr1:3732	ENSG00000237101		lncRNA	chr1:224219613-224
ENSG0000(	397	9.929339	chr1:3732	ENSG00000237193		Pseudoger	chr1:227482253-227
ENSG0000(	397	9.929339	chr1:3732	ENSG00000286719		lncRNA	chr1:224802959-224
ENSG0000(	397	9.929339	chr1:3732	LINC02813		lncRNA	chr1:224766324-224
ENSG0000(	397	9.929339	chr1:3732	ENSG00000278467		lncRNA	chr1:223994262-223
ENSG0000(	397	9.929339	chr1:3732	UBE2V1P13		Pseudoger	chr1:214612960-214
ENSG0000(	397	9.929339	chr1:3732	ENSG00000278180		Pseudoger	chr1:227490691-227
ENSG0000(	397	9.929339	chr1:3732	RNA5S12		smallRNA	chr1:228634871-228
ENSG0000(	397	9.929339	chr1:3732	RNA5S11		smallRNA	chr1:228632631-228
ENSG0000(	397	9.929339	chr1:3732	RPL7AP81		Pseudoger	chr1:220448516-220
ENSG0000(	397	9.929339	chr1:3732	snoU13		smallRNA	chr1:226304262-226
ENSG0000(	397	9.929339	chr1:3732	U3		smallRNA	chr1:219962686-219
ENSG0000(	397	9.929339	chr1:3732	ENSG00000236636		Pseudoger	chr1:227264776-227
ENSG0000(	397	9.929339	chr1:3732	snoU13		smallRNA	chr1:219987511-219
ENSG0000(	397	9.929339	chr1:3732	RNU1-141P		smallRNA	chr1:218129795-218
ENSG0000(	397	9.929339	chr1:3732	TMEM63A	NCGv7	protein_c	chr1:225845536-225
ENSG0000(	397	9.929339	chr1:3732	ENSG00000236773		Pseudoger	chr1:224175476-224
ENSG0000(	397	9.929339	chr1:3732	H3-3A-DT		lncRNA	chr1:226045561-226
ENSG0000(	397	9.929339	chr1:3732	ENSG00000272750		lncRNA	chr1:222658867-222
ENSG0000(	397	9.929339	chr1:3732	ENSG00000272823		lncRNA	chr1:220828676-220
ENSG0000(	397	9.929339	chr1:3732	SNORA36B		smallRNA	chr1:220200546-220
ENSG0000(	397	9.929339	chr1:3732	LAX1		protein_c	chr1:203765177-203
ENSG0000(	397	9.929339	chr1:3732	ENSG00000280157		TEC	chr1:228121523-228
ENSG0000(	397	9.929339	chr1:3732	snoU13		smallRNA	chr1:220137164-220
ENSG0000(	397	9.929339	chr1:3732	SNORA77		smallRNA	chr1:203729581-203
ENSG0000(	397	9.929339	chr1:3732	MRPS18BP1		Pseudoger	chr1:216201635-216
ENSG0000(	397	9.929339	chr1:3732	RNA5S5		smallRNA	chr1:228619232-228
ENSG0000(	397	9.929339	chr1:3732	RNA5S3		smallRNA	chr1:228614750-228
ENSG0000(	397	9.929339	chr1:3732	GJC2	DriverDB\	protein_c	chr1:228149930-228
ENSG0000(	397	9.929339	chr1:3732	ENSG00000286773		lncRNA	chr1:228329467-228

ENSG0000(	397	9.929339	chr1:3732	TGFB2-OT1		lncRNA	chr1:218442626-218
ENSG0000(	397	9.929339	chr1:3732	ENSG00000238232		lncRNA	chr1:219557192-219
ENSG0000(	397	9.929339	chr1:3732	ENSG00000287259		lncRNA	chr1:226827711-226
ENSG0000(	397	9.929339	chr1:3732	MARK1		protein_c	chr1:220528136-220
ENSG0000(	397	9.929339	chr1:3732	RNA5S9		smallRNA	chr1:228628148-228
ENSG0000(	397	9.929339	chr1:3732	RPS27P5		Pseudoger	chr1:226781501-226
ENSG0000(	397	9.929339	chr1:3732	RAB3GAP2		protein_c	chr1:220148293-220
ENSG0000(	397	9.929339	chr1:3732	LINC00210		lncRNA	chr1:217892900-217
ENSG0000(	397	9.929339	chr1:3732	ENSG00000275406		Pseudoger	chr1:226331999-226
ENSG0000(	397	9.929339	chr1:3732	MTARC2		protein_c	chr1:220748225-220
ENSG0000(	397	9.929339	chr1:3732	FAM177B	NCGv7	protein_c	chr1:222737202-222
ENSG0000(	397	9.929339	chr1:3732	CENPF	NCGv7	protein_c	chr1:214603195-214
ENSG0000(	397	9.929339	chr1:3732	PROX1		protein_c	chr1:213983181-214
ENSG0000(	397	9.929339	chr1:3732	RNA5S14		smallRNA	chr1:228639337-228
ENSG0000(	397	9.929339	chr1:3732	LINC02257		lncRNA	chr1:221880981-221
ENSG0000(	397	9.929339	chr1:3732	RNU6-1008P		smallRNA	chr1:224305380-224
ENSG0000(	397	9.929339	chr1:3732	ENSG00000287338		lncRNA	chr1:223091872-223
ENSG0000(	397	9.929339	chr1:3732	RHOU		protein_c	chr1:228735479-228
ENSG0000(	397	9.929339	chr1:3732	LINC01352		lncRNA	chr1:220829255-220
ENSG0000(	397	9.929339	chr1:3732	LINC02817		lncRNA	chr1:221330080-221
ENSG0000(	397	9.929339	chr1:3732	ENSG00000274895		lncRNA	chr1:213983793-213
ENSG0000(	397	9.929339	chr1:3732	CIAO2AP2		Pseudoger	chr1:228114997-228
ENSG0000(	397	9.929339	chr1:3732	ENSG00000234863		Pseudoger	chr1:220455154-220
ENSG0000(	397	9.929339	chr1:3732	ENSG00000286775		lncRNA	chr1:218046943-218
ENSG0000(	397	9.929339	chr1:3732	H2BU1		protein_c	chr1:228458103-228
ENSG0000(	397	9.929339	chr1:3732	TUBB8P10		Pseudoger	chr1:227493029-227
ENSG0000(	397	9.929339	chr1:3732	LINC01703		lncRNA	chr1:226083590-226
ENSG0000(	397	9.929339	chr1:3732	TGFB2		protein_c	chr1:218345336-218
ENSG0000(	397	9.929339	chr1:3732	ENSG00000237481		lncRNA	chr1:229319403-229
ENSG0000(	397	9.929339	chr1:3732	MIR320B2		smallRNA	chr1:224257004-224
ENSG0000(	397	9.929339	chr1:3732	LINC01710		lncRNA	chr1:218912757-218
ENSG0000(	397	9.929339	chr1:3732	ENSG00000277007		lncRNA	chr1:219270774-219
ENSG0000(	397	9.929339	chr1:3732	GPATCH2		protein_c	chr1:217426992-217
ENSG0000(	397	9.929339	chr1:3732	ENSG00000276997		lncRNA	chr1:222477252-222
ENSG0000(	397	9.929339	chr1:3732	FTH1P2		Pseudoger	chr1:228687415-228
ENSG0000(	397	9.929339	chr1:3732	LINC02809		lncRNA	chr1:228073909-228
ENSG0000(	397	9.929339	chr1:3732	ENSG00000287008		lncRNA	chr1:214946909-214
ENSG0000(	397	9.929339	chr1:3732	ENSG00000287205		lncRNA	chr1:227518738-227
ENSG0000(	397	9.929339	chr1:3732	SEPTIN14P17		Pseudoger	chr1:227980051-227
ENSG0000(	397	9.929339	chr1:3732	SNORA51		smallRNA	chr1:228652436-228
ENSG0000(	397	9.929339	chr1:3732	NDUFB1P2		Pseudoger	chr1:222945725-222
ENSG0000(	397	9.929339	chr1:3732	RNA5S1		smallRNA	chr1:228610268-228
ENSG0000(	397	9.929339	chr1:3732	LINC00538		lncRNA	chr1:213924749-213
ENSG0000(	397	9.929339	chr1:3732	ENSG00000231563		lncRNA	chr1:228407196-228
ENSG0000(	397	9.929339	chr1:3732	NSA2P1		Pseudoger	chr1:203656969-203
ENSG0000(	397	9.929339	chr1:3732	snoU13		smallRNA	chr1:222911844-222
ENSG0000(	397	9.929339	chr1:3732	OPTC		protein_c	chr1:203494153-203
ENSG0000(	397	9.929339	chr1:3732	PRELP		protein_c	chr1:203475806-203
ENSG0000(	397	9.929339	chr1:3732	LARP7P1		Pseudoger	chr1:203400266-203
ENSG0000(	397	9.929339	chr1:3732	RPL23AP15		Pseudoger	chr1:228449163-228
ENSG0000(	397	9.929339	chr1:3732	CICP13		Pseudoger	chr1:222468094-222

ENSG0000(	397	9.929339	chr1:3732	LINC02779	lncRNA	chr1:220485104-220
ENSG0000(	397	9.929339	chr1:3732	ENSG00000236230	lncRNA	chr1:222088806-222
ENSG0000(	397	9.929339	chr1:3732	ENSG00000287684	lncRNA	chr1:222743356-222
ENSG0000(	397	9.929339	chr1:3732	ENSG00000287676	lncRNA	chr1:219294982-219
ENSG0000(	397	9.929339	chr1:3732	RNA5S6	smallRNA	chr1:228621447-228
ENSG0000(	397	9.929339	chr1:3732	RNU6-403P	smallRNA	chr1:221837334-221
ENSG0000(	397	9.929339	chr1:3732	RNA5S2	smallRNA	chr1:228612509-228
ENSG0000(	397	9.929339	chr1:3732	USH2A-AS1	lncRNA	chr1:216194051-216
ENSG0000(	397	9.929339	chr1:3732	LINC02765	lncRNA	chr1:225447233-225
ENSG0000(	397	9.929339	chr1:3732	ACBD3-AS1	lncRNA	chr1:226148003-226
ENSG0000(	397	9.929339	chr1:3732	snoU13	smallRNA	chr1:226316061-226
ENSG0000(	397	9.929339	chr1:3732	MIR215	smallRNA	chr1:220117853-220
ENSG0000(	397	9.929339	chr1:3732	ENSG00000272167	lncRNA	chr1:214028891-214
ENSG0000(	397	9.929339	chr1:3732	ENSG00000271475	Pseudoger	chr1:228776312-228
ENSG0000(	397	9.929339	chr1:3732	PRELID3BP1	Pseudoger	chr1:220467954-220
ENSG0000(	397	9.929339	chr1:3732	ENSG00000286398	lncRNA	chr1:221549362-221
ENSG0000(	397	9.929339	chr1:3732	ENSG00000286389	lncRNA	chr1:227786753-227
ENSG0000(	397	9.929339	chr1:3732	TUBB8P9	Pseudoger	chr1:227506182-227
ENSG0000(	397	9.929339	chr1:3732	KCNK2	protein_c	chr1:215005775-215
ENSG0000(	397	9.929339	chr1:3732	DNAJB6P6	Pseudoger	chr1:224661173-224
ENSG0000(	397	9.929339	chr1:3732	RNA5S4	smallRNA	chr1:228616991-228
ENSG0000(	397	9.929339	chr1:3732	ENSG00000226211	Pseudoger	chr1:221133865-221
ENSG0000(	397	9.929339	chr1:3732	RNA5S17	smallRNA	chr1:228646040-228
ENSG0000(	397	9.929339	chr1:3732	ENSG00000286231	protein_c	chr1:220786990-220
ENSG0000(	397	9.929339	chr1:3732	ENSG00000286174	lncRNA	chr1:224703272-224
ENSG0000(	397	9.929339	chr1:3732	ACTBP11	Pseudoger	chr1:223863726-223
ENSG0000(	397	9.929339	chr1:3732	ENSG00000287895	lncRNA	chr1:228119149-228
ENSG0000(	397	9.929339	chr1:3732	RNA5S8	smallRNA	chr1:228625909-228
ENSG0000(	397	9.929339	chr1:3732	BTF3P9	Pseudoger	chr1:227434064-227
ENSG0000(	397	9.929339	chr1:3732	ENSG00000271399	Pseudoger	chr1:228858010-228
ENSG0000(	397	9.929339	chr1:3732	LINC01641	lncRNA	chr1:227393554-227
ENSG0000(	397	9.929339	chr1:3732	LINC02775	lncRNA	chr1:214051194-214
ENSG0000(	397	9.929339	chr1:3732	ENSG00000287315	lncRNA	chr1:228357012-228
ENSG0000(	397	9.929339	chr1:3732	ENSG00000287627	lncRNA	chr1:226538305-226
ENSG0000(	397	9.929339	chr1:3732	LINC02815	lncRNA	chr1:229022773-229
ENSG0000(	397	9.929339	chr1:3732	ENSG00000282418	lncRNA	chr1:225465021-225
ENSG0000(	397	9.929339	chr1:3732	RNA5S10	smallRNA	chr1:228630390-228
ENSG0000(	397	9.929339	chr1:3732	ENSG00000282265	lncRNA	chr1:215393646-215
ENSG0000(	397	9.929339	chr1:3732	snoU13	smallRNA	chr1:224336791-224
ENSG0000(	397	9.929339	chr1:3732	RNU6-791P	smallRNA	chr1:222503632-222
ENSG0000(	396	9.904328	chr16:531	ENSG00000276131	lncRNA	chr16:58392153-583
ENSG0000(	395	9.879317	chr11:76(	SLC5A12	protein_c	chr11:26667020-267
ENSG0000(	395	9.879317	chr11:76(	ENSG00000255454	Pseudoger	chr11:25630720-256
ENSG0000(	395	9.879317	chr11:76(	RPL36AP40	Pseudoger	chr11:25588475-255
ENSG0000(	395	9.879317	chr11:76(	ENSG00000255489	Pseudoger	chr11:24701275-247
ENSG0000(	395	9.879317	chr11:76(	ENSG00000255505	Pseudoger	chr11:26427051-264
ENSG0000(	395	9.879317	chr11:76(	FIBIN	protein_c	chr11:26994112-269
ENSG0000(	395	9.879317	chr11:76(	BBOX1-AS1	lncRNA	chr11:27047186-272
ENSG0000(	395	9.879317	chr11:76(	ENSG00000287227	lncRNA	chr11:26244640-262
ENSG0000(	395	9.879317	chr11:76(	MUC15	protein_c	chr11:26559032-265
ENSG0000(	395	9.879317	chr11:76(	LINC02699	lncRNA	chr11:25734757-257

ENSG0000(	395	9.879317	chr11:76(	ANO3		protein_c	chr11:26188842-266
ENSG0000(	395	9.879317	chr11:76(	ENSG00000255086		Pseudoger	chr11:26045987-260
ENSG0000(	395	9.879317	chr11:76(	BBOX1		protein_c	chr11:27040725-271
ENSG0000(	395	9.879317	chr11:76(	AC015820.1		smallRNA	chr11:25537278-255
ENSG0000(	395	9.879317	chr11:76(	ENSG00000255368		Pseudoger	chr11:25140533-251
ENSG0000(	395	9.879317	chr11:76(	Y_RNA		smallRNA	chr11:24455911-244
ENSG0000(	395	9.879317	chr22:22(	D86994.1		smallRNA	chr22:22697906-226
ENSG0000(	395	9.879317	chr11:76(	LINC02686		lncRNA	chr11:24235477-242
ENSG0000(	395	9.879317	chr11:76(	LUZP2	NCGv7	protein_c	chr11:24496970-250
ENSG0000(	395	9.879317	chr11:76(	ENSG00000254754		lncRNA	chr11:25924188-259
ENSG0000(	395	9.879317	chr11:76(	ANO3-AS1		lncRNA	chr11:26285578-263
ENSG0000(	394	9.854306	chr2:4707	TIA1	NCGv7	protein_c	chr2:70209444-7024
ENSG0000(	394	9.854306	chr2:4707	ENSG00000235495		lncRNA	chr2:67565604-6768
ENSG0000(	394	9.854306	chr2:4707	RNU6-548P		smallRNA	chr2:64994746-6499
ENSG0000(	394	9.854306	chr2:4707	RPS4XP5		Pseudoger	chr2:63642455-6364
ENSG0000(	394	9.854306	chr2:4707	LINC01828		lncRNA	chr2:67086446-6731
ENSG0000(	394	9.854306	chr2:4707	PCYOX1		protein_c	chr2:70257386-7028
ENSG0000(	394	9.854306	chr2:4707	AFTPH-DT		lncRNA	chr2:64522187-6452
ENSG0000(	394	9.854306	chr2:4707	LINC01873		lncRNA	chr2:66383306-6639
ENSG0000(	394	9.854306	chr2:4707	RNU6-1216P		smallRNA	chr2:69182877-6918
ENSG0000(	394	9.854306	chr2:4707	C2orf42		protein_c	chr2:70149885-7024
ENSG0000(	394	9.854306	chr2:4707	AC017084.1		smallRNA	chr2:70455530-7045
ENSG0000(	394	9.854306	chr2:4707	GKN2		protein_c	chr2:68945232-6895
ENSG0000(	394	9.854306	chr2:4707	AAK1		protein_c	chr2:69457997-6967
ENSG0000(	394	9.854306	chr2:4707	ENSG00000290118		lncRNA	chr2:64275361-6427
ENSG0000(	394	9.854306	chr2:4707	PLEK		protein_c	chr2:68365282-6839
ENSG0000(	394	9.854306	chr2:4707	PNO1		protein_c	chr2:68157888-6817
ENSG0000(	394	9.854306	chr2:4707	ENSG00000287123		lncRNA	chr2:64836985-6484
ENSG0000(	394	9.854306	chr2:4707	AC007365.2		smallRNA	chr2:64776104-6477
ENSG0000(	394	9.854306	chr2:4707	RN7SL635P		smallRNA	chr2:65545403-6554
ENSG0000(	394	9.854306	chr2:4707	SLC1A4		protein_c	chr2:64988477-6502
ENSG0000(	394	9.854306	chr2:4707	ENSG00000227293		lncRNA	chr2:66235377-6623
ENSG0000(	394	9.854306	chr2:4707	LINC02245		lncRNA	chr2:64901840-6505
ENSG0000(	394	9.854306	chr2:4707	B3GALNT1P1		Pseudoger	chr2:69597353-6959
ENSG0000(	394	9.854306	chr2:4707	ENSG00000214525		Pseudoger	chr2:68528241-6852
ENSG0000(	394	9.854306	chr2:4707	ENSG00000228079		lncRNA	chr2:64086353-6408
ENSG0000(	394	9.854306	chr2:4707	ENSG00000203395		lncRNA	chr2:68361214-6836
ENSG0000(	394	9.854306	chr2:4707	LINC02934		lncRNA	chr2:65436711-6620
ENSG0000(	394	9.854306	chr2:4707	ENSG00000281920		lncRNA	chr2:65623272-6562
ENSG0000(	394	9.854306	chr2:4707	CEP68		protein_c	chr2:65056354-6508
ENSG0000(	394	9.854306	chr2:4707	Y_RNA		smallRNA	chr2:64834056-6483
ENSG0000(	394	9.854306	chr2:4707	UGP2		protein_c	chr2:63840952-6389
ENSG0000(	394	9.854306	chr2:4707	MDH1		protein_c	chr2:63588609-6360
ENSG0000(	394	9.854306	chr2:4707	APLF		protein_c	chr2:68467572-6865
ENSG0000(	394	9.854306	chr2:4707	PROKR1		protein_c	chr2:68643579-6865
ENSG0000(	394	9.854306	chr2:4707	GKN1		protein_c	chr2:68974573-6898
ENSG0000(	394	9.854306	chr2:4707	ANTXR1		protein_c	chr2:69013176-6924
ENSG0000(	394	9.854306	chr2:4707	AC017083.1		smallRNA	chr2:68229926-6823
ENSG0000(	394	9.854306	chr2:4707	ENSG00000280257		TEC	chr2:65790039-6579
ENSG0000(	394	9.854306	chr2:4707	MEIS1	TAG;AC	protein_c	chr2:66433452-6657
ENSG0000(	394	9.854306	chr2:4707	NFU1		protein_c	chr2:69396113-6943

ENSG0000(	394	9.854306	chr2:4707	PCBP1	NCGv7	protein_c	chr2:70087477-7008
ENSG0000(	394	9.854306	chr2:4707	ENSG00000238012		lncRNA	chr2:64330481-6433
ENSG0000(	394	9.854306	chr2:4707	ENSG00000286347		lncRNA	chr2:69030042-6903
ENSG0000(	394	9.854306	chr2:4707	FBXO48		protein_c	chr2:68459422-6846
ENSG0000(	394	9.854306	chr2:4707	DNAJB12P1		Pseudoger	chr2:65500993-6550
ENSG0000(	394	9.854306	chr2:4707	VPS54		protein_c	chr2:63892146-6401
ENSG0000(	394	9.854306	chr2:4707	CSP1		Pseudoger	chr2:63717122-6371
ENSG0000(	394	9.854306	chr2:4707	ETAA1		protein_c	chr2:67397322-6741
ENSG0000(	394	9.854306	chr2:4707	SNRPG		protein_c	chr2:70281362-7029
ENSG0000(	394	9.854306	chr2:4707	ENSG00000235725		lncRNA	chr2:65589566-6564
ENSG0000(	394	9.854306	chr2:4707	GKN3P		Pseudoger	chr2:68921248-6892
ENSG0000(	394	9.854306	chr2:4707	LINC01888		lncRNA	chr2:68832014-6883
ENSG0000(	394	9.854306	chr2:4707	LINC01890		lncRNA	chr2:68822855-6883
ENSG0000(	394	9.854306	chr2:4707	KRT18P33		Pseudoger	chr2:65666695-6566
ENSG0000(	394	9.854306	chr2:4707	HNRNPA1P66		Pseudoger	chr2:63751697-6375
ENSG0000(	394	9.854306	chr2:4707	MRPL36P1		Pseudoger	chr2:70102551-7010
ENSG0000(	394	9.854306	chr2:4707	VDAC2P5		Pseudoger	chr2:65205108-6520
ENSG0000(	394	9.854306	chr2:4707	ENSG00000236605		lncRNA	chr2:67324627-6732
ENSG0000(	394	9.854306	chr2:4707	AC074391.2		smallRNA	chr2:66041250-6604
ENSG0000(	394	9.854306	chr2:4707	RN7SL604P		smallRNA	chr2:69516751-6951
ENSG0000(	394	9.854306	chr2:4707	ENSG00000233060		lncRNA	chr2:70089721-7009
ENSG0000(	394	9.854306	chr2:4707	PCBP1-AS1		lncRNA	chr2:69960104-7010
ENSG0000(	394	9.854306	chr2:4707	ENSG00000237217		Pseudoger	chr2:64450096-6445
ENSG0000(	394	9.854306	chr2:4707	ENSG00000284932		Pseudoger	chr2:68117026-6811
ENSG0000(	394	9.854306	chr2:4707	RN7SL211P		smallRNA	chr2:64906865-6490
ENSG0000(	394	9.854306	chr2:4707	LINC01628		lncRNA	chr2:66921510-6692
ENSG0000(	394	9.854306	chr2:4707	ASPRV1		protein_c	chr2:69960089-6996
ENSG0000(	394	9.854306	chr2:4707	LINC01797		lncRNA	chr2:66696190-6670
ENSG0000(	394	9.854306	chr2:4707	TGFA-IT1		lncRNA	chr2:70467385-7046
ENSG0000(	394	9.854306	chr2:4707	LINC01816		lncRNA	chr2:70124034-7013
ENSG0000(	394	9.854306	chr2:4707	PRELID1P6		Pseudoger	chr2:63622178-6362
ENSG0000(	394	9.854306	chr2:4707	ENSG00000232693		lncRNA	chr2:65373700-6538
ENSG0000(	394	9.854306	chr2:4707	ENSG00000289943		lncRNA	chr2:63839866-6384
ENSG0000(	394	9.854306	chr2:4707	SERTAD2	TAG;AC	protein_c	chr2:64631621-6475
ENSG0000(	394	9.854306	chr2:4707	TGFA	NCGv7	protein_c	chr2:70447284-7055
ENSG0000(	394	9.854306	chr2:4707	RNU6-100P		smallRNA	chr2:64578892-6457
ENSG0000(	394	9.854306	chr2:4707	Y_RNA		smallRNA	chr2:69334600-6933
ENSG0000(	394	9.854306	chr2:4707	ARHGAP25		protein_c	chr2:68679601-6882
ENSG0000(	394	9.854306	chr2:4707	RPL23AP37		Pseudoger	chr2:64347193-6434
ENSG0000(	394	9.854306	chr2:4707	PPIAP64		Pseudoger	chr2:68125265-6812
ENSG0000(	394	9.854306	chr2:4707	ENSG00000229229		lncRNA	chr2:70402934-7042
ENSG0000(	394	9.854306	chr2:4707	SPRED2		protein_c	chr2:65310851-6543
ENSG0000(	394	9.854306	chr2:4707	GFPT1		protein_c	chr2:69319780-6938
ENSG0000(	394	9.854306	chr2:4707	RPL11P1		Pseudoger	chr2:64960053-6496
ENSG0000(	394	9.854306	chr2:4707	RPS15AP15		Pseudoger	chr2:65511771-6551
ENSG0000(	394	9.854306	chr2:4707	ENSG00000289533		lncRNA	chr2:67331881-6734
ENSG0000(	394	9.854306	chr2:4707	MXD1		protein_c	chr2:69897688-6994
ENSG0000(	394	9.854306	chr2:4707	BMP10		protein_c	chr2:68860909-6887
ENSG0000(	394	9.854306	chr2:4707	ENSG00000286002		lncRNA	chr2:67677499-6768
ENSG0000(	394	9.854306	chr2:4707	FBXL12P1		Pseudoger	chr2:68023694-6802
ENSG0000(	394	9.854306	chr2:4707	RPL39P15		Pseudoger	chr2:70253855-7025

ENSG0000(	394	9.854306	chr2:4707	LINC01812	lncRNA	chr2:67796054-6782
ENSG0000(	394	9.854306	chr2:4707	WDR4P2	Pseudoger	chr2:68445710-6844
ENSG0000(	394	9.854306	chr2:4707	FAM136A	protein_c	chr2:70295975-7030
ENSG0000(	394	9.854306	chr2:4707	RPS10P9	Pseudoger	chr2:64665607-6466
ENSG0000(	394	9.854306	chr2:4707	DNAAF10	protein_c	chr2:68122936-6815
ENSG0000(	394	9.854306	chr2:4707	AC017083.3	smallRNA	chr2:68273104-6827
ENSG0000(	394	9.854306	chr2:4707	RPL36AP16	Pseudoger	chr2:69594741-6959
ENSG0000(	394	9.854306	chr2:4707	LINC01829	lncRNA	chr2:67123357-6739
ENSG0000(	394	9.854306	chr2:4707	BRD7P6	Pseudoger	chr2:70353010-7035
ENSG0000(	394	9.854306	chr2:4707	Vault	smallRNA	chr2:65555432-6555
ENSG0000(	394	9.854306	chr2:4707	ENSG00000279485	TEC	chr2:63517892-6351
ENSG0000(	394	9.854306	chr2:4707	PPP3R1	protein_c	chr2:68178857-6825
ENSG0000(	394	9.854306	chr2:4707	AC007386.4	lncRNA	chr2:65030727-6503
ENSG0000(	394	9.854306	chr2:4707	MEIS1-AS2	lncRNA	chr2:66439088-6644
ENSG0000(	394	9.854306	chr2:4707	PELI1	protein_c	chr2:64092652-6414
ENSG0000(	394	9.854306	chr2:4707	AFTPH	protein_c	chr2:64524299-6459
ENSG0000(	394	9.854306	chr2:4707	LGALSL	protein_c	chr2:64453969-6446
ENSG0000(	394	9.854306	chr2:4707	MIR3126	smallRNA	chr2:69103682-6910
ENSG0000(	394	9.854306	chr2:4707	ENSG00000233849	lncRNA	chr2:70301451-7030
ENSG0000(	394	9.854306	chr2:4707	LGALSL-DT	lncRNA	chr2:64395220-6445
ENSG0000(	394	9.854306	chr2:4707	CNRIP1	protein_c	chr2:68284171-6832
ENSG0000(	394	9.854306	chr2:4707	RPL27P6	Pseudoger	chr2:63684305-6368
ENSG0000(	394	9.854306	chr2:4707	SNRNP27	protein_c	chr2:69893956-6990
ENSG0000(	394	9.854306	chr2:4707	SNORA74	smallRNA	chr2:65158662-6515
ENSG0000(	394	9.854306	chr2:4707	snoU13	smallRNA	chr2:69955645-6995
ENSG0000(	394	9.854306	chr2:4707	ENSG00000225815	lncRNA	chr2:66327349-6632
ENSG0000(	394	9.854306	chr2:4707	RN7SL341P	smallRNA	chr2:64817378-6481
ENSG0000(	394	9.854306	chr2:4707	DNMT3AP1	Pseudoger	chr2:66820684-6682
ENSG0000(	394	9.854306	chr2:4707	ENSG00000271597	Pseudoger	chr2:69251818-6925
ENSG0000(	394	9.854306	chr2:4707	ENSG00000231024	lncRNA	chr2:69700192-6971
ENSG0000(	394	9.854306	chr2:4707	ENSG00000273763	Pseudoger	chr2:65203502-6522
ENSG0000(	394	9.854306	chr2:4707	AC118345.2	smallRNA	chr2:66239500-6623
ENSG0000(	394	9.854306	chr2:4707	ENSG00000226756	lncRNA	chr2:64644612-6464
ENSG0000(	394	9.854306	chr2:4707	LINC00309	lncRNA	chr2:64185078-6420
ENSG0000(	394	9.854306	chr2:4707	LINC01798	lncRNA	chr2:66574030-6673
ENSG0000(	394	9.854306	chr2:4707	LINC01799	lncRNA	chr2:66904436-6697
ENSG0000(	394	9.854306	chr2:4707	SNORA36C	smallRNA	chr2:69520043-6952
ENSG0000(	394	9.854306	chr2:4707	ENSG00000288986	lncRNA	chr2:64765463-6476
ENSG0000(	394	9.854306	chr2:4707	ENSG00000288932	lncRNA	chr2:64273709-6429
ENSG0000(	394	9.854306	chr2:4707	ANXA4	protein_c	chr2:69644425-6982
ENSG0000(	394	9.854306	chr2:4707	ENSG00000288869	lncRNA	chr2:69789544-6979
ENSG0000(	394	9.854306	chr2:4707	RAB1A	protein_c	chr2:65070696-6513
ENSG0000(	394	9.854306	chr2:4707	ACTR2	protein_c	chr2:65227788-6527
ENSG0000(	394	9.854306	chr2:4707	snoU13	smallRNA	chr2:69667359-6966
ENSG0000(	394	9.854306	chr2:4707	ENSG00000289156	lncRNA	chr2:68251603-6834
ENSG0000(	394	9.854306	chr2:4707	LINC02579	lncRNA	chr2:64606975-6461
ENSG0000(	394	9.854306	chr2:4707	LINC01800	lncRNA	chr2:64846130-6486
ENSG0000(	394	9.854306	chr2:4707	AC007389.4	smallRNA	chr2:65667256-6566
ENSG0000(	394	9.854306	chr2:4707	ENSG00000230355	Pseudoger	chr2:66881087-6688
ENSG0000(	394	9.854306	chr2:4707	ENSG00000289176	lncRNA	chr2:64337103-6434
ENSG0000(	394	9.854306	chr2:4707	MIR4778	smallRNA	chr2:66358249-6635

ENSG0000(	394	9.854306	chr2:4707	ENSG00000270354		Pseudoger	chr2:64208498-6420
ENSG0000(	394	9.854306	chr2:4707	GMCL1		protein_c	chr2:69829660-6988
ENSG0000(	394	9.854306	chr2:4707	MEIS1-AS3		lncRNA	chr2:66426735-6643
ENSG0000(	394	9.854306	chr2:4707	LINC02831		lncRNA	chr2:67562067-6762
ENSG0000(	394	9.854306	chr2:4707	ENSG00000234255		lncRNA	chr2:65439838-6545
ENSG0000(	394	9.854306	chr2:4707	C1D		protein_c	chr2:68041130-6811
ENSG0000(	394	9.854306	chr2:4707	ENSG00000289250		lncRNA	chr2:69663299-6966
ENSG0000(	394	9.854306	chr2:4707	ENSG00000225889		lncRNA	chr2:64143239-6425
ENSG0000(	394	9.854306	chr2:4707	RN7SL470P		smallRNA	chr2:70075018-7007
ENSG0000(	394	9.854306	chr2:4707	ENSG00000223859		lncRNA	chr2:67040546-6704
ENSG0000(	394	9.854306	chr2:4707	ENSG00000238201		lncRNA	chr2:64338067-6434
ENSG0000(	394	9.854306	chr2:4707	AC096664.3		smallRNA	chr2:63695393-6369
ENSG0000(	394	9.854306	chr2:4707	MIR1285-2		smallRNA	chr2:70252918-7025
ENSG0000(	394	9.854306	chr2:4707	ENSG00000273275		lncRNA	chr2:68179833-6818
ENSG0000(	394	9.854306	chr2:4707	LINC01805		lncRNA	chr2:64486353-6450
ENSG0000(	394	9.854306	chr2:4707	ENSG00000273064		lncRNA	chr2:68252870-6825
ENSG0000(	394	9.854306	chr2:4707	ENSG00000273398		protein_c	chr2:68131238-6826
ENSG0000(	394	9.854306	chr2:4707	ENSG00000275381		Pseudoger	chr2:69844509-6984
ENSG0000(	394	9.854306	chr2:4707	MIR4433		smallRNA	chr2:64340759-6434
ENSG0000(	394	9.854306	chr2:4707	snoU13		smallRNA	chr2:69912695-6991
ENSG0000(	394	9.854306	chr2:4707	RPL23AP92		Pseudoger	chr2:69873565-6987
ENSG0000(	391	9.779273	chr11:76(	AC084859.1		smallRNA	chr11:12963640-129
ENSG0000(	387	9.67923	chr11:76(	CALCA		protein_c	chr11:14966622-149
ENSG0000(	387	9.67923	chr11:76(	ENSG00000246225		lncRNA	chr11:22829380-229
ENSG0000(	387	9.67923	chr11:76(	C11orf58		protein_c	chr11:16613132-167
ENSG0000(	387	9.67923	chr11:76(	GALNT18		protein_c	chr11:11270877-116
ENSG0000(	387	9.67923	chr11:76(	ENSG00000255067		lncRNA	chr11:12848795-128
ENSG0000(	387	9.67923	chr11:76(	NUCB2		protein_c	chr11:17208153-173
ENSG0000(	387	9.67923	chr11:76(	SOX6		protein_c	chr11:15966449-167
ENSG0000(	387	9.67923	chr11:76(	HTATIP2		protein_c	chr11:20363685-203
ENSG0000(	387	9.67923	chr11:76(	ENSG00000254680		lncRNA	chr11:12261426-122
ENSG0000(	387	9.67923	chr11:76(	MIR3159		smallRNA	chr11:18387787-183
ENSG0000(	387	9.67923	chr11:76(	ENSG00000289976		lncRNA	chr11:11152351-111
ENSG0000(	387	9.67923	chr11:76(	ENSG00000254645		lncRNA	chr11:15701265-157
ENSG0000(	387	9.67923	chr11:76(	DBX1		protein_c	chr11:20156155-201
ENSG0000(	387	9.67923	chr11:76(	CSNK2A3		protein_c	chr11:11351942-113
ENSG0000(	387	9.67923	chr11:76(	ENSG00000254586		lncRNA	chr11:17695010-176
ENSG0000(	387	9.67923	chr11:76(	ENSG00000255088		Pseudoger	chr11:13758111-137
ENSG0000(	387	9.67923	chr11:76(	ZDHHC13		protein_c	chr11:19117099-191
ENSG0000(	387	9.67923	chr11:76(	AC090099.1		smallRNA	chr11:18183076-181
ENSG0000(	387	9.67923	chr11:76(	ENSG00000280288		TEC	chr11:11725332-117
ENSG0000(	387	9.67923	chr11:76(	ENSG00000240808		Pseudoger	chr11:17137976-171
ENSG0000(	387	9.67923	chr11:76(	CENPUP1		Pseudoger	chr11:13756027-137
ENSG0000(	387	9.67923	chr11:76(	SAA2-SAA4		protein_c	chr11:18231423-182
ENSG0000(	387	9.67923	chr11:76(	ENSG00000255074		Pseudoger	chr11:14440787-144
ENSG0000(	387	9.67923	chr11:76(	NAV2-AS4		lncRNA	chr11:19510890-195
ENSG0000(	387	9.67923	chr11:76(	ENSG00000240881		Pseudoger	chr11:23520322-235
ENSG0000(	387	9.67923	chr11:76(	RPS13	NCGv7	protein_c	chr11:17074388-170
ENSG0000(	387	9.67923	chr11:76(	LINC02682		lncRNA	chr11:15910528-159
ENSG0000(	387	9.67923	chr11:76(	RASSF10-DT		lncRNA	chr11:13001090-130
ENSG0000(	387	9.67923	chr11:76(	AC100767.1		smallRNA	chr11:23471644-234

ENSG0000(	387	9.67923	chr11:76(	ENSG00000254688	lncRNA	chr11:12822435-128
ENSG0000(	387	9.67923	chr11:76(	HPS5	protein_c	chr11:18278668-183
ENSG0000(	387	9.67923	chr11:76(	ENSG00000254861	lncRNA	chr11:23761330-238
ENSG0000(	387	9.67923	chr11:76(	MORF4L1P3	Pseudoger	chr11:14673606-146
ENSG0000(	387	9.67923	chr11:76(	SDHCP4	Pseudoger	chr11:17435672-174
ENSG0000(	387	9.67923	chr11:76(	CYP2R1	protein_c	chr11:14877440-148
ENSG0000(	387	9.67923	chr11:76(	ENSG00000255018	Pseudoger	chr11:13152200-131
ENSG0000(	387	9.67923	chr11:76(	NAV2-AS1	lncRNA	chr11:20119684-201
ENSG0000(	387	9.67923	chr11:76(	PTPN5	protein_c	chr11:18727928-187
ENSG0000(	387	9.67923	chr11:76(	IGSF22	protein_c	chr11:18704312-187
ENSG0000(	387	9.67923	chr11:76(	ENSG00000254906	lncRNA	chr11:20670425-206
ENSG0000(	387	9.67923	chr11:76(	ENSG00000254927	lncRNA	chr11:13826843-138
ENSG0000(	387	9.67923	chr11:76(	SPTY2D1	protein_c	chr11:18606403-186
ENSG0000(	387	9.67923	chr11:76(	ENSG00000279837	TEC	chr11:18601882-186
ENSG0000(	387	9.67923	chr11:76(	LINC02545	lncRNA	chr11:13844862-138
ENSG0000(	387	9.67923	chr11:76(	LINC02751	lncRNA	chr11:15552855-156
ENSG0000(	387	9.67923	chr11:76(	ENSG00000254957	Pseudoger	chr11:11135482-111
ENSG0000(	387	9.67923	chr11:76(	IGSF22-AS1	lncRNA	chr11:18706537-187
ENSG0000(	387	9.67923	chr11:76(	ENSG00000254983	lncRNA	chr11:12303533-123
ENSG0000(	387	9.67923	chr11:76(	SPTY2D1OS	protein_c	chr11:18588781-186
ENSG0000(	387	9.67923	chr11:76(	ENSG00000254878	lncRNA	chr11:16023190-160
ENSG0000(	387	9.67923	chr11:76(	NAV2-AS5	lncRNA	chr11:19502672-195
ENSG0000(	387	9.67923	chr11:76(	AC025300.1	smallRNA	chr11:12344767-123
ENSG0000(	387	9.67923	chr11:76(	MRGPRX12P	Pseudoger	chr11:18153277-181
ENSG0000(	387	9.67923	chr11:76(	ENSG00000254991	lncRNA	chr11:12066929-120
ENSG0000(	387	9.67923	chr11:76(	ENSG00000254695	lncRNA	chr11:15605484-157
ENSG0000(	387	9.67923	chr11:76(	MRGPRX3	protein_c	chr11:18120955-181
ENSG0000(	387	9.67923	chr11:76(	ENSG00000289944	lncRNA	chr11:21734212-217
ENSG0000(	387	9.67923	chr11:76(	MRGPRX10P	Pseudoger	chr11:18971746-189
ENSG0000(	387	9.67923	chr11:76(	MRGPRX11P	Pseudoger	chr11:18956055-189
ENSG0000(	387	9.67923	chr11:76(	AC116533.2	smallRNA	chr11:17054912-170
ENSG0000(	387	9.67923	chr11:76(	SLC17A6-DT	lncRNA	chr11:22283730-223
ENSG0000(	387	9.67923	chr11:76(	ENSG00000279266	TEC	chr11:20131730-201
ENSG0000(	387	9.67923	chr11:76(	GTF2H1	protein_c	chr11:18322295-183
ENSG0000(	387	9.67923	chr11:76(	ENSG00000254789	lncRNA	chr11:15571813-156
ENSG0000(	387	9.67923	chr11:76(	FAR1-IT1	lncRNA	chr11:13669327-136
ENSG0000(	387	9.67923	chr11:76(	ENSG00000254816	Pseudoger	chr11:21169748-211
ENSG0000(	387	9.67923	chr11:76(	MRGPRX4	protein_c	chr11:18172837-181
ENSG0000(	387	9.67923	chr11:76(	ENSG00000289116	lncRNA	chr11:15890568-158
ENSG0000(	387	9.67923	chr11:76(	ENSG00000254820	lncRNA	chr11:22113448-221
ENSG0000(	387	9.67923	chr11:76(	ENSG00000254847	lncRNA	chr11:12538083-125
ENSG0000(	387	9.67923	chr11:76(	MIR4486	smallRNA	chr11:19575310-195
ENSG0000(	387	9.67923	chr11:76(	SPON1-AS1	lncRNA	chr11:14262846-142
ENSG0000(	387	9.67923	chr11:76(	ENSG00000254583	Pseudoger	chr11:13478333-134
ENSG0000(	387	9.67923	chr11:76(	ENSG00000287962	lncRNA	chr11:22010402-221
ENSG0000(	387	9.67923	chr11:76(	INSC	protein_c	chr11:15112424-152
ENSG0000(	387	9.67923	chr11:76(	RNA5SP333	Pseudoger	chr11:18248389-182
ENSG0000(	387	9.67923	chr11:76(	NAV2-AS6	lncRNA	chr11:19710934-197
ENSG0000(	387	9.67923	chr11:76(	ENSG00000290957	lncRNA	chr11:17013998-170
ENSG0000(	387	9.67923	chr11:76(	ENSG00000271491	Pseudoger	chr11:23370116-233
ENSG0000(	387	9.67923	chr11:76(	RNU6-585P	smallRNA	chr11:16974421-169

ENSG0000(	387	9.67923	chr11:76(	RNA5SP338		Pseudoger	chr11:22688351-226
ENSG0000(	387	9.67923	chr11:76(	RNU7-49P		smallRNA	chr11:14478892-144
ENSG0000(	387	9.67923	chr11:76(	NAV2		protein_c	chr11:19350724-201
ENSG0000(	387	9.67923	chr11:76(	ENSG00000285562		lncRNA	chr11:18142341-181
ENSG0000(	387	9.67923	chr11:76(	RNA5SP334		Pseudoger	chr11:18266393-182
ENSG0000(	387	9.67923	chr11:76(	ENSG00000285545		lncRNA	chr11:17349053-173
ENSG0000(	387	9.67923	chr11:76(	LDHAL6A	NCGv7	protein_c	chr11:18455824-184
ENSG0000(	387	9.67923	chr11:76(	LDHC		protein_c	chr11:18412318-184
ENSG0000(	387	9.67923	chr11:76(	SAAL1		protein_c	chr11:18069935-181
ENSG0000(	387	9.67923	chr11:76(	SAA3P		Pseudoger	chr11:18112626-181
ENSG0000(	387	9.67923	chr21:32{	SNORA70		smallRNA	chr21:32841861-328
ENSG0000(	387	9.67923	chr11:76(	PLEKHA7		protein_c	chr11:16777297-170
ENSG0000(	387	9.67923	chr11:76(	SAA1		protein_c	chr11:18266260-182
ENSG0000(	387	9.67923	chr11:76(	ENSG00000270897		Pseudoger	chr11:11182107-111
ENSG0000(	387	9.67923	chr11:76(	E2F8		protein_c	chr11:19224063-192
ENSG0000(	387	9.67923	chr11:76(	ENSG00000254564		Pseudoger	chr11:23431677-234
ENSG0000(	387	9.67923	chr11:76(	HMGN2P36		Pseudoger	chr11:13610363-136
ENSG0000(	387	9.67923	chr11:76(	OR7E41P		Pseudoger	chr11:14987671-149
ENSG0000(	387	9.67923	chr11:76(	MRGPRX1		protein_c	chr11:18933499-189
ENSG0000(	387	9.67923	chr11:76(	USP47		protein_c	chr11:11841423-119
ENSG0000(	387	9.67923	chr11:76(	ENSG00000287898		lncRNA	chr11:17476595-174
ENSG0000(	387	9.67923	chr11:76(	ABCC8		protein_c	chr11:17392498-174
ENSG0000(	387	9.67923	chr11:76(	ENSG00000286382		lncRNA	chr11:20596522-205
ENSG0000(	387	9.67923	chr11:76(	COPB1		protein_c	chr11:14443440-145
ENSG0000(	387	9.67923	chr11:76(	PSMA1		protein_c	chr11:14504874-146
ENSG0000(	387	9.67923	chr11:76(	MYOD1	NCGv7	protein_c	chr11:17719571-177
ENSG0000(	387	9.67923	chr11:76(	NCR3LG1		protein_c	chr11:17351800-173
ENSG0000(	387	9.67923	chr11:76(	SERGEF		protein_c	chr11:17788048-180
ENSG0000(	387	9.67923	chr11:76(	ANO5		protein_c	chr11:21782659-222
ENSG0000(	387	9.67923	chr11:76(	KCNC1		protein_c	chr11:17734774-178
ENSG0000(	387	9.67923	chr11:76(	TPH1		protein_c	chr11:18017555-180
ENSG0000(	387	9.67923	chr11:76(	ENSG00000286521		lncRNA	chr11:12619326-126
ENSG0000(	387	9.67923	chr11:76(	USH1C		protein_c	chr11:17493895-175
ENSG0000(	387	9.67923	chr11:76(	OTOG		protein_c	chr11:17547259-176
ENSG0000(	387	9.67923	chr11:76(	CSRP3		protein_c	chr11:19182030-192
ENSG0000(	387	9.67923	chr11:76(	LINC00958		lncRNA	chr11:12961541-129
ENSG0000(	387	9.67923	chr11:76(	SNORD14A		smallRNA	chr11:17074654-170
ENSG0000(	387	9.67923	chr11:76(	TSG101		protein_c	chr11:18468336-185
ENSG0000(	387	9.67923	chr11:76(	KCNJ11		protein_c	chr11:17365172-173
ENSG0000(	387	9.67923	chr11:76(	RNU6-593P		smallRNA	chr11:17115652-171
ENSG0000(	387	9.67923	chr11:76(	RASSF10		protein_c	chr11:13009316-130
ENSG0000(	387	9.67923	chr11:76(	ENSG00000213779		Pseudoger	chr11:17193489-171
ENSG0000(	387	9.67923	chr11:76(	AC103974.1		smallRNA	chr11:18804408-188
ENSG0000(	387	9.67923	chr11:76(	AKR1B1P3		Pseudoger	chr11:16484084-164
ENSG0000(	387	9.67923	chr11:76(	snoMBII-202		smallRNA	chr11:15481808-154
ENSG0000(	387	9.67923	chr11:76(	CALCP		Pseudoger	chr11:14907518-149
ENSG0000(	387	9.67923	chr11:76(	ENSG00000287548		lncRNA	chr11:13463377-134
ENSG0000(	387	9.67923	chr11:76(	LINC02752		lncRNA	chr11:11020883-111
ENSG0000(	387	9.67923	chr11:76(	LINC02683		lncRNA	chr11:13921450-139
ENSG0000(	387	9.67923	chr11:76(	CALCB	NCGv7	protein_c	chr11:14904997-150
ENSG0000(	387	9.67923	chr11:76(	NAV2-AS2		lncRNA	chr11:20043767-200

ENSG0000(	387	9.67923	chr11:76(	THAP12P4		Pseudoger	chr11:23478068-234
ENSG0000(	387	9.67923	chr11:76(	LINC02547		lncRNA	chr11:12030875-120
ENSG0000(	387	9.67923	chr11:76(	MRGPRX7P		Pseudoger	chr11:18863558-188
ENSG0000(	387	9.67923	chr11:76(	ENSG00000254540		lncRNA	chr11:22361213-223
ENSG0000(	387	9.67923	chr11:76(	MRGPRX6P		Pseudoger	chr11:18886933-188
ENSG0000(	387	9.67923	chr11:76(	NAV2-AS3		lncRNA	chr11:19978699-199
ENSG0000(	387	9.67923	chr11:76(	MRGPRX13P		Pseudoger	chr11:18196657-181
ENSG0000(	387	9.67923	chr11:76(	ENSG00000290083		lncRNA	chr11:13276550-132
ENSG0000(	387	9.67923	chr11:76(	RNA5SP337		Pseudoger	chr11:21383801-213
ENSG0000(	387	9.67923	chr11:76(	AC116533.3		smallRNA	chr11:17007878-170
ENSG0000(	387	9.67923	chr11:76(	RPL39P26		Pseudoger	chr11:13685499-136
ENSG0000(	387	9.67923	chr11:76(	RNA5SP335		Pseudoger	chr11:19401872-194
ENSG0000(	387	9.67923	chr11:76(	SCARNA16		smallRNA	chr11:12904817-129
ENSG0000(	387	9.67923	chr11:76(	ARNTL	NCGv7	protein_c	chr11:13276652-133
ENSG0000(	387	9.67923	chr11:76(	TEAD1	TAG	protein_c	chr11:12674421-129
ENSG0000(	387	9.67923	chr11:76(	RN7SKP90		smallRNA	chr11:16842253-168
ENSG0000(	387	9.67923	chr11:76(	MICAL2		protein_c	chr11:12094008-123
ENSG0000(	387	9.67923	chr11:76(	Y_RNA		smallRNA	chr11:13686550-136
ENSG0000(	387	9.67923	chr11:76(	RRAS2	NCGv7;AC	protein_c	chr11:14277922-143
ENSG0000(	387	9.67923	chr11:76(	SLC25A51P4		Pseudoger	chr11:18209693-182
ENSG0000(	387	9.67923	chr11:76(	LDHA		protein_c	chr11:18394560-184
ENSG0000(	387	9.67923	chr11:76(	ST13P5		Pseudoger	chr11:18261982-182
ENSG0000(	387	9.67923	chr11:76(	SAA2		protein_c	chr11:18239223-182
ENSG0000(	387	9.67923	chr11:76(	MIR4299		smallRNA	chr11:11656651-116
ENSG0000(	387	9.67923	chr11:76(	NELL1		protein_c	chr11:20669551-215
ENSG0000(	387	9.67923	chr11:76(	SLC6A5		protein_c	chr11:20599594-206
ENSG0000(	387	9.67923	chr11:76(	RNU6-783P		smallRNA	chr11:23849778-238
ENSG0000(	387	9.67923	chr11:76(	PIK3C2A	NCGv7	protein_c	chr11:17077730-172
ENSG0000(	387	9.67923	chr11:76(	SLC25A51P4		lncRNA	chr11:18209138-182
ENSG0000(	387	9.67923	chr11:76(	SAA3P		lncRNA	chr11:18112472-181
ENSG0000(	387	9.67923	chr11:76(	RNA5SP332		Pseudoger	chr11:14134986-141
ENSG0000(	387	9.67923	chr11:76(	MIR4694		smallRNA	chr11:19760004-197
ENSG0000(	387	9.67923	chr11:76(	ENSG00000286960		lncRNA	chr11:15864452-158
ENSG0000(	387	9.67923	chr11:76(	MRGPRX5P		Pseudoger	chr11:18910436-189
ENSG0000(	387	9.67923	chr11:76(	ENSG00000256206		protein_c	chr11:14493783-145
ENSG0000(	387	9.67923	chr11:76(	UEVLD		protein_c	chr11:18529609-185
ENSG0000(	387	9.67923	chr11:76(	TMEM86A		protein_c	chr11:18693122-187
ENSG0000(	387	9.67923	chr11:76(	ENSG00000256006		lncRNA	chr11:18405609-184
ENSG0000(	387	9.67923	chr11:76(	ENSG00000225477		Pseudoger	chr11:21283779-212
ENSG0000(	387	9.67923	chr11:76(	RNA5SP336		Pseudoger	chr11:21000881-210
ENSG0000(	387	9.67923	chr11:76(	PTH		protein_c	chr11:13492054-134
ENSG0000(	387	9.67923	chr11:76(	PDE3B		protein_c	chr11:14643804-148
ENSG0000(	387	9.67923	chr11:76(	ENSG00000255558		lncRNA	chr11:13054615-131
ENSG0000(	387	9.67923	chr11:76(	ENSG00000255511		Pseudoger	chr11:18870989-188
ENSG0000(	387	9.67923	chr11:76(	SNORA1		smallRNA	chr11:19591156-195
ENSG0000(	387	9.67923	chr11:76(	H3P33		Pseudoger	chr11:11856490-118
ENSG0000(	387	9.67923	chr11:76(	PARVA		protein_c	chr11:12377563-125
ENSG0000(	387	9.67923	chr11:76(	ENSG00000255470		lncRNA	chr11:18140186-181
ENSG0000(	387	9.67923	chr11:76(	ENSG00000255462		lncRNA	chr11:11570084-115
ENSG0000(	387	9.67923	chr11:76(	ENSG00000255448		lncRNA	chr11:18000542-180
ENSG0000(	387	9.67923	chr11:76(	MRGPRX9P		Pseudoger	chr11:19029697-190

ENSG0000(	387	9.67923	chr11:76(	LINC02718		lncRNA	chr11:23154683-232
ENSG0000(	387	9.67923	chr11:76(	LINC02548		lncRNA	chr11:13784017-138
ENSG0000(	387	9.67923	chr11:76(	ENSG00000255401		Pseudoger	chr11:13488612-134
ENSG0000(	387	9.67923	chr11:76(	ENSG00000256361		Pseudoger	chr11:18511043-185
ENSG0000(	387	9.67923	chr11:76(	ENSG00000277803		Pseudoger	chr11:23886135-238
ENSG0000(	387	9.67923	chr11:76(	YWHABP2		Pseudoger	chr11:18490243-184
ENSG0000(	387	9.67923	chr11:76(	SAA4		protein_c	chr11:18231355-182
ENSG0000(	387	9.67923	chr11:76(	ENSG00000203258		lncRNA	chr11:12921186-129
ENSG0000(	387	9.67923	chr11:76(	FANCF	NCGv7;AC	protein_c	chr11:22622533-226
ENSG0000(	387	9.67923	chr11:76(	RN7SL188P		smallRNA	chr11:16576851-165
ENSG0000(	387	9.67923	chr11:76(	MRGPRX2		protein_c	chr11:19054455-190
ENSG0000(	387	9.67923	chr11:76(	SVIP		protein_c	chr11:22813799-228
ENSG0000(	387	9.67923	chr11:76(	ENSG00000286998		lncRNA	chr11:18761664-187
ENSG0000(	387	9.67923	chr11:76(	ENSG00000289499		lncRNA	chr11:18526957-185
ENSG0000(	387	9.67923	chr11:76(	RNA5SP331		Pseudoger	chr11:13907484-139
ENSG0000(	387	9.67923	chr11:76(	AC116533.1		smallRNA	chr11:17073952-170
ENSG0000(	387	9.67923	chr11:76(	MRGPRX8P		Pseudoger	chr11:18836304-188
ENSG0000(	387	9.67923	chr11:76(	ENSG00000286959		lncRNA	chr11:11781971-118
ENSG0000(	387	9.67923	chr11:76(	ENSG00000260196		lncRNA	chr11:17380649-173
ENSG0000(	387	9.67923	chr11:76(	PRMT3		protein_c	chr11:20387558-205
ENSG0000(	387	9.67923	chr11:76(	SPON1		protein_c	chr11:13962723-142
ENSG0000(	387	9.67923	chr11:76(	SRSF3P1		Pseudoger	chr11:18665050-186
ENSG0000(	387	9.67923	chr11:76(	ENSG00000256734		Pseudoger	chr11:18450112-184
ENSG0000(	387	9.67923	chr11:76(	ENSG00000256588		lncRNA	chr11:18507608-185
ENSG0000(	387	9.67923	chr11:76(	BTBD10		protein_c	chr11:13388008-134
ENSG0000(	387	9.67923	chr11:76(	GAS2		protein_c	chr11:22625509-228
ENSG0000(	387	9.67923	chr11:76(	LINC02989		lncRNA	chr11:12086891-120
ENSG0000(	387	9.67923	chr11:76(	MTCH1P2		Pseudoger	chr11:18590093-185
ENSG0000(	387	9.67923	chr11:76(	HIGD1AP5		Pseudoger	chr11:18106583-181
ENSG0000(	387	9.67923	chr11:76(	HMGB1P40		Pseudoger	chr11:20574186-205
ENSG0000(	387	9.67923	chr11:76(	RN7SKP151		smallRNA	chr11:13353208-133
ENSG0000(	387	9.67923	chr11:76(	ENSG00000255357		lncRNA	chr11:22492087-225
ENSG0000(	387	9.67923	chr11:76(	NAV2-IT1		lncRNA	chr11:19380484-193
ENSG0000(	387	9.67923	chr11:76(	ENSG00000255167		lncRNA	chr11:21260061-212
ENSG0000(	387	9.67923	chr11:76(	ENSG00000255351		lncRNA	chr11:11352426-113
ENSG0000(	387	9.67923	chr11:76(	RPL36AP37		Pseudoger	chr11:16974693-169
ENSG0000(	387	9.67923	chr11:76(	LINC02729		lncRNA	chr11:17695266-176
ENSG0000(	387	9.67923	chr11:76(	LINC02726		lncRNA	chr11:23730588-237
ENSG0000(	387	9.67923	chr11:76(	DKK3		protein_c	chr11:11956207-120
ENSG0000(	387	9.67923	chr11:76(	WIZP1		Pseudoger	chr11:23403805-234
ENSG0000(	387	9.67923	chr11:76(	ENSG00000255244		lncRNA	chr11:18934985-189
ENSG0000(	387	9.67923	chr11:76(	LINC01495		lncRNA	chr11:22445673-224
ENSG0000(	387	9.67923	chr11:76(	ENSG00000255309		Pseudoger	chr11:11759101-117
ENSG0000(	387	9.67923	chr11:76(	CSRP3-AS1		lncRNA	chr11:19196775-192
ENSG0000(	387	9.67923	chr11:76(	CCDC179		protein_c	chr11:22846922-228
ENSG0000(	387	9.67923	chr11:76(	ENSG00000255260		lncRNA	chr11:11243188-112
ENSG0000(	387	9.67923	chr11:76(	OR7E14P		Pseudoger	chr11:17052025-170
ENSG0000(	387	9.67923	chr11:76(	SNORD14B		smallRNA	chr11:17075779-170
ENSG0000(	387	9.67923	chr11:76(	ENSG00000197149		Pseudoger	chr11:17228279-172
ENSG0000(	387	9.67923	chr11:76(	FAR1		protein_c	chr11:13668668-137
ENSG0000(	387	9.67923	chr11:76(	SLC17A6		protein_c	chr11:22338381-223

ENSG0000(	387	9.67923	chr21:32{	ENSG00000224427		Pseudoger	chr21:32841496-328
ENSG0000(	387	9.67923	chr11:76(	ENSG00000255372		lncRNA	chr11:22261209-222
ENSG0000(	387	9.67923	chr11:76(	MTND5P21		Pseudoger	chr11:11239759-112
ENSG0000(	387	9.67923	chr11:76(	AC103794.1		smallRNA	chr11:16124316-161
ENSG0000(	387	9.67923	chr11:76(	GLTPP1		Pseudoger	chr11:18189000-181
ENSG0000(	387	9.67923	chr11:76(	ENSG00000255160		lncRNA	chr11:19299883-193
ENSG0000(	385	9.629208	chr15:69{	ENSG00000260104		Pseudoger	chr15:75249232-752
ENSG0000(	385	9.629208	chr15:69{	ENSG00000260152		Pseudoger	chr15:75114261-751
ENSG0000(	385	9.629208	chr15:69{	ENSG00000260103		Pseudoger	chr15:74478070-744
ENSG0000(	385	9.629208	chr15:69{	PHB1P20		Pseudoger	chr15:72450657-724
ENSG0000(	385	9.629208	chr15:69{	ENSG00000260144		Pseudoger	chr15:72605183-726
ENSG0000(	385	9.629208	chr15:69{	ENSG00000285729		lncRNA	chr15:72858354-728
ENSG0000(	385	9.629208	chr15:69{	ENSG00000261043		lncRNA	chr15:75759501-757
ENSG0000(	385	9.629208	chr15:69{	ENSG00000260165		Pseudoger	chr15:75480661-754
ENSG0000(	385	9.629208	chr15:69{	DNM1P33		Pseudoger	chr15:74062780-740
ENSG0000(	385	9.629208	chr15:69{	CPLX3		protein_c	chr15:74826627-748
ENSG0000(	385	9.629208	chr15:69{	ENSG00000260037		lncRNA	chr15:71818396-718
ENSG0000(	385	9.629208	chr15:69{	ENSG00000260919		lncRNA	chr15:74613194-746
ENSG0000(	385	9.629208	chr15:69{	ENSG00000291229		lncRNA	chr15:75299953-753
ENSG0000(	385	9.629208	chr15:69{	ENSG00000270036		lncRNA	chr15:76343642-763
ENSG0000(	385	9.629208	chr15:69{	AC108137.1		smallRNA	chr15:73891409-738
ENSG0000(	385	9.629208	chr15:69{	THSD4-AS1		lncRNA	chr15:71147650-711
ENSG0000(	385	9.629208	chr15:69{	ENSG00000261384		lncRNA	chr15:74303005-743
ENSG0000(	385	9.629208	chr15:69{	LARP6		protein_c	chr15:70829130-708
ENSG0000(	385	9.629208	chr15:69{	SENP8		protein_c	chr15:72114258-721
ENSG0000(	385	9.629208	chr15:69{	SNORD112		smallRNA	chr15:75817092-758
ENSG0000(	385	9.629208	chr15:69{	ENSG00000261281		Pseudoger	chr15:72682266-726
ENSG0000(	385	9.629208	chr15:69{	ENSG00000261232		Pseudoger	chr15:76472099-764
ENSG0000(	385	9.629208	chr15:69{	ARIH1	NCGv7	protein_c	chr15:72474330-726
ENSG0000(	385	9.629208	chr15:69{	NIFKP4		Pseudoger	chr15:75251743-752
ENSG0000(	385	9.629208	chr15:69{	FKBP1AP2		Pseudoger	chr15:73143236-731
ENSG0000(	385	9.629208	chr15:69{	ENSG00000259722		Pseudoger	chr15:77204578-772
ENSG0000(	385	9.629208	chr15:69{	ENSG00000259744		lncRNA	chr15:70848883-708
ENSG0000(	385	9.629208	chr15:69{	AC022872.1		smallRNA	chr15:72051251-720
ENSG0000(	385	9.629208	chr15:69{	HMGB1P6		Pseudoger	chr15:71164770-711
ENSG0000(	385	9.629208	chr15:69{	LINC02259		lncRNA	chr15:72608481-726
ENSG0000(	385	9.629208	chr15:69{	ENSG00000269951		lncRNA	chr15:77067654-770
ENSG0000(	385	9.629208	chr15:69{	ANP32BP1		Pseudoger	chr15:75321397-753
ENSG0000(	385	9.629208	chr15:69{	HEXA	NCGv7	protein_c	chr15:72340924-723
ENSG0000(	385	9.629208	chr15:69{	PKM		protein_c	chr15:72199029-722
ENSG0000(	385	9.629208	chr15:69{	ENSG00000259909		Pseudoger	chr15:72638821-726
ENSG0000(	385	9.629208	chr15:69{	ENSG00000259931		Pseudoger	chr15:75512770-755
ENSG0000(	385	9.629208	chr15:69{	ENSG00000261187		lncRNA	chr15:72465128-724
ENSG0000(	385	9.629208	chr15:69{	ENSG00000260173		lncRNA	chr15:72140504-721
ENSG0000(	385	9.629208	chr15:69{	ENSG00000260586		lncRNA	chr15:71332120-713
ENSG0000(	385	9.629208	chr15:69{	ADPGK-AS1		lncRNA	chr15:72782835-727
ENSG0000(	385	9.629208	chr15:69{	NRG4		protein_c	chr15:75935969-760
ENSG0000(	385	9.629208	chr15:69{	ISLR		protein_c	chr15:74173710-741
ENSG0000(	385	9.629208	chr15:69{	ENSG00000260685		Pseudoger	chr15:75950464-759
ENSG0000(	385	9.629208	chr15:69{	ENSG00000260672		lncRNA	chr15:72615810-726
ENSG0000(	385	9.629208	chr15:69{	THAP10		protein_c	chr15:70881342-708

ENSG0000(	385	9.629208	chr15:69!	AC021818.1		smallRNA	chr15:69785415-697
ENSG0000(	385	9.629208	chr15:69!	TMEM266		protein_c	chr15:76059958-762
ENSG0000(	385	9.629208	chr15:69!	INSYN1-AS1		lncRNA	chr15:73752317-737
ENSG0000(	385	9.629208	chr15:69!	ENSG00000291009		lncRNA	chr15:75775553-757
ENSG0000(	385	9.629208	chr15:69!	ENSG00000260483		Pseudoger	chr15:75022980-750
ENSG0000(	385	9.629208	chr15:69!	PPIAP47		Pseudoger	chr15:75746769-757
ENSG0000(	385	9.629208	chr15:69!	RCN2		protein_c	chr15:76931738-769
ENSG0000(	385	9.629208	chr15:69!	ENSG00000260534		lncRNA	chr15:72589691-725
ENSG0000(	385	9.629208	chr15:69!	EIF5A2P1		Pseudoger	chr15:72041194-720
ENSG0000(	385	9.629208	chr15:69!	LOXL1		protein_c	chr15:73925989-739
ENSG0000(	385	9.629208	chr15:69!	SNUPN		protein_c	chr15:75598083-756
ENSG0000(	385	9.629208	chr15:69!	ENSG00000287926		lncRNA	chr15:75225784-752
ENSG0000(	385	9.629208	chr15:69!	SIN3A	NCGv7	protein_c	chr15:75369379-754
ENSG0000(	385	9.629208	chr15:69!	ENSG00000260660		Pseudoger	chr15:75226401-752
ENSG0000(	385	9.629208	chr15:69!	PTPN9		protein_c	chr15:75463251-755
ENSG0000(	385	9.629208	chr15:69!	ENSG00000260624		lncRNA	chr15:73870949-738
ENSG0000(	385	9.629208	chr15:69!	ENSG00000259650		lncRNA	chr15:73335260-733
ENSG0000(	385	9.629208	chr15:69!	ENSG00000275527		lncRNA	chr15:74598919-745
ENSG0000(	385	9.629208	chr15:69!	DNM1P34		Pseudoger	chr15:75301642-753
ENSG0000(	385	9.629208	chr15:69!	HEXA-AS1		lncRNA	chr15:72376051-723
ENSG0000(	385	9.629208	chr15:69!	ENSG00000274937		lncRNA	chr15:74374678-743
ENSG0000(	385	9.629208	chr15:69!	ENSG00000260206		lncRNA	chr15:75636139-756
ENSG0000(	385	9.629208	chr15:69!	TBC1D21		protein_c	chr15:73873564-738
ENSG0000(	385	9.629208	chr15:69!	C15orf39		protein_c	chr15:75195643-752
ENSG0000(	385	9.629208	chr15:69!	ENSG00000260235		lncRNA	chr15:75624793-756
ENSG0000(	385	9.629208	chr15:69!	PPIAP46		Pseudoger	chr15:74350768-743
ENSG0000(	385	9.629208	chr15:69!	ISLR2		protein_c	chr15:74100311-741
ENSG0000(	385	9.629208	chr15:69!	ENSG00000260892		lncRNA	chr15:75676227-756
ENSG0000(	385	9.629208	chr15:69!	GOLGA6C		protein_c	chr15:75258334-752
ENSG0000(	385	9.629208	chr15:69!	ENSG00000275454		lncRNA	chr15:75639760-756
ENSG0000(	385	9.629208	chr15:69!	ENSG00000260269		lncRNA	chr15:75527150-756
ENSG0000(	385	9.629208	chr15:69!	FBXO22		protein_c	chr15:75903876-759
ENSG0000(	385	9.629208	chr15:69!	ENSG00000260274		lncRNA	chr15:75368057-753
ENSG0000(	385	9.629208	chr15:69!	ENSG00000287741		lncRNA	chr15:70556521-705
ENSG0000(	385	9.629208	chr15:69!	ENSG00000275645		lncRNA	chr15:75346744-753
ENSG0000(	385	9.629208	chr15:69!	CD276		protein_c	chr15:73683966-737
ENSG0000(	385	9.629208	chr15:69!	ENSG00000260288		lncRNA	chr15:75737820-757
ENSG0000(	385	9.629208	chr15:69!	ENSG00000260729		protein_c	chr15:72284727-723
ENSG0000(	385	9.629208	chr15:69!	CSK	TAG	protein_c	chr15:74782080-748
ENSG0000(	385	9.629208	chr15:69!	ENSG00000259652		lncRNA	chr15:77043680-770
ENSG0000(	385	9.629208	chr15:69!	ENSG00000279033		TEC	chr15:77484275-774
ENSG0000(	385	9.629208	chr15:69!	STOML1		protein_c	chr15:73978926-739
ENSG0000(	385	9.629208	chr15:69!	DNM1P49		Pseudoger	chr15:75791470-757
ENSG0000(	385	9.629208	chr15:69!	CYP11A1		protein_c	chr15:74337759-743
ENSG0000(	385	9.629208	chr15:69!	ENSG00000273025		protein_c	chr15:72266746-723
ENSG0000(	385	9.629208	chr15:69!	BBS4		protein_c	chr15:72686179-727
ENSG0000(	385	9.629208	chr15:69!	PML	NCGv7;AC	protein_c	chr15:73994673-740
ENSG0000(	385	9.629208	chr15:69!	MIR4513		smallRNA	chr15:74788672-747
ENSG0000(	385	9.629208	chr15:69!	CYP1A1		protein_c	chr15:74719542-747
ENSG0000(	385	9.629208	chr15:69!	ULK3		protein_c	chr15:74836118-748
ENSG0000(	385	9.629208	chr15:69!	GOLGA6D		protein_c	chr15:75282835-752

ENSG0000(	385	9.629208	chr15:69	CCDC33		protein_c	chr15:74202705-743
ENSG0000(	385	9.629208	chr15:69	CELF6		protein_c	chr15:72284727-723
ENSG0000(	385	9.629208	chr15:69	ENSG00000277749		lncRNA	chr15:74311516-743
ENSG0000(	385	9.629208	chr15:69	SCAMP2		protein_c	chr15:74843730-748
ENSG0000(	385	9.629208	chr15:69	CYP1A2		protein_c	chr15:74748845-747
ENSG0000(	385	9.629208	chr15:69	STRA6	AC	protein_c	chr15:74179466-742
ENSG0000(	385	9.629208	chr15:69	UACA	NCGv7	protein_c	chr15:70654554-707
ENSG0000(	385	9.629208	chr15:69	LRRC49		protein_c	chr15:70853239-710
ENSG0000(	385	9.629208	chr15:69	PARP6		protein_c	chr15:72241181-722
ENSG0000(	385	9.629208	chr15:69	LMAN1L		protein_c	chr15:74812716-748
ENSG0000(	385	9.629208	chr15:69	AC113208.1		smallRNA	chr15:75225871-752
ENSG0000(	385	9.629208	chr15:69	LINC02255		lncRNA	chr15:74379083-743
ENSG0000(	385	9.629208	chr15:69	ENSG00000287543		lncRNA	chr15:74040190-740
ENSG0000(	385	9.629208	chr15:69	ENSG00000278313		Pseudoger	chr15:72604399-726
ENSG0000(	385	9.629208	chr15:69	PPCDC		protein_c	chr15:75023586-751
ENSG0000(	385	9.629208	chr15:69	MAN2C1		protein_c	chr15:75355207-753
ENSG0000(	385	9.629208	chr15:69	ADPGK		protein_c	chr15:72751294-727
ENSG0000(	385	9.629208	chr15:69	ENSG00000287503		lncRNA	chr15:76263197-762
ENSG0000(	385	9.629208	chr15:69	TLE3		protein_c	chr15:70047790-700
ENSG0000(	385	9.629208	chr15:69	ENSG00000286696		lncRNA	chr15:74429445-744
ENSG0000(	385	9.629208	chr15:69	COMMD4		protein_c	chr15:75336020-753
ENSG0000(	385	9.629208	chr15:69	UBE2Q2		protein_c	chr15:75843307-759
ENSG0000(	385	9.629208	chr15:69	UBL7		protein_c	chr15:74445977-744
ENSG0000(	385	9.629208	chr15:69	PSTPIP1		protein_c	chr15:76993359-770
ENSG0000(	385	9.629208	chr15:69	GOLGA6A		protein_c	chr15:74069857-740
ENSG0000(	385	9.629208	chr15:69	COMMD4P2		Pseudoger	chr15:74097106-740
ENSG0000(	385	9.629208	chr15:69	ISL2		protein_c	chr15:76336773-763
ENSG0000(	385	9.629208	chr15:69	NEIL1		protein_c	chr15:75346955-753
ENSG0000(	385	9.629208	chr15:69	ETFA		protein_c	chr15:76188555-763
ENSG0000(	385	9.629208	chr15:69	SEMA7A		protein_c	chr15:74409289-744
ENSG0000(	385	9.629208	chr15:69	HMG20A		protein_c	chr15:77420412-774
ENSG0000(	385	9.629208	chr15:69	SCAPER	NCGv7	protein_c	chr15:76347904-769
ENSG0000(	385	9.629208	chr15:69	HCN4		protein_c	chr15:73319859-733
ENSG0000(	385	9.629208	chr15:69	CT62		lncRNA	chr15:71110244-711
ENSG0000(	385	9.629208	chr15:69	NR2E3		protein_c	chr15:71792638-718
ENSG0000(	385	9.629208	chr15:69	TSPAN3		protein_c	chr15:77041404-770
ENSG0000(	385	9.629208	chr15:69	ENSG00000278408		lncRNA	chr15:71547280-715
ENSG0000(	385	9.629208	chr15:69	ENSG00000261821		lncRNA	chr15:74365435-743
ENSG0000(	385	9.629208	chr15:69	ENSG00000288901		lncRNA	chr15:75129685-751
ENSG0000(	385	9.629208	chr15:69	ENSG00000259624		lncRNA	chr15:70768011-707
ENSG0000(	385	9.629208	chr15:69	ENSG00000261813		Pseudoger	chr15:74976240-749
ENSG0000(	385	9.629208	chr15:69	MYO9A	NCGv7	protein_c	chr15:71822291-721
ENSG0000(	385	9.629208	chr15:69	TMEM202-AS1		lncRNA	chr15:72407778-724
ENSG0000(	385	9.629208	chr15:69	GOLGA6B		protein_c	chr15:72654697-726
ENSG0000(	385	9.629208	chr15:69	ENSG00000274515		lncRNA	chr15:75645020-756
ENSG0000(	385	9.629208	chr15:69	ENSG00000259362		lncRNA	chr15:77525540-775
ENSG0000(	385	9.629208	chr15:69	ENSG00000259422		lncRNA	chr15:76174891-761
ENSG0000(	385	9.629208	chr15:69	NPM1P43		Pseudoger	chr15:73161779-731
ENSG0000(	385	9.629208	chr15:69	ENSG00000259452		Pseudoger	chr15:70791013-707
ENSG0000(	385	9.629208	chr15:69	KRT8P9		Pseudoger	chr15:70858714-708
ENSG0000(	385	9.629208	chr15:69	NPTN-IT1		lncRNA	chr15:73567012-735

ENSG0000(	385	9.629208	chr15:69!	SCARNA20		smallRNA	chr15:75121536-751
ENSG0000(	385	9.629208	chr15:69!	LINC02205		lncRNA	chr15:70503907-705
ENSG0000(	385	9.629208	chr15:69!	GEMIN8P1		Pseudoger	chr15:69803316-698
ENSG0000(	385	9.629208	chr15:69!	SALRNA3		lncRNA	chr15:70615547-706
ENSG0000(	385	9.629208	chr15:69!	NEO1		protein_c	chr15:73051710-733
ENSG0000(	385	9.629208	chr15:69!	ENSG00000259503		lncRNA	chr15:70321576-703
ENSG0000(	385	9.629208	chr15:69!	ENSG00000259514		lncRNA	chr15:76339609-763
ENSG0000(	385	9.629208	chr15:69!	ENSG00000259528		lncRNA	chr15:73255334-732
ENSG0000(	385	9.629208	chr15:69!	ENSG00000259532		lncRNA	chr15:70748932-707
ENSG0000(	385	9.629208	chr15:69!	KRT8P23		Pseudoger	chr15:76979245-769
ENSG0000(	385	9.629208	chr15:69!	TYRO3P		Pseudoger	chr15:76258986-762
ENSG0000(	385	9.629208	chr15:69!	MIR3713		smallRNA	chr15:76586647-765
ENSG0000(	385	9.629208	chr15:69!	RN7SL510P		smallRNA	chr15:75895040-758
ENSG0000(	385	9.629208	chr15:69!	AC108861.1		smallRNA	chr15:71475487-714
ENSG0000(	385	9.629208	chr15:69!	ENSG00000280309		TEC	chr15:74299503-743
ENSG0000(	385	9.629208	chr15:69!	LOXL1-AS1		lncRNA	chr15:73908071-739
ENSG0000(	385	9.629208	chr15:69!	ENSG00000261779		lncRNA	chr15:75211301-752
ENSG0000(	385	9.629208	chr15:69!	ENSG00000289525		lncRNA	chr15:69748674-697
ENSG0000(	385	9.629208	chr15:69!	ENSG00000274297		lncRNA	chr15:70758269-707
ENSG0000(	385	9.629208	chr15:69!	ENSG00000276807		lncRNA	chr15:73730048-737
ENSG0000(	385	9.629208	chr15:69!	ENSG00000276744		lncRNA	chr15:75452964-754
ENSG0000(	385	9.629208	chr15:69!	ENSG00000261775		lncRNA	chr15:74489602-745
ENSG0000(	385	9.629208	chr15:69!	ENSG00000261714		Pseudoger	chr15:75415249-754
ENSG0000(	385	9.629208	chr15:69!	ENSG00000261632		lncRNA	chr15:71972206-720
ENSG0000(	385	9.629208	chr15:69!	SALRNA2		lncRNA	chr15:70635249-706
ENSG0000(	385	9.629208	chr15:69!	ENSG00000259309		lncRNA	chr15:69820756-698
ENSG0000(	385	9.629208	chr15:69!	ENSG00000261606		lncRNA	chr15:74816223-748
ENSG0000(	385	9.629208	chr15:69!	ENSG00000261543		lncRNA	chr15:74152800-741
ENSG0000(	385	9.629208	chr15:69!	ENSG00000261460		lncRNA	chr15:72278867-723
ENSG0000(	385	9.629208	chr15:69!	LINC02204		lncRNA	chr15:70570958-705
ENSG0000(	385	9.629208	chr15:69!	ENSG00000278991		TEC	chr15:77063397-770
ENSG0000(	385	9.629208	chr15:69!	MRPS15P1		Pseudoger	chr15:73483196-734
ENSG0000(	385	9.629208	chr15:69!	ENSG00000259227		Pseudoger	chr15:70602124-706
ENSG0000(	385	9.629208	chr15:69!	ENSG00000259252		lncRNA	chr15:70195638-701
ENSG0000(	385	9.629208	chr15:69!	NPM1P42		Pseudoger	chr15:72899399-729
ENSG0000(	385	9.629208	chr15:69!	RNU2-65P		smallRNA	chr15:72045183-720
ENSG0000(	385	9.629208	chr15:69!	NPTN		protein_c	chr15:73560014-736
ENSG0000(	385	9.629208	chr15:69!	ARID3B	NCGv7	protein_c	chr15:74541206-745
ENSG0000(	385	9.629208	chr15:69!	ENSG00000230459		Pseudoger	chr15:77278465-772
ENSG0000(	385	9.629208	chr15:69!	RNA5SP399		Pseudoger	chr15:71858570-718
ENSG0000(	385	9.629208	chr15:69!	RN7SL319P		smallRNA	chr15:75785112-757
ENSG0000(	385	9.629208	chr15:69!	IMP3		protein_c	chr15:75639085-756
ENSG0000(	385	9.629208	chr15:69!	ENSG00000248540		lncRNA	chr15:74125915-741
ENSG0000(	385	9.629208	chr15:69!	ODF3L1		protein_c	chr15:75724041-757
ENSG0000(	385	9.629208	chr15:69!	RPL29P30		Pseudoger	chr15:70796597-708
ENSG0000(	385	9.629208	chr15:69!	GOLGA6EP		Pseudoger	chr15:75775594-757
ENSG0000(	385	9.629208	chr15:69!	HIGD2B		protein_c	chr15:72675798-726
ENSG0000(	385	9.629208	chr15:69!	RN7SKP217		smallRNA	chr15:76736641-767
ENSG0000(	385	9.629208	chr15:69!	MIR630		smallRNA	chr15:72587217-725
ENSG0000(	385	9.629208	chr15:69!	ENSG00000203392		lncRNA	chr15:75678548-756
ENSG0000(	385	9.629208	chr15:69!	ENSG00000243568		Pseudoger	chr15:72134641-721

ENSG0000(	385	9.629208	chr15:695	RPL12P35		Pseudoger	chr15:72379215-723
ENSG0000(	385	9.629208	chr15:695	MIR631		smallRNA	chr15:75353611-753
ENSG0000(	385	9.629208	chr15:695	RNU6-745P		smallRNA	chr15:70193236-701
ENSG0000(	385	9.629208	chr15:695	REC114		protein_c	chr15:73443164-735
ENSG0000(	385	9.629208	chr15:695	RN7SL489P		smallRNA	chr15:75268408-752
ENSG0000(	385	9.629208	chr15:695	ENSG00000212664		Pseudoger	chr15:71341158-713
ENSG0000(	385	9.629208	chr15:695	RPL5P3		Pseudoger	chr15:71063101-710
ENSG0000(	385	9.629208	chr15:695	MIR629		smallRNA	chr15:70079372-700
ENSG0000(	385	9.629208	chr15:695	UBL7-DT		lncRNA	chr15:74461248-745
ENSG0000(	385	9.629208	chr15:695	RPL13P4		Pseudoger	chr15:75388267-753
ENSG0000(	385	9.629208	chr15:695	SNX33		protein_c	chr15:75647912-756
ENSG0000(	385	9.629208	chr15:695	RN7SL278P		smallRNA	chr15:76976917-769
ENSG0000(	385	9.629208	chr15:695	CSPG4	NCGv7	protein_c	chr15:75674322-757
ENSG0000(	385	9.629208	chr15:695	RPL36AP45		Pseudoger	chr15:75186651-751
ENSG0000(	385	9.629208	chr15:695	DNM1P35		lncRNA	chr15:75727670-757
ENSG0000(	385	9.629208	chr15:695	INSYN1		protein_c	chr15:73735458-737
ENSG0000(	385	9.629208	chr15:695	CLK3	NCGv7	protein_c	chr15:74598500-746
ENSG0000(	385	9.629208	chr15:695	THSD4		protein_c	chr15:71096952-717
ENSG0000(	385	9.629208	chr15:695	EDC3		protein_c	chr15:74630558-746
ENSG0000(	385	9.629208	chr15:695	Y_RNA		smallRNA	chr15:74983274-749
ENSG0000(	385	9.629208	chr15:695	RN7SL853P		smallRNA	chr15:72664555-726
ENSG0000(	385	9.629208	chr15:695	SCAMP5		protein_c	chr15:74957219-750
ENSG0000(	385	9.629208	chr15:695	GRAMD2A		protein_c	chr15:72159806-721
ENSG0000(	385	9.629208	chr15:695	TMEM202		protein_c	chr15:72398302-724
ENSG0000(	385	9.629208	chr15:695	RN7SL429P		smallRNA	chr15:74072423-740
ENSG0000(	385	9.629208	chr15:695	RN7SL327P		smallRNA	chr15:75292684-752
ENSG0000(	385	9.629208	chr15:695	SNORD77		smallRNA	chr15:74490959-744
ENSG0000(	385	9.629208	chr15:695	RN7SL485P		smallRNA	chr15:72611820-726
ENSG0000(	385	9.629208	chr15:695	RPP25		protein_c	chr15:74954418-749
ENSG0000(	385	9.629208	chr15:695	COX5A		protein_c	chr15:74919791-749
ENSG0000(	385	9.629208	chr15:695	MPI		protein_c	chr15:74890005-749
ENSG0000(	385	9.629208	chr15:695	FAM219B		protein_c	chr15:74899992-749
ENSG0000(	385	9.629208	chr15:695	PEAK1		protein_c	chr15:77100656-774
ENSG0000(	384	9.604197	chr5:1466	snoU13		smallRNA	chr5:150543590-150
ENSG0000(	384	9.604197	chr5:1466	RPS14		protein_c	chr5:150442635-150
ENSG0000(	384	9.604197	chr5:1466	TIGD6		protein_c	chr5:149993118-150
ENSG0000(	384	9.604197	chr5:1466	ENSG00000270978		Pseudoger	chr5:151848886-151
ENSG0000(	384	9.604197	chr5:1466	IL17B		protein_c	chr5:149371324-149
ENSG0000(	384	9.604197	chr5:1466	MYOZ3	NCGv7	protein_c	chr5:150660882-150
ENSG0000(	384	9.604197	chr5:1466	ENSG00000271494		Pseudoger	chr5:151380341-151
ENSG0000(	384	9.604197	chr5:1466	ENSG00000271795		lncRNA	chr5:151509453-151
ENSG0000(	384	9.604197	chr5:1466	GRPEL2		protein_c	chr5:149345430-149
ENSG0000(	384	9.604197	chr5:1466	ATP6V1G1P5		Pseudoger	chr5:151319422-151
ENSG0000(	384	9.604197	chr5:1466	HTR4		protein_c	chr5:148451032-148
ENSG0000(	384	9.604197	chr5:1466	ENSG00000272112		lncRNA	chr5:151724831-151
ENSG0000(	384	9.604197	chr5:1466	ZNF300P1		Pseudoger	chr5:150930763-150
ENSG0000(	384	9.604197	chr5:1466	SLC36A1		protein_c	chr5:151437046-151
ENSG0000(	384	9.604197	chr5:1466	ENSG00000272239		lncRNA	chr5:147401760-147
ENSG0000(	384	9.604197	chr5:1466	MIR378A		smallRNA	chr5:149732825-149
ENSG0000(	384	9.604197	chr5:1466	DPYSL3		protein_c	chr5:147390808-147
ENSG0000(	384	9.604197	chr5:1466	CSNK1A1	NCGv7	protein_c	chr5:149492982-149

ENSG0000(	384	9.604197	chr5:146(	HMGXB3		protein_c	chr5:150000046-150
ENSG0000(	384	9.604197	chr5:146(	PDGFRB	NCGv7;AC	protein_c	chr5:150113839-150
ENSG0000(	384	9.604197	chr5:146(	CARMN		lncRNA	chr5:149406689-149
ENSG0000(	384	9.604197	chr5:146(	CDX1		protein_c	chr5:150166778-150
ENSG0000(	384	9.604197	chr5:146(	ENSG00000277866		Pseudoger	chr5:150978419-150
ENSG0000(	384	9.604197	chr5:146(	AC034205.1		smallRNA	chr5:151368444-151
ENSG0000(	384	9.604197	chr5:146(	CCDC69		protein_c	chr5:151181052-151
ENSG0000(	384	9.604197	chr5:146(	ENSG00000275871		lncRNA	chr5:149425771-149
ENSG0000(	384	9.604197	chr5:146(	IRGM		protein_c	chr5:150846521-150
ENSG0000(	384	9.604197	chr5:146(	ENSG00000275765		lncRNA	chr5:151769783-151
ENSG0000(	384	9.604197	chr5:146(	PPP2R2B-IT1		lncRNA	chr5:146914207-146
ENSG0000(	384	9.604197	chr5:146(	ENSG00000249518		Pseudoger	chr5:147851644-147
ENSG0000(	384	9.604197	chr5:146(	HMGN1P16		Pseudoger	chr5:148221360-148
ENSG0000(	384	9.604197	chr5:146(	LINC01470		lncRNA	chr5:152618965-153
ENSG0000(	384	9.604197	chr5:146(	SCGB3A2		protein_c	chr5:147870682-147
ENSG0000(	384	9.604197	chr5:146(	ANXA6		protein_c	chr5:151100706-151
ENSG0000(	384	9.604197	chr5:146(	ENSG00000274235		Pseudoger	chr5:150950109-150
ENSG0000(	384	9.604197	chr5:146(	snoU13		smallRNA	chr5:150029349-150
ENSG0000(	384	9.604197	chr5:146(	SPINK14		protein_c	chr5:148168546-148
ENSG0000(	384	9.604197	chr5:146(	GM2A		protein_c	chr5:151212150-151
ENSG0000(	384	9.604197	chr5:146(	AC021078.1		smallRNA	chr5:149604276-149
ENSG0000(	384	9.604197	chr5:146(	U3		smallRNA	chr5:149695749-149
ENSG0000(	384	9.604197	chr5:146(	SPINK1		protein_c	chr5:147824572-147
ENSG0000(	384	9.604197	chr5:146(	ATOX1-AS1		lncRNA	chr5:151753992-151
ENSG0000(	384	9.604197	chr5:146(	MIR584		smallRNA	chr5:149062313-149
ENSG0000(	384	9.604197	chr5:146(	ZNF300		protein_c	chr5:150894392-150
ENSG0000(	384	9.604197	chr5:146(	ENSG00000248647		lncRNA	chr5:149163955-149
ENSG0000(	384	9.604197	chr5:146(	ATOX1	DriverDB\	protein_c	chr5:151742316-151
ENSG0000(	384	9.604197	chr5:146(	RN7SL868P		smallRNA	chr5:149722070-149
ENSG0000(	384	9.604197	chr5:146(	RN7SL177P		smallRNA	chr5:153755382-153
ENSG0000(	384	9.604197	chr5:146(	ARHGEF37		protein_c	chr5:149551947-149
ENSG0000(	384	9.604197	chr5:146(	FBXO38		protein_c	chr5:148383935-148
ENSG0000(	384	9.604197	chr5:146(	SPINK7		protein_c	chr5:148312419-148
ENSG0000(	384	9.604197	chr5:146(	PCYOX1L		protein_c	chr5:149358037-149
ENSG0000(	384	9.604197	chr5:146(	GLRA1		protein_c	chr5:151822513-151
ENSG0000(	384	9.604197	chr5:146(	KRT8P48		Pseudoger	chr5:146706381-146
ENSG0000(	384	9.604197	chr5:146(	TNIP1		protein_c	chr5:151029945-151
ENSG0000(	384	9.604197	chr5:146(	G3BP1	NCGv7	protein_c	chr5:151771045-151
ENSG0000(	384	9.604197	chr5:146(	CSF1R	NCGv7;AC	protein_c	chr5:150053291-150
ENSG0000(	384	9.604197	chr5:146(	ENSG00000248696		Pseudoger	chr5:150014785-150
ENSG0000(	384	9.604197	chr5:146(	SPINK6		protein_c	chr5:148202794-148
ENSG0000(	384	9.604197	chr5:146(	SMIM3		protein_c	chr5:150778757-150
ENSG0000(	384	9.604197	chr5:146(	ENSG00000248362		lncRNA	chr5:147886086-147
ENSG0000(	384	9.604197	chr5:146(	C5orf46		protein_c	chr5:147880726-147
ENSG0000(	384	9.604197	chr5:146(	GRIA1		protein_c	chr5:153489615-153
ENSG0000(	384	9.604197	chr5:146(	PPARGC1B		protein_c	chr5:149730298-149
ENSG0000(	384	9.604197	chr5:146(	SLC26A2		protein_c	chr5:149960758-149
ENSG0000(	384	9.604197	chr5:146(	AFAP1L1		protein_c	chr5:149271859-149
ENSG0000(	384	9.604197	chr5:146(	MARCOL		protein_c	chr5:148221650-148
ENSG0000(	384	9.604197	chr5:146(	SPINK13		protein_c	chr5:148268180-148
ENSG0000(	384	9.604197	chr5:146(	RPL7P1		Pseudoger	chr5:150094302-150

ENSG0000(	384	9.604197	chr5:146(	FBXO38-DT		lncRNA	chr5:148088125-148
ENSG0000(	384	9.604197	chr5:146(	ARSI		protein_c	chr5:150296343-150
ENSG0000(	384	9.604197	chr5:146(	ENSG00000260581		lncRNA	chr5:151652275-151
ENSG0000(	384	9.604197	chr5:146(	ENSG00000253297		lncRNA	chr5:148644687-148
ENSG0000(	384	9.604197	chr5:146(	AC011357.1		smallRNA	chr5:146734835-146
ENSG0000(	384	9.604197	chr5:146(	ENSG00000253406		lncRNA	chr5:149216523-149
ENSG0000(	384	9.604197	chr5:146(	NMUR2	NCGv7	protein_c	chr5:152391546-152
ENSG0000(	384	9.604197	chr5:146(	DCTN4		protein_c	chr5:150708440-150
ENSG0000(	384	9.604197	chr5:146(	ENSG00000253472		Pseudoger	chr5:151378003-151
ENSG0000(	384	9.604197	chr5:146(	PDE6A		protein_c	chr5:149857953-149
ENSG0000(	384	9.604197	chr5:146(	SPINK5		protein_c	chr5:148025683-148
ENSG0000(	384	9.604197	chr5:146(	ENSG00000230551		lncRNA	chr5:149494314-149
ENSG0000(	384	9.604197	chr5:146(	GRPEL2-AS1		lncRNA	chr5:149348116-149
ENSG0000(	384	9.604197	chr5:146(	ENSG00000253852		lncRNA	chr5:150608428-150
ENSG0000(	384	9.604197	chr5:146(	ENSG00000253865		lncRNA	chr5:149372174-149
ENSG0000(	384	9.604197	chr5:146(	ENSG00000253897		Pseudoger	chr5:151366433-151
ENSG0000(	384	9.604197	chr5:146(	SPARC	AC	protein_c	chr5:151661096-151
ENSG0000(	384	9.604197	chr5:146(	LINC01933		lncRNA	chr5:151949571-152
ENSG0000(	384	9.604197	chr5:146(	RPL36AP20		Pseudoger	chr5:152495718-152
ENSG0000(	384	9.604197	chr5:146(	SLC36A2		protein_c	chr5:151314972-151
ENSG0000(	384	9.604197	chr5:146(	ENSG00000254298		lncRNA	chr5:151158106-151
ENSG0000(	384	9.604197	chr5:146(	SLC36A3		protein_c	chr5:151276358-151
ENSG0000(	384	9.604197	chr5:146(	NDST1-AS1		lncRNA	chr5:150475531-150
ENSG0000(	384	9.604197	chr5:146(	RPL29P14		Pseudoger	chr5:149545383-149
ENSG0000(	384	9.604197	chr5:146(	CLMAT3		lncRNA	chr5:151676945-151
ENSG0000(	384	9.604197	chr5:146(	RN7SL791P		smallRNA	chr5:146656403-146
ENSG0000(	384	9.604197	chr5:146(	JAKMIP2	NCGv7	protein_c	chr5:147585438-147
ENSG0000(	384	9.604197	chr5:146(	MIR143		smallRNA	chr5:149428918-149
ENSG0000(	384	9.604197	chr5:146(	ENSG00000261382		lncRNA	chr5:153901459-153
ENSG0000(	384	9.604197	chr5:146(	RPS20P4		Pseudoger	chr5:150021567-150
ENSG0000(	384	9.604197	chr5:146(	GPX3		protein_c	chr5:151020591-151
ENSG0000(	384	9.604197	chr5:146(	ABLIM3		protein_c	chr5:149141483-149
ENSG0000(	384	9.604197	chr5:146(	SH3TC2		protein_c	chr5:148923639-149
ENSG0000(	384	9.604197	chr5:146(	ENSG00000272411		lncRNA	chr5:148970340-148
ENSG0000(	384	9.604197	chr5:146(	MFAP3		protein_c	chr5:154038959-154
ENSG0000(	384	9.604197	chr5:146(	RN7SKP145		smallRNA	chr5:149116178-149
ENSG0000(	384	9.604197	chr5:146(	PDGFRL2P		Pseudoger	chr5:151173981-151
ENSG0000(	384	9.604197	chr5:146(	ENSG00000287323		lncRNA	chr5:149694237-149
ENSG0000(	384	9.604197	chr5:146(	RNA5SP198		Pseudoger	chr5:151875566-151
ENSG0000(	384	9.604197	chr5:146(	SH3TC2-DT		lncRNA	chr5:149063239-149
ENSG0000(	384	9.604197	chr5:146(	ENSG00000289430		lncRNA	chr5:148859611-148
ENSG0000(	384	9.604197	chr5:146(	MYOZ3-AS1		lncRNA	chr5:150670658-150
ENSG0000(	384	9.604197	chr5:146(	ADRB2		protein_c	chr5:148826611-148
ENSG0000(	384	9.604197	chr5:146(	SPINK9		protein_c	chr5:148321203-148
ENSG0000(	384	9.604197	chr5:146(	STK32A	DriverDBv	protein_c	chr5:147234963-147
ENSG0000(	384	9.604197	chr5:146(	CD74	NCGv7;AC	protein_c	chr5:150401637-150
ENSG0000(	384	9.604197	chr5:146(	SYNPO		protein_c	chr5:150601080-150
ENSG0000(	384	9.604197	chr5:146(	NDST1		protein_c	chr5:150485818-150
ENSG0000(	384	9.604197	chr5:146(	LINC01861		lncRNA	chr5:153887428-153
ENSG0000(	384	9.604197	chr5:146(	ENSG00000289970		lncRNA	chr5:151081195-151
ENSG0000(	384	9.604197	chr5:146(	ENSG00000251320		lncRNA	chr5:147887112-147

ENSG0000(	384	9.604197	chr5:146(	ENSG00000251330		lncRNA	chr5:148430159-148
ENSG0000(	384	9.604197	chr5:146(	ENSG00000285736		lncRNA	chr5:149324220-149
ENSG0000(	384	9.604197	chr5:146(	RNU6-588P		smallRNA	chr5:149606637-149
ENSG0000(	384	9.604197	chr5:146(	ENSG00000286749		lncRNA	chr5:152374998-152
ENSG0000(	384	9.604197	chr5:146(	ENSG00000286657		lncRNA	chr5:150621007-150
ENSG0000(	384	9.604197	chr5:146(	AC010295.1		smallRNA	chr5:154093304-154
ENSG0000(	384	9.604197	chr5:146(	RNA5SP197		Pseudoger	chr5:151477459-151
ENSG0000(	384	9.604197	chr5:146(	RPLP1P6		Pseudoger	chr5:151765859-151
ENSG0000(	384	9.604197	chr5:146(	RN7SKP232		smallRNA	chr5:151704289-151
ENSG0000(	384	9.604197	chr7:330(	SNORA5B		smallRNA	chr7:45105968-4510
ENSG0000(	384	9.604197	chr5:146(	Y_RNA		smallRNA	chr5:149940030-149
ENSG0000(	384	9.604197	chr5:146(	FAM114A2		protein_c	chr5:153990148-154
ENSG0000(	384	9.604197	chr5:146(	MFFP2		Pseudoger	chr5:149932014-149
ENSG0000(	384	9.604197	chr2:4707	RNU6-137P		smallRNA	chr2:42712740-4271
ENSG0000(	384	9.604197	chr5:146(	ENSG00000286331		lncRNA	chr5:150427904-150
ENSG0000(	384	9.604197	chr5:146(	CAMK2A		protein_c	chr5:150219491-150
ENSG0000(	384	9.604197	chr5:146(	ENSG00000286468		lncRNA	chr5:147093259-147
ENSG0000(	384	9.604197	chr5:146(	Y_RNA		smallRNA	chr5:150098406-150
ENSG0000(	384	9.604197	chr5:146(	JAKMIP2-AS1		lncRNA	chr5:147559994-147
ENSG0000(	384	9.604197	chr5:146(	EEF1GP2		Pseudoger	chr5:147922179-147
ENSG0000(	384	9.604197	chr5:146(	ENSG00000287630		lncRNA	chr5:147725551-147
ENSG0000(	384	9.604197	chr5:146(	ENSG00000288774		lncRNA	chr5:147874114-147
ENSG0000(	384	9.604197	chr5:146(	PGBD4P3		Pseudoger	chr5:148307298-148
ENSG0000(	384	9.604197	chr5:146(	SLC6A7		protein_c	chr5:150190062-150
ENSG0000(	384	9.604197	chr5:146(	FAT2	NCGv7	protein_c	chr5:151504092-151
ENSG0000(	384	9.604197	chr5:146(	TCOF1		protein_c	chr5:150357629-150
ENSG0000(	384	9.604197	chr5:146(	ENSG00000290991		lncRNA	chr5:151366299-151
ENSG0000(	384	9.604197	chr5:146(	ZNF300P1		lncRNA	chr5:150930456-150
ENSG0000(	384	9.604197	chr5:146(	RNU6-732P		smallRNA	chr5:149057554-149
ENSG0000(	384	9.604197	chr5:146(	RBM22		protein_c	chr5:150690792-150
ENSG0000(	384	9.604197	chr5:146(	ENSG00000283653		Pseudoger	chr5:148362777-148
ENSG0000(	384	9.604197	chr5:146(	STK32A-AS1		lncRNA	chr5:147180204-147
ENSG0000(	384	9.604197	chr5:146(	ENSG00000288081		lncRNA	chr5:151595264-151
ENSG0000(	382	9.554175	chr1:152	CORT		protein_c	chr1:10450031-1045
ENSG0000(	382	9.554175	chr1:152	C1orf127	NCGv7	protein_c	chr1:10946471-1098
ENSG0000(	382	9.554175	chr1:152	CENPS		protein_c	chr1:10430433-1044
ENSG0000(	382	9.554175	chr1:152	RNU6-537P		smallRNA	chr1:11152350-1115
ENSG0000(	382	9.554175	chr1:152	ENSG00000287727		lncRNA	chr1:10612222-1061
ENSG0000(	382	9.554175	chr1:152	ENSG00000226849		lncRNA	chr1:11068471-1107
ENSG0000(	382	9.554175	chr1:152	ENSG00000241326		lncRNA	chr1:9983141-99845
ENSG0000(	382	9.554175	chr1:152	RN7SL721P		smallRNA	chr1:10390002-1039
ENSG0000(	382	9.554175	chr1:152	CASZ1	NCGv7	protein_c	chr1:10636604-1079
ENSG0000(	382	9.554175	chr1:152	UBE4B		protein_c	chr1:10032832-1018
ENSG0000(	382	9.554175	chr1:152	AL713997.1		smallRNA	chr1:10998176-1099
ENSG0000(	382	9.554175	chr1:152	RN7SL614P		smallRNA	chr1:10616836-1061
ENSG0000(	382	9.554175	chr1:152	RNA5SP40		Pseudoger	chr1:9437669-94377
ENSG0000(	382	9.554175	chr1:152	PGD		protein_c	chr1:10398592-1042
ENSG0000(	382	9.554175	chr1:152	LINC01647		lncRNA	chr1:11609468-1161
ENSG0000(	382	9.554175	chr1:152	RBP7		protein_c	chr1:9997206-10016
ENSG0000(	382	9.554175	chr1:152	ENSG00000233268		Pseudoger	chr1:9660828-96626
ENSG0000(	382	9.554175	chr1:152	SLC2A5		protein_c	chr1:9035106-90884

ENSG0000(	382	9.554175	chr1:1522	LZIC		protein_c	chr1:9922113-99434
ENSG0000(	382	9.554175	chr1:1522	MZT1P1		Pseudoger	chr1:9780822-97812
ENSG0000(	382	9.554175	chr1:1522	CENPS-CORT		protein_c	chr1:10430102-1045
ENSG0000(	382	9.554175	chr1:1522	MIR34AHG		lncRNA	chr1:9148011-91989
ENSG0000(	382	9.554175	chr1:1522	EXOSC10-AS1		lncRNA	chr1:11099430-1110
ENSG0000(	382	9.554175	chr1:1522	ENSG00000228150		lncRNA	chr1:9942923-99499
ENSG0000(	382	9.554175	chr1:1522	ENSG00000223989		lncRNA	chr1:9848318-98501
ENSG0000(	382	9.554175	chr1:1522	LNCTAM34A		lncRNA	chr1:9182004-91962
ENSG0000(	382	9.554175	chr1:1522	ENSG00000224340		Pseudoger	chr1:10054445-1005
ENSG0000(	382	9.554175	chr1:1522	MIR5697		smallRNA	chr1:9967381-99674
ENSG0000(	382	9.554175	chr1:1522	MTCYBP45		Pseudoger	chr1:11425257-1142
ENSG0000(	382	9.554175	chr1:1522	RN7SKP269		smallRNA	chr1:9947318-99476
ENSG0000(	382	9.554175	chr1:1522	PGAM1P11		Pseudoger	chr1:10058671-1005
ENSG0000(	382	9.554175	chr1:1522	ENSG00000203469		lncRNA	chr1:10458555-1045
ENSG0000(	382	9.554175	chr1:1522	EXOSC10		protein_c	chr1:11066618-1109
ENSG0000(	382	9.554175	chr1:1522	DISP3	NCGv7	protein_c	chr1:11479155-1153
ENSG0000(	382	9.554175	chr1:1522	Y_RNA		smallRNA	chr1:10999862-1099
ENSG0000(	382	9.554175	chr1:1522	RNU6-828P		smallRNA	chr1:10163268-1016
ENSG0000(	382	9.554175	chr1:1522	ENSG00000235263		lncRNA	chr1:9501092-95034
ENSG0000(	382	9.554175	chr1:1522	SPSB1		protein_c	chr1:9292894-93695
ENSG0000(	382	9.554175	chr1:1522	SLC25A33		protein_c	chr1:9539465-95851
ENSG0000(	382	9.554175	chr1:1522	RN7SL451P		smallRNA	chr1:8979578-89798
ENSG0000(	382	9.554175	chr1:1522	MIR1273D		smallRNA	chr1:10227718-1022
ENSG0000(	382	9.554175	chr1:1522	PIK3CD-AS2		lncRNA	chr1:9672405-96875
ENSG0000(	382	9.554175	chr1:1522	FBXO44		protein_c	chr1:11654375-1166
ENSG0000(	382	9.554175	chr1:1522	MIR34A		smallRNA	chr1:9151668-91517
ENSG0000(	382	9.554175	chr1:1522	RN7SL731P		smallRNA	chr1:10306465-1030
ENSG0000(	382	9.554175	chr1:1522	CFL1P6		Pseudoger	chr1:10990978-1099
ENSG0000(	382	9.554175	chr1:1522	PIK3CD	NCGv7	protein_c	chr1:9629889-97291
ENSG0000(	382	9.554175	chr1:1522	ANGPTL7		protein_c	chr1:11189355-1119
ENSG0000(	382	9.554175	chr1:1522	PEX14		protein_c	chr1:10472288-1063
ENSG0000(	382	9.554175	chr1:1522	SCARNA16		smallRNA	chr1:9082696-90828
ENSG0000(	382	9.554175	chr1:1522	RNU6-291P		smallRNA	chr1:11226254-1122
ENSG0000(	382	9.554175	chr1:1522	HSPE1P24		Pseudoger	chr1:10895761-1089
ENSG0000(	382	9.554175	chr1:1522	ENSG00000289914		lncRNA	chr1:9162322-91629
ENSG0000(	382	9.554175	chr1:1522	UBE2V2P3		Pseudoger	chr1:11278616-1127
ENSG0000(	382	9.554175	chr1:1522	ENSG00000284735		lncRNA	chr1:10381906-1038
ENSG0000(	382	9.554175	chr1:1522	AL590639.1		Pseudoger	chr1:10018027-1001
ENSG0000(	382	9.554175	chr1:1522	ENSG00000280113		TEC	chr1:9826289-98282
ENSG0000(	382	9.554175	chr1:1522	RNU6-37P		smallRNA	chr1:10298966-1029
ENSG0000(	382	9.554175	chr1:1522	RPL39P6		Pseudoger	chr1:11232963-1123
ENSG0000(	382	9.554175	chr1:1522	SLC2A7		protein_c	chr1:9002973-90264
ENSG0000(	382	9.554175	chr1:1522	MASP2		protein_c	chr1:11022009-1104
ENSG0000(	382	9.554175	chr1:1522	ENSG00000284708		lncRNA	chr1:11623558-1164
ENSG0000(	382	9.554175	chr1:1522	ENSG00000290109		lncRNA	chr1:8970490-89712
ENSG0000(	382	9.554175	chr1:1522	LINC02606		lncRNA	chr1:9425094-94405
ENSG0000(	382	9.554175	chr1:1522	PIK3CD-AS1		lncRNA	chr1:9652610-96545
ENSG0000(	382	9.554175	chr1:1522	MAD2L2		protein_c	chr1:11658918-1169
ENSG0000(	382	9.554175	chr1:1522	FBXO2		protein_c	chr1:11637018-1165
ENSG0000(	382	9.554175	chr1:1522	SRM		protein_c	chr1:11054584-1106
ENSG0000(	382	9.554175	chr1:1522	ENSG00000284652		lncRNA	chr1:9421098-94228

ENSG0000(	382	9.554175	chr1:1522	ENSG00000284646		lncRNA	chr1:11311734-1131
ENSG0000(	382	9.554175	chr1:1522	ENSG00000284642		lncRNA	chr1:10395416-1039
ENSG0000(	382	9.554175	chr1:1522	GPR157		protein_c	chr1:9100305-91291
ENSG0000(	382	9.554175	chr1:1522	MTOR-AS1		lncRNA	chr1:11143898-1114
ENSG0000(	382	9.554175	chr1:1522	ENSG00000231181		Pseudoger	chr1:9576427-95769
ENSG0000(	382	9.554175	chr1:1522	DFFA		protein_c	chr1:10456522-1047
ENSG0000(	382	9.554175	chr1:1522	TMEM274P		Pseudoger	chr1:9950572-99607
ENSG0000(	382	9.554175	chr1:1522	MTOR	NCGv7;AC	protein_c	chr1:11106535-1126
ENSG0000(	382	9.554175	chr1:1522	ENSG00000285701		lncRNA	chr1:9900614-99080
ENSG0000(	382	9.554175	chr1:1522	H6PD		protein_c	chr1:9234774-92713
ENSG0000(	382	9.554175	chr1:1522	UBIAD1		protein_c	chr1:11273198-1129
ENSG0000(	382	9.554175	chr1:1522	Z98044.1		smallRNA	chr1:9278391-92785
ENSG0000(	382	9.554175	chr1:1522	TMEM201	NCGv7	protein_c	chr1:9588911-96148
ENSG0000(	382	9.554175	chr1:1522	snoU13		smallRNA	chr1:11169507-1116
ENSG0000(	382	9.554175	chr1:1522	ENSG00000271989		lncRNA	chr1:10429881-1043
ENSG0000(	382	9.554175	chr1:1522	CLSTN1		protein_c	chr1:9728926-98239
ENSG0000(	382	9.554175	chr1:1522	ENSG00000272078		lncRNA	chr1:10639241-1065
ENSG0000(	382	9.554175	chr1:1522	ENSG00000285833		lncRNA	chr1:11500803-1150
ENSG0000(	382	9.554175	chr1:1522	KIF1B	NCGv7	protein_c	chr1:10210570-1038
ENSG0000(	382	9.554175	chr1:1522	TARDBP		protein_c	chr1:11012344-1103
ENSG0000(	382	9.554175	chr1:1522	NMNAT1		protein_c	chr1:9943428-99855
ENSG0000(	382	9.554175	chr1:1522	CTNNBIP1		protein_c	chr1:9848276-99103
ENSG0000(	380	9.504153	chr2:4707	RN7SL140P		smallRNA	chr2:20175346-2017
ENSG0000(	379	9.479142	chr12:685	ENSG00000257512		Pseudoger	chr12:93314809-933
ENSG0000(	377	9.42912	chr2:7442	RPL27AP4		Pseudoger	chr2:106304755-106
ENSG0000(	374	9.354088	chr4:5048	ENSG00000213480		Pseudoger	chr4:121369433-121
ENSG0000(	373	9.329077	chr22:227	IGLVVI-22-1		Pseudoger	chr22:22700760-227
ENSG0000(	364	9.103978	chr12:583	PRELID2P1		Pseudoger	chr12:68957377-689
ENSG0000(	363	9.078967	chr2:7442	RNA5SP99		Pseudoger	chr2:81496214-8149
ENSG0000(	360	9.003934	chr2:3496	ENSG00000271868		lncRNA	chr2:3496956-34974
ENSG0000(	359	8.978924	chr2:4707	RNU6-1048P		smallRNA	chr2:43892690-4389
ENSG0000(	355	8.87888	chr6:105(	RIPOR2	NCGv7	protein_c	chr6:24804282-2504
ENSG0000(	355	8.87888	chr6:105(	ENSG00000282804		protein_c	chr6:24797373-2480
ENSG0000(	355	8.87888	chr6:105(	RNU6-391P		smallRNA	chr6:24365651-2436
ENSG0000(	355	8.87888	chr6:105(	ARMH2		protein_c	chr6:24797335-2479
ENSG0000(	355	8.87888	chr6:105(	ACOT13		protein_c	chr6:24667035-2470
ENSG0000(	355	8.87888	chr6:105(	KIAA0319		protein_c	chr6:24544104-2464
ENSG0000(	355	8.87888	chr6:105(	KRT8P43		Pseudoger	chr6:24598012-2459
ENSG0000(	355	8.87888	chr6:105(	TDP2		protein_c	chr6:24649979-2466
ENSG0000(	355	8.87888	chr6:105(	AL590084.1		smallRNA	chr6:25203253-2520
ENSG0000(	355	8.87888	chr6:105(	ENSG00000278128		Pseudoger	chr6:23854444-2385
ENSG0000(	355	8.87888	chr6:105(	HDGFL1		protein_c	chr6:22569566-2257
ENSG0000(	355	8.87888	chr6:105(	C6orf62	NCGv7	protein_c	chr6:24704861-2471
ENSG0000(	355	8.87888	chr6:105(	ENSG00000288887		lncRNA	chr6:24936045-2495
ENSG0000(	355	8.87888	chr6:105(	ENSG00000288851		lncRNA	chr6:24720322-2472
ENSG0000(	355	8.87888	chr6:105(	GMNN	AC	protein_c	chr6:24774931-2478
ENSG0000(	355	8.87888	chr6:105(	ENSG00000216718		Pseudoger	chr6:25261239-2526
ENSG0000(	355	8.87888	chr6:105(	LINC02828		lncRNA	chr6:24721658-2475
ENSG0000(	355	8.87888	chr6:105(	ALDH5A1	NCGv7	protein_c	chr6:24494867-2453
ENSG0000(	355	8.87888	chr6:105(	MTCO2P33		Pseudoger	chr6:24947880-2494
ENSG0000(	355	8.87888	chr6:105(	AL512428.1		smallRNA	chr6:24839967-2484

ENSG0000(	355	8.87888	chr6:105(	GPLD1		protein_c	chr6:24424565-2449
ENSG0000(	355	8.87888	chr6:105(	ENSG00000262400		Pseudoger	chr6:25152547-2515
ENSG0000(	355	8.87888	chr6:105(	ENSG00000217805		Pseudoger	chr6:25181359-2518
ENSG0000(	355	8.87888	chr6:105(	ASS1P1		Pseudoger	chr6:25023247-2502
ENSG0000(	355	8.87888	chr6:105(	SNORD46		smallRNA	chr6:24166273-2416
ENSG0000(	355	8.87888	chr6:105(	ENSG00000219681		Pseudoger	chr6:25218688-2521
ENSG0000(	355	8.87888	chr6:105(	ENSG00000229313		lncRNA	chr6:25041839-2505
ENSG0000(	355	8.87888	chr6:105(	KAAG1		lncRNA	chr6:24356903-2435
ENSG0000(	355	8.87888	chr6:105(	ENSG00000285801		lncRNA	chr6:25061796-2513
ENSG0000(	355	8.87888	chr6:105(	ENSG00000228772		lncRNA	chr6:22589137-2259
ENSG0000(	355	8.87888	chr6:105(	ENSG00000286633		lncRNA	chr6:25012985-2501
ENSG0000(	355	8.87888	chr6:105(	HNRNPA1P58		Pseudoger	chr6:24001824-2400
ENSG0000(	355	8.87888	chr6:105(	ENSG00000219682		Pseudoger	chr6:25140003-2514
ENSG0000(	355	8.87888	chr6:105(	DCDC2		protein_c	chr6:24171755-2435
ENSG0000(	355	8.87888	chr6:105(	ENSG00000272402		lncRNA	chr6:24706747-2470
ENSG0000(	355	8.87888	chr6:105(	ENSG00000272345		lncRNA	chr6:24700907-2470
ENSG0000(	355	8.87888	chr6:105(	MRS2	DriverDB\	protein_c	chr6:24402908-2442
ENSG0000(	355	8.87888	chr6:105(	ENSG00000220748		Pseudoger	chr6:23971879-2397
ENSG0000(	355	8.87888	chr6:105(	KATNBL1P5		Pseudoger	chr6:25248263-2524
ENSG0000(	355	8.87888	chr16:65(	AC023818.1		smallRNA	chr16:48421127-484
ENSG0000(	355	8.87888	chr6:105(	NRSN1		protein_c	chr6:24126186-2415
ENSG0000(	355	8.87888	chr6:105(	ENSG00000219453		Pseudoger	chr6:23649496-2364
ENSG0000(	355	8.87888	chr6:105(	RPL6P18		Pseudoger	chr6:23102680-2310
ENSG0000(	355	8.87888	chr6:105(	ENSG00000286954		lncRNA	chr6:22663507-2267
ENSG0000(	355	8.87888	chr6:105(	Y_RNA		smallRNA	chr6:25287432-2528
ENSG0000(	355	8.87888	chr6:105(	ENSG00000213972		Pseudoger	chr6:25272200-2527
ENSG0000(	355	8.87888	chr6:105(	SPTLC1P2		Pseudoger	chr6:23856698-2385
ENSG0000(	355	8.87888	chr6:105(	RN7SL334P		smallRNA	chr6:25031015-2503
ENSG0000(	355	8.87888	chr6:105(	ENSG00000290589		lncRNA	chr6:23854139-2385
ENSG0000(	355	8.87888	chr6:105(	ENSG00000218806		Pseudoger	chr6:24750671-2475
ENSG0000(	355	8.87888	chr6:105(	RNY5P5		smallRNA	chr6:25192413-2519
ENSG0000(	355	8.87888	chr6:105(	PPIAP29		Pseudoger	chr6:24976419-2497
ENSG0000(	355	8.87888	chr6:105(	ENSG00000289368		lncRNA	chr6:23344699-2358
ENSG0000(	355	8.87888	chr6:105(	ENSG00000223623		lncRNA	chr6:25053627-2505
ENSG0000(	355	8.87888	chr6:105(	RNU6-1060P		smallRNA	chr6:23124981-2312
ENSG0000(	355	8.87888	chr6:105(	ENSG00000233358		lncRNA	chr6:22744395-2303
ENSG0000(	355	8.87888	chr6:105(	PRL		protein_c	chr6:22287244-2230
ENSG0000(	355	8.87888	chr6:105(	ENSG00000235743		lncRNA	chr6:23337711-2340
ENSG0000(	354	8.853869	chr16:65(	ENSG00000277999		lncRNA	chr16:29272220-292
ENSG0000(	352	8.803847	chr19:10:	ENSG00000279753		TEC	chr19:1038727-1039
ENSG0000(	350	8.753825	chr17:70!	MIR4723		smallRNA	chr17:28360654-283
ENSG0000(	347	8.678792	chr6:105(	H4C4	NCGv7	protein_c	chr6:26188710-2618
ENSG0000(	346	8.653781	chr12:12(	RPL35AP28		Pseudoger	chr12:49863173-498
ENSG0000(	344	8.60376	chr22:22:	IGLV7-43		protein_c	chr22:22395018-223
ENSG0000(	343	8.578749	chr1:373	RNA5SP79		Pseudoger	chr1:230820250-230
ENSG0000(	343	8.578749	chr1:373	ENSG00000244137		lncRNA	chr1:230710698-230
ENSG0000(	343	8.578749	chr1:373	TRIM67		protein_c	chr1:231162058-231
ENSG0000(	343	8.578749	chr1:406	RN7SL148P		smallRNA	chr1:244103932-244
ENSG0000(	343	8.578749	chr1:373	RNU6-1211P		smallRNA	chr1:232700204-232
ENSG0000(	343	8.578749	chr1:373	FAM89A		protein_c	chr1:231018958-231
ENSG0000(	343	8.578749	chr1:373	LINC01745		lncRNA	chr1:232718071-232

ENSG0000(	343	8.578749	chr1:3732	MAP10		protein_c	chr1:232805416-232
ENSG0000(	343	8.578749	chr1:3732	HMGB1P26		Pseudoger	chr1:229705234-229
ENSG0000(	343	8.578749	chr1:3732	C1orf198		protein_c	chr1:230837119-230
ENSG0000(	343	8.578749	chr1:3732	ENSG00000286071		lncRNA	chr1:231767464-231
ENSG0000(	343	8.578749	chr1:3732	RNU5A-5P		smallRNA	chr1:231670635-231
ENSG0000(	343	8.578749	chr1:3732	snoU13		smallRNA	chr1:230895432-230
ENSG0000(	343	8.578749	chr1:3732	ENSG00000235817		Pseudoger	chr1:230612009-230
ENSG0000(	343	8.578749	chr1:3732	ENSG00000251508		Pseudoger	chr1:233295325-233
ENSG0000(	343	8.578749	chr1:3732	TRIM67-AS1		lncRNA	chr1:231184098-231
ENSG0000(	343	8.578749	chr1:3732	ENSG00000235152		lncRNA	chr1:232160091-232
ENSG0000(	343	8.578749	chr1:3732	TSNAX-DISC1		protein_c	chr1:231528653-231
ENSG0000(	343	8.578749	chr1:3732	NUP133-DT		lncRNA	chr1:229508369-229
ENSG0000(	343	8.578749	chr1:3732	GNPAT		protein_c	chr1:231241207-231
ENSG0000(	343	8.578749	chr1:3732	SIPA1L2	DriverDBv	protein_c	chr1:232397965-232
ENSG0000(	343	8.578749	chr1:3732	ENSG00000236372		lncRNA	chr1:232174932-232
ENSG0000(	343	8.578749	chr1:3732	ENSG00000223393		lncRNA	chr1:230868259-230
ENSG0000(	343	8.578749	chr1:3732	RNU4-21P		smallRNA	chr1:229535064-229
ENSG0000(	343	8.578749	chr1:3732	LINC01744		lncRNA	chr1:232727251-232
ENSG0000(	343	8.578749	chr1:3732	SPRTN		protein_c	chr1:231337104-231
ENSG0000(	343	8.578749	chr1:3732	ARV1		protein_c	chr1:230978981-231
ENSG0000(	343	8.578749	chr1:3732	AL844165.1		smallRNA	chr1:230988989-230
ENSG0000(	343	8.578749	chr1:3732	ENSG00000282564		lncRNA	chr1:230426491-230
ENSG0000(	343	8.578749	chr1:3732	LINC01737		lncRNA	chr1:230592660-230
ENSG0000(	343	8.578749	chr1:3732	TSNAX		protein_c	chr1:231528541-231
ENSG0000(	343	8.578749	chr1:3732	ENSG00000225656		lncRNA	chr1:230823641-230
ENSG0000(	343	8.578749	chr1:3732	ENSG00000229595		Pseudoger	chr1:231117831-231
ENSG0000(	343	8.578749	chr1:3732	ENSG00000213028		Pseudoger	chr1:229688999-229
ENSG0000(	343	8.578749	chr1:3732	HMGN2P19		Pseudoger	chr1:229570532-229
ENSG0000(	343	8.578749	chr1:3732	EXOC8	NCGv7	protein_c	chr1:231332753-231
ENSG0000(	343	8.578749	chr1:3732	MIR1182		smallRNA	chr1:231019828-231
ENSG0000(	343	8.578749	chr1:3732	ENSG00000287395		lncRNA	chr1:230889949-230
ENSG0000(	343	8.578749	chr1:3732	ENSG00000224436		Pseudoger	chr1:232221938-232
ENSG0000(	343	8.578749	chr1:3732	ENSG00000286774		lncRNA	chr1:232843386-232
ENSG0000(	343	8.578749	chr1:3732	ENSG00000224407		lncRNA	chr1:230280312-230
ENSG0000(	343	8.578749	chr1:3732	TAF5L		protein_c	chr1:229593134-229
ENSG0000(	343	8.578749	chr1:3732	RN7SL837P		smallRNA	chr1:230894141-230
ENSG0000(	343	8.578749	chr1:3732	AGT	DriverDBv	protein_c	chr1:230690776-230
ENSG0000(	343	8.578749	chr1:3732	RN7SL299P		smallRNA	chr1:232222866-232
ENSG0000(	343	8.578749	chr1:3732	RNU1-74P		smallRNA	chr1:232832017-232
ENSG0000(	343	8.578749	chr1:3732	LINC01682		lncRNA	chr1:229812917-229
ENSG0000(	343	8.578749	chr1:3732	SNRPD2P2		Pseudoger	chr1:231475956-231
ENSG0000(	343	8.578749	chr1:3732	RPS7P3		Pseudoger	chr1:233288868-233
ENSG0000(	343	8.578749	chr1:3732	LINC00582		lncRNA	chr1:231591292-231
ENSG0000(	343	8.578749	chr1:3732	ENSG00000227934		Pseudoger	chr1:231021611-231
ENSG0000(	343	8.578749	chr1:3732	ENSG00000227006		lncRNA	chr1:230258694-230
ENSG0000(	343	8.578749	chr1:3732	RN7SL467P		smallRNA	chr1:230729880-230
ENSG0000(	343	8.578749	chr1:3732	BX323860.1		smallRNA	chr1:230049182-230
ENSG0000(	343	8.578749	chr1:3732	ENSG00000287856		protein_c	chr1:231363797-231
ENSG0000(	343	8.578749	chr1:3732	MAP3K21	NCGv7	protein_c	chr1:233327724-233
ENSG0000(	343	8.578749	chr1:3732	TTC13		protein_c	chr1:230906243-230
ENSG0000(	343	8.578749	chr1:3732	GALNT2		protein_c	chr1:230057990-230

ENSG0000(	343	8.578749	chr1:3732	C1orf131		protein_c	chr1:231223763-231
ENSG0000(	343	8.578749	chr1:3732	ENSG00000288037		lncRNA	chr1:230878662-230
ENSG0000(	343	8.578749	chr1:3732	DISC1-IT1		lncRNA	chr1:231925834-231
ENSG0000(	343	8.578749	chr1:3732	RNA5SP78		Pseudoger	chr1:229549905-229
ENSG0000(	343	8.578749	chr1:3732	URB2	NCGv7	protein_c	chr1:229626247-229
ENSG0000(	343	8.578749	chr1:3732	PGBD5		protein_c	chr1:230314490-230
ENSG0000(	343	8.578749	chr1:3732	ENSG00000233461		lncRNA	chr1:231520729-231
ENSG0000(	343	8.578749	chr1:3732	ENSG00000232175		Pseudoger	chr1:232917235-232
ENSG0000(	343	8.578749	chr1:3732	PCNX2		protein_c	chr1:232983435-233
ENSG0000(	343	8.578749	chr1:3732	ENSG00000287450		Pseudoger	chr1:231339421-231
ENSG0000(	343	8.578749	chr1:3732	LINC01736		lncRNA	chr1:230002372-230
ENSG0000(	343	8.578749	chr1:3732	CAPN9	DriverDBv	protein_c	chr1:230747384-230
ENSG0000(	343	8.578749	chr1:3732	EGLN1		protein_c	chr1:231363751-231
ENSG0000(	343	8.578749	chr1:3732	ABCB10		protein_c	chr1:229516582-229
ENSG0000(	343	8.578749	chr1:3732	DISC1		protein_c	chr1:231626790-232
ENSG0000(	343	8.578749	chr1:3732	COG2		protein_c	chr1:230642481-230
ENSG0000(	343	8.578749	chr1:3732	NTPCR		protein_c	chr1:232950605-232
ENSG0000(	334	8.35365	chr2:7442	MIR4780		smallRNA	chr2:88082519-8808
ENSG0000(	333	8.328639	chr2:7442	IGKV1OR2-6		Pseudoger	chr2:97355058-9735
ENSG0000(	333	8.328639	chr2:7442	FAHD2B	NCGv7	protein_c	chr2:97083583-9709
ENSG0000(	333	8.328639	chr2:7442	ENSG00000277701		lncRNA	chr2:97281356-9729
ENSG0000(	333	8.328639	chr2:7442	IGKV2OR2-2		Pseudoger	chr2:97050729-9705
ENSG0000(	333	8.328639	chr2:7442	RN7SL313P		smallRNA	chr2:97100584-9710
ENSG0000(	333	8.328639	chr2:7442	GPAT2P2		Pseudoger	chr2:97081098-9708
ENSG0000(	333	8.328639	chr2:7442	IGKV2OR2-7D		Pseudoger	chr2:97335671-9733
ENSG0000(	333	8.328639	chr2:7442	IGKV2OR2-10		Pseudoger	chr2:97331533-9733
ENSG0000(	333	8.328639	chr2:7442	IGKV2OR2-1		Pseudoger	chr2:97046588-9704
ENSG0000(	333	8.328639	chr2:7442	IGKV1OR2-3		Pseudoger	chr2:97060128-9706
ENSG0000(	333	8.328639	chr15:695	ENSG00000288604		Pseudoger	chr15:79923142-799
ENSG0000(	333	8.328639	chr2:7442	ENSG00000279791		TEC	chr2:97094935-9709
ENSG0000(	333	8.328639	chr2:7442	IGKV1OR2-11		Pseudoger	chr2:97322137-9732
ENSG0000(	333	8.328639	chr2:7442	ANKRD36	NCGv7	protein_c	chr2:97113153-9726
ENSG0000(	333	8.328639	chr2:7442	IGKV3OR2-5		Pseudoger	chr2:97348898-9734
ENSG0000(	332	8.303628	chr15:695	RN7SL214P		smallRNA	chr15:77916723-779
ENSG0000(	327	8.178574	chr3:8573	BFSP2		protein_c	chr3:133400056-133
ENSG0000(	327	8.178574	chr3:8573	ENSG00000287617		lncRNA	chr3:125799887-125
ENSG0000(	327	8.178574	chr3:8573	LSAMP	NCGv7	protein_c	chr3:115802363-117
ENSG0000(	327	8.178574	chr3:8573	SOX14		protein_c	chr3:137764315-137
ENSG0000(	327	8.178574	chr3:8573	GCSAM		protein_c	chr3:112120839-112
ENSG0000(	327	8.178574	chr3:8573	SNRPCP11		Pseudoger	chr3:125816082-125
ENSG0000(	327	8.178574	chr3:8573	TMEM39A		protein_c	chr3:119428949-119
ENSG0000(	327	8.178574	chr3:8573	ENSG00000289358		lncRNA	chr3:109337740-109
ENSG0000(	327	8.178574	chr3:8573	ZBTB20-AS2		lncRNA	chr3:114684580-114
ENSG0000(	327	8.178574	chr3:8573	ENSG00000272967		lncRNA	chr3:119579212-119
ENSG0000(	327	8.178574	chr3:8573	TMPRSS7		protein_c	chr3:112034736-112
ENSG0000(	327	8.178574	chr3:8573	ROPN1		protein_c	chr3:123968521-123
ENSG0000(	327	8.178574	chr3:8573	SEC22A		protein_c	chr3:123201927-123
ENSG0000(	327	8.178574	chr3:8573	TOPBP1		protein_c	chr3:133598175-133
ENSG0000(	327	8.178574	chr3:8573	CSTA		protein_c	chr3:122325248-122
ENSG0000(	327	8.178574	chr3:8573	LINC02614		lncRNA	chr3:125827238-125
ENSG0000(	327	8.178574	chr3:8573	ENPP7P4		Pseudoger	chr3:125848223-125

ENSG0000(	327	8.178574	chr3:857:	ENSG00000289351		lncRNA	chr3:129230545-129
ENSG0000(	327	8.178574	chr3:857:	DPPA4	NCGv7;AC	protein_c	chr3:109326144-109
ENSG0000(	327	8.178574	chr3:857:	ENSG00000241257		Pseudoger	chr3:109364047-109
ENSG0000(	327	8.178574	chr3:857:	MIR1280		smallRNA	chr3:128362165-128
ENSG0000(	327	8.178574	chr3:857:	AC072031.1		smallRNA	chr3:121922098-121
ENSG0000(	327	8.178574	chr3:857:	LINC02035		lncRNA	chr3:122886941-122
ENSG0000(	327	8.178574	chr3:857:	MIR4788		smallRNA	chr3:134437827-134
ENSG0000(	327	8.178574	chr3:857:	AC083906.1		smallRNA	chr3:130033458-130
ENSG0000(	327	8.178574	chr3:857:	AC117401.1		smallRNA	chr3:124061994-124
ENSG0000(	327	8.178574	chr3:857:	CCDC191	DriverDB\	protein_c	chr3:113964137-114
ENSG0000(	327	8.178574	chr3:857:	RN7SL767P		smallRNA	chr3:113632704-113
ENSG0000(	327	8.178574	chr3:857:	PLS1	DriverDB\	protein_c	chr3:142596393-142
ENSG0000(	327	8.178574	chr3:857:	HMGB3P14		Pseudoger	chr3:134170487-134
ENSG0000(	327	8.178574	chr3:857:	ENSG00000273174		lncRNA	chr3:129123439-129
ENSG0000(	327	8.178574	chr3:857:	ENSG00000241526		Pseudoger	chr3:141724425-141
ENSG0000(	327	8.178574	chr3:857:	LINC01990		lncRNA	chr3:107430892-107
ENSG0000(	327	8.178574	chr3:857:	AC097103.1		smallRNA	chr3:139494618-139
ENSG0000(	327	8.178574	chr3:857:	ENSG00000273123		lncRNA	chr3:123715851-123
ENSG0000(	327	8.178574	chr3:857:	OR7E21P		Pseudoger	chr3:130034553-130
ENSG0000(	327	8.178574	chr3:857:	ENSG00000241490		lncRNA	chr3:114214313-114
ENSG0000(	327	8.178574	chr3:857:	ENSG00000289469		lncRNA	chr3:129184058-129
ENSG0000(	327	8.178574	chr3:857:	ENSG00000250983		Pseudoger	chr3:133546071-133
ENSG0000(	327	8.178574	chr3:857:	HSPA8P9		Pseudoger	chr3:137880295-137
ENSG0000(	327	8.178574	chr3:857:	YWHAQP6		Pseudoger	chr3:141600276-141
ENSG0000(	327	8.178574	chr3:857:	LINC00635		lncRNA	chr3:107840228-107
ENSG0000(	327	8.178574	chr3:857:	ENSG00000241439		Pseudoger	chr3:125958556-125
ENSG0000(	327	8.178574	chr3:857:	TDGF1P6		Pseudoger	chr3:136155549-136
ENSG0000(	327	8.178574	chr3:857:	EEF1A1P25		Pseudoger	chr3:138825063-138
ENSG0000(	327	8.178574	chr3:857:	ENSG00000241400		Pseudoger	chr3:141258418-141
ENSG0000(	327	8.178574	chr3:857:	LINC00903		lncRNA	chr3:116552473-116
ENSG0000(	327	8.178574	chr3:857:	POPDC2		protein_c	chr3:119636457-119
ENSG0000(	327	8.178574	chr3:857:	AC080008.1		smallRNA	chr3:124696938-124
ENSG0000(	327	8.178574	chr3:857:	SPICE1		protein_c	chr3:113442718-113
ENSG0000(	327	8.178574	chr3:857:	RYK	TAG	protein_c	chr3:134065303-134
ENSG0000(	327	8.178574	chr3:857:	CLDN18		protein_c	chr3:137998735-138
ENSG0000(	327	8.178574	chr3:857:	PARP9		protein_c	chr3:122527924-122
ENSG0000(	327	8.178574	chr3:857:	COX17		protein_c	chr3:119654513-119
ENSG0000(	327	8.178574	chr3:857:	CCDC54		protein_c	chr3:107377439-107
ENSG0000(	327	8.178574	chr3:857:	GUCA1C		protein_c	chr3:108907792-108
ENSG0000(	327	8.178574	chr3:857:	ENSG00000240895		lncRNA	chr3:110527482-110
ENSG0000(	327	8.178574	chr3:857:	SLC49A4		protein_c	chr3:122795069-122
ENSG0000(	327	8.178574	chr3:857:	SLC35A5	NCGv7	protein_c	chr3:112561709-112
ENSG0000(	327	8.178574	chr3:857:	ENSG00000203644		lncRNA	chr3:129847048-129
ENSG0000(	327	8.178574	chr3:857:	MIR5682		smallRNA	chr3:121049640-121
ENSG0000(	327	8.178574	chr3:857:	ZNF148	IntOGen-I	protein_c	chr3:125225669-125
ENSG0000(	327	8.178574	chr3:857:	ENSG00000272844		lncRNA	chr3:112990447-112
ENSG0000(	327	8.178574	chr3:857:	NMNAT3	DriverDB\	protein_c	chr3:139560180-139
ENSG0000(	327	8.178574	chr3:857:	LINC02042		lncRNA	chr3:112736447-112
ENSG0000(	327	8.178574	chr3:857:	TPRA1		protein_c	chr3:127571232-127
ENSG0000(	327	8.178574	chr3:857:	DNAJC13		protein_c	chr3:132417502-132
ENSG0000(	327	8.178574	chr3:857:	DBR1	NCGv7	protein_c	chr3:138160988-138

ENSG0000(	327	8.178574	chr3:857:	PLCXD2		protein_c	chr3:111674676-111
ENSG0000(	327	8.178574	chr3:857:	ENSG00000240890		Pseudoger	chr3:132386522-132
ENSG0000(	327	8.178574	chr3:857:	GPR156		protein_c	chr3:120164645-120
ENSG0000(	327	8.178574	chr3:857:	ENSG00000240882		Pseudoger	chr3:120306726-120
ENSG0000(	327	8.178574	chr3:857:	H2BP3		Pseudoger	chr3:114103249-114
ENSG0000(	327	8.178574	chr3:857:	NAP1L1P3		Pseudoger	chr3:120805866-120
ENSG0000(	327	8.178574	chr3:857:	LSAMP-AS1		lncRNA	chr3:116360024-116
ENSG0000(	327	8.178574	chr3:857:	RUVBL1		protein_c	chr3:128064778-128
ENSG0000(	327	8.178574	chr3:857:	C3orf85		protein_c	chr3:109118252-109
ENSG0000(	327	8.178574	chr3:857:	PDIA5		protein_c	chr3:123067025-123
ENSG0000(	327	8.178574	chr3:857:	AC092988.1		smallRNA	chr3:140403420-140
ENSG0000(	327	8.178574	chr3:857:	ENSG00000241219		lncRNA	chr3:113050912-113
ENSG0000(	327	8.178574	chr3:857:	CSP2		Pseudoger	chr3:107327830-107
ENSG0000(	327	8.178574	chr3:857:	B4GALT4	DriverDB\	protein_c	chr3:119211732-119
ENSG0000(	327	8.178574	chr3:857:	NDUFB4		protein_c	chr3:120596328-120
ENSG0000(	327	8.178574	chr3:857:	LINC02024		lncRNA	chr3:117678693-117
ENSG0000(	327	8.178574	chr3:857:	RN7SL582P		smallRNA	chr3:116582554-116
ENSG0000(	327	8.178574	chr3:857:	MYLK	NCGv7	protein_c	chr3:123610049-123
ENSG0000(	327	8.178574	chr3:857:	ARGFX		protein_c	chr3:121567949-121
ENSG0000(	327	8.178574	chr3:857:	ARHGAP31-AS1		lncRNA	chr3:119314293-119
ENSG0000(	327	8.178574	chr3:857:	FBXO40	NCGv7	protein_c	chr3:121593379-121
ENSG0000(	327	8.178574	chr3:857:	NAA50		protein_c	chr3:113716458-113
ENSG0000(	327	8.178574	chr3:857:	PIK3R4	NCGv7	protein_c	chr3:130678934-130
ENSG0000(	327	8.178574	chr3:857:	ENSG00000289324		lncRNA	chr3:125375372-125
ENSG0000(	327	8.178574	chr3:857:	MIR567		smallRNA	chr3:112112801-112
ENSG0000(	327	8.178574	chr3:857:	RNU6-726P		smallRNA	chr3:131092821-131
ENSG0000(	327	8.178574	chr3:857:	DTX3L		protein_c	chr3:122564338-122
ENSG0000(	327	8.178574	chr3:857:	CD80		protein_c	chr3:119524293-119
ENSG0000(	327	8.178574	chr3:857:	ENSG00000241546		Pseudoger	chr3:120041190-120
ENSG0000(	327	8.178574	chr3:857:	Y_RNA		smallRNA	chr3:110727021-110
ENSG0000(	327	8.178574	chr3:857:	ZDHHC23	DriverDB\	protein_c	chr3:113947901-113
ENSG0000(	327	8.178574	chr3:857:	ENSG00000261826		lncRNA	chr3:140865075-140
ENSG0000(	327	8.178574	chr3:857:	FSTL1		protein_c	chr3:120392293-120
ENSG0000(	327	8.178574	chr3:857:	ENSG00000248850		Pseudoger	chr3:126672106-126
ENSG0000(	327	8.178574	chr3:857:	USF3		protein_c	chr3:113648385-113
ENSG0000(	327	8.178574	chr3:857:	ENSG00000287366		lncRNA	chr3:120833936-120
ENSG0000(	327	8.178574	chr3:857:	NPM1P17		Pseudoger	chr3:137723774-137
ENSG0000(	327	8.178574	chr3:857:	MARK2P6		Pseudoger	chr3:128852112-128
ENSG0000(	327	8.178574	chr3:857:	RPL10P7		Pseudoger	chr3:119635526-119
ENSG0000(	327	8.178574	chr3:857:	DNAJB8-AS1		lncRNA	chr3:128463594-128
ENSG0000(	327	8.178574	chr3:857:	NUP210P3		Pseudoger	chr3:129323046-129
ENSG0000(	327	8.178574	chr3:857:	IL20RB-AS1		lncRNA	chr3:136959125-136
ENSG0000(	327	8.178574	chr3:857:	CIP2A	AC	protein_c	chr3:108549864-108
ENSG0000(	327	8.178574	chr3:857:	CNBP	NCGv7	protein_c	chr3:129167827-129
ENSG0000(	327	8.178574	chr3:857:	CCDC80		protein_c	chr3:112596797-112
ENSG0000(	327	8.178574	chr3:857:	RETNLB		protein_c	chr3:108743424-108
ENSG0000(	327	8.178574	chr3:857:	ENSG00000242029		lncRNA	chr3:109648107-109
ENSG0000(	327	8.178574	chr3:857:	ENSG00000289742		Pseudoger	chr3:112896631-112
ENSG0000(	327	8.178574	chr3:857:	AC092905.1		smallRNA	chr3:109497645-109
ENSG0000(	327	8.178574	chr3:857:	POLQ	NCGv7	protein_c	chr3:121431431-121
ENSG0000(	327	8.178574	chr3:857:	TRAT1		protein_c	chr3:108822770-108

ENSG0000(	327	8.178574	chr3:857:	ENSG00000242001		Pseudoger	chr3:125681305-125
ENSG0000(	327	8.178574	chr3:857:	PRR23B		protein_c	chr3:139019031-139
ENSG0000(	327	8.178574	chr3:857:	LRRC58		protein_c	chr3:120324509-120
ENSG0000(	327	8.178574	chr3:857:	ENSG00000250129		lncRNA	chr3:131053317-131
ENSG0000(	327	8.178574	chr3:857:	TEX55		protein_c	chr3:119146151-119
ENSG0000(	327	8.178574	chr3:857:	ENSG00000249417		lncRNA	chr3:141267353-141
ENSG0000(	327	8.178574	chr3:857:	ENSG00000287232		lncRNA	chr3:126213204-126
ENSG0000(	327	8.178574	chr3:857:	NECTIN3-AS1		lncRNA	chr3:110888384-111
ENSG0000(	327	8.178574	chr3:857:	RPS3AP14		Pseudoger	chr3:125795106-125
ENSG0000(	327	8.178574	chr3:857:	NPHP3-ACAD11		protein_c	chr3:132558142-132
ENSG0000(	327	8.178574	chr3:857:	COPB2	NCGv7	protein_c	chr3:139353946-139
ENSG0000(	327	8.178574	chr3:857:	RUVBL1-AS1		lncRNA	chr3:128075810-128
ENSG0000(	327	8.178574	chr3:857:	MTND3P6		Pseudoger	chr3:106896483-106
ENSG0000(	327	8.178574	chr3:857:	ENSG00000242199		Pseudoger	chr3:124733418-124
ENSG0000(	327	8.178574	chr3:857:	ZNF80		protein_c	chr3:114234631-114
ENSG0000(	327	8.178574	chr3:857:	RNU6-1200P		smallRNA	chr3:117544205-117
ENSG0000(	327	8.178574	chr3:857:	AC048346.1		smallRNA	chr3:140490052-140
ENSG0000(	327	8.178574	chr3:857:	SLC15A2	NCGv7	protein_c	chr3:121894401-121
ENSG0000(	327	8.178574	chr3:857:	ENSG00000251270		lncRNA	chr3:141115124-141
ENSG0000(	327	8.178574	chr3:857:	LINC02618		Pseudoger	chr3:141660536-141
ENSG0000(	327	8.178574	chr3:857:	WDR5B		protein_c	chr3:122411846-122
ENSG0000(	327	8.178574	chr3:857:	RNA5SP142		Pseudoger	chr3:137518134-137
ENSG0000(	327	8.178574	chr3:857:	AC078794.1		smallRNA	chr3:122926996-122
ENSG0000(	327	8.178574	chr3:857:	ENSG00000242103		Pseudoger	chr3:121356991-121
ENSG0000(	327	8.178574	chr3:857:	TRMT112P5		Pseudoger	chr3:139845078-139
ENSG0000(	327	8.178574	chr3:857:	RNU6ATAC15P		smallRNA	chr3:110551845-110
ENSG0000(	327	8.178574	chr3:857:	PIK3CB	NCGv7	protein_c	chr3:138652698-138
ENSG0000(	327	8.178574	chr3:857:	RNU6-425P		smallRNA	chr3:142145454-142
ENSG0000(	327	8.178574	chr3:857:	CD200	NCGv7	protein_c	chr3:112332347-112
ENSG0000(	327	8.178574	chr3:857:	ENSG00000241634		Pseudoger	chr3:108543367-108
ENSG0000(	327	8.178574	chr3:857:	ENSG00000289632		lncRNA	chr3:140488639-140
ENSG0000(	327	8.178574	chr3:857:	TBILA		lncRNA	chr3:112133423-112
ENSG0000(	327	8.178574	chr3:857:	TF		protein_c	chr3:133746040-133
ENSG0000(	327	8.178574	chr3:857:	NIP7P2		Pseudoger	chr3:132401253-132
ENSG0000(	327	8.178574	chr3:857:	SEC61A1		protein_c	chr3:128051641-128
ENSG0000(	327	8.178574	chr3:857:	RNU6-678P		smallRNA	chr3:133664935-133
ENSG0000(	327	8.178574	chr3:857:	RPS27P12		Pseudoger	chr3:129218093-129
ENSG0000(	327	8.178574	chr3:857:	RNA5SP141		Pseudoger	chr3:134783436-134
ENSG0000(	327	8.178574	chr3:857:	RNA5SP137		Pseudoger	chr3:125058304-125
ENSG0000(	327	8.178574	chr3:857:	snoU13		smallRNA	chr3:122750372-122
ENSG0000(	327	8.178574	chr3:857:	MTND4P16		Pseudoger	chr3:106894352-106
ENSG0000(	327	8.178574	chr3:857:	STAG1-DT		lncRNA	chr3:136752630-136
ENSG0000(	327	8.178574	chr3:857:	ENSG00000287421		lncRNA	chr3:106160417-106
ENSG0000(	327	8.178574	chr3:857:	ENSG00000261763		lncRNA	chr3:139678620-139
ENSG0000(	327	8.178574	chr3:857:	ENSG00000250934		lncRNA	chr3:126266747-126
ENSG0000(	327	8.178574	chr3:857:	ENSG00000241596		lncRNA	chr3:115658533-115
ENSG0000(	327	8.178574	chr3:857:	ISY1-RAB43		protein_c	chr3:129087575-129
ENSG0000(	327	8.178574	chr3:857:	ENSG00000287440		lncRNA	chr3:126312385-126
ENSG0000(	327	8.178574	chr3:857:	NEPRO		protein_c	chr3:113002444-113
ENSG0000(	327	8.178574	chr3:857:	ZBTB20-AS1		lncRNA	chr3:114351771-114
ENSG0000(	327	8.178574	chr3:857:	ENSG00000241777		Pseudoger	chr3:108725440-108

ENSG0000(	327	8.178574	chr3:857:	GTPBP8		protein_c	chr3:112990984-113
ENSG0000(	327	8.178574	chr3:857:	RNU6-1308P		smallRNA	chr3:107007568-107
ENSG0000(	327	8.178574	chr3:857:	NDUFS6P1		Pseudoger	chr3:135959275-135
ENSG0000(	327	8.178574	chr3:857:	CD47		protein_c	chr3:108043091-108
ENSG0000(	327	8.178574	chr3:857:	H1-10		protein_c	chr3:129314771-129
ENSG0000(	327	8.178574	chr3:857:	ENSG00000273486		lncRNA	chr3:136837338-136
ENSG0000(	327	8.178574	chr3:857:	ENSG00000261146		lncRNA	chr3:137791973-137
ENSG0000(	327	8.178574	chr3:857:	ENSG00000261159		lncRNA	chr3:128859716-128
ENSG0000(	327	8.178574	chr3:857:	ENSG00000261167		lncRNA	chr3:131455126-131
ENSG0000(	327	8.178574	chr3:857:	GP9		protein_c	chr3:129060779-129
ENSG0000(	327	8.178574	chr3:857:	COPB2-DT		lncRNA	chr3:139389761-139
ENSG0000(	327	8.178574	chr3:857:	ENSG00000273455		lncRNA	chr3:136087475-136
ENSG0000(	327	8.178574	chr3:857:	ENSG00000273454		lncRNA	chr3:123277353-123
ENSG0000(	327	8.178574	chr3:857:	ENSG00000273394		lncRNA	chr3:113947005-113
ENSG0000(	327	8.178574	chr3:857:	TPT1P3		Pseudoger	chr3:141709016-141
ENSG0000(	327	8.178574	chr3:857:	DPPA2	AC	protein_c	chr3:109293788-109
ENSG0000(	327	8.178574	chr3:857:	RNA5SP140		Pseudoger	chr3:133710076-133
ENSG0000(	327	8.178574	chr3:857:	ENSG00000273437		lncRNA	chr3:129163606-129
ENSG0000(	327	8.178574	chr3:857:	ENSG00000261364		lncRNA	chr3:109176438-109
ENSG0000(	327	8.178574	chr3:857:	ENSG00000289641		lncRNA	chr3:127227404-127
ENSG0000(	327	8.178574	chr3:857:	CDV3		protein_c	chr3:133573686-133
ENSG0000(	327	8.178574	chr3:857:	ENSG00000241889		Pseudoger	chr3:113885298-113
ENSG0000(	327	8.178574	chr3:857:	CD200R1		protein_c	chr3:112921205-112
ENSG0000(	327	8.178574	chr3:857:	MIR4796		smallRNA	chr3:114743445-114
ENSG0000(	327	8.178574	chr3:857:	TMEM108-AS1		lncRNA	chr3:133245603-133
ENSG0000(	327	8.178574	chr3:857:	ENSG00000251012		protein_c	chr3:119147375-119
ENSG0000(	327	8.178574	chr3:857:	ZBTB20-AS3		lncRNA	chr3:114873114-114
ENSG0000(	327	8.178574	chr3:857:	HNRNPA1P23		Pseudoger	chr3:122317609-122
ENSG0000(	327	8.178574	chr3:857:	ENSG00000240086		lncRNA	chr3:135138469-135
ENSG0000(	327	8.178574	chr3:857:	ENSG00000288557		lncRNA	chr3:106684388-106
ENSG0000(	327	8.178574	chr3:857:	NEPRO-AS1		lncRNA	chr3:113019468-113
ENSG0000(	327	8.178574	chr3:857:	SPSB4	DriverDB\	protein_c	chr3:141051347-141
ENSG0000(	327	8.178574	chr3:857:	MTND6P6		Pseudoger	chr3:106900593-106
ENSG0000(	327	8.178574	chr3:857:	ENSG00000288585		lncRNA	chr3:141449745-141
ENSG0000(	327	8.178574	chr3:857:	LINC02004		lncRNA	chr3:134313498-134
ENSG0000(	327	8.178574	chr3:857:	YBX1P3		Pseudoger	chr3:114930541-114
ENSG0000(	327	8.178574	chr3:857:	FCF1P3		Pseudoger	chr3:106848671-106
ENSG0000(	327	8.178574	chr3:857:	ENSG00000239994		lncRNA	chr3:119744139-119
ENSG0000(	327	8.178574	chr3:857:	ENSG00000239311		lncRNA	chr3:111466313-111
ENSG0000(	327	8.178574	chr3:857:	OR7E53P		Pseudoger	chr3:125734295-125
ENSG0000(	327	8.178574	chr3:857:	ENSG00000288896		lncRNA	chr3:115147775-115
ENSG0000(	327	8.178574	chr3:857:	MORC1-AS1		lncRNA	chr3:109101456-109
ENSG0000(	327	8.178574	chr3:857:	TMED10P2		Pseudoger	chr3:128538020-128
ENSG0000(	327	8.178574	chr3:857:	SIDT1		protein_c	chr3:113532555-113
ENSG0000(	327	8.178574	chr3:857:	RPL23AP41		Pseudoger	chr3:140902194-140
ENSG0000(	327	8.178574	chr3:857:	MCM2	DriverDB\	protein_c	chr3:127598410-127
ENSG0000(	327	8.178574	chr3:857:	ACKR4		protein_c	chr3:132597270-132
ENSG0000(	327	8.178574	chr3:857:	SNORA70		smallRNA	chr3:108574565-108
ENSG0000(	327	8.178574	chr3:857:	B4GALT4-AS1		lncRNA	chr3:119226486-119
ENSG0000(	327	8.178574	chr3:857:	ACAD11		protein_c	chr3:132558138-132
ENSG0000(	327	8.178574	chr3:857:	AC093004.1		smallRNA	chr3:130369227-130

ENSG0000(	327	8.178574	chr3:857(	MIR568	smallRNA	chr3:114316475-114
ENSG0000(	327	8.178574	chr3:857(	DIMT1P1	Pseudoger	chr3:109701456-109
ENSG0000(	327	8.178574	chr3:857(	RPL7L1P7	Pseudoger	chr3:139081654-139
ENSG0000(	327	8.178574	chr3:857(	ENSG00000288996	lncRNA	chr3:129161398-129
ENSG0000(	327	8.178574	chr3:857(	NCK1-DT	lncRNA	chr3:136835345-136
ENSG0000(	327	8.178574	chr3:857(	ENSG00000288111	lncRNA	chr3:130179511-130
ENSG0000(	327	8.178574	chr3:857(	MTCO1P29	Pseudoger	chr3:120722024-120
ENSG0000(	327	8.178574	chr3:857(	ENSG00000239268	lncRNA	chr3:117672154-117
ENSG0000(	327	8.178574	chr3:857(	BFSP2-AS1	lncRNA	chr3:133429269-133
ENSG0000(	327	8.178574	chr3:857(	LINC00636	lncRNA	chr3:107834586-107
ENSG0000(	327	8.178574	chr3:857(	ENSG00000251058	lncRNA	chr3:140461000-140
ENSG0000(	327	8.178574	chr3:857(	C3orf36	lncRNA	chr3:133928145-133
ENSG0000(	327	8.178574	chr3:857(	ENSG00000240393	Pseudoger	chr3:117674342-117
ENSG0000(	327	8.178574	chr3:857(	Y_RNA	smallRNA	chr3:122025195-122
ENSG0000(	327	8.178574	chr3:857(	FAM86HP	Pseudoger	chr3:130099258-130
ENSG0000(	327	8.178574	chr3:857(	AC083906.2	smallRNA	chr3:130096373-130
ENSG0000(	327	8.178574	chr3:857(	AF186996.2	smallRNA	chr3:125690256-125
ENSG0000(	327	8.178574	chr3:857(	ENSG00000239280	Pseudoger	chr3:113740823-113
ENSG0000(	327	8.178574	chr3:857(	Y_RNA	smallRNA	chr3:120210320-120
ENSG0000(	327	8.178574	chr3:857(	ENSG00000239288	Pseudoger	chr3:109241507-109
ENSG0000(	327	8.178574	chr3:857(	VPS26AP1	Pseudoger	chr3:113919222-113
ENSG0000(	327	8.178574	chr3:857(	ANAPC13	protein_c	chr3:134477706-134
ENSG0000(	327	8.178574	chr3:857(	LINC01471	lncRNA	chr3:127480690-127
ENSG0000(	327	8.178574	chr3:857(	ENSG00000272840	lncRNA	chr3:125774714-125
ENSG0000(	327	8.178574	chr3:857(	ENSG00000288868	lncRNA	chr3:121749202-121
ENSG0000(	327	8.178574	chr3:857(	HSPBAP1	protein_c	chr3:122739999-122
ENSG0000(	327	8.178574	chr3:857(	LINC01210	lncRNA	chr3:137771660-137
ENSG0000(	327	8.178574	chr3:857(	ENSG00000288769	lncRNA	chr3:127456778-127
ENSG0000(	327	8.178574	chr3:857(	ENSG00000270880	Pseudoger	chr3:113984037-113
ENSG0000(	327	8.178574	chr3:857(	MYLK-AS1	lncRNA	chr3:123585143-123
ENSG0000(	327	8.178574	chr3:857(	ENSG00000270492	Pseudoger	chr3:129334586-129
ENSG0000(	327	8.178574	chr3:857(	ENSG00000239804	Pseudoger	chr3:125787888-125
ENSG0000(	327	8.178574	chr3:857(	ENSG00000288713	lncRNA	chr3:124723524-124
ENSG0000(	327	8.178574	chr3:857(	ENSG00000288700	lncRNA	chr3:134485721-134
ENSG0000(	327	8.178574	chr3:857(	ENSG00000288662	lncRNA	chr3:119969044-120
ENSG0000(	327	8.178574	chr3:857(	RNU6-1174P	smallRNA	chr3:134780527-134
ENSG0000(	327	8.178574	chr3:857(	SNORD112	smallRNA	chr3:113137966-113
ENSG0000(	327	8.178574	chr3:857(	MTND1P16	Pseudoger	chr3:106901068-106
ENSG0000(	327	8.178574	chr3:857(	Y_RNA	smallRNA	chr3:125528244-125
ENSG0000(	327	8.178574	chr3:857(	PLS1-AS1	lncRNA	chr3:142654784-142
ENSG0000(	327	8.178574	chr3:857(	U8	smallRNA	chr3:135799694-135
ENSG0000(	327	8.178574	chr3:857(	ENSG00000270782	Pseudoger	chr3:120924612-120
ENSG0000(	327	8.178574	chr3:857(	RAB7A	protein_c	chr3:128693669-128
ENSG0000(	327	8.178574	chr3:857(	MTND4LP3	Pseudoger	chr3:106895729-106
ENSG0000(	327	8.178574	chr3:857(	ENSG00000270773	Pseudoger	chr3:129345411-129
ENSG0000(	327	8.178574	chr3:857(	ENSG00000288667	lncRNA	chr3:119978153-119
ENSG0000(	327	8.178574	chr3:857(	RNU6-232P	smallRNA	chr3:125388934-125
ENSG0000(	327	8.178574	chr3:857(	MARK2P8	Pseudoger	chr3:128748538-128
ENSG0000(	327	8.178574	chr3:857(	MGLL	protein_c	chr3:127689062-128
ENSG0000(	327	8.178574	chr3:857(	SIDT1-AS1	lncRNA	chr3:113588748-113
ENSG0000(	327	8.178574	chr3:857(	MBD4	protein_c	chr3:129430947-129

ENSG0000(	327	8.178574	chr3:857(	EEFSEC		protein_c	chr3:128153481-128
ENSG0000(	327	8.178574	chr3:857(	ENSG00000239432		Pseudoger	chr3:125756086-125
ENSG0000(	327	8.178574	chr3:857(	MRPS22		protein_c	chr3:139005806-139
ENSG0000(	327	8.178574	chr3:857(	PPP2R3A	NCGv7	protein_c	chr3:135965728-136
ENSG0000(	327	8.178574	chr3:857(	H3P12		Pseudoger	chr3:109409678-109
ENSG0000(	327	8.178574	chr3:857(	IGSF11-AS1		lncRNA	chr3:118943073-118
ENSG0000(	327	8.178574	chr3:857(	RN7SL752P		smallRNA	chr3:129591349-129
ENSG0000(	327	8.178574	chr3:857(	RFKP2		Pseudoger	chr3:112030931-112
ENSG0000(	327	8.178574	chr3:857(	ENSG00000239455		Pseudoger	chr3:107104911-107
ENSG0000(	327	8.178574	chr3:857(	CCDC54-AS1		lncRNA	chr3:107272611-107
ENSG0000(	327	8.178574	chr3:857(	ENSG00000288848		lncRNA	chr3:105869180-105
ENSG0000(	327	8.178574	chr3:857(	ENSG00000239482		lncRNA	chr3:112302478-112
ENSG0000(	327	8.178574	chr3:857(	RPS15AP16		Pseudoger	chr3:128798841-128
ENSG0000(	327	8.178574	chr3:857(	KBTBD12		protein_c	chr3:127915232-127
ENSG0000(	327	8.178574	chr3:857(	RNU6-789P		smallRNA	chr3:136721394-136
ENSG0000(	327	8.178574	chr3:857(	RNU1-100P		smallRNA	chr3:142420205-142
ENSG0000(	327	8.178574	chr3:857(	ENSG00000288806		lncRNA	chr3:123701293-123
ENSG0000(	327	8.178574	chr3:857(	ENSG00000239835		Pseudoger	chr3:120028740-120
ENSG0000(	327	8.178574	chr3:857(	RNU2-37P		smallRNA	chr3:128075073-128
ENSG0000(	327	8.178574	chr3:857(	PHB1P8		Pseudoger	chr3:119791829-119
ENSG0000(	327	8.178574	chr3:857(	FAM214BP1		Pseudoger	chr3:114231398-114
ENSG0000(	327	8.178574	chr3:857(	GK5		protein_c	chr3:142157527-142
ENSG0000(	327	8.178574	chr3:857(	TMCC1-DT		lncRNA	chr3:129893811-129
ENSG0000(	327	8.178574	chr3:857(	ALG1L	DriverDB\	Pseudoger	chr3:125929272-125
ENSG0000(	327	8.178574	chr3:857(	IFT122		protein_c	chr3:129429607-129
ENSG0000(	327	8.178574	chr3:857(	ENSG00000240787		Pseudoger	chr3:111570638-111
ENSG0000(	327	8.178574	chr3:857(	AF186996.1		smallRNA	chr3:125736216-125
ENSG0000(	327	8.178574	chr3:857(	AC063944.1		smallRNA	chr3:107365182-107
ENSG0000(	327	8.178574	chr3:857(	RHO	AC	protein_c	chr3:129528639-129
ENSG0000(	327	8.178574	chr3:857(	IL20RB	DriverDB\	protein_c	chr3:136946230-137
ENSG0000(	327	8.178574	chr3:857(	AC083908.1		smallRNA	chr3:131188311-131
ENSG0000(	327	8.178574	chr3:857(	MIR4446		smallRNA	chr3:113594876-113
ENSG0000(	327	8.178574	chr3:857(	ENSG00000240776		Pseudoger	chr3:113850237-113
ENSG0000(	327	8.178574	chr3:857(	AC112504.1		smallRNA	chr3:141787911-141
ENSG0000(	327	8.178574	chr3:857(	MSL2		protein_c	chr3:136148917-136
ENSG0000(	327	8.178574	chr3:857(	MIR4447		smallRNA	chr3:116850277-116
ENSG0000(	327	8.178574	chr3:857(	ENSG00000287784		lncRNA	chr3:127165506-127
ENSG0000(	327	8.178574	chr3:857(	ENSG00000240774		Pseudoger	chr3:120484171-120
ENSG0000(	327	8.178574	chr3:857(	PLCXD2-AS1		lncRNA	chr3:111676736-111
ENSG0000(	327	8.178574	chr3:857(	RN7SKP212		smallRNA	chr3:130811768-130
ENSG0000(	327	8.178574	chr3:857(	ENSG00000287795		lncRNA	chr3:115413476-115
ENSG0000(	327	8.178574	chr3:857(	snoU13		smallRNA	chr3:129074095-129
ENSG0000(	327	8.178574	chr3:857(	ENSG00000287805		lncRNA	chr3:113986835-113
ENSG0000(	327	8.178574	chr3:857(	ENSG00000240751		Pseudoger	chr3:113360267-113
ENSG0000(	327	8.178574	chr3:857(	RPN1	AC	protein_c	chr3:128619969-128
ENSG0000(	327	8.178574	chr3:857(	Y_RNA		smallRNA	chr3:125516979-125
ENSG0000(	327	8.178574	chr3:857(	ENSG00000289153		lncRNA	chr3:115757662-115
ENSG0000(	327	8.178574	chr3:857(	RN7SKP124		smallRNA	chr3:139584105-139
ENSG0000(	327	8.178574	chr3:857(	PSMC2P1		Pseudoger	chr3:132175402-132
ENSG0000(	327	8.178574	chr3:857(	ENSG00000272832		lncRNA	chr3:133543064-133
ENSG0000(	327	8.178574	chr3:857(	AC078785.1		smallRNA	chr3:113092444-113

ENSG0000(	327	8.178574	chr3:857:	ENSG00000233597		Pseudoger	chr3:142043623-142
ENSG0000(	327	8.178574	chr3:857:	KLF15		protein_c	chr3:126342635-126
ENSG0000(	327	8.178574	chr3:857:	CPNE4		protein_c	chr3:131533555-132
ENSG0000(	327	8.178574	chr3:857:	WDR5B-DT		lncRNA	chr3:122416200-122
ENSG0000(	327	8.178574	chr3:857:	BTLA	NCGv7	protein_c	chr3:112463966-112
ENSG0000(	327	8.178574	chr3:857:	snoU13		smallRNA	chr3:121591294-121
ENSG0000(	327	8.178574	chr3:857:	ENSG00000272678		lncRNA	chr3:123283593-123
ENSG0000(	327	8.178574	chr3:857:	COL6A4P2		Pseudoger	chr3:130212823-130
ENSG0000(	327	8.178574	chr3:857:	TIMMDC1-DT		lncRNA	chr3:119497678-119
ENSG0000(	327	8.178574	chr3:857:	ENSG00000272656		lncRNA	chr3:139349024-139
ENSG0000(	327	8.178574	chr3:857:	ENSG00000272609		lncRNA	chr3:138004649-138
ENSG0000(	327	8.178574	chr3:857:	CFAP100		protein_c	chr3:126394909-126
ENSG0000(	327	8.178574	chr3:857:	MTND2P14		Pseudoger	chr3:106902173-106
ENSG0000(	327	8.178574	chr3:857:	ENSG00000272597		lncRNA	chr3:107329430-107
ENSG0000(	327	8.178574	chr3:857:	OR7E130P		Pseudoger	chr3:125703349-125
ENSG0000(	327	8.178574	chr3:857:	MIR5092		smallRNA	chr3:125151465-125
ENSG0000(	327	8.178574	chr3:857:	snoU13		smallRNA	chr3:132280618-132
ENSG0000(	327	8.178574	chr3:857:	ENSG00000289134		lncRNA	chr3:119639782-119
ENSG0000(	327	8.178574	chr3:857:	KY		protein_c	chr3:134599923-134
ENSG0000(	327	8.178574	chr3:857:	ENSG00000240695		Pseudoger	chr3:136736500-136
ENSG0000(	327	8.178574	chr3:857:	ENSG00000288022		lncRNA	chr3:125061448-125
ENSG0000(	327	8.178574	chr3:857:	RNA5SP138		Pseudoger	chr3:126564565-126
ENSG0000(	327	8.178574	chr3:857:	ENSG00000240562		lncRNA	chr3:127489553-127
ENSG0000(	327	8.178574	chr3:857:	ENSG00000289069		lncRNA	chr3:110966981-110
ENSG0000(	327	8.178574	chr3:857:	ENSG00000287977		Pseudoger	chr3:122172405-122
ENSG0000(	327	8.178574	chr3:857:	Y_RNA		smallRNA	chr3:129818932-129
ENSG0000(	327	8.178574	chr3:857:	ZXDC		protein_c	chr3:126437601-126
ENSG0000(	327	8.178574	chr3:857:	UBA5		protein_c	chr3:132654446-132
ENSG0000(	327	8.178574	chr3:857:	ENSG00000249253		Pseudoger	chr3:130293337-130
ENSG0000(	327	8.178574	chr3:857:	SLC35G2	NCGv7	protein_c	chr3:136818647-136
ENSG0000(	327	8.178574	chr3:857:	MIR5002		smallRNA	chr3:124132929-124
ENSG0000(	327	8.178574	chr3:857:	SEMA5B	NCGv7	protein_c	chr3:122909082-123
ENSG0000(	327	8.178574	chr3:857:	RNU6-1236P		smallRNA	chr3:109225129-109
ENSG0000(	327	8.178574	chr3:857:	ATR	NCGv7	protein_c	chr3:142449007-142
ENSG0000(	327	8.178574	chr3:857:	ENSG00000249098		Pseudoger	chr3:130918226-130
ENSG0000(	327	8.178574	chr3:857:	Y_RNA		smallRNA	chr3:130914341-130
ENSG0000(	327	8.178574	chr3:857:	ENSG00000288074		lncRNA	chr3:115418862-115
ENSG0000(	327	8.178574	chr3:857:	ENSG00000288079		lncRNA	chr3:113259786-113
ENSG0000(	327	8.178574	chr3:857:	CCDC14		protein_c	chr3:123897305-123
ENSG0000(	327	8.178574	chr3:857:	SLC41A3-AS1		lncRNA	chr3:126083659-126
ENSG0000(	327	8.178574	chr3:857:	GAPDHP39		Pseudoger	chr3:138777832-138
ENSG0000(	327	8.178574	chr3:857:	SNORA33		smallRNA	chr3:133551186-133
ENSG0000(	327	8.178574	chr3:857:	ENSG00000249290		lncRNA	chr3:140449435-140
ENSG0000(	327	8.178574	chr3:857:	ISY1		protein_c	chr3:129127415-129
ENSG0000(	327	8.178574	chr3:857:	LRRC58-DT		Pseudoger	chr3:120349449-120
ENSG0000(	327	8.178574	chr3:857:	SLCO2A1		protein_c	chr3:133932701-134
ENSG0000(	327	8.178574	chr3:857:	PPIAP72		Pseudoger	chr3:138643874-138
ENSG0000(	327	8.178574	chr3:857:	PRR23C		protein_c	chr3:139042102-139
ENSG0000(	327	8.178574	chr3:857:	KRT8P36		Pseudoger	chr3:138101478-138
ENSG0000(	327	8.178574	chr3:857:	snoU13		smallRNA	chr3:125446061-125
ENSG0000(	327	8.178574	chr3:857:	MIR5704		smallRNA	chr3:131985855-131

ENSG0000(	327	8.178574	chr3:857:	ENSG00000289118		lncRNA	chr3:131771870-131
ENSG0000(	327	8.178574	chr3:857:	U1		smallRNA	chr3:126160283-126
ENSG0000(	327	8.178574	chr3:857:	RNA5SP143		Pseudoger	chr3:142591677-142
ENSG0000(	327	8.178574	chr3:857:	AC117422.1		smallRNA	chr3:126069064-126
ENSG0000(	327	8.178574	chr3:857:	ENSG00000249305		Pseudoger	chr3:140619694-140
ENSG0000(	327	8.178574	chr3:857:	RPL7P15		Pseudoger	chr3:124151960-124
ENSG0000(	327	8.178574	chr3:857:	ATP1B3	DriverDB\	protein_c	chr3:141876124-141
ENSG0000(	327	8.178574	chr3:857:	MIR4445		smallRNA	chr3:109602828-109
ENSG0000(	327	8.178574	chr3:857:	MIR544B		smallRNA	chr3:124732439-124
ENSG0000(	327	8.178574	chr3:857:	ITGB5		protein_c	chr3:124761948-124
ENSG0000(	327	8.178574	chr3:857:	GSK3B		protein_c	chr3:119821321-120
ENSG0000(	327	8.178574	chr3:857:	RNU6-143P		smallRNA	chr3:124407691-124
ENSG0000(	327	8.178574	chr3:857:	LINC01215		lncRNA	chr3:108125821-108
ENSG0000(	327	8.178574	chr3:857:	RNU6-1142P		smallRNA	chr3:129819777-129
ENSG0000(	327	8.178574	chr3:857:	ATP5MC1P3		Pseudoger	chr3:138889255-138
ENSG0000(	327	8.178574	chr3:857:	ENSG00000242222		Pseudoger	chr3:135925891-135
ENSG0000(	327	8.178574	chr3:857:	ENSG00000287207		lncRNA	chr3:122515006-122
ENSG0000(	327	8.178574	chr3:857:	ENSG00000250643		lncRNA	chr3:129954105-129
ENSG0000(	327	8.178574	chr3:857:	ENSG00000285908		lncRNA	chr3:133799440-133
ENSG0000(	327	8.178574	chr3:857:	DUBR		lncRNA	chr3:107220744-107
ENSG0000(	327	8.178574	chr3:857:	RAB43		protein_c	chr3:129087569-129
ENSG0000(	327	8.178574	chr3:857:	ENSG00000285943		protein_c	chr3:113361901-113
ENSG0000(	327	8.178574	chr3:857:	MTND5P16		Pseudoger	chr3:106898889-106
ENSG0000(	327	8.178574	chr3:857:	LINC02049		lncRNA	chr3:120833440-120
ENSG0000(	327	8.178574	chr3:857:	ENSG00000214301		Pseudoger	chr3:133490824-133
ENSG0000(	327	8.178574	chr3:857:	RN7SL172P		smallRNA	chr3:121653996-121
ENSG0000(	327	8.178574	chr3:857:	HSPA8P19		Pseudoger	chr3:132645831-132
ENSG0000(	327	8.178574	chr3:857:	DNAJB6P7		Pseudoger	chr3:125344085-125
ENSG0000(	327	8.178574	chr3:857:	ENSG00000243483		Pseudoger	chr3:113041390-113
ENSG0000(	327	8.178574	chr3:857:	OR7E93P		Pseudoger	chr3:125724539-125
ENSG0000(	327	8.178574	chr3:857:	ENSG00000250592		lncRNA	chr3:130899414-130
ENSG0000(	327	8.178574	chr3:857:	ENSG00000248459		Pseudoger	chr3:129998531-129
ENSG0000(	327	8.178574	chr3:857:	GRAMD1C		protein_c	chr3:113828182-113
ENSG0000(	327	8.178574	chr3:857:	ENSG00000279507		TEC	chr3:128914833-128
ENSG0000(	327	8.178574	chr3:857:	ENSG00000249820		Pseudoger	chr3:133409955-133
ENSG0000(	327	8.178574	chr3:857:	ENSG00000249725		lncRNA	chr3:133015004-133
ENSG0000(	327	8.178574	chr3:857:	NUDT16-DT		lncRNA	chr3:131325092-131
ENSG0000(	327	8.178574	chr3:857:	ENSG00000279349		TEC	chr3:112525548-112
ENSG0000(	327	8.178574	chr3:857:	RPL39P5		Pseudoger	chr3:134351852-134
ENSG0000(	327	8.178574	chr3:857:	HMGN1P9		Pseudoger	chr3:134385197-134
ENSG0000(	327	8.178574	chr3:857:	ACTG1P1		Pseudoger	chr3:139493809-139
ENSG0000(	327	8.178574	chr3:857:	C3orf56		protein_c	chr3:127193131-127
ENSG0000(	327	8.178574	chr3:857:	U3		smallRNA	chr3:109018910-109
ENSG0000(	327	8.178574	chr3:857:	CD96		protein_c	chr3:111292719-111
ENSG0000(	327	8.178574	chr3:857:	ZBTB20	NCGv7	protein_c	chr3:114314500-115
ENSG0000(	327	8.178574	chr3:857:	COPG1		protein_c	chr3:129249606-129
ENSG0000(	327	8.178574	chr3:857:	ENO1P3		Pseudoger	chr3:124862094-124
ENSG0000(	327	8.178574	chr3:857:	SNRPCP8		Pseudoger	chr3:130199708-130
ENSG0000(	327	8.178574	chr3:857:	TIGIT	NCGv7	protein_c	chr3:114276913-114
ENSG0000(	327	8.178574	chr3:857:	EFCAB12	DriverDB\	protein_c	chr3:129401321-129
ENSG0000(	327	8.178574	chr3:857:	ENSG00000280053		TEC	chr3:126973065-126

ENSG0000(	327	8.178574	chr3:857:	ENSG00000280042		TEC	chr3:124791119-124
ENSG0000(	327	8.178574	chr3:857:	ENSG00000243945		Pseudoger	chr3:109977540-109
ENSG0000(	327	8.178574	chr3:857:	LINC02044		lncRNA	chr3:113142350-113
ENSG0000(	327	8.178574	chr3:857:	RNU6-230P		smallRNA	chr3:125119097-125
ENSG0000(	327	8.178574	chr3:857:	EIF4BP8		Pseudoger	chr3:122660613-122
ENSG0000(	327	8.178574	chr3:857:	ENSG00000243886		Pseudoger	chr3:137535572-137
ENSG0000(	327	8.178574	chr3:857:	RN7SKP25		smallRNA	chr3:142673597-142
ENSG0000(	327	8.178574	chr3:857:	CFAP44-AS1		lncRNA	chr3:113403988-113
ENSG0000(	327	8.178574	chr3:857:	LINC02000		lncRNA	chr3:134055256-134
ENSG0000(	327	8.178574	chr3:857:	DRD3	NCGv7	protein_c	chr3:114127580-114
ENSG0000(	327	8.178574	chr3:857:	QTRT2		protein_c	chr3:114005833-114
ENSG0000(	327	8.178574	chr3:857:	ENSG00000243813		Pseudoger	chr3:121099108-121
ENSG0000(	327	8.178574	chr3:857:	EVA1CP6		Pseudoger	chr3:130048143-130
ENSG0000(	327	8.178574	chr3:857:	OR7E29P		Pseudoger	chr3:125712139-125
ENSG0000(	327	8.178574	chr3:857:	HMGB3P13		Pseudoger	chr3:134437605-134
ENSG0000(	327	8.178574	chr3:857:	ARMC8		protein_c	chr3:138187248-138
ENSG0000(	327	8.178574	chr3:857:	TIMMDC1		protein_c	chr3:119498547-119
ENSG0000(	327	8.178574	chr3:857:	HGD		protein_c	chr3:120628172-120
ENSG0000(	327	8.178574	chr3:857:	NPHP3		protein_c	chr3:132680609-132
ENSG0000(	327	8.178574	chr3:857:	CD86	NCGv7	protein_c	chr3:122055362-122
ENSG0000(	327	8.178574	chr3:857:	AMOTL2		protein_c	chr3:134355347-134
ENSG0000(	327	8.178574	chr3:857:	FAM162A		protein_c	chr3:122384161-122
ENSG0000(	327	8.178574	chr3:857:	KPNA1		protein_c	chr3:122421902-122
ENSG0000(	327	8.178574	chr3:857:	HNRNPA1P17		Pseudoger	chr3:108325520-108
ENSG0000(	327	8.178574	chr3:857:	PCCB		protein_c	chr3:136250340-136
ENSG0000(	327	8.178574	chr3:857:	CEP70	DriverDB\	protein_c	chr3:138494344-138
ENSG0000(	327	8.178574	chr3:857:	TUSC7		lncRNA	chr3:116709235-116
ENSG0000(	327	8.178574	chr3:857:	ENSG00000243081		lncRNA	chr3:112396647-112
ENSG0000(	327	8.178574	chr3:857:	POGLUT1	NCGv7	protein_c	chr3:119468963-119
ENSG0000(	327	8.178574	chr3:857:	RBP2		protein_c	chr3:139452884-139
ENSG0000(	327	8.178574	chr3:857:	RBP1	DriverDB\	protein_c	chr3:139517434-139
ENSG0000(	327	8.178574	chr3:857:	SLC25A36		protein_c	chr3:140941830-140
ENSG0000(	327	8.178574	chr3:857:	ENSG00000227267		Pseudoger	chr3:136055184-136
ENSG0000(	327	8.178574	chr3:857:	COL6A6	NCGv7	protein_c	chr3:130517177-130
ENSG0000(	327	8.178574	chr3:857:	GRK7	DriverDB\	protein_c	chr3:141763408-141
ENSG0000(	327	8.178574	chr3:857:	H1-10-AS1		lncRNA	chr3:129315392-129
ENSG0000(	327	8.178574	chr3:857:	ENSG00000249691		Pseudoger	chr3:134510531-134
ENSG0000(	327	8.178574	chr3:857:	BCL2L12P1		Pseudoger	chr3:131526447-131
ENSG0000(	327	8.178574	chr3:857:	RAD51AP1P1		Pseudoger	chr3:136899076-136
ENSG0000(	327	8.178574	chr3:857:	PRR23A		protein_c	chr3:139003962-139
ENSG0000(	327	8.178574	chr3:857:	RN7SL815P		smallRNA	chr3:115837868-115
ENSG0000(	327	8.178574	chr3:857:	ADCY5	NCGv7	protein_c	chr3:123282296-123
ENSG0000(	327	8.178574	chr3:857:	ENSG00000243276		lncRNA	chr3:118004819-118
ENSG0000(	327	8.178574	chr3:857:	LINC01205		lncRNA	chr3:109409990-109
ENSG0000(	327	8.178574	chr3:857:	PARP14		protein_c	chr3:122680839-122
ENSG0000(	327	8.178574	chr3:857:	ENSG00000214280		Pseudoger	chr3:139582928-139
ENSG0000(	327	8.178574	chr3:857:	ENSG00000279328		TEC	chr3:126432796-126
ENSG0000(	327	8.178574	chr3:857:	GSTO3P		Pseudoger	chr3:130827659-130
ENSG0000(	327	8.178574	chr3:857:	MTCO2P29		Pseudoger	chr3:120722458-120
ENSG0000(	327	8.178574	chr3:857:	AC121332.1		smallRNA	chr3:131111670-131
ENSG0000(	327	8.178574	chr3:857:	ENSG00000231305		lncRNA	chr3:128860620-128

ENSG0000(	327	8.178574	chr3:857:	MTCO1P35		Pseudoger	chr3:106900854-106
ENSG0000(	327	8.178574	chr3:857:	ENSG00000279277		lncRNA	chr3:108032456-108
ENSG0000(	327	8.178574	chr3:857:	PARP15		protein_c	chr3:122577628-122
ENSG0000(	327	8.178574	chr3:857:	ENSG00000279147		TEC	chr3:141936707-141
ENSG0000(	327	8.178574	chr3:857:	CEP63		protein_c	chr3:134485699-134
ENSG0000(	327	8.178574	chr3:857:	IQCB1	NCGv7	protein_c	chr3:121769761-121
ENSG0000(	327	8.178574	chr3:857:	GOLGB1	NCGv7	protein_c	chr3:121663199-121
ENSG0000(	327	8.178574	chr3:857:	FOXL2NB	DriverDB\	protein_c	chr3:138947217-138
ENSG0000(	327	8.178574	chr3:857:	ENSG00000248468		lncRNA	chr3:131502573-131
ENSG0000(	327	8.178574	chr3:857:	BPESC1		lncRNA	chr3:139104185-139
ENSG0000(	327	8.178574	chr3:857:	ENSG00000250543		lncRNA	chr3:139688403-139
ENSG0000(	327	8.178574	chr3:857:	ENSG00000290993		lncRNA	chr3:129382922-129
ENSG0000(	327	8.178574	chr3:857:	NT5C3AP2		Pseudoger	chr3:111633672-111
ENSG0000(	327	8.178574	chr3:857:	ALDH1L1-AS2		lncRNA	chr3:126180012-126
ENSG0000(	327	8.178574	chr3:857:	LINC02034		lncRNA	chr3:127537937-127
ENSG0000(	327	8.178574	chr3:857:	ENSG00000282950		lncRNA	chr3:120365993-120
ENSG0000(	327	8.178574	chr3:857:	SNORA24		smallRNA	chr3:128714571-128
ENSG0000(	327	8.178574	chr3:857:	ENSG00000282860		lncRNA	chr3:127571232-127
ENSG0000(	327	8.178574	chr3:857:	ENSG00000244932		Pseudoger	chr3:129381298-129
ENSG0000(	327	8.178574	chr3:857:	ENSG00000244740		Pseudoger	chr3:136205540-136
ENSG0000(	327	8.178574	chr3:857:	RPSAP29		Pseudoger	chr3:110682286-110
ENSG0000(	327	8.178574	chr3:857:	LINC00488		lncRNA	chr3:109178143-109
ENSG0000(	327	8.178574	chr3:857:	CFAP100-DT		lncRNA	chr3:126393032-126
ENSG0000(	327	8.178574	chr3:857:	NCK1		protein_c	chr3:136862208-136
ENSG0000(	327	8.178574	chr3:857:	TRH		protein_c	chr3:129974688-129
ENSG0000(	327	8.178574	chr3:857:	RN7SL724P		smallRNA	chr3:139609671-139
ENSG0000(	327	8.178574	chr3:857:	HCLS1		protein_c	chr3:121631399-121
ENSG0000(	327	8.178574	chr3:857:	NFYBP1		Pseudoger	chr3:109915976-109
ENSG0000(	327	8.178574	chr3:857:	RNU6-823P		smallRNA	chr3:128141875-128
ENSG0000(	327	8.178574	chr3:857:	ENSG00000244652		Pseudoger	chr3:106325737-106
ENSG0000(	327	8.178574	chr3:857:	MIX23		protein_c	chr3:122359591-122
ENSG0000(	327	8.178574	chr3:857:	KALRN	NCGv7	protein_c	chr3:124033369-124
ENSG0000(	327	8.178574	chr3:857:	COL6A5		protein_c	chr3:130345516-130
ENSG0000(	327	8.178574	chr3:857:	NUDT16L2P		Pseudoger	chr3:131361818-131
ENSG0000(	327	8.178574	chr3:857:	RNU6-1294P		smallRNA	chr3:142407657-142
ENSG0000(	327	8.178574	chr3:857:	LINC01391		lncRNA	chr3:138935189-138
ENSG0000(	327	8.178574	chr3:857:	ENSG00000284624		lncRNA	chr3:125766516-125
ENSG0000(	327	8.178574	chr3:857:	DNAJB8		protein_c	chr3:128462437-128
ENSG0000(	327	8.178574	chr3:857:	LINC02021		lncRNA	chr3:130111669-130
ENSG0000(	327	8.178574	chr3:857:	ENSG00000291293		lncRNA	chr3:106449775-106
ENSG0000(	327	8.178574	chr3:857:	ENSG00000283669		Pseudoger	chr3:112321140-112
ENSG0000(	327	8.178574	chr3:857:	GATA2	NCGv7;AC	protein_c	chr3:128479427-128
ENSG0000(	327	8.178574	chr3:857:	UROC1		protein_c	chr3:126481166-126
ENSG0000(	327	8.178574	chr3:857:	PLXND1	NCGv7	protein_c	chr3:129555214-129
ENSG0000(	327	8.178574	chr3:857:	CLSTN2-AS1		lncRNA	chr3:140505611-140
ENSG0000(	327	8.178574	chr3:857:	ENSG00000284095		TEC	chr3:130868004-130
ENSG0000(	327	8.178574	chr3:857:	CHCHD6	DriverDB\	protein_c	chr3:126704240-126
ENSG0000(	327	8.178574	chr3:857:	AC078855.1		smallRNA	chr3:108320392-108
ENSG0000(	327	8.178574	chr3:857:	MIR548I1		smallRNA	chr3:125790404-125
ENSG0000(	327	8.178574	chr3:857:	OR7E97P		Pseudoger	chr3:125747084-125
ENSG0000(	327	8.178574	chr3:857:	CLSTN2	NCGv7	protein_c	chr3:139935185-140

ENSG0000(	327	8.178574	chr3:857:	FAIM		protein_c	chr3:138608606-138
ENSG0000(	327	8.178574	chr3:857:	ESYT3		protein_c	chr3:138434586-138
ENSG0000(	327	8.178574	chr3:857:	MRAS	NCGv7;AC	protein_c	chr3:138347648-138
ENSG0000(	327	8.178574	chr3:857:	ENSG00000249869		Pseudoger	chr3:130013182-130
ENSG0000(	327	8.178574	chr3:857:	DZIP1L		protein_c	chr3:138061990-138
ENSG0000(	327	8.178574	chr3:857:	ENSG00000284660		Pseudoger	chr3:125766601-125
ENSG0000(	327	8.178574	chr3:857:	ENSG00000284731		Pseudoger	chr3:130002789-130
ENSG0000(	327	8.178574	chr3:857:	ENSG00000244561		Pseudoger	chr3:109396616-109
ENSG0000(	327	8.178574	chr3:857:	ENSG00000285836		lncRNA	chr3:113998782-114
ENSG0000(	327	8.178574	chr3:857:	EIF4E2P2		Pseudoger	chr3:115279125-115
ENSG0000(	327	8.178574	chr3:857:	RN7SL762P		smallRNA	chr3:120110498-120
ENSG0000(	327	8.178574	chr3:857:	GATA2-AS1		lncRNA	chr3:128489212-128
ENSG0000(	327	8.178574	chr3:857:	LINC02014		lncRNA	chr3:130089433-130
ENSG0000(	327	8.178574	chr3:857:	ITGB5-AS1		lncRNA	chr3:124781155-124
ENSG0000(	327	8.178574	chr3:857:	RN7SL698P		smallRNA	chr3:128785147-128
ENSG0000(	327	8.178574	chr3:857:	RNU7-47P		smallRNA	chr3:142801207-142
ENSG0000(	327	8.178574	chr3:857:	LINC02016		lncRNA	chr3:127322307-127
ENSG0000(	327	8.178574	chr3:857:	PPIAP15		Pseudoger	chr3:109471329-109
ENSG0000(	327	8.178574	chr3:857:	NME9		protein_c	chr3:138261437-138
ENSG0000(	327	8.178574	chr3:857:	ENSG00000244144		Pseudoger	chr3:112185480-112
ENSG0000(	327	8.178574	chr3:857:	RAB6B	DriverDB\	protein_c	chr3:133824235-133
ENSG0000(	327	8.178574	chr3:857:	ATP6V0CP2		Pseudoger	chr3:111478737-111
ENSG0000(	327	8.178574	chr3:857:	RN7SL397P		smallRNA	chr3:120121909-120
ENSG0000(	327	8.178574	chr3:857:	ENSG00000280399		TEC	chr3:138482065-138
ENSG0000(	327	8.178574	chr3:857:	ATP1B3-AS1		lncRNA	chr3:141918252-141
ENSG0000(	327	8.178574	chr3:857:	HMGN1P10		Pseudoger	chr3:136609050-136
ENSG0000(	327	8.178574	chr3:857:	RNU7-198P		smallRNA	chr3:136633148-136
ENSG0000(	327	8.178574	chr3:857:	MARK2P17		Pseudoger	chr3:128843564-128
ENSG0000(	327	8.178574	chr3:857:	ENSG00000244062		Pseudoger	chr3:133760300-133
ENSG0000(	327	8.178574	chr3:857:	GTF2E1		protein_c	chr3:120742637-120
ENSG0000(	327	8.178574	chr3:857:	ENSG00000244327		lncRNA	chr3:142465315-142
ENSG0000(	327	8.178574	chr3:857:	ENSG00000285631		lncRNA	chr3:130821184-130
ENSG0000(	327	8.178574	chr3:857:	C3orf22		protein_c	chr3:126526999-126
ENSG0000(	327	8.178574	chr3:857:	OR7E129P		Pseudoger	chr3:130021553-130
ENSG0000(	327	8.178574	chr3:857:	ENSG00000291096		lncRNA	chr3:125928689-125
ENSG0000(	327	8.178574	chr3:857:	SNORA7B		smallRNA	chr3:129397210-129
ENSG0000(	327	8.178574	chr3:857:	ENSG00000285558		protein_c	chr3:141738474-141
ENSG0000(	327	8.178574	chr3:857:	RASA2	NCGv7	protein_c	chr3:141487027-141
ENSG0000(	327	8.178574	chr3:857:	PXYLP1	DriverDB\	protein_c	chr3:141228726-141
ENSG0000(	327	8.178574	chr3:857:	TRIM42		protein_c	chr3:140678064-140
ENSG0000(	327	8.178574	chr3:857:	CHST13		protein_c	chr3:126524155-126
ENSG0000(	327	8.178574	chr3:857:	PISRT1		lncRNA	chr3:139232992-139
ENSG0000(	327	8.178574	chr3:857:	ENSG00000291081		lncRNA	chr3:130097782-130
ENSG0000(	327	8.178574	chr3:857:	SLC9C1	NCGv7	protein_c	chr3:112140898-112
ENSG0000(	327	8.178574	chr3:857:	EPHB1	NCGv7	protein_c	chr3:134795260-135
ENSG0000(	327	8.178574	chr3:857:	FAM86JP		lncRNA	chr3:125916609-125
ENSG0000(	327	8.178574	chr3:857:	H1-8	NCGv7	protein_c	chr3:129543175-129
ENSG0000(	327	8.178574	chr3:857:	ENSG00000285585		protein_c	chr3:119703076-119
ENSG0000(	327	8.178574	chr3:857:	ENSG00000244441		Pseudoger	chr3:120512850-120
ENSG0000(	327	8.178574	chr3:857:	AC092902.1		smallRNA	chr3:125832429-125
ENSG0000(	327	8.178574	chr3:857:	ENSG00000285600		lncRNA	chr3:127620106-127

ENSG0000(	327	8.178574	chr3:857	ENSG00000291042		lncRNA	chr3:133661926-133
ENSG0000(	327	8.178574	chr3:857	ENSG00000285619		lncRNA	chr3:128181402-128
ENSG0000(	327	8.178574	chr3:857	TMCC1		protein_c	chr3:129647792-129
ENSG0000(	327	8.178574	chr3:857	ENSG00000248557		Pseudoger	chr3:126624792-126
ENSG0000(	327	8.178574	chr3:857	ENSG00000243072		Pseudoger	chr3:125679573-125
ENSG0000(	327	8.178574	chr3:857	HACD2		protein_c	chr3:123490820-123
ENSG0000(	327	8.178574	chr3:857	HEG1		protein_c	chr3:124965710-125
ENSG0000(	327	8.178574	chr3:857	ENSG00000242659		lncRNA	chr3:113746872-113
ENSG0000(	327	8.178574	chr3:857	ENSG00000286660		lncRNA	chr3:118488876-118
ENSG0000(	327	8.178574	chr3:857	GSK3B-DT		lncRNA	chr3:120094895-120
ENSG0000(	327	8.178574	chr3:857	ENSG00000242613		Pseudoger	chr3:120388029-120
ENSG0000(	327	8.178574	chr3:857	FOXL2	NCGv7;AC	protein_c	chr3:138944224-138
ENSG0000(	327	8.178574	chr3:857	RNU6-1127P		smallRNA	chr3:119341834-119
ENSG0000(	327	8.178574	chr3:857	NUP210P1		Pseudoger	chr3:126660609-126
ENSG0000(	327	8.178574	chr3:857	ENSG00000286729		lncRNA	chr3:129277753-129
ENSG0000(	327	8.178574	chr3:857	POU5F1P6		Pseudoger	chr3:128674735-128
ENSG0000(	327	8.178574	chr3:857	ENSG00000286735		lncRNA	chr3:120448974-120
ENSG0000(	327	8.178574	chr3:857	GAP43		protein_c	chr3:115623510-115
ENSG0000(	327	8.178574	chr3:857	CFAP91		protein_c	chr3:119703022-119
ENSG0000(	327	8.178574	chr3:857	RCC2P4		Pseudoger	chr3:126766434-126
ENSG0000(	327	8.178574	chr3:857	ENSG00000242531		Pseudoger	chr3:122416882-122
ENSG0000(	327	8.178574	chr3:857	OR7E100P		Pseudoger	chr3:112524187-112
ENSG0000(	327	8.178574	chr3:857	ENSG00000248659		Pseudoger	chr3:129632019-129
ENSG0000(	327	8.178574	chr3:857	ASTE1		protein_c	chr3:131013875-131
ENSG0000(	327	8.178574	chr3:857	ENSG00000242479		Pseudoger	chr3:142450102-142
ENSG0000(	327	8.178574	chr3:857	ZBTB38		protein_c	chr3:141324213-141
ENSG0000(	327	8.178574	chr3:857	RPL7AP11		Pseudoger	chr3:121494110-121
ENSG0000(	327	8.178574	chr3:857	ENSG00000259976		lncRNA	chr3:114314501-114
ENSG0000(	327	8.178574	chr3:857	BZW1P2		Pseudoger	chr3:116645902-116
ENSG0000(	327	8.178574	chr3:857	ENSG00000251471		lncRNA	chr3:139837220-139
ENSG0000(	327	8.178574	chr3:857	ENSG00000290241		lncRNA	chr3:128850540-128
ENSG0000(	327	8.178574	chr3:857	CD200R1L	DriverDB	protein_c	chr3:112815709-112
ENSG0000(	327	8.178574	chr3:857	MTCO3P35		Pseudoger	chr3:106896891-106
ENSG0000(	327	8.178574	chr3:857	AC023593.1		smallRNA	chr3:127664704-127
ENSG0000(	327	8.178574	chr3:857	ENSG00000251448		lncRNA	chr3:126288123-126
ENSG0000(	327	8.178574	chr3:857	ENSG00000290242		lncRNA	chr3:128854556-128
ENSG0000(	327	8.178574	chr3:857	RPS26P21		Pseudoger	chr3:119298665-119
ENSG0000(	327	8.178574	chr3:857	RNF7		protein_c	chr3:141738249-141
ENSG0000(	327	8.178574	chr3:857	RNU6-1284P		smallRNA	chr3:136430084-136
ENSG0000(	327	8.178574	chr3:857	Y_RNA		smallRNA	chr3:106515897-106
ENSG0000(	327	8.178574	chr3:857	ENSG00000242816		lncRNA	chr3:117719859-117
ENSG0000(	327	8.178574	chr3:857	ENSG00000286584		lncRNA	chr3:119666232-119
ENSG0000(	327	8.178574	chr3:857	ENSG00000251447		Pseudoger	chr3:131537571-131
ENSG0000(	327	8.178574	chr3:857	ALCAM		protein_c	chr3:105366909-105
ENSG0000(	327	8.178574	chr3:857	CD200R1L-AS1		lncRNA	chr3:112802478-112
ENSG0000(	327	8.178574	chr3:857	ZBTB20-AS4		lncRNA	chr3:115100423-115
ENSG0000(	327	8.178574	chr3:857	RNY4P4		smallRNA	chr3:136588209-136
ENSG0000(	327	8.178574	chr3:857	ARHGAP31		protein_c	chr3:119294383-119
ENSG0000(	327	8.178574	chr3:857	ENSG00000276763		Pseudoger	chr3:108574683-108
ENSG0000(	327	8.178574	chr3:857	LINC00882		lncRNA	chr3:106630469-107
ENSG0000(	327	8.178574	chr3:857	HMCES		protein_c	chr3:129278828-129

ENSG0000(	327	8.178574	chr3:857:	NPHP3-AS1		lncRNA	chr3:132721750-132
ENSG0000(	327	8.178574	chr3:857:	Y_RNA		smallRNA	chr3:127202372-127
ENSG0000(	327	8.178574	chr3:857:	ENSG00000248773		lncRNA	chr3:140972744-140
ENSG0000(	327	8.178574	chr3:857:	ENSG00000260633		lncRNA	chr3:134347288-134
ENSG0000(	327	8.178574	chr3:857:	ENSG00000286988		lncRNA	chr3:139316157-139
ENSG0000(	327	8.178574	chr3:857:	ENSG00000249505		Pseudoger	chr3:129045763-129
ENSG0000(	327	8.178574	chr3:857:	ENSG00000287022		lncRNA	chr3:121394772-121
ENSG0000(	327	8.178574	chr3:857:	AC130888.1		smallRNA	chr3:130183523-130
ENSG0000(	327	8.178574	chr3:857:	ENSG00000287045		lncRNA	chr3:142656967-142
ENSG0000(	327	8.178574	chr3:857:	STAG1	NCGv7	protein_c	chr3:136336236-136
ENSG0000(	327	8.178574	chr3:857:	A4GNT	DriverDB\	protein_c	chr3:138123713-138
ENSG0000(	327	8.178574	chr3:857:	ENSG00000275348		Pseudoger	chr3:122482990-122
ENSG0000(	327	8.178574	chr3:857:	ACAD9-DT		lncRNA	chr3:128871913-128
ENSG0000(	327	8.178574	chr3:857:	LINC00901		lncRNA	chr3:116921431-116
ENSG0000(	327	8.178574	chr3:857:	ENSG00000260391		lncRNA	chr3:124723788-124
ENSG0000(	327	8.178574	chr3:857:	RASA2-IT1		lncRNA	chr3:141525133-141
ENSG0000(	327	8.178574	chr3:857:	RNU6-1047P		smallRNA	chr3:127240968-127
ENSG0000(	327	8.178574	chr3:857:	ENSG00000287143		lncRNA	chr3:127837436-127
ENSG0000(	327	8.178574	chr3:857:	INHCAP		Pseudoger	chr3:133688192-133
ENSG0000(	327	8.178574	chr3:857:	ENSG00000287155		lncRNA	chr3:141251004-141
ENSG0000(	327	8.178574	chr3:857:	RPL23AP40		Pseudoger	chr3:138796851-138
ENSG0000(	327	8.178574	chr3:857:	DUTP1		Pseudoger	chr3:125310881-125
ENSG0000(	327	8.178574	chr3:857:	ENSG00000242308		Pseudoger	chr3:112696908-112
ENSG0000(	327	8.178574	chr3:857:	ZBTB20-AS5		lncRNA	chr3:114445521-114
ENSG0000(	327	8.178574	chr3:857:	snoU13		smallRNA	chr3:129073296-129
ENSG0000(	327	8.178574	chr3:857:	ENSG00000286982		lncRNA	chr3:134774543-134
ENSG0000(	327	8.178574	chr3:857:	MYLK-AS2		lncRNA	chr3:123689644-123
ENSG0000(	327	8.178574	chr3:857:	RNA5SP139		Pseudoger	chr3:127963608-127
ENSG0000(	327	8.178574	chr3:857:	ENSG00000286806		lncRNA	chr3:128563316-128
ENSG0000(	327	8.178574	chr3:857:	ENSG00000248787		lncRNA	chr3:125907765-125
ENSG0000(	327	8.178574	chr3:857:	RPL6P9		Pseudoger	chr3:142580910-142
ENSG0000(	327	8.178574	chr3:857:	TXNRD3		protein_c	chr3:126571779-126
ENSG0000(	327	8.178574	chr3:857:	ENSG00000286822		lncRNA	chr3:141851549-141
ENSG0000(	327	8.178574	chr3:857:	CASR		protein_c	chr3:122183668-122
ENSG0000(	327	8.178574	chr3:857:	ALDH1L1-AS1		lncRNA	chr3:126103640-126
ENSG0000(	327	8.178574	chr3:857:	ENSG00000286827		lncRNA	chr3:120811530-120
ENSG0000(	327	8.178574	chr3:857:	ENSG00000290035		lncRNA	chr3:127218943-127
ENSG0000(	327	8.178574	chr3:857:	AC010207.1		smallRNA	chr3:134410044-134
ENSG0000(	327	8.178574	chr3:857:	ENSG00000286854		lncRNA	chr3:106367979-106
ENSG0000(	327	8.178574	chr6:105(	H1-4	NCGv7	protein_c	chr6:26156329-2615
ENSG0000(	327	8.178574	chr3:857:	ENSG00000286915		lncRNA	chr3:136778181-136
ENSG0000(	327	8.178574	chr3:857:	ENSG00000290001		lncRNA	chr3:129067449-129
ENSG0000(	327	8.178574	chr3:857:	ENSG00000286919		lncRNA	chr3:127274581-127
ENSG0000(	327	8.178574	chr3:857:	ALG1L2		protein_c	chr3:130081831-130
ENSG0000(	327	8.178574	chr3:857:	ENSG00000248790		lncRNA	chr3:139466430-139
ENSG0000(	327	8.178574	chr3:857:	ENSG00000286956		lncRNA	chr3:107046026-107
ENSG0000(	327	8.178574	chr3:857:	ENSG00000249540		Pseudoger	chr3:141307612-141
ENSG0000(	327	8.178574	chr3:857:	MYH15		protein_c	chr3:108380368-108
ENSG0000(	327	8.178574	chr3:857:	RNU4-62P		smallRNA	chr3:121655475-121
ENSG0000(	327	8.178574	chr3:857:	PRR20G		protein_c	chr3:127283783-127
ENSG0000(	327	8.178574	chr3:857:	STXBP5L	DriverDB\	protein_c	chr3:120908072-121

ENSG0000(	327	8.178574	chr3:857	ATP6V1A		protein_c	chr3:113747027-113
ENSG0000(	327	8.178574	chr3:857	LINC01565		lncRNA	chr3:128572000-128
ENSG0000(	327	8.178574	chr3:857	PLXNA1	DriverDB	protein_c	chr3:126982693-127
ENSG0000(	327	8.178574	chr3:857	ENSG00000286396		Pseudoger	chr3:138125695-138
ENSG0000(	327	8.178574	chr3:857	ILDR1	DriverDB	protein_c	chr3:121987323-122
ENSG0000(	327	8.178574	chr3:857	EAF2	NCGv7	protein_c	chr3:121835183-121
ENSG0000(	327	8.178574	chr3:857	ROPN1B		protein_c	chr3:125969160-125
ENSG0000(	327	8.178574	chr3:857	ENSG00000242968		Pseudoger	chr3:136808551-136
ENSG0000(	327	8.178574	chr3:857	SNORD112		smallRNA	chr3:122245766-122
ENSG0000(	327	8.178574	chr3:857	ACAD9		protein_c	chr3:128879596-128
ENSG0000(	327	8.178574	chr3:857	MTATP6P22		Pseudoger	chr3:106897671-106
ENSG0000(	327	8.178574	chr3:857	RPL32P3		Pseudoger	chr3:129396218-129
ENSG0000(	327	8.178574	chr3:857	SLC41A3		protein_c	chr3:126006357-126
ENSG0000(	327	8.178574	chr3:857	C3orf52		protein_c	chr3:112086335-112
ENSG0000(	327	8.178574	chr3:857	ENSG00000286492		lncRNA	chr3:111835723-111
ENSG0000(	327	8.178574	chr3:857	ZBED2		protein_c	chr3:111592900-111
ENSG0000(	327	8.178574	chr3:857	RNU6-736P		smallRNA	chr3:139388277-139
ENSG0000(	327	8.178574	chr3:857	CFAP44		protein_c	chr3:113286930-113
ENSG0000(	327	8.178574	chr3:857	SNX4		protein_c	chr3:125446650-125
ENSG0000(	327	8.178574	chr3:857	MRPL3		protein_c	chr3:131462212-131
ENSG0000(	327	8.178574	chr3:857	PODXL2	DriverDB	protein_c	chr3:127629185-127
ENSG0000(	327	8.178574	chr3:857	ENSG00000243016		Pseudoger	chr3:127221194-127
ENSG0000(	327	8.178574	chr3:857	UPK1B		protein_c	chr3:119173517-119
ENSG0000(	327	8.178574	chr3:857	EFCC1		protein_c	chr3:129001304-129
ENSG0000(	327	8.178574	chr3:857	CFAP92		protein_c	chr3:128909866-129
ENSG0000(	327	8.178574	chr3:857	PTMAP8		Pseudoger	chr3:117026698-117
ENSG0000(	327	8.178574	chr3:857	NEK11		protein_c	chr3:131026850-131
ENSG0000(	327	8.178574	chr3:857	RNU5E-8P		smallRNA	chr3:116965112-116
ENSG0000(	327	8.178574	chr3:857	ABTB1		protein_c	chr3:127672935-127
ENSG0000(	327	8.178574	chr3:857	ENPP7P3		Pseudoger	chr3:130167790-130
ENSG0000(	327	8.178574	chr3:857	FTH1P4		Pseudoger	chr3:128764466-128
ENSG0000(	327	8.178574	chr3:857	AC068754.1		smallRNA	chr3:122281853-122
ENSG0000(	327	8.178574	chr3:857	ENSG00000251579		Pseudoger	chr3:130927754-130
ENSG0000(	327	8.178574	chr3:857	ACP3		protein_c	chr3:132317369-132
ENSG0000(	327	8.178574	chr3:857	MARK3P3		Pseudoger	chr3:128848673-128
ENSG0000(	327	8.178574	chr3:857	RNU6-509P		smallRNA	chr3:141902924-141
ENSG0000(	327	8.178574	chr3:857	ATP2C1		protein_c	chr3:130850595-131
ENSG0000(	327	8.178574	chr3:857	NUDT16		protein_c	chr3:131381671-131
ENSG0000(	327	8.178574	chr3:857	UMPS	NCGv7	protein_c	chr3:124730433-124
ENSG0000(	327	8.178574	chr3:857	XRN1	NCGv7	protein_c	chr3:142306607-142
ENSG0000(	327	8.178574	chr3:857	NR1I2	NCGv7	protein_c	chr3:119780484-119
ENSG0000(	327	8.178574	chr3:857	ATG3	NCGv7	protein_c	chr3:112532510-112
ENSG0000(	327	8.178574	chr3:857	IGSF11	DriverDB	protein_c	chr3:118900557-119
ENSG0000(	327	8.178574	chr3:857	DZIP3		protein_c	chr3:108589705-108
ENSG0000(	327	8.178574	chr3:857	ADPRH		protein_c	chr3:119579268-119
ENSG0000(	327	8.178574	chr3:857	RABL3		protein_c	chr3:120684938-120
ENSG0000(	327	8.178574	chr3:857	Y_RNA		smallRNA	chr3:106688678-106
ENSG0000(	327	8.178574	chr3:857	PLA1A	NCGv7	protein_c	chr3:119597875-119
ENSG0000(	327	8.178574	chr3:857	BOC	NCGv7;AC	protein_c	chr3:113211003-113
ENSG0000(	327	8.178574	chr3:857	NECTIN3		protein_c	chr3:111070071-111
ENSG0000(	327	8.178574	chr3:857	ENSG00000250796		Pseudoger	chr3:128869624-128

ENSG0000(	327	8.178574	chr3:857:	TAGLN3		protein_c	chr3:111998739-112
ENSG0000(	327	8.178574	chr3:857:	ABHD10		protein_c	chr3:111979010-111
ENSG0000(	327	8.178574	chr3:857:	TFDP2	DriverDB\	protein_c	chr3:141944428-142
ENSG0000(	327	8.178574	chr3:857:	PHLDB2	DriverDB\	protein_c	chr3:111732497-111
ENSG0000(	327	8.178574	chr3:857:	ENSG00000248607		lncRNA	chr3:126056923-126
ENSG0000(	327	8.178574	chr3:857:	BBX	NCGv7	protein_c	chr3:107522936-107
ENSG0000(	327	8.178574	chr3:857:	CBLB	NCGv7;AC	protein_c	chr3:105655461-105
ENSG0000(	327	8.178574	chr3:857:	SRPRB		protein_c	chr3:133784023-133
ENSG0000(	327	8.178574	chr3:857:	TMEM108		protein_c	chr3:133038391-133
ENSG0000(	327	8.178574	chr3:857:	SNORA5		smallRNA	chr3:123814077-123
ENSG0000(	327	8.178574	chr3:857:	MUC13		protein_c	chr3:124905442-124
ENSG0000(	327	8.178574	chr3:857:	IFT57		protein_c	chr3:108160812-108
ENSG0000(	327	8.178574	chr3:857:	HHLA2	NCGv7	protein_c	chr3:108296529-108
ENSG0000(	327	8.178574	chr3:857:	MORC1		protein_c	chr3:108958248-109
ENSG0000(	327	8.178574	chr3:857:	ENSG00000242880		lncRNA	chr3:115147605-115
ENSG0000(	327	8.178574	chr3:857:	ALDH1L1		protein_c	chr3:126103562-126
ENSG0000(	327	8.178574	chr3:857:	FAM86JP		Pseudoger	chr3:125916624-125
ENSG0000(	327	8.178574	chr3:857:	RPL7P16		Pseudoger	chr3:132243528-132
ENSG0000(	327	8.178574	chr3:857:	SLC12A8		protein_c	chr3:125082636-125
ENSG0000(	327	8.178574	chr3:857:	KRT18P35		Pseudoger	chr3:141470634-141
ENSG0000(	327	8.178574	chr3:857:	OSBPL11		protein_c	chr3:125528858-125
ENSG0000(	327	8.178574	chr3:857:	TRPC1		protein_c	chr3:142724034-142
ENSG0000(	326	8.153563	chr2:744:	RP11-685N3.1		protein_c	chr2:87338477-8733
ENSG0000(	326	8.153563	chr2:744:	MALLP2		Pseudoger	chr2:88811633-8881
ENSG0000(	326	8.153563	chr2:744:	EIF2AK3-DT		lncRNA	chr2:88627539-8863
ENSG0000(	326	8.153563	chr2:744:	IGKV2D-30		protein_c	chr2:89936859-8993
ENSG0000(	326	8.153563	chr2:744:	ENSG00000225420		lncRNA	chr2:88538720-8857
ENSG0000(	326	8.153563	chr2:744:	AC233263.1		Pseudoger	chr2:87344236-8734
ENSG0000(	326	8.153563	chr2:744:	ENSG00000287670		lncRNA	chr2:88016780-8802
ENSG0000(	326	8.153563	chr2:744:	NDUFB4P7		Pseudoger	chr2:87968609-8796
ENSG0000(	326	8.153563	chr2:744:	RNY4P15		smallRNA	chr2:88229569-8822
ENSG0000(	326	8.153563	chr2:744:	RNU6-1007P		smallRNA	chr2:88414898-8841
ENSG0000(	326	8.153563	chr2:744:	RPL38P6		Pseudoger	chr2:88428078-8842
ENSG0000(	326	8.153563	chr2:744:	CYTOR		lncRNA	chr2:87454781-8763
ENSG0000(	326	8.153563	chr2:744:	PGBD4P5		Pseudoger	chr2:88922328-8892
ENSG0000(	326	8.153563	chr2:744:	TEX37		protein_c	chr2:88524649-8852
ENSG0000(	326	8.153563	chr2:744:	ANAPC1P4		Pseudoger	chr2:87700984-8773
ENSG0000(	326	8.153563	chr2:744:	RPS14P5		Pseudoger	chr2:87654890-8765
ENSG0000(	326	8.153563	chr2:744:	PLGLB2		protein_c	chr2:87748087-8775
ENSG0000(	326	8.153563	chr2:744:	EIF2AK3	NCGv7	protein_c	chr2:88556741-8869
ENSG0000(	326	8.153563	chr2:744:	ENSG00000224881		Pseudoger	chr2:87379880-8738
ENSG0000(	326	8.153563	chr2:744:	AC068279.1		Pseudoger	chr2:87369233-8736
ENSG0000(	326	8.153563	chr2:744:	IGKV6D-21		protein_c	chr2:90021567-9002
ENSG0000(	326	8.153563	chr2:744:	AC012671.3		smallRNA	chr2:88294592-8829
ENSG0000(	326	8.153563	chr2:744:	IGKV1D-27		Pseudoger	chr2:89968867-8996
ENSG0000(	326	8.153563	chr2:744:	ENSG00000288437		Pseudoger	chr2:88003008-8800
ENSG0000(	326	8.153563	chr2:744:	AC233263.2		Pseudoger	chr2:87369232-8736
ENSG0000(	326	8.153563	chr2:744:	IGKV1D-13		protein_c	chr2:90154073-9015
ENSG0000(	326	8.153563	chr2:744:	LINC01943		lncRNA	chr2:87439523-8745
ENSG0000(	326	8.153563	chr2:744:	SMYD1		protein_c	chr2:88067825-8811
ENSG0000(	326	8.153563	chr2:744:	ENSG00000273445		lncRNA	chr2:87477495-8747

ENSG0000(	326	8.153563	chr2:744؛	snoU13		smallRNA	chr2:88011102-8801
ENSG0000(	326	8.153563	chr2:744؛	KRCC1	NCGv7	protein_c	chr2:88027205-8806
ENSG0000(	326	8.153563	chr2:744؛	AC096579.1		smallRNA	chr2:88767318-8876
ENSG0000(	326	8.153563	chr2:744؛	IGKV1D-39		protein_c	chr2:89862482-8986
ENSG0000(	326	8.153563	chr2:744؛	snoU13		smallRNA	chr2:88307360-8830
ENSG0000(	326	8.153563	chr2:744؛	IGKV2-40		protein_c	chr2:89330116-8933
ENSG0000(	326	8.153563	chr2:744؛	ENSG00000284879		lncRNA	chr2:87455476-8776
ENSG0000(	326	8.153563	chr2:744؛	AC068279.2		Pseudoger	chr2:87359054-8735
ENSG0000(	326	8.153563	chr2:744؛	IGKV7-3		Pseudoger	chr2:88915081-8891
ENSG0000(	326	8.153563	chr2:744؛	ANAPC1P5		Pseudoger	chr2:87980714-8799
ENSG0000(	326	8.153563	chr2:744؛	AC012671.1		smallRNA	chr2:88462767-8846
ENSG0000(	326	8.153563	chr2:744؛	ENSG00000277852		Pseudoger	chr2:89203509-8920
ENSG0000(	326	8.153563	chr2:744؛	IGKV2D-10		Pseudoger	chr2:90179889-9018
ENSG0000(	326	8.153563	chr2:744؛	IGKV2D-19		Pseudoger	chr2:90046796-9004
ENSG0000(	326	8.153563	chr2:744؛	IGKV2D-29		protein_c	chr2:89947512-8994
ENSG0000(	326	8.153563	chr2:744؛	IGKJ3		protein_c	chr2:88861221-8886
ENSG0000(	326	8.153563	chr2:744؛	IGKJ1		protein_c	chr2:88861886-8886
ENSG0000(	326	8.153563	chr2:744؛	IGKV3D-31		Pseudoger	chr2:89929701-8993
ENSG0000(	326	8.153563	chr2:744؛	RNU2-63P		smallRNA	chr2:88016354-8801
ENSG0000(	326	8.153563	chr2:744؛	IGKV2-29		Pseudoger	chr2:89234174-8923
ENSG0000(	326	8.153563	chr2:744؛	AC012671.4		smallRNA	chr2:88374139-8837
ENSG0000(	326	8.153563	chr2:744؛	IGKV4-1		protein_c	chr2:88885397-8888
ENSG0000(	326	8.153563	chr2:744؛	IGKV5-2		protein_c	chr2:88897232-8889
ENSG0000(	326	8.153563	chr2:744؛	IGKV1D-35		Pseudoger	chr2:89895502-8989
ENSG0000(	326	8.153563	chr2:744؛	IGKV1D-17		protein_c	chr2:90082635-9008
ENSG0000(	326	8.153563	chr2:744؛	FABP1		protein_c	chr2:88122982-8812
ENSG0000(	326	8.153563	chr2:744؛	ENSG00000290802		lncRNA	chr2:88811186-8882
ENSG0000(	326	8.153563	chr2:744؛	IGKV3D-11		protein_c	chr2:90172802-9017
ENSG0000(	326	8.153563	chr2:744؛	IGKV6-21		protein_c	chr2:89159751-8916
ENSG0000(	326	8.153563	chr2:744؛	IGKV1-32		Pseudoger	chr2:89253571-8925
ENSG0000(	326	8.153563	chr2:744؛	IGKV1D-37		protein_c	chr2:89884740-8988
ENSG0000(	326	8.153563	chr2:744؛	IGKV3-34		Pseudoger	chr2:89275298-8927
ENSG0000(	326	8.153563	chr2:744؛	IGKV2D-26		protein_c	chr2:89985922-8998
ENSG0000(	326	8.153563	chr2:744؛	IGKV3D-20		protein_c	chr2:90038848-9003
ENSG0000(	326	8.153563	chr2:744؛	IGKV6D-41		protein_c	chr2:90069662-9007
ENSG0000(	326	8.153563	chr2:744؛	IGKV2-19		Pseudoger	chr2:89134975-8913
ENSG0000(	326	8.153563	chr2:744؛	IGKV2-23		Pseudoger	chr2:89172022-8917
ENSG0000(	326	8.153563	chr2:744؛	IGKV2-38		Pseudoger	chr2:89309898-8931
ENSG0000(	326	8.153563	chr2:744؛	IGKV1-22		Pseudoger	chr2:89170775-8917
ENSG0000(	326	8.153563	chr2:744؛	IGKV1-16		protein_c	chr2:89099859-8910
ENSG0000(	326	8.153563	chr2:744؛	IGKV3-15		protein_c	chr2:89085177-8908
ENSG0000(	326	8.153563	chr2:744؛	PAFAH1B1P1		Pseudoger	chr2:87565828-8756
ENSG0000(	326	8.153563	chr2:744؛	IGKV1D-12		protein_c	chr2:90159680-9016
ENSG0000(	326	8.153563	chr2:744؛	IGKV1-33		protein_c	chr2:89268001-8926
ENSG0000(	326	8.153563	chr2:744؛	RPIA	NCGv7	protein_c	chr2:88691673-8875
ENSG0000(	326	8.153563	chr2:744؛	WBP1P2		Pseudoger	chr2:87972656-8797
ENSG0000(	326	8.153563	chr2:744؛	ANKRD36BP2		Pseudoger	chr2:88782712-8880
ENSG0000(	326	8.153563	chr2:744؛	IGKV2D-14		Pseudoger	chr2:90121786-9012
ENSG0000(	326	8.153563	chr2:744؛	MRPL45P1		Pseudoger	chr2:88364695-8836
ENSG0000(	326	8.153563	chr2:744؛	IGKV2D-23		Pseudoger	chr2:90009402-9000
ENSG0000(	326	8.153563	chr2:744؛	MIR4435-1		smallRNA	chr2:87629755-8762

ENSG0000(	326	8.153563	chr2:7442	MIR4436A	smallRNA	chr2:88812370-8881
ENSG0000(	326	8.153563	chr2:7442	IGKV2D-18	Pseudoger	chr2:90052581-9005
ENSG0000(	326	8.153563	chr2:7442	IGKV2-18	Pseudoger	chr2:89128724-8912
ENSG0000(	326	8.153563	chr2:7442	IGKV1-8	protein_c	chr2:88992409-8899
ENSG0000(	326	8.153563	chr2:7442	IGKV2-26	Pseudoger	chr2:89196096-8919
ENSG0000(	326	8.153563	chr2:7442	IGKV2-30	protein_c	chr2:89244781-8924
ENSG0000(	326	8.153563	chr2:7442	IGKV3D-25	Pseudoger	chr2:89989987-8999
ENSG0000(	326	8.153563	chr2:7442	AC073416.1	Pseudoger	chr2:90099156-9009
ENSG0000(	326	8.153563	chr2:7442	IGKV2-28	protein_c	chr2:89221698-8922
ENSG0000(	326	8.153563	chr2:7442	IGKV1-39	protein_c	chr2:89319625-8932
ENSG0000(	326	8.153563	chr2:7442	IGKC	protein_c	chr2:88857161-8885
ENSG0000(	326	8.153563	chr2:7442	MTATP8P2	Pseudoger	chr2:87824942-8782
ENSG0000(	326	8.153563	chr2:7442	IGKJ5	protein_c	chr2:88860568-8886
ENSG0000(	326	8.153563	chr2:7442	IGKV2D-38	Pseudoger	chr2:89872463-8987
ENSG0000(	326	8.153563	chr2:7442	IGKJ4	protein_c	chr2:88860886-8886
ENSG0000(	326	8.153563	chr2:7442	IGKV2D-40	protein_c	chr2:89851791-8985
ENSG0000(	326	8.153563	chr2:7442	IGKV1-27	protein_c	chr2:89213423-8921
ENSG0000(	326	8.153563	chr2:7442	IGKV1D-33	protein_c	chr2:89913982-8991
ENSG0000(	326	8.153563	chr2:7442	IGKV1-35	Pseudoger	chr2:89286689-8928
ENSG0000(	326	8.153563	chr2:7442	ENSG00000289429	lncRNA	chr2:87659372-8768
ENSG0000(	326	8.153563	chr2:7442	IGKV3-20	protein_c	chr2:89142574-8914
ENSG0000(	326	8.153563	chr2:7442	IGKV2D-24	protein_c	chr2:90004797-9000
ENSG0000(	326	8.153563	chr2:7442	IGKV3-11	protein_c	chr2:89027171-8902
ENSG0000(	326	8.153563	chr2:7442	ENSG00000288734	lncRNA	chr2:88136919-8813
ENSG0000(	326	8.153563	chr2:7442	IGKV1-12	protein_c	chr2:89040224-8904
ENSG0000(	326	8.153563	chr2:7442	IGKV3-31	Pseudoger	chr2:89252211-8925
ENSG0000(	326	8.153563	chr2:7442	IGKV2D-28	protein_c	chr2:89959979-8996
ENSG0000(	326	8.153563	chr2:7442	IGKV1D-22	Pseudoger	chr2:90010741-9001
ENSG0000(	326	8.153563	chr2:7442	IGKV1-37	protein_c	chr2:89297264-8929
ENSG0000(	326	8.153563	chr2:7442	IGKV1-9	protein_c	chr2:89009982-8901
ENSG0000(	326	8.153563	chr2:7442	IGKV2-10	Pseudoger	chr2:89019992-8902
ENSG0000(	326	8.153563	chr2:7442	IGKV2-14	Pseudoger	chr2:89078010-8907
ENSG0000(	326	8.153563	chr2:7442	IGKV1D-8	protein_c	chr2:90220727-9022
ENSG0000(	326	8.153563	chr2:7442	IGKV3-25	Pseudoger	chr2:89192500-8919
ENSG0000(	326	8.153563	chr2:7442	IGKV3D-15	protein_c	chr2:90114838-9011
ENSG0000(	326	8.153563	chr2:7442	IGKV3-7	protein_c	chr2:88978468-8897
ENSG0000(	326	8.153563	chr2:7442	FOXI3	protein_c	chr2:88446787-8845
ENSG0000(	326	8.153563	chr2:7442	IGKV1-6	protein_c	chr2:88966262-8896
ENSG0000(	326	8.153563	chr2:7442	IGKV2-4	Pseudoger	chr2:88931666-8893
ENSG0000(	326	8.153563	chr2:7442	IGKV1D-32	Pseudoger	chr2:89928422-8992
ENSG0000(	326	8.153563	chr2:7442	IGKV3OR2-268	protein_c	chr2:87338511-8733
ENSG0000(	326	8.153563	chr2:7442	ENSG00000240040	lncRNA	chr2:88811186-8886
ENSG0000(	326	8.153563	chr2:7442	ANKRD36BP2	lncRNA	chr2:88765807-8880
ENSG0000(	326	8.153563	chr2:7442	IGKV1D-16	protein_c	chr2:90100236-9010
ENSG0000(	326	8.153563	chr2:7442	IGKV1D-43	protein_c	chr2:90209873-9021
ENSG0000(	326	8.153563	chr2:7442	IGKV1-17	protein_c	chr2:89117342-8911
ENSG0000(	326	8.153563	chr2:7442	RGPD2	protein_c	chr2:87755960-8782
ENSG0000(	326	8.153563	chr2:7442	IGKV3D-34	Pseudoger	chr2:89906757-8990
ENSG0000(	326	8.153563	chr2:7442	IGKV1-13	Pseudoger	chr2:89045995-8904
ENSG0000(	326	8.153563	chr2:7442	IGKV1-5	protein_c	chr2:88947301-8894
ENSG0000(	326	8.153563	chr2:7442	IGKV2-24	protein_c	chr2:89176328-8917

ENSG0000(	326	8.153563	chr2:7442	RNU6-568P	smallRNA	chr2:88367793-8836
ENSG0000(	326	8.153563	chr2:7442	ENSG00000290104	lncRNA	chr2:88864876-8886
ENSG0000(	326	8.153563	chr2:7442	IGKV2D-36	Pseudoger	chr2:89887022-8988
ENSG0000(	326	8.153563	chr2:7442	RNU6-1168P	smallRNA	chr2:88383494-8838
ENSG0000(	326	8.153563	chr2:7442	IGKV1D-42	protein_c	chr2:90190193-9019
ENSG0000(	326	8.153563	chr2:7442	IGKV2-36	Pseudoger	chr2:89295233-8929
ENSG0000(	326	8.153563	chr2:7442	THNSL2	protein_c	chr2:88170295-8818
ENSG0000(	324	8.103541	chr15:695	ENSG00000259622	Pseudoger	chr15:82010212-820
ENSG0000(	324	8.103541	chr15:695	EFL1	protein_c	chr15:82130206-822
ENSG0000(	324	8.103541	chr15:695	ENSG00000260836	protein_c	chr15:82536788-825
ENSG0000(	324	8.103541	chr15:695	ENSG00000290424	lncRNA	chr15:78752107-787
ENSG0000(	324	8.103541	chr15:695	ANKRD34C	protein_c	chr15:79282722-792
ENSG0000(	324	8.103541	chr15:695	ENSG00000273920	lncRNA	chr15:81335577-813
ENSG0000(	324	8.103541	chr15:695	ENSG00000260988	lncRNA	chr15:78141243-781
ENSG0000(	324	8.103541	chr15:695	ENSG00000290426	lncRNA	chr15:78660644-787
ENSG0000(	324	8.103541	chr15:695	ENSG00000289704	lncRNA	chr15:82160249-821
ENSG0000(	324	8.103541	chr15:695	ENSG00000259175	lncRNA	chr15:80554609-805
ENSG0000(	324	8.103541	chr15:695	ENSG00000261229	lncRNA	chr15:79843547-798
ENSG0000(	324	8.103541	chr15:695	ENSG00000289561	lncRNA	chr15:79365422-793
ENSG0000(	324	8.103541	chr15:695	CHRNA5	protein_c	chr15:78565520-785
ENSG0000(	324	8.103541	chr15:695	ST20-AS1	lncRNA	chr15:79922771-799
ENSG0000(	324	8.103541	chr15:695	ENSG00000290949	lncRNA	chr15:82519590-825
ENSG0000(	324	8.103541	chr15:695	ENSG00000277482	lncRNA	chr15:78299701-782
ENSG0000(	324	8.103541	chr15:695	ENSG00000261403	lncRNA	chr15:77954075-779
ENSG0000(	324	8.103541	chr15:695	RASGRF1	protein_c	chr15:78959906-790
ENSG0000(	324	8.103541	chr15:695	ENSG00000252690	lncRNA	chr15:82752884-827
ENSG0000(	324	8.103541	chr15:695	ENSG00000261762	lncRNA	chr15:78589123-785
ENSG0000(	324	8.103541	chr15:695	ENSG00000287408	lncRNA	chr15:79331591-793
ENSG0000(	324	8.103541	chr15:695	GOLGA6GP	Pseudoger	chr15:78727177-787
ENSG0000(	324	8.103541	chr15:695	LINGO1-AS1	lncRNA	chr15:77641764-776
ENSG0000(	324	8.103541	chr15:695	ENSG00000280359	TEC	chr15:77784610-777
ENSG0000(	324	8.103541	chr15:695	ENSG00000261244	lncRNA	chr15:77916522-779
ENSG0000(	324	8.103541	chr15:695	DNM1P38	Pseudoger	chr15:82531653-825
ENSG0000(	324	8.103541	chr15:695	ADAMTS7P3	Pseudoger	chr15:77976042-779
ENSG0000(	324	8.103541	chr15:695	ENSG00000273512	Pseudoger	chr15:78749833-787
ENSG0000(	324	8.103541	chr15:695	MIR549	smallRNA	chr15:80841978-808
ENSG0000(	324	8.103541	chr15:695	ENSG00000259649	lncRNA	chr15:80896191-809
ENSG0000(	324	8.103541	chr15:695	ENSG00000289703	lncRNA	chr15:82173391-821
ENSG0000(	324	8.103541	chr15:695	LINGO1-AS2	lncRNA	chr15:77660052-776
ENSG0000(	324	8.103541	chr15:695	CHRNB4	protein_c	chr15:78624111-787
ENSG0000(	324	8.103541	chr15:695	SH2D7	protein_c	chr15:78077808-781
ENSG0000(	324	8.103541	chr15:695	ENSG00000279394	TEC	chr15:79875920-798
ENSG0000(	324	8.103541	chr15:695	ENSG00000279758	TEC	chr15:82738386-827
ENSG0000(	324	8.103541	chr15:695	PSMA4	protein_c	chr15:78540405-785
ENSG0000(	324	8.103541	chr15:695	ENSG00000279719	TEC	chr15:79989905-799
ENSG0000(	324	8.103541	chr15:695	MIR184	smallRNA	chr15:79209788-792
ENSG0000(	324	8.103541	chr15:695	ENSG00000279705	TEC	chr15:80014157-800
ENSG0000(	324	8.103541	chr15:695	ENSG00000279694	TEC	chr15:78339208-783
ENSG0000(	324	8.103541	chr15:695	RN7SL410P	smallRNA	chr15:82478039-824
ENSG0000(	324	8.103541	chr15:695	ENSG00000286817	lncRNA	chr15:82749252-827
ENSG0000(	324	8.103541	chr15:695	UBE2Q2P6	Pseudoger	chr15:82445719-824

ENSG0000(	324	8.103541	chr15:695	ENSG00000286813	lncRNA	chr15:79894502-798
ENSG0000(	324	8.103541	chr15:695	ENSG00000279421	TEC	chr15:78599892-786
ENSG0000(	324	8.103541	chr15:695	CTSH	protein_c	chr15:78921058-789
ENSG0000(	324	8.103541	chr15:695	ENSG00000290948	lncRNA	chr15:82472993-825
ENSG0000(	324	8.103541	chr15:695	GOLGA6L9	protein_c	chr15:82430018-824
ENSG0000(	324	8.103541	chr15:695	ENSG00000279373	TEC	chr15:78537681-785
ENSG0000(	324	8.103541	chr15:695	ENSG00000258010	lncRNA	chr15:80433795-804
ENSG0000(	324	8.103541	chr15:695	ENSG00000275995	lncRNA	chr15:81403026-814
ENSG0000(	324	8.103541	chr15:695	RNU6-380P	smallRNA	chr15:80663772-806
ENSG0000(	324	8.103541	chr15:695	CSPG4P10	Pseudoger	chr15:82459400-824
ENSG0000(	324	8.103541	chr15:695	RNU6-415P	smallRNA	chr15:78898840-788
ENSG0000(	324	8.103541	chr15:695	CEMIP	protein_c	chr15:80779343-809
ENSG0000(	324	8.103541	chr15:695	CSPG4P13	Pseudoger	chr15:77894684-779
ENSG0000(	324	8.103541	chr15:695	FAH	protein_c	chr15:80152490-801
ENSG0000(	324	8.103541	chr15:695	ENSG00000259967	lncRNA	chr15:80165923-801
ENSG0000(	324	8.103541	chr15:695	ENSG00000259792	lncRNA	chr15:77993405-779
ENSG0000(	324	8.103541	chr15:695	ENSG00000279834	TEC	chr15:81060953-810
ENSG0000(	324	8.103541	chr15:695	RPL9P9	Pseudoger	chr15:82372255-824
ENSG0000(	324	8.103541	chr15:695	LINGO1	protein_c	chr15:77613027-778
ENSG0000(	324	8.103541	chr15:695	MEX3B	protein_c	chr15:82041778-820
ENSG0000(	324	8.103541	chr15:695	ENSG00000259692	lncRNA	chr15:81633426-820
ENSG0000(	324	8.103541	chr15:695	ADAMTS7P1	Pseudoger	chr15:82298553-823
ENSG0000(	324	8.103541	chr15:695	ENSG00000290947	lncRNA	chr15:82464708-824
ENSG0000(	324	8.103541	chr15:695	ENSG00000290946	lncRNA	chr15:82445733-824
ENSG0000(	324	8.103541	chr15:695	ENSG00000290944	lncRNA	chr15:82298663-823
ENSG0000(	324	8.103541	chr15:695	ENSG00000259707	Pseudoger	chr15:82691723-826
ENSG0000(	324	8.103541	chr15:695	GOLGA6FP	Pseudoger	chr15:77915184-779
ENSG0000(	324	8.103541	chr15:695	RP11-152F13.3	Pseudoger	chr15:82472993-825
ENSG0000(	324	8.103541	chr15:695	DNAJA4-DT	lncRNA	chr15:78250502-782
ENSG0000(	324	8.103541	chr15:695	RPL21P116	Pseudoger	chr15:78863247-788
ENSG0000(	324	8.103541	chr15:695	ENSG00000279235	TEC	chr15:78906127-789
ENSG0000(	324	8.103541	chr15:695	ENSG00000259770	Pseudoger	chr15:79944498-799
ENSG0000(	324	8.103541	chr15:695	RNU1-77P	smallRNA	chr15:82174224-821
ENSG0000(	324	8.103541	chr15:695	ENSG00000285974	lncRNA	chr15:82180743-821
ENSG0000(	324	8.103541	chr15:695	ENSG00000260674	Pseudoger	chr15:79743311-797
ENSG0000(	324	8.103541	chr15:695	AP3B2	protein_c	chr15:82659281-827
ENSG0000(	324	8.103541	chr15:695	ENSG00000260619	lncRNA	chr15:80990804-809
ENSG0000(	324	8.103541	chr15:695	ENSG00000287108	lncRNA	chr15:80769600-807
ENSG0000(	324	8.103541	chr15:695	RP13-608F4.6	Pseudoger	chr15:82544597-825
ENSG0000(	324	8.103541	chr15:695	ENSG00000238166	Pseudoger	chr15:78753114-787
ENSG0000(	324	8.103541	chr15:695	MESD	protein_c	chr15:80946289-809
ENSG0000(	324	8.103541	chr15:695	ACSBG1	protein_c	chr15:78167468-782
ENSG0000(	324	8.103541	chr15:695	ENSG00000286488	lncRNA	chr15:81315806-813
ENSG0000(	324	8.103541	chr15:695	ENSG00000259555	lncRNA	chr15:78625895-786
ENSG0000(	324	8.103541	chr15:695	ENSG00000259594	lncRNA	chr15:81554003-816
ENSG0000(	324	8.103541	chr15:695	IL16	protein_c	chr15:81159575-813
ENSG0000(	324	8.103541	chr15:695	AC026956.1	smallRNA	chr15:82044294-820
ENSG0000(	324	8.103541	chr15:695	LINC00927	lncRNA	chr15:80263068-803
ENSG0000(	324	8.103541	chr15:695	SAXO2	protein_c	chr15:82262810-822
ENSG0000(	324	8.103541	chr15:695	CHRNA3	protein_c	chr15:78593052-786
ENSG0000(	324	8.103541	chr15:695	ENSG00000259413	Pseudoger	chr15:78382573-783

ENSG0000(	324	8.103541	chr15:695	AC069082.1		smallRNA	chr15:79114293-791
ENSG0000(	324	8.103541	chr15:695	CTXND1		protein_c	chr15:80195481-802
ENSG0000(	324	8.103541	chr15:695	HNRNPCP3		Pseudoger	chr15:79236332-792
ENSG0000(	324	8.103541	chr15:695	ENSG00000259420		lncRNA	chr15:77568970-776
ENSG0000(	324	8.103541	chr15:695	FSD2		protein_c	chr15:82755362-828
ENSG0000(	324	8.103541	chr15:695	ENSG00000177699		lncRNA	chr15:78978889-789
ENSG0000(	324	8.103541	chr15:695	ENSG00000287872		lncRNA	chr15:82113384-821
ENSG0000(	324	8.103541	chr15:695	TMC3	NCGv7	protein_c	chr15:81331088-813
ENSG0000(	324	8.103541	chr15:695	RPL18P11		Pseudoger	chr15:78661047-786
ENSG0000(	324	8.103541	chr15:695	ENSG00000271725		lncRNA	chr15:81303215-813
ENSG0000(	324	8.103541	chr15:695	UBE2Q2P2		Pseudoger	chr15:82391844-824
ENSG0000(	324	8.103541	chr15:695	MTHFS		protein_c	chr15:79833585-798
ENSG0000(	324	8.103541	chr15:695	ADAMTS7		protein_c	chr15:78759206-788
ENSG0000(	324	8.103541	chr15:695	ABHD17C		protein_c	chr15:80679684-807
ENSG0000(	324	8.103541	chr15:695	IREB2		protein_c	chr15:78437431-785
ENSG0000(	324	8.103541	chr15:695	CIB2		protein_c	chr15:78104606-781
ENSG0000(	324	8.103541	chr15:695	ENSG00000259442		lncRNA	chr15:82744223-827
ENSG0000(	324	8.103541	chr15:695	TMED3		protein_c	chr15:79311112-794
ENSG0000(	324	8.103541	chr15:695	ENSG00000271983		lncRNA	chr15:80693216-806
ENSG0000(	324	8.103541	chr15:695	ANP32BP3		Pseudoger	chr15:81117635-811
ENSG0000(	324	8.103541	chr15:695	ENSG00000287813		lncRNA	chr15:80440294-804
ENSG0000(	324	8.103541	chr15:695	ENSG00000213527		Pseudoger	chr15:79751689-797
ENSG0000(	324	8.103541	chr15:695	ARNT2	NCGv7	protein_c	chr15:80404350-805
ENSG0000(	324	8.103541	chr15:695	MIR4514		smallRNA	chr15:80997417-809
ENSG0000(	324	8.103541	chr15:695	ENSG00000290665		lncRNA	chr15:77918420-779
ENSG0000(	324	8.103541	chr15:695	RNU6-667P		smallRNA	chr15:79817349-798
ENSG0000(	324	8.103541	chr15:695	ENSG00000290667		lncRNA	chr15:77987992-779
ENSG0000(	324	8.103541	chr15:695	ENSG00000270919		lncRNA	chr15:80580029-805
ENSG0000(	324	8.103541	chr15:695	ENSG00000290666		lncRNA	chr15:77941899-779
ENSG0000(	324	8.103541	chr15:695	ENSG00000288625		protein_c	chr15:81002142-810
ENSG0000(	324	8.103541	chr15:695	ENSG00000228141		Pseudoger	chr15:82710471-827
ENSG0000(	324	8.103541	chr15:695	ENSG00000291296		lncRNA	chr15:77914217-779
ENSG0000(	324	8.103541	chr15:695	ANKRD34C-AS1		lncRNA	chr15:79123939-792
ENSG0000(	324	8.103541	chr15:695	HYKK		protein_c	chr15:78507564-785
ENSG0000(	324	8.103541	chr15:695	ACTG1P17		Pseudoger	chr15:82725873-827
ENSG0000(	324	8.103541	chr15:695	ENSG00000288592		Pseudoger	chr15:79899031-798
ENSG0000(	324	8.103541	chr15:695	ENSG00000278603		Pseudoger	chr15:82472203-824
ENSG0000(	324	8.103541	chr15:695	ENSG00000278600		lncRNA	chr15:79920195-799
ENSG0000(	324	8.103541	chr15:695	ENSG00000271364		Pseudoger	chr15:79984151-799
ENSG0000(	324	8.103541	chr15:695	ENSG00000259322		lncRNA	chr15:78293286-782
ENSG0000(	324	8.103541	chr15:695	ENSG00000259229		Pseudoger	chr15:79758549-797
ENSG0000(	324	8.103541	chr15:695	ENSG00000288561		Pseudoger	chr15:78831041-788
ENSG0000(	324	8.103541	chr15:695	SNORD112		smallRNA	chr15:80142058-801
ENSG0000(	324	8.103541	chr15:695	AC105339.2		smallRNA	chr15:82726550-827
ENSG0000(	324	8.103541	chr15:695	AC027811.1		smallRNA	chr15:79338730-793
ENSG0000(	324	8.103541	chr15:695	CSPG4P8		Pseudoger	chr15:82459472-824
ENSG0000(	324	8.103541	chr15:695	hsa-mir-5572		smallRNA	chr15:80581103-805
ENSG0000(	324	8.103541	chr15:695	AC010724.1		smallRNA	chr15:82477123-824
ENSG0000(	324	8.103541	chr15:695	ST20-MTHFS		protein_c	chr15:79845150-799
ENSG0000(	324	8.103541	chr15:695	TMC3-AS1		lncRNA	chr15:81324338-815
ENSG0000(	324	8.103541	chr15:695	ENSG00000290664		lncRNA	chr15:77901661-779

ENSG0000(	324	8.103541	chr15:69!	CPEB1-AS1		lncRNA	chr15:82647770-826
ENSG0000(	324	8.103541	chr15:69!	RPS17		protein_c	chr15:82536750-825
ENSG0000(	324	8.103541	chr15:69!	ENSG00000259208		Pseudoger	chr15:79668342-796
ENSG0000(	324	8.103541	chr15:69!	RPL9P8		Pseudoger	chr15:82372255-824
ENSG0000(	324	8.103541	chr15:69!	ENSG00000272418		lncRNA	chr15:78280950-782
ENSG0000(	324	8.103541	chr15:69!	TBC1D2B		protein_c	chr15:77984036-780
ENSG0000(	324	8.103541	chr15:69!	COMMD4P1		Pseudoger	chr15:77941442-779
ENSG0000(	324	8.103541	chr15:69!	MINAR1	NCGv7	protein_c	chr15:79432336-794
ENSG0000(	324	8.103541	chr15:69!	WDR61		protein_c	chr15:78277835-782
ENSG0000(	324	8.103541	chr15:69!	LINC01583		lncRNA	chr15:82088569-820
ENSG0000(	324	8.103541	chr15:69!	RP13-608F4.8		lncRNA	chr15:82418651-824
ENSG0000(	324	8.103541	chr15:69!	ENSG00000278013		lncRNA	chr15:82540870-825
ENSG0000(	324	8.103541	chr15:69!	BCL2A1	AC	protein_c	chr15:79960892-799
ENSG0000(	324	8.103541	chr15:69!	ENSG00000259543		lncRNA	chr15:81427448-817
ENSG0000(	324	8.103541	chr15:69!	CFAP161		protein_c	chr15:81007033-811
ENSG0000(	324	8.103541	chr15:69!	CRABP1		protein_c	chr15:78340353-783
ENSG0000(	324	8.103541	chr15:69!	IDH3A		protein_c	chr15:78131498-781
ENSG0000(	324	8.103541	chr15:69!	ENSG00000259546		lncRNA	chr15:80896190-809
ENSG0000(	324	8.103541	chr15:69!	SNORA63		smallRNA	chr15:78091172-780
ENSG0000(	324	8.103541	chr15:69!	ENSG00000259548		lncRNA	chr15:79832466-798
ENSG0000(	324	8.103541	chr15:69!	MORF4L1		protein_c	chr15:78810487-788
ENSG0000(	324	8.103541	chr15:69!	ENSG00000277782		lncRNA	chr15:80999593-809
ENSG0000(	324	8.103541	chr15:69!	ENSG00000259562		Pseudoger	chr15:78290527-782
ENSG0000(	324	8.103541	chr15:69!	RN7SL61P		smallRNA	chr15:82478039-824
ENSG0000(	324	8.103541	chr15:69!	ENSG00000259213		lncRNA	chr15:77787193-777
ENSG0000(	324	8.103541	chr15:69!	ENSG00000278202		lncRNA	chr15:82418651-824
ENSG0000(	324	8.103541	chr15:69!	RP13-608F4.1		Pseudoger	chr15:82519590-825
ENSG0000(	324	8.103541	chr15:69!	TFDP1P3		Pseudoger	chr15:79559256-795
ENSG0000(	324	8.103541	chr15:69!	ST20		lncRNA	chr15:79898840-799
ENSG0000(	324	8.103541	chr15:69!	AC135995.1		smallRNA	chr15:82477123-824
ENSG0000(	324	8.103541	chr15:69!	CPEB1	NCGv7	protein_c	chr15:82543201-826
ENSG0000(	324	8.103541	chr15:69!	ENSG00000284709		Pseudoger	chr15:81385390-813
ENSG0000(	324	8.103541	chr15:69!	WHAMM		protein_c	chr15:82809628-828
ENSG0000(	324	8.103541	chr15:69!	SNHG21		lncRNA	chr15:82750564-827
ENSG0000(	324	8.103541	chr15:69!	TLNRD1		protein_c	chr15:81000923-810
ENSG0000(	324	8.103541	chr15:69!	STARD5		protein_c	chr15:81309053-813
ENSG0000(	324	8.103541	chr15:69!	MIR1827		smallRNA	chr15:78038531-780
ENSG0000(	324	8.103541	chr15:69!	ARNT2-DT		lncRNA	chr15:80344853-804
ENSG0000(	324	8.103541	chr15:69!	ENSG00000284803		lncRNA	chr15:82533175-825
ENSG0000(	324	8.103541	chr15:69!	ZFAND6		protein_c	chr15:80059568-801
ENSG0000(	324	8.103541	chr15:69!	DNAJA4		protein_c	chr15:78264086-782
ENSG0000(	324	8.103541	chr15:69!	GOLGA6L17P		Pseudoger	chr15:82519654-825
ENSG0000(	324	8.103541	chr15:69!	ENSG00000259474		lncRNA	chr15:78480057-784
ENSG0000(	322	8.053519	chr15:40!	ENSG00000259286		Pseudoger	chr15:68833830-688
ENSG0000(	322	8.053519	chr15:40!	LINC02206		lncRNA	chr15:66931537-669
ENSG0000(	322	8.053519	chr15:40!	ENSG00000259191		Pseudoger	chr15:69434466-694
ENSG0000(	322	8.053519	chr2:744:	KANSL3		protein_c	chr2:96593170-9664
ENSG0000(	322	8.053519	chr15:40!	ENSG00000259202		lncRNA	chr15:67142734-671
ENSG0000(	322	8.053519	chr15:40!	HMGN2P47		Pseudoger	chr15:67000814-670
ENSG0000(	322	8.053519	chr2:744:	LMAN2L		protein_c	chr2:96705929-9674
ENSG0000(	322	8.053519	chr2:744:	ENSG00000286654		lncRNA	chr2:96912549-9691

ENSG0000(	322	8.053519	chr2:744	ENSG00000278766		lncRNA	chr2:97421075-9743
ENSG0000(	322	8.053519	chr15:40	ENSG00000260007		protein_c	chr15:68184032-682
ENSG0000(	322	8.053519	chr15:40	PIAS1		protein_c	chr15:68054309-681
ENSG0000(	322	8.053519	chr15:40	ENSG00000259222		lncRNA	chr15:69080879-690
ENSG0000(	322	8.053519	chr2:744	ENSG00000235833		Pseudoger	chr2:97523949-9752
ENSG0000(	322	8.053519	chr15:40	KIF23-AS1		lncRNA	chr15:69396904-694
ENSG0000(	322	8.053519	chr15:40	LINC02896		lncRNA	chr15:69458522-694
ENSG0000(	322	8.053519	chr15:40	SPESP1		protein_c	chr15:68818221-689
ENSG0000(	322	8.053519	chr15:40	LINC01169		lncRNA	chr15:66582190-666
ENSG0000(	322	8.053519	chr15:40	IQCH		protein_c	chr15:67254786-675
ENSG0000(	322	8.053519	chr15:40	ENSG00000259457		lncRNA	chr15:69564724-695
ENSG0000(	322	8.053519	chr15:40	ENSG00000259437		lncRNA	chr15:66919811-669
ENSG0000(	322	8.053519	chr2:744	ENSG00000235480		lncRNA	chr2:96527940-9653
ENSG0000(	322	8.053519	chr15:40	AAGAB		protein_c	chr15:67200667-672
ENSG0000(	322	8.053519	chr15:40	IQCH-AS1		lncRNA	chr15:67290636-675
ENSG0000(	322	8.053519	chr15:40	NOX5		protein_c	chr15:68930504-690
ENSG0000(	322	8.053519	chr15:40	SNORA77		smallRNA	chr15:69325293-693
ENSG0000(	322	8.053519	chr15:40	ENSG00000259504		lncRNA	chr15:69278675-692
ENSG0000(	322	8.053519	chr15:40	ENSG00000259410		lncRNA	chr15:67832725-678
ENSG0000(	322	8.053519	chr15:40	HNRNPA1P5		Pseudoger	chr15:67627852-676
ENSG0000(	322	8.053519	chr15:40	ENSG00000259347		lncRNA	chr15:66984103-670
ENSG0000(	322	8.053519	chr2:744	FER1L5		protein_c	chr2:96642737-9670
ENSG0000(	322	8.053519	chr15:40	ENSG00000277152		lncRNA	chr15:66860303-668
ENSG0000(	322	8.053519	chr2:744	CNN2P11		Pseudoger	chr2:94725674-9472
ENSG0000(	322	8.053519	chr2:744	ITPRIPL1		protein_c	chr2:96325317-9633
ENSG0000(	322	8.053519	chr15:40	ENSG00000259645		lncRNA	chr15:69391192-693
ENSG0000(	322	8.053519	chr15:40	CORO2B		protein_c	chr15:68578993-687
ENSG0000(	322	8.053519	chr15:40	RNA5SP398		smallRNA	chr15:69367585-693
ENSG0000(	322	8.053519	chr15:40	RPS24P16		Pseudoger	chr15:67232446-672
ENSG0000(	322	8.053519	chr2:744	CNNM3		protein_c	chr2:96816245-9683
ENSG0000(	322	8.053519	chr2:744	ASTL		protein_c	chr2:96122818-9613
ENSG0000(	322	8.053519	chr15:40	EWSAT1		lncRNA	chr15:69072926-690
ENSG0000(	322	8.053519	chr15:40	ENSG00000245719		lncRNA	chr15:67834310-678
ENSG0000(	322	8.053519	chr2:744	ENSG00000289135		lncRNA	chr2:96815093-9681
ENSG0000(	322	8.053519	chr15:40	C15orf61		protein_c	chr15:67521131-675
ENSG0000(	322	8.053519	chr15:40	MIR4312		smallRNA	chr15:68801850-688
ENSG0000(	322	8.053519	chr2:744	snoU13		smallRNA	chr2:96981282-9698
ENSG0000(	322	8.053519	chr15:40	AC026992.1		smallRNA	chr15:69247109-692
ENSG0000(	322	8.053519	chr15:40	MAP2K5		protein_c	chr15:67542703-678
ENSG0000(	322	8.053519	chr15:40	KIF23		protein_c	chr15:69414246-694
ENSG0000(	322	8.053519	chr15:40	ITGA11		protein_c	chr15:68296532-684
ENSG0000(	322	8.053519	chr15:40	RPLP1		protein_c	chr15:69452814-694
ENSG0000(	322	8.053519	chr15:40	PAQR5		protein_c	chr15:69298912-694
ENSG0000(	322	8.053519	chr15:40	SMAD6	NCGv7	protein_c	chr15:66702236-667
ENSG0000(	322	8.053519	chr15:40	AC107871.1		smallRNA	chr15:68229672-682
ENSG0000(	322	8.053519	chr2:744	ENSG00000230343		Pseudoger	chr2:97034442-9703
ENSG0000(	322	8.053519	chr15:40	GLCE		protein_c	chr15:69160584-692
ENSG0000(	322	8.053519	chr15:40	ENSG00000260109		Pseudoger	chr15:67985059-679
ENSG0000(	322	8.053519	chr2:744	MIR3127		smallRNA	chr2:96798278-9679
ENSG0000(	322	8.053519	chr15:40	ANP32A		protein_c	chr15:68778535-688
ENSG0000(	322	8.053519	chr2:744	NCAPH		protein_c	chr2:96335766-9637

ENSG0000(	322	8.053519	chr15:409	DRAIC		lncRNA	chr15:69462921-698
ENSG0000(	322	8.053519	chr2:7442	STARD7		protein_c	chr2:96184859-9620
ENSG0000(	322	8.053519	chr2:7442	DUSP2	NCGv7	protein_c	chr2:96143169-9614
ENSG0000(	322	8.053519	chr2:7442	ENSG00000230747		lncRNA	chr2:96307263-9632
ENSG0000(	322	8.053519	chr15:409	U3		smallRNA	chr15:69457942-694
ENSG0000(	322	8.053519	chr15:409	ENSG00000270490		Pseudoger	chr15:66808404-668
ENSG0000(	322	8.053519	chr2:7442	IGKV2OR2-7		Pseudoger	chr2:97372532-9737
ENSG0000(	322	8.053519	chr15:409	MAP2K5-DT		lncRNA	chr15:67541072-675
ENSG0000(	322	8.053519	chr2:7442	ANKRD39		protein_c	chr2:96836611-9685
ENSG0000(	322	8.053519	chr15:409	FEM1B		protein_c	chr15:68277745-682
ENSG0000(	322	8.053519	chr15:409	CALML4		protein_c	chr15:68190705-682
ENSG0000(	322	8.053519	chr15:409	CLN6		protein_c	chr15:68206992-682
ENSG0000(	322	8.053519	chr2:7442	AC159540.1		lncRNA	chr2:97415474-9743
ENSG0000(	322	8.053519	chr15:409	CARS1P1		Pseudoger	chr15:68749501-687
ENSG0000(	322	8.053519	chr2:7442	ENSG00000228873		lncRNA	chr2:96145602-9614
ENSG0000(	322	8.053519	chr2:7442	ENSG00000248134		Pseudoger	chr2:97018343-9701
ENSG0000(	322	8.053519	chr2:7442	IGKV1OR2-9		Pseudoger	chr2:97386082-9738
ENSG0000(	322	8.053519	chr15:409	ENSG00000288068		lncRNA	chr15:68985795-689
ENSG0000(	322	8.053519	chr2:7442	CNNM4		protein_c	chr2:96760902-9681
ENSG0000(	322	8.053519	chr2:7442	RNA5SP101		Pseudoger	chr2:96956708-9695
ENSG0000(	322	8.053519	chr15:409	AC026512.1		smallRNA	chr15:69160651-691
ENSG0000(	322	8.053519	chr15:409	SKOR1		protein_c	chr15:67819704-678
ENSG0000(	322	8.053519	chr2:7442	TMEM127	NCGv7	protein_c	chr2:96248514-9626
ENSG0000(	322	8.053519	chr2:7442	IGKV2OR2-8		Pseudoger	chr2:97376674-9737
ENSG0000(	322	8.053519	chr15:409	SMAD3	NCGv7	protein_c	chr15:67063763-671
ENSG0000(	322	8.053519	chr15:409	NOX5		protein_c	chr15:69014695-690
ENSG0000(	322	8.053519	chr15:409	ENSG00000273851		Pseudoger	chr15:69022230-690
ENSG0000(	322	8.053519	chr2:7442	ENSG00000273634		Pseudoger	chr2:97008368-9700
ENSG0000(	322	8.053519	chr2:7442	ENSG00000275094		Pseudoger	chr2:97000436-9700
ENSG0000(	322	8.053519	chr15:409	ENSG00000260657		lncRNA	chr15:68267792-682
ENSG0000(	322	8.053519	chr15:409	ENSG00000274995		lncRNA	chr15:66740445-667
ENSG0000(	322	8.053519	chr15:409	ENSG00000285919		lncRNA	chr15:68880663-689
ENSG0000(	322	8.053519	chr15:409	HMGN2P40		Pseudoger	chr15:68254549-682
ENSG0000(	322	8.053519	chr2:7442	ADRA2B		protein_c	chr2:96112876-9611
ENSG0000(	322	8.053519	chr2:7442	CIAO1		protein_c	chr2:96266159-9627
ENSG0000(	322	8.053519	chr2:7442	STARD7-AS1		lncRNA	chr2:96208389-9624
ENSG0000(	322	8.053519	chr2:7442	ANKRD36B		protein_c	chr2:97492663-9758
ENSG0000(	322	8.053519	chr2:7442	SNRNP200	NCGv7	protein_c	chr2:96274338-9632
ENSG0000(	322	8.053519	chr15:409	ENSG00000259265		lncRNA	chr15:69037549-690
ENSG0000(	322	8.053519	chr2:7442	ARID5A		protein_c	chr2:96536743-9655
ENSG0000(	322	8.053519	chr2:7442	ENSG00000236847		Pseudoger	chr2:97035461-9703
ENSG0000(	322	8.053519	chr15:409	ENSG00000289998		lncRNA	chr15:68755025-687
ENSG0000(	322	8.053519	chr2:7442	AC159540.3		lncRNA	chr2:97404351-9740
ENSG0000(	322	8.053519	chr15:409	ENSG00000261702		Pseudoger	chr15:67974391-679
ENSG0000(	322	8.053519	chr2:7442	NEURL3		protein_c	chr2:96497646-9650
ENSG0000(	322	8.053519	chr15:409	RNU6-1		smallRNA	chr15:67839939-678
ENSG0000(	322	8.053519	chr2:7442	ANKRD23		protein_c	chr2:96824526-9685
ENSG0000(	322	8.053519	chr15:409	ENSG00000286770		lncRNA	chr15:68483202-684
ENSG0000(	322	8.053519	chr2:7442	CNNM3-DT		lncRNA	chr2:96812239-9681
ENSG0000(	322	8.053519	chr2:7442	SEMA4C		protein_c	chr2:96859718-9687
ENSG0000(	322	8.053519	chr15:409	Y_RNA		smallRNA	chr15:69406441-694

ENSG0000(	322	8.053519	chr15:40§	PAQR5-DT		lncRNA	chr15:69278328-692
ENSG0000(	322	8.053519	chr15:40§	RN7SL438P		smallRNA	chr15:69250527-692
ENSG0000(	322	8.053519	chr2:744¿	FAM178B		protein_c	chr2:96875882-9698
ENSG0000(	322	8.053519	chr2:744¿	TRIM43CP		Pseudoger	chr2:97025981-9703
ENSG0000(	312	7.80341	chr22:49¿	ENSG00000236867		Pseudoger	chr22:49817248-498
ENSG0000(	312	7.80341	chr16:65(	ENSG00000289491		protein_c	chr16:30610211-306
ENSG0000(	311	7.778399	chr3:857:	RPL23AP43		Pseudoger	chr3:32785646-3278
ENSG0000(	310	7.753388	chr14:67(	COX7A2P1		Pseudoger	chr14:67652300-676
ENSG0000(	309	7.728377	chr12:58:	RN7SL804P		smallRNA	chr12:69400903-694
ENSG0000(	304	7.603322	chr2:744¿	ST3GAL5-AS1		lncRNA	chr2:85889151-8589
ENSG0000(	304	7.603322	chr2:744¿	ENSG00000290846		lncRNA	chr2:91617160-9166
ENSG0000(	304	7.603322	chr2:744¿	LINC00342		lncRNA	chr2:95807052-9583
ENSG0000(	304	7.603322	chr2:744¿	RNF103-CH	DriverDB\	protein_c	chr2:86505668-8672
ENSG0000(	304	7.603322	chr2:744¿	ENSG00000286011		lncRNA	chr2:85418504-8542
ENSG0000(	304	7.603322	chr2:744¿	IGKV3D-7		protein_c	chr2:90234812-9023
ENSG0000(	304	7.603322	chr2:744¿	ENSG00000291176		lncRNA	chr2:96010526-9602
ENSG0000(	304	7.603322	chr2:744¿	LSP1P5		lncRNA	chr2:91587019-9166
ENSG0000(	304	7.603322	chr2:744¿	ENSG00000286036		lncRNA	chr2:97618638-9763
ENSG0000(	304	7.603322	chr2:744¿	ENSG00000286045		lncRNA	chr2:75710782-7572
ENSG0000(	304	7.603322	chr2:744¿	ENSG00000232502		Pseudoger	chr2:94760774-9476
ENSG0000(	304	7.603322	chr2:744¿	ANAPC1P2		lncRNA	chr2:87030675-8707
ENSG0000(	304	7.603322	chr2:744¿	ENSG00000235959		Pseudoger	chr2:95640181-9564
ENSG0000(	304	7.603322	chr2:744¿	ENSG00000291013		lncRNA	chr2:86861825-8689
ENSG0000(	304	7.603322	chr2:744¿	CD8B		protein_c	chr2:86815339-8686
ENSG0000(	304	7.603322	chr2:744¿	ENSG00000291024		lncRNA	chr2:94811046-9481
ENSG0000(	304	7.603322	chr2:744¿	ENSG00000286202		lncRNA	chr2:76691007-7669
ENSG0000(	304	7.603322	chr2:744¿	ENSG00000291025		lncRNA	chr2:94867486-9494
ENSG0000(	304	7.603322	chr2:744¿	ENSG00000232594		Pseudoger	chr2:94961047-9496
ENSG0000(	304	7.603322	chr2:744¿	C2orf92		protein_c	chr2:97664217-9770
ENSG0000(	304	7.603322	chr2:744¿	LINC01809		lncRNA	chr2:83522814-8352
ENSG0000(	304	7.603322	chr2:744¿	KMT5AP2		Pseudoger	chr2:91747940-9174
ENSG0000(	304	7.603322	chr2:744¿	RBM7P1		Pseudoger	chr2:80162428-8016
ENSG0000(	304	7.603322	chr2:744¿	GAPDHP57		Pseudoger	chr2:75455994-7545
ENSG0000(	304	7.603322	chr2:744¿	ENSG00000291126		lncRNA	chr2:95526651-9553
ENSG0000(	304	7.603322	chr2:744¿	ENSG00000236209		lncRNA	chr2:74919555-7492
ENSG0000(	304	7.603322	chr2:744¿	Y_RNA		smallRNA	chr2:86159956-8616
ENSG0000(	304	7.603322	chr2:744¿	ENSG00000224879		lncRNA	chr2:79158374-7918
ENSG0000(	304	7.603322	chr2:744¿	ENSG00000290897		lncRNA	chr2:95789654-9579
ENSG0000(	304	7.603322	chr2:744¿	ENSG00000236026		Pseudoger	chr2:95606924-9560
ENSG0000(	304	7.603322	chr2:744¿	TRIM51JP		Pseudoger	chr2:95574901-9558
ENSG0000(	304	7.603322	chr2:744¿	WDR54		protein_c	chr2:74421678-7442
ENSG0000(	304	7.603322	chr2:744¿	GCFC2		protein_c	chr2:75652000-7571
ENSG0000(	304	7.603322	chr2:744¿	KMT2CP1		lncRNA	chr2:91686102-9171
ENSG0000(	304	7.603322	chr2:744¿	ENSG00000290771		lncRNA	chr2:79135503-7913
ENSG0000(	304	7.603322	chr2:744¿	ENSG00000286932		lncRNA	chr2:77210587-7732
ENSG0000(	304	7.603322	chr2:744¿	ENSG00000286211		lncRNA	chr2:82831234-8286
ENSG0000(	304	7.603322	chr2:744¿	ENSG00000233444		Pseudoger	chr2:81666477-8166
ENSG0000(	304	7.603322	chr2:744¿	REEP1		protein_c	chr2:86213993-8633
ENSG0000(	304	7.603322	chr2:744¿	LINC01851		lncRNA	chr2:77915870-7791
ENSG0000(	304	7.603322	chr2:744¿	GPR160P1		Pseudoger	chr2:85686053-8568
ENSG0000(	304	7.603322	chr2:744¿	SFTPB		protein_c	chr2:85657314-8566

ENSG00000(	304	7.603322	chr2:7442	ENSG00000287628		lncRNA	chr2:85934535-8593
ENSG00000(	304	7.603322	chr2:7442	RNA5SP100		Pseudoger	chr2:91674857-9167
ENSG00000(	304	7.603322	chr2:7442	CTNNA2	NCGv7	protein_c	chr2:79185231-8064
ENSG00000(	304	7.603322	chr2:7442	IGKV1OR2-2		Pseudoger	chr2:92034522-9203
ENSG00000(	304	7.603322	chr2:7442	ENSG00000233447		Pseudoger	chr2:94974544-9497
ENSG00000(	304	7.603322	chr2:7442	ENSG00000287625		lncRNA	chr2:84926019-8496
ENSG00000(	304	7.603322	chr2:7442	ENSG00000289370		lncRNA	chr2:95206349-9520
ENSG00000(	304	7.603322	chr2:7442	ENSG00000287607		Pseudoger	chr2:91578478-9157
ENSG00000(	304	7.603322	chr2:7442	ATOH8		protein_c	chr2:85751344-8579
ENSG00000(	304	7.603322	chr2:7442	LRRTM4-AS1		lncRNA	chr2:76985965-7700
ENSG00000(	304	7.603322	chr2:7442	ENSG00000287687		lncRNA	chr2:74723873-7477
ENSG00000(	304	7.603322	chr2:7442	ENSG00000233275		Pseudoger	chr2:95434759-9543
ENSG00000(	304	7.603322	chr2:7442	ENSG00000287474		lncRNA	chr2:75799974-7620
ENSG00000(	304	7.603322	chr2:7442	ENSG00000289685		protein_c	chr2:95085391-9516
ENSG00000(	304	7.603322	chr2:7442	IGSF3P2		Pseudoger	chr2:91736726-9176
ENSG00000(	304	7.603322	chr2:7442	VWA3B		protein_c	chr2:98087116-9831
ENSG00000(	304	7.603322	chr2:7442	FAM95A		lncRNA	chr2:94755326-9479
ENSG00000(	304	7.603322	chr2:7442	SMC3P1		Pseudoger	chr2:99102018-9910
ENSG00000(	304	7.603322	chr2:7442	ENSG00000287362		lncRNA	chr2:95067074-9507
ENSG00000(	304	7.603322	chr2:7442	UBTFL3		Pseudoger	chr2:95625213-9562
ENSG00000(	304	7.603322	chr2:7442	RPS2P17		Pseudoger	chr2:84915868-8491
ENSG00000(	304	7.603322	chr2:7442	RPL12P18		Pseudoger	chr2:84874696-8487
ENSG00000(	304	7.603322	chr2:7442	UBTFL5		Pseudoger	chr2:95450310-9545
ENSG00000(	304	7.603322	chr2:7442	RPS24P6		Pseudoger	chr2:94912432-9491
ENSG00000(	304	7.603322	chr2:7442	POLR1A		protein_c	chr2:86020216-8610
ENSG00000(	304	7.603322	chr2:7442	ANKRD11P1		Pseudoger	chr2:81194337-8120
ENSG00000(	304	7.603322	chr2:7442	ENSG00000286227		lncRNA	chr2:79269905-7929
ENSG00000(	304	7.603322	chr2:7442	RNU4-8P		smallRNA	chr2:97664591-9766
ENSG00000(	304	7.603322	chr2:7442	TMEM131		protein_c	chr2:97756333-9799
ENSG00000(	304	7.603322	chr2:7442	AC113612.1		Pseudoger	chr2:90261265-9026
ENSG00000(	304	7.603322	chr2:7442	TOR1BP1		Pseudoger	chr2:74618856-7462
ENSG00000(	304	7.603322	chr2:7442	ENSG00000288858		lncRNA	chr2:85594864-8559
ENSG00000(	304	7.603322	chr2:7442	ENSG00000233850		lncRNA	chr2:95025193-9502
ENSG00000(	304	7.603322	chr2:7442	Y_RNA		smallRNA	chr2:85367585-8536
ENSG00000(	304	7.603322	chr2:7442	ENSG00000288960		lncRNA	chr2:95074906-9507
ENSG00000(	304	7.603322	chr2:7442	ENSG00000223977		Pseudoger	chr2:83218890-8321
ENSG00000(	304	7.603322	chr2:7442	ENSG00000233757		protein_c	chr2:95207521-9525
ENSG00000(	304	7.603322	chr2:7442	MGAT4A		protein_c	chr2:98619106-9873
ENSG00000(	304	7.603322	chr2:7442	OR7E102P		Pseudoger	chr2:95546531-9554
ENSG00000(	304	7.603322	chr2:7442	MAT2A		protein_c	chr2:85539168-8554
ENSG00000(	304	7.603322	chr2:7442	ENSG00000287931		lncRNA	chr2:87075653-8711
ENSG00000(	304	7.603322	chr2:7442	ENSG00000289076		lncRNA	chr2:84459572-8446
ENSG00000(	304	7.603322	chr2:7442	USP39		protein_c	chr2:85602856-8564
ENSG00000(	304	7.603322	chr2:7442	RNU6-827P		smallRNA	chr2:78882447-7888
ENSG00000(	304	7.603322	chr2:7442	CNN2P8		Pseudoger	chr2:94737337-9473
ENSG00000(	304	7.603322	chr2:7442	ENSG00000223917		Pseudoger	chr2:95486480-9548
ENSG00000(	304	7.603322	chr2:7442	MTCO3P45		Pseudoger	chr2:94900990-9490
ENSG00000(	304	7.603322	chr2:7442	ANAPC1P1		Pseudoger	chr2:86871301-8691
ENSG00000(	304	7.603322	chr2:7442	ENSG00000287763		lncRNA	chr2:87311460-8734
ENSG00000(	304	7.603322	chr2:7442	ENSG00000287749		lncRNA	chr2:76197855-7625
ENSG00000(	304	7.603322	chr2:7442	CENPNP1		Pseudoger	chr2:87221113-8722

ENSG0000(	304	7.603322	chr2:7442	VAMP5	protein_c	chr2:85584431-8559
ENSG0000(	304	7.603322	chr2:7442	U8	smallRNA	chr2:86347062-8634
ENSG0000(	304	7.603322	chr2:7442	RNF181	protein_c	chr2:85595725-8559
ENSG0000(	304	7.603322	chr2:7442	TMEM150A	protein_c	chr2:85598547-8560
ENSG0000(	304	7.603322	chr2:7442	C2orf68	protein_c	chr2:85605254-8561
ENSG0000(	304	7.603322	chr2:7442	AC073464.11	Pseudoger	chr2:94795808-9480
ENSG0000(	304	7.603322	chr2:7442	ENSG00000204745	Pseudoger	chr2:87125198-8719
ENSG0000(	304	7.603322	chr2:7442	REG1CP	Pseudoger	chr2:79135701-7913
ENSG0000(	304	7.603322	chr2:7442	RNU6-640P	smallRNA	chr2:86515204-8651
ENSG0000(	304	7.603322	chr2:7442	REG1B	protein_c	chr2:79085023-7908
ENSG0000(	304	7.603322	chr2:7442	ENSG00000290110	lncRNA	chr2:85328084-8532
ENSG0000(	304	7.603322	chr2:7442	DHFRP3	Pseudoger	chr2:82856826-8285
ENSG0000(	304	7.603322	chr2:7442	AC018696.7	Pseudoger	chr2:91561304-9156
ENSG0000(	304	7.603322	chr2:7442	CHMP4AP1	Pseudoger	chr2:81418723-8141
ENSG0000(	304	7.603322	chr2:7442	ST6GALNAC2P1	Pseudoger	chr2:84039885-8404
ENSG0000(	304	7.603322	chr2:7442	ENSG00000235463	Pseudoger	chr2:85068809-8506
ENSG0000(	304	7.603322	chr2:7442	RNU6-674P	smallRNA	chr2:85204926-8520
ENSG0000(	304	7.603322	chr2:7442	LINC01815	lncRNA	chr2:81461358-8146
ENSG0000(	304	7.603322	chr2:7442	RPL37P10	Pseudoger	chr2:83594956-8359
ENSG0000(	304	7.603322	chr2:7442	SNX18P14	Pseudoger	chr2:94786688-9478
ENSG0000(	304	7.603322	chr2:7442	RPS28P5	Pseudoger	chr2:74754670-7475
ENSG0000(	304	7.603322	chr2:7442	ENSG00000235584	lncRNA	chr2:95660588-9566
ENSG0000(	304	7.603322	chr2:7442	ATP5F1BP1	Pseudoger	chr2:98206151-9820
ENSG0000(	304	7.603322	chr2:7442	ENSG00000234837	Pseudoger	chr2:94750582-9475
ENSG0000(	304	7.603322	chr2:7442	RN7SKP203	smallRNA	chr2:76445079-7644
ENSG0000(	304	7.603322	chr2:7442	PARTICL	lncRNA	chr2:85537462-8553
ENSG0000(	304	7.603322	chr2:7442	MTND6P7	Pseudoger	chr2:82817538-8281
ENSG0000(	304	7.603322	chr2:7442	HMGN1P36	Pseudoger	chr2:97827248-9782
ENSG0000(	304	7.603322	chr2:7442	CYP4F32P	Pseudoger	chr2:94759259-9476
ENSG0000(	304	7.603322	chr2:7442	ENSG00000224719	Pseudoger	chr2:95502791-9550
ENSG0000(	304	7.603322	chr2:7442	ENSG00000222000	lncRNA	chr2:98331389-9835
ENSG0000(	304	7.603322	chr2:7442	ENSG00000290565	lncRNA	chr2:91686102-9171
ENSG0000(	304	7.603322	chr2:7442	LDHAP7	Pseudoger	chr2:84777259-8477
ENSG0000(	304	7.603322	chr2:7442	ENSG00000290575	lncRNA	chr2:91892472-9191
ENSG0000(	304	7.603322	chr2:7442	ENSG00000224731	lncRNA	chr2:80028012-8003
ENSG0000(	304	7.603322	chr2:7442	ENSG00000286260	lncRNA	chr2:78880713-7893
ENSG0000(	304	7.603322	chr2:7442	FAHD2CP	Pseudoger	chr2:96013730-9602
ENSG0000(	304	7.603322	chr2:7442	REG3A	protein_c	chr2:79157003-7915
ENSG0000(	304	7.603322	chr2:7442	ENSG00000286698	lncRNA	chr2:91759462-9176
ENSG0000(	304	7.603322	chr2:7442	IGKV1OR2-1	Pseudoger	chr2:91817771-9181
ENSG0000(	304	7.603322	chr2:7442	TMSB10	protein_c	chr2:84905656-8490
ENSG0000(	304	7.603322	chr2:7442	CAPG AC	protein_c	chr2:85394753-8541
ENSG0000(	304	7.603322	chr2:7442	Y_RNA	smallRNA	chr2:82307067-8230
ENSG0000(	304	7.603322	chr2:7442	RETSAT	protein_c	chr2:85341955-8535
ENSG0000(	304	7.603322	chr2:7442	SUCLA2P2	Pseudoger	chr2:76106016-7610
ENSG0000(	304	7.603322	chr2:7442	ENSG00000234877	lncRNA	chr2:78597911-7859
ENSG0000(	304	7.603322	chr2:7442	ENSG00000287172	lncRNA	chr2:76185020-7639
ENSG0000(	304	7.603322	chr2:7442	AC018690.1	smallRNA	chr2:99520103-9952
ENSG0000(	304	7.603322	chr2:7442	ENSG00000287130	lncRNA	chr2:76684975-7669
ENSG0000(	304	7.603322	chr2:7442	ENSG00000231781	lncRNA	chr2:81983272-8200
ENSG0000(	304	7.603322	chr2:7442	ENSG00000287026	lncRNA	chr2:76633365-7667

ENSG0000(	304	7.603322	chr2:744:	ENSG00000287025		lncRNA	chr2:76893894-7689
ENSG0000(	304	7.603322	chr2:744:	CHEK2P3		Pseudoger	chr2:91957436-9196
ENSG0000(	304	7.603322	chr2:744:	ENSG00000234903		Pseudoger	chr2:95468863-9546
ENSG0000(	304	7.603322	chr2:744:	LINC01291		lncRNA	chr2:74918148-7493
ENSG0000(	304	7.603322	chr2:744:	INPP4A	NCGv7	protein_c	chr2:98444854-9859
ENSG0000(	304	7.603322	chr2:744:	ENSG00000286883		lncRNA	chr2:74465339-7447
ENSG0000(	304	7.603322	chr2:744:	RNU4-84P		smallRNA	chr2:98782410-9878
ENSG0000(	304	7.603322	chr2:744:	ENSG00000233105		Pseudoger	chr2:94964473-9496
ENSG0000(	304	7.603322	chr2:744:	MRPL53		protein_c	chr2:74471982-7447
ENSG0000(	304	7.603322	chr2:744:	ENSG00000233037		Pseudoger	chr2:95542730-9554
ENSG0000(	304	7.603322	chr2:744:	ENSG00000290085		lncRNA	chr2:84969747-8497
ENSG0000(	304	7.603322	chr2:744:	FABP7P2		Pseudoger	chr2:95368507-9536
ENSG0000(	304	7.603322	chr2:744:	ENSG00000235147		Pseudoger	chr2:92006680-9200
ENSG0000(	304	7.603322	chr2:744:	MAL		protein_c	chr2:95025677-9505
ENSG0000(	304	7.603322	chr2:744:	ENSG00000286739		lncRNA	chr2:74715269-7480
ENSG0000(	304	7.603322	chr2:744:	ENSG00000235186		Pseudoger	chr2:94991880-9499
ENSG0000(	304	7.603322	chr2:744:	ENSG00000224585		Pseudoger	chr2:94971954-9497
ENSG0000(	304	7.603322	chr2:744:	RNU6-812P		smallRNA	chr2:78882628-7888
ENSG0000(	304	7.603322	chr2:744:	M1AP		protein_c	chr2:74557883-7464
ENSG0000(	304	7.603322	chr2:744:	RNA5SP98		Pseudoger	chr2:76772909-7677
ENSG0000(	304	7.603322	chr2:744:	ENSG00000272183		lncRNA	chr2:74501717-7450
ENSG0000(	304	7.603322	chr2:744:	ENSG00000270696		lncRNA	chr2:75660462-7566
ENSG0000(	304	7.603322	chr2:744:	ENSG00000270996		lncRNA	chr2:75719120-7572
ENSG0000(	304	7.603322	chr2:744:	IGKV1OR2-118		Pseudoger	chr2:90315365-9031
ENSG0000(	304	7.603322	chr2:744:	ENSG00000271003		Pseudoger	chr2:95607073-9561
ENSG0000(	304	7.603322	chr2:744:	ENSG00000271014		Pseudoger	chr2:85360798-8536
ENSG0000(	304	7.603322	chr2:744:	SUPT4H1P1		Pseudoger	chr2:75651288-7565
ENSG0000(	304	7.603322	chr2:744:	RNF103	NCGv7	protein_c	chr2:86603398-8662
ENSG0000(	304	7.603322	chr2:744:	DBF4P3		Pseudoger	chr2:87301658-8730
ENSG0000(	304	7.603322	chr2:744:	PNPP1		Pseudoger	chr2:76258034-7625
ENSG0000(	304	7.603322	chr2:744:	RNU6-685P		smallRNA	chr2:82268612-8226
ENSG0000(	304	7.603322	chr2:744:	USP21P2		Pseudoger	chr2:76260755-7626
ENSG0000(	304	7.603322	chr2:744:	ENSG00000271452		lncRNA	chr2:75669989-7567
ENSG0000(	304	7.603322	chr2:744:	Y_RNA		smallRNA	chr2:85332895-8533
ENSG0000(	304	7.603322	chr2:744:	CRLF3P3		Pseudoger	chr2:84031140-8403
ENSG0000(	304	7.603322	chr2:744:	Y_RNA		smallRNA	chr2:85460144-8546
ENSG0000(	304	7.603322	chr2:744:	TRIM64FP		Pseudoger	chr2:95514827-9552
ENSG0000(	304	7.603322	chr2:744:	NKAIN1P2		Pseudoger	chr2:91723023-9172
ENSG0000(	304	7.603322	chr2:744:	WBP1		protein_c	chr2:74458400-7446
ENSG0000(	304	7.603322	chr2:744:	ENSG00000270571		lncRNA	chr2:75154366-7518
ENSG0000(	304	7.603322	chr2:744:	PEBP1P2		Pseudoger	chr2:85341281-8534
ENSG0000(	304	7.603322	chr2:744:	TRABD2A		protein_c	chr2:84821650-8490
ENSG0000(	304	7.603322	chr2:744:	REV1		protein_c	chr2:99400475-9949
ENSG0000(	304	7.603322	chr2:744:	COX5B		protein_c	chr2:97646062-9764
ENSG0000(	304	7.603322	chr2:744:	MIR4264		smallRNA	chr2:79649294-7964
ENSG0000(	304	7.603322	chr2:744:	CCDC142		protein_c	chr2:74471986-7448
ENSG0000(	304	7.603322	chr2:744:	SEMA4F		protein_c	chr2:74654228-7468
ENSG0000(	304	7.603322	chr2:744:	RN7SL126P		smallRNA	chr2:85567664-8556
ENSG0000(	304	7.603322	chr2:744:	RN7SL830P		smallRNA	chr2:85532344-8553
ENSG0000(	304	7.603322	chr2:744:	ENSG00000266931		Pseudoger	chr2:87055658-8707
ENSG0000(	304	7.603322	chr2:744:	ENSG00000270470		Pseudoger	chr2:78437850-7843

ENSG0000(	304	7.603322	chr2:7442	KMT2CP5	Pseudoger	chr2:91696452-9171
ENSG0000(	304	7.603322	chr2:7442	RGPD1	protein_c	chr2:86913783-8701
ENSG0000(	304	7.603322	chr2:7442	MRPL35	protein_c	chr2:86199355-8621
ENSG0000(	304	7.603322	chr2:7442	IMMT	protein_c	chr2:86143932-8619
ENSG0000(	304	7.603322	chr2:7442	PTCD3	protein_c	chr2:86106223-8614
ENSG0000(	304	7.603322	chr2:7442	ENSG00000270193	Pseudoger	chr2:95616492-9561
ENSG0000(	304	7.603322	chr2:7442	ENSG00000270462	lncRNA	chr2:75697583-7569
ENSG0000(	304	7.603322	chr2:7442	snoU13	smallRNA	chr2:75635141-7563
ENSG0000(	304	7.603322	chr2:7442	NDUFB4P5	Pseudoger	chr2:86934462-8693
ENSG0000(	304	7.603322	chr2:7442	ACTR3BP2	Pseudoger	chr2:91940668-9194
ENSG0000(	304	7.603322	chr2:7442	RNU7-46P	smallRNA	chr2:98840675-9884
ENSG0000(	304	7.603322	chr2:7442	ENSG00000274028	Pseudoger	chr2:95588149-9558
ENSG0000(	304	7.603322	chr2:7442	CRACDL	protein_c	chr2:98793846-9893
ENSG0000(	304	7.603322	chr2:7442	INO80B-WBP1	protein_c	chr2:74455088-7446
ENSG0000(	304	7.603322	chr2:7442	AC027612.2	smallRNA	chr2:91763925-9176
ENSG0000(	304	7.603322	chr2:7442	ENSG00000275075	Pseudoger	chr2:91578478-9157
ENSG0000(	304	7.603322	chr2:7442	ZNF2	protein_c	chr2:95165432-9518
ENSG0000(	304	7.603322	chr2:7442	LSM3P3	Pseudoger	chr2:85102389-8510
ENSG0000(	304	7.603322	chr2:7442	VAMP8	protein_c	chr2:85561562-8558
ENSG0000(	304	7.603322	chr2:7442	RNU7-162P	smallRNA	chr2:85373454-8537
ENSG0000(	304	7.603322	chr2:7442	ENSG00000231062	lncRNA	chr2:95051395-9505
ENSG0000(	304	7.603322	chr2:7442	AC015971.1	smallRNA	chr2:86586140-8658
ENSG0000(	304	7.603322	chr2:7442	ENSG00000238162	Pseudoger	chr2:95485541-9548
ENSG0000(	304	7.603322	chr2:7442	ENSG00000275490	Pseudoger	chr2:90309229-9030
ENSG0000(	304	7.603322	chr2:7442	ENSG00000275497	Pseudoger	chr2:95633850-9563
ENSG0000(	304	7.603322	chr2:7442	TCF7L1-IT1	lncRNA	chr2:85186409-8518
ENSG0000(	304	7.603322	chr2:7442	ENSG00000275767	Pseudoger	chr2:91589464-9162
ENSG0000(	304	7.603322	chr2:7442	SLC2AXP1	Pseudoger	chr2:95196449-9519
ENSG0000(	304	7.603322	chr2:7442	ENSG00000273825	Pseudoger	chr2:91589469-9162
ENSG0000(	304	7.603322	chr2:7442	AC016670.1	smallRNA	chr2:80244623-8024
ENSG0000(	304	7.603322	chr2:7442	snoU109	smallRNA	chr2:75489576-7548
ENSG0000(	304	7.603322	chr2:7442	C2orf15	protein_c	chr2:99141707-9915
ENSG0000(	304	7.603322	chr2:7442	RNU7-96P	smallRNA	chr2:97913054-9791
ENSG0000(	304	7.603322	chr2:7442	ANKRD20A8P	Pseudoger	chr2:94791103-9485
ENSG0000(	304	7.603322	chr2:7442	ANKRD36C NCGv7	protein_c	chr2:95836919-9599
ENSG0000(	304	7.603322	chr2:7442	ENSG00000272564	lncRNA	chr2:85904279-8590
ENSG0000(	304	7.603322	chr2:7442	HK2-DT	lncRNA	chr2:74832655-7483
ENSG0000(	304	7.603322	chr2:7442	ENSG00000272913	lncRNA	chr2:95524873-9552
ENSG0000(	304	7.603322	chr2:7442	snoU13	smallRNA	chr2:75418846-7541
ENSG0000(	304	7.603322	chr2:7442	ENSG00000228272	lncRNA	chr2:84315108-8435
ENSG0000(	304	7.603322	chr2:7442	ENSG00000230975	lncRNA	chr2:80699388-8087
ENSG0000(	304	7.603322	chr2:7442	ENSG00000230964	Pseudoger	chr2:91578478-9157
ENSG0000(	304	7.603322	chr2:7442	ENSG00000273080	lncRNA	chr2:86195154-8619
ENSG0000(	304	7.603322	chr2:7442	ENSG00000273155	protein_c	chr2:99154998-9919
ENSG0000(	304	7.603322	chr2:7442	ENSG00000273196	lncRNA	chr2:85387074-8538
ENSG0000(	304	7.603322	chr2:7442	ENSG00000230968	lncRNA	chr2:77672215-7767
ENSG0000(	304	7.603322	chr2:7442	ENSG00000273305	lncRNA	chr2:95537969-9553
ENSG0000(	304	7.603322	chr2:7442	CYCSP6	Pseudoger	chr2:78412793-7841
ENSG0000(	304	7.603322	chr2:7442	TSGA10	protein_c	chr2:98997261-9915
ENSG0000(	304	7.603322	chr2:7442	ENSG00000265897	Pseudoger	chr2:90359808-9036
ENSG0000(	304	7.603322	chr2:7442	RN7SKP83	smallRNA	chr2:85820435-8582

ENSG0000(	304	7.603322	chr2:7442	LYG1		protein_c	chr2:99284238-9930
ENSG0000(	304	7.603322	chr2:7442	TGOLN2		protein_c	chr2:85318027-8532
ENSG0000(	304	7.603322	chr2:7442	TCF7L1	NCGv7	protein_c	chr2:85133392-8531
ENSG0000(	304	7.603322	chr2:7442	RNU6-1320P		smallRNA	chr2:94846533-9484
ENSG0000(	304	7.603322	chr2:7442	LBX2-AS1		lncRNA	chr2:74502552-7450
ENSG0000(	304	7.603322	chr2:7442	FUNDC2P2		Pseudoger	chr2:84290683-8429
ENSG0000(	304	7.603322	chr2:7442	DUXAP1		Pseudoger	chr2:84750769-8475
ENSG0000(	304	7.603322	chr2:7442	GXYLT1P7		Pseudoger	chr2:94734655-9473
ENSG0000(	304	7.603322	chr2:7442	RN7SL210P		smallRNA	chr2:96004565-9600
ENSG0000(	304	7.603322	chr2:7442	AC079117.2		smallRNA	chr2:77020336-7702
ENSG0000(	304	7.603322	chr2:7442	LRRTM1	NCGv7	protein_c	chr2:80288351-8030
ENSG0000(	304	7.603322	chr2:7442	PLGLB1		protein_c	chr2:87002559-8702
ENSG0000(	304	7.603322	chr2:7442	ENSG00000227120		Pseudoger	chr2:95436133-9543
ENSG0000(	304	7.603322	chr2:7442	ENSG00000227088		lncRNA	chr2:77652025-7829
ENSG0000(	304	7.603322	chr2:7442	TEKT4		protein_c	chr2:94871430-9487
ENSG0000(	304	7.603322	chr2:7442	COA5		protein_c	chr2:98599314-9860
ENSG0000(	304	7.603322	chr2:7442	RN7SL575P		smallRNA	chr2:95003547-9500
ENSG0000(	304	7.603322	chr2:7442	AC018696.5		Pseudoger	chr2:91544070-9154
ENSG0000(	304	7.603322	chr2:7442	PABPC1P6		Pseudoger	chr2:91877969-9188
ENSG0000(	304	7.603322	chr2:7442	SH2D6		protein_c	chr2:85418714-8543
ENSG0000(	304	7.603322	chr2:7442	ENSG00000229689		Pseudoger	chr2:95525345-9552
ENSG0000(	304	7.603322	chr2:7442	LYARP1		Pseudoger	chr2:82268427-8226
ENSG0000(	304	7.603322	chr2:7442	LBX2		protein_c	chr2:74497517-7450
ENSG0000(	304	7.603322	chr2:7442	HK2		protein_c	chr2:74834127-7489
ENSG0000(	304	7.603322	chr2:7442	ENSG00000246575		Pseudoger	chr2:85315041-8531
ENSG0000(	304	7.603322	chr2:7442	ENSG00000229494		lncRNA	chr2:78088729-7812
ENSG0000(	304	7.603322	chr2:7442	EIF5B		protein_c	chr2:99337371-9940
ENSG0000(	304	7.603322	chr2:7442	MITD1		protein_c	chr2:99161427-9918
ENSG0000(	304	7.603322	chr2:7442	ENSG00000229498		lncRNA	chr2:85815130-8582
ENSG0000(	304	7.603322	chr2:7442	CTNNA2-AS1		lncRNA	chr2:79492704-7951
ENSG0000(	304	7.603322	chr2:7442	SLC9B1P2		Pseudoger	chr2:91883076-9193
ENSG0000(	304	7.603322	chr2:7442	MTCYBP7		Pseudoger	chr2:82818131-8281
ENSG0000(	304	7.603322	chr2:7442	PROM2		protein_c	chr2:95274449-9529
ENSG0000(	304	7.603322	chr2:7442	RN7SL251P		smallRNA	chr2:85442495-8544
ENSG0000(	304	7.603322	chr2:7442	RPSAP22		Pseudoger	chr2:85490930-8549
ENSG0000(	304	7.603322	chr2:7442	ABCD1P5		Pseudoger	chr2:91840601-9184
ENSG0000(	304	7.603322	chr2:7442	CD8A		protein_c	chr2:86784610-8680
ENSG0000(	304	7.603322	chr2:7442	RMND5A		protein_c	chr2:86720291-8677
ENSG0000(	304	7.603322	chr2:7442	ENSG00000259848		Pseudoger	chr2:94886861-9489
ENSG0000(	304	7.603322	chr2:7442	CNGA3		protein_c	chr2:98346188-9839
ENSG0000(	304	7.603322	chr2:7442	ENSG00000230477		Pseudoger	chr2:75598071-7559
ENSG0000(	304	7.603322	chr2:7442	LIPT1		protein_c	chr2:99154955-9916
ENSG0000(	304	7.603322	chr2:7442	ENSG00000261600		lncRNA	chr2:91580336-9158
ENSG0000(	304	7.603322	chr2:7442	AC079117.3		smallRNA	chr2:77041778-7704
ENSG0000(	304	7.603322	chr2:7442	MTND4P25		Pseudoger	chr2:82814984-8281
ENSG0000(	304	7.603322	chr2:7442	LRRTM4	NCGv7	protein_c	chr2:76747685-7759
ENSG0000(	304	7.603322	chr2:7442	MRPL30		protein_c	chr2:99181152-9919
ENSG0000(	304	7.603322	chr2:7442	GNA13P1		Pseudoger	chr2:79573764-7957
ENSG0000(	304	7.603322	chr2:7442	LYG2		protein_c	chr2:99242246-9925
ENSG0000(	304	7.603322	chr2:7442	AC096753.1		smallRNA	chr2:79794270-7979
ENSG0000(	304	7.603322	chr2:7442	MIR5000		smallRNA	chr2:75090812-7509

ENSG0000(	304	7.603322	chr2:7442	ENSG00000227987		lncRNA	chr2:98346995-9835
ENSG0000(	304	7.603322	chr2:7442	SNRPEP11		Pseudoger	chr2:85262144-8526
ENSG0000(	304	7.603322	chr2:7442	AC018696.1		Pseudoger	chr2:91443388-9144
ENSG0000(	304	7.603322	chr2:7442	MIR4771-2		smallRNA	chr2:87194786-8719
ENSG0000(	304	7.603322	chr2:7442	ANAPC1P3		Pseudoger	chr2:87118534-8712
ENSG0000(	304	7.603322	chr2:7442	GPAT2		protein_c	chr2:96021946-9603
ENSG0000(	304	7.603322	chr12:685	MIR3652		smallRNA	chr12:103930425-10
ENSG0000(	304	7.603322	chr2:7442	MIR4779		smallRNA	chr2:86193026-8619
ENSG0000(	304	7.603322	chr2:7442	SUCLG1		protein_c	chr2:84423528-8446
ENSG0000(	304	7.603322	chr2:7442	SNORD94		smallRNA	chr2:86135870-8613
ENSG0000(	304	7.603322	chr2:7442	ENSG00000230083		Pseudoger	chr2:95590969-9559
ENSG0000(	304	7.603322	chr2:7442	AC092675.1		smallRNA	chr2:98296938-9829
ENSG0000(	304	7.603322	chr2:7442	DQX1		protein_c	chr2:74518131-7452
ENSG0000(	304	7.603322	chr2:7442	MRPS5		protein_c	chr2:95085369-9512
ENSG0000(	304	7.603322	chr2:7442	ZNF514		protein_c	chr2:95122087-9516
ENSG0000(	304	7.603322	chr2:7442	TRIM43		protein_c	chr2:95592001-9559
ENSG0000(	304	7.603322	chr2:7442	TRIM43B		protein_c	chr2:95477008-9548
ENSG0000(	304	7.603322	chr2:7442	REG3G		protein_c	chr2:79025686-7902
ENSG0000(	304	7.603322	chr2:7442	DRD5P1		Pseudoger	chr2:91684447-9168
ENSG0000(	304	7.603322	chr2:7442	RBX1P1		Pseudoger	chr2:82609652-8260
ENSG0000(	304	7.603322	chr2:7442	ENSG00000241962		protein_c	chr2:99141485-9932
ENSG0000(	304	7.603322	chr2:7442	RN7SL201P		smallRNA	chr2:81967079-8196
ENSG0000(	304	7.603322	chr2:7442	ENSG00000248821		Pseudoger	chr2:95413456-9541
ENSG0000(	304	7.603322	chr2:7442	RN7SL113P		smallRNA	chr2:85368282-8536
ENSG0000(	304	7.603322	chr2:7442	LSP1P4		Pseudoger	chr2:91636684-9165
ENSG0000(	304	7.603322	chr2:7442	LINC02611		lncRNA	chr2:98761098-9877
ENSG0000(	304	7.603322	chr2:7442	KCMF1		protein_c	chr2:84971093-8505
ENSG0000(	304	7.603322	chr2:7442	LINC01964		lncRNA	chr2:85061213-8506
ENSG0000(	304	7.603322	chr2:7442	AC113612.2		Pseudoger	chr2:90255285-9025
ENSG0000(	304	7.603322	chr2:7442	LINC01293		lncRNA	chr2:74940258-7494
ENSG0000(	304	7.603322	chr2:7442	ENSG00000276118		Pseudoger	chr2:91578478-9157
ENSG0000(	304	7.603322	chr2:7442	ENSG00000237308		Pseudoger	chr2:95496022-9549
ENSG0000(	304	7.603322	chr2:7442	ENSG00000213605		Pseudoger	chr2:86885075-8688
ENSG0000(	304	7.603322	chr2:7442	ENSG00000277747		Pseudoger	chr2:95460144-9546
ENSG0000(	304	7.603322	chr2:7442	RTKN		protein_c	chr2:74425835-7444
ENSG0000(	304	7.603322	chr2:7442	RNU7-64P		smallRNA	chr2:85441916-8544
ENSG0000(	304	7.603322	chr2:7442	KCNIP3	DriverDBv	protein_c	chr2:95297327-9538
ENSG0000(	304	7.603322	chr2:7442	FAHD2A		protein_c	chr2:95402708-9541
ENSG0000(	304	7.603322	chr2:7442	ACTR1B		protein_c	chr2:97655939-9766
ENSG0000(	304	7.603322	chr2:7442	ZAP70		protein_c	chr2:97713576-9773
ENSG0000(	304	7.603322	chr2:7442	ENSG00000277171		Pseudoger	chr2:79547151-7954
ENSG0000(	304	7.603322	chr2:7442	INO80B		protein_c	chr2:74455087-7445
ENSG0000(	304	7.603322	chr2:7442	MOGS		protein_c	chr2:74461057-7446
ENSG0000(	304	7.603322	chr2:7442	TTC31		protein_c	chr2:74483073-7449
ENSG0000(	304	7.603322	chr2:7442	PCGF1		protein_c	chr2:74505043-7450
ENSG0000(	304	7.603322	chr2:7442	TLX2		protein_c	chr2:74513463-7451
ENSG0000(	304	7.603322	chr2:7442	ENSG00000237498		lncRNA	chr2:82476825-8253
ENSG0000(	304	7.603322	chr2:7442	ENSG00000277095		Pseudoger	chr2:91589464-9162
ENSG0000(	304	7.603322	chr2:7442	AUP1		protein_c	chr2:74526645-7452
ENSG0000(	304	7.603322	chr2:7442	HTRA2	IntOGen-I	protein_c	chr2:74529596-7453
ENSG0000(	304	7.603322	chr2:7442	LOXL3		protein_c	chr2:74532258-7455

ENSG0000(	304	7.603322	chr2:744	DOK1		protein_c	chr2:74549026-7455
ENSG0000(	304	7.603322	chr2:744	POLE4		protein_c	chr2:74958643-7497
ENSG0000(	304	7.603322	chr2:744	TACR1		protein_c	chr2:75046463-7519
ENSG0000(	304	7.603322	chr2:744	EVA1A		protein_c	chr2:75469302-7556
ENSG0000(	304	7.603322	chr2:744	MRPL19		protein_c	chr2:75646783-7569
ENSG0000(	304	7.603322	chr2:744	GPAT2P1		Pseudoger	chr2:95792220-9580
ENSG0000(	304	7.603322	chr2:744	REG1A	NCGv7	protein_c	chr2:79120362-7912
ENSG0000(	304	7.603322	chr2:744	DNAH6		protein_c	chr2:84516455-8481
ENSG0000(	304	7.603322	chr2:744	UNC50		protein_c	chr2:98608579-9861
ENSG0000(	304	7.603322	chr2:744	ELMOD3		protein_c	chr2:85354394-8539
ENSG0000(	304	7.603322	chr2:744	ENSG00000278131		Pseudoger	chr2:91589494-9162
ENSG0000(	304	7.603322	chr2:744	ANAPC1P2		Pseudoger	chr2:87031815-8705
ENSG0000(	304	7.603322	chr2:744	KMT2CP2		Pseudoger	chr2:91696435-9171
ENSG0000(	304	7.603322	chr2:744	ENSG00000237293		lncRNA	chr2:75474453-7548
ENSG0000(	304	7.603322	chr2:744	ENSG00000283427		Pseudoger	chr2:91607493-9162
ENSG0000(	304	7.603322	chr2:744	ENSG00000283214		Pseudoger	chr2:91736724-9173
ENSG0000(	304	7.603322	chr2:744	ENSG00000283196		Pseudoger	chr2:91654920-9165
ENSG0000(	304	7.603322	chr2:744	KMT2CP4		Pseudoger	chr2:91696451-9171
ENSG0000(	304	7.603322	chr2:744	ENSG00000236431		Pseudoger	chr2:95536117-9553
ENSG0000(	304	7.603322	chr2:744	MTND5P27		Pseudoger	chr2:82815809-8281
ENSG0000(	304	7.603322	chr2:744	ENSG00000281904		lncRNA	chr2:90365736-9036
ENSG0000(	304	7.603322	chr2:744	LINC01955		lncRNA	chr2:87249095-8725
ENSG0000(	304	7.603322	chr2:744	YWHAQP5		Pseudoger	chr2:98694109-9869
ENSG0000(	304	7.603322	chr2:744	TVP23BP2		Pseudoger	chr2:74628328-7462
ENSG0000(	304	7.603322	chr2:744	RNU1-38P		smallRNA	chr2:85728194-8572
ENSG0000(	304	7.603322	chr2:744	RN7SKP164		smallRNA	chr2:76595413-7659
ENSG0000(	304	7.603322	chr2:744	ENSG00000236750		Pseudoger	chr2:95641634-9564
ENSG0000(	304	7.603322	chr2:744	SNORA19		smallRNA	chr2:86364136-8636
ENSG0000(	304	7.603322	chr2:744	U3		smallRNA	chr2:75627953-7562
ENSG0000(	304	7.603322	chr2:744	WBP1P1		Pseudoger	chr2:86930250-8693
ENSG0000(	304	7.603322	chr2:744	RNU6-1312P		smallRNA	chr2:83657735-8365
ENSG0000(	304	7.603322	chr2:744	GGT8P		Pseudoger	chr2:91775944-9178
ENSG0000(	304	7.603322	chr2:744	Y_RNA		smallRNA	chr2:85434507-8543
ENSG0000(	304	7.603322	chr2:744	ENSG00000237031		lncRNA	chr2:80572681-8061
ENSG0000(	304	7.603322	chr2:744	RNU6-561P		smallRNA	chr2:79636862-7963
ENSG0000(	304	7.603322	chr2:744	UBTFL6		Pseudoger	chr2:97636780-9763
ENSG0000(	304	7.603322	chr2:744	AC093162.1		smallRNA	chr2:85299034-8529
ENSG0000(	304	7.603322	chr2:744	MTCO1P48		Pseudoger	chr2:94899566-9490
ENSG0000(	304	7.603322	chr2:744	RPL38P2		Pseudoger	chr2:77788382-7779
ENSG0000(	304	7.603322	chr2:744	ENSG00000231331		Pseudoger	chr2:94953161-9495
ENSG0000(	304	7.603322	chr2:744	ENSG00000237085		Pseudoger	chr2:91859384-9185
ENSG0000(	304	7.603322	chr2:744	CHMP3-AS1		lncRNA	chr2:86562070-8661
ENSG0000(	304	7.603322	chr2:744	SOWAHCP5		Pseudoger	chr2:94861362-9486
ENSG0000(	304	7.603322	chr2:744	SNORD112		smallRNA	chr2:83858823-8385
ENSG0000(	304	7.603322	chr2:744	ENSG00000273306		lncRNA	chr2:99405218-9940
ENSG0000(	304	7.603322	chr2:744	GGCX		protein_c	chr2:85544720-8556
ENSG0000(	304	7.603322	chr2:744	EVA1A-AS		lncRNA	chr2:75524068-7554
ENSG0000(	304	7.603322	chr2:744	KDM3A		protein_c	chr2:86440647-8649
ENSG0000(	304	7.603322	chr2:744	ST3GAL5		protein_c	chr2:85837120-8590
ENSG0000(	304	7.603322	chr2:744	GNLY		protein_c	chr2:85685175-8569
ENSG0000(	304	7.603322	chr2:744	IGKV1OR1-1		Pseudoger	chr2:91486789-9148

ENSG0000(	304	7.603322	chr2:744	TXNDC9		protein_c	chr2:99318982-9934
ENSG0000(	304	7.603322	chr2:744	ENSG00000276362		Pseudoger	chr2:84850711-8485
ENSG0000(	304	7.603322	chr2:744	CHMP3		protein_c	chr2:86503430-8656
ENSG0000(	303	7.578312	chr2:4707	AC016727.1		smallRNA	chr2:61555360-6155
ENSG0000(	299	7.478268	chr16:209	RN7SL245P		smallRNA	chr16:22636455-226
ENSG0000(	298	7.453257	chr5:7979	RNU1-76P		smallRNA	chr5:7980146-79803
ENSG0000(	295	7.378224	chr16:65(	ENSG00000260953		lncRNA	chr16:29262273-292
ENSG0000(	295	7.378224	chr16:65(	ENSG00000289029		lncRNA	chr16:29288799-292
ENSG0000(	295	7.378224	chr16:65(	ENSG00000260413		Pseudoger	chr16:29312350-293
ENSG0000(	295	7.378224	chr16:291	snoU13		smallRNA	chr16:87314720-873
ENSG0000(	294	7.353213	chr17:709	ENSG00000265099		Pseudoger	chr17:20938023-209
ENSG0000(	292	7.303191	chr16:209	AC008731.1		Pseudoger	chr16:24919393-249
ENSG0000(	291	7.27818	chr6:105(	ENSG00000286368		lncRNA	chr6:21369690-2138
ENSG0000(	291	7.27818	chr6:105(	RN7SKP240		smallRNA	chr6:22085544-2208
ENSG0000(	291	7.27818	chr6:105(	BOLA2P3		Pseudoger	chr6:21602128-2160
ENSG0000(	291	7.27818	chr6:105(	NBAT1		lncRNA	chr6:22133205-2214
ENSG0000(	291	7.27818	chr6:105(	SOX4	DriverDB	protein_c	chr6:21593751-2159
ENSG0000(	291	7.27818	chr6:105(	CASC15		lncRNA	chr6:21664184-2265
ENSG0000(	291	7.27818	chr6:105(	ENSG00000280443		TEC	chr6:21822536-2182
ENSG0000(	291	7.27818	chr10:231	YWHAZP3		Pseudoger	chr10:23136924-231
ENSG0000(	291	7.27818	chr6:105(	ENSG00000283480		lncRNA	chr6:21528739-2159
ENSG0000(	291	7.27818	chr6:105(	ENSG00000219404		Pseudoger	chr6:22213306-2221
ENSG0000(	291	7.27818	chr6:105(	ENSG00000231754		lncRNA	chr6:21521671-2152
ENSG0000(	291	7.27818	chr6:105(	LINC00581		lncRNA	chr6:21485896-2152
ENSG0000(	290	7.253169	chr22:22(	ENSG00000223999		lncRNA	chr22:22692778-226
ENSG0000(	289	7.228159	chr1:4061	MIR3123		smallRNA	chr1:241132272-241
ENSG0000(	289	7.228159	chr1:4061	ENSG00000289114		lncRNA	chr1:235161321-235
ENSG0000(	289	7.228159	chr1:4061	RPS7P5		Pseudoger	chr1:240012646-240
ENSG0000(	289	7.228159	chr1:4061	MIR4428		smallRNA	chr1:237471119-237
ENSG0000(	289	7.228159	chr1:4061	ENSG00000237250		lncRNA	chr1:237862175-237
ENSG0000(	289	7.228159	chr1:4061	LGALS8		protein_c	chr1:236518000-236
ENSG0000(	289	7.228159	chr1:4061	ENSG00000231272		lncRNA	chr1:234261706-234
ENSG0000(	289	7.228159	chr1:4061	NID1	NCGv7	protein_c	chr1:235975830-236
ENSG0000(	289	7.228159	chr1:4061	MTND4P10		Pseudoger	chr1:235540053-235
ENSG0000(	289	7.228159	chr1:4061	RPL6P3		Pseudoger	chr1:241831935-241
ENSG0000(	289	7.228159	chr1:4061	LGALS8-AS1		lncRNA	chr1:236523052-236
ENSG0000(	289	7.228159	chr1:4061	ENSG00000289377		lncRNA	chr1:234771523-234
ENSG0000(	289	7.228159	chr1:4061	ENSG00000283377		Pseudoger	chr1:237942698-237
ENSG0000(	289	7.228159	chr1:4061	ENSG00000241475		lncRNA	chr1:234660271-234
ENSG0000(	289	7.228159	chr1:4061	MTND6P15		Pseudoger	chr1:237949214-237
ENSG0000(	289	7.228159	chr1:4061	ENSG00000228844		Pseudoger	chr1:240636599-240
ENSG0000(	289	7.228159	chr1:4061	ENSG00000286263		lncRNA	chr1:234811052-234
ENSG0000(	289	7.228159	chr1:4061	CHRM3-AS2		lncRNA	chr1:239703381-239
ENSG0000(	289	7.228159	chr1:4061	RN7SKP12		smallRNA	chr1:242188647-242
ENSG0000(	289	7.228159	chr1:4061	ENSG00000233332		lncRNA	chr1:234212606-234
ENSG0000(	289	7.228159	chr1:4061	TARBP1		protein_c	chr1:234391313-234
ENSG0000(	289	7.228159	chr1:4061	MIR4753		smallRNA	chr1:235190034-235
ENSG0000(	289	7.228159	chr1:4061	ENSG00000289628		lncRNA	chr1:237814959-237
ENSG0000(	289	7.228159	chr1:4061	ENSG00000228830		lncRNA	chr1:234607008-234
ENSG0000(	289	7.228159	chr1:4061	ZP4		protein_c	chr1:237877864-237
ENSG0000(	289	7.228159	chr1:4061	ENSG00000227962		Pseudoger	chr1:235336806-235

ENSG0000(	289	7.228159	chr1:4061	MTR		protein_c	chr1:236795260-236
ENSG0000(	289	7.228159	chr1:4061	ENSG00000236358		lncRNA	chr1:234357006-234
ENSG0000(	289	7.228159	chr1:4061	MIR4427		smallRNA	chr1:233624152-233
ENSG0000(	289	7.228159	chr1:4061	ENSG00000233735		lncRNA	chr1:240177839-240
ENSG0000(	289	7.228159	chr1:4061	RPSAP21		Pseudoger	chr1:236819634-236
ENSG0000(	289	7.228159	chr1:4061	ENSG00000286210		lncRNA	chr1:234535963-234
ENSG0000(	289	7.228159	chr1:4061	THAP12P8		Pseudoger	chr1:240769420-240
ENSG0000(	289	7.228159	chr1:4061	ENSG00000224525		lncRNA	chr1:242203555-242
ENSG0000(	289	7.228159	chr1:4061	KCNK1	NCGv7	protein_c	chr1:233614106-233
ENSG0000(	289	7.228159	chr1:4061	ENSG00000230404		lncRNA	chr1:234565298-234
ENSG0000(	289	7.228159	chr1:4061	MTND4LP21		Pseudoger	chr1:235541412-235
ENSG0000(	289	7.228159	chr1:4061	HNRNPA1P42		Pseudoger	chr1:240919653-240
ENSG0000(	289	7.228159	chr1:4061	ENSG00000286142		lncRNA	chr1:236123667-236
ENSG0000(	289	7.228159	chr1:4061	MIR1537		smallRNA	chr1:235853000-235
ENSG0000(	289	7.228159	chr1:4061	MTCYBP15		Pseudoger	chr1:237948017-237
ENSG0000(	289	7.228159	chr1:4061	EDARADD		protein_c	chr1:236348257-236
ENSG0000(	289	7.228159	chr1:4061	ENSG00000233519		lncRNA	chr1:240400671-240
ENSG0000(	289	7.228159	chr1:4061	LYST-AS1		lncRNA	chr1:235839483-235
ENSG0000(	289	7.228159	chr1:4061	ENSG00000230325		lncRNA	chr1:236540094-236
ENSG0000(	289	7.228159	chr1:4061	CHML		protein_c	chr1:241628851-241
ENSG0000(	289	7.228159	chr1:4061	RNU5E-2P		smallRNA	chr1:235863388-235
ENSG0000(	289	7.228159	chr1:4061	ENSG00000232166		Pseudoger	chr1:234284972-234
ENSG0000(	289	7.228159	chr1:4061	ENSG00000289305		lncRNA	chr1:233613570-233
ENSG0000(	289	7.228159	chr1:4061	KMO	NCGv7	protein_c	chr1:241532134-241
ENSG0000(	289	7.228159	chr1:4061	MIR4671		smallRNA	chr1:234306467-234
ENSG0000(	289	7.228159	chr1:4061	ENSG00000286109		lncRNA	chr1:233724454-233
ENSG0000(	289	7.228159	chr1:4061	MIPEPP2		Pseudoger	chr1:238777049-238
ENSG0000(	289	7.228159	chr1:4061	SLC35F3-AS1		lncRNA	chr1:234268583-234
ENSG0000(	289	7.228159	chr1:4061	LINC02961		lncRNA	chr1:234757619-234
ENSG0000(	289	7.228159	chr1:4061	ENSG00000228044		lncRNA	chr1:234646289-234
ENSG0000(	289	7.228159	chr1:4061	LINC01132		lncRNA	chr1:234724042-234
ENSG0000(	289	7.228159	chr1:4061	RN7SKP195		smallRNA	chr1:237120807-237
ENSG0000(	289	7.228159	chr1:4061	OPN3		protein_c	chr1:241590102-241
ENSG0000(	289	7.228159	chr1:4061	MTND5P18		Pseudoger	chr1:237949736-237
ENSG0000(	289	7.228159	chr1:4061	ENSG00000227854		Pseudoger	chr1:239052748-239
ENSG0000(	289	7.228159	chr1:4061	LINC02768		lncRNA	chr1:235957879-235
ENSG0000(	289	7.228159	chr1:4061	ENSG00000232989		Pseudoger	chr1:238268494-238
ENSG0000(	289	7.228159	chr1:4061	B3GALNT2		protein_c	chr1:235447190-235
ENSG0000(	289	7.228159	chr1:4061	KRT18P32		Pseudoger	chr1:238491358-238
ENSG0000(	289	7.228159	chr1:4061	LINC00184		lncRNA	chr1:234629311-234
ENSG0000(	289	7.228159	chr1:4061	WDR64	NCGv7	protein_c	chr1:241652278-241
ENSG0000(	289	7.228159	chr1:4061	LINC01354		lncRNA	chr1:234527887-234
ENSG0000(	289	7.228159	chr1:4061	RGS7	NCGv7	protein_c	chr1:240767636-241
ENSG0000(	289	7.228159	chr1:4061	RPS21P1		Pseudoger	chr1:235432985-235
ENSG0000(	289	7.228159	chr1:4061	ENSG00000258082		lncRNA	chr1:234978814-234
ENSG0000(	289	7.228159	chr1:4061	U8		smallRNA	chr1:234593275-234
ENSG0000(	289	7.228159	chr1:4061	RP11-293G6__A.2		lncRNA	chr1:235419515-235
ENSG0000(	289	7.228159	chr1:4061	ENSG00000243781		Pseudoger	chr1:237926831-237
ENSG0000(	289	7.228159	chr1:4061	MTND3P8		Pseudoger	chr1:235541759-235
ENSG0000(	289	7.228159	chr1:4061	ENSG00000285177		lncRNA	chr1:235366353-235
ENSG0000(	289	7.228159	chr1:4061	TBCE		protein_c	chr1:235328570-235

ENSG0000(	289	7.228159	chr1:4061	TOMM20		protein_c	chr1:235109341-235
ENSG0000(	289	7.228159	chr1:4061	GGPS1		protein_c	chr1:235327350-235
ENSG0000(	289	7.228159	chr1:4061	MT1HL1		protein_c	chr1:237004103-237
ENSG0000(	289	7.228159	chr1:4061	ENSG00000282097		lncRNA	chr1:234709383-234
ENSG0000(	289	7.228159	chr1:4061	TBCE		protein_c	chr1:235367360-235
ENSG0000(	289	7.228159	chr1:4061	COA6		protein_c	chr1:234373456-234
ENSG0000(	289	7.228159	chr1:4061	IRF2BP2	NCGv7	protein_c	chr1:234604269-234
ENSG0000(	289	7.228159	chr1:4061	GREM2		protein_c	chr1:240489573-240
ENSG0000(	289	7.228159	chr1:4061	ENO1P1		Pseudoger	chr1:236483165-236
ENSG0000(	289	7.228159	chr1:4061	FMN2	NCGv7	protein_c	chr1:240014348-240
ENSG0000(	289	7.228159	chr1:4061	ENSG00000283166		Pseudoger	chr1:237941452-237
ENSG0000(	289	7.228159	chr1:4061	ENSG00000227236		Pseudoger	chr1:235614674-235
ENSG0000(	289	7.228159	chr1:4061	ENSG00000231440		lncRNA	chr1:240588522-240
ENSG0000(	289	7.228159	chr1:4061	GNG4		protein_c	chr1:235547685-235
ENSG0000(	289	7.228159	chr1:4061	ENSG00000226919		lncRNA	chr1:240763334-240
ENSG0000(	289	7.228159	chr1:4061	ARID4B		protein_c	chr1:235131634-235
ENSG0000(	289	7.228159	chr1:4061	CFL1P4		Pseudoger	chr1:241993185-241
ENSG0000(	289	7.228159	chr1:4061	ADH5P3		Pseudoger	chr1:240170155-240
ENSG0000(	289	7.228159	chr1:4061	MTCO1P38		Pseudoger	chr1:237940762-237
ENSG0000(	289	7.228159	chr1:4061	ENSG00000238085		Pseudoger	chr1:240998451-240
ENSG0000(	289	7.228159	chr1:4061	LYST		protein_c	chr1:235661041-235
ENSG0000(	289	7.228159	chr1:4061	ENSG00000230026		Pseudoger	chr1:235361153-235
ENSG0000(	289	7.228159	chr1:4061	Y_RNA		smallRNA	chr1:240698320-240
ENSG0000(	289	7.228159	chr1:4061	YWHAQP9		Pseudoger	chr1:238107736-238
ENSG0000(	289	7.228159	chr1:4061	ENSG00000230015		lncRNA	chr1:240739131-240
ENSG0000(	289	7.228159	chr1:4061	LNCATV		lncRNA	chr1:234957342-234
ENSG0000(	289	7.228159	chr1:4061	RNU6-725P		smallRNA	chr1:238325687-238
ENSG0000(	289	7.228159	chr1:4061	RPL23AP23		Pseudoger	chr1:235295865-235
ENSG0000(	289	7.228159	chr1:4061	RPL35P1		Pseudoger	chr1:236981339-236
ENSG0000(	289	7.228159	chr1:4061	ENSG00000237845		lncRNA	chr1:235942553-235
ENSG0000(	289	7.228159	chr1:4061	ENSG00000237922		Pseudoger	chr1:236285976-236
ENSG0000(	289	7.228159	chr1:4061	SNORA14B		smallRNA	chr1:235127803-235
ENSG0000(	289	7.228159	chr1:4061	RNU6-1139P		smallRNA	chr1:242023949-242
ENSG0000(	289	7.228159	chr1:4061	ENSG00000233018		Pseudoger	chr1:235097187-235
ENSG0000(	289	7.228159	chr1:4061	SLC35F3		protein_c	chr1:233904676-234
ENSG0000(	289	7.228159	chr1:4061	ENSG00000228818		Pseudoger	chr1:240142670-240
ENSG0000(	289	7.228159	chr1:4061	RNU5F-8P		smallRNA	chr1:240653367-240
ENSG0000(	289	7.228159	chr1:4061	ENSG00000259776		lncRNA	chr1:239247808-239
ENSG0000(	289	7.228159	chr1:4061	RAC1P7		Pseudoger	chr1:234026851-234
ENSG0000(	289	7.228159	chr1:4061	LINC01139		lncRNA	chr1:238476542-238
ENSG0000(	289	7.228159	chr1:4061	ENSG00000215805		Pseudoger	chr1:239972787-239
ENSG0000(	289	7.228159	chr1:4061	RFKP1		Pseudoger	chr1:240823006-240
ENSG0000(	289	7.228159	chr1:4061	MAP1LC3C		protein_c	chr1:241995490-241
ENSG0000(	289	7.228159	chr1:4061	RPL23AP20		Pseudoger	chr1:241916123-241
ENSG0000(	289	7.228159	chr1:4061	ENSG00000289057		lncRNA	chr1:234599568-234
ENSG0000(	289	7.228159	chr1:4061	RNA5SP81		smallRNA	chr1:242134272-242
ENSG0000(	289	7.228159	chr1:4061	ENSG00000273058		lncRNA	chr1:236536162-236
ENSG0000(	289	7.228159	chr1:4061	EXO1		protein_c	chr1:241847967-241
ENSG0000(	289	7.228159	chr1:4061	ERO1B	DriverDBv	protein_c	chr1:236214681-236
ENSG0000(	289	7.228159	chr1:4061	ENSG00000270710		Pseudoger	chr1:235565761-235
ENSG0000(	289	7.228159	chr1:4061	ENSG00000272362		lncRNA	chr1:234644666-234

ENSG0000(	289	7.228159	chr1:4061	COA6-AS1		lncRNA	chr1:234372807-234
ENSG0000(	289	7.228159	chr1:4061	HEATR1	NCGv7	protein_c	chr1:236549005-236
ENSG0000(	289	7.228159	chr1:4061	ENSG00000288723		lncRNA	chr1:241722926-241
ENSG0000(	289	7.228159	chr1:4061	RNY4P16		smallRNA	chr1:234837973-234
ENSG0000(	289	7.228159	chr1:4061	MTCO3P46		Pseudoger	chr1:235542159-235
ENSG0000(	289	7.228159	chr1:4061	BECN2	DriverDB\	protein_c	chr1:241957767-241
ENSG0000(	289	7.228159	chr1:4061	ENSG00000234464		lncRNA	chr1:238238943-238
ENSG0000(	289	7.228159	chr1:4061	ENSG00000287633		lncRNA	chr1:234550542-234
ENSG0000(	289	7.228159	chr1:4061	ENSG00000288760		lncRNA	chr1:234531338-234
ENSG0000(	289	7.228159	chr1:4061	ENSG00000272865		lncRNA	chr1:242147230-242
ENSG0000(	289	7.228159	chr1:4061	ENSG00000226014		Pseudoger	chr1:240549867-240
ENSG0000(	289	7.228159	chr1:4061	RNU6-968P		smallRNA	chr1:235915415-235
ENSG0000(	289	7.228159	chr1:4061	ENSG00000287738		lncRNA	chr1:241453751-241
ENSG0000(	289	7.228159	chr1:4061	ENSG00000286496		lncRNA	chr1:241357343-241
ENSG0000(	289	7.228159	chr1:4061	ENSG00000231979		lncRNA	chr1:239915439-239
ENSG0000(	289	7.228159	chr1:4061	ENSG00000277099		Pseudoger	chr1:242060490-242
ENSG0000(	289	7.228159	chr1:4061	LDHAP2		Pseudoger	chr1:235738005-235
ENSG0000(	289	7.228159	chr1:4061	Y_RNA		smallRNA	chr1:240154651-240
ENSG0000(	289	7.228159	chr1:4061	ENSG00000288099		lncRNA	chr1:237013373-237
ENSG0000(	289	7.228159	chr1:4061	RBM34		protein_c	chr1:235131183-235
ENSG0000(	289	7.228159	chr1:4061	Y_RNA		smallRNA	chr1:236060677-236
ENSG0000(	289	7.228159	chr1:4061	ENSG00000234872		Pseudoger	chr1:240654241-240
ENSG0000(	289	7.228159	chr1:4061	ENSG00000235605		Pseudoger	chr1:234356704-234
ENSG0000(	289	7.228159	chr1:4061	ENSG00000287921		lncRNA	chr1:233904104-233
ENSG0000(	289	7.228159	chr1:4061	GPR137B		protein_c	chr1:236142505-236
ENSG0000(	289	7.228159	chr1:4061	ACTN2		protein_c	chr1:236664141-236
ENSG0000(	289	7.228159	chr1:4061	MTND6P14		Pseudoger	chr1:235537520-235
ENSG0000(	289	7.228159	chr1:4061	MTCYBP14		Pseudoger	chr1:235519971-235
ENSG0000(	289	7.228159	chr1:4061	RNU4-77P		smallRNA	chr1:233448626-233
ENSG0000(	289	7.228159	chr1:4061	ENSG00000231877		lncRNA	chr1:238485445-238
ENSG0000(	289	7.228159	chr1:4061	ENSG00000287177		lncRNA	chr1:238842767-238
ENSG0000(	289	7.228159	chr1:4061	RPL36P6		Pseudoger	chr1:241305580-241
ENSG0000(	289	7.228159	chr1:4061	RYR2		protein_c	chr1:237042184-237
ENSG0000(	289	7.228159	chr1:4061	ENSG00000225486		Pseudoger	chr1:233836080-233
ENSG0000(	289	7.228159	chr1:4061	Y_RNA		smallRNA	chr1:240341385-240
ENSG0000(	289	7.228159	chr1:4061	ENSG00000287513		lncRNA	chr1:241640555-241
ENSG0000(	289	7.228159	chr1:4061	ENSG00000287516		lncRNA	chr1:241413716-241
ENSG0000(	289	7.228159	chr1:4061	ENSG00000287102		lncRNA	chr1:240007524-240
ENSG0000(	289	7.228159	chr1:4061	CHRM3		protein_c	chr1:239386565-239
ENSG0000(	289	7.228159	chr1:4061	ENSG00000273367		lncRNA	chr1:234372186-234
ENSG0000(	289	7.228159	chr1:4061	FH	NCGv7;AC	protein_c	chr1:241497511-241
ENSG0000(	289	7.228159	chr1:4061	RNU2-70P		smallRNA	chr1:236267780-236
ENSG0000(	289	7.228159	chr1:4061	LINC02971		lncRNA	chr1:234957231-234
ENSG0000(	289	7.228159	chr1:4061	ENSG00000235371		Pseudoger	chr1:236110061-236
ENSG0000(	289	7.228159	chr1:4061	ENSG00000286666		lncRNA	chr1:233844621-233
ENSG0000(	289	7.228159	chr1:4061	ENSG00000224359		lncRNA	chr1:240530452-240
ENSG0000(	289	7.228159	chr1:4061	ENSG00000273416		lncRNA	chr1:235104180-235
ENSG0000(	289	7.228159	chr1:4061	CHRM3-AS1		lncRNA	chr1:239898016-239
ENSG0000(	289	7.228159	chr1:4061	TUBB8P6		Pseudoger	chr1:242057085-242
ENSG0000(	289	7.228159	chr1:4061	MTND5P19		Pseudoger	chr1:235538052-235
ENSG0000(	289	7.228159	chr1:4061	SNORA25		smallRNA	chr1:237555040-237

ENSG0000(	289	7.228159	chr1:4061	ENSG00000230628		lncRNA	chr1:234669523-234
ENSG0000(	289	7.228159	chr1:4061	snoU13		smallRNA	chr1:236300980-236
ENSG0000(	289	7.228159	chr1:4061	ENSG00000287589		lncRNA	chr1:239205138-239
ENSG0000(	289	7.228159	chr1:4061	ENSG00000287423		lncRNA	chr1:233527544-233
ENSG0000(	288	7.203148	chr2:4707	MIR4432		smallRNA	chr2:60387362-6038
ENSG0000(	286	7.153126	chr16:209	KDM8		protein_c	chr16:27203508-272
ENSG0000(	286	7.153126	chr16:209	KATNIP		protein_c	chr16:27550133-277
ENSG0000(	286	7.153126	chr16:209	ENSG00000261329		lncRNA	chr16:27678940-277
ENSG0000(	286	7.153126	chr16:209	ENSG00000283662		lncRNA	chr16:28258686-282
ENSG0000(	286	7.153126	chr16:209	SNORA25		smallRNA	chr16:28179098-281
ENSG0000(	286	7.153126	chr16:209	CLN3		protein_c	chr16:28474111-284
ENSG0000(	286	7.153126	chr16:209	CDC37P2		Pseudoger	chr16:28413703-284
ENSG0000(	286	7.153126	chr16:209	LINC02129		lncRNA	chr16:27158451-271
ENSG0000(	286	7.153126	chr16:209	ENSG00000261482		lncRNA	chr16:27066928-270
ENSG0000(	286	7.153126	chr16:209	ENSG00000274092		lncRNA	chr16:27313387-273
ENSG0000(	286	7.153126	chr16:209	NPIPB6		protein_c	chr16:28342517-283
ENSG0000(	286	7.153126	chr16:209	Y_RNA		smallRNA	chr16:27722284-277
ENSG0000(	286	7.153126	chr16:209	APOBR	NCGv7	protein_c	chr16:28494643-284
ENSG0000(	286	7.153126	chr16:209	NPIPB7		protein_c	chr16:28456372-284
ENSG0000(	286	7.153126	chr16:209	RNU6-1241P		smallRNA	chr16:27998295-279
ENSG0000(	286	7.153126	chr16:209	EIF3CL		protein_c	chr16:28379579-284
ENSG0000(	286	7.153126	chr16:209	IL21R-AS1		lncRNA	chr16:27447669-274
ENSG0000(	286	7.153126	chr16:209	LINC02195		lncRNA	chr16:26584755-265
ENSG0000(	286	7.153126	chr16:209	C16orf82		protein_c	chr16:27066927-270
ENSG0000(	286	7.153126	chr16:209	snoU13		smallRNA	chr16:28442666-284
ENSG0000(	286	7.153126	chr16:209	ENSG00000246465		lncRNA	chr16:28284885-282
ENSG0000(	286	7.153126	chr16:209	RNU6-159P		smallRNA	chr16:27863251-278
ENSG0000(	286	7.153126	chr16:209	ENSG00000271623		Pseudoger	chr16:28364700-283
ENSG0000(	286	7.153126	chr16:209	NSMCE1-DT		lncRNA	chr16:27268205-272
ENSG0000(	286	7.153126	chr16:209	ENSG00000286712		lncRNA	chr16:26546802-266
ENSG0000(	286	7.153126	chr16:209	ENSG00000275654		Pseudoger	chr16:28451644-284
ENSG0000(	286	7.153126	chr16:209	ENSG00000260580		lncRNA	chr16:26721874-267
ENSG0000(	286	7.153126	chr16:209	TPRKBP2		Pseudoger	chr16:28111173-281
ENSG0000(	286	7.153126	chr16:209	Y_RNA		smallRNA	chr16:28183318-281
ENSG0000(	286	7.153126	chr16:209	ENSG00000259940		lncRNA	chr16:27213308-272
ENSG0000(	286	7.153126	chr16:209	IL27		protein_c	chr16:28499362-285
ENSG0000(	286	7.153126	chr16:209	AC009035.1		smallRNA	chr16:26736229-267
ENSG0000(	286	7.153126	chr16:209	ENSG00000270118		lncRNA	chr16:28454141-284
ENSG0000(	286	7.153126	chr16:209	SBK1		protein_c	chr16:28259246-283
ENSG0000(	286	7.153126	chr16:209	ENSG00000277191		Pseudoger	chr16:28341437-283
ENSG0000(	286	7.153126	chr16:209	GAPDHP35		Pseudoger	chr16:28239693-282
ENSG0000(	286	7.153126	chr16:209	ENSG00000273553		lncRNA	chr16:27687182-276
ENSG0000(	286	7.153126	chr16:209	NSMCE1		protein_c	chr16:27224994-272
ENSG0000(	286	7.153126	chr16:209	IL4R	NCGv7	protein_c	chr16:27313668-273
ENSG0000(	286	7.153126	chr16:209	XPO6		protein_c	chr16:28097976-282
ENSG0000(	286	7.153126	chr16:209	ENSG00000261832		protein_c	chr16:28456371-284
ENSG0000(	286	7.153126	chr16:209	ENSG00000261736		lncRNA	chr16:27643199-276
ENSG0000(	286	7.153126	chr16:209	IL21R	AC	protein_c	chr16:27402174-274
ENSG0000(	286	7.153126	chr16:209	EEF1A1P38		Pseudoger	chr16:27133483-271
ENSG0000(	286	7.153126	chr16:209	GTF3C1		protein_c	chr16:27459555-275
ENSG0000(	286	7.153126	chr16:209	GSG1L		protein_c	chr16:27787528-280

ENSG0000(	286	7.153126	chr16:209	AC016597.1	smallRNA	chr16:27661900-276
ENSG0000(	285	7.128115	chr2:4707	RPS12P3	Pseudoger	chr2:60938204-6093
ENSG0000(	285	7.128115	chr2:4707	MIR4431	smallRNA	chr2:52702522-5270
ENSG0000(	285	7.128115	chr2:4707	ENSG00000233891	lncRNA	chr2:59238703-5973
ENSG0000(	285	7.128115	chr2:4707	ENSG00000273063	lncRNA	chr2:58241349-5824
ENSG0000(	285	7.128115	chr2:4707	ENSG00000270335	Pseudoger	chr2:62348948-6235
ENSG0000(	285	7.128115	chr2:4707	PSAT1P2	Pseudoger	chr2:62552463-6255
ENSG0000(	285	7.128115	chr2:4707	RNU6-612P	smallRNA	chr2:60719640-6071
ENSG0000(	285	7.128115	chr2:4707	GGCTP3	Pseudoger	chr2:52474073-5247
ENSG0000(	285	7.128115	chr2:4707	ENSG00000287344	lncRNA	chr2:51977787-5202
ENSG0000(	285	7.128115	chr2:4707	CRYGGP	Pseudoger	chr2:51775258-5177
ENSG0000(	285	7.128115	chr2:4707	ENSG00000285519	lncRNA	chr2:54768492-5480
ENSG0000(	285	7.128115	chr2:4707	AC019198.1	smallRNA	chr2:55472744-5547
ENSG0000(	285	7.128115	chr2:4707	AC008064.1	smallRNA	chr2:53651401-5365
ENSG0000(	285	7.128115	chr2:4707	BTF3P5	Pseudoger	chr2:55435156-5543
ENSG0000(	285	7.128115	chr2:4707	ENSG00000289247	lncRNA	chr2:61854376-6185
ENSG0000(	285	7.128115	chr2:4707	PPP4R3B-DT	lncRNA	chr2:55617869-5561
ENSG0000(	285	7.128115	chr2:4707	ENSG00000287640	lncRNA	chr2:60383141-6038
ENSG0000(	285	7.128115	chr2:4707	RNU7-81P	smallRNA	chr2:54850289-5485
ENSG0000(	285	7.128115	chr2:4707	RNU6-775P	smallRNA	chr2:55451004-5545
ENSG0000(	285	7.128115	chr2:4707	ENSG00000270437	Pseudoger	chr2:63083008-6308
ENSG0000(	285	7.128115	chr2:4707	RNA5SP94	Pseudoger	chr2:59694762-5969
ENSG0000(	285	7.128115	chr2:4707	EHBP1-AS1	lncRNA	chr2:62957326-6304
ENSG0000(	285	7.128115	chr2:4707	LINC01813	lncRNA	chr2:56077417-5609
ENSG0000(	285	7.128115	chr2:4707	RPS24P7	Pseudoger	chr2:61803143-6180
ENSG0000(	285	7.128115	chr2:4707	ZNF863P	Pseudoger	chr2:52071355-5207
ENSG0000(	285	7.128115	chr2:4707	ENSG00000236498	lncRNA	chr2:61868432-6188
ENSG0000(	285	7.128115	chr2:4707	ENSG00000270447	Pseudoger	chr2:59514890-5951
ENSG0000(	285	7.128115	chr2:4707	RPL27P5	Pseudoger	chr2:63108118-6310
ENSG0000(	285	7.128115	chr2:4707	MIR3682	smallRNA	chr2:53849122-5384
ENSG0000(	285	7.128115	chr2:4707	ENSG00000232668	Pseudoger	chr2:52883243-5288
ENSG0000(	285	7.128115	chr2:4707	LINC01867	lncRNA	chr2:52370602-5239
ENSG0000(	285	7.128115	chr2:4707	Y_RNA	smallRNA	chr2:62726636-6272
ENSG0000(	285	7.128115	chr2:4707	ENSG00000240401	lncRNA	chr2:55282350-5534
ENSG0000(	285	7.128115	chr2:4707	ENSG00000286360	lncRNA	chr2:61878940-6188
ENSG0000(	285	7.128115	chr2:4707	ENSG00000267520	lncRNA	chr2:60925909-6093
ENSG0000(	285	7.128115	chr2:4707	AC093165.1	smallRNA	chr2:55108710-5510
ENSG0000(	285	7.128115	chr2:4707	C2orf74	protein_c	chr2:61145068-6116
ENSG0000(	285	7.128115	chr2:4707	RSL24D1P2	Pseudoger	chr2:62561058-6256
ENSG0000(	285	7.128115	chr2:4707	RPL21P37	Pseudoger	chr2:62532583-6253
ENSG0000(	285	7.128115	chr2:4707	ENSG00000289065	lncRNA	chr2:54115268-5411
ENSG0000(	285	7.128115	chr2:4707	SPTBN1-AS1	lncRNA	chr2:54516048-5454
ENSG0000(	285	7.128115	chr2:4707	MIR5192	smallRNA	chr2:62205826-6220
ENSG0000(	285	7.128115	chr2:4707	CCDC12P1	Pseudoger	chr2:51926882-5192
ENSG0000(	285	7.128115	chr2:4707	ENSG00000228541	lncRNA	chr2:62296246-6246
ENSG0000(	285	7.128115	chr2:4707	ENSG00000273302	lncRNA	chr2:61199979-6120
ENSG0000(	285	7.128115	chr2:4707	CDPF1P1	Pseudoger	chr2:55224280-5522
ENSG0000(	285	7.128115	chr2:4707	RNU6-221P	smallRNA	chr2:55456106-5545
ENSG0000(	285	7.128115	chr2:4707	RN7SL51P	smallRNA	chr2:62262389-6226
ENSG0000(	285	7.128115	chr2:4707	NONOP2	Pseudoger	chr2:60936819-6093
ENSG0000(	285	7.128115	chr2:4707	LINC01122	lncRNA	chr2:58427738-5906

ENSG0000(	285	7.128115	chr2:4707	ENSG00000231815	lncRNA	chr2:59434552-5944
ENSG0000(	285	7.128115	chr2:4707	MTFR2P1	Pseudoger	chr2:63232453-6323
ENSG0000(	285	7.128115	chr2:4707	RPL37P13	Pseudoger	chr2:62507545-6250
ENSG0000(	285	7.128115	chr2:4707	REL-DT	lncRNA	chr2:60823069-6088
ENSG0000(	285	7.128115	chr2:4707	ENSG00000232604	lncRNA	chr2:52864235-5286
ENSG0000(	285	7.128115	chr2:4707	ENSG00000223897	Pseudoger	chr2:53486144-5348
ENSG0000(	285	7.128115	chr2:4707	ENSG00000234624	Pseudoger	chr2:61416887-6141
ENSG0000(	285	7.128115	chr2:4707	ENSG00000228033	lncRNA	chr2:52722671-5296
ENSG0000(	285	7.128115	chr2:4707	ENSG00000229839	lncRNA	chr2:62069447-6214
ENSG0000(	285	7.128115	chr2:4707	ENSG00000229831	Pseudoger	chr2:61820208-6182
ENSG0000(	285	7.128115	chr2:4707	ENSG00000270569	Pseudoger	chr2:57429548-5743
ENSG0000(	285	7.128115	chr2:4707	snoU13	smallRNA	chr2:57016061-5701
ENSG0000(	285	7.128115	chr2:4707	RPL21P30	Pseudoger	chr2:54029552-5403
ENSG0000(	285	7.128115	chr2:4707	RPS29P10	Pseudoger	chr2:61589498-6158
ENSG0000(	285	7.128115	chr2:4707	ENSG00000271657	Pseudoger	chr2:62168862-6217
ENSG0000(	285	7.128115	chr2:4707	ENSG00000234943	lncRNA	chr2:54545368-5454
ENSG0000(	285	7.128115	chr2:4707	RN7SL361P	smallRNA	chr2:60640705-6064
ENSG0000(	285	7.128115	chr2:4707	ENSG00000289410	lncRNA	chr2:61728808-6176
ENSG0000(	285	7.128115	chr2:4707	PPIAP63	Pseudoger	chr2:56750300-5675
ENSG0000(	285	7.128115	chr2:4707	MIR217HG	lncRNA	chr2:55963191-5604
ENSG0000(	285	7.128115	chr2:4707	RN7SL632P	smallRNA	chr2:60831665-6083
ENSG0000(	285	7.128115	chr2:4707	EIF3FP3	Pseudoger	chr2:58251440-5825
ENSG0000(	285	7.128115	chr2:4707	ACTG1P22	Pseudoger	chr2:57755428-5776
ENSG0000(	285	7.128115	chr2:4707	EIF2S2P7	Pseudoger	chr2:57048350-5704
ENSG0000(	285	7.128115	chr2:4707	SPTBN1-AS2	lncRNA	chr2:54661011-5468
ENSG0000(	285	7.128115	chr2:4707	ENSG00000289855	lncRNA	chr2:61764544-6176
ENSG0000(	285	7.128115	chr2:4707	RPL21P33	Pseudoger	chr2:60852260-6085
ENSG0000(	285	7.128115	chr2:4707	IFITM3P9	Pseudoger	chr2:60682873-6068
ENSG0000(	285	7.128115	chr2:4707	ENSG00000233953	lncRNA	chr2:60495686-6049
ENSG0000(	285	7.128115	chr2:4707	ENSG00000289529	lncRNA	chr2:58429434-5847
ENSG0000(	285	7.128115	chr2:4707	EML6-AS1	lncRNA	chr2:54747103-5475
ENSG0000(	285	7.128115	chr2:4707	ENSG00000231043	Pseudoger	chr2:58460292-5846
ENSG0000(	285	7.128115	chr2:4707	ENSG00000271146	Pseudoger	chr2:61249780-6125
ENSG0000(	285	7.128115	chr2:4707	MIR4432HG	lncRNA	chr2:60336446-6043
ENSG0000(	285	7.128115	chr2:4707	ENSG00000271243	Pseudoger	chr2:61575774-6157
ENSG0000(	285	7.128115	chr2:4707	RN7SL18P	smallRNA	chr2:62491178-6249
ENSG0000(	285	7.128115	chr2:4707	HMGB1P31	Pseudoger	chr2:54051334-5405
ENSG0000(	285	7.128115	chr2:4707	ENSG00000233251	lncRNA	chr2:56173534-5618
ENSG0000(	285	7.128115	chr2:4707	PPP4R3B	protein_c	chr2:55547292-5561
ENSG0000(	285	7.128115	chr2:4707	LINC01795	lncRNA	chr2:58275532-5829
ENSG0000(	285	7.128115	chr2:4707	RNU6-634P	smallRNA	chr2:55499950-5550
ENSG0000(	285	7.128115	chr2:4707	ENSG00000289606	lncRNA	chr2:55235605-5523
ENSG0000(	285	7.128115	chr2:4707	ENSG00000286604	lncRNA	chr2:59778685-5979
ENSG0000(	285	7.128115	chr2:4707	ENSG00000287875	lncRNA	chr2:58040211-5804
ENSG0000(	285	7.128115	chr2:4707	ENSG00000285857	lncRNA	chr2:61527340-6152
ENSG0000(	285	7.128115	chr2:4707	ATP1B3P1	Pseudoger	chr2:60734895-6073
ENSG0000(	285	7.128115	chr2:4707	ENSG00000271889	lncRNA	chr2:61151433-6116
ENSG0000(	285	7.128115	chr2:4707	ENSG00000285611	lncRNA	chr2:60057601-6007
ENSG0000(	285	7.128115	chr2:4707	ENSG00000289627	lncRNA	chr2:54581577-5458
ENSG0000(	285	7.128115	chr2:4707	AC007179.2	smallRNA	chr2:59532984-5953
ENSG0000(	285	7.128115	chr2:4707	ENSG00000285673	lncRNA	chr2:59014354-5927

ENSG0000(	285	7.128115	chr2:4707	ENSG00000286480		lncRNA	chr2:63106879-6319
ENSG0000(	285	7.128115	chr2:4707	ENSG00000241114		Pseudoger	chr2:54079974-5408
ENSG0000(	285	7.128115	chr2:4707	ENSG00000226605		lncRNA	chr2:62826064-6285
ENSG0000(	285	7.128115	chr2:4707	SCARNA16		smallRNA	chr2:53470447-5347
ENSG0000(	285	7.128115	chr2:4707	snoU13		smallRNA	chr2:53839725-5383
ENSG0000(	285	7.128115	chr2:4707	snoU13		smallRNA	chr2:62264995-6226
ENSG0000(	285	7.128115	chr2:4707	USP34-DT		lncRNA	chr2:61471188-6148
ENSG0000(	285	7.128115	chr2:4707	ENSG00000226622		lncRNA	chr2:62533681-6266
ENSG0000(	285	7.128115	chr2:4707	RPS20P9		Pseudoger	chr2:62939916-6294
ENSG0000(	285	7.128115	chr2:4707	RNU7-172P		smallRNA	chr2:54166944-5416
ENSG0000(	285	7.128115	chr2:4707	ENSG00000277498		Pseudoger	chr2:61858137-6186
ENSG0000(	285	7.128115	chr2:4707	RPSAP26		Pseudoger	chr2:62146413-6214
ENSG0000(	285	7.128115	chr2:4707	RPL31P30		Pseudoger	chr2:61856695-6185
ENSG0000(	285	7.128115	chr2:4707	ENSG00000274769		lncRNA	chr2:61115787-6116
ENSG0000(	285	7.128115	chr2:4707	ENSG00000285755		lncRNA	chr2:57289648-5738
ENSG0000(	285	7.128115	chr2:4707	DBIL5P2		Pseudoger	chr2:63117851-6311
ENSG0000(	285	7.128115	chr2:4707	ENSG00000272180		lncRNA	chr2:55952158-5618
ENSG0000(	285	7.128115	chr2:4707	ENSG00000290071		lncRNA	chr2:55137264-5513
ENSG0000(	285	7.128115	chr2:4707	ENSG00000272156		lncRNA	chr2:54082554-5408
ENSG0000(	285	7.128115	chr2:4707	AC073215.1		smallRNA	chr2:58062581-5806
ENSG0000(	285	7.128115	chr2:4707	ENSG00000271955		lncRNA	chr2:59218680-6010
ENSG0000(	285	7.128115	chr2:4707	ENSG00000271894		lncRNA	chr2:56147630-5638
ENSG0000(	285	7.128115	chr2:4707	RPL23AP32		Pseudoger	chr2:54529343-5452
ENSG0000(	285	7.128115	chr2:4707	ENSG00000286524		lncRNA	chr2:62817764-6281
ENSG0000(	285	7.128115	chr2:4707	ENSG00000236837		lncRNA	chr2:52494688-5250
ENSG0000(	285	7.128115	chr2:4707	ENSG00000230840		Pseudoger	chr2:51925692-5192
ENSG0000(	285	7.128115	chr2:4707	FTH1P6		Pseudoger	chr2:52629743-5263
ENSG0000(	285	7.128115	chr2:4707	C2orf73		protein_c	chr2:54330034-5438
ENSG0000(	285	7.128115	chr2:4707	PNPT1		protein_c	chr2:55634061-5569
ENSG0000(	285	7.128115	chr2:4707	RTN4	NCGv7	protein_c	chr2:54972187-5511
ENSG0000(	285	7.128115	chr2:4707	CCDC88A	NCGv7	protein_c	chr2:55287842-5541
ENSG0000(	285	7.128115	chr2:4707	EFEMP1		protein_c	chr2:55865967-5592
ENSG0000(	285	7.128115	chr2:4707	PUS10		protein_c	chr2:60940222-6101
ENSG0000(	285	7.128115	chr2:4707	PAPOLG		protein_c	chr2:60756253-6080
ENSG0000(	285	7.128115	chr2:4707	RPL26P13		Pseudoger	chr2:60711484-6071
ENSG0000(	285	7.128115	chr2:4707	ENSG00000213486		Pseudoger	chr2:61710076-6171
ENSG0000(	285	7.128115	chr2:4707	RNU4-51P		smallRNA	chr2:60911303-6091
ENSG0000(	285	7.128115	chr2:4707	SNORD78		smallRNA	chr2:57544535-5754
ENSG0000(	285	7.128115	chr2:4707	ACYP2		protein_c	chr2:53970838-5430
ENSG0000(	285	7.128115	chr2:4707	SNORA12		smallRNA	chr2:55565703-5556
ENSG0000(	285	7.128115	chr2:4707	RNA5SP95		Pseudoger	chr2:60998752-6099
ENSG0000(	285	7.128115	chr2:4707	AC007131.3		smallRNA	chr2:59241621-5924
ENSG0000(	285	7.128115	chr2:4707	USP34		protein_c	chr2:61187463-6147
ENSG0000(	285	7.128115	chr2:4707	CHAC2		protein_c	chr2:53767804-5377
ENSG0000(	285	7.128115	chr2:4707	CCT4		protein_c	chr2:61868085-6188
ENSG0000(	285	7.128115	chr2:4707	RPS27A	NCGv7	protein_c	chr2:55231903-5523
ENSG0000(	285	7.128115	chr2:4707	WDPCP		protein_c	chr2:63119559-6382
ENSG0000(	285	7.128115	chr2:4707	EHBP1		protein_c	chr2:62673851-6304
ENSG0000(	285	7.128115	chr2:4707	OTX1		protein_c	chr2:63050057-6305
ENSG0000(	285	7.128115	chr2:4707	RNA5SP93		Pseudoger	chr2:56235217-5623
ENSG0000(	285	7.128115	chr2:4707	RNU1-32P		smallRNA	chr2:60384605-6038

ENSG0000(	285	7.128115	chr2:4707	B3GNT2		protein_c	chr2:62196115-6222
ENSG0000(	285	7.128115	chr2:4707	SPTBN1		protein_c	chr2:54456317-5467
ENSG0000(	285	7.128115	chr2:4707	CCDC85A		protein_c	chr2:56183990-5638
ENSG0000(	285	7.128115	chr2:4707	BCL11A	NCGv7;AC	protein_c	chr2:60450520-6055
ENSG0000(	285	7.128115	chr2:4707	COMMD1		protein_c	chr2:61888724-6214
ENSG0000(	285	7.128115	chr2:4707	PEX13		protein_c	chr2:61017225-6105
ENSG0000(	285	7.128115	chr2:4707	SANBR		protein_c	chr2:61065871-6113
ENSG0000(	285	7.128115	chr2:4707	CLHC1		protein_c	chr2:55172547-5523
ENSG0000(	285	7.128115	chr2:4707	PRORSD1P		Pseudoger	chr2:55282319-5528
ENSG0000(	285	7.128115	chr2:4707	CFAP36		protein_c	chr2:55519604-5554
ENSG0000(	285	7.128115	chr2:4707	XPO1	NCGv7;AC	protein_c	chr2:61476032-6153
ENSG0000(	285	7.128115	chr2:4707	ENSG00000203327		lncRNA	chr2:55214387-5521
ENSG0000(	285	7.128115	chr2:4707	EML6		protein_c	chr2:54723499-5497
ENSG0000(	285	7.128115	chr2:4707	MIR216A		smallRNA	chr2:55988950-5598
ENSG0000(	285	7.128115	chr2:4707	RN7SKP208		smallRNA	chr2:55951654-5595
ENSG0000(	285	7.128115	chr2:4707	AHSA2P		Pseudoger	chr2:61177418-6119
ENSG0000(	285	7.128115	chr2:4707	ASB3		protein_c	chr2:53532672-5386
ENSG0000(	285	7.128115	chr2:4707	TSPYL6		protein_c	chr2:54253178-5425
ENSG0000(	285	7.128115	chr2:4707	MTIF2		protein_c	chr2:55236595-5526
ENSG0000(	285	7.128115	chr2:4707	Y_RNA		smallRNA	chr2:52297995-5229
ENSG0000(	285	7.128115	chr2:4707	CRTC1P1		Pseudoger	chr2:52570648-5257
ENSG0000(	285	7.128115	chr2:4707	MIR217		smallRNA	chr2:55982967-5598
ENSG0000(	285	7.128115	chr2:4707	ERLEC1		protein_c	chr2:53787009-5383
ENSG0000(	285	7.128115	chr2:4707	MIR216B		smallRNA	chr2:56000714-5600
ENSG0000(	285	7.128115	chr2:4707	GPR75		protein_c	chr2:53852912-5385
ENSG0000(	285	7.128115	chr2:4707	PSME4		protein_c	chr2:53864069-5397
ENSG0000(	285	7.128115	chr2:4707	RNU6-997P		smallRNA	chr2:53570374-5357
ENSG0000(	285	7.128115	chr2:4707	FAM161A		protein_c	chr2:61824848-6185
ENSG0000(	285	7.128115	chr2:4707	FANCL		protein_c	chr2:58159243-5824
ENSG0000(	285	7.128115	chr2:4707	REL	NCGv7;AC	protein_c	chr2:60881491-6093
ENSG0000(	285	7.128115	chr2:4707	C2orf74-DT		lncRNA	chr2:61141592-6114
ENSG0000(	285	7.128115	chr2:4707	RNU6-508P		smallRNA	chr2:59647621-5964
ENSG0000(	285	7.128115	chr2:4707	RNU6-433P		smallRNA	chr2:55014418-5501
ENSG0000(	285	7.128115	chr2:4707	VRK2		protein_c	chr2:57907629-5815
ENSG0000(	285	7.128115	chr2:4707	LINC01793		lncRNA	chr2:59217708-5927
ENSG0000(	285	7.128115	chr2:4707	TMEM17		protein_c	chr2:62500218-6251
ENSG0000(	285	7.128115	chr2:4707	RNU6-1145P		smallRNA	chr2:61605616-6160
ENSG0000(	285	7.128115	chr2:4707	Y_RNA		smallRNA	chr2:55286018-5528
ENSG0000(	285	7.128115	chr2:4707	SNORA70B		smallRNA	chr2:61417244-6141
ENSG0000(	283	7.078093	chr2:4707	YIPF4		protein_c	chr2:32277904-3231
ENSG0000(	283	7.078093	chr2:4707	RN7SL96P		smallRNA	chr2:38936880-3893
ENSG0000(	283	7.078093	chr2:4707	ENSG00000269210		lncRNA	chr2:38959287-3896
ENSG0000(	283	7.078093	chr2:4707	ENSG00000273035		lncRNA	chr2:39323328-3932
ENSG0000(	283	7.078093	chr2:4707	EPCAM	AC	protein_c	chr2:47345158-4738
ENSG0000(	283	7.078093	chr2:4707	MRPL50P1		Pseudoger	chr2:35724759-3572
ENSG0000(	283	7.078093	chr2:4707	ENSG00000234579		lncRNA	chr2:30986939-3099
ENSG0000(	283	7.078093	chr2:4707	ENSG00000227938		lncRNA	chr2:28448167-2845
ENSG0000(	283	7.078093	chr2:4707	LTBP1		protein_c	chr2:32946953-3339
ENSG0000(	283	7.078093	chr2:4707	snoZ247		smallRNA	chr2:41734377-4173
ENSG0000(	283	7.078093	chr2:4707	ENSG00000230118		Pseudoger	chr2:30746444-3074
ENSG0000(	283	7.078093	chr2:4707	RNA5SP90		Pseudoger	chr2:31228312-3122

ENSG0000(	283	7.078093	chr2:4707	MCFD2		protein_c	chr2:46901870-4694
ENSG0000(	283	7.078093	chr2:4707	LINC01914		lncRNA	chr2:41931599-4193
ENSG0000(	283	7.078093	chr2:4707	AC016907.1		smallRNA	chr2:30066124-3006
ENSG0000(	283	7.078093	chr2:4707	ENSG00000287658		lncRNA	chr2:30343222-3034
ENSG0000(	283	7.078093	chr2:4707	RNA5SP92		Pseudoger	chr2:33332898-3333
ENSG0000(	283	7.078093	chr2:4707	RN7SL602P		smallRNA	chr2:34809253-3480
ENSG0000(	283	7.078093	chr2:4707	AC073255.1		smallRNA	chr2:30432353-3043
ENSG0000(	283	7.078093	chr2:4707	AC010739.1		smallRNA	chr2:41596386-4159
ENSG0000(	283	7.078093	chr2:4707	ENSG00000278957		TEC	chr2:44927914-4492
ENSG0000(	283	7.078093	chr2:4707	RNU6-1185P		smallRNA	chr2:39393522-3939
ENSG0000(	283	7.078093	chr2:4707	AC074091.1		smallRNA	chr2:27581889-2758
ENSG0000(	283	7.078093	chr2:4707	BIRC6-AS1		lncRNA	chr2:32377631-3237
ENSG0000(	283	7.078093	chr2:4707	MAP4K3	NCGv7	protein_c	chr2:39249266-3943
ENSG0000(	283	7.078093	chr2:4707	RHOQ		protein_c	chr2:46541806-4658
ENSG0000(	283	7.078093	chr2:4707	FAM98A		protein_c	chr2:33532744-3359
ENSG0000(	283	7.078093	chr2:4707	RNU6-986P		smallRNA	chr2:27475494-2747
ENSG0000(	283	7.078093	chr2:4707	LINC01820		lncRNA	chr2:46392291-4639
ENSG0000(	283	7.078093	chr2:4707	FOSL2-AS1		lncRNA	chr2:28384409-2839
ENSG0000(	283	7.078093	chr2:4707	ENSG00000279254		TEC	chr2:46668870-4667
ENSG0000(	283	7.078093	chr2:4707	YPEL5		protein_c	chr2:30146941-3016
ENSG0000(	283	7.078093	chr2:4707	ENSG00000279544		TEC	chr2:32563328-3256
ENSG0000(	283	7.078093	chr2:4707	ATL2		protein_c	chr2:38293954-3837
ENSG0000(	283	7.078093	chr2:4707	SRBD1		protein_c	chr2:45388680-4561
ENSG0000(	283	7.078093	chr2:4707	SNORD53_SNORD92		smallRNA	chr2:28927983-2892
ENSG0000(	283	7.078093	chr2:4707	ENSG00000274159		lncRNA	chr2:32548675-3254
ENSG0000(	283	7.078093	chr2:4707	ENSG00000225402		Pseudoger	chr2:37816915-3781
ENSG0000(	283	7.078093	chr2:4707	TTC7A		protein_c	chr2:46915869-4707
ENSG0000(	283	7.078093	chr2:4707	SUPT7L	NCGv7	protein_c	chr2:27650809-2766
ENSG0000(	283	7.078093	chr2:4707	EPCAM-DT		lncRNA	chr2:47192405-4734
ENSG0000(	283	7.078093	chr2:4707	AL121652.2		smallRNA	chr2:31810195-3181
ENSG0000(	283	7.078093	chr2:4707	ENSG00000270210		lncRNA	chr2:28425945-2842
ENSG0000(	283	7.078093	chr2:4707	CRIPT		protein_c	chr2:46616416-4663
ENSG0000(	283	7.078093	chr2:4707	ENSG00000289727		lncRNA	chr2:32233386-3226
ENSG0000(	283	7.078093	chr2:4707	ZNF512		protein_c	chr2:27582969-2762
ENSG0000(	283	7.078093	chr2:4707	AL121652.1		smallRNA	chr2:31919862-3191
ENSG0000(	283	7.078093	chr2:4707	ENSG00000225284		lncRNA	chr2:38861720-3886
ENSG0000(	283	7.078093	chr2:4707	Y_RNA		smallRNA	chr2:28972414-2897
ENSG0000(	283	7.078093	chr2:4707	LINC01118		lncRNA	chr2:46698940-4682
ENSG0000(	283	7.078093	chr2:4707	ENSG00000273165		lncRNA	chr2:31852976-3185
ENSG0000(	283	7.078093	chr2:4707	DHX57		protein_c	chr2:38797729-3887
ENSG0000(	283	7.078093	chr2:4707	CDC42EP3		protein_c	chr2:37641882-3773
ENSG0000(	283	7.078093	chr2:4707	RNU6-1116P		smallRNA	chr2:37435510-3743
ENSG0000(	283	7.078093	chr2:4707	MRPL33		protein_c	chr2:27771717-2798
ENSG0000(	283	7.078093	chr2:4707	HSPE1P13		Pseudoger	chr2:39098149-3909
ENSG0000(	283	7.078093	chr2:4707	RN7SKP119		smallRNA	chr2:47359505-4735
ENSG0000(	283	7.078093	chr2:4707	SLC8A1		protein_c	chr2:40097270-4061
ENSG0000(	283	7.078093	chr2:4707	TOGARAM2		protein_c	chr2:28956611-2905
ENSG0000(	283	7.078093	chr2:4707	LINC02580		lncRNA	chr2:43092530-4321
ENSG0000(	283	7.078093	chr2:4707	LDHAP3		Pseudoger	chr2:41819747-4182
ENSG0000(	283	7.078093	chr2:4707	ENSG00000284608		lncRNA	chr2:46429195-4648
ENSG0000(	283	7.078093	chr2:4707	ENSG00000229224		lncRNA	chr2:29088649-2909

ENSG0000(	283	7.078093	chr2:4707	DPY30		protein_c	chr2:31867809-3203
ENSG0000(	283	7.078093	chr2:4707	ENSG00000270422		Pseudoger	chr2:31651381-3165
ENSG0000(	283	7.078093	chr2:4707	ENSG00000225943		Pseudoger	chr2:30077093-3007
ENSG0000(	283	7.078093	chr2:4707	RPL36AP14		Pseudoger	chr2:46256860-4625
ENSG0000(	283	7.078093	chr2:4707	MEMO1		protein_c	chr2:31865060-3201
ENSG0000(	283	7.078093	chr2:4707	CAPN13		protein_c	chr2:30722771-3082
ENSG0000(	283	7.078093	chr2:4707	ENSG00000289013		lncRNA	chr2:40394673-4039
ENSG0000(	283	7.078093	chr2:4707	SNORD92		smallRNA	chr2:28913664-2891
ENSG0000(	283	7.078093	chr2:4707	LINC01936		lncRNA	chr2:30346623-3036
ENSG0000(	283	7.078093	chr2:4707	H2ACP2		Pseudoger	chr2:33056333-3306
ENSG0000(	283	7.078093	chr2:4707	Y_RNA		smallRNA	chr2:42637961-4263
ENSG0000(	283	7.078093	chr2:4707	ENSG00000224058		Pseudoger	chr2:47731402-4773
ENSG0000(	283	7.078093	chr2:4707	RPL7P12		Pseudoger	chr2:38231568-3823
ENSG0000(	283	7.078093	chr2:4707	SLC4A1AP		protein_c	chr2:27663426-2769
ENSG0000(	283	7.078093	chr2:4707	ENSG00000271443		Pseudoger	chr2:38535258-3853
ENSG0000(	283	7.078093	chr2:4707	SNORD75		smallRNA	chr2:42440377-4244
ENSG0000(	283	7.078093	chr2:4707	ENSG00000278908		TEC	chr2:30677762-3067
ENSG0000(	283	7.078093	chr2:4707	ENSG00000233978		lncRNA	chr2:43041193-4304
ENSG0000(	283	7.078093	chr2:4707	AC069303.1		smallRNA	chr2:33786038-3378
ENSG0000(	283	7.078093	chr2:4707	ENSG00000273006		lncRNA	chr2:38193348-3819
ENSG0000(	283	7.078093	chr2:4707	ENSG00000289082		lncRNA	chr2:42795326-4279
ENSG0000(	283	7.078093	chr2:4707	ENSG00000236213		lncRNA	chr2:37562486-3764
ENSG0000(	283	7.078093	chr2:4707	WDR43		protein_c	chr2:28894667-2894
ENSG0000(	283	7.078093	chr2:4707	SPDYA		protein_c	chr2:28782517-2885
ENSG0000(	283	7.078093	chr2:4707	PLB1	NCGv7	protein_c	chr2:28457145-2864
ENSG0000(	283	7.078093	chr2:4707	RMDN2-AS1		lncRNA	chr2:37949911-3806
ENSG0000(	283	7.078093	chr2:4707	SMIM7P1		Pseudoger	chr2:35219377-3521
ENSG0000(	283	7.078093	chr2:4707	EHD3		protein_c	chr2:31234152-3126
ENSG0000(	283	7.078093	chr2:4707	MIR559		smallRNA	chr2:47377675-4737
ENSG0000(	283	7.078093	chr2:4707	ENSG00000233862		lncRNA	chr2:30051066-3014
ENSG0000(	283	7.078093	chr2:4707	Y_RNA		smallRNA	chr2:39128826-3912
ENSG0000(	283	7.078093	chr2:4707	ENSG00000233845		lncRNA	chr2:47035279-4704
ENSG0000(	283	7.078093	chr2:4707	ENSG00000287145		lncRNA	chr2:41716133-4173
ENSG0000(	283	7.078093	chr2:4707	ENSG00000229160		lncRNA	chr2:38132637-3813
ENSG0000(	283	7.078093	chr2:4707	MIR4765		smallRNA	chr2:32635255-3263
ENSG0000(	283	7.078093	chr2:4707	ENSG00000289326		lncRNA	chr2:27752831-2775
ENSG0000(	283	7.078093	chr2:4707	GCKR		protein_c	chr2:27496839-2752
ENSG0000(	283	7.078093	chr2:4707	LCLAT1		protein_c	chr2:30447226-3064
ENSG0000(	283	7.078093	chr2:4707	ENSG00000286415		lncRNA	chr2:33599442-3365
ENSG0000(	283	7.078093	chr2:4707	MSH2	NCGv7;AC	protein_c	chr2:47403067-4766
ENSG0000(	283	7.078093	chr2:4707	ENSG00000236572		lncRNA	chr2:37744333-3774
ENSG0000(	283	7.078093	chr2:4707	ENSG00000286963		lncRNA	chr2:29319554-2935
ENSG0000(	283	7.078093	chr2:4707	PCARE		protein_c	chr2:29060976-2907
ENSG0000(	283	7.078093	chr2:4707	KRT18P52		Pseudoger	chr2:31822591-3182
ENSG0000(	283	7.078093	chr2:4707	PPM1B-DT		lncRNA	chr2:44167625-4416
ENSG0000(	283	7.078093	chr2:4707	ENSG00000287316		lncRNA	chr2:37466781-3752
ENSG0000(	283	7.078093	chr2:4707	SLC3A1		protein_c	chr2:44275458-4432
ENSG0000(	283	7.078093	chr2:4707	ENSG00000287387		lncRNA	chr2:43219849-4322
ENSG0000(	283	7.078093	chr2:4707	ENSG00000272027		lncRNA	chr2:34692290-3470
ENSG0000(	283	7.078093	chr2:4707	CEBPZ		protein_c	chr2:37201612-3723
ENSG0000(	283	7.078093	chr2:4707	FBXO11	NCGv7;AC	protein_c	chr2:47789316-4790

ENSG0000(	283	7.078093	chr2:4707	ENSG00000286728	lncRNA	chr2:45169616-4521
ENSG0000(	283	7.078093	chr2:4707	RNU6-566P	smallRNA	chr2:44154789-4415
ENSG0000(	283	7.078093	chr2:4707	SIX3	protein_c	chr2:44941702-4494
ENSG0000(	283	7.078093	chr2:4707	ENSG00000228563	lncRNA	chr2:31526942-3156
ENSG0000(	283	7.078093	chr2:4707	RPS27AP7	Pseudoger	chr2:47883455-4788
ENSG0000(	283	7.078093	chr2:4707	SOS1-IT1	lncRNA	chr2:38992279-3899
ENSG0000(	283	7.078093	chr2:4707	RNA5SP91	Pseudoger	chr2:33285769-3328
ENSG0000(	283	7.078093	chr2:4707	Y_RNA	smallRNA	chr2:28927243-2892
ENSG0000(	283	7.078093	chr2:4707	ENSG00000229695	Pseudoger	chr2:43680465-4368
ENSG0000(	283	7.078093	chr2:4707	ENSG00000287313	lncRNA	chr2:38408719-3841
ENSG0000(	283	7.078093	chr2:4707	RNA5SP89	Pseudoger	chr2:28683976-2868
ENSG0000(	283	7.078093	chr2:4707	ENSG00000237320	lncRNA	chr2:34998273-3500
ENSG0000(	283	7.078093	chr2:4707	RPS12P4	Pseudoger	chr2:41850203-4185
ENSG0000(	283	7.078093	chr2:4707	SIX3-AS1	lncRNA	chr2:44940153-4494
ENSG0000(	283	7.078093	chr2:4707	AC067957.1	smallRNA	chr2:44782046-4478
ENSG0000(	283	7.078093	chr2:4707	ENSG00000235267	Pseudoger	chr2:27455156-2745
ENSG0000(	283	7.078093	chr2:4707	ENSG00000286796	lncRNA	chr2:43128819-4313
ENSG0000(	283	7.078093	chr2:4707	PREPL	protein_c	chr2:44316281-4436
ENSG0000(	283	7.078093	chr2:4707	BIRC6-AS2	lncRNA	chr2:32557273-3257
ENSG0000(	283	7.078093	chr2:4707	ENSG00000273269	protein_c	chr2:47065941-4717
ENSG0000(	283	7.078093	chr2:4707	DYNC2LI1	protein_c	chr2:43774039-4381
ENSG0000(	283	7.078093	chr2:4707	ENSG00000272054	lncRNA	chr2:37208875-3721
ENSG0000(	283	7.078093	chr2:4707	THUMPD2	protein_c	chr2:39736060-3977
ENSG0000(	283	7.078093	chr2:4707	RNU6-958P	smallRNA	chr2:43408307-4340
ENSG0000(	283	7.078093	chr2:4707	RNU6-951P	smallRNA	chr2:38147415-3814
ENSG0000(	283	7.078093	chr2:4707	AL121652.3	smallRNA	chr2:31823018-3182
ENSG0000(	283	7.078093	chr2:4707	PDSS1P2	Pseudoger	chr2:44166266-4416
ENSG0000(	283	7.078093	chr2:4707	PPM1B	protein_c	chr2:44167969-4424
ENSG0000(	283	7.078093	chr2:4707	ENSG00000289272	lncRNA	chr2:44228188-4422
ENSG0000(	283	7.078093	chr2:4707	CYP1B1	protein_c	chr2:38066973-3810
ENSG0000(	283	7.078093	chr2:4707	BCYRN1	smallRNA	chr2:47335315-4733
ENSG0000(	283	7.078093	chr2:4707	RPL36AP15	Pseudoger	chr2:47797826-4779
ENSG0000(	283	7.078093	chr2:4707	LINC00486	lncRNA	chr2:32927085-3294
ENSG0000(	283	7.078093	chr2:4707	RNU6-577P	smallRNA	chr2:36867398-3686
ENSG0000(	283	7.078093	chr2:4707	MIR548AD	smallRNA	chr2:35471405-3547
ENSG0000(	283	7.078093	chr2:4707	ENSG00000228925	lncRNA	chr2:46899275-4690
ENSG0000(	283	7.078093	chr2:4707	RPS13P3	Pseudoger	chr2:42469817-4247
ENSG0000(	283	7.078093	chr2:4707	MIR4263	smallRNA	chr2:27996367-2799
ENSG0000(	283	7.078093	chr2:4707	SULT6B1	protein_c	chr2:37167820-3719
ENSG0000(	283	7.078093	chr2:4707	ENSG00000273090	lncRNA	chr2:36513255-3651
ENSG0000(	283	7.078093	chr2:4707	CCDC121	protein_c	chr2:27625638-2762
ENSG0000(	283	7.078093	chr2:4707	ABCG5	protein_c	chr2:43812472-4383
ENSG0000(	283	7.078093	chr2:4707	CALM2	protein_c	chr2:47160084-4717
ENSG0000(	283	7.078093	chr2:4707	CRIM1-DT	lncRNA	chr2:36354744-3635
ENSG0000(	283	7.078093	chr2:4707	ENSG00000287468	lncRNA	chr2:40620667-4063
ENSG0000(	283	7.078093	chr2:4707	ENSG00000213620	Pseudoger	chr2:31290762-3129
ENSG0000(	283	7.078093	chr2:4707	EML4-AS1	lncRNA	chr2:42143238-4217
ENSG0000(	283	7.078093	chr2:4707	RPLP0P6	Pseudoger	chr2:38481851-3848
ENSG0000(	283	7.078093	chr2:4707	RN7SKP66	smallRNA	chr2:43772120-4377
ENSG0000(	283	7.078093	chr2:4707	SLC25A5P2	Pseudoger	chr2:33839782-3384
ENSG0000(	283	7.078093	chr2:4707	LINC01318	lncRNA	chr2:34067226-3406

ENSG0000(	283	7.078093	chr2:4707	SNORD53		smallRNA	chr2:28927067-2892
ENSG0000(	283	7.078093	chr2:4707	LINC00211		lncRNA	chr2:37820498-3787
ENSG0000(	283	7.078093	chr2:4707	GAPDHP25		Pseudoger	chr2:38285410-3828
ENSG0000(	283	7.078093	chr2:4707	ENSG00000287255		lncRNA	chr2:40511921-4054
ENSG0000(	283	7.078093	chr2:4707	NLRC4		protein_c	chr2:32224453-3226
ENSG0000(	283	7.078093	chr2:4707	MSH2-OT1		lncRNA	chr2:47527008-4753
ENSG0000(	283	7.078093	chr2:4707	LBH		protein_c	chr2:30231534-3032
ENSG0000(	283	7.078093	chr2:4707	HNRNPA1P57		Pseudoger	chr2:41143780-4115
ENSG0000(	283	7.078093	chr2:4707	PPP1CB		protein_c	chr2:28751640-2880
ENSG0000(	283	7.078093	chr2:4707	DDX50P1		Pseudoger	chr2:32201600-3220
ENSG0000(	283	7.078093	chr2:4707	ENSG00000197644		lncRNA	chr2:29899597-2990
ENSG0000(	283	7.078093	chr2:4707	SNORA67		smallRNA	chr2:39283657-3928
ENSG0000(	283	7.078093	chr2:4707	LINC01320		lncRNA	chr2:33706886-3473
ENSG0000(	283	7.078093	chr2:4707	LINC01883		lncRNA	chr2:38431294-3843
ENSG0000(	283	7.078093	chr2:4707	ENSG00000279519		TEC	chr2:36839922-3684
ENSG0000(	283	7.078093	chr2:4707	EIF2AK2		protein_c	chr2:37099210-3715
ENSG0000(	283	7.078093	chr2:4707	ATP6V0E1P3		Pseudoger	chr2:33602041-3360
ENSG0000(	283	7.078093	chr2:4707	AC011748.1		smallRNA	chr2:34403664-3440
ENSG0000(	283	7.078093	chr2:4707	ENSG00000259080		lncRNA	chr2:27583046-2763
ENSG0000(	283	7.078093	chr2:4707	NME2P2		Pseudoger	chr2:47705468-4770
ENSG0000(	283	7.078093	chr2:4707	ENSG00000286519		lncRNA	chr2:45173722-4517
ENSG0000(	283	7.078093	chr2:4707	RNU6-647P		smallRNA	chr2:32214456-3221
ENSG0000(	283	7.078093	chr2:4707	SNORD112		smallRNA	chr2:32991259-3299
ENSG0000(	283	7.078093	chr2:4707	ENSG00000289545		lncRNA	chr2:37489453-3760
ENSG0000(	283	7.078093	chr2:4707	LINC01833		lncRNA	chr2:44921077-4493
ENSG0000(	283	7.078093	chr2:4707	GPN1		protein_c	chr2:27628247-2765
ENSG0000(	283	7.078093	chr2:4707	ENSG00000253515		lncRNA	chr2:46429229-4650
ENSG0000(	283	7.078093	chr2:4707	HEATR5B		protein_c	chr2:36968383-3708
ENSG0000(	283	7.078093	chr2:4707	ENSG00000273233		lncRNA	chr2:28810281-2881
ENSG0000(	283	7.078093	chr2:4707	EML4	NCGv7;AC	protein_c	chr2:42169353-4233
ENSG0000(	283	7.078093	chr2:4707	LRPPRC		protein_c	chr2:43886224-4399
ENSG0000(	283	7.078093	chr2:4707	IFT172		protein_c	chr2:27444377-2748
ENSG0000(	283	7.078093	chr2:4707	ABCG8	NCGv7	protein_c	chr2:43831942-4388
ENSG0000(	283	7.078093	chr2:4707	CAMKMT		protein_c	chr2:44361947-4477
ENSG0000(	283	7.078093	chr2:4707	ENSG00000271228		lncRNA	chr2:32013061-3201
ENSG0000(	283	7.078093	chr2:4707	RNU6-846P		smallRNA	chr2:36959362-3695
ENSG0000(	283	7.078093	chr2:4707	GALM		protein_c	chr2:38666081-3874
ENSG0000(	283	7.078093	chr2:4707	ENSG00000225156		lncRNA	chr2:44954664-4496
ENSG0000(	283	7.078093	chr2:4707	HNRNPLL		protein_c	chr2:38561969-3860
ENSG0000(	283	7.078093	chr2:4707	ARL14EPP1		Pseudoger	chr2:37148530-3714
ENSG0000(	283	7.078093	chr2:4707	PRKCE-AS1		lncRNA	chr2:45674701-4567
ENSG0000(	283	7.078093	chr2:4707	ENSG00000229013		Pseudoger	chr2:35471716-3547
ENSG0000(	283	7.078093	chr2:4707	MTA3		protein_c	chr2:42494569-4275
ENSG0000(	283	7.078093	chr2:4707	C2orf16		protein_c	chr2:27537386-2758
ENSG0000(	283	7.078093	chr2:4707	ENSG00000235653		Pseudoger	chr2:39929110-3992
ENSG0000(	283	7.078093	chr2:4707	ENSG00000225187		lncRNA	chr2:47067822-4707
ENSG0000(	283	7.078093	chr2:4707	ENSG00000234936		lncRNA	chr2:43229573-4323
ENSG0000(	283	7.078093	chr2:4707	PRKD3		protein_c	chr2:37250502-3732
ENSG0000(	283	7.078093	chr2:4707	LINC01913		lncRNA	chr2:41860155-4189
ENSG0000(	283	7.078093	chr2:4707	ALK	NCGv7;AC	protein_c	chr2:29192774-2992
ENSG0000(	283	7.078093	chr2:4707	ENSG00000270640		lncRNA	chr2:28396815-2839

ENSG0000(	283	7.078093	chr2:4707	CLIP4		protein_c	chr2:29097705-2919
ENSG0000(	283	7.078093	chr2:4707	ENSG00000285542		protein_c	chr2:44168851-4432
ENSG0000(	283	7.078093	chr2:4707	BIRC6-AS2		Pseudoger	chr2:32557703-3255
ENSG0000(	283	7.078093	chr2:4707	ENSG00000231054		lncRNA	chr2:45168583-4516
ENSG0000(	283	7.078093	chr2:4707	RACK1P2		Pseudoger	chr2:36656322-3665
ENSG0000(	283	7.078093	chr2:4707	ENSG00000226398		lncRNA	chr2:42015625-4202
ENSG0000(	283	7.078093	chr2:4707	RN7SL414P		smallRNA	chr2:45569201-4556
ENSG0000(	283	7.078093	chr2:4707	C1GALT1C1L		protein_c	chr2:43675151-4367
ENSG0000(	283	7.078093	chr2:4707	VDAC1P13		Pseudoger	chr2:42463139-4246
ENSG0000(	283	7.078093	chr2:4707	ENSG00000285577		lncRNA	chr2:33274465-3328
ENSG0000(	283	7.078093	chr2:4707	LINC01946		lncRNA	chr2:31793823-3180
ENSG0000(	283	7.078093	chr2:4707	ENSG00000215263		Pseudoger	chr2:42532766-4253
ENSG0000(	283	7.078093	chr2:4707	RNU6-939P		smallRNA	chr2:37331770-3733
ENSG0000(	283	7.078093	chr2:4707	ENSG00000232518		lncRNA	chr2:38668202-3867
ENSG0000(	283	7.078093	chr2:4707	CEBPZOS		protein_c	chr2:37196488-3721
ENSG0000(	283	7.078093	chr2:4707	PPIAP62		Pseudoger	chr2:47939738-4794
ENSG0000(	283	7.078093	chr2:4707	MORN2		protein_c	chr2:38875976-3892
ENSG0000(	283	7.078093	chr2:4707	HNRNPA1P61		Pseudoger	chr2:33636502-3363
ENSG0000(	283	7.078093	chr2:4707	FTOP1		Pseudoger	chr2:42797225-4279
ENSG0000(	283	7.078093	chr2:4707	ENSG00000231156		lncRNA	chr2:45013214-4501
ENSG0000(	283	7.078093	chr2:4707	ENSG00000272814		lncRNA	chr2:46956615-4695
ENSG0000(	283	7.078093	chr2:4707	STPG4		protein_c	chr2:47045538-4715
ENSG0000(	283	7.078093	chr2:4707	ENSG00000288535		lncRNA	chr2:35263711-3528
ENSG0000(	283	7.078093	chr2:4707	ENSG00000288886		lncRNA	chr2:42792299-4279
ENSG0000(	283	7.078093	chr2:4707	ENSG00000226523		Pseudoger	chr2:42680088-4268
ENSG0000(	283	7.078093	chr2:4707	SIX2	NCGv7	protein_c	chr2:45005182-4500
ENSG0000(	283	7.078093	chr2:4707	FEZ2		protein_c	chr2:36531805-3664
ENSG0000(	283	7.078093	chr2:4707	LINC01119		lncRNA	chr2:46816697-4685
ENSG0000(	283	7.078093	chr2:4707	ENSG00000288553		lncRNA	chr2:29841187-2995
ENSG0000(	283	7.078093	chr2:4707	AL121655.1		smallRNA	chr2:32088304-3208
ENSG0000(	283	7.078093	chr2:4707	ENSG00000227292		lncRNA	chr2:38203363-3823
ENSG0000(	283	7.078093	chr2:4707	TTC27		protein_c	chr2:32628032-3282
ENSG0000(	283	7.078093	chr2:4707	LINC00486		lncRNA	chr2:32825359-3292
ENSG0000(	283	7.078093	chr2:4707	LINC01121		lncRNA	chr2:45164816-4532
ENSG0000(	283	7.078093	chr2:4707	RN7SL817P		smallRNA	chr2:46448226-4644
ENSG0000(	283	7.078093	chr2:4707	LINC02898		lncRNA	chr2:41935368-4195
ENSG0000(	283	7.078093	chr2:4707	CDKL4		protein_c	chr2:39168045-3924
ENSG0000(	283	7.078093	chr2:4707	FNDC4		protein_c	chr2:27491883-2749
ENSG0000(	283	7.078093	chr2:4707	LINC01126		lncRNA	chr2:43227210-4322
ENSG0000(	283	7.078093	chr2:4707	ENSG00000288937		lncRNA	chr2:32039839-3204
ENSG0000(	283	7.078093	chr2:4707	VIT	NCGv7	protein_c	chr2:36696690-3681
ENSG0000(	283	7.078093	chr2:4707	AC097506.1		smallRNA	chr2:33927384-3392
ENSG0000(	283	7.078093	chr2:4707	ENSG00000230979		Pseudoger	chr2:47690716-4769
ENSG0000(	283	7.078093	chr2:4707	LINC01460		lncRNA	chr2:27705786-2771
ENSG0000(	283	7.078093	chr2:4707	TTC39DP		Pseudoger	chr2:38763534-3876
ENSG0000(	283	7.078093	chr2:4707	ENSG00000232696		lncRNA	chr2:46078015-4607
ENSG0000(	283	7.078093	chr2:4707	RBKS		protein_c	chr2:27781379-2789
ENSG0000(	283	7.078093	chr2:4707	NDUFAF7		protein_c	chr2:37231631-3725
ENSG0000(	283	7.078093	chr2:4707	HAAO		protein_c	chr2:42767089-4279
ENSG0000(	283	7.078093	chr2:4707	ENSG00000219391		Pseudoger	chr2:44065894-4406
ENSG0000(	283	7.078093	chr2:4707	SOCS5		protein_c	chr2:46698952-4678

ENSG0000(	283	7.078093	chr2:4707	AC106899.1		smallRNA	chr2:29503907-2950
ENSG0000(	283	7.078093	chr2:4707	Y_RNA		smallRNA	chr2:43620878-4362
ENSG0000(	283	7.078093	chr2:4707	PRKCE	AC	protein_c	chr2:45651345-4618
ENSG0000(	283	7.078093	chr2:4707	SPAST		protein_c	chr2:32063556-3215
ENSG0000(	283	7.078093	chr2:4707	KCNG3		protein_c	chr2:42442017-4249
ENSG0000(	283	7.078093	chr2:4707	TRMT61B		protein_c	chr2:28849821-2887
ENSG0000(	283	7.078093	chr2:4707	RASGRP3		protein_c	chr2:33436324-3356
ENSG0000(	283	7.078093	chr2:4707	SLC30A6	NCGv7	protein_c	chr2:32165841-3222
ENSG0000(	283	7.078093	chr2:4707	CYP1B1-AS1		lncRNA	chr2:38073447-3823
ENSG0000(	283	7.078093	chr2:4707	MYG1P1		Pseudoger	chr2:27896116-2789
ENSG0000(	283	7.078093	chr2:4707	ENSG00000285925		lncRNA	chr2:37339957-3734
ENSG0000(	283	7.078093	chr2:4707	KRTCAP2P1		Pseudoger	chr2:44996413-4499
ENSG0000(	283	7.078093	chr2:4707	LINC01819		lncRNA	chr2:42972255-4304
ENSG0000(	283	7.078093	chr2:4707	EPAS1	NCGv7	protein_c	chr2:46293667-4638
ENSG0000(	283	7.078093	chr2:4707	ENSG00000276517		lncRNA	chr2:32526504-3252
ENSG0000(	283	7.078093	chr2:4707	LINC02613		lncRNA	chr2:38406527-3851
ENSG0000(	283	7.078093	chr2:4707	THADA		protein_c	chr2:43230851-4359
ENSG0000(	283	7.078093	chr2:4707	ENSG00000285754		lncRNA	chr2:34134371-3422
ENSG0000(	283	7.078093	chr2:4707	AC009305.2		smallRNA	chr2:31035553-3103
ENSG0000(	283	7.078093	chr2:4707	COX7A2L		protein_c	chr2:42333546-4242
ENSG0000(	283	7.078093	chr2:4707	ENSG00000285898		lncRNA	chr2:40591285-4067
ENSG0000(	283	7.078093	chr2:4707	Y_RNA		smallRNA	chr2:32945339-3294
ENSG0000(	283	7.078093	chr2:4707	PIGF		protein_c	chr2:46580937-4661
ENSG0000(	283	7.078093	chr2:4707	H3P5		Pseudoger	chr2:30209995-3021
ENSG0000(	283	7.078093	chr2:4707	SOS1	NCGv7	protein_c	chr2:38962206-3912
ENSG0000(	283	7.078093	chr2:4707	ENSG00000290100		lncRNA	chr2:37617328-3766
ENSG0000(	283	7.078093	chr2:4707	RNU6-198P		smallRNA	chr2:39082589-3908
ENSG0000(	283	7.078093	chr2:4707	ENSG00000276334		lncRNA	chr2:32521927-3252
ENSG0000(	283	7.078093	chr2:4707	SRSF7		protein_c	chr2:38743599-3875
ENSG0000(	283	7.078093	chr2:4707	PPP1CB-DT		lncRNA	chr2:28707511-2875
ENSG0000(	283	7.078093	chr2:4707	RMDN2		protein_c	chr2:37923187-3806
ENSG0000(	283	7.078093	chr2:4707	BIRC6	NCGv7	protein_c	chr2:32357023-3261
ENSG0000(	283	7.078093	chr2:4707	QPCT		protein_c	chr2:37342827-3737
ENSG0000(	283	7.078093	chr2:4707	RNU6-242P		smallRNA	chr2:43091388-4309
ENSG0000(	283	7.078093	chr2:4707	CRIM1		protein_c	chr2:36355778-3655
ENSG0000(	283	7.078093	chr2:4707	ENSG00000272754		lncRNA	chr2:32321638-3232
ENSG0000(	283	7.078093	chr2:4707	STRN	IntOGen-I	protein_c	chr2:36837698-3696
ENSG0000(	283	7.078093	chr2:4707	SNORA64		smallRNA	chr2:30187434-3018
ENSG0000(	283	7.078093	chr2:4707	MSH6	NCGv7;AC	protein_c	chr2:47695530-4781
ENSG0000(	283	7.078093	chr2:4707	ENSG00000280154		TEC	chr2:30408170-3040
ENSG0000(	283	7.078093	chr2:4707	SLC30A6-DT		lncRNA	chr2:32165041-3216
ENSG0000(	283	7.078093	chr2:4707	RNU6-851P		smallRNA	chr2:38884560-3888
ENSG0000(	283	7.078093	chr2:4707	CHORDC1P1		Pseudoger	chr2:42826322-4282
ENSG0000(	283	7.078093	chr2:4707	ATP6V1E2	DriverDB\	protein_c	chr2:46490750-4654
ENSG0000(	283	7.078093	chr2:4707	ENSG00000226548		lncRNA	chr2:46852020-4685
ENSG0000(	283	7.078093	chr2:4707	PLEKHH2		protein_c	chr2:43637260-4376
ENSG0000(	283	7.078093	chr2:4707	ZFP36L2	NCGv7	protein_c	chr2:43222402-4322
ENSG0000(	283	7.078093	chr2:4707	ENSG00000232153		lncRNA	chr2:34732287-3482
ENSG0000(	283	7.078093	chr2:4707	MYADML		Pseudoger	chr2:33722721-3372
ENSG0000(	283	7.078093	chr2:4707	NPLP1		Pseudoger	chr2:38769265-3877
ENSG0000(	283	7.078093	chr2:4707	SNORA36		smallRNA	chr2:27642043-2764

ENSG0000(	283	7.078093	chr2:4707	PRKD3-DT		lncRNA	chr2:37325340-3732
ENSG0000(	283	7.078093	chr2:4707	RNU6-1117P		smallRNA	chr2:35471605-3547
ENSG0000(	283	7.078093	chr2:4707	ENSG00000285984		lncRNA	chr2:30887626-3089
ENSG0000(	283	7.078093	chr2:4707	SLC8A1-AS1		lncRNA	chr2:39786453-4025
ENSG0000(	283	7.078093	chr2:4707	TMEM178A		protein_c	chr2:39664982-3971
ENSG0000(	283	7.078093	chr2:4707	ENSG00000223522		lncRNA	chr2:28307063-2831
ENSG0000(	283	7.078093	chr2:4707	GEMIN6		protein_c	chr2:38751534-3878
ENSG0000(	283	7.078093	chr2:4707	ENSG00000226994		lncRNA	chr2:34799850-3518
ENSG0000(	283	7.078093	chr2:4707	MAP4K3-DT		lncRNA	chr2:39436530-3966
ENSG0000(	283	7.078093	chr2:4707	ENSG00000288707		lncRNA	chr2:43995985-4399
ENSG0000(	283	7.078093	chr2:4707	FOSL2		protein_c	chr2:28392448-2841
ENSG0000(	283	7.078093	chr2:4707	Y_RNA		smallRNA	chr2:41857271-4185
ENSG0000(	283	7.078093	chr2:4707	RNU4-63P		smallRNA	chr2:41871271-4187
ENSG0000(	283	7.078093	chr2:4707	LINC02583		lncRNA	chr2:46429190-4644
ENSG0000(	283	7.078093	chr2:4707	RPL12P19		Pseudoger	chr2:44270621-4427
ENSG0000(	283	7.078093	chr2:4707	ENSG00000231336		lncRNA	chr2:46166789-4616
ENSG0000(	283	7.078093	chr2:4707	GPATCH11		protein_c	chr2:37084518-3709
ENSG0000(	283	7.078093	chr2:4707	FAM133EP		Pseudoger	chr2:28015777-2801
ENSG0000(	283	7.078093	chr2:4707	RPL26P15		Pseudoger	chr2:46003942-4600
ENSG0000(	283	7.078093	chr2:4707	ENSG00000226087		lncRNA	chr2:47225781-4724
ENSG0000(	283	7.078093	chr2:4707	ENSG00000289003		lncRNA	chr2:39480783-3948
ENSG0000(	283	7.078093	chr2:4707	ARHGEF33		protein_c	chr2:38889875-3897
ENSG0000(	283	7.078093	chr2:4707	RPL23AP34		Pseudoger	chr2:28308161-2830
ENSG0000(	283	7.078093	chr2:4707	ENSG00000233230		lncRNA	chr2:47905678-4790
ENSG0000(	283	7.078093	chr2:4707	TMEM247		protein_c	chr2:46479565-4648
ENSG0000(	283	7.078093	chr2:4707	SRD5A2		protein_c	chr2:31522480-3158
ENSG0000(	283	7.078093	chr2:4707	CAPN14		protein_c	chr2:31173056-3123
ENSG0000(	283	7.078093	chr2:4707	KRTCAP3		protein_c	chr2:27442366-2744
ENSG0000(	283	7.078093	chr2:4707	ENSG00000288994		lncRNA	chr2:38342476-3834
ENSG0000(	283	7.078093	chr2:4707	BABAM2	NCGv7	protein_c	chr2:27889941-2833
ENSG0000(	283	7.078093	chr2:4707	RN7SL516P		smallRNA	chr2:29681029-2968
ENSG0000(	283	7.078093	chr2:4707	GALNT14		protein_c	chr2:30910467-3115
ENSG0000(	283	7.078093	chr2:4707	XDH	NCGv7	protein_c	chr2:31334321-3141
ENSG0000(	283	7.078093	chr2:4707	snoU13		smallRNA	chr2:44239568-4423
ENSG0000(	283	7.078093	chr2:4707	RHOQ-AS1		lncRNA	chr2:46568256-4658
ENSG0000(	283	7.078093	chr2:4707	KCNK12		protein_c	chr2:47509290-4757
ENSG0000(	283	7.078093	chr2:4707	RNU6-688P		smallRNA	chr2:47781379-4778
ENSG0000(	283	7.078093	chr2:4707	AK2P2		Pseudoger	chr2:31823413-3182
ENSG0000(	283	7.078093	chr2:4707	AC105398.1		smallRNA	chr2:29081270-2908
ENSG0000(	283	7.078093	chr2:4707	ENSG00000280276		TEC	chr2:38121935-3812
ENSG0000(	283	7.078093	chr2:4707	ENSG00000288992		lncRNA	chr2:40450663-4045
ENSG0000(	283	7.078093	chr2:4707	ENSG00000230737		lncRNA	chr2:29890371-2989
ENSG0000(	283	7.078093	chr2:4707	RPL31P16		Pseudoger	chr2:37194382-3719
ENSG0000(	283	7.078093	chr2:4707	LINC01794		lncRNA	chr2:40746481-4076
ENSG0000(	283	7.078093	chr2:4707	ASS1P2		Pseudoger	chr2:38810432-3881
ENSG0000(	283	7.078093	chr2:4707	ENSG00000230730		lncRNA	chr2:28633282-2866
ENSG0000(	283	7.078093	chr2:4707	OXER1		protein_c	chr2:42762499-4276
ENSG0000(	283	7.078093	chr2:4707	MIR558		smallRNA	chr2:32532153-3253
ENSG0000(	283	7.078093	chr2:4707	SNRPGP7		Pseudoger	chr2:28460256-2846
ENSG0000(	283	7.078093	chr2:4707	PKDCC		protein_c	chr2:42048021-4205
ENSG0000(	282	7.053082	chr16:65(	ENSG00000259807		lncRNA	chr16:29215385-292

ENSG0000(	282	7.053082	chr6:105(	HIST1H4B		protein_c	chr6:26026896-2602
ENSG0000(	282	7.053082	chr3:857:	ENSG00000242911		Pseudoger	chr3:94935760-9493
ENSG0000(	282	7.053082	chr16:65(	ENSG00000273582		lncRNA	chr16:29225594-292
ENSG0000(	282	7.053082	chr16:65(	ENSG00000279106		TEC	chr16:29204633-292
ENSG0000(	281	7.028071	chr1:4061	ENSG00000270818		Pseudoger	chr1:242882066-242
ENSG0000(	281	7.028071	chr1:4061	ENSG00000289439		lncRNA	chr1:244308554-244
ENSG0000(	281	7.028071	chr1:4061	ENSG00000291216		lncRNA	chr1:243029512-243
ENSG0000(	281	7.028071	chr18:31{	SNORD58C		smallRNA	chr18:49489245-494
ENSG0000(	281	7.028071	chr1:4061	ADSS2		protein_c	chr1:244408494-244
ENSG0000(	281	7.028071	chr1:4061	ENSG00000286486		lncRNA	chr1:244087306-244
ENSG0000(	281	7.028071	chr1:4061	RPL10AP5		Pseudoger	chr1:242365189-242
ENSG0000(	281	7.028071	chr1:4061	CICP21		Pseudoger	chr1:243049782-243
ENSG0000(	281	7.028071	chr14:88{	RNU4-92P		smallRNA	chr14:88820297-888
ENSG0000(	281	7.028071	chr1:4061	ENSG00000213690		Pseudoger	chr1:242376923-242
ENSG0000(	281	7.028071	chr1:4061	SEPTIN14P21		Pseudoger	chr1:243047698-243
ENSG0000(	281	7.028071	chr1:4061	LINC01347		lncRNA	chr1:243056307-243
ENSG0000(	281	7.028071	chr1:4061	FCF1P7		Pseudoger	chr1:243267257-243
ENSG0000(	281	7.028071	chr1:4061	CEP170	NCGv7	protein_c	chr1:243124428-243
ENSG0000(	281	7.028071	chr1:4061	ENSG00000237759		lncRNA	chr1:244107365-244
ENSG0000(	281	7.028071	chr1:4061	ENSG00000279774		TEC	chr1:244064330-244
ENSG0000(	281	7.028071	chr1:4061	ENSG00000253326		Pseudoger	chr1:243054861-243
ENSG0000(	281	7.028071	chr1:4061	ENSG00000282317		lncRNA	chr1:244731024-244
ENSG0000(	281	7.028071	chr1:4061	PLD5		protein_c	chr1:242082986-242
ENSG0000(	281	7.028071	chr1:4061	ENSG00000227230		lncRNA	chr1:243135898-243
ENSG0000(	281	7.028071	chr1:4061	FABP7P1		Pseudoger	chr1:243624666-243
ENSG0000(	281	7.028071	chr1:4061	SDCCAG8	NCGv7	protein_c	chr1:243256034-243
ENSG0000(	281	7.028071	chr1:4061	AL445675.1		smallRNA	chr1:242897870-242
ENSG0000(	281	7.028071	chr1:4061	ENSG00000229960		lncRNA	chr1:244068820-244
ENSG0000(	281	7.028071	chr1:4061	AKT3-IT1		lncRNA	chr1:243793205-243
ENSG0000(	281	7.028071	chr1:4061	AKT3	NCGv7	protein_c	chr1:243488233-243
ENSG0000(	281	7.028071	chr1:4061	C1orf100	NCGv7	protein_c	chr1:244352635-244
ENSG0000(	281	7.028071	chr1:4061	LINC01347		Pseudoger	chr1:243087710-243
ENSG0000(	281	7.028071	chr1:4061	AC099757.1		smallRNA	chr1:244589546-244
ENSG0000(	281	7.028071	chr1:4061	ENSG00000277704		Pseudoger	chr1:242890247-242
ENSG0000(	281	7.028071	chr1:4061	LINC02774		lncRNA	chr1:243917402-244
ENSG0000(	281	7.028071	chr1:4061	ENSG00000289055		lncRNA	chr1:244047342-244
ENSG0000(	281	7.028071	chr1:4061	ENSG00000232184		lncRNA	chr1:243702857-243
ENSG0000(	281	7.028071	chr1:4061	RNU6-747P		smallRNA	chr1:243081156-243
ENSG0000(	281	7.028071	chr1:4061	ENSG00000287531		lncRNA	chr1:244184953-244
ENSG0000(	281	7.028071	chr1:4061	ENSG00000236031		lncRNA	chr1:243545532-243
ENSG0000(	281	7.028071	chr1:4061	DESI2		protein_c	chr1:244653103-244
ENSG0000(	281	7.028071	chr1:4061	ENSG00000270859		Pseudoger	chr1:242671140-242
ENSG0000(	281	7.028071	chr1:4061	ZBTB18	DriverDB\	protein_c	chr1:244048547-244
ENSG0000(	281	7.028071	chr1:4061	CATSPERE		protein_c	chr1:244454377-244
ENSG0000(	281	7.028071	chr1:4061	RSL24D1P4		Pseudoger	chr1:242772620-242
ENSG0000(	281	7.028071	chr1:4061	ENSG00000226750		Pseudoger	chr1:242345558-242
ENSG0000(	281	7.028071	chr1:4061	ENSG00000234116		lncRNA	chr1:243005845-243
ENSG0000(	281	7.028071	chr1:4061	ENSG00000232085		lncRNA	chr1:243164638-243
ENSG0000(	281	7.028071	chr1:4061	MIR4677		smallRNA	chr1:243346176-243
ENSG0000(	281	7.028071	chr12:68{	ENSG00000258313		lncRNA	chr12:95387890-953
ENSG0000(	281	7.028071	chr1:4061	CYCSP5		Pseudoger	chr1:244598391-244

ENSG0000(	281	7.028071	chr1:4061	ENSG00000240963		lncRNA	chr1:244375100-244
ENSG0000(	281	7.028071	chr1:4061	TGIF2P1		Pseudoger	chr1:244394976-244
ENSG0000(	281	7.028071	chr1:4061	ENSG00000232059		Pseudoger	chr1:244694432-244
ENSG0000(	281	7.028071	chr1:4061	ENSG00000284188		protein_c	chr1:244729701-244
ENSG0000(	281	7.028071	chr6:105(	ENSG00000234427		lncRNA	chr6:11810602-1181
ENSG0000(	281	7.028071	chr1:4061	ENSG00000230199		Pseudoger	chr1:242975005-242
ENSG0000(	280	7.00306	chr12:68	RPL41P5		Pseudoger	chr12:93083598-930
ENSG0000(	278	6.953038	chr12:58	ENSG00000257835		lncRNA	chr12:77379770-773
ENSG0000(	278	6.953038	chr12:58	MIR1252		smallRNA	chr12:79419257-794
ENSG0000(	278	6.953038	chr12:58	RNU7-106P		smallRNA	chr12:80068691-800
ENSG0000(	278	6.953038	chr12:58	ENSG00000257879		lncRNA	chr12:78426826-784
ENSG0000(	278	6.953038	chr12:58	ENSG00000257875		Pseudoger	chr12:82468456-824
ENSG0000(	278	6.953038	chr12:58	ENSG00000243071		Pseudoger	chr12:76660405-766
ENSG0000(	278	6.953038	chr12:58	ENSG00000278011		lncRNA	chr12:88601862-886
ENSG0000(	278	6.953038	chr12:58	RPL23AP68		Pseudoger	chr12:87169985-871
ENSG0000(	278	6.953038	chr12:58	RPS4XP15		Pseudoger	chr12:87944885-879
ENSG0000(	278	6.953038	chr12:58	ENSG00000257872		Pseudoger	chr12:76137425-761
ENSG0000(	278	6.953038	chr12:58	ENSG00000257124		lncRNA	chr12:83661009-836
ENSG0000(	278	6.953038	chr12:58	Y_RNA		smallRNA	chr12:88430442-884
ENSG0000(	278	6.953038	chr12:58	ENSG00000287030		lncRNA	chr12:76589476-766
ENSG0000(	278	6.953038	chr12:58	SNORA3		smallRNA	chr12:84183324-841
ENSG0000(	278	6.953038	chr12:58	ENSG00000257764		lncRNA	chr12:69353493-693
ENSG0000(	278	6.953038	chr12:58	AC131213.1		smallRNA	chr12:72790451-727
ENSG0000(	278	6.953038	chr12:58	RPL6P25		Pseudoger	chr12:83151331-831
ENSG0000(	278	6.953038	chr12:58	TRHDE-AS1		lncRNA	chr12:72249964-722
ENSG0000(	278	6.953038	chr12:58	MRS2P2		Pseudoger	chr12:71849228-718
ENSG0000(	278	6.953038	chr12:58	NAP1L1	NCGv7	protein_c	chr12:76036585-760
ENSG0000(	278	6.953038	chr12:58	ENSG00000257729		lncRNA	chr12:84154434-842
ENSG0000(	278	6.953038	chr12:58	ENSG00000198923		Pseudoger	chr12:75964799-759
ENSG0000(	278	6.953038	chr12:58	LINC02821		lncRNA	chr12:70180338-702
ENSG0000(	278	6.953038	chr12:58	NUP107-DT		lncRNA	chr12:68674371-686
ENSG0000(	278	6.953038	chr12:58	CHCHD3P2		Pseudoger	chr12:72610678-726
ENSG0000(	278	6.953038	chr12:58	ENSG00000257777		Pseudoger	chr12:75600047-756
ENSG0000(	278	6.953038	chr12:58	ENSG00000257894		lncRNA	chr12:79341205-795
ENSG0000(	278	6.953038	chr12:58	CAPS2-AS1		lncRNA	chr12:75234740-752
ENSG0000(	278	6.953038	chr12:58	snoU13		smallRNA	chr12:77162926-771
ENSG0000(	278	6.953038	chr12:58	RPL7P43		Pseudoger	chr12:76721534-767
ENSG0000(	278	6.953038	chr12:58	ENSG00000289384		lncRNA	chr12:88381483-884
ENSG0000(	278	6.953038	chr12:58	ENSG00000287132		lncRNA	chr12:70239114-702
ENSG0000(	278	6.953038	chr12:58	AKIRIN1P1		Pseudoger	chr12:80561017-805
ENSG0000(	278	6.953038	chr12:58	LINC01490		lncRNA	chr12:80763151-807
ENSG0000(	278	6.953038	chr12:58	AC078917.1		smallRNA	chr12:79348319-793
ENSG0000(	278	6.953038	chr12:58	ENSG00000256325		lncRNA	chr12:68828118-688
ENSG0000(	278	6.953038	chr12:58	RAP1B		protein_c	chr12:68610855-686
ENSG0000(	278	6.953038	chr12:58	RN7SL734P		smallRNA	chr12:75957834-759
ENSG0000(	278	6.953038	chr12:58	C1GALT1P1		Pseudoger	chr12:69284397-692
ENSG0000(	278	6.953038	chr12:58	LINC02445		lncRNA	chr12:73758657-738
ENSG0000(	278	6.953038	chr12:58	CPM	DriverDB	protein_c	chr12:68842197-689
ENSG0000(	278	6.953038	chr12:58	MDM2	NCGv7;AC	protein_c	chr12:68808177-688
ENSG0000(	278	6.953038	chr12:58	ZDHHC17		protein_c	chr12:76764103-768
ENSG0000(	278	6.953038	chr12:58	TSPAN8		protein_c	chr12:71125085-714

ENSG0000(	278	6.953038	chr12:58:	MIR4699		smallRNA	chr12:81158388-811
ENSG0000(	278	6.953038	chr12:58:	MRPS6P4		Pseudoger	chr12:89319364-893
ENSG0000(	278	6.953038	chr12:58:	ENSG00000289369		lncRNA	chr12:86737397-867
ENSG0000(	278	6.953038	chr12:58:	ENSG00000257897		Pseudoger	chr12:86015697-860
ENSG0000(	278	6.953038	chr12:58:	ENSG00000257752		Pseudoger	chr12:88057876-880
ENSG0000(	278	6.953038	chr12:58:	LRRIQ1	NCGv7	protein_c	chr12:85036314-852
ENSG0000(	278	6.953038	chr12:58:	AC083811.1		smallRNA	chr12:82342596-823
ENSG0000(	278	6.953038	chr12:58:	ENSG00000257837		lncRNA	chr12:82505211-825
ENSG0000(	278	6.953038	chr12:58:	ZFC3H1		protein_c	chr12:71609599-716
ENSG0000(	278	6.953038	chr12:58:	ENSG00000256678		Pseudoger	chr12:68805011-688
ENSG0000(	278	6.953038	chr12:58:	KCNMB4		protein_c	chr12:70366290-704
ENSG0000(	278	6.953038	chr12:58:	NTS		protein_c	chr12:85874295-858
ENSG0000(	278	6.953038	chr12:58:	CCDC59		protein_c	chr12:82223681-823
ENSG0000(	278	6.953038	chr12:58:	ENSG00000271259		lncRNA	chr12:89371820-893
ENSG0000(	278	6.953038	chr12:58:	ENSG00000257113		Pseudoger	chr12:74274952-742
ENSG0000(	278	6.953038	chr12:58:	PRANCR		lncRNA	chr12:69901918-702
ENSG0000(	278	6.953038	chr12:58:	ENSG00000257855		Pseudoger	chr12:85567878-855
ENSG0000(	278	6.953038	chr12:58:	C12orf29		protein_c	chr12:88033846-880
ENSG0000(	278	6.953038	chr12:58:	PTPRB	NCGv7	protein_c	chr12:70515870-706
ENSG0000(	278	6.953038	chr12:58:	SNRPGP20		Pseudoger	chr12:79989014-799
ENSG0000(	278	6.953038	chr12:58:	ENSG00000243164		Pseudoger	chr12:73648666-736
ENSG0000(	278	6.953038	chr12:58:	RAB3IP	DriverDB\	protein_c	chr12:69738860-698
ENSG0000(	278	6.953038	chr12:58:	BEST3		protein_c	chr12:69643360-696
ENSG0000(	278	6.953038	chr12:58:	ENSG00000256664		Pseudoger	chr12:68841946-688
ENSG0000(	278	6.953038	chr12:58:	PHLDA1-DT		lncRNA	chr12:76032658-760
ENSG0000(	278	6.953038	chr12:58:	TMTC2	NCGv7	protein_c	chr12:82686880-831
ENSG0000(	278	6.953038	chr12:58:	LINC02426		lncRNA	chr12:81953719-819
ENSG0000(	278	6.953038	chr12:58:	ENSG00000257823		Pseudoger	chr12:75305452-753
ENSG0000(	278	6.953038	chr12:58:	ENSG00000274979		lncRNA	chr12:69326574-693
ENSG0000(	278	6.953038	chr12:58:	LRRC10		protein_c	chr12:69608564-696
ENSG0000(	278	6.953038	chr12:58:	ENSG00000258205		Pseudoger	chr12:87746567-877
ENSG0000(	278	6.953038	chr12:58:	ENSG00000258088		lncRNA	chr12:75694010-756
ENSG0000(	278	6.953038	chr12:58:	ENSG00000258215		Pseudoger	chr12:80860955-808
ENSG0000(	278	6.953038	chr12:58:	LINC02424		lncRNA	chr12:78326680-783
ENSG0000(	278	6.953038	chr12:58:	ENSG00000258225		lncRNA	chr12:78052181-780
ENSG0000(	278	6.953038	chr12:58:	ENSG00000257429		lncRNA	chr12:80583683-805
ENSG0000(	278	6.953038	chr12:58:	ENSG00000257410		lncRNA	chr12:71793855-717
ENSG0000(	278	6.953038	chr12:58:	ENSG00000258084		lncRNA	chr12:78352519-784
ENSG0000(	278	6.953038	chr12:58:	ENSG00000258235		lncRNA	chr12:72727923-727
ENSG0000(	278	6.953038	chr12:58:	ENSG00000285191		lncRNA	chr12:84147304-841
ENSG0000(	278	6.953038	chr12:58:	METTL25		protein_c	chr12:82358528-824
ENSG0000(	278	6.953038	chr12:58:	RPL10P13		Pseudoger	chr12:75688794-756
ENSG0000(	278	6.953038	chr12:58:	CAPS2		protein_c	chr12:75275979-753
ENSG0000(	278	6.953038	chr12:58:	ENSG00000258294		lncRNA	chr12:73115957-731
ENSG0000(	278	6.953038	chr12:58:	ENSG00000258304		Pseudoger	chr12:76115711-761
ENSG0000(	278	6.953038	chr12:58:	ENSG00000258320		Pseudoger	chr12:74170445-741
ENSG0000(	278	6.953038	chr12:58:	ENSG00000258077		lncRNA	chr12:75563202-759
ENSG0000(	278	6.953038	chr12:58:	RPS26P45		Pseudoger	chr12:69422656-694
ENSG0000(	278	6.953038	chr12:58:	LINC02394		lncRNA	chr12:74039024-740
ENSG0000(	278	6.953038	chr12:58:	ENSG00000277247		lncRNA	chr12:70570969-705
ENSG0000(	278	6.953038	chr12:58:	ENSG00000258338		lncRNA	chr12:83171590-831

ENSG0000(	278	6.953038	chr12:58:	ENSG00000258358		Pseudoger	chr12:84448625-844
ENSG0000(	278	6.953038	chr12:58:	ENSG00000277223		lncRNA	chr12:71448405-714
ENSG0000(	278	6.953038	chr12:58:	ENSG00000258073		Pseudoger	chr12:84939524-849
ENSG0000(	278	6.953038	chr12:58:	ENSG00000258066		lncRNA	chr12:77775783-777
ENSG0000(	278	6.953038	chr12:58:	ENSG00000257386		lncRNA	chr12:74538145-745
ENSG0000(	278	6.953038	chr12:58:	ENSG00000258375		Pseudoger	chr12:81545326-815
ENSG0000(	278	6.953038	chr12:58:	ENSG00000258064		protein_c	chr12:71674204-716
ENSG0000(	278	6.953038	chr12:58:	ENSG00000258206		Pseudoger	chr12:85781892-857
ENSG0000(	278	6.953038	chr12:58:	ENSG00000257431		lncRNA	chr12:82512677-825
ENSG0000(	278	6.953038	chr12:58:	ENSG00000258053		lncRNA	chr12:71047402-711
ENSG0000(	278	6.953038	chr12:58:	ENSG00000258090		Pseudoger	chr12:76757191-767
ENSG0000(	278	6.953038	chr12:58:	CCNG2P1		Pseudoger	chr12:75257635-752
ENSG0000(	278	6.953038	chr12:58:	ENSG00000188646		Pseudoger	chr12:74663774-746
ENSG0000(	278	6.953038	chr12:58:	ENSG00000257515		lncRNA	chr12:71709171-717
ENSG0000(	278	6.953038	chr12:58:	ENSG00000258115		lncRNA	chr12:72046149-720
ENSG0000(	278	6.953038	chr12:58:	LINC02444		lncRNA	chr12:73159190-732
ENSG0000(	278	6.953038	chr12:58:	MKRN9P		Pseudoger	chr12:87782163-877
ENSG0000(	278	6.953038	chr12:58:	ENSG00000258091		Pseudoger	chr12:75926213-759
ENSG0000(	278	6.953038	chr12:58:	KITLG		protein_c	chr12:88492793-885
ENSG0000(	278	6.953038	chr12:58:	LINC02373		lncRNA	chr12:69449470-694
ENSG0000(	278	6.953038	chr12:58:	GLIPR1-AS1		lncRNA	chr12:75483454-754
ENSG0000(	278	6.953038	chr12:58:	ENSG00000257474		lncRNA	chr12:79540203-795
ENSG0000(	278	6.953038	chr12:58:	ENSG00000258140		lncRNA	chr12:69212108-692
ENSG0000(	278	6.953038	chr12:58:	ENSG00000258162		lncRNA	chr12:81378042-815
ENSG0000(	278	6.953038	chr12:58:	PPFIA2-AS1		lncRNA	chr12:81270669-813
ENSG0000(	278	6.953038	chr12:58:	RPL7P42		Pseudoger	chr12:68684595-686
ENSG0000(	278	6.953038	chr12:58:	ENSG00000258168		lncRNA	chr12:70468080-705
ENSG0000(	278	6.953038	chr12:58:	ENSG00000258170		lncRNA	chr12:82481118-824
ENSG0000(	278	6.953038	chr12:58:	PPP1R12A-AS1		lncRNA	chr12:79934901-799
ENSG0000(	278	6.953038	chr12:58:	ENSG00000258173		Pseudoger	chr12:87753414-877
ENSG0000(	278	6.953038	chr12:58:	ENSG00000258178		lncRNA	chr12:85958686-859
ENSG0000(	278	6.953038	chr12:58:	ENSG00000257564		Pseudoger	chr12:78793526-787
ENSG0000(	278	6.953038	chr12:58:	ENSG00000258179		Pseudoger	chr12:87820654-878
ENSG0000(	278	6.953038	chr12:58:	ENSG00000257454		lncRNA	chr12:71034122-711
ENSG0000(	278	6.953038	chr12:58:	PHLDA1-AS1		lncRNA	chr12:76030494-760
ENSG0000(	278	6.953038	chr12:58:	ENSG00000258185		lncRNA	chr12:86599578-868
ENSG0000(	278	6.953038	chr12:58:	ENSG00000257434		lncRNA	chr12:75020969-750
ENSG0000(	278	6.953038	chr12:58:	ENSG00000258193		Pseudoger	chr12:81998632-819
ENSG0000(	278	6.953038	chr12:58:	GLIPR1L2	NCGv7	protein_c	chr12:75391089-754
ENSG0000(	278	6.953038	chr12:58:	RNU7-120P		smallRNA	chr12:89282223-892
ENSG0000(	278	6.953038	chr12:58:	ENSG00000257910		lncRNA	chr12:76878193-768
ENSG0000(	278	6.953038	chr12:58:	ENSG00000257941		Pseudoger	chr12:76057538-760
ENSG0000(	278	6.953038	chr12:58:	YEATS4	NCGv7;AC	protein_c	chr12:69359710-693
ENSG0000(	278	6.953038	chr12:58:	RPL7P38		Pseudoger	chr12:80028893-800
ENSG0000(	278	6.953038	chr12:58:	ENSG00000257682		lncRNA	chr12:73203815-732
ENSG0000(	278	6.953038	chr12:58:	PPP1R12A		protein_c	chr12:79773563-799
ENSG0000(	278	6.953038	chr12:58:	LINC02458		lncRNA	chr12:89010681-893
ENSG0000(	278	6.953038	chr12:58:	ENSG00000257265		lncRNA	chr12:71007773-710
ENSG0000(	278	6.953038	chr12:58:	PTPRR		protein_c	chr12:70638073-709
ENSG0000(	278	6.953038	chr12:58:	ENSG00000257940		lncRNA	chr12:87816486-878
ENSG0000(	278	6.953038	chr12:58:	ENSG00000286043		lncRNA	chr12:82673070-826

ENSG0000(	278	6.953038	chr12:583	ENSG00000257241	lncRNA	chr12:69946543-699
ENSG0000(	278	6.953038	chr12:583	AC083810.1	smallRNA	chr12:82264943-822
ENSG0000(	278	6.953038	chr12:583	PRXL2AP1	Pseudoger	chr12:78130337-781
ENSG0000(	278	6.953038	chr12:583	LNCOG	lncRNA	chr12:76259836-763
ENSG0000(	278	6.953038	chr2:7442	RPL34P8	Pseudoger	chr2:111675026-111
ENSG0000(	278	6.953038	chr12:583	ENSG00000257526	lncRNA	chr12:76559870-766
ENSG0000(	278	6.953038	chr12:583	ENSG00000257199	Pseudoger	chr12:76984079-769
ENSG0000(	278	6.953038	chr12:583	ENSG00000257191	lncRNA	chr12:78960258-790
ENSG0000(	278	6.953038	chr12:583	ENSG00000257183	lncRNA	chr12:74248637-742
ENSG0000(	278	6.953038	chr12:583	ENSG00000286259	lncRNA	chr12:75649195-756
ENSG0000(	278	6.953038	chr12:583	ENSG00000257181	lncRNA	chr12:68841288-688
ENSG0000(	278	6.953038	chr12:583	ENSG00000286608	lncRNA	chr12:89353798-894
ENSG0000(	278	6.953038	chr12:583	AC027288.2	smallRNA	chr12:79462464-794
ENSG0000(	278	6.953038	chr12:583	CEP290	protein_c	chr12:88049016-881
ENSG0000(	278	6.953038	chr12:583	LINC02258	lncRNA	chr12:87795733-878
ENSG0000(	278	6.953038	chr12:583	RASSF9	protein_c	chr12:85800703-858
ENSG0000(	278	6.953038	chr12:583	ENSG00000257165	lncRNA	chr12:78448995-785
ENSG0000(	278	6.953038	chr12:583	ENSG00000247131	lncRNA	chr12:69713633-697
ENSG0000(	278	6.953038	chr12:583	ENSG00000289595	lncRNA	chr12:68804873-688
ENSG0000(	278	6.953038	chr12:583	ENSG00000271327	lncRNA	chr12:89367807-893
ENSG0000(	278	6.953038	chr12:583	ENSG00000258052	protein_c	chr12:69801669-698
ENSG0000(	278	6.953038	chr12:583	H3P35	Pseudoger	chr12:72431722-724
ENSG0000(	278	6.953038	chr12:583	VENTXP3	Pseudoger	chr12:74292324-742
ENSG0000(	278	6.953038	chr12:583	PPP1R12A-AS2	lncRNA	chr12:79690144-797
ENSG0000(	278	6.953038	chr12:583	ENSG00000258044	lncRNA	chr12:79823778-798
ENSG0000(	278	6.953038	chr12:583	ENSG00000274021	lncRNA	chr12:89351015-893
ENSG0000(	278	6.953038	chr12:583	MGAT4C	protein_c	chr12:85955666-868
ENSG0000(	278	6.953038	chr12:583	ENSG00000273987	lncRNA	chr12:75333798-753
ENSG0000(	278	6.953038	chr12:583	LINC02820	lncRNA	chr12:85318060-853
ENSG0000(	278	6.953038	chr12:583	ATXN7L3B	protein_c	chr12:74537835-745
ENSG0000(	278	6.953038	chr12:583	ENSG00000258026	lncRNA	chr12:81094371-811
ENSG0000(	278	6.953038	chr12:583	ENSG00000257329	lncRNA	chr12:75964440-759
ENSG0000(	278	6.953038	chr12:583	ENSG00000257998	Pseudoger	chr12:74728038-747
ENSG0000(	278	6.953038	chr12:583	ENSG00000257587	lncRNA	chr12:73906940-739
ENSG0000(	278	6.953038	chr12:583	ENSG00000257991	Pseudoger	chr12:71835034-718
ENSG0000(	278	6.953038	chr12:583	NOP56P3	Pseudoger	chr12:79558782-795
ENSG0000(	278	6.953038	chr12:583	ENSG00000257948	lncRNA	chr12:78794132-788
ENSG0000(	278	6.953038	chr12:583	ENSG00000277130	lncRNA	chr12:79818784-798
ENSG0000(	278	6.953038	chr12:583	ENSG00000286476	lncRNA	chr12:77129178-771
ENSG0000(	278	6.953038	chr12:583	ENSG00000257609	Pseudoger	chr12:81868368-818
ENSG0000(	278	6.953038	chr12:583	RN7SL696P	smallRNA	chr12:79524877-795
ENSG0000(	278	6.953038	chr12:583	ENSG00000257629	Pseudoger	chr12:89027757-890
ENSG0000(	278	6.953038	chr12:583	ENSG00000257323	Pseudoger	chr12:74656561-746
ENSG0000(	278	6.953038	chr12:583	FAHD2P1	Pseudoger	chr12:70671918-706
ENSG0000(	278	6.953038	chr12:583	CYCSP30	Pseudoger	chr12:87750142-877
ENSG0000(	278	6.953038	chr12:583	ENSG00000257296	Pseudoger	chr12:84671598-846
ENSG0000(	278	6.953038	chr12:583	BBS10	protein_c	chr12:76344474-763
ENSG0000(	278	6.953038	chr12:583	LINC02464	lncRNA	chr12:77219603-773
ENSG0000(	278	6.953038	chr12:583	ENSG00000257289	Pseudoger	chr12:68913804-689
ENSG0000(	278	6.953038	chr12:583	ALX1	protein_c	chr12:85280220-853
ENSG0000(	278	6.953038	chr12:583	ENSG00000257604	Pseudoger	chr12:79500119-795

ENSG0000(	278	6.953038	chr12:58:	YWHAQP7		Pseudoger	chr12:76619386-766
ENSG0000(	278	6.953038	chr12:58:	RPL10P12		Pseudoger	chr12:68626870-686
ENSG0000(	278	6.953038	chr12:58:	C12orf50		protein_c	chr12:87980035-880
ENSG0000(	278	6.953038	chr12:58:	LGR5		protein_c	chr12:71439798-715
ENSG0000(	278	6.953038	chr12:58:	LIN7A	NCGv7	protein_c	chr12:80792520-809
ENSG0000(	278	6.953038	chr12:58:	AC139931.1		smallRNA	chr12:69117568-691
ENSG0000(	278	6.953038	chr12:58:	RN7SKP261		smallRNA	chr12:80343515-803
ENSG0000(	278	6.953038	chr12:58:	AC136188.1		smallRNA	chr12:73899870-738
ENSG0000(	278	6.953038	chr12:58:	ENSG00000281333		lncRNA	chr12:88580531-886
ENSG0000(	278	6.953038	chr12:58:	RNU6-1012P		smallRNA	chr12:73679188-736
ENSG0000(	278	6.953038	chr12:58:	NAV3	NCGv7	protein_c	chr12:77324641-782
ENSG0000(	278	6.953038	chr12:58:	PTPRQ		protein_c	chr12:80405994-806
ENSG0000(	278	6.953038	chr12:58:	TRHDE	NCGv7	protein_c	chr12:72087266-726
ENSG0000(	278	6.953038	chr12:58:	ATP5PDP4		Pseudoger	chr12:68642519-686
ENSG0000(	278	6.953038	chr12:58:	AC131012.1		smallRNA	chr12:75724453-757
ENSG0000(	278	6.953038	chr12:58:	ENSG00000280049		TEC	chr12:87041916-870
ENSG0000(	278	6.953038	chr12:58:	OSBPL8	NCGv7	protein_c	chr12:76351797-765
ENSG0000(	278	6.953038	chr12:58:	RNU7-4P		smallRNA	chr12:68911921-689
ENSG0000(	278	6.953038	chr12:58:	ENSG00000279551		TEC	chr12:69660034-696
ENSG0000(	278	6.953038	chr12:58:	RAB21		protein_c	chr12:71754863-718
ENSG0000(	278	6.953038	chr12:58:	MIR3913-2		smallRNA	chr12:69584722-695
ENSG0000(	278	6.953038	chr12:58:	RNA5SP363		Pseudoger	chr12:79878305-798
ENSG0000(	278	6.953038	chr12:58:	PAWR		protein_c	chr12:79574979-796
ENSG0000(	278	6.953038	chr12:58:	LYZ		protein_c	chr12:69348381-693
ENSG0000(	278	6.953038	chr12:58:	RNU6-1271P		smallRNA	chr12:76139567-761
ENSG0000(	278	6.953038	chr2:4707	RNU6-961P		smallRNA	chr2:20175805-2017
ENSG0000(	278	6.953038	chr12:58:	FRS2	DriverDB\	protein_c	chr12:69470349-695
ENSG0000(	278	6.953038	chr12:58:	RNU6-977P		smallRNA	chr12:83021890-830
ENSG0000(	278	6.953038	chr12:58:	AC010196.1		smallRNA	chr12:86745068-867
ENSG0000(	278	6.953038	chr12:58:	CCT2	DriverDB\	protein_c	chr12:69585426-696
ENSG0000(	278	6.953038	chr12:58:	TSPAN19	DriverDB\	protein_c	chr12:85014311-850
ENSG0000(	278	6.953038	chr12:58:	ENSG00000279530		TEC	chr12:70321542-703
ENSG0000(	278	6.953038	chr12:58:	RNU1-117P		smallRNA	chr12:88581639-885
ENSG0000(	278	6.953038	chr12:58:	ENSG00000289283		lncRNA	chr12:70317612-703
ENSG0000(	278	6.953038	chr12:58:	SYT1	AC	protein_c	chr12:78863993-794
ENSG0000(	278	6.953038	chr12:58:	KCNC2		protein_c	chr12:75040077-752
ENSG0000(	278	6.953038	chr12:58:	TMEM19		protein_c	chr12:71686082-717
ENSG0000(	278	6.953038	chr12:58:	MYF5		protein_c	chr12:80716912-807
ENSG0000(	278	6.953038	chr12:58:	PHLDA1	NCGv7	protein_c	chr12:76025447-760
ENSG0000(	278	6.953038	chr12:58:	KRR1		protein_c	chr12:75490863-755
ENSG0000(	278	6.953038	chr12:58:	MIR617		smallRNA	chr12:80832533-808
ENSG0000(	278	6.953038	chr12:58:	TMTC3	NCGv7	protein_c	chr12:88142296-881
ENSG0000(	278	6.953038	chr12:58:	MYF6		protein_c	chr12:80707634-807
ENSG0000(	278	6.953038	chr12:58:	AC021887.1		smallRNA	chr12:85647253-856
ENSG0000(	278	6.953038	chr12:58:	CPSF6	DriverDB\	protein_c	chr12:69239569-692
ENSG0000(	278	6.953038	chr12:58:	ENSG00000288530		lncRNA	chr12:71582293-715
ENSG0000(	278	6.953038	chr12:58:	RNA5SP364		Pseudoger	chr12:88051701-880
ENSG0000(	278	6.953038	chr12:58:	THAP2		protein_c	chr12:71664301-716
ENSG0000(	278	6.953038	chr12:58:	SLC6A15	NCGv7	protein_c	chr12:84859491-849
ENSG0000(	278	6.953038	chr12:58:	AC090015.1		smallRNA	chr12:74017775-740
ENSG0000(	278	6.953038	chr12:58:	ACSS3		protein_c	chr12:80936414-812

ENSG0000(	278	6.953038	chr12:58:	ENSG00000289040		lncRNA	chr12:77723756-777
ENSG0000(	278	6.953038	chr12:58:	AC133749.1		smallRNA	chr12:68956406-689
ENSG0000(	278	6.953038	chr12:58:	RNU4-65P		smallRNA	chr12:70443784-704
ENSG0000(	278	6.953038	chr12:58:	ENSG00000289174		lncRNA	chr12:88781256-888
ENSG0000(	278	6.953038	chr12:58:	ENSG00000289173		lncRNA	chr12:68610197-686
ENSG0000(	278	6.953038	chr12:58:	U8		smallRNA	chr12:73764279-737
ENSG0000(	278	6.953038	chr12:58:	AC027288.1		smallRNA	chr12:79481048-794
ENSG0000(	278	6.953038	chr12:58:	MIR618		smallRNA	chr12:80935736-809
ENSG0000(	278	6.953038	chr12:58:	DUSP6		protein_c	chr12:89347235-893
ENSG0000(	278	6.953038	chr12:58:	GLIPR1		protein_c	chr12:75480753-755
ENSG0000(	278	6.953038	chr12:58:	NUP107	DriverDB\	protein_c	chr12:68686951-687
ENSG0000(	278	6.953038	chr12:58:	RN7SKP172		smallRNA	chr12:76315751-763
ENSG0000(	278	6.953038	chr12:58:	GLIPR1L1		protein_c	chr12:75334670-753
ENSG0000(	278	6.953038	chr12:58:	CNOT2	DriverDB\	protein_c	chr12:70243002-703
ENSG0000(	278	6.953038	chr12:58:	MYRFL		protein_c	chr12:69825227-699
ENSG0000(	278	6.953038	chr12:58:	TPH2		protein_c	chr12:71938845-721
ENSG0000(	278	6.953038	chr12:58:	OTOGL	NCGv7	protein_c	chr12:80099537-803
ENSG0000(	278	6.953038	chr12:58:	E2F7		protein_c	chr12:77021248-770
ENSG0000(	278	6.953038	chr12:58:	KRT8P39		Pseudoger	chr12:68705634-687
ENSG0000(	278	6.953038	chr12:58:	ENSG00000289309		lncRNA	chr12:83888705-841
ENSG0000(	278	6.953038	chr12:58:	LINC02882		lncRNA	chr12:73820315-744
ENSG0000(	278	6.953038	chr7:330(	AC006483.1		smallRNA	chr7:5528103-55281
ENSG0000(	278	6.953038	chr12:58:	SNORA70		smallRNA	chr12:75689152-756
ENSG0000(	278	6.953038	chr12:58:	SNORA17		smallRNA	chr12:71639355-716
ENSG0000(	278	6.953038	chr12:58:	TBC1D15	AC	protein_c	chr12:71839707-719
ENSG0000(	278	6.953038	chr12:58:	PPFIA2		protein_c	chr12:81257975-817
ENSG0000(	278	6.953038	chr12:58:	SLC35E3	DriverDB\	protein_c	chr12:68746125-687
ENSG0000(	278	6.953038	chr12:58:	SNORA70G		smallRNA	chr12:68627234-686
ENSG0000(	278	6.953038	chr12:58:	ENSG00000289218		lncRNA	chr12:84986786-849
ENSG0000(	278	6.953038	chr12:58:	Y_RNA		smallRNA	chr12:70906917-709
ENSG0000(	278	6.953038	chr12:58:	ENSG00000288941		lncRNA	chr12:83456715-834
ENSG0000(	278	6.953038	chr12:58:	ENSG00000230291		Pseudoger	chr12:80102899-801
ENSG0000(	278	6.953038	chr12:58:	CSRP2		protein_c	chr12:76858709-768
ENSG0000(	276	6.903016	chr6:340!	UST		protein_c	chr6:148747030-149
ENSG0000(	276	6.903016	chr6:340!	snoU13		smallRNA	chr6:150164584-150
ENSG0000(	276	6.903016	chr6:340!	NUP43		protein_c	chr6:149724315-149
ENSG0000(	276	6.903016	chr6:340!	LRP11		protein_c	chr6:149818757-149
ENSG0000(	276	6.903016	chr6:340!	SASH1		protein_c	chr6:148272304-148
ENSG0000(	276	6.903016	chr16:65(	ENSG00000284685		lncRNA	chr16:29139379-293
ENSG0000(	276	6.903016	chr6:340!	PCMT1		protein_c	chr6:149749443-149
ENSG0000(	276	6.903016	chr6:340!	ULBP1		protein_c	chr6:149963943-149
ENSG0000(	276	6.903016	chr6:340!	ENSG00000219553		Pseudoger	chr6:149491567-149
ENSG0000(	276	6.903016	chr16:65(	ENSG00000261552		lncRNA	chr16:28989140-289
ENSG0000(	276	6.903016	chr6:340!	ENSG00000287473		lncRNA	chr6:148155120-148
ENSG0000(	276	6.903016	chr6:340!	CCT7P1		Pseudoger	chr6:149879962-149
ENSG0000(	276	6.903016	chr16:65(	MIR4517		smallRNA	chr16:28958583-289
ENSG0000(	276	6.903016	chr6:340!	Y_RNA		smallRNA	chr6:149631437-149
ENSG0000(	276	6.903016	chr6:340!	BTBD10P2		Pseudoger	chr6:149794763-149
ENSG0000(	276	6.903016	chr6:340!	RNU1-33P		smallRNA	chr6:145463238-145
ENSG0000(	276	6.903016	chr6:340!	ENSG00000273997		Pseudoger	chr6:147406531-147
ENSG0000(	276	6.903016	chr16:65(	ENSG00000260908		Pseudoger	chr16:29053732-291

ENSG0000(	276	6.903016	chr16:65(	ENSG00000284649		Pseudoger	chr16:29113050-291
ENSG0000(	276	6.903016	chr6:340!	ULBP3		protein_c	chr6:150061053-150
ENSG0000(	276	6.903016	chr6:340!	ENSG00000288551		lncRNA	chr6:145749917-145
ENSG0000(	276	6.903016	chr6:340!	LATS1	NCGv7	protein_c	chr6:149658153-149
ENSG0000(	276	6.903016	chr6:340!	SHPRH	NCGv7	protein_c	chr6:145864245-145
ENSG0000(	276	6.903016	chr6:340!	ENSG00000281021		lncRNA	chr6:149576089-149
ENSG0000(	276	6.903016	chr6:340!	ENSG00000228408		lncRNA	chr6:149217926-149
ENSG0000(	276	6.903016	chr6:340!	GRM1	NCGv7;AC	protein_c	chr6:146027646-146
ENSG0000(	276	6.903016	chr6:340!	TAB2-AS1		lncRNA	chr6:149243299-149
ENSG0000(	276	6.903016	chr6:340!	RNA5SP222		Pseudoger	chr6:146340420-146
ENSG0000(	276	6.903016	chr6:340!	SNRPEP6		Pseudoger	chr6:148567378-148
ENSG0000(	276	6.903016	chr6:340!	RP1-12G14.6		lncRNA	chr6:149575465-149
ENSG0000(	276	6.903016	chr6:340!	RN7SL234P		smallRNA	chr6:149291352-149
ENSG0000(	276	6.903016	chr6:340!	RNU6-734P		smallRNA	chr6:146639700-146
ENSG0000(	276	6.903016	chr6:340!	ENSG00000217612		Pseudoger	chr6:145489630-145
ENSG0000(	276	6.903016	chr6:340!	ENSG00000219487		Pseudoger	chr6:149112009-149
ENSG0000(	276	6.903016	chr6:340!	ENSG00000285991		protein_c	chr6:149817937-149
ENSG0000(	276	6.903016	chr6:340!	RAET1K		Pseudoger	chr6:150000090-150
ENSG0000(	276	6.903016	chr6:340!	ENSG00000289045		lncRNA	chr6:149497715-149
ENSG0000(	276	6.903016	chr6:340!	RAET1E-AS1		lncRNA	chr6:149863494-149
ENSG0000(	276	6.903016	chr6:340!	RAET1L		protein_c	chr6:150018334-150
ENSG0000(	276	6.903016	chr6:340!	ENSG00000283608		lncRNA	chr6:149246095-149
ENSG0000(	276	6.903016	chr6:340!	ENSG00000235168		lncRNA	chr6:149561152-149
ENSG0000(	276	6.903016	chr16:65(	ENSG00000284671		Pseudoger	chr16:29075022-291
ENSG0000(	276	6.903016	chr6:340!	LUADT1		lncRNA	chr6:147158925-147
ENSG0000(	276	6.903016	chr6:340!	ENSG00000233330		lncRNA	chr6:149591755-149
ENSG0000(	276	6.903016	chr6:340!	UST-AS1		lncRNA	chr6:148955628-148
ENSG0000(	276	6.903016	chr6:340!	ADGB-DT		lncRNA	chr6:146594516-146
ENSG0000(	276	6.903016	chr6:340!	ENSG00000270638		lncRNA	chr6:145735570-145
ENSG0000(	276	6.903016	chr6:340!	ENSG00000230205		lncRNA	chr6:148133809-148
ENSG0000(	276	6.903016	chr6:340!	FUNDC2P3		Pseudoger	chr6:146171315-146
ENSG0000(	276	6.903016	chr6:340!	ENSG00000288056		lncRNA	chr6:145784406-145
ENSG0000(	276	6.903016	chr6:340!	YAP1P1		Pseudoger	chr6:147406889-147
ENSG0000(	276	6.903016	chr6:340!	ENSG00000227748		lncRNA	chr6:146854983-146
ENSG0000(	276	6.903016	chr6:340!	FABP12P1		Pseudoger	chr6:149304107-149
ENSG0000(	276	6.903016	chr6:340!	SUMO4		protein_c	chr6:149400262-149
ENSG0000(	276	6.903016	chr6:340!	ENSG00000287976		lncRNA	chr6:148001716-148
ENSG0000(	276	6.903016	chr6:340!	ENSG00000216811		Pseudoger	chr6:146364931-146
ENSG0000(	276	6.903016	chr6:340!	SAMD5		protein_c	chr6:147508690-147
ENSG0000(	276	6.903016	chr6:340!	RNU6-1222P		smallRNA	chr6:148278672-148
ENSG0000(	276	6.903016	chr16:65(	ENSG00000291188		lncRNA	chr16:29114717-292
ENSG0000(	276	6.903016	chr6:340!	AL033378.1		smallRNA	chr6:148524153-148
ENSG0000(	276	6.903016	chr6:340!	SNORA2		smallRNA	chr6:149594625-149
ENSG0000(	276	6.903016	chr16:65(	ENSG00000260367		lncRNA	chr16:28973962-289
ENSG0000(	276	6.903016	chr6:340!	ENSG00000285889		lncRNA	chr6:149797317-149
ENSG0000(	276	6.903016	chr16:65(	ENSG00000290677		lncRNA	chr16:29103657-291
ENSG0000(	276	6.903016	chr6:340!	STXBP5-AS1		lncRNA	chr6:146824539-147
ENSG0000(	276	6.903016	chr6:340!	ENSG00000216906		Pseudoger	chr6:149904243-149
ENSG0000(	276	6.903016	chr6:340!	ADGB		protein_c	chr6:146598967-146
ENSG0000(	276	6.903016	chr6:340!	ENSG00000285598		lncRNA	chr6:145296545-145
ENSG0000(	276	6.903016	chr6:340!	RPS18P9		Pseudoger	chr6:149592687-149

ENSG0000(	276	6.903016	chr6:340!	ENSG00000289359		lncRNA	chr6:149140235-149
ENSG0000(	276	6.903016	chr16:65(	NFATC2IP-AS1		lncRNA	chr16:28952816-289
ENSG0000(	276	6.903016	chr6:340!	ENSG00000235972		lncRNA	chr6:150040547-150
ENSG0000(	276	6.903016	chr6:340!	FBXO30		protein_c	chr6:145793502-145
ENSG0000(	276	6.903016	chr6:340!	ENSG00000273132		lncRNA	chr6:149852462-149
ENSG0000(	276	6.903016	chr16:65(	NPIPB10P		Pseudoger	chr16:29038655-290
ENSG0000(	276	6.903016	chr16:65(	SPNS1		protein_c	chr16:28974221-289
ENSG0000(	276	6.903016	chr6:340!	RAET1E		protein_c	chr6:149883179-149
ENSG0000(	276	6.903016	chr6:340!	BTF3P10		Pseudoger	chr6:149977922-149
ENSG0000(	276	6.903016	chr6:340!	ENSG00000216621		Pseudoger	chr6:149934527-149
ENSG0000(	276	6.903016	chr6:340!	RAET1M		Pseudoger	chr6:150032769-150
ENSG0000(	276	6.903016	chr6:340!	RAET1G		protein_c	chr6:149916878-149
ENSG0000(	276	6.903016	chr6:340!	RAET1K		lncRNA	chr6:149998019-150
ENSG0000(	276	6.903016	chr6:340!	STXBP5		protein_c	chr6:147204417-147
ENSG0000(	276	6.903016	chr6:340!	ZC3H12D		protein_c	chr6:149446795-149
ENSG0000(	276	6.903016	chr6:340!	ENSG00000278899		lncRNA	chr6:149717621-149
ENSG0000(	276	6.903016	chr16:65(	ENSG00000289080		lncRNA	chr16:29145438-291
ENSG0000(	276	6.903016	chr6:340!	EPM2A-DT		lncRNA	chr6:145799409-145
ENSG0000(	276	6.903016	chr6:340!	ULBP2		protein_c	chr6:149942014-149
ENSG0000(	276	6.903016	chr6:340!	ENSG00000272397		lncRNA	chr6:146948528-146
ENSG0000(	276	6.903016	chr6:340!	ENSG00000231760		lncRNA	chr6:149796151-149
ENSG0000(	276	6.903016	chr6:340!	GINM1		protein_c	chr6:149566294-149
ENSG0000(	276	6.903016	chr16:65(	snoU13		smallRNA	chr16:29066255-290
ENSG0000(	276	6.903016	chr6:340!	ENSG00000280184		TEC	chr6:145789270-145
ENSG0000(	276	6.903016	chr6:340!	UST-AS2		lncRNA	chr6:149027700-149
ENSG0000(	276	6.903016	chr6:340!	KATNBL1P6		Pseudoger	chr6:146802359-146
ENSG0000(	276	6.903016	chr16:65(	ENSG00000260517		lncRNA	chr16:29139661-292
ENSG0000(	276	6.903016	chr6:340!	ENSG00000219298		Pseudoger	chr6:149977471-149
ENSG0000(	276	6.903016	chr6:340!	ENSG00000227681		lncRNA	chr6:147660703-147
ENSG0000(	276	6.903016	chr16:65(	NFATC2IP		protein_c	chr16:28950807-289
ENSG0000(	276	6.903016	chr6:340!	PPIL4	NCGv7	protein_c	chr6:149504495-149
ENSG0000(	276	6.903016	chr6:340!	ENSG00000234675		lncRNA	chr6:148017422-148
ENSG0000(	276	6.903016	chr6:340!	RPSAP40		Pseudoger	chr6:148647707-148
ENSG0000(	276	6.903016	chr6:340!	RAB32		protein_c	chr6:146543833-146
ENSG0000(	276	6.903016	chr6:340!	CYP51A1P3		Pseudoger	chr6:148478693-148
ENSG0000(	276	6.903016	chr6:340!	AL356739.1		smallRNA	chr6:145348758-145
ENSG0000(	276	6.903016	chr6:340!	ENSG00000270828		lncRNA	chr6:145736911-145
ENSG0000(	276	6.903016	chr6:340!	TAB2	DriverDB\	protein_c	chr6:149218641-149
ENSG0000(	276	6.903016	chr6:340!	KATNA1		protein_c	chr6:149594873-149
ENSG0000(	276	6.903016	chr6:340!	RAET1E-AS1		lncRNA	chr6:149884431-149
ENSG0000(	276	6.903016	chr6:340!	EPM2A		protein_c	chr6:145382535-145
ENSG0000(	276	6.903016	chr6:340!	ENSG00000226249		lncRNA	chr6:147741434-147
ENSG0000(	276	6.903016	chr16:65(	RRN3P2		lncRNA	chr16:29074976-291
ENSG0000(	276	6.903016	chr6:340!	ENSG00000215094		Pseudoger	chr6:149951833-149
ENSG0000(	276	6.903016	chr6:340!	ENSG00000273719		Pseudoger	chr6:146638844-146
ENSG0000(	276	6.903016	chr16:65(	LAT		protein_c	chr16:28984826-289
ENSG0000(	276	6.903016	chr6:340!	AL603766.1		smallRNA	chr6:149176707-149
ENSG0000(	276	6.903016	chr6:340!	ENSG00000224658		lncRNA	chr6:148237585-148
ENSG0000(	276	6.903016	chr6:340!	PHB1P1		Pseudoger	chr6:150042546-150
ENSG0000(	276	6.903016	chr6:340!	RNU7-3P		smallRNA	chr6:149516956-149
ENSG0000(	276	6.903016	chr16:65(	ENSG00000261067		lncRNA	chr16:28974804-289

ENSG0000(	273	6.827984	chr5:103(	ENSG00000279028		lncRNA	chr5:140966212-140
ENSG0000(	273	6.827984	chr22:227	IGLVI-20		Pseudoger	chr22:22715290-227
ENSG0000(	269	6.72794	chr5:103(	SNORA74		smallRNA	chr5:139276180-139
ENSG0000(	268	6.702929	chr16:65(	ENSG00000289756		Pseudoger	chr16:28620063-286
ENSG0000(	268	6.702929	chr16:65(	ENSG00000289754		lncRNA	chr16:28537040-286
ENSG0000(	268	6.702929	chr16:65(	MIR4721		smallRNA	chr16:28843919-288
ENSG0000(	268	6.702929	chr16:65(	SNORA43		smallRNA	chr16:28881480-288
ENSG0000(	268	6.702929	chr16:65(	ENSG00000289755		lncRNA	chr16:28605587-286
ENSG0000(	268	6.702929	chr16:65(	NPIPB8		protein_c	chr16:28637654-286
ENSG0000(	268	6.702929	chr16:65(	ENSG00000278528		Pseudoger	chr16:28773824-287
ENSG0000(	268	6.702929	chr16:65(	ENSG00000271495		Pseudoger	chr16:28548331-285
ENSG0000(	268	6.702929	chr16:65(	ATP2A1-AS1		lncRNA	chr16:28877938-288
ENSG0000(	268	6.702929	chr16:65(	ATXN2L		protein_c	chr16:28822999-288
ENSG0000(	268	6.702929	chr16:65(	ENSG00000275807		lncRNA	chr16:28822431-288
ENSG0000(	268	6.702929	chr16:65(	CDC37P1		Pseudoger	chr16:28700294-287
ENSG0000(	268	6.702929	chr16:65(	ENSG00000240634		Pseudoger	chr16:28813980-288
ENSG0000(	268	6.702929	chr16:65(	ENSG00000278725		Pseudoger	chr16:28602340-286
ENSG0000(	268	6.702929	chr16:65(	snoU13		smallRNA	chr16:28672233-286
ENSG0000(	268	6.702929	chr16:65(	CD19		protein_c	chr16:28931965-289
ENSG0000(	268	6.702929	chr16:65(	TUFM		protein_c	chr16:28842411-288
ENSG0000(	268	6.702929	chr16:65(	ENSG00000261419		Pseudoger	chr16:28659696-287
ENSG0000(	268	6.702929	chr16:65(	RABEP2		protein_c	chr16:28904421-289
ENSG0000(	268	6.702929	chr16:65(	ENSG00000251417		lncRNA	chr16:28802743-288
ENSG0000(	268	6.702929	chr16:65(	EIF3C		protein_c	chr16:28688558-287
ENSG0000(	268	6.702929	chr16:65(	snoU13		smallRNA	chr16:28786410-287
ENSG0000(	268	6.702929	chr16:65(	SH2B1		protein_c	chr16:28846600-288
ENSG0000(	268	6.702929	chr16:65(	NPIPB9	DriverDB\	protein_c	chr16:28751787-287
ENSG0000(	268	6.702929	chr16:65(	ATP2A1		protein_c	chr16:28878405-289
ENSG0000(	268	6.702929	chr16:65(	NUPR1		protein_c	chr16:28532708-285
ENSG0000(	268	6.702929	chr16:65(	ENSG00000260570		lncRNA	chr16:28830612-288
ENSG0000(	268	6.702929	chr16:65(	ENSG00000288656		lncRNA	chr16:28592055-286
ENSG0000(	268	6.702929	chr16:65(	ENSG00000275441		lncRNA	chr16:28553707-285
ENSG0000(	268	6.702929	chr16:65(	ENSG00000270424		Pseudoger	chr16:28749959-287
ENSG0000(	268	6.702929	chr16:65(	ENSG00000261766		lncRNA	chr16:28862166-288
ENSG0000(	268	6.702929	chr16:65(	ENSG00000285020		Pseudoger	chr16:28612131-286
ENSG0000(	268	6.702929	chr16:65(	SULT1A1		protein_c	chr16:28605196-286
ENSG0000(	268	6.702929	chr16:65(	SGF29		protein_c	chr16:28553915-285
ENSG0000(	268	6.702929	chr16:65(	SULT1A2		protein_c	chr16:28591943-285
ENSG0000(	268	6.702929	chr16:65(	ENSG00000260796		lncRNA	chr16:28820570-288
ENSG0000(	268	6.702929	chr16:65(	ENSG00000278665		Pseudoger	chr16:28599241-286
ENSG0000(	267	6.677918	chr16:65(	RNU7-199P		smallRNA	chr16:31267437-312
ENSG0000(	267	6.677918	chr12:12(	ENSG00000257083		lncRNA	chr12:66950754-670
ENSG0000(	267	6.677918	chr16:531	ENSG00000279123		TEC	chr16:70778433-707
ENSG0000(	267	6.677918	chr12:12(	GGTA2P		Pseudoger	chr12:67265842-672
ENSG0000(	267	6.677918	chr12:12(	OSBPL9P5		Pseudoger	chr12:66563524-665
ENSG0000(	267	6.677918	chr12:12(	OSBPL9P4		Pseudoger	chr12:66563967-665
ENSG0000(	267	6.677918	chr5:169(	EEF1A1P19		Pseudoger	chr5:43495073-4349
ENSG0000(	267	6.677918	chr12:12(	ENSG00000279310		TEC	chr12:67065018-670
ENSG0000(	267	6.677918	chr12:12(	RAB11AP2		Pseudoger	chr12:67131567-671
ENSG0000(	266	6.652907	chr16:209	DCTN5		protein_c	chr16:23641466-236
ENSG0000(	266	6.652907	chr16:209	ENSG00000277041		Pseudoger	chr16:22537478-225

ENSG0000(	266	6.652907	chr2:4707	FSHR	NCGv7	protein_c	chr2:48962157-4915
ENSG0000(	266	6.652907	chr16:209	LINC01567		lncRNA	chr16:24661422-246
ENSG0000(	266	6.652907	chr2:4707	FOXN2		protein_c	chr2:48314637-4837
ENSG0000(	266	6.652907	chr2:4707	STON1		protein_c	chr2:48529383-4859
ENSG0000(	266	6.652907	chr16:209	LCMT1		protein_c	chr16:25111731-251
ENSG0000(	266	6.652907	chr16:209	NDUFAB1		protein_c	chr16:23581014-235
ENSG0000(	266	6.652907	chr16:209	HS3ST4		protein_c	chr16:25691959-261
ENSG0000(	266	6.652907	chr16:209	CACNG3		protein_c	chr16:24256335-243
ENSG0000(	266	6.652907	chr2:4707	ENSG00000285548		lncRNA	chr2:50324643-5034
ENSG0000(	266	6.652907	chr2:4707	AC007682.3		smallRNA	chr2:50961245-5096
ENSG0000(	266	6.652907	chr16:209	snoU13		smallRNA	chr16:21889567-218
ENSG0000(	266	6.652907	chr16:209	ENSG00000278912		TEC	chr16:23696665-236
ENSG0000(	266	6.652907	chr16:209	ENSG00000290675		lncRNA	chr16:21939756-219
ENSG0000(	266	6.652907	chr16:209	ENSG00000290674		lncRNA	chr16:21901552-219
ENSG0000(	266	6.652907	chr16:209	PRKCB	NCGv7	protein_c	chr16:23835983-242
ENSG0000(	266	6.652907	chr16:209	ERN2	NCGv7	protein_c	chr16:23690310-237
ENSG0000(	266	6.652907	chr16:209	ENSG00000280400		TEC	chr16:23061767-230
ENSG0000(	266	6.652907	chr16:209	ENSG00000274460		lncRNA	chr16:21950218-219
ENSG0000(	266	6.652907	chr16:209	NPIPB5		protein_c	chr16:22479121-225
ENSG0000(	266	6.652907	chr16:209	ENSG00000287739		lncRNA	chr16:22170309-221
ENSG0000(	266	6.652907	chr2:4707	RNU6-282P		smallRNA	chr2:48501922-4850
ENSG0000(	266	6.652907	chr16:209	ENSG00000261266		lncRNA	chr16:23687049-236
ENSG0000(	266	6.652907	chr16:209	SCML2P2		Pseudoger	chr16:25069570-250
ENSG0000(	266	6.652907	chr16:209	ENSG00000261669		lncRNA	chr16:24803451-248
ENSG0000(	266	6.652907	chr16:209	RNU7-24P		smallRNA	chr16:24400056-244
ENSG0000(	266	6.652907	chr16:209	EEF2K	NCGv7	protein_c	chr16:22206278-222
ENSG0000(	266	6.652907	chr2:4707	MTCO1P42		Pseudoger	chr2:50588690-5058
ENSG0000(	266	6.652907	chr16:209	ZKSCAN2-DT		lncRNA	chr16:25257952-252
ENSG0000(	266	6.652907	chr16:209	snoU13		smallRNA	chr16:22481474-224
ENSG0000(	266	6.652907	chr16:209	ENSG00000261113		lncRNA	chr16:22286708-222
ENSG0000(	266	6.652907	chr16:209	ENSG00000261090		lncRNA	chr16:23017723-230
ENSG0000(	266	6.652907	chr16:209	OTOA		protein_c	chr16:21663968-217
ENSG0000(	266	6.652907	chr2:4707	CTBP2P5		Pseudoger	chr2:48915267-4891
ENSG0000(	266	6.652907	chr16:209	SCNN1G		protein_c	chr16:23182745-232
ENSG0000(	266	6.652907	chr16:209	OTOAP1		Pseudoger	chr16:22546964-225
ENSG0000(	266	6.652907	chr16:209	SLC7A5P2		Pseudoger	chr16:21519830-215
ENSG0000(	266	6.652907	chr16:209	ENSG00000257639		Pseudoger	chr16:21532945-215
ENSG0000(	266	6.652907	chr16:209	LINC02890		lncRNA	chr16:22612543-226
ENSG0000(	266	6.652907	chr16:209	ZKSCAN2		protein_c	chr16:25236001-252
ENSG0000(	266	6.652907	chr16:209	PDZD9		protein_c	chr16:21983865-220
ENSG0000(	266	6.652907	chr16:209	SNORA1		smallRNA	chr16:24333415-243
ENSG0000(	266	6.652907	chr16:209	ENSG00000287809		lncRNA	chr16:21511635-215
ENSG0000(	266	6.652907	chr16:209	ENSG00000260973		lncRNA	chr16:22610531-226
ENSG0000(	266	6.652907	chr16:209	ABCA3P1		Pseudoger	chr16:21938865-219
ENSG0000(	266	6.652907	chr2:4707	ENSG00000230773		lncRNA	chr2:47924181-4831
ENSG0000(	266	6.652907	chr16:209	LINC02191		lncRNA	chr16:25419812-254
ENSG0000(	266	6.652907	chr2:4707	RPL7P13		Pseudoger	chr2:49878623-4987
ENSG0000(	266	6.652907	chr16:209	SLC5A11		protein_c	chr16:24845841-249
ENSG0000(	266	6.652907	chr16:209	ENSG00000261723		lncRNA	chr16:23605841-236
ENSG0000(	266	6.652907	chr16:209	MFSD13B		Pseudoger	chr16:22382436-224
ENSG0000(	266	6.652907	chr16:209	AC008938.1		smallRNA	chr16:24438988-244

ENSG0000(	266	6.652907	chr16:209	HS3ST2		protein_c	chr16:22814162-229
ENSG0000(	266	6.652907	chr16:209	PALB2	NCGv7;AC	protein_c	chr16:23603160-236
ENSG0000(	266	6.652907	chr2:4707	AC007682.2		smallRNA	chr2:50943292-5094
ENSG0000(	266	6.652907	chr16:209	ENSG00000260482		lncRNA	chr16:23670011-236
ENSG0000(	266	6.652907	chr2:4707	LHCGR		protein_c	chr2:48686774-4875
ENSG0000(	266	6.652907	chr2:4707	PPP1R21		protein_c	chr2:48440598-4851
ENSG0000(	266	6.652907	chr16:209	ENSG00000279202		TEC	chr16:24098239-240
ENSG0000(	266	6.652907	chr16:209	ENSG00000259955		lncRNA	chr16:25238318-252
ENSG0000(	266	6.652907	chr2:4707	RNU6-439P		smallRNA	chr2:49233794-4923
ENSG0000(	266	6.652907	chr2:4707	GTF2A1L		protein_c	chr2:48617798-4873
ENSG0000(	266	6.652907	chr16:209	GGA2		protein_c	chr16:23463542-235
ENSG0000(	266	6.652907	chr16:209	SCARNA6		smallRNA	chr16:21587627-215
ENSG0000(	266	6.652907	chr2:4707	TPT1P11		Pseudoger	chr2:48632856-4863
ENSG0000(	266	6.652907	chr16:209	RRN3P1		Pseudoger	chr16:21797750-218
ENSG0000(	266	6.652907	chr16:209	ENSG00000286808		lncRNA	chr16:22331039-223
ENSG0000(	266	6.652907	chr16:209	ARHGAP17		protein_c	chr16:24919389-250
ENSG0000(	266	6.652907	chr16:209	OTOAP1		lncRNA	chr16:22545698-225
ENSG0000(	266	6.652907	chr16:209	RRN3P3		lncRNA	chr16:22418672-224
ENSG0000(	266	6.652907	chr16:209	CCNYL7		Pseudoger	chr16:23500905-235
ENSG0000(	266	6.652907	chr16:209	RN7SKP23		TEC	chr16:23412380-234
ENSG0000(	266	6.652907	chr16:209	ENSG00000260566		lncRNA	chr16:23061406-230
ENSG0000(	266	6.652907	chr16:209	ENSG00000231876		lncRNA	chr16:26302064-263
ENSG0000(	266	6.652907	chr2:4707	ENSG00000287867		lncRNA	chr2:51441959-5145
ENSG0000(	266	6.652907	chr16:209	ENSG00000275494		lncRNA	chr16:25106568-251
ENSG0000(	266	6.652907	chr16:209	ENSG00000259782		lncRNA	chr16:23446446-234
ENSG0000(	266	6.652907	chr2:4707	AC007560.2		smallRNA	chr2:50798964-5079
ENSG0000(	266	6.652907	chr2:4707	ENSG00000231918		lncRNA	chr2:51032601-5240
ENSG0000(	266	6.652907	chr16:209	ENSG00000275445		lncRNA	chr16:22007480-220
ENSG0000(	266	6.652907	chr16:209	HSPE1P16		Pseudoger	chr16:26062750-260
ENSG0000(	266	6.652907	chr2:4707	VN1R18P		Pseudoger	chr2:47989625-4799
ENSG0000(	266	6.652907	chr16:209	RNU6-1005P		smallRNA	chr16:21642054-216
ENSG0000(	266	6.652907	chr16:209	SMG1P1		Pseudoger	chr16:22437401-224
ENSG0000(	266	6.652907	chr2:4707	ENSG00000282998		lncRNA	chr2:49202126-4945
ENSG0000(	266	6.652907	chr16:209	ENSG00000279756		TEC	chr16:23753689-237
ENSG0000(	266	6.652907	chr16:209	RN7SKP23		smallRNA	chr16:23413584-234
ENSG0000(	266	6.652907	chr2:4707	ENSG00000282890		lncRNA	chr2:48809340-4941
ENSG0000(	266	6.652907	chr16:209	LCMT1-AS1		lncRNA	chr16:25085168-251
ENSG0000(	266	6.652907	chr16:209	ENSG00000279276		TEC	chr16:23962951-239
ENSG0000(	266	6.652907	chr16:209	ENSG00000261596		lncRNA	chr16:21626742-216
ENSG0000(	266	6.652907	chr16:209	MIR548W		smallRNA	chr16:26025237-260
ENSG0000(	266	6.652907	chr2:4707	SNORA75		smallRNA	chr2:49888868-4988
ENSG0000(	266	6.652907	chr16:209	USP31	NCGv7	protein_c	chr16:23061406-231
ENSG0000(	266	6.652907	chr16:209	UQCRC2		protein_c	chr16:21953288-219
ENSG0000(	266	6.652907	chr16:209	SNORA75		smallRNA	chr16:23441841-234
ENSG0000(	266	6.652907	chr16:209	RNU6-196P		smallRNA	chr16:21642294-216
ENSG0000(	266	6.652907	chr16:209	SUB1P4		Pseudoger	chr16:23537061-235
ENSG0000(	266	6.652907	chr2:4707	RNU4-49P		smallRNA	chr2:48340687-4834
ENSG0000(	266	6.652907	chr16:209	ENSG00000260255		Pseudoger	chr16:25824710-258
ENSG0000(	266	6.652907	chr16:209	LINC02194		lncRNA	chr16:24236104-242
ENSG0000(	266	6.652907	chr16:209	ENSG00000260277		lncRNA	chr16:22083256-220
ENSG0000(	266	6.652907	chr16:209	ENSG00000260306		lncRNA	chr16:21794095-217

ENSG0000(	266	6.652907	chr16:20(	CDR2		protein_c	chr16:22345936-224
ENSG0000(	266	6.652907	chr16:20(	ENSG00000260136		lncRNA	chr16:23452758-234
ENSG0000(	266	6.652907	chr16:20(	SDR42E2		protein_c	chr16:22162492-221
ENSG0000(	266	6.652907	chr2:4707	AC009234.2		smallRNA	chr2:50696172-5069
ENSG0000(	266	6.652907	chr16:20(	IGSF6		protein_c	chr16:21639550-216
ENSG0000(	266	6.652907	chr16:20(	ENSG00000263326		Pseudoger	chr16:25071490-250
ENSG0000(	266	6.652907	chr16:20(	AQP8		protein_c	chr16:25215731-252
ENSG0000(	266	6.652907	chr16:20(	ENSG00000285882		lncRNA	chr16:25787987-260
ENSG0000(	266	6.652907	chr16:20(	ENSG00000261583		lncRNA	chr16:23711990-237
ENSG0000(	266	6.652907	chr16:20(	ENSG00000260072		lncRNA	chr16:24472798-244
ENSG0000(	266	6.652907	chr16:20(	RNA5SP405		Pseudoger	chr16:26028912-260
ENSG0000(	266	6.652907	chr16:20(	ENSG00000291179		lncRNA	chr16:25031695-250
ENSG0000(	266	6.652907	chr2:4707	ENSG00000282828		lncRNA	chr2:49563388-4959
ENSG0000(	266	6.652907	chr16:20(	ENSG00000260395		Pseudoger	chr16:21998310-219
ENSG0000(	266	6.652907	chr16:20(	LCMT1-AS2		lncRNA	chr16:25140577-251
ENSG0000(	266	6.652907	chr16:20(	ENSG00000260635		lncRNA	chr16:22365121-223
ENSG0000(	266	6.652907	chr16:20(	HMGN2P3		Pseudoger	chr16:26032539-260
ENSG0000(	266	6.652907	chr16:20(	SCNN1B		protein_c	chr16:23278231-233
ENSG0000(	266	6.652907	chr16:20(	EARS2		protein_c	chr16:23520754-235
ENSG0000(	266	6.652907	chr16:20(	SMG1P4		Pseudoger	chr16:21879338-219
ENSG0000(	266	6.652907	chr2:4707	NRXN1		protein_c	chr2:49918503-5122
ENSG0000(	266	6.652907	chr16:20(	METTL9		protein_c	chr16:21597218-216
ENSG0000(	266	6.652907	chr16:20(	COG7		protein_c	chr16:23388493-234
ENSG0000(	266	6.652907	chr16:20(	CYCSP39		Pseudoger	chr16:25454339-254
ENSG0000(	266	6.652907	chr16:20(	LINC02175		lncRNA	chr16:25066937-250
ENSG0000(	266	6.652907	chr2:4707	KNOP1P3		Pseudoger	chr2:51511058-5151
ENSG0000(	266	6.652907	chr16:20(	MOSMO		protein_c	chr16:22007638-220
ENSG0000(	266	6.652907	chr16:20(	ENSG00000291060		lncRNA	chr16:22437008-224
ENSG0000(	266	6.652907	chr16:20(	PLK1	NCGv7;AC	protein_c	chr16:23677656-236
ENSG0000(	266	6.652907	chr16:20(	ENSG00000260714		Pseudoger	chr16:25015978-250
ENSG0000(	266	6.652907	chr16:20(	VWA3A	DriverDB\	protein_c	chr16:22092538-221
ENSG0000(	266	6.652907	chr2:4707	STON1-GTF2A1L		protein_c	chr2:48529925-4877
ENSG0000(	266	6.652907	chr2:4707	ENSG00000279956		protein_c	chr2:48632291-4875
ENSG0000(	266	6.652907	chr16:20(	CDR2-DT		lncRNA	chr16:22374859-223
ENSG0000(	266	6.652907	chr16:20(	ENSG00000283213		lncRNA	chr16:22806289-228
ENSG0000(	266	6.652907	chr16:20(	ENSG00000260741		lncRNA	chr16:23194878-232
ENSG0000(	266	6.652907	chr16:20(	ENSG00000260751		lncRNA	chr16:23568673-235
ENSG0000(	266	6.652907	chr16:20(	NPIPB4		protein_c	chr16:21834569-218
ENSG0000(	266	6.652907	chr2:4707	AC007402.1		smallRNA	chr2:51435122-5143
ENSG0000(	266	6.652907	chr2:4707	PPP1R21-DT		lncRNA	chr2:48440043-4844
ENSG0000(	266	6.652907	chr2:4707	ENSG00000238165		Pseudoger	chr2:50829442-5082
ENSG0000(	266	6.652907	chr16:20(	ENSG00000273956		lncRNA	chr16:21526824-215
ENSG0000(	266	6.652907	chr2:4707	ENSG00000286412		lncRNA	chr2:51011777-5101
ENSG0000(	266	6.652907	chr16:20(	UBFD1		protein_c	chr16:23557721-235
ENSG0000(	266	6.652907	chr16:20(	TNRC6A	NCGv7	protein_c	chr16:24610209-248
ENSG0000(	266	6.652907	chr2:4707	ENSG00000283058		lncRNA	chr2:50620963-5063
ENSG0000(	266	6.652907	chr16:20(	RBBP6	NCGv7	protein_c	chr16:24537693-245
ENSG0000(	266	6.652907	chr16:20(	ENSG00000279554		TEC	chr16:24208105-242
ENSG0000(	266	6.652907	chr16:20(	CHP2		protein_c	chr16:23755026-237
ENSG0000(	266	6.652907	chr16:20(	ENSG00000262700		Pseudoger	chr16:25093387-250
ENSG0000(	266	6.652907	chr16:20(	RN7SL557P		smallRNA	chr16:25185207-251

ENSG0000(	266	6.652907	chr16:20‹	ENSG00000289171		lncRNA	chr16:24550351-245
ENSG0000(	266	6.652907	chr16:20‹	POLR3E	NCGv7	protein_c	chr16:22297375-223
ENSG0000(	266	6.652907	chr16:20‹	RRN3P3		Pseudoger	chr16:22429796-224
ENSG0000(	266	6.652907	chr16:20‹	ENSG00000262587		Pseudoger	chr16:25031881-250
ENSG0000(	266	6.652907	chr16:20‹	ENSG00000285613		lncRNA	chr16:23167577-231
ENSG0000(	266	6.652907	chr16:20‹	ENSG00000228480		Pseudoger	chr16:26354796-263
ENSG0000(	266	6.652907	chr16:20‹	ENSG00000276721		Pseudoger	chr16:21833516-218
ENSG0000(	266	6.652907	chr2:4707	ELOBP3		Pseudoger	chr2:48780602-4878
ENSG0000(	266	6.652907	chr2:4707	RN7SKP224		smallRNA	chr2:48217575-4821
ENSG0000(	266	6.652907	chr16:20‹	ENSG00000291066		lncRNA	chr16:21796106-218
ENSG0000(	266	6.652907	chr16:20‹	ENSG00000291262		lncRNA	chr16:21879773-218
ENSG0000(	266	6.652907	chr16:20‹	ENSG00000271609		Pseudoger	chr16:21538487-215
ENSG0000(	262	6.552863	chr12:68‡	SNRPF		protein_c	chr12:95858952-959
ENSG0000(	262	6.552863	chr12:68‡	PLXNC1		protein_c	chr12:94148577-943
ENSG0000(	262	6.552863	chr12:68‡	APPL2		protein_c	chr12:105173297-10
ENSG0000(	262	6.552863	chr12:68‡	LINC02399		lncRNA	chr12:89947693-899
ENSG0000(	262	6.552863	chr12:68‡	C12orf75-AS1		lncRNA	chr12:105304867-10
ENSG0000(	262	6.552863	chr12:68‡	FAM222A		protein_c	chr12:109713825-10
ENSG0000(	262	6.552863	chr12:68‡	CBX3P5		Pseudoger	chr12:95286318-952
ENSG0000(	262	6.552863	chr12:68‡	ENSG00000257746		lncRNA	chr12:93090480-931
ENSG0000(	262	6.552863	chr12:68‡	MMAB	NCGv7	protein_c	chr12:109553715-10
ENSG0000(	262	6.552863	chr12:68‡	ENSG00000277738		lncRNA	chr12:91634887-916
ENSG0000(	262	6.552863	chr12:68‡	ENSG00000287957		lncRNA	chr12:106867177-10
ENSG0000(	262	6.552863	chr12:68‡	ENSG00000257737		lncRNA	chr12:103654780-10
ENSG0000(	262	6.552863	chr12:68‡	ENSG00000257732		lncRNA	chr12:104262314-10
ENSG0000(	262	6.552863	chr12:68‡	FOXN4		protein_c	chr12:109277978-10
ENSG0000(	262	6.552863	chr12:68‡	ENSG00000277715		lncRNA	chr12:106250759-10
ENSG0000(	262	6.552863	chr12:68‡	snoU13		smallRNA	chr12:101789815-10
ENSG0000(	262	6.552863	chr12:68‡	AMDHD1		protein_c	chr12:95943331-959
ENSG0000(	262	6.552863	chr12:68‡	MIR135A2		smallRNA	chr12:97563812-975
ENSG0000(	262	6.552863	chr12:68‡	AC010209.1		smallRNA	chr12:100655722-10
ENSG0000(	262	6.552863	chr12:68‡	USP44	NCGv7	protein_c	chr12:95516560-955
ENSG0000(	262	6.552863	chr12:68‡	IGF1		protein_c	chr12:102395874-10
ENSG0000(	262	6.552863	chr12:68‡	ENSG00000285030		Pseudoger	chr12:95885169-958
ENSG0000(	262	6.552863	chr12:68‡	ENSG00000257715		lncRNA	chr12:96025323-960
ENSG0000(	262	6.552863	chr12:68‡	ENSG00000286197		lncRNA	chr12:103518334-10
ENSG0000(	262	6.552863	chr12:68‡	PWP1		protein_c	chr12:107685799-10
ENSG0000(	262	6.552863	chr12:68‡	GALNT4		protein_c	chr12:89519412-895
ENSG0000(	262	6.552863	chr16:65(	ENSG00000257691		Pseudoger	chr16:29475064-294
ENSG0000(	262	6.552863	chr12:68‡	UHRF1BP1L-DT		lncRNA	chr12:100143058-10
ENSG0000(	262	6.552863	chr12:68‡	ENSG00000270506		Pseudoger	chr12:93035261-930
ENSG0000(	262	6.552863	chr12:68‡	snoMe28S-Am2634		smallRNA	chr12:97552137-975
ENSG0000(	262	6.552863	chr12:68‡	DRAM1		protein_c	chr12:101877580-10
ENSG0000(	262	6.552863	chr12:68‡	GOLGA2P5		lncRNA	chr12:100156357-10
ENSG0000(	262	6.552863	chr12:68‡	ENSG00000257711		lncRNA	chr12:106678583-10
ENSG0000(	262	6.552863	chr12:68‡	ENSG00000257703		lncRNA	chr12:103079634-10
ENSG0000(	262	6.552863	chr12:68‡	ENSG00000257611		Pseudoger	chr12:106170004-10
ENSG0000(	262	6.552863	chr12:68‡	ASCL1	AC	protein_c	chr12:102957674-10
ENSG0000(	262	6.552863	chr12:68‡	WASHC4		protein_c	chr12:105107324-10
ENSG0000(	262	6.552863	chr12:68‡	MIR619		smallRNA	chr12:108836908-10
ENSG0000(	262	6.552863	chr12:68‡	ALDH1L2		protein_c	chr12:105019784-10

ENSG0000(	262	6.552863	chr12:68‹	ENSG00000290576		lncRNA	chr12:100173196-10
ENSG0000(	262	6.552863	chr12:68‹	snoU13		smallRNA	chr12:93350616-933
ENSG0000(	262	6.552863	chr12:68‹	ENSG00000257579		lncRNA	chr12:107610034-10
ENSG0000(	262	6.552863	chr12:68‹	ENSG00000257580		Pseudoger	chr12:98317741-983
ENSG0000(	262	6.552863	chr12:68‹	TDG	NCGv7	protein_c	chr12:103965822-10
ENSG0000(	262	6.552863	chr12:68‹	STAB2		protein_c	chr12:103587273-10
ENSG0000(	262	6.552863	chr12:68‹	GAS2L3		protein_c	chr12:100573683-10
ENSG0000(	262	6.552863	chr12:68‹	NEDD1		protein_c	chr12:96907224-969
ENSG0000(	262	6.552863	chr12:68‹	SLC41A2		protein_c	chr12:104801801-10
ENSG0000(	262	6.552863	chr12:68‹	ISCU		protein_c	chr12:108562582-10
ENSG0000(	262	6.552863	chr12:68‹	SYCP3		protein_c	chr12:101728648-10
ENSG0000(	262	6.552863	chr12:68‹	PAH		protein_c	chr12:102836889-10
ENSG0000(	262	6.552863	chr12:68‹	ENSG00000257681		lncRNA	chr12:103746315-10
ENSG0000(	262	6.552863	chr12:68‹	CKAP4		protein_c	chr12:106237881-10
ENSG0000(	262	6.552863	chr12:68‹	ALKBH2		protein_c	chr12:109088188-10
ENSG0000(	262	6.552863	chr12:68‹	SCYL2	NCGv7	protein_c	chr12:100267140-10
ENSG0000(	262	6.552863	chr12:68‹	RNU4-32P		smallRNA	chr12:109567975-10
ENSG0000(	262	6.552863	chr12:68‹	ENSG00000279798		TEC	chr12:97563257-975
ENSG0000(	262	6.552863	chr12:68‹	AC078819.1		smallRNA	chr12:103985111-10
ENSG0000(	262	6.552863	chr12:68‹	SLC25A3		protein_c	chr12:98593591-986
ENSG0000(	262	6.552863	chr12:68‹	SLC5A8		protein_c	chr12:101155493-10
ENSG0000(	262	6.552863	chr12:68‹	Y_RNA		smallRNA	chr12:100257688-10
ENSG0000(	262	6.552863	chr12:68‹	SART3	AC	protein_c	chr12:108522214-10
ENSG0000(	262	6.552863	chr12:68‹	ASCL4		protein_c	chr12:107774704-10
ENSG0000(	262	6.552863	chr12:68‹	UNG		protein_c	chr12:109097597-10
ENSG0000(	262	6.552863	chr12:68‹	ENSG00000255655		lncRNA	chr12:109445410-10
ENSG0000(	262	6.552863	chr12:68‹	EEF1B2P4		Pseudoger	chr12:106901283-10
ENSG0000(	262	6.552863	chr12:68‹	FAM222A-AS1		lncRNA	chr12:109734166-10
ENSG0000(	262	6.552863	chr12:68‹	ENSG00000257121		Pseudoger	chr12:95100531-951
ENSG0000(	262	6.552863	chr12:68‹	ATP2B1-AS1		lncRNA	chr12:89708959-897
ENSG0000(	262	6.552863	chr12:68‹	ENSG00000186076		Pseudoger	chr12:93640822-936
ENSG0000(	262	6.552863	chr12:68‹	AC126177.1		smallRNA	chr12:107799635-10
ENSG0000(	262	6.552863	chr12:68‹	ACACB		protein_c	chr12:109116587-10
ENSG0000(	262	6.552863	chr12:68‹	CLLU1		lncRNA	chr12:92421531-924
ENSG0000(	262	6.552863	chr12:68‹	ENSG00000257129		Pseudoger	chr12:107910403-10
ENSG0000(	262	6.552863	chr12:68‹	PGAM1P5		Pseudoger	chr12:95551582-956
ENSG0000(	262	6.552863	chr12:68‹	LINC02822		lncRNA	chr12:90576402-908
ENSG0000(	262	6.552863	chr12:68‹	ENSG00000257157		Pseudoger	chr12:97679879-976
ENSG0000(	262	6.552863	chr12:68‹	RN7SKP263		smallRNA	chr12:94008739-940
ENSG0000(	262	6.552863	chr12:68‹	TMPO-AS1		lncRNA	chr12:98512973-985
ENSG0000(	262	6.552863	chr12:68‹	ENSG00000257169		Pseudoger	chr12:96386818-963
ENSG0000(	262	6.552863	chr16:65(	MIR3680-2		smallRNA	chr16:29599179-295
ENSG0000(	262	6.552863	chr12:68‹	LINC02385		lncRNA	chr12:104170395-10
ENSG0000(	262	6.552863	chr12:68‹	ENSG00000257194		lncRNA	chr12:90107739-901
ENSG0000(	262	6.552863	chr12:68‹	HNRNPA1P50		Pseudoger	chr12:92883773-928
ENSG0000(	262	6.552863	chr12:68‹	ENSG00000257202		lncRNA	chr12:101923410-10
ENSG0000(	262	6.552863	chr12:68‹	NACAP8		Pseudoger	chr12:93124063-931
ENSG0000(	262	6.552863	chr12:68‹	PRDM4		protein_c	chr12:107732871-10
ENSG0000(	262	6.552863	chr12:68‹	CENPCP1		Pseudoger	chr12:89500093-895
ENSG0000(	262	6.552863	chr12:68‹	USP30		protein_c	chr12:109023089-10
ENSG0000(	262	6.552863	chr12:68‹	ENSG00000257221		lncRNA	chr12:108628668-10

ENSG0000(	262	6.552863	chr12:68!	PLEKHG7		protein_c	chr12:92702843-927
ENSG0000(	262	6.552863	chr12:68!	NTN4	NCGv7	protein_c	chr12:95657807-957
ENSG0000(	262	6.552863	chr12:68!	ENSG00000240441		Pseudoger	chr12:107719599-10
ENSG0000(	262	6.552863	chr12:68!	ENSG00000280088		TEC	chr12:100032325-10
ENSG0000(	262	6.552863	chr12:68!	NUAK1	NCGv7	protein_c	chr12:106063345-10
ENSG0000(	262	6.552863	chr12:68!	USP30-AS1		lncRNA	chr12:109052344-10
ENSG0000(	262	6.552863	chr12:68!	NOPCHAP1		protein_c	chr12:104986316-10
ENSG0000(	262	6.552863	chr12:68!	TMEM263		protein_c	chr12:106955719-10
ENSG0000(	262	6.552863	chr16:65(	SNX29P2		Pseudoger	chr16:29355787-293
ENSG0000(	262	6.552863	chr12:68!	ENSG00000256139		lncRNA	chr12:109111218-10
ENSG0000(	262	6.552863	chr12:68!	ABTB3		protein_c	chr12:107318421-10
ENSG0000(	262	6.552863	chr12:68!	KRT19P2		Pseudoger	chr12:94834147-948
ENSG0000(	262	6.552863	chr12:68!	ENSG00000216285		Pseudoger	chr12:104030779-10
ENSG0000(	262	6.552863	chr12:68!	UBE3B		protein_c	chr12:109477402-10
ENSG0000(	262	6.552863	chr12:68!	RN7SL176P		smallRNA	chr12:100158497-10
ENSG0000(	262	6.552863	chr12:68!	AC021052.1		smallRNA	chr12:96443480-964
ENSG0000(	262	6.552863	chr12:68!	ANO4	NCGv7	protein_c	chr12:100717526-10
ENSG0000(	262	6.552863	chr12:68!	VEZT		protein_c	chr12:95217746-953
ENSG0000(	262	6.552863	chr12:68!	CRADD	NCGv7	protein_c	chr12:93677375-938
ENSG0000(	262	6.552863	chr12:68!	WSCD2	NCGv7	protein_c	chr12:108129288-10
ENSG0000(	262	6.552863	chr12:68!	ACTR6		protein_c	chr12:100199122-10
ENSG0000(	262	6.552863	chr12:68!	NUP37		protein_c	chr12:102073103-10
ENSG0000(	262	6.552863	chr12:68!	RPL30P12		Pseudoger	chr12:106910757-10
ENSG0000(	262	6.552863	chr12:68!	Y_RNA		smallRNA	chr12:101446825-10
ENSG0000(	262	6.552863	chr12:68!	LINC01486		lncRNA	chr12:109354083-10
ENSG0000(	262	6.552863	chr12:68!	ENSG00000288744		lncRNA	chr12:103965893-10
ENSG0000(	262	6.552863	chr16:65(	NPIPB12		protein_c	chr16:29484690-295
ENSG0000(	262	6.552863	chr12:68!	ENSG00000280110		TEC	chr12:108778912-10
ENSG0000(	262	6.552863	chr12:68!	RNU6-36P		smallRNA	chr12:97721716-977
ENSG0000(	262	6.552863	chr12:68!	RMST		lncRNA	chr12:97430884-975
ENSG0000(	262	6.552863	chr12:68!	SELPLG		protein_c	chr12:108621895-10
ENSG0000(	262	6.552863	chr12:68!	ENSG00000257222		lncRNA	chr12:102063355-10
ENSG0000(	262	6.552863	chr12:68!	ENSG00000257548		lncRNA	chr12:107093298-10
ENSG0000(	262	6.552863	chr12:68!	ANKS1B		protein_c	chr12:98726457-999
ENSG0000(	262	6.552863	chr12:68!	ENSG00000257415		Pseudoger	chr12:103503381-10
ENSG0000(	262	6.552863	chr12:68!	ENSG00000257426		Pseudoger	chr12:107899638-10
ENSG0000(	262	6.552863	chr12:68!	RN7SL88P		smallRNA	chr12:96101953-961
ENSG0000(	262	6.552863	chr12:68!	RNU6-1068P		smallRNA	chr12:101411357-10
ENSG0000(	262	6.552863	chr12:68!	CLLU1-AS1		lncRNA	chr12:92420094-924
ENSG0000(	262	6.552863	chr12:68!	LINC02397		lncRNA	chr12:92466451-924
ENSG0000(	262	6.552863	chr12:68!	RPL21P106		Pseudoger	chr12:92168400-921
ENSG0000(	262	6.552863	chr16:65(	BOLA2-SMG1P6		protein_c	chr16:29443230-294
ENSG0000(	262	6.552863	chr16:65(	snoU13		smallRNA	chr16:29539262-295
ENSG0000(	262	6.552863	chr12:68!	ENSG00000257438		lncRNA	chr12:106103163-10
ENSG0000(	262	6.552863	chr12:68!	SETP7		Pseudoger	chr12:107271394-10
ENSG0000(	262	6.552863	chr12:68!	ENSG00000257458		lncRNA	chr12:98931682-989
ENSG0000(	262	6.552863	chr12:68!	MIR4303		smallRNA	chr12:97995383-979
ENSG0000(	262	6.552863	chr12:68!	ENSG00000257470		lncRNA	chr12:97024021-971
ENSG0000(	262	6.552863	chr12:68!	MRPL2P1		Pseudoger	chr12:89752913-897
ENSG0000(	262	6.552863	chr12:68!	ENSG00000266923		lncRNA	chr12:92421531-924
ENSG0000(	262	6.552863	chr12:68!	ENSG00000257489		Pseudoger	chr12:100180553-10

ENSG0000(	262	6.552863	chr12:68!	ENSG00000241917		Pseudoger	chr12:101936216-10
ENSG0000(	262	6.552863	chr12:68!	RNU7-184P		smallRNA	chr12:103114034-10
ENSG0000(	262	6.552863	chr12:68!	EID3		protein_c	chr12:104303739-10
ENSG0000(	262	6.552863	chr12:68!	PAFAH1B2P2		Pseudoger	chr12:97644345-977
ENSG0000(	262	6.552863	chr16:65(	ENSG00000257506		Pseudoger	chr16:29478879-294
ENSG0000(	262	6.552863	chr12:68!	C12orf73		protein_c	chr12:103940763-10
ENSG0000(	262	6.552863	chr12:68!	CFAP54	NCGv7	protein_c	chr12:96489571-968
ENSG0000(	262	6.552863	chr12:68!	ENSG00000290003		lncRNA	chr12:106349054-10
ENSG0000(	262	6.552863	chr12:68!	ENSG00000257514		lncRNA	chr12:101646720-10
ENSG0000(	262	6.552863	chr12:68!	NDUFA12		protein_c	chr12:94895297-950
ENSG0000(	262	6.552863	chr12:68!	ENSG00000257543		lncRNA	chr12:101408372-10
ENSG0000(	262	6.552863	chr12:68!	ENSG00000257545		lncRNA	chr12:106495958-10
ENSG0000(	262	6.552863	chr12:68!	CHST11	NCGv7	protein_c	chr12:104455295-10
ENSG0000(	262	6.552863	chr12:68!	ENSG00000241744		Pseudoger	chr12:101228141-10
ENSG0000(	262	6.552863	chr12:68!	LINC01619		lncRNA	chr12:91984976-921
ENSG0000(	262	6.552863	chr12:68!	RNU6-172P		smallRNA	chr12:101796410-10
ENSG0000(	262	6.552863	chr12:68!	ENSG00000257252		lncRNA	chr12:93316722-933
ENSG0000(	262	6.552863	chr12:68!	CORO1C		protein_c	chr12:108645109-10
ENSG0000(	262	6.552863	chr12:68!	ENSG00000286049		lncRNA	chr12:91423781-914
ENSG0000(	262	6.552863	chr12:68!	ENSG00000257254		Pseudoger	chr12:102513251-10
ENSG0000(	262	6.552863	chr12:68!	ENSG00000288102		lncRNA	chr12:90617759-908
ENSG0000(	262	6.552863	chr12:68!	ENSG00000279939		TEC	chr12:89544203-895
ENSG0000(	262	6.552863	chr12:68!	AC068641.1		smallRNA	chr12:89592827-895
ENSG0000(	262	6.552863	chr12:68!	ENSG00000286056		lncRNA	chr12:98420448-984
ENSG0000(	262	6.552863	chr12:68!	ENSG00000257281		Pseudoger	chr12:108281103-10
ENSG0000(	262	6.552863	chr12:68!	ENSG00000257754		lncRNA	chr12:103819610-10
ENSG0000(	262	6.552863	chr12:68!	Y_RNA		smallRNA	chr12:93021606-930
ENSG0000(	262	6.552863	chr12:68!	PARPBP		protein_c	chr12:102120183-10
ENSG0000(	262	6.552863	chr12:68!	DAO	NCGv7	protein_c	chr12:108858932-10
ENSG0000(	262	6.552863	chr12:68!	RPS27P23		Pseudoger	chr12:101424441-10
ENSG0000(	262	6.552863	chr12:68!	ENSG00000288092		lncRNA	chr12:103013362-10
ENSG0000(	262	6.552863	chr12:68!	RNA5SP369		Pseudoger	chr12:101836820-10
ENSG0000(	262	6.552863	chr16:65(	SMG1P2		Pseudoger	chr16:29527568-295
ENSG0000(	262	6.552863	chr12:68!	ENSG00000257322		lncRNA	chr12:93003390-932
ENSG0000(	262	6.552863	chr12:68!	KCTD10		protein_c	chr12:109448655-10
ENSG0000(	262	6.552863	chr12:68!	MVK		protein_c	chr12:109573255-10
ENSG0000(	262	6.552863	chr12:68!	ENSG00000257325		lncRNA	chr12:100852331-10
ENSG0000(	262	6.552863	chr12:68!	ENSG00000241556		Pseudoger	chr12:95467397-954
ENSG0000(	262	6.552863	chr12:68!	ENSG00000257327		lncRNA	chr12:103841451-10
ENSG0000(	262	6.552863	chr12:68!	LINC02413		lncRNA	chr12:92999218-930
ENSG0000(	262	6.552863	chr12:68!	ENSG00000257360		Pseudoger	chr12:94140077-941
ENSG0000(	262	6.552863	chr12:68!	RNU6-768P		smallRNA	chr12:101199271-10
ENSG0000(	262	6.552863	chr12:68!	ENSG00000257392		Pseudoger	chr12:107887730-10
ENSG0000(	262	6.552863	chr12:68!	ENSG00000257398		lncRNA	chr12:107809483-10
ENSG0000(	262	6.552863	chr12:68!	ENSG00000257400		lncRNA	chr12:94491546-944
ENSG0000(	262	6.552863	chr12:68!	ENSG00000257283		lncRNA	chr12:93894965-939
ENSG0000(	262	6.552863	chr12:68!	ENSG00000257878		lncRNA	chr12:95996460-960
ENSG0000(	262	6.552863	chr12:68!	LINC02410		lncRNA	chr12:95803097-958
ENSG0000(	262	6.552863	chr12:68!	RNA5SP367		Pseudoger	chr12:101472339-10
ENSG0000(	262	6.552863	chr12:68!	DCN		protein_c	chr12:91140484-911
ENSG0000(	262	6.552863	chr12:68!	RNU7-169P		smallRNA	chr12:108668448-10

ENSG0000(	262	6.552863	chr12:68!	ENSG00000258230	Pseudoger	chr12:101773114-10
ENSG0000(	262	6.552863	chr12:68!	SPIC	protein_c	chr12:101475336-10
ENSG0000(	262	6.552863	chr12:68!	MYO1H NCGv7	protein_c	chr12:109347900-10
ENSG0000(	262	6.552863	chr12:68!	RN7SL330P	smallRNA	chr12:94361220-943
ENSG0000(	262	6.552863	chr12:68!	YPEL5P3	Pseudoger	chr12:96060024-960
ENSG0000(	262	6.552863	chr12:68!	AC190387.1	smallRNA	chr12:108987555-10
ENSG0000(	262	6.552863	chr12:68!	MIR1251	smallRNA	chr12:97491900-974
ENSG0000(	262	6.552863	chr12:68!	LINC02452	lncRNA	chr12:96160692-961
ENSG0000(	262	6.552863	chr12:68!	ENSG00000258262	Pseudoger	chr12:92530460-925
ENSG0000(	262	6.552863	chr12:68!	ENSG00000258271	Pseudoger	chr12:93529560-935
ENSG0000(	262	6.552863	chr12:68!	CMKLR1 NCGv7	protein_c	chr12:108288044-10
ENSG0000(	262	6.552863	chr12:68!	ENSG00000258272	lncRNA	chr12:96422326-964
ENSG0000(	262	6.552863	chr12:68!	CRADD-AS1	lncRNA	chr12:93707791-937
ENSG0000(	262	6.552863	chr12:68!	RN7SL179P	smallRNA	chr12:98524476-985
ENSG0000(	262	6.552863	chr12:68!	NENFP2	Pseudoger	chr12:101963901-10
ENSG0000(	262	6.552863	chr12:68!	BRWD1P2	Pseudoger	chr12:89919437-899
ENSG0000(	262	6.552863	chr12:68!	ENSG00000289949	lncRNA	chr12:96131433-961
ENSG0000(	262	6.552863	chr12:68!	ENSG00000258302	lncRNA	chr12:89561129-895
ENSG0000(	262	6.552863	chr12:68!	ENSG00000258303	lncRNA	chr12:93836167-938
ENSG0000(	262	6.552863	chr12:68!	HELLPAR	lncRNA	chr12:102197585-10
ENSG0000(	262	6.552863	chr12:68!	ENSG00000258308	lncRNA	chr12:101954992-10
ENSG0000(	262	6.552863	chr12:68!	ENSG00000258312	lncRNA	chr12:98113014-982
ENSG0000(	262	6.552863	chr12:68!	AC010203.1	smallRNA	chr12:100157256-10
ENSG0000(	262	6.552863	chr12:68!	SNRPF-DT	lncRNA	chr12:95791733-958
ENSG0000(	262	6.552863	chr12:68!	snoU13	smallRNA	chr12:93265629-932
ENSG0000(	262	6.552863	chr12:68!	ENSG00000258355	lncRNA	chr12:106245613-10
ENSG0000(	262	6.552863	chr12:68!	NSA2P2	Pseudoger	chr12:94521090-945
ENSG0000(	262	6.552863	chr12:68!	ENSG00000286903	lncRNA	chr12:105236338-10
ENSG0000(	262	6.552863	chr12:68!	ENSG00000258224	Pseudoger	chr12:91871122-918
ENSG0000(	262	6.552863	chr12:68!	RNU6-247P	smallRNA	chr12:95792529-957
ENSG0000(	262	6.552863	chr12:68!	ENSG00000258216	lncRNA	chr12:89712048-901
ENSG0000(	262	6.552863	chr16:65(	NPIPB11	protein_c	chr16:29381310-294
ENSG0000(	262	6.552863	chr12:68!	SUCLG2P2	Pseudoger	chr12:94548241-945
ENSG0000(	262	6.552863	chr12:68!	RN7SL483P	smallRNA	chr12:94515476-945
ENSG0000(	262	6.552863	chr12:68!	PRDM4-AS1	lncRNA	chr12:107736555-10
ENSG0000(	262	6.552863	chr12:68!	MIR492	smallRNA	chr12:94834398-948
ENSG0000(	262	6.552863	chr12:68!	ENSG00000258142	Pseudoger	chr12:102230027-10
ENSG0000(	262	6.552863	chr12:68!	ENSG00000258148	Pseudoger	chr12:91193709-911
ENSG0000(	262	6.552863	chr16:65(	ENSG00000258150	Pseudoger	chr16:29465222-294
ENSG0000(	262	6.552863	chr12:68!	HSPE1P4	Pseudoger	chr12:101873316-10
ENSG0000(	262	6.552863	chr12:68!	ENSG00000286678	lncRNA	chr12:107219764-10
ENSG0000(	262	6.552863	chr12:68!	AC069227.1	smallRNA	chr12:102926514-10
ENSG0000(	262	6.552863	chr12:68!	LINC00485	lncRNA	chr12:102809280-10
ENSG0000(	262	6.552863	chr12:68!	LINC02412	lncRNA	chr12:93173470-931
ENSG0000(	262	6.552863	chr12:68!	ENSG00000258172	lncRNA	chr12:94272150-942
ENSG0000(	262	6.552863	chr16:65(	ENSG00000279228	Pseudoger	chr16:29466069-294
ENSG0000(	262	6.552863	chr12:68!	RNU6-1183P	smallRNA	chr12:101871742-10
ENSG0000(	262	6.552863	chr12:68!	ENSG00000289437	lncRNA	chr12:95260099-952
ENSG0000(	262	6.552863	chr12:68!	RN7SKP11	smallRNA	chr12:96427200-964
ENSG0000(	262	6.552863	chr12:68!	RNU6-148P	smallRNA	chr12:89754059-897
ENSG0000(	262	6.552863	chr12:68!	ENSG00000258177	lncRNA	chr12:96222797-962

ENSG0000(	262	6.552863	chr12:68E	SNORD74		smallRNA	chr12:93266017-932
ENSG0000(	262	6.552863	chr12:68E	ENSG00000286907		lncRNA	chr12:92008052-920
ENSG0000(	262	6.552863	chr12:68E	LINC02392		lncRNA	chr12:90280894-903
ENSG0000(	262	6.552863	chr16:65(	ENSG00000279583		TEC	chr16:29595130-295
ENSG0000(	262	6.552863	chr12:68E	RN7SKP250		smallRNA	chr12:109662863-10
ENSG0000(	262	6.552863	chr12:68E	ENSG00000258204		Pseudoger	chr12:98794485-987
ENSG0000(	262	6.552863	chr12:68E	RNU4-24P		smallRNA	chr12:96303375-963
ENSG0000(	262	6.552863	chr12:68E	MIR4495		smallRNA	chr12:97939056-979
ENSG0000(	262	6.552863	chr12:68E	ENSG00000271382		lncRNA	chr12:93945041-939
ENSG0000(	262	6.552863	chr12:68E	ENSG00000258365		lncRNA	chr12:94277758-942
ENSG0000(	262	6.552863	chr12:68E	DEPDC4	NCGv7	protein_c	chr12:100203669-10
ENSG0000(	262	6.552863	chr16:65(	SLX1B		protein_c	chr16:29454533-294
ENSG0000(	262	6.552863	chr12:68E	ENSG00000279148		TEC	chr12:100026248-10
ENSG0000(	262	6.552863	chr12:68E	ENSG00000279190		TEC	chr12:97150081-971
ENSG0000(	262	6.552863	chr12:68E	Y_RNA		smallRNA	chr12:107650789-10
ENSG0000(	262	6.552863	chr16:65(	ENSG00000198106		lncRNA	chr16:29291220-293
ENSG0000(	262	6.552863	chr12:68E	TMCC3		protein_c	chr12:94567122-946
ENSG0000(	262	6.552863	chr12:68E	CCDC38	NCGv7	protein_c	chr12:95867048-959
ENSG0000(	262	6.552863	chr12:68E	RBMS2P1		Pseudoger	chr12:94423744-944
ENSG0000(	262	6.552863	chr12:68E	SNORD74		smallRNA	chr12:107374747-10
ENSG0000(	262	6.552863	chr12:68E	ENSG00000279176		TEC	chr12:104552073-10
ENSG0000(	262	6.552863	chr12:68E	SLC17A8		protein_c	chr12:100357074-10
ENSG0000(	262	6.552863	chr12:68E	AC138123.1		smallRNA	chr12:93097251-930
ENSG0000(	262	6.552863	chr12:68E	HCFC2	NCGv7	protein_c	chr12:104064531-10
ENSG0000(	262	6.552863	chr12:68E	RFX4		protein_c	chr12:106583004-10
ENSG0000(	262	6.552863	chr12:68E	CDK17		protein_c	chr12:96278261-964
ENSG0000(	262	6.552863	chr12:68E	ENSG00000289557		lncRNA	chr12:104644173-10
ENSG0000(	262	6.552863	chr12:68E	AC013283.1		smallRNA	chr12:98605874-986
ENSG0000(	262	6.552863	chr12:68E	MIR331		smallRNA	chr12:95308420-953
ENSG0000(	262	6.552863	chr12:68E	TXNRD1		protein_c	chr12:104215779-10
ENSG0000(	262	6.552863	chr12:68E	RPL18AP3		Pseudoger	chr12:104265309-10
ENSG0000(	262	6.552863	chr12:68E	LINC02456		lncRNA	chr12:102257213-10
ENSG0000(	262	6.552863	chr12:68E	C12orf75		protein_c	chr12:105235290-10
ENSG0000(	262	6.552863	chr12:68E	EEA1		protein_c	chr12:92770637-929
ENSG0000(	262	6.552863	chr12:68E	RNU6-101P		smallRNA	chr12:101765406-10
ENSG0000(	262	6.552863	chr12:68E	RIC8B		protein_c	chr12:106774621-10
ENSG0000(	262	6.552863	chr12:68E	SOCS2-AS1		lncRNA	chr12:93542022-935
ENSG0000(	262	6.552863	chr12:68E	LINC01498		lncRNA	chr12:108434130-10
ENSG0000(	262	6.552863	chr12:68E	CRY1		protein_c	chr12:106991364-10
ENSG0000(	262	6.552863	chr12:68E	ENSG00000289510		lncRNA	chr12:95336664-954
ENSG0000(	262	6.552863	chr12:68E	ENSG00000282964		lncRNA	chr12:102953289-10
ENSG0000(	262	6.552863	chr12:68E	FICD		protein_c	chr12:108515277-10
ENSG0000(	262	6.552863	chr12:68E	ENSG00000286520		lncRNA	chr12:105699168-10
ENSG0000(	262	6.552863	chr12:68E	NT5DC3		protein_c	chr12:103770453-10
ENSG0000(	262	6.552863	chr12:68E	RNU6-361P		smallRNA	chr12:108926435-10
ENSG0000(	262	6.552863	chr12:68E	PPIAP8		Pseudoger	chr12:98590723-985
ENSG0000(	262	6.552863	chr12:68E	RNU4-41P		smallRNA	chr12:98417896-984
ENSG0000(	262	6.552863	chr12:68E	ENSG00000289476		lncRNA	chr12:95305929-953
ENSG0000(	262	6.552863	chr12:68E	FGD6		protein_c	chr12:95076749-952
ENSG0000(	262	6.552863	chr12:68E	IKBIP		protein_c	chr12:98613405-986
ENSG0000(	262	6.552863	chr12:68E	C12orf42		protein_c	chr12:103237591-10

ENSG0000(	262	6.552863	chr12:68§	ENSG00000286410		lncRNA	chr12:104858838-10
ENSG0000(	262	6.552863	chr12:68§	LINC02453		lncRNA	chr12:98485544-985
ENSG0000(	262	6.552863	chr12:68§	GARIN6		protein_c	chr12:99647753-996
ENSG0000(	262	6.552863	chr12:68§	EEF1A1P33		Pseudoger	chr12:96900697-969
ENSG0000(	262	6.552863	chr12:68§	POC1B-GALNT4		protein_c	chr12:89519408-895
ENSG0000(	262	6.552863	chr12:68§	GOLGA2P5		Pseudoger	chr12:100156621-10
ENSG0000(	262	6.552863	chr12:68§	RN7SL793P		smallRNA	chr12:102217320-10
ENSG0000(	262	6.552863	chr12:68§	ENSG00000287454		lncRNA	chr12:96229563-962
ENSG0000(	262	6.552863	chr12:68§	ENSG00000286434		lncRNA	chr12:97155234-971
ENSG0000(	262	6.552863	chr12:68§	TCP11L2	NCGv7	protein_c	chr12:106301929-10
ENSG0000(	262	6.552863	chr12:68§	CCER1	NCGv7	protein_c	chr12:90905622-909
ENSG0000(	262	6.552863	chr16:65(	SULT1A4		protein_c	chr16:29459913-294
ENSG0000(	262	6.552863	chr12:68§	RNA5SP368		Pseudoger	chr12:101779814-10
ENSG0000(	262	6.552863	chr12:68§	RNA5SP370		Pseudoger	chr12:104125236-10
ENSG0000(	262	6.552863	chr12:68§	UHRF1BP1L		protein_c	chr12:100028455-10
ENSG0000(	262	6.552863	chr12:68§	AC011313.1		smallRNA	chr12:105327906-10
ENSG0000(	262	6.552863	chr12:68§	ENSG00000289605		lncRNA	chr12:91276241-916
ENSG0000(	262	6.552863	chr12:68§	BTG1-DT		lncRNA	chr12:92145573-922
ENSG0000(	262	6.552863	chr12:68§	ENSG00000237774		Pseudoger	chr12:95125760-951
ENSG0000(	262	6.552863	chr12:68§	RNA5SP372		Pseudoger	chr12:109059986-10
ENSG0000(	262	6.552863	chr12:68§	CHPT1	NCGv7	protein_c	chr12:101696947-10
ENSG0000(	262	6.552863	chr12:68§	MRPL42		protein_c	chr12:93467514-935
ENSG0000(	262	6.552863	chr12:68§	GNPTAB	NCGv7	protein_c	chr12:101745499-10
ENSG0000(	262	6.552863	chr12:68§	RNA5SP371		Pseudoger	chr12:107486511-10
ENSG0000(	262	6.552863	chr12:68§	MIR3685		smallRNA	chr12:95309923-953
ENSG0000(	262	6.552863	chr12:68§	Y_RNA		smallRNA	chr12:92170353-921
ENSG0000(	262	6.552863	chr12:68§	ENSG00000258131		lncRNA	chr12:96985656-971
ENSG0000(	262	6.552863	chr12:68§	LINC02404		lncRNA	chr12:91876924-918
ENSG0000(	262	6.552863	chr12:68§	MIR5700		smallRNA	chr12:94561789-945
ENSG0000(	262	6.552863	chr12:68§	snoU13		smallRNA	chr12:92544892-925
ENSG0000(	262	6.552863	chr16:65(	ENSG00000278078		Pseudoger	chr16:29380242-293
ENSG0000(	262	6.552863	chr12:68§	SNORA40		lncRNA	chr12:108778144-10
ENSG0000(	262	6.552863	chr12:68§	BTG1	NCGv7;AC	protein_c	chr12:92140278-921
ENSG0000(	262	6.552863	chr16:65(	SMG1P6		Pseudoger	chr16:29425800-294
ENSG0000(	262	6.552863	chr12:68§	PIGAP1		Pseudoger	chr12:100578744-10
ENSG0000(	262	6.552863	chr12:68§	ENSG00000274598		lncRNA	chr12:108833721-10
ENSG0000(	262	6.552863	chr12:68§	GLT8D2	NCGv7	protein_c	chr12:103988987-10
ENSG0000(	262	6.552863	chr12:68§	EPYC	NCGv7	protein_c	chr12:90963682-910
ENSG0000(	262	6.552863	chr12:68§	ARL1		protein_c	chr12:101393116-10
ENSG0000(	262	6.552863	chr12:68§	RNU6-1329P		smallRNA	chr12:92952639-929
ENSG0000(	262	6.552863	chr12:68§	HAL		protein_c	chr12:95972662-959
ENSG0000(	262	6.552863	chr12:68§	LSM3P2		Pseudoger	chr12:94590821-945
ENSG0000(	262	6.552863	chr12:68§	SSH1	NCGv7	protein_c	chr12:108778191-10
ENSG0000(	262	6.552863	chr12:68§	snoU13		smallRNA	chr12:101358748-10
ENSG0000(	262	6.552863	chr12:68§	ENSG00000274560		lncRNA	chr12:101696002-10
ENSG0000(	262	6.552863	chr12:68§	CASC18		lncRNA	chr12:105704203-10
ENSG0000(	262	6.552863	chr12:68§	POC1B-AS1		lncRNA	chr12:89524594-895
ENSG0000(	262	6.552863	chr12:68§	C12orf42-AS1		lncRNA	chr12:103151834-10
ENSG0000(	262	6.552863	chr12:68§	AC090051.1		smallRNA	chr12:104951760-10
ENSG0000(	262	6.552863	chr12:68§	ENSG00000257863		lncRNA	chr12:91901458-919
ENSG0000(	262	6.552863	chr12:68§	ENSG00000289344		lncRNA	chr12:107660410-10

ENSG0000(	262	6.552863	chr12:68{	Y_RNA		smallRNA	chr12:101719808-10
ENSG0000(	262	6.552863	chr16:65(	SLX1B-SULT1A4		lncRNA	chr16:29455105-294
ENSG0000(	262	6.552863	chr12:68{	TMPO		protein_c	chr12:98515579-985
ENSG0000(	262	6.552863	chr12:68{	MYBPC1		protein_c	chr12:101568353-10
ENSG0000(	262	6.552863	chr12:68{	ENSG00000257890		lncRNA	chr12:106050961-10
ENSG0000(	262	6.552863	chr12:68{	TMEM263-DT		lncRNA	chr12:106954029-10
ENSG0000(	262	6.552863	chr12:68{	ENSG00000257787		lncRNA	chr12:90809207-908
ENSG0000(	262	6.552863	chr12:68{	ATP2B1		protein_c	chr12:89588049-897
ENSG0000(	262	6.552863	chr18:951	RNU2-27P		smallRNA	chr18:9518119-9518
ENSG0000(	262	6.552863	chr12:68{	ENSG00000257766		lncRNA	chr12:103668575-10
ENSG0000(	262	6.552863	chr12:68{	snoU13		smallRNA	chr12:103720942-10
ENSG0000(	262	6.552863	chr12:68{	METAP2		protein_c	chr12:95473520-955
ENSG0000(	262	6.552863	chr12:68{	KRT18P20		Pseudoger	chr12:104976749-10
ENSG0000(	262	6.552863	chr12:68{	RNU6-808P		smallRNA	chr12:95290692-952
ENSG0000(	262	6.552863	chr12:68{	LINC02391		lncRNA	chr12:92247697-923
ENSG0000(	262	6.552863	chr12:68{	LINC02401		lncRNA	chr12:103547751-10
ENSG0000(	262	6.552863	chr12:68{	AC068643.1		smallRNA	chr12:103238398-10
ENSG0000(	262	6.552863	chr12:68{	APAF1		protein_c	chr12:98645290-987
ENSG0000(	262	6.552863	chr12:68{	KERA		protein_c	chr12:91050491-910
ENSG0000(	262	6.552863	chr12:68{	LUM	NCGv7	protein_c	chr12:91102629-911
ENSG0000(	262	6.552863	chr12:68{	WASHC3		protein_c	chr12:102012840-10
ENSG0000(	262	6.552863	chr12:68{	NFYB		protein_c	chr12:104117086-10
ENSG0000(	262	6.552863	chr12:68{	LTA4H	NCGv7	protein_c	chr12:96000753-960
ENSG0000(	262	6.552863	chr12:68{	SOCS2		protein_c	chr12:93569814-935
ENSG0000(	262	6.552863	chr12:68{	RN7SL737P		smallRNA	chr12:93509487-935
ENSG0000(	262	6.552863	chr12:68{	PMCH		protein_c	chr12:102196459-10
ENSG0000(	262	6.552863	chr12:68{	ELK3	AC	protein_c	chr12:96194375-962
ENSG0000(	262	6.552863	chr12:68{	UBE2N		protein_c	chr12:93405673-934
ENSG0000(	262	6.552863	chr16:65(	BOLA2		protein_c	chr16:29453590-294
ENSG0000(	262	6.552863	chr12:68{	ENSG00000287202		lncRNA	chr12:108532074-10
ENSG0000(	262	6.552863	chr12:68{	POC1B		protein_c	chr12:89419718-895
ENSG0000(	262	6.552863	chr12:68{	POLR3B		protein_c	chr12:106357748-10
ENSG0000(	262	6.552863	chr12:68{	TRPV4		protein_c	chr12:109783087-10
ENSG0000(	262	6.552863	chr12:68{	MTERF2		protein_c	chr12:106977277-10
ENSG0000(	262	6.552863	chr12:68{	RN7SL630P		smallRNA	chr12:93831076-938
ENSG0000(	262	6.552863	chr12:68{	TMEM119		protein_c	chr12:108589851-10
ENSG0000(	262	6.552863	chr12:68{	ST13P3		Pseudoger	chr12:106015897-10
ENSG0000(	262	6.552863	chr12:68{	U8		smallRNA	chr12:103592112-10
ENSG0000(	262	6.552863	chr12:68{	Y_RNA		smallRNA	chr12:96979528-969
ENSG0000(	262	6.552863	chr12:68{	Y_RNA		smallRNA	chr12:95275770-952
ENSG0000(	262	6.552863	chr12:68{	ENSG00000271177		Pseudoger	chr12:100525186-10
ENSG0000(	262	6.552863	chr12:68{	RNU6-735P		smallRNA	chr12:95438737-954
ENSG0000(	262	6.552863	chr12:68{	LINC02823		lncRNA	chr12:91327045-913
ENSG0000(	262	6.552863	chr16:65(	snoU13		smallRNA	chr16:29437497-294
ENSG0000(	262	6.552863	chr12:68{	ENSG00000257995		lncRNA	chr12:90617759-906
ENSG0000(	262	6.552863	chr12:68{	ENSG00000258007		lncRNA	chr12:101038420-10
ENSG0000(	262	6.552863	chr12:68{	ENSG00000258012		Pseudoger	chr12:92858079-928
ENSG0000(	262	6.552863	chr12:68{	Y_RNA		smallRNA	chr12:93460725-934
ENSG0000(	262	6.552863	chr12:68{	ENSG00000287675		lncRNA	chr12:99985045-999
ENSG0000(	262	6.552863	chr12:68{	NR2C1		protein_c	chr12:95020229-950
ENSG0000(	262	6.552863	chr16:65(	ENSG00000260335		Pseudoger	chr16:29449191-294

ENSG0000(	262	6.552863	chr12:68	ENSG00000287714		lncRNA	chr12:92026447-920
ENSG0000(	262	6.552863	chr16:65(	ENSG00000290678		lncRNA	chr16:29464967-294
ENSG0000(	262	6.552863	chr12:68	MIR3922		smallRNA	chr12:104591633-10
ENSG0000(	262	6.552863	chr12:68	SNX5P2		Pseudoger	chr12:101066734-10
ENSG0000(	262	6.552863	chr12:68	SLC9A7P1		Pseudoger	chr12:98453835-984
ENSG0000(	262	6.552863	chr12:68	ENSG00000258072		Pseudoger	chr12:107903270-10
ENSG0000(	262	6.552863	chr12:68	ENSG00000258035		lncRNA	chr12:94167528-941
ENSG0000(	262	6.552863	chr12:68	ENSG00000258039		lncRNA	chr12:99093359-991
ENSG0000(	262	6.552863	chr12:68	RNA5SP365		Pseudoger	chr12:89726936-897
ENSG0000(	262	6.552863	chr12:68	CEP83	NCGv7	protein_c	chr12:94306449-944
ENSG0000(	262	6.552863	chr12:68	CEP83-DT		lncRNA	chr12:94460003-944
ENSG0000(	262	6.552863	chr16:65(	ENSG00000290679		lncRNA	chr16:29467161-294
ENSG0000(	262	6.552863	chr12:68	NUDT4		protein_c	chr12:93377883-934
ENSG0000(	262	6.552863	chr12:68	LINC00615		lncRNA	chr12:90918023-909
ENSG0000(	262	6.552863	chr16:65(	ENSG00000290680		lncRNA	chr16:29350746-293
ENSG0000(	262	6.552863	chr12:68	ENSG00000257994		Pseudoger	chr12:103505411-10
ENSG0000(	262	6.552863	chr12:68	ENSG00000287237		lncRNA	chr12:94260548-942
ENSG0000(	262	6.552863	chr12:68	ENSG00000258111		Pseudoger	chr12:104514029-10
ENSG0000(	262	6.552863	chr12:68	UTP20	NCGv7	protein_c	chr12:101280105-10
ENSG0000(	262	6.552863	chr12:68	HSP90B1		protein_c	chr12:103930107-10
ENSG0000(	262	6.552863	chr12:68	ENSG00000286258		lncRNA	chr12:104171600-10
ENSG0000(	262	6.552863	chr12:68	TTC41P		Pseudoger	chr12:103843749-10
ENSG0000(	262	6.552863	chr12:68	RPS4XP1		Pseudoger	chr12:100009052-10
ENSG0000(	262	6.552863	chr12:68	SNORA53		smallRNA	chr12:98599635-985
ENSG0000(	262	6.552863	chr12:68	ENSG00000258125		lncRNA	chr12:91680131-916
ENSG0000(	262	6.552863	chr12:68	RNA5SP366		Pseudoger	chr12:99083783-990
ENSG0000(	262	6.552863	chr12:68	ENSG00000257918		lncRNA	chr12:106714924-10
ENSG0000(	262	6.552863	chr12:68	LINC02409		lncRNA	chr12:97272692-972
ENSG0000(	262	6.552863	chr12:68	snoU13		smallRNA	chr12:100402145-10
ENSG0000(	262	6.552863	chr12:68	RNU7-94P		smallRNA	chr12:106282927-10
ENSG0000(	262	6.552863	chr12:68	Y_RNA		smallRNA	chr12:104851824-10
ENSG0000(	262	6.552863	chr12:68	NR1H4	NCGv7	protein_c	chr12:100473708-10
ENSG0000(	262	6.552863	chr12:68	Y_RNA		smallRNA	chr12:95617689-956
ENSG0000(	262	6.552863	chr12:68	ENSG00000289387		lncRNA	chr12:105762150-10
ENSG0000(	262	6.552863	chr12:68	RNU5E-5P		smallRNA	chr12:101466705-10
ENSG0000(	262	6.552863	chr12:68	ENSG00000257943		Pseudoger	chr12:95311312-954
ENSG0000(	262	6.552863	chr12:68	ENSG00000257951		Pseudoger	chr12:107880581-10
ENSG0000(	262	6.552863	chr12:68	ENSG00000279630		TEC	chr12:109020833-10
ENSG0000(	262	6.552863	chr12:68	Y_RNA		smallRNA	chr12:108738484-10
ENSG0000(	261	6.527853	chr2:4707	MIR3681		smallRNA	chr2:12199130-1219
ENSG0000(	261	6.527853	chr19:132	SNORD35B		smallRNA	chr19:49497720-494
ENSG0000(	260	6.502842	chr9:1333	SNORD36A		smallRNA	chr9:133350456-133
ENSG0000(	260	6.502842	chr9:1333	SNORD36C		smallRNA	chr9:133350847-133
ENSG0000(	260	6.502842	chr11:76(	Y_RNA		smallRNA	chr11:8943380-8943
ENSG0000(	260	6.502842	chr16:65(	RNY4P3		smallRNA	chr16:50098264-500
ENSG0000(	260	6.502842	chr9:1333	SNORD36B		smallRNA	chr9:133350095-133
ENSG0000(	259	6.477831	chr19:132	RPL39P34		Pseudoger	chr19:52564076-525
ENSG0000(	258	6.45282	chr16:531	AC010543.1		smallRNA	chr16:57874384-578
ENSG0000(	257	6.427809	chr22:22(	IGLV3-19		protein_c	chr22:22720623-227
ENSG0000(	257	6.427809	chr22:22(	IGLV2-18		protein_c	chr22:22734607-227
ENSG0000(	257	6.427809	chr22:22(	ENSG00000278667		Pseudoger	chr22:22730725-227

ENSG0000(	257	6.427809	chr22:22(	IGLV3-17		Pseudoger	chr22:22738944-227
ENSG0000(	257	6.427809	chr22:22(	IGLV3-21		protein_c	chr22:22711689-227
ENSG0000(	257	6.427809	chr22:22(	D87007.1		smallRNA	chr22:22734575-227
ENSG0000(	256	6.402798	chr11:76(	RPL23AP65		Pseudoger	chr11:9606142-9606
ENSG0000(	255	6.377787	chr3:857:	AC106827.2		smallRNA	chr3:65941247-6594
ENSG0000(	253	6.327765	chr6:105(	ENSG00000290032		lncRNA	chr6:27138293-2713
ENSG0000(	252	6.302754	chr16:20!	ENSG00000263029		Pseudoger	chr16:16260866-162
ENSG0000(	252	6.302754	chr16:20!	ENSG00000286849		lncRNA	chr16:17405678-174
ENSG0000(	252	6.302754	chr16:20!	ENSG00000279415		TEC	chr16:17227023-172
ENSG0000(	252	6.302754	chr16:20!	ENSG00000285850		lncRNA	chr16:17453470-174
ENSG0000(	252	6.302754	chr1:152:	RNU1-7P		smallRNA	chr1:8206434-82065
ENSG0000(	252	6.302754	chr10:26!	ENSG00000271220		Pseudoger	chr10:28812899-288
ENSG0000(	252	6.302754	chr16:20!	ENSG00000260405		Pseudoger	chr16:17096202-170
ENSG0000(	252	6.302754	chr1:152:	ENSG00000229305		Pseudoger	chr1:8189824-81927
ENSG0000(	252	6.302754	chr16:20!	ENSG00000263335		lncRNA	chr16:15726674-157
ENSG0000(	252	6.302754	chr16:20!	RN7SL90P		smallRNA	chr16:16637255-166
ENSG0000(	252	6.302754	chr1:152:	ENO1		protein_c	chr1:8861000-88791
ENSG0000(	252	6.302754	chr1:152:	ENSG00000288816		lncRNA	chr1:8191692-81949
ENSG0000(	252	6.302754	chr16:20!	MIR3670-2		smallRNA	chr16:16306370-163
ENSG0000(	252	6.302754	chr16:20!	CEP20		protein_c	chr16:15865719-158
ENSG0000(	252	6.302754	chr16:20!	ENSG00000260242		lncRNA	chr16:18002806-181
ENSG0000(	252	6.302754	chr16:20!	ENSG00000260126		lncRNA	chr16:16499533-166
ENSG0000(	252	6.302754	chr16:20!	ENSG00000279410		TEC	chr16:17192863-171
ENSG0000(	252	6.302754	chr16:20!	ENSG00000275927		lncRNA	chr16:17445825-174
ENSG0000(	252	6.302754	chr16:20!	ENSG00000259929		lncRNA	chr16:17933189-181
ENSG0000(	252	6.302754	chr16:20!	ENSG00000263065		lncRNA	chr16:15741151-157
ENSG0000(	252	6.302754	chr16:20!	MYH11	IntOGen-I	protein_c	chr16:15703135-158
ENSG0000(	252	6.302754	chr16:20!	ENSG00000277770		Pseudoger	chr16:15358978-153
ENSG0000(	252	6.302754	chr16:20!	NOMO3		protein_c	chr16:16232506-163
ENSG0000(	252	6.302754	chr16:20!	BMERB1		protein_c	chr16:15434475-156
ENSG0000(	252	6.302754	chr1:152:	ENSG00000270282		Pseudoger	chr1:8512653-85130
ENSG0000(	252	6.302754	chr1:152:	CA6		protein_c	chr1:8945867-89750
ENSG0000(	252	6.302754	chr16:20!	ENSG00000261181		Pseudoger	chr16:16394912-163
ENSG0000(	252	6.302754	chr16:20!	ENSG00000279620		TEC	chr16:17249674-172
ENSG0000(	252	6.302754	chr16:20!	MPV17L	DriverDB\	protein_c	chr16:15395754-154
ENSG0000(	252	6.302754	chr1:152:	Y_RNA		smallRNA	chr1:8796574-87966
ENSG0000(	252	6.302754	chr16:20!	ENSG00000261130		protein_c	chr16:15395754-155
ENSG0000(	252	6.302754	chr16:20!	PKD1P2		Pseudoger	chr16:16356224-163
ENSG0000(	252	6.302754	chr1:152:	RERE-AS1		lncRNA	chr1:8424645-84350
ENSG0000(	252	6.302754	chr1:152:	RNU6-304P		smallRNA	chr1:8883427-88835
ENSG0000(	252	6.302754	chr1:152:	Y_RNA		smallRNA	chr1:8328067-83281
ENSG0000(	252	6.302754	chr16:20!	ENSG00000280429		TEC	chr16:15790975-157
ENSG0000(	252	6.302754	chr16:20!	ENSG00000261748		Pseudoger	chr16:16952502-169
ENSG0000(	252	6.302754	chr16:20!	ENSG00000260945		lncRNA	chr16:17540331-175
ENSG0000(	252	6.302754	chr16:20!	ENSG00000260933		Pseudoger	chr16:18170356-181
ENSG0000(	252	6.302754	chr1:152:	RN7SL729P		smallRNA	chr1:8275124-82753
ENSG0000(	252	6.302754	chr16:20!	ABCC1		protein_c	chr16:15949138-161
ENSG0000(	252	6.302754	chr16:20!	RPL15P20		Pseudoger	chr16:15931697-159
ENSG0000(	252	6.302754	chr16:20!	MARF1		protein_c	chr16:15594387-156
ENSG0000(	252	6.302754	chr16:20!	MIR3179-2		lncRNA	chr16:16300150-163
ENSG0000(	252	6.302754	chr16:20!	XYLT1		protein_c	chr16:17101769-174

ENSG0000(	252	6.302754	chr16:209	ENSG00000280283		TEC	chr16:17961597-179
ENSG0000(	252	6.302754	chr16:209	NPIPA5		protein_c	chr16:15363628-153
ENSG0000(	252	6.302754	chr16:209	ENSG00000277920		Pseudoger	chr16:16351545-163
ENSG0000(	252	6.302754	chr16:209	NPIPA9		protein_c	chr16:16317444-163
ENSG0000(	252	6.302754	chr16:209	ENSG00000257769		lncRNA	chr16:15608474-156
ENSG0000(	252	6.302754	chr1:1522	SLC45A1		protein_c	chr1:8318114-83441
ENSG0000(	252	6.302754	chr1:1522	SNORA77		smallRNA	chr1:8511795-85119
ENSG0000(	252	6.302754	chr1:1522	ENO1-AS1		lncRNA	chr1:8878835-88798
ENSG0000(	252	6.302754	chr16:209	ENSG00000261555		lncRNA	chr16:17825252-178
ENSG0000(	252	6.302754	chr16:209	ENSG00000279729		TEC	chr16:16760905-167
ENSG0000(	252	6.302754	chr1:1522	RNU6-991P		smallRNA	chr1:8292157-82922
ENSG0000(	252	6.302754	chr16:209	ENSG00000287905		lncRNA	chr16:16612378-166
ENSG0000(	252	6.302754	chr16:209	ENSG00000261448		lncRNA	chr16:17134504-171
ENSG0000(	252	6.302754	chr1:1522	LINC01714		lncRNA	chr1:8201518-82152
ENSG0000(	252	6.302754	chr16:209	ENSG00000261362		Pseudoger	chr16:16541217-165
ENSG0000(	252	6.302754	chr1:1522	ENSG00000233645		lncRNA	chr1:8218190-82205
ENSG0000(	252	6.302754	chr16:209	NPIPA7		protein_c	chr16:16379140-163
ENSG0000(	252	6.302754	chr16:209	ENSG00000278994		TEC	chr16:18201501-182
ENSG0000(	252	6.302754	chr16:209	hsa-mir-3180-3		smallRNA	chr16:16309875-163
ENSG0000(	252	6.302754	chr16:209	PKD1P1		Pseudoger	chr16:16310341-163
ENSG0000(	252	6.302754	chr1:1522	ENSG00000228423		lncRNA	chr1:8805860-88070
ENSG0000(	252	6.302754	chr16:209	ENSG00000262332		lncRNA	chr16:16223027-162
ENSG0000(	252	6.302754	chr16:209	ABCC6		protein_c	chr16:16149565-162
ENSG0000(	252	6.302754	chr16:209	ENSG00000262171		lncRNA	chr16:15885029-158
ENSG0000(	252	6.302754	chr16:209	RPL17P40		Pseudoger	chr16:16094192-160
ENSG0000(	252	6.302754	chr16:209	ENSG00000257366		lncRNA	chr16:16308542-163
ENSG0000(	252	6.302754	chr1:1522	ENSG00000232208		Pseudoger	chr1:8907393-89077
ENSG0000(	252	6.302754	chr16:209	MIR484		smallRNA	chr16:15643294-156
ENSG0000(	252	6.302754	chr16:209	NDE1		protein_c	chr16:15643267-157
ENSG0000(	252	6.302754	chr16:209	ENSG00000279490		TEC	chr16:17082295-170
ENSG0000(	252	6.302754	chr1:1522	ENSG00000226545		Pseudoger	chr1:8798475-87995
ENSG0000(	252	6.302754	chr1:1522	HMGN2P17		Pseudoger	chr1:8893409-88941
ENSG0000(	252	6.302754	chr1:1522	RERE	AC	protein_c	chr1:8352397-88489
ENSG0000(	252	6.302754	chr16:209	TCERG1P2		Pseudoger	chr16:17094351-170
ENSG0000(	252	6.302754	chr16:209	ENSG00000262380		lncRNA	chr16:15683290-156
ENSG0000(	252	6.302754	chr16:209	RPL7P47		Pseudoger	chr16:17932720-179
ENSG0000(	252	6.302754	chr1:1522	RPL7P7		Pseudoger	chr1:8786211-87869
ENSG0000(	252	6.302754	chr16:209	Y_RNA		smallRNA	chr16:16314112-163
ENSG0000(	252	6.302754	chr1:1522	RPL23AP19		Pseudoger	chr1:8831007-88313
ENSG0000(	252	6.302754	chr1:1522	ENSG00000232663		Pseudoger	chr1:8909742-89099
ENSG0000(	252	6.302754	chr1:1522	RPL7P11		Pseudoger	chr1:8750430-87510
ENSG0000(	252	6.302754	chr16:209	ENSG00000280206		lncRNA	chr16:15701237-157
ENSG0000(	252	6.302754	chr16:209	ENSG00000262848		lncRNA	chr16:16290135-162
ENSG0000(	252	6.302754	chr16:209	RNU6-213P		smallRNA	chr16:15877163-158
ENSG0000(	249	6.227721	chr11:76(	CTR9		protein_c	chr11:10751246-108
ENSG0000(	249	6.227721	chr11:76(	MIR5691		smallRNA	chr11:9090312-9090
ENSG0000(	249	6.227721	chr11:76(	SNORA3		smallRNA	chr11:8684227-8684
ENSG0000(	249	6.227721	chr11:76(	ENSG00000271758		lncRNA	chr11:7704628-7882
ENSG0000(	249	6.227721	chr11:76(	ENSG00000275612		lncRNA	chr11:7698951-7699
ENSG0000(	249	6.227721	chr11:76(	ENSG00000286561		lncRNA	chr11:9982753-1000
ENSG0000(	249	6.227721	chr11:76(	IRAG1	NCGv7	protein_c	chr11:10573091-106

ENSG0000(	249	6.227721	chr11:76(	KRT8P41		Pseudoger	chr11:9094377-9095
ENSG0000(	249	6.227721	chr11:76(	ENSG00000289919		lncRNA	chr11:10899428-108
ENSG0000(	249	6.227721	chr11:76(	Y_RNA		smallRNA	chr11:8877417-8877
ENSG0000(	249	6.227721	chr11:76(	Y_RNA		smallRNA	chr11:10725903-107
ENSG0000(	249	6.227721	chr11:76(	ENSG00000289714		Pseudoger	chr11:7950420-7950
ENSG0000(	249	6.227721	chr11:76(	SNORA23		smallRNA	chr11:9428773-9428
ENSG0000(	249	6.227721	chr11:76(	ENSG00000239470		Pseudoger	chr11:9660438-9660
ENSG0000(	249	6.227721	chr11:76(	ZBED5		protein_c	chr11:10812074-108
ENSG0000(	249	6.227721	chr11:76(	SNORD97		smallRNA	chr11:10801467-108
ENSG0000(	249	6.227721	chr11:76(	snoU13		smallRNA	chr11:9578028-9578
ENSG0000(	249	6.227721	chr11:76(	ADM		protein_c	chr11:10305073-103
ENSG0000(	249	6.227721	chr11:76(	OR5P4P		Pseudoger	chr11:7745990-7746
ENSG0000(	249	6.227721	chr11:76(	ENSG00000286935		lncRNA	chr11:9089283-9090
ENSG0000(	249	6.227721	chr11:76(	IPO7	NCGv7	protein_c	chr11:9384652-9448
ENSG0000(	249	6.227721	chr11:76(	RN7SKP50		smallRNA	chr11:9680924-9681
ENSG0000(	249	6.227721	chr11:76(	ENSG00000289518		lncRNA	chr11:9613503-9614
ENSG0000(	249	6.227721	chr11:76(	ENSG00000254921		lncRNA	chr11:8035202-8039
ENSG0000(	249	6.227721	chr11:76(	DENND5A		protein_c	chr11:9138825-9265
ENSG0000(	249	6.227721	chr11:76(	TUB-AS1		lncRNA	chr11:8060038-8069
ENSG0000(	249	6.227721	chr11:76(	C11orf16		protein_c	chr11:8920076-8933
ENSG0000(	249	6.227721	chr11:76(	ENSG00000279391		TEC	chr11:8075573-8077
ENSG0000(	249	6.227721	chr11:76(	OR10AB1P		Pseudoger	chr11:7728400-7729
ENSG0000(	249	6.227721	chr11:76(	RNU6-943P		smallRNA	chr11:7889182-7889
ENSG0000(	249	6.227721	chr11:76(	SCARNA20		smallRNA	chr11:8555016-8555
ENSG0000(	249	6.227721	chr11:76(	IRAG1-AS1		lncRNA	chr11:10541258-105
ENSG0000(	249	6.227721	chr11:76(	RNA5SP330		Pseudoger	chr11:8845263-8845
ENSG0000(	249	6.227721	chr11:76(	ENSG00000255476		lncRNA	chr11:9839143-9929
ENSG0000(	249	6.227721	chr11:76(	ENSG00000246308		lncRNA	chr11:10809199-108
ENSG0000(	249	6.227721	chr11:76(	ENSG00000255463		Pseudoger	chr11:9631008-9631
ENSG0000(	249	6.227721	chr11:76(	SWAP70	NCGv7;AC	protein_c	chr11:9664077-9752
ENSG0000(	249	6.227721	chr11:76(	LYVE1		protein_c	chr11:10556966-106
ENSG0000(	249	6.227721	chr11:76(	AMPD3		protein_c	chr11:10308313-105
ENSG0000(	249	6.227721	chr11:76(	CASC23		lncRNA	chr11:8011278-8016
ENSG0000(	249	6.227721	chr11:76(	WEE1		protein_c	chr11:9573670-9593
ENSG0000(	249	6.227721	chr11:76(	ZBED5-AS1		lncRNA	chr11:10858179-109
ENSG0000(	249	6.227721	chr11:76(	ASCL3		protein_c	chr11:8937578-8943
ENSG0000(	249	6.227721	chr11:76(	RNU7-28P		smallRNA	chr11:9843815-9843
ENSG0000(	249	6.227721	chr11:76(	RPL7AP55		Pseudoger	chr11:7783697-7784
ENSG0000(	249	6.227721	chr11:76(	ENSG00000276668		lncRNA	chr11:7699562-7699
ENSG0000(	249	6.227721	chr11:76(	TMEM41B		protein_c	chr11:9280654-9314
ENSG0000(	249	6.227721	chr11:76(	AKIP1		protein_c	chr11:8911139-8920
ENSG0000(	249	6.227721	chr11:76(	DENND2B	NCGv7	protein_c	chr11:8693351-8910
ENSG0000(	249	6.227721	chr11:76(	RPL27A		protein_c	chr11:8682788-8714
ENSG0000(	249	6.227721	chr11:76(	TRIM66		protein_c	chr11:8612037-8682
ENSG0000(	249	6.227721	chr11:76(	OR5P1P		Pseudoger	chr11:7772890-7773
ENSG0000(	249	6.227721	chr11:76(	LMO1	NCGv7;AC	protein_c	chr11:8224309-8268
ENSG0000(	249	6.227721	chr11:76(	RIC3		protein_c	chr11:8106056-8169
ENSG0000(	249	6.227721	chr11:76(	TUB		protein_c	chr11:8019244-8106
ENSG0000(	249	6.227721	chr11:76(	CYB5R2		protein_c	chr11:7665100-7677
ENSG0000(	249	6.227721	chr11:76(	TMEM9B		protein_c	chr11:8947202-8965
ENSG0000(	249	6.227721	chr11:76(	ENSG00000250041		lncRNA	chr11:10884158-108

ENSG0000(	249	6.227721	chr11:76(	NRIP3		protein_c	chr11:8965682-9005
ENSG0000(	249	6.227721	chr11:76(	SCUBE2		protein_c	chr11:9019476-9138
ENSG0000(	249	6.227721	chr11:76(	EIF3F		protein_c	chr11:7970251-8001
ENSG0000(	249	6.227721	chr11:76(	RIC3-DT		lncRNA	chr11:8169167-8178
ENSG0000(	249	6.227721	chr11:76(	SBF2		protein_c	chr11:9776776-1030
ENSG0000(	249	6.227721	chr11:76(	ENSG00000290809		lncRNA	chr11:7726436-7729
ENSG0000(	249	6.227721	chr11:76(	STK33		protein_c	chr11:8391868-8594
ENSG0000(	249	6.227721	chr11:76(	OR5P3		protein_c	chr11:7824818-7830
ENSG0000(	249	6.227721	chr11:76(	ENSG00000255097		lncRNA	chr11:9242448-9245
ENSG0000(	249	6.227721	chr11:76(	ADM-DT		lncRNA	chr11:10302657-103
ENSG0000(	249	6.227721	chr11:76(	COX6CP5		Pseudoger	chr11:7977481-7977
ENSG0000(	249	6.227721	chr11:76(	ENSG00000254665		lncRNA	chr11:8693357-8696
ENSG0000(	249	6.227721	chr11:76(	OR5P2		protein_c	chr11:7795974-7796
ENSG0000(	249	6.227721	chr11:76(	OVCH2		protein_c	chr11:7689437-7706
ENSG0000(	249	6.227721	chr11:76(	ENSG00000254707		lncRNA	chr11:7705288-7709
ENSG0000(	249	6.227721	chr11:76(	ENSG00000254719		Pseudoger	chr11:10272052-102
ENSG0000(	249	6.227721	chr11:76(	ENSG00000254765		Pseudoger	chr11:9958744-9959
ENSG0000(	249	6.227721	chr11:76(	TMEM9B-AS1		lncRNA	chr11:8964502-8977
ENSG0000(	249	6.227721	chr11:76(	ENSG00000254865		lncRNA	chr11:10149192-101
ENSG0000(	249	6.227721	chr11:76(	PRR13P2		Pseudoger	chr11:9311284-9311
ENSG0000(	249	6.227721	chr11:76(	ENSG00000254900		lncRNA	chr11:8679089-8680
ENSG0000(	249	6.227721	chr11:76(	ENSG00000254951		lncRNA	chr11:7754393-7905
ENSG0000(	249	6.227721	chr11:76(	NLRP10		protein_c	chr11:7957537-7965
ENSG0000(	249	6.227721	chr11:76(	ZNF143	NCGv7	protein_c	chr11:9460319-9528
ENSG0000(	249	6.227721	chr11:76(	ENSG00000254397		lncRNA	chr11:9430356-9433
ENSG0000(	249	6.227721	chr11:76(	OR10A3		protein_c	chr11:7937171-7941
ENSG0000(	249	6.227721	chr11:76(	RNU6ATAC33P		smallRNA	chr11:10399192-103
ENSG0000(	249	6.227721	chr11:76(	SBF2-AS1		lncRNA	chr11:9758268-9811
ENSG0000(	249	6.227721	chr11:76(	ENSG00000268403		lncRNA	chr11:9459549-9460
ENSG0000(	249	6.227721	chr11:76(	EIF4G2		protein_c	chr11:10797050-108
ENSG0000(	249	6.227721	chr11:76(	DENND2B-AS1		lncRNA	chr11:8768778-8810
ENSG0000(	249	6.227721	chr11:76(	RNF141		protein_c	chr11:10511673-105
ENSG0000(	249	6.227721	chr11:76(	LINC02709		lncRNA	chr11:9754770-9759
ENSG0000(	249	6.227721	chr11:76(	NRIP3-DT		lncRNA	chr11:9004093-9067
ENSG0000(	249	6.227721	chr11:76(	OR5E1P		Pseudoger	chr11:7848700-7849
ENSG0000(	249	6.227721	chr11:76(	OR10A6		protein_c	chr11:7924592-7931
ENSG0000(	249	6.227721	chr11:76(	RN7SL56P		smallRNA	chr11:9580906-9581
ENSG0000(	247	6.1777	chr2:7442	snoU13		smallRNA	chr2:112271271-112
ENSG0000(	246	6.152689	chr12:583	ENSG00000256708		Pseudoger	chr12:68143318-681
ENSG0000(	246	6.152689	chr12:583	IFNG-AS1		lncRNA	chr12:67989445-682
ENSG0000(	246	6.152689	chr12:583	ENSG00000256835		Pseudoger	chr12:67978107-679
ENSG0000(	246	6.152689	chr12:583	HNRNPA1P70		Pseudoger	chr12:68035767-680
ENSG0000(	246	6.152689	chr12:583	CAND1		protein_c	chr12:67269358-673
ENSG0000(	246	6.152689	chr12:583	ENSG00000256917		Pseudoger	chr12:68487687-684
ENSG0000(	246	6.152689	chr7:330(	TRGV6		Pseudoger	chr7:38340700-3834
ENSG0000(	246	6.152689	chr12:583	IL26		protein_c	chr12:68201349-682
ENSG0000(	246	6.152689	chr12:583	IFNG	AC	protein_c	chr12:68154768-681
ENSG0000(	246	6.152689	chr12:583	MDM1	DriverDB\	protein_c	chr12:68272443-683
ENSG0000(	246	6.152689	chr12:583	ENSG00000273824		lncRNA	chr12:68426331-684
ENSG0000(	246	6.152689	chr12:583	ENSG00000235872		lncRNA	chr12:67658991-676
ENSG0000(	246	6.152689	chr12:583	IL22	NCGv7	protein_c	chr12:68248242-682

ENSG0000(	246	6.152689	chr12:583	RPL39P28		Pseudoger	chr12:68001394-680
ENSG0000(	246	6.152689	chr12:583	ENSG00000286563		lncRNA	chr12:67329014-673
ENSG0000(	246	6.152689	chr12:583	DYRK2		protein_c	chr12:67648338-676
ENSG0000(	246	6.152689	chr12:583	ENSG00000279505		TEC	chr12:67753701-677
ENSG0000(	246	6.152689	chr12:583	LINC02420		lncRNA	chr12:67440998-674
ENSG0000(	246	6.152689	chr12:583	NTAN1P3		Pseudoger	chr12:67424272-674
ENSG0000(	246	6.152689	chr12:583	MRPL40P1		Pseudoger	chr12:67351436-673
ENSG0000(	246	6.152689	chr12:583	ENSG00000256075		Pseudoger	chr12:68473741-684
ENSG0000(	246	6.152689	chr12:583	LINC02421		lncRNA	chr12:67709047-677
ENSG0000(	246	6.152689	chr12:583	LINC02442		lncRNA	chr12:67571197-675
ENSG0000(	246	6.152689	chr12:583	LINC02408		lncRNA	chr12:67443105-675
ENSG0000(	246	6.152689	chr12:583	ENSG00000256273		lncRNA	chr12:68344664-683
ENSG0000(	246	6.152689	chr12:583	LINC01479		lncRNA	chr12:67929235-679
ENSG0000(	246	6.152689	chr12:583	RPSAP12		Pseudoger	chr12:68552995-685
ENSG0000(	246	6.152689	chr12:583	LINC02384		lncRNA	chr12:68332888-684
ENSG0000(	242	6.052645	chr16:65(	VN1R65P		Pseudoger	chr16:31554105-315
ENSG0000(	241	6.027634	chr6:105(	ENSG00000282988		protein_c	chr6:26195595-2619
ENSG0000(	241	6.027634	chr6:105(	GUSBP2		Pseudoger	chr6:26878572-2688
ENSG0000(	241	6.027634	chr6:105(	H1-1		protein_c	chr6:26017032-2601
ENSG0000(	241	6.027634	chr6:105(	H2BC11	NCGv7	protein_c	chr6:27125897-2713
ENSG0000(	241	6.027634	chr6:105(	GUSBP2		lncRNA	chr6:26871484-2695
ENSG0000(	241	6.027634	chr6:105(	H4C7		protein_c	chr6:26246611-2624
ENSG0000(	241	6.027634	chr6:105(	ENSG00000283064		lncRNA	chr6:26160765-2617
ENSG0000(	241	6.027634	chr6:105(	H2AC8		protein_c	chr6:26216921-2621
ENSG0000(	241	6.027634	chr6:105(	CMAHP		Pseudoger	chr6:25061626-2545
ENSG0000(	241	6.027634	chr6:105(	H2BC12	NCGv7	protein_c	chr6:27146361-2714
ENSG0000(	241	6.027634	chr6:105(	MIR3143		smallRNA	chr6:27147626-2714
ENSG0000(	241	6.027634	chr6:105(	ENSG00000272810		lncRNA	chr6:26013241-2601
ENSG0000(	241	6.027634	chr6:105(	ENSG00000290789		lncRNA	chr6:26272021-2627
ENSG0000(	241	6.027634	chr6:105(	H3C2	NCGv7;AC	protein_c	chr6:26031589-2603
ENSG0000(	241	6.027634	chr6:105(	SLC17A1		protein_c	chr6:25782915-2583
ENSG0000(	241	6.027634	chr6:105(	H1-3	NCGv7	protein_c	chr6:26234212-2623
ENSG0000(	241	6.027634	chr6:105(	HCG11		lncRNA	chr6:26521709-2652
ENSG0000(	241	6.027634	chr6:105(	H2AC10P		Pseudoger	chr6:26272193-2627
ENSG0000(	241	6.027634	chr6:105(	LINC00240		lncRNA	chr6:26956932-2705
ENSG0000(	241	6.027634	chr6:105(	BTN2A2		protein_c	chr6:26383096-2639
ENSG0000(	241	6.027634	chr6:105(	RNU6-987P		smallRNA	chr6:25383599-2538
ENSG0000(	241	6.027634	chr6:105(	ENSG00000287050		lncRNA	chr6:26324705-2633
ENSG0000(	241	6.027634	chr6:105(	H2BC2P		Pseudoger	chr6:25731782-2573
ENSG0000(	241	6.027634	chr6:105(	U91328.20		lncRNA	chr6:25990761-2599
ENSG0000(	241	6.027634	chr6:105(	ENSG00000285571		lncRNA	chr6:26677003-2668
ENSG0000(	241	6.027634	chr6:105(	ENSG00000289447		lncRNA	chr6:26285352-2628
ENSG0000(	241	6.027634	chr6:105(	H4C5	NCGv7	protein_c	chr6:26204610-2620
ENSG0000(	241	6.027634	chr6:105(	ENSG00000286933		lncRNA	chr6:25632673-2564
ENSG0000(	241	6.027634	chr6:105(	CARMIL1	DriverDBv	protein_c	chr6:25279078-2562
ENSG0000(	241	6.027634	chr6:105(	BTN2A3P		Pseudoger	chr6:26422119-2643
ENSG0000(	241	6.027634	chr6:105(	BTN1A1		protein_c	chr6:26500303-2651
ENSG0000(	241	6.027634	chr6:105(	RNU6-502P		smallRNA	chr6:26519351-2651
ENSG0000(	241	6.027634	chr6:105(	ENSG00000217275		Pseudoger	chr6:26202156-2620
ENSG0000(	241	6.027634	chr6:105(	SLC17A3		protein_c	chr6:25833066-2588
ENSG0000(	241	6.027634	chr6:105(	SLC17A4		protein_c	chr6:25754699-2578

ENSG0000(	241	6.027634	chr6:105(	VN1R11P		Pseudoger	chr6:27083280-2708
ENSG0000(	241	6.027634	chr6:105(	H3C4	NCGv7	protein_c	chr6:26196784-2619
ENSG0000(	241	6.027634	chr6:105(	H4C9	AC	protein_c	chr6:27139282-2713
ENSG0000(	241	6.027634	chr6:105(	VN1R12P		Pseudoger	chr6:27033435-2703
ENSG0000(	241	6.027634	chr6:105(	VN1R13P		Pseudoger	chr6:27060365-2706
ENSG0000(	241	6.027634	chr6:105(	ENSG00000284607		lncRNA	chr6:26569324-2657
ENSG0000(	241	6.027634	chr6:105(	POM121L6P		Pseudoger	chr6:26896952-2689
ENSG0000(	241	6.027634	chr6:105(	H2BC5	NCGv7	protein_c	chr6:26158122-2617
ENSG0000(	241	6.027634	chr6:105(	HMGN4		protein_c	chr6:26538366-2654
ENSG0000(	241	6.027634	chr6:105(	H4C8	NCGv7	protein_c	chr6:26277609-2628
ENSG0000(	241	6.027634	chr6:105(	BTN1A1P1		Pseudoger	chr6:26477872-2647
ENSG0000(	241	6.027634	chr6:105(	H2BC1		protein_c	chr6:25726777-2572
ENSG0000(	241	6.027634	chr6:105(	H1-12P		Pseudoger	chr6:26195554-2619
ENSG0000(	241	6.027634	chr6:105(	H3C7		protein_c	chr6:26250142-2625
ENSG0000(	241	6.027634	chr6:105(	H3C9P		Pseudoger	chr6:26321876-2632
ENSG0000(	241	6.027634	chr6:105(	H2AC2P		Pseudoger	chr6:25732497-2573
ENSG0000(	241	6.027634	chr6:105(	H3C5P		Pseudoger	chr6:26219045-2621
ENSG0000(	241	6.027634	chr6:105(	ENSG00000270935		Pseudoger	chr6:27268113-2726
ENSG0000(	241	6.027634	chr6:105(	H3C6		protein_c	chr6:26224199-2622
ENSG0000(	241	6.027634	chr6:105(	H2AC4		protein_c	chr6:26033092-2603
ENSG0000(	241	6.027634	chr6:105(	H2BC10		protein_c	chr6:26272931-2627
ENSG0000(	241	6.027634	chr6:105(	H4C1		protein_c	chr6:26021649-2602
ENSG0000(	241	6.027634	chr6:105(	TRNAI2		smallRNA	chr6:27020335-2702
ENSG0000(	241	6.027634	chr6:105(	HFE		protein_c	chr6:26087281-2609
ENSG0000(	241	6.027634	chr6:105(	AL160037.1		smallRNA	chr6:25407895-2540
ENSG0000(	241	6.027634	chr6:105(	ABT1		protein_c	chr6:26596953-2660
ENSG0000(	241	6.027634	chr6:105(	H3C8	NCGv7	protein_c	chr6:26270918-2627
ENSG0000(	241	6.027634	chr6:105(	H4C3		protein_c	chr6:26103933-2610
ENSG0000(	241	6.027634	chr6:105(	H2AC6	NCGv7	protein_c	chr6:26124145-2613
ENSG0000(	241	6.027634	chr6:105(	snoU13		smallRNA	chr6:25435280-2543
ENSG0000(	241	6.027634	chr6:105(	H2BP5		Pseudoger	chr6:25885419-2588
ENSG0000(	241	6.027634	chr6:105(	H2BC4		protein_c	chr6:26114873-2612
ENSG0000(	241	6.027634	chr6:105(	H2AC7	NCGv7	protein_c	chr6:26198784-2619
ENSG0000(	241	6.027634	chr6:105(	ENSG00000290009		lncRNA	chr6:27178342-2717
ENSG0000(	241	6.027634	chr6:105(	H2AC11	NCGv7	protein_c	chr6:27133043-2713
ENSG0000(	241	6.027634	chr6:105(	H2AC12		protein_c	chr6:27147106-2714
ENSG0000(	241	6.027634	chr6:105(	ZNF322		protein_c	chr6:26634383-2665
ENSG0000(	241	6.027634	chr6:105(	H2BC6		protein_c	chr6:26172059-2618
ENSG0000(	241	6.027634	chr6:105(	H2AC1		protein_c	chr6:25723397-2572
ENSG0000(	241	6.027634	chr6:105(	SCGN		protein_c	chr6:25652201-2570
ENSG0000(	241	6.027634	chr6:105(	PRSS16		protein_c	chr6:27247701-2725
ENSG0000(	241	6.027634	chr6:105(	H2BC9	NCGv7	protein_c	chr6:26251614-2625
ENSG0000(	241	6.027634	chr6:105(	ENSG00000272558		lncRNA	chr6:25983812-2599
ENSG0000(	241	6.027634	chr6:105(	PRELID1P2		Pseudoger	chr6:25678769-2567
ENSG0000(	241	6.027634	chr6:105(	LINC02980		lncRNA	chr6:25992641-2600
ENSG0000(	241	6.027634	chr6:105(	ENSG00000272468		lncRNA	chr6:27122657-2712
ENSG0000(	241	6.027634	chr6:105(	ENSG00000290055		lncRNA	chr6:26474337-2647
ENSG0000(	241	6.027634	chr6:105(	ENSG00000289117		lncRNA	chr6:26157477-2615
ENSG0000(	241	6.027634	chr6:105(	VN1R14P		Pseudoger	chr6:26630554-2663
ENSG0000(	241	6.027634	chr6:105(	ENSG00000287966		lncRNA	chr6:27018935-2702
ENSG0000(	241	6.027634	chr6:105(	BTN3A2	NCGv7	protein_c	chr6:26365159-2637

ENSG0000(	241	6.027634	chr6:105(	ENSG00000271071		Pseudoger	chr6:26611903-2661
ENSG0000(	241	6.027634	chr6:105(	H3P26		Pseudoger	chr6:26016379-2601
ENSG0000(	241	6.027634	chr6:105(	AL021917.1		smallRNA	chr6:26365476-2636
ENSG0000(	241	6.027634	chr6:105(	LARP1P1		Pseudoger	chr6:26164072-2616
ENSG0000(	241	6.027634	chr6:105(	ENSG00000291193		lncRNA	chr6:25732433-2573
ENSG0000(	241	6.027634	chr6:105(	H3C1		protein_c	chr6:26020451-2602
ENSG0000(	241	6.027634	chr6:105(	RNU2-62P		smallRNA	chr6:27109790-2710
ENSG0000(	241	6.027634	chr6:105(	ENSG00000261584		lncRNA	chr6:26686241-2668
ENSG0000(	241	6.027634	chr6:105(	ENSG00000272312		lncRNA	chr6:27001208-2700
ENSG0000(	241	6.027634	chr6:105(	RPL10P2		Pseudoger	chr6:27211244-2721
ENSG0000(	241	6.027634	chr6:105(	H1-2	NCGv7	protein_c	chr6:26055740-2605
ENSG0000(	241	6.027634	chr6:105(	RNU6-1259P		smallRNA	chr6:26352971-2635
ENSG0000(	241	6.027634	chr6:105(	H2AC5P		Pseudoger	chr6:26043989-2604
ENSG0000(	241	6.027634	chr6:105(	H2BC3	NCGv7	protein_c	chr6:26043227-2604
ENSG0000(	241	6.027634	chr6:105(	BTN3A3		protein_c	chr6:26440472-2645
ENSG0000(	241	6.027634	chr6:105(	H1-6		protein_c	chr6:26107412-2610
ENSG0000(	241	6.027634	chr6:105(	ENSG00000275846		lncRNA	chr6:26602733-2660
ENSG0000(	241	6.027634	chr6:105(	H2AC3P		Pseudoger	chr6:25882026-2588
ENSG0000(	241	6.027634	chr6:105(	BTN3A1		protein_c	chr6:26402237-2641
ENSG0000(	241	6.027634	chr6:105(	ENSG00000290217		protein_c	chr6:25652210-2573
ENSG0000(	241	6.027634	chr6:105(	SLC17A2		protein_c	chr6:25912754-2593
ENSG0000(	241	6.027634	chr6:105(	TRIM38		protein_c	chr6:25962802-2599
ENSG0000(	241	6.027634	chr6:105(	BTN2A3P		lncRNA	chr6:26421391-2643
ENSG0000(	241	6.027634	chr6:105(	BTN2A1		protein_c	chr6:26457904-2647
ENSG0000(	239	5.977612	chr14:417	FKBP1BP1		Pseudoger	chr14:41742639-417
ENSG0000(	237	5.92759	chr3:857:	ENSG00000277143		Pseudoger	chr3:158336430-158
ENSG0000(	235	5.877568	chr8:127(	ENSG00000253135		Pseudoger	chr8:42128774-4212
ENSG0000(	234	5.852557	chr17:70:	PPIAP53		Pseudoger	chr17:15506866-155
ENSG0000(	232	5.802536	chr5:103(	IL13		protein_c	chr5:132656263-132
ENSG0000(	232	5.802536	chr5:103(	LINC02999		lncRNA	chr5:134429051-134
ENSG0000(	232	5.802536	chr5:103(	PPP2CA		protein_c	chr5:134194035-134
ENSG0000(	232	5.802536	chr5:103(	PITX1		protein_c	chr5:135027734-135
ENSG0000(	232	5.802536	chr5:103(	ENSG00000280420		TEC	chr5:134522613-134
ENSG0000(	232	5.802536	chr5:103(	ENSG00000270892		Pseudoger	chr5:134863091-134
ENSG0000(	232	5.802536	chr5:103(	ENSG00000288758		lncRNA	chr5:133239988-133
ENSG0000(	232	5.802536	chr5:103(	C5orf15		protein_c	chr5:133955510-133
ENSG0000(	232	5.802536	chr5:103(	SEC24A		protein_c	chr5:134648785-134
ENSG0000(	232	5.802536	chr5:103(	TXNDC15		protein_c	chr5:134874371-134
ENSG0000(	232	5.802536	chr5:103(	AC009009.1		smallRNA	chr5:135713119-135
ENSG0000(	232	5.802536	chr5:103(	SKP1		protein_c	chr5:134148935-134
ENSG0000(	232	5.802536	chr5:103(	ENSG00000250994		lncRNA	chr5:134436686-134
ENSG0000(	232	5.802536	chr5:103(	MACROH2A1		protein_c	chr5:135334381-135
ENSG0000(	232	5.802536	chr5:103(	AC114296.1		smallRNA	chr5:135804505-135
ENSG0000(	232	5.802536	chr5:103(	IL5		protein_c	chr5:132541445-132
ENSG0000(	232	5.802536	chr5:103(	ENSG00000231585		lncRNA	chr5:132011448-132
ENSG0000(	232	5.802536	chr5:103(	IRF1	NCGv7	protein_c	chr5:132440440-132
ENSG0000(	232	5.802536	chr5:103(	RAD50		protein_c	chr5:132556019-132
ENSG0000(	232	5.802536	chr5:103(	ENSG00000230612		lncRNA	chr5:132688681-132
ENSG0000(	232	5.802536	chr5:103(	IL4		protein_c	chr5:132673986-132
ENSG0000(	232	5.802536	chr5:103(	LINC02900		lncRNA	chr5:135236234-135
ENSG0000(	232	5.802536	chr5:103(	PITX1-AS1		lncRNA	chr5:135033280-135

ENSG0000(	232	5.802536	chr5:103(	ENSG00000249639		lncRNA	chr5:135450613-135
ENSG0000(	232	5.802536	chr5:103(	ENSG00000251243		Pseudoger	chr5:133720495-133
ENSG0000(	232	5.802536	chr5:103(	C5orf24		protein_c	chr5:134845680-134
ENSG0000(	232	5.802536	chr5:103(	ENSG00000279584		TEC	chr5:131797415-131
ENSG0000(	232	5.802536	chr5:103(	ENSG00000249478		lncRNA	chr5:133243764-133
ENSG0000(	232	5.802536	chr5:103(	ENSG00000227616		Pseudoger	chr5:132147491-132
ENSG0000(	232	5.802536	chr5:103(	Y_RNA		smallRNA	chr5:132468147-132
ENSG0000(	232	5.802536	chr5:103(	AC005195.1		smallRNA	chr5:133625845-133
ENSG0000(	232	5.802536	chr5:103(	PPP2CA-DT		lncRNA	chr5:134226410-134
ENSG0000(	232	5.802536	chr5:103(	KIF3A		protein_c	chr5:132692628-132
ENSG0000(	232	5.802536	chr5:103(	LINC01843		lncRNA	chr5:134506552-134
ENSG0000(	232	5.802536	chr5:103(	SLC25A48-AS1		lncRNA	chr5:135648584-135
ENSG0000(	232	5.802536	chr5:103(	PDLIM4		protein_c	chr5:132257696-132
ENSG0000(	232	5.802536	chr5:103(	ENSG00000280104		TEC	chr5:133457805-133
ENSG0000(	232	5.802536	chr5:103(	snoU13		smallRNA	chr5:133850857-133
ENSG0000(	232	5.802536	chr5:103(	Y_RNA		smallRNA	chr5:132945342-132
ENSG0000(	232	5.802536	chr5:103(	ENSG00000272772		protein_c	chr5:134157088-134
ENSG0000(	232	5.802536	chr5:103(	ENSG00000224015		lncRNA	chr5:132179234-132
ENSG0000(	232	5.802536	chr5:103(	ENSG00000271128		Pseudoger	chr5:134081914-134
ENSG0000(	232	5.802536	chr5:103(	RNU6-456P		smallRNA	chr5:134500178-134
ENSG0000(	232	5.802536	chr5:103(	ENSG00000243648		Pseudoger	chr5:134422838-134
ENSG0000(	232	5.802536	chr5:103(	MIR3936		smallRNA	chr5:132365490-132
ENSG0000(	232	5.802536	chr5:103(	ENSG00000223539		Pseudoger	chr5:135742462-135
ENSG0000(	232	5.802536	chr5:103(	MIR1289-2		smallRNA	chr5:133427596-133
ENSG0000(	232	5.802536	chr5:103(	ACSL6-AS1		lncRNA	chr5:132003592-132
ENSG0000(	232	5.802536	chr5:103(	ENSG00000279691		TEC	chr5:133052769-133
ENSG0000(	232	5.802536	chr5:103(	NEUROG1		protein_c	chr5:135534282-135
ENSG0000(	232	5.802536	chr5:103(	ENSG00000283782		protein_c	chr5:132410832-132
ENSG0000(	232	5.802536	chr5:103(	TH2LCRR		lncRNA	chr5:132630589-132
ENSG0000(	232	5.802536	chr5:103(	JADE2	DriverDB\	protein_c	chr5:134524312-134
ENSG0000(	232	5.802536	chr5:103(	MIR5692C1		smallRNA	chr5:135802985-135
ENSG0000(	232	5.802536	chr5:103(	ENSG00000234758		lncRNA	chr5:131944408-131
ENSG0000(	232	5.802536	chr5:103(	CAMLG		protein_c	chr5:134738495-134
ENSG0000(	232	5.802536	chr5:103(	ENSG00000270021		lncRNA	chr5:135399280-135
ENSG0000(	232	5.802536	chr5:103(	ENSG00000250167		lncRNA	chr5:135476913-135
ENSG0000(	232	5.802536	chr5:103(	RNU6-1311P		smallRNA	chr5:134634839-134
ENSG0000(	232	5.802536	chr5:103(	ENSG00000251616		lncRNA	chr5:133111055-133
ENSG0000(	232	5.802536	chr5:103(	Y_RNA		smallRNA	chr5:132993099-132
ENSG0000(	232	5.802536	chr5:103(	AFF4	NCGv7	protein_c	chr5:132875395-132
ENSG0000(	232	5.802536	chr5:103(	ENSG00000248753		lncRNA	chr5:135120526-135
ENSG0000(	232	5.802536	chr5:103(	TCF7	NCGv7	protein_c	chr5:134114681-134
ENSG0000(	232	5.802536	chr5:103(	ENSG00000279197		TEC	chr5:133574829-133
ENSG0000(	232	5.802536	chr5:103(	ENSG00000248245		lncRNA	chr5:133160438-133
ENSG0000(	232	5.802536	chr5:103(	MIR3936HG		lncRNA	chr5:132311285-132
ENSG0000(	232	5.802536	chr5:103(	CDKL3		protein_c	chr5:134286350-134
ENSG0000(	232	5.802536	chr5:103(	ENSG00000273345		protein_c	chr5:134196905-134
ENSG0000(	232	5.802536	chr5:103(	EEF1A1P50		Pseudoger	chr5:132942932-132
ENSG0000(	232	5.802536	chr5:103(	MTND5P11		Pseudoger	chr5:134924648-134
ENSG0000(	232	5.802536	chr5:103(	ENSG00000279799		TEC	chr5:134905235-134
ENSG0000(	232	5.802536	chr5:103(	AC011431.2		smallRNA	chr5:135801229-135
ENSG0000(	232	5.802536	chr5:103(	ENSG00000248648		Pseudoger	chr5:133003119-133

ENSG0000(	232	5.802536	chr5:103(	MTND4P12		Pseudoger	chr5:134926660-134
ENSG0000(	232	5.802536	chr5:103(	SHROOM1		protein_c	chr5:132822141-132
ENSG0000(	232	5.802536	chr5:103(	ENSG00000248482		lncRNA	chr5:135124380-135
ENSG0000(	232	5.802536	chr5:103(	snoZ6		smallRNA	chr5:132080066-132
ENSG0000(	232	5.802536	chr5:103(	ENSG00000248559		lncRNA	chr5:134399495-134
ENSG0000(	232	5.802536	chr5:103(	LEAP2		protein_c	chr5:132873444-132
ENSG0000(	232	5.802536	chr5:103(	UQCRQ	DriverDBv	protein_c	chr5:132866630-132
ENSG0000(	232	5.802536	chr5:103(	GDF9		protein_c	chr5:132861181-132
ENSG0000(	232	5.802536	chr5:103(	SEPTIN8		protein_c	chr5:132750819-132
ENSG0000(	232	5.802536	chr5:103(	ENSG00000287054		lncRNA	chr5:133025847-133
ENSG0000(	232	5.802536	chr5:103(	ENSG00000250244		lncRNA	chr5:133256492-133
ENSG0000(	232	5.802536	chr5:103(	IL3		protein_c	chr5:132060655-132
ENSG0000(	232	5.802536	chr5:103(	ACSL6	NCGv7	protein_c	chr5:131949973-132
ENSG0000(	232	5.802536	chr5:103(	ENSG00000235775		Pseudoger	chr5:132163603-132
ENSG0000(	232	5.802536	chr5:103(	ENSG00000273217		protein_c	chr5:131425891-131
ENSG0000(	232	5.802536	chr5:103(	CCNI2		protein_c	chr5:132747426-132
ENSG0000(	232	5.802536	chr5:103(	AFF4-DT		lncRNA	chr5:132963770-132
ENSG0000(	232	5.802536	chr5:103(	ENSG00000229738		Pseudoger	chr5:132987652-132
ENSG0000(	232	5.802536	chr5:103(	ATP6V0E1P1		Pseudoger	chr5:132948493-132
ENSG0000(	232	5.802536	chr5:103(	MTND3P25		Pseudoger	chr5:134928393-134
ENSG0000(	232	5.802536	chr5:103(	PCBD2		protein_c	chr5:134905120-135
ENSG0000(	232	5.802536	chr5:103(	IRF1-AS1		lncRNA	chr5:132410636-132
ENSG0000(	232	5.802536	chr5:103(	ENSG00000224431		Pseudoger	chr5:132199456-132
ENSG0000(	232	5.802536	chr5:103(	ENSG00000272203		lncRNA	chr5:132817248-132
ENSG0000(	232	5.802536	chr5:103(	MIR4461		smallRNA	chr5:134928039-134
ENSG0000(	232	5.802536	chr5:103(	SLC22A5	DriverDBv	protein_c	chr5:132369710-132
ENSG0000(	232	5.802536	chr5:103(	ENSG00000281938		protein_c	chr5:131807143-132
ENSG0000(	232	5.802536	chr5:103(	VDAC1		protein_c	chr5:133971871-134
ENSG0000(	232	5.802536	chr5:103(	FSTL4		protein_c	chr5:133196455-133
ENSG0000(	232	5.802536	chr5:103(	SLC22A4		protein_c	chr5:132294394-132
ENSG0000(	232	5.802536	chr5:103(	RNA5SP192		Pseudoger	chr5:132848577-132
ENSG0000(	232	5.802536	chr5:103(	MTCYBP18		Pseudoger	chr5:134923303-134
ENSG0000(	232	5.802536	chr5:103(	ENSG00000286408		lncRNA	chr5:133051065-133
ENSG0000(	232	5.802536	chr5:103(	RNU6-1164P		smallRNA	chr5:134701172-134
ENSG0000(	232	5.802536	chr5:103(	MTND6P4		Pseudoger	chr5:134924123-134
ENSG0000(	232	5.802536	chr5:103(	P4HA2-AS1		lncRNA	chr5:132184876-132
ENSG0000(	232	5.802536	chr5:103(	CDKN2AIPNL		protein_c	chr5:134402065-134
ENSG0000(	232	5.802536	chr5:103(	ENSG00000250378		lncRNA	chr5:135812667-135
ENSG0000(	232	5.802536	chr5:103(	RNU6-757P		smallRNA	chr5:134715483-134
ENSG0000(	232	5.802536	chr5:103(	SOWAHA		protein_c	chr5:132813302-132
ENSG0000(	232	5.802536	chr5:103(	ENSG00000279469		TEC	chr5:134394360-134
ENSG0000(	232	5.802536	chr5:103(	MTND4LP30		Pseudoger	chr5:134928030-134
ENSG0000(	232	5.802536	chr2:4707	ENSG00000279884		TEC	chr2:174545385-174
ENSG0000(	232	5.802536	chr5:103(	P4HA2		protein_c	chr5:132190147-132
ENSG0000(	232	5.802536	chr5:103(	CXCL14		protein_c	chr5:135570679-135
ENSG0000(	232	5.802536	chr5:103(	EPIST		lncRNA	chr5:135038831-135
ENSG0000(	232	5.802536	chr5:103(	SLC25A48		protein_c	chr5:135476913-135
ENSG0000(	232	5.802536	chr5:103(	WSPAR		lncRNA	chr5:133913677-133
ENSG0000(	232	5.802536	chr5:103(	DDX46		protein_c	chr5:134758771-134
ENSG0000(	232	5.802536	chr5:103(	ENSG00000250409		lncRNA	chr5:133387773-133
ENSG0000(	232	5.802536	chr5:103(	DCANP1		protein_c	chr5:135444214-135

ENSG0000(	232	5.802536	chr5:103(	HSPA4	NCGv7;AC	protein_c	chr5:133052013-133
ENSG0000(	232	5.802536	chr5:103(	LINC02863		lncRNA	chr5:132419416-132
ENSG0000(	232	5.802536	chr5:103(	CSF2		protein_c	chr5:132073789-132
ENSG0000(	232	5.802536	chr5:103(	ENSG00000239615		Pseudoger	chr5:134267421-134
ENSG0000(	232	5.802536	chr5:103(	TIFAB		protein_c	chr5:135444226-135
ENSG0000(	232	5.802536	chr5:103(	ZCCHC10		protein_c	chr5:132996985-133
ENSG0000(	232	5.802536	chr5:103(	UBE2B		protein_c	chr5:134371184-134
ENSG0000(	232	5.802536	chr5:103(	CATSPER3		protein_c	chr5:134967907-135
ENSG0000(	232	5.802536	chr5:103(	ENSG00000271737		lncRNA	chr5:134004834-134
ENSG0000(	232	5.802536	chr5:103(	RPL6P15		Pseudoger	chr5:133172218-133
ENSG0000(	232	5.802536	chr5:103(	SAR1B		protein_c	chr5:134601149-134
ENSG0000(	232	5.802536	chr5:103(	MIR3661		smallRNA	chr5:134225757-134
ENSG0000(	232	5.802536	chr5:103(	FNIP1		protein_c	chr5:131641714-131
ENSG0000(	232	5.802536	chr5:103(	ENSG00000241179		Pseudoger	chr5:134804778-134
ENSG0000(	232	5.802536	chr5:103(	RN7SL541P		smallRNA	chr5:134517810-134
ENSG0000(	232	5.802536	chr5:103(	MEIKIN		protein_c	chr5:131806990-131
ENSG0000(	232	5.802536	chr5:103(	ENSG00000277619		lncRNA	chr5:135034521-135
ENSG0000(	229	5.727503	chr22:231	ENSG00000214093		Pseudoger	chr22:31559483-315
ENSG0000(	229	5.727503	chr18:31(	MIR4529		smallRNA	chr18:55479221-554
ENSG0000(	227	5.677481	chr3:857(	PLSCR4	NCGv7	protein_c	chr3:146192335-146
ENSG0000(	227	5.677481	chr3:857(	U3		smallRNA	chr3:146953959-146
ENSG0000(	227	5.677481	chr3:857(	RPL21P71		Pseudoger	chr3:147276768-147
ENSG0000(	227	5.677481	chr3:857(	SLC9A9-AS2		lncRNA	chr3:143381338-143
ENSG0000(	227	5.677481	chr3:857(	ENSG00000243818		lncRNA	chr3:142912116-142
ENSG0000(	227	5.677481	chr3:857(	ENSG00000243822		Pseudoger	chr3:149357169-149
ENSG0000(	227	5.677481	chr3:857(	ENSG00000240521		lncRNA	chr3:148850933-148
ENSG0000(	227	5.677481	chr3:857(	MED28P2		Pseudoger	chr3:149099499-149
ENSG0000(	227	5.677481	chr3:857(	ENSG00000240888		lncRNA	chr3:143313067-143
ENSG0000(	227	5.677481	chr3:857(	ENSG00000244468		lncRNA	chr3:149284782-149
ENSG0000(	227	5.677481	chr3:857(	PAQR9		protein_c	chr3:142949164-142
ENSG0000(	227	5.677481	chr3:857(	ENSG00000241358		Pseudoger	chr3:146391363-146
ENSG0000(	227	5.677481	chr3:857(	HLTF-AS1		lncRNA	chr3:149086332-149
ENSG0000(	227	5.677481	chr3:857(	ZIC4	NCGv7	protein_c	chr3:147386046-147
ENSG0000(	227	5.677481	chr3:857(	PLOD2		protein_c	chr3:146035139-146
ENSG0000(	227	5.677481	chr3:857(	LNCSRLR		lncRNA	chr3:146066344-146
ENSG0000(	227	5.677481	chr3:857(	CPHL1P		Pseudoger	chr3:149229356-149
ENSG0000(	227	5.677481	chr3:857(	GAPDHP47		Pseudoger	chr3:143503275-143
ENSG0000(	227	5.677481	chr3:857(	ENSG00000279320		TEC	chr3:146504570-146
ENSG0000(	227	5.677481	chr3:857(	CHST2		protein_c	chr3:143119771-143
ENSG0000(	227	5.677481	chr3:857(	ENSG00000240095		lncRNA	chr3:145961755-145
ENSG0000(	227	5.677481	chr3:857(	ENSG00000268129		lncRNA	chr3:143000907-143
ENSG0000(	227	5.677481	chr3:857(	ZIC1	NCGv7	protein_c	chr3:147393422-147
ENSG0000(	227	5.677481	chr3:857(	U2SURP	NCGv7	protein_c	chr3:142964497-143
ENSG0000(	227	5.677481	chr3:857(	ENSG00000239661		lncRNA	chr3:147844123-147
ENSG0000(	227	5.677481	chr3:857(	RNA5SP144		Pseudoger	chr3:144186905-144
ENSG0000(	227	5.677481	chr3:857(	ENSG00000243635		Pseudoger	chr3:104502700-104
ENSG0000(	227	5.677481	chr3:857(	ENSG00000243620		lncRNA	chr3:146909685-147
ENSG0000(	227	5.677481	chr3:857(	HLTF	NCGv7	protein_c	chr3:149030127-149
ENSG0000(	227	5.677481	chr3:857(	ENSG00000243596		Pseudoger	chr3:146342996-146
ENSG0000(	227	5.677481	chr3:857(	ENSG00000240950		Pseudoger	chr3:142827096-142
ENSG0000(	227	5.677481	chr3:857(	TM4SF18	NCGv7	protein_c	chr3:149318498-149

ENSG0000(	227	5.677481	chr3:857:	HPS3		protein_c	chr3:149129638-149
ENSG0000(	227	5.677481	chr3:857:	GYG1		protein_c	chr3:148991408-149
ENSG0000(	227	5.677481	chr3:857:	CPA3		protein_c	chr3:148865296-148
ENSG0000(	227	5.677481	chr3:857:	PLSCR2	NCGv7	protein_c	chr3:146391421-146
ENSG0000(	227	5.677481	chr3:857:	PCOLCE2	DriverDB\	protein_c	chr3:142815922-142
ENSG0000(	227	5.677481	chr3:857:	ENSG00000289451		lncRNA	chr3:147688945-147
ENSG0000(	227	5.677481	chr3:857:	ENSG00000244650		lncRNA	chr3:148160911-148
ENSG0000(	227	5.677481	chr3:857:	CPB1		protein_c	chr3:148791102-148
ENSG0000(	227	5.677481	chr3:857:	ENSG00000235110		Pseudoger	chr3:143855739-143
ENSG0000(	227	5.677481	chr3:857:	PLSCR5-AS1		lncRNA	chr3:146589602-146
ENSG0000(	227	5.677481	chr3:857:	ENSG00000243733		lncRNA	chr3:143111802-143
ENSG0000(	227	5.677481	chr3:857:	ENSG00000243415		lncRNA	chr3:146064042-146
ENSG0000(	227	5.677481	chr3:857:	PLSCR5		protein_c	chr3:146576555-146
ENSG0000(	227	5.677481	chr3:857:	ENSG00000248374		Pseudoger	chr3:143620528-143
ENSG0000(	227	5.677481	chr3:857:	LINC02045		lncRNA	chr3:148192872-148
ENSG0000(	227	5.677481	chr3:857:	ST13P15		Pseudoger	chr3:143517123-143
ENSG0000(	227	5.677481	chr3:857:	ENSG00000285660		lncRNA	chr3:148090341-148
ENSG0000(	227	5.677481	chr3:857:	RPL21P39		Pseudoger	chr3:145824257-145
ENSG0000(	227	5.677481	chr3:857:	Y_RNA		smallRNA	chr3:149094353-149
ENSG0000(	227	5.677481	chr3:857:	SLC9A9-AS1		lncRNA	chr3:143342246-143
ENSG0000(	227	5.677481	chr3:857:	ENSG00000244358		lncRNA	chr3:145939912-145
ENSG0000(	227	5.677481	chr3:857:	ENSG00000241592		Pseudoger	chr3:142960372-142
ENSG0000(	227	5.677481	chr3:857:	PLSCR1		protein_c	chr3:146515180-146
ENSG0000(	227	5.677481	chr3:857:	HNRNPA1P20		Pseudoger	chr3:148166344-148
ENSG0000(	227	5.677481	chr3:857:	PAQR9-AS1		lncRNA	chr3:142960650-143
ENSG0000(	227	5.677481	chr3:857:	RNU6-428P		smallRNA	chr3:146571477-146
ENSG0000(	227	5.677481	chr3:857:	DIPK2A		protein_c	chr3:143971823-144
ENSG0000(	227	5.677481	chr3:857:	AGTR1		protein_c	chr3:148697784-148
ENSG0000(	227	5.677481	chr3:857:	ENSG00000289986		lncRNA	chr3:144350307-144
ENSG0000(	227	5.677481	chr3:857:	RNU6-505P		smallRNA	chr3:146996607-146
ENSG0000(	227	5.677481	chr3:857:	ENSG00000286714		lncRNA	chr3:149224138-149
ENSG0000(	227	5.677481	chr3:857:	LINC02032		lncRNA	chr3:148076432-148
ENSG0000(	227	5.677481	chr3:857:	LINC02046		lncRNA	chr3:148279682-148
ENSG0000(	227	5.677481	chr3:857:	ENSG00000280397		TEC	chr3:144442211-144
ENSG0000(	227	5.677481	chr3:857:	AC092925.1		smallRNA	chr3:147911406-147
ENSG0000(	227	5.677481	chr3:857:	LARP7P4		Pseudoger	chr3:145523538-145
ENSG0000(	227	5.677481	chr3:857:	GM2AP1		Pseudoger	chr3:145684572-145
ENSG0000(	227	5.677481	chr3:857:	RPL38P1		Pseudoger	chr3:148695994-148
ENSG0000(	227	5.677481	chr3:857:	ENSG00000241679		lncRNA	chr3:143123362-143
ENSG0000(	227	5.677481	chr3:857:	ENSG00000287198		lncRNA	chr3:148922703-148
ENSG0000(	227	5.677481	chr3:857:	PBX2P1		Pseudoger	chr3:143176327-143
ENSG0000(	227	5.677481	chr3:857:	NPM1P28		Pseudoger	chr3:147590996-147
ENSG0000(	227	5.677481	chr3:857:	UBQLN4P1		Pseudoger	chr3:148985868-148
ENSG0000(	227	5.677481	chr3:857:	HMGB1P30		Pseudoger	chr3:148824782-148
ENSG0000(	227	5.677481	chr3:857:	ENSG00000239922		lncRNA	chr3:147939905-148
ENSG0000(	227	5.677481	chr3:857:	ENSG00000261051		lncRNA	chr3:146059585-146
ENSG0000(	227	5.677481	chr3:857:	AC018450.1		smallRNA	chr3:143165648-143
ENSG0000(	227	5.677481	chr3:857:	CP		protein_c	chr3:149162410-149
ENSG0000(	227	5.677481	chr3:857:	ENSG00000285798		lncRNA	chr3:148028046-148
ENSG0000(	227	5.677481	chr3:857:	ENSG00000241792		lncRNA	chr3:148093175-148
ENSG0000(	227	5.677481	chr3:857:	ENSG00000285557		lncRNA	chr3:148368384-148

ENSG0000(	227	5.677481	chr3:857:	AC092958.1		smallRNA	chr3:148080833-148
ENSG0000(	227	5.677481	chr3:857:	ZIC4-AS1		lncRNA	chr3:147386967-147
ENSG0000(	227	5.677481	chr3:857:	LINC02010		lncRNA	chr3:146921795-146
ENSG0000(	227	5.677481	chr3:857:	SLC9A9		protein_c	chr3:143265222-143
ENSG0000(	226	5.65247	chr7:330(	ENSG00000234457		Pseudoger	chr7:36305631-3630
ENSG0000(	225	5.627459	chr16:20!	SMG1P3		Pseudoger	chr16:21447078-215
ENSG0000(	225	5.627459	chr16:20!	ENSG00000290192		lncRNA	chr16:21385801-214
ENSG0000(	225	5.627459	chr16:20!	ENSG00000276548		Pseudoger	chr16:21401089-214
ENSG0000(	225	5.627459	chr16:20!	snoU13		smallRNA	chr16:21457326-214
ENSG0000(	225	5.627459	chr16:20!	ENSG00000291073		lncRNA	chr16:21446683-215
ENSG0000(	225	5.627459	chr16:20!	NPIPB3		protein_c	chr16:21402237-214
ENSG0000(	225	5.627459	chr16:20!	SNX29P1		Pseudoger	chr16:21376648-213
ENSG0000(	224	5.602448	chr2:744:	ENSG00000230958		Pseudoger	chr2:111865798-111
ENSG0000(	224	5.602448	chr2:744:	ENSG00000272861		lncRNA	chr2:105249404-105
ENSG0000(	224	5.602448	chr2:744:	LINC02946		lncRNA	chr2:105600703-105
ENSG0000(	224	5.602448	chr2:744:	MTLN		protein_c	chr2:110211529-110
ENSG0000(	224	5.602448	chr2:744:	UXS1		protein_c	chr2:106093308-106
ENSG0000(	224	5.602448	chr2:744:	ENSG00000290732		lncRNA	chr2:110231106-110
ENSG0000(	224	5.602448	chr2:744:	ENSG00000236555		Pseudoger	chr2:112346011-112
ENSG0000(	224	5.602448	chr2:744:	GCC2	NCGv7	protein_c	chr2:108449107-108
ENSG0000(	224	5.602448	chr2:744:	ENSG00000227992		Pseudoger	chr2:111203964-111
ENSG0000(	224	5.602448	chr2:744:	ENSG00000226905		Pseudoger	chr2:106963193-106
ENSG0000(	224	5.602448	chr2:744:	ENSG00000235319		lncRNA	chr2:105324210-105
ENSG0000(	224	5.602448	chr2:744:	SEPTIN10		protein_c	chr2:109542799-109
ENSG0000(	224	5.602448	chr2:744:	ENSG00000286904		lncRNA	chr2:112039378-112
ENSG0000(	224	5.602448	chr2:744:	ZC3H6		protein_c	chr2:112275597-112
ENSG0000(	224	5.602448	chr2:744:	ENSG00000261760		Pseudoger	chr2:110245756-110
ENSG0000(	224	5.602448	chr2:744:	DBF4P2		Pseudoger	chr2:111558413-111
ENSG0000(	224	5.602448	chr2:744:	NDUFB4P6		Pseudoger	chr2:112286057-112
ENSG0000(	224	5.602448	chr2:744:	GACAT1		lncRNA	chr2:107754112-107
ENSG0000(	224	5.602448	chr2:744:	CHCHD5		protein_c	chr2:112584240-112
ENSG0000(	224	5.602448	chr2:744:	ENSG00000229682		Pseudoger	chr2:106368209-106
ENSG0000(	224	5.602448	chr2:744:	ENSG00000229730		Pseudoger	chr2:110012265-110
ENSG0000(	224	5.602448	chr2:744:	ENSG00000290731		lncRNA	chr2:109933161-109
ENSG0000(	224	5.602448	chr2:744:	MIR4436B2		smallRNA	chr2:110284853-110
ENSG0000(	224	5.602448	chr2:744:	CD8B2		protein_c	chr2:106487364-106
ENSG0000(	224	5.602448	chr2:744:	TGFBRAP1		protein_c	chr2:105264391-105
ENSG0000(	224	5.602448	chr2:744:	RTRAFP1		Pseudoger	chr2:111940302-111
ENSG0000(	224	5.602448	chr2:744:	LINC01106		lncRNA	chr2:110375138-110
ENSG0000(	224	5.602448	chr2:744:	LINC01886		lncRNA	chr2:107529292-107
ENSG0000(	224	5.602448	chr2:744:	RGPD4		protein_c	chr2:107826892-107
ENSG0000(	224	5.602448	chr2:744:	FHL2	AC	protein_c	chr2:105357712-105
ENSG0000(	224	5.602448	chr2:744:	ENSG00000290733		lncRNA	chr2:110402914-110
ENSG0000(	224	5.602448	chr2:744:	ENSG00000184115		Pseudoger	chr2:110397406-110
ENSG0000(	224	5.602448	chr2:744:	EDAR	NCGv7	protein_c	chr2:108894471-108
ENSG0000(	224	5.602448	chr2:744:	GPAA1P1		Pseudoger	chr2:109984058-109
ENSG0000(	224	5.602448	chr2:744:	SLC20A1-DT		lncRNA	chr2:112641832-112
ENSG0000(	224	5.602448	chr2:744:	POLR1B		protein_c	chr2:112541915-112
ENSG0000(	224	5.602448	chr2:744:	ENSG00000291125		lncRNA	chr2:106382110-106
ENSG0000(	224	5.602448	chr2:744:	RPS21P2		Pseudoger	chr2:106288803-106
ENSG0000(	224	5.602448	chr2:744:	ENSG00000286769		lncRNA	chr2:107698737-107

ENSG0000(	224	5.602448	chr2:744	C2orf49-DT	lncRNA	chr2:105333935-105
ENSG0000(	224	5.602448	chr2:744	SULT1C2P1	lncRNA	chr2:108322238-108
ENSG0000(	224	5.602448	chr2:744	LIMS4	protein_c	chr2:110445883-110
ENSG0000(	224	5.602448	chr2:744	LIMS3	protein_c	chr2:109898428-109
ENSG0000(	224	5.602448	chr2:744	MIR4435-2HG	lncRNA	chr2:111006015-111
ENSG0000(	224	5.602448	chr2:744	SRSF3P4	Pseudoger	chr2:106253231-106
ENSG0000(	224	5.602448	chr2:744	RPL22P11	Pseudoger	chr2:109737139-109
ENSG0000(	224	5.602448	chr2:744	LINC01885	lncRNA	chr2:107382715-107
ENSG0000(	224	5.602448	chr2:744	RPL37P12	Pseudoger	chr2:109552536-109
ENSG0000(	224	5.602448	chr2:744	RPL22P10	Pseudoger	chr2:106247369-106
ENSG0000(	224	5.602448	chr2:744	RPL22P8	Pseudoger	chr2:107914948-107
ENSG0000(	224	5.602448	chr2:744	CENPNP2	Pseudoger	chr2:111742586-111
ENSG0000(	224	5.602448	chr2:744	SULT1C2P2	Pseudoger	chr2:108317725-108
ENSG0000(	224	5.602448	chr2:744	ZBTB45P1	Pseudoger	chr2:109986939-109
ENSG0000(	224	5.602448	chr2:744	ENSG00000270190	lncRNA	chr2:111266868-111
ENSG0000(	224	5.602448	chr2:744	SMIM12P1	Pseudoger	chr2:108423612-108
ENSG0000(	224	5.602448	chr2:744	ENSG00000234162	lncRNA	chr2:105874509-105
ENSG0000(	224	5.602448	chr2:744	AC140479.1	smallRNA	chr2:110301312-110
ENSG0000(	224	5.602448	chr2:744	SNRPGP9	Pseudoger	chr2:109251422-109
ENSG0000(	224	5.602448	chr2:744	ENSG00000286545	lncRNA	chr2:112239621-112
ENSG0000(	224	5.602448	chr2:744	ENSG00000273471	lncRNA	chr2:110386411-110
ENSG0000(	224	5.602448	chr2:744	SULT1C4	protein_c	chr2:108377911-108
ENSG0000(	224	5.602448	chr2:744	SULT1C2	protein_c	chr2:108288639-108
ENSG0000(	224	5.602448	chr2:744	ENSG00000273595	Pseudoger	chr2:106207394-106
ENSG0000(	224	5.602448	chr2:744	ENSG00000226991	Pseudoger	chr2:110358790-110
ENSG0000(	224	5.602448	chr2:744	SOWAHC	protein_c	chr2:109614364-109
ENSG0000(	224	5.602448	chr2:744	ENSG00000237666	Pseudoger	chr2:106470799-106
ENSG0000(	224	5.602448	chr2:744	SULT1C2P1	Pseudoger	chr2:108322353-108
ENSG0000(	224	5.602448	chr2:744	ENSG00000233648	Pseudoger	chr2:108694750-108
ENSG0000(	224	5.602448	chr2:744	ENSG00000230499	lncRNA	chr2:111195963-111
ENSG0000(	224	5.602448	chr2:744	ENSG00000228251	lncRNA	chr2:112590796-112
ENSG0000(	224	5.602448	chr2:744	LINC01123	lncRNA	chr2:109987063-109
ENSG0000(	224	5.602448	chr2:744	SRSF3P6	Pseudoger	chr2:109732731-109
ENSG0000(	224	5.602448	chr2:744	ENSG00000230650	Pseudoger	chr2:110360608-110
ENSG0000(	224	5.602448	chr2:744	RGPD4-AS1	lncRNA	chr2:107823063-107
ENSG0000(	224	5.602448	chr2:744	ACOXL-AS1	lncRNA	chr2:111098345-111
ENSG0000(	224	5.602448	chr2:744	ST6GAL2-IT1	lncRNA	chr2:106822923-106
ENSG0000(	224	5.602448	chr2:744	ENSG00000283283	Pseudoger	chr2:110022447-110
ENSG0000(	224	5.602448	chr2:744	ENSG00000238273	lncRNA	chr2:105363038-105
ENSG0000(	224	5.602448	chr2:744	ENSG00000289202	lncRNA	chr2:110678205-110
ENSG0000(	224	5.602448	chr2:744	ENSG00000227294	lncRNA	chr2:106834464-106
ENSG0000(	224	5.602448	chr2:744	PLGLA	Pseudoger	chr2:106382171-106
ENSG0000(	224	5.602448	chr2:744	GMCL1P2	Pseudoger	chr2:108367116-108
ENSG0000(	224	5.602448	chr2:744	ENSG00000230696	lncRNA	chr2:109594693-109
ENSG0000(	224	5.602448	chr9:197	snoU13	smallRNA	chr9:130449854-130
ENSG0000(	224	5.602448	chr2:744	AC013268.1	smallRNA	chr2:110126732-110
ENSG0000(	224	5.602448	chr2:744	LINC01594	lncRNA	chr2:108167125-108
ENSG0000(	224	5.602448	chr2:744	PAFAH1B1P2	Pseudoger	chr2:111383752-111
ENSG0000(	224	5.602448	chr2:744	ENSG00000283167	Pseudoger	chr2:110321402-110
ENSG0000(	224	5.602448	chr2:744	RPS14P4	Pseudoger	chr2:111295629-111
ENSG0000(	224	5.602448	chr2:744	SETD6P1	Pseudoger	chr2:108226015-108

ENSG0000(	224	5.602448	chr2:744	ENSG00000186148		Pseudoger	chr2:109947672-109
ENSG0000(	224	5.602448	chr2:744	ENSG00000233494		Pseudoger	chr2:107902997-107
ENSG0000(	224	5.602448	chr2:744	LIMS1-AS1		lncRNA	chr2:108676795-108
ENSG0000(	224	5.602448	chr2:744	ACTP1		Pseudoger	chr2:108099110-108
ENSG0000(	224	5.602448	chr2:744	ENSG00000227270		Pseudoger	chr2:107903964-107
ENSG0000(	224	5.602448	chr2:744	GPAA1P2		Pseudoger	chr2:110386377-110
ENSG0000(	224	5.602448	chr2:744	ENSG00000233339		lncRNA	chr2:106179542-106
ENSG0000(	224	5.602448	chr2:744	ENSG00000280878		lncRNA	chr2:111468810-111
ENSG0000(	224	5.602448	chr2:744	ENSG00000230603		Pseudoger	chr2:109665209-109
ENSG0000(	224	5.602448	chr2:744	NCK2		protein_c	chr2:105744912-105
ENSG0000(	224	5.602448	chr2:744	SH3RF3-AS1		lncRNA	chr2:109127327-109
ENSG0000(	224	5.602448	chr2:744	MIR4267		smallRNA	chr2:110069961-110
ENSG0000(	224	5.602448	chr2:744	ACTR1AP1		Pseudoger	chr2:110184054-110
ENSG0000(	224	5.602448	chr2:744	GCC2-AS1		lncRNA	chr2:108507515-108
ENSG0000(	224	5.602448	chr2:744	AC108868.1		protein_c	chr2:106483272-106
ENSG0000(	224	5.602448	chr2:744	SRSF3P5		Pseudoger	chr2:107920763-107
ENSG0000(	224	5.602448	chr2:744	ENSG00000284337		protein_c	chr2:109898432-109
ENSG0000(	224	5.602448	chr2:744	ENSG00000232001		lncRNA	chr2:106521563-106
ENSG0000(	224	5.602448	chr2:744	ACOXL	NCGv7	protein_c	chr2:110732539-111
ENSG0000(	224	5.602448	chr2:744	MIR4771-1		smallRNA	chr2:111771061-111
ENSG0000(	224	5.602448	chr2:744	RPL10P5		Pseudoger	chr2:108712262-108
ENSG0000(	224	5.602448	chr2:744	ZC3H8		protein_c	chr2:112211529-112
ENSG0000(	224	5.602448	chr2:744	LINC01789		lncRNA	chr2:107254691-107
ENSG0000(	224	5.602448	chr2:744	ENSG00000287141		lncRNA	chr2:112061084-112
ENSG0000(	224	5.602448	chr2:744	FBLN7		protein_c	chr2:112138385-112
ENSG0000(	224	5.602448	chr2:744	LINC01593		lncRNA	chr2:108049200-108
ENSG0000(	224	5.602448	chr2:744	ENSG00000243389		lncRNA	chr2:112589040-112
ENSG0000(	224	5.602448	chr2:744	RANBP2	NCGv7	protein_c	chr2:108719482-108
ENSG0000(	224	5.602448	chr2:744	MERTK	TAG;AC	protein_c	chr2:111898607-112
ENSG0000(	224	5.602448	chr2:744	SLC20A1		protein_c	chr2:112645939-112
ENSG0000(	224	5.602448	chr2:744	ENSG00000287308		lncRNA	chr2:105601146-105
ENSG0000(	224	5.602448	chr2:744	CCDC138		protein_c	chr2:108786757-108
ENSG0000(	224	5.602448	chr2:744	SLC30A6P1		Pseudoger	chr2:111952144-111
ENSG0000(	224	5.602448	chr2:744	ENSG00000271590		lncRNA	chr2:111210995-111
ENSG0000(	224	5.602448	chr2:744	ENSG00000290119		lncRNA	chr2:105790035-105
ENSG0000(	224	5.602448	chr2:744	ENSG00000229118		lncRNA	chr2:111265283-111
ENSG0000(	224	5.602448	chr2:744	ENSG00000235522		lncRNA	chr2:105846534-105
ENSG0000(	224	5.602448	chr2:744	ENSG00000225744		lncRNA	chr2:111607841-111
ENSG0000(	224	5.602448	chr2:744	ANAPC1P6		Pseudoger	chr2:106369724-106
ENSG0000(	224	5.602448	chr2:744	SLC5A7		protein_c	chr2:107986523-108
ENSG0000(	224	5.602448	chr2:744	RPL22P12		Pseudoger	chr2:110633947-110
ENSG0000(	224	5.602448	chr2:744	MALL		protein_c	chr2:110083870-110
ENSG0000(	224	5.602448	chr2:744	TMEM87B		protein_c	chr2:112055269-112
ENSG0000(	224	5.602448	chr2:744	BMS1P19		Pseudoger	chr2:109667128-109
ENSG0000(	224	5.602448	chr2:744	EEF1E1P1		Pseudoger	chr2:111887914-111
ENSG0000(	224	5.602448	chr2:744	RGPD6		protein_c	chr2:110513802-110
ENSG0000(	224	5.602448	chr2:744	RGPD3	NCGv7	protein_c	chr2:106391290-106
ENSG0000(	224	5.602448	chr2:744	SULT1C3		protein_c	chr2:108239968-108
ENSG0000(	224	5.602448	chr2:744	ILRUNP1		Pseudoger	chr2:106344566-106
ENSG0000(	224	5.602448	chr2:744	ENSG00000231505		lncRNA	chr2:106521903-106
ENSG0000(	224	5.602448	chr2:744	MIR4436B1		smallRNA	chr2:110086433-110

ENSG0000(	224	5.602448	chr2:744	ENSG00000257207	protein_c	chr2:110402934-110
ENSG0000(	224	5.602448	chr2:744	ENSG00000231536	lncRNA	chr2:110610916-110
ENSG0000(	224	5.602448	chr2:744	BUB1	protein_c	chr2:110637528-110
ENSG0000(	224	5.602448	chr2:744	LIMS1	protein_c	chr2:108533671-108
ENSG0000(	224	5.602448	chr2:744	RPL39P16	Pseudoger	chr2:108878308-108
ENSG0000(	224	5.602448	chr2:744	ECRG4	protein_c	chr2:106063246-106
ENSG0000(	224	5.602448	chr2:744	RGPD8	protein_c	chr2:112368369-112
ENSG0000(	224	5.602448	chr2:744	ENSG00000231583	Pseudoger	chr2:110500100-110
ENSG0000(	224	5.602448	chr2:744	ENSG00000235881	lncRNA	chr2:110709575-110
ENSG0000(	224	5.602448	chr2:744	BCL2L11 NCGv7	protein_c	chr2:111119378-111
ENSG0000(	224	5.602448	chr2:744	ZBTB45P2	Pseudoger	chr2:110383112-110
ENSG0000(	224	5.602448	chr2:744	MIR4265	smallRNA	chr2:109141490-109
ENSG0000(	224	5.602448	chr2:744	ENSG00000282304	lncRNA	chr2:110245591-110
ENSG0000(	224	5.602448	chr2:744	ENSG00000285016	lncRNA	chr2:111429324-111
ENSG0000(	224	5.602448	chr2:744	ENSG00000282033	lncRNA	chr2:109758799-109
ENSG0000(	224	5.602448	chr2:744	TTL AC	protein_c	chr2:112482156-112
ENSG0000(	224	5.602448	chr2:744	MIR4435-2	smallRNA	chr2:111321012-111
ENSG0000(	224	5.602448	chr2:744	ENSG00000231747	Pseudoger	chr2:112621809-112
ENSG0000(	224	5.602448	chr2:744	PPP1R2P5	Pseudoger	chr2:106940880-106
ENSG0000(	224	5.602448	chr2:744	RGPD5	protein_c	chr2:109792758-109
ENSG0000(	224	5.602448	chr2:744	ANAPC1	protein_c	chr2:111611639-111
ENSG0000(	224	5.602448	chr2:744	ENSG00000286218	lncRNA	chr2:106933739-106
ENSG0000(	224	5.602448	chr2:744	ENSG00000227574	lncRNA	chr2:110449242-110
ENSG0000(	224	5.602448	chr2:744	SOCAR	lncRNA	chr2:111491272-111
ENSG0000(	224	5.602448	chr2:744	ENSG00000235721	Pseudoger	chr2:110007675-110
ENSG0000(	224	5.602448	chr2:744	WASF1P1	Pseudoger	chr2:108276812-108
ENSG0000(	224	5.602448	chr2:744	MIR4266	smallRNA	chr2:109313571-109
ENSG0000(	224	5.602448	chr2:744	AC012360.1	protein_c	chr2:105251302-105
ENSG0000(	224	5.602448	chr2:744	ST6GAL2 NCGv7	protein_c	chr2:106801600-106
ENSG0000(	224	5.602448	chr2:744	ENSG00000225588	lncRNA	chr2:107362282-107
ENSG0000(	224	5.602448	chr2:744	ENSG00000227842	Pseudoger	chr2:111717508-111
ENSG0000(	224	5.602448	chr2:744	MALLP1	Pseudoger	chr2:110279606-110
ENSG0000(	224	5.602448	chr2:744	Y_RNA	smallRNA	chr2:112579484-112
ENSG0000(	224	5.602448	chr2:744	LINC01918	lncRNA	chr2:105144113-105
ENSG0000(	224	5.602448	chr2:744	GPR45	protein_c	chr2:105241743-105
ENSG0000(	224	5.602448	chr2:744	ENSG00000283684	lncRNA	chr2:109937438-109
ENSG0000(	224	5.602448	chr2:744	EEF1A1P12	Pseudoger	chr2:106697331-106
ENSG0000(	224	5.602448	chr2:744	ENSG00000278962	TEC	chr2:112188364-112
ENSG0000(	224	5.602448	chr2:744	RPL5P9	Pseudoger	chr2:110967834-110
ENSG0000(	224	5.602448	chr2:744	VINAC1P	Pseudoger	chr2:112439312-112
ENSG0000(	224	5.602448	chr2:744	C2orf49	protein_c	chr2:105337532-105
ENSG0000(	224	5.602448	chr2:744	NPHP1	protein_c	chr2:110122311-110
ENSG0000(	221	5.527415	chr16:65(	ABHD17AP8	Pseudoger	chr16:32199902-322
ENSG0000(	221	5.527415	chr16:65(	PHB1P21	Pseudoger	chr16:52934813-529
ENSG0000(	221	5.527415	chr5:103(	RNU6-460P	smallRNA	chr5:138116573-138
ENSG0000(	221	5.527415	chr5:103(	ENSG00000289267	lncRNA	chr5:138463990-138
ENSG0000(	221	5.527415	chr16:65(	ENSG00000280454	Pseudoger	chr16:54114697-541
ENSG0000(	221	5.527415	chr16:65(	ENSG00000260312	lncRNA	chr16:33857935-338
ENSG0000(	221	5.527415	chr5:103(	RN7SKP246	smallRNA	chr5:144535354-144
ENSG0000(	221	5.527415	chr16:65(	ENSG00000260311	lncRNA	chr16:32663925-326
ENSG0000(	221	5.527415	chr16:65(	RPL23AP72	Pseudoger	chr16:47150132-471

ENSG0000(	221	5.527415	chr16:65(	CASC22		lncRNA	chr16:52258564-522
ENSG0000(	221	5.527415	chr16:65(	YPEL3		protein_c	chr16:30092314-300
ENSG0000(	221	5.527415	chr16:65(	ENSG00000260308		Pseudoger	chr16:33771035-337
ENSG0000(	221	5.527415	chr16:65(	CKBP1		Pseudoger	chr16:46842632-468
ENSG0000(	221	5.527415	chr5:103(	TRPC6P2		Pseudoger	chr5:145728360-145
ENSG0000(	221	5.527415	chr16:65(	ENSG00000260899		lncRNA	chr16:30740667-307
ENSG0000(	221	5.527415	chr5:103(	HARS2		protein_c	chr5:140691430-140
ENSG0000(	221	5.527415	chr5:103(	ENSG00000289155		lncRNA	chr5:138543464-138
ENSG0000(	221	5.527415	chr16:65(	PABPC1P13		Pseudoger	chr16:32439069-324
ENSG0000(	221	5.527415	chr16:65(	ENSG00000260900		Pseudoger	chr16:33059936-330
ENSG0000(	221	5.527415	chr16:65(	IGHV3OR16-8		protein_c	chr16:33009175-330
ENSG0000(	221	5.527415	chr5:103(	SMAD5-AS1		lncRNA	chr5:136129507-136
ENSG0000(	221	5.527415	chr5:103(	ENSG00000279375		lncRNA	chr5:141191599-141
ENSG0000(	221	5.527415	chr5:103(	ENSG00000249429		lncRNA	chr5:143489855-143
ENSG0000(	221	5.527415	chr16:65(	ACTR3BP3		Pseudoger	chr16:32379071-323
ENSG0000(	221	5.527415	chr16:65(	AC135050.1		smallRNA	chr16:31109230-311
ENSG0000(	221	5.527415	chr5:103(	HNRNPA0		protein_c	chr5:137745651-137
ENSG0000(	221	5.527415	chr16:65(	AC023824.1		smallRNA	chr16:35208506-352
ENSG0000(	221	5.527415	chr5:103(	AC005370.1		smallRNA	chr5:142650000-142
ENSG0000(	221	5.527415	chr16:65(	ASPHD1	NCGv7	protein_c	chr16:29900375-299
ENSG0000(	221	5.527415	chr16:65(	ENSG00000260845		lncRNA	chr16:32735909-327
ENSG0000(	221	5.527415	chr16:65(	ENSG00000289181		lncRNA	chr16:29709156-297
ENSG0000(	221	5.527415	chr16:65(	FRG2HP		Pseudoger	chr16:35335277-353
ENSG0000(	221	5.527415	chr16:65(	SEZ6L2		protein_c	chr16:29871159-298
ENSG0000(	221	5.527415	chr16:65(	MIR762		smallRNA	chr16:30893903-308
ENSG0000(	221	5.527415	chr16:65(	ENSG00000260847		lncRNA	chr16:32188333-321
ENSG0000(	221	5.527415	chr5:103(	SNORA74A		smallRNA	chr5:139278781-139
ENSG0000(	221	5.527415	chr16:65(	ENSG00000260850		lncRNA	chr16:51354456-515
ENSG0000(	221	5.527415	chr5:103(	RNA5SP196		Pseudoger	chr5:146586099-146
ENSG0000(	221	5.527415	chr16:65(	ENSG00000280607		lncRNA	chr16:29808679-298
ENSG0000(	221	5.527415	chr5:103(	ENSG00000289306		lncRNA	chr5:142149435-142
ENSG0000(	221	5.527415	chr5:103(	ANKRD49P3		Pseudoger	chr5:136857628-136
ENSG0000(	221	5.527415	chr16:65(	RNA5SP416		Pseudoger	chr16:35751574-357
ENSG0000(	221	5.527415	chr16:65(	FBXL19-AS1		lncRNA	chr16:30919319-309
ENSG0000(	221	5.527415	chr5:103(	PCDHB2		protein_c	chr5:141094607-141
ENSG0000(	221	5.527415	chr5:103(	ENSG00000272154		lncRNA	chr5:141100242-141
ENSG0000(	221	5.527415	chr5:103(	ENSG00000279558		lncRNA	chr5:141078076-141
ENSG0000(	221	5.527415	chr16:65(	LINC02184		lncRNA	chr16:34978744-349
ENSG0000(	221	5.527415	chr16:65(	ENSG00000260857		lncRNA	chr16:35504370-355
ENSG0000(	221	5.527415	chr16:65(	ENSG00000260605		lncRNA	chr16:51035118-510
ENSG0000(	221	5.527415	chr16:65(	ABHD17AP7		Pseudoger	chr16:32726615-327
ENSG0000(	221	5.527415	chr16:65(	ENSG00000260866		Pseudoger	chr16:32388135-324
ENSG0000(	221	5.527415	chr5:103(	ENSG00000287527		lncRNA	chr5:141822076-141
ENSG0000(	221	5.527415	chr16:65(	ENSG00000289307		lncRNA	chr16:54929495-549
ENSG0000(	221	5.527415	chr16:65(	SLX1A		protein_c	chr16:30193875-301
ENSG0000(	221	5.527415	chr16:65(	IRX5		protein_c	chr16:54930865-549
ENSG0000(	221	5.527415	chr16:65(	ENSG00000290953		lncRNA	chr16:31994821-319
ENSG0000(	221	5.527415	chr5:103(	VTRNA2-1		smallRNA	chr5:136080470-136
ENSG0000(	221	5.527415	chr16:65(	FRG2IP		Pseudoger	chr16:35405747-354
ENSG0000(	221	5.527415	chr16:65(	HNRNPA1L3		protein_c	chr16:51553436-516
[truncated: 2,440,438 more chars]
