# Supplementary material for: MUC20 regulated by extrachromosomal circular DNA attenuates proteasome inhibitor resistance of multiple myeloma by modulating cuproptosis
Source: J Exp Clin Cancer Res. 2024 Mar 5;43:68. doi: 10.1186/s13046-024-02972-6 (PMC10913264; doi:10.1186/s13046-024-02972-6)
Supplement: Supplementary file 2 — Additional file 2: Supplementary Table S1. Demographic characteristics of HDs and patients with MM. Supplementary Table S2. Demographic characteristics of patients with NDMM and RRMM. Supplementary Table S8. DEED-amplified encoding genes both in PI-resistant KAS-6/1 and U266 cells. [file 13046_2024_2972_MOESM2_ESM.zip › Supplementary Table S6.pdf]

GeneID	bp_evid	ences_Nor	eccDNA_II	ene_symbo	rDriverG	ne_bioty	GeneLocus
ENSG0000(	3757	83.90337	chr1:1043	RPSAP65		Pseudoger	chr1:63262113-6326
ENSG0000(	3019	67.42195	chr1:2197	CRIP1P3		Pseudoger	chr1:202096759-202
ENSG0000(	2922	65.25569	chr1:2197	ENSG00000227579		lncRNA	chr1:177392667-177
ENSG0000(	2922	65.25569	chr1:2197	LINC01741		lncRNA	chr1:177700524-177
ENSG0000(	2922	65.25569	chr1:2197	TEX35		protein_c	chr1:178513109-178
ENSG0000(	2922	65.25569	chr1:2197	SCARNA3		smallRNA	chr1:175968398-175
ENSG0000(	2922	65.25569	chr1:2197	CLEC20A		protein_c	chr1:178479240-178
ENSG0000(	2922	65.25569	chr1:2197	SEC16B	DriverDBv	protein_c	chr1:177923956-177
ENSG0000(	2922	65.25569	chr1:2197	MORF4L1P7		Pseudoger	chr1:176367699-176
ENSG0000(	2922	65.25569	chr1:2197	ENSG00000286754		lncRNA	chr1:176829128-176
ENSG0000(	2922	65.25569	chr1:2197	CRYZL2P		Pseudoger	chr1:178006136-178
ENSG0000(	2922	65.25569	chr1:2197	LINC02803		lncRNA	chr1:175904762-175
ENSG0000(	2922	65.25569	chr1:2197	ENSG00000285910		lncRNA	chr1:178511563-178
ENSG0000(	2922	65.25569	chr1:2197	BRINP2		protein_c	chr1:177170958-177
ENSG0000(	2922	65.25569	chr1:2197	COP1-DT		lncRNA	chr1:176207646-176
ENSG0000(	2922	65.25569	chr1:2197	MIR488		smallRNA	chr1:177029363-177
ENSG0000(	2922	65.25569	chr1:2197	ASTN1	NCGv7	protein_c	chr1:176857302-177
ENSG0000(	2922	65.25569	chr1:2197	ENSG00000228686		lncRNA	chr1:176017277-176
ENSG0000(	2922	65.25569	chr1:2197	RASAL2-AS1		lncRNA	chr1:178090677-178
ENSG0000(	2922	65.25569	chr1:2197	PTP4A1P7		Pseudoger	chr1:176616273-176
ENSG0000(	2922	65.25569	chr1:2197	LINC01645		lncRNA	chr1:177351560-177
ENSG0000(	2922	65.25569	chr1:2197	COP1		protein_c	chr1:175944831-176
ENSG0000(	2922	65.25569	chr1:2197	C1orf220		lncRNA	chr1:178542752-178
ENSG0000(	2922	65.25569	chr1:2197	ENSG00000232463		Pseudoger	chr1:176231200-176
ENSG0000(	2922	65.25569	chr1:2197	ENSG00000231020		Pseudoger	chr1:176305672-176
ENSG0000(	2922	65.25569	chr1:2197	CRYZL2P-SEC16B		lncRNA	chr1:177928788-178
ENSG0000(	2922	65.25569	chr1:2197	RNU2-12P		smallRNA	chr1:176243862-176
ENSG0000(	2922	65.25569	chr1:2197	AL021398.1		smallRNA	chr1:177186360-177
ENSG0000(	2922	65.25569	chr1:2197	ENSG00000270575		Pseudoger	chr1:178194342-178
ENSG0000(	2922	65.25569	chr1:2197	RPS29P5		Pseudoger	chr1:175921975-175
ENSG0000(	2922	65.25569	chr1:2197	ENSG00000227815		Pseudoger	chr1:176272483-176
ENSG0000(	2922	65.25569	chr1:2197	ENSG00000276563		Pseudoger	chr1:178017127-178
ENSG0000(	2922	65.25569	chr1:2197	RASAL2		protein_c	chr1:178094104-178
ENSG0000(	2922	65.25569	chr1:2197	AL122019.1		smallRNA	chr1:177552870-177
ENSG0000(	2922	65.25569	chr1:2197	ENSG00000213058		Pseudoger	chr1:178411616-178
ENSG0000(	2922	65.25569	chr1:2197	PAPPA2		protein_c	chr1:176463171-176
ENSG0000(	2922	65.25569	chr1:2197	RNA5SP69		Pseudoger	chr1:178560913-178
ENSG0000(	2916	65.1217	chr1:2197	APOBEC4		protein_c	chr1:183646275-183
ENSG0000(	2916	65.1217	chr1:2197	ENSG00000289581		lncRNA	chr1:183874511-183
ENSG0000(	2916	65.1217	chr1:2197	RNU7-183P		smallRNA	chr1:185434244-185
ENSG0000(	2916	65.1217	chr1:2197	RNU6-152P		smallRNA	chr1:182327068-182
ENSG0000(	2916	65.1217	chr1:2197	FTH1P25		Pseudoger	chr1:185071567-185
ENSG0000(	2916	65.1217	chr1:2197	ENSG00000285638		lncRNA	chr1:190878145-191
ENSG0000(	2916	65.1217	chr1:2197	ENSG00000289589		lncRNA	chr1:181086644-181
ENSG0000(	2916	65.1217	chr1:2197	ENSG00000223847		Pseudoger	chr1:187714243-187
ENSG0000(	2916	65.1217	chr1:2197	RN7SL230P		smallRNA	chr1:179900262-179
ENSG0000(	2916	65.1217	chr1:2197	U6		smallRNA	chr1:180758722-180
ENSG0000(	2916	65.1217	chr1:2197	ENSG00000285986		Pseudoger	chr1:196850283-196
ENSG0000(	2916	65.1217	chr1:2197	ENSG00000238061		Pseudoger	chr1:185280844-185
ENSG0000(	2916	65.1217	chr1:2197	HMGN1P4		Pseudoger	chr1:182942115-182

ENSG0000(	2916	65.1217	chr1:2197	ENSG00000287929		lncRNA	chr1:183252263-183
ENSG0000(	2916	65.1217	chr1:2197	RPS27AP5		Pseudoger	chr1:192716183-192
ENSG0000(	2916	65.1217	chr1:2197	RPL23AP22		Pseudoger	chr1:193756815-193
ENSG0000(	2916	65.1217	chr1:2197	ENSG00000288950		lncRNA	chr1:193482540-193
ENSG0000(	2916	65.1217	chr1:2197	ENSG00000230470		lncRNA	chr1:184408337-184
ENSG0000(	2916	65.1217	chr1:2197	MRPS21P3		Pseudoger	chr1:197363817-197
ENSG0000(	2916	65.1217	chr1:2197	ENSG00000273384		lncRNA	chr1:178651706-178
ENSG0000(	2916	65.1217	chr1:2197	GLRX2		protein_c	chr1:193090866-193
ENSG0000(	2916	65.1217	chr1:2197	ENSG00000225982		lncRNA	chr1:182086551-182
ENSG0000(	2916	65.1217	chr1:2197	ENSG00000231714		lncRNA	chr1:194350943-194
ENSG0000(	2916	65.1217	chr1:2197	ENSG00000232750		Pseudoger	chr1:179035309-179
ENSG0000(	2916	65.1217	chr1:2197	OVAAL		lncRNA	chr1:180509380-180
ENSG0000(	2916	65.1217	chr1:2197	ENSG00000289573		lncRNA	chr1:182149703-182
ENSG0000(	2916	65.1217	chr1:2197	LINC01350		lncRNA	chr1:185558371-185
ENSG0000(	2916	65.1217	chr1:2197	LINC01035		lncRNA	chr1:188905688-189
ENSG0000(	2916	65.1217	chr1:2197	ENSG00000241505		lncRNA	chr1:190480379-190
ENSG0000(	2916	65.1217	chr1:2197	CFHR1		protein_c	chr1:196819731-196
ENSG0000(	2916	65.1217	chr1:2197	AL596220.1		protein_c	chr1:186394991-186
ENSG0000(	2916	65.1217	chr1:2197	EIF1P3		Pseudoger	chr1:182336001-182
ENSG0000(	2916	65.1217	chr1:2197	ENSG00000273004		lncRNA	chr1:185317779-185
ENSG0000(	2916	65.1217	chr1:2197	TOR1AIP2		protein_c	chr1:179839967-179
ENSG0000(	2916	65.1217	chr1:2197	ENSG00000286655		lncRNA	chr1:184080657-184
ENSG0000(	2916	65.1217	chr1:2197	RN7SL374P		smallRNA	chr1:179364313-179
ENSG0000(	2916	65.1217	chr1:2197	KIAA1614		protein_c	chr1:180912897-180
ENSG0000(	2916	65.1217	chr1:2197	ENSG00000289732		Pseudoger	chr1:183587174-183
ENSG0000(	2916	65.1217	chr1:2197	DHX9	NCGv7	protein_c	chr1:182839347-182
ENSG0000(	2916	65.1217	chr1:2197	RNASEL		protein_c	chr1:182573634-182
ENSG0000(	2916	65.1217	chr1:2197	RGS8		protein_c	chr1:182641816-182
ENSG0000(	2916	65.1217	chr1:2197	KRT18P28		Pseudoger	chr1:182959074-182
ENSG0000(	2916	65.1217	chr1:2197	ENSG00000238108		Pseudoger	chr1:190797524-190
ENSG0000(	2916	65.1217	chr1:2197	STX6		protein_c	chr1:180972712-181
ENSG0000(	2916	65.1217	chr1:2197	GLUL		protein_c	chr1:182378098-182
ENSG0000(	2916	65.1217	chr1:2197	ENSG00000271558		Pseudoger	chr1:187506838-187
ENSG0000(	2916	65.1217	chr1:2197	BRINP3	NCGv7	protein_c	chr1:190097658-190
ENSG0000(	2916	65.1217	chr1:2197	ENSG00000235582		Pseudoger	chr1:197735636-197
ENSG0000(	2916	65.1217	chr1:2197	CFHR3		protein_c	chr1:196774813-196
ENSG0000(	2916	65.1217	chr1:2197	LINC01699		lncRNA	chr1:181236388-181
ENSG0000(	2916	65.1217	chr1:2197	UCHL5	NCGv7	protein_c	chr1:193012250-193
ENSG0000(	2916	65.1217	chr1:2197	ENSG00000272906		lncRNA	chr1:179881607-179
ENSG0000(	2916	65.1217	chr1:2197	Y_RNA		smallRNA	chr1:185630428-185
ENSG0000(	2916	65.1217	chr1:2197	ENSG00000234041		Pseudoger	chr1:179137764-179
ENSG0000(	2916	65.1217	chr1:2197	PDC-AS1		lncRNA	chr1:186423481-186
ENSG0000(	2916	65.1217	chr1:2197	RO60	NCGv7	protein_c	chr1:193059454-193
ENSG0000(	2916	65.1217	chr1:2197	LINC01344		lncRNA	chr1:182096338-182
ENSG0000(	2916	65.1217	chr1:2197	CEP350		protein_c	chr1:179954674-180
ENSG0000(	2916	65.1217	chr1:2197	ENSG00000243155		lncRNA	chr1:180944042-180
ENSG0000(	2916	65.1217	chr1:2197	NPL		protein_c	chr1:182789293-182
ENSG0000(	2916	65.1217	chr1:2197	ENSG00000286378		lncRNA	chr1:184664282-184
ENSG0000(	2916	65.1217	chr1:2197	COX5BP8		Pseudoger	chr1:179255733-179
ENSG0000(	2916	65.1217	chr1:2197	ENSG00000286372		lncRNA	chr1:183372870-183
ENSG0000(	2916	65.1217	chr1:2197	LINC01686		lncRNA	chr1:182615254-182

ENSG0000(	2916	65.1217	chr1:2197	AL357932.1		smallRNA	chr1:195126681-195
ENSG0000(	2916	65.1217	chr1:2197	ENSG00000270443		Pseudoger	chr1:182433893-182
ENSG0000(	2916	65.1217	chr1:2197	ENSG00000225359		lncRNA	chr1:181190471-181
ENSG0000(	2916	65.1217	chr1:2197	RN7SKP156		smallRNA	chr1:188155839-188
ENSG0000(	2916	65.1217	chr1:2197	EIF4A1P11		Pseudoger	chr1:179201705-179
ENSG0000(	2916	65.1217	chr1:2197	PTGS2		protein_c	chr1:186671791-186
ENSG0000(	2916	65.1217	chr1:2197	SMG7-AS1		lncRNA	chr1:183460874-183
ENSG0000(	2916	65.1217	chr1:2197	Y_RNA		smallRNA	chr1:197685640-197
ENSG0000(	2916	65.1217	chr1:2197	ENSG00000289697		protein_c	chr1:196651852-196
ENSG0000(	2916	65.1217	chr1:2197	LAMC1	NCGv7	protein_c	chr1:183023420-183
ENSG0000(	2916	65.1217	chr1:2197	ENSG00000226640		lncRNA	chr1:193678894-193
ENSG0000(	2916	65.1217	chr1:2197	NIBAN1		protein_c	chr1:184790724-184
ENSG0000(	2916	65.1217	chr1:2197	SNORD112		smallRNA	chr1:184677934-184
ENSG0000(	2916	65.1217	chr1:2197	ENSG00000286285		lncRNA	chr1:193457422-193
ENSG0000(	2916	65.1217	chr1:2197	RN7SKP229		smallRNA	chr1:181839473-181
ENSG0000(	2916	65.1217	chr1:2197	ENSG00000226570		Pseudoger	chr1:182955390-182
ENSG0000(	2916	65.1217	chr1:2197	SLC4A1APP2		Pseudoger	chr1:187706561-187
ENSG0000(	2916	65.1217	chr1:2197	LINC01036		lncRNA	chr1:187070700-187
ENSG0000(	2916	65.1217	chr1:2197	Y_RNA		smallRNA	chr1:185257911-185
ENSG0000(	2916	65.1217	chr1:2197	ENSG00000150732		Pseudoger	chr1:188067298-188
ENSG0000(	2916	65.1217	chr1:2197	ENSG00000230260		lncRNA	chr1:197437976-197
ENSG0000(	2916	65.1217	chr1:2197	LINC01732		lncRNA	chr1:181174484-181
ENSG0000(	2916	65.1217	chr1:2197	RGS18	NCGv7	protein_c	chr1:192158462-192
ENSG0000(	2916	65.1217	chr1:2197	PACERR		lncRNA	chr1:186680601-186
ENSG0000(	2916	65.1217	chr1:2197	ENSG00000261250		lncRNA	chr1:179543201-179
ENSG0000(	2916	65.1217	chr1:2197	ENSG00000288574		lncRNA	chr1:181808927-181
ENSG0000(	2916	65.1217	chr1:2197	GAPDHP75		Pseudoger	chr1:189132350-189
ENSG0000(	2916	65.1217	chr1:2197	ENSG00000232036		Pseudoger	chr1:184566511-184
ENSG0000(	2916	65.1217	chr1:2197	ZBTB41		protein_c	chr1:197153682-197
ENSG0000(	2916	65.1217	chr1:2197	ENSG00000237283		lncRNA	chr1:188705623-188
ENSG0000(	2916	65.1217	chr1:2197	RNU6-1240P		smallRNA	chr1:186311825-186
ENSG0000(	2916	65.1217	chr1:2197	TPR	NCGv7;AC	protein_c	chr1:186311652-186
ENSG0000(	2916	65.1217	chr1:2197	ENSG00000224691		lncRNA	chr1:186176814-186
ENSG0000(	2916	65.1217	chr1:2197	VDAC1P4		Pseudoger	chr1:180434800-180
ENSG0000(	2916	65.1217	chr1:2197	ENSG00000285847		lncRNA	chr1:184607599-184
ENSG0000(	2916	65.1217	chr1:2197	LAMC1-AS1		lncRNA	chr1:183138402-183
ENSG0000(	2916	65.1217	chr1:2197	ENSG00000237861		Pseudoger	chr1:197222222-197
ENSG0000(	2916	65.1217	chr1:2197	ENSG00000288562		lncRNA	chr1:186624700-186
ENSG0000(	2916	65.1217	chr1:2197	COLGALT2		protein_c	chr1:183929854-184
ENSG0000(	2916	65.1217	chr1:2197	AL358354.1		smallRNA	chr1:180545832-180
ENSG0000(	2916	65.1217	chr1:2197	LINC01031		lncRNA	chr1:193304745-193
ENSG0000(	2916	65.1217	chr1:2197	MIR3121		smallRNA	chr1:180438314-180
ENSG0000(	2916	65.1217	chr1:2197	U3		smallRNA	chr1:193731858-193
ENSG0000(	2916	65.1217	chr1:2197	ACBD6		protein_c	chr1:180269653-180
ENSG0000(	2916	65.1217	chr1:2197	ARPC5		protein_c	chr1:183620846-183
ENSG0000(	2916	65.1217	chr1:2197	AL513344.1		smallRNA	chr1:182756919-182
ENSG0000(	2916	65.1217	chr1:2197	ENSG00000261182		lncRNA	chr1:188218400-188
ENSG0000(	2916	65.1217	chr1:2197	RNA5SP70		Pseudoger	chr1:181771566-181
ENSG0000(	2916	65.1217	chr1:2197	LINC01701		lncRNA	chr1:189775465-189
ENSG0000(	2916	65.1217	chr1:2197	RN7SL654P		smallRNA	chr1:184335658-184
ENSG0000(	2916	65.1217	chr1:2197	B3GALT2		protein_c	chr1:193178730-193

ENSG0000(	2916	65.1217	chr1:2197	SOAT1	NCGv7	protein_c	chr1:179293714-179
ENSG0000(	2916	65.1217	chr1:2197	LAMC2	TAG	protein_c	chr1:183186238-183
ENSG0000(	2916	65.1217	chr1:2197	HNRNPA1P46		Pseudoger	chr1:191146025-191
ENSG0000(	2916	65.1217	chr1:2197	AXDND1		protein_c	chr1:179365720-179
ENSG0000(	2916	65.1217	chr1:2197	TDRD5	NCGv7	protein_c	chr1:179591613-179
ENSG0000(	2916	65.1217	chr1:2197	RNU6-41P		smallRNA	chr1:182982212-182
ENSG0000(	2916	65.1217	chr1:2197	ENSG00000273198		lncRNA	chr1:186521773-186
ENSG0000(	2916	65.1217	chr1:2197	IER5		protein_c	chr1:181088700-181
ENSG0000(	2916	65.1217	chr1:2197	ENSG00000270994		Pseudoger	chr1:183709305-183
ENSG0000(	2916	65.1217	chr1:2197	RNU7-13P		smallRNA	chr1:184821428-184
ENSG0000(	2916	65.1217	chr1:2197	YPEL5P1		Pseudoger	chr1:182182730-182
ENSG0000(	2916	65.1217	chr1:2197	HNRNPA1P54		Pseudoger	chr1:179447602-179
ENSG0000(	2916	65.1217	chr1:2197	MEF2AP1		Pseudoger	chr1:179447578-179
ENSG0000(	2916	65.1217	chr1:2197	ENSG00000232309		lncRNA	chr1:182127297-182
ENSG0000(	2916	65.1217	chr1:2197	MCRIP2P2		Pseudoger	chr1:185435839-185
ENSG0000(	2916	65.1217	chr1:2197	ENSG00000261060		lncRNA	chr1:179590372-179
ENSG0000(	2916	65.1217	chr1:2197	MIR1278		smallRNA	chr1:193136503-193
ENSG0000(	2916	65.1217	chr1:2197	AL359853.1		smallRNA	chr1:179710250-179
ENSG0000(	2916	65.1217	chr1:2197	ENSG00000289432		lncRNA	chr1:179017289-179
ENSG0000(	2916	65.1217	chr1:2197	PTPN2P1		Pseudoger	chr1:178746683-178
ENSG0000(	2916	65.1217	chr1:2197	KIAA1614-AS1		lncRNA	chr1:180949699-180
ENSG0000(	2916	65.1217	chr1:2197	LINC01633		lncRNA	chr1:184999710-185
ENSG0000(	2916	65.1217	chr1:2197	TSEN15		protein_c	chr1:184051651-184
ENSG0000(	2916	65.1217	chr1:2197	ENSG00000243062		lncRNA	chr1:179730191-179
ENSG0000(	2916	65.1217	chr1:2197	MR1		protein_c	chr1:181033374-181
ENSG0000(	2916	65.1217	chr1:2197	ENSG00000238270		lncRNA	chr1:189868001-189
ENSG0000(	2916	65.1217	chr1:2197	LINC02770		lncRNA	chr1:191823432-192
ENSG0000(	2916	65.1217	chr1:2197	TEDDM1		protein_c	chr1:182398117-182
ENSG0000(	2916	65.1217	chr1:2197	ENSG00000285718		lncRNA	chr1:194785517-194
ENSG0000(	2916	65.1217	chr1:2197	LINC00272		lncRNA	chr1:182407621-182
ENSG0000(	2916	65.1217	chr1:2197	ENSG00000225006		lncRNA	chr1:188508538-188
ENSG0000(	2916	65.1217	chr1:2197	ENSG00000271269		Pseudoger	chr1:182733792-182
ENSG0000(	2916	65.1217	chr1:2197	snoU109		smallRNA	chr1:193057281-193
ENSG0000(	2916	65.1217	chr1:2197	ZNF101P2		Pseudoger	chr1:192993449-192
ENSG0000(	2916	65.1217	chr1:2197	RALGPS2-AS1		lncRNA	chr1:178724306-178
ENSG0000(	2916	65.1217	chr1:2197	ENSG00000251520		Pseudoger	chr1:180964511-180
ENSG0000(	2916	65.1217	chr1:2197	ENSG00000228238		Pseudoger	chr1:186578279-186
ENSG0000(	2916	65.1217	chr1:2197	BRINP3-DT		lncRNA	chr1:190478551-190
ENSG0000(	2916	65.1217	chr1:2197	ENSG00000231791		lncRNA	chr1:184329071-184
ENSG0000(	2916	65.1217	chr1:2197	MIR4424		smallRNA	chr1:178677749-178
ENSG0000(	2916	65.1217	chr1:2197	ENSG00000224901		lncRNA	chr1:197757319-197
ENSG0000(	2916	65.1217	chr1:2197	ENSG00000235083		Pseudoger	chr1:188671353-188
ENSG0000(	2916	65.1217	chr1:2197	ENSG00000228191		Pseudoger	chr1:179271116-179
ENSG0000(	2916	65.1217	chr1:2197	AL136987.1		smallRNA	chr1:192491128-192
ENSG0000(	2916	65.1217	chr1:2197	ENSG00000228167		Pseudoger	chr1:194718795-194
ENSG0000(	2916	65.1217	chr1:2197	C1orf21-DT		lncRNA	chr1:184385753-184
ENSG0000(	2916	65.1217	chr1:2197	ENSG00000286966		lncRNA	chr1:183613537-183
ENSG0000(	2916	65.1217	chr1:2197	LINC02818		lncRNA	chr1:179829609-179
ENSG0000(	2916	65.1217	chr1:2197	ENSG00000289099		lncRNA	chr1:183605128-183
ENSG0000(	2916	65.1217	chr1:2197	CACNA1E	NCGv7	protein_c	chr1:181317690-181
ENSG0000(	2916	65.1217	chr1:2197	ENSG00000285894		lncRNA	chr1:188013642-188

ENSG0000(	2916	65.1217	chr1:2197	CLPTM1LP1		Pseudoger	chr1:189035961-189
ENSG0000(	2916	65.1217	chr1:2197	RPSAP16		Pseudoger	chr1:179968686-179
ENSG0000(	2916	65.1217	chr1:2197	ENSG00000224810		lncRNA	chr1:182062677-182
ENSG0000(	2916	65.1217	chr1:2197	ENSG00000229407		lncRNA	chr1:179816184-179
ENSG0000(	2916	65.1217	chr1:2197	SETP10		Pseudoger	chr1:179183734-179
ENSG0000(	2916	65.1217	chr1:2197	AL450304.1		smallRNA	chr1:182959485-182
ENSG0000(	2916	65.1217	chr1:2197	TOR3A		protein_c	chr1:179082070-179
ENSG0000(	2916	65.1217	chr1:2197	ENSG00000224278		Pseudoger	chr1:188242139-188
ENSG0000(	2916	65.1217	chr1:2197	AL590085.1		smallRNA	chr1:180827895-180
ENSG0000(	2916	65.1217	chr1:2197	GS1-279B7.1		Pseudoger	chr1:185321157-185
ENSG0000(	2916	65.1217	chr1:2197	ASPM	NCGv7	protein_c	chr1:197084121-197
ENSG0000(	2916	65.1217	chr1:2197	ENSG00000270711		Pseudoger	chr1:180970837-180
ENSG0000(	2916	65.1217	chr1:2197	ENSG00000238054		lncRNA	chr1:188869474-188
ENSG0000(	2916	65.1217	chr1:2197	RGS2		protein_c	chr1:192809039-192
ENSG0000(	2916	65.1217	chr1:2197	KCNT2	NCGv7	protein_c	chr1:196225779-196
ENSG0000(	2916	65.1217	chr1:2197	ENSG00000287472		lncRNA	chr1:189868381-189
ENSG0000(	2916	65.1217	chr1:2197	RPL22P24		Pseudoger	chr1:185171335-185
ENSG0000(	2916	65.1217	chr1:2197	ENSG00000227141		Pseudoger	chr1:179586705-179
ENSG0000(	2916	65.1217	chr1:2197	ENSG00000228664		Pseudoger	chr1:182328497-182
ENSG0000(	2916	65.1217	chr1:2197	ENSG00000273844		Pseudoger	chr1:196970495-196
ENSG0000(	2916	65.1217	chr1:2197	FAM20B		protein_c	chr1:179025804-179
ENSG0000(	2916	65.1217	chr1:2197	ANGPTL1		protein_c	chr1:178849535-178
ENSG0000(	2916	65.1217	chr1:2197	TEDDM2P		Pseudoger	chr1:182441577-182
ENSG0000(	2916	65.1217	chr1:2197	ENSG00000261729		lncRNA	chr1:185646463-185
ENSG0000(	2916	65.1217	chr1:2197	ENSG00000225711		Pseudoger	chr1:179220938-179
ENSG0000(	2916	65.1217	chr1:2197	CFHR4		protein_c	chr1:196888014-196
ENSG0000(	2916	65.1217	chr1:2197	ENSG00000261831		lncRNA	chr1:179926641-179
ENSG0000(	2916	65.1217	chr1:2197	RGS13		protein_c	chr1:192636138-192
ENSG0000(	2916	65.1217	chr1:2197	RALGPS2		protein_c	chr1:178725165-178
ENSG0000(	2916	65.1217	chr1:2197	ENSG00000225811		lncRNA	chr1:190264898-190
ENSG0000(	2916	65.1217	chr1:2197	SNORA67		smallRNA	chr1:179201487-179
ENSG0000(	2916	65.1217	chr1:2197	RNA5SP71		smallRNA	chr1:182944365-182
ENSG0000(	2916	65.1217	chr1:2197	ENSG00000223344		lncRNA	chr1:191858707-191
ENSG0000(	2916	65.1217	chr1:2197	NPHS2		protein_c	chr1:179550539-179
ENSG0000(	2916	65.1217	chr1:2197	ENSG00000237011		lncRNA	chr1:193684246-193
ENSG0000(	2916	65.1217	chr1:2197	ENSG00000226814		Pseudoger	chr1:192800571-192
ENSG0000(	2916	65.1217	chr1:2197	QSOX1		protein_c	chr1:180154869-180
ENSG0000(	2916	65.1217	chr1:2197	ENSG00000274702		Pseudoger	chr1:187632166-187
ENSG0000(	2916	65.1217	chr1:2197	RPL5P5		Pseudoger	chr1:185226808-185
ENSG0000(	2916	65.1217	chr1:2197	Y_RNA		smallRNA	chr1:180519016-180
ENSG0000(	2916	65.1217	chr1:2197	ENSG00000227240		lncRNA	chr1:193473224-194
ENSG0000(	2916	65.1217	chr1:2197	DENND1B		protein_c	chr1:197504748-197
ENSG0000(	2916	65.1217	chr1:2197	ENSG00000233583		Pseudoger	chr1:185262286-185
ENSG0000(	2916	65.1217	chr1:2197	EEF1A1P32		Pseudoger	chr1:197688760-197
ENSG0000(	2916	65.1217	chr1:2197	CDC73	NCGv7;AC	protein_c	chr1:193121983-193
ENSG0000(	2916	65.1217	chr1:2197	MIR4735		smallRNA	chr1:196582413-196
ENSG0000(	2916	65.1217	chr1:2197	LINC01688		lncRNA	chr1:182712862-182
ENSG0000(	2916	65.1217	chr1:2197	SEPTIN14P12		Pseudoger	chr1:197138748-197
ENSG0000(	2916	65.1217	chr1:2197	RPS3AP8		Pseudoger	chr1:183266602-183
ENSG0000(	2916	65.1217	chr1:2197	ENSG00000233196		Pseudoger	chr1:186580515-186
ENSG0000(	2916	65.1217	chr1:2197	ENSG00000236025		Pseudoger	chr1:190781787-190

ENSG0000(	2916	65.1217	chr1:2197	Y_RNA		smallRNA	chr1:185251313-185
ENSG0000(	2916	65.1217	chr1:2197	CRB1	NCGv7	protein_c	chr1:197268204-197
ENSG0000(	2916	65.1217	chr1:2197	CFHR5		protein_c	chr1:196975010-197
ENSG0000(	2916	65.1217	chr1:2197	ZNF648		protein_c	chr1:182054570-182
ENSG0000(	2916	65.1217	chr1:2197	TOR1AIP1		protein_c	chr1:179882042-179
ENSG0000(	2916	65.1217	chr1:2197	ENSG00000289995		lncRNA	chr1:192609359-192
ENSG0000(	2916	65.1217	chr1:2197	TRMT1L		protein_c	chr1:185118101-185
ENSG0000(	2916	65.1217	chr1:2197	Y_RNA		smallRNA	chr1:185266535-185
ENSG0000(	2916	65.1217	chr1:2197	RNF2		protein_c	chr1:185045526-185
ENSG0000(	2916	65.1217	chr1:2197	ENSG00000228687		Pseudoger	chr1:192796533-192
ENSG0000(	2916	65.1217	chr1:2197	RGL1	NCGv7	protein_c	chr1:183636085-183
ENSG0000(	2916	65.1217	chr1:2197	LHX4		protein_c	chr1:180230264-180
ENSG0000(	2916	65.1217	chr1:2197	RNU6-983P		smallRNA	chr1:194488103-194
ENSG0000(	2916	65.1217	chr1:2197	ODR4		protein_c	chr1:186375838-186
ENSG0000(	2916	65.1217	chr1:2197	LINC01720		lncRNA	chr1:190624890-190
ENSG0000(	2916	65.1217	chr1:2197	HMCN1	NCGv7	protein_c	chr1:185734391-186
ENSG0000(	2916	65.1217	chr1:2197	ENSG00000287452		lncRNA	chr1:181962889-181
ENSG0000(	2916	65.1217	chr1:2197	LINC01724		lncRNA	chr1:196044883-196
ENSG0000(	2916	65.1217	chr1:2197	RGSL1		protein_c	chr1:182409192-182
ENSG0000(	2916	65.1217	chr1:2197	Y_RNA		smallRNA	chr1:184171714-184
ENSG0000(	2916	65.1217	chr1:2197	FAM163A		protein_c	chr1:179743291-179
ENSG0000(	2916	65.1217	chr1:2197	RGS16		protein_c	chr1:182598623-182
ENSG0000(	2916	65.1217	chr1:2197	Y_RNA		smallRNA	chr1:185634073-185
ENSG0000(	2916	65.1217	chr1:2197	XPR1		protein_c	chr1:180632022-180
ENSG0000(	2916	65.1217	chr1:2197	ENSG00000261817		lncRNA	chr1:180117140-180
ENSG0000(	2916	65.1217	chr1:2197	SNORA67		smallRNA	chr1:179196473-179
ENSG0000(	2916	65.1217	chr1:2197	SNORA63		smallRNA	chr1:178753654-178
ENSG0000(	2916	65.1217	chr1:2197	ENSG00000227554		lncRNA	chr1:183754418-183
ENSG0000(	2916	65.1217	chr1:2197	CFHR2	NCGv7	protein_c	chr1:196943738-196
ENSG0000(	2916	65.1217	chr1:2197	RN7SKP126		smallRNA	chr1:192875686-192
ENSG0000(	2916	65.1217	chr1:2197	ENSG00000290127		lncRNA	chr1:197201486-197
ENSG0000(	2916	65.1217	chr1:2197	ENSG00000279838		TEC	chr1:185292384-185
ENSG0000(	2916	65.1217	chr1:2197	F13B		protein_c	chr1:197038741-197
ENSG0000(	2916	65.1217	chr1:2197	PLA2G4A		protein_c	chr1:186828949-186
ENSG0000(	2916	65.1217	chr1:2197	RGS1		protein_c	chr1:192575763-192
ENSG0000(	2916	65.1217	chr1:2197	ABL2	NCGv7;AC	protein_c	chr1:179099330-179
ENSG0000(	2916	65.1217	chr1:2197	SHCBP1L		protein_c	chr1:182899865-182
ENSG0000(	2916	65.1217	chr1:2197	NMNAT2		protein_c	chr1:183248237-183
ENSG0000(	2916	65.1217	chr1:2197	AL137800.1		smallRNA	chr1:183510675-183
ENSG0000(	2916	65.1217	chr1:2197	OCLM		protein_c	chr1:186400572-186
ENSG0000(	2916	65.1217	chr1:2197	ENSG00000287364		lncRNA	chr1:195747024-195
ENSG0000(	2916	65.1217	chr1:2197	DHX9-AS1		lncRNA	chr1:182837185-182
ENSG0000(	2916	65.1217	chr1:2197	RPS3AP9		Pseudoger	chr1:188694320-188
ENSG0000(	2916	65.1217	chr1:2197	ENSG00000223450		Pseudoger	chr1:180000438-180
ENSG0000(	2916	65.1217	chr1:2197	RNA5SP73		Pseudoger	chr1:189666149-189
ENSG0000(	2916	65.1217	chr1:2197	LINC01680		lncRNA	chr1:191221159-191
ENSG0000(	2916	65.1217	chr1:2197	ENSG00000230987		Pseudoger	chr1:189989570-189
ENSG0000(	2916	65.1217	chr1:2197	RNU5F-2P		smallRNA	chr1:179576268-179
ENSG0000(	2916	65.1217	chr1:2197	C1orf21		protein_c	chr1:184387029-184
ENSG0000(	2916	65.1217	chr1:2197	Y_RNA		smallRNA	chr1:184315658-184
ENSG0000(	2916	65.1217	chr1:2197	ENSG00000288078		lncRNA	chr1:186224472-186

ENSG0000(	2916	65.1217	chr1:2197	ENSG00000279401		TEC	chr1:185518651-185
ENSG0000(	2916	65.1217	chr1:2197	FAM204BP		Pseudoger	chr1:197746751-197
ENSG0000(	2916	65.1217	chr1:2197	SWT1		protein_c	chr1:185157080-185
ENSG0000(	2916	65.1217	chr1:2197	ENSG00000236792		Pseudoger	chr1:192247505-192
ENSG0000(	2916	65.1217	chr1:2197	ENSG00000261642		lncRNA	chr1:191151510-191
ENSG0000(	2916	65.1217	chr1:2197	EEF1A1P14		Pseudoger	chr1:194188967-194
ENSG0000(	2916	65.1217	chr1:2197	CFH		protein_c	chr1:196651754-196
ENSG0000(	2916	65.1217	chr1:2197	ENSG00000271187		Pseudoger	chr1:191179521-191
ENSG0000(	2916	65.1217	chr1:2197	ENSG00000290066		lncRNA	chr1:184629542-184
ENSG0000(	2916	65.1217	chr1:2197	ENSG00000260360		lncRNA	chr1:179953184-179
ENSG0000(	2916	65.1217	chr1:2197	EDEM3		protein_c	chr1:184690237-184
ENSG0000(	2916	65.1217	chr1:2197	PDC		protein_c	chr1:186443566-186
ENSG0000(	2916	65.1217	chr1:2197	ENSG00000226723		Pseudoger	chr1:192246708-192
ENSG0000(	2916	65.1217	chr1:2197	LINC02816		lncRNA	chr1:180906651-180
ENSG0000(	2916	65.1217	chr1:2197	RGS21		protein_c	chr1:192316992-192
ENSG0000(	2916	65.1217	chr1:2197	ENSG00000285280		lncRNA	chr1:192167786-192
ENSG0000(	2916	65.1217	chr1:2197	ENSG00000236069		lncRNA	chr1:192517190-192
ENSG0000(	2916	65.1217	chr1:2197	RNA5SP72		Pseudoger	chr1:185014951-185
ENSG0000(	2916	65.1217	chr1:2197	IVNS1ABP		protein_c	chr1:185296388-185
ENSG0000(	2916	65.1217	chr1:2197	PRG4	NCGv7	protein_c	chr1:186296279-186
ENSG0000(	2916	65.1217	chr1:2197	SMG7	NCGv7	protein_c	chr1:183472216-183
ENSG0000(	2916	65.1217	chr1:2197	FDPSP1		Pseudoger	chr1:187563061-187
ENSG0000(	2916	65.1217	chr1:2197	NCF2	NCGv7	protein_c	chr1:183554461-183
ENSG0000(	2910	64.9877	chr1:2197	LINC00862		lncRNA	chr1:200253419-200
ENSG0000(	2910	64.9877	chr1:2197	LINC02789		lncRNA	chr1:199148598-199
ENSG0000(	2910	64.9877	chr1:2197	ENSG00000227747		Pseudoger	chr1:198949016-198
ENSG0000(	2910	64.9877	chr1:2197	ENSG00000287989		lncRNA	chr1:198450666-198
ENSG0000(	2910	64.9877	chr1:2197	LINC01222		lncRNA	chr1:199006040-199
ENSG0000(	2910	64.9877	chr1:2197	AL450244.1		smallRNA	chr1:199615188-199
ENSG0000(	2910	64.9877	chr1:2197	NR5A2		protein_c	chr1:200027614-200
ENSG0000(	2910	64.9877	chr1:2197	ENSG00000229220		Pseudoger	chr1:200147531-200
ENSG0000(	2910	64.9877	chr1:2197	ENSG00000286541		lncRNA	chr1:199040501-199
ENSG0000(	2910	64.9877	chr1:2197	RPL23AP16		Pseudoger	chr1:199371877-199
ENSG0000(	2910	64.9877	chr1:2197	ENSG00000273093		lncRNA	chr1:200315435-200
ENSG0000(	2910	64.9877	chr1:2197	NEK7		protein_c	chr1:198156994-198
ENSG0000(	2910	64.9877	chr1:2197	ATP6V1G3		protein_c	chr1:198523222-198
ENSG0000(	2910	64.9877	chr1:2197	AC096633.1		smallRNA	chr1:200144834-200
ENSG0000(	2910	64.9877	chr1:2197	PEBP1P3		Pseudoger	chr1:198679139-198
ENSG0000(	2910	64.9877	chr1:2197	ENSG00000229747		Pseudoger	chr1:199908921-199
ENSG0000(	2910	64.9877	chr1:2197	LHX9		protein_c	chr1:197911902-197
ENSG0000(	2910	64.9877	chr1:2197	MIR181A1HG		lncRNA	chr1:198777861-198
ENSG0000(	2910	64.9877	chr1:2197	MIR181A1		smallRNA	chr1:198859044-198
ENSG0000(	2910	64.9877	chr1:2197	ZNF281		protein_c	chr1:200404940-200
ENSG0000(	2910	64.9877	chr1:2197	MIR181B1		smallRNA	chr1:198858873-198
ENSG0000(	2910	64.9877	chr1:2197	RNU6-570P		smallRNA	chr1:200054061-200
ENSG0000(	2910	64.9877	chr1:2197	PTPRC	NCGv7;AC	protein_c	chr1:198638457-198
ENSG0000(	2910	64.9877	chr1:2197	ENSG00000290125		lncRNA	chr1:199932233-199
ENSG0000(	2910	64.9877	chr1:2197	AC105941.1		smallRNA	chr1:199197080-199
ENSG0000(	2910	64.9877	chr1:2197	C1orf53		protein_c	chr1:197902630-197
ENSG0000(	2910	64.9877	chr1:2197	ENSG00000231984		Pseudoger	chr1:199752491-199
ENSG0000(	2910	64.9877	chr1:2197	RNU6-778P		smallRNA	chr1:199888159-199

ENSG0000(	2910	64.9877	chr1:2197	ENSG00000261573		lncRNA	chr1:198657553-198
ENSG0000(	2910	64.9877	chr1:2197	ENSG00000213045		Pseudoger	chr1:200329161-200
ENSG0000(	2910	64.9877	chr1:2197	PRR13P1		Pseudoger	chr1:198197779-198
ENSG0000(	2910	64.9877	chr1:2197	ENSG00000225172		lncRNA	chr1:198973379-198
ENSG0000(	2910	64.9877	chr1:2197	LINC01221		lncRNA	chr1:199016133-199
ENSG0000(	2910	64.9877	chr1:2197	ENSG00000223881		lncRNA	chr1:198597724-198
ENSG0000(	2910	64.9877	chr1:2197	CCNQP1		Pseudoger	chr1:200213678-200
ENSG0000(	2910	64.9877	chr1:2197	RNU6-609P		smallRNA	chr1:200014689-200
ENSG0000(	2910	64.9877	chr1:2197	ENSG00000230623		lncRNA	chr1:200333193-200
ENSG0000(	2910	64.9877	chr1:2197	EEF1A1P44		Pseudoger	chr1:199387141-199
ENSG0000(	2910	64.9877	chr1:2197	ENSG00000282849		lncRNA	chr1:200478020-200
ENSG0000(	2910	64.9877	chr1:2197	RNU6-716P		smallRNA	chr1:200008505-200
ENSG0000(	2910	64.9877	chr1:2197	ENSG00000228530		Pseudoger	chr1:199876978-199
ENSG0000(	2904	64.85371	chr1:2197	SNORA70		smallRNA	chr1:201978461-201
ENSG0000(	2879	64.29539	chr1:2197	TNNI1		protein_c	chr1:201403768-201
ENSG0000(	2879	64.29539	chr1:2197	ARL8A		protein_c	chr1:202133404-202
ENSG0000(	2879	64.29539	chr1:2197	LAD1		protein_c	chr1:201380833-201
ENSG0000(	2879	64.29539	chr1:2197	LGR6		protein_c	chr1:202193799-202
ENSG0000(	2879	64.29539	chr1:2197	IPO9		protein_c	chr1:201829149-201
ENSG0000(	2879	64.29539	chr1:2197	RPS10P7		Pseudoger	chr1:201518703-201
ENSG0000(	2879	64.29539	chr1:2197	CSRP1-AS1		lncRNA	chr1:201507241-201
ENSG0000(	2879	64.29539	chr1:2197	PTPRVP		Pseudoger	chr1:202168051-202
ENSG0000(	2879	64.29539	chr1:2197	RPL34P6		Pseudoger	chr1:200863808-200
ENSG0000(	2879	64.29539	chr1:2197	ENSG00000236390		lncRNA	chr1:201673105-201
ENSG0000(	2879	64.29539	chr1:2197	KIF14	AC	protein_c	chr1:200551497-200
ENSG0000(	2879	64.29539	chr1:2197	KIF21B	NCGv7	protein_c	chr1:200969390-201
ENSG0000(	2879	64.29539	chr1:2197	ENSG00000232626		Pseudoger	chr1:202438396-202
ENSG0000(	2879	64.29539	chr1:2197	CAMSAP2		protein_c	chr1:200738893-200
ENSG0000(	2879	64.29539	chr1:2197	MIR1231		smallRNA	chr1:201808611-201
ENSG0000(	2879	64.29539	chr1:2197	SNORA70		smallRNA	chr1:202527310-202
ENSG0000(	2879	64.29539	chr1:2197	IPO9-AS1		lncRNA	chr1:201688259-201
ENSG0000(	2879	64.29539	chr1:2197	ENSG00000229191		lncRNA	chr1:201023949-201
ENSG0000(	2879	64.29539	chr1:2197	snoU13		smallRNA	chr1:202231664-202
ENSG0000(	2879	64.29539	chr1:2197	CACNA1S		protein_c	chr1:201039512-201
ENSG0000(	2879	64.29539	chr1:2197	PKP1		protein_c	chr1:201283452-201
ENSG0000(	2879	64.29539	chr1:2197	ENSG00000282221		lncRNA	chr1:201399633-201
ENSG0000(	2879	64.29539	chr1:2197	TNNT2		protein_c	chr1:201359008-201
ENSG0000(	2879	64.29539	chr1:2197	DDX59		protein_c	chr1:200623896-200
ENSG0000(	2879	64.29539	chr1:2197	ENSG00000235121		lncRNA	chr1:201723294-201
ENSG0000(	2879	64.29539	chr1:2197	GPR25		protein_c	chr1:200872981-200
ENSG0000(	2879	64.29539	chr1:2197	U6		smallRNA	chr1:202410108-202
ENSG0000(	2879	64.29539	chr1:2197	NAV1	NCGv7	protein_c	chr1:201539127-201
ENSG0000(	2879	64.29539	chr1:2197	PHLDA3		protein_c	chr1:201464278-201
ENSG0000(	2879	64.29539	chr1:2197	ENSG00000229821		lncRNA	chr1:201222113-201
ENSG0000(	2879	64.29539	chr1:2197	MIR5191		smallRNA	chr1:201719508-201
ENSG0000(	2879	64.29539	chr1:2197	ASCL5		protein_c	chr1:201113943-201
ENSG0000(	2879	64.29539	chr1:2197	ELF3	NCGv7	protein_c	chr1:202007945-202
ENSG0000(	2879	64.29539	chr1:2197	UBE2T		protein_c	chr1:202331544-202
ENSG0000(	2879	64.29539	chr1:2197	PPP1R12B		protein_c	chr1:202348699-202
ENSG0000(	2879	64.29539	chr1:2197	RPL10P4		Pseudoger	chr1:201978642-201
ENSG0000(	2879	64.29539	chr1:2197	GPR37L1		protein_c	chr1:202122886-202

ENSG0000(	2879	64.29539	chr1:2197	ENSG00000223774		lncRNA	chr1:201893842-201
ENSG0000(	2879	64.29539	chr1:2197	RNU6-501P		smallRNA	chr1:201733406-201
ENSG0000(	2879	64.29539	chr1:2197	DDX59-AS1		lncRNA	chr1:200669507-200
ENSG0000(	2879	64.29539	chr1:2197	RNPEP		protein_c	chr1:201982372-202
ENSG0000(	2879	64.29539	chr1:2197	ENSG00000224818		lncRNA	chr1:201464383-201
ENSG0000(	2879	64.29539	chr1:2197	ENSG00000286600		lncRNA	chr1:201359018-201
ENSG0000(	2879	64.29539	chr1:2197	RNU6-704P		smallRNA	chr1:200933345-200
ENSG0000(	2879	64.29539	chr1:2197	PTPN7		protein_c	chr1:202147013-202
ENSG0000(	2879	64.29539	chr1:2197	ENSG00000249007		lncRNA	chr1:202011370-202
ENSG0000(	2879	64.29539	chr1:2197	ENSG00000234132		lncRNA	chr1:201031136-201
ENSG0000(	2879	64.29539	chr1:2197	ENSG00000227048		Pseudoger	chr1:201428975-201
ENSG0000(	2879	64.29539	chr1:2197	ENSG00000235811		Pseudoger	chr1:202039851-202
ENSG0000(	2879	64.29539	chr1:2197	CSRP1		protein_c	chr1:201483530-201
ENSG0000(	2879	64.29539	chr1:2197	ENSG00000232296		Pseudoger	chr1:202028606-202
ENSG0000(	2879	64.29539	chr1:2197	INAVA		protein_c	chr1:200891048-200
ENSG0000(	2879	64.29539	chr1:2197	MROH3P		Pseudoger	chr1:200917460-200
ENSG0000(	2879	64.29539	chr1:2197	ELF3-AS1		lncRNA	chr1:201995696-202
ENSG0000(	2879	64.29539	chr1:2197	TIMM17A	NCGv7	protein_c	chr1:201955503-201
ENSG0000(	2879	64.29539	chr1:2197	IGFN1	NCGv7	protein_c	chr1:201190824-201
ENSG0000(	2879	64.29539	chr1:2197	SHISA4	NCGv7	protein_c	chr1:201888680-201
ENSG0000(	2879	64.29539	chr1:2197	TMEM9		protein_c	chr1:201134772-201
ENSG0000(	2879	64.29539	chr1:2197	LMOD1		protein_c	chr1:201896456-201
ENSG0000(	2879	64.29539	chr1:2197	CYCSP4		Pseudoger	chr1:202369526-202
ENSG0000(	2879	64.29539	chr1:2197	ENSG00000236439		Pseudoger	chr1:202471864-202
ENSG0000(	2879	64.29539	chr1:2197	snoU13		smallRNA	chr1:202197628-202
ENSG0000(	2858	63.82641	chr1:104?	COX6B1P7		Pseudoger	chr1:68282388-6828
ENSG0000(	2852	63.69241	chr1:2197	TMCC2		protein_c	chr1:205227946-205
ENSG0000(	2852	63.69241	chr1:2197	TMEM81		protein_c	chr1:205083129-205
ENSG0000(	2852	63.69241	chr1:2197	ENSG00000225620		lncRNA	chr1:202632428-202
ENSG0000(	2852	63.69241	chr1:2197	ENSG00000286572		lncRNA	chr1:204064533-204
ENSG0000(	2852	63.69241	chr1:2197	LEMD1-AS1		lncRNA	chr1:205373252-205
ENSG0000(	2852	63.69241	chr1:2197	PPFIA4	DriverDB\	protein_c	chr1:203026491-203
ENSG0000(	2852	63.69241	chr1:2197	KLHDC8A		protein_c	chr1:205336061-205
ENSG0000(	2852	63.69241	chr1:2197	ENSG00000237848		Pseudoger	chr1:204394541-204
ENSG0000(	2852	63.69241	chr1:2197	SLC25A39P1		Pseudoger	chr1:202796030-202
ENSG0000(	2852	63.69241	chr1:2197	NSA2P1		Pseudoger	chr1:203656969-203
ENSG0000(	2852	63.69241	chr1:2197	ACTG1P25		Pseudoger	chr1:202861754-202
ENSG0000(	2852	63.69241	chr1:2197	ENSG00000224671		lncRNA	chr1:203144694-203
ENSG0000(	2852	63.69241	chr1:2197	ETNK2		protein_c	chr1:204131062-204
ENSG0000(	2852	63.69241	chr1:2197	RNA5SP74		Pseudoger	chr1:204562413-204
ENSG0000(	2852	63.69241	chr1:2197	MYOG		protein_c	chr1:203083129-203
ENSG0000(	2852	63.69241	chr1:2197	PIK3C2B	NCGv7	protein_c	chr1:204422628-204
ENSG0000(	2852	63.69241	chr1:2197	PLEKHA6		protein_c	chr1:204218853-204
ENSG0000(	2852	63.69241	chr1:2197	FMOD		protein_c	chr1:203340628-203
ENSG0000(	2852	63.69241	chr1:2197	MYBPH		protein_c	chr1:203167811-203
ENSG0000(	2852	63.69241	chr1:2197	ENSG00000261065		lncRNA	chr1:204131062-204
ENSG0000(	2852	63.69241	chr1:2197	CHI3L1		protein_c	chr1:203178931-203
ENSG0000(	2852	63.69241	chr1:2197	NPM1P40		Pseudoger	chr1:203255743-203
ENSG0000(	2852	63.69241	chr1:2197	MGAT4FP		Pseudoger	chr1:202986557-202
ENSG0000(	2852	63.69241	chr1:2197	HNRNPA1P59		Pseudoger	chr1:202911812-202
ENSG0000(	2852	63.69241	chr1:2197	SYT2	DriverDB\	protein_c	chr1:202590596-202

ENSG0000(	2852	63.69241	chr1:2197	CBX1P3		Pseudoger	chr1:203954640-203
ENSG0000(	2852	63.69241	chr1:2197	RNA5SP75		Pseudoger	chr1:204707320-204
ENSG0000(	2852	63.69241	chr1:2197	ENSG00000234775		lncRNA	chr1:203115468-203
ENSG0000(	2852	63.69241	chr1:2197	MDM4	NCGv7;AC	protein_c	chr1:204516379-204
ENSG0000(	2852	63.69241	chr1:2197	ENSG00000236108		Pseudoger	chr1:205134646-205
ENSG0000(	2852	63.69241	chr1:2197	SOX13		protein_c	chr1:204073115-204
ENSG0000(	2852	63.69241	chr1:2197	MGAT4EP		Pseudoger	chr1:202820266-202
ENSG0000(	2852	63.69241	chr1:2197	SNORD112		smallRNA	chr1:204904747-204
ENSG0000(	2852	63.69241	chr1:2197	ZC3H11A	NCGv7	protein_c	chr1:203795623-203
ENSG0000(	2852	63.69241	chr1:2197	CHIT1		protein_c	chr1:203212827-203
ENSG0000(	2852	63.69241	chr1:2197	AL583832.1		smallRNA	chr1:205062252-205
ENSG0000(	2852	63.69241	chr1:2197	DSTYK		protein_c	chr1:205142505-205
ENSG0000(	2852	63.69241	chr1:2197	ENSG00000228153		lncRNA	chr1:204663872-204
ENSG0000(	2852	63.69241	chr1:2197	ENSG00000290909		lncRNA	chr1:202987277-202
ENSG0000(	2852	63.69241	chr1:2197	LEMD1-DT		lncRNA	chr1:205455929-205
ENSG0000(	2852	63.69241	chr1:2197	GOLT1A	DriverDB\	protein_c	chr1:204198163-204
ENSG0000(	2852	63.69241	chr1:2197	ENSG00000287197		lncRNA	chr1:204822664-204
ENSG0000(	2852	63.69241	chr1:2197	ATP2B4		protein_c	chr1:203626832-203
ENSG0000(	2852	63.69241	chr1:2197	REN		protein_c	chr1:204154819-204
ENSG0000(	2852	63.69241	chr1:2197	RABIF		protein_c	chr1:202878282-202
ENSG0000(	2852	63.69241	chr1:2197	KISS1		protein_c	chr1:204190341-204
ENSG0000(	2852	63.69241	chr1:2197	ENSG00000227417		Pseudoger	chr1:203805621-203
ENSG0000(	2852	63.69241	chr1:2197	ENSG00000286383		lncRNA	chr1:203996532-204
ENSG0000(	2852	63.69241	chr1:2197	LAX1		protein_c	chr1:203765177-203
ENSG0000(	2852	63.69241	chr1:2197	ENSG00000231691		lncRNA	chr1:204276901-204
ENSG0000(	2852	63.69241	chr1:2197	LINC00628		lncRNA	chr1:204368431-204
ENSG0000(	2852	63.69241	chr1:2197	AL161793.1		smallRNA	chr1:204653102-204
ENSG0000(	2852	63.69241	chr1:2197	NUAK2	AC	protein_c	chr1:205302063-205
ENSG0000(	2852	63.69241	chr1:2197	CDK18		protein_c	chr1:205504596-205
ENSG0000(	2852	63.69241	chr1:2197	ENSG00000213041		Pseudoger	chr1:205202191-205
ENSG0000(	2852	63.69241	chr1:2197	ENSG00000240710		lncRNA	chr1:204603035-204
ENSG0000(	2852	63.69241	chr1:2197	NFASC		protein_c	chr1:204828651-205
ENSG0000(	2852	63.69241	chr1:2197	ENSG00000225522		Pseudoger	chr1:204183006-204
ENSG0000(	2852	63.69241	chr1:2197	SCARNA20		smallRNA	chr1:204727991-204
ENSG0000(	2852	63.69241	chr1:2197	Y_RNA		smallRNA	chr1:202914880-202
ENSG0000(	2852	63.69241	chr1:2197	ENSG00000288925		lncRNA	chr1:203290128-203
ENSG0000(	2852	63.69241	chr1:2197	AC096645.1		smallRNA	chr1:203999245-203
ENSG0000(	2852	63.69241	chr1:2197	PCAT6		lncRNA	chr1:202810850-202
ENSG0000(	2852	63.69241	chr1:2197	SNRPE		protein_c	chr1:203861599-203
ENSG0000(	2852	63.69241	chr1:2197	LEMD1		protein_c	chr1:205381378-205
ENSG0000(	2852	63.69241	chr1:2197	ENSG00000260021		lncRNA	chr1:202810238-202
ENSG0000(	2852	63.69241	chr1:2197	SNRPGP10		Pseudoger	chr1:205351247-205
ENSG0000(	2852	63.69241	chr1:2197	BTG2-DT		lncRNA	chr1:203298758-203
ENSG0000(	2852	63.69241	chr1:2197	ENSG00000288644		protein_c	chr1:203802094-203
ENSG0000(	2852	63.69241	chr1:2197	ADORA1		protein_c	chr1:203090654-203
ENSG0000(	2852	63.69241	chr1:2197	MIR135B		smallRNA	chr1:205448302-205
ENSG0000(	2852	63.69241	chr1:2197	ENSG00000235449		Pseudoger	chr1:202767229-202
ENSG0000(	2852	63.69241	chr1:2197	RBBP5		protein_c	chr1:205086142-205
ENSG0000(	2852	63.69241	chr1:2197	KLHL12		protein_c	chr1:202891116-202
ENSG0000(	2852	63.69241	chr1:2197	ENSG00000236779		Pseudoger	chr1:204528845-204
ENSG0000(	2852	63.69241	chr1:2197	RPL35AP5		Pseudoger	chr1:203835585-203

ENSG0000(	2852	63.69241	chr1:2197	TUBA5P		Pseudoger	chr1:202852991-202
ENSG0000(	2852	63.69241	chr1:2197	CNTN2	AC	protein_c	chr1:205042937-205
ENSG0000(	2852	63.69241	chr1:2197	ADIPOR1		protein_c	chr1:202940826-202
ENSG0000(	2852	63.69241	chr1:2197	CYB5R1		protein_c	chr1:202961873-202
ENSG0000(	2852	63.69241	chr1:2197	RNU6-487P		smallRNA	chr1:203318996-203
ENSG0000(	2852	63.69241	chr1:2197	LRRN2		protein_c	chr1:204617170-204
ENSG0000(	2852	63.69241	chr1:2197	LINC01353		lncRNA	chr1:203273221-203
ENSG0000(	2852	63.69241	chr1:2197	ENSG00000271580		Pseudoger	chr1:205091163-205
ENSG0000(	2852	63.69241	chr1:2197	BTG2	NCGv7	protein_c	chr1:203305491-203
ENSG0000(	2852	63.69241	chr1:2197	HSPE1P6		Pseudoger	chr1:203903723-203
ENSG0000(	2852	63.69241	chr1:2197	LARP7P1		Pseudoger	chr1:203400266-203
ENSG0000(	2852	63.69241	chr1:2197	OPTC		protein_c	chr1:203494153-203
ENSG0000(	2852	63.69241	chr1:2197	LINC00303		lncRNA	chr1:204032447-204
ENSG0000(	2852	63.69241	chr1:2197	ENSG00000231547		Pseudoger	chr1:202999738-203
ENSG0000(	2852	63.69241	chr1:2197	PRELP		protein_c	chr1:203475806-203
ENSG0000(	2852	63.69241	chr1:2197	PPP1R15B		protein_c	chr1:204396492-204
ENSG0000(	2852	63.69241	chr1:2197	KDM5B	AC	protein_c	chr1:202724495-202
ENSG0000(	2852	63.69241	chr1:2197	ZBED6		protein_c	chr1:203795623-203
ENSG0000(	2852	63.69241	chr1:2197	ERLNC1		lncRNA	chr1:204141404-204
ENSG0000(	2852	63.69241	chr1:2197	SNORA77		smallRNA	chr1:203729581-203
ENSG0000(	2852	63.69241	chr1:2197	PPP1R15B-AS1		lncRNA	chr1:204377850-204
ENSG0000(	2852	63.69241	chr1:2197	ENSG00000229657		Pseudoger	chr1:204946608-204
ENSG0000(	2852	63.69241	chr1:2197	ENSG00000229652		Pseudoger	chr1:203353365-203
ENSG0000(	2852	63.69241	chr1:2197	ENSG00000291234		lncRNA	chr1:202851828-202
ENSG0000(	2852	63.69241	chr1:2197	ENSG00000219133		Pseudoger	chr1:204346776-204
ENSG0000(	2852	63.69241	chr1:2197	KRT8P29		Pseudoger	chr1:203872574-203
ENSG0000(	2852	63.69241	chr1:2197	ENSG00000240219		lncRNA	chr1:204626775-204
ENSG0000(	2852	63.69241	chr1:2197	BLACAT1		protein_c	chr1:205434885-205
ENSG0000(	2852	63.69241	chr1:2197	ENSG00000288934		lncRNA	chr1:204411365-204
ENSG0000(	2852	63.69241	chr1:2197	TMEM183A		protein_c	chr1:203007374-203
ENSG0000(	2852	63.69241	chr1:2197	TMCC2-AS1		lncRNA	chr1:205233821-205
ENSG0000(	2852	63.69241	chr1:2197	ENSG00000226862		lncRNA	chr1:202604268-202
ENSG0000(	2832	63.24576	chr1:2197	ENSG00000232261		Pseudoger	chr1:171751543-171
ENSG0000(	2832	63.24576	chr1:2197	ENSG00000226552		Pseudoger	chr1:171083565-171
ENSG0000(	2832	63.24576	chr1:2197	ENSG00000225243		lncRNA	chr1:171199244-171
ENSG0000(	2832	63.24576	chr1:2197	snoU13		smallRNA	chr1:171481907-171
ENSG0000(	2832	63.24576	chr1:2197	RNU6-773P		smallRNA	chr1:171519816-171
ENSG0000(	2832	63.24576	chr1:2197	Y_RNA		smallRNA	chr1:171253906-171
ENSG0000(	2832	63.24576	chr1:2197	MROH9		protein_c	chr1:170935526-171
ENSG0000(	2832	63.24576	chr1:2197	FMO6P		Pseudoger	chr1:171137740-171
ENSG0000(	2832	63.24576	chr1:2197	FMO3		protein_c	chr1:171090901-171
ENSG0000(	2832	63.24576	chr1:2197	RN7SL425P		smallRNA	chr1:171492411-171
ENSG0000(	2832	63.24576	chr1:2197	PFN1P1		Pseudoger	chr1:171670517-171
ENSG0000(	2832	63.24576	chr1:2197	PRRC2C	DriverDB\	protein_c	chr1:171485530-171
ENSG0000(	2832	63.24576	chr1:2197	Y_RNA		smallRNA	chr1:171814512-171
ENSG0000(	2832	63.24576	chr1:2197	ENSG00000236741		Pseudoger	chr1:171762074-171
ENSG0000(	2832	63.24576	chr1:2197	ENSG00000213060		Pseudoger	chr1:171803517-171
ENSG0000(	2832	63.24576	chr1:2197	SRP14P4		Pseudoger	chr1:171345105-171
ENSG0000(	2832	63.24576	chr1:2197	VAMP4	NCGv7	protein_c	chr1:171700160-171
ENSG0000(	2832	63.24576	chr1:2197	ENSG00000224600		Pseudoger	chr1:171824610-171
ENSG0000(	2832	63.24576	chr1:2197	FMO2		protein_c	chr1:171185249-171

ENSG0000(	2832	63.24576	chr1:2197	ENSG00000271459		Pseudoger	chr1:171755803-171
ENSG0000(	2832	63.24576	chr1:2197	FMO4		protein_c	chr1:171314183-171
ENSG0000(	2832	63.24576	chr1:2197	BX284613.1		smallRNA	chr1:171041347-171
ENSG0000(	2832	63.24576	chr1:2197	CYCSP53		Pseudoger	chr1:171444699-171
ENSG0000(	2832	63.24576	chr1:2197	ENSG00000231424		lncRNA	chr1:170748573-171
ENSG0000(	2832	63.24576	chr1:2197	RPL4P3		Pseudoger	chr1:171683128-171
ENSG0000(	2832	63.24576	chr1:2197	METTL13		protein_c	chr1:171781660-171
ENSG0000(	2832	63.24576	chr1:2197	FMO1		protein_c	chr1:171248471-171
ENSG0000(	2832	63.24576	chr1:2197	ENSG00000271811		lncRNA	chr1:170667381-170
ENSG0000(	2832	63.24576	chr1:2197	RNU6-290P		smallRNA	chr1:171418644-171
ENSG0000(	2832	63.24576	chr1:2197	SCARNA20		smallRNA	chr1:171768070-171
ENSG0000(	2832	63.24576	chr1:2197	ENSG00000235303		lncRNA	chr1:170598854-170
ENSG0000(	2832	63.24576	chr1:2197	HMGB1P11		Pseudoger	chr1:171270954-171
ENSG0000(	2832	63.24576	chr1:2197	MYOC		protein_c	chr1:171635417-171
ENSG0000(	2832	63.24576	chr1:2197	MIR1295A		smallRNA	chr1:171101728-171
ENSG0000(	2832	63.24576	chr1:2197	PRRX1	NCGv7	protein_c	chr1:170662728-170
ENSG0000(	2832	63.24576	chr1:2197	MYOCOS		protein_c	chr1:171600621-171
ENSG0000(	2832	63.24576	chr1:2197	GM2AP2		Pseudoger	chr1:171392229-171
ENSG0000(	2820	62.97777	chr1:1043	LINC00466		lncRNA	chr1:63159083-6331
ENSG0000(	2820	62.97777	chr1:1043	ENSG00000213703		Pseudoger	chr1:62641122-6264
ENSG0000(	2820	62.97777	chr1:1043	ENSG00000229225		lncRNA	chr1:63078081-6307
ENSG0000(	2820	62.97777	chr1:1043	DOCK7		protein_c	chr1:62454298-6268
ENSG0000(	2820	62.97777	chr1:1043	ANGPTL3		protein_c	chr1:62597520-6260
ENSG0000(	2820	62.97777	chr1:1043	RNA5SP49		Pseudoger	chr1:63186336-6318
ENSG0000(	2820	62.97777	chr1:1043	Y_RNA		smallRNA	chr1:63338263-6333
ENSG0000(	2820	62.97777	chr1:1043	AL138847.1		protein_c	chr1:62607766-6260
ENSG0000(	2820	62.97777	chr1:1043	CFL1P3		Pseudoger	chr1:63843196-6384
ENSG0000(	2820	62.97777	chr1:1043	DOCK7-DT		lncRNA	chr1:62688482-6271
ENSG0000(	2820	62.97777	chr1:1043	ATG4C		protein_c	chr1:62784132-6286
ENSG0000(	2820	62.97777	chr1:1043	DLEU2L		lncRNA	chr1:63547082-6355
ENSG0000(	2820	62.97777	chr1:1043	ITGB3BP		protein_c	chr1:63440770-6359
ENSG0000(	2820	62.97777	chr1:1043	ENSG00000237163		Pseudoger	chr1:62905180-6290
ENSG0000(	2820	62.97777	chr1:1043	ENSG00000228734		lncRNA	chr1:63249920-6325
ENSG0000(	2820	62.97777	chr1:1043	LINC01739		lncRNA	chr1:62975751-6302
ENSG0000(	2820	62.97777	chr1:1043	ENSG00000270549		Pseudoger	chr1:62530636-6253
ENSG0000(	2820	62.97777	chr1:1043	ENSG00000203605		lncRNA	chr1:63139250-6316
ENSG0000(	2820	62.97777	chr1:1043	ENSG00000286455		lncRNA	chr1:63011197-6301
ENSG0000(	2820	62.97777	chr1:1043	ALG6		protein_c	chr1:63367575-6343
ENSG0000(	2820	62.97777	chr1:1043	ENSG00000234318		lncRNA	chr1:62896009-6290
ENSG0000(	2820	62.97777	chr1:1043	ENSG00000227485		lncRNA	chr1:63024207-6302
ENSG0000(	2820	62.97777	chr1:1043	PGM1		protein_c	chr1:63593411-6366
ENSG0000(	2820	62.97777	chr1:1043	ENSG00000236674		Pseudoger	chr1:63359823-6336
ENSG0000(	2820	62.97777	chr1:1043	FOXD3-AS1		lncRNA	chr1:63320878-6332
ENSG0000(	2820	62.97777	chr1:1043	EFCAB7		protein_c	chr1:63523372-6357
ENSG0000(	2820	62.97777	chr1:1043	ENSG00000286429		lncRNA	chr1:63487957-6350
ENSG0000(	2820	62.97777	chr1:1043	ENSG00000177452		Pseudoger	chr1:63788721-6378
ENSG0000(	2820	62.97777	chr1:1043	FOXD3		protein_c	chr1:63322567-6332
ENSG0000(	2820	62.97777	chr1:1043	RNU7-123P		smallRNA	chr1:63536711-6353
ENSG0000(	2820	62.97777	chr1:1043	RN7SL488P		smallRNA	chr1:63529617-6352
ENSG0000(	2820	62.97777	chr1:1043	RN7SL130P		smallRNA	chr1:63655743-6365
ENSG0000(	2820	62.97777	chr1:1043	USP1		protein_c	chr1:62436297-6245

ENSG0000(	2820	62.97777	chr1:104:	ENSG00000278967		TEC	chr1:62607766-6260
ENSG0000(	2772	61.90581	chr1:2197	SLC25A38P1		Pseudoger	chr1:172748560-172
ENSG0000(	2772	61.90581	chr1:2197	SNORD112		smallRNA	chr1:172348143-172
ENSG0000(	2772	61.90581	chr1:2197	C1orf105		protein_c	chr1:172420685-172
ENSG0000(	2772	61.90581	chr1:2197	DNM3		protein_c	chr1:171817887-172
ENSG0000(	2772	61.90581	chr1:2197	SUCO		protein_c	chr1:172532349-172
ENSG0000(	2772	61.90581	chr1:2197	ENSG00000287336		lncRNA	chr1:172210711-172
ENSG0000(	2772	61.90581	chr1:2197	MIR214		smallRNA	chr1:172138798-172
ENSG0000(	2772	61.90581	chr1:2197	ENSG00000224228		lncRNA	chr1:172775905-173
ENSG0000(	2772	61.90581	chr1:2197	DNM3-IT1		lncRNA	chr1:171864187-171
ENSG0000(	2772	61.90581	chr1:2197	AIMP1P2		Pseudoger	chr1:172885947-172
ENSG0000(	2772	61.90581	chr1:2197	MIR199A2		smallRNA	chr1:172144535-172
ENSG0000(	2772	61.90581	chr1:2197	FASLG		protein_c	chr1:172659103-172
ENSG0000(	2772	61.90581	chr1:2197	ENSG00000224000		lncRNA	chr1:172906900-172
ENSG0000(	2772	61.90581	chr1:2197	ENSG00000279061		TEC	chr1:172752586-172
ENSG0000(	2772	61.90581	chr1:2197	TNFSF18		protein_c	chr1:173039202-173
ENSG0000(	2772	61.90581	chr1:2197	DNM3OS		lncRNA	chr1:172138397-172
ENSG0000(	2772	61.90581	chr1:2197	RNU6-157P		smallRNA	chr1:172366540-172
ENSG0000(	2772	61.90581	chr1:2197	PIGC	NCGv7	protein_c	chr1:172370189-172
ENSG0000(	2772	61.90581	chr1:2197	RNU6-693P		smallRNA	chr1:172613428-172
ENSG0000(	2742	61.23583	chr1:104:	RNU6-414P		smallRNA	chr1:61816419-6181
ENSG0000(	2742	61.23583	chr1:104:	RNU6-371P		smallRNA	chr1:62298149-6229
ENSG0000(	2742	61.23583	chr1:104:	AC099791.1		smallRNA	chr1:61629031-6162
ENSG0000(	2742	61.23583	chr1:104:	L1TD1		protein_c	chr1:62194849-6221
ENSG0000(	2742	61.23583	chr1:104:	ENSG00000223920		Pseudoger	chr1:61654194-6165
ENSG0000(	2742	61.23583	chr1:104:	MIR3116-1		smallRNA	chr1:62078786-6207
ENSG0000(	2742	61.23583	chr1:104:	RNU6-1177P		smallRNA	chr1:61852499-6185
ENSG0000(	2742	61.23583	chr1:104:	ENSG00000287224		lncRNA	chr1:61588049-6158
ENSG0000(	2742	61.23583	chr1:104:	KANK4		protein_c	chr1:62236165-6231
ENSG0000(	2742	61.23583	chr1:104:	LAMTOR5P1		Pseudoger	chr1:62038842-6203
ENSG0000(	2742	61.23583	chr1:104:	PATJ-DT		lncRNA	chr1:61741998-6174
ENSG0000(	2742	61.23583	chr1:104:	ENSG00000237227		Pseudoger	chr1:62208136-6220
ENSG0000(	2742	61.23583	chr1:104:	PATJ	NCGv7	protein_c	chr1:61742477-6217
ENSG0000(	2742	61.23583	chr1:104:	RPS15AP7		Pseudoger	chr1:62190522-6219
ENSG0000(	2742	61.23583	chr1:104:	Y_RNA		smallRNA	chr1:62211557-6221
ENSG0000(	2742	61.23583	chr1:104:	RN7SL180P		smallRNA	chr1:62072448-6207
ENSG0000(	2742	61.23583	chr1:104:	PIGPP2		Pseudoger	chr1:62189131-6218
ENSG0000(	2742	61.23583	chr1:104:	TM2D1		protein_c	chr1:61681046-6172
ENSG0000(	2718	60.69985	chr1:2197	PYDC5		protein_c	chr1:158999971-159
ENSG0000(	2718	60.69985	chr1:2197	ENSG00000288775		lncRNA	chr1:159776325-159
ENSG0000(	2718	60.69985	chr1:2197	CRPP1		Pseudoger	chr1:159704983-159
ENSG0000(	2718	60.69985	chr1:2197	OR10J2P		Pseudoger	chr1:159279041-159
ENSG0000(	2718	60.69985	chr1:2197	ENSG00000198358		lncRNA	chr1:160932465-160
ENSG0000(	2718	60.69985	chr1:2197	ENSG00000227741		lncRNA	chr1:160202199-160
ENSG0000(	2718	60.69985	chr1:2197	SMIM42		protein_c	chr1:158127287-158
ENSG0000(	2718	60.69985	chr1:2197	SLAMF1	NCGv7	protein_c	chr1:160608106-160
ENSG0000(	2718	60.69985	chr1:2197	RP11-226L15.5		lncRNA	chr1:160024953-160
ENSG0000(	2718	60.69985	chr1:2197	ENSG00000228863		lncRNA	chr1:160670778-160
ENSG0000(	2718	60.69985	chr1:2197	ATP1A4		protein_c	chr1:160151586-160
ENSG0000(	2718	60.69985	chr1:2197	SUMO1P3		Pseudoger	chr1:160317403-160
ENSG0000(	2718	60.69985	chr1:2197	DUSP23	DriverDB\	protein_c	chr1:159780932-159

ENSG0000(	2718	60.69985	chr1:2197	FCER1A	NCGv7	protein_c	chr1:159289714-159
ENSG0000(	2718	60.69985	chr1:2197	OR10J6P		Pseudoger	chr1:159598298-159
ENSG0000(	2718	60.69985	chr1:2197	ITLN2		protein_c	chr1:160945025-160
ENSG0000(	2718	60.69985	chr1:2197	EI24P2		Pseudoger	chr1:158454198-158
ENSG0000(	2718	60.69985	chr1:2197	F11R	DriverDB\	protein_c	chr1:160995211-161
ENSG0000(	2718	60.69985	chr1:2197	OR10X1		protein_c	chr1:158578919-158
ENSG0000(	2718	60.69985	chr1:2197	APCS		protein_c	chr1:159587826-159
ENSG0000(	2718	60.69985	chr1:2197	CRP	NCGv7	protein_c	chr1:159712289-159
ENSG0000(	2718	60.69985	chr1:2197	RNA5SP60		Pseudoger	chr1:159178473-159
ENSG0000(	2718	60.69985	chr1:2197	OR10AE1P		Pseudoger	chr1:159581620-159
ENSG0000(	2718	60.69985	chr1:2197	OR6K3		protein_c	chr1:158716327-158
ENSG0000(	2718	60.69985	chr1:2197	OR2AQ1P		Pseudoger	chr1:158796014-158
ENSG0000(	2718	60.69985	chr1:2197	OR6N1		protein_c	chr1:158747814-158
ENSG0000(	2718	60.69985	chr1:2197	OR10J5		protein_c	chr1:159535078-159
ENSG0000(	2718	60.69985	chr1:2197	HSP90AA3P		Pseudoger	chr1:158523672-158
ENSG0000(	2718	60.69985	chr1:2197	OR6P1		protein_c	chr1:158560606-158
ENSG0000(	2718	60.69985	chr1:2197	SNORD64		smallRNA	chr1:159851906-159
ENSG0000(	2718	60.69985	chr1:2197	OR6Y1		protein_c	chr1:158544550-158
ENSG0000(	2718	60.69985	chr1:2197	CD48		protein_c	chr1:160678746-160
ENSG0000(	2718	60.69985	chr1:2197	AIM2		protein_c	chr1:159061599-159
ENSG0000(	2718	60.69985	chr1:2197	OR10J7P		Pseudoger	chr1:159351093-159
ENSG0000(	2718	60.69985	chr1:2197	LINC01704		lncRNA	chr1:158131983-158
ENSG0000(	2718	60.69985	chr1:2197	ENSG00000287040		lncRNA	chr1:159900475-159
ENSG0000(	2718	60.69985	chr1:2197	OR10J9P		Pseudoger	chr1:159405423-159
ENSG0000(	2718	60.69985	chr1:2197	NHLH1		protein_c	chr1:160367071-160
ENSG0000(	2718	60.69985	chr1:2197	PPIAP37		Pseudoger	chr1:160848010-160
ENSG0000(	2718	60.69985	chr1:2197	RPSAP18		Pseudoger	chr1:160266340-160
ENSG0000(	2718	60.69985	chr1:2197	AL359753.1		smallRNA	chr1:159059249-159
ENSG0000(	2718	60.69985	chr1:2197	LINC01133		lncRNA	chr1:159958035-159
ENSG0000(	2718	60.69985	chr1:2197	ENSG00000289484		lncRNA	chr1:159501664-159
ENSG0000(	2718	60.69985	chr1:2197	SLAMF7		protein_c	chr1:160739057-160
ENSG0000(	2718	60.69985	chr1:2197	ENSG00000234425		lncRNA	chr1:160537073-160
ENSG0000(	2718	60.69985	chr1:2197	SNHG28		lncRNA	chr1:159834480-159
ENSG0000(	2718	60.69985	chr1:2197	hsa-mir-4259		smallRNA	chr1:159899979-159
ENSG0000(	2718	60.69985	chr1:2197	CD84		protein_c	chr1:160541095-160
ENSG0000(	2718	60.69985	chr1:2197	AL590560.1		protein_c	chr1:159910094-159
ENSG0000(	2718	60.69985	chr1:2197	ENSG00000235226		Pseudoger	chr1:159759170-159
ENSG0000(	2718	60.69985	chr1:2197	LINC02819		lncRNA	chr1:159466321-159
ENSG0000(	2718	60.69985	chr1:2197	ENSG00000236656		lncRNA	chr1:158474454-158
ENSG0000(	2718	60.69985	chr1:2197	ENSG00000229914		Pseudoger	chr1:158195633-158
ENSG0000(	2718	60.69985	chr1:2197	AL138930.2		smallRNA	chr1:160545648-160
ENSG0000(	2718	60.69985	chr1:2197	SETP9		Pseudoger	chr1:160670148-160
ENSG0000(	2718	60.69985	chr1:2197	OR10R2	NCGv7	protein_c	chr1:158472220-158
ENSG0000(	2718	60.69985	chr1:2197	PYHIN5P		Pseudoger	chr1:158878746-158
ENSG0000(	2718	60.69985	chr1:2197	KCNJ10		protein_c	chr1:159998651-160
ENSG0000(	2718	60.69985	chr1:2197	OR10K1		protein_c	chr1:158461574-158
ENSG0000(	2718	60.69985	chr1:2197	IFI16		protein_c	chr1:158999968-159
ENSG0000(	2718	60.69985	chr1:2197	OR6N2		protein_c	chr1:158774222-158
ENSG0000(	2718	60.69985	chr1:2197	ENSG00000237409		Pseudoger	chr1:160020300-160
ENSG0000(	2718	60.69985	chr1:2197	PYHIN1		protein_c	chr1:158930796-158
ENSG0000(	2718	60.69985	chr1:2197	MNDA		protein_c	chr1:158831351-158

ENSG0000(	2718	60.69985	chr1:2197	KIRREL1		protein_c	chr1:157993273-158
ENSG0000(	2718	60.69985	chr1:2197	ENSG00000225279		lncRNA	chr1:160062461-160
ENSG0000(	2718	60.69985	chr1:2197	KIRREL1-IT1		lncRNA	chr1:158025550-158
ENSG0000(	2718	60.69985	chr1:2197	SPTA1	NCGv7	protein_c	chr1:158610704-158
ENSG0000(	2718	60.69985	chr1:2197	ENSG00000272668		lncRNA	chr1:159854870-159
ENSG0000(	2718	60.69985	chr1:2197	ENSG00000256029		protein_c	chr1:159834474-159
ENSG0000(	2718	60.69985	chr1:2197	ATP1A2		protein_c	chr1:160115759-160
ENSG0000(	2718	60.69985	chr1:2197	OR10Z1		protein_c	chr1:158605268-158
ENSG0000(	2718	60.69985	chr1:2197	OR10T2		protein_c	chr1:158398522-158
ENSG0000(	2718	60.69985	chr1:2197	ACKR1		protein_c	chr1:159203307-159
ENSG0000(	2718	60.69985	chr1:2197	ENSG00000275801		Pseudoger	chr1:160775954-160
ENSG0000(	2718	60.69985	chr1:2197	OR10T1P		Pseudoger	chr1:158445068-158
ENSG0000(	2718	60.69985	chr1:2197	ENSG00000176320		lncRNA	chr1:158197922-158
ENSG0000(	2718	60.69985	chr1:2197	PEX19		protein_c	chr1:160276807-160
ENSG0000(	2718	60.69985	chr1:2197	NCSTN	NCGv7	protein_c	chr1:160343294-160
ENSG0000(	2718	60.69985	chr1:2197	VANGL2		protein_c	chr1:160400564-160
ENSG0000(	2718	60.69985	chr1:2197	ENSG00000258465		protein_c	chr1:160216800-160
ENSG0000(	2718	60.69985	chr1:2197	SLAMF6		protein_c	chr1:160485030-160
ENSG0000(	2718	60.69985	chr1:2197	ENSG00000233691		Pseudoger	chr1:160935537-160
ENSG0000(	2718	60.69985	chr1:2197	RNU4-42P		smallRNA	chr1:160392768-160
ENSG0000(	2718	60.69985	chr1:2197	OR6K2		protein_c	chr1:158699678-158
ENSG0000(	2718	60.69985	chr1:2197	CADM3-AS1		lncRNA	chr1:159194325-159
ENSG0000(	2718	60.69985	chr1:2197	ENSG00000290105		lncRNA	chr1:159890207-159
ENSG0000(	2718	60.69985	chr1:2197	ENSG00000279430		TEC	chr1:159910094-159
ENSG0000(	2718	60.69985	chr1:2197	OR10K2		protein_c	chr1:158418210-158
ENSG0000(	2718	60.69985	chr1:2197	COPA		protein_c	chr1:160288594-160
ENSG0000(	2718	60.69985	chr1:2197	CD244		protein_c	chr1:160830160-160
ENSG0000(	2718	60.69985	chr1:2197	OR10R1P		Pseudoger	chr1:158514785-158
ENSG0000(	2718	60.69985	chr1:2197	CFAP45	NCGv7	protein_c	chr1:159872364-159
ENSG0000(	2718	60.69985	chr1:2197	CASQ1		protein_c	chr1:160190575-160
ENSG0000(	2718	60.69985	chr1:2197	PIGM	DriverDBv	protein_c	chr1:160024953-160
ENSG0000(	2718	60.69985	chr1:2197	Y_RNA		smallRNA	chr1:160326104-160
ENSG0000(	2718	60.69985	chr1:2197	ENSG00000228560		lncRNA	chr1:159346166-159
ENSG0000(	2718	60.69985	chr1:2197	OR10J1		protein_c	chr1:159437845-159
ENSG0000(	2718	60.69985	chr1:2197	PEA15		protein_c	chr1:160205380-160
ENSG0000(	2718	60.69985	chr1:2197	OR6K5P		Pseudoger	chr1:158742146-158
ENSG0000(	2718	60.69985	chr1:2197	RAD1P2		Pseudoger	chr1:159081133-159
ENSG0000(	2718	60.69985	chr1:2197	HMGN1P5		Pseudoger	chr1:158266753-158
ENSG0000(	2718	60.69985	chr1:2197	OR10J3		protein_c	chr1:159313720-159
ENSG0000(	2718	60.69985	chr1:2197	AL121985.1		Pseudoger	chr1:160697970-160
ENSG0000(	2718	60.69985	chr1:2197	ENSG00000276632		Pseudoger	chr1:159649151-159
ENSG0000(	2718	60.69985	chr1:2197	OR10R3P		Pseudoger	chr1:158491219-158
ENSG0000(	2718	60.69985	chr1:2197	OR6K4P		Pseudoger	chr1:158724113-158
ENSG0000(	2718	60.69985	chr1:2197	IGSF9	DriverDBv	protein_c	chr1:159927039-159
ENSG0000(	2718	60.69985	chr1:2197	OR6K6		protein_c	chr1:158754720-158
ENSG0000(	2718	60.69985	chr1:2197	ENSG00000232188		lncRNA	chr1:160931739-160
ENSG0000(	2718	60.69985	chr1:2197	OR10AA1P		Pseudoger	chr1:158808399-158
ENSG0000(	2718	60.69985	chr1:2197	MPTX1		Pseudoger	chr1:159276503-159
ENSG0000(	2718	60.69985	chr1:2197	ELL2P1		Pseudoger	chr1:158175850-158
ENSG0000(	2718	60.69985	chr1:2197	CADM3		protein_c	chr1:159171609-159
ENSG0000(	2718	60.69985	chr1:2197	SLAMF9		protein_c	chr1:159951492-159

ENSG0000(	2718	60.69985	chr1:2197	DCAF8-DT		lncRNA	chr1:160261731-160
ENSG0000(	2718	60.69985	chr1:2197	KCNJ9	NCGv7	protein_c	chr1:160081538-160
ENSG0000(	2718	60.69985	chr1:2197	OR10J8P		Pseudoger	chr1:159366161-159
ENSG0000(	2718	60.69985	chr1:2197	ENSG00000231100		Pseudoger	chr1:159557368-159
ENSG0000(	2718	60.69985	chr1:2197	IGSF8		protein_c	chr1:160091340-160
ENSG0000(	2718	60.69985	chr1:2197	OR6K1P		Pseudoger	chr1:158694539-158
ENSG0000(	2718	60.69985	chr1:2197	LY9		protein_c	chr1:160796074-160
ENSG0000(	2718	60.69985	chr1:2197	FCRL6		protein_c	chr1:159800511-159
ENSG0000(	2718	60.69985	chr1:2197	SLAMF8		protein_c	chr1:159826811-159
ENSG0000(	2718	60.69985	chr1:2197	OR10J4		protein_c	chr1:159432204-159
ENSG0000(	2718	60.69985	chr1:2197	AL121987.1		smallRNA	chr1:160205377-160
ENSG0000(	2718	60.69985	chr1:2197	VSIG8		protein_c	chr1:159854316-159
ENSG0000(	2718	60.69985	chr1:2197	ENSG00000273933		Pseudoger	chr1:159972548-159
ENSG0000(	2718	60.69985	chr1:2197	ENSG00000274562		Pseudoger	chr1:160776975-160
ENSG0000(	2718	60.69985	chr1:2197	CD1D	NCGv7	protein_c	chr1:158178030-158
ENSG0000(	2718	60.69985	chr1:2197	CD1A	NCGv7	protein_c	chr1:158254424-158
ENSG0000(	2718	60.69985	chr1:2197	TAGLN2		protein_c	chr1:159918107-159
ENSG0000(	2718	60.69985	chr1:2197	ITLN1	NCGv7	protein_c	chr1:160876540-160
ENSG0000(	2718	60.69985	chr1:2197	AL138930.1		smallRNA	chr1:160503376-160
ENSG0000(	2718	60.69985	chr1:2197	CD1C		protein_c	chr1:158289923-158
ENSG0000(	2718	60.69985	chr1:2197	ENSG00000213080		Pseudoger	chr1:160894980-160
ENSG0000(	2718	60.69985	chr1:2197	DCAF8		protein_c	chr1:160215715-160
ENSG0000(	2718	60.69985	chr1:2197	CD1B	NCGv7	protein_c	chr1:158327951-158
ENSG0000(	2718	60.69985	chr1:2197	CD1E		protein_c	chr1:158353696-158
ENSG0000(	2619	58.48893	chr1:2197	MIR5187		smallRNA	chr1:161227186-161
ENSG0000(	2619	58.48893	chr1:2197	ENSG00000288670		lncRNA	chr1:161368022-161
ENSG0000(	2619	58.48893	chr1:2197	ENSG00000277882		Pseudoger	chr1:161411597-161
ENSG0000(	2619	58.48893	chr1:2197	CFAP126		protein_c	chr1:161364733-161
ENSG0000(	2619	58.48893	chr1:2197	ADAMTS4		protein_c	chr1:161184302-161
ENSG0000(	2619	58.48893	chr1:2197	ENSG00000283317		lncRNA	chr1:161433444-161
ENSG0000(	2619	58.48893	chr1:2197	SDHC	NCGv7;AC	protein_c	chr1:161314381-161
ENSG0000(	2619	58.48893	chr1:2197	DEDD	DriverDB\	protein_c	chr1:161120974-161
ENSG0000(	2619	58.48893	chr1:2197	MPZ		protein_c	chr1:161304735-161
ENSG0000(	2619	58.48893	chr1:2197	TOMM40L		protein_c	chr1:161225939-161
ENSG0000(	2619	58.48893	chr1:2197	NDUFS2		protein_c	chr1:161197104-161
ENSG0000(	2619	58.48893	chr1:2197	APOA2		protein_c	chr1:161222292-161
ENSG0000(	2619	58.48893	chr1:2197	FCER1G		protein_c	chr1:161215234-161
ENSG0000(	2619	58.48893	chr1:2197	ENSG00000283696		lncRNA	chr1:161399409-161
ENSG0000(	2619	58.48893	chr1:2197	ENSG00000289141		lncRNA	chr1:161389547-161
ENSG0000(	2619	58.48893	chr1:2197	B4GALT3	NCGv7	protein_c	chr1:161171310-161
ENSG0000(	2619	58.48893	chr1:2197	RP11-122G18.8		lncRNA	chr1:161374762-161
ENSG0000(	2619	58.48893	chr1:2197	NIT1		protein_c	chr1:161118086-161
ENSG0000(	2619	58.48893	chr1:2197	ENSG00000288093		lncRNA	chr1:161399998-161
ENSG0000(	2619	58.48893	chr1:2197	PFDN2	DriverDB\	protein_c	chr1:161100556-161
ENSG0000(	2619	58.48893	chr1:2197	GLRX5P2		Pseudoger	chr1:161034834-161
ENSG0000(	2619	58.48893	chr1:2197	NR1I3	DriverDB\	protein_c	chr1:161229666-161
ENSG0000(	2619	58.48893	chr1:2197	USP21	DriverDB\	protein_c	chr1:161159450-161
ENSG0000(	2619	58.48893	chr1:2197	ENSG00000270149		protein_c	chr1:160997957-161
ENSG0000(	2619	58.48893	chr1:2197	NECTIN4-AS1		lncRNA	chr1:161084465-161
ENSG0000(	2619	58.48893	chr1:2197	ENSG00000289121		lncRNA	chr1:161046027-161
ENSG0000(	2619	58.48893	chr1:2197	RRM2P2		Pseudoger	chr1:161378707-161

ENSG0000(	2619	58.48893	chr1:2197	PPOX	DriverDB\	protein_c	chr1:161166056-161
ENSG0000(	2619	58.48893	chr1:2197	ENSG00000215840		Pseudoger	chr1:161406068-161
ENSG0000(	2619	58.48893	chr1:2197	ENSG00000290115		lncRNA	chr1:161165695-161
ENSG0000(	2619	58.48893	chr1:2197	UFC1	DriverDB\	protein_c	chr1:161152776-161
ENSG0000(	2619	58.48893	chr1:2197	NECTIN4	DriverDB\	protein_c	chr1:161070998-161
ENSG0000(	2619	58.48893	chr1:2197	AL591806.1		protein_c	chr1:161065865-161
ENSG0000(	2619	58.48893	chr1:2197	ENSG00000289106		lncRNA	chr1:161364221-161
ENSG0000(	2619	58.48893	chr1:2197	ENSG00000224985		lncRNA	chr1:161153760-161
ENSG0000(	2619	58.48893	chr1:2197	USF1		protein_c	chr1:161039251-161
ENSG0000(	2619	58.48893	chr1:2197	RNU6-481P		smallRNA	chr1:161401289-161
ENSG0000(	2619	58.48893	chr1:2197	KLHDC9	DriverDB\	protein_c	chr1:161098361-161
ENSG0000(	2619	58.48893	chr1:2197	ARHGAP30		protein_c	chr1:161046946-161
ENSG0000(	2619	58.48893	chr1:2197	AL590714.1		protein_c	chr1:161220370-161
ENSG0000(	2619	58.48893	chr1:2197	ACA64		smallRNA	chr1:161141208-161
ENSG0000(	2619	58.48893	chr1:2197	TSTD1	DriverDB\	protein_c	chr1:161037631-161
ENSG0000(	2619	58.48893	chr1:2197	PCP4L1		protein_c	chr1:161258745-161
ENSG0000(	2576	57.52863	chr1:104:	ENSG00000227556		Pseudoger	chr1:78317157-7831
ENSG0000(	2570	57.39464	chr1:2197	ENSG00000227094		lncRNA	chr1:162316852-162
ENSG0000(	2570	57.39464	chr1:2197	NOS1AP		protein_c	chr1:162069691-162
ENSG0000(	2570	57.39464	chr1:2197	ENSG00000229808		Pseudoger	chr1:161890833-161
ENSG0000(	2570	57.39464	chr1:2197	RN7SL466P		smallRNA	chr1:161735808-161
ENSG0000(	2570	57.39464	chr1:2197	FCGR2A		protein_c	chr1:161505430-161
ENSG0000(	2570	57.39464	chr1:2197	RPL31P11		Pseudoger	chr1:161683695-161
ENSG0000(	2570	57.39464	chr1:2197	DUSP12	AC	protein_c	chr1:161749758-161
ENSG0000(	2570	57.39464	chr1:2197	RPS23P9		Pseudoger	chr1:161617992-161
ENSG0000(	2570	57.39464	chr1:2197	ENSG00000289768		protein_c	chr1:161544807-161
ENSG0000(	2570	57.39464	chr1:2197	ENSG00000234211		lncRNA	chr1:161671978-161
ENSG0000(	2570	57.39464	chr1:2197	Y_RNA		smallRNA	chr1:161699506-161
ENSG0000(	2570	57.39464	chr1:2197	FCGR2B	NCGv7;AC	protein_c	chr1:161663143-161
ENSG0000(	2570	57.39464	chr1:2197	ATF6-DT		lncRNA	chr1:161749452-161
ENSG0000(	2570	57.39464	chr1:2197	ENSG00000285636		lncRNA	chr1:162146709-162
ENSG0000(	2570	57.39464	chr1:2197	ENSG00000254706		protein_c	chr1:162365407-162
ENSG0000(	2570	57.39464	chr1:2197	FCRLA		protein_c	chr1:161706972-161
ENSG0000(	2570	57.39464	chr1:2197	SPATA46		protein_c	chr1:162373203-162
ENSG0000(	2570	57.39464	chr1:2197	HSPA6		protein_c	chr1:161524540-161
ENSG0000(	2570	57.39464	chr1:2197	HSPA7		Pseudoger	chr1:161606291-161
ENSG0000(	2570	57.39464	chr1:2197	C1orf226		protein_c	chr1:162378841-162
ENSG0000(	2570	57.39464	chr1:2197	MIR4654		smallRNA	chr1:162157107-162
ENSG0000(	2570	57.39464	chr1:2197	ENSG00000283360		lncRNA	chr1:161403409-161
ENSG0000(	2570	57.39464	chr1:2197	ENSG00000289273		lncRNA	chr1:161518705-161
ENSG0000(	2570	57.39464	chr1:2197	MIR556		smallRNA	chr1:162342546-162
ENSG0000(	2570	57.39464	chr1:2197	ENSG00000224515		lncRNA	chr1:161556290-161
ENSG0000(	2570	57.39464	chr1:2197	FCGR3A	NCGv7	protein_c	chr1:161541759-161
ENSG0000(	2570	57.39464	chr1:2197	ENSG00000273112		lncRNA	chr1:161513176-161
ENSG0000(	2570	57.39464	chr1:2197	FCGR2C		protein_c	chr1:161581339-161
ENSG0000(	2570	57.39464	chr1:2197	OLFML2B		protein_c	chr1:161983192-162
ENSG0000(	2570	57.39464	chr1:2197	RPS23P10		Pseudoger	chr1:161536571-161
ENSG0000(	2570	57.39464	chr1:2197	FCGR3B		protein_c	chr1:161623196-161
ENSG0000(	2570	57.39464	chr1:2197	ATF6		protein_c	chr1:161766298-161
ENSG0000(	2570	57.39464	chr1:2197	FCRLB	DriverDB\	protein_c	chr1:161721544-161
ENSG0000(	2570	57.39464	chr1:2197	RNA5SP61		Pseudoger	chr1:162338643-162

ENSG0000(	2570	57.39464	chr1:2197	ENSG00000227818	lncRNA	chr1:162039016-162
ENSG0000(	2566	57.30531	chr1:1043	NEXN-AS1	lncRNA	chr1:77881348-7788
ENSG0000(	2566	57.30531	chr1:1043	ENSG00000233099	lncRNA	chr1:77346046-7734
ENSG0000(	2566	57.30531	chr1:1043	RNA5SP22	Pseudoger	chr1:78094807-7809
ENSG0000(	2566	57.30531	chr1:1043	LINC02567	lncRNA	chr1:76758124-7677
ENSG0000(	2566	57.30531	chr1:1043	ENSG00000261213	lncRNA	chr1:75122518-7512
ENSG0000(	2566	57.30531	chr1:1043	MIGA1	protein_c	chr1:77779624-7787
ENSG0000(	2566	57.30531	chr1:1043	ENSG00000233894	lncRNA	chr1:74468195-7446
ENSG0000(	2566	57.30531	chr1:1043	LINC02792	lncRNA	chr1:79325008-7934
ENSG0000(	2566	57.30531	chr1:1043	ENSG00000235011	lncRNA	chr1:79323769-7932
ENSG0000(	2566	57.30531	chr1:1043	ENSG00000285928	lncRNA	chr1:78022565-7802
ENSG0000(	2566	57.30531	chr1:1043	RNU6-1102P	smallRNA	chr1:78088988-7808
ENSG0000(	2566	57.30531	chr1:1043	PIGK	protein_c	chr1:77088989-7721
ENSG0000(	2566	57.30531	chr1:1043	RNFT1P2	Pseudoger	chr1:78170481-7817
ENSG0000(	2566	57.30531	chr1:1043	ENSG00000213579	Pseudoger	chr1:75582099-7558
ENSG0000(	2566	57.30531	chr1:1043	AC096951.1	smallRNA	chr1:77007744-7700
ENSG0000(	2566	57.30531	chr1:1043	ENSG00000287870	lncRNA	chr1:77248633-7725
ENSG0000(	2566	57.30531	chr1:1043	RN7SL370P	smallRNA	chr1:77645324-7764
ENSG0000(	2566	57.30531	chr1:1043	SLC44A5 NCGv7	protein_c	chr1:75202129-7561
ENSG0000(	2566	57.30531	chr1:1043	MSH4	protein_c	chr1:75796882-7591
ENSG0000(	2566	57.30531	chr1:1043	ENSG00000288543	lncRNA	chr1:77067920-7707
ENSG0000(	2566	57.30531	chr1:1043	ENSG00000224493	Pseudoger	chr1:75521562-7552
ENSG0000(	2566	57.30531	chr1:1043	MGC27382	lncRNA	chr1:78229599-7836
ENSG0000(	2566	57.30531	chr1:1043	ENSG00000282898	lncRNA	chr1:79323769-7932
ENSG0000(	2566	57.30531	chr1:1043	ENSG00000223905	Pseudoger	chr1:76353583-7635
ENSG0000(	2566	57.30531	chr1:1043	IFI44	protein_c	chr1:78649796-7866
ENSG0000(	2566	57.30531	chr1:1043	ASB17	protein_c	chr1:75918873-7593
ENSG0000(	2566	57.30531	chr1:1043	AK5	protein_c	chr1:77282019-7755
ENSG0000(	2566	57.30531	chr1:1043	ENSG00000235400	Pseudoger	chr1:78749073-7875
ENSG0000(	2566	57.30531	chr1:1043	FPGT-TNNI3K	protein_c	chr1:74198235-7454
ENSG0000(	2566	57.30531	chr1:1043	SNORD45A	smallRNA	chr1:75787889-7578
ENSG0000(	2566	57.30531	chr1:1043	TNNI3K	protein_c	chr1:74235387-7454
ENSG0000(	2566	57.30531	chr1:1043	ENSG00000229943	lncRNA	chr1:74963314-7496
ENSG0000(	2566	57.30531	chr1:1043	CRYZ	protein_c	chr1:74705482-7473
ENSG0000(	2566	57.30531	chr1:1043	RNU7-8P	smallRNA	chr1:77420325-7742
ENSG0000(	2566	57.30531	chr1:1043	RNU6-503P	smallRNA	chr1:75538015-7553
ENSG0000(	2566	57.30531	chr1:1043	RNU6-161P	smallRNA	chr1:76753135-7675
ENSG0000(	2566	57.30531	chr1:1043	ENSG00000230027	lncRNA	chr1:76041691-7606
ENSG0000(	2566	57.30531	chr1:1043	ENSG00000225605	lncRNA	chr1:75926454-7601
ENSG0000(	2566	57.30531	chr1:1043	HSPE1P25	Pseudoger	chr1:77853355-7785
ENSG0000(	2566	57.30531	chr1:1043	ENSG00000238015	Pseudoger	chr1:78666272-7866
ENSG0000(	2566	57.30531	chr1:1043	SNORD45B	smallRNA	chr1:75789477-7578
ENSG0000(	2566	57.30531	chr1:1043	ERICH3	protein_c	chr1:74568117-7467
ENSG0000(	2566	57.30531	chr1:1043	ACTG1P21	Pseudoger	chr1:77773865-7777
ENSG0000(	2566	57.30531	chr1:1043	USP33	protein_c	chr1:77695987-7775
ENSG0000(	2566	57.30531	chr1:1043	RNU6-622P	smallRNA	chr1:75183045-7518
ENSG0000(	2566	57.30531	chr1:1043	RNA5SP20	Pseudoger	chr1:77614869-7761
ENSG0000(	2566	57.30531	chr1:1043	NSRP1P1	Pseudoger	chr1:77847110-7784
ENSG0000(	2566	57.30531	chr1:1043	AC104837.1	smallRNA	chr1:78687060-7868
ENSG0000(	2566	57.30531	chr1:1043	ERICH3-AS1	lncRNA	chr1:74577430-7462
ENSG0000(	2566	57.30531	chr1:1043	ENSG00000224149	lncRNA	chr1:75129974-7513

ENSG0000(	2566	57.30531	chr1:1043	ENSG00000213561		Pseudoger	chr1:78043383-7804
ENSG0000(	2566	57.30531	chr1:1043	AC104458.1		smallRNA	chr1:76718140-7671
ENSG0000(	2566	57.30531	chr1:1043	SNORD45C		smallRNA	chr1:75787072-7578
ENSG0000(	2566	57.30531	chr1:1043	DLSTP1		Pseudoger	chr1:75743423-7574
ENSG0000(	2566	57.30531	chr1:1043	RNA5SP23		Pseudoger	chr1:78375164-7837
ENSG0000(	2566	57.30531	chr1:1043	RNA5SP21		Pseudoger	chr1:77779904-7777
ENSG0000(	2566	57.30531	chr1:1043	LRRC53		protein_c	chr1:74469376-7451
ENSG0000(	2566	57.30531	chr1:1043	ACADM		protein_c	chr1:75724431-7578
ENSG0000(	2566	57.30531	chr1:1043	ENSG00000226084		Pseudoger	chr1:77129114-7712
ENSG0000(	2566	57.30531	chr1:1043	ENSG00000230863		Pseudoger	chr1:75641178-7572
ENSG0000(	2566	57.30531	chr1:1043	ENSG00000224127		lncRNA	chr1:75127830-7513
ENSG0000(	2566	57.30531	chr1:1043	ENSG00000237324		lncRNA	chr1:74341579-7437
ENSG0000(	2566	57.30531	chr1:1043	FUBP1	NCGv7;AC	protein_c	chr1:77944055-7797
ENSG0000(	2566	57.30531	chr1:1043	ST6GALNAC5		protein_c	chr1:76867480-7706
ENSG0000(	2566	57.30531	chr1:1043	NEXN		protein_c	chr1:77888513-7794
ENSG0000(	2566	57.30531	chr1:1043	PTGFR	NCGv7	protein_c	chr1:78303884-7854
ENSG0000(	2566	57.30531	chr1:1043	ENSG00000273338		lncRNA	chr1:78004346-7800
ENSG0000(	2566	57.30531	chr1:1043	ENSG00000287647		lncRNA	chr1:77431314-7743
ENSG0000(	2566	57.30531	chr1:1043	ENSG00000213560		Pseudoger	chr1:78091499-7809
ENSG0000(	2566	57.30531	chr1:1043	LHX8		protein_c	chr1:75128434-7516
ENSG0000(	2566	57.30531	chr1:1043	ENSG00000272864		lncRNA	chr1:74698769-7469
ENSG0000(	2566	57.30531	chr1:1043	AC095030.1		smallRNA	chr1:77391422-7739
ENSG0000(	2566	57.30531	chr1:1043	TPI1P1		Pseudoger	chr1:76699789-7670
ENSG0000(	2566	57.30531	chr1:1043	ADGRL4		protein_c	chr1:78889764-7928
ENSG0000(	2566	57.30531	chr1:1043	DNAJB4		protein_c	chr1:77979175-7801
ENSG0000(	2566	57.30531	chr1:1043	GIPC2		protein_c	chr1:77979542-7813
ENSG0000(	2566	57.30531	chr1:1043	ENSG00000289212		lncRNA	chr1:77219520-7722
ENSG0000(	2566	57.30531	chr1:1043	RABGGTB		protein_c	chr1:75786197-7579
ENSG0000(	2566	57.30531	chr1:1043	IFI44L	NCGv7	protein_c	chr1:78619902-7864
ENSG0000(	2566	57.30531	chr1:1043	AC093430.1		smallRNA	chr1:79135133-7913
ENSG0000(	2566	57.30531	chr1:1043	ENSG00000272855		lncRNA	chr1:76636877-7663
ENSG0000(	2566	57.30531	chr1:1043	ENSG00000228187		Pseudoger	chr1:77194825-7719
ENSG0000(	2566	57.30531	chr1:1043	ST6GALNAC(	NCGv7	protein_c	chr1:76074746-7663
ENSG0000(	2566	57.30531	chr1:1043	ZZZ3		protein_c	chr1:77562416-7768
ENSG0000(	2566	57.30531	chr1:1043	ENSG00000288822		lncRNA	chr1:79488644-7949
ENSG0000(	2566	57.30531	chr1:1043	AL445464.1		smallRNA	chr1:75842632-7584
ENSG0000(	2566	57.30531	chr1:1043	ENSG00000219201		Pseudoger	chr1:77810861-7781
ENSG0000(	2566	57.30531	chr1:1043	TYW3		protein_c	chr1:74733152-7476
ENSG0000(	2566	57.30531	chr1:1043	PSAT1P3		Pseudoger	chr1:79054945-7905
ENSG0000(	2560	57.17131	chr1:1043	SRSF11		protein_c	chr1:70205682-7025
ENSG0000(	2560	57.17131	chr1:1043	ENSG00000228988		lncRNA	chr1:70218589-7022
ENSG0000(	2560	57.17131	chr1:1043	RNA5SP50		Pseudoger	chr1:73749517-7374
ENSG0000(	2560	57.17131	chr1:1043	CTH	DriverDBv	protein_c	chr1:70411218-7043
ENSG0000(	2560	57.17131	chr1:1043	LRRC7-AS1		lncRNA	chr1:70013982-7003
ENSG0000(	2560	57.17131	chr1:1043	ENSG00000269933		lncRNA	chr1:71005854-7100
ENSG0000(	2560	57.17131	chr1:1043	PIN1P1		Pseudoger	chr1:69919322-6992
ENSG0000(	2560	57.17131	chr1:1043	LINC01707		lncRNA	chr1:69055838-6922
ENSG0000(	2560	57.17131	chr1:1043	RN7SL538P		smallRNA	chr1:69879592-6987
ENSG0000(	2560	57.17131	chr1:1043	ENSG00000231985		lncRNA	chr1:71570956-7157
ENSG0000(	2560	57.17131	chr1:1043	LINC02797		lncRNA	chr1:72793104-7285
ENSG0000(	2560	57.17131	chr1:1043	NEGR1		protein_c	chr1:71395943-7228

ENSG0000(	2560	57.17131	chr1:1043	RN7SKP19		smallRNA	chr1:73191604-7319
ENSG0000(	2560	57.17131	chr1:1043	RNU6-1246P		smallRNA	chr1:72717663-7271
ENSG0000(	2560	57.17131	chr1:1043	MIR186		smallRNA	chr1:71067631-7106
ENSG0000(	2560	57.17131	chr1:1043	HHLA3		lncRNA	chr1:70354786-7038
ENSG0000(	2560	57.17131	chr1:1043	RN7SL242P		smallRNA	chr1:70180146-7018
ENSG0000(	2560	57.17131	chr1:1043	LINC02791		lncRNA	chr1:69215835-6924
ENSG0000(	2560	57.17131	chr1:1043	FPGT		protein_c	chr1:74198238-7423
ENSG0000(	2560	57.17131	chr1:1043	ENSG00000285473		lncRNA	chr1:68974010-6902
ENSG0000(	2560	57.17131	chr1:1043	ENSG00000287453		lncRNA	chr1:69551848-6956
ENSG0000(	2560	57.17131	chr1:1043	LINC01788		lncRNA	chr1:70706441-7078
ENSG0000(	2560	57.17131	chr1:1043	HHLA3-AS1		lncRNA	chr1:70359562-7036
ENSG0000(	2560	57.17131	chr1:1043	ENSG00000285778		lncRNA	chr1:73787370-7391
ENSG0000(	2560	57.17131	chr1:1043	SGO1P1		Pseudoger	chr1:69606855-6960
ENSG0000(	2560	57.17131	chr1:1043	LRRC7	NCGv7	protein_c	chr1:69567922-7015
ENSG0000(	2560	57.17131	chr1:1043	RNU4ATAC8P		smallRNA	chr1:73883713-7388
ENSG0000(	2560	57.17131	chr1:1043	ENSG00000235782		lncRNA	chr1:70947379-7095
ENSG0000(	2560	57.17131	chr1:1043	PTGER3		protein_c	chr1:70852353-7104
ENSG0000(	2560	57.17131	chr1:1043	ENSG00000225087		lncRNA	chr1:72636547-7289
ENSG0000(	2560	57.17131	chr1:1043	ENSG00000271992		lncRNA	chr1:70445071-7044
ENSG0000(	2560	57.17131	chr1:1043	ZRANB2		protein_c	chr1:71063291-7108
ENSG0000(	2560	57.17131	chr1:1043	AL360297.1		smallRNA	chr1:71141975-7114
ENSG0000(	2560	57.17131	chr1:1043	LINC01758		lncRNA	chr1:69433255-6943
ENSG0000(	2560	57.17131	chr1:1043	LINC02238		lncRNA	chr1:73635216-7371
ENSG0000(	2560	57.17131	chr1:1043	ENSG00000226208		lncRNA	chr1:70715933-7072
ENSG0000(	2560	57.17131	chr1:1043	KRT8P21		Pseudoger	chr1:73104792-7310
ENSG0000(	2560	57.17131	chr1:1043	CASP3P1		Pseudoger	chr1:70660657-7066
ENSG0000(	2560	57.17131	chr1:1043	ENSG00000286863		lncRNA	chr1:72283170-7275
ENSG0000(	2560	57.17131	chr1:1043	CHORDC1P5		Pseudoger	chr1:70530526-7053
ENSG0000(	2560	57.17131	chr1:1043	ZRANB2-AS1		lncRNA	chr1:71048855-7106
ENSG0000(	2560	57.17131	chr1:1043	LRRC40		protein_c	chr1:70144805-7020
ENSG0000(	2560	57.17131	chr1:1043	ENSG00000287283		lncRNA	chr1:69706950-6971
ENSG0000(	2560	57.17131	chr1:1043	NEGR1-IT1		lncRNA	chr1:71794232-7183
ENSG0000(	2560	57.17131	chr1:1043	LINC02796		lncRNA	chr1:72765031-7279
ENSG0000(	2560	57.17131	chr1:1043	ENSG00000226324		Pseudoger	chr1:71367054-7136
ENSG0000(	2560	57.17131	chr1:1043	LRRIQ3	NCGv7	protein_c	chr1:74026015-7419
ENSG0000(	2560	57.17131	chr1:1043	LINC01360		lncRNA	chr1:73305609-7335
ENSG0000(	2560	57.17131	chr1:1043	ENSG00000271618		Pseudoger	chr1:71738173-7173
ENSG0000(	2560	57.17131	chr1:1043	ENSG00000280317		TEC	chr1:72979014-7297
ENSG0000(	2560	57.17131	chr1:1043	GDI2P2		Pseudoger	chr1:72274552-7227
ENSG0000(	2560	57.17131	chr1:1043	ANKRD13C		protein_c	chr1:70258999-7035
ENSG0000(	2560	57.17131	chr1:1043	ZRANB2-DT		lncRNA	chr1:71081324-7148
ENSG0000(	2560	57.17131	chr1:1043	RPL31P12		Pseudoger	chr1:72301472-7230
ENSG0000(	2556	57.08198	chr1:2197	PBX1-AS1		lncRNA	chr1:164769116-164
ENSG0000(	2556	57.08198	chr1:2197	LMX1A	NCGv7	protein_c	chr1:165201867-165
ENSG0000(	2556	57.08198	chr1:2197	ENSG00000225755		Pseudoger	chr1:163237214-163
ENSG0000(	2556	57.08198	chr1:2197	CCDC190		protein_c	chr1:162824458-162
ENSG0000(	2556	57.08198	chr1:2197	ENSG00000289408		lncRNA	chr1:164900169-164
ENSG0000(	2556	57.08198	chr1:2197	NUF2	DriverDBv	protein_c	chr1:163266576-163
ENSG0000(	2556	57.08198	chr1:2197	RGS5		protein_c	chr1:163111121-163
ENSG0000(	2556	57.08198	chr1:2197	ENSG00000225122		Pseudoger	chr1:163422405-163
ENSG0000(	2556	57.08198	chr1:2197	SLAMF6P1		Pseudoger	chr1:162445549-162

ENSG0000(	2556	57.08198	chr1:2197	UAP1	NCGv7	protein_c	chr1:162561722-162
ENSG0000(	2556	57.08198	chr1:2197	RGS4		protein_c	chr1:163068775-163
ENSG0000(	2556	57.08198	chr1:2197	RNA5SP62		Pseudoger	chr1:163468496-163
ENSG0000(	2556	57.08198	chr1:2197	SH2D1B		protein_c	chr1:162395268-162
ENSG0000(	2556	57.08198	chr1:2197	U3		smallRNA	chr1:163923670-163
ENSG0000(	2556	57.08198	chr1:2197	SNORD112		smallRNA	chr1:163385865-163
ENSG0000(	2556	57.08198	chr1:2197	RGS5-AS1		lncRNA	chr1:163161675-163
ENSG0000(	2556	57.08198	chr1:2197	ENSG00000228289		Pseudoger	chr1:163769339-163
ENSG0000(	2556	57.08198	chr1:2197	LMX1A-AS2		lncRNA	chr1:165210627-165
ENSG0000(	2556	57.08198	chr1:2197	RNU6-171P		smallRNA	chr1:164639565-164
ENSG0000(	2556	57.08198	chr1:2197	RGS5		lncRNA	chr1:163244505-163
ENSG0000(	2556	57.08198	chr1:2197	RN7SL861P		smallRNA	chr1:162777730-162
ENSG0000(	2556	57.08198	chr1:2197	RNU5F-6P		smallRNA	chr1:164351273-164
ENSG0000(	2556	57.08198	chr1:2197	UHMK1		protein_c	chr1:162497251-162
ENSG0000(	2556	57.08198	chr1:2197	Y_RNA		smallRNA	chr1:164854231-164
ENSG0000(	2556	57.08198	chr1:2197	ENSG00000272574		lncRNA	chr1:162593103-162
ENSG0000(	2556	57.08198	chr1:2197	ENSG00000227667		Pseudoger	chr1:162979551-162
ENSG0000(	2556	57.08198	chr1:2197	NMNAT1P2		Pseudoger	chr1:164343005-164
ENSG0000(	2556	57.08198	chr1:2197	ENSG00000271917		lncRNA	chr1:164828436-164
ENSG0000(	2556	57.08198	chr1:2197	DDR2	NCGv7	protein_c	chr1:162631373-162
ENSG0000(	2556	57.08198	chr1:2197	UQCRBP2		Pseudoger	chr1:162541332-162
ENSG0000(	2556	57.08198	chr1:2197	AL390119.1		smallRNA	chr1:164983902-164
ENSG0000(	2556	57.08198	chr1:2197	ENSG00000237756		lncRNA	chr1:163259850-163
ENSG0000(	2556	57.08198	chr1:2197	ENSG00000269887		lncRNA	chr1:164680085-164
ENSG0000(	2556	57.08198	chr1:2197	RNA5SP63		Pseudoger	chr1:163509484-163
ENSG0000(	2556	57.08198	chr1:2197	PBX1	NCGv7;AC	protein_c	chr1:164555584-164
ENSG0000(	2556	57.08198	chr1:2197	LMX1A-AS1		lncRNA	chr1:165215951-165
ENSG0000(	2556	57.08198	chr1:2197	RNU6-755P		smallRNA	chr1:164980035-164
ENSG0000(	2556	57.08198	chr1:2197	HMGB3P6		Pseudoger	chr1:164356767-164
ENSG0000(	2556	57.08198	chr1:2197	ENSG00000230739		Pseudoger	chr1:162824795-162
ENSG0000(	2556	57.08198	chr1:2197	HSD17B7		protein_c	chr1:162790702-162
ENSG0000(	2556	57.08198	chr1:2197	RPL35AP7		Pseudoger	chr1:164921318-164
ENSG0000(	2556	57.08198	chr1:2197	UAP1-DT		lncRNA	chr1:162560227-162
ENSG0000(	2556	57.08198	chr1:2197	ENSG00000289713		Pseudoger	chr1:162441192-162
ENSG0000(	2556	57.08198	chr1:2197	SNORD112		smallRNA	chr1:165072473-165
ENSG0000(	2548	56.90332	chr1:104:	ENSG00000289407		lncRNA	chr1:45583238-4558
ENSG0000(	2548	56.90332	chr1:104:	TXN2P1		Pseudoger	chr1:84085741-8408
ENSG0000(	2547	56.88099	chr1:2197	LRRC52		protein_c	chr1:165544000-165
ENSG0000(	2547	56.88099	chr1:2197	ENSG00000273365		lncRNA	chr1:165706556-165
ENSG0000(	2547	56.88099	chr1:2197	ENSG00000236206		lncRNA	chr1:165598356-165
ENSG0000(	2547	56.88099	chr1:2197	LRRC52-AS1		lncRNA	chr1:165476833-165
ENSG0000(	2547	56.88099	chr1:2197	PRELID1P7		Pseudoger	chr1:165497724-165
ENSG0000(	2547	56.88099	chr1:2197	ALDH9A1		protein_c	chr1:165662216-165
ENSG0000(	2547	56.88099	chr1:2197	ENSG00000215838		Pseudoger	chr1:165698750-165
ENSG0000(	2547	56.88099	chr1:2197	ENSG00000230659		Pseudoger	chr1:165819353-165
ENSG0000(	2547	56.88099	chr1:2197	ENSG00000237783		Pseudoger	chr1:165581613-165
ENSG0000(	2547	56.88099	chr1:2197	Y_RNA		smallRNA	chr1:165662585-165
ENSG0000(	2547	56.88099	chr1:2197	RXRG		protein_c	chr1:165400922-165
ENSG0000(	2547	56.88099	chr1:2197	TMCO1		protein_c	chr1:165724293-165
ENSG0000(	2547	56.88099	chr1:2197	ENSG00000230175		Pseudoger	chr1:165671256-165
ENSG0000(	2547	56.88099	chr1:2197	ENSG00000225272		Pseudoger	chr1:165676310-165

ENSG00000	2547	56.88099	chr1:2197	MGST3		protein_c	chr1:165631213-165
ENSG00000	2547	56.88099	chr1:2197	TMCO1-AS1		lncRNA	chr1:165768929-165
ENSG00000	2541	56.74699	chr1:1142	ATP6V1E1P1		Pseudogen	chr1:42903232-4290
ENSG00000	2534	56.59067	chr1:1043	ENSG00000285409		lncRNA	chr1:79967733-8005
ENSG00000	2534	56.59067	chr1:1043	ENSG00000227062		Pseudogen	chr1:80464301-8046
ENSG00000	2534	56.59067	chr1:1043	MTND2P30		Pseudogen	chr1:81080790-8108
ENSG00000	2534	56.59067	chr1:1043	TTLL7-IT1		lncRNA	chr1:83979118-8398
ENSG00000	2534	56.59067	chr1:1043	AL606519.1		smallRNA	chr1:80329379-8032
ENSG00000	2534	56.59067	chr1:1043	ENSG00000285782		lncRNA	chr1:83397555-8342
ENSG00000	2534	56.59067	chr1:1043	LINC01712		lncRNA	chr1:83445967-8348
ENSG00000	2534	56.59067	chr1:1043	ADH5P2		Pseudogen	chr1:79521080-7952
ENSG00000	2534	56.59067	chr1:1043	SYDE2		protein_c	chr1:85156889-8520
ENSG00000	2534	56.59067	chr1:1043	ENSG00000285851		lncRNA	chr1:84498350-8455
ENSG00000	2534	56.59067	chr1:1043	ENSG00000234953		lncRNA	chr1:81513880-8155
ENSG00000	2534	56.59067	chr1:1043	ENSG00000237076		lncRNA	chr1:83766417-8380
ENSG00000	2534	56.59067	chr1:1043	CCN1		protein_c	chr1:85580761-8558
ENSG00000	2534	56.59067	chr1:1043	MCOLN2		protein_c	chr1:84925583-8499
ENSG00000	2534	56.59067	chr1:1043	DDAH1		protein_c	chr1:85318481-8557
ENSG00000	2534	56.59067	chr1:1043	ENSG00000260322		lncRNA	chr1:80114943-8011
ENSG00000	2534	56.59067	chr1:1043	LINC01725		lncRNA	chr1:83575776-8386
ENSG00000	2534	56.59067	chr1:1043	TTLL7		protein_c	chr1:83865024-8399
ENSG00000	2534	56.59067	chr1:1043	LINC01361		lncRNA	chr1:82970820-8298
ENSG00000	2534	56.59067	chr1:1043	UOX		Pseudogen	chr1:84363706-8439
ENSG00000	2534	56.59067	chr1:1043	ENSG00000230285		lncRNA	chr1:85599131-8560
ENSG00000	2534	56.59067	chr1:1043	ENSG00000282057		lncRNA	chr1:85482281-8557
ENSG00000	2534	56.59067	chr1:1043	NEDD8P1		Pseudogen	chr1:84244334-8424
ENSG00000	2534	56.59067	chr1:1043	RPL7P10		Pseudogen	chr1:81098267-8109
ENSG00000	2534	56.59067	chr1:1043	BCL10	NCGv7;AC	protein_c	chr1:85265776-8527
ENSG00000	2534	56.59067	chr1:1043	DNAI3		protein_c	chr1:84999147-8513
ENSG00000	2534	56.59067	chr1:1043	C1orf52		protein_c	chr1:85249953-8525
ENSG00000	2534	56.59067	chr1:1043	PRKACB		protein_c	chr1:84078062-8423
ENSG00000	2534	56.59067	chr1:1043	ENSG00000285325		lncRNA	chr1:84785427-8478
ENSG00000	2534	56.59067	chr1:1043	RN7SKP247		smallRNA	chr1:81251789-8125
ENSG00000	2534	56.59067	chr1:1043	SPATA1		protein_c	chr1:84506300-8456
ENSG00000	2534	56.59067	chr1:1043	ENSG00000233290		lncRNA	chr1:82212413-8284
ENSG00000	2534	56.59067	chr1:1043	MED28P8		Pseudogen	chr1:81557121-8155
ENSG00000	2534	56.59067	chr1:1043	LINC01555		lncRNA	chr1:84628230-8463
ENSG00000	2534	56.59067	chr1:1043	HMGB1P18		Pseudogen	chr1:80283352-8028
ENSG00000	2534	56.59067	chr1:1043	HNRNPA1P64		Pseudogen	chr1:80451083-8045
ENSG00000	2534	56.59067	chr1:1043	Y_RNA		smallRNA	chr1:85435175-8543
ENSG00000	2534	56.59067	chr1:1043	LINC01362		lncRNA	chr1:82903183-8316
ENSG00000	2534	56.59067	chr1:1043	ENSG00000235756		Pseudogen	chr1:80092103-8009
ENSG00000	2534	56.59067	chr1:1043	ENSG00000236676		lncRNA	chr1:81585941-8162
ENSG00000	2534	56.59067	chr1:1043	ENSG00000285201		lncRNA	chr1:84038529-8406
ENSG00000	2534	56.59067	chr1:1043	ENSG00000232622		Pseudogen	chr1:84636158-8463
ENSG00000	2534	56.59067	chr1:1043	ENSG00000227960		lncRNA	chr1:81505099-8150
ENSG00000	2534	56.59067	chr1:1043	ENSG00000289881		lncRNA	chr1:84614068-8462
ENSG00000	2534	56.59067	chr1:1043	ENSG00000285179		lncRNA	chr1:81209834-8122
ENSG00000	2534	56.59067	chr1:1043	GNG5		protein_c	chr1:84498323-8450
ENSG00000	2534	56.59067	chr1:1043	AL590113.1		smallRNA	chr1:85284610-8528
ENSG00000	2534	56.59067	chr1:1043	ENSG00000280099		TEC	chr1:85152487-8515

ENSG0000(	2534	56.59067	chr1:104:	ENSG00000234683		Pseudoger	chr1:81596157-8159
ENSG0000(	2534	56.59067	chr1:104:	ARID3BP1		Pseudoger	chr1:81501794-8150
ENSG0000(	2534	56.59067	chr1:104:	HNRNPA3P14		Pseudoger	chr1:81426456-8142
ENSG0000(	2534	56.59067	chr1:104:	ENSG00000285361		lncRNA	chr1:84477039-8447
ENSG0000(	2534	56.59067	chr1:104:	ENSG00000285374		lncRNA	chr1:84607099-8461
ENSG0000(	2534	56.59067	chr1:104:	LPAR3	NCGv7	protein_c	chr1:84811602-8489
ENSG0000(	2534	56.59067	chr1:104:	DNASE2B		protein_c	chr1:84398484-8441
ENSG0000(	2534	56.59067	chr1:104:	PRKACB-DT		lncRNA	chr1:84076331-8407
ENSG0000(	2534	56.59067	chr1:104:	MIR4423		smallRNA	chr1:85133794-8513
ENSG0000(	2534	56.59067	chr1:104:	CTBS		protein_c	chr1:84549611-8457
ENSG0000(	2534	56.59067	chr1:104:	ST13P20		Pseudoger	chr1:81721693-8172
ENSG0000(	2534	56.59067	chr1:104:	ENSG00000235089		Pseudoger	chr1:81208568-8120
ENSG0000(	2534	56.59067	chr1:104:	MCOLN3		protein_c	chr1:85018082-8504
ENSG0000(	2534	56.59067	chr1:104:	ENSG00000277670		Pseudoger	chr1:80124004-8012
ENSG0000(	2534	56.59067	chr1:104:	SNORD81		smallRNA	chr1:85592280-8559
ENSG0000(	2534	56.59067	chr1:104:	ENSG00000273264		lncRNA	chr1:85467295-8546
ENSG0000(	2534	56.59067	chr1:104:	SAMD13		protein_c	chr1:84298366-8438
ENSG0000(	2534	56.59067	chr1:104:	ENSG00000249237		Pseudoger	chr1:84344678-8434
ENSG0000(	2534	56.59067	chr1:104:	AC104169.1		smallRNA	chr1:84793068-8479
ENSG0000(	2534	56.59067	chr1:104:	Y_RNA		smallRNA	chr1:85264296-8526
ENSG0000(	2534	56.59067	chr1:104:	AL035706.1		smallRNA	chr1:83793877-8379
ENSG0000(	2534	56.59067	chr1:104:	SSX2IP		protein_c	chr1:84643706-8469
ENSG0000(	2534	56.59067	chr1:104:	SNORA2		smallRNA	chr1:84277321-8427
ENSG0000(	2534	56.59067	chr1:104:	ENSG00000272691		lncRNA	chr1:85578500-8557
ENSG0000(	2534	56.59067	chr1:104:	ENSG00000229486		Pseudoger	chr1:84015865-8401
ENSG0000(	2534	56.59067	chr1:104:	ENSG00000234108		Pseudoger	chr1:80495903-8049
ENSG0000(	2534	56.59067	chr1:104:	BCL10-AS1		lncRNA	chr1:85276388-8544
ENSG0000(	2534	56.59067	chr1:104:	ENSG00000224326		lncRNA	chr1:80534978-8058
ENSG0000(	2534	56.59067	chr1:104:	ENSG00000284882		lncRNA	chr1:84574114-8458
ENSG0000(	2534	56.59067	chr1:104:	RPF1		protein_c	chr1:84479259-8449
ENSG0000(	2534	56.59067	chr1:104:	LINC01781		lncRNA	chr1:80535755-8064
ENSG0000(	2534	56.59067	chr1:104:	ENSG00000225598		lncRNA	chr1:80373364-8037
ENSG0000(	2534	56.59067	chr1:104:	ADGRL2	NCGv7	protein_c	chr1:81306147-8199
ENSG0000(	2511	56.07702	chr1:104:	ENSG00000233877		Pseudoger	chr1:64941979-6494
ENSG0000(	2511	56.07702	chr1:104:	Y_RNA		smallRNA	chr1:64066640-6406
ENSG0000(	2511	56.07702	chr1:104:	RNU6-387P		smallRNA	chr1:67417214-6741
ENSG0000(	2511	56.07702	chr1:104:	AL157407.1		smallRNA	chr1:68058177-6805
ENSG0000(	2511	56.07702	chr1:104:	RPS29P7		Pseudoger	chr1:65154480-6515
ENSG0000(	2511	56.07702	chr1:104:	RN7SL854P		smallRNA	chr1:65761060-6576
ENSG0000(	2511	56.07702	chr1:104:	ENSG00000290094		lncRNA	chr1:65310302-6531
ENSG0000(	2511	56.07702	chr1:104:	RNU7-62P		smallRNA	chr1:64384398-6438
ENSG0000(	2511	56.07702	chr1:104:	ENSG00000238139		Pseudoger	chr1:67561073-6756
ENSG0000(	2511	56.07702	chr1:104:	DNAI4		protein_c	chr1:66812885-6692
ENSG0000(	2511	56.07702	chr1:104:	PDE4B		protein_c	chr1:65792514-6637
ENSG0000(	2511	56.07702	chr1:104:	DNAJB6P4		Pseudoger	chr1:67278052-6727
ENSG0000(	2511	56.07702	chr1:104:	ENSG00000275678		lncRNA	chr1:67121605-6712
ENSG0000(	2511	56.07702	chr1:104:	AL109843.1		smallRNA	chr1:67239440-6723
ENSG0000(	2511	56.07702	chr1:104:	RNU4ATAC4P		smallRNA	chr1:67267601-6726
ENSG0000(	2511	56.07702	chr1:104:	ENSG00000285407		lncRNA	chr1:68679202-6894
ENSG0000(	2511	56.07702	chr1:104:	LINC01702		lncRNA	chr1:67522299-6753
ENSG0000(	2511	56.07702	chr1:104:	SNORA31		smallRNA	chr1:67102645-6710

ENSG0000(	2511	56.07702	chr1:1043	snoU13		smallRNA	chr1:65571549-6557
ENSG0000(	2511	56.07702	chr1:1043	INSL5		protein_c	chr1:66797740-6680
ENSG0000(	2511	56.07702	chr1:1043	DYNLT5		protein_c	chr1:66752459-6677
ENSG0000(	2511	56.07702	chr1:1043	COX6CP13		Pseudoger	chr1:65298755-6529
ENSG0000(	2511	56.07702	chr1:1043	MIR101-1		smallRNA	chr1:65058434-6505
ENSG0000(	2511	56.07702	chr1:1043	RNU4-88P		smallRNA	chr1:66094461-6609
ENSG0000(	2511	56.07702	chr1:1043	RNU6-1031P		smallRNA	chr1:67541127-6754
ENSG0000(	2511	56.07702	chr1:1043	GNG12-AS1		lncRNA	chr1:67832293-6820
ENSG0000(	2511	56.07702	chr1:1043	MIR4794		smallRNA	chr1:64579847-6457
ENSG0000(	2511	56.07702	chr1:1043	ENSG00000229440		Pseudoger	chr1:68381441-6838
ENSG0000(	2511	56.07702	chr1:1043	ENSG00000289394		lncRNA	chr1:66925327-6692
ENSG0000(	2511	56.07702	chr1:1043	ENSG00000285041		lncRNA	chr1:68633701-6864
ENSG0000(	2511	56.07702	chr1:1043	MRPS21P1		Pseudoger	chr1:65092392-6509
ENSG0000(	2511	56.07702	chr1:1043	MIR1262		smallRNA	chr1:68183518-6818
ENSG0000(	2511	56.07702	chr1:1043	ENSG00000235804		Pseudoger	chr1:65077413-6507
ENSG0000(	2511	56.07702	chr1:1043	ENSG00000229294		lncRNA	chr1:65279456-6530
ENSG0000(	2511	56.07702	chr1:1043	RN7SL392P		smallRNA	chr1:67656833-6765
ENSG0000(	2511	56.07702	chr1:1043	ENSG00000248458		lncRNA	chr1:66665864-6667
ENSG0000(	2511	56.07702	chr1:1043	DIRAS3		protein_c	chr1:68045886-6805
ENSG0000(	2511	56.07702	chr1:1043	MIR3671		smallRNA	chr1:65057755-6505
ENSG0000(	2511	56.07702	chr1:1043	IL23R		protein_c	chr1:67138907-6725
ENSG0000(	2511	56.07702	chr1:1043	LEPROT		protein_c	chr1:65420587-6543
ENSG0000(	2511	56.07702	chr1:1043	LINC01359		lncRNA	chr1:64972225-6500
ENSG0000(	2511	56.07702	chr1:1043	ENSG00000285079		lncRNA	chr1:65703962-6571
ENSG0000(	2511	56.07702	chr1:1043	ENSG00000233589		lncRNA	chr1:68479129-6848
ENSG0000(	2511	56.07702	chr1:1043	AL354978.1		smallRNA	chr1:66730967-6673
ENSG0000(	2511	56.07702	chr1:1043	SERBP1		protein_c	chr1:67407810-6743
ENSG0000(	2511	56.07702	chr1:1043	SLC2A3P2		Pseudoger	chr1:64984608-6498
ENSG0000(	2511	56.07702	chr1:1043	RNU6-809P		smallRNA	chr1:64028894-6402
ENSG0000(	2511	56.07702	chr1:1043	RP4-535B20.1		lncRNA	chr1:65066627-6506
ENSG0000(	2511	56.07702	chr1:1043	MIER1		protein_c	chr1:66924895-6698
ENSG0000(	2511	56.07702	chr1:1043	ENSG00000235055		Pseudoger	chr1:68043330-6804
ENSG0000(	2511	56.07702	chr1:1043	DEPDC1	DriverDBv	protein_c	chr1:68474152-6849
ENSG0000(	2511	56.07702	chr1:1043	ENSG00000224570		Pseudoger	chr1:65576129-6557
ENSG0000(	2511	56.07702	chr1:1043	PDE4B-AS1		lncRNA	chr1:66042500-6605
ENSG0000(	2511	56.07702	chr1:1043	C1orf141		protein_c	chr1:67092165-6723
ENSG0000(	2511	56.07702	chr1:1043	ENSG00000284928		lncRNA	chr1:64186791-6419
ENSG0000(	2511	56.07702	chr1:1043	IL12RB2		protein_c	chr1:67307364-6739
ENSG0000(	2511	56.07702	chr1:1043	CTBP2P8		Pseudoger	chr1:68161761-6816
ENSG0000(	2511	56.07702	chr1:1043	LEPR	NCGv7	protein_c	chr1:65420652-6564
ENSG0000(	2511	56.07702	chr1:1043	ROR1-AS1		lncRNA	chr1:64094379-6417
ENSG0000(	2511	56.07702	chr1:1043	SGIP1		protein_c	chr1:66533267-6675
ENSG0000(	2511	56.07702	chr1:1043	SLC35D1		protein_c	chr1:66999350-6705
ENSG0000(	2511	56.07702	chr1:1043	DNAJC6		protein_c	chr1:65248219-6541
ENSG0000(	2511	56.07702	chr1:1043	RNU6-1176P		smallRNA	chr1:65022968-6502
ENSG0000(	2511	56.07702	chr1:1043	RNU6-586P		smallRNA	chr1:67196140-6719
ENSG0000(	2511	56.07702	chr1:1043	RNU7-80P		smallRNA	chr1:67772593-6777
ENSG0000(	2511	56.07702	chr1:1043	JAK1	NCGv7;AC	protein_c	chr1:64833223-6506
ENSG0000(	2511	56.07702	chr1:1043	ENSG00000237852		lncRNA	chr1:65486406-6549
ENSG0000(	2511	56.07702	chr1:1043	MIR3117		smallRNA	chr1:66628440-6662
ENSG0000(	2511	56.07702	chr1:1043	ENSG00000272506		lncRNA	chr1:65003470-6500

ENSG0000(	2511	56.07702	chr1:104:	ENSG00000231080		lncRNA	chr1:66826942-6682
ENSG0000(	2511	56.07702	chr1:104:	ENSG00000234784		Pseudoger	chr1:64918443-6491
ENSG0000(	2511	56.07702	chr1:104:	TXNP2		Pseudoger	chr1:68514375-6851
ENSG0000(	2511	56.07702	chr1:104:	GNG12		protein_c	chr1:67701475-6783
ENSG0000(	2511	56.07702	chr1:104:	ARL5AP3		Pseudoger	chr1:68049360-6804
ENSG0000(	2511	56.07702	chr1:104:	ENSG00000288804		lncRNA	chr1:65067808-6506
ENSG0000(	2511	56.07702	chr1:104:	ROR1		protein_c	chr1:63774017-6418
ENSG0000(	2511	56.07702	chr1:104:	UBE2U		protein_c	chr1:64203623-6426
ENSG0000(	2511	56.07702	chr1:104:	RPS7P4		Pseudoger	chr1:68242474-6824
ENSG0000(	2511	56.07702	chr1:104:	CACHD1	NCGv7	protein_c	chr1:64470129-6469
ENSG0000(	2511	56.07702	chr1:104:	DEPDC1-AS1		lncRNA	chr1:68496676-6853
ENSG0000(	2511	56.07702	chr1:104:	RPE65		protein_c	chr1:68428822-6844
ENSG0000(	2511	56.07702	chr1:104:	RNU2-15P		smallRNA	chr1:65415816-6541
ENSG0000(	2511	56.07702	chr1:104:	AK4		protein_c	chr1:65147549-6523
ENSG0000(	2511	56.07702	chr1:104:	HNRNPCP9		Pseudoger	chr1:67660155-6766
ENSG0000(	2511	56.07702	chr1:104:	WLS	NCGv7	protein_c	chr1:68098473-6823
ENSG0000(	2511	56.07702	chr1:104:	GADD45A		protein_c	chr1:67685201-6768
ENSG0000(	2511	56.07702	chr1:104:	ELOCP18		Pseudoger	chr1:68375327-6837
ENSG0000(	2511	56.07702	chr1:104:	RAVER2		protein_c	chr1:64745075-6483
ENSG0000(	2488	55.56337	chr1:104:	Y_RNA		smallRNA	chr1:89020246-8902
ENSG0000(	2476	55.29538	chr1:104:	ENSG00000227034		Pseudoger	chr1:99008218-9900
ENSG0000(	2468	55.11672	chr1:104:	ENSG00000228086		lncRNA	chr1:100462399-100
ENSG0000(	2468	55.11672	chr1:104:	RN7SL440P		smallRNA	chr1:94150738-9415
ENSG0000(	2468	55.11672	chr1:104:	WDR82P2		Pseudoger	chr1:91534666-9153
ENSG0000(	2468	55.11672	chr1:104:	MFSD14A		protein_c	chr1:100038095-100
ENSG0000(	2468	55.11672	chr1:104:	ENSG00000237954		lncRNA	chr1:95356229-9538
ENSG0000(	2468	55.11672	chr1:104:	GFI1	TAG;AC	protein_c	chr1:92473043-9248
ENSG0000(	2468	55.11672	chr1:104:	RNU4-75P		smallRNA	chr1:99784740-9978
ENSG0000(	2468	55.11672	chr1:104:	RNA5SP53		Pseudoger	chr1:93488333-9348
ENSG0000(	2468	55.11672	chr1:104:	NFU1P2		Pseudoger	chr1:98077000-9807
ENSG0000(	2468	55.11672	chr1:104:	FRRS1		protein_c	chr1:99703970-9976
ENSG0000(	2468	55.11672	chr1:104:	ENSG00000260464		lncRNA	chr1:93847174-9384
ENSG0000(	2468	55.11672	chr1:104:	RN7SKP270		smallRNA	chr1:96695856-9669
ENSG0000(	2468	55.11672	chr1:104:	ENSG00000236098		lncRNA	chr1:94318479-9432
ENSG0000(	2468	55.11672	chr1:104:	AGL		protein_c	chr1:99850361-9992
ENSG0000(	2468	55.11672	chr1:104:	ENSG00000215871		Pseudoger	chr1:100331804-100
ENSG0000(	2468	55.11672	chr1:104:	SNX7	NCGv7	protein_c	chr1:98661701-9876
ENSG0000(	2468	55.11672	chr1:104:	Y_RNA		smallRNA	chr1:93027410-9302
ENSG0000(	2468	55.11672	chr1:104:	ENSG00000288810		lncRNA	chr1:98047173-9804
ENSG0000(	2468	55.11672	chr1:104:	LINC02609		lncRNA	chr1:90769086-9085
ENSG0000(	2468	55.11672	chr1:104:	FEN1P1		Pseudoger	chr1:91328369-9132
ENSG0000(	2468	55.11672	chr1:104:	snoU13		smallRNA	chr1:94151418-9415
ENSG0000(	2468	55.11672	chr1:104:	MTCO1P21		Pseudoger	chr1:93927714-9392
ENSG0000(	2468	55.11672	chr1:104:	SNORA66		smallRNA	chr1:92838018-9283
ENSG0000(	2468	55.11672	chr1:104:	ENSG00000228084		lncRNA	chr1:99968382-9996
ENSG0000(	2468	55.11672	chr1:104:	ENSG00000287797		lncRNA	chr1:92978265-9298
ENSG0000(	2468	55.11672	chr1:104:	ENSG00000229567		Pseudoger	chr1:93278961-9327
ENSG0000(	2468	55.11672	chr1:104:	ENSG00000271252		lncRNA	chr1:95743096-9575
ENSG0000(	2468	55.11672	chr1:104:	RPL5	NCGv7;AC	protein_c	chr1:92832013-9284
ENSG0000(	2468	55.11672	chr1:104:	ARHGAP29		protein_c	chr1:94148988-9427
ENSG0000(	2468	55.11672	chr1:104:	RNU6-750P		smallRNA	chr1:99978939-9997

ENSG0000(	2468	55.11672	chr1:104(	SEC63P1		Pseudoger	chr1:97545701-9754
ENSG0000(	2468	55.11672	chr1:104(	ABCA4		protein_c	chr1:93992834-9412
ENSG0000(	2468	55.11672	chr1:104(	ENSG00000233482		lncRNA	chr1:94145111-9414
ENSG0000(	2468	55.11672	chr1:104(	ENSG00000226952		Pseudoger	chr1:100099239-100
ENSG0000(	2468	55.11672	chr1:104(	PRKAR1AP1		Pseudoger	chr1:92125301-9212
ENSG0000(	2468	55.11672	chr1:104(	SNORA66		smallRNA	chr1:92840719-9284
ENSG0000(	2468	55.11672	chr1:104(	AC104457.1		smallRNA	chr1:100378682-100
ENSG0000(	2468	55.11672	chr1:104(	BRDT	NCGv7	protein_c	chr1:91949343-9201
ENSG0000(	2468	55.11672	chr1:104(	ARHGAP29-AS1		lncRNA	chr1:94247819-9441
ENSG0000(	2468	55.11672	chr1:104(	ENSG00000229635		Pseudoger	chr1:93384487-9338
ENSG0000(	2468	55.11672	chr1:104(	BCAR3		protein_c	chr1:93561741-9384
ENSG0000(	2468	55.11672	chr1:104(	RN7SL692P		smallRNA	chr1:92974829-9297
ENSG0000(	2468	55.11672	chr1:104(	ENSG00000288826		lncRNA	chr1:100036632-100
ENSG0000(	2468	55.11672	chr1:104(	RPL7P9		Pseudoger	chr1:96678874-9667
ENSG0000(	2468	55.11672	chr1:104(	FNBP1L	NCGv7	protein_c	chr1:93448118-9355
ENSG0000(	2468	55.11672	chr1:104(	SASS6		protein_c	chr1:100083563-100
ENSG0000(	2468	55.11672	chr1:104(	AL356479.1		smallRNA	chr1:95504976-9550
ENSG0000(	2468	55.11672	chr1:104(	RN7SL831P		smallRNA	chr1:96583209-9658
ENSG0000(	2468	55.11672	chr1:104(	ENSG00000228852		lncRNA	chr1:95243167-9527
ENSG0000(	2468	55.11672	chr1:104(	DBT		protein_c	chr1:100186919-100
ENSG0000(	2468	55.11672	chr1:104(	ENSG00000272094		lncRNA	chr1:90860550-9086
ENSG0000(	2468	55.11672	chr1:104(	MIR378G		smallRNA	chr1:94745860-9474
ENSG0000(	2468	55.11672	chr1:104(	LINC02607		lncRNA	chr1:95510059-9578
ENSG0000(	2468	55.11672	chr1:104(	ENSG00000225923		Pseudoger	chr1:96390652-9639
ENSG0000(	2468	55.11672	chr1:104(	MIR137HG		lncRNA	chr1:97933474-9804
ENSG0000(	2468	55.11672	chr1:104(	BRI3P1		Pseudoger	chr1:100213293-100
ENSG0000(	2468	55.11672	chr1:104(	MIR760		smallRNA	chr1:93846832-9384
ENSG0000(	2468	55.11672	chr1:104(	CCNJP2		Pseudoger	chr1:92755794-9275
ENSG0000(	2468	55.11672	chr1:104(	MTCO3P21		Pseudoger	chr1:93924743-9392
ENSG0000(	2468	55.11672	chr1:104(	ENSG00000223906		lncRNA	chr1:100344477-100
ENSG0000(	2468	55.11672	chr1:104(	ENSG00000289544		lncRNA	chr1:92961858-9296
ENSG0000(	2468	55.11672	chr1:104(	BCAS2P2		Pseudoger	chr1:100393033-100
ENSG0000(	2468	55.11672	chr1:104(	TLCD4-RWDD3		protein_c	chr1:95117923-9524
ENSG0000(	2468	55.11672	chr1:104(	SLC35A3		protein_c	chr1:99969351-1000
ENSG0000(	2468	55.11672	chr1:104(	ENSG00000287919		lncRNA	chr1:95282233-9528
ENSG0000(	2468	55.11672	chr1:104(	ENSG00000241073		lncRNA	chr1:100057990-100
ENSG0000(	2468	55.11672	chr1:104(	RN7SL235P		smallRNA	chr1:91939269-9193
ENSG0000(	2468	55.11672	chr1:104(	HSP90B3P		Pseudoger	chr1:91642516-9164
ENSG0000(	2468	55.11672	chr1:104(	RPL7AP17		Pseudoger	chr1:100586649-100
ENSG0000(	2468	55.11672	chr1:104(	DIPK1A		protein_c	chr1:92832737-9296
ENSG0000(	2468	55.11672	chr1:104(	MTATP6P13		Pseudoger	chr1:93925406-9392
ENSG0000(	2468	55.11672	chr1:104(	MIR553		smallRNA	chr1:100281241-100
ENSG0000(	2468	55.11672	chr1:104(	TRMT13		protein_c	chr1:100133150-100
ENSG0000(	2468	55.11672	chr1:104(	H3P3		Pseudoger	chr1:92749175-9274
ENSG0000(	2468	55.11672	chr1:104(	ENSG00000230287		Pseudoger	chr1:100249090-100
ENSG0000(	2468	55.11672	chr1:104(	Y_RNA		smallRNA	chr1:93385711-9338
ENSG0000(	2468	55.11672	chr1:104(	CCDC18-AS1		lncRNA	chr1:93262186-9334
ENSG0000(	2468	55.11672	chr1:104(	ALG14		protein_c	chr1:94974405-9507
ENSG0000(	2468	55.11672	chr1:104(	NDUFS5P2		Pseudoger	chr1:96584422-9658
ENSG0000(	2468	55.11672	chr1:104(	PLPPR5-AS1		lncRNA	chr1:99004276-9924
ENSG0000(	2468	55.11672	chr1:104(	ENSG00000225297		Pseudoger	chr1:93199755-9319

ENSG0000	2468	55.11672	chr1:104	AC093577.1		smallRNA	chr1:92982237-9298
ENSG0000	2468	55.11672	chr1:104	Y_RNA		smallRNA	chr1:99791662-9979
ENSG0000	2468	55.11672	chr1:104	C1orf146		protein_c	chr1:92217915-9224
ENSG0000	2468	55.11672	chr1:104	ENSG00000223787		Pseudoge	chr1:92580476-9258
ENSG0000	2468	55.11672	chr1:104	ENSG00000283761		protein_c	chr1:99970011-1000
ENSG0000	2468	55.11672	chr1:104	ENSG00000285922		lncRNA	chr1:98052077-9805
ENSG0000	2468	55.11672	chr1:104	ENSG00000223675		lncRNA	chr1:94585556-9459
ENSG0000	2468	55.11672	chr1:104	TMED5		protein_c	chr1:93149742-9318
ENSG0000	2468	55.11672	chr1:104	SLC44A3-AS1		Pseudoge	chr1:94613814-9485
ENSG0000	2468	55.11672	chr1:104	DR1		protein_c	chr1:93345907-9336
ENSG0000	2468	55.11672	chr1:104	BCAR3-AS1		lncRNA	chr1:93591966-9361
ENSG0000	2468	55.11672	chr1:104	CNN3		protein_c	chr1:94896949-9492
ENSG0000	2468	55.11672	chr1:104	PLPPR5		protein_c	chr1:98890245-9922
ENSG0000	2468	55.11672	chr1:104	DNTTIP2		protein_c	chr1:93866284-9387
ENSG0000	2468	55.11672	chr1:104	EVI5	AC	protein_c	chr1:92508696-9279
ENSG0000	2468	55.11672	chr1:104	HMGB3P9		Pseudoge	chr1:92647048-9264
ENSG0000	2468	55.11672	chr1:104	F3		protein_c	chr1:94529173-9454
ENSG0000	2468	55.11672	chr1:104	AL451010.1		smallRNA	chr1:92229256-9222
ENSG0000	2468	55.11672	chr1:104	ABCD3		protein_c	chr1:94418389-9451
ENSG0000	2468	55.11672	chr1:104	DPYD-IT1		lncRNA	chr1:97394154-9742
ENSG0000	2468	55.11672	chr1:104	SNORD21		smallRNA	chr1:92837289-9283
ENSG0000	2468	55.11672	chr1:104	RPL26P9		Pseudoge	chr1:97585862-9758
ENSG0000	2468	55.11672	chr1:104	PTBP2		protein_c	chr1:96721665-9682
ENSG0000	2468	55.11672	chr1:104	ENSG00000259946		lncRNA	chr1:97967005-9796
ENSG0000	2468	55.11672	chr1:104	LINC01776		lncRNA	chr1:98210747-9827
ENSG0000	2468	55.11672	chr1:104	GCLM		protein_c	chr1:93885199-9390
ENSG0000	2468	55.11672	chr1:104	RPL23AP90		Pseudoge	chr1:100196816-100
ENSG0000	2468	55.11672	chr1:104	RWDD3-DT		lncRNA	chr1:95161676-9523
ENSG0000	2468	55.11672	chr1:104	AC092812.1		smallRNA	chr1:95886540-9588
ENSG0000	2468	55.11672	chr1:104	TGFBR3	NCGv7	protein_c	chr1:91680343-9190
ENSG0000	2468	55.11672	chr1:104	Y_RNA		smallRNA	chr1:95125511-9512
ENSG0000	2468	55.11672	chr1:104	ENSG00000229052		Pseudoge	chr1:92930696-9293
ENSG0000	2468	55.11672	chr1:104	AL160056.1		smallRNA	chr1:98373385-9837
ENSG0000	2468	55.11672	chr1:104	LINC01650		lncRNA	chr1:95351251-9535
ENSG0000	2468	55.11672	chr1:104	ALG14-AS1		lncRNA	chr1:95061596-9506
ENSG0000	2468	55.11672	chr1:104	ENSG00000289483		lncRNA	chr1:92028938-9202
ENSG0000	2468	55.11672	chr1:104	LINC02790		lncRNA	chr1:95937901-9602
ENSG0000	2468	55.11672	chr1:104	PLPPR4		protein_c	chr1:99264292-9930
ENSG0000	2468	55.11672	chr1:104	UBE2WP1		Pseudoge	chr1:96418594-9641
ENSG0000	2468	55.11672	chr1:104	MTCO2P21		Pseudoge	chr1:93926615-9392
ENSG0000	2468	55.11672	chr1:104	ENSG00000229067		Pseudoge	chr1:91600171-9160
ENSG0000	2468	55.11672	chr1:104	RNU1-130P		smallRNA	chr1:96225901-9622
ENSG0000	2468	55.11672	chr1:104	KATNBL1P2		Pseudoge	chr1:94650544-9465
ENSG0000	2468	55.11672	chr1:104	GAPDHP29		Pseudoge	chr1:94302038-9430
ENSG0000	2468	55.11672	chr1:104	ENSG00000286692		lncRNA	chr1:94417743-9441
ENSG0000	2468	55.11672	chr1:104	ENSG00000233129		Pseudoge	chr1:93934479-9393
ENSG0000	2468	55.11672	chr1:104	SLC44A3	NCGv7	protein_c	chr1:94820342-9489
ENSG0000	2468	55.11672	chr1:104	MTF2		protein_c	chr1:93079235-9313
ENSG0000	2468	55.11672	chr1:104	ENSG00000225505		Pseudoge	chr1:92732000-9273
ENSG0000	2468	55.11672	chr1:104	BARHL2		protein_c	chr1:90711539-9071
ENSG0000	2468	55.11672	chr1:104	RTCA-AS1		lncRNA	chr1:100251528-100

ENSG0000(	2468	55.11672	chr1:104:	ENSG00000288736		lncRNA	chr1:94541937-9455
ENSG0000(	2468	55.11672	chr1:104:	RNU6-970P		smallRNA	chr1:92969604-9296
ENSG0000(	2468	55.11672	chr1:104:	ACTBP12		Pseudoger	chr1:92229018-9222
ENSG0000(	2468	55.11672	chr1:104:	CDC7		protein_c	chr1:91500851-9152
ENSG0000(	2468	55.11672	chr1:104:	RN7SKP123		smallRNA	chr1:93026252-9302
ENSG0000(	2468	55.11672	chr1:104:	RNU6-210P		smallRNA	chr1:93010257-9301
ENSG0000(	2468	55.11672	chr1:104:	LINC01761		lncRNA	chr1:95474737-9547
ENSG0000(	2468	55.11672	chr1:104:	GAPDHP46		Pseudoger	chr1:92114803-9211
ENSG0000(	2468	55.11672	chr1:104:	HMGB3P10		Pseudoger	chr1:99698242-9969
ENSG0000(	2468	55.11672	chr1:104:	RNU4-59P		smallRNA	chr1:92700819-9270
ENSG0000(	2468	55.11672	chr1:104:	TLCD4		protein_c	chr1:95117355-9519
ENSG0000(	2468	55.11672	chr1:104:	ENSG00000226773		Pseudoger	chr1:92203148-9220
ENSG0000(	2468	55.11672	chr1:104:	AL451051.1		smallRNA	chr1:99829465-9982
ENSG0000(	2468	55.11672	chr1:104:	RN7SL653P		smallRNA	chr1:91829776-9183
ENSG0000(	2468	55.11672	chr1:104:	EPHX4		protein_c	chr1:92029985-9206
ENSG0000(	2468	55.11672	chr1:104:	LRRC39		protein_c	chr1:100148448-100
ENSG0000(	2468	55.11672	chr1:104:	RWDD3		protein_c	chr1:95234210-9524
ENSG0000(	2468	55.11672	chr1:104:	ZNF644		protein_c	chr1:90915298-9102
ENSG0000(	2468	55.11672	chr1:104:	RTCA	NCGv7	protein_c	chr1:100266216-100
ENSG0000(	2468	55.11672	chr1:104:	BTBD8		protein_c	chr1:92080305-9218
ENSG0000(	2468	55.11672	chr1:104:	ENSG00000285530		lncRNA	chr1:100220488-100
ENSG0000(	2468	55.11672	chr1:104:	LPCAT2BP		Pseudoger	chr1:92066306-9206
ENSG0000(	2468	55.11672	chr1:104:	RPL36AP11		Pseudoger	chr1:93190740-9319
ENSG0000(	2468	55.11672	chr1:104:	RN7SL824P		smallRNA	chr1:92402391-9240
ENSG0000(	2468	55.11672	chr1:104:	CCDC18		protein_c	chr1:93179919-9327
ENSG0000(	2468	55.11672	chr1:104:	MTND3P21		Pseudoger	chr1:93924386-9392
ENSG0000(	2468	55.11672	chr1:104:	RPAP2		protein_c	chr1:92299059-9240
ENSG0000(	2468	55.11672	chr1:104:	LINC02788		lncRNA	chr1:90835660-9084
ENSG0000(	2468	55.11672	chr1:104:	DPYD-AS2		lncRNA	chr1:97796921-9779
ENSG0000(	2468	55.11672	chr1:104:	CDC14A		protein_c	chr1:100345001-100
ENSG0000(	2468	55.11672	chr1:104:	LINC01760		lncRNA	chr1:95310928-9531
ENSG0000(	2468	55.11672	chr1:104:	HFM1		protein_c	chr1:91260766-9140
ENSG0000(	2468	55.11672	chr1:104:	EEF1A1P11		Pseudoger	chr1:96446930-9644
ENSG0000(	2468	55.11672	chr1:104:	DPYD	NCGv7	protein_c	chr1:97077743-9799
ENSG0000(	2468	55.11672	chr1:104:	SETSIP		protein_c	chr1:92074533-9207
ENSG0000(	2468	55.11672	chr1:104:	LINC01787		lncRNA	chr1:96254069-9637
ENSG0000(	2468	55.11672	chr1:104:	ENSG00000231992		lncRNA	chr1:95120147-9513
ENSG0000(	2468	55.11672	chr1:104:	ENSG00000287076		lncRNA	chr1:90719576-9072
ENSG0000(	2468	55.11672	chr1:104:	DPYD-AS1		lncRNA	chr1:97095923-9732
ENSG0000(	2468	55.11672	chr1:104:	ENSG00000250890		Pseudoger	chr1:93926032-9392
ENSG0000(	2468	55.11672	chr1:104:	PHKA1P1		Pseudoger	chr1:90892992-9089
ENSG0000(	2468	55.11672	chr1:104:	ENSG00000280040		TEC	chr1:98660388-9866
ENSG0000(	2468	55.11672	chr1:104:	AL592205.1		smallRNA	chr1:96902699-9690
ENSG0000(	2468	55.11672	chr1:104:	CNN3-DT		lncRNA	chr1:94927361-9496
ENSG0000(	2468	55.11672	chr1:104:	SNORA51		smallRNA	chr1:92846059-9284
ENSG0000(	2468	55.11672	chr1:104:	MTND4P11		Pseudoger	chr1:93922574-9392
ENSG0000(	2468	55.11672	chr1:104:	RNU6-1318P		smallRNA	chr1:100000637-100
ENSG0000(	2468	55.11672	chr1:104:	ENSG00000232918		Pseudoger	chr1:94406395-9440
ENSG0000(	2468	55.11672	chr1:104:	ENSG00000273487		lncRNA	chr1:92189237-9219
ENSG0000(	2468	55.11672	chr1:104:	GPR88		protein_c	chr1:100538139-100
ENSG0000(	2468	55.11672	chr1:104:	GLMN		protein_c	chr1:92246402-9229

ENSG0000(	2468	55.11672	chr1:1043	RPL5P6		Pseudoger	chr1:91023919-9102
ENSG0000(	2468	55.11672	chr1:1043	ENSG00000270911		Pseudoger	chr1:97855575-9785
ENSG0000(	2468	55.11672	chr1:1043	LINC01763		lncRNA	chr1:90851122-9085
ENSG0000(	2468	55.11672	chr1:1043	AL592205.2		smallRNA	chr1:96935545-9693
ENSG0000(	2468	55.11672	chr1:1043	CHCHD2P5		Pseudoger	chr1:93921268-9392
ENSG0000(	2468	55.11672	chr1:1043	LINC01708		lncRNA	chr1:99472332-9960
ENSG0000(	2468	55.11672	chr1:1043	ENSG00000230718		Pseudoger	chr1:97774669-9777
ENSG0000(	2468	55.11672	chr1:1043	ENSG00000231996		Pseudoger	chr1:99842610-9984
ENSG0000(	2468	55.11672	chr1:1043	Y_RNA		smallRNA	chr1:91261625-9126
ENSG0000(	2468	55.11672	chr1:1043	PALMD		protein_c	chr1:99646113-9969
ENSG0000(	2468	55.11672	chr1:1043	ENSG00000233983		Pseudoger	chr1:99464378-9946
ENSG0000(	2467	55.09438	chr1:2197	RPS3AP10		Pseudoger	chr1:166022215-166
ENSG0000(	2467	55.09438	chr1:2197	MAEL		protein_c	chr1:166975582-167
ENSG0000(	2467	55.09438	chr1:2197	UCK2		protein_c	chr1:165827614-165
ENSG0000(	2467	55.09438	chr1:2197	ILDR2		protein_c	chr1:166895711-166
ENSG0000(	2467	55.09438	chr1:2197	DUTP6		Pseudoger	chr1:166868748-166
ENSG0000(	2467	55.09438	chr1:2197	ENSG00000230898		lncRNA	chr1:166147782-166
ENSG0000(	2467	55.09438	chr1:2197	RNA5SP64		Pseudoger	chr1:166042244-166
ENSG0000(	2467	55.09438	chr1:2197	ENSG00000271527		Pseudoger	chr1:165941235-165
ENSG0000(	2467	55.09438	chr1:2197	ENSG00000229588		lncRNA	chr1:166165852-166
ENSG0000(	2467	55.09438	chr1:2197	ENSG00000225325		lncRNA	chr1:166387727-166
ENSG0000(	2467	55.09438	chr1:2197	POGK	DriverDBv	protein_c	chr1:166839447-166
ENSG0000(	2467	55.09438	chr1:2197	FMO11P		Pseudoger	chr1:166763334-166
ENSG0000(	2467	55.09438	chr1:2197	FMO7P		Pseudoger	chr1:166474745-166
ENSG0000(	2467	55.09438	chr1:2197	CNN2P10		Pseudoger	chr1:166796266-166
ENSG0000(	2467	55.09438	chr1:2197	RNA5SP65		Pseudoger	chr1:167005959-167
ENSG0000(	2467	55.09438	chr1:2197	RPL4P2		Pseudoger	chr1:166747379-166
ENSG0000(	2467	55.09438	chr1:2197	ENSG00000236364		lncRNA	chr1:165889725-165
ENSG0000(	2467	55.09438	chr1:2197	FAM78B	NCGv7	protein_c	chr1:166057426-166
ENSG0000(	2467	55.09438	chr1:2197	snoU13		smallRNA	chr1:167041435-167
ENSG0000(	2467	55.09438	chr1:2197	TADA1	DriverDBv	protein_c	chr1:166856510-166
ENSG0000(	2467	55.09438	chr1:2197	ENSG00000215835		Pseudoger	chr1:166275629-166
ENSG0000(	2467	55.09438	chr1:2197	LINC01675		lncRNA	chr1:166474879-166
ENSG0000(	2467	55.09438	chr1:2197	MIR921		smallRNA	chr1:166154743-166
ENSG0000(	2467	55.09438	chr1:2197	FMO10P		Pseudoger	chr1:166665885-166
ENSG0000(	2467	55.09438	chr1:2197	FAM78B-AS1		lncRNA	chr1:166081183-166
ENSG0000(	2467	55.09438	chr1:2197	FMO9P		Pseudoger	chr1:166612470-166
ENSG0000(	2467	55.09438	chr1:2197	FMO9P		lncRNA	chr1:166603916-166
ENSG0000(	2467	55.09438	chr1:2197	FMO8P		Pseudoger	chr1:166566178-166
ENSG0000(	2465	55.04972	chr1:2197	ENSG00000234604		Pseudoger	chr1:169474060-169
ENSG0000(	2459	54.91572	chr1:1043	VCAM1		protein_c	chr1:100719742-100
ENSG0000(	2459	54.91572	chr1:1043	SOD2P1		Pseudoger	chr1:103100143-103
ENSG0000(	2459	54.91572	chr1:1043	EXTL2		protein_c	chr1:100872372-100
ENSG0000(	2459	54.91572	chr1:1043	AMY1A		protein_c	chr1:103655760-103
ENSG0000(	2459	54.91572	chr1:1043	AMYP1		Pseudoger	chr1:103713723-103
ENSG0000(	2459	54.91572	chr1:1043	SLC30A7		protein_c	chr1:100896076-100
ENSG0000(	2459	54.91572	chr1:1043	RNPC3-DT		lncRNA	chr1:103415980-103
ENSG0000(	2459	54.91572	chr1:1043	RPL36AP12		Pseudoger	chr1:100651947-100
ENSG0000(	2459	54.91572	chr1:1043	ENSG00000230864		lncRNA	chr1:102763322-102
ENSG0000(	2459	54.91572	chr1:1043	ENSG00000285525		lncRNA	chr1:100628230-100
ENSG0000(	2459	54.91572	chr1:1043	DNAJA1P5		Pseudoger	chr1:101893105-101

ENSG0000(	2459	54.91572	chr1:104:	ENSG00000289192		lncRNA	chr1:101964793-101
ENSG0000(	2459	54.91572	chr1:104:	ENSG00000235795		lncRNA	chr1:100995473-100
ENSG0000(	2459	54.91572	chr1:104:	RPSAP19		Pseudoger	chr1:101786340-101
ENSG0000(	2459	54.91572	chr1:104:	RP11-347K2.2		lncRNA	chr1:103418079-103
ENSG0000(	2459	54.91572	chr1:104:	ENSG00000271578		Pseudoger	chr1:101190520-101
ENSG0000(	2459	54.91572	chr1:104:	RNU6-352P		smallRNA	chr1:101859851-101
ENSG0000(	2459	54.91572	chr1:104:	DPH5-DT		lncRNA	chr1:101025844-101
ENSG0000(	2459	54.91572	chr1:104:	FTLP17		Pseudoger	chr1:104153306-104
ENSG0000(	2459	54.91572	chr1:104:	RNPC3		protein_c	chr1:103525691-103
ENSG0000(	2459	54.91572	chr1:104:	S1PR1-DT		lncRNA	chr1:101234555-101
ENSG0000(	2459	54.91572	chr1:104:	ENSG00000225191		Pseudoger	chr1:103926567-103
ENSG0000(	2459	54.91572	chr1:104:	LINC01307		lncRNA	chr1:101323337-101
ENSG0000(	2459	54.91572	chr1:104:	ENSG00000271277		Pseudoger	chr1:101882516-101
ENSG0000(	2459	54.91572	chr1:104:	AMY2B	NCGv7	protein_c	chr1:103553815-103
ENSG0000(	2459	54.91572	chr1:104:	S1PR1	NCGv7	protein_c	chr1:101236865-101
ENSG0000(	2459	54.91572	chr1:104:	AC093157.1		protein_c	chr1:100990205-100
ENSG0000(	2459	54.91572	chr1:104:	LINC01349		lncRNA	chr1:100627049-100
ENSG0000(	2459	54.91572	chr1:104:	RNU6-965P		smallRNA	chr1:101728642-101
ENSG0000(	2459	54.91572	chr1:104:	ENSG00000233359		lncRNA	chr1:102199739-102
ENSG0000(	2459	54.91572	chr1:104:	SCARNA16		smallRNA	chr1:101133153-101
ENSG0000(	2459	54.91572	chr1:104:	LINC01709		lncRNA	chr1:101639509-101
ENSG0000(	2459	54.91572	chr1:104:	ENSG00000230759		lncRNA	chr1:103414879-103
ENSG0000(	2459	54.91572	chr1:104:	snoU13		smallRNA	chr1:101228664-101
ENSG0000(	2459	54.91572	chr1:104:	ENSG00000228399		Pseudoger	chr1:101256274-101
ENSG0000(	2459	54.91572	chr1:104:	DPH5		protein_c	chr1:100989623-101
ENSG0000(	2459	54.91572	chr1:104:	ENSG00000215869		Pseudoger	chr1:104072983-104
ENSG0000(	2459	54.91572	chr1:104:	ENSG00000289355		lncRNA	chr1:101150560-101
ENSG0000(	2459	54.91572	chr1:104:	ENSG00000234441		Pseudoger	chr1:103668071-103
ENSG0000(	2459	54.91572	chr1:104:	AMY1C		protein_c	chr1:103745323-103
ENSG0000(	2459	54.91572	chr1:104:	HNRNPA1P68		Pseudoger	chr1:100941017-100
ENSG0000(	2459	54.91572	chr1:104:	COL11A1	NCGv7	protein_c	chr1:102876467-103
ENSG0000(	2459	54.91572	chr1:104:	PPIAP7		Pseudoger	chr1:101270875-101
ENSG0000(	2459	54.91572	chr1:104:	RP11-347K2.1		lncRNA	chr1:103414879-103
ENSG0000(	2459	54.91572	chr1:104:	OLFM3	NCGv7	protein_c	chr1:101802560-101
ENSG0000(	2459	54.91572	chr1:104:	ENSG00000273204		lncRNA	chr1:100894928-100
ENSG0000(	2459	54.91572	chr1:104:	RN7SKP285		smallRNA	chr1:103523562-103
ENSG0000(	2459	54.91572	chr1:104:	AMY2A		protein_c	chr1:103617427-103
ENSG0000(	2459	54.91572	chr1:104:	ACTG1P4		Pseudoger	chr1:103569553-103
ENSG0000(	2459	54.91572	chr1:104:	AMY1B		protein_c	chr1:103687415-103
ENSG0000(	2456	54.84873	chr1:104:	ENSG00000230735		lncRNA	chr1:89629725-8967
ENSG0000(	2456	54.84873	chr1:104:	SH3GLB1	NCGv7	protein_c	chr1:86704570-8674
ENSG0000(	2456	54.84873	chr1:104:	COL24A1		protein_c	chr1:85729233-8615
ENSG0000(	2456	54.84873	chr1:104:	ENSG00000235251		Pseudoger	chr1:87044935-8704
ENSG0000(	2456	54.84873	chr1:104:	RNU6-695P		smallRNA	chr1:90253456-9025
ENSG0000(	2456	54.84873	chr1:104:	ENSG00000284637		Pseudoger	chr1:89203280-8920
ENSG0000(	2456	54.84873	chr1:104:	ENSG00000288629		protein_c	chr1:89579592-8957
ENSG0000(	2456	54.84873	chr1:104:	ENSG00000231349		Pseudoger	chr1:86404176-8640
ENSG0000(	2456	54.84873	chr1:104:	CAPNS1P1		Pseudoger	chr1:89394033-8939
ENSG0000(	2456	54.84873	chr1:104:	ENSG00000267734		lncRNA	chr1:86932199-8693
ENSG0000(	2456	54.84873	chr1:104:	GBP1P1		Pseudoger	chr1:89410319-8942
ENSG0000(	2456	54.84873	chr1:104:	GBP1P1		lncRNA	chr1:89407679-8942

ENSG0000(	2456	54.84873	chr1:104:	PKN2		protein_c	chr1:88684222-8883
ENSG0000(	2456	54.84873	chr1:104:	LINC02795		lncRNA	chr1:86288704-8632
ENSG0000(	2456	54.84873	chr1:104:	ENSG00000287406		lncRNA	chr1:90242088-9028
ENSG0000(	2456	54.84873	chr1:104:	ENSG00000267561		protein_c	chr1:86993009-8716
ENSG0000(	2456	54.84873	chr1:104:	LRRC8D		protein_c	chr1:89821014-8993
ENSG0000(	2456	54.84873	chr1:104:	ENSG00000287015		lncRNA	chr1:90045998-9004
ENSG0000(	2456	54.84873	chr1:104:	ENSG00000284734		lncRNA	chr1:89198714-8920
ENSG0000(	2456	54.84873	chr1:104:	CLCA3P		Pseudoger	chr1:86634276-8665
ENSG0000(	2456	54.84873	chr1:104:	ENSG00000235308		Pseudoger	chr1:88923370-8892
ENSG0000(	2456	54.84873	chr1:104:	RBMXL1		protein_c	chr1:88979456-8899
ENSG0000(	2456	54.84873	chr1:104:	ENSG00000233235		Pseudoger	chr1:89324522-8933
ENSG0000(	2456	54.84873	chr1:104:	ENSG00000231613		lncRNA	chr1:89788914-8979
ENSG0000(	2456	54.84873	chr1:104:	GBP5		protein_c	chr1:89256189-8927
ENSG0000(	2456	54.84873	chr1:104:	LRRC8D-DT		lncRNA	chr1:89820174-8982
ENSG0000(	2456	54.84873	chr1:104:	CLCA1		protein_c	chr1:86468368-8650
ENSG0000(	2456	54.84873	chr1:104:	CLCA4		protein_c	chr1:86547078-8658
ENSG0000(	2456	54.84873	chr1:104:	RNA5SP52		Pseudoger	chr1:87453240-8745
ENSG0000(	2456	54.84873	chr1:104:	ENSG00000272672		lncRNA	chr1:89939601-8994
ENSG0000(	2456	54.84873	chr1:104:	ENSG00000226394		Pseudoger	chr1:89661212-8966
ENSG0000(	2456	54.84873	chr1:104:	PKN2-AS1		lncRNA	chr1:87620803-8868
ENSG0000(	2456	54.84873	chr1:104:	GBP7		protein_c	chr1:89131742-8917
ENSG0000(	2456	54.84873	chr1:104:	ENSG00000230721		Pseudoger	chr1:86784913-8678
ENSG0000(	2456	54.84873	chr1:104:	RP4-604K5.2		lncRNA	chr1:86943685-8694
ENSG0000(	2456	54.84873	chr1:104:	AL356270.1		smallRNA	chr1:86606270-8660
ENSG0000(	2456	54.84873	chr1:104:	ENSG00000237568		lncRNA	chr1:89260582-8926
ENSG0000(	2456	54.84873	chr1:104:	LMO4	AC	protein_c	chr1:87328880-8734
ENSG0000(	2456	54.84873	chr1:104:	ENSG00000238081		Pseudoger	chr1:89289676-8929
ENSG0000(	2456	54.84873	chr1:104:	HS2ST1	NCGv7	protein_c	chr1:86914635-8710
ENSG0000(	2456	54.84873	chr1:104:	RNU6-125P		smallRNA	chr1:88816779-8881
ENSG0000(	2456	54.84873	chr1:104:	ENSG00000230053		Pseudoger	chr1:88498309-8849
ENSG0000(	2456	54.84873	chr1:104:	LRRC8B		protein_c	chr1:89524829-8959
ENSG0000(	2456	54.84873	chr1:104:	ENSG00000284846		lncRNA	chr1:86821558-8683
ENSG0000(	2456	54.84873	chr1:104:	AL139139.1		smallRNA	chr1:87151298-8715
ENSG0000(	2456	54.84873	chr1:104:	GEMIN8P4		Pseudoger	chr1:89993593-8999
ENSG0000(	2456	54.84873	chr1:104:	ENSG00000286802		lncRNA	chr1:89128432-8914
ENSG0000(	2456	54.84873	chr1:104:	GBP4		protein_c	chr1:89181144-8919
ENSG0000(	2456	54.84873	chr1:104:	RNA5SP51		Pseudoger	chr1:85883680-8588
ENSG0000(	2456	54.84873	chr1:104:	ENSG00000286758		lncRNA	chr1:88462936-8846
ENSG0000(	2456	54.84873	chr1:104:	LINC02787		lncRNA	chr1:90510910-9053
ENSG0000(	2456	54.84873	chr1:104:	LRRC8C		protein_c	chr1:89633072-8976
ENSG0000(	2456	54.84873	chr1:104:	ENSG00000271949		protein_c	chr1:89633140-8993
ENSG0000(	2456	54.84873	chr1:104:	ENSG00000229505		Pseudoger	chr1:86029854-8603
ENSG0000(	2456	54.84873	chr1:104:	U3		smallRNA	chr1:90657750-9065
ENSG0000(	2456	54.84873	chr1:104:	RN7SKP272		smallRNA	chr1:89987713-8998
ENSG0000(	2456	54.84873	chr1:104:	GBP2		protein_c	chr1:89106132-8915
ENSG0000(	2456	54.84873	chr1:104:	ZNHIT6	AC	protein_c	chr1:85649417-8570
ENSG0000(	2456	54.84873	chr1:104:	ENSG00000270507		Pseudoger	chr1:88313153-8831
ENSG0000(	2456	54.84873	chr1:104:	ZNF326		protein_c	chr1:89995110-9003
ENSG0000(	2456	54.84873	chr1:104:	ENSG00000225568		Pseudoger	chr1:87045875-8704
ENSG0000(	2456	54.84873	chr1:104:	CDCA4P2		Pseudoger	chr1:86552625-8655
ENSG0000(	2456	54.84873	chr1:104:	GBP3		protein_c	chr1:89006679-8902

ENSG0000(	2456	54.84873	chr1:104:	CLCA4-AS1		lncRNA	chr1:86569024-8670
ENSG0000(	2456	54.84873	chr1:104:	ENSG00000286548		lncRNA	chr1:89427533-8952
ENSG0000(	2456	54.84873	chr1:104:	ELOCP19		Pseudoger	chr1:88829102-8882
ENSG0000(	2456	54.84873	chr1:104:	snoU13		smallRNA	chr1:89768212-8976
ENSG0000(	2456	54.84873	chr1:104:	KYAT3		protein_c	chr1:88935773-8899
ENSG0000(	2456	54.84873	chr1:104:	GTF2B	NCGv7	protein_c	chr1:88852633-8889
ENSG0000(	2456	54.84873	chr1:104:	GBP1		protein_c	chr1:89051882-8906
ENSG0000(	2456	54.84873	chr1:104:	CLCA2		protein_c	chr1:86424171-8645
ENSG0000(	2456	54.84873	chr1:104:	ENSG00000289582		lncRNA	chr1:89127160-8912
ENSG0000(	2456	54.84873	chr1:104:	SELENOF		protein_c	chr1:86862445-8691
ENSG0000(	2456	54.84873	chr1:104:	PTGES3P1		Pseudoger	chr1:89104285-8910
ENSG0000(	2456	54.84873	chr1:104:	LRRC8C-DT		lncRNA	chr1:89581291-8963
ENSG0000(	2456	54.84873	chr1:104:	CLCA3P		lncRNA	chr1:86634273-8665
ENSG0000(	2456	54.84873	chr1:104:	LINC01140		lncRNA	chr1:87129765-8716
ENSG0000(	2456	54.84873	chr1:104:	LINC01364		lncRNA	chr1:87353521-8737
ENSG0000(	2456	54.84873	chr1:104:	ENSG00000289712		Pseudoger	chr1:89236843-8923
ENSG0000(	2456	54.84873	chr1:104:	ENSG00000279778		TEC	chr1:87805286-8780
ENSG0000(	2456	54.84873	chr1:104:	RPL36AP10		Pseudoger	chr1:88577880-8857
ENSG0000(	2456	54.84873	chr1:104:	GBP6		protein_c	chr1:89364059-8938
ENSG0000(	2456	54.84873	chr1:104:	RN7SL583P		smallRNA	chr1:88477831-8847
ENSG0000(	2456	54.84873	chr1:104:	ODF2L		protein_c	chr1:86346824-8639
ENSG0000(	2456	54.84873	chr1:104:	LINC02801		lncRNA	chr1:87212669-8726
ENSG0000(	2456	54.84873	chr1:104:	ENSG00000287372		lncRNA	chr1:90388193-9042
ENSG0000(	2445	54.60307	chr1:2197	ISCUP1		Pseudoger	chr1:170211010-170
ENSG0000(	2445	54.60307	chr1:2197	MIR557		smallRNA	chr1:168375524-168
ENSG0000(	2445	54.60307	chr1:2197	ATP1B1		protein_c	chr1:169105697-169
ENSG0000(	2445	54.60307	chr1:2197	ENSG00000289466		TEC	chr1:169112164-169
ENSG0000(	2445	54.60307	chr1:2197	RPL7AP21		Pseudoger	chr1:168578653-168
ENSG0000(	2445	54.60307	chr1:2197	ENSG00000232959		lncRNA	chr1:170024077-170
ENSG0000(	2445	54.60307	chr1:2197	NME7		protein_c	chr1:169132531-169
ENSG0000(	2445	54.60307	chr1:2197	AL049798.1		smallRNA	chr1:168747796-168
ENSG0000(	2445	54.60307	chr1:2197	AL354732.1		smallRNA	chr1:170370213-170
ENSG0000(	2445	54.60307	chr1:2197	ENSG00000283255		lncRNA	chr1:168401483-168
ENSG0000(	2445	54.60307	chr1:2197	GORAB	DriverDB\	protein_c	chr1:170531819-170
ENSG0000(	2445	54.60307	chr1:2197	MIR3119-2		smallRNA	chr1:170151378-170
ENSG0000(	2445	54.60307	chr1:2197	XCL1	DriverDB\	protein_c	chr1:168576605-168
ENSG0000(	2445	54.60307	chr1:2197	ENSG00000288139		Pseudoger	chr1:169915004-169
ENSG0000(	2445	54.60307	chr1:2197	SIGLEC30P		Pseudoger	chr1:170115636-170
ENSG0000(	2445	54.60307	chr1:2197	LINC01681		lncRNA	chr1:170173865-170
ENSG0000(	2445	54.60307	chr1:2197	SLC19A2	DriverDB\	protein_c	chr1:169463909-169
ENSG0000(	2445	54.60307	chr1:2197	NTMT2		protein_c	chr1:170145959-170
ENSG0000(	2445	54.60307	chr1:2197	METTL18		protein_c	chr1:169792529-169
ENSG0000(	2445	54.60307	chr1:2197	RNA5SP66		Pseudoger	chr1:169067264-169
ENSG0000(	2445	54.60307	chr1:2197	BLZF1	DriverDB\	protein_c	chr1:169367970-169
ENSG0000(	2445	54.60307	chr1:2197	F5	NCGv7	protein_c	chr1:169511951-169
ENSG0000(	2445	54.60307	chr1:2197	SELP	NCGv7	protein_c	chr1:169588849-169
ENSG0000(	2445	54.60307	chr1:2197	CCDC181		protein_c	chr1:169394870-169
ENSG0000(	2445	54.60307	chr1:2197	XCL2	NCGv7	protein_c	chr1:168540768-168
ENSG0000(	2445	54.60307	chr1:2197	RN7SL269P		smallRNA	chr1:169957944-169
ENSG0000(	2445	54.60307	chr1:2197	RPL29P7		Pseudoger	chr1:168938467-168
ENSG0000(	2445	54.60307	chr1:2197	ENSG00000213062		lncRNA	chr1:169486076-169

ENSG0000(	2445	54.60307	chr1:2197	LINC00970		lncRNA	chr1:168903905-169
ENSG0000(	2445	54.60307	chr1:2197	MRPS10P1		Pseudoger	chr1:169990067-169
ENSG0000(	2445	54.60307	chr1:2197	SELL		protein_c	chr1:169690665-169
ENSG0000(	2445	54.60307	chr1:2197	RPL7AP19		Pseudoger	chr1:168542737-168
ENSG0000(	2445	54.60307	chr1:2197	LINC01142		lncRNA	chr1:170271395-170
ENSG0000(	2445	54.60307	chr1:2197	KIFAP3		protein_c	chr1:169921326-170
ENSG0000(	2445	54.60307	chr1:2197	RN7SL333P		smallRNA	chr1:169859756-169
ENSG0000(	2445	54.60307	chr1:2197	HAUS4P1		Pseudoger	chr1:170369223-170
ENSG0000(	2445	54.60307	chr1:2197	SELE		protein_c	chr1:169722640-169
ENSG0000(	2445	54.60307	chr1:2197	ENSG00000287282		lncRNA	chr1:169059576-169
ENSG0000(	2445	54.60307	chr1:2197	GORAB-AS1		lncRNA	chr1:170460453-170
ENSG0000(	2445	54.60307	chr1:2197	ENSG00000287831		lncRNA	chr1:168898633-168
ENSG0000(	2445	54.60307	chr1:2197	ENSG00000235575		lncRNA	chr1:169310665-169
ENSG0000(	2445	54.60307	chr1:2197	ENSG00000230704		lncRNA	chr1:169762929-169
ENSG0000(	2445	54.60307	chr1:2197	SUMO1P2		Pseudoger	chr1:168898136-168
ENSG0000(	2445	54.60307	chr1:2197	ENSG00000228697		lncRNA	chr1:168400829-168
ENSG0000(	2445	54.60307	chr1:2197	LINC00626		lncRNA	chr1:168784012-168
ENSG0000(	2445	54.60307	chr1:2197	C1orf112	DriverDB\	protein_c	chr1:169662007-169
ENSG0000(	2445	54.60307	chr1:2197	DPT		protein_c	chr1:168695468-168
ENSG0000(	2445	54.60307	chr1:2197	QRSL1P1		Pseudoger	chr1:168449672-168
ENSG0000(	2445	54.60307	chr1:2197	ENSG00000235736		lncRNA	chr1:168763365-168
ENSG0000(	2445	54.60307	chr1:2197	ENSG00000285622		lncRNA	chr1:168695874-169
ENSG0000(	2445	54.60307	chr1:2197	ENSG00000237707		lncRNA	chr1:169104124-169
ENSG0000(	2445	54.60307	chr1:2197	SCYL3		protein_c	chr1:169849631-169
ENSG0000(	2445	54.60307	chr1:2197	ENSG00000225545		lncRNA	chr1:170587249-170
ENSG0000(	2433	54.33508	chr1:1043	ENSG00000290536		lncRNA	chr1:58047889-5804
ENSG0000(	2433	54.33508	chr1:1043	MYSM1		protein_c	chr1:58643440-5870
ENSG0000(	2433	54.33508	chr1:1043	FGGY-DT		lncRNA	chr1:59131932-5929
ENSG0000(	2433	54.33508	chr1:1043	ENSG00000270457		lncRNA	chr1:59289303-5928
ENSG0000(	2433	54.33508	chr1:1043	ENSG00000225475		Pseudoger	chr1:56619409-5661
ENSG0000(	2433	54.33508	chr1:1043	PHB1P3		Pseudoger	chr1:58999676-5900
ENSG0000(	2433	54.33508	chr1:1043	ENSG00000284808		lncRNA	chr1:61481087-6153
ENSG0000(	2433	54.33508	chr1:1043	ENSG00000226883		lncRNA	chr1:59754747-5978
ENSG0000(	2433	54.33508	chr1:1043	TACSTD2	AC	protein_c	chr1:58575433-5857
ENSG0000(	2433	54.33508	chr1:1043	MIR4711		smallRNA	chr1:59733227-5973
ENSG0000(	2433	54.33508	chr1:1043	ENSG00000272226		lncRNA	chr1:58812808-5881
ENSG0000(	2433	54.33508	chr1:1043	ENSG00000231740		lncRNA	chr1:58838448-5885
ENSG0000(	2433	54.33508	chr1:1043	Y_RNA		smallRNA	chr1:58722279-5872
ENSG0000(	2433	54.33508	chr1:1043	HNRNPA1P6		Pseudoger	chr1:58048175-5804
ENSG0000(	2433	54.33508	chr1:1043	ENSG00000283445		lncRNA	chr1:58715609-5877
ENSG0000(	2433	54.33508	chr1:1043	JUN	NCGv7;AC	protein_c	chr1:58776845-5878
ENSG0000(	2433	54.33508	chr1:1043	AL137855.1		smallRNA	chr1:57757084-5775
ENSG0000(	2433	54.33508	chr1:1043	ENSG00000230546		Pseudoger	chr1:58084419-5808
ENSG0000(	2433	54.33508	chr1:1043	C8A		protein_c	chr1:56854768-5691
ENSG0000(	2433	54.33508	chr1:1043	NFIA		protein_c	chr1:60865259-6146
ENSG0000(	2433	54.33508	chr1:1043	OMA1	NCGv7	protein_c	chr1:58415384-5854
ENSG0000(	2433	54.33508	chr1:1043	RPL21P23		Pseudoger	chr1:56538452-5653
ENSG0000(	2433	54.33508	chr1:1043	DAB1	NCGv7	protein_c	chr1:56994778-5854
ENSG0000(	2433	54.33508	chr1:1043	ENSG00000286918		lncRNA	chr1:58546168-5856
ENSG0000(	2433	54.33508	chr1:1043	AC096534.1		smallRNA	chr1:61083455-6108
ENSG0000(	2433	54.33508	chr1:1043	ENSG00000241042		lncRNA	chr1:59054397-5905

ENSG0000(	2433	54.33508	chr1:104:	RN7SL713P		smallRNA	chr1:58565629-5856
ENSG0000(	2433	54.33508	chr1:104:	ENSG00000235215		lncRNA	chr1:59055999-5907
ENSG0000(	2433	54.33508	chr1:104:	ENSG00000231252		lncRNA	chr1:60659631-6086
ENSG0000(	2433	54.33508	chr1:104:	PLPP3		protein_c	chr1:56494761-5664
ENSG0000(	2433	54.33508	chr1:104:	RPS26P15		Pseudoger	chr1:58056133-5805
ENSG0000(	2433	54.33508	chr1:104:	PRKAA2		protein_c	chr1:56645314-5671
ENSG0000(	2433	54.33508	chr1:104:	ENSG00000270209		Pseudoger	chr1:58552913-5855
ENSG0000(	2433	54.33508	chr1:104:	NFIA-AS2		lncRNA	chr1:60912675-6105
ENSG0000(	2433	54.33508	chr1:104:	DAB1-AS1		lncRNA	chr1:57860532-5788
ENSG0000(	2433	54.33508	chr1:104:	RPL23AP85		Pseudoger	chr1:56585612-5658
ENSG0000(	2433	54.33508	chr1:104:	ENSG00000235038		lncRNA	chr1:58060139-5808
ENSG0000(	2433	54.33508	chr1:104:	LINC01358		lncRNA	chr1:58933643-5924
ENSG0000(	2433	54.33508	chr1:104:	C1orf87		protein_c	chr1:59987269-6007
ENSG0000(	2433	54.33508	chr1:104:	C8B		protein_c	chr1:56929207-5697
ENSG0000(	2433	54.33508	chr1:104:	AL136985.1		smallRNA	chr1:58764849-5876
ENSG0000(	2433	54.33508	chr1:104:	ENSG00000290013		lncRNA	chr1:58784270-5878
ENSG0000(	2433	54.33508	chr1:104:	LINC02778		lncRNA	chr1:60114875-6014
ENSG0000(	2433	54.33508	chr1:104:	LINC02777		lncRNA	chr1:58882868-5893
ENSG0000(	2433	54.33508	chr1:104:	NFIA-AS1		lncRNA	chr1:61248945-6125
ENSG0000(	2433	54.33508	chr1:104:	ENSG00000227935		lncRNA	chr1:57386576-5738
ENSG0000(	2433	54.33508	chr1:104:	FGGY		protein_c	chr1:59296638-5981
ENSG0000(	2433	54.33508	chr1:104:	ENSG00000229913		lncRNA	chr1:56823679-5682
ENSG0000(	2433	54.33508	chr1:104:	PGBD4P8		Pseudoger	chr1:60097415-6009
ENSG0000(	2433	54.33508	chr1:104:	ENSG00000233216		Pseudoger	chr1:58228682-5822
ENSG0000(	2433	54.33508	chr1:104:	ENSG00000236341		lncRNA	chr1:56963886-5699
ENSG0000(	2433	54.33508	chr1:104:	RN7SL475P		smallRNA	chr1:59974759-5997
ENSG0000(	2433	54.33508	chr1:104:	LINC01748		lncRNA	chr1:60515716-6064
ENSG0000(	2433	54.33508	chr1:104:	FYB2	NCGv7	protein_c	chr1:56718789-5681
ENSG0000(	2433	54.33508	chr1:104:	LINC01135		lncRNA	chr1:58784384-5890
ENSG0000(	2433	54.33508	chr1:104:	HOOK1		protein_c	chr1:59814786-5987
ENSG0000(	2433	54.33508	chr1:104:	CYP2J2		protein_c	chr1:59893308-5992
ENSG0000(	2433	54.33508	chr1:104:	AL161740.1		smallRNA	chr1:56966280-5696
ENSG0000(	2433	54.33508	chr1:104:	RPS20P5		Pseudoger	chr1:57605847-5760
ENSG0000(	2433	54.33508	chr1:104:	ENSG00000185839		Pseudoger	chr1:58630841-5863
ENSG0000(	2432	54.31275	chr1:219	snoU13		smallRNA	chr1:173281077-173
ENSG0000(	2432	54.31275	chr1:219	GOT2P2		Pseudoger	chr1:173141100-173
ENSG0000(	2432	54.31275	chr1:219	ENSG00000232751		Pseudoger	chr1:173351689-173
ENSG0000(	2432	54.31275	chr1:219	ENSG00000226375		lncRNA	chr1:173174300-173
ENSG0000(	2432	54.31275	chr1:219	TNFSF4		protein_c	chr1:173183731-173
ENSG0000(	2427	54.20108	chr1:104:	AC119674.1		smallRNA	chr1:56377333-5637
ENSG0000(	2427	54.20108	chr1:104:	ENSG00000284686		protein_c	chr1:56173433-5652
ENSG0000(	2427	54.20108	chr1:104:	ENSG00000235612		lncRNA	chr1:56145721-5615
ENSG0000(	2427	54.20108	chr1:104:	LINC01767		lncRNA	chr1:56414918-5641
ENSG0000(	2427	54.20108	chr1:104:	LINC01753		lncRNA	chr1:55915603-5594
ENSG0000(	2427	54.20108	chr1:104:	RPSAP20		Pseudoger	chr1:56207567-5620
ENSG0000(	2427	54.20108	chr1:104:	ENSG00000260971		lncRNA	chr1:56154545-5647
ENSG0000(	2427	54.20108	chr1:104:	LINC01755		lncRNA	chr1:55868254-5595
ENSG0000(	2427	54.20108	chr1:104:	PIGQP1		Pseudoger	chr1:55938714-5593
ENSG0000(	2423	54.11175	chr1:104:	MIR4781		smallRNA	chr1:54054079-5405
ENSG0000(	2414	53.91076	chr1:219	ENSG00000289426		lncRNA	chr1:173637713-173
ENSG0000(	2414	53.91076	chr1:219	ENSG00000231615		Pseudoger	chr1:173362397-173

ENSG0000(	2414	53.91076	chr1:2197	PRDX6-AS1		lncRNA	chr1:173417793-173
ENSG0000(	2414	53.91076	chr1:2197	KLHL20		protein_c	chr1:173714941-173
ENSG0000(	2414	53.91076	chr1:2197	PRDX6		protein_c	chr1:173477330-173
ENSG0000(	2414	53.91076	chr1:2197	ENSG00000238272		lncRNA	chr1:173555251-173
ENSG0000(	2414	53.91076	chr1:2197	ENSG00000285777		protein_c	chr1:173596060-173
ENSG0000(	2414	53.91076	chr1:2197	SLC9C2	NCGv7	protein_c	chr1:173500460-173
ENSG0000(	2414	53.91076	chr1:2197	TEX50		protein_c	chr1:173635338-173
ENSG0000(	2414	53.91076	chr1:2197	ANKRD45		protein_c	chr1:173608336-173
ENSG0000(	2414	53.91076	chr1:2197	snoU13		smallRNA	chr1:173660097-173
ENSG0000(	2414	53.91076	chr1:2197	ENSG00000225591		Pseudoger	chr1:173741674-173
ENSG0000(	2404	53.68743	chr1:1043	LINC01676		lncRNA	chr1:105587575-105
ENSG0000(	2404	53.68743	chr1:1043	ENSG00000285981		lncRNA	chr1:104998406-105
ENSG0000(	2404	53.68743	chr1:1043	ENSG00000237897		Pseudoger	chr1:105890693-105
ENSG0000(	2404	53.68743	chr1:1043	ENSG00000232952		Pseudoger	chr1:105891739-105
ENSG0000(	2404	53.68743	chr1:1043	CDK4P1		Pseudoger	chr1:105433994-105
ENSG0000(	2404	53.68743	chr1:1043	ENSG00000237480		lncRNA	chr1:105956694-106
ENSG0000(	2404	53.68743	chr1:1043	SEPTIN2P1		Pseudoger	chr1:105698039-105
ENSG0000(	2404	53.68743	chr1:1043	LINC01677		lncRNA	chr1:105927620-106
ENSG0000(	2404	53.68743	chr1:1043	ENSG00000230932		Pseudoger	chr1:106080801-106
ENSG0000(	2401	53.62044	chr1:1043	RNU5E-6P		smallRNA	chr1:44819883-4481
ENSG0000(	2395	53.48644	chr1:1043	LRP8		protein_c	chr1:53242364-5332
ENSG0000(	2395	53.48644	chr1:1043	ENSG00000230953		Pseudoger	chr1:52920422-5292
ENSG0000(	2395	53.48644	chr1:1043	TUBBP10		Pseudoger	chr1:52994726-5299
ENSG0000(	2395	53.48644	chr1:1043	SHISAL2A		protein_c	chr1:52633168-5266
ENSG0000(	2395	53.48644	chr1:1043	LRP8-DT		lncRNA	chr1:53328233-5333
ENSG0000(	2395	53.48644	chr1:1043	ENSG00000232762		lncRNA	chr1:53304536-5330
ENSG0000(	2395	53.48644	chr1:1043	MIR5095		smallRNA	chr1:52934930-5293
ENSG0000(	2395	53.48644	chr1:1043	ENSG00000236723		lncRNA	chr1:53209783-5321
ENSG0000(	2395	53.48644	chr1:1043	ZYG11A		protein_c	chr1:52842511-5289
ENSG0000(	2395	53.48644	chr1:1043	ENSG00000277397		Pseudoger	chr1:53180921-5318
ENSG0000(	2395	53.48644	chr1:1043	PODN		protein_c	chr1:53062052-5308
ENSG0000(	2395	53.48644	chr1:1043	MIR1273G		smallRNA	chr1:52940314-5294
ENSG0000(	2395	53.48644	chr1:1043	CPT2		protein_c	chr1:53196792-5321
ENSG0000(	2395	53.48644	chr1:1043	ECHDC2		protein_c	chr1:52895910-5292
ENSG0000(	2395	53.48644	chr1:1043	MIR1273F		smallRNA	chr1:52928674-5292
ENSG0000(	2395	53.48644	chr1:1043	ENSG00000226938		lncRNA	chr1:53348488-5334
ENSG0000(	2395	53.48644	chr1:1043	CZIB-DT		lncRNA	chr1:53220663-5322
ENSG0000(	2395	53.48644	chr1:1043	ENSG00000285954		lncRNA	chr1:53344031-5336
ENSG0000(	2395	53.48644	chr1:1043	RNU6-969P		smallRNA	chr1:52805108-5280
ENSG0000(	2395	53.48644	chr1:1043	ENSG00000242391		lncRNA	chr1:52881216-5288
ENSG0000(	2395	53.48644	chr1:1043	ENSG00000234578		lncRNA	chr1:53267935-5326
ENSG0000(	2395	53.48644	chr1:1043	COA7		protein_c	chr1:52684449-5269
ENSG0000(	2395	53.48644	chr1:1043	H3P2		Pseudoger	chr1:52943536-5294
ENSG0000(	2395	53.48644	chr1:1043	MAGOH		protein_c	chr1:53226900-5323
ENSG0000(	2395	53.48644	chr1:1043	CZIB		protein_c	chr1:53214099-5322
ENSG0000(	2395	53.48644	chr1:1043	SLC1A7		protein_c	chr1:53087179-5314
ENSG0000(	2395	53.48644	chr1:1043	NDUFS5P3		Pseudoger	chr1:52709122-5270
ENSG0000(	2395	53.48644	chr1:1043	LINC02812		lncRNA	chr1:53366656-5336
ENSG0000(	2395	53.48644	chr1:1043	ZYG11B		protein_c	chr1:52726453-5282
ENSG0000(	2395	53.48644	chr1:1043	RNU2-30P		smallRNA	chr1:52754322-5275
ENSG0000(	2395	53.48644	chr1:1043	HIGD1AP11		Pseudoger	chr1:53073110-5307

ENSG0000(	2395	53.48644	chr1:104	RRAS2P1		Pseudoger	chr1:52993201-5299
ENSG0000(	2395	53.48644	chr1:104	ENSG00000235563		lncRNA	chr1:53114576-5311
ENSG0000(	2395	53.48644	chr1:104	RN7SL62P		smallRNA	chr1:52714399-5271
ENSG0000(	2395	53.48644	chr1:104	ENSG00000231866		Pseudoger	chr1:52925249-5292
ENSG0000(	2395	53.48644	chr1:104	ENSG00000228838		lncRNA	chr1:53288024-5328
ENSG0000(	2395	53.48644	chr1:104	SCP2		protein_c	chr1:52927276-5305
ENSG0000(	2395	53.48644	chr1:104	ENSG00000232993		lncRNA	chr1:53069938-5308
ENSG0000(	2395	53.48644	chr1:104	RP5-1024G6.8		lncRNA	chr1:53242364-5324
ENSG0000(	2395	53.48644	chr1:104	RPS13P2		Pseudoger	chr1:52772194-5277
ENSG0000(	2395	53.48644	chr1:104	MAGOH-DT		lncRNA	chr1:53238550-5324
ENSG0000(	2372	52.97279	chr1:104	HNRNPA3P12		Pseudoger	chr1:53974969-5397
ENSG0000(	2372	52.97279	chr1:104	MRPL37		protein_c	chr1:54184041-5422
ENSG0000(	2372	52.97279	chr1:104	TTC22		protein_c	chr1:54779712-5480
ENSG0000(	2372	52.97279	chr1:104	MTCO2P34		Pseudoger	chr1:55372710-5537
ENSG0000(	2372	52.97279	chr1:104	ENSG00000232245		lncRNA	chr1:54416256-5442
ENSG0000(	2372	52.97279	chr1:104	GYG1P3		Pseudoger	chr1:55222379-5522
ENSG0000(	2372	52.97279	chr1:104	LRRC42		protein_c	chr1:53946085-5396
ENSG0000(	2372	52.97279	chr1:104	USP24		protein_c	chr1:55066359-5521
ENSG0000(	2372	52.97279	chr1:104	AL353898.1		Pseudoger	chr1:54015654-5401
ENSG0000(	2372	52.97279	chr1:104	HSPB11		protein_c	chr1:53916574-5394
ENSG0000(	2372	52.97279	chr1:104	BSND		protein_c	chr1:54998933-5501
ENSG0000(	2372	52.97279	chr1:104	ENSG00000256407		protein_c	chr1:54132686-5420
ENSG0000(	2372	52.97279	chr1:104	ENSG00000230728		lncRNA	chr1:54621477-5462
ENSG0000(	2372	52.97279	chr1:104	LEXM		protein_c	chr1:54806063-5484
ENSG0000(	2372	52.97279	chr1:104	ENSG00000280378		TEC	chr1:54033126-5403
ENSG0000(	2372	52.97279	chr1:104	MIR4422		smallRNA	chr1:55225641-5522
ENSG0000(	2372	52.97279	chr1:104	SNORD112		smallRNA	chr1:54525386-5452
ENSG0000(	2372	52.97279	chr1:104	TMEM61		protein_c	chr1:54980628-5499
ENSG0000(	2372	52.97279	chr1:104	LDLRAD1	NCGv7	protein_c	chr1:54007298-5401
ENSG0000(	2372	52.97279	chr1:104	YIPF1		protein_c	chr1:53851719-5388
ENSG0000(	2372	52.97279	chr1:104	ENSG00000287724		lncRNA	chr1:54874672-5488
ENSG0000(	2372	52.97279	chr1:104	NDC1		protein_c	chr1:53765478-5383
ENSG0000(	2372	52.97279	chr1:104	AC099796.1		smallRNA	chr1:54504668-5450
ENSG0000(	2372	52.97279	chr1:104	ENSG00000237453		lncRNA	chr1:54792885-5479
ENSG0000(	2372	52.97279	chr1:104	TCEANC2		protein_c	chr1:54053584-5411
ENSG0000(	2372	52.97279	chr1:104	ENSG00000280425		lncRNA	chr1:54137746-5414
ENSG0000(	2372	52.97279	chr1:104	PCSK9		protein_c	chr1:55039447-5506
ENSG0000(	2372	52.97279	chr1:104	ENSG00000234810		lncRNA	chr1:55329288-5607
ENSG0000(	2372	52.97279	chr1:104	GLIS1		protein_c	chr1:53506237-5373
ENSG0000(	2372	52.97279	chr1:104	DHCR24		protein_c	chr1:54849627-5488
ENSG0000(	2372	52.97279	chr1:104	AL353898.2		Pseudoger	chr1:54033143-5403
ENSG0000(	2372	52.97279	chr1:104	ENSG00000225183		Pseudoger	chr1:54089856-5409
ENSG0000(	2372	52.97279	chr1:104	SSBP3-AS1		lncRNA	chr1:54236440-5423
ENSG0000(	2372	52.97279	chr1:104	ENSG00000288527		Pseudoger	chr1:53441268-5344
ENSG0000(	2372	52.97279	chr1:104	DMRTB1		protein_c	chr1:53459399-5346
ENSG0000(	2372	52.97279	chr1:104	LINC02784		lncRNA	chr1:54516412-5451
ENSG0000(	2372	52.97279	chr1:104	PARS2		protein_c	chr1:54756898-5476
ENSG0000(	2372	52.97279	chr1:104	ENSG00000229687		Pseudoger	chr1:53841547-5384
ENSG0000(	2372	52.97279	chr1:104	RNU6-830P		smallRNA	chr1:55398514-5539
ENSG0000(	2372	52.97279	chr1:104	SSBP3		protein_c	chr1:54225433-5441
ENSG0000(	2372	52.97279	chr1:104	TMEM59		protein_c	chr1:54026681-5405

ENSG0000(	2372	52.97279	chr1:104:	HNRNPA1P63		Pseudoger	chr1:54536796-5453
ENSG0000(	2372	52.97279	chr1:104:	ENSG00000225632		lncRNA	chr1:54285404-5428
ENSG0000(	2372	52.97279	chr1:104:	CYB5RL		protein_c	chr1:54169651-5420
ENSG0000(	2372	52.97279	chr1:104:	CDCP2		protein_c	chr1:54132687-5415
ENSG0000(	2372	52.97279	chr1:104:	MROH7		protein_c	chr1:54641754-5471
ENSG0000(	2372	52.97279	chr1:104:	AL049745.1		smallRNA	chr1:53828792-5382
ENSG0000(	2372	52.97279	chr1:104:	GOT2P1		Pseudoger	chr1:55367466-5536
ENSG0000(	2372	52.97279	chr1:104:	AL357673.1		protein_c	chr1:54169660-5417
ENSG0000(	2372	52.97279	chr1:104:	ENSG00000237173		Pseudoger	chr1:54524824-5452
ENSG0000(	2372	52.97279	chr1:104:	AL353898.3		Pseudoger	chr1:54099968-5410
ENSG0000(	2372	52.97279	chr1:104:	ENSG00000242396		lncRNA	chr1:54886812-5497
ENSG0000(	2372	52.97279	chr1:104:	ENSG00000284601		lncRNA	chr1:54974900-5498
ENSG0000(	2372	52.97279	chr1:104:	RP4-758J24.5		lncRNA	chr1:54026683-5402
ENSG0000(	2372	52.97279	chr1:104:	TTC4	NCGv7	protein_c	chr1:54715861-5474
ENSG0000(	2372	52.97279	chr1:104:	MROH7-TTC4		protein_c	chr1:54641786-5474
ENSG0000(	2372	52.97279	chr1:104:	ACOT11		protein_c	chr1:54542257-5463
ENSG0000(	2372	52.97279	chr1:104:	RNU7-95P		smallRNA	chr1:53688749-5368
ENSG0000(	2372	52.97279	chr1:104:	ENSG00000279049		Pseudoger	chr1:54099968-5410
ENSG0000(	2372	52.97279	chr1:104:	RN7SKP291		smallRNA	chr1:55376526-5537
ENSG0000(	2372	52.97279	chr1:104:	MIR4422HG		lncRNA	chr1:55217645-5532
ENSG0000(	2372	52.97279	chr1:104:	ENSG00000287582		lncRNA	chr1:54514417-5451
ENSG0000(	2372	52.97279	chr1:104:	FAM151A		protein_c	chr1:54609181-5462
ENSG0000(	2372	52.97279	chr1:104:	DIO1		protein_c	chr1:53891239-5391
ENSG0000(	2372	52.97279	chr1:104:	SNORA58		smallRNA	chr1:53771018-5377
ENSG0000(	2372	52.97279	chr1:104:	Y_RNA		smallRNA	chr1:55484871-5548
ENSG0000(	2372	52.97279	chr1:104:	ENSG00000233271		lncRNA	chr1:54980950-5499
ENSG0000(	2372	52.97279	chr1:104:	DHCR24-DT		lncRNA	chr1:54887563-5488
ENSG0000(	2367	52.86113	chr1:114:	AL390776.1		smallRNA	chr1:44333105-4433
ENSG0000(	2364	52.79413	chr1:104:	ENSG00000287661		lncRNA	chr1:48552991-4855
ENSG0000(	2364	52.79413	chr1:104:	EPS15	NCGv7;AC	protein_c	chr1:51354263-5151
ENSG0000(	2364	52.79413	chr1:104:	CFL1P2		Pseudoger	chr1:51157788-5115
ENSG0000(	2364	52.79413	chr1:104:	ENSG00000231413		lncRNA	chr1:48078787-4808
ENSG0000(	2364	52.79413	chr1:104:	ENSG00000223390		lncRNA	chr1:52033391-5204
ENSG0000(	2364	52.79413	chr1:104:	NRDC		protein_c	chr1:51789191-5187
ENSG0000(	2364	52.79413	chr1:104:	SKINT1L		Pseudoger	chr1:48161799-4817
ENSG0000(	2364	52.79413	chr1:104:	ORC1		protein_c	chr1:52372829-5240
ENSG0000(	2364	52.79413	chr1:104:	ENSG00000279324		TEC	chr1:49994318-4999
ENSG0000(	2364	52.79413	chr1:104:	ENSG00000232514		Pseudoger	chr1:48497263-4849
ENSG0000(	2364	52.79413	chr1:104:	CYP46A4P		Pseudoger	chr1:48089368-4808
ENSG0000(	2364	52.79413	chr1:104:	LINC01562		lncRNA	chr1:51195095-5123
ENSG0000(	2364	52.79413	chr1:104:	ENSG00000272491		lncRNA	chr1:48227888-4822
ENSG0000(	2364	52.79413	chr1:104:	SNORA26		smallRNA	chr1:51724775-5172
ENSG0000(	2364	52.79413	chr1:104:	TXNDC12-AS1		lncRNA	chr1:52050918-5205
ENSG0000(	2364	52.79413	chr1:104:	RNF11		protein_c	chr1:51236273-5127
ENSG0000(	2364	52.79413	chr1:104:	ENSG00000228407		Pseudoger	chr1:52160261-5216
ENSG0000(	2364	52.79413	chr1:104:	CDKN2C	NCGv7;AC	protein_c	chr1:50960745-5097
ENSG0000(	2364	52.79413	chr1:104:	OSBPL9		protein_c	chr1:51577179-5179
ENSG0000(	2364	52.79413	chr1:104:	RNU6-877P		smallRNA	chr1:51382308-5138
ENSG0000(	2364	52.79413	chr1:104:	TSEN15P2		Pseudoger	chr1:51859778-5186
ENSG0000(	2364	52.79413	chr1:104:	AL645730.2		protein_c	chr1:49994318-4999
ENSG0000(	2364	52.79413	chr1:104:	TXNDC12		protein_c	chr1:52020131-5205

ENSG0000(	2364	52.79413	chr1:104:	FAF1-AS1		lncRNA	chr1:50461469-5047
ENSG0000(	2364	52.79413	chr1:104:	SPATA6	NCGv7	protein_c	chr1:48295373-4847
ENSG0000(	2364	52.79413	chr1:104:	AL109659.1		protein_c	chr1:48103634-4810
ENSG0000(	2364	52.79413	chr1:104:	ENSG00000279214		TEC	chr1:48262230-4826
ENSG0000(	2364	52.79413	chr1:104:	RNU6-1026P		smallRNA	chr1:50582404-5058
ENSG0000(	2364	52.79413	chr1:104:	PHB1P12		Pseudoger	chr1:50780340-5078
ENSG0000(	2364	52.79413	chr1:104:	FCF1P6		Pseudoger	chr1:50405430-5040
ENSG0000(	2364	52.79413	chr1:104:	SLC5A9		protein_c	chr1:48222685-4824
ENSG0000(	2364	52.79413	chr1:104:	ENSG00000236004		Pseudoger	chr1:52189916-5219
ENSG0000(	2364	52.79413	chr1:104:	AL645730.1		smallRNA	chr1:49982215-4998
ENSG0000(	2364	52.79413	chr1:104:	TTC39A		protein_c	chr1:51287258-5134
ENSG0000(	2364	52.79413	chr1:104:	CC2D1B		protein_c	chr1:52345723-5236
ENSG0000(	2364	52.79413	chr1:104:	HMGB1P45		Pseudoger	chr1:50398825-5039
ENSG0000(	2364	52.79413	chr1:104:	RN7SL788P		smallRNA	chr1:52150105-5215
ENSG0000(	2364	52.79413	chr1:104:	PDCL3P6		Pseudoger	chr1:52179848-5218
ENSG0000(	2364	52.79413	chr1:104:	EEF1GP7		Pseudoger	chr1:52573114-5257
ENSG0000(	2364	52.79413	chr1:104:	RNU6-1253P		smallRNA	chr1:50750296-5075
ENSG0000(	2364	52.79413	chr1:104:	EPS15-AS1		lncRNA	chr1:51518288-5156
ENSG0000(	2364	52.79413	chr1:104:	ENSG00000272100		lncRNA	chr1:52353487-5235
ENSG0000(	2364	52.79413	chr1:104:	snoU13		smallRNA	chr1:52411442-5241
ENSG0000(	2364	52.79413	chr1:104:	RNA5SP48		Pseudoger	chr1:51973410-5197
ENSG0000(	2364	52.79413	chr1:104:	ENSG00000285839		protein_c	chr1:52020153-5203
ENSG0000(	2364	52.79413	chr1:104:	AGBL4-AS1		lncRNA	chr1:49257411-4926
ENSG0000(	2364	52.79413	chr1:104:	DNAJC19P7		Pseudoger	chr1:52252062-5225
ENSG0000(	2364	52.79413	chr1:104:	PRPF38A		protein_c	chr1:52404602-5242
ENSG0000(	2364	52.79413	chr1:104:	ENSG00000279096		TEC	chr1:48102068-4810
ENSG0000(	2364	52.79413	chr1:104:	ENSG00000230828		Pseudoger	chr1:50114937-5011
ENSG0000(	2364	52.79413	chr1:104:	BEND5		protein_c	chr1:48727519-4877
ENSG0000(	2364	52.79413	chr1:104:	TUT4		protein_c	chr1:52408282-5255
ENSG0000(	2364	52.79413	chr1:104:	ENSG00000235105		Pseudoger	chr1:48435967-4843
ENSG0000(	2364	52.79413	chr1:104:	ENSG00000238140		lncRNA	chr1:51461721-5146
ENSG0000(	2364	52.79413	chr1:104:	ENSG00000266993		lncRNA	chr1:51793934-5179
ENSG0000(	2364	52.79413	chr1:104:	AL589663.1		smallRNA	chr1:51900920-5190
ENSG0000(	2364	52.79413	chr1:104:	MIR4421		smallRNA	chr1:51059837-5105
ENSG0000(	2364	52.79413	chr1:104:	ENSG00000284645		lncRNA	chr1:50252569-5025
ENSG0000(	2364	52.79413	chr1:104:	ZNF859P		Pseudoger	chr1:49841821-4984
ENSG0000(	2364	52.79413	chr1:104:	Y_RNA		smallRNA	chr1:51865633-5186
ENSG0000(	2364	52.79413	chr1:104:	ENSG00000272371		lncRNA	chr1:52554818-5255
ENSG0000(	2364	52.79413	chr1:104:	PPP1R8P1		Pseudoger	chr1:48325080-4832
ENSG0000(	2364	52.79413	chr1:104:	CALR4P		Pseudoger	chr1:51561866-5159
ENSG0000(	2364	52.79413	chr1:104:	ENSG00000232027		Pseudoger	chr1:51372270-5137
ENSG0000(	2364	52.79413	chr1:104:	ENSG00000236434		lncRNA	chr1:51264916-5126
ENSG0000(	2364	52.79413	chr1:104:	RNU6-1281P		smallRNA	chr1:51538625-5153
ENSG0000(	2364	52.79413	chr1:104:	ENSG00000233407		lncRNA	chr1:50206084-5022
ENSG0000(	2364	52.79413	chr1:104:	Y_RNA		smallRNA	chr1:51107222-5110
ENSG0000(	2364	52.79413	chr1:104:	MTND2P29		Pseudoger	chr1:50017092-5001
ENSG0000(	2364	52.79413	chr1:104:	MIR761		smallRNA	chr1:51836341-5183
ENSG0000(	2364	52.79413	chr1:104:	ENSG00000290466		lncRNA	chr1:48096092-4816
ENSG0000(	2364	52.79413	chr1:104:	ENSG00000234080		Pseudoger	chr1:50326131-5032
ENSG0000(	2364	52.79413	chr1:104:	RP5-850O15.4		lncRNA	chr1:50437028-5043
ENSG0000(	2364	52.79413	chr1:104:	ENSG00000223429		Pseudoger	chr1:52162186-5216

ENSG0000(	2364	52.79413	chr1:104	TTC39A-AS1		lncRNA	chr1:51329654-5133
ENSG0000(	2364	52.79413	chr1:104	ENSG00000287078		lncRNA	chr1:52365443-5236
ENSG0000(	2364	52.79413	chr1:104	ENSG00000233406		Pseudoger	chr1:51250603-5125
ENSG0000(	2364	52.79413	chr1:104	ANAPC10P1		Pseudoger	chr1:52253621-5225
ENSG0000(	2364	52.79413	chr1:104	GPX7		protein_c	chr1:52602371-5260
ENSG0000(	2364	52.79413	chr1:104	ENSG00000284700		lncRNA	chr1:50423609-5042
ENSG0000(	2364	52.79413	chr1:104	LINC02808		lncRNA	chr1:50229662-5032
ENSG0000(	2364	52.79413	chr1:104	Y_RNA		smallRNA	chr1:50499758-5049
ENSG0000(	2364	52.79413	chr1:104	PLA2G12AP1		Pseudoger	chr1:52368677-5236
ENSG0000(	2364	52.79413	chr1:104	ENSG00000286597		lncRNA	chr1:48926021-4893
ENSG0000(	2364	52.79413	chr1:104	ENSG00000223720		lncRNA	chr1:48172972-4820
ENSG0000(	2364	52.79413	chr1:104	ENSG00000229032		Pseudoger	chr1:51980473-5198
ENSG0000(	2364	52.79413	chr1:104	AGBL4-IT1		lncRNA	chr1:49374201-4947
ENSG0000(	2364	52.79413	chr1:104	ENSG00000237478		Pseudoger	chr1:49691262-4969
ENSG0000(	2364	52.79413	chr1:104	FAF1		protein_c	chr1:50437028-5096
ENSG0000(	2364	52.79413	chr1:104	GAPDHP51		Pseudoger	chr1:51707138-5170
ENSG0000(	2364	52.79413	chr1:104	RNU4-61P		smallRNA	chr1:48447936-4844
ENSG0000(	2364	52.79413	chr1:104	LINC02794		lncRNA	chr1:48050659-4809
ENSG0000(	2364	52.79413	chr1:104	snoU13		smallRNA	chr1:51231253-5123
ENSG0000(	2364	52.79413	chr1:104	AL162430.1		smallRNA	chr1:51190883-5119
ENSG0000(	2364	52.79413	chr1:104	AGBL4		protein_c	chr1:48532854-5002
ENSG0000(	2364	52.79413	chr1:104	C1orf185	NCGv7	protein_c	chr1:51102221-5114
ENSG0000(	2364	52.79413	chr1:104	RN7SL290P		smallRNA	chr1:51995740-5199
ENSG0000(	2364	52.79413	chr1:104	ENSG00000272175		lncRNA	chr1:51801028-5180
ENSG0000(	2364	52.79413	chr1:104	ELAVL4-AS1		lncRNA	chr1:50174306-5017
ENSG0000(	2364	52.79413	chr1:104	SLC25A6P3		Pseudoger	chr1:51709062-5170
ENSG0000(	2364	52.79413	chr1:104	BTF3L4		protein_c	chr1:52056199-5209
ENSG0000(	2364	52.79413	chr1:104	ENSG00000229846		lncRNA	chr1:49025595-4918
ENSG0000(	2364	52.79413	chr1:104	RAB3B		protein_c	chr1:51907956-5199
ENSG0000(	2364	52.79413	chr1:104	KTI12		protein_c	chr1:52032103-5203
ENSG0000(	2364	52.79413	chr1:104	RNU6-723P		smallRNA	chr1:48344209-4834
ENSG0000(	2364	52.79413	chr1:104	AL162430.2		smallRNA	chr1:51188463-5118
ENSG0000(	2364	52.79413	chr1:104	DMRTA2		protein_c	chr1:50417550-5042
ENSG0000(	2364	52.79413	chr1:104	ZFYVE9		protein_c	chr1:52142089-5234
ENSG0000(	2364	52.79413	chr1:104	snoU13		smallRNA	chr1:49151392-4915
ENSG0000(	2364	52.79413	chr1:104	ENSG00000290102		lncRNA	chr1:50967883-5096
ENSG0000(	2364	52.79413	chr1:104	MRPS6P2		Pseudoger	chr1:50846468-5084
ENSG0000(	2364	52.79413	chr1:104	ENSG00000291246		lncRNA	chr1:48164710-4818
ENSG0000(	2364	52.79413	chr1:104	ELAVL4	NCGv7	protein_c	chr1:50024029-5020
ENSG0000(	2362	52.74947	chr1:104	SLC25A3P1		Pseudoger	chr1:53413149-5344
ENSG0000(	2360	52.7048	chr1:219	ENSG00000260990		lncRNA	chr1:175307218-175
ENSG0000(	2360	52.7048	chr1:219	ENSG00000237249		Pseudoger	chr1:174892417-174
ENSG0000(	2360	52.7048	chr1:219	SNORD78		smallRNA	chr1:173865622-173
ENSG0000(	2360	52.7048	chr1:219	CACYBP		protein_c	chr1:174999163-175
ENSG0000(	2360	52.7048	chr1:219	TNR	NCGv7	protein_c	chr1:175315194-175
ENSG0000(	2360	52.7048	chr1:219	ENSG00000289425		lncRNA	chr1:174967328-174
ENSG0000(	2360	52.7048	chr1:219	snoU13		smallRNA	chr1:174200129-174
ENSG0000(	2360	52.7048	chr1:219	ENTR1P2		Pseudoger	chr1:175044626-175
ENSG0000(	2360	52.7048	chr1:219	CENPL	DriverDB\	protein_c	chr1:173799550-173
ENSG0000(	2360	52.7048	chr1:219	SERPINC1		protein_c	chr1:173903800-173
ENSG0000(	2360	52.7048	chr1:219	RNA5SP68		Pseudoger	chr1:173969318-173

ENSG0000(	2360	52.7048	chr1:2197	RN7SKP160		smallRNA	chr1:173791548-173
ENSG0000(	2360	52.7048	chr1:2197	GAS5-AS1		lncRNA	chr1:173862473-173
ENSG0000(	2360	52.7048	chr1:2197	DARS2		protein_c	chr1:173824653-173
ENSG0000(	2360	52.7048	chr1:2197	MRPS14	NCGv7	protein_c	chr1:175010789-175
ENSG0000(	2360	52.7048	chr1:2197	TNN	NCGv7	protein_c	chr1:175067833-175
ENSG0000(	2360	52.7048	chr1:2197	BANF1P4		Pseudoger	chr1:174756850-174
ENSG0000(	2360	52.7048	chr1:2197	LINC01657		lncRNA	chr1:175877343-175
ENSG0000(	2360	52.7048	chr1:2197	RC3H1		protein_c	chr1:173931084-174
ENSG0000(	2360	52.7048	chr1:2197	TNR-IT1		lncRNA	chr1:175538775-175
ENSG0000(	2360	52.7048	chr1:2197	KIAA0040		protein_c	chr1:175156986-175
ENSG0000(	2360	52.7048	chr1:2197	RABGAP1L-IT1		lncRNA	chr1:174896958-174
ENSG0000(	2360	52.7048	chr1:2197	Y_RNA		smallRNA	chr1:175022479-175
ENSG0000(	2360	52.7048	chr1:2197	RPS29P4		Pseudoger	chr1:175297080-175
ENSG0000(	2360	52.7048	chr1:2197	GPR52		protein_c	chr1:174447964-174
ENSG0000(	2360	52.7048	chr1:2197	ZBTB37	DriverDBv	protein_c	chr1:173868082-173
ENSG0000(	2360	52.7048	chr1:2197	ENSG00000235869		Pseudoger	chr1:174922107-174
ENSG0000(	2360	52.7048	chr1:2197	RC3H1-DT		lncRNA	chr1:174022509-174
ENSG0000(	2360	52.7048	chr1:2197	ENSG00000287697		lncRNA	chr1:174998353-174
ENSG0000(	2360	52.7048	chr1:2197	RABGAP1L-AS1		lncRNA	chr1:174934947-174
ENSG0000(	2360	52.7048	chr1:2197	NDUFAF4P4		Pseudoger	chr1:174849667-174
ENSG0000(	2360	52.7048	chr1:2197	RABGAP1L		protein_c	chr1:174159410-174
ENSG0000(	2360	52.7048	chr1:2197	AL022400.1		smallRNA	chr1:174348265-174
ENSG0000(	2360	52.7048	chr1:2197	ENSG00000237317		Pseudoger	chr1:174367105-174
ENSG0000(	2360	52.7048	chr1:2197	RNU6-307P		smallRNA	chr1:174996524-174
ENSG0000(	2360	52.7048	chr1:2197	RC3H1-IT1		lncRNA	chr1:174009267-174
ENSG0000(	2360	52.7048	chr1:2197	Y_RNA		smallRNA	chr1:173808489-173
ENSG0000(	2360	52.7048	chr1:2197	RABGAP1L-DT		lncRNA	chr1:174110268-174
ENSG0000(	2360	52.7048	chr1:2197	GAS5		lncRNA	chr1:173858559-173
ENSG0000(	2360	52.7048	chr1:2197	RNA5SP67		Pseudoger	chr1:173921070-173
ENSG0000(	2360	52.7048	chr1:2197	RPL30P1		Pseudoger	chr1:174090136-174
ENSG0000(	2360	52.7048	chr1:2197	ENSG00000230687		lncRNA	chr1:175203228-175
ENSG0000(	2349	52.45914	chr1:1142	OOSP1P1		Pseudoger	chr1:44155028-4415
ENSG0000(	2349	52.45914	chr1:1142	KDM4A-AS1		lncRNA	chr1:43685123-4370
ENSG0000(	2349	52.45914	chr1:1142	ARMH1		protein_c	chr1:44674692-4472
ENSG0000(	2349	52.45914	chr1:1142	HYI-AS1		lncRNA	chr1:43453927-4345
ENSG0000(	2349	52.45914	chr1:1142	KLF17		protein_c	chr1:44118821-4413
ENSG0000(	2349	52.45914	chr1:1142	SHMT1P1		Pseudoger	chr1:43850300-4385
ENSG0000(	2349	52.45914	chr1:1142	RNF220		protein_c	chr1:44405194-4465
ENSG0000(	2349	52.45914	chr1:1142	ENSG00000284989		protein_c	chr1:43650149-4393
ENSG0000(	2349	52.45914	chr1:1142	KLF18		protein_c	chr1:44137821-4414
ENSG0000(	2349	52.45914	chr1:1142	DPH2		protein_c	chr1:43970000-4397
ENSG0000(	2349	52.45914	chr1:1142	ENSG00000227994		Pseudoger	chr1:44172506-4417
ENSG0000(	2349	52.45914	chr1:1142	KDM4A		protein_c	chr1:43650149-4370
ENSG0000(	2349	52.45914	chr1:1142	CCDC24		protein_c	chr1:43991359-4399
ENSG0000(	2349	52.45914	chr1:1142	Y_RNA		smallRNA	chr1:44153385-4415
ENSG0000(	2349	52.45914	chr1:1142	RNU5D-1		smallRNA	chr1:44731055-4473
ENSG0000(	2349	52.45914	chr1:1142	RN7SL479P		smallRNA	chr1:44117100-4411
ENSG0000(	2349	52.45914	chr1:1142	ENSG00000285649		lncRNA	chr1:43968351-4397
ENSG0000(	2349	52.45914	chr1:1142	DMAP1		protein_c	chr1:44213455-4422
ENSG0000(	2349	52.45914	chr1:1142	ENSG00000271329		Pseudoger	chr1:44187943-4418
ENSG0000(	2349	52.45914	chr1:1142	SZT2-AS1		lncRNA	chr1:43447776-4344

ENSG0000(	2349	52.45914	chr1:1142	ENSG00000230615		lncRNA	chr1:44030414-4411
ENSG0000(	2349	52.45914	chr1:1142	RNU6-369P		smallRNA	chr1:44390722-4439
ENSG0000(	2349	52.45914	chr1:1142	RNU5F-1		smallRNA	chr1:44721786-4472
ENSG0000(	2349	52.45914	chr1:1142	RNU6-1058P		smallRNA	chr1:43716467-4371
ENSG0000(	2349	52.45914	chr1:1142	MIR5584		smallRNA	chr1:44545493-4454
ENSG0000(	2349	52.45914	chr1:1142	ERI3-IT1		lncRNA	chr1:44243408-4424
ENSG0000(	2349	52.45914	chr1:1142	B4GALT2		protein_c	chr1:43978943-4399
ENSG0000(	2349	52.45914	chr1:1142	TMEM53		protein_c	chr1:44635238-4467
ENSG0000(	2349	52.45914	chr1:1142	ST3GAL3-AS1		lncRNA	chr1:43709392-4372
ENSG0000(	2349	52.45914	chr1:1142	ENSG00000233674		Pseudoger	chr1:43743471-4374
ENSG0000(	2349	52.45914	chr1:1142	ATP6V0B	NCGv7	protein_c	chr1:43974487-4397
ENSG0000(	2349	52.45914	chr1:1142	ENSG00000233514		Pseudoger	chr1:44122153-4412
ENSG0000(	2349	52.45914	chr1:1142	HYI		protein_c	chr1:43450989-4345
ENSG0000(	2349	52.45914	chr1:1142	SLC6A9	NCGv7	protein_c	chr1:43991500-4403
ENSG0000(	2349	52.45914	chr1:1142	PTPRF		protein_c	chr1:43525187-4362
ENSG0000(	2349	52.45914	chr1:1142	RP11-7O11.3		lncRNA	chr1:43944370-4394
ENSG0000(	2349	52.45914	chr1:1142	ENSG00000226804		Pseudoger	chr1:44150594-4415
ENSG0000(	2349	52.45914	chr1:1142	ST3GAL3		protein_c	chr1:43705824-4393
ENSG0000(	2349	52.45914	chr1:1142	KRT8P47		Pseudoger	chr1:44103306-4410
ENSG0000(	2349	52.45914	chr1:1142	ERI3		protein_c	chr1:44221070-4435
ENSG0000(	2349	52.45914	chr1:1142	ENSG00000227163		Pseudoger	chr1:44087958-4408
ENSG0000(	2349	52.45914	chr1:1142	IPO13		protein_c	chr1:43946950-4396
ENSG0000(	2349	52.45914	chr1:1142	ARTN		protein_c	chr1:43933320-4393
ENSG0000(	2331	52.05716	chr1:1043	ZSWIM5		protein_c	chr1:45016399-4530
ENSG0000(	2331	52.05716	chr1:1043	ATP6V0E1P4		Pseudoger	chr1:47550196-4755
ENSG0000(	2331	52.05716	chr1:1043	ENSG00000288208		protein_c	chr1:45329262-4549
ENSG0000(	2331	52.05716	chr1:1043	MRPS17P1		Pseudoger	chr1:44988705-4499
ENSG0000(	2331	52.05716	chr1:1043	EFCAB14		protein_c	chr1:46674659-4671
ENSG0000(	2331	52.05716	chr1:1043	UROD		protein_c	chr1:45010950-4501
ENSG0000(	2331	52.05716	chr1:1043	CCNB1IP1P1		Pseudoger	chr1:44958557-4495
ENSG0000(	2331	52.05716	chr1:1043	PLK3		protein_c	chr1:44800377-4480
ENSG0000(	2331	52.05716	chr1:1043	KNCN		protein_c	chr1:46545641-4655
ENSG0000(	2331	52.05716	chr1:1043	DMBX1		protein_c	chr1:46489836-4651
ENSG0000(	2331	52.05716	chr1:1043	RPS15AP11		Pseudoger	chr1:44780331-4478
ENSG0000(	2331	52.05716	chr1:1043	CYP4X1		protein_c	chr1:47023669-4705
ENSG0000(	2331	52.05716	chr1:1043	CYP4A44P		Pseudoger	chr1:47002995-4700
ENSG0000(	2331	52.05716	chr1:1043	LINC01144		lncRNA	chr1:45303910-4530
ENSG0000(	2331	52.05716	chr1:1043	CYP4A22		protein_c	chr1:47137435-4714
ENSG0000(	2331	52.05716	chr1:1043	PDZK1IP1 AC		protein_c	chr1:47183582-4719
ENSG0000(	2331	52.05716	chr1:1043	IPP		protein_c	chr1:45694324-4575
ENSG0000(	2331	52.05716	chr1:1043	TAL1	NCGv7;AC	protein_c	chr1:47216290-4723
ENSG0000(	2331	52.05716	chr1:1043	RPL6P1		Pseudoger	chr1:45781277-4578
ENSG0000(	2331	52.05716	chr1:1043	CMPK1		protein_c	chr1:47333790-4739
ENSG0000(	2331	52.05716	chr1:1043	CYP4A26P		Pseudoger	chr1:46967679-4696
ENSG0000(	2331	52.05716	chr1:1043	ENSG00000271355		Pseudoger	chr1:47483698-4748
ENSG0000(	2331	52.05716	chr1:1043	snoU13		smallRNA	chr1:45358652-4535
ENSG0000(	2331	52.05716	chr1:1043	ENSG00000226957		lncRNA	chr1:46046818-4604
ENSG0000(	2331	52.05716	chr1:1043	HMGB1P48		Pseudoger	chr1:45530927-4553
ENSG0000(	2331	52.05716	chr1:1043	ENSG00000227857		lncRNA	chr1:46134531-4613
ENSG0000(	2331	52.05716	chr1:1043	ENSG00000291138		lncRNA	chr1:46433827-4648
ENSG0000(	2331	52.05716	chr1:1043	BTBD19		protein_c	chr1:44808523-4481

ENSG0000(	2331	52.05716	chr1:104:	FAAHP1		Pseudoger	chr1:46432129-4644
ENSG0000(	2331	52.05716	chr1:104:	RPL21P24		Pseudoger	chr1:47497894-4749
ENSG0000(	2331	52.05716	chr1:104:	PPIAP35		Pseudoger	chr1:44988234-4498
ENSG0000(	2331	52.05716	chr1:104:	SNORD55		smallRNA	chr1:44775864-4477
ENSG0000(	2331	52.05716	chr1:104:	MMACHC	NCGv7	protein_c	chr1:45500300-4551
ENSG0000(	2331	52.05716	chr1:104:	UQCRH		protein_c	chr1:46303698-4631
ENSG0000(	2331	52.05716	chr1:104:	ENSG00000226499		Pseudoger	chr1:44843921-4484
ENSG0000(	2331	52.05716	chr1:104:	TOE1		protein_c	chr1:45340052-4534
ENSG0000(	2331	52.05716	chr1:104:	CYP4A43P		Pseudoger	chr1:46994382-4699
ENSG0000(	2331	52.05716	chr1:104:	NASP		protein_c	chr1:45583846-4561
ENSG0000(	2331	52.05716	chr1:104:	MTND1P34		Pseudoger	chr1:47164510-4716
ENSG0000(	2331	52.05716	chr1:104:	CYP4A11	NCGv7	protein_c	chr1:46929177-4694
ENSG0000(	2331	52.05716	chr1:104:	RPS15AP10		Pseudoger	chr1:45645816-4564
ENSG0000(	2331	52.05716	chr1:104:	CYP4A22-AS1		lncRNA	chr1:47096653-4717
ENSG0000(	2331	52.05716	chr1:104:	FOXE3		protein_c	chr1:47416285-4741
ENSG0000(	2331	52.05716	chr1:104:	ATPAF1		protein_c	chr1:46632737-4667
ENSG0000(	2331	52.05716	chr1:104:	FLJ00388		protein_c	chr1:47761307-4776
ENSG0000(	2331	52.05716	chr1:104:	TMEM275		protein_c	chr1:46532166-4654
ENSG0000(	2331	52.05716	chr1:104:	ENSG00000226252		lncRNA	chr1:47225797-4723
ENSG0000(	2331	52.05716	chr1:104:	MUTYH	NCGv7;AC	protein_c	chr1:45329163-4534
ENSG0000(	2331	52.05716	chr1:104:	DYNLT4		protein_c	chr1:44805893-4480
ENSG0000(	2331	52.05716	chr1:104:	OSTCP5		Pseudoger	chr1:45069977-4507
ENSG0000(	2331	52.05716	chr1:104:	TMEM69		protein_c	chr1:45688181-4569
ENSG0000(	2331	52.05716	chr1:104:	SNORD38B		smallRNA	chr1:44778390-4477
ENSG0000(	2331	52.05716	chr1:104:	ENSG00000290041		lncRNA	chr1:44807524-4480
ENSG0000(	2331	52.05716	chr1:104:	GPBP1L1		protein_c	chr1:45627304-4568
ENSG0000(	2331	52.05716	chr1:104:	CCDC17		protein_c	chr1:45620044-4562
ENSG0000(	2331	52.05716	chr1:104:	HECTD3		protein_c	chr1:45002540-4501
ENSG0000(	2331	52.05716	chr1:104:	ENSG00000280836		Pseudoger	chr1:45581219-4558
ENSG0000(	2331	52.05716	chr1:104:	FOXD2		protein_c	chr1:47438044-4744
ENSG0000(	2331	52.05716	chr1:104:	RPL7AP16		Pseudoger	chr1:45651039-4565
ENSG0000(	2331	52.05716	chr1:104:	CYP4A27P		Pseudoger	chr1:47000898-4700
ENSG0000(	2331	52.05716	chr1:104:	HPDL		protein_c	chr1:45326895-4532
ENSG0000(	2331	52.05716	chr1:104:	LINC01738		lncRNA	chr1:47688463-4770
ENSG0000(	2331	52.05716	chr1:104:	SNORD38A		smallRNA	chr1:44777843-4477
ENSG0000(	2331	52.05716	chr1:104:	TESK2		protein_c	chr1:45343883-4549
ENSG0000(	2331	52.05716	chr1:104:	MKNK1-AS1		lncRNA	chr1:46538611-4657
ENSG0000(	2331	52.05716	chr1:104:	ENSG00000236476		Pseudoger	chr1:46742329-4674
ENSG0000(	2331	52.05716	chr1:104:	ENSG00000281133		Pseudoger	chr1:45580892-4558
ENSG0000(	2331	52.05716	chr1:104:	EIF2B3		protein_c	chr1:44850522-4498
ENSG0000(	2331	52.05716	chr1:104:	FOXD2-AS1		lncRNA	chr1:47432133-4743
ENSG0000(	2331	52.05716	chr1:104:	LINC00853		lncRNA	chr1:47179250-4718
ENSG0000(	2331	52.05716	chr1:104:	ENSG00000223814		lncRNA	chr1:47761132-4776
ENSG0000(	2331	52.05716	chr1:104:	PIK3R3	NCGv7	protein_c	chr1:46040140-4613
ENSG0000(	2331	52.05716	chr1:104:	CYP4Z2P		Pseudoger	chr1:46843178-4690
ENSG0000(	2331	52.05716	chr1:104:	CCDC163		protein_c	chr1:45493866-4550
ENSG0000(	2331	52.05716	chr1:104:	P3R3URF		protein_c	chr1:46175486-4617
ENSG0000(	2331	52.05716	chr1:104:	TSPAN1	AC	protein_c	chr1:46175073-4618
ENSG0000(	2331	52.05716	chr1:104:	NENFP1		Pseudoger	chr1:46665910-4666
ENSG0000(	2331	52.05716	chr1:104:	CYP4Z1	NCGv7	protein_c	chr1:47067231-4711
ENSG0000(	2331	52.05716	chr1:104:	AL591415.1		smallRNA	chr1:47621093-4762

ENSG0000(	2331	52.05716	chr1:1043	TEX38		protein_c	chr1:46668855-4667
ENSG0000(	2331	52.05716	chr1:1043	RAD54L		protein_c	chr1:46246461-4627
ENSG0000(	2331	52.05716	chr1:1043	PRDX1		protein_c	chr1:45510914-4554
ENSG0000(	2331	52.05716	chr1:1043	MAST2		protein_c	chr1:45786987-4603
ENSG0000(	2331	52.05716	chr1:1043	TUBAP8		Pseudoger	chr1:46891639-4689
ENSG0000(	2331	52.05716	chr1:1043	ENSG00000225721		lncRNA	chr1:44759037-4477
ENSG0000(	2331	52.05716	chr1:1043	AL136380.1		smallRNA	chr1:44913068-4491
ENSG0000(	2331	52.05716	chr1:1043	CYP4B1	NCGv7	protein_c	chr1:46757838-4681
ENSG0000(	2331	52.05716	chr1:1043	ENSG00000225028		lncRNA	chr1:47818066-4782
ENSG0000(	2331	52.05716	chr1:1043	ENSG00000233114		Pseudoger	chr1:46104950-4610
ENSG0000(	2331	52.05716	chr1:1043	POMGNT1		protein_c	chr1:46188683-4622
ENSG0000(	2331	52.05716	chr1:1043	MOB3C		protein_c	chr1:46607719-4661
ENSG0000(	2331	52.05716	chr1:1043	BEST4		protein_c	chr1:44783585-4478
ENSG0000(	2331	52.05716	chr1:1043	AL592294.1		smallRNA	chr1:45232218-4523
ENSG0000(	2331	52.05716	chr1:1043	LINC01389		lncRNA	chr1:47380928-4740
ENSG0000(	2331	52.05716	chr1:1043	TUBAP9		Pseudoger	chr1:47074778-4707
ENSG0000(	2331	52.05716	chr1:1043	ENSG00000225779		Pseudoger	chr1:46370586-4637
ENSG0000(	2331	52.05716	chr1:1043	FAAH		protein_c	chr1:46394317-4641
ENSG0000(	2331	52.05716	chr1:1043	EFCAB14-AS1		lncRNA	chr1:46674036-4669
ENSG0000(	2331	52.05716	chr1:1043	ENSG00000290081		lncRNA	chr1:47180175-4718
ENSG0000(	2331	52.05716	chr1:1043	ENSG00000281112		Pseudoger	chr1:45592722-4559
ENSG0000(	2331	52.05716	chr1:1043	NSUN4		protein_c	chr1:46340789-4636
ENSG0000(	2331	52.05716	chr1:1043	KIF2C		protein_c	chr1:44739818-4476
ENSG0000(	2331	52.05716	chr1:1043	RPS8		protein_c	chr1:44775251-4477
ENSG0000(	2331	52.05716	chr1:1043	LURAP1		protein_c	chr1:46203334-4622
ENSG0000(	2331	52.05716	chr1:1043	TRABD2B		protein_c	chr1:47760528-4799
ENSG0000(	2331	52.05716	chr1:1043	AL356458.1		smallRNA	chr1:47502504-4750
ENSG0000(	2331	52.05716	chr1:1043	AKR1A1		protein_c	chr1:45550543-4557
ENSG0000(	2331	52.05716	chr1:1043	RNA5SP47		Pseudoger	chr1:44932323-4493
ENSG0000(	2331	52.05716	chr1:1043	P3R3URF-PIK3R3		protein_c	chr1:46043661-4617
ENSG0000(	2331	52.05716	chr1:1043	MKNK1	NCGv7	protein_c	chr1:46557407-4661
ENSG0000(	2331	52.05716	chr1:1043	CYP4Z2P		lncRNA	chr1:46843095-4690
ENSG0000(	2331	52.05716	chr1:1043	PPIAP36		Pseudoger	chr1:45415020-4541
ENSG0000(	2331	52.05716	chr1:1043	TMA16P2		Pseudoger	chr1:45846994-4584
ENSG0000(	2331	52.05716	chr1:1043	LINC01398		lncRNA	chr1:46446600-4645
ENSG0000(	2331	52.05716	chr1:1043	ENSG00000230896		lncRNA	chr1:45694684-4569
ENSG0000(	2331	52.05716	chr1:1043	PTCH2	AC	protein_c	chr1:44819844-4484
ENSG0000(	2331	52.05716	chr1:1043	ENSG00000281825		Pseudoger	chr1:45605657-4560
ENSG0000(	2331	52.05716	chr1:1043	STIL	NCGv7;AC	protein_c	chr1:47250139-4731
ENSG0000(	2331	52.05716	chr1:1043	AL359473.1		smallRNA	chr1:45033969-4503
ENSG0000(	2331	52.05716	chr1:1043	SNORD46		smallRNA	chr1:44776490-4477
ENSG0000(	2331	52.05716	chr1:1043	LRRC41		protein_c	chr1:46261196-4630
ENSG0000(	2312	51.63284	chr17:674	ENSG00000277382		lncRNA	chr17:76709760-767
ENSG0000(	2305	51.47651	chr1:1142	AL645859.1		smallRNA	chr1:29253542-2925
ENSG0000(	2274	50.7842	chr1:2197	ENSG00000261000		lncRNA	chr1:206503948-206
ENSG0000(	2274	50.7842	chr1:2197	PM20D1	NCGv7	protein_c	chr1:205828025-205
ENSG0000(	2274	50.7842	chr1:2197	DYRK3-AS1		lncRNA	chr1:206634184-206
ENSG0000(	2274	50.7842	chr1:2197	IKBKE	AC	protein_c	chr1:206470476-206
ENSG0000(	2274	50.7842	chr1:2197	MFSD4A		protein_c	chr1:205568885-205
ENSG0000(	2274	50.7842	chr1:2197	RAB29		protein_c	chr1:205767986-205
ENSG0000(	2274	50.7842	chr1:2197	ENSG00000287432		lncRNA	chr1:206147163-206

ENSG0000(	2274	50.7842	chr1:2197	SNORD60		smallRNA	chr1:206080238-206
ENSG0000(	2274	50.7842	chr1:2197	RPL7AP20		Pseudoger	chr1:206528915-206
ENSG0000(	2274	50.7842	chr1:2197	EIF2D		protein_c	chr1:206571292-206
ENSG0000(	2274	50.7842	chr1:2197	FAM72A	AC	protein_c	chr1:206186178-206
ENSG0000(	2274	50.7842	chr1:2197	DYRK3		protein_c	chr1:206635536-206
ENSG0000(	2274	50.7842	chr1:2197	ENSG00000285417		Pseudoger	chr1:206035252-206
ENSG0000(	2274	50.7842	chr1:2197	SNORD112		smallRNA	chr1:206681963-206
ENSG0000(	2274	50.7842	chr1:2197	SLC45A3	IntOGen-I	protein_c	chr1:205657851-205
ENSG0000(	2274	50.7842	chr1:2197	CTSE		protein_c	chr1:206009146-206
ENSG0000(	2274	50.7842	chr1:2197	ENSG00000279946		TEC	chr1:206541758-206
ENSG0000(	2274	50.7842	chr1:2197	ELK4	NCGv7;AC	protein_c	chr1:205597556-205
ENSG0000(	2274	50.7842	chr1:2197	ENSG00000229509		Pseudoger	chr1:206333327-206
ENSG0000(	2274	50.7842	chr1:2197	ENSG00000227687		lncRNA	chr1:205935128-205
ENSG0000(	2274	50.7842	chr1:2197	RP11-31207.2		lncRNA	chr1:206203345-206
ENSG0000(	2274	50.7842	chr1:2197	RNU6-418P		smallRNA	chr1:205595041-205
ENSG0000(	2274	50.7842	chr1:2197	SNORA72		smallRNA	chr1:205731221-205
ENSG0000(	2274	50.7842	chr1:2197	ENSG00000286619		lncRNA	chr1:205813322-205
ENSG0000(	2274	50.7842	chr1:2197	RNU2-19P		smallRNA	chr1:205566716-205
ENSG0000(	2274	50.7842	chr1:2197	SLC41A1		protein_c	chr1:205789094-205
ENSG0000(	2274	50.7842	chr1:2197	SRGAP2		protein_c	chr1:206342846-206
ENSG0000(	2274	50.7842	chr1:2197	ENSG00000236889		lncRNA	chr1:206175059-206
ENSG0000(	2274	50.7842	chr1:2197	NUCKS1		protein_c	chr1:205712822-205
ENSG0000(	2274	50.7842	chr1:2197	RPL22P4		Pseudoger	chr1:206160886-206
ENSG0000(	2274	50.7842	chr1:2197	ENSG00000285521		lncRNA	chr1:205775559-205
ENSG0000(	2274	50.7842	chr1:2197	AVPR1B-DT		lncRNA	chr1:206117782-206
ENSG0000(	2274	50.7842	chr1:2197	RASSF5		protein_c	chr1:206507531-206
ENSG0000(	2274	50.7842	chr1:2197	AVPR1B	DriverDB\	protein_c	chr1:206106935-206
ENSG0000(	2274	50.7842	chr1:2197	SLC26A9	DriverDB\	protein_c	chr1:205913048-205
ENSG0000(	2274	50.7842	chr1:2197	RHEX		protein_c	chr1:206053172-206
ENSG0000(	2274	50.7842	chr1:2197	C1orf147		lncRNA	chr1:206491116-206
ENSG0000(	2274	50.7842	chr1:2197	ENSG00000236942		Pseudoger	chr1:205625483-205
ENSG0000(	2271	50.71721	chr1:1142	MED8-AS1		lncRNA	chr1:43385113-4338
ENSG0000(	2271	50.71721	chr1:1142	ENSG00000235002		Pseudoger	chr1:42412398-4241
ENSG0000(	2271	50.71721	chr1:1142	RNU6-870P		smallRNA	chr1:43023549-4302
ENSG0000(	2271	50.71721	chr1:1142	ENSG00000288955		lncRNA	chr1:42924460-4292
ENSG0000(	2271	50.71721	chr1:1142	ENSG00000234917		lncRNA	chr1:42678735-4268
ENSG0000(	2271	50.71721	chr1:1142	RIMKLA	DriverDB\	protein_c	chr1:42380792-4242
ENSG0000(	2271	50.71721	chr1:1142	AL451006.1		smallRNA	chr1:41759141-4175
ENSG0000(	2271	50.71721	chr1:1142	GUCA2B		protein_c	chr1:42153410-4215
ENSG0000(	2271	50.71721	chr1:1142	MKRN8P		Pseudoger	chr1:42891094-4289
ENSG0000(	2271	50.71721	chr1:1142	CFAP57		protein_c	chr1:43172330-4325
ENSG0000(	2271	50.71721	chr1:1142	ENSG00000287113		lncRNA	chr1:43348288-4334
ENSG0000(	2271	50.71721	chr1:1142	FOXJ3		protein_c	chr1:42176539-4233
ENSG0000(	2271	50.71721	chr1:1142	RNU6-536P		smallRNA	chr1:42569033-4256
ENSG0000(	2271	50.71721	chr1:1142	RNA5SP46		Pseudoger	chr1:43196417-4319
ENSG0000(	2271	50.71721	chr1:1142	CCDC30		protein_c	chr1:42463221-4265
ENSG0000(	2271	50.71721	chr1:1142	ENSG00000287587		lncRNA	chr1:42036143-4205
ENSG0000(	2271	50.71721	chr1:1142	ZNF691-DT		lncRNA	chr1:42832522-4284
ENSG0000(	2271	50.71721	chr1:1142	SVBP		protein_c	chr1:42807052-4281
ENSG0000(	2271	50.71721	chr1:1142	CLDN19		protein_c	chr1:42733093-4274
ENSG0000(	2271	50.71721	chr1:1142	ENSG00000227527		lncRNA	chr1:42335386-4233

ENSG0000(	2271	50.71721	chr1:1142	C1orf50		protein_c	chr1:42767245-4277
ENSG0000(	2271	50.71721	chr1:1142	GUCA2A		protein_c	chr1:42162690-4216
ENSG0000(	2271	50.71721	chr1:1142	MED8		protein_c	chr1:43383917-4338
ENSG0000(	2271	50.71721	chr1:1142	ERMAP		protein_c	chr1:42817122-4284
ENSG0000(	2271	50.71721	chr1:1142	C1orf50-AS1		lncRNA	chr1:42775813-4277
ENSG0000(	2271	50.71721	chr1:1142	ZNF691		protein_c	chr1:42846573-4285
ENSG0000(	2271	50.71721	chr1:1142	HNRNPFP1		Pseudoger	chr1:42040597-4204
ENSG0000(	2271	50.71721	chr1:1142	RNU6-880P		smallRNA	chr1:42991438-4299
ENSG0000(	2271	50.71721	chr1:1142	SLC2A1-DT		lncRNA	chr1:42959049-4299
ENSG0000(	2271	50.71721	chr1:1142	TMEM125		protein_c	chr1:43269983-4327
ENSG0000(	2271	50.71721	chr1:1142	ATP6V0CP4		Pseudoger	chr1:42952202-4295
ENSG0000(	2271	50.71721	chr1:1142	ENSG00000285728		lncRNA	chr1:42658687-4268
ENSG0000(	2271	50.71721	chr1:1142	TMSB4XP1		Pseudoger	chr1:42500205-4250
ENSG0000(	2271	50.71721	chr1:1142	C1orf210		protein_c	chr1:43281877-4328
ENSG0000(	2271	50.71721	chr1:1142	P3H1		protein_c	chr1:42746335-4276
ENSG0000(	2271	50.71721	chr1:1142	PPCS		protein_c	chr1:42456117-4247
ENSG0000(	2271	50.71721	chr1:1142	ENSG00000277513		Pseudoger	chr1:43104086-4310
ENSG0000(	2271	50.71721	chr1:1142	SLC2A1		protein_c	chr1:42925353-4295
ENSG0000(	2271	50.71721	chr1:1142	ENSG00000283580		protein_c	chr1:42767292-4279
ENSG0000(	2271	50.71721	chr1:1142	ENSG00000228776		Pseudoger	chr1:42140635-4214
ENSG0000(	2271	50.71721	chr1:1142	RPS3AP11		Pseudoger	chr1:42491739-4249
ENSG0000(	2271	50.71721	chr1:1142	ENSG00000236180		Pseudoger	chr1:42570970-4257
ENSG0000(	2271	50.71721	chr1:1142	EBNA1BP2		protein_c	chr1:43164175-4327
ENSG0000(	2271	50.71721	chr1:1142	YBX1	AC	protein_c	chr1:42682418-4270
ENSG0000(	2271	50.71721	chr1:1142	TIE1		protein_c	chr1:43300982-4332
ENSG0000(	2271	50.71721	chr1:1142	CDC20	NCGv7	protein_c	chr1:43358981-4336
ENSG0000(	2271	50.71721	chr1:1142	ELOVL1		protein_c	chr1:43363398-4336
ENSG0000(	2271	50.71721	chr1:1142	CDC20-DT		lncRNA	chr1:43354684-4335
ENSG0000(	2271	50.71721	chr1:1142	ZMYND12		protein_c	chr1:42430329-4245
ENSG0000(	2271	50.71721	chr1:1142	SZT2		protein_c	chr1:43389882-4345
ENSG0000(	2271	50.71721	chr1:1142	FAM183A		protein_c	chr1:43145153-4315
ENSG0000(	2271	50.71721	chr1:1142	PPIH		protein_c	chr1:42658335-4267
ENSG0000(	2271	50.71721	chr1:1142	MPL	NCGv7;AC	protein_c	chr1:43337818-4335
ENSG0000(	2271	50.71721	chr1:1142	TMEM269		protein_c	chr1:42784991-4281
ENSG0000(	2271	50.71721	chr1:1142	ENSG00000233708		Pseudoger	chr1:42886597-4288
ENSG0000(	2271	50.71721	chr1:1142	ENSG00000288772		lncRNA	chr1:43368180-4336
ENSG0000(	2271	50.71721	chr1:1142	ENSG00000283973		lncRNA	chr1:42959065-4296
ENSG0000(	2270	50.69487	chr2:3094	RNA5-8SP5		Pseudoger	chr2:132253154-132
ENSG0000(	2243	50.0919	chr1:2197	ENSG00000227722		Pseudoger	chr1:168317497-168
ENSG0000(	2243	50.0919	chr1:2197	TBX19		protein_c	chr1:168280877-168
ENSG0000(	2243	50.0919	chr1:2197	SFT2D2		protein_c	chr1:168225938-168
ENSG0000(	2243	50.0919	chr1:2197	POU2F1		protein_c	chr1:167220876-167
ENSG0000(	2243	50.0919	chr1:2197	ENSG00000272033		lncRNA	chr1:167379108-167
ENSG0000(	2243	50.0919	chr1:2197	ENSG00000237131		Pseudoger	chr1:168215405-168
ENSG0000(	2243	50.0919	chr1:2197	RCSD1		protein_c	chr1:167630093-167
ENSG0000(	2243	50.0919	chr1:2197	MIR1255B2		smallRNA	chr1:167998660-167
ENSG0000(	2243	50.0919	chr1:2197	ENSG00000231029		Pseudoger	chr1:167591392-167
ENSG0000(	2243	50.0919	chr1:2197	ENSG00000232194		lncRNA	chr1:167820406-167
ENSG0000(	2243	50.0919	chr1:2197	ENSG00000233411		lncRNA	chr1:167457383-167
ENSG0000(	2243	50.0919	chr1:2197	ENSG00000287218		lncRNA	chr1:167455195-167
ENSG0000(	2243	50.0919	chr1:2197	ADCY10	DriverDB\	protein_c	chr1:167809386-167

ENSG0000(	2243	50.0919	chr1:2197	ENSG00000250762		Pseudoger	chr1:167819898-167
ENSG0000(	2243	50.0919	chr1:2197	RNU6-1310P		smallRNA	chr1:168263375-168
ENSG0000(	2243	50.0919	chr1:2197	MPC2	DriverDB\	protein_c	chr1:167916675-167
ENSG0000(	2243	50.0919	chr1:2197	GPA33		protein_c	chr1:167052836-167
ENSG0000(	2243	50.0919	chr1:2197	ANKRD36BP1		Pseudoger	chr1:168245565-168
ENSG0000(	2243	50.0919	chr1:2197	STYXL2		protein_c	chr1:167094075-167
ENSG0000(	2243	50.0919	chr1:2197	GPR161		protein_c	chr1:168079542-168
ENSG0000(	2243	50.0919	chr1:2197	MPZL1		protein_c	chr1:167721192-167
ENSG0000(	2243	50.0919	chr1:2197	TIPRL		protein_c	chr1:168178962-168
ENSG0000(	2243	50.0919	chr1:2197	POU2F1-DT		lncRNA	chr1:167219822-167
ENSG0000(	2243	50.0919	chr1:2197	AKR1D1P1		Pseudoger	chr1:167519536-167
ENSG0000(	2243	50.0919	chr1:2197	CREG1	DriverDB\	protein_c	chr1:167529117-167
ENSG0000(	2243	50.0919	chr1:2197	GCSHP5		Pseudoger	chr1:168055901-168
ENSG0000(	2243	50.0919	chr1:2197	RPL34P1		Pseudoger	chr1:168210616-168
ENSG0000(	2243	50.0919	chr1:2197	DCAF6		protein_c	chr1:167935783-168
ENSG0000(	2243	50.0919	chr1:2197	ENSG00000227907		lncRNA	chr1:167052551-167
ENSG0000(	2243	50.0919	chr1:2197	ENSG00000241666		lncRNA	chr1:167627385-167
ENSG0000(	2243	50.0919	chr1:2197	CD247		protein_c	chr1:167425027-167
ENSG0000(	2243	50.0919	chr1:2197	LINC01363		lncRNA	chr1:167175363-167
ENSG0000(	2243	50.0919	chr1:2197	ENSG00000273160		lncRNA	chr1:167457742-167
ENSG0000(	2243	50.0919	chr1:2197	ENSG00000213068		Pseudoger	chr1:167162423-167
ENSG0000(	2241	50.04723	chr1:1142	ENSG00000287743		lncRNA	chr1:40659848-4066
ENSG0000(	2241	50.04723	chr1:1142	CITED4		protein_c	chr1:40861054-4086
ENSG0000(	2241	50.04723	chr1:1142	PPT1		protein_c	chr1:40072710-4009
ENSG0000(	2241	50.04723	chr1:1142	NFYC		protein_c	chr1:40691648-4077
ENSG0000(	2241	50.04723	chr1:1142	ENSG00000230881		lncRNA	chr1:41535443-4153
ENSG0000(	2241	50.04723	chr1:1142	ZFP69		protein_c	chr1:40477290-4049
ENSG0000(	2241	50.04723	chr1:1142	HPCAL4		protein_c	chr1:39678648-3969
ENSG0000(	2241	50.04723	chr1:1142	ENSG00000286640		Pseudoger	chr1:41302938-4130
ENSG0000(	2241	50.04723	chr1:1142	ENSG00000284895		protein_c	chr1:41585306-4162
ENSG0000(	2241	50.04723	chr1:1142	BMP8B		protein_c	chr1:39757182-3978
ENSG0000(	2241	50.04723	chr1:1142	ENSG00000229528		lncRNA	chr1:40863914-4087
ENSG0000(	2241	50.04723	chr1:1142	BMP8B-AS1		lncRNA	chr1:39779969-3978
ENSG0000(	2241	50.04723	chr1:1142	Y_RNA		smallRNA	chr1:39944890-3994
ENSG0000(	2241	50.04723	chr1:1142	ENSG00000213172		Pseudoger	chr1:40364766-4036
ENSG0000(	2241	50.04723	chr1:1142	ZFP69B		protein_c	chr1:40450102-4046
ENSG0000(	2241	50.04723	chr1:1142	TRIT1		protein_c	chr1:39838110-3988
ENSG0000(	2241	50.04723	chr1:1142	ENSG00000286668		lncRNA	chr1:40939294-4094
ENSG0000(	2241	50.04723	chr1:1142	NFYC-AS1		lncRNA	chr1:40690380-4069
ENSG0000(	2241	50.04723	chr1:1142	ENSG00000225333		Pseudoger	chr1:39718028-3971
ENSG0000(	2241	50.04723	chr1:1142	ENSG00000227311		Pseudoger	chr1:40333078-4033
ENSG0000(	2241	50.04723	chr1:1142	FOXO6		protein_c	chr1:41361922-4138
ENSG0000(	2241	50.04723	chr1:1142	EXO5		protein_c	chr1:40508741-4051
ENSG0000(	2241	50.04723	chr1:1142	ENSG00000230638		Pseudoger	chr1:41542069-4154
ENSG0000(	2241	50.04723	chr1:1142	ENSG00000228940		Pseudoger	chr1:40938104-4093
ENSG0000(	2241	50.04723	chr1:1142	ENSG00000261798		lncRNA	chr1:39788976-3979
ENSG0000(	2241	50.04723	chr1:1142	EDN2		protein_c	chr1:41478775-4148
ENSG0000(	2241	50.04723	chr1:1142	MIR30E		smallRNA	chr1:40754355-4075
ENSG0000(	2241	50.04723	chr1:1142	PPIE		protein_c	chr1:39692182-3976
ENSG0000(	2241	50.04723	chr1:1142	RNA5SP45		Pseudoger	chr1:41466937-4146
ENSG0000(	2241	50.04723	chr1:1142	ENSG00000284719		lncRNA	chr1:39799419-3980

ENSG0000(	2241	50.04723	chr1:1142	ZMPSTE24		protein_c	chr1:40258041-4029
ENSG0000(	2241	50.04723	chr1:1142	CAP1		protein_c	chr1:40040233-4007
ENSG0000(	2241	50.04723	chr1:1142	Y_RNA		smallRNA	chr1:39881566-3988
ENSG0000(	2241	50.04723	chr1:1142	RPL23AP17		Pseudoger	chr1:41098638-4109
ENSG0000(	2241	50.04723	chr1:1142	HIVEP3		protein_c	chr1:41506365-4203
ENSG0000(	2241	50.04723	chr1:1142	ENSG00000236505		Pseudoger	chr1:40563534-4056
ENSG0000(	2241	50.04723	chr1:1142	ENSG00000260920		lncRNA	chr1:40464319-4046
ENSG0000(	2241	50.04723	chr1:1142	MFSD2A		protein_c	chr1:39955112-3996
ENSG0000(	2241	50.04723	chr1:1142	ZNF684		protein_c	chr1:40531573-4054
ENSG0000(	2241	50.04723	chr1:1142	ENSG00000279667		TEC	chr1:40473055-4047
ENSG0000(	2241	50.04723	chr1:1142	ENSG00000229213		Pseudoger	chr1:39795843-3979
ENSG0000(	2241	50.04723	chr1:1142	KCNQ4	DriverDBv	protein_c	chr1:40783787-4084
ENSG0000(	2241	50.04723	chr1:1142	RIMS3		protein_c	chr1:40620680-4066
ENSG0000(	2241	50.04723	chr1:1142	ENSG00000287400		lncRNA	chr1:41241772-4133
ENSG0000(	2241	50.04723	chr1:1142	MYCL	NCGv7;AC	protein_c	chr1:39895426-3990
ENSG0000(	2241	50.04723	chr1:1142	ZMPSTE24-DT		lncRNA	chr1:40256333-4025
ENSG0000(	2241	50.04723	chr1:1142	RPL36AP9		Pseudoger	chr1:41264550-4126
ENSG0000(	2241	50.04723	chr1:1142	TMCO2		protein_c	chr1:40245947-4025
ENSG0000(	2241	50.04723	chr1:1142	ENSG00000237899		lncRNA	chr1:40669089-4068
ENSG0000(	2241	50.04723	chr1:1142	ENSG00000231296		Pseudoger	chr1:40262672-4026
ENSG0000(	2241	50.04723	chr1:1142	SCMH1-DT		lncRNA	chr1:41242373-4128
ENSG0000(	2241	50.04723	chr1:1142	ENSG00000228477		Pseudoger	chr1:39962680-3996
ENSG0000(	2241	50.04723	chr1:1142	COL9A2	NCGv7	protein_c	chr1:40300489-4031
ENSG0000(	2241	50.04723	chr1:1142	UBE2V1P8		Pseudoger	chr1:40942251-4094
ENSG0000(	2241	50.04723	chr1:1142	EXO5-DT		lncRNA	chr1:40493157-4050
ENSG0000(	2241	50.04723	chr1:1142	SLFNL1-AS1		lncRNA	chr1:41014590-4104
ENSG0000(	2241	50.04723	chr1:1142	CTPS1		protein_c	chr1:40979300-4101
ENSG0000(	2241	50.04723	chr1:1142	RNU7-121P		smallRNA	chr1:39723566-3972
ENSG0000(	2241	50.04723	chr1:1142	SLFNL1		protein_c	chr1:41015589-4102
ENSG0000(	2241	50.04723	chr1:1142	ENSG00000284677		lncRNA	chr1:40436199-4045
ENSG0000(	2241	50.04723	chr1:1142	ENSG00000290111		lncRNA	chr1:40394763-4039
ENSG0000(	2241	50.04723	chr1:1142	ENSG00000238186		lncRNA	chr1:40515754-4051
ENSG0000(	2241	50.04723	chr1:1142	RNU6-1237P		smallRNA	chr1:40177843-4017
ENSG0000(	2241	50.04723	chr1:1142	FOXO6-AS1		lncRNA	chr1:41375004-4137
ENSG0000(	2241	50.04723	chr1:1142	LINC02811		lncRNA	chr1:39801414-3981
ENSG0000(	2241	50.04723	chr1:1142	GTF2F2P2		Pseudoger	chr1:40593633-4059
ENSG0000(	2241	50.04723	chr1:1142	SCMH1		protein_c	chr1:41027202-4124
ENSG0000(	2241	50.04723	chr1:1142	ENSG00000289711		Pseudoger	chr1:39733327-3973
ENSG0000(	2241	50.04723	chr1:1142	ENSG00000291157		lncRNA	chr1:41302911-4130
ENSG0000(	2241	50.04723	chr1:1142	OAZ1P1		Pseudoger	chr1:40132764-4013
ENSG0000(	2241	50.04723	chr1:1142	RN7SL326P		smallRNA	chr1:40804846-4080
ENSG0000(	2241	50.04723	chr1:1142	MIR30C1		smallRNA	chr1:40757284-4075
ENSG0000(	2241	50.04723	chr1:1142	RLF		protein_c	chr1:40161387-4024
ENSG0000(	2241	50.04723	chr1:1142	ENSG00000286838		lncRNA	chr1:40559666-4058
ENSG0000(	2241	50.04723	chr1:1142	SMAP2		protein_c	chr1:40344850-4042
ENSG0000(	2241	50.04723	chr1:1142	MYCL-AS1		lncRNA	chr1:39897745-3989
ENSG0000(	2241	50.04723	chr1:1142	ENSG00000227278		lncRNA	chr1:40514461-4051
ENSG0000(	2241	50.04723	chr1:1142	OXCT2	NCGv7	protein_c	chr1:39769523-3977
ENSG0000(	2240	50.0249	chr2:3094	MIR4783		smallRNA	chr2:127423537-127
ENSG0000(	2231	49.8239	chr2:3094	ENSG00000237856		lncRNA	chr2:123065321-123
ENSG0000(	2231	49.8239	chr2:3094	ENSG00000270815		Pseudoger	chr2:124778454-124

ENSG0000(	2231	49.8239	chr2:309<	LINC01889		lncRNA	chr2:125710969-125
ENSG0000(	2231	49.8239	chr2:309<	snosnR60_Z15		smallRNA	chr2:125128832-125
ENSG0000(	2231	49.8239	chr2:309<	ELOAP1		Pseudoger	chr2:123695302-123
ENSG0000(	2231	49.8239	chr2:309<	ENSG00000286206		lncRNA	chr2:125665331-125
ENSG0000(	2231	49.8239	chr2:309<	RN7SKP102		smallRNA	chr2:123869256-123
ENSG0000(	2231	49.8239	chr2:309<	RNU6-259P		smallRNA	chr2:124989219-124
ENSG0000(	2231	49.8239	chr2:309<	LINC01826		lncRNA	chr2:123066151-123
ENSG0000(	2231	49.8239	chr2:309<	PSMD14P1		Pseudoger	chr2:123762171-123
ENSG0000(	2231	49.8239	chr2:309<	ENSG00000226708		lncRNA	chr2:123443266-123
ENSG0000(	2231	49.8239	chr2:309<	AC092646.3		smallRNA	chr2:123682107-123
ENSG0000(	2231	49.8239	chr2:309<	ENSG00000287854		lncRNA	chr2:123421486-123
ENSG0000(	2231	49.8239	chr2:309<	ENSG00000224410		Pseudoger	chr2:125823108-125
ENSG0000(	2231	49.8239	chr2:309<	MTND5P22		Pseudoger	chr2:124680722-124
ENSG0000(	2231	49.8239	chr2:309<	ENSG00000279874		TEC	chr2:123850522-123
ENSG0000(	2231	49.8239	chr2:309<	ENSG00000286384		lncRNA	chr2:124695242-124
ENSG0000(	2231	49.8239	chr2:309<	RNA5SP102		Pseudoger	chr2:124932915-124
ENSG0000(	2231	49.8239	chr2:309<	ENSG00000287871		lncRNA	chr2:122503366-122
ENSG0000(	2231	49.8239	chr2:309<	CNTNAP5-DT		lncRNA	chr2:124011984-124
ENSG0000(	2231	49.8239	chr2:309<	CNTNAP5		protein_c	chr2:124025287-124
ENSG0000(	2229	49.77924	chr1:104:	MIR197		smallRNA	chr1:109598893-109
ENSG0000(	2229	49.77924	chr1:114:	PRDX3P2		Pseudoger	chr1:28526318-2852
ENSG0000(	2220	49.57825	chr2:309<	RNU6-617P		smallRNA	chr2:131602790-131
ENSG0000(	2220	49.57825	chr2:309<	ENSG00000287414		lncRNA	chr2:132010195-132
ENSG0000(	2220	49.57825	chr2:309<	NEK2P4		Pseudoger	chr2:131177618-131
ENSG0000(	2220	49.57825	chr2:309<	Y_RNA		smallRNA	chr2:128350829-128
ENSG0000(	2220	49.57825	chr2:309<	RNU6-848P		smallRNA	chr2:130622838-130
ENSG0000(	2220	49.57825	chr2:309<	PLAC9P1		lncRNA	chr2:129893985-129
ENSG0000(	2220	49.57825	chr2:309<	MTND4P27		Pseudoger	chr2:130278757-130
ENSG0000(	2220	49.57825	chr2:309<	CYP4F30P		Pseudoger	chr2:130681229-130
ENSG0000(	2220	49.57825	chr2:309<	RNU6-473P		smallRNA	chr2:130497743-130
ENSG0000(	2220	49.57825	chr2:309<	GNAQP1		Pseudoger	chr2:131423801-131
ENSG0000(	2220	49.57825	chr2:309<	ZNF285CP		Pseudoger	chr2:132309309-132
ENSG0000(	2220	49.57825	chr2:309<	FAM201B		Pseudoger	chr2:132352722-132
ENSG0000(	2220	49.57825	chr2:309<	MTCO2P7		Pseudoger	chr2:130275592-130
ENSG0000(	2220	49.57825	chr2:309<	ZRANB3	NCGv7	protein_c	chr2:135136916-135
ENSG0000(	2220	49.57825	chr2:309<	LCT-AS1		lncRNA	chr2:135820191-135
ENSG0000(	2220	49.57825	chr2:309<	PLAC9P1		Pseudoger	chr2:129923558-129
ENSG0000(	2220	49.57825	chr2:309<	RN7SL701P		smallRNA	chr2:131874631-131
ENSG0000(	2220	49.57825	chr2:309<	UBXN4		protein_c	chr2:135741734-135
ENSG0000(	2220	49.57825	chr2:309<	MTCYBP8		Pseudoger	chr2:130282679-130
ENSG0000(	2220	49.57825	chr2:309<	C2orf27A		lncRNA	chr2:131647990-131
ENSG0000(	2220	49.57825	chr2:309<	ENSG00000272769		lncRNA	chr2:132345616-132
ENSG0000(	2220	49.57825	chr2:309<	CDC27P1		Pseudoger	chr2:132262328-132
ENSG0000(	2220	49.57825	chr2:309<	GPR39		protein_c	chr2:132416805-132
ENSG0000(	2220	49.57825	chr2:309<	NCKAP5		protein_c	chr2:132671788-133
ENSG0000(	2220	49.57825	chr2:309<	ENSG00000217289		Pseudoger	chr2:129992889-129
ENSG0000(	2220	49.57825	chr2:309<	RNU6-579P		smallRNA	chr2:133333829-133
ENSG0000(	2220	49.57825	chr2:309<	ENSG00000226886		Pseudoger	chr2:132037787-132
ENSG0000(	2220	49.57825	chr2:309<	ENSG00000225448		Pseudoger	chr2:131181153-131
ENSG0000(	2220	49.57825	chr2:309<	RAB6C-AS1		Pseudoger	chr2:129968258-129
ENSG0000(	2220	49.57825	chr2:309<	ENSG00000228471		Pseudoger	chr2:130082092-130

ENSG00000	2220	49.57825	chr2:3094	ENSG00000235615		lncRNA	chr2:131964731-131
ENSG00000	2220	49.57825	chr2:3094	NF1P8		Pseudoger	chr2:131189834-131
ENSG00000	2220	49.57825	chr2:3094	ENSG00000244337		lncRNA	chr2:131958277-131
ENSG00000	2220	49.57825	chr2:3094	FAR2P1		Pseudoger	chr2:130028309-130
ENSG00000	2220	49.57825	chr2:3094	MAP3K19	NCGv7	protein_c	chr2:134964485-135
ENSG00000	2220	49.57825	chr2:3094	FAR2P4		Pseudoger	chr2:131296843-131
ENSG00000	2220	49.57825	chr2:3094	ENSG00000289974		lncRNA	chr2:136119456-136
ENSG00000	2220	49.57825	chr2:3094	G3BP1P1		Pseudoger	chr2:135510546-135
ENSG00000	2220	49.57825	chr2:3094	LINC01856		lncRNA	chr2:129923177-129
ENSG00000	2220	49.57825	chr2:3094	ENSG00000227632		Pseudoger	chr2:130202312-130
ENSG00000	2220	49.57825	chr2:3094	POTEI		protein_c	chr2:130459455-130
ENSG00000	2220	49.57825	chr2:3094	POTEKP		Pseudoger	chr2:131591752-131
ENSG00000	2220	49.57825	chr2:3094	LINC01854		lncRNA	chr2:129242173-129
ENSG00000	2220	49.57825	chr2:3094	ENSG00000274353		Pseudoger	chr2:131821987-131
ENSG00000	2220	49.57825	chr2:3094	ENSG00000271179		Pseudoger	chr2:132080290-132
ENSG00000	2220	49.57825	chr2:3094	PLEKHB2		protein_c	chr2:131104847-131
ENSG00000	2220	49.57825	chr2:3094	RNA5SP103		Pseudoger	chr2:128445081-128
ENSG00000	2220	49.57825	chr2:3094	RHOQP3		Pseudoger	chr2:130212870-130
ENSG00000	2220	49.57825	chr2:3094	ENSG00000287463		lncRNA	chr2:134718188-134
ENSG00000	2220	49.57825	chr2:3094	LCT		protein_c	chr2:135787850-135
ENSG00000	2220	49.57825	chr2:3094	MTCO1P7		Pseudoger	chr2:130273908-130
ENSG00000	2220	49.57825	chr2:3094	RAB3GAP1		protein_c	chr2:135052289-135
ENSG00000	2220	49.57825	chr2:3094	CCNT2		protein_c	chr2:134918235-134
ENSG00000	2220	49.57825	chr2:3094	ENSG00000284619		Pseudoger	chr2:129988158-129
ENSG00000	2220	49.57825	chr2:3094	ENSG00000230803		lncRNA	chr2:132285795-132
ENSG00000	2220	49.57825	chr2:3094	KLF2P3		Pseudoger	chr2:130692243-130
ENSG00000	2220	49.57825	chr2:3094	RNU6-175P		smallRNA	chr2:132406752-132
ENSG00000	2220	49.57825	chr2:3094	ENSG00000228721		Pseudoger	chr2:132911096-132
ENSG00000	2220	49.57825	chr2:3094	AC013269.1		Pseudoger	chr2:130463386-130
ENSG00000	2220	49.57825	chr2:3094	ENSG00000291278		lncRNA	chr2:130034788-130
ENSG00000	2220	49.57825	chr2:3094	SMIM39		protein_c	chr2:131035092-131
ENSG00000	2220	49.57825	chr2:3094	ARHGEF4-AS1		lncRNA	chr2:130830978-130
ENSG00000	2220	49.57825	chr2:3094	ARHGAP42P2		Pseudoger	chr2:130006200-130
ENSG00000	2220	49.57825	chr2:3094	NCKAP5-AS2		lncRNA	chr2:133264968-133
ENSG00000	2220	49.57825	chr2:3094	RNA5SP104		Pseudoger	chr2:134231195-134
ENSG00000	2220	49.57825	chr2:3094	ENSG00000225341		lncRNA	chr2:130530275-130
ENSG00000	2220	49.57825	chr2:3094	ENSG00000276460		Pseudoger	chr2:131398439-131
ENSG00000	2220	49.57825	chr2:3094	ENSG00000286833		lncRNA	chr2:132347468-132
ENSG00000	2220	49.57825	chr2:3094	ARHGEF4		protein_c	chr2:130836914-131
ENSG00000	2220	49.57825	chr2:3094	KLF2P2		Pseudoger	chr2:130427731-130
ENSG00000	2220	49.57825	chr2:3094	MTND1P29		Pseudoger	chr2:130270440-130
ENSG00000	2220	49.57825	chr2:3094	ENSG00000286697		lncRNA	chr2:134028608-134
ENSG00000	2220	49.57825	chr2:3094	ENSG00000284604		lncRNA	chr2:131303037-131
ENSG00000	2220	49.57825	chr2:3094	ENSG00000282944		lncRNA	chr2:130681228-130
ENSG00000	2220	49.57825	chr2:3094	MED15P3		Pseudoger	chr2:131395217-131
ENSG00000	2220	49.57825	chr2:3094	POTEF		protein_c	chr2:130073535-130
ENSG00000	2220	49.57825	chr2:3094	NOC2LP2		Pseudoger	chr2:131442644-131
ENSG00000	2220	49.57825	chr2:3094	ENSG00000225594		Pseudoger	chr2:132087289-132
ENSG00000	2220	49.57825	chr2:3094	ENSG00000225819		lncRNA	chr2:130515997-130
ENSG00000	2220	49.57825	chr2:3094	ENSG00000288031		Pseudoger	chr2:131692392-131
ENSG00000	2220	49.57825	chr2:3094	PPIAP65		Pseudoger	chr2:129496297-129

ENSG0000(	2220	49.57825	chr2:309<	LINC02572	lncRNA	chr2:129825126-129
ENSG0000(	2220	49.57825	chr2:309<	RPL19P4	Pseudoger	chr2:130293822-130
ENSG0000(	2220	49.57825	chr2:309<	KLF2P1	Pseudoger	chr2:130036958-130
ENSG0000(	2220	49.57825	chr2:309<	CYP4F27P	Pseudoger	chr2:130048426-130
ENSG0000(	2220	49.57825	chr2:309<	ENSG00000237532	lncRNA	chr2:128580491-128
ENSG0000(	2220	49.57825	chr2:309<	MTCYBP10	Pseudoger	chr2:131369062-131
ENSG0000(	2220	49.57825	chr2:309<	NCKAP5-AS1	lncRNA	chr2:132915557-132
ENSG0000(	2220	49.57825	chr2:309<	MTND6P10	Pseudoger	chr2:131369785-131
ENSG0000(	2220	49.57825	chr2:309<	ISCA1P6	Pseudoger	chr2:128518788-128
ENSG0000(	2220	49.57825	chr2:309<	TUBA3E	protein_c	chr2:130191745-130
ENSG0000(	2220	49.57825	chr2:309<	TMEM163	protein_c	chr2:134455759-134
ENSG0000(	2220	49.57825	chr2:309<	MGAT5	protein_c	chr2:134119983-134
ENSG0000(	2220	49.57825	chr2:309<	MED15P5	Pseudoger	chr2:130251568-130
ENSG0000(	2220	49.57825	chr2:309<	SMPD4BP	Pseudoger	chr2:131492813-131
ENSG0000(	2220	49.57825	chr2:309<	ENSG00000284171	Pseudoger	chr2:130278444-130
ENSG0000(	2220	49.57825	chr2:309<	FAM168B	protein_c	chr2:131047876-131
ENSG0000(	2220	49.57825	chr2:309<	ENSG00000284659	Pseudoger	chr2:131419665-131
ENSG0000(	2220	49.57825	chr2:309<	CFC1B	protein_c	chr2:130521197-130
ENSG0000(	2220	49.57825	chr2:309<	MZT2B	protein_c	chr2:130181737-130
ENSG0000(	2220	49.57825	chr2:309<	MTCO1P18	Pseudoger	chr2:131382643-131
ENSG0000(	2220	49.57825	chr2:309<	ENSG00000230065	lncRNA	chr2:132660526-132
ENSG0000(	2220	49.57825	chr2:309<	LINC01120	lncRNA	chr2:131402778-131
ENSG0000(	2220	49.57825	chr2:309<	MTATP6P4	Pseudoger	chr2:131380916-131
ENSG0000(	2220	49.57825	chr2:309<	GRAMD4P8	Pseudoger	chr2:131646704-131
ENSG0000(	2220	49.57825	chr2:309<	RAB6C	protein_c	chr2:129979666-129
ENSG0000(	2220	49.57825	chr2:309<	ENSG00000289534	lncRNA	chr2:134201818-134
ENSG0000(	2220	49.57825	chr2:309<	MED15P4	Pseudoger	chr2:131535187-131
ENSG0000(	2220	49.57825	chr2:309<	CCDC74B	protein_c	chr2:130139287-130
ENSG0000(	2220	49.57825	chr2:309<	MTND5P23	Pseudoger	chr2:131370306-131
ENSG0000(	2220	49.57825	chr2:309<	PRSS40A	Pseudoger	chr2:130571659-130
ENSG0000(	2220	49.57825	chr2:309<	SCARNA4	smallRNA	chr2:130929762-130
ENSG0000(	2220	49.57825	chr2:309<	MIR663B	smallRNA	chr2:132256966-132
ENSG0000(	2220	49.57825	chr2:309<	MIR5590	smallRNA	chr2:134857820-134
ENSG0000(	2220	49.57825	chr2:309<	ANKRD30BINCGv7	protein_c	chr2:132147591-132
ENSG0000(	2220	49.57825	chr2:309<	EDDM3CP	Pseudoger	chr2:134319654-134
ENSG0000(	2220	49.57825	chr2:309<	TOMM40P4	Pseudoger	chr2:131723253-131
ENSG0000(	2220	49.57825	chr2:309<	ENSG00000283303	lncRNA	chr2:131363364-131
ENSG0000(	2220	49.57825	chr2:309<	AC013718.1	Pseudoger	chr2:134456196-134
ENSG0000(	2220	49.57825	chr2:309<	NOC2LP1	Pseudoger	chr2:130229379-130
ENSG0000(	2220	49.57825	chr2:309<	CCDC74A	protein_c	chr2:131527675-131
ENSG0000(	2220	49.57825	chr2:309<	ENSG00000284003	Pseudoger	chr2:131373647-131
ENSG0000(	2220	49.57825	chr2:309<	VDAC2P4	Pseudoger	chr2:134797169-134
ENSG0000(	2220	49.57825	chr2:309<	TEKT4P3	Pseudoger	chr2:130313934-130
ENSG0000(	2220	49.57825	chr2:309<	ENSG00000229797	lncRNA	chr2:130742959-130
ENSG0000(	2220	49.57825	chr2:309<	AC104405.1	smallRNA	chr2:132523985-132
ENSG0000(	2220	49.57825	chr2:309<	AC103564.9	Pseudoger	chr2:131776567-131
ENSG0000(	2220	49.57825	chr2:309<	MIR128-1	smallRNA	chr2:135665397-135
ENSG0000(	2220	49.57825	chr2:309<	ENSG00000232408	lncRNA	chr2:130583549-130
ENSG0000(	2220	49.57825	chr2:309<	RNU6-127P	smallRNA	chr2:131229607-131
ENSG0000(	2220	49.57825	chr2:309<	SSBP3P2	Pseudoger	chr2:131352214-131
ENSG0000(	2220	49.57825	chr2:309<	ENSG00000238277	lncRNA	chr2:129063846-129

ENSG0000(	2220	49.57825	chr2:309⁄	CDRT15P4		Pseudoger	chr2:131766187-131
ENSG0000(	2220	49.57825	chr2:309⁄	ENSG00000224352		Pseudoger	chr2:130330142-130
ENSG0000(	2220	49.57825	chr2:309⁄	POTEE		protein_c	chr2:131209536-131
ENSG0000(	2220	49.57825	chr2:309⁄	ENSG00000286026		lncRNA	chr2:129990456-130
ENSG0000(	2220	49.57825	chr2:309⁄	ENSG00000232760		Pseudoger	chr2:131833783-131
ENSG0000(	2220	49.57825	chr2:309⁄	MTND5P29		Pseudoger	chr2:130280299-130
ENSG0000(	2220	49.57825	chr2:309⁄	RNU6-1049P		smallRNA	chr2:130109204-130
ENSG0000(	2220	49.57825	chr2:309⁄	RN7SKP154		smallRNA	chr2:133391109-133
ENSG0000(	2220	49.57825	chr2:309⁄	PTPN18		protein_c	chr2:130356045-130
ENSG0000(	2220	49.57825	chr2:309⁄	ENSG00000290654		lncRNA	chr2:131279290-131
ENSG0000(	2220	49.57825	chr2:309⁄	MTCO3P7		Pseudoger	chr2:130277184-130
ENSG0000(	2220	49.57825	chr2:309⁄	ENSG00000285819		lncRNA	chr2:131191641-131
ENSG0000(	2220	49.57825	chr2:309⁄	MTND2P18		Pseudoger	chr2:131384461-131
ENSG0000(	2220	49.57825	chr2:309⁄	PRSS40B		lncRNA	chr2:130539095-130
ENSG0000(	2220	49.57825	chr2:309⁄	KLF2P4		Pseudoger	chr2:131299220-131
ENSG0000(	2220	49.57825	chr2:309⁄	FAR2P2		Pseudoger	chr2:130419071-130
ENSG0000(	2220	49.57825	chr2:309⁄	AMER3		protein_c	chr2:130755540-130
ENSG0000(	2220	49.57825	chr2:309⁄	PRSS40A		lncRNA	chr2:130570829-130
ENSG0000(	2220	49.57825	chr2:309⁄	RAB6C-AS1		lncRNA	chr2:129966592-129
ENSG0000(	2220	49.57825	chr2:309⁄	CDRT15P3		lncRNA	chr2:131794961-131
ENSG0000(	2220	49.57825	chr2:309⁄	ENSG00000290616		lncRNA	chr2:131733656-131
ENSG0000(	2220	49.57825	chr2:309⁄	LINC01087		lncRNA	chr2:131637025-131
ENSG0000(	2220	49.57825	chr2:309⁄	MIR3679		smallRNA	chr2:134127125-134
ENSG0000(	2220	49.57825	chr2:309⁄	ENSG00000227745		Pseudoger	chr2:132132856-132
ENSG0000(	2220	49.57825	chr2:309⁄	NBEAP2		Pseudoger	chr2:131722417-131
ENSG0000(	2220	49.57825	chr2:309⁄	LYPD1		protein_c	chr2:132643286-132
ENSG0000(	2220	49.57825	chr2:309⁄	ENSG00000229203		lncRNA	chr2:131829801-131
ENSG0000(	2220	49.57825	chr2:309⁄	POTEJ		protein_c	chr2:130611440-130
ENSG0000(	2220	49.57825	chr2:309⁄	R3HDM1		protein_c	chr2:135531455-135
ENSG0000(	2220	49.57825	chr2:309⁄	YBX1P7		Pseudoger	chr2:132488554-132
ENSG0000(	2220	49.57825	chr2:309⁄	ENSG00000286957		lncRNA	chr2:129877896-129
ENSG0000(	2220	49.57825	chr2:309⁄	RHOQP2		Pseudoger	chr2:131460999-131
ENSG0000(	2220	49.57825	chr2:309⁄	ENSG00000290902		lncRNA	chr2:132304806-132
ENSG0000(	2220	49.57825	chr2:309⁄	ENSG00000290591		lncRNA	chr2:130012065-130
ENSG0000(	2220	49.57825	chr2:309⁄	ENSG00000290599		lncRNA	chr2:130429827-130
ENSG0000(	2220	49.57825	chr2:309⁄	MED15P9		Pseudoger	chr2:130135978-130
ENSG0000(	2220	49.57825	chr2:309⁄	snoU13		smallRNA	chr2:135065632-135
ENSG0000(	2220	49.57825	chr2:309⁄	LINC01945		lncRNA	chr2:131983937-131
ENSG0000(	2220	49.57825	chr2:309⁄	ENSG00000290594		lncRNA	chr2:131758031-131
ENSG0000(	2220	49.57825	chr2:309⁄	CDRT15P3		Pseudoger	chr2:131795136-131
ENSG0000(	2220	49.57825	chr2:309⁄	MED15P8		Pseudoger	chr2:131201142-131
ENSG0000(	2220	49.57825	chr2:309⁄	ENSG00000228098		Pseudoger	chr2:134816362-134
ENSG0000(	2220	49.57825	chr2:309⁄	ENSG00000290596		lncRNA	chr2:130416755-130
ENSG0000(	2220	49.57825	chr2:309⁄	ENSG00000279512		TEC	chr2:133117004-133
ENSG0000(	2220	49.57825	chr2:309⁄	MTND2P22		Pseudoger	chr2:130272479-130
ENSG0000(	2220	49.57825	chr2:309⁄	ENSG00000250207		Pseudoger	chr2:129992602-129
ENSG0000(	2220	49.57825	chr2:309⁄	MTCO2P18		Pseudoger	chr2:131381840-131
ENSG0000(	2220	49.57825	chr2:309⁄	SNORA40		smallRNA	chr2:135136628-135
ENSG0000(	2220	49.57825	chr2:309⁄	ENSG00000286058		lncRNA	chr2:130026845-130
ENSG0000(	2220	49.57825	chr2:309⁄	IMP4	NCGv7	protein_c	chr2:130342877-130
ENSG0000(	2220	49.57825	chr2:309⁄	ENSG00000234398		Pseudoger	chr2:130245971-130

ENSG0000(	2220	49.57825	chr2:3094	ENSG00000286068	lncRNA	chr2:132685224-132
ENSG0000(	2220	49.57825	chr2:3094	ENSG00000223430	lncRNA	chr2:133554315-133
ENSG0000(	2220	49.57825	chr2:3094	MTND4P21	Pseudoger	chr2:131372294-131
ENSG0000(	2220	49.57825	chr2:3094	AC140481.3	Pseudoger	chr2:130656999-130
ENSG0000(	2220	49.57825	chr2:3094	RN7SKP103	smallRNA	chr2:132523617-132
ENSG0000(	2220	49.57825	chr2:3094	GPR148	protein_c	chr2:130729070-130
ENSG0000(	2220	49.57825	chr2:3094	RN7SKP93	smallRNA	chr2:133596091-133
ENSG0000(	2220	49.57825	chr2:3094	MTND1P26	Pseudoger	chr2:131385700-131
ENSG0000(	2220	49.57825	chr2:3094	HS6ST1	protein_c	chr2:128236716-128
ENSG0000(	2220	49.57825	chr2:3094	CYP4F62P	Pseudoger	chr2:130439285-130
ENSG0000(	2220	49.57825	chr2:3094	MTND3P18	Pseudoger	chr2:131379743-131
ENSG0000(	2220	49.57825	chr2:3094	ACMSD	protein_c	chr2:134838616-134
ENSG0000(	2220	49.57825	chr2:3094	ENSG00000289374	lncRNA	chr2:129443088-129
ENSG0000(	2220	49.57825	chr2:3094	Y_RNA	smallRNA	chr2:135810169-135
ENSG0000(	2220	49.57825	chr2:3094	ENSG00000273073	lncRNA	chr2:131461821-131
ENSG0000(	2220	49.57825	chr2:3094	POTEF-AS1	lncRNA	chr2:130107684-130
ENSG0000(	2220	49.57825	chr2:3094	RNU6-1132P	smallRNA	chr2:132152243-132
ENSG0000(	2220	49.57825	chr2:3094	RNU6-512P	smallRNA	chr2:135656477-135
ENSG0000(	2220	49.57825	chr2:3094	MIR4784	smallRNA	chr2:131491160-131
ENSG0000(	2220	49.57825	chr2:3094	AC018804.1	smallRNA	chr2:130183443-130
ENSG0000(	2220	49.57825	chr2:3094	CYCSP8	Pseudoger	chr2:130776646-130
ENSG0000(	2220	49.57825	chr2:3094	MZT2A	protein_c	chr2:131464900-131
ENSG0000(	2220	49.57825	chr2:3094	ENSG00000286208	lncRNA	chr2:131555007-131
ENSG0000(	2220	49.57825	chr2:3094	ENSG00000284706	Pseudoger	chr2:131335502-131
ENSG0000(	2220	49.57825	chr2:3094	MED15P9	lncRNA	chr2:130129621-130
ENSG0000(	2220	49.57825	chr2:3094	PRSS40B	Pseudoger	chr2:130539839-130
ENSG0000(	2220	49.57825	chr2:3094	MTND3P15	Pseudoger	chr2:130278036-130
ENSG0000(	2220	49.57825	chr2:3094	RAB6D	protein_c	chr2:131360492-131
ENSG0000(	2220	49.57825	chr2:3094	ARHGAP42P1	Pseudoger	chr2:131328176-131
ENSG0000(	2220	49.57825	chr2:3094	TUBA3D	protein_c	chr2:131476119-131
ENSG0000(	2220	49.57825	chr2:3094	FAR2P3	Pseudoger	chr2:130690133-130
ENSG0000(	2220	49.57825	chr2:3094	MCM6	protein_c	chr2:135839626-135
ENSG0000(	2220	49.57825	chr2:3094	snoU13	smallRNA	chr2:129427422-129
ENSG0000(	2220	49.57825	chr2:3094	NCKAP5-IT1	lncRNA	chr2:133431666-133
ENSG0000(	2220	49.57825	chr2:3094	AC011755.1	protein_c	chr2:133117004-133
ENSG0000(	2220	49.57825	chr2:3094	CFC1	protein_c	chr2:130592165-130
ENSG0000(	2220	49.57825	chr2:3094	MTATP6P7	Pseudoger	chr2:130276507-130
ENSG0000(	2220	49.57825	chr2:3094	MTCO3P18	Pseudoger	chr2:131380153-131
ENSG0000(	2220	49.57825	chr2:3094	SMPD4	protein_c	chr2:130151392-130
ENSG0000(	2220	49.57825	chr2:3094	CYP4F30P	lncRNA	chr2:130680050-130
ENSG0000(	2220	49.57825	chr2:3094	MTND6P8	Pseudoger	chr2:130282085-130
ENSG0000(	2220	49.57825	chr2:3094	RPL22P7	Pseudoger	chr2:129939848-129
ENSG0000(	2220	49.57825	chr2:3094	CCDC115	protein_c	chr2:130337933-130
ENSG0000(	2220	49.57825	chr2:3094	ENSG00000290700	lncRNA	chr2:130693282-130
ENSG0000(	2220	49.57825	chr2:3094	CCNT2-AS1	lncRNA	chr2:134735464-134
ENSG0000(	2214	49.44425	chr2:3094	ENSG00000286515	lncRNA	chr2:126154583-126
ENSG0000(	2213	49.42192	chr1:1043	AC114491.1	smallRNA	chr1:107448469-107
ENSG0000(	2208	49.31026	chr2:3094	ENSG00000232101	Pseudoger	chr2:128151154-128
ENSG0000(	2208	49.31026	chr2:3094	ENSG00000286145	lncRNA	chr2:127294212-127
ENSG0000(	2208	49.31026	chr2:3094	ENSG00000272789	lncRNA	chr2:127625997-127
ENSG0000(	2208	49.31026	chr2:3094	SLC6A14P3	Pseudoger	chr2:126311466-126

ENSG0000(	2208	49.31026	chr2:309<	CYP27C1		protein_c	chr2:127183832-127
ENSG0000(	2208	49.31026	chr2:309<	ENSG00000260634		lncRNA	chr2:127023537-127
ENSG0000(	2208	49.31026	chr2:309<	LIMS2		protein_c	chr2:127638381-127
ENSG0000(	2208	49.31026	chr2:309<	ENSG00000286873		lncRNA	chr2:127888829-127
ENSG0000(	2208	49.31026	chr2:309<	RNU6-675P		smallRNA	chr2:126702839-126
ENSG0000(	2208	49.31026	chr2:309<	ZFP91P1		Pseudoger	chr2:127840606-127
ENSG0000(	2208	49.31026	chr2:309<	ENSG00000286400		lncRNA	chr2:127141976-127
ENSG0000(	2208	49.31026	chr2:309<	WDR33		protein_c	chr2:127701027-127
ENSG0000(	2208	49.31026	chr2:309<	RPS26P19		Pseudoger	chr2:127846736-127
ENSG0000(	2208	49.31026	chr2:309<	ENSG00000290113		lncRNA	chr2:126220632-126
ENSG0000(	2208	49.31026	chr2:309<	SAP130		protein_c	chr2:127941217-128
ENSG0000(	2208	49.31026	chr2:309<	RPL21P34		Pseudoger	chr2:128217588-128
ENSG0000(	2208	49.31026	chr2:309<	ENSG00000289982		lncRNA	chr2:128090621-128
ENSG0000(	2208	49.31026	chr2:309<	BIN1		protein_c	chr2:127048027-127
ENSG0000(	2208	49.31026	chr2:309<	ENSG00000235774		lncRNA	chr2:126308494-126
ENSG0000(	2208	49.31026	chr2:309<	RNU6-395P		smallRNA	chr2:127845236-127
ENSG0000(	2208	49.31026	chr2:309<	PROC		protein_c	chr2:127418427-127
ENSG0000(	2208	49.31026	chr2:309<	ENSG00000231731		lncRNA	chr2:127455394-127
ENSG0000(	2208	49.31026	chr2:309<	MAP3K2		protein_c	chr2:127298668-127
ENSG0000(	2208	49.31026	chr2:309<	UGGT1		protein_c	chr2:128091200-128
ENSG0000(	2208	49.31026	chr2:309<	GYPC	NCGv7	protein_c	chr2:126656133-126
ENSG0000(	2208	49.31026	chr2:309<	RNY4P7		smallRNA	chr2:127798903-127
ENSG0000(	2208	49.31026	chr2:309<	ENSG00000204399		Pseudoger	chr2:127931327-127
ENSG0000(	2208	49.31026	chr2:309<	ENSG00000234044		Pseudoger	chr2:128067194-128
ENSG0000(	2208	49.31026	chr2:309<	MAP3K2-DT		lncRNA	chr2:127388173-127
ENSG0000(	2208	49.31026	chr2:309<	RNU6-1147P		smallRNA	chr2:127316873-127
ENSG0000(	2208	49.31026	chr2:309<	MYO7B		protein_c	chr2:127535683-127
ENSG0000(	2208	49.31026	chr2:309<	GPR17		protein_c	chr2:127645864-127
ENSG0000(	2208	49.31026	chr2:309<	POLR2D	NCGv7	protein_c	chr2:127843553-127
ENSG0000(	2208	49.31026	chr2:309<	Y_RNA		smallRNA	chr2:128067800-128
ENSG0000(	2208	49.31026	chr2:309<	NIFKP9		Pseudoger	chr2:127170011-127
ENSG0000(	2208	49.31026	chr2:309<	ENSG00000260163		lncRNA	chr2:127025211-127
ENSG0000(	2208	49.31026	chr2:309<	RNU7-182P		smallRNA	chr2:127017421-127
ENSG0000(	2208	49.31026	chr2:309<	ENSG00000286971		lncRNA	chr2:127024866-127
ENSG0000(	2208	49.31026	chr2:309<	SRMP3		Pseudoger	chr2:128139792-128
ENSG0000(	2208	49.31026	chr2:309<	ENSG00000235128		Pseudoger	chr2:126679622-126
ENSG0000(	2208	49.31026	chr2:309<	RNU4-48P		smallRNA	chr2:127472234-127
ENSG0000(	2208	49.31026	chr2:309<	TEX51		protein_c	chr2:126898864-126
ENSG0000(	2208	49.31026	chr2:309<	YWHAZP2		Pseudoger	chr2:126557435-126
ENSG0000(	2208	49.31026	chr2:309<	AMMECR1L		protein_c	chr2:127861630-127
ENSG0000(	2208	49.31026	chr2:309<	DYNLT3P2		Pseudoger	chr2:128199901-128
ENSG0000(	2208	49.31026	chr2:309<	RN7SL206P		smallRNA	chr2:128122081-128
ENSG0000(	2208	49.31026	chr2:309<	ERCC3	NCGv7;AC	protein_c	chr2:127257290-127
ENSG0000(	2208	49.31026	chr2:309<	LINC01941		lncRNA	chr2:126109761-126
ENSG0000(	2208	49.31026	chr2:309<	RPL14P6		Pseudoger	chr2:128203361-128
ENSG0000(	2208	49.31026	chr2:309<	ENSG00000272667		lncRNA	chr2:127886556-127
ENSG0000(	2208	49.31026	chr2:309<	SFT2D3		protein_c	chr2:127701497-127
ENSG0000(	2208	49.31026	chr2:309<	ENSG00000288524		lncRNA	chr2:126640708-126
ENSG0000(	2208	49.31026	chr2:309<	Y_RNA		smallRNA	chr2:127829747-127
ENSG0000(	2208	49.31026	chr2:309<	IWS1	NCGv7	protein_c	chr2:127436207-127
ENSG0000(	2208	49.31026	chr2:309<	WBP11P2		Pseudoger	chr2:127247258-127

ENSG0000(	2208	49.31026	chr2:3094	ENSG00000287742		lncRNA	chr2:127406731-127
ENSG0000(	2188	48.86361	chr1:1142	ENSG00000234481		lncRNA	chr1:36769812-3677
ENSG0000(	2188	48.86361	chr1:1142	PHC2	NCGv7	protein_c	chr1:33323623-3343
ENSG0000(	2188	48.86361	chr1:1142	HSPD1P14		Pseudoger	chr1:33838523-3384
ENSG0000(	2188	48.86361	chr1:1142	MIR552		smallRNA	chr1:34669599-3466
ENSG0000(	2188	48.86361	chr1:1142	RPL5P4		Pseudoger	chr1:35350722-3535
ENSG0000(	2188	48.86361	chr1:1142	ZSCAN20		protein_c	chr1:33472645-3350
ENSG0000(	2188	48.86361	chr1:1142	AGO4	NCGv7	protein_c	chr1:35808016-3585
ENSG0000(	2188	48.86361	chr1:1142	RN7SKP16		smallRNA	chr1:33336566-3333
ENSG0000(	2188	48.86361	chr1:1142	CSMD2	NCGv7	protein_c	chr1:33513998-3416
ENSG0000(	2188	48.86361	chr1:1142	STK40		protein_c	chr1:36339624-3638
ENSG0000(	2188	48.86361	chr1:1142	AL121988.1		smallRNA	chr1:34778561-3477
ENSG0000(	2188	48.86361	chr1:1142	AL138837.1		smallRNA	chr1:33442025-3344
ENSG0000(	2188	48.86361	chr1:1142	ENSG00000271741		protein_c	chr1:34981533-3503
ENSG0000(	2188	48.86361	chr1:1142	ENSG00000230163		lncRNA	chr1:34850694-3485
ENSG0000(	2188	48.86361	chr1:1142	A3GALT2		protein_c	chr1:33306766-3332
ENSG0000(	2188	48.86361	chr1:1142	GJA4		protein_c	chr1:34792999-3479
ENSG0000(	2188	48.86361	chr1:1142	CSMD2-AS1		lncRNA	chr1:33868953-3389
ENSG0000(	2188	48.86361	chr1:1142	RN7SL131P		smallRNA	chr1:36191915-3619
ENSG0000(	2188	48.86361	chr1:1142	ZMYM4-AS1		lncRNA	chr1:35358822-3536
ENSG0000(	2188	48.86361	chr1:1142	AC115286.1		smallRNA	chr1:34175866-3417
ENSG0000(	2188	48.86361	chr1:1142	HMGB4		protein_c	chr1:33860475-3386
ENSG0000(	2188	48.86361	chr1:1142	ENSG00000232335		lncRNA	chr1:35739389-3574
ENSG0000(	2188	48.86361	chr1:1142	MIR3605		smallRNA	chr1:33332393-3333
ENSG0000(	2188	48.86361	chr1:1142	Y_RNA		smallRNA	chr1:35195969-3519
ENSG0000(	2188	48.86361	chr1:1142	ENSG00000235907		Pseudoger	chr1:33512008-3351
ENSG0000(	2188	48.86361	chr1:1142	SNORA62		smallRNA	chr1:35310274-3531
ENSG0000(	2188	48.86361	chr1:1142	CFAP97P1		Pseudoger	chr1:35873270-3587
ENSG0000(	2188	48.86361	chr1:1142	ADPRS		protein_c	chr1:36088892-3609
ENSG0000(	2188	48.86361	chr1:1142	ENSG00000255811		lncRNA	chr1:34761426-3478
ENSG0000(	2188	48.86361	chr1:1142	ENSG00000284705		lncRNA	chr1:36703953-3671
ENSG0000(	2188	48.86361	chr1:1142	SNORD112		smallRNA	chr1:34943756-3494
ENSG0000(	2188	48.86361	chr1:1142	GRIK3	NCGv7	protein_c	chr1:36795527-3703
ENSG0000(	2188	48.86361	chr1:1142	ZMYM6		protein_c	chr1:34986165-3503
ENSG0000(	2188	48.86361	chr1:1142	ZMYM1		protein_c	chr1:35032172-3511
ENSG0000(	2188	48.86361	chr1:1142	ENSG00000286899		lncRNA	chr1:35908980-3591
ENSG0000(	2188	48.86361	chr1:1142	SMIM12		protein_c	chr1:34712737-3485
ENSG0000(	2188	48.86361	chr1:1142	ENSG00000225313		lncRNA	chr1:33307348-3334
ENSG0000(	2188	48.86361	chr1:1142	RN7SL503P		smallRNA	chr1:35292200-3529
ENSG0000(	2188	48.86361	chr1:1142	ZNF362	NCGv7	protein_c	chr1:33256492-3330
ENSG0000(	2188	48.86361	chr1:1142	PHC2-AS1		lncRNA	chr1:33350352-3336
ENSG0000(	2188	48.86361	chr1:1142	TFAP2E		protein_c	chr1:35573314-3559
ENSG0000(	2188	48.86361	chr1:1142	GJB5		protein_c	chr1:34755047-3475
ENSG0000(	2188	48.86361	chr1:1142	MAP7D1		protein_c	chr1:36155579-3618
ENSG0000(	2188	48.86361	chr1:1142	ENSG00000271554		lncRNA	chr1:35992109-3601
ENSG0000(	2188	48.86361	chr1:1142	RNY5P1		smallRNA	chr1:35427816-3542
ENSG0000(	2188	48.86361	chr1:1142	PSMB2		protein_c	chr1:35599541-3564
ENSG0000(	2188	48.86361	chr1:1142	ZMYM4		protein_c	chr1:35268709-3542
ENSG0000(	2188	48.86361	chr1:1142	SNORA63		smallRNA	chr1:36418450-3641
ENSG0000(	2188	48.86361	chr1:1142	OSCP1		protein_c	chr1:36415827-3645
ENSG0000(	2188	48.86361	chr1:1142	MRPS15		protein_c	chr1:36455718-3646

ENSG0000(	2188	48.86361	chr1:1142	RNU4-27P		smallRNA	chr1:36402721-3640
ENSG0000(	2188	48.86361	chr1:1142	AL513327.1		Pseudoger	chr1:33299374-3330
ENSG0000(	2188	48.86361	chr1:1142	ENSG00000284640		lncRNA	chr1:35141515-3514
ENSG0000(	2188	48.86361	chr1:1142	ENSG00000284720		lncRNA	chr1:36768122-3676
ENSG0000(	2188	48.86361	chr1:1142	ENSG00000284773		protein_c	chr1:34974356-3498
ENSG0000(	2188	48.86361	chr1:1142	COL8A2		protein_c	chr1:36095239-3612
ENSG0000(	2188	48.86361	chr1:1142	DLGAP3		protein_c	chr1:34865436-3492
ENSG0000(	2188	48.86361	chr1:1142	SFPQ	NCGv7;AC	protein_c	chr1:35176378-3519
ENSG0000(	2188	48.86361	chr1:1142	C1orf94		protein_c	chr1:34166883-3421
ENSG0000(	2188	48.86361	chr1:1142	EVA1B		protein_c	chr1:36322030-3632
ENSG0000(	2188	48.86361	chr1:1142	ENSG00000287703		lncRNA	chr1:34640157-3468
ENSG0000(	2188	48.86361	chr1:1142	ENSG00000284721		lncRNA	chr1:33194788-3320
ENSG0000(	2188	48.86361	chr1:1142	ENSG00000271914		lncRNA	chr1:35929720-3593
ENSG0000(	2188	48.86361	chr1:1142	NCDN		protein_c	chr1:35557473-3556
ENSG0000(	2188	48.86361	chr1:1142	EFCAB14P1		Pseudoger	chr1:35122022-3512
ENSG0000(	2188	48.86361	chr1:1142	KIAA0319L		protein_c	chr1:35393883-3555
ENSG0000(	2188	48.86361	chr1:1142	SH3D21		protein_c	chr1:36306368-3632
ENSG0000(	2188	48.86361	chr1:1142	GJB3		protein_c	chr1:34781214-3478
ENSG0000(	2188	48.86361	chr1:1142	UBE2V2P4		Pseudoger	chr1:36241898-3624
ENSG0000(	2188	48.86361	chr1:1142	C1orf216		protein_c	chr1:35713877-3571
ENSG0000(	2188	48.86361	chr1:1142	RPL12P45		Pseudoger	chr1:35053468-3505
ENSG0000(	2188	48.86361	chr1:1142	CLSPN		protein_c	chr1:35720218-3576
ENSG0000(	2188	48.86361	chr1:1142	TEKT2		protein_c	chr1:36084094-3608
ENSG0000(	2188	48.86361	chr1:1142	AGO1		protein_c	chr1:35869808-3593
ENSG0000(	2188	48.86361	chr1:1142	AGO3		protein_c	chr1:35930718-3607
ENSG0000(	2188	48.86361	chr1:1142	TLR12P		Pseudoger	chr1:33466249-3346
ENSG0000(	2188	48.86361	chr1:1142	GPR199P		Pseudoger	chr1:34975699-3497
ENSG0000(	2188	48.86361	chr1:1142	ENSG00000286379		lncRNA	chr1:36329630-3633
ENSG0000(	2188	48.86361	chr1:1142	TFAP2E-AS1		lncRNA	chr1:35569807-3557
ENSG0000(	2188	48.86361	chr1:1142	ENSG00000232862		Pseudoger	chr1:36080066-3608
ENSG0000(	2188	48.86361	chr1:1142	LSM10		protein_c	chr1:36391238-3639
ENSG0000(	2188	48.86361	chr1:1142	TMEM35B		protein_c	chr1:34981380-3498
ENSG0000(	2188	48.86361	chr1:1142	ENSG00000236274		Pseudoger	chr1:35509742-3551
ENSG0000(	2188	48.86361	chr1:1142	RNA5SP42		Pseudoger	chr1:34112949-3411
ENSG0000(	2188	48.86361	chr1:1142	GJB4		protein_c	chr1:34759740-3476
ENSG0000(	2188	48.86361	chr1:1142	FTLP18		Pseudoger	chr1:36630335-3663
ENSG0000(	2188	48.86361	chr1:1142	TRAPPC3		protein_c	chr1:36136570-3615
ENSG0000(	2188	48.86361	chr1:1142	THRAP3	NCGv7;AC	protein_c	chr1:36224432-3630
ENSG0000(	2188	48.86361	chr1:1142	RN7SL281P		smallRNA	chr1:35706025-3570
ENSG0000(	2188	48.86361	chr1:1142	RN7SL136P		smallRNA	chr1:35264222-3526
ENSG0000(	2188	48.86361	chr1:1142	ENSG00000270241		Pseudoger	chr1:34276859-3427
ENSG0000(	2188	48.86361	chr1:1142	CSF3R	NCGv7;AC	protein_c	chr1:36466043-3648
ENSG0000(	2188	48.86361	chr1:1142	ENSG00000270115		lncRNA	chr1:33261212-3326
ENSG0000(	2187	48.84127	chr1:1142	ENSG00000226438		lncRNA	chr1:39249838-3925
ENSG0000(	2187	48.84127	chr1:1142	PABPC4	NCGv7	protein_c	chr1:39560709-3957
ENSG0000(	2187	48.84127	chr1:1142	HSPE1P8		Pseudoger	chr1:39304294-3930
ENSG0000(	2187	48.84127	chr1:1142	HEYL		protein_c	chr1:39623435-3963
ENSG0000(	2187	48.84127	chr1:1142	SNORA55		smallRNA	chr1:39567374-3956
ENSG0000(	2187	48.84127	chr1:1142	BMP8A		protein_c	chr1:39491636-3952
ENSG0000(	2187	48.84127	chr1:1142	PPIEL		lncRNA	chr1:39522280-3955
ENSG0000(	2187	48.84127	chr1:1142	PABPC4-AS1		lncRNA	chr1:39565052-3957

ENSG0000(	2187	48.84127	chr1:1142	NT5C1A		protein_c	chr1:39651229-3967
ENSG0000(	2187	48.84127	chr1:1142	ENSG00000225903		lncRNA	chr1:39633416-3963
ENSG0000(	2187	48.84127	chr1:1142	PPIEL		Pseudoger	chr1:39531838-3955
ENSG0000(	2187	48.84127	chr1:1142	OXCT2P1		Pseudoger	chr1:39514956-3951
ENSG0000(	2181	48.70728	chr1:1043	ENSG00000241720		lncRNA	chr1:109725820-109
ENSG0000(	2181	48.70728	chr1:1043	GSTM4		protein_c	chr1:109656099-109
ENSG0000(	2181	48.70728	chr1:1043	GPSM2		protein_c	chr1:108875350-108
ENSG0000(	2181	48.70728	chr1:1043	RNU6V		smallRNA	chr1:109591534-109
ENSG0000(	2181	48.70728	chr1:1043	CHIAP2		Pseudoger	chr1:111280060-111
ENSG0000(	2181	48.70728	chr1:1043	AHCYL1	AC	protein_c	chr1:109984765-110
ENSG0000(	2181	48.70728	chr1:1043	AMPD2		protein_c	chr1:109616104-109
ENSG0000(	2181	48.70728	chr1:1043	KCNC4		protein_c	chr1:110210314-110
ENSG0000(	2181	48.70728	chr1:1043	LRIF1		protein_c	chr1:110947190-110
ENSG0000(	2181	48.70728	chr1:1043	RNU6-792P		smallRNA	chr1:111490317-111
ENSG0000(	2181	48.70728	chr1:1043	AL390036.1		smallRNA	chr1:108018653-108
ENSG0000(	2181	48.70728	chr1:1043	SLC16A4	NCGv7	protein_c	chr1:110362851-110
ENSG0000(	2181	48.70728	chr1:1043	WDR77		protein_c	chr1:111439890-111
ENSG0000(	2181	48.70728	chr1:1043	ENSG00000238122		lncRNA	chr1:108261196-108
ENSG0000(	2181	48.70728	chr1:1043	SLC25A24P2		Pseudoger	chr1:108383736-108
ENSG0000(	2181	48.70728	chr1:1043	PRPF38B	NCGv7	protein_c	chr1:108692310-108
ENSG0000(	2181	48.70728	chr1:1043	ENSG00000228076		Pseudoger	chr1:108766841-108
ENSG0000(	2181	48.70728	chr1:1043	CYB561D1		protein_c	chr1:109494052-109
ENSG0000(	2181	48.70728	chr1:1043	ENSG00000229283		lncRNA	chr1:111317600-111
ENSG0000(	2181	48.70728	chr1:1043	LINC01768		lncRNA	chr1:109828355-109
ENSG0000(	2181	48.70728	chr1:1043	ATP5PB		protein_c	chr1:111448864-111
ENSG0000(	2181	48.70728	chr1:1043	GSTM3		protein_c	chr1:109733932-109
ENSG0000(	2181	48.70728	chr1:1043	VAV3-AS1		lncRNA	chr1:107964443-107
ENSG0000(	2181	48.70728	chr1:1043	RAP1A	AC	protein_c	chr1:111542218-111
ENSG0000(	2181	48.70728	chr1:1043	RANP5		Pseudoger	chr1:109046828-109
ENSG0000(	2181	48.70728	chr1:1043	ELAPOR1		protein_c	chr1:109113679-109
ENSG0000(	2181	48.70728	chr1:1043	VAV3	TAG;AC	protein_c	chr1:107571161-107
ENSG0000(	2181	48.70728	chr1:1043	PGBP		Pseudoger	chr1:111382860-111
ENSG0000(	2181	48.70728	chr1:1043	ENSG00000251484		Pseudoger	chr1:109103535-109
ENSG0000(	2181	48.70728	chr1:1043	PIFO		protein_c	chr1:111346600-111
ENSG0000(	2181	48.70728	chr1:1043	CEPT1		protein_c	chr1:111139479-111
ENSG0000(	2181	48.70728	chr1:1043	ENSG00000228703		lncRNA	chr1:109628417-109
ENSG0000(	2181	48.70728	chr1:1043	LINC02785		lncRNA	chr1:108040263-108
ENSG0000(	2181	48.70728	chr1:1043	LAMTOR5	AC	protein_c	chr1:110401249-110
ENSG0000(	2181	48.70728	chr1:1043	ENSG00000226483		Pseudoger	chr1:108508574-108
ENSG0000(	2181	48.70728	chr1:1043	ENSG00000280186		TEC	chr1:108200413-108
ENSG0000(	2181	48.70728	chr1:1043	ST13P21		Pseudoger	chr1:108502358-108
ENSG0000(	2181	48.70728	chr1:1043	SLC6A17-AS1		lncRNA	chr1:110165948-110
ENSG0000(	2181	48.70728	chr1:1043	STXBP3		protein_c	chr1:108746674-108
ENSG0000(	2181	48.70728	chr1:1043	SORT1		protein_c	chr1:109309568-109
ENSG0000(	2181	48.70728	chr1:1043	PSRC1		protein_c	chr1:109279556-109
ENSG0000(	2181	48.70728	chr1:1043	RNU7-122P		smallRNA	chr1:109207794-109
ENSG0000(	2181	48.70728	chr1:1043	GNAT2		protein_c	chr1:109603091-109
ENSG0000(	2181	48.70728	chr1:1043	ENSG00000228665		Pseudoger	chr1:109030067-109
ENSG0000(	2181	48.70728	chr1:1043	CHIA		protein_c	chr1:111290851-111
ENSG0000(	2181	48.70728	chr1:1043	INKA2-AS1		lncRNA	chr1:111739579-111
ENSG0000(	2181	48.70728	chr1:1043	SNORA25		smallRNA	chr1:110272484-110

ENSG0000(	2181	48.70728	chr1:104:	Y_RNA		smallRNA	chr1:111446798-111
ENSG0000(	2181	48.70728	chr1:104:	GSTM1	DriverDB\	protein_c	chr1:109687814-109
ENSG0000(	2181	48.70728	chr1:104:	SPATA42		lncRNA	chr1:108857217-108
ENSG0000(	2181	48.70728	chr1:104:	RBM15-AS1		lncRNA	chr1:110286375-110
ENSG0000(	2181	48.70728	chr1:104:	SLC25A24P1		Pseudoger	chr1:108273139-108
ENSG0000(	2181	48.70728	chr1:104:	RNU6-151P		smallRNA	chr1:111650431-111
ENSG0000(	2181	48.70728	chr1:104:	GSTM5	NCGv7	protein_c	chr1:109711780-109
ENSG0000(	2181	48.70728	chr1:104:	TMIGD3		protein_c	chr1:111483348-111
ENSG0000(	2181	48.70728	chr1:104:	NBPF6		protein_c	chr1:108450282-108
ENSG0000(	2181	48.70728	chr1:104:	CYMP-AS1		lncRNA	chr1:110487680-110
ENSG0000(	2181	48.70728	chr1:104:	KCND3-AS1		lncRNA	chr1:111909336-111
ENSG0000(	2181	48.70728	chr1:104:	SLC16A4-AS1		lncRNA	chr1:110370154-110
ENSG0000(	2181	48.70728	chr1:104:	ENSG00000290547		lncRNA	chr1:108272943-108
ENSG0000(	2181	48.70728	chr1:104:	ENSG00000232971		lncRNA	chr1:108734256-108
ENSG0000(	2181	48.70728	chr1:104:	ENSG00000258634		lncRNA	chr1:110058340-110
ENSG0000(	2181	48.70728	chr1:104:	TAF13		protein_c	chr1:109062496-109
ENSG0000(	2181	48.70728	chr1:104:	CHIAP2		lncRNA	chr1:111280059-111
ENSG0000(	2181	48.70728	chr1:104:	ENSG00000289612		lncRNA	chr1:107140235-107
ENSG0000(	2181	48.70728	chr1:104:	ENSG00000282887		lncRNA	chr1:110472543-110
ENSG0000(	2181	48.70728	chr1:104:	TMEM167B-DT		lncRNA	chr1:109087971-109
ENSG0000(	2181	48.70728	chr1:104:	FNDC7		protein_c	chr1:108712908-108
ENSG0000(	2181	48.70728	chr1:104:	PSMA5		protein_c	chr1:109399042-109
ENSG0000(	2181	48.70728	chr1:104:	KCNA10		protein_c	chr1:110517217-110
ENSG0000(	2181	48.70728	chr1:104:	AL365361.1		smallRNA	chr1:110652942-110
ENSG0000(	2181	48.70728	chr1:104:	DDX20	NCGv7	protein_c	chr1:111754832-111
ENSG0000(	2181	48.70728	chr1:104:	ENSG00000284830		lncRNA	chr1:111745299-111
ENSG0000(	2181	48.70728	chr1:104:	ENSG00000261654		lncRNA	chr1:110936369-110
ENSG0000(	2181	48.70728	chr1:104:	CSF1	AC	protein_c	chr1:109910242-109
ENSG0000(	2181	48.70728	chr1:104:	ATXN7L2		protein_c	chr1:109483479-109
ENSG0000(	2181	48.70728	chr1:104:	STRIP1		protein_c	chr1:110031577-110
ENSG0000(	2181	48.70728	chr1:104:	CHI3L2		protein_c	chr1:111200771-111
ENSG0000(	2181	48.70728	chr1:104:	KCND3-IT1		lncRNA	chr1:111853762-111
ENSG0000(	2181	48.70728	chr1:104:	KCNA2		protein_c	chr1:110519837-110
ENSG0000(	2181	48.70728	chr1:104:	LINC01160		lncRNA	chr1:111599655-111
ENSG0000(	2181	48.70728	chr1:104:	ENSG00000270066		lncRNA	chr1:109100193-109
ENSG0000(	2181	48.70728	chr1:104:	INKA2		protein_c	chr1:111680630-111
ENSG0000(	2181	48.70728	chr1:104:	ENSG00000282852		Pseudoger	chr1:110256754-110
ENSG0000(	2181	48.70728	chr1:104:	ENSG00000270976		Pseudoger	chr1:106780223-106
ENSG0000(	2181	48.70728	chr1:104:	ENSG00000260246		lncRNA	chr1:109693117-109
ENSG0000(	2181	48.70728	chr1:104:	ENSG00000237349		Pseudoger	chr1:108986963-108
ENSG0000(	2181	48.70728	chr1:104:	LINC01397		lncRNA	chr1:110082651-110
ENSG0000(	2181	48.70728	chr1:104:	ENSG00000235526		lncRNA	chr1:110177643-110
ENSG0000(	2181	48.70728	chr1:104:	ENSG00000273010		lncRNA	chr1:110963302-110
ENSG0000(	2181	48.70728	chr1:104:	ENSG00000270342		Pseudoger	chr1:106544342-106
ENSG0000(	2181	48.70728	chr1:104:	ENSG00000244716		Pseudoger	chr1:108992282-108
ENSG0000(	2181	48.70728	chr1:104:	RBM15	NCGv7;AC	protein_c	chr1:110338506-110
ENSG0000(	2181	48.70728	chr1:104:	KCNA3		protein_c	chr1:110653560-110
ENSG0000(	2181	48.70728	chr1:104:	ENSG00000283999		lncRNA	chr1:110473756-110
ENSG0000(	2181	48.70728	chr1:104:	NDUFA4P1		Pseudoger	chr1:107505203-107
ENSG0000(	2181	48.70728	chr1:104:	DENND2D		protein_c	chr1:111185969-111
ENSG0000(	2181	48.70728	chr1:104:	RNA5SP54		Pseudoger	chr1:111041834-111

ENSG0000(	2181	48.70728	chr1:104:	ENSG00000243960	lncRNA	chr1:111438638-111
ENSG0000(	2181	48.70728	chr1:104:	ENSG00000232240	Pseudoger	chr1:111323833-111
ENSG0000(	2181	48.70728	chr1:104:	ENSG00000260948	lncRNA	chr1:111431046-111
ENSG0000(	2181	48.70728	chr1:104:	CELSR2	protein_c	chr1:109249539-109
ENSG0000(	2181	48.70728	chr1:104:	ENSG00000273221	lncRNA	chr1:111184415-111
ENSG0000(	2181	48.70728	chr1:104:	PROK1	protein_c	chr1:110451149-110
ENSG0000(	2181	48.70728	chr1:104:	ENSG00000272982	lncRNA	chr1:111181374-111
ENSG0000(	2181	48.70728	chr1:104:	CD53	protein_c	chr1:110871188-110
ENSG0000(	2181	48.70728	chr1:104:	NBPF4	protein_c	chr1:108222464-108
ENSG0000(	2181	48.70728	chr1:104:	CLCC1	protein_c	chr1:108881885-108
ENSG0000(	2181	48.70728	chr1:104:	CHIAP3	Pseudoger	chr1:111353275-111
ENSG0000(	2181	48.70728	chr1:104:	GPR61	protein_c	chr1:109539872-109
ENSG0000(	2181	48.70728	chr1:104:	C1orf162	protein_c	chr1:111473792-111
ENSG0000(	2181	48.70728	chr1:104:	ALX3	protein_c	chr1:110059870-110
ENSG0000(	2181	48.70728	chr1:104:	DRAM2	protein_c	chr1:111117163-111
ENSG0000(	2181	48.70728	chr1:104:	ENSG00000270380	lncRNA	chr1:110456505-110
ENSG0000(	2181	48.70728	chr1:104:	MYBPHL	protein_c	chr1:109292365-109
ENSG0000(	2181	48.70728	chr1:104:	AKNAD1	protein_c	chr1:108815898-108
ENSG0000(	2181	48.70728	chr1:104:	NBPF5P	Pseudoger	chr1:108376119-108
ENSG0000(	2181	48.70728	chr1:104:	ENSG00000232811	lncRNA	chr1:110943467-110
ENSG0000(	2181	48.70728	chr1:104:	PGBP	lncRNA	chr1:111384519-111
ENSG0000(	2181	48.70728	chr1:104:	FAM102B	protein_c	chr1:108560089-108
ENSG0000(	2181	48.70728	chr1:104:	NTNG1	protein_c	chr1:107140007-107
ENSG0000(	2181	48.70728	chr1:104:	CCNT2P1	Pseudoger	chr1:111007700-111
ENSG0000(	2181	48.70728	chr1:104:	KCNC4-DT	lncRNA	chr1:110208834-110
ENSG0000(	2181	48.70728	chr1:104:	CYMP	Pseudoger	chr1:110480752-110
ENSG0000(	2181	48.70728	chr1:104:	ENSG00000290126	lncRNA	chr1:108690627-108
ENSG0000(	2181	48.70728	chr1:104:	OR11I1P	Pseudoger	chr1:110853939-110
ENSG0000(	2181	48.70728	chr1:104:	SYPL2	protein_c	chr1:109466546-109
ENSG0000(	2181	48.70728	chr1:104:	ENSG00000260879	lncRNA	chr1:108199926-108
ENSG0000(	2181	48.70728	chr1:104:	ENSG00000288803	lncRNA	chr1:110680508-110
ENSG0000(	2181	48.70728	chr1:104:	CHIAP1	Pseudoger	chr1:111250254-111
ENSG0000(	2181	48.70728	chr1:104:	ENSG00000235005	lncRNA	chr1:109884176-109
ENSG0000(	2181	48.70728	chr1:104:	ENSG00000224698	lncRNA	chr1:108420689-108
ENSG0000(	2181	48.70728	chr1:104:	WDR47	protein_c	chr1:108970214-109
ENSG0000(	2181	48.70728	chr1:104:	MTATP6P14	Pseudoger	chr1:106802755-106
ENSG0000(	2181	48.70728	chr1:104:	EPS8L3	protein_c	chr1:109750080-109
ENSG0000(	2181	48.70728	chr1:104:	ENSG00000254942	lncRNA	chr1:109539906-109
ENSG0000(	2181	48.70728	chr1:104:	LAMTOR5-AS1	lncRNA	chr1:110347116-110
ENSG0000(	2181	48.70728	chr1:104:	Y_RNA	smallRNA	chr1:110764408-110
ENSG0000(	2181	48.70728	chr1:104:	RPL7P8	Pseudoger	chr1:109651370-109
ENSG0000(	2181	48.70728	chr1:104:	GSTM2	protein_c	chr1:109668022-109
ENSG0000(	2181	48.70728	chr1:104:	SLC6A17	protein_c	chr1:110150494-110
ENSG0000(	2181	48.70728	chr1:104:	NRBF2P3	Pseudoger	chr1:110848077-110
ENSG0000(	2181	48.70728	chr1:104:	ENSG00000290117	lncRNA	chr1:109546610-109
ENSG0000(	2181	48.70728	chr1:104:	ENSG00000283354	Pseudoger	chr1:108495475-108
ENSG0000(	2181	48.70728	chr1:104:	UBE2FP3	Pseudoger	chr1:111437514-111
ENSG0000(	2181	48.70728	chr1:104:	ENSG00000290552	lncRNA	chr1:108375838-108
ENSG0000(	2181	48.70728	chr1:104:	NDUFA5P10	Pseudoger	chr1:109810642-109
ENSG0000(	2181	48.70728	chr1:104:	HENMT1	protein_c	chr1:108648290-108
ENSG0000(	2181	48.70728	chr1:104:	ENSG00000225113	lncRNA	chr1:109596225-109

ENSG0000(	2181	48.70728	chr1:1043	ADORA3		protein_c	chr1:111499429-111
ENSG0000(	2181	48.70728	chr1:1043	UBL4B		protein_c	chr1:110112443-110
ENSG0000(	2181	48.70728	chr1:1043	HIGD1AP12		Pseudoger	chr1:111380291-111
ENSG0000(	2181	48.70728	chr1:1043	ENSG00000261055		lncRNA	chr1:109895973-109
ENSG0000(	2181	48.70728	chr1:1043	LINC01661		lncRNA	chr1:106818224-106
ENSG0000(	2181	48.70728	chr1:1043	PRMT6		protein_c	chr1:107056674-107
ENSG0000(	2181	48.70728	chr1:1043	SARS1		protein_c	chr1:109213918-109
ENSG0000(	2181	48.70728	chr1:1043	SLC25A24		protein_c	chr1:108134043-108
ENSG0000(	2181	48.70728	chr1:1043	AL591042.1		smallRNA	chr1:107776174-107
ENSG0000(	2181	48.70728	chr1:1043	TMEM167B		protein_c	chr1:109090764-109
ENSG0000(	2181	48.70728	chr1:1043	CFAP276		protein_c	chr1:109105951-109
ENSG0000(	2181	48.70728	chr1:1043	ENSG00000285923		lncRNA	chr1:108661533-108
ENSG0000(	2181	48.70728	chr1:1043	GNAI3		protein_c	chr1:109548615-109
ENSG0000(	2181	48.70728	chr1:1043	AMIGO1		protein_c	chr1:109504178-109
ENSG0000(	2181	48.70728	chr1:1043	OVGP1		protein_c	chr1:111414319-111
ENSG0000(	2181	48.70728	chr1:1043	KRT18P57		Pseudoger	chr1:111648291-111
ENSG0000(	2181	48.70728	chr1:1043	MTCO1P14		Pseudoger	chr1:106804474-106
ENSG0000(	2179	48.66261	chr1:1142	KPNA6		protein_c	chr1:32108056-3217
ENSG0000(	2179	48.66261	chr1:1142	LINC01226		lncRNA	chr1:31506226-3158
ENSG0000(	2179	48.66261	chr1:1142	FAM167B		protein_c	chr1:32247222-3224
ENSG0000(	2179	48.66261	chr1:1142	PEF1		protein_c	chr1:31629866-3164
ENSG0000(	2179	48.66261	chr1:1142	ENSG00000264078		lncRNA	chr1:31644694-3164
ENSG0000(	2179	48.66261	chr1:1142	RBBP4		protein_c	chr1:32651142-3268
ENSG0000(	2179	48.66261	chr1:1142	ENSG00000269967		lncRNA	chr1:31851913-3192
ENSG0000(	2179	48.66261	chr1:1142	KIAA1522		protein_c	chr1:32741830-3277
ENSG0000(	2179	48.66261	chr1:1142	ENSG00000278966		TEC	chr1:32973553-3297
ENSG0000(	2179	48.66261	chr1:1142	PTP4A2	AC	protein_c	chr1:31906421-3194
ENSG0000(	2179	48.66261	chr1:1142	CCDC28B		protein_c	chr1:32200595-3220
ENSG0000(	2179	48.66261	chr1:1142	SYNC		protein_c	chr1:32679906-3270
ENSG0000(	2179	48.66261	chr1:1142	TMEM234		protein_c	chr1:32214472-3222
ENSG0000(	2179	48.66261	chr1:1142	LCK	NCGv7;AC	protein_c	chr1:32251244-3228
ENSG0000(	2179	48.66261	chr1:1142	ENSG00000291132		lncRNA	chr1:32231656-3224
ENSG0000(	2179	48.66261	chr1:1142	ENSG00000203620		lncRNA	chr1:31842019-3185
ENSG0000(	2179	48.66261	chr1:1142	MARCKSL1		protein_c	chr1:32333839-3233
ENSG0000(	2179	48.66261	chr1:1142	ENSG00000229044		lncRNA	chr1:31333067-3134
ENSG0000(	2179	48.66261	chr1:1142	DCDC2B		protein_c	chr1:32209089-3221
ENSG0000(	2179	48.66261	chr1:1142	LRRC37A12P		Pseudoger	chr1:32423214-3242
ENSG0000(	2179	48.66261	chr1:1142	Y_RNA		smallRNA	chr1:32286452-3228
ENSG0000(	2179	48.66261	chr1:1142	FNDC5		protein_c	chr1:32862268-3287
ENSG0000(	2179	48.66261	chr1:1142	MIR5585		smallRNA	chr1:32086949-3208
ENSG0000(	2179	48.66261	chr1:1142	TSSK3		protein_c	chr1:32351521-3236
ENSG0000(	2179	48.66261	chr1:1142	ZBTB8A		protein_c	chr1:32539427-3260
ENSG0000(	2179	48.66261	chr1:1142	BSDC1		protein_c	chr1:32364633-3239
ENSG0000(	2179	48.66261	chr1:1142	ENSG00000284702		lncRNA	chr1:31972189-3198
ENSG0000(	2179	48.66261	chr1:1142	ENSG00000287691		lncRNA	chr1:32925454-3295
ENSG0000(	2179	48.66261	chr1:1142	IQCC		protein_c	chr1:32205671-3220
ENSG0000(	2179	48.66261	chr1:1142	AC114494.1		protein_c	chr1:31429345-3142
ENSG0000(	2179	48.66261	chr1:1142	SNRNP40		protein_c	chr1:31259568-3129
ENSG0000(	2179	48.66261	chr1:1142	MTMR9LP		Pseudoger	chr1:32231847-3224
ENSG0000(	2179	48.66261	chr1:1142	Y_RNA		smallRNA	chr1:32639951-3264
ENSG0000(	2179	48.66261	chr1:1142	HCRTR1		protein_c	chr1:31617686-3163

ENSG0000(	2179	48.66261	chr1:1142	ADGRB2		protein_c	chr1:31727117-3176
ENSG0000(	2179	48.66261	chr1:1142	ENSG00000278997		TEC	chr1:33141871-3314
ENSG0000(	2179	48.66261	chr1:1142	ENSG00000254553		protein_c	chr1:32465057-3260
ENSG0000(	2179	48.66261	chr1:1142	FAM229A		protein_c	chr1:32361270-3236
ENSG0000(	2179	48.66261	chr1:1142	SERINC2		protein_c	chr1:31409565-3143
ENSG0000(	2179	48.66261	chr1:1142	ENSG00000224409		lncRNA	chr1:32717734-3272
ENSG0000(	2179	48.66261	chr1:1142	AK2		protein_c	chr1:33007986-3308
ENSG0000(	2179	48.66261	chr1:1142	ZBTB8B		protein_c	chr1:32465072-3249
ENSG0000(	2179	48.66261	chr1:1142	ENSG00000270850		Pseudoger	chr1:32421979-3242
ENSG0000(	2179	48.66261	chr1:1142	ENSG00000288678		protein_c	chr1:31919563-3191
ENSG0000(	2179	48.66261	chr1:1142	EEF1A1P46		Pseudoger	chr1:31487589-3148
ENSG0000(	2179	48.66261	chr1:1142	AL136115.1		protein_c	chr1:31913573-3191
ENSG0000(	2179	48.66261	chr1:1142	ENSG00000233775		lncRNA	chr1:32349194-3235
ENSG0000(	2179	48.66261	chr1:1142	RP11-439L8.3		lncRNA	chr1:31506240-3150
ENSG0000(	2179	48.66261	chr1:1142	AL031602.1		smallRNA	chr1:32926294-3292
ENSG0000(	2179	48.66261	chr1:1142	ZCCHC17		protein_c	chr1:31296982-3136
ENSG0000(	2179	48.66261	chr1:1142	ENSG00000290045		lncRNA	chr1:32240526-3224
ENSG0000(	2179	48.66261	chr1:1142	FABP3		protein_c	chr1:31365253-3137
ENSG0000(	2179	48.66261	chr1:1142	HPCA		protein_c	chr1:32885994-3289
ENSG0000(	2179	48.66261	chr1:1142	ENSG00000236065		lncRNA	chr1:32987075-3303
ENSG0000(	2179	48.66261	chr1:1142	TMEM54		protein_c	chr1:32894594-3290
ENSG0000(	2179	48.66261	chr1:1142	AZIN2		protein_c	chr1:33081104-3312
ENSG0000(	2179	48.66261	chr1:1142	GAPDHP20		Pseudoger	chr1:32402109-3240
ENSG0000(	2179	48.66261	chr1:1142	ENSG00000228634		lncRNA	chr1:31933020-3193
ENSG0000(	2179	48.66261	chr1:1142	TINAGL1		protein_c	chr1:31576485-3158
ENSG0000(	2179	48.66261	chr1:1142	RN7SL122P		smallRNA	chr1:32457835-3245
ENSG0000(	2179	48.66261	chr1:1142	ENSG00000254545		lncRNA	chr1:31789130-3179
ENSG0000(	2179	48.66261	chr1:1142	YARS1		protein_c	chr1:32775237-3281
ENSG0000(	2179	48.66261	chr1:1142	SPOCD1		protein_c	chr1:31790422-3181
ENSG0000(	2179	48.66261	chr1:1142	ENSG00000250135		lncRNA	chr1:32170733-3217
ENSG0000(	2179	48.66261	chr1:1142	TMEM39B		protein_c	chr1:32072031-3210
ENSG0000(	2179	48.66261	chr1:1142	KHDRBS1		protein_c	chr1:32013868-3206
ENSG0000(	2179	48.66261	chr1:1142	ZBTB8OS		protein_c	chr1:32600172-3265
ENSG0000(	2179	48.66261	chr1:1142	MIR4254		smallRNA	chr1:31758660-3175
ENSG0000(	2179	48.66261	chr1:1142	RNU6-40P		smallRNA	chr1:31497577-3149
ENSG0000(	2179	48.66261	chr1:1142	FKSG48		protein_c	chr1:32973667-3297
ENSG0000(	2179	48.66261	chr1:1142	COL16A1		protein_c	chr1:31652263-3170
ENSG0000(	2179	48.66261	chr1:1142	HDAC1	NCGv7;AC	protein_c	chr1:32292083-3233
ENSG0000(	2179	48.66261	chr1:1142	ENSG00000279179		TEC	chr1:33162851-3316
ENSG0000(	2179	48.66261	chr1:1142	ENSG00000224066		lncRNA	chr1:32204769-3220
ENSG0000(	2179	48.66261	chr1:1142	ENSG00000229447		Pseudoger	chr1:31263245-3126
ENSG0000(	2179	48.66261	chr1:1142	ENSG00000217644		Pseudoger	chr1:32979947-3298
ENSG0000(	2179	48.66261	chr1:1142	PEF1-AS1		lncRNA	chr1:31644049-3166
ENSG0000(	2179	48.66261	chr1:1142	ENSG00000239670		Pseudoger	chr1:32986952-3298
ENSG0000(	2179	48.66261	chr1:1142	EIF3I	AC	protein_c	chr1:32221077-3224
ENSG0000(	2179	48.66261	chr1:1142	S100PBP		protein_c	chr1:32816767-3285
ENSG0000(	2179	48.66261	chr1:1142	ENSG00000203325		lncRNA	chr1:32052291-3207
ENSG0000(	2179	48.66261	chr1:1142	RNF19B		protein_c	chr1:32936445-3296
ENSG0000(	2179	48.66261	chr1:1142	TRIM62		protein_c	chr1:33145399-3318
ENSG0000(	2179	48.66261	chr1:1142	TXLNA		protein_c	chr1:32179675-3219
ENSG0000(	2179	48.66261	chr1:1142	ENSG00000229167		lncRNA	chr1:31571585-3157

ENSG0000(	2170	48.46162	chr1:1142	SNRPEP7		Pseudoger	chr1:27211265-2721
ENSG0000(	2162	48.28296	chr2:3094	MTND4LP12		Pseudoger	chr2:143098123-143
ENSG0000(	2162	48.28296	chr2:3094	MTND6P11		Pseudoger	chr2:143094233-143
ENSG0000(	2162	48.28296	chr2:3094	MTND5P24		Pseudoger	chr2:143094789-143
ENSG0000(	2162	48.28296	chr2:3094	UBE2V1P14		Pseudoger	chr2:142543418-142
ENSG0000(	2162	48.28296	chr2:3094	MTND3P9		Pseudoger	chr2:143098484-143
ENSG0000(	2162	48.28296	chr2:3094	MTCYBP11		Pseudoger	chr2:143093056-143
ENSG0000(	2162	48.28296	chr2:3094	RRN3P4		Pseudoger	chr2:142854095-142
ENSG0000(	2162	48.28296	chr2:3094	MTATP6P5		Pseudoger	chr2:143099936-143
ENSG0000(	2162	48.28296	chr2:3094	SFXN4P1		Pseudoger	chr2:142947300-142
ENSG0000(	2162	48.28296	chr2:3094	MTND4P22		Pseudoger	chr2:143096839-143
ENSG0000(	2162	48.28296	chr2:3094	AC013444.2		smallRNA	chr2:143005700-143
ENSG0000(	2162	48.28296	chr2:3094	ENSG00000244125		lncRNA	chr2:142131178-142
ENSG0000(	2162	48.28296	chr2:3094	AC016706.1		smallRNA	chr2:142312940-142
ENSG0000(	2162	48.28296	chr2:3094	MTCO3P5		Pseudoger	chr2:143098898-143
ENSG0000(	2162	48.28296	chr2:3094	ENSG00000257284		lncRNA	chr2:143162078-143
ENSG0000(	2162	48.28296	chr2:3094	AC012003.1		smallRNA	chr2:141997377-141
ENSG0000(	2162	48.28296	chr2:3094	MTCO2P5		Pseudoger	chr2:143100930-143
ENSG0000(	2162	48.28296	chr2:3094	KYNU		protein_c	chr2:142877657-143
ENSG0000(	2148	47.9703	chr1:1142	snoU13		smallRNA	chr1:25346274-2534
ENSG0000(	2148	47.9703	chr1:1142	AL033528.1		smallRNA	chr1:25911749-2591
ENSG0000(	2148	47.9703	chr1:1142	ENSG00000272432		lncRNA	chr1:25247837-2524
ENSG0000(	2148	47.9703	chr1:1142	SCARNA18		smallRNA	chr1:26006216-2600
ENSG0000(	2148	47.9703	chr1:1142	ENSG00000231953		lncRNA	chr1:25208139-2520
ENSG0000(	2148	47.9703	chr1:1142	SYF2	NCGv7	protein_c	chr1:25222276-2523
ENSG0000(	2148	47.9703	chr1:1142	ENSG00000272478		lncRNA	chr1:25831913-2583
ENSG0000(	2148	47.9703	chr1:1142	ENSG00000284602		lncRNA	chr1:25232586-2523
ENSG0000(	2148	47.9703	chr1:1142	MAN1C1		protein_c	chr1:25616791-2578
ENSG0000(	2148	47.9703	chr1:1142	MTFR1L	NCGv7	protein_c	chr1:25818640-2583
ENSG0000(	2148	47.9703	chr1:1142	AL031284.1		smallRNA	chr1:25406231-2540
ENSG0000(	2148	47.9703	chr1:1142	PDIK1L		protein_c	chr1:26111165-2612
ENSG0000(	2148	47.9703	chr1:1142	ENSG00000227312		Pseudoger	chr1:24563627-2456
ENSG0000(	2148	47.9703	chr1:1142	RSRP1		protein_c	chr1:25242249-2533
ENSG0000(	2148	47.9703	chr1:1142	RHD	DriverDBv	protein_c	chr1:25272393-2533
ENSG0000(	2148	47.9703	chr1:1142	ENSG00000236528		lncRNA	chr1:25859580-2586
ENSG0000(	2148	47.9703	chr1:1142	NCMAP-DT		lncRNA	chr1:24538802-2455
ENSG0000(	2148	47.9703	chr1:1142	SCARNA17		smallRNA	chr1:26006006-2600
ENSG0000(	2148	47.9703	chr1:1142	PAQR7		protein_c	chr1:25861484-2587
ENSG0000(	2148	47.9703	chr1:1142	ENSG00000278572		Pseudoger	chr1:26218581-2622
ENSG0000(	2148	47.9703	chr1:1142	RN7SL857P		smallRNA	chr1:24529455-2452
ENSG0000(	2148	47.9703	chr1:1142	ENSG00000259984		Pseudoger	chr1:25336429-2533
ENSG0000(	2148	47.9703	chr1:1142	SDHDP6		Pseudoger	chr1:25294164-2529
ENSG0000(	2148	47.9703	chr1:1142	LINC02793		lncRNA	chr1:25041136-2504
ENSG0000(	2148	47.9703	chr1:1142	STMN1	AC	protein_c	chr1:25884181-2590
ENSG0000(	2148	47.9703	chr1:1142	ENSG00000261025		lncRNA	chr1:24968423-2497
ENSG0000(	2148	47.9703	chr1:1142	ENSG00000284657		lncRNA	chr1:25239494-2524
ENSG0000(	2148	47.9703	chr1:1142	MIR3917		smallRNA	chr1:25906362-2590
ENSG0000(	2148	47.9703	chr1:1142	FAM110D		protein_c	chr1:26159079-2616
ENSG0000(	2148	47.9703	chr1:1142	Y_RNA		smallRNA	chr1:25877496-2587
ENSG0000(	2148	47.9703	chr1:1142	RNU6-1208P		smallRNA	chr1:24777873-2477
ENSG0000(	2148	47.9703	chr1:1142	ENSG00000284699		lncRNA	chr1:24704894-2471

ENSG0000(	2148	47.9703	chr1:1142	SLC30A2		protein_c	chr1:26037252-2604
ENSG0000(	2148	47.9703	chr1:1142	ENSG00000284309		lncRNA	chr1:26046665-2604
ENSG0000(	2148	47.9703	chr1:1142	ZNF593		protein_c	chr1:26169908-2617
ENSG0000(	2148	47.9703	chr1:1142	CLIC4		protein_c	chr1:24745382-2484
ENSG0000(	2148	47.9703	chr1:1142	IFITM3P7		Pseudoger	chr1:25125053-2512
ENSG0000(	2148	47.9703	chr1:1142	ENSG00000233478		lncRNA	chr1:25644544-2565
ENSG0000(	2148	47.9703	chr1:1142	RNU6-110P		smallRNA	chr1:25964197-2596
ENSG0000(	2148	47.9703	chr1:1142	CNKSR1	NCGv7	protein_c	chr1:26177484-2618
ENSG0000(	2148	47.9703	chr1:1142	ZPLD2P		Pseudoger	chr1:26209741-2622
ENSG0000(	2148	47.9703	chr1:1142	TMEM50A		protein_c	chr1:25338317-2536
ENSG0000(	2148	47.9703	chr1:1142	RHCE		protein_c	chr1:25362249-2543
ENSG0000(	2148	47.9703	chr1:1142	PAFAH2		protein_c	chr1:25959767-2599
ENSG0000(	2148	47.9703	chr1:1142	RUNX3	AC	protein_c	chr1:24899511-2496
ENSG0000(	2148	47.9703	chr1:1142	EXTL1		protein_c	chr1:26019884-2603
ENSG0000(	2148	47.9703	chr1:1142	snoU13		smallRNA	chr1:24666983-2466
ENSG0000(	2148	47.9703	chr1:1142	ENSG00000238084		Pseudoger	chr1:25398721-2539
ENSG0000(	2148	47.9703	chr1:1142	ENSG00000223624		Pseudoger	chr1:25888970-2588
ENSG0000(	2148	47.9703	chr1:1142	LDLRAP1		protein_c	chr1:25543606-2556
ENSG0000(	2148	47.9703	chr1:1142	Y_RNA		smallRNA	chr1:24625411-2462
ENSG0000(	2148	47.9703	chr1:1142	ENSG00000233755		lncRNA	chr1:25043707-2511
ENSG0000(	2148	47.9703	chr1:1142	CATSPER4		protein_c	chr1:26190561-2620
ENSG0000(	2148	47.9703	chr1:1142	AUNIP		protein_c	chr1:25831913-2585
ENSG0000(	2148	47.9703	chr1:1142	SELENON		protein_c	chr1:25800193-2581
ENSG0000(	2148	47.9703	chr1:1142	RNU6-1171P		smallRNA	chr1:25340971-2534
ENSG0000(	2148	47.9703	chr1:1142	TRIM63		protein_c	chr1:26051301-2606
ENSG0000(	2148	47.9703	chr1:1142	AL391650.1		smallRNA	chr1:26071578-2607
ENSG0000(	2148	47.9703	chr1:1142	SNRPFP2		Pseudoger	chr1:25887360-2588
ENSG0000(	2148	47.9703	chr1:1142	MACO1		protein_c	chr1:25430858-2550
ENSG0000(	2148	47.9703	chr1:1142	ENSG00000225643		lncRNA	chr1:25581478-2559
ENSG0000(	2148	47.9703	chr1:1142	ZNF593OS		protein_c	chr1:26169516-2617
ENSG0000(	2148	47.9703	chr1:1142	C1orf232		protein_c	chr1:26164101-2616
ENSG0000(	2148	47.9703	chr1:1142	ENSG00000255054		protein_c	chr1:25811470-2582
ENSG0000(	2148	47.9703	chr1:1142	SRRM1		protein_c	chr1:24631716-2467
ENSG0000(	2148	47.9703	chr1:1142	NCMAP		protein_c	chr1:24556087-2460
ENSG0000(	2148	47.9703	chr1:1142	ENSG00000261349		Pseudoger	chr1:25266102-2526
ENSG0000(	2148	47.9703	chr1:1142	RUNX3-AS1		lncRNA	chr1:24961345-2496
ENSG0000(	2148	47.9703	chr1:1142	MIR4425		smallRNA	chr1:25023503-2502
ENSG0000(	2148	47.9703	chr1:1142	ENSG00000228172		lncRNA	chr1:25816749-2582
ENSG0000(	2146	47.92564	chr1:1142	ENSG00000225616		Pseudoger	chr1:28982278-2898
ENSG0000(	2138	47.74698	chr1:1142	MIR5581		smallRNA	chr1:37500935-3750
ENSG0000(	2133	47.63532	chr1:2197	ENSG00000270708		Pseudoger	chr1:220220291-220
ENSG0000(	2132	47.61298	chr1:1142	ENSG00000243659		Pseudoger	chr1:27311240-2731
ENSG0000(	2132	47.61298	chr1:1142	ENSG00000274944		protein_c	chr1:38864501-3888
ENSG0000(	2132	47.61298	chr1:1142	SNORD99		smallRNA	chr1:28578749-2857
ENSG0000(	2132	47.61298	chr1:1142	ENSG00000289452		lncRNA	chr1:26620707-2662
ENSG0000(	2132	47.61298	chr1:1142	ENSG00000229985		Pseudoger	chr1:27176751-2717
ENSG0000(	2132	47.61298	chr1:1142	RP11-242O24.5		lncRNA	chr1:29152489-2915
ENSG0000(	2132	47.61298	chr1:1142	ENSG00000284676		lncRNA	chr1:29755175-2979
ENSG0000(	2132	47.61298	chr1:1142	ENSG00000226698		lncRNA	chr1:26876133-2687
ENSG0000(	2132	47.61298	chr1:1142	OPRD1	NCGv7	protein_c	chr1:28812170-2887
ENSG0000(	2132	47.61298	chr1:1142	AL645944.1		smallRNA	chr1:29884902-2988

ENSG0000(	2132	47.61298	chr1:1142	RPL32P6		Pseudoger	chr1:26983628-2698
ENSG0000(	2132	47.61298	chr1:1142	ENSG00000271398		lncRNA	chr1:28247144-2824
ENSG0000(	2132	47.61298	chr1:1142	SH3BGRL3		protein_c	chr1:26280086-2628
ENSG0000(	2132	47.61298	chr1:1142	ENSG00000270031		lncRNA	chr1:27819983-2782
ENSG0000(	2132	47.61298	chr1:1142	WDTC1	NCGv7	protein_c	chr1:27234632-2730
ENSG0000(	2132	47.61298	chr1:1142	ATP5IF1		protein_c	chr1:28236109-2824
ENSG0000(	2132	47.61298	chr1:1142	RP1-212P9.2		lncRNA	chr1:28867575-2887
ENSG0000(	2132	47.61298	chr1:1142	MED18		protein_c	chr1:28329002-2833
ENSG0000(	2132	47.61298	chr1:1142	RPS6KA1		protein_c	chr1:26529761-2657
ENSG0000(	2132	47.61298	chr1:1142	THEMIS2		protein_c	chr1:27872543-2788
ENSG0000(	2132	47.61298	chr1:1142	SESN2	NCGv7	protein_c	chr1:28259518-2828
ENSG0000(	2132	47.61298	chr1:1142	RSPO1		protein_c	chr1:37611350-3763
ENSG0000(	2132	47.61298	chr1:1142	SYTL1	NCGv7	protein_c	chr1:27342020-2735
ENSG0000(	2132	47.61298	chr1:1142	DPPA2P2		Pseudoger	chr1:26519354-2652
ENSG0000(	2132	47.61298	chr1:1142	GPN2		protein_c	chr1:26876132-2689
ENSG0000(	2132	47.61298	chr1:1142	FCN3		protein_c	chr1:27369110-2737
ENSG0000(	2132	47.61298	chr1:1142	MAP3K6	NCGv7	protein_c	chr1:27355184-2736
ENSG0000(	2132	47.61298	chr1:1142	ENSG00000270103		lncRNA	chr1:28648600-2864
ENSG0000(	2132	47.61298	chr1:1142	ENSG00000273637		lncRNA	chr1:38839333-3887
ENSG0000(	2132	47.61298	chr1:1142	SRSF4		protein_c	chr1:29147743-2918
ENSG0000(	2132	47.61298	chr1:1142	ENSG00000237934		lncRNA	chr1:29223933-2922
ENSG0000(	2132	47.61298	chr1:1142	RNU6-753P		smallRNA	chr1:38396659-3839
ENSG0000(	2132	47.61298	chr1:1142	ZDHHC18		protein_c	chr1:26826688-2685
ENSG0000(	2132	47.61298	chr1:1142	ENSG00000270605		lncRNA	chr1:28239509-2824
ENSG0000(	2132	47.61298	chr1:1142	RNA5SP44		Pseudoger	chr1:39154164-3915
ENSG0000(	2132	47.61298	chr1:1142	ENSG00000269971		lncRNA	chr1:27773858-2777
ENSG0000(	2132	47.61298	chr1:1142	snoU13		smallRNA	chr1:37750202-3775
ENSG0000(	2132	47.61298	chr1:1142	RNU7-29P		smallRNA	chr1:28144156-2814
ENSG0000(	2132	47.61298	chr1:1142	ENSG00000260063		lncRNA	chr1:26692132-2669
ENSG0000(	2132	47.61298	chr1:1142	ACTG1P20		Pseudoger	chr1:27325329-2732
ENSG0000(	2132	47.61298	chr1:1142	C1orf109		protein_c	chr1:37681570-3769
ENSG0000(	2132	47.61298	chr1:1142	RRAGC	IntOGen-I	protein_c	chr1:38838198-3885
ENSG0000(	2132	47.61298	chr1:1142	RNA5SP43		Pseudoger	chr1:37264677-3726
ENSG0000(	2132	47.61298	chr1:1142	ENSG00000223944		lncRNA	chr1:37154761-3732
ENSG0000(	2132	47.61298	chr1:1142	GPR3		protein_c	chr1:27392622-2739
ENSG0000(	2132	47.61298	chr1:1142	RNU6-510P		smallRNA	chr1:37991462-3799
ENSG0000(	2132	47.61298	chr1:1142	RNU6-584P		smallRNA	chr1:37885023-3788
ENSG0000(	2132	47.61298	chr1:1142	ENSG00000233728		lncRNA	chr1:37799720-3780
ENSG0000(	2132	47.61298	chr1:1142	Y_RNA		smallRNA	chr1:26593940-2659
ENSG0000(	2132	47.61298	chr1:1142	ENSG00000286433		lncRNA	chr1:27827812-2783
ENSG0000(	2132	47.61298	chr1:1142	ENSG00000289576		lncRNA	chr1:28116812-2812
ENSG0000(	2132	47.61298	chr1:1142	RN7SL501P		smallRNA	chr1:26763624-2676
ENSG0000(	2132	47.61298	chr1:1142	ENSG00000290123		lncRNA	chr1:28234080-2823
ENSG0000(	2132	47.61298	chr1:1142	ENSG00000289554		lncRNA	chr1:26881109-2688
ENSG0000(	2132	47.61298	chr1:1142	LIN28A	AC	protein_c	chr1:26410817-2642
ENSG0000(	2132	47.61298	chr1:1142	NR0B2		protein_c	chr1:26911489-2691
ENSG0000(	2132	47.61298	chr1:1142	IFI6		protein_c	chr1:27666064-2767
ENSG0000(	2132	47.61298	chr1:1142	AHDC1		protein_c	chr1:27534035-2760
ENSG0000(	2132	47.61298	chr1:1142	DNAJC8		protein_c	chr1:28199456-2823
ENSG0000(	2132	47.61298	chr1:1142	MIR4255		smallRNA	chr1:37161563-3716
ENSG0000(	2132	47.61298	chr1:1142	INPP5B		protein_c	chr1:37860697-3794

ENSG0000(	2132	47.61298	chr1:1142	MECR		protein_c	chr1:29192657-2923
ENSG0000(	2132	47.61298	chr1:1142	ENSG00000223583		Pseudoger	chr1:26454653-2645
ENSG0000(	2132	47.61298	chr1:1142	MTF1		protein_c	chr1:37809574-3785
ENSG0000(	2132	47.61298	chr1:1142	CHMP1AP1		Pseudoger	chr1:27686810-2768
ENSG0000(	2132	47.61298	chr1:1142	RN7SL559P		smallRNA	chr1:28031886-2803
ENSG0000(	2132	47.61298	chr1:1142	CEP85		protein_c	chr1:26234200-2627
ENSG0000(	2132	47.61298	chr1:1142	GJA9		protein_c	chr1:38874069-3888
ENSG0000(	2132	47.61298	chr1:1142	RAB42	NCGv7	protein_c	chr1:28592200-2859
ENSG0000(	2132	47.61298	chr1:1142	NUDC		protein_c	chr1:26900238-2694
ENSG0000(	2132	47.61298	chr1:1142	SNHG12		lncRNA	chr1:28578538-2858
ENSG0000(	2132	47.61298	chr1:1142	MIR3659		smallRNA	chr1:38089231-3808
ENSG0000(	2132	47.61298	chr1:1142	C1orf122		protein_c	chr1:37806979-3780
ENSG0000(	2132	47.61298	chr1:1142	ENSG00000284632		lncRNA	chr1:38754216-3881
ENSG0000(	2132	47.61298	chr1:1142	NDUFS5		protein_c	chr1:39026318-3903
ENSG0000(	2132	47.61298	chr1:1142	ENSG00000284650		lncRNA	chr1:37133489-3713
ENSG0000(	2132	47.61298	chr1:1142	LINC02786		lncRNA	chr1:38129464-3814
ENSG0000(	2132	47.61298	chr1:1142	ENSG00000223589		Pseudoger	chr1:38080572-3808
ENSG0000(	2132	47.61298	chr1:1142	PHACTR4		protein_c	chr1:28369582-2850
ENSG0000(	2132	47.61298	chr1:1142	LINC02574		lncRNA	chr1:27660328-2766
ENSG0000(	2132	47.61298	chr1:1142	DNALI1		protein_c	chr1:37556919-3756
ENSG0000(	2132	47.61298	chr1:1142	SNIP1		protein_c	chr1:37534449-3755
ENSG0000(	2132	47.61298	chr1:1142	ENSG00000229820		Pseudoger	chr1:28453541-2845
ENSG0000(	2132	47.61298	chr1:1142	MEAF6		protein_c	chr1:37489993-3751
ENSG0000(	2132	47.61298	chr1:1142	ZC3H12A	NCGv7	protein_c	chr1:37474580-3748
ENSG0000(	2132	47.61298	chr1:1142	SNORA63		smallRNA	chr1:37884237-3788
ENSG0000(	2132	47.61298	chr1:1142	SCARNA24		smallRNA	chr1:28689665-2868
ENSG0000(	2132	47.61298	chr1:1142	ENSG00000287244		lncRNA	chr1:27724822-2772
ENSG0000(	2132	47.61298	chr1:1142	SMPDL3B		protein_c	chr1:27935000-2795
ENSG0000(	2132	47.61298	chr1:1142	DHDDS		protein_c	chr1:26432282-2647
ENSG0000(	2132	47.61298	chr1:1142	POU3F1		protein_c	chr1:38043829-3804
ENSG0000(	2132	47.61298	chr1:1142	ENSG00000233427		lncRNA	chr1:28870483-2887
ENSG0000(	2132	47.61298	chr1:1142	LINC01756		lncRNA	chr1:29329620-2935
ENSG0000(	2132	47.61298	chr1:1142	ENSG00000238231		Pseudoger	chr1:27990158-2799
ENSG0000(	2132	47.61298	chr1:1142	SNHG3		lncRNA	chr1:28505980-2851
ENSG0000(	2132	47.61298	chr1:1142	AL109927.1		protein_c	chr1:27850574-2785
ENSG0000(	2132	47.61298	chr1:1142	NPM1P39		Pseudoger	chr1:27206930-2720
ENSG0000(	2132	47.61298	chr1:1142	ENSG00000228943		Pseudoger	chr1:28109739-2811
ENSG0000(	2132	47.61298	chr1:1142	TAF12		protein_c	chr1:28587829-2864
ENSG0000(	2132	47.61298	chr1:1142	RNU6-949P		smallRNA	chr1:27675603-2767
ENSG0000(	2132	47.61298	chr1:1142	ENSG00000241169		lncRNA	chr1:27457198-2745
ENSG0000(	2132	47.61298	chr1:1142	RN7SL490P		smallRNA	chr1:26348465-2634
ENSG0000(	2132	47.61298	chr1:1142	RRAGC-DT		lncRNA	chr1:38859912-3896
ENSG0000(	2132	47.61298	chr1:1142	EIF1P2		Pseudoger	chr1:38958275-3895
ENSG0000(	2132	47.61298	chr1:1142	Y_RNA		smallRNA	chr1:28422555-2842
ENSG0000(	2132	47.61298	chr1:1142	AL929472.1		protein_c	chr1:37826560-3782
ENSG0000(	2132	47.61298	chr1:1142	OSTCP2		Pseudoger	chr1:26985897-2698
ENSG0000(	2132	47.61298	chr1:1142	EPB41	NCGv7	protein_c	chr1:28887091-2912
ENSG0000(	2132	47.61298	chr1:1142	ZNF683		protein_c	chr1:26361634-2637
ENSG0000(	2132	47.61298	chr1:1142	ACTN4P2		Pseudoger	chr1:37776670-3777
ENSG0000(	2132	47.61298	chr1:1142	ENSG00000237749		Pseudoger	chr1:37556247-3755
ENSG0000(	2132	47.61298	chr1:1142	MYCBP		protein_c	chr1:38862493-3887

ENSG0000(	2132	47.61298	chr1:114‹	ZC3H12A-DT		lncRNA	chr1:37350934-3747
ENSG0000(	2132	47.61298	chr1:114‹	EPHA10	DriverDB\	protein_c	chr1:37713880-3776
ENSG0000(	2132	47.61298	chr1:114‹	PTAFR		protein_c	chr1:28147166-2819
ENSG0000(	2132	47.61298	chr1:114‹	FHL3		protein_c	chr1:37996770-3800
ENSG0000(	2132	47.61298	chr1:114‹	ENSG00000237429		lncRNA	chr1:27525805-2753
ENSG0000(	2132	47.61298	chr1:114‹	MACF1	NCGv7	protein_c	chr1:39081316-3948
ENSG0000(	2132	47.61298	chr1:114‹	SF3A3		protein_c	chr1:37956975-3799
ENSG0000(	2132	47.61298	chr1:114‹	RNU6-636P		smallRNA	chr1:37203610-3720
ENSG0000(	2132	47.61298	chr1:114‹	RPL12P13		Pseudoger	chr1:26980165-2698
ENSG0000(	2132	47.61298	chr1:114‹	ENSG00000228176		lncRNA	chr1:29708851-2970
ENSG0000(	2132	47.61298	chr1:114‹	RCC1	NCGv7	protein_c	chr1:28505943-2853
ENSG0000(	2132	47.61298	chr1:114‹	ENSG00000225011		Pseudoger	chr1:29488193-2949
ENSG0000(	2132	47.61298	chr1:114‹	UTP11		protein_c	chr1:38009258-3802
ENSG0000(	2132	47.61298	chr1:114‹	Y_RNA		smallRNA	chr1:28985710-2898
ENSG0000(	2132	47.61298	chr1:114‹	ENSG00000270733		Pseudoger	chr1:26263041-2626
ENSG0000(	2132	47.61298	chr1:114‹	ENSG00000284748		lncRNA	chr1:37596126-3760
ENSG0000(	2132	47.61298	chr1:114‹	RPS29P6		Pseudoger	chr1:37330852-3733
ENSG0000(	2132	47.61298	chr1:114‹	SNORA73B		smallRNA	chr1:28508559-2850
ENSG0000(	2132	47.61298	chr1:114‹	MIR1976		smallRNA	chr1:26554542-2655
ENSG0000(	2132	47.61298	chr1:114‹	ENSG00000214812		Pseudoger	chr1:28120449-2812
ENSG0000(	2132	47.61298	chr1:114‹	HMGN2		protein_c	chr1:26472440-2647
ENSG0000(	2132	47.61298	chr1:114‹	TMEM200B		protein_c	chr1:29119429-2912
ENSG0000(	2132	47.61298	chr1:114‹	AL353354.1		protein_c	chr1:28200502-2820
ENSG0000(	2132	47.61298	chr1:114‹	HSPA5P1		Pseudoger	chr1:38708931-3871
ENSG0000(	2132	47.61298	chr1:114‹	MANEAL		protein_c	chr1:37793847-3780
ENSG0000(	2132	47.61298	chr1:114‹	ENSG00000225854		Pseudoger	chr1:26326688-2632
ENSG0000(	2132	47.61298	chr1:114‹	ENSG00000227050		lncRNA	chr1:27938875-2796
ENSG0000(	2132	47.61298	chr1:114‹	ENSG00000287810		Pseudoger	chr1:26817300-2681
ENSG0000(	2132	47.61298	chr1:114‹	RPEP3		Pseudoger	chr1:27739954-2774
ENSG0000(	2132	47.61298	chr1:114‹	ENSG00000231207		Pseudoger	chr1:27389468-2739
ENSG0000(	2132	47.61298	chr1:114‹	ENSG00000225750		lncRNA	chr1:29144494-2914
ENSG0000(	2132	47.61298	chr1:114‹	TRNP1		protein_c	chr1:26993692-2700
ENSG0000(	2132	47.61298	chr1:114‹	RNU6-48P		smallRNA	chr1:27325219-2732
ENSG0000(	2132	47.61298	chr1:114‹	CD52		protein_c	chr1:26317958-2632
ENSG0000(	2132	47.61298	chr1:114‹	CDCA8		protein_c	chr1:37692481-3770
ENSG0000(	2132	47.61298	chr1:114‹	GNL2		protein_c	chr1:37566816-3759
ENSG0000(	2132	47.61298	chr1:114‹	Y_RNA		smallRNA	chr1:28881726-2888
ENSG0000(	2132	47.61298	chr1:114‹	CRYBG2		protein_c	chr1:26321698-2636
ENSG0000(	2132	47.61298	chr1:114‹	ENSG00000230955		lncRNA	chr1:37860697-3786
ENSG0000(	2132	47.61298	chr1:114‹	FGR	TAG;AC	protein_c	chr1:27612064-2763
ENSG0000(	2132	47.61298	chr1:114‹	ENSG00000231344		Pseudoger	chr1:27739091-2773
ENSG0000(	2132	47.61298	chr1:114‹	ENSG00000235912		Pseudoger	chr1:27649419-2764
ENSG0000(	2132	47.61298	chr1:114‹	SPCS2P4		Pseudoger	chr1:28095742-2809
ENSG0000(	2132	47.61298	chr1:114‹	ENSG00000279443		TEC	chr1:28544460-2854
ENSG0000(	2132	47.61298	chr1:114‹	RNU6-605P		smallRNA	chr1:38926870-3892
ENSG0000(	2132	47.61298	chr1:114‹	FAM76A		protein_c	chr1:27725961-2776
ENSG0000(	2132	47.61298	chr1:114‹	ENSG00000235673		Pseudoger	chr1:37840986-3784
ENSG0000(	2132	47.61298	chr1:114‹	YRDC		protein_c	chr1:37802945-3780
ENSG0000(	2132	47.61298	chr1:114‹	CHCHD3P3		Pseudoger	chr1:27200834-2720
ENSG0000(	2132	47.61298	chr1:114‹	RHBDL2		protein_c	chr1:38885807-3894
ENSG0000(	2132	47.61298	chr1:114‹	TENT5B		protein_c	chr1:27005020-2701

ENSG0000(	2132	47.61298	chr1:1142	WASF2		protein_c	chr1:27404230-2749
ENSG0000(	2132	47.61298	chr1:1142	KDF1		protein_c	chr1:26949562-2696
ENSG0000(	2132	47.61298	chr1:1142	EYA3		protein_c	chr1:27970344-2808
ENSG0000(	2132	47.61298	chr1:1142	XKR8		protein_c	chr1:27959588-2796
ENSG0000(	2132	47.61298	chr1:1142	RN7SL165P		smallRNA	chr1:26814822-2681
ENSG0000(	2132	47.61298	chr1:1142	RNU6-424P		smallRNA	chr1:27693731-2769
ENSG0000(	2132	47.61298	chr1:1142	DHDDS-AS1		lncRNA	chr1:26462756-2646
ENSG0000(	2132	47.61298	chr1:1142	ENSG00000225886		lncRNA	chr1:27669468-2770
ENSG0000(	2132	47.61298	chr1:1142	UBXN11		protein_c	chr1:26281328-2631
ENSG0000(	2132	47.61298	chr1:1142	SFN		protein_c	chr1:26863149-2686
ENSG0000(	2132	47.61298	chr1:1142	ENSG00000287422		lncRNA	chr1:39226670-3923
ENSG0000(	2132	47.61298	chr1:1142	LINC01685		lncRNA	chr1:38474825-3851
ENSG0000(	2132	47.61298	chr1:1142	GMEB1		protein_c	chr1:28668778-2871
ENSG0000(	2132	47.61298	chr1:1142	SCARNA1		smallRNA	chr1:27834401-2783
ENSG0000(	2132	47.61298	chr1:1142	TRNAU1AP		protein_c	chr1:28553085-2857
ENSG0000(	2132	47.61298	chr1:1142	ENSG00000289291		lncRNA	chr1:28736044-2873
ENSG0000(	2132	47.61298	chr1:1142	Y_RNA		smallRNA	chr1:27255464-2725
ENSG0000(	2132	47.61298	chr1:1142	GPATCH3		protein_c	chr1:26890488-2690
ENSG0000(	2132	47.61298	chr1:1142	ENSG00000227416		Pseudoger	chr1:37782457-3778
ENSG0000(	2132	47.61298	chr1:1142	RNU6ATAC27P		smallRNA	chr1:28481362-2848
ENSG0000(	2132	47.61298	chr1:1142	Y_RNA		smallRNA	chr1:37737955-3773
ENSG0000(	2132	47.61298	chr1:1142	ENSG00000229247		Pseudoger	chr1:26640377-2664
ENSG0000(	2132	47.61298	chr1:1142	ENSG00000270927		Pseudoger	chr1:29904865-2990
ENSG0000(	2132	47.61298	chr1:1142	AC092265.1		smallRNA	chr1:29644545-2964
ENSG0000(	2132	47.61298	chr1:1142	YTHDF2	NCGv7	protein_c	chr1:28736621-2876
ENSG0000(	2132	47.61298	chr1:1142	Y_RNA		smallRNA	chr1:38950825-3895
ENSG0000(	2132	47.61298	chr1:1142	RN7SL679P		smallRNA	chr1:26593246-2659
ENSG0000(	2132	47.61298	chr1:1142	MIR3659HG		lncRNA	chr1:38047314-3811
ENSG0000(	2132	47.61298	chr1:1142	AL139151.1		smallRNA	chr1:29432481-2943
ENSG0000(	2132	47.61298	chr1:1142	snoU13		smallRNA	chr1:28517476-2851
ENSG0000(	2132	47.61298	chr1:1142	CD164L2		protein_c	chr1:27379176-2738
ENSG0000(	2132	47.61298	chr1:1142	ENSG00000286552		lncRNA	chr1:38149544-3816
ENSG0000(	2132	47.61298	chr1:1142	snoU13		smallRNA	chr1:26642286-2664
ENSG0000(	2132	47.61298	chr1:1142	LINC01343		lncRNA	chr1:38209034-3821
ENSG0000(	2132	47.61298	chr1:1142	RNU6-1245P		smallRNA	chr1:27824538-2782
ENSG0000(	2132	47.61298	chr1:1142	PTPRU	NCGv7	protein_c	chr1:29236516-2932
ENSG0000(	2132	47.61298	chr1:1142	FTH1P1		Pseudoger	chr1:37544763-3754
ENSG0000(	2132	47.61298	chr1:1142	PIGV		protein_c	chr1:26787054-2680
ENSG0000(	2132	47.61298	chr1:1142	TAF12-DT		lncRNA	chr1:28643228-2864
ENSG0000(	2132	47.61298	chr1:1142	ENSG00000275350		Pseudoger	chr1:38383838-3838
ENSG0000(	2132	47.61298	chr1:1142	ENSG00000290006		lncRNA	chr1:26692586-2669
ENSG0000(	2132	47.61298	chr1:1142	RNU6-608P		smallRNA	chr1:39120940-3912
ENSG0000(	2132	47.61298	chr1:1142	AL353354.2		protein_c	chr1:28200559-2820
ENSG0000(	2132	47.61298	chr1:1142	TMEM222		protein_c	chr1:27322145-2733
ENSG0000(	2132	47.61298	chr1:1142	RNU6-176P		smallRNA	chr1:28142737-2814
ENSG0000(	2132	47.61298	chr1:1142	STX12		protein_c	chr1:27773219-2782
ENSG0000(	2132	47.61298	chr1:1142	RPA2		protein_c	chr1:27891524-2791
ENSG0000(	2132	47.61298	chr1:1142	AKIRIN1	DriverDB\	protein_c	chr1:38991276-3900
ENSG0000(	2132	47.61298	chr1:1142	SLC9A1		protein_c	chr1:27098809-2716
ENSG0000(	2132	47.61298	chr1:1142	ARID1A	NCGv7;AC	protein_c	chr1:26693236-2678
ENSG0000(	2132	47.61298	chr1:1142	WDTC1-DT		lncRNA	chr1:27229106-2723

ENSG0000(	2132	47.61298	chr1:114	ENSG00000287987		lncRNA	chr1:38193619-3821
ENSG0000(	2132	47.61298	chr1:114	ENSG00000235069		Pseudoger	chr1:26647447-2664
ENSG0000(	2132	47.61298	chr1:114	PPP1R8		protein_c	chr1:27830782-2785
ENSG0000(	2106	47.03234	chr2:309	ENSG00000234455		lncRNA	chr2:121081437-121
ENSG0000(	2106	47.03234	chr2:309	ENSG00000236255		lncRNA	chr2:117833937-117
ENSG0000(	2106	47.03234	chr2:309	ENSG00000229774		lncRNA	chr2:120686635-120
ENSG0000(	2106	47.03234	chr2:309	ENSG00000279227		TEC	chr2:118014174-118
ENSG0000(	2106	47.03234	chr2:309	CLASP1-AS1		lncRNA	chr2:121530422-121
ENSG0000(	2106	47.03234	chr2:309	SCTR-AS1		lncRNA	chr2:119476428-119
ENSG0000(	2106	47.03234	chr2:309	RPL12P15		Pseudoger	chr2:121658475-121
ENSG0000(	2106	47.03234	chr2:309	LINC01101		TEC	chr2:120464335-120
ENSG0000(	2106	47.03234	chr2:309	STEAP3		protein_c	chr2:119223831-119
ENSG0000(	2106	47.03234	chr2:309	EPB41L5		protein_c	chr2:120013077-120
ENSG0000(	2106	47.03234	chr2:309	EN1	NCGv7	protein_c	chr2:118842171-118
ENSG0000(	2106	47.03234	chr2:309	PTPN4		protein_c	chr2:119759922-119
ENSG0000(	2106	47.03234	chr2:309	AC013275.1		smallRNA	chr2:119570900-119
ENSG0000(	2106	47.03234	chr2:309	SCTR		protein_c	chr2:119439843-119
ENSG0000(	2106	47.03234	chr2:309	CFAP221		protein_c	chr2:119544432-119
ENSG0000(	2106	47.03234	chr2:309	AC016764.1		smallRNA	chr2:121035073-121
ENSG0000(	2106	47.03234	chr2:309	ENSG00000236145		lncRNA	chr2:121178327-121
ENSG0000(	2106	47.03234	chr2:309	ENSG00000237614		lncRNA	chr2:120542905-120
ENSG0000(	2106	47.03234	chr2:309	INHBB		protein_c	chr2:120346136-120
ENSG0000(	2106	47.03234	chr2:309	MTND5P28		Pseudoger	chr2:120215181-120
ENSG0000(	2106	47.03234	chr2:309	snoU13		smallRNA	chr2:121705967-121
ENSG0000(	2106	47.03234	chr2:309	TMEM37		protein_c	chr2:119429901-119
ENSG0000(	2106	47.03234	chr2:309	ENSG00000271709		lncRNA	chr2:120866378-120
ENSG0000(	2106	47.03234	chr2:309	THORLNC		lncRNA	chr2:118132128-118
ENSG0000(	2106	47.03234	chr2:309	TFCP2L1		protein_c	chr2:121216587-121
ENSG0000(	2106	47.03234	chr2:309	ENSG00000235840		lncRNA	chr2:120319007-120
ENSG0000(	2106	47.03234	chr2:309	DDX18	NCGv7	protein_c	chr2:117814691-117
ENSG0000(	2106	47.03234	chr2:309	ENSG00000270798		Pseudoger	chr2:121743479-121
ENSG0000(	2106	47.03234	chr2:309	ENSG00000272895		lncRNA	chr2:117998745-117
ENSG0000(	2106	47.03234	chr2:309	RN7SL111P		smallRNA	chr2:118016382-118
ENSG0000(	2106	47.03234	chr2:309	NIFK		protein_c	chr2:121726945-121
ENSG0000(	2106	47.03234	chr2:309	RPL17P15		Pseudoger	chr2:119698623-119
ENSG0000(	2106	47.03234	chr2:309	RN7SL468P		smallRNA	chr2:119169822-119
ENSG0000(	2106	47.03234	chr2:309	LINC01956		lncRNA	chr2:118766965-118
ENSG0000(	2106	47.03234	chr2:309	NIFK-AS1		lncRNA	chr2:121649320-121
ENSG0000(	2106	47.03234	chr2:309	ENSG00000238207		lncRNA	chr2:117754139-117
ENSG0000(	2106	47.03234	chr2:309	MTND4LP14		Pseudoger	chr2:120213344-120
ENSG0000(	2106	47.03234	chr2:309	TSN		protein_c	chr2:121737103-121
ENSG0000(	2106	47.03234	chr2:309	RPS17P7		Pseudoger	chr2:121309995-121
ENSG0000(	2106	47.03234	chr2:309	TMEM185B		protein_c	chr2:120217479-120
ENSG0000(	2106	47.03234	chr2:309	NPM1P32		Pseudoger	chr2:121708512-121
ENSG0000(	2106	47.03234	chr2:309	C2orf76		protein_c	chr2:119302225-119
ENSG0000(	2106	47.03234	chr2:309	Y_RNA		smallRNA	chr2:121040610-121
ENSG0000(	2106	47.03234	chr2:309	ENSG00000235066		lncRNA	chr2:117995397-118
ENSG0000(	2106	47.03234	chr2:309	MTATP6P26		Pseudoger	chr2:120211054-120
ENSG0000(	2106	47.03234	chr2:309	ENSG00000224967		Pseudoger	chr2:117934025-117
ENSG0000(	2106	47.03234	chr2:309	ENSG00000289091		lncRNA	chr2:120255848-120
ENSG0000(	2106	47.03234	chr2:309	RPL27P7		Pseudoger	chr2:119993199-119

ENSG0000(	2106	47.03234	chr2:309	Y_RNA		smallRNA	chr2:120651273-120
ENSG0000(	2106	47.03234	chr2:309	ENSG00000259094		lncRNA	chr2:118949306-118
ENSG0000(	2106	47.03234	chr2:309	CLASP1	NCGv7	protein_c	chr2:121337776-121
ENSG0000(	2106	47.03234	chr2:309	snoU13		smallRNA	chr2:119917318-119
ENSG0000(	2106	47.03234	chr2:309	GLI2	AC	protein_c	chr2:120735623-120
ENSG0000(	2106	47.03234	chr2:309	ENSG00000290590		lncRNA	chr2:117860333-117
ENSG0000(	2106	47.03234	chr2:309	MTND3P10		Pseudoger	chr2:120212935-120
ENSG0000(	2106	47.03234	chr2:309	ENSG00000224789		lncRNA	chr2:120174885-120
ENSG0000(	2106	47.03234	chr2:309	MTND4P26		Pseudoger	chr2:120213631-120
ENSG0000(	2106	47.03234	chr2:309	STEAP3-AS1		lncRNA	chr2:119244422-119
ENSG0000(	2106	47.03234	chr2:309	RNU4ATAC		smallRNA	chr2:121530881-121
ENSG0000(	2106	47.03234	chr2:309	ENSG00000279721		TEC	chr2:121688203-121
ENSG0000(	2106	47.03234	chr2:309	ENSG00000232140		lncRNA	chr2:120552481-120
ENSG0000(	2106	47.03234	chr2:309	C1QL2	NCGv7	protein_c	chr2:119156243-119
ENSG0000(	2106	47.03234	chr2:309	MTCO3P43		Pseudoger	chr2:120211727-120
ENSG0000(	2106	47.03234	chr2:309	DBI	NCGv7	protein_c	chr2:119366924-119
ENSG0000(	2106	47.03234	chr2:309	FLJ14816		protein_c	chr2:120464335-120
ENSG0000(	2106	47.03234	chr2:309	TMEM177		protein_c	chr2:119679167-119
ENSG0000(	2106	47.03234	chr2:309	CCDC93		protein_c	chr2:117915478-118
ENSG0000(	2106	47.03234	chr2:309	HTR5BP		Pseudoger	chr2:117859427-117
ENSG0000(	2106	47.03234	chr2:309	ENSG00000286481		lncRNA	chr2:121902501-122
ENSG0000(	2106	47.03234	chr2:309	Y_RNA		smallRNA	chr2:121603073-121
ENSG0000(	2106	47.03234	chr2:309	Y_RNA		smallRNA	chr2:120192245-120
ENSG0000(	2106	47.03234	chr2:309	MARCO		protein_c	chr2:118942194-118
ENSG0000(	2106	47.03234	chr2:309	INSIG2		protein_c	chr2:118088452-118
ENSG0000(	2106	47.03234	chr2:309	RALB	TAG	protein_c	chr2:120240064-120
ENSG0000(	2106	47.03234	chr2:309	LINC01823		lncRNA	chr2:121779133-121
ENSG0000(	2098	46.85368	chr2:309	CXCR4	NCGv7;AC	protein_c	chr2:136114349-136
ENSG0000(	2098	46.85368	chr2:309	HNMT		protein_c	chr2:137964020-138
ENSG0000(	2098	46.85368	chr2:309	LINC01832		lncRNA	chr2:138101878-138
ENSG0000(	2098	46.85368	chr2:309	DARS1-AS1		lncRNA	chr2:135985124-136
ENSG0000(	2098	46.85368	chr2:309	RNA5SP105		Pseudoger	chr2:137512098-137
ENSG0000(	2098	46.85368	chr2:309	UBBP1		Pseudoger	chr2:136329441-136
ENSG0000(	2098	46.85368	chr2:309	ENSG00000237262		lncRNA	chr2:136077892-136
ENSG0000(	2098	46.85368	chr2:309	THSD7B	NCGv7	protein_c	chr2:136765545-137
ENSG0000(	2098	46.85368	chr2:309	Y_RNA		smallRNA	chr2:137715332-137
ENSG0000(	2098	46.85368	chr2:309	AC010146.2		smallRNA	chr2:136544371-136
ENSG0000(	2098	46.85368	chr2:309	AC020601.1		lncRNA	chr2:137878754-137
ENSG0000(	2098	46.85368	chr2:309	HNRNPKP2		Pseudoger	chr2:136199114-136
ENSG0000(	2098	46.85368	chr2:309	SMC4P1		Pseudoger	chr2:136544712-136
ENSG0000(	2098	46.85368	chr2:309	AC092786.1		smallRNA	chr2:136645108-136
ENSG0000(	2098	46.85368	chr2:309	RN7SKP141		smallRNA	chr2:136390332-136
ENSG0000(	2098	46.85368	chr2:309	ENSG00000279024		TEC	chr2:136230753-136
ENSG0000(	2098	46.85368	chr2:309	MANEALP1		Pseudoger	chr2:135897955-135
ENSG0000(	2098	46.85368	chr2:309	DARS1		protein_c	chr2:135905881-135
ENSG0000(	2098	46.85368	chr2:309	RPL15P5		Pseudoger	chr2:138278574-138
ENSG0000(	2093	46.74201	chr1:219	KCNK2		protein_c	chr1:215005775-215
ENSG0000(	2093	46.74201	chr1:219	ENSG00000272167		lncRNA	chr1:214028891-214
ENSG0000(	2093	46.74201	chr1:219	SMYD2		protein_c	chr1:214281102-214
ENSG0000(	2093	46.74201	chr1:219	USH2A-AS2		lncRNA	chr1:216072465-216
ENSG0000(	2093	46.74201	chr1:219	LINC00538		lncRNA	chr1:213924749-213

ENSG0000(	2093	46.74201	chr1:2197	ENSG00000223869		Pseudoger	chr1:214943123-214
ENSG0000(	2093	46.74201	chr1:2197	MRPS18BP1		Pseudoger	chr1:216201635-216
ENSG0000(	2093	46.74201	chr1:2197	CENPF	NCGv7	protein_c	chr1:214603195-214
ENSG0000(	2093	46.74201	chr1:2197	LINC02775		lncRNA	chr1:214051194-214
ENSG0000(	2093	46.74201	chr1:2197	ABHD17AP3		Pseudoger	chr1:214605470-214
ENSG0000(	2093	46.74201	chr1:2197	AL592063.1		smallRNA	chr1:213609696-213
ENSG0000(	2093	46.74201	chr1:2197	UBE2V1P13		Pseudoger	chr1:214612960-214
ENSG0000(	2093	46.74201	chr1:2197	ENSG00000229242		lncRNA	chr1:215886582-215
ENSG0000(	2093	46.74201	chr1:2197	PTPN14	NCGv7	protein_c	chr1:214348700-214
ENSG0000(	2093	46.74201	chr1:2197	USH2A	NCGv7	protein_c	chr1:215622891-216
ENSG0000(	2093	46.74201	chr1:2197	ENSG00000274895		lncRNA	chr1:213983793-213
ENSG0000(	2093	46.74201	chr1:2197	ENSG00000287008		lncRNA	chr1:214946909-214
ENSG0000(	2093	46.74201	chr1:2197	VDAC1P10		Pseudoger	chr1:215376484-215
ENSG0000(	2093	46.74201	chr1:2197	PROX1-AS1		lncRNA	chr1:213817751-213
ENSG0000(	2093	46.74201	chr1:2197	ENSG00000228255		lncRNA	chr1:213731416-213
ENSG0000(	2093	46.74201	chr1:2197	PROX1		protein_c	chr1:213983181-214
ENSG0000(	2093	46.74201	chr1:2197	KCTD3		protein_c	chr1:215567304-215
ENSG0000(	2093	46.74201	chr1:2197	ENSG00000282265		lncRNA	chr1:215393646-215
ENSG0000(	2093	46.74201	chr1:2197	USH2A-AS1		lncRNA	chr1:216194051-216
ENSG0000(	2093	46.74201	chr1:2197	ENSG00000228470		lncRNA	chr1:214344172-214
ENSG0000(	2093	46.74201	chr1:2197	RPL31P13		Pseudoger	chr1:213428708-213
ENSG0000(	2093	46.74201	chr1:2197	KRT18P12		Pseudoger	chr1:214532195-214
ENSG0000(	2093	46.74201	chr1:2197	SNORD116		smallRNA	chr1:215630026-215
ENSG0000(	2093	46.74201	chr1:2197	ENSG00000213036		Pseudoger	chr1:214482813-214
ENSG0000(	2093	46.74201	chr1:2197	ENSG00000225233		lncRNA	chr1:213492288-213
ENSG0000(	2093	46.74201	chr1:2197	GAPDHP24		Pseudoger	chr1:214870734-214
ENSG0000(	2090	46.67502	chr2:3094	AC012353.1		smallRNA	chr2:141638844-141
ENSG0000(	2088	46.63035	chr1:2197	H2BU2P		Pseudoger	chr1:228464213-228
ENSG0000(	2087	46.60802	chr1:1043	PDZK1		protein_c	chr1:145670851-145
ENSG0000(	2087	46.60802	chr1:1043	ANKRD35		protein_c	chr1:145866559-145
ENSG0000(	2087	46.60802	chr1:1043	ENSG00000244619		lncRNA	chr1:145892846-145
ENSG0000(	2087	46.60802	chr1:1043	WI2-925H4.1		lncRNA	chr1:145601945-145
ENSG0000(	2087	46.60802	chr1:1043	LIX1L		protein_c	chr1:145933422-145
ENSG0000(	2087	46.60802	chr1:1043	ANKRD34A		protein_c	chr1:145959440-145
ENSG0000(	2087	46.60802	chr1:1043	NUDT17		protein_c	chr1:145845629-145
ENSG0000(	2087	46.60802	chr1:1043	RBM8A	NCGv7	protein_c	chr1:145921555-145
ENSG0000(	2087	46.60802	chr1:1043	POLR3C		protein_c	chr1:145824052-145
ENSG0000(	2087	46.60802	chr1:1043	ENSG00000287374		lncRNA	chr1:145475606-145
ENSG0000(	2087	46.60802	chr1:1043	ENSG00000278431		lncRNA	chr1:145961387-145
ENSG0000(	2087	46.60802	chr1:1043	RNF115		protein_c	chr1:145738867-145
ENSG0000(	2087	46.60802	chr1:1043	GPR89A		protein_c	chr1:145607987-145
ENSG0000(	2087	46.60802	chr1:1043	ENSG00000289565		protein_c	chr1:145917713-145
ENSG0000(	2087	46.60802	chr1:1043	LIX1L-AS1		lncRNA	chr1:145926589-145
ENSG0000(	2087	46.60802	chr1:1043	PIAS3		protein_c	chr1:145848521-145
ENSG0000(	2087	46.60802	chr1:1043	GNRHR2		Pseudoger	chr1:145919012-145
ENSG0000(	2087	46.60802	chr1:1043	PEX11B		protein_c	chr1:145911349-145
ENSG0000(	2087	46.60802	chr1:1043	CD160		protein_c	chr1:145719470-145
ENSG0000(	2087	46.60802	chr1:1043	ITGA10		protein_c	chr1:145891207-145
ENSG0000(	2086	46.58569	chr2:3094	RNF14P1		Pseudoger	chr2:138412066-138
ENSG0000(	2086	46.58569	chr2:3094	YY1P2		Pseudoger	chr2:138897151-138
ENSG0000(	2086	46.58569	chr2:3094	RNU6-904P		smallRNA	chr2:141167257-141

ENSG0000(	2086	46.58569	chr2:309<	MRPS18BP2		Pseudoger	chr2:139668547-139
ENSG0000(	2086	46.58569	chr2:309<	ENSG00000241772		lncRNA	chr2:138569090-138
ENSG0000(	2086	46.58569	chr2:309<	LRRC57P1		Pseudoger	chr2:140898423-140
ENSG0000(	2086	46.58569	chr2:309<	ENSG00000223554		lncRNA	chr2:139824896-139
ENSG0000(	2086	46.58569	chr2:309<	NXPH2		protein_c	chr2:138669157-138
ENSG0000(	2086	46.58569	chr2:309<	COPRSP1		Pseudoger	chr2:140683360-140
ENSG0000(	2086	46.58569	chr2:309<	RPL9P13		Pseudoger	chr2:139732708-139
ENSG0000(	2086	46.58569	chr2:309<	ENSG00000230569		lncRNA	chr2:138470656-138
ENSG0000(	2086	46.58569	chr2:309<	MTCO1P44		Pseudoger	chr2:140216997-140
ENSG0000(	2086	46.58569	chr2:309<	MTND2P19		Pseudoger	chr2:140217651-140
ENSG0000(	2086	46.58569	chr2:309<	RN7SKP286		smallRNA	chr2:138863597-138
ENSG0000(	2086	46.58569	chr2:309<	AHCYP4		Pseudoger	chr2:138901433-138
ENSG0000(	2086	46.58569	chr2:309<	ENSG00000226939		lncRNA	chr2:139366165-139
ENSG0000(	2086	46.58569	chr2:309<	ENSG00000287147		lncRNA	chr2:138917204-138
ENSG0000(	2086	46.58569	chr2:309<	SNORA72		smallRNA	chr2:139511424-139
ENSG0000(	2086	46.58569	chr2:309<	ENSG00000286778		lncRNA	chr2:139323921-139
ENSG0000(	2086	46.58569	chr2:309<	ENSG00000229131		lncRNA	chr2:139469775-139
ENSG0000(	2086	46.58569	chr2:309<	RN7SL283P		smallRNA	chr2:139982684-139
ENSG0000(	2086	46.58569	chr2:309<	MTND1P27		Pseudoger	chr2:140220584-140
ENSG0000(	2086	46.58569	chr2:309<	SPOPL		protein_c	chr2:138501770-138
ENSG0000(	2086	46.58569	chr2:309<	YWHAEP5		Pseudoger	chr2:138288029-138
ENSG0000(	2086	46.58569	chr2:309<	LINC02631		lncRNA	chr2:138601599-138
ENSG0000(	2086	46.58569	chr2:309<	RPS16P3		Pseudoger	chr2:141208082-141
ENSG0000(	2086	46.58569	chr2:309<	ENSG00000260059		lncRNA	chr2:138599663-138
ENSG0000(	2086	46.58569	chr2:309<	IDI1P1		Pseudoger	chr2:138307319-138
ENSG0000(	2086	46.58569	chr2:309<	LRP1B	NCGv7	protein_c	chr2:140231423-142
ENSG0000(	2086	46.58569	chr2:309<	Y_RNA		smallRNA	chr2:140992543-140
ENSG0000(	2086	46.58569	chr2:309<	LINC01853		lncRNA	chr2:140103583-140
ENSG0000(	2086	46.58569	chr2:309<	AC078851.2		smallRNA	chr2:139826692-139
ENSG0000(	2086	46.58569	chr2:309<	SPOPL-DT		lncRNA	chr2:138418284-138
ENSG0000(	2078	46.40703	chr1:2197	LINC01719		lncRNA	chr1:146052565-146
ENSG0000(	2078	46.40703	chr1:2197	ENSG00000280778		protein_c	chr1:145927257-145
ENSG0000(	2078	46.40703	chr1:2197	POLR3GL		protein_c	chr1:145964689-145
ENSG0000(	2078	46.40703	chr1:2197	RNVU1-6		smallRNA	chr1:146052080-146
ENSG0000(	2078	46.40703	chr1:2197	HJV		protein_c	chr1:146017467-146
ENSG0000(	2078	46.40703	chr1:2197	ENSG00000287190		lncRNA	chr1:146050440-146
ENSG0000(	2078	46.40703	chr1:2197	TXNIP	NCGv7	protein_c	chr1:145992434-145
ENSG0000(	2073	46.29536	chr1:1043	RNVU1-7		smallRNA	chr1:145465617-145
ENSG0000(	2071	46.2507	chr1:2197	HYDIN2		Pseudoger	chr1:146875321-146
ENSG0000(	2071	46.2507	chr1:2197	PDZK1P1		Pseudoger	chr1:147994301-148
ENSG0000(	2071	46.2507	chr1:2197	ENSG00000276509		lncRNA	chr1:146235805-146
ENSG0000(	2071	46.2507	chr1:2197	PDIA3P1		Pseudoger	chr1:147172744-147
ENSG0000(	2071	46.2507	chr1:2197	NOTCH2NLA		protein_c	chr1:146146202-146
ENSG0000(	2071	46.2507	chr1:2197	ENSG00000274415		lncRNA	chr1:147757185-147
ENSG0000(	2071	46.2507	chr1:2197	GPR89B		protein_c	chr1:147928393-147
ENSG0000(	2071	46.2507	chr1:2197	BCL9	NCGv7;AC	protein_c	chr1:147541501-147
ENSG0000(	2071	46.2507	chr1:2197	GJA8		protein_c	chr1:147902795-147
ENSG0000(	2071	46.2507	chr1:2197	RPL7AP15		Pseudoger	chr1:147223554-147
ENSG0000(	2071	46.2507	chr1:2197	LINC02804		lncRNA	chr1:148013203-148
ENSG0000(	2071	46.2507	chr1:2197	PFN1P8		Pseudoger	chr1:146957117-146
ENSG0000(	2071	46.2507	chr1:2197	RP11-337C18.9		lncRNA	chr1:147175602-147

ENSG0000(	2071	46.2507	chr1:2197	NOTCH2NLR		protein_c	chr1:146155128-146
ENSG0000(	2071	46.2507	chr1:2197	ENSG00000227242		Pseudoger	chr1:147019656-147
ENSG0000(	2071	46.2507	chr1:2197	ACP6		protein_c	chr1:147629652-147
ENSG0000(	2071	46.2507	chr1:2197	ENSG00000213226		Pseudoger	chr1:147319110-147
ENSG0000(	2071	46.2507	chr1:2197	NOTCH2NLB		protein_c	chr1:146149342-146
ENSG0000(	2071	46.2507	chr1:2197	ENSG00000234225		lncRNA	chr1:147001931-147
ENSG0000(	2071	46.2507	chr1:2197	ENSG00000234190		lncRNA	chr1:147777590-147
ENSG0000(	2071	46.2507	chr1:2197	ENSG00000290705		lncRNA	chr1:147993862-148
ENSG0000(	2071	46.2507	chr1:2197	ENSG00000227139		lncRNA	chr1:147697794-147
ENSG0000(	2071	46.2507	chr1:2197	Y_RNA		smallRNA	chr1:147420199-147
ENSG0000(	2071	46.2507	chr1:2197	ENSG00000289419		lncRNA	chr1:147608331-147
ENSG0000(	2071	46.2507	chr1:2197	ENSG00000273059		lncRNA	chr1:148011799-148
ENSG0000(	2071	46.2507	chr1:2197	NBPF13P		Pseudoger	chr1:147099482-147
ENSG0000(	2071	46.2507	chr1:2197	ENSG00000225603		Pseudoger	chr1:147050817-147
ENSG0000(	2071	46.2507	chr1:2197	RN7SL261P		smallRNA	chr1:147689256-147
ENSG0000(	2071	46.2507	chr1:2197	RNVU1-8		smallRNA	chr1:147079746-147
ENSG0000(	2071	46.2507	chr1:2197	NOTCH2NLC		protein_c	chr1:146148864-146
ENSG0000(	2071	46.2507	chr1:2197	OR13Z1P		Pseudoger	chr1:147419053-147
ENSG0000(	2071	46.2507	chr1:2197	ENSG00000286185		protein_c	chr1:146069621-146
ENSG0000(	2071	46.2507	chr1:2197	NBPF10		protein_c	chr1:146075000-146
ENSG0000(	2071	46.2507	chr1:2197	ENSG00000223728		Pseudoger	chr1:147840962-147
ENSG0000(	2071	46.2507	chr1:2197	RNVU1-8		smallRNA	chr1:147084616-147
ENSG0000(	2071	46.2507	chr1:2197	ENSG00000237188		lncRNA	chr1:147172755-147
ENSG0000(	2071	46.2507	chr1:2197	SSBL4P		Pseudoger	chr1:147082338-147
ENSG0000(	2071	46.2507	chr1:2197	OR13Z3P		Pseudoger	chr1:147482238-147
ENSG0000(	2071	46.2507	chr1:2197	ENSG00000288626		protein_c	chr1:147611590-147
ENSG0000(	2071	46.2507	chr1:2197	CHD1L	TAG;AC	protein_c	chr1:147242654-147
ENSG0000(	2071	46.2507	chr1:2197	LINC00624		lncRNA	chr1:147258885-147
ENSG0000(	2071	46.2507	chr1:2197	OR13Z2P		Pseudoger	chr1:147445579-147
ENSG0000(	2071	46.2507	chr1:2197	GJA5		protein_c	chr1:147756199-147
ENSG0000(	2071	46.2507	chr1:2197	GPR89C		protein_c	chr1:147953335-147
ENSG0000(	2071	46.2507	chr1:2197	FMO5	DriverDBv	protein_c	chr1:147175351-147
ENSG0000(	2071	46.2507	chr1:2197	PRKAB2	NCGv7	protein_c	chr1:147155106-147
ENSG0000(	2071	46.2507	chr1:2197	CCT8P1		Pseudoger	chr1:147203276-147
ENSG0000(	2071	46.2507	chr1:2197	RP11-458D21.6		lncRNA	chr1:146237251-146
ENSG0000(	2071	46.2507	chr1:2197	RNU1-129P		smallRNA	chr1:148014417-148
ENSG0000(	2061	46.02737	chr1:2197	LINC02767		lncRNA	chr1:207959292-207
ENSG0000(	2061	46.02737	chr1:2197	IL10		protein_c	chr1:206767602-206
ENSG0000(	2061	46.02737	chr1:2197	C4BPAP1		Pseudoger	chr1:207165496-207
ENSG0000(	2061	46.02737	chr1:2197	CD34		protein_c	chr1:207880972-207
ENSG0000(	2061	46.02737	chr1:2197	CD46		protein_c	chr1:207752037-207
ENSG0000(	2061	46.02737	chr1:2197	CR2		protein_c	chr1:207453024-207
ENSG0000(	2061	46.02737	chr1:2197	CD46P1		Pseudoger	chr1:207645234-207
ENSG0000(	2061	46.02737	chr1:2197	ENSG00000224114		Pseudoger	chr1:206695837-206
ENSG0000(	2061	46.02737	chr1:2197	C4BPAP2		Pseudoger	chr1:207225798-207
ENSG0000(	2061	46.02737	chr1:2197	IL19		protein_c	chr1:206770764-206
ENSG0000(	2061	46.02737	chr1:2197	ENSG00000285239		lncRNA	chr1:207709024-207
ENSG0000(	2061	46.02737	chr1:2197	ENSG00000243636		lncRNA	chr1:207179296-207
ENSG0000(	2061	46.02737	chr1:2197	ENSG00000283044		lncRNA	chr1:207401691-207
ENSG0000(	2061	46.02737	chr1:2197	CDCA4P3		Pseudoger	chr1:207658454-207
ENSG0000(	2061	46.02737	chr1:2197	ENSG00000286198		lncRNA	chr1:208244966-208

ENSG0000(	2061	46.02737	chr1:2197	LINC02942		lncRNA	chr1:207240122-207
ENSG0000(	2061	46.02737	chr1:2197	snoU13		smallRNA	chr1:207038694-207
ENSG0000(	2061	46.02737	chr1:2197	ENSG00000271680		Pseudoger	chr1:206905928-206
ENSG0000(	2061	46.02737	chr1:2197	ENSG00000226843		Pseudoger	chr1:208255290-208
ENSG0000(	2061	46.02737	chr1:2197	YOD1		protein_c	chr1:207043849-207
ENSG0000(	2061	46.02737	chr1:2197	CD55		protein_c	chr1:207321519-207
ENSG0000(	2061	46.02737	chr1:2197	Y_RNA		smallRNA	chr1:206747980-206
ENSG0000(	2061	46.02737	chr1:2197	PIGR	DriverDBv	protein_c	chr1:206928522-206
ENSG0000(	2061	46.02737	chr1:2197	IL24		protein_c	chr1:206897443-206
ENSG0000(	2061	46.02737	chr1:2197	CR1	NCGv7	protein_c	chr1:207496147-207
ENSG0000(	2061	46.02737	chr1:2197	IL20		protein_c	chr1:206865623-206
ENSG0000(	2061	46.02737	chr1:2197	CR1L		protein_c	chr1:207645113-207
ENSG0000(	2061	46.02737	chr1:2197	ENSG00000287220		lncRNA	chr1:208106102-208
ENSG0000(	2061	46.02737	chr1:2197	ENSG00000226565		Pseudoger	chr1:207150205-207
ENSG0000(	2061	46.02737	chr1:2197	FCAMR		protein_c	chr1:206957965-206
ENSG0000(	2061	46.02737	chr1:2197	FCMR		protein_c	chr1:206903317-206
ENSG0000(	2061	46.02737	chr1:2197	ENSG00000289071		lncRNA	chr1:207822903-207
ENSG0000(	2061	46.02737	chr1:2197	PFKFB2	DriverDBv	protein_c	chr1:207034366-207
ENSG0000(	2061	46.02737	chr1:2197	ENSG00000226945		Pseudoger	chr1:206907619-206
ENSG0000(	2061	46.02737	chr1:2197	C4BPA		protein_c	chr1:207104233-207
ENSG0000(	2061	46.02737	chr1:2197	C4BPB	DriverDBv	protein_c	chr1:207088860-207
ENSG0000(	2061	46.02737	chr1:2197	ENSG00000285719		lncRNA	chr1:207909992-207
ENSG0000(	2061	46.02737	chr1:2197	AL691452.1		smallRNA	chr1:207515285-207
ENSG0000(	2061	46.02737	chr1:2197	MIR29B2CHG		lncRNA	chr1:207801518-207
ENSG0000(	2061	46.02737	chr1:2197	CDCA4P4		Pseudoger	chr1:207762584-207
ENSG0000(	2061	46.02737	chr1:2197	ENSG00000236911		lncRNA	chr1:207551925-207
ENSG0000(	2061	46.02737	chr1:2197	ENSG00000275392		lncRNA	chr1:207127010-207
ENSG0000(	2061	46.02737	chr1:2197	C1orf116		protein_c	chr1:207018522-207
ENSG0000(	2061	46.02737	chr1:2197	MAPKAPK2		protein_c	chr1:206684905-206
ENSG0000(	2061	46.02737	chr1:2197	PLXNA2	NCGv7	protein_c	chr1:208022242-208
ENSG0000(	2057	45.93804	chr2:3094	POLR2DP1		Pseudoger	chr2:112995517-112
ENSG0000(	2050	45.78171	chr1:1142	ENSG00000226166		Pseudoger	chr1:12692909-1269
ENSG0000(	2031	45.3574	chr1:2197	RP11-353N4.3		Pseudoger	chr1:149693026-149
ENSG0000(	2017	45.04474	chr1:2197	ETV3L		protein_c	chr1:157092043-157
ENSG0000(	2017	45.04474	chr1:2197	ENSG00000289593		lncRNA	chr1:156504660-156
ENSG0000(	2017	45.04474	chr1:2197	HDGF		protein_c	chr1:156742109-156
ENSG0000(	2017	45.04474	chr1:2197	ENSG00000233712		Pseudoger	chr1:157636300-157
ENSG0000(	2017	45.04474	chr1:2197	MRPS21P2		Pseudoger	chr1:157861197-157
ENSG0000(	2017	45.04474	chr1:2197	CRABP2		protein_c	chr1:156699606-156
ENSG0000(	2017	45.04474	chr1:2197	ISG20L2		protein_c	chr1:156721891-156
ENSG0000(	2017	45.04474	chr1:2197	PRCC	AC	protein_c	chr1:156750610-156
ENSG0000(	2017	45.04474	chr1:2197	FCRL5		protein_c	chr1:157513377-157
ENSG0000(	2017	45.04474	chr1:2197	ENSG00000272405		lncRNA	chr1:156641666-156
ENSG0000(	2017	45.04474	chr1:2197	CD5L		protein_c	chr1:157830911-157
ENSG0000(	2017	45.04474	chr1:2197	ENSG00000285570		lncRNA	chr1:156689676-156
ENSG0000(	2017	45.04474	chr1:2197	MRPL24		protein_c	chr1:156737303-156
ENSG0000(	2017	45.04474	chr1:2197	METTL25B		protein_c	chr1:156728442-156
ENSG0000(	2017	45.04474	chr1:2197	AL365181.1		smallRNA	chr1:156587856-156
ENSG0000(	2017	45.04474	chr1:2197	ENSG00000286151		Pseudoger	chr1:157400927-157
ENSG0000(	2017	45.04474	chr1:2197	ENSG00000272971		lncRNA	chr1:156614742-156
ENSG0000(	2017	45.04474	chr1:2197	MIR765		smallRNA	chr1:156936131-156

ENSG0000(	2017	45.04474	chr1:2197	FCRL2		protein_c	chr1:157745733-157
ENSG0000(	2017	45.04474	chr1:2197	RP11-85G21.2		lncRNA	chr1:157287703-157
ENSG0000(	2017	45.04474	chr1:2197	FCRL4	NCGv7	protein_c	chr1:157573747-157
ENSG0000(	2017	45.04474	chr1:2197	ENSG00000286005		Pseudoger	chr1:157437484-157
ENSG0000(	2017	45.04474	chr1:2197	LRRC71		protein_c	chr1:156920632-156
ENSG0000(	2017	45.04474	chr1:2197	ENSG00000286073		Pseudoger	chr1:157287267-157
ENSG0000(	2017	45.04474	chr1:2197	FCRL3		protein_c	chr1:157674321-157
ENSG0000(	2017	45.04474	chr1:2197	GPATCH4	NCGv7	protein_c	chr1:156594301-156
ENSG0000(	2017	45.04474	chr1:2197	ENSG00000236731		Pseudoger	chr1:157629939-157
ENSG0000(	2017	45.04474	chr1:2197	CYCSP52		Pseudoger	chr1:157128362-157
ENSG0000(	2017	45.04474	chr1:2197	ENSG00000229953		lncRNA	chr1:156646507-156
ENSG0000(	2017	45.04474	chr1:2197	FCRL1	NCGv7	protein_c	chr1:157794403-157
ENSG0000(	2017	45.04474	chr1:2197	ENSG00000228239		lncRNA	chr1:157232231-157
ENSG0000(	2017	45.04474	chr1:2197	ENSG00000231700		Pseudoger	chr1:157709086-157
ENSG0000(	2017	45.04474	chr1:2197	NAXE		protein_c	chr1:156591756-156
ENSG0000(	2017	45.04474	chr1:2197	RN7SL612P		smallRNA	chr1:156985757-156
ENSG0000(	2017	45.04474	chr1:2197	INSRR		protein_c	chr1:156840063-156
ENSG0000(	2017	45.04474	chr1:2197	Y_RNA		smallRNA	chr1:156484098-156
ENSG0000(	2017	45.04474	chr1:2197	SH2D2A		protein_c	chr1:156806243-156
ENSG0000(	2017	45.04474	chr1:2197	IQGAP3	DriverDB\	protein_c	chr1:156525405-156
ENSG0000(	2017	45.04474	chr1:2197	ENSG00000290592		lncRNA	chr1:157171116-157
ENSG0000(	2017	45.04474	chr1:2197	TTC24		protein_c	chr1:156579723-156
ENSG0000(	2017	45.04474	chr1:2197	ENSG00000229961		Pseudoger	chr1:157182860-157
ENSG0000(	2017	45.04474	chr1:2197	MEF2D	NCGv7	protein_c	chr1:156463727-156
ENSG0000(	2017	45.04474	chr1:2197	ETV3	NCGv7;AC	protein_c	chr1:157121191-157
ENSG0000(	2017	45.04474	chr1:2197	NES	NCGv7	protein_c	chr1:156668763-156
ENSG0000(	2017	45.04474	chr1:2197	ENSG00000287624		lncRNA	chr1:156768105-156
ENSG0000(	2017	45.04474	chr1:2197	LINC02772		lncRNA	chr1:157273760-157
ENSG0000(	2017	45.04474	chr1:2197	VDAC1P9		Pseudoger	chr1:157724180-157
ENSG0000(	2017	45.04474	chr1:2197	ENSG00000284592		Pseudoger	chr1:157204779-157
ENSG0000(	2017	45.04474	chr1:2197	BCAN-AS1		lncRNA	chr1:156637783-156
ENSG0000(	2017	45.04474	chr1:2197	snoU13		smallRNA	chr1:156529330-156
ENSG0000(	2017	45.04474	chr1:2197	ENSG00000237588		lncRNA	chr1:156687695-156
ENSG0000(	2017	45.04474	chr1:2197	ENSG00000260460		lncRNA	chr1:156509854-156
ENSG0000(	2017	45.04474	chr1:2197	HAPLN2		protein_c	chr1:156619331-156
ENSG0000(	2017	45.04474	chr1:2197	ARHGEF11	NCGv7	protein_c	chr1:156934840-157
ENSG0000(	2017	45.04474	chr1:2197	ENSG00000223356		lncRNA	chr1:156712212-156
ENSG0000(	2017	45.04474	chr1:2197	KRT8P45		Pseudoger	chr1:157073257-157
ENSG0000(	2017	45.04474	chr1:2197	NTRK1	NCGv7;AC	protein_c	chr1:156815640-156
ENSG0000(	2017	45.04474	chr1:2197	ENSG00000236957		Pseudoger	chr1:157925065-157
ENSG0000(	2017	45.04474	chr1:2197	ENSG00000288835		lncRNA	chr1:156503083-156
ENSG0000(	2017	45.04474	chr1:2197	PEAR1	NCGv7	protein_c	chr1:156893698-156
ENSG0000(	2017	45.04474	chr1:2197	ENSG00000227217		lncRNA	chr1:157691762-157
ENSG0000(	2017	45.04474	chr1:2197	ENSG00000291226		lncRNA	chr1:157925974-157
ENSG0000(	2017	45.04474	chr1:2197	BCAN		protein_c	chr1:156641390-156
ENSG0000(	2017	45.04474	chr1:2197	SMU1P1		Pseudoger	chr1:157059232-157
ENSG0000(	2006	44.79908	chr1:2197	ENSG00000289700		protein_c	chr1:209779629-209
ENSG0000(	2006	44.79908	chr1:2197	ENSG00000223649		lncRNA	chr1:211492255-211
ENSG0000(	2006	44.79908	chr1:2197	ENSG00000234004		Pseudoger	chr1:211173488-211
ENSG0000(	2006	44.79908	chr1:2197	UBBP2		Pseudoger	chr1:217850403-217
ENSG0000(	2006	44.79908	chr1:2197	ENSG00000288738		lncRNA	chr1:211605839-211

ENSG0000(	2006	44.79908	chr1:2197	LINC02608		lncRNA	chr1:212180141-212
ENSG0000(	2006	44.79908	chr1:2197	LINC02771		lncRNA	chr1:212466699-212
ENSG0000(	2006	44.79908	chr1:2197	RP11-61J19.4		lncRNA	chr1:212557833-212
ENSG0000(	2006	44.79908	chr1:2197	ADORA2BP1		Pseudoger	chr1:209744373-209
ENSG0000(	2006	44.79908	chr1:2197	Y_RNA		smallRNA	chr1:211803017-211
ENSG0000(	2006	44.79908	chr1:2197	ENSG00000236905		Pseudoger	chr1:212824027-212
ENSG0000(	2006	44.79908	chr1:2197	AL356310.1		smallRNA	chr1:211653456-211
ENSG0000(	2006	44.79908	chr1:2197	FDPSP8		Pseudoger	chr1:211660189-211
ENSG0000(	2006	44.79908	chr1:2197	GARIN4		protein_c	chr1:212624474-212
ENSG0000(	2006	44.79908	chr1:2197	FLVCR1-DT		lncRNA	chr1:212852105-212
ENSG0000(	2006	44.79908	chr1:2197	SNX25P1		Pseudoger	chr1:211417025-211
ENSG0000(	2006	44.79908	chr1:2197	ENSG00000287902		lncRNA	chr1:209531216-209
ENSG0000(	2006	44.79908	chr1:2197	ATF3	AC	protein_c	chr1:212565334-212
ENSG0000(	2006	44.79908	chr1:2197	ENSG00000235862		lncRNA	chr1:212624284-212
ENSG0000(	2006	44.79908	chr1:2197	ENSG00000229016		Pseudoger	chr1:218301262-218
ENSG0000(	2006	44.79908	chr1:2197	ENSG00000277007		lncRNA	chr1:219270774-219
ENSG0000(	2006	44.79908	chr1:2197	LINC02769		lncRNA	chr1:208626741-208
ENSG0000(	2006	44.79908	chr1:2197	RPS6KC1		protein_c	chr1:213051233-213
ENSG0000(	2006	44.79908	chr1:2197	TATDN3		protein_c	chr1:212791828-212
ENSG0000(	2006	44.79908	chr1:2197	RNU5A-8P		smallRNA	chr1:210374154-210
ENSG0000(	2006	44.79908	chr1:2197	SNORA26		smallRNA	chr1:212025561-212
ENSG0000(	2006	44.79908	chr1:2197	ENSG00000229832		lncRNA	chr1:212357418-212
ENSG0000(	2006	44.79908	chr1:2197	TGFB2-AS1		lncRNA	chr1:218344190-218
ENSG0000(	2006	44.79908	chr1:2197	ENSG00000287033		lncRNA	chr1:211376804-211
ENSG0000(	2006	44.79908	chr1:2197	MIR205HG		lncRNA	chr1:209428817-209
ENSG0000(	2006	44.79908	chr1:2197	ESRRG	NCGv7	protein_c	chr1:216503246-217
ENSG0000(	2006	44.79908	chr1:2197	ENSG00000279333		TEC	chr1:210678315-210
ENSG0000(	2006	44.79908	chr1:2197	ATP5MC2P1		Pseudoger	chr1:209267798-209
ENSG0000(	2006	44.79908	chr1:2197	LINC02869		lncRNA	chr1:218459265-218
ENSG0000(	2006	44.79908	chr1:2197	LINC00210		lncRNA	chr1:217892900-217
ENSG0000(	2006	44.79908	chr1:2197	NEK2-DT		lncRNA	chr1:211675749-211
ENSG0000(	2006	44.79908	chr1:2197	ENSG00000223842		lncRNA	chr1:219222248-219
ENSG0000(	2006	44.79908	chr1:2197	RD3		protein_c	chr1:211476522-211
ENSG0000(	2006	44.79908	chr1:2197	ENSG00000226036		Pseudoger	chr1:212647296-212
ENSG0000(	2006	44.79908	chr1:2197	LINC01693		lncRNA	chr1:211639440-211
ENSG0000(	2006	44.79908	chr1:2197	BPNT2P1		Pseudoger	chr1:210462345-210
ENSG0000(	2006	44.79908	chr1:2197	LINC01696		lncRNA	chr1:209325392-209
ENSG0000(	2006	44.79908	chr1:2197	PRELID1P5		Pseudoger	chr1:211207239-211
ENSG0000(	2006	44.79908	chr1:2197	ENSG00000286775		lncRNA	chr1:218046943-218
ENSG0000(	2006	44.79908	chr1:2197	ENSG00000235182		Pseudoger	chr1:212853280-212
ENSG0000(	2006	44.79908	chr1:2197	ENSG00000287046		lncRNA	chr1:209107503-209
ENSG0000(	2006	44.79908	chr1:2197	SLC30A1		protein_c	chr1:211571568-211
ENSG0000(	2006	44.79908	chr1:2197	G0S2		protein_c	chr1:209675412-209
ENSG0000(	2006	44.79908	chr1:2197	BATF3		protein_c	chr1:212686417-212
ENSG0000(	2006	44.79908	chr1:2197	LPGAT1		protein_c	chr1:211743457-211
ENSG0000(	2006	44.79908	chr1:2197	RN7SL344P		smallRNA	chr1:211792113-211
ENSG0000(	2006	44.79908	chr1:2197	RNU6-423P		smallRNA	chr1:212692264-212
ENSG0000(	2006	44.79908	chr1:2197	ENSG00000288007		lncRNA	chr1:212559363-212
ENSG0000(	2006	44.79908	chr1:2197	ENSG00000287354		lncRNA	chr1:210362861-210
ENSG0000(	2006	44.79908	chr1:2197	SERTAD4-AS1		lncRNA	chr1:210231456-210
ENSG0000(	2006	44.79908	chr1:2197	ENSG00000261314		lncRNA	chr1:211583015-211

ENSG0000(	2006	44.79908	chr1:2197	TGFB2-OT1		lncRNA	chr1:218442626-218
ENSG0000(	2006	44.79908	chr1:2197	ENSG00000287343		lncRNA	chr1:209987333-209
ENSG0000(	2006	44.79908	chr1:2197	RPS26P13		Pseudoger	chr1:208697369-208
ENSG0000(	2006	44.79908	chr1:2197	LINC00467		lncRNA	chr1:211382736-211
ENSG0000(	2006	44.79908	chr1:2197	SPATA17-AS1		lncRNA	chr1:217781198-217
ENSG0000(	2006	44.79908	chr1:2197	LINC01710		lncRNA	chr1:218912757-218
ENSG0000(	2006	44.79908	chr1:2197	KCNH1-IT1		lncRNA	chr1:211132588-211
ENSG0000(	2006	44.79908	chr1:2197	LINC01717		lncRNA	chr1:208728665-208
ENSG0000(	2006	44.79908	chr1:2197	SPATA17		protein_c	chr1:217631324-217
ENSG0000(	2006	44.79908	chr1:2197	ENSG00000282718		lncRNA	chr1:212916787-212
ENSG0000(	2006	44.79908	chr1:2197	ENSG00000230063		lncRNA	chr1:212297448-212
ENSG0000(	2006	44.79908	chr1:2197	ENSG00000232537		lncRNA	chr1:209147220-209
ENSG0000(	2006	44.79908	chr1:2197	LYPLAL1		protein_c	chr1:219173869-219
ENSG0000(	2006	44.79908	chr1:2197	LAMB3		protein_c	chr1:209614870-209
ENSG0000(	2006	44.79908	chr1:2197	SPATA45		protein_c	chr1:212830141-212
ENSG0000(	2006	44.79908	chr1:2197	ENSG00000228081		Pseudoger	chr1:209173014-209
ENSG0000(	2006	44.79908	chr1:2197	TGFB2		protein_c	chr1:218345336-218
ENSG0000(	2006	44.79908	chr1:2197	GPATCH2		protein_c	chr1:217426992-217
ENSG0000(	2006	44.79908	chr1:2197	ENSG00000224260		lncRNA	chr1:209528455-209
ENSG0000(	2006	44.79908	chr1:2197	MIR3122		smallRNA	chr1:212077613-212
ENSG0000(	2006	44.79908	chr1:2197	U3		smallRNA	chr1:218541691-218
ENSG0000(	2006	44.79908	chr1:2197	LINC01774		lncRNA	chr1:208972454-208
ENSG0000(	2006	44.79908	chr1:2197	RCOR3		protein_c	chr1:211258377-211
ENSG0000(	2006	44.79908	chr1:2197	ENSG00000228792		lncRNA	chr1:211635865-211
ENSG0000(	2006	44.79908	chr1:2197	ENSG00000234915		lncRNA	chr1:212299495-212
ENSG0000(	2006	44.79908	chr1:2197	ENSG00000230714		lncRNA	chr1:218031835-218
ENSG0000(	2006	44.79908	chr1:2197	RIMKLBP2		Pseudoger	chr1:219199914-219
ENSG0000(	2006	44.79908	chr1:2197	LPGAT1-AS1		lncRNA	chr1:211829636-211
ENSG0000(	2006	44.79908	chr1:2197	SNORA16B		smallRNA	chr1:212352816-212
ENSG0000(	2006	44.79908	chr1:2197	RPL23AP18		Pseudoger	chr1:212309051-212
ENSG0000(	2006	44.79908	chr1:2197	ENSG00000278684		Pseudoger	chr1:209001338-209
ENSG0000(	2006	44.79908	chr1:2197	ENSG00000223375		Pseudoger	chr1:218338541-218
ENSG0000(	2006	44.79908	chr1:2197	NEK2	NCGv7	protein_c	chr1:211658657-211
ENSG0000(	2006	44.79908	chr1:2197	RNU1-141P		smallRNA	chr1:218129795-218
ENSG0000(	2006	44.79908	chr1:2197	TRAF5	NCGv7	protein_c	chr1:211326615-211
ENSG0000(	2006	44.79908	chr1:2197	SERTAD4	DriverDB\	protein_c	chr1:210232796-210
ENSG0000(	2006	44.79908	chr1:2197	PACC1		protein_c	chr1:212363928-212
ENSG0000(	2006	44.79908	chr1:2197	LINC01740		lncRNA	chr1:212545694-212
ENSG0000(	2006	44.79908	chr1:2197	CAMK1G		protein_c	chr1:209583714-209
ENSG0000(	2006	44.79908	chr1:2197	LYPLAL1-DT		lncRNA	chr1:218976672-219
ENSG0000(	2006	44.79908	chr1:2197	NSL1		protein_c	chr1:212726153-212
ENSG0000(	2006	44.79908	chr1:2197	NENF	DriverDB\	protein_c	chr1:212432920-212
ENSG0000(	2006	44.79908	chr1:2197	LINC02773		lncRNA	chr1:212653305-212
ENSG0000(	2006	44.79908	chr1:2197	LINC01653		lncRNA	chr1:218043505-218
ENSG0000(	2006	44.79908	chr1:2197	C1orf74		protein_c	chr1:209779208-209
ENSG0000(	2006	44.79908	chr1:2197	TRAF3IP3	NCGv7	protein_c	chr1:209756032-209
ENSG0000(	2006	44.79908	chr1:2197	ENSG00000287445		lncRNA	chr1:212430269-212
ENSG0000(	2006	44.79908	chr1:2197	ENSG00000226868		lncRNA	chr1:211715928-211
ENSG0000(	2006	44.79908	chr1:2197	PPP2R5A	NCGv7	protein_c	chr1:212285410-212
ENSG0000(	2006	44.79908	chr1:2197	ENSG00000287157		lncRNA	chr1:210386657-210
ENSG0000(	2006	44.79908	chr1:2197	HSD11B1-AS1		lncRNA	chr1:209661356-209

ENSG0000(	2006	44.79908	chr1:2197	ENSG00000287676		lncRNA	chr1:219294982-219
ENSG0000(	2006	44.79908	chr1:2197	LINC01735		lncRNA	chr1:208606564-208
ENSG0000(	2006	44.79908	chr1:2197	RPL21P28		Pseudoger	chr1:212051524-212
ENSG0000(	2006	44.79908	chr1:2197	AC092017.1		smallRNA	chr1:211210989-211
ENSG0000(	2006	44.79908	chr1:2197	VASH2		protein_c	chr1:212950520-212
ENSG0000(	2006	44.79908	chr1:2197	ENSG00000229983		lncRNA	chr1:212168207-212
ENSG0000(	2006	44.79908	chr1:2197	ENSG00000233455		Pseudoger	chr1:210303684-210
ENSG0000(	2006	44.79908	chr1:2197	RN7SKP98		smallRNA	chr1:212099521-212
ENSG0000(	2006	44.79908	chr1:2197	RRP15		protein_c	chr1:218285293-218
ENSG0000(	2006	44.79908	chr1:2197	ENSG00000286213		lncRNA	chr1:212504178-212
ENSG0000(	2006	44.79908	chr1:2197	KCNH1		protein_c	chr1:210676823-211
ENSG0000(	2006	44.79908	chr1:2197	ENSG00000233626		Pseudoger	chr1:211936249-211
ENSG0000(	2006	44.79908	chr1:2197	ENSG00000283952		protein_c	chr1:211082872-211
ENSG0000(	2006	44.79908	chr1:2197	FLVCR1		protein_c	chr1:212858275-212
ENSG0000(	2006	44.79908	chr1:2197	IPO8P1		Pseudoger	chr1:210859177-210
ENSG0000(	2006	44.79908	chr1:2197	NXNP1		Pseudoger	chr1:218881600-218
ENSG0000(	2006	44.79908	chr1:2197	TFDP1P1		Pseudoger	chr1:209232196-209
ENSG0000(	2006	44.79908	chr1:2197	MIR4260		smallRNA	chr1:209623444-209
ENSG0000(	2006	44.79908	chr1:2197	ST13P19		Pseudoger	chr1:210265636-210
ENSG0000(	2006	44.79908	chr1:2197	DTL	NCGv7	protein_c	chr1:212035553-212
ENSG0000(	2006	44.79908	chr1:2197	INTS7	NCGv7;AC	protein_c	chr1:211940399-212
ENSG0000(	2006	44.79908	chr1:2197	HSD11B1		protein_c	chr1:209686178-209
ENSG0000(	2006	44.79908	chr1:2197	HHAT	NCGv7	protein_c	chr1:210328252-210
ENSG0000(	2006	44.79908	chr1:2197	SYT14		protein_c	chr1:209900923-210
ENSG0000(	2006	44.79908	chr1:2197	IRF6	NCGv7	protein_c	chr1:209785617-209
ENSG0000(	2006	44.79908	chr1:2197	LINC01698		lncRNA	chr1:209367662-209
ENSG0000(	2006	44.79908	chr1:2197	ANGEL2		protein_c	chr1:212992182-213
ENSG0000(	2006	44.79908	chr1:2197	UTP25		protein_c	chr1:209827972-209
ENSG0000(	2006	44.79908	chr1:2197	RN7SL512P		smallRNA	chr1:212615708-212
ENSG0000(	2006	44.79908	chr1:2197	ENSG00000284376		lncRNA	chr1:211108445-211
ENSG0000(	2006	44.79908	chr1:2197	ARPC3P2		Pseudoger	chr1:211442274-211
ENSG0000(	2006	44.79908	chr1:2197	ENSG00000236317		Pseudoger	chr1:212855175-212
ENSG0000(	2001	44.68742	chr1:1043	RNU1-137P		smallRNA	chr1:145431527-145
ENSG0000(	2000	44.66509	chr1:1043	ENSG00000237503		Pseudoger	chr1:143846097-143
ENSG0000(	1999	44.64275	chr1:2197	ABHD17AP1		Pseudoger	chr1:148146394-148
ENSG0000(	1999	44.64275	chr1:2197	RNVU1-9		smallRNA	chr1:148038753-148
ENSG0000(	1999	44.64275	chr1:2197	ENSG00000224335		Pseudoger	chr1:148234273-148
ENSG0000(	1999	44.64275	chr1:2197	RNVU1-10		smallRNA	chr1:148362370-148
ENSG0000(	1999	44.64275	chr1:2197	RNVU1-2		smallRNA	chr1:148385829-148
ENSG0000(	1999	44.64275	chr1:2197	LINC01731		lncRNA	chr1:148271884-148
ENSG0000(	1999	44.64275	chr1:2197	LINC02805		lncRNA	chr1:148156139-148
ENSG0000(	1999	44.64275	chr1:2197	RNVU1-11		smallRNA	chr1:148388490-148
ENSG0000(	1999	44.64275	chr1:2197	NBPF24		protein_c	chr1:148102151-148
ENSG0000(	1999	44.64275	chr1:2197	ABHD17AP2		Pseudoger	chr1:148146395-148
ENSG0000(	1999	44.64275	chr1:2197	XXyac-YX155B6.6		lncRNA	chr1:148162787-148
ENSG0000(	1999	44.64275	chr1:2197	ENSG00000224481		lncRNA	chr1:148295895-148
ENSG0000(	1999	44.64275	chr1:2197	XXyac-YX155B6.2		Pseudoger	chr1:148080598-148
ENSG0000(	1999	44.64275	chr1:2197	RNU1-13P		smallRNA	chr1:148388490-148
ENSG0000(	1999	44.64275	chr1:2197	RNU1-135P		smallRNA	chr1:148385829-148
ENSG0000(	1999	44.64275	chr1:2197	RNU1-122P		smallRNA	chr1:148334612-148
ENSG0000(	1999	44.64275	chr1:2197	PFN1P5		Pseudoger	chr1:148129480-148

ENSG0000(	1999	44.64275	chr1:2197	RNU1-120P		smallRNA	chr1:148263476-148
ENSG0000(	1999	44.64275	chr1:2197	RNVU1-1		smallRNA	chr1:148362370-148
ENSG0000(	1999	44.64275	chr1:2197	RP6-206I17.2		lncRNA	chr1:148402453-148
ENSG0000(	1999	44.64275	chr1:2197	RP6-206I17.4		Pseudoger	chr1:148435103-148
ENSG0000(	1999	44.64275	chr1:2197	RNVU1-12		smallRNA	chr1:148402715-148
ENSG0000(	1999	44.64275	chr1:2197	ENSG00000272824		lncRNA	chr1:148358245-148
ENSG0000(	1999	44.64275	chr1:2197	RP11-495P10.7		lncRNA	chr1:148295792-148
ENSG0000(	1999	44.64275	chr1:2197	ENSG00000227700		Pseudoger	chr1:148246169-148
ENSG0000(	1999	44.64275	chr1:2197	ENSG00000235988		Pseudoger	chr1:148317683-148
ENSG0000(	1999	44.64275	chr1:2197	RP11-495P10.8		lncRNA	chr1:148290890-148
ENSG0000(	1999	44.64275	chr1:2197	RP6-206I17.3		lncRNA	chr1:148435062-148
ENSG0000(	1999	44.64275	chr1:2197	LINC02806		lncRNA	chr1:148295180-148
ENSG0000(	1999	44.64275	chr1:2197	RP4-565E6.1		lncRNA	chr1:148162787-148
ENSG0000(	1999	44.64275	chr1:2197	RP11-289I10.3		Pseudoger	chr1:148511083-148
ENSG0000(	1999	44.64275	chr1:2197	ENSG00000225871		Pseudoger	chr1:148435105-148
ENSG0000(	1999	44.64275	chr1:2197	PFN1P4		Pseudoger	chr1:148129497-148
ENSG0000(	1999	44.64275	chr1:2197	RNA5SP58		smallRNA	chr1:148193716-148
ENSG0000(	1999	44.64275	chr1:2197	ENSG00000291232		lncRNA	chr1:148402516-148
ENSG0000(	1999	44.64275	chr1:2197	RNA5SP57		smallRNA	chr1:148193716-148
ENSG0000(	1999	44.64275	chr1:2197	PDE4DIPP6		Pseudoger	chr1:148415258-148
ENSG0000(	1999	44.64275	chr1:2197	Y_RNA		smallRNA	chr1:148330271-148
ENSG0000(	1999	44.64275	chr1:2197	BX842679.1		protein_(	chr1:148159688-148
ENSG0000(	1999	44.64275	chr1:2197	ENSG00000228626		Pseudoger	chr1:148288001-148
ENSG0000(	1999	44.64275	chr1:2197	RNVU1-3		smallRNA	chr1:148402715-148
ENSG0000(	1999	44.64275	chr1:2197	PPIAL4G	NCGv7	protein_(	chr1:148482548-148
ENSG0000(	1996	44.57576	chr1:2197	AGT	DriverDB\	protein_(	chr1:230690776-230
ENSG0000(	1996	44.57576	chr1:2197	HLX-AS1		lncRNA	chr1:220832763-220
ENSG0000(	1996	44.57576	chr1:2197	RNA5S10		smallRNA	chr1:228630390-228
ENSG0000(	1996	44.57576	chr1:2197	LINC01736		lncRNA	chr1:230002372-230
ENSG0000(	1996	44.57576	chr1:2197	ENSG00000235817		Pseudoger	chr1:230612009-230
ENSG0000(	1996	44.57576	chr1:2197	TUBB8P9		Pseudoger	chr1:227506182-227
ENSG0000(	1996	44.57576	chr1:2197	RNA5S7		smallRNA	chr1:228623667-228
ENSG0000(	1996	44.57576	chr1:2197	RNA5S17		smallRNA	chr1:228646040-228
ENSG0000(	1996	44.57576	chr1:2197	URB2	NCGv7	protein_(	chr1:229626247-229
ENSG0000(	1996	44.57576	chr1:2197	ENSG00000288862		lncRNA	chr1:229407361-229
ENSG0000(	1996	44.57576	chr1:2197	snoU13		smallRNA	chr1:220137164-220
ENSG0000(	1996	44.57576	chr1:2197	ITPKB-AS1		lncRNA	chr1:226668897-226
ENSG0000(	1996	44.57576	chr1:2197	RNA5S13		smallRNA	chr1:228637096-228
ENSG0000(	1996	44.57576	chr1:2197	TRIM67-AS1		lncRNA	chr1:231184098-231
ENSG0000(	1996	44.57576	chr1:2197	TRIM67		protein_(	chr1:231162058-231
ENSG0000(	1996	44.57576	chr1:2197	TAF5L		protein_(	chr1:229593134-229
ENSG0000(	1996	44.57576	chr1:2197	C1orf198		protein_(	chr1:230837119-230
ENSG0000(	1996	44.57576	chr1:2197	CIAO2AP2		Pseudoger	chr1:228114997-228
ENSG0000(	1996	44.57576	chr1:2197	ENSG00000228525		Pseudoger	chr1:226958069-226
ENSG0000(	1996	44.57576	chr1:2197	ENSG00000234863		Pseudoger	chr1:220455154-220
ENSG0000(	1996	44.57576	chr1:2197	ABCB10		protein_(	chr1:229516582-229
ENSG0000(	1996	44.57576	chr1:2197	RPS27P5		Pseudoger	chr1:226781501-226
ENSG0000(	1996	44.57576	chr1:2197	LYPLAL1-AS1		lncRNA	chr1:219409039-219
ENSG0000(	1996	44.57576	chr1:2197	ENSG00000236230		lncRNA	chr1:222088806-222
ENSG0000(	1996	44.57576	chr1:2197	COG2		protein_(	chr1:230642481-230
ENSG0000(	1996	44.57576	chr1:2197	CAPN9	DriverDB\	protein_(	chr1:230747384-230

ENSG0000(	1996	44.57576	chr1:2197	ENSG00000228729		Pseudoger	chr1:227234269-227
ENSG0000(	1996	44.57576	chr1:2197	RNA5S1		smallRNA	chr1:228610268-228
ENSG0000(	1996	44.57576	chr1:2197	RNA5S8		smallRNA	chr1:228625909-228
ENSG0000(	1996	44.57576	chr1:2197	MIXL1		protein_c	chr1:226223618-226
ENSG0000(	1996	44.57576	chr1:2197	ENSG00000286719		lncRNA	chr1:224802959-224
ENSG0000(	1996	44.57576	chr1:2197	RNU4-57P		smallRNA	chr1:223373822-223
ENSG0000(	1996	44.57576	chr1:2197	RNA5SP19		Pseudoger	chr1:228555793-228
ENSG0000(	1996	44.57576	chr1:2197	RNU4-21P		smallRNA	chr1:229535064-229
ENSG0000(	1996	44.57576	chr1:2197	SNORA51		smallRNA	chr1:228652436-228
ENSG0000(	1996	44.57576	chr1:2197	BROX		protein_c	chr1:222712553-222
ENSG0000(	1996	44.57576	chr1:2197	RNA5S4		smallRNA	chr1:228616991-228
ENSG0000(	1996	44.57576	chr1:2197	RNU6-1008P		smallRNA	chr1:224305380-224
ENSG0000(	1996	44.57576	chr1:2197	ENSG00000287627		lncRNA	chr1:226538305-226
ENSG0000(	1996	44.57576	chr1:2197	RNA5S5		smallRNA	chr1:228619232-228
ENSG0000(	1996	44.57576	chr1:2197	ZC3H11B		protein_c	chr1:219608012-219
ENSG0000(	1996	44.57576	chr1:2197	ENSG00000272750		lncRNA	chr1:222658867-222
ENSG0000(	1996	44.57576	chr1:2197	ENSG00000228625		lncRNA	chr1:227178333-227
ENSG0000(	1996	44.57576	chr1:2197	ZNF847P		Pseudoger	chr1:227696892-227
ENSG0000(	1996	44.57576	chr1:2197	BTNL10	DriverDB\	Pseudoger	chr1:228510425-228
ENSG0000(	1996	44.57576	chr1:2197	ENSG00000286773		lncRNA	chr1:228329467-228
ENSG0000(	1996	44.57576	chr1:2197	ENSG00000225656		lncRNA	chr1:230823641-230
ENSG0000(	1996	44.57576	chr1:2197	RPL23AP15		Pseudoger	chr1:228449163-228
ENSG0000(	1996	44.57576	chr1:2197	RNA5SP79		Pseudoger	chr1:230820250-230
ENSG0000(	1996	44.57576	chr1:2197	C1orf115	DriverDB\	protein_c	chr1:220690363-220
ENSG0000(	1996	44.57576	chr1:2197	ENSG00000272823		lncRNA	chr1:220828676-220
ENSG0000(	1996	44.57576	chr1:2197	RAB3GAP2		protein_c	chr1:220148293-220
ENSG0000(	1996	44.57576	chr1:2197	BPNT1		protein_c	chr1:220057482-220
ENSG0000(	1996	44.57576	chr1:2197	LINC02815		lncRNA	chr1:229022773-229
ENSG0000(	1996	44.57576	chr1:2197	ENSG00000282418		lncRNA	chr1:225465021-225
ENSG0000(	1996	44.57576	chr1:2197	ENSG00000282564		lncRNA	chr1:230426491-230
ENSG0000(	1996	44.57576	chr1:2197	ENSG00000279306		TEC	chr1:228486188-228
ENSG0000(	1996	44.57576	chr1:2197	ENSG00000242757		Pseudoger	chr1:227430526-227
ENSG0000(	1996	44.57576	chr1:2197	LINC01703		lncRNA	chr1:226083590-226
ENSG0000(	1996	44.57576	chr1:2197	ENSG00000255835		protein_c	chr1:225886696-225
ENSG0000(	1996	44.57576	chr1:2197	RNA5S3		smallRNA	chr1:228614750-228
ENSG0000(	1996	44.57576	chr1:2197	ACBD3		protein_c	chr1:226144679-226
ENSG0000(	1996	44.57576	chr1:2197	MIR3620		smallRNA	chr1:228097263-228
ENSG0000(	1996	44.57576	chr1:2197	LINC02779		lncRNA	chr1:220485104-220
ENSG0000(	1996	44.57576	chr1:2197	LINC02813		lncRNA	chr1:224766324-224
ENSG0000(	1996	44.57576	chr1:2197	RPS15AP12		Pseudoger	chr1:220143964-220
ENSG0000(	1996	44.57576	chr1:2197	snoU13		smallRNA	chr1:219987511-219
ENSG0000(	1996	44.57576	chr1:2197	STUM		protein_c	chr1:226548764-226
ENSG0000(	1996	44.57576	chr1:2197	IBA57-DT		lncRNA	chr1:228164086-228
ENSG0000(	1996	44.57576	chr1:2197	RAB4A		protein_c	chr1:229271062-229
ENSG0000(	1996	44.57576	chr1:2197	LINC01352		lncRNA	chr1:220829255-220
ENSG0000(	1996	44.57576	chr1:2197	ENSG00000238232		lncRNA	chr1:219557192-219
ENSG0000(	1996	44.57576	chr1:2197	XRCC6P3		Pseudoger	chr1:220313945-220
ENSG0000(	1996	44.57576	chr1:2197	ENSG00000276997		lncRNA	chr1:222477252-222
ENSG0000(	1996	44.57576	chr1:2197	RN7SKP276		smallRNA	chr1:229410500-229
ENSG0000(	1996	44.57576	chr1:2197	CDKN2AIPNLP1		Pseudoger	chr1:226493188-226
ENSG0000(	1996	44.57576	chr1:2197	MIA3		protein_c	chr1:222618097-222

ENSG0000(	1996	44.57576	chr1:2197	RAB4A-AS1		lncRNA	chr1:229256892-229
ENSG0000(	1996	44.57576	chr1:2197	DISP1	NCGv7	protein_c	chr1:222815022-223
ENSG0000(	1996	44.57576	chr1:2197	MORF4L1P1		Pseudoger	chr1:220253570-220
ENSG0000(	1996	44.57576	chr1:2197	WNT3A		protein_c	chr1:228006998-228
ENSG0000(	1996	44.57576	chr1:2197	H3-3A-DT		lncRNA	chr1:226045561-226
ENSG0000(	1996	44.57576	chr1:2197	H3-4		protein_c	chr1:228424845-228
ENSG0000(	1996	44.57576	chr1:2197	AIDA		protein_c	chr1:222668013-222
ENSG0000(	1996	44.57576	chr1:2197	RNF187		protein_c	chr1:228487382-228
ENSG0000(	1996	44.57576	chr1:2197	AL592310.1		smallRNA	chr1:227645280-227
ENSG0000(	1996	44.57576	chr1:2197	ENSG00000288999		lncRNA	chr1:223846081-223
ENSG0000(	1996	44.57576	chr1:2197	RPL7AP81		Pseudoger	chr1:220448516-220
ENSG0000(	1996	44.57576	chr1:2197	MTARC1		protein_c	chr1:220786352-220
ENSG0000(	1996	44.57576	chr1:2197	PGBD5		protein_c	chr1:230314490-230
ENSG0000(	1996	44.57576	chr1:2197	ENSG00000290989		lncRNA	chr1:223992743-224
ENSG0000(	1996	44.57576	chr1:2197	ENSG00000287684		lncRNA	chr1:222743356-222
ENSG0000(	1996	44.57576	chr1:2197	GJC2	DriverDB\	protein_c	chr1:228149930-228
ENSG0000(	1996	44.57576	chr1:2197	ENSG00000230331		Pseudoger	chr1:229425020-229
ENSG0000(	1996	44.57576	chr1:2197	BTF3P9		Pseudoger	chr1:227434064-227
ENSG0000(	1996	44.57576	chr1:2197	ENSG00000280157		TEC	chr1:228121523-228
ENSG0000(	1996	44.57576	chr1:2197	ENSG00000291068		lncRNA	chr1:223951394-223
ENSG0000(	1996	44.57576	chr1:2197	LINC02257		lncRNA	chr1:221880981-221
ENSG0000(	1996	44.57576	chr1:2197	RN7SL276P		smallRNA	chr1:222708237-222
ENSG0000(	1996	44.57576	chr1:2197	TUBB8P10		Pseudoger	chr1:227493029-227
ENSG0000(	1996	44.57576	chr1:2197	TMEM78		lncRNA	chr1:229249636-229
ENSG0000(	1996	44.57576	chr1:2197	RNA5SP76		Pseudoger	chr1:219761789-219
ENSG0000(	1996	44.57576	chr1:2197	SEPTIN7P13		Pseudoger	chr1:223995895-224
ENSG0000(	1996	44.57576	chr1:2197	ENSG00000289142		lncRNA	chr1:220878225-220
ENSG0000(	1996	44.57576	chr1:2197	ENSG00000244137		lncRNA	chr1:230710698-230
ENSG0000(	1996	44.57576	chr1:2197	DUSP5P1		Pseudoger	chr1:228650241-228
ENSG0000(	1996	44.57576	chr1:2197	LINC02809		lncRNA	chr1:228073909-228
ENSG0000(	1996	44.57576	chr1:2197	RNA5S16		smallRNA	chr1:228643809-228
ENSG0000(	1996	44.57576	chr1:2197	ENSG00000288674		protein_c	chr1:226870184-226
ENSG0000(	1996	44.57576	chr1:2197	FAM133FP		Pseudoger	chr1:227598424-227
ENSG0000(	1996	44.57576	chr1:2197	ENSG00000248322		lncRNA	chr1:225936411-225
ENSG0000(	1996	44.57576	chr1:2197	TAF1A-AS1		lncRNA	chr1:222589825-222
ENSG0000(	1996	44.57576	chr1:2197	LINC02474		lncRNA	chr1:221966341-221
ENSG0000(	1996	44.57576	chr1:2197	ENSG00000242861		lncRNA	chr1:225840883-225
ENSG0000(	1996	44.57576	chr1:2197	AL596330.1		smallRNA	chr1:224722467-224
ENSG0000(	1996	44.57576	chr1:2197	RNA5SP77		Pseudoger	chr1:227561181-227
ENSG0000(	1996	44.57576	chr1:2197	ENSG00000231563		lncRNA	chr1:228407196-228
ENSG0000(	1996	44.57576	chr1:2197	RNA5S12		smallRNA	chr1:228634871-228
ENSG0000(	1996	44.57576	chr1:2197	RNA5S11		smallRNA	chr1:228632631-228
ENSG0000(	1996	44.57576	chr1:2197	ENSG00000237481		lncRNA	chr1:229319403-229
ENSG0000(	1996	44.57576	chr1:2197	DNAH14	NCGv7	protein_c	chr1:224896262-225
ENSG0000(	1996	44.57576	chr1:2197	HMGB1P26		Pseudoger	chr1:229705234-229
ENSG0000(	1996	44.57576	chr1:2197	HLX		protein_c	chr1:220879431-220
ENSG0000(	1996	44.57576	chr1:2197	LIN9		protein_c	chr1:226231149-226
ENSG0000(	1996	44.57576	chr1:2197	FTH1P2		Pseudoger	chr1:228687415-228
ENSG0000(	1996	44.57576	chr1:2197	OBSCN	NCGv7	protein_c	chr1:228208044-228
ENSG0000(	1996	44.57576	chr1:2197	PRSS38	NCGv7	protein_c	chr1:227815675-227
ENSG0000(	1996	44.57576	chr1:2197	TRIM11		protein_c	chr1:228393673-228

ENSG0000(	1996	44.57576	chr1:2197	ENAH		protein_c	chr1:225486765-225
ENSG0000(	1996	44.57576	chr1:2197	ITPKB-IT1		lncRNA	chr1:226656080-226
ENSG0000(	1996	44.57576	chr1:2197	RNU6-403P		smallRNA	chr1:221837334-221
ENSG0000(	1996	44.57576	chr1:2197	CCSAP		protein_c	chr1:229321011-229
ENSG0000(	1996	44.57576	chr1:2197	ENSG00000287895		lncRNA	chr1:228119149-228
ENSG0000(	1996	44.57576	chr1:2197	EPRS1	NCGv7	protein_c	chr1:219968600-220
ENSG0000(	1996	44.57576	chr1:2197	AL844165.1		smallRNA	chr1:230988989-230
ENSG0000(	1996	44.57576	chr1:2197	ENSG00000226211		Pseudoger	chr1:221133865-221
ENSG0000(	1996	44.57576	chr1:2197	PRELID3BP1		Pseudoger	chr1:220467954-220
ENSG0000(	1996	44.57576	chr1:2197	MIR215		smallRNA	chr1:220117853-220
ENSG0000(	1996	44.57576	chr1:2197	ENSG00000224407		lncRNA	chr1:230280312-230
ENSG0000(	1996	44.57576	chr1:2197	RHOU		protein_c	chr1:228735479-228
ENSG0000(	1996	44.57576	chr1:2197	TP53BP2	NCGv7	protein_c	chr1:223779893-223
ENSG0000(	1996	44.57576	chr1:2197	ENSG00000289602		lncRNA	chr1:225653285-225
ENSG0000(	1996	44.57576	chr1:2197	ACTA1		protein_c	chr1:229430365-229
ENSG0000(	1996	44.57576	chr1:2197	WNT9A		protein_c	chr1:227918656-227
ENSG0000(	1996	44.57576	chr1:2197	EPHX1	DriverDBv	protein_c	chr1:225810124-225
ENSG0000(	1996	44.57576	chr1:2197	ENSG00000270094		lncRNA	chr1:228394290-228
ENSG0000(	1996	44.57576	chr1:2197	ENSG00000287395		lncRNA	chr1:230889949-230
ENSG0000(	1996	44.57576	chr1:2197	ENSG00000226927		lncRNA	chr1:220359731-220
ENSG0000(	1996	44.57576	chr1:2197	MIR5008		smallRNA	chr1:227941590-227
ENSG0000(	1996	44.57576	chr1:2197	ENSG00000226920		lncRNA	chr1:229440284-229
ENSG0000(	1996	44.57576	chr1:2197	MARK1		protein_c	chr1:220528136-220
ENSG0000(	1996	44.57576	chr1:2197	NUCKS1P1		Pseudoger	chr1:227410617-227
ENSG0000(	1996	44.57576	chr1:2197	QRSL1P2		Pseudoger	chr1:222261833-222
ENSG0000(	1996	44.57576	chr1:2197	HHIPL2		protein_c	chr1:222522258-222
ENSG0000(	1996	44.57576	chr1:2197	ENSG00000289962		lncRNA	chr1:225467092-225
ENSG0000(	1996	44.57576	chr1:2197	DUSP10		protein_c	chr1:221701424-221
ENSG0000(	1996	44.57576	chr1:2197	SUSD4	DriverDBv	protein_c	chr1:223220819-223
ENSG0000(	1996	44.57576	chr1:2197	snoU13		smallRNA	chr1:230895432-230
ENSG0000(	1996	44.57576	chr1:2197	ISCA1P2		Pseudoger	chr1:229042171-229
ENSG0000(	1996	44.57576	chr1:2197	LINC02814		lncRNA	chr1:229087114-229
ENSG0000(	1996	44.57576	chr1:2197	LEFTY1		protein_c	chr1:225886282-225
ENSG0000(	1996	44.57576	chr1:2197	ENSG00000236773		Pseudoger	chr1:224175476-224
ENSG0000(	1996	44.57576	chr1:2197	LBR		protein_c	chr1:225401502-225
ENSG0000(	1996	44.57576	chr1:2197	LEFTY3P		Pseudoger	chr1:225803148-225
ENSG0000(	1996	44.57576	chr1:2197	ENSG00000286174		lncRNA	chr1:224703272-224
ENSG0000(	1996	44.57576	chr1:2197	PARP1		protein_c	chr1:226360210-226
ENSG0000(	1996	44.57576	chr1:2197	PSEN2		protein_c	chr1:226870184-226
ENSG0000(	1996	44.57576	chr1:2197	ENSG00000269934		lncRNA	chr1:228238241-228
ENSG0000(	1996	44.57576	chr1:2197	RN7SL467P		smallRNA	chr1:230729880-230
ENSG0000(	1996	44.57576	chr1:2197	PHB1P11		Pseudoger	chr1:223856579-223
ENSG0000(	1996	44.57576	chr1:2197	TTC13		protein_c	chr1:230906243-230
ENSG0000(	1996	44.57576	chr1:2197	ENSG00000286421		lncRNA	chr1:221555550-221
ENSG0000(	1996	44.57576	chr1:2197	GALNT2		protein_c	chr1:230057990-230
ENSG0000(	1996	44.57576	chr1:2197	ACTBP11		Pseudoger	chr1:223863726-223
ENSG0000(	1996	44.57576	chr1:2197	ENSG00000271475		Pseudoger	chr1:228776312-228
ENSG0000(	1996	44.57576	chr1:2197	SEPTIN14P17		Pseudoger	chr1:227980051-227
ENSG0000(	1996	44.57576	chr1:2197	CICP5		Pseudoger	chr1:223947605-223
ENSG0000(	1996	44.57576	chr1:2197	ENSG00000227585		Pseudoger	chr1:221549786-221
ENSG0000(	1996	44.57576	chr1:2197	RNU6-791P		smallRNA	chr1:222503632-222

ENSG0000(	1996	44.57576	chr1:2197	ENSG00000286398		lncRNA	chr1:221549362-221
ENSG0000(	1996	44.57576	chr1:2197	C1orf131		protein_c	chr1:231223763-231
ENSG0000(	1996	44.57576	chr1:2197	ENSG00000227496		lncRNA	chr1:225700264-225
ENSG0000(	1996	44.57576	chr1:2197	ENSG00000286389		lncRNA	chr1:227786753-227
ENSG0000(	1996	44.57576	chr1:2197	PYCR2		protein_c	chr1:225919877-225
ENSG0000(	1996	44.57576	chr1:2197	ENSG00000287205		lncRNA	chr1:227518738-227
ENSG0000(	1996	44.57576	chr1:2197	RN7SL837P		smallRNA	chr1:230894141-230
ENSG0000(	1996	44.57576	chr1:2197	ENSG00000223393		lncRNA	chr1:230868259-230
ENSG0000(	1996	44.57576	chr1:2197	LINC01737		lncRNA	chr1:230592660-230
ENSG0000(	1996	44.57576	chr1:2197	ZNF678		protein_c	chr1:227563543-227
ENSG0000(	1996	44.57576	chr1:2197	ENSG00000270287		Pseudoger	chr1:226411615-226
ENSG0000(	1996	44.57576	chr1:2197	ENSG00000237101		lncRNA	chr1:224219613-224
ENSG0000(	1996	44.57576	chr1:2197	TMEM63A	NCGv7	protein_c	chr1:225845536-225
ENSG0000(	1996	44.57576	chr1:2197	FAM177B	NCGv7	protein_c	chr1:222737202-222
ENSG0000(	1996	44.57576	chr1:2197	ENSG00000260505		lncRNA	chr1:220401122-220
ENSG0000(	1996	44.57576	chr1:2197	snoU13		smallRNA	chr1:226316061-226
ENSG0000(	1996	44.57576	chr1:2197	ENSG00000229595		Pseudoger	chr1:231117831-231
ENSG0000(	1996	44.57576	chr1:2197	ENSG00000233706		lncRNA	chr1:226992140-226
ENSG0000(	1996	44.57576	chr1:2197	ENSG00000286231		protein_c	chr1:220786990-220
ENSG0000(	1996	44.57576	chr1:2197	ENSG00000226601		lncRNA	chr1:223181144-223
ENSG0000(	1996	44.57576	chr1:2197	RNU6-1304P		smallRNA	chr1:225741275-225
ENSG0000(	1996	44.57576	chr1:2197	RN7SKP165		smallRNA	chr1:226445937-226
ENSG0000(	1996	44.57576	chr1:2197	ENSG00000229742		Pseudoger	chr1:224297646-224
ENSG0000(	1996	44.57576	chr1:2197	CICP26		Pseudoger	chr1:227975390-227
ENSG0000(	1996	44.57576	chr1:2197	ENSG00000287532		lncRNA	chr1:227123895-227
ENSG0000(	1996	44.57576	chr1:2197	AURKAP1		Pseudoger	chr1:220266706-220
ENSG0000(	1996	44.57576	chr1:2197	ENSG00000227625		Pseudoger	chr1:228134785-228
ENSG0000(	1996	44.57576	chr1:2197	RNA5SP78		Pseudoger	chr1:229549905-229
ENSG0000(	1996	44.57576	chr1:2197	NDUFA3P3		Pseudoger	chr1:225964179-225
ENSG0000(	1996	44.57576	chr1:2197	ARV1		protein_c	chr1:230978981-231
ENSG0000(	1996	44.57576	chr1:2197	ENSG00000233920		lncRNA	chr1:229223457-229
ENSG0000(	1996	44.57576	chr1:2197	HDAC1P2		Pseudoger	chr1:220625740-220
ENSG0000(	1996	44.57576	chr1:2197	RNU6-1319P		smallRNA	chr1:223976146-223
ENSG0000(	1996	44.57576	chr1:2197	NUP133-DT		lncRNA	chr1:229508369-229
ENSG0000(	1996	44.57576	chr1:2197	H2AW		protein_c	chr1:228434777-228
ENSG0000(	1996	44.57576	chr1:2197	ENSG00000288037		lncRNA	chr1:230878662-230
ENSG0000(	1996	44.57576	chr1:2197	TAF1A		protein_c	chr1:222557902-222
ENSG0000(	1996	44.57576	chr1:2197	ENSG00000287525		lncRNA	chr1:227280449-227
ENSG0000(	1996	44.57576	chr1:2197	SLC30A10		protein_c	chr1:219685427-219
ENSG0000(	1996	44.57576	chr1:2197	ENSG00000227711		Pseudoger	chr1:227509028-227
ENSG0000(	1996	44.57576	chr1:2197	RPLP0P5		Pseudoger	chr1:220316667-220
ENSG0000(	1996	44.57576	chr1:2197	CNIH3-AS2		lncRNA	chr1:224608130-224
ENSG0000(	1996	44.57576	chr1:2197	RNA5S2		smallRNA	chr1:228612509-228
ENSG0000(	1996	44.57576	chr1:2197	RN7SKP49		smallRNA	chr1:224107282-224
ENSG0000(	1996	44.57576	chr1:2197	RNU6-1248P		smallRNA	chr1:223690051-223
ENSG0000(	1996	44.57576	chr1:2197	ENSG00000226643		lncRNA	chr1:222452738-222
ENSG0000(	1996	44.57576	chr1:2197	RNA5SP18		Pseudoger	chr1:228647912-228
ENSG0000(	1996	44.57576	chr1:2197	ENSG00000223570		Pseudoger	chr1:226188870-226
ENSG0000(	1996	44.57576	chr1:2197	ENSG00000236846		lncRNA	chr1:223144049-223
ENSG0000(	1996	44.57576	chr1:2197	AC092765.1		smallRNA	chr1:222013007-222
ENSG0000(	1996	44.57576	chr1:2197	RNU6-180P		smallRNA	chr1:229383128-229

ENSG0000(	1996	44.57576	chr1:2197	ENSG00000278180		Pseudoger	chr1:227490691-227
ENSG0000(	1996	44.57576	chr1:2197	SNAP47		protein_c	chr1:227728200-227
ENSG0000(	1996	44.57576	chr1:2197	SNORA36B		smallRNA	chr1:220200546-220
ENSG0000(	1996	44.57576	chr1:2197	C1orf35		protein_c	chr1:228100726-228
ENSG0000(	1996	44.57576	chr1:2197	RN7SL464P		smallRNA	chr1:220571743-220
ENSG0000(	1996	44.57576	chr1:2197	MIR194-1		smallRNA	chr1:220118157-220
ENSG0000(	1996	44.57576	chr1:2197	MIR1182		smallRNA	chr1:231019828-231
ENSG0000(	1996	44.57576	chr1:2197	H3-3A	NCGv7;AC	protein_c	chr1:226061851-226
ENSG0000(	1996	44.57576	chr1:2197	TLR5	NCGv7	protein_c	chr1:223109404-223
ENSG0000(	1996	44.57576	chr1:2197	COQ8A		protein_c	chr1:226940286-226
ENSG0000(	1996	44.57576	chr1:2197	ENSG00000232436		lncRNA	chr1:221508559-221
ENSG0000(	1996	44.57576	chr1:2197	ENSG00000229399		Pseudoger	chr1:222641414-222
ENSG0000(	1996	44.57576	chr1:2197	CNIH3-AS1		lncRNA	chr1:224717504-224
ENSG0000(	1996	44.57576	chr1:2197	snoU13		smallRNA	chr1:224336791-224
ENSG0000(	1996	44.57576	chr1:2197	MIR320B2		smallRNA	chr1:224257004-224
ENSG0000(	1996	44.57576	chr1:2197	FBXO28	NCGv7	protein_c	chr1:224114111-224
ENSG0000(	1996	44.57576	chr1:2197	ENSG00000227006		lncRNA	chr1:230258694-230
ENSG0000(	1996	44.57576	chr1:2197	ENSG00000289341		lncRNA	chr1:225999615-226
ENSG0000(	1996	44.57576	chr1:2197	MTARC2		protein_c	chr1:220748225-220
ENSG0000(	1996	44.57576	chr1:2197	ENSG00000289348		lncRNA	chr1:226349171-226
ENSG0000(	1996	44.57576	chr1:2197	DEGS1		protein_c	chr1:224175756-224
ENSG0000(	1996	44.57576	chr1:2197	snoU13		smallRNA	chr1:226304262-226
ENSG0000(	1996	44.57576	chr1:2197	RNA5S6		smallRNA	chr1:228621447-228
ENSG0000(	1996	44.57576	chr1:2197	SNAP47-AS1		lncRNA	chr1:227743831-227
ENSG0000(	1996	44.57576	chr1:2197	SDE2		protein_c	chr1:225982702-225
ENSG0000(	1996	44.57576	chr1:2197	AKR1B1P1		Pseudoger	chr1:224574434-224
ENSG0000(	1996	44.57576	chr1:2197	RNA5S9		smallRNA	chr1:228628148-228
ENSG0000(	1996	44.57576	chr1:2197	RNA5S14		smallRNA	chr1:228639337-228
ENSG0000(	1996	44.57576	chr1:2197	AC096643.1		smallRNA	chr1:219663377-219
ENSG0000(	1996	44.57576	chr1:2197	MIR4742		smallRNA	chr1:224398227-224
ENSG0000(	1996	44.57576	chr1:2197	ENSG00000289880		lncRNA	chr1:222742640-222
ENSG0000(	1996	44.57576	chr1:2197	H2BU1		protein_c	chr1:228458103-228
ENSG0000(	1996	44.57576	chr1:2197	ARF1		protein_c	chr1:228082708-228
ENSG0000(	1996	44.57576	chr1:2197	ENSG00000227934		Pseudoger	chr1:231021611-231
ENSG0000(	1996	44.57576	chr1:2197	LINC01655		lncRNA	chr1:221819842-221
ENSG0000(	1996	44.57576	chr1:2197	LINC02817		lncRNA	chr1:221330080-221
ENSG0000(	1996	44.57576	chr1:2197	YBX1P9		Pseudoger	chr1:226318015-226
ENSG0000(	1996	44.57576	chr1:2197	ENSG00000287259		lncRNA	chr1:226827711-226
ENSG0000(	1996	44.57576	chr1:2197	ENSG00000278467		lncRNA	chr1:223994262-223
ENSG0000(	1996	44.57576	chr1:2197	ENSG00000290037		lncRNA	chr1:229431366-229
ENSG0000(	1996	44.57576	chr1:2197	CAPN2		protein_c	chr1:223701593-223
ENSG0000(	1996	44.57576	chr1:2197	RNA5S15		smallRNA	chr1:228641568-228
ENSG0000(	1996	44.57576	chr1:2197	MRPL55		protein_c	chr1:228106679-228
ENSG0000(	1996	44.57576	chr1:2197	OBSCN-AS1		lncRNA	chr1:228203503-228
ENSG0000(	1996	44.57576	chr1:2197	WDR26		protein_c	chr1:224385146-224
ENSG0000(	1996	44.57576	chr1:2197	SNORA72		smallRNA	chr1:224179641-224
ENSG0000(	1996	44.57576	chr1:2197	ENSG00000275406		Pseudoger	chr1:226331999-226
ENSG0000(	1996	44.57576	chr1:2197	snoU13		smallRNA	chr1:222911844-222
ENSG0000(	1996	44.57576	chr1:2197	TRIM17		protein_c	chr1:228407935-228
ENSG0000(	1996	44.57576	chr1:2197	CCDC185		protein_c	chr1:223393415-223
ENSG0000(	1996	44.57576	chr1:2197	SNRPEP10		Pseudoger	chr1:223644110-223

ENSG0000(	1996	44.57576	chr1:2197	HMGN2P19		Pseudoger	chr1:229570532-229
ENSG0000(	1996	44.57576	chr1:2197	ENSG00000236636		Pseudoger	chr1:227264776-227
ENSG0000(	1996	44.57576	chr1:2197	ENSG00000226349		lncRNA	chr1:225710968-225
ENSG0000(	1996	44.57576	chr1:2197	ENSG00000271399		Pseudoger	chr1:228858010-228
ENSG0000(	1996	44.57576	chr1:2197	CNIH4		protein_c	chr1:224356858-224
ENSG0000(	1996	44.57576	chr1:2197	RNU6ATAC35P		lncRNA	chr1:220825620-220
ENSG0000(	1996	44.57576	chr1:2197	RPS3AP7		Pseudoger	chr1:226438564-226
ENSG0000(	1996	44.57576	chr1:2197	JMJD4		protein_c	chr1:227730425-227
ENSG0000(	1996	44.57576	chr1:2197	LINC01682		lncRNA	chr1:229812917-229
ENSG0000(	1996	44.57576	chr1:2197	GUK1		protein_c	chr1:228139962-228
ENSG0000(	1996	44.57576	chr1:2197	NVL		protein_c	chr1:224227334-224
ENSG0000(	1996	44.57576	chr1:2197	ENSG00000229930		Pseudoger	chr1:224030704-224
ENSG0000(	1996	44.57576	chr1:2197	LINC01641		lncRNA	chr1:227393554-227
ENSG0000(	1996	44.57576	chr1:2197	SRP9		protein_c	chr1:225777813-225
ENSG0000(	1996	44.57576	chr1:2197	MIR4666A		smallRNA	chr1:228462074-228
ENSG0000(	1996	44.57576	chr1:2197	DNAJB6P6		Pseudoger	chr1:224661173-224
ENSG0000(	1996	44.57576	chr1:2197	ENSG00000287315		lncRNA	chr1:228357012-228
ENSG0000(	1996	44.57576	chr1:2197	CDC42BPA		protein_c	chr1:226989865-227
ENSG0000(	1996	44.57576	chr1:2197	ENSG00000237193		Pseudoger	chr1:227482253-227
ENSG0000(	1996	44.57576	chr1:2197	BX323860.1		smallRNA	chr1:230049182-230
ENSG0000(	1996	44.57576	chr1:2197	CNIH3		protein_c	chr1:224434660-224
ENSG0000(	1996	44.57576	chr1:2197	IBA57		protein_c	chr1:228165804-228
ENSG0000(	1996	44.57576	chr1:2197	ENSG00000287338		lncRNA	chr1:223091872-223
ENSG0000(	1996	44.57576	chr1:2197	ENSG00000270104		lncRNA	chr1:228384114-228
ENSG0000(	1996	44.57576	chr1:2197	ITPKB	NCGv7	protein_c	chr1:226631690-226
ENSG0000(	1996	44.57576	chr1:2197	ENSG00000213028		Pseudoger	chr1:229688999-229
ENSG0000(	1996	44.57576	chr1:2197	NUP133	NCGv7	protein_c	chr1:229440259-229
ENSG0000(	1996	44.57576	chr1:2197	ENSG00000270110		lncRNA	chr1:228295549-228
ENSG0000(	1996	44.57576	chr1:2197	LINC02765		lncRNA	chr1:225447233-225
ENSG0000(	1996	44.57576	chr1:2197	U3		smallRNA	chr1:219962686-219
ENSG0000(	1996	44.57576	chr1:2197	FAM89A		protein_c	chr1:231018958-231
ENSG0000(	1996	44.57576	chr1:2197	CICP13		Pseudoger	chr1:222468094-222
ENSG0000(	1996	44.57576	chr1:2197	IARS2		protein_c	chr1:220094132-220
ENSG0000(	1996	44.57576	chr1:2197	ENSG00000232628		lncRNA	chr1:224208741-224
ENSG0000(	1996	44.57576	chr1:2197	NDUFB1P2		Pseudoger	chr1:222945725-222
ENSG0000(	1996	44.57576	chr1:2197	ACBD3-AS1		lncRNA	chr1:226148003-226
ENSG0000(	1996	44.57576	chr1:2197	LINC01705		lncRNA	chr1:222010825-222
ENSG0000(	1996	44.57576	chr1:2197	ENSG00000270598		Pseudoger	chr1:226127178-226
ENSG0000(	1996	44.57576	chr1:2197	LEFTY2		protein_c	chr1:225936598-225
ENSG0000(	1996	44.57576	chr1:2197	SNX2P1		Pseudoger	chr1:220207618-220
ENSG0000(	1993	44.50876	chr2:3094	UBBP3		Pseudoger	chr2:148863564-148
ENSG0000(	1984	44.30777	chr1:2197	ENSG00000233461		lncRNA	chr1:231520729-231
ENSG0000(	1984	44.30777	chr1:2197	LINC00582		lncRNA	chr1:231591292-231
ENSG0000(	1984	44.30777	chr1:2197	RNU5A-5P		smallRNA	chr1:231670635-231
ENSG0000(	1984	44.30777	chr1:2197	RNA5SP80		Pseudoger	chr1:231281414-231
ENSG0000(	1984	44.30777	chr1:2197	EXOC8	NCGv7	protein_c	chr1:231332753-231
ENSG0000(	1984	44.30777	chr1:2197	GNPAT		protein_c	chr1:231241207-231
ENSG0000(	1984	44.30777	chr1:2197	EGLN1		protein_c	chr1:231363751-231
ENSG0000(	1984	44.30777	chr1:2197	SNRPD2P2		Pseudoger	chr1:231475956-231
ENSG0000(	1984	44.30777	chr1:2197	ENSG00000287450		Pseudoger	chr1:231339421-231
ENSG0000(	1984	44.30777	chr1:2197	ENSG00000287856		protein_c	chr1:231363797-231

ENSG0000(	1984	44.30777	chr1:2197	TSNAX		protein_c	chr1:231528541-231
ENSG0000(	1984	44.30777	chr1:2197	SPRTN		protein_c	chr1:231337104-231
ENSG0000(	1981	44.24077	chr2:3094	ENSG00000270190		lncRNA	chr2:111266868-111
ENSG0000(	1981	44.24077	chr2:3094	PGM5P4		Pseudoger	chr2:113541937-113
ENSG0000(	1981	44.24077	chr2:3094	MIR4435-2HG		lncRNA	chr2:111006015-111
ENSG0000(	1981	44.24077	chr2:3094	RNU6-1180P		smallRNA	chr2:113086805-113
ENSG0000(	1981	44.24077	chr2:3094	DPP10-AS3		lncRNA	chr2:114828432-114
ENSG0000(	1981	44.24077	chr2:3094	HMGN2P23		Pseudoger	chr2:112981166-112
ENSG0000(	1981	44.24077	chr2:3094	ENSG00000287451		lncRNA	chr2:115835129-115
ENSG0000(	1981	44.24077	chr2:3094	CENPNP2		Pseudoger	chr2:111742586-111
ENSG0000(	1981	44.24077	chr2:3094	RTRAFP1		Pseudoger	chr2:111940302-111
ENSG0000(	1981	44.24077	chr2:3094	FOXD4L1	IntOGen-I	protein_c	chr2:113498665-113
ENSG0000(	1981	44.24077	chr2:3094	IGKV1OR2-108		protein_c	chr2:113406396-113
ENSG0000(	1981	44.24077	chr2:3094	RGPD8		protein_c	chr2:112368369-112
ENSG0000(	1981	44.24077	chr2:3094	CKAP2L		protein_c	chr2:112736349-112
ENSG0000(	1981	44.24077	chr2:3094	ENSG00000233479		lncRNA	chr2:113669166-113
ENSG0000(	1981	44.24077	chr2:3094	ENSG00000225744		lncRNA	chr2:111607841-111
ENSG0000(	1981	44.24077	chr2:3094	DPP10-AS2		lncRNA	chr2:114833830-114
ENSG0000(	1981	44.24077	chr2:3094	ENSG00000243389		lncRNA	chr2:112589040-112
ENSG0000(	1981	44.24077	chr2:3094	ACTR3		protein_c	chr2:113890063-113
ENSG0000(	1981	44.24077	chr2:3094	SLC35F5		protein_c	chr2:113705011-113
ENSG0000(	1981	44.24077	chr2:3094	ENSG00000230958		Pseudoger	chr2:111865798-111
ENSG0000(	1981	44.24077	chr2:3094	TTL	AC	protein_c	chr2:112482156-112
ENSG0000(	1981	44.24077	chr2:3094	ENSG00000286776		lncRNA	chr2:115079354-115
ENSG0000(	1981	44.24077	chr2:3094	RNU6-744P		smallRNA	chr2:113681111-113
ENSG0000(	1981	44.24077	chr2:3094	RPS14P4		Pseudoger	chr2:111295629-111
ENSG0000(	1981	44.24077	chr2:3094	ENSG00000279267		TEC	chr2:113605867-113
ENSG0000(	1981	44.24077	chr2:3094	AC110769.1		smallRNA	chr2:113990010-113
ENSG0000(	1981	44.24077	chr2:3094	RPL34P8		Pseudoger	chr2:111675026-111
ENSG0000(	1981	44.24077	chr2:3094	NT5DC4		protein_c	chr2:112721020-112
ENSG0000(	1981	44.24077	chr2:3094	RABL2A		protein_c	chr2:113627229-113
ENSG0000(	1981	44.24077	chr2:3094	SLC20A1		protein_c	chr2:112645939-112
ENSG0000(	1981	44.24077	chr2:3094	RP11-65I12.1		lncRNA	chr2:113240730-113
ENSG0000(	1981	44.24077	chr2:3094	FBLN7		protein_c	chr2:112138385-112
ENSG0000(	1981	44.24077	chr2:3094	ACOXL-AS1		lncRNA	chr2:111098345-111
ENSG0000(	1981	44.24077	chr2:3094	SNRPA1P1		Pseudoger	chr2:113657908-113
ENSG0000(	1981	44.24077	chr2:3094	ZC3H8		protein_c	chr2:112211529-112
ENSG0000(	1981	44.24077	chr2:3094	WASH9P		Pseudoger	chr2:113588555-113
ENSG0000(	1981	44.24077	chr2:3094	LINC02936		lncRNA	chr2:113677702-113
ENSG0000(	1981	44.24077	chr2:3094	CDK8P2		Pseudoger	chr2:112932625-112
ENSG0000(	1981	44.24077	chr2:3094	XIAPP3		Pseudoger	chr2:112853328-112
ENSG0000(	1981	44.24077	chr2:3094	ACTR3-AS1		lncRNA	chr2:113831049-113
ENSG0000(	1981	44.24077	chr2:3094	IL1A	NCGv7	protein_c	chr2:112773925-112
ENSG0000(	1981	44.24077	chr2:3094	DBF4P2		Pseudoger	chr2:111558413-111
ENSG0000(	1981	44.24077	chr2:3094	SOCAR		lncRNA	chr2:111491272-111
ENSG0000(	1981	44.24077	chr2:3094	snoU13		smallRNA	chr2:112271271-112
ENSG0000(	1981	44.24077	chr2:3094	LINC01191		lncRNA	chr2:113970719-114
ENSG0000(	1981	44.24077	chr2:3094	RPSAP23		Pseudoger	chr2:115468056-115
ENSG0000(	1981	44.24077	chr2:3094	ENSG00000278962		TEC	chr2:112188364-112
ENSG0000(	1981	44.24077	chr2:3094	ENSG00000234174		lncRNA	chr2:113171535-113
ENSG0000(	1981	44.24077	chr2:3094	ENSG00000229118		lncRNA	chr2:111265283-111

ENSG0000(	1981	44.24077	chr2:309⁄	LINC01961		lncRNA	chr2:113512222-113
ENSG0000(	1981	44.24077	chr2:309⁄	RN7SL297P		smallRNA	chr2:111930175-111
ENSG0000(	1981	44.24077	chr2:309⁄	ENSG00000287937		lncRNA	chr2:112817076-112
ENSG0000(	1981	44.24077	chr2:309⁄	WASH2P		Pseudoger	chr2:113588550-113
ENSG0000(	1981	44.24077	chr2:309⁄	ENSG00000235242		lncRNA	chr2:114122274-114
ENSG0000(	1981	44.24077	chr2:309⁄	FAM138B		lncRNA	chr2:113577382-113
ENSG0000(	1981	44.24077	chr2:309⁄	NDUFB4P6		Pseudoger	chr2:112286057-112
ENSG0000(	1981	44.24077	chr2:309⁄	snoU13		smallRNA	chr2:114281084-114
ENSG0000(	1981	44.24077	chr2:309⁄	BCL2L11	NCGv7	protein_c	chr2:111119378-111
ENSG0000(	1981	44.24077	chr2:309⁄	DDX11L2		Pseudoger	chr2:113599036-113
ENSG0000(	1981	44.24077	chr2:309⁄	ANAPC1		protein_c	chr2:111611639-111
ENSG0000(	1981	44.24077	chr2:309⁄	ENSG00000287165		lncRNA	chr2:113582354-113
ENSG0000(	1981	44.24077	chr2:309⁄	TMEM87B		protein_c	chr2:112055269-112
ENSG0000(	1981	44.24077	chr2:309⁄	IL1F10		protein_c	chr2:113067970-113
ENSG0000(	1981	44.24077	chr2:309⁄	IL1B	NCGv7	protein_c	chr2:112829751-112
ENSG0000(	1981	44.24077	chr2:309⁄	ENSG00000244063		lncRNA	chr2:113829390-113
ENSG0000(	1981	44.24077	chr2:309⁄	ENSG00000289035		lncRNA	chr2:113370392-113
ENSG0000(	1981	44.24077	chr2:309⁄	MIR4435-2		smallRNA	chr2:111321012-111
ENSG0000(	1981	44.24077	chr2:309⁄	AC079753.1		smallRNA	chr2:112844311-112
ENSG0000(	1981	44.24077	chr2:309⁄	ENSG00000236555		Pseudoger	chr2:112346011-112
ENSG0000(	1981	44.24077	chr2:309⁄	SLC30A6P1		Pseudoger	chr2:111952144-111
ENSG0000(	1981	44.24077	chr2:309⁄	MIR1302-3		smallRNA	chr2:113582959-113
ENSG0000(	1981	44.24077	chr2:309⁄	DDX11L17		Pseudoger	chr2:113599784-113
ENSG0000(	1981	44.24077	chr2:309⁄	ENSG00000286545		lncRNA	chr2:112239621-112
ENSG0000(	1981	44.24077	chr2:309⁄	ENSG00000227992		Pseudoger	chr2:111203964-111
ENSG0000(	1981	44.24077	chr2:309⁄	ENSG00000280228		TEC	chr2:112840328-112
ENSG0000(	1981	44.24077	chr2:309⁄	ENSG00000227842		Pseudoger	chr2:111717508-111
ENSG0000(	1981	44.24077	chr2:309⁄	VINAC1P		Pseudoger	chr2:112439312-112
ENSG0000(	1981	44.24077	chr2:309⁄	LINC02966		lncRNA	chr2:113325009-113
ENSG0000(	1981	44.24077	chr2:309⁄	ENSG00000286904		lncRNA	chr2:112039378-112
ENSG0000(	1981	44.24077	chr2:309⁄	ENSG00000286513		lncRNA	chr2:114114679-114
ENSG0000(	1981	44.24077	chr2:309⁄	Y_RNA		smallRNA	chr2:112579484-112
ENSG0000(	1981	44.24077	chr2:309⁄	DPP10-AS1		lncRNA	chr2:115126622-115
ENSG0000(	1981	44.24077	chr2:309⁄	ENSG00000272563		lncRNA	chr2:113432600-113
ENSG0000(	1981	44.24077	chr2:309⁄	PAFAH1B1P2		Pseudoger	chr2:111383752-111
ENSG0000(	1981	44.24077	chr2:309⁄	ZC3H6		protein_c	chr2:112275597-112
ENSG0000(	1981	44.24077	chr2:309⁄	PGM5P4-AS1		lncRNA	chr2:113526836-113
ENSG0000(	1981	44.24077	chr2:309⁄	ENSG00000279957		TEC	chr2:113904086-113
ENSG0000(	1981	44.24077	chr2:309⁄	SEPHS1P7		Pseudoger	chr2:114260050-114
ENSG0000(	1981	44.24077	chr2:309⁄	IL36B		protein_c	chr2:113022089-113
ENSG0000(	1981	44.24077	chr2:309⁄	MERTK	TAG;AC	protein_c	chr2:111898607-112
ENSG0000(	1981	44.24077	chr2:309⁄	IL37		protein_c	chr2:112911165-112
ENSG0000(	1981	44.24077	chr2:309⁄	MIR4782		smallRNA	chr2:113721290-113
ENSG0000(	1981	44.24077	chr2:309⁄	PSD4		protein_c	chr2:113157325-113
ENSG0000(	1981	44.24077	chr2:309⁄	snoU13		smallRNA	chr2:113848133-113
ENSG0000(	1981	44.24077	chr2:309⁄	MIR4771-1		smallRNA	chr2:111771061-111
ENSG0000(	1981	44.24077	chr2:309⁄	PAX8-AS1		lncRNA	chr2:113211421-113
ENSG0000(	1981	44.24077	chr2:309⁄	CBWD2		protein_c	chr2:113437691-113
ENSG0000(	1981	44.24077	chr2:309⁄	ENSG00000230499		lncRNA	chr2:111195963-111
ENSG0000(	1981	44.24077	chr2:309⁄	U3		smallRNA	chr2:114005441-114
ENSG0000(	1981	44.24077	chr2:309⁄	ENSG00000228251		lncRNA	chr2:112590796-112

ENSG0000(	1981	44.24077	chr2:309	DPP10		protein_c	chr2:114442299-115
ENSG0000(	1981	44.24077	chr2:309	ENSG00000243179		lncRNA	chr2:113979909-113
ENSG0000(	1981	44.24077	chr2:309	AC016745.1		protein_c	chr2:113447415-113
ENSG0000(	1981	44.24077	chr2:309	ENSG00000270019		lncRNA	chr2:113888203-113
ENSG0000(	1981	44.24077	chr2:309	RPL5P9		Pseudoger	chr2:110967834-110
ENSG0000(	1981	44.24077	chr2:309	ACRP1		Pseudoger	chr2:113667478-113
ENSG0000(	1981	44.24077	chr2:309	IL36G		protein_c	chr2:112973203-112
ENSG0000(	1981	44.24077	chr2:309	ENSG00000231747		Pseudoger	chr2:112621809-112
ENSG0000(	1981	44.24077	chr2:309	ENSG00000285016		lncRNA	chr2:111429324-111
ENSG0000(	1981	44.24077	chr2:309	IL36RN		protein_c	chr2:113058638-113
ENSG0000(	1981	44.24077	chr2:309	CHCHD5		protein_c	chr2:112584240-112
ENSG0000(	1981	44.24077	chr2:309	ENSG00000280878		lncRNA	chr2:111468810-111
ENSG0000(	1981	44.24077	chr2:309	ENSG00000287141		lncRNA	chr2:112061084-112
ENSG0000(	1981	44.24077	chr2:309	RPL23AP7		Pseudoger	chr2:113610502-113
ENSG0000(	1981	44.24077	chr2:309	PAX8	AC	protein_c	chr2:113215997-113
ENSG0000(	1981	44.24077	chr2:309	EEF1E1P1		Pseudoger	chr2:111887914-111
ENSG0000(	1981	44.24077	chr2:309	RNU2-41P		smallRNA	chr2:114420125-114
ENSG0000(	1981	44.24077	chr2:309	SLC20A1-DT		lncRNA	chr2:112641832-112
ENSG0000(	1981	44.24077	chr2:309	WASH2P		lncRNA	chr2:113583593-113
ENSG0000(	1981	44.24077	chr2:309	IL36A	NCGv7	protein_c	chr2:113005459-113
ENSG0000(	1981	44.24077	chr2:309	ENSG00000271590		lncRNA	chr2:111210995-111
ENSG0000(	1981	44.24077	chr2:309	POLR1B		protein_c	chr2:112541915-112
ENSG0000(	1981	44.24077	chr2:309	IL1RN		protein_c	chr2:113099315-113
ENSG0000(	1979	44.1961	chr1:104	RP11-289H16.1		lncRNA	chr1:144917169-144
ENSG0000(	1979	44.1961	chr1:104	FCGR1CP		Pseudoger	chr1:143874793-143
ENSG0000(	1979	44.1961	chr1:104	ENSG00000271644		Pseudoger	chr1:144965024-144
ENSG0000(	1979	44.1961	chr1:104	RNA5SP59		Pseudoger	chr1:143439604-143
ENSG0000(	1979	44.1961	chr1:104	ENSG00000270962		Pseudoger	chr1:143784376-143
ENSG0000(	1979	44.1961	chr1:104	FAM72C		protein_c	chr1:143944179-143
ENSG0000(	1979	44.1961	chr1:104	RNVU1-15		smallRNA	chr1:144412575-144
ENSG0000(	1979	44.1961	chr1:104	RNVU1-18		smallRNA	chr1:143729407-143
ENSG0000(	1979	44.1961	chr1:104	RPL22P5		Pseudoger	chr1:143929994-143
ENSG0000(	1979	44.1961	chr1:104	LINC01691		lncRNA	chr1:121573946-121
ENSG0000(	1979	44.1961	chr1:104	ENSG00000271439		Pseudoger	chr1:144401068-144
ENSG0000(	1979	44.1961	chr1:104	CR812485.1		smallRNA	chr1:143828767-143
ENSG0000(	1979	44.1961	chr1:104	ENSG00000223779		Pseudoger	chr1:143745249-143
ENSG0000(	1979	44.1961	chr1:104	RPL22P6		Pseudoger	chr1:143929994-143
ENSG0000(	1979	44.1961	chr1:104	ENSG00000227193		lncRNA	chr1:143905552-143
ENSG0000(	1979	44.1961	chr1:104	HIST2H3DP1		Pseudoger	chr1:143905555-143
ENSG0000(	1979	44.1961	chr1:104	RP11-403I13.7		lncRNA	chr1:143790010-143
ENSG0000(	1979	44.1961	chr1:104	ENSG00000223612		Pseudoger	chr1:145233001-145
ENSG0000(	1979	44.1961	chr1:104	PFN1P12		Pseudoger	chr1:143619994-143
ENSG0000(	1979	44.1961	chr1:104	RP11-343N15.5		lncRNA	chr1:121391395-121
ENSG0000(	1979	44.1961	chr1:104	RNVU1-16		smallRNA	chr1:145281115-145
ENSG0000(	1979	44.1961	chr1:104	RP6-137J22.3		Pseudoger	chr1:145269604-145
ENSG0000(	1979	44.1961	chr1:104	RNVU1-17		smallRNA	chr1:143699456-143
ENSG0000(	1979	44.1961	chr1:104	LINC02798		lncRNA	chr1:121396754-121
ENSG0000(	1979	44.1961	chr1:104	RP11-666A1.3		Pseudoger	chr1:144418677-144
ENSG0000(	1979	44.1961	chr1:104	PFN1P6		Pseudoger	chr1:144442605-144
ENSG0000(	1979	44.1961	chr1:104	PPIAL4E		protein_c	chr1:144372874-144
ENSG0000(	1979	44.1961	chr1:104	H2BP1		Pseudoger	chr1:143904287-143

ENSG0000(	1979	44.1961	chr1:104	ENSG00000223495		Pseudoger	chr1:143498784-143
ENSG0000(	1979	44.1961	chr1:104	ENSG00000264145		Pseudoger	chr1:143449570-143
ENSG0000(	1979	44.1961	chr1:104	ENSG00000203825		Pseudoger	chr1:143541768-143
ENSG0000(	1979	44.1961	chr1:104	FAM72D	NCGv7	protein_c	chr1:143955287-143
ENSG0000(	1979	44.1961	chr1:104	EMBP1		lncRNA	chr1:121519103-121
ENSG0000(	1979	44.1961	chr1:114	RN7SKP269		smallRNA	chr1:9947318-99476
ENSG0000(	1979	44.1961	chr1:104	FAM72B		protein_c	chr1:143955289-143
ENSG0000(	1979	44.1961	chr1:104	NKAIN1P1		Pseudoger	chr1:143487771-143
ENSG0000(	1979	44.1961	chr1:104	RNU1-143P		smallRNA	chr1:143791542-143
ENSG0000(	1979	44.1961	chr1:104	H3-7		protein_c	chr1:143894544-143
ENSG0000(	1979	44.1961	chr1:104	ENSG00000228826		lncRNA	chr1:121494329-121
ENSG0000(	1979	44.1961	chr1:104	DRD5P2		Pseudoger	chr1:143449275-143
ENSG0000(	1979	44.1961	chr1:104	ENSG00000275129		Pseudoger	chr1:144965024-144
ENSG0000(	1979	44.1961	chr1:104	RP11-666A1.5		lncRNA	chr1:144418122-144
ENSG0000(	1979	44.1961	chr1:104	FCGR1BP		Pseudoger	chr1:143876113-143
ENSG0000(	1979	44.1961	chr1:104	ENSG00000229002		Pseudoger	chr1:144472534-144
ENSG0000(	1979	44.1961	chr1:104	KMT2CP1		Pseudoger	chr1:143461247-143
ENSG0000(	1979	44.1961	chr1:104	ENSG00000230186		lncRNA	chr1:143905487-143
ENSG0000(	1979	44.1961	chr1:104	SRGAP2-AS1		lncRNA	chr1:121360156-121
ENSG0000(	1979	44.1961	chr1:104	KMT2CP3		Pseudoger	chr1:143461220-143
ENSG0000(	1979	44.1961	chr1:104	MTIF2P1		Pseudoger	chr1:121502344-121
ENSG0000(	1979	44.1961	chr1:104	H2BP1		lncRNA	chr1:143894527-143
ENSG0000(	1979	44.1961	chr1:104	RP11-403I13.9		lncRNA	chr1:143811359-143
ENSG0000(	1979	44.1961	chr1:104	LINC02799		lncRNA	chr1:143499186-143
ENSG0000(	1979	44.1961	chr1:104	ENSG00000290735		lncRNA	chr1:143875171-143
ENSG0000(	1979	44.1961	chr1:104	AL109844.1		smallRNA	chr1:143828767-143
ENSG0000(	1979	44.1961	chr1:104	SRGAP2C		Pseudoger	chr1:121365263-121
ENSG0000(	1979	44.1961	chr1:104	RP11-439A17.9		lncRNA	chr1:143877730-143
ENSG0000(	1979	44.1961	chr1:104	LSP1P5		lncRNA	chr1:143401427-143
ENSG0000(	1979	44.1961	chr1:104	ENSG00000272583		lncRNA	chr1:121518365-121
ENSG0000(	1979	44.1961	chr1:104	ENSG00000277702		Pseudoger	chr1:143419624-143
ENSG0000(	1979	44.1961	chr1:104	RP11-14N7.2		lncRNA	chr1:143401429-143
ENSG0000(	1979	44.1961	chr1:104	AL732363.1		smallRNA	chr1:143541645-143
ENSG0000(	1979	44.1961	chr1:104	ENSG00000289318		lncRNA	chr1:143972669-143
ENSG0000(	1979	44.1961	chr1:104	FAM91A3P		Pseudoger	chr1:143766540-143
ENSG0000(	1979	44.1961	chr1:104	EMBP1		Pseudoger	chr1:121519345-121
ENSG0000(	1979	44.1961	chr1:104	RNU1-92P		smallRNA	chr1:143720510-143
ENSG0000(	1979	44.1961	chr1:104	NBPF17P		Pseudoger	chr1:143595216-143
ENSG0000(	1979	44.1961	chr1:104	ENSG00000232721		lncRNA	chr1:143735983-143
ENSG0000(	1979	44.1961	chr1:104	RP11-439A17.7		lncRNA	chr1:143972630-143
ENSG0000(	1979	44.1961	chr1:104	RNU1-114P		smallRNA	chr1:143652050-143
ENSG0000(	1979	44.1961	chr1:104	RP11-439A17.10		lncRNA	chr1:143874925-143
ENSG0000(	1977	44.15144	chr1:219	NUDT4B		protein_c	chr1:148748773-148
ENSG0000(	1977	44.15144	chr1:219	ENSG00000271546		Pseudoger	chr1:148795796-148
ENSG0000(	1977	44.15144	chr1:219	SEC22B		protein_c	chr1:148770173-148
ENSG0000(	1977	44.15144	chr1:219	ENSG00000254539		lncRNA	chr1:149048575-149
ENSG0000(	1977	44.15144	chr1:219	NUDT4P2		Pseudoger	chr1:148748952-148
ENSG0000(	1977	44.15144	chr1:219	SEC22B2P		Pseudoger	chr1:148772639-148
ENSG0000(	1977	44.15144	chr1:219	ENSG00000272755		lncRNA	chr1:148865453-148
ENSG0000(	1977	44.15144	chr1:219	PFN1P3		Pseudoger	chr1:149084616-149
ENSG0000(	1977	44.15144	chr1:219	NBPF25P		Pseudoger	chr1:149058924-149

ENSG0000(	1977	44.15144	chr1:2197	ENSG00000291233		lncRNA	chr1:148889429-148
ENSG0000(	1977	44.15144	chr1:2197	RNU2-38P		smallRNA	chr1:148939738-148
ENSG0000(	1977	44.15144	chr1:2197	NBPF9		protein_c	chr1:149054026-149
ENSG0000(	1977	44.15144	chr1:2197	PDE4DIP	NCGv7;AC	protein_c	chr1:148808139-149
ENSG0000(	1977	44.15144	chr1:2197	SEC22B3P		Pseudoger	chr1:148772639-148
ENSG0000(	1977	44.15144	chr1:2197	ENSG00000289642		lncRNA	chr1:149013782-149
ENSG0000(	1977	44.15144	chr1:2197	ENSG00000255148		lncRNA	chr1:149018670-149
ENSG0000(	1977	44.15144	chr1:2197	RN7SKP88		smallRNA	chr1:148839482-148
ENSG0000(	1977	44.15144	chr1:2197	AL590452.1		protein_c	chr1:148893126-148
ENSG0000(	1977	44.15144	chr1:2197	PFN1P2		Pseudoger	chr1:149084616-149
ENSG0000(	1977	44.15144	chr1:2197	ENSG00000254913		lncRNA	chr1:149006308-149
ENSG0000(	1977	44.15144	chr1:2197	PDE4DIPP2		Pseudoger	chr1:148808504-149
ENSG0000(	1977	44.15144	chr1:2197	ENSG00000290999		lncRNA	chr1:148962571-149
ENSG0000(	1977	44.15144	chr1:2197	AL138796.1		smallRNA	chr1:149007076-149
ENSG0000(	1977	44.15144	chr1:2197	SEC22B4P		Pseudoger	chr1:148772639-148
ENSG0000(	1977	44.15144	chr1:2197	RNU1-59P		smallRNA	chr1:149162782-149
ENSG0000(	1977	44.15144	chr1:2197	RNU6-1071P		smallRNA	chr1:148739378-148
ENSG0000(	1970	43.99511	chr1:1043	RNVU1-13		smallRNA	chr1:120850818-120
ENSG0000(	1970	43.99511	chr1:1043	RNVU1-5		smallRNA	chr1:120942599-120
ENSG0000(	1970	43.99511	chr1:1043	RP3-328E19.4		Pseudoger	chr1:120844645-120
ENSG0000(	1970	43.99511	chr1:1043	RP3-328E19.5		Pseudoger	chr1:120861538-120
ENSG0000(	1970	43.99511	chr1:1043	RNVU1-19		smallRNA	chr1:120850819-120
ENSG0000(	1970	43.99511	chr1:1043	RP11-763B22.10		Pseudoger	chr1:120861539-120
ENSG0000(	1970	43.99511	chr1:1043	RP11-277L2.6		Pseudoger	chr1:120861539-120
ENSG0000(	1956	43.68246	chr1:1142	ENSG00000232768		Pseudoger	chr1:31050872-3105
ENSG0000(	1956	43.68246	chr1:1142	ENSG00000231949		lncRNA	chr1:30415825-3042
ENSG0000(	1956	43.68246	chr1:1142	ENSG00000229607		lncRNA	chr1:30810378-3081
ENSG0000(	1956	43.68246	chr1:1142	ENSG00000237329		Pseudoger	chr1:31036734-3103
ENSG0000(	1956	43.68246	chr1:1142	ENSG00000233372		lncRNA	chr1:30140263-3014
ENSG0000(	1956	43.68246	chr1:1142	ENSG00000235143		lncRNA	chr1:30858158-3086
ENSG0000(	1956	43.68246	chr1:1142	ENSG00000231251		Pseudoger	chr1:30226523-3022
ENSG0000(	1956	43.68246	chr1:1142	ENSG00000236335		lncRNA	chr1:30409560-3041
ENSG0000(	1956	43.68246	chr1:1142	MIR4420		smallRNA	chr1:30739156-3073
ENSG0000(	1956	43.68246	chr1:1142	RN7SKP91		smallRNA	chr1:30843823-3084
ENSG0000(	1956	43.68246	chr1:1142	ENSG00000225142		Pseudoger	chr1:31108188-3110
ENSG0000(	1956	43.68246	chr1:1142	SNORD85		smallRNA	chr1:30968164-3096
ENSG0000(	1956	43.68246	chr1:1142	MATN1-AS1		lncRNA	chr1:30718504-3072
ENSG0000(	1956	43.68246	chr1:1142	SELENOWP1		Pseudoger	chr1:31094987-3109
ENSG0000(	1956	43.68246	chr1:1142	SNORD103B		smallRNA	chr1:30949117-3094
ENSG0000(	1956	43.68246	chr1:1142	LINC01778		lncRNA	chr1:30824217-3083
ENSG0000(	1956	43.68246	chr1:1142	LINC01648		lncRNA	chr1:30013952-3003
ENSG0000(	1956	43.68246	chr1:1142	SNORD103A		smallRNA	chr1:30935688-3093
ENSG0000(	1956	43.68246	chr1:1142	PUM1		protein_c	chr1:30931506-3106
ENSG0000(	1956	43.68246	chr1:1142	NKAIN1		protein_c	chr1:31179745-3123
ENSG0000(	1956	43.68246	chr1:1142	ENSG00000289710		protein_c	chr1:30576655-3057
ENSG0000(	1956	43.68246	chr1:1142	SDC3		protein_c	chr1:30869466-3090
ENSG0000(	1956	43.68246	chr1:1142	MATN1		protein_c	chr1:30711277-3072
ENSG0000(	1956	43.68246	chr1:1142	LAPTM5		protein_c	chr1:30732469-3075
ENSG0000(	1956	43.68246	chr1:1142	ENSG00000287510		lncRNA	chr1:30731693-3073
ENSG0000(	1954	43.63779	chr2:3094	ENSG00000224869		Pseudoger	chr2:116726453-116
ENSG0000(	1954	43.63779	chr2:3094	ENSG00000271667		Pseudoger	chr2:116687218-116

ENSG0000(	1954	43.63779	chr2:309<	MTCYBP39		Pseudoger	chr2:116751158-116
ENSG0000(	1954	43.63779	chr2:309<	MTND2P21		Pseudoger	chr2:117025077-117
ENSG0000(	1954	43.63779	chr2:309<	ENSG00000235871		Pseudoger	chr2:116818086-116
ENSG0000(	1954	43.63779	chr2:309<	ENSG00000227291		Pseudoger	chr2:117180892-117
ENSG0000(	1954	43.63779	chr2:309<	ENSG00000286370		lncRNA	chr2:117536387-117
ENSG0000(	1954	43.63779	chr2:309<	MTCO1P43		Pseudoger	chr2:117026537-117
ENSG0000(	1954	43.63779	chr2:309<	RNU7-190P		smallRNA	chr2:117139715-117
ENSG0000(	1954	43.63779	chr2:309<	MTND1P28		Pseudoger	chr2:117023918-117
ENSG0000(	1947	43.48146	chr1:2197	ENSG00000235152		lncRNA	chr1:232160091-232
ENSG0000(	1947	43.48146	chr1:2197	LINC01354		lncRNA	chr1:234527887-234
ENSG0000(	1947	43.48146	chr1:2197	ENSG00000289057		lncRNA	chr1:234599568-234
ENSG0000(	1947	43.48146	chr1:2197	COA6-AS1		lncRNA	chr1:234372807-234
ENSG0000(	1947	43.48146	chr1:2197	RNU6-1211P		smallRNA	chr1:232700204-232
ENSG0000(	1947	43.48146	chr1:2197	ENSG00000236372		lncRNA	chr1:232174932-232
ENSG0000(	1947	43.48146	chr1:2197	PCNX2		protein_c	chr1:232983435-233
ENSG0000(	1947	43.48146	chr1:2197	ENSG00000286210		lncRNA	chr1:234535963-234
ENSG0000(	1947	43.48146	chr1:2197	KCNK1	NCGv7	protein_c	chr1:233614106-233
ENSG0000(	1947	43.48146	chr1:2197	NTPCR		protein_c	chr1:232950605-232
ENSG0000(	1947	43.48146	chr1:2197	RNU4-77P		smallRNA	chr1:233448626-233
ENSG0000(	1947	43.48146	chr1:2197	SIPA1L2	DriverDBv	protein_c	chr1:232397965-232
ENSG0000(	1947	43.48146	chr1:2197	ENSG00000286666		lncRNA	chr1:233844621-233
ENSG0000(	1947	43.48146	chr1:2197	U8		smallRNA	chr1:234593275-234
ENSG0000(	1947	43.48146	chr1:2197	ENSG00000236358		lncRNA	chr1:234357006-234
ENSG0000(	1947	43.48146	chr1:2197	SLC35F3		protein_c	chr1:233904676-234
ENSG0000(	1947	43.48146	chr1:2197	MIR4427		smallRNA	chr1:233624152-233
ENSG0000(	1947	43.48146	chr1:2197	MIR4671		smallRNA	chr1:234306467-234
ENSG0000(	1947	43.48146	chr1:2197	ENSG00000286071		lncRNA	chr1:231767464-231
ENSG0000(	1947	43.48146	chr1:2197	ENSG00000287921		lncRNA	chr1:233904104-233
ENSG0000(	1947	43.48146	chr1:2197	ENSG00000251508		Pseudoger	chr1:233295325-233
ENSG0000(	1947	43.48146	chr1:2197	ENSG00000233332		lncRNA	chr1:234212606-234
ENSG0000(	1947	43.48146	chr1:2197	LINC01744		lncRNA	chr1:232727251-232
ENSG0000(	1947	43.48146	chr1:2197	RN7SL299P		smallRNA	chr1:232222866-232
ENSG0000(	1947	43.48146	chr1:2197	ENSG00000235605		Pseudoger	chr1:234356704-234
ENSG0000(	1947	43.48146	chr1:2197	ENSG00000286774		lncRNA	chr1:232843386-232
ENSG0000(	1947	43.48146	chr1:2197	ENSG00000286109		lncRNA	chr1:233724454-233
ENSG0000(	1947	43.48146	chr1:2197	ENSG00000288760		lncRNA	chr1:234531338-234
ENSG0000(	1947	43.48146	chr1:2197	TARBP1		protein_c	chr1:234391313-234
ENSG0000(	1947	43.48146	chr1:2197	ENSG00000287423		lncRNA	chr1:233527544-233
ENSG0000(	1947	43.48146	chr1:2197	ENSG00000228830		lncRNA	chr1:234607008-234
ENSG0000(	1947	43.48146	chr1:2197	RNU1-74P		smallRNA	chr1:232832017-232
ENSG0000(	1947	43.48146	chr1:2197	ENSG00000287633		lncRNA	chr1:234550542-234
ENSG0000(	1947	43.48146	chr1:2197	ENSG00000232166		Pseudoger	chr1:234284972-234
ENSG0000(	1947	43.48146	chr1:2197	ENSG00000232175		Pseudoger	chr1:232917235-232
ENSG0000(	1947	43.48146	chr1:2197	DISC1-IT1		lncRNA	chr1:231925834-231
ENSG0000(	1947	43.48146	chr1:2197	LINC01745		lncRNA	chr1:232718071-232
ENSG0000(	1947	43.48146	chr1:2197	RAC1P7		Pseudoger	chr1:234026851-234
ENSG0000(	1947	43.48146	chr1:2197	ENSG00000230404		lncRNA	chr1:234565298-234
ENSG0000(	1947	43.48146	chr1:2197	MAP3K21	NCGv7	protein_c	chr1:233327724-233
ENSG0000(	1947	43.48146	chr1:2197	ENSG00000224436		Pseudoger	chr1:232221938-232
ENSG0000(	1947	43.48146	chr1:2197	COA6		protein_c	chr1:234373456-234
ENSG0000(	1947	43.48146	chr1:2197	ENSG00000289305		lncRNA	chr1:233613570-233

ENSG0000(	1947	43.48146	chr1:2197	IRF2BP2	NCGv7	protein_c	chr1:234604269-234
ENSG0000(	1947	43.48146	chr1:2197	TSNAX-DISC1		protein_c	chr1:231528653-231
ENSG0000(	1947	43.48146	chr1:2197	SLC35F3-AS1		lncRNA	chr1:234268583-234
ENSG0000(	1947	43.48146	chr1:2197	ENSG00000273367		lncRNA	chr1:234372186-234
ENSG0000(	1947	43.48146	chr1:2197	ENSG00000225486		Pseudoger	chr1:233836080-233
ENSG0000(	1947	43.48146	chr1:2197	MAP10		protein_c	chr1:232805416-232
ENSG0000(	1947	43.48146	chr1:2197	ENSG00000231272		lncRNA	chr1:234261706-234
ENSG0000(	1947	43.48146	chr1:2197	DISC1		protein_c	chr1:231626790-232
ENSG0000(	1947	43.48146	chr1:2197	RPS7P3		Pseudoger	chr1:233288868-233
ENSG0000(	1901	42.45417	chr1:2197	RPRD2		protein_c	chr1:150363091-150
ENSG0000(	1901	42.45417	chr1:2197	ENSG00000285818		Pseudoger	chr1:152079557-152
ENSG0000(	1901	42.45417	chr1:2197	LCE1D	NCGv7	protein_c	chr1:152796721-152
ENSG0000(	1901	42.45417	chr1:2197	H3C15		protein_c	chr1:149852608-149
ENSG0000(	1901	42.45417	chr1:2197	TMOD4		protein_c	chr1:151169986-151
ENSG0000(	1901	42.45417	chr1:2197	CREB3L4	NCGv7	protein_c	chr1:153967534-153
ENSG0000(	1901	42.45417	chr1:2197	TCHHL1		protein_c	chr1:152084141-152
ENSG0000(	1901	42.45417	chr1:2197	SCNM1		protein_c	chr1:151156664-151
ENSG0000(	1901	42.45417	chr1:2197	LYSMD1		protein_c	chr1:151159748-151
ENSG0000(	1901	42.45417	chr1:2197	ZNF687-AS1		lncRNA	chr1:151279678-151
ENSG0000(	1901	42.45417	chr1:2197	SPTLC1P4		Pseudoger	chr1:152077952-152
ENSG0000(	1901	42.45417	chr1:2197	VPS72		protein_c	chr1:151176304-151
ENSG0000(	1901	42.45417	chr1:2197	LINC01527		lncRNA	chr1:152930040-152
ENSG0000(	1901	42.45417	chr1:2197	TNFAIP8L2		protein_c	chr1:151156649-151
ENSG0000(	1901	42.45417	chr1:2197	ENSG00000229699		lncRNA	chr1:153174518-153
ENSG0000(	1901	42.45417	chr1:2197	HRNR	NCGv7	protein_c	chr1:152212076-152
ENSG0000(	1901	42.45417	chr1:2197	ENSG00000290792		lncRNA	chr1:149844498-149
ENSG0000(	1901	42.45417	chr1:2197	LCE1A		protein_c	chr1:152827473-152
ENSG0000(	1901	42.45417	chr1:2197	ENSG00000290791		lncRNA	chr1:149842875-149
ENSG0000(	1901	42.45417	chr1:2197	ENSG00000285867		lncRNA	chr1:153586813-153
ENSG0000(	1901	42.45417	chr1:2197	S100A6		protein_c	chr1:153534599-153
ENSG0000(	1901	42.45417	chr1:2197	ENSG00000290790		lncRNA	chr1:149676889-149
ENSG0000(	1901	42.45417	chr1:2197	MRPS21	NCGv7	protein_c	chr1:150293861-150
ENSG0000(	1901	42.45417	chr1:2197	H2BC19P		Pseudoger	chr1:149843041-149
ENSG0000(	1901	42.45417	chr1:2197	RPL6P31		Pseudoger	chr1:150053864-150
ENSG0000(	1901	42.45417	chr1:2197	ENSG00000223861		Pseudoger	chr1:151557446-151
ENSG0000(	1901	42.45417	chr1:2197	LCE3B		protein_c	chr1:152613811-152
ENSG0000(	1901	42.45417	chr1:2197	LCE3D		protein_c	chr1:152579381-152
ENSG0000(	1901	42.45417	chr1:2197	ENSG00000271853		lncRNA	chr1:153626332-153
ENSG0000(	1901	42.45417	chr1:2197	APH1A		protein_c	chr1:150265399-150
ENSG0000(	1901	42.45417	chr1:2197	PRPF3	NCGv7	protein_c	chr1:150321479-150
ENSG0000(	1901	42.45417	chr1:2197	ENSG00000289457		lncRNA	chr1:150561466-150
ENSG0000(	1901	42.45417	chr1:2197	SPRR2A		protein_c	chr1:153056120-153
ENSG0000(	1901	42.45417	chr1:2197	LCE2C		protein_c	chr1:152675279-152
ENSG0000(	1901	42.45417	chr1:2197	ENSG00000275296		Pseudoger	chr1:149754303-149
ENSG0000(	1901	42.45417	chr1:2197	CTSS	NCGv7	protein_c	chr1:150730079-150
ENSG0000(	1901	42.45417	chr1:2197	RP11-353N4.4		lncRNA	chr1:149701425-149
ENSG0000(	1901	42.45417	chr1:2197	LCE2A		protein_c	chr1:152698345-152
ENSG0000(	1901	42.45417	chr1:2197	RIIAD1		protein_c	chr1:151710433-151
ENSG0000(	1901	42.45417	chr1:2197	LCE4A	NCGv7	protein_c	chr1:152708160-152
ENSG0000(	1901	42.45417	chr1:2197	S100A10		protein_c	chr1:151982915-151
ENSG0000(	1901	42.45417	chr1:2197	ENSG00000232536		lncRNA	chr1:151540516-151

ENSG0000(	1901	42.45417	chr1:2197	SNORA31		smallRNA	chr1:153012482-153
ENSG0000(	1901	42.45417	chr1:2197	RN7SL600P		smallRNA	chr1:150568973-150
ENSG0000(	1901	42.45417	chr1:2197	C1orf54		protein_c	chr1:150268200-150
ENSG0000(	1901	42.45417	chr1:2197	AL358813.1		Pseudoger	chr1:149717832-149
ENSG0000(	1901	42.45417	chr1:2197	CA14		protein_c	chr1:150257251-150
ENSG0000(	1901	42.45417	chr1:2197	FCGR1A		protein_c	chr1:149782671-149
ENSG0000(	1901	42.45417	chr1:2197	NPR1		protein_c	chr1:153678688-153
ENSG0000(	1901	42.45417	chr1:2197	KRT8P28		Pseudoger	chr1:151949523-151
ENSG0000(	1901	42.45417	chr1:2197	C2CD4D-AS1		lncRNA	chr1:151841877-151
ENSG0000(	1901	42.45417	chr1:2197	LCE2D		protein_c	chr1:152663380-152
ENSG0000(	1901	42.45417	chr1:2197	RNU1-68P		smallRNA	chr1:149700151-149
ENSG0000(	1901	42.45417	chr1:2197	ENSG00000259357		lncRNA	chr1:150965245-150
ENSG0000(	1901	42.45417	chr1:2197	CYCSP51		Pseudoger	chr1:150903896-150
ENSG0000(	1901	42.45417	chr1:2197	BNIPL	DriverDBv	protein_c	chr1:151036321-151
ENSG0000(	1901	42.45417	chr1:2197	S100A11		protein_c	chr1:152032506-152
ENSG0000(	1901	42.45417	chr1:2197	AL589765.1		protein_c	chr1:151710433-151
ENSG0000(	1901	42.45417	chr1:2197	SMCP		protein_c	chr1:152878322-152
ENSG0000(	1901	42.45417	chr1:2197	IVL		protein_c	chr1:152908546-152
ENSG0000(	1901	42.45417	chr1:2197	OAZ3		protein_c	chr1:151762899-151
ENSG0000(	1901	42.45417	chr1:2197	C1orf56		protein_c	chr1:151047751-151
ENSG0000(	1901	42.45417	chr1:2197	POGZ		protein_c	chr1:151402724-151
ENSG0000(	1901	42.45417	chr1:2197	ARNT	NCGv7;AC	protein_c	chr1:150809713-150
ENSG0000(	1901	42.45417	chr1:2197	MRPL9		protein_c	chr1:151759647-151
ENSG0000(	1901	42.45417	chr1:2197	SEMA6C		protein_c	chr1:151131685-151
ENSG0000(	1901	42.45417	chr1:2197	ENSA		protein_c	chr1:150600851-150
ENSG0000(	1901	42.45417	chr1:2197	CERS2		protein_c	chr1:150960583-150
ENSG0000(	1901	42.45417	chr1:2197	SELENBP1		protein_c	chr1:151364304-151
ENSG0000(	1901	42.45417	chr1:2197	ANXA9		protein_c	chr1:150982249-150
ENSG0000(	1901	42.45417	chr1:2197	MINDY1		protein_c	chr1:150996549-151
ENSG0000(	1901	42.45417	chr1:2197	ANP32E		protein_c	chr1:150218417-150
ENSG0000(	1901	42.45417	chr1:2197	PIP5K1A		protein_c	chr1:151197949-151
ENSG0000(	1901	42.45417	chr1:2197	PI4KB		protein_c	chr1:151291797-151
ENSG0000(	1901	42.45417	chr1:2197	RFX5		protein_c	chr1:151340640-151
ENSG0000(	1901	42.45417	chr1:2197	S100A14		protein_c	chr1:153614255-153
ENSG0000(	1901	42.45417	chr1:2197	CTSK		protein_c	chr1:150794880-150
ENSG0000(	1901	42.45417	chr1:2197	S100A13		protein_c	chr1:153618787-153
ENSG0000(	1901	42.45417	chr1:2197	FLG-AS1		lncRNA	chr1:152168125-152
ENSG0000(	1901	42.45417	chr1:2197	ENSG00000243613		lncRNA	chr1:153746851-153
ENSG0000(	1901	42.45417	chr1:2197	PUDPP2		Pseudoger	chr1:152124016-152
ENSG0000(	1901	42.45417	chr1:2197	LCEP1		Pseudoger	chr1:152744299-152
ENSG0000(	1901	42.45417	chr1:2197	RFX5-AS1		lncRNA	chr1:151346938-151
ENSG0000(	1901	42.45417	chr1:2197	HORMAD1	NCGv7	protein_c	chr1:150698060-150
ENSG0000(	1901	42.45417	chr1:2197	GOLPH3L		protein_c	chr1:150646230-150
ENSG0000(	1901	42.45417	chr1:2197	GABPB2		protein_c	chr1:151070578-151
ENSG0000(	1901	42.45417	chr1:2197	SNAPIN		protein_c	chr1:153658703-153
ENSG0000(	1901	42.45417	chr1:2197	GATAD2B		protein_c	chr1:153789030-153
ENSG0000(	1901	42.45417	chr1:2197	ILF2		protein_c	chr1:153661788-153
ENSG0000(	1901	42.45417	chr1:2197	INTS3	AC	protein_c	chr1:153728050-153
ENSG0000(	1901	42.45417	chr1:2197	FLG	NCGv7	protein_c	chr1:152302165-152
ENSG0000(	1901	42.45417	chr1:2197	ENSG00000236940		Pseudoger	chr1:151757659-151
ENSG0000(	1901	42.45417	chr1:2197	SLC39A1		protein_c	chr1:153959099-153

ENSG0000(	1901	42.45417	chr1:2197	S100A7	NCGv7;AC	protein_c	chr1:153457744-153
ENSG0000(	1901	42.45417	chr1:2197	SLC27A3		protein_c	chr1:153774354-153
ENSG0000(	1901	42.45417	chr1:2197	S100A5		protein_c	chr1:153537147-153
ENSG0000(	1901	42.45417	chr1:2197	THEM5	DriverDB\	protein_c	chr1:151847101-151
ENSG0000(	1901	42.45417	chr1:2197	U3		smallRNA	chr1:153998041-153
ENSG0000(	1901	42.45417	chr1:2197	Y_RNA		smallRNA	chr1:150882451-150
ENSG0000(	1901	42.45417	chr1:2197	ENSG00000290074		lncRNA	chr1:150579917-150
ENSG0000(	1901	42.45417	chr1:2197	S100A8	NCGv7;AC	protein_c	chr1:153390032-153
ENSG0000(	1901	42.45417	chr1:2197	RAB13		protein_c	chr1:153981617-153
ENSG0000(	1901	42.45417	chr1:2197	H4C15		protein_c	chr1:149854045-149
ENSG0000(	1901	42.45417	chr1:2197	JTB		protein_c	chr1:153974269-153
ENSG0000(	1901	42.45417	chr1:2197	S100A4	AC	protein_c	chr1:153543613-153
ENSG0000(	1901	42.45417	chr1:2197	RN7SL473P		smallRNA	chr1:150566564-150
ENSG0000(	1901	42.45417	chr1:2197	CRNN		protein_c	chr1:152409243-152
ENSG0000(	1901	42.45417	chr1:2197	FLG2		protein_c	chr1:152348735-152
ENSG0000(	1901	42.45417	chr1:2197	RPS29P29		Pseudoger	chr1:151111912-151
ENSG0000(	1901	42.45417	chr1:2197	MCL1	NCGv7;AC	protein_c	chr1:150560895-150
ENSG0000(	1901	42.45417	chr1:2197	ADAMTSL4		protein_c	chr1:150549369-150
ENSG0000(	1901	42.45417	chr1:2197	OTUD7B		protein_c	chr1:149937812-150
ENSG0000(	1901	42.45417	chr1:2197	LCE7A		protein_c	chr1:152859996-152
ENSG0000(	1901	42.45417	chr1:2197	H4C14		protein_c	chr1:149832657-149
ENSG0000(	1901	42.45417	chr1:2197	RN7SL480P		smallRNA	chr1:150211632-150
ENSG0000(	1901	42.45417	chr1:2197	ENSG00000236713		Pseudoger	chr1:150780272-150
ENSG0000(	1901	42.45417	chr1:2197	S100A7L2		Pseudoger	chr1:153437058-153
ENSG0000(	1901	42.45417	chr1:2197	SNORA44		smallRNA	chr1:151527831-151
ENSG0000(	1901	42.45417	chr1:2197	SPRR1A		protein_c	chr1:152984081-152
ENSG0000(	1901	42.45417	chr1:2197	SPRR1B		protein_c	chr1:153031203-153
ENSG0000(	1901	42.45417	chr1:2197	RN7SL444P		smallRNA	chr1:151300667-151
ENSG0000(	1901	42.45417	chr1:2197	ENSG00000232937		lncRNA	chr1:151765709-151
ENSG0000(	1901	42.45417	chr1:2197	S100A3		protein_c	chr1:153547329-153
ENSG0000(	1901	42.45417	chr1:2197	TDRKH	DriverDB\	protein_c	chr1:151770107-151
ENSG0000(	1901	42.45417	chr1:2197	RNU2-17P		smallRNA	chr1:150236967-150
ENSG0000(	1901	42.45417	chr1:2197	LCEP4		Pseudoger	chr1:152644393-152
ENSG0000(	1901	42.45417	chr1:2197	S100A12		protein_c	chr1:153373711-153
ENSG0000(	1901	42.45417	chr1:2197	S100A9		protein_c	chr1:153357854-153
ENSG0000(	1901	42.45417	chr1:2197	ENSG00000273481		lncRNA	chr1:151327949-151
ENSG0000(	1901	42.45417	chr1:2197	NBPF18P		Pseudoger	chr1:152018662-152
ENSG0000(	1901	42.45417	chr1:2197	PGLYRP4		protein_c	chr1:153330120-153
ENSG0000(	1901	42.45417	chr1:2197	SPRR2D		protein_c	chr1:153039732-153
ENSG0000(	1901	42.45417	chr1:2197	ENSG00000223599		Pseudoger	chr1:153852106-153
ENSG0000(	1901	42.45417	chr1:2197	CDC42SE1		protein_c	chr1:151050971-151
ENSG0000(	1901	42.45417	chr1:2197	SPRR3		protein_c	chr1:153001747-153
ENSG0000(	1901	42.45417	chr1:2197	ENSG00000234262		Pseudoger	chr1:153023962-153
ENSG0000(	1901	42.45417	chr1:2197	CRCT1		protein_c	chr1:152514482-152
ENSG0000(	1901	42.45417	chr1:2197	SETDB1	NCGv7;AC	protein_c	chr1:150926263-150
ENSG0000(	1901	42.45417	chr1:2197	LCE1B		protein_c	chr1:152811971-152
ENSG0000(	1901	42.45417	chr1:2197	SNX27		protein_c	chr1:151612006-151
ENSG0000(	1901	42.45417	chr1:2197	CGN	DriverDB\	protein_c	chr1:151510510-151
ENSG0000(	1901	42.45417	chr1:2197	TARS2		protein_c	chr1:150487414-150
ENSG0000(	1901	42.45417	chr1:2197	ZNF687	NCGv7	protein_c	chr1:151281618-151
ENSG0000(	1901	42.45417	chr1:2197	ECM1		protein_c	chr1:150508062-150

ENSG0000(	1901	42.45417	chr1:2197	SF3B4		protein_c	chr1:149923317-149
ENSG0000(	1901	42.45417	chr1:2197	TUFT1		protein_c	chr1:151540305-151
ENSG0000(	1901	42.45417	chr1:2197	RORC	DriverDBv	protein_c	chr1:151806071-151
ENSG0000(	1901	42.45417	chr1:2197	PRUNE1		protein_c	chr1:151008420-151
ENSG0000(	1901	42.45417	chr1:2197	RP11-277L2.3		lncRNA	chr1:149607448-149
ENSG0000(	1901	42.45417	chr1:2197	S100A2		protein_c	chr1:153561108-153
ENSG0000(	1901	42.45417	chr1:2197	MIR4257		smallRNA	chr1:150551929-150
ENSG0000(	1901	42.45417	chr1:2197	SPRR2B	NCGv7	protein_c	chr1:153070226-153
ENSG0000(	1901	42.45417	chr1:2197	LCE1F		protein_c	chr1:152775140-152
ENSG0000(	1901	42.45417	chr1:2197	LINGO4		protein_c	chr1:151800264-151
ENSG0000(	1901	42.45417	chr1:2197	S100A16		protein_c	chr1:153606886-153
ENSG0000(	1901	42.45417	chr1:2197	MLLT11	DriverDBv	protein_c	chr1:151060397-151
ENSG0000(	1901	42.45417	chr1:2197	ENSG00000289935		lncRNA	chr1:153945551-153
ENSG0000(	1901	42.45417	chr1:2197	ENSG00000233222		lncRNA	chr1:153750983-153
ENSG0000(	1901	42.45417	chr1:2197	LAPTM4BP1		Pseudoger	chr1:153379821-153
ENSG0000(	1901	42.45417	chr1:2197	LCE1C		protein_c	chr1:152804832-152
ENSG0000(	1901	42.45417	chr1:2197	ENSG00000227045		lncRNA	chr1:151701026-151
ENSG0000(	1901	42.45417	chr1:2197	ENSG00000233030		lncRNA	chr1:149785659-149
ENSG0000(	1901	42.45417	chr1:2197	ENSG00000275557		lncRNA	chr1:149607765-149
ENSG0000(	1901	42.45417	chr1:2197	HIST2H2BC		Pseudoger	chr1:149850193-149
ENSG0000(	1901	42.45417	chr1:2197	VPS45	NCGv7	protein_c	chr1:150067279-150
ENSG0000(	1901	42.45417	chr1:2197	RN7SL44P		smallRNA	chr1:153500467-153
ENSG0000(	1901	42.45417	chr1:2197	TDRKH-AS1		lncRNA	chr1:151790804-151
ENSG0000(	1901	42.45417	chr1:2197	C1orf68		protein_c	chr1:152719522-152
ENSG0000(	1901	42.45417	chr1:2197	LINC02988		lncRNA	chr1:150173049-150
ENSG0000(	1901	42.45417	chr1:2197	SNORA40		smallRNA	chr1:150600539-150
ENSG0000(	1901	42.45417	chr1:2197	HIST2H2AA3		protein_c	chr1:149841933-149
ENSG0000(	1901	42.45417	chr1:2197	ENSG00000272030		lncRNA	chr1:153631438-153
ENSG0000(	1901	42.45417	chr1:2197	RNU6-160P		smallRNA	chr1:153331622-153
ENSG0000(	1901	42.45417	chr1:2197	ENSG00000250734		lncRNA	chr1:151612038-151
ENSG0000(	1901	42.45417	chr1:2197	SPRR5		protein_c	chr1:152947206-152
ENSG0000(	1901	42.45417	chr1:2197	HMGN3P1		Pseudoger	chr1:152399577-152
ENSG0000(	1901	42.45417	chr1:2197	ENSG00000226500		Pseudoger	chr1:149754301-149
ENSG0000(	1901	42.45417	chr1:2197	ENSG00000231073		lncRNA	chr1:150973123-150
ENSG0000(	1901	42.45417	chr1:2197	Y_RNA		smallRNA	chr1:153726252-153
ENSG0000(	1901	42.45417	chr1:2197	ENSG00000231827		Pseudoger	chr1:153795173-153
ENSG0000(	1901	42.45417	chr1:2197	H3C13		protein_c	chr1:149813225-149
ENSG0000(	1901	42.45417	chr1:2197	SV2A		protein_c	chr1:149903318-149
ENSG0000(	1901	42.45417	chr1:2197	ENSG00000285184		lncRNA	chr1:150045660-150
ENSG0000(	1901	42.45417	chr1:2197	ENSG00000269489		lncRNA	chr1:151798054-151
ENSG0000(	1901	42.45417	chr1:2197	ENSG00000238279		lncRNA	chr1:153533430-153
ENSG0000(	1901	42.45417	chr1:2197	H2AC21		protein_c	chr1:149887469-149
ENSG0000(	1901	42.45417	chr1:2197	AL358813.3		smallRNA	chr1:149737309-149
ENSG0000(	1901	42.45417	chr1:2197	RN7SL372P		smallRNA	chr1:153704088-153
ENSG0000(	1901	42.45417	chr1:2197	LINC00869		Pseudoger	chr1:149655747-149
ENSG0000(	1901	42.45417	chr1:2197	LORICRIN		protein_c	chr1:153259687-153
ENSG0000(	1901	42.45417	chr1:2197	RPLP0P4		Pseudoger	chr1:153225080-153
ENSG0000(	1901	42.45417	chr1:2197	S100A7A	NCGv7	protein_c	chr1:153416520-153
ENSG0000(	1901	42.45417	chr1:2197	LCE1E		protein_c	chr1:152786214-152
ENSG0000(	1901	42.45417	chr1:2197	ENSG00000269621		lncRNA	chr1:151755541-151
ENSG0000(	1901	42.45417	chr1:2197	H2AC20	NCGv7	protein_c	chr1:149886918-149

ENSG0000(	1901	42.45417	chr1:2197	Y_RNA		smallRNA	chr1:151841736-151
ENSG0000(	1901	42.45417	chr1:2197	LCE5A		protein_c	chr1:152510803-152
ENSG0000(	1901	42.45417	chr1:2197	RP11-277L2.5		lncRNA	chr1:149618320-149
ENSG0000(	1901	42.45417	chr1:2197	S100A7P1		Pseudoger	chr1:153427020-153
ENSG0000(	1901	42.45417	chr1:2197	ENSG00000291199		lncRNA	chr1:153793937-153
ENSG0000(	1901	42.45417	chr1:2197	SPRR2F		protein_c	chr1:153112121-153
ENSG0000(	1901	42.45417	chr1:2197	FALEC		lncRNA	chr1:150515757-150
ENSG0000(	1901	42.45417	chr1:2197	ENSG00000284738		lncRNA	chr1:153923284-153
ENSG0000(	1901	42.45417	chr1:2197	ENSG00000273026		lncRNA	chr1:153966516-153
ENSG0000(	1901	42.45417	chr1:2197	DENND4B	NCGv7	protein_c	chr1:153929501-153
ENSG0000(	1901	42.45417	chr1:2197	SPRR4		protein_c	chr1:152970648-152
ENSG0000(	1901	42.45417	chr1:2197	PGLYRP3		protein_c	chr1:153297116-153
ENSG0000(	1901	42.45417	chr1:2197	ENSG00000231416		Pseudoger	chr1:153995632-153
ENSG0000(	1901	42.45417	chr1:2197	RNU6-884P		smallRNA	chr1:151022746-151
ENSG0000(	1901	42.45417	chr1:2197	ENSG00000236327		Pseudoger	chr1:153890595-153
ENSG0000(	1901	42.45417	chr1:2197	ENSG00000268288		lncRNA	chr1:151766486-151
ENSG0000(	1901	42.45417	chr1:2197	H2AC19		protein_c	chr1:149851061-149
ENSG0000(	1901	42.45417	chr1:2197	ENSG00000284964		Pseudoger	chr1:149607467-149
ENSG0000(	1901	42.45417	chr1:2197	MTMR11		protein_c	chr1:149928651-149
ENSG0000(	1901	42.45417	chr1:2197	RP11-277L2.4		lncRNA	chr1:149606196-149
ENSG0000(	1901	42.45417	chr1:2197	CRTC2		protein_c	chr1:153947669-153
ENSG0000(	1901	42.45417	chr1:2197	ENSG00000288880		lncRNA	chr1:150629814-150
ENSG0000(	1901	42.45417	chr1:2197	ENSG00000285641		protein_c	chr1:153975850-153
ENSG0000(	1901	42.45417	chr1:2197	ENSG00000285651		lncRNA	chr1:151885251-151
ENSG0000(	1901	42.45417	chr1:2197	CHTOP		protein_c	chr1:153633982-153
ENSG0000(	1901	42.45417	chr1:2197	SPRR2C		Pseudoger	chr1:153140491-153
ENSG0000(	1901	42.45417	chr1:2197	S100A1	NCGv7	protein_c	chr1:153627926-153
ENSG0000(	1901	42.45417	chr1:2197	ENSG00000229021		lncRNA	chr1:151994531-152
ENSG0000(	1901	42.45417	chr1:2197	RNY4P25		smallRNA	chr1:151439000-151
ENSG0000(	1901	42.45417	chr1:2197	SPRR2G		protein_c	chr1:153149582-153
ENSG0000(	1901	42.45417	chr1:2197	LCE2B		protein_c	chr1:152686123-152
ENSG0000(	1901	42.45417	chr1:2197	ENSG00000249602		lncRNA	chr1:151763384-151
ENSG0000(	1901	42.45417	chr1:2197	snoU13		smallRNA	chr1:150261694-150
ENSG0000(	1901	42.45417	chr1:2197	C2CD4D	DriverDB\	protein_c	chr1:151837818-151
ENSG0000(	1901	42.45417	chr1:2197	RNU6-1042P		smallRNA	chr1:150701866-150
ENSG0000(	1901	42.45417	chr1:2197	PLEKHO1		protein_c	chr1:150149183-150
ENSG0000(	1901	42.45417	chr1:2197	CIART		protein_c	chr1:150282543-150
ENSG0000(	1901	42.45417	chr1:2197	ENSG00000282386		lncRNA	chr1:153964361-153
ENSG0000(	1901	42.45417	chr1:2197	ENSG00000276110		lncRNA	chr1:150255095-150
ENSG0000(	1901	42.45417	chr1:2197	PSMD4		protein_c	chr1:151254709-151
ENSG0000(	1901	42.45417	chr1:2197	TCHH	NCGv7	protein_c	chr1:152106317-152
ENSG0000(	1901	42.45417	chr1:2197	PSMB4		protein_c	chr1:151399560-151
ENSG0000(	1901	42.45417	chr1:2197	RNU6-662P		smallRNA	chr1:151747597-151
ENSG0000(	1901	42.45417	chr1:2197	LCE3E		protein_c	chr1:152565654-152
ENSG0000(	1901	42.45417	chr1:2197	CELF3		protein_c	chr1:151700058-151
ENSG0000(	1901	42.45417	chr1:2197	LCE3A		protein_c	chr1:152622834-152
ENSG0000(	1901	42.45417	chr1:2197	Y_RNA		smallRNA	chr1:153785720-153
ENSG0000(	1901	42.45417	chr1:2197	THEM4		protein_c	chr1:151870866-151
ENSG0000(	1901	42.45417	chr1:2197	PRR9		protein_c	chr1:153217584-153
ENSG0000(	1901	42.45417	chr1:2197	LINC02962		lncRNA	chr1:152205858-152
ENSG0000(	1901	42.45417	chr1:2197	MIR554		smallRNA	chr1:151545796-151

ENSG0000(	1901	42.45417	chr1:2197	ENSG00000261168		lncRNA	chr1:151130075-151
ENSG0000(	1901	42.45417	chr1:2197	ENSG00000224645		lncRNA	chr1:151340648-151
ENSG0000(	1901	42.45417	chr1:2197	CTXND2		protein_c	chr1:150887136-150
ENSG0000(	1901	42.45417	chr1:2197	BOLA1		protein_c	chr1:149887890-149
ENSG0000(	1901	42.45417	chr1:2197	ENSG00000289288		lncRNA	chr1:151146793-151
ENSG0000(	1901	42.45417	chr1:2197	LCEP3		Pseudoger	chr1:152656332-152
ENSG0000(	1901	42.45417	chr1:2197	snoU13		smallRNA	chr1:153754124-153
ENSG0000(	1901	42.45417	chr1:2197	RP11-353N4.1		lncRNA	chr1:149621576-149
ENSG0000(	1901	42.45417	chr1:2197	ENSG00000226716		lncRNA	chr1:152122534-152
ENSG0000(	1901	42.45417	chr1:2197	RP11-196G18.23		lncRNA	chr1:149831312-149
ENSG0000(	1901	42.45417	chr1:2197	LCE3C		protein_c	chr1:152600234-152
ENSG0000(	1901	42.45417	chr1:2197	H3C14		protein_c	chr1:149840687-149
ENSG0000(	1901	42.45417	chr1:2197	GEMIN2P1		Pseudoger	chr1:153717303-153
ENSG0000(	1901	42.45417	chr1:2197	AL356356.1		protein_c	chr1:150549421-150
ENSG0000(	1901	42.45417	chr1:2197	JTB-DT		lncRNA	chr1:153977727-153
ENSG0000(	1901	42.45417	chr1:2197	RNU6-1062P		smallRNA	chr1:151629324-151
ENSG0000(	1901	42.45417	chr1:2197	ENSG00000285779		protein_c	chr1:153959151-153
ENSG0000(	1901	42.45417	chr1:2197	ENSG00000286581		lncRNA	chr1:151944890-151
ENSG0000(	1901	42.45417	chr1:2197	ADAMTSL4-AS1		lncRNA	chr1:150560202-150
ENSG0000(	1901	42.45417	chr1:2197	RPS27	NCGv7	protein_c	chr1:153990762-153
ENSG0000(	1901	42.45417	chr1:2197	S100A15A		Pseudoger	chr1:153396591-153
ENSG0000(	1901	42.45417	chr1:2197	KPRP		protein_c	chr1:152758025-152
ENSG0000(	1901	42.45417	chr1:2197	RNU6-179P		smallRNA	chr1:154039916-154
ENSG0000(	1901	42.45417	chr1:2197	RNVU1-20		smallRNA	chr1:149636766-149
ENSG0000(	1901	42.45417	chr1:2197	ENSG00000289041		lncRNA	chr1:150281114-150
ENSG0000(	1901	42.45417	chr1:2197	ENSG00000289062		lncRNA	chr1:152897800-152
ENSG0000(	1901	42.45417	chr1:2197	H2BC21		protein_c	chr1:149884459-149
ENSG0000(	1901	42.45417	chr1:2197	RNU6-1309P		smallRNA	chr1:150812591-150
ENSG0000(	1901	42.45417	chr1:2197	LCEP2		Pseudoger	chr1:152737518-152
ENSG0000(	1901	42.45417	chr1:2197	RPTN		protein_c	chr1:152153595-152
ENSG0000(	1901	42.45417	chr1:2197	H2BC18		protein_c	chr1:149782689-149
ENSG0000(	1901	42.45417	chr1:2197	LCE6A		protein_c	chr1:152842856-152
ENSG0000(	1901	42.45417	chr1:2197	PDE4DIPP4		lncRNA	chr1:149677473-149
ENSG0000(	1901	42.45417	chr1:2197	LELP1		protein_c	chr1:153203430-153
ENSG0000(	1901	42.45417	chr1:2197	AL358813.2		protein_c	chr1:149704411-149
ENSG0000(	1901	42.45417	chr1:2197	RPS27AP6		Pseudoger	chr1:150881236-150
ENSG0000(	1901	42.45417	chr1:2197	SPRR2E		protein_c	chr1:153093135-153
ENSG0000(	1901	42.45417	chr1:2197	ADAMTSL4-AS2		lncRNA	chr1:150548562-150
ENSG0000(	1901	42.45417	chr1:2197	UBE2D3P3		Pseudoger	chr1:150800473-150
ENSG0000(	1894	42.29784	chr1:1043	ENSG00000229911		Pseudoger	chr1:118712999-118
ENSG0000(	1894	42.29784	chr1:1043	REG4		protein_c	chr1:119794017-119
ENSG0000(	1894	42.29784	chr1:1043	RNU1-75P		smallRNA	chr1:119331397-119
ENSG0000(	1894	42.29784	chr1:1043	ENSG00000226172		lncRNA	chr1:119000344-119
ENSG0000(	1894	42.29784	chr1:1043	RNU6-465P		smallRNA	chr1:120126974-120
ENSG0000(	1894	42.29784	chr1:1043	NBPF7P		Pseudoger	chr1:119834870-119
ENSG0000(	1894	42.29784	chr1:1043	RBMX2P3		Pseudoger	chr1:119084998-119
ENSG0000(	1894	42.29784	chr1:1043	GAPDHP23		Pseudoger	chr1:119462029-119
ENSG0000(	1894	42.29784	chr1:1043	PSMC1P12		Pseudoger	chr1:118614333-118
ENSG0000(	1894	42.29784	chr1:1043	HSD3B2		protein_c	chr1:119414931-119
ENSG0000(	1894	42.29784	chr1:1043	RP5-1042I8.7		lncRNA	chr1:119909255-119
ENSG0000(	1894	42.29784	chr1:1043	WARS2-IT1		lncRNA	chr1:119047405-119

ENSG0000(	1894	42.29784	chr1:104:	WARS2-AS1		lncRNA	chr1:119140391-119
ENSG0000(	1894	42.29784	chr1:104:	snoU13		smallRNA	chr1:119259041-119
ENSG0000(	1894	42.29784	chr1:104:	HSD3BP2		Pseudoger	chr1:119439001-119
ENSG0000(	1894	42.29784	chr1:104:	HSD3B1		protein_c	chr1:119507198-119
ENSG0000(	1894	42.29784	chr1:104:	RPS3AP12		Pseudoger	chr1:119126539-119
ENSG0000(	1894	42.29784	chr1:104:	HSD3BP4		Pseudoger	chr1:119564066-119
ENSG0000(	1894	42.29784	chr1:104:	PFN1P9		Pseudoger	chr1:119853316-119
ENSG0000(	1894	42.29784	chr1:104:	RP6-42F4.1		lncRNA	chr1:120150758-120
ENSG0000(	1894	42.29784	chr1:104:	HMGCS2		protein_c	chr1:119748002-119
ENSG0000(	1894	42.29784	chr1:104:	HSD3BP3		Pseudoger	chr1:119538509-119
ENSG0000(	1894	42.29784	chr1:104:	GAPDHP32		Pseudoger	chr1:119533749-119
ENSG0000(	1894	42.29784	chr1:104:	GAPDHP58		Pseudoger	chr1:119495836-119
ENSG0000(	1894	42.29784	chr1:104:	HAO2-IT1		lncRNA	chr1:119368946-119
ENSG0000(	1894	42.29784	chr1:104:	LINC01780		lncRNA	chr1:119327399-119
ENSG0000(	1894	42.29784	chr1:104:	GAPDHP33		Pseudoger	chr1:119596167-119
ENSG0000(	1894	42.29784	chr1:104:	LINC00622		lncRNA	chr1:119597702-119
ENSG0000(	1894	42.29784	chr1:104:	WARS2		protein_c	chr1:119031216-119
ENSG0000(	1894	42.29784	chr1:104:	ENSG00000227712		lncRNA	chr1:119230313-119
ENSG0000(	1894	42.29784	chr1:104:	HAO2		protein_c	chr1:119368779-119
ENSG0000(	1894	42.29784	chr1:104:	RNA5SP56		Pseudoger	chr1:118264372-118
ENSG0000(	1894	42.29784	chr1:104:	NOTCH2P1		Pseudoger	chr1:119886304-119
ENSG0000(	1894	42.29784	chr1:104:	ENSG00000273406		lncRNA	chr1:120076616-120
ENSG0000(	1894	42.29784	chr1:104:	HSD3BP1		Pseudoger	chr1:119467221-119
ENSG0000(	1894	42.29784	chr1:104:	GAPDHP74		Pseudoger	chr1:119434166-119
ENSG0000(	1894	42.29784	chr1:104:	NOTCH2	NCGv7;AC	protein_c	chr1:119911553-120
ENSG0000(	1894	42.29784	chr1:104:	ZNF697	NCGv7	protein_c	chr1:119619377-119
ENSG0000(	1894	42.29784	chr1:104:	ADAM30		protein_c	chr1:119893533-119
ENSG0000(	1894	42.29784	chr1:104:	ENSG00000287980		lncRNA	chr1:118185349-118
ENSG0000(	1894	42.29784	chr1:104:	RPL6P2		Pseudoger	chr1:119219314-119
ENSG0000(	1894	42.29784	chr1:104:	TBX15		protein_c	chr1:118883046-118
ENSG0000(	1894	42.29784	chr1:104:	HSD3BP5		Pseudoger	chr1:119601340-119
ENSG0000(	1894	42.29784	chr1:104:	GAPDHP27		Pseudoger	chr1:119558755-119
ENSG0000(	1894	42.29784	chr1:104:	ENSG00000239216		lncRNA	chr1:119000618-119
ENSG0000(	1894	42.29784	chr1:104:	PHGDH	NCGv7	protein_c	chr1:119648411-119
ENSG0000(	1879	41.96285	chr1:219	RN7SL431P		smallRNA	chr1:154166247-154
ENSG0000(	1871	41.78419	chr1:219	AQP10		protein_c	chr1:154321090-154
ENSG0000(	1871	41.78419	chr1:219	RIT1	NCGv7;AC	protein_c	chr1:155897808-155
ENSG0000(	1871	41.78419	chr1:219	RNU6-106P		smallRNA	chr1:155358712-155
ENSG0000(	1871	41.78419	chr1:219	PKLR		protein_c	chr1:155289293-155
ENSG0000(	1871	41.78419	chr1:219	EFNA4		protein_c	chr1:155063737-155
ENSG0000(	1871	41.78419	chr1:219	GBAP1		lncRNA	chr1:155213821-155
ENSG0000(	1871	41.78419	chr1:219	KCNN3	NCGv7	protein_c	chr1:154697455-154
ENSG0000(	1871	41.78419	chr1:219	RNU6-121P		smallRNA	chr1:154297650-154
ENSG0000(	1871	41.78419	chr1:219	HCN3		protein_c	chr1:155277463-155
ENSG0000(	1871	41.78419	chr1:219	C1orf43		protein_c	chr1:154206696-154
ENSG0000(	1871	41.78419	chr1:219	POU5F1P4		Pseudoger	chr1:155433178-155
ENSG0000(	1871	41.78419	chr1:219	ENSG00000278694		Pseudoger	chr1:154312462-154
ENSG0000(	1871	41.78419	chr1:219	PSMD8P1		Pseudoger	chr1:154414369-154
ENSG0000(	1871	41.78419	chr1:219	ENSG00000271267		Pseudoger	chr1:155566050-155
ENSG0000(	1871	41.78419	chr1:219	HAX1	NCGv7;AC	protein_c	chr1:154272355-154
ENSG0000(	1871	41.78419	chr1:219	THBS3-AS1		lncRNA	chr1:155194996-155

ENSG0000(	1871	41.78419	chr1:2197	MIR5698		smallRNA	chr1:154104521-154
ENSG0000(	1871	41.78419	chr1:2197	AL713999.1		smallRNA	chr1:155236749-155
ENSG0000(	1871	41.78419	chr1:2197	ATP8B2	NCGv7	protein_c	chr1:154325525-154
ENSG0000(	1871	41.78419	chr1:2197	RUSC1-AS1		lncRNA	chr1:155316863-155
ENSG0000(	1871	41.78419	chr1:2197	ENSG00000271380		lncRNA	chr1:154961825-154
ENSG0000(	1871	41.78419	chr1:2197	MIR190B		smallRNA	chr1:154193665-154
ENSG0000(	1871	41.78419	chr1:2197	ADAM15		protein_c	chr1:155050566-155
ENSG0000(	1871	41.78419	chr1:2197	ASH1L-AS1		lncRNA	chr1:155562026-155
ENSG0000(	1871	41.78419	chr1:2197	RNU6-239P		smallRNA	chr1:154295503-154
ENSG0000(	1871	41.78419	chr1:2197	SYT11		protein_c	chr1:155859567-155
ENSG0000(	1871	41.78419	chr1:2197	SNORD59		smallRNA	chr1:154288460-154
ENSG0000(	1871	41.78419	chr1:2197	SEMA4A		protein_c	chr1:156147366-156
ENSG0000(	1871	41.78419	chr1:2197	TPM3	NCGv7;AC	protein_c	chr1:154155308-154
ENSG0000(	1871	41.78419	chr1:2197	NUP210L	NCGv7	protein_c	chr1:153992685-154
ENSG0000(	1871	41.78419	chr1:2197	RAB25		protein_c	chr1:156061160-156
ENSG0000(	1871	41.78419	chr1:2197	AL355388.1		smallRNA	chr1:156077373-156
ENSG0000(	1871	41.78419	chr1:2197	ENSG00000246203		Pseudoger	chr1:155614726-155
ENSG0000(	1871	41.78419	chr1:2197	ENSG00000287839		lncRNA	chr1:155626755-155
ENSG0000(	1871	41.78419	chr1:2197	KHDC4		protein_c	chr1:155913045-155
ENSG0000(	1871	41.78419	chr1:2197	MUC1	DriverDBv	protein_c	chr1:155185824-155
ENSG0000(	1871	41.78419	chr1:2197	RHBG	NCGv7	protein_c	chr1:156369211-156
ENSG0000(	1871	41.78419	chr1:2197	DAP3		protein_c	chr1:155687960-155
ENSG0000(	1871	41.78419	chr1:2197	ENSG00000236263		lncRNA	chr1:155211151-155
ENSG0000(	1871	41.78419	chr1:2197	RNU7-150P		smallRNA	chr1:155143271-155
ENSG0000(	1871	41.78419	chr1:2197	VHLL		protein_c	chr1:156298624-156
ENSG0000(	1871	41.78419	chr1:2197	CLK2		protein_c	chr1:155262868-155
ENSG0000(	1871	41.78419	chr1:2197	AL606500.1		protein_c	chr1:154612591-154
ENSG0000(	1871	41.78419	chr1:2197	UBAP2L		protein_c	chr1:154220179-154
ENSG0000(	1871	41.78419	chr1:2197	ENSG00000233875		lncRNA	chr1:154579065-154
ENSG0000(	1871	41.78419	chr1:2197	PMF1-BGLAP		protein_c	chr1:156212982-156
ENSG0000(	1871	41.78419	chr1:2197	MIR92B		smallRNA	chr1:155195177-155
ENSG0000(	1871	41.78419	chr1:2197	PAQR6		protein_c	chr1:156243320-156
ENSG0000(	1871	41.78419	chr1:2197	RNU6-1297P		smallRNA	chr1:155419397-155
ENSG0000(	1871	41.78419	chr1:2197	ARHGEF2-AS2		lncRNA	chr1:155978799-155
ENSG0000(	1871	41.78419	chr1:2197	ASH1L-IT1		lncRNA	chr1:155396010-155
ENSG0000(	1871	41.78419	chr1:2197	SHE	NCGv7	protein_c	chr1:154469772-154
ENSG0000(	1871	41.78419	chr1:2197	RPSAP17		Pseudoger	chr1:154378207-154
ENSG0000(	1871	41.78419	chr1:2197	ENSG00000273088		protein_c	chr1:155169409-155
ENSG0000(	1871	41.78419	chr1:2197	DPM3		protein_c	chr1:155139891-155
ENSG0000(	1871	41.78419	chr1:2197	ENSG00000286391		lncRNA	chr1:154564855-154
ENSG0000(	1871	41.78419	chr1:2197	MSTO1		protein_c	chr1:155563235-155
ENSG0000(	1871	41.78419	chr1:2197	MIR9-1HG		lncRNA	chr1:156404250-156
ENSG0000(	1871	41.78419	chr1:2197	SNORA58		smallRNA	chr1:154259727-154
ENSG0000(	1871	41.78419	chr1:2197	MSTO2P		Pseudoger	chr1:155745829-155
ENSG0000(	1871	41.78419	chr1:2197	GBA		protein_c	chr1:155234452-155
ENSG0000(	1871	41.78419	chr1:2197	AL589685.1		Pseudoger	chr1:156323511-156
ENSG0000(	1871	41.78419	chr1:2197	RNU4-19P		smallRNA	chr1:155894281-155
ENSG0000(	1871	41.78419	chr1:2197	SCARNA4		smallRNA	chr1:155925958-155
ENSG0000(	1871	41.78419	chr1:2197	LAMTOR2		protein_c	chr1:156054782-156
ENSG0000(	1871	41.78419	chr1:2197	ENSG00000227673		lncRNA	chr1:155710098-155
ENSG0000(	1871	41.78419	chr1:2197	ARHGEF2	AC	protein_c	chr1:155946851-156

ENSG0000(	1871	41.78419	chr1:2197	GON4L		protein_c	chr1:155749659-155
ENSG0000(	1871	41.78419	chr1:2197	ENSG00000270361		lncRNA	chr1:154937370-154
ENSG0000(	1871	41.78419	chr1:2197	ASH1L	NCGv7	protein_c	chr1:155335268-155
ENSG0000(	1871	41.78419	chr1:2197	SCAMP3		protein_c	chr1:155255979-155
ENSG0000(	1871	41.78419	chr1:2197	RPS7P2		Pseudoger	chr1:154078866-154
ENSG0000(	1871	41.78419	chr1:2197	DAP3P1		Pseudoger	chr1:155586644-155
ENSG0000(	1871	41.78419	chr1:2197	EFNA1		protein_c	chr1:155127876-155
ENSG0000(	1871	41.78419	chr1:2197	ENSG00000251246		protein_c	chr1:155063748-155
ENSG0000(	1871	41.78419	chr1:2197	ENSG00000273110		lncRNA	chr1:154480012-154
ENSG0000(	1871	41.78419	chr1:2197	SNORA42		smallRNA	chr1:155919909-155
ENSG0000(	1871	41.78419	chr1:2197	MEX3A	DriverDBv	protein_c	chr1:156072013-156
ENSG0000(	1871	41.78419	chr1:2197	ENSG00000234937		Pseudoger	chr1:155845367-155
ENSG0000(	1871	41.78419	chr1:2197	Y_RNA		smallRNA	chr1:155120490-155
ENSG0000(	1871	41.78419	chr1:2197	SNORA26		smallRNA	chr1:156192063-156
ENSG0000(	1871	41.78419	chr1:2197	ENSG00000237920		Pseudoger	chr1:154376966-154
ENSG0000(	1871	41.78419	chr1:2197	ARHGEF2-AS1		lncRNA	chr1:155991390-156
ENSG0000(	1871	41.78419	chr1:2197	IL6R-AS1		lncRNA	chr1:154402328-154
ENSG0000(	1871	41.78419	chr1:2197	ENSG00000232519		lncRNA	chr1:155609776-155
ENSG0000(	1871	41.78419	chr1:2197	UBE2Q1-AS1		lncRNA	chr1:154553609-154
ENSG0000(	1871	41.78419	chr1:2197	MIR9-1		smallRNA	chr1:156420331-156
ENSG0000(	1871	41.78419	chr1:2197	GLMP		protein_c	chr1:156290089-156
ENSG0000(	1871	41.78419	chr1:2197	ENSG00000287064		lncRNA	chr1:154671593-154
ENSG0000(	1871	41.78419	chr1:2197	BGLAP		protein_c	chr1:156242184-156
ENSG0000(	1871	41.78419	chr1:2197	CKS1B	AC	protein_c	chr1:154974653-154
ENSG0000(	1871	41.78419	chr1:2197	MTX1		protein_c	chr1:155208695-155
ENSG0000(	1871	41.78419	chr1:2197	DCST1-AS1		lncRNA	chr1:155045191-155
ENSG0000(	1871	41.78419	chr1:2197	THBS3		protein_c	chr1:155195588-155
ENSG0000(	1871	41.78419	chr1:2197	snoU13		smallRNA	chr1:155415558-155
ENSG0000(	1871	41.78419	chr1:2197	RXFP4		protein_c	chr1:155941638-155
ENSG0000(	1871	41.78419	chr1:2197	ENSG00000237390		lncRNA	chr1:156388226-156
ENSG0000(	1871	41.78419	chr1:2197	ENSG00000289103		lncRNA	chr1:155000799-155
ENSG0000(	1871	41.78419	chr1:2197	SLC50A1		protein_c	chr1:155135344-155
ENSG0000(	1871	41.78419	chr1:2197	SMG5	NCGv7	protein_c	chr1:156249224-156
ENSG0000(	1871	41.78419	chr1:2197	EFNA3		protein_c	chr1:155078837-155
ENSG0000(	1871	41.78419	chr1:2197	MIR4258		smallRNA	chr1:154975693-154
ENSG0000(	1871	41.78419	chr1:2197	YY1AP1	AC	protein_c	chr1:155659443-155
ENSG0000(	1871	41.78419	chr1:2197	SLC25A44		protein_c	chr1:156193932-156
ENSG0000(	1871	41.78419	chr1:2197	PMF1		protein_c	chr1:156212993-156
ENSG0000(	1871	41.78419	chr1:2197	MIR555		smallRNA	chr1:155346350-155
ENSG0000(	1871	41.78419	chr1:2197	ENSG00000223503		Pseudoger	chr1:155590601-155
ENSG0000(	1871	41.78419	chr1:2197	TRIM46	DriverDBv	protein_c	chr1:155173787-155
ENSG0000(	1871	41.78419	chr1:2197	SHC1		protein_c	chr1:154962298-154
ENSG0000(	1871	41.78419	chr1:2197	GBAP1		Pseudoger	chr1:155214368-155
ENSG0000(	1871	41.78419	chr1:2197	KRTCAP2		protein_c	chr1:155169408-155
ENSG0000(	1871	41.78419	chr1:2197	TSACC		protein_c	chr1:156337314-156
ENSG0000(	1871	41.78419	chr1:2197	CCT3		protein_c	chr1:156308968-156
ENSG0000(	1871	41.78419	chr1:2197	HMGN2P18		Pseudoger	chr1:155148544-155
ENSG0000(	1871	41.78419	chr1:2197	TMEM79		protein_c	chr1:156282935-156
ENSG0000(	1871	41.78419	chr1:2197	SSR2		protein_c	chr1:156009048-156
ENSG0000(	1871	41.78419	chr1:2197	RUSC1		protein_c	chr1:155320894-155
ENSG0000(	1871	41.78419	chr1:2197	FDPS	AC	protein_c	chr1:155308748-155

ENSG0000(	1871	41.78419	chr1:2197	CHRNB2	NCGv7	protein_c	chr1:154567778-154
ENSG0000(	1871	41.78419	chr1:2197	AL590431.1		smallRNA	chr1:154254445-154
ENSG0000(	1871	41.78419	chr1:2197	FLAD1		protein_c	chr1:154983338-154
ENSG0000(	1871	41.78419	chr1:2197	UBE2Q1		protein_c	chr1:154548577-154
ENSG0000(	1871	41.78419	chr1:2197	IL6R		protein_c	chr1:154405193-154
ENSG0000(	1871	41.78419	chr1:2197	ADAR		protein_c	chr1:154581695-154
ENSG0000(	1871	41.78419	chr1:2197	LMNA		protein_c	chr1:156082573-156
ENSG0000(	1871	41.78419	chr1:2197	FAM189B	AC	protein_c	chr1:155247205-155
ENSG0000(	1871	41.78419	chr1:2197	PBXIP1		protein_c	chr1:154944076-154
ENSG0000(	1871	41.78419	chr1:2197	DCST2	DriverDBv	protein_c	chr1:155018520-155
ENSG0000(	1871	41.78419	chr1:2197	TDRD10	NCGv7	protein_c	chr1:154502219-154
ENSG0000(	1871	41.78419	chr1:2197	CFAP141		protein_c	chr1:154199085-154
ENSG0000(	1871	41.78419	chr1:2197	ZBTB7B	NCGv7	protein_c	chr1:155002630-155
ENSG0000(	1871	41.78419	chr1:2197	ENSG00000285677		lncRNA	chr1:156001953-156
ENSG0000(	1871	41.78419	chr1:2197	MTX1P1		Pseudoger	chr1:155230975-155
ENSG0000(	1871	41.78419	chr1:2197	PMVK		protein_c	chr1:154924740-154
ENSG0000(	1871	41.78419	chr1:2197	PYGO2		protein_c	chr1:154957026-154
ENSG0000(	1871	41.78419	chr1:2197	LENEP	DriverDBv	protein_c	chr1:154993586-154
ENSG0000(	1871	41.78419	chr1:2197	RNU7-57P		smallRNA	chr1:154338743-154
ENSG0000(	1871	41.78419	chr1:2197	DCST1	DriverDBv	protein_c	chr1:155033824-155
ENSG0000(	1871	41.78419	chr1:2197	UBQLN4		protein_c	chr1:156035299-156
ENSG0000(	1867	41.69486	chr1:1043	AL157904.1		smallRNA	chr1:116905150-116
ENSG0000(	1867	41.69486	chr1:1043	PTGFRN	NCGv7	protein_c	chr1:116909916-116
ENSG0000(	1867	41.69486	chr1:1043	TTF2		protein_c	chr1:117060326-117
ENSG0000(	1867	41.69486	chr1:1043	RNA5SP55		Pseudoger	chr1:116962347-116
ENSG0000(	1867	41.69486	chr1:1043	AL355794.1		smallRNA	chr1:116592106-116
ENSG0000(	1867	41.69486	chr1:1043	RPS15AP9		Pseudoger	chr1:117138229-117
ENSG0000(	1867	41.69486	chr1:1043	MAN1A2	NCGv7	protein_c	chr1:117367449-117
ENSG0000(	1867	41.69486	chr1:1043	FTH1P22		Pseudoger	chr1:116775104-116
ENSG0000(	1867	41.69486	chr1:1043	SPAG17	NCGv7	protein_c	chr1:117953590-118
ENSG0000(	1867	41.69486	chr1:1043	ENSG00000279513		TEC	chr1:117493515-117
ENSG0000(	1867	41.69486	chr1:1043	LINC02868		lncRNA	chr1:116694112-116
ENSG0000(	1867	41.69486	chr1:1043	ENSG00000270719		Pseudoger	chr1:117700029-117
ENSG0000(	1867	41.69486	chr1:1043	ENSG00000271427		lncRNA	chr1:117364899-117
ENSG0000(	1867	41.69486	chr1:1043	GDAP2		protein_c	chr1:117863485-117
ENSG0000(	1867	41.69486	chr1:1043	CD2		protein_c	chr1:116754430-116
ENSG0000(	1867	41.69486	chr1:1043	ENSG00000272715		lncRNA	chr1:116909149-116
ENSG0000(	1867	41.69486	chr1:1043	LINC01525		lncRNA	chr1:117272182-117
ENSG0000(	1867	41.69486	chr1:1043	CD101-AS1		lncRNA	chr1:117025482-117
ENSG0000(	1867	41.69486	chr1:1043	CD101		protein_c	chr1:117001750-117
ENSG0000(	1867	41.69486	chr1:1043	IGSF3		protein_c	chr1:116574399-116
ENSG0000(	1867	41.69486	chr1:1043	NEFHP1		Pseudoger	chr1:116739981-116
ENSG0000(	1867	41.69486	chr1:1043	MIR320B1		smallRNA	chr1:116671749-116
ENSG0000(	1867	41.69486	chr1:1043	VDAC2P3		Pseudoger	chr1:117640812-117
ENSG0000(	1867	41.69486	chr1:1043	MIR942		smallRNA	chr1:117094643-117
ENSG0000(	1867	41.69486	chr1:1043	VTCN1		protein_c	chr1:117143587-117
ENSG0000(	1867	41.69486	chr1:1043	PNRC2P1		Pseudoger	chr1:117778087-117
ENSG0000(	1867	41.69486	chr1:1043	TRIM45		protein_c	chr1:117111060-117
ENSG0000(	1867	41.69486	chr1:1043	GAPDHP64		Pseudoger	chr1:116713833-116
ENSG0000(	1867	41.69486	chr1:1043	TENT5C-DT		lncRNA	chr1:117596832-117
ENSG0000(	1867	41.69486	chr1:1043	WDR3		protein_c	chr1:117929720-117

ENSG0000(	1867	41.69486	chr1:104:	TENT5C	NCGv7;AC	protein_c	chr1:117606048-117
ENSG0000(	1867	41.69486	chr1:104:	VPS25P1		Pseudoger	chr1:117549415-117
ENSG0000(	1867	41.69486	chr1:104:	SNORA40		smallRNA	chr1:117688621-117
ENSG0000(	1858	41.49387	chr2:309	AC013268.1		smallRNA	chr2:110126732-110
ENSG0000(	1849	41.29287	chr1:104:	ENSG00000287103		lncRNA	chr1:115356664-115
ENSG0000(	1849	41.29287	chr1:104:	LINC01762		lncRNA	chr1:116423724-116
ENSG0000(	1849	41.29287	chr1:104:	CNOT7P2		Pseudoger	chr1:115564601-115
ENSG0000(	1849	41.29287	chr1:104:	ENSG00000286276		lncRNA	chr1:116493350-116
ENSG0000(	1849	41.29287	chr1:104:	VANGL1	NCGv7	protein_c	chr1:115641970-115
ENSG0000(	1849	41.29287	chr1:104:	MAB21L3		protein_c	chr1:116111399-116
ENSG0000(	1849	41.29287	chr1:104:	SNORA42		smallRNA	chr1:115621872-115
ENSG0000(	1849	41.29287	chr1:104:	AL512638.1		smallRNA	chr1:115485651-115
ENSG0000(	1849	41.29287	chr1:104:	ENSG00000271143		Pseudoger	chr1:115916497-115
ENSG0000(	1849	41.29287	chr1:104:	ENSG00000226973		Pseudoger	chr1:115471941-115
ENSG0000(	1849	41.29287	chr1:104:	ENSG00000270631		Pseudoger	chr1:115577229-115
ENSG0000(	1849	41.29287	chr1:104:	NGF		protein_c	chr1:115285904-115
ENSG0000(	1849	41.29287	chr1:104:	NAP1L4P1		Pseudoger	chr1:116532936-116
ENSG0000(	1849	41.29287	chr1:104:	NHLH2		protein_c	chr1:115836377-115
ENSG0000(	1849	41.29287	chr1:104:	Y_RNA		smallRNA	chr1:116452536-116
ENSG0000(	1849	41.29287	chr1:104:	ENSG00000287217		lncRNA	chr1:116289237-116
ENSG0000(	1849	41.29287	chr1:104:	ENSG00000224950		lncRNA	chr1:116493016-116
ENSG0000(	1849	41.29287	chr1:104:	ENSG00000230381		lncRNA	chr1:116429049-116
ENSG0000(	1849	41.29287	chr1:104:	AL136376.1		protein_c	chr1:116373268-116
ENSG0000(	1849	41.29287	chr1:104:	U3		smallRNA	chr1:116278606-116
ENSG0000(	1849	41.29287	chr1:104:	SLC22A15		protein_c	chr1:115976513-116
ENSG0000(	1849	41.29287	chr1:104:	CASQ2		protein_c	chr1:115700021-115
ENSG0000(	1849	41.29287	chr1:104:	RN7SL420P		smallRNA	chr1:115606471-115
ENSG0000(	1849	41.29287	chr1:104:	RNU6-817P		smallRNA	chr1:116413766-116
ENSG0000(	1849	41.29287	chr1:104:	ATP1A1-AS1		lncRNA	chr1:116378437-116
ENSG0000(	1849	41.29287	chr1:104:	ENSG00000274468		Pseudoger	chr1:115479428-115
ENSG0000(	1849	41.29287	chr1:104:	LINC01779		lncRNA	chr1:116164209-116
ENSG0000(	1849	41.29287	chr1:104:	NGF-AS1		lncRNA	chr1:115283034-115
ENSG0000(	1849	41.29287	chr1:104:	ENSG00000237993		lncRNA	chr1:116013813-116
ENSG0000(	1849	41.29287	chr1:104:	MIR548AC		smallRNA	chr1:116560024-116
ENSG0000(	1849	41.29287	chr1:104:	LINC01649		lncRNA	chr1:115904855-115
ENSG0000(	1849	41.29287	chr1:104:	ELOCP20		Pseudoger	chr1:115556826-115
ENSG0000(	1849	41.29287	chr1:104:	ENSG00000285698		lncRNA	chr1:115270767-115
ENSG0000(	1849	41.29287	chr1:104:	HNRNPA1P43		Pseudoger	chr1:115856910-115
ENSG0000(	1849	41.29287	chr1:104:	CD58	NCGv7	protein_c	chr1:116514534-116
ENSG0000(	1849	41.29287	chr1:104:	ATP1A1	NCGv7;AC	protein_c	chr1:116372668-116
ENSG0000(	1848	41.27054	chr2:309	INPP4A	NCGv7	protein_c	chr2:98444854-9859
ENSG0000(	1848	41.27054	chr2:309	AC092675.1		smallRNA	chr2:98296938-9829
ENSG0000(	1848	41.27054	chr2:309	ENSG00000222000		lncRNA	chr2:98331389-9835
ENSG0000(	1848	41.27054	chr2:309	ATP5F1BP1		Pseudoger	chr2:98206151-9820
ENSG0000(	1848	41.27054	chr2:309	ENSG00000227987		lncRNA	chr2:98346995-9835
ENSG0000(	1848	41.27054	chr2:309	CNGA3		protein_c	chr2:98346188-9839
ENSG0000(	1832	40.91322	chr1:114	PPIAP34		Pseudoger	chr1:22322840-2232
ENSG0000(	1832	40.91322	chr1:114	RP1-224A6.9		lncRNA	chr1:22100613-2210
ENSG0000(	1832	40.91322	chr1:114	LINC01355		lncRNA	chr1:23281307-2328
ENSG0000(	1832	40.91322	chr1:114	ID3	NCGv7	protein_c	chr1:23557926-2355
ENSG0000(	1832	40.91322	chr1:114	ENSG00000289014		lncRNA	chr1:23167098-2316

ENSG0000(	1832	40.91322	chr1:1142	MIR4418		smallRNA	chr1:22266239-2226
ENSG0000(	1832	40.91322	chr1:1142	ENSG00000284726		lncRNA	chr1:23297797-2330
ENSG0000(	1832	40.91322	chr1:1142	ENSG00000289694		protein_c	chr1:22025142-2209
ENSG0000(	1832	40.91322	chr1:1142	CDC42-AS1		lncRNA	chr1:22028317-2205
ENSG0000(	1832	40.91322	chr1:1142	ENSG00000289692		protein_c	chr1:22636506-2264
ENSG0000(	1832	40.91322	chr1:1142	LINC00339		lncRNA	chr1:22024558-2203
ENSG0000(	1832	40.91322	chr1:1142	MPHOSPH6P1		Pseudoger	chr1:22068340-2206
ENSG0000(	1832	40.91322	chr1:1142	RNU6-135P		smallRNA	chr1:23163953-2316
ENSG0000(	1832	40.91322	chr1:1142	ZBTB40-IT1		lncRNA	chr1:22517474-2251
ENSG0000(	1832	40.91322	chr1:1142	ENSG00000229010		Pseudoger	chr1:23140325-2314
ENSG0000(	1832	40.91322	chr1:1142	Y_RNA		smallRNA	chr1:23370254-2337
ENSG0000(	1832	40.91322	chr1:1142	ENSG00000235052		lncRNA	chr1:23549139-2355
ENSG0000(	1832	40.91322	chr1:1142	ENSG00000232482		Pseudoger	chr1:23410832-2341
ENSG0000(	1832	40.91322	chr1:1142	WNT4		protein_c	chr1:22117313-2214
ENSG0000(	1832	40.91322	chr1:1142	MIR3115		smallRNA	chr1:23044305-2304
ENSG0000(	1832	40.91322	chr1:1142	C1QB		protein_c	chr1:22652762-2266
ENSG0000(	1832	40.91322	chr1:1142	C1QA		protein_c	chr1:22635077-2263
ENSG0000(	1832	40.91322	chr1:1142	ENSG00000285794		Pseudoger	chr1:22030527-2203
ENSG0000(	1832	40.91322	chr1:1142	TEX46		protein_c	chr1:23010834-2301
ENSG0000(	1832	40.91322	chr1:1142	ZNF436		protein_c	chr1:23359448-2336
ENSG0000(	1832	40.91322	chr1:1142	HTR1D		protein_c	chr1:23191895-2321
ENSG0000(	1832	40.91322	chr1:1142	TCEA3		protein_c	chr1:23380909-2342
ENSG0000(	1832	40.91322	chr1:1142	ASAP3		protein_c	chr1:23428563-2348
ENSG0000(	1832	40.91322	chr1:1142	CDC42	NCGv7	protein_c	chr1:22052627-2210
ENSG0000(	1832	40.91322	chr1:1142	EPHA8		protein_c	chr1:22563489-2260
ENSG0000(	1832	40.91322	chr1:1142	MIR4419A		smallRNA	chr1:23057858-2305
ENSG0000(	1832	40.91322	chr1:1142	HNRNPR		protein_c	chr1:23303771-2334
ENSG0000(	1832	40.91322	chr1:1142	MIR4684		smallRNA	chr1:22719517-2271
ENSG0000(	1832	40.91322	chr1:1142	ENSG00000271420		lncRNA	chr1:23378380-2337
ENSG0000(	1832	40.91322	chr1:1142	ENSG00000285873		lncRNA	chr1:22142850-2215
ENSG0000(	1832	40.91322	chr1:1142	MIR4253		smallRNA	chr1:22863159-2286
ENSG0000(	1832	40.91322	chr1:1142	ENSG00000225952		lncRNA	chr1:22835713-2283
ENSG0000(	1832	40.91322	chr1:1142	ZBTB40		protein_c	chr1:22428838-2253
ENSG0000(	1832	40.91322	chr1:1142	C1QC		protein_c	chr1:22643014-2264
ENSG0000(	1832	40.91322	chr1:1142	ENSG00000240553		lncRNA	chr1:23020147-2308
ENSG0000(	1832	40.91322	chr1:1142	KDM1A	NCGv7	protein_c	chr1:23019443-2308
ENSG0000(	1832	40.91322	chr1:1142	RNU6-514P		smallRNA	chr1:23162704-2316
ENSG0000(	1832	40.91322	chr1:1142	CDC42-IT1		lncRNA	chr1:22059197-2206
ENSG0000(	1832	40.91322	chr1:1142	LINC01635		lncRNA	chr1:22023990-2202
ENSG0000(	1832	40.91322	chr1:1142	EPHB2		protein_c	chr1:22710839-2292
ENSG0000(	1832	40.91322	chr1:1142	AL611946.1		smallRNA	chr1:22748817-2274
ENSG0000(	1832	40.91322	chr1:1142	ENSG00000279625		TEC	chr1:22364630-2236
ENSG0000(	1832	40.91322	chr1:1142	LACTBL1		protein_c	chr1:22953043-2297
ENSG0000(	1832	40.91322	chr1:1142	ENSG00000215381		Pseudoger	chr1:23244765-2324
ENSG0000(	1832	40.91322	chr1:1142	ZNF436-AS1		lncRNA	chr1:23368939-2337
ENSG0000(	1832	40.91322	chr1:1142	LUZP1	NCGv7	protein_c	chr1:23084030-2317
ENSG0000(	1832	40.91322	chr1:1142	E2F2	NCGv7	protein_c	chr1:23506438-2353
ENSG0000(	1819	40.6229	chr1:1043	NUTF2P4		Pseudoger	chr1:112748095-112
ENSG0000(	1816	40.5559	chr1:1142	ENSG00000280222		TEC	chr1:18109389-1811
ENSG0000(	1816	40.5559	chr1:1142	ENSG00000238142		lncRNA	chr1:16887577-1688
ENSG0000(	1816	40.5559	chr1:1142	ENSG00000272084		lncRNA	chr1:19072110-1907

ENSG0000(	1816	40.5559	chr1:1142	ENSG00000289402		lncRNA	chr1:20486358-2048
ENSG0000(	1816	40.5559	chr1:1142	RN7SL386P		smallRNA	chr1:21987481-2198
ENSG0000(	1816	40.5559	chr1:1142	RNU6-1022P		smallRNA	chr1:21987816-2198
ENSG0000(	1816	40.5559	chr1:1142	ENSG00000270728		Pseudoger	chr1:19297080-1929
ENSG0000(	1816	40.5559	chr1:1142	ENSG00000228549		lncRNA	chr1:16870945-1688
ENSG0000(	1816	40.5559	chr1:1142	MRTO4		protein_c	chr1:19251805-1926
ENSG0000(	1816	40.5559	chr1:1142	AKR7A2		protein_c	chr1:19303965-1931
ENSG0000(	1816	40.5559	chr1:1142	RPS4XP4		Pseudoger	chr1:20525989-2052
ENSG0000(	1816	40.5559	chr1:1142	FAM43B		protein_c	chr1:20552573-2055
ENSG0000(	1816	40.5559	chr1:1142	PDE4DIPP10		Pseudoger	chr1:21411460-2141
ENSG0000(	1816	40.5559	chr1:1142	RP13-279N23.2		protein_c	chr1:18849273-1892
ENSG0000(	1816	40.5559	chr1:1142	PPP1R11P1		Pseudoger	chr1:21397987-2139
ENSG0000(	1816	40.5559	chr1:1142	AC004824.1		smallRNA	chr1:17413631-1741
ENSG0000(	1816	40.5559	chr1:1142	RP5-930J4.4		lncRNA	chr1:20742987-2074
ENSG0000(	1816	40.5559	chr1:1142	RNF186		protein_c	chr1:19814029-1981
ENSG0000(	1816	40.5559	chr1:1142	ACTL8		protein_c	chr1:17755333-1782
ENSG0000(	1816	40.5559	chr1:1142	PADI2	AC	protein_c	chr1:17066761-1711
ENSG0000(	1816	40.5559	chr1:1142	RCC2-AS1		lncRNA	chr1:17406760-1740
ENSG0000(	1816	40.5559	chr1:1142	ENSG00000286898		lncRNA	chr1:16976302-1697
ENSG0000(	1816	40.5559	chr1:1142	SDHB	NCGv7;AC	protein_c	chr1:17018722-1705
ENSG0000(	1816	40.5559	chr1:1142	RCC2		protein_c	chr1:17406760-1743
ENSG0000(	1816	40.5559	chr1:1142	MIR3972		smallRNA	chr1:17277889-1727
ENSG0000(	1816	40.5559	chr1:1142	MFAP2		protein_c	chr1:16974502-1698
ENSG0000(	1816	40.5559	chr1:1142	KLHDC7A		protein_c	chr1:18480930-1848
ENSG0000(	1816	40.5559	chr1:1142	AKR7L		protein_c	chr1:19265982-1927
ENSG0000(	1816	40.5559	chr1:1142	MIR4695		smallRNA	chr1:18883202-1888
ENSG0000(	1816	40.5559	chr1:1142	IGSF21		protein_c	chr1:18107798-1837
ENSG0000(	1816	40.5559	chr1:1142	PADI6		protein_c	chr1:17372196-1740
ENSG0000(	1816	40.5559	chr1:1142	ENSG00000236073		Pseudoger	chr1:21073639-2107
ENSG0000(	1816	40.5559	chr1:1142	TAS1R2		protein_c	chr1:18839599-1885
ENSG0000(	1816	40.5559	chr1:1142	PLA2G2D		protein_c	chr1:20111939-2011
ENSG0000(	1816	40.5559	chr1:1142	PINK1-AS		lncRNA	chr1:20642657-2065
ENSG0000(	1816	40.5559	chr1:1142	KIF17		protein_c	chr1:20664014-2071
ENSG0000(	1816	40.5559	chr1:1142	ECE1		protein_c	chr1:21217247-2134
ENSG0000(	1816	40.5559	chr1:1142	ENSG00000288636		protein_c	chr1:17005068-1701
ENSG0000(	1816	40.5559	chr1:1142	EMC1-AS1		lncRNA	chr1:19210348-1924
ENSG0000(	1816	40.5559	chr1:1142	ENSG00000236009		lncRNA	chr1:21415898-2141
ENSG0000(	1816	40.5559	chr1:1142	RN7SL304P		smallRNA	chr1:19970969-1997
ENSG0000(	1816	40.5559	chr1:1142	Y_RNA		smallRNA	chr1:17158197-1715
ENSG0000(	1816	40.5559	chr1:1142	PLA2G2F		protein_c	chr1:20139323-2015
ENSG0000(	1816	40.5559	chr1:1142	NBL1	NCGv7	protein_c	chr1:19596979-1965
ENSG0000(	1816	40.5559	chr1:1142	AC004824.2		protein_c	chr1:17329012-1734
ENSG0000(	1816	40.5559	chr1:1142	LINC01654		lncRNA	chr1:18065657-1807
ENSG0000(	1816	40.5559	chr1:1142	CELA3B		protein_c	chr1:21977022-2199
ENSG0000(	1816	40.5559	chr1:1142	ENSG00000226526		lncRNA	chr1:16978926-1700
ENSG0000(	1816	40.5559	chr1:1142	ENSG00000235241		Pseudoger	chr1:16889095-1688
ENSG0000(	1816	40.5559	chr1:1142	ENSG00000283234		Pseudoger	chr1:21950679-2195
ENSG0000(	1816	40.5559	chr1:1142	DYNLL1P3		Pseudoger	chr1:18513118-1851
ENSG0000(	1816	40.5559	chr1:1142	IGSF21-AS1		lncRNA	chr1:18166929-1817
ENSG0000(	1816	40.5559	chr1:1142	ENSG00000226487		lncRNA	chr1:20412304-2041
ENSG0000(	1816	40.5559	chr1:1142	ENSG00000284743		lncRNA	chr1:20478779-2048

ENSG0000(	1816	40.5559	chr1:1142	RN7SL768P	smallRNA	chr1:22003585-2200
ENSG0000(	1816	40.5559	chr1:1142	RN7SL85P	smallRNA	chr1:19319805-1932
ENSG0000(	1816	40.5559	chr1:1142	ENSG00000272426	lncRNA	chr1:16904339-1690
ENSG0000(	1816	40.5559	chr1:1142	PADI4	protein_c	chr1:17308195-1736
ENSG0000(	1816	40.5559	chr1:1142	ENSG00000282843	lncRNA	chr1:17193232-1720
ENSG0000(	1816	40.5559	chr1:1142	ATP13A2	protein_c	chr1:16985958-1701
ENSG0000(	1816	40.5559	chr1:1142	ENSG00000225387	lncRNA	chr1:18385829-1838
ENSG0000(	1816	40.5559	chr1:1142	ENSG00000225478	Pseudoger	chr1:18595414-1859
ENSG0000(	1816	40.5559	chr1:1142	ENSG00000235432	Pseudoger	chr1:20692734-2069
ENSG0000(	1816	40.5559	chr1:1142	RNF186-AS1	lncRNA	chr1:19814367-1981
ENSG0000(	1816	40.5559	chr1:1142	RNU1-2	smallRNA	chr1:16895980-1689
ENSG0000(	1816	40.5559	chr1:1142	RNU7-200P	smallRNA	chr1:20841241-2084
ENSG0000(	1816	40.5559	chr1:1142	ENSG00000226396	Pseudoger	chr1:19608114-1960
ENSG0000(	1816	40.5559	chr1:1142	AL031005.1	smallRNA	chr1:21176566-2117
ENSG0000(	1816	40.5559	chr1:1142	ENSG00000284710	lncRNA	chr1:20272018-2027
ENSG0000(	1816	40.5559	chr1:1142	AL391357.1	Pseudoger	chr1:20650363-2065
ENSG0000(	1816	40.5559	chr1:1142	HTR6	protein_c	chr1:19664875-1968
ENSG0000(	1816	40.5559	chr1:1142	LINC01757	lncRNA	chr1:20243095-2024
ENSG0000(	1816	40.5559	chr1:1142	ALDH4A1	protein_c	chr1:18871430-1890
ENSG0000(	1816	40.5559	chr1:1142	VWA5B1	protein_c	chr1:20290875-2035
ENSG0000(	1816	40.5559	chr1:1142	CAMK2N1	protein_c	chr1:20482391-2048
ENSG0000(	1816	40.5559	chr1:1142	CDA	protein_c	chr1:20589086-2061
ENSG0000(	1816	40.5559	chr1:1142	UBXN10	protein_c	chr1:20186096-2019
ENSG0000(	1816	40.5559	chr1:1142	RNU6-1099P	smallRNA	chr1:19305076-1930
ENSG0000(	1816	40.5559	chr1:1142	PINK1	protein_c	chr1:20633458-2065
ENSG0000(	1816	40.5559	chr1:1142	ENSG00000226664	lncRNA	chr1:20294211-2032
ENSG0000(	1816	40.5559	chr1:1142	TMCO4	protein_c	chr1:19682240-1979
ENSG0000(	1816	40.5559	chr1:1142	RNU6-776P	smallRNA	chr1:22010985-2201
ENSG0000(	1816	40.5559	chr1:1142	MICOS10	protein_c	chr1:19484403-1962
ENSG0000(	1816	40.5559	chr1:1142	AKR7A3	protein_c	chr1:19282573-1928
ENSG0000(	1816	40.5559	chr1:1142	CROCCP5	Pseudoger	chr1:21434318-2143
ENSG0000(	1816	40.5559	chr1:1142	RN7SL186P	smallRNA	chr1:22010650-2201
ENSG0000(	1816	40.5559	chr1:1142	ENSG00000232037	Pseudoger	chr1:21908098-2190
ENSG0000(	1816	40.5559	chr1:1142	ENSG00000231978	Pseudoger	chr1:21768269-2176
ENSG0000(	1816	40.5559	chr1:1142	DDOST	protein_c	chr1:20651767-2066
ENSG0000(	1816	40.5559	chr1:1142	HSPE1P27	Pseudoger	chr1:21161475-2116
ENSG0000(	1816	40.5559	chr1:1142	PAX7 AC	protein_c	chr1:18630846-1874
ENSG0000(	1816	40.5559	chr1:1142	ECE1-AS1	lncRNA	chr1:21293290-2129
ENSG0000(	1816	40.5559	chr1:1142	SLC66A1	protein_c	chr1:19312326-1932
ENSG0000(	1816	40.5559	chr1:1142	PFN1P10	Pseudoger	chr1:21459756-2146
ENSG0000(	1816	40.5559	chr1:1142	MICOS10-DT	lncRNA	chr1:19591802-1959
ENSG0000(	1816	40.5559	chr1:1142	ENSG00000236936	lncRNA	chr1:21266082-2126
ENSG0000(	1816	40.5559	chr1:1142	ALPL	protein_c	chr1:21509397-2157
ENSG0000(	1816	40.5559	chr1:1142	PADI3	protein_c	chr1:17249098-1728
ENSG0000(	1816	40.5559	chr1:1142	ENSG00000284653	lncRNA	chr1:18015712-1804
ENSG0000(	1816	40.5559	chr1:1142	RN7SL421P	smallRNA	chr1:21978951-2197
ENSG0000(	1816	40.5559	chr1:1142	ENSG00000286064	Pseudoger	chr1:19260521-1926
ENSG0000(	1816	40.5559	chr1:1142	snoU13	smallRNA	chr1:19532170-1953
ENSG0000(	1816	40.5559	chr1:1142	LDLRAD2	protein_c	chr1:21812265-2182
ENSG0000(	1816	40.5559	chr1:1142	MICOS10-NBL1	protein_c	chr1:19597067-1965
ENSG0000(	1816	40.5559	chr1:1142	PADI1	protein_c	chr1:17205128-1724

ENSG0000(	1816	40.5559	chr1:1142	SH2D5		protein_c	chr1:20719731-2073
ENSG0000(	1816	40.5559	chr1:1142	LINC02596		lncRNA	chr1:21586472-2159
ENSG0000(	1816	40.5559	chr1:1142	LINC02810		lncRNA	chr1:17717625-1774
ENSG0000(	1816	40.5559	chr1:1142	EMC1		protein_c	chr1:19215660-1925
ENSG0000(	1816	40.5559	chr1:1142	HS6ST1P1		Pseudoger	chr1:21428303-2142
ENSG0000(	1816	40.5559	chr1:1142	AL137127.1		smallRNA	chr1:19083552-1908
ENSG0000(	1816	40.5559	chr1:1142	ENSG00000289715		protein_c	chr1:21547404-2155
ENSG0000(	1816	40.5559	chr1:1142	EIF4G3		protein_c	chr1:20806292-2117
ENSG0000(	1816	40.5559	chr1:1142	AL359815.1		smallRNA	chr1:21592411-2159
ENSG0000(	1816	40.5559	chr1:1142	RAP1GAP		protein_c	chr1:21596221-2166
ENSG0000(	1816	40.5559	chr1:1142	PLA2G5		protein_c	chr1:20028179-2009
ENSG0000(	1816	40.5559	chr1:1142	PLA2G2C		protein_c	chr1:20161253-2018
ENSG0000(	1816	40.5559	chr1:1142	RN7SL277P		smallRNA	chr1:19424384-1942
ENSG0000(	1816	40.5559	chr1:1142	MUL1		protein_c	chr1:20499448-2050
ENSG0000(	1816	40.5559	chr1:1142	ENSG00000290122		lncRNA	chr1:16905199-1690
ENSG0000(	1816	40.5559	chr1:1142	UBR4		protein_c	chr1:19074510-1921
ENSG0000(	1816	40.5559	chr1:1142	ENSG00000227066		lncRNA	chr1:20154171-2016
ENSG0000(	1816	40.5559	chr1:1142	MIR1290		smallRNA	chr1:18897071-1889
ENSG0000(	1816	40.5559	chr1:1142	HP1BP3		protein_c	chr1:20740266-2078
ENSG0000(	1816	40.5559	chr1:1142	USP48		protein_c	chr1:21678298-2178
ENSG0000(	1816	40.5559	chr1:1142	LINC01141		lncRNA	chr1:20360579-2043
ENSG0000(	1816	40.5559	chr1:1142	IFFO2		protein_c	chr1:18904280-1895
ENSG0000(	1816	40.5559	chr1:1142	MIR1256		smallRNA	chr1:20988314-2098
ENSG0000(	1816	40.5559	chr1:1142	ENSG00000290096		lncRNA	chr1:17439186-1743
ENSG0000(	1816	40.5559	chr1:1142	LINC02783		lncRNA	chr1:17189783-1719
ENSG0000(	1816	40.5559	chr1:1142	HSPG2	NCGv7	protein_c	chr1:21822244-2193
ENSG0000(	1816	40.5559	chr1:1142	NBPF3		protein_c	chr1:21440128-2148
ENSG0000(	1816	40.5559	chr1:1142	CELA3A		protein_c	chr1:22001657-2201
ENSG0000(	1816	40.5559	chr1:1142	ENSG00000233069		lncRNA	chr1:20732880-2073
ENSG0000(	1816	40.5559	chr1:1142	ARHGEF10I	NCGv7	protein_c	chr1:17539698-1769
ENSG0000(	1816	40.5559	chr1:1142	RPS15AP6		Pseudoger	chr1:21003550-2100
ENSG0000(	1816	40.5559	chr1:1142	RNU4-28P		smallRNA	chr1:19510593-1951
ENSG0000(	1816	40.5559	chr1:1142	NBPF2P		Pseudoger	chr1:21424625-2142
ENSG0000(	1816	40.5559	chr1:1142	PLA2G2A		protein_c	chr1:19975431-1998
ENSG0000(	1816	40.5559	chr1:1142	PLA2G2E		protein_c	chr1:19920009-1992
ENSG0000(	1816	40.5559	chr1:1142	ENSG00000284641		lncRNA	chr1:20476222-2047
ENSG0000(	1816	40.5559	chr1:1142	snoU13		smallRNA	chr1:17449763-1744
ENSG0000(	1816	40.5559	chr1:1142	CAPZB		protein_c	chr1:19338775-1948
ENSG0000(	1816	40.5559	chr1:1142	ENSG00000285959		lncRNA	chr1:21983606-2203
ENSG0000(	1816	40.5559	chr1:1142	OTUD3		protein_c	chr1:19882395-1991
ENSG0000(	1816	40.5559	chr1:1142	ENSG00000287192		lncRNA	chr1:21177054-2117
ENSG0000(	1812	40.46657	chr2:3094	MIR4265		smallRNA	chr2:109141490-109
ENSG0000(	1808	40.37724	chr1:1142	ELOA-AS1		lncRNA	chr1:23706901-2377
ENSG0000(	1808	40.37724	chr1:1142	GRHL3-AS1		lncRNA	chr1:24307556-2432
ENSG0000(	1808	40.37724	chr1:1142	snoU13		smallRNA	chr1:23882255-2388
ENSG0000(	1808	40.37724	chr1:1142	ELOA		protein_c	chr1:23743448-2376
ENSG0000(	1808	40.37724	chr1:1142	LYPLA2		protein_c	chr1:23791145-2379
ENSG0000(	1808	40.37724	chr1:1142	IFNLR1		protein_c	chr1:24154168-2418
ENSG0000(	1808	40.37724	chr1:1142	LINC02800		lncRNA	chr1:24200240-2421
ENSG0000(	1808	40.37724	chr1:1142	CNR2	AC	protein_c	chr1:23870515-2391
ENSG0000(	1808	40.37724	chr1:1142	RCAN3AS		lncRNA	chr1:24496254-2453

ENSG0000(	1808	40.37724	chr1:1142	ENSG00000289835		lncRNA	chr1:23790609-2379
ENSG0000(	1808	40.37724	chr1:1142	ENSG00000285802		lncRNA	chr1:23576436-2359
ENSG0000(	1808	40.37724	chr1:1142	PNRC2		protein_c	chr1:23956839-2396
ENSG0000(	1808	40.37724	chr1:1142	RP5-886K2.1		Pseudoger	chr1:23705801-2370
ENSG0000(	1808	40.37724	chr1:1142	RN7SL24P		smallRNA	chr1:23881794-2388
ENSG0000(	1808	40.37724	chr1:1142	ENSG00000225315		lncRNA	chr1:24040835-2408
ENSG0000(	1808	40.37724	chr1:1142	MDS2	AC	lncRNA	chr1:23581495-2364
ENSG0000(	1808	40.37724	chr1:1142	ENSG00000232557		lncRNA	chr1:23907111-2390
ENSG0000(	1808	40.37724	chr1:1142	HMGCL		protein_c	chr1:23801885-2383
ENSG0000(	1808	40.37724	chr1:1142	GALE		protein_c	chr1:23795599-2380
ENSG0000(	1808	40.37724	chr1:1142	ENSG00000288982		lncRNA	chr1:24476362-2447
ENSG0000(	1808	40.37724	chr1:1142	RCAN3		protein_c	chr1:24502351-2454
ENSG0000(	1808	40.37724	chr1:1142	EEF1A1P48		Pseudoger	chr1:23670294-2368
ENSG0000(	1808	40.37724	chr1:1142	RPL36P5		Pseudoger	chr1:24007881-2400
ENSG0000(	1808	40.37724	chr1:1142	RN7SL532P		smallRNA	chr1:23736610-2373
ENSG0000(	1808	40.37724	chr1:1142	IL22RA1		protein_c	chr1:24119771-2414
ENSG0000(	1808	40.37724	chr1:1142	BTBD6P1		Pseudoger	chr1:23901471-2390
ENSG0000(	1808	40.37724	chr1:1142	FUCA1		protein_c	chr1:23845077-2386
ENSG0000(	1808	40.37724	chr1:1142	NIPAL3		protein_c	chr1:24415802-2447
ENSG0000(	1808	40.37724	chr1:1142	MIR378F		smallRNA	chr1:23929070-2392
ENSG0000(	1808	40.37724	chr1:1142	RPL11		protein_c	chr1:23691742-2369
ENSG0000(	1808	40.37724	chr1:1142	AL590683.1		smallRNA	chr1:24227917-2422
ENSG0000(	1808	40.37724	chr1:1142	MYOM3	NCGv7	protein_c	chr1:24056035-2411
ENSG0000(	1808	40.37724	chr1:1142	H3P1		Pseudoger	chr1:23949016-2394
ENSG0000(	1808	40.37724	chr1:1142	PITHD1		protein_c	chr1:23778418-2378
ENSG0000(	1808	40.37724	chr1:1142	MYOM3-AS1		lncRNA	chr1:24066774-2408
ENSG0000(	1808	40.37724	chr1:1142	STPG1		protein_c	chr1:24356999-2441
ENSG0000(	1808	40.37724	chr1:1142	GRHL3		protein_c	chr1:24199558-2436
ENSG0000(	1808	40.37724	chr1:1142	SRSF10		protein_c	chr1:23964347-2398
ENSG0000(	1808	40.37724	chr1:1142	AL590683.2		smallRNA	chr1:24279767-2427
ENSG0000(	1795	40.08692	chr1:1142	RNU6-1265P		smallRNA	chr1:14124233-1412
ENSG0000(	1769	39.50627	chr1:1043	ENSG00000225075		lncRNA	chr1:112693688-112
ENSG0000(	1769	39.50627	chr1:1043	BCL2L15		protein_c	chr1:113876816-113
ENSG0000(	1769	39.50627	chr1:1043	MTND5P20		Pseudoger	chr1:113576757-113
ENSG0000(	1769	39.50627	chr1:1043	MOV10	NCGv7	protein_c	chr1:112673141-112
ENSG0000(	1769	39.50627	chr1:1043	RHOC	AC	protein_c	chr1:112701127-112
ENSG0000(	1769	39.50627	chr1:1043	RN7SL432P		smallRNA	chr1:114697629-114
ENSG0000(	1769	39.50627	chr1:1043	snoU13		smallRNA	chr1:112371004-112
ENSG0000(	1769	39.50627	chr1:1043	MRPL57P1		Pseudoger	chr1:114279011-114
ENSG0000(	1769	39.50627	chr1:1043	CTTNBP2NL		protein_c	chr1:112396214-112
ENSG0000(	1769	39.50627	chr1:1043	snoU13		smallRNA	chr1:112195502-112
ENSG0000(	1769	39.50627	chr1:1043	ENSG00000228040		Pseudoger	chr1:112890767-112
ENSG0000(	1769	39.50627	chr1:1043	AKR7A2P1		Pseudoger	chr1:112923423-112
ENSG0000(	1769	39.50627	chr1:1043	MRPL53P1		Pseudoger	chr1:112625906-112
ENSG0000(	1769	39.50627	chr1:1043	HIPK1-AS1		lncRNA	chr1:113924000-113
ENSG0000(	1769	39.50627	chr1:1043	LINC01750		lncRNA	chr1:111989770-111
ENSG0000(	1769	39.50627	chr1:1043	PPM1J-DT		lncRNA	chr1:112715672-112
ENSG0000(	1769	39.50627	chr1:1043	LRIG2	NCGv7	protein_c	chr1:113073198-113
ENSG0000(	1769	39.50627	chr1:1043	SLC16A1-AS1		lncRNA	chr1:112956415-113
ENSG0000(	1769	39.50627	chr1:1043	ENSG00000271419		Pseudoger	chr1:114353986-114
ENSG0000(	1769	39.50627	chr1:1043	TRIM33	NCGv7;AC	protein_c	chr1:114392790-114

ENSG0000(	1769	39.50627	chr1:104:	SYCP1		protein_c	chr1:114854863-114
ENSG0000(	1769	39.50627	chr1:104:	PPM1J	NCGv7	protein_c	chr1:112709994-112
ENSG0000(	1769	39.50627	chr1:104:	LINC01357		lncRNA	chr1:112849821-112
ENSG0000(	1769	39.50627	chr1:104:	ENSG00000226984		Pseudoger	chr1:114459934-114
ENSG0000(	1769	39.50627	chr1:104:	ST7L		protein_c	chr1:112523514-112
ENSG0000(	1769	39.50627	chr1:104:	ENSG00000232499		Pseudoger	chr1:113449700-113
ENSG0000(	1769	39.50627	chr1:104:	HIPK1		protein_c	chr1:113929324-113
ENSG0000(	1769	39.50627	chr1:104:	LINC01765		lncRNA	chr1:115099580-115
ENSG0000(	1769	39.50627	chr1:104:	Y_RNA		smallRNA	chr1:114727720-114
ENSG0000(	1769	39.50627	chr1:104:	SLC16A1		protein_c	chr1:112911847-112
ENSG0000(	1769	39.50627	chr1:104:	ENSG00000232895		lncRNA	chr1:114206427-114
ENSG0000(	1769	39.50627	chr1:104:	ENSG00000273483		lncRNA	chr1:112517799-112
ENSG0000(	1769	39.50627	chr1:104:	AP4B1		protein_c	chr1:113894194-113
ENSG0000(	1769	39.50627	chr1:104:	WNT2B		protein_c	chr1:112466541-112
ENSG0000(	1769	39.50627	chr1:104:	PTPN22	NCGv7	protein_c	chr1:113813811-113
ENSG0000(	1769	39.50627	chr1:104:	CAPZA1		protein_c	chr1:112619805-112
ENSG0000(	1769	39.50627	chr1:104:	SYT6		protein_c	chr1:114089291-114
ENSG0000(	1769	39.50627	chr1:104:	TSHB		protein_c	chr1:115029826-115
ENSG0000(	1769	39.50627	chr1:104:	TSPAN2		protein_c	chr1:115048011-115
ENSG0000(	1769	39.50627	chr1:104:	TAFA3		protein_c	chr1:112718905-112
ENSG0000(	1769	39.50627	chr1:104:	LINC01356		lncRNA	chr1:112820170-112
ENSG0000(	1769	39.50627	chr1:104:	AP4B1-AS1		lncRNA	chr1:113856635-113
ENSG0000(	1769	39.50627	chr1:104:	AMPD1		protein_c	chr1:114673090-114
ENSG0000(	1769	39.50627	chr1:104:	BCAS2		protein_c	chr1:114567557-114
ENSG0000(	1769	39.50627	chr1:104:	ENSG00000236887		Pseudoger	chr1:113198825-113
ENSG0000(	1769	39.50627	chr1:104:	snoU13		smallRNA	chr1:112652588-112
ENSG0000(	1769	39.50627	chr1:104:	OLFML3		protein_c	chr1:113979391-114
ENSG0000(	1769	39.50627	chr1:104:	PHTF1		protein_c	chr1:113696831-113
ENSG0000(	1769	39.50627	chr1:104:	KCND3		protein_c	chr1:111770662-111
ENSG0000(	1769	39.50627	chr1:104:	ENSG00000287807		lncRNA	chr1:112978610-112
ENSG0000(	1769	39.50627	chr1:104:	DENND2C		protein_c	chr1:114582848-114
ENSG0000(	1769	39.50627	chr1:104:	Y_RNA		smallRNA	chr1:114490724-114
ENSG0000(	1769	39.50627	chr1:104:	DCLRE1B		protein_c	chr1:113904619-113
ENSG0000(	1769	39.50627	chr1:104:	EIF2S2P5		Pseudoger	chr1:114468315-114
ENSG0000(	1769	39.50627	chr1:104:	ENSG00000233839		Pseudoger	chr1:113168994-113
ENSG0000(	1769	39.50627	chr1:104:	NRAS	NCGv7;AC	protein_c	chr1:114704469-114
ENSG0000(	1769	39.50627	chr1:104:	CSDE1	NCGv7	protein_c	chr1:114716913-114
ENSG0000(	1769	39.50627	chr2:309‹	CBX3P6		Pseudoger	chr2:154940541-154
ENSG0000(	1769	39.50627	chr1:104:	RSBN1	NCGv7	protein_c	chr1:113761832-113
ENSG0000(	1769	39.50627	chr1:104:	LRIG2-DT		lncRNA	chr1:113011687-113
ENSG0000(	1769	39.50627	chr1:104:	RLIMP2		Pseudoger	chr1:113125321-113
ENSG0000(	1769	39.50627	chr1:104:	SIKE1		protein_c	chr1:114769479-114
ENSG0000(	1769	39.50627	chr1:104:	ENSG00000232450		Pseudoger	chr1:113698884-113
ENSG0000(	1769	39.50627	chr1:104:	ENSG00000282048		lncRNA	chr1:114032377-114
ENSG0000(	1769	39.50627	chr1:104:	MAGI3		protein_c	chr1:113390515-113
ENSG0000(	1769	39.50627	chr1:104:	PKMP1		Pseudoger	chr1:114535995-114
ENSG0000(	1769	39.50627	chr1:104:	NR1H5P		Pseudoger	chr1:114837227-114
ENSG0000(	1769	39.50627	chr1:104:	RNU7-70P		smallRNA	chr1:112634719-112
ENSG0000(	1769	39.50627	chr1:104:	LINC02884		lncRNA	chr1:112176836-112
ENSG0000(	1769	39.50627	chr1:104:	TXNP3		Pseudoger	chr1:112363281-112
ENSG0000(	1769	39.50627	chr1:104:	ENSG00000271810		protein_c	chr1:112702614-112

ENSG0000(	1769	39.50627	chr1:1043	ENSG00000270780		Pseudoger	chr1:114001433-114
ENSG0000(	1769	39.50627	chr1:1043	ENSG00000231128		lncRNA	chr1:113812379-113
ENSG0000(	1761	39.32761	chr2:3094	METAP2P1		Pseudoger	chr2:145826285-145
ENSG0000(	1761	39.32761	chr2:3094	ENSG00000233842		lncRNA	chr2:145872209-145
ENSG0000(	1761	39.32761	chr2:3094	ENSG00000228655		lncRNA	chr2:143295421-143
ENSG0000(	1761	39.32761	chr2:3094	ENSG00000257226		lncRNA	chr2:143765940-143
ENSG0000(	1761	39.32761	chr2:3094	LINC01966		lncRNA	chr2:144877734-144
ENSG0000(	1761	39.32761	chr2:3094	RPL17P12		Pseudoger	chr2:146194296-146
ENSG0000(	1761	39.32761	chr2:3094	ENSG00000283118		lncRNA	chr2:144444848-144
ENSG0000(	1761	39.32761	chr2:3094	ARHGAP15-AS1		lncRNA	chr2:143629133-143
ENSG0000(	1761	39.32761	chr2:3094	AC009951.1		Pseudoger	chr2:144461112-144
ENSG0000(	1761	39.32761	chr2:3094	Y_RNA		smallRNA	chr2:143897263-143
ENSG0000(	1761	39.32761	chr2:3094	ENSG00000226218		lncRNA	chr2:145600907-145
ENSG0000(	1761	39.32761	chr2:3094	ENSG00000279166		TEC	chr2:144494265-144
ENSG0000(	1761	39.32761	chr2:3094	RPL6P5		Pseudoger	chr2:145337230-145
ENSG0000(	1761	39.32761	chr2:3094	SGCEP1		Pseudoger	chr2:144754776-144
ENSG0000(	1761	39.32761	chr2:3094	ENSG00000236356		lncRNA	chr2:143799467-143
ENSG0000(	1761	39.32761	chr2:3094	OTX2P2		Pseudoger	chr2:146477427-146
ENSG0000(	1761	39.32761	chr2:3094	RNU7-2P		smallRNA	chr2:146145156-146
ENSG0000(	1761	39.32761	chr2:3094	ENSG00000235435		lncRNA	chr2:145294277-145
ENSG0000(	1761	39.32761	chr2:3094	ARHGAP15		protein_c	chr2:143091362-143
ENSG0000(	1761	39.32761	chr2:3094	ZEB2-AS1		lncRNA	chr2:144517978-144
ENSG0000(	1761	39.32761	chr2:3094	ENSG00000225107		lncRNA	chr2:145569294-145
ENSG0000(	1761	39.32761	chr2:3094	AC009951.2		Pseudoger	chr2:144450668-144
ENSG0000(	1761	39.32761	chr2:3094	TEX41		lncRNA	chr2:144666312-145
ENSG0000(	1761	39.32761	chr2:3094	ENSG00000283994		lncRNA	chr2:143601517-143
ENSG0000(	1761	39.32761	chr2:3094	LINC02993		lncRNA	chr2:144523541-144
ENSG0000(	1761	39.32761	chr1:1142	CROCCP4		Pseudoger	chr1:16750233-1675
ENSG0000(	1761	39.32761	chr2:3094	PABPC1P2		Pseudoger	chr2:146587506-146
ENSG0000(	1761	39.32761	chr2:3094	GTDC1		protein_c	chr2:143938068-144
ENSG0000(	1761	39.32761	chr2:3094	ZEB2		protein_c	chr2:144364364-144
ENSG0000(	1761	39.32761	chr2:3094	ENSG00000286225		lncRNA	chr2:145572662-145
ENSG0000(	1761	39.32761	chr2:3094	ENSG00000234940		lncRNA	chr2:144688413-144
ENSG0000(	1761	39.32761	chr2:3094	ENSG00000232377		lncRNA	chr2:143937073-143
ENSG0000(	1761	39.32761	chr2:3094	ENSG00000271583		Pseudoger	chr2:146833501-146
ENSG0000(	1761	39.32761	chr2:3094	LINC01412		lncRNA	chr2:144521868-144
ENSG0000(	1761	39.32761	chr2:3094	ENSG00000257277		lncRNA	chr2:143674315-143
ENSG0000(	1761	39.32761	chr2:3094	ENSG00000279876		TEC	chr2:145874520-145
ENSG0000(	1761	39.32761	chr2:3094	snR65		smallRNA	chr2:145335070-145
ENSG0000(	1759	39.28294	chr2:3094	AC009297.1		smallRNA	chr2:154231718-154
ENSG0000(	1759	39.28294	chr2:3094	ENSG00000287900		lncRNA	chr2:154688296-154
ENSG0000(	1759	39.28294	chr2:3094	LINC01850		lncRNA	chr2:153337705-153
ENSG0000(	1759	39.28294	chr2:3094	ATP5PBP4		Pseudoger	chr2:153158938-153
ENSG0000(	1759	39.28294	chr2:3094	RPL23AP29		Pseudoger	chr2:153370612-153
ENSG0000(	1759	39.28294	chr2:3094	ENSG00000224612		lncRNA	chr2:153168399-153
ENSG0000(	1759	39.28294	chr2:3094	PHB1P4		Pseudoger	chr2:154286589-154
ENSG0000(	1759	39.28294	chr2:3094	ENSG00000226338		Pseudoger	chr2:153208086-153
ENSG0000(	1759	39.28294	chr2:3094	RNA5SP107		Pseudoger	chr2:154590407-154
ENSG0000(	1759	39.28294	chr2:3094	ENSG00000224675		lncRNA	chr2:154435853-154
ENSG0000(	1759	39.28294	chr2:3094	TUBAP13		Pseudoger	chr2:153420677-153
ENSG0000(	1759	39.28294	chr2:3094	GALNT13	NCGv7	protein_c	chr2:153871922-154

ENSG0000(	1759	39.28294	chr2:309	AC008166.1		smallRNA	chr2:154146105-154
ENSG0000(	1759	39.28294	chr2:309	RPRM		protein_c	chr2:153477338-153
ENSG0000(	1759	39.28294	chr2:309	ENSG00000227400		lncRNA	chr2:153421616-153
ENSG0000(	1759	39.28294	chr2:309	SNORD56		smallRNA	chr2:153446813-153
ENSG0000(	1759	39.28294	chr2:309	DNAJA1P2		Pseudoger	chr2:153761450-153
ENSG0000(	1759	39.28294	chr2:309	ENSG00000238004		lncRNA	chr2:154459857-154
ENSG0000(	1759	39.28294	chr2:309	KCNJ3		protein_c	chr2:154697855-154
ENSG0000(	1756	39.21595	chr1:114	RPL12P14		Pseudoger	chr1:15792796-1579
ENSG0000(	1756	39.21595	chr1:114	PRAMEF35P		Pseudoger	chr1:13104403-1310
ENSG0000(	1756	39.21595	chr1:114	ENSG00000289380		lncRNA	chr1:13757778-1375
ENSG0000(	1756	39.21595	chr1:114	RP11-219C24.6		Pseudoger	chr1:13305955-1330
ENSG0000(	1756	39.21595	chr1:114	AL359771.1		smallRNA	chr1:13623902-1362
ENSG0000(	1756	39.21595	chr1:114	MIR5697		smallRNA	chr1:9967381-99674
ENSG0000(	1756	39.21595	chr1:114	CD24P1		Pseudoger	chr1:15614643-1561
ENSG0000(	1756	39.21595	chr1:114	ENSG00000178715		Pseudoger	chr1:15828232-1582
ENSG0000(	1756	39.21595	chr1:114	ZBTB2P1		Pseudoger	chr1:15226373-1522
ENSG0000(	1756	39.21595	chr1:114	KIF1B	NCGv7	protein_c	chr1:10210570-1038
ENSG0000(	1756	39.21595	chr1:114	RNA5SP40		Pseudoger	chr1:9437669-94377
ENSG0000(	1756	39.21595	chr1:114	ENSG00000241326		lncRNA	chr1:9983141-99845
ENSG0000(	1756	39.21595	chr1:114	AL590639.1		Pseudoger	chr1:10018027-1001
ENSG0000(	1756	39.21595	chr1:114	TBC1D3P6		Pseudoger	chr1:15989871-1599
ENSG0000(	1756	39.21595	chr1:114	CTNNBIP1		protein_c	chr1:9848276-99103
ENSG0000(	1756	39.21595	chr1:114	FAM131C2P		Pseudoger	chr1:16035178-1604
ENSG0000(	1756	39.21595	chr1:114	PRAMEF30P		Pseudoger	chr1:12838125-1284
ENSG0000(	1756	39.21595	chr1:114	MST1L		Pseudoger	chr1:16757232-1676
ENSG0000(	1756	39.21595	chr1:114	ENSG00000270914		Pseudoger	chr1:12017216-1201
ENSG0000(	1756	39.21595	chr1:114	PDE4DIPP8		Pseudoger	chr1:16548651-1655
ENSG0000(	1756	39.21595	chr1:114	AADACL3		protein_c	chr1:12716110-1272
ENSG0000(	1756	39.21595	chr1:114	MASP2		protein_c	chr1:11022009-1104
ENSG0000(	1756	39.21595	chr1:114	RNA5SP41		Pseudoger	chr1:13623184-1362
ENSG0000(	1756	39.21595	chr1:114	KAZN		protein_c	chr1:13892792-1511
ENSG0000(	1756	39.21595	chr1:114	MTCYBP45		Pseudoger	chr1:11425257-1142
ENSG0000(	1756	39.21595	chr1:114	RN7SL721P		smallRNA	chr1:10390002-1039
ENSG0000(	1756	39.21595	chr1:114	CROCCP3		Pseudoger	chr1:16474396-1649
ENSG0000(	1756	39.21595	chr1:114	SNORA70		smallRNA	chr1:12221148-1222
ENSG0000(	1756	39.21595	chr1:114	PRAMEF6		protein_c	chr1:12938472-1294
ENSG0000(	1756	39.21595	chr1:114	RN7SL649P		smallRNA	chr1:12036742-1203
ENSG0000(	1756	39.21595	chr1:114	ENSG00000224340		Pseudoger	chr1:10054445-1005
ENSG0000(	1756	39.21595	chr1:114	TBCAP2		Pseudoger	chr1:14692129-1469
ENSG0000(	1756	39.21595	chr1:114	ENSG00000290849		lncRNA	chr1:16687339-1669
ENSG0000(	1756	39.21595	chr1:114	ENSG00000275503		Pseudoger	chr1:15989140-1598
ENSG0000(	1756	39.21595	chr1:114	PRAMEF28P		Pseudoger	chr1:13342528-1334
ENSG0000(	1756	39.21595	chr1:114	PRAMEF8		protein_c	chr1:13281035-1328
ENSG0000(	1756	39.21595	chr1:114	ENSG00000223989		lncRNA	chr1:9848318-98501
ENSG0000(	1756	39.21595	chr1:114	PGAM1P11		Pseudoger	chr1:10058671-1005
ENSG0000(	1756	39.21595	chr1:114	LINC01772		lncRNA	chr1:16460948-1646
ENSG0000(	1756	39.21595	chr1:114	SPEN	NCGv7	protein_c	chr1:15836095-1594
ENSG0000(	1756	39.21595	chr1:114	HNRNPCL1	NCGv7	protein_c	chr1:12847377-1284
ENSG0000(	1756	39.21595	chr1:114	KAZN-AS1		lncRNA	chr1:14338825-1441
ENSG0000(	1756	39.21595	chr1:114	MFFP1		Pseudoger	chr1:15191828-1519
ENSG0000(	1756	39.21595	chr1:114	CLCNKB		protein_c	chr1:16040252-1605

ENSG0000(	1756	39.21595	chr1:1142	ENSG00000236045		lncRNA	chr1:15334166-1533
ENSG0000(	1756	39.21595	chr1:1142	MZT1P1		Pseudoger	chr1:9780822-97812
ENSG0000(	1756	39.21595	chr1:1142	MIR1273D		smallRNA	chr1:10227718-1022
ENSG0000(	1756	39.21595	chr1:1142	FHAD1-AS1		lncRNA	chr1:15326680-1534
ENSG0000(	1756	39.21595	chr1:1142	RNU6ATAC18P		smallRNA	chr1:12569972-1257
ENSG0000(	1756	39.21595	chr1:1142	CENPS		protein_c	chr1:10430433-1044
ENSG0000(	1756	39.21595	chr1:1142	CFL1P6		Pseudoger	chr1:10990978-1099
ENSG0000(	1756	39.21595	chr1:1142	AL355149.1		smallRNA	chr1:16548914-1654
ENSG0000(	1756	39.21595	chr1:1142	C1orf127	NCGv7	protein_c	chr1:10946471-1098
ENSG0000(	1756	39.21595	chr1:1142	ENSG00000234607		Pseudoger	chr1:15969632-1597
ENSG0000(	1756	39.21595	chr1:1142	RNU7-179P		smallRNA	chr1:15608078-1560
ENSG0000(	1756	39.21595	chr1:1142	CFAP107		protein_c	chr1:12746200-1276
ENSG0000(	1756	39.21595	chr1:1142	PRAMEF4	NCGv7	protein_c	chr1:12879212-1288
ENSG0000(	1756	39.21595	chr1:1142	ENSG00000223643		lncRNA	chr1:16851257-1685
ENSG0000(	1756	39.21595	chr1:1142	ENSG00000285853		lncRNA	chr1:16515034-1652
ENSG0000(	1756	39.21595	chr1:1142	RP11-248D7.2		Pseudoger	chr1:13245863-1324
ENSG0000(	1756	39.21595	chr1:1142	SZRD1		protein_c	chr1:16352575-1639
ENSG0000(	1756	39.21595	chr1:1142	ENSG00000259961		lncRNA	chr1:13513220-1351
ENSG0000(	1756	39.21595	chr1:1142	RSC1A1		protein_c	chr1:15659713-1566
ENSG0000(	1756	39.21595	chr1:1142	ENSG00000283773		Pseudoger	chr1:16642767-1664
ENSG0000(	1756	39.21595	chr1:1142	ENSG00000290851		lncRNA	chr1:16754910-1677
ENSG0000(	1756	39.21595	chr1:1142	ENSG00000290850		lncRNA	chr1:16740280-1675
ENSG0000(	1756	39.21595	chr1:1142	CELA2B		protein_c	chr1:15465909-1549
ENSG0000(	1756	39.21595	chr1:1142	C1orf167		protein_c	chr1:11761787-1178
ENSG0000(	1756	39.21595	chr1:1142	LINC01783		lncRNA	chr1:16533886-1653
ENSG0000(	1756	39.21595	chr1:1142	SPATA21		protein_c	chr1:16387117-1643
ENSG0000(	1756	39.21595	chr1:1142	BRWD1P1		Pseudoger	chr1:13555001-1355
ENSG0000(	1756	39.21595	chr1:1142	ENSG00000224174		lncRNA	chr1:16520694-1652
ENSG0000(	1756	39.21595	chr1:1142	CROCCP2		Pseudoger	chr1:16618969-1665
ENSG0000(	1756	39.21595	chr1:1142	ENSG00000285833		lncRNA	chr1:11500803-1150
ENSG0000(	1756	39.21595	chr1:1142	NECAP2		protein_c	chr1:16440721-1646
ENSG0000(	1756	39.21595	chr1:1142	CROCCP3		lncRNA	chr1:16467436-1649
ENSG0000(	1756	39.21595	chr1:1142	PLOD1		protein_c	chr1:11934205-1197
ENSG0000(	1756	39.21595	chr1:1142	ENSG00000270620		Pseudoger	chr1:15917698-1591
ENSG0000(	1756	39.21595	chr1:1142	FAM131C		protein_c	chr1:16057769-1607
ENSG0000(	1756	39.21595	chr1:1142	RNU6-37P		smallRNA	chr1:10298966-1029
ENSG0000(	1756	39.21595	chr1:1142	ENSG00000231181		Pseudoger	chr1:9576427-95769
ENSG0000(	1756	39.21595	chr1:1142	ARHGEF19		protein_c	chr1:16197854-1621
ENSG0000(	1756	39.21595	chr1:1142	HSPE1P24		Pseudoger	chr1:10895761-1089
ENSG0000(	1756	39.21595	chr1:1142	CHCHD2P6		Pseudoger	chr1:15604597-1560
ENSG0000(	1756	39.21595	chr1:1142	MTOR	NCGv7;AC	protein_c	chr1:11106535-1126
ENSG0000(	1756	39.21595	chr1:1142	Y_RNA		smallRNA	chr1:10999862-1099
ENSG0000(	1756	39.21595	chr1:1142	RPL10P17		Pseudoger	chr1:12220794-1222
ENSG0000(	1756	39.21595	chr1:1142	PRAMEF13		protein_c	chr1:13341907-1334
ENSG0000(	1756	39.21595	chr1:1142	ENSG00000284652		lncRNA	chr1:9421098-94228
ENSG0000(	1756	39.21595	chr1:1142	ESPNP		Pseudoger	chr1:16692280-1672
ENSG0000(	1756	39.21595	chr1:1142	PGD		protein_c	chr1:10398592-1042
ENSG0000(	1756	39.21595	chr1:1142	CTRC		protein_c	chr1:15438442-1544
ENSG0000(	1756	39.21595	chr1:1142	LZIC		protein_c	chr1:9922113-99434
ENSG0000(	1756	39.21595	chr1:1142	PEX14		protein_c	chr1:10472288-1063
ENSG0000(	1756	39.21595	chr1:1142	ENSG00000231606		lncRNA	chr1:14221887-1430

ENSG0000(	1756	39.21595	chr1:1142	PRAMEF29P		Pseudoger	chr1:12926162-1292
ENSG0000(	1756	39.21595	chr1:1142	RNU1-4		smallRNA	chr1:16740516-1674
ENSG0000(	1756	39.21595	chr1:1142	AL355149.2		protein_c	chr1:16539066-1653
ENSG0000(	1756	39.21595	chr1:1142	RBP7		protein_c	chr1:9997206-10016
ENSG0000(	1756	39.21595	chr1:1142	ENSG00000284646		lncRNA	chr1:11311734-1131
ENSG0000(	1756	39.21595	chr1:1142	ENSG00000233268		Pseudoger	chr1:9660828-96626
ENSG0000(	1756	39.21595	chr1:1142	HNRNPCL2		protein_c	chr1:13115488-1311
ENSG0000(	1756	39.21595	chr1:1142	ENSG00000285646		lncRNA	chr1:11907940-1191
ENSG0000(	1756	39.21595	chr1:1142	ENSG00000285701		lncRNA	chr1:9900614-99080
ENSG0000(	1756	39.21595	chr1:1142	ENSG00000291077		lncRNA	chr1:16215907-1621
ENSG0000(	1756	39.21595	chr1:1142	AL021920.2		protein_c	chr1:16733952-1673
ENSG0000(	1756	39.21595	chr1:1142	TNFRSF1B	NCGv7;AC	protein_c	chr1:12166991-1220
ENSG0000(	1756	39.21595	chr1:1142	C1orf167-AS1		lncRNA	chr1:11777077-1177
ENSG0000(	1756	39.21595	chr1:1142	PIK3CD-AS1		lncRNA	chr1:9652610-96545
ENSG0000(	1756	39.21595	chr1:1142	ARHGEF19-AS1		lncRNA	chr1:16197854-1619
ENSG0000(	1756	39.21595	chr1:1142	RN7SL614P		smallRNA	chr1:10616836-1061
ENSG0000(	1756	39.21595	chr1:1142	SPEN-AS1		lncRNA	chr1:15834474-1584
ENSG0000(	1756	39.21595	chr1:1142	ENSG00000284708		lncRNA	chr1:11623558-1164
ENSG0000(	1756	39.21595	chr1:1142	SBF1P2		Pseudoger	chr1:11877770-1188
ENSG0000(	1756	39.21595	chr1:1142	snoU13		smallRNA	chr1:12739736-1273
ENSG0000(	1756	39.21595	chr1:1142	CROCCP2		lncRNA	chr1:16618253-1664
ENSG0000(	1756	39.21595	chr1:1142	ENSG00000237445		lncRNA	chr1:13657311-1365
ENSG0000(	1756	39.21595	chr1:1142	ENSG00000288927		lncRNA	chr1:12618389-1261
ENSG0000(	1756	39.21595	chr1:1142	ENSG00000233078		lncRNA	chr1:16006160-1600
ENSG0000(	1756	39.21595	chr1:1142	ENSG00000279151		lncRNA	chr1:16701546-1670
ENSG0000(	1756	39.21595	chr1:1142	PIK3CD-AS2		lncRNA	chr1:9672405-96875
ENSG0000(	1756	39.21595	chr1:1142	FBXO42		protein_c	chr1:16246840-1635
ENSG0000(	1756	39.21595	chr1:1142	ENSG00000284735		lncRNA	chr1:10381906-1038
ENSG0000(	1756	39.21595	chr1:1142	AGTRAP		protein_c	chr1:11736084-1175
ENSG0000(	1756	39.21595	chr1:1142	MST1P2		Pseudoger	chr1:16645622-1665
ENSG0000(	1756	39.21595	chr1:1142	SNORA59A	AC	smallRNA	chr1:12507246-1250
ENSG0000(	1756	39.21595	chr1:1142	NBPF1	NCGv7	protein_c	chr1:16562319-1661
ENSG0000(	1756	39.21595	chr1:1142	EFHD2	NCGv7	protein_c	chr1:15409888-1543
ENSG0000(	1756	39.21595	chr1:1142	MTHFR	NCGv7	protein_c	chr1:11785723-1180
ENSG0000(	1756	39.21595	chr1:1142	SLC25A34-AS1		lncRNA	chr1:15740048-1574
ENSG0000(	1756	39.21595	chr1:1142	SCARNA11		smallRNA	chr1:13696070-1369
ENSG0000(	1756	39.21595	chr1:1142	DDI2		protein_c	chr1:15617458-1566
ENSG0000(	1756	39.21595	chr1:1142	ENSG00000231353		Pseudoger	chr1:15988182-1598
ENSG0000(	1756	39.21595	chr1:1142	PRAMEF34P		Pseudoger	chr1:13095179-1309
ENSG0000(	1756	39.21595	chr1:1142	SCARNA21		smallRNA	chr1:15542165-1554
ENSG0000(	1756	39.21595	chr1:1142	RNU6-771P		smallRNA	chr1:13279125-1327
ENSG0000(	1756	39.21595	chr1:1142	RNU1-3		smallRNA	chr1:16666785-1666
ENSG0000(	1756	39.21595	chr1:1142	UQCRHL		protein_c	chr1:15807169-1580
ENSG0000(	1756	39.21595	chr1:1142	MT1XP1		Pseudoger	chr1:16241213-1624
ENSG0000(	1756	39.21595	chr1:1142	ENSG00000282740		lncRNA	chr1:16739938-1675
ENSG0000(	1756	39.21595	chr1:1142	ENSG00000261135		lncRNA	chr1:16514645-1651
ENSG0000(	1756	39.21595	chr1:1142	ENSG00000237301		lncRNA	chr1:15586136-1560
ENSG0000(	1756	39.21595	chr1:1142	LINC02606		lncRNA	chr1:9425094-94405
ENSG0000(	1756	39.21595	chr1:1142	CLCN6		protein_c	chr1:11806096-1184
ENSG0000(	1756	39.21595	chr1:1142	EIF1AXP1		Pseudoger	chr1:16685621-1668
ENSG0000(	1756	39.21595	chr1:1142	ENSG00000282143		lncRNA	chr1:16656879-1666

ENSG0000(	1756	39.21595	chr1:1142	PRAMEF32P		Pseudoger	chr1:13273539-1327
ENSG0000(	1756	39.21595	chr1:1142	ENSG00000235263		lncRNA	chr1:9501092-95034
ENSG0000(	1756	39.21595	chr1:1142	LINC01647		lncRNA	chr1:11609468-1161
ENSG0000(	1756	39.21595	chr1:1142	MTOR-AS1		lncRNA	chr1:11143898-1114
ENSG0000(	1756	39.21595	chr1:1142	TMEM201	NCGv7	protein_c	chr1:9588911-96148
ENSG0000(	1756	39.21595	chr1:1142	ANO7L1		Pseudoger	chr1:16216469-1622
ENSG0000(	1756	39.21595	chr1:1142	CELA2A		protein_c	chr1:15456728-1547
ENSG0000(	1756	39.21595	chr1:1142	VPS13D		protein_c	chr1:12230030-1251
ENSG0000(	1756	39.21595	chr1:1142	XX-FW84067D5.2		Pseudoger	chr1:13305955-1330
ENSG0000(	1756	39.21595	chr1:1142	RNU6-537P		smallRNA	chr1:11152350-1115
ENSG0000(	1756	39.21595	chr1:1142	CLCNKA		protein_c	chr1:16018875-1603
ENSG0000(	1756	39.21595	chr1:1142	EPHA2	NCGv7;AC	protein_c	chr1:16124337-1615
ENSG0000(	1756	39.21595	chr1:1142	ENSG00000284642		lncRNA	chr1:10395416-1039
ENSG0000(	1756	39.21595	chr1:1142	FBLIM1		protein_c	chr1:15756607-1578
ENSG0000(	1756	39.21595	chr1:1142	TMEM82		protein_c	chr1:15742499-1574
ENSG0000(	1756	39.21595	chr1:1142	RNU5E-1		smallRNA	chr1:11908152-1190
ENSG0000(	1756	39.21595	chr1:1142	SLC25A34		protein_c	chr1:15736258-1574
ENSG0000(	1756	39.21595	chr1:1142	RNU6-1072P		smallRNA	chr1:12922554-1292
ENSG0000(	1756	39.21595	chr1:1142	ENSG00000224621		lncRNA	chr1:16159266-1616
ENSG0000(	1756	39.21595	chr1:1142	DRAXIN		protein_c	chr1:11691710-1172
ENSG0000(	1756	39.21595	chr1:1142	DHRS3		protein_c	chr1:12567910-1261
ENSG0000(	1756	39.21595	chr1:1142	SRARP		protein_c	chr1:16004236-1600
ENSG0000(	1756	39.21595	chr1:1142	RNU6-291P		smallRNA	chr1:11226254-1122
ENSG0000(	1756	39.21595	chr1:1142	PDPN		protein_c	chr1:13583465-1361
ENSG0000(	1756	39.21595	chr1:1142	DFFA		protein_c	chr1:10456522-1047
ENSG0000(	1756	39.21595	chr1:1142	RP13-221M14.2		lncRNA	chr1:13079329-1307
ENSG0000(	1756	39.21595	chr1:1142	ENSG00000285604		lncRNA	chr1:12088441-1209
ENSG0000(	1756	39.21595	chr1:1142	LRRC38		protein_c	chr1:13474973-1351
ENSG0000(	1756	39.21595	chr1:1142	FHAD1	DriverDBv	protein_c	chr1:15236521-1540
ENSG0000(	1756	39.21595	chr1:1142	snoU13		smallRNA	chr1:15910897-1591
ENSG0000(	1756	39.21595	chr1:1142	PRAMEF10		protein_c	chr1:12892896-1289
ENSG0000(	1756	39.21595	chr1:1142	TMEM274P		Pseudoger	chr1:9950572-99607
ENSG0000(	1756	39.21595	chr1:1142	ENSG00000271742		lncRNA	chr1:15682873-1568
ENSG0000(	1756	39.21595	chr1:1142	PRAMEF14		protein_c	chr1:13341892-1334
ENSG0000(	1756	39.21595	chr1:1142	C1orf134		protein_c	chr1:16228873-1622
ENSG0000(	1756	39.21595	chr1:1142	ZBTB17		protein_c	chr1:15941869-1597
ENSG0000(	1756	39.21595	chr1:1142	ENSG00000226849		lncRNA	chr1:11068471-1107
ENSG0000(	1756	39.21595	chr1:1142	TMEM51		protein_c	chr1:15152532-1522
ENSG0000(	1756	39.21595	chr1:1142	snoU13		smallRNA	chr1:11169507-1116
ENSG0000(	1756	39.21595	chr1:1142	ENSG00000272482		lncRNA	chr1:12618900-1261
ENSG0000(	1756	39.21595	chr1:1142	AL713997.1		smallRNA	chr1:10998176-1099
ENSG0000(	1756	39.21595	chr1:1142	RNU6-777P		smallRNA	chr1:12077881-1207
ENSG0000(	1756	39.21595	chr1:1142	PLEKHM2		protein_c	chr1:15684320-1573
ENSG0000(	1756	39.21595	chr1:1142	MIR3675		smallRNA	chr1:16858949-1685
ENSG0000(	1756	39.21595	chr1:1142	NMNAT1		protein_c	chr1:9943428-99855
ENSG0000(	1756	39.21595	chr1:1142	SRM		protein_c	chr1:11054584-1106
ENSG0000(	1756	39.21595	chr1:1142	LINC02766		lncRNA	chr1:12525716-1253
ENSG0000(	1756	39.21595	chr1:1142	EFHD2-AS1		lncRNA	chr1:15402979-1540
ENSG0000(	1756	39.21595	chr1:1142	ENSG00000228150		lncRNA	chr1:9942923-99499
ENSG0000(	1756	39.21595	chr1:1142	ENSG00000288398		lncRNA	chr1:16228674-1623
ENSG0000(	1756	39.21595	chr1:1142	NPPB		protein_c	chr1:11857464-1185

ENSG0000(	1756	39.21595	chr1:1142	ENSG00000203469		lncRNA	chr1:10458555-1045
ENSG0000(	1756	39.21595	chr1:1142	AADACL4	NCGv7	protein_c	chr1:12644085-1266
ENSG0000(	1756	39.21595	chr1:1142	UBIAD1		protein_c	chr1:11273198-1129
ENSG0000(	1756	39.21595	chr1:1142	AGMAT		protein_c	chr1:15571699-1558
ENSG0000(	1756	39.21595	chr1:1142	PRAMEF19		protein_c	chr1:13368431-1337
ENSG0000(	1756	39.21595	chr1:1142	TARDBP		protein_c	chr1:11012344-1103
ENSG0000(	1756	39.21595	chr1:1142	TNFRSF8	NCGv7	protein_c	chr1:12063303-1214
ENSG0000(	1756	39.21595	chr1:1142	FBXO2		protein_c	chr1:11637018-1165
ENSG0000(	1756	39.21595	chr1:1142	PRAMEF21		protein_c	chr1:13410450-1342
ENSG0000(	1756	39.21595	chr1:1142	HSPB7		protein_c	chr1:16014028-1601
ENSG0000(	1756	39.21595	chr1:1142	TMEM51-AS1		lncRNA	chr1:15111815-1515
ENSG0000(	1756	39.21595	chr1:1142	EPHA2-AS1		lncRNA	chr1:16155176-1615
ENSG0000(	1756	39.21595	chr1:1142	LINC01784		lncRNA	chr1:12822686-1282
ENSG0000(	1756	39.21595	chr1:1142	PRAMEF20		protein_c	chr1:13410450-1342
ENSG0000(	1756	39.21595	chr1:1142	CORT		protein_c	chr1:10450031-1045
ENSG0000(	1756	39.21595	chr1:1142	PRAMEF18		protein_c	chr1:13369067-1337
ENSG0000(	1756	39.21595	chr1:1142	UBE2V2P3		Pseudoger	chr1:11278616-1127
ENSG0000(	1756	39.21595	chr1:1142	PRAMEF7		protein_c	chr1:12916610-1292
ENSG0000(	1756	39.21595	chr1:1142	CLSTN1		protein_c	chr1:9728926-98239
ENSG0000(	1756	39.21595	chr1:1142	PIK3CD	NCGv7	protein_c	chr1:9629889-97291
ENSG0000(	1756	39.21595	chr1:1142	ENSG00000272510		lncRNA	chr1:15565611-1556
ENSG0000(	1756	39.21595	chr1:1142	PRAMEF11		protein_c	chr1:12824610-1283
ENSG0000(	1756	39.21595	chr1:1142	PRAMEF17		protein_c	chr1:13389632-1339
ENSG0000(	1756	39.21595	chr1:1142	RP13-221M14.3		Pseudoger	chr1:13095174-1309
ENSG0000(	1756	39.21595	chr1:1142	SLC25A33		protein_c	chr1:9539465-95851
ENSG0000(	1756	39.21595	chr1:1142	AL137798.1		protein_c	chr1:16673003-1667
ENSG0000(	1756	39.21595	chr1:1142	ENSG00000271732		lncRNA	chr1:16617391-1661
ENSG0000(	1756	39.21595	chr1:1142	RNU6-828P		smallRNA	chr1:10163268-1016
ENSG0000(	1756	39.21595	chr1:1142	SPSB1		protein_c	chr1:9292894-93695
ENSG0000(	1756	39.21595	chr1:1142	ENSG00000287756		lncRNA	chr1:14774469-1477
ENSG0000(	1756	39.21595	chr1:1142	PRAMEF16		protein_c	chr1:13389628-1339
ENSG0000(	1756	39.21595	chr1:1142	UBE4B		protein_c	chr1:10032832-1018
ENSG0000(	1756	39.21595	chr1:1142	PRAMEF9		protein_c	chr1:13315581-1332
ENSG0000(	1756	39.21595	chr1:1142	RN7SL731P		smallRNA	chr1:10306465-1030
ENSG0000(	1756	39.21595	chr1:1142	ENSG00000237938		lncRNA	chr1:15720312-1573
ENSG0000(	1756	39.21595	chr1:1142	FBXO44		protein_c	chr1:11654375-1166
ENSG0000(	1756	39.21595	chr1:1142	CPLANE2		protein_c	chr1:16231692-1623
ENSG0000(	1756	39.21595	chr1:1142	CASP9	NCGv7	protein_c	chr1:15490832-1552
ENSG0000(	1756	39.21595	chr1:1142	PDE4DIPP9		Pseudoger	chr1:16855407-1685
ENSG0000(	1756	39.21595	chr1:1142	PRAMEF26		protein_c	chr1:13148905-1315
ENSG0000(	1756	39.21595	chr1:1142	ENSG00000280114		Pseudoger	chr1:16681097-1668
ENSG0000(	1756	39.21595	chr1:1142	ENSG00000287384		lncRNA	chr1:11979533-1198
ENSG0000(	1756	39.21595	chr1:1142	PRAMEF2		protein_c	chr1:12857086-1286
ENSG0000(	1756	39.21595	chr1:1142	AL021920.1		smallRNA	chr1:16681255-1668
ENSG0000(	1756	39.21595	chr1:1142	RPL39P6		Pseudoger	chr1:11232963-1123
ENSG0000(	1756	39.21595	chr1:1142	AL121992.1		smallRNA	chr1:15684472-1568
ENSG0000(	1756	39.21595	chr1:1142	MFN2		protein_c	chr1:11980181-1201
ENSG0000(	1756	39.21595	chr1:1142	RPL23AP89		Pseudoger	chr1:12080293-1208
ENSG0000(	1756	39.21595	chr1:1142	NPPA		protein_c	chr1:11845709-1184
ENSG0000(	1756	39.21595	chr1:1142	MIR4632		smallRNA	chr1:12191713-1219
ENSG0000(	1756	39.21595	chr1:1142	ENSG00000280113		TEC	chr1:9826289-98282

ENSG0000(	1756	39.21595	chr1:114ž	Y_RNA		smallRNA	chr1:12024012-1202
ENSG0000(	1756	39.21595	chr1:114ž	DNAJC16		protein_c	chr1:15526813-1559
ENSG0000(	1756	39.21595	chr1:114ž	KIAA2013		protein_c	chr1:11919591-1192
ENSG0000(	1756	39.21595	chr1:114ž	EXOSC10-AS1		lncRNA	chr1:11099430-1110
ENSG0000(	1756	39.21595	chr1:114ž	DISP3	NCGv7	protein_c	chr1:11479155-1153
ENSG0000(	1756	39.21595	chr1:114ž	EXOSC10		protein_c	chr1:11066618-1109
ENSG0000(	1756	39.21595	chr1:114ž	ENSG00000287727		lncRNA	chr1:10612222-1061
ENSG0000(	1756	39.21595	chr1:114ž	PRAMEF5		protein_c	chr1:13254198-1326
ENSG0000(	1756	39.21595	chr1:114ž	RNU5E-4P		smallRNA	chr1:11909808-1190
ENSG0000(	1756	39.21595	chr1:114ž	CASZ1	NCGv7	protein_c	chr1:10636604-1079
ENSG0000(	1756	39.21595	chr1:114ž	ENSG00000271989		lncRNA	chr1:10429881-1043
ENSG0000(	1756	39.21595	chr1:114ž	PRDM2	NCGv7	protein_c	chr1:13700188-1382
ENSG0000(	1756	39.21595	chr1:114ž	PRAMEF1		protein_c	chr1:12791397-1279
ENSG0000(	1756	39.21595	chr1:114ž	RPL22P3		Pseudoger	chr1:16369150-1636
ENSG0000(	1756	39.21595	chr1:114ž	ENSG00000272078		lncRNA	chr1:10639241-1065
ENSG0000(	1756	39.21595	chr1:114ž	FBXO6		protein_c	chr1:11664200-1167
ENSG0000(	1756	39.21595	chr1:114ž	MAD2L2		protein_c	chr1:11658918-1169
ENSG0000(	1756	39.21595	chr1:114ž	TMEM51-AS2		lncRNA	chr1:15164344-1517
ENSG0000(	1756	39.21595	chr1:114ž	RNU1-1		smallRNA	chr1:16514122-1651
ENSG0000(	1756	39.21595	chr1:114ž	PRAMEF12		protein_c	chr1:12773738-1277
ENSG0000(	1756	39.21595	chr1:114ž	ANGPTL7		protein_c	chr1:11189355-1119
ENSG0000(	1756	39.21595	chr1:114ž	MIIP		protein_c	chr1:12019466-1203
ENSG0000(	1756	39.21595	chr1:114ž	CENPS-CORT		protein_c	chr1:10430102-1045
ENSG0000(	1756	39.21595	chr1:114ž	RP11-219C24.10		lncRNA	chr1:13324039-1332
ENSG0000(	1755	39.19361	chr2:309	RNU6-692P		smallRNA	chr2:147408342-147
ENSG0000(	1755	39.19361	chr2:309	AC013406.1		smallRNA	chr2:147466630-147
ENSG0000(	1755	39.19361	chr2:309	RNU6-715P		smallRNA	chr2:147495438-147
ENSG0000(	1755	39.19361	chr2:309	ENSG00000287847		lncRNA	chr2:147150707-147
ENSG0000(	1755	39.19361	chr2:309	snoU13		smallRNA	chr2:147323971-147
ENSG0000(	1755	39.19361	chr2:309	RNU6-1275P		smallRNA	chr2:147877422-147
ENSG0000(	1755	39.19361	chr2:309	ENSG00000229143		Pseudoger	chr2:147810346-147
ENSG0000(	1755	39.19361	chr2:309	LINC01911		lncRNA	chr2:146682289-146
ENSG0000(	1755	39.19361	chr2:309	Y_RNA		smallRNA	chr2:147472133-147
ENSG0000(	1755	39.19361	chr2:309	RNA5SP106		Pseudoger	chr2:147713455-147
ENSG0000(	1755	39.19361	chr2:309	ENSG00000223911		lncRNA	chr2:147899401-147
ENSG0000(	1745	38.97029	chr1:2197	LINC02961		lncRNA	chr1:234757619-234
ENSG0000(	1745	38.97029	chr1:2197	ENSG00000289377		lncRNA	chr1:234771523-234
ENSG0000(	1745	38.97029	chr1:2197	RNY4P16		smallRNA	chr1:234837973-234
ENSG0000(	1745	38.97029	chr1:2197	ENSG00000230628		lncRNA	chr1:234669523-234
ENSG0000(	1745	38.97029	chr1:2197	LINC01132		lncRNA	chr1:234724042-234
ENSG0000(	1745	38.97029	chr1:2197	ENSG00000241475		lncRNA	chr1:234660271-234
ENSG0000(	1745	38.97029	chr1:2197	LINC00184		lncRNA	chr1:234629311-234
ENSG0000(	1745	38.97029	chr1:2197	ENSG00000282097		lncRNA	chr1:234709383-234
ENSG0000(	1745	38.97029	chr1:2197	ENSG00000272362		lncRNA	chr1:234644666-234
ENSG0000(	1745	38.97029	chr1:2197	ENSG00000228044		lncRNA	chr1:234646289-234
ENSG0000(	1743	38.92562	chr2:309	ENSG00000286611		lncRNA	chr2:170820066-170
ENSG0000(	1732	38.67997	chr2:309	RPL37P12		Pseudoger	chr2:109552536-109
ENSG0000(	1732	38.67997	chr2:309	ENSG00000231536		lncRNA	chr2:110610916-110
ENSG0000(	1732	38.67997	chr2:309	ENSG00000184115		Pseudoger	chr2:110397406-110
ENSG0000(	1732	38.67997	chr2:309	ENSG00000283167		Pseudoger	chr2:110321402-110
ENSG0000(	1732	38.67997	chr2:309	SRSF3P6		Pseudoger	chr2:109732731-109

ENSG0000C	1732	38.67997	chr2:3094	GPAA1P1		Pseudoger	chr2:109984058-109
ENSG0000C	1732	38.67997	chr2:3094	MALLP1		Pseudoger	chr2:110279606-110
ENSG0000C	1732	38.67997	chr2:3094	LINC01123		lncRNA	chr2:109987063-109
ENSG0000C	1732	38.67997	chr2:3094	RPL22P11		Pseudoger	chr2:109737139-109
ENSG0000C	1732	38.67997	chr2:3094	GMCL1P2		Pseudoger	chr2:108367116-108
ENSG0000C	1732	38.67997	chr1:1142	ENSG00000226545		Pseudoger	chr1:8798475-87995
ENSG0000C	1732	38.67997	chr2:3094	ACTR1AP1		Pseudoger	chr2:110184054-110
ENSG0000C	1732	38.67997	chr2:3094	MIR4436B2		smallRNA	chr2:110284853-110
ENSG0000C	1732	38.67997	chr2:3094	ENSG00000229730		Pseudoger	chr2:110012265-110
ENSG0000C	1732	38.67997	chr1:1142	SLC2A7		protein_c	chr1:9002973-90264
ENSG0000C	1732	38.67997	chr1:1142	RPL7P11		Pseudoger	chr1:8750430-87510
ENSG0000C	1732	38.67997	chr2:3094	ENSG00000282033		lncRNA	chr2:109758799-109
ENSG0000C	1732	38.67997	chr1:1142	ENO1		protein_c	chr1:8861000-88791
ENSG0000C	1732	38.67997	chr2:3094	GCC2-AS1		lncRNA	chr2:108507515-108
ENSG0000C	1732	38.67997	chr1:1142	ENSG00000232663		Pseudoger	chr1:8909742-89099
ENSG0000C	1732	38.67997	chr1:1142	HMGN2P17		Pseudoger	chr1:8893409-88941
ENSG0000C	1732	38.67997	chr1:1142	ENSG00000228423		lncRNA	chr1:8805860-88070
ENSG0000C	1732	38.67997	chr1:1142	ENSG00000289914		lncRNA	chr1:9162322-91629
ENSG0000C	1732	38.67997	chr2:3094	ENSG00000186148		Pseudoger	chr2:109947672-109
ENSG0000C	1732	38.67997	chr2:3094	ENSG00000261760		Pseudoger	chr2:110245756-110
ENSG0000C	1732	38.67997	chr2:3094	SH3RF3-AS1		lncRNA	chr2:109127327-109
ENSG0000C	1732	38.67997	chr2:3094	LIMS1		protein_c	chr2:108533671-108
ENSG0000C	1732	38.67997	chr1:1142	ENSG00000232208		Pseudoger	chr1:8907393-89077
ENSG0000C	1732	38.67997	chr2:3094	LIMS4		protein_c	chr2:110445883-110
ENSG0000C	1732	38.67997	chr2:3094	ACOXL	NCGv7	protein_c	chr2:110732539-111
ENSG0000C	1732	38.67997	chr2:3094	RANBP2	NCGv7	protein_c	chr2:108719482-108
ENSG0000C	1732	38.67997	chr1:1142	CA6		protein_c	chr1:8945867-89750
ENSG0000C	1732	38.67997	chr2:3094	ENSG00000289202		lncRNA	chr2:110678205-110
ENSG0000C	1732	38.67997	chr2:3094	MIR4436B1		smallRNA	chr2:110086433-110
ENSG0000C	1732	38.67997	chr2:3094	BUB1		protein_c	chr2:110637528-110
ENSG0000C	1732	38.67997	chr2:3094	BMS1P19		Pseudoger	chr2:109667128-109
ENSG0000C	1732	38.67997	chr1:1142	SCARNA16		smallRNA	chr1:9082696-90828
ENSG0000C	1732	38.67997	chr1:1142	LNCTAM34A		lncRNA	chr1:9182004-91962
ENSG0000C	1732	38.67997	chr2:3094	ENSG00000282304		lncRNA	chr2:110245591-110
ENSG0000C	1732	38.67997	chr2:3094	SULT1C2P1		lncRNA	chr2:108322238-108
ENSG0000C	1732	38.67997	chr2:3094	ZBTB45P1		Pseudoger	chr2:109986939-109
ENSG0000C	1732	38.67997	chr2:3094	SULT1C2P1		Pseudoger	chr2:108322353-108
ENSG0000C	1732	38.67997	chr2:3094	RPL39P16		Pseudoger	chr2:108878308-108
ENSG0000C	1732	38.67997	chr2:3094	RGPD5		protein_c	chr2:109792758-109
ENSG0000C	1732	38.67997	chr2:3094	ENSG00000290732		lncRNA	chr2:110231106-110
ENSG0000C	1732	38.67997	chr1:1142	GPR157		protein_c	chr1:9100305-91291
ENSG0000C	1732	38.67997	chr2:3094	AC140479.1		smallRNA	chr2:110301312-110
ENSG0000C	1732	38.67997	chr2:3094	SULT1C2		protein_c	chr2:108288639-108
ENSG0000C	1732	38.67997	chr2:3094	ENSG00000257207		protein_c	chr2:110402934-110
ENSG0000C	1732	38.67997	chr2:3094	ENSG00000273471		lncRNA	chr2:110386411-110
ENSG0000C	1732	38.67997	chr2:3094	ENSG00000231583		Pseudoger	chr2:110500100-110
ENSG0000C	1732	38.67997	chr1:1142	Y_RNA		smallRNA	chr1:8796574-87966
ENSG0000C	1732	38.67997	chr2:3094	SULT1C4		protein_c	chr2:108377911-108
ENSG0000C	1732	38.67997	chr2:3094	ENSG00000230603		Pseudoger	chr2:109665209-109
ENSG0000C	1732	38.67997	chr2:3094	MIR4267		smallRNA	chr2:110069961-110
ENSG0000C	1732	38.67997	chr2:3094	RGPD6		protein_c	chr2:110513802-110

ENSG0000(	1732	38.67997	chr2:309⁄	ENSG00000230650		Pseudoger	chr2:110360608-110
ENSG0000(	1732	38.67997	chr1:1142	ENO1-AS1		lncRNA	chr1:8878835-88798
ENSG0000(	1732	38.67997	chr1:1142	RNU6-304P		smallRNA	chr1:8883427-88835
ENSG0000(	1732	38.67997	chr2:309⁄	SULT1C2P2		Pseudoger	chr2:108317725-108
ENSG0000(	1732	38.67997	chr1:1142	MIR34AHG		lncRNA	chr1:9148011-91989
ENSG0000(	1732	38.67997	chr2:309⁄	ENSG00000230696		lncRNA	chr2:109594693-109
ENSG0000(	1732	38.67997	chr2:309⁄	ENSG00000284337		protein_c	chr2:109898432-109
ENSG0000(	1732	38.67997	chr1:1142	RPL23AP19		Pseudoger	chr1:8831007-88313
ENSG0000(	1732	38.67997	chr2:309⁄	SOWAHC		protein_c	chr2:109614364-109
ENSG0000(	1732	38.67997	chr2:309⁄	ENSG00000235881		lncRNA	chr2:110709575-110
ENSG0000(	1732	38.67997	chr2:309⁄	SMIM12P1		Pseudoger	chr2:108423612-108
ENSG0000(	1732	38.67997	chr2:309⁄	SNRPGP9		Pseudoger	chr2:109251422-109
ENSG0000(	1732	38.67997	chr2:309⁄	ENSG00000227574		lncRNA	chr2:110449242-110
ENSG0000(	1732	38.67997	chr2:309⁄	MIR4266		smallRNA	chr2:109313571-109
ENSG0000(	1732	38.67997	chr2:309⁄	ENSG00000290733		lncRNA	chr2:110402914-110
ENSG0000(	1732	38.67997	chr2:309⁄	ENSG00000290731		lncRNA	chr2:109933161-109
ENSG0000(	1732	38.67997	chr2:309⁄	LIMS3		protein_c	chr2:109898428-109
ENSG0000(	1732	38.67997	chr2:309⁄	SETD6P1		Pseudoger	chr2:108226015-108
ENSG0000(	1732	38.67997	chr2:309⁄	ENSG00000233648		Pseudoger	chr2:108694750-108
ENSG0000(	1732	38.67997	chr1:1142	RPL7P7		Pseudoger	chr1:8786211-87869
ENSG0000(	1732	38.67997	chr1:1142	RN7SL451P		smallRNA	chr1:8979578-89798
ENSG0000(	1732	38.67997	chr2:309⁄	ZBTB45P2		Pseudoger	chr2:110383112-110
ENSG0000(	1732	38.67997	chr1:1142	MIR34A		smallRNA	chr1:9151668-91517
ENSG0000(	1732	38.67997	chr2:309⁄	WASF1P1		Pseudoger	chr2:108276812-108
ENSG0000(	1732	38.67997	chr2:309⁄	SULT1C3		protein_c	chr2:108239968-108
ENSG0000(	1732	38.67997	chr2:309⁄	MALL		protein_c	chr2:110083870-110
ENSG0000(	1732	38.67997	chr2:309⁄	NPHP1		protein_c	chr2:110122311-110
ENSG0000(	1732	38.67997	chr2:309⁄	LIMS1-AS1		lncRNA	chr2:108676795-108
ENSG0000(	1732	38.67997	chr2:309⁄	ENSG00000283283		Pseudoger	chr2:110022447-110
ENSG0000(	1732	38.67997	chr2:309⁄	GCC2	NCGv7	protein_c	chr2:108449107-108
ENSG0000(	1732	38.67997	chr2:309⁄	GPAA1P2		Pseudoger	chr2:110386377-110
ENSG0000(	1732	38.67997	chr1:1142	H6PD		protein_c	chr1:9234774-92713
ENSG0000(	1732	38.67997	chr2:309⁄	ENSG00000226991		Pseudoger	chr2:110358790-110
ENSG0000(	1732	38.67997	chr2:309⁄	RPL10P5		Pseudoger	chr2:108712262-108
ENSG0000(	1732	38.67997	chr2:309⁄	CCDC138		protein_c	chr2:108786757-108
ENSG0000(	1732	38.67997	chr1:1142	Z98044.1		smallRNA	chr1:9278391-92785
ENSG0000(	1732	38.67997	chr1:1142	ENSG00000290109		lncRNA	chr1:8970490-89712
ENSG0000(	1732	38.67997	chr2:309⁄	ENSG00000235721		Pseudoger	chr2:110007675-110
ENSG0000(	1732	38.67997	chr2:309⁄	LINC01106		lncRNA	chr2:110375138-110
ENSG0000(	1732	38.67997	chr1:1142	SLC2A5		protein_c	chr1:9035106-90884
ENSG0000(	1732	38.67997	chr2:309⁄	RPL22P12		Pseudoger	chr2:110633947-110
ENSG0000(	1732	38.67997	chr2:309⁄	ENSG00000283684		lncRNA	chr2:109937438-109
ENSG0000(	1732	38.67997	chr2:309⁄	SEPTIN10		protein_c	chr2:109542799-109
ENSG0000(	1732	38.67997	chr2:309⁄	MTLN		protein_c	chr2:110211529-110
ENSG0000(	1732	38.67997	chr2:309⁄	EDAR	NCGv7	protein_c	chr2:108894471-108
ENSG0000(	1726	38.54597	chr2:309⁄	FAM8A3P		Pseudoger	chr2:149320593-149
ENSG0000(	1726	38.54597	chr2:309⁄	ENSG00000236885		lncRNA	chr2:151114811-151
ENSG0000(	1726	38.54597	chr2:309⁄	HNRNPDLP2		Pseudoger	chr2:158419955-158
ENSG0000(	1726	38.54597	chr2:309⁄	RNA5SP109		Pseudoger	chr2:162496936-162
ENSG0000(	1726	38.54597	chr2:309⁄	ENSG00000224331		Pseudoger	chr2:164687287-164
ENSG0000(	1726	38.54597	chr2:309⁄	RN7SL124P		smallRNA	chr2:151372003-151

ENSG00000	1726	38.54597	chr2:3094	ENSG00000289488		lncRNA	chr2:158545074-158
ENSG00000	1726	38.54597	chr2:3094	Y_RNA		smallRNA	chr2:166123494-166
ENSG00000	1726	38.54597	chr2:3094	AC108057.1		smallRNA	chr2:157015172-157
ENSG00000	1726	38.54597	chr2:3094	MBD5		protein_c	chr2:148021011-148
ENSG00000	1726	38.54597	chr2:3094	ENSG00000236841		lncRNA	chr2:162159762-162
ENSG00000	1726	38.54597	chr2:3094	ENSG00000286167		lncRNA	chr2:148909406-148
ENSG00000	1726	38.54597	chr2:3094	ARL5A		protein_c	chr2:151788984-151
ENSG00000	1726	38.54597	chr2:3094	LYPD6		protein_c	chr2:149329985-149
ENSG00000	1726	38.54597	chr2:3094	LINC01876		lncRNA	chr2:156011530-156
ENSG00000	1726	38.54597	chr2:3094	ENSG00000286530		lncRNA	chr2:157033837-157
ENSG00000	1726	38.54597	chr2:3094	BAZ2B-AS1		lncRNA	chr2:159615292-159
ENSG00000	1726	38.54597	chr2:3094	EIF3EP2		Pseudoger	chr2:162088259-162
ENSG00000	1726	38.54597	chr2:3094	NR4A2	NCGv7	protein_c	chr2:156324437-156
ENSG00000	1726	38.54597	chr2:3094	RNU2-9P		smallRNA	chr2:148825691-148
ENSG00000	1726	38.54597	chr2:3094	LINC01958		lncRNA	chr2:156655224-156
ENSG00000	1726	38.54597	chr2:3094	SLC4A10	NCGv7	protein_c	chr2:161424332-161
ENSG00000	1726	38.54597	chr2:3094	LINC01931		lncRNA	chr2:149745648-149
ENSG00000	1726	38.54597	chr2:3094	ENSG00000277998		Pseudoger	chr2:164573741-164
ENSG00000	1726	38.54597	chr2:3094	ENSG00000270776		Pseudoger	chr2:152539197-152
ENSG00000	1726	38.54597	chr2:3094	RNU6-601P		smallRNA	chr2:149610668-149
ENSG00000	1726	38.54597	chr2:3094	ENSG00000288089		lncRNA	chr2:165433438-165
ENSG00000	1726	38.54597	chr2:3094	SCN1A		protein_c	chr2:165984641-166
ENSG00000	1726	38.54597	chr2:3094	ENSG00000225214		lncRNA	chr2:152098328-152
ENSG00000	1726	38.54597	chr2:3094	TANK-AS1		lncRNA	chr2:161096231-161
ENSG00000	1726	38.54597	chr2:3094	RPEP5		Pseudoger	chr2:161931199-161
ENSG00000	1726	38.54597	chr2:3094	PKP4	NCGv7	protein_c	chr2:158456952-158
ENSG00000	1726	38.54597	chr2:3094	CACNB4		protein_c	chr2:151832771-152
ENSG00000	1726	38.54597	chr2:3094	SCN3A	NCGv7	protein_c	chr2:165087526-165
ENSG00000	1726	38.54597	chr2:3094	ENSG00000270893		Pseudoger	chr2:159444117-159
ENSG00000	1726	38.54597	chr2:3094	RBMS1		protein_c	chr2:160272151-160
ENSG00000	1726	38.54597	chr2:3094	PLA2R1	NCGv7	protein_c	chr2:159932006-160
ENSG00000	1726	38.54597	chr2:3094	RPL7AP22		Pseudoger	chr2:158548425-158
ENSG00000	1726	38.54597	chr2:3094	ENSG00000287048		lncRNA	chr2:156788731-156
ENSG00000	1726	38.54597	chr2:3094	LYPD6B		protein_c	chr2:149038107-149
ENSG00000	1726	38.54597	chr2:3094	CCDC148		protein_c	chr2:158171073-158
ENSG00000	1726	38.54597	chr2:3094	ENSG00000287085		lncRNA	chr2:152450162-152
ENSG00000	1726	38.54597	chr2:3094	ENSG00000286679		lncRNA	chr2:155525424-155
ENSG00000	1726	38.54597	chr2:3094	GALNT3		protein_c	chr2:165747588-165
ENSG00000	1726	38.54597	chr2:3094	NEB	NCGv7	protein_c	chr2:151485336-151
ENSG00000	1726	38.54597	chr2:3094	AC105402.1		smallRNA	chr2:148891878-148
ENSG00000	1726	38.54597	chr2:3094	ERMN		protein_c	chr2:157318631-157
ENSG00000	1726	38.54597	chr2:3094	RNU6-580P		smallRNA	chr2:159027036-159
ENSG00000	1726	38.54597	chr2:3094	GALNT5		protein_c	chr2:157257705-157
ENSG00000	1726	38.54597	chr2:3094	LY75-CD30	IntOGen-I	protein_c	chr2:159771851-159
ENSG00000	1726	38.54597	chr2:3094	RPL30P2		Pseudoger	chr2:152131555-152
ENSG00000	1726	38.54597	chr2:3094	XIRP2	NCGv7	protein_c	chr2:166888480-167
ENSG00000	1726	38.54597	chr2:3094	TIMM8AP1		Pseudoger	chr2:162077357-162
ENSG00000	1726	38.54597	chr2:3094	CAPZA1P2		Pseudoger	chr2:159462417-159
ENSG00000	1726	38.54597	chr2:3094	Y_RNA		smallRNA	chr2:159536244-159
ENSG00000	1726	38.54597	chr2:3094	AC009495.1		smallRNA	chr2:165814215-165
ENSG00000	1726	38.54597	chr2:3094	ENSG00000287091		lncRNA	chr2:159797288-159

ENSG0000(	1726	38.54597	chr2:3094	RNU6-436P		smallRNA	chr2:157912772-157
ENSG0000(	1726	38.54597	chr2:3094	EPC2		protein_c	chr2:148644440-148
ENSG0000(	1726	38.54597	chr2:3094	TANK		protein_c	chr2:161136908-161
ENSG0000(	1726	38.54597	chr2:3094	SCN7A		protein_c	chr2:166403573-166
ENSG0000(	1726	38.54597	chr2:3094	RN7SKP152		smallRNA	chr2:166180652-166
ENSG0000(	1726	38.54597	chr2:3094	ENSG00000224076		lncRNA	chr2:161422659-161
ENSG0000(	1726	38.54597	chr2:3094	RND3		protein_c	chr2:150468195-150
ENSG0000(	1726	38.54597	chr2:3094	ENSG00000228064		lncRNA	chr2:150982506-151
ENSG0000(	1726	38.54597	chr2:3094	MARCHF7		protein_c	chr2:159712457-159
ENSG0000(	1726	38.54597	chr2:3094	ENSG00000289474		lncRNA	chr2:148881726-148
ENSG0000(	1726	38.54597	chr2:3094	LY75		protein_c	chr2:159803355-159
ENSG0000(	1726	38.54597	chr2:3094	RNU6-627P		smallRNA	chr2:163288995-163
ENSG0000(	1726	38.54597	chr2:3094	PRPF40A		protein_c	chr2:152651593-152
ENSG0000(	1726	38.54597	chr2:3094	PKP4-AS1		lncRNA	chr2:158658337-158
ENSG0000(	1726	38.54597	chr2:3094	ENSG00000233397		lncRNA	chr2:162092232-162
ENSG0000(	1726	38.54597	chr2:3094	UPP2-IT1		lncRNA	chr2:158127957-158
ENSG0000(	1726	38.54597	chr2:3094	AC092841.1		smallRNA	chr2:161918782-161
ENSG0000(	1726	38.54597	chr2:3094	RIF1		protein_c	chr2:151409883-151
ENSG0000(	1726	38.54597	chr2:3094	ENSG00000234932		Pseudoger	chr2:152850653-152
ENSG0000(	1726	38.54597	chr2:3094	ORC4		protein_c	chr2:147930396-148
ENSG0000(	1726	38.54597	chr2:3094	LINC02612		lncRNA	chr2:150612381-150
ENSG0000(	1726	38.54597	chr2:3094	ENSG00000237220		lncRNA	chr2:150566134-150
ENSG0000(	1726	38.54597	chr2:3094	TBR1	NCGv7	protein_c	chr2:161416297-161
ENSG0000(	1726	38.54597	chr2:3094	ARL6IP6		protein_c	chr2:152717647-152
ENSG0000(	1726	38.54597	chr2:3094	LINC01817		lncRNA	chr2:150234892-150
ENSG0000(	1726	38.54597	chr2:3094	ENSG00000283657		Pseudoger	chr2:166414489-166
ENSG0000(	1726	38.54597	chr2:3094	SCN2A		protein_c	chr2:165194993-165
ENSG0000(	1726	38.54597	chr2:3094	RNA5SP111		Pseudoger	chr2:164895677-164
ENSG0000(	1726	38.54597	chr2:3094	RNU2-21P		smallRNA	chr2:158935798-158
ENSG0000(	1726	38.54597	chr2:3094	ENSG00000229195		lncRNA	chr2:165794857-165
ENSG0000(	1726	38.54597	chr2:3094	MTCYBP9		Pseudoger	chr2:155314066-155
ENSG0000(	1726	38.54597	chr2:3094	ENSG00000235192		lncRNA	chr2:165794851-165
ENSG0000(	1726	38.54597	chr2:3094	RN7SKP281		smallRNA	chr2:157298807-157
ENSG0000(	1726	38.54597	chr2:3094	TTC21B-AS1		lncRNA	chr2:165933857-165
ENSG0000(	1726	38.54597	chr2:3094	USP12P2		Pseudoger	chr2:148295656-148
ENSG0000(	1726	38.54597	chr2:3094	ENSG00000226266		lncRNA	chr2:159670708-159
ENSG0000(	1726	38.54597	chr2:3094	ENSG00000279844		TEC	chr2:160849313-160
ENSG0000(	1726	38.54597	chr2:3094	KRT18P46		Pseudoger	chr2:161580072-161
ENSG0000(	1726	38.54597	chr2:3094	RPS20P13		Pseudoger	chr2:148870024-148
ENSG0000(	1726	38.54597	chr2:3094	Y_RNA		smallRNA	chr2:152284350-152
ENSG0000(	1726	38.54597	chr2:3094	PSMD14-DT		lncRNA	chr2:161222785-161
ENSG0000(	1726	38.54597	chr2:3094	ENSG00000286207		lncRNA	chr2:152635268-152
ENSG0000(	1726	38.54597	chr2:3094	ENSG00000232337		Pseudoger	chr2:161048606-161
ENSG0000(	1726	38.54597	chr2:3094	OR7E90P		Pseudoger	chr2:158874642-158
ENSG0000(	1726	38.54597	chr2:3094	LINC01806		lncRNA	chr2:161244720-161
ENSG0000(	1726	38.54597	chr2:3094	UBQLN4P2		Pseudoger	chr2:152876820-152
ENSG0000(	1726	38.54597	chr2:3094	RNU6-932P		smallRNA	chr2:157985789-157
ENSG0000(	1726	38.54597	chr2:3094	snoU13		smallRNA	chr2:156422195-156
ENSG0000(	1726	38.54597	chr2:3094	MXRA7P1		Pseudoger	chr2:161340816-161
ENSG0000(	1726	38.54597	chr2:3094	RPL7P61		Pseudoger	chr2:163152251-163
ENSG0000(	1726	38.54597	chr2:3094	UPP2		protein_c	chr2:157876702-158

ENSG0000(	1726	38.54597	chr2:309<	LINC01818		lncRNA	chr2:150169474-150
ENSG0000(	1726	38.54597	chr2:309<	MTCO1P45		Pseudoger	chr2:155263458-155
ENSG0000(	1726	38.54597	chr2:309<	ENSG00000288066		lncRNA	chr2:151796688-151
ENSG0000(	1726	38.54597	chr2:309<	RNA5SP108		Pseudoger	chr2:161409554-161
ENSG0000(	1726	38.54597	chr2:309<	SNORA48		smallRNA	chr2:148260574-148
ENSG0000(	1726	38.54597	chr2:309<	ENSG00000286621		lncRNA	chr2:158847619-158
ENSG0000(	1726	38.54597	chr2:309<	MTND5P30		Pseudoger	chr2:155311697-155
ENSG0000(	1726	38.54597	chr2:309<	MTA3P1		Pseudoger	chr2:157961410-157
ENSG0000(	1726	38.54597	chr2:309<	ENSG00000225813		Pseudoger	chr2:161281902-161
ENSG0000(	1726	38.54597	chr2:309<	GPD2		protein_c	chr2:156435290-156
ENSG0000(	1726	38.54597	chr2:309<	STAM2	NCGv7	protein_c	chr2:152116801-152
ENSG0000(	1726	38.54597	chr2:309<	SLC38A11		protein_c	chr2:164894354-164
ENSG0000(	1726	38.54597	chr2:309<	RN7SL423P		smallRNA	chr2:160877547-160
ENSG0000(	1726	38.54597	chr2:309<	AC009299.1		smallRNA	chr2:161350217-161
ENSG0000(	1726	38.54597	chr2:309<	ENSG00000288091		lncRNA	chr2:161577192-161
ENSG0000(	1726	38.54597	chr2:309<	AC009480.1		smallRNA	chr2:147940980-147
ENSG0000(	1726	38.54597	chr2:309<	ENSG00000281469		TEC	chr2:148044380-148
ENSG0000(	1726	38.54597	chr2:309<	COBLL1		protein_c	chr2:164653624-164
ENSG0000(	1726	38.54597	chr2:309<	ENSG00000285155		lncRNA	chr2:160141981-160
ENSG0000(	1726	38.54597	chr2:309<	RPS3AP13		Pseudoger	chr2:159689217-159
ENSG0000(	1726	38.54597	chr2:309<	AC110086.1		smallRNA	chr2:164423525-164
ENSG0000(	1726	38.54597	chr2:309<	GSTM3P2		Pseudoger	chr2:159056005-159
ENSG0000(	1726	38.54597	chr2:309<	MIR4785		smallRNA	chr2:160407810-160
ENSG0000(	1726	38.54597	chr2:309<	DAPL1		protein_c	chr2:158795317-158
ENSG0000(	1726	38.54597	chr2:309<	ENSG00000286234		lncRNA	chr2:152176020-152
ENSG0000(	1726	38.54597	chr2:309<	LINC01920		lncRNA	chr2:150552532-150
ENSG0000(	1726	38.54597	chr2:309<	CD302		protein_c	chr2:159768628-159
ENSG0000(	1726	38.54597	chr2:309<	MMADHC		protein_c	chr2:149569637-149
ENSG0000(	1726	38.54597	chr2:309<	KCNH7		protein_c	chr2:162371407-162
ENSG0000(	1726	38.54597	chr2:309<	snoZ5		smallRNA	chr2:158534200-158
ENSG0000(	1726	38.54597	chr2:309<	LINC02478		lncRNA	chr2:160257716-160
ENSG0000(	1726	38.54597	chr2:309<	ENSG00000280105		TEC	chr2:161433874-161
ENSG0000(	1726	38.54597	chr2:309<	RBM43		protein_c	chr2:151247940-151
ENSG0000(	1726	38.54597	chr2:309<	RPL17P13		Pseudoger	chr2:149439897-149
ENSG0000(	1726	38.54597	chr2:309<	SCN1A-AS1		lncRNA	chr2:165957188-166
ENSG0000(	1726	38.54597	chr2:309<	AHCTF1P1		Pseudoger	chr2:161500885-161
ENSG0000(	1726	38.54597	chr2:309<	FMNL2		protein_c	chr2:152335174-152
ENSG0000(	1726	38.54597	chr2:309<	FAP		protein_c	chr2:162170684-162
ENSG0000(	1726	38.54597	chr2:309<	ENSG00000251621		lncRNA	chr2:161424015-161
ENSG0000(	1726	38.54597	chr2:309<	ENSG00000286889		lncRNA	chr2:156803339-156
ENSG0000(	1726	38.54597	chr2:309<	snoU13		smallRNA	chr2:161558544-161
ENSG0000(	1726	38.54597	chr2:309<	ACVR2A	NCGv7	protein_c	chr2:147844517-147
ENSG0000(	1726	38.54597	chr2:309<	MTND2P20		Pseudoger	chr2:155264163-155
ENSG0000(	1726	38.54597	chr2:309<	RMRPP3		smallRNA	chr2:160396829-160
ENSG0000(	1726	38.54597	chr2:309<	FAM133DP		Pseudoger	chr2:157379724-157
ENSG0000(	1726	38.54597	chr2:309<	RNU6-546P		smallRNA	chr2:155330421-155
ENSG0000(	1726	38.54597	chr2:309<	ENSG00000271320		lncRNA	chr2:157917712-157
ENSG0000(	1726	38.54597	chr2:309<	MIR4773-1		smallRNA	chr2:151368334-151
ENSG0000(	1726	38.54597	chr2:309<	AC019201.1		smallRNA	chr2:157441550-157
ENSG0000(	1726	38.54597	chr2:309<	ENSG00000286335		lncRNA	chr2:149170561-149
ENSG0000(	1726	38.54597	chr2:309<	SCN9A	NCGv7	protein_c	chr2:166195185-166

ENSG0000(	1726	38.54597	chr2:309∠	ENSG00000232411		lncRNA	chr2:165833048-165
ENSG0000(	1726	38.54597	chr2:309∠	AC013731.1		smallRNA	chr2:157855270-157
ENSG0000(	1726	38.54597	chr2:309∠	MMADHC-DT		lncRNA	chr2:149587196-150
ENSG0000(	1726	38.54597	chr2:309∠	ENSG00000228586		lncRNA	chr2:158685903-158
ENSG0000(	1726	38.54597	chr2:309∠	ENSG00000223642		lncRNA	chr2:159386367-159
ENSG0000(	1726	38.54597	chr2:309∠	KCNH7-AS1		lncRNA	chr2:162768936-162
ENSG0000(	1726	38.54597	chr2:309∠	ENSG00000226946		Pseudoger	chr2:161568502-161
ENSG0000(	1726	38.54597	chr2:309∠	ENSG00000286081		lncRNA	chr2:150150633-150
ENSG0000(	1726	38.54597	chr2:309∠	RN7SL455P		smallRNA	chr2:165149195-165
ENSG0000(	1726	38.54597	chr2:309∠	DPP4		protein_c	chr2:161992245-162
ENSG0000(	1726	38.54597	chr2:309∠	RNU6-1001P		smallRNA	chr2:155089439-155
ENSG0000(	1726	38.54597	chr2:309∠	ENSG00000236283		lncRNA	chr2:164840661-165
ENSG0000(	1726	38.54597	chr2:309∠	OR7E89P		Pseudoger	chr2:158853755-158
ENSG0000(	1726	38.54597	chr2:309∠	ENSG00000225182		Pseudoger	chr2:166889502-166
ENSG0000(	1726	38.54597	chr2:309∠	XIRP2-AS1		lncRNA	chr2:167123904-167
ENSG0000(	1726	38.54597	chr2:309∠	ENSG00000227055		Pseudoger	chr2:159812234-159
ENSG0000(	1726	38.54597	chr2:309∠	ENSG00000222031		lncRNA	chr2:151001220-151
ENSG0000(	1726	38.54597	chr2:309∠	ENSG00000281772		TEC	chr2:148062154-148
ENSG0000(	1726	38.54597	chr2:309∠	snoU13		smallRNA	chr2:156410144-156
ENSG0000(	1726	38.54597	chr2:309∠	FIGN		protein_c	chr2:163593396-163
ENSG0000(	1726	38.54597	chr2:309∠	KIF5C-AS1		lncRNA	chr2:148866470-148
ENSG0000(	1726	38.54597	chr2:309∠	MTND4P28		Pseudoger	chr2:155311023-155
ENSG0000(	1726	38.54597	chr2:309∠	ENSG00000283228		protein_c	chr2:151802651-151
ENSG0000(	1726	38.54597	chr2:309∠	WDSUB1		protein_c	chr2:159235798-159
ENSG0000(	1726	38.54597	chr2:309∠	MTND6P9		Pseudoger	chr2:155313440-155
ENSG0000(	1726	38.54597	chr2:309∠	NMI		protein_c	chr2:151270470-151
ENSG0000(	1726	38.54597	chr2:309∠	PRPS1P1		Pseudoger	chr2:164213539-164
ENSG0000(	1726	38.54597	chr2:309∠	CCDC148-AS1		lncRNA	chr2:158166650-158
ENSG0000(	1726	38.54597	chr2:309∠	TNFAIP6	NCGv7	protein_c	chr2:151357592-151
ENSG0000(	1726	38.54597	chr2:309∠	TTC21B	NCGv7	protein_c	chr2:165857475-165
ENSG0000(	1726	38.54597	chr2:309∠	CSRNP3		protein_c	chr2:165469647-165
ENSG0000(	1726	38.54597	chr2:309∠	TXNP5		Pseudoger	chr2:149068596-149
ENSG0000(	1726	38.54597	chr2:309∠	snoU13		smallRNA	chr2:166358730-166
ENSG0000(	1726	38.54597	chr2:309∠	GCA		protein_c	chr2:162318840-162
ENSG0000(	1726	38.54597	chr2:309∠	OR7E28P		Pseudoger	chr2:158862311-158
ENSG0000(	1726	38.54597	chr2:309∠	RPLP0P7		Pseudoger	chr2:156777706-156
ENSG0000(	1726	38.54597	chr2:309∠	PTP4A1P1		Pseudoger	chr2:158065545-158
ENSG0000(	1726	38.54597	chr2:309∠	ITGB6		protein_c	chr2:160099667-160
ENSG0000(	1726	38.54597	chr2:309∠	IFIH1		protein_c	chr2:162267074-162
ENSG0000(	1726	38.54597	chr2:309∠	GCG		protein_c	chr2:162142882-162
ENSG0000(	1726	38.54597	chr2:309∠	ATP5F1AP2		Pseudoger	chr2:155269633-155
ENSG0000(	1726	38.54597	chr2:309∠	ENSG00000231675		Pseudoger	chr2:158703287-158
ENSG0000(	1726	38.54597	chr2:309∠	FABP5P10		Pseudoger	chr2:151186188-151
ENSG0000(	1726	38.54597	chr2:309∠	MAPRE1P3		Pseudoger	chr2:165285253-165
ENSG0000(	1726	38.54597	chr2:309∠	ACVR1	NCGv7	protein_c	chr2:157736251-157
ENSG0000(	1726	38.54597	chr2:309∠	USP8P2		Pseudoger	chr2:148872253-148
ENSG0000(	1726	38.54597	chr2:309∠	SNORA70F		smallRNA	chr2:164687643-164
ENSG0000(	1726	38.54597	chr2:309∠	DPP4-DT		lncRNA	chr2:162073256-162
ENSG0000(	1726	38.54597	chr2:309∠	HEBP2P1		Pseudoger	chr2:156033247-156
ENSG0000(	1726	38.54597	chr2:309∠	ENSG00000237844		lncRNA	chr2:163743913-164
ENSG0000(	1726	38.54597	chr2:309∠	ENSG00000270557		lncRNA	chr2:157877683-157

ENSG0000(	1726	38.54597	chr2:309<	ENSG00000234584		lncRNA	chr2:157725708-157
ENSG0000(	1726	38.54597	chr2:309<	AC011308.1		protein_c	chr2:156496085-156
ENSG0000(	1726	38.54597	chr2:309<	CYP2C56P		Pseudoger	chr2:164321933-164
ENSG0000(	1726	38.54597	chr2:309<	ENSG00000232359		lncRNA	chr2:150595102-150
ENSG0000(	1726	38.54597	chr2:309<	RNA5SP110		Pseudoger	chr2:164858432-164
ENSG0000(	1726	38.54597	chr2:309<	ACVR1C		protein_c	chr2:157526767-157
ENSG0000(	1726	38.54597	chr2:309<	CYTIP		protein_c	chr2:157414619-157
ENSG0000(	1726	38.54597	chr2:309<	BTF3L4P2		Pseudoger	chr2:159003975-159
ENSG0000(	1726	38.54597	chr2:309<	TANC1		protein_c	chr2:158968640-159
ENSG0000(	1726	38.54597	chr2:309<	NUDCP1		Pseudoger	chr2:152389937-152
ENSG0000(	1726	38.54597	chr2:309<	RPS29P8		Pseudoger	chr2:148595158-148
ENSG0000(	1726	38.54597	chr2:309<	BAZ2B	NCGv7	protein_c	chr2:159318979-159
ENSG0000(	1726	38.54597	chr2:309<	ENSG00000230991		lncRNA	chr2:154964308-154
ENSG0000(	1726	38.54597	chr2:309<	ENSG00000282440		lncRNA	chr2:156305108-156
ENSG0000(	1726	38.54597	chr2:309<	GRB14		protein_c	chr2:164492417-164
ENSG0000(	1726	38.54597	chr2:309<	PSMD14		protein_c	chr2:161308425-161
ENSG0000(	1726	38.54597	chr2:309<	RN7SL393P		smallRNA	chr2:158447164-158
ENSG0000(	1726	38.54597	chr2:309<	CDK7P1		Pseudoger	chr2:157210847-157
ENSG0000(	1722	38.45664	chr2:309<	ENSG00000225588		lncRNA	chr2:107362282-107
ENSG0000(	1722	38.45664	chr2:309<	LINC01885		lncRNA	chr2:107382715-107
ENSG0000(	1722	38.45664	chr2:309<	SLC5A7		protein_c	chr2:107986523-108
ENSG0000(	1722	38.45664	chr2:309<	SRSF3P5		Pseudoger	chr2:107920763-107
ENSG0000(	1722	38.45664	chr2:309<	PPP1R2P5		Pseudoger	chr2:106940880-106
ENSG0000(	1722	38.45664	chr2:309<	LINC01593		lncRNA	chr2:108049200-108
ENSG0000(	1722	38.45664	chr2:309<	RPL22P8		Pseudoger	chr2:107914948-107
ENSG0000(	1722	38.45664	chr2:309<	LINC01789		lncRNA	chr2:107254691-107
ENSG0000(	1722	38.45664	chr2:309<	RGPD4		protein_c	chr2:107826892-107
ENSG0000(	1722	38.45664	chr2:309<	LINC01886		lncRNA	chr2:107529292-107
ENSG0000(	1722	38.45664	chr2:309<	ENSG00000286218		lncRNA	chr2:106933739-106
ENSG0000(	1722	38.45664	chr2:309<	ENSG00000233494		Pseudoger	chr2:107902997-107
ENSG0000(	1722	38.45664	chr2:309<	LINC01594		lncRNA	chr2:108167125-108
ENSG0000(	1722	38.45664	chr2:309<	GACAT1		lncRNA	chr2:107754112-107
ENSG0000(	1722	38.45664	chr2:309<	ENSG00000227270		Pseudoger	chr2:107903964-107
ENSG0000(	1722	38.45664	chr2:309<	RGPD4-AS1		lncRNA	chr2:107823063-107
ENSG0000(	1722	38.45664	chr2:309<	ACTP1		Pseudoger	chr2:108099110-108
ENSG0000(	1722	38.45664	chr2:309<	ENSG00000286769		lncRNA	chr2:107698737-107
ENSG0000(	1722	38.45664	chr2:309<	ST6GAL2-IT1		lncRNA	chr2:106822923-106
ENSG0000(	1722	38.45664	chr2:309<	ENSG00000227294		lncRNA	chr2:106834464-106
ENSG0000(	1722	38.45664	chr2:309<	ENSG00000226905		Pseudoger	chr2:106963193-106
ENSG0000(	1722	38.45664	chr2:309<	ST6GAL2	NCGv7	protein_c	chr2:106801600-106
ENSG0000(	1691	37.76433	chr2:309<	AC068706.1		smallRNA	chr2:176383427-176
ENSG0000(	1691	37.76433	chr2:309<	CIR1		protein_c	chr2:174348022-174
ENSG0000(	1691	37.76433	chr2:309<	RPS2P18		Pseudoger	chr2:173297629-173
ENSG0000(	1691	37.76433	chr2:309<	MGAT4A		protein_c	chr2:98619106-9873
ENSG0000(	1691	37.76433	chr2:309<	RNU4-84P		smallRNA	chr2:98782410-9878
ENSG0000(	1691	37.76433	chr2:309<	HOXD4		protein_c	chr2:176151550-176
ENSG0000(	1691	37.76433	chr2:309<	AC096649.4		smallRNA	chr2:175330373-175
ENSG0000(	1691	37.76433	chr2:309<	HOXD12		protein_c	chr2:176099795-176
ENSG0000(	1691	37.76433	chr2:309<	ENSG00000289349		lncRNA	chr2:175594179-175
ENSG0000(	1691	37.76433	chr2:309<	CDCA7		protein_c	chr2:173354820-173
ENSG0000(	1691	37.76433	chr2:309<	ENSG00000222043		lncRNA	chr2:177264359-177

ENSG0000(	1691	37.76433	chr2:309⁴	RNU6ATAC14P		smallRNA	chr2:176664676-176
ENSG0000(	1691	37.76433	chr2:309⁴	ENSG00000279884		TEC	chr2:174545385-174
ENSG0000(	1691	37.76433	chr2:309⁴	Y_RNA		smallRNA	chr2:177194983-177
ENSG0000(	1691	37.76433	chr2:309⁴	ENSG00000279160		TEC	chr2:176189919-176
ENSG0000(	1691	37.76433	chr2:309⁴	OLA1		protein_c	chr2:174072447-174
ENSG0000(	1691	37.76433	chr2:309⁴	AC068706.2		smallRNA	chr2:176382232-176
ENSG0000(	1691	37.76433	chr2:309⁴	ENSG00000271825		lncRNA	chr2:177300600-177
ENSG0000(	1691	37.76433	chr2:309⁴	UNC50		protein_c	chr2:98608579-9861
ENSG0000(	1691	37.76433	chr2:309⁴	ENSG00000289296		lncRNA	chr2:173965488-173
ENSG0000(	1691	37.76433	chr2:309⁴	LINC01116		lncRNA	chr2:176611437-176
ENSG0000(	1691	37.76433	chr2:309⁴	RPL5P7		Pseudoger	chr2:173871653-173
ENSG0000(	1691	37.76433	chr2:309⁴	MIR10B		smallRNA	chr2:176150303-176
ENSG0000(	1691	37.76433	chr2:309⁴	AC007435.1		smallRNA	chr2:175034517-175
ENSG0000(	1691	37.76433	chr2:309⁴	Y_RNA		smallRNA	chr2:174264878-174
ENSG0000(	1691	37.76433	chr2:309⁴	ENSG00000270799		Pseudoger	chr2:174171891-174
ENSG0000(	1691	37.76433	chr2:309⁴	NFE2L2	NCGv7;AC	protein_c	chr2:177218667-177
ENSG0000(	1691	37.76433	chr2:309⁴	GPR155		protein_c	chr2:174431571-174
ENSG0000(	1691	37.76433	chr2:309⁴	HOXD-AS2		lncRNA	chr2:176121611-176
ENSG0000(	1691	37.76433	chr2:309⁴	CHRNA1		protein_c	chr2:174747592-174
ENSG0000(	1691	37.76433	chr2:309⁴	HOXD1		protein_c	chr2:176188668-176
ENSG0000(	1691	37.76433	chr2:309⁴	LNPK		protein_c	chr2:175923882-176
ENSG0000(	1691	37.76433	chr2:309⁴	ENSG00000227098		lncRNA	chr2:176830839-176
ENSG0000(	1691	37.76433	chr2:309⁴	SP3		protein_c	chr2:173880850-173
ENSG0000(	1691	37.76433	chr2:309⁴	RPS15AP14		Pseudoger	chr2:175184215-175
ENSG0000(	1691	37.76433	chr2:309⁴	H3P7		Pseudoger	chr2:177344443-177
ENSG0000(	1691	37.76433	chr2:309⁴	ENSG00000237016		Pseudoger	chr2:173575110-173
ENSG0000(	1691	37.76433	chr2:309⁴	GPR155-DT		lncRNA	chr2:174487380-174
ENSG0000(	1691	37.76433	chr2:309⁴	ENSG00000237655		lncRNA	chr2:177603089-177
ENSG0000(	1691	37.76433	chr2:309⁴	MAP3K20		protein_c	chr2:173075435-173
ENSG0000(	1691	37.76433	chr2:309⁴	RAPGEF4		protein_c	chr2:172735274-173
ENSG0000(	1691	37.76433	chr2:309⁴	ENSG00000218175		Pseudoger	chr2:176200908-176
ENSG0000(	1691	37.76433	chr2:309⁴	RPA3P1		Pseudoger	chr2:174501952-174
ENSG0000(	1691	37.76433	chr2:309⁴	ATP5MC3		protein_c	chr2:175176258-175
ENSG0000(	1691	37.76433	chr2:309⁴	AGPS		protein_c	chr2:177392746-177
ENSG0000(	1691	37.76433	chr2:309⁴	ENSG00000229750		lncRNA	chr2:175167904-175
ENSG0000(	1691	37.76433	chr2:309⁴	RPSAP24		Pseudoger	chr2:174043008-174
ENSG0000(	1691	37.76433	chr2:309⁴	ENSG00000272729		lncRNA	chr2:176164164-176
ENSG0000(	1691	37.76433	chr2:309⁴	ENSG00000229066		lncRNA	chr2:175257250-175
ENSG0000(	1691	37.76433	chr2:309⁴	SCRN3		protein_c	chr2:174395730-174
ENSG0000(	1691	37.76433	chr2:309⁴	EXTL2P1		Pseudoger	chr2:175842887-175
ENSG0000(	1691	37.76433	chr2:309⁴	ENSG00000237617		Pseudoger	chr2:173705948-173
ENSG0000(	1691	37.76433	chr2:309⁴	HOXD13	NCGv7;AC	protein_c	chr2:176092721-176
ENSG0000(	1691	37.76433	chr2:309⁴	ENSG00000229779		lncRNA	chr2:175904371-175
ENSG0000(	1691	37.76433	chr2:309⁴	RNU6-5P		smallRNA	chr2:174557966-174
ENSG0000(	1691	37.76433	chr2:309⁴	HOXD11	NCGv7;AC	protein_c	chr2:176104216-176
ENSG0000(	1691	37.76433	chr2:309⁴	HOXD10		protein_c	chr2:176108790-176
ENSG0000(	1691	37.76433	chr2:309⁴	HOXD9	AC	protein_c	chr2:176122719-176
ENSG0000(	1691	37.76433	chr2:309⁴	CHN1	AC	protein_c	chr2:174798809-175
ENSG0000(	1691	37.76433	chr2:309⁴	HAGLROS		lncRNA	chr2:176177717-176
ENSG0000(	1691	37.76433	chr2:309⁴	MTX2		protein_c	chr2:176269395-176
ENSG0000(	1691	37.76433	chr2:309⁴	STUB1P1		Pseudoger	chr2:177177695-177

ENSG0000(	1691	37.76433	chr2:309<	RNU6-1290P		smallRNA	chr2:175029769-175
ENSG0000(	1691	37.76433	chr2:309<	EVX2		protein_c	chr2:176077472-176
ENSG0000(	1691	37.76433	chr2:309<	JPT1P1		Pseudoger	chr2:173431349-173
ENSG0000(	1691	37.76433	chr2:309<	PPIAP66		Pseudoger	chr2:173485865-173
ENSG0000(	1691	37.76433	chr2:309<	RPSAP25		Pseudoger	chr2:176242111-176
ENSG0000(	1691	37.76433	chr2:309<	ALDH7A1P2		Pseudoger	chr2:172893763-172
ENSG0000(	1691	37.76433	chr2:309<	AC019046.1		smallRNA	chr2:173061732-173
ENSG0000(	1691	37.76433	chr2:309<	ENSG00000229434		lncRNA	chr2:176506245-176
ENSG0000(	1691	37.76433	chr2:309<	RNA5SP112		Pseudoger	chr2:177138699-177
ENSG0000(	1691	37.76433	chr2:309<	Y_RNA		smallRNA	chr2:175176499-175
ENSG0000(	1691	37.76433	chr2:309<	TTC30A		protein_c	chr2:177612999-177
ENSG0000(	1691	37.76433	chr2:309<	MIR933		smallRNA	chr2:175167633-175
ENSG0000(	1691	37.76433	chr2:309<	ENSG00000230552		lncRNA	chr2:176723614-176
ENSG0000(	1691	37.76433	chr2:309<	RNU6-763P		smallRNA	chr2:175022214-175
ENSG0000(	1691	37.76433	chr2:309<	AC009336.1		smallRNA	chr2:176108643-176
ENSG0000(	1691	37.76433	chr2:309<	MAP3K20-AS1		lncRNA	chr2:173166446-173
ENSG0000(	1691	37.76433	chr2:309<	KRT8P40		Pseudoger	chr2:177197985-177
ENSG0000(	1691	37.76433	chr2:309<	RNU6-187P		smallRNA	chr2:176929985-176
ENSG0000(	1691	37.76433	chr2:309<	ENSG00000237798		lncRNA	chr2:174575227-174
ENSG0000(	1691	37.76433	chr2:309<	HNRNPA1P39		Pseudoger	chr2:174310015-174
ENSG0000(	1691	37.76433	chr2:309<	MIR4444-1		smallRNA	chr2:177212726-177
ENSG0000(	1691	37.76433	chr2:309<	ENSG00000270460		lncRNA	chr2:173811076-173
ENSG0000(	1691	37.76433	chr2:309<	ENSG00000236231		lncRNA	chr2:176524879-176
ENSG0000(	1691	37.76433	chr2:309<	ENSG00000271996		lncRNA	chr2:177306373-177
ENSG0000(	1691	37.76433	chr2:309<	ENSG00000223777		Pseudoger	chr2:176809426-176
ENSG0000(	1691	37.76433	chr2:309<	H3P6		Pseudoger	chr2:174719908-174
ENSG0000(	1691	37.76433	chr2:309<	HOXD8		protein_c	chr2:176129694-176
ENSG0000(	1691	37.76433	chr2:309<	SP9		protein_c	chr2:174334954-174
ENSG0000(	1691	37.76433	chr2:309<	DNAJC19P5		Pseudoger	chr2:177229191-177
ENSG0000(	1691	37.76433	chr2:309<	LINC01960		lncRNA	chr2:174025280-174
ENSG0000(	1691	37.76433	chr2:309<	FUCA1P1		Pseudoger	chr2:176804987-176
ENSG0000(	1691	37.76433	chr2:309<	ENSG00000236391		Pseudoger	chr2:173338219-173
ENSG0000(	1691	37.76433	chr2:309<	ENSG00000280414		TEC	chr2:174334438-174
ENSG0000(	1691	37.76433	chr2:309<	ENSG00000279205		TEC	chr2:176822030-176
ENSG0000(	1691	37.76433	chr2:309<	CBY1P1		Pseudoger	chr2:173477996-173
ENSG0000(	1691	37.76433	chr2:309<	YWHAQP5		Pseudoger	chr2:98694109-9869
ENSG0000(	1691	37.76433	chr2:309<	LRRC2P1		Pseudoger	chr2:174299324-174
ENSG0000(	1691	37.76433	chr2:309<	LINC01117		lncRNA	chr2:176495255-176
ENSG0000(	1691	37.76433	chr2:309<	RPL21P31		Pseudoger	chr2:175033778-175
ENSG0000(	1691	37.76433	chr2:309<	ENSG00000273258		lncRNA	chr2:174011856-174
ENSG0000(	1691	37.76433	chr2:309<	ATF2		protein_c	chr2:175072250-175
ENSG0000(	1691	37.76433	chr2:309<	ENSG00000235047		lncRNA	chr2:175897336-175
ENSG0000(	1691	37.76433	chr2:309<	snoU13		smallRNA	chr2:177345858-177
ENSG0000(	1691	37.76433	chr2:309<	HOXD3		protein_c	chr2:176136612-176
ENSG0000(	1691	37.76433	chr2:309<	RN7SL65P		smallRNA	chr2:174240142-174
ENSG0000(	1691	37.76433	chr2:309<	HAGLR		lncRNA	chr2:176164051-176
ENSG0000(	1691	37.76433	chr2:309<	HNRNPA3	NCGv7	protein_c	chr2:177212694-177
ENSG0000(	1691	37.76433	chr2:309<	ENSG00000229337		lncRNA	chr2:177111036-177
ENSG0000(	1691	37.76433	chr2:309<	LINC02611		lncRNA	chr2:98761098-9877
ENSG0000(	1691	37.76433	chr2:309<	ENSG00000236501		lncRNA	chr2:176989418-177
ENSG0000(	1691	37.76433	chr2:309<	ENSG00000236449		lncRNA	chr2:174547141-174

ENSG0000(	1691	37.76433	chr2:309<	MIR3128		smallRNA	chr2:177255945-177
ENSG0000(	1691	37.76433	chr2:309<	COA5		protein_c	chr2:98599314-9860
ENSG0000(	1691	37.76433	chr2:309<	ENSG00000271151		lncRNA	chr2:173968351-173
ENSG0000(	1691	37.76433	chr2:309<	Y_RNA		smallRNA	chr2:174977803-174
ENSG0000(	1691	37.76433	chr2:309<	ENSG00000213963		lncRNA	chr2:177271125-177
ENSG0000(	1691	37.76433	chr2:309<	PPIAP67		Pseudoger	chr2:176338637-176
ENSG0000(	1691	37.76433	chr2:309<	WIPF1		protein_c	chr2:174559572-174
ENSG0000(	1691	37.76433	chr2:309<	ENSG00000290018		lncRNA	chr2:174719821-174
ENSG0000(	1691	37.76433	chr2:309<	TTC30B		protein_c	chr2:177548998-177
ENSG0000(	1691	37.76433	chr2:309<	LINC01305		lncRNA	chr2:174326027-174
ENSG0000(	1676	37.42934	chr1:1142	RN7SL729P		smallRNA	chr1:8275124-82753
ENSG0000(	1676	37.42934	chr1:1142	LINC01714		lncRNA	chr1:8201518-82152
ENSG0000(	1676	37.42934	chr1:1142	RERE	AC	protein_c	chr1:8352397-88489
ENSG0000(	1676	37.42934	chr1:1142	RERE-AS1		lncRNA	chr1:8424645-84350
ENSG0000(	1676	37.42934	chr1:1142	RNU1-7P		smallRNA	chr1:8206434-82065
ENSG0000(	1676	37.42934	chr1:1142	SLC45A1		protein_c	chr1:8318114-83441
ENSG0000(	1676	37.42934	chr1:1142	SNORA77		smallRNA	chr1:8511795-85119
ENSG0000(	1676	37.42934	chr1:1142	ENSG00000233645		lncRNA	chr1:8218190-82205
ENSG0000(	1676	37.42934	chr1:1142	ENSG00000270282		Pseudoger	chr1:8512653-85130
ENSG0000(	1676	37.42934	chr1:1142	Y_RNA		smallRNA	chr1:8328067-83281
ENSG0000(	1676	37.42934	chr1:1142	RNU6-991P		smallRNA	chr1:8292157-82922
ENSG0000(	1669	37.27302	chr1:1142	ENSG00000229305		Pseudoger	chr1:8189824-81927
ENSG0000(	1669	37.27302	chr1:1142	Y_RNA		smallRNA	chr1:7982881-79829
ENSG0000(	1669	37.27302	chr1:1142	TNFRSF9		protein_c	chr1:7915871-79431
ENSG0000(	1669	37.27302	chr1:1142	ENSG00000284716		lncRNA	chr1:7998187-79999
ENSG0000(	1669	37.27302	chr1:1142	RPL7AP18		Pseudoger	chr1:8057245-80584
ENSG0000(	1669	37.27302	chr1:1142	ENSG00000284747		lncRNA	chr1:7991134-80053
ENSG0000(	1669	37.27302	chr1:1142	ERRFI1	NCGv7	protein_c	chr1:8004404-80263
ENSG0000(	1669	37.27302	chr1:1142	ENSG00000288816		lncRNA	chr1:8191692-81949
ENSG0000(	1669	37.27302	chr1:1142	ERRFI1-DT		lncRNA	chr1:8026738-81227
ENSG0000(	1669	37.27302	chr1:1142	PARK7	TAG;AC	protein_c	chr1:7954291-79855
ENSG0000(	1669	37.27302	chr1:1142	ENSG00000270330		Pseudoger	chr1:7942372-79427
ENSG0000(	1655	36.96036	chr2:309<	C2orf15		protein_c	chr2:99141707-9915
ENSG0000(	1655	36.96036	chr2:309<	ENSG00000230393		lncRNA	chr2:100104919-100
ENSG0000(	1655	36.96036	chr2:309<	MITD1		protein_c	chr2:99161427-9918
ENSG0000(	1655	36.96036	chr2:309<	TXNDC9		protein_c	chr2:99318982-9934
ENSG0000(	1655	36.96036	chr2:309<	EIF5B		protein_c	chr2:99337371-9940
ENSG0000(	1655	36.96036	chr2:309<	ENSG00000241962		protein_c	chr2:99141485-9932
ENSG0000(	1655	36.96036	chr2:309<	SMC3P1		Pseudoger	chr2:99102018-9910
ENSG0000(	1655	36.96036	chr2:309<	MRPL30		protein_c	chr2:99181152-9919
ENSG0000(	1655	36.96036	chr2:309<	ENSG00000273306		lncRNA	chr2:99405218-9940
ENSG0000(	1655	36.96036	chr2:309<	CRACDL		protein_c	chr2:98793846-9893
ENSG0000(	1655	36.96036	chr2:309<	RNU7-46P		smallRNA	chr2:98840675-9884
ENSG0000(	1655	36.96036	chr2:309<	LIPT1		protein_c	chr2:99154955-9916
ENSG0000(	1655	36.96036	chr2:309<	AFF3	NCGv7;AC	protein_c	chr2:99545419-1001
ENSG0000(	1655	36.96036	chr2:309<	LYG1		protein_c	chr2:99284238-9930
ENSG0000(	1655	36.96036	chr2:309<	TSGA10		protein_c	chr2:98997261-9915
ENSG0000(	1655	36.96036	chr2:309<	REV1		protein_c	chr2:99400475-9949
ENSG0000(	1655	36.96036	chr2:309<	AC018690.1		smallRNA	chr2:99520103-9952
ENSG0000(	1655	36.96036	chr2:309<	LYG2		protein_c	chr2:99242246-9925
ENSG0000(	1655	36.96036	chr2:309<	ENSG00000273155		protein_c	chr2:99154998-9919

ENSG0000(	1654	36.93803	chr2:309⁴	RPL21P38		Pseudoger	chr2:171587093-171
ENSG0000(	1654	36.93803	chr2:309⁴	ENSG00000229827		Pseudoger	chr2:169577371-169
ENSG0000(	1654	36.93803	chr2:309⁴	GORASP2		protein_c	chr2:170928464-170
ENSG0000(	1654	36.93803	chr2:309⁴	AC010092.1		smallRNA	chr2:170916209-170
ENSG0000(	1654	36.93803	chr2:309⁴	METTL5		protein_c	chr2:169810081-169
ENSG0000(	1654	36.93803	chr2:309⁴	DYNC1I2		protein_c	chr2:171687409-171
ENSG0000(	1654	36.93803	chr2:309⁴	SSB	NCGv7	protein_c	chr2:169791933-169
ENSG0000(	1654	36.93803	chr2:309⁴	ERICH2-DT		lncRNA	chr2:170700368-170
ENSG0000(	1654	36.93803	chr2:309⁴	Y_RNA		smallRNA	chr2:170783167-170
ENSG0000(	1654	36.93803	chr2:309⁴	RPS15P4		Pseudoger	chr2:171517270-171
ENSG0000(	1654	36.93803	chr2:309⁴	RN7SL95P		smallRNA	chr2:168440906-168
ENSG0000(	1654	36.93803	chr2:309⁴	LINC01124		lncRNA	chr2:170712451-170
ENSG0000(	1654	36.93803	chr2:309⁴	RPS26P20		Pseudoger	chr2:171374931-171
ENSG0000(	1654	36.93803	chr2:309⁴	ENSG00000286557		lncRNA	chr2:172323260-172
ENSG0000(	1654	36.93803	chr2:309⁴	DLX2		protein_c	chr2:172099438-172
ENSG0000(	1654	36.93803	chr2:309⁴	DHRS9		protein_c	chr2:169064789-169
ENSG0000(	1654	36.93803	chr2:309⁴	SLC25A12		protein_c	chr2:171783405-171
ENSG0000(	1654	36.93803	chr2:309⁴	ABCB11		protein_c	chr2:168915498-169
ENSG0000(	1654	36.93803	chr2:309⁴	ENSG00000234061		Pseudoger	chr2:171492905-171
ENSG0000(	1654	36.93803	chr2:309⁴	FASTKD1		protein_c	chr2:169528508-169
ENSG0000(	1654	36.93803	chr2:309⁴	RNU6-182P		smallRNA	chr2:171856566-171
ENSG0000(	1654	36.93803	chr2:309⁴	STK39		protein_c	chr2:167954020-168
ENSG0000(	1654	36.93803	chr2:309⁴	RNU6-1006P		smallRNA	chr2:170119996-170
ENSG0000(	1654	36.93803	chr2:309⁴	KLHL23		protein_c	chr2:169694488-169
ENSG0000(	1654	36.93803	chr2:309⁴	TLK1		protein_c	chr2:170990823-171
ENSG0000(	1654	36.93803	chr2:309⁴	DCAF17		protein_c	chr2:171434217-171
ENSG0000(	1654	36.93803	chr2:309⁴	CYBRD1		protein_c	chr2:171522247-171
ENSG0000(	1654	36.93803	chr2:309⁴	MYO3B		protein_c	chr2:170178145-170
ENSG0000(	1654	36.93803	chr2:309⁴	PPIG	NCGv7	protein_c	chr2:169584342-169
ENSG0000(	1654	36.93803	chr2:309⁴	ENSG00000289413		lncRNA	chr2:172091379-172
ENSG0000(	1654	36.93803	chr2:309⁴	ENSG00000286115		lncRNA	chr2:170890393-170
ENSG0000(	1654	36.93803	chr2:309⁴	PDK1		protein_c	chr2:172555373-172
ENSG0000(	1654	36.93803	chr2:309⁴	B3GALT1		protein_c	chr2:167293001-167
ENSG0000(	1654	36.93803	chr2:309⁴	CTAGE14P		Pseudoger	chr2:167713663-167
ENSG0000(	1654	36.93803	chr2:309⁴	CFAP210		protein_c	chr2:169645425-169
ENSG0000(	1654	36.93803	chr2:309⁴	ITGA6		protein_c	chr2:172427354-172
ENSG0000(	1654	36.93803	chr2:309⁴	CYB5AP2		Pseudoger	chr2:169790093-169
ENSG0000(	1654	36.93803	chr2:309⁴	ENSG00000230104		Pseudoger	chr2:172674212-172
ENSG0000(	1654	36.93803	chr2:309⁴	CERS6		protein_c	chr2:168456249-168
ENSG0000(	1654	36.93803	chr2:309⁴	ENSG00000251569		protein_c	chr2:169479480-169
ENSG0000(	1654	36.93803	chr2:309⁴	U3		smallRNA	chr2:169816279-169
ENSG0000(	1654	36.93803	chr2:309⁴	PDK1-AS1		lncRNA	chr2:172480840-172
ENSG0000(	1654	36.93803	chr2:309⁴	DLX1		protein_c	chr2:172084740-172
ENSG0000(	1654	36.93803	chr2:309⁴	UBR3	NCGv7	protein_c	chr2:169827454-170
ENSG0000(	1654	36.93803	chr2:309⁴	KLHL41		protein_c	chr2:169509702-169
ENSG0000(	1654	36.93803	chr2:309⁴	ITGA6-AS1		lncRNA	chr2:172464262-172
ENSG0000(	1654	36.93803	chr2:309⁴	METAP1D		protein_c	chr2:171999943-172
ENSG0000(	1654	36.93803	chr2:309⁴	PHF5GP		Pseudoger	chr2:168231921-168
ENSG0000(	1654	36.93803	chr2:309⁴	ENSG00000232555		lncRNA	chr2:172137345-172
ENSG0000(	1654	36.93803	chr2:309⁴	NOSTRIN		protein_c	chr2:168786539-168
ENSG0000(	1654	36.93803	chr2:309⁴	ENSG00000288048		lncRNA	chr2:172234230-172

ENSG00000	1654	36.93803	chr2:3094	RNU6-766P		smallRNA	chr2:168606390-168
ENSG00000	1654	36.93803	chr2:3094	G6PC2		protein_c	chr2:168901291-168
ENSG00000	1654	36.93803	chr2:3094	SPC25		protein_c	chr2:168834132-168
ENSG00000	1654	36.93803	chr2:3094	NSA2P5		Pseudoger	chr2:170077224-170
ENSG00000	1654	36.93803	chr2:3094	PTCHD3P2		Pseudoger	chr2:169767185-169
ENSG00000	1654	36.93803	chr2:3094	RAPGEF4-AS1		lncRNA	chr2:172677141-172
ENSG00000	1654	36.93803	chr2:3094	ENSG00000213981		lncRNA	chr2:170640374-170
ENSG00000	1654	36.93803	chr2:3094	METTL8		protein_c	chr2:171315746-171
ENSG00000	1654	36.93803	chr2:3094	GAD1		protein_c	chr2:170813213-170
ENSG00000	1654	36.93803	chr2:3094	EIF2S2P4		Pseudoger	chr2:170751805-170
ENSG00000	1654	36.93803	chr2:3094	ENSG00000235321		lncRNA	chr2:169100743-169
ENSG00000	1654	36.93803	chr2:3094	ENSG00000228222		lncRNA	chr2:167293171-167
ENSG00000	1654	36.93803	chr2:3094	B3GALT1-AS1		lncRNA	chr2:167814757-167
ENSG00000	1654	36.93803	chr2:3094	SNORA51		smallRNA	chr2:169727872-169
ENSG00000	1654	36.93803	chr2:3094	ENSG00000288958		lncRNA	chr2:172081990-172
ENSG00000	1654	36.93803	chr2:3094	PHOSPHO2		protein_c	chr2:169694454-169
ENSG00000	1654	36.93803	chr2:3094	HMGB1P4		Pseudoger	chr2:170601662-170
ENSG00000	1654	36.93803	chr2:3094	ERICH2		protein_c	chr2:170766878-170
ENSG00000	1654	36.93803	chr2:3094	RN7SL813P		smallRNA	chr2:168451324-168
ENSG00000	1654	36.93803	chr2:3094	MYO3B-AS1		lncRNA	chr2:170332085-170
ENSG00000	1654	36.93803	chr2:3094	CERS6-AS1		lncRNA	chr2:168771951-168
ENSG00000	1654	36.93803	chr2:3094	ENSG00000235934		lncRNA	chr2:170814686-170
ENSG00000	1654	36.93803	chr2:3094	BBS5		protein_c	chr2:169479480-169
ENSG00000	1654	36.93803	chr2:3094	snoU13		smallRNA	chr2:172691299-172
ENSG00000	1654	36.93803	chr2:3094	MIR4774		smallRNA	chr2:168582943-168
ENSG00000	1654	36.93803	chr2:3094	ENSG00000226963		lncRNA	chr2:172427774-172
ENSG00000	1654	36.93803	chr2:3094	ENSG00000278924		TEC	chr2:172423807-172
ENSG00000	1654	36.93803	chr2:3094	snoU13		smallRNA	chr2:172156029-172
ENSG00000	1654	36.93803	chr2:3094	UBE2V1P6		Pseudoger	chr2:169115195-169
ENSG00000	1654	36.93803	chr2:3094	DLX2-DT		lncRNA	chr2:172103006-172
ENSG00000	1654	36.93803	chr2:3094	snoU13		smallRNA	chr2:171146330-171
ENSG00000	1654	36.93803	chr2:3094	SP5		protein_c	chr2:170715337-170
ENSG00000	1654	36.93803	chr2:3094	DAP3P2		Pseudoger	chr2:171491422-171
ENSG00000	1654	36.93803	chr2:3094	LRP2		protein_c	chr2:169127109-169
ENSG00000	1654	36.93803	chr2:3094	RNU7-148P		smallRNA	chr2:167631480-167
ENSG00000	1654	36.93803	chr2:3094	Y_RNA		smallRNA	chr2:172558151-172
ENSG00000	1654	36.93803	chr2:3094	ENSG00000226072		Pseudoger	chr2:169658132-169
ENSG00000	1632	36.44671	chr1:1142	ENSG00000236948		lncRNA	chr1:5561709-56682
ENSG00000	1632	36.44671	chr1:1142	RNU1-8P		smallRNA	chr1:7219360-72195
ENSG00000	1632	36.44671	chr1:1142	ENSG00000284739		lncRNA	chr1:4963954-49732
ENSG00000	1632	36.44671	chr1:1142	TNFRSF25		protein_c	chr1:6460786-64661
ENSG00000	1632	36.44671	chr1:1142	ENSG00000284744		lncRNA	chr1:6767954-67700
ENSG00000	1632	36.44671	chr1:1142	ENSG00000229519		Pseudoger	chr1:6547905-65486
ENSG00000	1632	36.44671	chr1:1142	RNF207-AS1		lncRNA	chr1:6204840-62057
ENSG00000	1632	36.44671	chr1:1142	THAP3		protein_c	chr1:6624868-66355
ENSG00000	1632	36.44671	chr1:1142	ENSG00000260972		lncRNA	chr1:5492978-54946
ENSG00000	1632	36.44671	chr1:1142	AL356693.1		smallRNA	chr1:5815871-58159
ENSG00000	1632	36.44671	chr1:1142	LINC01672		lncRNA	chr1:6724637-67300
ENSG00000	1632	36.44671	chr1:1142	MIR4689		smallRNA	chr1:5862672-58627
ENSG00000	1632	36.44671	chr1:1142	CAMTA1	NCGv7;AC	protein_c	chr1:6785454-77697
ENSG00000	1632	36.44671	chr1:1142	ENSG00000285629		protein_c	chr1:6159430-61977

ENSG0000(	1632	36.44671	chr1:1142	ENSG00000269978		lncRNA	chr1:7700704-77009
ENSG0000(	1632	36.44671	chr1:1142	RNF207	NCGv7	protein_c	chr1:6205475-62212
ENSG0000(	1632	36.44671	chr1:1142	CAMTA1-AS2		lncRNA	chr1:7382487-73897
ENSG0000(	1632	36.44671	chr1:1142	GPR153		protein_c	chr1:6247353-62610
ENSG0000(	1632	36.44671	chr1:1142	DNAJC11		protein_c	chr1:6634168-67019
ENSG0000(	1632	36.44671	chr1:1142	ENSG00000289893		lncRNA	chr1:5733358-57714
ENSG0000(	1632	36.44671	chr1:1142	ACOT7		protein_c	chr1:6264269-63937
ENSG0000(	1632	36.44671	chr1:1142	ZBTB48		protein_c	chr1:6579994-65892
ENSG0000(	1632	36.44671	chr1:1142	KLHL21		protein_c	chr1:6590724-66146
ENSG0000(	1632	36.44671	chr1:1142	ENSG00000236266		lncRNA	chr1:7810242-78273
ENSG0000(	1632	36.44671	chr1:1142	PLEKHG5		protein_c	chr1:6467122-65200
ENSG0000(	1632	36.44671	chr1:1142	NOL9	NCGv7	protein_c	chr1:6521347-65545
ENSG0000(	1632	36.44671	chr1:1142	ENSG00000283356		Pseudoger	chr1:5554747-55548
ENSG0000(	1632	36.44671	chr1:1142	RPL22	NCGv7;AC	protein_c	chr1:6185020-62093
ENSG0000(	1632	36.44671	chr1:1142	NPHP4		protein_c	chr1:5862811-59924
ENSG0000(	1632	36.44671	chr1:1142	ENSG00000284616		lncRNA	chr1:5301928-53073
ENSG0000(	1632	36.44671	chr1:1142	CAMTA1-DT		lncRNA	chr1:6783892-67848
ENSG0000(	1632	36.44671	chr1:1142	ENSG00000231868		lncRNA	chr1:6443034-64470
ENSG0000(	1632	36.44671	chr1:1142	ENSG00000227950		Pseudoger	chr1:6834333-68346
ENSG0000(	1632	36.44671	chr1:1142	ICMT		protein_c	chr1:6221193-62359
ENSG0000(	1632	36.44671	chr1:1142	CHD5	NCGv7	protein_c	chr1:6101787-61803
ENSG0000(	1632	36.44671	chr1:1142	PHF13		protein_c	chr1:6613731-66240
ENSG0000(	1632	36.44671	chr1:1142	ESPN		protein_c	chr1:6424776-64613
ENSG0000(	1632	36.44671	chr1:1142	ENSG00000270035		lncRNA	chr1:7698303-76988
ENSG0000(	1632	36.44671	chr1:1142	MIR4252		smallRNA	chr1:6429834-64298
ENSG0000(	1632	36.44671	chr1:1142	ENSG00000237365		lncRNA	chr1:7008376-70142
ENSG0000(	1632	36.44671	chr1:1142	AL356261.1		smallRNA	chr1:5893839-58939
ENSG0000(	1632	36.44671	chr1:1142	ICMT-DT		lncRNA	chr1:6234692-62394
ENSG0000(	1632	36.44671	chr1:1142	TAS1R1		protein_c	chr1:6555307-65797
ENSG0000(	1632	36.44671	chr1:1142	HES3		protein_c	chr1:6244179-62455
ENSG0000(	1632	36.44671	chr1:1142	ENSG00000284692		lncRNA	chr1:5478736-54930
ENSG0000(	1632	36.44671	chr1:1142	UTS2		protein_c	chr1:7843083-78535
ENSG0000(	1632	36.44671	chr1:1142	LINC02781		lncRNA	chr1:4973381-49815
ENSG0000(	1632	36.44671	chr1:1142	PER3	NCGv7	protein_c	chr1:7784291-78451
ENSG0000(	1632	36.44671	chr1:1142	HES2		protein_c	chr1:6412418-64246
ENSG0000(	1632	36.44671	chr1:1142	RNU6-731P		smallRNA	chr1:6540854-65409
ENSG0000(	1632	36.44671	chr1:1142	ENSG00000270171		lncRNA	chr1:7693124-76948
ENSG0000(	1632	36.44671	chr1:1142	LINC02782		lncRNA	chr1:5086459-50908
ENSG0000(	1632	36.44671	chr1:1142	MIR4417		smallRNA	chr1:5564071-55641
ENSG0000(	1632	36.44671	chr1:1142	CAMTA1-IT1		lncRNA	chr1:7368942-73702
ENSG0000(	1632	36.44671	chr1:1142	ENSG00000284666		lncRNA	chr1:5480787-54820
ENSG0000(	1632	36.44671	chr1:1142	KCNAB2		protein_c	chr1:5990927-61011
ENSG0000(	1632	36.44671	chr1:1142	ENSG00000271746		lncRNA	chr1:6393555-63943
ENSG0000(	1632	36.44671	chr1:1142	ENSG00000269925		lncRNA	chr1:7776383-77767
ENSG0000(	1632	36.44671	chr1:1142	snoU13		smallRNA	chr1:6752719-67528
ENSG0000(	1632	36.44671	chr1:1142	VAMP3		protein_c	chr1:7771296-77814
ENSG0000(	1632	36.44671	chr1:1142	CAMTA1-AS1		lncRNA	chr1:7441096-74415
ENSG0000(	1613	36.02239	chr2:3094	ENSG00000273595		Pseudoger	chr2:106207394-106
ENSG0000(	1613	36.02239	chr2:3094	LINC01159		lncRNA	chr2:104865407-104
ENSG0000(	1613	36.02239	chr2:3094	RN7SL360P		smallRNA	chr2:100600866-100
ENSG0000(	1613	36.02239	chr2:3094	RN7SL548P		smallRNA	chr2:101093569-101

ENSG00000	1613	36.02239	chr2:3094	IL1R1-AS1		lncRNA	chr2:102172621-102
ENSG00000	1613	36.02239	chr2:3094	PDCL3		protein_c	chr2:100562993-100
ENSG00000	1613	36.02239	chr2:3094	CHST10	NCGv7	protein_c	chr2:100391860-100
ENSG00000	1613	36.02239	chr2:3094	MRPS9-AS2		lncRNA	chr2:104936239-105
ENSG00000	1613	36.02239	chr2:3094	IL1RL1	NCGv7	protein_c	chr2:102311502-102
ENSG00000	1613	36.02239	chr2:3094	HMGN2P22		Pseudoger	chr2:100591150-100
ENSG00000	1613	36.02239	chr2:3094	ENSG00000290119		lncRNA	chr2:105790035-105
ENSG00000	1613	36.02239	chr2:3094	IL18R1		protein_c	chr2:102311529-102
ENSG00000	1613	36.02239	chr2:3094	LINC01935		lncRNA	chr2:102967408-102
ENSG00000	1613	36.02239	chr2:3094	IL1RL2		protein_c	chr2:102187006-102
ENSG00000	1613	36.02239	chr2:3094	RFX8		protein_c	chr2:101397359-101
ENSG00000	1613	36.02239	chr2:3094	IL1R1		protein_c	chr2:102064544-102
ENSG00000	1613	36.02239	chr2:3094	LINC01965		lncRNA	chr2:103874236-104
ENSG00000	1613	36.02239	chr2:3094	AC010884.2		smallRNA	chr2:104992902-104
ENSG00000	1613	36.02239	chr2:3094	ENSG00000227157		lncRNA	chr2:104406471-104
ENSG00000	1613	36.02239	chr2:3094	SRSF3P4		Pseudoger	chr2:106253231-106
ENSG00000	1613	36.02239	chr2:3094	FLJ20373		protein_c	chr2:101892493-101
ENSG00000	1613	36.02239	chr2:3094	RN7SL611P		smallRNA	chr2:100556050-100
ENSG00000	1613	36.02239	chr2:3094	SLC9A4		protein_c	chr2:102473226-102
ENSG00000	1613	36.02239	chr2:3094	IL18RAP	NCGv7	protein_c	chr2:102418689-102
ENSG00000	1613	36.02239	chr2:3094	ENSG00000289498		lncRNA	chr2:104860752-104
ENSG00000	1613	36.02239	chr2:3094	PRCPP1		Pseudoger	chr2:101520834-101
ENSG00000	1613	36.02239	chr2:3094	ENSG00000270699		Pseudoger	chr2:100377421-100
ENSG00000	1613	36.02239	chr2:3094	IL1R2		protein_c	chr2:101991960-102
ENSG00000	1613	36.02239	chr2:3094	LINC01870		lncRNA	chr2:101479390-101
ENSG00000	1613	36.02239	chr2:3094	NMS		protein_c	chr2:100470482-100
ENSG00000	1613	36.02239	chr2:3094	ENSG00000223947		lncRNA	chr2:100993676-101
ENSG00000	1613	36.02239	chr2:3094	ENSG00000233339		lncRNA	chr2:106179542-106
ENSG00000	1613	36.02239	chr2:3094	ARPP19P2		Pseudoger	chr2:100509277-100
ENSG00000	1613	36.02239	chr2:3094	PLGLA		Pseudoger	chr2:106382171-106
ENSG00000	1613	36.02239	chr2:3094	TBC1D8		protein_c	chr2:101007228-101
ENSG00000	1613	36.02239	chr2:3094	CD8B2		protein_c	chr2:106487364-106
ENSG00000	1613	36.02239	chr2:3094	PANTR1		lncRNA	chr2:104764932-104
ENSG00000	1613	36.02239	chr2:3094	ENSG00000232597		lncRNA	chr2:103855829-103
ENSG00000	1613	36.02239	chr2:3094	ENSG00000287308		lncRNA	chr2:105601146-105
ENSG00000	1613	36.02239	chr2:3094	ENSG00000287135		lncRNA	chr2:104928803-104
ENSG00000	1613	36.02239	chr2:3094	SNORD89		smallRNA	chr2:101272936-101
ENSG00000	1613	36.02239	chr2:3094	ENSG00000229682		Pseudoger	chr2:106368209-106
ENSG00000	1613	36.02239	chr2:3094	MIR5696		smallRNA	chr2:101309450-101
ENSG00000	1613	36.02239	chr2:3094	SLC9A2		protein_c	chr2:102619553-102
ENSG00000	1613	36.02239	chr2:3094	ENSG00000279904		TEC	chr2:104706612-104
ENSG00000	1613	36.02239	chr2:3094	snoU13		smallRNA	chr2:100577469-100
ENSG00000	1613	36.02239	chr2:3094	LINC01796		lncRNA	chr2:102873183-102
ENSG00000	1613	36.02239	chr2:3094	TMEM182		protein_c	chr2:102736905-103
ENSG00000	1613	36.02239	chr2:3094	ANAPC1P6		Pseudoger	chr2:106369724-106
ENSG00000	1613	36.02239	chr2:3094	LINC02946		lncRNA	chr2:105600703-105
ENSG00000	1613	36.02239	chr2:3094	POU3F3	NCGv7	protein_c	chr2:104853287-104
ENSG00000	1613	36.02239	chr2:3094	CAPZBP1		Pseudoger	chr2:103623630-103
ENSG00000	1613	36.02239	chr2:3094	ENSG00000288565		lncRNA	chr2:104656135-104
ENSG00000	1613	36.02239	chr2:3094	LINC01831		lncRNA	chr2:104412145-104
ENSG00000	1613	36.02239	chr2:3094	U3		smallRNA	chr2:104532773-104

ENSG00000	1613	36.02239	chr2:3094	ENSG00000288948		lncRNA	chr2:101797890-101
ENSG00000	1613	36.02239	chr2:3094	SNORA72		smallRNA	chr2:104733234-104
ENSG00000	1613	36.02239	chr2:3094	ENSG00000237666		Pseudogen	chr2:106470799-106
ENSG00000	1613	36.02239	chr2:3094	ENSG00000235522		lncRNA	chr2:105846534-105
ENSG00000	1613	36.02239	chr2:3094	RNF149		protein_c	chr2:101271219-101
ENSG00000	1613	36.02239	chr2:3094	LINC01918		lncRNA	chr2:105144113-105
ENSG00000	1613	36.02239	chr2:3094	ENSG00000227680		lncRNA	chr2:102987323-102
ENSG00000	1613	36.02239	chr2:3094	NPAS2-AS1		lncRNA	chr2:100970767-100
ENSG00000	1613	36.02239	chr2:3094	ENSG00000228968		lncRNA	chr2:104125268-104
ENSG00000	1613	36.02239	chr2:3094	MRPS9-AS1		lncRNA	chr2:105092368-105
ENSG00000	1613	36.02239	chr2:3094	ENSG00000286290		lncRNA	chr2:103362082-103
ENSG00000	1613	36.02239	chr2:3094	ENSG00000227623		Pseudogen	chr2:103275904-103
ENSG00000	1613	36.02239	chr2:3094	LINC01102		lncRNA	chr2:104430130-104
ENSG00000	1613	36.02239	chr2:3094	CREG2		protein_c	chr2:101345550-101
ENSG00000	1613	36.02239	chr2:3094	AC013402.1		smallRNA	chr2:104568817-104
ENSG00000	1613	36.02239	chr2:3094	FAM183DP		Pseudogen	chr2:102249857-102
ENSG00000	1613	36.02239	chr2:3094	LINC01127		lncRNA	chr2:101962052-101
ENSG00000	1613	36.02239	chr2:3094	ENSG00000287771		lncRNA	chr2:102037963-102
ENSG00000	1613	36.02239	chr2:3094	ENSG00000287832		lncRNA	chr2:100642444-100
ENSG00000	1613	36.02239	chr2:3094	CRLF3P1		Pseudogen	chr2:103494241-103
ENSG00000	1613	36.02239	chr2:3094	RPL23AP27		Pseudogen	chr2:104678223-104
ENSG00000	1613	36.02239	chr2:3094	ENSG00000228488		lncRNA	chr2:100722221-100
ENSG00000	1613	36.02239	chr2:3094	LINC01103		lncRNA	chr2:104488458-104
ENSG00000	1613	36.02239	chr2:3094	ECRG4		protein_c	chr2:106063246-106
ENSG00000	1613	36.02239	chr2:3094	ENSG00000231505		lncRNA	chr2:106521903-106
ENSG00000	1613	36.02239	chr2:3094	RPS6P3		Pseudogen	chr2:101509530-101
ENSG00000	1613	36.02239	chr2:3094	ENSG00000269707		lncRNA	chr2:104853285-104
ENSG00000	1613	36.02239	chr2:3094	RALBP1P2		Pseudogen	chr2:100425348-100
ENSG00000	1613	36.02239	chr2:3094	ENSG00000229209		lncRNA	chr2:103109759-103
ENSG00000	1613	36.02239	chr2:3094	ENSG00000238273		lncRNA	chr2:105363038-105
ENSG00000	1613	36.02239	chr2:3094	CYCSP7		Pseudogen	chr2:100370255-100
ENSG00000	1613	36.02239	chr2:3094	MFSD9		protein_c	chr2:102714630-102
ENSG00000	1613	36.02239	chr2:3094	TGFBRAP1		protein_c	chr2:105264391-105
ENSG00000	1613	36.02239	chr2:3094	MRPS9		protein_c	chr2:105038069-105
ENSG00000	1613	36.02239	chr2:3094	GPR45		protein_c	chr2:105241743-105
ENSG00000	1613	36.02239	chr2:3094	C2orf49		protein_c	chr2:105337532-105
ENSG00000	1613	36.02239	chr2:3094	LINC01849		lncRNA	chr2:100603752-100
ENSG00000	1613	36.02239	chr2:3094	TBC1D8-AS1		lncRNA	chr2:101151660-101
ENSG00000	1613	36.02239	chr2:3094	RGPD3	NCGv7	protein_c	chr2:106391290-106
ENSG00000	1613	36.02239	chr2:3094	ENSG00000272861		lncRNA	chr2:105249404-105
ENSG00000	1613	36.02239	chr2:3094	AC108868.1		protein_c	chr2:106483272-106
ENSG00000	1613	36.02239	chr2:3094	MIR4772		smallRNA	chr2:102432289-102
ENSG00000	1613	36.02239	chr2:3094	ENSG00000235319		lncRNA	chr2:105324210-105
ENSG00000	1613	36.02239	chr2:3094	ENSG00000236525		lncRNA	chr2:102433957-102
ENSG00000	1613	36.02239	chr2:3094	EEF1A1P12		Pseudogen	chr2:106697331-106
ENSG00000	1613	36.02239	chr2:3094	ENSG00000286737		lncRNA	chr2:100739958-100
ENSG00000	1613	36.02239	chr2:3094	C2orf49-DT		lncRNA	chr2:105333935-105
ENSG00000	1613	36.02239	chr2:3094	ENSG00000231626		lncRNA	chr2:104580821-104
ENSG00000	1613	36.02239	chr2:3094	AC012360.1		protein_c	chr2:105251302-105
ENSG00000	1613	36.02239	chr2:3094	LONRF2		protein_c	chr2:100271875-100
ENSG00000	1613	36.02239	chr2:3094	ENSG00000291125		lncRNA	chr2:106382110-106

ENSG00000	1613	36.02239	chr2:3094	NPAS2		protein_c	chr2:100820139-100
ENSG00000	1613	36.02239	chr2:3094	BBIP1P1		Pseudogen	chr2:101068378-101
ENSG00000	1613	36.02239	chr2:3094	FHL2	AC	protein_c	chr2:105357712-105
ENSG00000	1613	36.02239	chr2:3094	ENSG00000226058		Pseudogen	chr2:101375587-101
ENSG00000	1613	36.02239	chr2:3094	ENSG00000234162		lncRNA	chr2:105874509-105
ENSG00000	1613	36.02239	chr2:3094	LINC01104		lncRNA	chr2:100208254-100
ENSG00000	1613	36.02239	chr2:3094	MAP4K4		protein_c	chr2:101696850-101
ENSG00000	1613	36.02239	chr2:3094	NCK2		protein_c	chr2:105744912-105
ENSG00000	1613	36.02239	chr2:3094	ENSG00000236109		lncRNA	chr2:104583138-104
ENSG00000	1613	36.02239	chr2:3094	SDR42E1P5		Pseudogen	chr2:102411243-102
ENSG00000	1613	36.02239	chr2:3094	ENSG00000287793		lncRNA	chr2:104423039-104
ENSG00000	1613	36.02239	chr2:3094	NANOGNBP1		Pseudogen	chr2:100660199-100
ENSG00000	1613	36.02239	chr2:3094	AHCYP3		Pseudogen	chr2:104395002-104
ENSG00000	1613	36.02239	chr2:3094	RPL27AP4		Pseudogen	chr2:106304755-106
ENSG00000	1613	36.02239	chr2:3094	ENSG00000232034		lncRNA	chr2:100822661-100
ENSG00000	1613	36.02239	chr2:3094	ENSG00000236141		lncRNA	chr2:103856970-103
ENSG00000	1613	36.02239	chr2:3094	ENSG00000228528		lncRNA	chr2:104702650-104
ENSG00000	1613	36.02239	chr2:3094	CNOT11		protein_c	chr2:101252886-101
ENSG00000	1613	36.02239	chr2:3094	LINC01114		lncRNA	chr2:104746638-104
ENSG00000	1613	36.02239	chr2:3094	LINC01868		lncRNA	chr2:100669892-100
ENSG00000	1613	36.02239	chr2:3094	RPS21P2		Pseudogen	chr2:106288803-106
ENSG00000	1613	36.02239	chr2:3094	ENSG00000232001		lncRNA	chr2:106521563-106
ENSG00000	1613	36.02239	chr2:3094	ENSG00000289077		lncRNA	chr2:100818723-100
ENSG00000	1613	36.02239	chr2:3094	UXS1		protein_c	chr2:106093308-106
ENSG00000	1613	36.02239	chr2:3094	ILRUNP1		Pseudogen	chr2:106344566-106
ENSG00000	1613	36.02239	chr2:3094	ENSG00000221849		Pseudogen	chr2:103527620-103
ENSG00000	1613	36.02239	chr2:3094	ENSG00000234389		lncRNA	chr2:102438713-102
ENSG00000	1613	36.02239	chr2:3094	ENSG00000286047		lncRNA	chr2:101535601-101
ENSG00000	1613	36.02239	chr2:3094	RPL22P10		Pseudogen	chr2:106247369-106
ENSG00000	1613	36.02239	chr2:3094	ENSG00000286101		lncRNA	chr2:101551592-101
ENSG00000	1613	36.02239	chr2:3094	HMGB3P11		Pseudogen	chr2:104771282-104
ENSG00000	1613	36.02239	chr2:3094	ENSG00000289167		lncRNA	chr2:101961603-101
ENSG00000	1613	36.02239	chr2:3094	ENSG00000230690		lncRNA	chr2:104659337-104
ENSG00000	1613	36.02239	chr2:3094	RPL31	NCGv7	protein_c	chr2:101002229-101
ENSG00000	1589	35.48641	chr2:3094	MTCO3P45		Pseudogen	chr2:94900990-9490
ENSG00000	1573	35.12909	chr2:3094	RPL38P6		Pseudogen	chr2:88428078-8842
ENSG00000	1547	34.54844	chr2:3094	Y_RNA		smallRNA	chr2:85367585-8536
ENSG00000	1547	34.54844	chr2:3094	ENSG00000271014		Pseudogen	chr2:85360798-8536
ENSG00000	1547	34.54844	chr2:3094	PEBP1P2		Pseudogen	chr2:85341281-8534
ENSG00000	1547	34.54844	chr2:3094	SNRPEP11		Pseudogen	chr2:85262144-8526
ENSG00000	1547	34.54844	chr2:3094	AC093162.1		smallRNA	chr2:85299034-8529
ENSG00000	1547	34.54844	chr2:3094	RETSAT		protein_c	chr2:85341955-8535
ENSG00000	1547	34.54844	chr2:3094	TGOLN2		protein_c	chr2:85318027-8532
ENSG00000	1547	34.54844	chr2:3094	Y_RNA		smallRNA	chr2:85332895-8533
ENSG00000	1547	34.54844	chr2:3094	RN7SL113P		smallRNA	chr2:85368282-8536
ENSG00000	1547	34.54844	chr2:3094	RNU7-162P		smallRNA	chr2:85373454-8537
ENSG00000	1547	34.54844	chr2:3094	ENSG00000246575		Pseudogen	chr2:85315041-8531
ENSG00000	1547	34.54844	chr2:3094	ENSG00000290110		lncRNA	chr2:85328084-8532
ENSG00000	1533	34.23579	chr2:3094	DBF4P3		Pseudogen	chr2:87301658-8730
ENSG00000	1523	34.01246	chr1:1142	ENSG00000238260		lncRNA	chr1:3622857-36247
ENSG00000	1523	34.01246	chr2:3094	IGKV3-34		Pseudogen	chr2:89275298-8927

ENSG0000(	1523	34.01246	chr2:309	IGKV3D-31		Pseudoger	chr2:89929701-8993
ENSG0000(	1523	34.01246	chr1:114	TPRG1L	NCGv7	protein_c	chr1:3625015-36301
ENSG0000(	1523	34.01246	chr2:309	IGKV3-15		protein_c	chr2:89085177-8908
ENSG0000(	1523	34.01246	chr2:309	IGKV1-32		Pseudoger	chr2:89253571-8925
ENSG0000(	1523	34.01246	chr2:309	ENSG00000240040		lncRNA	chr2:88811186-8886
ENSG0000(	1523	34.01246	chr2:309	IGKV2-18		Pseudoger	chr2:89128724-8912
ENSG0000(	1523	34.01246	chr2:309	IGKV2-24		protein_c	chr2:89176328-8917
ENSG0000(	1523	34.01246	chr2:309	ANKRD36BP2		Pseudoger	chr2:88782712-8880
ENSG0000(	1523	34.01246	chr2:309	RPIA	NCGv7	protein_c	chr2:88691673-8875
ENSG0000(	1523	34.01246	chr2:309	IGKV7-3		Pseudoger	chr2:88915081-8891
ENSG0000(	1523	34.01246	chr2:309	RNU6-1168P		smallRNA	chr2:88383494-8838
ENSG0000(	1523	34.01246	chr1:114	ENSG00000284703		lncRNA	chr1:4012921-40195
ENSG0000(	1523	34.01246	chr2:309	RNU6-568P		smallRNA	chr2:88367793-8836
ENSG0000(	1523	34.01246	chr2:309	NDUFB4P7		Pseudoger	chr2:87968609-8796
ENSG0000(	1523	34.01246	chr2:309	IGKV1-9		protein_c	chr2:89009982-8901
ENSG0000(	1523	34.01246	chr1:114	EEF1DP6		Pseudoger	chr1:4175528-41758
ENSG0000(	1523	34.01246	chr1:114	ENSG00000227169		lncRNA	chr1:4551735-45521
ENSG0000(	1523	34.01246	chr1:114	TP73-AS2		lncRNA	chr1:3712200-37142
ENSG0000(	1523	34.01246	chr2:309	AC073416.1		Pseudoger	chr2:90099156-9009
ENSG0000(	1523	34.01246	chr2:309	ENSG00000287670		lncRNA	chr2:88016780-8802
ENSG0000(	1523	34.01246	chr1:114	SMIM1		protein_c	chr1:3772749-37759
ENSG0000(	1523	34.01246	chr1:114	LINC01134		lncRNA	chr1:3900347-39172
ENSG0000(	1523	34.01246	chr2:309	IGKV6D-21		protein_c	chr2:90021567-9002
ENSG0000(	1523	34.01246	chr2:309	IGKV1D-13		protein_c	chr2:90154073-9015
ENSG0000(	1523	34.01246	chr2:309	IGKV3D-15		protein_c	chr2:90114838-9011
ENSG0000(	1523	34.01246	chr2:309	IGKV2-10		Pseudoger	chr2:89019992-8902
ENSG0000(	1523	34.01246	chr2:309	IGKV1D-16		protein_c	chr2:90100236-9010
ENSG0000(	1523	34.01246	chr2:309	IGKV2-28		protein_c	chr2:89221698-8922
ENSG0000(	1523	34.01246	chr2:309	IGKV2-14		Pseudoger	chr2:89078010-8907
ENSG0000(	1523	34.01246	chr2:309	IGKV2-29		Pseudoger	chr2:89234174-8923
ENSG0000(	1523	34.01246	chr1:114	LINC01345		lncRNA	chr1:3944547-39490
ENSG0000(	1523	34.01246	chr2:309	IGKV1D-17		protein_c	chr2:90082635-9008
ENSG0000(	1523	34.01246	chr2:309	ENSG00000284879		lncRNA	chr2:87455476-8776
ENSG0000(	1523	34.01246	chr2:309	ANKRD36BP2		lncRNA	chr2:88765807-8880
ENSG0000(	1523	34.01246	chr2:309	IGKV1-33		protein_c	chr2:89268001-8926
ENSG0000(	1523	34.01246	chr2:309	IGKV1-37		protein_c	chr2:89297264-8929
ENSG0000(	1523	34.01246	chr2:309	IGKV2D-30		protein_c	chr2:89936859-8993
ENSG0000(	1523	34.01246	chr2:309	IGKJ3		protein_c	chr2:88861221-8886
ENSG0000(	1523	34.01246	chr2:309	IGKV2D-29		protein_c	chr2:89947512-8994
ENSG0000(	1523	34.01246	chr2:309	IGKJ1		protein_c	chr2:88861886-8886
ENSG0000(	1523	34.01246	chr2:309	IGKV4-1		protein_c	chr2:88885397-8888
ENSG0000(	1523	34.01246	chr2:309	IGKV1D-8		protein_c	chr2:90220727-9022
ENSG0000(	1523	34.01246	chr2:309	IGKV5-2		protein_c	chr2:88897232-8889
ENSG0000(	1523	34.01246	chr2:309	IGKV6-21		protein_c	chr2:89159751-8916
ENSG0000(	1523	34.01246	chr2:309	snoU13		smallRNA	chr2:88011102-8801
ENSG0000(	1523	34.01246	chr2:309	IGKV1-6		protein_c	chr2:88966262-8896
ENSG0000(	1523	34.01246	chr2:309	IGKV3-11		protein_c	chr2:89027171-8902
ENSG0000(	1523	34.01246	chr2:309	IGKV2D-26		protein_c	chr2:89985922-8998
ENSG0000(	1523	34.01246	chr2:309	IGKV2-40		protein_c	chr2:89330116-8933
ENSG0000(	1523	34.01246	chr2:309	IGKV3-31		Pseudoger	chr2:89252211-8925
ENSG0000(	1523	34.01246	chr2:309	IGKV3D-20		protein_c	chr2:90038848-9003

ENSG0000(	1523	34.01246	chr2:309	ENSG00000288734	lncRNA	chr2:88136919-8813
ENSG0000(	1523	34.01246	chr2:309	IGKV6D-41	protein_c	chr2:90069662-9007
ENSG0000(	1523	34.01246	chr2:309	IGKV3D-11	protein_c	chr2:90172802-9017
ENSG0000(	1523	34.01246	chr2:309	IGKV1D-42	protein_c	chr2:90190193-9019
ENSG0000(	1523	34.01246	chr2:309	IGKV2-30	protein_c	chr2:89244781-8924
ENSG0000(	1523	34.01246	chr2:309	IGKV2D-36	Pseudoger	chr2:89887022-8988
ENSG0000(	1523	34.01246	chr2:309	IGKV1-22	Pseudoger	chr2:89170775-8917
ENSG0000(	1523	34.01246	chr2:309	IGKV2D-10	Pseudoger	chr2:90179889-9018
ENSG0000(	1523	34.01246	chr2:309	ENSG00000273445	lncRNA	chr2:87477495-8747
ENSG0000(	1523	34.01246	chr2:309	IGKJ5	protein_c	chr2:88860568-8886
ENSG0000(	1523	34.01246	chr2:309	IGKC	protein_c	chr2:88857161-8885
ENSG0000(	1523	34.01246	chr2:309	IGKV3-20	protein_c	chr2:89142574-8914
ENSG0000(	1523	34.01246	chr2:309	MTATP8P2	Pseudoger	chr2:87824942-8782
ENSG0000(	1523	34.01246	chr2:309	RGPD2	protein_c	chr2:87755960-8782
ENSG0000(	1523	34.01246	chr1:114	LINC01346	lncRNA	chr1:3940486-39552
ENSG0000(	1523	34.01246	chr2:309	RPS14P5	Pseudoger	chr2:87654890-8765
ENSG0000(	1523	34.01246	chr2:309	IGKV2D-38	Pseudoger	chr2:89872463-8987
ENSG0000(	1523	34.01246	chr2:309	IGKV2D-40	protein_c	chr2:89851791-8985
ENSG0000(	1523	34.01246	chr2:309	IGKV1D-22	Pseudoger	chr2:90010741-9001
ENSG0000(	1523	34.01246	chr2:309	MIR4780	smallRNA	chr2:88082519-8808
ENSG0000(	1523	34.01246	chr2:309	RP11-685N3.1	protein_c	chr2:87338477-8733
ENSG0000(	1523	34.01246	chr2:309	IGKV2D-28	protein_c	chr2:89959979-8996
ENSG0000(	1523	34.01246	chr2:309	IGKV1-8	protein_c	chr2:88992409-8899
ENSG0000(	1523	34.01246	chr2:309	IGKV1D-33	protein_c	chr2:89913982-8991
ENSG0000(	1523	34.01246	chr2:309	IGKV3-25	Pseudoger	chr2:89192500-8919
ENSG0000(	1523	34.01246	chr2:309	IGKV1D-43	protein_c	chr2:90209873-9021
ENSG0000(	1523	34.01246	chr2:309	FOXI3	protein_c	chr2:88446787-8845
ENSG0000(	1523	34.01246	chr2:309	IGKV1D-35	Pseudoger	chr2:89895502-8989
ENSG0000(	1523	34.01246	chr2:309	IGKV1D-32	Pseudoger	chr2:89928422-8992
ENSG0000(	1523	34.01246	chr2:309	IGKV2-23	Pseudoger	chr2:89172022-8917
ENSG0000(	1523	34.01246	chr1:114	ENSG00000272153	lncRNA	chr1:3785008-37855
ENSG0000(	1523	34.01246	chr1:114	DFFB	protein_c	chr1:3857267-38854
ENSG0000(	1523	34.01246	chr2:309	IGKV1-12	protein_c	chr2:89040224-8904
ENSG0000(	1523	34.01246	chr2:309	PAFAH1B1P1	Pseudoger	chr2:87565828-8756
ENSG0000(	1523	34.01246	chr2:309	WBP1P2	Pseudoger	chr2:87972656-8797
ENSG0000(	1523	34.01246	chr2:309	RNU6-1007P	smallRNA	chr2:88414898-8841
ENSG0000(	1523	34.01246	chr2:309	IGKJ4	protein_c	chr2:88860886-8886
ENSG0000(	1523	34.01246	chr2:309	ENSG00000225420	lncRNA	chr2:88538720-8857
ENSG0000(	1523	34.01246	chr2:309	IGKV2-38	Pseudoger	chr2:89309898-8931
ENSG0000(	1523	34.01246	chr2:309	IGKV1D-12	protein_c	chr2:90159680-9016
ENSG0000(	1523	34.01246	chr2:309	PGBD4P5	Pseudoger	chr2:88922328-8892
ENSG0000(	1523	34.01246	chr1:114	C1orf174	protein_c	chr1:3889125-39002
ENSG0000(	1523	34.01246	chr2:309	CYTOR	lncRNA	chr2:87454781-8763
ENSG0000(	1523	34.01246	chr2:309	AC068279.1	Pseudoger	chr2:87369233-8736
ENSG0000(	1523	34.01246	chr2:309	IGKV1D-37	protein_c	chr2:89884740-8988
ENSG0000(	1523	34.01246	chr2:309	AC233263.1	Pseudoger	chr2:87344236-8734
ENSG0000(	1523	34.01246	chr2:309	ENSG00000277852	Pseudoger	chr2:89203509-8920
ENSG0000(	1523	34.01246	chr2:309	IGKV2D-23	Pseudoger	chr2:90009402-9000
ENSG0000(	1523	34.01246	chr2:309	ENSG00000288437	Pseudoger	chr2:88003008-8800
ENSG0000(	1523	34.01246	chr1:114	AL691523.1	smallRNA	chr1:3884064-38841
ENSG0000(	1523	34.01246	chr2:309	FABP1	protein_c	chr2:88122982-8812

ENSG0000(	1523	34.01246	chr2:309⁄	AC012671.1		smallRNA	chr2:88462767-8846
ENSG0000(	1523	34.01246	chr2:309⁄	MRPL45P1		Pseudoger	chr2:88364695-8836
ENSG0000(	1523	34.01246	chr2:309⁄	IGKV1-35		Pseudoger	chr2:89286689-8928
ENSG0000(	1523	34.01246	chr2:309⁄	ANAPC1P4		Pseudoger	chr2:87700984-8773
ENSG0000(	1523	34.01246	chr1:114⁄	LRRC47	NCGv7	protein_c	chr1:3778559-37964
ENSG0000(	1523	34.01246	chr1:114⁄	TP73-AS1		Pseudoger	chr1:3735511-37473
ENSG0000(	1523	34.01246	chr2:309⁄	IGKV1-27		protein_c	chr2:89213423-8921
ENSG0000(	1523	34.01246	chr2:309⁄	THNSL2		protein_c	chr2:88170295-8818
ENSG0000(	1523	34.01246	chr2:309⁄	IGKV1D-27		Pseudoger	chr2:89968867-8996
ENSG0000(	1523	34.01246	chr2:309⁄	AC233263.2		Pseudoger	chr2:87369232-8736
ENSG0000(	1523	34.01246	chr2:309⁄	IGKV2D-19		Pseudoger	chr2:90046796-9004
ENSG0000(	1523	34.01246	chr2:309⁄	IGKV2-36		Pseudoger	chr2:89295233-8929
ENSG0000(	1523	34.01246	chr2:309⁄	IGKV2D-14		Pseudoger	chr2:90121786-9012
ENSG0000(	1523	34.01246	chr2:309⁄	ENSG00000224881		Pseudoger	chr2:87379880-8738
ENSG0000(	1523	34.01246	chr1:114⁄	MIR551A		smallRNA	chr1:3560695-35607
ENSG0000(	1523	34.01246	chr2:309⁄	AC012671.3		smallRNA	chr2:88294592-8829
ENSG0000(	1523	34.01246	chr1:114⁄	LINC02780		lncRNA	chr1:3976133-40127
ENSG0000(	1523	34.01246	chr1:114⁄	CEP104		protein_c	chr1:3812086-38573
ENSG0000(	1523	34.01246	chr2:309⁄	MIR4435-1		smallRNA	chr2:87629755-8762
ENSG0000(	1523	34.01246	chr2:309⁄	ENSG00000290104		lncRNA	chr2:88864876-8886
ENSG0000(	1523	34.01246	chr2:309⁄	MALLP2		Pseudoger	chr2:88811633-8881
ENSG0000(	1523	34.01246	chr2:309⁄	AC068279.2		Pseudoger	chr2:87359054-8735
ENSG0000(	1523	34.01246	chr2:309⁄	PLGLB2		protein_c	chr2:87748087-8775
ENSG0000(	1523	34.01246	chr2:309⁄	IGKV3-7		protein_c	chr2:88978468-8897
ENSG0000(	1523	34.01246	chr2:309⁄	MIR4436A		smallRNA	chr2:88812370-8881
ENSG0000(	1523	34.01246	chr2:309⁄	snoU13		smallRNA	chr2:88307360-8830
ENSG0000(	1523	34.01246	chr2:309⁄	AC096579.1		smallRNA	chr2:88767318-8876
ENSG0000(	1523	34.01246	chr2:309⁄	ENSG00000290802		lncRNA	chr2:88811186-8882
ENSG0000(	1523	34.01246	chr1:114⁄	TP73-AS3		lncRNA	chr1:3658938-36687
ENSG0000(	1523	34.01246	chr2:309⁄	AC012671.4		smallRNA	chr2:88374139-8837
ENSG0000(	1523	34.01246	chr2:309⁄	IGKV1-16		protein_c	chr2:89099859-8910
ENSG0000(	1523	34.01246	chr1:114⁄	ENSG00000287586		lncRNA	chr1:4730211-47349
ENSG0000(	1523	34.01246	chr2:309⁄	IGKV2-4		Pseudoger	chr2:88931666-8893
ENSG0000(	1523	34.01246	chr1:114⁄	WRAP73	NCGv7	protein_c	chr1:3630767-36527
ENSG0000(	1523	34.01246	chr2:309⁄	IGKV2-19		Pseudoger	chr2:89134975-8913
ENSG0000(	1523	34.01246	chr2:309⁄	TEX37		protein_c	chr2:88524649-8852
ENSG0000(	1523	34.01246	chr2:309⁄	IGKV1-5		protein_c	chr2:88947301-8894
ENSG0000(	1523	34.01246	chr2:309⁄	ANAPC1P5		Pseudoger	chr2:87980714-8799
ENSG0000(	1523	34.01246	chr2:309⁄	IGKV1-39		protein_c	chr2:89319625-8932
ENSG0000(	1523	34.01246	chr2:309⁄	IGKV1D-39		protein_c	chr2:89862482-8986
ENSG0000(	1523	34.01246	chr2:309⁄	EIF2AK3-DT		lncRNA	chr2:88627539-8863
ENSG0000(	1523	34.01246	chr1:114⁄	LINC01777		lncRNA	chr1:4412027-44246
ENSG0000(	1523	34.01246	chr2:309⁄	IGKV2-26		Pseudoger	chr2:89196096-8919
ENSG0000(	1523	34.01246	chr2:309⁄	IGKV2D-24		protein_c	chr2:90004797-9000
ENSG0000(	1523	34.01246	chr2:309⁄	IGKV1-17		protein_c	chr2:89117342-8911
ENSG0000(	1523	34.01246	chr2:309⁄	IGKV3D-25		Pseudoger	chr2:89989987-8999
ENSG0000(	1523	34.01246	chr1:114⁄	CCDC27		protein_c	chr1:3746460-37716
ENSG0000(	1523	34.01246	chr1:114⁄	ENSG00000284694		lncRNA	chr1:4479131-44843
ENSG0000(	1523	34.01246	chr2:309⁄	IGKV3D-34		Pseudoger	chr2:89906757-8990
ENSG0000(	1523	34.01246	chr2:309⁄	IGKV2D-18		Pseudoger	chr2:90052581-9005
ENSG0000(	1523	34.01246	chr2:309⁄	ENSG00000289429		lncRNA	chr2:87659372-8768

ENSG0000(	1523	34.01246	chr1:1142	AJAP1	NCGv7	protein_c	chr1:4654609-47925
ENSG0000(	1523	34.01246	chr2:3094	EIF2AK3	NCGv7	protein_c	chr2:88556741-8869
ENSG0000(	1523	34.01246	chr1:1142	RN7SL574P		smallRNA	chr1:3782815-37831
ENSG0000(	1523	34.01246	chr2:3094	LINC01943		lncRNA	chr2:87439523-8745
ENSG0000(	1523	34.01246	chr2:3094	RNY4P15		smallRNA	chr2:88229569-8822
ENSG0000(	1523	34.01246	chr2:3094	RNU2-63P		smallRNA	chr2:88016354-8801
ENSG0000(	1523	34.01246	chr2:3094	IGKV1-13		Pseudoger	chr2:89045995-8904
ENSG0000(	1523	34.01246	chr1:1142	LINC01646		lncRNA	chr1:4571481-45940
ENSG0000(	1523	34.01246	chr2:3094	SMYD1		protein_c	chr2:88067825-8811
ENSG0000(	1523	34.01246	chr1:1142	TP73	NCGv7;AC	protein_c	chr1:3652516-37362
ENSG0000(	1523	34.01246	chr2:3094	KRCC1	NCGv7	protein_c	chr2:88027205-8806
ENSG0000(	1523	34.01246	chr2:3094	IGKV3OR2-268		protein_c	chr2:87338511-8733
ENSG0000(	1511	33.74447	chr2:3094	ENSG00000280037		TEC	chr2:73761269-7376
ENSG0000(	1511	33.74447	chr2:3094	ALMS1	NCGv7	protein_c	chr2:73385758-7362
ENSG0000(	1511	33.74447	chr2:3094	ANKRD11P1		Pseudoger	chr2:81194337-8120
ENSG0000(	1511	33.74447	chr2:3094	ENSG00000286260		lncRNA	chr2:78880713-7893
ENSG0000(	1511	33.74447	chr2:3094	ST6GALNAC2P1		Pseudoger	chr2:84039885-8404
ENSG0000(	1511	33.74447	chr2:3094	SLC4A5	NCGv7	protein_c	chr2:74216242-7434
ENSG0000(	1511	33.74447	chr2:3094	ENSG00000290771		lncRNA	chr2:79135503-7913
ENSG0000(	1511	33.74447	chr2:3094	ENSG00000229494		lncRNA	chr2:78088729-7812
ENSG0000(	1511	33.74447	chr2:3094	MIR5000		smallRNA	chr2:75090812-7509
ENSG0000(	1511	33.74447	chr2:3094	U3		smallRNA	chr2:75627953-7562
ENSG0000(	1511	33.74447	chr2:3094	RNU6-827P		smallRNA	chr2:78882447-7888
ENSG0000(	1511	33.74447	chr2:3094	FNBP1P1		Pseudoger	chr2:74120680-7412
ENSG0000(	1511	33.74447	chr2:3094	DCTN1	NCGv7	protein_c	chr2:74361154-7439
ENSG0000(	1511	33.74447	chr2:3094	ENSG00000159239		protein_c	chr2:74393836-7442
ENSG0000(	1511	33.74447	chr2:3094	WDR54		protein_c	chr2:74421678-7442
ENSG0000(	1511	33.74447	chr2:3094	NAT8B		protein_c	chr2:73700576-7370
ENSG0000(	1511	33.74447	chr2:3094	RBM7P1		Pseudoger	chr2:80162428-8016
ENSG0000(	1511	33.74447	chr2:3094	ENSG00000287435		lncRNA	chr2:73305941-7330
ENSG0000(	1511	33.74447	chr2:3094	ENSG00000287250		lncRNA	chr2:73823154-7382
ENSG0000(	1511	33.74447	chr2:3094	ENSG00000286227		lncRNA	chr2:79269905-7929
ENSG0000(	1511	33.74447	chr2:3094	RTKN		protein_c	chr2:74425835-7444
ENSG0000(	1511	33.74447	chr2:3094	GAPDHP57		Pseudoger	chr2:75455994-7545
ENSG0000(	1511	33.74447	chr2:3094	C2orf81		protein_c	chr2:74414176-7442
ENSG0000(	1511	33.74447	chr2:3094	MRPL53		protein_c	chr2:74471982-7447
ENSG0000(	1511	33.74447	chr2:3094	WBP1		protein_c	chr2:74458400-7446
ENSG0000(	1511	33.74447	chr2:3094	ENSG00000287474		lncRNA	chr2:75799974-7620
ENSG0000(	1511	33.74447	chr2:3094	ENSG00000286244		lncRNA	chr2:73834422-7385
ENSG0000(	1511	33.74447	chr2:3094	LDHAP7		Pseudoger	chr2:84777259-8477
ENSG0000(	1511	33.74447	chr2:3094	MOB1A		protein_c	chr2:74152528-7417
ENSG0000(	1511	33.74447	chr2:3094	ENSG00000236209		lncRNA	chr2:74919555-7492
ENSG0000(	1511	33.74447	chr2:3094	ENSG00000286045		lncRNA	chr2:75710782-7572
ENSG0000(	1511	33.74447	chr2:3094	ENSG00000286211		lncRNA	chr2:82831234-8286
ENSG0000(	1511	33.74447	chr2:3094	DGUOK		protein_c	chr2:73926826-7395
ENSG0000(	1511	33.74447	chr2:3094	LBX2		protein_c	chr2:74497517-7450
ENSG0000(	1511	33.74447	chr2:3094	ENSG00000286202		lncRNA	chr2:76691007-7669
ENSG0000(	1511	33.74447	chr2:3094	RPS2P17		Pseudoger	chr2:84915868-8491
ENSG0000(	1511	33.74447	chr2:3094	RPL12P18		Pseudoger	chr2:84874696-8487
ENSG0000(	1511	33.74447	chr2:3094	RPL38P2		Pseudoger	chr2:77788382-7779
ENSG0000(	1511	33.74447	chr2:3094	ENSG00000276362		Pseudoger	chr2:84850711-8485

ENSG0000(	1511	33.74447	chr2:309<	GCFC2		protein_c	chr2:75652000-7571
ENSG0000(	1511	33.74447	chr2:309<	TOR1BP1		Pseudoger	chr2:74618856-7462
ENSG0000(	1511	33.74447	chr2:309<	ALMS1P1		lncRNA	chr2:73641083-7369
ENSG0000(	1511	33.74447	chr2:309<	SUCLG1		protein_c	chr2:84423528-8446
ENSG0000(	1511	33.74447	chr2:309<	LOXL3		protein_c	chr2:74532258-7455
ENSG0000(	1511	33.74447	chr2:309<	ENSG00000234521		Pseudoger	chr2:74407797-7441
ENSG0000(	1511	33.74447	chr2:309<	MIR4264		smallRNA	chr2:79649294-7964
ENSG0000(	1511	33.74447	chr2:309<	REG1A	NCGv7	protein_c	chr2:79120362-7912
ENSG0000(	1511	33.74447	chr2:309<	TRABD2A		protein_c	chr2:84821650-8490
ENSG0000(	1511	33.74447	chr2:309<	LINC01851		lncRNA	chr2:77915870-7791
ENSG0000(	1511	33.74447	chr2:309<	PNPP1		Pseudoger	chr2:76258034-7625
ENSG0000(	1511	33.74447	chr2:309<	REG3G		protein_c	chr2:79025686-7902
ENSG0000(	1511	33.74447	chr2:309<	TPRKB		protein_c	chr2:73729104-7373
ENSG0000(	1511	33.74447	chr2:309<	NAT8		protein_c	chr2:73640723-7364
ENSG0000(	1511	33.74447	chr2:309<	DQX1		protein_c	chr2:74518131-7452
ENSG0000(	1511	33.74447	chr2:309<	DUSP11		protein_c	chr2:73761782-7378
ENSG0000(	1511	33.74447	chr2:309<	CRLF3P3		Pseudoger	chr2:84031140-8403
ENSG0000(	1511	33.74447	chr2:309<	PCGF1		protein_c	chr2:74505043-7450
ENSG0000(	1511	33.74447	chr2:309<	TTC31		protein_c	chr2:74483073-7449
ENSG0000(	1511	33.74447	chr2:309<	RNA5SP97		Pseudoger	chr2:73968123-7396
ENSG0000(	1511	33.74447	chr2:309<	ENSG00000234877		lncRNA	chr2:78597911-7859
ENSG0000(	1511	33.74447	chr2:309<	MOGS		protein_c	chr2:74461057-7446
ENSG0000(	1511	33.74447	chr2:309<	INO80B		protein_c	chr2:74455087-7445
ENSG0000(	1511	33.74447	chr2:309<	ENSG00000290085		lncRNA	chr2:84969747-8497
ENSG0000(	1511	33.74447	chr2:309<	ENSG00000287687		lncRNA	chr2:74723873-7477
ENSG0000(	1511	33.74447	chr2:309<	REG1B		protein_c	chr2:79085023-7908
ENSG0000(	1511	33.74447	chr2:309<	MRPL19		protein_c	chr2:75646783-7569
ENSG0000(	1511	33.74447	chr2:309<	STAMBP		protein_c	chr2:73828916-7387
ENSG0000(	1511	33.74447	chr2:309<	DOK1		protein_c	chr2:74549026-7455
ENSG0000(	1511	33.74447	chr2:309<	snoU109		smallRNA	chr2:75489576-7548
ENSG0000(	1511	33.74447	chr2:309<	TCF7L1	NCGv7	protein_c	chr2:85133392-8531
ENSG0000(	1511	33.74447	chr2:309<	POLE4		protein_c	chr2:74958643-7497
ENSG0000(	1511	33.74447	chr2:309<	TACR1		protein_c	chr2:75046463-7519
ENSG0000(	1511	33.74447	chr2:309<	HTRA2	IntOGen-I	protein_c	chr2:74529596-7453
ENSG0000(	1511	33.74447	chr2:309<	DUXAP1		Pseudoger	chr2:84750769-8475
ENSG0000(	1511	33.74447	chr2:309<	ENSG00000277171		Pseudoger	chr2:79547151-7954
ENSG0000(	1511	33.74447	chr2:309<	TMSB10		protein_c	chr2:84905656-8490
ENSG0000(	1511	33.74447	chr2:309<	LBX2-AS1		lncRNA	chr2:74502552-7450
ENSG0000(	1511	33.74447	chr2:309<	AUP1		protein_c	chr2:74526645-7452
ENSG0000(	1511	33.74447	chr2:309<	ENSG00000233444		Pseudoger	chr2:81666477-8166
ENSG0000(	1511	33.74447	chr2:309<	TLX2		protein_c	chr2:74513463-7451
ENSG0000(	1511	33.74447	chr2:309<	RNU6-111P		smallRNA	chr2:73298683-7329
ENSG0000(	1511	33.74447	chr2:309<	REG3A		protein_c	chr2:79157003-7915
ENSG0000(	1511	33.74447	chr2:309<	EVA1A		protein_c	chr2:75469302-7556
ENSG0000(	1511	33.74447	chr2:309<	ENSG00000227088		lncRNA	chr2:77652025-7829
ENSG0000(	1511	33.74447	chr2:309<	ENSG00000284902		lncRNA	chr2:73469525-7347
ENSG0000(	1511	33.74447	chr2:309<	ENSG00000228272		lncRNA	chr2:84315108-8435
ENSG0000(	1511	33.74447	chr2:309<	LINC01291		lncRNA	chr2:74918148-7493
ENSG0000(	1511	33.74447	chr2:309<	snoU13		smallRNA	chr2:75635141-7563
ENSG0000(	1511	33.74447	chr2:309<	LINC01815		lncRNA	chr2:81461358-8146
ENSG0000(	1511	33.74447	chr2:309<	ENSG00000237031		lncRNA	chr2:80572681-8061

ENSG0000(	1511	33.74447	chr2:309<	TET3	NCGv7	protein_c	chr2:73984910-7410
ENSG0000(	1511	33.74447	chr2:309<	HMGA1P8		Pseudoger	chr2:74418122-7441
ENSG0000(	1511	33.74447	chr2:309<	SUCLA2P2		Pseudoger	chr2:76106016-7610
ENSG0000(	1511	33.74447	chr2:309<	C2orf78		protein_c	chr2:73784183-7381
ENSG0000(	1511	33.74447	chr2:309<	ENSG00000217702		lncRNA	chr2:74123965-7413
ENSG0000(	1511	33.74447	chr2:309<	ENSG00000285068		lncRNA	chr2:73352610-7338
ENSG0000(	1511	33.74447	chr2:309<	MTHFD2	NCGv7	protein_c	chr2:74186172-7421
ENSG0000(	1511	33.74447	chr2:309<	CTNNA2	NCGv7	protein_c	chr2:79185231-8064
ENSG0000(	1511	33.74447	chr2:309<	ENSG00000287749		lncRNA	chr2:76197855-7625
ENSG0000(	1511	33.74447	chr2:309<	BOLA3		protein_c	chr2:74135400-7414
ENSG0000(	1511	33.74447	chr2:309<	HK2		protein_c	chr2:74834127-7489
ENSG0000(	1511	33.74447	chr2:309<	ENSG00000264324		protein_c	chr2:74211604-7436
ENSG0000(	1511	33.74447	chr2:309<	M1AP		protein_c	chr2:74557883-7464
ENSG0000(	1511	33.74447	chr2:309<	ENSG00000235499		lncRNA	chr2:73985132-7398
ENSG0000(	1511	33.74447	chr2:309<	NOTO		protein_c	chr2:73202574-7321
ENSG0000(	1511	33.74447	chr2:309<	ENSG00000287625		lncRNA	chr2:84926019-8496
ENSG0000(	1511	33.74447	chr2:309<	LRRTM4-AS1		lncRNA	chr2:76985965-7700
ENSG0000(	1511	33.74447	chr2:309<	FUNDC2P2		Pseudoger	chr2:84290683-8429
ENSG0000(	1511	33.74447	chr2:309<	RN7SL201P		smallRNA	chr2:81967079-8196
ENSG0000(	1511	33.74447	chr2:309<	CTNNA2-AS1		lncRNA	chr2:79492704-7951
ENSG0000(	1511	33.74447	chr2:309<	MTCYBP7		Pseudoger	chr2:82818131-8281
ENSG0000(	1511	33.74447	chr2:309<	AC096546.1		smallRNA	chr2:73488579-7348
ENSG0000(	1511	33.74447	chr2:309<	LRRTM1	NCGv7	protein_c	chr2:80288351-8030
ENSG0000(	1511	33.74447	chr2:309<	ENSG00000235463		Pseudoger	chr2:85068809-8506
ENSG0000(	1511	33.74447	chr2:309<	AC016670.1		smallRNA	chr2:80244623-8024
ENSG0000(	1511	33.74447	chr2:309<	RPSAP28		Pseudoger	chr2:73370019-7337
ENSG0000(	1511	33.74447	chr2:309<	RNU6-685P		smallRNA	chr2:82268612-8226
ENSG0000(	1511	33.74447	chr2:309<	DNAH6		protein_c	chr2:84516455-8481
ENSG0000(	1511	33.74447	chr2:309<	NECAP1P2		Pseudoger	chr2:74350069-7435
ENSG0000(	1511	33.74447	chr2:309<	FBXO41		protein_c	chr2:73254682-7328
ENSG0000(	1511	33.74447	chr2:309<	ALMS1P1		Pseudoger	chr2:73671030-7368
ENSG0000(	1511	33.74447	chr2:309<	CYCSP6		Pseudoger	chr2:78412793-7841
ENSG0000(	1511	33.74447	chr2:309<	ACTG2		protein_c	chr2:73892314-7391
ENSG0000(	1511	33.74447	chr2:309<	REG1CP		Pseudoger	chr2:79135701-7913
ENSG0000(	1511	33.74447	chr2:309<	INO80B-WBP1		protein_c	chr2:74455088-7446
ENSG0000(	1511	33.74447	chr2:309<	ENSG00000287172		lncRNA	chr2:76185020-7639
ENSG0000(	1511	33.74447	chr2:309<	LSM3P3		Pseudoger	chr2:85102389-8510
ENSG0000(	1511	33.74447	chr2:309<	ENSG00000289076		lncRNA	chr2:84459572-8446
ENSG0000(	1511	33.74447	chr2:309<	LINC01293		lncRNA	chr2:74940258-7494
ENSG0000(	1511	33.74447	chr2:309<	ENSG00000223977		Pseudoger	chr2:83218890-8321
ENSG0000(	1511	33.74447	chr2:309<	LINC01964		lncRNA	chr2:85061213-8506
ENSG0000(	1511	33.74447	chr2:309<	Y_RNA		smallRNA	chr2:82307067-8230
ENSG0000(	1511	33.74447	chr2:309<	snoU13		smallRNA	chr2:75418846-7541
ENSG0000(	1511	33.74447	chr2:309<	TAF13P2		Pseudoger	chr2:74351134-7435
ENSG0000(	1511	33.74447	chr2:309<	ENSG00000273245		lncRNA	chr2:73750256-7375
ENSG0000(	1511	33.74447	chr2:309<	KRT18P26		Pseudoger	chr2:74306728-7430
ENSG0000(	1511	33.74447	chr2:309<	AC079117.3		smallRNA	chr2:77041778-7704
ENSG0000(	1511	33.74447	chr2:309<	LYARP1		Pseudoger	chr2:82268427-8226
ENSG0000(	1511	33.74447	chr2:309<	ENSG00000270996		lncRNA	chr2:75719120-7572
ENSG0000(	1511	33.74447	chr2:309<	RNA5SP98		Pseudoger	chr2:76772909-7677
ENSG0000(	1511	33.74447	chr2:309<	RPL37P10		Pseudoger	chr2:83594956-8359

ENSG0000(	1511	33.74447	chr2:309<	ALMS1-IT1		lncRNA	chr2:73456764-7345
ENSG0000(	1511	33.74447	chr2:309<	ENSG00000287026		lncRNA	chr2:76633365-7667
ENSG0000(	1511	33.74447	chr2:309<	DGUOK-AS1		lncRNA	chr2:73947322-7398
ENSG0000(	1511	33.74447	chr2:309<	CCDC142		protein_c	chr2:74471986-7448
ENSG0000(	1511	33.74447	chr2:309<	AC096753.1		smallRNA	chr2:79794270-7979
ENSG0000(	1511	33.74447	chr2:309<	TVP23BP2		Pseudoger	chr2:74628328-7462
ENSG0000(	1511	33.74447	chr2:309<	TCF7L1-IT1		lncRNA	chr2:85186409-8518
ENSG0000(	1511	33.74447	chr2:309<	ENSG00000270470		Pseudoger	chr2:78437850-7843
ENSG0000(	1511	33.74447	chr2:309<	CHMP4AP1		Pseudoger	chr2:81418723-8141
ENSG0000(	1511	33.74447	chr2:309<	BOLA3-DT		lncRNA	chr2:74148007-7415
ENSG0000(	1511	33.74447	chr2:309<	ENSG00000286623		lncRNA	chr2:74415422-7441
ENSG0000(	1511	33.74447	chr2:309<	ENSG00000230975		lncRNA	chr2:80699388-8087
ENSG0000(	1511	33.74447	chr2:309<	ENSG00000231781		lncRNA	chr2:81983272-8200
ENSG0000(	1511	33.74447	chr2:309<	ENSG00000286883		lncRNA	chr2:74465339-7447
ENSG0000(	1511	33.74447	chr2:309<	ENSG00000270462		lncRNA	chr2:75697583-7569
ENSG0000(	1511	33.74447	chr2:309<	MTND5P27		Pseudoger	chr2:82815809-8281
ENSG0000(	1511	33.74447	chr2:309<	DCTN1-AS1		lncRNA	chr2:74385474-7439
ENSG0000(	1511	33.74447	chr2:309<	ENSG00000237498		lncRNA	chr2:82476825-8253
ENSG0000(	1511	33.74447	chr2:309<	USP21P2		Pseudoger	chr2:76260755-7626
ENSG0000(	1511	33.74447	chr2:309<	RNU6-812P		smallRNA	chr2:78882628-7888
ENSG0000(	1511	33.74447	chr2:309<	ENSG00000279070		lncRNA	chr2:74196698-7419
ENSG0000(	1511	33.74447	chr2:309<	ENSG00000237293		lncRNA	chr2:75474453-7548
ENSG0000(	1511	33.74447	chr2:309<	ENSG00000279201		TEC	chr2:74191388-7419
ENSG0000(	1511	33.74447	chr2:309<	SMYD5		protein_c	chr2:73214222-7322
ENSG0000(	1511	33.74447	chr2:309<	ENSG00000287130		lncRNA	chr2:76684975-7669
ENSG0000(	1511	33.74447	chr2:309<	RNA5SP99		Pseudoger	chr2:81496214-8149
ENSG0000(	1511	33.74447	chr2:309<	KCMF1		protein_c	chr2:84971093-8505
ENSG0000(	1511	33.74447	chr2:309<	RN7SKP164		smallRNA	chr2:76595413-7659
ENSG0000(	1511	33.74447	chr2:309<	EGR4		protein_c	chr2:73290929-7329
ENSG0000(	1511	33.74447	chr2:309<	RNU6-1312P		smallRNA	chr2:83657735-8365
ENSG0000(	1511	33.74447	chr2:309<	ENSG00000224879		lncRNA	chr2:79158374-7918
ENSG0000(	1511	33.74447	chr2:309<	ENSG00000224731		lncRNA	chr2:80028012-8003
ENSG0000(	1511	33.74447	chr2:309<	ENSG00000286932		lncRNA	chr2:77210587-7732
ENSG0000(	1511	33.74447	chr2:309<	GNA13P1		Pseudoger	chr2:79573764-7957
ENSG0000(	1511	33.74447	chr2:309<	ENSG00000272183		lncRNA	chr2:74501717-7450
ENSG0000(	1511	33.74447	chr2:309<	SNORD112		smallRNA	chr2:83858823-8385
ENSG0000(	1511	33.74447	chr2:309<	PRADC1		protein_c	chr2:73228010-7323
ENSG0000(	1511	33.74447	chr2:309<	RBX1P1		Pseudoger	chr2:82609652-8260
ENSG0000(	1511	33.74447	chr2:309<	MTND4P25		Pseudoger	chr2:82814984-8281
ENSG0000(	1511	33.74447	chr2:309<	SEMA4F		protein_c	chr2:74654228-7468
ENSG0000(	1511	33.74447	chr2:309<	CCT7		protein_c	chr2:73233420-7325
ENSG0000(	1511	33.74447	chr2:309<	LRRTM4	NCGv7	protein_c	chr2:76747685-7759
ENSG0000(	1511	33.74447	chr2:309<	SUPT4H1P1		Pseudoger	chr2:75651288-7565
ENSG0000(	1511	33.74447	chr2:309<	RNU6-542P		smallRNA	chr2:74319399-7431
ENSG0000(	1511	33.74447	chr2:309<	EVA1A-AS		lncRNA	chr2:75524068-7554
ENSG0000(	1511	33.74447	chr2:309<	RPS28P5		Pseudoger	chr2:74754670-7475
ENSG0000(	1511	33.74447	chr2:309<	ENSG00000271452		lncRNA	chr2:75669989-7567
ENSG0000(	1511	33.74447	chr2:309<	AC079117.2		smallRNA	chr2:77020336-7702
ENSG0000(	1511	33.74447	chr2:309<	ENSG00000279181		TEC	chr2:73998337-7399
ENSG0000(	1511	33.74447	chr2:309<	DHFRP3		Pseudoger	chr2:82856826-8285
ENSG0000(	1511	33.74447	chr2:309<	ENSG00000230968		lncRNA	chr2:77672215-7767

ENSG0000(	1511	33.74447	chr2:309<	ENSG00000271536		Pseudoger	chr2:73877566-7387
ENSG0000(	1511	33.74447	chr2:309<	RNU6-561P		smallRNA	chr2:79636862-7963
ENSG0000(	1511	33.74447	chr2:309<	ENSG00000286739		lncRNA	chr2:74715269-7480
ENSG0000(	1511	33.74447	chr2:309<	RN7SKP203		smallRNA	chr2:76445079-7644
ENSG0000(	1511	33.74447	chr2:309<	ENSG00000230477		Pseudoger	chr2:75598071-7559
ENSG0000(	1511	33.74447	chr2:309<	ENSG00000270571		lncRNA	chr2:75154366-7518
ENSG0000(	1511	33.74447	chr2:309<	LINC01809		lncRNA	chr2:83522814-8352
ENSG0000(	1511	33.74447	chr2:309<	ENSG00000270696		lncRNA	chr2:75660462-7566
ENSG0000(	1511	33.74447	chr2:309<	MTND6P7		Pseudoger	chr2:82817538-8281
ENSG0000(	1511	33.74447	chr2:309<	RNU6-674P		smallRNA	chr2:85204926-8520
ENSG0000(	1511	33.74447	chr2:309<	ENSG00000287025		lncRNA	chr2:76893894-7689
ENSG0000(	1511	33.74447	chr2:309<	HK2-DT		lncRNA	chr2:74832655-7483
ENSG0000(	1505	33.61048	chr2:309<	IGKV3OR2-5		Pseudoger	chr2:97348898-9734
ENSG0000(	1505	33.61048	chr2:309<	ANAPC1P1		Pseudoger	chr2:86871301-8691
ENSG0000(	1505	33.61048	chr2:309<	KMT2CP1		lncRNA	chr2:91686102-9171
ENSG0000(	1505	33.61048	chr2:309<	STARD7		protein_c	chr2:96184859-9620
ENSG0000(	1505	33.61048	chr2:309<	ENSG00000234903		Pseudoger	chr2:95468863-9546
ENSG0000(	1505	33.61048	chr2:309<	ELMOD3		protein_c	chr2:85354394-8539
ENSG0000(	1505	33.61048	chr2:309<	GXYLT1P7		Pseudoger	chr2:94734655-9473
ENSG0000(	1505	33.61048	chr2:309<	ENSG00000270193		Pseudoger	chr2:95616492-9561
ENSG0000(	1505	33.61048	chr2:309<	GNLY		protein_c	chr2:85685175-8569
ENSG0000(	1505	33.61048	chr2:309<	ENSG00000230964		Pseudoger	chr2:91578478-9157
ENSG0000(	1505	33.61048	chr2:309<	CNNM3-DT		lncRNA	chr2:96812239-9681
ENSG0000(	1505	33.61048	chr2:309<	ENSG00000287763		lncRNA	chr2:87311460-8734
ENSG0000(	1505	33.61048	chr2:309<	IGKV1OR2-11		Pseudoger	chr2:97322137-9732
ENSG0000(	1505	33.61048	chr2:309<	AC027612.2		smallRNA	chr2:91763925-9176
ENSG0000(	1505	33.61048	chr2:309<	ENSG00000234837		Pseudoger	chr2:94750582-9475
ENSG0000(	1505	33.61048	chr2:309<	ENSG00000275497		Pseudoger	chr2:95633850-9563
ENSG0000(	1505	33.61048	chr2:309<	GGCX		protein_c	chr2:85544720-8556
ENSG0000(	1505	33.61048	chr2:309<	RNU7-96P		smallRNA	chr2:97913054-9791
ENSG0000(	1505	33.61048	chr2:309<	ENSG00000275490		Pseudoger	chr2:90309229-9030
ENSG0000(	1505	33.61048	chr2:309<	RNF103-CH	DriverDBv	protein_c	chr2:86505668-8672
ENSG0000(	1505	33.61048	chr2:309<	DUSP2	NCGv7	protein_c	chr2:96143169-9614
ENSG0000(	1505	33.61048	chr2:309<	CHEK2P3		Pseudoger	chr2:91957436-9196
ENSG0000(	1505	33.61048	chr2:309<	CENPNP1		Pseudoger	chr2:87221113-8722
ENSG0000(	1505	33.61048	chr2:309<	ENSG00000229689		Pseudoger	chr2:95525345-9552
ENSG0000(	1505	33.61048	chr2:309<	ENSG00000273305		lncRNA	chr2:95537969-9553
ENSG0000(	1505	33.61048	chr2:309<	SNRNP200	NCGv7	protein_c	chr2:96274338-9632
ENSG0000(	1505	33.61048	chr2:309<	CIAO1		protein_c	chr2:96266159-9627
ENSG0000(	1505	33.61048	chr2:309<	TRIM43		protein_c	chr2:95592001-9559
ENSG0000(	1505	33.61048	chr2:309<	TRIM43B		protein_c	chr2:95477008-9548
ENSG0000(	1505	33.61048	chr2:309<	ENSG00000287628		lncRNA	chr2:85934535-8593
ENSG0000(	1505	33.61048	chr2:309<	ANAPC1P2		lncRNA	chr2:87030675-8707
ENSG0000(	1505	33.61048	chr2:309<	MAL		protein_c	chr2:95025677-9505
ENSG0000(	1505	33.61048	chr2:309<	ADRA2B		protein_c	chr2:96112876-9611
ENSG0000(	1505	33.61048	chr2:309<	SOWAHCP5		Pseudoger	chr2:94861362-9486
ENSG0000(	1505	33.61048	chr2:309<	NKAIN1P2		Pseudoger	chr2:91723023-9172
ENSG0000(	1505	33.61048	chr2:309<	ENSG00000233447		Pseudoger	chr2:94974544-9497
ENSG0000(	1505	33.61048	chr2:309<	IGKV1OR2-9		Pseudoger	chr2:97386082-9738
ENSG0000(	1505	33.61048	chr2:309<	NCAPH		protein_c	chr2:96335766-9637
ENSG0000(	1505	33.61048	chr2:309<	ENSG00000274028		Pseudoger	chr2:95588149-9558

ENSG0000(	1505	33.61048	chr2:309⁄	RNU7-64P		smallRNA	chr2:85441916-8544
ENSG0000(	1505	33.61048	chr2:309⁄	ENSG00000232594		Pseudoger	chr2:94961047-9496
ENSG0000(	1505	33.61048	chr2:309⁄	IGKV1OR2-3		Pseudoger	chr2:97060128-9706
ENSG0000(	1505	33.61048	chr2:309⁄	ENSG00000275767		Pseudoger	chr2:91589464-9162
ENSG0000(	1505	33.61048	chr2:309⁄	SLC2AXP1		Pseudoger	chr2:95196449-9519
ENSG0000(	1505	33.61048	chr2:309⁄	ENSG00000286698		lncRNA	chr2:91759462-9176
ENSG0000(	1505	33.61048	chr2:309⁄	STARD7-AS1		lncRNA	chr2:96208389-9624
ENSG0000(	1505	33.61048	chr2:309⁄	UBTFL3		Pseudoger	chr2:95625213-9562
ENSG0000(	1505	33.61048	chr2:309⁄	ZNF514		protein_c	chr2:95122087-9516
ENSG0000(	1505	33.61048	chr2:309⁄	MRPS5		protein_c	chr2:95085369-9512
ENSG0000(	1505	33.61048	chr2:309⁄	IGKV2OR2-1		Pseudoger	chr2:97046588-9704
ENSG0000(	1505	33.61048	chr2:309⁄	COX5B		protein_c	chr2:97646062-9764
ENSG0000(	1505	33.61048	chr2:309⁄	LSP1P5		lncRNA	chr2:91587019-9166
ENSG0000(	1505	33.61048	chr2:309⁄	IGKV3D-7		protein_c	chr2:90234812-9023
ENSG0000(	1505	33.61048	chr2:309⁄	KMT2CP2		Pseudoger	chr2:91696435-9171
ENSG0000(	1505	33.61048	chr2:309⁄	RN7SL210P		smallRNA	chr2:96004565-9600
ENSG0000(	1505	33.61048	chr2:309⁄	CD8B		protein_c	chr2:86815339-8686
ENSG0000(	1505	33.61048	chr2:309⁄	ENSG00000289370		lncRNA	chr2:95206349-9520
ENSG0000(	1505	33.61048	chr2:309⁄	ENSG00000279791		TEC	chr2:97094935-9709
ENSG0000(	1505	33.61048	chr2:309⁄	TRIM64FP		Pseudoger	chr2:95514827-9552
ENSG0000(	1505	33.61048	chr2:309⁄	FAHD2CP		Pseudoger	chr2:96013730-9602
ENSG0000(	1505	33.61048	chr2:309⁄	VAMP8		protein_c	chr2:85561562-8558
ENSG0000(	1505	33.61048	chr2:309⁄	ENSG00000232502		Pseudoger	chr2:94760774-9476
ENSG0000(	1505	33.61048	chr2:309⁄	PARTICL		lncRNA	chr2:85537462-8553
ENSG0000(	1505	33.61048	chr2:309⁄	ENSG00000265897		Pseudoger	chr2:90359808-9036
ENSG0000(	1505	33.61048	chr2:309⁄	FAHD2B	NCGv7	protein_c	chr2:97083583-9709
ENSG0000(	1505	33.61048	chr2:309⁄	TRIM43CP		Pseudoger	chr2:97025981-9703
ENSG0000(	1505	33.61048	chr2:309⁄	ST3GAL5-AS1		lncRNA	chr2:85889151-8589
ENSG0000(	1505	33.61048	chr2:309⁄	ENSG00000290897		lncRNA	chr2:95789654-9579
ENSG0000(	1505	33.61048	chr2:309⁄	PROM2		protein_c	chr2:95274449-9529
ENSG0000(	1505	33.61048	chr2:309⁄	ANKRD36	NCGv7	protein_c	chr2:97113153-9726
ENSG0000(	1505	33.61048	chr2:309⁄	TMEM127	NCGv7	protein_c	chr2:96248514-9626
ENSG0000(	1505	33.61048	chr2:309⁄	KMT5AP2		Pseudoger	chr2:91747940-9174
ENSG0000(	1505	33.61048	chr2:309⁄	ST3GAL5		protein_c	chr2:85837120-8590
ENSG0000(	1505	33.61048	chr2:309⁄	ASTL		protein_c	chr2:96122818-9613
ENSG0000(	1505	33.61048	chr2:309⁄	ENSG00000286654		lncRNA	chr2:96912549-9691
ENSG0000(	1505	33.61048	chr2:309⁄	GPAT2P2		Pseudoger	chr2:97081098-9708
ENSG0000(	1505	33.61048	chr2:309⁄	ENSG00000230747		lncRNA	chr2:96307263-9632
ENSG0000(	1505	33.61048	chr2:309⁄	IGKV2OR2-7D		Pseudoger	chr2:97335671-9733
ENSG0000(	1505	33.61048	chr2:309⁄	CNNM3		protein_c	chr2:96816245-9683
ENSG0000(	1505	33.61048	chr2:309⁄	SEMA4C		protein_c	chr2:96859718-9687
ENSG0000(	1505	33.61048	chr2:309⁄	ENSG00000271003		Pseudoger	chr2:95607073-9561
ENSG0000(	1505	33.61048	chr2:309⁄	ENSG00000286011		lncRNA	chr2:85418504-8542
ENSG0000(	1505	33.61048	chr2:309⁄	IGKV1OR2-118		Pseudoger	chr2:90315365-9031
ENSG0000(	1505	33.61048	chr2:309⁄	snoU13		smallRNA	chr2:96981282-9698
ENSG0000(	1505	33.61048	chr2:309⁄	ENSG00000286036		lncRNA	chr2:97618638-9763
ENSG0000(	1505	33.61048	chr2:309⁄	ENSG00000290575		lncRNA	chr2:91892472-9191
ENSG0000(	1505	33.61048	chr2:309⁄	AC159540.3		lncRNA	chr2:97404351-9740
ENSG0000(	1505	33.61048	chr2:309⁄	MIR4779		smallRNA	chr2:86193026-8619
ENSG0000(	1505	33.61048	chr2:309⁄	CNN2P8		Pseudoger	chr2:94737337-9473
ENSG0000(	1505	33.61048	chr2:309⁄	ENSG00000224585		Pseudoger	chr2:94971954-9497

ENSG0000(	1505	33.61048	chr2:309<	ANKRD39		protein_c	chr2:96836611-9685
ENSG0000(	1505	33.61048	chr2:309<	ENSG00000283214		Pseudoger	chr2:91736724-9173
ENSG0000(	1505	33.61048	chr2:309<	ENSG00000283427		Pseudoger	chr2:91607493-9162
ENSG0000(	1505	33.61048	chr2:309<	ENSG00000277701		lncRNA	chr2:97281356-9729
ENSG0000(	1505	33.61048	chr2:309<	FAM178B		protein_c	chr2:96875882-9698
ENSG0000(	1505	33.61048	chr2:309<	PABPC1P6		Pseudoger	chr2:91877969-9188
ENSG0000(	1505	33.61048	chr2:309<	ENSG00000283196		Pseudoger	chr2:91654920-9165
ENSG0000(	1505	33.61048	chr2:309<	ENSG00000236750		Pseudoger	chr2:95641634-9564
ENSG0000(	1505	33.61048	chr2:309<	ENSG00000277747		Pseudoger	chr2:95460144-9546
ENSG0000(	1505	33.61048	chr2:309<	C2orf92		protein_c	chr2:97664217-9770
ENSG0000(	1505	33.61048	chr2:309<	MAT2A		protein_c	chr2:85539168-8554
ENSG0000(	1505	33.61048	chr2:309<	VAMP5		protein_c	chr2:85584431-8559
ENSG0000(	1505	33.61048	chr2:309<	ZNF2		protein_c	chr2:95165432-9518
ENSG0000(	1505	33.61048	chr2:309<	RNU4-8P		smallRNA	chr2:97664591-9766
ENSG0000(	1505	33.61048	chr2:309<	ENSG00000275094		Pseudoger	chr2:97000436-9700
ENSG0000(	1505	33.61048	chr2:309<	POLR1A		protein_c	chr2:86020216-8610
ENSG0000(	1505	33.61048	chr2:309<	REEP1		protein_c	chr2:86213993-8633
ENSG0000(	1505	33.61048	chr2:309<	AC018696.1		Pseudoger	chr2:91443388-9144
ENSG0000(	1505	33.61048	chr2:309<	SFTPB		protein_c	chr2:85657314-8566
ENSG0000(	1505	33.61048	chr2:309<	ENSG00000273080		lncRNA	chr2:86195154-8619
ENSG0000(	1505	33.61048	chr2:309<	ENSG00000228873		lncRNA	chr2:96145602-9614
ENSG0000(	1505	33.61048	chr2:309<	ENSG00000275075		Pseudoger	chr2:91578478-9157
ENSG0000(	1505	33.61048	chr2:309<	USP39		protein_c	chr2:85602856-8564
ENSG0000(	1505	33.61048	chr2:309<	LINC01955		lncRNA	chr2:87249095-8725
ENSG0000(	1505	33.61048	chr2:309<	C2orf68		protein_c	chr2:85605254-8561
ENSG0000(	1505	33.61048	chr2:309<	RNF103	NCGv7	protein_c	chr2:86603398-8662
ENSG0000(	1505	33.61048	chr2:309<	ENSG00000224719		Pseudoger	chr2:95502791-9550
ENSG0000(	1505	33.61048	chr2:309<	RNA5SP101		Pseudoger	chr2:96956708-9695
ENSG0000(	1505	33.61048	chr2:309<	CNN2P11		Pseudoger	chr2:94725674-9472
ENSG0000(	1505	33.61048	chr2:309<	ENSG00000289135		lncRNA	chr2:96815093-9681
ENSG0000(	1505	33.61048	chr2:309<	TMEM150A		protein_c	chr2:85598547-8560
ENSG0000(	1505	33.61048	chr2:309<	ENSG00000291176		lncRNA	chr2:96010526-9602
ENSG0000(	1505	33.61048	chr2:309<	RNF181		protein_c	chr2:85595725-8559
ENSG0000(	1505	33.61048	chr2:309<	ENSG00000290565		lncRNA	chr2:91686102-9171
ENSG0000(	1505	33.61048	chr2:309<	KMT2CP4		Pseudoger	chr2:91696451-9171
ENSG0000(	1505	33.61048	chr2:309<	CHMP3-AS1		lncRNA	chr2:86562070-8661
ENSG0000(	1505	33.61048	chr2:309<	RNA5SP100		Pseudoger	chr2:91674857-9167
ENSG0000(	1505	33.61048	chr2:309<	ENSG00000259848		Pseudoger	chr2:94886861-9489
ENSG0000(	1505	33.61048	chr2:309<	DRD5P1		Pseudoger	chr2:91684447-9168
ENSG0000(	1505	33.61048	chr2:309<	AC159540.1		lncRNA	chr2:97415474-9743
ENSG0000(	1505	33.61048	chr2:309<	ENSG00000235584		lncRNA	chr2:95660588-9566
ENSG0000(	1505	33.61048	chr2:309<	SNX18P14		Pseudoger	chr2:94786688-9478
ENSG0000(	1505	33.61048	chr2:309<	ENSG00000235833		Pseudoger	chr2:97523949-9752
ENSG0000(	1505	33.61048	chr2:309<	RN7SL575P		smallRNA	chr2:95003547-9500
ENSG0000(	1505	33.61048	chr2:309<	ENSG00000233850		lncRNA	chr2:95025193-9502
ENSG0000(	1505	33.61048	chr2:309<	ENSG00000287607		Pseudoger	chr2:91578478-9157
ENSG0000(	1505	33.61048	chr2:309<	GPR160P1		Pseudoger	chr2:85686053-8568
ENSG0000(	1505	33.61048	chr2:309<	ENSG00000236847		Pseudoger	chr2:97035461-9703
ENSG0000(	1505	33.61048	chr2:309<	OR7E102P		Pseudoger	chr2:95546531-9554
ENSG0000(	1505	33.61048	chr2:309<	Y_RNA		smallRNA	chr2:85434507-8543
ENSG0000(	1505	33.61048	chr2:309<	CHMP3		protein_c	chr2:86503430-8656

ENSG0000	1505	33.61048	chr2:309	CNNM4		protein_c	chr2:96760902-9681
ENSG0000	1505	33.61048	chr2:309	GPAT2P1		Pseudogen	chr2:95792220-9580
ENSG0000	1505	33.61048	chr2:309	KMT2CP5		Pseudogen	chr2:91696452-9171
ENSG0000	1505	33.61048	chr2:309	UBTFL6		Pseudogen	chr2:97636780-9763
ENSG0000	1505	33.61048	chr2:309	ENSG00000272564		lncRNA	chr2:85904279-8590
ENSG0000	1505	33.61048	chr2:309	ENSG00000290846		lncRNA	chr2:91617160-9166
ENSG0000	1505	33.61048	chr2:309	KDM3A		protein_c	chr2:86440647-8649
ENSG0000	1505	33.61048	chr2:309	ENSG00000233757		protein_c	chr2:95207521-9525
ENSG0000	1505	33.61048	chr2:309	Y_RNA		smallRNA	chr2:85460144-8546
ENSG0000	1505	33.61048	chr2:309	RN7SL830P		smallRNA	chr2:85532344-8553
ENSG0000	1505	33.61048	chr2:309	ACTR3BP2		Pseudogen	chr2:91940668-9194
ENSG0000	1505	33.61048	chr2:309	ENSG00000266931		Pseudogen	chr2:87055658-8707
ENSG0000	1505	33.61048	chr2:309	ANKRD23		protein_c	chr2:96824526-9685
ENSG0000	1505	33.61048	chr2:309	ENSG00000248821		Pseudogen	chr2:95413456-9541
ENSG0000	1505	33.61048	chr2:309	NEURL3		protein_c	chr2:96497646-9650
ENSG0000	1505	33.61048	chr2:309	IGKV1OR2-6		Pseudogen	chr2:97355058-9735
ENSG0000	1505	33.61048	chr2:309	RGPD1		protein_c	chr2:86913783-8701
ENSG0000	1505	33.61048	chr2:309	TMEM131		protein_c	chr2:97756333-9799
ENSG0000	1505	33.61048	chr2:309	RN7SL126P		smallRNA	chr2:85567664-8556
ENSG0000	1505	33.61048	chr2:309	ITPRIPL1		protein_c	chr2:96325317-9633
ENSG0000	1505	33.61048	chr2:309	ENSG00000248134		Pseudogen	chr2:97018343-9701
ENSG0000	1505	33.61048	chr2:309	FER1L5		protein_c	chr2:96642737-9670
ENSG0000	1505	33.61048	chr2:309	HMGN1P36		Pseudogen	chr2:97827248-9782
ENSG0000	1505	33.61048	chr2:309	ENSG00000237308		Pseudogen	chr2:95496022-9549
ENSG0000	1505	33.61048	chr2:309	U8		smallRNA	chr2:86347062-8634
ENSG0000	1505	33.61048	chr2:309	ENSG00000273196		lncRNA	chr2:85387074-8538
ENSG0000	1505	33.61048	chr2:309	AC113612.1		Pseudogen	chr2:90261265-9026
ENSG0000	1505	33.61048	chr2:309	ENSG00000213605		Pseudogen	chr2:86885075-8688
ENSG0000	1505	33.61048	chr2:309	ENSG00000278131		Pseudogen	chr2:91589494-9162
ENSG0000	1505	33.61048	chr2:309	SNORA19		smallRNA	chr2:86364136-8636
ENSG0000	1505	33.61048	chr2:309	ENSG00000288960		lncRNA	chr2:95074906-9507
ENSG0000	1505	33.61048	chr2:309	TEKT4		protein_c	chr2:94871430-9487
ENSG0000	1505	33.61048	chr2:309	AC113612.2		Pseudogen	chr2:90255285-9025
ENSG0000	1505	33.61048	chr2:309	UBTFL5		Pseudogen	chr2:95450310-9545
ENSG0000	1505	33.61048	chr2:309	ATOH8		protein_c	chr2:85751344-8579
ENSG0000	1505	33.61048	chr2:309	ARID5A		protein_c	chr2:96536743-9655
ENSG0000	1505	33.61048	chr2:309	AC018696.7		Pseudogen	chr2:91561304-9156
ENSG0000	1505	33.61048	chr2:309	IGKV2OR2-2		Pseudogen	chr2:97050729-9705
ENSG0000	1505	33.61048	chr2:309	GPAT2		protein_c	chr2:96021946-9603
ENSG0000	1505	33.61048	chr2:309	ENSG00000231062		lncRNA	chr2:95051395-9505
ENSG0000	1505	33.61048	chr2:309	ENSG00000231331		Pseudogen	chr2:94953161-9495
ENSG0000	1505	33.61048	chr2:309	ACTR1B		protein_c	chr2:97655939-9766
ENSG0000	1505	33.61048	chr2:309	TRIM51JP		Pseudogen	chr2:95574901-9558
ENSG0000	1505	33.61048	chr2:309	LMAN2L		protein_c	chr2:96705929-9674
ENSG0000	1505	33.61048	chr2:309	ZAP70		protein_c	chr2:97713576-9773
ENSG0000	1505	33.61048	chr2:309	RN7SL251P		smallRNA	chr2:85442495-8544
ENSG0000	1505	33.61048	chr2:309	ANKRD36C	NCGv7	protein_c	chr2:95836919-9599
ENSG0000	1505	33.61048	chr2:309	CAPG	AC	protein_c	chr2:85394753-8541
ENSG0000	1505	33.61048	chr2:309	ENSG00000287362		lncRNA	chr2:95067074-9507
ENSG0000	1505	33.61048	chr2:309	SH2D6		protein_c	chr2:85418714-8543
ENSG0000	1505	33.61048	chr2:309	KANSL3		protein_c	chr2:96593170-9664

ENSG0000(	1505	33.61048	chr2:309<	WBP1P1		Pseudoger	chr2:86930250-8693
ENSG0000(	1505	33.61048	chr2:309<	AC015971.1		smallRNA	chr2:86586140-8658
ENSG0000(	1505	33.61048	chr2:309<	ANAPC1P3		Pseudoger	chr2:87118534-8712
ENSG0000(	1505	33.61048	chr2:309<	ENSG00000230083		Pseudoger	chr2:95590969-9559
ENSG0000(	1505	33.61048	chr2:309<	ENSG00000272913		lncRNA	chr2:95524873-9552
ENSG0000(	1505	33.61048	chr2:309<	ENSG00000273634		Pseudoger	chr2:97008368-9700
ENSG0000(	1505	33.61048	chr2:309<	ENSG00000227120		Pseudoger	chr2:95436133-9543
ENSG0000(	1505	33.61048	chr2:309<	ENSG00000235959		Pseudoger	chr2:95640181-9564
ENSG0000(	1505	33.61048	chr2:309<	MRPL35		protein_c	chr2:86199355-8621
ENSG0000(	1505	33.61048	chr2:309<	ENSG00000278766		lncRNA	chr2:97421075-9743
ENSG0000(	1505	33.61048	chr2:309<	IMMT		protein_c	chr2:86143932-8619
ENSG0000(	1505	33.61048	chr2:309<	ENSG00000230343		Pseudoger	chr2:97034442-9703
ENSG0000(	1505	33.61048	chr2:309<	RPSAP22		Pseudoger	chr2:85490930-8549
ENSG0000(	1505	33.61048	chr2:309<	PTCD3		protein_c	chr2:86106223-8614
ENSG0000(	1505	33.61048	chr2:309<	ENSG00000236431		Pseudoger	chr2:95536117-9553
ENSG0000(	1505	33.61048	chr2:309<	GGT8P		Pseudoger	chr2:91775944-9178
ENSG0000(	1505	33.61048	chr2:309<	FAM95A		lncRNA	chr2:94755326-9479
ENSG0000(	1505	33.61048	chr2:309<	ENSG00000273825		Pseudoger	chr2:91589469-9162
ENSG0000(	1505	33.61048	chr2:309<	ENSG00000261600		lncRNA	chr2:91580336-9158
ENSG0000(	1505	33.61048	chr2:309<	RMND5A		protein_c	chr2:86720291-8677
ENSG0000(	1505	33.61048	chr2:309<	CD8A		protein_c	chr2:86784610-8680
ENSG0000(	1505	33.61048	chr2:309<	ANKRD36B		protein_c	chr2:97492663-9758
ENSG0000(	1505	33.61048	chr2:309<	ANAPC1P2		Pseudoger	chr2:87031815-8705
ENSG0000(	1505	33.61048	chr2:309<	ENSG00000223917		Pseudoger	chr2:95486480-9548
ENSG0000(	1505	33.61048	chr2:309<	ENSG00000238162		Pseudoger	chr2:95485541-9548
ENSG0000(	1505	33.61048	chr2:309<	ENSG00000291013		lncRNA	chr2:86861825-8689
ENSG0000(	1505	33.61048	chr2:309<	ENSG00000237085		Pseudoger	chr2:91859384-9185
ENSG0000(	1505	33.61048	chr2:309<	ENSG00000235147		Pseudoger	chr2:92006680-9200
ENSG0000(	1505	33.61048	chr2:309<	ENSG00000233275		Pseudoger	chr2:95434759-9543
ENSG0000(	1505	33.61048	chr2:309<	ENSG00000277095		Pseudoger	chr2:91589464-9162
ENSG0000(	1505	33.61048	chr2:309<	KCNIP3	DriverDB\	protein_c	chr2:95297327-9538
ENSG0000(	1505	33.61048	chr2:309<	IGKV2OR2-10		Pseudoger	chr2:97331533-9733
ENSG0000(	1505	33.61048	chr2:309<	ENSG00000291024		lncRNA	chr2:94811046-9481
ENSG0000(	1505	33.61048	chr2:309<	ENSG00000276118		Pseudoger	chr2:91578478-9157
ENSG0000(	1505	33.61048	chr2:309<	IGKV2OR2-7		Pseudoger	chr2:97372532-9737
ENSG0000(	1505	33.61048	chr2:309<	IGKV1OR1-1		Pseudoger	chr2:91486789-9148
ENSG0000(	1505	33.61048	chr2:309<	ENSG00000281904		lncRNA	chr2:90365736-9036
ENSG0000(	1505	33.61048	chr2:309<	ENSG00000291025		lncRNA	chr2:94867486-9494
ENSG0000(	1505	33.61048	chr2:309<	FAHD2A		protein_c	chr2:95402708-9541
ENSG0000(	1505	33.61048	chr2:309<	NDUFB4P5		Pseudoger	chr2:86934462-8693
ENSG0000(	1505	33.61048	chr2:309<	Y_RNA		smallRNA	chr2:86159956-8616
ENSG0000(	1505	33.61048	chr2:309<	IGSF3P2		Pseudoger	chr2:91736726-9176
ENSG0000(	1505	33.61048	chr2:309<	ENSG00000289685		protein_c	chr2:95085391-9516
ENSG0000(	1505	33.61048	chr2:309<	ENSG00000235186		Pseudoger	chr2:94991880-9499
ENSG0000(	1505	33.61048	chr2:309<	FABP7P2		Pseudoger	chr2:95368507-9536
ENSG0000(	1505	33.61048	chr2:309<	ENSG00000235480		lncRNA	chr2:96527940-9653
ENSG0000(	1505	33.61048	chr2:309<	ENSG00000291126		lncRNA	chr2:95526651-9553
ENSG0000(	1505	33.61048	chr2:309<	ENSG00000288858		lncRNA	chr2:85594864-8559
ENSG0000(	1505	33.61048	chr2:309<	ENSG00000233037		Pseudoger	chr2:95542730-9554
ENSG0000(	1505	33.61048	chr2:309<	IGKV2OR2-8		Pseudoger	chr2:97376674-9737
ENSG0000(	1505	33.61048	chr2:309<	PLGLB1		protein_c	chr2:87002559-8702

ENSG0000(	1505	33.61048	chr2:309<	AC018696.5		Pseudoger	chr2:91544070-9154
ENSG0000(	1505	33.61048	chr2:309<	MIR3127		smallRNA	chr2:96798278-9679
ENSG0000(	1505	33.61048	chr2:309<	RPS24P6		Pseudoger	chr2:94912432-9491
ENSG0000(	1505	33.61048	chr2:309<	RN7SL313P		smallRNA	chr2:97100584-9710
ENSG0000(	1505	33.61048	chr2:309<	RNU6-640P		smallRNA	chr2:86515204-8651
ENSG0000(	1505	33.61048	chr2:309<	RNU1-38P		smallRNA	chr2:85728194-8572
ENSG0000(	1505	33.61048	chr2:309<	IGKV1OR2-1		Pseudoger	chr2:91817771-9181
ENSG0000(	1505	33.61048	chr2:309<	AC073464.11		Pseudoger	chr2:94795808-9480
ENSG0000(	1505	33.61048	chr2:309<	CYP4F32P		Pseudoger	chr2:94759259-9476
ENSG0000(	1505	33.61048	chr2:309<	RN7SKP83		smallRNA	chr2:85820435-8582
ENSG0000(	1505	33.61048	chr2:309<	ANKRD20A8P		Pseudoger	chr2:94791103-9485
ENSG0000(	1505	33.61048	chr2:309<	LINC00342		lncRNA	chr2:95807052-9583
ENSG0000(	1505	33.61048	chr2:309<	RNU6-1320P		smallRNA	chr2:94846533-9484
ENSG0000(	1505	33.61048	chr2:309<	MTCO1P48		Pseudoger	chr2:94899566-9490
ENSG0000(	1505	33.61048	chr2:309<	ABCD1P5		Pseudoger	chr2:91840601-9184
ENSG0000(	1505	33.61048	chr2:309<	ENSG00000204745		Pseudoger	chr2:87125198-8719
ENSG0000(	1505	33.61048	chr2:309<	ENSG00000287931		lncRNA	chr2:87075653-8711
ENSG0000(	1505	33.61048	chr2:309<	MIR4771-2		smallRNA	chr2:87194786-8719
ENSG0000(	1505	33.61048	chr2:309<	IGKV1OR2-2		Pseudoger	chr2:92034522-9203
ENSG0000(	1505	33.61048	chr2:309<	ENSG00000236026		Pseudoger	chr2:95606924-9560
ENSG0000(	1505	33.61048	chr2:309<	SLC9B1P2		Pseudoger	chr2:91883076-9193
ENSG0000(	1505	33.61048	chr2:309<	ENSG00000233105		Pseudoger	chr2:94964473-9496
ENSG0000(	1505	33.61048	chr2:309<	ENSG00000229498		lncRNA	chr2:85815130-8582
ENSG0000(	1505	33.61048	chr2:309<	LSP1P4		Pseudoger	chr2:91636684-9165
ENSG0000(	1505	33.61048	chr2:309<	SNORD94		smallRNA	chr2:86135870-8613
ENSG0000(	1503	33.56581	chr2:309<	RNU6-105P		smallRNA	chr2:71379755-7137
ENSG0000(	1503	33.56581	chr2:309<	ENSG00000281195		lncRNA	chr2:71373938-7137
ENSG0000(	1503	33.56581	chr2:309<	ENSG00000278060		lncRNA	chr2:72932974-7293
ENSG0000(	1503	33.56581	chr2:309<	SPR		protein_c	chr2:72887382-7289
ENSG0000(	1503	33.56581	chr2:309<	DYSF	NCGv7	protein_c	chr2:71453561-7168
ENSG0000(	1503	33.56581	chr2:309<	PAIP2B		protein_c	chr2:71182738-7122
ENSG0000(	1503	33.56581	chr2:309<	ENSG00000233870		Pseudoger	chr2:71178587-7117
ENSG0000(	1503	33.56581	chr2:309<	MCEE		protein_c	chr2:71109684-7113
ENSG0000(	1503	33.56581	chr2:309<	EMX1		protein_c	chr2:72916260-7293
ENSG0000(	1503	33.56581	chr2:309<	ZNF638	NCGv7	protein_c	chr2:71276561-7143
ENSG0000(	1503	33.56581	chr2:309<	NAGK		protein_c	chr2:71064344-7107
ENSG0000(	1503	33.56581	chr2:309<	RPS15AP13		Pseudoger	chr2:72288785-7228
ENSG0000(	1503	33.56581	chr2:309<	ENSG00000272702		lncRNA	chr2:73113018-7311
ENSG0000(	1503	33.56581	chr2:309<	MPHOSPH10		protein_c	chr2:71130310-7115
ENSG0000(	1503	33.56581	chr2:309<	ENSG00000289463		lncRNA	chr2:71419974-7142
ENSG0000(	1503	33.56581	chr2:309<	RPS20P10		Pseudoger	chr2:71984182-7198
ENSG0000(	1503	33.56581	chr2:309<	SNORD78		smallRNA	chr2:72760534-7276
ENSG0000(	1503	33.56581	chr2:309<	ENSG00000272735		lncRNA	chr2:71067519-7106
ENSG0000(	1503	33.56581	chr2:309<	RAB11FIP5		protein_c	chr2:73073382-7315
ENSG0000(	1503	33.56581	chr2:309<	SFXN5		protein_c	chr2:72942036-7307
ENSG0000(	1503	33.56581	chr2:309<	EXOC6B		protein_c	chr2:72175984-7282
ENSG0000(	1503	33.56581	chr2:309<	RNU2-39P		smallRNA	chr2:72721806-7272
ENSG0000(	1503	33.56581	chr2:309<	CYP26B1		protein_c	chr2:72129238-7214
ENSG0000(	1503	33.56581	chr2:309<	AC007881.1		smallRNA	chr2:71125523-7112
ENSG0000(	1503	33.56581	chr2:309<	ENSG00000288065		lncRNA	chr2:71112877-7111
ENSG0000(	1503	33.56581	chr2:309<	OR7E62P		Pseudoger	chr2:71055527-7105

ENSG0000(	1503	33.56581	chr2:309‹	ENSG00000289327		lncRNA	chr2:71453081-7145
ENSG0000(	1503	33.56581	chr2:309‹	ENSG00000289615		lncRNA	chr2:72144651-7214
ENSG0000(	1479	33.02983	chr2:309‹	AC007682.2		smallRNA	chr2:50943292-5094
ENSG0000(	1468	32.78417	chr2:309‹	ENSG00000289326		lncRNA	chr2:27752831-2775
ENSG0000(	1468	32.78417	chr2:309‹	GCKR		protein_c	chr2:27496839-2752
ENSG0000(	1468	32.78417	chr2:309‹	ENSG00000259080		lncRNA	chr2:27583046-2763
ENSG0000(	1468	32.78417	chr2:309‹	RPL23AP34		Pseudoger	chr2:28308161-2830
ENSG0000(	1468	32.78417	chr2:309‹	FNDC4		protein_c	chr2:27491883-2749
ENSG0000(	1468	32.78417	chr2:309‹	SUPT7L	NCGv7	protein_c	chr2:27650809-2766
ENSG0000(	1468	32.78417	chr2:309‹	MYG1P1		Pseudoger	chr2:27896116-2789
ENSG0000(	1468	32.78417	chr2:309‹	MRPL33		protein_c	chr2:27771717-2798
ENSG0000(	1468	32.78417	chr2:309‹	SNORA36		smallRNA	chr2:27642043-2764
ENSG0000(	1468	32.78417	chr2:309‹	BABAM2	NCGv7	protein_c	chr2:27889941-2833
ENSG0000(	1468	32.78417	chr2:309‹	RNU6-986P		smallRNA	chr2:27475494-2747
ENSG0000(	1468	32.78417	chr2:309‹	KRTCAP3		protein_c	chr2:27442366-2744
ENSG0000(	1468	32.78417	chr2:309‹	MIR4263		smallRNA	chr2:27996367-2799
ENSG0000(	1468	32.78417	chr2:309‹	ENSG00000270640		lncRNA	chr2:28396815-2839
ENSG0000(	1468	32.78417	chr2:309‹	IFT172		protein_c	chr2:27444377-2748
ENSG0000(	1468	32.78417	chr2:309‹	ENSG00000235267		Pseudoger	chr2:27455156-2745
ENSG0000(	1468	32.78417	chr2:309‹	RBKS		protein_c	chr2:27781379-2789
ENSG0000(	1468	32.78417	chr2:309‹	FOSL2-AS1		lncRNA	chr2:28384409-2839
ENSG0000(	1468	32.78417	chr2:309‹	SLC4A1AP		protein_c	chr2:27663426-2769
ENSG0000(	1468	32.78417	chr2:309‹	FAM133EP		Pseudoger	chr2:28015777-2801
ENSG0000(	1468	32.78417	chr2:309‹	C2orf16		protein_c	chr2:27537386-2758
ENSG0000(	1468	32.78417	chr2:309‹	ZNF512		protein_c	chr2:27582969-2762
ENSG0000(	1468	32.78417	chr2:309‹	ENSG00000223522		lncRNA	chr2:28307063-2831
ENSG0000(	1468	32.78417	chr2:309‹	CCDC121		protein_c	chr2:27625638-2762
ENSG0000(	1468	32.78417	chr2:309‹	LINC01460		lncRNA	chr2:27705786-2771
ENSG0000(	1468	32.78417	chr2:309‹	AC074091.1		smallRNA	chr2:27581889-2758
ENSG0000(	1468	32.78417	chr2:309‹	FOSL2		protein_c	chr2:28392448-2841
ENSG0000(	1468	32.78417	chr2:309‹	GPN1		protein_c	chr2:27628247-2765
ENSG0000(	1463	32.67251	chr2:309‹	RNU6-1216P		smallRNA	chr2:69182877-6918
ENSG0000(	1463	32.67251	chr2:309‹	ENSG00000275381		Pseudoger	chr2:69844509-6984
ENSG0000(	1463	32.67251	chr2:309‹	AC007040.2		smallRNA	chr2:71012331-7101
ENSG0000(	1463	32.67251	chr2:309‹	ENSG00000233060		lncRNA	chr2:70089721-7009
ENSG0000(	1463	32.67251	chr2:309‹	MOB4P1		Pseudoger	chr2:70810798-7081
ENSG0000(	1463	32.67251	chr2:309‹	ENSG00000229229		lncRNA	chr2:70402934-7042
ENSG0000(	1463	32.67251	chr2:309‹	FAM136A		protein_c	chr2:70295975-7030
ENSG0000(	1463	32.67251	chr2:309‹	snoU13		smallRNA	chr2:70930340-7093
ENSG0000(	1463	32.67251	chr2:309‹	RPL39P15		Pseudoger	chr2:70253855-7025
ENSG0000(	1463	32.67251	chr2:309‹	snoU13		smallRNA	chr2:69955645-6995
ENSG0000(	1463	32.67251	chr2:309‹	ENSG00000271597		Pseudoger	chr2:69251818-6925
ENSG0000(	1463	32.67251	chr2:309‹	ENSG00000228384		lncRNA	chr2:70994510-7100
ENSG0000(	1463	32.67251	chr2:309‹	BRD7P6		Pseudoger	chr2:70353010-7035
ENSG0000(	1463	32.67251	chr2:309‹	ASPRV1		protein_c	chr2:69960089-6996
ENSG0000(	1463	32.67251	chr2:309‹	PCBP1-AS1		lncRNA	chr2:69960104-7010
ENSG0000(	1463	32.67251	chr2:309‹	ENSG00000233849		lncRNA	chr2:70301451-7030
ENSG0000(	1463	32.67251	chr2:309‹	B3GALNT1P1		Pseudoger	chr2:69597353-6959
ENSG0000(	1463	32.67251	chr2:309‹	AC007881.2		smallRNA	chr2:71039307-7103
ENSG0000(	1463	32.67251	chr2:309‹	GFPT1		protein_c	chr2:69319780-6938
ENSG0000(	1463	32.67251	chr2:309‹	RPL36AP16		Pseudoger	chr2:69594741-6959

ENSG00000	1463	32.67251	chr2:3094	SNORA36C		smallRNA	chr2:69520043-6952
ENSG00000	1463	32.67251	chr2:3094	LINC01816		lncRNA	chr2:70124034-7013
ENSG00000	1463	32.67251	chr2:3094	ANXA4		protein_c	chr2:69644425-6982
ENSG00000	1463	32.67251	chr2:3094	MXD1		protein_c	chr2:69897688-6994
ENSG00000	1463	32.67251	chr2:3094	CLEC4F		protein_c	chr2:70808643-7082
ENSG00000	1463	32.67251	chr2:3094	ADD2	NCGv7	protein_c	chr2:70607618-7076
ENSG00000	1463	32.67251	chr2:3094	MIR1285-2		smallRNA	chr2:70252918-7025
ENSG00000	1463	32.67251	chr2:3094	ENSG00000289250		lncRNA	chr2:69663299-6966
ENSG00000	1463	32.67251	chr2:3094	NFU1		protein_c	chr2:69396113-6943
ENSG00000	1463	32.67251	chr2:3094	PCBP1	NCGv7	protein_c	chr2:70087477-7008
ENSG00000	1463	32.67251	chr2:3094	MRPL36P1		Pseudogen	chr2:70102551-7010
ENSG00000	1463	32.67251	chr2:3094	SNRPG		protein_c	chr2:70281362-7029
ENSG00000	1463	32.67251	chr2:3094	SNRNP27		protein_c	chr2:69893956-6990
ENSG00000	1463	32.67251	chr2:3094	ENSG00000288869		lncRNA	chr2:69789544-6979
ENSG00000	1463	32.67251	chr2:3094	RNA5SP96		Pseudogen	chr2:69181897-6918
ENSG00000	1463	32.67251	chr2:3094	AC007040.3		smallRNA	chr2:71013089-7101
ENSG00000	1463	32.67251	chr2:3094	RN7SL470P		smallRNA	chr2:70075018-7007
ENSG00000	1463	32.67251	chr2:3094	ANKRD53		protein_c	chr2:70978380-7098
ENSG00000	1463	32.67251	chr2:3094	GMCL1		protein_c	chr2:69829660-6988
ENSG00000	1463	32.67251	chr2:3094	ATP6V1B1		protein_c	chr2:70935900-7096
ENSG00000	1463	32.67251	chr2:3094	RN7SL160P		smallRNA	chr2:70965571-7096
ENSG00000	1463	32.67251	chr2:3094	TEX261		protein_c	chr2:70968325-7099
ENSG00000	1463	32.67251	chr2:3094	ENSG00000235035		lncRNA	chr2:70687142-7069
ENSG00000	1463	32.67251	chr2:3094	FIGLA		protein_c	chr2:70777310-7079
ENSG00000	1463	32.67251	chr2:3094	ATP6V1B1-AS1		lncRNA	chr2:70941817-7094
ENSG00000	1463	32.67251	chr2:3094	LINC01143		lncRNA	chr2:70887871-7088
ENSG00000	1463	32.67251	chr2:3094	AAK1		protein_c	chr2:69457997-6967
ENSG00000	1463	32.67251	chr2:3094	OR7E91P		Pseudogen	chr2:71029028-7102
ENSG00000	1463	32.67251	chr2:3094	HMGN2P21		Pseudogen	chr2:70803157-7080
ENSG00000	1463	32.67251	chr2:3094	snoU13		smallRNA	chr2:69912695-6991
ENSG00000	1463	32.67251	chr2:3094	ENSG00000231024		lncRNA	chr2:69700192-6971
ENSG00000	1463	32.67251	chr2:3094	RN7SL604P		smallRNA	chr2:69516751-6951
ENSG00000	1463	32.67251	chr2:3094	snoU13		smallRNA	chr2:69667359-6966
ENSG00000	1463	32.67251	chr2:3094	TGFA	NCGv7	protein_c	chr2:70447284-7055
ENSG00000	1463	32.67251	chr2:3094	RPL23AP92		Pseudogen	chr2:69873565-6987
ENSG00000	1463	32.67251	chr2:3094	TGFA-IT1		lncRNA	chr2:70467385-7046
ENSG00000	1463	32.67251	chr2:3094	TIA1	NCGv7	protein_c	chr2:70209444-7024
ENSG00000	1463	32.67251	chr2:3094	OR7E91P		lncRNA	chr2:71024127-7102
ENSG00000	1463	32.67251	chr2:3094	PCYOX1		protein_c	chr2:70257386-7028
ENSG00000	1463	32.67251	chr2:3094	Y_RNA		smallRNA	chr2:69334600-6933
ENSG00000	1463	32.67251	chr2:3094	ELOCP21		Pseudogen	chr2:70955899-7095
ENSG00000	1463	32.67251	chr2:3094	ENSG00000258881		protein_c	chr2:70939318-7099
ENSG00000	1463	32.67251	chr2:3094	ENSG00000286979		lncRNA	chr2:70629657-7064
ENSG00000	1463	32.67251	chr2:3094	AC017084.1		smallRNA	chr2:70455530-7045
ENSG00000	1463	32.67251	chr2:3094	ENSG00000236469		lncRNA	chr2:71002531-7106
ENSG00000	1463	32.67251	chr2:3094	C2orf42		protein_c	chr2:70149885-7024
ENSG00000	1463	32.67251	chr2:3094	OR7E46P		Pseudogen	chr2:71037685-7103
ENSG00000	1463	32.67251	chr2:3094	AC007040.1		smallRNA	chr2:71017024-7101
ENSG00000	1463	32.67251	chr2:3094	CD207		protein_c	chr2:70830211-7083
ENSG00000	1463	32.67251	chr2:3094	VAX2		protein_c	chr2:70900576-7096
ENSG00000	1458	32.56085	chr2:3094	ENSG00000270289		Pseudogen	chr2:180473137-180

ENSG0000(	1458	32.56085	chr2:3094	MIR4437		smallRNA	chr2:181305593-181
ENSG0000(	1458	32.56085	chr2:3094	KRT18P29		Pseudoger	chr2:181961212-181
ENSG0000(	1458	32.56085	chr2:3094	ENSG00000234595		lncRNA	chr2:181683113-181
ENSG0000(	1458	32.56085	chr2:3094	ENSG00000238171		lncRNA	chr2:181076051-181
ENSG0000(	1458	32.56085	chr2:3094	SCHLAP1		lncRNA	chr2:180692104-180
ENSG0000(	1458	32.56085	chr2:3094	ITPRID2		protein_c	chr2:181891730-181
ENSG0000(	1458	32.56085	chr2:3094	SAP18P2		Pseudoger	chr2:181694442-181
ENSG0000(	1458	32.56085	chr2:3094	RPL27AP3		Pseudoger	chr2:180938830-180
ENSG0000(	1458	32.56085	chr2:3094	ENSG00000289389		lncRNA	chr2:181305836-181
ENSG0000(	1458	32.56085	chr2:3094	ENSG00000225570		lncRNA	chr2:181690380-181
ENSG0000(	1458	32.56085	chr2:3094	CERKL		protein_c	chr2:181535041-181
ENSG0000(	1458	32.56085	chr2:3094	ENSG00000226681		lncRNA	chr2:181422154-181
ENSG0000(	1458	32.56085	chr2:3094	ENSG00000287536		lncRNA	chr2:182278684-182
ENSG0000(	1458	32.56085	chr2:3094	LINC01934		lncRNA	chr2:181086076-181
ENSG0000(	1458	32.56085	chr2:3094	NEUROD1		protein_c	chr2:181668295-181
ENSG0000(	1458	32.56085	chr2:3094	AC064837.1		smallRNA	chr2:182088429-182
ENSG0000(	1458	32.56085	chr2:3094	FTH1P20		Pseudoger	chr2:180872867-180
ENSG0000(	1458	32.56085	chr2:3094	UBE2E3-DT		lncRNA	chr2:180979427-180
ENSG0000(	1458	32.56085	chr2:3094	RN7SL267P		smallRNA	chr2:182314284-182
ENSG0000(	1458	32.56085	chr2:3094	ITGA4	NCGv7	protein_c	chr2:181457202-181
ENSG0000(	1458	32.56085	chr2:3094	AC009478.2		smallRNA	chr2:180617118-180
ENSG0000(	1458	32.56085	chr2:3094	RNA5SP113		Pseudoger	chr2:182048822-182
ENSG0000(	1458	32.56085	chr2:3094	ENSG00000225258		lncRNA	chr2:180571712-180
ENSG0000(	1458	32.56085	chr2:3094	UBE2E3		protein_c	chr2:180967248-181
ENSG0000(	1458	32.56085	chr2:3094	ITPRID2-DT		lncRNA	chr2:181887851-181
ENSG0000(	1458	32.56085	chr2:3094	RNU6ATAC19P		smallRNA	chr2:181738606-181
ENSG0000(	1458	32.56085	chr2:3094	PDE1A		protein_c	chr2:182139968-182
ENSG0000(	1458	32.56085	chr2:3094	PPP1R1C		protein_c	chr2:181954241-182
ENSG0000(	1458	32.56085	chr2:3094	ENSG00000279559		TEC	chr2:180801227-180
ENSG0000(	1453	32.44919	chr2:3094	ENSG00000288886		lncRNA	chr2:42792299-4279
ENSG0000(	1453	32.44919	chr2:3094	EML4-AS1		lncRNA	chr2:42143238-4217
ENSG0000(	1453	32.44919	chr2:3094	ENSG00000287145		lncRNA	chr2:41716133-4173
ENSG0000(	1453	32.44919	chr2:3094	ENSG00000289082		lncRNA	chr2:42795326-4279
ENSG0000(	1453	32.44919	chr2:3094	ENSG00000215263		Pseudoger	chr2:42532766-4253
ENSG0000(	1453	32.44919	chr2:3094	Y_RNA		smallRNA	chr2:41857271-4185
ENSG0000(	1453	32.44919	chr2:3094	SNORD75		smallRNA	chr2:42440377-4244
ENSG0000(	1453	32.44919	chr2:3094	KCNG3		protein_c	chr2:42442017-4249
ENSG0000(	1453	32.44919	chr2:3094	snoZ247		smallRNA	chr2:41734377-4173
ENSG0000(	1453	32.44919	chr2:3094	RPS13P3		Pseudoger	chr2:42469817-4247
ENSG0000(	1453	32.44919	chr2:3094	LINC01914		lncRNA	chr2:41931599-4193
ENSG0000(	1453	32.44919	chr2:3094	RNU6-137P		smallRNA	chr2:42712740-4271
ENSG0000(	1453	32.44919	chr2:3094	HAAO		protein_c	chr2:42767089-4279
ENSG0000(	1453	32.44919	chr2:3094	CHORDC1P1		Pseudoger	chr2:42826322-4282
ENSG0000(	1453	32.44919	chr2:3094	Y_RNA		smallRNA	chr2:42637961-4263
ENSG0000(	1453	32.44919	chr2:3094	RPS12P4		Pseudoger	chr2:41850203-4185
ENSG0000(	1453	32.44919	chr2:3094	MTA3		protein_c	chr2:42494569-4275
ENSG0000(	1453	32.44919	chr2:3094	COX7A2L		protein_c	chr2:42333546-4242
ENSG0000(	1453	32.44919	chr2:3094	PKDCC		protein_c	chr2:42048021-4205
ENSG0000(	1453	32.44919	chr2:3094	LINC02898		lncRNA	chr2:41935368-4195
ENSG0000(	1453	32.44919	chr2:3094	LDHAP3		Pseudoger	chr2:41819747-4182
ENSG0000(	1453	32.44919	chr2:3094	VDAC1P13		Pseudoger	chr2:42463139-4246

ENSG0000(	1453	32.44919	chr2:309⁴	OXER1		protein_c	chr2:42762499-4276
ENSG0000(	1453	32.44919	chr2:309⁴	ENSG00000226523		Pseudoger	chr2:42680088-4268
ENSG0000(	1453	32.44919	chr2:309⁴	ENSG00000226398		lncRNA	chr2:42015625-4202
ENSG0000(	1453	32.44919	chr2:309⁴	LINC01913		lncRNA	chr2:41860155-4189
ENSG0000(	1453	32.44919	chr2:309⁴	EML4	NCGv7;AC	protein_c	chr2:42169353-4233
ENSG0000(	1453	32.44919	chr2:309⁴	RNU4-63P		smallRNA	chr2:41871271-4187
ENSG0000(	1453	32.44919	chr2:309⁴	FTOP1		Pseudoger	chr2:42797225-4279
ENSG0000(	1451	32.40452	chr2:309⁴	ENSG00000230355		Pseudoger	chr2:66881087-6688
ENSG0000(	1451	32.40452	chr2:309⁴	VWA3B		protein_c	chr2:98087116-9831
ENSG0000(	1450	32.38219	chr2:309⁴	RNU6-282P		smallRNA	chr2:48501922-4850
ENSG0000(	1448	32.33752	chr2:309⁴	ZC3H15		protein_c	chr2:186486253-186
ENSG0000(	1448	32.33752	chr2:309⁴	DNAJC10		protein_c	chr2:182716255-182
ENSG0000(	1448	32.33752	chr2:309⁴	RNU6-989P		smallRNA	chr2:187270698-187
ENSG0000(	1448	32.33752	chr2:309⁴	RPL23AP33		Pseudoger	chr2:184902111-184
ENSG0000(	1448	32.33752	chr2:309⁴	AC068718.2		smallRNA	chr2:187742587-187
ENSG0000(	1448	32.33752	chr2:309⁴	ZNF804A	NCGv7	protein_c	chr2:184598529-184
ENSG0000(	1448	32.33752	chr2:309⁴	MED28P3		Pseudoger	chr2:186364598-186
ENSG0000(	1448	32.33752	chr2:309⁴	ENSG00000286797		lncRNA	chr2:185425063-185
ENSG0000(	1448	32.33752	chr2:309⁴	ENSG00000234172		lncRNA	chr2:183904529-183
ENSG0000(	1448	32.33752	chr2:309⁴	FRZB		protein_c	chr2:182833275-182
ENSG0000(	1448	32.33752	chr2:309⁴	IMPDH1P7		Pseudoger	chr2:187001876-187
ENSG0000(	1448	32.33752	chr2:309⁴	CALCRL		protein_c	chr2:187341964-187
ENSG0000(	1448	32.33752	chr2:309⁴	SNORA77		smallRNA	chr2:183064235-183
ENSG0000(	1448	32.33752	chr2:309⁴	ZSWIM2		protein_c	chr2:186827475-186
ENSG0000(	1448	32.33752	chr2:309⁴	KRT8P10		Pseudoger	chr2:183071040-183
ENSG0000(	1448	32.33752	chr2:309⁴	GAPDHP59		Pseudoger	chr2:187415552-187
ENSG0000(	1448	32.33752	chr2:309⁴	ENSG00000286807		lncRNA	chr2:183997372-184
ENSG0000(	1448	32.33752	chr2:309⁴	Y_RNA		smallRNA	chr2:183022859-183
ENSG0000(	1448	32.33752	chr2:309⁴	ST13P2		Pseudoger	chr2:187825341-187
ENSG0000(	1448	32.33752	chr2:309⁴	ENSG00000286879		lncRNA	chr2:183897950-183
ENSG0000(	1448	32.33752	chr2:309⁴	FSIP2	NCGv7	protein_c	chr2:185738804-185
ENSG0000(	1448	32.33752	chr2:309⁴	ENSG00000259915		lncRNA	chr2:186354570-186
ENSG0000(	1448	32.33752	chr2:309⁴	RNA5SP114		Pseudoger	chr2:188276732-188
ENSG0000(	1448	32.33752	chr2:309⁴	ENSG00000286980		lncRNA	chr2:184204372-184
ENSG0000(	1448	32.33752	chr2:309⁴	ENSG00000289205		lncRNA	chr2:188033794-188
ENSG0000(	1448	32.33752	chr2:309⁴	LIN28AP1		Pseudoger	chr2:183260642-183
ENSG0000(	1448	32.33752	chr2:309⁴	ENSG00000213115		Pseudoger	chr2:188227189-188
ENSG0000(	1448	32.33752	chr2:309⁴	CACYBPP2		Pseudoger	chr2:183607442-183
ENSG0000(	1448	32.33752	chr2:309⁴	NUP35		protein_c	chr2:183117513-183
ENSG0000(	1448	32.33752	chr2:309⁴	DUSP19		protein_c	chr2:183078559-183
ENSG0000(	1448	32.33752	chr2:309⁴	ENSG00000224643		lncRNA	chr2:183083405-183
ENSG0000(	1448	32.33752	chr2:309⁴	LINC01473		lncRNA	chr2:186032884-186
ENSG0000(	1448	32.33752	chr2:309⁴	ENSG00000227227		lncRNA	chr2:186641339-186
ENSG0000(	1448	32.33752	chr2:309⁴	ELF2P4		Pseudoger	chr2:185547135-185
ENSG0000(	1448	32.33752	chr2:309⁴	U8		smallRNA	chr2:185661774-185
ENSG0000(	1448	32.33752	chr2:309⁴	MIR548AE1		smallRNA	chr2:184378975-184
ENSG0000(	1448	32.33752	chr2:309⁴	snoU13		smallRNA	chr2:183949333-183
ENSG0000(	1448	32.33752	chr2:309⁴	ITGAV	NCGv7	protein_c	chr2:186590010-186
ENSG0000(	1448	32.33752	chr2:309⁴	ENSG00000283839		lncRNA	chr2:184593577-184
ENSG0000(	1448	32.33752	chr2:309⁴	RPL21P32		Pseudoger	chr2:185956587-185
ENSG0000(	1448	32.33752	chr2:309⁴	ENSG00000286152		lncRNA	chr2:184049467-184

ENSG0000(	1448	32.33752	chr2:309⁄	ENSG00000272800		lncRNA	chr2:183178806-183
ENSG0000(	1448	32.33752	chr2:309⁄	ENSG00000226410		Pseudoger	chr2:186162949-186
ENSG0000(	1448	32.33752	chr2:309⁄	ENSG00000287621		lncRNA	chr2:183253652-183
ENSG0000(	1448	32.33752	chr2:309⁄	Y_RNA		smallRNA	chr2:182828124-182
ENSG0000(	1448	32.33752	chr2:309⁄	RN7SKP42		smallRNA	chr2:187122562-187
ENSG0000(	1448	32.33752	chr2:309⁄	MIR561		smallRNA	chr2:188297492-188
ENSG0000(	1448	32.33752	chr2:309⁄	RPL23AP35		Pseudoger	chr2:186261419-186
ENSG0000(	1448	32.33752	chr2:309⁄	RPL31P15		Pseudoger	chr2:182788098-182
ENSG0000(	1448	32.33752	chr2:309⁄	NCKAP1		protein_c	chr2:182909115-183
ENSG0000(	1448	32.33752	chr2:309⁄	DPRXP1		Pseudoger	chr2:186488624-186
ENSG0000(	1448	32.33752	chr2:309⁄	FAM171B	NCGv7	protein_c	chr2:186694060-186
ENSG0000(	1448	32.33752	chr2:309⁄	FSIP2-AS1		lncRNA	chr2:185652374-185
ENSG0000(	1448	32.33752	chr2:309⁄	TFPI		protein_c	chr2:187464230-187
ENSG0000(	1448	32.33752	chr2:309⁄	FSIP2-AS2		lncRNA	chr2:185719874-185
ENSG0000(	1448	32.33752	chr2:309⁄	RNU6-1122P		smallRNA	chr2:182873841-182
ENSG0000(	1448	32.33752	chr2:309⁄	ENSG00000287129		lncRNA	chr2:185950469-185
ENSG0000(	1448	32.33752	chr2:309⁄	CALCRL-AS1		lncRNA	chr2:187003220-187
ENSG0000(	1446	32.29286	chr2:309⁄	AC008064.1		smallRNA	chr2:53651401-5365
ENSG0000(	1442	32.20353	chr2:309⁄	TTN-AS1		lncRNA	chr2:178521183-178
ENSG0000(	1442	32.20353	chr2:309⁄	PJVK		protein_c	chr2:178451346-178
ENSG0000(	1442	32.20353	chr2:309⁄	API5P2		Pseudoger	chr2:177997273-177
ENSG0000(	1442	32.20353	chr2:309⁄	ENSG00000236664		lncRNA	chr2:177953111-178
ENSG0000(	1442	32.20353	chr2:309⁄	CCDC141		protein_c	chr2:178829757-179
ENSG0000(	1442	32.20353	chr2:309⁄	NUDCP2		Pseudoger	chr2:178454716-178
ENSG0000(	1442	32.20353	chr2:309⁄	RNU5E-9P		smallRNA	chr2:178142548-178
ENSG0000(	1442	32.20353	chr2:309⁄	CWC22	NCGv7	protein_c	chr2:179944876-180
ENSG0000(	1442	32.20353	chr2:309⁄	RBM45		protein_c	chr2:178112424-178
ENSG0000(	1442	32.20353	chr2:309⁄	CHROMR		lncRNA	chr2:178413635-178
ENSG0000(	1442	32.20353	chr2:309⁄	TTN		protein_c	chr2:178525989-178
ENSG0000(	1442	32.20353	chr2:309⁄	SDHDP5		Pseudoger	chr2:178017993-178
ENSG0000(	1442	32.20353	chr2:309⁄	ENSG00000271011		lncRNA	chr2:178577103-178
ENSG0000(	1442	32.20353	chr2:309⁄	ENSG00000279598		TEC	chr2:178554561-178
ENSG0000(	1442	32.20353	chr2:309⁄	ZNF385B		protein_c	chr2:179441982-179
ENSG0000(	1442	32.20353	chr2:309⁄	ENSG00000267784		lncRNA	chr2:178723457-178
ENSG0000(	1442	32.20353	chr2:309⁄	ENSG00000270277		lncRNA	chr2:178548884-178
ENSG0000(	1442	32.20353	chr2:309⁄	RNU7-104P		smallRNA	chr2:178831371-178
ENSG0000(	1442	32.20353	chr2:309⁄	ACA59		smallRNA	chr2:179023257-179
ENSG0000(	1442	32.20353	chr2:309⁄	ENSG00000227241		Pseudoger	chr2:178105637-178
ENSG0000(	1442	32.20353	chr2:309⁄	ENSG00000271401		lncRNA	chr2:178644717-178
ENSG0000(	1442	32.20353	chr2:309⁄	ENSG00000271141		lncRNA	chr2:178616581-178
ENSG0000(	1442	32.20353	chr2:309⁄	snoU13		smallRNA	chr2:179125536-179
ENSG0000(	1442	32.20353	chr2:309⁄	RPS6P2		Pseudoger	chr2:179001756-179
ENSG0000(	1442	32.20353	chr2:309⁄	TXNL4AP1		Pseudoger	chr2:179814251-179
ENSG0000(	1442	32.20353	chr2:309⁄	MIR1258		smallRNA	chr2:179860836-179
ENSG0000(	1442	32.20353	chr2:309⁄	PRKRA		protein_c	chr2:178431292-178
ENSG0000(	1442	32.20353	chr2:309⁄	CYCTP		Pseudoger	chr2:178092503-178
ENSG0000(	1442	32.20353	chr2:309⁄	PLEKHA3		protein_c	chr2:178480457-178
ENSG0000(	1442	32.20353	chr2:309⁄	PDE11A-AS1		lncRNA	chr2:177653419-177
ENSG0000(	1442	32.20353	chr2:309⁄	SESTD1		protein_c	chr2:179101678-179
ENSG0000(	1442	32.20353	chr2:309⁄	ENSG00000237477		lncRNA	chr2:179273831-179
ENSG0000(	1442	32.20353	chr2:309⁄	SNORA43		smallRNA	chr2:179934401-179

ENSG0000(	1442	32.20353	chr2:309<	RAD52P1	Pseudoger	chr2:179399728-179
ENSG0000(	1442	32.20353	chr2:309<	OSBPL6	protein_c	chr2:178194481-178
ENSG0000(	1442	32.20353	chr2:309<	ENSG00000287149	lncRNA	chr2:178828349-179
ENSG0000(	1442	32.20353	chr2:309<	FKBP7	protein_c	chr2:178463664-178
ENSG0000(	1442	32.20353	chr2:309<	RNU6-629P	smallRNA	chr2:178038902-178
ENSG0000(	1442	32.20353	chr2:309<	ENSG00000270956	lncRNA	chr2:178541125-178
ENSG0000(	1442	32.20353	chr2:309<	ENSG00000270574	lncRNA	chr2:178578790-178
ENSG0000(	1442	32.20353	chr2:309<	PDE11A	protein_c	chr2:177623244-178
ENSG0000(	1434	32.02487	chr2:309<	WDR4P2	Pseudoger	chr2:68445710-6844
ENSG0000(	1434	32.02487	chr2:309<	MIR3126	smallRNA	chr2:69103682-6910
ENSG0000(	1434	32.02487	chr2:309<	LINC01828	lncRNA	chr2:67086446-6731
ENSG0000(	1434	32.02487	chr2:309<	APLF	protein_c	chr2:68467572-6865
ENSG0000(	1434	32.02487	chr2:309<	ENSG00000284932	Pseudoger	chr2:68117026-6811
ENSG0000(	1434	32.02487	chr2:309<	PNO1	protein_c	chr2:68157888-6817
ENSG0000(	1434	32.02487	chr2:309<	ARHGAP25	protein_c	chr2:68679601-6882
ENSG0000(	1434	32.02487	chr2:309<	LINC01799	lncRNA	chr2:66904436-6697
ENSG0000(	1434	32.02487	chr2:309<	ENSG00000236605	lncRNA	chr2:67324627-6732
ENSG0000(	1434	32.02487	chr2:309<	ENSG00000286002	lncRNA	chr2:67677499-6768
ENSG0000(	1434	32.02487	chr2:309<	GKN1	protein_c	chr2:68974573-6898
ENSG0000(	1434	32.02487	chr2:309<	ENSG00000273398	protein_c	chr2:68131238-6826
ENSG0000(	1434	32.02487	chr2:309<	AC017083.3	smallRNA	chr2:68273104-6827
ENSG0000(	1434	32.02487	chr2:309<	BMP10	protein_c	chr2:68860909-6887
ENSG0000(	1434	32.02487	chr2:309<	PROKR1	protein_c	chr2:68643579-6865
ENSG0000(	1434	32.02487	chr2:309<	PLEK	protein_c	chr2:68365282-6839
ENSG0000(	1434	32.02487	chr2:309<	C1D	protein_c	chr2:68041130-6811
ENSG0000(	1434	32.02487	chr2:309<	ENSG00000286347	lncRNA	chr2:69030042-6903
ENSG0000(	1434	32.02487	chr2:309<	LINC01890	lncRNA	chr2:68822855-6883
ENSG0000(	1434	32.02487	chr2:309<	ANTXR1	protein_c	chr2:69013176-6924
ENSG0000(	1434	32.02487	chr2:309<	ENSG00000273275	lncRNA	chr2:68179833-6818
ENSG0000(	1434	32.02487	chr2:309<	ENSG00000289533	lncRNA	chr2:67331881-6734
ENSG0000(	1434	32.02487	chr2:309<	FBXO48	protein_c	chr2:68459422-6846
ENSG0000(	1434	32.02487	chr2:309<	GKN2	protein_c	chr2:68945232-6895
ENSG0000(	1434	32.02487	chr2:309<	DNMT3AP1	Pseudoger	chr2:66820684-6682
ENSG0000(	1434	32.02487	chr2:309<	PPIAP64	Pseudoger	chr2:68125265-6812
ENSG0000(	1434	32.02487	chr2:309<	PPP3R1	protein_c	chr2:68178857-6825
ENSG0000(	1434	32.02487	chr2:309<	ENSG00000203395	lncRNA	chr2:68361214-6836
ENSG0000(	1434	32.02487	chr2:309<	LINC02831	lncRNA	chr2:67562067-6762
ENSG0000(	1434	32.02487	chr2:309<	CNRIP1	protein_c	chr2:68284171-6832
ENSG0000(	1434	32.02487	chr2:309<	GKN3P	Pseudoger	chr2:68921248-6892
ENSG0000(	1434	32.02487	chr2:309<	ETAA1	protein_c	chr2:67397322-6741
ENSG0000(	1434	32.02487	chr2:309<	ENSG00000273064	lncRNA	chr2:68252870-6825
ENSG0000(	1434	32.02487	chr2:309<	ENSG00000223859	lncRNA	chr2:67040546-6704
ENSG0000(	1434	32.02487	chr2:309<	AC017083.1	smallRNA	chr2:68229926-6823
ENSG0000(	1434	32.02487	chr2:309<	LINC01812	lncRNA	chr2:67796054-6782
ENSG0000(	1434	32.02487	chr2:309<	LINC01628	lncRNA	chr2:66921510-6692
ENSG0000(	1434	32.02487	chr2:309<	ENSG00000289156	lncRNA	chr2:68251603-6834
ENSG0000(	1434	32.02487	chr2:309<	DNAAF10	protein_c	chr2:68122936-6815
ENSG0000(	1434	32.02487	chr2:309<	LINC01888	lncRNA	chr2:68832014-6883
ENSG0000(	1434	32.02487	chr2:309<	ENSG00000235495	lncRNA	chr2:67565604-6768
ENSG0000(	1434	32.02487	chr2:309<	FBXL12P1	Pseudoger	chr2:68023694-6802
ENSG0000(	1434	32.02487	chr2:309<	LINC01829	lncRNA	chr2:67123357-6739

ENSG0000(	1434	32.02487	chr2:309⁄	ENSG00000214525		Pseudoger	chr2:68528241-6852
ENSG0000(	1424	31.80154	chr2:309⁄	ENSG00000235056		lncRNA	chr2:195003711-195
ENSG0000(	1424	31.80154	chr2:309⁄	SEC61GP1		Pseudoger	chr2:193730555-193
ENSG0000(	1424	31.80154	chr2:309⁄	ENSG00000272211		lncRNA	chr2:196151263-196
ENSG0000(	1424	31.80154	chr2:309⁄	ENSG00000282836		lncRNA	chr2:196638422-196
ENSG0000(	1424	31.80154	chr2:309⁄	LINC01827		lncRNA	chr2:195569628-195
ENSG0000(	1424	31.80154	chr2:309⁄	RPS17P8		Pseudoger	chr2:192774082-192
ENSG0000(	1424	31.80154	chr2:309⁄	ENSG00000224099		lncRNA	chr2:195448532-195
ENSG0000(	1424	31.80154	chr2:309⁄	LINC01825		lncRNA	chr2:195532945-195
ENSG0000(	1424	31.80154	chr2:309⁄	RNU6-169P		smallRNA	chr2:195514036-195
ENSG0000(	1424	31.80154	chr2:309⁄	ENSG00000271893		lncRNA	chr2:195451778-195
ENSG0000(	1424	31.80154	chr2:309⁄	ENSG00000225394		Pseudoger	chr2:194454581-194
ENSG0000(	1424	31.80154	chr2:309⁄	DNAH7	NCGv7	protein_c	chr2:195737703-196
ENSG0000(	1424	31.80154	chr2:309⁄	SLC44A3P1		Pseudoger	chr2:193256483-193
ENSG0000(	1424	31.80154	chr2:309⁄	ENSG00000286868		lncRNA	chr2:193867722-193
ENSG0000(	1424	31.80154	chr2:309⁄	SNORD59		smallRNA	chr2:194664107-194
ENSG0000(	1424	31.80154	chr2:309⁄	HNRNPA1P47		Pseudoger	chr2:194187379-194
ENSG0000(	1424	31.80154	chr2:309⁄	RN7SL820P		smallRNA	chr2:196289409-196
ENSG0000(	1424	31.80154	chr2:309⁄	C2orf66		protein_c	chr2:196804417-196
ENSG0000(	1424	31.80154	chr2:309⁄	HECW2-AS1		lncRNA	chr2:196260024-196
ENSG0000(	1424	31.80154	chr2:309⁄	GTF3C3		protein_c	chr2:196763035-196
ENSG0000(	1424	31.80154	chr2:309⁄	ENSG00000224670		Pseudoger	chr2:194027919-194
ENSG0000(	1424	31.80154	chr2:309⁄	CCDC150		protein_c	chr2:196639554-196
ENSG0000(	1424	31.80154	chr2:309⁄	LINC01790		lncRNA	chr2:194730595-194
ENSG0000(	1424	31.80154	chr2:309⁄	ENSG00000232227		lncRNA	chr2:192644102-192
ENSG0000(	1424	31.80154	chr2:309⁄	PCGEM1		lncRNA	chr2:192749845-192
ENSG0000(	1424	31.80154	chr2:309⁄	SCARNA16		smallRNA	chr2:196686148-196
ENSG0000(	1424	31.80154	chr2:309⁄	STK17B		protein_c	chr2:196133583-196
ENSG0000(	1424	31.80154	chr2:309⁄	GLULP6		Pseudoger	chr2:194129354-194
ENSG0000(	1424	31.80154	chr2:309⁄	ENSG00000286539		lncRNA	chr2:194587925-194
ENSG0000(	1424	31.80154	chr2:309⁄	HECW2	NCGv7	protein_c	chr2:196189099-196
ENSG0000(	1424	31.80154	chr2:309⁄	ENSG00000260142		lncRNA	chr2:193898367-193
ENSG0000(	1424	31.80154	chr2:309⁄	SLC39A10		protein_c	chr2:195575977-195
ENSG0000(	1424	31.80154	chr2:309⁄	LINC01821		lncRNA	chr2:194344190-194
ENSG0000(	1424	31.80154	chr2:309⁄	ENSG00000286358		lncRNA	chr2:193170704-193
ENSG0000(	1424	31.80154	chr2:309⁄	DNAJC17P1		Pseudoger	chr2:193276668-193
ENSG0000(	1424	31.80154	chr2:309⁄	ENSG00000227878		Pseudoger	chr2:196063312-196
ENSG0000(	1424	31.80154	chr2:309⁄	ENSG00000229395		lncRNA	chr2:192629919-192
ENSG0000(	1424	31.80154	chr2:309⁄	AC073973.1		smallRNA	chr2:194558174-194
ENSG0000(	1424	31.80154	chr2:309⁄	AHCYP5		Pseudoger	chr2:195582794-195
ENSG0000(	1424	31.80154	chr2:309⁄	snoU13		smallRNA	chr2:196215219-196
ENSG0000(	1424	31.80154	chr2:309⁄	RNU6-915P		smallRNA	chr2:195670372-195
ENSG0000(	1424	31.80154	chr2:309⁄	ENSG00000280176		TEC	chr2:196831975-196
ENSG0000(	1424	31.80154	chr2:309⁄	E2F3P2		Pseudoger	chr2:195994259-195
ENSG0000(	1421	31.73454	chr2:309⁄	ENSG00000288064		lncRNA	chr2:191045458-191
ENSG0000(	1421	31.73454	chr2:309⁄	DNAJB1P1		Pseudoger	chr2:191881182-191
ENSG0000(	1421	31.73454	chr2:309⁄	HMGB1P27		Pseudoger	chr2:191174233-191
ENSG0000(	1421	31.73454	chr2:309⁄	ENSG00000288582		lncRNA	chr2:191733428-191
ENSG0000(	1421	31.73454	chr2:309⁄	GLS		protein_c	chr2:190880821-190
ENSG0000(	1421	31.73454	chr2:309⁄	MYO1B	NCGv7	protein_c	chr2:191245185-191
ENSG0000(	1421	31.73454	chr2:309⁄	CAVIN2		protein_c	chr2:191834310-191

ENSG0000(	1421	31.73454	chr2:309<	STAT4-AS1		lncRNA	chr2:191021526-191
ENSG0000(	1421	31.73454	chr2:309<	MYO1B-AS1		lncRNA	chr2:191229165-191
ENSG0000(	1421	31.73454	chr2:309<	ENSG00000235852		lncRNA	chr2:190880797-190
ENSG0000(	1421	31.73454	chr2:309<	ENSG00000225884		lncRNA	chr2:191792797-191
ENSG0000(	1421	31.73454	chr2:309<	ENSG00000288900		lncRNA	chr2:191677213-191
ENSG0000(	1421	31.73454	chr2:309<	RNU6-959P		smallRNA	chr2:191121950-191
ENSG0000(	1421	31.73454	chr2:309<	ENSG00000280083		TEC	chr2:191154118-191
ENSG0000(	1421	31.73454	chr2:309<	STAT4	NCGv7	protein_c	chr2:191029576-191
ENSG0000(	1421	31.73454	chr2:309<	STAT1		protein_c	chr2:190908460-191
ENSG0000(	1421	31.73454	chr2:309<	ENSG00000230686		lncRNA	chr2:191017954-191
ENSG0000(	1421	31.73454	chr2:309<	RNU6-1045P		smallRNA	chr2:191370938-191
ENSG0000(	1421	31.73454	chr2:309<	RAB1AP1		Pseudoger	chr2:190992639-190
ENSG0000(	1421	31.73454	chr2:309<	NABP1	NCGv7	protein_c	chr2:191678068-191
ENSG0000(	1415	31.60055	chr2:309<	MARS2		protein_c	chr2:197705369-197
ENSG0000(	1415	31.60055	chr2:309<	ENSG00000287858		lncRNA	chr2:197640441-197
ENSG0000(	1415	31.60055	chr2:309<	HNRNPA3P15		Pseudoger	chr2:197014990-197
ENSG0000(	1415	31.60055	chr2:309<	ANKRD44-DT		lncRNA	chr2:197311393-197
ENSG0000(	1415	31.60055	chr2:309<	SF3B1	NCGv7;AC	protein_c	chr2:197388515-197
ENSG0000(	1415	31.60055	chr2:309<	ENSG00000231699		Pseudoger	chr2:197533803-197
ENSG0000(	1415	31.60055	chr2:309<	RFTN2		protein_c	chr2:197568224-197
ENSG0000(	1415	31.60055	chr2:309<	ATP5MC2P3		Pseudoger	chr2:197263111-197
ENSG0000(	1415	31.60055	chr2:309<	MOB4		protein_c	chr2:197515571-197
ENSG0000(	1415	31.60055	chr2:309<	HSPE1		protein_c	chr2:197500140-197
ENSG0000(	1415	31.60055	chr2:309<	PGAP1		protein_c	chr2:196833004-196
ENSG0000(	1415	31.60055	chr2:309<	NPM1P46		Pseudoger	chr2:197379701-197
ENSG0000(	1415	31.60055	chr2:309<	RPL4P7		Pseudoger	chr2:197028711-197
ENSG0000(	1415	31.60055	chr2:309<	SNORA4		smallRNA	chr2:197404718-197
ENSG0000(	1415	31.60055	chr2:309<	ENSG00000222017		lncRNA	chr2:197693106-197
ENSG0000(	1415	31.60055	chr2:309<	ANKRD44-IT1		lncRNA	chr2:197250858-197
ENSG0000(	1415	31.60055	chr2:309<	HSPD1		protein_c	chr2:197486584-197
ENSG0000(	1415	31.60055	chr2:309<	HSPE1-MOB4		protein_c	chr2:197500413-197
ENSG0000(	1415	31.60055	chr2:309<	ANKRD44-AS1		lncRNA	chr2:197197927-197
ENSG0000(	1415	31.60055	chr2:309<	ANKRD44		protein_c	chr2:196967017-197
ENSG0000(	1415	31.60055	chr2:309<	RNU6-1029P		smallRNA	chr2:197447745-197
ENSG0000(	1415	31.60055	chr2:309<	COQ10B	NCGv7	protein_c	chr2:197453493-197
ENSG0000(	1408	31.44422	chr2:309<	GULP1		protein_c	chr2:188291669-188
ENSG0000(	1408	31.44422	chr2:309<	ASNSD1		protein_c	chr2:189661385-189
ENSG0000(	1408	31.44422	chr2:309<	KDM3AP1		Pseudoger	chr2:189486480-189
ENSG0000(	1408	31.44422	chr2:309<	OSGEPL1-AS1		lncRNA	chr2:189762704-189
ENSG0000(	1408	31.44422	chr2:309<	MSTN		protein_c	chr2:190055700-190
ENSG0000(	1408	31.44422	chr2:309<	AC008122.1		smallRNA	chr2:189884764-189
ENSG0000(	1408	31.44422	chr2:309<	PMS1	NCGv7;AC	protein_c	chr2:189784085-189
ENSG0000(	1408	31.44422	chr2:309<	WDR75	NCGv7	protein_c	chr2:189441446-189
ENSG0000(	1408	31.44422	chr2:309<	ASDURF		protein_c	chr2:189661452-189
ENSG0000(	1408	31.44422	chr2:309<	AC118063.1		smallRNA	chr2:189311671-189
ENSG0000(	1408	31.44422	chr2:309<	Y_RNA		smallRNA	chr2:189753464-189
ENSG0000(	1408	31.44422	chr2:309<	HIBCH		protein_c	chr2:190189735-190
ENSG0000(	1408	31.44422	chr2:309<	ENSG00000273240		lncRNA	chr2:189763859-189
ENSG0000(	1408	31.44422	chr2:309<	HNRNPCP2		Pseudoger	chr2:189923336-189
ENSG0000(	1408	31.44422	chr2:309<	MIR3129		smallRNA	chr2:189133036-189
ENSG0000(	1408	31.44422	chr2:309<	MIR1245A		smallRNA	chr2:188978092-188

ENSG0000	1408	31.44422	chr2:309	ENSG00000276828		Pseudoger	chr2:189625443-189
ENSG0000	1408	31.44422	chr2:309	RNF11P1		Pseudoger	chr2:189930277-189
ENSG0000	1408	31.44422	chr2:309	LINC01090		lncRNA	chr2:187712816-188
ENSG0000	1408	31.44422	chr2:309	RN7SKP179		smallRNA	chr2:190553264-190
ENSG0000	1408	31.44422	chr2:309	C2orf88		protein_c	chr2:189879609-190
ENSG0000	1408	31.44422	chr2:309	NAB1		protein_c	chr2:190646746-190
ENSG0000	1408	31.44422	chr2:309	COL5A2		protein_c	chr2:189031898-189
ENSG0000	1408	31.44422	chr2:309	INPP1		protein_c	chr2:190343570-190
ENSG0000	1408	31.44422	chr2:309	ENSG00000223523		lncRNA	chr2:188598791-188
ENSG0000	1408	31.44422	chr2:309	ENSG00000287418		lncRNA	chr2:189537384-189
ENSG0000	1408	31.44422	chr2:309	ENSG00000288866		lncRNA	chr2:189439780-189
ENSG0000	1408	31.44422	chr2:309	ENSG00000228073		lncRNA	chr2:189095063-189
ENSG0000	1408	31.44422	chr2:309	TERF1P6		Pseudoger	chr2:189917254-189
ENSG0000	1408	31.44422	chr2:309	DIRC1		lncRNA	chr2:188734155-188
ENSG0000	1408	31.44422	chr2:309	ENSG00000228509		lncRNA	chr2:190672297-190
ENSG0000	1408	31.44422	chr2:309	SLC40A1		protein_c	chr2:189560590-189
ENSG0000	1408	31.44422	chr2:309	NEMP2-DT		lncRNA	chr2:190534855-190
ENSG0000	1408	31.44422	chr2:309	ANKAR		protein_c	chr2:189674290-189
ENSG0000	1408	31.44422	chr2:309	COL3A1	NCGv7	protein_c	chr2:188974373-189
ENSG0000	1408	31.44422	chr2:309	MFSD6		protein_c	chr2:190408355-190
ENSG0000	1408	31.44422	chr2:309	ORMDL1		protein_c	chr2:189770267-189
ENSG0000	1408	31.44422	chr2:309	OSGEPL1		protein_c	chr2:189746660-189
ENSG0000	1408	31.44422	chr2:309	ENSG00000290089		lncRNA	chr2:190457656-190
ENSG0000	1408	31.44422	chr2:309	ENSG00000272979		lncRNA	chr2:190454092-190
ENSG0000	1408	31.44422	chr2:309	NEMP2		protein_c	chr2:190504338-190
ENSG0000	1408	31.44422	chr2:309	ENSG00000284052		lncRNA	chr2:190607660-190
ENSG0000	1408	31.44422	chr2:309	KRT18P19		Pseudoger	chr2:189311263-189
ENSG0000	1408	31.44422	chr2:309	ENSG00000286165		protein_c	chr2:189661519-189
ENSG0000	1401	31.28789	chr2:309	SLC8A1		protein_c	chr2:40097270-4061
ENSG0000	1401	31.28789	chr2:309	MORN2		protein_c	chr2:38875976-3892
ENSG0000	1401	31.28789	chr2:309	GEMIN6		protein_c	chr2:38751534-3878
ENSG0000	1401	31.28789	chr2:309	TMEM178A		protein_c	chr2:39664982-3971
ENSG0000	1401	31.28789	chr2:309	ENSG00000273035		lncRNA	chr2:39323328-3932
ENSG0000	1401	31.28789	chr2:309	ENSG00000288992		lncRNA	chr2:40450663-4045
ENSG0000	1401	31.28789	chr2:309	AC010739.1		smallRNA	chr2:41596386-4159
ENSG0000	1401	31.28789	chr2:309	ENSG00000269210		lncRNA	chr2:38959287-3896
ENSG0000	1401	31.28789	chr2:309	RNU6-851P		smallRNA	chr2:38884560-3888
ENSG0000	1401	31.28789	chr2:309	HNRNPA1P57		Pseudoger	chr2:41143780-4115
ENSG0000	1401	31.28789	chr2:309	ENSG00000285898		lncRNA	chr2:40591285-4067
ENSG0000	1401	31.28789	chr2:309	ARHGEF33		protein_c	chr2:38889875-3897
ENSG0000	1401	31.28789	chr2:309	ENSG00000225284		lncRNA	chr2:38861720-3886
ENSG0000	1401	31.28789	chr2:309	RNU6-198P		smallRNA	chr2:39082589-3908
ENSG0000	1401	31.28789	chr2:309	ENSG00000289003		lncRNA	chr2:39480783-3948
ENSG0000	1401	31.28789	chr2:309	MAP4K3-DT		lncRNA	chr2:39436530-3966
ENSG0000	1401	31.28789	chr2:309	ENSG00000289013		lncRNA	chr2:40394673-4039
ENSG0000	1401	31.28789	chr2:309	MAP4K3	NCGv7	protein_c	chr2:39249266-3943
ENSG0000	1401	31.28789	chr2:309	DHX57		protein_c	chr2:38797729-3887
ENSG0000	1401	31.28789	chr2:309	CDKL4		protein_c	chr2:39168045-3924
ENSG0000	1401	31.28789	chr2:309	TTC39DP		Pseudoger	chr2:38763534-3876
ENSG0000	1401	31.28789	chr2:309	RNU6-1185P		smallRNA	chr2:39393522-3939
ENSG0000	1401	31.28789	chr2:309	RN7SL96P		smallRNA	chr2:38936880-3893

ENSG0000(	1401	31.28789	chr2:309⁄	THUMPD2		protein_c	chr2:39736060-3977
ENSG0000(	1401	31.28789	chr2:309⁄	ASS1P2		Pseudoger	chr2:38810432-3881
ENSG0000(	1401	31.28789	chr2:309⁄	ENSG00000232518		lncRNA	chr2:38668202-3867
ENSG0000(	1401	31.28789	chr2:309⁄	NPLP1		Pseudoger	chr2:38769265-3877
ENSG0000(	1401	31.28789	chr2:309⁄	Y_RNA		smallRNA	chr2:39128826-3912
ENSG0000(	1401	31.28789	chr2:309⁄	LINC01794		lncRNA	chr2:40746481-4076
ENSG0000(	1401	31.28789	chr2:309⁄	SLC8A1-AS1		lncRNA	chr2:39786453-4025
ENSG0000(	1401	31.28789	chr2:309⁄	ENSG00000287255		lncRNA	chr2:40511921-4054
ENSG0000(	1401	31.28789	chr2:309⁄	HSPE1P13		Pseudoger	chr2:39098149-3909
ENSG0000(	1401	31.28789	chr2:309⁄	SRSF7		protein_c	chr2:38743599-3875
ENSG0000(	1401	31.28789	chr2:309⁄	ENSG00000235653		Pseudoger	chr2:39929110-3992
ENSG0000(	1401	31.28789	chr2:309⁄	SOS1	NCGv7	protein_c	chr2:38962206-3912
ENSG0000(	1401	31.28789	chr2:309⁄	SNORA67		smallRNA	chr2:39283657-3928
ENSG0000(	1401	31.28789	chr2:309⁄	ENSG00000287468		lncRNA	chr2:40620667-4063
ENSG0000(	1401	31.28789	chr2:309⁄	SOS1-IT1		lncRNA	chr2:38992279-3899
ENSG0000(	1401	31.28789	chr2:309⁄	GALM		protein_c	chr2:38666081-3874
ENSG0000(	1399	31.24323	chr2:309⁄	LRPPRC		protein_c	chr2:43886224-4399
ENSG0000(	1399	31.24323	chr2:309⁄	LINC01833		lncRNA	chr2:44921077-4493
ENSG0000(	1399	31.24323	chr2:309⁄	ENSG00000289272		lncRNA	chr2:44228188-4422
ENSG0000(	1399	31.24323	chr2:309⁄	ABCG5		protein_c	chr2:43812472-4383
ENSG0000(	1399	31.24323	chr2:309⁄	ENSG00000225156		lncRNA	chr2:44954664-4496
ENSG0000(	1399	31.24323	chr2:309⁄	SIX3		protein_c	chr2:44941702-4494
ENSG0000(	1399	31.24323	chr2:309⁄	AC067957.1		smallRNA	chr2:44782046-4478
ENSG0000(	1399	31.24323	chr2:309⁄	SLC3A1		protein_c	chr2:44275458-4432
ENSG0000(	1399	31.24323	chr2:309⁄	PREPL		protein_c	chr2:44316281-4436
ENSG0000(	1399	31.24323	chr2:309⁄	SIX2	NCGv7	protein_c	chr2:45005182-4500
ENSG0000(	1399	31.24323	chr2:309⁄	DYNC2LI1		protein_c	chr2:43774039-4381
ENSG0000(	1399	31.24323	chr2:309⁄	PPM1B		protein_c	chr2:44167969-4424
ENSG0000(	1399	31.24323	chr2:309⁄	ENSG00000285542		protein_c	chr2:44168851-4432
ENSG0000(	1399	31.24323	chr2:309⁄	PLCL1		protein_c	chr2:197804593-198
ENSG0000(	1399	31.24323	chr2:309⁄	KRTCAP2P1		Pseudoger	chr2:44996413-4499
ENSG0000(	1399	31.24323	chr2:309⁄	snoU13		smallRNA	chr2:44239568-4423
ENSG0000(	1399	31.24323	chr2:309⁄	RNU6-1048P		smallRNA	chr2:43892690-4389
ENSG0000(	1399	31.24323	chr2:309⁄	SIX3-AS1		lncRNA	chr2:44940153-4494
ENSG0000(	1399	31.24323	chr2:309⁄	PLEKHH2		protein_c	chr2:43637260-4376
ENSG0000(	1399	31.24323	chr2:309⁄	RN7SKP66		smallRNA	chr2:43772120-4377
ENSG0000(	1399	31.24323	chr2:309⁄	ENSG00000231156		lncRNA	chr2:45013214-4501
ENSG0000(	1399	31.24323	chr2:309⁄	AC019330.2		smallRNA	chr2:198566645-198
ENSG0000(	1399	31.24323	chr2:309⁄	C1GALT1C1L		protein_c	chr2:43675151-4367
ENSG0000(	1399	31.24323	chr2:309⁄	ENSG00000286728		lncRNA	chr2:45169616-4521
ENSG0000(	1399	31.24323	chr2:309⁄	RPL12P19		Pseudoger	chr2:44270621-4427
ENSG0000(	1399	31.24323	chr2:309⁄	ENSG00000278957		TEC	chr2:44927914-4492
ENSG0000(	1399	31.24323	chr2:309⁄	ENSG00000257045		lncRNA	chr2:199325310-199
ENSG0000(	1399	31.24323	chr2:309⁄	ENSG00000237035		lncRNA	chr2:198187802-198
ENSG0000(	1399	31.24323	chr2:309⁄	ENSG00000219391		Pseudoger	chr2:44065894-4406
ENSG0000(	1399	31.24323	chr2:309⁄	RNU6-566P		smallRNA	chr2:44154789-4415
ENSG0000(	1399	31.24323	chr2:309⁄	ENSG00000229695		Pseudoger	chr2:43680465-4368
ENSG0000(	1399	31.24323	chr2:309⁄	ENSG00000230407		Pseudoger	chr2:199108584-199
ENSG0000(	1399	31.24323	chr2:309⁄	BOLL		protein_c	chr2:197726879-197
ENSG0000(	1399	31.24323	chr2:309⁄	ENSG00000225421		lncRNA	chr2:198493242-198
ENSG0000(	1399	31.24323	chr2:309⁄	ENSG00000231054		lncRNA	chr2:45168583-4516

ENSG0000(	1399	31.24323	chr2:309<	ENSG00000231557		lncRNA	chr2:198882573-199
ENSG0000(	1399	31.24323	chr2:309<	ENSG00000286519		lncRNA	chr2:45173722-4517
ENSG0000(	1399	31.24323	chr2:309<	CAMKMT		protein_c	chr2:44361947-4477
ENSG0000(	1399	31.24323	chr2:309<	ABCG8	NCGv7	protein_c	chr2:43831942-4388
ENSG0000(	1399	31.24323	chr2:309<	RNU7-147P		smallRNA	chr2:199191059-199
ENSG0000(	1399	31.24323	chr2:309<	LINC01121		lncRNA	chr2:45164816-4532
ENSG0000(	1399	31.24323	chr2:309<	ENSG00000288707		lncRNA	chr2:43995985-4399
ENSG0000(	1399	31.24323	chr2:309<	ENSG00000286020		lncRNA	chr2:197817423-197
ENSG0000(	1399	31.24323	chr2:309<	PPM1B-DT		lncRNA	chr2:44167625-4416
ENSG0000(	1399	31.24323	chr2:309<	PDSS1P2		Pseudoger	chr2:44166266-4416
ENSG0000(	1399	31.24323	chr2:309<	LINC01923		lncRNA	chr2:198299276-198
ENSG0000(	1395	31.1539	chr2:309<	CAVIN2-AS1		lncRNA	chr2:191846534-192
ENSG0000(	1395	31.1539	chr2:309<	TMEFF2		protein_c	chr2:191949043-192
ENSG0000(	1387	30.97524	chr2:309<	SCARNA16		smallRNA	chr2:53470447-5347
ENSG0000(	1386	30.95291	chr2:309<	LINC01119		lncRNA	chr2:46816697-4685
ENSG0000(	1386	30.95291	chr2:309<	ENSG00000224058		Pseudoger	chr2:47731402-4773
ENSG0000(	1386	30.95291	chr2:309<	MIR559		smallRNA	chr2:47377675-4737
ENSG0000(	1386	30.95291	chr2:309<	ENSG00000230979		Pseudoger	chr2:47690716-4769
ENSG0000(	1386	30.95291	chr2:309<	PIGF		protein_c	chr2:46580937-4661
ENSG0000(	1386	30.95291	chr2:309<	CALM2		protein_c	chr2:47160084-4717
ENSG0000(	1386	30.95291	chr2:309<	ENSG00000226548		lncRNA	chr2:46852020-4685
ENSG0000(	1386	30.95291	chr2:309<	MCFD2		protein_c	chr2:46901870-4694
ENSG0000(	1386	30.95291	chr2:309<	LINC02583		lncRNA	chr2:46429190-4644
ENSG0000(	1386	30.95291	chr2:309<	ENSG00000233230		lncRNA	chr2:47905678-4790
ENSG0000(	1386	30.95291	chr2:309<	MSH2-OT1		lncRNA	chr2:47527008-4753
ENSG0000(	1386	30.95291	chr2:309<	ENSG00000279254		TEC	chr2:46668870-4667
ENSG0000(	1386	30.95291	chr2:309<	ENSG00000232696		lncRNA	chr2:46078015-4607
ENSG0000(	1386	30.95291	chr2:309<	LINC01820		lncRNA	chr2:46392291-4639
ENSG0000(	1386	30.95291	chr2:309<	ENSG00000226087		lncRNA	chr2:47225781-4724
ENSG0000(	1386	30.95291	chr2:309<	RPL36AP14		Pseudoger	chr2:46256860-4625
ENSG0000(	1386	30.95291	chr2:309<	RHOQ-AS1		lncRNA	chr2:46568256-4658
ENSG0000(	1386	30.95291	chr2:309<	KCNK12		protein_c	chr2:47509290-4757
ENSG0000(	1386	30.95291	chr2:309<	EPAS1	NCGv7	protein_c	chr2:46293667-4638
ENSG0000(	1386	30.95291	chr2:309<	MSH6	NCGv7;AC	protein_c	chr2:47695530-4781
ENSG0000(	1386	30.95291	chr2:309<	MSH2	NCGv7;AC	protein_c	chr2:47403067-4766
ENSG0000(	1386	30.95291	chr2:309<	VN1R18P		Pseudoger	chr2:47989625-4799
ENSG0000(	1386	30.95291	chr2:309<	NME2P2		Pseudoger	chr2:47705468-4770
ENSG0000(	1386	30.95291	chr2:309<	EPCAM-DT		lncRNA	chr2:47192405-4734
ENSG0000(	1386	30.95291	chr2:309<	RPL26P15		Pseudoger	chr2:46003942-4600
ENSG0000(	1386	30.95291	chr2:309<	ENSG00000225187		lncRNA	chr2:47067822-4707
ENSG0000(	1386	30.95291	chr2:309<	PPIAP62		Pseudoger	chr2:47939738-4794
ENSG0000(	1386	30.95291	chr2:309<	RN7SKP119		smallRNA	chr2:47359505-4735
ENSG0000(	1386	30.95291	chr2:309<	BCYRN1		smallRNA	chr2:47335315-4733
ENSG0000(	1386	30.95291	chr2:309<	RPS27AP7		Pseudoger	chr2:47883455-4788
ENSG0000(	1386	30.95291	chr2:309<	ENSG00000253515		lncRNA	chr2:46429229-4650
ENSG0000(	1386	30.95291	chr2:309<	PRKCE	AC	protein_c	chr2:45651345-4618
ENSG0000(	1386	30.95291	chr2:309<	PRKCE-AS1		lncRNA	chr2:45674701-4567
ENSG0000(	1386	30.95291	chr2:309<	SOCS5		protein_c	chr2:46698952-4678
ENSG0000(	1386	30.95291	chr2:309<	LINC01118		lncRNA	chr2:46698940-4682
ENSG0000(	1386	30.95291	chr2:309<	ENSG00000273269		protein_c	chr2:47065941-4717
ENSG0000(	1386	30.95291	chr2:309<	FBXO11	NCGv7;AC	protein_c	chr2:47789316-4790

ENSG0000(	1386	30.95291	chr2:309<	ENSG00000284608		lncRNA	chr2:46429195-4648
ENSG0000(	1386	30.95291	chr2:309<	TMEM247		protein_c	chr2:46479565-4648
ENSG0000(	1386	30.95291	chr2:309<	ENSG00000233845		lncRNA	chr2:47035279-4704
ENSG0000(	1386	30.95291	chr2:309<	RN7SL414P		smallRNA	chr2:45569201-4556
ENSG0000(	1386	30.95291	chr2:309<	ENSG00000228925		lncRNA	chr2:46899275-4690
ENSG0000(	1386	30.95291	chr2:309<	RHOQ		protein_c	chr2:46541806-4658
ENSG0000(	1386	30.95291	chr2:309<	SRBD1		protein_c	chr2:45388680-4561
ENSG0000(	1386	30.95291	chr2:309<	ENSG00000272814		lncRNA	chr2:46956615-4695
ENSG0000(	1386	30.95291	chr2:309<	RNU6-688P		smallRNA	chr2:47781379-4778
ENSG0000(	1386	30.95291	chr2:309<	RN7SL817P		smallRNA	chr2:46448226-4644
ENSG0000(	1386	30.95291	chr2:309<	STPG4		protein_c	chr2:47045538-4715
ENSG0000(	1386	30.95291	chr2:309<	TTC7A		protein_c	chr2:46915869-4707
ENSG0000(	1386	30.95291	chr2:309<	EPCAM	AC	protein_c	chr2:47345158-4738
ENSG0000(	1386	30.95291	chr2:309<	ENSG00000231336		lncRNA	chr2:46166789-4616
ENSG0000(	1386	30.95291	chr2:309<	CRIPT		protein_c	chr2:46616416-4663
ENSG0000(	1386	30.95291	chr2:309<	RPL36AP15		Pseudoger	chr2:47797826-4779
ENSG0000(	1386	30.95291	chr2:309<	ATP6V1E2	DriverDB\	protein_c	chr2:46490750-4654
ENSG0000(	1383	30.88591	chr2:309<	ENSG00000274769		lncRNA	chr2:61115787-6116
ENSG0000(	1383	30.88591	chr2:309<	RNU1-32P		smallRNA	chr2:60384605-6038
ENSG0000(	1383	30.88591	chr2:309<	ENSG00000271889		lncRNA	chr2:61151433-6116
ENSG0000(	1383	30.88591	chr2:309<	REL-DT		lncRNA	chr2:60823069-6088
ENSG0000(	1383	30.88591	chr2:309<	ENSG00000271955		lncRNA	chr2:59218680-6010
ENSG0000(	1383	30.88591	chr2:309<	RNU6-508P		smallRNA	chr2:59647621-5964
ENSG0000(	1383	30.88591	chr2:309<	IFITM3P9		Pseudoger	chr2:60682873-6068
ENSG0000(	1383	30.88591	chr2:309<	LINC01793		lncRNA	chr2:59217708-5927
ENSG0000(	1383	30.88591	chr2:309<	RNA5SP95		Pseudoger	chr2:60998752-6099
ENSG0000(	1383	30.88591	chr2:309<	AC007131.3		smallRNA	chr2:59241621-5924
ENSG0000(	1383	30.88591	chr2:309<	ENSG00000233953		lncRNA	chr2:60495686-6049
ENSG0000(	1383	30.88591	chr2:309<	RPL26P13		Pseudoger	chr2:60711484-6071
ENSG0000(	1383	30.88591	chr2:309<	PAPOLG		protein_c	chr2:60756253-6080
ENSG0000(	1383	30.88591	chr2:309<	ENSG00000287640		lncRNA	chr2:60383141-6038
ENSG0000(	1383	30.88591	chr2:309<	NONOP2		Pseudoger	chr2:60936819-6093
ENSG0000(	1383	30.88591	chr2:309<	ENSG00000286604		lncRNA	chr2:59778685-5979
ENSG0000(	1383	30.88591	chr2:309<	AHSA2P		Pseudoger	chr2:61177418-6119
ENSG0000(	1383	30.88591	chr2:309<	ENSG00000285611		lncRNA	chr2:60057601-6007
ENSG0000(	1383	30.88591	chr2:309<	ENSG00000270447		Pseudoger	chr2:59514890-5951
ENSG0000(	1383	30.88591	chr2:309<	ENSG00000285673		lncRNA	chr2:59014354-5927
ENSG0000(	1383	30.88591	chr2:309<	RPS12P3		Pseudoger	chr2:60938204-6093
ENSG0000(	1383	30.88591	chr2:309<	SANBR		protein_c	chr2:61065871-6113
ENSG0000(	1383	30.88591	chr2:309<	RN7SL632P		smallRNA	chr2:60831665-6083
ENSG0000(	1383	30.88591	chr2:309<	RN7SL361P		smallRNA	chr2:60640705-6064
ENSG0000(	1383	30.88591	chr2:309<	REL	NCGv7;AC	protein_c	chr2:60881491-6093
ENSG0000(	1383	30.88591	chr2:309<	ENSG00000231815		lncRNA	chr2:59434552-5944
ENSG0000(	1383	30.88591	chr2:309<	RPL21P33		Pseudoger	chr2:60852260-6085
ENSG0000(	1383	30.88591	chr2:309<	PUS10		protein_c	chr2:60940222-6101
ENSG0000(	1383	30.88591	chr2:309<	MIR4432HG		lncRNA	chr2:60336446-6043
ENSG0000(	1383	30.88591	chr2:309<	RNU4-51P		smallRNA	chr2:60911303-6091
ENSG0000(	1383	30.88591	chr2:309<	PEX13		protein_c	chr2:61017225-6105
ENSG0000(	1383	30.88591	chr2:309<	AC007179.2		smallRNA	chr2:59532984-5953
ENSG0000(	1383	30.88591	chr2:309<	ENSG00000233891		lncRNA	chr2:59238703-5973
ENSG0000(	1383	30.88591	chr2:309<	MIR4432		smallRNA	chr2:60387362-6038

ENSG0000(	1383	30.88591	chr2:309⁄	RNA5SP94		Pseudoger	chr2:59694762-5969
ENSG0000(	1383	30.88591	chr2:309⁄	C2orf74-DT		lncRNA	chr2:61141592-6114
ENSG0000(	1383	30.88591	chr2:309⁄	BCL11A	NCGv7;AC	protein_c	chr2:60450520-6055
ENSG0000(	1383	30.88591	chr2:309⁄	ATP1B3P1		Pseudoger	chr2:60734895-6073
ENSG0000(	1383	30.88591	chr2:309⁄	RNU6-612P		smallRNA	chr2:60719640-6071
ENSG0000(	1383	30.88591	chr2:309⁄	C2orf74		protein_c	chr2:61145068-6116
ENSG0000(	1383	30.88591	chr2:309⁄	ENSG00000267520		lncRNA	chr2:60925909-6093
ENSG0000(	1380	30.81891	chr2:309⁄	LINC02580		lncRNA	chr2:43092530-4321
ENSG0000(	1380	30.81891	chr2:309⁄	LINC01126		lncRNA	chr2:43227210-4322
ENSG0000(	1380	30.81891	chr2:309⁄	TPT1P11		Pseudoger	chr2:48632856-4863
ENSG0000(	1380	30.81891	chr2:309⁄	STON1-GTF2A1L		protein_c	chr2:48529925-4877
ENSG0000(	1380	30.81891	chr2:309⁄	ENSG00000234936		lncRNA	chr2:43229573-4323
ENSG0000(	1380	30.81891	chr2:309⁄	PPP1R21		protein_c	chr2:48440598-4851
ENSG0000(	1380	30.81891	chr2:309⁄	RNU6-958P		smallRNA	chr2:43408307-4340
ENSG0000(	1380	30.81891	chr2:309⁄	Y_RNA		smallRNA	chr2:43620878-4362
ENSG0000(	1380	30.81891	chr2:309⁄	PPP1R21-DT		lncRNA	chr2:48440043-4844
ENSG0000(	1380	30.81891	chr2:309⁄	RNU4-49P		smallRNA	chr2:48340687-4834
ENSG0000(	1380	30.81891	chr2:309⁄	THADA		protein_c	chr2:43230851-4359
ENSG0000(	1380	30.81891	chr2:309⁄	GTF2A1L		protein_c	chr2:48617798-4873
ENSG0000(	1380	30.81891	chr2:309⁄	LINC01819		lncRNA	chr2:42972255-4304
ENSG0000(	1380	30.81891	chr2:309⁄	RN7SKP224		smallRNA	chr2:48217575-4821
ENSG0000(	1380	30.81891	chr2:309⁄	LHCGR		protein_c	chr2:48686774-4875
ENSG0000(	1380	30.81891	chr2:309⁄	ENSG00000279956		protein_c	chr2:48632291-4875
ENSG0000(	1380	30.81891	chr2:309⁄	ENSG00000233978		lncRNA	chr2:43041193-4304
ENSG0000(	1380	30.81891	chr2:309⁄	ELOBP3		Pseudoger	chr2:48780602-4878
ENSG0000(	1380	30.81891	chr2:309⁄	RNU6-242P		smallRNA	chr2:43091388-4309
ENSG0000(	1380	30.81891	chr2:309⁄	STON1		protein_c	chr2:48529383-4859
ENSG0000(	1380	30.81891	chr2:309⁄	ENSG00000287387		lncRNA	chr2:43219849-4322
ENSG0000(	1380	30.81891	chr2:309⁄	ZFP36L2	NCGv7	protein_c	chr2:43222402-4322
ENSG0000(	1380	30.81891	chr2:309⁄	FOXN2		protein_c	chr2:48314637-4837
ENSG0000(	1380	30.81891	chr2:309⁄	ENSG00000286796		lncRNA	chr2:43128819-4313
ENSG0000(	1373	30.66258	chr2:309⁄	GGCTP3		Pseudoger	chr2:52474073-5247
ENSG0000(	1373	30.66258	chr2:309⁄	RNU6-439P		smallRNA	chr2:49233794-4923
ENSG0000(	1373	30.66258	chr2:309⁄	ASB3		protein_c	chr2:53532672-5386
ENSG0000(	1373	30.66258	chr2:309⁄	LINC01867		lncRNA	chr2:52370602-5239
ENSG0000(	1373	30.66258	chr2:309⁄	ENSG00000272156		lncRNA	chr2:54082554-5408
ENSG0000(	1373	30.66258	chr2:309⁄	CHAC2		protein_c	chr2:53767804-5377
ENSG0000(	1373	30.66258	chr2:309⁄	ENSG00000231918		lncRNA	chr2:51032601-5240
ENSG0000(	1373	30.66258	chr2:309⁄	HMGB1P31		Pseudoger	chr2:54051334-5405
ENSG0000(	1373	30.66258	chr2:309⁄	FTH1P6		Pseudoger	chr2:52629743-5263
ENSG0000(	1373	30.66258	chr2:309⁄	ENSG00000287344		lncRNA	chr2:51977787-5202
ENSG0000(	1373	30.66258	chr2:309⁄	ENSG00000223897		Pseudoger	chr2:53486144-5348
ENSG0000(	1373	30.66258	chr2:309⁄	ENSG00000286412		lncRNA	chr2:51011777-5101
ENSG0000(	1373	30.66258	chr2:309⁄	ENSG00000282890		lncRNA	chr2:48809340-4941
ENSG0000(	1373	30.66258	chr2:309⁄	ENSG00000289065		lncRNA	chr2:54115268-5411
ENSG0000(	1373	30.66258	chr2:309⁄	NRXN1		protein_c	chr2:49918503-5122
ENSG0000(	1373	30.66258	chr2:309⁄	MTCO1P42		Pseudoger	chr2:50588690-5058
ENSG0000(	1373	30.66258	chr2:309⁄	CRTC1P1		Pseudoger	chr2:52570648-5257
ENSG0000(	1373	30.66258	chr2:309⁄	ENSG00000236837		lncRNA	chr2:52494688-5250
ENSG0000(	1373	30.66258	chr2:309⁄	ENSG00000238165		Pseudoger	chr2:50829442-5082
ENSG0000(	1373	30.66258	chr2:309⁄	AC007560.2		smallRNA	chr2:50798964-5079

ENSG0000(	1373	30.66258	chr2:309⁄	ENSG00000228033		lncRNA	chr2:52722671-5296
ENSG0000(	1373	30.66258	chr2:309⁄	ERLEC1		protein_c	chr2:53787009-5383
ENSG0000(	1373	30.66258	chr2:309⁄	ENSG00000282998		lncRNA	chr2:49202126-4945
ENSG0000(	1373	30.66258	chr2:309⁄	KNOP1P3		Pseudoger	chr2:51511058-5151
ENSG0000(	1373	30.66258	chr2:309⁄	ENSG00000232604		lncRNA	chr2:52864235-5286
ENSG0000(	1373	30.66258	chr2:309⁄	RPL21P30		Pseudoger	chr2:54029552-5403
ENSG0000(	1373	30.66258	chr2:309⁄	CTBP2P5		Pseudoger	chr2:48915267-4891
ENSG0000(	1373	30.66258	chr2:309⁄	ZNF863P		Pseudoger	chr2:52071355-5207
ENSG0000(	1373	30.66258	chr2:309⁄	ENSG00000282828		lncRNA	chr2:49563388-4959
ENSG0000(	1373	30.66258	chr2:309⁄	CRYGGP		Pseudoger	chr2:51775258-5177
ENSG0000(	1373	30.66258	chr2:309⁄	FSHR	NCGv7	protein_c	chr2:48962157-4915
ENSG0000(	1373	30.66258	chr2:309⁄	ENSG00000232668		Pseudoger	chr2:52883243-5288
ENSG0000(	1373	30.66258	chr2:309⁄	AC007682.3		smallRNA	chr2:50961245-5096
ENSG0000(	1373	30.66258	chr2:309⁄	SNORA75		smallRNA	chr2:49888868-4988
ENSG0000(	1373	30.66258	chr2:309⁄	GPR75		protein_c	chr2:53852912-5385
ENSG0000(	1373	30.66258	chr2:309⁄	CCDC12P1		Pseudoger	chr2:51926882-5192
ENSG0000(	1373	30.66258	chr2:309⁄	PSME4		protein_c	chr2:53864069-5397
ENSG0000(	1373	30.66258	chr2:309⁄	MIR3682		smallRNA	chr2:53849122-5384
ENSG0000(	1373	30.66258	chr2:309⁄	RNU6-997P		smallRNA	chr2:53570374-5357
ENSG0000(	1373	30.66258	chr2:309⁄	MIR4431		smallRNA	chr2:52702522-5270
ENSG0000(	1373	30.66258	chr2:309⁄	ENSG00000285548		lncRNA	chr2:50324643-5034
ENSG0000(	1373	30.66258	chr2:309⁄	ENSG00000230840		Pseudoger	chr2:51925692-5192
ENSG0000(	1373	30.66258	chr2:309⁄	snoU13		smallRNA	chr2:53839725-5383
ENSG0000(	1373	30.66258	chr2:309⁄	ENSG00000287867		lncRNA	chr2:51441959-5145
ENSG0000(	1373	30.66258	chr2:309⁄	AC007402.1		smallRNA	chr2:51435122-5143
ENSG0000(	1373	30.66258	chr2:309⁄	AC009234.2		smallRNA	chr2:50696172-5069
ENSG0000(	1373	30.66258	chr2:309⁄	ENSG00000241114		Pseudoger	chr2:54079974-5408
ENSG0000(	1373	30.66258	chr2:309⁄	ENSG00000283058		lncRNA	chr2:50620963-5063
ENSG0000(	1373	30.66258	chr2:309⁄	RPL7P13		Pseudoger	chr2:49878623-4987
ENSG0000(	1373	30.66258	chr2:309⁄	Y_RNA		smallRNA	chr2:52297995-5229
ENSG0000(	1370	30.59558	chr2:309⁄	ENSG00000230773		lncRNA	chr2:47924181-4831
ENSG0000(	1367	30.52859	chr2:309⁄	AC007389.4		smallRNA	chr2:65667256-6566
ENSG0000(	1358	30.32759	chr1:2197	LINC02971		lncRNA	chr1:234957231-234
ENSG0000(	1358	30.32759	chr1:2197	MIR4753		smallRNA	chr1:235190034-235
ENSG0000(	1358	30.32759	chr1:2197	SNORA25		smallRNA	chr1:237555040-237
ENSG0000(	1358	30.32759	chr1:2197	LYST		protein_c	chr1:235661041-235
ENSG0000(	1358	30.32759	chr1:2197	ENSG00000237250		lncRNA	chr1:237862175-237
ENSG0000(	1358	30.32759	chr1:2197	MTR		protein_c	chr1:236795260-236
ENSG0000(	1358	30.32759	chr1:2197	LGALS8		protein_c	chr1:236518000-236
ENSG0000(	1358	30.32759	chr1:2197	RNU5E-2P		smallRNA	chr1:235863388-235
ENSG0000(	1358	30.32759	chr1:2197	snoU13		smallRNA	chr1:236300980-236
ENSG0000(	1358	30.32759	chr1:2197	NID1	NCGv7	protein_c	chr1:235975830-236
ENSG0000(	1358	30.32759	chr1:2197	LINC02768		lncRNA	chr1:235957879-235
ENSG0000(	1358	30.32759	chr1:2197	TOMM20		protein_c	chr1:235109341-235
ENSG0000(	1358	30.32759	chr1:2197	ENSG00000230325		lncRNA	chr1:236540094-236
ENSG0000(	1358	30.32759	chr1:2197	KRT18P32		Pseudoger	chr1:238491358-238
ENSG0000(	1358	30.32759	chr1:2197	MTND3P8		Pseudoger	chr1:235541759-235
ENSG0000(	1358	30.32759	chr1:2197	TBCE		protein_c	chr1:235328570-235
ENSG0000(	1358	30.32759	chr1:2197	ERO1B	DriverDB\	protein_c	chr1:236214681-236
ENSG0000(	1358	30.32759	chr1:2197	ARID4B		protein_c	chr1:235131634-235
ENSG0000(	1358	30.32759	chr1:2197	ENSG00000273058		lncRNA	chr1:236536162-236

ENSG0000(	1358	30.32759	chr1:2197	ENSG00000234464		lncRNA	chr1:238238943-238
ENSG0000(	1358	30.32759	chr1:2197	RBM34		protein_c	chr1:235131183-235
ENSG0000(	1358	30.32759	chr1:2197	MTCO3P46		Pseudoger	chr1:235542159-235
ENSG0000(	1358	30.32759	chr1:2197	ENSG00000289114		lncRNA	chr1:235161321-235
ENSG0000(	1358	30.32759	chr1:2197	RPL35P1		Pseudoger	chr1:236981339-236
ENSG0000(	1358	30.32759	chr1:2197	ENSG00000227962		Pseudoger	chr1:235336806-235
ENSG0000(	1358	30.32759	chr1:2197	LDHAP2		Pseudoger	chr1:235738005-235
ENSG0000(	1358	30.32759	chr1:2197	LNCATV		lncRNA	chr1:234957342-234
ENSG0000(	1358	30.32759	chr1:2197	MT1HL1		protein_c	chr1:237004103-237
ENSG0000(	1358	30.32759	chr1:2197	ZP4		protein_c	chr1:237877864-237
ENSG0000(	1358	30.32759	chr1:2197	ENSG00000227236		Pseudoger	chr1:235614674-235
ENSG0000(	1358	30.32759	chr1:2197	MTND4LP21		Pseudoger	chr1:235541412-235
ENSG0000(	1358	30.32759	chr1:2197	ENSG00000283377		Pseudoger	chr1:237942698-237
ENSG0000(	1358	30.32759	chr1:2197	ENSG00000270710		Pseudoger	chr1:235565761-235
ENSG0000(	1358	30.32759	chr1:2197	ENSG00000235371		Pseudoger	chr1:236110061-236
ENSG0000(	1358	30.32759	chr1:2197	LINC01139		lncRNA	chr1:238476542-238
ENSG0000(	1358	30.32759	chr1:2197	RPSAP21		Pseudoger	chr1:236819634-236
ENSG0000(	1358	30.32759	chr1:2197	ENSG00000232989		Pseudoger	chr1:238268494-238
ENSG0000(	1358	30.32759	chr1:2197	YWHAQP9		Pseudoger	chr1:238107736-238
ENSG0000(	1358	30.32759	chr1:2197	RPS21P1		Pseudoger	chr1:235432985-235
ENSG0000(	1358	30.32759	chr1:2197	ENSG00000230026		Pseudoger	chr1:235361153-235
ENSG0000(	1358	30.32759	chr1:2197	TBCE		protein_c	chr1:235367360-235
ENSG0000(	1358	30.32759	chr1:2197	ENO1P1		Pseudoger	chr1:236483165-236
ENSG0000(	1358	30.32759	chr1:2197	ENSG00000289628		lncRNA	chr1:237814959-237
ENSG0000(	1358	30.32759	chr1:2197	ENSG00000233018		Pseudoger	chr1:235097187-235
ENSG0000(	1358	30.32759	chr1:2197	LGALS8-AS1		lncRNA	chr1:236523052-236
ENSG0000(	1358	30.32759	chr1:2197	MTND5P19		Pseudoger	chr1:235538052-235
ENSG0000(	1358	30.32759	chr1:2197	ENSG00000285177		lncRNA	chr1:235366353-235
ENSG0000(	1358	30.32759	chr1:2197	MTCYBP15		Pseudoger	chr1:237948017-237
ENSG0000(	1358	30.32759	chr1:2197	ENSG00000273416		lncRNA	chr1:235104180-235
ENSG0000(	1358	30.32759	chr1:2197	MTCO1P38		Pseudoger	chr1:237940762-237
ENSG0000(	1358	30.32759	chr1:2197	ENSG00000283166		Pseudoger	chr1:237941452-237
ENSG0000(	1358	30.32759	chr1:2197	ENSG00000237922		Pseudoger	chr1:236285976-236
ENSG0000(	1358	30.32759	chr1:2197	GNG4		protein_c	chr1:235547685-235
ENSG0000(	1358	30.32759	chr1:2197	Y_RNA		smallRNA	chr1:236060677-236
ENSG0000(	1358	30.32759	chr1:2197	ENSG00000286263		lncRNA	chr1:234811052-234
ENSG0000(	1358	30.32759	chr1:2197	MTND4P10		Pseudoger	chr1:235540053-235
ENSG0000(	1358	30.32759	chr1:2197	GGPS1		protein_c	chr1:235327350-235
ENSG0000(	1358	30.32759	chr1:2197	MTCYBP14		Pseudoger	chr1:235519971-235
ENSG0000(	1358	30.32759	chr1:2197	EDARADD		protein_c	chr1:236348257-236
ENSG0000(	1358	30.32759	chr1:2197	RNU6-968P		smallRNA	chr1:235915415-235
ENSG0000(	1358	30.32759	chr1:2197	RN7SKP195		smallRNA	chr1:237120807-237
ENSG0000(	1358	30.32759	chr1:2197	ENSG00000288099		lncRNA	chr1:237013373-237
ENSG0000(	1358	30.32759	chr1:2197	RPL23AP23		Pseudoger	chr1:235295865-235
ENSG0000(	1358	30.32759	chr1:2197	MTND6P15		Pseudoger	chr1:237949214-237
ENSG0000(	1358	30.32759	chr1:2197	HEATR1	NCGv7	protein_c	chr1:236549005-236
ENSG0000(	1358	30.32759	chr1:2197	RNU6-725P		smallRNA	chr1:238325687-238
ENSG0000(	1358	30.32759	chr1:2197	RYR2		protein_c	chr1:237042184-237
ENSG0000(	1358	30.32759	chr1:2197	ENSG00000243781		Pseudoger	chr1:237926831-237
ENSG0000(	1358	30.32759	chr1:2197	ENSG00000258082		lncRNA	chr1:234978814-234
ENSG0000(	1358	30.32759	chr1:2197	B3GALNT2		protein_c	chr1:235447190-235

ENSG0000(	1358	30.32759	chr1:2197	MTND6P14		Pseudoger	chr1:235537520-235
ENSG0000(	1358	30.32759	chr1:2197	MIR1537		smallRNA	chr1:235853000-235
ENSG0000(	1358	30.32759	chr1:2197	ENSG00000286142		lncRNA	chr1:236123667-236
ENSG0000(	1358	30.32759	chr1:2197	SNORA14B		smallRNA	chr1:235127803-235
ENSG0000(	1358	30.32759	chr1:2197	MIR4428		smallRNA	chr1:237471119-237
ENSG0000(	1358	30.32759	chr1:2197	ENSG00000237845		lncRNA	chr1:235942553-235
ENSG0000(	1358	30.32759	chr1:2197	RP11-293G6__A.2		lncRNA	chr1:235419515-235
ENSG0000(	1358	30.32759	chr1:2197	RN7SL668P		smallRNA	chr1:234904186-234
ENSG0000(	1358	30.32759	chr1:2197	ACTN2		protein_c	chr1:236664141-236
ENSG0000(	1358	30.32759	chr1:2197	MTND5P18		Pseudoger	chr1:237949736-237
ENSG0000(	1358	30.32759	chr1:2197	LYST-AS1		lncRNA	chr1:235839483-235
ENSG0000(	1358	30.32759	chr1:2197	ENSG00000231877		lncRNA	chr1:238485445-238
ENSG0000(	1358	30.32759	chr1:2197	RNU2-70P		smallRNA	chr1:236267780-236
ENSG0000(	1358	30.32759	chr1:2197	GPR137B		protein_c	chr1:236142505-236
ENSG0000(	1356	30.28293	chr2:3094	RPS27A	NCGv7	protein_c	chr2:55231903-5523
ENSG0000(	1356	30.28293	chr2:3094	ENSG00000290071		lncRNA	chr2:55137264-5513
ENSG0000(	1356	30.28293	chr2:3094	Y_RNA		smallRNA	chr2:55286018-5528
ENSG0000(	1356	30.28293	chr2:3094	MTIF2		protein_c	chr2:55236595-5526
ENSG0000(	1356	30.28293	chr2:3094	SPTBN1-AS1		lncRNA	chr2:54516048-5454
ENSG0000(	1356	30.28293	chr2:3094	RTN4	NCGv7	protein_c	chr2:54972187-5511
ENSG0000(	1356	30.28293	chr2:3094	SNORA12		smallRNA	chr2:55565703-5556
ENSG0000(	1356	30.28293	chr2:3094	SPTBN1		protein_c	chr2:54456317-5467
ENSG0000(	1356	30.28293	chr2:3094	ENSG00000289627		lncRNA	chr2:54581577-5458
ENSG0000(	1356	30.28293	chr2:3094	RNU6-221P		smallRNA	chr2:55456106-5545
ENSG0000(	1356	30.28293	chr2:3094	RNU6-433P		smallRNA	chr2:55014418-5501
ENSG0000(	1356	30.28293	chr2:3094	ENSG00000240401		lncRNA	chr2:55282350-5534
ENSG0000(	1356	30.28293	chr2:3094	C2orf73		protein_c	chr2:54330034-5438
ENSG0000(	1356	30.28293	chr2:3094	RNU6-775P		smallRNA	chr2:55451004-5545
ENSG0000(	1356	30.28293	chr2:3094	ENSG00000285519		lncRNA	chr2:54768492-5480
ENSG0000(	1356	30.28293	chr2:3094	CFAP36		protein_c	chr2:55519604-5554
ENSG0000(	1356	30.28293	chr2:3094	BTF3P5		Pseudoger	chr2:55435156-5543
ENSG0000(	1356	30.28293	chr2:3094	RNU7-172P		smallRNA	chr2:54166944-5416
ENSG0000(	1356	30.28293	chr2:3094	ENSG00000289606		lncRNA	chr2:55235605-5523
ENSG0000(	1356	30.28293	chr2:3094	EML6-AS1		lncRNA	chr2:54747103-5475
ENSG0000(	1356	30.28293	chr2:3094	RPL23AP32		Pseudoger	chr2:54529343-5452
ENSG0000(	1356	30.28293	chr2:3094	SPTBN1-AS2		lncRNA	chr2:54661011-5468
ENSG0000(	1356	30.28293	chr2:3094	PPP4R3B		protein_c	chr2:55547292-5561
ENSG0000(	1356	30.28293	chr2:3094	RNU6-634P		smallRNA	chr2:55499950-5550
ENSG0000(	1356	30.28293	chr2:3094	EML6		protein_c	chr2:54723499-5497
ENSG0000(	1356	30.28293	chr2:3094	CCDC88A	NCGv7	protein_c	chr2:55287842-5541
ENSG0000(	1356	30.28293	chr2:3094	ENSG00000203327		lncRNA	chr2:55214387-5521
ENSG0000(	1356	30.28293	chr2:3094	CLHC1		protein_c	chr2:55172547-5523
ENSG0000(	1356	30.28293	chr2:3094	PRORSD1P		Pseudoger	chr2:55282319-5528
ENSG0000(	1356	30.28293	chr2:3094	TSPYL6		protein_c	chr2:54253178-5425
ENSG0000(	1356	30.28293	chr2:3094	ACYP2		protein_c	chr2:53970838-5430
ENSG0000(	1356	30.28293	chr2:3094	RNU7-81P		smallRNA	chr2:54850289-5485
ENSG0000(	1356	30.28293	chr2:3094	CDPF1P1		Pseudoger	chr2:55224280-5522
ENSG0000(	1356	30.28293	chr2:3094	ENSG00000234943		lncRNA	chr2:54545368-5454
ENSG0000(	1356	30.28293	chr2:3094	AC019198.1		smallRNA	chr2:55472744-5547
ENSG0000(	1356	30.28293	chr2:3094	PPP4R3B-DT		lncRNA	chr2:55617869-5561
ENSG0000(	1356	30.28293	chr2:3094	PNPT1		protein_c	chr2:55634061-5569

ENSG0000(	1356	30.28293	chr2:309<	AC093165.1		smallRNA	chr2:55108710-5510
ENSG0000(	1354	30.23826	chr2:309<	B3GNT2		protein_c	chr2:62196115-6222
ENSG0000(	1354	30.23826	chr2:309<	LINC02934		lncRNA	chr2:65436711-6620
ENSG0000(	1354	30.23826	chr2:309<	CSP1		Pseudoger	chr2:63717122-6371
ENSG0000(	1354	30.23826	chr2:309<	MDH1		protein_c	chr2:63588609-6360
ENSG0000(	1354	30.23826	chr2:309<	ENSG00000271657		Pseudoger	chr2:62168862-6217
ENSG0000(	1354	30.23826	chr2:309<	RNU6-100P		smallRNA	chr2:64578892-6457
ENSG0000(	1354	30.23826	chr2:309<	LINC02579		lncRNA	chr2:64606975-6461
ENSG0000(	1354	30.23826	chr2:309<	SERTAD2	TAG;AC	protein_c	chr2:64631621-6475
ENSG0000(	1354	30.23826	chr2:309<	SLC1A4		protein_c	chr2:64988477-6502
ENSG0000(	1354	30.23826	chr2:309<	ENSG00000226605		lncRNA	chr2:62826064-6285
ENSG0000(	1354	30.23826	chr2:309<	ENSG00000280257		TEC	chr2:65790039-6579
ENSG0000(	1354	30.23826	chr2:309<	RN7SL18P		smallRNA	chr2:62491178-6249
ENSG0000(	1354	30.23826	chr2:309<	LGALSL-DT		lncRNA	chr2:64395220-6445
ENSG0000(	1354	30.23826	chr2:309<	SPRED2		protein_c	chr2:65310851-6543
ENSG0000(	1354	30.23826	chr2:309<	ENSG00000289943		lncRNA	chr2:63839866-6384
ENSG0000(	1354	30.23826	chr2:309<	ENSG00000279485		TEC	chr2:63517892-6351
ENSG0000(	1354	30.23826	chr2:309<	ENSG00000213486		Pseudoger	chr2:61710076-6171
ENSG0000(	1354	30.23826	chr2:309<	ENSG00000229831		Pseudoger	chr2:61820208-6182
ENSG0000(	1354	30.23826	chr2:309<	ENSG00000226622		lncRNA	chr2:62533681-6266
ENSG0000(	1354	30.23826	chr2:309<	LINC01805		lncRNA	chr2:64486353-6450
ENSG0000(	1354	30.23826	chr2:309<	RSL24D1P2		Pseudoger	chr2:62561058-6256
ENSG0000(	1354	30.23826	chr2:309<	RN7SL341P		smallRNA	chr2:64817378-6481
ENSG0000(	1354	30.23826	chr2:309<	ACA59		smallRNA	chr2:63883249-6388
ENSG0000(	1354	30.23826	chr2:309<	HNRNPA1P66		Pseudoger	chr2:63751697-6375
ENSG0000(	1354	30.23826	chr2:309<	LINC01873		lncRNA	chr2:66383306-6639
ENSG0000(	1354	30.23826	chr2:309<	ENSG00000273763		Pseudoger	chr2:65203502-6522
ENSG0000(	1354	30.23826	chr2:309<	ENSG00000271243		Pseudoger	chr2:61575774-6157
ENSG0000(	1354	30.23826	chr2:309<	ENSG00000235725		lncRNA	chr2:65589566-6564
ENSG0000(	1354	30.23826	chr2:309<	DBIL5P2		Pseudoger	chr2:63117851-6311
ENSG0000(	1354	30.23826	chr2:309<	LINC01797		lncRNA	chr2:66696190-6670
ENSG0000(	1354	30.23826	chr2:309<	ENSG00000238201		lncRNA	chr2:64338067-6434
ENSG0000(	1354	30.23826	chr2:309<	ENSG00000228541		lncRNA	chr2:62296246-6246
ENSG0000(	1354	30.23826	chr2:309<	AC074391.2		smallRNA	chr2:66041250-6604
ENSG0000(	1354	30.23826	chr2:309<	AFTPH-DT		lncRNA	chr2:64522187-6452
ENSG0000(	1354	30.23826	chr2:309<	ENSG00000229839		lncRNA	chr2:62069447-6214
ENSG0000(	1354	30.23826	chr2:309<	AFTPH		protein_c	chr2:64524299-6459
ENSG0000(	1354	30.23826	chr2:309<	LINC01800		lncRNA	chr2:64846130-6486
ENSG0000(	1354	30.23826	chr2:309<	RPSAP26		Pseudoger	chr2:62146413-6214
ENSG0000(	1354	30.23826	chr2:309<	LGALSL		protein_c	chr2:64453969-6446
ENSG0000(	1354	30.23826	chr2:309<	ENSG00000285857		lncRNA	chr2:61527340-6152
ENSG0000(	1354	30.23826	chr2:309<	ENSG00000281920		lncRNA	chr2:65623272-6562
ENSG0000(	1354	30.23826	chr2:309<	SNORA70B		smallRNA	chr2:61417244-6141
ENSG0000(	1354	30.23826	chr2:309<	ENSG00000234255		lncRNA	chr2:65439838-6545
ENSG0000(	1354	30.23826	chr2:309<	Y_RNA		smallRNA	chr2:62726636-6272
ENSG0000(	1354	30.23826	chr2:309<	ENSG00000270354		Pseudoger	chr2:64208498-6420
ENSG0000(	1354	30.23826	chr2:309<	LINC00309		lncRNA	chr2:64185078-6420
ENSG0000(	1354	30.23826	chr2:309<	AC096664.3		smallRNA	chr2:63695393-6369
ENSG0000(	1354	30.23826	chr2:309<	DNAJB12P1		Pseudoger	chr2:65500993-6550
ENSG0000(	1354	30.23826	chr2:309<	ENSG00000277498		Pseudoger	chr2:61858137-6186
ENSG0000(	1354	30.23826	chr2:309<	ENSG00000270335		Pseudoger	chr2:62348948-6235

ENSG0000(	1354	30.23826	chr2:309⁄	RPS10P9	Pseudoger	chr2:64665607-6466
ENSG0000(	1354	30.23826	chr2:309⁄	RPL21P37	Pseudoger	chr2:62532583-6253
ENSG0000(	1354	30.23826	chr2:309⁄	AC007365.2	smallRNA	chr2:64776104-6477
ENSG0000(	1354	30.23826	chr2:309⁄	RNU6-1145P	smallRNA	chr2:61605616-6160
ENSG0000(	1354	30.23826	chr2:309⁄	ENSG00000287123	lncRNA	chr2:64836985-6484
ENSG0000(	1354	30.23826	chr2:309⁄	RPL11P1	Pseudoger	chr2:64960053-6496
ENSG0000(	1354	30.23826	chr2:309⁄	ENSG00000270437	Pseudoger	chr2:63083008-6308
ENSG0000(	1354	30.23826	chr2:309⁄	ENSG00000288986	lncRNA	chr2:64765463-6476
ENSG0000(	1354	30.23826	chr2:309⁄	MIR5192	smallRNA	chr2:62205826-6220
ENSG0000(	1354	30.23826	chr2:309⁄	LINC02245	lncRNA	chr2:64901840-6505
ENSG0000(	1354	30.23826	chr2:309⁄	SNORA74	smallRNA	chr2:65158662-6515
ENSG0000(	1354	30.23826	chr2:309⁄	RPS20P9	Pseudoger	chr2:62939916-6294
ENSG0000(	1354	30.23826	chr2:309⁄	ENSG00000225815	lncRNA	chr2:66327349-6632
ENSG0000(	1354	30.23826	chr2:309⁄	RPS24P7	Pseudoger	chr2:61803143-6180
ENSG0000(	1354	30.23826	chr2:309⁄	ENSG00000289176	lncRNA	chr2:64337103-6434
ENSG0000(	1354	30.23826	chr2:309⁄	ENSG00000286360	lncRNA	chr2:61878940-6188
ENSG0000(	1354	30.23826	chr2:309⁄	PSAT1P2	Pseudoger	chr2:62552463-6255
ENSG0000(	1354	30.23826	chr2:309⁄	PELI1	protein_c	chr2:64092652-6414
ENSG0000(	1354	30.23826	chr2:309⁄	ENSG00000289855	lncRNA	chr2:61764544-6176
ENSG0000(	1354	30.23826	chr2:309⁄	RPS15AP15	Pseudoger	chr2:65511771-6551
ENSG0000(	1354	30.23826	chr2:309⁄	snoU13	smallRNA	chr2:62264995-6226
ENSG0000(	1354	30.23826	chr2:309⁄	RPL27P5	Pseudoger	chr2:63108118-6310
ENSG0000(	1354	30.23826	chr2:309⁄	RPS29P10	Pseudoger	chr2:61589498-6158
ENSG0000(	1354	30.23826	chr2:309⁄	PRELID1P6	Pseudoger	chr2:63622178-6362
ENSG0000(	1354	30.23826	chr2:309⁄	ENSG00000225889	lncRNA	chr2:64143239-6425
ENSG0000(	1354	30.23826	chr2:309⁄	ENSG00000289247	lncRNA	chr2:61854376-6185
ENSG0000(	1354	30.23826	chr2:309⁄	ENSG00000271146	Pseudoger	chr2:61249780-6125
ENSG0000(	1354	30.23826	chr2:309⁄	ENSG00000228079	lncRNA	chr2:64086353-6408
ENSG0000(	1354	30.23826	chr2:309⁄	MIR4433	smallRNA	chr2:64340759-6434
ENSG0000(	1354	30.23826	chr2:309⁄	RPL31P30	Pseudoger	chr2:61856695-6185
ENSG0000(	1354	30.23826	chr2:309⁄	ENSG00000226756	lncRNA	chr2:64644612-6464
ENSG0000(	1354	30.23826	chr2:309⁄	RAB1A	protein_c	chr2:65070696-6513
ENSG0000(	1354	30.23826	chr2:309⁄	ENSG00000290118	lncRNA	chr2:64275361-6427
ENSG0000(	1354	30.23826	chr2:309⁄	snoU13	smallRNA	chr2:64908587-6490
ENSG0000(	1354	30.23826	chr2:309⁄	CEP68	protein_c	chr2:65056354-6508
ENSG0000(	1354	30.23826	chr2:309⁄	USP34-DT	lncRNA	chr2:61471188-6148
ENSG0000(	1354	30.23826	chr2:309⁄	KRT18P33	Pseudoger	chr2:65666695-6566
ENSG0000(	1354	30.23826	chr2:309⁄	ENSG00000288932	lncRNA	chr2:64273709-6429
ENSG0000(	1354	30.23826	chr2:309⁄	EHBP1-AS1	lncRNA	chr2:62957326-6304
ENSG0000(	1354	30.23826	chr2:309⁄	EHBP1	protein_c	chr2:62673851-6304
ENSG0000(	1354	30.23826	chr2:309⁄	ENSG00000289410	lncRNA	chr2:61728808-6176
ENSG0000(	1354	30.23826	chr2:309⁄	MTFR2P1	Pseudoger	chr2:63232453-6323
ENSG0000(	1354	30.23826	chr2:309⁄	LINC01798	lncRNA	chr2:66574030-6673
ENSG0000(	1354	30.23826	chr2:309⁄	COMMD1	protein_c	chr2:61888724-6214
ENSG0000(	1354	30.23826	chr2:309⁄	MEIS1-AS2	lncRNA	chr2:66439088-6644
ENSG0000(	1354	30.23826	chr2:309⁄	OTX1	protein_c	chr2:63050057-6305
ENSG0000(	1354	30.23826	chr2:309⁄	ENSG00000237217	Pseudoger	chr2:64450096-6445
ENSG0000(	1354	30.23826	chr2:309⁄	ACTR2	protein_c	chr2:65227788-6527
ENSG0000(	1354	30.23826	chr2:309⁄	RPS4XP5	Pseudoger	chr2:63642455-6364
ENSG0000(	1354	30.23826	chr2:309⁄	RN7SL51P	smallRNA	chr2:62262389-6226
ENSG0000(	1354	30.23826	chr2:309⁄	TMEM17	protein_c	chr2:62500218-6251

ENSG0000(	1354	30.23826	chr2:309‹	MEIS1-AS3		lncRNA	chr2:66426735-6643
ENSG0000(	1354	30.23826	chr2:309‹	RPL27P6		Pseudoger	chr2:63684305-6368
ENSG0000(	1354	30.23826	chr2:309‹	UGP2		protein_c	chr2:63840952-6389
ENSG0000(	1354	30.23826	chr2:309‹	AC007386.4		lncRNA	chr2:65030727-6503
ENSG0000(	1354	30.23826	chr2:309‹	ENSG00000232693		lncRNA	chr2:65373700-6538
ENSG0000(	1354	30.23826	chr2:309‹	VDAC2P5		Pseudoger	chr2:65205108-6520
ENSG0000(	1354	30.23826	chr2:309‹	RPL23AP37		Pseudoger	chr2:64347193-6434
ENSG0000(	1354	30.23826	chr2:309‹	RN7SL635P		smallRNA	chr2:65545403-6554
ENSG0000(	1354	30.23826	chr2:309‹	AC118345.2		smallRNA	chr2:66239500-6623
ENSG0000(	1354	30.23826	chr2:309‹	XPO1	NCGv7;AC	protein_c	chr2:61476032-6153
ENSG0000(	1354	30.23826	chr2:309‹	ENSG00000273302		lncRNA	chr2:61199979-6120
ENSG0000(	1354	30.23826	chr2:309‹	ENSG00000227293		lncRNA	chr2:66235377-6623
ENSG0000(	1354	30.23826	chr2:309‹	ENSG00000286480		lncRNA	chr2:63106879-6319
ENSG0000(	1354	30.23826	chr2:309‹	AC016727.1		smallRNA	chr2:61555360-6155
ENSG0000(	1354	30.23826	chr2:309‹	MEIS1	TAG;AC	protein_c	chr2:66433452-6657
ENSG0000(	1354	30.23826	chr2:309‹	VPS54		protein_c	chr2:63892146-6401
ENSG0000(	1354	30.23826	chr2:309‹	Vault		smallRNA	chr2:65555432-6555
ENSG0000(	1354	30.23826	chr2:309‹	Y_RNA		smallRNA	chr2:64834056-6483
ENSG0000(	1354	30.23826	chr2:309‹	WDPCP		protein_c	chr2:63119559-6382
ENSG0000(	1354	30.23826	chr2:309‹	RN7SL211P		smallRNA	chr2:64906865-6490
ENSG0000(	1354	30.23826	chr2:309‹	MIR4778		smallRNA	chr2:66358249-6635
ENSG0000(	1354	30.23826	chr2:309‹	FAM161A		protein_c	chr2:61824848-6185
ENSG0000(	1354	30.23826	chr2:309‹	RPL37P13		Pseudoger	chr2:62507545-6250
ENSG0000(	1354	30.23826	chr2:309‹	RNU6-548P		smallRNA	chr2:64994746-6499
ENSG0000(	1354	30.23826	chr2:309‹	ENSG00000234624		Pseudoger	chr2:61416887-6141
ENSG0000(	1354	30.23826	chr2:309‹	ENSG00000286524		lncRNA	chr2:62817764-6281
ENSG0000(	1354	30.23826	chr2:309‹	USP34		protein_c	chr2:61187463-6147
ENSG0000(	1354	30.23826	chr2:309‹	CCT4		protein_c	chr2:61868085-6188
ENSG0000(	1354	30.23826	chr2:309‹	ENSG00000236498		lncRNA	chr2:61868432-6188
ENSG0000(	1354	30.23826	chr2:309‹	ENSG00000238012		lncRNA	chr2:64330481-6433
ENSG0000(	1341	29.94794	chr2:309‹	ACTG1P22		Pseudoger	chr2:57755428-5776
ENSG0000(	1341	29.94794	chr2:309‹	FANCL		protein_c	chr2:58159243-5824
ENSG0000(	1341	29.94794	chr2:309‹	EIF2S2P7		Pseudoger	chr2:57048350-5704
ENSG0000(	1341	29.94794	chr2:309‹	EFEMP1		protein_c	chr2:55865967-5592
ENSG0000(	1341	29.94794	chr2:309‹	MIR217HG		lncRNA	chr2:55963191-5604
ENSG0000(	1341	29.94794	chr2:309‹	PPIAP63		Pseudoger	chr2:56750300-5675
ENSG0000(	1341	29.94794	chr2:309‹	LINC01813		lncRNA	chr2:56077417-5609
ENSG0000(	1341	29.94794	chr2:309‹	LINC01795		lncRNA	chr2:58275532-5829
ENSG0000(	1341	29.94794	chr2:309‹	VRK2		protein_c	chr2:57907629-5815
ENSG0000(	1341	29.94794	chr2:309‹	SNORD78		smallRNA	chr2:57544535-5754
ENSG0000(	1341	29.94794	chr2:309‹	RNA5SP93		Pseudoger	chr2:56235217-5623
ENSG0000(	1341	29.94794	chr2:309‹	CCDC85A		protein_c	chr2:56183990-5638
ENSG0000(	1341	29.94794	chr2:309‹	ENSG00000233251		lncRNA	chr2:56173534-5618
ENSG0000(	1341	29.94794	chr2:309‹	ENSG00000231043		Pseudoger	chr2:58460292-5846
ENSG0000(	1341	29.94794	chr2:309‹	ENSG00000272180		lncRNA	chr2:55952158-5618
ENSG0000(	1341	29.94794	chr2:309‹	ENSG00000270569		Pseudoger	chr2:57429548-5743
ENSG0000(	1341	29.94794	chr2:309‹	MIR217		smallRNA	chr2:55982967-5598
ENSG0000(	1341	29.94794	chr2:309‹	AC073215.1		smallRNA	chr2:58062581-5806
ENSG0000(	1341	29.94794	chr2:309‹	ENSG00000289529		lncRNA	chr2:58429434-5847
ENSG0000(	1341	29.94794	chr2:309‹	RN7SKP208		smallRNA	chr2:55951654-5595
ENSG0000(	1341	29.94794	chr2:309‹	snoU13		smallRNA	chr2:57016061-5701

ENSG0000(	1341	29.94794	chr2:309⁄	ENSG00000273063		lncRNA	chr2:58241349-5824
ENSG0000(	1341	29.94794	chr2:309⁄	EIF3FP3		Pseudoger	chr2:58251440-5825
ENSG0000(	1341	29.94794	chr2:309⁄	MIR216A		smallRNA	chr2:55988950-5598
ENSG0000(	1341	29.94794	chr2:309⁄	MIR216B		smallRNA	chr2:56000714-5600
ENSG0000(	1341	29.94794	chr2:309⁄	ENSG00000285755		lncRNA	chr2:57289648-5738
ENSG0000(	1341	29.94794	chr2:309⁄	ENSG00000271894		lncRNA	chr2:56147630-5638
ENSG0000(	1341	29.94794	chr2:309⁄	ENSG00000287875		lncRNA	chr2:58040211-5804
ENSG0000(	1341	29.94794	chr2:309⁄	LINC01122		lncRNA	chr2:58427738-5906
ENSG0000(	1328	29.65762	chr2:309⁄	CYP1B1		protein_c	chr2:38066973-3810
ENSG0000(	1328	29.65762	chr2:309⁄	RNU6-951P		smallRNA	chr2:38147415-3814
ENSG0000(	1328	29.65762	chr2:309⁄	LINC01883		lncRNA	chr2:38431294-3843
ENSG0000(	1328	29.65762	chr2:309⁄	ENSG00000235586		lncRNA	chr2:38601598-3860
ENSG0000(	1328	29.65762	chr2:309⁄	RPLP0P6		Pseudoger	chr2:38481851-3848
ENSG0000(	1328	29.65762	chr2:309⁄	ENSG00000227292		lncRNA	chr2:38203363-3823
ENSG0000(	1328	29.65762	chr2:309⁄	HNRNPLL		protein_c	chr2:38561969-3860
ENSG0000(	1328	29.65762	chr2:309⁄	RMDN2		protein_c	chr2:37923187-3806
ENSG0000(	1328	29.65762	chr2:309⁄	ENSG00000229160		lncRNA	chr2:38132637-3813
ENSG0000(	1328	29.65762	chr2:309⁄	ENSG00000287313		lncRNA	chr2:38408719-3841
ENSG0000(	1328	29.65762	chr2:309⁄	ENSG00000288994		lncRNA	chr2:38342476-3834
ENSG0000(	1328	29.65762	chr2:309⁄	CYP1B1-AS1		lncRNA	chr2:38073447-3823
ENSG0000(	1328	29.65762	chr2:309⁄	ENSG00000280276		TEC	chr2:38121935-3812
ENSG0000(	1328	29.65762	chr2:309⁄	LINC02613		lncRNA	chr2:38406527-3851
ENSG0000(	1328	29.65762	chr2:309⁄	ENSG00000271443		Pseudoger	chr2:38535258-3853
ENSG0000(	1328	29.65762	chr2:309⁄	RMDN2-AS1		lncRNA	chr2:37949911-3806
ENSG0000(	1328	29.65762	chr2:309⁄	ENSG00000273006		lncRNA	chr2:38193348-3819
ENSG0000(	1328	29.65762	chr2:309⁄	GAPDHP25		Pseudoger	chr2:38285410-3828
ENSG0000(	1328	29.65762	chr2:309⁄	RPL7P12		Pseudoger	chr2:38231568-3823
ENSG0000(	1328	29.65762	chr2:309⁄	ATL2		protein_c	chr2:38293954-3837
ENSG0000(	1319	29.45662	chr2:309⁄	SATB2-AS1		lncRNA	chr2:199457689-199
ENSG0000(	1319	29.45662	chr2:309⁄	SATB2	NCGv7	protein_c	chr2:199269505-199
ENSG0000(	1310	29.25563	chr1:114⁄	ENSG00000272088		lncRNA	chr1:3487246-34876
ENSG0000(	1310	29.25563	chr1:114⁄	MEGF6		protein_c	chr1:3487951-36115
ENSG0000(	1306	29.1663	chr2:309⁄	FTCDNL1		protein_c	chr2:199760544-199
ENSG0000(	1306	29.1663	chr2:309⁄	SPATS2L		protein_c	chr2:200305881-200
ENSG0000(	1306	29.1663	chr2:309⁄	AOX1	NCGv7	protein_c	chr2:200586014-200
ENSG0000(	1306	29.1663	chr2:309⁄	AC105381.1		smallRNA	chr2:200387175-200
ENSG0000(	1306	29.1663	chr2:309⁄	ENSG00000287027		lncRNA	chr2:200086870-200
ENSG0000(	1306	29.1663	chr2:309⁄	ENSG00000227004		Pseudoger	chr2:199872172-199
ENSG0000(	1306	29.1663	chr2:309⁄	TYW5		protein_c	chr2:199928913-199
ENSG0000(	1306	29.1663	chr2:309⁄	ENSG00000232732		lncRNA	chr2:199867396-199
ENSG0000(	1306	29.1663	chr2:309⁄	RN7SL717P		smallRNA	chr2:199901083-199
ENSG0000(	1306	29.1663	chr2:309⁄	C2orf69		protein_c	chr2:199911293-199
ENSG0000(	1306	29.1663	chr2:309⁄	LINC01877		lncRNA	chr2:199608062-199
ENSG0000(	1306	29.1663	chr2:309⁄	MAIP1		protein_c	chr2:199955317-200
ENSG0000(	1306	29.1663	chr2:309⁄	ENSG00000287299		lncRNA	chr2:200390666-200
ENSG0000(	1306	29.1663	chr2:309⁄	Y_RNA		smallRNA	chr2:200373175-200
ENSG0000(	1306	29.1663	chr2:309⁄	SEPHS1P6		Pseudoger	chr2:199659551-199
ENSG0000(	1306	29.1663	chr2:309⁄	SGO2		protein_c	chr2:200510008-200
ENSG0000(	1306	29.1663	chr2:309⁄	KCTD18		protein_c	chr2:200488958-200
ENSG0000(	1278	28.54099	chr1:114⁄	RER1		protein_c	chr1:2391775-24054
ENSG0000(	1278	28.54099	chr1:114⁄	PEX10		protein_c	chr1:2403964-24137

ENSG0000(	1278	28.54099	chr1:1142	ENSG00000285945		lncRNA	chr1:2768091-27847
ENSG0000(	1278	28.54099	chr1:1142	ENSG00000226286		lncRNA	chr1:3132927-31337
ENSG0000(	1278	28.54099	chr1:1142	PRXL2B		protein_c	chr1:2586491-25914
ENSG0000(	1278	28.54099	chr1:1142	TNFRSF14	NCGv7;AC	protein_c	chr1:2555639-25653
ENSG0000(	1278	28.54099	chr1:1142	PANK4		protein_c	chr1:2508537-25265
ENSG0000(	1278	28.54099	chr1:1142	ENSG00000287828		lncRNA	chr1:3313052-33231
ENSG0000(	1278	28.54099	chr1:1142	ENSG00000234396		lncRNA	chr1:2212523-22207
ENSG0000(	1278	28.54099	chr1:1142	ENSG00000272420		lncRNA	chr1:2363061-23636
ENSG0000(	1278	28.54099	chr1:1142	MORN1	NCGv7	protein_c	chr1:2321253-23917
ENSG0000(	1278	28.54099	chr1:1142	ARHGEF16		protein_c	chr1:3454665-34811
ENSG0000(	1278	28.54099	chr1:1142	ENSG00000272235		lncRNA	chr1:3306636-33100
ENSG0000(	1278	28.54099	chr1:1142	ENSG00000283259		lncRNA	chr1:2773603-27764
ENSG0000(	1278	28.54099	chr1:1142	SKI	NCGv7;AC	protein_c	chr1:2227388-23102
ENSG0000(	1278	28.54099	chr1:1142	ENSG00000287356		lncRNA	chr1:2315040-23230
ENSG0000(	1278	28.54099	chr1:1142	ENSG00000225931		TEC	chr1:2566410-25698
ENSG0000(	1278	28.54099	chr1:1142	ENSG00000233234		lncRNA	chr1:2811850-28126
ENSG0000(	1278	28.54099	chr1:1142	ENSG00000231630		lncRNA	chr1:2814432-28149
ENSG0000(	1278	28.54099	chr1:1142	HES5		protein_c	chr1:2528745-25302
ENSG0000(	1278	28.54099	chr1:1142	ACTRT2		protein_c	chr1:3021467-30229
ENSG0000(	1278	28.54099	chr1:1142	ENSG00000286518		lncRNA	chr1:3367815-33739
ENSG0000(	1278	28.54099	chr1:1142	ENSG00000272161		lncRNA	chr1:2326201-23266
ENSG0000(	1278	28.54099	chr1:1142	MMEL1-AS1		lncRNA	chr1:2632568-26366
ENSG0000(	1278	28.54099	chr1:1142	ENSG00000279839		TEC	chr1:3205988-32086
ENSG0000(	1278	28.54099	chr1:1142	ENSG00000287396		lncRNA	chr1:2814056-28177
ENSG0000(	1278	28.54099	chr1:1142	ENSG00000228037		lncRNA	chr1:2581560-25845
ENSG0000(	1278	28.54099	chr1:1142	ENSG00000269896		Pseudoger	chr1:2350414-23528
ENSG0000(	1278	28.54099	chr1:1142	ENSG00000224387		lncRNA	chr1:2492300-24932
ENSG0000(	1278	28.54099	chr1:1142	MMEL1	NCGv7	protein_c	chr1:2590639-26330
ENSG0000(	1278	28.54099	chr1:1142	PLCH2	NCGv7	protein_c	chr1:2425980-25055
ENSG0000(	1278	28.54099	chr1:1142	PRDM16	NCGv7;AC	protein_c	chr1:3069168-34386
ENSG0000(	1278	28.54099	chr1:1142	ENSG00000284745		lncRNA	chr1:2960658-29687
ENSG0000(	1278	28.54099	chr1:1142	ENSG00000272449		lncRNA	chr1:2530064-25474
ENSG0000(	1278	28.54099	chr1:1142	ENSG00000289610		lncRNA	chr1:2585610-25860
ENSG0000(	1278	28.54099	chr1:1142	ENSG00000229393		lncRNA	chr1:2493437-24944
ENSG0000(	1278	28.54099	chr1:1142	PRDM16-DT		lncRNA	chr1:3059611-30690
ENSG0000(	1278	28.54099	chr1:1142	TNFRSF14-AS1		lncRNA	chr1:2549920-25570
ENSG0000(	1275	28.47399	chr2:3094	ENSG00000230286		lncRNA	chr2:26950308-2700
ENSG0000(	1275	28.47399	chr2:3094	CIB4		protein_c	chr2:26581205-2664
ENSG0000(	1275	28.47399	chr2:3094	RNU6-942P		smallRNA	chr2:26042675-2604
ENSG0000(	1275	28.47399	chr2:3094	NDUFB4P4		Pseudoger	chr2:25886964-2588
ENSG0000(	1275	28.47399	chr2:3094	PTGES3P2		Pseudoger	chr2:25822469-2582
ENSG0000(	1275	28.47399	chr2:3094	OST4		protein_c	chr2:27070472-2707
ENSG0000(	1275	28.47399	chr2:3094	ENSG00000271936		lncRNA	chr2:24825610-2482
ENSG0000(	1275	28.47399	chr2:3094	RPS2P15		Pseudoger	chr2:26101317-2610
ENSG0000(	1275	28.47399	chr2:3094	RNA5SP88		Pseudoger	chr2:24564630-2456
ENSG0000(	1275	28.47399	chr2:3094	ENSG00000236099		Pseudoger	chr2:27158563-2715
ENSG0000(	1275	28.47399	chr2:3094	SCARNA21		smallRNA	chr2:24273614-2427
ENSG0000(	1275	28.47399	chr2:3094	PTRHD1		protein_c	chr2:24789728-2479
ENSG0000(	1275	28.47399	chr2:3094	LINC02923		lncRNA	chr2:23375229-2338
ENSG0000(	1275	28.47399	chr2:3094	LINC01381		lncRNA	chr2:25204313-2520
ENSG0000(	1275	28.47399	chr2:3094	SMARCE1P6		Pseudoger	chr2:26149204-2615

ENSG0000(	1275	28.47399	chr2:309<	GAREM2		protein_c	chr2:26173088-2618
ENSG0000(	1275	28.47399	chr2:309<	ENSG00000242628		lncRNA	chr2:24210650-2422
ENSG0000(	1275	28.47399	chr2:309<	ENSG00000286829		lncRNA	chr2:24402995-2441
ENSG0000(	1275	28.47399	chr2:309<	SNORD14		smallRNA	chr2:24969388-2496
ENSG0000(	1275	28.47399	chr2:309<	DRC1		protein_c	chr2:26401920-2645
ENSG0000(	1275	28.47399	chr2:309<	ENSG00000279526		TEC	chr2:23616499-2361
ENSG0000(	1275	28.47399	chr2:309<	DPYSL5		protein_c	chr2:26847747-2695
ENSG0000(	1275	28.47399	chr2:309<	TRMT112P6		Pseudoger	chr2:26028208-2602
ENSG0000(	1275	28.47399	chr2:309<	RNU6-936P		smallRNA	chr2:24676309-2467
ENSG0000(	1275	28.47399	chr2:309<	RPS13P4		Pseudoger	chr2:23900693-2390
ENSG0000(	1275	28.47399	chr2:309<	KCNK3		protein_c	chr2:26692722-2673
ENSG0000(	1275	28.47399	chr2:309<	RAB10		protein_c	chr2:26034084-2613
ENSG0000(	1275	28.47399	chr2:309<	DTNB-AS1		lncRNA	chr2:25421117-2542
ENSG0000(	1275	28.47399	chr2:309<	AGBL5-AS1		lncRNA	chr2:27049683-2705
ENSG0000(	1275	28.47399	chr2:309<	AGBL5	NCGv7	protein_c	chr2:27042364-2707
ENSG0000(	1275	28.47399	chr2:309<	EFR3B		protein_c	chr2:25042076-2515
ENSG0000(	1275	28.47399	chr2:309<	KIF3C		protein_c	chr2:25926598-2598
ENSG0000(	1275	28.47399	chr2:309<	MFSD2B		protein_c	chr2:24010081-2406
ENSG0000(	1275	28.47399	chr2:309<	HADHA		protein_c	chr2:26190635-2624
ENSG0000(	1275	28.47399	chr2:309<	MAPRE3	NCGv7	protein_c	chr2:26970637-2702
ENSG0000(	1275	28.47399	chr2:309<	CAD	AC	protein_c	chr2:27217369-2724
ENSG0000(	1275	28.47399	chr2:309<	ENSG00000272056		lncRNA	chr2:27053618-2705
ENSG0000(	1275	28.47399	chr2:309<	ABHD1	DriverDBv	protein_c	chr2:27123789-2713
ENSG0000(	1275	28.47399	chr2:309<	ASXL2	NCGv7	protein_c	chr2:25733753-2587
ENSG0000(	1275	28.47399	chr2:309<	CGREF1		protein_c	chr2:27098889-2711
ENSG0000(	1275	28.47399	chr2:309<	ENSG00000286645		lncRNA	chr2:25000147-2501
ENSG0000(	1275	28.47399	chr2:309<	GTF3C2-AS1		lncRNA	chr2:27335520-2734
ENSG0000(	1275	28.47399	chr2:309<	SDHCP3		Pseudoger	chr2:23943846-2394
ENSG0000(	1275	28.47399	chr2:309<	ENSG00000225378		lncRNA	chr2:26671254-2667
ENSG0000(	1275	28.47399	chr2:309<	FTH1P3		Pseudoger	chr2:27392784-2739
ENSG0000(	1275	28.47399	chr2:309<	DNAJC27-AS1		lncRNA	chr2:24971390-2503
ENSG0000(	1275	28.47399	chr2:309<	ENSG00000232642		lncRNA	chr2:24165884-2417
ENSG0000(	1275	28.47399	chr2:309<	ENSG00000227133		Pseudoger	chr2:26160023-2616
ENSG0000(	1275	28.47399	chr2:309<	PPM1G		protein_c	chr2:27381195-2740
ENSG0000(	1275	28.47399	chr2:309<	UBXN2A		protein_c	chr2:23927285-2400
ENSG0000(	1275	28.47399	chr2:309<	SNX17		protein_c	chr2:27370496-2737
ENSG0000(	1275	28.47399	chr2:309<	PPIL1P1		Pseudoger	chr2:26162056-2616
ENSG0000(	1275	28.47399	chr2:309<	NCOA1	IntOGen-I	protein_c	chr2:24491254-2477
ENSG0000(	1275	28.47399	chr2:309<	EMP2P1		Pseudoger	chr2:26140263-2614
ENSG0000(	1275	28.47399	chr2:309<	NRBP1		protein_c	chr2:27427790-2744
ENSG0000(	1275	28.47399	chr2:309<	PGAM1P6		Pseudoger	chr2:23872268-2387
ENSG0000(	1275	28.47399	chr2:309<	PREB		protein_c	chr2:27130756-2713
ENSG0000(	1275	28.47399	chr2:309<	SLC5A6		protein_c	chr2:27199587-2721
ENSG0000(	1275	28.47399	chr2:309<	MIR1301		smallRNA	chr2:25328640-2532
ENSG0000(	1275	28.47399	chr2:309<	EMILIN1		protein_c	chr2:27078615-2708
ENSG0000(	1275	28.47399	chr2:309<	RPL37P11		Pseudoger	chr2:26678219-2667
ENSG0000(	1275	28.47399	chr2:309<	ATRAID		protein_c	chr2:27212041-2721
ENSG0000(	1275	28.47399	chr2:309<	RN7SL610P		smallRNA	chr2:23996797-2399
ENSG0000(	1275	28.47399	chr2:309<	CENPO		protein_c	chr2:24793136-2482
ENSG0000(	1275	28.47399	chr2:309<	CDKN2AIPNLP2		Pseudoger	chr2:26827169-2682
ENSG0000(	1275	28.47399	chr2:309<	TRIM54		protein_c	chr2:27282429-2730

ENSG0000(	1275	28.47399	chr2:309<	DTNB		protein_c	chr2:25377198-2567
ENSG0000(	1275	28.47399	chr2:309<	RNU6-370P		smallRNA	chr2:24045835-2404
ENSG0000(	1275	28.47399	chr2:309<	ENSG00000283031		lncRNA	chr2:23667208-2368
ENSG0000(	1275	28.47399	chr2:309<	ADCY3	NCGv7	protein_c	chr2:24819169-2492
ENSG0000(	1275	28.47399	chr2:309<	KHK		protein_c	chr2:27086747-2710
ENSG0000(	1275	28.47399	chr2:309<	ITSN2		protein_c	chr2:24202864-2436
ENSG0000(	1275	28.47399	chr2:309<	WDCP	AC	protein_c	chr2:24029347-2404
ENSG0000(	1275	28.47399	chr2:309<	TPM3P7		Pseudoger	chr2:25809925-2581
ENSG0000(	1275	28.47399	chr2:309<	ADGRF3		protein_c	chr2:26308173-2634
ENSG0000(	1275	28.47399	chr2:309<	RPL36AP13		Pseudoger	chr2:24334512-2433
ENSG0000(	1275	28.47399	chr2:309<	RN7SL856P		smallRNA	chr2:25058032-2505
ENSG0000(	1275	28.47399	chr2:309<	HADHB		protein_c	chr2:26243170-2629
ENSG0000(	1275	28.47399	chr2:309<	FAM166C		protein_c	chr2:26562585-2657
ENSG0000(	1275	28.47399	chr2:309<	ENSG00000224361		lncRNA	chr2:23507043-2352
ENSG0000(	1275	28.47399	chr2:309<	RPS13P5		Pseudoger	chr2:24968958-2496
ENSG0000(	1275	28.47399	chr2:309<	SELENOI		protein_c	chr2:26308547-2639
ENSG0000(	1275	28.47399	chr2:309<	ARNILA		lncRNA	chr2:25369136-2537
ENSG0000(	1275	28.47399	chr2:309<	DNAJC5G		protein_c	chr2:27275433-2728
ENSG0000(	1275	28.47399	chr2:309<	ZNF513		protein_c	chr2:27377235-2738
ENSG0000(	1275	28.47399	chr2:309<	CENPA		protein_c	chr2:26764289-2680
ENSG0000(	1275	28.47399	chr2:309<	OTOF		protein_c	chr2:26457203-2655
ENSG0000(	1275	28.47399	chr2:309<	PFN4		protein_c	chr2:24114809-2412
ENSG0000(	1275	28.47399	chr2:309<	AC010150.2		smallRNA	chr2:25722192-2572
ENSG0000(	1275	28.47399	chr2:309<	PRR30		protein_c	chr2:27136848-2713
ENSG0000(	1275	28.47399	chr2:309<	POMC		protein_c	chr2:25160853-2516
ENSG0000(	1275	28.47399	chr2:309<	AGBL5-IT1		lncRNA	chr2:27061038-2706
ENSG0000(	1275	28.47399	chr2:309<	DNAJC27		protein_c	chr2:24943636-2497
ENSG0000(	1275	28.47399	chr2:309<	TP53I3		protein_c	chr2:24077433-2408
ENSG0000(	1275	28.47399	chr2:309<	KLHL29		protein_c	chr2:23385179-2370
ENSG0000(	1275	28.47399	chr2:309<	ENSG00000276087		protein_c	chr2:24124366-2419
ENSG0000(	1275	28.47399	chr2:309<	DNMT3A	NCGv7;AC	protein_c	chr2:25227855-2534
ENSG0000(	1275	28.47399	chr2:309<	TMEM214		protein_c	chr2:27032910-2704
ENSG0000(	1275	28.47399	chr2:309<	ENSG00000218682		Pseudoger	chr2:25856461-2585
ENSG0000(	1275	28.47399	chr2:309<	UCN		protein_c	chr2:27307400-2730
ENSG0000(	1275	28.47399	chr2:309<	ATAD2B		protein_c	chr2:23748664-2392
ENSG0000(	1275	28.47399	chr2:309<	SF3B6		protein_c	chr2:24067586-2407
ENSG0000(	1275	28.47399	chr2:309<	ENSG00000223754		lncRNA	chr2:24199839-2420
ENSG0000(	1275	28.47399	chr2:309<	Y_RNA		smallRNA	chr2:25008845-2500
ENSG0000(	1275	28.47399	chr2:309<	SUCLA2P3		Pseudoger	chr2:25079901-2508
ENSG0000(	1275	28.47399	chr2:309<	HMGN2P20		Pseudoger	chr2:24330402-2433
ENSG0000(	1275	28.47399	chr2:309<	TCF23		protein_c	chr2:27149004-2715
ENSG0000(	1275	28.47399	chr2:309<	FAM228A		protein_c	chr2:24175053-2420
ENSG0000(	1275	28.47399	chr2:309<	MAPRE3-AS1		lncRNA	chr2:26984776-2701
ENSG0000(	1275	28.47399	chr2:309<	FAM228B		protein_c	chr2:24076526-2416
ENSG0000(	1275	28.47399	chr2:309<	UQCRHP2		Pseudoger	chr2:25994794-2599
ENSG0000(	1275	28.47399	chr2:309<	ENSG00000289567		lncRNA	chr2:25362232-2536
ENSG0000(	1275	28.47399	chr2:309<	ENSG00000286707		lncRNA	chr2:26298570-2630
ENSG0000(	1275	28.47399	chr2:309<	ENSG00000272148		lncRNA	chr2:27062428-2706
ENSG0000(	1275	28.47399	chr2:309<	GTF3C2-AS2		lncRNA	chr2:27356246-2736
ENSG0000(	1275	28.47399	chr2:309<	FKBP1B	DriverDB\	protein_c	chr2:24049701-2406
ENSG0000(	1275	28.47399	chr2:309<	EIF2B4		protein_c	chr2:27364352-2737

ENSG0000(	1275	28.47399	chr2:309	Y_RNA		smallRNA	chr2:25697076-2569
ENSG0000(	1275	28.47399	chr2:309	GTF3C2		protein_c	chr2:27325849-2735
ENSG0000(	1275	28.47399	chr2:309	MPV17		protein_c	chr2:27309492-2732
ENSG0000(	1275	28.47399	chr2:309	SLC30A3		protein_c	chr2:27253684-2727
ENSG0000(	1275	28.47399	chr2:309	SLC35F6		protein_c	chr2:26764284-2678
ENSG0000(	1250	27.91568	chr2:309	CEBPZ		protein_c	chr2:37201612-3723
ENSG0000(	1250	27.91568	chr2:309	ARL14EPP1		Pseudoger	chr2:37148530-3714
ENSG0000(	1250	27.91568	chr2:309	SULT6B1		protein_c	chr2:37167820-3719
ENSG0000(	1250	27.91568	chr2:309	FEZ2		protein_c	chr2:36531805-3664
ENSG0000(	1250	27.91568	chr2:309	CDC42EP3		protein_c	chr2:37641882-3773
ENSG0000(	1250	27.91568	chr2:309	ENSG00000279519		TEC	chr2:36839922-3684
ENSG0000(	1250	27.91568	chr2:309	RNU6-846P		smallRNA	chr2:36959362-3695
ENSG0000(	1250	27.91568	chr2:309	ENSG00000273090		lncRNA	chr2:36513255-3651
ENSG0000(	1250	27.91568	chr2:309	RNU6-1116P		smallRNA	chr2:37435510-3743
ENSG0000(	1250	27.91568	chr2:309	ENSG00000285925		lncRNA	chr2:37339957-3734
ENSG0000(	1250	27.91568	chr2:309	RNU6-577P		smallRNA	chr2:36867398-3686
ENSG0000(	1250	27.91568	chr2:309	PRKD3		protein_c	chr2:37250502-3732
ENSG0000(	1250	27.91568	chr2:309	ENSG00000290100		lncRNA	chr2:37617328-3766
ENSG0000(	1250	27.91568	chr2:309	PRKD3-DT		lncRNA	chr2:37325340-3732
ENSG0000(	1250	27.91568	chr2:309	CEBPZOS		protein_c	chr2:37196488-3721
ENSG0000(	1250	27.91568	chr2:309	GPATCH11		protein_c	chr2:37084518-3709
ENSG0000(	1250	27.91568	chr2:309	ENSG00000289545		lncRNA	chr2:37489453-3760
ENSG0000(	1250	27.91568	chr2:309	ENSG00000225402		Pseudoger	chr2:37816915-3781
ENSG0000(	1250	27.91568	chr2:309	HEATR5B		protein_c	chr2:36968383-3708
ENSG0000(	1250	27.91568	chr2:309	RACK1P2		Pseudoger	chr2:36656322-3665
ENSG0000(	1250	27.91568	chr2:309	STRN	IntOGen-I	protein_c	chr2:36837698-3696
ENSG0000(	1250	27.91568	chr2:309	ENSG00000287316		lncRNA	chr2:37466781-3752
ENSG0000(	1250	27.91568	chr2:309	NDUFAF7		protein_c	chr2:37231631-3725
ENSG0000(	1250	27.91568	chr2:309	RPL31P16		Pseudoger	chr2:37194382-3719
ENSG0000(	1250	27.91568	chr2:309	ENSG00000272054		lncRNA	chr2:37208875-3721
ENSG0000(	1250	27.91568	chr2:309	LINC00211		lncRNA	chr2:37820498-3787
ENSG0000(	1250	27.91568	chr2:309	EIF2AK2		protein_c	chr2:37099210-3715
ENSG0000(	1250	27.91568	chr2:309	RNU6-939P		smallRNA	chr2:37331770-3733
ENSG0000(	1250	27.91568	chr2:309	ENSG00000236213		lncRNA	chr2:37562486-3764
ENSG0000(	1250	27.91568	chr2:309	QPCT		protein_c	chr2:37342827-3737
ENSG0000(	1250	27.91568	chr2:309	ENSG00000236572		lncRNA	chr2:37744333-3774
ENSG0000(	1250	27.91568	chr2:309	VIT	NCGv7	protein_c	chr2:36696690-3681
ENSG0000(	1235	27.58069	chr1:219	MIPEPP2		Pseudoger	chr1:238777049-238
ENSG0000(	1235	27.58069	chr1:219	ENSG00000227854		Pseudoger	chr1:239052748-239
ENSG0000(	1235	27.58069	chr1:219	ENSG00000259776		lncRNA	chr1:239247808-239
ENSG0000(	1235	27.58069	chr1:219	ENSG00000287177		lncRNA	chr1:238842767-238
ENSG0000(	1235	27.58069	chr1:219	ENSG00000287589		lncRNA	chr1:239205138-239
ENSG0000(	1221	27.26804	chr2:309	MIR548AD		smallRNA	chr2:35471405-3547
ENSG0000(	1221	27.26804	chr2:309	CRIM1-DT		lncRNA	chr2:36354744-3635
ENSG0000(	1221	27.26804	chr2:309	ENSG00000237320		lncRNA	chr2:34998273-3500
ENSG0000(	1221	27.26804	chr2:309	RN7SL602P		smallRNA	chr2:34809253-3480
ENSG0000(	1221	27.26804	chr2:309	ENSG00000288535		lncRNA	chr2:35263711-3528
ENSG0000(	1221	27.26804	chr2:309	ENSG00000232153		lncRNA	chr2:34732287-3482
ENSG0000(	1221	27.26804	chr2:309	ENSG00000229013		Pseudoger	chr2:35471716-3547
ENSG0000(	1221	27.26804	chr2:309	MRPL50P1		Pseudoger	chr2:35724759-3572
ENSG0000(	1221	27.26804	chr2:309	ENSG00000272027		lncRNA	chr2:34692290-3470

ENSG0000(	1221	27.26804	chr2:309²	RNU6-1117P		smallRNA	chr2:35471605-3547
ENSG0000(	1221	27.26804	chr2:309²	AC011748.1		smallRNA	chr2:34403664-3440
ENSG0000(	1221	27.26804	chr2:309²	RPL21P36		Pseudoger	chr2:36299388-3629
ENSG0000(	1221	27.26804	chr2:309²	ENSG00000226994		lncRNA	chr2:34799850-3518
ENSG0000(	1221	27.26804	chr2:309²	CRIM1		protein_c	chr2:36355778-3655
ENSG0000(	1221	27.26804	chr2:309²	SMIM7P1		Pseudoger	chr2:35219377-3521
ENSG0000(	1208	26.97771	chr2:309²	BIRC6	NCGv7	protein_c	chr2:32357023-3261
ENSG0000(	1208	26.97771	chr2:309²	ENSG00000276517		lncRNA	chr2:32526504-3252
ENSG0000(	1208	26.97771	chr2:309²	ENSG00000270422		Pseudoger	chr2:31651381-3165
ENSG0000(	1208	26.97771	chr2:309²	AL121652.2		smallRNA	chr2:31810195-3181
ENSG0000(	1208	26.97771	chr2:309²	KRT18P52		Pseudoger	chr2:31822591-3182
ENSG0000(	1208	26.97771	chr2:309²	LINC01946		lncRNA	chr2:31793823-3180
ENSG0000(	1208	26.97771	chr2:309²	ENSG00000289727		lncRNA	chr2:32233386-3226
ENSG0000(	1208	26.97771	chr2:309²	SNORD112		smallRNA	chr2:32991259-3299
ENSG0000(	1208	26.97771	chr2:309²	RNA5SP90		Pseudoger	chr2:31228312-3122
ENSG0000(	1208	26.97771	chr2:309²	ENSG00000273165		lncRNA	chr2:31852976-3185
ENSG0000(	1208	26.97771	chr2:309²	ENSG00000286415		lncRNA	chr2:33599442-3365
ENSG0000(	1208	26.97771	chr2:309²	ENSG00000228563		lncRNA	chr2:31526942-3156
ENSG0000(	1208	26.97771	chr2:309²	NLRC4		protein_c	chr2:32224453-3226
ENSG0000(	1208	26.97771	chr2:309²	H2ACP2		Pseudoger	chr2:33056333-3306
ENSG0000(	1208	26.97771	chr2:309²	ENSG00000274159		lncRNA	chr2:32548675-3254
ENSG0000(	1208	26.97771	chr2:309²	RNA5SP91		Pseudoger	chr2:33285769-3328
ENSG0000(	1208	26.97771	chr2:309²	AK2P2		Pseudoger	chr2:31823413-3182
ENSG0000(	1208	26.97771	chr2:309²	ENSG00000279544		TEC	chr2:32563328-3256
ENSG0000(	1208	26.97771	chr2:309²	RNA5SP92		Pseudoger	chr2:33332898-3333
ENSG0000(	1208	26.97771	chr2:309²	GALNT14		protein_c	chr2:30910467-3115
ENSG0000(	1208	26.97771	chr2:309²	AC009305.2		smallRNA	chr2:31035553-3103
ENSG0000(	1208	26.97771	chr2:309²	LINC00486		lncRNA	chr2:32927085-3294
ENSG0000(	1208	26.97771	chr2:309²	MIR558		smallRNA	chr2:32532153-3253
ENSG0000(	1208	26.97771	chr2:309²	XDH	NCGv7	protein_c	chr2:31334321-3141
ENSG0000(	1208	26.97771	chr2:309²	LINC01318		lncRNA	chr2:34067226-3406
ENSG0000(	1208	26.97771	chr2:309²	ENSG00000271228		lncRNA	chr2:32013061-3201
ENSG0000(	1208	26.97771	chr2:309²	MYADML		Pseudoger	chr2:33722721-3372
ENSG0000(	1208	26.97771	chr2:309²	ENSG00000276334		lncRNA	chr2:32521927-3252
ENSG0000(	1208	26.97771	chr2:309²	ENSG00000234579		lncRNA	chr2:30986939-3099
ENSG0000(	1208	26.97771	chr2:309²	ENSG00000230118		Pseudoger	chr2:30746444-3074
ENSG0000(	1208	26.97771	chr2:309²	BIRC6-AS2		Pseudoger	chr2:32557703-3255
ENSG0000(	1208	26.97771	chr2:309²	AC069303.1		smallRNA	chr2:33786038-3378
ENSG0000(	1208	26.97771	chr2:309²	ENSG00000272754		lncRNA	chr2:32321638-3232
ENSG0000(	1208	26.97771	chr2:309²	BIRC6-AS2		lncRNA	chr2:32557273-3257
ENSG0000(	1208	26.97771	chr2:309²	ENSG00000213620		Pseudoger	chr2:31290762-3129
ENSG0000(	1208	26.97771	chr2:309²	CAPN13		protein_c	chr2:30722771-3082
ENSG0000(	1208	26.97771	chr2:309²	HNRNPA1P61		Pseudoger	chr2:33636502-3363
ENSG0000(	1208	26.97771	chr2:309²	MEMO1		protein_c	chr2:31865060-3201
ENSG0000(	1208	26.97771	chr2:309²	DPY30		protein_c	chr2:31867809-3203
ENSG0000(	1208	26.97771	chr2:309²	AL121652.3		smallRNA	chr2:31823018-3182
ENSG0000(	1208	26.97771	chr2:309²	ENSG00000288937		lncRNA	chr2:32039839-3204
ENSG0000(	1208	26.97771	chr2:309²	AL121652.1		smallRNA	chr2:31919862-3191
ENSG0000(	1208	26.97771	chr2:309²	CAPN14		protein_c	chr2:31173056-3123
ENSG0000(	1208	26.97771	chr2:309²	RNU6-647P		smallRNA	chr2:32214456-3221
ENSG0000(	1208	26.97771	chr2:309²	ENSG00000285577		lncRNA	chr2:33274465-3328

ENSG0000(	1208	26.97771	chr2:309<	LINC00486		lncRNA	chr2:32825359-3292
ENSG0000(	1208	26.97771	chr2:309<	LTBP1		protein_c	chr2:32946953-3339
ENSG0000(	1208	26.97771	chr2:309<	SLC25A5P2		Pseudoger	chr2:33839782-3384
ENSG0000(	1208	26.97771	chr2:309<	EHD3		protein_c	chr2:31234152-3126
ENSG0000(	1208	26.97771	chr2:309<	MIR4765		smallRNA	chr2:32635255-3263
ENSG0000(	1208	26.97771	chr2:309<	LINC01320		lncRNA	chr2:33706886-3473
ENSG0000(	1208	26.97771	chr2:309<	TTC27		protein_c	chr2:32628032-3282
ENSG0000(	1208	26.97771	chr2:309<	SPAST		protein_c	chr2:32063556-3215
ENSG0000(	1208	26.97771	chr2:309<	Y_RNA		smallRNA	chr2:32945339-3294
ENSG0000(	1208	26.97771	chr2:309<	YIPF4		protein_c	chr2:32277904-3231
ENSG0000(	1208	26.97771	chr2:309<	FAM98A		protein_c	chr2:33532744-3359
ENSG0000(	1208	26.97771	chr2:309<	AC097506.1		smallRNA	chr2:33927384-3392
ENSG0000(	1208	26.97771	chr2:309<	BIRC6-AS1		lncRNA	chr2:32377631-3237
ENSG0000(	1208	26.97771	chr2:309<	AL121655.1		smallRNA	chr2:32088304-3208
ENSG0000(	1208	26.97771	chr2:309<	ATP6V0E1P3		Pseudoger	chr2:33602041-3360
ENSG0000(	1208	26.97771	chr2:309<	RASGRP3		protein_c	chr2:33436324-3356
ENSG0000(	1208	26.97771	chr2:309<	SLC30A6	NCGv7	protein_c	chr2:32165841-3222
ENSG0000(	1208	26.97771	chr2:309<	SRD5A2		protein_c	chr2:31522480-3158
ENSG0000(	1208	26.97771	chr2:309<	DDX50P1		Pseudoger	chr2:32201600-3220
ENSG0000(	1208	26.97771	chr2:309<	ENSG00000285984		lncRNA	chr2:30887626-3089
ENSG0000(	1208	26.97771	chr2:309<	ENSG00000285754		lncRNA	chr2:34134371-3422
ENSG0000(	1208	26.97771	chr2:309<	SLC30A6-DT		lncRNA	chr2:32165041-3216
ENSG0000(	1201	26.82138	chr2:309<	ENSG00000278908		TEC	chr2:30677762-3067
ENSG0000(	1191	26.59806	chr8:412(	NRBF2P4		Pseudoger	chr8:107983116-107
ENSG0000(	1188	26.53106	chr2:309<	LCLAT1		protein_c	chr2:30447226-3064
ENSG0000(	1188	26.53106	chr2:309<	ENSG00000287658		lncRNA	chr2:30343222-3034
ENSG0000(	1188	26.53106	chr2:309<	LINC01936		lncRNA	chr2:30346623-3036
ENSG0000(	1188	26.53106	chr2:309<	AC073255.1		smallRNA	chr2:30432353-3043
ENSG0000(	1188	26.53106	chr2:309<	LBH		protein_c	chr2:30231534-3032
ENSG0000(	1188	26.53106	chr2:309<	ENSG00000280154		TEC	chr2:30408170-3040
ENSG0000(	1182	26.39707	chr2:309<	Y_RNA		smallRNA	chr2:28927243-2892
ENSG0000(	1182	26.39707	chr2:309<	SNRPGP7		Pseudoger	chr2:28460256-2846
ENSG0000(	1182	26.39707	chr2:309<	ENSG00000270210		lncRNA	chr2:28425945-2842
ENSG0000(	1182	26.39707	chr2:309<	ALK	NCGv7;AC	protein_c	chr2:29192774-2992
ENSG0000(	1182	26.39707	chr2:309<	SNORD53		smallRNA	chr2:28927067-2892
ENSG0000(	1182	26.39707	chr2:309<	RNA5SP89		Pseudoger	chr2:28683976-2868
ENSG0000(	1182	26.39707	chr2:309<	SNORA64		smallRNA	chr2:30187434-3018
ENSG0000(	1182	26.39707	chr2:309<	ENSG00000229224		lncRNA	chr2:29088649-2909
ENSG0000(	1182	26.39707	chr2:309<	SNORD92		smallRNA	chr2:28913664-2891
ENSG0000(	1182	26.39707	chr2:309<	AC106899.1		smallRNA	chr2:29503907-2950
ENSG0000(	1182	26.39707	chr2:309<	YPEL5		protein_c	chr2:30146941-3016
ENSG0000(	1182	26.39707	chr2:309<	ENSG00000227938		lncRNA	chr2:28448167-2845
ENSG0000(	1182	26.39707	chr2:309<	WDR43		protein_c	chr2:28894667-2894
ENSG0000(	1182	26.39707	chr2:309<	PLB1	NCGv7	protein_c	chr2:28457145-2864
ENSG0000(	1182	26.39707	chr2:309<	SPDYA		protein_c	chr2:28782517-2885
ENSG0000(	1182	26.39707	chr2:309<	CLIP4		protein_c	chr2:29097705-2919
ENSG0000(	1182	26.39707	chr2:309<	PPP1CB-DT		lncRNA	chr2:28707511-2875
ENSG0000(	1182	26.39707	chr2:309<	RN7SL516P		smallRNA	chr2:29681029-2968
ENSG0000(	1182	26.39707	chr2:309<	Y_RNA		smallRNA	chr2:28972414-2897
ENSG0000(	1182	26.39707	chr2:309<	PPP1CB		protein_c	chr2:28751640-2880
ENSG0000(	1182	26.39707	chr2:309<	H3P5		Pseudoger	chr2:30209995-3021

ENSG00000	1182	26.39707	chr2:3094	TOGARAM2		protein_c	chr2:28956611-2905
ENSG00000	1182	26.39707	chr2:3094	TRMT61B		protein_c	chr2:28849821-2887
ENSG00000	1182	26.39707	chr2:3094	ENSG00000197644		lncRNA	chr2:29899597-2990
ENSG00000	1182	26.39707	chr2:3094	ENSG00000233862		lncRNA	chr2:30051066-3014
ENSG00000	1182	26.39707	chr2:3094	ENSG00000225943		Pseudoger	chr2:30077093-3007
ENSG00000	1182	26.39707	chr2:3094	PCARE		protein_c	chr2:29060976-2907
ENSG00000	1182	26.39707	chr2:3094	ENSG00000273233		lncRNA	chr2:28810281-2881
ENSG00000	1182	26.39707	chr2:3094	AC105398.1		smallRNA	chr2:29081270-2908
ENSG00000	1182	26.39707	chr2:3094	AC016907.1		smallRNA	chr2:30066124-3006
ENSG00000	1182	26.39707	chr2:3094	ENSG00000286963		lncRNA	chr2:29319554-2935
ENSG00000	1182	26.39707	chr2:3094	ENSG00000288553		lncRNA	chr2:29841187-2995
ENSG00000	1182	26.39707	chr2:3094	ENSG00000230730		lncRNA	chr2:28633282-2866
ENSG00000	1182	26.39707	chr2:3094	SNORD53_SNORD92		smallRNA	chr2:28927983-2892
ENSG00000	1182	26.39707	chr2:3094	ENSG00000230737		lncRNA	chr2:29890371-2989
ENSG00000	1101	24.58813	chr6:1039	ENSG00000238079		Pseudoger	chr6:108372772-108
ENSG00000	1088	24.29781	chr2:3094	ENSG00000232719		lncRNA	chr2:201101382-201
ENSG00000	1088	24.29781	chr2:3094	RPL17P10		Pseudoger	chr2:201102089-201
ENSG00000	1088	24.29781	chr2:3094	NDUFB3		protein_c	chr2:201071433-201
ENSG00000	1088	24.29781	chr2:3094	CFLAR-AS1		lncRNA	chr2:201140278-201
ENSG00000	1088	24.29781	chr2:3094	TRAK2		protein_c	chr2:201377207-201
ENSG00000	1088	24.29781	chr2:3094	ENSG00000183308		lncRNA	chr2:200963263-201
ENSG00000	1088	24.29781	chr2:3094	ENSG00000291279		lncRNA	chr2:200740821-200
ENSG00000	1088	24.29781	chr2:3094	AOX3P		Pseudoger	chr2:200678233-200
ENSG00000	1088	24.29781	chr2:3094	RNU6-312P		smallRNA	chr2:200881715-200
ENSG00000	1088	24.29781	chr2:3094	AOX3P-AOX2P		lncRNA	chr2:200695723-200
ENSG00000	1088	24.29781	chr2:3094	MTND3P16		Pseudoger	chr2:201553688-201
ENSG00000	1088	24.29781	chr2:3094	LINC01792		lncRNA	chr2:200711535-200
ENSG00000	1088	24.29781	chr2:3094	ORC2		protein_c	chr2:200908977-200
ENSG00000	1088	24.29781	chr2:3094	MTND4LP13		Pseudoger	chr2:201214653-201
ENSG00000	1088	24.29781	chr2:3094	PPIL3		protein_c	chr2:200870907-200
ENSG00000	1088	24.29781	chr2:3094	UBE2V1P11		Pseudoger	chr2:201824548-201
ENSG00000	1088	24.29781	chr2:3094	BZW1-AS1		lncRNA	chr2:200780495-200
ENSG00000	1088	24.29781	chr2:3094	Y_RNA		smallRNA	chr2:201808681-201
ENSG00000	1088	24.29781	chr2:3094	RNU7-45P		smallRNA	chr2:201141904-201
ENSG00000	1088	24.29781	chr2:3094	ALS2		protein_c	chr2:201700267-201
ENSG00000	1088	24.29781	chr2:3094	CLK1		protein_c	chr2:200853009-200
ENSG00000	1088	24.29781	chr2:3094	CASP10	NCGv7	protein_c	chr2:201182872-201
ENSG00000	1088	24.29781	chr2:3094	RPL23AP30		Pseudoger	chr2:201066691-201
ENSG00000	1088	24.29781	chr2:3094	RNU6-31P		smallRNA	chr2:200830009-200
ENSG00000	1088	24.29781	chr2:3094	ENSG00000289698		lncRNA	chr2:201273053-201
ENSG00000	1088	24.29781	chr2:3094	RNU6-1206P		smallRNA	chr2:201079842-201
ENSG00000	1088	24.29781	chr2:3094	CFLAR	AC	protein_c	chr2:201116154-201
ENSG00000	1088	24.29781	chr2:3094	RNU6-440P		smallRNA	chr2:201927439-201
ENSG00000	1088	24.29781	chr2:3094	HNRNPA1P35		Pseudoger	chr2:201063089-201
ENSG00000	1088	24.29781	chr2:3094	TMEM237		protein_c	chr2:201620184-201
ENSG00000	1088	24.29781	chr2:3094	MTND4LP16		Pseudoger	chr2:201554096-201
ENSG00000	1088	24.29781	chr2:3094	AC005037.2		smallRNA	chr2:200843186-200
ENSG00000	1088	24.29781	chr2:3094	ENSG00000290906		lncRNA	chr2:200762308-200
ENSG00000	1088	24.29781	chr2:3094	BICD1P1		Pseudoger	chr2:200844240-200
ENSG00000	1088	24.29781	chr2:3094	CASP8	NCGv7;AC	protein_c	chr2:201233443-201
ENSG00000	1088	24.29781	chr2:3094	MTND4P23		Pseudoger	chr2:201213298-201

ENSG0000(	1088	24.29781	chr2:309⁄	ENSG00000289026		lncRNA	chr2:202032072-202
ENSG0000(	1088	24.29781	chr2:309⁄	BZW1		protein_c	chr2:200810594-200
ENSG0000(	1088	24.29781	chr2:309⁄	STRADB		protein_c	chr2:201387858-201
ENSG0000(	1088	24.29781	chr2:309⁄	MPP4		protein_c	chr2:201644870-201
ENSG0000(	1088	24.29781	chr2:309⁄	RPL38P5		Pseudoger	chr2:201162484-201
ENSG0000(	1088	24.29781	chr2:309⁄	RNA5SP115		Pseudoger	chr2:200849038-200
ENSG0000(	1088	24.29781	chr2:309⁄	AC007358.1		smallRNA	chr2:201953257-201
ENSG0000(	1088	24.29781	chr2:309⁄	RNU6-762P		smallRNA	chr2:200899020-200
ENSG0000(	1088	24.29781	chr2:309⁄	MTCO3P16		Pseudoger	chr2:201552859-201
ENSG0000(	1088	24.29781	chr2:309⁄	CDK15		protein_c	chr2:201790461-201
ENSG0000(	1088	24.29781	chr2:309⁄	RNU6-651P		smallRNA	chr2:201646716-201
ENSG0000(	1088	24.29781	chr2:309⁄	AC007242.1		smallRNA	chr2:201841129-201
ENSG0000(	1088	24.29781	chr2:309⁄	MTND5P25		Pseudoger	chr2:201212283-201
ENSG0000(	1088	24.29781	chr2:309⁄	Y_RNA		smallRNA	chr2:200863152-200
ENSG0000(	1088	24.29781	chr2:309⁄	ENO1P4		Pseudoger	chr2:201621646-201
ENSG0000(	1088	24.29781	chr2:309⁄	FAM126B	NCGv7	protein_c	chr2:200973718-201
ENSG0000(	1088	24.29781	chr2:309⁄	SCYL2P1		Pseudoger	chr2:201410544-201
ENSG0000(	1088	24.29781	chr2:309⁄	FLACC1		protein_c	chr2:201288271-201
ENSG0000(	1088	24.29781	chr2:309⁄	ENSG00000287133		lncRNA	chr2:201643398-201
ENSG0000(	1088	24.29781	chr2:309⁄	MTND5P31		Pseudoger	chr2:201556892-201
ENSG0000(	1088	24.29781	chr2:309⁄	IMPDH1P10		Pseudoger	chr2:201137516-201
ENSG0000(	1088	24.29781	chr2:309⁄	MTND4P29		Pseudoger	chr2:201554384-201
ENSG0000(	1088	24.29781	chr2:309⁄	ENSG00000290905		lncRNA	chr2:200695734-200
ENSG0000(	1088	24.29781	chr2:309⁄	NIF3L1		protein_c	chr2:200889327-200
ENSG0000(	1088	24.29781	chr2:309⁄	C2CD6	NCGv7	protein_c	chr2:201487421-201
ENSG0000(	1088	24.29781	chr2:309⁄	RPS2P16		Pseudoger	chr2:201762737-201
ENSG0000(	1088	24.29781	chr2:309⁄	AOX2P		Pseudoger	chr2:200738608-200
ENSG0000(	1088	24.29781	chr2:309⁄	Y_RNA		smallRNA	chr2:201423513-201
ENSG0000(	1088	24.29781	chr2:309⁄	RNU1-133P		smallRNA	chr2:200775584-200
ENSG0000(	1088	24.29781	chr2:309⁄	RN7SL694P		smallRNA	chr2:200927544-200
ENSG0000(	1088	24.29781	chr2:309⁄	ENSG00000234431		lncRNA	chr2:201166965-201
ENSG0000(	1088	24.29781	chr2:309⁄	MTATP6P16		Pseudoger	chr2:201550524-201
ENSG0000(	1088	24.29781	chr2:309⁄	MTCO2P16		Pseudoger	chr2:201549631-201
ENSG0000(	1020	22.77919	chr7:158ٰ	ENSG00000224136		Pseudoger	chr7:117882859-117
ENSG0000(	1010	22.55587	chr6:103ٰ	AL136446.1		smallRNA	chr6:114277385-114
ENSG0000(	1006	22.46654	chr11:58ٰ	RNU6-1123P		smallRNA	chr11:119656311-11
ENSG0000(	997	22.26555	chr12:171	ENSG00000274737		lncRNA	chr12:47817451-478
ENSG0000(	989	22.08689	chr2:309⁄	ENSG00000271629		Pseudoger	chr2:21198518-2119
ENSG0000(	989	22.08689	chr2:309⁄	ENSG00000270100		lncRNA	chr2:20678254-2067
ENSG0000(	989	22.08689	chr2:309⁄	ENSG00000280390		TEC	chr2:21023496-2102
ENSG0000(	989	22.08689	chr2:309⁄	Y_RNA		smallRNA	chr2:20480844-2048
ENSG0000(	989	22.08689	chr2:309⁄	NDUFAF2P1		Pseudoger	chr2:20529467-2052
ENSG0000(	989	22.08689	chr2:309⁄	CISD1P1		Pseudoger	chr2:19826249-1982
ENSG0000(	989	22.08689	chr2:309⁄	TTC32		protein_c	chr2:19896631-1990
ENSG0000(	989	22.08689	chr2:309⁄	RNA5SP86		Pseudoger	chr2:20401627-2040
ENSG0000(	989	22.08689	chr2:309⁄	TTC32-DT		lncRNA	chr2:19902022-1990
ENSG0000(	989	22.08689	chr2:309⁄	RNU6-961P		smallRNA	chr2:20175805-2017
ENSG0000(	989	22.08689	chr2:309⁄	ENSG00000269976		lncRNA	chr2:20586248-2058
ENSG0000(	989	22.08689	chr2:309⁄	RPS16P2		Pseudoger	chr2:20155618-2015
ENSG0000(	989	22.08689	chr2:309⁄	RNA5SP87		Pseudoger	chr2:22338886-2233
ENSG0000(	989	22.08689	chr2:309⁄	NUTF2P8		Pseudoger	chr2:21362292-2136

ENSG0000(	989	22.08689	chr2:309⁄	LINC01808		lncRNA	chr2:19468997-1952
ENSG0000(	989	22.08689	chr2:309⁄	LDAH		protein_c	chr2:20684014-2082
ENSG0000(	989	22.08689	chr2:309⁄	APOB	NCGv7	protein_c	chr2:21001429-2104
ENSG0000(	989	22.08689	chr2:309⁄	ENSG00000290108		lncRNA	chr2:20446571-2044
ENSG0000(	989	22.08689	chr2:309⁄	LINC01822		lncRNA	chr2:21687430-2171
ENSG0000(	989	22.08689	chr2:309⁄	SDC1		protein_c	chr2:20200797-2022
ENSG0000(	989	22.08689	chr2:309⁄	ENSG00000235537		lncRNA	chr2:21607465-2163
ENSG0000(	989	22.08689	chr2:309⁄	ENSG00000233714		lncRNA	chr2:23347654-2335
ENSG0000(	989	22.08689	chr2:309⁄	HS1BP3	NCGv7	protein_c	chr2:20560448-2065
ENSG0000(	989	22.08689	chr2:309⁄	WDR35		protein_c	chr2:19910263-1999
ENSG0000(	989	22.08689	chr2:309⁄	RPS25P3		Pseudoger	chr2:20606280-2060
ENSG0000(	989	22.08689	chr2:309⁄	ENSG00000287956		lncRNA	chr2:21094101-2109
ENSG0000(	989	22.08689	chr2:309⁄	ENSG00000228999		lncRNA	chr2:21932662-2253
ENSG0000(	989	22.08689	chr2:309⁄	ENSG00000228950		lncRNA	chr2:20451042-2045
ENSG0000(	989	22.08689	chr2:309⁄	LINC00954		lncRNA	chr2:19868860-1988
ENSG0000(	989	22.08689	chr2:309⁄	ENSG00000228538		lncRNA	chr2:21638068-2164
ENSG0000(	989	22.08689	chr2:309⁄	LINC01376		lncRNA	chr2:18986451-1934
ENSG0000(	989	22.08689	chr2:309⁄	RNU7-113P		smallRNA	chr2:20246326-2024
ENSG0000(	989	22.08689	chr2:309⁄	LINC01884		lncRNA	chr2:22508129-2254
ENSG0000(	989	22.08689	chr2:309⁄	MIR4757		smallRNA	chr2:19348429-1934
ENSG0000(	989	22.08689	chr2:309⁄	TDRD15		protein_c	chr2:21123968-2114
ENSG0000(	989	22.08689	chr2:309⁄	DRG1P1		Pseudoger	chr2:20239892-2024
ENSG0000(	989	22.08689	chr2:309⁄	HS1BP3-IT1		lncRNA	chr2:20590775-2059
ENSG0000(	989	22.08689	chr2:309⁄	AC007041.1		smallRNA	chr2:20353642-2035
ENSG0000(	989	22.08689	chr2:309⁄	ENSG00000232451		lncRNA	chr2:23018125-2319
ENSG0000(	989	22.08689	chr2:309⁄	RN7SL140P		smallRNA	chr2:20175346-2017
ENSG0000(	989	22.08689	chr2:309⁄	ENSG00000261012		lncRNA	chr2:20999313-2100
ENSG0000(	989	22.08689	chr2:309⁄	RN7SKP27		smallRNA	chr2:22861552-2286
ENSG0000(	989	22.08689	chr2:309⁄	ENSG00000231204		lncRNA	chr2:21317660-2156
ENSG0000(	989	22.08689	chr2:309⁄	SLC7A15P		Pseudoger	chr2:20386386-2039
ENSG0000(	989	22.08689	chr2:309⁄	LAPTM4A-DT		lncRNA	chr2:20052114-2005
ENSG0000(	989	22.08689	chr2:309⁄	ENSG00000233005		lncRNA	chr2:21221169-2197
ENSG0000(	989	22.08689	chr2:309⁄	RHOB	NCGv7	protein_c	chr2:20447074-2044
ENSG0000(	989	22.08689	chr2:309⁄	GDF7		protein_c	chr2:20667144-2067
ENSG0000(	989	22.08689	chr2:309⁄	OSR1		protein_c	chr2:19351485-1935
ENSG0000(	989	22.08689	chr2:309⁄	ENSG00000223530		lncRNA	chr2:23330664-2333
ENSG0000(	989	22.08689	chr2:309⁄	ENSG00000223634		lncRNA	chr2:23357516-2336
ENSG0000(	989	22.08689	chr2:309⁄	ENSG00000223691		Pseudoger	chr2:22188336-2219
ENSG0000(	989	22.08689	chr2:309⁄	RN7SL117P		smallRNA	chr2:21922993-2192
ENSG0000(	989	22.08689	chr2:309⁄	LINC02850		lncRNA	chr2:20859771-2086
ENSG0000(	989	22.08689	chr2:309⁄	LINC01830		lncRNA	chr2:22377594-2248
ENSG0000(	989	22.08689	chr2:309⁄	ENSG00000234378		lncRNA	chr2:20063856-2010
ENSG0000(	989	22.08689	chr2:309⁄	ENSG00000235911		lncRNA	chr2:19711715-1971
ENSG0000(	989	22.08689	chr2:309⁄	WDR35-DT		lncRNA	chr2:19990209-2000
ENSG0000(	989	22.08689	chr2:309⁄	LAPTM4A	NCGv7	protein_c	chr2:20032650-2005
ENSG0000(	989	22.08689	chr2:309⁄	ENSG00000287284		lncRNA	chr2:18939697-1894
ENSG0000(	989	22.08689	chr2:309⁄	ENSG00000234189		Pseudoger	chr2:22317053-2231
ENSG0000(	989	22.08689	chr2:309⁄	ENSG00000234597		lncRNA	chr2:19458220-1946
ENSG0000(	989	22.08689	chr2:309⁄	MATN3		protein_c	chr2:19992052-2001
ENSG0000(	989	22.08689	chr2:309⁄	ENSG00000227047		lncRNA	chr2:20499571-2050
ENSG0000(	989	22.08689	chr2:309⁄	PUM2		protein_c	chr2:20248691-2035

ENSG0000(	985	21.99756	chr13:279	MIR623		smallRNA	chr13:99356131-993
ENSG0000(	980	21.88589	chr7:1588	ENSG00000240499		lncRNA	chr7:122328469-122
ENSG0000(	980	21.88589	chr7:1588	WASL-DT		lncRNA	chr7:123749068-123
ENSG0000(	980	21.88589	chr7:1588	ENSG00000226680		lncRNA	chr7:123069249-123
ENSG0000(	980	21.88589	chr7:1588	ENSG00000230520		Pseudoger	chr7:118950386-118
ENSG0000(	980	21.88589	chr7:1588	ASB15-AS1		lncRNA	chr7:123584859-123
ENSG0000(	980	21.88589	chr7:1588	FEZF1-AS1		lncRNA	chr7:122303658-122
ENSG0000(	980	21.88589	chr7:1588	RNU6-517P		smallRNA	chr7:121194948-121
ENSG0000(	980	21.88589	chr7:1588	WNT16		protein_c	chr7:121325367-121
ENSG0000(	980	21.88589	chr7:1588	ENSG00000226636		Pseudoger	chr7:122159300-122
ENSG0000(	980	21.88589	chr7:1588	RPS26P31		Pseudoger	chr7:122681315-122
ENSG0000(	980	21.88589	chr7:1588	ENSG00000225795		Pseudoger	chr7:122676580-122
ENSG0000(	980	21.88589	chr7:1588	LYPLA1P1		Pseudoger	chr7:123230120-123
ENSG0000(	980	21.88589	chr7:1588	HMGN1P18		Pseudoger	chr7:121050927-121
ENSG0000(	980	21.88589	chr7:1588	FAM3C		protein_c	chr7:121348878-121
ENSG0000(	980	21.88589	chr7:1588	GTF3AP6		Pseudoger	chr7:118880103-118
ENSG0000(	980	21.88589	chr7:1588	PNPT1P2		Pseudoger	chr7:121842368-121
ENSG0000(	980	21.88589	chr7:1588	ENSG00000234985		Pseudoger	chr7:121440834-121
ENSG0000(	980	21.88589	chr7:1588	ENSG00000234418		lncRNA	chr7:122144405-122
ENSG0000(	980	21.88589	chr7:1588	IQUB	DriverDBv	protein_c	chr7:123452193-123
ENSG0000(	980	21.88589	chr7:1588	ENSG00000289578		lncRNA	chr7:121450948-121
ENSG0000(	980	21.88589	chr7:1588	RNA5SP241		Pseudoger	chr7:121083700-121
ENSG0000(	980	21.88589	chr7:1588	ENSG00000270516		Pseudoger	chr7:119178177-119
ENSG0000(	980	21.88589	chr7:1588	AC006926.1		smallRNA	chr7:118462499-118
ENSG0000(	980	21.88589	chr7:1588	ENSG00000234826		lncRNA	chr7:117998858-118
ENSG0000(	980	21.88589	chr7:1588	RNU6-581P		smallRNA	chr7:120672871-120
ENSG0000(	980	21.88589	chr7:1588	ASB15	NCGv7	protein_c	chr7:123567010-123
ENSG0000(	980	21.88589	chr7:1588	RNU7-154P		smallRNA	chr7:122081720-122
ENSG0000(	980	21.88589	chr7:1588	CYCSP19		Pseudoger	chr7:121398452-121
ENSG0000(	980	21.88589	chr7:1588	ENSG00000213302		Pseudoger	chr7:122234531-122
ENSG0000(	980	21.88589	chr7:1588	WNT2	NCGv7;AC	protein_c	chr7:117275451-117
ENSG0000(	980	21.88589	chr7:1588	ANKRD7		protein_c	chr7:118214669-118
ENSG0000(	980	21.88589	chr7:1588	TSPAN12	DriverDBv	protein_c	chr7:120787320-120
ENSG0000(	980	21.88589	chr7:1588	CPED1		protein_c	chr7:120988697-121
ENSG0000(	980	21.88589	chr7:1588	PTPRZ1		protein_c	chr7:121873089-122
ENSG0000(	980	21.88589	chr7:1588	ANKRD49P4		Pseudoger	chr7:117439982-117
ENSG0000(	980	21.88589	chr7:1588	WASL		protein_c	chr7:123681943-123
ENSG0000(	980	21.88589	chr7:1588	HYAL4		protein_c	chr7:123828983-123
ENSG0000(	980	21.88589	chr7:1588	AC004875.1		smallRNA	chr7:121574669-121
ENSG0000(	980	21.88589	chr7:1588	ENSG00000289345		lncRNA	chr7:123580881-123
ENSG0000(	980	21.88589	chr7:1588	ENSG00000232524		lncRNA	chr7:123456629-123
ENSG0000(	980	21.88589	chr7:1588	ENSG00000231295		lncRNA	chr7:120746738-120
ENSG0000(	980	21.88589	chr7:1588	CFTR		protein_c	chr7:117287120-117
ENSG0000(	980	21.88589	chr7:1588	CFTR-AS1		lncRNA	chr7:117560733-117
ENSG0000(	980	21.88589	chr7:1588	ENSG00000233417		lncRNA	chr7:120141016-120
ENSG0000(	980	21.88589	chr7:1588	ENSG00000286390		lncRNA	chr7:117332761-117
ENSG0000(	980	21.88589	chr7:1588	AASS		protein_c	chr7:122064583-122
ENSG0000(	980	21.88589	chr7:1588	U1		smallRNA	chr7:120005976-120
ENSG0000(	980	21.88589	chr7:1588	ENSG00000227371		Pseudoger	chr7:121419072-121
ENSG0000(	980	21.88589	chr7:1588	RNF148		protein_c	chr7:122701668-122
ENSG0000(	980	21.88589	chr7:1588	CADPS2		protein_c	chr7:122318411-122

ENSG0000(	980	21.88589	chr7:158{	ING3		protein_c	chr7:120950763-120
ENSG0000(	980	21.88589	chr7:158{	RNA5SP240		Pseudoger	chr7:120981426-120
ENSG0000(	980	21.88589	chr7:158{	KCND2		protein_c	chr7:120273175-120
ENSG0000(	980	21.88589	chr7:158{	FEZF1	DriverDBv	protein_c	chr7:122301303-122
ENSG0000(	980	21.88589	chr7:158{	ENSG00000287827		lncRNA	chr7:118511942-118
ENSG0000(	980	21.88589	chr7:158{	SLC13A1	NCGv7	protein_c	chr7:123113531-123
ENSG0000(	980	21.88589	chr7:158{	ENSG00000227573		Pseudoger	chr7:122849746-122
ENSG0000(	980	21.88589	chr7:158{	RN7SKP277		smallRNA	chr7:121736443-121
ENSG0000(	980	21.88589	chr7:158{	RNU6-296P		smallRNA	chr7:123457988-123
ENSG0000(	980	21.88589	chr7:158{	HYAL6P		Pseudoger	chr7:123814139-123
ENSG0000(	980	21.88589	chr7:158{	ENSG00000287554		lncRNA	chr7:121304657-121
ENSG0000(	980	21.88589	chr7:158{	ENSG00000233969		lncRNA	chr7:120166443-120
ENSG0000(	980	21.88589	chr7:158{	LMOD2		protein_c	chr7:123655866-123
ENSG0000(	980	21.88589	chr7:158{	CTTNBP2		protein_c	chr7:117710651-117
ENSG0000(	980	21.88589	chr7:158{	ENSG00000234001		Pseudoger	chr7:117586207-117
ENSG0000(	980	21.88589	chr7:158{	ENSG00000227743		lncRNA	chr7:121643334-121
ENSG0000(	980	21.88589	chr7:158{	LINC02476		lncRNA	chr7:119495024-119
ENSG0000(	980	21.88589	chr7:158{	ASZ1	NCGv7	protein_c	chr7:117363222-117
ENSG0000(	980	21.88589	chr7:158{	RNU6-11P		smallRNA	chr7:123790605-123
ENSG0000(	980	21.88589	chr7:158{	LSM8		protein_c	chr7:118184144-118
ENSG0000(	980	21.88589	chr7:158{	ENSG00000083622		lncRNA	chr7:117604791-117
ENSG0000(	980	21.88589	chr7:158{	RNF133		protein_c	chr7:122697735-122
ENSG0000(	980	21.88589	chr7:158{	TAS2R16		protein_c	chr7:122994704-122
ENSG0000(	980	21.88589	chr7:158{	ENSG00000237974		Pseudoger	chr7:117487737-117
ENSG0000(	980	21.88589	chr7:158{	AC091320.2		smallRNA	chr7:119484325-119
ENSG0000(	980	21.88589	chr7:158{	NDUFA5	NCGv7	protein_c	chr7:123536997-123
ENSG0000(	969	21.64023	chr6:103{	RPF2		protein_c	chr6:110982015-111
ENSG0000(	969	21.64023	chr6:103{	ENSG00000219150		Pseudoger	chr6:110706362-110
ENSG0000(	969	21.64023	chr6:103{	GSTM2P1		Pseudoger	chr6:111046868-111
ENSG0000(	969	21.64023	chr6:103{	ENSG00000233908		Pseudoger	chr6:109288440-109
ENSG0000(	969	21.64023	chr6:103{	ENSG00000289256		lncRNA	chr6:111873588-111
ENSG0000(	969	21.64023	chr6:103{	FCF1P5		Pseudoger	chr6:111353702-111
ENSG0000(	969	21.64023	chr6:103{	LNCPOIR		lncRNA	chr6:114477350-114
ENSG0000(	969	21.64023	chr6:103{	LINC02518		lncRNA	chr6:113428540-113
ENSG0000(	969	21.64023	chr6:103{	ENSG00000289961		lncRNA	chr6:113345749-113
ENSG0000(	969	21.64023	chr6:103{	ENSG00000260188		lncRNA	chr6:110477907-110
ENSG0000(	969	21.64023	chr6:103{	ENSG00000232299		lncRNA	chr6:112476538-112
ENSG0000(	969	21.64023	chr6:103{	TUBE1		protein_c	chr6:112070663-112
ENSG0000(	969	21.64023	chr6:103{	ENSG00000218872		Pseudoger	chr6:108856400-108
ENSG0000(	969	21.64023	chr6:103{	ENSG00000220506		Pseudoger	chr6:111494991-111
ENSG0000(	969	21.64023	chr6:103{	ENSG00000260273		lncRNA	chr6:109382795-109
ENSG0000(	969	21.64023	chr6:103{	SESN1		protein_c	chr6:108984309-109
ENSG0000(	969	21.64023	chr6:103{	ENSG00000219329		Pseudoger	chr6:110923566-110
ENSG0000(	969	21.64023	chr6:103{	AL357519.1		smallRNA	chr6:113704833-113
ENSG0000(	969	21.64023	chr6:103{	FCF1P10		Pseudoger	chr6:113010937-113
ENSG0000(	969	21.64023	chr6:103{	PTCHD3P3		Pseudoger	chr6:109288571-109
ENSG0000(	969	21.64023	chr6:103{	RPL7P28		Pseudoger	chr6:109327175-109
ENSG0000(	969	21.64023	chr6:103{	AL357514.1		smallRNA	chr6:112532182-112
ENSG0000(	969	21.64023	chr6:103{	AL365214.1		smallRNA	chr6:112361848-112
ENSG0000(	969	21.64023	chr6:103{	BRD7P4		Pseudoger	chr6:111430260-111
ENSG0000(	969	21.64023	chr6:103{	AL357519.2		smallRNA	chr6:113602915-113

ENSG0000(	969	21.64023	chr6:1039	ENSG00000237234		lncRNA	chr6:112154765-112
ENSG0000(	969	21.64023	chr6:1039	RPSAP45		Pseudoger	chr6:112355841-112
ENSG0000(	969	21.64023	chr6:1039	ENSG00000255389		lncRNA	chr6:111599875-111
ENSG0000(	969	21.64023	chr6:1039	ENSG00000289925		lncRNA	chr6:113635183-113
ENSG0000(	969	21.64023	chr6:1039	ZPR1P1		Pseudoger	chr6:108782126-108
ENSG0000(	969	21.64023	chr6:1039	ENSG00000219559		Pseudoger	chr6:110562175-110
ENSG0000(	969	21.64023	chr6:1039	ENSG00000219758		Pseudoger	chr6:113839279-113
ENSG0000(	969	21.64023	chr6:1039	MARCKS		protein_c	chr6:113857345-113
ENSG0000(	969	21.64023	chr6:1039	ENSG00000289847		lncRNA	chr6:110530684-110
ENSG0000(	969	21.64023	chr6:1039	HDAC2	NCGv7	protein_c	chr6:113933028-114
ENSG0000(	969	21.64023	chr6:1039	KRT18P65		Pseudoger	chr6:112361437-112
ENSG0000(	969	21.64023	chr6:1039	ENSG00000289198		lncRNA	chr6:113855537-113
ENSG0000(	969	21.64023	chr6:1039	SMPD2		protein_c	chr6:109440724-109
ENSG0000(	969	21.64023	chr6:1039	ENSG00000286691		lncRNA	chr6:110020011-110
ENSG0000(	969	21.64023	chr6:1039	RFPL4B		protein_c	chr6:112347330-112
ENSG0000(	969	21.64023	chr6:1039	RN7SL617P		smallRNA	chr6:110439999-110
ENSG0000(	969	21.64023	chr6:1039	LINC00222		lncRNA	chr6:108751654-108
ENSG0000(	969	21.64023	chr6:1039	CCDC162P		Pseudoger	chr6:109165831-109
ENSG0000(	969	21.64023	chr6:1039	DDO		protein_c	chr6:110391771-110
ENSG0000(	969	21.64023	chr7:3057	SNORA42		smallRNA	chr7:6016877-60170
ENSG0000(	969	21.64023	chr6:1039	FAM229B		protein_c	chr6:112087591-112
ENSG0000(	969	21.64023	chr6:1039	AMD1		protein_c	chr6:110874770-110
ENSG0000(	969	21.64023	chr6:1039	ENSG00000287728		lncRNA	chr6:113129707-113
ENSG0000(	969	21.64023	chr6:1039	snoU13		smallRNA	chr6:110963466-110
ENSG0000(	969	21.64023	chr6:1039	ENSG00000286616		lncRNA	chr6:111505307-111
ENSG0000(	969	21.64023	chr6:1039	HDAC2-AS2		lncRNA	chr6:113969701-114
ENSG0000(	969	21.64023	chr6:1039	RNU6-906P		smallRNA	chr6:111008270-111
ENSG0000(	969	21.64023	chr6:1039	ATP5MFP2		Pseudoger	chr6:108907615-108
ENSG0000(	969	21.64023	chr6:1039	MICAL1		protein_c	chr6:109444062-109
ENSG0000(	969	21.64023	chr6:1039	RNU6-1226P		smallRNA	chr6:112196440-112
ENSG0000(	969	21.64023	chr6:1039	REV3L-IT1		lncRNA	chr6:111360641-111
ENSG0000(	969	21.64023	chr6:1039	CD164		protein_c	chr6:109366514-109
ENSG0000(	969	21.64023	chr6:1039	ENSG00000286914		lncRNA	chr6:112392363-112
ENSG0000(	969	21.64023	chr6:1039	CDC40		protein_c	chr6:110180141-110
ENSG0000(	969	21.64023	chr6:1039	ENSG00000226079		lncRNA	chr6:114523443-114
ENSG0000(	969	21.64023	chr6:1039	LAMA4-AS1		lncRNA	chr6:112236093-112
ENSG0000(	969	21.64023	chr6:1039	ENSG00000271498		Pseudoger	chr6:112825939-112
ENSG0000(	969	21.64023	chr6:1039	snoU13		smallRNA	chr6:109291255-109
ENSG0000(	969	21.64023	chr6:1039	ENSG00000271607		Pseudoger	chr6:110863860-110
ENSG0000(	969	21.64023	chr6:1039	ENSG00000281613		protein_c	chr6:112236806-112
ENSG0000(	969	21.64023	chr6:1039	ENSG00000287268		lncRNA	chr6:110341973-110
ENSG0000(	969	21.64023	chr6:1039	LINC02527		lncRNA	chr6:111900305-111
ENSG0000(	969	21.64023	chr6:1039	PA2G4P5		Pseudoger	chr6:112616703-112
ENSG0000(	969	21.64023	chr6:1039	ENSG00000271730		lncRNA	chr6:108998482-108
ENSG0000(	969	21.64023	chr6:1039	ENSG00000271208		lncRNA	chr6:112234165-112
ENSG0000(	969	21.64023	chr6:1039	MROCKI		lncRNA	chr6:113868013-113
ENSG0000(	969	21.64023	chr6:1039	ENSG00000271789		lncRNA	chr6:111297126-111
ENSG0000(	969	21.64023	chr6:1039	ENSG00000270934		Pseudoger	chr6:110598093-110
ENSG0000(	969	21.64023	chr6:1039	ARMC2		protein_c	chr6:108848416-108
ENSG0000(	969	21.64023	chr6:1039	ENSG00000231912		lncRNA	chr6:113791829-113
ENSG0000(	969	21.64023	chr6:1039	RNA5SP213		Pseudoger	chr6:114220681-114

ENSG0000(	969	21.64023	chr6:1039	RNU6-653P		smallRNA	chr6:109059509-109
ENSG0000(	969	21.64023	chr6:1039	SNORA40		smallRNA	chr6:110848546-110
ENSG0000(	969	21.64023	chr6:1039	LINC02880		lncRNA	chr6:113904132-113
ENSG0000(	969	21.64023	chr6:1039	LINC02541		lncRNA	chr6:113616927-113
ENSG0000(	969	21.64023	chr6:1039	MFSD4B-DT		lncRNA	chr6:111227747-111
ENSG0000(	969	21.64023	chr6:1039	snoU13		smallRNA	chr6:112476726-112
ENSG0000(	969	21.64023	chr6:1039	AL357515.1		smallRNA	chr6:110969773-110
ENSG0000(	969	21.64023	chr6:1039	RNU6-1115P		smallRNA	chr6:110856417-110
ENSG0000(	969	21.64023	chr6:1039	CNN2P9		Pseudoger	chr6:110858239-110
ENSG0000(	969	21.64023	chr6:1039	ENSG00000231559		Pseudoger	chr6:108817680-108
ENSG0000(	969	21.64023	chr6:1039	ENSG00000213150		Pseudoger	chr6:110645699-110
ENSG0000(	969	21.64023	chr6:1039	GPR6		protein_c	chr6:109978256-109
ENSG0000(	969	21.64023	chr6:1039	ENSG00000288916		lncRNA	chr6:113586188-113
ENSG0000(	969	21.64023	chr6:1039	AL109947.1		smallRNA	chr6:109434174-109
ENSG0000(	969	21.64023	chr6:1039	TRAF3IP2-AS1		lncRNA	chr6:111483459-111
ENSG0000(	969	21.64023	chr6:1039	TRAF3IP2		protein_c	chr6:111555381-111
ENSG0000(	969	21.64023	chr6:1039	snoU13		smallRNA	chr6:111646327-111
ENSG0000(	969	21.64023	chr6:1039	RNU6-1163P		smallRNA	chr6:112971493-112
ENSG0000(	969	21.64023	chr6:1039	RNU6-957P		smallRNA	chr6:110722250-110
ENSG0000(	969	21.64023	chr6:1039	MFSD4B		protein_c	chr6:111259327-111
ENSG0000(	969	21.64023	chr6:1039	ENSG00000270661		lncRNA	chr6:112217640-112
ENSG0000(	969	21.64023	chr6:1039	ENSG00000286511		lncRNA	chr6:108798929-108
ENSG0000(	969	21.64023	chr6:1039	LAMA4	NCGv7	protein_c	chr6:112107931-112
ENSG0000(	969	21.64023	chr6:1039	CCN6	NCGv7	protein_c	chr6:112054075-112
ENSG0000(	969	21.64023	chr6:1039	Y_RNA		smallRNA	chr6:109305494-109
ENSG0000(	969	21.64023	chr6:1039	ENSG00000288560		lncRNA	chr6:112988311-113
ENSG0000(	969	21.64023	chr6:1039	RNU6-960P		smallRNA	chr6:111091213-111
ENSG0000(	969	21.64023	chr6:1039	SLC16A10		protein_c	chr6:111087503-111
ENSG0000(	969	21.64023	chr6:1039	FIG4		protein_c	chr6:109690609-109
ENSG0000(	969	21.64023	chr6:1039	ZBTB24		protein_c	chr6:109460632-109
ENSG0000(	969	21.64023	chr6:1039	ENSG00000272356		lncRNA	chr6:111309203-111
ENSG0000(	969	21.64023	chr6:1039	ENSG00000223811		lncRNA	chr6:113357003-113
ENSG0000(	969	21.64023	chr6:1039	WASF1		protein_c	chr6:110099819-110
ENSG0000(	969	21.64023	chr6:1039	ZBTB24-DT		lncRNA	chr6:109483638-109
ENSG0000(	969	21.64023	chr6:1039	ARMC2-AS1		lncRNA	chr6:108922976-108
ENSG0000(	969	21.64023	chr6:1039	AL513123.1		smallRNA	chr6:113514987-113
ENSG0000(	969	21.64023	chr6:1039	snoU13		smallRNA	chr6:113840814-113
ENSG0000(	969	21.64023	chr6:1039	ENSG00000217041		Pseudoger	chr6:110700562-110
ENSG0000(	969	21.64023	chr6:1039	HS3ST5		protein_c	chr6:114055596-114
ENSG0000(	969	21.64023	chr6:1039	U3		smallRNA	chr6:113781315-113
ENSG0000(	969	21.64023	chr6:1039	SOCS5P5		Pseudoger	chr6:113222166-113
ENSG0000(	969	21.64023	chr6:1039	RPSAP43		Pseudoger	chr6:114084168-114
ENSG0000(	969	21.64023	chr6:1039	PPIL6		protein_c	chr6:109390215-109
ENSG0000(	969	21.64023	chr6:1039	FEM1AP3		Pseudoger	chr6:112365704-112
ENSG0000(	969	21.64023	chr6:1039	METTL24		protein_c	chr6:110243940-110
ENSG0000(	969	21.64023	chr6:1039	RPS27AP11		Pseudoger	chr6:113581501-113
ENSG0000(	969	21.64023	chr6:1039	ENSG00000216663		Pseudoger	chr6:112325753-112
ENSG0000(	969	21.64023	chr6:1039	AK9		protein_c	chr6:109492855-109
ENSG0000(	969	21.64023	chr6:1039	REV3L	NCGv7	protein_c	chr6:111299028-111
ENSG0000(	969	21.64023	chr6:1039	AL109947.2		smallRNA	chr6:109504387-109
ENSG0000(	969	21.64023	chr6:1039	NUDT19P3		Pseudoger	chr6:114019621-114

ENSG0000(	969	21.64023	chr6:1039	DNAJA1P4		Pseudoger	chr6:114349483-114
ENSG0000(	969	21.64023	chr6:1039	CEP57L1		protein_c	chr6:109095110-109
ENSG0000(	969	21.64023	chr6:1039	SLC22A16		protein_c	chr6:110424687-110
ENSG0000(	969	21.64023	chr6:1039	FYN	NCGv7;AC	protein_c	chr6:111660332-111
ENSG0000(	969	21.64023	chr6:1039	ENSG00000236347		lncRNA	chr6:113531118-113
ENSG0000(	969	21.64023	chr6:1039	CDK19		protein_c	chr6:110609978-110
ENSG0000(	969	21.64023	chr6:1039	GTF3C6		protein_c	chr6:110958706-110
ENSG0000(	956	21.34991	chr2:3094	AC125238.1		smallRNA	chr2:203764707-203
ENSG0000(	954	21.30525	chr2:3094	RPL7P14		Pseudoger	chr2:203040108-203
ENSG0000(	946	21.12659	chr17:739	ENSG00000264007		lncRNA	chr17:29621617-296
ENSG0000(	936	20.90326	chr2:3094	ENSG00000256458		lncRNA	chr2:203328459-203
ENSG0000(	936	20.90326	chr2:3094	ENSG00000273456		lncRNA	chr2:202374769-202
ENSG0000(	936	20.90326	chr2:3094	BMPR2	NCGv7	protein_c	chr2:202376327-202
ENSG0000(	936	20.90326	chr2:3094	ENSG00000287524		lncRNA	chr2:204668863-204
ENSG0000(	936	20.90326	chr2:3094	MTND4P30		Pseudoger	chr2:202617983-202
ENSG0000(	936	20.90326	chr2:3094	ENSG00000289490		protein_c	chr2:202377112-202
ENSG0000(	936	20.90326	chr2:3094	RNU6-474P		smallRNA	chr2:203782037-203
ENSG0000(	936	20.90326	chr2:3094	DSTNP5		Pseudoger	chr2:204306842-204
ENSG0000(	936	20.90326	chr2:3094	ENSG00000235951		Pseudoger	chr2:204065706-204
ENSG0000(	936	20.90326	chr2:3094	RPL39P14		Pseudoger	chr2:202231696-202
ENSG0000(	936	20.90326	chr2:3094	MTCO2P17		Pseudoger	chr2:202614853-202
ENSG0000(	936	20.90326	chr2:3094	NPM1P33		Pseudoger	chr2:203772631-203
ENSG0000(	936	20.90326	chr2:3094	H3P8		Pseudoger	chr2:202505012-202
ENSG0000(	936	20.90326	chr2:3094	CYP20A1		protein_c	chr2:203238977-203
ENSG0000(	936	20.90326	chr2:3094	RPL12P16		Pseudoger	chr2:203190780-203
ENSG0000(	936	20.90326	chr2:3094	KRT8P15		Pseudoger	chr2:202840331-202
ENSG0000(	936	20.90326	chr2:3094	ENSG00000233459		Pseudoger	chr2:203634577-203
ENSG0000(	936	20.90326	chr2:3094	RN7SL753P		smallRNA	chr2:202333439-202
ENSG0000(	936	20.90326	chr2:3094	ENSG00000225304		Pseudoger	chr2:204379095-204
ENSG0000(	936	20.90326	chr2:3094	MRPL50P2		Pseudoger	chr2:203315390-203
ENSG0000(	936	20.90326	chr2:3094	RPL23AP36		Pseudoger	chr2:203074685-203
ENSG0000(	936	20.90326	chr2:3094	MTCO1P54		Pseudoger	chr2:202619185-202
ENSG0000(	936	20.90326	chr2:3094	PSMA2P3		Pseudoger	chr2:202179297-202
ENSG0000(	936	20.90326	chr2:3094	SNORD11		smallRNA	chr2:202293049-202
ENSG0000(	936	20.90326	chr2:3094	snoU13		smallRNA	chr2:202081875-202
ENSG0000(	936	20.90326	chr2:3094	ENSG00000233031		Pseudoger	chr2:203672175-203
ENSG0000(	936	20.90326	chr2:3094	SNORD11B		smallRNA	chr2:202291332-202
ENSG0000(	936	20.90326	chr2:3094	ENSG00000228513		Pseudoger	chr2:203061314-203
ENSG0000(	936	20.90326	chr2:3094	MTND3P17		Pseudoger	chr2:202617284-202
ENSG0000(	936	20.90326	chr2:3094	KRT18P39		Pseudoger	chr2:203764295-203
ENSG0000(	936	20.90326	chr2:3094	RN7SL670P		smallRNA	chr2:203259604-203
ENSG0000(	936	20.90326	chr2:3094	MTND4LP17		Pseudoger	chr2:202617695-202
ENSG0000(	936	20.90326	chr2:3094	CARF	NCGv7	protein_c	chr2:202912214-202
ENSG0000(	936	20.90326	chr2:3094	KRT8P52		Pseudoger	chr2:202924609-202
ENSG0000(	936	20.90326	chr2:3094	CD28	NCGv7	protein_c	chr2:203706475-203
ENSG0000(	936	20.90326	chr2:3094	ENSG00000270664		Pseudoger	chr2:202995194-202
ENSG0000(	936	20.90326	chr2:3094	ICA1L		protein_c	chr2:202773150-202
ENSG0000(	936	20.90326	chr2:3094	FZD7		protein_c	chr2:202033855-202
ENSG0000(	936	20.90326	chr2:3094	ICOS		protein_c	chr2:203936763-203
ENSG0000(	936	20.90326	chr2:3094	ENSG00000231903		lncRNA	chr2:202178660-202
ENSG0000(	936	20.90326	chr2:3094	RN7SL40P		smallRNA	chr2:202357121-202

ENSG0000(	936	20.90326	chr2:3094	ENSG00000234714		lncRNA	chr2:204090457-204
ENSG0000(	936	20.90326	chr2:3094	FAM117B	NCGv7	protein_c	chr2:202634969-202
ENSG0000(	936	20.90326	chr2:3094	DAZAP2P1		Pseudoger	chr2:202201384-202
ENSG0000(	936	20.90326	chr2:3094	MTCO1P17		Pseudoger	chr2:202614214-202
ENSG0000(	936	20.90326	chr2:3094	SNORD70		smallRNA	chr2:202278108-202
ENSG0000(	936	20.90326	chr2:3094	ENSG00000237843		lncRNA	chr2:204473834-204
ENSG0000(	936	20.90326	chr2:3094	MTATP6P17		Pseudoger	chr2:202615769-202
ENSG0000(	936	20.90326	chr2:3094	ENSG00000272966		lncRNA	chr2:202336739-202
ENSG0000(	936	20.90326	chr2:3094	PIMREGP1		Pseudoger	chr2:202336024-202
ENSG0000(	936	20.90326	chr2:3094	MTCO3P17		Pseudoger	chr2:202616700-202
ENSG0000(	936	20.90326	chr2:3094	ENSG00000286223		lncRNA	chr2:202302122-202
ENSG0000(	936	20.90326	chr2:3094	NOP58	NCGv7	protein_c	chr2:202265736-202
ENSG0000(	936	20.90326	chr2:3094	ENSG00000240761		Pseudoger	chr2:202773720-202
ENSG0000(	936	20.90326	chr2:3094	CTLA4		protein_c	chr2:203853888-203
ENSG0000(	936	20.90326	chr2:3094	SUMO1		protein_c	chr2:202206182-202
ENSG0000(	936	20.90326	chr2:3094	KIAA2012-AS1		lncRNA	chr2:202075410-202
ENSG0000(	936	20.90326	chr2:3094	KIAA2012		protein_c	chr2:202073255-202
ENSG0000(	936	20.90326	chr2:3094	RPL13AP12		Pseudoger	chr2:202520401-202
ENSG0000(	936	20.90326	chr2:3094	ENSG00000237271		lncRNA	chr2:204219984-204
ENSG0000(	936	20.90326	chr2:3094	ENSG00000289294		lncRNA	chr2:203237261-203
ENSG0000(	936	20.90326	chr2:3094	WDR12	NCGv7	protein_c	chr2:202874261-203
ENSG0000(	936	20.90326	chr2:3094	ENSG00000287041		lncRNA	chr2:202137570-202
ENSG0000(	936	20.90326	chr2:3094	ABI2		protein_c	chr2:203328280-203
ENSG0000(	936	20.90326	chr2:3094	SNORA1		smallRNA	chr2:203052285-203
ENSG0000(	936	20.90326	chr2:3094	ENSG00000273209		lncRNA	chr2:202032770-202
ENSG0000(	936	20.90326	chr2:3094	RAPH1		protein_c	chr2:203394345-203
ENSG0000(	936	20.90326	chr2:3094	SNORD70		smallRNA	chr2:202276431-202
ENSG0000(	936	20.90326	chr2:3094	NBEAL1		protein_c	chr2:203014608-203
ENSG0000(	936	20.90326	chr2:3094	ENSG00000236634		lncRNA	chr2:204470076-204
ENSG0000(	925	20.6576	chr6:1039	AL358133.1		smallRNA	chr6:69621373-6962
ENSG0000(	922	20.59061	chr6:1039	RNU6-975P		smallRNA	chr6:73464073-7346
ENSG0000(	921	20.56827	chr6:1039	TPI1P3		Pseudoger	chr6:116038756-116
ENSG0000(	919	20.52361	chr20:212	ENSG00000236028		lncRNA	chr20:46901143-469
ENSG0000(	914	20.41194	chr7:1588	ENSG00000219445		lncRNA	chr7:125229579-125
ENSG0000(	914	20.41194	chr7:1588	ENSG00000243574		lncRNA	chr7:124274671-124
ENSG0000(	914	20.41194	chr7:1588	SSU72P8		protein_c	chr7:124476371-124
ENSG0000(	914	20.41194	chr7:1588	ENSG00000280347		TEC	chr7:127359785-127
ENSG0000(	914	20.41194	chr7:1588	GRM8-AS1		lncRNA	chr7:127215127-127
ENSG0000(	914	20.41194	chr7:1588	ENSG00000227249		Pseudoger	chr7:126511135-126
ENSG0000(	914	20.41194	chr7:1588	ENSG00000230820		Pseudoger	chr7:126980967-126
ENSG0000(	914	20.41194	chr7:1588	PRELID3BP10		Pseudoger	chr7:127295620-127
ENSG0000(	914	20.41194	chr7:1588	POT1	NCGv7;AC	protein_c	chr7:124822386-124
ENSG0000(	914	20.41194	chr7:1588	ENSG00000242593		lncRNA	chr7:124032126-124
ENSG0000(	914	20.41194	chr7:1588	ENSG00000279419		TEC	chr7:124742312-124
ENSG0000(	914	20.41194	chr7:1588	ENSG00000287702		lncRNA	chr7:126495312-126
ENSG0000(	914	20.41194	chr7:1588	ENSG00000287568		lncRNA	chr7:126533665-126
ENSG0000(	914	20.41194	chr7:1588	POT1-AS1		lncRNA	chr7:124929873-125
ENSG0000(	914	20.41194	chr7:1588	MIR592		smallRNA	chr7:127058088-127
ENSG0000(	914	20.41194	chr7:1588	AC003968.1		smallRNA	chr7:126040229-126
ENSG0000(	914	20.41194	chr7:1588	ENSG00000224138		lncRNA	chr7:127350128-127
ENSG0000(	914	20.41194	chr7:1588	SND1-DT		lncRNA	chr7:127644685-127

ENSG0000(	914	20.41194	chr7:158{	GCC1		protein_c	chr7:127580628-127
ENSG0000(	914	20.41194	chr7:158{	GRM8	NCGv7	protein_c	chr7:126438598-127
ENSG0000(	914	20.41194	chr7:158{	ENSG00000241921		lncRNA	chr7:126378970-126
ENSG0000(	914	20.41194	chr7:158{	ENSG00000213296		Pseudoger	chr7:124480524-124
ENSG0000(	914	20.41194	chr7:158{	LINC02830		lncRNA	chr7:125151326-125
ENSG0000(	914	20.41194	chr7:158{	ENSG00000279265		TEC	chr7:127349833-127
ENSG0000(	914	20.41194	chr7:158{	ENSG00000213291		Pseudoger	chr7:125300504-125
ENSG0000(	914	20.41194	chr7:158{	ENSG00000224981		Pseudoger	chr7:126868767-126
ENSG0000(	914	20.41194	chr7:158{	SPAM1	NCGv7	protein_c	chr7:123925237-123
ENSG0000(	914	20.41194	chr7:158{	RNU6-102P		smallRNA	chr7:124647719-124
ENSG0000(	914	20.41194	chr7:158{	FSCN3		protein_c	chr7:127591409-127
ENSG0000(	914	20.41194	chr7:158{	GPR37		protein_c	chr7:124743885-124
ENSG0000(	914	20.41194	chr7:158{	PAX4	AC	protein_c	chr7:127610292-127
ENSG0000(	914	20.41194	chr7:158{	PPIAP93		Pseudoger	chr7:125345825-125
ENSG0000(	914	20.41194	chr7:158{	ZNF800		protein_c	chr7:127346790-127
ENSG0000(	914	20.41194	chr7:158{	ENSG00000197462		lncRNA	chr7:125917871-125
ENSG0000(	914	20.41194	chr7:158{	ENSG00000205898		Pseudoger	chr7:125159974-125
ENSG0000(	914	20.41194	chr7:158{	ENSG00000241324		lncRNA	chr7:124337380-124
ENSG0000(	914	20.41194	chr7:158{	EEF1GP1		Pseudoger	chr7:125033453-125
ENSG0000(	914	20.41194	chr7:158{	LINC03012		lncRNA	chr7:127476883-127
ENSG0000(	914	20.41194	chr7:158{	ARF5		protein_c	chr7:127588386-127
ENSG0000(	914	20.41194	chr7:158{	ENSG00000241345		lncRNA	chr7:123994622-124
ENSG0000(	914	20.41194	chr7:158{	ENSG00000225583		Pseudoger	chr7:123932132-123
ENSG0000(	914	20.41194	chr7:158{	TMEM229A		protein_c	chr7:124030921-124
ENSG0000(	914	20.41194	chr7:158{	C7orf77		lncRNA	chr7:124777292-124
ENSG0000(	914	20.41194	chr7:158{	ENSG00000234071		Pseudoger	chr7:125344969-125
ENSG0000(	908	20.27795	chr8:284:	RNA5SP263		Pseudoger	chr8:32256496-3225
ENSG0000(	901	20.12162	chr6:103(	RSPH4A		protein_c	chr6:116616479-116
ENSG0000(	901	20.12162	chr6:103(	RWDD1		protein_c	chr6:116571409-116
ENSG0000(	901	20.12162	chr6:103(	COL10A1		protein_c	chr6:116118909-116
ENSG0000(	901	20.12162	chr6:103(	RNU6-475P		smallRNA	chr6:114866873-114
ENSG0000(	901	20.12162	chr6:103(	KRT18P22		Pseudoger	chr6:116457323-116
ENSG0000(	901	20.12162	chr6:103(	DSE	NCGv7	protein_c	chr6:116254173-116
ENSG0000(	901	20.12162	chr6:103(	RN7SKP18		smallRNA	chr6:117299364-117
ENSG0000(	901	20.12162	chr6:103(	FRK		protein_c	chr6:115931149-116
ENSG0000(	901	20.12162	chr6:103(	DCBLD1		protein_c	chr6:117453817-117
ENSG0000(	901	20.12162	chr6:103(	CALHM4		protein_c	chr6:116529013-116
ENSG0000(	901	20.12162	chr6:103(	ENSG00000287253		lncRNA	chr6:117262243-117
ENSG0000(	901	20.12162	chr6:103(	ENSG00000230202		Pseudoger	chr6:117998975-117
ENSG0000(	901	20.12162	chr6:103(	RN7SKP51		smallRNA	chr6:117301455-117
ENSG0000(	901	20.12162	chr6:103(	RAP1BP3		Pseudoger	chr6:117431591-117
ENSG0000(	901	20.12162	chr6:103(	ENSG00000226181		lncRNA	chr6:117451130-117
ENSG0000(	901	20.12162	chr6:103(	VGLL2		protein_c	chr6:117265558-117
ENSG0000(	901	20.12162	chr6:103(	ENSG00000219619		Pseudoger	chr6:115358498-115
ENSG0000(	901	20.12162	chr6:103(	RNA5SP214		Pseudoger	chr6:117060682-117
ENSG0000(	901	20.12162	chr6:103(	RPS29P13		Pseudoger	chr6:117048670-117
ENSG0000(	901	20.12162	chr6:103(	KPNA5		protein_c	chr6:116681187-116
ENSG0000(	901	20.12162	chr6:103(	ENSG00000233558		Pseudoger	chr6:116258493-116
ENSG0000(	901	20.12162	chr6:103(	ENSG00000287097		lncRNA	chr6:114822994-115
ENSG0000(	901	20.12162	chr6:103(	CALHM5		protein_c	chr6:116511639-116
ENSG0000(	901	20.12162	chr6:103(	ENSG00000285446		protein_c	chr6:116399395-116

ENSG0000(	901	20.12162	chr6:1039	ENSG00000236326		lncRNA	chr6:116244187-116
ENSG0000(	901	20.12162	chr6:1039	CALHM6-AS1		lncRNA	chr6:116460739-116
ENSG0000(	901	20.12162	chr6:1039	NIP7P3		Pseudoger	chr6:116137058-116
ENSG0000(	901	20.12162	chr6:1039	NT5DC1		protein_c	chr6:116100851-116
ENSG0000(	901	20.12162	chr6:1039	ENSG00000289304		lncRNA	chr6:116569604-116
ENSG0000(	901	20.12162	chr6:1039	NUS1		protein_c	chr6:117675469-117
ENSG0000(	901	20.12162	chr6:1039	ZUP1		protein_c	chr6:116635618-116
ENSG0000(	901	20.12162	chr6:1039	ENSG00000282218		protein_c	chr6:117318211-117
ENSG0000(	901	20.12162	chr6:1039	TSPYL4	NCGv7	protein_c	chr6:116249964-116
ENSG0000(	901	20.12162	chr6:1039	GPRC6A		protein_c	chr6:116792085-116
ENSG0000(	901	20.12162	chr6:1039	TRAPPC3L		protein_c	chr6:116494989-116
ENSG0000(	901	20.12162	chr6:1039	ENSG00000289372		lncRNA	chr6:117658838-117
ENSG0000(	901	20.12162	chr6:1039	ENSG00000289376		lncRNA	chr6:115901795-116
ENSG0000(	901	20.12162	chr6:1039	ENSG00000287933		lncRNA	chr6:116033901-116
ENSG0000(	901	20.12162	chr6:1039	RNU6-253P		smallRNA	chr6:117457734-117
ENSG0000(	901	20.12162	chr6:1039	AL132671.1		smallRNA	chr6:117445390-117
ENSG0000(	901	20.12162	chr6:1039	LINC02534		lncRNA	chr6:115633540-115
ENSG0000(	901	20.12162	chr6:1391	ENSG00000289851		lncRNA	chr6:146914424-146
ENSG0000(	901	20.12162	chr6:1039	ENSG00000234117		lncRNA	chr6:116492297-116
ENSG0000(	901	20.12162	chr6:1039	Z84488.1		smallRNA	chr6:116457732-116
ENSG0000(	901	20.12162	chr6:1039	FAM162B		protein_c	chr6:116752197-116
ENSG0000(	901	20.12162	chr6:1039	TSPYL1	NCGv7	protein_c	chr6:116267760-116
ENSG0000(	901	20.12162	chr6:1039	NEPNP		Pseudoger	chr6:117633706-117
ENSG0000(	901	20.12162	chr6:1039	GOPC	NCGv7;AC	protein_c	chr6:117560269-117
ENSG0000(	901	20.12162	chr6:1039	ROS1	NCGv7;AC	protein_c	chr6:117287353-117
ENSG0000(	901	20.12162	chr6:1039	CALHM6	NCGv7	protein_c	chr6:116461370-116
ENSG0000(	901	20.12162	chr6:1039	CBX3P9		Pseudoger	chr6:116453014-116
ENSG0000(	901	20.12162	chr6:1039	RFX6	NCGv7	protein_c	chr6:116877212-116
ENSG0000(	896	20.00996	chr6:1039	RN7SL797P		smallRNA	chr6:96282567-9628
ENSG0000(	893	19.94296	chr6:1039	RNU6-194P		smallRNA	chr6:119327281-119
ENSG0000(	884	19.74197	chr6:1039	KHDC3L	NCGv7	protein_c	chr6:73362658-7336
ENSG0000(	884	19.74197	chr6:1039	CGAS		protein_c	chr6:73413515-7345
ENSG0000(	884	19.74197	chr6:1039	ENSG00000219736		Pseudoger	chr6:74849077-7485
ENSG0000(	884	19.74197	chr6:1039	snoU13		smallRNA	chr6:73393913-7339
ENSG0000(	884	19.74197	chr6:1039	ENSG00000224583		lncRNA	chr6:74642384-7465
ENSG0000(	884	19.74197	chr6:1039	TXNP7		Pseudoger	chr6:74004520-7400
ENSG0000(	884	19.74197	chr6:1039	ENSG00000289286		lncRNA	chr6:73570461-7357
ENSG0000(	884	19.74197	chr6:1039	AC019205.2		smallRNA	chr6:73432984-7343
ENSG0000(	884	19.74197	chr6:1039	EEF1A1-AS1		lncRNA	chr6:73523618-7357
ENSG0000(	884	19.74197	chr6:1039	OOEP-AS1		lncRNA	chr6:73369704-7338
ENSG0000(	884	19.74197	chr6:1039	OOEP		protein_c	chr6:73368555-7339
ENSG0000(	884	19.74197	chr6:1039	CD109-AS1		lncRNA	chr6:73693903-7369
ENSG0000(	884	19.74197	chr6:1039	Metazoa_SRP		smallRNA	chr6:73487592-7348
ENSG0000(	884	19.74197	chr6:1039	CD109		protein_c	chr6:73695785-7382
ENSG0000(	884	19.74197	chr6:1039	PAICSP3		Pseudoger	chr6:73327524-7332
ENSG0000(	884	19.74197	chr6:1039	SLC17A5	NCGv7	protein_c	chr6:73593379-7365
ENSG0000(	884	19.74197	chr6:1039	DPPA5		protein_c	chr6:73353063-7335
ENSG0000(	884	19.74197	chr6:1039	SDCBP2P1		Pseudoger	chr6:73322837-7332
ENSG0000(	884	19.74197	chr6:1039	RPL39P3		Pseudoger	chr6:73373108-7337
ENSG0000(	884	19.74197	chr6:1039	ENSG00000218483		Pseudoger	chr6:74610449-7461
ENSG0000(	884	19.74197	chr6:1039	EEF1A1	NCGv7;AC	protein_c	chr6:73489308-7352

ENSG0000(	884	19.74197	chr6:1039	RPS6P8		Pseudoger	chr6:73391038-7339
ENSG0000(	884	19.74197	chr6:1039	RPS27P15		Pseudoger	chr6:73618346-7361
ENSG0000(	884	19.74197	chr6:1039	ENSG00000290002		lncRNA	chr6:73388408-7338
ENSG0000(	884	19.74197	chr6:1039	DDX43		protein_c	chr6:73394828-7341
ENSG0000(	884	19.74197	chr6:1039	FAM136FP		Pseudoger	chr6:74282266-7428
ENSG0000(	884	19.74197	chr6:1039	AL603910.1		smallRNA	chr6:73477058-7347
ENSG0000(	884	19.74197	chr6:1039	ENSG00000272243		lncRNA	chr6:74530248-7473
ENSG0000(	884	19.74197	chr6:1039	MTO1		protein_c	chr6:73461578-7350
ENSG0000(	884	19.74197	chr6:1039	ENSG00000223967		Pseudoger	chr6:73526744-7352
ENSG0000(	884	19.74197	chr6:1039	ENSG00000279790		TEC	chr6:73971346-7397
ENSG0000(	884	19.74197	chr6:1039	AL590684.1		smallRNA	chr6:74719746-7471
ENSG0000(	884	19.74197	chr6:1039	ENSG00000223786		lncRNA	chr6:74069451-7469
ENSG0000(	878	19.60797	chr6:1039	ENSG00000234206		lncRNA	chr6:107509803-107
ENSG0000(	878	19.60797	chr6:1039	ENSG00000271608		Pseudoger	chr6:108551018-108
ENSG0000(	878	19.60797	chr6:1039	ENSG00000272476		lncRNA	chr6:107957413-107
ENSG0000(	878	19.60797	chr6:1039	MTRES1		protein_c	chr6:107028199-107
ENSG0000(	878	19.60797	chr6:1039	RPS24P12		Pseudoger	chr6:107229759-107
ENSG0000(	878	19.60797	chr6:1039	CRYBG1		protein_c	chr6:106360717-106
ENSG0000(	878	19.60797	chr6:1039	RNU6-770P		smallRNA	chr6:108392073-108
ENSG0000(	878	19.60797	chr6:1039	RPL23AP50		Pseudoger	chr6:107931800-107
ENSG0000(	878	19.60797	chr6:1039	ENSG00000224987		Pseudoger	chr6:107985089-107
ENSG0000(	878	19.60797	chr6:1039	ENSG00000233511		Pseudoger	chr6:108252214-108
ENSG0000(	878	19.60797	chr6:1039	ENSG00000280135		TEC	chr6:107697299-107
ENSG0000(	878	19.60797	chr6:1039	RNU6-117P		smallRNA	chr6:106738948-106
ENSG0000(	878	19.60797	chr6:1039	PRDM1	NCGv7;AC	protein_c	chr6:105993463-106
ENSG0000(	878	19.60797	chr6:1039	NR2E1		protein_c	chr6:108166022-108
ENSG0000(	878	19.60797	chr6:1039	snoU13		smallRNA	chr6:108243455-108
ENSG0000(	878	19.60797	chr6:1039	RN7SL47P		smallRNA	chr6:106283415-106
ENSG0000(	878	19.60797	chr6:1039	RNU6-344P		smallRNA	chr6:106304176-106
ENSG0000(	878	19.60797	chr6:1039	RNA5SP211		Pseudoger	chr6:106449381-106
ENSG0000(	878	19.60797	chr6:1039	ENSG00000227072		Pseudoger	chr6:108387512-108
ENSG0000(	878	19.60797	chr6:1039	SOBP		protein_c	chr6:107490106-107
ENSG0000(	878	19.60797	chr6:1039	SNORA73		smallRNA	chr6:107985659-107
ENSG0000(	878	19.60797	chr6:1039	OSTM1-AS1		lncRNA	chr6:108123457-108
ENSG0000(	878	19.60797	chr6:1039	RPL21P65		Pseudoger	chr6:106642463-106
ENSG0000(	878	19.60797	chr6:1039	SNX3		protein_c	chr6:108211222-108
ENSG0000(	878	19.60797	chr6:1039	SCML4		protein_c	chr6:107702154-107
ENSG0000(	878	19.60797	chr6:1039	QRSL1		protein_c	chr6:106629578-106
ENSG0000(	878	19.60797	chr6:1039	LINC02532		lncRNA	chr6:106705328-106
ENSG0000(	878	19.60797	chr6:1039	ENSG00000279498		TEC	chr6:108359084-108
ENSG0000(	878	19.60797	chr6:1039	RNU6-527P		smallRNA	chr6:106607716-106
ENSG0000(	878	19.60797	chr6:1039	BEND3		protein_c	chr6:107065182-107
ENSG0000(	878	19.60797	chr6:1039	AFG1L		protein_c	chr6:108294991-108
ENSG0000(	878	19.60797	chr6:1039	ENSG00000276620		Pseudoger	chr6:107192300-107
ENSG0000(	878	19.60797	chr6:1039	RNU6-1144P		smallRNA	chr6:108292766-108
ENSG0000(	878	19.60797	chr6:1039	RN7SKP211		smallRNA	chr6:105904373-105
ENSG0000(	878	19.60797	chr6:1039	Y_RNA		smallRNA	chr6:106454828-106
ENSG0000(	878	19.60797	chr6:1039	FOXO3	NCGv7	protein_c	chr6:108559835-108
ENSG0000(	878	19.60797	chr6:1039	ENSG00000230248		lncRNA	chr6:108275642-108
ENSG0000(	878	19.60797	chr6:1039	RNU6-437P		smallRNA	chr6:107930088-107
ENSG0000(	878	19.60797	chr6:1039	OSTM1		protein_c	chr6:108029245-108

ENSG0000(	878	19.60797	chr6:1039	ATG5		protein_c	chr6:106045423-106
ENSG0000(	878	19.60797	chr6:1039	LINC02526		lncRNA	chr6:106695535-106
ENSG0000(	878	19.60797	chr6:1039	RNA5SP212		Pseudoger	chr6:108252401-108
ENSG0000(	878	19.60797	chr6:1039	ENSG00000269919		lncRNA	chr6:106100140-106
ENSG0000(	878	19.60797	chr6:1039	ENSG00000279398		TEC	chr6:108178871-108
ENSG0000(	878	19.60797	chr6:1039	RNU6-1299P		smallRNA	chr6:107133071-107
ENSG0000(	878	19.60797	chr6:1039	ENSG00000287044		lncRNA	chr6:108441880-108
ENSG0000(	878	19.60797	chr6:1039	RPL3P7		Pseudoger	chr6:108004357-108
ENSG0000(	878	19.60797	chr6:1039	MIR587		smallRNA	chr6:106784125-106
ENSG0000(	878	19.60797	chr6:1039	PDSS2	NCGv7	protein_c	chr6:107152562-107
ENSG0000(	878	19.60797	chr6:1039	SEC63		protein_c	chr6:107867756-107
ENSG0000(	878	19.60797	chr6:1039	ENSG00000286562		lncRNA	chr6:108261288-108
ENSG0000(	878	19.60797	chr6:1039	RTN4IP1		protein_c	chr6:106570771-106
ENSG0000(	878	19.60797	chr6:1039	Y_RNA		smallRNA	chr6:106420706-106
ENSG0000(	878	19.60797	chr6:1039	RPL36AP24		Pseudoger	chr6:108318079-108
ENSG0000(	878	19.60797	chr6:1039	ENSG00000289433		lncRNA	chr6:107595351-107
ENSG0000(	878	19.60797	chr6:1039	ENSG00000233941		lncRNA	chr6:106358566-106
ENSG0000(	878	19.60797	chr6:1039	SUMO2P8		Pseudoger	chr6:108618000-108
ENSG0000(	878	19.60797	chr6:1039	ENSG00000289020		lncRNA	chr6:106702878-106
ENSG0000(	878	19.60797	chr6:1039	ENSG00000282408		lncRNA	chr6:106451496-106
ENSG0000(	878	19.60797	chr6:1039	MTHFD2P3		Pseudoger	chr6:107985811-107
ENSG0000(	878	19.60797	chr6:1039	Y_RNA		smallRNA	chr6:108060818-108
ENSG0000(	874	19.51864	chr6:1039	Y_RNA		smallRNA	chr6:52979158-5297
ENSG0000(	874	19.51864	chr6:1039	RN7SK		smallRNA	chr6:52995620-5299
ENSG0000(	874	19.51864	chr6:1039	ENSG00000291006		lncRNA	chr6:52664366-5266
ENSG0000(	874	19.51864	chr6:1039	TRAM2-AS1		lncRNA	chr6:52576787-5264
ENSG0000(	874	19.51864	chr6:1039	ENSG00000270382		Pseudoger	chr6:52657373-5265
ENSG0000(	874	19.51864	chr6:1039	GSTA3		protein_c	chr6:52896639-5290
ENSG0000(	874	19.51864	chr6:1039	GSTA6P		Pseudoger	chr6:52805613-5281
ENSG0000(	874	19.51864	chr6:1039	ENSG00000288646		protein_c	chr6:53065602-5306
ENSG0000(	874	19.51864	chr6:1039	ENSG00000291036		lncRNA	chr6:52939726-5297
ENSG0000(	874	19.51864	chr6:1039	ENSG00000288014		Pseudoger	chr6:52970650-5297
ENSG0000(	874	19.51864	chr6:1039	TRAM2		protein_c	chr6:52497408-5257
ENSG0000(	874	19.51864	chr6:1039	GSTA2		protein_c	chr6:52750087-5276
ENSG0000(	874	19.51864	chr6:1039	ENSG00000216775		Pseudoger	chr6:52665274-5266
ENSG0000(	874	19.51864	chr6:1039	GSTA12P		Pseudoger	chr6:52765280-5277
ENSG0000(	874	19.51864	chr6:1039	RN7SK		smallRNA	chr6:52995621-5299
ENSG0000(	874	19.51864	chr6:1039	ENSG00000289276		lncRNA	chr6:52364084-5236
ENSG0000(	874	19.51864	chr6:1039	TMEM14A		protein_c	chr6:52671113-5268
ENSG0000(	874	19.51864	chr6:1039	EFHC1		protein_c	chr6:52362123-5252
ENSG0000(	874	19.51864	chr6:1039	RN7SL244P		smallRNA	chr6:53090961-5309
ENSG0000(	874	19.51864	chr6:1039	GSTA9P		Pseudoger	chr6:52939906-5295
ENSG0000(	874	19.51864	chr6:1039	GSTA11P		Pseudoger	chr6:52847910-5287
ENSG0000(	874	19.51864	chr6:1039	FBXO9	NCGv7	protein_c	chr6:53051991-5310
ENSG0000(	874	19.51864	chr6:1039	GSTA4	NCGv7	protein_c	chr6:52977948-5299
ENSG0000(	874	19.51864	chr6:1039	GSTA10P		Pseudoger	chr6:52873014-5288
ENSG0000(	874	19.51864	chr6:1039	ENSG00000288614		protein_c	chr6:53041266-5304
ENSG0000(	874	19.51864	chr6:1039	GSTA8P		Pseudoger	chr6:52687930-5270
ENSG0000(	874	19.51864	chr6:1039	ENSG00000261745		lncRNA	chr6:53125644-5312
ENSG0000(	874	19.51864	chr6:1039	GSTA5		protein_c	chr6:52831655-5284
ENSG0000(	874	19.51864	chr6:1039	CILK1	NCGv7	protein_c	chr6:53001279-5306

ENSG0000(	874	19.51864	chr6:1039	GSTA7P	Pseudoger	chr6:52739590-5274
ENSG0000(	874	19.51864	chr6:1039	GSTA1	protein_c	chr6:52791371-5280
ENSG0000(	874	19.51864	chr6:1039	SREK1IP1P2	Pseudoger	chr6:52688661-5268
ENSG0000(	874	19.51864	chr6:1039	PAQR8	protein_c	chr6:52361421-5240
ENSG0000(	872	19.47398	chr6:1039	ENSG00000274844	Pseudoger	chr6:60826185-6082
ENSG0000(	872	19.47398	chr6:1039	RP11-452D24.1	Pseudoger	chr6:61060454-6106
ENSG0000(	872	19.47398	chr6:1039	PRIM2BP	Pseudoger	chr6:60400251-6054
ENSG0000(	872	19.47398	chr6:1039	ADGRB3-DT	lncRNA	chr6:68627879-6863
ENSG0000(	872	19.47398	chr6:1039	AL356131.1	smallRNA	chr6:61180831-6118
ENSG0000(	872	19.47398	chr6:1039	GAPDHP15	Pseudoger	chr6:57967687-5796
ENSG0000(	872	19.47398	chr6:1039	ENSG00000262651	Pseudoger	chr6:63612761-6361
ENSG0000(	872	19.47398	chr6:1039	SPTLC1P3	Pseudoger	chr6:63227485-6322
ENSG0000(	872	19.47398	chr6:1039	MIR30A	smallRNA	chr6:71403551-7140
ENSG0000(	872	19.47398	chr6:1039	GAPDHP42	Pseudoger	chr6:69745871-6974
ENSG0000(	872	19.47398	chr6:1039	KCNQ5-AS1	lncRNA	chr6:73130646-7314
ENSG0000(	872	19.47398	chr6:1039	ENSG00000287598	lncRNA	chr6:60789805-6094
ENSG0000(	872	19.47398	chr6:1039	ENSG00000218834	Pseudoger	chr6:68598204-6859
ENSG0000(	872	19.47398	chr6:1039	ENSG00000218274	Pseudoger	chr6:63395007-6339
ENSG0000(	872	19.47398	chr6:1039	ENSG00000243828	Pseudoger	chr6:64728837-6473
ENSG0000(	872	19.47398	chr6:1039	FKBP1C	protein_c	chr6:63211446-6321
ENSG0000(	872	19.47398	chr6:1039	ENSG00000218617	Pseudoger	chr6:63392222-6339
ENSG0000(	872	19.47398	chr6:1039	KHDC1	protein_c	chr6:73241314-7331
ENSG0000(	872	19.47398	chr6:1039	ENSG00000287939	lncRNA	chr6:71028582-7105
ENSG0000(	872	19.47398	chr6:1039	ENSG00000271761	lncRNA	chr6:57902609-5790
ENSG0000(	872	19.47398	chr6:1039	AL445256.1	smallRNA	chr6:72226707-7222
ENSG0000(	872	19.47398	chr6:1039	SNORD65	smallRNA	chr6:67210408-6721
ENSG0000(	872	19.47398	chr6:1039	ENSG00000214558	protein_c	chr6:65301476-6530
ENSG0000(	872	19.47398	chr6:1039	RNU7-48P	smallRNA	chr6:70513294-7051
ENSG0000(	872	19.47398	chr6:1039	ENSG00000279289	TEC	chr6:71386852-7139
ENSG0000(	872	19.47398	chr6:1039	ENSG00000217483	Pseudoger	chr6:72522641-7252
ENSG0000(	872	19.47398	chr6:1039	RPL7AP34	Pseudoger	chr6:63548708-6354
ENSG0000(	872	19.47398	chr6:1039	SLC25A6P6	Pseudoger	chr6:70663502-7066
ENSG0000(	872	19.47398	chr6:1039	DHFRP5	Pseudoger	chr6:62460940-6246
ENSG0000(	872	19.47398	chr6:1039	ENSG00000287300	lncRNA	chr6:70345919-7035
ENSG0000(	872	19.47398	chr6:1039	ENSG00000220030	Pseudoger	chr6:61240897-6124
ENSG0000(	872	19.47398	chr6:1039	ENSG00000253809	lncRNA	chr6:70222758-7024
ENSG0000(	872	19.47398	chr6:1039	ENSG00000285963	lncRNA	chr6:71532070-7153
ENSG0000(	872	19.47398	chr6:1039	RBBP4P4	Pseudoger	chr6:58119741-5812
ENSG0000(	872	19.47398	chr6:1039	ENSG00000286564	lncRNA	chr6:68223667-6824
ENSG0000(	872	19.47398	chr6:1039	RPSAP41	Pseudoger	chr6:73290403-7329
ENSG0000(	872	19.47398	chr6:1039	ENSG00000276127	Pseudoger	chr6:71251758-7125
ENSG0000(	872	19.47398	chr6:1039	OGFRL1	protein_c	chr6:71288811-7130
ENSG0000(	872	19.47398	chr6:1039	NUFIP1P1	Pseudoger	chr6:66093431-6609
ENSG0000(	872	19.47398	chr6:1039	KNOP1P4	Pseudoger	chr6:72986313-7298
ENSG0000(	872	19.47398	chr6:1039	RBPMS2P1	Pseudoger	chr6:73157517-7315
ENSG0000(	872	19.47398	chr6:1039	LINC01610	lncRNA	chr6:70394880-7040
ENSG0000(	872	19.47398	chr6:1039	AL121931.1	smallRNA	chr6:62726835-6272
ENSG0000(	872	19.47398	chr6:1039	ENSG00000288088	lncRNA	chr6:68332019-6857
ENSG0000(	872	19.47398	chr6:1039	LINC00680	lncRNA	chr6:57946074-5796
ENSG0000(	872	19.47398	chr6:1039	BECN1P2	Pseudoger	chr6:71075564-7107
ENSG0000(	872	19.47398	chr6:1039	GCNT1P4	Pseudoger	chr6:63857441-6385

ENSG0000(	872	19.47398	chr6:103(	PHF3	NCGv7	protein_(	chr6:63635802-6377
ENSG0000(	872	19.47398	chr6:103(	ENSG00000232295		lncRNA	chr6:71221457-7132
ENSG0000(	872	19.47398	chr6:103(	RIMS1	NCGv7	protein_(	chr6:71886550-7240
ENSG0000(	872	19.47398	chr6:103(	ENSG00000232120		lncRNA	chr6:64377795-6441
ENSG0000(	872	19.47398	chr6:103(	NPM1P37		Pseudoger	chr6:69705287-6970
ENSG0000(	872	19.47398	chr6:103(	MIR4282		smallRNA	chr6:72967687-7296
ENSG0000(	872	19.47398	chr6:103(	ENSG00000219575		Pseudoger	chr6:71550958-7155
ENSG0000(	872	19.47398	chr6:103(	SCAT8		lncRNA	chr6:63805797-6382
ENSG0000(	872	19.47398	chr6:103(	AL158051.1		smallRNA	chr6:69214730-6921
ENSG0000(	872	19.47398	chr6:103(	ENSG00000275773		lncRNA	chr6:60723148-6072
ENSG0000(	872	19.47398	chr6:103(	FAM135A-AS1		lncRNA	chr6:70412828-7041
ENSG0000(	872	19.47398	chr6:103(	RBBP4P3		Pseudoger	chr6:60873360-6087
ENSG0000(	872	19.47398	chr6:103(	ENSG00000272541		lncRNA	chr6:57855891-5785
ENSG0000(	872	19.47398	chr6:103(	FAM135A		protein_(	chr6:70412941-7056
ENSG0000(	872	19.47398	chr6:103(	COL19A1		protein_(	chr6:69866556-7021
ENSG0000(	872	19.47398	chr6:103(	ENSG00000262803		Pseudoger	chr6:70693839-7069
ENSG0000(	872	19.47398	chr6:103(	RNU4-66P		smallRNA	chr6:71652474-7165
ENSG0000(	872	19.47398	chr6:103(	ENSG00000181514		Pseudoger	chr6:72598451-7259
ENSG0000(	872	19.47398	chr6:103(	KHDRBS2-OT1		lncRNA	chr6:61630233-6168
ENSG0000(	872	19.47398	chr6:103(	LINC02549		lncRNA	chr6:68226972-6832
ENSG0000(	872	19.47398	chr6:103(	KRT19P1		Pseudoger	chr6:71584721-7158
ENSG0000(	872	19.47398	chr6:103(	ENSG00000243501		protein_(	chr6:73209746-7326
ENSG0000(	872	19.47398	chr6:103(	GUSBP4		Pseudoger	chr6:57919784-5793
ENSG0000(	872	19.47398	chr6:103(	ENSG00000227706		lncRNA	chr6:67878316-6788
ENSG0000(	872	19.47398	chr6:103(	SDHAF4		protein_(	chr6:70566917-7058
ENSG0000(	872	19.47398	chr6:103(	ENSG00000287557		lncRNA	chr6:67888977-6799
ENSG0000(	872	19.47398	chr6:103(	ENSG00000218048		Pseudoger	chr6:63440766-6344
ENSG0000(	872	19.47398	chr6:103(	ENSG00000232389		Pseudoger	chr6:70608234-7060
ENSG0000(	872	19.47398	chr6:103(	ADGRB3	NCGv7	protein_(	chr6:68635282-6939
ENSG0000(	872	19.47398	chr6:103(	RPL37P15		Pseudoger	chr6:70098390-7009
ENSG0000(	872	19.47398	chr6:103(	ENSG00000218520		Pseudoger	chr6:63229087-6323
ENSG0000(	872	19.47398	chr6:103(	KHDC1-AS1		lncRNA	chr6:73263212-7330
ENSG0000(	872	19.47398	chr6:103(	ENSG00000288712		protein_(	chr6:68635890-6863
ENSG0000(	872	19.47398	chr6:103(	ENSG00000230597		lncRNA	chr6:71328942-7132
ENSG0000(	872	19.47398	chr6:103(	ENSG00000218813		Pseudoger	chr6:63797189-6379
ENSG0000(	872	19.47398	chr6:103(	POM121L14P		Pseudoger	chr6:57937458-5793
ENSG0000(	872	19.47398	chr6:103(	AL606923.1		smallRNA	chr6:68493439-6849
ENSG0000(	872	19.47398	chr6:103(	AL109612.1		smallRNA	chr6:65467004-6546
ENSG0000(	872	19.47398	chr6:103(	ENSG00000289911		lncRNA	chr6:63378892-6356
ENSG0000(	872	19.47398	chr6:103(	LGSN	NCGv7	protein_(	chr6:63275951-6331
ENSG0000(	872	19.47398	chr6:103(	AL589736.1		smallRNA	chr6:62683178-6268
ENSG0000(	872	19.47398	chr6:103(	ENSG00000286723		lncRNA	chr6:57880071-5788
ENSG0000(	872	19.47398	chr6:103(	ENSG00000218732		Pseudoger	chr6:72316599-7231
ENSG0000(	872	19.47398	chr6:103(	PRIM2		protein_(	chr6:57314805-6054
ENSG0000(	872	19.47398	chr6:103(	SMAP1		protein_(	chr6:70667776-7086
ENSG0000(	872	19.47398	chr6:103(	MIR30C2		smallRNA	chr6:71376960-7137
ENSG0000(	872	19.47398	chr6:103(	EIF3EP1		Pseudoger	chr6:73291962-7329
ENSG0000(	872	19.47398	chr6:103(	ENSG00000283352		lncRNA	chr6:57919912-5796
ENSG0000(	872	19.47398	chr6:103(	ENSG00000270509		Pseudoger	chr6:67933485-6793
ENSG0000(	872	19.47398	chr6:103(	ADH5P4		Pseudoger	chr6:65836930-6583
ENSG0000(	872	19.47398	chr6:103(	LINC00680		Pseudoger	chr6:57959029-5796

ENSG0000(	872	19.47398	chr6:1039	AC002485.1		smallRNA	chr6:66359425-6635
ENSG0000(	872	19.47398	chr6:1039	ENSG00000271111		Pseudoger	chr6:67456346-6745
ENSG0000(	872	19.47398	chr6:1039	AL590874.1		smallRNA	chr6:67149509-6714
ENSG0000(	872	19.47398	chr6:1039	ENSG00000266680		lncRNA	chr6:63571005-6357
ENSG0000(	872	19.47398	chr6:1039	AL590558.1		smallRNA	chr6:62983084-6298
ENSG0000(	872	19.47398	chr6:1039	ENSG00000285838		lncRNA	chr6:68040290-6809
ENSG0000(	872	19.47398	chr6:1039	ENSG00000285976		protein_c	chr6:63572472-6358
ENSG0000(	872	19.47398	chr6:1039	HNRNPDP2		Pseudoger	chr6:64631205-6463
ENSG0000(	872	19.47398	chr6:1039	KHDC1P1		Pseudoger	chr6:73209083-7321
ENSG0000(	872	19.47398	chr6:1039	ENSG00000225096		lncRNA	chr6:57961438-5843
ENSG0000(	872	19.47398	chr6:1039	EEF1B2P5		Pseudoger	chr6:63480134-6348
ENSG0000(	872	19.47398	chr6:1039	ENSG00000275046		Pseudoger	chr6:58386799-5838
ENSG0000(	872	19.47398	chr6:1039	KHDRBS2	NCGv7	protein_c	chr6:61679961-6228
ENSG0000(	872	19.47398	chr6:1039	PTP4A1	AC	protein_c	chr6:63521746-6358
ENSG0000(	872	19.47398	chr6:1039	LINC01626		lncRNA	chr6:71450834-7145
ENSG0000(	872	19.47398	chr6:1039	KCNQ5-DT		lncRNA	chr6:72614386-7262
ENSG0000(	872	19.47398	chr6:1039	AC019205.1		smallRNA	chr6:73263008-7326
ENSG0000(	872	19.47398	chr6:1039	ENSG00000287679		lncRNA	chr6:61870097-6189
ENSG0000(	872	19.47398	chr6:1039	ENSG00000290597		lncRNA	chr6:60353963-6054
ENSG0000(	872	19.47398	chr6:1039	LINC00472		lncRNA	chr6:71343427-7142
ENSG0000(	872	19.47398	chr6:1039	COL9A1		protein_c	chr6:70216040-7030
ENSG0000(	872	19.47398	chr6:1039	EYS	NCGv7	protein_c	chr6:63719980-6570
ENSG0000(	872	19.47398	chr6:1039	ENSG00000266579		lncRNA	chr6:60386769-6039
ENSG0000(	872	19.47398	chr6:1039	KCNQ5	NCGv7	protein_c	chr6:72621792-7319
ENSG0000(	872	19.47398	chr6:1039	PGAM1P10		Pseudoger	chr6:73055097-7305
ENSG0000(	872	19.47398	chr6:1039	KHDC1L		protein_c	chr6:73223544-7322
ENSG0000(	872	19.47398	chr6:1039	ENSG00000223504		lncRNA	chr6:68055351-6806
ENSG0000(	872	19.47398	chr6:1039	ENSG00000286680		lncRNA	chr6:67884041-6798
ENSG0000(	872	19.47398	chr6:1039	RNA5SP208		Pseudoger	chr6:67467231-6746
ENSG0000(	872	19.47398	chr6:1039	U3		smallRNA	chr6:71126894-7112
ENSG0000(	872	19.47398	chr6:1039	ENSG00000269966		lncRNA	chr6:71295173-7141
ENSG0000(	872	19.47398	chr6:1039	LMBRD1		protein_c	chr6:69672757-6986
ENSG0000(	872	19.47398	chr6:1039	Y_RNA		smallRNA	chr6:67625356-6762
ENSG0000(	872	19.47398	chr6:1039	ENSG00000262566		Pseudoger	chr6:62611153-6261
ENSG0000(	872	19.47398	chr6:1039	ENSG00000217477		Pseudoger	chr6:63193072-6319
ENSG0000(	872	19.47398	chr6:1039	ENSG00000216687		Pseudoger	chr6:58071720-5807
ENSG0000(	872	19.47398	chr6:1039	ENSG00000272316		lncRNA	chr6:57908560-5791
ENSG0000(	872	19.47398	chr6:1039	ENSG00000289611		lncRNA	chr6:68840383-6884
ENSG0000(	872	19.47398	chr6:1039	AL354933.1		smallRNA	chr6:71585089-7158
ENSG0000(	872	19.47398	chr6:1039	RNU6-280P		smallRNA	chr6:67546651-6754
ENSG0000(	872	19.47398	chr6:1039	RNU7-66P		smallRNA	chr6:66728843-6672
ENSG0000(	872	19.47398	chr6:1039	RPL9P18		Pseudoger	chr6:63615827-6361
ENSG0000(	872	19.47398	chr6:1039	ENSG00000287380		lncRNA	chr6:71238743-7124
ENSG0000(	872	19.47398	chr6:1039	ENSG00000270521		Pseudoger	chr6:66710242-6671
ENSG0000(	872	19.47398	chr6:1039	LYPLA1P3		Pseudoger	chr6:71165076-7116
ENSG0000(	872	19.47398	chr6:1039	SLC25A51P1		Pseudoger	chr6:65788417-6578
ENSG0000(	872	19.47398	chr6:1039	ENSG00000217067		Pseudoger	chr6:62547427-6254
ENSG0000(	872	19.47398	chr6:1039	ENSG00000271967		lncRNA	chr6:70596438-7059
ENSG0000(	872	19.47398	chr6:1039	NDUFAB1P1		Pseudoger	chr6:70734503-7073
ENSG0000(	872	19.47398	chr6:1039	AL109922.1		smallRNA	chr6:64974393-6497
ENSG0000(	872	19.47398	chr6:1039	RNU6-411P		smallRNA	chr6:71182612-7118

ENSG0000(	872	19.47398	chr6:1039	GAPDHP41		Pseudoger	chr6:60719222-6072
ENSG0000(	872	19.47398	chr6:1039	B3GAT2		protein_c	chr6:70856679-7095
ENSG0000(	872	19.47398	chr6:1039	AL035467.1		smallRNA	chr6:71538884-7153
ENSG0000(	867	19.36232	chr6:1039	BMP5	NCGv7	protein_c	chr6:55753653-5587
ENSG0000(	867	19.36232	chr6:1039	ELOVL5		protein_c	chr6:53267398-5334
ENSG0000(	867	19.36232	chr6:1039	ENSG00000249379		lncRNA	chr6:53503109-5350
ENSG0000(	867	19.36232	chr6:1039	RPA3P2		Pseudoger	chr6:53378503-5337
ENSG0000(	867	19.36232	chr6:1039	HMGCLL1		protein_c	chr6:55434373-5557
ENSG0000(	867	19.36232	chr6:1039	RAB23	TAG;AC	protein_c	chr6:57186992-5722
ENSG0000(	867	19.36232	chr6:1039	RPL17P26		Pseudoger	chr6:56871191-5687
ENSG0000(	867	19.36232	chr6:1039	ENSG00000271367		lncRNA	chr6:53350158-5335
ENSG0000(	867	19.36232	chr6:1039	GCLC		protein_c	chr6:53497341-5361
ENSG0000(	867	19.36232	chr6:1039	BAG2		protein_c	chr6:57172326-5718
ENSG0000(	867	19.36232	chr6:1039	CLNS1AP1		Pseudoger	chr6:54485169-5448
ENSG0000(	867	19.36232	chr6:1039	KIAA1586		protein_c	chr6:57046532-5705
ENSG0000(	867	19.36232	chr6:1039	FAM83B	NCGv7	protein_c	chr6:54846771-5494
ENSG0000(	867	19.36232	chr6:1039	MIR548U		smallRNA	chr6:57390132-5739
ENSG0000(	867	19.36232	chr6:1039	NANOGP3		Pseudoger	chr6:53418452-5341
ENSG0000(	867	19.36232	chr6:1039	MLIP		protein_c	chr6:53929982-5426
ENSG0000(	867	19.36232	chr6:1039	RNU6-1023P		smallRNA	chr6:54786387-5478
ENSG0000(	867	19.36232	chr6:1039	ENSG00000231683		lncRNA	chr6:53561289-5361
ENSG0000(	867	19.36232	chr6:1039	RPS16P5		Pseudoger	chr6:53336943-5333
ENSG0000(	867	19.36232	chr6:1039	DST-AS1		lncRNA	chr6:56843928-5686
ENSG0000(	867	19.36232	chr6:1039	U3		smallRNA	chr6:53147808-5314
ENSG0000(	867	19.36232	chr6:1039	RNU6-626P		smallRNA	chr6:56945730-5694
ENSG0000(	867	19.36232	chr6:1039	AL591034.1		smallRNA	chr6:53456447-5345
ENSG0000(	867	19.36232	chr6:1039	ENSG00000271338		Pseudoger	chr6:53381519-5338
ENSG0000(	867	19.36232	chr6:1039	ZNF451	NCGv7	protein_c	chr6:57086844-5717
ENSG0000(	867	19.36232	chr6:1039	SOD1P1		Pseudoger	chr6:53196720-5319
ENSG0000(	867	19.36232	chr6:1039	ZNF451-AS1		lncRNA	chr6:57114894-5717
ENSG0000(	867	19.36232	chr6:1039	TINAG-AS1		lncRNA	chr6:54365335-5436
ENSG0000(	867	19.36232	chr6:1039	ENSG00000218713		Pseudoger	chr6:53206598-5320
ENSG0000(	867	19.36232	chr6:1039	GCM1		protein_c	chr6:53126961-5314
ENSG0000(	867	19.36232	chr6:1039	LRRC1		protein_c	chr6:53794497-5392
ENSG0000(	867	19.36232	chr6:1039	HCRTR2		protein_c	chr6:55106460-5528
ENSG0000(	867	19.36232	chr6:1039	TINAG		protein_c	chr6:54307859-5439
ENSG0000(	867	19.36232	chr6:1039	MLIP-AS1		lncRNA	chr6:53978549-5407
ENSG0000(	867	19.36232	chr6:1039	MIR5685		smallRNA	chr6:53276993-5327
ENSG0000(	867	19.36232	chr6:1039	RPL10P10		Pseudoger	chr6:54602487-5460
ENSG0000(	867	19.36232	chr6:1039	ENSG00000227602		lncRNA	chr6:56331788-5633
ENSG0000(	867	19.36232	chr6:1039	ENSG00000287745		lncRNA	chr6:54190206-5419
ENSG0000(	867	19.36232	chr6:1039	MLIP-IT1		lncRNA	chr6:53998890-5400
ENSG0000(	867	19.36232	chr6:1039	ENSG00000224984		lncRNA	chr6:54840118-5484
ENSG0000(	867	19.36232	chr6:1039	RN7SKP256		smallRNA	chr6:53415294-5341
ENSG0000(	867	19.36232	chr6:1039	RNU1-136P		smallRNA	chr6:53219261-5321
ENSG0000(	867	19.36232	chr6:1039	BEND6		protein_c	chr6:56955107-5702
ENSG0000(	867	19.36232	chr6:1039	FTH1P15		Pseudoger	chr6:57004520-5700
ENSG0000(	867	19.36232	chr6:1039	RPSAP44		Pseudoger	chr6:54624605-5462
ENSG0000(	867	19.36232	chr6:1039	ENSG00000236740		lncRNA	chr6:53930022-5399
ENSG0000(	867	19.36232	chr6:1039	ENSG00000220725		Pseudoger	chr6:55680642-5568
ENSG0000(	867	19.36232	chr6:1039	RCC2P7		Pseudoger	chr6:56431950-5643

ENSG0000(	867	19.36232	chr6:1039	KRASP1		Pseudoger	chr6:54770583-5477
ENSG0000(	867	19.36232	chr6:1039	DST	NCGv7	protein_c	chr6:56457987-5695
ENSG0000(	867	19.36232	chr6:1039	RNU6-464P		smallRNA	chr6:53153795-5315
ENSG0000(	867	19.36232	chr6:1039	OSTCP6		Pseudoger	chr6:56975606-5697
ENSG0000(	867	19.36232	chr6:1039	ENSG00000227885		lncRNA	chr6:53739266-5379
ENSG0000(	867	19.36232	chr6:1039	RPL31P28		Pseudoger	chr6:53354715-5335
ENSG0000(	867	19.36232	chr6:1039	KLHL31		protein_c	chr6:53647916-5366
ENSG0000(	867	19.36232	chr6:1039	COL21A1	NCGv7	protein_c	chr6:56056590-5639
ENSG0000(	867	19.36232	chr6:1039	LINC01564		lncRNA	chr6:53616471-5370
ENSG0000(	867	19.36232	chr6:1039	AL137008.1		smallRNA	chr6:56432379-5643
ENSG0000(	867	19.36232	chr6:1039	RPL31P33		Pseudoger	chr6:53368670-5336
ENSG0000(	867	19.36232	chr6:1039	ERHP2		Pseudoger	chr6:54016479-5401
ENSG0000(	867	19.36232	chr6:1039	ENSG00000271218		lncRNA	chr6:53918974-5392
ENSG0000(	867	19.36232	chr6:1039	NPM1P36		Pseudoger	chr6:55939790-5594
ENSG0000(	867	19.36232	chr6:1039	GFRAL	NCGv7	protein_c	chr6:55327469-5540
ENSG0000(	867	19.36232	chr6:1039	MRPL30P1		Pseudoger	chr6:57029521-5702
ENSG0000(	867	19.36232	chr6:1039	DHFRP6		Pseudoger	chr6:56276529-5627
ENSG0000(	867	19.36232	chr6:1039	ENSG00000261116		lncRNA	chr6:54943167-5494
ENSG0000(	867	19.36232	chr6:1039	HMGB1P20		Pseudoger	chr6:53235621-5323
ENSG0000(	843	18.82633	chr7:1588	ST7		protein_c	chr7:116953238-117
ENSG0000(	843	18.82633	chr7:1588	ENSG00000228368		lncRNA	chr7:116965846-116
ENSG0000(	843	18.82633	chr7:1588	AC002066.2		smallRNA	chr7:116345855-116
ENSG0000(	843	18.82633	chr7:1588	POLR2DP2		Pseudoger	chr7:115503367-115
ENSG0000(	843	18.82633	chr7:1588	ENSG00000237813		lncRNA	chr7:116238260-116
ENSG0000(	843	18.82633	chr7:1588	ENSG00000227532		Pseudoger	chr7:117187548-117
ENSG0000(	843	18.82633	chr7:1588	Y_RNA		smallRNA	chr7:115207894-115
ENSG0000(	843	18.82633	chr7:1588	MIPEPP1		Pseudoger	chr7:112735166-112
ENSG0000(	843	18.82633	chr7:1588	Y_RNA		smallRNA	chr7:116909877-116
ENSG0000(	843	18.82633	chr7:1588	ZNF277-AS1		lncRNA	chr7:112328189-112
ENSG0000(	843	18.82633	chr7:1588	ENSG00000243243		lncRNA	chr7:116237929-116
ENSG0000(	843	18.82633	chr7:1588	ENSG00000279288		TEC	chr7:113075124-113
ENSG0000(	843	18.82633	chr7:1588	ENSG00000225457		lncRNA	chr7:113100663-113
ENSG0000(	843	18.82633	chr7:1588	ENSG00000287592		lncRNA	chr7:113486407-113
ENSG0000(	843	18.82633	chr7:1588	LINC01392		lncRNA	chr7:115061537-115
ENSG0000(	843	18.82633	chr7:1588	ENSG00000288640		protein_c	chr7:112450460-112
ENSG0000(	843	18.82633	chr7:1588	ENSG00000279086		TEC	chr7:116209234-116
ENSG0000(	843	18.82633	chr7:1588	MDFIC	NCGv7	protein_c	chr7:114922094-115
ENSG0000(	843	18.82633	chr7:1588	RNA5SP239		Pseudoger	chr7:116944286-116
ENSG0000(	843	18.82633	chr7:1588	snoZ185		smallRNA	chr7:116433214-116
ENSG0000(	843	18.82633	chr7:1588	IFRD1		protein_c	chr7:112422887-112
ENSG0000(	843	18.82633	chr7:1588	NPM1P14		Pseudoger	chr7:112520488-112
ENSG0000(	843	18.82633	chr7:1588	TES	DriverDBv	protein_c	chr7:116210506-116
ENSG0000(	843	18.82633	chr7:1588	ENSG00000243345		lncRNA	chr7:115789729-115
ENSG0000(	843	18.82633	chr7:1588	ENSG00000180019		Pseudoger	chr7:112446086-112
ENSG0000(	843	18.82633	chr7:1588	ENSG00000223646		lncRNA	chr7:112616440-112
ENSG0000(	843	18.82633	chr7:1588	MTCYBP6		Pseudoger	chr7:117264393-117
ENSG0000(	843	18.82633	chr7:1588	ENSG00000237870		lncRNA	chr7:116275606-116
ENSG0000(	843	18.82633	chr7:1588	MIR3666		smallRNA	chr7:114653345-114
ENSG0000(	843	18.82633	chr7:1588	SMIM30		protein_c	chr7:113116718-113
ENSG0000(	843	18.82633	chr7:1588	TPM3P1		Pseudoger	chr7:116972165-116
ENSG0000(	843	18.82633	chr7:1588	Y_RNA		smallRNA	chr7:115833273-115

ENSG0000(	843	18.82633	chr7:158(	MTND4P6		Pseudoger	chr7:117263917-117
ENSG0000(	843	18.82633	chr7:158(	RAC1P6		Pseudoger	chr7:115136475-115
ENSG0000(	843	18.82633	chr7:158(	RPL36P13		Pseudoger	chr7:114297114-114
ENSG0000(	843	18.82633	chr7:158(	AC020606.1		smallRNA	chr7:114629855-114
ENSG0000(	843	18.82633	chr7:158(	ST7-AS2		lncRNA	chr7:117072072-117
ENSG0000(	843	18.82633	chr7:158(	RNA5SP238		Pseudoger	chr7:114613787-114
ENSG0000(	843	18.82633	chr7:158(	ENSG00000270997		Pseudoger	chr7:113415689-113
ENSG0000(	843	18.82633	chr7:158(	MTCYBP24		Pseudoger	chr7:112374324-112
ENSG0000(	843	18.82633	chr7:158(	ENSG00000288635		protein_c	chr7:116954391-117
ENSG0000(	843	18.82633	chr7:158(	BMT2		protein_c	chr7:112819147-112
ENSG0000(	843	18.82633	chr7:158(	ENSG00000235945		Pseudoger	chr7:116873454-116
ENSG0000(	843	18.82633	chr7:158(	ENSG00000287829		lncRNA	chr7:117091678-117
ENSG0000(	843	18.82633	chr7:158(	ENSG00000224595		lncRNA	chr7:114414244-114
ENSG0000(	843	18.82633	chr7:158(	COMETT		lncRNA	chr7:116563594-116
ENSG0000(	843	18.82633	chr7:158(	PPP1R3A	NCGv7	protein_c	chr7:113876777-114
ENSG0000(	843	18.82633	chr7:158(	ST7-AS1		lncRNA	chr7:116952446-116
ENSG0000(	843	18.82633	chr7:158(	ENSG00000242072		lncRNA	chr7:115647461-115
ENSG0000(	843	18.82633	chr7:158(	TMEM168		protein_c	chr7:112762377-112
ENSG0000(	843	18.82633	chr7:158(	ENSG00000230785		Pseudoger	chr7:117262918-117
ENSG0000(	843	18.82633	chr7:158(	ENSG00000288634		protein_c	chr7:112450487-112
ENSG0000(	843	18.82633	chr7:158(	ENSG00000240973		lncRNA	chr7:115679345-115
ENSG0000(	843	18.82633	chr7:158(	CAPZA2		protein_c	chr7:116811070-116
ENSG0000(	843	18.82633	chr7:158(	GPR85		protein_c	chr7:113078331-113
ENSG0000(	843	18.82633	chr7:158(	LSMEM1		protein_c	chr7:112480853-112
ENSG0000(	843	18.82633	chr7:158(	LINC01393		lncRNA	chr7:115030564-115
ENSG0000(	843	18.82633	chr7:158(	ENSG00000235427		lncRNA	chr7:116542718-116
ENSG0000(	843	18.82633	chr7:158(	MTND6P24		Pseudoger	chr7:112373733-112
ENSG0000(	843	18.82633	chr7:158(	ENSG00000278894		TEC	chr7:113451072-113
ENSG0000(	843	18.82633	chr7:158(	MTND5P8		Pseudoger	chr7:112372647-112
ENSG0000(	843	18.82633	chr7:158(	MET	NCGv7;AC	protein_c	chr7:116672196-116
ENSG0000(	843	18.82633	chr7:158(	CAV1	AC	protein_c	chr7:116524994-116
ENSG0000(	843	18.82633	chr7:158(	CAV2		protein_c	chr7:116287380-116
ENSG0000(	843	18.82633	chr7:158(	TFEC		protein_c	chr7:115935148-116
ENSG0000(	843	18.82633	chr7:158(	ENSG00000282859		lncRNA	chr7:114560961-114
ENSG0000(	843	18.82633	chr7:158(	ST7-OT4		lncRNA	chr7:116953899-117
ENSG0000(	843	18.82633	chr7:158(	SNORA25		smallRNA	chr7:115581315-115
ENSG0000(	843	18.82633	chr7:158(	FOXP2	NCGv7	protein_c	chr7:114086327-114
ENSG0000(	843	18.82633	chr7:158(	HRAT17		lncRNA	chr7:112953282-112
ENSG0000(	843	18.82633	chr7:158(	ENSG00000289630		lncRNA	chr7:113118666-113
ENSG0000(	839	18.737	chr6:103(	MIR3144		smallRNA	chr6:120015179-120
ENSG0000(	839	18.737	chr6:103(	ENSG00000220447		Pseudoger	chr6:121381472-121
ENSG0000(	839	18.737	chr6:103(	MAN1A1		protein_c	chr6:119177205-119
ENSG0000(	839	18.737	chr6:103(	ENSG00000287992		lncRNA	chr6:120840733-120
ENSG0000(	839	18.737	chr6:103(	SSXP10		Pseudoger	chr6:118589070-118
ENSG0000(	839	18.737	chr6:103(	FAM184A		protein_c	chr6:118959763-119
ENSG0000(	839	18.737	chr6:103(	MCM9		protein_c	chr6:118813442-118
ENSG0000(	839	18.737	chr6:103(	ASF1A		protein_c	chr6:118894152-118
ENSG0000(	839	18.737	chr6:103(	CEP85L		protein_c	chr6:118460772-118
ENSG0000(	839	18.737	chr6:103(	ENSG00000220326		Pseudoger	chr6:121641009-121
ENSG0000(	839	18.737	chr6:103(	HMGB3P18		Pseudoger	chr6:121858179-121
ENSG0000(	839	18.737	chr6:103(	RNU4-35P		smallRNA	chr6:121453981-121

ENSG0000(	839	18.737	chr6:1039	ENSG00000217139		Pseudoger	chr6:121682925-121
ENSG0000(	839	18.737	chr6:1039	ENSG00000286540		lncRNA	chr6:120462511-120
ENSG0000(	839	18.737	chr6:1039	SLC25A5P7		Pseudoger	chr6:121653795-121
ENSG0000(	839	18.737	chr6:1039	PLN		protein_c	chr6:118548296-118
ENSG0000(	839	18.737	chr6:1039	BRD7P3		Pseudoger	chr6:118501430-118
ENSG0000(	839	18.737	chr6:1039	SELENOKP3		Pseudoger	chr6:118757518-118
ENSG0000(	839	18.737	chr6:1039	COX6A1P3		Pseudoger	chr6:120781220-120
ENSG0000(	839	18.737	chr6:1039	GJA1		protein_c	chr6:121435595-121
ENSG0000(	839	18.737	chr6:1039	RNU6-214P		smallRNA	chr6:120526294-120
ENSG0000(	839	18.737	chr6:1039	ENSG00000216316		Pseudoger	chr6:119269133-119
ENSG0000(	839	18.737	chr6:1039	Y_RNA		smallRNA	chr6:121378797-121
ENSG0000(	839	18.737	chr6:1039	ENSG00000272714		Pseudoger	chr6:121389324-121
ENSG0000(	839	18.737	chr6:1039	ENSG00000287100		lncRNA	chr6:119349886-119
ENSG0000(	839	18.737	chr6:1039	ENSG00000220139		Pseudoger	chr6:119159297-119
ENSG0000(	839	18.737	chr6:1039	ENSG00000289871		lncRNA	chr6:121857813-121
ENSG0000(	839	18.737	chr6:1039	ENSG00000253194		lncRNA	chr6:118934770-119
ENSG0000(	839	18.737	chr6:1039	TBC1D32	NCGv7	protein_c	chr6:121079494-121
ENSG0000(	839	18.737	chr6:1039	ENSG00000219784		Pseudoger	chr6:121478273-121
ENSG0000(	839	18.737	chr6:1039	ENSG00000290064		lncRNA	chr6:121619478-121
ENSG0000(	839	18.737	chr6:1039	ENSG00000286339		lncRNA	chr6:118565660-118
ENSG0000(	839	18.737	chr6:1039	RNU2-8P		smallRNA	chr6:121580332-121
ENSG0000(	839	18.737	chr6:1039	MIR548B		smallRNA	chr6:119069047-119
ENSG0000(	839	18.737	chr6:1039	Y_RNA		smallRNA	chr6:119057880-119
ENSG0000(	839	18.737	chr6:1039	SLC35F1	NCGv7	protein_c	chr6:117907264-118
ENSG0000(	839	18.737	chr6:1039	RNU6-1286P		smallRNA	chr6:121354354-121
ENSG0000(	839	18.737	chr6:1039	RPL23AP48		Pseudoger	chr6:121679972-121
ENSG0000(	839	18.737	chr6:1039	ENSG00000216809		Pseudoger	chr6:118452469-118
ENSG0000(	839	18.737	chr6:1039	RNU4-76P		smallRNA	chr6:121542486-121
ENSG0000(	839	18.737	chr6:1039	RNA5SP215		Pseudoger	chr6:120686621-120
ENSG0000(	833	18.60301	chr6:1039	ENSG00000279453		TEC	chr6:122436789-122
ENSG0000(	833	18.60301	chr6:1039	ENSG00000287258		lncRNA	chr6:122562172-122
ENSG0000(	833	18.60301	chr6:1039	PKIB		protein_c	chr6:122471931-122
ENSG0000(	833	18.60301	chr6:1039	RN7SL564P		smallRNA	chr6:122745274-122
ENSG0000(	833	18.60301	chr6:1039	RNU1-18P		smallRNA	chr6:122211648-122
ENSG0000(	833	18.60301	chr6:1039	ENSG00000277408		Pseudoger	chr6:122531123-122
ENSG0000(	833	18.60301	chr6:1039	FABP7		protein_c	chr6:122779716-122
ENSG0000(	833	18.60301	chr6:1039	SMPDL3A		protein_c	chr6:122789049-122
ENSG0000(	833	18.60301	chr6:1039	ENSG00000287818		lncRNA	chr6:122711887-122
ENSG0000(	833	18.60301	chr6:1039	ENSG00000272472		lncRNA	chr6:122643388-122
ENSG0000(	833	18.60301	chr6:1039	ENSG00000285652		lncRNA	chr6:122975198-122
ENSG0000(	833	18.60301	chr6:1039	ATP5MGP2		Pseudoger	chr6:122859678-122
ENSG0000(	833	18.60301	chr6:1039	SERINC1		protein_c	chr6:122443351-122
ENSG0000(	833	18.60301	chr6:1039	CLVS2		protein_c	chr6:122996235-123
ENSG0000(	833	18.60301	chr6:1039	ENSG00000275339		Pseudoger	chr6:122454358-122
ENSG0000(	833	18.60301	chr6:1039	ENSG00000237321		lncRNA	chr6:123823240-123
ENSG0000(	833	18.60301	chr6:1039	ENSG00000279114		TEC	chr6:122471923-122
ENSG0000(	833	18.60301	chr6:1039	ENSG00000285691		lncRNA	chr6:123519697-123
ENSG0000(	833	18.60301	chr6:1039	ENSG00000285941		lncRNA	chr6:123589711-123
ENSG0000(	833	18.60301	chr6:1039	TRDN-AS1		lncRNA	chr6:123389421-123
ENSG0000(	833	18.60301	chr6:1039	AL354936.1		smallRNA	chr6:123740434-123
ENSG0000(	833	18.60301	chr6:1039	TRDN		protein_c	chr6:123216339-123

ENSG0000(	833	18.60301	chr6:1039	HSF2		protein_c	chr6:122399551-122
ENSG0000(	820	18.31269	chr6:1039	ENSG00000277797		lncRNA	chr6:81551686-8155
ENSG0000(	820	18.31269	chr6:1039	TENT5A		protein_c	chr6:81491439-8175
ENSG0000(	820	18.31269	chr6:1039	ENSG00000260574		lncRNA	chr6:81969453-8196
ENSG0000(	820	18.31269	chr6:1039	ENSG00000286875		lncRNA	chr6:82353861-8236
ENSG0000(	820	18.31269	chr6:1039	TPBG		protein_c	chr6:82363206-8236
ENSG0000(	820	18.31269	chr6:1039	ENSG00000219702		Pseudoger	chr6:81764211-8176
ENSG0000(	820	18.31269	chr6:1039	RNA5SP210		Pseudoger	chr6:81622200-8162
ENSG0000(	820	18.31269	chr6:1039	RNU6-130P		smallRNA	chr6:82210338-8221
ENSG0000(	820	18.31269	chr6:1039	ENSG00000226089		lncRNA	chr6:81527102-8153
ENSG0000(	820	18.31269	chr6:1039	LINC01526		lncRNA	chr6:81813286-8181
ENSG0000(	820	18.31269	chr6:1039	ENSG00000232031		lncRNA	chr6:81724722-8173
ENSG0000(	820	18.31269	chr6:1039	ENSG00000220537		Pseudoger	chr6:82263996-8226
ENSG0000(	820	18.31269	chr6:1039	SNORA70		smallRNA	chr6:81764024-8176
ENSG0000(	820	18.31269	chr6:1039	LINC02542		lncRNA	chr6:81844602-8216
ENSG0000(	820	18.31269	chr6:1039	IBTK		protein_c	chr6:82169986-8224
ENSG0000(	818	18.26802	chr6:1039	HTR1E	NCGv7	protein_c	chr6:86937528-8701
ENSG0000(	818	18.26802	chr6:1039	RCN1P1		Pseudoger	chr6:87121693-8712
ENSG0000(	818	18.26802	chr6:1039	CGA		protein_c	chr6:87085498-8709
ENSG0000(	818	18.26802	chr6:1039	ORC3		protein_c	chr6:87590067-8766
ENSG0000(	818	18.26802	chr6:1039	ENSG00000288021		lncRNA	chr6:85949690-8599
ENSG0000(	818	18.26802	chr6:1039	AKIRIN2	AC	protein_c	chr6:87674860-8770
ENSG0000(	818	18.26802	chr6:1039	MRAP2		protein_c	chr6:84033772-8409
ENSG0000(	818	18.26802	chr6:1039	snoU13		smallRNA	chr6:87720915-8772
ENSG0000(	818	18.26802	chr6:1039	NT5E		protein_c	chr6:85449584-8549
ENSG0000(	818	18.26802	chr6:1039	CNR1		protein_c	chr6:88139864-8816
ENSG0000(	818	18.26802	chr6:1039	RN7SL183P		smallRNA	chr6:87845650-8784
ENSG0000(	818	18.26802	chr6:1039	SNX14		protein_c	chr6:85504776-8559
ENSG0000(	818	18.26802	chr6:1039	SPACA1		protein_c	chr6:88047841-8806
ENSG0000(	818	18.26802	chr6:1039	CEP162		protein_c	chr6:84124241-8422
ENSG0000(	818	18.26802	chr6:1039	TBX18	NCGv7	protein_c	chr6:84687351-8476
ENSG0000(	818	18.26802	chr6:1039	RNU6-444P		smallRNA	chr6:87488445-8748
ENSG0000(	818	18.26802	chr6:1039	SYNCRIP	NCGv7	protein_c	chr6:85607779-8564
ENSG0000(	818	18.26802	chr6:1039	ENSG00000286871		lncRNA	chr6:88378280-8838
ENSG0000(	818	18.26802	chr6:1039	ENSG00000288009		lncRNA	chr6:89130887-8914
ENSG0000(	818	18.26802	chr6:1039	ENSG00000272008		lncRNA	chr6:87151159-8715
ENSG0000(	818	18.26802	chr6:1039	RIPPLY2		protein_c	chr6:83853360-8385
ENSG0000(	818	18.26802	chr6:1039	ENSG00000216902		Pseudoger	chr6:85257328-8525
ENSG0000(	818	18.26802	chr6:1039	ENSG00000287294		lncRNA	chr6:84769010-8477
ENSG0000(	818	18.26802	chr6:1039	Y_RNA		smallRNA	chr6:87826173-8782
ENSG0000(	818	18.26802	chr6:1039	SNHG5		lncRNA	chr6:85650491-8567
ENSG0000(	818	18.26802	chr6:1039	Y_RNA		smallRNA	chr6:85777639-8577
ENSG0000(	818	18.26802	chr6:1039	C6orf163		protein_c	chr6:87344813-8736
ENSG0000(	818	18.26802	chr6:1039	SMIM8		protein_c	chr6:87322583-8739
ENSG0000(	818	18.26802	chr6:1039	DUTP5		Pseudoger	chr6:85426657-8542
ENSG0000(	818	18.26802	chr6:1039	RNGTT		protein_c	chr6:88609897-8896
ENSG0000(	818	18.26802	chr6:1039	ENSG00000271114		Pseudoger	chr6:87891424-8789
ENSG0000(	818	18.26802	chr6:1039	ENSG00000279616		TEC	chr6:87356831-8735
ENSG0000(	818	18.26802	chr6:1039	CFAP206		protein_c	chr6:87407972-8746
ENSG0000(	818	18.26802	chr6:1039	ENSG00000286278		lncRNA	chr6:88047056-8804
ENSG0000(	818	18.26802	chr6:1039	ENSG00000217060		Pseudoger	chr6:85286076-8529

ENSG0000(	818	18.26802	chr6:1039	GJB7		protein_c	chr6:87282980-8732
ENSG0000(	818	18.26802	chr6:1039	ENSG00000216523		Pseudoger	chr6:84966397-8496
ENSG0000(	818	18.26802	chr6:1039	ENSG00000287705		lncRNA	chr6:83852312-8385
ENSG0000(	818	18.26802	chr6:1039	ENSG00000280277		TEC	chr6:88964527-8896
ENSG0000(	818	18.26802	chr6:1039	ENSG00000279565		TEC	chr6:88172261-8817
ENSG0000(	818	18.26802	chr6:1039	AL138919.1		smallRNA	chr6:87847545-8784
ENSG0000(	818	18.26802	chr6:1039	MTHFD2P2		Pseudoger	chr6:87000045-8700
ENSG0000(	818	18.26802	chr6:1039	TBX18-AS1		lncRNA	chr6:84687712-8470
ENSG0000(	818	18.26802	chr6:1039	ENSG00000271793		protein_c	chr6:85504776-8567
ENSG0000(	818	18.26802	chr6:1039	PNRC1-DT		lncRNA	chr6:89080164-8908
ENSG0000(	818	18.26802	chr6:1039	HSPD1P10		Pseudoger	chr6:87298772-8730
ENSG0000(	818	18.26802	chr6:1039	ENSG00000280232		TEC	chr6:85498441-8549
ENSG0000(	818	18.26802	chr6:1039	ENSG00000228679		lncRNA	chr6:83983728-8400
ENSG0000(	818	18.26802	chr6:1039	ENSG00000216324		Pseudoger	chr6:87425795-8742
ENSG0000(	818	18.26802	chr6:1039	RN7SL336P		smallRNA	chr6:89090946-8909
ENSG0000(	818	18.26802	chr6:1039	ENSG00000237874		lncRNA	chr6:83728055-8373
ENSG0000(	818	18.26802	chr6:1039	SMARCE1P2		Pseudoger	chr6:84429584-8443
ENSG0000(	818	18.26802	chr6:1039	RNU4-12P		smallRNA	chr6:85993021-8599
ENSG0000(	818	18.26802	chr6:1039	SLC35A1		protein_c	chr6:87470623-8751
ENSG0000(	818	18.26802	chr6:1039	LINC02535		lncRNA	chr6:85387219-8539
ENSG0000(	818	18.26802	chr6:1039	PM20D2		protein_c	chr6:89146055-8916
ENSG0000(	818	18.26802	chr6:1039	CYB5R4		protein_c	chr6:83859656-8396
ENSG0000(	818	18.26802	chr6:1039	RN7SL643P		smallRNA	chr6:86435852-8643
ENSG0000(	818	18.26802	chr6:1039	RN7SKP209		smallRNA	chr6:87083233-8708
ENSG0000(	818	18.26802	chr6:1039	ENSG00000218766		Pseudoger	chr6:85868953-8586
ENSG0000(	818	18.26802	chr6:1039	ENSG00000220291		Pseudoger	chr6:85155667-8516
ENSG0000(	818	18.26802	chr6:1039	PNRC1	NCGv7	protein_c	chr6:89080751-8908
ENSG0000(	818	18.26802	chr6:1039	ACTBP8		Pseudoger	chr6:88275882-8827
ENSG0000(	818	18.26802	chr6:1039	ZNF292	NCGv7	protein_c	chr6:87151803-8726
ENSG0000(	818	18.26802	chr6:1039	LINC01611		lncRNA	chr6:84421028-8455
ENSG0000(	818	18.26802	chr6:1039	ENSG00000219190		Pseudoger	chr6:86768522-8676
ENSG0000(	818	18.26802	chr6:1039	ENSG00000220240		Pseudoger	chr6:86897547-8689
ENSG0000(	818	18.26802	chr6:1039	ENSG00000213204		protein_c	chr6:87408012-8751
ENSG0000(	818	18.26802	chr6:1039	RARS2		protein_c	chr6:87513459-8759
ENSG0000(	818	18.26802	chr6:1039	AL391416.1		smallRNA	chr6:83367706-8336
ENSG0000(	818	18.26802	chr6:1039	TPT1P6		Pseudoger	chr6:85427453-8542
ENSG0000(	818	18.26802	chr6:1039	ENSG00000234426		lncRNA	chr6:88177804-8844
ENSG0000(	818	18.26802	chr6:1039	ENSG00000220960		Pseudoger	chr6:89059628-8906
ENSG0000(	818	18.26802	chr6:1039	ST13P16		Pseudoger	chr6:87444395-8744
ENSG0000(	818	18.26802	chr6:1039	ENSG00000218561		Pseudoger	chr6:86729708-8673
ENSG0000(	818	18.26802	chr6:1039	KRT18P64		Pseudoger	chr6:85287789-8528
ENSG0000(	818	18.26802	chr6:1039	SRSF12		protein_c	chr6:89095959-8911
ENSG0000(	818	18.26802	chr6:1039	RNU4-72P		smallRNA	chr6:85751581-8575
ENSG0000(	818	18.26802	chr6:1039	ENSG00000219500		Pseudoger	chr6:86432244-8643
ENSG0000(	818	18.26802	chr6:1039	NDUFA5P9		Pseudoger	chr6:86214237-8621
ENSG0000(	818	18.26802	chr6:1039	ENSG00000219951		Pseudoger	chr6:83392416-8339
ENSG0000(	818	18.26802	chr6:1039	ENSG00000289931		lncRNA	chr6:88963824-8896
ENSG0000(	818	18.26802	chr6:1039	PKMP3		Pseudoger	chr6:85659892-8566
ENSG0000(	818	18.26802	chr6:1039	RPL7P27		Pseudoger	chr6:86086415-8608
ENSG0000(	818	18.26802	chr6:1039	SNORA73		smallRNA	chr6:88714242-8871
ENSG0000(	818	18.26802	chr6:1039	AL391417.1		smallRNA	chr6:86473487-8647

ENSG0000(	818	18.26802	chr6:1039	SNAP91	protein_c	chr6:83552880-8370
ENSG0000(	818	18.26802	chr6:1039	ENSG00000220130	Pseudoger	chr6:89001548-8900
ENSG0000(	818	18.26802	chr6:1039	ENSG00000217334	Pseudoger	chr6:85731371-8573
ENSG0000(	818	18.26802	chr6:1039	ENSG00000219604	Pseudoger	chr6:84689292-8468
ENSG0000(	818	18.26802	chr6:1039	ENSG00000217769	Pseudoger	chr6:86897118-8689
ENSG0000(	818	18.26802	chr6:1039	RAB1AP2	Pseudoger	chr6:86170447-8617
ENSG0000(	818	18.26802	chr6:1039	ENSG00000218313	Pseudoger	chr6:87173368-8717
ENSG0000(	818	18.26802	chr6:1039	PRSS35	protein_c	chr6:83512534-8352
ENSG0000(	818	18.26802	chr6:1039	ENSG00000220131	Pseudoger	chr6:88265158-8826
ENSG0000(	818	18.26802	chr6:1039	SMIM11P1	Pseudoger	chr6:85735744-8573
ENSG0000(	818	18.26802	chr6:1039	RNU2-61P	smallRNA	chr6:89063500-8906
ENSG0000(	818	18.26802	chr6:1039	ENSG00000217776	Pseudoger	chr6:87070156-8707
ENSG0000(	818	18.26802	chr6:1039	RPL7P29	Pseudoger	chr6:86970466-8697
ENSG0000(	818	18.26802	chr6:1039	LINC02857	lncRNA	chr6:84019204-8402
ENSG0000(	818	18.26802	chr6:1039	ENSG00000218793	Pseudoger	chr6:87441165-8744
ENSG0000(	818	18.26802	chr6:1039	GABRR1	protein_c	chr6:89177504-8923
ENSG0000(	814	18.17869	chr6:1039	ENSG00000233967	lncRNA	chr6:80441295-8046
ENSG0000(	814	18.17869	chr6:1039	ENSG00000279022	TEC	chr6:80440730-8044
ENSG0000(	814	18.17869	chr6:1039	ENSG00000181705	Pseudoger	chr6:80499196-8049
ENSG0000(	814	18.17869	chr6:1039	LINCMD1	lncRNA	chr6:52146814-5215
ENSG0000(	814	18.17869	chr6:1039	MIR206	smallRNA	chr6:52144349-5214
ENSG0000(	814	18.17869	chr6:1039	AL590391.1	smallRNA	chr6:52061615-5206
ENSG0000(	814	18.17869	chr6:1039	MIR133B	smallRNA	chr6:52148923-5214
ENSG0000(	814	18.17869	chr6:1039	SLC25A20P1	Pseudoger	chr6:52246460-5224
ENSG0000(	814	18.17869	chr6:1039	IL17F	protein_c	chr6:52236681-5224
ENSG0000(	814	18.17869	chr6:1039	ENSG00000216352	Pseudoger	chr6:80555841-8055
ENSG0000(	814	18.17869	chr6:1039	IL17A	protein_c	chr6:52186375-5219
ENSG0000(	814	18.17869	chr6:1039	RN7SL580P	smallRNA	chr6:51975660-5197
ENSG0000(	814	18.17869	chr6:1039	MCM3	protein_c	chr6:52264014-5228
ENSG0000(	814	18.17869	chr6:1039	ENSG00000260645	lncRNA	chr6:80466958-8046
ENSG0000(	814	18.17869	chr6:1039	RPSAP72	Pseudoger	chr6:80470071-8047
ENSG0000(	812	18.13403	chr6:1039	PGM3	protein_c	chr6:83147324-8319
ENSG0000(	812	18.13403	chr6:1039	LAP3P1	Pseudoger	chr6:82924829-8292
ENSG0000(	812	18.13403	chr6:1039	RWDD2A	protein_c	chr6:83193357-8319
ENSG0000(	812	18.13403	chr6:1039	ENSG00000227215	lncRNA	chr6:82932601-8293
ENSG0000(	812	18.13403	chr6:1039	UBE3D	protein_c	chr6:82892390-8306
ENSG0000(	812	18.13403	chr6:1039	ME1	protein_c	chr6:83210402-8343
ENSG0000(	812	18.13403	chr6:1039	DOP1A	protein_c	chr6:83067666-8317
ENSG0000(	811	18.11169	chr2:3094	PARD3B	protein_c	chr2:204545475-205
ENSG0000(	811	18.11169	chr2:3094	ENSG00000225216	lncRNA	chr2:205756469-205
ENSG0000(	811	18.11169	chr2:3094	NRP2	protein_c	chr2:205681990-205
ENSG0000(	811	18.11169	chr2:3094	ENSG00000223890	Pseudoger	chr2:205253304-205
ENSG0000(	811	18.11169	chr2:3094	RN7SKP178	smallRNA	chr2:205900630-205
ENSG0000(	803	17.93303	chr2:3094	ENSG00000231896	lncRNA	chr2:208469850-208
ENSG0000(	803	17.93303	chr2:3094	ENSG00000225111	lncRNA	chr2:207868578-207
ENSG0000(	803	17.93303	chr2:3094	ENSG00000234902	lncRNA	chr2:207186717-207
ENSG0000(	803	17.93303	chr2:3094	MYOSLID	lncRNA	chr2:207166120-207
ENSG0000(	803	17.93303	chr2:3094	ENSG00000289113	lncRNA	chr2:209299674-209
ENSG0000(	803	17.93303	chr2:3094	LINC01857	lncRNA	chr2:207662375-207
ENSG0000(	803	17.93303	chr2:3094	Y_RNA	smallRNA	chr2:207687960-207
ENSG0000(	803	17.93303	chr2:3094	ENSG00000231955	lncRNA	chr2:206115547-206

ENSG0000(	803	17.93303	chr2:309<	CMKLR2		protein_c	chr2:206175316-206
ENSG0000(	803	17.93303	chr2:309<	PKP4P1		Pseudoger	chr2:209178966-209
ENSG0000(	803	17.93303	chr2:309<	ENSG00000271074		Pseudoger	chr2:211104934-211
ENSG0000(	803	17.93303	chr2:309<	HMGN1P6		Pseudoger	chr2:206259628-206
ENSG0000(	803	17.93303	chr2:309<	ENSG00000287662		lncRNA	chr2:208885755-208
ENSG0000(	803	17.93303	chr2:309<	ENSG00000223725		lncRNA	chr2:207239864-207
ENSG0000(	803	17.93303	chr2:309<	TPT1P2		Pseudoger	chr2:208214724-208
ENSG0000(	803	17.93303	chr2:309<	CRYGB		protein_c	chr2:208142573-208
ENSG0000(	803	17.93303	chr2:309<	ENSG00000231653		lncRNA	chr2:206640073-206
ENSG0000(	803	17.93303	chr2:309<	RNA5SP118		Pseudoger	chr2:209721913-209
ENSG0000(	803	17.93303	chr2:309<	LANCL1-AS1		lncRNA	chr2:210324759-210
ENSG0000(	803	17.93303	chr2:309<	FAM237A		protein_c	chr2:206642487-206
ENSG0000(	803	17.93303	chr2:309<	HNRNPA1P51		Pseudoger	chr2:206419292-206
ENSG0000(	803	17.93303	chr2:309<	IDH1-AS1		lncRNA	chr2:208255214-208
ENSG0000(	803	17.93303	chr2:309<	RNA5SP116		Pseudoger	chr2:208054161-208
ENSG0000(	803	17.93303	chr2:309<	ENSG00000260171		lncRNA	chr2:206821190-206
ENSG0000(	803	17.93303	chr2:309<	ENSG00000225610		lncRNA	chr2:205989585-206
ENSG0000(	803	17.93303	chr2:309<	MTCO1P46		Pseudoger	chr2:211778273-211
ENSG0000(	803	17.93303	chr2:309<	MIR1302-4		smallRNA	chr2:207269275-207
ENSG0000(	803	17.93303	chr2:309<	RNA5SP117		Pseudoger	chr2:208920547-208
ENSG0000(	803	17.93303	chr2:309<	ENSG00000289422		lncRNA	chr2:208064701-208
ENSG0000(	803	17.93303	chr2:309<	ENSG00000224529		Pseudoger	chr2:208135394-208
ENSG0000(	803	17.93303	chr2:309<	CRYGC		protein_c	chr2:208128137-208
ENSG0000(	803	17.93303	chr2:309<	FZD5		protein_c	chr2:207762598-207
ENSG0000(	803	17.93303	chr2:309<	CCNYL1		protein_c	chr2:207711540-207
ENSG0000(	803	17.93303	chr2:309<	ENSG00000286568		lncRNA	chr2:207217532-207
ENSG0000(	803	17.93303	chr2:309<	PPIAP68		Pseudoger	chr2:205949091-205
ENSG0000(	803	17.93303	chr2:309<	MTND2P23		Pseudoger	chr2:211777206-211
ENSG0000(	803	17.93303	chr2:309<	Y_RNA		smallRNA	chr2:206701065-206
ENSG0000(	803	17.93303	chr2:309<	SNAI1P1		Pseudoger	chr2:209808804-209
ENSG0000(	803	17.93303	chr2:309<	ZDBF2	NCGv7	protein_c	chr2:206274663-206
ENSG0000(	803	17.93303	chr2:309<	CRYGFP		Pseudoger	chr2:209145241-209
ENSG0000(	803	17.93303	chr2:309<	RN7SKP260		smallRNA	chr2:206260066-206
ENSG0000(	803	17.93303	chr2:309<	RPE	NCGv7	protein_c	chr2:210002565-210
ENSG0000(	803	17.93303	chr2:309<	Vault		smallRNA	chr2:206007880-206
ENSG0000(	803	17.93303	chr2:309<	H3P9		Pseudoger	chr2:208466238-208
ENSG0000(	803	17.93303	chr2:309<	SNORA41		smallRNA	chr2:206162228-206
ENSG0000(	803	17.93303	chr2:309<	Y_RNA		smallRNA	chr2:206025593-206
ENSG0000(	803	17.93303	chr2:309<	MIR2355		smallRNA	chr2:207109987-207
ENSG0000(	803	17.93303	chr2:309<	ENSG00000228577		lncRNA	chr2:206606497-206
ENSG0000(	803	17.93303	chr2:309<	RPL27P8		Pseudoger	chr2:206040432-206
ENSG0000(	803	17.93303	chr2:309<	RPL12P17		Pseudoger	chr2:208057248-208
ENSG0000(	803	17.93303	chr2:309<	ENSG00000290929		lncRNA	chr2:208108154-208
ENSG0000(	803	17.93303	chr2:309<	KANSL1L-AS1		lncRNA	chr2:210030572-210
ENSG0000(	803	17.93303	chr2:309<	RPL9P14		Pseudoger	chr2:208031383-208
ENSG0000(	803	17.93303	chr2:309<	CRYGEP		Pseudoger	chr2:208108233-208
ENSG0000(	803	17.93303	chr2:309<	ENSG00000224342		lncRNA	chr2:207173634-207
ENSG0000(	803	17.93303	chr2:309<	INO80D		protein_c	chr2:205993721-206
ENSG0000(	803	17.93303	chr2:309<	EEF1B2		protein_c	chr2:206159585-206
ENSG0000(	803	17.93303	chr2:309<	ADAM23		protein_c	chr2:206443532-206
ENSG0000(	803	17.93303	chr2:309<	C2orf80		protein_c	chr2:208165343-208

ENSG0000(	803	17.93303	chr2:309⁄	PIKFYVE		protein_c	chr2:208266255-208
ENSG0000(	803	17.93303	chr2:309⁄	LINC01802		lncRNA	chr2:207260445-207
ENSG0000(	803	17.93303	chr2:309⁄	ACADL		protein_c	chr2:210187126-210
ENSG0000(	803	17.93303	chr2:309⁄	LANCL1		protein_c	chr2:210427131-210
ENSG0000(	803	17.93303	chr2:309⁄	SNORD51		smallRNA	chr2:206161878-206
ENSG0000(	803	17.93303	chr2:309⁄	DYTN		protein_c	chr2:206651621-206
ENSG0000(	803	17.93303	chr2:309⁄	CRYGD		protein_c	chr2:208121607-208
ENSG0000(	803	17.93303	chr2:309⁄	MEAF6P1		Pseudoger	chr2:209178804-209
ENSG0000(	803	17.93303	chr2:309⁄	AC012362.1		smallRNA	chr2:208369561-208
ENSG0000(	803	17.93303	chr2:309⁄	Y_RNA		smallRNA	chr2:210265963-210
ENSG0000(	803	17.93303	chr2:309⁄	CREB1	NCGv7;AC	protein_c	chr2:207529737-207
ENSG0000(	803	17.93303	chr2:309⁄	KLF7		protein_c	chr2:207074137-207
ENSG0000(	803	17.93303	chr2:309⁄	ACER2P1		Pseudoger	chr2:206410087-206
ENSG0000(	803	17.93303	chr2:309⁄	MAP2	IntOGen-I	protein_c	chr2:209424047-209
ENSG0000(	803	17.93303	chr2:309⁄	PLEKHM3	NCGv7	protein_c	chr2:207821288-208
ENSG0000(	803	17.93303	chr2:309⁄	ENSG00000229321		lncRNA	chr2:206798325-206
ENSG0000(	803	17.93303	chr2:309⁄	ENSG00000232409		Pseudoger	chr2:208516908-208
ENSG0000(	803	17.93303	chr2:309⁄	RPSAP27		Pseudoger	chr2:208208135-208
ENSG0000(	803	17.93303	chr2:309⁄	IDH1	NCGv7;AC	protein_c	chr2:208236229-208
ENSG0000(	803	17.93303	chr2:309⁄	MDH1B		protein_c	chr2:206737763-206
ENSG0000(	803	17.93303	chr2:309⁄	FASTKD2		protein_c	chr2:206765357-206
ENSG0000(	803	17.93303	chr2:309⁄	KLF7-IT1		lncRNA	chr2:207120884-207
ENSG0000(	803	17.93303	chr2:309⁄	MYL6BP1		Pseudoger	chr2:208269343-208
ENSG0000(	803	17.93303	chr2:309⁄	RNU6-360P		smallRNA	chr2:208019638-208
ENSG0000(	803	17.93303	chr2:309⁄	INO80D-AS2		lncRNA	chr2:206084605-206
ENSG0000(	803	17.93303	chr2:309⁄	MIR4775		smallRNA	chr2:207754807-207
ENSG0000(	803	17.93303	chr2:309⁄	CPO		protein_c	chr2:206939518-206
ENSG0000(	803	17.93303	chr2:309⁄	RPS27P10		Pseudoger	chr2:211298941-211
ENSG0000(	803	17.93303	chr2:309⁄	MYL1		protein_c	chr2:210290150-210
ENSG0000(	803	17.93303	chr2:309⁄	ATP5POP1		Pseudoger	chr2:206236236-206
ENSG0000(	803	17.93303	chr2:309⁄	RNA5SP119		Pseudoger	chr2:211535653-211
ENSG0000(	803	17.93303	chr2:309⁄	CMKLR2-AS		lncRNA	chr2:206203543-206
ENSG0000(	803	17.93303	chr2:309⁄	CPS1-IT1		lncRNA	chr2:210617571-210
ENSG0000(	803	17.93303	chr2:309⁄	KANSL1L		protein_c	chr2:210021421-210
ENSG0000(	803	17.93303	chr2:309⁄	AC006994.3		smallRNA	chr2:210171296-210
ENSG0000(	803	17.93303	chr2:309⁄	NDUFS1		protein_c	chr2:206114817-206
ENSG0000(	803	17.93303	chr2:309⁄	ENSG00000279317		lncRNA	chr2:210171499-210
ENSG0000(	803	17.93303	chr2:309⁄	MIR3130-1		smallRNA	chr2:206783234-206
ENSG0000(	803	17.93303	chr2:309⁄	CPS1	NCGv7	protein_c	chr2:210477682-210
ENSG0000(	803	17.93303	chr2:309⁄	RPL6P6		Pseudoger	chr2:210088226-210
ENSG0000(	803	17.93303	chr2:309⁄	PTH2R		protein_c	chr2:208359714-208
ENSG0000(	803	17.93303	chr2:309⁄	ENSG00000272807		lncRNA	chr2:210028417-210
ENSG0000(	803	17.93303	chr2:309⁄	RN7SKP200		smallRNA	chr2:206221315-206
ENSG0000(	803	17.93303	chr2:309⁄	UNC80	NCGv7	protein_c	chr2:209771832-209
ENSG0000(	803	17.93303	chr2:309⁄	AC016697.3		smallRNA	chr2:208233444-208
ENSG0000(	803	17.93303	chr2:309⁄	GCSHP3		Pseudoger	chr2:206116110-206
ENSG0000(	803	17.93303	chr2:309⁄	ARPC1BP1		Pseudoger	chr2:208360631-208
ENSG0000(	803	17.93303	chr2:309⁄	ENSG00000288732		lncRNA	chr2:210548375-210
ENSG0000(	803	17.93303	chr2:309⁄	CRYGA		protein_c	chr2:208160740-208
ENSG0000(	803	17.93303	chr2:309⁄	RPS29P9		Pseudoger	chr2:207654781-207
ENSG0000(	803	17.93303	chr2:309⁄	PPP1R14BP2		Pseudoger	chr2:207681590-207

ENSG0000(	803	17.93303	chr2:309<	ENSG00000225916		lncRNA	chr2:207226949-207
ENSG0000(	803	17.93303	chr2:309<	HSPA8P6		Pseudoger	chr2:209072597-209
ENSG0000(	803	17.93303	chr2:309<	ENSG00000272851		lncRNA	chr2:207753872-207
ENSG0000(	803	17.93303	chr2:309<	METTL21A		protein_c	chr2:207580631-207
ENSG0000(	803	17.93303	chr2:309<	RNU6-664P		smallRNA	chr2:207680533-207
ENSG0000(	803	17.93303	chr2:309<	snoU13		smallRNA	chr2:208074061-208
ENSG0000(	803	17.93303	chr2:309<	VPS26CP1		Pseudoger	chr2:206686517-206
ENSG0000(	803	17.93303	chr2:309<	ENSG00000240440		lncRNA	chr2:207235299-207
ENSG0000(	803	17.93303	chr2:309<	ENSG00000286249		lncRNA	chr2:209353977-209
ENSG0000(	793	17.70971	chr2:309<	ENSG00000234938		lncRNA	chr2:215530447-215
ENSG0000(	793	17.70971	chr2:309<	ENSG00000223373		lncRNA	chr2:212911003-212
ENSG0000(	793	17.70971	chr2:309<	RPL10P6		Pseudoger	chr2:214847128-214
ENSG0000(	793	17.70971	chr2:309<	ABCA12		protein_c	chr2:214931542-215
ENSG0000(	793	17.70971	chr2:309<	ENSG00000227769		lncRNA	chr2:215004782-215
ENSG0000(	793	17.70971	chr2:309<	SPAG16		protein_c	chr2:213284389-214
ENSG0000(	793	17.70971	chr2:309<	FN1	IntOGen-I	protein_c	chr2:215360440-215
ENSG0000(	793	17.70971	chr2:309<	LINC01878		lncRNA	chr2:212795300-212
ENSG0000(	793	17.70971	chr2:309<	ENSAP3		Pseudoger	chr2:214697727-214
ENSG0000(	793	17.70971	chr2:309<	ENSG00000289002		lncRNA	chr2:213188417-213
ENSG0000(	793	17.70971	chr2:309<	LINC01953		lncRNA	chr2:213155806-213
ENSG0000(	793	17.70971	chr2:309<	PCED1CP		Pseudoger	chr2:212832161-212
ENSG0000(	793	17.70971	chr2:309<	RPL5P8		Pseudoger	chr2:214280638-214
ENSG0000(	793	17.70971	chr2:309<	VWC2L-IT1		lncRNA	chr2:214510196-214
ENSG0000(	793	17.70971	chr2:309<	snoU13		smallRNA	chr2:215302766-215
ENSG0000(	793	17.70971	chr2:309<	SNORA70		smallRNA	chr2:214846947-214
ENSG0000(	793	17.70971	chr2:309<	LINC02862		lncRNA	chr2:215274992-215
ENSG0000(	793	17.70971	chr2:309<	ENSG00000273118		lncRNA	chr2:212581357-213
ENSG0000(	793	17.70971	chr2:309<	ENSG00000270659		lncRNA	chr2:213151972-213
ENSG0000(	793	17.70971	chr2:309<	MIR4438		smallRNA	chr2:213758067-213
ENSG0000(	793	17.70971	chr2:309<	IKZF2	NCGv7	protein_c	chr2:212999691-213
ENSG0000(	793	17.70971	chr2:309<	MIR548F2		smallRNA	chr2:212426263-212
ENSG0000(	793	17.70971	chr2:309<	ENSG00000227824		lncRNA	chr2:215869923-215
ENSG0000(	793	17.70971	chr2:309<	ENSG00000216721		Pseudoger	chr2:215804731-215
ENSG0000(	793	17.70971	chr2:309<	AC122136.1		smallRNA	chr2:215878833-215
ENSG0000(	793	17.70971	chr2:309<	VWC2L		protein_c	chr2:214411054-214
ENSG0000(	793	17.70971	chr2:309<	SPAG16-DT		lncRNA	chr2:213276552-213
ENSG0000(	793	17.70971	chr2:309<	ENSG00000288791		lncRNA	chr2:215758930-215
ENSG0000(	793	17.70971	chr2:309<	AC093865.1		smallRNA	chr2:212763570-212
ENSG0000(	793	17.70971	chr2:309<	ENSG00000197585		lncRNA	chr2:214241676-214
ENSG0000(	793	17.70971	chr2:309<	ENSG00000286836		lncRNA	chr2:215178791-215
ENSG0000(	793	17.70971	chr2:309<	LINC01614		lncRNA	chr2:215718043-215
ENSG0000(	793	17.70971	chr2:309<	ENSG00000237525		lncRNA	chr2:215533133-215
ENSG0000(	793	17.70971	chr2:309<	ENSG00000286965		lncRNA	chr2:212932894-212
ENSG0000(	793	17.70971	chr2:309<	LINC00607		lncRNA	chr2:215611563-215
ENSG0000(	793	17.70971	chr2:309<	ENSG00000228618		lncRNA	chr2:215476667-215
ENSG0000(	793	17.70971	chr2:309<	FN1-DT		lncRNA	chr2:215436253-215
ENSG0000(	793	17.70971	chr2:309<	ENSG00000225166		lncRNA	chr2:215453688-215
ENSG0000(	793	17.70971	chr2:309<	ENSG00000272519		lncRNA	chr2:213266995-213
ENSG0000(	793	17.70971	chr2:309<	BARD1	NCGv7;AC	protein_c	chr2:214725646-214
ENSG0000(	793	17.70971	chr2:309<	ENSG00000227981		lncRNA	chr2:215579407-215
ENSG0000(	793	17.70971	chr2:309<	MIR4776-2		smallRNA	chr2:212926257-212

ENSG0000(	793	17.70971	chr2:309	ERBB4	NCGv7;AC	protein_c	chr2:211375717-212
ENSG0000(	793	17.70971	chr2:309	ATIC	AC	protein_c	chr2:215311956-215
ENSG0000(	793	17.70971	chr2:309	SNHG31		lncRNA	chr2:214810181-214
ENSG0000(	782	17.46405	chr5:164(	SNORA13		smallRNA	chr5:112161485-112
ENSG0000(	780	17.41938	chr3:380	SCN10A	NCGv7	protein_c	chr3:38696802-3881
ENSG0000(	780	17.41938	chr3:380	ENSG00000231243		lncRNA	chr3:38823902-3882
ENSG0000(	780	17.41938	chr3:380	AC116038.2		smallRNA	chr3:38800296-3880
ENSG0000(	774	17.28539	chr2:309	TMEM169		protein_c	chr2:216081866-216
ENSG0000(	774	17.28539	chr2:309	SMARCAL1-AS1		lncRNA	chr2:216385288-216
ENSG0000(	774	17.28539	chr2:309	SMARCAL1		protein_c	chr2:216412383-216
ENSG0000(	774	17.28539	chr2:309	ENSG00000226276		lncRNA	chr2:215939308-215
ENSG0000(	774	17.28539	chr2:309	PSMB3P2		Pseudoger	chr2:216610342-216
ENSG0000(	774	17.28539	chr2:309	POLHP1		Pseudoger	chr2:216156342-216
ENSG0000(	774	17.28539	chr2:309	RPL31P14		Pseudoger	chr2:216785774-216
ENSG0000(	774	17.28539	chr2:309	Y_RNA		smallRNA	chr2:216351403-216
ENSG0000(	774	17.28539	chr2:309	IGFBP2		protein_c	chr2:216632828-216
ENSG0000(	774	17.28539	chr2:309	LINC01963		lncRNA	chr2:216217045-216
ENSG0000(	774	17.28539	chr2:309	PECR		protein_c	chr2:215996329-216
ENSG0000(	774	17.28539	chr2:309	RPL37A		protein_c	chr2:216498825-216
ENSG0000(	774	17.28539	chr2:309	LINC01280		lncRNA	chr2:216590426-216
ENSG0000(	774	17.28539	chr2:309	RNA5SP120		Pseudoger	chr2:216793489-216
ENSG0000(	774	17.28539	chr2:309	MARCHF4		protein_c	chr2:216257865-216
ENSG0000(	774	17.28539	chr2:309	MREG		protein_c	chr2:215942584-216
ENSG0000(	774	17.28539	chr2:309	ENSG00000231092		lncRNA	chr2:216259595-216
ENSG0000(	774	17.28539	chr2:309	ENSG00000279348		TEC	chr2:216211404-216
ENSG0000(	774	17.28539	chr2:309	RPL37A-DT		lncRNA	chr2:216479030-216
ENSG0000(	774	17.28539	chr2:309	ENSG00000236478		Pseudoger	chr2:216174896-216
ENSG0000(	774	17.28539	chr2:309	XRCC5	NCGv7	protein_c	chr2:216107464-216
ENSG0000(	774	17.28539	chr2:309	FABP5P14		Pseudoger	chr2:216805737-216
ENSG0000(	774	17.28539	chr2:309	IGFBP5		protein_c	chr2:216672105-216
ENSG0000(	774	17.28539	chr2:309	ENSG00000225794		lncRNA	chr2:216594359-216
ENSG0000(	774	17.28539	chr2:309	ENSG00000290060		lncRNA	chr2:216395085-216
ENSG0000(	774	17.28539	chr2:309	ENSG00000241520		lncRNA	chr2:216483032-216
ENSG0000(	774	17.28539	chr2:309	ENSG00000233581		lncRNA	chr2:216303325-216
ENSG0000(	771	17.21839	chr6:1391	ENSG00000237851		lncRNA	chr6:142788123-142
ENSG0000(	771	17.21839	chr6:1391	ENSG00000227192		lncRNA	chr6:143039425-143
ENSG0000(	771	17.21839	chr6:1391	LINC01277		lncRNA	chr6:142966293-143
ENSG0000(	771	17.21839	chr6:1391	HIVEP2-DT		lncRNA	chr6:142946406-142
ENSG0000(	771	17.21839	chr6:1391	HIVEP2		protein_c	chr6:142751469-142
ENSG0000(	771	17.21839	chr6:1391	ENSG00000233138		lncRNA	chr6:142748443-142
ENSG0000(	771	17.21839	chr6:1391	ENSG00000225752		lncRNA	chr6:143094034-143
ENSG0000(	771	17.21839	chr6:1391	ENSG00000287084		lncRNA	chr6:142671972-142
ENSG0000(	765	17.0844	chr6:103	RN7SKP116		smallRNA	chr6:47715283-4771
ENSG0000(	765	17.0844	chr6:103	EEF1A1P42		Pseudoger	chr6:49358185-4936
ENSG0000(	765	17.0844	chr6:103	GLYATL3		protein_c	chr6:49499923-4952
ENSG0000(	765	17.0844	chr6:103	CENPQ		protein_c	chr6:49463370-4949
ENSG0000(	765	17.0844	chr6:103	ENSG00000217631		Pseudoger	chr6:49273600-4927
ENSG0000(	765	17.0844	chr6:103	AL391538.1		smallRNA	chr6:48849923-4885
ENSG0000(	765	17.0844	chr6:103	ENSG00000286811		lncRNA	chr6:48069213-4811
ENSG0000(	765	17.0844	chr6:103	ENSG00000287137		lncRNA	chr6:48754649-4879
ENSG0000(	765	17.0844	chr6:103	PTCHD4	NCGv7	protein_c	chr6:47856673-4811

ENSG0000(	765	17.0844	chr6:1039	ENSG00000216616		Pseudoger	chr6:47752828-4775
ENSG0000(	765	17.0844	chr6:1039	MMUT		protein_c	chr6:49430360-4946
ENSG0000(	765	17.0844	chr6:1039	ENSG00000278736		Pseudoger	chr6:48701491-4870
ENSG0000(	765	17.0844	chr6:1039	OPN5		protein_c	chr6:47781982-4783
ENSG0000(	765	17.0844	chr6:1039	ENSG00000218337		Pseudoger	chr6:49077712-4907
ENSG0000(	765	17.0844	chr6:1039	ADGRF2	NCGv7	Pseudoger	chr6:47656487-4769
ENSG0000(	765	17.0844	chr6:1039	RBMXP1		Pseudoger	chr6:48213604-4821
ENSG0000(	765	17.0844	chr6:1039	ENSG00000226594		lncRNA	chr6:47729827-4774
ENSG0000(	765	17.0844	chr6:1039	RNU7-65P		smallRNA	chr6:49344800-4934
ENSG0000(	765	17.0844	chr6:1039	HNRNPA3P4		Pseudoger	chr6:48149203-4815
ENSG0000(	765	17.0844	chr6:1039	RNU1-105P		smallRNA	chr6:47823390-4782
ENSG0000(	765	17.0844	chr6:1039	ENSG00000271162		Pseudoger	chr6:48952070-4895
ENSG0000(	765	17.0844	chr6:1039	ENSG00000274867		Pseudoger	chr6:49115537-4911
ENSG0000(	765	17.0844	chr6:1039	ENSG00000216813		Pseudoger	chr6:47562622-4756
ENSG0000(	765	17.0844	chr6:1039	ADGRF4		protein_c	chr6:47685864-4772
ENSG0000(	762	17.0174	chr5:1646	ENSG00000249282		Pseudoger	chr5:17601882-1760
ENSG0000(	761	16.99507	chr6:1039	HMGN3		protein_c	chr6:79201245-7923
ENSG0000(	761	16.99507	chr6:1039	ENSG00000237174		lncRNA	chr6:75357214-7539
ENSG0000(	761	16.99507	chr6:1039	ENSG00000230309		lncRNA	chr6:78604467-7860
ENSG0000(	761	16.99507	chr6:1039	MEI4		protein_c	chr6:77650274-7792
ENSG0000(	761	16.99507	chr6:1039	snoU13		smallRNA	chr6:75865964-7586
ENSG0000(	761	16.99507	chr6:1039	ENSG00000218029		Pseudoger	chr6:79354443-7935
ENSG0000(	761	16.99507	chr6:1039	ENSG00000218418		Pseudoger	chr6:80064286-8007
ENSG0000(	761	16.99507	chr6:1039	PHIP	NCGv7	protein_c	chr6:78934419-7907
ENSG0000(	761	16.99507	chr6:1039	ENSG00000286340		lncRNA	chr6:79077841-7914
ENSG0000(	761	16.99507	chr6:1039	ENSG00000262048		Pseudoger	chr6:79406240-7940
ENSG0000(	761	16.99507	chr6:1039	ENSG00000285401		Pseudoger	chr6:75744275-7574
ENSG0000(	761	16.99507	chr6:1039	FILIP1		protein_c	chr6:75291859-7549
ENSG0000(	761	16.99507	chr6:1039	ELOVL4		protein_c	chr6:79914814-7994
ENSG0000(	761	16.99507	chr6:1039	ENSG00000217512		Pseudoger	chr6:79067692-7906
ENSG0000(	761	16.99507	chr6:1039	H3P27		Pseudoger	chr6:75586122-7558
ENSG0000(	761	16.99507	chr6:1039	HMGN3-AS1		lncRNA	chr6:79233699-7923
ENSG0000(	761	16.99507	chr6:1039	IRAK1BP1		protein_c	chr6:78867551-7894
ENSG0000(	761	16.99507	chr6:1039	U3		smallRNA	chr6:75398861-7539
ENSG0000(	761	16.99507	chr6:1039	ENSG00000280511		lncRNA	chr6:76561328-7656
ENSG0000(	761	16.99507	chr6:1039	SH3BGRL2		protein_c	chr6:79631329-7970
ENSG0000(	761	16.99507	chr6:1039	COL12A1	NCGv7	protein_c	chr6:75084326-7520
ENSG0000(	761	16.99507	chr6:1039	ENSG00000220154		Pseudoger	chr6:79278105-7927
ENSG0000(	761	16.99507	chr6:1039	ENSG00000233835		lncRNA	chr6:79871873-7987
ENSG0000(	761	16.99507	chr6:1039	UBE2V1P15		Pseudoger	chr6:75465123-7546
ENSG0000(	761	16.99507	chr6:1039	DBIP1		Pseudoger	chr6:79436908-7943
ENSG0000(	761	16.99507	chr6:1039	U6		smallRNA	chr6:76557653-7655
ENSG0000(	761	16.99507	chr6:1039	MIR4463		smallRNA	chr6:75428407-7542
ENSG0000(	761	16.99507	chr6:1039	RPS6P7		Pseudoger	chr6:77496119-7749
ENSG0000(	761	16.99507	chr6:1039	Y_RNA		smallRNA	chr6:79301963-7930
ENSG0000(	761	16.99507	chr6:1039	RNU6-248P		smallRNA	chr6:76092834-7609
ENSG0000(	761	16.99507	chr6:1039	MYO6		protein_c	chr6:75749201-7591
ENSG0000(	761	16.99507	chr6:1039	HMGB1P39		Pseudoger	chr6:75319101-7531
ENSG0000(	761	16.99507	chr6:1039	LINC01621		lncRNA	chr6:79803574-7983
ENSG0000(	761	16.99507	chr6:1039	LCA5		protein_c	chr6:79484991-7953
ENSG0000(	761	16.99507	chr6:1039	ENSG00000288071		lncRNA	chr6:80046715-8008

ENSG0000(	761	16.99507	chr6:1039	ENSG00000261970		Pseudoger	chr6:79552794-7955
ENSG0000(	761	16.99507	chr6:1039	AL132875.1		smallRNA	chr6:79826793-7982
ENSG0000(	761	16.99507	chr6:1039	ENSG00000217786		Pseudoger	chr6:79326568-7932
ENSG0000(	761	16.99507	chr6:1039	RNU6-261P		smallRNA	chr6:76446988-7644
ENSG0000(	761	16.99507	chr6:1039	LCAL1		lncRNA	chr6:79307669-7931
ENSG0000(	761	16.99507	chr6:1039	AL356776.1		smallRNA	chr6:78961496-7896
ENSG0000(	761	16.99507	chr6:1039	AK4P5		Pseudoger	chr6:80077729-8007
ENSG0000(	761	16.99507	chr6:1039	ENSG00000231762		Pseudoger	chr6:76660486-7666
ENSG0000(	761	16.99507	chr6:1039	SNORD112		smallRNA	chr6:77937201-7793
ENSG0000(	761	16.99507	chr6:1039	ENSG00000220918		Pseudoger	chr6:79854684-7985
ENSG0000(	761	16.99507	chr6:1039	ENSG00000285586		lncRNA	chr6:79868402-7987
ENSG0000(	761	16.99507	chr6:1039	SENP6		protein_c	chr6:75601509-7571
ENSG0000(	761	16.99507	chr6:1039	RPL26P20		Pseudoger	chr6:75499705-7550
ENSG0000(	761	16.99507	chr6:1039	TTK	NCGv7	protein_c	chr6:80003887-8004
ENSG0000(	761	16.99507	chr6:1039	ENSG00000217488		Pseudoger	chr6:75610958-7561
ENSG0000(	761	16.99507	chr6:1039	ENSG00000279659		TEC	chr6:79573877-7957
ENSG0000(	761	16.99507	chr6:1039	BCKDHB	AC	protein_c	chr6:80106647-8034
ENSG0000(	761	16.99507	chr6:1039	ENSG00000231533		lncRNA	chr6:79420172-7952
ENSG0000(	761	16.99507	chr6:1039	TMEM30A	NCGv7	protein_c	chr6:75252924-7528
ENSG0000(	761	16.99507	chr6:1039	RNU1-34P		smallRNA	chr6:75473738-7547
ENSG0000(	761	16.99507	chr6:1039	ENSG00000272129		lncRNA	chr6:80355424-8035
ENSG0000(	761	16.99507	chr7:1588	SDHDP2		Pseudoger	chr7:135444461-135
ENSG0000(	761	16.99507	chr6:1039	ENSG00000272137		lncRNA	chr6:79561132-7956
ENSG0000(	761	16.99507	chr6:1039	ENSG00000229495		lncRNA	chr6:78809715-7881
ENSG0000(	761	16.99507	chr6:1039	TMEM30A-DT		lncRNA	chr6:75284992-7531
ENSG0000(	761	16.99507	chr6:1039	RNA5SP209		Pseudoger	chr6:75865239-7586
ENSG0000(	761	16.99507	chr6:1039	Y_RNA		smallRNA	chr6:75928513-7592
ENSG0000(	761	16.99507	chr6:1039	RN7SKP163		smallRNA	chr6:75654970-7565
ENSG0000(	761	16.99507	chr6:1039	IMPG1		protein_c	chr6:75921114-7607
ENSG0000(	761	16.99507	chr6:1039	ENSG00000271945		lncRNA	chr6:76774966-7709
ENSG0000(	761	16.99507	chr6:1039	ENSG00000276064		Pseudoger	chr6:77343557-7734
ENSG0000(	761	16.99507	chr6:1039	RPL17P25		Pseudoger	chr6:80374015-8037
ENSG0000(	761	16.99507	chr6:1039	RNU6-155P		smallRNA	chr6:75768059-7576
ENSG0000(	761	16.99507	chr6:1039	ENSG00000287811		lncRNA	chr6:79537185-7953
ENSG0000(	761	16.99507	chr6:1039	HTR1B		protein_c	chr6:77460924-7746
ENSG0000(	761	16.99507	chr6:1039	GAPDHP63		Pseudoger	chr6:79953005-7995
ENSG0000(	761	16.99507	chr6:1039	ENSG00000287816		lncRNA	chr6:79947810-7998
ENSG0000(	761	16.99507	chr6:1039	ENSG00000238156		lncRNA	chr6:75454944-7545
ENSG0000(	761	16.99507	chr6:1039	LINC02540		lncRNA	chr6:76521640-7659
ENSG0000(	761	16.99507	chr6:1039	RNU6-1338P		smallRNA	chr6:75593055-7559
ENSG0000(	761	16.99507	chr6:1039	RNU6-1016P		smallRNA	chr6:75644084-7564
ENSG0000(	761	16.99507	chr6:1039	COX7A2		protein_c	chr6:75237675-7525
ENSG0000(	761	16.99507	chr6:1039	RPL35AP18		Pseudoger	chr6:79964215-7996
ENSG0000(	758	16.92807	chr6:1039	MTATP6P25		Pseudoger	chr6:91726810-9172
ENSG0000(	758	16.92807	chr6:1039	ENSG00000219240		Pseudoger	chr6:89886156-8988
ENSG0000(	758	16.92807	chr6:1039	LYRM2		protein_c	chr6:89568144-8963
ENSG0000(	758	16.92807	chr6:1039	RN7SL11P		smallRNA	chr6:89478141-8947
ENSG0000(	758	16.92807	chr6:1039	ENSG00000226455		lncRNA	chr6:89950116-8995
ENSG0000(	758	16.92807	chr6:1039	U3		smallRNA	chr6:92119522-9211
ENSG0000(	758	16.92807	chr6:1039	ENSG00000237027		lncRNA	chr6:89560875-8956
ENSG0000(	758	16.92807	chr6:1039	DNAJC19P6		Pseudoger	chr6:89797598-8979

ENSG0000(	758	16.92807	chr6:1039	MDN1		protein_c	chr6:89642498-8981
ENSG0000(	758	16.92807	chr6:1039	ENSG00000219867		Pseudoger	chr6:91815843-9181
ENSG0000(	758	16.92807	chr6:1039	RPL5P19		Pseudoger	chr6:92013842-9201
ENSG0000(	758	16.92807	chr6:1039	Y_RNA		smallRNA	chr6:89898283-8989
ENSG0000(	758	16.92807	chr6:1039	ATF1P1		Pseudoger	chr6:92887251-9288
ENSG0000(	758	16.92807	chr6:1039	AL359987.1		smallRNA	chr6:92644741-9264
ENSG0000(	758	16.92807	chr6:1039	MAP3K7	AC	protein_c	chr6:90513573-9058
ENSG0000(	758	16.92807	chr6:1039	ENSG00000287789		lncRNA	chr6:90073051-9007
ENSG0000(	758	16.92807	chr6:1039	RPL22P14		Pseudoger	chr6:89876544-8987
ENSG0000(	758	16.92807	chr6:1039	GJA10		protein_c	chr6:89894469-8992
ENSG0000(	758	16.92807	chr6:1039	CASC6		lncRNA	chr6:91557292-9169
ENSG0000(	758	16.92807	chr6:1039	U3		smallRNA	chr6:92790453-9279
ENSG0000(	758	16.92807	chr6:1039	ENSG00000224605		lncRNA	chr6:91390313-9139
ENSG0000(	758	16.92807	chr6:1039	AL391559.1		smallRNA	chr6:90446772-9044
ENSG0000(	758	16.92807	chr6:1039	MIR4643		smallRNA	chr6:91521660-9152
ENSG0000(	758	16.92807	chr6:1039	BACH2	NCGv7	protein_c	chr6:89926528-9029
ENSG0000(	758	16.92807	chr6:1039	RN7SKP110		smallRNA	chr6:90001222-9000
ENSG0000(	758	16.92807	chr6:1039	PIMREGP3		Pseudoger	chr6:89814709-8981
ENSG0000(	758	16.92807	chr6:1039	ANKRD6		protein_c	chr6:89433152-8963
ENSG0000(	758	16.92807	chr6:1039	ENSG00000289988		lncRNA	chr6:90376124-9037
ENSG0000(	758	16.92807	chr6:1039	RRAGD		protein_c	chr6:89364616-8941
ENSG0000(	758	16.92807	chr6:1039	MIR4464		smallRNA	chr6:90312742-9031
ENSG0000(	758	16.92807	chr6:1039	ENSG00000288101		lncRNA	chr6:92631066-9263
ENSG0000(	758	16.92807	chr6:1039	GABRR2		protein_c	chr6:89254464-8931
ENSG0000(	758	16.92807	chr6:1039	Y_RNA		smallRNA	chr6:89841329-8984
ENSG0000(	758	16.92807	chr6:1039	CASP8AP2		protein_c	chr6:89829894-8987
ENSG0000(	758	16.92807	chr6:1039	ENSG00000285961		lncRNA	chr6:92002610-9218
ENSG0000(	758	16.92807	chr6:1039	LINC02531		lncRNA	chr6:92723003-9272
ENSG0000(	758	16.92807	chr6:1039	AL589947.1		smallRNA	chr6:92812041-9281
ENSG0000(	758	16.92807	chr6:1039	ENSG00000261038		lncRNA	chr6:92387841-9238
ENSG0000(	758	16.92807	chr6:1039	MTCO1P56		Pseudoger	chr6:91727282-9172
ENSG0000(	758	16.92807	chr6:1039	ENSG00000260271		lncRNA	chr6:90295507-9036
ENSG0000(	758	16.92807	chr6:1039	TUBB3P1		Pseudoger	chr6:89301783-8930
ENSG0000(	758	16.92807	chr6:1039	RN7SL415P		smallRNA	chr6:91739609-9173
ENSG0000(	758	16.92807	chr6:1039	NACAP7		Pseudoger	chr6:89422099-8942
ENSG0000(	758	16.92807	chr6:1039	snoU13		smallRNA	chr6:89823295-8982
ENSG0000(	758	16.92807	chr6:1039	MTND4LP19		Pseudoger	chr6:91727159-9172
ENSG0000(	758	16.92807	chr6:1039	MDN1-AS1		lncRNA	chr6:89673469-8967
ENSG0000(	758	16.92807	chr6:1039	ENSG00000218730		Pseudoger	chr6:90116700-9011
ENSG0000(	758	16.92807	chr6:1039	UBE2J1		protein_c	chr6:89326625-8935
ENSG0000(	756	16.8834	chr6:1039	ACTG1P9		Pseudoger	chr6:46204729-4620
ENSG0000(	756	16.8834	chr6:1039	RCAN2	NCGv7	protein_c	chr6:46220736-4649
ENSG0000(	756	16.8834	chr6:1039	RUNX2-AS1		lncRNA	chr6:45573346-4557
ENSG0000(	756	16.8834	chr6:1039	CLIC5	NCGv7	protein_c	chr6:45880827-4608
ENSG0000(	756	16.8834	chr6:1039	ENSG00000219384		Pseudoger	chr6:45158870-4515
ENSG0000(	756	16.8834	chr6:1039	RPL36P10		Pseudoger	chr6:46438310-4643
ENSG0000(	756	16.8834	chr6:1039	CD2AP-DT		lncRNA	chr6:47477208-4747
ENSG0000(	756	16.8834	chr6:1039	RNU6-754P		smallRNA	chr6:46018745-4601
ENSG0000(	756	16.8834	chr6:1039	Y_RNA		smallRNA	chr6:47489067-4748
ENSG0000(	756	16.8834	chr6:1039	ENPP4		protein_c	chr6:46129989-4614
ENSG0000(	756	16.8834	chr6:1039	MIR586		smallRNA	chr6:45197674-4519

ENSG0000(	756	16.8834	chr6:1039	TDRD6-AS1		lncRNA	chr6:46670444-4668
ENSG0000(	756	16.8834	chr6:1039	CYP39A1		protein_c	chr6:46549580-4665
ENSG0000(	756	16.8834	chr6:1039	ENSG00000231769		lncRNA	chr6:46096004-4612
ENSG0000(	756	16.8834	chr6:1039	ENSG00000287485		lncRNA	chr6:47374561-4739
ENSG0000(	756	16.8834	chr6:1039	ENSG00000271857		lncRNA	chr6:45421079-4542
ENSG0000(	756	16.8834	chr6:1039	RCAN2-DT		lncRNA	chr6:46492052-4660
ENSG0000(	756	16.8834	chr6:1039	SLC25A27		protein_c	chr6:46652915-4667
ENSG0000(	756	16.8834	chr6:1039	ADGRF5-AS1		lncRNA	chr6:46903471-4690
ENSG0000(	756	16.8834	chr6:1039	ADGRF1	AC	protein_c	chr6:46997708-4704
ENSG0000(	756	16.8834	chr6:1039	ANKRD66		protein_c	chr6:46746933-4675
ENSG0000(	756	16.8834	chr6:1039	CD2AP		protein_c	chr6:47477789-4762
ENSG0000(	756	16.8834	chr6:1039	B3GNTL1P2		Pseudoger	chr6:47368943-4736
ENSG0000(	756	16.8834	chr6:1039	SUPT3H		protein_c	chr6:44809317-4537
ENSG0000(	756	16.8834	chr6:1039	MEP1A	NCGv7	protein_c	chr6:46793389-4683
ENSG0000(	756	16.8834	chr6:1039	ENSG00000236164		lncRNA	chr6:46758296-4676
ENSG0000(	756	16.8834	chr6:1039	TDRD6		protein_c	chr6:46687875-4670
ENSG0000(	756	16.8834	chr6:1039	TNFRSF21		protein_c	chr6:47231532-4730
ENSG0000(	756	16.8834	chr6:1039	RNU6-515P		smallRNA	chr6:45646104-4564
ENSG0000(	756	16.8834	chr6:1039	PLA2G7		protein_c	chr6:46704201-4673
ENSG0000(	756	16.8834	chr6:1039	NUDT19P4		Pseudoger	chr6:44898711-4489
ENSG0000(	756	16.8834	chr6:1039	ADGRF5		protein_c	chr6:46852522-4695
ENSG0000(	756	16.8834	chr6:1039	ENSG00000276156		Pseudoger	chr6:45097496-4509
ENSG0000(	756	16.8834	chr6:1039	RBM22P4		Pseudoger	chr6:45098021-4509
ENSG0000(	756	16.8834	chr6:1039	ENPP5		protein_c	chr6:46159185-4617
ENSG0000(	756	16.8834	chr6:1039	AL355353.1		smallRNA	chr6:47462851-4746
ENSG0000(	756	16.8834	chr6:1039	ENSG00000286417		lncRNA	chr6:44727921-4483
ENSG0000(	756	16.8834	chr6:1039	RUNX2	TAG	protein_c	chr6:45328157-4566
ENSG0000(	752	16.79407	chr6:1039	ENSG00000219757		Pseudoger	chr6:102453367-102
ENSG0000(	752	16.79407	chr6:1039	ENSG00000220370		Pseudoger	chr6:94344737-9434
ENSG0000(	752	16.79407	chr6:1039	RPL35P3		Pseudoger	chr6:105302453-105
ENSG0000(	752	16.79407	chr6:1039	BVES-AS1		lncRNA	chr6:105136308-105
ENSG0000(	752	16.79407	chr6:1039	ENSG00000271042		Pseudoger	chr6:99082449-9908
ENSG0000(	752	16.79407	chr6:1039	RPL7AP35		Pseudoger	chr6:105298149-105
ENSG0000(	752	16.79407	chr6:1039	NPM1P10		Pseudoger	chr6:104025540-104
ENSG0000(	752	16.79407	chr6:1039	ENSG00000271099		Pseudoger	chr6:104860449-104
ENSG0000(	752	16.79407	chr6:1039	USP45		protein_c	chr6:99432325-9952
ENSG0000(	752	16.79407	chr6:1039	LIN28B-AS1		lncRNA	chr6:104864464-104
ENSG0000(	752	16.79407	chr6:1039	KRT18P50		Pseudoger	chr6:95991107-9599
ENSG0000(	752	16.79407	chr6:1039	ENSG00000287578		lncRNA	chr6:95917143-9601
ENSG0000(	752	16.79407	chr6:1039	RNU4-70P		smallRNA	chr6:96649453-9664
ENSG0000(	752	16.79407	chr6:1039	ENSG00000287683		lncRNA	chr6:93416951-9367
ENSG0000(	752	16.79407	chr6:1039	SIM1-AS1		lncRNA	chr6:100427118-100
ENSG0000(	752	16.79407	chr6:1039	ENSG00000271860		lncRNA	chr6:97283303-9840
ENSG0000(	752	16.79407	chr6:1039	ENSG00000219755		Pseudoger	chr6:99575712-9957
ENSG0000(	752	16.79407	chr6:1039	ENSG00000220695		Pseudoger	chr6:100530276-100
ENSG0000(	752	16.79407	chr6:1039	ENSG00000236920		lncRNA	chr6:98210020-9821
ENSG0000(	752	16.79407	chr6:1039	MCHR2	NCGv7	protein_c	chr6:99918519-9999
ENSG0000(	752	16.79407	chr6:1039	ENSG00000290011		lncRNA	chr6:104941624-104
ENSG0000(	752	16.79407	chr6:1039	HACE1		protein_c	chr6:104728094-104
ENSG0000(	752	16.79407	chr6:1039	NDUFAF4		protein_c	chr6:96889315-9689
ENSG0000(	752	16.79407	chr6:1039	EEF1GP6		Pseudoger	chr6:96750824-9675

ENSG0000(	752	16.79407	chr6:103(	HEY2		protein_c	chr6:125747664-125
ENSG0000(	752	16.79407	chr6:103(	PREP		protein_c	chr6:105273218-105
ENSG0000(	752	16.79407	chr6:103(	ENSG00000235099		lncRNA	chr6:93070387-9310
ENSG0000(	752	16.79407	chr6:103(	ENSG00000216378		Pseudoger	chr6:100177209-100
ENSG0000(	752	16.79407	chr6:103(	MIR548AI		smallRNA	chr6:99124609-9912
ENSG0000(	752	16.79407	chr6:103(	EIF4EBP2P3		Pseudoger	chr6:98179499-9817
ENSG0000(	752	16.79407	chr6:103(	ENSG00000283010		lncRNA	chr6:98829967-9883
ENSG0000(	752	16.79407	chr6:103(	ENSG00000287616		lncRNA	chr6:104487095-104
ENSG0000(	752	16.79407	chr6:103(	ENSG00000287499		lncRNA	chr6:102350271-102
ENSG0000(	752	16.79407	chr6:103(	MMS22L	NCGv7;AC	protein_c	chr6:97142161-9728
ENSG0000(	752	16.79407	chr6:103(	NCOA7		protein_c	chr6:125781161-125
ENSG0000(	752	16.79407	chr6:103(	snoU13		smallRNA	chr6:100957160-100
ENSG0000(	752	16.79407	chr6:103(	ENSG00000286299		lncRNA	chr6:125268087-125
ENSG0000(	752	16.79407	chr6:103(	PRDX2P4		Pseudoger	chr6:100136064-100
ENSG0000(	752	16.79407	chr6:103(	RPS7P8		Pseudoger	chr6:96648489-9664
ENSG0000(	752	16.79407	chr6:103(	COPS5P1		Pseudoger	chr6:93091976-9309
ENSG0000(	752	16.79407	chr6:103(	UFL1		protein_c	chr6:96521595-9655
ENSG0000(	752	16.79407	chr6:103(	RN7SKP56		smallRNA	chr6:125875168-125
ENSG0000(	752	16.79407	chr6:103(	FHL5		protein_c	chr6:96562548-9661
ENSG0000(	752	16.79407	chr6:103(	GPR63		protein_c	chr6:96794125-9683
ENSG0000(	752	16.79407	chr6:103(	FBXL4		protein_c	chr6:98868535-9894
ENSG0000(	752	16.79407	chr6:103(	TPD52L1		protein_c	chr6:125119049-125
ENSG0000(	752	16.79407	chr6:103(	CCNC	NCGv7	protein_c	chr6:99542387-9956
ENSG0000(	752	16.79407	chr6:103(	PRDM13	AC	protein_c	chr6:99606774-9961
ENSG0000(	752	16.79407	chr6:103(	RNU6-897P		smallRNA	chr6:104766822-104
ENSG0000(	752	16.79407	chr6:103(	FAXC		protein_c	chr6:99271168-9935
ENSG0000(	752	16.79407	chr6:103(	SIM1		protein_c	chr6:100385009-100
ENSG0000(	752	16.79407	chr6:103(	ASCC3	NCGv7	protein_c	chr6:100508194-100
ENSG0000(	752	16.79407	chr6:103(	BVES		protein_c	chr6:105096822-105
ENSG0000(	752	16.79407	chr6:103(	PNISR-AS1		lncRNA	chr6:99424911-9943
ENSG0000(	752	16.79407	chr6:103(	RNF217	NCGv7	protein_c	chr6:124962437-125
ENSG0000(	752	16.79407	chr6:103(	ENSG00000231628		lncRNA	chr6:105279016-105
ENSG0000(	752	16.79407	chr6:103(	HINT3		protein_c	chr6:125956770-125
ENSG0000(	752	16.79407	chr6:103(	HDDC2		protein_c	chr6:125219962-125
ENSG0000(	752	16.79407	chr6:103(	ENSG00000260000		lncRNA	chr6:100881450-100
ENSG0000(	752	16.79407	chr6:103(	COQ3		protein_c	chr6:99369401-9939
ENSG0000(	752	16.79407	chr6:103(	HEY2-AS1		lncRNA	chr6:125577545-125
ENSG0000(	752	16.79407	chr6:103(	ENSG00000226207		lncRNA	chr6:97710953-9771
ENSG0000(	752	16.79407	chr6:103(	AL359709.2		smallRNA	chr6:105196853-105
ENSG0000(	752	16.79407	chr6:103(	ENSG00000279403		TEC	chr6:94397664-9439
ENSG0000(	752	16.79407	chr6:103(	RNF217-AS	AC	lncRNA	chr6:124644434-124
ENSG0000(	752	16.79407	chr6:103(	ENSG00000187472		Pseudoger	chr6:98780257-9878
ENSG0000(	752	16.79407	chr6:103(	POPDC3		protein_c	chr6:105157900-105
ENSG0000(	752	16.79407	chr6:103(	PNISR		protein_c	chr6:99397629-9942
ENSG0000(	752	16.79407	chr6:103(	GRIK2	NCGv7	protein_c	chr6:100962701-102
ENSG0000(	752	16.79407	chr6:103(	RN7SL509P		smallRNA	chr6:96914701-9691
ENSG0000(	752	16.79407	chr6:103(	ENSG00000226409		lncRNA	chr6:125370034-125
ENSG0000(	752	16.79407	chr6:103(	RNU6-1106P		smallRNA	chr6:105045562-105
ENSG0000(	752	16.79407	chr6:103(	LIN28B	AC	protein_c	chr6:104936616-105
ENSG0000(	752	16.79407	chr6:103(	ENSG00000231143		lncRNA	chr6:94163920-9419
ENSG0000(	752	16.79407	chr6:103(	ACTG1P18		Pseudoger	chr6:101430411-101

ENSG0000(	752	16.79407	chr6:1039	ENSG00000286084		lncRNA	chr6:105403207-105
ENSG0000(	752	16.79407	chr6:1039	ENSG00000218536		Pseudoger	chr6:102078872-102
ENSG0000(	752	16.79407	chr6:1039	LINC02836		lncRNA	chr6:105612667-105
ENSG0000(	752	16.79407	chr6:1039	ENSG00000217120		Pseudoger	chr6:103002514-103
ENSG0000(	752	16.79407	chr6:1039	ENSG00000217331		Pseudoger	chr6:96672129-9667
ENSG0000(	752	16.79407	chr6:1039	Y_RNA		smallRNA	chr6:99642237-9964
ENSG0000(	752	16.79407	chr6:1039	RNA5SP216		Pseudoger	chr6:125979812-125
ENSG0000(	752	16.79407	chr6:1039	TSTD3		protein_c	chr6:99520976-9958
ENSG0000(	752	16.79407	chr6:1039	ENSG00000270484		Pseudoger	chr6:94442030-9444
ENSG0000(	752	16.79407	chr6:1039	MANEA-DT		lncRNA	chr6:95575183-9557
ENSG0000(	752	16.79407	chr6:1039	BDH2P1		Pseudoger	chr6:99174744-9917
ENSG0000(	752	16.79407	chr6:1039	ENSG00000289178		lncRNA	chr6:94546175-9473
ENSG0000(	752	16.79407	chr6:1039	TYMSP1		Pseudoger	chr6:96653274-9665
ENSG0000(	752	16.79407	chr6:1039	KLHL32		protein_c	chr6:96924620-9714
ENSG0000(	752	16.79407	chr6:1039	SNORA18		smallRNA	chr6:93879106-9387
ENSG0000(	752	16.79407	chr6:1039	EPHA7	NCGv7	protein_c	chr6:93240020-9341
ENSG0000(	752	16.79407	chr6:1039	LINC02523		lncRNA	chr6:125674353-125
ENSG0000(	752	16.79407	chr6:1039	ENSG00000287397		lncRNA	chr6:101452917-101
ENSG0000(	752	16.79407	chr6:1039	AL359709.1		smallRNA	chr6:105181149-105
ENSG0000(	752	16.79407	chr6:1039	ENSG00000289501		lncRNA	chr6:98832905-9883
ENSG0000(	752	16.79407	chr6:1039	NPM1P38		Pseudoger	chr6:100050372-100
ENSG0000(	752	16.79407	chr6:1039	ENSG00000224384		lncRNA	chr6:96785137-9679
ENSG0000(	752	16.79407	chr6:1039	UFL1-AS1		lncRNA	chr6:96199840-9652
ENSG0000(	752	16.79407	chr6:1039	POU3F2		protein_c	chr6:98834574-9883
ENSG0000(	752	16.79407	chr6:1039	SNORA33		smallRNA	chr6:103583135-103
ENSG0000(	752	16.79407	chr6:1039	ENSG00000270987		Pseudoger	chr6:100889603-100
ENSG0000(	752	16.79407	chr6:1039	ENSG00000219549		Pseudoger	chr6:95630115-9563
ENSG0000(	752	16.79407	chr6:1039	MIR2113		smallRNA	chr6:98024531-9802
ENSG0000(	752	16.79407	chr6:1039	ENSG00000284999		lncRNA	chr6:105679378-105
ENSG0000(	752	16.79407	chr6:1039	ENSG00000227535		lncRNA	chr6:104831129-104
ENSG0000(	752	16.79407	chr6:1039	CYCSP17		Pseudoger	chr6:95504182-9550
ENSG0000(	752	16.79407	chr6:1039	ENSG00000219088		Pseudoger	chr6:105666326-105
ENSG0000(	752	16.79407	chr6:1039	MCHR2-AS1		lncRNA	chr6:99993934-1001
ENSG0000(	752	16.79407	chr6:1039	ENSG00000288085		lncRNA	chr6:93720163-9374
ENSG0000(	752	16.79407	chr6:1039	NKAIN2		protein_c	chr6:123803865-124
ENSG0000(	752	16.79407	chr6:1039	NCOA7-AS1		lncRNA	chr6:125797856-125
ENSG0000(	752	16.79407	chr6:1039	ENSG00000229600		lncRNA	chr6:93886900-9388
ENSG0000(	752	16.79407	chr6:1039	ENSG00000288084		lncRNA	chr6:103866023-103
ENSG0000(	752	16.79407	chr6:1039	ENSG00000275716		Pseudoger	chr6:100093311-100
ENSG0000(	752	16.79407	chr6:1039	ENSG00000289081		lncRNA	chr6:93419821-9342
ENSG0000(	752	16.79407	chr6:1039	R3HDM2P2		Pseudoger	chr6:104017633-104
ENSG0000(	752	16.79407	chr6:1039	ENSG00000218173		Pseudoger	chr6:104687241-104
ENSG0000(	752	16.79407	chr6:1039	ENSG00000280451		TEC	chr6:93811825-9381
ENSG0000(	752	16.79407	chr6:1039	MANEA		protein_c	chr6:95577485-9560
ENSG0000(	752	16.79407	chr6:1039	MTCYBP36		Pseudoger	chr6:94446740-9444
ENSG0000(	752	16.79407	chr6:1039	FUT9		protein_c	chr6:96015974-9621
ENSG0000(	751	16.77174	chr6:1039	ENSG00000216613		Pseudoger	chr6:136419847-136
ENSG0000(	748	16.70474	chr3:3804	HSPA8P18		Pseudoger	chr3:19940269-1994
ENSG0000(	748	16.70474	chr3:3804	RNY4P22		smallRNA	chr3:20192782-2019
ENSG0000(	748	16.70474	chr3:3804	SAP18P3		Pseudoger	chr3:20007852-2000
ENSG0000(	748	16.70474	chr3:3804	SGO1	DriverDBv	protein_c	chr3:20160593-2018

ENSG0000(	748	16.70474	chr3:380<	EFHB		protein_c	chr3:19879472-1994
ENSG0000(	748	16.70474	chr3:380<	RAB5A		protein_c	chr3:19947097-1998
ENSG0000(	748	16.70474	chr3:380<	RNU6-815P		smallRNA	chr3:20507644-2050
ENSG0000(	748	16.70474	chr3:380<	ENSG00000282987		lncRNA	chr3:21006730-2122
ENSG0000(	748	16.70474	chr3:380<	KAT2B		protein_c	chr3:20040446-2015
ENSG0000(	748	16.70474	chr3:380<	RNU6-822P		smallRNA	chr3:20268965-2026
ENSG0000(	748	16.70474	chr3:380<	MIR3135A		smallRNA	chr3:20137565-2013
ENSG0000(	748	16.70474	chr3:380<	RPL39P18		Pseudoger	chr3:20012452-2001
ENSG0000(	748	16.70474	chr3:380<	PP2D1		protein_c	chr3:19979961-2001
ENSG0000(	748	16.70474	chr3:380<	AC104441.1		smallRNA	chr3:21138263-2113
ENSG0000(	748	16.70474	chr3:380<	RNU4-85P		smallRNA	chr3:19996803-1999
ENSG0000(	748	16.70474	chr3:380<	SGO1-AS1		lncRNA	chr3:20174244-2114
ENSG0000(	748	16.70474	chr3:380<	ENSG00000289138		lncRNA	chr3:19951702-1995
ENSG0000(	748	16.70474	chr3:380<	ENSG00000261734		lncRNA	chr3:20388249-2039
ENSG0000(	746	16.66008	chr6:1039	ENSG00000290804		lncRNA	chr6:49946021-4995
ENSG0000(	746	16.66008	chr6:1039	DEFB112		protein_c	chr6:50042099-5004
ENSG0000(	746	16.66008	chr6:1039	ENSG00000226733		lncRNA	chr6:50093389-5018
ENSG0000(	746	16.66008	chr6:1039	ENSG00000226707		lncRNA	chr6:49823712-4982
ENSG0000(	746	16.66008	chr6:1039	RPS17P5		Pseudoger	chr6:50857255-5085
ENSG0000(	746	16.66008	chr6:1039	DEFB110		protein_c	chr6:50009138-5002
ENSG0000(	746	16.66008	chr6:1039	SNORD66		smallRNA	chr6:51464690-5146
ENSG0000(	746	16.66008	chr6:1039	ENSG00000232702		Pseudoger	chr6:51410081-5141
ENSG0000(	746	16.66008	chr6:1039	ENSG00000286405		lncRNA	chr6:50411844-5041
ENSG0000(	746	16.66008	chr6:1039	DEFB133		Pseudoger	chr6:49946101-4994
ENSG0000(	746	16.66008	chr6:1039	CRISP3		protein_c	chr6:49727376-4974
ENSG0000(	746	16.66008	chr6:1039	C6orf141		protein_c	chr6:49550666-4956
ENSG0000(	746	16.66008	chr6:1039	TFAP2D	NCGv7	protein_c	chr6:50713526-5077
ENSG0000(	746	16.66008	chr6:1039	ENSG00000279127		TEC	chr6:49721931-4972
ENSG0000(	746	16.66008	chr6:1039	FTH1P5		Pseudoger	chr6:50912712-5091
ENSG0000(	746	16.66008	chr6:1039	ENSG00000230472		lncRNA	chr6:50587607-5063
ENSG0000(	746	16.66008	chr6:1039	DEFB114		protein_c	chr6:49960249-4996
ENSG0000(	746	16.66008	chr6:1039	ENSG00000214641		Pseudoger	chr6:51537155-5153
ENSG0000(	746	16.66008	chr6:1039	DEFB113		protein_c	chr6:49968677-4996
ENSG0000(	746	16.66008	chr6:1039	PGK2		protein_c	chr6:49785660-4978
ENSG0000(	746	16.66008	chr6:1039	ENSG00000216913		Pseudoger	chr6:50897615-5089
ENSG0000(	746	16.66008	chr6:1039	PKHD1	NCGv7	protein_c	chr6:51615299-5208
ENSG0000(	746	16.66008	chr6:1039	ENSG00000235122		lncRNA	chr6:49714325-4982
ENSG0000(	746	16.66008	chr6:1039	TFAP2B		protein_c	chr6:50818723-5084
ENSG0000(	746	16.66008	chr6:1039	CRISP2		protein_c	chr6:49692358-4971
ENSG0000(	746	16.66008	chr6:1039	CRISP1		protein_c	chr6:49834257-4987
ENSG0000(	746	16.66008	chr6:1039	CYP2AC1P		Pseudoger	chr6:49565924-4958
ENSG0000(	746	16.66008	chr6:1039	ENSG00000233470		lncRNA	chr6:50514035-5052
ENSG0000(	746	16.66008	chr6:1039	ENSG00000270306		Pseudoger	chr6:51385282-5138
ENSG0000(	746	16.66008	chr6:1039	RHAG		protein_c	chr6:49605175-4963
ENSG0000(	746	16.66008	chr6:1039	ENSG00000228689		lncRNA	chr6:51599723-5162
ENSG0000(	744	16.61541	chr2:309<	RNU5E-7P		smallRNA	chr2:15864935-1586
ENSG0000(	744	16.61541	chr2:309<	RN7SKP168		smallRNA	chr2:17122841-1712
ENSG0000(	744	16.61541	chr2:309<	ENSG00000224604		Pseudoger	chr2:16853820-1685
ENSG0000(	744	16.61541	chr2:309<	LINC00276		lncRNA	chr2:13710531-1440
ENSG0000(	744	16.61541	chr2:309<	GEN1		protein_c	chr2:17753858-1778
ENSG0000(	744	16.61541	chr2:309<	DDX1		protein_c	chr2:15591178-1563

ENSG0000(	744	16.61541	chr2:309<	RPS26P18		Pseudoger	chr2:15397435-1539
ENSG0000(	744	16.61541	chr2:309<	ENSG00000228876		lncRNA	chr2:16224047-1633
ENSG0000(	744	16.61541	chr2:309<	SNORA40		smallRNA	chr2:16199203-1619
ENSG0000(	744	16.61541	chr2:309<	SNORA40		smallRNA	chr2:18040606-1804
ENSG0000(	744	16.61541	chr2:309<	GACAT3		lncRNA	chr2:16013928-1608
ENSG0000(	744	16.61541	chr2:309<	ENSG00000232444		lncRNA	chr2:16316324-1631
ENSG0000(	744	16.61541	chr2:309<	LINC01866		lncRNA	chr2:16970034-1697
ENSG0000(	744	16.61541	chr2:309<	ENSG00000289364		lncRNA	chr2:16452112-1647
ENSG0000(	744	16.61541	chr2:309<	ENSG00000224194		lncRNA	chr2:15668684-1568
ENSG0000(	744	16.61541	chr2:309<	PSMC1P10		Pseudoger	chr2:17385091-1738
ENSG0000(	744	16.61541	chr2:309<	ENSG00000224400		lncRNA	chr2:16354256-1643
ENSG0000(	744	16.61541	chr2:309<	ENSG00000271315		Pseudoger	chr2:16149251-1614
ENSG0000(	744	16.61541	chr2:309<	ENSG00000223536		lncRNA	chr2:16728121-1676
ENSG0000(	744	16.61541	chr2:309<	RAD51AP2		protein_c	chr2:17510579-1751
ENSG0000(	744	16.61541	chr2:309<	ENSG00000279663		TEC	chr2:16541690-1654
ENSG0000(	744	16.61541	chr2:309<	MYCN	NCGv7;AC	protein_c	chr2:15940550-1594
ENSG0000(	744	16.61541	chr2:309<	ZFYVE9P2		Pseudoger	chr2:17284292-1728
ENSG0000(	744	16.61541	chr2:309<	RPLP1P5		Pseudoger	chr2:15869939-1587
ENSG0000(	744	16.61541	chr2:309<	RNU6-1288P		smallRNA	chr2:14274622-1427
ENSG0000(	744	16.61541	chr2:309<	ENSG00000226764		lncRNA	chr2:15997049-1606
ENSG0000(	744	16.61541	chr2:309<	ENSG00000237326		lncRNA	chr2:15801747-1581
ENSG0000(	744	16.61541	chr2:309<	MYCNUT		lncRNA	chr2:15920399-1593
ENSG0000(	744	16.61541	chr2:309<	ENSG00000231266		lncRNA	chr2:16227027-1622
ENSG0000(	744	16.61541	chr2:309<	ENSG00000237633		lncRNA	chr2:16523176-1655
ENSG0000(	744	16.61541	chr2:309<	LINC01804		lncRNA	chr2:15690782-1574
ENSG0000(	744	16.61541	chr2:309<	ENSG00000287291		lncRNA	chr2:14705668-1499
ENSG0000(	744	16.61541	chr2:309<	ENSG00000226041		lncRNA	chr2:16202430-1620
ENSG0000(	744	16.61541	chr2:309<	ENSG00000234022		lncRNA	chr2:15564170-1557
ENSG0000(	744	16.61541	chr2:309<	CYRIA		protein_c	chr2:16549459-1666
ENSG0000(	744	16.61541	chr2:309<	ENSG00000229370		lncRNA	chr2:13537673-1360
ENSG0000(	744	16.61541	chr2:309<	MYCNOS		lncRNA	chr2:15918350-1594
ENSG0000(	744	16.61541	chr2:309<	NBAS		protein_c	chr2:15166916-1556
ENSG0000(	744	16.61541	chr2:309<	KCNS3		protein_c	chr2:17877847-1836
ENSG0000(	744	16.61541	chr2:309<	VSNL1		protein_c	chr2:17539126-1765
ENSG0000(	744	16.61541	chr2:309<	LRATD1		protein_c	chr2:14632700-1465
ENSG0000(	744	16.61541	chr2:309<	ENSG00000236989		lncRNA	chr2:16085222-1610
ENSG0000(	744	16.61541	chr2:309<	ENSG00000228505		lncRNA	chr2:14616428-1463
ENSG0000(	744	16.61541	chr2:309<	snoU13		smallRNA	chr2:15903669-1590
ENSG0000(	744	16.61541	chr2:309<	ENSG00000285354		lncRNA	chr2:16294445-1630
ENSG0000(	744	16.61541	chr2:309<	MSGN1		protein_c	chr2:17816460-1781
ENSG0000(	744	16.61541	chr2:309<	SMC6		protein_c	chr2:17663812-1780
ENSG0000(	744	16.61541	chr2:309<	RN7SL104P		smallRNA	chr2:15950690-1595
ENSG0000(	744	16.61541	chr2:309<	ENSG00000235127		lncRNA	chr2:14886647-1490
ENSG0000(	744	16.61541	chr2:309<	ENSG00000227718		lncRNA	chr2:13723048-1375
ENSG0000(	736	16.43675	chr5:164(	ENSG00000249977		Pseudoger	chr5:97449358-9744
ENSG0000(	733	16.36975	chr7:158{	IRS3P		Pseudoger	chr7:100570131-100
ENSG0000(	733	16.36975	chr7:158{	EPO		protein_c	chr7:100720468-100
ENSG0000(	733	16.36975	chr7:158{	GIGYF1		protein_c	chr7:100679507-100
ENSG0000(	733	16.36975	chr7:158{	RN7SL416P		smallRNA	chr7:100530364-100
ENSG0000(	733	16.36975	chr7:158{	LRCH4		protein_c	chr7:100574011-100
ENSG0000(	733	16.36975	chr7:158{	SAP25		protein_c	chr7:100572228-100

ENSG0000(	733	16.36975	chr7:158{	ENSG00000289886		lncRNA	chr7:100569732-100
ENSG0000(	733	16.36975	chr7:158{	ZAN	NCGv7	protein_c	chr7:100733595-100
ENSG0000(	733	16.36975	chr7:158{	AGFG2	DriverDB\	protein_c	chr7:100539203-100
ENSG0000(	733	16.36975	chr7:158{	ACTL6B	NCGv7	protein_c	chr7:100643097-100
ENSG0000(	733	16.36975	chr7:158{	ENSG00000225807		lncRNA	chr7:100509416-100
ENSG0000(	733	16.36975	chr7:158{	GNB2		protein_c	chr7:100673567-100
ENSG0000(	733	16.36975	chr7:158{	POP7		protein_c	chr7:100706121-100
ENSG0000(	733	16.36975	chr7:158{	ENSG00000274272		lncRNA	chr7:100572232-100
ENSG0000(	733	16.36975	chr7:158{	TFR2	DriverDB\	protein_c	chr7:100620416-100
ENSG0000(	733	16.36975	chr7:158{	PCOLCE-AS1		lncRNA	chr7:100589402-100
ENSG0000(	733	16.36975	chr7:158{	MOSPD3		protein_c	chr7:100612102-100
ENSG0000(	733	16.36975	chr7:158{	PCOLCE		protein_c	chr7:100602363-100
ENSG0000(	733	16.36975	chr7:158{	FBXO24	DriverDB\	protein_c	chr7:100583982-100
ENSG0000(	727	16.23576	chr5:1937	MIR4637		smallRNA	chr5:14825929-1482
ENSG0000(	725	16.19109	chr6:1391	LINC01625		lncRNA	chr6:139435636-139
ENSG0000(	725	16.19109	chr6:1391	ENSG00000218565		Pseudoger	chr6:139338018-139
ENSG0000(	725	16.19109	chr6:1391	TXLNB		protein_c	chr6:139240061-139
ENSG0000(	725	16.19109	chr6:1391	CITED2	NCGv7	protein_c	chr6:139371807-139
ENSG0000(	723	16.14643	chr2:309<	NT5C1B		protein_c	chr2:18562871-1858
ENSG0000(	723	16.14643	chr2:309<	ENSG00000287849		lncRNA	chr2:18492216-1851
ENSG0000(	723	16.14643	chr2:309<	NT5C1B-RDH14		protein_c	chr2:18555545-1858
ENSG0000(	723	16.14643	chr2:309<	RNU6-1215P		smallRNA	chr2:18583367-1858
ENSG0000(	723	16.14643	chr2:309<	RDH14		protein_c	chr2:18554723-1856
ENSG0000(	723	16.14643	chr2:309<	ENSG00000287881		lncRNA	chr2:18403967-1850
ENSG0000(	723	16.14643	chr2:309<	ENSG00000260331		lncRNA	chr2:18547386-1854
ENSG0000(	723	16.14643	chr2:309<	ENSG00000224626		lncRNA	chr2:18784807-1878
ENSG0000(	715	15.96777	chr6:1391	ENSG00000205695		Pseudoger	chr6:139659928-139
ENSG0000(	715	15.96777	chr6:1391	ENSG00000287820		lncRNA	chr6:139938864-139
ENSG0000(	715	15.96777	chr6:1391	ENSG00000225148		lncRNA	chr6:139856104-139
ENSG0000(	715	15.96777	chr6:1391	FILNC1		lncRNA	chr6:139677639-139
ENSG0000(	715	15.96777	chr6:1391	ENSG00000226571		lncRNA	chr6:139271362-139
ENSG0000(	715	15.96777	chr6:1391	ATP5PBP6		Pseudoger	chr6:139614438-139
ENSG0000(	714	15.94544	chr6:103{	ENSG00000260212		Pseudoger	chr6:127659424-127
ENSG0000(	714	15.94544	chr5:164(	ENSG00000274832		Pseudoger	chr5:17669283-1766
ENSG0000(	714	15.94544	chr6:103{	ENSG00000219699		Pseudoger	chr6:127659690-127
ENSG0000(	713	15.9231	chr7:158{	MIR5090		smallRNA	chr7:102465742-102
ENSG0000(	713	15.9231	chr6:103{	ENSG00000218986		Pseudoger	chr6:39353747-3935
ENSG0000(	712	15.90077	chr3:380<	ENSG00000238267		Pseudoger	chr3:39338129-3933
ENSG0000(	712	15.90077	chr3:380<	CX3CR1	NCGv7	protein_c	chr3:39263495-3928
ENSG0000(	712	15.90077	chr3:380<	HNRNPA1P21		Pseudoger	chr3:39334979-3933
ENSG0000(	712	15.90077	chr3:380<	MOBP		protein_c	chr3:39467198-3952
ENSG0000(	712	15.90077	chr3:380<	ENSG00000270367		Pseudoger	chr3:39285985-3928
ENSG0000(	712	15.90077	chr3:380<	ENSG00000286781		lncRNA	chr3:39425342-3944
ENSG0000(	712	15.90077	chr3:380<	AC116038.1		smallRNA	chr3:38907314-3890
ENSG0000(	712	15.90077	chr3:380<	SNORA6		smallRNA	chr3:39408389-3940
ENSG0000(	712	15.90077	chr3:380<	XIRP1		protein_c	chr3:39183210-3919
ENSG0000(	712	15.90077	chr3:380<	TTC21A		protein_c	chr3:39107680-3913
ENSG0000(	712	15.90077	chr3:380<	RPSA		protein_c	chr3:39406716-3942
ENSG0000(	712	15.90077	chr3:380<	ENSG00000287620		lncRNA	chr3:39177761-3917
ENSG0000(	712	15.90077	chr3:380<	EEF1A1P24		Pseudoger	chr3:39358545-3935
ENSG0000(	712	15.90077	chr3:380<	ENSG00000283849		lncRNA	chr3:39152906-3915

ENSG00000	712	15.90077	chr3:3804	CCR8		protein_c	chr3:39329709-3933
ENSG00000	712	15.90077	chr3:3804	RNU6-1227P		smallRNA	chr3:39025987-3902
ENSG00000	712	15.90077	chr3:3804	SNORA62		smallRNA	chr3:39411054-3941
ENSG00000	712	15.90077	chr3:3804	DSTNP4		Pseudoger	chr3:39214199-3921
ENSG00000	712	15.90077	chr3:3804	NFU1P1		Pseudoger	chr3:39643638-3964
ENSG00000	712	15.90077	chr3:3804	ENSG00000284669		lncRNA	chr3:39148281-3917
ENSG00000	712	15.90077	chr3:3804	ENSG00000287958		lncRNA	chr3:39232531-3926
ENSG00000	712	15.90077	chr3:3804	ENSG00000287995		lncRNA	chr3:39213069-3921
ENSG00000	712	15.90077	chr3:3804	ENSG00000289279		lncRNA	chr3:39519506-3952
ENSG00000	712	15.90077	chr3:3804	ENSG00000287780		lncRNA	chr3:39292556-3938
ENSG00000	712	15.90077	chr3:3804	CSRNP1		protein_c	chr3:39141855-3915
ENSG00000	712	15.90077	chr3:3804	GORASP1	NCGv7	protein_c	chr3:39095222-3910
ENSG00000	712	15.90077	chr3:3804	WDR48		protein_c	chr3:39052013-3909
ENSG00000	712	15.90077	chr3:3804	RNU4-56P		smallRNA	chr3:39970125-3997
ENSG00000	712	15.90077	chr3:3804	AC099332.1		smallRNA	chr3:39417480-3941
ENSG00000	712	15.90077	chr3:3804	SCN11A	NCGv7	protein_c	chr3:38845764-3905
ENSG00000	712	15.90077	chr3:3804	ENSG00000285885		lncRNA	chr3:39494837-3950
ENSG00000	712	15.90077	chr3:3804	SLC25A38		protein_c	chr3:39383370-3939
ENSG00000	710	15.85611	chr6:1391	RNA5SP220		Pseudoger	chr6:140158591-140
ENSG00000	710	15.85611	chr6:1391	RN7SKP106		smallRNA	chr6:141486141-141
ENSG00000	710	15.85611	chr6:1391	MIR4465		smallRNA	chr6:140683814-140
ENSG00000	710	15.85611	chr6:1391	ENSG00000288714		lncRNA	chr6:140148490-140
ENSG00000	710	15.85611	chr6:1391	ENSG00000236366		lncRNA	chr6:142526455-142
ENSG00000	710	15.85611	chr6:1391	NMBR	NCGv7	protein_c	chr6:142058330-142
ENSG00000	710	15.85611	chr6:1391	ENSG00000275138		Pseudoger	chr6:141019788-141
ENSG00000	710	15.85611	chr6:1391	VTA1		protein_c	chr6:142147162-142
ENSG00000	710	15.85611	chr6:1391	AL360007.1		smallRNA	chr6:142262643-142
ENSG00000	710	15.85611	chr6:1391	NMBR-AS1		lncRNA	chr6:142088233-142
ENSG00000	710	15.85611	chr6:1391	ENSG00000216548		Pseudoger	chr6:140922457-140
ENSG00000	710	15.85611	chr6:1391	LINC02941		lncRNA	chr6:139976352-140
ENSG00000	710	15.85611	chr6:1391	RPS3AP23		Pseudoger	chr6:141635650-141
ENSG00000	710	15.85611	chr6:1391	ENSG00000270983		Pseudoger	chr6:142062717-142
ENSG00000	710	15.85611	chr6:1391	GJE1		protein_c	chr6:142132925-142
ENSG00000	710	15.85611	chr6:1391	ENSG00000259828		lncRNA	chr6:141447011-141
ENSG00000	710	15.85611	chr6:1391	ADGRG6	NCGv7	protein_c	chr6:142301854-142
ENSG00000	710	15.85611	chr6:1391	LINC02919		lncRNA	chr6:142251847-142
ENSG00000	710	15.85611	chr6:1391	AL356137.1		smallRNA	chr6:140660623-140
ENSG00000	710	15.85611	chr6:1391	ENSG00000286452		lncRNA	chr6:141403240-141
ENSG00000	710	15.85611	chr6:1391	MIR3668		smallRNA	chr6:140205252-140
ENSG00000	710	15.85611	chr6:1391	ENSG00000234147		lncRNA	chr6:140575812-140
ENSG00000	710	15.85611	chr6:1391	RPS3AP24		Pseudoger	chr6:140761529-140
ENSG00000	709	15.83377	chr5:1646	ENSG00000253940		Pseudoger	chr5:31065970-3106
ENSG00000	709	15.83377	chr5:1646	LINC02061		lncRNA	chr5:32646564-3265
ENSG00000	709	15.83377	chr5:1646	RPL5P14		Pseudoger	chr5:31847271-3184
ENSG00000	709	15.83377	chr5:1646	Y_RNA		smallRNA	chr5:32134738-3213
ENSG00000	709	15.83377	chr5:1646	ADH5P5		Pseudoger	chr5:23980587-2398
ENSG00000	709	15.83377	chr5:1646	ENSG00000230829		Pseudoger	chr5:32221932-3222
ENSG00000	709	15.83377	chr5:1646	ENSG00000279531		TEC	chr5:25425412-2542
ENSG00000	709	15.83377	chr5:1646	RNU6-760P		smallRNA	chr5:31832439-3183
ENSG00000	709	15.83377	chr5:1646	ENSG00000226400		Pseudoger	chr5:28927029-2892
ENSG00000	709	15.83377	chr5:1646	ENSG00000286961		lncRNA	chr5:22754227-2276

ENSG0000(	709	15.83377	chr5:164(	UBL5P1		Pseudoger	chr5:29600676-2960
ENSG0000(	709	15.83377	chr5:164(	RNU6-363P		smallRNA	chr5:31595565-3159
ENSG0000(	709	15.83377	chr5:164(	ENSG00000248378		lncRNA	chr5:31743988-3174
ENSG0000(	709	15.83377	chr5:164(	LINC02109		lncRNA	chr5:28809331-2921
ENSG0000(	709	15.83377	chr5:164(	RNU6-1079P		smallRNA	chr5:32234766-3223
ENSG0000(	709	15.83377	chr5:164(	LINC02064		lncRNA	chr5:29304305-2939
ENSG0000(	709	15.83377	chr5:164(	ENSG00000251654		lncRNA	chr5:25218973-2529
ENSG0000(	709	15.83377	chr5:164(	ENSG00000254138		lncRNA	chr5:31093977-3126
ENSG0000(	709	15.83377	chr5:164(	Y_RNA		smallRNA	chr5:24910594-2491
ENSG0000(	709	15.83377	chr5:164(	ENSG00000226432		Pseudoger	chr5:24170370-2417
ENSG0000(	709	15.83377	chr5:164(	ENSG00000248426		Pseudoger	chr5:26669711-2666
ENSG0000(	709	15.83377	chr5:164(	ENSG00000248813		lncRNA	chr5:31741833-3174
ENSG0000(	709	15.83377	chr5:164(	Y_RNA		smallRNA	chr5:23994502-2399
ENSG0000(	709	15.83377	chr5:164(	RNU6-358P		smallRNA	chr5:31820564-3182
ENSG0000(	709	15.83377	chr5:164(	ENSG00000253104		lncRNA	chr5:22139247-2214
ENSG0000(	709	15.83377	chr5:164(	ENSG00000279869		TEC	chr5:25716271-2571
ENSG0000(	709	15.83377	chr5:164(	ENSG00000248624		lncRNA	chr5:31657218-3166
ENSG0000(	709	15.83377	chr5:164(	ENSG00000290530		lncRNA	chr5:22142352-2215
ENSG0000(	709	15.83377	chr5:164(	GOLPH3-DT		lncRNA	chr5:32173960-3217
ENSG0000(	709	15.83377	chr5:164(	ENSG00000279923		TEC	chr5:31558573-3155
ENSG0000(	709	15.83377	chr5:164(	ENSG00000248878		Pseudoger	chr5:31725539-3172
ENSG0000(	709	15.83377	chr5:164(	LINC02899		lncRNA	chr5:23951348-2417
ENSG0000(	709	15.83377	chr5:164(	ENSG00000248687		Pseudoger	chr5:29104880-2910
ENSG0000(	709	15.83377	chr5:164(	ENSG00000287940		lncRNA	chr5:30733557-3074
ENSG0000(	709	15.83377	chr5:164(	ENSG00000230571		Pseudoger	chr5:26669346-2666
ENSG0000(	709	15.83377	chr5:164(	LINC02211		lncRNA	chr5:25187800-2532
ENSG0000(	709	15.83377	chr5:164(	ENSG00000248605		lncRNA	chr5:25404733-2544
ENSG0000(	709	15.83377	chr5:164(	ENSG00000233787		Pseudoger	chr5:29881709-2988
ENSG0000(	709	15.83377	chr5:164(	ENSG00000289601		lncRNA	chr5:31092322-3124
ENSG0000(	709	15.83377	chr5:164(	ENSG00000260761		lncRNA	chr5:25319834-2532
ENSG0000(	709	15.83377	chr5:164(	ENSG00000287471		lncRNA	chr5:22212476-2221
ENSG0000(	709	15.83377	chr5:164(	ENSG00000280305		TEC	chr5:29979669-2998
ENSG0000(	709	15.83377	chr5:164(	C5orf22		protein_c	chr5:31532287-3155
ENSG0000(	709	15.83377	chr5:164(	AC138951.1		Pseudoger	chr5:21493583-2149
ENSG0000(	709	15.83377	chr5:164(	CDH10	NCGv7	protein_c	chr5:24487100-2464
ENSG0000(	709	15.83377	chr5:164(	RN7SL572P		smallRNA	chr5:23201445-2320
ENSG0000(	709	15.83377	chr5:164(	SNORA18		smallRNA	chr5:29070496-2907
ENSG0000(	709	15.83377	chr5:164(	ENSG00000251294		lncRNA	chr5:24554018-2461
ENSG0000(	709	15.83377	chr5:164(	ENSG00000272130		lncRNA	chr5:21569755-2157
ENSG0000(	709	15.83377	chr5:164(	ENSG00000248591		Pseudoger	chr5:23456468-2345
ENSG0000(	709	15.83377	chr5:164(	ENSG00000276098		Pseudoger	chr5:29516005-2951
ENSG0000(	709	15.83377	chr5:164(	CDH6	NCGv7	protein_c	chr5:31193686-3132
ENSG0000(	709	15.83377	chr5:164(	PMCHL1		Pseudoger	chr5:22142373-2214
ENSG0000(	709	15.83377	chr5:164(	ENSG00000279671		TEC	chr5:25349841-2535
ENSG0000(	709	15.83377	chr5:164(	RNU6-909P		smallRNA	chr5:28624657-2862
ENSG0000(	709	15.83377	chr5:164(	ENSG00000250984		Pseudoger	chr5:29795938-2979
ENSG0000(	709	15.83377	chr5:164(	BTG4P1		Pseudoger	chr5:24786842-2478
ENSG0000(	709	15.83377	chr5:164(	ENSG00000248490		lncRNA	chr5:27406530-2743
ENSG0000(	709	15.83377	chr5:164(	ENSG00000287176		lncRNA	chr5:30545177-3069
ENSG0000(	709	15.83377	chr5:164(	RNU6-374P		smallRNA	chr5:25701217-2570
ENSG0000(	709	15.83377	chr5:164(	CDH9	NCGv7	protein_c	chr5:26880597-2712

ENSG0000(	709	15.83377	chr5:164(	DROSHA	NCGv7	protein_c	chr5:31400494-3153
ENSG0000(	709	15.83377	chr5:164(	ENSG00000277878		Pseudoger	chr5:28927298-2892
ENSG0000(	709	15.83377	chr5:164(	GOLPH3	AC	protein_c	chr5:32124716-3217
ENSG0000(	709	15.83377	chr5:164(	ENSG00000279107		TEC	chr5:21493436-2149
ENSG0000(	709	15.83377	chr5:164(	LINC02239		lncRNA	chr5:24835280-2484
ENSG0000(	709	15.83377	chr5:164(	ENSG00000287199		lncRNA	chr5:24478516-2448
ENSG0000(	709	15.83377	chr5:164(	SUB1	DriverDBv	protein_c	chr5:32531633-3260
ENSG0000(	709	15.83377	chr5:164(	ENSG00000251033		lncRNA	chr5:26711551-2674
ENSG0000(	709	15.83377	chr5:164(	RPL19P11		Pseudoger	chr5:31053565-3105
ENSG0000(	709	15.83377	chr5:164(	NPR3	NCGv7	protein_c	chr5:32689070-3279
ENSG0000(	709	15.83377	chr5:164(	SNORA40		smallRNA	chr5:23972188-2397
ENSG0000(	709	15.83377	chr5:164(	MSNP1		Pseudoger	chr5:25909503-2591
ENSG0000(	709	15.83377	chr5:164(	AC026703.2		smallRNA	chr5:32781538-3278
ENSG0000(	709	15.83377	chr5:164(	ENSG00000253766		lncRNA	chr5:21616262-2177
ENSG0000(	709	15.83377	chr5:164(	ENSG00000241739		Pseudoger	chr5:31840492-3184
ENSG0000(	709	15.83377	chr5:164(	ZFR		protein_c	chr5:32354350-3244
ENSG0000(	709	15.83377	chr5:164(	LINC02228		lncRNA	chr5:25087450-2519
ENSG0000(	709	15.83377	chr5:164(	AC010455.1		smallRNA	chr5:27986175-2798
ENSG0000(	709	15.83377	chr5:164(	LINC02103		lncRNA	chr5:28285964-2828
ENSG0000(	709	15.83377	chr5:164(	ENSG00000286134		lncRNA	chr5:24135160-2427
ENSG0000(	709	15.83377	chr5:164(	CCNB3P1		Pseudoger	chr5:26739753-2674
ENSG0000(	709	15.83377	chr5:164(	ENSG00000249142		Pseudoger	chr5:32522758-3252
ENSG0000(	709	15.83377	chr5:164(	SNORD29		smallRNA	chr5:24811481-2481
ENSG0000(	709	15.83377	chr5:164(	ENSG00000224282		Pseudoger	chr5:31822561-3182
ENSG0000(	709	15.83377	chr5:164(	ENSG00000286432		lncRNA	chr5:28614613-2865
ENSG0000(	709	15.83377	chr5:164(	ENSG00000249404		lncRNA	chr5:23013048-2301
ENSG0000(	709	15.83377	chr5:164(	RNU4-43P		smallRNA	chr5:26012889-2601
ENSG0000(	709	15.83377	chr5:164(	ENSG00000271035		Pseudoger	chr5:28897785-2889
ENSG0000(	709	15.83377	chr5:164(	ENSG00000250079		Pseudoger	chr5:28213359-2821
ENSG0000(	709	15.83377	chr5:164(	ENSG00000249289		Pseudoger	chr5:23329427-2332
ENSG0000(	709	15.83377	chr5:164(	HPRT1P2		Pseudoger	chr5:30248350-3024
ENSG0000(	709	15.83377	chr5:164(	AC008949.1		smallRNA	chr5:32379407-3237
ENSG0000(	709	15.83377	chr5:164(	snoU13		smallRNA	chr5:31824579-3182
ENSG0000(	709	15.83377	chr5:164(	RNU6-378P		smallRNA	chr5:32309662-3230
ENSG0000(	709	15.83377	chr5:164(	ENSG00000286238		lncRNA	chr5:24772144-2485
ENSG0000(	709	15.83377	chr5:164(	ENSG00000249688		lncRNA	chr5:31754219-3175
ENSG0000(	709	15.83377	chr5:164(	ENSG00000270430		Pseudoger	chr5:29105568-2910
ENSG0000(	709	15.83377	chr5:164(	ENSG00000250694		lncRNA	chr5:24352309-2435
ENSG0000(	709	15.83377	chr5:164(	MIR4279		smallRNA	chr5:31936102-3193
ENSG0000(	709	15.83377	chr5:164(	ENSG00000250524		lncRNA	chr5:24881943-2488
ENSG0000(	709	15.83377	chr5:164(	AC025458.1		smallRNA	chr5:31880035-3188
ENSG0000(	709	15.83377	chr5:164(	ENSG00000249114		lncRNA	chr5:31738269-3173
ENSG0000(	709	15.83377	chr5:164(	ENSG00000250703		Pseudoger	chr5:26670334-2667
ENSG0000(	709	15.83377	chr5:164(	ENSG00000250332		Pseudoger	chr5:23303565-2330
ENSG0000(	709	15.83377	chr5:164(	ENSG00000249099		lncRNA	chr5:26382519-2639
ENSG0000(	709	15.83377	chr5:164(	ENSG00000286454		lncRNA	chr5:24729379-2473
ENSG0000(	709	15.83377	chr5:164(	ENSG00000250764		lncRNA	chr5:32103445-3212
ENSG0000(	709	15.83377	chr5:164(	RN7SKP207		smallRNA	chr5:29948828-2994
ENSG0000(	709	15.83377	chr5:164(	TRPC6P9		Pseudoger	chr5:23688927-2368
ENSG0000(	709	15.83377	chr5:164(	Y_RNA		smallRNA	chr5:23299155-2329
ENSG0000(	709	15.83377	chr5:164(	ENSG00000250453		lncRNA	chr5:28548135-2880

ENSG00000	709	15.83377	chr5:1646	ENSG00000286741		lncRNA	chr5:32653321-3265
ENSG00000	709	15.83377	chr5:1646	MTMR12		protein_c	chr5:32226994-3231
ENSG00000	709	15.83377	chr5:1646	CDH12	NCGv7	protein_c	chr5:21750673-2285
ENSG00000	709	15.83377	chr5:1646	LSP1P3		Pseudoger	chr5:28926816-2892
ENSG00000	709	15.83377	chr5:1646	MIR579		smallRNA	chr5:32394378-3239
ENSG00000	709	15.83377	chr5:1646	TRPC6P6		Pseudoger	chr5:24749374-2474
ENSG00000	709	15.83377	chr5:1646	HSPD1P1		Pseudoger	chr5:21882585-2188
ENSG00000	709	15.83377	chr5:1646	ENSG00000250319		Pseudoger	chr5:23262159-2326
ENSG00000	709	15.83377	chr5:1646	ENSG00000250164		lncRNA	chr5:31747644-3174
ENSG00000	709	15.83377	chr5:1646	ENSG00000285042		lncRNA	chr5:25188059-2518
ENSG00000	709	15.83377	chr5:1646	DUX4L51		Pseudoger	chr5:31249879-3125
ENSG00000	709	15.83377	chr5:1646	TPT1P5		Pseudoger	chr5:31908361-3190
ENSG00000	709	15.83377	chr5:1646	PRDM9	NCGv7;AC	protein_c	chr5:23443586-2352
ENSG00000	709	15.83377	chr5:1646	ENSG00000286204		lncRNA	chr5:26473505-2648
ENSG00000	709	15.83377	chr5:1646	AC140132.1		smallRNA	chr5:21734211-2173
ENSG00000	709	15.83377	chr5:1646	GCNT1P2		Pseudoger	chr5:22580225-2258
ENSG00000	709	15.83377	chr5:1646	ENSG00000250118		Pseudoger	chr5:30365405-3036
ENSG00000	709	15.83377	chr5:1646	ENSG00000270495		Pseudoger	chr5:28808538-2880
ENSG00000	709	15.83377	chr5:1646	ENSG00000249562		Pseudoger	chr5:24578294-2457
ENSG00000	709	15.83377	chr5:1646	ENSG00000249774		Pseudoger	chr5:31923367-3192
ENSG00000	709	15.83377	chr5:1646	ENSG00000286182		lncRNA	chr5:29239922-2926
ENSG00000	709	15.83377	chr5:1646	ENSG00000249744		Pseudoger	chr5:29848684-2984
ENSG00000	709	15.83377	chr5:1646	PDZD2	NCGv7	protein_c	chr5:31639131-3211
ENSG00000	709	15.83377	chr5:1646	RNU6-738P		smallRNA	chr5:27111569-2711
ENSG00000	709	15.83377	chr5:1646	Y_RNA		smallRNA	chr5:31505684-3150
ENSG00000	709	15.83377	chr5:1646	PURPL		lncRNA	chr5:27217714-2749
ENSG00000	709	15.83377	chr5:1646	ENSG00000286625		lncRNA	chr5:25963839-2599
ENSG00000	709	15.83377	chr5:1646	SUCLG2P4		Pseudoger	chr5:29001760-2900
ENSG00000	704	15.72211	chr6:1391	ENSG00000280184		TEC	chr6:145789270-145
ENSG00000	704	15.72211	chr6:1391	ENSG00000257065		protein_c	chr6:143807092-143
ENSG00000	704	15.72211	chr6:1391	ENSG00000217195		Pseudoger	chr6:144706733-144
ENSG00000	704	15.72211	chr6:1391	SF3B5		protein_c	chr6:144094884-144
ENSG00000	704	15.72211	chr6:1391	ENSG00000217231		Pseudoger	chr6:144036618-144
ENSG00000	704	15.72211	chr6:1391	LUADT1		lncRNA	chr6:147158925-147
ENSG00000	704	15.72211	chr6:1391	STXBP5		protein_c	chr6:147204417-147
ENSG00000	704	15.72211	chr6:1391	ENSG00000270890		Pseudoger	chr6:143858062-143
ENSG00000	704	15.72211	chr6:1391	EPM2A		protein_c	chr6:145382535-145
ENSG00000	704	15.72211	chr6:1391	ADGB-DT		lncRNA	chr6:146594516-146
ENSG00000	704	15.72211	chr6:1391	ENSG00000272397		lncRNA	chr6:146948528-146
ENSG00000	704	15.72211	chr6:1391	HYMAI		lncRNA	chr6:144004916-144
ENSG00000	704	15.72211	chr6:1391	UTRN		protein_c	chr6:144285335-144
ENSG00000	704	15.72211	chr6:1391	GRM1	NCGv7;AC	protein_c	chr6:146027646-146
ENSG00000	704	15.72211	chr6:1391	PHACTR2	DriverDBv	protein_c	chr6:143536845-143
ENSG00000	704	15.72211	chr6:1391	SHPRH	NCGv7	protein_c	chr6:145864245-145
ENSG00000	704	15.72211	chr6:1391	ENSG00000219409		Pseudoger	chr6:144397959-144
ENSG00000	704	15.72211	chr6:1391	ENSG00000216475		Pseudoger	chr6:144257034-144
ENSG00000	704	15.72211	chr6:1391	RNA5SP221		Pseudoger	chr6:143449809-143
ENSG00000	704	15.72211	chr6:1391	EPM2A-DT		lncRNA	chr6:145799409-145
ENSG00000	704	15.72211	chr6:1391	ENSG00000217648		Pseudoger	chr6:143342246-143
ENSG00000	704	15.72211	chr6:1391	FUCA2	DriverDBv	protein_c	chr6:143494812-143
ENSG00000	704	15.72211	chr6:1391	ENSG00000288836		lncRNA	chr6:144139977-144

ENSG0000(	704	15.72211	chr6:1391	ENSG00000217612		Pseudoger	chr6:145489630-145
ENSG0000(	704	15.72211	chr6:1391	ENSG00000216642		Pseudoger	chr6:143327275-143
ENSG0000(	704	15.72211	chr6:1391	ENSG00000273997		Pseudoger	chr6:147406531-147
ENSG0000(	704	15.72211	chr6:1391	PHACTR2-AS1		lncRNA	chr6:143554325-143
ENSG0000(	704	15.72211	chr6:1391	ENSG00000216811		Pseudoger	chr6:146364931-146
ENSG0000(	704	15.72211	chr6:1391	ENSG00000280148		protein_c	chr6:143857318-143
ENSG0000(	704	15.72211	chr6:1391	ENSG00000289850		lncRNA	chr6:144922315-144
ENSG0000(	704	15.72211	chr6:1391	ENSG00000288551		lncRNA	chr6:145749917-145
ENSG0000(	704	15.72211	chr6:1391	PEX3	DriverDBv	protein_c	chr6:143450805-143
ENSG0000(	704	15.72211	chr6:1391	AIG1		protein_c	chr6:143060496-143
ENSG0000(	704	15.72211	chr6:1391	TPT1P4		Pseudoger	chr6:144200447-144
ENSG0000(	704	15.72211	chr6:1391	YAP1P1		Pseudoger	chr6:147406889-147
ENSG0000(	704	15.72211	chr6:1391	TUBB8P2		Pseudoger	chr6:143436216-143
ENSG0000(	704	15.72211	chr6:1391	ENSG00000270638		lncRNA	chr6:145735570-145
ENSG0000(	704	15.72211	chr6:1391	ENSG00000285598		lncRNA	chr6:145296545-145
ENSG0000(	704	15.72211	chr6:1391	ADAT2		protein_c	chr6:143422832-143
ENSG0000(	704	15.72211	chr6:1391	STXBP5-AS1		lncRNA	chr6:146824539-147
ENSG0000(	704	15.72211	chr6:1391	RAB32		protein_c	chr6:146543833-146
ENSG0000(	704	15.72211	chr6:1391	FBXO30		protein_c	chr6:145793502-145
ENSG0000(	704	15.72211	chr6:1391	PLAGL1		protein_c	chr6:143940300-144
ENSG0000(	704	15.72211	chr6:1391	RNU1-33P		smallRNA	chr6:145463238-145
ENSG0000(	704	15.72211	chr6:1391	ENSG00000227748		lncRNA	chr6:146854983-146
ENSG0000(	704	15.72211	chr6:1391	RNA5SP222		Pseudoger	chr6:146340420-146
ENSG0000(	704	15.72211	chr6:1391	STX11		protein_c	chr6:144150487-144
ENSG0000(	704	15.72211	chr6:1391	FUNDC2P3		Pseudoger	chr6:146171315-146
ENSG0000(	704	15.72211	chr6:1391	ENSG00000270655		Pseudoger	chr6:143386581-143
ENSG0000(	704	15.72211	chr6:1391	ADGB		protein_c	chr6:146598967-146
ENSG0000(	704	15.72211	chr6:1391	RNU6-734P		smallRNA	chr6:146639700-146
ENSG0000(	704	15.72211	chr6:1391	ENSG00000273719		Pseudoger	chr6:146638844-146
ENSG0000(	704	15.72211	chr6:1391	ENSG00000217495		Pseudoger	chr6:143298770-143
ENSG0000(	704	15.72211	chr6:1391	ENSG00000225311		lncRNA	chr6:144311699-144
ENSG0000(	704	15.72211	chr6:1391	ENSG00000270828		lncRNA	chr6:145736911-145
ENSG0000(	704	15.72211	chr6:1391	ZC2HC1B		protein_c	chr6:143864436-143
ENSG0000(	704	15.72211	chr6:1391	ENSG00000288056		lncRNA	chr6:145784406-145
ENSG0000(	704	15.72211	chr6:1391	MRPL42P3		Pseudoger	chr6:144136450-144
ENSG0000(	704	15.72211	chr6:1391	KATNBL1P6		Pseudoger	chr6:146802359-146
ENSG0000(	704	15.72211	chr6:1391	LTV1	DriverDBv	protein_c	chr6:143843338-143
ENSG0000(	704	15.72211	chr6:1391	ENSG00000278206		lncRNA	chr6:143484979-143
ENSG0000(	704	15.72211	chr6:1391	AL356739.1		smallRNA	chr6:145348758-145
ENSG0000(	704	15.72211	chr6:1391	ENSG00000220739		Pseudoger	chr6:144708106-144
ENSG0000(	702	15.67745	chr3:380	OR7E66P		Pseudoger	chr3:75347833-7534
ENSG0000(	700	15.63278	chr12:171	ENSG00000274902		lncRNA	chr12:47731908-477
ENSG0000(	695	15.52112	chr5:164	ENSG00000233974		Pseudoger	chr5:21481327-2148
ENSG0000(	695	15.52112	chr5:164	ENSG00000286751		lncRNA	chr5:20967211-2103
ENSG0000(	695	15.52112	chr5:164	ENSG00000248286		Pseudoger	chr5:21196720-2119
ENSG0000(	695	15.52112	chr5:164	GUSBP1		lncRNA	chr5:21341833-2158
ENSG0000(	695	15.52112	chr5:164	ENSG00000249359		lncRNA	chr5:21323873-2134
ENSG0000(	695	15.52112	chr5:164	GUSBP1		Pseudoger	chr5:21459560-2150
ENSG0000(	692	15.45412	chr3:380	ENSG00000289351		lncRNA	chr3:129230545-129
ENSG0000(	692	15.45412	chr3:380	SNORA24		smallRNA	chr3:128714571-128
ENSG0000(	692	15.45412	chr3:380	ENSG00000291081		lncRNA	chr3:130097782-130

ENSG00000	692	15.45412	chr3:3804	FTH1P4		Pseudogen	chr3:128764466-128
ENSG00000	692	15.45412	chr3:3804	RNU6-726P		smallRNA	chr3:131092821-131
ENSG00000	692	15.45412	chr3:3804	ENSG00000290242		lncRNA	chr3:128854556-128
ENSG00000	692	15.45412	chr3:3804	AC130888.1		smallRNA	chr3:130183523-130
ENSG00000	692	15.45412	chr3:3804	MBD4		protein_c	chr3:129430947-129
ENSG00000	692	15.45412	chr3:3804	AC083908.1		smallRNA	chr3:131188311-131
ENSG00000	692	15.45412	chr3:3804	ACAD9		protein_c	chr3:128879596-128
ENSG00000	692	15.45412	chr3:3804	ENSG00000285631		lncRNA	chr3:130821184-130
ENSG00000	692	15.45412	chr3:3804	ENSG00000290001		lncRNA	chr3:129067449-129
ENSG00000	692	15.45412	chr3:3804	MARK3P3		Pseudogen	chr3:128848673-128
ENSG00000	692	15.45412	chr3:3804	ENSG00000248659		Pseudogen	chr3:129632019-129
ENSG00000	692	15.45412	chr3:3804	RHO	AC	protein_c	chr3:129528639-129
ENSG00000	692	15.45412	chr3:3804	ENSG00000288996		lncRNA	chr3:129161398-129
ENSG00000	692	15.45412	chr3:3804	Y_RNA		smallRNA	chr3:129818932-129
ENSG00000	692	15.45412	chr3:3804	OR7E21P		Pseudogen	chr3:130034553-130
ENSG00000	692	15.45412	chr3:3804	MARK2P17		Pseudogen	chr3:128843564-128
ENSG00000	692	15.45412	chr3:3804	Y_RNA		smallRNA	chr3:130914341-130
ENSG00000	692	15.45412	chr3:3804	ENSG00000290241		lncRNA	chr3:128850540-128
ENSG00000	692	15.45412	chr3:3804	ENSG00000249869		Pseudogen	chr3:130013182-130
ENSG00000	692	15.45412	chr3:3804	RPS27P12		Pseudogen	chr3:129218093-129
ENSG00000	692	15.45412	chr3:3804	RPS15AP16		Pseudogen	chr3:128798841-128
ENSG00000	692	15.45412	chr3:3804	COL6A4P2		Pseudogen	chr3:130212823-130
ENSG00000	692	15.45412	chr3:3804	HMCES		protein_c	chr3:129278828-129
ENSG00000	692	15.45412	chr3:3804	IFT122		protein_c	chr3:129429607-129
ENSG00000	692	15.45412	chr3:3804	AC121332.1		smallRNA	chr3:131111670-131
ENSG00000	692	15.45412	chr3:3804	GP9		protein_c	chr3:129060779-129
ENSG00000	692	15.45412	chr3:3804	NUDT16		protein_c	chr3:131381671-131
ENSG00000	692	15.45412	chr3:3804	POU5F1P6		Pseudogen	chr3:128674735-128
ENSG00000	692	15.45412	chr3:3804	ALG1L2		protein_c	chr3:130081831-130
ENSG00000	692	15.45412	chr3:3804	CNBP	NCGv7	protein_c	chr3:129167827-129
ENSG00000	692	15.45412	chr3:3804	RPN1	AC	protein_c	chr3:128619969-128
ENSG00000	692	15.45412	chr3:3804	ENSG00000273174		lncRNA	chr3:129123439-129
ENSG00000	692	15.45412	chr3:3804	ENSG00000261159		lncRNA	chr3:128859716-128
ENSG00000	692	15.45412	chr3:3804	ENSG00000251579		Pseudogen	chr3:130927754-130
ENSG00000	692	15.45412	chr3:3804	snoU13		smallRNA	chr3:129074095-129
ENSG00000	692	15.45412	chr3:3804	LINC02021		lncRNA	chr3:130111669-130
ENSG00000	692	15.45412	chr3:3804	ASTE1		protein_c	chr3:131013875-131
ENSG00000	692	15.45412	chr3:3804	LINC01565		lncRNA	chr3:128572000-128
ENSG00000	692	15.45412	chr3:3804	AC083906.1		smallRNA	chr3:130033458-130
ENSG00000	692	15.45412	chr3:3804	ENSG00000286729		lncRNA	chr3:129277753-129
ENSG00000	692	15.45412	chr3:3804	RAB7A		protein_c	chr3:128693669-128
ENSG00000	692	15.45412	chr3:3804	MIR5704		smallRNA	chr3:131985855-131
ENSG00000	692	15.45412	chr3:3804	ISY1		protein_c	chr3:129127415-129
ENSG00000	692	15.45412	chr3:3804	ENSG00000270773		Pseudogen	chr3:129345411-129
ENSG00000	692	15.45412	chr3:3804	ENSG00000279507		TEC	chr3:128914833-128
ENSG00000	692	15.45412	chr3:3804	ENSG00000290993		lncRNA	chr3:129382922-129
ENSG00000	692	15.45412	chr3:3804	ENSG00000288111		lncRNA	chr3:130179511-130
ENSG00000	692	15.45412	chr3:3804	SNORA7B		smallRNA	chr3:129397210-129
ENSG00000	692	15.45412	chr3:3804	RAB43		protein_c	chr3:129087569-129
ENSG00000	692	15.45412	chr3:3804	ENSG00000231305		lncRNA	chr3:128860620-128
ENSG00000	692	15.45412	chr3:3804	ENPP7P3		Pseudogen	chr3:130167790-130

ENSG0000(	692	15.45412	chr3:380<	EFCAB12	DriverDB\	protein_c	chr3:129401321-129
ENSG0000(	692	15.45412	chr3:380<	COPG1		protein_c	chr3:129249606-129
ENSG0000(	692	15.45412	chr3:380<	EFCC1		protein_c	chr3:129001304-129
ENSG0000(	692	15.45412	chr3:380<	TRH		protein_c	chr3:129974688-129
ENSG0000(	692	15.45412	chr3:380<	CFAP92		protein_c	chr3:128909866-129
ENSG0000(	692	15.45412	chr3:380<	TMCC1		protein_c	chr3:129647792-129
ENSG0000(	692	15.45412	chr3:380<	NEK11		protein_c	chr3:131026850-131
ENSG0000(	692	15.45412	chr3:380<	MRPL3		protein_c	chr3:131462212-131
ENSG0000(	692	15.45412	chr3:380<	ENSG00000250643		lncRNA	chr3:129954105-129
ENSG0000(	692	15.45412	chr3:380<	ENSG00000261167		lncRNA	chr3:131455126-131
ENSG0000(	692	15.45412	chr3:380<	ATP2C1		protein_c	chr3:130850595-131
ENSG0000(	692	15.45412	chr3:380<	ENSG00000270492		Pseudoger	chr3:129334586-129
ENSG0000(	692	15.45412	chr3:380<	MARK2P8		Pseudoger	chr3:128748538-128
ENSG0000(	692	15.45412	chr3:380<	ENSG00000249505		Pseudoger	chr3:129045763-129
ENSG0000(	692	15.45412	chr3:380<	COL6A5		protein_c	chr3:130345516-130
ENSG0000(	692	15.45412	chr3:380<	RNU6-1142P		smallRNA	chr3:129819777-129
ENSG0000(	692	15.45412	chr3:380<	ENSG00000203644		lncRNA	chr3:129847048-129
ENSG0000(	692	15.45412	chr3:380<	FAM86HP		Pseudoger	chr3:130099258-130
ENSG0000(	692	15.45412	chr3:380<	H1-10-AS1		lncRNA	chr3:129315392-129
ENSG0000(	692	15.45412	chr3:380<	COL6A6	NCGv7	protein_c	chr3:130517177-130
ENSG0000(	692	15.45412	chr3:380<	DNAJB8-AS1		lncRNA	chr3:128463594-128
ENSG0000(	692	15.45412	chr3:380<	AC083906.2		smallRNA	chr3:130096373-130
ENSG0000(	692	15.45412	chr3:380<	ENSG00000289118		lncRNA	chr3:131771870-131
ENSG0000(	692	15.45412	chr3:380<	NUDT16L2P		Pseudoger	chr3:131361818-131
ENSG0000(	692	15.45412	chr3:380<	OR7E129P		Pseudoger	chr3:130021553-130
ENSG0000(	692	15.45412	chr3:380<	ENSG00000250129		lncRNA	chr3:131053317-131
ENSG0000(	692	15.45412	chr3:380<	RN7SL752P		smallRNA	chr3:129591349-129
ENSG0000(	692	15.45412	chr3:380<	RN7SL698P		smallRNA	chr3:128785147-128
ENSG0000(	692	15.45412	chr3:380<	ENSG00000284731		Pseudoger	chr3:130002789-130
ENSG0000(	692	15.45412	chr3:380<	NUDT16-DT		lncRNA	chr3:131325092-131
ENSG0000(	692	15.45412	chr3:380<	ENSG00000249253		Pseudoger	chr3:130293337-130
ENSG0000(	692	15.45412	chr3:380<	EVA1CP6		Pseudoger	chr3:130048143-130
ENSG0000(	692	15.45412	chr3:380<	ENSG00000250796		Pseudoger	chr3:128869624-128
ENSG0000(	692	15.45412	chr3:380<	TMED10P2		Pseudoger	chr3:128538020-128
ENSG0000(	692	15.45412	chr3:380<	ENSG00000251447		Pseudoger	chr3:131537571-131
ENSG0000(	692	15.45412	chr3:380<	ISY1-RAB43		protein_c	chr3:129087575-129
ENSG0000(	692	15.45412	chr3:380<	SNRPCP8		Pseudoger	chr3:130199708-130
ENSG0000(	692	15.45412	chr3:380<	ACAD9-DT		lncRNA	chr3:128871913-128
ENSG0000(	692	15.45412	chr3:380<	MARK2P6		Pseudoger	chr3:128852112-128
ENSG0000(	692	15.45412	chr3:380<	ENSG00000244932		Pseudoger	chr3:129381298-129
ENSG0000(	692	15.45412	chr3:380<	GSTO3P		Pseudoger	chr3:130827659-130
ENSG0000(	692	15.45412	chr3:380<	ENSG00000250592		lncRNA	chr3:130899414-130
ENSG0000(	692	15.45412	chr3:380<	RN7SKP212		smallRNA	chr3:130811768-130
ENSG0000(	692	15.45412	chr3:380<	GATA2	NCGv7;AC	protein_c	chr3:128479427-128
ENSG0000(	692	15.45412	chr3:380<	DNAJB8		protein_c	chr3:128462437-128
ENSG0000(	692	15.45412	chr3:380<	ENSG00000248468		lncRNA	chr3:131502573-131
ENSG0000(	692	15.45412	chr3:380<	TMCC1-DT		lncRNA	chr3:129893811-129
ENSG0000(	692	15.45412	chr3:380<	snoU13		smallRNA	chr3:129073296-129
ENSG0000(	692	15.45412	chr3:380<	ENSG00000289469		lncRNA	chr3:129184058-129
ENSG0000(	692	15.45412	chr3:380<	H1-8	NCGv7	protein_c	chr3:129543175-129
ENSG0000(	692	15.45412	chr3:380<	PIK3R4	NCGv7	protein_c	chr3:130678934-130

ENSG00000	692	15.45412	chr3:3804	ENSG00000248459		Pseudoger	chr3:129998531-129
ENSG00000	692	15.45412	chr3:3804	PLXND1	NCGv7	protein_c	chr3:129555214-129
ENSG00000	692	15.45412	chr3:3804	NUP210P3		Pseudoger	chr3:129323046-129
ENSG00000	692	15.45412	chr3:3804	BCL2L12P1		Pseudoger	chr3:131526447-131
ENSG00000	692	15.45412	chr3:3804	H1-10		protein_c	chr3:129314771-129
ENSG00000	692	15.45412	chr3:3804	LINC02014		lncRNA	chr3:130089433-130
ENSG00000	692	15.45412	chr3:3804	ENSG00000273437		lncRNA	chr3:129163606-129
ENSG00000	692	15.45412	chr3:3804	AC093004.1		smallRNA	chr3:130369227-130
ENSG00000	692	15.45412	chr3:3804	ENSG00000284095		TEC	chr3:130868004-130
ENSG00000	692	15.45412	chr3:3804	GATA2-AS1		lncRNA	chr3:128489212-128
ENSG00000	692	15.45412	chr3:3804	RPL32P3		Pseudoger	chr3:129396218-129
ENSG00000	692	15.45412	chr3:3804	ENSG00000249098		Pseudoger	chr3:130918226-130
ENSG00000	692	15.45412	chr3:3804	ENSG00000286806		lncRNA	chr3:128563316-128
ENSG00000	688	15.36479	chr3:3804	RPL7P16		Pseudoger	chr3:132243528-132
ENSG00000	688	15.36479	chr3:3804	CPNE4		protein_c	chr3:131533555-132
ENSG00000	688	15.36479	chr3:3804	PSMC2P1		Pseudoger	chr3:132175402-132
ENSG00000	688	15.36479	chr3:3804	NPHP3-ACAD11		protein_c	chr3:132558142-132
ENSG00000	688	15.36479	chr3:3804	ACP3		protein_c	chr3:132317369-132
ENSG00000	688	15.36479	chr3:3804	HSPA8P19		Pseudoger	chr3:132645831-132
ENSG00000	688	15.36479	chr3:3804	NPHP3		protein_c	chr3:132680609-132
ENSG00000	688	15.36479	chr3:3804	BFSP2		protein_c	chr3:133400056-133
ENSG00000	688	15.36479	chr3:3804	NPHP3-AS1		lncRNA	chr3:132721750-132
ENSG00000	688	15.36479	chr3:3804	ENSG00000214301		Pseudoger	chr3:133490824-133
ENSG00000	688	15.36479	chr3:3804	DNAJC13		protein_c	chr3:132417502-132
ENSG00000	688	15.36479	chr3:3804	UBA5		protein_c	chr3:132654446-132
ENSG00000	688	15.36479	chr3:3804	TMEM108		protein_c	chr3:133038391-133
ENSG00000	688	15.36479	chr3:3804	ENSG00000250983		Pseudoger	chr3:133546071-133
ENSG00000	688	15.36479	chr3:3804	CDV3		protein_c	chr3:133573686-133
ENSG00000	688	15.36479	chr3:3804	ACAD11		protein_c	chr3:132558138-132
ENSG00000	688	15.36479	chr3:3804	snoU13		smallRNA	chr3:132280618-132
ENSG00000	688	15.36479	chr3:3804	NIP7P2		Pseudoger	chr3:132401253-132
ENSG00000	688	15.36479	chr3:3804	TMEM108-AS1		lncRNA	chr3:133245603-133
ENSG00000	688	15.36479	chr3:3804	ENSG00000240890		Pseudoger	chr3:132386522-132
ENSG00000	688	15.36479	chr3:3804	ACKR4		protein_c	chr3:132597270-132
ENSG00000	688	15.36479	chr3:3804	ENSG00000249725		lncRNA	chr3:133015004-133
ENSG00000	688	15.36479	chr3:3804	ENSG00000249820		Pseudoger	chr3:133409955-133
ENSG00000	688	15.36479	chr3:3804	SNORA33		smallRNA	chr3:133551186-133
ENSG00000	688	15.36479	chr3:3804	ENSG00000272832		lncRNA	chr3:133543064-133
ENSG00000	688	15.36479	chr3:3804	BFSP2-AS1		lncRNA	chr3:133429269-133
ENSG00000	686	15.32012	chr5:1646	snoU13		smallRNA	chr5:18843897-1884
ENSG00000	686	15.32012	chr5:1646	Y_RNA		smallRNA	chr5:16623004-1662
ENSG00000	686	15.32012	chr5:1646	TAF11L9		protein_c	chr5:17593798-1759
ENSG00000	686	15.32012	chr5:1646	CDH18-AS1		lncRNA	chr5:20305565-2034
ENSG00000	686	15.32012	chr5:1646	RN7SL58P		smallRNA	chr5:18547312-1854
ENSG00000	686	15.32012	chr5:1646	RNU6-1003P		smallRNA	chr5:17240611-1724
ENSG00000	686	15.32012	chr5:1646	ENSG00000248766		Pseudoger	chr5:20158662-2015
ENSG00000	686	15.32012	chr5:1646	ENSG00000248160		Pseudoger	chr5:17647712-1764
ENSG00000	686	15.32012	chr5:1646	ENSG00000249199		lncRNA	chr5:17367588-1737
ENSG00000	686	15.32012	chr5:1646	FTH1P10		Pseudoger	chr5:17353695-1735
ENSG00000	686	15.32012	chr5:1646	RN7SKP133		smallRNA	chr5:17345616-1734
ENSG00000	686	15.32012	chr5:1646	RPL36AP21		Pseudoger	chr5:18049232-1804

ENSG0000(	686	15.32012	chr5:164(	TAF11L5		protein_c	chr5:17525235-1752
ENSG0000(	686	15.32012	chr5:164(	RP11-432M8.8		Pseudoger	chr5:17604757-1760
ENSG0000(	686	15.32012	chr5:164(	TAF11L12		protein_c	chr5:17610496-1761
ENSG0000(	686	15.32012	chr5:164(	TAF11L10		protein_c	chr5:17597232-1759
ENSG0000(	686	15.32012	chr5:164(	ENSG00000250667		Pseudoger	chr5:17495658-1749
ENSG0000(	686	15.32012	chr5:164(	LINC02218		protein_c	chr5:17444010-1748
ENSG0000(	686	15.32012	chr5:164(	H3Y2		protein_c	chr5:17490967-1749
ENSG0000(	686	15.32012	chr5:164(	CDH18	NCGv7	protein_c	chr5:19471296-2057
ENSG0000(	686	15.32012	chr5:164(	TAF11L6		protein_c	chr5:17528669-1752
ENSG0000(	686	15.32012	chr5:164(	ENSG00000271892		lncRNA	chr5:17107360-1710
ENSG0000(	686	15.32012	chr5:164(	UBE2V1P12		Pseudoger	chr5:18886622-1888
ENSG0000(	686	15.32012	chr5:164(	TAF11L2		protein_c	chr5:17498231-1749
ENSG0000(	686	15.32012	chr5:164(	ENSG00000249174		lncRNA	chr5:18965861-1914
ENSG0000(	686	15.32012	chr5:164(	ENSG00000248422		Pseudoger	chr5:17598432-1759
ENSG0000(	686	15.32012	chr5:164(	H3P17		Pseudoger	chr5:17502043-1750
ENSG0000(	686	15.32012	chr5:164(	LINC02111		lncRNA	chr5:17378906-1738
ENSG0000(	686	15.32012	chr5:164(	H3Y1		protein_c	chr5:17654868-1765
ENSG0000(	686	15.32012	chr5:164(	BASP1	DriverDB\	protein_c	chr5:17065598-1727
ENSG0000(	686	15.32012	chr5:164(	TAF11L3		protein_c	chr5:17518367-1751
ENSG0000(	686	15.32012	chr5:164(	TAF11L8		protein_c	chr5:17590364-1759
ENSG0000(	686	15.32012	chr5:164(	ENSG00000249927		Pseudoger	chr5:18893902-1889
ENSG0000(	686	15.32012	chr5:164(	DCAF13P2		Pseudoger	chr5:17202274-1720
ENSG0000(	686	15.32012	chr5:164(	ENSG00000248471		Pseudoger	chr5:17642962-1764
ENSG0000(	686	15.32012	chr5:164(	ENSG00000248542		Pseudoger	chr5:17649046-1764
ENSG0000(	686	15.32012	chr5:164(	H3P18		Pseudoger	chr5:17511833-1751
ENSG0000(	686	15.32012	chr5:164(	RNA5SP180		Pseudoger	chr5:17156971-1715
ENSG0000(	686	15.32012	chr5:164(	MYO10		protein_c	chr5:16661907-1693
ENSG0000(	686	15.32012	chr5:164(	HSPD1P15		Pseudoger	chr5:19233366-1923
ENSG0000(	686	15.32012	chr5:164(	AC118463.1		smallRNA	chr5:19735510-1973
ENSG0000(	686	15.32012	chr5:164(	ENSG00000250415		lncRNA	chr5:16617225-1662
ENSG0000(	686	15.32012	chr5:164(	H3P19		Pseudoger	chr5:17614781-1761
ENSG0000(	686	15.32012	chr5:164(	BASP1-AS1		lncRNA	chr5:17089296-1721
ENSG0000(	686	15.32012	chr5:164(	RPL32P14		Pseudoger	chr5:19041009-1904
ENSG0000(	686	15.32012	chr5:164(	SNORD81		smallRNA	chr5:18236123-1823
ENSG0000(	686	15.32012	chr5:164(	ENSG00000250088		Pseudoger	chr5:17587290-1758
ENSG0000(	686	15.32012	chr5:164(	ENSG00000251487		lncRNA	chr5:19035197-1903
ENSG0000(	686	15.32012	chr5:164(	ENSG00000249285		Pseudoger	chr5:18908866-1890
ENSG0000(	686	15.32012	chr5:164(	LINC02217		lncRNA	chr5:17403889-1744
ENSG0000(	686	15.32012	chr5:164(	ENSG00000248308		Pseudoger	chr5:20830166-2083
ENSG0000(	686	15.32012	chr5:164(	TAF11L7		protein_c	chr5:17585162-1758
ENSG0000(	686	15.32012	chr5:164(	ENSG00000214132		Pseudoger	chr5:20304045-2030
ENSG0000(	686	15.32012	chr5:164(	ENSG00000248337		Pseudoger	chr5:17654373-1765
ENSG0000(	686	15.32012	chr5:164(	TAF11L14		protein_c	chr5:17634460-1763
ENSG0000(	686	15.32012	chr5:164(	RNU6-660P		smallRNA	chr5:16941843-1694
ENSG0000(	686	15.32012	chr5:164(	ENSG00000185296		Pseudoger	chr5:17306381-1730
ENSG0000(	686	15.32012	chr5:164(	TAF11L4		protein_c	chr5:17521801-1752
ENSG0000(	686	15.32012	chr5:164(	H3P22		Pseudoger	chr5:17670168-1767
ENSG0000(	686	15.32012	chr5:164(	RNA5SP179		Pseudoger	chr5:16853994-1685
ENSG0000(	686	15.32012	chr5:164(	Y_RNA		smallRNA	chr5:17117576-1711
ENSG0000(	686	15.32012	chr5:164(	RETREG1	DriverDB\	protein_c	chr5:16473038-1661
ENSG0000(	686	15.32012	chr5:164(	LINC02223		lncRNA	chr5:17684584-1795

ENSG0000(	686	15.32012	chr5:164(	LINC02241		lncRNA	chr5:20611806-2093
ENSG0000(	686	15.32012	chr5:164(	TAF11L13		protein_c	chr5:17632088-1763
ENSG0000(	686	15.32012	chr5:164(	H3P20		Pseudoger	chr5:17620533-1762
ENSG0000(	686	15.32012	chr5:164(	LINC02100		lncRNA	chr5:18704433-1874
ENSG0000(	686	15.32012	chr5:164(	H3P21		Pseudoger	chr5:17625551-1762
ENSG0000(	686	15.32012	chr5:164(	RETREG1-AS1		lncRNA	chr5:16615926-1668
ENSG0000(	686	15.32012	chr5:164(	LINC02146		lncRNA	chr5:20606710-2061
ENSG0000(	686	15.32012	chr5:164(	ENSG00000248223		lncRNA	chr5:17353910-1735
ENSG0000(	686	15.32012	chr5:164(	RPS26P28		Pseudoger	chr5:16902294-1690
ENSG0000(	681	15.20846	chr3:380⁄	IMPDH1P8		Pseudoger	chr3:15878047-1587
ENSG0000(	681	15.20846	chr3:380⁄	BTD		protein_c	chr3:15601341-1572
ENSG0000(	681	15.20846	chr3:380⁄	ENSG00000287042		lncRNA	chr3:15860112-1613
ENSG0000(	681	15.20846	chr3:380⁄	MIR563		smallRNA	chr3:15873771-1587
ENSG0000(	681	15.20846	chr3:380⁄	ZNF385D		protein_c	chr3:21412218-2237
ENSG0000(	681	15.20846	chr3:380⁄	PLCL2		protein_c	chr3:16802651-1709
ENSG0000(	681	15.20846	chr3:380⁄	PLCL2-AS1		lncRNA	chr3:17042742-1704
ENSG0000(	681	15.20846	chr3:380⁄	DAZL		protein_c	chr3:16586792-1667
ENSG0000(	681	15.20846	chr3:380⁄	ENSG00000287377		lncRNA	chr3:16361348-1637
ENSG0000(	681	15.20846	chr3:380⁄	ENSG00000229271		lncRNA	chr3:16647666-1669
ENSG0000(	681	15.20846	chr3:380⁄	ENSG00000286352		lncRNA	chr3:17742952-1774
ENSG0000(	681	15.20846	chr3:380⁄	RNU6-138P		smallRNA	chr3:18954943-1895
ENSG0000(	681	15.20846	chr3:380⁄	ENSG00000269391		lncRNA	chr3:16524800-1653
ENSG0000(	681	15.20846	chr3:380⁄	DPH3	NCGv7	protein_c	chr3:16257061-1626
ENSG0000(	681	15.20846	chr3:380⁄	VENTXP7		Pseudoger	chr3:21405737-2140
ENSG0000(	681	15.20846	chr3:380⁄	ENSG00000272477		lncRNA	chr3:18408680-1840
ENSG0000(	681	15.20846	chr3:380⁄	AC023798.1		smallRNA	chr3:21829738-2182
ENSG0000(	681	15.20846	chr3:380⁄	SATB1-AS1		lncRNA	chr3:18445024-1892
ENSG0000(	681	15.20846	chr3:380⁄	ENSG00000285654		lncRNA	chr3:16252123-1625
ENSG0000(	681	15.20846	chr3:380⁄	ENSG00000279465		TEC	chr3:15731877-1573
ENSG0000(	681	15.20846	chr3:380⁄	ANKRD28		protein_c	chr3:15667236-1585
ENSG0000(	681	15.20846	chr3:380⁄	ENSG00000233133		Pseudoger	chr3:17706058-1770
ENSG0000(	681	15.20846	chr3:380⁄	ENSG00000272498		lncRNA	chr3:16339308-1633
ENSG0000(	681	15.20846	chr3:380⁄	OXNAD1	NCGv7	protein_c	chr3:16265160-1635
ENSG0000(	681	15.20846	chr3:380⁄	LINC00690		lncRNA	chr3:16524771-1654
ENSG0000(	681	15.20846	chr3:380⁄	PDCL3P3		Pseudoger	chr3:17877455-1787
ENSG0000(	681	15.20846	chr3:380⁄	ENSG00000226238		lncRNA	chr3:17990023-1799
ENSG0000(	681	15.20846	chr3:380⁄	ENSG00000231780		Pseudoger	chr3:17333126-1733
ENSG0000(	681	15.20846	chr3:380⁄	ZNF385D-AS2		lncRNA	chr3:21942566-2197
ENSG0000(	681	15.20846	chr3:380⁄	ENSG00000286689		lncRNA	chr3:15970003-1597
ENSG0000(	681	15.20846	chr3:380⁄	KCNH8		protein_c	chr3:19148510-1953
ENSG0000(	681	15.20846	chr3:380⁄	AC104183.1		smallRNA	chr3:21388533-2138
ENSG0000(	681	15.20846	chr3:380⁄	ENSG00000271964		lncRNA	chr3:16314439-1631
ENSG0000(	681	15.20846	chr3:380⁄	AC010139.1		smallRNA	chr3:16619521-1661
ENSG0000(	681	15.20846	chr3:380⁄	ENSG00000272529		lncRNA	chr3:16345126-1634
ENSG0000(	681	15.20846	chr3:380⁄	MIR3134		smallRNA	chr3:15697298-1569
ENSG0000(	681	15.20846	chr3:380⁄	ENSG00000285731		lncRNA	chr3:17141015-1720
ENSG0000(	681	15.20846	chr3:380⁄	MIR3714		smallRNA	chr3:16933196-1693
ENSG0000(	681	15.20846	chr3:380⁄	SATB1	IntOGen-I	protein_c	chr3:18345377-1844
ENSG0000(	681	15.20846	chr3:380⁄	ENSG00000288050		lncRNA	chr3:16444540-1644
ENSG0000(	681	15.20846	chr3:380⁄	RAD23BP1		Pseudoger	chr3:18538673-1853
ENSG0000(	681	15.20846	chr3:380⁄	ENSG00000287069		lncRNA	chr3:19389185-1970

ENSG0000(	681	15.20846	chr3:380<	ENSG00000270739		Pseudoger	chr3:16275060-1627
ENSG0000(	681	15.20846	chr3:380<	GALNT15		protein_c	chr3:16174680-1623
ENSG0000(	681	15.20846	chr3:380<	ENSG00000270409		Pseudoger	chr3:15732252-1573
ENSG0000(	681	15.20846	chr3:380<	RFTN1	NCGv7	protein_c	chr3:16313574-1651
ENSG0000(	681	15.20846	chr3:380<	U7		smallRNA	chr3:17664963-1766
ENSG0000(	681	15.20846	chr3:380<	TBC1D5		protein_c	chr3:17157162-1844
ENSG0000(	681	15.20846	chr3:380<	CDYLP1		Pseudoger	chr3:16692745-1669
ENSG0000(	681	15.20846	chr3:380<	HACL1		protein_c	chr3:15560699-1560
ENSG0000(	681	15.20846	chr3:380<	MIR4791		smallRNA	chr3:19314848-1931
ENSG0000(	681	15.20846	chr3:380<	BALR6		lncRNA	chr3:18013226-1804
ENSG0000(	681	15.20846	chr3:380<	ENSG00000227309		Pseudoger	chr3:17465150-1746
ENSG0000(	681	15.20846	chr3:380<	ENSG00000213383		Pseudoger	chr3:17871729-1787
ENSG0000(	681	15.20846	chr3:380<	HMGB1P5		Pseudoger	chr3:22381720-2238
ENSG0000(	681	15.20846	chr3:380<	AC090644.1		smallRNA	chr3:17135650-1713
ENSG0000(	681	15.20846	chr3:380<	ZNF385D-AS1		lncRNA	chr3:21542789-2157
ENSG0000(	681	15.20846	chr3:380<	ENSG00000290787		lncRNA	chr3:15732827-1573
ENSG0000(	681	15.20846	chr3:380<	RN7SL4P		smallRNA	chr3:15738515-1573
ENSG0000(	672	15.00747	chr3:380<	ENSG00000242545		lncRNA	chr3:59464330-5951
ENSG0000(	672	15.00747	chr3:380<	ENSG00000243903		lncRNA	chr3:59380024-5938
ENSG0000(	672	15.00747	chr3:380<	ENSG00000241804		Pseudoger	chr3:59535863-5953
ENSG0000(	671	14.98514	chr6:1039	KRT8P42		Pseudoger	chr6:134296997-134
ENSG0000(	671	14.98514	chr6:1039	LINC03004		lncRNA	chr6:137657998-137
ENSG0000(	671	14.98514	chr6:1039	BCLAF1	NCGv7	protein_c	chr6:136256627-136
ENSG0000(	671	14.98514	chr6:1039	MYB	NCGv7;AC	protein_c	chr6:135181308-135
ENSG0000(	671	14.98514	chr6:1039	RPL35AP3		Pseudoger	chr6:136973930-136
ENSG0000(	671	14.98514	chr6:1039	ENSG00000289312		lncRNA	chr6:136290014-136
ENSG0000(	671	14.98514	chr6:1039	AL357060.1		smallRNA	chr6:137716948-137
ENSG0000(	671	14.98514	chr6:1039	MAP3K5-AS1		lncRNA	chr6:136629066-136
ENSG0000(	671	14.98514	chr6:1039	7SK		smallRNA	chr6:136545192-136
ENSG0000(	671	14.98514	chr6:1039	TNFAIP3	NCGv7;AC	protein_c	chr6:137867214-137
ENSG0000(	671	14.98514	chr6:1039	Y_RNA		smallRNA	chr6:135474504-135
ENSG0000(	671	14.98514	chr6:1039	ENSG00000288054		lncRNA	chr6:135882780-135
ENSG0000(	671	14.98514	chr6:1039	ENSG00000240056		lncRNA	chr6:134941392-134
ENSG0000(	671	14.98514	chr6:1039	MAP3K5-AS2		lncRNA	chr6:136784045-136
ENSG0000(	671	14.98514	chr6:1039	MIR548A2		smallRNA	chr6:135239160-135
ENSG0000(	671	14.98514	chr6:1039	RPSAP42		Pseudoger	chr6:137995270-137
ENSG0000(	671	14.98514	chr6:1039	AHI1-DT		lncRNA	chr6:135497422-135
ENSG0000(	671	14.98514	chr6:1039	ENSG00000236389		lncRNA	chr6:134706060-134
ENSG0000(	671	14.98514	chr6:1039	ENSG00000235399		lncRNA	chr6:136995170-136
ENSG0000(	671	14.98514	chr6:1039	NDUFS5P1		Pseudoger	chr6:136475862-136
ENSG0000(	671	14.98514	chr6:1039	SNORA27		smallRNA	chr6:136855698-136
ENSG0000(	671	14.98514	chr6:1039	ALDH8A1		protein_c	chr6:134917393-134
ENSG0000(	671	14.98514	chr6:1039	HBS1L		protein_c	chr6:134960378-135
ENSG0000(	671	14.98514	chr6:1039	LINC02524		lncRNA	chr6:135628787-135
ENSG0000(	671	14.98514	chr6:1039	AL512290.1		smallRNA	chr6:135977811-135
ENSG0000(	671	14.98514	chr6:1039	AHI1	NCGv7;AC	protein_c	chr6:135283407-135
ENSG0000(	671	14.98514	chr6:1039	ENSG00000220412		Pseudoger	chr6:137705423-137
ENSG0000(	671	14.98514	chr6:1039	FAM8A6P		Pseudoger	chr6:134603564-134
ENSG0000(	671	14.98514	chr6:1039	ENSG00000286887		lncRNA	chr6:134296301-134
ENSG0000(	671	14.98514	chr6:1039	ENSG00000234084		lncRNA	chr6:135301568-135
ENSG0000(	671	14.98514	chr6:1039	SGK1	NCGv7;AC	protein_c	chr6:134169248-134

ENSG0000(	671	14.98514	chr6:1039	ENSG00000287974		lncRNA	chr6:134345688-134
ENSG0000(	671	14.98514	chr6:1039	ENSG00000227723		lncRNA	chr6:134636489-134
ENSG0000(	671	14.98514	chr6:1039	GAPDHP73		Pseudoger	chr6:135619165-135
ENSG0000(	671	14.98514	chr6:1039	LINC03002		lncRNA	chr6:134520163-134
ENSG0000(	671	14.98514	chr6:1039	ENSG00000277973		Pseudoger	chr6:136206478-136
ENSG0000(	671	14.98514	chr6:1039	HMGA1P7		Pseudoger	chr6:134115235-134
ENSG0000(	671	14.98514	chr6:1039	Y_RNA		smallRNA	chr6:136934765-136
ENSG0000(	671	14.98514	chr6:1039	ENSG00000286313		lncRNA	chr6:135854053-135
ENSG0000(	671	14.98514	chr6:1039	RN7SL408P		smallRNA	chr6:134133573-134
ENSG0000(	671	14.98514	chr6:1039	IL20RA		protein_c	chr6:136999971-137
ENSG0000(	671	14.98514	chr6:1039	ENSG00000287094		lncRNA	chr6:135323399-135
ENSG0000(	671	14.98514	chr6:1039	IL22RA2		protein_c	chr6:137143820-137
ENSG0000(	671	14.98514	chr6:1039	MEMO1P2		Pseudoger	chr6:134897874-134
ENSG0000(	671	14.98514	chr6:1039	COX5BP2		Pseudoger	chr6:136034553-136
ENSG0000(	671	14.98514	chr6:1039	ENSG00000220660		Pseudoger	chr6:136364129-136
ENSG0000(	671	14.98514	chr6:1039	MAP7	NCGv7	protein_c	chr6:136342281-136
ENSG0000(	671	14.98514	chr6:1039	ENSG00000283265		lncRNA	chr6:137693068-137
ENSG0000(	671	14.98514	chr6:1039	LINC01010		lncRNA	chr6:134343307-134
ENSG0000(	671	14.98514	chr6:1039	SNORD112		smallRNA	chr6:137540400-137
ENSG0000(	671	14.98514	chr6:1039	PDE7B	NCGv7	protein_c	chr6:135851701-136
ENSG0000(	671	14.98514	chr6:1039	OLIG3		protein_c	chr6:137492199-137
ENSG0000(	671	14.98514	chr6:1039	NHEG1		lncRNA	chr6:136982165-136
ENSG0000(	671	14.98514	chr6:1039	MTFR2		protein_c	chr6:136231024-136
ENSG0000(	671	14.98514	chr6:1039	Y_RNA		smallRNA	chr6:137784374-137
ENSG0000(	671	14.98514	chr6:1039	CHCHD2P4		Pseudoger	chr6:134393142-134
ENSG0000(	671	14.98514	chr6:1039	ENSG00000232876		lncRNA	chr6:135055033-135
ENSG0000(	671	14.98514	chr6:1039	MAP3K5	NCGv7	protein_c	chr6:136557046-136
ENSG0000(	671	14.98514	chr6:1039	RNA5SP219		Pseudoger	chr6:136630243-136
ENSG0000(	671	14.98514	chr6:1039	ENSG00000290029		lncRNA	chr6:134373662-134
ENSG0000(	671	14.98514	chr6:1039	ENSG00000236378		lncRNA	chr6:135807148-135
ENSG0000(	671	14.98514	chr6:1039	LINC02528		lncRNA	chr6:137943079-137
ENSG0000(	671	14.98514	chr6:1039	ENSG00000224374		lncRNA	chr6:135259996-135
ENSG0000(	671	14.98514	chr6:1039	ENSG00000216519		Pseudoger	chr6:136317961-136
ENSG0000(	671	14.98514	chr6:1039	SLC2A12		protein_c	chr6:133987581-134
ENSG0000(	671	14.98514	chr6:1039	PERP		protein_c	chr6:138088505-138
ENSG0000(	671	14.98514	chr6:1039	AL360178.1		smallRNA	chr6:135914755-135
ENSG0000(	671	14.98514	chr6:1039	BTF3L4P3		Pseudoger	chr6:137543897-137
ENSG0000(	671	14.98514	chr6:1039	ENSG00000287393		lncRNA	chr6:137900585-137
ENSG0000(	671	14.98514	chr6:1039	Y_RNA		smallRNA	chr6:134283092-134
ENSG0000(	671	14.98514	chr6:1039	snoU13		smallRNA	chr6:134206411-134
ENSG0000(	671	14.98514	chr6:1039	LINC02539		lncRNA	chr6:137730170-137
ENSG0000(	671	14.98514	chr6:1039	ENSG00000260418		lncRNA	chr6:136335714-136
ENSG0000(	671	14.98514	chr6:1039	SLC35D3		protein_c	chr6:136922301-136
ENSG0000(	671	14.98514	chr6:1039	ENSG00000287413		lncRNA	chr6:134074123-134
ENSG0000(	671	14.98514	chr6:1039	HMGB1P17		Pseudoger	chr6:135636086-135
ENSG0000(	671	14.98514	chr6:1039	WAKMAR2		lncRNA	chr6:137823673-137
ENSG0000(	671	14.98514	chr6:1039	CT69		lncRNA	chr6:134428239-134
ENSG0000(	671	14.98514	chr6:1039	IFNGR1	IntOGen-I	protein_c	chr6:137197483-137
ENSG0000(	671	14.98514	chr6:1039	LINC02865		lncRNA	chr6:137945366-137
ENSG0000(	671	14.98514	chr6:1039	ENSG00000229722		lncRNA	chr6:134606299-134
ENSG0000(	671	14.98514	chr6:1039	MYB-AS1		lncRNA	chr6:135195083-135

ENSG0000(	671	14.98514	chr6:1039	ENSG00000278744		Pseudoger	chr6:134009033-134
ENSG0000(	671	14.98514	chr6:1039	RPS29P32		Pseudoger	chr6:134211450-134
ENSG0000(	671	14.98514	chr6:1039	MAP7-AS1		lncRNA	chr6:136550661-136
ENSG0000(	671	14.98514	chr6:1039	PEX7		protein_c	chr6:136822564-136
ENSG0000(	671	14.98514	chr6:1039	RNA5SP218		Pseudoger	chr6:134257035-134
ENSG0000(	671	14.98514	chr6:1039	ENSG00000237596		lncRNA	chr6:135991936-136
ENSG0000(	671	14.98514	chr6:1039	RPL7AP37		Pseudoger	chr6:136900233-136
ENSG0000(	670	14.9628	chr5:1937	ENSG00000248699		Pseudoger	chr5:15450701-1545
ENSG0000(	670	14.9628	chr5:1937	ENSG00000271980		lncRNA	chr5:10264597-1026
ENSG0000(	670	14.9628	chr5:1937	RNA5SP177		Pseudoger	chr5:9760907-97610
ENSG0000(	670	14.9628	chr5:1937	RNU6-429P		smallRNA	chr5:11027200-1102
ENSG0000(	670	14.9628	chr5:1937	CTD-2350J17.1		lncRNA	chr5:15602189-1561
ENSG0000(	670	14.9628	chr5:1937	ENSG00000250619		lncRNA	chr5:9001774-90459
ENSG0000(	670	14.9628	chr5:1937	SEPHS2P1		Pseudoger	chr5:14960232-1496
ENSG0000(	670	14.9628	chr5:1937	AC016575.1		smallRNA	chr5:14910558-1491
ENSG0000(	670	14.9628	chr5:1937	ENSG00000272417		lncRNA	chr5:10203600-1020
ENSG0000(	670	14.9628	chr5:1937	CCT5		protein_c	chr5:10249929-1026
ENSG0000(	670	14.9628	chr5:1937	ATPSCKMT		protein_c	chr5:10225507-1024
ENSG0000(	670	14.9628	chr5:1937	ROPN1L-AS1		lncRNA	chr5:10440983-1044
ENSG0000(	670	14.9628	chr5:1937	ENSG00000279979		TEC	chr5:10675317-1067
ENSG0000(	670	14.9628	chr5:1937	ENSG00000251423		lncRNA	chr5:13860303-1390
ENSG0000(	670	14.9628	chr5:1937	LINC02220		lncRNA	chr5:12914068-1303
ENSG0000(	670	14.9628	chr5:1937	ENSG00000286556		lncRNA	chr5:9789295-98126
ENSG0000(	670	14.9628	chr5:1937	ENSG00000280016		TEC	chr5:10082571-1008
ENSG0000(	670	14.9628	chr5:1937	ENSG00000280017		TEC	chr5:10541454-1054
ENSG0000(	670	14.9628	chr5:1937	ENSG00000260515		lncRNA	chr5:10195121-1019
ENSG0000(	670	14.9628	chr5:1937	ENSG00000251548		Pseudoger	chr5:10664495-1066
ENSG0000(	670	14.9628	chr5:1937	DNAH5	DriverDB\	protein_c	chr5:13690328-1401
ENSG0000(	670	14.9628	chr5:1937	ENSG00000271998		lncRNA	chr5:10195187-1019
ENSG0000(	670	14.9628	chr5:1937	ENSG00000279635		TEC	chr5:8980404-89806
ENSG0000(	670	14.9628	chr5:1937	U8		smallRNA	chr5:15110786-1511
ENSG0000(	670	14.9628	chr5:1937	TRIO	NCGv7;AC	protein_c	chr5:14143342-1453
ENSG0000(	670	14.9628	chr5:1937	ENSG00000250576		lncRNA	chr5:10775058-1077
ENSG0000(	670	14.9628	chr5:1937	ENSG00000289112		lncRNA	chr5:16465865-1646
ENSG0000(	670	14.9628	chr5:1937	ENSG00000243744		Pseudoger	chr5:13637919-1363
ENSG0000(	670	14.9628	chr5:1937	NENFP3		Pseudoger	chr5:13553654-1355
ENSG0000(	670	14.9628	chr5:1937	OTULINL		protein_c	chr5:14581792-1461
ENSG0000(	670	14.9628	chr5:1937	SNORD123		smallRNA	chr5:9548836-95489
ENSG0000(	670	14.9628	chr5:1937	DAP		protein_c	chr5:10679230-1076
ENSG0000(	670	14.9628	chr5:1937	CMBL	DriverDB\	protein_c	chr5:10275875-1030
ENSG0000(	670	14.9628	chr5:1937	ROPN1L		protein_c	chr5:10441524-1047
ENSG0000(	670	14.9628	chr5:1937	RPS23P5		Pseudoger	chr5:13250235-1325
ENSG0000(	670	14.9628	chr5:1937	EEF1A1P13		Pseudoger	chr5:14651941-1465
ENSG0000(	670	14.9628	chr5:1937	ENSG00000280369		TEC	chr5:10342813-1034
ENSG0000(	670	14.9628	chr5:1937	ENSG00000280368		TEC	chr5:10171030-1017
ENSG0000(	670	14.9628	chr5:1937	ENSG00000259802		lncRNA	chr5:10323547-1035
ENSG0000(	670	14.9628	chr5:1937	ENSG00000251112		Pseudoger	chr5:12794100-1279
ENSG0000(	670	14.9628	chr5:1937	MARCHF6		protein_c	chr5:10353695-1044
ENSG0000(	670	14.9628	chr5:1937	ENSG00000248525		lncRNA	chr5:9621377-96584
ENSG0000(	670	14.9628	chr5:1937	DAP-DT		lncRNA	chr5:10761065-1077
ENSG0000(	670	14.9628	chr5:1937	ENSG00000286441		lncRNA	chr5:14970909-1499

ENSG0000(	670	14.9628	chr5:1937	LINC02112		lncRNA	chr5:9641305-99038
ENSG0000(	670	14.9628	chr5:1937	LINC01513		lncRNA	chr5:10479371-1048
ENSG0000(	670	14.9628	chr5:1937	MIR887		smallRNA	chr5:15935182-1593
ENSG0000(	670	14.9628	chr5:1937	ENSG00000286970		lncRNA	chr5:14728587-1473
ENSG0000(	670	14.9628	chr5:1937	CTNND2	NCGv7;AC	protein_c	chr5:10971836-1190
ENSG0000(	670	14.9628	chr5:1937	ANKRD33B	DriverDB\	protein_c	chr5:10564070-1065
ENSG0000(	670	14.9628	chr5:1937	MARK2P5		Pseudoger	chr5:15384220-1538
ENSG0000(	670	14.9628	chr5:1937	TAS2R1		protein_c	chr5:9627347-97123
ENSG0000(	670	14.9628	chr5:1937	ENSG00000279755		TEC	chr5:10064243-1006
ENSG0000(	670	14.9628	chr5:1937	LINC02150		lncRNA	chr5:16373361-1644
ENSG0000(	670	14.9628	chr5:1937	ENSG00000280449		TEC	chr5:10168783-1016
ENSG0000(	670	14.9628	chr5:1937	MARCHF11-DT		lncRNA	chr5:16180238-1618
ENSG0000(	670	14.9628	chr5:1937	RPL30P7		Pseudoger	chr5:10488820-1048
ENSG0000(	670	14.9628	chr5:1937	ENSG00000278865		TEC	chr5:10676096-1067
ENSG0000(	670	14.9628	chr5:1937	ANKH		protein_c	chr5:14704800-1487
ENSG0000(	670	14.9628	chr5:1937	OTULIN		protein_c	chr5:14664664-1469
ENSG0000(	670	14.9628	chr5:1937	AC106771.1		smallRNA	chr5:12599623-1259
ENSG0000(	670	14.9628	chr5:1937	ENSG00000279011		TEC	chr5:10057502-1005
ENSG0000(	670	14.9628	chr5:1937	MIR4636		smallRNA	chr5:9053816-90538
ENSG0000(	670	14.9628	chr5:1937	SEMA5A	NCGv7	protein_c	chr5:9035033-95460
ENSG0000(	670	14.9628	chr5:1937	ENSG00000271715		lncRNA	chr5:10269489-1026
ENSG0000(	670	14.9628	chr5:1937	ENSG00000248145		lncRNA	chr5:13144000-1319
ENSG0000(	670	14.9628	chr5:1937	LINC02221		lncRNA	chr5:9854371-98999
ENSG0000(	670	14.9628	chr5:1937	UQCRBP3		Pseudoger	chr5:14874400-1487
ENSG0000(	670	14.9628	chr5:1937	ENSG00000249053		Pseudoger	chr5:12794731-1279
ENSG0000(	670	14.9628	chr5:1937	MARCHF11-AS1		lncRNA	chr5:16129114-1614
ENSG0000(	670	14.9628	chr5:1937	ENSG00000278874		TEC	chr5:10070531-1007
ENSG0000(	670	14.9628	chr5:1937	ENSG00000279450		TEC	chr5:10556582-1055
ENSG0000(	670	14.9628	chr5:1937	ANKRD33B-AS1		lncRNA	chr5:10627260-1062
ENSG0000(	670	14.9628	chr5:1937	CCT6P2		Pseudoger	chr5:14639426-1464
ENSG0000(	670	14.9628	chr5:1937	Y_RNA		smallRNA	chr5:10292041-1029
ENSG0000(	670	14.9628	chr5:1937	ENSG00000250001		lncRNA	chr5:9519853-95231
ENSG0000(	670	14.9628	chr5:1937	Y_RNA		smallRNA	chr5:10337277-1033
ENSG0000(	670	14.9628	chr5:1937	RNU6-679P		smallRNA	chr5:12297399-1229
ENSG0000(	670	14.9628	chr5:1937	NACAP6		Pseudoger	chr5:16192088-1619
ENSG0000(	670	14.9628	chr5:1937	ENSG00000249737		lncRNA	chr5:16428536-1643
ENSG0000(	670	14.9628	chr5:1937	ENSG00000248791		Pseudoger	chr5:14620020-1462
ENSG0000(	670	14.9628	chr5:1937	ENSG00000248968		lncRNA	chr5:10248325-1024
ENSG0000(	670	14.9628	chr5:1937	ANKH-DT		lncRNA	chr5:14872332-1487
ENSG0000(	670	14.9628	chr5:1937	ENSG00000248783		lncRNA	chr5:12553890-1257
ENSG0000(	670	14.9628	chr5:1937	FBXL7		protein_c	chr5:15500180-1593
ENSG0000(	670	14.9628	chr5:1937	HNRNPKP5		Pseudoger	chr5:14877667-1487
ENSG0000(	670	14.9628	chr5:1937	SEMA5A-AS1		lncRNA	chr5:9511333-95180
ENSG0000(	670	14.9628	chr5:1937	OTULIN-DT		lncRNA	chr5:14661808-1466
ENSG0000(	670	14.9628	chr5:1937	ENSG00000286377		lncRNA	chr5:15425758-1549
ENSG0000(	670	14.9628	chr5:1937	SNHG18		lncRNA	chr5:9546170-95507
ENSG0000(	670	14.9628	chr5:1937	LINC01194		lncRNA	chr5:12574830-1280
ENSG0000(	670	14.9628	chr5:1937	LINC02213		lncRNA	chr5:10504996-1052
ENSG0000(	670	14.9628	chr5:1937	RNA5SP178		Pseudoger	chr5:16033229-1603
ENSG0000(	670	14.9628	chr5:1937	ENSG00000251196		Pseudoger	chr5:10652211-1065
ENSG0000(	670	14.9628	chr5:1937	ZNF622		protein_c	chr5:16451519-1646

ENSG0000(	670	14.9628	chr5:1937	ENSG00000248537		lncRNA	chr5:9363275-94224
ENSG0000(	670	14.9628	chr5:1937	ENSG00000288967		lncRNA	chr5:12624975-1262
ENSG0000(	670	14.9628	chr5:1937	ENSG00000249807		lncRNA	chr5:10137138-1013
ENSG0000(	670	14.9628	chr5:1937	ENSG00000287581		lncRNA	chr5:14356886-1435
ENSG0000(	670	14.9628	chr5:1937	LINC02212		lncRNA	chr5:10493291-1050
ENSG0000(	670	14.9628	chr5:1937	MARCHF11	NCGv7	protein_c	chr5:16067139-1618
ENSG0000(	670	14.9628	chr5:1937	RBBP4P1		Pseudoger	chr5:14797125-1479
ENSG0000(	670	14.9628	chr5:1937	ENSG00000249166		lncRNA	chr5:10493291-1049
ENSG0000(	670	14.9628	chr5:1937	ENSG00000279698		TEC	chr5:9498401-94987
ENSG0000(	670	14.9628	chr5:1937	LINC02149		lncRNA	chr5:15112120-1526
ENSG0000(	670	14.9628	chr5:1937	AC016553.1		smallRNA	chr5:13174765-1317
ENSG0000(	669	14.94047	chr7:1588	POLR2J3		protein_c	chr7:102562133-102
ENSG0000(	669	14.94047	chr7:1588	ENSG00000270249		protein_c	chr7:102541501-102
ENSG0000(	669	14.94047	chr7:1588	SPDYE2		protein_c	chr7:102551226-102
ENSG0000(	669	14.94047	chr7:1588	AC105052.1		smallRNA	chr7:102606366-102
ENSG0000(	669	14.94047	chr7:1588	POLR2J3		protein_c	chr7:102537918-102
ENSG0000(	669	14.94047	chr7:1588	UPK3BL2		protein_c	chr7:102537919-102
ENSG0000(	666	14.87347	chr10:81(	RPS12P17		Pseudoger	chr10:69161530-691
ENSG0000(	664	14.82881	chr12:171	SNORD112		smallRNA	chr12:62117887-621
ENSG0000(	664	14.82881	chr12:171	KRT8P19		Pseudoger	chr12:61879318-618
ENSG0000(	664	14.82881	chr12:171	RPL21P104		Pseudoger	chr12:61775923-617
ENSG0000(	664	14.82881	chr12:171	DUX4L52		Pseudoger	chr12:61600067-616
ENSG0000(	664	14.82881	chr12:171	RPS3P6		Pseudoger	chr12:62021570-620
ENSG0000(	664	14.82881	chr12:171	ENSG00000286402		lncRNA	chr12:61668302-617
ENSG0000(	664	14.82881	chr12:171	ENSG00000257880		lncRNA	chr12:62139999-621
ENSG0000(	663	14.80648	chr7:1588	RN7SL750P		smallRNA	chr7:100821029-100
ENSG0000(	663	14.80648	chr7:1588	LRWD1	NCGv7	protein_c	chr7:102464956-102
ENSG0000(	663	14.80648	chr7:1588	ENSG00000261535		lncRNA	chr7:102153355-102
ENSG0000(	663	14.80648	chr7:1588	COL26A1		protein_c	chr7:101362875-101
ENSG0000(	663	14.80648	chr7:1588	ORAI2		protein_c	chr7:102433106-102
ENSG0000(	663	14.80648	chr7:1588	RNU6-1322P		smallRNA	chr7:105332790-105
ENSG0000(	663	14.80648	chr7:1588	ENSG00000289956		lncRNA	chr7:103152899-103
ENSG0000(	663	14.80648	chr7:1588	ALKBH4		protein_c	chr7:102456238-102
ENSG0000(	663	14.80648	chr7:1588	ENSG00000272219		lncRNA	chr7:101960116-101
ENSG0000(	663	14.80648	chr7:1588	SPDYE6		protein_c	chr7:102345746-102
ENSG0000(	663	14.80648	chr7:1588	FBXL13		protein_c	chr7:102812838-103
ENSG0000(	663	14.80648	chr7:1588	ENSG00000290830		lncRNA	chr7:102364162-102
ENSG0000(	663	14.80648	chr7:1588	ENSG00000259294		lncRNA	chr7:101822247-101
ENSG0000(	663	14.80648	chr7:1588	ENSG00000224415		Pseudoger	chr7:103141349-103
ENSG0000(	663	14.80648	chr7:1588	SLC12A9		protein_c	chr7:100826820-100
ENSG0000(	663	14.80648	chr7:1588	ENSG00000278683		Pseudoger	chr7:101273322-101
ENSG0000(	663	14.80648	chr7:1588	FIS1		protein_c	chr7:101239458-101
ENSG0000(	663	14.80648	chr7:1588	EPHB4	NCGv7	protein_c	chr7:100802565-100
ENSG0000(	663	14.80648	chr7:1588	AZGP1P2		Pseudoger	chr7:101287482-101
ENSG0000(	663	14.80648	chr7:1588	SRRT	NCGv7	protein_c	chr7:100875103-100
ENSG0000(	663	14.80648	chr7:1588	RPL36P12		Pseudoger	chr7:105244652-105
ENSG0000(	663	14.80648	chr7:1588	ENSG00000270764		Pseudoger	chr7:105189190-105
ENSG0000(	663	14.80648	chr7:1588	ENSG00000242154		lncRNA	chr7:105304277-105
ENSG0000(	663	14.80648	chr7:1588	SLC26A5-AS1		lncRNA	chr7:103445207-103
ENSG0000(	663	14.80648	chr7:1588	ENSG00000223886		Pseudoger	chr7:105530209-105
ENSG0000(	663	14.80648	chr7:1588	RN7SKP86		smallRNA	chr7:103484208-103

ENSG0000(	663	14.80648	chr7:158{	LHFPL3	DriverDB\	protein_c	chr7:104328603-104
ENSG0000(	663	14.80648	chr7:158{	NAPEPLD		protein_c	chr7:103099776-103
ENSG0000(	663	14.80648	chr7:158{	ENSG00000288914		protein_c	chr7:105040858-105
ENSG0000(	663	14.80648	chr7:158{	ENSG00000279724		Pseudoger	chr7:102188599-102
ENSG0000(	663	14.80648	chr7:158{	RASA4	DriverDB\	protein_c	chr7:102579646-102
ENSG0000(	663	14.80648	chr7:158{	Y_RNA		smallRNA	chr7:103434994-103
ENSG0000(	663	14.80648	chr7:158{	RN7SL8P		smallRNA	chr7:104911917-104
ENSG0000(	663	14.80648	chr7:158{	DNAJC2		protein_c	chr7:103312289-103
ENSG0000(	663	14.80648	chr7:158{	ENSG00000279168		lncRNA	chr7:102579104-102
ENSG0000(	663	14.80648	chr7:158{	SERPINE1		protein_c	chr7:101127104-101
ENSG0000(	663	14.80648	chr7:158{	RNU6-1136P		smallRNA	chr7:102834605-102
ENSG0000(	663	14.80648	chr7:158{	SNORA48		smallRNA	chr7:102194076-102
ENSG0000(	663	14.80648	chr7:158{	ENSG00000280004		Pseudoger	chr7:102186819-102
ENSG0000(	663	14.80648	chr7:158{	SPDYE2B		protein_c	chr7:102650319-102
ENSG0000(	663	14.80648	chr7:158{	RASA4DP		Pseudoger	chr7:102681836-102
ENSG0000(	663	14.80648	chr7:158{	PSMC2		protein_c	chr7:103328570-103
ENSG0000(	663	14.80648	chr7:158{	PMPCB		protein_c	chr7:103297435-103
ENSG0000(	663	14.80648	chr7:158{	SH2B2		protein_c	chr7:102285091-102
ENSG0000(	663	14.80648	chr7:158{	AP1S1	NCGv7	protein_c	chr7:101154456-101
ENSG0000(	663	14.80648	chr7:158{	ENSG00000286830		lncRNA	chr7:102699228-102
ENSG0000(	663	14.80648	chr7:158{	MIR5480		smallRNA	chr7:102405742-102
ENSG0000(	663	14.80648	chr7:158{	AC005086.4		Pseudoger	chr7:102188599-102
ENSG0000(	663	14.80648	chr7:158{	DGAT2L7P		Pseudoger	chr7:101201809-101
ENSG0000(	663	14.80648	chr7:158{	MUC12	NCGv7	protein_c	chr7:100972000-101
ENSG0000(	663	14.80648	chr7:158{	DPY19L2P2		lncRNA	chr7:103175133-103
ENSG0000(	663	14.80648	chr7:158{	EIF4BP6		Pseudoger	chr7:104667749-104
ENSG0000(	663	14.80648	chr7:158{	AC005088.1		smallRNA	chr7:102238321-102
ENSG0000(	663	14.80648	chr7:158{	MIR4285		smallRNA	chr7:102293103-102
ENSG0000(	663	14.80648	chr7:158{	RN7SKP198		smallRNA	chr7:102857450-102
ENSG0000(	663	14.80648	chr7:158{	LRRC17	NCGv7	protein_c	chr7:102913000-102
ENSG0000(	663	14.80648	chr7:158{	IFT22		protein_c	chr7:101310914-101
ENSG0000(	663	14.80648	chr7:158{	ENSG00000280404		Pseudoger	chr7:102161120-102
ENSG0000(	663	14.80648	chr7:158{	EMSLR		lncRNA	chr7:101308270-101
ENSG0000(	663	14.80648	chr7:158{	VGF		protein_c	chr7:101162509-101
ENSG0000(	663	14.80648	chr7:158{	PRKRIP1		protein_c	chr7:102363872-102
ENSG0000(	663	14.80648	chr7:158{	ENSG00000239480		lncRNA	chr7:102426818-102
ENSG0000(	663	14.80648	chr7:158{	ENSG00000288749		lncRNA	chr7:101302767-101
ENSG0000(	663	14.80648	chr7:158{	ENSG00000170409		Pseudoger	chr7:102327256-102
ENSG0000(	663	14.80648	chr7:158{	YBX1P2		Pseudoger	chr7:105582258-105
ENSG0000(	663	14.80648	chr7:158{	TRIM56		protein_c	chr7:101085481-101
ENSG0000(	663	14.80648	chr7:158{	MUC17	NCGv7	protein_c	chr7:101020072-101
ENSG0000(	663	14.80648	chr7:158{	LINC01004		lncRNA	chr7:104950315-105
ENSG0000(	663	14.80648	chr7:158{	LHFPL3-AS2		lncRNA	chr7:104894628-104
ENSG0000(	663	14.80648	chr7:158{	ENSG00000205236		protein_c	chr7:102582523-102
ENSG0000(	663	14.80648	chr7:158{	ENSG00000278586		Pseudoger	chr7:102264706-102
ENSG0000(	663	14.80648	chr7:158{	CUX1	NCGv7;AC	protein_c	chr7:101815904-102
ENSG0000(	663	14.80648	chr7:158{	MUC12-AS1		lncRNA	chr7:101014319-101
ENSG0000(	663	14.80648	chr7:158{	ENSG00000289360		protein_c	chr7:105040848-105
ENSG0000(	663	14.80648	chr7:158{	NAT16		protein_c	chr7:101170496-101
ENSG0000(	663	14.80648	chr7:158{	SLC26A5		protein_c	chr7:103352730-103
ENSG0000(	663	14.80648	chr7:158{	SLC12A9-AS1		lncRNA	chr7:100837314-100

ENSG0000(	663	14.80648	chr7:1588	CRYZP1		Pseudoger	chr7:103088664-103
ENSG0000(	663	14.80648	chr7:1588	DPY19L2P2		Pseudoger	chr7:103175343-103
ENSG0000(	663	14.80648	chr7:1588	ENSG00000237606		Pseudoger	chr7:104826336-104
ENSG0000(	663	14.80648	chr7:1588	ARMC10		protein_c	chr7:103074881-103
ENSG0000(	663	14.80648	chr7:1588	RINT1	AC	protein_c	chr7:105532169-105
ENSG0000(	663	14.80648	chr7:1588	RASA4B		protein_c	chr7:102479976-102
ENSG0000(	663	14.80648	chr7:1588	TRIP6	AC	protein_c	chr7:100867387-100
ENSG0000(	663	14.80648	chr7:1588	LNCPRESS1		lncRNA	chr7:101299558-101
ENSG0000(	663	14.80648	chr7:1588	LINC01007		lncRNA	chr7:101562779-101
ENSG0000(	663	14.80648	chr7:1588	LHFPL3-AS1		lncRNA	chr7:104738597-104
ENSG0000(	663	14.80648	chr7:1588	AC005086.2		Pseudoger	chr7:102186819-102
ENSG0000(	663	14.80648	chr7:1588	Y_RNA		smallRNA	chr7:103433461-103
ENSG0000(	663	14.80648	chr7:1588	ENSG00000267645		protein_c	chr7:102637049-102
ENSG0000(	663	14.80648	chr7:1588	ENSG00000236226		lncRNA	chr7:103030104-103
ENSG0000(	663	14.80648	chr7:1588	POLR2J2		protein_c	chr7:102665368-102
ENSG0000(	663	14.80648	chr7:1588	SRPK2		protein_c	chr7:105110704-105
ENSG0000(	663	14.80648	chr7:1588	AC005086.1		Pseudoger	chr7:102161120-102
ENSG0000(	663	14.80648	chr7:1588	RN7SL549P		smallRNA	chr7:100906299-100
ENSG0000(	663	14.80648	chr7:1588	SNORD112		smallRNA	chr7:104971287-104
ENSG0000(	663	14.80648	chr7:1588	ENSG00000239486		Pseudoger	chr7:102380465-102
ENSG0000(	663	14.80648	chr7:1588	KMT2E-AS1		lncRNA	chr7:105013277-105
ENSG0000(	663	14.80648	chr7:1588	RPSAP46		Pseudoger	chr7:101203614-101
ENSG0000(	663	14.80648	chr7:1588	ZNHIT1		protein_c	chr7:101218165-101
ENSG0000(	663	14.80648	chr7:1588	AC093668.2		Pseudoger	chr7:102479732-102
ENSG0000(	663	14.80648	chr7:1588	AC093668.1		smallRNA	chr7:102507203-102
ENSG0000(	663	14.80648	chr7:1588	ENSG00000272918		lncRNA	chr7:105102838-105
ENSG0000(	663	14.80648	chr7:1588	UFSP1	DriverDBv	protein_c	chr7:100888721-100
ENSG0000(	663	14.80648	chr7:1588	PLOD3	DriverDBv	protein_c	chr7:101205977-101
ENSG0000(	663	14.80648	chr7:1588	ENSG00000233683		Pseudoger	chr7:101388868-101
ENSG0000(	663	14.80648	chr7:1588	RPS29P16		Pseudoger	chr7:103348601-103
ENSG0000(	663	14.80648	chr7:1588	FAM185A		protein_c	chr7:102748971-102
ENSG0000(	663	14.80648	chr7:1588	ENSG00000289613		lncRNA	chr7:103315169-103
ENSG0000(	663	14.80648	chr7:1588	RELN	NCGv7	protein_c	chr7:103471381-103
ENSG0000(	663	14.80648	chr7:1588	RWDD4P1		Pseudoger	chr7:105301522-105
ENSG0000(	663	14.80648	chr7:1588	ORC5	DriverDBv	protein_c	chr7:104126341-104
ENSG0000(	663	14.80648	chr7:1588	NFE4		lncRNA	chr7:102973483-102
ENSG0000(	663	14.80648	chr7:1588	ENSG00000239969		Pseudoger	chr7:102375808-102
ENSG0000(	663	14.80648	chr7:1588	RNU6-1104P		smallRNA	chr7:101269938-101
ENSG0000(	663	14.80648	chr7:1588	MOGAT3	DriverDBv	protein_c	chr7:101195007-101
ENSG0000(	663	14.80648	chr7:1588	RPL23AP95		Pseudoger	chr7:103152007-103
ENSG0000(	663	14.80648	chr7:1588	RPL7AP39		Pseudoger	chr7:102755146-102
ENSG0000(	663	14.80648	chr7:1588	AC005086.3		Pseudoger	chr7:102162296-102
ENSG0000(	663	14.80648	chr7:1588	PUS7		protein_c	chr7:105439661-105
ENSG0000(	663	14.80648	chr7:1588	ACHE	DriverDBv	protein_c	chr7:100889994-100
ENSG0000(	663	14.80648	chr7:1588	MYL10		protein_c	chr7:101613330-101
ENSG0000(	663	14.80648	chr7:1588	CLDN15	DriverDBv	protein_c	chr7:101232092-101
ENSG0000(	663	14.80648	chr7:1588	ENSG00000271482		Pseudoger	chr7:105204600-105
ENSG0000(	663	14.80648	chr7:1588	POLR2J		protein_c	chr7:102473128-102
ENSG0000(	663	14.80648	chr7:1588	Y_RNA		smallRNA	chr7:102336869-102
ENSG0000(	663	14.80648	chr7:1588	ENSG00000279482		TEC	chr7:103161947-103
ENSG0000(	663	14.80648	chr7:1588	UPK3BL1		protein_c	chr7:102637025-102

ENSG0000(	663	14.80648	chr7:158{	AC093668.3		smallRNA	chr7:102480080-102
ENSG0000(	663	14.80648	chr7:158{	AC073127.1		smallRNA	chr7:103014256-103
ENSG0000(	663	14.80648	chr7:158{	RPS29P15		Pseudoger	chr7:100928370-100
ENSG0000(	663	14.80648	chr7:158{	MIR4467		smallRNA	chr7:102471469-102
ENSG0000(	663	14.80648	chr7:158{	KMT2E	NCGv7	protein_c	chr7:104940943-105
ENSG0000(	663	14.80648	chr7:158{	AC125387.1		smallRNA	chr7:101682015-101
ENSG0000(	663	14.80648	chr7:158{	EFCAB10		protein_c	chr7:105565120-105
ENSG0000(	663	14.80648	chr7:158{	ENSG00000272604		lncRNA	chr7:105571083-105
ENSG0000(	663	14.80648	chr7:158{	RN7SKP54		smallRNA	chr7:101058299-101
ENSG0000(	663	14.80648	chr7:158{	S100A11P1		Pseudoger	chr7:103262000-103
ENSG0000(	663	14.80648	chr7:158{	MIR4653		smallRNA	chr7:101159473-101
ENSG0000(	663	14.80648	chr7:158{	PMS2P12		Pseudoger	chr7:102337316-102
ENSG0000(	661	14.76181	chr6:103{	PRELID1P1		Pseudoger	chr6:126643488-126
ENSG0000(	661	14.76181	chr6:103{	STX7		protein_c	chr6:132445867-132
ENSG0000(	661	14.76181	chr6:103{	MOXD1		protein_c	chr6:132296055-132
ENSG0000(	661	14.76181	chr6:103{	ENSG00000219302		Pseudoger	chr6:129819637-129
ENSG0000(	661	14.76181	chr6:103{	Y_RNA		smallRNA	chr6:128584390-128
ENSG0000(	661	14.76181	chr6:103{	RNA5SP217		Pseudoger	chr6:127317883-127
ENSG0000(	661	14.76181	chr6:103{	EPB41L2		protein_c	chr6:130839347-131
ENSG0000(	661	14.76181	chr6:103{	ENSG00000216917		Pseudoger	chr6:131780721-131
ENSG0000(	661	14.76181	chr6:103{	ENSG00000286438		lncRNA	chr6:133061240-133
ENSG0000(	661	14.76181	chr6:103{	ENSG00000229923		lncRNA	chr6:130697312-130
ENSG0000(	661	14.76181	chr6:103{	THEMIS		protein_c	chr6:127708072-127
ENSG0000(	661	14.76181	chr6:103{	RNU6-200P		smallRNA	chr6:126590287-126
ENSG0000(	661	14.76181	chr6:103{	MRPS17P5		Pseudoger	chr6:127909833-127
ENSG0000(	661	14.76181	chr6:103{	SNORD100		smallRNA	chr6:132816802-132
ENSG0000(	661	14.76181	chr6:103{	HMGB1P13		Pseudoger	chr6:132868218-132
ENSG0000(	661	14.76181	chr6:103{	ENPP1		protein_c	chr6:131808016-131
ENSG0000(	661	14.76181	chr6:103{	ENSG00000219666		Pseudoger	chr6:129783769-129
ENSG0000(	661	14.76181	chr6:103{	MIR548H5		smallRNA	chr6:131792172-131
ENSG0000(	661	14.76181	chr6:103{	TRMT11		protein_c	chr6:125986479-126
ENSG0000(	661	14.76181	chr6:103{	RBM11P1		Pseudoger	chr6:132764660-132
ENSG0000(	661	14.76181	chr6:103{	RPL5P21		Pseudoger	chr6:129756298-129
ENSG0000(	661	14.76181	chr6:103{	SNORD101		smallRNA	chr6:132815307-132
ENSG0000(	661	14.76181	chr6:103{	C6orf58		protein_c	chr6:127519455-127
ENSG0000(	661	14.76181	chr6:103{	TAAR3P		Pseudoger	chr6:132608252-132
ENSG0000(	661	14.76181	chr6:103{	TAAR7P		Pseudoger	chr6:132559024-132
ENSG0000(	661	14.76181	chr6:103{	B3GALNT2P1		Pseudoger	chr6:129800908-129
ENSG0000(	661	14.76181	chr6:103{	ENSG00000220694		Pseudoger	chr6:127435636-127
ENSG0000(	661	14.76181	chr6:103{	ENSG00000288529		protein_c	chr6:133953304-133
ENSG0000(	661	14.76181	chr6:103{	MTCYBP4		Pseudoger	chr6:133150568-133
ENSG0000(	661	14.76181	chr6:103{	ENSG00000234484		lncRNA	chr6:132752675-132
ENSG0000(	661	14.76181	chr6:103{	ENSG00000220110		Pseudoger	chr6:128639188-128
ENSG0000(	661	14.76181	chr6:103{	RPL21P67		Pseudoger	chr6:131469059-131
ENSG0000(	661	14.76181	chr6:103{	LINC00326		lncRNA	chr6:132954257-133
ENSG0000(	661	14.76181	chr6:103{	LINC02536		lncRNA	chr6:127664554-127
ENSG0000(	661	14.76181	chr6:103{	ENSG00000226149		lncRNA	chr6:129439485-129
ENSG0000(	661	14.76181	chr6:103{	LAMA2	NCGv7	protein_c	chr6:128883138-129
ENSG0000(	661	14.76181	chr6:103{	BMPR1AP1		Pseudoger	chr6:129157523-129
ENSG0000(	661	14.76181	chr6:103{	ENSG00000220522		Pseudoger	chr6:127416535-127
ENSG0000(	661	14.76181	chr6:103{	RSPO3	NCGv7	protein_c	chr6:127118671-127

ENSG0000(	661	14.76181	chr6:1039	RPL5P18		Pseudoger	chr6:127362513-127
ENSG0000(	661	14.76181	chr6:1039	ENSG00000290067		lncRNA	chr6:131217724-131
ENSG0000(	661	14.76181	chr6:1039	ENSG00000255330		protein_c	chr6:127438406-127
ENSG0000(	661	14.76181	chr6:1039	L3MBTL3		protein_c	chr6:130013699-130
ENSG0000(	661	14.76181	chr6:1039	EEF1A1P36		Pseudoger	chr6:132271982-132
ENSG0000(	661	14.76181	chr6:1039	KIAA0408		protein_c	chr6:127438406-127
ENSG0000(	661	14.76181	chr6:1039	SELENOKP2		Pseudoger	chr6:131819803-131
ENSG0000(	661	14.76181	chr6:1039	RN7SKP245		smallRNA	chr6:131820334-131
ENSG0000(	661	14.76181	chr6:1039	snoU13		smallRNA	chr6:128613201-128
ENSG0000(	661	14.76181	chr6:1039	AL137251.1		smallRNA	chr6:130434265-130
ENSG0000(	661	14.76181	chr6:1039	SLC18B1		protein_c	chr6:132769370-132
ENSG0000(	661	14.76181	chr6:1039	TAAR1		protein_c	chr6:132643312-132
ENSG0000(	661	14.76181	chr6:1039	TAAR8		protein_c	chr6:132552672-132
ENSG0000(	661	14.76181	chr6:1039	TAAR6		protein_c	chr6:132570322-132
ENSG0000(	661	14.76181	chr6:1039	ENSG00000234567		lncRNA	chr6:133452857-133
ENSG0000(	661	14.76181	chr6:1039	TAAR9		protein_c	chr6:132538277-132
ENSG0000(	661	14.76181	chr6:1039	TAAR2		protein_c	chr6:132617022-132
ENSG0000(	661	14.76181	chr6:1039	ARHGAP18		protein_c	chr6:129576132-129
ENSG0000(	661	14.76181	chr6:1039	ENSG00000237115		Pseudoger	chr6:131825981-131
ENSG0000(	661	14.76181	chr6:1039	RPL23AP46		Pseudoger	chr6:132997551-132
ENSG0000(	661	14.76181	chr6:1039	ENSG00000219284		Pseudoger	chr6:127632958-127
ENSG0000(	661	14.76181	chr6:1039	TMEM244		protein_c	chr6:129831244-129
ENSG0000(	661	14.76181	chr6:1039	FTH1P26		Pseudoger	chr6:133676729-133
ENSG0000(	661	14.76181	chr6:1039	ENSG00000280155		TEC	chr6:132130252-132
ENSG0000(	661	14.76181	chr6:1039	MED23	NCGv7	protein_c	chr6:131573966-131
ENSG0000(	661	14.76181	chr6:1039	YAP1P3		Pseudoger	chr6:126627484-126
ENSG0000(	661	14.76181	chr6:1039	TCF21	NCGv7	protein_c	chr6:133889113-133
ENSG0000(	661	14.76181	chr6:1039	CCN2	AC	protein_c	chr6:131948176-131
ENSG0000(	661	14.76181	chr6:1039	ARG1		protein_c	chr6:131470832-131
ENSG0000(	661	14.76181	chr6:1039	RNF146		protein_c	chr6:127266726-127
ENSG0000(	661	14.76181	chr6:1039	CENPW	AC	protein_c	chr6:126340115-126
ENSG0000(	661	14.76181	chr6:1039	AKAP7		protein_c	chr6:131135467-131
ENSG0000(	661	14.76181	chr6:1039	TMEM200A		protein_c	chr6:130366017-130
ENSG0000(	661	14.76181	chr6:1039	RNU6-861P		smallRNA	chr6:129436875-129
ENSG0000(	661	14.76181	chr6:1039	ENSG00000233351		lncRNA	chr6:129479615-129
ENSG0000(	661	14.76181	chr6:1039	TBPL1	DriverDBv	protein_c	chr6:133952170-133
ENSG0000(	661	14.76181	chr6:1039	SAMD3		protein_c	chr6:130144315-130
ENSG0000(	661	14.76181	chr6:1039	ENSG00000286663		lncRNA	chr6:132599972-132
ENSG0000(	661	14.76181	chr6:1039	VNN1		protein_c	chr6:132680849-132
ENSG0000(	661	14.76181	chr6:1039	VNN2		protein_c	chr6:132743870-132
ENSG0000(	661	14.76181	chr6:1039	TARID		lncRNA	chr6:133502252-133
ENSG0000(	661	14.76181	chr6:1039	PTPRK-AS1		lncRNA	chr6:128027886-128
ENSG0000(	661	14.76181	chr6:1039	RPL21P66		Pseudoger	chr6:132518830-132
ENSG0000(	661	14.76181	chr6:1039	ENSG00000289262		lncRNA	chr6:131294417-131
ENSG0000(	661	14.76181	chr6:1039	RPS12		protein_c	chr6:132814569-132
ENSG0000(	661	14.76181	chr6:1039	MESTP1		Pseudoger	chr6:128928995-128
ENSG0000(	661	14.76181	chr6:1039	ECHDC1		protein_c	chr6:127288712-127
ENSG0000(	661	14.76181	chr6:1039	ENSG00000287731		lncRNA	chr6:126719560-126
ENSG0000(	661	14.76181	chr6:1039	YWHAZP4		Pseudoger	chr6:127355756-127
ENSG0000(	661	14.76181	chr6:1039	ENSG00000218857		Pseudoger	chr6:131184325-131
ENSG0000(	661	14.76181	chr6:1039	CTAGE9		protein_c	chr6:131708441-131

ENSG0000(	661	14.76181	chr6:1039	Z97352.1		smallRNA	chr6:129747737-129
ENSG0000(	661	14.76181	chr6:1039	Y_RNA		smallRNA	chr6:128761717-128
ENSG0000(	661	14.76181	chr6:1039	SMLR1		protein_c	chr6:130827406-130
ENSG0000(	661	14.76181	chr6:1039	SOGA3		protein_c	chr6:127472806-127
ENSG0000(	661	14.76181	chr6:1039	ENSG00000272428		lncRNA	chr6:133540784-133
ENSG0000(	661	14.76181	chr6:1039	LINC01312		lncRNA	chr6:133821147-133
ENSG0000(	661	14.76181	chr6:1039	HSPE1P21		Pseudoger	chr6:133510386-133
ENSG0000(	661	14.76181	chr6:1039	Y_RNA		smallRNA	chr6:130573996-130
ENSG0000(	661	14.76181	chr6:1039	CCNG1P1		Pseudoger	chr6:132698783-132
ENSG0000(	661	14.76181	chr6:1039	ENSG00000218187		Pseudoger	chr6:127654860-127
ENSG0000(	661	14.76181	chr6:1039	ENSG00000224733		lncRNA	chr6:128500527-128
ENSG0000(	661	14.76181	chr6:1039	MIR548AJ1		smallRNA	chr6:132115192-132
ENSG0000(	661	14.76181	chr6:1039	ENSG00000223542		lncRNA	chr6:133435077-133
ENSG0000(	661	14.76181	chr6:1039	ENSG00000279960		TEC	chr6:132891924-132
ENSG0000(	661	14.76181	chr6:1039	SNORA33		smallRNA	chr6:132817219-132
ENSG0000(	661	14.76181	chr6:1039	LINC01013		lncRNA	chr6:131901848-132
ENSG0000(	661	14.76181	chr6:1039	ENSG00000289190		lncRNA	chr6:128520728-128
ENSG0000(	661	14.76181	chr6:1039	MIR588		smallRNA	chr6:126484631-126
ENSG0000(	661	14.76181	chr6:1039	ENSG00000286215		Pseudoger	chr6:126304223-127
ENSG0000(	661	14.76181	chr6:1039	RNU4-18P		smallRNA	chr6:131642818-131
ENSG0000(	661	14.76181	chr6:1039	VNN3P		Pseudoger	chr6:132722784-132
ENSG0000(	661	14.76181	chr6:1039	HLFP1		Pseudoger	chr6:132674885-132
ENSG0000(	661	14.76181	chr6:1039	PPP1R14BP5		Pseudoger	chr6:126257921-126
ENSG0000(	661	14.76181	chr6:1039	OR2A4		protein_c	chr6:131699644-131
ENSG0000(	661	14.76181	chr6:1039	TAAR4P		Pseudoger	chr6:132594398-132
ENSG0000(	661	14.76181	chr6:1039	EYA4	NCGv7	protein_c	chr6:133240514-133
ENSG0000(	661	14.76181	chr6:1039	ENPP3		protein_c	chr6:131628442-131
ENSG0000(	661	14.76181	chr6:1039	RPS4XP9		Pseudoger	chr6:126683036-126
ENSG0000(	661	14.76181	chr6:1039	ENSG00000288977		lncRNA	chr6:131125625-131
ENSG0000(	661	14.76181	chr6:1039	TAAR3P		lncRNA	chr6:132608225-132
ENSG0000(	661	14.76181	chr6:1039	ENSG00000227678		lncRNA	chr6:130133410-130
ENSG0000(	661	14.76181	chr6:1039	TAAR5		protein_c	chr6:132588592-132
ENSG0000(	661	14.76181	chr6:1039	PTPRK	NCGv7	protein_c	chr6:127968785-128
ENSG0000(	661	14.76181	chr6:1039	EEF1DP5		Pseudoger	chr6:128580113-128
ENSG0000(	660	14.73948	chr3:3804	MIR544B		smallRNA	chr3:124732439-124
ENSG0000(	660	14.73948	chr3:3804	ENSG00000288806		lncRNA	chr3:123701293-123
ENSG0000(	660	14.73948	chr3:3804	AC080008.1		smallRNA	chr3:124696938-124
ENSG0000(	660	14.73948	chr3:3804	KALRN	NCGv7	protein_c	chr3:124033369-124
ENSG0000(	660	14.73948	chr3:3804	ENSG00000273123		lncRNA	chr3:123715851-123
ENSG0000(	660	14.73948	chr3:3804	CCDC14		protein_c	chr3:123897305-123
ENSG0000(	660	14.73948	chr3:3804	RNU6-143P		smallRNA	chr3:124407691-124
ENSG0000(	660	14.73948	chr3:3804	MYLK-AS2		lncRNA	chr3:123689644-123
ENSG0000(	660	14.73948	chr3:3804	ENSG00000260391		lncRNA	chr3:124723788-124
ENSG0000(	660	14.73948	chr3:3804	ROPN1		protein_c	chr3:123968521-123
ENSG0000(	660	14.73948	chr3:3804	AC117401.1		smallRNA	chr3:124061994-124
ENSG0000(	660	14.73948	chr3:3804	SNORA5		smallRNA	chr3:123814077-123
ENSG0000(	660	14.73948	chr3:3804	RPL7P15		Pseudoger	chr3:124151960-124
ENSG0000(	660	14.73948	chr3:3804	ENSG00000288713		lncRNA	chr3:124723524-124
ENSG0000(	660	14.73948	chr3:3804	MIR5002		smallRNA	chr3:124132929-124
ENSG0000(	660	14.73948	chr3:3804	UMPS	NCGv7	protein_c	chr3:124730433-124
ENSG0000(	660	14.73948	chr3:3804	ENSG00000242199		Pseudoger	chr3:124733418-124

ENSG0000(	659	14.71715	chr6:1039	SMIM28		protein_c	chr6:138377905-138
ENSG0000(	659	14.71715	chr6:1039	ENSG00000231329		lncRNA	chr6:139144204-139
ENSG0000(	659	14.71715	chr6:1039	MIR3145		smallRNA	chr6:138435213-138
ENSG0000(	659	14.71715	chr6:1039	ENSG00000220600		Pseudoger	chr6:138878899-138
ENSG0000(	659	14.71715	chr6:1039	ABRACL		protein_c	chr6:139028745-139
ENSG0000(	659	14.71715	chr6:1039	ACKR4P1		Pseudoger	chr6:138822747-138
ENSG0000(	659	14.71715	chr6:1039	PBOV1		protein_c	chr6:138215986-138
ENSG0000(	659	14.71715	chr6:1039	ENSG00000274594		Pseudoger	chr6:138464099-138
ENSG0000(	659	14.71715	chr6:1039	ECT2L	NCGv7;AC	protein_c	chr6:138795911-138
ENSG0000(	659	14.71715	chr6:1039	CCDC28A-AS1		lncRNA	chr6:138725211-138
ENSG0000(	659	14.71715	chr6:1039	ENSG00000272446		lncRNA	chr6:139159157-139
ENSG0000(	659	14.71715	chr6:1039	HEBP2		protein_c	chr6:138403531-138
ENSG0000(	659	14.71715	chr6:1039	HECA		protein_c	chr6:139135080-139
ENSG0000(	659	14.71715	chr6:1039	MARCKSL1P2		Pseudoger	chr6:138402585-138
ENSG0000(	659	14.71715	chr6:1039	NHSL1		protein_c	chr6:138422043-138
ENSG0000(	659	14.71715	chr6:1039	ARFGEF3		protein_c	chr6:138161939-138
ENSG0000(	659	14.71715	chr6:1039	ENSG00000218499		Pseudoger	chr6:138393373-138
ENSG0000(	659	14.71715	chr6:1039	CCDC28A	NCGv7;AC	protein_c	chr6:138773769-138
ENSG0000(	659	14.71715	chr6:1039	NHSL1-AS1		lncRNA	chr6:138692548-138
ENSG0000(	659	14.71715	chr6:1039	MTCH1P1		Pseudoger	chr6:138650226-138
ENSG0000(	659	14.71715	chr6:1039	REPS1	DriverDBv	protein_c	chr6:138903493-138
ENSG0000(	659	14.71715	chr6:1039	RNU6-427P		smallRNA	chr6:138859027-138
ENSG0000(	654	14.60548	chr12:171	ENSG00000257989		lncRNA	chr12:52092485-521
ENSG0000(	654	14.60548	chr12:171	VDR		protein_c	chr12:47841537-479
ENSG0000(	654	14.60548	chr12:171	ENSG00000257964		lncRNA	chr12:49576840-495
ENSG0000(	654	14.60548	chr12:171	ENSG00000257955		lncRNA	chr12:48019771-480
ENSG0000(	654	14.60548	chr12:171	DDN-AS1		lncRNA	chr12:48998367-490
ENSG0000(	654	14.60548	chr12:171	PRPF40B	NCGv7	protein_c	chr12:49568218-496
ENSG0000(	654	14.60548	chr12:171	ENSG00000278126		lncRNA	chr12:51201684-512
ENSG0000(	654	14.60548	chr12:171	ENSG00000257985		lncRNA	chr12:48011304-480
ENSG0000(	654	14.60548	chr12:171	ENSG00000272822		protein_c	chr12:48903503-489
ENSG0000(	654	14.60548	chr12:171	ENSG00000260122		lncRNA	chr12:51809705-518
ENSG0000(	654	14.60548	chr12:171	RPS10P20		Pseudoger	chr12:48487946-484
ENSG0000(	654	14.60548	chr12:171	ENSG00000257905		Pseudoger	chr12:47593208-475
ENSG0000(	654	14.60548	chr12:171	DDX23	NCGv7	protein_c	chr12:48829756-488
ENSG0000(	654	14.60548	chr12:171	ENSG00000240399		Pseudoger	chr12:48054813-480
ENSG0000(	654	14.60548	chr12:171	KRT82		protein_c	chr12:52393931-524
ENSG0000(	654	14.60548	chr12:171	RNU6-199P		smallRNA	chr12:51162901-511
ENSG0000(	654	14.60548	chr12:171	Y_RNA		smallRNA	chr12:50939927-509
ENSG0000(	654	14.60548	chr12:171	ASIC1		protein_c	chr12:50057548-500
ENSG0000(	654	14.60548	chr12:171	KRT71		protein_c	chr12:52543909-525
ENSG0000(	654	14.60548	chr12:171	SENP1		protein_c	chr12:48042897-481
ENSG0000(	654	14.60548	chr12:171	PRKAG1	NCGv7	protein_c	chr12:49002274-490
ENSG0000(	654	14.60548	chr12:171	ENSG00000257954		Pseudoger	chr12:49389516-493
ENSG0000(	654	14.60548	chr12:171	ENSG00000269514		lncRNA	chr12:48198357-482
ENSG0000(	654	14.60548	chr12:171	ENDOU		protein_c	chr12:47709734-477
ENSG0000(	654	14.60548	chr12:171	TMDD1		protein_c	chr12:51813921-518
ENSG0000(	654	14.60548	chr12:171	ENSG00000257378		lncRNA	chr12:49954639-499
ENSG0000(	654	14.60548	chr12:171	Y_RNA		smallRNA	chr12:49132853-491
ENSG0000(	654	14.60548	chr12:171	CSRNP2		protein_c	chr12:51061205-510
ENSG0000(	654	14.60548	chr12:171	ENSG00000274156		lncRNA	chr12:50934942-509

ENSG0000(	654	14.60548	chr12:171	ENSG00000258181		lncRNA	chr12:47248124-472
ENSG0000(	654	14.60548	chr12:171	SLC11A2		protein_c	chr12:50979401-510
ENSG0000(	654	14.60548	chr12:171	RAPGEF3	NCGv7	protein_c	chr12:47734363-477
ENSG0000(	654	14.60548	chr12:171	RNU6-1093P		smallRNA	chr12:50256238-502
ENSG0000(	654	14.60548	chr12:171	OR8S1		protein_c	chr12:48525632-485
ENSG0000(	654	14.60548	chr12:171	ENSG00000258203		lncRNA	chr12:48005277-480
ENSG0000(	654	14.60548	chr12:171	KRT84	NCGv7	protein_c	chr12:52377812-523
ENSG0000(	654	14.60548	chr12:171	SLC48A1		protein_c	chr12:47753916-477
ENSG0000(	654	14.60548	chr12:171	RNU6-600P		smallRNA	chr12:48892130-488
ENSG0000(	654	14.60548	chr12:171	PHB1P18		Pseudoger	chr12:48168847-481
ENSG0000(	654	14.60548	chr12:171	Y_RNA		smallRNA	chr12:51156868-511
ENSG0000(	654	14.60548	chr12:171	RNU6-238P		smallRNA	chr12:50656973-506
ENSG0000(	654	14.60548	chr12:171	TAMALIN		protein_c	chr12:52006946-520
ENSG0000(	654	14.60548	chr12:171	KRT77		protein_c	chr12:52689626-527
ENSG0000(	654	14.60548	chr12:171	BIN2		protein_c	chr12:51281038-513
ENSG0000(	654	14.60548	chr12:171	RNU6-834P		smallRNA	chr12:49593104-495
ENSG0000(	654	14.60548	chr12:171	ENSG00000274797		lncRNA	chr12:50953924-509
ENSG0000(	654	14.60548	chr12:171	AQP6		protein_c	chr12:49967194-499
ENSG0000(	654	14.60548	chr12:171	LINC02416		lncRNA	chr12:47353754-473
ENSG0000(	654	14.60548	chr12:171	ENSG00000257346		lncRNA	chr12:49090208-490
ENSG0000(	654	14.60548	chr12:171	PFKM		protein_c	chr12:48105139-481
ENSG0000(	654	14.60548	chr12:171	Y_RNA		smallRNA	chr12:50743568-507
ENSG0000(	654	14.60548	chr12:171	ENSG00000288710		protein_c	chr12:49002274-490
ENSG0000(	654	14.60548	chr12:171	RNU6-1273P		smallRNA	chr12:51037499-510
ENSG0000(	654	14.60548	chr12:171	ENSG00000257925		lncRNA	chr12:47237734-472
ENSG0000(	654	14.60548	chr12:171	SNORA64		smallRNA	chr12:47346166-473
ENSG0000(	654	14.60548	chr12:171	ENSG00000272368		lncRNA	chr12:50112197-501
ENSG0000(	654	14.60548	chr12:171	SMIM41		protein_c	chr12:52079704-521
ENSG0000(	654	14.60548	chr12:171	FMNL3	NCGv7	protein_c	chr12:49636499-497
ENSG0000(	654	14.60548	chr12:171	RNU6-1203P		smallRNA	chr12:48081319-480
ENSG0000(	654	14.60548	chr12:171	KRT89P		Pseudoger	chr12:52341197-523
ENSG0000(	654	14.60548	chr12:171	RNU6-940P		smallRNA	chr12:48975543-489
ENSG0000(	654	14.60548	chr12:171	ADCY6		protein_c	chr12:48766194-487
ENSG0000(	654	14.60548	chr12:171	ENSG00000278385		lncRNA	chr12:47905122-479
ENSG0000(	654	14.60548	chr12:171	DDN		protein_c	chr12:48995149-489
ENSG0000(	654	14.60548	chr12:171	AQP5		protein_c	chr12:49961872-499
ENSG0000(	654	14.60548	chr12:171	AC078864.2		smallRNA	chr12:52113925-521
ENSG0000(	654	14.60548	chr12:171	ENSG00000257464		Pseudoger	chr12:49442424-494
ENSG0000(	654	14.60548	chr12:171	RACGAP1		protein_c	chr12:49976923-500
ENSG0000(	654	14.60548	chr12:171	LINC02156		lncRNA	chr12:47377638-474
ENSG0000(	654	14.60548	chr12:171	TEX49		protein_c	chr12:48727435-487
ENSG0000(	654	14.60548	chr12:171	ENSG00000261586		lncRNA	chr12:51817899-518
ENSG0000(	654	14.60548	chr12:171	LARP4		protein_c	chr12:50392383-504
ENSG0000(	654	14.60548	chr12:171	RPAP3-DT		lncRNA	chr12:47706058-477
ENSG0000(	654	14.60548	chr12:171	DHH		protein_c	chr12:49086656-490
ENSG0000(	654	14.60548	chr12:171	ENSG00000257531		Pseudoger	chr12:50217662-502
ENSG0000(	654	14.60548	chr12:171	KRT75		protein_c	chr12:52424070-524
ENSG0000(	654	14.60548	chr12:171	KCNH3		protein_c	chr12:49539030-495
ENSG0000(	654	14.60548	chr12:171	LINC02396		lncRNA	chr12:49908882-499
ENSG0000(	654	14.60548	chr12:171	COX14		protein_c	chr12:50112082-501
ENSG0000(	654	14.60548	chr12:171	KRT73		protein_c	chr12:52607570-526

ENSG0000(	654	14.60548	chr12:171	KRT5		protein_c	chr12:52514575-525
ENSG0000(	654	14.60548	chr12:171	KRT86		protein_c	chr12:52249300-523
ENSG0000(	654	14.60548	chr12:171	ENSG00000257298		lncRNA	chr12:50185580-501
ENSG0000(	654	14.60548	chr12:171	SMAGP		protein_c	chr12:51244558-512
ENSG0000(	654	14.60548	chr12:171	KRT6C		protein_c	chr12:52468516-524
ENSG0000(	654	14.60548	chr12:171	WNT1	TAG;AC	protein_c	chr12:48978322-489
ENSG0000(	654	14.60548	chr12:171	KRT74		protein_c	chr12:52565782-525
ENSG0000(	654	14.60548	chr12:171	KRT72		protein_c	chr12:52585589-526
ENSG0000(	654	14.60548	chr12:171	DNAJC22		protein_c	chr12:49346888-493
ENSG0000(	654	14.60548	chr12:171	RNU6-769P		smallRNA	chr12:50633273-506
ENSG0000(	654	14.60548	chr12:171	ENSG00000287537		lncRNA	chr12:49536677-495
ENSG0000(	654	14.60548	chr12:171	ACVR1B	NCGv7	protein_c	chr12:51951699-519
ENSG0000(	654	14.60548	chr12:171	ENSG00000257735		lncRNA	chr12:48350945-484
ENSG0000(	654	14.60548	chr12:171	KRT7		protein_c	chr12:52232520-522
ENSG0000(	654	14.60548	chr12:171	KRT87P		Pseudoger	chr12:52250466-522
ENSG0000(	654	14.60548	chr12:171	PCED1B	NCGv7	protein_c	chr12:47079603-472
ENSG0000(	654	14.60548	chr12:171	FAIM2		protein_c	chr12:49866896-499
ENSG0000(	654	14.60548	chr12:171	TFCP2	AC	protein_c	chr12:51093656-511
ENSG0000(	654	14.60548	chr12:171	LSM6P2		Pseudoger	chr12:49770960-497
ENSG0000(	654	14.60548	chr12:171	TROAP		protein_c	chr12:49323236-493
ENSG0000(	654	14.60548	chr12:171	KRT85		protein_c	chr12:52360006-523
ENSG0000(	654	14.60548	chr12:171	FAM186B	DriverDB\	protein_c	chr12:49582885-496
ENSG0000(	654	14.60548	chr12:171	RNU6-574P		smallRNA	chr12:51987657-519
ENSG0000(	654	14.60548	chr12:171	FAM186A	IntOGen-I	protein_c	chr12:50326230-503
ENSG0000(	654	14.60548	chr12:171	LINC02874		lncRNA	chr12:52205392-522
ENSG0000(	654	14.60548	chr12:171	PRPH		protein_c	chr12:49295147-492
ENSG0000(	654	14.60548	chr12:171	snoU13		smallRNA	chr12:48960738-489
ENSG0000(	654	14.60548	chr12:171	KRT83		protein_c	chr12:52314301-523
ENSG0000(	654	14.60548	chr12:171	OR7E47P		Pseudoger	chr12:52107278-521
ENSG0000(	654	14.60548	chr12:171	CCDC184		protein_c	chr12:48183644-481
ENSG0000(	654	14.60548	chr12:171	ENSG00000271018		Pseudoger	chr12:50756892-507
ENSG0000(	654	14.60548	chr12:171	ENSG00000290967		lncRNA	chr12:52344219-523
ENSG0000(	654	14.60548	chr12:171	KRT87P		lncRNA	chr12:52250544-522
ENSG0000(	654	14.60548	chr12:171	OR7E47P		lncRNA	chr12:52084744-521
ENSG0000(	654	14.60548	chr12:171	OR11M1P		Pseudoger	chr12:48627879-486
ENSG0000(	654	14.60548	chr12:171	DAZAP2		protein_c	chr12:51238724-512
ENSG0000(	654	14.60548	chr12:171	TMPRSS12		protein_c	chr12:50842920-508
ENSG0000(	654	14.60548	chr12:171	ENSG00000258051		Pseudoger	chr12:48160245-481
ENSG0000(	654	14.60548	chr12:171	MIR4701		smallRNA	chr12:48771975-487
ENSG0000(	654	14.60548	chr12:171	ENSG00000257500		lncRNA	chr12:52407580-524
ENSG0000(	654	14.60548	chr12:171	ENSG00000280054		TEC	chr12:47728151-477
ENSG0000(	654	14.60548	chr12:171	ASB8		protein_c	chr12:48147789-481
ENSG0000(	654	14.60548	chr12:171	ENSG00000257807		Pseudoger	chr12:47484689-474
ENSG0000(	654	14.60548	chr12:171	RPL35AP28		Pseudoger	chr12:49863173-498
ENSG0000(	654	14.60548	chr12:171	Y_RNA		smallRNA	chr12:48809911-488
ENSG0000(	654	14.60548	chr12:171	OR5BJ1P		Pseudoger	chr12:48394510-483
ENSG0000(	654	14.60548	chr12:171	HIGD1C		protein_c	chr12:50952538-509
ENSG0000(	654	14.60548	chr12:171	ENSG00000277173		lncRNA	chr12:47768529-477
ENSG0000(	654	14.60548	chr12:171	BCDIN3D-AS1		lncRNA	chr12:49827913-498
ENSG0000(	654	14.60548	chr12:171	OR5BS1P		protein_c	chr12:48559882-485
ENSG0000(	654	14.60548	chr12:171	LINC02395		lncRNA	chr12:49900311-499

ENSG0000(	654	14.60548	chr12:171	ENSG00000273765		lncRNA	chr12:48360920-483
ENSG0000(	654	14.60548	chr12:171	ATF1	NCGv7;AC	protein_c	chr12:50763710-508
ENSG0000(	654	14.60548	chr12:171	SPATS2		protein_c	chr12:49366584-495
ENSG0000(	654	14.60548	chr12:171	ENSG00000285102		Pseudoger	chr12:52275821-522
ENSG0000(	654	14.60548	chr12:171	NR4A1		protein_c	chr12:52022832-520
ENSG0000(	654	14.60548	chr12:171	SNORA2B		smallRNA	chr12:48667457-486
ENSG0000(	654	14.60548	chr12:171	ATG101		protein_c	chr12:52069246-520
ENSG0000(	654	14.60548	chr12:171	TUBA1B		protein_c	chr12:49127782-491
ENSG0000(	654	14.60548	chr12:171	OR5BK1P		Pseudoger	chr12:48355792-483
ENSG0000(	654	14.60548	chr12:171	SLC4A8-AS1		lncRNA	chr12:51421956-514
ENSG0000(	654	14.60548	chr12:171	TROAP-AS1		lncRNA	chr12:49292631-493
ENSG0000(	654	14.60548	chr12:171	ZNF641		protein_c	chr12:48337180-483
ENSG0000(	654	14.60548	chr12:171	LALBA		protein_c	chr12:48567684-485
ENSG0000(	654	14.60548	chr12:171	CACNB3		protein_c	chr12:48813794-488
ENSG0000(	654	14.60548	chr12:171	ARF3		protein_c	chr12:48935723-489
ENSG0000(	654	14.60548	chr12:171	RPAP3		protein_c	chr12:47661249-477
ENSG0000(	654	14.60548	chr12:171	ENSG00000276454		lncRNA	chr12:47265665-472
ENSG0000(	654	14.60548	chr12:171	ENSG00000258101		lncRNA	chr12:49232790-492
ENSG0000(	654	14.60548	chr12:171	FKBP11		protein_c	chr12:48921518-489
ENSG0000(	654	14.60548	chr12:171	ADCY6-DT		lncRNA	chr12:48789147-487
ENSG0000(	654	14.60548	chr12:171	ENSG00000257663		lncRNA	chr12:52076841-520
ENSG0000(	654	14.60548	chr12:171	PHB1P19		Pseudoger	chr12:51124099-511
ENSG0000(	654	14.60548	chr12:171	ENSG00000278842		Pseudoger	chr12:50286244-502
ENSG0000(	654	14.60548	chr12:171	TMEM106C		protein_c	chr12:47963569-479
ENSG0000(	654	14.60548	chr12:171	ENSG00000271547		Pseudoger	chr12:48800576-488
ENSG0000(	654	14.60548	chr12:171	ENSG00000205537		lncRNA	chr12:47882649-479
ENSG0000(	654	14.60548	chr12:171	KRT7-AS		lncRNA	chr12:52245048-522
ENSG0000(	654	14.60548	chr12:171	KRT128P		Pseudoger	chr12:52630339-526
ENSG0000(	654	14.60548	chr12:171	KRT81		protein_c	chr12:52285913-522
ENSG0000(	654	14.60548	chr12:171	ENSG00000271490		Pseudoger	chr12:50806335-508
ENSG0000(	654	14.60548	chr12:171	ENSG00000287051		lncRNA	chr12:52298856-523
ENSG0000(	654	14.60548	chr12:171	PPIAP45		Pseudoger	chr12:47341614-473
ENSG0000(	654	14.60548	chr12:171	ENSG00000257653		lncRNA	chr12:48766194-487
ENSG0000(	654	14.60548	chr12:171	METTL7AP1		Pseudoger	chr12:52229093-522
ENSG0000(	654	14.60548	chr12:171	OR8T1P		Pseudoger	chr12:48442030-484
ENSG0000(	654	14.60548	chr12:171	ENSG00000257256		lncRNA	chr12:50219604-502
ENSG0000(	654	14.60548	chr12:171	ENSG00000289695		protein_c	chr12:50924969-509
ENSG0000(	654	14.60548	chr12:171	MIR4494		smallRNA	chr12:47364186-473
ENSG0000(	654	14.60548	chr12:171	HIGD1AP9		Pseudoger	chr12:49619334-496
ENSG0000(	654	14.60548	chr12:171	ENSG00000276691		lncRNA	chr12:47788426-477
ENSG0000(	654	14.60548	chr12:171	OR8S21P		Pseudoger	chr12:48417150-484
ENSG0000(	654	14.60548	chr12:171	ENSG00000291295		lncRNA	chr12:49293252-492
ENSG0000(	654	14.60548	chr12:171	SNORA2A		smallRNA	chr12:48656648-486
ENSG0000(	654	14.60548	chr12:171	ENSG00000226138		Pseudoger	chr12:48085062-480
ENSG0000(	654	14.60548	chr12:171	ENSG00000244266		Pseudoger	chr12:50365652-503
ENSG0000(	654	14.60548	chr12:171	ENSG00000257253		lncRNA	chr12:49861207-498
ENSG0000(	654	14.60548	chr12:171	KRT6A		protein_c	chr12:52487176-524
ENSG0000(	654	14.60548	chr12:171	ENSG00000258121		lncRNA	chr12:48483287-485
ENSG0000(	654	14.60548	chr12:171	AQP5-AS1		lncRNA	chr12:49951512-499
ENSG0000(	654	14.60548	chr12:171	ENSG00000257570		Pseudoger	chr12:49709237-497
ENSG0000(	654	14.60548	chr12:171	KMT2D	NCGv7	protein_c	chr12:49018975-490

ENSG0000(	654	14.60548	chr12:171	RHEBL1		protein_c	chr12:49064676-490
ENSG0000(	654	14.60548	chr12:171	TUBA1A		protein_c	chr12:49184686-491
ENSG0000(	654	14.60548	chr12:171	TUBA1C	NCGv7	protein_c	chr12:49188736-492
ENSG0000(	654	14.60548	chr12:171	NCKAP5L		protein_c	chr12:49791146-498
ENSG0000(	654	14.60548	chr12:171	AQP2		protein_c	chr12:49950737-499
ENSG0000(	654	14.60548	chr12:171	WNT10B	AC	protein_c	chr12:48965340-489
ENSG0000(	654	14.60548	chr12:171	GPD1		protein_c	chr12:50103982-501
ENSG0000(	654	14.60548	chr12:171	ANKRD33		protein_c	chr12:51888009-518
ENSG0000(	654	14.60548	chr12:171	ENSG00000268069		lncRNA	chr12:47784923-477
ENSG0000(	654	14.60548	chr12:171	ENSG00000257700		lncRNA	chr12:52692605-526
ENSG0000(	654	14.60548	chr12:171	PARK7P1		Pseudoger	chr12:49595148-495
ENSG0000(	654	14.60548	chr12:171	CCNT1		protein_c	chr12:48688458-487
ENSG0000(	654	14.60548	chr12:171	AC078864.1		smallRNA	chr12:52110634-521
ENSG0000(	654	14.60548	chr12:171	KRT80		protein_c	chr12:52168996-521
ENSG0000(	654	14.60548	chr12:171	RN7SL519P		smallRNA	chr12:50841500-508
ENSG0000(	654	14.60548	chr12:171	IFITM3P6		Pseudoger	chr12:47135498-471
ENSG0000(	654	14.60548	chr12:171	KRT1		protein_c	chr12:52674736-526
ENSG0000(	654	14.60548	chr12:171	PCED1B-AS1		lncRNA	chr12:47205893-472
ENSG0000(	654	14.60548	chr12:171	MIR1293		smallRNA	chr12:50234142-502
ENSG0000(	654	14.60548	chr12:171	KRT6B		protein_c	chr12:52446651-524
ENSG0000(	654	14.60548	chr12:171	ADI1P3		Pseudoger	chr12:47463087-474
ENSG0000(	654	14.60548	chr12:171	ENSG00000276814		lncRNA	chr12:48039784-480
ENSG0000(	654	14.60548	chr12:171	METTL7A	NCGv7	protein_c	chr12:50923472-509
ENSG0000(	654	14.60548	chr12:171	ENSG00000197376		Pseudoger	chr12:48507354-485
ENSG0000(	654	14.60548	chr12:171	POU6F1		protein_c	chr12:51186936-512
ENSG0000(	654	14.60548	chr12:171	ENSG00000271621		Pseudoger	chr12:50847925-508
ENSG0000(	654	14.60548	chr12:171	ENSG00000276390		lncRNA	chr12:47699401-476
ENSG0000(	654	14.60548	chr12:171	ENSG00000271596		Pseudoger	chr12:50949117-509
ENSG0000(	654	14.60548	chr12:171	SNORA34		smallRNA	chr12:48654382-486
ENSG0000(	654	14.60548	chr12:171	KRT73-AS1		lncRNA	chr12:52601467-526
ENSG0000(	654	14.60548	chr12:171	LINC02354		lncRNA	chr12:47826854-478
ENSG0000(	654	14.60548	chr12:171	HNRNPA3P10		Pseudoger	chr12:51712469-517
ENSG0000(	654	14.60548	chr12:171	MIR4698		smallRNA	chr12:47187812-471
ENSG0000(	654	14.60548	chr12:171	CCDC65		protein_c	chr12:48904110-489
ENSG0000(	654	14.60548	chr12:171	KRT88P		Pseudoger	chr12:52263948-522
ENSG0000(	654	14.60548	chr12:171	C1QL4		protein_c	chr12:49332409-493
ENSG0000(	654	14.60548	chr12:171	MCRS1	NCGv7	protein_c	chr12:49556544-495
ENSG0000(	654	14.60548	chr12:171	ENSG00000275228		lncRNA	chr12:48327942-483
ENSG0000(	654	14.60548	chr12:171	ENSG00000277672		Pseudoger	chr12:49075039-490
ENSG0000(	654	14.60548	chr12:171	RPL32P27		Pseudoger	chr12:48903418-489
ENSG0000(	654	14.60548	chr12:171	ENSG00000258232		lncRNA	chr12:49265156-492
ENSG0000(	654	14.60548	chr12:171	AMIGO2		protein_c	chr12:47075707-470
ENSG0000(	654	14.60548	chr12:171	SMARCD1	NCGv7	protein_c	chr12:50085200-501
ENSG0000(	654	14.60548	chr12:171	ENSG00000257830		lncRNA	chr12:52274647-522
ENSG0000(	654	14.60548	chr12:171	H1-7		protein_c	chr12:48328980-483
ENSG0000(	654	14.60548	chr12:171	ENSG00000258021		Pseudoger	chr12:51900565-519
ENSG0000(	654	14.60548	chr12:171	ENSG00000257829		lncRNA	chr12:52306616-523
ENSG0000(	654	14.60548	chr12:171	C12orf54		protein_c	chr12:48482498-484
ENSG0000(	654	14.60548	chr12:171	ENSG00000258253		lncRNA	chr12:52380460-524
ENSG0000(	654	14.60548	chr12:171	ENSG00000258283		lncRNA	chr12:48995150-489
ENSG0000(	654	14.60548	chr12:171	ENSG00000258017		lncRNA	chr12:49127782-491

ENSG0000(	654	14.60548	chr12:171	ENSG00000260473		lncRNA	chr12:51815043-518
ENSG0000(	654	14.60548	chr12:171	ENSG00000279875		TEC	chr12:48189349-481
ENSG0000(	654	14.60548	chr12:171	POLR2KP1		Pseudoger	chr12:49594091-495
ENSG0000(	654	14.60548	chr12:171	KRT2		protein_c	chr12:52644558-526
ENSG0000(	654	14.60548	chr12:171	ENSG00000258234		lncRNA	chr12:48231098-482
ENSG0000(	654	14.60548	chr12:171	ENSG00000279840		TEC	chr12:47960497-479
ENSG0000(	654	14.60548	chr12:171	ANP32D	AC	protein_c	chr12:48472559-484
ENSG0000(	654	14.60548	chr12:171	COL2A1	NCGv7	protein_c	chr12:47972967-480
ENSG0000(	654	14.60548	chr12:171	ENSG00000257848		Pseudoger	chr12:48171590-481
ENSG0000(	654	14.60548	chr12:171	KRT90P		Pseudoger	chr12:52411742-524
ENSG0000(	654	14.60548	chr12:171	OR10AD1		protein_c	chr12:48202339-482
ENSG0000(	654	14.60548	chr12:171	Y_RNA		smallRNA	chr12:48927939-489
ENSG0000(	654	14.60548	chr12:171	RND1	TAG	protein_c	chr12:48857145-488
ENSG0000(	654	14.60548	chr12:171	OR5BT1P		Pseudoger	chr12:48385417-483
ENSG0000(	654	14.60548	chr12:171	DIP2B		protein_c	chr12:50504985-507
ENSG0000(	654	14.60548	chr12:171	KANSL2		protein_c	chr12:48653211-486
ENSG0000(	654	14.60548	chr12:171	U6		smallRNA	chr12:51014134-510
ENSG0000(	654	14.60548	chr12:171	ENSG00000258273		Pseudoger	chr12:48333755-483
ENSG0000(	654	14.60548	chr12:171	BCDIN3D		protein_c	chr12:49836043-498
ENSG0000(	654	14.60548	chr12:171	LIMA1		protein_c	chr12:50175788-502
ENSG0000(	654	14.60548	chr12:171	LETMD1	AC	protein_c	chr12:51047962-510
ENSG0000(	654	14.60548	chr12:171	FIGNL2-DT		lncRNA	chr12:51848223-518
ENSG0000(	654	14.60548	chr12:171	ENSG00000274124		lncRNA	chr12:48152817-481
ENSG0000(	654	14.60548	chr12:171	LMBR1L		protein_c	chr12:49097136-491
ENSG0000(	654	14.60548	chr12:171	SLC4A8		protein_c	chr12:51391317-515
ENSG0000(	654	14.60548	chr12:171	CERS5		protein_c	chr12:50129289-501
ENSG0000(	654	14.60548	chr12:171	NR4A1AS		lncRNA	chr12:52058459-520
ENSG0000(	654	14.60548	chr12:171	CELA1	NCGv7	protein_c	chr12:51328442-513
ENSG0000(	654	14.60548	chr12:171	GALNT6		protein_c	chr12:51351247-513
ENSG0000(	654	14.60548	chr12:171	SCN8A	DriverDB	protein_c	chr12:51590266-518
ENSG0000(	654	14.60548	chr12:171	FIGNL2		protein_c	chr12:51817899-518
ENSG0000(	654	14.60548	chr12:171	TMBIM6	NCGv7	protein_c	chr12:49707725-497
ENSG0000(	654	14.60548	chr12:171	ACVRL1		protein_c	chr12:51906908-519
ENSG0000(	654	14.60548	chr12:171	HDAC7	NCGv7	protein_c	chr12:47782722-478
ENSG0000(	654	14.60548	chr12:171	LINC00592		lncRNA	chr12:52164115-522
ENSG0000(	654	14.60548	chr12:171	RPL35AP29		Pseudoger	chr12:51106817-511
ENSG0000(	653	14.58315	chr3:3804	ENSG00000291042		lncRNA	chr3:133661926-133
ENSG0000(	653	14.58315	chr3:3804	CEP63		protein_c	chr3:134485699-134
ENSG0000(	653	14.58315	chr4:4108	RPL6P14		Pseudoger	chr4:104886118-104
ENSG0000(	653	14.58315	chr3:3804	RPL39P5		Pseudoger	chr3:134351852-134
ENSG0000(	653	14.58315	chr3:3804	RYK	TAG	protein_c	chr3:134065303-134
ENSG0000(	653	14.58315	chr3:3804	SRPRB		protein_c	chr3:133784023-133
ENSG0000(	653	14.58315	chr3:3804	RNU6-678P		smallRNA	chr3:133664935-133
ENSG0000(	653	14.58315	chr3:3804	RNU6-1174P		smallRNA	chr3:134780527-134
ENSG0000(	653	14.58315	chr3:3804	ENSG00000286982		lncRNA	chr3:134774543-134
ENSG0000(	653	14.58315	chr3:3804	ANAPC13		protein_c	chr3:134477706-134
ENSG0000(	653	14.58315	chr3:3804	ENSG00000260633		lncRNA	chr3:134347288-134
ENSG0000(	653	14.58315	chr3:3804	TOPBP1		protein_c	chr3:133598175-133
ENSG0000(	653	14.58315	chr3:3804	LINC02004		lncRNA	chr3:134313498-134
ENSG0000(	653	14.58315	chr3:3804	ENSG00000249691		Pseudoger	chr3:134510531-134
ENSG0000(	653	14.58315	chr3:3804	RAB6B	DriverDB	protein_c	chr3:133824235-133

ENSG0000(	653	14.58315	chr3:380⁴	INHCAP		Pseudoger	chr3:133688192-133
ENSG0000(	653	14.58315	chr3:380⁴	RNA5SP140		Pseudoger	chr3:133710076-133
ENSG0000(	653	14.58315	chr3:380⁴	RNA5SP141		Pseudoger	chr3:134783436-134
ENSG0000(	653	14.58315	chr3:380⁴	KY		protein_c	chr3:134599923-134
ENSG0000(	653	14.58315	chr3:380⁴	SLCO2A1		protein_c	chr3:133932701-134
ENSG0000(	653	14.58315	chr3:380⁴	MIR4788		smallRNA	chr3:134437827-134
ENSG0000(	653	14.58315	chr3:380⁴	C3orf36		lncRNA	chr3:133928145-133
ENSG0000(	653	14.58315	chr3:380⁴	ENSG00000244062		Pseudoger	chr3:133760300-133
ENSG0000(	653	14.58315	chr3:380⁴	LINC02000		lncRNA	chr3:134055256-134
ENSG0000(	653	14.58315	chr3:380⁴	AMOTL2		protein_c	chr3:134355347-134
ENSG0000(	653	14.58315	chr3:380⁴	AC010207.1		smallRNA	chr3:134410044-134
ENSG0000(	653	14.58315	chr3:380⁴	ENSG00000288700		lncRNA	chr3:134485721-134
ENSG0000(	653	14.58315	chr3:380⁴	HMGN1P9		Pseudoger	chr3:134385197-134
ENSG0000(	653	14.58315	chr3:380⁴	TF		protein_c	chr3:133746040-133
ENSG0000(	653	14.58315	chr3:380⁴	ENSG00000285908		lncRNA	chr3:133799440-133
ENSG0000(	653	14.58315	chr3:380⁴	HMGB3P14		Pseudoger	chr3:134170487-134
ENSG0000(	653	14.58315	chr3:380⁴	HMGB3P13		Pseudoger	chr3:134437605-134
ENSG0000(	652	14.56082	chr5:164(	AC034236.2		smallRNA	chr5:116030018-116
ENSG0000(	646	14.42682	chr19:60(	GAPDHP38		Pseudoger	chr19:46558641-465
ENSG0000(	640	14.29283	chr12:171	ENSG00000258133		Pseudoger	chr12:58394596-583
ENSG0000(	636	14.2035	chr3:380⁴	LAPTM4BP2		Pseudoger	chr3:72884234-7288
ENSG0000(	632	14.11417	chr6:103(	GTPBP2	DriverDB\	protein_c	chr6:43605316-4362
ENSG0000(	632	14.11417	chr6:103(	RSPH9	DriverDB\	protein_c	chr6:43645036-4367
ENSG0000(	632	14.11417	chr6:103(	ENSG00000220614		Pseudoger	chr6:43328134-4332
ENSG0000(	632	14.11417	chr6:103(	ENSG00000272114		lncRNA	chr6:43770429-4377
ENSG0000(	632	14.11417	chr6:103(	LINC01512		lncRNA	chr6:43891045-4393
ENSG0000(	632	14.11417	chr6:103(	RPL12P47		Pseudoger	chr6:43310231-4331
ENSG0000(	632	14.11417	chr6:103(	AL109615.1		smallRNA	chr6:44013001-4401
ENSG0000(	632	14.11417	chr6:103(	ENSG00000271754		lncRNA	chr6:43519180-4351
ENSG0000(	632	14.11417	chr6:103(	VEGFA	DriverDB\	protein_c	chr6:43770184-4378
ENSG0000(	632	14.11417	chr6:103(	SLC35B2		protein_c	chr6:44254096-4425
ENSG0000(	632	14.11417	chr6:103(	RPS2P28		Pseudoger	chr6:43363479-4336
ENSG0000(	632	14.11417	chr6:103(	TCTE1		protein_c	chr6:44278734-4429
ENSG0000(	632	14.11417	chr6:103(	ENSG00000289609		lncRNA	chr6:43990798-4399
ENSG0000(	632	14.11417	chr6:103(	TTBK1		protein_c	chr6:43243481-4328
ENSG0000(	632	14.11417	chr6:103(	MYMX		protein_c	chr6:44216926-4421
ENSG0000(	632	14.11417	chr6:103(	SCIRT		lncRNA	chr6:43931572-4407
ENSG0000(	632	14.11417	chr6:103(	ENSG00000287055		lncRNA	chr6:43370026-4342
ENSG0000(	632	14.11417	chr6:103(	ENSG00000226558		Pseudoger	chr6:43364220-4336
ENSG0000(	632	14.11417	chr6:103(	SLC29A1	DriverDB\	protein_c	chr6:44219553-4423
ENSG0000(	632	14.11417	chr6:103(	CRIP3		protein_c	chr6:43299710-4330
ENSG0000(	632	14.11417	chr6:103(	MRPS18A	DriverDB\	protein_c	chr6:43671202-4368
ENSG0000(	632	14.11417	chr6:103(	DNPH1	DriverDB\	protein_c	chr6:43225629-4322
ENSG0000(	632	14.11417	chr6:103(	ENSG00000219470		Pseudoger	chr6:43538822-4353
ENSG0000(	632	14.11417	chr6:103(	MRPL14	DriverDB\	protein_c	chr6:44113451-4412
ENSG0000(	632	14.11417	chr6:103(	CUL9	NCGv7	protein_c	chr6:43182184-4322
ENSG0000(	632	14.11417	chr6:103(	SRF		protein_c	chr6:43171269-4318
ENSG0000(	632	14.11417	chr6:103(	CDC5L	NCGv7;AC	protein_c	chr6:44387706-4445
ENSG0000(	632	14.11417	chr6:103(	PTK7	DriverDB\	protein_c	chr6:43076307-4316
ENSG0000(	632	14.11417	chr6:103(	ENSG00000287266		lncRNA	chr6:43403815-4340
ENSG0000(	632	14.11417	chr6:103(	KLC4-AS1		lncRNA	chr6:43074331-4307

ENSG0000(	632	14.11417	chr6:1039	HSP90AB1	NCGv7	protein_c	chr6:44246166-4425
ENSG0000(	632	14.11417	chr6:1039	MIR4642		smallRNA	chr6:44435641-4443
ENSG0000(	632	14.11417	chr6:1039	NFKBIE	NCGv7	protein_c	chr6:44258166-4426
ENSG0000(	632	14.11417	chr6:1039	TJAP1	DriverDBv	protein_c	chr6:43477523-4350
ENSG0000(	632	14.11417	chr6:1039	MAD2L1BP	DriverDBv	protein_c	chr6:43629540-4364
ENSG0000(	632	14.11417	chr6:1039	POLH-AS1		lncRNA	chr6:43588230-4359
ENSG0000(	632	14.11417	chr6:1039	ENSG00000279076		TEC	chr6:44551577-4455
ENSG0000(	632	14.11417	chr6:1039	YIPF3	DriverDBv	protein_c	chr6:43511832-4351
ENSG0000(	632	14.11417	chr6:1039	SPATS1		protein_c	chr6:44342650-4438
ENSG0000(	632	14.11417	chr6:1039	ENSG00000236961		lncRNA	chr6:43722786-4373
ENSG0000(	632	14.11417	chr6:1039	ENSG00000272442		protein_c	chr6:44273194-4437
ENSG0000(	632	14.11417	chr6:1039	ENSG00000231881		lncRNA	chr6:44058792-4408
ENSG0000(	632	14.11417	chr6:1039	ENSG00000237530		lncRNA	chr6:44513262-4452
ENSG0000(	632	14.11417	chr6:1039	SCARNA15		smallRNA	chr6:43544144-4354
ENSG0000(	632	14.11417	chr6:1039	ENSG00000223469		lncRNA	chr6:43851757-4385
ENSG0000(	632	14.11417	chr6:1039	AARS2	DriverDBv	protein_c	chr6:44298731-4431
ENSG0000(	632	14.11417	chr6:1039	ABCC10		protein_c	chr6:43427366-4345
ENSG0000(	632	14.11417	chr6:1039	CAPN11		protein_c	chr6:44158811-4418
ENSG0000(	632	14.11417	chr6:1039	XPO5		protein_c	chr6:43522334-4357
ENSG0000(	632	14.11417	chr6:1039	SLC22A7		protein_c	chr6:43295694-4330
ENSG0000(	632	14.11417	chr6:1039	POLH	DriverDBv	protein_c	chr6:43576185-4362
ENSG0000(	632	14.11417	chr6:1039	POLR1C	DriverDBv	protein_c	chr6:43509702-4356
ENSG0000(	632	14.11417	chr6:1039	TMEM63B		protein_c	chr6:44126914-4415
ENSG0000(	632	14.11417	chr6:1039	ENSG00000283573		lncRNA	chr6:43803193-4384
ENSG0000(	632	14.11417	chr6:1039	ENSG00000183239		Pseudoger	chr6:44089242-4408
ENSG0000(	632	14.11417	chr6:1039	LINC02537		lncRNA	chr6:43844878-4385
ENSG0000(	632	14.11417	chr6:1039	ENSG00000218107		Pseudoger	chr6:43705949-4370
ENSG0000(	632	14.11417	chr6:1039	MIR4647		smallRNA	chr6:44254206-4425
ENSG0000(	632	14.11417	chr6:1039	TMEM151B		protein_c	chr6:44270450-4430
ENSG0000(	632	14.11417	chr6:1039	ENSG00000287562		lncRNA	chr6:44090787-4409
ENSG0000(	632	14.11417	chr6:1039	DLK2		protein_c	chr6:43450352-4345
ENSG0000(	632	14.11417	chr6:1039	LRRC73		protein_c	chr6:43506968-4351
ENSG0000(	632	14.11417	chr6:1039	RNU6-1113P		smallRNA	chr6:43474186-4347
ENSG0000(	632	14.11417	chr6:1039	C6orf223		lncRNA	chr6:44000580-4400
ENSG0000(	632	14.11417	chr6:1039	ZNF318		protein_c	chr6:43307134-4336
ENSG0000(	632	14.11417	chr6:1039	ENSG00000245261		lncRNA	chr6:43213801-4322
ENSG0000(	631	14.09183	chr12:171	MIRLET7I		smallRNA	chr12:62603686-626
ENSG0000(	627	14.0025	chr12:171	ENSG00000286351		lncRNA	chr12:59102144-591
ENSG0000(	627	14.0025	chr12:171	ENSG00000257308		Pseudoger	chr12:61231159-612
ENSG0000(	627	14.0025	chr12:171	ENSG00000258214		lncRNA	chr12:59366218-594
ENSG0000(	627	14.0025	chr12:171	LRIG3-DT		lncRNA	chr12:58920639-590
ENSG0000(	627	14.0025	chr12:171	SLC16A7		protein_c	chr12:59596029-597
ENSG0000(	627	14.0025	chr12:171	LINC02448		lncRNA	chr12:59523278-595
ENSG0000(	627	14.0025	chr12:171	Y_RNA		smallRNA	chr12:60341824-603
ENSG0000(	627	14.0025	chr12:171	RNU6-279P		smallRNA	chr12:59369051-593
ENSG0000(	627	14.0025	chr12:171	ENSG00000257288		Pseudoger	chr12:59052980-590
ENSG0000(	627	14.0025	chr12:171	ENSG00000257865		Pseudoger	chr12:60418983-604
ENSG0000(	627	14.0025	chr12:171	AC108721.2		smallRNA	chr12:59528059-595
ENSG0000(	627	14.0025	chr12:171	LRIG3	NCGv7;AC	protein_c	chr12:58872149-589
ENSG0000(	627	14.0025	chr12:171	ENSG00000257146		Pseudoger	chr12:59719698-597
ENSG0000(	627	14.0025	chr12:171	RPS6P22		Pseudoger	chr12:59020169-590

ENSG0000(	627	14.0025	chr12:171	ENSG00000258184		Pseudoger	chr12:60215994-602
ENSG0000(	627	14.0025	chr12:171	ENSG00000287014		lncRNA	chr12:60150187-602
ENSG0000(	627	14.0025	chr12:171	RNU4-20P		smallRNA	chr12:59586793-595
ENSG0000(	627	14.0025	chr12:171	ENSG00000237176		Pseudoger	chr12:59812102-598
ENSG0000(	627	14.0025	chr12:171	RNU6-871P		smallRNA	chr12:59450673-594
ENSG0000(	627	14.0025	chr12:171	ENSG00000258231		lncRNA	chr12:58544124-588
ENSG0000(	627	14.0025	chr12:171	ENSG00000257294		lncRNA	chr12:60092864-600
ENSG0000(	627	14.0025	chr12:171	AC091517.1		smallRNA	chr12:60026771-600
ENSG0000(	627	14.0025	chr12:171	LINC02388		lncRNA	chr12:58565959-587
ENSG0000(	627	14.0025	chr12:171	ENSG00000286279		lncRNA	chr12:59322745-594
ENSG0000(	627	14.0025	chr12:171	AC108721.1		smallRNA	chr12:59528177-595
ENSG0000(	627	14.0025	chr12:171	SNORA19		smallRNA	chr12:60363822-603
ENSG0000(	625	13.95784	chr10:81(	ENSG00000226426		lncRNA	chr10:64036345-640
ENSG0000(	615	13.73451	chr1:1142	ENSG00000271806		lncRNA	chr1:2141084-21452
ENSG0000(	615	13.73451	chr1:1142	FAAP20		protein_c	chr1:2184461-22127
ENSG0000(	615	13.73451	chr1:1142	PRKCZ-AS1		lncRNA	chr1:2181794-21843
ENSG0000(	614	13.71218	chr4:4108	SNORA3		smallRNA	chr4:73263960-7326
ENSG0000(	610	13.62285	chr2:3094	AC011994.1		smallRNA	chr2:11607404-1160
ENSG0000(	610	13.62285	chr2:3094	ENSG00000145063		lncRNA	chr2:11105317-1113
ENSG0000(	610	13.62285	chr2:3094	RPL6P4		Pseudoger	chr2:11102142-1110
ENSG0000(	610	13.62285	chr2:3094	ENSG00000232056		lncRNA	chr2:10847577-1085
ENSG0000(	610	13.62285	chr2:3094	SNORA80B		smallRNA	chr2:10446714-1044
ENSG0000(	610	13.62285	chr2:3094	MIR3681		smallRNA	chr2:12199130-1219
ENSG0000(	610	13.62285	chr2:3094	ENSG00000261117		lncRNA	chr2:12715415-1271
ENSG0000(	610	13.62285	chr2:3094	MIR4429		smallRNA	chr2:11540605-1154
ENSG0000(	610	13.62285	chr2:3094	SNORD18		smallRNA	chr2:12030303-1203
ENSG0000(	610	13.62285	chr2:3094	ENSG00000272275		lncRNA	chr2:10767875-1077
ENSG0000(	610	13.62285	chr2:3094	E2F6		protein_c	chr2:11444375-1146
ENSG0000(	610	13.62285	chr2:3094	ENSG00000230790		lncRNA	chr2:11740997-1174
ENSG0000(	610	13.62285	chr2:3094	LINC00570		lncRNA	chr2:11372612-1140
ENSG0000(	610	13.62285	chr2:3094	ENSG00000276411		lncRNA	chr2:11673964-1167
ENSG0000(	610	13.62285	chr2:3094	RN7SL832P		lncRNA	chr2:10690344-1069
ENSG0000(	610	13.62285	chr2:3094	LINC01954		lncRNA	chr2:10844224-1088
ENSG0000(	610	13.62285	chr2:3094	NTSR2		protein_c	chr2:11658178-1167
ENSG0000(	610	13.62285	chr2:3094	ENSG00000228496		lncRNA	chr2:11681434-1168
ENSG0000(	610	13.62285	chr2:3094	LINC03037		lncRNA	chr2:11357515-1136
ENSG0000(	610	13.62285	chr2:3094	ENSG00000236162		lncRNA	chr2:10717773-1072
ENSG0000(	610	13.62285	chr2:3094	CDK8P1		Pseudoger	chr2:11665369-1166
ENSG0000(	610	13.62285	chr2:3094	GREB1		protein_c	chr2:11482341-1164
ENSG0000(	610	13.62285	chr2:3094	ROCK2		protein_c	chr2:11179759-1134
ENSG0000(	610	13.62285	chr2:3094	LPIN1		protein_c	chr2:11677595-1182
ENSG0000(	610	13.62285	chr2:3094	ENSG00000290030		lncRNA	chr2:12276707-1227
ENSG0000(	610	13.62285	chr2:3094	RNU7-138P		smallRNA	chr2:10744186-1074
ENSG0000(	610	13.62285	chr2:3094	MIR3681HG		lncRNA	chr2:11833926-1270
ENSG0000(	610	13.62285	chr2:3094	ODC1	NCGv7	protein_c	chr2:10439968-1044
ENSG0000(	610	13.62285	chr2:3094	ENSG00000285569		lncRNA	chr2:11405508-1142
ENSG0000(	610	13.62285	chr2:3094	ENSG00000270488		Pseudoger	chr2:10874291-1087
ENSG0000(	610	13.62285	chr2:3094	AIDAP1		Pseudoger	chr2:11308025-1130
ENSG0000(	610	13.62285	chr2:3094	RNA5SP84		Pseudoger	chr2:11517397-1151
ENSG0000(	610	13.62285	chr2:3094	ENSG00000223360		lncRNA	chr2:12598987-1260
ENSG0000(	610	13.62285	chr2:3094	SLC66A3		protein_c	chr2:11155198-1117

ENSG0000(	610	13.62285	chr2:309<	RNU6-843P		smallRNA	chr2:12411398-1241
ENSG0000(	610	13.62285	chr2:309<	RNU2-13P		smallRNA	chr2:11561194-1156
ENSG0000(	610	13.62285	chr2:309<	RN7SL674P		smallRNA	chr2:11584773-1158
ENSG0000(	610	13.62285	chr2:309<	MIR3125		smallRNA	chr2:12737367-1273
ENSG0000(	610	13.62285	chr2:309<	C2orf50		protein_c	chr2:11133128-1115
ENSG0000(	610	13.62285	chr2:309<	MIR548S		smallRNA	chr2:11767444-1176
ENSG0000(	610	13.62285	chr2:309<	ENSG00000286427		lncRNA	chr2:12702764-1271
ENSG0000(	610	13.62285	chr2:309<	RNU7-176P		smallRNA	chr2:10815485-1081
ENSG0000(	610	13.62285	chr2:309<	ODC1-DT		lncRNA	chr2:10448654-1045
ENSG0000(	610	13.62285	chr2:309<	ENSG00000231403		lncRNA	chr2:11388023-1139
ENSG0000(	610	13.62285	chr2:309<	KCNF1		protein_c	chr2:10911934-1091
ENSG0000(	610	13.62285	chr2:309<	ENSG00000234818		lncRNA	chr2:10589166-1060
ENSG0000(	610	13.62285	chr2:309<	NOL10		protein_c	chr2:10562347-1068
ENSG0000(	610	13.62285	chr2:309<	MIR4262		smallRNA	chr2:11836933-1183
ENSG0000(	610	13.62285	chr2:309<	RNA5SP85		smallRNA	chr2:11561661-1156
ENSG0000(	610	13.62285	chr2:309<	ENSG00000203643		lncRNA	chr2:11721619-1172
ENSG0000(	610	13.62285	chr2:309<	ENSG00000289856		lncRNA	chr2:12189604-1219
ENSG0000(	610	13.62285	chr2:309<	TRIB2	AC	protein_c	chr2:12716910-1274
ENSG0000(	610	13.62285	chr2:309<	PPIAP60		Pseudoger	chr2:11351627-1135
ENSG0000(	610	13.62285	chr2:309<	ATP6V1C2		protein_c	chr2:10721100-1078
ENSG0000(	610	13.62285	chr2:309<	PDIA6		protein_c	chr2:10783391-1083
ENSG0000(	610	13.62285	chr2:309<	ENSG00000225649		lncRNA	chr2:12780593-1300
ENSG0000(	610	13.62285	chr2:309<	RNU6-1081P		smallRNA	chr2:11233988-1123
ENSG0000(	610	13.62285	chr2:309<	ENSG00000285876		lncRNA	chr2:13000953-1333
ENSG0000(	609	13.60052	chr12:171	SNORA74		smallRNA	chr12:42347061-423
ENSG0000(	608	13.57819	chr3:380<	ENSG00000279328		TEC	chr3:126432796-126
ENSG0000(	608	13.57819	chr3:380<	ENSG00000242816		lncRNA	chr3:117719859-117
ENSG0000(	608	13.57819	chr3:380<	POGLUT1	NCGv7	protein_c	chr3:119468963-119
ENSG0000(	608	13.57819	chr3:380<	AC023593.1		smallRNA	chr3:127664704-127
ENSG0000(	608	13.57819	chr3:380<	ENSG00000288868		lncRNA	chr3:121749202-121
ENSG0000(	608	13.57819	chr3:380<	RPS26P21		Pseudoger	chr3:119298665-119
ENSG0000(	608	13.57819	chr3:380<	RNU2-37P		smallRNA	chr3:128075073-128
ENSG0000(	608	13.57819	chr3:380<	KLF15		protein_c	chr3:126342635-126
ENSG0000(	608	13.57819	chr3:380<	B4GALT4-AS1		lncRNA	chr3:119226486-119
ENSG0000(	608	13.57819	chr3:380<	TPRA1		protein_c	chr3:127571232-127
ENSG0000(	608	13.57819	chr3:380<	ZNF148	IntOGen-I	protein_c	chr3:125225669-125
ENSG0000(	608	13.57819	chr3:380<	MYLK-AS1		lncRNA	chr3:123585143-123
ENSG0000(	608	13.57819	chr6:1391	ENSG00000237593		Pseudoger	chr6:154216931-154
ENSG0000(	608	13.57819	chr3:380<	MIR548I1		smallRNA	chr3:125790404-125
ENSG0000(	608	13.57819	chr3:380<	MIR1280		smallRNA	chr3:128362165-128
ENSG0000(	608	13.57819	chr3:380<	ENSG00000243016		Pseudoger	chr3:127221194-127
ENSG0000(	608	13.57819	chr3:380<	TXNRD3		protein_c	chr3:126571779-126
ENSG0000(	608	13.57819	chr3:380<	ENSG00000239432		Pseudoger	chr3:125756086-125
ENSG0000(	608	13.57819	chr3:380<	ENSG00000289641		lncRNA	chr3:127227404-127
ENSG0000(	608	13.57819	chr3:380<	NAP1L1P3		Pseudoger	chr3:120805866-120
ENSG0000(	608	13.57819	chr3:380<	ENSG00000289134		lncRNA	chr3:119639782-119
ENSG0000(	608	13.57819	chr3:380<	ENSG00000272840		lncRNA	chr3:125774714-125
ENSG0000(	608	13.57819	chr3:380<	ENSG00000273454		lncRNA	chr3:123277353-123
ENSG0000(	608	13.57819	chr3:380<	SLC41A3-AS1		lncRNA	chr3:126083659-126
ENSG0000(	608	13.57819	chr3:380<	MTCO2P29		Pseudoger	chr3:120722458-120
ENSG0000(	608	13.57819	chr3:380<	SLC15A2	NCGv7	protein_c	chr3:121894401-121

ENSG00000	608	13.57819	chr3:3804	RNU4-62P		smallRNA	chr3:121655475-121
ENSG00000	608	13.57819	chr3:3804	ENSG00000240393		Pseudogen	chr3:117674342-117
ENSG00000	608	13.57819	chr3:3804	AC068754.1		smallRNA	chr3:122281853-122
ENSG00000	608	13.57819	chr3:3804	NUP210P1		Pseudogen	chr3:126660609-126
ENSG00000	608	13.57819	chr3:3804	LINC02035		lncRNA	chr3:122886941-122
ENSG00000	608	13.57819	chr3:3804	SNORD112		smallRNA	chr3:122245766-122
ENSG00000	608	13.57819	chr3:3804	HCLS1		protein_c	chr3:121631399-121
ENSG00000	608	13.57819	chr3:3804	RNA5SP137		Pseudogen	chr3:125058304-125
ENSG00000	608	13.57819	chr3:3804	ALDH1L1-AS1		lncRNA	chr3:126103640-126
ENSG00000	608	13.57819	chr3:3804	ENSG00000289324		lncRNA	chr3:125375372-125
ENSG00000	608	13.57819	chr6:1391	SCAF8		protein_c	chr6:154733378-154
ENSG00000	608	13.57819	chr3:3804	FSTL1		protein_c	chr3:120392293-120
ENSG00000	608	13.57819	chr3:3804	ENSG00000272967		lncRNA	chr3:119579212-119
ENSG00000	608	13.57819	chr3:3804	LRRC58		protein_c	chr3:120324509-120
ENSG00000	608	13.57819	chr3:3804	ENSG00000284660		Pseudogen	chr3:125766601-125
ENSG00000	608	13.57819	chr3:3804	HNRNPA1P23		Pseudogen	chr3:122317609-122
ENSG00000	608	13.57819	chr6:1391	ENSG00000231316		Pseudogen	chr6:154605616-154
ENSG00000	608	13.57819	chr3:3804	AC078794.1		smallRNA	chr3:122926996-122
ENSG00000	608	13.57819	chr3:3804	AC092902.1		smallRNA	chr3:125832429-125
ENSG00000	608	13.57819	chr3:3804	OR7E29P		Pseudogen	chr3:125712139-125
ENSG00000	608	13.57819	chr3:3804	ENSG00000243072		Pseudogen	chr3:125679573-125
ENSG00000	608	13.57819	chr3:3804	DTX3L		protein_c	chr3:122564338-122
ENSG00000	608	13.57819	chr3:3804	TEX55		protein_c	chr3:119146151-119
ENSG00000	608	13.57819	chr3:3804	ENSG00000290035		lncRNA	chr3:127218943-127
ENSG00000	608	13.57819	chr3:3804	ENSG00000239268		lncRNA	chr3:117672154-117
ENSG00000	608	13.57819	chr3:3804	SLC12A8		protein_c	chr3:125082636-125
ENSG00000	608	13.57819	chr3:3804	ENSG00000243276		lncRNA	chr3:118004819-118
ENSG00000	608	13.57819	chr3:3804	RNU6-230P		smallRNA	chr3:125119097-125
ENSG00000	608	13.57819	chr6:1391	MTRES1P1		Pseudogen	chr6:154549517-154
ENSG00000	608	13.57819	chr3:3804	CFAP100		protein_c	chr3:126394909-126
ENSG00000	608	13.57819	chr3:3804	FBXO40	NCGv7	protein_c	chr3:121593379-121
ENSG00000	608	13.57819	chr3:3804	WDR5B		protein_c	chr3:122411846-122
ENSG00000	608	13.57819	chr3:3804	OR7E97P		Pseudogen	chr3:125747084-125
ENSG00000	608	13.57819	chr3:3804	Y_RNA		smallRNA	chr3:125528244-125
ENSG00000	608	13.57819	chr3:3804	FAM162A		protein_c	chr3:122384161-122
ENSG00000	608	13.57819	chr3:3804	ENSG00000244441		Pseudogen	chr3:120512850-120
ENSG00000	608	13.57819	chr6:1391	ENSG00000288520		protein_c	chr6:154159828-154
ENSG00000	608	13.57819	chr3:3804	LINC02034		lncRNA	chr3:127537937-127
ENSG00000	608	13.57819	chr3:3804	RNA5SP139		Pseudogen	chr3:127963608-127
ENSG00000	608	13.57819	chr3:3804	OR7E130P		Pseudogen	chr3:125703349-125
ENSG00000	608	13.57819	chr3:3804	ENSG00000242103		Pseudogen	chr3:121356991-121
ENSG00000	608	13.57819	chr3:3804	ENSG00000243813		Pseudogen	chr3:121099108-121
ENSG00000	608	13.57819	chr3:3804	RNU6-1047P		smallRNA	chr3:127240968-127
ENSG00000	608	13.57819	chr3:3804	ENSG00000240774		Pseudogen	chr3:120484171-120
ENSG00000	608	13.57819	chr3:3804	ARHGAP31-AS1		lncRNA	chr3:119314293-119
ENSG00000	608	13.57819	chr3:3804	ENSG00000286660		lncRNA	chr3:118488876-118
ENSG00000	608	13.57819	chr3:3804	TIMMDC1		protein_c	chr3:119498547-119
ENSG00000	608	13.57819	chr3:3804	AF186996.1		smallRNA	chr3:125736216-125
ENSG00000	608	13.57819	chr3:3804	AC072031.1		smallRNA	chr3:121922098-121
ENSG00000	608	13.57819	chr3:3804	GOLGB1	NCGv7	protein_c	chr3:121663199-121
ENSG00000	608	13.57819	chr3:3804	IQCB1	NCGv7	protein_c	chr3:121769761-121

ENSG0000(	608	13.57819	chr3:3804	HGD		protein_c	chr3:120628172-120
ENSG0000(	608	13.57819	chr3:3804	CD86	NCGv7	protein_c	chr3:122055362-122
ENSG0000(	608	13.57819	chr3:3804	PARP15		protein_c	chr3:122577628-122
ENSG0000(	608	13.57819	chr3:3804	PARP14		protein_c	chr3:122680839-122
ENSG0000(	608	13.57819	chr3:3804	ADCY5	NCGv7	protein_c	chr3:123282296-123
ENSG0000(	608	13.57819	chr3:3804	ARGFX		protein_c	chr3:121567949-121
ENSG0000(	608	13.57819	chr3:3804	ENSG00000282950		lncRNA	chr3:120365993-120
ENSG0000(	608	13.57819	chr3:3804	ZXDC		protein_c	chr3:126437601-126
ENSG0000(	608	13.57819	chr3:3804	OR7E93P		Pseudoger	chr3:125724539-125
ENSG0000(	608	13.57819	chr6:1391	IPCEF1		protein_c	chr6:154154496-154
ENSG0000(	608	13.57819	chr3:3804	RPL7AP11		Pseudoger	chr3:121494110-121
ENSG0000(	608	13.57819	chr3:3804	MGLL		protein_c	chr3:127689062-128
ENSG0000(	608	13.57819	chr3:3804	snoU13		smallRNA	chr3:125446061-125
ENSG0000(	608	13.57819	chr3:3804	ENSG00000282860		lncRNA	chr3:127571232-127
ENSG0000(	608	13.57819	chr3:3804	ENSG00000248850		Pseudoger	chr3:126672106-126
ENSG0000(	608	13.57819	chr3:3804	MCM2	DriverDBv	protein_c	chr3:127598410-127
ENSG0000(	608	13.57819	chr6:1391	U8		smallRNA	chr6:154905081-154
ENSG0000(	608	13.57819	chr3:3804	U1		smallRNA	chr3:126160283-126
ENSG0000(	608	13.57819	chr3:3804	ENSG00000291096		lncRNA	chr3:125928689-125
ENSG0000(	608	13.57819	chr3:3804	ENSG00000287617		lncRNA	chr3:125799887-125
ENSG0000(	608	13.57819	chr3:3804	RNA5SP138		Pseudoger	chr3:126564565-126
ENSG0000(	608	13.57819	chr3:3804	LINC01471		lncRNA	chr3:127480690-127
ENSG0000(	608	13.57819	chr3:3804	LINC02024		lncRNA	chr3:117678693-117
ENSG0000(	608	13.57819	chr6:1391	CNKSR3		protein_c	chr6:154387515-154
ENSG0000(	608	13.57819	chr3:3804	GTF2E1		protein_c	chr3:120742637-120
ENSG0000(	608	13.57819	chr3:3804	ENSG00000285585		protein_c	chr3:119703076-119
ENSG0000(	608	13.57819	chr6:1391	ENSG00000218757		Pseudoger	chr6:154845074-154
ENSG0000(	608	13.57819	chr3:3804	HEG1		protein_c	chr3:124965710-125
ENSG0000(	608	13.57819	chr3:3804	MUC13		protein_c	chr3:124905442-124
ENSG0000(	608	13.57819	chr3:3804	ENSG00000248787		lncRNA	chr3:125907765-125
ENSG0000(	608	13.57819	chr3:3804	KPNA1		protein_c	chr3:122421902-122
ENSG0000(	608	13.57819	chr3:3804	ENSG00000240882		Pseudoger	chr3:120306726-120
ENSG0000(	608	13.57819	chr3:3804	RUVBL1-AS1		lncRNA	chr3:128075810-128
ENSG0000(	608	13.57819	chr3:3804	PODXL2	DriverDBv	protein_c	chr3:127629185-127
ENSG0000(	608	13.57819	chr3:3804	UPK1B		protein_c	chr3:119173517-119
ENSG0000(	608	13.57819	chr3:3804	ENSG00000280042		TEC	chr3:124791119-124
ENSG0000(	608	13.57819	chr3:3804	ENSG00000287784		lncRNA	chr3:127165506-127
ENSG0000(	608	13.57819	chr3:3804	CD80		protein_c	chr3:119524293-119
ENSG0000(	608	13.57819	chr3:3804	ENSG00000248607		lncRNA	chr3:126056923-126
ENSG0000(	608	13.57819	chr3:3804	B4GALT4	DriverDBv	protein_c	chr3:119211732-119
ENSG0000(	608	13.57819	chr6:1391	ENSG00000275419		Pseudoger	chr6:154542552-154
ENSG0000(	608	13.57819	chr3:3804	POPDC2		protein_c	chr3:119636457-119
ENSG0000(	608	13.57819	chr3:3804	CSTA		protein_c	chr3:122325248-122
ENSG0000(	608	13.57819	chr3:3804	SEC22A		protein_c	chr3:123201927-123
ENSG0000(	608	13.57819	chr3:3804	ENSG00000242001		Pseudoger	chr3:125681305-125
ENSG0000(	608	13.57819	chr3:3804	Y_RNA		smallRNA	chr3:122025195-122
ENSG0000(	608	13.57819	chr3:3804	RPL10P7		Pseudoger	chr3:119635526-119
ENSG0000(	608	13.57819	chr3:3804	ENSG00000288022		lncRNA	chr3:125061448-125
ENSG0000(	608	13.57819	chr3:3804	Y_RNA		smallRNA	chr3:125516979-125
ENSG0000(	608	13.57819	chr3:3804	ENSG00000285619		lncRNA	chr3:128181402-128
ENSG0000(	608	13.57819	chr3:3804	ENSG00000251448		lncRNA	chr3:126288123-126

ENSG00000	608	13.57819	chr3:3804	ENSG00000270782		Pseudoger	chr3:120924612-120
ENSG00000	608	13.57819	chr3:3804	ENSG00000239994		lncRNA	chr3:119744139-119
ENSG00000	608	13.57819	chr3:3804	SEC61A1		protein_c	chr3:128051641-128
ENSG00000	608	13.57819	chr3:3804	ABTB1		protein_c	chr3:127672935-127
ENSG00000	608	13.57819	chr3:3804	EIF4BP8		Pseudoger	chr3:122660613-122
ENSG00000	608	13.57819	chr3:3804	PLXNA1	DriverDBv	protein_c	chr3:126982693-127
ENSG00000	608	13.57819	chr3:3804	FAM86JP		Pseudoger	chr3:125916624-125
ENSG00000	608	13.57819	chr3:3804	FAM86JP		lncRNA	chr3:125916609-125
ENSG00000	608	13.57819	chr3:3804	ARHGAP31		protein_c	chr3:119294383-119
ENSG00000	608	13.57819	chr3:3804	COX17		protein_c	chr3:119654513-119
ENSG00000	608	13.57819	chr3:3804	ENSG00000248557		Pseudoger	chr3:126624792-126
ENSG00000	608	13.57819	chr3:3804	RPS3AP14		Pseudoger	chr3:125795106-125
ENSG00000	608	13.57819	chr3:3804	RNU6-1127P		smallRNA	chr3:119341834-119
ENSG00000	608	13.57819	chr3:3804	AF186996.2		smallRNA	chr3:125690256-125
ENSG00000	608	13.57819	chr3:3804	MYLK	NCGv7	protein_c	chr3:123610049-123
ENSG00000	608	13.57819	chr3:3804	ENSG00000285600		lncRNA	chr3:127620106-127
ENSG00000	608	13.57819	chr3:3804	NDUFB4		protein_c	chr3:120596328-120
ENSG00000	608	13.57819	chr3:3804	ROPN1B		protein_c	chr3:125969160-125
ENSG00000	608	13.57819	chr3:3804	CASR		protein_c	chr3:122183668-122
ENSG00000	608	13.57819	chr3:3804	KBTBD12		protein_c	chr3:127915232-127
ENSG00000	608	13.57819	chr3:3804	PDIA5		protein_c	chr3:123067025-123
ENSG00000	608	13.57819	chr3:3804	PARP9		protein_c	chr3:122527924-122
ENSG00000	608	13.57819	chr3:3804	SNX4		protein_c	chr3:125446650-125
ENSG00000	608	13.57819	chr3:3804	UROC1		protein_c	chr3:126481166-126
ENSG00000	608	13.57819	chr3:3804	ENSG00000275348		Pseudoger	chr3:122482990-122
ENSG00000	608	13.57819	chr3:3804	CHCHD6	DriverDBv	protein_c	chr3:126704240-126
ENSG00000	608	13.57819	chr3:3804	SLC41A3		protein_c	chr3:126006357-126
ENSG00000	608	13.57819	chr3:3804	ENSG00000287440		lncRNA	chr3:126312385-126
ENSG00000	608	13.57819	chr3:3804	LRRC58-DT		Pseudoger	chr3:120349449-120
ENSG00000	608	13.57819	chr6:1391	RNU6-824P		smallRNA	chr6:154825176-154
ENSG00000	608	13.57819	chr6:1391	OPRM1		protein_c	chr6:154010496-154
ENSG00000	608	13.57819	chr3:3804	RUVBL1		protein_c	chr3:128064778-128
ENSG00000	608	13.57819	chr6:1391	ENSG00000274451		Pseudoger	chr6:155004835-155
ENSG00000	608	13.57819	chr3:3804	MIX23		protein_c	chr3:122359591-122
ENSG00000	608	13.57819	chr3:3804	ENSG00000241546		Pseudoger	chr3:120041190-120
ENSG00000	608	13.57819	chr3:3804	RNU6-232P		smallRNA	chr3:125388934-125
ENSG00000	608	13.57819	chr3:3804	ILDR1	DriverDBv	protein_c	chr3:121987323-122
ENSG00000	608	13.57819	chr3:3804	EAF2	NCGv7	protein_c	chr3:121835183-121
ENSG00000	608	13.57819	chr3:3804	STXBP5L	DriverDBv	protein_c	chr3:120908072-121
ENSG00000	608	13.57819	chr3:3804	Y_RNA		smallRNA	chr3:127202372-127
ENSG00000	608	13.57819	chr3:3804	GPR156		protein_c	chr3:120164645-120
ENSG00000	608	13.57819	chr3:3804	ALG1L	DriverDBv	Pseudoger	chr3:125929272-125
ENSG00000	608	13.57819	chr3:3804	RN7SL172P		smallRNA	chr3:121653996-121
ENSG00000	608	13.57819	chr3:3804	ENSG00000287977		Pseudoger	chr3:122172405-122
ENSG00000	608	13.57819	chr3:3804	ENSG00000272678		lncRNA	chr3:123283593-123
ENSG00000	608	13.57819	chr6:1391	ENSG00000205625		Pseudoger	chr6:154706643-154
ENSG00000	608	13.57819	chr3:3804	OSBPL11		protein_c	chr3:125528858-125
ENSG00000	608	13.57819	chr3:3804	ALDH1L1		protein_c	chr3:126103562-126
ENSG00000	608	13.57819	chr3:3804	ENSG00000288667		lncRNA	chr3:119978153-119
ENSG00000	608	13.57819	chr3:3804	C3orf56		protein_c	chr3:127193131-127
ENSG00000	608	13.57819	chr3:3804	ENSG00000288662		lncRNA	chr3:119969044-120

ENSG0000(	608	13.57819	chr3:380∠	LINC02016		lncRNA	chr3:127322307-127
ENSG0000(	608	13.57819	chr3:380∠	ENSG00000250934		lncRNA	chr3:126266747-126
ENSG0000(	608	13.57819	chr3:380∠	ENSG00000287022		lncRNA	chr3:121394772-121
ENSG0000(	608	13.57819	chr3:380∠	AC117422.1		smallRNA	chr3:126069064-126
ENSG0000(	608	13.57819	chr3:380∠	GSK3B		protein_c	chr3:119821321-120
ENSG0000(	608	13.57819	chr3:380∠	ITGB5		protein_c	chr3:124761948-124
ENSG0000(	608	13.57819	chr3:380∠	ENSG00000286919		lncRNA	chr3:127274581-127
ENSG0000(	608	13.57819	chr3:380∠	ITGB5-AS1		lncRNA	chr3:124781155-124
ENSG0000(	608	13.57819	chr3:380∠	DNAJB6P7		Pseudoger	chr3:125344085-125
ENSG0000(	608	13.57819	chr3:380∠	TMEM39A		protein_c	chr3:119428949-119
ENSG0000(	608	13.57819	chr3:380∠	ENSG00000241439		Pseudoger	chr3:125958556-125
ENSG0000(	608	13.57819	chr6:1391	MIR1273C		smallRNA	chr6:154853360-154
ENSG0000(	608	13.57819	chr3:380∠	C3orf22		protein_c	chr3:126526999-126
ENSG0000(	608	13.57819	chr3:380∠	WDR5B-DT		lncRNA	chr3:122416200-122
ENSG0000(	608	13.57819	chr3:380∠	OR7E53P		Pseudoger	chr3:125734295-125
ENSG0000(	608	13.57819	chr3:380∠	RN7SL762P		smallRNA	chr3:120110498-120
ENSG0000(	608	13.57819	chr3:380∠	ENSG00000288769		lncRNA	chr3:127456778-127
ENSG0000(	608	13.57819	chr3:380∠	SEMA5B	NCGv7	protein_c	chr3:122909082-123
ENSG0000(	608	13.57819	chr3:380∠	HACD2		protein_c	chr3:123490820-123
ENSG0000(	608	13.57819	chr3:380∠	CHST13		protein_c	chr3:126524155-126
ENSG0000(	608	13.57819	chr3:380∠	MTCO1P29		Pseudoger	chr3:120722024-120
ENSG0000(	608	13.57819	chr3:380∠	ALDH1L1-AS2		lncRNA	chr3:126180012-126
ENSG0000(	608	13.57819	chr3:380∠	EEFSEC		protein_c	chr3:128153481-128
ENSG0000(	608	13.57819	chr3:380∠	PHB1P8		Pseudoger	chr3:119791829-119
ENSG0000(	608	13.57819	chr3:380∠	Y_RNA		smallRNA	chr3:120210320-120
ENSG0000(	608	13.57819	chr3:380∠	TIMMDC1-DT		lncRNA	chr3:119497678-119
ENSG0000(	608	13.57819	chr3:380∠	POLQ	NCGv7	protein_c	chr3:121431431-121
ENSG0000(	608	13.57819	chr3:380∠	GSK3B-DT		lncRNA	chr3:120094895-120
ENSG0000(	608	13.57819	chr3:380∠	CFAP100-DT		lncRNA	chr3:126393032-126
ENSG0000(	608	13.57819	chr3:380∠	ENSG00000287232		lncRNA	chr3:126213204-126
ENSG0000(	608	13.57819	chr3:380∠	MIR5092		smallRNA	chr3:125151465-125
ENSG0000(	608	13.57819	chr6:1391	RPS4XP8		Pseudoger	chr6:154576309-154
ENSG0000(	608	13.57819	chr6:1391	ENSG00000287260		lncRNA	chr6:154510770-154
ENSG0000(	608	13.57819	chr3:380∠	SLC49A4		protein_c	chr3:122795069-122
ENSG0000(	608	13.57819	chr3:380∠	RNU6-823P		smallRNA	chr3:128141875-128
ENSG0000(	608	13.57819	chr3:380∠	ENSG00000286735		lncRNA	chr3:120448974-120
ENSG0000(	608	13.57819	chr3:380∠	IGSF11-AS1		lncRNA	chr3:118943073-118
ENSG0000(	608	13.57819	chr3:380∠	RCC2P4		Pseudoger	chr3:126766434-126
ENSG0000(	608	13.57819	chr3:380∠	ENSG00000287366		lncRNA	chr3:120833936-120
ENSG0000(	608	13.57819	chr3:380∠	RNU6-1200P		smallRNA	chr3:117544205-117
ENSG0000(	608	13.57819	chr3:380∠	snoU13		smallRNA	chr3:121591294-121
ENSG0000(	608	13.57819	chr3:380∠	ENSG00000242531		Pseudoger	chr3:122416882-122
ENSG0000(	608	13.57819	chr3:380∠	RN7SL397P		smallRNA	chr3:120121909-120
ENSG0000(	608	13.57819	chr6:1391	HMGB3P19		Pseudoger	chr6:153938433-153
ENSG0000(	608	13.57819	chr3:380∠	LINC02614		lncRNA	chr3:125827238-125
ENSG0000(	608	13.57819	chr3:380∠	ENPP7P4		Pseudoger	chr3:125848223-125
ENSG0000(	608	13.57819	chr3:380∠	CFAP91		protein_c	chr3:119703022-119
ENSG0000(	608	13.57819	chr3:380∠	PRR20G		protein_c	chr3:127283783-127
ENSG0000(	608	13.57819	chr3:380∠	MIR5682		smallRNA	chr3:121049640-121
ENSG0000(	608	13.57819	chr3:380∠	ENO1P3		Pseudoger	chr3:124862094-124
ENSG0000(	608	13.57819	chr3:380∠	ENSG00000287207		lncRNA	chr3:122515006-122

ENSG0000(	608	13.57819	chr6:1391	AL121952.1		smallRNA	chr6:154881282-154
ENSG0000(	608	13.57819	chr3:380⁴	PLA1A	NCGv7	protein_c	chr3:119597875-119
ENSG0000(	608	13.57819	chr3:380⁴	ENSG00000242613		Pseudoger	chr3:120388029-120
ENSG0000(	608	13.57819	chr3:380⁴	NR1I2	NCGv7	protein_c	chr3:119780484-119
ENSG0000(	608	13.57819	chr3:380⁴	HSPBAP1		protein_c	chr3:122739999-122
ENSG0000(	608	13.57819	chr6:1391	ENSG00000286553		lncRNA	chr6:154477982-154
ENSG0000(	608	13.57819	chr3:380⁴	LINC02049		lncRNA	chr3:120833440-120
ENSG0000(	608	13.57819	chr3:380⁴	IGSF11	DriverDB\	protein_c	chr3:118900557-119
ENSG0000(	608	13.57819	chr3:380⁴	snoU13		smallRNA	chr3:122750372-122
ENSG0000(	608	13.57819	chr3:380⁴	ADPRH		protein_c	chr3:119579268-119
ENSG0000(	608	13.57819	chr3:380⁴	ENSG00000284624		lncRNA	chr3:125766516-125
ENSG0000(	608	13.57819	chr3:380⁴	ENSG00000286584		lncRNA	chr3:119666232-119
ENSG0000(	608	13.57819	chr3:380⁴	RABL3		protein_c	chr3:120684938-120
ENSG0000(	608	13.57819	chr3:380⁴	ENSG00000286827		lncRNA	chr3:120811530-120
ENSG0000(	608	13.57819	chr3:380⁴	SNRPCP11		Pseudoger	chr3:125816082-125
ENSG0000(	608	13.57819	chr3:380⁴	ENSG00000239804		Pseudoger	chr3:125787888-125
ENSG0000(	608	13.57819	chr3:380⁴	ENSG00000287143		lncRNA	chr3:127837436-127
ENSG0000(	608	13.57819	chr3:380⁴	DUTP1		Pseudoger	chr3:125310881-125
ENSG0000(	608	13.57819	chr3:380⁴	ENSG00000251012		protein_c	chr3:119147375-119
ENSG0000(	608	13.57819	chr3:380⁴	ENSG00000240562		lncRNA	chr3:127489553-127
ENSG0000(	608	13.57819	chr3:380⁴	ENSG00000239835		Pseudoger	chr3:120028740-120
ENSG0000(	608	13.57819	chr3:380⁴	ENSG00000280053		TEC	chr3:126973065-126
ENSG0000(	606	13.53352	chr4:4108	GAPDHP56		Pseudoger	chr4:130503357-130
ENSG0000(	599	13.37719	chr12:171	ENSG00000286591		lncRNA	chr12:41951493-420
ENSG0000(	599	13.37719	chr12:171	ENSG00000257813		Pseudoger	chr12:43569769-435
ENSG0000(	599	13.37719	chr12:171	RNU6-713P		smallRNA	chr12:40554161-405
ENSG0000(	599	13.37719	chr12:171	ENSG00000257376		Pseudoger	chr12:42431665-424
ENSG0000(	599	13.37719	chr12:171	ENSG00000257239		lncRNA	chr12:41829898-419
ENSG0000(	599	13.37719	chr12:171	LINC02451		lncRNA	chr12:42646583-426
ENSG0000(	599	13.37719	chr12:171	ENSG00000271379		Pseudoger	chr12:42069935-420
ENSG0000(	599	13.37719	chr12:171	AC079603.1		smallRNA	chr12:43291495-432
ENSG0000(	599	13.37719	chr12:171	ENSG00000271508		Pseudoger	chr12:43633093-436
ENSG0000(	599	13.37719	chr12:171	PPHLN1		protein_c	chr12:42238447-424
ENSG0000(	599	13.37719	chr12:171	RNA5SP360		Pseudoger	chr12:41658676-416
ENSG0000(	599	13.37719	chr12:171	MUC19		protein_c	chr12:40393395-405
ENSG0000(	599	13.37719	chr12:171	ENSG00000271272		Pseudoger	chr12:42929848-429
ENSG0000(	599	13.37719	chr12:171	ENSG00000257674		Pseudoger	chr12:42286911-422
ENSG0000(	599	13.37719	chr12:171	ENSG00000257680		Pseudoger	chr12:40728811-407
ENSG0000(	599	13.37719	chr12:171	ENSG00000257687		Pseudoger	chr12:42627008-426
ENSG0000(	599	13.37719	chr12:171	RN7SL10P		smallRNA	chr12:42242215-422
ENSG0000(	599	13.37719	chr12:171	LINC02400		lncRNA	chr12:41764144-417
ENSG0000(	599	13.37719	chr12:171	YAF2		protein_c	chr12:42157104-422
ENSG0000(	599	13.37719	chr12:171	CNTN1	NCGv7	protein_c	chr12:40692439-410
ENSG0000(	599	13.37719	chr12:171	ENSG00000241251		Pseudoger	chr12:43560784-435
ENSG0000(	599	13.37719	chr12:171	ENSG00000289310		lncRNA	chr12:40229956-402
ENSG0000(	599	13.37719	chr12:171	PDZRN4	NCGv7	protein_c	chr12:41188320-415
ENSG0000(	599	13.37719	chr12:171	Y_RNA		smallRNA	chr12:42454720-424
ENSG0000(	599	13.37719	chr3:380⁴	snoU13		smallRNA	chr3:101201832-101
ENSG0000(	599	13.37719	chr12:171	LINC02461		lncRNA	chr12:43155315-431
ENSG0000(	599	13.37719	chr12:171	ENSG00000258068		lncRNA	chr12:42485353-424
ENSG0000(	599	13.37719	chr12:171	LINC02402		lncRNA	chr12:42615221-426

ENSG0000(	599	13.37719	chr12:171	ENSG00000257128		Pseudoger	chr12:41911256-419
ENSG0000(	599	13.37719	chr12:171	ENSG00000258167		lncRNA	chr12:40395853-404
ENSG0000(	599	13.37719	chr12:171	MTND2P17		Pseudoger	chr12:41698495-416
ENSG0000(	599	13.37719	chr12:171	ENSG00000257225		lncRNA	chr12:42459366-424
ENSG0000(	599	13.37719	chr12:171	ZCRB1	NCGv7	protein_c	chr12:42312086-423
ENSG0000(	599	13.37719	chr12:171	ENSG00000229899		Pseudoger	chr12:40286289-402
ENSG0000(	599	13.37719	chr12:171	PRICKLE1		protein_c	chr12:42456757-425
ENSG0000(	599	13.37719	chr12:171	RPS27P21		Pseudoger	chr12:42678873-426
ENSG0000(	599	13.37719	chr12:171	AC023513.1		smallRNA	chr12:42079761-420
ENSG0000(	599	13.37719	chr12:171	GXYLT1	NCGv7	protein_c	chr12:42081845-421
ENSG0000(	599	13.37719	chr12:171	MRPS36P5		Pseudoger	chr12:43054089-430
ENSG0000(	599	13.37719	chr12:171	MTND1P24		Pseudoger	chr12:41699391-417
ENSG0000(	599	13.37719	chr12:171	ENSG00000274682		lncRNA	chr12:40978744-409
ENSG0000(	599	13.37719	chr12:171	ENSG00000257405		Pseudoger	chr12:42636815-426
ENSG0000(	599	13.37719	chr12:171	ADAMTS20	NCGv7	protein_c	chr12:43353866-435
ENSG0000(	599	13.37719	chr12:171	ENSG00000274943		lncRNA	chr12:42361267-423
ENSG0000(	599	13.37719	chr12:171	ENSG00000257228		lncRNA	chr12:41409467-414
ENSG0000(	599	13.37719	chr12:171	LINC02450		lncRNA	chr12:42687195-427
ENSG0000(	599	13.37719	chr12:171	RNU6-249P		smallRNA	chr12:42398550-423
ENSG0000(	599	13.37719	chr12:171	ENSG00000257373		lncRNA	chr12:42979254-429
ENSG0000(	599	13.37719	chr12:171	ENSG00000257849		lncRNA	chr12:42966122-429
ENSG0000(	597	13.33253	chr12:171	SNORD83		smallRNA	chr12:64575665-645
ENSG0000(	597	13.33253	chr12:171	RNU1-83P		smallRNA	chr12:62850738-628
ENSG0000(	597	13.33253	chr6:1391	ENSG00000284615		lncRNA	chr6:152112759-152
ENSG0000(	597	13.33253	chr12:171	snoU13		smallRNA	chr12:64355762-643
ENSG0000(	597	13.33253	chr6:1391	ENSG00000233330		lncRNA	chr6:149591755-149
ENSG0000(	597	13.33253	chr12:171	ATP6V1E1P3		Pseudoger	chr12:64338178-643
ENSG0000(	597	13.33253	chr12:171	PPM1H		protein_c	chr12:62643994-629
ENSG0000(	597	13.33253	chr6:1391	ENSG00000230205		lncRNA	chr6:148133809-148
ENSG0000(	597	13.33253	chr6:1391	IYD		protein_c	chr6:150368892-150
ENSG0000(	597	13.33253	chr6:1391	MTND4P13		Pseudoger	chr6:153668879-153
ENSG0000(	597	13.33253	chr6:1391	RPSAP40		Pseudoger	chr6:148647707-148
ENSG0000(	597	13.33253	chr6:1391	ENSG00000216621		Pseudoger	chr6:149934527-149
ENSG0000(	597	13.33253	chr6:1391	ENSG00000233823		lncRNA	chr6:151813276-151
ENSG0000(	597	13.33253	chr6:1391	ENSG00000215094		Pseudoger	chr6:149951833-149
ENSG0000(	597	13.33253	chr12:171	Y_RNA		smallRNA	chr12:62935701-629
ENSG0000(	597	13.33253	chr6:1391	ENSG00000219298		Pseudoger	chr6:149977471-149
ENSG0000(	597	13.33253	chr6:1391	ENSG00000213121		lncRNA	chr6:153231320-153
ENSG0000(	597	13.33253	chr12:171	snoU13		smallRNA	chr12:62651175-626
ENSG0000(	597	13.33253	chr12:171	MIRLET7IHG		lncRNA	chr12:62602752-626
ENSG0000(	597	13.33253	chr6:1391	ZBTB2		protein_c	chr6:151364115-151
ENSG0000(	597	13.33253	chr12:171	ENSG00000255817		lncRNA	chr12:64108763-641
ENSG0000(	597	13.33253	chr6:1391	RAET1L		protein_c	chr6:150018334-150
ENSG0000(	597	13.33253	chr6:1391	ENSG00000287473		lncRNA	chr6:148155120-148
ENSG0000(	597	13.33253	chr6:1391	RAET1E-AS1		lncRNA	chr6:149884431-149
ENSG0000(	597	13.33253	chr12:171	ENSG00000243024		Pseudoger	chr12:64222333-643
ENSG0000(	597	13.33253	chr6:1391	ENSG00000223598		lncRNA	chr6:151088103-151
ENSG0000(	597	13.33253	chr6:1391	MTRF1L		protein_c	chr6:152987362-153
ENSG0000(	597	13.33253	chr6:1391	FBXO5	NCGv7	protein_c	chr6:152970519-152
ENSG0000(	597	13.33253	chr6:1391	MTCO2P31		Pseudoger	chr6:153665707-153
ENSG0000(	597	13.33253	chr6:1391	ULBP1		protein_c	chr6:149963943-149

ENSG0000(	597	13.33253	chr6:1391	RMND1		protein_c	chr6:151398898-151
ENSG0000(	597	13.33253	chr6:1391	UST		protein_c	chr6:148747030-149
ENSG0000(	597	13.33253	chr6:1391	SASH1		protein_c	chr6:148272304-148
ENSG0000(	597	13.33253	chr6:1391	ENSG00000289359		lncRNA	chr6:149140235-149
ENSG0000(	597	13.33253	chr6:1391	ARL4AP5		Pseudoger	chr6:150934968-150
ENSG0000(	597	13.33253	chr6:1391	AL080276.1		smallRNA	chr6:153052462-153
ENSG0000(	597	13.33253	chr6:1391	MTCO3P31		Pseudoger	chr6:153667318-153
ENSG0000(	597	13.33253	chr12:171	AC084357.1		smallRNA	chr12:63830754-638
ENSG0000(	597	13.33253	chr6:1391	RAET1E		protein_c	chr6:149883179-149
ENSG0000(	597	13.33253	chr6:1391	AL603766.1		smallRNA	chr6:149176707-149
ENSG0000(	597	13.33253	chr6:1391	AL133269.1		smallRNA	chr6:152856666-152
ENSG0000(	597	13.33253	chr6:1391	ENSG00000216906		Pseudoger	chr6:149904243-149
ENSG0000(	597	13.33253	chr12:171	RNU6-1009P		smallRNA	chr12:64204793-642
ENSG0000(	597	13.33253	chr6:1391	AL033378.1		smallRNA	chr6:148524153-148
ENSG0000(	597	13.33253	chr6:1391	SSR1P1		Pseudoger	chr6:150384307-150
ENSG0000(	597	13.33253	chr6:1391	HSPA8P15		Pseudoger	chr6:151411259-151
ENSG0000(	597	13.33253	chr6:1391	PPIL4	NCGv7	protein_c	chr6:149504495-149
ENSG0000(	597	13.33253	chr6:1391	TAB2-AS1		lncRNA	chr6:149243299-149
ENSG0000(	597	13.33253	chr6:1391	ENSG00000281021		lncRNA	chr6:149576089-149
ENSG0000(	597	13.33253	chr6:1391	ENSG00000219553		Pseudoger	chr6:149491567-149
ENSG0000(	597	13.33253	chr6:1391	ENSG00000235972		lncRNA	chr6:150040547-150
ENSG0000(	597	13.33253	chr6:1391	RN7SL234P		smallRNA	chr6:149291352-149
ENSG0000(	597	13.33253	chr12:171	XPOT		protein_c	chr12:64404392-644
ENSG0000(	597	13.33253	chr6:1391	RN7SKP268		smallRNA	chr6:151319635-151
ENSG0000(	597	13.33253	chr6:1391	RNU6-1247P		smallRNA	chr6:151280515-151
ENSG0000(	597	13.33253	chr6:1391	ENSG00000219622		Pseudoger	chr6:150504811-150
ENSG0000(	597	13.33253	chr6:1391	snoU13		smallRNA	chr6:150164584-150
ENSG0000(	597	13.33253	chr6:1391	PHB1P1		Pseudoger	chr6:150042546-150
ENSG0000(	597	13.33253	chr6:1391	PDCL3P5		Pseudoger	chr6:150827663-150
ENSG0000(	597	13.33253	chr6:1391	AL358134.2		smallRNA	chr6:153419021-153
ENSG0000(	597	13.33253	chr6:1391	ENSG00000285889		lncRNA	chr6:149797317-149
ENSG0000(	597	13.33253	chr12:171	PABPC1P4		Pseudoger	chr12:63822021-638
ENSG0000(	597	13.33253	chr6:1391	RPL32P16		Pseudoger	chr6:151099529-151
ENSG0000(	597	13.33253	chr6:1391	ENSG00000226249		lncRNA	chr6:147741434-147
ENSG0000(	597	13.33253	chr12:171	ENSG00000255629		lncRNA	chr12:64146388-641
ENSG0000(	597	13.33253	chr6:1391	RNA5SP225		Pseudoger	chr6:153420438-153
ENSG0000(	597	13.33253	chr6:1391	BTF3P10		Pseudoger	chr6:149977922-149
ENSG0000(	597	13.33253	chr6:1391	ENSG00000213087		Pseudoger	chr6:151225550-151
ENSG0000(	597	13.33253	chr12:171	ENSG00000249753		lncRNA	chr12:63623788-637
ENSG0000(	597	13.33253	chr12:171	RXYLT1-AS1		lncRNA	chr12:63804739-638
ENSG0000(	597	13.33253	chr6:1391	ENSG00000226193		lncRNA	chr6:152402398-152
ENSG0000(	597	13.33253	chr6:1391	FABP12P1		Pseudoger	chr6:149304107-149
ENSG0000(	597	13.33253	chr6:1391	RNU6-300P		smallRNA	chr6:151201341-151
ENSG0000(	597	13.33253	chr6:1391	ULBP2		protein_c	chr6:149942014-149
ENSG0000(	597	13.33253	chr12:171	ENSG00000279444		TEC	chr12:63151065-631
ENSG0000(	597	13.33253	chr6:1391	AKAP12		protein_c	chr6:151239967-151
ENSG0000(	597	13.33253	chr6:1391	RNU6-896P		smallRNA	chr6:153681447-153
ENSG0000(	597	13.33253	chr12:171	ENSG00000256314		lncRNA	chr12:64654060-646
ENSG0000(	597	13.33253	chr12:171	C12orf56		protein_c	chr12:64264762-643
ENSG0000(	597	13.33253	chr6:1391	SYNE1	NCGv7	protein_c	chr6:152121687-152
ENSG0000(	597	13.33253	chr6:1391	ULBP3		protein_c	chr6:150061053-150

ENSG0000(	597	13.33253	chr6:1391	LATS1	NCGv7	protein_c	chr6:149658153-149
ENSG0000(	597	13.33253	chr12:171	ENSG00000257005		Pseudoger	chr12:63651627-636
ENSG0000(	597	13.33253	chr6:1391	BTBD10P2		Pseudoger	chr6:149794763-149
ENSG0000(	597	13.33253	chr12:171	RSL24D1P5		Pseudoger	chr12:63055275-630
ENSG0000(	597	13.33253	chr6:1391	CYP51A1P3		Pseudoger	chr6:148478693-148
ENSG0000(	597	13.33253	chr12:171	ENSG00000288665		lncRNA	chr12:64497968-645
ENSG0000(	597	13.33253	chr6:1391	MTND4LP20		Pseudoger	chr6:153668589-153
ENSG0000(	597	13.33253	chr6:1391	RAET1M		Pseudoger	chr6:150032769-150
ENSG0000(	597	13.33253	chr6:1391	ENSG00000278899		lncRNA	chr6:149717621-149
ENSG0000(	597	13.33253	chr6:1391	ENSG00000226599		lncRNA	chr6:150650772-150
ENSG0000(	597	13.33253	chr6:1391	ENSG00000219487		Pseudoger	chr6:149112009-149
ENSG0000(	597	13.33253	chr6:1391	ENSG00000232891		lncRNA	chr6:150600834-150
ENSG0000(	597	13.33253	chr12:171	ENSG00000256399		Pseudoger	chr12:63887379-638
ENSG0000(	597	13.33253	chr12:171	ENSG00000258117		lncRNA	chr12:63292625-633
ENSG0000(	597	13.33253	chr6:1391	AL133260.1		smallRNA	chr6:151039916-151
ENSG0000(	597	13.33253	chr6:1391	RNY4P20		smallRNA	chr6:151298841-151
ENSG0000(	597	13.33253	chr12:171	ENSG00000255886		lncRNA	chr12:64038562-640
ENSG0000(	597	13.33253	chr12:171	KICS2		protein_c	chr12:64186316-642
ENSG0000(	597	13.33253	chr6:1391	RNU7-3P		smallRNA	chr6:149516956-149
ENSG0000(	597	13.33253	chr12:171	ENSG00000255583		Pseudoger	chr12:63684748-637
ENSG0000(	597	13.33253	chr6:1391	RAET1K		Pseudoger	chr6:150000090-150
ENSG0000(	597	13.33253	chr6:1391	Y_RNA		smallRNA	chr6:151378000-151
ENSG0000(	597	13.33253	chr12:171	SRGAP1		protein_c	chr12:63843761-641
ENSG0000(	597	13.33253	chr12:171	GAPDHP44		Pseudoger	chr12:62755227-627
ENSG0000(	597	13.33253	chr6:1391	RAET1K		lncRNA	chr6:149998019-150
ENSG0000(	597	13.33253	chr6:1391	SNORA2		smallRNA	chr6:149594625-149
ENSG0000(	597	13.33253	chr12:171	DPY19L2		protein_c	chr12:63558913-636
ENSG0000(	597	13.33253	chr12:171	RASSF3-DT		lncRNA	chr12:64599078-646
ENSG0000(	597	13.33253	chr6:1391	RAET1E-AS1		lncRNA	chr6:149863494-149
ENSG0000(	597	13.33253	chr6:1391	RPS18P9		Pseudoger	chr6:149592687-149
ENSG0000(	597	13.33253	chr6:1391	ENSG00000224658		lncRNA	chr6:148237585-148
ENSG0000(	597	13.33253	chr6:1391	PPP1R14C		protein_c	chr6:150143044-150
ENSG0000(	597	13.33253	chr6:1391	RNU6-813P		smallRNA	chr6:151615773-151
ENSG0000(	597	13.33253	chr12:171	RPL14P1		Pseudoger	chr12:62965325-629
ENSG0000(	597	13.33253	chr12:171	MON2		protein_c	chr12:62466817-626
ENSG0000(	597	13.33253	chr6:1391	ENSG00000273132		lncRNA	chr6:149852462-149
ENSG0000(	597	13.33253	chr6:1391	UST-AS2		lncRNA	chr6:149027700-149
ENSG0000(	597	13.33253	chr6:1391	ENSG00000231760		lncRNA	chr6:149796151-149
ENSG0000(	597	13.33253	chr12:171	LINC01465		lncRNA	chr12:62601751-626
ENSG0000(	597	13.33253	chr6:1391	ENSG00000234675		lncRNA	chr6:148017422-148
ENSG0000(	597	13.33253	chr6:1391	RNU4-7P		smallRNA	chr6:150326623-150
ENSG0000(	597	13.33253	chr6:1391	SNRPEP6		Pseudoger	chr6:148567378-148
ENSG0000(	597	13.33253	chr12:171	OOEPP2		Pseudoger	chr12:64266413-642
ENSG0000(	597	13.33253	chr12:171	RPL32P26		Pseudoger	chr12:62658823-626
ENSG0000(	597	13.33253	chr6:1391	ESR1	NCGv7	protein_c	chr6:151656691-152
ENSG0000(	597	13.33253	chr6:1391	RNA5SP224		Pseudoger	chr6:153159220-153
ENSG0000(	597	13.33253	chr6:1391	GINM1		protein_c	chr6:149566294-149
ENSG0000(	597	13.33253	chr6:1391	MYCT1		protein_c	chr6:152697897-152
ENSG0000(	597	13.33253	chr6:1391	PLEKHG1		protein_c	chr6:150599883-150
ENSG0000(	597	13.33253	chr6:1391	SUMO4		protein_c	chr6:149400262-149
ENSG0000(	597	13.33253	chr6:1391	ENSG00000289045		lncRNA	chr6:149497715-149

ENSG0000(	597	13.33253	chr6:1391	ENSG00000287976		lncRNA	chr6:148001716-148
ENSG0000(	597	13.33253	chr12:171	ENSG00000257235		lncRNA	chr12:63004338-630
ENSG0000(	597	13.33253	chr12:171	ENSG00000290896		lncRNA	chr12:63682523-637
ENSG0000(	597	13.33253	chr12:171	HNRNPA1P69		Pseudoger	chr12:63271404-632
ENSG0000(	597	13.33253	chr12:171	MIR548C		smallRNA	chr12:64622509-646
ENSG0000(	597	13.33253	chr6:1391	ENSG00000283608		lncRNA	chr6:149246095-149
ENSG0000(	597	13.33253	chr6:1391	RNA5SP223		Pseudoger	chr6:152410275-152
ENSG0000(	597	13.33253	chr12:171	RPL36AP41		Pseudoger	chr12:63871739-638
ENSG0000(	597	13.33253	chr6:1391	ENSG00000287914		lncRNA	chr6:152091712-152
ENSG0000(	597	13.33253	chr12:171	RNU6-399P		smallRNA	chr12:62545582-625
ENSG0000(	597	13.33253	chr6:1391	ENSG00000231883		lncRNA	chr6:151196681-151
ENSG0000(	597	13.33253	chr6:1391	NUP43		protein_c	chr6:149724315-149
ENSG0000(	597	13.33253	chr6:1391	MTHFD1L		protein_c	chr6:150865679-151
ENSG0000(	597	13.33253	chr6:1391	LRP11		protein_c	chr6:149818757-149
ENSG0000(	597	13.33253	chr6:1391	CCDC170		protein_c	chr6:151494017-151
ENSG0000(	597	13.33253	chr6:1391	TAB2	DriverDBv	protein_c	chr6:149218641-149
ENSG0000(	597	13.33253	chr6:1391	PCMT1		protein_c	chr6:149749443-149
ENSG0000(	597	13.33253	chr12:171	RXYLT1		protein_c	chr12:63779833-638
ENSG0000(	597	13.33253	chr6:1391	ENSG00000285991		protein_c	chr6:149817937-149
ENSG0000(	597	13.33253	chr6:1391	KATNA1		protein_c	chr6:149594873-149
ENSG0000(	597	13.33253	chr12:171	AVPR1A		protein_c	chr12:63142759-631
ENSG0000(	597	13.33253	chr6:1391	HSPD1P16		Pseudoger	chr6:152705735-152
ENSG0000(	597	13.33253	chr6:1391	RGS17		protein_c	chr6:153004459-153
ENSG0000(	597	13.33253	chr6:1391	ENSG00000220745		Pseudoger	chr6:152959359-152
ENSG0000(	597	13.33253	chr12:171	ENSG00000256571		lncRNA	chr12:63878787-638
ENSG0000(	597	13.33253	chr12:171	ENSG00000275180		lncRNA	chr12:62603366-626
ENSG0000(	597	13.33253	chr12:171	ENSG00000257779		Pseudoger	chr12:63002469-630
ENSG0000(	597	13.33253	chr12:171	ENSG00000225195		lncRNA	chr12:64628344-646
ENSG0000(	597	13.33253	chr6:1391	NANOGP11		lncRNA	chr6:152545930-152
ENSG0000(	597	13.33253	chr6:1391	MTND3P20		Pseudoger	chr6:153668177-153
ENSG0000(	597	13.33253	chr12:171	ENSG00000255780		Pseudoger	chr12:64032412-640
ENSG0000(	597	13.33253	chr12:171	ENSG00000287618		lncRNA	chr12:64099415-641
ENSG0000(	597	13.33253	chr12:171	ENSG00000255566		Pseudoger	chr12:64442510-644
ENSG0000(	597	13.33253	chr6:1391	ENSG00000224029		lncRNA	chr6:150624677-150
ENSG0000(	597	13.33253	chr6:1391	snoU13		smallRNA	chr6:151347027-151
ENSG0000(	597	13.33253	chr12:171	USP15		protein_c	chr12:62260338-624
ENSG0000(	597	13.33253	chr12:171	ENSG00000277895		lncRNA	chr12:64451591-644
ENSG0000(	597	13.33253	chr6:1391	TUBB4BP7		Pseudoger	chr6:152898047-152
ENSG0000(	597	13.33253	chr6:1391	AL358134.1		smallRNA	chr6:153344113-153
ENSG0000(	597	13.33253	chr6:1391	UST-AS1		lncRNA	chr6:148955628-148
ENSG0000(	597	13.33253	chr6:1391	MTATP6P31		Pseudoger	chr6:153666627-153
ENSG0000(	597	13.33253	chr6:1391	ENSG00000227627		lncRNA	chr6:152983331-152
ENSG0000(	597	13.33253	chr12:171	RNU6-595P		smallRNA	chr12:62334091-623
ENSG0000(	597	13.33253	chr6:1391	RNU6-302P		smallRNA	chr6:150939222-150
ENSG0000(	597	13.33253	chr12:171	ENSG00000257568		lncRNA	chr12:62482349-624
ENSG0000(	597	13.33253	chr12:171	TBK1	NCGv7	protein_c	chr12:64452090-645
ENSG0000(	597	13.33253	chr12:171	ENSG00000240027		Pseudoger	chr12:63760358-637
ENSG0000(	597	13.33253	chr6:1391	ENSG00000242246		Pseudoger	chr6:153666466-153
ENSG0000(	597	13.33253	chr6:1391	Y_RNA		smallRNA	chr6:149631437-149
ENSG0000(	597	13.33253	chr12:171	LDHAL6CP		Pseudoger	chr12:63003553-630
ENSG0000(	597	13.33253	chr12:171	ENSG00000256199		lncRNA	chr12:64507166-645

ENSG0000(	597	13.33253	chr12:171	TAFA2		protein_c	chr12:61708273-622
ENSG0000(	597	13.33253	chr6:1391	ENSG00000228408		lncRNA	chr6:149217926-149
ENSG0000(	597	13.33253	chr6:1391	ENSG00000237312		lncRNA	chr6:153304595-153
ENSG0000(	597	13.33253	chr6:1391	RP1-12G14.6		lncRNA	chr6:149575465-149
ENSG0000(	597	13.33253	chr6:1391	LINC02840		lncRNA	chr6:152754903-152
ENSG0000(	597	13.33253	chr6:1391	ENSG00000218426		Pseudoger	chr6:153282287-153
ENSG0000(	597	13.33253	chr6:1391	RNU6-1222P		smallRNA	chr6:148278672-148
ENSG0000(	597	13.33253	chr6:1391	CCT7P1		Pseudoger	chr6:149879962-149
ENSG0000(	597	13.33253	chr6:1391	ZC3H12D		protein_c	chr6:149446795-149
ENSG0000(	597	13.33253	chr6:1391	ENSG00000232290		lncRNA	chr6:151054897-151
ENSG0000(	597	13.33253	chr6:1391	ENSG00000235168		lncRNA	chr6:149561152-149
ENSG0000(	597	13.33253	chr6:1391	ARMT1		protein_c	chr6:151452258-151
ENSG0000(	597	13.33253	chr6:1391	VIP		protein_c	chr6:152750797-152
ENSG0000(	597	13.33253	chr6:1391	SYNE1-AS1		lncRNA	chr6:152380546-152
ENSG0000(	597	13.33253	chr12:171	KLF17P1		Pseudoger	chr12:62234390-622
ENSG0000(	597	13.33253	chr6:1391	RAET1G		protein_c	chr6:149916878-149
ENSG0000(	597	13.33253	chr12:171	RNU5A-7P		smallRNA	chr12:64228560-642
ENSG0000(	596	13.3102	chr4:733(	MIR5096		smallRNA	chr4:78820752-7882
ENSG0000(	596	13.3102	chr1:175(	AL359983.1		smallRNA	chr1:245370334-245
ENSG0000(	589	13.15387	chr7:158(	AC008085.1		smallRNA	chr7:131852680-131
ENSG0000(	589	13.15387	chr7:158(	snosnR60_Z15		smallRNA	chr7:131916235-131
ENSG0000(	589	13.15387	chr7:158(	CAPZA1P4		Pseudoger	chr7:131892616-131
ENSG0000(	589	13.15387	chr7:158(	FLJ40288		lncRNA	chr7:132648794-132
ENSG0000(	589	13.15387	chr7:158(	ENSG00000236386		Pseudoger	chr7:131893822-131
ENSG0000(	589	13.15387	chr7:158(	NDUFB9P2		Pseudoger	chr7:131753746-131
ENSG0000(	589	13.15387	chr7:158(	ENSG00000235429		Pseudoger	chr7:133315009-133
ENSG0000(	589	13.15387	chr7:158(	COX5BP3		Pseudoger	chr7:133727368-133
ENSG0000(	589	13.15387	chr7:158(	RPS3AP27		Pseudoger	chr7:133732493-133
ENSG0000(	589	13.15387	chr7:158(	AC083875.1		smallRNA	chr7:133262640-133
ENSG0000(	589	13.15387	chr7:158(	ENSG00000229532		Pseudoger	chr7:132086266-132
ENSG0000(	589	13.15387	chr7:158(	SNORD46		smallRNA	chr7:132753023-132
ENSG0000(	589	13.15387	chr7:158(	PLXNA4		protein_c	chr7:132123340-132
ENSG0000(	589	13.15387	chr7:158(	CHCHD3		protein_c	chr7:132784870-133
ENSG0000(	589	13.15387	chr7:158(	U6		smallRNA	chr7:133082829-133
ENSG0000(	589	13.15387	chr7:158(	ENSG00000283041		Pseudoger	chr7:133034607-133
ENSG0000(	589	13.15387	chr7:158(	ENSG00000225144		lncRNA	chr7:132264152-132
ENSG0000(	589	13.15387	chr7:158(	ENSG00000225881		lncRNA	chr7:132758970-132
ENSG0000(	589	13.15387	chr12:14(	PRELID2P1		Pseudoger	chr12:68957377-689
ENSG0000(	589	13.15387	chr7:158(	EXOC4		protein_c	chr7:133253073-134
ENSG0000(	589	13.15387	chr7:158(	ENSG00000224865		lncRNA	chr7:131897289-131
ENSG0000(	589	13.15387	chr7:158(	ENSG00000223436		lncRNA	chr7:132352334-132
ENSG0000(	589	13.15387	chr7:158(	ENSG00000227197		lncRNA	chr7:132830693-132
ENSG0000(	589	13.15387	chr7:158(	ST13P7		Pseudoger	chr7:133169416-133
ENSG0000(	588	13.13154	chr1:175(	C1orf100	NCGv7	protein_c	chr1:244352635-244
ENSG0000(	588	13.13154	chr1:175(	TGIF2P1		Pseudoger	chr1:244394976-244
ENSG0000(	588	13.13154	chr1:175(	ZBTB18	DriverDB\	protein_c	chr1:244048547-244
ENSG0000(	588	13.13154	chr1:175(	ENSG00000240963		lncRNA	chr1:244375100-244
ENSG0000(	588	13.13154	chr1:175(	HNRNPU	NCGv7	protein_c	chr1:244840638-244
ENSG0000(	588	13.13154	chr1:175(	RNU6-999P		smallRNA	chr1:245043928-245
ENSG0000(	588	13.13154	chr1:175(	ENSG00000289439		lncRNA	chr1:244308554-244
ENSG0000(	588	13.13154	chr1:175(	ENSG00000272195		lncRNA	chr1:244969350-244

ENSG0000(	588	13.13154	chr1:175{	ENSG00000284188	protein_c	chr1:244729701-244
ENSG0000(	588	13.13154	chr1:175{	RNU6-1089P	smallRNA	chr1:245023015-245
ENSG0000(	588	13.13154	chr1:175{	RN7SKP55	smallRNA	chr1:244943910-244
ENSG0000(	588	13.13154	chr1:175{	ENSG00000287601	lncRNA	chr1:244834374-244
ENSG0000(	588	13.13154	chr1:175{	ENSG00000286486	lncRNA	chr1:244087306-244
ENSG0000(	588	13.13154	chr1:175{	RNU6-947P	smallRNA	chr1:244954858-244
ENSG0000(	588	13.13154	chr1:175{	KIF26B-AS1	lncRNA	chr1:245206444-245
ENSG0000(	588	13.13154	chr1:175{	ENSG00000273175	lncRNA	chr1:244863782-244
ENSG0000(	588	13.13154	chr1:175{	ENSG00000287531	lncRNA	chr1:244184953-244
ENSG0000(	588	13.13154	chr1:175{	AC099757.1	smallRNA	chr1:244589546-244
ENSG0000(	588	13.13154	chr1:175{	ENSG00000229960	lncRNA	chr1:244068820-244
ENSG0000(	588	13.13154	chr1:175{	ENSG00000279774	TEC	chr1:244064330-244
ENSG0000(	588	13.13154	chr1:175{	ENSG00000282317	lncRNA	chr1:244731024-244
ENSG0000(	588	13.13154	chr1:175{	RNU1-132P	smallRNA	chr1:245133927-245
ENSG0000(	588	13.13154	chr1:175{	CYCSP5	Pseudoger	chr1:244598391-244
ENSG0000(	588	13.13154	chr1:175{	ENSG00000232059	Pseudoger	chr1:244694432-244
ENSG0000(	588	13.13154	chr1:175{	DESI2	protein_c	chr1:244653103-244
ENSG0000(	588	13.13154	chr1:175{	ADSS2	protein_c	chr1:244408494-244
ENSG0000(	588	13.13154	chr1:175{	ENSG00000223353	Pseudoger	chr1:245123471-245
ENSG0000(	588	13.13154	chr1:175{	COX20	protein_c	chr1:244835616-244
ENSG0000(	588	13.13154	chr1:175{	EFCAB2	protein_c	chr1:244969682-245
ENSG0000(	588	13.13154	chr1:175{	ENSG00000289055	lncRNA	chr1:244047342-244
ENSG0000(	588	13.13154	chr1:175{	DNAJC19P8	Pseudoger	chr1:245282831-245
ENSG0000(	588	13.13154	chr1:175{	RN7SL148P	smallRNA	chr1:244103932-244
ENSG0000(	588	13.13154	chr1:175{	CATSPERE	protein_c	chr1:244454377-244
ENSG0000(	588	13.13154	chr1:175{	ENSG00000237759	lncRNA	chr1:244107365-244
ENSG0000(	587	13.1092	chr7:158{	RPL35P5	Pseudoger	chr7:66606738-6660
ENSG0000(	586	13.08687	chr12:171	ENSG00000275481	lncRNA	chr12:46388856-463
ENSG0000(	586	13.08687	chr12:171	ENSG00000290959	lncRNA	chr12:43739635-437
ENSG0000(	586	13.08687	chr12:171	ENSG00000289229	lncRNA	chr12:45728313-457
ENSG0000(	586	13.08687	chr12:171	ENSG00000287386	lncRNA	chr12:45164186-451
ENSG0000(	586	13.08687	chr12:171	ENSG00000278475	lncRNA	chr12:45789189-457
ENSG0000(	586	13.08687	chr12:171	ENSG00000257896	Pseudoger	chr12:43738829-437
ENSG0000(	586	13.08687	chr12:171	OR7A19P	Pseudoger	chr12:46592573-465
ENSG0000(	586	13.08687	chr12:171	LINC00938	lncRNA	chr12:45718046-457
ENSG0000(	586	13.08687	chr12:171	ENSG00000257864	Pseudoger	chr12:44248432-442
ENSG0000(	586	13.08687	chr12:171	ENSG00000257368	Pseudoger	chr12:45444766-454
ENSG0000(	586	13.08687	chr12:171	RNA5SP361	Pseudoger	chr12:45117771-451
ENSG0000(	586	13.08687	chr12:171	ENSG00000257496	lncRNA	chr12:46384233-464
ENSG0000(	586	13.08687	chr12:171	ENSG00000275286	lncRNA	chr12:44498616-444
ENSG0000(	586	13.08687	chr12:171	Y_RNA	smallRNA	chr12:44880868-448
ENSG0000(	586	13.08687	chr12:171	SLC38A4	protein_c	chr12:46764761-468
ENSG0000(	586	13.08687	chr12:171	DBX2-AS1	lncRNA	chr12:45050901-451
ENSG0000(	586	13.08687	chr12:171	ENSG00000257947	lncRNA	chr12:44244394-442
ENSG0000(	586	13.08687	chr12:171	ENSG00000289046	lncRNA	chr12:45990924-459
ENSG0000(	586	13.08687	chr12:171	TMEM117	protein_c	chr12:43835967-443
ENSG0000(	586	13.08687	chr12:171	RN7SL246P	smallRNA	chr12:45874029-458
ENSG0000(	586	13.08687	chr12:171	ENSG00000286671	lncRNA	chr12:44499961-445
ENSG0000(	586	13.08687	chr12:171	RACGAP1P1	Pseudoger	chr12:45063473-450
ENSG0000(	586	13.08687	chr12:171	RNU6-671P	smallRNA	chr12:45554590-455
ENSG0000(	586	13.08687	chr12:171	ANO6	protein_c	chr12:45215987-454

ENSG0000(	586	13.08687	chr12:171	TWF1		protein_c	chr12:43793723-438
ENSG0000(	586	13.08687	chr12:171	SCAF11		protein_c	chr12:45919131-459
ENSG0000(	586	13.08687	chr12:171	ENSG00000257738		Pseudoger	chr12:45200817-452
ENSG0000(	586	13.08687	chr12:171	SLC38A2		protein_c	chr12:46358188-463
ENSG0000(	586	13.08687	chr12:171	SLC38A4-AS1		lncRNA	chr12:46383433-468
ENSG0000(	586	13.08687	chr12:171	MARK3P1		Pseudoger	chr12:46682071-466
ENSG0000(	586	13.08687	chr12:171	ENSG00000239397		Pseudoger	chr12:46004038-460
ENSG0000(	586	13.08687	chr12:171	NELL2		protein_c	chr12:44508275-449
ENSG0000(	586	13.08687	chr12:171	KNOP1P2		Pseudoger	chr12:45880950-458
ENSG0000(	586	13.08687	chr12:171	ENSG00000278896		TEC	chr12:46404644-464
ENSG0000(	586	13.08687	chr12:171	PLEKHA8P1		Pseudoger	chr12:45139782-452
ENSG0000(	586	13.08687	chr12:171	SSBL3P		Pseudoger	chr12:45071769-450
ENSG0000(	586	13.08687	chr12:171	ZNF75BP		Pseudoger	chr12:44008620-440
ENSG0000(	586	13.08687	chr12:171	DBX2		protein_c	chr12:45014672-450
ENSG0000(	586	13.08687	chr12:171	EEF1A1P17		Pseudoger	chr12:43659827-436
ENSG0000(	586	13.08687	chr12:171	ENSG00000272369		lncRNA	chr12:46537502-466
ENSG0000(	586	13.08687	chr12:171	PUS7L		protein_c	chr12:43718992-437
ENSG0000(	586	13.08687	chr12:171	IRAK4	NCGv7	protein_c	chr12:43758944-437
ENSG0000(	586	13.08687	chr12:171	SLC38A1		protein_c	chr12:46183063-462
ENSG0000(	586	13.08687	chr12:171	ENSG00000257657		lncRNA	chr12:45475520-456
ENSG0000(	586	13.08687	chr12:171	SLC38A2-AS1		lncRNA	chr12:46371463-463
ENSG0000(	586	13.08687	chr12:171	ENSG00000291253		lncRNA	chr12:43736628-437
ENSG0000(	586	13.08687	chr12:171	ENSG00000274591		lncRNA	chr12:46239106-462
ENSG0000(	586	13.08687	chr12:171	ARID2	NCGv7;AC	protein_c	chr12:45729706-459
ENSG0000(	586	13.08687	chr12:171	ENSG00000274723		lncRNA	chr12:46970504-469
ENSG0000(	586	13.08687	chr12:171	ENSG00000278351		lncRNA	chr12:45256473-452
ENSG0000(	584	13.04221	chr3:380	CEP97	DriverDB	protein_c	chr3:101724534-101
ENSG0000(	584	13.04221	chr3:380	HHLA2	NCGv7	protein_c	chr3:108296529-108
ENSG0000(	584	13.04221	chr3:380	Y_RNA		smallRNA	chr3:101647421-101
ENSG0000(	584	13.04221	chr3:380	SRRM1P2		Pseudoger	chr3:83937045-8393
ENSG0000(	584	13.04221	chr3:380	MORC1		protein_c	chr3:108958248-109
ENSG0000(	584	13.04221	chr3:380	WDR82P1		Pseudoger	chr3:94937086-9493
ENSG0000(	584	13.04221	chr3:380	IFT57		protein_c	chr3:108160812-108
ENSG0000(	584	13.04221	chr3:380	ENSG00000242190		lncRNA	chr3:81762706-8176
ENSG0000(	584	13.04221	chr3:380	THAP12P2		Pseudoger	chr3:86012561-8601
ENSG0000(	584	13.04221	chr3:380	POU1F1		protein_c	chr3:87259404-8727
ENSG0000(	584	13.04221	chr3:380	Y_RNA		smallRNA	chr3:101458530-101
ENSG0000(	584	13.04221	chr3:380	ENSG00000240107		Pseudoger	chr3:103538635-103
ENSG0000(	584	13.04221	chr3:380	GBE1	NCGv7	protein_c	chr3:81489703-8176
ENSG0000(	584	13.04221	chr3:380	MTATP6P22		Pseudoger	chr3:106897671-106
ENSG0000(	584	13.04221	chr3:380	TOMM70		protein_c	chr3:100363431-100
ENSG0000(	584	13.04221	chr3:380	Y_RNA		smallRNA	chr3:106688678-106
ENSG0000(	584	13.04221	chr3:380	ACTR3P3		Pseudoger	chr3:101163094-101
ENSG0000(	584	13.04221	chr3:380	OR5H5P		Pseudoger	chr3:98197211-9819
ENSG0000(	584	13.04221	chr3:380	CADM2	NCGv7	protein_c	chr3:84958989-8607
ENSG0000(	584	13.04221	chr3:380	RN7SL647P		smallRNA	chr3:78115158-7811
ENSG0000(	584	13.04221	chr3:380	MTND1P16		Pseudoger	chr3:106901068-106
ENSG0000(	584	13.04221	chr3:380	ZNF654		protein_c	chr3:88059255-8814
ENSG0000(	584	13.04221	chr3:380	LINC00879		lncRNA	chr3:94937980-9516
ENSG0000(	584	13.04221	chr3:380	ENSG00000243945		Pseudoger	chr3:109977540-109
ENSG0000(	584	13.04221	chr3:380	FCF1P3		Pseudoger	chr3:106848671-106

ENSG0000(	584	13.04221	chr3:380<	ENSG00000242145		lncRNA	chr3:81208193-8120
ENSG0000(	584	13.04221	chr3:380<	ABCF2P1		Pseudoger	chr3:88317156-8831
ENSG0000(	584	13.04221	chr3:380<	ENSG00000240573		lncRNA	chr3:78087404-7809
ENSG0000(	584	13.04221	chr3:380<	TMEM45A		protein_c	chr3:100492619-100
ENSG0000(	584	13.04221	chr3:380<	ENSG00000290819		lncRNA	chr3:98100793-9810
ENSG0000(	584	13.04221	chr3:380<	RNU6-865P		smallRNA	chr3:100952627-100
ENSG0000(	584	13.04221	chr3:380<	ST3GAL6	NCGv7	protein_c	chr3:98732236-9882
ENSG0000(	584	13.04221	chr3:380<	ABI3BP		protein_c	chr3:100749156-100
ENSG0000(	584	13.04221	chr3:380<	ENSG00000240572		Pseudoger	chr3:99326377-9932
ENSG0000(	584	13.04221	chr3:380<	ENSG00000244652		Pseudoger	chr3:106325737-106
ENSG0000(	584	13.04221	chr3:380<	GPR15		protein_c	chr3:98531978-9853
ENSG0000(	584	13.04221	chr3:380<	Y_RNA		smallRNA	chr3:101628310-101
ENSG0000(	584	13.04221	chr3:380<	BBX	NCGv7	protein_c	chr3:107522936-107
ENSG0000(	584	13.04221	chr3:380<	ENSG00000240827		Pseudoger	chr3:98620065-9862
ENSG0000(	584	13.04221	chr3:380<	CBLB	NCGv7;AC	protein_c	chr3:105655461-105
ENSG0000(	584	13.04221	chr3:380<	RNU6-873P		smallRNA	chr3:87742543-8774
ENSG0000(	584	13.04221	chr3:380<	NIT2		protein_c	chr3:100334739-100
ENSG0000(	584	13.04221	chr3:380<	ARL6		protein_c	chr3:97764521-9780
ENSG0000(	584	13.04221	chr3:380<	MTND6P6		Pseudoger	chr3:106900593-106
ENSG0000(	584	13.04221	chr3:380<	TRMT10C	NCGv7	protein_c	chr3:101561868-101
ENSG0000(	584	13.04221	chr3:380<	ENSG00000277746		Pseudoger	chr3:86816740-8681
ENSG0000(	584	13.04221	chr3:380<	Y_RNA		smallRNA	chr3:93967926-9396
ENSG0000(	584	13.04221	chr3:380<	NDUFA5P5		Pseudoger	chr3:88601061-8860
ENSG0000(	584	13.04221	chr3:380<	ENSG00000286516		lncRNA	chr3:95916726-9591
ENSG0000(	584	13.04221	chr3:380<	ENSG00000240752		Pseudoger	chr3:78758974-7875
ENSG0000(	584	13.04221	chr3:380<	ENSG00000288557		lncRNA	chr3:106684388-106
ENSG0000(	584	13.04221	chr3:380<	ENSG00000240241		lncRNA	chr3:78266940-7829
ENSG0000(	584	13.04221	chr3:380<	ENSG00000288105		lncRNA	chr3:79411714-7947
ENSG0000(	584	13.04221	chr3:380<	ENSG00000286447		lncRNA	chr3:96810838-9681
ENSG0000(	584	13.04221	chr3:380<	LINC02025		lncRNA	chr3:84881984-8489
ENSG0000(	584	13.04221	chr3:380<	RPL18AP8		Pseudoger	chr3:96619304-9661
ENSG0000(	584	13.04221	chr3:380<	NDUFA4P2		Pseudoger	chr3:103240987-103
ENSG0000(	584	13.04221	chr3:380<	OR5H4P		Pseudoger	chr3:98222044-9822
ENSG0000(	584	13.04221	chr3:380<	Y_RNA		smallRNA	chr3:101662060-101
ENSG0000(	584	13.04221	chr3:380<	RPSAP29		Pseudoger	chr3:110682286-110
ENSG0000(	584	13.04221	chr3:380<	OR5H7P		Pseudoger	chr3:98238352-9823
ENSG0000(	584	13.04221	chr3:380<	ENSG00000286329		lncRNA	chr3:80602716-8067
ENSG0000(	584	13.04221	chr3:380<	RNU6-511P		smallRNA	chr3:93977029-9397
ENSG0000(	584	13.04221	chr3:380<	ZBTB11		protein_c	chr3:101648889-101
ENSG0000(	584	13.04221	chr3:380<	LINC02027		lncRNA	chr3:80993811-8110
ENSG0000(	584	13.04221	chr3:380<	RPL24		protein_c	chr3:101681091-101
ENSG0000(	584	13.04221	chr3:380<	UBFD1P1		Pseudoger	chr3:98434034-9843
ENSG0000(	584	13.04221	chr3:380<	RNU6-461P		smallRNA	chr3:102755064-102
ENSG0000(	584	13.04221	chr3:380<	HMGB1P38		Pseudoger	chr3:79957566-7995
ENSG0000(	584	13.04221	chr3:380<	TFG	IntOGen-I	protein_c	chr3:100709295-100
ENSG0000(	584	13.04221	chr3:380<	DUBR		lncRNA	chr3:107220744-107
ENSG0000(	584	13.04221	chr3:380<	ENSG00000249474		lncRNA	chr3:101823793-101
ENSG0000(	584	13.04221	chr3:380<	MTND3P6		Pseudoger	chr3:106896483-106
ENSG0000(	584	13.04221	chr3:380<	HMGN1P7		Pseudoger	chr3:93988634-9398
ENSG0000(	584	13.04221	chr3:380<	MTND2P14		Pseudoger	chr3:106902173-106
ENSG0000(	584	13.04221	chr3:380<	Y_RNA		smallRNA	chr3:106515897-106

ENSG0000(	584	13.04221	chr3:380<	RNU6-1236P	smallRNA	chr3:109225129-109
ENSG0000(	584	13.04221	chr3:380<	Y_RNA	smallRNA	chr3:110727021-110
ENSG0000(	584	13.04221	chr3:380<	AC117444.1	smallRNA	chr3:96666194-9666
ENSG0000(	584	13.04221	chr3:380<	RBBP4P2	Pseudoger	chr3:94075912-9407
ENSG0000(	584	13.04221	chr3:380<	MTHFD2P1	Pseudoger	chr3:95654423-9568
ENSG0000(	584	13.04221	chr3:380<	ENSG00000279658	lncRNA	chr3:97836986-9787
ENSG0000(	584	13.04221	chr3:380<	snoZ40	smallRNA	chr3:86125216-8612
ENSG0000(	584	13.04221	chr3:380<	FAM136CP	Pseudoger	chr3:101414228-101
ENSG0000(	584	13.04221	chr3:380<	NFYBP1	Pseudoger	chr3:109915976-109
ENSG0000(	584	13.04221	chr3:380<	ENSG00000285635	protein_c	chr3:98521133-9858
ENSG0000(	584	13.04221	chr3:380<	OR5AC4P	Pseudoger	chr3:98104754-9810
ENSG0000(	584	13.04221	chr3:380<	CCDC54-AS1	lncRNA	chr3:107272611-107
ENSG0000(	584	13.04221	chr3:380<	OR5BM1P	Pseudoger	chr3:98053374-9805
ENSG0000(	584	13.04221	chr3:380<	ENSG00000271671	Pseudoger	chr3:96244732-9624
ENSG0000(	584	13.04221	chr3:380<	KRT8P25	Pseudoger	chr3:87323268-8732
ENSG0000(	584	13.04221	chr3:380<	CADM2-AS2	lncRNA	chr3:85799987-8582
ENSG0000(	584	13.04221	chr3:380<	SETP6	Pseudoger	chr3:81612238-8161
ENSG0000(	584	13.04221	chr3:380<	ARL13B	protein_c	chr3:93980139-9405
ENSG0000(	584	13.04221	chr3:380<	RNU6-217P	smallRNA	chr3:77811741-7781
ENSG0000(	584	13.04221	chr3:380<	ENSG00000287273	lncRNA	chr3:84940619-8494
ENSG0000(	584	13.04221	chr3:380<	ZNF90P1	Pseudoger	chr3:101357292-101
ENSG0000(	584	13.04221	chr3:380<	ACTG1P13	Pseudoger	chr3:99215224-9921
ENSG0000(	584	13.04221	chr3:380<	ENSG00000251088	lncRNA	chr3:98233651-9845
ENSG0000(	584	13.04221	chr3:380<	ENSG00000241754	lncRNA	chr3:102621061-102
ENSG0000(	584	13.04221	chr3:380<	WWP1P1	Pseudoger	chr3:98657802-9866
ENSG0000(	584	13.04221	chr3:380<	DCBLD2	protein_c	chr3:98795941-9890
ENSG0000(	584	13.04221	chr3:380<	PPATP1	Pseudoger	chr3:87051192-8705
ENSG0000(	584	13.04221	chr3:380<	ROBO1	protein_c	chr3:78597239-7976
ENSG0000(	584	13.04221	chr3:380<	ENSG00000241280	lncRNA	chr3:101960358-101
ENSG0000(	584	13.04221	chr3:380<	ENSG00000282527	lncRNA	chr3:97800732-9782
ENSG0000(	584	13.04221	chr3:380<	ENSG00000287378	lncRNA	chr3:100132903-100
ENSG0000(	584	13.04221	chr3:380<	PDCL3P4	Pseudoger	chr3:101686827-101
ENSG0000(	584	13.04221	chr3:380<	ENSG00000241776	Pseudoger	chr3:84691681-8469
ENSG0000(	584	13.04221	chr3:380<	ENSG00000287421	lncRNA	chr3:106160417-106
ENSG0000(	584	13.04221	chr3:380<	ENSG00000241777	Pseudoger	chr3:108725440-108
ENSG0000(	584	13.04221	chr3:380<	ALCAM	protein_c	chr3:105366909-105
ENSG0000(	584	13.04221	chr3:380<	ZPLD1	protein_c	chr3:102099244-102
ENSG0000(	584	13.04221	chr3:380<	SNORD61	smallRNA	chr3:100221521-100
ENSG0000(	584	13.04221	chr3:380<	DUSP12P1	Pseudoger	chr3:100221268-100
ENSG0000(	584	13.04221	chr3:380<	ENSG00000241634	Pseudoger	chr3:108543367-108
ENSG0000(	584	13.04221	chr3:380<	LINC02070	lncRNA	chr3:86481943-8649
ENSG0000(	584	13.04221	chr3:380<	PPIAP15	Pseudoger	chr3:109471329-109
ENSG0000(	584	13.04221	chr3:380<	DIMT1P1	Pseudoger	chr3:109701456-109
ENSG0000(	584	13.04221	chr3:380<	AC134729.1	smallRNA	chr3:77926705-7792
ENSG0000(	584	13.04221	chr3:380<	ENSG00000286956	lncRNA	chr3:107046026-107
ENSG0000(	584	13.04221	chr3:380<	FILIP1L NCGv7	protein_c	chr3:99828811-1001
ENSG0000(	584	13.04221	chr3:380<	OSBPL9P1	Pseudoger	chr3:80163327-8016
ENSG0000(	584	13.04221	chr3:380<	ENSG00000291293	lncRNA	chr3:106449775-106
ENSG0000(	584	13.04221	chr3:380<	VGLL3	protein_c	chr3:86876388-8699
ENSG0000(	584	13.04221	chr3:380<	OR5K3	protein_c	chr3:98390666-9839
ENSG0000(	584	13.04221	chr3:380<	HNRNPA3P8	Pseudoger	chr3:80216261-8021

ENSG0000(	584	13.04221	chr3:380<	OR5H14		protein_c	chr3:98147479-9815
ENSG0000(	584	13.04221	chr3:380<	LNP1		protein_c	chr3:100401532-100
ENSG0000(	584	13.04221	chr3:380<	MIR3921		smallRNA	chr3:99964314-9996
ENSG0000(	584	13.04221	chr3:380<	AC117479.1		smallRNA	chr3:79584592-7958
ENSG0000(	584	13.04221	chr3:380<	RNU6-26P		smallRNA	chr3:98804978-9880
ENSG0000(	584	13.04221	chr3:380<	LINC00635		lncRNA	chr3:107840228-107
ENSG0000(	584	13.04221	chr3:380<	AC108697.1		smallRNA	chr3:96433315-9643
ENSG0000(	584	13.04221	chr3:380<	ENSG00000286854		lncRNA	chr3:106367979-106
ENSG0000(	584	13.04221	chr3:380<	snoU13		smallRNA	chr3:101502155-101
ENSG0000(	584	13.04221	chr3:380<	PROS1		protein_c	chr3:93873051-9398
ENSG0000(	584	13.04221	chr3:380<	RPL7AP23		Pseudoger	chr3:82319172-8232
ENSG0000(	584	13.04221	chr3:380<	ENSG00000239767		lncRNA	chr3:86258936-8626
ENSG0000(	584	13.04221	chr3:380<	CBX5P1		Pseudoger	chr3:88095408-8809
ENSG0000(	584	13.04221	chr3:380<	ENSG00000287053		lncRNA	chr3:81359739-8136
ENSG0000(	584	13.04221	chr3:380<	ENSG00000276763		Pseudoger	chr3:108574683-108
ENSG0000(	584	13.04221	chr3:380<	ENSG00000241593		lncRNA	chr3:81246579-8129
ENSG0000(	584	13.04221	chr3:380<	LINC00506		lncRNA	chr3:87089129-8715
ENSG0000(	584	13.04221	chr3:380<	ENSG00000287158		lncRNA	chr3:83953639-8405
ENSG0000(	584	13.04221	chr3:380<	CMSS1		protein_c	chr3:99817837-1001
ENSG0000(	584	13.04221	chr3:380<	ENSG00000241257		Pseudoger	chr3:109364047-109
ENSG0000(	584	13.04221	chr3:380<	ENSG00000251172		Pseudoger	chr3:97481177-9748
ENSG0000(	584	13.04221	chr3:380<	MTND4P16		Pseudoger	chr3:106894352-106
ENSG0000(	584	13.04221	chr3:380<	RCC2P5		Pseudoger	chr3:96753185-9675
ENSG0000(	584	13.04221	chr3:380<	EPHA3	NCGv7	protein_c	chr3:89107621-8948
ENSG0000(	584	13.04221	chr3:380<	RDUR		lncRNA	chr3:101940859-101
ENSG0000(	584	13.04221	chr3:380<	SNORA70		smallRNA	chr3:108574565-108
ENSG0000(	584	13.04221	chr3:380<	RAP1BP2		Pseudoger	chr3:104063039-104
ENSG0000(	584	13.04221	chr3:380<	LINC00488		lncRNA	chr3:109178143-109
ENSG0000(	584	13.04221	chr3:380<	GABRR3		protein_c	chr3:97985102-9803
ENSG0000(	584	13.04221	chr3:380<	ENSG00000242029		lncRNA	chr3:109648107-109
ENSG0000(	584	13.04221	chr3:380<	SNORA31		smallRNA	chr3:100646121-100
ENSG0000(	584	13.04221	chr3:380<	PDLIM1P4		Pseudoger	chr3:98782188-9878
ENSG0000(	584	13.04221	chr3:380<	LINC01205		lncRNA	chr3:109409990-109
ENSG0000(	584	13.04221	chr3:380<	RPL38P4		Pseudoger	chr3:98525061-9852
ENSG0000(	584	13.04221	chr3:380<	CSNKA2IP		protein_c	chr3:88338416-8846
ENSG0000(	584	13.04221	chr3:380<	Y_RNA		smallRNA	chr3:101955077-101
ENSG0000(	584	13.04221	chr3:380<	ENSG00000286567		lncRNA	chr3:83924157-8393
ENSG0000(	584	13.04221	chr3:380<	ZBTB11-AS1		lncRNA	chr3:101676424-101
ENSG0000(	584	13.04221	chr3:380<	RN7SL751P		smallRNA	chr3:78938005-7893
ENSG0000(	584	13.04221	chr3:380<	MTCO2P6		Pseudoger	chr3:89587886-8958
ENSG0000(	584	13.04221	chr3:380<	AC055723.1		smallRNA	chr3:99691480-9969
ENSG0000(	584	13.04221	chr3:380<	ENSG00000244561		Pseudoger	chr3:109396616-109
ENSG0000(	584	13.04221	chr3:380<	ENSG00000288004		lncRNA	chr3:83384445-8343
ENSG0000(	584	13.04221	chr3:380<	SENP7		protein_c	chr3:101324205-101
ENSG0000(	584	13.04221	chr3:380<	APOOP2		Pseudoger	chr3:87594838-8759
ENSG0000(	584	13.04221	chr3:380<	ENSG00000240895		lncRNA	chr3:110527482-110
ENSG0000(	584	13.04221	chr3:380<	RNU1-43P		smallRNA	chr3:103159960-103
ENSG0000(	584	13.04221	chr3:380<	VTI1BP1		Pseudoger	chr3:100225374-100
ENSG0000(	584	13.04221	chr3:380<	GUCA1C		protein_c	chr3:108907792-108
ENSG0000(	584	13.04221	chr3:380<	CCDC54		protein_c	chr3:107377439-107
ENSG0000(	584	13.04221	chr3:380<	ENSG00000242009		lncRNA	chr3:81840514-8195

ENSG0000	584	13.04221	chr3:380	HSPE1P19		Pseudoger	chr3:90261414-9026
ENSG0000	584	13.04221	chr3:380	ENSG00000248839		lncRNA	chr3:98522570-9852
ENSG0000	584	13.04221	chr3:380	DPPA4	NCGv7;AC	protein_c	chr3:109326144-109
ENSG0000	584	13.04221	chr3:380	C3orf85		protein_c	chr3:109118252-109
ENSG0000	584	13.04221	chr3:380	LINC01215		lncRNA	chr3:108125821-108
ENSG0000	584	13.04221	chr3:380	CSP2		Pseudoger	chr3:107327830-107
ENSG0000	584	13.04221	chr3:380	ENSG00000241815		Pseudoger	chr3:96566357-9656
ENSG0000	584	13.04221	chr3:380	GAPDHP50		Pseudoger	chr3:89047442-8904
ENSG0000	584	13.04221	chr3:380	AC016970.1		smallRNA	chr3:104054708-104
ENSG0000	584	13.04221	chr3:380	ENSG00000282978		Pseudoger	chr3:101457241-101
ENSG0000	584	13.04221	chr3:380	RNU7-108P		smallRNA	chr3:94098507-9409
ENSG0000	584	13.04221	chr3:380	LINC02077		lncRNA	chr3:78038705-7804
ENSG0000	584	13.04221	chr3:380	RIOX2	AC	protein_c	chr3:97941818-9797
ENSG0000	584	13.04221	chr3:380	MIR3923		smallRNA	chr3:79507887-7950
ENSG0000	584	13.04221	chr3:380	MTND4LP3		Pseudoger	chr3:106895729-106
ENSG0000	584	13.04221	chr3:380	ENSG00000244464		lncRNA	chr3:99598064-9963
ENSG0000	584	13.04221	chr3:380	ENSG00000287682		lncRNA	chr3:101878333-101
ENSG0000	584	13.04221	chr3:380	CYP51A1P1		Pseudoger	chr3:82806515-8280
ENSG0000	584	13.04221	chr3:380	ENSG00000249225		lncRNA	chr3:98099908-9814
ENSG0000	584	13.04221	chr3:380	TBC1D23		protein_c	chr3:100260992-100
ENSG0000	584	13.04221	chr3:380	MIR4795		smallRNA	chr3:87226189-8722
ENSG0000	584	13.04221	chr3:380	Y_RNA		smallRNA	chr3:88431191-8843
ENSG0000	584	13.04221	chr3:380	RNU6-1263P		smallRNA	chr3:98908459-9890
ENSG0000	584	13.04221	chr3:380	TMEM30CP		Pseudoger	chr3:100185687-100
ENSG0000	584	13.04221	chr3:380	MIR5688		smallRNA	chr3:85385710-8538
ENSG0000	584	13.04221	chr3:380	MTCO1P35		Pseudoger	chr3:106900854-106
ENSG0000	584	13.04221	chr3:380	MIR548AB		smallRNA	chr3:103524033-103
ENSG0000	584	13.04221	chr3:380	ENSG00000286602		lncRNA	chr3:98573061-9857
ENSG0000	584	13.04221	chr3:380	ENSG00000285614		lncRNA	chr3:97973012-9797
ENSG0000	584	13.04221	chr3:380	RPS18P6		Pseudoger	chr3:95171920-9517
ENSG0000	584	13.04221	chr3:380	OR5H6		protein_c	chr3:98263252-9826
ENSG0000	584	13.04221	chr3:380	ENSG00000242299		Pseudoger	chr3:101576489-101
ENSG0000	584	13.04221	chr3:380	ENSG00000244281		Pseudoger	chr3:81366990-8136
ENSG0000	584	13.04221	chr3:380	EPHA6	NCGv7	protein_c	chr3:96814581-9776
ENSG0000	584	13.04221	chr3:380	ENSG00000213431		Pseudoger	chr3:100910975-100
ENSG0000	584	13.04221	chr3:380	U3		smallRNA	chr3:90030284-9003
ENSG0000	584	13.04221	chr3:380	AC021660.1		smallRNA	chr3:98623949-9862
ENSG0000	584	13.04221	chr3:380	IMPG2	NCGv7	protein_c	chr3:101222546-101
ENSG0000	584	13.04221	chr3:380	CIP2A	AC	protein_c	chr3:108549864-108
ENSG0000	584	13.04221	chr3:380	RETNLB		protein_c	chr3:108743424-108
ENSG0000	584	13.04221	chr3:380	PCNP		protein_c	chr3:101574180-101
ENSG0000	584	13.04221	chr3:380	FAM172BP		Pseudoger	chr3:101521891-101
ENSG0000	584	13.04221	chr3:380	RNU6ATAC6P		smallRNA	chr3:87910220-8791
ENSG0000	584	13.04221	chr3:380	TRAT1		protein_c	chr3:108822770-108
ENSG0000	584	13.04221	chr3:380	CADM2-AS1		lncRNA	chr3:85992183-8602
ENSG0000	584	13.04221	chr3:380	DPPA2	AC	protein_c	chr3:109293788-109
ENSG0000	584	13.04221	chr3:380	LINC01990		lncRNA	chr3:107430892-107
ENSG0000	584	13.04221	chr3:380	ENSG00000261292		lncRNA	chr3:97759523-9776
ENSG0000	584	13.04221	chr3:380	MIR4445		smallRNA	chr3:109602828-109
ENSG0000	584	13.04221	chr3:380	ENSG00000243296		lncRNA	chr3:99500347-9950
ENSG0000	584	13.04221	chr3:380	LINC00971		lncRNA	chr3:84638405-8488

ENSG0000(	584	13.04221	chr3:380∠	RN7SKP61		smallRNA	chr3:78161698-7816
ENSG0000(	584	13.04221	chr3:380∠	HNRNPKP4		Pseudoger	chr3:96349554-9635
ENSG0000(	584	13.04221	chr3:380∠	DHFR2		protein_c	chr3:94047836-9406
ENSG0000(	584	13.04221	chr3:380∠	NSUN3		protein_c	chr3:94062980-9413
ENSG0000(	584	13.04221	chr3:380∠	RNU6ATAC15P		smallRNA	chr3:110551845-110
ENSG0000(	584	13.04221	chr3:380∠	CGGBP1		protein_c	chr3:88051944-8814
ENSG0000(	584	13.04221	chr3:380∠	ARMC10P1		Pseudoger	chr3:94506766-9450
ENSG0000(	584	13.04221	chr3:380∠	AC092905.1		smallRNA	chr3:109497645-109
ENSG0000(	584	13.04221	chr3:380∠	H3P12		Pseudoger	chr3:109409678-109
ENSG0000(	584	13.04221	chr3:380∠	AC107025.1		smallRNA	chr3:84496576-8449
ENSG0000(	584	13.04221	chr3:380∠	ENSG00000239572		lncRNA	chr3:87641412-8779
ENSG0000(	584	13.04221	chr3:380∠	AC078855.1		smallRNA	chr3:108320392-108
ENSG0000(	584	13.04221	chr3:380∠	CPOX		protein_c	chr3:98579446-9859
ENSG0000(	584	13.04221	chr3:380∠	CLDND1	DriverDB\	protein_c	chr3:98497912-9852
ENSG0000(	584	13.04221	chr3:380∠	MRPS17P3		Pseudoger	chr3:78525101-7852
ENSG0000(	584	13.04221	chr3:380∠	OR5K1		protein_c	chr3:98463201-9847
ENSG0000(	584	13.04221	chr3:380∠	LINC00973		lncRNA	chr3:98981058-9898
ENSG0000(	584	13.04221	chr3:380∠	ENSG00000289078		lncRNA	chr3:98191359-9819
ENSG0000(	584	13.04221	chr3:380∠	RN7SKP284		smallRNA	chr3:86362132-8636
ENSG0000(	584	13.04221	chr3:380∠	ENSG00000242828		lncRNA	chr3:80761042-8078
ENSG0000(	584	13.04221	chr3:380∠	CDV3P1		Pseudoger	chr3:96776945-9677
ENSG0000(	584	13.04221	chr3:380∠	CHMP2B	NCGv7	protein_c	chr3:87227271-8725
ENSG0000(	584	13.04221	chr3:380∠	ENSG00000240404		Pseudoger	chr3:81612790-8161
ENSG0000(	584	13.04221	chr3:380∠	MTCO3P35		Pseudoger	chr3:106896891-106
ENSG0000(	584	13.04221	chr3:380∠	OR5H1		protein_c	chr3:98130721-9813
ENSG0000(	584	13.04221	chr3:380∠	RNU6-712P		smallRNA	chr3:90184634-9018
ENSG0000(	584	13.04221	chr3:380∠	AC132660.1		smallRNA	chr3:84905679-8490
ENSG0000(	584	13.04221	chr3:380∠	ICE2P2		Pseudoger	chr3:88948142-8895
ENSG0000(	584	13.04221	chr3:380∠	RNU6-1094P		smallRNA	chr3:96608814-9660
ENSG0000(	584	13.04221	chr3:380∠	LINC00882		lncRNA	chr3:106630469-107
ENSG0000(	584	13.04221	chr3:380∠	ENSG00000288848		lncRNA	chr3:105869180-105
ENSG0000(	584	13.04221	chr3:380∠	LINC02050		lncRNA	chr3:80764862-8078
ENSG0000(	584	13.04221	chr3:380∠	ENSG00000279277		lncRNA	chr3:108032456-108
ENSG0000(	584	13.04221	chr3:380∠	AC078828.1		smallRNA	chr3:99366801-9936
ENSG0000(	584	13.04221	chr3:380∠	RPL32P7		Pseudoger	chr3:101599243-101
ENSG0000(	584	13.04221	chr3:380∠	GMFBP1		Pseudoger	chr3:100665050-100
ENSG0000(	584	13.04221	chr3:380∠	ENSG00000285780		lncRNA	chr3:89283016-8931
ENSG0000(	584	13.04221	chr3:380∠	OR5H15		protein_c	chr3:98166696-9816
ENSG0000(	584	13.04221	chr3:380∠	CD47		protein_c	chr3:108043091-108
ENSG0000(	584	13.04221	chr3:380∠	AC063944.1		smallRNA	chr3:107365182-107
ENSG0000(	584	13.04221	chr3:380∠	OR5K2		protein_c	chr3:98497681-9849
ENSG0000(	584	13.04221	chr3:380∠	ENSG00000261364		lncRNA	chr3:109176438-109
ENSG0000(	584	13.04221	chr3:380∠	MTCO1P6		Pseudoger	chr3:89588714-8959
ENSG0000(	584	13.04221	chr3:380∠	RNU6-1308P		smallRNA	chr3:107007568-107
ENSG0000(	584	13.04221	chr3:380∠	RNU2-28P		smallRNA	chr3:81509476-8150
ENSG0000(	584	13.04221	chr3:380∠	OR5H3P		Pseudoger	chr3:98207423-9820
ENSG0000(	584	13.04221	chr3:380∠	LINC00636		lncRNA	chr3:107834586-107
ENSG0000(	584	13.04221	chr3:380∠	DZIP3		protein_c	chr3:108589705-108
ENSG0000(	584	13.04221	chr3:380∠	ENSG00000242911		Pseudoger	chr3:94935760-9493
ENSG0000(	584	13.04221	chr3:380∠	ENSG00000288780		lncRNA	chr3:87959768-8796
ENSG0000(	584	13.04221	chr3:380∠	OR5K4		protein_c	chr3:98353854-9835

ENSG00000	584	13.04221	chr3:3804	OR5AC2		protein_c	chr3:98087173-9808
ENSG00000	584	13.04221	chr3:3804	PSMC1P6		Pseudogen	chr3:87630301-8763
ENSG00000	584	13.04221	chr3:3804	OR5AC1		protein_c	chr3:98064472-9806
ENSG00000	584	13.04221	chr3:3804	ST3GAL6-AS1		lncRNA	chr3:98706236-9873
ENSG00000	584	13.04221	chr3:3804	RNU6-1256P		smallRNA	chr3:101615826-101
ENSG00000	584	13.04221	chr3:3804	OR5H8		protein_c	chr3:98306752-9831
ENSG00000	584	13.04221	chr3:3804	ENSG00000239462		lncRNA	chr3:98902045-9901
ENSG00000	584	13.04221	chr3:3804	NXPE3		protein_c	chr3:101779177-101
ENSG00000	584	13.04221	chr3:3804	ENSG00000239455		Pseudogen	chr3:107104911-107
ENSG00000	584	13.04221	chr3:3804	AC108739.1		smallRNA	chr3:96166817-9616
ENSG00000	584	13.04221	chr3:3804	ENSG00000243089		lncRNA	chr3:99507187-9952
ENSG00000	584	13.04221	chr3:3804	ADGRG7	DriverDBv	protein_c	chr3:100609601-100
ENSG00000	584	13.04221	chr3:3804	ENSG00000243635		Pseudogen	chr3:104502700-104
ENSG00000	584	13.04221	chr3:3804	COL8A1		protein_c	chr3:99638475-9979
ENSG00000	584	13.04221	chr3:3804	MYH15		protein_c	chr3:108380368-108
ENSG00000	584	13.04221	chr3:3804	PROS2P		Pseudogen	chr3:90202316-9025
ENSG00000	584	13.04221	chr3:3804	C3orf38		protein_c	chr3:88149959-8816
ENSG00000	584	13.04221	chr3:3804	OR5H2		protein_c	chr3:98282505-9828
ENSG00000	584	13.04221	chr3:3804	ENSG00000272597		lncRNA	chr3:107329430-107
ENSG00000	584	13.04221	chr3:3804	MORC1-AS1		lncRNA	chr3:109101456-109
ENSG00000	584	13.04221	chr3:3804	POU5F1P7		Pseudogen	chr3:98119191-9811
ENSG00000	584	13.04221	chr3:3804	LINC02008		lncRNA	chr3:81986138-8246
ENSG00000	584	13.04221	chr3:3804	MTND5P16		Pseudogen	chr3:106898889-106
ENSG00000	584	13.04221	chr3:3804	ENSG00000239288		Pseudogen	chr3:109241507-109
ENSG00000	584	13.04221	chr3:3804	ENSG00000289509		lncRNA	chr3:98262714-9832
ENSG00000	584	13.04221	chr3:3804	AC108696.1		smallRNA	chr3:84290112-8429
ENSG00000	584	13.04221	chr3:3804	CRYBG3		protein_c	chr3:97822011-9794
ENSG00000	584	13.04221	chr3:3804	NFKBIZ	NCGv7	protein_c	chr3:101827991-101
ENSG00000	584	13.04221	chr3:3804	ENSG00000289358		lncRNA	chr3:109337740-109
ENSG00000	584	13.04221	chr3:3804	TUBBP11		Pseudogen	chr3:103630661-103
ENSG00000	584	13.04221	chr3:3804	ENSG00000289629		lncRNA	chr3:101595787-101
ENSG00000	584	13.04221	chr3:3804	ENSG00000242586		Pseudogen	chr3:80442081-8044
ENSG00000	584	13.04221	chr3:3804	ENSG00000288654		protein_c	chr3:88058139-8805
ENSG00000	584	13.04221	chr3:3804	RNU6-1129P		smallRNA	chr3:86235321-8623
ENSG00000	584	13.04221	chr3:3804	RNU6-488P		smallRNA	chr3:93843764-9384
ENSG00000	584	13.04221	chr3:3804	HNRNPA1P17		Pseudogen	chr3:108325520-108
ENSG00000	584	13.04221	chr3:3804	HTR1F		protein_c	chr3:87792706-8799
ENSG00000	584	13.04221	chr3:3804	STX19		protein_c	chr3:94014365-9402
ENSG00000	584	13.04221	chr3:3804	U3		smallRNA	chr3:109018910-109
ENSG00000	584	13.04221	chr3:3804	ENSG00000273374		lncRNA	chr3:99802699-9980
ENSG00000	583	13.01987	chr3:3804	PCCB		protein_c	chr3:136250340-136
ENSG00000	583	13.01987	chr3:3804	SOX14		protein_c	chr3:137764315-137
ENSG00000	583	13.01987	chr3:3804	NDUFS6P1		Pseudogen	chr3:135959275-135
ENSG00000	583	13.01987	chr3:3804	ENSG00000272609		lncRNA	chr3:138004649-138
ENSG00000	583	13.01987	chr3:3804	SLC35G2	NCGv7	protein_c	chr3:136818647-136
ENSG00000	583	13.01987	chr3:3804	EPHB1	NCGv7	protein_c	chr3:134795260-135
ENSG00000	583	13.01987	chr3:3804	ENSG00000240695		Pseudogen	chr3:136736500-136
ENSG00000	583	13.01987	chr3:3804	HSPA8P9		Pseudogen	chr3:137880295-137
ENSG00000	583	13.01987	chr3:3804	ENSG00000273486		lncRNA	chr3:136837338-136
ENSG00000	583	13.01987	chr3:3804	ENSG00000242222		Pseudogen	chr3:135925891-135
ENSG00000	583	13.01987	chr3:3804	U8		smallRNA	chr3:135799694-135

ENSG0000(	583	13.01987	chr3:3804	RNU7-198P		smallRNA	chr3:136633148-136
ENSG0000(	583	13.01987	chr3:3804	NPM1P17		Pseudoger	chr3:137723774-137
ENSG0000(	583	13.01987	chr3:3804	RNA5SP142		Pseudoger	chr3:137518134-137
ENSG0000(	583	13.01987	chr3:3804	NCK1		protein_c	chr3:136862208-136
ENSG0000(	583	13.01987	chr3:3804	RAD51AP1P1		Pseudoger	chr3:136899076-136
ENSG0000(	583	13.01987	chr3:3804	STAG1-DT		lncRNA	chr3:136752630-136
ENSG0000(	583	13.01987	chr3:3804	ENSG00000273455		lncRNA	chr3:136087475-136
ENSG0000(	583	13.01987	chr3:3804	TDGF1P6		Pseudoger	chr3:136155549-136
ENSG0000(	583	13.01987	chr3:3804	STAG1	NCGv7	protein_c	chr3:136336236-136
ENSG0000(	583	13.01987	chr3:3804	ENSG00000286915		lncRNA	chr3:136778181-136
ENSG0000(	583	13.01987	chr3:3804	ENSG00000243886		Pseudoger	chr3:137535572-137
ENSG0000(	583	13.01987	chr3:3804	RNU6-789P		smallRNA	chr3:136721394-136
ENSG0000(	583	13.01987	chr3:3804	PPP2R3A	NCGv7	protein_c	chr3:135965728-136
ENSG0000(	583	13.01987	chr3:3804	RNU6-1284P		smallRNA	chr3:136430084-136
ENSG0000(	583	13.01987	chr3:3804	RNY4P4		smallRNA	chr3:136588209-136
ENSG0000(	583	13.01987	chr3:3804	ENSG00000240086		lncRNA	chr3:135138469-135
ENSG0000(	583	13.01987	chr3:3804	NCK1-DT		lncRNA	chr3:136835345-136
ENSG0000(	583	13.01987	chr3:3804	ENSG00000227267		Pseudoger	chr3:136055184-136
ENSG0000(	583	13.01987	chr3:3804	ENSG00000261146		lncRNA	chr3:137791973-137
ENSG0000(	583	13.01987	chr3:3804	IL20RB	DriverDBv	protein_c	chr3:136946230-137
ENSG0000(	583	13.01987	chr3:3804	ENSG00000244740		Pseudoger	chr3:136205540-136
ENSG0000(	583	13.01987	chr3:3804	MSL2		protein_c	chr3:136148917-136
ENSG0000(	583	13.01987	chr3:3804	ENSG00000242968		Pseudoger	chr3:136808551-136
ENSG0000(	583	13.01987	chr3:3804	HMGN1P10		Pseudoger	chr3:136609050-136
ENSG0000(	583	13.01987	chr3:3804	IL20RB-AS1		lncRNA	chr3:136959125-136
ENSG0000(	583	13.01987	chr3:3804	LINC01210		lncRNA	chr3:137771660-137
ENSG0000(	579	12.93054	chr4:7339	CCDC158		protein_c	chr4:76312997-7642
ENSG0000(	579	12.93054	chr4:7339	ENSG00000271676		Pseudoger	chr4:77112495-7711
ENSG0000(	579	12.93054	chr4:7339	ENSG00000287401		lncRNA	chr4:76240740-7631
ENSG0000(	579	12.93054	chr4:7339	SNORD75		smallRNA	chr4:77702746-7770
ENSG0000(	579	12.93054	chr6:1391	SAMD5		protein_c	chr6:147508690-147
ENSG0000(	579	12.93054	chr4:7339	CXCL1	TAG;AC	protein_c	chr4:73869393-7387
ENSG0000(	579	12.93054	chr4:7339	RCHY1		protein_c	chr4:75479033-7551
ENSG0000(	579	12.93054	chr4:7339	STBD1		protein_c	chr4:76306733-7631
ENSG0000(	579	12.93054	chr4:7339	CCNI	NCGv7	protein_c	chr4:77047155-7707
ENSG0000(	579	12.93054	chr4:7339	ENSG00000250735		lncRNA	chr4:75401195-7542
ENSG0000(	579	12.93054	chr4:7339	MTHFD2L		protein_c	chr4:74114174-7430
ENSG0000(	579	12.93054	chr4:7339	PF4		protein_c	chr4:73980811-7398
ENSG0000(	579	12.93054	chr4:7339	ENSG00000250532		lncRNA	chr4:74418917-7444
ENSG0000(	579	12.93054	chr4:7339	ENSG00000289586		lncRNA	chr4:76908814-7694
ENSG0000(	579	12.93054	chr4:7339	ENSG00000287375		lncRNA	chr4:74881174-7488
ENSG0000(	579	12.93054	chr4:7339	ENSG00000288019		lncRNA	chr4:74076533-7407
ENSG0000(	579	12.93054	chr4:7339	SDAD1		protein_c	chr4:75940950-7599
ENSG0000(	579	12.93054	chr4:7339	USO1		protein_c	chr4:75724577-7581
ENSG0000(	579	12.93054	chr4:7339	CCNG2		protein_c	chr4:77157207-7743
ENSG0000(	579	12.93054	chr4:7339	PPEF2		protein_c	chr4:75859864-7590
ENSG0000(	579	12.93054	chr4:7339	SCARB2		protein_c	chr4:76158737-7623
ENSG0000(	579	12.93054	chr4:7339	SEPTIN11		protein_c	chr4:76949751-7704
ENSG0000(	579	12.93054	chr4:7339	G3BP2	NCGv7	protein_c	chr4:75641849-7572
ENSG0000(	579	12.93054	chr4:7339	CXCL8		protein_c	chr4:73740519-7374
ENSG0000(	579	12.93054	chr4:7339	CXCL9		protein_c	chr4:76001275-7600

ENSG0000(	579	12.93054	chr4:7339	RASSF6	NCGv7	protein_c	chr4:73571550-7362
ENSG0000(	579	12.93054	chr4:7339	NUP54	NCGv7	protein_c	chr4:76107562-7614
ENSG0000(	579	12.93054	chr4:7339	NAAA	AC	protein_c	chr4:75913660-7594
ENSG0000(	579	12.93054	chr4:7339	CXCL13		protein_c	chr4:77511753-7761
ENSG0000(	579	12.93054	chr4:7339	ENSG00000249036		lncRNA	chr4:77394491-7749
ENSG0000(	579	12.93054	chr4:7339	ENSG00000248646		lncRNA	chr4:75361207-7543
ENSG0000(	579	12.93054	chr4:7339	RN7SL218P		smallRNA	chr4:74011578-7401
ENSG0000(	579	12.93054	chr4:7339	PPBP		protein_c	chr4:73986439-7398
ENSG0000(	579	12.93054	chr4:7339	ENSG00000248165		lncRNA	chr4:74993877-7503
ENSG0000(	579	12.93054	chr4:7339	ENSG00000289241		lncRNA	chr4:74099403-7409
ENSG0000(	579	12.93054	chr4:7339	CXCL10		protein_c	chr4:76021118-7602
ENSG0000(	579	12.93054	chr4:7339	PARM1-AS1		lncRNA	chr4:74955974-7497
ENSG0000(	579	12.93054	chr4:7339	EREG		protein_c	chr4:74365145-7438
ENSG0000(	579	12.93054	chr4:7339	CXCL6		protein_c	chr4:73836640-7384
ENSG0000(	579	12.93054	chr4:7339	CXCL11		protein_c	chr4:76033682-7604
ENSG0000(	579	12.93054	chr4:7339	ENSG00000250006		Pseudoger	chr4:77311397-7731
ENSG0000(	579	12.93054	chr4:7339	MIR548AH		smallRNA	chr4:76575551-7657
ENSG0000(	579	12.93054	chr4:7339	LINC02483		lncRNA	chr4:75354076-7536
ENSG0000(	579	12.93054	chr4:7339	ART3		protein_c	chr4:76011184-7611
ENSG0000(	579	12.93054	chr4:7339	PPBPP1		Pseudoger	chr4:73847866-7384
ENSG0000(	579	12.93054	chr4:7339	RPL36P8		Pseudoger	chr4:76036750-7603
ENSG0000(	579	12.93054	chr4:7339	PARM1	AC	protein_c	chr4:74933095-7505
ENSG0000(	579	12.93054	chr4:7339	UMLILO		lncRNA	chr4:73710302-7371
ENSG0000(	579	12.93054	chr4:7339	SOWAHB		protein_c	chr4:76894152-7689
ENSG0000(	579	12.93054	chr4:7339	AC104687.1		smallRNA	chr4:76853964-7685
ENSG0000(	579	12.93054	chr4:7339	RNU6-145P		smallRNA	chr4:76532222-7653
ENSG0000(	579	12.93054	chr4:7339	ENSG00000289515		lncRNA	chr4:76305887-7630
ENSG0000(	579	12.93054	chr4:7339	ALB	NCGv7	protein_c	chr4:73397114-7342
ENSG0000(	579	12.93054	chr4:7339	AC112249.1		smallRNA	chr4:76493247-7649
ENSG0000(	579	12.93054	chr4:7339	SNX5P1		Pseudoger	chr4:76344550-7634
ENSG0000(	579	12.93054	chr4:7339	ENSG00000249051		Pseudoger	chr4:73777636-7377
ENSG0000(	579	12.93054	chr4:7339	ENSG00000250560		Pseudoger	chr4:75194867-7519
ENSG0000(	579	12.93054	chr4:7339	ENSG00000289496		lncRNA	chr4:77076049-7707
ENSG0000(	579	12.93054	chr4:7339	ENSG00000289530		lncRNA	chr4:73706323-7370
ENSG0000(	579	12.93054	chr4:7339	PPBPP2		Pseudoger	chr4:74054041-7405
ENSG0000(	579	12.93054	chr4:7339	CXCL3	AC	protein_c	chr4:74036589-7403
ENSG0000(	579	12.93054	chr4:7339	ENSG00000249278		Pseudoger	chr4:76509284-7650
ENSG0000(	579	12.93054	chr4:7339	ENSG00000251185		lncRNA	chr4:75269068-7536
ENSG0000(	579	12.93054	chr4:7339	PPBPP2		lncRNA	chr4:74054038-7405
ENSG0000(	579	12.93054	chr4:7339	ENSG00000248831		Pseudoger	chr4:77350370-7735
ENSG0000(	579	12.93054	chr4:7339	ENSG00000288888		lncRNA	chr4:77056585-7705
ENSG0000(	579	12.93054	chr6:1391	ENSG00000227681		lncRNA	chr6:147660703-147
ENSG0000(	579	12.93054	chr4:7339	ENSG00000289443		lncRNA	chr4:77048964-7705
ENSG0000(	579	12.93054	chr4:7339	ENSG00000251017		Pseudoger	chr4:74085995-7408
ENSG0000(	579	12.93054	chr4:7339	ENSG00000251454		lncRNA	chr4:75341279-7535
ENSG0000(	579	12.93054	chr4:7339	ENSG00000250315		Pseudoger	chr4:75101477-7510
ENSG0000(	579	12.93054	chr4:7339	ENSG00000287037		lncRNA	chr4:73997933-7400
ENSG0000(	579	12.93054	chr4:7339	CXCL5		protein_c	chr4:73995642-7399
ENSG0000(	579	12.93054	chr4:7339	ENSG00000286074		lncRNA	chr4:76148561-7620
ENSG0000(	579	12.93054	chr4:7339	BTC		protein_c	chr4:74744759-7479
ENSG0000(	579	12.93054	chr4:7339	ODAPH		protein_c	chr4:75556048-7556

ENSG0000(	579	12.93054	chr4:7339	RNU2-16P		smallRNA	chr4:75829454-7582
ENSG0000(	579	12.93054	chr4:7339	TXNP6		Pseudoger	chr4:76958860-7695
ENSG0000(	579	12.93054	chr4:7339	FAM47E		protein_c	chr4:76214040-7628
ENSG0000(	579	12.93054	chr4:7339	AC097470.1		smallRNA	chr4:74326886-7432
ENSG0000(	579	12.93054	chr4:7339	ENSG00000242727		Pseudoger	chr4:76891034-7689
ENSG0000(	579	12.93054	chr4:7339	ENSG00000224218		lncRNA	chr4:76758554-7680
ENSG0000(	579	12.93054	chr4:7339	ENSG00000288796		protein_c	chr4:73981074-7398
ENSG0000(	579	12.93054	chr4:7339	FAM47E-STBD1		protein_c	chr4:76251721-7631
ENSG0000(	579	12.93054	chr4:7339	RPL7P17		Pseudoger	chr4:77082403-7708
ENSG0000(	579	12.93054	chr4:7339	HNRNPA1P55		Pseudoger	chr4:73938604-7393
ENSG0000(	579	12.93054	chr4:7339	HSPE1P23		Pseudoger	chr4:74917822-7491
ENSG0000(	579	12.93054	chr4:7339	RPL36AP18		Pseudoger	chr4:76401251-7640
ENSG0000(	579	12.93054	chr4:7339	ENSG00000249970		Pseudoger	chr4:73543822-7354
ENSG0000(	579	12.93054	chr4:7339	LINC02499		lncRNA	chr4:73508803-7353
ENSG0000(	579	12.93054	chr4:7339	SDAD1-AS1		lncRNA	chr4:75980790-7600
ENSG0000(	579	12.93054	chr4:7339	AREG	NCGv7	protein_c	chr4:74445136-7445
ENSG0000(	579	12.93054	chr4:7339	PF4V1		protein_c	chr4:73853296-7385
ENSG0000(	579	12.93054	chr4:7339	ENSG00000270669		Pseudoger	chr4:77216416-7721
ENSG0000(	579	12.93054	chr4:7339	SNORD50		smallRNA	chr4:76402076-7640
ENSG0000(	579	12.93054	chr4:7339	AC112719.1		smallRNA	chr4:76095408-7609
ENSG0000(	579	12.93054	chr4:7339	CXCL2	AC	protein_c	chr4:74097040-7409
ENSG0000(	579	12.93054	chr4:7339	ENSG00000229717		lncRNA	chr4:75822966-7583
ENSG0000(	579	12.93054	chr4:7339	AFP		protein_c	chr4:73431138-7345
ENSG0000(	579	12.93054	chr4:7339	CDKL2		protein_c	chr4:75576496-7563
ENSG0000(	579	12.93054	chr4:7339	THAP6		protein_c	chr4:75513946-7555
ENSG0000(	579	12.93054	chr4:7339	CXCL1P1		Pseudoger	chr4:73944011-7394
ENSG0000(	579	12.93054	chr4:7339	SHROOM3-AS1		lncRNA	chr4:76708853-7680
ENSG0000(	579	12.93054	chr4:7339	AFM	NCGv7	protein_c	chr4:73481745-7350
ENSG0000(	579	12.93054	chr4:7339	RNU6-1187P		smallRNA	chr4:77150328-7715
ENSG0000(	579	12.93054	chr4:7339	SHROOM3	NCGv7	protein_c	chr4:76435229-7678
ENSG0000(	579	12.93054	chr4:7339	ENSG00000270244		Pseudoger	chr4:76886029-7688
ENSG0000(	579	12.93054	chr4:7339	MIR4450		smallRNA	chr4:76573568-7657
ENSG0000(	579	12.93054	chr4:7339	ENSG00000269559		lncRNA	chr4:74156511-7415
ENSG0000(	579	12.93054	chr4:7339	LINC02562		lncRNA	chr4:75081702-7508
ENSG0000(	579	12.93054	chr4:7339	RNU6-1000P		smallRNA	chr4:76356610-7635
ENSG0000(	579	12.93054	chr4:7339	EPGN		protein_c	chr4:74308470-7431
ENSG0000(	579	12.93054	chr4:7339	Y_RNA		smallRNA	chr4:75662121-7566
ENSG0000(	573	12.79655	chr3:3804	OR7E55P		Pseudoger	chr3:75370448-7537
ENSG0000(	573	12.79655	chr3:3804	ENSG00000287398		lncRNA	chr3:75214510-7524
ENSG0000(	573	12.79655	chr3:3804	BZW1P2		Pseudoger	chr3:116645902-116
ENSG0000(	573	12.79655	chr3:3804	ENSG00000242364		Pseudoger	chr3:73648399-7364
ENSG0000(	573	12.79655	chr3:3804	LINC00903		lncRNA	chr3:116552473-116
ENSG0000(	573	12.79655	chr3:3804	ENSG00000285943		protein_c	chr3:113361901-113
ENSG0000(	573	12.79655	chr3:3804	CLUHP10		Pseudoger	chr3:75629993-7563
ENSG0000(	573	12.79655	chr3:3804	LINC02018		lncRNA	chr3:75435232-7553
ENSG0000(	573	12.79655	chr3:3804	EBLN2		protein_c	chr3:73061659-7306
ENSG0000(	573	12.79655	chr3:3804	ROBO2	NCGv7	protein_c	chr3:75906695-7764
ENSG0000(	573	12.79655	chr3:3804	snoU13		smallRNA	chr3:73081987-7308
ENSG0000(	573	12.79655	chr3:3804	RN7SL271P		smallRNA	chr3:71827177-7182
ENSG0000(	573	12.79655	chr3:3804	UBE2Q2P9		Pseudoger	chr3:72006774-7200
ENSG0000(	573	12.79655	chr3:3804	RNU6-281P		smallRNA	chr3:70808350-7080

ENSG0000(	573	12.79655	chr3:380⁄	ENSG00000260670		lncRNA	chr3:72321051-7232
ENSG0000(	573	12.79655	chr3:380⁄	ATP6V0CP2		Pseudoger	chr3:111478737-111
ENSG0000(	573	12.79655	chr3:380⁄	ENSG00000244144		Pseudoger	chr3:112185480-112
ENSG0000(	573	12.79655	chr3:380⁄	ZNF717	NCGv7	protein_c	chr3:75678660-7578
ENSG0000(	573	12.79655	chr3:380⁄	ZBTB20-AS2		lncRNA	chr3:114684580-114
ENSG0000(	573	12.79655	chr3:380⁄	EIF4E2P2		Pseudoger	chr3:115279125-115
ENSG0000(	573	12.79655	chr3:380⁄	LINC00901		lncRNA	chr3:116921431-116
ENSG0000(	573	12.79655	chr3:380⁄	RN7SL294P		smallRNA	chr3:75182756-7518
ENSG0000(	573	12.79655	chr3:380⁄	SIDT1		protein_c	chr3:113532555-113
ENSG0000(	573	12.79655	chr3:380⁄	ENSG00000239280		Pseudoger	chr3:113740823-113
ENSG0000(	573	12.79655	chr3:380⁄	AC096971.1		smallRNA	chr3:70878660-7087
ENSG0000(	573	12.79655	chr3:380⁄	PTMAP8		Pseudoger	chr3:117026698-117
ENSG0000(	573	12.79655	chr3:380⁄	EIF4E3		protein_c	chr3:71675414-7175
ENSG0000(	573	12.79655	chr3:380⁄	SHQ1	NCGv7	protein_c	chr3:72749277-7286
ENSG0000(	573	12.79655	chr3:380⁄	VDAC1P7		Pseudoger	chr3:77316752-7731
ENSG0000(	573	12.79655	chr3:380⁄	ENSG00000240751		Pseudoger	chr3:113360267-113
ENSG0000(	573	12.79655	chr3:380⁄	ENSG00000287595		lncRNA	chr3:72738906-7274
ENSG0000(	573	12.79655	chr3:380⁄	ENSG00000259976		lncRNA	chr3:114314501-114
ENSG0000(	573	12.79655	chr3:380⁄	CD200	NCGv7	protein_c	chr3:112332347-112
ENSG0000(	573	12.79655	chr3:380⁄	CCDC80		protein_c	chr3:112596797-112
ENSG0000(	573	12.79655	chr3:380⁄	ENSG00000241219		lncRNA	chr3:113050912-113
ENSG0000(	573	12.79655	chr3:380⁄	SNRPCP10		Pseudoger	chr3:75521803-7552
ENSG0000(	573	12.79655	chr3:380⁄	ENSG00000287567		lncRNA	chr3:77567508-7757
ENSG0000(	573	12.79655	chr3:380⁄	RN7SL92P		smallRNA	chr3:75713016-7571
ENSG0000(	573	12.79655	chr3:380⁄	ENSG00000278934		TEC	chr3:73624410-7362
ENSG0000(	573	12.79655	chr3:380⁄	ENSG00000282912		Pseudoger	chr3:75624899-7562
ENSG0000(	573	12.79655	chr3:380⁄	ENSG00000270562		lncRNA	chr3:71584943-7158
ENSG0000(	573	12.79655	chr3:380⁄	AKR1B1P2		Pseudoger	chr3:74135959-7413
ENSG0000(	573	12.79655	chr3:380⁄	snoU13		smallRNA	chr3:72572965-7257
ENSG0000(	573	12.79655	chr3:380⁄	NEPRO-AS1		lncRNA	chr3:113019468-113
ENSG0000(	573	12.79655	chr3:380⁄	SNORD112		smallRNA	chr3:113137966-113
ENSG0000(	573	12.79655	chr3:380⁄	ENSG00000239482		lncRNA	chr3:112302478-112
ENSG0000(	573	12.79655	chr3:380⁄	RNU2-64P		smallRNA	chr3:73110992-7311
ENSG0000(	573	12.79655	chr3:380⁄	MIR1324		smallRNA	chr3:75630763-7563
ENSG0000(	573	12.79655	chr3:380⁄	FAM214BP1		Pseudoger	chr3:114231398-114
ENSG0000(	573	12.79655	chr3:380⁄	OR7E22P		Pseudoger	chr3:75356467-7535
ENSG0000(	573	12.79655	chr3:380⁄	PROK2		protein_c	chr3:71771655-7178
ENSG0000(	573	12.79655	chr3:380⁄	NT5C3AP2		Pseudoger	chr3:111633672-111
ENSG0000(	573	12.79655	chr3:380⁄	ENSG00000290410		lncRNA	chr3:75540049-7557
ENSG0000(	573	12.79655	chr3:380⁄	ENSG00000277855		lncRNA	chr3:71581721-7162
ENSG0000(	573	12.79655	chr3:380⁄	ENSG00000273461		lncRNA	chr3:72061061-7206
ENSG0000(	573	12.79655	chr3:380⁄	MIR1284		smallRNA	chr3:71541970-7154
ENSG0000(	573	12.79655	chr3:380⁄	ENSG00000285708		protein_c	chr3:70959226-7175
ENSG0000(	573	12.79655	chr3:380⁄	RNU7-19P		smallRNA	chr3:73027808-7302
ENSG0000(	573	12.79655	chr3:380⁄	ENSG00000241537		Pseudoger	chr3:72772539-7277
ENSG0000(	573	12.79655	chr3:380⁄	FAM86DP		lncRNA	chr3:75413292-7543
ENSG0000(	573	12.79655	chr3:380⁄	ZDHHC23	DriverDB\	protein_c	chr3:113947901-113
ENSG0000(	573	12.79655	chr3:380⁄	ENSG00000287131		lncRNA	chr3:71785428-7178
ENSG0000(	573	12.79655	chr3:380⁄	GCSAM		protein_c	chr3:112120839-112
ENSG0000(	573	12.79655	chr3:380⁄	AC099326.1		smallRNA	chr3:69920125-6992
ENSG0000(	573	12.79655	chr3:380⁄	ENSG00000239383		Pseudoger	chr3:75590298-7559

ENSG0000(	573	12.79655	chr3:380⁄	AC026877.1		smallRNA	chr3:76417896-7641
ENSG0000(	573	12.79655	chr3:380⁄	ZBTB20-AS1		lncRNA	chr3:114351771-114
ENSG0000(	573	12.79655	chr3:380⁄	PHLDB2	DriverDB\	protein_c	chr3:111732497-111
ENSG0000(	573	12.79655	chr3:380⁄	AC078785.1		smallRNA	chr3:113092444-113
ENSG0000(	573	12.79655	chr3:380⁄	LINC00960		lncRNA	chr3:75672251-7574
ENSG0000(	573	12.79655	chr3:380⁄	ENSG00000243081		lncRNA	chr3:112396647-112
ENSG0000(	573	12.79655	chr3:380⁄	LINC00870		lncRNA	chr3:72151236-7220
ENSG0000(	573	12.79655	chr3:380⁄	SIDT1-AS1		lncRNA	chr3:113588748-113
ENSG0000(	573	12.79655	chr3:380⁄	ENSG00000289742		Pseudoger	chr3:112896631-112
ENSG0000(	573	12.79655	chr3:380⁄	OR7E121P		Pseudoger	chr3:75598652-7559
ENSG0000(	573	12.79655	chr3:380⁄	DUX4L26		Pseudoger	chr3:75668931-7567
ENSG0000(	573	12.79655	chr3:380⁄	AGGF1P3		Pseudoger	chr3:75653561-7565
ENSG0000(	573	12.79655	chr3:380⁄	RN7SL767P		smallRNA	chr3:113632704-113
ENSG0000(	573	12.79655	chr3:380⁄	OR7E100P		Pseudoger	chr3:112524187-112
ENSG0000(	573	12.79655	chr3:380⁄	PSMD12P1		Pseudoger	chr3:72876737-7287
ENSG0000(	573	12.79655	chr3:380⁄	ENSG00000276471		lncRNA	chr3:72080564-7208
ENSG0000(	573	12.79655	chr3:380⁄	ENSG00000241490		lncRNA	chr3:114214313-114
ENSG0000(	573	12.79655	chr3:380⁄	TAGLN3		protein_c	chr3:111998739-112
ENSG0000(	573	12.79655	chr3:380⁄	ABHD10		protein_c	chr3:111979010-111
ENSG0000(	573	12.79655	chr3:380⁄	RNU5E-8P		smallRNA	chr3:116965112-116
ENSG0000(	573	12.79655	chr3:380⁄	CFAP44		protein_c	chr3:113286930-113
ENSG0000(	573	12.79655	chr3:380⁄	ENSG00000291222		lncRNA	chr3:75356437-7536
ENSG0000(	573	12.79655	chr3:380⁄	TIGIT	NCGv7	protein_c	chr3:114276913-114
ENSG0000(	573	12.79655	chr3:380⁄	ENSG00000239311		lncRNA	chr3:111466313-111
ENSG0000(	573	12.79655	chr3:380⁄	ENSG00000223738		Pseudoger	chr3:74101349-7410
ENSG0000(	573	12.79655	chr3:380⁄	PDZRN3-AS1		lncRNA	chr3:73621713-7362
ENSG0000(	573	12.79655	chr3:380⁄	CD96		protein_c	chr3:111292719-111
ENSG0000(	573	12.79655	chr3:380⁄	ENSG00000288679		lncRNA	chr3:70613598-7061
ENSG0000(	573	12.79655	chr3:380⁄	LSAMP	NCGv7	protein_c	chr3:115802363-117
ENSG0000(	573	12.79655	chr3:380⁄	ZNF80		protein_c	chr3:114234631-114
ENSG0000(	573	12.79655	chr3:380⁄	ENSG00000242618		lncRNA	chr3:72202944-7220
ENSG0000(	573	12.79655	chr3:380⁄	RN7SL418P		smallRNA	chr3:69769489-6976
ENSG0000(	573	12.79655	chr3:380⁄	ENSG00000273394		lncRNA	chr3:113947005-113
ENSG0000(	573	12.79655	chr3:380⁄	MIR4273		smallRNA	chr3:75738280-7573
ENSG0000(	573	12.79655	chr3:380⁄	BOC	NCGv7;AC	protein_c	chr3:113211003-113
ENSG0000(	573	12.79655	chr3:380⁄	AC133041.1		smallRNA	chr3:75549797-7554
ENSG0000(	573	12.79655	chr3:380⁄	RFKP2		Pseudoger	chr3:112030931-112
ENSG0000(	573	12.79655	chr3:380⁄	ENSG00000241596		lncRNA	chr3:115658533-115
ENSG0000(	573	12.79655	chr3:380⁄	CD200R1L	DriverDB\	protein_c	chr3:112815709-112
ENSG0000(	573	12.79655	chr3:380⁄	RARRES2P1		Pseudoger	chr3:75658534-7565
ENSG0000(	573	12.79655	chr3:380⁄	LSP1P2		Pseudoger	chr3:75395188-7539
ENSG0000(	573	12.79655	chr3:380⁄	ENSG00000239350		Pseudoger	chr3:72085109-7208
ENSG0000(	573	12.79655	chr3:380⁄	TUSC7		lncRNA	chr3:116709235-116
ENSG0000(	573	12.79655	chr3:380⁄	ZBTB20	NCGv7	protein_c	chr3:114314500-115
ENSG0000(	573	12.79655	chr3:380⁄	ATG3	NCGv7	protein_c	chr3:112532510-112
ENSG0000(	573	12.79655	chr3:380⁄	ENSG00000244345		lncRNA	chr3:72504806-7255
ENSG0000(	573	12.79655	chr3:380⁄	FOXP1-AS1		lncRNA	chr3:71289769-7130
ENSG0000(	573	12.79655	chr3:380⁄	LINC02047		lncRNA	chr3:73999805-7400
ENSG0000(	573	12.79655	chr3:380⁄	MIR4796		smallRNA	chr3:114743445-114
ENSG0000(	573	12.79655	chr3:380⁄	AC112219.1		smallRNA	chr3:72262322-7226
ENSG0000(	573	12.79655	chr3:380⁄	CCDC137P		Pseudoger	chr3:72086888-7208

ENSG00000	573	12.79655	chr3:3804	ENSG00000286492		lncRNA	chr3:111835723-111
ENSG00000	573	12.79655	chr3:3804	LINC02044		lncRNA	chr3:113142350-113
ENSG00000	573	12.79655	chr3:3804	HMGB1P36		Pseudogen	chr3:70753854-7075
ENSG00000	573	12.79655	chr3:3804	LSAMP-AS1		lncRNA	chr3:116360024-116
ENSG00000	573	12.79655	chr3:3804	RNU6-557P		smallRNA	chr3:73092148-7309
ENSG00000	573	12.79655	chr3:3804	ENSG00000243483		Pseudogen	chr3:113041390-113
ENSG00000	573	12.79655	chr3:3804	FOXP1-IT1		lncRNA	chr3:71570255-7157
ENSG00000	573	12.79655	chr3:3804	UQCRHP4		Pseudogen	chr3:70140217-7014
ENSG00000	573	12.79655	chr3:3804	SAMMSON		lncRNA	chr3:69999550-7051
ENSG00000	573	12.79655	chr3:3804	MIR4447		smallRNA	chr3:116850277-116
ENSG00000	573	12.79655	chr3:3804	ENSG00000239568		Pseudogen	chr3:72583370-7258
ENSG00000	573	12.79655	chr3:3804	ENSG00000279349		TEC	chr3:112525548-112
ENSG00000	573	12.79655	chr3:3804	ENSG00000242880		lncRNA	chr3:115147605-115
ENSG00000	573	12.79655	chr3:3804	ENSG00000272774		lncRNA	chr3:72178279-7217
ENSG00000	573	12.79655	chr3:3804	TBILA		lncRNA	chr3:112133423-112
ENSG00000	573	12.79655	chr3:3804	FAM86DP		Pseudogen	chr3:75422961-7543
ENSG00000	573	12.79655	chr3:3804	ENSG00000288793		lncRNA	chr3:76580318-7658
ENSG00000	573	12.79655	chr3:3804	ENSG00000287882		lncRNA	chr3:72301616-7231
ENSG00000	573	12.79655	chr3:3804	ENSG00000270880		Pseudogen	chr3:113984037-113
ENSG00000	573	12.79655	chr3:3804	CAP1P1		Pseudogen	chr3:76434018-7643
ENSG00000	573	12.79655	chr3:3804	CFAP44-AS1		lncRNA	chr3:113403988-113
ENSG00000	573	12.79655	chr3:3804	FRG2C		protein_c	chr3:75664328-7566
ENSG00000	573	12.79655	chr3:3804	Y_RNA		smallRNA	chr3:74166052-7416
ENSG00000	573	12.79655	chr3:3804	UNC93B3		Pseudogen	chr3:75619506-7562
ENSG00000	573	12.79655	chr3:3804	ENSG00000288896		lncRNA	chr3:115147775-115
ENSG00000	573	12.79655	chr3:3804	ENSG00000240787		Pseudogen	chr3:111570638-111
ENSG00000	573	12.79655	chr3:3804	GXYLT2		protein_c	chr3:72888046-7299
ENSG00000	573	12.79655	chr3:3804	HNRNPA3P6		Pseudogen	chr3:75214631-7521
ENSG00000	573	12.79655	chr3:3804	TMPRSS7		protein_c	chr3:112034736-112
ENSG00000	573	12.79655	chr3:3804	ZBED2		protein_c	chr3:111592900-111
ENSG00000	573	12.79655	chr3:3804	AC128653.1		smallRNA	chr3:74534783-7453
ENSG00000	573	12.79655	chr3:3804	ENPP7P2		Pseudogen	chr3:75446769-7549
ENSG00000	573	12.79655	chr3:3804	RPS3AP15		Pseudogen	chr3:75542149-7554
ENSG00000	573	12.79655	chr3:3804	GAP43		protein_c	chr3:115623510-115
ENSG00000	573	12.79655	chr3:3804	SLC35A5	NCGv7	protein_c	chr3:112561709-112
ENSG00000	573	12.79655	chr3:3804	PDZRN3	NCGv7	protein_c	chr3:73382431-7362
ENSG00000	573	12.79655	chr3:3804	RNU7-119P		smallRNA	chr3:73593940-7359
ENSG00000	573	12.79655	chr3:3804	ZBTB20-AS4		lncRNA	chr3:115100423-115
ENSG00000	573	12.79655	chr3:3804	RNU1-62P		smallRNA	chr3:72576691-7257
ENSG00000	573	12.79655	chr3:3804	YBX1P3		Pseudogen	chr3:114930541-114
ENSG00000	573	12.79655	chr3:3804	ENSG00000283669		Pseudogen	chr3:112321140-112
ENSG00000	573	12.79655	chr3:3804	USF3		protein_c	chr3:113648385-113
ENSG00000	573	12.79655	chr3:3804	MIR567		smallRNA	chr3:112112801-112
ENSG00000	573	12.79655	chr3:3804	MDFIC2		protein_c	chr3:70194479-7031
ENSG00000	573	12.79655	chr3:3804	CD200R1L-AS1		lncRNA	chr3:112802478-112
ENSG00000	573	12.79655	chr3:3804	ENSG00000272844		lncRNA	chr3:112990447-112
ENSG00000	573	12.79655	chr3:3804	FOXP1	NCGv7;AC	protein_c	chr3:70954693-7158
ENSG00000	573	12.79655	chr3:3804	AC117481.1		smallRNA	chr3:75346139-7534
ENSG00000	573	12.79655	chr3:3804	AC134025.1		smallRNA	chr3:70251643-7025
ENSG00000	573	12.79655	chr3:3804	MITF	NCGv7;AC	protein_c	chr3:69739456-6996
ENSG00000	573	12.79655	chr3:3804	MIR568		smallRNA	chr3:114316475-114

ENSG0000(	573	12.79655	chr3:380<	RNU6-386P		smallRNA	chr3:77092057-7709
ENSG0000(	573	12.79655	chr3:380<	MIR4444-1		smallRNA	chr3:75214476-7521
ENSG0000(	573	12.79655	chr3:380<	ENSG00000290077		lncRNA	chr3:71501711-7150
ENSG0000(	573	12.79655	chr3:380<	RNU6-1270P		smallRNA	chr3:73241789-7324
ENSG0000(	573	12.79655	chr3:380<	ATP6V1A		protein_c	chr3:113747027-113
ENSG0000(	573	12.79655	chr3:380<	PLCXD2		protein_c	chr3:111674676-111
ENSG0000(	573	12.79655	chr3:380<	LINC02005		lncRNA	chr3:73805754-7390
ENSG0000(	573	12.79655	chr3:380<	VPS26AP1		Pseudoger	chr3:113919222-113
ENSG0000(	573	12.79655	chr3:380<	GPATCH11P1		Pseudoger	chr3:73182501-7318
ENSG0000(	573	12.79655	chr3:380<	LINC02042		lncRNA	chr3:112736447-112
ENSG0000(	573	12.79655	chr3:380<	RPL23AP49		Pseudoger	chr3:75624275-7567
ENSG0000(	573	12.79655	chr3:380<	SLC9C1	NCGv7	protein_c	chr3:112140898-112
ENSG0000(	573	12.79655	chr3:380<	QTRT2		protein_c	chr3:114005833-114
ENSG0000(	573	12.79655	chr3:380<	C3orf52		protein_c	chr3:112086335-112
ENSG0000(	573	12.79655	chr3:380<	H2BP3		Pseudoger	chr3:114103249-114
ENSG0000(	573	12.79655	chr3:380<	DRD3	NCGv7	protein_c	chr3:114127580-114
ENSG0000(	573	12.79655	chr3:380<	NIPA2P2		Pseudoger	chr3:75084155-7508
ENSG0000(	573	12.79655	chr3:380<	MYLKP1		Pseudoger	chr3:75328549-7533
ENSG0000(	573	12.79655	chr3:380<	ENSG00000242659		lncRNA	chr3:113746872-113
ENSG0000(	573	12.79655	chr3:380<	ENSG00000288079		lncRNA	chr3:113259786-113
ENSG0000(	573	12.79655	chr3:380<	NECTIN3-AS1		lncRNA	chr3:110888384-111
ENSG0000(	573	12.79655	chr3:380<	MIR4446		smallRNA	chr3:113594876-113
ENSG0000(	573	12.79655	chr3:380<	NECTIN3		protein_c	chr3:111070071-111
ENSG0000(	573	12.79655	chr3:380<	Y_RNA		smallRNA	chr3:75964980-7596
ENSG0000(	573	12.79655	chr3:380<	ENSG00000274387		lncRNA	chr3:71567863-7157
ENSG0000(	573	12.79655	chr3:380<	ENSG00000289069		lncRNA	chr3:110966981-110
ENSG0000(	573	12.79655	chr3:380<	BTLA	NCGv7	protein_c	chr3:112463966-112
ENSG0000(	573	12.79655	chr3:380<	ENSG00000241889		Pseudoger	chr3:113885298-113
ENSG0000(	573	12.79655	chr3:380<	ENSG00000242953		Pseudoger	chr3:75395404-7539
ENSG0000(	573	12.79655	chr3:380<	CNTN3		protein_c	chr3:74262568-7461
ENSG0000(	573	12.79655	chr3:380<	ENSG00000240776		Pseudoger	chr3:113850237-113
ENSG0000(	573	12.79655	chr3:380<	ENSG00000288074		lncRNA	chr3:115418862-115
ENSG0000(	573	12.79655	chr3:380<	ENSG00000285836		lncRNA	chr3:113998782-114
ENSG0000(	573	12.79655	chr3:380<	ENSG00000289153		lncRNA	chr3:115757662-115
ENSG0000(	573	12.79655	chr3:380<	PPP4R2		protein_c	chr3:72996803-7306
ENSG0000(	573	12.79655	chr3:380<	ALG1L6P		Pseudoger	chr3:75415070-7542
ENSG0000(	573	12.79655	chr3:380<	ZBTB20-AS5		lncRNA	chr3:114445521-114
ENSG0000(	573	12.79655	chr3:380<	PLCXD2-AS1		lncRNA	chr3:111676736-111
ENSG0000(	573	12.79655	chr3:380<	EVA1CP5		Pseudoger	chr3:75384059-7539
ENSG0000(	573	12.79655	chr3:380<	ENSG00000287669		lncRNA	chr3:70617766-7062
ENSG0000(	573	12.79655	chr3:380<	GPR27		protein_c	chr3:71753855-7175
ENSG0000(	573	12.79655	chr3:380<	ENSG00000242308		Pseudoger	chr3:112696908-112
ENSG0000(	573	12.79655	chr3:380<	FTH1P23		Pseudoger	chr3:72929044-7292
ENSG0000(	573	12.79655	chr3:380<	RN7SL582P		smallRNA	chr3:116582554-116
ENSG0000(	573	12.79655	chr3:380<	LINC00877		lncRNA	chr3:71943592-7227
ENSG0000(	573	12.79655	chr3:380<	ENSG00000272710		Pseudoger	chr3:75579978-7558
ENSG0000(	573	12.79655	chr3:380<	GRAMD1C		protein_c	chr3:113828182-113
ENSG0000(	573	12.79655	chr3:380<	AC139453.1		smallRNA	chr3:75369259-7536
ENSG0000(	573	12.79655	chr3:380<	RYBP	NCGv7	protein_c	chr3:72371825-7244
ENSG0000(	573	12.79655	chr3:380<	COX6CP6		Pseudoger	chr3:70751123-7075
ENSG0000(	573	12.79655	chr3:380<	CD200R1		protein_c	chr3:112921205-112

ENSG0000(	573	12.79655	chr3:380<	ENSG00000287805		lncRNA	chr3:113986835-113
ENSG0000(	573	12.79655	chr3:380<	GTPBP8		protein_c	chr3:112990984-113
ENSG0000(	573	12.79655	chr3:380<	RN7SL815P		smallRNA	chr3:115837868-115
ENSG0000(	573	12.79655	chr3:380<	ENSG00000271034		Pseudoger	chr3:70125070-7012
ENSG0000(	573	12.79655	chr3:380<	ENSG00000287795		lncRNA	chr3:115413476-115
ENSG0000(	573	12.79655	chr3:380<	RNA5SP136		Pseudoger	chr3:72691705-7269
ENSG0000(	573	12.79655	chr3:380<	SPICE1		protein_c	chr3:113442718-113
ENSG0000(	573	12.79655	chr3:380<	NAA50		protein_c	chr3:113716458-113
ENSG0000(	573	12.79655	chr3:380<	CCDC191	DriverDB\	protein_c	chr3:113964137-114
ENSG0000(	573	12.79655	chr3:380<	ZBTB20-AS3		lncRNA	chr3:114873114-114
ENSG0000(	573	12.79655	chr3:380<	NEPRO		protein_c	chr3:113002444-113
ENSG0000(	572	12.77421	chr3:380<	ENSG00000272656		lncRNA	chr3:139349024-139
ENSG0000(	572	12.77421	chr3:380<	ACTG1P1		Pseudoger	chr3:139493809-139
ENSG0000(	572	12.77421	chr3:380<	NMNAT3	DriverDB\	protein_c	chr3:139560180-139
ENSG0000(	572	12.77421	chr3:380<	RPL23AP40		Pseudoger	chr3:138796851-138
ENSG0000(	572	12.77421	chr3:380<	ENSG00000261763		lncRNA	chr3:139678620-139
ENSG0000(	572	12.77421	chr3:380<	RN7SKP124		smallRNA	chr3:139584105-139
ENSG0000(	572	12.77421	chr3:380<	ENSG00000214280		Pseudoger	chr3:139582928-139
ENSG0000(	572	12.77421	chr3:380<	PRR23A		protein_c	chr3:139003962-139
ENSG0000(	572	12.77421	chr3:380<	KRT8P36		Pseudoger	chr3:138101478-138
ENSG0000(	572	12.77421	chr3:380<	FOXL2NB	DriverDB\	protein_c	chr3:138947217-138
ENSG0000(	572	12.77421	chr3:380<	DZIP1L		protein_c	chr3:138061990-138
ENSG0000(	572	12.77421	chr3:380<	ARMC8		protein_c	chr3:138187248-138
ENSG0000(	572	12.77421	chr3:380<	ENSG00000286988		lncRNA	chr3:139316157-139
ENSG0000(	572	12.77421	chr3:380<	A4GNT	DriverDB\	protein_c	chr3:138123713-138
ENSG0000(	572	12.77421	chr3:380<	LINC01391		lncRNA	chr3:138935189-138
ENSG0000(	572	12.77421	chr3:380<	MRPS22		protein_c	chr3:139005806-139
ENSG0000(	572	12.77421	chr3:380<	ENSG00000248790		lncRNA	chr3:139466430-139
ENSG0000(	572	12.77421	chr3:380<	GAPDHP39		Pseudoger	chr3:138777832-138
ENSG0000(	572	12.77421	chr3:380<	CEP70	DriverDB\	protein_c	chr3:138494344-138
ENSG0000(	572	12.77421	chr3:380<	PRR23B		protein_c	chr3:139019031-139
ENSG0000(	572	12.77421	chr3:380<	RBP1	DriverDB\	protein_c	chr3:139517434-139
ENSG0000(	572	12.77421	chr3:380<	EEF1A1P25		Pseudoger	chr3:138825063-138
ENSG0000(	572	12.77421	chr3:380<	RBP2		protein_c	chr3:139452884-139
ENSG0000(	572	12.77421	chr3:380<	FOXL2	NCGv7;AC	protein_c	chr3:138944224-138
ENSG0000(	572	12.77421	chr3:380<	ENSG00000286396		Pseudoger	chr3:138125695-138
ENSG0000(	572	12.77421	chr3:380<	PPIAP72		Pseudoger	chr3:138643874-138
ENSG0000(	572	12.77421	chr3:380<	COPB2	NCGv7	protein_c	chr3:139353946-139
ENSG0000(	572	12.77421	chr3:380<	PRR23C		protein_c	chr3:139042102-139
ENSG0000(	572	12.77421	chr3:380<	ENSG00000250543		lncRNA	chr3:139688403-139
ENSG0000(	572	12.77421	chr3:380<	PIK3CB	NCGv7	protein_c	chr3:138652698-138
ENSG0000(	572	12.77421	chr3:380<	COPB2-DT		lncRNA	chr3:139389761-139
ENSG0000(	572	12.77421	chr3:380<	CLDN18		protein_c	chr3:137998735-138
ENSG0000(	572	12.77421	chr3:380<	NME9		protein_c	chr3:138261437-138
ENSG0000(	572	12.77421	chr3:380<	AC097103.1		smallRNA	chr3:139494618-139
ENSG0000(	572	12.77421	chr3:380<	RN7SL724P		smallRNA	chr3:139609671-139
ENSG0000(	572	12.77421	chr3:380<	FAIM		protein_c	chr3:138608606-138
ENSG0000(	572	12.77421	chr3:380<	DBR1	NCGv7	protein_c	chr3:138160988-138
ENSG0000(	572	12.77421	chr3:380<	ESYT3		protein_c	chr3:138434586-138
ENSG0000(	572	12.77421	chr3:380<	RNU6-736P		smallRNA	chr3:139388277-139
ENSG0000(	572	12.77421	chr3:380<	ATP5MC1P3		Pseudoger	chr3:138889255-138

ENSG0000(	572	12.77421	chr3:380⁴	ENSG00000280399		TEC	chr3:138482065-138
ENSG0000(	572	12.77421	chr3:380⁴	BPESC1		lncRNA	chr3:139104185-139
ENSG0000(	572	12.77421	chr3:380⁴	RPL7L1P7		Pseudoger	chr3:139081654-139
ENSG0000(	572	12.77421	chr3:380⁴	MRAS	NCGv7;AC	protein_c	chr3:138347648-138
ENSG0000(	572	12.77421	chr3:380⁴	PISRT1		lncRNA	chr3:139232992-139
ENSG0000(	570	12.72955	chr3:380⁴	MARK2P14		Pseudoger	chr3:11906527-1191
ENSG0000(	570	12.72955	chr3:380⁴	ENSG00000272263		lncRNA	chr3:12832219-1283
ENSG0000(	570	12.72955	chr3:380⁴	NUP210P2		Pseudoger	chr3:11900011-1190
ENSG0000(	570	12.72955	chr3:380⁴	CYCSP12		Pseudoger	chr3:11878830-1187
ENSG0000(	570	12.72955	chr3:380⁴	snoU13		smallRNA	chr3:12632734-1263
ENSG0000(	570	12.72955	chr3:380⁴	LINC02022		lncRNA	chr3:12877522-1288
ENSG0000(	570	12.72955	chr3:380⁴	ENSG00000271716		lncRNA	chr3:11607184-1161
ENSG0000(	570	12.72955	chr3:380⁴	AC026166.1		smallRNA	chr3:12071038-1207
ENSG0000(	570	12.72955	chr3:380⁴	CRIP1P1		Pseudoger	chr3:12673699-1267
ENSG0000(	570	12.72955	chr3:380⁴	MKRN2OS	DriverDB\	protein_c	chr3:12514934-1256
ENSG0000(	570	12.72955	chr3:380⁴	SNORA7A		smallRNA	chr3:12840312-1284
ENSG0000(	570	12.72955	chr3:380⁴	ENSG00000272483		lncRNA	chr3:11611602-1161
ENSG0000(	570	12.72955	chr3:380⁴	CHCHD4P4		Pseudoger	chr3:11234256-1123
ENSG0000(	570	12.72955	chr3:380⁴	ENSG00000235240		Pseudoger	chr3:11488300-1148
ENSG0000(	570	12.72955	chr3:380⁴	ENSG00000225026		Pseudoger	chr3:12328003-1232
ENSG0000(	570	12.72955	chr3:380⁴	RN7SL147P		smallRNA	chr3:11952790-1195
ENSG0000(	570	12.72955	chr3:380⁴	ENSG00000270531		Pseudoger	chr3:11529488-1152
ENSG0000(	570	12.72955	chr3:380⁴	RNA5SP123		Pseudoger	chr3:12511095-1251
ENSG0000(	570	12.72955	chr3:380⁴	MTCO1P5		Pseudoger	chr3:12165299-1216
ENSG0000(	570	12.72955	chr3:380⁴	FANCD2P2		Pseudoger	chr3:11859674-1189
ENSG0000(	570	12.72955	chr3:380⁴	ENSG00000226621		Pseudoger	chr3:11290406-1129
ENSG0000(	570	12.72955	chr3:380⁴	ATG7	NCGv7	protein_c	chr3:11272309-1155
ENSG0000(	570	12.72955	chr3:380⁴	ACTG1P12		Pseudoger	chr3:12070195-1207
ENSG0000(	570	12.72955	chr3:380⁴	KRT18P17		Pseudoger	chr3:12787393-1278
ENSG0000(	570	12.72955	chr3:380⁴	GSTM5P1		Pseudoger	chr3:12257801-1225
ENSG0000(	570	12.72955	chr3:380⁴	RNU6-377P		smallRNA	chr3:12514706-1251
ENSG0000(	570	12.72955	chr3:380⁴	TAMM41	NCGv7	protein_c	chr3:11790442-1184
ENSG0000(	570	12.72955	chr3:380⁴	ENSG00000288952		lncRNA	chr3:12178694-1223
ENSG0000(	570	12.72955	chr3:380⁴	ENSG00000251038		Pseudoger	chr3:11909634-1191
ENSG0000(	570	12.72955	chr3:380⁴	PPARG	NCGv7;AC	protein_c	chr3:12287368-1243
ENSG0000(	570	12.72955	chr3:380⁴	RAF1	NCGv7;AC	protein_c	chr3:12582101-1266
ENSG0000(	570	12.72955	chr3:380⁴	ENSG00000250939		Pseudoger	chr3:12850659-1285
ENSG0000(	570	12.72955	chr3:380⁴	SYN2	NCGv7	protein_c	chr3:12004388-1219
ENSG0000(	570	12.72955	chr3:380⁴	RPL32		protein_c	chr3:12834485-1284
ENSG0000(	570	12.72955	chr3:380⁴	CAND2		protein_c	chr3:12796472-1283
ENSG0000(	570	12.72955	chr3:380⁴	TIMP4		protein_c	chr3:12153068-1215
ENSG0000(	570	12.72955	chr3:380⁴	VGLL4		protein_c	chr3:11556069-1177
ENSG0000(	570	12.72955	chr3:380⁴	ENSG00000289809		protein_c	chr3:12825768-1287
ENSG0000(	570	12.72955	chr3:380⁴	ENSG00000290072		lncRNA	chr3:12664310-1266
ENSG0000(	570	12.72955	chr3:380⁴	RNU6-404P		smallRNA	chr3:12501623-1250
ENSG0000(	570	12.72955	chr3:380⁴	TSEN2	DriverDB\	protein_c	chr3:12484421-1254
ENSG0000(	570	12.72955	chr3:380⁴	TMEM40	NCGv7	protein_c	chr3:12733528-1276
ENSG0000(	570	12.72955	chr3:380⁴	MKRN2	DriverDB\	protein_c	chr3:12557057-1258
ENSG0000(	568	12.68488	chr3:380⁴	RPLP0P8		Pseudoger	chr3:68633706-6863
ENSG0000(	568	12.68488	chr3:380⁴	ENSG00000241884		lncRNA	chr3:67296300-6730
ENSG0000(	568	12.68488	chr3:380⁴	ENSG00000240568		Pseudoger	chr3:68192398-6819

ENSG00000	568	12.68488	chr3:3804	MIR3136		smallRNA	chr3:69048958-6904
ENSG00000	568	12.68488	chr3:3804	ENSG00000287873		lncRNA	chr3:66997168-6699
ENSG00000	568	12.68488	chr3:3804	ENSG00000240842		lncRNA	chr3:69564106-6957
ENSG00000	568	12.68488	chr3:3804	TAFA1		protein_c	chr3:68004247-6854
ENSG00000	568	12.68488	chr3:3804	SUCLG2-DT		lncRNA	chr3:67654669-6794
ENSG00000	568	12.68488	chr3:3804	AC113171.1		smallRNA	chr3:67828172-6782
ENSG00000	568	12.68488	chr3:3804	RBM43P1		Pseudoger	chr3:69471452-6947
ENSG00000	568	12.68488	chr3:3804	FRMD4B		protein_c	chr3:69168782-6954
ENSG00000	568	12.68488	chr3:3804	SUCLG2		protein_c	chr3:67360460-6765
ENSG00000	568	12.68488	chr3:3804	AC096922.1		smallRNA	chr3:68634916-6863
ENSG00000	568	12.68488	chr3:3804	RNA5SP135		Pseudoger	chr3:68882967-6888
ENSG00000	568	12.68488	chr3:3804	TMF1		protein_c	chr3:69019827-6905
ENSG00000	568	12.68488	chr3:3804	LMOD3		protein_c	chr3:69106065-6912
ENSG00000	568	12.68488	chr3:3804	ENSG00000286967		lncRNA	chr3:68500613-6850
ENSG00000	568	12.68488	chr3:3804	UBA3		protein_c	chr3:69054730-6908
ENSG00000	568	12.68488	chr3:3804	EOGT-DT		lncRNA	chr3:69013941-6905
ENSG00000	568	12.68488	chr3:3804	ENSG00000240265		Pseudoger	chr3:69535664-6953
ENSG00000	568	12.68488	chr3:3804	NDUFB4P1		Pseudoger	chr3:67513940-6751
ENSG00000	568	12.68488	chr3:3804	COPS8P2		Pseudoger	chr3:68145004-6814
ENSG00000	568	12.68488	chr3:3804	KBTBD8	NCGv7	protein_c	chr3:66998307-6701
ENSG00000	568	12.68488	chr3:3804	ENSG00000241667		lncRNA	chr3:69591341-6959
ENSG00000	568	12.68488	chr3:3804	PSMC1P1		Pseudoger	chr3:68635705-6863
ENSG00000	568	12.68488	chr3:3804	ARL6IP5		protein_c	chr3:69084937-6910
ENSG00000	568	12.68488	chr3:3804	TAFA4		protein_c	chr3:68731766-6895
ENSG00000	568	12.68488	chr3:3804	EOGT		protein_c	chr3:68975217-6901
ENSG00000	567	12.66255	chr17:674	RN7SKP180		smallRNA	chr17:72663823-726
ENSG00000	567	12.66255	chr2:3094	CYS1	NCGv7	protein_c	chr2:10056473-1008
ENSG00000	567	12.66255	chr2:3094	ENSG00000285872		lncRNA	chr2:10287704-1030
ENSG00000	567	12.66255	chr2:3094	RN7SL66P		smallRNA	chr2:10280631-1028
ENSG00000	567	12.66255	chr2:3094	SNORA2		smallRNA	chr2:10155072-1015
ENSG00000	567	12.66255	chr2:3094	TAF1B		protein_c	chr2:9843443-99344
ENSG00000	567	12.66255	chr2:3094	ENSG00000260077		lncRNA	chr2:10039092-1004
ENSG00000	567	12.66255	chr2:3094	ENSG00000271787		lncRNA	chr2:10054421-1005
ENSG00000	567	12.66255	chr2:3094	ENSG00000188525		lncRNA	chr2:10001757-1000
ENSG00000	567	12.66255	chr2:3094	SNORA51		smallRNA	chr2:10296047-1029
ENSG00000	567	12.66255	chr2:3094	MIR4261		smallRNA	chr2:10192614-1019
ENSG00000	567	12.66255	chr2:3094	HPCAL1		protein_c	chr2:10302889-1042
ENSG00000	567	12.66255	chr2:3094	ENSG00000233502		lncRNA	chr2:10083781-1008
ENSG00000	567	12.66255	chr2:3094	SNORA26		smallRNA	chr2:10090205-1009
ENSG00000	567	12.66255	chr2:3094	ENSG00000287305		lncRNA	chr2:9939088-99508
ENSG00000	567	12.66255	chr2:3094	GRHL1		protein_c	chr2:9951693-10002
ENSG00000	567	12.66255	chr2:3094	RRM2		protein_c	chr2:10120698-1021
ENSG00000	567	12.66255	chr2:3094	ENSG00000269973		lncRNA	chr2:9936360-99395
ENSG00000	567	12.66255	chr2:3094	ENSG00000260476		lncRNA	chr2:10021578-1002
ENSG00000	567	12.66255	chr2:3094	ENSG00000289033		lncRNA	chr2:9996244-10009
ENSG00000	567	12.66255	chr2:3094	KLF11		protein_c	chr2:10042849-1005
ENSG00000	562	12.55089	chr1:1758	ENSG00000231979		lncRNA	chr1:239915439-239
ENSG00000	562	12.55089	chr1:1758	RN7SKP12		smallRNA	chr1:242188647-242
ENSG00000	562	12.55089	chr1:1758	CHRM3-AS1		lncRNA	chr1:239898016-239
ENSG00000	562	12.55089	chr1:1758	ENSG00000228844		Pseudoger	chr1:240636599-240
ENSG00000	562	12.55089	chr1:1758	MIR3123		smallRNA	chr1:241132272-241

ENSG0000(	562	12.55089	chr1:175{	Y_RNA		smallRNA	chr1:240698320-240
ENSG0000(	562	12.55089	chr1:175{	CFL1P4		Pseudoger	chr1:241993185-241
ENSG0000(	562	12.55089	chr1:175{	RGS7	NCGv7	protein_c	chr1:240767636-241
ENSG0000(	562	12.55089	chr1:175{	LINC01347		Pseudoger	chr1:243087710-243
ENSG0000(	562	12.55089	chr1:175{	RNU5F-8P		smallRNA	chr1:240653367-240
ENSG0000(	562	12.55089	chr1:175{	CEP170	NCGv7	protein_c	chr1:243124428-243
ENSG0000(	562	12.55089	chr1:175{	ENSG00000231612		lncRNA	chr1:245673732-245
ENSG0000(	562	12.55089	chr1:175{	ENSG00000232085		lncRNA	chr1:243164638-243
ENSG0000(	562	12.55089	chr1:175{	MAP1LC3C		protein_c	chr1:241995490-241
ENSG0000(	562	12.55089	chr1:175{	ENSG00000272865		lncRNA	chr1:242147230-242
ENSG0000(	562	12.55089	chr1:175{	EXO1		protein_c	chr1:241847967-241
ENSG0000(	562	12.55089	chr1:175{	ADH5P3		Pseudoger	chr1:240170155-240
ENSG0000(	562	12.55089	chr1:175{	ENSG00000232184		lncRNA	chr1:243702857-243
ENSG0000(	562	12.55089	chr1:175{	Y_RNA		smallRNA	chr1:240341385-240
ENSG0000(	562	12.55089	chr1:175{	THAP12P8		Pseudoger	chr1:240769420-240
ENSG0000(	562	12.55089	chr1:175{	ENSG00000234872		Pseudoger	chr1:240654241-240
ENSG0000(	562	12.55089	chr1:175{	ENSG00000213690		Pseudoger	chr1:242376923-242
ENSG0000(	562	12.55089	chr1:175{	SDCCAG8	NCGv7	protein_c	chr1:243256034-243
ENSG0000(	562	12.55089	chr1:175{	WDR64	NCGv7	protein_c	chr1:241652278-241
ENSG0000(	562	12.55089	chr1:175{	ENSG00000228818		Pseudoger	chr1:240142670-240
ENSG0000(	562	12.55089	chr1:175{	SEPTIN14P21		Pseudoger	chr1:243047698-243
ENSG0000(	562	12.55089	chr1:175{	AKT3-IT1		lncRNA	chr1:243793205-243
ENSG0000(	562	12.55089	chr1:175{	OPN3		protein_c	chr1:241590102-241
ENSG0000(	562	12.55089	chr1:175{	ENSG00000233519		lncRNA	chr1:240400671-240
ENSG0000(	562	12.55089	chr1:175{	ENSG00000270859		Pseudoger	chr1:242671140-242
ENSG0000(	562	12.55089	chr1:175{	ENSG00000277704		Pseudoger	chr1:242890247-242
ENSG0000(	562	12.55089	chr1:175{	ENSG00000230199		Pseudoger	chr1:242975005-242
ENSG0000(	562	12.55089	chr1:175{	ENSG00000270818		Pseudoger	chr1:242882066-242
ENSG0000(	562	12.55089	chr1:175{	ENSG00000277099		Pseudoger	chr1:242060490-242
ENSG0000(	562	12.55089	chr1:175{	HNRNPA1P42		Pseudoger	chr1:240919653-240
ENSG0000(	562	12.55089	chr1:175{	ENSG00000230015		lncRNA	chr1:240739131-240
ENSG0000(	562	12.55089	chr1:175{	ENSG00000233735		lncRNA	chr1:240177839-240
ENSG0000(	562	12.55089	chr1:175{	CHRM3-AS2		lncRNA	chr1:239703381-239
ENSG0000(	562	12.55089	chr1:175{	ENSG00000288723		lncRNA	chr1:241722926-241
ENSG0000(	562	12.55089	chr1:175{	RPS7P5		Pseudoger	chr1:240012646-240
ENSG0000(	562	12.55089	chr1:175{	FH	NCGv7;AC	protein_c	chr1:241497511-241
ENSG0000(	562	12.55089	chr1:175{	RNA5SP81		smallRNA	chr1:242134272-242
ENSG0000(	562	12.55089	chr1:175{	ENSG00000234116		lncRNA	chr1:243005845-243
ENSG0000(	562	12.55089	chr1:175{	KMO	NCGv7	protein_c	chr1:241532134-241
ENSG0000(	562	12.55089	chr1:175{	AKT3	NCGv7	protein_c	chr1:243488233-243
ENSG0000(	562	12.55089	chr1:175{	FMN2	NCGv7	protein_c	chr1:240014348-240
ENSG0000(	562	12.55089	chr1:175{	RNU6-1139P		smallRNA	chr1:242023949-242
ENSG0000(	562	12.55089	chr1:175{	RPL6P3		Pseudoger	chr1:241831935-241
ENSG0000(	562	12.55089	chr1:175{	ENSG00000238085		Pseudoger	chr1:240998451-240
ENSG0000(	562	12.55089	chr1:175{	ENSG00000215805		Pseudoger	chr1:239972787-239
ENSG0000(	562	12.55089	chr1:175{	ENSG00000231440		lncRNA	chr1:240588522-240
ENSG0000(	562	12.55089	chr1:175{	RNU6-747P		smallRNA	chr1:243081156-243
ENSG0000(	562	12.55089	chr1:175{	RSL24D1P4		Pseudoger	chr1:242772620-242
ENSG0000(	562	12.55089	chr1:175{	ENSG00000286496		lncRNA	chr1:241357343-241
ENSG0000(	562	12.55089	chr1:175{	RFKP1		Pseudoger	chr1:240823006-240
ENSG0000(	562	12.55089	chr1:175{	KIF26B		protein_c	chr1:245154985-245

ENSG0000(	562	12.55089	chr1:175{	CHRM3		protein_c	chr1:239386565-239
ENSG0000(	562	12.55089	chr1:175{	ENSG00000226014		Pseudoger	chr1:240549867-240
ENSG0000(	562	12.55089	chr1:175{	CICP21		Pseudoger	chr1:243049782-243
ENSG0000(	562	12.55089	chr1:175{	ENSG00000224525		lncRNA	chr1:242203555-242
ENSG0000(	562	12.55089	chr1:175{	ENSG00000287516		lncRNA	chr1:241413716-241
ENSG0000(	562	12.55089	chr1:175{	ENSG00000238224		lncRNA	chr1:245614773-245
ENSG0000(	562	12.55089	chr1:175{	Y_RNA		smallRNA	chr1:240154651-240
ENSG0000(	562	12.55089	chr1:175{	AL445675.1		smallRNA	chr1:242897870-242
ENSG0000(	562	12.55089	chr1:175{	RPL10AP5		Pseudoger	chr1:242365189-242
ENSG0000(	562	12.55089	chr1:175{	MIR4677		smallRNA	chr1:243346176-243
ENSG0000(	562	12.55089	chr1:175{	ENSG00000287738		lncRNA	chr1:241453751-241
ENSG0000(	562	12.55089	chr1:175{	RPL36P6		Pseudoger	chr1:241305580-241
ENSG0000(	562	12.55089	chr1:175{	CHML		protein_c	chr1:241628851-241
ENSG0000(	562	12.55089	chr1:175{	FCF1P7		Pseudoger	chr1:243267257-243
ENSG0000(	562	12.55089	chr1:175{	TUBB8P6		Pseudoger	chr1:242057085-242
ENSG0000(	562	12.55089	chr1:175{	ENSG00000224359		lncRNA	chr1:240530452-240
ENSG0000(	562	12.55089	chr1:175{	GREM2		protein_c	chr1:240489573-240
ENSG0000(	562	12.55089	chr1:175{	ENSG00000287513		lncRNA	chr1:241640555-241
ENSG0000(	562	12.55089	chr1:175{	RPL23AP20		Pseudoger	chr1:241916123-241
ENSG0000(	562	12.55089	chr1:175{	ENSG00000226750		Pseudoger	chr1:242345558-242
ENSG0000(	562	12.55089	chr1:175{	ENSG00000253326		Pseudoger	chr1:243054861-243
ENSG0000(	562	12.55089	chr1:175{	BECN2	DriverDB\	protein_c	chr1:241957767-241
ENSG0000(	562	12.55089	chr1:175{	ENSG00000236031		lncRNA	chr1:243545532-243
ENSG0000(	562	12.55089	chr1:175{	FABP7P1		Pseudoger	chr1:243624666-243
ENSG0000(	562	12.55089	chr1:175{	ENSG00000226919		lncRNA	chr1:240763334-240
ENSG0000(	562	12.55089	chr1:175{	LINC02774		lncRNA	chr1:243917402-244
ENSG0000(	562	12.55089	chr1:175{	ENSG00000287102		lncRNA	chr1:240007524-240
ENSG0000(	562	12.55089	chr1:175{	LINC01347		lncRNA	chr1:243056307-243
ENSG0000(	562	12.55089	chr1:175{	ENSG00000291216		lncRNA	chr1:243029512-243
ENSG0000(	562	12.55089	chr1:175{	ENSG00000227230		lncRNA	chr1:243135898-243
ENSG0000(	562	12.55089	chr1:175{	PLD5		protein_c	chr1:242082986-242
ENSG0000(	561	12.52856	chr4:733(	ENSG00000289379		lncRNA	chr4:77820363-7782
ENSG0000(	561	12.52856	chr4:733(	ENSG00000249072		Pseudoger	chr4:78008512-7800
ENSG0000(	561	12.52856	chr4:733(	HIGD1AP13		Pseudoger	chr4:78648954-7864
ENSG0000(	561	12.52856	chr4:733(	Y_RNA		smallRNA	chr4:78632273-7863
ENSG0000(	561	12.52856	chr4:733(	ENSG00000239793		Pseudoger	chr4:78768499-7876
ENSG0000(	561	12.52856	chr4:733(	SERBP1P5		Pseudoger	chr4:78180866-7818
ENSG0000(	561	12.52856	chr4:733(	ENSG00000287632		lncRNA	chr4:78669690-7869
ENSG0000(	561	12.52856	chr4:733(	ENSG00000250214		Pseudoger	chr4:77864508-7786
ENSG0000(	561	12.52856	chr4:733(	BMP2K-DT		lncRNA	chr4:78773654-7877
ENSG0000(	561	12.52856	chr4:733(	HNRNPA1P56		Pseudoger	chr4:77987860-7798
ENSG0000(	561	12.52856	chr6:103(	ENSG00000219453		Pseudoger	chr6:23649496-2364
ENSG0000(	561	12.52856	chr4:733(	MICOS10P4		Pseudoger	chr4:78379197-7837
ENSG0000(	561	12.52856	chr4:733(	ENSG00000248128		Pseudoger	chr4:78003143-7800
ENSG0000(	561	12.52856	chr4:733(	snoU13		smallRNA	chr4:78640100-7864
ENSG0000(	561	12.52856	chr4:733(	AC093897.1		smallRNA	chr4:78188336-7818
ENSG0000(	561	12.52856	chr4:733(	ENSG00000248926		Pseudoger	chr4:77958000-7795
ENSG0000(	561	12.52856	chr4:733(	CNOT6L		protein_c	chr4:77713387-7781
ENSG0000(	561	12.52856	chr4:733(	MRPL1		protein_c	chr4:77862830-7795
ENSG0000(	561	12.52856	chr4:733(	FRAS1		protein_c	chr4:78057323-7854
ENSG0000(	561	12.52856	chr4:733(	ANXA3		protein_c	chr4:78551747-7861

ENSG0000(	561	12.52856	chr4:733(	HMGB1P44		Pseudoger	chr4:77963940-7796
ENSG0000(	561	12.52856	chr4:733(	LINC01094		lncRNA	chr4:78638780-7868
ENSG0000(	559	12.48389	chr4:410(	COX18		protein_c	chr4:73052362-7306
ENSG0000(	559	12.48389	chr4:410(	ANKRD17-DT		lncRNA	chr4:73259209-7331
ENSG0000(	559	12.48389	chr4:410(	NPFFR2		protein_c	chr4:72031902-7214
ENSG0000(	559	12.48389	chr4:410(	ANKRD17	NCGv7	protein_c	chr4:73073376-7325
ENSG0000(	559	12.48389	chr4:410(	ENSG00000250877		lncRNA	chr4:72323028-7233
ENSG0000(	559	12.48389	chr4:410(	HMGA1P2		Pseudoger	chr4:73098822-7309
ENSG0000(	559	12.48389	chr4:410(	ENSG00000249976		Pseudoger	chr4:73337233-7333
ENSG0000(	559	12.48389	chr4:410(	HNRNPA1P67		Pseudoger	chr4:72807267-7280
ENSG0000(	559	12.48389	chr4:410(	RNU6ATAC5P		smallRNA	chr4:73026748-7302
ENSG0000(	559	12.48389	chr4:410(	RNU4ATAC9P		smallRNA	chr4:72965178-7296
ENSG0000(	559	12.48389	chr4:410(	ADAMTS3	NCGv7	protein_c	chr4:72280969-7256
ENSG0000(	558	12.46156	chr12:31(	ENSG00000276292		lncRNA	chr12:118037869-11
ENSG0000(	553	12.3499	chr11:62(	ENSG00000267811		lncRNA	chr11:62771120-627
ENSG0000(	553	12.3499	chr11:62(	CHRM1		protein_c	chr11:62908679-629
ENSG0000(	553	12.3499	chr11:62(	SCGB2A1		protein_c	chr11:62208673-622
ENSG0000(	553	12.3499	chr11:62(	STX5		protein_c	chr11:62806860-628
ENSG0000(	553	12.3499	chr11:62(	SCGB1D2		protein_c	chr11:62242239-622
ENSG0000(	553	12.3499	chr11:62(	WDR74	NCGv7	protein_c	chr11:62832342-628
ENSG0000(	553	12.3499	chr11:62(	TMEM223		protein_c	chr11:62771629-627
ENSG0000(	553	12.3499	chr11:62(	SNHG1		lncRNA	chr11:62851978-628
ENSG0000(	553	12.3499	chr11:62(	SLC3A2	AC	protein_c	chr11:62856004-628
ENSG0000(	553	12.3499	chr11:62(	ENSG00000250659		lncRNA	chr11:62537312-625
ENSG0000(	553	12.3499	chr11:62(	NXF1	NCGv7	protein_c	chr11:62792123-628
ENSG0000(	553	12.3499	chr11:62(	AP003064.1		smallRNA	chr11:62463635-624
ENSG0000(	553	12.3499	chr11:62(	B3GAT3		protein_c	chr11:62615296-626
ENSG0000(	553	12.3499	chr11:62(	UQCC3		protein_c	chr11:62670273-626
ENSG0000(	553	12.3499	chr11:62(	INCENP		protein_c	chr11:62123998-621
ENSG0000(	553	12.3499	chr11:62(	EML3		protein_c	chr11:62602218-626
ENSG0000(	553	12.3499	chr11:62(	ROM1		protein_c	chr11:62611722-626
ENSG0000(	553	12.3499	chr11:62(	UBXN1		protein_c	chr11:62676498-626
ENSG0000(	553	12.3499	chr11:62(	MTA2		protein_c	chr11:62593214-626
ENSG0000(	553	12.3499	chr11:62(	RN7SL119P		smallRNA	chr11:62816830-628
ENSG0000(	553	12.3499	chr11:62(	SCGB2A2		protein_c	chr11:62270158-622
ENSG0000(	553	12.3499	chr11:62(	ENSG00000285388		Pseudoger	chr11:62936427-629
ENSG0000(	553	12.3499	chr11:62(	TMEM179B		protein_c	chr11:62787402-627
ENSG0000(	553	12.3499	chr11:62(	GANAB		protein_c	chr11:62624826-626
ENSG0000(	553	12.3499	chr11:62(	BSCL2		protein_c	chr11:62689289-627
ENSG0000(	553	12.3499	chr11:62(	SCGB1D4		protein_c	chr11:62296281-622
ENSG0000(	553	12.3499	chr11:62(	POLR2G		protein_c	chr11:62761565-627
ENSG0000(	553	12.3499	chr11:62(	ENSG00000254404		lncRNA	chr11:62213427-622
ENSG0000(	553	12.3499	chr11:62(	ZBTB3		protein_c	chr11:62748319-627
ENSG0000(	553	12.3499	chr11:62(	AP003064.2		smallRNA	chr11:62372656-623
ENSG0000(	553	12.3499	chr11:62(	STX5-DT		lncRNA	chr11:62832234-628
ENSG0000(	553	12.3499	chr11:62(	LRRN4CL		protein_c	chr11:62686406-626
ENSG0000(	553	12.3499	chr11:62(	ENSG00000255446		lncRNA	chr11:62421845-624
ENSG0000(	553	12.3499	chr11:62(	AHNAK	NCGv7	protein_c	chr11:62433542-625
ENSG0000(	553	12.3499	chr11:62(	EEF1G		protein_c	chr11:62559596-625
ENSG0000(	553	12.3499	chr11:62(	INTS5	AC	protein_c	chr11:62646848-626
ENSG0000(	553	12.3499	chr11:62(	ENSG00000254424		Pseudoger	chr11:62072882-620

ENSG0000(	553	12.3499	chr11:62(	SNORA57		smallRNA	chr11:62665422-626
ENSG0000(	553	12.3499	chr11:62(	ENSG00000285656		lncRNA	chr11:62116470-621
ENSG0000(	553	12.3499	chr11:62(	SCGB1D1		protein_c	chr11:62190216-621
ENSG0000(	553	12.3499	chr11:62(	LINC02733		lncRNA	chr11:62049863-620
ENSG0000(	553	12.3499	chr11:62(	ENSG00000257002		lncRNA	chr11:62909546-629
ENSG0000(	553	12.3499	chr11:62(	TAF6L		protein_c	chr11:62771357-627
ENSG0000(	553	12.3499	chr11:62(	ENSG00000255118		lncRNA	chr11:62336911-623
ENSG0000(	553	12.3499	chr11:62(	SCGB1A1		protein_c	chr11:62405103-624
ENSG0000(	553	12.3499	chr11:62(	SLC22A6		protein_c	chr11:62936385-629
ENSG0000(	553	12.3499	chr11:62(	NPM1P35		Pseudoger	chr11:62330946-623
ENSG0000(	553	12.3499	chr11:62(	ENSG00000254964		lncRNA	chr11:62606161-626
ENSG0000(	553	12.3499	chr11:62(	TTC9C		protein_c	chr11:62728069-627
ENSG0000(	553	12.3499	chr11:62(	ENSG00000255432		protein_c	chr11:62649694-626
ENSG0000(	553	12.3499	chr11:62(	ENSG00000269176		lncRNA	chr11:62786023-627
ENSG0000(	553	12.3499	chr11:62(	ENSG00000255126		lncRNA	chr11:62391516-623
ENSG0000(	553	12.3499	chr11:62(	ENSG00000257058		lncRNA	chr11:62545999-625
ENSG0000(	553	12.3499	chr11:62(	ENSG00000269463		lncRNA	chr11:62807682-628
ENSG0000(	553	12.3499	chr11:62(	RNU6-118P		smallRNA	chr11:62815966-628
ENSG0000(	553	12.3499	chr11:62(	RN7SL259P		smallRNA	chr11:62935984-629
ENSG0000(	553	12.3499	chr11:62(	RNU2-2P		smallRNA	chr11:62841619-628
ENSG0000(	553	12.3499	chr11:62(	ASRGL1		protein_c	chr11:62337448-623
ENSG0000(	553	12.3499	chr11:62(	ENSG00000289194		lncRNA	chr11:62153730-621
ENSG0000(	553	12.3499	chr11:62(	LBHD1		protein_c	chr11:62662817-626
ENSG0000(	553	12.3499	chr11:62(	GNG3	NCGv7	protein_c	chr11:62707676-627
ENSG0000(	553	12.3499	chr11:62(	ENSG00000255508		protein_c	chr11:62559603-625
ENSG0000(	553	12.3499	chr11:62(	TEX54		protein_c	chr11:62832319-628
ENSG0000(	553	12.3499	chr11:62(	HNRNPUL2		protein_c	chr11:62712630-627
ENSG0000(	553	12.3499	chr11:62(	RCC2P6		Pseudoger	chr11:62371146-623
ENSG0000(	553	12.3499	chr11:62(	ENSG00000289562		lncRNA	chr11:62574174-625
ENSG0000(	553	12.3499	chr11:62(	C11orf98		protein_c	chr11:62662817-626
ENSG0000(	553	12.3499	chr11:62(	TUT1		protein_c	chr11:62575045-625
ENSG0000(	553	12.3499	chr11:62(	EEF1DP8		Pseudoger	chr11:62169293-621
ENSG0000(	553	12.3499	chr11:62(	HNRNPUL2-BSCL2		protein_c	chr11:62690275-627
ENSG0000(	553	12.3499	chr11:62(	CSKMT		protein_c	chr11:62665309-626
ENSG0000(	552	12.32756	chr12:14(	MIR331		smallRNA	chr12:95308420-953
ENSG0000(	551	12.30523	chr6:1039	ENSG00000274256		Pseudoger	chr6:41250851-4125
ENSG0000(	551	12.30523	chr6:1039	ENSG00000227516		lncRNA	chr6:41868622-4186
ENSG0000(	551	12.30523	chr6:1039	ENSG00000279284		TEC	chr6:41381392-4138
ENSG0000(	551	12.30523	chr6:1039	RPL32P15		Pseudoger	chr6:41308166-4130
ENSG0000(	551	12.30523	chr6:1039	RPL24P4		Pseudoger	chr6:42956345-4295
ENSG0000(	551	12.30523	chr6:1039	LINC01276		lncRNA	chr6:41499033-4151
ENSG0000(	551	12.30523	chr6:1039	GUCA1A		protein_c	chr6:42173364-4218
ENSG0000(	551	12.30523	chr6:1039	NPM1P51		Pseudoger	chr6:41666906-4166
ENSG0000(	551	12.30523	chr6:1039	ENSG00000231102		lncRNA	chr6:41720396-4173
ENSG0000(	551	12.30523	chr6:1039	ENSG00000223946		lncRNA	chr6:42030053-4203
ENSG0000(	551	12.30523	chr6:1039	LINC02976		lncRNA	chr6:42940364-4294
ENSG0000(	551	12.30523	chr6:1039	TOMM6		protein_c	chr6:41787662-4178
ENSG0000(	551	12.30523	chr6:1039	ATP6V0CP3		Pseudoger	chr6:42727234-4272
ENSG0000(	551	12.30523	chr6:1039	MRPS10		protein_c	chr6:42206807-4221
ENSG0000(	551	12.30523	chr6:1039	PGC		protein_c	chr6:41736711-4175
ENSG0000(	551	12.30523	chr6:1039	ENSG00000269387		lncRNA	chr6:41764292-4176

ENSG0000(	551	12.30523	chr6:1039	TREML3P		Pseudoger	chr6:41209634-4121
ENSG0000(	551	12.30523	chr6:1039	RN7SL403P		smallRNA	chr6:43036198-4303
ENSG0000(	551	12.30523	chr6:1039	USP49	DriverDB\	protein_c	chr6:41789896-4189
ENSG0000(	551	12.30523	chr6:1039	RNA5SP207		Pseudoger	chr6:41239520-4123
ENSG0000(	551	12.30523	chr6:1039	ENSG00000289216		lncRNA	chr6:42191657-4219
ENSG0000(	551	12.30523	chr6:1039	GUCA1ANB		protein_c	chr6:42155406-4216
ENSG0000(	551	12.30523	chr6:1039	RPL36AP5		Pseudoger	chr6:42499710-4250
ENSG0000(	551	12.30523	chr6:1039	NCR2		protein_c	chr6:41335608-4135
ENSG0000(	551	12.30523	chr6:1039	ENSG00000268745		lncRNA	chr6:41791410-4179
ENSG0000(	551	12.30523	chr6:1039	AL136967.1		smallRNA	chr6:41355449-4135
ENSG0000(	551	12.30523	chr6:1039	ENSG00000272223		lncRNA	chr6:43033897-4303
ENSG0000(	551	12.30523	chr6:1039	snoU13		smallRNA	chr6:42505500-4250
ENSG0000(	551	12.30523	chr6:1039	TREML4		protein_c	chr6:41228339-4123
ENSG0000(	551	12.30523	chr6:1039	CUL7	NCGv7;AC	protein_c	chr6:43037617-4305
ENSG0000(	551	12.30523	chr6:1039	C6orf226		protein_c	chr6:42890265-4289
ENSG0000(	551	12.30523	chr6:1039	CNPY3	DriverDB\	protein_c	chr6:42929480-4293
ENSG0000(	551	12.30523	chr6:1039	MED20	DriverDB\	protein_c	chr6:41905354-4192
ENSG0000(	551	12.30523	chr6:1039	C6orf132		protein_c	chr6:42092233-4214
ENSG0000(	551	12.30523	chr6:1039	MIR4641		smallRNA	chr6:41598723-4159
ENSG0000(	551	12.30523	chr6:1039	MRPL2		protein_c	chr6:43054029-4305
ENSG0000(	551	12.30523	chr6:1039	Y_RNA		smallRNA	chr6:41917416-4191
ENSG0000(	551	12.30523	chr6:1039	ENSG00000287825		lncRNA	chr6:42927686-4292
ENSG0000(	551	12.30523	chr6:1039	PPP2R5D		protein_c	chr6:42984553-4301
ENSG0000(	551	12.30523	chr6:1039	BICRAL	DriverDB\	protein_c	chr6:42746958-4286
ENSG0000(	551	12.30523	chr6:1039	PRPH2		protein_c	chr6:42696598-4272
ENSG0000(	551	12.30523	chr6:1039	TBCC	DriverDB\	protein_c	chr6:42744498-4274
ENSG0000(	551	12.30523	chr6:1039	GUCA1B		protein_c	chr6:42183284-4219
ENSG0000(	551	12.30523	chr6:1039	ENSG00000124593		protein_c	chr6:41780349-4179
ENSG0000(	551	12.30523	chr6:1039	UBR2	DriverDB\	protein_c	chr6:42564029-4269
ENSG0000(	551	12.30523	chr6:1039	BYSL		protein_c	chr6:41921499-4193
ENSG0000(	551	12.30523	chr6:1039	RNU6-643P		smallRNA	chr6:41302466-4130
ENSG0000(	551	12.30523	chr6:1039	RNU6-890P		smallRNA	chr6:42664162-4266
ENSG0000(	551	12.30523	chr6:1039	TAF8		protein_c	chr6:42050513-4208
ENSG0000(	551	12.30523	chr6:1039	RNU6-761P		smallRNA	chr6:42018408-4201
ENSG0000(	551	12.30523	chr6:1039	SNORA8		smallRNA	chr6:41832854-4183
ENSG0000(	551	12.30523	chr6:1039	FRS3		protein_c	chr6:41770176-4178
ENSG0000(	551	12.30523	chr6:1039	KLC4	DriverDB\	protein_c	chr6:43040777-4307
ENSG0000(	551	12.30523	chr6:1039	RPL7L1	DriverDB\	protein_c	chr6:42879616-4288
ENSG0000(	551	12.30523	chr6:1039	PEX6		protein_c	chr6:42963865-4297
ENSG0000(	551	12.30523	chr6:1039	FOXP4	DriverDB\	protein_c	chr6:41546381-4160
ENSG0000(	551	12.30523	chr6:1039	ENSG00000288010		lncRNA	chr6:42893761-4290
ENSG0000(	551	12.30523	chr6:1039	ENSG00000290563		lncRNA	chr6:41208713-4121
ENSG0000(	551	12.30523	chr6:1039	PRICKLE4		protein_c	chr6:41780782-4178
ENSG0000(	551	12.30523	chr6:1039	TRERF1	NCGv7	protein_c	chr6:42224931-4245
ENSG0000(	551	12.30523	chr6:1039	RRP36	DriverDB\	protein_c	chr6:43021623-4303
ENSG0000(	551	12.30523	chr6:1039	ENSG00000290147		protein_c	chr6:42155406-4218
ENSG0000(	551	12.30523	chr6:1039	ENSG00000280371		TEC	chr6:41405819-4140
ENSG0000(	551	12.30523	chr6:1039	KLHDC3	DriverDB\	protein_c	chr6:43014103-4302
ENSG0000(	551	12.30523	chr6:1039	TREM1		protein_c	chr6:41267926-4128
ENSG0000(	551	12.30523	chr6:1039	ENSG00000288721		protein_c	chr6:41793314-4192
ENSG0000(	551	12.30523	chr6:1039	TREML5P		Pseudoger	chr6:41247369-4124

ENSG0000(	551	12.30523	chr6:1039	PTCRA		protein_c	chr6:42915989-4292
ENSG0000(	551	12.30523	chr6:1039	ENSG00000218809		Pseudoger	chr6:41269875-4127
ENSG0000(	551	12.30523	chr6:1039	FOXP4-AS1		lncRNA	chr6:41452889-4154
ENSG0000(	551	12.30523	chr6:1039	CCND3	NCGv7;AC	protein_c	chr6:41934934-4205
ENSG0000(	551	12.30523	chr6:1039	ENSG00000278745		Pseudoger	chr6:41252696-4125
ENSG0000(	551	12.30523	chr6:1039	MEA1		protein_c	chr6:43011143-4301
ENSG0000(	551	12.30523	chr6:1039	U3		smallRNA	chr6:42412200-4241
ENSG0000(	551	12.30523	chr6:1039	ENSG00000290049		lncRNA	chr6:42980542-4298
ENSG0000(	551	12.30523	chr6:1039	TREML2		protein_c	chr6:41189749-4120
ENSG0000(	551	12.30523	chr6:1039	TFEB	NCGv7;AC	protein_c	chr6:41683978-4173
ENSG0000(	551	12.30523	chr6:1039	GNMT		protein_c	chr6:42960754-4296
ENSG0000(	551	12.30523	chr6:1039	ENSG00000288564		protein_c	chr6:43051066-4305
ENSG0000(	551	12.30523	chr6:1039	MDFI		protein_c	chr6:41636882-4165
ENSG0000(	547	12.2159	chr6:1391	MIR1202		smallRNA	chr6:155946797-155
ENSG0000(	547	12.2159	chr6:1391	ENSG00000289830		lncRNA	chr6:156395883-156
ENSG0000(	547	12.2159	chr6:1391	TIAM2		protein_c	chr6:154832697-155
ENSG0000(	547	12.2159	chr6:1391	RNU7-152P		smallRNA	chr6:155654963-155
ENSG0000(	547	12.2159	chr6:1391	ENSG00000287092		lncRNA	chr6:155911195-156
ENSG0000(	547	12.2159	chr6:1391	CLDN20	NCGv7	protein_c	chr6:155264013-155
ENSG0000(	547	12.2159	chr6:1391	NOX3		protein_c	chr6:155395368-155
ENSG0000(	547	12.2159	chr6:1391	ENSG00000232529		lncRNA	chr6:155380511-155
ENSG0000(	547	12.2159	chr6:1391	ENSG00000285797		lncRNA	chr6:156350579-156
ENSG0000(	547	12.2159	chr6:1391	ENSG00000227360		lncRNA	chr6:156493226-156
ENSG0000(	547	12.2159	chr6:1391	ENSG00000235381		lncRNA	chr6:155253139-155
ENSG0000(	547	12.2159	chr6:1391	SNORD28		smallRNA	chr6:156378750-156
ENSG0000(	547	12.2159	chr6:1391	TFB1M		protein_c	chr6:155256134-155
ENSG0000(	544	12.1489	chr6:1391	MIR3918		smallRNA	chr6:158764661-158
ENSG0000(	544	12.1489	chr6:1391	RSPH3		protein_c	chr6:158972871-159
ENSG0000(	544	12.1489	chr6:1391	ENSG00000287591		lncRNA	chr6:158296671-158
ENSG0000(	544	12.1489	chr6:1391	TATDN2P2		Pseudoger	chr6:158609706-158
ENSG0000(	544	12.1489	chr6:1391	RNU6-293P		smallRNA	chr6:158926466-158
ENSG0000(	544	12.1489	chr6:1391	TULP4		protein_c	chr6:158232236-158
ENSG0000(	544	12.1489	chr6:1391	ENSG00000285492		lncRNA	chr6:159051674-159
ENSG0000(	544	12.1489	chr6:1391	DYNLT1		protein_c	chr6:158636474-158
ENSG0000(	544	12.1489	chr6:1391	ENSG00000285159		lncRNA	chr6:158853588-158
ENSG0000(	544	12.1489	chr6:1391	SRP72P2		Pseudoger	chr6:158237336-158
ENSG0000(	544	12.1489	chr6:1391	ENSG00000238019		lncRNA	chr6:158397885-158
ENSG0000(	544	12.1489	chr6:1391	CACYBPP3		Pseudoger	chr6:158515605-158
ENSG0000(	544	12.1489	chr6:1391	ENSG00000288845		lncRNA	chr6:159045152-159
ENSG0000(	544	12.1489	chr6:1391	TMEM181		protein_c	chr6:158536436-158
ENSG0000(	544	12.1489	chr6:1391	ENSG00000226032		lncRNA	chr6:159042192-159
ENSG0000(	544	12.1489	chr6:1391	AMZ2P2		Pseudoger	chr6:158725747-158
ENSG0000(	544	12.1489	chr6:1391	OSTCP1		Pseudoger	chr6:158841117-158
ENSG0000(	544	12.1489	chr6:1391	AL590703.1		smallRNA	chr6:158174984-158
ENSG0000(	544	12.1489	chr6:1391	ENSG00000274023		lncRNA	chr6:158282841-158
ENSG0000(	544	12.1489	chr6:1391	SERAC1		protein_c	chr6:158109519-158
ENSG0000(	544	12.1489	chr6:1391	ENSG00000224478		lncRNA	chr6:159094093-159
ENSG0000(	544	12.1489	chr6:1391	RN7SL173P		smallRNA	chr6:158437797-158
ENSG0000(	544	12.1489	chr6:1391	TAGAP		protein_c	chr6:159034468-159
ENSG0000(	544	12.1489	chr6:1391	SYTL3		protein_c	chr6:158650014-158
ENSG0000(	544	12.1489	chr6:1391	EZR	NCGv7;AC	protein_c	chr6:158765741-158

ENSG0000(	544	12.1489	chr6:1391	TAGAP-AS1		lncRNA	chr6:158988178-159
ENSG0000(	544	12.1489	chr6:1391	LINC02901		lncRNA	chr6:158869848-158
ENSG0000(	544	12.1489	chr6:1391	ENSG00000213076		Pseudoger	chr6:158921271-158
ENSG0000(	544	12.1489	chr6:1391	GTF2H5		protein_c	chr6:158168350-158
ENSG0000(	544	12.1489	chr6:1391	EZR-AS1		lncRNA	chr6:158817979-158
ENSG0000(	542	12.10424	chr10:81(	ZNF503	NCGv7	protein_c	chr10:75397830-754
ENSG0000(	542	12.10424	chr10:81(	PLA2G12B		protein_c	chr10:72934762-729
ENSG0000(	542	12.10424	chr10:81(	ENSG00000280238		TEC	chr10:73791218-737
ENSG0000(	542	12.10424	chr10:81(	ENSG00000224500		lncRNA	chr10:77313941-773
ENSG0000(	542	12.10424	chr10:81(	COMTD1		protein_c	chr10:75233641-752
ENSG0000(	542	12.10424	chr10:81(	ANXA7		protein_c	chr10:73375101-734
ENSG0000(	542	12.10424	chr10:81(	FAM149B1		protein_c	chr10:73168119-732
ENSG0000(	542	12.10424	chr10:81(	ASCC1		protein_c	chr10:72096032-722
ENSG0000(	542	12.10424	chr10:81(	ENSG00000270808		Pseudoger	chr10:73740538-737
ENSG0000(	542	12.10424	chr10:81(	OIT3		protein_c	chr10:72893584-729
ENSG0000(	542	12.10424	chr10:81(	GLUD1P3		lncRNA	chr10:73730562-737
ENSG0000(	542	12.10424	chr10:81(	ENSG00000237768		lncRNA	chr10:73071295-730
ENSG0000(	542	12.10424	chr10:81(	MYOZ1		protein_c	chr10:73631612-736
ENSG0000(	542	12.10424	chr10:81(	RN7SL518P		smallRNA	chr10:76242024-762
ENSG0000(	542	12.10424	chr10:81(	ENSG00000285810		lncRNA	chr10:75041876-750
ENSG0000(	542	12.10424	chr10:81(	MIR4676		smallRNA	chr10:72721029-727
ENSG0000(	542	12.10424	chr10:81(	ENSG00000228280		lncRNA	chr10:75742740-757
ENSG0000(	542	12.10424	chr10:81(	Y_RNA		smallRNA	chr10:72220752-722
ENSG0000(	542	12.10424	chr10:81(	SNORA11		smallRNA	chr10:73126080-731
ENSG0000(	542	12.10424	chr10:81(	TIMM9P1		Pseudoger	chr10:74344550-743
ENSG0000(	542	12.10424	chr10:81(	SYNPO2L-AS1		lncRNA	chr10:73653980-736
ENSG0000(	542	12.10424	chr10:81(	AC012047.1		smallRNA	chr10:76127251-761
ENSG0000(	542	12.10424	chr10:81(	ENSG00000280166		TEC	chr10:72730557-727
ENSG0000(	542	12.10424	chr10:81(	VDAC2		protein_c	chr10:75210154-752
ENSG0000(	542	12.10424	chr10:81(	ENSG00000272627		lncRNA	chr10:72766560-727
ENSG0000(	542	12.10424	chr10:81(	ENSG00000230526		lncRNA	chr10:71878356-718
ENSG0000(	542	12.10424	chr10:81(	VSIR		protein_c	chr10:71747556-717
ENSG0000(	542	12.10424	chr10:81(	SPOCK2		protein_c	chr10:72059034-720
ENSG0000(	542	12.10424	chr10:81(	MICU1		protein_c	chr10:72367340-726
ENSG0000(	542	12.10424	chr10:81(	PPP3CB		protein_c	chr10:73436433-734
ENSG0000(	542	12.10424	chr10:81(	ENSG00000272692		lncRNA	chr10:75409142-754
ENSG0000(	542	12.10424	chr10:81(	SAMD8		protein_c	chr10:75099586-751
ENSG0000(	542	12.10424	chr10:81(	KAT6B	NCGv7;AC	protein_c	chr10:74824927-750
ENSG0000(	542	12.10424	chr10:81(	ENSG00000286715		lncRNA	chr10:75592644-756
ENSG0000(	542	12.10424	chr10:81(	BMS1P4		Pseudoger	chr10:73717115-737
ENSG0000(	542	12.10424	chr10:81(	Y_RNA		smallRNA	chr10:73082494-730
ENSG0000(	542	12.10424	chr10:81(	ADK		protein_c	chr10:74151202-747
ENSG0000(	542	12.10424	chr10:81(	CFAP70	NCGv7	protein_c	chr10:73253762-733
ENSG0000(	542	12.10424	chr10:81(	MCU		protein_c	chr10:72692143-728
ENSG0000(	542	12.10424	chr10:81(	ATP5MC1P8		Pseudoger	chr10:76836568-768
ENSG0000(	542	12.10424	chr10:81(	AL513185.1		smallRNA	chr10:72576724-725
ENSG0000(	542	12.10424	chr10:81(	RNU6-805P		smallRNA	chr10:72391964-723
ENSG0000(	542	12.10424	chr10:81(	DUSP8P5		Pseudoger	chr10:73731824-737
ENSG0000(	542	12.10424	chr10:81(	RMRPP1		smallRNA	chr10:73774322-737
ENSG0000(	542	12.10424	chr10:81(	ENSG00000230609		Pseudoger	chr10:72560264-725
ENSG0000(	542	12.10424	chr10:81(	HMGA1P5		Pseudoger	chr10:75276376-752

ENSG0000(	542	12.10424	chr10:81(	VCL	protein_c	chr10:73995193-741
ENSG0000(	542	12.10424	chr10:81(	ENSG00000272630	lncRNA	chr10:73098044-731
ENSG0000(	542	12.10424	chr10:81(	NDUFA8P1	Pseudoger	chr10:74641410-746
ENSG0000(	542	12.10424	chr10:81(	BMS1P4	lncRNA	chr10:73699151-737
ENSG0000(	542	12.10424	chr10:81(	RNU7-38P	smallRNA	chr10:71844735-718
ENSG0000(	542	12.10424	chr10:81(	ENSG00000288823	lncRNA	chr10:73780624-737
ENSG0000(	542	12.10424	chr10:81(	ENSG00000279088	TEC	chr10:73742962-737
ENSG0000(	542	12.10424	chr10:81(	DUSP29	protein_c	chr10:75037472-750
ENSG0000(	542	12.10424	chr10:81(	snoU13	smallRNA	chr10:73301781-733
ENSG0000(	542	12.10424	chr10:81(	USP54	protein_c	chr10:73497538-736
ENSG0000(	542	12.10424	chr10:81(	AL353731.1	smallRNA	chr10:73428667-734
ENSG0000(	542	12.10424	chr10:81(	RPL26P6	Pseudoger	chr10:73422259-734
ENSG0000(	542	12.10424	chr10:81(	ENSG00000273248	lncRNA	chr10:75408537-754
ENSG0000(	542	12.10424	chr10:81(	PPP3CB-AS1	lncRNA	chr10:73495525-735
ENSG0000(	542	12.10424	chr10:81(	PSAP	protein_c	chr10:71816298-718
ENSG0000(	542	12.10424	chr10:81(	DDIT4	protein_c	chr10:72273920-722
ENSG0000(	542	12.10424	chr5:164(	PSMC1P5	Pseudoger	chr5:107195156-107
ENSG0000(	542	12.10424	chr10:81(	C10orf55	lncRNA	chr10:73909969-739
ENSG0000(	542	12.10424	chr10:81(	SNORA36	smallRNA	chr10:72180858-721
ENSG0000(	542	12.10424	chr10:81(	NDST2	protein_c	chr10:73801911-738
ENSG0000(	542	12.10424	chr10:81(	RNU6-883P	smallRNA	chr10:73529051-735
ENSG0000(	542	12.10424	chr10:81(	DNAJC9-AS1	lncRNA	chr10:73247360-732
ENSG0000(	542	12.10424	chr10:81(	ENSG00000234149	lncRNA	chr10:75003055-750
ENSG0000(	542	12.10424	chr10:81(	ENSG00000223428	Pseudoger	chr10:75288333-752
ENSG0000(	542	12.10424	chr10:81(	Metazoa_SRP	smallRNA	chr10:72582803-725
ENSG0000(	542	12.10424	chr10:81(	Y_RNA	smallRNA	chr10:74551392-745
ENSG0000(	542	12.10424	chr10:81(	ENSG00000289362	lncRNA	chr10:71551031-715
ENSG0000(	542	12.10424	chr10:81(	SNORA31	smallRNA	chr10:76835377-768
ENSG0000(	542	12.10424	chr10:81(	RNU6-833P	smallRNA	chr10:73279062-732
ENSG0000(	542	12.10424	chr10:81(	ENSG00000290737	lncRNA	chr10:73715843-737
ENSG0000(	542	12.10424	chr10:81(	ENSG00000282915	lncRNA	chr10:72467749-724
ENSG0000(	542	12.10424	chr10:81(	ENSG00000278992	TEC	chr10:76270206-762
ENSG0000(	542	12.10424	chr10:81(	SPA17P1	Pseudoger	chr10:75382393-753
ENSG0000(	542	12.10424	chr5:164(	ENSG00000250145	Pseudoger	chr5:106543066-106
ENSG0000(	542	12.10424	chr10:81(	LRMDA	protein_c	chr10:75431624-765
ENSG0000(	542	12.10424	chr10:81(	ENSG00000288559	lncRNA	chr10:73247342-732
ENSG0000(	542	12.10424	chr10:81(	KCNMA1-AS1	lncRNA	chr10:76888044-769
ENSG0000(	542	12.10424	chr10:81(	ENSG00000272599	lncRNA	chr10:73124573-731
ENSG0000(	542	12.10424	chr10:81(	ENSG00000279502	TEC	chr10:72756398-727
ENSG0000(	542	12.10424	chr10:81(	P4HA1	protein_c	chr10:73007217-730
ENSG0000(	542	12.10424	chr10:81(	MIR606	smallRNA	chr10:75552458-755
ENSG0000(	542	12.10424	chr10:81(	ENSG00000238215	Pseudoger	chr10:72643011-726
ENSG0000(	542	12.10424	chr10:81(	ECD	protein_c	chr10:73130155-731
ENSG0000(	542	12.10424	chr10:81(	ENSG00000229990	lncRNA	chr10:73841833-738
ENSG0000(	542	12.10424	chr10:81(	CHST3	protein_c	chr10:71964395-720
ENSG0000(	542	12.10424	chr10:81(	ZSWIM8-AS1	lncRNA	chr10:73796514-738
ENSG0000(	542	12.10424	chr10:81(	PLAU	protein_c	chr10:73909177-739
ENSG0000(	542	12.10424	chr10:81(	CAMK2G-AS1	lncRNA	chr10:73813518-738
ENSG0000(	542	12.10424	chr10:81(	CAMK2G	protein_c	chr10:73812501-738
ENSG0000(	542	12.10424	chr10:81(	Y_RNA	smallRNA	chr10:73346936-733
ENSG0000(	542	12.10424	chr10:81(	ENSG00000236842	lncRNA	chr10:75430571-754

ENSG0000(	542	12.10424	chr10:81(	DUSP13	protein_c	chr10:75094432-751
ENSG0000(	542	12.10424	chr10:81(	ENSG00000279689	TEC	chr10:73769264-737
ENSG0000(	542	12.10424	chr10:81(	ANAPC16	protein_c	chr10:72216000-722
ENSG0000(	542	12.10424	chr10:81(	PPIAP13	Pseudoger	chr10:75089248-750
ENSG0000(	542	12.10424	chr10:81(	SYNPO2L	protein_c	chr10:73644881-736
ENSG0000(	542	12.10424	chr10:81(	NUDT13	protein_c	chr10:73110375-731
ENSG0000(	542	12.10424	chr10:81(	AL392111.1	smallRNA	chr10:75184958-751
ENSG0000(	542	12.10424	chr10:81(	DNAJB12	protein_c	chr10:72332830-723
ENSG0000(	542	12.10424	chr10:81(	MSS51	protein_c	chr10:73423579-734
ENSG0000(	542	12.10424	chr10:81(	MRPS16	protein_c	chr10:73248843-732
ENSG0000(	542	12.10424	chr10:81(	ENSG00000230575	lncRNA	chr10:76437408-764
ENSG0000(	542	12.10424	chr10:81(	ENSG00000289506	lncRNA	chr10:72272288-722
ENSG0000(	542	12.10424	chr10:81(	RPL39P25	Pseudoger	chr10:75269392-752
ENSG0000(	542	12.10424	chr10:81(	NPM1P24	Pseudoger	chr10:72917641-729
ENSG0000(	542	12.10424	chr5:164(	SNORA31	smallRNA	chr5:106546665-106
ENSG0000(	542	12.10424	chr10:81(	BMS1P4-AGAP5	lncRNA	chr10:73674295-737
ENSG0000(	542	12.10424	chr8:412(	RN7SL685P	smallRNA	chr8:100957883-100
ENSG0000(	542	12.10424	chr10:81(	RPL17P50	Pseudoger	chr10:73005833-730
ENSG0000(	542	12.10424	chr10:81(	Y_RNA	smallRNA	chr10:73192223-731
ENSG0000(	542	12.10424	chr10:81(	AP3M1	protein_c	chr10:74120255-741
ENSG0000(	542	12.10424	chr10:81(	ENSG00000232342	lncRNA	chr10:74506081-745
ENSG0000(	542	12.10424	chr10:81(	ENSG00000272748	lncRNA	chr10:74821610-748
ENSG0000(	542	12.10424	chr10:81(	RNU6-673P	smallRNA	chr10:76260800-762
ENSG0000(	542	12.10424	chr10:81(	POLR3DP1	Pseudoger	chr10:74654956-746
ENSG0000(	542	12.10424	chr10:81(	RPL15P14	Pseudoger	chr10:72189941-721
ENSG0000(	542	12.10424	chr10:81(	RPSAP6	Pseudoger	chr10:74371535-743
ENSG0000(	542	12.10424	chr10:81(	ENSG00000232646	Pseudoger	chr10:73065126-730
ENSG0000(	542	12.10424	chr10:81(	SEC24C	protein_c	chr10:73744372-737
ENSG0000(	542	12.10424	chr10:81(	DNAJC9	protein_c	chr10:73183362-732
ENSG0000(	542	12.10424	chr10:81(	ZNF503-AS1	lncRNA	chr10:75243568-753
ENSG0000(	542	12.10424	chr10:81(	MRPL35P3	Pseudoger	chr10:74527584-745
ENSG0000(	542	12.10424	chr10:81(	SNX19P4	Pseudoger	chr10:72483245-724
ENSG0000(	542	12.10424	chr10:81(	ENSG00000279814	TEC	chr10:75450081-754
ENSG0000(	542	12.10424	chr10:81(	ENSG00000270087	lncRNA	chr10:75279725-754
ENSG0000(	542	12.10424	chr10:81(	EIF4A2P2	Pseudoger	chr10:73199826-732
ENSG0000(	542	12.10424	chr10:81(	ENSG00000226163	lncRNA	chr10:72501746-725
ENSG0000(	542	12.10424	chr10:81(	Y_RNA	smallRNA	chr10:72654165-726
ENSG0000(	542	12.10424	chr10:81(	RPS26P41	Pseudoger	chr10:73537803-735
ENSG0000(	542	12.10424	chr10:81(	ZNF503-AS2	lncRNA	chr10:75401519-754
ENSG0000(	542	12.10424	chr10:81(	C10orf105	protein_c	chr10:71711701-717
ENSG0000(	542	12.10424	chr10:81(	RAB5CP1	Pseudoger	chr10:74423435-744
ENSG0000(	542	12.10424	chr10:81(	FAM32CP	Pseudoger	chr10:74744386-747
ENSG0000(	542	12.10424	chr10:81(	DNAJC9-AS1	lncRNA	chr10:73252791-732
ENSG0000(	542	12.10424	chr10:81(	ZSWIM8	protein_c	chr10:73785606-738
ENSG0000(	542	12.10424	chr10:81(	AGAP5	protein_c	chr10:73674287-736
ENSG0000(	542	12.10424	chr10:81(	ENSG00000268584	lncRNA	chr10:73625996-736
ENSG0000(	542	12.10424	chr5:164(	LINC01950	lncRNA	chr5:106815197-107
ENSG0000(	542	12.10424	chr10:81(	ENSG00000272140	lncRNA	chr10:73703735-737
ENSG0000(	542	12.10424	chr10:81(	ENSG00000268659	lncRNA	chr10:75642476-756
ENSG0000(	542	12.10424	chr10:81(	ENSG00000225761	lncRNA	chr10:74005137-740
ENSG0000(	542	12.10424	chr10:81(	HMGN2P34	Pseudoger	chr10:72636497-726

ENSG0000(	542	12.10424	chr10:81(	KCNMA1-AS2	lncRNA	chr10:77147652-771
ENSG0000(	542	12.10424	chr5:164(	ENSG00000251204	Pseudoger	chr5:106415576-106
ENSG0000(	542	12.10424	chr10:81(	ENSG00000269256	lncRNA	chr10:76558350-765
ENSG0000(	542	12.10424	chr10:81(	RPS26P42	Pseudoger	chr10:75125217-751
ENSG0000(	542	12.10424	chr10:81(	CHCHD1	protein_c	chr10:73782047-737
ENSG0000(	542	12.10424	chr10:81(	ENSG00000227186	lncRNA	chr10:75023475-750
ENSG0000(	542	12.10424	chr10:81(	ENSG00000272791	lncRNA	chr10:73630556-736
ENSG0000(	542	12.10424	chr10:81(	FUT11	protein_c	chr10:73772276-737
ENSG0000(	542	12.10424	chr10:81(	ENSG00000233144	lncRNA	chr10:73381433-733
ENSG0000(	542	12.10424	chr10:81(	ENSG00000272916	protein_c	chr10:73796514-738
ENSG0000(	542	12.10424	chr10:81(	ENSG00000289592	lncRNA	chr10:71888499-718
ENSG0000(	542	12.10424	chr10:81(	ENSG00000272988	lncRNA	chr10:72053294-720
ENSG0000(	542	12.10424	chr10:81(	GLUD1P3	Pseudoger	chr10:73731176-737
ENSG0000(	542	12.10424	chr10:81(	AL731568.1	smallRNA	chr10:75823686-758
ENSG0000(	542	12.10424	chr10:81(	RNA5SP320	Pseudoger	chr10:73706490-737
ENSG0000(	541	12.08191	chr4:410{	ENSG00000251411	Pseudoger	chr4:86913266-8691
ENSG0000(	541	12.08191	chr4:410{	ENSG00000248984	lncRNA	chr4:91108023-9111
ENSG0000(	541	12.08191	chr4:410{	ENSG00000251095	lncRNA	chr4:89410960-8972
ENSG0000(	541	12.08191	chr2:309{	ENSG00000244260	lncRNA	chr2:9671875-97084
ENSG0000(	541	12.08191	chr4:410{	ENSG00000250908	lncRNA	chr4:93318623-9331
ENSG0000(	541	12.08191	chr4:410{	BTF3P13	Pseudoger	chr4:98740742-9874
ENSG0000(	541	12.08191	chr4:410{	Y_RNA	smallRNA	chr4:83636196-8363
ENSG0000(	541	12.08191	chr4:410{	NDUFS5P4	Pseudoger	chr4:98976800-9897
ENSG0000(	541	12.08191	chr4:410{	KPNA2P1	Pseudoger	chr4:80079532-8008
ENSG0000(	541	12.08191	chr4:410{	ABT1P1	Pseudoger	chr4:99022311-9902
ENSG0000(	541	12.08191	chr4:410{	RN7SKP28	smallRNA	chr4:96348738-9634
ENSG0000(	541	12.08191	chr4:410{	ADH7	protein_c	chr4:99412261-9943
ENSG0000(	541	12.08191	chr2:309{	ENSG00000243491	lncRNA	chr2:9757496-97703
ENSG0000(	541	12.08191	chr4:410{	ENSG00000274238	lncRNA	chr4:89743792-8974
ENSG0000(	541	12.08191	chr4:410{	UNC5C-AS1	lncRNA	chr4:95549129-9555
ENSG0000(	541	12.08191	chr4:410{	TSPAN5	protein_c	chr4:98470367-9865
ENSG0000(	541	12.08191	chr4:410{	RN7SL127P	smallRNA	chr4:78898855-7889
ENSG0000(	541	12.08191	chr4:410{	ENSG00000286490	lncRNA	chr4:82612184-8261
ENSG0000(	541	12.08191	chr4:410{	LINC02267	lncRNA	chr4:96310701-9681
ENSG0000(	541	12.08191	chr4:410{	RNU6-1059P	smallRNA	chr4:95868667-9586
ENSG0000(	541	12.08191	chr2:309{	snoU13	smallRNA	chr2:9532242-95323
ENSG0000(	541	12.08191	chr4:410{	ENSG00000249001	lncRNA	chr4:87568035-8773
ENSG0000(	541	12.08191	chr4:410{	HPSE	protein_c	chr4:83292461-8333
ENSG0000(	541	12.08191	chr4:410{	ENSG00000287841	lncRNA	chr4:96841995-9685
ENSG0000(	541	12.08191	chr4:410{	BIN2P1	Pseudoger	chr4:82275071-8227
ENSG0000(	541	12.08191	chr4:410{	ENSG00000263923	lncRNA	chr4:98928897-9899
ENSG0000(	541	12.08191	chr4:410{	AC019131.1	smallRNA	chr4:98929923-9892
ENSG0000(	541	12.08191	chr4:410{	PCNAP1	Pseudoger	chr4:99160514-9916
ENSG0000(	541	12.08191	chr4:410{	ENSG00000290776	lncRNA	chr4:79587302-7958
ENSG0000(	541	12.08191	chr4:410{	ADH5	protein_c	chr4:99070978-9908
ENSG0000(	541	12.08191	chr4:410{	RNU6-112P	smallRNA	chr4:88275205-8827
ENSG0000(	541	12.08191	chr4:410{	snoR442	smallRNA	chr4:82949168-8294
ENSG0000(	541	12.08191	chr4:410{	LNCPRESS2	lncRNA	chr4:92268767-9227
ENSG0000(	541	12.08191	chr4:410{	COQ2	protein_c	chr4:83261536-8328
ENSG0000(	541	12.08191	chr4:410{	RNU5A-2P	smallRNA	chr4:81334303-8133
ENSG0000(	541	12.08191	chr2:309{	Y_RNA	smallRNA	chr2:9615267-96153

ENSG0000(	541	12.08191	chr4:4108	SLC10A6		protein_c	chr4:86823468-8684
ENSG0000(	541	12.08191	chr4:4108	SCD5		protein_c	chr4:82629539-8279
ENSG0000(	541	12.08191	chr4:4108	ENSG00000251523		lncRNA	chr4:98496364-9850
ENSG0000(	541	12.08191	chr4:4108	NPM1P41		Pseudoger	chr4:82010665-8201
ENSG0000(	541	12.08191	chr4:4108	ENSG00000287181		lncRNA	chr4:88352222-8835
ENSG0000(	541	12.08191	chr4:4108	TBCAP3		Pseudoger	chr4:98909537-9890
ENSG0000(	541	12.08191	chr4:4108	MTND1P19		Pseudoger	chr4:92702345-9270
ENSG0000(	541	12.08191	chr4:4108	Y_RNA		smallRNA	chr4:98173784-9817
ENSG0000(	541	12.08191	chr4:4108	ENSG00000276542		lncRNA	chr4:89748283-8974
ENSG0000(	541	12.08191	chr4:4108	AC098870.1		smallRNA	chr4:85910478-8591
ENSG0000(	541	12.08191	chr4:4108	ENSG00000251401		Pseudoger	chr4:90682996-9068
ENSG0000(	541	12.08191	chr4:4108	ENSG00000246090		lncRNA	chr4:99088805-9930
ENSG0000(	541	12.08191	chr4:4108	ENSG00000286189		lncRNA	chr4:84970180-8497
ENSG0000(	541	12.08191	chr4:4108	ADH6		protein_c	chr4:99202638-9921
ENSG0000(	541	12.08191	chr4:4108	PRKG2-AS1		lncRNA	chr4:81164922-8119
ENSG0000(	541	12.08191	chr4:4108	THAP9-AS1		lncRNA	chr4:82893009-8290
ENSG0000(	541	12.08191	chr4:4108	MIR5705		smallRNA	chr4:87300495-8730
ENSG0000(	541	12.08191	chr4:4108	ENSG00000287239		lncRNA	chr4:89995223-8999
ENSG0000(	541	12.08191	chr4:4108	CFAP299		protein_c	chr4:80335730-8096
ENSG0000(	541	12.08191	chr4:4108	THAP9		protein_c	chr4:82900684-8291
ENSG0000(	541	12.08191	chr4:4108	ENSG00000249049		lncRNA	chr4:91319034-9132
ENSG0000(	541	12.08191	chr4:4108	ENSG00000249052		lncRNA	chr4:91887886-9190
ENSG0000(	541	12.08191	chr4:4108	COX5BP1		Pseudoger	chr4:81919995-8192
ENSG0000(	541	12.08191	chr4:4108	ENSG00000274154		Pseudoger	chr4:83369416-8336
ENSG0000(	541	12.08191	chr4:4108	BMP2K		protein_c	chr4:78776342-7891
ENSG0000(	541	12.08191	chr4:4108	MTTP		protein_c	chr4:99564081-9962
ENSG0000(	541	12.08191	chr4:4108	ENSG00000251285		Pseudoger	chr4:88220569-8822
ENSG0000(	541	12.08191	chr4:4108	LIN54		protein_c	chr4:82909973-8301
ENSG0000(	541	12.08191	chr4:4108	ENSG00000242318		Pseudoger	chr4:98409290-9840
ENSG0000(	541	12.08191	chr4:4108	ENSG00000248749		lncRNA	chr4:84371393-8438
ENSG0000(	541	12.08191	chr4:4108	IBSP		protein_c	chr4:87799554-8781
ENSG0000(	541	12.08191	chr4:4108	ENSG00000248750		Pseudoger	chr4:92884663-9288
ENSG0000(	541	12.08191	chr4:4108	ENSG00000287512		lncRNA	chr4:98961083-9896
ENSG0000(	541	12.08191	chr2:3094	YWHAQ	AC	protein_c	chr2:9583967-96309
ENSG0000(	541	12.08191	chr4:4108	ENOPH1		protein_c	chr4:82430590-8246
ENSG0000(	541	12.08191	chr4:4108	ENSG00000248725		Pseudoger	chr4:85246157-8524
ENSG0000(	541	12.08191	chr4:4108	HNRNPA3P13		Pseudoger	chr4:82128535-8212
ENSG0000(	541	12.08191	chr4:4108	RNU1-36P		smallRNA	chr4:88000237-8800
ENSG0000(	541	12.08191	chr4:4108	PCAT4		lncRNA	chr4:79827471-7987
ENSG0000(	541	12.08191	chr4:4108	PIGY	NCGv7	protein_c	chr4:88520998-8852
ENSG0000(	541	12.08191	chr4:4108	PPM1K-DT		lncRNA	chr4:88284507-8834
ENSG0000(	541	12.08191	chr4:4108	HSD17B13		protein_c	chr4:87303789-8732
ENSG0000(	541	12.08191	chr4:4108	ENSG00000254044		lncRNA	chr4:97334635-9763
ENSG0000(	541	12.08191	chr4:4108	RNU6-499P		smallRNA	chr4:82174547-8217
ENSG0000(	541	12.08191	chr4:4108	ENSG00000286618		lncRNA	chr4:87974385-8800
ENSG0000(	541	12.08191	chr4:4108	ENSG00000250572		lncRNA	chr4:87261931-8726
ENSG0000(	541	12.08191	chr4:4108	C4orf54		protein_c	chr4:99636529-9965
ENSG0000(	541	12.08191	chr4:4108	WDFY3-AS1		lncRNA	chr4:84796614-8481
ENSG0000(	541	12.08191	chr4:4108	AC034154.1		smallRNA	chr4:97486806-9748
ENSG0000(	541	12.08191	chr4:4108	ENSG00000248511		lncRNA	chr4:92297251-9230
ENSG0000(	541	12.08191	chr4:4108	RPL6P13		Pseudoger	chr4:86870191-8687

ENSG0000(	541	12.08191	chr4:410{	ENSG00000287552		lncRNA	chr4:94315486-9434
ENSG0000(	541	12.08191	chr4:410{	ENSG00000250677		Pseudoger	chr4:83237303-8323
ENSG0000(	541	12.08191	chr4:410{	ENSG00000280056		TEC	chr4:92260367-9226
ENSG0000(	541	12.08191	chr4:410{	ENSG00000250655		Pseudoger	chr4:96212279-9621
ENSG0000(	541	12.08191	chr2:309	IAH1		protein_c	chr2:9473658-94965
ENSG0000(	541	12.08191	chr4:410{	PRDM8-AS1		lncRNA	chr4:80182637-8019
ENSG0000(	541	12.08191	chr4:410{	snoU13		smallRNA	chr4:87768485-8776
ENSG0000(	541	12.08191	chr4:410{	HERC3		protein_c	chr4:88523810-8870
ENSG0000(	541	12.08191	chr4:410{	ENSG00000248676		lncRNA	chr4:99594799-9962
ENSG0000(	541	12.08191	chr4:410{	MIR3684		smallRNA	chr4:98997387-9899
ENSG0000(	541	12.08191	chr4:410{	SMARCAD1-DT		lncRNA	chr4:94117792-9420
ENSG0000(	541	12.08191	chr4:410{	SNORD42		smallRNA	chr4:82402638-8240
ENSG0000(	541	12.08191	chr4:410{	NUDT9		protein_c	chr4:87422573-8745
ENSG0000(	541	12.08191	chr4:410{	ADH1B	NCGv7	protein_c	chr4:99304971-9935
ENSG0000(	541	12.08191	chr4:410{	RNU6-818P		smallRNA	chr4:88201703-8820
ENSG0000(	541	12.08191	chr4:410{	GPRIN3		protein_c	chr4:89236383-8930
ENSG0000(	541	12.08191	chr4:410{	ENSG00000227304		Pseudoger	chr4:82494786-8249
ENSG0000(	541	12.08191	chr4:410{	RNU6-34P		smallRNA	chr4:96152297-9615
ENSG0000(	541	12.08191	chr4:410{	BMPR1B		protein_c	chr4:94757955-9515
ENSG0000(	541	12.08191	chr4:410{	RAP1GDS1	NCGv7;AC	protein_c	chr4:98261384-9844
ENSG0000(	541	12.08191	chr4:410{	WDFY3-AS2		lncRNA	chr4:84965534-8501
ENSG0000(	541	12.08191	chr4:410{	MMRN1		protein_c	chr4:89879532-8995
ENSG0000(	541	12.08191	chr4:410{	TECRP1		Pseudoger	chr4:86949669-8695
ENSG0000(	541	12.08191	chr4:410{	ENSG00000287392		lncRNA	chr4:92183235-9226
ENSG0000(	541	12.08191	chr4:410{	ENSG00000248627		lncRNA	chr4:92833685-9283
ENSG0000(	541	12.08191	chr4:410{	AFF1	IntOGen-I	protein_c	chr4:86935002-8714
ENSG0000(	541	12.08191	chr4:410{	GK2		protein_c	chr4:79406361-7940
ENSG0000(	541	12.08191	chr4:410{	ENSG00000286978		lncRNA	chr4:88358945-8836
ENSG0000(	541	12.08191	chr4:410{	HMGB3P15		Pseudoger	chr4:94195940-9419
ENSG0000(	541	12.08191	chr4:410{	TIGD2		protein_c	chr4:89111533-8911
ENSG0000(	541	12.08191	chr4:410{	C4orf17		protein_c	chr4:99511012-9954
ENSG0000(	541	12.08191	chr4:410{	ENSG00000251399		lncRNA	chr4:79596542-7959
ENSG0000(	541	12.08191	chr4:410{	GPAT3		protein_c	chr4:83535914-8360
ENSG0000(	541	12.08191	chr4:410{	CCSER1		protein_c	chr4:90127394-9160
ENSG0000(	541	12.08191	chr4:410{	PMPCAP1		Pseudoger	chr4:92182477-9218
ENSG0000(	541	12.08191	chr4:410{	HERC5	DriverDB\	protein_c	chr4:88457119-8850
ENSG0000(	541	12.08191	chr4:410{	LINC02994		lncRNA	chr4:83796436-8429
ENSG0000(	541	12.08191	chr4:410{	RN7SKP48		smallRNA	chr4:85100496-8510
ENSG0000(	541	12.08191	chr4:410{	ENSG00000241853		Pseudoger	chr4:88163579-8816
ENSG0000(	541	12.08191	chr4:410{	ARHGAP24		protein_c	chr4:85475150-8600
ENSG0000(	541	12.08191	chr4:410{	FAM13A		protein_c	chr4:88725955-8911
ENSG0000(	541	12.08191	chr4:410{	HERC3		protein_c	chr4:88592434-8870
ENSG0000(	541	12.08191	chr4:410{	HERC6		protein_c	chr4:88378739-8844
ENSG0000(	541	12.08191	chr4:410{	COPS4		protein_c	chr4:83034447-8307
ENSG0000(	541	12.08191	chr4:410{	FGF5	NCGv7;AC	protein_c	chr4:80266639-8033
ENSG0000(	541	12.08191	chr4:410{	HNRNPD	NCGv7	protein_c	chr4:82352498-8237
ENSG0000(	541	12.08191	chr2:309	ENSG00000271855		lncRNA	chr2:9555899-95567
ENSG0000(	541	12.08191	chr4:410{	ATOH1		protein_c	chr4:93828753-9383
ENSG0000(	541	12.08191	chr4:410{	PRKG2		protein_c	chr4:81087370-8121
ENSG0000(	541	12.08191	chr4:410{	ENSG00000253170		lncRNA	chr4:97120701-9713
ENSG0000(	541	12.08191	chr4:410{	RASGEF1B		protein_c	chr4:81426393-8204

ENSG0000(	541	12.08191	chr4:410{	SEC31A		protein_c	chr4:82818509-8290
ENSG0000(	541	12.08191	chr4:410{	PLAC8	AC	protein_c	chr4:83090048-8313
ENSG0000(	541	12.08191	chr4:410{	SMARCAD1		protein_c	chr4:94207611-9429
ENSG0000(	541	12.08191	chr4:410{	TRMT10A		protein_c	chr4:99546709-9956
ENSG0000(	541	12.08191	chr4:410{	RPL3P13		Pseudoger	chr4:84544304-8454
ENSG0000(	541	12.08191	chr4:410{	LINC01088		lncRNA	chr4:78939485-7930
ENSG0000(	541	12.08191	chr4:410{	ENSG00000277695		lncRNA	chr4:89747802-8975
ENSG0000(	541	12.08191	chr4:410{	LINC00575		lncRNA	chr4:82610974-8262
ENSG0000(	541	12.08191	chr2:309<	ADAM17		protein_c	chr2:9488486-95567
ENSG0000(	541	12.08191	chr4:410{	ENSG00000270842		Pseudoger	chr4:82775461-8277
ENSG0000(	541	12.08191	chr4:410{	CHCHD2P7		Pseudoger	chr4:87785920-8778
ENSG0000(	541	12.08191	chr4:410{	GRID2		protein_c	chr4:92303966-9381
ENSG0000(	541	12.08191	chr4:410{	ENSG00000255723		lncRNA	chr4:87317170-8734
ENSG0000(	541	12.08191	chr4:410{	ENSG00000248113		Pseudoger	chr4:82580117-8258
ENSG0000(	541	12.08191	chr4:410{	ENSG00000249960		Pseudoger	chr4:82571137-8257
ENSG0000(	541	12.08191	chr4:410{	SNORA75		smallRNA	chr4:79843102-7984
ENSG0000(	541	12.08191	chr4:410{	SPARCL1		protein_c	chr4:87473335-8753
ENSG0000(	541	12.08191	chr4:410{	DSPP	NCGv7	protein_c	chr4:87608529-8761
ENSG0000(	541	12.08191	chr4:410{	DMP1		protein_c	chr4:87650280-8766
ENSG0000(	541	12.08191	chr4:410{	Y_RNA		smallRNA	chr4:87412228-8741
ENSG0000(	541	12.08191	chr4:410{	EIF4E	AC	protein_c	chr4:98879276-9892
ENSG0000(	541	12.08191	chr4:410{	HSP90AB3P		Pseudoger	chr4:87891843-8789
ENSG0000(	541	12.08191	chr4:410{	RPL30P5		Pseudoger	chr4:83502699-8350
ENSG0000(	541	12.08191	chr4:410{	ENSG00000248196		Pseudoger	chr4:87166844-8716
ENSG0000(	541	12.08191	chr4:410{	RNU6-469P		smallRNA	chr4:84886386-8488
ENSG0000(	541	12.08191	chr4:410{	UNC5C	NCGv7	protein_c	chr4:95162504-9554
ENSG0000(	541	12.08191	chr4:410{	ENSG00000251647		Pseudoger	chr4:83377363-8337
ENSG0000(	541	12.08191	chr4:410{	ENSG00000285458		protein_c	chr4:86886472-8693
ENSG0000(	541	12.08191	chr4:410{	ENSG00000288563		lncRNA	chr4:89582507-8970
ENSG0000(	541	12.08191	chr4:410{	RN7SL681P		smallRNA	chr4:87386886-8738
ENSG0000(	541	12.08191	chr4:410{	ENSG00000214559		lncRNA	chr4:98251688-9826
ENSG0000(	541	12.08191	chr4:410{	AC083829.1		smallRNA	chr4:88493933-8849
ENSG0000(	541	12.08191	chr4:410{	PIGY-DT		lncRNA	chr4:88523826-8852
ENSG0000(	541	12.08191	chr2:309<	ENSG00000240960		Pseudoger	chr2:9746704-97468
ENSG0000(	541	12.08191	chr4:410{	MAPK10-AS1		lncRNA	chr4:86117912-8621
ENSG0000(	541	12.08191	chr4:410{	ENSG00000250057		lncRNA	chr4:83233512-8324
ENSG0000(	541	12.08191	chr4:410{	ENSG00000289034		lncRNA	chr4:88006143-8800
ENSG0000(	541	12.08191	chr4:410{	ENSG00000273156		lncRNA	chr4:82344876-8234
ENSG0000(	541	12.08191	chr4:410{	MIR575		smallRNA	chr4:82753337-8275
ENSG0000(	541	12.08191	chr4:410{	MEPE		protein_c	chr4:87821398-8784
ENSG0000(	541	12.08191	chr4:410{	RPL5P12		Pseudoger	chr4:98025390-9802
ENSG0000(	541	12.08191	chr4:410{	FAM177A1P1		Pseudoger	chr4:98955982-9895
ENSG0000(	541	12.08191	chr4:410{	NAP1L5		protein_c	chr4:88695913-8869
ENSG0000(	541	12.08191	chr4:410{	ADH1C		protein_c	chr4:99336497-9935
ENSG0000(	541	12.08191	chr4:410{	Y_RNA		smallRNA	chr4:87743952-8774
ENSG0000(	541	12.08191	chr4:410{	GAPDHP60		Pseudoger	chr4:87207092-8720
ENSG0000(	541	12.08191	chr4:410{	VAMP9P		Pseudoger	chr4:82284971-8234
ENSG0000(	541	12.08191	chr4:410{	ENSG00000270480		Pseudoger	chr4:82691737-8269
ENSG0000(	541	12.08191	chr4:410{	NAA11		protein_c	chr4:79225694-7932
ENSG0000(	541	12.08191	chr4:410{	HNRNPD-DT		lncRNA	chr4:82374142-8238
ENSG0000(	541	12.08191	chr2:309<	ENSG00000288819		lncRNA	chr2:218368113-218

ENSG0000(	541	12.08191	chr4:410{	RNU6-907P		smallRNA	chr4:89130852-8913
ENSG0000(	541	12.08191	chr2:309	ENSG00000240687		lncRNA	chr2:9638707-97129
ENSG0000(	541	12.08191	chr4:410{	RNU7-149P		smallRNA	chr4:98966815-9896
ENSG0000(	541	12.08191	chr4:410{	ENSG00000249764		Pseudoger	chr4:98713804-9871
ENSG0000(	541	12.08191	chr4:410{	ENSG00000249755		Pseudoger	chr4:88527160-8852
ENSG0000(	541	12.08191	chr4:410{	TSPAN5-DT		lncRNA	chr4:98658894-9866
ENSG0000(	541	12.08191	chr4:410{	ENSG00000249171		lncRNA	chr4:83668510-8373
ENSG0000(	541	12.08191	chr2:309	RNU4-73P		smallRNA	chr2:9740643-97407
ENSG0000(	541	12.08191	chr4:410{	MTCYBP44		Pseudoger	chr4:81733385-8173
ENSG0000(	541	12.08191	chr4:410{	NCOA4P2		Pseudoger	chr4:88508591-8851
ENSG0000(	541	12.08191	chr4:410{	PRDM8	AC	protein_c	chr4:80183879-8020
ENSG0000(	541	12.08191	chr4:410{	ADH1A		protein_c	chr4:99276369-9929
ENSG0000(	541	12.08191	chr4:410{	BMP3		protein_c	chr4:81030708-8105
ENSG0000(	541	12.08191	chr4:410{	HNRNPDL	NCGv7	protein_c	chr4:82422565-8243
ENSG0000(	541	12.08191	chr4:410{	DUTP8		Pseudoger	chr4:98048555-9804
ENSG0000(	541	12.08191	chr4:410{	ENSG00000249951		lncRNA	chr4:94675245-9470
ENSG0000(	541	12.08191	chr4:410{	RNU6-774P		smallRNA	chr4:84233657-8423
ENSG0000(	541	12.08191	chr4:410{	TMSB4XP8		Pseudoger	chr4:90838903-9083
ENSG0000(	541	12.08191	chr4:410{	ENSG00000270720		lncRNA	chr4:89119284-8911
ENSG0000(	541	12.08191	chr4:410{	KRT19P6		Pseudoger	chr4:91885046-9188
ENSG0000(	541	12.08191	chr4:410{	MAPK10		protein_c	chr4:85990007-8659
ENSG0000(	541	12.08191	chr4:410{	RPL30P6		Pseudoger	chr4:95644952-9564
ENSG0000(	541	12.08191	chr4:410{	ENSG00000214980		Pseudoger	chr4:84244003-8424
ENSG0000(	541	12.08191	chr4:410{	RPL7AP26		Pseudoger	chr4:82490823-8249
ENSG0000(	541	12.08191	chr4:410{	METAP1		protein_c	chr4:98995659-9906
ENSG0000(	541	12.08191	chr4:410{	RN7SL552P		smallRNA	chr4:84687728-8468
ENSG0000(	541	12.08191	chr4:410{	RN7SKP248		smallRNA	chr4:90370123-9037
ENSG0000(	541	12.08191	chr4:410{	TMEM150C		protein_c	chr4:82483170-8256
ENSG0000(	541	12.08191	chr4:410{	HELQ		protein_c	chr4:83407343-8345
ENSG0000(	541	12.08191	chr4:410{	ABCG2	NCGv7	protein_c	chr4:88090150-8823
ENSG0000(	541	12.08191	chr4:410{	PKD2	NCGv7	protein_c	chr4:88007635-8807
ENSG0000(	541	12.08191	chr4:410{	RPL35AP11		Pseudoger	chr4:94369833-9437
ENSG0000(	541	12.08191	chr4:410{	SNCA-AS1		lncRNA	chr4:89836408-8984
ENSG0000(	541	12.08191	chr4:410{	HSD17B11		protein_c	chr4:87336515-8739
ENSG0000(	541	12.08191	chr4:410{	ENSG00000279013		TEC	chr4:91603275-9160
ENSG0000(	541	12.08191	chr4:410{	MRPS18C		protein_c	chr4:83455932-8346
ENSG0000(	541	12.08191	chr4:410{	ENSG00000286035		lncRNA	chr4:83075957-8308
ENSG0000(	541	12.08191	chr4:410{	ABRAXAS1		protein_c	chr4:83459517-8352
ENSG0000(	541	12.08191	chr4:410{	ENSG00000250300		Pseudoger	chr4:99469598-9949
ENSG0000(	541	12.08191	chr4:410{	RNU6-1298P		smallRNA	chr4:88226729-8822
ENSG0000(	541	12.08191	chr4:410{	RPSAP39		Pseudoger	chr4:80161129-8016
ENSG0000(	541	12.08191	chr4:410{	CRYZP2		Pseudoger	chr4:97916353-9791
ENSG0000(	541	12.08191	chr4:410{	RACK1P3		Pseudoger	chr4:92821986-9282
ENSG0000(	541	12.08191	chr4:410{	SPP1		protein_c	chr4:87975667-8798
ENSG0000(	541	12.08191	chr4:410{	ENSG00000248401		Pseudoger	chr4:83247179-8324
ENSG0000(	541	12.08191	chr4:410{	ENSG00000232327		Pseudoger	chr4:80386178-8038
ENSG0000(	541	12.08191	chr4:410{	IGBP1P4		Pseudoger	chr4:82401578-8240
ENSG0000(	541	12.08191	chr4:410{	KLHL8	NCGv7	protein_c	chr4:87160103-8724
ENSG0000(	541	12.08191	chr4:410{	SNCA		protein_c	chr4:89700345-8983
ENSG0000(	541	12.08191	chr4:410{	PYURF		protein_c	chr4:88520998-8852
ENSG0000(	541	12.08191	chr4:410{	Y_RNA		smallRNA	chr4:82944738-8294

ENSG0000(	541	12.08191	chr4:410{	LINC00989		lncRNA	chr4:79491802-7962
ENSG0000(	541	12.08191	chr4:410{	SLC25A14P1		Pseudoger	chr4:83477524-8347
ENSG0000(	541	12.08191	chr4:410{	HPGDS		protein_c	chr4:94298535-9434
ENSG0000(	541	12.08191	chr4:410{	ANTXR2		protein_c	chr4:79901146-8012
ENSG0000(	541	12.08191	chr4:410{	PDLIM5	NCGv7	protein_c	chr4:94451857-9466
ENSG0000(	541	12.08191	chr4:410{	ADH4		protein_c	chr4:99123657-9915
ENSG0000(	541	12.08191	chr4:410{	PDHA2		protein_c	chr4:95840093-9584
ENSG0000(	541	12.08191	chr4:410{	STPG2		protein_c	chr4:97184093-9814
ENSG0000(	541	12.08191	chr4:410{	LINC02469		lncRNA	chr4:79663761-7969
ENSG0000(	541	12.08191	chr4:410{	PAQR3		protein_c	chr4:78887127-7893
ENSG0000(	541	12.08191	chr4:410{	RN7SKP244		smallRNA	chr4:88583666-8858
ENSG0000(	541	12.08191	chr4:410{	COX7A2P2		Pseudoger	chr4:96902801-9690
ENSG0000(	541	12.08191	chr4:410{	PTPN13	NCGv7	protein_c	chr4:86594315-8681
ENSG0000(	541	12.08191	chr4:410{	RN7SKP96		smallRNA	chr4:86336318-8633
ENSG0000(	541	12.08191	chr4:410{	WDFY3	NCGv7	protein_c	chr4:84668765-8496
ENSG0000(	541	12.08191	chr4:410{	STPG2-AS1		lncRNA	chr4:97366681-9749
ENSG0000(	541	12.08191	chr4:410{	ENSG00000289532		lncRNA	chr4:98259390-9826
ENSG0000(	541	12.08191	chr4:410{	ENSG00000289480		lncRNA	chr4:82348252-8234
ENSG0000(	541	12.08191	chr4:410{	C4orf36		protein_c	chr4:86876205-8689
ENSG0000(	541	12.08191	chr4:410{	BMPR1B-DT		lncRNA	chr4:94743668-9475
ENSG0000(	541	12.08191	chr4:410{	ENSG00000249262		Pseudoger	chr4:87410644-8741
ENSG0000(	541	12.08191	chr4:410{	PPM1K		protein_c	chr4:88257620-8828
ENSG0000(	541	12.08191	chr4:410{	Y_RNA		smallRNA	chr4:88330176-8833
ENSG0000(	541	12.08191	chr4:410{	OR7E94P		Pseudoger	chr4:79587757-7958
ENSG0000(	541	12.08191	chr4:410{	ENSG00000272777		lncRNA	chr4:99067256-9906
ENSG0000(	541	12.08191	chr4:410{	RNU6-33P		smallRNA	chr4:88684848-8868
ENSG0000(	541	12.08191	chr4:410{	ENSG00000284968		lncRNA	chr4:86924630-8693
ENSG0000(	541	12.08191	chr4:410{	MIR4452		smallRNA	chr4:86542482-8654
ENSG0000(	541	12.08191	chr4:410{	FAM13A-AS1		lncRNA	chr4:88709298-8873
ENSG0000(	541	12.08191	chr4:410{	RNU6ATAC31P		smallRNA	chr4:88206427-8820
ENSG0000(	541	12.08191	chr4:410{	RNU6-615P		smallRNA	chr4:83003412-8300
ENSG0000(	541	12.08191	chr4:410{	CDS1		protein_c	chr4:84583127-8465
ENSG0000(	541	12.08191	chr2:309	ENSG00000239300		lncRNA	chr2:9501466-95124
ENSG0000(	541	12.08191	chr4:410{	MIR4451		smallRNA	chr4:85722468-8572
ENSG0000(	541	12.08191	chr4:410{	NKX6-1		protein_c	chr4:84491985-8449
ENSG0000(	541	12.08191	chr4:410{	ENSG00000272856		lncRNA	chr4:87460807-8746
ENSG0000(	541	12.08191	chr4:410{	RNA5SP164		Pseudoger	chr4:93820171-9382
ENSG0000(	541	12.08191	chr4:410{	ENSG00000289186		lncRNA	chr4:82566385-8257
ENSG0000(	541	12.08191	chr4:410{	SNORA31		smallRNA	chr4:81928313-8192
ENSG0000(	541	12.08191	chr4:410{	ENSG00000250202		Pseudoger	chr4:86876338-8687
ENSG0000(	535	11.94791	chr6:103	H2AC17	NCGv7	protein_c	chr6:27892699-2789
ENSG0000(	534	11.92558	chr11:46	OR2AL1P		Pseudoger	chr11:105194440-10
ENSG0000(	532	11.88091	chr2:309	EIF1P7		Pseudoger	chr2:9271292-92716
ENSG0000(	532	11.88091	chr2:309	SNRPEP5		Pseudoger	chr2:8603082-86033
ENSG0000(	532	11.88091	chr2:309	ENSG00000242136		lncRNA	chr2:9103440-91051
ENSG0000(	532	11.88091	chr2:309	ENSG00000287119		lncRNA	chr2:7671330-76757
ENSG0000(	532	11.88091	chr2:309	ENSG00000289191		lncRNA	chr2:8281464-82825
ENSG0000(	532	11.88091	chr2:309	LINC01814		lncRNA	chr2:8461703-85929
ENSG0000(	532	11.88091	chr2:309	CMPK2		protein_c	chr2:6840570-68666
ENSG0000(	532	11.88091	chr2:309	ENSG00000213774		Pseudoger	chr2:7324748-73250
ENSG0000(	532	11.88091	chr2:309	MIR7515HG		lncRNA	chr2:6615389-66511

ENSG0000(	532	11.88091	chr2:309<	RSAD2		protein_c	chr2:6865557-68982
ENSG0000(	532	11.88091	chr2:309<	LINC01871		lncRNA	chr2:7725682-77322
ENSG0000(	532	11.88091	chr2:309<	KIDINS220		protein_c	chr2:8721081-88376
ENSG0000(	532	11.88091	chr2:309<	NRIR		lncRNA	chr2:6819463-68404
ENSG0000(	532	11.88091	chr2:309<	ENSG00000226506		lncRNA	chr2:7886767-78997
ENSG0000(	532	11.88091	chr2:309<	ENSG00000286851		lncRNA	chr2:6649706-66508
ENSG0000(	532	11.88091	chr2:309<	ENSG00000286857		lncRNA	chr2:7937282-79534
ENSG0000(	532	11.88091	chr2:309<	ENSG00000244310		lncRNA	chr2:9110457-91169
ENSG0000(	532	11.88091	chr2:309<	ENSG00000261104		lncRNA	chr2:9106593-91098
ENSG0000(	532	11.88091	chr2:309<	LINC00298		lncRNA	chr2:7922425-82781
ENSG0000(	532	11.88091	chr2:309<	ENSG00000232979		Pseudoger	chr2:7735645-77357
ENSG0000(	532	11.88091	chr2:309<	ENSG00000271947		lncRNA	chr2:6905724-69063
ENSG0000(	532	11.88091	chr2:309<	LINC02973		lncRNA	chr2:7260871-72615
ENSG0000(	532	11.88091	chr2:309<	LINC00487		lncRNA	chr2:6728177-67703
ENSG0000(	532	11.88091	chr2:309<	ENSG00000290023		lncRNA	chr2:9004095-90046
ENSG0000(	532	11.88091	chr2:309<	ENSG00000230515		lncRNA	chr2:7671604-76722
ENSG0000(	532	11.88091	chr2:309<	ENSG00000290050		lncRNA	chr2:7748569-77492
ENSG0000(	532	11.88091	chr2:309<	GRASLND		lncRNA	chr2:6911754-69187
ENSG0000(	532	11.88091	chr2:309<	ASAP2	NCGv7	protein_c	chr2:9206765-94056
ENSG0000(	532	11.88091	chr2:309<	RNF144A		protein_c	chr2:6917412-70682
ENSG0000(	532	11.88091	chr2:309<	RNU6ATAC37P		smallRNA	chr2:7576841-75769
ENSG0000(	532	11.88091	chr2:309<	ENSG00000290092		lncRNA	chr2:7876670-78917
ENSG0000(	532	11.88091	chr2:309<	ENSG00000240980		lncRNA	chr2:9143166-91461
ENSG0000(	532	11.88091	chr2:309<	snoU13		smallRNA	chr2:8978895-89789
ENSG0000(	532	11.88091	chr2:309<	ENSG00000223884		lncRNA	chr2:7045329-70779
ENSG0000(	532	11.88091	chr2:309<	HMGB1P25		Pseudoger	chr2:9018293-90188
ENSG0000(	532	11.88091	chr2:309<	ENSG00000229740		lncRNA	chr2:8139335-81449
ENSG0000(	532	11.88091	chr2:309<	ENSG00000229727		lncRNA	chr2:7383227-74505
ENSG0000(	532	11.88091	chr2:309<	LINC00299		lncRNA	chr2:7988683-84882
ENSG0000(	532	11.88091	chr2:309<	RPL30P3		Pseudoger	chr2:9081395-90817
ENSG0000(	532	11.88091	chr2:309<	ID2	AC	protein_c	chr2:8678845-86844
ENSG0000(	532	11.88091	chr2:309<	ENSG00000229405		Pseudoger	chr2:7736438-77370
ENSG0000(	532	11.88091	chr2:309<	MBOAT2	NCGv7	protein_c	chr2:8852690-90037
ENSG0000(	532	11.88091	chr2:309<	ITGB1BP1		protein_c	chr2:9403475-94235
ENSG0000(	532	11.88091	chr2:309<	CPSF3		protein_c	chr2:9423651-94731
ENSG0000(	532	11.88091	chr2:309<	RN7SKP112		smallRNA	chr2:7141227-71415
ENSG0000(	532	11.88091	chr2:309<	ENSG00000285591		lncRNA	chr2:8488420-85050
ENSG0000(	532	11.88091	chr2:309<	ID2-AS1		lncRNA	chr2:8666636-86818
ENSG0000(	532	11.88091	chr7:158ε	RN7SL81P		smallRNA	chr7:128761337-128
ENSG0000(	532	11.88091	chr2:309<	ENSG00000231083		lncRNA	chr2:8600892-86229
ENSG0000(	529	11.81392	chr10:81(	RPL23AP61		Pseudoger	chr10:46063249-460
ENSG0000(	528	11.79158	chr8:412(	RPS12P15		Pseudoger	chr8:102503753-102
ENSG0000(	523	11.67992	chr2:309<	ENSG00000231597		lncRNA	chr2:216854389-216
ENSG0000(	523	11.67992	chr2:309<	TNP1		protein_c	chr2:216859458-216
ENSG0000(	523	11.67992	chr2:309<	LINC01921		lncRNA	chr2:216859948-216
ENSG0000(	523	11.67992	chr2:309<	ENSG00000237479		lncRNA	chr2:216866332-216
ENSG0000(	523	11.67992	chr5:164(	ENSG00000249153		lncRNA	chr5:85848502-8584
ENSG0000(	523	11.67992	chr2:309<	ENSG00000236295		lncRNA	chr2:216870441-216
ENSG0000(	521	11.63526	chr7:158ε	ENSG00000242162		Pseudoger	chr7:129066021-129
ENSG0000(	521	11.63526	chr7:158ε	ENSG00000280828		Pseudoger	chr7:128533652-128
ENSG0000(	521	11.63526	chr7:158ε	snoU13		smallRNA	chr7:129061531-129

ENSG0000(	521	11.63526	chr7:1588	FLNC-AS1		lncRNA	chr7:128850162-128
ENSG0000(	521	11.63526	chr7:1588	ENSG00000273270		lncRNA	chr7:128524016-128
ENSG0000(	521	11.63526	chr7:1588	ENSG00000271553		lncRNA	chr7:128667043-128
ENSG0000(	521	11.63526	chr7:1588	AC018635.1		smallRNA	chr7:128290004-128
ENSG0000(	521	11.63526	chr7:1588	ENSG00000286380		lncRNA	chr7:129763060-129
ENSG0000(	521	11.63526	chr7:1588	ENSG00000242078		lncRNA	chr7:129783370-129
ENSG0000(	521	11.63526	chr7:1588	ENSG00000241493		Pseudoger	chr7:128669087-128
ENSG0000(	521	11.63526	chr7:1588	ENSG00000271344		lncRNA	chr7:128690451-128
ENSG0000(	521	11.63526	chr7:1588	ENSG00000243302		Pseudoger	chr7:128651185-128
ENSG0000(	521	11.63526	chr7:1588	HILPDA		protein_c	chr7:128455849-128
ENSG0000(	521	11.63526	chr7:1588	Y_RNA		smallRNA	chr7:129164849-129
ENSG0000(	521	11.63526	chr7:1588	RNU1-72P		smallRNA	chr7:129484504-129
ENSG0000(	521	11.63526	chr7:1588	RNA5SP245		Pseudoger	chr7:130027277-130
ENSG0000(	521	11.63526	chr7:1588	Y_RNA		smallRNA	chr7:129961989-129
ENSG0000(	521	11.63526	chr7:1588	ENSG00000286722		lncRNA	chr7:129366827-129
ENSG0000(	521	11.63526	chr7:1588	RNU6-177P		smallRNA	chr7:128627172-128
ENSG0000(	521	11.63526	chr7:1588	snoU13		smallRNA	chr7:130344415-130
ENSG0000(	521	11.63526	chr7:1588	RN7SL306P		smallRNA	chr7:128970734-128
ENSG0000(	521	11.63526	chr7:1588	ENSG00000273329		lncRNA	chr7:129604548-129
ENSG0000(	521	11.63526	chr7:1588	Y_RNA		smallRNA	chr7:129954693-129
ENSG0000(	521	11.63526	chr7:1588	SMKR1		protein_c	chr7:129502531-129
ENSG0000(	521	11.63526	chr7:1588	TMEM209		protein_c	chr7:130164713-130
ENSG0000(	521	11.63526	chr7:1588	CICP14		Pseudoger	chr7:128655962-128
ENSG0000(	521	11.63526	chr7:1588	KCP	DriverDBv	protein_c	chr7:128862042-128
ENSG0000(	521	11.63526	chr7:1588	MIR335		smallRNA	chr7:130496111-130
ENSG0000(	521	11.63526	chr7:1588	LEP		protein_c	chr7:128241278-128
ENSG0000(	521	11.63526	chr7:1588	GARIN1B		protein_c	chr7:128709061-128
ENSG0000(	521	11.63526	chr7:1588	ENSG00000270992		Pseudoger	chr7:130255902-130
ENSG0000(	521	11.63526	chr7:1588	AC025594.1		smallRNA	chr7:128906772-128
ENSG0000(	521	11.63526	chr7:1588	ENSG00000242588		lncRNA	chr7:128531707-128
ENSG0000(	521	11.63526	chr7:1588	ATP6V1FNB		protein_c	chr7:128866330-128
ENSG0000(	521	11.63526	chr7:1588	SNRPGP3		Pseudoger	chr7:129477875-129
ENSG0000(	521	11.63526	chr7:1588	CYCSP20		Pseudoger	chr7:129117513-129
ENSG0000(	521	11.63526	chr7:1588	ENSG00000243230		lncRNA	chr7:129209775-129
ENSG0000(	521	11.63526	chr7:1588	MIR96		smallRNA	chr7:129774692-129
ENSG0000(	521	11.63526	chr7:1588	RNU7-27P		smallRNA	chr7:128344081-128
ENSG0000(	521	11.63526	chr7:1588	ENSG00000270823		lncRNA	chr7:130495794-130
ENSG0000(	521	11.63526	chr7:1588	RNU7-54P		smallRNA	chr7:128443449-128
ENSG0000(	521	11.63526	chr7:1588	TSPAN33		protein_c	chr7:129144884-129
ENSG0000(	521	11.63526	chr7:1588	SND1	NCGv7	protein_c	chr7:127652194-128
ENSG0000(	521	11.63526	chr7:1588	MIR182		smallRNA	chr7:129770383-129
ENSG0000(	521	11.63526	chr7:1588	HILPDA-AS1		lncRNA	chr7:128455937-128
ENSG0000(	521	11.63526	chr7:1588	CPA5		protein_c	chr7:130344816-130
ENSG0000(	521	11.63526	chr7:1588	SSMEM1		protein_c	chr7:130206344-130
ENSG0000(	521	11.63526	chr7:1588	PRRT4		protein_c	chr7:128350325-128
ENSG0000(	521	11.63526	chr7:1588	RNA5SP242		Pseudoger	chr7:128697439-128
ENSG0000(	521	11.63526	chr7:1588	CPA2		protein_c	chr7:130266863-130
ENSG0000(	521	11.63526	chr7:1588	METTL2B		protein_c	chr7:128476729-128
ENSG0000(	521	11.63526	chr7:1588	MIR129-1		smallRNA	chr7:128207871-128
ENSG0000(	521	11.63526	chr7:1588	ENSG00000224163		Pseudoger	chr7:128912732-128
ENSG0000(	521	11.63526	chr7:1588	MIR183		smallRNA	chr7:129774905-129

ENSG0000(	521	11.63526	chr7:158{	AHCYL2	NCGv7	protein_c	chr7:129225030-129
ENSG0000(	521	11.63526	chr7:158{	ENSG00000213280		Pseudoger	chr7:128570241-128
ENSG0000(	521	11.63526	chr7:158{	RNA5SP244		Pseudoger	chr7:129756266-129
ENSG0000(	521	11.63526	chr7:158{	SND1-IT1		lncRNA	chr7:127997597-128
ENSG0000(	521	11.63526	chr7:158{	IMP3P2		Pseudoger	chr7:128693450-128
ENSG0000(	521	11.63526	chr7:158{	ENSG00000270953		lncRNA	chr7:130507660-130
ENSG0000(	521	11.63526	chr7:158{	ENSG00000240579		Pseudoger	chr7:127857852-127
ENSG0000(	521	11.63526	chr7:158{	ENSG00000272915		lncRNA	chr7:128264526-128
ENSG0000(	521	11.63526	chr7:158{	ENSG00000273184		lncRNA	chr7:128455840-128
ENSG0000(	521	11.63526	chr7:158{	ENSG00000241573		Pseudoger	chr7:129096027-129
ENSG0000(	521	11.63526	chr7:158{	GARIN1A		protein_c	chr7:128671693-128
ENSG0000(	521	11.63526	chr7:158{	EFCAB3P1		Pseudoger	chr7:128466563-128
ENSG0000(	521	11.63526	chr7:158{	ENSG00000240571		lncRNA	chr7:130173718-130
ENSG0000(	521	11.63526	chr7:158{	MESTIT1		lncRNA	chr7:130486042-130
ENSG0000(	521	11.63526	chr7:158{	MIR593		smallRNA	chr7:128081861-128
ENSG0000(	521	11.63526	chr7:158{	ENSG00000241102		Pseudoger	chr7:129095301-129
ENSG0000(	521	11.63526	chr7:158{	ENSG00000242261		Pseudoger	chr7:128306649-128
ENSG0000(	521	11.63526	chr7:158{	RBM28		protein_c	chr7:128297685-128
ENSG0000(	521	11.63526	chr7:158{	CDC26P1		Pseudoger	chr7:129410113-129
ENSG0000(	521	11.63526	chr7:158{	KLHDC10		protein_c	chr7:130070534-130
ENSG0000(	521	11.63526	chr7:158{	RNU7-16P		smallRNA	chr7:129405635-129
ENSG0000(	521	11.63526	chr7:158{	ATP6V1F		protein_c	chr7:128862856-128
ENSG0000(	521	11.63526	chr7:158{	TPI1P2		Pseudoger	chr7:129055223-129
ENSG0000(	521	11.63526	chr7:158{	ENSG00000290319		lncRNA	chr7:129780410-129
ENSG0000(	521	11.63526	chr7:158{	ENSG00000230715		Pseudoger	chr7:128652841-128
ENSG0000(	521	11.63526	chr7:158{	ENSG00000289434		lncRNA	chr7:128221388-128
ENSG0000(	521	11.63526	chr7:158{	SMO	NCGv7;AC	protein_c	chr7:129188633-129
ENSG0000(	521	11.63526	chr7:158{	CCDC136		protein_c	chr7:128790757-128
ENSG0000(	521	11.63526	chr7:158{	ENSG00000230626		Pseudoger	chr7:129126518-129
ENSG0000(	521	11.63526	chr7:158{	UBE2H		protein_c	chr7:129830732-129
ENSG0000(	521	11.63526	chr7:158{	UBE2H-DT		lncRNA	chr7:129953234-130
ENSG0000(	521	11.63526	chr7:158{	CALU	DriverDBv	protein_c	chr7:128739292-128
ENSG0000(	521	11.63526	chr7:158{	ENSG00000275106		TEC	chr7:128952527-128
ENSG0000(	521	11.63526	chr7:158{	CPA4		protein_c	chr7:130293134-130
ENSG0000(	521	11.63526	chr7:158{	LRRC4		protein_c	chr7:128027071-128
ENSG0000(	521	11.63526	chr7:158{	AC073320.1		smallRNA	chr7:129985647-129
ENSG0000(	521	11.63526	chr7:158{	FLNC		protein_c	chr7:128830406-128
ENSG0000(	521	11.63526	chr7:158{	STRIP2		protein_c	chr7:129434432-129
ENSG0000(	521	11.63526	chr7:158{	OPN1SW		protein_c	chr7:128772485-128
ENSG0000(	521	11.63526	chr7:158{	IRF5	NCGv7	protein_c	chr7:128937457-128
ENSG0000(	521	11.63526	chr7:158{	LINC03008		lncRNA	chr7:130141707-130
ENSG0000(	521	11.63526	chr7:158{	ODCP		Pseudoger	chr7:129028889-129
ENSG0000(	521	11.63526	chr7:158{	RNA5SP243		Pseudoger	chr7:128697710-128
ENSG0000(	521	11.63526	chr7:158{	snoU13		smallRNA	chr7:130319279-130
ENSG0000(	521	11.63526	chr7:158{	MEST	DriverDBv	protein_c	chr7:130486171-130
ENSG0000(	521	11.63526	chr7:158{	ZC3HC1	NCGv7	protein_c	chr7:130018287-130
ENSG0000(	521	11.63526	chr7:158{	CPA1		protein_c	chr7:130380339-130
ENSG0000(	521	11.63526	chr7:158{	ENSG00000243679		Pseudoger	chr7:128653969-128
ENSG0000(	521	11.63526	chr7:158{	IMPDH1		protein_c	chr7:128392277-128
ENSG0000(	521	11.63526	chr7:158{	ENSG00000288881		lncRNA	chr7:129642758-129
ENSG0000(	521	11.63526	chr7:158{	ENSG00000229413		Pseudoger	chr7:128653690-128

ENSG0000(	521	11.63526	chr7:158{	NRF1		protein_c	chr7:129611720-129
ENSG0000(	521	11.63526	chr7:158{	TNPO3		protein_c	chr7:128954180-129
ENSG0000(	521	11.63526	chr7:158{	ENSG00000228700		Pseudoger	chr7:128433422-128
ENSG0000(	521	11.63526	chr7:158{	CEP41		protein_c	chr7:130393771-130
ENSG0000(	521	11.63526	chr7:158{	ENSG00000259920		lncRNA	chr7:130481491-130
ENSG0000(	520	11.61292	chr6:103{	TDRG1		lncRNA	chr6:40334775-4038
ENSG0000(	520	11.61292	chr6:103{	AL031905.1		smallRNA	chr6:38359148-3835
ENSG0000(	520	11.61292	chr6:103{	SNORA8		smallRNA	chr6:38822307-3882
ENSG0000(	520	11.61292	chr6:103{	ENSG00000279942		TEC	chr6:37567716-3757
ENSG0000(	520	11.61292	chr6:103{	LINC02520		lncRNA	chr6:37507348-3753
ENSG0000(	520	11.61292	chr6:103{	ENSG00000218521		Pseudoger	chr6:38002832-3800
ENSG0000(	520	11.61292	chr6:103{	CMTR1	NCGv7	protein_c	chr6:37433219-3748
ENSG0000(	520	11.61292	chr6:103{	LINC00951		lncRNA	chr6:40344346-4038
ENSG0000(	520	11.61292	chr6:103{	DNAH8	NCGv7	protein_c	chr6:38715311-3903
ENSG0000(	520	11.61292	chr6:103{	CCDC167		protein_c	chr6:37482938-3749
ENSG0000(	520	11.61292	chr6:103{	BTBD9		protein_c	chr6:38168451-3864
ENSG0000(	520	11.61292	chr6:103{	MIR4462		smallRNA	chr6:37555365-3755
ENSG0000(	520	11.61292	chr6:103{	GLO1	AC	protein_c	chr6:38675925-3870
ENSG0000(	520	11.61292	chr6:103{	MOCS1		protein_c	chr6:39899578-3993
ENSG0000(	520	11.61292	chr6:103{	KCNK17		protein_c	chr6:39299001-3931
ENSG0000(	520	11.61292	chr6:103{	DNAH8-DT		lncRNA	chr6:38714051-3871
ENSG0000(	520	11.61292	chr6:103{	ENSG00000227920		lncRNA	chr6:37545145-3755
ENSG0000(	520	11.61292	chr6:103{	ZFAND3		protein_c	chr6:37819727-3815
ENSG0000(	520	11.61292	chr6:103{	ENSG00000281969		TEC	chr6:39818751-3982
ENSG0000(	520	11.61292	chr6:103{	ANKRD18EP		Pseudoger	chr6:39110321-3911
ENSG0000(	520	11.61292	chr6:103{	TFGP1		Pseudoger	chr6:38587323-3858
ENSG0000(	520	11.61292	chr6:103{	RNU1-87P		smallRNA	chr6:37915573-3791
ENSG0000(	520	11.61292	chr6:103{	E2F4P1		Pseudoger	chr6:39553811-3955
ENSG0000(	520	11.61292	chr6:103{	DAAM2		protein_c	chr6:39792298-3990
ENSG0000(	520	11.61292	chr6:103{	SNORD45		smallRNA	chr6:38207274-3820
ENSG0000(	520	11.61292	chr6:103{	ZFAND3-DT		lncRNA	chr6:37815777-3781
ENSG0000(	520	11.61292	chr6:103{	BTBD9-AS1		lncRNA	chr6:38481692-3848
ENSG0000(	520	11.61292	chr6:103{	RNU1-54P		smallRNA	chr6:39620345-3962
ENSG0000(	520	11.61292	chr6:103{	RN7SL465P		smallRNA	chr6:38744086-3874
ENSG0000(	520	11.61292	chr6:103{	ENSG00000227131		lncRNA	chr6:40271566-4027
ENSG0000(	520	11.61292	chr6:103{	ENSG00000229559		Pseudoger	chr6:37543553-3754
ENSG0000(	520	11.61292	chr6:103{	DAAM2-AS1		lncRNA	chr6:39881804-3990
ENSG0000(	520	11.61292	chr6:103{	KCNK16		protein_c	chr6:39314698-3932
ENSG0000(	520	11.61292	chr6:103{	KCNK5		protein_c	chr6:39188971-3922
ENSG0000(	520	11.61292	chr6:103{	KIF6		protein_c	chr6:39329990-3972
ENSG0000(	520	11.61292	chr6:103{	ENSG00000220076		Pseudoger	chr6:38928031-3892
ENSG0000(	520	11.61292	chr6:103{	Y_RNA		smallRNA	chr6:38565950-3856
ENSG0000(	520	11.61292	chr6:103{	ENSG00000220556		Pseudoger	chr6:39039603-3903
ENSG0000(	520	11.61292	chr6:103{	ENSG00000236075		lncRNA	chr6:40501631-4050
ENSG0000(	520	11.61292	chr6:103{	TUBBP9		Pseudoger	chr6:39934595-4000
ENSG0000(	520	11.61292	chr6:103{	RNU6-250P		smallRNA	chr6:40407853-4040
ENSG0000(	520	11.61292	chr6:103{	DNAH8-AS1		lncRNA	chr6:38923029-3895
ENSG0000(	520	11.61292	chr6:103{	GLP1R		protein_c	chr6:39048781-3909
ENSG0000(	520	11.61292	chr6:103{	ENSG00000180211		Pseudoger	chr6:39958414-3995
ENSG0000(	520	11.61292	chr6:103{	SAYSD1		protein_c	chr6:39104063-3911
ENSG0000(	520	11.61292	chr6:103{	RN7SL285P		smallRNA	chr6:37832922-3783

ENSG0000(	520	11.61292	chr6:1039	ENSG00000226454		lncRNA	chr6:40505507-4052
ENSG0000(	520	11.61292	chr6:1039	ENSG00000219273		Pseudoger	chr6:38762905-3876
ENSG0000(	520	11.61292	chr6:1039	MDGA1		protein_c	chr6:37630679-3769
ENSG0000(	519	11.59059	chr5:1646	ENSG00000254310		Pseudoger	chr5:78753596-7875
ENSG0000(	518	11.56826	chr12:171	AVIL	DriverDB\	protein_c	chr12:57797376-578
ENSG0000(	518	11.56826	chr12:171	GLS2	NCGv7	protein_c	chr12:56470944-564
ENSG0000(	518	11.56826	chr12:171	AGAP2	AC	protein_c	chr12:57723761-577
ENSG0000(	518	11.56826	chr12:171	ENSG00000257159		Pseudoger	chr12:57967058-579
ENSG0000(	518	11.56826	chr12:171	R3HDM2		protein_c	chr12:57253762-574
ENSG0000(	518	11.56826	chr12:171	CDK4	NCGv7;AC	protein_c	chr12:57747727-577
ENSG0000(	518	11.56826	chr12:171	RN7SKP65		smallRNA	chr12:57977965-579
ENSG0000(	518	11.56826	chr12:171	TSPAN31	DriverDB\	protein_c	chr12:57738013-577
ENSG0000(	518	11.56826	chr12:171	MIP		protein_c	chr12:56449502-564
ENSG0000(	518	11.56826	chr12:171	B4GALNT1		protein_c	chr12:57623409-576
ENSG0000(	518	11.56826	chr12:171	ENSG00000276272		lncRNA	chr12:56411944-564
ENSG0000(	518	11.56826	chr12:171	RPL21P103		Pseudoger	chr12:58244378-582
ENSG0000(	518	11.56826	chr12:171	LRP1-AS		lncRNA	chr12:57144620-571
ENSG0000(	518	11.56826	chr12:171	ARHGEF25	NCGv7	protein_c	chr12:57610180-576
ENSG0000(	518	11.56826	chr12:171	ENSG00000257342		lncRNA	chr12:57694132-577
ENSG0000(	518	11.56826	chr12:171	NDUFA4L2		protein_c	chr12:57234903-572
ENSG0000(	518	11.56826	chr12:171	AC023237.1		smallRNA	chr12:57121662-571
ENSG0000(	518	11.56826	chr12:171	PRIM1		protein_c	chr12:56731296-567
ENSG0000(	518	11.56826	chr12:171	OS9	NCGv7	protein_c	chr12:57693841-577
ENSG0000(	518	11.56826	chr12:171	SLC26A10		Pseudoger	chr12:57619527-576
ENSG0000(	518	11.56826	chr12:171	ENSG00000285133		protein_c	chr12:57517712-575
ENSG0000(	518	11.56826	chr12:171	ENSG00000287200		lncRNA	chr12:57457596-574
ENSG0000(	518	11.56826	chr12:171	ENSG00000273805		lncRNA	chr12:57894232-578
ENSG0000(	518	11.56826	chr12:171	DCTN2		protein_c	chr12:57529633-575
ENSG0000(	518	11.56826	chr12:171	ENSG00000287715		lncRNA	chr12:56987734-569
ENSG0000(	518	11.56826	chr12:171	EEF1AKMT3	DriverDB\	protein_c	chr12:57771492-577
ENSG0000(	518	11.56826	chr12:171	SNORA48		smallRNA	chr12:56861375-568
ENSG0000(	518	11.56826	chr12:171	RBMS2		protein_c	chr12:56521820-565
ENSG0000(	518	11.56826	chr12:171	BAZ2A	NCGv7	protein_c	chr12:56595596-566
ENSG0000(	518	11.56826	chr12:171	LRP1	NCGv7	protein_c	chr12:57128483-572
ENSG0000(	518	11.56826	chr12:171	ENSG00000279411		TEC	chr12:58103907-581
ENSG0000(	518	11.56826	chr12:171	INHBC		protein_c	chr12:57434784-574
ENSG0000(	518	11.56826	chr12:171	DDIT3	NCGv7;AC	protein_c	chr12:57516588-575
ENSG0000(	518	11.56826	chr12:171	ARHGAP9		protein_c	chr12:57472264-574
ENSG0000(	518	11.56826	chr12:171	RNU6-1083P		smallRNA	chr12:57806154-578
ENSG0000(	518	11.56826	chr12:171	CTDSP2	DriverDB\	protein_c	chr12:57819927-578
ENSG0000(	518	11.56826	chr12:171	TSFM	DriverDB\	protein_c	chr12:57782761-578
ENSG0000(	518	11.56826	chr12:171	MARCHF9		protein_c	chr12:57755103-577
ENSG0000(	518	11.56826	chr12:171	ENSG00000273536		Pseudoger	chr12:56840973-568
ENSG0000(	518	11.56826	chr12:171	INHBE		protein_c	chr12:57452323-574
ENSG0000(	518	11.56826	chr12:171	APOF		protein_c	chr12:56360568-563
ENSG0000(	518	11.56826	chr12:171	MIR26A2		smallRNA	chr12:57824609-578
ENSG0000(	518	11.56826	chr12:171	RN7SL312P		smallRNA	chr12:57513917-575
ENSG0000(	518	11.56826	chr12:171	RDH16		protein_c	chr12:56951431-569
ENSG0000(	518	11.56826	chr12:171	GPR182		protein_c	chr12:56994492-569
ENSG0000(	518	11.56826	chr12:171	ENSG00000245651		lncRNA	chr12:57869834-578
ENSG0000(	518	11.56826	chr12:171	ZBTB39		protein_c	chr12:56998836-570

ENSG0000(	518	11.56826	chr12:171	TAC3		protein_c	chr12:57010000-570
ENSG0000(	518	11.56826	chr12:171	RNU6-594P		smallRNA	chr12:57511064-575
ENSG0000(	518	11.56826	chr12:171	LINC02403		lncRNA	chr12:58087892-580
ENSG0000(	518	11.56826	chr12:171	ENSG00000274569		Pseudoger	chr12:56413647-564
ENSG0000(	518	11.56826	chr12:171	ENSG00000287908		protein_c	chr12:57615280-576
ENSG0000(	518	11.56826	chr12:171	SPRYD4		protein_c	chr12:56468578-564
ENSG0000(	518	11.56826	chr12:171	SNORD59A		smallRNA	chr12:56645027-566
ENSG0000(	518	11.56826	chr12:171	ENSG00000269903		lncRNA	chr12:57814494-578
ENSG0000(	518	11.56826	chr12:171	RN7SL809P		smallRNA	chr12:56670452-566
ENSG0000(	518	11.56826	chr12:171	MBD6	NCGv7	protein_c	chr12:57520710-575
ENSG0000(	518	11.56826	chr12:171	R3HDM2-DT		lncRNA	chr12:57431116-574
ENSG0000(	518	11.56826	chr12:171	PIP4K2C		protein_c	chr12:57591174-576
ENSG0000(	518	11.56826	chr12:171	ATP23	DriverDB\	protein_c	chr12:57906039-579
ENSG0000(	518	11.56826	chr12:171	ENSG00000270039		lncRNA	chr12:57803838-578
ENSG0000(	518	11.56826	chr12:171	ENSG00000224713		lncRNA	chr12:57612118-576
ENSG0000(	518	11.56826	chr12:171	STAT6	NCGv7	protein_c	chr12:57095408-571
ENSG0000(	518	11.56826	chr12:171	APONP		Pseudoger	chr12:56376818-563
ENSG0000(	518	11.56826	chr12:171	NAB2	NCGv7	protein_c	chr12:57089043-570
ENSG0000(	518	11.56826	chr12:171	NEMP1		protein_c	chr12:57055643-570
ENSG0000(	518	11.56826	chr12:171	MYO1A		protein_c	chr12:57028517-570
ENSG0000(	518	11.56826	chr12:171	MARS1		protein_c	chr12:57475445-575
ENSG0000(	518	11.56826	chr12:171	MIR616		smallRNA	chr12:57519163-575
ENSG0000(	518	11.56826	chr12:171	STAC3		protein_c	chr12:57243453-572
ENSG0000(	518	11.56826	chr12:171	NACA	AC	protein_c	chr12:56712305-567
ENSG0000(	518	11.56826	chr12:171	DTX3		protein_c	chr12:57604622-576
ENSG0000(	518	11.56826	chr12:171	snoU13		smallRNA	chr12:57668944-576
ENSG0000(	518	11.56826	chr12:171	ENSG00000278400		Pseudoger	chr12:56941174-569
ENSG0000(	518	11.56826	chr12:171	ENSG00000285625		protein_c	chr12:56714612-567
ENSG0000(	518	11.56826	chr12:171	ENSG00000285528		protein_c	chr12:56449700-564
ENSG0000(	518	11.56826	chr12:171	PTGES3		protein_c	chr12:56663341-566
ENSG0000(	518	11.56826	chr12:171	ENSG00000257668		Pseudoger	chr12:57983795-579
ENSG0000(	518	11.56826	chr12:171	TIMELESS		protein_c	chr12:56416363-564
ENSG0000(	518	11.56826	chr12:171	RNU6-343P		smallRNA	chr12:56588518-565
ENSG0000(	518	11.56826	chr12:171	SDR9C7		protein_c	chr12:56923133-569
ENSG0000(	518	11.56826	chr12:171	AGAP2-AS1		lncRNA	chr12:57726271-577
ENSG0000(	518	11.56826	chr12:171	Y_RNA		smallRNA	chr12:57227974-572
ENSG0000(	518	11.56826	chr12:171	ATP5F1B	NCGv7	protein_c	chr12:56638175-566
ENSG0000(	518	11.56826	chr12:171	HSPD1P4		Pseudoger	chr12:56511002-565
ENSG0000(	518	11.56826	chr12:171	ENSG00000257921		protein_c	chr12:57772660-577
ENSG0000(	518	11.56826	chr12:171	AC025165.1		smallRNA	chr12:57774249-577
ENSG0000(	518	11.56826	chr12:171	ENSG00000257953		lncRNA	chr12:57837092-578
ENSG0000(	518	11.56826	chr12:171	SHMT2		protein_c	chr12:57229573-572
ENSG0000(	518	11.56826	chr12:171	HSD17B6		protein_c	chr12:56752161-567
ENSG0000(	518	11.56826	chr12:171	RPL13AP23		Pseudoger	chr12:57674665-576
ENSG0000(	518	11.56826	chr12:171	METTL1	DriverDB\	protein_c	chr12:57768471-577
ENSG0000(	518	11.56826	chr12:171	KIF5A		protein_c	chr12:57546026-575
ENSG0000(	518	11.56826	chr12:171	ENSG00000258830		protein_c	chr12:57249609-572
ENSG0000(	518	11.56826	chr12:171	ENSG00000276727		lncRNA	chr12:57229498-572
ENSG0000(	518	11.56826	chr12:171	RNU6-879P		smallRNA	chr12:57415296-574
ENSG0000(	518	11.56826	chr12:171	ENSG00000278399		Pseudoger	chr12:56981211-569
ENSG0000(	518	11.56826	chr12:171	GIHCG		lncRNA	chr12:57930115-579

ENSG0000(	518	11.56826	chr12:171	ENSG00000258816		Pseudoger	chr12:56855694-568
ENSG0000(	518	11.56826	chr12:171	NXPH4		protein_c	chr12:57216794-572
ENSG0000(	518	11.56826	chr12:171	ENSG00000279134		TEC	chr12:58094914-580
ENSG0000(	518	11.56826	chr12:171	MIR1228		smallRNA	chr12:57194504-571
ENSG0000(	518	11.56826	chr12:171	ENSG00000259099		Pseudoger	chr12:56380361-563
ENSG0000(	518	11.56826	chr12:171	ENSG00000258679		Pseudoger	chr12:56822985-568
ENSG0000(	518	11.56826	chr12:171	GLI1	NCGv7;AC	protein_c	chr12:57459785-574
ENSG0000(	518	11.56826	chr12:171	CYP27B1	DriverDBv	protein_c	chr12:57762334-577
ENSG0000(	516	11.52359	chr6:1391	FNDC1-IT1		lncRNA	chr6:159240786-159
ENSG0000(	516	11.52359	chr6:1391	FNDC1-AS1		lncRNA	chr6:159165899-159
ENSG0000(	516	11.52359	chr6:1391	FNDC1		protein_c	chr6:159169400-159
ENSG0000(	515	11.50126	chr5:164(	ANKRD34B		protein_c	chr5:80556755-8057
ENSG0000(	515	11.50126	chr5:164(	ATG10		protein_c	chr5:81972023-8227
ENSG0000(	515	11.50126	chr5:164(	RPS2P25		Pseudoger	chr5:85762559-8576
ENSG0000(	515	11.50126	chr5:164(	RASGRF2	NCGv7	protein_c	chr5:80960363-8123
ENSG0000(	515	11.50126	chr5:164(	ENSG00000249857		lncRNA	chr5:82940458-8294
ENSG0000(	515	11.50126	chr5:164(	ENSG00000249100		lncRNA	chr5:82765404-8276
ENSG0000(	515	11.50126	chr5:164(	ENSG00000251374		Pseudoger	chr5:82265157-8226
ENSG0000(	515	11.50126	chr5:164(	RN7SKP295		smallRNA	chr5:83685866-8368
ENSG0000(	515	11.50126	chr5:164(	SERINC5		protein_c	chr5:80111651-8025
ENSG0000(	515	11.50126	chr5:164(	CKMT2		protein_c	chr5:81233320-8126
ENSG0000(	515	11.50126	chr5:164(	ENSG00000288741		lncRNA	chr5:80256200-8025
ENSG0000(	515	11.50126	chr5:164(	ZCCHC9		protein_c	chr5:81301587-8131
ENSG0000(	515	11.50126	chr5:164(	ENSG00000243385		Pseudoger	chr5:83201229-8320
ENSG0000(	515	11.50126	chr5:164(	ENSG00000289317		lncRNA	chr5:80407323-8040
ENSG0000(	515	11.50126	chr5:164(	SCARNA18		smallRNA	chr5:83064204-8306
ENSG0000(	515	11.50126	chr5:164(	PPIAP11		Pseudoger	chr5:82009602-8201
ENSG0000(	515	11.50126	chr5:164(	RASGRF2-AS1		lncRNA	chr5:80947694-8096
ENSG0000(	515	11.50126	chr5:164(	ENSG00000248667		lncRNA	chr5:85420028-8542
ENSG0000(	515	11.50126	chr5:164(	RPL5P17		Pseudoger	chr5:85464434-8546
ENSG0000(	515	11.50126	chr5:164(	RNU6-448P		smallRNA	chr5:84196883-8419
ENSG0000(	515	11.50126	chr5:164(	RBBP4P6		Pseudoger	chr5:85190629-8519
ENSG0000(	515	11.50126	chr5:164(	RNU4-11P		smallRNA	chr5:83803554-8380
ENSG0000(	515	11.50126	chr5:164(	EDIL3		protein_c	chr5:83940554-8438
ENSG0000(	515	11.50126	chr5:164(	AC010595.1		smallRNA	chr5:85634412-8563
ENSG0000(	515	11.50126	chr5:164(	ST13P12		Pseudoger	chr5:82968888-8297
ENSG0000(	515	11.50126	chr5:164(	RPL5P16		Pseudoger	chr5:82777797-8277
ENSG0000(	515	11.50126	chr5:164(	RPS23		protein_c	chr5:82273320-8227
ENSG0000(	515	11.50126	chr5:164(	RPS27AP9		Pseudoger	chr5:80498534-8049
ENSG0000(	515	11.50126	chr5:164(	MSH3	NCGv7	protein_c	chr5:80654652-8087
ENSG0000(	515	11.50126	chr5:164(	ENSG00000250874		lncRNA	chr5:85663232-8566
ENSG0000(	515	11.50126	chr5:164(	ACOT12		protein_c	chr5:81329996-8139
ENSG0000(	515	11.50126	chr5:164(	XRCC4		protein_c	chr5:83077498-8335
ENSG0000(	515	11.50126	chr5:164(	FAM151B		protein_c	chr5:80487969-8054
ENSG0000(	515	11.50126	chr5:164(	DHFR		protein_c	chr5:80626226-8065
ENSG0000(	515	11.50126	chr5:164(	AC114969.1		smallRNA	chr5:81978250-8197
ENSG0000(	515	11.50126	chr5:164(	DBIP2		Pseudoger	chr5:80603383-8060
ENSG0000(	515	11.50126	chr5:164(	ENSG00000249842		lncRNA	chr5:85446974-8544
ENSG0000(	515	11.50126	chr5:164(	VCAN-AS1		lncRNA	chr5:83531352-8358
ENSG0000(	515	11.50126	chr5:164(	ENSG00000287938		lncRNA	chr5:81817531-8184
ENSG0000(	515	11.50126	chr5:164(	RBMX2P5		Pseudoger	chr5:80331573-8033

ENSG0000(	515	11.50126	chr5:164(	ATG10-IT1		lncRNA	chr5:81991995-8199
ENSG0000(	515	11.50126	chr5:164(	EDIL3-DT		lncRNA	chr5:84382424-8449
ENSG0000(	515	11.50126	chr5:164(	ENSG00000244076		Pseudoger	chr5:80315671-8031
ENSG0000(	515	11.50126	chr5:164(	ENSG00000249023		lncRNA	chr5:85429702-8543
ENSG0000(	515	11.50126	chr5:164(	ENSG00000251066		Pseudoger	chr5:85549409-8554
ENSG0000(	515	11.50126	chr5:164(	LINC01338		lncRNA	chr5:82807475-8286
ENSG0000(	515	11.50126	chr5:164(	AC026700.1		smallRNA	chr5:85528044-8552
ENSG0000(	515	11.50126	chr5:164(	COQ10BP2		Pseudoger	chr5:83279921-8328
ENSG0000(	515	11.50126	chr5:164(	ATG10-AS1		lncRNA	chr5:82073055-8207
ENSG0000(	515	11.50126	chr5:164(	ENSG00000248170		Pseudoger	chr5:85237681-8523
ENSG0000(	515	11.50126	chr5:164(	ENSG00000248870		lncRNA	chr5:82586776-8258
ENSG0000(	515	11.50126	chr5:164(	ENSG00000250253		Pseudoger	chr5:85210060-8521
ENSG0000(	515	11.50126	chr5:164(	ENSG00000242858		Pseudoger	chr5:83746388-8374
ENSG0000(	515	11.50126	chr5:164(	PPIAP79		Pseudoger	chr5:84999812-8500
ENSG0000(	515	11.50126	chr5:164(	snoU13		smallRNA	chr5:80239487-8023
ENSG0000(	515	11.50126	chr5:164(	LINC01337		lncRNA	chr5:80608623-8062
ENSG0000(	515	11.50126	chr5:164(	ATP6AP1L		Pseudoger	chr5:82279462-8238
ENSG0000(	515	11.50126	chr5:164(	SEM1P1		Pseudoger	chr5:81892490-8189
ENSG0000(	515	11.50126	chr5:164(	ENSG00000248967		Pseudoger	chr5:80746339-8074
ENSG0000(	515	11.50126	chr5:164(	ENSG00000249349		lncRNA	chr5:85112342-8511
ENSG0000(	515	11.50126	chr5:164(	HNRNPA1P12		Pseudoger	chr5:80359080-8036
ENSG0000(	515	11.50126	chr5:164(	ENSG00000248569		Pseudoger	chr5:80351021-8035
ENSG0000(	515	11.50126	chr5:164(	ENSG00000184188		Pseudoger	chr5:80299678-8030
ENSG0000(	515	11.50126	chr5:164(	ENSG00000248105		Pseudoger	chr5:82824884-8282
ENSG0000(	515	11.50126	chr5:164(	KRT18P45		Pseudoger	chr5:80288449-8028
ENSG0000(	515	11.50126	chr5:164(	ENSG00000249664		lncRNA	chr5:83012285-8301
ENSG0000(	515	11.50126	chr5:164(	ENSG00000249655		lncRNA	chr5:80630313-8063
ENSG0000(	515	11.50126	chr5:164(	ENSG00000244630		Pseudoger	chr5:80855507-8085
ENSG0000(	515	11.50126	chr5:164(	FAM151B-DT		lncRNA	chr5:80411231-8048
ENSG0000(	515	11.50126	chr5:164(	ENSG00000248794		Pseudoger	chr5:81242330-8124
ENSG0000(	515	11.50126	chr5:164(	CKMT2-AS1		lncRNA	chr5:81201341-8130
ENSG0000(	515	11.50126	chr5:164(	TMEM167A		protein_c	chr5:83052846-8307
ENSG0000(	515	11.50126	chr5:164(	ENSG00000248112		lncRNA	chr5:82919376-8292
ENSG0000(	515	11.50126	chr5:164(	SPZ1		protein_c	chr5:80319625-8032
ENSG0000(	515	11.50126	chr5:164(	RN7SL378P		smallRNA	chr5:82078427-8207
ENSG0000(	515	11.50126	chr5:164(	ENSG00000249483		lncRNA	chr5:81851601-8185
ENSG0000(	515	11.50126	chr5:164(	HAPLN1	NCGv7	protein_c	chr5:83637805-8372
ENSG0000(	515	11.50126	chr5:164(	RNU6-211P		smallRNA	chr5:80365726-8036
ENSG0000(	515	11.50126	chr5:164(	ENSG00000251675		lncRNA	chr5:80128361-8014
ENSG0000(	515	11.50126	chr5:164(	FTH1P9		Pseudoger	chr5:83426676-8342
ENSG0000(	515	11.50126	chr5:164(	ENSG00000286721		lncRNA	chr5:81408517-8141
ENSG0000(	515	11.50126	chr5:164(	ENSG00000248393		lncRNA	chr5:82545862-8254
ENSG0000(	515	11.50126	chr5:164(	RNU6-620P		smallRNA	chr5:83703448-8370
ENSG0000(	515	11.50126	chr5:164(	VCAN	NCGv7	protein_c	chr5:83471618-8358
ENSG0000(	515	11.50126	chr5:164(	SSBP2	DriverDBv	protein_c	chr5:81412804-8175
ENSG0000(	515	11.50126	chr5:164(	ZP3P1		Pseudoger	chr5:83875166-8387
ENSG0000(	515	11.50126	chr5:164(	SNORA31		smallRNA	chr5:80306235-8030
ENSG0000(	515	11.50126	chr5:164(	ENSG00000249792		lncRNA	chr5:85212958-8521
ENSG0000(	515	11.50126	chr5:164(	AC109496.1		smallRNA	chr5:83973796-8397
ENSG0000(	515	11.50126	chr5:164(	ZFYVE16		protein_c	chr5:80408013-8048
ENSG0000(	515	11.50126	chr5:164(	RPL7P24		Pseudoger	chr5:80500332-8050

ENSG0000(	515	11.50126	chr5:164(	ENSG00000251001		Pseudoger	chr5:80997183-8099
ENSG0000(	515	11.50126	chr5:164(	ENSG00000249772		lncRNA	chr5:81113385-8111
ENSG0000(	515	11.50126	chr5:164(	ENSG00000271862		lncRNA	chr5:83049376-8305
ENSG0000(	514	11.47893	chr5:164(	ENSG00000290550		lncRNA	chr5:86282460-8629
ENSG0000(	514	11.47893	chr5:164(	ENSG00000248363		lncRNA	chr5:86353803-8636
ENSG0000(	514	11.47893	chr5:164(	RNU6-804P		smallRNA	chr5:86663144-8666
ENSG0000(	514	11.47893	chr5:164(	MIR3607		smallRNA	chr5:86620506-8662
ENSG0000(	514	11.47893	chr5:164(	COX7C	NCGv7	protein_c	chr5:86617928-8662
ENSG0000(	514	11.47893	chr5:164(	LINC02059		lncRNA	chr5:86746818-8674
ENSG0000(	514	11.47893	chr5:164(	PTP4A1P4		Pseudoger	chr5:86087526-8608
ENSG0000(	514	11.47893	chr5:164(	NBPF22P		Pseudoger	chr5:86282766-8629
ENSG0000(	514	11.47893	chr5:164(	ENSG00000251585		Pseudoger	chr5:86402465-8640
ENSG0000(	514	11.47893	chr5:164(	ENSG00000250124		lncRNA	chr5:86380660-8638
ENSG0000(	514	11.47893	chr5:164(	ENSG00000248195		lncRNA	chr5:86335024-8633
ENSG0000(	514	11.47893	chr5:164(	ENSG00000280009		TEC	chr5:86305233-8630
ENSG0000(	514	11.47893	chr5:164(	ENSG00000248701		lncRNA	chr5:86797685-8680
ENSG0000(	514	11.47893	chr5:164(	ENSG00000242477		Pseudoger	chr5:86884231-8688
ENSG0000(	513	11.45659	chr5:164(	ENSG00000251093		lncRNA	chr5:91226475-9122
ENSG0000(	512	11.43426	chr8:412(	AC107890.1		smallRNA	chr8:113407527-113
ENSG0000(	511	11.41193	chr8:412(	RN7SKP226		smallRNA	chr8:128220504-128
ENSG0000(	510	11.3896	chr8:412(	ENSG00000270682		Pseudoger	chr8:97788210-9778
ENSG0000(	509	11.36726	chr2:309⁄	PIK3CDP1		Pseudoger	chr2:6495877-64967
ENSG0000(	509	11.36726	chr2:309⁄	LINC01824		lncRNA	chr2:6495651-65118
ENSG0000(	507	11.3226	chr5:164(	THBS4		protein_c	chr5:79991311-8008
ENSG0000(	507	11.3226	chr5:164(	HMGB1P35		Pseudoger	chr5:77146568-7714
ENSG0000(	507	11.3226	chr5:164(	HOMER1		protein_c	chr5:79372636-7951
ENSG0000(	507	11.3226	chr3:380⁄	ENSG00000223398		Pseudoger	chr3:13460424-1346
ENSG0000(	507	11.3226	chr5:164(	SNORA47		smallRNA	chr5:77080434-7708
ENSG0000(	507	11.3226	chr5:164(	ENSG00000288846		lncRNA	chr5:79543786-7954
ENSG0000(	507	11.3226	chr5:164(	AC008581.1		smallRNA	chr5:77137967-7713
ENSG0000(	507	11.3226	chr5:164(	F2RL1		protein_c	chr5:76818933-7683
ENSG0000(	507	11.3226	chr5:164(	ENSG00000250258		lncRNA	chr5:79774348-7981
ENSG0000(	507	11.3226	chr5:164(	ENSG00000214890		Pseudoger	chr5:79000112-7900
ENSG0000(	507	11.3226	chr5:164(	RPS2P24		Pseudoger	chr5:77545397-7754
ENSG0000(	507	11.3226	chr5:164(	MTX3		protein_c	chr5:79976716-7999
ENSG0000(	507	11.3226	chr5:164(	AC024568.1		smallRNA	chr5:78056624-7805
ENSG0000(	507	11.3226	chr5:164(	AGGF1	NCGv7	protein_c	chr5:77029251-7706
ENSG0000(	507	11.3226	chr5:164(	ENSG00000289924		lncRNA	chr5:76818312-7681
ENSG0000(	507	11.3226	chr5:164(	ENSG00000253558		Pseudoger	chr5:78041888-7804
ENSG0000(	507	11.3226	chr5:164(	WDR41		protein_c	chr5:77425970-7762
ENSG0000(	507	11.3226	chr5:164(	RPL7AP32		Pseudoger	chr5:79529302-7953
ENSG0000(	507	11.3226	chr5:164(	ENSG00000285000		protein_c	chr5:77030902-7715
ENSG0000(	507	11.3226	chr5:164(	PDE8B		protein_c	chr5:77210449-7742
ENSG0000(	507	11.3226	chr5:164(	ENSG00000250330		Pseudoger	chr5:79229904-7923
ENSG0000(	507	11.3226	chr5:164(	TRMT112P2		Pseudoger	chr5:80033171-8003
ENSG0000(	507	11.3226	chr5:164(	ARSB		protein_c	chr5:78777209-7898
ENSG0000(	507	11.3226	chr5:164(	ENSG00000251605		Pseudoger	chr5:78307503-7830
ENSG0000(	507	11.3226	chr5:164(	ENSG00000213755		Pseudoger	chr5:79510434-7951
ENSG0000(	507	11.3226	chr5:164(	JMY		protein_c	chr5:79236131-7932
ENSG0000(	507	11.3226	chr5:164(	CMYA5		protein_c	chr5:79689836-7980
ENSG0000(	507	11.3226	chr5:164(	RPS3AP20		Pseudoger	chr5:79284132-7928

ENSG0000(	507	11.3226	chr5:164(	TENT2		protein_c	chr5:79612120-7968
ENSG0000(	507	11.3226	chr5:164(	ACTBP2		Pseudoger	chr5:77784881-7778
ENSG0000(	507	11.3226	chr3:380	ENSG00000238138		Pseudoger	chr3:13460294-1346
ENSG0000(	507	11.3226	chr5:164(	ENSG00000253572		lncRNA	chr5:77942757-7794
ENSG0000(	507	11.3226	chr5:164(	ENSG00000249829		Pseudoger	chr5:79191549-7919
ENSG0000(	507	11.3226	chr5:164(	THBS4-AS1		lncRNA	chr5:80052374-8008
ENSG0000(	507	11.3226	chr5:164(	BHMT		protein_c	chr5:79111809-7913
ENSG0000(	507	11.3226	chr5:164(	ENSG00000284762		protein_c	chr5:77086732-7742
ENSG0000(	507	11.3226	chr5:164(	CRHBP		protein_c	chr5:76953045-7698
ENSG0000(	507	11.3226	chr5:164(	LHFPL2		protein_c	chr5:78485215-7877
ENSG0000(	507	11.3226	chr5:164(	ALDH7A1P1		Pseudoger	chr5:77290268-7729
ENSG0000(	507	11.3226	chr5:164(	DMGDH		protein_c	chr5:78997564-7923
ENSG0000(	507	11.3226	chr5:164(	BHMT2	NCGv7	protein_c	chr5:79069767-7909
ENSG0000(	507	11.3226	chr5:164(	Y_RNA		smallRNA	chr5:79534853-7953
ENSG0000(	507	11.3226	chr5:164(	ENSG00000238254		Pseudoger	chr5:79849552-7984
ENSG0000(	507	11.3226	chr5:164(	RNU6-183P		smallRNA	chr5:78314916-7831
ENSG0000(	507	11.3226	chr5:164(	SCAMP1-AS1		lncRNA	chr5:78342333-7836
ENSG0000(	507	11.3226	chr5:164(	ENSG00000250615		lncRNA	chr5:77073881-7707
ENSG0000(	507	11.3226	chr5:164(	RNY3P1		smallRNA	chr5:79170234-7917
ENSG0000(	507	11.3226	chr5:164(	LINC01455		lncRNA	chr5:79936579-7996
ENSG0000(	507	11.3226	chr5:164(	S100Z		protein_c	chr5:76850001-7692
ENSG0000(	507	11.3226	chr5:164(	OTP		protein_c	chr5:77628712-7763
ENSG0000(	507	11.3226	chr5:164(	TBCA		protein_c	chr5:77691166-7786
ENSG0000(	507	11.3226	chr5:164(	AP3B1		protein_c	chr5:78000522-7829
ENSG0000(	507	11.3226	chr5:164(	SCAMP1		protein_c	chr5:78360611-7848
ENSG0000(	507	11.3226	chr5:164(	ZBED3		protein_c	chr5:77072072-7708
ENSG0000(	507	11.3226	chr5:164(	F2R		protein_c	chr5:76716126-7673
ENSG0000(	507	11.3226	chr5:164(	snoU13		smallRNA	chr5:76784096-7678
ENSG0000(	507	11.3226	chr5:164(	Y_RNA		smallRNA	chr5:78317036-7831
ENSG0000(	507	11.3226	chr5:164(	RN7SL208P		smallRNA	chr5:76836902-7683
ENSG0000(	507	11.3226	chr5:164(	ZBED3-AS1		lncRNA	chr5:77086688-7716
ENSG0000(	507	11.3226	chr5:164(	SNORA18		smallRNA	chr5:79220942-7922
ENSG0000(	507	11.3226	chr5:164(	RPL7P23		Pseudoger	chr5:77582376-7758
ENSG0000(	507	11.3226	chr5:164(	HMGB1P21		Pseudoger	chr5:78644265-7864
ENSG0000(	507	11.3226	chr5:164(	ENSG00000286818		lncRNA	chr5:79840797-7984
ENSG0000(	507	11.3226	chr5:164(	RNU6ATAC36P		smallRNA	chr5:76948791-7694
ENSG0000(	507	11.3226	chr5:164(	ENSG00000254170		Pseudoger	chr5:78708734-7871
ENSG0000(	507	11.3226	chr5:164(	RBX1P2		Pseudoger	chr5:80019609-8001
ENSG0000(	507	11.3226	chr3:380	SNORD5		smallRNA	chr3:25555543-2555
ENSG0000(	507	11.3226	chr5:164(	ATP6V1G1P6		Pseudoger	chr5:78222184-7822
ENSG0000(	507	11.3226	chr5:164(	ENSG00000287162		lncRNA	chr5:77958198-7795
ENSG0000(	506	11.30027	chr5:164(	ENSG00000250158		lncRNA	chr5:96247776-9627
ENSG0000(	506	11.30027	chr5:164(	LINC02234		lncRNA	chr5:97840912-9792
ENSG0000(	506	11.30027	chr5:164(	SPATA9		protein_c	chr5:95652181-9569
ENSG0000(	506	11.30027	chr5:164(	LIX1		protein_c	chr5:97091867-9714
ENSG0000(	506	11.30027	chr5:164(	MRPS35P2		Pseudoger	chr5:98402542-9840
ENSG0000(	506	11.30027	chr5:164(	RGMB-AS1		lncRNA	chr5:98769618-9877
ENSG0000(	506	11.30027	chr5:164(	GLRX		protein_c	chr5:95751319-9582
ENSG0000(	506	11.30027	chr5:164(	CSNK1A1P3		Pseudoger	chr5:98833382-9883
ENSG0000(	506	11.30027	chr5:164(	ENSG00000248758		lncRNA	chr5:97188090-9720
ENSG0000(	506	11.30027	chr5:164(	LNPEP		protein_c	chr5:96935394-9703

ENSG0000(	506	11.30027	chr5:164(	ENSG00000248734		lncRNA	chr5:96784777-9678
ENSG0000(	506	11.30027	chr5:164(	RNU6-402P		smallRNA	chr5:98889731-9888
ENSG0000(	506	11.30027	chr5:164(	ENSG00000286953		lncRNA	chr5:97799404-9788
ENSG0000(	506	11.30027	chr5:164(	FABP5P5		Pseudoger	chr5:95973041-9597
ENSG0000(	506	11.30027	chr5:164(	LIX1-AS1		lncRNA	chr5:97089075-9743
ENSG0000(	506	11.30027	chr5:164(	RTRAFP2		Pseudoger	chr5:95440044-9544
ENSG0000(	506	11.30027	chr5:164(	AC022142.1		smallRNA	chr5:98639097-9863
ENSG0000(	506	11.30027	chr5:164(	ENSG00000247121		lncRNA	chr5:96814028-9693
ENSG0000(	506	11.30027	chr5:164(	SETP22		Pseudoger	chr5:97056402-9705
ENSG0000(	506	11.30027	chr5:164(	ENSG00000251054		lncRNA	chr5:97223371-9722
ENSG0000(	506	11.30027	chr5:164(	ENSG00000248236		Pseudoger	chr5:99594329-9959
ENSG0000(	506	11.30027	chr5:164(	MCTP1-AS1		lncRNA	chr5:94979151-9498
ENSG0000(	506	11.30027	chr5:164(	MTCO2P24		Pseudoger	chr5:98409675-9841
ENSG0000(	506	11.30027	chr5:164(	ENSG00000289337		lncRNA	chr5:96165522-9617
ENSG0000(	506	11.30027	chr5:164(	LINC01340		lncRNA	chr5:97504663-9769
ENSG0000(	506	11.30027	chr5:164(	MTCO1P24		Pseudoger	chr5:98410235-9841
ENSG0000(	506	11.30027	chr5:164(	ENSG00000272021		lncRNA	chr5:95849309-9584
ENSG0000(	506	11.30027	chr5:164(	ENSG00000249444		Pseudoger	chr5:99499070-9949
ENSG0000(	506	11.30027	chr5:164(	ENSG00000251193		Pseudoger	chr5:97737894-9773
ENSG0000(	506	11.30027	chr5:164(	ENSG00000248928		Pseudoger	chr5:99594880-9959
ENSG0000(	506	11.30027	chr5:164(	ENSG00000241597		Pseudoger	chr5:98954394-9895
ENSG0000(	506	11.30027	chr5:164(	MTCYBP40		Pseudoger	chr5:97677547-9767
ENSG0000(	506	11.30027	chr5:164(	ENSG00000286828		lncRNA	chr5:97183827-9721
ENSG0000(	506	11.30027	chr5:164(	GPR150		protein_c	chr5:95620087-9562
ENSG0000(	506	11.30027	chr5:164(	LINC01846		lncRNA	chr5:98085866-9816
ENSG0000(	506	11.30027	chr5:164(	ENSG00000250240		lncRNA	chr5:95701249-9573
ENSG0000(	506	11.30027	chr5:164(	RGMB		protein_c	chr5:98768650-9879
ENSG0000(	506	11.30027	chr5:164(	GUSBP8		Pseudoger	chr5:99532628-9953
ENSG0000(	506	11.30027	chr5:164(	KRT8P32		Pseudoger	chr5:98392070-9839
ENSG0000(	506	11.30027	chr5:164(	RFESD		protein_c	chr5:95646754-9568
ENSG0000(	506	11.30027	chr5:164(	ENSG00000249746		lncRNA	chr5:96213263-9621
ENSG0000(	506	11.30027	chr5:164(	PSME2P1		Pseudoger	chr5:98213402-9821
ENSG0000(	506	11.30027	chr5:164(	RIOK2	NCGv7	protein_c	chr5:97160867-9718
ENSG0000(	506	11.30027	chr5:164(	ENSG00000206356		Pseudoger	chr5:99522311-9952
ENSG0000(	506	11.30027	chr5:164(	ENSG00000279232		lncRNA	chr5:98792861-9879
ENSG0000(	506	11.30027	chr5:164(	DDX18P4		Pseudoger	chr5:98679402-9868
ENSG0000(	506	11.30027	chr5:164(	CHD1-DT		lncRNA	chr5:98929163-9899
ENSG0000(	506	11.30027	chr5:164(	PCSK1		protein_c	chr5:96390333-9643
ENSG0000(	506	11.30027	chr5:164(	ELL2		protein_c	chr5:95885098-9596
ENSG0000(	506	11.30027	chr5:164(	ENSG00000249761		Pseudoger	chr5:97336858-9733
ENSG0000(	506	11.30027	chr5:164(	Y_RNA		smallRNA	chr5:96962656-9696
ENSG0000(	506	11.30027	chr5:164(	ENSG00000227836		Pseudoger	chr5:96942299-9694
ENSG0000(	506	11.30027	chr5:164(	RNU1-73P		smallRNA	chr5:97175950-9717
ENSG0000(	506	11.30027	chr5:164(	ENSG00000249101		Pseudoger	chr5:98025965-9802
ENSG0000(	506	11.30027	chr5:164(	TTC37		protein_c	chr5:95461755-9555
ENSG0000(	506	11.30027	chr5:164(	RNU6-524P		smallRNA	chr5:96210121-9621
ENSG0000(	506	11.30027	chr5:164(	GGCTP1		Pseudoger	chr5:95834424-9583
ENSG0000(	506	11.30027	chr5:164(	Y_RNA		smallRNA	chr5:98936638-9893
ENSG0000(	506	11.30027	chr5:164(	HSPD1P11		Pseudoger	chr5:95768999-9577
ENSG0000(	506	11.30027	chr5:164(	MIR583HG		lncRNA	chr5:96050115-9621
ENSG0000(	506	11.30027	chr5:164(	AC020900.2		smallRNA	chr5:96694531-9669

ENSG0000(	506	11.30027	chr5:164€	ENSG00000290580		lncRNA	chr5:99489559-9949
ENSG0000(	506	11.30027	chr5:164€	RPL7P18		Pseudoger	chr5:94825961-9482
ENSG0000(	506	11.30027	chr5:164€	ENSG00000250362		lncRNA	chr5:95861786-9587
ENSG0000(	506	11.30027	chr5:164€	ARSK		protein_c	chr5:95555101-9560
ENSG0000(	506	11.30027	chr5:164€	RHOBTB3		protein_c	chr5:95713522-9582
ENSG0000(	506	11.30027	chr5:164€	CRLF3P2		Pseudoger	chr5:99948295-9994
ENSG0000(	506	11.30027	chr5:164€	ERAP1		protein_c	chr5:96760810-9680
ENSG0000(	506	11.30027	chr5:164€	ENSG00000251314		lncRNA	chr5:95962001-9663
ENSG0000(	506	11.30027	chr5:164€	ERAP2		protein_c	chr5:96875986-9691
ENSG0000(	506	11.30027	chr5:164€	ENSG00000272109		lncRNA	chr5:96803688-9680
ENSG0000(	506	11.30027	chr5:164€	MIR583		smallRNA	chr5:96079138-9607
ENSG0000(	506	11.30027	chr5:164€	LINC01554		lncRNA	chr5:95838245-9586
ENSG0000(	506	11.30027	chr5:164€	AC020900.1		smallRNA	chr5:96590192-9659
ENSG0000(	506	11.30027	chr5:164€	RNU6-308P		smallRNA	chr5:95521369-9552
ENSG0000(	506	11.30027	chr5:164€	YTHDF1P1		Pseudoger	chr5:97368776-9737
ENSG0000(	506	11.30027	chr5:164€	ENSG00000250955		lncRNA	chr5:95964999-9598
ENSG0000(	506	11.30027	chr5:164€	ENSG00000251409		lncRNA	chr5:95835521-9585
ENSG0000(	506	11.30027	chr5:164€	CTBP2P4		Pseudoger	chr5:98576341-9857
ENSG0000(	506	11.30027	chr5:164€	CAST	NCGv7	protein_c	chr5:96525267-9677
ENSG0000(	506	11.30027	chr5:164€	MCTP1		protein_c	chr5:94703690-9528
ENSG0000(	506	11.30027	chr5:164€	ENSG00000249135		Pseudoger	chr5:98203059-9820
ENSG0000(	506	11.30027	chr5:164€	CHD1	NCGv7	protein_c	chr5:98853985-9892
ENSG0000(	506	11.30027	chr5:164€	ENSG00000176183		Pseudoger	chr5:98338744-9833
ENSG0000(	506	11.30027	chr5:164€	ENSG00000249180		lncRNA	chr5:96741079-9674
ENSG0000(	506	11.30027	chr5:164€	FAM81B		protein_c	chr5:95391366-9545
ENSG0000(	506	11.30027	chr5:164€	ENSG00000249175		lncRNA	chr5:94788789-9479
ENSG0000(	506	11.30027	chr5:164€	LINC02113		lncRNA	chr5:99549432-9957
ENSG0000(	505	11.27793	chr12:171	ENSG00000258344		lncRNA	chr12:54276631-543
ENSG0000(	505	11.27793	chr12:171	OLA1P3		Pseudoger	chr12:55870048-558
ENSG0000(	505	11.27793	chr12:171	ENSG00000285692		lncRNA	chr12:53531752-536
ENSG0000(	505	11.27793	chr8:412€	ENSG00000253949		lncRNA	chr8:108581062-108
ENSG0000(	505	11.27793	chr12:171	KRT18		protein_c	chr12:52948871-529
ENSG0000(	505	11.27793	chr12:171	HOXC5		protein_c	chr12:54033050-540
ENSG0000(	505	11.27793	chr12:171	ENSG00000258311		protein_c	chr12:55716036-557
ENSG0000(	505	11.27793	chr12:171	ENSG00000258345		lncRNA	chr12:56118968-561
ENSG0000(	505	11.27793	chr8:412€	LINC01609		lncRNA	chr8:111093263-111
ENSG0000(	505	11.27793	chr12:171	RARG		protein_c	chr12:53210567-532
ENSG0000(	505	11.27793	chr12:171	OR6C70		protein_c	chr12:55469200-554
ENSG0000(	505	11.27793	chr8:412€	LINC01608		lncRNA	chr8:110899996-111
ENSG0000(	505	11.27793	chr8:412€	PGAM1P13		Pseudoger	chr8:107647279-107
ENSG0000(	505	11.27793	chr12:171	RN7SL390P		smallRNA	chr12:54255631-542
ENSG0000(	505	11.27793	chr12:171	ENSG00000291180		lncRNA	chr12:54076838-540
ENSG0000(	505	11.27793	chr12:171	MUCL1		protein_c	chr12:54830518-548
ENSG0000(	505	11.27793	chr12:171	OR6C75		protein_c	chr12:55362975-553
ENSG0000(	505	11.27793	chr8:412€	ENSG00000253271		Pseudoger	chr8:117055818-117
ENSG0000(	505	11.27793	chr8:412€	ANGPT1	NCGv7	protein_c	chr8:107249482-107
ENSG0000(	505	11.27793	chr12:171	ENSG00000288663		lncRNA	chr12:53295492-533
ENSG0000(	505	11.27793	chr12:171	ENSG00000258554		lncRNA	chr12:55966838-559
ENSG0000(	505	11.27793	chr12:171	ATF7-NPFF		protein_c	chr12:53506691-536
ENSG0000(	505	11.27793	chr8:412€	AARD	DriverDB\	protein_c	chr8:116938207-116
ENSG0000(	505	11.27793	chr12:171	ENSG00000248576		lncRNA	chr12:54163139-541

ENSG0000(	505	11.27793	chr12:171	IL23A		protein_c	chr12:56334174-563
ENSG0000(	505	11.27793	chr8:412(	RNU7-84P		smallRNA	chr8:106729795-106
ENSG0000(	505	11.27793	chr12:171	CALCOCO1	NCGv7	protein_c	chr12:53708517-537
ENSG0000(	505	11.27793	chr8:412(	ENSG00000253207		lncRNA	chr8:113600310-113
ENSG0000(	505	11.27793	chr8:412(	SAMD12		protein_c	chr8:118189455-118
ENSG0000(	505	11.27793	chr8:412(	ENSG00000253956		lncRNA	chr8:110334743-110
ENSG0000(	505	11.27793	chr12:171	MIR148B		smallRNA	chr12:54337216-543
ENSG0000(	505	11.27793	chr8:412(	PKHD1L1	NCGv7	protein_c	chr8:109362461-109
ENSG0000(	505	11.27793	chr12:171	TNS2		protein_c	chr12:53046969-530
ENSG0000(	505	11.27793	chr12:171	KRT78		protein_c	chr12:52837804-528
ENSG0000(	505	11.27793	chr12:171	SP7		protein_c	chr12:53326575-533
ENSG0000(	505	11.27793	chr8:412(	ENSG00000253122		lncRNA	chr8:110609241-110
ENSG0000(	505	11.27793	chr8:412(	AURKBP1		Pseudoger	chr8:108131100-108
ENSG0000(	505	11.27793	chr12:171	GTSF1		protein_c	chr12:54455950-544
ENSG0000(	505	11.27793	chr12:171	Y_RNA		smallRNA	chr12:54099191-540
ENSG0000(	505	11.27793	chr12:171	ATF7		protein_c	chr12:53507856-536
ENSG0000(	505	11.27793	chr8:412(	RPS26P35		Pseudoger	chr8:118761856-118
ENSG0000(	505	11.27793	chr12:171	ENSG00000258317		lncRNA	chr12:56120033-561
ENSG0000(	505	11.27793	chr12:171	RN7SKP289		smallRNA	chr12:53816185-538
ENSG0000(	505	11.27793	chr12:171	KRT8		protein_c	chr12:52897187-529
ENSG0000(	505	11.27793	chr12:171	ENSG00000273046		lncRNA	chr12:54017110-540
ENSG0000(	505	11.27793	chr8:412(	COLEC10		protein_c	chr8:118995452-119
ENSG0000(	505	11.27793	chr12:171	OR6C71P		Pseudoger	chr12:55376824-553
ENSG0000(	505	11.27793	chr8:412(	RAD21-AS1		lncRNA	chr8:116874424-116
ENSG0000(	505	11.27793	chr8:412(	ENSG00000253107		Pseudoger	chr8:110555408-110
ENSG0000(	505	11.27793	chr12:171	ENSG00000258921		lncRNA	chr12:55638912-556
ENSG0000(	505	11.27793	chr12:171	OR9K2	NCGv7	protein_c	chr12:55126406-551
ENSG0000(	505	11.27793	chr12:171	ENSG00000273049		protein_c	chr12:53985845-540
ENSG0000(	505	11.27793	chr12:171	STAT2		protein_c	chr12:56341597-563
ENSG0000(	505	11.27793	chr6:1039	TMEM217		protein_c	chr6:37212180-3725
ENSG0000(	505	11.27793	chr12:171	KRT76		protein_c	chr12:52768155-527
ENSG0000(	505	11.27793	chr12:171	PA2G4	AC	protein_c	chr12:56104537-561
ENSG0000(	505	11.27793	chr12:171	OR6C5P		Pseudoger	chr12:55311835-553
ENSG0000(	505	11.27793	chr12:171	HOXC8		protein_c	chr12:54008985-540
ENSG0000(	505	11.27793	chr8:412(	LINC00536		lncRNA	chr8:115950511-116
ENSG0000(	505	11.27793	chr12:171	KRT4		protein_c	chr12:52806549-528
ENSG0000(	505	11.27793	chr12:171	MFSD5		protein_c	chr12:53251251-532
ENSG0000(	505	11.27793	chr12:171	PYM1		protein_c	chr12:55901413-559
ENSG0000(	505	11.27793	chr12:171	METTL7B		protein_c	chr12:55681736-556
ENSG0000(	505	11.27793	chr12:171	ENSG00000258763		lncRNA	chr12:55434734-555
ENSG0000(	505	11.27793	chr8:412(	H2AZP7		Pseudoger	chr8:111621458-111
ENSG0000(	505	11.27793	chr8:412(	ENSG00000253398		lncRNA	chr8:119419910-119
ENSG0000(	505	11.27793	chr12:171	PSMB3P1		Pseudoger	chr12:55654558-556
ENSG0000(	505	11.27793	chr6:1039	RPL12P2		Pseudoger	chr6:37091314-3709
ENSG0000(	505	11.27793	chr12:171	KRT127P		Pseudoger	chr12:52751978-527
ENSG0000(	505	11.27793	chr8:412(	RNA5SP275		Pseudoger	chr8:107884494-107
ENSG0000(	505	11.27793	chr12:171	ENSG00000289854		lncRNA	chr12:54358489-543
ENSG0000(	505	11.27793	chr6:1039	SNORD112		smallRNA	chr6:37183597-3718
ENSG0000(	505	11.27793	chr12:171	RNU6-950P		smallRNA	chr12:54317781-543
ENSG0000(	505	11.27793	chr12:171	ENSG00000258199		lncRNA	chr12:56162359-561
ENSG0000(	505	11.27793	chr12:171	RNU1-69P		smallRNA	chr12:55874678-558

ENSG0000(	505	11.27793	chr8:412(	RNU6-12P		smallRNA	chr8:118976513-118
ENSG0000(	505	11.27793	chr8:412(	ENSG00000253499		Pseudoger	chr8:114318400-114
ENSG0000(	505	11.27793	chr12:171	ENSG00000270175		lncRNA	chr12:53500151-535
ENSG0000(	505	11.27793	chr8:412(	ENSG00000289767		protein_c	chr8:109539726-109
ENSG0000(	505	11.27793	chr12:171	RPL7P41		Pseudoger	chr12:52885429-528
ENSG0000(	505	11.27793	chr8:412(	ENSG00000253489		lncRNA	chr8:111179193-111
ENSG0000(	505	11.27793	chr12:171	FLJ12825		lncRNA	chr12:54058254-541
ENSG0000(	505	11.27793	chr8:412(	ENSG00000253622		lncRNA	chr8:117128455-117
ENSG0000(	505	11.27793	chr12:171	PCBP2-OT1		lncRNA	chr12:53464468-534
ENSG0000(	505	11.27793	chr8:412(	RN7SL228P		smallRNA	chr8:117017946-117
ENSG0000(	505	11.27793	chr12:171	ENSG00000260030		lncRNA	chr12:53754358-537
ENSG0000(	505	11.27793	chr12:171	OR6C69P		Pseudoger	chr12:55262539-552
ENSG0000(	505	11.27793	chr12:171	ENSG00000213470		Pseudoger	chr12:53632788-536
ENSG0000(	505	11.27793	chr8:412(	ENSG00000253657		Pseudoger	chr8:108572643-108
ENSG0000(	505	11.27793	chr8:412(	ENSG00000253672		lncRNA	chr8:116402543-116
ENSG0000(	505	11.27793	chr12:171	GPR84-AS1		lncRNA	chr12:54353792-544
ENSG0000(	505	11.27793	chr12:171	SPRYD3		protein_c	chr12:53064316-530
ENSG0000(	505	11.27793	chr12:171	IGFBP6		protein_c	chr12:53097436-531
ENSG0000(	505	11.27793	chr8:412(	TAGLN2P1		Pseudoger	chr8:106697427-106
ENSG0000(	505	11.27793	chr8:412(	ENSG00000289382		lncRNA	chr8:116652014-116
ENSG0000(	505	11.27793	chr12:171	SOAT2		protein_c	chr12:53103486-531
ENSG0000(	505	11.27793	chr12:171	MIR615		smallRNA	chr12:54033950-540
ENSG0000(	505	11.27793	chr8:412(	TRMT10BP1		Pseudoger	chr8:108652373-108
ENSG0000(	505	11.27793	chr12:171	SARNP		protein_c	chr12:55752463-558
ENSG0000(	505	11.27793	chr8:412(	RPSAP48		Pseudoger	chr8:110105076-110
ENSG0000(	505	11.27793	chr8:412(	ENSG00000275905		Pseudoger	chr8:106980967-106
ENSG0000(	505	11.27793	chr12:171	ENSG00000249388		lncRNA	chr12:54081736-541
ENSG0000(	505	11.27793	chr12:171	MIR196A2		smallRNA	chr12:53991738-539
ENSG0000(	505	11.27793	chr12:171	MYL6		protein_c	chr12:56158346-561
ENSG0000(	505	11.27793	chr12:171	ENSG00000270458		Pseudoger	chr12:55662337-556
ENSG0000(	505	11.27793	chr12:171	CISTR		lncRNA	chr12:53746337-537
ENSG0000(	505	11.27793	chr8:412(	SNORD112		smallRNA	chr8:109596243-109
ENSG0000(	505	11.27793	chr12:171	ENSG00000260597		lncRNA	chr12:54019910-540
ENSG0000(	505	11.27793	chr12:171	OR10P1		protein_c	chr12:55636892-556
ENSG0000(	505	11.27793	chr8:412(	AP000428.1		smallRNA	chr8:107254407-107
ENSG0000(	505	11.27793	chr8:412(	TMEM74		protein_c	chr8:108606850-108
ENSG0000(	505	11.27793	chr8:412(	CSMD3	NCGv7	protein_c	chr8:112222928-113
ENSG0000(	505	11.27793	chr8:412(	KCNV1		protein_c	chr8:109963636-109
ENSG0000(	505	11.27793	chr8:412(	TNFRSF11E	DriverDBv	protein_c	chr8:118923557-118
ENSG0000(	505	11.27793	chr8:412(	MED30		protein_c	chr8:117520713-117
ENSG0000(	505	11.27793	chr8:412(	SLC30A8		protein_c	chr8:116950273-117
ENSG0000(	505	11.27793	chr8:412(	RAD21	NCGv7;AC	protein_c	chr8:116845934-116
ENSG0000(	505	11.27793	chr12:171	ENSG00000260470		lncRNA	chr12:54145069-541
ENSG0000(	505	11.27793	chr12:171	GPR84-AS1		lncRNA	chr12:54353661-544
ENSG0000(	505	11.27793	chr12:171	AAAS		protein_c	chr12:53307456-533
ENSG0000(	505	11.27793	chr12:171	VDAC1P5		Pseudoger	chr12:54802746-548
ENSG0000(	505	11.27793	chr12:171	CBX5		protein_c	chr12:54230942-542
ENSG0000(	505	11.27793	chr12:171	ENSG00000283536		protein_c	chr12:53241900-532
ENSG0000(	505	11.27793	chr12:171	OR6C66P		Pseudoger	chr12:55388445-553
ENSG0000(	505	11.27793	chr12:171	KRT3		protein_c	chr12:52789685-527
ENSG0000(	505	11.27793	chr8:412(	SNORA31		smallRNA	chr8:117313528-117

ENSG0000(	505	11.27793	chr12:171	VTI1BP3		Pseudoger	chr12:53131350-531
ENSG0000(	505	11.27793	chr8:4126	SAMD12-AS1		lncRNA	chr8:118620498-118
ENSG0000(	505	11.27793	chr12:171	OR10AE3P		Pseudoger	chr12:55646195-556
ENSG0000(	505	11.27793	chr12:171	EIF4B		protein_c	chr12:53006282-530
ENSG0000(	505	11.27793	chr12:171	ENSG00000258260		lncRNA	chr12:56267793-562
ENSG0000(	505	11.27793	chr12:171	KRT125P		Pseudoger	chr12:52729517-527
ENSG0000(	505	11.27793	chr8:4126	ABRA		protein_c	chr8:106759483-106
ENSG0000(	505	11.27793	chr6:1039	RN7SL273P		smallRNA	chr6:37361185-3736
ENSG0000(	505	11.27793	chr8:4126	TRHR		protein_c	chr8:109086585-109
ENSG0000(	505	11.27793	chr12:171	ENSG00000289965		lncRNA	chr12:55709985-557
ENSG0000(	505	11.27793	chr12:171	ENSG00000275488		Pseudoger	chr12:53574632-535
ENSG0000(	505	11.27793	chr12:171	CS		protein_c	chr12:56271699-563
ENSG0000(	505	11.27793	chr12:171	TMEM198B		Pseudoger	chr12:55829608-558
ENSG0000(	505	11.27793	chr12:171	PMEL		protein_c	chr12:55954105-559
ENSG0000(	505	11.27793	chr12:171	KRT79		protein_c	chr12:52821408-528
ENSG0000(	505	11.27793	chr8:4126	ENSG00000289094		lncRNA	chr8:108195494-108
ENSG0000(	505	11.27793	chr12:171	OR6U2P		Pseudoger	chr12:55611623-556
ENSG0000(	505	11.27793	chr8:4126	ENSG00000253796		lncRNA	chr8:108871128-109
ENSG0000(	505	11.27793	chr12:171	SP1		protein_c	chr12:53380176-534
ENSG0000(	505	11.27793	chr12:171	HIGD1AP1		Pseudoger	chr12:53142053-531
ENSG0000(	505	11.27793	chr8:4126	RPS10P16		Pseudoger	chr8:117523271-117
ENSG0000(	505	11.27793	chr8:4126	LINC02237		lncRNA	chr8:111376639-111
ENSG0000(	505	11.27793	chr12:171	PRR13		protein_c	chr12:53441678-534
ENSG0000(	505	11.27793	chr12:171	OR6C72P		Pseudoger	chr12:55283750-552
ENSG0000(	505	11.27793	chr12:171	OR6C1		protein_c	chr12:55314343-553
ENSG0000(	505	11.27793	chr12:171	OR6C3		protein_c	chr12:55330043-553
ENSG0000(	505	11.27793	chr12:171	OR6C65		protein_c	chr12:55400529-554
ENSG0000(	505	11.27793	chr12:171	OR6C68		protein_c	chr12:55492378-554
ENSG0000(	505	11.27793	chr8:4126	CARS1P2		Pseudoger	chr8:114791653-114
ENSG0000(	505	11.27793	chr12:171	ENSG00000289154		lncRNA	chr12:54278612-542
ENSG0000(	505	11.27793	chr12:171	HOXC13-AS		lncRNA	chr12:53935328-539
ENSG0000(	505	11.27793	chr8:4126	EIF3E	NCGv7;AC	protein_c	chr8:108162787-108
ENSG0000(	505	11.27793	chr12:171	ENSG00000290754		lncRNA	chr12:55608043-556
ENSG0000(	505	11.27793	chr12:171	OR6C76		protein_c	chr12:55426254-554
ENSG0000(	505	11.27793	chr12:171	ARL2BPP2		Pseudoger	chr12:52763405-527
ENSG0000(	505	11.27793	chr12:171	RNU7-40P		smallRNA	chr12:56352387-563
ENSG0000(	505	11.27793	chr8:4126	ENSG00000253717		lncRNA	chr8:110766863-110
ENSG0000(	505	11.27793	chr6:1039	TBC1D22B		protein_c	chr6:37257772-3733
ENSG0000(	505	11.27793	chr12:171	ERBB3	NCGv7;AC	protein_c	chr12:56076799-561
ENSG0000(	505	11.27793	chr8:4126	RNA5SP276		Pseudoger	chr8:116022686-116
ENSG0000(	505	11.27793	chr12:171	DGKA	NCGv7	protein_c	chr12:55927319-559
ENSG0000(	505	11.27793	chr6:1039	ENSG00000275550		Pseudoger	chr6:37295584-3729
ENSG0000(	505	11.27793	chr8:4126	RN7SL826P		smallRNA	chr8:117163968-117
ENSG0000(	505	11.27793	chr12:171	CD63-AS1		lncRNA	chr12:55729094-557
ENSG0000(	505	11.27793	chr8:4126	Y_RNA		smallRNA	chr8:113950886-113
ENSG0000(	505	11.27793	chr8:4126	EMC2	DriverDB\	protein_c	chr8:108443601-108
ENSG0000(	505	11.27793	chr12:171	ZNF385A		protein_c	chr12:54369133-543
ENSG0000(	505	11.27793	chr8:4126	SNORA32		smallRNA	chr8:119388348-119
ENSG0000(	505	11.27793	chr12:171	ENSG00000290813		lncRNA	chr12:55376742-553
ENSG0000(	505	11.27793	chr8:4126	TRPS1	NCGv7	protein_c	chr8:115408496-115
ENSG0000(	505	11.27793	chr12:171	ITGA5		protein_c	chr12:54395261-544

ENSG0000(	505	11.27793	chr8:412(	ENSG00000241385		Pseudoger	chr8:109899246-109
ENSG0000(	505	11.27793	chr12:171	DCD	AC	protein_c	chr12:54644589-546
ENSG0000(	505	11.27793	chr12:171	ENSG00000290814		lncRNA	chr12:55385837-553
ENSG0000(	505	11.27793	chr12:171	OR6C64P		Pseudoger	chr12:55522593-555
ENSG0000(	505	11.27793	chr8:412(	ENSG00000253754		lncRNA	chr8:108226200-108
ENSG0000(	505	11.27793	chr8:412(	ENSG00000255402		lncRNA	chr8:109973943-109
ENSG0000(	505	11.27793	chr12:171	ENSG00000237493		Pseudoger	chr12:55980432-559
ENSG0000(	505	11.27793	chr12:171	ZC3H10		protein_c	chr12:56118260-561
ENSG0000(	505	11.27793	chr12:171	RNU6-333P		smallRNA	chr12:53138545-531
ENSG0000(	505	11.27793	chr12:171	OR9R1P		Pseudoger	chr12:55158847-551
ENSG0000(	505	11.27793	chr8:412(	RNU2-67P		smallRNA	chr8:109873913-109
ENSG0000(	505	11.27793	chr12:171	ATP5MC2		protein_c	chr12:53632726-536
ENSG0000(	505	11.27793	chr8:412(	AF130343.1		smallRNA	chr8:116287351-116
ENSG0000(	505	11.27793	chr12:171	ENSG00000257379		lncRNA	chr12:53441741-534
ENSG0000(	505	11.27793	chr12:171	ENSG00000257384		Pseudoger	chr12:55732366-557
ENSG0000(	505	11.27793	chr12:171	OR9K1P		Pseudoger	chr12:55113077-551
ENSG0000(	505	11.27793	chr12:171	ENSG00000257389		Pseudoger	chr12:52837403-528
ENSG0000(	505	11.27793	chr12:171	ENSG00000257824		lncRNA	chr12:54543111-545
ENSG0000(	505	11.27793	chr12:171	ENSG00000257390		protein_c	chr12:55757275-558
ENSG0000(	505	11.27793	chr8:412(	ENSG00000287949		lncRNA	chr8:107857589-107
ENSG0000(	505	11.27793	chr12:171	RPL41		protein_c	chr12:56116590-561
ENSG0000(	505	11.27793	chr8:412(	ENSG00000287942		lncRNA	chr8:111699519-111
ENSG0000(	505	11.27793	chr8:412(	ENSG00000286946		lncRNA	chr8:112446575-112
ENSG0000(	505	11.27793	chr12:171	AC009779.1		smallRNA	chr12:55554995-555
ENSG0000(	505	11.27793	chr12:171	SMUG1	NCGv7	protein_c	chr12:54121277-541
ENSG0000(	505	11.27793	chr12:171	IKZF4	DriverDB\	protein_c	chr12:56007659-560
ENSG0000(	505	11.27793	chr12:171	ENSG00000257616		Pseudoger	chr12:52730425-527
ENSG0000(	505	11.27793	chr8:412(	ENSG00000286937		lncRNA	chr8:113438334-113
ENSG0000(	505	11.27793	chr12:171	HOXC12		protein_c	chr12:53954903-539
ENSG0000(	505	11.27793	chr12:171	HOXC10		protein_c	chr12:53985065-539
ENSG0000(	505	11.27793	chr12:171	AC107016.2		smallRNA	chr12:52951708-529
ENSG0000(	505	11.27793	chr12:171	NFE2		protein_c	chr12:54292111-543
ENSG0000(	505	11.27793	chr12:171	LINC01154		lncRNA	chr12:54428303-544
ENSG0000(	505	11.27793	chr12:171	SNORD81		smallRNA	chr12:53791305-537
ENSG0000(	505	11.27793	chr12:171	KRT126P		Pseudoger	chr12:52715567-527
ENSG0000(	505	11.27793	chr12:171	ENSG00000144785		protein_c	chr12:56285916-563
ENSG0000(	505	11.27793	chr8:412(	NUDCD1	DriverDB\	protein_c	chr8:109240919-109
ENSG0000(	505	11.27793	chr12:171	MIR3198-2		smallRNA	chr12:54231397-542
ENSG0000(	505	11.27793	chr12:171	RN7SL770P		smallRNA	chr12:56195147-561
ENSG0000(	505	11.27793	chr12:171	CDK2		protein_c	chr12:55966781-559
ENSG0000(	505	11.27793	chr8:412(	HMGB1P46		Pseudoger	chr8:107173200-107
ENSG0000(	505	11.27793	chr8:412(	CCN3	AC	protein_c	chr8:119416446-119
ENSG0000(	505	11.27793	chr12:171	HOXC4	DriverDB\	protein_c	chr12:54016931-540
ENSG0000(	505	11.27793	chr12:171	LACRT		protein_c	chr12:54630811-546
ENSG0000(	505	11.27793	chr8:412(	ENSG00000250267		lncRNA	chr8:109298598-109
ENSG0000(	505	11.27793	chr8:412(	ENSG00000254278		lncRNA	chr8:119062942-119
ENSG0000(	505	11.27793	chr8:412(	RNU4-37P		smallRNA	chr8:112148742-112
ENSG0000(	505	11.27793	chr12:171	AMHR2		protein_c	chr12:53423855-534
ENSG0000(	505	11.27793	chr6:103(	PIM1	NCGv7;AC	protein_c	chr6:37170152-3717
ENSG0000(	505	11.27793	chr12:171	OR6C2		protein_c	chr12:55444069-554
ENSG0000(	505	11.27793	chr12:171	OR6C6		protein_c	chr12:55293988-552

ENSG0000(	505	11.27793	chr12:171	OR6C7P		Pseudoger	chr12:55342841-553
ENSG0000(	505	11.27793	chr12:171	TNS2-AS1		lncRNA	chr12:53012884-530
ENSG0000(	505	11.27793	chr8:412(	TRPS1-AS1		lncRNA	chr8:115509602-115
ENSG0000(	505	11.27793	chr12:171	CD63		protein_c	chr12:55725323-557
ENSG0000(	505	11.27793	chr6:103(	TMEM217B		protein_c	chr6:37212181-3725
ENSG0000(	505	11.27793	chr12:171	ENSG00000257509		lncRNA	chr12:55761550-557
ENSG0000(	505	11.27793	chr12:171	DNAJC14		protein_c	chr12:55820960-558
ENSG0000(	505	11.27793	chr12:171	ENSG00000257343		Pseudoger	chr12:52746596-527
ENSG0000(	505	11.27793	chr8:412(	NDUFB9P3		Pseudoger	chr8:110809394-110
ENSG0000(	505	11.27793	chr12:171	RNF41		protein_c	chr12:56202179-562
ENSG0000(	505	11.27793	chr12:171	MYL6B		protein_c	chr12:56152256-561
ENSG0000(	505	11.27793	chr12:171	ENSG00000257350		Pseudoger	chr12:55122668-551
ENSG0000(	505	11.27793	chr8:412(	AC024996.1		smallRNA	chr8:112475601-112
ENSG0000(	505	11.27793	chr12:171	ENSG00000257634		lncRNA	chr12:54682973-546
ENSG0000(	505	11.27793	chr12:171	ENSG00000286069		lncRNA	chr12:53739611-538
ENSG0000(	505	11.27793	chr12:171	OR6C4		protein_c	chr12:55549602-555
ENSG0000(	505	11.27793	chr8:412(	MIR2053		smallRNA	chr8:112643493-112
ENSG0000(	505	11.27793	chr12:171	OR2AP1		protein_c	chr12:55572468-555
ENSG0000(	505	11.27793	chr12:171	HOXC11	NCGv7;AC	protein_c	chr12:53973126-539
ENSG0000(	505	11.27793	chr8:412(	ENY2	DriverDB	protein_c	chr8:109334324-109
ENSG0000(	505	11.27793	chr8:412(	AC103863.1		smallRNA	chr8:116136773-116
ENSG0000(	505	11.27793	chr12:171	SCAT2		lncRNA	chr12:54262615-542
ENSG0000(	505	11.27793	chr8:412(	ENSG00000227514		Pseudoger	chr8:113376669-113
ENSG0000(	505	11.27793	chr12:171	RN7SL744P		smallRNA	chr12:54344610-543
ENSG0000(	505	11.27793	chr12:171	ENSG00000257449		lncRNA	chr12:56029649-560
ENSG0000(	505	11.27793	chr12:171	ENSG00000257740		lncRNA	chr12:56308868-563
ENSG0000(	505	11.27793	chr8:412(	SERPINA15P		Pseudoger	chr8:111380603-111
ENSG0000(	505	11.27793	chr8:412(	ENSG00000254247		Pseudoger	chr8:118982579-118
ENSG0000(	505	11.27793	chr12:171	OR6C74		protein_c	chr12:55247198-552
ENSG0000(	505	11.27793	chr12:171	EIF4A1P4		Pseudoger	chr12:53153272-531
ENSG0000(	505	11.27793	chr12:171	FAM242C		lncRNA	chr12:54085132-541
ENSG0000(	505	11.27793	chr12:171	HOTAIR	AC	lncRNA	chr12:53962308-539
ENSG0000(	505	11.27793	chr12:171	HOXC-AS1		lncRNA	chr12:53999022-540
ENSG0000(	505	11.27793	chr12:171	SUOX		protein_c	chr12:55997180-560
ENSG0000(	505	11.27793	chr8:412(	MTCO1P47		Pseudoger	chr8:110933263-110
ENSG0000(	505	11.27793	chr12:171	SLC39A5		protein_c	chr12:56230049-562
ENSG0000(	505	11.27793	chr8:412(	ENSG00000287819		lncRNA	chr8:113432224-113
ENSG0000(	505	11.27793	chr12:171	TARBP2		protein_c	chr12:53500921-535
ENSG0000(	505	11.27793	chr8:412(	ENSG00000225885		lncRNA	chr8:118282139-118
ENSG0000(	505	11.27793	chr12:171	ZNF740		protein_c	chr12:53180704-531
ENSG0000(	505	11.27793	chr12:171	ANKRD52		protein_c	chr12:56237807-562
ENSG0000(	505	11.27793	chr12:171	ESYT1		protein_c	chr12:56118250-561
ENSG0000(	505	11.27793	chr12:171	MYG1		protein_c	chr12:53299695-533
ENSG0000(	505	11.27793	chr12:171	GPR84		protein_c	chr12:54362445-543
ENSG0000(	505	11.27793	chr12:171	CSAD		protein_c	chr12:53157663-531
ENSG0000(	505	11.27793	chr12:171	NPFF		protein_c	chr12:53506688-535
ENSG0000(	505	11.27793	chr12:171	NABP2		protein_c	chr12:56222015-562
ENSG0000(	505	11.27793	chr12:171	ITGB7	NCGv7	protein_c	chr12:53191323-532
ENSG0000(	505	11.27793	chr12:171	MAP3K12	NCGv7	protein_c	chr12:53479669-535
ENSG0000(	505	11.27793	chr12:171	SMARCC2		protein_c	chr12:56162359-561
ENSG0000(	505	11.27793	chr12:171	RPS26		protein_c	chr12:56041351-560

ENSG0000(	505	11.27793	chr8:412(	ENSG00000287826		lncRNA	chr8:116289622-116
ENSG0000(	505	11.27793	chr12:171	HOXC13	NCGv7;AC	protein_c	chr12:53938831-539
ENSG0000(	505	11.27793	chr12:171	ENSG00000257475		lncRNA	chr12:53012104-530
ENSG0000(	505	11.27793	chr12:171	PDE1B		protein_c	chr12:54549601-545
ENSG0000(	505	11.27793	chr12:171	ORMDL2		protein_c	chr12:55818041-558
ENSG0000(	505	11.27793	chr12:171	ENSG00000257404		Pseudoger	chr12:52782650-527
ENSG0000(	505	11.27793	chr12:171	PFDN5		protein_c	chr12:53295291-532
ENSG0000(	505	11.27793	chr8:412(	MAPK6P5		Pseudoger	chr8:109470131-109
ENSG0000(	505	11.27793	chr12:171	MMP19		protein_c	chr12:55835433-558
ENSG0000(	505	11.27793	chr12:171	NCKAP1L		protein_c	chr12:54497752-545
ENSG0000(	505	11.27793	chr12:171	NEUROD4		protein_c	chr12:55019974-550
ENSG0000(	505	11.27793	chr8:412(	ENSG00000254037		Pseudoger	chr8:119480279-119
ENSG0000(	505	11.27793	chr12:171	GSTP1P1		Pseudoger	chr12:55900300-559
ENSG0000(	505	11.27793	chr12:171	GLYCAM1		Pseudoger	chr12:54608187-546
ENSG0000(	505	11.27793	chr12:171	ENSG00000257411		protein_c	chr12:56101331-561
ENSG0000(	505	11.27793	chr12:171	MYL6B-AS1		lncRNA	chr12:56150796-561
ENSG0000(	505	11.27793	chr8:412(	snoU13		smallRNA	chr8:112563875-112
ENSG0000(	505	11.27793	chr12:171	HOXC6	NCGv7	protein_c	chr12:53990624-540
ENSG0000(	505	11.27793	chr8:412(	MIR3610		smallRNA	chr8:116874728-116
ENSG0000(	505	11.27793	chr12:171	LINC02381		lncRNA	chr12:54126019-541
ENSG0000(	505	11.27793	chr12:171	OR10U1P		Pseudoger	chr12:55193882-551
ENSG0000(	505	11.27793	chr12:171	OR6C73P		Pseudoger	chr12:55396340-553
ENSG0000(	505	11.27793	chr8:412(	ENSG00000286766		lncRNA	chr8:107499773-107
ENSG0000(	505	11.27793	chr12:171	ENSG00000257808		lncRNA	chr12:53159586-531
ENSG0000(	505	11.27793	chr12:171	HOXC-AS3		lncRNA	chr12:53981509-539
ENSG0000(	505	11.27793	chr12:171	MYG1-AS1		lncRNA	chr12:53298655-533
ENSG0000(	505	11.27793	chr12:171	ENSG00000273973		lncRNA	chr12:55929170-559
ENSG0000(	505	11.27793	chr6:103(	snoU13		smallRNA	chr6:37251204-3725
ENSG0000(	505	11.27793	chr12:171	CNPY2		protein_c	chr12:56309842-563
ENSG0000(	505	11.27793	chr8:412(	ENSG00000286282		lncRNA	chr8:119350291-119
ENSG0000(	505	11.27793	chr8:412(	ENSG00000286308		lncRNA	chr8:117265272-117
ENSG0000(	505	11.27793	chr12:171	CNPY2-AS1		lncRNA	chr12:56300075-563
ENSG0000(	505	11.27793	chr12:171	HOXC9		protein_c	chr12:53994895-540
ENSG0000(	505	11.27793	chr8:412(	RPS17P14		Pseudoger	chr8:108378831-108
ENSG0000(	505	11.27793	chr12:171	ENSG00000273890		lncRNA	chr12:56010091-560
ENSG0000(	505	11.27793	chr12:171	HNRNPA1	AC	protein_c	chr12:54280193-542
ENSG0000(	505	11.27793	chr12:171	ENSG00000257534		lncRNA	chr12:54162065-541
ENSG0000(	505	11.27793	chr12:171	ENSG00000257550		lncRNA	chr12:53513891-535
ENSG0000(	505	11.27793	chr12:171	HOXC-AS2		lncRNA	chr12:53993810-539
ENSG0000(	505	11.27793	chr8:412(	EBAG9		protein_c	chr8:109539711-109
ENSG0000(	505	11.27793	chr8:412(	UTP23	DriverDBv	protein_c	chr8:116766505-116
ENSG0000(	505	11.27793	chr12:171	PAN2		protein_c	chr12:56316223-563
ENSG0000(	505	11.27793	chr12:171	ENSG00000250654		Pseudoger	chr12:54079267-540
ENSG0000(	505	11.27793	chr8:412(	MAL2-AS1		lncRNA	chr8:119214625-119
ENSG0000(	505	11.27793	chr12:171	BLOC1S1		protein_c	chr12:55716038-557
ENSG0000(	505	11.27793	chr12:171	BTBD10P1		Pseudoger	chr12:52748776-527
ENSG0000(	505	11.27793	chr8:412(	ENSG00000287208		lncRNA	chr8:107186997-107
ENSG0000(	505	11.27793	chr8:412(	EXT1	NCGv7;AC	protein_c	chr8:117794490-118
ENSG0000(	505	11.27793	chr12:171	GDF11		protein_c	chr12:55743122-557
ENSG0000(	505	11.27793	chr12:171	ENSG00000257553		lncRNA	chr12:56104614-561
ENSG0000(	505	11.27793	chr12:171	RAB5B		protein_c	chr12:55973913-559

ENSG0000(	505	11.27793	chr12:171	ITGA7		protein_c	chr12:55684568-557
ENSG0000(	505	11.27793	chr8:412(	ENSG00000255099		Pseudoger	chr8:109777912-109
ENSG0000(	505	11.27793	chr12:171	RDH5		protein_c	chr12:55720367-557
ENSG0000(	505	11.27793	chr12:171	COQ10A		protein_c	chr12:56266890-562
ENSG0000(	505	11.27793	chr8:412(	SYBU-AS1		lncRNA	chr8:109644115-109
ENSG0000(	505	11.27793	chr8:412(	ENSG00000254339		lncRNA	chr8:114282037-114
ENSG0000(	505	11.27793	chr8:412(	RSPO2	NCGv7	protein_c	chr8:107899316-108
ENSG0000(	505	11.27793	chr8:412(	MAL2	DriverDB\	protein_c	chr8:119165034-119
ENSG0000(	505	11.27793	chr12:171	PHC1P1		Pseudoger	chr12:55411727-554
ENSG0000(	505	11.27793	chr8:412(	EIF3H		protein_c	chr8:116642130-116
ENSG0000(	505	11.27793	chr12:171	PPP1R1A		protein_c	chr12:54575387-545
ENSG0000(	505	11.27793	chr8:412(	RPL30P16		Pseudoger	chr8:112538889-112
ENSG0000(	505	11.27793	chr12:171	TESPA1		protein_c	chr12:54948015-549
ENSG0000(	505	11.27793	chr12:171	ESPL1	NCGv7;AC	protein_c	chr12:53268299-532
ENSG0000(	505	11.27793	chr12:171	OR10A7	NCGv7	protein_c	chr12:55221025-552
ENSG0000(	505	11.27793	chr12:171	PCBP2	NCGv7	protein_c	chr12:53452102-534
ENSG0000(	505	11.27793	chr6:103(	ENSG00000286672		lncRNA	chr6:37301210-3730
ENSG0000(	505	11.27793	chr6:103(	RNF8		protein_c	chr6:37353979-3739
ENSG0000(	505	11.27793	chr8:412(	ENSG00000287289		lncRNA	chr8:107160354-107
ENSG0000(	505	11.27793	chr8:412(	SYBU		protein_c	chr8:109573978-109
ENSG0000(	505	11.27793	chr12:171	ENSG00000257870		lncRNA	chr12:55009746-550
ENSG0000(	505	11.27793	chr12:171	COPZ1		protein_c	chr12:54301202-543
ENSG0000(	505	11.27793	chr8:412(	EEF1A1P37		Pseudoger	chr8:111225585-111
ENSG0000(	504	11.2556	chr17:42(	MIR196A1		smallRNA	chr17:48632490-486
ENSG0000(	504	11.2556	chr10:81(	Y_RNA		smallRNA	chr10:32056746-320
ENSG0000(	503	11.23327	chr6:1391	ENSG00000285642		lncRNA	chr6:158000109-158
ENSG0000(	503	11.23327	chr6:1391	SYNJ2-IT1		lncRNA	chr6:158001107-158
ENSG0000(	503	11.23327	chr6:1391	HSPE1P26		Pseudoger	chr6:157924402-157
ENSG0000(	503	11.23327	chr6:1391	RNU6-786P		smallRNA	chr6:157934168-157
ENSG0000(	503	11.23327	chr6:1391	SYNJ2		protein_c	chr6:157981863-158
ENSG0000(	502	11.21094	chr3:380(	BRK1		protein_c	chr3:10115675-1012
ENSG0000(	502	11.21094	chr3:380(	SRGAP3-AS4		lncRNA	chr3:9249742-92575
ENSG0000(	502	11.21094	chr3:380(	ENSG00000287086		lncRNA	chr3:10149986-1015
ENSG0000(	502	11.21094	chr3:380(	EMC3		protein_c	chr3:9962682-10011
ENSG0000(	502	11.21094	chr3:380(	AC018506.1		smallRNA	chr3:9426257-94263
ENSG0000(	502	11.21094	chr3:380(	snoU13		smallRNA	chr3:10141150-1014
ENSG0000(	502	11.21094	chr3:380(	ENSG00000228351		lncRNA	chr3:8079322-81255
ENSG0000(	502	11.21094	chr3:380(	ENSG00000288550		protein_c	chr3:9917116-99453
ENSG0000(	502	11.21094	chr3:380(	IL17RC		protein_c	chr3:9917074-99336
ENSG0000(	502	11.21094	chr3:380(	RNU6-882P		smallRNA	chr3:9919051-99191
ENSG0000(	502	11.21094	chr3:380(	IL17RE		protein_c	chr3:9902612-99164
ENSG0000(	502	11.21094	chr3:380(	OGG1		protein_c	chr3:9749944-97882
ENSG0000(	502	11.21094	chr3:380(	ENSG00000286962		lncRNA	chr3:10914196-1097
ENSG0000(	502	11.21094	chr3:380(	ENSG00000269886		lncRNA	chr3:9812762-98130
ENSG0000(	502	11.21094	chr3:380(	GHRLOS		lncRNA	chr3:10285754-1029
ENSG0000(	502	11.21094	chr3:380(	TTLL3		protein_c	chr3:9808086-98551
ENSG0000(	502	11.21094	chr3:380(	PGAM1P4		Pseudoger	chr3:9348443-93491
ENSG0000(	502	11.21094	chr3:380(	ARPC4-TTLL3		protein_c	chr3:9793082-98354
ENSG0000(	502	11.21094	chr3:380(	OXTR		protein_c	chr3:8750381-87696
ENSG0000(	502	11.21094	chr3:380(	ENSG00000287623		lncRNA	chr3:9100803-91040
ENSG0000(	502	11.21094	chr3:380(	ENSG00000291007		lncRNA	chr3:8682943-86937

ENSG0000(	502	11.21094	chr3:380⁴	CRELD1		protein_c	chr3:9933793-99454
ENSG0000(	502	11.21094	chr3:380⁴	PRRT3	NCGv7	protein_c	chr3:9945542-99524
ENSG0000(	502	11.21094	chr3:380⁴	MTMR14		protein_c	chr3:9649433-97023
ENSG0000(	502	11.21094	chr3:380⁴	ENSG00000269982		lncRNA	chr3:9958717-99625
ENSG0000(	502	11.21094	chr3:380⁴	ENSG00000254485		lncRNA	chr3:9292588-93633
ENSG0000(	502	11.21094	chr3:380⁴	ENSG00000285906		lncRNA	chr3:11193439-1122
ENSG0000(	502	11.21094	chr3:380⁴	FANCD2OS		protein_c	chr3:10081317-1010
ENSG0000(	502	11.21094	chr3:380⁴	ENSG00000291078		lncRNA	chr3:10014235-1002
ENSG0000(	502	11.21094	chr3:380⁴	ENSG00000230599		lncRNA	chr3:10767464-1077
ENSG0000(	502	11.21094	chr3:380⁴	CIDECP1		Pseudoger	chr3:10017490-1002
ENSG0000(	502	11.21094	chr3:380⁴	DUSP5P2		Pseudoger	chr3:9600748-96027
ENSG0000(	502	11.21094	chr3:380⁴	TADA3		protein_c	chr3:9779967-97930
ENSG0000(	502	11.21094	chr3:380⁴	LMCD1-AS1		lncRNA	chr3:7951263-86119
ENSG0000(	502	11.21094	chr3:380⁴	AC034187.3		smallRNA	chr3:8700011-87000
ENSG0000(	502	11.21094	chr3:380⁴	ENSG00000287878		lncRNA	chr3:9693182-97213
ENSG0000(	502	11.21094	chr3:380⁴	ARPC4		protein_c	chr3:9792495-98071
ENSG0000(	502	11.21094	chr3:380⁴	PRRT3-AS1		lncRNA	chr3:9947404-99547
ENSG0000(	502	11.21094	chr3:380⁴	ENSG00000272410		protein_c	chr3:10249372-1028
ENSG0000(	502	11.21094	chr3:380⁴	RNU4ATAC17P		smallRNA	chr3:8364529-83646
ENSG0000(	502	11.21094	chr3:380⁴	SLC6A1-AS1		lncRNA	chr3:11006098-1101
ENSG0000(	502	11.21094	chr3:380⁴	JAGN1		protein_c	chr3:9890574-98943
ENSG0000(	502	11.21094	chr3:380⁴	ENSG00000289763		protein_c	chr3:10249042-1024
ENSG0000(	502	11.21094	chr3:380⁴	FANCD2	NCGv7;AC	protein_c	chr3:10026370-1010
ENSG0000(	502	11.21094	chr3:380⁴	CPNE9		protein_c	chr3:9703826-97299
ENSG0000(	502	11.21094	chr3:380⁴	ENSG00000287964		lncRNA	chr3:11060973-1109
ENSG0000(	502	11.21094	chr3:380⁴	HRH1		protein_c	chr3:11137093-1126
ENSG0000(	502	11.21094	chr3:380⁴	MIR378B		smallRNA	chr3:10330229-1033
ENSG0000(	502	11.21094	chr3:380⁴	snoU13		smallRNA	chr3:10133385-1013
ENSG0000(	502	11.21094	chr3:380⁴	SNORA43		smallRNA	chr3:8931495-89316
ENSG0000(	502	11.21094	chr3:380⁴	SRGAP3-AS2		lncRNA	chr3:9192493-91944
ENSG0000(	502	11.21094	chr3:380⁴	CYCSP10		Pseudoger	chr3:10000647-1000
ENSG0000(	502	11.21094	chr3:380⁴	AC034187.1		smallRNA	chr3:8696049-86961
ENSG0000(	502	11.21094	chr3:380⁴	GHRL		protein_c	chr3:10285666-1029
ENSG0000(	502	11.21094	chr3:380⁴	AC090841.1		smallRNA	chr3:10481963-1048
ENSG0000(	502	11.21094	chr3:380⁴	AC034187.4		smallRNA	chr3:8695314-86953
ENSG0000(	502	11.21094	chr3:380⁴	TATDN2		protein_c	chr3:10248023-1028
ENSG0000(	502	11.21094	chr3:380⁴	IRAK2		protein_c	chr3:10164919-1024
ENSG0000(	502	11.21094	chr3:380⁴	SEC13		protein_c	chr3:10293131-1032
ENSG0000(	502	11.21094	chr3:380⁴	ENSG00000224884		lncRNA	chr3:8573726-85931
ENSG0000(	502	11.21094	chr3:380⁴	ATP2B2		protein_c	chr3:10324023-1070
ENSG0000(	502	11.21094	chr3:380⁴	hsa-mir-548ba		smallRNA	chr3:8771191-87712
ENSG0000(	502	11.21094	chr3:380⁴	SLC6A1		protein_c	chr3:10992186-1103
ENSG0000(	502	11.21094	chr3:380⁴	ATP2B2-IT1		lncRNA	chr3:10566255-1057
ENSG0000(	502	11.21094	chr3:380⁴	AC034187.5		smallRNA	chr3:8696177-86962
ENSG0000(	502	11.21094	chr3:380⁴	SLC6A11	NCGv7	protein_c	chr3:10816201-1094
ENSG0000(	502	11.21094	chr3:380⁴	ENSG00000231401		lncRNA	chr3:8359344-83664
ENSG0000(	502	11.21094	chr3:380⁴	THUMPD3-AS1		lncRNA	chr3:9349689-93985
ENSG0000(	502	11.21094	chr3:380⁴	MIR885		smallRNA	chr3:10394489-1039
ENSG0000(	502	11.21094	chr3:380⁴	EMC3-AS1		Pseudoger	chr3:9987904-99943
ENSG0000(	502	11.21094	chr3:380⁴	ENSG00000206567		lncRNA	chr3:10004048-1001
ENSG0000(	502	11.21094	chr3:380⁴	SRGAP3-AS3		lncRNA	chr3:9216895-92195

ENSG0000(	502	11.21094	chr3:380⁄	CIDEC		protein_c	chr3:9866711-98802
ENSG0000(	502	11.21094	chr3:380⁄	VHL	NCGv7;AC	protein_c	chr3:10141778-1015
ENSG0000(	502	11.21094	chr3:380⁄	THUMPD3		protein_c	chr3:9362971-93867
ENSG0000(	502	11.21094	chr3:380⁄	LINC00606		lncRNA	chr3:10759350-1076
ENSG0000(	502	11.21094	chr3:380⁄	OR7E122P		Pseudoger	chr3:8688143-86891
ENSG0000(	502	11.21094	chr3:380⁄	RNU6-670P		smallRNA	chr3:10037552-1003
ENSG0000(	502	11.21094	chr3:380⁄	SETD5		protein_c	chr3:9397615-94792
ENSG0000(	502	11.21094	chr3:380⁄	SRGAP3-AS1		lncRNA	chr3:9014123-90159
ENSG0000(	502	11.21094	chr3:380⁄	SSUH2		protein_c	chr3:8619386-87450
ENSG0000(	502	11.21094	chr3:380⁄	ENSG00000288969		lncRNA	chr3:11183856-1118
ENSG0000(	502	11.21094	chr3:380⁄	CAMK1		protein_c	chr3:9757347-97699
ENSG0000(	502	11.21094	chr3:380⁄	RAD18		protein_c	chr3:8775402-89637
ENSG0000(	502	11.21094	chr3:380⁄	LINC00852		lncRNA	chr3:10284419-1028
ENSG0000(	502	11.21094	chr3:380⁄	LHFPL4		protein_c	chr3:9498361-95538
ENSG0000(	502	11.21094	chr3:380⁄	SRGAP3	IntOGen-I	protein_c	chr3:8980591-93630
ENSG0000(	502	11.21094	chr3:380⁄	RPUSD3		protein_c	chr3:9837849-98446
ENSG0000(	502	11.21094	chr3:380⁄	BRPF1	NCGv7	protein_c	chr3:9731729-97480
ENSG0000(	502	11.21094	chr3:380⁄	ATP2B2-IT2		lncRNA	chr3:10626015-1062
ENSG0000(	502	11.21094	chr3:380⁄	ENSG00000291224		lncRNA	chr3:9986885-10006
ENSG0000(	502	11.21094	chr3:380⁄	RNU6-814P		smallRNA	chr3:10217212-1021
ENSG0000(	502	11.21094	chr3:380⁄	CAV3		protein_c	chr3:8733802-88418
ENSG0000(	502	11.21094	chr3:380⁄	AC069276.1		smallRNA	chr3:8006477-80065
ENSG0000(	502	11.21094	chr3:380⁄	CYCSP11		Pseudoger	chr3:10057973-1005
ENSG0000(	502	11.21094	chr3:380⁄	LMCD1	NCGv7	protein_c	chr3:8501807-85746
ENSG0000(	500	11.16627	chr8:412(	ZFPM2	NCGv7	protein_c	chr8:104590733-105
ENSG0000(	500	11.16627	chr8:412(	GAPDHP62		Pseudoger	chr8:100550724-100
ENSG0000(	500	11.16627	chr8:412(	Y_RNA		smallRNA	chr8:100185138-100
ENSG0000(	500	11.16627	chr8:412(	ENSG00000253420		lncRNA	chr8:105662352-105
ENSG0000(	500	11.16627	chr8:412(	ZFPM2-AS1		lncRNA	chr8:105546089-106
ENSG0000(	500	11.16627	chr8:412(	MAILR		lncRNA	chr8:102864271-103
ENSG0000(	500	11.16627	chr8:412(	ENSG00000289653		protein_c	chr8:102858046-102
ENSG0000(	500	11.16627	chr8:412(	ANKRD46	DriverDB\	protein_c	chr8:100509752-100
ENSG0000(	500	11.16627	chr8:412(	ENSG00000254492		Pseudoger	chr8:102154550-102
ENSG0000(	500	11.16627	chr8:412(	RN7SKP85		smallRNA	chr8:98921233-9892
ENSG0000(	500	11.16627	chr8:412(	AP003471.3		smallRNA	chr8:104435453-104
ENSG0000(	500	11.16627	chr8:412(	snoU13		smallRNA	chr8:103665295-103
ENSG0000(	500	11.16627	chr8:412(	ENSG00000253923		Pseudoger	chr8:102417979-102
ENSG0000(	500	11.16627	chr8:412(	ENSG00000253912		Pseudoger	chr8:99804159-9980
ENSG0000(	500	11.16627	chr8:412(	MIR548A3		smallRNA	chr8:104484369-104
ENSG0000(	500	11.16627	chr8:412(	ENSG00000253526		lncRNA	chr8:105826570-105
ENSG0000(	500	11.16627	chr8:412(	ENSG00000253562		lncRNA	chr8:99091738-9909
ENSG0000(	500	11.16627	chr8:412(	ENSG00000236583		Pseudoger	chr8:99233048-9923
ENSG0000(	500	11.16627	chr8:412(	RPS20P23		Pseudoger	chr8:100776512-100
ENSG0000(	500	11.16627	chr8:412(	RNU7-177P		smallRNA	chr8:97773978-9777
ENSG0000(	500	11.16627	chr8:412(	ENSG00000260368		lncRNA	chr8:100913247-100
ENSG0000(	500	11.16627	chr8:412(	AP001205.1		smallRNA	chr8:100707878-100
ENSG0000(	500	11.16627	chr8:412(	ENSG00000283959		lncRNA	chr8:102656241-102
ENSG0000(	500	11.16627	chr8:412(	ENSG00000253395		lncRNA	chr8:101122145-101
ENSG0000(	500	11.16627	chr8:412(	MIR5680		smallRNA	chr8:102125432-102
ENSG0000(	500	11.16627	chr8:412(	AP003356.1		smallRNA	chr8:102733635-102
ENSG0000(	500	11.16627	chr8:412(	ENSG00000271830		lncRNA	chr8:103481266-103

ENSG0000(	500	11.16627	chr8:412(	MTCO1P4		Pseudoger	chr8:103088796-103
ENSG0000(	500	11.16627	chr8:412(	ENSG00000253942		Pseudoger	chr8:101223941-101
ENSG0000(	500	11.16627	chr8:412(	MIR4471		smallRNA	chr8:100382763-100
ENSG0000(	500	11.16627	chr8:412(	ENSG00000254236		lncRNA	chr8:103020187-103
ENSG0000(	500	11.16627	chr8:412(	ENSG00000254084		lncRNA	chr8:101411873-101
ENSG0000(	500	11.16627	chr8:412(	ENSG00000286337		lncRNA	chr8:103319392-103
ENSG0000(	500	11.16627	chr8:412(	OXR1	DriverDB\	protein_c	chr8:106270144-106
ENSG0000(	500	11.16627	chr8:412(	RPL19P14		Pseudoger	chr8:98782055-9878
ENSG0000(	500	11.16627	chr8:412(	MIR599		smallRNA	chr8:99536636-9953
ENSG0000(	500	11.16627	chr8:412(	ENSG00000254021		lncRNA	chr8:106265435-106
ENSG0000(	500	11.16627	chr8:412(	RNU6-1224P		smallRNA	chr8:102533241-102
ENSG0000(	500	11.16627	chr8:412(	ENSG00000254024		lncRNA	chr8:101461177-101
ENSG0000(	500	11.16627	chr8:412(	AP003550.1		smallRNA	chr8:103092624-103
ENSG0000(	500	11.16627	chr8:412(	ENSG00000253539		lncRNA	chr8:99796615-9979
ENSG0000(	500	11.16627	chr8:412(	RRM2B	DriverDB\	protein_c	chr8:102204502-102
ENSG0000(	500	11.16627	chr8:412(	ENSG00000287479		lncRNA	chr8:101275425-101
ENSG0000(	500	11.16627	chr8:412(	UFM1P3		Pseudoger	chr8:100165226-100
ENSG0000(	500	11.16627	chr8:412(	ENSG00000254364		lncRNA	chr8:100492528-100
ENSG0000(	500	11.16627	chr8:412(	ENSG00000243171		Pseudoger	chr8:105287830-105
ENSG0000(	500	11.16627	chr8:412(	Y_RNA		smallRNA	chr8:99527826-9952
ENSG0000(	500	11.16627	chr8:412(	ENSG00000253629		lncRNA	chr8:101686547-101
ENSG0000(	500	11.16627	chr8:412(	BAALC-AS2		lncRNA	chr8:103132959-103
ENSG0000(	500	11.16627	chr8:412(	RPL5P24		Pseudoger	chr8:102923371-102
ENSG0000(	500	11.16627	chr8:412(	RNU6-914P		smallRNA	chr8:98192205-9819
ENSG0000(	500	11.16627	chr8:412(	ENSG00000253415		Pseudoger	chr8:98904603-9890
ENSG0000(	500	11.16627	chr12:171	RNA5SP358		Pseudoger	chr12:38161466-381
ENSG0000(	500	11.16627	chr8:412(	snoU13		smallRNA	chr8:101847256-101
ENSG0000(	500	11.16627	chr8:412(	ENSG00000253532		lncRNA	chr8:99119352-9912
ENSG0000(	500	11.16627	chr8:412(	ENSG00000278914		TEC	chr8:98391401-9839
ENSG0000(	500	11.16627	chr8:412(	ENSG00000254112		Pseudoger	chr8:98633547-9863
ENSG0000(	500	11.16627	chr8:412(	RPS26P6		Pseudoger	chr8:100895771-100
ENSG0000(	500	11.16627	chr8:412(	ENSG00000260640		lncRNA	chr8:97241742-9724
ENSG0000(	500	11.16627	chr8:412(	DCSTAMP	NCGv7	protein_c	chr8:104339087-104
ENSG0000(	500	11.16627	chr8:412(	VPS13B-DT		lncRNA	chr8:98957568-9901
ENSG0000(	500	11.16627	chr8:412(	DCAF13	DriverDB\	protein_c	chr8:103414714-103
ENSG0000(	500	11.16627	chr8:412(	SLC25A32		protein_c	chr8:103398635-103
ENSG0000(	500	11.16627	chr8:412(	CTHRC1		protein_c	chr8:103371538-103
ENSG0000(	500	11.16627	chr8:412(	ENSG00000270861		Pseudoger	chr8:97853021-9785
ENSG0000(	500	11.16627	chr8:412(	OXR1-AS1		lncRNA	chr8:106520474-106
ENSG0000(	500	11.16627	chr8:412(	FZD6	DriverDB\	protein_c	chr8:103298433-103
ENSG0000(	500	11.16627	chr8:412(	COX6C	DriverDB\	protein_c	chr8:99873200-9989
ENSG0000(	500	11.16627	chr8:412(	HSPE1P14		Pseudoger	chr8:102451763-102
ENSG0000(	500	11.16627	chr8:412(	MTCO2P4		Pseudoger	chr8:103091263-103
ENSG0000(	500	11.16627	chr8:412(	ENSG00000271882		lncRNA	chr8:101208148-101
ENSG0000(	500	11.16627	chr8:412(	SUMO2P19		Pseudoger	chr8:102242040-102
ENSG0000(	500	11.16627	chr8:412(	BAALC-AS1		lncRNA	chr8:103153394-103
ENSG0000(	500	11.16627	chr8:412(	ZNF706	DriverDB\	protein_c	chr8:101177878-101
ENSG0000(	500	11.16627	chr8:412(	BAALC	AC	protein_c	chr8:103140713-103
ENSG0000(	500	11.16627	chr8:412(	YWHAZ	DriverDB\	protein_c	chr8:100916523-100
ENSG0000(	500	11.16627	chr8:412(	SNORD77		smallRNA	chr8:100004583-100
ENSG0000(	500	11.16627	chr8:412(	OSR2	NCGv7	protein_c	chr8:98944403-9895

ENSG0000(	500	11.16627	chr8:412(	MIR3151		smallRNA	chr8:103154614-103
ENSG0000(	500	11.16627	chr8:412(	AP001572.1		smallRNA	chr8:103851599-103
ENSG0000(	500	11.16627	chr8:412(	ENSG00000253997		Pseudoger	chr8:104990308-104
ENSG0000(	500	11.16627	chr8:412(	KCNS2		protein_c	chr8:98426958-9843
ENSG0000(	500	11.16627	chr8:412(	MATN2		protein_c	chr8:97868840-9803
ENSG0000(	500	11.16627	chr8:412(	RGS22		protein_c	chr8:99960936-1001
ENSG0000(	500	11.16627	chr8:412(	VPS13B	NCGv7	protein_c	chr8:99013266-9987
ENSG0000(	500	11.16627	chr8:412(	POLR2K	DriverDB\	protein_c	chr8:100150623-100
ENSG0000(	500	11.16627	chr8:412(	RPL30	DriverDB\	protein_c	chr8:98024851-9804
ENSG0000(	500	11.16627	chr8:412(	RIDA	DriverDB\	protein_c	chr8:98102344-9811
ENSG0000(	500	11.16627	chr8:412(	FBXO43	DriverDB\	protein_c	chr8:100133351-100
ENSG0000(	500	11.16627	chr8:412(	RNU7-67P		smallRNA	chr8:101307367-101
ENSG0000(	500	11.16627	chr8:412(	ENSG00000289048		lncRNA	chr8:101491259-101
ENSG0000(	500	11.16627	chr8:412(	MTND1P5		Pseudoger	chr8:103085651-103
ENSG0000(	500	11.16627	chr8:412(	ENSG00000272037		lncRNA	chr8:102256392-102
ENSG0000(	500	11.16627	chr8:412(	SNX31	NCGv7	protein_c	chr8:100572889-100
ENSG0000(	500	11.16627	chr8:412(	MIR1273A		smallRNA	chr8:100023982-100
ENSG0000(	500	11.16627	chr8:412(	UBR5-DT		lncRNA	chr8:102239386-102
ENSG0000(	500	11.16627	chr8:412(	PTMAP15		Pseudoger	chr8:103559099-103
ENSG0000(	500	11.16627	chr8:412(	ENSG00000288752		lncRNA	chr8:98045653-9804
ENSG0000(	500	11.16627	chr8:412(	ENSG00000288945		lncRNA	chr8:103451131-103
ENSG0000(	500	11.16627	chr8:412(	ENSG00000239344		Pseudoger	chr8:103768281-103
ENSG0000(	500	11.16627	chr8:412(	Y_RNA		smallRNA	chr8:100158868-100
ENSG0000(	500	11.16627	chr8:412(	RPL12P24		Pseudoger	chr8:105737280-105
ENSG0000(	500	11.16627	chr8:412(	ENSG00000253282		lncRNA	chr8:101287445-101
ENSG0000(	500	11.16627	chr8:412(	SPAG1	DriverDB\	protein_c	chr8:100157906-100
ENSG0000(	500	11.16627	chr8:412(	RNU6ATAC41P		smallRNA	chr8:100739802-100
ENSG0000(	500	11.16627	chr8:412(	U3		smallRNA	chr8:97358265-9735
ENSG0000(	500	11.16627	chr8:412(	ENSG00000254041		lncRNA	chr8:105142860-105
ENSG0000(	500	11.16627	chr8:412(	ENSG00000253217		lncRNA	chr8:100337595-100
ENSG0000(	500	11.16627	chr8:412(	NCALD		protein_c	chr8:101686542-102
ENSG0000(	500	11.16627	chr8:412(	UBR5	NCGv7;AC	protein_c	chr8:102252273-102
ENSG0000(	500	11.16627	chr8:412(	ENSG00000237704		Pseudoger	chr8:99340305-9934
ENSG0000(	500	11.16627	chr8:412(	ENSG00000253740		lncRNA	chr8:100618581-100
ENSG0000(	500	11.16627	chr8:412(	POU5F1P2		Pseudoger	chr8:102621509-102
ENSG0000(	500	11.16627	chr8:412(	AC104986.1		smallRNA	chr8:98999284-9899
ENSG0000(	500	11.16627	chr8:412(	Y_RNA		smallRNA	chr8:100755101-100
ENSG0000(	500	11.16627	chr8:412(	ENSG00000254141		lncRNA	chr8:105669991-105
ENSG0000(	500	11.16627	chr8:412(	DUXAP2		Pseudoger	chr8:101366417-101
ENSG0000(	500	11.16627	chr8:412(	RN7SL563P		smallRNA	chr8:101397635-101
ENSG0000(	500	11.16627	chr8:412(	MTDH	DriverDB\	protein_c	chr8:97644184-9773
ENSG0000(	500	11.16627	chr8:412(	AC012564.1		smallRNA	chr8:104755106-104
ENSG0000(	500	11.16627	chr8:412(	DPYS	NCGv7	protein_c	chr8:104330324-104
ENSG0000(	500	11.16627	chr8:412(	ENSG00000261670		lncRNA	chr8:103464389-103
ENSG0000(	500	11.16627	chr8:412(	ENSG00000253468		Pseudoger	chr8:98412882-9841
ENSG0000(	500	11.16627	chr8:412(	ENSG00000253851		lncRNA	chr8:103228425-103
ENSG0000(	500	11.16627	chr8:412(	LINC01181		lncRNA	chr8:103121032-103
ENSG0000(	500	11.16627	chr8:412(	ENSG00000253842		lncRNA	chr8:100427559-100
ENSG0000(	500	11.16627	chr8:412(	Y_RNA		smallRNA	chr8:102950467-102
ENSG0000(	500	11.16627	chr8:412(	ENSG00000229625		lncRNA	chr8:98961428-9896
ENSG0000(	500	11.16627	chr8:412(	ENSG00000253180		Pseudoger	chr8:99003407-9900

ENSG0000(	500	11.16627	chr8:412(	LINC02933		lncRNA	chr8:103383078-103
ENSG0000(	500	11.16627	chr8:412(	MIR875		smallRNA	chr8:99536786-9953
ENSG0000(	500	11.16627	chr8:412(	ENSG00000253477		lncRNA	chr8:103483398-103
ENSG0000(	500	11.16627	chr8:412(	ENSG00000253355		lncRNA	chr8:101128987-101
ENSG0000(	500	11.16627	chr8:412(	RPS23P1		Pseudoger	chr8:97865054-9786
ENSG0000(	500	11.16627	chr8:412(	ENSG00000225017		Pseudoger	chr8:99346052-9934
ENSG0000(	500	11.16627	chr8:412(	RN7SL350P		smallRNA	chr8:99613786-9961
ENSG0000(	500	11.16627	chr8:412(	PDCL3P2		Pseudoger	chr8:101871830-101
ENSG0000(	500	11.16627	chr8:412(	PABPC1	NCGv7	protein_c	chr8:100685816-100
ENSG0000(	500	11.16627	chr8:412(	ENSG00000253824		lncRNA	chr8:100380486-100
ENSG0000(	500	11.16627	chr8:412(	TSPYL5	DriverDB\	protein_c	chr8:97273488-9727
ENSG0000(	500	11.16627	chr8:412(	ENSG00000253153		Pseudoger	chr8:101562008-101
ENSG0000(	500	11.16627	chr8:412(	AP003471.2		smallRNA	chr8:104425359-104
ENSG0000(	500	11.16627	chr8:412(	snoU13		smallRNA	chr8:102388526-102
ENSG0000(	500	11.16627	chr8:412(	LRP12		protein_c	chr8:104489231-104
ENSG0000(	500	11.16627	chr8:412(	ENSG00000243939		Pseudoger	chr8:104981164-104
ENSG0000(	500	11.16627	chr8:412(	ENSG00000287654		lncRNA	chr8:97643366-9764
ENSG0000(	500	11.16627	chr8:412(	ENSG00000253991		Pseudoger	chr8:101528723-101
ENSG0000(	500	11.16627	chr8:412(	ENSG00000285982		protein_c	chr8:103398658-103
ENSG0000(	500	11.16627	chr8:412(	SNORA72		smallRNA	chr8:98042086-9804
ENSG0000(	500	11.16627	chr8:412(	ENSG00000235683		Pseudoger	chr8:99695957-9969
ENSG0000(	500	11.16627	chr8:412(	RNU4-83P		smallRNA	chr8:100831728-100
ENSG0000(	500	11.16627	chr8:412(	TMCC1P1		Pseudoger	chr8:105194990-105
ENSG0000(	500	11.16627	chr8:412(	ENSG00000179577		lncRNA	chr8:104419512-104
ENSG0000(	500	11.16627	chr8:412(	ENSG00000240271		Pseudoger	chr8:105011107-105
ENSG0000(	500	11.16627	chr8:412(	ADI1P2		Pseudoger	chr8:102688317-102
ENSG0000(	500	11.16627	chr8:412(	SLC16A14P1		Pseudoger	chr8:106215763-106
ENSG0000(	500	11.16627	chr8:412(	RNF19A	DriverDB\	protein_c	chr8:100257060-100
ENSG0000(	500	11.16627	chr8:412(	ZNNT1		lncRNA	chr8:101166805-101
ENSG0000(	500	11.16627	chr8:412(	RPL30-AS1		lncRNA	chr8:98041726-9804
ENSG0000(	500	11.16627	chr8:412(	ODF1		protein_c	chr8:102551589-102
ENSG0000(	500	11.16627	chr8:412(	Y_RNA		smallRNA	chr8:105929126-105
ENSG0000(	500	11.16627	chr8:412(	AP003354.1		smallRNA	chr8:102960813-102
ENSG0000(	500	11.16627	chr8:412(	ENSG00000253385		lncRNA	chr8:102854455-102
ENSG0000(	500	11.16627	chr8:412(	RN7SKP249		smallRNA	chr8:101137959-101
ENSG0000(	500	11.16627	chr8:412(	FLJ42969		lncRNA	chr8:101034868-101
ENSG0000(	500	11.16627	chr8:412(	Y_RNA		smallRNA	chr8:100962935-100
ENSG0000(	500	11.16627	chr8:412(	ENSG00000289284		lncRNA	chr8:100688004-100
ENSG0000(	500	11.16627	chr8:412(	ENSG00000272321		lncRNA	chr8:98436669-9843
ENSG0000(	500	11.16627	chr8:412(	ENSG00000253911		lncRNA	chr8:98603253-9860
ENSG0000(	500	11.16627	chr8:412(	ENSG00000271930		lncRNA	chr8:98943595-9894
ENSG0000(	500	11.16627	chr8:412(	RNU6-1092P		smallRNA	chr8:100683488-100
ENSG0000(	500	11.16627	chr8:412(	RNU6-748P		smallRNA	chr8:98462944-9846
ENSG0000(	500	11.16627	chr8:412(	ENSG00000271560		Pseudoger	chr8:101337496-101
ENSG0000(	500	11.16627	chr8:412(	LINC02844		lncRNA	chr8:101261541-101
ENSG0000(	500	11.16627	chr8:412(	ENSG00000254644		Pseudoger	chr8:103568603-103
ENSG0000(	500	11.16627	chr8:412(	RIMS2	NCGv7	protein_c	chr8:103500610-104
ENSG0000(	500	11.16627	chr8:412(	GASAL1		lncRNA	chr8:102805517-102
ENSG0000(	500	11.16627	chr8:412(	RNU6-703P		smallRNA	chr8:98048856-9804
ENSG0000(	500	11.16627	chr8:412(	ENSG00000253666		lncRNA	chr8:100475667-100
ENSG0000(	500	11.16627	chr8:412(	RNU6-1011P		smallRNA	chr8:103384961-103

ENSG0000(	500	11.16627	chr8:412(	KLF10		protein_c	chr8:102648784-102
ENSG0000(	500	11.16627	chr8:412(	ENSG00000253263		lncRNA	chr8:102891876-102
ENSG0000(	500	11.16627	chr8:412(	LAPTM4B	NCGv7;AC	protein_c	chr8:97775057-9785
ENSG0000(	500	11.16627	chr8:412(	ENSG00000279138		TEC	chr8:104478539-104
ENSG0000(	500	11.16627	chr8:412(	ENSG00000253633		lncRNA	chr8:102528740-102
ENSG0000(	500	11.16627	chr8:412(	SUMO2P18		Pseudoger	chr8:97858049-9785
ENSG0000(	500	11.16627	chr8:412(	MRPL57P7		Pseudoger	chr8:98906260-9890
ENSG0000(	500	11.16627	chr8:412(	ENSG00000253737		lncRNA	chr8:101139665-101
ENSG0000(	500	11.16627	chr8:412(	STK3		protein_c	chr8:98371228-9894
ENSG0000(	500	11.16627	chr8:412(	NDUFA5P2		Pseudoger	chr8:104566086-104
ENSG0000(	500	11.16627	chr8:412(	LINC02845		lncRNA	chr8:101387299-101
ENSG0000(	500	11.16627	chr8:412(	NIPAL2	DriverDB\	protein_c	chr8:98189826-9829
ENSG0000(	500	11.16627	chr8:412(	Y_RNA		smallRNA	chr8:97772313-9777
ENSG0000(	500	11.16627	chr8:412(	POP1		protein_c	chr8:98117293-9815
ENSG0000(	500	11.16627	chr8:412(	NPM1P52		Pseudoger	chr8:103016744-103
ENSG0000(	500	11.16627	chr8:412(	GRHL2	NCGv7	protein_c	chr8:101492439-101
ENSG0000(	500	11.16627	chr8:412(	AZIN1	NCGv7	protein_c	chr8:102826111-102
ENSG0000(	500	11.16627	chr8:412(	ATP6V1C1	DriverDB\	protein_c	chr8:103021063-103
ENSG0000(	500	11.16627	chr8:412(	ERICH5		protein_c	chr8:98064522-9809
ENSG0000(	500	11.16627	chr8:412(	ENSG00000283157		lncRNA	chr8:104699479-104
ENSG0000(	500	11.16627	chr8:412(	RNU6ATAC8P		smallRNA	chr8:102421197-102
ENSG0000(	500	11.16627	chr8:412(	ENSG00000213750		Pseudoger	chr8:97624203-9762
ENSG0000(	500	11.16627	chr8:412(	NACA4P		Pseudoger	chr8:101361794-101
ENSG0000(	500	11.16627	chr8:412(	ENSG00000287952		lncRNA	chr8:99893373-9989
ENSG0000(	498	11.12161	chr3:380<	AC099545.1		smallRNA	chr3:63334787-6333
ENSG0000(	498	11.12161	chr3:380<	PRICKLE2-AS1		lncRNA	chr3:64099273-6410
ENSG0000(	498	11.12161	chr3:380<	PPIAP71		Pseudoger	chr3:60732144-6073
ENSG0000(	498	11.12161	chr3:380<	PXK		protein_c	chr3:58332880-5842
ENSG0000(	498	11.12161	chr3:380<	U3		smallRNA	chr3:64071808-6407
ENSG0000(	498	11.12161	chr3:380<	ENSG00000225673		Pseudoger	chr3:60730055-6073
ENSG0000(	498	11.12161	chr3:380<	PDHB	NCGv7	protein_c	chr3:58427630-5843
ENSG0000(	498	11.12161	chr3:380<	SNTN		protein_c	chr3:63652675-6367
ENSG0000(	498	11.12161	chr3:380<	ENSG00000225662		Pseudoger	chr3:62779176-6277
ENSG0000(	498	11.12161	chr3:380<	ENSG00000271174		Pseudoger	chr3:66622760-6662
ENSG0000(	498	11.12161	chr3:380<	ENSG00000236722		Pseudoger	chr3:58511310-5851
ENSG0000(	498	11.12161	chr3:380<	ENSG00000287649		lncRNA	chr3:59809269-5981
ENSG0000(	498	11.12161	chr3:380<	ADAMTS9-AS1		lncRNA	chr3:64561322-6459
ENSG0000(	498	11.12161	chr3:380<	ENSG00000272360		lncRNA	chr3:58490537-5849
ENSG0000(	498	11.12161	chr3:380<	RNU6-787P		smallRNA	chr3:66293114-6629
ENSG0000(	498	11.12161	chr3:380<	FHIT	NCGv7;AC	protein_c	chr3:59747277-6125
ENSG0000(	498	11.12161	chr3:380<	SYNPR-AS1		lncRNA	chr3:63423596-6355
ENSG0000(	498	11.12161	chr3:380<	PRICKLE2-AS1		lncRNA	chr3:64067964-6410
ENSG0000(	498	11.12161	chr3:380<	ADAMTS9-AS2		lncRNA	chr3:64684909-6505
ENSG0000(	498	11.12161	chr3:380<	ENSG00000224479		Pseudoger	chr3:63741827-6382
ENSG0000(	498	11.12161	chr3:380<	PRICKLE2-AS2		lncRNA	chr3:64103470-6410
ENSG0000(	498	11.12161	chr3:380<	ACOX2		protein_c	chr3:58505136-5853
ENSG0000(	498	11.12161	chr3:380<	FAM3D-AS1		lncRNA	chr3:58607080-5864
ENSG0000(	498	11.12161	chr3:380<	FAM107A		protein_c	chr3:58564117-5862
ENSG0000(	498	11.12161	chr3:380<	PTPRG-AS1		lncRNA	chr3:62221249-6236
ENSG0000(	498	11.12161	chr3:380<	CFAP20DC-AS1		lncRNA	chr3:58824437-5901
ENSG0000(	498	11.12161	chr3:380<	MAGI1-IT1		lncRNA	chr3:65872815-6595

ENSG0000(	498	11.12161	chr3:380⁄	CFAP20DC-DT		lncRNA	chr3:59050164-5975
ENSG0000(	498	11.12161	chr3:380⁄	SCAANT1		lncRNA	chr3:63911518-6391
ENSG0000(	498	11.12161	chr3:380⁄	LINC00994		lncRNA	chr3:64078361-6408
ENSG0000(	498	11.12161	chr3:380⁄	ENSG00000273493		lncRNA	chr3:58329965-5833
ENSG0000(	498	11.12161	chr3:380⁄	RN7SL482P		smallRNA	chr3:66323855-6632
ENSG0000(	498	11.12161	chr3:380⁄	KCTD6		protein_c	chr3:58492096-5850
ENSG0000(	498	11.12161	chr3:380⁄	ABHD6		protein_c	chr3:58237532-5829
ENSG0000(	498	11.12161	chr3:380⁄	RNU2-10P		smallRNA	chr3:62118300-6211
ENSG0000(	498	11.12161	chr3:380⁄	Y_RNA		smallRNA	chr3:61739127-6173
ENSG0000(	498	11.12161	chr3:380⁄	FEZF2		protein_c	chr3:62369681-6237
ENSG0000(	498	11.12161	chr3:380⁄	AC104331.1		smallRNA	chr3:65232900-6523
ENSG0000(	498	11.12161	chr3:380⁄	RPP14		protein_c	chr3:58306245-5832
ENSG0000(	498	11.12161	chr3:380⁄	ID2B		Pseudoger	chr3:62124204-6212
ENSG0000(	498	11.12161	chr3:380⁄	CFAP20DC		protein_c	chr3:58717365-5905
ENSG0000(	498	11.12161	chr3:380⁄	MAGI1-AS1		lncRNA	chr3:65893816-6592
ENSG0000(	498	11.12161	chr3:380⁄	UBL5P3		Pseudoger	chr3:63203218-6320
ENSG0000(	498	11.12161	chr3:380⁄	RPL17P17		Pseudoger	chr3:65843890-6584
ENSG0000(	498	11.12161	chr3:380⁄	PRICKLE2-DT		lncRNA	chr3:64445231-6445
ENSG0000(	498	11.12161	chr3:380⁄	ENSG00000226082		Pseudoger	chr3:62926371-6292
ENSG0000(	498	11.12161	chr3:380⁄	RNU6-139P		smallRNA	chr3:62814382-6281
ENSG0000(	498	11.12161	chr3:380⁄	ENSG00000270059		lncRNA	chr3:65359268-6535
ENSG0000(	498	11.12161	chr3:380⁄	ENSG00000288039		lncRNA	chr3:66780076-6682
ENSG0000(	498	11.12161	chr3:380⁄	ENSG00000243384		lncRNA	chr3:58572744-5857
ENSG0000(	498	11.12161	chr3:380⁄	ENSG00000203647		Pseudoger	chr3:66008770-6600
ENSG0000(	498	11.12161	chr3:380⁄	HTD2		protein_c	chr3:58306262-5832
ENSG0000(	498	11.12161	chr3:380⁄	ADAMTS9	NCGv7	protein_c	chr3:64515654-6468
ENSG0000(	498	11.12161	chr3:380⁄	PRICKLE2	NCGv7	protein_c	chr3:64092236-6444
ENSG0000(	498	11.12161	chr3:380⁄	PSMD6		protein_c	chr3:64010550-6402
ENSG0000(	498	11.12161	chr3:380⁄	ATXN7		protein_c	chr3:63863155-6400
ENSG0000(	498	11.12161	chr3:380⁄	THOC7	NCGv7	protein_c	chr3:63833870-6386
ENSG0000(	498	11.12161	chr3:380⁄	C3orf49		protein_c	chr3:63819299-6384
ENSG0000(	498	11.12161	chr3:380⁄	SYNPR		protein_c	chr3:63228315-6361
ENSG0000(	498	11.12161	chr3:380⁄	KRT8P35		Pseudoger	chr3:63096374-6309
ENSG0000(	498	11.12161	chr3:380⁄	PTPRG		protein_c	chr3:61561569-6229
ENSG0000(	498	11.12161	chr3:380⁄	CADPS		protein_c	chr3:62398346-6287
ENSG0000(	498	11.12161	chr3:380⁄	ENSG00000275607		Pseudoger	chr3:58379217-5837
ENSG0000(	498	11.12161	chr3:380⁄	ENSG00000272182		lncRNA	chr3:58428255-5842
ENSG0000(	498	11.12161	chr3:380⁄	RNA5SP134		Pseudoger	chr3:63545440-6354
ENSG0000(	498	11.12161	chr3:380⁄	ENSG00000272181		lncRNA	chr3:64019508-6401
ENSG0000(	498	11.12161	chr3:380⁄	LRIG1	NCGv7	protein_c	chr3:66378797-6650
ENSG0000(	498	11.12161	chr3:380⁄	RPL10AP6		Pseudoger	chr3:61742455-6174
ENSG0000(	498	11.12161	chr3:380⁄	PPIAP70		Pseudoger	chr3:60690225-6069
ENSG0000(	498	11.12161	chr3:380⁄	PRDX3P4		Pseudoger	chr3:64174401-6417
ENSG0000(	498	11.12161	chr3:380⁄	RNU6-739P		smallRNA	chr3:64511755-6451
ENSG0000(	498	11.12161	chr3:380⁄	PSMD6-AS2		lncRNA	chr3:64004022-6401
ENSG0000(	498	11.12161	chr3:380⁄	ENSG00000271843		lncRNA	chr3:64008082-6400
ENSG0000(	498	11.12161	chr3:380⁄	MAGI1		protein_c	chr3:65353525-6603
ENSG0000(	498	11.12161	chr3:380⁄	LINC02040		lncRNA	chr3:65174916-6519
ENSG0000(	498	11.12161	chr3:380⁄	RN7SL863P		smallRNA	chr3:62796126-6279
ENSG0000(	498	11.12161	chr3:380⁄	U3		smallRNA	chr3:60856390-6085
ENSG0000(	498	11.12161	chr3:380⁄	AC106827.2		smallRNA	chr3:65941247-6594

ENSG00000	498	11.12161	chr3:3804	RPL21P41		Pseudoger	chr3:66637149-6663
ENSG00000	498	11.12161	chr3:3804	Y_RNA		smallRNA	chr3:66003839-6600
ENSG00000	498	11.12161	chr3:3804	PRICKLE2-AS3		lncRNA	chr3:64187544-6420
ENSG00000	498	11.12161	chr3:3804	ILF2P1		Pseudoger	chr3:65687301-6568
ENSG00000	498	11.12161	chr3:3804	ENSG00000240097		Pseudoger	chr3:60616822-6061
ENSG00000	498	11.12161	chr3:3804	C3orf14		protein_c	chr3:62319015-6233
ENSG00000	498	11.12161	chr3:3804	THOC7-AS1		lncRNA	chr3:63860645-6386
ENSG00000	498	11.12161	chr3:3804	ENSG00000285738		lncRNA	chr3:66794041-6679
ENSG00000	498	11.12161	chr3:3804	PSMD6-AS1		lncRNA	chr3:64011964-6401
ENSG00000	498	11.12161	chr3:3804	LINC00698		lncRNA	chr3:62950430-6312
ENSG00000	498	11.12161	chr3:3804	FAM3D		protein_c	chr3:58633946-5866
ENSG00000	497	11.09927	chr5:1646	AC008394.1		protein_c	chr5:87216606-8723
ENSG00000	497	11.09927	chr5:1646	ENSG00000287862		lncRNA	chr5:87863703-8814
ENSG00000	497	11.09927	chr5:1646	MEF2C	NCGv7;AC	protein_c	chr5:88717117-8890
ENSG00000	497	11.09927	chr5:1646	RNU6-606P		smallRNA	chr5:87299687-8729
ENSG00000	497	11.09927	chr5:1646	ENSG00000289462		lncRNA	chr5:88140403-8814
ENSG00000	497	11.09927	chr5:1646	ENSG00000271904		lncRNA	chr5:88433892-8849
ENSG00000	497	11.09927	chr5:1646	RNA5SP187		Pseudoger	chr5:88274258-8827
ENSG00000	497	11.09927	chr5:1646	LINC02060		lncRNA	chr5:88408982-8843
ENSG00000	497	11.09927	chr5:1646	RN7SKP34		smallRNA	chr5:87102603-8710
ENSG00000	497	11.09927	chr5:1646	LINC00461		lncRNA	chr5:88507546-8869
ENSG00000	497	11.09927	chr5:1646	RASA1	NCGv7	protein_c	chr5:87267883-8739
ENSG00000	497	11.09927	chr5:1646	ENSG00000285190		lncRNA	chr5:87412342-8749
ENSG00000	497	11.09927	chr5:1646	ENSG00000250306		Pseudoger	chr5:88382948-8838
ENSG00000	497	11.09927	chr5:1646	CCNH		protein_c	chr5:87318416-8741
ENSG00000	497	11.09927	chr5:1646	LINC02144		lncRNA	chr5:87665345-8773
ENSG00000	497	11.09927	chr5:1646	ENSG00000289184		lncRNA	chr5:88143177-8814
ENSG00000	497	11.09927	chr5:1646	TMEM161B-DT		lncRNA	chr5:88268864-8843
ENSG00000	497	11.09927	chr5:1646	TMEM161B		protein_c	chr5:88189633-8826
ENSG00000	497	11.09927	chr5:1646	ENSG00000250377		lncRNA	chr5:88691757-8869
ENSG00000	497	11.09927	chr5:1646	RNU6-727P		smallRNA	chr5:87420341-8742
ENSG00000	497	11.09927	chr5:1646	MEF2C-AS2		lncRNA	chr5:88676014-8877
ENSG00000	497	11.09927	chr5:1646	RN7SL629P		smallRNA	chr5:87294191-8729
ENSG00000	497	11.09927	chr5:1646	ENSG00000287783		lncRNA	chr5:87767608-8777
ENSG00000	497	11.09927	chr5:1646	ENSG00000250555		lncRNA	chr5:88692651-8869
ENSG00000	497	11.09927	chr5:1646	SNORA70		smallRNA	chr5:88382772-8838
ENSG00000	497	11.09927	chr5:1646	MIR4280		smallRNA	chr5:87114879-8711
ENSG00000	497	11.09927	chr5:1646	RPS3AP22		Pseudoger	chr5:88381957-8838
ENSG00000	497	11.09927	chr5:1646	ENSG00000249061		lncRNA	chr5:86967321-8713
ENSG00000	497	11.09927	chr5:1646	ENSG00000289008		lncRNA	chr5:88685191-8868
ENSG00000	497	11.09927	chr5:1646	LINC02488		lncRNA	chr5:87662040-8770
ENSG00000	497	11.09927	chr5:1646	H3P23		Pseudoger	chr5:88602780-8860
ENSG00000	496	11.07694	chr6:1039	ENSG00000270666		Pseudoger	chr6:27515039-2751
ENSG00000	494	11.03228	chr6:1391	SNX9		protein_c	chr6:157700387-157
ENSG00000	494	11.03228	chr6:1391	TMEM242-DT		lncRNA	chr6:157323964-157
ENSG00000	494	11.03228	chr6:1391	ZDHHC14		protein_c	chr6:157381133-157
ENSG00000	494	11.03228	chr6:1391	ENSG00000271551		lncRNA	chr6:156776360-156
ENSG00000	494	11.03228	chr6:1391	ENSG00000288910		lncRNA	chr6:157060632-157
ENSG00000	494	11.03228	chr6:1391	RP11-193H22.2		Pseudoger	chr6:157577087-157
ENSG00000	494	11.03228	chr6:1391	ENSG00000218631		Pseudoger	chr6:157372556-157
ENSG00000	494	11.03228	chr6:1391	ENSG00000213078		Pseudoger	chr6:157365990-157

ENSG0000(	494	11.03228	chr6:1391	H3P28		Pseudoger	chr6:156662050-156
ENSG0000(	494	11.03228	chr6:1391	ENSG00000270487		lncRNA	chr6:156780327-156
ENSG0000(	494	11.03228	chr6:1391	LDHAL6FP		Pseudoger	chr6:157299045-157
ENSG0000(	494	11.03228	chr6:1391	ENSG00000286482		lncRNA	chr6:157377062-157
ENSG0000(	494	11.03228	chr6:1391	snoU13		smallRNA	chr6:157291311-157
ENSG0000(	494	11.03228	chr6:1391	ENSG00000271265		lncRNA	chr6:156774217-156
ENSG0000(	494	11.03228	chr6:1391	ENSG00000233044		lncRNA	chr6:157107727-157
ENSG0000(	494	11.03228	chr6:1391	ENSG00000234361		lncRNA	chr6:157829143-157
ENSG0000(	494	11.03228	chr6:1391	MIR3692		smallRNA	chr6:157529132-157
ENSG0000(	494	11.03228	chr6:1391	ARID1B	NCGv7	protein_c	chr6:156776020-157
ENSG0000(	494	11.03228	chr6:1391	SNX9-AS1		lncRNA	chr6:157885114-157
ENSG0000(	494	11.03228	chr6:1391	MIR4466		smallRNA	chr6:156779678-156
ENSG0000(	494	11.03228	chr6:1391	ENSG00000229502		lncRNA	chr6:157872571-157
ENSG0000(	494	11.03228	chr6:1391	ENSG00000218596		Pseudoger	chr6:156976294-156
ENSG0000(	494	11.03228	chr6:1391	TMEM242		protein_c	chr6:157289025-157
ENSG0000(	492	10.98761	chr17:739	ENSG00000275185		lncRNA	chr17:30899110-308
ENSG0000(	491	10.96528	chr7:1588	ENSG00000226045		Pseudoger	chr7:130645225-130
ENSG0000(	490	10.94295	chr3:380	CCDC66	NCGv7	protein_c	chr3:56557161-5662
ENSG0000(	490	10.94295	chr3:380	ARF4-AS1		lncRNA	chr3:57597531-5760
ENSG0000(	490	10.94295	chr3:380	FLNB		protein_c	chr3:58008398-5817
ENSG0000(	490	10.94295	chr3:380	ERC2-IT1		lncRNA	chr3:55657206-5565
ENSG0000(	490	10.94295	chr3:380	RNU6-1181P		smallRNA	chr3:57512450-5751
ENSG0000(	490	10.94295	chr3:380	DENND6A-AS1		lncRNA	chr3:57628810-5765
ENSG0000(	490	10.94295	chr3:380	RNU6-483P		smallRNA	chr3:57548838-5754
ENSG0000(	490	10.94295	chr3:380	ARF4		protein_c	chr3:57571363-5759
ENSG0000(	490	10.94295	chr3:380	PDE12		protein_c	chr3:57556274-5756
ENSG0000(	490	10.94295	chr3:380	ENSG00000229863		Pseudoger	chr3:57134142-5713
ENSG0000(	490	10.94295	chr3:380	APPL1		protein_c	chr3:57227726-5727
ENSG0000(	490	10.94295	chr3:380	DNAH12	NCGv7	protein_c	chr3:57293699-5754
ENSG0000(	490	10.94295	chr3:380	DENND6A		protein_c	chr3:57625454-5769
ENSG0000(	490	10.94295	chr3:380	RN7SKP45		smallRNA	chr3:56072104-5607
ENSG0000(	490	10.94295	chr3:380	RNF7P1		Pseudoger	chr3:57519669-5752
ENSG0000(	490	10.94295	chr3:380	ENSG00000283148		lncRNA	chr3:58174478-5817
ENSG0000(	490	10.94295	chr3:380	DNASE1L3		protein_c	chr3:58192257-5821
ENSG0000(	490	10.94295	chr3:380	DENND6A-DT		lncRNA	chr3:57693205-5769
ENSG0000(	490	10.94295	chr5:1646	AC004769.1		smallRNA	chr5:130277625-130
ENSG0000(	490	10.94295	chr3:380	MIR3938		smallRNA	chr3:55852492-5585
ENSG0000(	490	10.94295	chr3:380	ARHGEF3	NCGv7	protein_c	chr3:56727418-5707
ENSG0000(	490	10.94295	chr3:380	TASOR		protein_c	chr3:56620132-5668
ENSG0000(	490	10.94295	chr3:380	ENSG00000283511		Pseudoger	chr3:58180009-5818
ENSG0000(	490	10.94295	chr3:380	ENSG00000272202		lncRNA	chr3:57078943-5708
ENSG0000(	490	10.94295	chr3:380	SLMAP		protein_c	chr3:57755450-5793
ENSG0000(	490	10.94295	chr3:380	HESX1		protein_c	chr3:57197838-5722
ENSG0000(	490	10.94295	chr3:380	ENSG00000237186		Pseudoger	chr3:57530345-5753
ENSG0000(	490	10.94295	chr3:380	SPATA12		protein_c	chr3:57060664-5707
ENSG0000(	490	10.94295	chr3:380	RNA5SP133		Pseudoger	chr3:56185104-5618
ENSG0000(	490	10.94295	chr3:380	IL17RD		protein_c	chr3:57089982-5717
ENSG0000(	490	10.94295	chr3:380	PPIAP16		Pseudoger	chr3:57941818-5794
ENSG0000(	490	10.94295	chr3:380	PDHA1P1		Pseudoger	chr3:57800056-5780
ENSG0000(	490	10.94295	chr3:380	Y_RNA		smallRNA	chr3:57699773-5769
ENSG0000(	490	10.94295	chr3:380	snoU13		smallRNA	chr3:57470145-5747

ENSG0000(	490	10.94295	chr3:380⁴	FLNB-AS1		lncRNA	chr3:58162547-5817
ENSG0000(	490	10.94295	chr3:380⁴	ENSG00000286952		lncRNA	chr3:57293083-5730
ENSG0000(	490	10.94295	chr3:380⁴	RNU6ATAC26P		smallRNA	chr3:57619447-5761
ENSG0000(	490	10.94295	chr3:380⁴	ENSG00000287502		lncRNA	chr3:57555581-5755
ENSG0000(	490	10.94295	chr3:380⁴	ENSG00000240777		lncRNA	chr3:55610603-5561
ENSG0000(	490	10.94295	chr3:380⁴	ARHGEF3-AS1		lncRNA	chr3:56940040-5696
ENSG0000(	490	10.94295	chr3:380⁴	Y_RNA		smallRNA	chr3:57493667-5749
ENSG0000(	490	10.94295	chr3:380⁴	RNU6-108P		smallRNA	chr3:57536252-5753
ENSG0000(	490	10.94295	chr3:380⁴	ASB14		protein_c	chr3:57268342-5729
ENSG0000(	489	10.92061	chr6:1039	UNC5CL		protein_c	chr6:41026895-4103
ENSG0000(	489	10.92061	chr6:1039	NFYA	DriverDB\	protein_c	chr6:41072974-4110
ENSG0000(	489	10.92061	chr5:164(	LINC02161		lncRNA	chr5:89581209-8967
ENSG0000(	489	10.92061	chr6:1039	LRFN2	NCGv7	protein_c	chr6:40391591-4058
ENSG0000(	489	10.92061	chr6:1039	ENSG00000237947		lncRNA	chr6:40713411-4071
ENSG0000(	489	10.92061	chr5:164(	ENSG00000288740		lncRNA	chr5:89301289-8930
ENSG0000(	489	10.92061	chr6:1039	TREM2		protein_c	chr6:41158506-4116
ENSG0000(	489	10.92061	chr6:1039	ENSG00000290034		lncRNA	chr6:41154450-4117
ENSG0000(	489	10.92061	chr5:164(	MEF2C-AS1		lncRNA	chr5:88883328-8946
ENSG0000(	489	10.92061	chr6:1039	APOBEC2	NCGv7	protein_c	chr6:41053202-4106
ENSG0000(	489	10.92061	chr6:1039	TSPO2		protein_c	chr6:41042467-4104
ENSG0000(	489	10.92061	chr6:1039	ENSG00000287678		lncRNA	chr6:41080624-4110
ENSG0000(	489	10.92061	chr6:1039	OARD1	DriverDB\	protein_c	chr6:41033627-4109
ENSG0000(	489	10.92061	chr6:1039	TREML1		protein_c	chr6:41149337-4115
ENSG0000(	489	10.92061	chr6:1039	ADCY10P1		Pseudoger	chr6:41101022-4113
ENSG0000(	489	10.92061	chr6:1039	ADCY10P1		lncRNA	chr6:41101034-4114
ENSG0000(	489	10.92061	chr5:164(	ENSG00000250831		lncRNA	chr5:89466537-8947
ENSG0000(	487	10.87595	chr11:62(	KLC2-AS1		lncRNA	chr11:66264777-662
ENSG0000(	487	10.87595	chr11:62(	OVOL1		protein_c	chr11:65787063-657
ENSG0000(	487	10.87595	chr11:62(	ESRRA	IntOGen-I	protein_c	chr11:64305497-643
ENSG0000(	487	10.87595	chr11:62(	GPR137		protein_c	chr11:64270062-642
ENSG0000(	487	10.87595	chr11:62(	SNX32		protein_c	chr11:65833834-658
ENSG0000(	487	10.87595	chr11:62(	RNASEH2C		protein_c	chr11:65714005-657
ENSG0000(	487	10.87595	chr11:62(	TRMT112		protein_c	chr11:64316460-643
ENSG0000(	487	10.87595	chr11:62(	MUS81		protein_c	chr11:65857126-658
ENSG0000(	487	10.87595	chr11:62(	MAP3K11		protein_c	chr11:65597756-656
ENSG0000(	487	10.87595	chr11:62(	KAT5		protein_c	chr11:65711996-657
ENSG0000(	487	10.87595	chr11:62(	BAD		protein_c	chr11:64269830-642
ENSG0000(	487	10.87595	chr11:62(	RELA	NCGv7	protein_c	chr11:65653599-656
ENSG0000(	487	10.87595	chr11:62(	CFL1		protein_c	chr11:65823022-658
ENSG0000(	487	10.87595	chr11:62(	KCNK7		protein_c	chr11:65592836-655
ENSG0000(	487	10.87595	chr11:62(	ATP5MGP1		Pseudoger	chr11:63834667-638
ENSG0000(	487	10.87595	chr11:62(	EHBP1L1		protein_c	chr11:65576046-655
ENSG0000(	487	10.87595	chr11:62(	EFEMP2		protein_c	chr11:65866441-658
ENSG0000(	487	10.87595	chr11:62(	MACROD1	AC	protein_c	chr11:63998558-641
ENSG0000(	487	10.87595	chr11:62(	CDC42BPG	NCGv7	protein_c	chr11:64823052-648
ENSG0000(	487	10.87595	chr11:62(	KLC2-AS2		lncRNA	chr11:66267635-662
ENSG0000(	487	10.87595	chr11:62(	TIGD3	NCGv7	protein_c	chr11:65354751-653
ENSG0000(	487	10.87595	chr11:62(	LGALS12		protein_c	chr11:63506052-635
ENSG0000(	487	10.87595	chr11:62(	RTN3		protein_c	chr11:63681446-637
ENSG0000(	487	10.87595	chr11:62(	PLAAT4		protein_c	chr11:63536808-635
ENSG0000(	487	10.87595	chr11:62(	ENSG00000203520		lncRNA	chr11:63616308-636

ENSG0000(	487	10.87595	chr11:62(	PLAAT2		protein_c	chr11:63552770-635
ENSG0000(	487	10.87595	chr11:62(	ENSG00000286756		lncRNA	chr11:65487884-654
ENSG0000(	487	10.87595	chr11:62(	ENSG00000286816		lncRNA	chr11:64486136-644
ENSG0000(	487	10.87595	chr11:62(	FLRT1		protein_c	chr11:64035931-641
ENSG0000(	487	10.87595	chr11:62(	DPF2		protein_c	chr11:65333843-653
ENSG0000(	487	10.87595	chr11:62(	MEN1	NCGv7;AC	protein_c	chr11:64803510-648
ENSG0000(	487	10.87595	chr11:62(	PRDX5		protein_c	chr11:64318121-643
ENSG0000(	487	10.87595	chr11:62(	FRMD8	NCGv7	protein_c	chr11:65386621-654
ENSG0000(	487	10.87595	chr11:62(	SPDYC		protein_c	chr11:65170233-651
ENSG0000(	487	10.87595	chr11:62(	ENSG00000173727		Pseudoger	chr11:65455269-654
ENSG0000(	487	10.87595	chr11:62(	CAPN1		protein_c	chr11:65180566-652
ENSG0000(	487	10.87595	chr11:62(	POLA2		protein_c	chr11:65261920-653
ENSG0000(	487	10.87595	chr11:62(	PDCL2P2		Pseudoger	chr11:65160194-651
ENSG0000(	487	10.87595	chr11:62(	CTSW		protein_c	chr11:65879809-658
ENSG0000(	487	10.87595	chr11:62(	FIBP		protein_c	chr11:65883740-658
ENSG0000(	487	10.87595	chr11:62(	RN7SL596P		smallRNA	chr11:63797788-637
ENSG0000(	487	10.87595	chr11:62(	PPP1R14B		protein_c	chr11:64244479-642
ENSG0000(	487	10.87595	chr11:62(	STIP1		protein_c	chr11:64185272-642
ENSG0000(	487	10.87595	chr11:62(	ENSG00000256100		protein_c	chr11:63974620-639
ENSG0000(	487	10.87595	chr11:62(	ZNHIT2		protein_c	chr11:65116403-651
ENSG0000(	487	10.87595	chr11:62(	ZNRD2		protein_c	chr11:65570460-655
ENSG0000(	487	10.87595	chr11:62(	ENSG00000256341		lncRNA	chr11:64118272-641
ENSG0000(	487	10.87595	chr11:62(	FKBP2		protein_c	chr11:64241003-642
ENSG0000(	487	10.87595	chr11:62(	ENSG00000291174		lncRNA	chr11:65455257-654
ENSG0000(	487	10.87595	chr11:62(	VEGFB		protein_c	chr11:64234584-642
ENSG0000(	487	10.87595	chr11:62(	ENSG00000255320		lncRNA	chr11:66244717-662
ENSG0000(	487	10.87595	chr11:62(	ENSG00000256116		lncRNA	chr11:64229214-642
ENSG0000(	487	10.87595	chr11:62(	RNU6-1306P		smallRNA	chr11:63882587-638
ENSG0000(	487	10.87595	chr11:62(	ENSG00000287412		lncRNA	chr11:63495484-635
ENSG0000(	487	10.87595	chr11:62(	SART1		protein_c	chr11:65961728-659
ENSG0000(	487	10.87595	chr11:62(	TMEM151A		protein_c	chr11:66291894-662
ENSG0000(	487	10.87595	chr11:62(	ENSG00000255557		lncRNA	chr11:65745729-657
ENSG0000(	487	10.87595	chr11:62(	TSGA10IP		protein_c	chr11:65945480-659
ENSG0000(	487	10.87595	chr11:62(	ARL2		protein_c	chr11:65014160-650
ENSG0000(	487	10.87595	chr11:62(	SLC22A12		protein_c	chr11:64590641-646
ENSG0000(	487	10.87595	chr11:62(	ENSG00000232500		lncRNA	chr11:64500846-645
ENSG0000(	487	10.87595	chr11:62(	ENSG00000289339		lncRNA	chr11:65575330-655
ENSG0000(	487	10.87595	chr11:62(	ENSG00000287917		lncRNA	chr11:65305345-653
ENSG0000(	487	10.87595	chr11:62(	RPS6KA4		protein_c	chr11:64359148-643
ENSG0000(	487	10.87595	chr11:62(	ZFPL1		protein_c	chr11:65084210-650
ENSG0000(	487	10.87595	chr11:62(	ENSG00000255173		lncRNA	chr11:65117157-651
ENSG0000(	487	10.87595	chr11:62(	MIR4489		smallRNA	chr11:65649192-656
ENSG0000(	487	10.87595	chr11:62(	SYVN1		protein_c	chr11:65121780-651
ENSG0000(	487	10.87595	chr11:62(	PLAAT3		protein_c	chr11:63573195-636
ENSG0000(	487	10.87595	chr11:62(	ENSG00000289259		lncRNA	chr11:65471472-654
ENSG0000(	487	10.87595	chr11:62(	SLC25A45	NCGv7	protein_c	chr11:65375192-653
ENSG0000(	487	10.87595	chr11:62(	ENSG00000275598		Pseudoger	chr11:63469376-634
ENSG0000(	487	10.87595	chr11:62(	ENSG00000289231		lncRNA	chr11:65353024-653
ENSG0000(	487	10.87595	chr11:62(	KCNK4-TEX40		lncRNA	chr11:64291722-643
ENSG0000(	487	10.87595	chr11:62(	RP11-869B15.1		lncRNA	chr11:64784921-647
ENSG0000(	487	10.87595	chr11:62(	AP5B1		protein_c	chr11:65773898-657

ENSG0000(	487	10.87595	chr11:62(	SF1-DT		lncRNA	chr11:64778954-647
ENSG0000(	487	10.87595	chr11:62(	AP001362.1		protein_(	chr11:65591194-655
ENSG0000(	487	10.87595	chr11:62(	ENSG00000257086		lncRNA	chr11:64246939-642
ENSG0000(	487	10.87595	chr11:62(	CCDC88B		protein_(	chr11:64340204-643
ENSG0000(	487	10.87595	chr11:62(	ENSG00000289486		lncRNA	chr11:64184892-641
ENSG0000(	487	10.87595	chr11:62(	SIPA1		protein_(	chr11:65638101-656
ENSG0000(	487	10.87595	chr11:62(	IMMP1LP1		Pseudoger	chr11:63632233-636
ENSG0000(	487	10.87595	chr11:62(	SNRPGP19		Pseudoger	chr11:65514403-655
ENSG0000(	487	10.87595	chr11:62(	C11orf68		protein_(	chr11:65916810-659
ENSG0000(	487	10.87595	chr11:62(	FOSL1	TAG;AC	protein_(	chr11:65892049-659
ENSG0000(	487	10.87595	chr11:62(	ENSG00000245156		lncRNA	chr11:66269832-662
ENSG0000(	487	10.87595	chr11:62(	CCDC85B		protein_(	chr11:65890673-658
ENSG0000(	487	10.87595	chr11:62(	ENSG00000254501		lncRNA	chr11:65110714-651
ENSG0000(	487	10.87595	chr11:62(	ENSG00000286459		lncRNA	chr11:66043298-660
ENSG0000(	487	10.87595	chr11:62(	ENSG00000289883		lncRNA	chr11:65476515-654
ENSG0000(	487	10.87595	chr11:62(	ENSG00000256789		lncRNA	chr11:63637677-636
ENSG0000(	487	10.87595	chr11:62(	AP001266.1		Pseudoger	chr11:65777621-657
ENSG0000(	487	10.87595	chr11:62(	ENSG00000289560		lncRNA	chr11:66311859-663
ENSG0000(	487	10.87595	chr11:62(	ENSG00000287821		lncRNA	chr11:65260996-652
ENSG0000(	487	10.87595	chr11:62(	ENSG00000256824		lncRNA	chr11:64035970-641
ENSG0000(	487	10.87595	chr11:62(	ENSG00000286264		protein_(	chr11:64241095-642
ENSG0000(	487	10.87595	chr11:62(	ENSG00000278952		TEC	chr11:65118310-651
ENSG0000(	487	10.87595	chr11:62(	SCYL1		protein_(	chr11:65525077-655
ENSG0000(	487	10.87595	chr11:62(	SLC22A20P		Pseudoger	chr11:65213840-652
ENSG0000(	487	10.87595	chr11:62(	ENSG00000270117		lncRNA	chr11:65498008-654
ENSG0000(	487	10.87595	chr11:62(	TALAM1		lncRNA	chr11:65499312-655
ENSG0000(	487	10.87595	chr11:62(	PPP1R14B-AS1		lncRNA	chr11:64245838-642
ENSG0000(	487	10.87595	chr11:62(	ENSG00000254461		lncRNA	chr11:66259567-662
ENSG0000(	487	10.87595	chr11:62(	HIGD1AP10		Pseudoger	chr11:65145691-651
ENSG0000(	487	10.87595	chr11:62(	ENSG00000237363		Pseudoger	chr11:64531044-645
ENSG0000(	487	10.87595	chr11:62(	ENSG00000279093		TEC	chr11:65561484-655
ENSG0000(	487	10.87595	chr11:62(	DNAJC4		protein_(	chr11:64230278-642
ENSG0000(	487	10.87595	chr11:62(	SNX15		protein_(	chr11:65027439-650
ENSG0000(	487	10.87595	chr11:62(	ATG2A	NCGv7	protein_(	chr11:64894546-649
ENSG0000(	487	10.87595	chr11:62(	EHD1		protein_(	chr11:64851642-648
ENSG0000(	487	10.87595	chr11:62(	NRXN2	NCGv7	protein_(	chr11:64606174-647
ENSG0000(	487	10.87595	chr11:62(	CATSPERZ		protein_(	chr11:64300358-643
ENSG0000(	487	10.87595	chr11:62(	NAA40		protein_(	chr11:63938959-639
ENSG0000(	487	10.87595	chr11:62(	MIR1237		smallRNA	chr11:64368602-643
ENSG0000(	487	10.87595	chr11:62(	ENSG00000255404		lncRNA	chr11:65795946-657
ENSG0000(	487	10.87595	chr11:62(	GPHA2		protein_(	chr11:64934471-649
ENSG0000(	487	10.87595	chr11:62(	KCNK4		protein_(	chr11:64291302-643
ENSG0000(	487	10.87595	chr11:62(	TRPT1		protein_(	chr11:64223799-642
ENSG0000(	487	10.87595	chr11:62(	NUDT22		protein_(	chr11:64225941-642
ENSG0000(	487	10.87595	chr11:62(	FERMT3		protein_(	chr11:64205926-642
ENSG0000(	487	10.87595	chr11:62(	PLCB3		protein_(	chr11:64251530-642
ENSG0000(	487	10.87595	chr11:62(	MRPL49		protein_(	chr11:65122183-651
ENSG0000(	487	10.87595	chr11:62(	ENSG00000285816		protein_(	chr11:65261928-653
ENSG0000(	487	10.87595	chr11:62(	CDC42EP2		protein_(	chr11:65314866-653
ENSG0000(	487	10.87595	chr11:62(	FAU		protein_(	chr11:65120630-651
ENSG0000(	487	10.87595	chr11:62(	TM7SF2		protein_(	chr11:65111845-651

ENSG0000(	487	10.87595	chr11:62(	ZFTA		protein_c	chr11:63759892-637
ENSG0000(	487	10.87595	chr11:62(	RN7SL309P		smallRNA	chr11:65695535-656
ENSG0000(	487	10.87595	chr11:62(	LINC02736		lncRNA	chr11:65487241-654
ENSG0000(	487	10.87595	chr11:62(	MALAT1	AC	lncRNA	chr11:65497688-655
ENSG0000(	487	10.87595	chr11:62(	MIR4690		smallRNA	chr11:65636310-656
ENSG0000(	487	10.87595	chr11:62(	ENSG00000254452		lncRNA	chr11:66276779-662
ENSG0000(	487	10.87595	chr11:62(	RNU2-23P		smallRNA	chr11:65147584-651
ENSG0000(	487	10.87595	chr11:62(	TMEM262		protein_c	chr11:65084979-650
ENSG0000(	487	10.87595	chr11:62(	NRXN2-AS1		lncRNA	chr11:64646399-646
ENSG0000(	487	10.87595	chr11:62(	ENSG00000289058		lncRNA	chr11:64687682-646
ENSG0000(	487	10.87595	chr11:62(	CDCA5		protein_c	chr11:65066300-650
ENSG0000(	487	10.87595	chr11:62(	KRT8P26		Pseudoger	chr11:65726939-657
ENSG0000(	487	10.87595	chr11:62(	FAM89B		protein_c	chr11:65572349-655
ENSG0000(	487	10.87595	chr11:62(	PGAM1P8		Pseudoger	chr11:65174117-651
ENSG0000(	487	10.87595	chr11:62(	MIR194-2HG		lncRNA	chr11:64889560-648
ENSG0000(	487	10.87595	chr11:62(	ATL3		protein_c	chr11:63624087-636
ENSG0000(	487	10.87595	chr11:62(	RN7SL114P		smallRNA	chr11:65049777-650
ENSG0000(	487	10.87595	chr11:62(	LINC02724		lncRNA	chr11:64449074-644
ENSG0000(	487	10.87595	chr11:62(	LINC02723		lncRNA	chr11:64394342-643
ENSG0000(	487	10.87595	chr11:62(	ENSG00000231492		lncRNA	chr11:64420311-644
ENSG0000(	487	10.87595	chr11:62(	ENSG00000288852		lncRNA	chr11:64183353-641
ENSG0000(	487	10.87595	chr11:62(	ARL2-SNX15		protein_c	chr11:65014182-650
ENSG0000(	487	10.87595	chr11:62(	RELA-DT		lncRNA	chr11:65662988-656
ENSG0000(	487	10.87595	chr11:62(	ENSG00000230835		Pseudoger	chr11:64881535-648
ENSG0000(	487	10.87595	chr11:62(	DRAP1		protein_c	chr11:65919274-659
ENSG0000(	487	10.87595	chr11:62(	COX8A		protein_c	chr11:63974620-639
ENSG0000(	487	10.87595	chr11:62(	CATSPER1		protein_c	chr11:66016752-660
ENSG0000(	487	10.87595	chr11:62(	ENSG00000256481		lncRNA	chr11:64081690-640
ENSG0000(	487	10.87595	chr11:62(	BANF1	TAG	protein_c	chr11:66002228-660
ENSG0000(	487	10.87595	chr11:62(	ENSG00000255038		lncRNA	chr11:66067277-660
ENSG0000(	487	10.87595	chr11:62(	PLAAT5		protein_c	chr11:63461404-634
ENSG0000(	487	10.87595	chr11:62(	ENSG00000290061		lncRNA	chr11:65492897-654
ENSG0000(	487	10.87595	chr11:62(	PCNX3		protein_c	chr11:65615776-656
ENSG0000(	487	10.87595	chr11:62(	snoU13		smallRNA	chr11:66053144-660
ENSG0000(	487	10.87595	chr11:62(	MIR192		smallRNA	chr11:64891137-648
ENSG0000(	487	10.87595	chr11:62(	CST6		protein_c	chr11:66012008-660
ENSG0000(	487	10.87595	chr11:62(	ENSG00000255478		lncRNA	chr11:65367438-653
ENSG0000(	487	10.87595	chr11:62(	RCOR2		protein_c	chr11:63911230-639
ENSG0000(	487	10.87595	chr11:62(	OTUB1	DriverDBv	protein_c	chr11:63985853-640
ENSG0000(	487	10.87595	chr11:62(	RASGRP2	NCGv7	protein_c	chr11:64726911-647
ENSG0000(	487	10.87595	chr11:62(	YIF1A		protein_c	chr11:66284580-662
ENSG0000(	487	10.87595	chr11:62(	GAL3ST3		protein_c	chr11:66040765-660
ENSG0000(	487	10.87595	chr11:62(	PPP2R5B		protein_c	chr11:64917553-649
ENSG0000(	487	10.87595	chr11:62(	RAB1B		protein_c	chr11:66268590-662
ENSG0000(	487	10.87595	chr11:62(	ZNRD2-DT		lncRNA	chr11:65568482-655
ENSG0000(	487	10.87595	chr11:62(	PACS1		protein_c	chr11:66070272-662
ENSG0000(	487	10.87595	chr11:62(	snoU13		smallRNA	chr11:66152621-661
ENSG0000(	487	10.87595	chr11:62(	PYGM		protein_c	chr11:64746389-647
ENSG0000(	487	10.87595	chr11:62(	CAPN1-AS1		lncRNA	chr11:65177606-651
ENSG0000(	487	10.87595	chr11:62(	NEAT1	AC	lncRNA	chr11:65422774-654
ENSG0000(	487	10.87595	chr11:62(	RNU6-45P		smallRNA	chr11:63970470-639

ENSG00000	487	10.87595	chr11:620	MARK2	NCGv7	protein_c	chr11:63838928-639
ENSG00000	487	10.87595	chr11:620	KLC2		protein_c	chr11:66257294-662
ENSG00000	487	10.87595	chr11:620	SPINDOC		protein_c	chr11:63813456-638
ENSG00000	487	10.87595	chr11:620	ENSG00000271100		Pseudoger	chr11:63698596-636
ENSG00000	487	10.87595	chr11:620	VPS51	NCGv7	protein_c	chr11:65089324-651
ENSG00000	487	10.87595	chr11:620	LTBP3		protein_c	chr11:65538559-655
ENSG00000	487	10.87595	chr11:620	EEF1A1P18		Pseudoger	chr11:65025390-650
ENSG00000	487	10.87595	chr11:620	ENSG00000236935		lncRNA	chr11:64325050-643
ENSG00000	487	10.87595	chr11:620	MAP4K2		protein_c	chr11:64784918-648
ENSG00000	487	10.87595	chr11:620	CNIH2		protein_c	chr11:66278175-662
ENSG00000	487	10.87595	chr11:620	SAC3D1		protein_c	chr11:65040901-650
ENSG00000	487	10.87595	chr11:620	OVOL1-AS1		lncRNA	chr11:65789051-657
ENSG00000	487	10.87595	chr11:620	SLC22A11	NCGv7	protein_c	chr11:64555690-645
ENSG00000	487	10.87595	chr11:620	NAALADL1		protein_c	chr11:65044818-650
ENSG00000	487	10.87595	chr11:620	SF1	NCGv7	protein_c	chr11:64764606-647
ENSG00000	487	10.87595	chr11:620	ENSG00000290026		lncRNA	chr11:65574399-655
ENSG00000	487	10.87595	chr11:620	EIF1AD		protein_c	chr11:65996545-660
ENSG00000	487	10.87595	chr11:620	ENSG00000254867		Pseudoger	chr11:65983679-659
ENSG00000	487	10.87595	chr11:620	SF3B2	NCGv7	protein_c	chr11:66050729-660
ENSG00000	487	10.87595	chr11:620	BATF2		protein_c	chr11:64987945-649
ENSG00000	487	10.87595	chr11:620	MAJIN		protein_c	chr11:64937517-649
ENSG00000	487	10.87595	chr11:620	Y_RNA		smallRNA	chr11:64296037-642
ENSG00000	487	10.87595	chr11:620	ENSG00000290057		lncRNA	chr11:65421448-654
ENSG00000	486	10.85362	chr3:3804	ENSG00000242568		lncRNA	chr3:55335462-5535
ENSG00000	486	10.85362	chr3:3804	LINC02017		lncRNA	chr3:55178172-5518
ENSG00000	486	10.85362	chr3:3804	ENSG00000239991		lncRNA	chr3:55166910-5517
ENSG00000	486	10.85362	chr5:1646	ENSG00000251419		Pseudoger	chr5:75831255-7583
ENSG00000	486	10.85362	chr5:1646	SNRPCP2		Pseudoger	chr5:76376675-7637
ENSG00000	486	10.85362	chr5:1646	SAP18P1		Pseudoger	chr5:76075531-7607
ENSG00000	486	10.85362	chr5:1646	ANKDD1B		protein_c	chr5:75611182-7568
ENSG00000	486	10.85362	chr5:1646	BIN2P2		Pseudoger	chr5:75910565-7591
ENSG00000	486	10.85362	chr5:1646	RNU6-680P		smallRNA	chr5:75709495-7570
ENSG00000	486	10.85362	chr5:1646	ENSG00000251342		Pseudoger	chr5:76078666-7607
ENSG00000	486	10.85362	chr5:1646	ENSG00000250574		Pseudoger	chr5:76510440-7651
ENSG00000	486	10.85362	chr5:1646	IQGAP2	NCGv7	protein_c	chr5:76403285-7670
ENSG00000	486	10.85362	chr5:1646	HMGN2P4		Pseudoger	chr5:76242024-7624
ENSG00000	486	10.85362	chr3:3804	ESRG		lncRNA	chr3:54632122-5463
ENSG00000	486	10.85362	chr3:3804	WNT5A	AC	protein_c	chr3:55465715-5549
ENSG00000	486	10.85362	chr5:1646	RNA5SP186		Pseudoger	chr5:76091708-7609
ENSG00000	486	10.85362	chr3:3804	ENSG00000242775		lncRNA	chr3:55360443-5536
ENSG00000	486	10.85362	chr3:3804	LINC02030		lncRNA	chr3:55226862-5530
ENSG00000	486	10.85362	chr3:3804	RPS15P5		Pseudoger	chr3:54626215-5462
ENSG00000	486	10.85362	chr5:1646	F2RL2		protein_c	chr5:76615482-7662
ENSG00000	486	10.85362	chr3:3804	ERC2		protein_c	chr3:55508311-5646
ENSG00000	486	10.85362	chr3:3804	ENSG00000242317		lncRNA	chr3:55493830-5550
ENSG00000	486	10.85362	chr3:3804	CACNA2D3-AS1		lncRNA	chr3:54874605-5490
ENSG00000	486	10.85362	chr5:1646	POC5	NCGv7	protein_c	chr5:75674124-7571
ENSG00000	486	10.85362	chr5:1646	ENSG00000251668		Pseudoger	chr5:76170930-7617
ENSG00000	486	10.85362	chr5:1646	ENSG00000249713		lncRNA	chr5:76606608-7660
ENSG00000	486	10.85362	chr5:1646	ENSG00000249301		Pseudoger	chr5:75910283-7591
ENSG00000	486	10.85362	chr5:1646	ENSG00000225407		lncRNA	chr5:76691439-7671

ENSG0000(	486	10.85362	chr5:164(	RNU7-175P		smallRNA	chr5:75538598-7553
ENSG0000(	486	10.85362	chr3:380<	WNT5A-AS1		lncRNA	chr3:55487699-5548
ENSG0000(	486	10.85362	chr5:164(	ENSG00000248881		lncRNA	chr5:75598482-7559
ENSG0000(	486	10.85362	chr5:164(	RAP1BL		Pseudoger	chr5:76173629-7617
ENSG0000(	486	10.85362	chr5:164(	PDCD5P2		Pseudoger	chr5:76280956-7628
ENSG0000(	486	10.85362	chr5:164(	ENSG00000272040		lncRNA	chr5:75608817-7560
ENSG0000(	486	10.85362	chr5:164(	SLC25A5P9		Pseudoger	chr5:75752570-7575
ENSG0000(	486	10.85362	chr5:164(	POLK		protein_c	chr5:75511756-7560
ENSG0000(	486	10.85362	chr5:164(	SV2C-AS1		lncRNA	chr5:76081946-7608
ENSG0000(	486	10.85362	chr3:380<	LRTM1	NCGv7	protein_c	chr3:54918231-5496
ENSG0000(	486	10.85362	chr5:164(	SV2C		protein_c	chr5:76083383-7635
ENSG0000(	486	10.85362	chr5:164(	ENSG00000250348		lncRNA	chr5:76285542-7631
ENSG0000(	486	10.85362	chr5:164(	ENSG00000228367		Pseudoger	chr5:75920670-7592
ENSG0000(	485	10.83128	chr12:149	RPS27P23		Pseudoger	chr12:101424441-10
ENSG0000(	485	10.83128	chr2:309<	AC010887.1		smallRNA	chr2:217176498-217
ENSG0000(	483	10.78662	chr12:149	ENSG00000258365		lncRNA	chr12:94277758-942
ENSG0000(	483	10.78662	chr12:149	ENSG00000258271		Pseudoger	chr12:93529560-935
ENSG0000(	483	10.78662	chr12:149	RPL21P106		Pseudoger	chr12:92168400-921
ENSG0000(	483	10.78662	chr12:149	ENSG00000257322		lncRNA	chr12:93003390-932
ENSG0000(	483	10.78662	chr12:149	ENSG00000257194		lncRNA	chr12:90107739-901
ENSG0000(	483	10.78662	chr12:149	LSM3P2		Pseudoger	chr12:94590821-945
ENSG0000(	483	10.78662	chr12:149	LINC02822		lncRNA	chr12:90576402-908
ENSG0000(	483	10.78662	chr12:149	ENSG00000257995		lncRNA	chr12:90617759-906
ENSG0000(	483	10.78662	chr12:149	MIR492		smallRNA	chr12:94834398-948
ENSG0000(	483	10.78662	chr12:149	ENSG00000271382		lncRNA	chr12:93945041-939
ENSG0000(	483	10.78662	chr12:149	PLXNC1		protein_c	chr12:94148577-943
ENSG0000(	483	10.78662	chr12:149	ENSG00000258262		Pseudoger	chr12:92530460-925
ENSG0000(	483	10.78662	chr12:149	LINC02404		lncRNA	chr12:91876924-918
ENSG0000(	483	10.78662	chr12:149	RPL41P5		Pseudoger	chr12:93083598-930
ENSG0000(	483	10.78662	chr12:149	ENSG00000237774		Pseudoger	chr12:95125760-951
ENSG0000(	483	10.78662	chr12:149	ENSG00000287237		lncRNA	chr12:94260548-942
ENSG0000(	483	10.78662	chr12:149	LINC01619		lncRNA	chr12:91984976-921
ENSG0000(	483	10.78662	chr12:149	ENSG00000257878		lncRNA	chr12:95996460-960
ENSG0000(	483	10.78662	chr12:149	SNORD74		smallRNA	chr12:93266017-932
ENSG0000(	483	10.78662	chr12:149	USP44	NCGv7	protein_c	chr12:95516560-955
ENSG0000(	483	10.78662	chr12:149	ENSG00000257252		lncRNA	chr12:93316722-933
ENSG0000(	483	10.78662	chr12:149	HNRNPA1P50		Pseudoger	chr12:92883773-928
ENSG0000(	483	10.78662	chr12:149	ENSG00000257863		lncRNA	chr12:91901458-919
ENSG0000(	483	10.78662	chr12:149	ENSG00000266923		lncRNA	chr12:92421531-924
ENSG0000(	483	10.78662	chr12:149	ENSG00000257283		lncRNA	chr12:93894965-939
ENSG0000(	483	10.78662	chr12:149	ENSG00000288102		lncRNA	chr12:90617759-908
ENSG0000(	483	10.78662	chr12:149	AC138123.1		smallRNA	chr12:93097251-930
ENSG0000(	483	10.78662	chr12:149	ENSG00000257943		Pseudoger	chr12:95311312-954
ENSG0000(	483	10.78662	chr12:149	CBX3P5		Pseudoger	chr12:95286318-952
ENSG0000(	483	10.78662	chr12:149	NACAP8		Pseudoger	chr12:93124063-931
ENSG0000(	483	10.78662	chr12:149	KRT19P2		Pseudoger	chr12:94834147-948
ENSG0000(	483	10.78662	chr12:149	PGAM1P5		Pseudoger	chr12:95551582-956
ENSG0000(	483	10.78662	chr12:149	ENSG00000257121		Pseudoger	chr12:95100531-951
ENSG0000(	483	10.78662	chr12:149	ENSG00000258012		Pseudoger	chr12:92858079-928
ENSG0000(	483	10.78662	chr12:149	LINC02823		lncRNA	chr12:91327045-913
ENSG0000(	483	10.78662	chr12:149	RNU6-808P		smallRNA	chr12:95290692-952

ENSG0000(	483	10.78662	chr12:149	BTG1	NCGv7;AC	protein_c	chr12:92140278-921
ENSG0000(	483	10.78662	chr12:149	LUM	NCGv7	protein_c	chr12:91102629-911
ENSG0000(	483	10.78662	chr12:149	SOCS2		protein_c	chr12:93569814-935
ENSG0000(	483	10.78662	chr12:149	ENSG00000289605		lncRNA	chr12:91276241-916
ENSG0000(	483	10.78662	chr12:149	ENSG00000258313		lncRNA	chr12:95387890-953
ENSG0000(	483	10.78662	chr12:149	RBMS2P1		Pseudoger	chr12:94423744-944
ENSG0000(	483	10.78662	chr12:149	ENSG00000257400		lncRNA	chr12:94491546-944
ENSG0000(	483	10.78662	chr12:149	NR2C1		protein_c	chr12:95020229-950
ENSG0000(	483	10.78662	chr12:149	ENSG00000289510		lncRNA	chr12:95336664-954
ENSG0000(	483	10.78662	chr12:149	CCDC38	NCGv7	protein_c	chr12:95867048-959
ENSG0000(	483	10.78662	chr12:149	ENSG00000289476		lncRNA	chr12:95305929-953
ENSG0000(	483	10.78662	chr12:149	VEZT		protein_c	chr12:95217746-953
ENSG0000(	483	10.78662	chr12:149	ENSG00000289437		lncRNA	chr12:95260099-952
ENSG0000(	483	10.78662	chr12:149	LINC02399		lncRNA	chr12:89947693-899
ENSG0000(	483	10.78662	chr12:149	Y_RNA		smallRNA	chr12:95617689-956
ENSG0000(	483	10.78662	chr12:149	RN7SL630P		smallRNA	chr12:93831076-938
ENSG0000(	483	10.78662	chr12:149	ENSG00000258125		lncRNA	chr12:91680131-916
ENSG0000(	483	10.78662	chr12:149	ENSG00000258172		lncRNA	chr12:94272150-942
ENSG0000(	483	10.78662	chr12:149	LINC02412		lncRNA	chr12:93173470-931
ENSG0000(	483	10.78662	chr12:149	CLLU1-AS1		lncRNA	chr12:92420094-924
ENSG0000(	483	10.78662	chr12:149	LINC02397		lncRNA	chr12:92466451-924
ENSG0000(	483	10.78662	chr12:149	KERA		protein_c	chr12:91050491-910
ENSG0000(	483	10.78662	chr12:149	SNRPF		protein_c	chr12:95858952-959
ENSG0000(	483	10.78662	chr12:149	CRADD	NCGv7	protein_c	chr12:93677375-938
ENSG0000(	483	10.78662	chr12:149	ENSG00000257787		lncRNA	chr12:90809207-908
ENSG0000(	483	10.78662	chr12:149	ENSG00000270506		Pseudoger	chr12:93035261-930
ENSG0000(	483	10.78662	chr12:149	AMDHD1		protein_c	chr12:95943331-959
ENSG0000(	483	10.78662	chr12:149	ENSG00000258303		lncRNA	chr12:93836167-938
ENSG0000(	483	10.78662	chr12:149	snoU13		smallRNA	chr12:93265629-932
ENSG0000(	483	10.78662	chr12:149	ENSG00000258148		Pseudoger	chr12:91193709-911
ENSG0000(	483	10.78662	chr12:149	EEA1		protein_c	chr12:92770637-929
ENSG0000(	483	10.78662	chr12:149	ENSG00000257746		lncRNA	chr12:93090480-931
ENSG0000(	483	10.78662	chr12:149	ENSG00000257512		Pseudoger	chr12:93314809-933
ENSG0000(	483	10.78662	chr12:149	LINC02413		lncRNA	chr12:92999218-930
ENSG0000(	483	10.78662	chr12:149	TMCC3		protein_c	chr12:94567122-946
ENSG0000(	483	10.78662	chr12:149	RNU6-735P		smallRNA	chr12:95438737-954
ENSG0000(	483	10.78662	chr12:149	ENSG00000257360		Pseudoger	chr12:94140077-941
ENSG0000(	483	10.78662	chr12:149	ENSG00000285030		Pseudoger	chr12:95885169-958
ENSG0000(	483	10.78662	chr12:149	DCN		protein_c	chr12:91140484-911
ENSG0000(	483	10.78662	chr12:149	ENSG00000258035		lncRNA	chr12:94167528-941
ENSG0000(	483	10.78662	chr12:149	ENSG00000286049		lncRNA	chr12:91423781-914
ENSG0000(	483	10.78662	chr12:149	snoU13		smallRNA	chr12:92544892-925
ENSG0000(	483	10.78662	chr12:149	ENSG00000258224		Pseudoger	chr12:91871122-918
ENSG0000(	483	10.78662	chr12:149	CLLU1		lncRNA	chr12:92421531-924
ENSG0000(	483	10.78662	chr12:149	MIR5700		smallRNA	chr12:94561789-945
ENSG0000(	483	10.78662	chr2:3094	MIR3131		smallRNA	chr2:219058688-219
ENSG0000(	483	10.78662	chr12:149	CRADD-AS1		lncRNA	chr12:93707791-937
ENSG0000(	483	10.78662	chr12:149	NSA2P2		Pseudoger	chr12:94521090-945
ENSG0000(	483	10.78662	chr12:149	NTN4	NCGv7	protein_c	chr12:95657807-957
ENSG0000(	483	10.78662	chr12:149	BRWD1P2		Pseudoger	chr12:89919437-899
ENSG0000(	483	10.78662	chr12:149	ENSG00000287714		lncRNA	chr12:92026447-920

ENSG0000(	483	10.78662	chr12:149	ENSG00000241556		Pseudoger	chr12:95467397-954
ENSG0000(	483	10.78662	chr12:149	SNRPF-DT		lncRNA	chr12:95791733-958
ENSG0000(	483	10.78662	chr12:149	snoU13		smallRNA	chr12:93350616-933
ENSG0000(	483	10.78662	chr12:149	SUCLG2P2		Pseudoger	chr12:94548241-945
ENSG0000(	483	10.78662	chr12:149	MIR3685		smallRNA	chr12:95309923-953
ENSG0000(	483	10.78662	chr12:149	LINC02410		lncRNA	chr12:95803097-958
ENSG0000(	483	10.78662	chr12:149	Y_RNA		smallRNA	chr12:93021606-930
ENSG0000(	483	10.78662	chr12:149	RN7SKP263		smallRNA	chr12:94008739-940
ENSG0000(	483	10.78662	chr12:149	Y_RNA		smallRNA	chr12:95275770-952
ENSG0000(	483	10.78662	chr12:149	LINC02392		lncRNA	chr12:90280894-903
ENSG0000(	483	10.78662	chr12:149	ENSG00000286907		lncRNA	chr12:92008052-920
ENSG0000(	483	10.78662	chr12:149	EPYC	NCGv7	protein_c	chr12:90963682-910
ENSG0000(	483	10.78662	chr12:149	HAL		protein_c	chr12:95972662-959
ENSG0000(	483	10.78662	chr12:149	RNU6-1329P		smallRNA	chr12:92952639-929
ENSG0000(	483	10.78662	chr12:149	METAP2		protein_c	chr12:95473520-955
ENSG0000(	483	10.78662	chr12:149	NDUFA12		protein_c	chr12:94895297-950
ENSG0000(	483	10.78662	chr12:149	Y_RNA		smallRNA	chr12:93460725-934
ENSG0000(	483	10.78662	chr12:149	FGD6		protein_c	chr12:95076749-952
ENSG0000(	483	10.78662	chr12:149	RNU6-247P		smallRNA	chr12:95792529-957
ENSG0000(	483	10.78662	chr12:149	RN7SL330P		smallRNA	chr12:94361220-943
ENSG0000(	483	10.78662	chr12:149	SOCS2-AS1		lncRNA	chr12:93542022-935
ENSG0000(	483	10.78662	chr12:149	MRPL42		protein_c	chr12:93467514-935
ENSG0000(	483	10.78662	chr12:149	UBE2N		protein_c	chr12:93405673-934
ENSG0000(	483	10.78662	chr12:149	ENSG00000277738		lncRNA	chr12:91634887-916
ENSG0000(	483	10.78662	chr12:149	CEP83-DT		lncRNA	chr12:94460003-944
ENSG0000(	483	10.78662	chr12:149	BTG1-DT		lncRNA	chr12:92145573-922
ENSG0000(	483	10.78662	chr12:149	CCER1	NCGv7	protein_c	chr12:90905622-909
ENSG0000(	483	10.78662	chr12:149	RN7SL483P		smallRNA	chr12:94515476-945
ENSG0000(	483	10.78662	chr12:149	RN7SL737P		smallRNA	chr12:93509487-935
ENSG0000(	483	10.78662	chr12:149	PLEKHG7		protein_c	chr12:92702843-927
ENSG0000(	483	10.78662	chr12:149	Y_RNA		smallRNA	chr12:92170353-921
ENSG0000(	483	10.78662	chr12:149	LINC00615		lncRNA	chr12:90918023-909
ENSG0000(	483	10.78662	chr12:149	CEP83	NCGv7	protein_c	chr12:94306449-944
ENSG0000(	483	10.78662	chr12:149	LINC02391		lncRNA	chr12:92247697-923
ENSG0000(	483	10.78662	chr12:149	ENSG00000186076		Pseudoger	chr12:93640822-936
ENSG0000(	483	10.78662	chr12:149	NUDT4		protein_c	chr12:93377883-934
ENSG0000(	481	10.74195	chr5:1646	ENSG00000234292		lncRNA	chr5:91280097-9128
ENSG0000(	481	10.74195	chr5:1646	ENSG00000285618		lncRNA	chr5:91132303-9114
ENSG0000(	481	10.74195	chr5:1646	POU5F2		protein_c	chr5:93733220-9374
ENSG0000(	481	10.74195	chr5:1646	ENSG00000287180		lncRNA	chr5:93621683-9367
ENSG0000(	481	10.74195	chr5:1646	AC120120.1		smallRNA	chr5:92405946-9240
ENSG0000(	481	10.74195	chr5:1646	LYSMD3		protein_c	chr5:90515611-9052
ENSG0000(	481	10.74195	chr5:1646	ENSG00000251023		lncRNA	chr5:93860669-9386
ENSG0000(	481	10.74195	chr5:1646	CCT7P2		Pseudoger	chr5:92889387-9289
ENSG0000(	481	10.74195	chr5:1646	ENSG00000289274		lncRNA	chr5:93598593-9360
ENSG0000(	481	10.74195	chr5:1646	LDHBP3		Pseudoger	chr5:92840807-9284
ENSG0000(	481	10.74195	chr5:1646	MTND6P3		Pseudoger	chr5:94568929-9456
ENSG0000(	481	10.74195	chr5:1646	ENSG00000249169		lncRNA	chr5:92654848-9267
ENSG0000(	481	10.74195	chr5:1646	CETN3		protein_c	chr5:90392257-9040
ENSG0000(	481	10.74195	chr5:1646	ENSG00000248588		lncRNA	chr5:92823935-9284
ENSG0000(	481	10.74195	chr5:1646	MBLAC2	DriverDB\	protein_c	chr5:90458209-9047

ENSG0000(	481	10.74195	chr5:164(	RNU4-90P		smallRNA	chr5:91270727-9127
ENSG0000(	481	10.74195	chr5:164(	PCBP2P3		Pseudoger	chr5:91843687-9184
ENSG0000(	481	10.74195	chr5:164(	MIR2277		smallRNA	chr5:93620696-9362
ENSG0000(	481	10.74195	chr5:164(	ENSG00000212930		lncRNA	chr5:91223419-9122
ENSG0000(	481	10.74195	chr5:164(	ENSG00000241059		lncRNA	chr5:91355380-9135
ENSG0000(	481	10.74195	chr5:164(	TMEM251P1		Pseudoger	chr5:91030990-9103
ENSG0000(	481	10.74195	chr5:164(	ENSG00000240388		Pseudoger	chr5:91354478-9135
ENSG0000(	481	10.74195	chr5:164(	ENSG00000286638		lncRNA	chr5:91280229-9128
ENSG0000(	481	10.74195	chr5:164(	AC026781.1		smallRNA	chr5:92717432-9271
ENSG0000(	481	10.74195	chr5:164(	MTND5P12		Pseudoger	chr5:94567461-9456
ENSG0000(	481	10.74195	chr5:164(	ADGRV1	NCGv7	protein_c	chr5:90529344-9116
ENSG0000(	481	10.74195	chr5:164(	NR2F1-AS1		lncRNA	chr5:93360779-9358
ENSG0000(	481	10.74195	chr5:164(	POLD2P1		Pseudoger	chr5:93267429-9326
ENSG0000(	481	10.74195	chr5:164(	RAB5CP2		Pseudoger	chr5:91476382-9147
ENSG0000(	481	10.74195	chr5:164(	ARRDC3		protein_c	chr5:91368631-9138
ENSG0000(	481	10.74195	chr5:164(	POLR3G		protein_c	chr5:90471748-9051
ENSG0000(	481	10.74195	chr5:164(	ENSG00000214942		lncRNA	chr5:89900664-8999
ENSG0000(	481	10.74195	chr5:164(	ENSG00000255647		lncRNA	chr5:90410000-9041
ENSG0000(	481	10.74195	chr5:164(	MIR3660		smallRNA	chr5:90016621-9001
ENSG0000(	481	10.74195	chr5:164(	ENSG00000251361		lncRNA	chr5:93019663-9306
ENSG0000(	481	10.74195	chr5:164(	AC093311.1		smallRNA	chr5:94578769-9457
ENSG0000(	481	10.74195	chr5:164(	NR2F1		protein_c	chr5:93583222-9359
ENSG0000(	481	10.74195	chr5:164(	LINC02058		lncRNA	chr5:92907180-9293
ENSG0000(	481	10.74195	chr5:164(	ARRDC3-AS1		lncRNA	chr5:91380349-9161
ENSG0000(	481	10.74195	chr5:164(	MTCYBP35		Pseudoger	chr5:94569540-9457
ENSG0000(	481	10.74195	chr5:164(	ENSG00000272406		lncRNA	chr5:93741640-9374
ENSG0000(	481	10.74195	chr5:164(	KIAA0825		protein_c	chr5:94150851-9461
ENSG0000(	481	10.74195	chr5:164(	ENSG00000249776		lncRNA	chr5:92410256-9266
ENSG0000(	481	10.74195	chr5:164(	FAM172A		protein_c	chr5:93617725-9411
ENSG0000(	481	10.74195	chr5:164(	NPM1P27		Pseudoger	chr5:93682838-9368
ENSG0000(	481	10.74195	chr5:164(	ENSG00000249984		lncRNA	chr5:92675956-9268
ENSG0000(	481	10.74195	chr5:164(	ENSG00000232578		Pseudoger	chr5:94592771-9459
ENSG0000(	481	10.74195	chr5:164(	LINC01339		lncRNA	chr5:90153052-9029
ENSG0000(	481	10.74195	chr5:164(	ENSG00000286509		lncRNA	chr5:93088856-9309
ENSG0000(	481	10.74195	chr5:164(	ENSG00000278905		TEC	chr5:93600357-9360
ENSG0000(	481	10.74195	chr5:164(	SLF1		protein_c	chr5:94618669-9473
ENSG0000(	481	10.74195	chr5:164(	ENSG00000253321		lncRNA	chr5:90353037-9035
ENSG0000(	481	10.74195	chr5:164(	ENSG00000287447		lncRNA	chr5:93790505-9380
ENSG0000(	481	10.74195	chr5:164(	ENSG00000260871		lncRNA	chr5:90388468-9038
ENSG0000(	481	10.74195	chr5:164(	ENSG00000270133		lncRNA	chr5:94611906-9461
ENSG0000(	481	10.74195	chr5:164(	LUCAT1		lncRNA	chr5:91054834-9131
ENSG0000(	481	10.74195	chr5:164(	ENSG00000286577		lncRNA	chr5:94111720-9417
ENSG0000(	481	10.74195	chr5:164(	ENSG00000286121		lncRNA	chr5:91642643-9179
ENSG0000(	480	10.71962	chr10:81(	RPA2P2		Pseudoger	chr10:81136540-811
ENSG0000(	480	10.71962	chr10:81(	LINC02655		lncRNA	chr10:80649797-806
ENSG0000(	480	10.71962	chr10:81(	TSPAN14-AS1		lncRNA	chr10:80529592-805
ENSG0000(	480	10.71962	chr10:81(	FARSBP1		Pseudoger	chr10:80776165-807
ENSG0000(	480	10.71962	chr10:81(	ENSG00000233703		Pseudoger	chr10:80749632-807
ENSG0000(	480	10.71962	chr10:81(	SH2D4B		protein_c	chr10:80537902-806
ENSG0000(	480	10.71962	chr10:81(	RPS7P9		Pseudoger	chr10:80716483-807
ENSG0000(	480	10.71962	chr10:81(	WARS2P1		Pseudoger	chr10:80940665-809

ENSG0000(	480	10.71962	chr10:81(	AL356154.1		smallRNA	chr10:81144652-811
ENSG0000(	476	10.63029	chr10:81(	ENSG00000277981		Pseudoger	chr10:77738246-777
ENSG0000(	476	10.63029	chr10:81(	NUTM2B		protein_c	chr10:79703227-797
ENSG0000(	476	10.63029	chr10:81(	ENSG00000229569		lncRNA	chr10:79000484-790
ENSG0000(	476	10.63029	chr10:81(	ENSG00000280355		TEC	chr10:79681973-796
ENSG0000(	476	10.63029	chr10:81(	RPS24		protein_c	chr10:78033760-780
ENSG0000(	476	10.63029	chr10:81(	H2AZP5		Pseudoger	chr10:77953495-779
ENSG0000(	476	10.63029	chr10:81(	AC074323.1		smallRNA	chr10:78750602-787
ENSG0000(	476	10.63029	chr10:81(	RPS12P18		Pseudoger	chr10:79544087-795
ENSG0000(	476	10.63029	chr10:81(	KCNMA1		protein_c	chr10:76869601-776
ENSG0000(	476	10.63029	chr10:81(	ENSG00000271985		Pseudoger	chr10:79526508-795
ENSG0000(	476	10.63029	chr10:81(	ENSG00000288090		lncRNA	chr10:78288059-782
ENSG0000(	476	10.63029	chr10:81(	DLG5-AS1		lncRNA	chr10:77927372-779
ENSG0000(	476	10.63029	chr10:81(	ENSG00000228748		lncRNA	chr10:77782866-777
ENSG0000(	476	10.63029	chr10:81(	GNAI2P2		Pseudoger	chr10:78068623-780
ENSG0000(	476	10.63029	chr10:81(	SFTPA3P		Pseudoger	chr10:79595294-795
ENSG0000(	476	10.63029	chr10:81(	MBL3P		Pseudoger	chr10:79582298-795
ENSG0000(	476	10.63029	chr10:81(	ENSG00000230229		lncRNA	chr10:78352597-783
ENSG0000(	476	10.63029	chr10:81(	ENSG00000229543		lncRNA	chr10:78293842-783
ENSG0000(	476	10.63029	chr10:81(	ZMIZ1-AS1		lncRNA	chr10:78943328-790
ENSG0000(	476	10.63029	chr10:81(	ENSG00000235705		Pseudoger	chr10:79507597-795
ENSG0000(	476	10.63029	chr10:81(	KCNMA1-AS3		lncRNA	chr10:77350851-773
ENSG0000(	476	10.63029	chr10:81(	EIF5AL1		protein_c	chr10:79512533-795
ENSG0000(	476	10.63029	chr10:81(	IMPDH1P5		Pseudoger	chr10:77780337-777
ENSG0000(	476	10.63029	chr10:81(	RNA5SP321		Pseudoger	chr10:77587049-775
ENSG0000(	476	10.63029	chr10:81(	ENSG00000224886		Pseudoger	chr10:79684494-796
ENSG0000(	476	10.63029	chr10:81(	RNU6-1266P		smallRNA	chr10:77776951-777
ENSG0000(	476	10.63029	chr10:81(	NUTM2B-AS1		lncRNA	chr10:79661394-798
ENSG0000(	476	10.63029	chr10:81(	ENSG00000244733		lncRNA	chr10:79660891-796
ENSG0000(	476	10.63029	chr10:81(	ENSG00000204049		lncRNA	chr10:77866875-778
ENSG0000(	476	10.63029	chr10:81(	ENSG00000213514		Pseudoger	chr10:77730766-777
ENSG0000(	476	10.63029	chr10:81(	COX6CP15		Pseudoger	chr10:77433572-774
ENSG0000(	476	10.63029	chr10:81(	ZMIZ1	NCGv7	protein_c	chr10:79068966-793
ENSG0000(	476	10.63029	chr10:81(	NUTM2E		protein_c	chr10:79826739-798
ENSG0000(	476	10.63029	chr10:81(	ENSG00000272447		lncRNA	chr10:79825902-798
ENSG0000(	476	10.63029	chr10:81(	ENSG00000282952		lncRNA	chr10:78538391-785
ENSG0000(	476	10.63029	chr10:81(	LINC02679		lncRNA	chr10:79628757-796
ENSG0000(	476	10.63029	chr10:81(	PPIF		protein_c	chr10:79347469-793
ENSG0000(	476	10.63029	chr10:81(	LINC00595		lncRNA	chr10:78179185-785
ENSG0000(	476	10.63029	chr10:81(	ENSG00000282863		lncRNA	chr10:78179174-786
ENSG0000(	476	10.63029	chr10:81(	RN7SL284P		smallRNA	chr10:77912274-779
ENSG0000(	476	10.63029	chr10:81(	POLR3A		protein_c	chr10:77953148-780
ENSG0000(	476	10.63029	chr10:81(	ENSG00000235426		lncRNA	chr10:79382328-794
ENSG0000(	476	10.63029	chr10:81(	DLG5		protein_c	chr10:77790791-779
ENSG0000(	476	10.63029	chr10:81(	SNORA71		smallRNA	chr10:78367507-783
ENSG0000(	476	10.63029	chr10:81(	ENSG00000228683		lncRNA	chr10:78696062-786
ENSG0000(	476	10.63029	chr10:81(	SFTPA2		protein_c	chr10:79555852-795
ENSG0000(	476	10.63029	chr10:81(	ENSG00000283096		lncRNA	chr10:78176928-781
ENSG0000(	476	10.63029	chr10:81(	ZCCHC24	NCGv7	protein_c	chr10:79382325-794
ENSG0000(	476	10.63029	chr10:81(	ENSG00000272489		lncRNA	chr10:79663192-796
ENSG0000(	476	10.63029	chr10:81(	ENSG00000241317		lncRNA	chr10:79504073-795

ENSG0000	476	10.63029	chr10:81	TPRX1P1		Pseudoger	chr10:79499666-794
ENSG0000	476	10.63029	chr10:81	SFTPA1		protein_c	chr10:79610939-796
ENSG0000	475	10.60796	chr12:171	ENSG00000286682		lncRNA	chr12:64820179-648
ENSG0000	475	10.60796	chr12:171	ENSG00000280320		TEC	chr12:64696191-646
ENSG0000	475	10.60796	chr12:171	RASSF3		protein_c	chr12:64507001-646
ENSG0000	475	10.60796	chr12:171	snoU13		smallRNA	chr12:64760868-647
ENSG0000	475	10.60796	chr12:171	GNS		protein_c	chr12:64713445-647
ENSG0000	475	10.60796	chr12:171	ENSG00000280181		TEC	chr12:64709458-647
ENSG0000	472	10.54096	chr3:380	EAF1		protein_c	chr3:15427598-1545
ENSG0000	472	10.54096	chr3:380	RNU6-454P		smallRNA	chr3:15339749-1533
ENSG0000	472	10.54096	chr3:380	FGD5	IntOGen-I	protein_c	chr3:14810853-1493
ENSG0000	472	10.54096	chr3:380	ENSG00000230172		lncRNA	chr3:14764952-1476
ENSG0000	472	10.54096	chr3:380	COL6A4P1		lncRNA	chr3:15165275-1520
ENSG0000	472	10.54096	chr3:380	ENSG00000283392		lncRNA	chr3:14602035-1464
ENSG0000	472	10.54096	chr3:380	snoU13		smallRNA	chr3:15316620-1531
ENSG0000	472	10.54096	chr3:380	ENSG00000289750		lncRNA	chr3:15048548-1509
ENSG0000	472	10.54096	chr3:380	CCDC174		protein_c	chr3:14651762-1467
ENSG0000	472	10.54096	chr3:380	RN7SL110P		smallRNA	chr3:15506625-1550
ENSG0000	472	10.54096	chr3:380	LINC02922		lncRNA	chr3:14648194-1464
ENSG0000	472	10.54096	chr3:380	ENSG00000288077		lncRNA	chr3:15501439-1550
ENSG0000	472	10.54096	chr3:380	LINC02011		lncRNA	chr3:14799331-1481
ENSG0000	472	10.54096	chr3:380	RHBDF1P1		Pseudoger	chr3:14572852-1457
ENSG0000	472	10.54096	chr3:380	ENSG00000228874		Pseudoger	chr3:15131848-1513
ENSG0000	472	10.54096	chr3:380	COLQ		protein_c	chr3:15450133-1552
ENSG0000	472	10.54096	chr3:380	METTL6		protein_c	chr3:15381275-1544
ENSG0000	472	10.54096	chr3:380	AC090954.1		smallRNA	chr3:15134383-1513
ENSG0000	472	10.54096	chr3:380	RNU6-1024P		smallRNA	chr3:15433110-1543
ENSG0000	472	10.54096	chr3:380	HMGN2P7		Pseudoger	chr3:15375373-1537
ENSG0000	472	10.54096	chr3:380	NR2C2		protein_c	chr3:14947583-1505
ENSG0000	472	10.54096	chr3:380	COL6A4P1		Pseudoger	chr3:15151833-1518
ENSG0000	472	10.54096	chr3:380	EAF1-AS1		lncRNA	chr3:15436171-1545
ENSG0000	472	10.54096	chr3:380	RBSN		protein_c	chr3:15070073-1509
ENSG0000	472	10.54096	chr3:380	ENSG00000290932		lncRNA	chr3:15151843-1515
ENSG0000	472	10.54096	chr3:380	C3orf20		protein_c	chr3:14675141-1477
ENSG0000	472	10.54096	chr3:380	RNU6-905P		smallRNA	chr3:14530982-1453
ENSG0000	472	10.54096	chr3:380	CAPN7		protein_c	chr3:15206152-1525
ENSG0000	472	10.54096	chr3:380	SH3BP5-AS1		lncRNA	chr3:15254184-1526
ENSG0000	472	10.54096	chr3:380	SH3BP5		protein_c	chr3:15254353-1534
ENSG0000	472	10.54096	chr3:380	Y_RNA		smallRNA	chr3:15237000-1523
ENSG0000	472	10.54096	chr3:380	ENSG00000285969		lncRNA	chr3:14791534-1479
ENSG0000	472	10.54096	chr3:380	RPS3AP53		Pseudoger	chr3:15141380-1514
ENSG0000	472	10.54096	chr3:380	FGD5-AS1		lncRNA	chr3:14920347-1494
ENSG0000	472	10.54096	chr3:380	MIR4270		smallRNA	chr3:15496239-1549
ENSG0000	472	10.54096	chr3:380	MRPS25		protein_c	chr3:15009611-1506
ENSG0000	471	10.51863	chr2:309	LINC02832		lncRNA	chr2:219685381-219
ENSG0000	471	10.51863	chr2:309	SLC23A3		protein_c	chr2:219161465-219
ENSG0000	471	10.51863	chr5:164	SELENOTP2		Pseudoger	chr5:127176696-127
ENSG0000	471	10.51863	chr2:309	ENSG00000269068		lncRNA	chr2:219559083-219
ENSG0000	471	10.51863	chr5:164	BOLA3P3		Pseudoger	chr5:126663337-126
ENSG0000	471	10.51863	chr2:309	TUBA4A		protein_c	chr2:219249710-219
ENSG0000	471	10.51863	chr2:309	VIL1		protein_c	chr2:218419121-218

ENSG0000(	471	10.51863	chr2:309⁄	DNPEP-AS1		lncRNA	chr2:219388496-219
ENSG0000(	471	10.51863	chr2:309⁄	AAMP		protein_c	chr2:218264129-218
ENSG0000(	471	10.51863	chr2:309⁄	PNKD	NCGv7	protein_c	chr2:218269651-218
ENSG0000(	471	10.51863	chr5:164(	CTXN3		protein_c	chr5:127649082-127
ENSG0000(	471	10.51863	chr5:164(	ENSG00000282925		lncRNA	chr5:127588746-127
ENSG0000(	471	10.51863	chr5:164(	ENSG00000250602		lncRNA	chr5:126372477-126
ENSG0000(	471	10.51863	chr2:309⁄	SLC4A3		protein_c	chr2:219627394-219
ENSG0000(	471	10.51863	chr2:309⁄	ENSG00000224414		Pseudoger	chr2:217693895-217
ENSG0000(	471	10.51863	chr2:309⁄	PLCD4		protein_c	chr2:218607855-218
ENSG0000(	471	10.51863	chr2:309⁄	ZNF142		protein_c	chr2:218633329-218
ENSG0000(	471	10.51863	chr2:309⁄	PRKAG3		protein_c	chr2:218822308-218
ENSG0000(	471	10.51863	chr2:309⁄	CCDC140		lncRNA	chr2:222298147-222
ENSG0000(	471	10.51863	chr2:309⁄	WNT6		protein_c	chr2:218859805-218
ENSG0000(	471	10.51863	chr5:164(	ENSG00000249577		Pseudoger	chr5:127465822-127
ENSG0000(	471	10.51863	chr2:309⁄	RPL23AP28		Pseudoger	chr2:222044525-222
ENSG0000(	471	10.51863	chr2:309⁄	ENSG00000268896		lncRNA	chr2:219547211-219
ENSG0000(	471	10.51863	chr2:309⁄	RPL23AP31		Pseudoger	chr2:218841599-218
ENSG0000(	471	10.51863	chr2:309⁄	OBSL1		protein_c	chr2:219550728-219
ENSG0000(	471	10.51863	chr2:309⁄	RPL23P4		Pseudoger	chr2:219697799-219
ENSG0000(	471	10.51863	chr2:309⁄	ENSG00000287498		lncRNA	chr2:217061067-217
ENSG0000(	471	10.51863	chr5:164(	LMNB1		protein_c	chr5:126776623-126
ENSG0000(	471	10.51863	chr2:309⁄	ZFAND2B		protein_c	chr2:219195237-219
ENSG0000(	471	10.51863	chr2:309⁄	TNS1		protein_c	chr2:217799588-218
ENSG0000(	471	10.51863	chr5:164(	AC010235.1		smallRNA	chr5:126196835-126
ENSG0000(	471	10.51863	chr2:309⁄	LLPHP3		Pseudoger	chr2:221944223-221
ENSG0000(	471	10.51863	chr2:309⁄	ENSG00000287529		lncRNA	chr2:219198640-219
ENSG0000(	471	10.51863	chr2:309⁄	ENSG00000235337		lncRNA	chr2:220127546-220
ENSG0000(	471	10.51863	chr2:309⁄	CATIP	NCGv7	protein_c	chr2:218356857-218
ENSG0000(	471	10.51863	chr2:309⁄	LINC01494		lncRNA	chr2:218900811-218
ENSG0000(	471	10.51863	chr2:309⁄	ENSG00000286272		lncRNA	chr2:220811618-220
ENSG0000(	471	10.51863	chr2:309⁄	MIR26B		smallRNA	chr2:218402646-218
ENSG0000(	471	10.51863	chr2:309⁄	ENSG00000267919		lncRNA	chr2:219645090-219
ENSG0000(	471	10.51863	chr2:309⁄	CXCR2P1		Pseudoger	chr2:218060339-218
ENSG0000(	471	10.51863	chr2:309⁄	ENSG00000284820		protein_c	chr2:219209772-219
ENSG0000(	471	10.51863	chr2:309⁄	CDK5R2	AC	protein_c	chr2:218959666-218
ENSG0000(	471	10.51863	chr2:309⁄	ENSG00000236451		lncRNA	chr2:220450679-220
ENSG0000(	471	10.51863	chr2:309⁄	ENSG00000268603		lncRNA	chr2:219497611-219
ENSG0000(	471	10.51863	chr2:309⁄	ASIC4-AS1		lncRNA	chr2:219482073-219
ENSG0000(	471	10.51863	chr2:309⁄	HMGB1P9		Pseudoger	chr2:218200434-218
ENSG0000(	471	10.51863	chr2:309⁄	ENSG00000224090		lncRNA	chr2:219002215-219
ENSG0000(	471	10.51863	chr2:309⁄	CXCR2	NCGv7	protein_c	chr2:218125289-218
ENSG0000(	471	10.51863	chr2:309⁄	CHPF		protein_c	chr2:219538954-219
ENSG0000(	471	10.51863	chr2:309⁄	TNS1-AS1		lncRNA	chr2:217978707-217
ENSG0000(	471	10.51863	chr2:309⁄	DNPEP		protein_c	chr2:219372043-219
ENSG0000(	471	10.51863	chr5:164(	MRPS5P3		Pseudoger	chr5:127143082-127
ENSG0000(	471	10.51863	chr2:309⁄	ENSG00000272944		lncRNA	chr2:221572506-221
ENSG0000(	471	10.51863	chr2:309⁄	ABCB6		protein_c	chr2:219209772-219
ENSG0000(	471	10.51863	chr2:309⁄	INHA		protein_c	chr2:219569162-219
ENSG0000(	471	10.51863	chr2:309⁄	LINC00608		lncRNA	chr2:218975393-218
ENSG0000(	471	10.51863	chr2:309⁄	CNPPD1		protein_c	chr2:219171897-219
ENSG0000(	471	10.51863	chr2:309⁄	ARPC2		protein_c	chr2:218217141-218

ENSG0000(	471	10.51863	chr5:164(	RPSAP37		Pseudoger	chr5:125966777-125
ENSG0000(	471	10.51863	chr2:309⁄	CYP27A1		protein_c	chr2:218781749-218
ENSG0000(	471	10.51863	chr5:164(	ENSG00000250956		Pseudoger	chr5:127179756-127
ENSG0000(	471	10.51863	chr2:309⁄	ENSG00000232089		Pseudoger	chr2:221162771-221
ENSG0000(	471	10.51863	chr2:309⁄	ENSG00000288902		lncRNA	chr2:219947834-220
ENSG0000(	471	10.51863	chr2:309⁄	ENSG00000288898		lncRNA	chr2:218909158-218
ENSG0000(	471	10.51863	chr5:164(	PRRC1		protein_c	chr5:127517640-127
ENSG0000(	471	10.51863	chr5:164(	C5orf63		protein_c	chr5:127045235-127
ENSG0000(	471	10.51863	chr2:309⁄	GPBAR1		protein_c	chr2:218259496-218
ENSG0000(	471	10.51863	chr2:309⁄	TMEM198		protein_c	chr2:219543663-219
ENSG0000(	471	10.51863	chr2:309⁄	CT75		lncRNA	chr2:222318471-222
ENSG0000(	471	10.51863	chr2:309⁄	TMEM256P2		Pseudoger	chr2:221532668-221
ENSG0000(	471	10.51863	chr2:309⁄	PAX3	NCGv7;AC	protein_c	chr2:222199887-222
ENSG0000(	471	10.51863	chr2:309⁄	TTLL4		protein_c	chr2:218710835-218
ENSG0000(	471	10.51863	chr2:309⁄	USP37		protein_c	chr2:218450251-218
ENSG0000(	471	10.51863	chr2:309⁄	DNAJB2	NCGv7	protein_c	chr2:219279342-219
ENSG0000(	471	10.51863	chr2:309⁄	WNT10A	AC	protein_c	chr2:218880852-218
ENSG0000(	471	10.51863	chr2:309⁄	TMBIM1		protein_c	chr2:218274197-218
ENSG0000(	471	10.51863	chr2:309⁄	DIRC3		lncRNA	chr2:217284019-217
ENSG0000(	471	10.51863	chr2:309⁄	ENSG00000234446		lncRNA	chr2:221609324-221
ENSG0000(	471	10.51863	chr2:309⁄	ENSG00000228973		lncRNA	chr2:219625059-219
ENSG0000(	471	10.51863	chr2:309⁄	HSPA9P1		Pseudoger	chr2:221961737-221
ENSG0000(	471	10.51863	chr2:309⁄	RNU6-642P		smallRNA	chr2:218916184-218
ENSG0000(	471	10.51863	chr2:309⁄	DNAJB6P3		Pseudoger	chr2:219684030-219
ENSG0000(	471	10.51863	chr2:309⁄	RN7SKP43		smallRNA	chr2:217251556-217
ENSG0000(	471	10.51863	chr2:309⁄	ENSG00000234638		lncRNA	chr2:219425071-219
ENSG0000(	471	10.51863	chr5:164(	HNRNPKP1		Pseudoger	chr5:127511464-127
ENSG0000(	471	10.51863	chr5:164(	ENSG00000271766		lncRNA	chr5:127478295-127
ENSG0000(	471	10.51863	chr2:309⁄	TUBA4B		protein_c	chr2:219253243-219
ENSG0000(	471	10.51863	chr2:309⁄	RESP18	NCGv7	protein_c	chr2:219327407-219
ENSG0000(	471	10.51863	chr2:309⁄	RPL7L1P9		Pseudoger	chr2:217760547-217
ENSG0000(	471	10.51863	chr5:164(	ENSG00000279446		TEC	chr5:127073587-127
ENSG0000(	471	10.51863	chr2:309⁄	MIR153-1		smallRNA	chr2:219294111-219
ENSG0000(	471	10.51863	chr2:309⁄	ENSG00000261338		lncRNA	chr2:218255319-218
ENSG0000(	471	10.51863	chr2:309⁄	ENSG00000280323		TEC	chr2:219427477-219
ENSG0000(	471	10.51863	chr5:164(	ALDH7A1		protein_c	chr5:126531200-126
ENSG0000(	471	10.51863	chr5:164(	PHAX	NCGv7	protein_c	chr5:126600925-126
ENSG0000(	471	10.51863	chr2:309⁄	ENSG00000225911		Pseudoger	chr2:220048822-220
ENSG0000(	471	10.51863	chr2:309⁄	LINC01803		lncRNA	chr2:219903900-219
ENSG0000(	471	10.51863	chr5:164(	ENSG00000230929		Pseudoger	chr5:126628019-126
ENSG0000(	471	10.51863	chr2:309⁄	RNU6-136P		smallRNA	chr2:218589214-218
ENSG0000(	471	10.51863	chr2:309⁄	STK16		protein_c	chr2:219245455-219
ENSG0000(	471	10.51863	chr2:309⁄	ENSG00000272555		lncRNA	chr2:218818690-218
ENSG0000(	471	10.51863	chr2:309⁄	ANKZF1		protein_c	chr2:219229783-219
ENSG0000(	471	10.51863	chr2:309⁄	IHH		protein_c	chr2:219054424-219
ENSG0000(	471	10.51863	chr2:309⁄	CRYBA2		protein_c	chr2:218990189-218
ENSG0000(	471	10.51863	chr2:309⁄	FEV	NCGv7;AC	protein_c	chr2:218981087-218
ENSG0000(	471	10.51863	chr2:309⁄	STK36		protein_c	chr2:218672069-218
ENSG0000(	471	10.51863	chr2:309⁄	RNF25		protein_c	chr2:218663892-218
ENSG0000(	471	10.51863	chr5:164(	ENSG00000283897		lncRNA	chr5:127215159-127
ENSG0000(	471	10.51863	chr5:164(	ENSG00000286615		lncRNA	chr5:127231959-127

ENSG0000(		471	10.51863	chr2:3094	CXCR1		protein_c	chr2:218162841-218
ENSG0000(		471	10.51863	chr2:3094	ENSG00000286606		lncRNA	chr2:219065308-219
ENSG0000(		471	10.51863	chr5:1646	RNU6-963P		smallRNA	chr5:126553302-126
ENSG0000(		471	10.51863	chr2:3094	CDK5R2-AS1		lncRNA	chr2:218944629-218
ENSG0000(		471	10.51863	chr2:3094	SLC11A1		protein_c	chr2:218382029-218
ENSG0000(		471	10.51863	chr2:3094	EPHA4	NCGv7	protein_c	chr2:221418027-221
ENSG0000(		471	10.51863	chr2:3094	BCS1L		protein_c	chr2:218658764-218
ENSG0000(		471	10.51863	chr2:3094	CATIP-AS2		lncRNA	chr2:218326889-218
ENSG0000(		471	10.51863	chr2:3094	RUFY4		protein_c	chr2:218034960-218
ENSG0000(		471	10.51863	chr2:3094	ENSG00000224819		lncRNA	chr2:220776818-220
ENSG0000(		471	10.51863	chr2:3094	RN7SL764P		smallRNA	chr2:219102434-219
ENSG0000(		471	10.51863	chr2:3094	snoU13		smallRNA	chr2:218184979-218
ENSG0000(		471	10.51863	chr5:1646	LMNB1-DT		lncRNA	chr5:126751963-126
ENSG0000(		471	10.51863	chr5:1646	AC011416.1		smallRNA	chr5:127260936-127
ENSG0000(		471	10.51863	chr5:1646	CUL1P1		Pseudoger	chr5:127784618-127
ENSG0000(		471	10.51863	chr2:3094	PTPRN		protein_c	chr2:219289623-219
ENSG0000(		471	10.51863	chr5:1646	MARCHF3		protein_c	chr5:126867714-127
ENSG0000(		471	10.51863	chr2:3094	DES		protein_c	chr2:219418377-219
ENSG0000(		471	10.51863	chr5:1646	RNU6-752P		smallRNA	chr5:126755316-126
ENSG0000(		471	10.51863	chr2:3094	snoU13		smallRNA	chr2:218328580-218
ENSG0000(		471	10.51863	chr2:3094	CATIP-AS1		lncRNA	chr2:218366661-218
ENSG0000(		471	10.51863	chr5:1646	ENSG00000248799		lncRNA	chr5:127651693-127
ENSG0000(		471	10.51863	chr2:3094	ASIC4		protein_c	chr2:219514170-219
ENSG0000(		471	10.51863	chr2:3094	SPEG	NCGv7	protein_c	chr2:219434843-219
ENSG0000(		471	10.51863	chr2:3094	MIR3132		smallRNA	chr2:219549073-219
ENSG0000(		471	10.51863	chr2:3094	KRT8P30		Pseudoger	chr2:218953401-218
ENSG0000(		471	10.51863	chr2:3094	U3		smallRNA	chr2:218619801-218
ENSG0000(		471	10.51863	chr2:3094	CFAP65		protein_c	chr2:219002846-219
ENSG0000(		471	10.51863	chr2:3094	ENSG00000280537		protein_c	chr2:219075329-219
ENSG0000(		471	10.51863	chr5:1646	TEX43		protein_c	chr5:126631705-126
ENSG0000(		471	10.51863	chr2:3094	GLB1L		protein_c	chr2:219236598-219
ENSG0000(		471	10.51863	chr5:1646	HSPE1P10		Pseudoger	chr5:126737438-126
ENSG0000(		471	10.51863	chr2:3094	ENSG00000273361		lncRNA	chr2:218398743-218
ENSG0000(		471	10.51863	chr2:3094	MIR375		smallRNA	chr2:219001640-219
ENSG0000(		471	10.51863	chr2:3094	AC019051.1		smallRNA	chr2:220476983-220
ENSG0000(		471	10.51863	chr2:3094	MIR4268		smallRNA	chr2:219906502-219
ENSG0000(		471	10.51863	chr5:1646	MEGF10	DriverDBv	protein_c	chr5:127290796-127
ENSG0000(		471	10.51863	chr2:3094	RPL23P5		Pseudoger	chr2:221592441-221
ENSG0000(		471	10.51863	chr2:3094	RN7SKP213		smallRNA	chr2:219590687-219
ENSG0000(		471	10.51863	chr2:3094	CTDSP1		protein_c	chr2:218398256-218
ENSG0000(		471	10.51863	chr2:3094	CNOT9	NCGv7	protein_c	chr2:218568580-218
ENSG0000(		471	10.51863	chr2:3094	STK11IP	NCGv7	protein_c	chr2:219597857-219
ENSG0000(		471	10.51863	chr2:3094	RN7SKP38		smallRNA	chr2:218500729-218
ENSG0000(		471	10.51863	chr2:3094	GMPPA		protein_c	chr2:219498865-219
ENSG0000(		471	10.51863	chr2:3094	ENSG00000288658		protein_c	chr2:222314148-222
ENSG0000(		471	10.51863	chr5:1646	ENSG00000249904		Pseudoger	chr5:125886285-125
ENSG0000(		471	10.51863	chr2:3094	AC068946.1		smallRNA	chr2:219224023-219
ENSG0000(		471	10.51863	chr2:3094	ENSG00000291236		lncRNA	chr2:218059155-218
ENSG0000(		471	10.51863	chr5:1646	ENSG00000249950		Pseudoger	chr5:125862746-125
ENSG0000(		471	10.51863	chr2:3094	ENSG00000230432		lncRNA	chr2:219299002-219
ENSG0000(		471	10.51863	chr5:1646	GRAMD2B		protein_c	chr5:126360132-126

ENSG0000(	471	10.51863	chr2:309<	ENSG00000239498	lncRNA	chr2:220105656-220
ENSG0000(	471	10.51863	chr2:309<	DIRC3-AS1	lncRNA	chr2:216694404-217
ENSG0000(	471	10.51863	chr2:309<	ENSG00000286143	protein_c	chr2:219491867-219
ENSG0000(	471	10.51863	chr5:164(	ENSG00000250080	Pseudoger	chr5:127170535-127
ENSG0000(	471	10.51863	chr2:309<	ENSG00000290000	lncRNA	chr2:221286244-221
ENSG0000(	471	10.51863	chr2:309<	ENSG00000290093	lncRNA	chr2:219177068-219
ENSG0000(	471	10.51863	chr2:309<	CT75	Pseudoger	chr2:222318275-222
ENSG0000(	471	10.51863	chr2:309<	AC114765.3	smallRNA	chr2:220191878-220
ENSG0000(	471	10.51863	chr2:309<	ATG9A	protein_c	chr2:219219380-219
ENSG0000(	471	10.51863	chr2:309<	ENSG00000232789	Pseudoger	chr2:219358870-219
ENSG0000(	471	10.51863	chr2:309<	ENSG00000286154	lncRNA	chr2:218799729-218
ENSG0000(	471	10.51863	chr2:309<	NHEJ1	protein_c	chr2:219069355-219
ENSG0000(	471	10.51863	chr2:309<	SPEGNB	protein_c	chr2:219496131-219
ENSG0000(	471	10.51863	chr2:309<	RETREG2	protein_c	chr2:219176225-219
ENSG0000(	471	10.51863	chr2:309<	AC079834.2	smallRNA	chr2:221519564-221
ENSG0000(	471	10.51863	chr2:309<	ENSG00000286199	lncRNA	chr2:219904983-219
ENSG0000(	471	10.51863	chr2:309<	ENSG00000286170	lncRNA	chr2:221306290-221
ENSG0000(	471	10.51863	chr5:164(	LINC02039	lncRNA	chr5:126179565-126
ENSG0000(	471	10.51863	chr2:309<	RPL19P5	Pseudoger	chr2:218354075-218
ENSG0000(	471	10.51863	chr5:164(	ENSG00000279118	TEC	chr5:126496279-126
ENSG0000(	469	10.47396	chr5:164(	ANKRD31	protein_c	chr5:75068275-7523
ENSG0000(	469	10.47396	chr5:164(	PTCD2	protein_c	chr5:72320367-7236
ENSG0000(	469	10.47396	chr5:164(	AC099522.1	Pseudoger	chr5:73446256-7344
ENSG0000(	469	10.47396	chr5:164(	ENSG00000289639	lncRNA	chr5:74868003-7486
ENSG0000(	469	10.47396	chr5:164(	ENSG00000261269	lncRNA	chr5:72439903-7244
ENSG0000(	469	10.47396	chr5:164(	BTF3-DT	lncRNA	chr5:73497549-7349
ENSG0000(	469	10.47396	chr5:164(	FCHO2-DT	lncRNA	chr5:72955206-7295
ENSG0000(	469	10.47396	chr5:164(	RNU6-658P	smallRNA	chr5:74725099-7472
ENSG0000(	469	10.47396	chr5:164(	TNPO1-DT	lncRNA	chr5:72794405-7281
ENSG0000(	469	10.47396	chr5:164(	FCHO2	protein_c	chr5:72956041-7309
ENSG0000(	469	10.47396	chr5:164(	SUMO2P5	Pseudoger	chr5:75225424-7522
ENSG0000(	469	10.47396	chr5:164(	HEXB	protein_c	chr5:74640023-7472
ENSG0000(	469	10.47396	chr5:164(	ENSG00000242198	Pseudoger	chr5:75374463-7537
ENSG0000(	469	10.47396	chr5:164(	Y_RNA	smallRNA	chr5:73100073-7310
ENSG0000(	469	10.47396	chr5:164(	ENSG00000239517	Pseudoger	chr5:74931662-7493
ENSG0000(	469	10.47396	chr5:164(	TMEM171	protein_c	chr5:73120569-7313
ENSG0000(	469	10.47396	chr5:164(	LINC01332	lncRNA	chr5:74327995-7433
ENSG0000(	469	10.47396	chr5:164(	ENSG00000259968	lncRNA	chr5:73952940-7395
ENSG0000(	469	10.47396	chr5:164(	LINC02056	lncRNA	chr5:72574120-7266
ENSG0000(	469	10.47396	chr5:164(	ENSG00000249856	lncRNA	chr5:74917726-7502
ENSG0000(	469	10.47396	chr5:164(	FOXD1	protein_c	chr5:73444827-7344
ENSG0000(	469	10.47396	chr5:164(	GCNT4	protein_c	chr5:75025346-7505
ENSG0000(	469	10.47396	chr5:164(	FUNDC2P1	Pseudoger	chr5:73508685-7350
ENSG0000(	469	10.47396	chr5:164(	ENSG00000244061	Pseudoger	chr5:72381794-7238
ENSG0000(	469	10.47396	chr5:164(	RN7SL814P	smallRNA	chr5:74317986-7431
ENSG0000(	469	10.47396	chr5:164(	YBX1P5	Pseudoger	chr5:72417489-7241
ENSG0000(	469	10.47396	chr5:164(	ZNF366	protein_c	chr5:72439903-7250
ENSG0000(	469	10.47396	chr5:164(	LINC01385	lncRNA	chr5:73451498-7345
ENSG0000(	469	10.47396	chr5:164(	RPL27AP5	Pseudoger	chr5:74990189-7499
ENSG0000(	469	10.47396	chr5:164(	LINC01331	lncRNA	chr5:74111690-7453
ENSG0000(	469	10.47396	chr5:164(	ENSG00000289535	lncRNA	chr5:74948146-7497

ENSG0000(	469	10.47396	chr5:164(	LINC02122		lncRNA	chr5:74084068-7410
ENSG0000(	469	10.47396	chr5:164(	ENSG00000287701		lncRNA	chr5:74899115-7490
ENSG0000(	469	10.47396	chr5:164(	ENSG00000249157		Pseudoger	chr5:75006713-7500
ENSG0000(	469	10.47396	chr5:164(	ENSG00000247372		lncRNA	chr5:75320155-7533
ENSG0000(	469	10.47396	chr5:164(	LINC01335		lncRNA	chr5:74258689-7432
ENSG0000(	469	10.47396	chr5:164(	RNU7-196P		smallRNA	chr5:73828012-7382
ENSG0000(	469	10.47396	chr5:164(	ENSG00000251613		lncRNA	chr5:72687112-7277
ENSG0000(	469	10.47396	chr5:164(	ENSG00000271926		lncRNA	chr5:72953635-7295
ENSG0000(	469	10.47396	chr5:164(	ENSG00000251467		Pseudoger	chr5:72996920-7299
ENSG0000(	469	10.47396	chr5:164(	ENSG00000288883		lncRNA	chr5:74294413-7430
ENSG0000(	469	10.47396	chr5:164(	ENSG00000250071		Pseudoger	chr5:74798994-7480
ENSG0000(	469	10.47396	chr5:164(	CHP1P1		Pseudoger	chr5:73020700-7302
ENSG0000(	469	10.47396	chr5:164(	FAM169A		protein_c	chr5:74777574-7486
ENSG0000(	469	10.47396	chr5:164(	H2BL1P		Pseudoger	chr5:72733833-7273
ENSG0000(	469	10.47396	chr5:164(	ENSG00000250747		Pseudoger	chr5:73152416-7315
ENSG0000(	469	10.47396	chr5:164(	ANKRA2		protein_c	chr5:73552190-7356
ENSG0000(	469	10.47396	chr5:164(	FOXD1-AS1		lncRNA	chr5:73446357-7344
ENSG0000(	469	10.47396	chr5:164(	LINC02230		lncRNA	chr5:73337906-7333
ENSG0000(	469	10.47396	chr5:164(	UTP15		protein_c	chr5:73565443-7358
ENSG0000(	469	10.47396	chr5:164(	ENC1	NCGv7	protein_c	chr5:74627406-7464
ENSG0000(	469	10.47396	chr5:164(	ENSG00000249293		lncRNA	chr5:73778039-7378
ENSG0000(	469	10.47396	chr5:164(	SNORA40		smallRNA	chr5:74882656-7488
ENSG0000(	469	10.47396	chr5:164(	ENSG00000184084		Pseudoger	chr5:73803296-7380
ENSG0000(	469	10.47396	chr5:164(	TMEM174	DriverDBv	protein_c	chr5:73173193-7317
ENSG0000(	469	10.47396	chr5:164(	RPL35AP13		Pseudoger	chr5:72878868-7287
ENSG0000(	469	10.47396	chr5:164(	RPL7P22		Pseudoger	chr5:72725419-7272
ENSG0000(	469	10.47396	chr5:164(	ENSG00000287090		lncRNA	chr5:73195314-7320
ENSG0000(	469	10.47396	chr5:164(	CERT1		protein_c	chr5:75356345-7551
ENSG0000(	469	10.47396	chr5:164(	HMGCR		protein_c	chr5:75336329-7536
ENSG0000(	469	10.47396	chr5:164(	ENSG00000196390		Pseudoger	chr5:74929880-7493
ENSG0000(	469	10.47396	chr5:164(	LINC01333		lncRNA	chr5:74321293-7434
ENSG0000(	469	10.47396	chr5:164(	MRPS27		protein_c	chr5:72214953-7232
ENSG0000(	469	10.47396	chr5:164(	RN7SL153P		smallRNA	chr5:72314399-7231
ENSG0000(	469	10.47396	chr5:164(	AC106732.1		smallRNA	chr5:74271682-7427
ENSG0000(	469	10.47396	chr5:164(	LINC01386		lncRNA	chr5:73454187-7347
ENSG0000(	469	10.47396	chr5:164(	NSA2		protein_c	chr5:74766991-7478
ENSG0000(	469	10.47396	chr5:164(	ENSG00000249149		Pseudoger	chr5:73382384-7341
ENSG0000(	469	10.47396	chr5:164(	ENSG00000251599		lncRNA	chr5:73132008-7315
ENSG0000(	469	10.47396	chr5:164(	TNPO1	NCGv7	protein_c	chr5:72816312-7291
ENSG0000(	469	10.47396	chr5:164(	RNU6-1330P		smallRNA	chr5:74779309-7477
ENSG0000(	469	10.47396	chr5:164(	Y_RNA		smallRNA	chr5:72768702-7276
ENSG0000(	469	10.47396	chr5:164(	AC116345.1		smallRNA	chr5:73154111-7315
ENSG0000(	469	10.47396	chr5:164(	MIR4804		smallRNA	chr5:72878591-7287
ENSG0000(	469	10.47396	chr5:164(	ARHGEF28		protein_c	chr5:73626158-7394
ENSG0000(	469	10.47396	chr5:164(	GFM2		protein_c	chr5:74721206-7476
ENSG0000(	469	10.47396	chr5:164(	FAM169A-AS1		lncRNA	chr5:74865893-7486
ENSG0000(	469	10.47396	chr5:164(	ENSG00000249743		lncRNA	chr5:73213930-7329
ENSG0000(	469	10.47396	chr5:164(	BTF3		protein_c	chr5:73498408-7350
ENSG0000(	466	10.40697	chr10:81(	C1DP2		Pseudoger	chr10:80031968-800
ENSG0000(	466	10.40697	chr10:81(	ENSG00000229616		Pseudoger	chr10:80017713-800
ENSG0000(	466	10.40697	chr10:81(	ENSG00000234382		Pseudoger	chr10:80233664-802

ENSG0000(	466	10.40697	chr10:81(	MAT1A		protein_c	chr10:80271820-802
ENSG0000(	466	10.40697	chr10:81(	PRXL2A		protein_c	chr10:80407829-804
ENSG0000(	466	10.40697	chr10:81(	DYDC2		protein_c	chr10:80344745-803
ENSG0000(	466	10.40697	chr10:81(	ENSG00000279399		TEC	chr10:80091411-800
ENSG0000(	466	10.40697	chr11:62(	ENSG00000256041		Pseudoger	chr11:63032503-630
ENSG0000(	466	10.40697	chr10:81(	EIF5AP4		Pseudoger	chr10:80247219-802
ENSG0000(	466	10.40697	chr11:62(	CCND2P1		Pseudoger	chr11:63243085-632
ENSG0000(	466	10.40697	chr11:62(	ENSG00000256181		Pseudoger	chr11:63265836-632
ENSG0000(	466	10.40697	chr11:62(	SLC22A10		protein_c	chr11:63268022-633
ENSG0000(	466	10.40697	chr10:81(	TSPAN14		protein_c	chr10:80454265-805
ENSG0000(	466	10.40697	chr10:81(	DYDC1		protein_c	chr10:80336105-803
ENSG0000(	466	10.40697	chr10:81(	SFTPD		protein_c	chr10:79937467-799
ENSG0000(	466	10.40697	chr10:81(	ENSG00000279359		TEC	chr10:80249710-802
ENSG0000(	466	10.40697	chr10:81(	ZNF519P1		Pseudoger	chr10:80297720-802
ENSG0000(	466	10.40697	chr10:81(	RPL22P18		Pseudoger	chr10:80125941-801
ENSG0000(	466	10.40697	chr11:62(	TUBAP7		Pseudoger	chr11:63046785-630
ENSG0000(	466	10.40697	chr10:81(	BMS1P21		Pseudoger	chr10:79906605-799
ENSG0000(	466	10.40697	chr10:81(	SFTPD-AS1		lncRNA	chr10:79968213-799
ENSG0000(	466	10.40697	chr10:81(	TMEM254-AS1		lncRNA	chr10:80046860-800
ENSG0000(	466	10.40697	chr10:81(	ZNRF2P3		Pseudoger	chr10:79981839-799
ENSG0000(	466	10.40697	chr10:81(	ENSG00000231569		Pseudoger	chr10:87408044-874
ENSG0000(	466	10.40697	chr10:81(	LINC00857		lncRNA	chr10:80207372-802
ENSG0000(	466	10.40697	chr10:81(	ANXA11		protein_c	chr10:80150889-802
ENSG0000(	466	10.40697	chr10:81(	DPY19L2P5		Pseudoger	chr10:79982910-799
ENSG0000(	466	10.40697	chr10:81(	RPS12P2		Pseudoger	chr10:80215851-802
ENSG0000(	466	10.40697	chr10:81(	C1DP3		Pseudoger	chr10:80024737-800
ENSG0000(	466	10.40697	chr11:62(	SLC22A9		protein_c	chr11:63369785-634
ENSG0000(	466	10.40697	chr10:81(	CTSLP6		Pseudoger	chr10:79870375-798
ENSG0000(	466	10.40697	chr11:62(	SLC22A25		protein_c	chr11:63158437-632
ENSG0000(	466	10.40697	chr10:81(	MBL1P		Pseudoger	chr10:79920178-799
ENSG0000(	466	10.40697	chr10:81(	ENSG00000283913		lncRNA	chr10:79904898-799
ENSG0000(	466	10.40697	chr10:81(	TMEM254		protein_c	chr10:80078646-800
ENSG0000(	466	10.40697	chr11:62(	SLC22A8	NCGv7	protein_c	chr11:62989154-630
ENSG0000(	466	10.40697	chr10:81(	ENSG00000282432		Pseudoger	chr10:80053428-800
ENSG0000(	466	10.40697	chr10:81(	NPAP1P2		Pseudoger	chr10:79860573-798
ENSG0000(	466	10.40697	chr11:62(	RPL29P22		Pseudoger	chr11:63115880-631
ENSG0000(	466	10.40697	chr10:81(	ENSG00000235858		lncRNA	chr10:80333771-803
ENSG0000(	466	10.40697	chr11:62(	SLC22A24		protein_c	chr11:63079940-631
ENSG0000(	466	10.40697	chr10:81(	PGGT1BP2		Pseudoger	chr10:79892148-798
ENSG0000(	466	10.40697	chr10:81(	C1DP4		Pseudoger	chr10:80040621-800
ENSG0000(	466	10.40697	chr10:81(	PLAC9		protein_c	chr10:80131682-801
ENSG0000(	465	10.38463	chr2:309(	RNU6-619P		smallRNA	chr2:222498291-222
ENSG0000(	463	10.33997	chr5:164(	FEM1C		protein_c	chr5:115520908-115
ENSG0000(	463	10.33997	chr5:164(	ENSG00000270779		Pseudoger	chr5:110908284-110
ENSG0000(	463	10.33997	chr5:164(	STARD4		protein_c	chr5:111496033-111
ENSG0000(	463	10.33997	chr5:164(	MIR548F3		smallRNA	chr5:110513829-110
ENSG0000(	463	10.33997	chr5:164(	SLC25A46		protein_c	chr5:110738136-110
ENSG0000(	463	10.33997	chr5:164(	PTMAP2		Pseudoger	chr5:118973796-118
ENSG0000(	463	10.33997	chr5:164(	ENSG00000250928		Pseudoger	chr5:119011471-119
ENSG0000(	463	10.33997	chr5:164(	ZNF474		protein_c	chr5:122129546-122
ENSG0000(	463	10.33997	chr5:164(	ENSG00000243304		Pseudoger	chr5:115264669-115

ENSG0000(	463	10.33997	chr5:164(	ENSG00000248827		Pseudoger	chr5:107724961-107
ENSG0000(	463	10.33997	chr5:164(	LOX	IntOGen-I	protein_c	chr5:122063195-122
ENSG0000(	463	10.33997	chr5:164(	COMMD10		protein_c	chr5:116085016-116
ENSG0000(	463	10.33997	chr5:164(	ENSG00000289497		lncRNA	chr5:115364197-115
ENSG0000(	463	10.33997	chr5:164(	RAB9BP1		Pseudoger	chr5:105099473-105
ENSG0000(	463	10.33997	chr5:164(	MTND3P19		Pseudoger	chr5:100050719-100
ENSG0000(	463	10.33997	chr5:164(	ENSG00000288008		lncRNA	chr5:124993408-124
ENSG0000(	463	10.33997	chr5:164(	ENSG00000232633		lncRNA	chr5:113323028-113
ENSG0000(	463	10.33997	chr5:164(	ENSG00000248876		lncRNA	chr5:109470843-109
ENSG0000(	463	10.33997	chr5:164(	LAMTOR3P2		Pseudoger	chr5:119132651-119
ENSG0000(	463	10.33997	chr5:164(	PGGT1B		protein_c	chr5:115204012-115
ENSG0000(	463	10.33997	chr5:164(	CCDC112		protein_c	chr5:115267190-115
ENSG0000(	463	10.33997	chr5:164(	CSNK1G3		protein_c	chr5:123512177-123
ENSG0000(	463	10.33997	chr5:164(	ENSG00000248443		lncRNA	chr5:124829472-124
ENSG0000(	463	10.33997	chr5:164(	TNFAIP8	NCGv7	protein_c	chr5:119268692-119
ENSG0000(	463	10.33997	chr5:164(	OR7H2P		Pseudoger	chr5:101816475-101
ENSG0000(	463	10.33997	chr5:164(	ENSG00000248350		Pseudoger	chr5:112363456-112
ENSG0000(	463	10.33997	chr5:164(	CTNNA1P1		Pseudoger	chr5:115389643-115
ENSG0000(	463	10.33997	chr5:164(	MTCYBP22		Pseudoger	chr5:100045928-100
ENSG0000(	463	10.33997	chr5:164(	MTND6P22		Pseudoger	chr5:100046450-100
ENSG0000(	463	10.33997	chr5:164(	MTCO2P22		Pseudoger	chr5:100052859-100
ENSG0000(	463	10.33997	chr5:164(	TMEM232	DriverDB\	protein_c	chr5:110289233-110
ENSG0000(	463	10.33997	chr5:164(	PRELID3BP8		Pseudoger	chr5:120658077-120
ENSG0000(	463	10.33997	chr5:164(	SRFBP1		protein_c	chr5:121961975-122
ENSG0000(	463	10.33997	chr5:164(	ENSG00000286338		lncRNA	chr5:102664610-102
ENSG0000(	463	10.33997	chr5:164(	RNU6-644P		smallRNA	chr5:116188455-116
ENSG0000(	463	10.33997	chr5:164(	SRP19		protein_c	chr5:112861188-112
ENSG0000(	463	10.33997	chr5:164(	ENSG00000229855		lncRNA	chr5:121164687-121
ENSG0000(	463	10.33997	chr5:164(	ENSG00000288766		lncRNA	chr5:123511177-123
ENSG0000(	463	10.33997	chr5:164(	PRDM6-AS1		lncRNA	chr5:123087248-123
ENSG0000(	463	10.33997	chr5:164(	RP11-395P13.5		lncRNA	chr5:125382859-125
ENSG0000(	463	10.33997	chr5:164(	XBP1P1		Pseudoger	chr5:112885094-112
ENSG0000(	463	10.33997	chr5:164(	RNA5SP188		Pseudoger	chr5:102131007-102
ENSG0000(	463	10.33997	chr5:164(	LINC02201		lncRNA	chr5:122628952-122
ENSG0000(	463	10.33997	chr5:164(	SLCO4C1		protein_c	chr5:102233986-102
ENSG0000(	463	10.33997	chr5:164(	ENSG00000286111		lncRNA	chr5:125493261-125
ENSG0000(	463	10.33997	chr5:164(	RN7SL802P		smallRNA	chr5:101581830-101
ENSG0000(	463	10.33997	chr5:164(	ZRSR2P1		Pseudoger	chr5:112891610-112
ENSG0000(	463	10.33997	chr5:164(	RP11-395P13.2		lncRNA	chr5:125368568-125
ENSG0000(	463	10.33997	chr5:164(	HMGN2P27		Pseudoger	chr5:116218026-116
ENSG0000(	463	10.33997	chr5:164(	MTND4P35		Pseudoger	chr5:100048988-100
ENSG0000(	463	10.33997	chr5:164(	snoU13		smallRNA	chr5:119069297-119
ENSG0000(	463	10.33997	chr5:164(	RN7SKP117		smallRNA	chr5:125350868-125
ENSG0000(	463	10.33997	chr5:164(	SEMA6A-AS1		lncRNA	chr5:116447547-116
ENSG0000(	463	10.33997	chr5:164(	ENSG00000249787		lncRNA	chr5:100399047-100
ENSG0000(	463	10.33997	chr5:164(	ENSG00000287794		Pseudoger	chr5:122373512-122
ENSG0000(	463	10.33997	chr5:164(	LINC01957		lncRNA	chr5:114576041-114
ENSG0000(	463	10.33997	chr5:164(	TMED7-TI(	DriverDB\	protein_c	chr5:115578642-115
ENSG0000(	463	10.33997	chr5:164(	ENSG00000249791		lncRNA	chr5:115188563-115
ENSG0000(	463	10.33997	chr5:164(	LINC00491		lncRNA	chr5:102604220-102
ENSG0000(	463	10.33997	chr5:164(	ATG12		protein_c	chr5:115828200-115

ENSG0000(	463	10.33997	chr5:164(	SEPTIN7P10	Pseudoger	chr5:119126782-119
ENSG0000(	463	10.33997	chr5:164(	ENSG00000251214	lncRNA	chr5:124459912-124
ENSG0000(	463	10.33997	chr5:164(	ENSG00000250678	Pseudoger	chr5:119010060-119
ENSG0000(	463	10.33997	chr5:164(	RPL35AP15	Pseudoger	chr5:117301104-117
ENSG0000(	463	10.33997	chr5:164(	DCP2	protein_c	chr5:112976702-113
ENSG0000(	463	10.33997	chr5:164(	ENSG00000249318	lncRNA	chr5:111265809-111
ENSG0000(	463	10.33997	chr5:164(	PPIC-AS1	lncRNA	chr5:123036271-123
ENSG0000(	463	10.33997	chr5:164(	ENSG00000273957	Pseudoger	chr5:100388853-100
ENSG0000(	463	10.33997	chr10:81(	ENSG00000288918	lncRNA	chr10:82987781-829
ENSG0000(	463	10.33997	chr5:164(	ENSG00000250949	lncRNA	chr5:117031200-117
ENSG0000(	463	10.33997	chr5:164(	RNU2-49P	smallRNA	chr5:115774319-115
ENSG0000(	463	10.33997	chr5:164(	ENSG00000279772	TEC	chr5:123508736-123
ENSG0000(	463	10.33997	chr5:164(	MAN2A1-DT	lncRNA	chr5:109687802-109
ENSG0000(	463	10.33997	chr5:164(	PRDM6 AC	protein_c	chr5:123089241-123
ENSG0000(	463	10.33997	chr5:164(	LINC02148	lncRNA	chr5:118282575-118
ENSG0000(	463	10.33997	chr5:164(	MGC32805	lncRNA	chr5:122436497-122
ENSG0000(	463	10.33997	chr5:164(	RNU4-69P	smallRNA	chr5:120710698-120
ENSG0000(	463	10.33997	chr5:164(	snoU13	smallRNA	chr5:123009542-123
ENSG0000(	463	10.33997	chr5:164(	ENSG00000272265	lncRNA	chr5:116078110-116
ENSG0000(	463	10.33997	chr5:164(	RN7SL255P	smallRNA	chr5:104260533-104
ENSG0000(	463	10.33997	chr5:164(	ENSG00000248268	lncRNA	chr5:111277517-111
ENSG0000(	463	10.33997	chr5:164(	ENSG00000185641	Pseudoger	chr5:116051917-116
ENSG0000(	463	10.33997	chr5:164(	ENSG00000249944	Pseudoger	chr5:115489634-115
ENSG0000(	463	10.33997	chr5:164(	ENSG00000248203	lncRNA	chr5:103246048-103
ENSG0000(	463	10.33997	chr5:164(	RNU6-482P	smallRNA	chr5:112778363-112
ENSG0000(	463	10.33997	chr5:164(	TSSK1B	protein_c	chr5:113432553-113
ENSG0000(	463	10.33997	chr5:164(	ENSG00000250383	lncRNA	chr5:108818041-108
ENSG0000(	463	10.33997	chr5:164(	MTCO3P22	Pseudoger	chr5:100051133-100
ENSG0000(	463	10.33997	chr5:164(	CAMK4	protein_c	chr5:111223653-111
ENSG0000(	463	10.33997	chr5:164(	KRT18P16	Pseudoger	chr5:123636110-123
ENSG0000(	463	10.33997	chr5:164(	RNU6-47P	smallRNA	chr5:109014834-109
ENSG0000(	463	10.33997	chr5:164(	ENSG00000250847	Pseudoger	chr5:121195980-121
ENSG0000(	463	10.33997	chr5:164(	TRIM36	protein_c	chr5:115124762-115
ENSG0000(	463	10.33997	chr5:164(	AC113407.1	smallRNA	chr5:100050365-100
ENSG0000(	463	10.33997	chr5:164(	SNCAIP	protein_c	chr5:122311354-122
ENSG0000(	463	10.33997	chr10:81(	ENSG00000285739	lncRNA	chr10:82207620-822
ENSG0000(	463	10.33997	chr5:164(	MTND4LP5	Pseudoger	chr5:100050359-100
ENSG0000(	463	10.33997	chr5:164(	SNX24	protein_c	chr5:122843439-123
ENSG0000(	463	10.33997	chr5:164(	ENSG00000246316	lncRNA	chr5:114475339-114
ENSG0000(	463	10.33997	chr5:164(	HMGB1P29	Pseudoger	chr5:124220579-124
ENSG0000(	463	10.33997	chr5:164(	MACIR	protein_c	chr5:103258763-103
ENSG0000(	463	10.33997	chr5:164(	MTATP6P2	Pseudoger	chr5:100051920-100
ENSG0000(	463	10.33997	chr5:164(	LINC01170	lncRNA	chr5:124059794-124
ENSG0000(	463	10.33997	chr5:164(	ENSG00000251627	Pseudoger	chr5:110983563-110
ENSG0000(	463	10.33997	chr5:164(	AC093267.1	smallRNA	chr5:122776431-122
ENSG0000(	463	10.33997	chr5:164(	AC008536.1	smallRNA	chr5:112905638-112
ENSG0000(	463	10.33997	chr5:164(	LINC02214	lncRNA	chr5:116742991-116
ENSG0000(	463	10.33997	chr5:164(	ENSG00000286839	lncRNA	chr5:113738106-113
ENSG0000(	463	10.33997	chr5:164(	Y_RNA	smallRNA	chr5:123510436-123
ENSG0000(	463	10.33997	chr5:164(	ENSG00000248600	lncRNA	chr5:124452277-124
ENSG0000(	463	10.33997	chr5:164(	ENSG00000251456	lncRNA	chr5:124734618-124

ENSG0000(	463	10.33997	chr5:164(	EEF1A1P20		Pseudoger	chr5:99996547-9999
ENSG0000(	463	10.33997	chr5:164(	RN7SKP57		smallRNA	chr5:111719769-111
ENSG0000(	463	10.33997	chr5:164(	ENSG00000289054		lncRNA	chr5:122326610-122
ENSG0000(	463	10.33997	chr5:164(	HMGN1P14		Pseudoger	chr5:111846050-111
ENSG0000(	463	10.33997	chr5:164(	HSD17B4		protein_c	chr5:119452465-119
ENSG0000(	463	10.33997	chr5:164(	ENSG00000251293		lncRNA	chr5:120245448-120
ENSG0000(	463	10.33997	chr5:164(	ENSG00000249433		Pseudoger	chr5:115493557-115
ENSG0000(	463	10.33997	chr5:164(	ENSG00000250803		protein_c	chr5:122129622-122
ENSG0000(	463	10.33997	chr5:164(	ENSG00000249426		lncRNA	chr5:118760474-118
ENSG0000(	463	10.33997	chr5:164(	AC012603.1		smallRNA	chr5:109685580-109
ENSG0000(	463	10.33997	chr5:164(	ENSG00000249959		lncRNA	chr5:107699392-107
ENSG0000(	463	10.33997	chr10:81(	ENSG00000286876		lncRNA	chr10:83708841-837
ENSG0000(	463	10.33997	chr5:164(	ENSG00000286882		lncRNA	chr5:109165289-109
ENSG0000(	463	10.33997	chr5:164(	RNU6-334P		smallRNA	chr5:104780288-104
ENSG0000(	463	10.33997	chr5:164(	ENSG00000253776		lncRNA	chr5:104773641-104
ENSG0000(	463	10.33997	chr5:164(	ENSG00000250806		lncRNA	chr5:100654112-100
ENSG0000(	463	10.33997	chr5:164(	ENSG00000272139		lncRNA	chr5:122311297-122
ENSG0000(	463	10.33997	chr5:164(	AP3S1		protein_c	chr5:115841592-115
ENSG0000(	463	10.33997	chr5:164(	DTWD2	NCGv7	protein_c	chr5:118836074-118
ENSG0000(	463	10.33997	chr5:164(	HMGB3P17		Pseudoger	chr5:123468781-123
ENSG0000(	463	10.33997	chr5:164(	APC	NCGv7;AC	protein_c	chr5:112707498-112
ENSG0000(	463	10.33997	chr5:164(	ENSG00000279860		TEC	chr5:121363002-121
ENSG0000(	463	10.33997	chr5:164(	NREP		protein_c	chr5:111662621-111
ENSG0000(	463	10.33997	chr5:164(	WDR36		protein_c	chr5:111092321-111
ENSG0000(	463	10.33997	chr5:164(	TUBAP15		Pseudoger	chr5:119652523-119
ENSG0000(	463	10.33997	chr5:164(	ENSG00000250728		Pseudoger	chr5:111155244-111
ENSG0000(	463	10.33997	chr10:81(	RNU6-129P		smallRNA	chr10:83764358-837
ENSG0000(	463	10.33997	chr5:164(	HMGB1P22		Pseudoger	chr5:125265528-125
ENSG0000(	463	10.33997	chr5:164(	ENSG00000247311		lncRNA	chr5:122114598-122
ENSG0000(	463	10.33997	chr5:164(	RN7SL782P		smallRNA	chr5:107734775-107
ENSG0000(	463	10.33997	chr5:164(	ENSG00000260686		lncRNA	chr5:122832356-122
ENSG0000(	463	10.33997	chr5:164(	ENSG00000290027		lncRNA	chr5:112521511-112
ENSG0000(	463	10.33997	chr5:164(	ENSG00000288965		lncRNA	chr5:111238810-111
ENSG0000(	463	10.33997	chr5:164(	ENSG00000249017		lncRNA	chr5:102500541-102
ENSG0000(	463	10.33997	chr5:164(	ENSG00000249021		lncRNA	chr5:115691462-115
ENSG0000(	463	10.33997	chr5:164(	FAM170A	DriverDB\	protein_c	chr5:119629558-119
ENSG0000(	463	10.33997	chr5:164(	KCNN2	NCGv7	protein_c	chr5:114055926-114
ENSG0000(	463	10.33997	chr5:164(	RNU6-718P		smallRNA	chr5:120337549-120
ENSG0000(	463	10.33997	chr5:164(	RP11-395P13.4		lncRNA	chr5:125376834-125
ENSG0000(	463	10.33997	chr5:164(	TMED7		protein_c	chr5:115613210-115
ENSG0000(	463	10.33997	chr5:164(	ENSG00000286745		lncRNA	chr5:118581242-118
ENSG0000(	463	10.33997	chr5:164(	RN7SL174P		smallRNA	chr5:119306344-119
ENSG0000(	463	10.33997	chr5:164(	ENSG00000271797		lncRNA	chr5:115262505-115
ENSG0000(	463	10.33997	chr5:164(	EIF3KP1		Pseudoger	chr5:103032376-103
ENSG0000(	463	10.33997	chr5:164(	ENSG00000248296		lncRNA	chr5:124492775-124
ENSG0000(	463	10.33997	chr5:164(	RN7SL689P		smallRNA	chr5:123022489-123
ENSG0000(	463	10.33997	chr5:164(	ENSG00000248867		Pseudoger	chr5:109448349-109
ENSG0000(	463	10.33997	chr5:164(	NUDT12	DriverDB\	protein_c	chr5:103548855-103
ENSG0000(	463	10.33997	chr5:164(	TICAM2		protein_c	chr5:115578496-115
ENSG0000(	463	10.33997	chr5:164(	ENSG00000250882		lncRNA	chr5:112173570-112
ENSG0000(	463	10.33997	chr5:164(	LINC02208		lncRNA	chr5:118000253-118

ENSG0000(	463	10.33997	chr5:164(	ENSG00000249476		lncRNA	chr5:109237120-109
ENSG0000(	463	10.33997	chr5:164(	MAN2A1	NCGv7	protein_c	chr5:109689927-109
ENSG0000(	463	10.33997	chr10:81(	NRG3-AS1		lncRNA	chr10:82228985-822
ENSG0000(	463	10.33997	chr10:81(	ENSG00000223565		Pseudoger	chr10:83735506-837
ENSG0000(	463	10.33997	chr10:81(	NRG3	NCGv7	protein_c	chr10:81875194-829
ENSG0000(	463	10.33997	chr5:164(	RNA5SP190		Pseudoger	chr5:119445716-119
ENSG0000(	463	10.33997	chr5:164(	ENSG00000285809		lncRNA	chr5:117313085-117
ENSG0000(	463	10.33997	chr5:164(	ENSG00000251477		Pseudoger	chr5:117719240-117
ENSG0000(	463	10.33997	chr5:164(	DDX43P1		Pseudoger	chr5:115974513-115
ENSG0000(	463	10.33997	chr5:164(	FER	TAG	protein_c	chr5:108747841-109
ENSG0000(	463	10.33997	chr10:81(	AL096864.1		smallRNA	chr10:82050262-820
ENSG0000(	463	10.33997	chr5:164(	ENSG00000250242		lncRNA	chr5:115087892-115
ENSG0000(	463	10.33997	chr5:164(	GJA1P1		Pseudoger	chr5:109051315-109
ENSG0000(	463	10.33997	chr5:164(	ENSG00000247402		lncRNA	chr5:102605635-102
ENSG0000(	463	10.33997	chr5:164(	MIR5706		smallRNA	chr5:119154637-119
ENSG0000(	463	10.33997	chr5:164(	ENSG00000213661		Pseudoger	chr5:121616247-121
ENSG0000(	463	10.33997	chr5:164(	ENSG00000213663		Pseudoger	chr5:119547131-119
ENSG0000(	463	10.33997	chr5:164(	ENSG00000244245		Pseudoger	chr5:108593609-108
ENSG0000(	463	10.33997	chr5:164(	ENSG00000248927		lncRNA	chr5:120781218-120
ENSG0000(	463	10.33997	chr5:164(	ENSG00000251099		Pseudoger	chr5:111572236-111
ENSG0000(	463	10.33997	chr10:81(	ENSG00000287358		lncRNA	chr10:81870778-818
ENSG0000(	463	10.33997	chr5:164(	ENSG00000271918		lncRNA	chr5:116083807-116
ENSG0000(	463	10.33997	chr5:164(	ENSG00000253613		lncRNA	chr5:111076921-111
ENSG0000(	463	10.33997	chr5:164(	SNORA51		smallRNA	chr5:110684794-110
ENSG0000(	463	10.33997	chr5:164(	LINC02216		lncRNA	chr5:118575575-118
ENSG0000(	463	10.33997	chr5:164(	CEP120		protein_c	chr5:123344890-123
ENSG0000(	463	10.33997	chr5:164(	ENSG00000250567		lncRNA	chr5:103408941-103
ENSG0000(	463	10.33997	chr5:164(	ENSG00000242814		Pseudoger	chr5:124808828-124
ENSG0000(	463	10.33997	chr5:164(	SEMA6A-AS2		lncRNA	chr5:116574482-116
ENSG0000(	463	10.33997	chr5:164(	ENSG00000251135		Pseudoger	chr5:121040090-121
ENSG0000(	463	10.33997	chr5:164(	MCC	AC	protein_c	chr5:113022106-113
ENSG0000(	463	10.33997	chr5:164(	RNU1-140P		smallRNA	chr5:104098874-104
ENSG0000(	463	10.33997	chr5:164(	ENSG00000213655		Pseudoger	chr5:123236079-123
ENSG0000(	463	10.33997	chr5:164(	PPIC		protein_c	chr5:123023250-123
ENSG0000(	463	10.33997	chr5:164(	HMGB3P16		Pseudoger	chr5:112452703-112
ENSG0000(	463	10.33997	chr5:164(	RN7SKP62		smallRNA	chr5:100733058-100
ENSG0000(	463	10.33997	chr5:164(	ENSG00000251076		lncRNA	chr5:112228283-112
ENSG0000(	463	10.33997	chr5:164(	LINC02115		lncRNA	chr5:103528434-103
ENSG0000(	463	10.33997	chr5:164(	HMGN1P15		Pseudoger	chr5:115289036-115
ENSG0000(	463	10.33997	chr5:164(	LINC01023		lncRNA	chr5:108725707-108
ENSG0000(	463	10.33997	chr5:164(	PRR16		protein_c	chr5:120464300-120
ENSG0000(	463	10.33997	chr5:164(	NREP-AS1		lncRNA	chr5:111912508-112
ENSG0000(	463	10.33997	chr5:164(	RN7SKP68		smallRNA	chr5:102302504-102
ENSG0000(	463	10.33997	chr5:164(	ENSG00000286503		lncRNA	chr5:108382156-108
ENSG0000(	463	10.33997	chr10:81(	ENSG00000229458		lncRNA	chr10:82224213-822
ENSG0000(	463	10.33997	chr5:164(	AC008967.1		smallRNA	chr5:111555895-111
ENSG0000(	463	10.33997	chr5:164(	PGAM5P1		Pseudoger	chr5:109884610-109
ENSG0000(	463	10.33997	chr5:164(	RN7SL711P		smallRNA	chr5:123070782-123
ENSG0000(	463	10.33997	chr5:164(	snoU13		smallRNA	chr5:122796125-122
ENSG0000(	463	10.33997	chr5:164(	ENSG00000248752		lncRNA	chr5:125333369-126
ENSG0000(	463	10.33997	chr5:164(	EPB41L4A-AS1		lncRNA	chr5:112160526-112

ENSG0000(	463	10.33997	chr5:164(	ENSG00000258864		protein_c	chr5:112827213-112
ENSG0000(	463	10.33997	chr5:164(	RP11-395P13.3		lncRNA	chr5:125370165-125
ENSG0000(	463	10.33997	chr5:164(	snoU13		smallRNA	chr5:119610202-119
ENSG0000(	463	10.33997	chr5:164(	ENSG00000251132		lncRNA	chr5:115031273-115
ENSG0000(	463	10.33997	chr5:164(	ENSG00000249600		Pseudoger	chr5:121671373-121
ENSG0000(	463	10.33997	chr5:164(	RN7SKP230		smallRNA	chr5:109699500-109
ENSG0000(	463	10.33997	chr5:164(	SEMA6A	NCGv7	protein_c	chr5:116443555-116
ENSG0000(	463	10.33997	chr5:164(	MIR548P		smallRNA	chr5:100816482-100
ENSG0000(	463	10.33997	chr5:164(	ENSG00000249112		lncRNA	chr5:124707827-124
ENSG0000(	463	10.33997	chr5:164(	BCLAF1P1		Pseudoger	chr5:110948029-110
ENSG0000(	463	10.33997	chr5:164(	ENSG00000249150		lncRNA	chr5:116819220-116
ENSG0000(	463	10.33997	chr10:81(	RNU6-478P		smallRNA	chr10:83067962-830
ENSG0000(	463	10.33997	chr5:164(	ZNF474-AS1		lncRNA	chr5:122154496-122
ENSG0000(	463	10.33997	chr5:164(	LINC01848		lncRNA	chr5:109883182-109
ENSG0000(	463	10.33997	chr5:164(	MTCO1P22		Pseudoger	chr5:100053686-100
ENSG0000(	463	10.33997	chr5:164(	ENSG00000248428		lncRNA	chr5:110970951-111
ENSG0000(	463	10.33997	chr5:164(	LINC02215		lncRNA	chr5:118596188-118
ENSG0000(	463	10.33997	chr5:164(	SLCO6A1		protein_c	chr5:102371774-102
ENSG0000(	463	10.33997	chr5:164(	HMGN1P13		Pseudoger	chr5:111572102-111
ENSG0000(	463	10.33997	chr5:164(	ENSG00000249621		lncRNA	chr5:122321291-122
ENSG0000(	463	10.33997	chr5:164(	RPL23AP44		Pseudoger	chr5:121575848-121
ENSG0000(	463	10.33997	chr5:164(	ENSG00000261036		lncRNA	chr5:120345907-120
ENSG0000(	463	10.33997	chr10:81(	LINC02650		lncRNA	chr10:83672406-836
ENSG0000(	463	10.33997	chr5:164(	RNU7-34P		smallRNA	chr5:118758211-118
ENSG0000(	463	10.33997	chr5:164(	RNU6-1119P		smallRNA	chr5:100153672-100
ENSG0000(	463	10.33997	chr5:164(	KRT8P33		Pseudoger	chr5:123400922-123
ENSG0000(	463	10.33997	chr5:164(	H3P24		Pseudoger	chr5:115770586-115
ENSG0000(	463	10.33997	chr5:164(	CBX3P3		Pseudoger	chr5:112777630-112
ENSG0000(	463	10.33997	chr5:164(	RNU6-373P		smallRNA	chr5:119007571-119
ENSG0000(	463	10.33997	chr5:164(	RPS17P2		Pseudoger	chr5:116716243-116
ENSG0000(	463	10.33997	chr5:164(	Y_RNA		smallRNA	chr5:100021804-100
ENSG0000(	463	10.33997	chr5:164(	YTHDC2		protein_c	chr5:113513694-113
ENSG0000(	463	10.33997	chr5:164(	AC027320.1		smallRNA	chr5:119257340-119
ENSG0000(	463	10.33997	chr5:164(	ENSG00000289912		lncRNA	chr5:100903394-100
ENSG0000(	463	10.33997	chr5:164(	LINC02240		lncRNA	chr5:124808981-125
ENSG0000(	463	10.33997	chr5:164(	ARL14EPL		protein_c	chr5:116032324-116
ENSG0000(	463	10.33997	chr5:164(	FAM174A	DriverDBv	protein_c	chr5:100535374-100
ENSG0000(	463	10.33997	chr5:164(	AK3P4		Pseudoger	chr5:115402380-115
ENSG0000(	463	10.33997	chr5:164(	ARGFXP1		Pseudoger	chr5:122675795-122
ENSG0000(	463	10.33997	chr5:164(	RPL7L1P4		Pseudoger	chr5:117778826-117
ENSG0000(	463	10.33997	chr5:164(	SNX2		protein_c	chr5:122775079-122
ENSG0000(	463	10.33997	chr10:81(	RNU6-441P		smallRNA	chr10:82340771-823
ENSG0000(	463	10.33997	chr5:164(	ENSG00000250194		Pseudoger	chr5:125074665-125
ENSG0000(	463	10.33997	chr5:164(	EFNA5		protein_c	chr5:107376894-107
ENSG0000(	463	10.33997	chr5:164(	RNA5SP189		Pseudoger	chr5:105922994-105
ENSG0000(	463	10.33997	chr5:164(	RPS14P8		Pseudoger	chr5:116562562-116
ENSG0000(	463	10.33997	chr5:164(	LINC02147		lncRNA	chr5:117730515-118
ENSG0000(	463	10.33997	chr10:81(	AL356140.1		smallRNA	chr10:83437034-834
ENSG0000(	463	10.33997	chr5:164(	PAM	DriverDBv	protein_c	chr5:102753981-103
ENSG0000(	463	10.33997	chr5:164(	ENSG00000250650		Pseudoger	chr5:117019548-117
ENSG0000(	463	10.33997	chr5:164(	RN7SKP89		smallRNA	chr5:114253513-114

ENSG0000(	463	10.33997	chr5:164(	PPIP5K2		protein_c	chr5:103120149-103
ENSG0000(	463	10.33997	chr5:164(	RNU6-701P		smallRNA	chr5:119138859-119
ENSG0000(	463	10.33997	chr5:164(	GIN1		protein_c	chr5:103086000-103
ENSG0000(	463	10.33997	chr5:164(	ENSG00000251367		lncRNA	chr5:109467353-109
ENSG0000(	463	10.33997	chr5:164(	ENSG00000251421		lncRNA	chr5:124395603-124
ENSG0000(	463	10.33997	chr5:164(	CCT5P1		Pseudoger	chr5:115512077-115
ENSG0000(	463	10.33997	chr5:164(	LINC00492		lncRNA	chr5:102581368-102
ENSG0000(	463	10.33997	chr5:164(	LINC00992		lncRNA	chr5:117415509-117
ENSG0000(	463	10.33997	chr5:164(	RP11-395P13.6		lncRNA	chr5:125395379-125
ENSG0000(	463	10.33997	chr5:164(	ENSG00000249068		Pseudoger	chr5:109840128-109
ENSG0000(	463	10.33997	chr5:164(	ZNF608	NCGv7	protein_c	chr5:124636913-124
ENSG0000(	463	10.33997	chr5:164(	ENSG00000289458		lncRNA	chr5:124497294-124
ENSG0000(	463	10.33997	chr5:164(	ENSG00000248261		lncRNA	chr5:102141893-102
ENSG0000(	463	10.33997	chr5:164(	ENSG00000249261		lncRNA	chr5:124868972-124
ENSG0000(	463	10.33997	chr5:164(	FBXL17	NCGv7	protein_c	chr5:107859035-108
ENSG0000(	463	10.33997	chr5:164(	LINC02200		lncRNA	chr5:112628436-112
ENSG0000(	463	10.33997	chr5:164(	DMXL1-DT		lncRNA	chr5:119006347-119
ENSG0000(	463	10.33997	chr5:164(	ENSG00000249495		Pseudoger	chr5:101976296-101
ENSG0000(	463	10.33997	chr5:164(	RACK1P1		Pseudoger	chr5:108784098-108
ENSG0000(	463	10.33997	chr5:164(	TSLP		protein_c	chr5:111070062-111
ENSG0000(	463	10.33997	chr5:164(	ENSG00000283462		lncRNA	chr5:103880129-103
ENSG0000(	463	10.33997	chr5:164(	FABP5P6		Pseudoger	chr5:119555250-119
ENSG0000(	463	10.33997	chr5:164(	ENSG00000250438		lncRNA	chr5:124469591-124
ENSG0000(	463	10.33997	chr5:164(	ENSG00000250441		Pseudoger	chr5:108894347-108
ENSG0000(	463	10.33997	chr5:164(	FTMT		protein_c	chr5:121851882-121
ENSG0000(	463	10.33997	chr5:164(	ENSG00000278958		TEC	chr5:104434772-104
ENSG0000(	463	10.33997	chr5:164(	AC034236.1		smallRNA	chr5:116043171-116
ENSG0000(	463	10.33997	chr5:164(	ENSG00000251187		lncRNA	chr5:112192020-112
ENSG0000(	463	10.33997	chr5:164(	ENSG00000248440		lncRNA	chr5:109497877-109
ENSG0000(	463	10.33997	chr5:164(	ENSG00000248709		lncRNA	chr5:115738978-115
ENSG0000(	463	10.33997	chr5:164(	ENSG00000249916		lncRNA	chr5:122369762-122
ENSG0000(	463	10.33997	chr5:164(	ENSG00000234259		Pseudoger	chr5:119680197-119
ENSG0000(	463	10.33997	chr5:164(	DMXL1	NCGv7	protein_c	chr5:119037772-119
ENSG0000(	463	10.33997	chr5:164(	REEP5	NCGv7	protein_c	chr5:112876385-112
ENSG0000(	463	10.33997	chr5:164(	LINCADL		lncRNA	chr5:115956571-115
ENSG0000(	463	10.33997	chr5:164(	ENSG00000253584		lncRNA	chr5:104917492-105
ENSG0000(	463	10.33997	chr5:164(	RPS3AP21		Pseudoger	chr5:111192226-111
ENSG0000(	463	10.33997	chr5:164(	RN7SKP122		smallRNA	chr5:107810629-107
ENSG0000(	463	10.33997	chr5:164(	NIHCOLE		lncRNA	chr5:104079847-104
ENSG0000(	463	10.33997	chr5:164(	RNU4ATAC13P		smallRNA	chr5:113493835-113
ENSG0000(	463	10.33997	chr5:164(	TRIM36-IT1		lncRNA	chr5:115148764-115
ENSG0000(	463	10.33997	chr5:164(	ENSG00000250015		lncRNA	chr5:116302354-116
ENSG0000(	463	10.33997	chr5:164(	LVRN		protein_c	chr5:115962454-116
ENSG0000(	463	10.33997	chr5:164(	GUSBP19		Pseudoger	chr5:100375700-100
ENSG0000(	463	10.33997	chr5:164(	ENSG00000251574		lncRNA	chr5:104383298-105
ENSG0000(	463	10.33997	chr5:164(	Y_RNA		smallRNA	chr5:108891437-108
ENSG0000(	463	10.33997	chr5:164(	PDZPH1P		Pseudoger	chr5:103430406-103
ENSG0000(	463	10.33997	chr5:164(	STARD4-AS1		lncRNA	chr5:111510396-111
ENSG0000(	463	10.33997	chr10:81(	MARK2P15		Pseudoger	chr10:83311628-833
ENSG0000(	463	10.33997	chr5:164(	GUSBP7		Pseudoger	chr5:100062275-100
ENSG0000(	463	10.33997	chr5:164(	ENSG00000248853		Pseudoger	chr5:121059244-121

ENSG0000(	463	10.33997	chr5:164(	CDO1		protein_c	chr5:115804733-115
ENSG0000(	463	10.33997	chr5:164(	EPB41L4A		protein_c	chr5:112142441-112
ENSG0000(	463	10.33997	chr5:164(	ENSG00000289260		lncRNA	chr5:108727866-108
ENSG0000(	463	10.33997	chr5:164(	PJA2		protein_c	chr5:109334713-109
ENSG0000(	463	10.33997	chr5:164(	FAM174A-DT		lncRNA	chr5:100428124-100
ENSG0000(	463	10.33997	chr5:164(	KRT18P42		Pseudoger	chr5:109588338-109
ENSG0000(	463	10.33997	chr5:164(	EPB41L4A-DT		lncRNA	chr5:112419583-112
ENSG0000(	463	10.33997	chr5:164(	ENSG00000251014		Pseudoger	chr5:112546973-112
ENSG0000(	463	10.33997	chr5:164(	ST8SIA4		protein_c	chr5:100806933-100
ENSG0000(	463	10.33997	chr5:164(	MTND5P10		Pseudoger	chr5:100046977-100
ENSG0000(	463	10.33997	chr5:164(	TICAM2-AS1		lncRNA	chr5:115602057-115
ENSG0000(	463	10.33997	chr5:164(	ENSG00000288890		lncRNA	chr5:123619519-123
ENSG0000(	463	10.33997	chr5:164(	snoU13		smallRNA	chr5:119468959-119
ENSG0000(	460	10.27297	chr10:81(	MIR1296		smallRNA	chr10:63372957-633
ENSG0000(	460	10.27297	chr7:158(	ENSG00000279483		Pseudoger	chr7:137513859-137
ENSG0000(	460	10.27297	chr20:21(	SNORD36		smallRNA	chr20:48135943-481
ENSG0000(	455	10.16131	chr2:309(	ENSG00000228585		Pseudoger	chr2:5313740-53141
ENSG0000(	455	10.16131	chr2:309(	NPM1P48		Pseudoger	chr2:4514204-45150
ENSG0000(	455	10.16131	chr2:309(	ENSG00000234275		lncRNA	chr2:6258348-63417
ENSG0000(	455	10.16131	chr2:309(	SILC1		lncRNA	chr2:5932543-60035
ENSG0000(	455	10.16131	chr2:309(	ENSG00000242540		lncRNA	chr2:5696220-57196
ENSG0000(	455	10.16131	chr2:309(	SOX11	NCGv7	protein_c	chr2:5692384-57013
ENSG0000(	455	10.16131	chr2:309(	LINC01810		lncRNA	chr2:5810641-58126
ENSG0000(	455	10.16131	chr2:309(	LINC01247		lncRNA	chr2:6366010-63754
ENSG0000(	455	10.16131	chr2:309(	ENSG00000230090		lncRNA	chr2:5618327-56911
ENSG0000(	455	10.16131	chr2:309(	SNORA31		smallRNA	chr2:4827001-48270
ENSG0000(	455	10.16131	chr2:309(	ENSG00000289300		lncRNA	chr2:5071437-50734
ENSG0000(	455	10.16131	chr2:309(	LINC01248		lncRNA	chr2:5602505-56914
ENSG0000(	455	10.16131	chr2:309(	LINC01249		lncRNA	chr2:4628216-46564
ENSG0000(	455	10.16131	chr2:309(	ENSG00000232835		lncRNA	chr2:5549780-55560
ENSG0000(	455	10.16131	chr2:309(	ENSG00000236106		lncRNA	chr2:5726253-57303
ENSG0000(	455	10.16131	chr2:309(	RNU6-649P		smallRNA	chr2:4945277-49453
ENSG0000(	451	10.07198	chr4:410(	MTND5P5		Pseudoger	chr4:101972423-101
ENSG0000(	451	10.07198	chr4:410(	LINC01216		lncRNA	chr4:100660279-100
ENSG0000(	451	10.07198	chr4:410(	H2AZ1-DT		lncRNA	chr4:99950006-1001
ENSG0000(	451	10.07198	chr4:410(	ENSG00000251309		lncRNA	chr4:101976894-102
ENSG0000(	451	10.07198	chr4:410(	LINC01217		lncRNA	chr4:100778582-100
ENSG0000(	451	10.07198	chr4:410(	ENSG00000279098		TEC	chr4:99942081-9994
ENSG0000(	451	10.07198	chr4:410(	MIR1255A		smallRNA	chr4:101330302-101
ENSG0000(	451	10.07198	chr4:410(	AC121157.1		smallRNA	chr4:100129945-100
ENSG0000(	451	10.07198	chr4:410(	FLJ20021		lncRNA	chr4:101347752-101
ENSG0000(	451	10.07198	chr4:410(	BANK1		protein_c	chr4:101411286-102
ENSG0000(	451	10.07198	chr4:410(	ENSG00000286124		lncRNA	chr4:101640946-101
ENSG0000(	451	10.07198	chr7:158(	RN7SKP223		smallRNA	chr7:138091254-138
ENSG0000(	451	10.07198	chr4:410(	ENSG00000250403		Pseudoger	chr4:99990737-9999
ENSG0000(	451	10.07198	chr4:410(	DDIT4L		protein_c	chr4:100185870-100
ENSG0000(	451	10.07198	chr4:410(	RNU6-462P		smallRNA	chr4:101723876-101
ENSG0000(	451	10.07198	chr4:410(	DDIT4L-AS1		lncRNA	chr4:100190033-100
ENSG0000(	451	10.07198	chr4:410(	DNAJB14		protein_c	chr4:99896248-9994
ENSG0000(	451	10.07198	chr4:410(	ENSG00000279913		TEC	chr4:99844138-9984
ENSG0000(	451	10.07198	chr4:410(	H2AZ1		protein_c	chr4:99948086-9995

ENSG0000(	451	10.07198	chr4:410{	LINC01218		lncRNA	chr4:100812255-100
ENSG0000(	451	10.07198	chr4:410{	EMCN		protein_c	chr4:100395341-100
ENSG0000(	451	10.07198	chr4:410{	DYNLL1P6		Pseudoger	chr4:100041841-100
ENSG0000(	451	10.07198	chr4:410{	LAMTOR3		protein_c	chr4:99878336-9989
ENSG0000(	451	10.07198	chr4:410{	ENSG00000286150		lncRNA	chr4:100421655-100
ENSG0000(	451	10.07198	chr4:410{	DAPP1		protein_c	chr4:99816827-9987
ENSG0000(	451	10.07198	chr4:410{	PPP3CA	NCGv7	protein_c	chr4:101023409-101
ENSG0000(	450	10.04964	chr12:171	CLUHP8		Pseudoger	chr12:38082046-380
ENSG0000(	450	10.04964	chr12:171	ENSG00000258119		lncRNA	chr12:38544177-385
ENSG0000(	450	10.04964	chr3:380<	LSM3		protein_c	chr3:14178817-1420
ENSG0000(	450	10.04964	chr12:171	ABCD2		protein_c	chr12:39550033-396
ENSG0000(	450	10.04964	chr12:171	TUBB8P5		Pseudoger	chr12:38201566-382
ENSG0000(	450	10.04964	chr3:380<	HDAC11		protein_c	chr3:13479724-1350
ENSG0000(	450	10.04964	chr12:171	SLC2A13	NCGv7	protein_c	chr12:39755025-401
ENSG0000(	450	10.04964	chr12:171	RNA5SP359		Pseudoger	chr12:38163403-381
ENSG0000(	450	10.04964	chr3:380<	TPRXL		Pseudoger	chr3:13937273-1408
ENSG0000(	450	10.04964	chr3:380<	CHCHD4		protein_c	chr3:14112077-1412
ENSG0000(	450	10.04964	chr12:171	AK6P2		Pseudoger	chr12:38078529-380
ENSG0000(	450	10.04964	chr12:171	AC067735.1		smallRNA	chr12:38913240-389
ENSG0000(	450	10.04964	chr3:380<	HDAC11-AS1		lncRNA	chr3:13476982-1348
ENSG0000(	450	10.04964	chr12:171	ENSG00000257237		Pseudoger	chr12:39058028-390
ENSG0000(	450	10.04964	chr3:380<	ENSG00000231362		Pseudoger	chr3:13052967-1305
ENSG0000(	450	10.04964	chr3:380<	XPC	NCGv7;AC	protein_c	chr3:14145147-1417
ENSG0000(	450	10.04964	chr12:171	LRRK2-DT		lncRNA	chr12:40186009-402
ENSG0000(	450	10.04964	chr3:380<	SLC6A6		protein_c	chr3:14402576-1448
ENSG0000(	450	10.04964	chr3:380<	ENSG00000287059		lncRNA	chr3:14038647-1404
ENSG0000(	450	10.04964	chr3:380<	WNT7A		protein_c	chr3:13816258-1388
ENSG0000(	450	10.04964	chr3:380<	FBLN2	NCGv7	protein_c	chr3:13549125-1363
ENSG0000(	450	10.04964	chr3:380<	TMEM43		protein_c	chr3:14125015-1414
ENSG0000(	450	10.04964	chr3:380<	VN1R20P		Pseudoger	chr3:13926813-1392
ENSG0000(	450	10.04964	chr3:380<	XPC-AS1		lncRNA	chr3:14144637-1416
ENSG0000(	450	10.04964	chr12:171	CPNE8		protein_c	chr12:38646822-389
ENSG0000(	450	10.04964	chr12:171	KIF21A	NCGv7	protein_c	chr12:39293228-394
ENSG0000(	450	10.04964	chr12:171	LRRK2	NCGv7	protein_c	chr12:40196744-403
ENSG0000(	450	10.04964	chr7:158{	RN7SKP187		smallRNA	chr7:112288623-112
ENSG0000(	450	10.04964	chr12:171	Y_RNA		smallRNA	chr12:38274448-382
ENSG0000(	450	10.04964	chr12:171	ENSG00000287442		lncRNA	chr12:38155476-381
ENSG0000(	450	10.04964	chr3:380<	IQSEC1		protein_c	chr3:12897043-1328
ENSG0000(	450	10.04964	chr12:171	CPNE8-AS1		lncRNA	chr12:38906451-389
ENSG0000(	450	10.04964	chr3:380<	AC093611.2		smallRNA	chr3:13717918-1371
ENSG0000(	450	10.04964	chr3:380<	LINC00620		lncRNA	chr3:13650696-1374
ENSG0000(	450	10.04964	chr12:171	RPL7AP74		Pseudoger	chr12:39466442-394
ENSG0000(	450	10.04964	chr3:380<	AC093611.1		smallRNA	chr3:13738060-1373
ENSG0000(	450	10.04964	chr12:171	ENSG00000257292		Pseudoger	chr12:38205098-382
ENSG0000(	450	10.04964	chr12:171	ENSG00000286576		lncRNA	chr12:39447404-394
ENSG0000(	450	10.04964	chr3:380<	FGD5P1		Pseudoger	chr3:13934255-1393
ENSG0000(	450	10.04964	chr12:171	AC121336.1		smallRNA	chr12:39807095-398
ENSG0000(	450	10.04964	chr12:171	AC117372.1		smallRNA	chr12:38321566-383
ENSG0000(	450	10.04964	chr3:380<	LINC01267		lncRNA	chr3:14348451-1435
ENSG0000(	450	10.04964	chr12:171	AC121334.1		smallRNA	chr12:39443048-394
ENSG0000(	450	10.04964	chr3:380<	GRIP2		protein_c	chr3:14489107-1455

ENSG0000(	450	10.04964	chr12:171	LINC02471		lncRNA	chr12:40156113-402
ENSG0000(	450	10.04964	chr12:171	SNORA22		smallRNA	chr12:39819750-398
ENSG0000(	450	10.04964	chr12:171	NF1P12		Pseudoger	chr12:38206231-382
ENSG0000(	450	10.04964	chr3:380	RNA5SP124		Pseudoger	chr3:14394648-1439
ENSG0000(	450	10.04964	chr3:380	FGD5P1		lncRNA	chr3:13933056-1393
ENSG0000(	450	10.04964	chr12:171	LINC02406		lncRNA	chr12:39087347-391
ENSG0000(	450	10.04964	chr3:380	VN1R21P		Pseudoger	chr3:14089107-1408
ENSG0000(	450	10.04964	chr3:380	ENSG00000268279		protein_c	chr3:14135217-1414
ENSG0000(	450	10.04964	chr12:171	ENSG00000285732		lncRNA	chr12:39611819-396
ENSG0000(	450	10.04964	chr12:171	C12orf40		protein_c	chr12:39626167-399
ENSG0000(	450	10.04964	chr12:171	ENSG00000257645		Pseudoger	chr12:38532895-385
ENSG0000(	450	10.04964	chr7:158	ZNF277		protein_c	chr7:112206695-112
ENSG0000(	450	10.04964	chr12:171	ENSG00000270718		Pseudoger	chr12:39170646-391
ENSG0000(	450	10.04964	chr6:103	RPSAP2		Pseudoger	chr6:28732017-2873
ENSG0000(	450	10.04964	chr12:171	ALG10B		protein_c	chr12:38316762-383
ENSG0000(	450	10.04964	chr3:380	ENSG00000290044		lncRNA	chr3:14144198-1414
ENSG0000(	450	10.04964	chr12:171	ENSG00000270273		Pseudoger	chr12:39539581-395
ENSG0000(	450	10.04964	chr12:171	RPL30P13		Pseudoger	chr12:40068243-400
ENSG0000(	450	10.04964	chr12:171	ENSG00000257586		Pseudoger	chr12:38994803-389
ENSG0000(	450	10.04964	chr3:380	ENSG00000255021		lncRNA	chr3:14272373-1430
ENSG0000(	450	10.04964	chr3:380	NUP210	DriverDBv	protein_c	chr3:13316235-1342
ENSG0000(	450	10.04964	chr12:171	LINC02555		lncRNA	chr12:40140926-401
ENSG0000(	449	10.02731	chr10:81(	RGR	AC	protein_c	chr10:84230666-842
ENSG0000(	449	10.02731	chr10:81(	RPL12P29		Pseudoger	chr10:84389170-843
ENSG0000(	449	10.02731	chr10:81(	LINC00858		lncRNA	chr10:84267747-842
ENSG0000(	449	10.02731	chr10:81(	HMGN2P8		Pseudoger	chr10:84081429-840
ENSG0000(	449	10.02731	chr10:81(	LRIT2		protein_c	chr10:84220571-842
ENSG0000(	449	10.02731	chr10:81(	C10orf99		protein_c	chr10:84173801-841
ENSG0000(	449	10.02731	chr10:81(	AC091487.1		smallRNA	chr10:84863265-848
ENSG0000(	449	10.02731	chr10:81(	RPS3AP5		Pseudoger	chr10:84560443-845
ENSG0000(	449	10.02731	chr10:81(	ENSG00000233258		lncRNA	chr10:83911654-839
ENSG0000(	449	10.02731	chr10:81(	CERNA2		lncRNA	chr10:84167228-841
ENSG0000(	449	10.02731	chr10:81(	ENSG00000271933		lncRNA	chr10:84138420-841
ENSG0000(	449	10.02731	chr10:81(	LRIT1		protein_c	chr10:84231520-842
ENSG0000(	449	10.02731	chr10:81(	CACYBPP1		Pseudoger	chr10:84454679-844
ENSG0000(	449	10.02731	chr10:81(	ATOH7		protein_c	chr10:68230595-682
ENSG0000(	449	10.02731	chr10:81(	CDHR1	NCGv7	protein_c	chr10:84194537-842
ENSG0000(	449	10.02731	chr10:81(	GHITM		protein_c	chr10:84139482-841
ENSG0000(	449	10.02731	chr10:81(	RNU1-65P		smallRNA	chr10:84003332-840
ENSG0000(	449	10.02731	chr10:81(	CCSER2		protein_c	chr10:84328586-845
ENSG0000(	449	10.02731	chr10:81(	TNPO1P1		Pseudoger	chr10:84390908-843
ENSG0000(	448	10.00498	chr7:158	RNU6-417P		smallRNA	chr7:63011463-6301
ENSG0000(	447	9.982647	chr8:412	CPQ	NCGv7	protein_c	chr8:96645242-9714
ENSG0000(	447	9.982647	chr8:412	Y_RNA		smallRNA	chr8:96309221-9630
ENSG0000(	447	9.982647	chr8:412	GDF6		protein_c	chr8:96142333-9616
ENSG0000(	447	9.982647	chr7:158	ASB4		protein_c	chr7:95478444-9554
ENSG0000(	447	9.982647	chr7:158	ENSG00000237160		Pseudoger	chr7:95348718-9534
ENSG0000(	447	9.982647	chr8:412	AC116154.1		smallRNA	chr8:95678838-9567
ENSG0000(	447	9.982647	chr7:158	ENSG00000279067		TEC	chr7:96118647-9611
ENSG0000(	447	9.982647	chr8:412	ENSG00000253872		lncRNA	chr8:95505406-9552
ENSG0000(	447	9.982647	chr8:412	MTERF3	NCGv7	protein_c	chr8:96239398-9626

ENSG0000(	447	9.982647	chr8:412(	ENSG00000270077		lncRNA	chr8:97144170-9714
ENSG0000(	447	9.982647	chr8:412(	ENSG00000253105		lncRNA	chr8:96370800-9638
ENSG0000(	447	9.982647	chr8:412(	SDC2		protein_c	chr8:96493813-9661
ENSG0000(	447	9.982647	chr7:1588	PON2		protein_c	chr7:95404862-9543
ENSG0000(	447	9.982647	chr8:412(	ENSG00000248762		lncRNA	chr8:95986108-9599
ENSG0000(	447	9.982647	chr7:1588	PON3		protein_c	chr7:95359872-9539
ENSG0000(	447	9.982647	chr7:1588	ENSG00000233942		lncRNA	chr7:95471835-9547
ENSG0000(	447	9.982647	chr7:1588	MIR591		smallRNA	chr7:96219662-9621
ENSG0000(	447	9.982647	chr7:1588	DYNC1I1	NCGv7	protein_c	chr7:95772506-9611
ENSG0000(	447	9.982647	chr7:1588	GABPAP		Pseudoger	chr7:63893286-6389
ENSG0000(	447	9.982647	chr8:412(	UQCRB-AS1		lncRNA	chr8:96235427-9623
ENSG0000(	447	9.982647	chr8:412(	SRSF3P2		Pseudoger	chr8:95947781-9594
ENSG0000(	447	9.982647	chr7:1588	ENSG00000228751		Pseudoger	chr7:95350164-9535
ENSG0000(	447	9.982647	chr8:412(	GAPDHP30		Pseudoger	chr8:96068729-9606
ENSG0000(	447	9.982647	chr8:412(	ENSG00000271349		Pseudoger	chr8:96126967-9612
ENSG0000(	447	9.982647	chr7:1588	PDK4-AS1		lncRNA	chr7:95545191-9561
ENSG0000(	447	9.982647	chr8:412(	UQCRB		protein_c	chr8:96222947-9623
ENSG0000(	447	9.982647	chr8:412(	PTDSS1	NCGv7	protein_c	chr8:96261902-9633
ENSG0000(	447	9.982647	chr7:1588	PDK4		protein_c	chr7:95583499-9559
ENSG0000(	447	9.982647	chr8:412(	ENSG00000270131		lncRNA	chr8:96140572-9614
ENSG0000(	447	9.982647	chr7:1588	ENSG00000273138		lncRNA	chr7:95416108-9541
ENSG0000(	447	9.982647	chr8:412(	RNU6-690P		smallRNA	chr8:95599114-9559
ENSG0000(	447	9.982647	chr7:1588	ENSG00000279525		TEC	chr7:95542145-9554
ENSG0000(	447	9.982647	chr8:412(	ENSG00000272249		lncRNA	chr8:97132835-9713
ENSG0000(	447	9.982647	chr8:412(	RNU6-1172P		smallRNA	chr8:96431054-9643
ENSG0000(	447	9.982647	chr7:1588	ENSG00000290101		lncRNA	chr7:95396497-9539
ENSG0000(	445	9.937982	chr7:1588	CYREN		protein_c	chr7:135092363-135
ENSG0000(	445	9.937982	chr7:1588	AGBL3	DriverDBv	protein_c	chr7:134986508-135
ENSG0000(	445	9.937982	chr7:1588	ENSG00000271522		lncRNA	chr7:130790208-130
ENSG0000(	445	9.937982	chr7:1588	MKLN1-AS		lncRNA	chr7:131309469-131
ENSG0000(	445	9.937982	chr7:1588	ENSG00000271204		lncRNA	chr7:130930209-130
ENSG0000(	445	9.937982	chr7:1588	ENSG00000286458		lncRNA	chr7:134816543-134
ENSG0000(	445	9.937982	chr7:1588	ENSG00000273489		lncRNA	chr7:131493964-131
ENSG0000(	445	9.937982	chr7:1588	AKR1B1		protein_c	chr7:134442356-134
ENSG0000(	445	9.937982	chr7:1588	AC083862.1		protein_c	chr7:135148045-135
ENSG0000(	445	9.937982	chr7:1588	SLC13A4	DriverDBv	protein_c	chr7:135681231-135
ENSG0000(	445	9.937982	chr7:1588	ENSG00000287733		lncRNA	chr7:135170816-135
ENSG0000(	445	9.937982	chr7:1588	H4P1		Pseudoger	chr7:130823205-130
ENSG0000(	445	9.937982	chr7:1588	ENSG00000272941		lncRNA	chr7:135168403-135
ENSG0000(	445	9.937982	chr7:1588	PODXL		protein_c	chr7:131500262-131
ENSG0000(	445	9.937982	chr7:1588	AKR1B15		protein_c	chr7:134549110-134
ENSG0000(	445	9.937982	chr7:1588	ENSG00000224545		Pseudoger	chr7:131520137-131
ENSG0000(	445	9.937982	chr7:1588	ENSG00000224375		lncRNA	chr7:134684144-134
ENSG0000(	445	9.937982	chr7:1588	MKLN1		protein_c	chr7:131110096-131
ENSG0000(	445	9.937982	chr7:1588	RNF14P4		Pseudoger	chr7:135128444-135
ENSG0000(	445	9.937982	chr7:1588	CNOT4		protein_c	chr7:135361795-135
ENSG0000(	445	9.937982	chr7:1588	ENSG00000236395		Pseudoger	chr7:131702269-131
ENSG0000(	445	9.937982	chr7:1588	SLC35B4		protein_c	chr7:134289332-134
ENSG0000(	445	9.937982	chr7:1588	RNA5SP246		Pseudoger	chr7:130602795-130
ENSG0000(	445	9.937982	chr7:1588	ENSG00000232716		Pseudoger	chr7:130840204-130
ENSG0000(	445	9.937982	chr7:1588	NUP205		protein_c	chr7:135557917-135

ENSG0000(	445	9.937982	chr7:158{	ENSG00000236238		Pseudoger	chr7:131665507-131
ENSG0000(	445	9.937982	chr7:158{	LRGUK	DriverDB\	protein_c	chr7:134127299-134
ENSG0000(	445	9.937982	chr7:158{	ENSG00000273319		lncRNA	chr7:130936464-130
ENSG0000(	445	9.937982	chr7:158{	STMP1		protein_c	chr7:135662496-135
ENSG0000(	445	9.937982	chr7:158{	CALD1	NCGv7	protein_c	chr7:134744252-134
ENSG0000(	445	9.937982	chr7:158{	RNU6-1154P		smallRNA	chr7:135665599-135
ENSG0000(	445	9.937982	chr7:158{	KLF14		protein_c	chr7:130730697-130
ENSG0000(	445	9.937982	chr7:158{	TMEM140	AC	protein_c	chr7:135148072-135
ENSG0000(	445	9.937982	chr7:158{	LINC-PINT		lncRNA	chr7:130791264-131
ENSG0000(	445	9.937982	chr7:158{	AC091736.1		smallRNA	chr7:135798006-135
ENSG0000(	445	9.937982	chr7:158{	ENSG00000273219		lncRNA	chr7:135704537-135
ENSG0000(	445	9.937982	chr7:158{	AKR1B10	NCGv7	protein_c	chr7:134527567-134
ENSG0000(	445	9.937982	chr7:158{	SLC23A4P		Pseudoger	chr7:135270017-135
ENSG0000(	445	9.937982	chr7:158{	ENSG00000229177		lncRNA	chr7:134346071-134
ENSG0000(	445	9.937982	chr7:158{	ENSG00000271414		Pseudoger	chr7:135650972-135
ENSG0000(	445	9.937982	chr7:158{	TSGA13		protein_c	chr7:130668643-130
ENSG0000(	445	9.937982	chr7:158{	LINC00513		lncRNA	chr7:130853720-130
ENSG0000(	445	9.937982	chr7:158{	EEF1B2P6		Pseudoger	chr7:131661952-131
ENSG0000(	445	9.937982	chr7:158{	ENSG00000289175		lncRNA	chr7:134953657-134
ENSG0000(	445	9.937982	chr7:158{	ENSG00000233287		Pseudoger	chr7:131242590-131
ENSG0000(	445	9.937982	chr7:158{	ENSG00000229858		Pseudoger	chr7:130822868-130
ENSG0000(	445	9.937982	chr7:158{	STRA8		protein_c	chr7:135231979-135
ENSG0000(	445	9.937982	chr7:158{	ENSG00000290805		lncRNA	chr7:135246113-135
ENSG0000(	445	9.937982	chr7:158{	FAM180A		protein_c	chr7:135728348-135
ENSG0000(	445	9.937982	chr7:158{	WDR91		protein_c	chr7:135183839-135
ENSG0000(	445	9.937982	chr7:158{	TUBB3P2		Pseudoger	chr7:134734898-134
ENSG0000(	445	9.937982	chr7:158{	ENSG00000287547		lncRNA	chr7:130668852-130
ENSG0000(	445	9.937982	chr7:158{	RNU6-1010P		smallRNA	chr7:131054886-131
ENSG0000(	445	9.937982	chr7:158{	BPGM		protein_c	chr7:134646811-134
ENSG0000(	445	9.937982	chr7:158{	ENSG00000231098		lncRNA	chr7:134284500-134
ENSG0000(	445	9.937982	chr7:158{	ENSG00000273297		lncRNA	chr7:134368737-134
ENSG0000(	445	9.937982	chr7:158{	ENSG00000231794		lncRNA	chr7:135198401-135
ENSG0000(	445	9.937982	chr7:158{	ENSG00000223718		Pseudoger	chr7:135660039-135
ENSG0000(	443	9.893317	chr12:14(	MIR4303		smallRNA	chr12:97995383-979
ENSG0000(	443	9.893317	chr12:14(	CFAP54	NCGv7	protein_c	chr12:96489571-968
ENSG0000(	443	9.893317	chr12:14(	AC013283.1		smallRNA	chr12:98605874-986
ENSG0000(	443	9.893317	chr12:14(	Y_RNA		smallRNA	chr12:96979528-969
ENSG0000(	443	9.893317	chr12:14(	RNU6-36P		smallRNA	chr12:97721716-977
ENSG0000(	443	9.893317	chr12:14(	TMPO-AS1		lncRNA	chr12:98512973-985
ENSG0000(	443	9.893317	chr12:14(	SLC25A3		protein_c	chr12:98593591-986
ENSG0000(	443	9.893317	chr12:14(	CDK17		protein_c	chr12:96278261-964
ENSG0000(	443	9.893317	chr12:14(	RN7SL179P		smallRNA	chr12:98524476-985
ENSG0000(	443	9.893317	chr12:14(	MIR4495		smallRNA	chr12:97939056-979
ENSG0000(	443	9.893317	chr12:14(	NEDD1		protein_c	chr12:96907224-969
ENSG0000(	443	9.893317	chr12:14(	ENSG00000257470		lncRNA	chr12:97024021-971
ENSG0000(	443	9.893317	chr12:14(	YPEL5P3		Pseudoger	chr12:96060024-960
ENSG0000(	443	9.893317	chr12:14(	LINC02453		lncRNA	chr12:98485544-985
ENSG0000(	443	9.893317	chr12:14(	LINC02409		lncRNA	chr12:97272692-972
ENSG0000(	443	9.893317	chr12:14(	ENSG00000257715		lncRNA	chr12:96025323-960
ENSG0000(	443	9.893317	chr12:14(	snoMe28S-Am2634		smallRNA	chr12:97552137-975
ENSG0000(	443	9.893317	chr12:14(	ENSG00000258177		lncRNA	chr12:96222797-962

ENSG0000(	443	9.893317	chr12:149	ENSG00000279798		TEC	chr12:97563257-975
ENSG0000(	443	9.893317	chr12:149	RNU4-24P		smallRNA	chr12:96303375-963
ENSG0000(	443	9.893317	chr12:149	TMPO		protein_	chr12:98515579-985
ENSG0000(	443	9.893317	chr12:149	ENSG00000257169		Pseudoger	chr12:96386818-963
ENSG0000(	443	9.893317	chr12:149	PPIAP8		Pseudoger	chr12:98590723-985
ENSG0000(	443	9.893317	chr12:149	LTA4H	NCGv7	protein_	chr12:96000753-960
ENSG0000(	443	9.893317	chr12:149	ELK3	AC	protein_	chr12:96194375-962
ENSG0000(	443	9.893317	chr12:149	RN7SKP11		smallRNA	chr12:96427200-964
ENSG0000(	443	9.893317	chr12:149	ENSG00000258272		lncRNA	chr12:96422326-964
ENSG0000(	443	9.893317	chr12:149	APAF1		protein_	chr12:98645290-987
ENSG0000(	443	9.893317	chr12:149	RMST		lncRNA	chr12:97430884-975
ENSG0000(	443	9.893317	chr12:149	ENSG00000286434		lncRNA	chr12:97155234-971
ENSG0000(	443	9.893317	chr12:149	ENSG00000257580		Pseudoger	chr12:98317741-983
ENSG0000(	443	9.893317	chr12:149	RNU4-41P		smallRNA	chr12:98417896-984
ENSG0000(	443	9.893317	chr12:149	RN7SL88P		smallRNA	chr12:96101953-961
ENSG0000(	443	9.893317	chr12:149	ENSG00000258131		lncRNA	chr12:96985656-971
ENSG0000(	443	9.893317	chr12:149	PAFAH1B2P2		Pseudoger	chr12:97644345-977
ENSG0000(	443	9.893317	chr12:149	LINC02452		lncRNA	chr12:96160692-961
ENSG0000(	443	9.893317	chr12:149	IKBIP		protein_	chr12:98613405-986
ENSG0000(	443	9.893317	chr12:149	ENSG00000258312		lncRNA	chr12:98113014-982
ENSG0000(	443	9.893317	chr12:149	ENSG00000286056		lncRNA	chr12:98420448-984
ENSG0000(	443	9.893317	chr12:149	EEF1A1P33		Pseudoger	chr12:96900697-969
ENSG0000(	443	9.893317	chr12:149	ENSG00000279190		TEC	chr12:97150081-971
ENSG0000(	443	9.893317	chr12:149	ENSG00000287454		lncRNA	chr12:96229563-962
ENSG0000(	443	9.893317	chr12:149	ENSG00000258204		Pseudoger	chr12:98794485-987
ENSG0000(	443	9.893317	chr12:149	MIR1251		smallRNA	chr12:97491900-974
ENSG0000(	443	9.893317	chr12:149	SNORA53		smallRNA	chr12:98599635-985
ENSG0000(	443	9.893317	chr12:149	SLC9A7P1		Pseudoger	chr12:98453835-984
ENSG0000(	443	9.893317	chr12:149	AC021052.1		smallRNA	chr12:96443480-964
ENSG0000(	443	9.893317	chr12:149	MIR135A2		smallRNA	chr12:97563812-975
ENSG0000(	443	9.893317	chr12:149	ENSG00000257157		Pseudoger	chr12:97679879-976
ENSG0000(	443	9.893317	chr12:149	ENSG00000289949		lncRNA	chr12:96131433-961
ENSG0000(	443	9.893317	chr17:739	ENSG00000264174		lncRNA	chr17:34725509-347
ENSG0000(	442	9.870984	chr7:1588	TMEM248		protein_	chr7:66921225-6695
ENSG0000(	442	9.870984	chr7:1588	ENSG00000179342		Pseudoger	chr7:66505155-6650
ENSG0000(	442	9.870984	chr7:1588	ENSG00000179131		Pseudoger	chr7:66914581-6691
ENSG0000(	442	9.870984	chr7:1588	ENSG00000286456		lncRNA	chr7:64574673-6461
ENSG0000(	442	9.870984	chr13:279	ENSG00000286334		lncRNA	chr13:97278768-972
ENSG0000(	442	9.870984	chr7:1588	SNORA22		smallRNA	chr7:65755526-6575
ENSG0000(	442	9.870984	chr13:279	RPL7AP61		Pseudoger	chr13:97901653-979
ENSG0000(	442	9.870984	chr13:279	AMMECR1LP1		Pseudoger	chr13:96573659-965
ENSG0000(	442	9.870984	chr7:1588	STAG3L4		Pseudoger	chr7:67305987-6730
ENSG0000(	442	9.870984	chr7:1588	VN1R42P		Pseudoger	chr7:64933273-6493
ENSG0000(	442	9.870984	chr7:1588	ENSG00000232546		Pseudoger	chr7:66848496-6685
ENSG0000(	442	9.870984	chr7:1588	ENSG00000282381		lncRNA	chr7:65269359-6531
ENSG0000(	442	9.870984	chr13:279	MTND6P18		Pseudoger	chr13:95693983-956
ENSG0000(	442	9.870984	chr7:1588	INTS4P2		Pseudoger	chr7:65647823-6571
ENSG0000(	442	9.870984	chr7:1588	YWHAEP1		Pseudoger	chr7:64433830-6443
ENSG0000(	442	9.870984	chr7:1588	BNIP3P42		Pseudoger	chr7:64551498-6455
ENSG0000(	442	9.870984	chr13:279	CLDN10-AS1		lncRNA	chr13:95479444-955
ENSG0000(	442	9.870984	chr13:279	RNF113B		protein_	chr13:98175785-981

ENSG0000(	442	9.870984	chr7:158{	ZNF680P1		Pseudoger	chr7:64469185-6447
ENSG0000(	442	9.870984	chr13:27{	ENSG00000286416		lncRNA	chr13:97405169-974
ENSG0000(	442	9.870984	chr13:27{	MBNL2	NCGv7	protein_c	chr13:97221434-973
ENSG0000(	442	9.870984	chr7:158{	ENSG00000228019		Pseudoger	chr7:67307605-6730
ENSG0000(	442	9.870984	chr13:27{	HSP90AB6P		Pseudoger	chr13:96883842-968
ENSG0000(	442	9.870984	chr7:158{	AC006480.1		smallRNA	chr7:67356680-6735
ENSG0000(	442	9.870984	chr13:27{	RAP2A		protein_c	chr13:97434169-974
ENSG0000(	442	9.870984	chr7:158{	ENSG00000287580		lncRNA	chr7:64801958-6480
ENSG0000(	442	9.870984	chr7:158{	ENSG00000189316		lncRNA	chr7:64888527-6489
ENSG0000(	442	9.870984	chr7:158{	ENSG00000273448		lncRNA	chr7:67333047-6733
ENSG0000(	442	9.870984	chr7:158{	VN1R40P		Pseudoger	chr7:64443802-6444
ENSG0000(	442	9.870984	chr7:158{	SBDS	NCGv7;AC	protein_c	chr7:66987680-6699
ENSG0000(	442	9.870984	chr7:158{	RNU6-912P		smallRNA	chr7:65814672-6581
ENSG0000(	442	9.870984	chr7:158{	ASL	NCGv7	protein_c	chr7:66075800-6609
ENSG0000(	442	9.870984	chr7:158{	MIR4650-1		smallRNA	chr7:67114322-6711
ENSG0000(	442	9.870984	chr7:158{	ENSG00000286342		lncRNA	chr7:64947571-6495
ENSG0000(	442	9.870984	chr7:158{	SNORA15		smallRNA	chr7:65760052-6576
ENSG0000(	442	9.870984	chr13:27{	MTCYBP3		Pseudoger	chr13:95694569-956
ENSG0000(	442	9.870984	chr7:158{	ENSG00000224172		Pseudoger	chr7:64735933-6473
ENSG0000(	442	9.870984	chr7:158{	RN7SL43P		smallRNA	chr7:66980120-6698
ENSG0000(	442	9.870984	chr7:158{	ENSG00000275833		Pseudoger	chr7:65764535-6576
ENSG0000(	442	9.870984	chr13:27{	ENSG00000270725		lncRNA	chr13:97939356-979
ENSG0000(	442	9.870984	chr13:27{	SLC15A1	NCGv7	protein_c	chr13:98683801-987
ENSG0000(	442	9.870984	chr13:27{	SNORD112		smallRNA	chr13:96965941-969
ENSG0000(	442	9.870984	chr13:27{	OXGR1		protein_c	chr13:96985713-969
ENSG0000(	442	9.870984	chr7:158{	ZNF138		protein_c	chr7:64794388-6483
ENSG0000(	442	9.870984	chr13:27{	snoU13		smallRNA	chr13:97057379-970
ENSG0000(	442	9.870984	chr13:27{	CYCSP35		Pseudoger	chr13:98629686-986
ENSG0000(	442	9.870984	chr7:158{	SNORA63		smallRNA	chr7:65327396-6532
ENSG0000(	442	9.870984	chr7:158{	ENSG00000249319		protein_c	chr7:66087761-6615
ENSG0000(	442	9.870984	chr7:158{	RNU6-1254P		smallRNA	chr7:66891188-6689
ENSG0000(	442	9.870984	chr7:158{	ENSG00000234338		Pseudoger	chr7:64835280-6483
ENSG0000(	442	9.870984	chr7:158{	ENSG00000289177		lncRNA	chr7:66845003-6684
ENSG0000(	442	9.870984	chr7:158{	AC092685.1		smallRNA	chr7:65263414-6526
ENSG0000(	442	9.870984	chr13:27{	FTLP8		Pseudoger	chr13:97986950-979
ENSG0000(	442	9.870984	chr7:158{	LINC03011		lncRNA	chr7:66493607-6649
ENSG0000(	442	9.870984	chr13:27{	TULP3P1		Pseudoger	chr13:96940149-969
ENSG0000(	442	9.870984	chr7:158{	MTDHP1		Pseudoger	chr7:64942143-6494
ENSG0000(	442	9.870984	chr7:158{	ZNF273		protein_c	chr7:64870172-6493
ENSG0000(	442	9.870984	chr7:158{	ENSG00000236529		lncRNA	chr7:65840055-6584
ENSG0000(	442	9.870984	chr7:158{	TYW1		protein_c	chr7:66995173-6723
ENSG0000(	442	9.870984	chr7:158{	ENSG00000226767		Pseudoger	chr7:65508773-6550
ENSG0000(	442	9.870984	chr7:158{	CCT6P3		lncRNA	chr7:65038354-6507
ENSG0000(	442	9.870984	chr7:158{	AC073089.1		smallRNA	chr7:67139071-6713
ENSG0000(	442	9.870984	chr13:27{	RN7SL164P		smallRNA	chr13:96169545-961
ENSG0000(	442	9.870984	chr7:158{	ENSG00000234185		lncRNA	chr7:66119603-6616
ENSG0000(	442	9.870984	chr7:158{	RNU6-313P		smallRNA	chr7:66344304-6634
ENSG0000(	442	9.870984	chr7:158{	CCT6P3		Pseudoger	chr7:65064999-6507
ENSG0000(	442	9.870984	chr7:158{	RNU6-96P		smallRNA	chr7:66395191-6639
ENSG0000(	442	9.870984	chr13:27{	RN7SL60P		smallRNA	chr13:98671980-986
ENSG0000(	442	9.870984	chr7:158{	GTF2IP14		Pseudoger	chr7:65084103-6510

ENSG0000(	442	9.870984	chr13:279	IPO5	NCGv7	protein_c	chr13:97953658-980
ENSG0000(	442	9.870984	chr17:739	RN7SL458P		smallRNA	chr17:38409799-384
ENSG0000(	442	9.870984	chr7:1588	ENSG00000289108		lncRNA	chr7:65219563-6525
ENSG0000(	442	9.870984	chr7:1588	LINC03006		Pseudoger	chr7:65770473-6577
ENSG0000(	442	9.870984	chr7:1588	ZNF92		protein_c	chr7:65373799-6540
ENSG0000(	442	9.870984	chr7:1588	ENSG00000236928		Pseudoger	chr7:66434634-6643
ENSG0000(	442	9.870984	chr13:279	ENSG00000226134		lncRNA	chr13:97675011-976
ENSG0000(	442	9.870984	chr7:1588	VKORC1L1		protein_c	chr7:65873074-6595
ENSG0000(	442	9.870984	chr7:1588	ENSG00000234500		Pseudoger	chr7:66511556-6654
ENSG0000(	442	9.870984	chr7:1588	PMS2P4		Pseudoger	chr7:67295608-6729
ENSG0000(	442	9.870984	chr7:1588	ENSG00000289015		lncRNA	chr7:66681258-6668
ENSG0000(	442	9.870984	chr13:279	RN7SKP7		smallRNA	chr13:96949249-969
ENSG0000(	442	9.870984	chr7:1588	SEPHS1P1		Pseudoger	chr7:64852397-6485
ENSG0000(	442	9.870984	chr7:1588	INTS4P1		Pseudoger	chr7:65141241-6521
ENSG0000(	442	9.870984	chr7:1588	KCTD7	NCGv7	protein_c	chr7:66628881-6664
ENSG0000(	442	9.870984	chr13:279	FARP1		protein_c	chr13:98142562-984
ENSG0000(	442	9.870984	chr7:1588	SNORA63		smallRNA	chr7:65326719-6532
ENSG0000(	442	9.870984	chr7:1588	ENSG00000284461		protein_c	chr7:66628958-6681
ENSG0000(	442	9.870984	chr7:1588	ZNF117		protein_c	chr7:64971772-6500
ENSG0000(	442	9.870984	chr7:1588	ENSG00000226824		lncRNA	chr7:66654513-6668
ENSG0000(	442	9.870984	chr7:1588	RABGEF1	NCGv7	protein_c	chr7:66682164-6681
ENSG0000(	442	9.870984	chr7:1588	ENSG00000290090		lncRNA	chr7:66114830-6611
ENSG0000(	442	9.870984	chr7:1588	LINC03006		lncRNA	chr7:65647010-6577
ENSG0000(	442	9.870984	chr13:279	ENSG00000286544		lncRNA	chr13:97725172-977
ENSG0000(	442	9.870984	chr7:1588	ENSG00000177418		Pseudoger	chr7:66556889-6655
ENSG0000(	442	9.870984	chr13:279	ENSG00000269189		lncRNA	chr13:98233650-982
ENSG0000(	442	9.870984	chr13:279	DNAJC3-DT		lncRNA	chr13:95648711-956
ENSG0000(	442	9.870984	chr13:279	ENSG00000276809		lncRNA	chr13:95744726-957
ENSG0000(	442	9.870984	chr13:279	snoU13		smallRNA	chr13:97885809-978
ENSG0000(	442	9.870984	chr7:1588	SNORA15		smallRNA	chr7:65070538-6507
ENSG0000(	442	9.870984	chr7:1588	snoU13		smallRNA	chr7:66790354-6679
ENSG0000(	442	9.870984	chr13:279	STK24-AS1		lncRNA	chr13:98577244-985
ENSG0000(	442	9.870984	chr13:279	snR65		smallRNA	chr13:95714677-957
ENSG0000(	442	9.870984	chr7:1588	ENSG00000224365		Pseudoger	chr7:66004017-6600
ENSG0000(	442	9.870984	chr13:279	UGGT2		protein_c	chr13:95801580-960
ENSG0000(	442	9.870984	chr13:279	DNAJC3		protein_c	chr13:95677139-957
ENSG0000(	442	9.870984	chr13:279	STK24		protein_c	chr13:98445185-985
ENSG0000(	442	9.870984	chr7:1588	GTF2IP5		Pseudoger	chr7:65773620-6580
ENSG0000(	442	9.870984	chr7:1588	snoU13		smallRNA	chr7:66434507-6643
ENSG0000(	442	9.870984	chr7:1588	SAPCD2P3		Pseudoger	chr7:66556216-6655
ENSG0000(	442	9.870984	chr7:1588	ENSG00000275400		Pseudoger	chr7:66553805-6655
ENSG0000(	442	9.870984	chr13:279	HS6ST3		protein_c	chr13:96090107-968
ENSG0000(	442	9.870984	chr13:279	CALM2P4		Pseudoger	chr13:98640814-986
ENSG0000(	442	9.870984	chr7:1588	ENSG00000239985		Pseudoger	chr7:65038372-6503
ENSG0000(	442	9.870984	chr7:1588	CRCP		protein_c	chr7:66114604-6615
ENSG0000(	442	9.870984	chr7:1588	LINC00174		lncRNA	chr7:66376044-6649
ENSG0000(	442	9.870984	chr7:1588	AC068533.1		smallRNA	chr7:66056234-6605
ENSG0000(	442	9.870984	chr7:1588	INTS4P1		lncRNA	chr7:65141032-6523
ENSG0000(	442	9.870984	chr7:1588	ZNF107		protein_c	chr7:64666099-6471
ENSG0000(	442	9.870984	chr13:279	ENSG00000275919		Pseudoger	chr13:98871742-988
ENSG0000(	442	9.870984	chr7:1588	ERV3-1		protein_c	chr7:64990356-6500

ENSG0000(	442	9.870984	chr13:279	LINC00456		lncRNA	chr13:97168751-971
ENSG0000(	442	9.870984	chr7:1588	ENSG00000232906		Pseudoger	chr7:65355934-6535
ENSG0000(	442	9.870984	chr7:1588	LINC02604		lncRNA	chr7:66902857-6690
ENSG0000(	442	9.870984	chr7:1588	SNORA22		smallRNA	chr7:65065999-6506
ENSG0000(	442	9.870984	chr7:1588	ENSG00000233383		Pseudoger	chr7:67089257-6708
ENSG0000(	442	9.870984	chr11:469	SNORD56		smallRNA	chr11:90118391-901
ENSG0000(	442	9.870984	chr7:1588	ENSG00000277206		Pseudoger	chr7:64768156-6476
ENSG0000(	442	9.870984	chr13:279	HMGN1P24		Pseudoger	chr13:95809310-958
ENSG0000(	442	9.870984	chr7:1588	ENSG00000224669		Pseudoger	chr7:64582733-6458
ENSG0000(	442	9.870984	chr7:1588	ENSG00000271064		Pseudoger	chr7:66748838-6674
ENSG0000(	442	9.870984	chr13:279	NUS1P4		Pseudoger	chr13:98610292-986
ENSG0000(	442	9.870984	chr7:1588	ENSG00000290553		lncRNA	chr7:65647864-6571
ENSG0000(	442	9.870984	chr7:1588	ENSG00000244510		Pseudoger	chr7:66480394-6649
ENSG0000(	442	9.870984	chr13:279	RN7SKP8		smallRNA	chr13:98203835-982
ENSG0000(	442	9.870984	chr7:1588	ENSG00000287317		lncRNA	chr7:64651655-6465
ENSG0000(	442	9.870984	chr7:1588	EEF1DP4		Pseudoger	chr7:64862999-6486
ENSG0000(	442	9.870984	chr7:1588	ENSG00000213642		Pseudoger	chr7:64569428-6457
ENSG0000(	442	9.870984	chr7:1588	ENSG00000272831		lncRNA	chr7:66739829-6674
ENSG0000(	442	9.870984	chr13:279	ENSG00000269599		lncRNA	chr13:98329655-983
ENSG0000(	442	9.870984	chr7:1588	ENSG00000227113		Pseudoger	chr7:65075023-6507
ENSG0000(	442	9.870984	chr7:1588	AC104057.1		protein_c	chr7:65647167-6564
ENSG0000(	442	9.870984	chr7:1588	RSL24D1P3		Pseudoger	chr7:65335174-6533
ENSG0000(	442	9.870984	chr13:279	DZIP1		protein_c	chr13:95578202-956
ENSG0000(	442	9.870984	chr7:1588	ENSG00000237026		Pseudoger	chr7:65235790-6523
ENSG0000(	442	9.870984	chr13:279	PSMA6P4		Pseudoger	chr13:97475742-974
ENSG0000(	442	9.870984	chr13:279	FARP1-AS1		lncRNA	chr13:98435405-984
ENSG0000(	442	9.870984	chr7:1588	GTF2IP23		Pseudoger	chr7:66880708-6688
ENSG0000(	442	9.870984	chr7:1588	ENSG00000229886		Pseudoger	chr7:66025126-6603
ENSG0000(	442	9.870984	chr7:1588	ENSG00000291124		lncRNA	chr7:65751037-6576
ENSG0000(	442	9.870984	chr7:1588	ENSG00000235421		Pseudoger	chr7:65525629-6555
ENSG0000(	442	9.870984	chr7:1588	Y_RNA		smallRNA	chr7:67297653-6729
ENSG0000(	442	9.870984	chr7:1588	ENSG00000277544		Pseudoger	chr7:65290429-6529
ENSG0000(	442	9.870984	chr13:279	ENSG00000272329		lncRNA	chr13:96948018-969
ENSG0000(	442	9.870984	chr7:1588	ENSG00000276650		Pseudoger	chr7:64425623-6442
ENSG0000(	442	9.870984	chr7:1588	PMS2P4		lncRNA	chr7:67139961-6730
ENSG0000(	442	9.870984	chr13:279	MIR3170		smallRNA	chr13:98208524-982
ENSG0000(	442	9.870984	chr7:1588	BNIP3P11		Pseudoger	chr7:64678954-6468
ENSG0000(	442	9.870984	chr7:1588	ENSG00000205596		Pseudoger	chr7:66070904-6607
ENSG0000(	442	9.870984	chr13:279	ENSG00000276704		lncRNA	chr13:97437268-974
ENSG0000(	442	9.870984	chr7:1588	ENSG00000235349		Pseudoger	chr7:64566814-6456
ENSG0000(	442	9.870984	chr7:1588	SKP1P1		Pseudoger	chr7:66423405-6642
ENSG0000(	442	9.870984	chr7:1588	ENSG00000230386		Pseudoger	chr7:65617082-6561
ENSG0000(	442	9.870984	chr7:1588	CCT6P1		Pseudoger	chr7:65751142-6576
ENSG0000(	442	9.870984	chr7:1588	ENSG00000229064		Pseudoger	chr7:65247608-6524
ENSG0000(	442	9.870984	chr13:279	ENSG00000271437		Pseudoger	chr13:98553243-985
ENSG0000(	442	9.870984	chr7:1588	HNRNPCP7		Pseudoger	chr7:64500825-6450
ENSG0000(	442	9.870984	chr7:1588	ZNF680	IntOGen-I	protein_c	chr7:64519878-6456
ENSG0000(	442	9.870984	chr7:1588	GTF2IP9		Pseudoger	chr7:66407288-6640
ENSG0000(	442	9.870984	chr7:1588	ENSG00000238124		lncRNA	chr7:65463071-6546
ENSG0000(	442	9.870984	chr7:1588	ENSG00000276475		Pseudoger	chr7:64590599-6459
ENSG0000(	442	9.870984	chr7:1588	RNU6-1229P		smallRNA	chr7:65023204-6502

ENSG0000(	442	9.870984	chr7:1588	GUSB	NCGv7	protein_c	chr7:65960684-6598
ENSG0000(	442	9.870984	chr7:1588	TPST1	DriverDBv	protein_c	chr7:66205317-6642
ENSG0000(	442	9.870984	chr13:279	MTND5P2		Pseudoger	chr13:95692538-956
ENSG0000(	442	9.870984	chr7:1588	ENSG00000291136		lncRNA	chr7:66526088-6659
ENSG0000(	442	9.870984	chr7:1588	ENSG00000287869		lncRNA	chr7:64867427-6487
ENSG0000(	442	9.870984	chr7:1588	ENSG00000244657		Pseudoger	chr7:66485095-6648
ENSG0000(	442	9.870984	chr7:1588	STAG3L4		lncRNA	chr7:67302621-6736
ENSG0000(	442	9.870984	chr7:1588	ENSG00000279785		TEC	chr7:66474556-6647
ENSG0000(	442	9.870984	chr13:279	DOCK9-AS1		lncRNA	chr13:98832084-988
ENSG0000(	442	9.870984	chr13:279	RNA5SP37		Pseudoger	chr13:97362972-973
ENSG0000(	442	9.870984	chr7:1588	RABGEF1P2		Pseudoger	chr7:66427949-6645
ENSG0000(	442	9.870984	chr7:1588	GTF2IRD1P1		Pseudoger	chr7:66815836-6683
ENSG0000(	442	9.870984	chr13:279	ENSG00000276573		lncRNA	chr13:97435946-974
ENSG0000(	442	9.870984	chr7:1588	GTF2IRD1P1		lncRNA	chr7:66809993-6684
ENSG0000(	442	9.870984	chr7:1588	RNU6-973P		smallRNA	chr7:65859660-6585
ENSG0000(	441	9.848652	chr10:81(	RPL17P35		Pseudoger	chr10:64620374-646
ENSG0000(	441	9.848652	chr10:81(	LINC00844		lncRNA	chr10:58999482-590
ENSG0000(	441	9.848652	chr10:81(	snoU13		smallRNA	chr10:66615237-666
ENSG0000(	441	9.848652	chr10:81(	ENSG00000235140		lncRNA	chr10:59578467-596
ENSG0000(	441	9.848652	chr10:81(	FAM13C		protein_c	chr10:59246130-593
ENSG0000(	441	9.848652	chr10:81(	ENSG00000289834		lncRNA	chr10:61900107-619
ENSG0000(	441	9.848652	chr10:81(	ENSG00000224301		lncRNA	chr10:63123929-631
ENSG0000(	441	9.848652	chr10:81(	AC022538.1		smallRNA	chr10:65710060-657
ENSG0000(	441	9.848652	chr10:81(	TMEM26-AS1		lncRNA	chr10:61452639-614
ENSG0000(	441	9.848652	chr7:1588	ENSG00000224448		Pseudoger	chr7:99638242-9963
ENSG0000(	441	9.848652	chr10:81(	ENSG00000285871		lncRNA	chr10:68896920-689
ENSG0000(	441	9.848652	chr10:81(	LINC01553		lncRNA	chr10:59955430-599
ENSG0000(	441	9.848652	chr10:81(	RPL12P8		Pseudoger	chr10:67874332-678
ENSG0000(	441	9.848652	chr10:81(	ENSG00000273360		lncRNA	chr10:66079243-661
ENSG0000(	441	9.848652	chr10:81(	ENSG00000287969		lncRNA	chr10:59357007-593
ENSG0000(	441	9.848652	chr10:81(	DNA2		protein_c	chr10:68414064-684
ENSG0000(	441	9.848652	chr10:81(	RNU6-571P		smallRNA	chr10:68911653-689
ENSG0000(	441	9.848652	chr10:81(	RNU6-1250P		smallRNA	chr10:67808502-678
ENSG0000(	441	9.848652	chr10:81(	ENSG00000277056		Pseudoger	chr10:66837457-668
ENSG0000(	441	9.848652	chr10:81(	DNAJC19P1		Pseudoger	chr10:67833763-678
ENSG0000(	441	9.848652	chr10:81(	PBLD		protein_c	chr10:68282660-683
ENSG0000(	441	9.848652	chr10:81(	ENSG00000288052		lncRNA	chr10:61493843-614
ENSG0000(	441	9.848652	chr10:81(	AKR1B10P1		Pseudoger	chr10:67750284-677
ENSG0000(	441	9.848652	chr10:81(	CTNNA3	NCGv7	protein_c	chr10:65912457-677
ENSG0000(	441	9.848652	chr10:81(	RPL7AP51		Pseudoger	chr10:67334123-673
ENSG0000(	441	9.848652	chr10:81(	ENSG00000286373		lncRNA	chr10:63630672-636
ENSG0000(	441	9.848652	chr10:81(	ENSG00000270421		Pseudoger	chr10:63358745-633
ENSG0000(	441	9.848652	chr10:81(	snoR442		smallRNA	chr10:66494699-664
ENSG0000(	441	9.848652	chr10:81(	ENSG00000228566		lncRNA	chr10:63664664-646
ENSG0000(	441	9.848652	chr10:81(	ENSG00000225299		lncRNA	chr10:67052609-670
ENSG0000(	441	9.848652	chr10:81(	SUPV3L1		protein_c	chr10:69180234-692
ENSG0000(	441	9.848652	chr10:81(	EGR2	NCGv7	protein_c	chr10:62811996-628
ENSG0000(	441	9.848652	chr10:81(	NEK4P3		Pseudoger	chr10:65054460-650
ENSG0000(	441	9.848652	chr10:81(	KRT19P4		Pseudoger	chr10:68260557-682
ENSG0000(	441	9.848652	chr10:81(	LINC02621		lncRNA	chr10:62289521-623
ENSG0000(	441	9.848652	chr10:81(	RNA5SP319		Pseudoger	chr10:68461407-684

ENSG0000(	441	9.848652	chr10:81(	CABCOCO1		protein_c	chr10:61662929-617
ENSG0000(	441	9.848652	chr10:81(	DDX21		protein_c	chr10:68956135-689
ENSG0000(	441	9.848652	chr10:81(	RNU6-697P		smallRNA	chr10:68846805-688
ENSG0000(	441	9.848652	chr10:81(	ENSG00000270352		Pseudoger	chr10:63658074-636
ENSG0000(	441	9.848652	chr10:81(	ENSG00000272592		lncRNA	chr10:61684892-616
ENSG0000(	441	9.848652	chr10:81(	CCDC6	NCGv7;AC	protein_c	chr10:59788747-599
ENSG0000(	441	9.848652	chr10:81(	ENSG00000224412		Pseudoger	chr10:63489461-634
ENSG0000(	441	9.848652	chr10:81(	ENSG00000289989		lncRNA	chr10:60944378-609
ENSG0000(	441	9.848652	chr10:81(	ENSG00000289487		lncRNA	chr10:62814433-629
ENSG0000(	441	9.848652	chr10:81(	LINC00845		lncRNA	chr10:61001088-611
ENSG0000(	441	9.848652	chr10:81(	snoU13		smallRNA	chr10:68712958-687
ENSG0000(	441	9.848652	chr10:81(	AC067751.1		smallRNA	chr10:62658185-626
ENSG0000(	441	9.848652	chr10:81(	VPS26A		protein_c	chr10:69123512-691
ENSG0000(	441	9.848652	chr10:81(	SLC25A16		protein_c	chr10:68477998-685
ENSG0000(	441	9.848652	chr10:81(	STOX1		protein_c	chr10:68827531-688
ENSG0000(	441	9.848652	chr10:81(	ANK3-DT		lncRNA	chr10:60734342-607
ENSG0000(	441	9.848652	chr10:81(	RN7SKP196		smallRNA	chr10:58869184-588
ENSG0000(	441	9.848652	chr10:81(	ENSG00000282906		lncRNA	chr10:65123667-651
ENSG0000(	441	9.848652	chr10:81(	CCAR1	NCGv7	protein_c	chr10:68721012-687
ENSG0000(	441	9.848652	chr10:81(	ENSG00000235356		lncRNA	chr10:65271319-653
ENSG0000(	441	9.848652	chr10:81(	TRAF6P1		Pseudoger	chr10:59136625-591
ENSG0000(	441	9.848652	chr10:81(	RPL26P27		Pseudoger	chr10:68499532-684
ENSG0000(	441	9.848652	chr10:81(	DDX50	NCGv7	protein_c	chr10:68901286-689
ENSG0000(	441	9.848652	chr10:81(	TMEM14DP		Pseudoger	chr10:68544489-685
ENSG0000(	441	9.848652	chr10:81(	ENSG00000260400		lncRNA	chr10:68698500-687
ENSG0000(	441	9.848652	chr10:81(	RPL21P92		Pseudoger	chr10:67793770-677
ENSG0000(	441	9.848652	chr10:81(	ENSG00000236556		lncRNA	chr10:59282147-592
ENSG0000(	441	9.848652	chr10:81(	ALDH7A1P4		Pseudoger	chr10:62741208-627
ENSG0000(	441	9.848652	chr10:81(	PRELID1P3		Pseudoger	chr10:63427297-634
ENSG0000(	441	9.848652	chr10:81(	SIRT1	AC	protein_c	chr10:67884656-679
ENSG0000(	441	9.848652	chr10:81(	KIFBP		protein_c	chr10:68988803-690
ENSG0000(	441	9.848652	chr10:81(	CDK1	AC	protein_c	chr10:60778331-607
ENSG0000(	441	9.848652	chr10:81(	LINC01515		lncRNA	chr10:65570338-658
ENSG0000(	441	9.848652	chr10:81(	HNRNPH3	NCGv7	protein_c	chr10:68331174-683
ENSG0000(	441	9.848652	chr10:81(	RPL26P29		Pseudoger	chr10:68424560-684
ENSG0000(	441	9.848652	chr10:81(	FAM133CP		Pseudoger	chr10:58715554-587
ENSG0000(	441	9.848652	chr10:81(	TET1	NCGv7	protein_c	chr10:68560337-686
ENSG0000(	441	9.848652	chr10:81(	MYL6P3		Pseudoger	chr10:65169438-651
ENSG0000(	441	9.848652	chr10:81(	Y_RNA		smallRNA	chr10:68503152-685
ENSG0000(	441	9.848652	chr10:81(	CYP2C61P		Pseudoger	chr10:64762883-647
ENSG0000(	441	9.848652	chr10:81(	RN7SKP202		smallRNA	chr10:68158079-681
ENSG0000(	441	9.848652	chr10:81(	AL671972.1		smallRNA	chr10:62116215-621
ENSG0000(	441	9.848652	chr10:81(	AL356741.1		smallRNA	chr10:67970273-679
ENSG0000(	441	9.848652	chr10:81(	ANK3	NCGv7	protein_c	chr10:60026298-607
ENSG0000(	441	9.848652	chr10:81(	RPS3AP38		Pseudoger	chr10:67960702-679
ENSG0000(	441	9.848652	chr10:81(	SRGN		protein_c	chr10:69088103-691
ENSG0000(	441	9.848652	chr10:81(	HERC4		protein_c	chr10:67921899-680
ENSG0000(	441	9.848652	chr10:81(	MYPN		protein_c	chr10:68087897-682
ENSG0000(	441	9.848652	chr10:81(	DNAJC12		protein_c	chr10:67796669-678
ENSG0000(	441	9.848652	chr10:81(	ENSG00000232682		lncRNA	chr10:60050668-600
ENSG0000(	441	9.848652	chr10:81(	RN7SL220P		smallRNA	chr10:68007720-680

ENSG0000(	441	9.848652	chr10:81(	MRLN		protein_c	chr10:59736692-597
ENSG0000(	441	9.848652	chr10:81(	RTKN2		protein_c	chr10:62183035-622
ENSG0000(	441	9.848652	chr10:81(	AC067742.1		smallRNA	chr10:61926936-619
ENSG0000(	441	9.848652	chr10:81(	ENSG00000282121		lncRNA	chr10:60139912-601
ENSG0000(	441	9.848652	chr10:81(	RPLP1P10		Pseudoger	chr10:59088701-590
ENSG0000(	441	9.848652	chr10:81(	RPL7AP50		Pseudoger	chr10:63902451-639
ENSG0000(	441	9.848652	chr10:81(	ENSG00000289325		protein_c	chr10:66926308-669
ENSG0000(	441	9.848652	chr10:81(	MRPL50P4		Pseudoger	chr10:59551404-595
ENSG0000(	441	9.848652	chr10:81(	DBF4P1		Pseudoger	chr10:64168959-641
ENSG0000(	441	9.848652	chr10:81(	JMJD1C-AS1		lncRNA	chr10:63465229-634
ENSG0000(	441	9.848652	chr10:81(	Y_RNA		smallRNA	chr10:68735720-687
ENSG0000(	441	9.848652	chr10:81(	RN7SL394P		smallRNA	chr10:67825310-678
ENSG0000(	441	9.848652	chr10:81(	RN7SL591P		smallRNA	chr10:62350009-623
ENSG0000(	441	9.848652	chr10:81(	RN7SL373P		smallRNA	chr10:68966016-689
ENSG0000(	441	9.848652	chr10:81(	ACTBP14		Pseudoger	chr10:69022778-690
ENSG0000(	441	9.848652	chr10:81(	RHOBTB1		protein_c	chr10:60869438-610
ENSG0000(	441	9.848652	chr10:81(	NRBF2		protein_c	chr10:63133247-631
ENSG0000(	441	9.848652	chr10:81(	SNORD98		smallRNA	chr10:68755172-687
ENSG0000(	441	9.848652	chr10:81(	MIR1254-1		smallRNA	chr10:68759318-687
ENSG0000(	441	9.848652	chr10:81(	LINC02625		lncRNA	chr10:61781745-618
ENSG0000(	441	9.848652	chr10:81(	RPS3AP37		Pseudoger	chr10:68674356-686
ENSG0000(	441	9.848652	chr10:81(	POU5F1P5		Pseudoger	chr10:68010205-680
ENSG0000(	441	9.848652	chr10:81(	ENSG00000272892		lncRNA	chr10:67849525-678
ENSG0000(	441	9.848652	chr10:81(	RUFY2		protein_c	chr10:68341107-684
ENSG0000(	441	9.848652	chr10:81(	ENSG00000233724		Pseudoger	chr10:65248032-652
ENSG0000(	441	9.848652	chr10:81(	ENSG00000270494		Pseudoger	chr10:68717311-687
ENSG0000(	441	9.848652	chr10:81(	REEP3		protein_c	chr10:63521401-636
ENSG0000(	441	9.848652	chr10:81(	ADO		protein_c	chr10:62804720-628
ENSG0000(	441	9.848652	chr10:81(	SLC16A9		protein_c	chr10:59650764-597
ENSG0000(	441	9.848652	chr10:81(	PHYHIPL		protein_c	chr10:59176643-592
ENSG0000(	441	9.848652	chr10:81(	TFAM		protein_c	chr10:58385345-583
ENSG0000(	441	9.848652	chr10:81(	MED28P1		Pseudoger	chr10:68991137-689
ENSG0000(	441	9.848652	chr10:81(	Y_RNA		smallRNA	chr10:60081741-600
ENSG0000(	441	9.848652	chr10:81(	RNU2-72P		smallRNA	chr10:60912765-609
ENSG0000(	441	9.848652	chr10:81(	JMJD1C	NCGv7	protein_c	chr10:63167221-635
ENSG0000(	441	9.848652	chr10:81(	ARL4AP1		Pseudoger	chr10:60684505-606
ENSG0000(	441	9.848652	chr10:81(	TATDN1P1		Pseudoger	chr10:63222155-632
ENSG0000(	441	9.848652	chr10:81(	ZNF365		protein_c	chr10:62374192-624
ENSG0000(	441	9.848652	chr10:81(	ENSG00000288011		lncRNA	chr10:62339553-623
ENSG0000(	441	9.848652	chr10:81(	MRPL35P2		Pseudoger	chr10:63634317-636
ENSG0000(	441	9.848652	chr10:81(	TMEM26		protein_c	chr10:61406642-614
ENSG0000(	441	9.848652	chr10:81(	LRRTM3		protein_c	chr10:66926036-671
ENSG0000(	441	9.848652	chr10:81(	ENSG00000285837		protein_c	chr10:62374192-626
ENSG0000(	441	9.848652	chr10:81(	BICC1		protein_c	chr10:58512872-588
ENSG0000(	441	9.848652	chr10:81(	LINC02929		lncRNA	chr10:62520448-626
ENSG0000(	441	9.848652	chr10:81(	ANXA2P3		Pseudoger	chr10:64825572-648
ENSG0000(	441	9.848652	chr10:81(	LINC02640		lncRNA	chr10:68233251-682
ENSG0000(	441	9.848652	chr10:81(	ENSG00000238280		lncRNA	chr10:62682652-628
ENSG0000(	441	9.848652	chr10:81(	ARID5B	NCGv7	protein_c	chr10:61901684-620
ENSG0000(	441	9.848652	chr10:81(	RNU6-543P		smallRNA	chr10:63110139-631
ENSG0000(	441	9.848652	chr10:81(	LINC02671		lncRNA	chr10:64901136-650

ENSG0000(	441	9.848652	chr10:81(	COX20P1		Pseudoger	chr10:68632371-686
ENSG0000(	440	9.826319	chr5:164(	ENSG00000249894		lncRNA	chr5:67800740-6789
ENSG0000(	440	9.826319	chr5:164(	PPIAP78		Pseudoger	chr5:66572057-6657
ENSG0000(	440	9.826319	chr5:164(	ENSG00000248664		lncRNA	chr5:69113109-6916
ENSG0000(	440	9.826319	chr5:164(	ENSG00000239870		Pseudoger	chr5:67608654-6760
ENSG0000(	440	9.826319	chr5:164(	ERBIN-DT		lncRNA	chr5:65924629-6592
ENSG0000(	440	9.826319	chr5:164(	ENSG00000248803		Pseudoger	chr5:66622868-6662
ENSG0000(	440	9.826319	chr5:164(	SHISAL2B		protein_c	chr5:64690442-6471
ENSG0000(	440	9.826319	chr5:164(	ENSG00000291221		lncRNA	chr5:70462244-7047
ENSG0000(	440	9.826319	chr5:164(	LINC02065		lncRNA	chr5:66205120-6620
ENSG0000(	440	9.826319	chr5:164(	snoU13		smallRNA	chr5:69539161-6953
ENSG0000(	440	9.826319	chr5:164(	RPEP1		Pseudoger	chr5:65251127-6525
ENSG0000(	440	9.826319	chr5:164(	TRAPPC13		protein_c	chr5:65625004-6566
ENSG0000(	440	9.826319	chr5:164(	GUSBP15		Pseudoger	chr5:70516387-7055
ENSG0000(	440	9.826319	chr5:164(	ENSG00000269983		lncRNA	chr5:70449636-7045
ENSG0000(	440	9.826319	chr5:164(	snoU13		smallRNA	chr5:66231129-6623
ENSG0000(	440	9.826319	chr5:164(	SREK1		protein_c	chr5:66139971-6618
ENSG0000(	440	9.826319	chr5:164(	NAIPP2		Pseudoger	chr5:70094659-7012
ENSG0000(	440	9.826319	chr5:164(	Y_RNA		smallRNA	chr5:65123369-6512
ENSG0000(	440	9.826319	chr5:164(	RNU6-540P		smallRNA	chr5:65604661-6560
ENSG0000(	440	9.826319	chr5:164(	ENSG00000286314		lncRNA	chr5:65965373-6596
ENSG0000(	440	9.826319	chr5:164(	ENSG00000253744		lncRNA	chr5:66144156-6614
ENSG0000(	440	9.826319	chr5:164(	RP11-1415C14.1		Pseudoger	chr5:70214942-7021
ENSG0000(	440	9.826319	chr5:164(	LINC02242		lncRNA	chr5:67632266-6764
ENSG0000(	440	9.826319	chr5:164(	MAST4	NCGv7	protein_c	chr5:66596380-6716
ENSG0000(	440	9.826319	chr5:164(	BCL9P1		Pseudoger	chr5:67636382-6763
ENSG0000(	440	9.826319	chr5:164(	ERBIN	NCGv7	protein_c	chr5:65883128-6608
ENSG0000(	440	9.826319	chr5:164(	RN7SL616P		smallRNA	chr5:69478993-6947
ENSG0000(	440	9.826319	chr5:164(	CDH12P3		Pseudoger	chr5:70132679-7013
ENSG0000(	440	9.826319	chr5:164(	RN7SL169P		smallRNA	chr5:64539504-6453
ENSG0000(	440	9.826319	chr5:164(	U8		smallRNA	chr5:68873954-6887
ENSG0000(	440	9.826319	chr5:164(	RNU6-1232P		smallRNA	chr5:68159061-6815
ENSG0000(	440	9.826319	chr5:164(	SERF1B		protein_c	chr5:70025247-7004
ENSG0000(	440	9.826319	chr5:164(	ENSG00000251158		Pseudoger	chr5:69898867-6990
ENSG0000(	440	9.826319	chr5:164(	SMN2		protein_c	chr5:70049638-7007
ENSG0000(	440	9.826319	chr5:164(	VWA8P1		Pseudoger	chr5:68854910-6885
ENSG0000(	440	9.826319	chr5:164(	RP11-497H16.6		Pseudoger	chr5:70508079-7051
ENSG0000(	440	9.826319	chr5:164(	MAST4-AS1		lncRNA	chr5:67001383-6700
ENSG0000(	440	9.826319	chr5:164(	SLC30A5		protein_c	chr5:69093991-6913
ENSG0000(	440	9.826319	chr5:164(	NDUFB9P1		Pseudoger	chr5:69349936-6935
ENSG0000(	440	9.826319	chr5:164(	RNU6-724P		smallRNA	chr5:69530613-6953
ENSG0000(	440	9.826319	chr5:164(	CDH12P2		Pseudoger	chr5:69985204-6998
ENSG0000(	440	9.826319	chr5:164(	ENSG00000253333		Pseudoger	chr5:70495100-7050
ENSG0000(	440	9.826319	chr5:164(	GUSBP3		Pseudoger	chr5:69640266-6967
ENSG0000(	440	9.826319	chr5:164(	RN7SL476P		smallRNA	chr5:69455575-6945
ENSG0000(	440	9.826319	chr5:164(	OCLN		protein_c	chr5:69492292-6955
ENSG0000(	440	9.826319	chr5:164(	ENSG00000250978		lncRNA	chr5:67463809-6747
ENSG0000(	440	9.826319	chr5:164(	ENSG00000248288		Pseudoger	chr5:65209921-6521
ENSG0000(	440	9.826319	chr5:164(	ENSG00000285151		lncRNA	chr5:70055820-7005
ENSG0000(	440	9.826319	chr5:164(	CDK7		protein_c	chr5:69234795-6927
ENSG0000(	440	9.826319	chr5:164(	ENSG00000290556		lncRNA	chr5:69607099-6962

ENSG0000(	440	9.826319	chr5:164(	ENSG00000286062		lncRNA	chr5:66350148-6644
ENSG0000(	440	9.826319	chr5:164(	ENSG00000248769		Pseudoger	chr5:69653248-6965
ENSG0000(	440	9.826319	chr5:164(	CFL1P5		Pseudoger	chr5:69313371-6931
ENSG0000(	440	9.826319	chr5:164(	ENSG00000291220		lncRNA	chr5:70415396-7044
ENSG0000(	440	9.826319	chr5:164(	ENSG00000249721		Pseudoger	chr5:67692812-6769
ENSG0000(	440	9.826319	chr5:164(	LINC02198		lncRNA	chr5:68970692-6903
ENSG0000(	440	9.826319	chr5:164(	ENSG00000248884		lncRNA	chr5:68430339-6843
ENSG0000(	440	9.826319	chr5:164(	snoU13		smallRNA	chr5:69175824-6917
ENSG0000(	440	9.826319	chr5:164(	LINC02219		lncRNA	chr5:68189876-6819
ENSG0000(	440	9.826319	chr5:164(	NAIPP3		Pseudoger	chr5:69618313-6962
ENSG0000(	440	9.826319	chr5:164(	ENSG00000280187		TEC	chr5:69186359-6918
ENSG0000(	440	9.826319	chr5:164(	MARVELD2		protein_c	chr5:69415065-6944
ENSG0000(	440	9.826319	chr5:164(	MRPS36		protein_c	chr5:69217760-6923
ENSG0000(	440	9.826319	chr5:164(	ENSG00000250421		lncRNA	chr5:67699429-6790
ENSG0000(	440	9.826319	chr5:164(	GTF2H2B		Pseudoger	chr5:70415352-7044
ENSG0000(	440	9.826319	chr5:164(	GUSBP14		Pseudoger	chr5:70219918-7025
ENSG0000(	440	9.826319	chr5:164(	GUSBP13		Pseudoger	chr5:69875271-6992
ENSG0000(	440	9.826319	chr5:164(	RP11-497H16.4		Pseudoger	chr5:70529371-7053
ENSG0000(	440	9.826319	chr5:164(	RP11-1415C14.2		Pseudoger	chr5:70232992-7023
ENSG0000(	440	9.826319	chr5:164(	ENSG00000254701		Pseudoger	chr5:70197255-7020
ENSG0000(	440	9.826319	chr5:164(	AC010376.1		smallRNA	chr5:67967445-6796
ENSG0000(	440	9.826319	chr5:164(	NAIPP1		Pseudoger	chr5:70473448-7047
ENSG0000(	440	9.826319	chr5:164(	SNORA50		smallRNA	chr5:69160806-6916
ENSG0000(	440	9.826319	chr5:164(	ENSG00000250081		lncRNA	chr5:65486444-6548
ENSG0000(	440	9.826319	chr5:164(	ENSG00000250138		Pseudoger	chr5:69631963-6963
ENSG0000(	440	9.826319	chr5:164(	PPWD1		protein_c	chr5:65563236-6558
ENSG0000(	440	9.826319	chr5:164(	ENSG00000290560		lncRNA	chr5:69639459-6967
ENSG0000(	440	9.826319	chr5:164(	SNORA76		smallRNA	chr5:65961183-6596
ENSG0000(	440	9.826319	chr5:164(	NLN	NCGv7	protein_c	chr5:65722205-6587
ENSG0000(	440	9.826319	chr5:164(	ENSG00000250066		lncRNA	chr5:68963246-6896
ENSG0000(	440	9.826319	chr5:164(	TRIM23	NCGv7	protein_c	chr5:65589690-6562
ENSG0000(	440	9.826319	chr5:164(	ENSG00000249588		lncRNA	chr5:68523878-6853
ENSG0000(	440	9.826319	chr5:164(	ADAMTS6	NCGv7	protein_c	chr5:65148738-6548
ENSG0000(	440	9.826319	chr5:164(	ENSG00000251391		lncRNA	chr5:66298394-6639
ENSG0000(	440	9.826319	chr5:164(	LINC02997		lncRNA	chr5:67379378-6780
ENSG0000(	440	9.826319	chr5:164(	LINC02229		lncRNA	chr5:66507380-6651
ENSG0000(	440	9.826319	chr5:164(	CCNB1	AC	protein_c	chr5:69167135-6917
ENSG0000(	440	9.826319	chr5:164(	SUMO2P4		Pseudoger	chr5:69068925-6906
ENSG0000(	440	9.826319	chr5:164(	RPS27P14		Pseudoger	chr5:69469883-6947
ENSG0000(	440	9.826319	chr5:164(	CENPK		protein_c	chr5:65517766-6556
ENSG0000(	440	9.826319	chr5:164(	RGS7BP		protein_c	chr5:64506015-6461
ENSG0000(	440	9.826319	chr5:164(	RAD17	NCGv7	protein_c	chr5:69369293-6941
ENSG0000(	440	9.826319	chr5:164(	PIK3R1	NCGv7;AC	protein_c	chr5:68215740-6830
ENSG0000(	440	9.826319	chr5:164(	ENSG00000250669		Pseudoger	chr5:67690033-6769
ENSG0000(	440	9.826319	chr5:164(	CENPH		protein_c	chr5:69189574-6921
ENSG0000(	440	9.826319	chr5:164(	ENSG00000286647		lncRNA	chr5:67356663-6736
ENSG0000(	440	9.826319	chr5:164(	CTC-498J12.1		lncRNA	chr5:69038518-6904
ENSG0000(	440	9.826319	chr5:164(	GTF2H2C		protein_c	chr5:69560191-6959
ENSG0000(	440	9.826319	chr5:164(	ENSG00000273860		Pseudoger	chr5:67855735-6785
ENSG0000(	440	9.826319	chr5:164(	CHCHD2P2		Pseudoger	chr5:69333929-6933
ENSG0000(	440	9.826319	chr5:164(	ENSG00000253801		Pseudoger	chr5:68087161-6808

ENSG0000(	440	9.826319	chr5:164(	EEF1B2P2	Pseudoger	chr5:68159218-6815
ENSG0000(	440	9.826319	chr5:164(	RN7SL9P	smallRNA	chr5:70074846-7007
ENSG0000(	440	9.826319	chr5:164(	RP11-497H16.2	Pseudoger	chr5:70487602-7049
ENSG0000(	440	9.826319	chr5:164(	ENSG00000249295	lncRNA	chr5:69477472-6950
ENSG0000(	440	9.826319	chr5:164(	CWC27	protein_c	chr5:64766368-6510
ENSG0000(	440	9.826319	chr5:164(	MAST4-IT1	lncRNA	chr5:66662331-6666
ENSG0000(	440	9.826319	chr5:164(	ENSG00000248359	lncRNA	chr5:68508223-6856
ENSG0000(	440	9.826319	chr5:164(	ENSG00000285999	lncRNA	chr5:66085405-6609
ENSG0000(	440	9.826319	chr5:164(	CCDC125	protein_c	chr5:69280175-6933
ENSG0000(	440	9.826319	chr5:164(	SHLD3	protein_c	chr5:65625027-6563
ENSG0000(	440	9.826319	chr5:164(	RNU6-294P	smallRNA	chr5:64573569-6457
ENSG0000(	440	9.826319	chr5:164(	AC079467.1	smallRNA	chr5:67464522-6746
ENSG0000(	440	9.826319	chr5:164(	TILRLS	lncRNA	chr5:67268022-6727
ENSG0000(	440	9.826319	chr5:164(	SGTB	protein_c	chr5:65665928-6572
ENSG0000(	440	9.826319	chr5:164(	ENSG00000249335	lncRNA	chr5:68792609-6904
ENSG0000(	440	9.826319	chr5:164(	AK6	protein_c	chr5:69350984-6937
ENSG0000(	440	9.826319	chr5:164(	CD180	protein_c	chr5:67179613-6719
ENSG0000(	440	9.826319	chr5:164(	RN7SL103P	smallRNA	chr5:69160036-6916
ENSG0000(	440	9.826319	chr5:164(	ENSG00000251648	Pseudoger	chr5:65732973-6573
ENSG0000(	440	9.826319	chr5:164(	MRPL49P1	Pseudoger	chr5:64674853-6467
ENSG0000(	440	9.826319	chr5:164(	SREK1IP1	protein_c	chr5:64718148-6476
ENSG0000(	440	9.826319	chr5:164(	TAF9	protein_c	chr5:69362026-6937
ENSG0000(	440	9.826319	chr5:164(	ENSG00000205644	Pseudoger	chr5:65415353-6541
ENSG0000(	439	9.803987	chr11:469	MTMR12P1	Pseudoger	chr11:101208905-10
ENSG0000(	439	9.803987	chr11:469	MTCO2P15	Pseudoger	chr11:103403512-10
ENSG0000(	439	9.803987	chr11:469	NPAT	protein_c	chr11:108157215-10
ENSG0000(	439	9.803987	chr7:158	ENSG00000231153	Pseudoger	chr7:95018407-9501
ENSG0000(	439	9.803987	chr11:469	BIRC2 AC	protein_c	chr11:102347211-10
ENSG0000(	439	9.803987	chr7:158	GRPEL2P3	Pseudoger	chr7:94782886-9478
ENSG0000(	439	9.803987	chr11:469	ENSG00000270202	Pseudoger	chr11:111107060-11
ENSG0000(	439	9.803987	chr11:469	ENSG00000243777	Pseudoger	chr11:102295060-10
ENSG0000(	439	9.803987	chr11:469	LINC02719	lncRNA	chr11:106112459-10
ENSG0000(	439	9.803987	chr11:469	CWC15	protein_c	chr11:94962620-949
ENSG0000(	439	9.803987	chr11:469	ENSG00000277984	Pseudoger	chr11:95057282-950
ENSG0000(	439	9.803987	chr11:469	ZC3H12C	protein_c	chr11:110093392-11
ENSG0000(	439	9.803987	chr11:469	AP003558.1	smallRNA	chr11:99424938-994
ENSG0000(	439	9.803987	chr11:469	RNA5SP345	Pseudoger	chr11:95840017-958
ENSG0000(	439	9.803987	chr11:469	RNU6-654P	smallRNA	chr11:109005517-10
ENSG0000(	439	9.803987	chr11:469	ENSG00000254730	Pseudoger	chr11:108727741-10
ENSG0000(	439	9.803987	chr11:469	DYNC2H1	protein_c	chr11:103109410-10
ENSG0000(	439	9.803987	chr11:469	MMP10	protein_c	chr11:102770502-10
ENSG0000(	439	9.803987	chr11:469	AP001282.1	smallRNA	chr11:106801349-10
ENSG0000(	439	9.803987	chr11:469	ENSG00000254599	lncRNA	chr11:98676391-986
ENSG0000(	439	9.803987	chr11:469	ENSG00000287245	lncRNA	chr11:109946581-10
ENSG0000(	439	9.803987	chr11:469	ENSG00000254587	lncRNA	chr11:96590317-969
ENSG0000(	439	9.803987	chr11:469	ENSG00000260008	lncRNA	chr11:102107886-10
ENSG0000(	439	9.803987	chr11:469	ENSG00000284715	Pseudoger	chr11:104975356-10
ENSG0000(	439	9.803987	chr11:469	CASP4	protein_c	chr11:104942866-10
ENSG0000(	439	9.803987	chr7:158	ATP5PBP2	Pseudoger	chr7:94738652-9473
ENSG0000(	439	9.803987	chr11:469	SLN	protein_c	chr11:107707378-10
ENSG0000(	439	9.803987	chr11:469	ENSG00000255605	lncRNA	chr11:95698086-957

ENSG0000(	439	9.803987	chr7:158{	PPP1R9A-AS1		lncRNA	chr7:95035731-9521
ENSG0000(	439	9.803987	chr11:46{	ENSG00000254758		Pseudoger	chr11:107642887-10
ENSG0000(	439	9.803987	chr11:46{	RNA5SP348		smallRNA	chr11:104252579-10
ENSG0000(	439	9.803987	chr11:46{	RNU6-952P		smallRNA	chr11:102313722-10
ENSG0000(	439	9.803987	chr7:158{	PPP1R9A	NCGv7	protein_c	chr7:94907202-9529
ENSG0000(	439	9.803987	chr11:46{	ENSG00000289383		lncRNA	chr11:105181864-10
ENSG0000(	439	9.803987	chr11:46{	MTATP6P15		Pseudoger	chr11:103402588-10
ENSG0000(	439	9.803987	chr11:46{	ENSG00000254506		Pseudoger	chr11:101584295-10
ENSG0000(	439	9.803987	chr11:46{	Y_RNA		smallRNA	chr11:108084913-10
ENSG0000(	439	9.803987	chr11:46{	RPS2P39		Pseudoger	chr11:108690289-10
ENSG0000(	439	9.803987	chr7:158{	RNU6-956P		smallRNA	chr7:94712409-9471
ENSG0000(	439	9.803987	chr11:46{	ENSG00000255065		Pseudoger	chr11:106310045-10
ENSG0000(	439	9.803987	chr11:46{	ENSG00000270449		Pseudoger	chr11:104682383-10
ENSG0000(	439	9.803987	chr7:158{	ENSG00000237729		Pseudoger	chr7:93669826-9367
ENSG0000(	439	9.803987	chr11:46{	ENSG00000270423		Pseudoger	chr11:109985243-10
ENSG0000(	439	9.803987	chr11:46{	ENSG00000287028		lncRNA	chr11:111097155-11
ENSG0000(	439	9.803987	chr11:46{	ENSG00000270753		Pseudoger	chr11:97086160-970
ENSG0000(	439	9.803987	chr11:46{	MMP3		protein_c	chr11:102835801-10
ENSG0000(	439	9.803987	chr11:46{	ARHGAP42-AS1		lncRNA	chr11:100684162-10
ENSG0000(	439	9.803987	chr7:158{	RPS3AP25		Pseudoger	chr7:94695027-9469
ENSG0000(	439	9.803987	chr11:46{	CASP4LP		Pseudoger	chr11:104903453-10
ENSG0000(	439	9.803987	chr11:46{	ENSG00000255149		Pseudoger	chr11:110876994-11
ENSG0000(	439	9.803987	chr11:46{	LINC02737		lncRNA	chr11:96508425-965
ENSG0000(	439	9.803987	chr11:46{	ENSG00000287846		Pseudoger	chr11:106106398-10
ENSG0000(	439	9.803987	chr11:46{	ENSG00000290498		lncRNA	chr11:106250019-10
ENSG0000(	439	9.803987	chr11:46{	ENSG00000254555		Pseudoger	chr11:97908253-979
ENSG0000(	439	9.803987	chr11:46{	ENSG00000290773		lncRNA	chr11:102752123-10
ENSG0000(	439	9.803987	chr7:158{	RNU6-1328P		smallRNA	chr7:94495299-9449
ENSG0000(	439	9.803987	chr11:46{	RNU6-277P		smallRNA	chr11:105974826-10
ENSG0000(	439	9.803987	chr11:46{	AP005718.1		smallRNA	chr11:108469455-10
ENSG0000(	439	9.803987	chr7:158{	ENSG00000225898		Pseudoger	chr7:93777839-9377
ENSG0000(	439	9.803987	chr11:46{	ENSG00000290797		lncRNA	chr11:105092469-10
ENSG0000(	439	9.803987	chr11:46{	CNTN5		protein_c	chr11:99020949-100
ENSG0000(	439	9.803987	chr11:46{	ENSG00000285696		lncRNA	chr11:108142458-10
ENSG0000(	439	9.803987	chr7:158{	VPS50	DriverDB\	protein_c	chr7:93232340-9336
ENSG0000(	439	9.803987	chr11:46{	ENSG00000270578		Pseudoger	chr11:95145437-951
ENSG0000(	439	9.803987	chr11:46{	RNU7-159P		smallRNA	chr11:102903892-10
ENSG0000(	439	9.803987	chr11:46{	ENSG00000255336		Pseudoger	chr11:105246880-10
ENSG0000(	439	9.803987	chr11:46{	snoU13		smallRNA	chr11:102056963-10
ENSG0000(	439	9.803987	chr11:46{	AP000673.1		smallRNA	chr11:105933954-10
ENSG0000(	439	9.803987	chr11:46{	RN7SKP115		smallRNA	chr11:100839630-10
ENSG0000(	439	9.803987	chr7:158{	SAMD9L	NCGv7	protein_c	chr7:93130056-9314
ENSG0000(	439	9.803987	chr7:158{	ENSG00000278959		TEC	chr7:93954044-9395
ENSG0000(	439	9.803987	chr11:46{	ENSG00000255528		lncRNA	chr11:109002465-10
ENSG0000(	439	9.803987	chr11:46{	LINC02732		lncRNA	chr11:110355130-11
ENSG0000(	439	9.803987	chr11:46{	ENSG00000277459		lncRNA	chr11:102109827-10
ENSG0000(	439	9.803987	chr11:46{	RN7SKP53		smallRNA	chr11:99120176-991
ENSG0000(	439	9.803987	chr11:46{	RPS17P15		Pseudoger	chr11:111105547-11
ENSG0000(	439	9.803987	chr11:46{	ENSG00000254422		lncRNA	chr11:102229851-10
ENSG0000(	439	9.803987	chr11:46{	ANGPTL5		protein_c	chr11:101890674-10
ENSG0000(	439	9.803987	chr7:158{	MIR653		smallRNA	chr7:93482760-9348

ENSG0000(	439	9.803987	chr11:469	ENSG00000256035		Pseudoger	chr11:102806948-10
ENSG0000(	439	9.803987	chr11:469	PLS1P1		Pseudoger	chr11:101765935-10
ENSG0000(	439	9.803987	chr11:469	MMP1		protein_c	chr11:102789401-10
ENSG0000(	439	9.803987	chr7:1588	TFPI2-DT		lncRNA	chr7:93890913-9389
ENSG0000(	439	9.803987	chr11:469	BUD13P1		Pseudoger	chr11:95143637-951
ENSG0000(	439	9.803987	chr11:469	ENSG00000255376		Pseudoger	chr11:96642671-966
ENSG0000(	439	9.803987	chr11:469	C11orf87		protein_c	chr11:109422190-10
ENSG0000(	439	9.803987	chr11:469	CASP12		protein_c	chr11:104885718-10
ENSG0000(	439	9.803987	chr11:469	ENSG00000285878		lncRNA	chr11:103252217-10
ENSG0000(	439	9.803987	chr11:469	KDM4D		protein_c	chr11:94973709-949
ENSG0000(	439	9.803987	chr11:469	RPA2P3		Pseudoger	chr11:100336856-10
ENSG0000(	439	9.803987	chr11:469	ENSG00000270969		Pseudoger	chr11:107585901-10
ENSG0000(	439	9.803987	chr11:469	MIR4693		smallRNA	chr11:103849906-10
ENSG0000(	439	9.803987	chr11:469	AASDHPPT		protein_c	chr11:106075501-10
ENSG0000(	439	9.803987	chr11:469	ENSG00000239861		Pseudoger	chr11:103045237-10
ENSG0000(	439	9.803987	chr11:469	MTND2P26		Pseudoger	chr11:103406213-10
ENSG0000(	439	9.803987	chr7:1588	TFPI2		protein_c	chr7:93885396-9389
ENSG0000(	439	9.803987	chr7:1588	BET1	DriverDBv	protein_c	chr7:93962762-9400
ENSG0000(	439	9.803987	chr11:469	HSPD1P13		Pseudoger	chr11:105706299-10
ENSG0000(	439	9.803987	chr11:469	RNU4-55P		smallRNA	chr11:105824634-10
ENSG0000(	439	9.803987	chr11:469	ENSG00000225678		Pseudoger	chr11:102751070-10
ENSG0000(	439	9.803987	chr11:469	ATM	NCGv7;AC	protein_c	chr11:108223044-10
ENSG0000(	439	9.803987	chr11:469	CASP1P2		Pseudoger	chr11:105063345-10
ENSG0000(	439	9.803987	chr11:469	AP003730.1		smallRNA	chr11:97912797-979
ENSG0000(	439	9.803987	chr11:469	MTMR2		protein_c	chr11:95821766-959
ENSG0000(	439	9.803987	chr11:469	ENSG00000254569		Pseudoger	chr11:104873264-10
ENSG0000(	439	9.803987	chr11:469	SRSF8		protein_c	chr11:95066919-950
ENSG0000(	439	9.803987	chr11:469	PGR	NCGv7	protein_c	chr11:101029624-10
ENSG0000(	439	9.803987	chr11:469	CUL5		protein_c	chr11:108008898-10
ENSG0000(	439	9.803987	chr11:469	LINC02553		lncRNA	chr11:97222644-972
ENSG0000(	439	9.803987	chr11:469	CASP1P1		Pseudoger	chr11:105122661-10
ENSG0000(	439	9.803987	chr11:469	MIR3920		smallRNA	chr11:101519820-10
ENSG0000(	439	9.803987	chr11:469	ENSG00000254890		Pseudoger	chr11:109486968-10
ENSG0000(	439	9.803987	chr11:469	C11orf65		protein_c	chr11:108308519-10
ENSG0000(	439	9.803987	chr11:469	CEP57		protein_c	chr11:95789965-958
ENSG0000(	439	9.803987	chr11:469	MTND1P36		Pseudoger	chr11:103407441-10
ENSG0000(	439	9.803987	chr11:469	AMOTL1	NCGv7	protein_c	chr11:94706431-948
ENSG0000(	439	9.803987	chr11:469	ARHGAP42		protein_c	chr11:100687288-10
ENSG0000(	439	9.803987	chr11:469	SRSF8BP		Pseudoger	chr11:95037482-950
ENSG0000(	439	9.803987	chr11:469	ENSG00000270868		Pseudoger	chr11:102306112-10
ENSG0000(	439	9.803987	chr11:469	TFAMP2		Pseudoger	chr11:109907004-10
ENSG0000(	439	9.803987	chr11:469	CARD16		protein_c	chr11:105041326-10
ENSG0000(	439	9.803987	chr11:469	RNA5SP350		Pseudoger	chr11:111040098-11
ENSG0000(	439	9.803987	chr11:469	ENSG00000287802		lncRNA	chr11:106465985-10
ENSG0000(	439	9.803987	chr11:469	ENSG00000290078		lncRNA	chr11:110328408-11
ENSG0000(	439	9.803987	chr11:469	ENSG00000240652		Pseudoger	chr11:107908420-10
ENSG0000(	439	9.803987	chr11:469	ENSG00000255548		lncRNA	chr11:103945548-10
ENSG0000(	439	9.803987	chr7:1588	MIR489		smallRNA	chr7:93483936-9348
ENSG0000(	439	9.803987	chr11:469	ENSG00000255653		Pseudoger	chr11:95011858-950
ENSG0000(	439	9.803987	chr11:469	ENSG00000273600		Pseudoger	chr11:95756959-957
ENSG0000(	439	9.803987	chr11:469	MTCO3P15		Pseudoger	chr11:103402013-10

ENSG0000(	439	9.803987	chr11:469	Y_RNA		smallRNA	chr11:95473272-954
ENSG0000(	439	9.803987	chr7:1588	PON1	NCGv7	protein_c	chr7:95297676-9532
ENSG0000(	439	9.803987	chr11:469	JRKL		protein_c	chr11:96389989-965
ENSG0000(	439	9.803987	chr7:1588	COL1A2	NCGv7	protein_c	chr7:94394895-9443
ENSG0000(	439	9.803987	chr11:469	AP001925.1		protein_c	chr11:108259528-10
ENSG0000(	439	9.803987	chr11:469	KBTBD3		protein_c	chr11:106051098-10
ENSG0000(	439	9.803987	chr11:469	ENSG00000288833		lncRNA	chr11:102346213-10
ENSG0000(	439	9.803987	chr11:469	PPIAP43		Pseudoger	chr11:100666459-10
ENSG0000(	439	9.803987	chr11:469	SNORD39		smallRNA	chr11:109263494-10
ENSG0000(	439	9.803987	chr11:469	LINC02713		lncRNA	chr11:97878475-979
ENSG0000(	439	9.803987	chr11:469	TRPC6	NCGv7	protein_c	chr11:101451564-10
ENSG0000(	439	9.803987	chr11:469	ENSG00000256254		Pseudoger	chr11:102766801-10
ENSG0000(	439	9.803987	chr11:469	ASS1P13		Pseudoger	chr11:107176286-10
ENSG0000(	439	9.803987	chr11:469	RAB39A		protein_c	chr11:107928448-10
ENSG0000(	439	9.803987	chr7:1588	ENSG00000285964		lncRNA	chr7:94311138-9434
ENSG0000(	439	9.803987	chr11:469	MAML2	NCGv7;AC	protein_c	chr11:95976598-963
ENSG0000(	439	9.803987	chr11:469	ENSG00000257012		Pseudoger	chr11:95040411-950
ENSG0000(	439	9.803987	chr11:469	BOLA3P1		Pseudoger	chr11:102880492-10
ENSG0000(	439	9.803987	chr11:469	ENSG00000276521		Pseudoger	chr11:102963460-10
ENSG0000(	439	9.803987	chr11:469	ENSG00000203334		lncRNA	chr11:108957718-10
ENSG0000(	439	9.803987	chr7:1588	RN7SKP129		smallRNA	chr7:94801514-9480
ENSG0000(	439	9.803987	chr11:469	MMP8		protein_c	chr11:102711796-10
ENSG0000(	439	9.803987	chr11:469	CCDC82		protein_c	chr11:96349241-963
ENSG0000(	439	9.803987	chr11:469	MMP27		protein_c	chr11:102691487-10
ENSG0000(	439	9.803987	chr11:469	CEP126		protein_c	chr11:101915010-10
ENSG0000(	439	9.803987	chr7:1588	ENSG00000236453		lncRNA	chr7:94022833-9406
ENSG0000(	439	9.803987	chr7:1588	ENSG00000285090		lncRNA	chr7:94278680-9439
ENSG0000(	439	9.803987	chr7:1588	RNU4-16P		smallRNA	chr7:95098227-9509
ENSG0000(	439	9.803987	chr11:469	ST13P11		Pseudoger	chr11:94913047-949
ENSG0000(	439	9.803987	chr11:469	ARHGAP20		protein_c	chr11:110577042-11
ENSG0000(	439	9.803987	chr11:469	MMP20		protein_c	chr11:102576832-10
ENSG0000(	439	9.803987	chr11:469	LINC02550		lncRNA	chr11:111091932-11
ENSG0000(	439	9.803987	chr11:469	FAM76B		protein_c	chr11:95768953-957
ENSG0000(	439	9.803987	chr11:469	GUCY1A2	NCGv7	protein_c	chr11:106674019-10
ENSG0000(	439	9.803987	chr11:469	CWF19L2		protein_c	chr11:107326360-10
ENSG0000(	439	9.803987	chr11:469	MSANTD4		protein_c	chr11:105995623-10
ENSG0000(	439	9.803987	chr11:469	ENSG00000285813		lncRNA	chr11:105995185-10
ENSG0000(	439	9.803987	chr7:1588	PEG10		protein_c	chr7:94656325-9466
ENSG0000(	439	9.803987	chr11:469	EXPH5	NCGv7	protein_c	chr11:108505435-10
ENSG0000(	439	9.803987	chr11:469	SMARCE1P1		Pseudoger	chr11:107403404-10
ENSG0000(	439	9.803987	chr11:469	MTCO1P15		Pseudoger	chr11:103404309-10
ENSG0000(	439	9.803987	chr11:469	RPSAP50		Pseudoger	chr11:109982192-10
ENSG0000(	439	9.803987	chr11:469	WTAPP1		Pseudoger	chr11:102746968-10
ENSG0000(	439	9.803987	chr11:469	MIR1260B		smallRNA	chr11:96341438-963
ENSG0000(	439	9.803987	chr11:469	RNA5SP349		Pseudoger	chr11:109120878-10
ENSG0000(	439	9.803987	chr11:469	ENSG00000254824		Pseudoger	chr11:103409580-10
ENSG0000(	439	9.803987	chr11:469	ENSG00000260966		lncRNA	chr11:103050687-10
ENSG0000(	439	9.803987	chr11:469	RDX		protein_c	chr11:109864295-11
ENSG0000(	439	9.803987	chr11:469	FDX1	NCGv7	protein_c	chr11:110429948-11
ENSG0000(	439	9.803987	chr11:469	ENSG00000287556		lncRNA	chr11:111013452-11
ENSG0000(	439	9.803987	chr11:469	ENSG00000254433		lncRNA	chr11:106085990-10

ENSG0000(	439	9.803987	chr11:469	ENSG00000256469		lncRNA	chr11:94874052-949
ENSG0000(	439	9.803987	chr11:469	BIRC3	NCGv7;AC	protein_c	chr11:102317450-10
ENSG0000(	439	9.803987	chr11:469	ENSG00000255467		lncRNA	chr11:108105074-10
ENSG0000(	439	9.803987	chr7:1588	ENSG00000278388		Pseudoger	chr7:93914987-9391
ENSG0000(	439	9.803987	chr11:469	ENSG00000254939		Pseudoger	chr11:98565074-985
ENSG0000(	439	9.803987	chr11:469	AC015600.1		smallRNA	chr11:100794441-10
ENSG0000(	439	9.803987	chr11:469	snoU13		smallRNA	chr11:102058305-10
ENSG0000(	439	9.803987	chr7:1588	HEPACAM2	NCGv7	protein_c	chr7:93188534-9322
ENSG0000(	439	9.803987	chr11:469	ENSG00000288528		lncRNA	chr11:102316173-10
ENSG0000(	439	9.803987	chr11:469	ENSG00000285842		lncRNA	chr11:95571040-957
ENSG0000(	439	9.803987	chr11:469	snoU13		smallRNA	chr11:100934274-10
ENSG0000(	439	9.803987	chr11:469	ALKBH8		protein_c	chr11:107502727-10
ENSG0000(	439	9.803987	chr11:469	HNRNPA1P60		Pseudoger	chr11:110788026-11
ENSG0000(	439	9.803987	chr11:469	SESN3		protein_c	chr11:95165513-952
ENSG0000(	439	9.803987	chr11:469	ENSG00000274486		Pseudoger	chr11:95030549-950
ENSG0000(	439	9.803987	chr11:469	ENSG00000281655		lncRNA	chr11:102641078-10
ENSG0000(	439	9.803987	chr11:469	LINC02552		lncRNA	chr11:104445868-10
ENSG0000(	439	9.803987	chr11:469	ENSG00000255380		Pseudoger	chr11:98130239-981
ENSG0000(	439	9.803987	chr11:469	MMP12	AC	protein_c	chr11:102862736-10
ENSG0000(	439	9.803987	chr11:469	ENSG00000286345		lncRNA	chr11:106241974-10
ENSG0000(	439	9.803987	chr7:1588	snoU13		smallRNA	chr7:95276586-9527
ENSG0000(	439	9.803987	chr11:469	LNCRNA-IUR		lncRNA	chr11:95150539-952
ENSG0000(	439	9.803987	chr7:1588	ARF1P1		Pseudoger	chr7:94833904-9483
ENSG0000(	439	9.803987	chr11:469	ENSG00000255210		Pseudoger	chr11:110049528-11
ENSG0000(	439	9.803987	chr11:469	ENSG00000213252		Pseudoger	chr11:106826392-10
ENSG0000(	439	9.803987	chr11:469	ENSG00000255028		lncRNA	chr11:109355085-10
ENSG0000(	439	9.803987	chr11:469	ENSG00000254830		lncRNA	chr11:98938912-989
ENSG0000(	439	9.803987	chr11:469	CARD18		protein_c	chr11:105137714-10
ENSG0000(	439	9.803987	chr11:469	TMEM123-DT		lncRNA	chr11:102452919-10
ENSG0000(	439	9.803987	chr11:469	MMP13	NCGv7	protein_c	chr11:102942995-10
ENSG0000(	439	9.803987	chr11:469	SLC35F2		protein_c	chr11:107790991-10
ENSG0000(	439	9.803987	chr7:1588	SAMD9		protein_c	chr7:93099513-9311
ENSG0000(	439	9.803987	chr11:469	ENSG00000282834		Pseudoger	chr11:95203727-952
ENSG0000(	439	9.803987	chr7:1588	CALCR	NCGv7	protein_c	chr7:93424486-9357
ENSG0000(	439	9.803987	chr11:469	RPL32P25		Pseudoger	chr11:95963219-959
ENSG0000(	439	9.803987	chr11:469	ENSG00000288012		lncRNA	chr11:108008678-10
ENSG0000(	439	9.803987	chr11:469	Y_RNA		smallRNA	chr11:108229503-10
ENSG0000(	439	9.803987	chr11:469	RNA5SP347		Pseudoger	chr11:97657464-976
ENSG0000(	439	9.803987	chr11:469	PGR-AS1		lncRNA	chr11:101129077-10
ENSG0000(	439	9.803987	chr11:469	KDM4F		protein_c	chr11:95049422-950
ENSG0000(	439	9.803987	chr11:469	CASP1		protein_c	chr11:105025397-10
ENSG0000(	439	9.803987	chr11:469	CASP5		protein_c	chr11:104994235-10
ENSG0000(	439	9.803987	chr11:469	LINC02715		lncRNA	chr11:109741625-10
ENSG0000(	439	9.803987	chr11:469	ACAT1		protein_c	chr11:108116695-10
ENSG0000(	439	9.803987	chr11:469	RNA5SP346		Pseudoger	chr11:96474572-964
ENSG0000(	439	9.803987	chr11:469	ENDOD1		protein_c	chr11:95089846-951
ENSG0000(	439	9.803987	chr11:469	MMP7	DriverDBv	protein_c	chr11:102520508-10
ENSG0000(	439	9.803987	chr11:469	JRKL-AS1		lncRNA	chr11:96447132-965
ENSG0000(	439	9.803987	chr11:469	ENSG00000285921		lncRNA	chr11:96092374-961
ENSG0000(	439	9.803987	chr11:469	ENSG00000250390		lncRNA	chr11:95482406-954
ENSG0000(	439	9.803987	chr7:1588	BET1-AS1		lncRNA	chr7:93969442-9401

ENSG0000(	439	9.803987	chr11:469	TMEM123		protein_c	chr11:102396332-10
ENSG0000(	439	9.803987	chr11:469	CFAP300		protein_c	chr11:102047437-10
ENSG0000(	439	9.803987	chr11:469	ENSG00000288255		Pseudoger	chr11:105055567-10
ENSG0000(	439	9.803987	chr11:469	POGLUT3		protein_c	chr11:108472112-10
ENSG0000(	439	9.803987	chr11:469	ENSG00000254702		Pseudoger	chr11:107736009-10
ENSG0000(	439	9.803987	chr11:469	CASP4LP		lncRNA	chr11:104901549-10
ENSG0000(	439	9.803987	chr11:469	RN7SL222P		smallRNA	chr11:100641978-10
ENSG0000(	439	9.803987	chr11:469	ENSG00000274584		Pseudoger	chr11:106824025-10
ENSG0000(	439	9.803987	chr7:1588	NDUFAF4P2		Pseudoger	chr7:93844789-9384
ENSG0000(	439	9.803987	chr11:469	DCUN1D5		protein_c	chr11:103050686-10
ENSG0000(	439	9.803987	chr11:469	CARD17		Pseudoger	chr11:105092486-10
ENSG0000(	439	9.803987	chr11:469	ENSG00000255483		Pseudoger	chr11:107600379-10
ENSG0000(	439	9.803987	chr11:469	ENSG00000254987		lncRNA	chr11:103675994-10
ENSG0000(	439	9.803987	chr11:469	CYCSP29		Pseudoger	chr11:108822333-10
ENSG0000(	439	9.803987	chr11:469	ENSG00000255482		lncRNA	chr11:102467255-10
ENSG0000(	439	9.803987	chr11:469	ENSG00000271600		Pseudoger	chr11:102308557-10
ENSG0000(	439	9.803987	chr11:469	GRIA4		protein_c	chr11:105609535-10
ENSG0000(	439	9.803987	chr11:469	YAP1	AC	protein_c	chr11:102110447-10
ENSG0000(	439	9.803987	chr7:1588	MIR4652		smallRNA	chr7:93716928-9371
ENSG0000(	439	9.803987	chr7:1588	CASD1		protein_c	chr7:94509219-9455
ENSG0000(	439	9.803987	chr7:1588	GNGT1		protein_c	chr7:93591573-9391
ENSG0000(	439	9.803987	chr7:1588	SGCE		protein_c	chr7:94524204-9465
ENSG0000(	439	9.803987	chr11:469	ELMOD1		protein_c	chr11:107591091-10
ENSG0000(	439	9.803987	chr11:469	DDX10	NCGv7;AC	protein_c	chr11:108665058-10
ENSG0000(	439	9.803987	chr7:1588	ENSG00000236938		lncRNA	chr7:94071759-9407
ENSG0000(	439	9.803987	chr7:1588	HINT1P2		Pseudoger	chr7:95018163-9501
ENSG0000(	439	9.803987	chr11:469	ENSG00000271390		lncRNA	chr11:111089870-11
ENSG0000(	439	9.803987	chr7:1588	GNG11		protein_c	chr7:93921735-9392
ENSG0000(	439	9.803987	chr11:469	ENSG00000289232		lncRNA	chr11:106579494-10
ENSG0000(	439	9.803987	chr11:469	ENSG00000256916		lncRNA	chr11:102606916-10
ENSG0000(	439	9.803987	chr11:469	MED28P5		Pseudoger	chr11:97000274-970
ENSG0000(	439	9.803987	chr11:469	KDM4E		protein_c	chr11:95025258-950
ENSG0000(	439	9.803987	chr11:469	ENSG00000261098		lncRNA	chr11:107312132-10
ENSG0000(	439	9.803987	chr11:469	ENSG00000254482		Pseudoger	chr11:109637468-10
ENSG0000(	439	9.803987	chr11:469	ENSG00000254811		Pseudoger	chr11:106264315-10
ENSG0000(	439	9.803987	chr11:469	ENSG00000289465		lncRNA	chr11:109803905-11
ENSG0000(	439	9.803987	chr11:469	PDGFD		protein_c	chr11:103907189-10
ENSG0000(	439	9.803987	chr11:469	DDI1		protein_c	chr11:104036640-10
ENSG0000(	438	9.781654	chr2:3094	RN7SL531P		smallRNA	chr2:4029681-40299
ENSG0000(	438	9.781654	chr2:3094	ENSG00000229550		lncRNA	chr2:4136644-41407
ENSG0000(	438	9.781654	chr2:3094	AC068292.1		smallRNA	chr2:4228428-42285
ENSG0000(	437	9.759322	chr3:3804	MIR128-2		smallRNA	chr3:35744476-3574
ENSG0000(	437	9.759322	chr3:3804	ENSG00000228171		Pseudoger	chr3:23389661-2338
ENSG0000(	437	9.759322	chr3:3804	NKIRAS1		protein_c	chr3:23889951-2394
ENSG0000(	437	9.759322	chr3:3804	ENSG00000281100		TEC	chr3:36823151-3682
ENSG0000(	437	9.759322	chr3:3804	RPL31P20		Pseudoger	chr3:24430748-2443
ENSG0000(	437	9.759322	chr6:1039	SNORD32B		smallRNA	chr6:29582249-2958
ENSG0000(	437	9.759322	chr3:3804	THRB	TAG	protein_c	chr3:24117153-2449
ENSG0000(	437	9.759322	chr3:3804	HMGB3P12		Pseudoger	chr3:26216322-2621
ENSG0000(	437	9.759322	chr3:3804	ENSG00000227549		lncRNA	chr3:30521664-3052
ENSG0000(	437	9.759322	chr3:3804	AC104306.2		smallRNA	chr3:32515726-3251

ENSG0000(	437	9.759322	chr3:380<	LINC00691		lncRNA	chr3:24096269-2410
ENSG0000(	437	9.759322	chr3:380<	MESTP4		Pseudoger	chr3:29087396-2908
ENSG0000(	437	9.759322	chr3:380<	KIAA1143P2		Pseudoger	chr3:27703129-2770
ENSG0000(	437	9.759322	chr3:380<	ARPP21-AS1		lncRNA	chr3:35650197-3565
ENSG0000(	437	9.759322	chr3:380<	ENSG00000283563		protein_c	chr3:28349178-2976
ENSG0000(	437	9.759322	chr3:380<	ENSG00000290076		lncRNA	chr3:37862435-3786
ENSG0000(	437	9.759322	chr3:380<	LINC01811		lncRNA	chr3:33956972-3467
ENSG0000(	437	9.759322	chr3:380<	ENSG00000289420		lncRNA	chr3:35421084-3542
ENSG0000(	437	9.759322	chr3:380<	H3P11		Pseudoger	chr3:31269007-3126
ENSG0000(	437	9.759322	chr3:380<	RPS20P15		Pseudoger	chr3:27462506-2746
ENSG0000(	437	9.759322	chr3:380<	AC137674.1		smallRNA	chr3:30138248-3013
ENSG0000(	437	9.759322	chr3:380<	DLEC1P1		Pseudoger	chr3:38325237-3832
ENSG0000(	437	9.759322	chr3:380<	UBE2FP1		Pseudoger	chr3:37143512-3714
ENSG0000(	437	9.759322	chr3:380<	NR1D2	NCGv7	protein_c	chr3:23945286-2398
ENSG0000(	437	9.759322	chr3:380<	TMPPE		protein_c	chr3:33090421-3309
ENSG0000(	437	9.759322	chr3:380<	RARB		protein_c	chr3:24687887-2559
ENSG0000(	437	9.759322	chr3:380<	Y_RNA		smallRNA	chr3:27547757-2754
ENSG0000(	437	9.759322	chr3:380<	ENSG00000237982		Pseudoger	chr3:38270283-3827
ENSG0000(	437	9.759322	chr3:380<	AC098650.1		smallRNA	chr3:29369337-2936
ENSG0000(	437	9.759322	chr3:380<	TOP2B		protein_c	chr3:25597978-2566
ENSG0000(	437	9.759322	chr3:380<	PDCD6IP		protein_c	chr3:33798571-3386
ENSG0000(	437	9.759322	chr3:380<	LINC01967		lncRNA	chr3:27916774-2805
ENSG0000(	437	9.759322	chr3:380<	ENSG00000228112		Pseudoger	chr3:33727250-3372
ENSG0000(	437	9.759322	chr3:380<	RRBP1P2		Pseudoger	chr3:22811390-2281
ENSG0000(	437	9.759322	chr3:380<	SEC13P1		Pseudoger	chr3:33033863-3303
ENSG0000(	437	9.759322	chr3:380<	OXSM		protein_c	chr3:25782917-2579
ENSG0000(	437	9.759322	chr3:380<	RNU6ATAC4P		smallRNA	chr3:36968191-3696
ENSG0000(	437	9.759322	chr3:380<	ZCWPW2	NCGv7	protein_c	chr3:28348721-2853
ENSG0000(	437	9.759322	chr3:380<	NIFKP7		Pseudoger	chr3:32063974-3206
ENSG0000(	437	9.759322	chr3:380<	snoU13		smallRNA	chr3:32172656-3217
ENSG0000(	437	9.759322	chr3:380<	RBMS3-AS2		lncRNA	chr3:29526251-2964
ENSG0000(	437	9.759322	chr3:380<	ENSG00000227498		lncRNA	chr3:34524818-3454
ENSG0000(	437	9.759322	chr3:380<	ENSG00000224618		Pseudoger	chr3:30626423-3062
ENSG0000(	437	9.759322	chr3:380<	KRT8P18		Pseudoger	chr3:35215705-3521
ENSG0000(	437	9.759322	chr3:380<	RFC3P1		Pseudoger	chr3:36169767-3617
ENSG0000(	437	9.759322	chr3:380<	Y_RNA		smallRNA	chr3:32045873-3204
ENSG0000(	437	9.759322	chr3:380<	DCLK3		protein_c	chr3:36712421-3676
ENSG0000(	437	9.759322	chr3:380<	ITGA9-AS1		lncRNA	chr3:37693655-3786
ENSG0000(	437	9.759322	chr3:380<	ENSG00000290046		lncRNA	chr3:37176387-3720
ENSG0000(	437	9.759322	chr3:380<	RANP7		Pseudoger	chr3:22904135-2290
ENSG0000(	437	9.759322	chr3:380<	ENSG00000230119		Pseudoger	chr3:33190233-3319
ENSG0000(	437	9.759322	chr3:380<	RNU6-922P		smallRNA	chr3:23613661-2361
ENSG0000(	437	9.759322	chr3:380<	RNA5SP126		Pseudoger	chr3:25352284-2535
ENSG0000(	437	9.759322	chr3:380<	Y_RNA		smallRNA	chr3:38125292-3812
ENSG0000(	437	9.759322	chr3:380<	ENSG00000289450		lncRNA	chr3:30373063-3046
ENSG0000(	437	9.759322	chr3:380<	AC092038.1		smallRNA	chr3:23399500-2339
ENSG0000(	437	9.759322	chr3:380<	TRANK1		protein_c	chr3:36826819-3694
ENSG0000(	437	9.759322	chr3:380<	RPSAP11		Pseudoger	chr3:32190747-3219
ENSG0000(	437	9.759322	chr3:380<	CMC1		protein_c	chr3:28241584-2832
ENSG0000(	437	9.759322	chr3:380<	NEK10		protein_c	chr3:27106484-2736
ENSG0000(	437	9.759322	chr3:380<	SDAD1P3		Pseudoger	chr3:33771963-3377

ENSG0000(	437	9.759322	chr3:380<	TCEA1P2		Pseudoger	chr3:37275693-3727
ENSG0000(	437	9.759322	chr3:380<	DLEC1	NCGv7	protein_c	chr3:38039205-3812
ENSG0000(	437	9.759322	chr3:380<	KRT18P15		Pseudoger	chr3:32258940-3226
ENSG0000(	437	9.759322	chr3:380<	AC099535.1		smallRNA	chr3:27362566-2736
ENSG0000(	437	9.759322	chr3:380<	ITGA9		protein_c	chr3:37452115-3782
ENSG0000(	437	9.759322	chr3:380<	UBE2E1		protein_c	chr3:23805955-2389
ENSG0000(	437	9.759322	chr3:380<	EOMES	NCGv7	protein_c	chr3:27715949-2772
ENSG0000(	437	9.759322	chr3:380<	APRG1		lncRNA	chr3:37381062-3744
ENSG0000(	437	9.759322	chr3:380<	STAC		protein_c	chr3:36380503-3654
ENSG0000(	437	9.759322	chr3:380<	ENSG00000272149		lncRNA	chr3:33144104-3314
ENSG0000(	437	9.759322	chr3:380<	ENSG00000270377		Pseudoger	chr3:32243847-3224
ENSG0000(	437	9.759322	chr3:380<	ZNF860		protein_c	chr3:31981750-3199
ENSG0000(	437	9.759322	chr3:380<	ENSG00000271943		Pseudoger	chr3:27632942-2763
ENSG0000(	437	9.759322	chr3:380<	NBPF21P		Pseudoger	chr3:36616006-3663
ENSG0000(	437	9.759322	chr3:380<	AC097639.1		smallRNA	chr3:32259728-3225
ENSG0000(	437	9.759322	chr3:380<	RPL24P7		Pseudoger	chr3:23134372-2313
ENSG0000(	437	9.759322	chr3:380<	CTDSPL		protein_c	chr3:37861880-3798
ENSG0000(	437	9.759322	chr3:380<	SLC22A14		protein_c	chr3:38282294-3831
ENSG0000(	437	9.759322	chr3:380<	NPM1P23		Pseudoger	chr3:24096512-2409
ENSG0000(	437	9.759322	chr3:380<	AC092798.1		smallRNA	chr3:25768660-2576
ENSG0000(	437	9.759322	chr3:380<	CNOT10-AS1		lncRNA	chr3:32730635-3273
ENSG0000(	437	9.759322	chr3:380<	OSBPL10-AS1		lncRNA	chr3:31704058-3172
ENSG0000(	437	9.759322	chr3:380<	GOLGA4		protein_c	chr3:37243191-3736
ENSG0000(	437	9.759322	chr3:380<	ENSG00000289460		lncRNA	chr3:33275247-3327
ENSG0000(	437	9.759322	chr3:380<	RPL21P40		Pseudoger	chr3:32030196-3203
ENSG0000(	437	9.759322	chr3:380<	NDUFAF4P3		Pseudoger	chr3:37789921-3779
ENSG0000(	437	9.759322	chr3:380<	SALL4P5		Pseudoger	chr3:22989823-2299
ENSG0000(	437	9.759322	chr3:380<	RNA5SP127		Pseudoger	chr3:31228715-3122
ENSG0000(	437	9.759322	chr3:380<	AC092038.2		smallRNA	chr3:23480497-2348
ENSG0000(	437	9.759322	chr3:380<	RNU6-235P		smallRNA	chr3:38303773-3830
ENSG0000(	437	9.759322	chr3:380<	SLC4A7		protein_c	chr3:27372721-2748
ENSG0000(	437	9.759322	chr3:380<	ENSG00000234723		Pseudoger	chr3:22670201-2267
ENSG0000(	437	9.759322	chr3:380<	UBE2E1-AS1		lncRNA	chr3:23804024-2380
ENSG0000(	437	9.759322	chr3:380<	RPL21P135		Pseudoger	chr3:37748110-3774
ENSG0000(	437	9.759322	chr3:380<	MTND4LP9		Pseudoger	chr3:29797652-2979
ENSG0000(	437	9.759322	chr3:380<	RBMS3-AS3		lncRNA	chr3:29054570-2929
ENSG0000(	437	9.759322	chr3:380<	FECHP1		Pseudoger	chr3:34872641-3487
ENSG0000(	437	9.759322	chr3:380<	RN7SL216P		smallRNA	chr3:25020163-2502
ENSG0000(	437	9.759322	chr3:380<	ARL4AP4		Pseudoger	chr3:23878981-2387
ENSG0000(	437	9.759322	chr3:380<	GADL1		protein_c	chr3:30726197-3089
ENSG0000(	437	9.759322	chr3:380<	U3		smallRNA	chr3:30304322-3030
ENSG0000(	437	9.759322	chr3:380<	Y_RNA		smallRNA	chr3:37093133-3709
ENSG0000(	437	9.759322	chr3:380<	CLASP2	NCGv7	protein_c	chr3:33496245-3371
ENSG0000(	437	9.759322	chr3:380<	LINC00692		lncRNA	chr3:25858525-2587
ENSG0000(	437	9.759322	chr3:380<	ACAA1		protein_c	chr3:38103129-3813
ENSG0000(	437	9.759322	chr3:380<	RPL36AP17		Pseudoger	chr3:35871866-3587
ENSG0000(	437	9.759322	chr3:380<	THRB-AS1		lncRNA	chr3:24494087-2468
ENSG0000(	437	9.759322	chr3:380<	THRB-IT1		lncRNA	chr3:24455136-2445
ENSG0000(	437	9.759322	chr3:380<	XYLB		protein_c	chr3:38346760-3842
ENSG0000(	437	9.759322	chr3:380<	RBMS3		protein_c	chr3:28574791-3001
ENSG0000(	437	9.759322	chr3:380<	RPL29P11		Pseudoger	chr3:37016523-3701

ENSG0000(	437	9.759322	chr3:380<	DYNC1LI1		protein_c	chr3:32525974-3257
ENSG0000(	437	9.759322	chr3:380<	RPEP2		Pseudoger	chr3:26015669-2601
ENSG0000(	437	9.759322	chr3:380<	ENSG00000288926		lncRNA	chr3:31530910-3153
ENSG0000(	437	9.759322	chr3:380<	STT3B		protein_c	chr3:31532638-3163
ENSG0000(	437	9.759322	chr3:380<	PLCD1	NCGv7	protein_c	chr3:38007496-3802
ENSG0000(	437	9.759322	chr3:380<	ENSG00000288972		lncRNA	chr3:37817408-3781
ENSG0000(	437	9.759322	chr3:380<	RNA5SP129		Pseudoger	chr3:37341649-3734
ENSG0000(	437	9.759322	chr3:380<	ENSG00000230807		Pseudoger	chr3:27486247-2748
ENSG0000(	437	9.759322	chr3:380<	Y_RNA		smallRNA	chr3:23273807-2327
ENSG0000(	437	9.759322	chr3:380<	UBE2E2-DT		lncRNA	chr3:23194929-2320
ENSG0000(	437	9.759322	chr3:380<	SUSD5		protein_c	chr3:33150043-3321
ENSG0000(	437	9.759322	chr3:380<	AZI2	NCGv7	protein_c	chr3:28315003-2834
ENSG0000(	437	9.759322	chr3:380<	ARPP21		protein_c	chr3:35638945-3579
ENSG0000(	437	9.759322	chr3:380<	Y_RNA		smallRNA	chr3:38014568-3801
ENSG0000(	437	9.759322	chr3:380<	TGFBR2	NCGv7	protein_c	chr3:30606601-3069
ENSG0000(	437	9.759322	chr3:380<	OSBPL10		protein_c	chr3:31657912-3207
ENSG0000(	437	9.759322	chr3:380<	HSPD1P6		Pseudoger	chr3:36767117-3678
ENSG0000(	437	9.759322	chr3:380<	RNU6-1301P		smallRNA	chr3:37140327-3714
ENSG0000(	437	9.759322	chr3:380<	H3P10		Pseudoger	chr3:25441164-2544
ENSG0000(	437	9.759322	chr3:380<	MLH1	NCGv7;AC	protein_c	chr3:36993350-3705
ENSG0000(	437	9.759322	chr3:380<	ENSG00000289130		lncRNA	chr3:24137832-2429
ENSG0000(	437	9.759322	chr3:380<	AC114877.1		smallRNA	chr3:26753937-2675
ENSG0000(	437	9.759322	chr3:380<	NGLY1		protein_c	chr3:25718944-2579
ENSG0000(	437	9.759322	chr3:380<	ENSG00000236732		Pseudoger	chr3:32151083-3215
ENSG0000(	437	9.759322	chr3:380<	RNU7-73P		smallRNA	chr3:37573151-3757
ENSG0000(	437	9.759322	chr3:380<	CMTM8		protein_c	chr3:32238679-3237
ENSG0000(	437	9.759322	chr3:380<	CMTM6		protein_c	chr3:32481312-3250
ENSG0000(	437	9.759322	chr3:380<	RPL30P4		Pseudoger	chr3:32635222-3263
ENSG0000(	437	9.759322	chr3:380<	PPP2R2DP1		Pseudoger	chr3:38051759-3805
ENSG0000(	437	9.759322	chr3:380<	RNU6-243P		smallRNA	chr3:35256840-3525
ENSG0000(	437	9.759322	chr3:380<	PRADC1P1		Pseudoger	chr3:36976316-3697
ENSG0000(	437	9.759322	chr3:380<	PDCD6IP-DT		lncRNA	chr3:33793644-3379
ENSG0000(	437	9.759322	chr3:380<	EIF3KP2		Pseudoger	chr3:24555834-2455
ENSG0000(	437	9.759322	chr3:380<	GLB1		protein_c	chr3:32996609-3309
ENSG0000(	437	9.759322	chr3:380<	LINC01980		lncRNA	chr3:27797552-2793
ENSG0000(	437	9.759322	chr3:380<	MYD88	NCGv7;AC	protein_c	chr3:38138478-3814
ENSG0000(	437	9.759322	chr3:380<	CCR4	NCGv7	protein_c	chr3:32951644-3295
ENSG0000(	437	9.759322	chr3:380<	ENSG00000261468		lncRNA	chr3:30697661-3069
ENSG0000(	437	9.759322	chr3:380<	LINC01985		lncRNA	chr3:30518833-3052
ENSG0000(	437	9.759322	chr3:380<	AC020626.1		smallRNA	chr3:23767611-2376
ENSG0000(	437	9.759322	chr3:380<	EXOG		protein_c	chr3:38496127-3854
ENSG0000(	437	9.759322	chr3:380<	CFL1P7		Pseudoger	chr3:25324174-2532
ENSG0000(	437	9.759322	chr3:380<	LRRC3B		protein_c	chr3:26622772-2671
ENSG0000(	437	9.759322	chr3:380<	LRRFIP2		protein_c	chr3:37052626-3718
ENSG0000(	437	9.759322	chr3:380<	CNN2P6		Pseudoger	chr3:31181751-3118
ENSG0000(	437	9.759322	chr3:380<	VILL	NCGv7	protein_c	chr3:37988059-3800
ENSG0000(	437	9.759322	chr3:380<	OXSR1		protein_c	chr3:38165089-3825
ENSG0000(	437	9.759322	chr3:380<	SLC22A13		protein_c	chr3:38265812-3827
ENSG0000(	437	9.759322	chr3:380<	CNOT10		protein_c	chr3:32685145-3277
ENSG0000(	437	9.759322	chr3:380<	AC137674.2		smallRNA	chr3:30138960-3013
ENSG0000(	437	9.759322	chr3:380<	ENSG00000287774		lncRNA	chr3:30292502-3030

ENSG0000(	437	9.759322	chr3:380<	ENSG00000272334		lncRNA	chr3:36973117-3697
ENSG0000(	437	9.759322	chr3:380<	MIR4792		smallRNA	chr3:24521362-2452
ENSG0000(	437	9.759322	chr3:380<	UBE2D3P2		Pseudoger	chr3:37134658-3713
ENSG0000(	437	9.759322	chr3:380<	RNU6-342P		smallRNA	chr3:27265580-2726
ENSG0000(	437	9.759322	chr3:380<	ENSG00000272554		lncRNA	chr3:24496259-2452
ENSG0000(	437	9.759322	chr3:380<	CIAO2AP1		Pseudoger	chr3:28427963-2842
ENSG0000(	437	9.759322	chr3:380<	ACVR2B-AS1		lncRNA	chr3:38451027-3845
ENSG0000(	437	9.759322	chr3:380<	MIR4442		smallRNA	chr3:25664873-2566
ENSG0000(	437	9.759322	chr3:380<	ENSG00000230480		Pseudoger	chr3:28447725-2844
ENSG0000(	437	9.759322	chr3:380<	RNU1-96P		smallRNA	chr3:27513871-2751
ENSG0000(	437	9.759322	chr3:380<	AC099540.1		smallRNA	chr3:31395937-3139
ENSG0000(	437	9.759322	chr3:380<	ENSG00000287981		lncRNA	chr3:34152979-3415
ENSG0000(	437	9.759322	chr3:380<	CRTAP		protein_c	chr3:33114014-3314
ENSG0000(	437	9.759322	chr3:380<	CMTM7		protein_c	chr3:32391698-3248
ENSG0000(	437	9.759322	chr3:380<	SUMO2P10		Pseudoger	chr3:33077204-3307
ENSG0000(	437	9.759322	chr3:380<	ENSG00000236452		lncRNA	chr3:34203244-3426
ENSG0000(	437	9.759322	chr3:380<	FBXL2		protein_c	chr3:33277025-3340
ENSG0000(	437	9.759322	chr3:380<	RNA5SP128		Pseudoger	chr3:33491983-3349
ENSG0000(	437	9.759322	chr3:380<	VENTXP4		Pseudoger	chr3:26346681-2634
ENSG0000(	437	9.759322	chr3:380<	LINC02033		lncRNA	chr3:36819276-3682
ENSG0000(	437	9.759322	chr3:380<	hsa-mir-466		smallRNA	chr3:31161704-3116
ENSG0000(	437	9.759322	chr3:380<	ENSG00000287641		lncRNA	chr3:34692820-3469
ENSG0000(	437	9.759322	chr3:380<	RPS12P5		Pseudoger	chr3:29390848-2939
ENSG0000(	437	9.759322	chr3:380<	UBP1		protein_c	chr3:33388336-3344
ENSG0000(	437	9.759322	chr3:380<	COX6CP10		Pseudoger	chr3:33554636-3355
ENSG0000(	437	9.759322	chr3:380<	SCN5A	NCGv7	protein_c	chr3:38548057-3864
ENSG0000(	437	9.759322	chr3:380<	ENSG00000287348		lncRNA	chr3:27369568-2738
ENSG0000(	437	9.759322	chr3:380<	ENSG00000271653		lncRNA	chr3:37216779-3721
ENSG0000(	437	9.759322	chr3:380<	RN7SKP227		smallRNA	chr3:36685040-3668
ENSG0000(	437	9.759322	chr3:380<	MIR26A1		smallRNA	chr3:37969404-3796
ENSG0000(	437	9.759322	chr3:380<	RPL15		protein_c	chr3:23916591-2392
ENSG0000(	437	9.759322	chr3:380<	SUGT1P2		Pseudoger	chr3:32752910-3275
ENSG0000(	437	9.759322	chr3:380<	RN7SL296P		smallRNA	chr3:33103008-3310
ENSG0000(	437	9.759322	chr3:380<	GOLGA4-AS1		lncRNA	chr3:37241789-3724
ENSG0000(	437	9.759322	chr3:380<	LRRC3B-AS1		lncRNA	chr3:26614261-2662
ENSG0000(	437	9.759322	chr3:380<	SNORA25		smallRNA	chr3:32037526-3203
ENSG0000(	437	9.759322	chr3:380<	ENSG00000226955		Pseudoger	chr3:32592972-3259
ENSG0000(	437	9.759322	chr3:380<	LINC01981		lncRNA	chr3:27830886-2783
ENSG0000(	437	9.759322	chr3:380<	EPM2AIP1		protein_c	chr3:36985043-3699
ENSG0000(	437	9.759322	chr3:380<	MICOS10P3		Pseudoger	chr3:27214816-2721
ENSG0000(	437	9.759322	chr3:380<	DDTP1		Pseudoger	chr3:38515448-3851
ENSG0000(	437	9.759322	chr3:380<	RNU7-110P		smallRNA	chr3:33414150-3341
ENSG0000(	437	9.759322	chr3:380<	TRIM71		protein_c	chr3:32817997-3289
ENSG0000(	437	9.759322	chr3:380<	ENSG00000271993		lncRNA	chr3:37182107-3718
ENSG0000(	437	9.759322	chr3:380<	ACVR2B	NCGv7	protein_c	chr3:38453890-3849
ENSG0000(	437	9.759322	chr3:380<	snoU13		smallRNA	chr3:37196741-3719
ENSG0000(	437	9.759322	chr3:380<	RPL18AP7		Pseudoger	chr3:38526802-3852
ENSG0000(	437	9.759322	chr3:380<	RPL32P11		Pseudoger	chr3:25749488-2574
ENSG0000(	437	9.759322	chr3:380<	Y_RNA		smallRNA	chr3:38505480-3850
ENSG0000(	437	9.759322	chr3:380<	RNU6-788P		smallRNA	chr3:23641489-2364
ENSG0000(	437	9.759322	chr3:380<	LINC02084		lncRNA	chr3:27712910-2771

ENSG0000(	437	9.759322	chr3:380	GPD1L		protein_c	chr3:32105689-3216
ENSG0000(	437	9.759322	chr3:380	IGBP1P3		Pseudoger	chr3:32620903-3262
ENSG0000(	437	9.759322	chr3:380	TAF9BP1		Pseudoger	chr3:25754925-2575
ENSG0000(	437	9.759322	chr3:380	ENSG00000231449		Pseudoger	chr3:37196204-3719
ENSG0000(	437	9.759322	chr3:380	ENSG00000213849		Pseudoger	chr3:32507955-3250
ENSG0000(	437	9.759322	chr3:380	AC097361.1		smallRNA	chr3:29011242-2901
ENSG0000(	437	9.759322	chr3:380	ENSG00000234073		Pseudoger	chr3:36880184-3688
ENSG0000(	437	9.759322	chr3:380	RBMS3-AS1		lncRNA	chr3:29926811-2993
ENSG0000(	437	9.759322	chr3:380	RNA5SP125		Pseudoger	chr3:25025007-2502
ENSG0000(	437	9.759322	chr3:380	RPL23AP43		Pseudoger	chr3:32785646-3278
ENSG0000(	437	9.759322	chr3:380	THRAP3P1		Pseudoger	chr3:31452832-3145
ENSG0000(	437	9.759322	chr3:380	UBE2E2		protein_c	chr3:23203020-2359
ENSG0000(	437	9.759322	chr3:380	CRIP1P2		Pseudoger	chr3:25686896-2568
ENSG0000(	437	9.759322	chr3:380	RN7SL859P		smallRNA	chr3:27419338-2741
ENSG0000(	437	9.759322	chr3:380	ENSG00000223968		Pseudoger	chr3:27624202-2762
ENSG0000(	437	9.759322	chr3:380	ENSG00000261572		lncRNA	chr3:32236688-3223
ENSG0000(	437	9.759322	chr3:380	ENSG00000232167		Pseudoger	chr3:27525353-2752
ENSG0000(	437	9.759322	chr3:380	ZNF587P1		Pseudoger	chr3:31794470-3179
ENSG0000(	436	9.736989	chr20:21	RN7SKP100		smallRNA	chr20:43063326-430
ENSG0000(	434	9.692324	chr17:73	SNORD124		smallRNA	chr17:40027542-400
ENSG0000(	433	9.669991	chr7:158	CCM2		protein_c	chr7:44999475-4507
ENSG0000(	433	9.669991	chr7:158	UPP1	DriverDB	protein_c	chr7:48088628-4810
ENSG0000(	433	9.669991	chr7:158	TUBBP6		Pseudoger	chr7:55645620-5564
ENSG0000(	433	9.669991	chr7:158	PKD1L1-AS1		lncRNA	chr7:47795291-4781
ENSG0000(	433	9.669991	chr7:158	RNU1-14P		smallRNA	chr7:53366058-5336
ENSG0000(	433	9.669991	chr7:158	NACAD		protein_c	chr7:45080437-4508
ENSG0000(	433	9.669991	chr7:158	ENSG00000226999		Pseudoger	chr7:45534523-4553
ENSG0000(	433	9.669991	chr7:158	ENSG00000237210		Pseudoger	chr7:55244247-5524
ENSG0000(	433	9.669991	chr7:158	POM121L12		protein_c	chr7:53035633-5303
ENSG0000(	433	9.669991	chr7:158	SEC61G-DT		lncRNA	chr7:54759313-5481
ENSG0000(	433	9.669991	chr7:158	RNU6-326P		smallRNA	chr7:45843634-4584
ENSG0000(	433	9.669991	chr7:158	EGFR-AS1		lncRNA	chr7:55179750-5518
ENSG0000(	433	9.669991	chr7:158	HUS1		protein_c	chr7:47963288-4797
ENSG0000(	433	9.669991	chr7:158	MRPL42P4		Pseudoger	chr7:47026128-4702
ENSG0000(	433	9.669991	chr7:158	ENSG00000229403		lncRNA	chr7:51614251-5163
ENSG0000(	433	9.669991	chr7:158	ENSG00000287953		lncRNA	chr7:54185071-5425
ENSG0000(	433	9.669991	chr7:158	SNORA73		smallRNA	chr7:54865818-5486
ENSG0000(	433	9.669991	chr7:158	ENSG00000237551		Pseudoger	chr7:96283357-9628
ENSG0000(	433	9.669991	chr7:158	RNU6-532P		smallRNA	chr7:96330638-9633
ENSG0000(	433	9.669991	chr7:158	COBL	DriverDB	protein_c	chr7:51016212-5131
ENSG0000(	433	9.669991	chr7:158	TBRG4		protein_c	chr7:45100100-4511
ENSG0000(	433	9.669991	chr7:158	CICP20		Pseudoger	chr7:45816216-4581
ENSG0000(	433	9.669991	chr7:158	ENSG00000225018		Pseudoger	chr7:55876738-5587
ENSG0000(	433	9.669991	chr7:158	SEC61G	NCGv7	protein_c	chr7:54752250-5475
ENSG0000(	433	9.669991	chr7:158	LANCL2	DriverDB	protein_c	chr7:55365337-5543
ENSG0000(	433	9.669991	chr7:158	EPS15P1		Pseudoger	chr7:46781373-4678
ENSG0000(	433	9.669991	chr7:158	ENSG00000229192		lncRNA	chr7:47000620-4707
ENSG0000(	433	9.669991	chr7:158	SGO1P2		Pseudoger	chr7:52891837-5289
ENSG0000(	433	9.669991	chr7:158	ENSG00000235620		lncRNA	chr7:50274790-5027
ENSG0000(	433	9.669991	chr7:158	ENSG00000286908		lncRNA	chr7:52493152-5250
ENSG0000(	433	9.669991	chr7:158	ENSG00000230680		lncRNA	chr7:46477822-4648

ENSG0000(	433	9.669991	chr7:158{	ENSG00000228005		lncRNA	chr7:50141540-5014
ENSG0000(	433	9.669991	chr7:158{	SRSF8CP		Pseudoger	chr7:47052793-4705
ENSG0000(	433	9.669991	chr7:158{	AC084368.1		smallRNA	chr7:96306760-9630
ENSG0000(	433	9.669991	chr7:158{	ENSG00000228735		lncRNA	chr7:54576052-5457
ENSG0000(	433	9.669991	chr7:158{	ENSG00000233539		lncRNA	chr7:46673785-4675
ENSG0000(	433	9.669991	chr7:158{	RPL31P35		Pseudoger	chr7:54656358-5465
ENSG0000(	433	9.669991	chr7:158{	PSPHP1		Pseudoger	chr7:55764797-5577
ENSG0000(	433	9.669991	chr7:158{	ENSG00000275834		Pseudoger	chr7:96306810-9630
ENSG0000(	433	9.669991	chr7:158{	ENSG00000286404		lncRNA	chr7:54721724-5473
ENSG0000(	433	9.669991	chr7:158{	SNORA22		smallRNA	chr7:56055365-5605
ENSG0000(	433	9.669991	chr7:158{	SLC25A5P3		Pseudoger	chr7:54419444-5442
ENSG0000(	433	9.669991	chr7:158{	ENSG00000275875		Pseudoger	chr7:55741525-5574
ENSG0000(	433	9.669991	chr7:158{	SNHG15		lncRNA	chr7:44983019-4498
ENSG0000(	433	9.669991	chr7:158{	ENSG00000251378		Pseudoger	chr7:45818582-4581
ENSG0000(	433	9.669991	chr7:158{	ENSG00000289251		lncRNA	chr7:45187005-4519
ENSG0000(	433	9.669991	chr7:158{	ENSG00000223829		lncRNA	chr7:46969644-4702
ENSG0000(	433	9.669991	chr7:158{	VSTM2A		protein_c	chr7:54542325-5457
ENSG0000(	433	9.669991	chr7:158{	RN7SKP218		smallRNA	chr7:53490148-5349
ENSG0000(	433	9.669991	chr7:158{	DDC-AS1		lncRNA	chr7:50531759-5054
ENSG0000(	433	9.669991	chr7:158{	ENSG00000227080		Pseudoger	chr7:51388430-5138
ENSG0000(	433	9.669991	chr7:158{	ENSG00000286995		lncRNA	chr7:49524208-4958
ENSG0000(	433	9.669991	chr7:158{	RNU6-1091P		smallRNA	chr7:50435380-5043
ENSG0000(	433	9.669991	chr7:158{	SEM1	DriverDB\	protein_c	chr7:96481626-9670
ENSG0000(	433	9.669991	chr7:158{	FIGNL1	NCGv7	protein_c	chr7:50444128-5054
ENSG0000(	433	9.669991	chr7:158{	DDC		protein_c	chr7:50458436-5056
ENSG0000(	433	9.669991	chr7:158{	TNS3		protein_c	chr7:47275154-4758
ENSG0000(	433	9.669991	chr7:158{	LINC01447		lncRNA	chr7:47608465-4762
ENSG0000(	433	9.669991	chr7:158{	CICP12		Pseudoger	chr7:55798034-5579
ENSG0000(	433	9.669991	chr7:158{	CICP17		Pseudoger	chr7:51382284-5138
ENSG0000(	433	9.669991	chr7:158{	GDI2P1		Pseudoger	chr7:48902556-4890
ENSG0000(	433	9.669991	chr7:158{	ENSG00000231317		Pseudoger	chr7:55656768-5567
ENSG0000(	433	9.669991	chr7:158{	LINC02854		lncRNA	chr7:53926676-5394
ENSG0000(	433	9.669991	chr7:158{	ENSG00000286987		lncRNA	chr7:51773836-5178
ENSG0000(	433	9.669991	chr7:158{	ENSG00000285741		lncRNA	chr7:51471717-5172
ENSG0000(	433	9.669991	chr7:158{	ENSG00000230936		Pseudoger	chr7:55342316-5534
ENSG0000(	433	9.669991	chr7:158{	ENSG00000229762		Pseudoger	chr7:55810779-5581
ENSG0000(	433	9.669991	chr7:158{	ENSG00000234686		lncRNA	chr7:49230137-4925
ENSG0000(	433	9.669991	chr7:158{	HMGN1P19		Pseudoger	chr7:46634614-4663
ENSG0000(	433	9.669991	chr7:158{	RNU6-364P		smallRNA	chr7:96341140-9634
ENSG0000(	433	9.669991	chr7:158{	ENSG00000279578		TEC	chr7:48847766-4885
ENSG0000(	433	9.669991	chr7:158{	SNORA5B		smallRNA	chr7:45105968-4510
ENSG0000(	433	9.669991	chr7:158{	ABCA13	NCGv7	protein_c	chr7:48171458-4864
ENSG0000(	433	9.669991	chr7:158{	ELK1P1		Pseudoger	chr7:45391626-4539
ENSG0000(	433	9.669991	chr7:158{	ENSG00000286658		lncRNA	chr7:50641725-5064
ENSG0000(	433	9.669991	chr7:158{	CICP11		Pseudoger	chr7:55736779-5573
ENSG0000(	433	9.669991	chr7:158{	MYO1G	NCGv7	protein_c	chr7:44962662-4497
ENSG0000(	433	9.669991	chr7:158{	RN7SL64P		smallRNA	chr7:55892726-5589
ENSG0000(	433	9.669991	chr7:158{	snoU13		smallRNA	chr7:52323798-5232
ENSG0000(	433	9.669991	chr7:158{	SNORA5A		smallRNA	chr7:45104349-4510
ENSG0000(	433	9.669991	chr7:158{	FTLP15		Pseudoger	chr7:45997540-4599
ENSG0000(	433	9.669991	chr7:158{	PKD1L1		protein_c	chr7:47740202-4794

ENSG0000(	433	9.669991	chr7:1588	SLC25A13	DriverDB\	protein_c	chr7:96120220-9632
ENSG0000(	433	9.669991	chr7:1588	IGFBP1		protein_c	chr7:45888360-4589
ENSG0000(	433	9.669991	chr7:1588	ENSG00000228897		Pseudoger	chr7:51386363-5138
ENSG0000(	433	9.669991	chr7:1588	ENSG00000279104		TEC	chr7:49760897-4976
ENSG0000(	433	9.669991	chr7:1588	RNU7-76P		smallRNA	chr7:45975377-4597
ENSG0000(	433	9.669991	chr7:1588	ENSG00000260997		lncRNA	chr7:44958999-4496
ENSG0000(	433	9.669991	chr7:1588	ADCY1	NCGv7	protein_c	chr7:45574140-4572
ENSG0000(	433	9.669991	chr7:1588	SUN3	DriverDB\	protein_c	chr7:47987148-4802
ENSG0000(	433	9.669991	chr7:1588	RNU6-1125P		smallRNA	chr7:54621025-5462
ENSG0000(	433	9.669991	chr7:1588	HAUS6P1		Pseudoger	chr7:53187388-5318
ENSG0000(	433	9.669991	chr7:1588	C7orf57		protein_c	chr7:48035511-4806
ENSG0000(	433	9.669991	chr7:1588	ENSG00000288525		Pseudoger	chr7:54676217-5467
ENSG0000(	433	9.669991	chr7:1588	ENSG00000273720		Pseudoger	chr7:55743073-5574
ENSG0000(	433	9.669991	chr7:1588	ELDR		lncRNA	chr7:55235965-5525
ENSG0000(	433	9.669991	chr7:1588	ENSG00000231681		lncRNA	chr7:50202001-5026
ENSG0000(	433	9.669991	chr7:1588	ENSG00000272768		lncRNA	chr7:44884953-4488
ENSG0000(	433	9.669991	chr7:1588	ENSG00000226838		Pseudoger	chr7:45816557-4582
ENSG0000(	433	9.669991	chr7:1588	SNORA4		smallRNA	chr7:50935350-5093
ENSG0000(	433	9.669991	chr7:1588	ENSG00000228173		lncRNA	chr7:48660125-4866
ENSG0000(	433	9.669991	chr7:1588	GRB10		protein_c	chr7:50590063-5079
ENSG0000(	433	9.669991	chr7:1588	VWC2		protein_c	chr7:49773638-4992
ENSG0000(	433	9.669991	chr7:1588	FKBP9P1		lncRNA	chr7:55681074-5568
ENSG0000(	433	9.669991	chr7:1588	RNU2-29P		smallRNA	chr7:53776136-5377
ENSG0000(	433	9.669991	chr7:1588	GNL2P1		Pseudoger	chr7:49942251-4994
ENSG0000(	433	9.669991	chr7:1588	ENSG00000286738		lncRNA	chr7:45268681-4538
ENSG0000(	433	9.669991	chr7:1588	TTC4P1		Pseudoger	chr7:45999621-4600
ENSG0000(	433	9.669991	chr7:1588	ENSG00000291207		lncRNA	chr7:45723780-4576
ENSG0000(	433	9.669991	chr7:1588	ENSG00000291208		lncRNA	chr7:45769060-4581
ENSG0000(	433	9.669991	chr7:1588	ENSG00000233960		lncRNA	chr7:52165235-5219
ENSG0000(	433	9.669991	chr7:1588	ENSG00000240355		lncRNA	chr7:46476457-4647
ENSG0000(	433	9.669991	chr7:1588	ENSG00000285165		lncRNA	chr7:50388489-5040
ENSG0000(	433	9.669991	chr7:1588	PSPH	DriverDB\	protein_c	chr7:56011051-5605
ENSG0000(	433	9.669991	chr7:1588	SNORA31		smallRNA	chr7:52269437-5226
ENSG0000(	433	9.669991	chr7:1588	NIPSNAP2	DriverDB\	protein_c	chr7:55951793-5600
ENSG0000(	433	9.669991	chr7:1588	CDC14C		protein_c	chr7:48924547-4892
ENSG0000(	433	9.669991	chr7:1588	ENSG00000225705		Pseudoger	chr7:48846426-4885
ENSG0000(	433	9.669991	chr7:1588	RNU6-389P		smallRNA	chr7:55685977-5568
ENSG0000(	433	9.669991	chr7:1588	ENSG00000249773		protein_c	chr7:55887277-5595
ENSG0000(	433	9.669991	chr7:1588	SPATA48		protein_c	chr7:50095883-5015
ENSG0000(	433	9.669991	chr7:1588	ROBO2P1		Pseudoger	chr7:51600286-5160
ENSG0000(	433	9.669991	chr7:1588	ENSG00000290107		lncRNA	chr7:45690599-4569
ENSG0000(	433	9.669991	chr7:1588	FKBP9P1		Pseudoger	chr7:55682652-5571
ENSG0000(	433	9.669991	chr7:1588	AC004458.1		smallRNA	chr7:96468331-9646
ENSG0000(	433	9.669991	chr7:1588	ENSG00000230160		Pseudoger	chr7:44919669-4492
ENSG0000(	433	9.669991	chr7:1588	SUMO2P3		Pseudoger	chr7:55732144-5573
ENSG0000(	433	9.669991	chr7:1588	ENSG00000228204		lncRNA	chr7:50866747-5102
ENSG0000(	433	9.669991	chr7:1588	VSTM2A-OT1		lncRNA	chr7:54556970-5457
ENSG0000(	433	9.669991	chr7:1588	ENSG00000232072		lncRNA	chr7:46890625-4704
ENSG0000(	433	9.669991	chr7:1588	ENSG00000227499		Pseudoger	chr7:55877912-5587
ENSG0000(	433	9.669991	chr7:1588	SNORA5C		smallRNA	chr7:45104906-4510
ENSG0000(	433	9.669991	chr7:1588	ENSG00000218586		Pseudoger	chr7:54933699-5493

ENSG0000(	433	9.669991	chr7:1588	LINC01446		lncRNA	chr7:53655508-5381
ENSG0000(	433	9.669991	chr7:1588	RAMP3		protein_c	chr7:45157791-4518
ENSG0000(	433	9.669991	chr7:1588	ENSG00000234105		lncRNA	chr7:53514992-5351
ENSG0000(	433	9.669991	chr7:1588	ENSG00000235738		Pseudoger	chr7:55797946-5579
ENSG0000(	433	9.669991	chr7:1588	ENSG00000225507		Pseudoger	chr7:47956793-4795
ENSG0000(	433	9.669991	chr7:1588	RAC1P9		Pseudoger	chr7:53779783-5378
ENSG0000(	433	9.669991	chr7:1588	ENSG00000228627		lncRNA	chr7:53559214-5357
ENSG0000(	433	9.669991	chr7:1588	HAUS6P3		Pseudoger	chr7:53862233-5386
ENSG0000(	433	9.669991	chr7:1588	ENSG00000275295		Pseudoger	chr7:45303620-4530
ENSG0000(	433	9.669991	chr7:1588	LINC02838		lncRNA	chr7:48708327-4871
ENSG0000(	433	9.669991	chr7:1588	MIR4657		smallRNA	chr7:44881748-4488
ENSG0000(	433	9.669991	chr7:1588	ENSG00000290114		lncRNA	chr7:50450421-5045
ENSG0000(	433	9.669991	chr7:1588	RPL7L1P2		Pseudoger	chr7:51259429-5126
ENSG0000(	433	9.669991	chr7:1588	RNU6-241P		smallRNA	chr7:45789585-4578
ENSG0000(	433	9.669991	chr7:1588	ENSG00000225537		lncRNA	chr7:45460712-4554
ENSG0000(	433	9.669991	chr7:1588	CALM1P2		Pseudoger	chr7:55259809-5526
ENSG0000(	433	9.669991	chr7:1588	RNU6-1126P		smallRNA	chr7:55789368-5578
ENSG0000(	433	9.669991	chr7:1588	CCDC201		protein_c	chr7:45859994-4587
ENSG0000(	433	9.669991	chr7:1588	ENSG00000232418		Pseudoger	chr7:53787167-5378
ENSG0000(	433	9.669991	chr7:1588	ENSG00000236046		lncRNA	chr7:50839363-5084
ENSG0000(	433	9.669991	chr7:1588	LINC01445		lncRNA	chr7:54330670-5445
ENSG0000(	433	9.669991	chr7:1588	LINC00525		lncRNA	chr7:47761476-4776
ENSG0000(	433	9.669991	chr7:1588	LINC02902		lncRNA	chr7:47655244-4766
ENSG0000(	433	9.669991	chr7:1588	ENSG00000229628		lncRNA	chr7:45990905-4600
ENSG0000(	433	9.669991	chr7:1588	RNU7-188P		smallRNA	chr7:96377857-9637
ENSG0000(	433	9.669991	chr7:1588	ENSG00000287039		lncRNA	chr7:51849086-5186
ENSG0000(	433	9.669991	chr7:1588	RRBP1P1		Pseudoger	chr7:53002420-5300
ENSG0000(	433	9.669991	chr7:1588	ZPBP	DriverDB\	protein_c	chr7:49850421-5012
ENSG0000(	433	9.669991	chr7:1588	EGFR	NCGv7;AC	protein_c	chr7:55019017-5521
ENSG0000(	433	9.669991	chr7:1588	MRPS17	DriverDB\	protein_c	chr7:55951877-5595
ENSG0000(	433	9.669991	chr7:1588	ENSG00000229459		lncRNA	chr7:46261064-4629
ENSG0000(	433	9.669991	chr7:1588	SEPTIN7P2		Pseudoger	chr7:45736787-4576
ENSG0000(	433	9.669991	chr7:1588	DDX43P2		Pseudoger	chr7:49258493-4925
ENSG0000(	433	9.669991	chr7:1588	ENSG00000286315		lncRNA	chr7:47252139-4725
ENSG0000(	433	9.669991	chr7:1588	RNF138P2		Pseudoger	chr7:53316149-5331
ENSG0000(	433	9.669991	chr7:1588	ENSG00000287521		lncRNA	chr7:50093279-5009
ENSG0000(	433	9.669991	chr7:1588	ZNF619P1		Pseudoger	chr7:46144937-4614
ENSG0000(	433	9.669991	chr7:1588	VOPP1-DT		lncRNA	chr7:55573171-5558
ENSG0000(	433	9.669991	chr7:1588	RN7SL292P		smallRNA	chr7:51716658-5171
ENSG0000(	433	9.669991	chr7:1588	ENSG00000233977		lncRNA	chr7:55592074-5559
ENSG0000(	433	9.669991	chr7:1588	ENSG00000237471		lncRNA	chr7:45940449-4598
ENSG0000(	433	9.669991	chr7:1588	ZNF713	DriverDB\	protein_c	chr7:55887456-5594
ENSG0000(	433	9.669991	chr7:1588	ENSG00000288798		lncRNA	chr7:51327615-5135
ENSG0000(	433	9.669991	chr7:1588	ENSG00000228085		Pseudoger	chr7:50843578-5084
ENSG0000(	433	9.669991	chr7:1588	VOPP1	DriverDB\	protein_c	chr7:55436056-5557
ENSG0000(	433	9.669991	chr7:1588	ENSG00000280920		lncRNA	chr7:54201224-5420
ENSG0000(	433	9.669991	chr7:1588	CDC42P2		Pseudoger	chr7:55638274-5563
ENSG0000(	433	9.669991	chr7:1588	SEPTIN14		protein_c	chr7:55793540-5586
ENSG0000(	433	9.669991	chr7:1588	PURB		protein_c	chr7:44876299-4488
ENSG0000(	433	9.669991	chr7:1588	ENSG00000231394		lncRNA	chr7:55593777-5559
ENSG0000(	433	9.669991	chr7:1588	IGFBP3		protein_c	chr7:45912245-4592

ENSG0000(	433	9.669991	chr7:158{	ENSG00000237760	lncRNA	chr7:46302120-4634
ENSG0000(	433	9.669991	chr7:158{	snoU13	smallRNA	chr7:54359892-5435
ENSG0000(	430	9.602994	chr17:73(	ENSG00000267547	lncRNA	chr17:35403837-354
ENSG0000(	429	9.580661	chr7:158{	AC069285.1	smallRNA	chr7:63068087-6306
ENSG0000(	429	9.580661	chr7:158{	snoU2_19	smallRNA	chr7:63175940-6317
ENSG0000(	429	9.580661	chr7:158{	SAPCD2P4	Pseudoger	chr7:63113635-6311
ENSG0000(	429	9.580661	chr7:158{	PHKG1P1	Pseudoger	chr7:63233035-6323
ENSG0000(	429	9.580661	chr7:158{	ENSG00000223889	Pseudoger	chr7:63393749-6339
ENSG0000(	429	9.580661	chr7:158{	ZNF90P3	Pseudoger	chr7:63087070-6308
ENSG0000(	429	9.580661	chr7:158{	ARAFP2	Pseudoger	chr7:63404842-6340
ENSG0000(	429	9.580661	chr7:158{	VN1R31P	Pseudoger	chr7:63367427-6336
ENSG0000(	429	9.580661	chr7:158{	MIR4283-2	smallRNA	chr7:63621090-6362
ENSG0000(	429	9.580661	chr7:158{	ENSG00000225451	Pseudoger	chr7:63876341-6387
ENSG0000(	429	9.580661	chr7:158{	ENSG00000234387	lncRNA	chr7:63394277-6339
ENSG0000(	429	9.580661	chr7:158{	TNRC18P2	Pseudoger	chr7:63567743-6358
ENSG0000(	429	9.580661	chr7:158{	ENSG00000229301	lncRNA	chr7:63354457-6335
ENSG0000(	429	9.580661	chr7:158{	SEPTIN7P5	Pseudoger	chr7:63495799-6350
ENSG0000(	429	9.580661	chr7:158{	ENSG00000232165	Pseudoger	chr7:63345840-6334
ENSG0000(	429	9.580661	chr7:158{	SEPTIN7P4	Pseudoger	chr7:63241590-6324
ENSG0000(	429	9.580661	chr7:158{	SLC25A1P2	Pseudoger	chr7:63398046-6339
ENSG0000(	429	9.580661	chr7:158{	ENSG00000236638	Pseudoger	chr7:63263065-6326
ENSG0000(	429	9.580661	chr7:158{	ENSG00000290558	lncRNA	chr7:63291552-6330
ENSG0000(	429	9.580661	chr7:158{	ZNF733P	Pseudoger	chr7:63291518-6330
ENSG0000(	429	9.580661	chr7:158{	ENSG00000226411	Pseudoger	chr7:63699390-6370
ENSG0000(	429	9.580661	chr7:158{	VN1R32P	Pseudoger	chr7:63377329-6337
ENSG0000(	429	9.580661	chr7:158{	PHKG1P2	Pseudoger	chr7:63509303-6351
ENSG0000(	429	9.580661	chr7:158{	ENSG00000227148	lncRNA	chr7:63044827-6305
ENSG0000(	429	9.580661	chr7:158{	ENSG00000227923	Pseudoger	chr7:63354033-6335
ENSG0000(	429	9.580661	chr7:158{	ENSG00000236574	Pseudoger	chr7:63843314-6384
ENSG0000(	429	9.580661	chr7:158{	RN7SL855P	smallRNA	chr7:63611445-6361
ENSG0000(	429	9.580661	chr7:158{	ZNF734P	Pseudoger	chr7:63449819-6345
ENSG0000(	429	9.580661	chr7:158{	CICP24	Pseudoger	chr7:63768257-6377
ENSG0000(	429	9.580661	chr7:158{	ENSG00000233454	Pseudoger	chr7:63057453-6306
ENSG0000(	429	9.580661	chr7:158{	ARAFP3	Pseudoger	chr7:63342711-6334
ENSG0000(	429	9.580661	chr7:158{	ENSG00000227305	lncRNA	chr7:63393745-6342
ENSG0000(	429	9.580661	chr7:158{	ENSG00000268181	lncRNA	chr7:63396341-6339
ENSG0000(	429	9.580661	chr7:158{	ENSG00000232817	lncRNA	chr7:63388808-6339
ENSG0000(	429	9.580661	chr7:158{	ENSG00000226587	lncRNA	chr7:63326674-6335
ENSG0000(	429	9.580661	chr7:158{	ENSG00000244550	lncRNA	chr7:63233115-6324
ENSG0000(	429	9.580661	chr7:158{	ENSG00000230132	Pseudoger	chr7:63480598-6348
ENSG0000(	429	9.580661	chr7:158{	ENSG00000227397	Pseudoger	chr7:63789909-6379
ENSG0000(	429	9.580661	chr7:158{	ENSG00000233918	Pseudoger	chr7:62275360-6227
ENSG0000(	429	9.580661	chr7:158{	ENSG00000227545	Pseudoger	chr7:63349154-6335
ENSG0000(	429	9.580661	chr7:158{	VN1R33P	Pseudoger	chr7:63401385-6340
ENSG0000(	429	9.580661	chr7:158{	SLC29A4P2	Pseudoger	chr7:63556598-6356
ENSG0000(	429	9.580661	chr7:158{	ENSG00000230600	lncRNA	chr7:63380466-6338
ENSG0000(	429	9.580661	chr7:158{	ENSG00000226401	Pseudoger	chr7:63632608-6363
ENSG0000(	429	9.580661	chr7:158{	ENSG00000237572	Pseudoger	chr7:63209219-6321
ENSG0000(	429	9.580661	chr7:158{	ENSG00000230000	lncRNA	chr7:63348861-6335
ENSG0000(	429	9.580661	chr7:158{	SEPTIN14P1	Pseudoger	chr7:63117501-6315
ENSG0000(	428	9.558329	chr13:27(	MIR4306	smallRNA	chr13:99643059-996

ENSG0000(	428	9.558329	chr13:279	GAPDHP22		Pseudoger	chr13:99190579-991
ENSG0000(	428	9.558329	chr13:279	GPR18		protein_c	chr13:99254732-992
ENSG0000(	428	9.558329	chr13:279	LINC01039		lncRNA	chr13:99577112-995
ENSG0000(	428	9.558329	chr13:279	DOCK9		protein_c	chr13:98793429-990
ENSG0000(	428	9.558329	chr13:279	ENSG00000280710		lncRNA	chr13:99498737-995
ENSG0000(	428	9.558329	chr13:279	HMGB3P4		Pseudoger	chr13:99372173-993
ENSG0000(	428	9.558329	chr13:279	RN7SKP9		smallRNA	chr13:99205708-992
ENSG0000(	428	9.558329	chr13:279	RNY3P6		smallRNA	chr13:99536319-995
ENSG0000(	428	9.558329	chr13:279	GPR183	NCGv7	protein_c	chr13:99294539-993
ENSG0000(	428	9.558329	chr13:279	RPL7L1P12		Pseudoger	chr13:98949719-989
ENSG0000(	428	9.558329	chr13:279	UBAC2	NCGv7	protein_c	chr13:99200774-993
ENSG0000(	428	9.558329	chr13:279	DOCK9-DT		lncRNA	chr13:99087819-990
ENSG0000(	428	9.558329	chr13:279	ENSG00000288581		lncRNA	chr13:99835813-998
ENSG0000(	428	9.558329	chr13:279	SNORA25		smallRNA	chr13:99783857-997
ENSG0000(	428	9.558329	chr13:279	ENSG00000290577		lncRNA	chr13:99404684-994
ENSG0000(	428	9.558329	chr13:279	TM9SF2		protein_c	chr13:99446311-995
ENSG0000(	428	9.558329	chr13:279	ENSG00000285448		lncRNA	chr13:99429211-994
ENSG0000(	428	9.558329	chr13:279	AL583784.1		smallRNA	chr13:99453396-994
ENSG0000(	428	9.558329	chr13:279	CFL1P8		Pseudoger	chr13:99582706-995
ENSG0000(	428	9.558329	chr13:279	RNU6-83P		smallRNA	chr13:99025234-990
ENSG0000(	428	9.558329	chr13:279	CLYBL-AS1		lncRNA	chr13:99726245-997
ENSG0000(	428	9.558329	chr13:279	LINC01232		lncRNA	chr13:99486962-994
ENSG0000(	428	9.558329	chr13:279	CCR12P		Pseudoger	chr13:99407837-994
ENSG0000(	428	9.558329	chr13:279	snoU13		smallRNA	chr13:99784054-997
ENSG0000(	428	9.558329	chr13:279	ENSG00000287746		lncRNA	chr13:99584014-995
ENSG0000(	428	9.558329	chr13:279	H2AZP3		Pseudoger	chr13:99215372-992
ENSG0000(	428	9.558329	chr13:279	RPS6P23		Pseudoger	chr13:99173593-991
ENSG0000(	428	9.558329	chr13:279	CLYBL-AS2		lncRNA	chr13:99690081-996
ENSG0000(	428	9.558329	chr13:279	LINC00449		lncRNA	chr13:99499727-995
ENSG0000(	428	9.558329	chr13:279	UBAC2-AS1		lncRNA	chr13:99181223-992
ENSG0000(	426	9.513664	chr5:1646	GUSBP16		Pseudoger	chr5:70751184-7079
ENSG0000(	426	9.513664	chr21:360	RIMKLBP1		Pseudoger	chr21:36050214-360
ENSG0000(	426	9.513664	chr5:1646	AC145141.2		smallRNA	chr5:71387276-7138
ENSG0000(	426	9.513664	chr5:1646	NAIPP4		Pseudoger	chr5:71102898-7112
ENSG0000(	426	9.513664	chr5:1646	CDH12P1		Pseudoger	chr5:70860285-7086
ENSG0000(	426	9.513664	chr5:1646	GTF2H2		protein_c	chr5:71035016-7106
ENSG0000(	426	9.513664	chr5:1646	RP11-1198D22.3		Pseudoger	chr5:71215306-7121
ENSG0000(	426	9.513664	chr5:1646	BDP1		protein_c	chr5:71455651-7156
ENSG0000(	426	9.513664	chr5:1646	ENSG00000285804		lncRNA	chr5:72087782-7210
ENSG0000(	426	9.513664	chr5:1646	SERF1A		protein_c	chr5:70900669-7091
ENSG0000(	426	9.513664	chr5:1646	MCCC2		protein_c	chr5:71579531-7165
ENSG0000(	426	9.513664	chr5:1646	AC143336.1		smallRNA	chr5:71614984-7161
ENSG0000(	426	9.513664	chr5:1646	SMN1		protein_c	chr5:70925030-7095
ENSG0000(	426	9.513664	chr5:1646	ENSG00000285204		lncRNA	chr5:70931244-7093
ENSG0000(	426	9.513664	chr5:1646	ENSG00000253536		Pseudoger	chr5:71475761-7147
ENSG0000(	426	9.513664	chr5:1646	ENSG00000289810		lncRNA	chr5:71032670-7103
ENSG0000(	426	9.513664	chr5:1646	ENSG00000253985		lncRNA	chr5:71372676-7137
ENSG0000(	426	9.513664	chr5:1646	MAP1B	DriverDB\	protein_c	chr5:72107234-7220
ENSG0000(	426	9.513664	chr5:1646	GUSBP9		Pseudoger	chr5:71197646-7120
ENSG0000(	426	9.513664	chr5:1646	PMCHL2		Pseudoger	chr5:71375830-7137
ENSG0000(	426	9.513664	chr5:1646	LINC02197		lncRNA	chr5:71337182-7144

ENSG0000(	426	9.513664	chr5:164€	ENSG00000288349		Pseudoger	chr5:70775506-7077
ENSG0000(	426	9.513664	chr5:164€	OCLNP1		Pseudoger	chr5:71074225-7109
ENSG0000(	426	9.513664	chr5:164€	CDH12P4		Pseudoger	chr5:71132993-7113
ENSG0000(	426	9.513664	chr5:164€	MIR4803		smallRNA	chr5:72169467-7216
ENSG0000(	426	9.513664	chr5:164€	GUSBP17		Pseudoger	chr5:71220356-7125
ENSG0000(	426	9.513664	chr5:164€	ENSG00000249981		lncRNA	chr5:71445616-7144
ENSG0000(	426	9.513664	chr5:164€	PMCHL2		lncRNA	chr5:71375786-7138
ENSG0000(	426	9.513664	chr5:164€	NAIP		protein_c	chr5:70968166-7102
ENSG0000(	426	9.513664	chr5:164€	ENSG00000278824		Pseudoger	chr5:71754378-7175
ENSG0000(	426	9.513664	chr5:164€	HMGN1P12		Pseudoger	chr5:71537652-7153
ENSG0000(	426	9.513664	chr5:164€	snoU13		smallRNA	chr5:71088632-7108
ENSG0000(	426	9.513664	chr5:164€	RP11-1198D22.2		Pseudoger	chr5:71233327-7123
ENSG0000(	426	9.513664	chr5:164€	CARTPT		protein_c	chr5:71719275-7172
ENSG0000(	425	9.491331	chr7:158€	COPS6		protein_c	chr7:100088969-100
ENSG0000(	425	9.491331	chr7:158€	CYP3A7-CYP3A51P		protein_c	chr7:99684957-9973
ENSG0000(	425	9.491331	chr7:158€	BRI3	DriverDB\	protein_c	chr7:98252379-9831
ENSG0000(	425	9.491331	chr7:158€	RN7SKP104		smallRNA	chr7:97598933-9759
ENSG0000(	425	9.491331	chr7:158€	ENSG00000286742		lncRNA	chr7:92647906-9266
ENSG0000(	425	9.491331	chr12:14€	GOLGA2P5		Pseudoger	chr12:100156621-10
ENSG0000(	425	9.491331	chr7:158€	ENSG00000287932		lncRNA	chr7:92638198-9264
ENSG0000(	425	9.491331	chr7:158€	TMEM225B		protein_c	chr7:99598267-9961
ENSG0000(	425	9.491331	chr12:14€	ENSG00000258039		lncRNA	chr12:99093359-991
ENSG0000(	425	9.491331	chr7:158€	PEX1		protein_c	chr7:92487020-9252
ENSG0000(	425	9.491331	chr12:14€	ENSG00000241744		Pseudoger	chr12:101228141-10
ENSG0000(	425	9.491331	chr7:158€	LMTK2	DriverDB\	protein_c	chr7:98106862-9820
ENSG0000(	425	9.491331	chr7:158€	DLX6		protein_c	chr7:97005553-9701
ENSG0000(	425	9.491331	chr7:158€	AC069294.1		smallRNA	chr7:99713037-9971
ENSG0000(	425	9.491331	chr7:158€	RNU6-10P		smallRNA	chr7:92701708-9270
ENSG0000(	425	9.491331	chr7:158€	BHLHA15		protein_c	chr7:98211439-9821
ENSG0000(	425	9.491331	chr12:14€	UHRF1BP1L-DT		lncRNA	chr12:100143058-10
ENSG0000(	425	9.491331	chr7:158€	BAIAP2L1	DriverDB\	protein_c	chr7:98291650-9840
ENSG0000(	425	9.491331	chr7:158€	HMGB3P21		Pseudoger	chr7:97135015-9713
ENSG0000(	425	9.491331	chr12:14€	ENSG00000279148		TEC	chr12:100026248-10
ENSG0000(	425	9.491331	chr7:158€	MCM7	NCGv7	protein_c	chr7:100092728-100
ENSG0000(	425	9.491331	chr7:158€	snoU13		smallRNA	chr7:98884990-9888
ENSG0000(	425	9.491331	chr7:158€	ENSG00000235713		Pseudoger	chr7:99992397-9999
ENSG0000(	425	9.491331	chr7:158€	RPS3AP29		Pseudoger	chr7:97898347-9789
ENSG0000(	425	9.491331	chr7:158€	ENSG00000232097		Pseudoger	chr7:97938439-9793
ENSG0000(	425	9.491331	chr7:158€	ENSG00000228335		Pseudoger	chr7:99442890-9944
ENSG0000(	425	9.491331	chr7:158€	Y_RNA		smallRNA	chr7:99936610-9993
ENSG0000(	425	9.491331	chr7:158€	CDK6-AS1		lncRNA	chr7:92836367-9291
ENSG0000(	425	9.491331	chr7:158€	ZSCAN21		protein_c	chr7:100049774-100
ENSG0000(	425	9.491331	chr12:14€	NR1H4	NCGv7	protein_c	chr12:100473708-10
ENSG0000(	425	9.491331	chr7:158€	ENSG00000232032		Pseudoger	chr7:97908256-9790
ENSG0000(	425	9.491331	chr7:158€	CCZ1P1		Pseudoger	chr7:97969005-9797
ENSG0000(	425	9.491331	chr7:158€	ZNF655		protein_c	chr7:99558406-9957
ENSG0000(	425	9.491331	chr7:158€	ARPC1B	NCGv7	protein_c	chr7:99374249-9939
ENSG0000(	425	9.491331	chr7:158€	ENSG00000237640		lncRNA	chr7:99929392-9994
ENSG0000(	425	9.491331	chr7:158€	MYH16		Pseudoger	chr7:99238829-9931
ENSG0000(	425	9.491331	chr7:158€	ENSG00000272950		lncRNA	chr7:98322853-9832
ENSG0000(	425	9.491331	chr7:158€	ENSG00000284292		protein_c	chr7:99325879-9939

ENSG0000(	425	9.491331	chr7:158{	ZNF3	DriverDB\	protein_c	chr7:100064033-100
ENSG0000(	425	9.491331	chr7:158{	MBLAC1		protein_c	chr7:100126785-100
ENSG0000(	425	9.491331	chr7:158{	CYP3A51P		Pseudoger	chr7:99685145-9970
ENSG0000(	425	9.491331	chr12:149	ACTR6		protein_c	chr12:100199122-10
ENSG0000(	425	9.491331	chr7:158{	CDK6	NCGv7;AC	protein_c	chr7:92604921-9283
ENSG0000(	425	9.491331	chr12:149	RN7SL176P		smallRNA	chr12:100158497-10
ENSG0000(	425	9.491331	chr12:149	ENSG00000290576		lncRNA	chr12:100173196-10
ENSG0000(	425	9.491331	chr7:158{	MIR3609		smallRNA	chr7:98881650-9888
ENSG0000(	425	9.491331	chr12:149	ENSG00000257543		lncRNA	chr12:101408372-10
ENSG0000(	425	9.491331	chr12:149	ENSG00000258007		lncRNA	chr12:101038420-10
ENSG0000(	425	9.491331	chr7:158{	ENSG00000284523		lncRNA	chr7:99252452-9932
ENSG0000(	425	9.491331	chr7:158{	ENSG00000226744		Pseudoger	chr7:97870167-9787
ENSG0000(	425	9.491331	chr12:149	ENSG00000257489		Pseudoger	chr12:100180553-10
ENSG0000(	425	9.491331	chr12:149	ENSG00000287675		lncRNA	chr12:99985045-999
ENSG0000(	425	9.491331	chr7:158{	AZGP1P1		Pseudoger	chr7:99980762-9998
ENSG0000(	425	9.491331	chr7:158{	TRIM4		protein_c	chr7:99876958-9991
ENSG0000(	425	9.491331	chr7:158{	RPL7AP40		Pseudoger	chr7:97200708-9720
ENSG0000(	425	9.491331	chr12:149	UHRF1BP1L		protein_c	chr12:100028455-10
ENSG0000(	425	9.491331	chr7:158{	ENSG00000223402		Pseudoger	chr7:98478551-9847
ENSG0000(	425	9.491331	chr7:158{	MIR25		smallRNA	chr7:100093560-100
ENSG0000(	425	9.491331	chr12:149	ANO4	NCGv7	protein_c	chr12:100717526-10
ENSG0000(	425	9.491331	chr12:149	ANKS1B		protein_c	chr12:98726457-999
ENSG0000(	425	9.491331	chr7:158{	ARPC1A	DriverDB\	protein_c	chr7:99325898-9936
ENSG0000(	425	9.491331	chr12:149	DEPDC4	NCGv7	protein_c	chr12:100203669-10
ENSG0000(	425	9.491331	chr7:158{	ENSG00000244055		lncRNA	chr7:92457564-9249
ENSG0000(	425	9.491331	chr7:158{	TRAPPC14		protein_c	chr7:100154420-100
ENSG0000(	425	9.491331	chr7:158{	GAL3ST4		protein_c	chr7:100159244-100
ENSG0000(	425	9.491331	chr7:158{	ENSG00000288889		lncRNA	chr7:98616014-9861
ENSG0000(	425	9.491331	chr12:149	snoU13		smallRNA	chr12:100402145-10
ENSG0000(	425	9.491331	chr12:149	Y_RNA		smallRNA	chr12:101446825-10
ENSG0000(	425	9.491331	chr7:158{	FAM200A		protein_c	chr7:99546300-9955
ENSG0000(	425	9.491331	chr12:149	GOLGA2P5		lncRNA	chr12:100156357-10
ENSG0000(	425	9.491331	chr12:149	ENSG00000257325		lncRNA	chr12:100852331-10
ENSG0000(	425	9.491331	chr7:158{	TAC1	AC	protein_c	chr7:97732084-9774
ENSG0000(	425	9.491331	chr7:158{	OR2AE1		protein_c	chr7:99876062-9987
ENSG0000(	425	9.491331	chr7:158{	TMEM130		protein_c	chr7:98846488-9887
ENSG0000(	425	9.491331	chr7:158{	ENSG00000260445		Pseudoger	chr7:99869841-9986
ENSG0000(	425	9.491331	chr7:158{	CYP3A43		protein_c	chr7:99828013-9986
ENSG0000(	425	9.491331	chr7:158{	RN7SL13P		smallRNA	chr7:98023729-9802
ENSG0000(	425	9.491331	chr7:158{	AC079781.1		smallRNA	chr7:97905972-9790
ENSG0000(	425	9.491331	chr7:158{	RN7SL7P		smallRNA	chr7:92971004-9297
ENSG0000(	425	9.491331	chr7:158{	OCM2		protein_c	chr7:97984687-9799
ENSG0000(	425	9.491331	chr7:158{	MIR5692A1		smallRNA	chr7:97963658-9796
ENSG0000(	425	9.491331	chr7:158{	DLX5	AC	protein_c	chr7:97020396-9702
ENSG0000(	425	9.491331	chr12:149	Y_RNA		smallRNA	chr12:100257688-10
ENSG0000(	425	9.491331	chr12:149	RNA5SP366		Pseudoger	chr12:99083783-990
ENSG0000(	425	9.491331	chr7:158{	RBM48		protein_c	chr7:92528773-9254
ENSG0000(	425	9.491331	chr7:158{	SNRPCP9		Pseudoger	chr7:97885868-9788
ENSG0000(	425	9.491331	chr7:158{	OR7E38P		Pseudoger	chr7:97966090-9796
ENSG0000(	425	9.491331	chr7:158{	ENSG00000286923		lncRNA	chr7:99997690-1000
ENSG0000(	425	9.491331	chr7:158{	ASNS	DriverDB\	protein_c	chr7:97851677-9787

ENSG0000(	425	9.491331	chr7:158{	ZKSCAN1	DriverDB\	protein_c	chr7:100015572-10(
ENSG0000(	425	9.491331	chr7:158{	CYP3A5	DriverDB\	protein_c	chr7:99648194-9967
ENSG0000(	425	9.491331	chr7:158{	ENSG00000286921		lncRNA	chr7:99974976-9997
ENSG0000(	425	9.491331	chr7:158{	ENSG00000284840		Pseudoger	chr7:99032894-9903
ENSG0000(	425	9.491331	chr12:149	PIGAP1		Pseudoger	chr12:100578744-10
ENSG0000(	425	9.491331	chr7:158{	PTCD1		protein_c	chr7:99416739-9946
ENSG0000(	425	9.491331	chr7:158{	OR7E7P		Pseudoger	chr7:97946987-9794
ENSG0000(	425	9.491331	chr7:158{	AC004745.1		smallRNA	chr7:97291514-9729
ENSG0000(	425	9.491331	chr12:149	ENSG00000257458		lncRNA	chr12:98931682-989
ENSG0000(	425	9.491331	chr12:149	SLC5A8		protein_c	chr12:101155493-10
ENSG0000(	425	9.491331	chr12:149	ENSG00000280088		TEC	chr12:100032325-10
ENSG0000(	425	9.491331	chr7:158{	FAM133B		protein_c	chr7:92560758-9259
ENSG0000(	425	9.491331	chr7:158{	RNU6-393P		smallRNA	chr7:98794718-9879
ENSG0000(	425	9.491331	chr7:158{	CPSF4		protein_c	chr7:99438922-9945
ENSG0000(	425	9.491331	chr7:158{	SNORA40		smallRNA	chr7:99952033-9995
ENSG0000(	425	9.491331	chr7:158{	ZNF394	DriverDB\	protein_c	chr7:99473877-9950
ENSG0000(	425	9.491331	chr7:158{	SDHAF3		protein_c	chr7:97117698-9718
ENSG0000(	425	9.491331	chr7:158{	ENSG00000278819		Pseudoger	chr7:92540268-9255
ENSG0000(	425	9.491331	chr7:158{	ATP5MF-PTCD1		protein_c	chr7:99419749-9946
ENSG0000(	425	9.491331	chr7:158{	DLX6-AS1		lncRNA	chr7:96955141-9701
ENSG0000(	425	9.491331	chr7:158{	AP1S2P1		Pseudoger	chr7:97437518-9743
ENSG0000(	425	9.491331	chr7:158{	RN7SL478P		smallRNA	chr7:97998325-9799
ENSG0000(	425	9.491331	chr12:149	snoU13		smallRNA	chr12:101358748-10
ENSG0000(	425	9.491331	chr7:158{	GJC3		protein_c	chr7:99923266-9992
ENSG0000(	425	9.491331	chr7:158{	AZGP1	NCGv7	protein_c	chr7:99966720-9997
ENSG0000(	425	9.491331	chr12:149	ARL1		protein_c	chr12:101393116-10
ENSG0000(	425	9.491331	chr7:158{	RN7SL252P		smallRNA	chr7:96940070-9694
ENSG0000(	425	9.491331	chr7:158{	CYP3A52P		Pseudoger	chr7:99872168-9987
ENSG0000(	425	9.491331	chr7:158{	ENSG00000270453		Pseudoger	chr7:92442077-9244
ENSG0000(	425	9.491331	chr12:149	AC010209.1		smallRNA	chr12:100655722-10
ENSG0000(	425	9.491331	chr7:158{	AZGP1P1		lncRNA	chr7:99980741-9998
ENSG0000(	425	9.491331	chr7:158{	ENSG00000284627		Pseudoger	chr7:97928082-9792
ENSG0000(	425	9.491331	chr7:158{	ZKSCAN5	DriverDB\	protein_c	chr7:99504662-9953
ENSG0000(	425	9.491331	chr7:158{	RPL7P60		Pseudoger	chr7:100139629-10(
ENSG0000(	425	9.491331	chr7:158{	KPNA7		protein_c	chr7:99173572-9925
ENSG0000(	425	9.491331	chr7:158{	TAF6	DriverDB\	protein_c	chr7:100106876-10(
ENSG0000(	425	9.491331	chr12:149	UTP20	NCGv7	protein_c	chr12:101280105-10
ENSG0000(	425	9.491331	chr7:158{	ERVW-1		protein_c	chr7:92468380-9247
ENSG0000(	425	9.491331	chr7:158{	ZNF789	DriverDB\	protein_c	chr7:99472890-9950
ENSG0000(	425	9.491331	chr7:158{	SMURF1	DriverDB\	protein_c	chr7:99027440-9914
ENSG0000(	425	9.491331	chr7:158{	ENSG00000242798		lncRNA	chr7:100115214-10(
ENSG0000(	425	9.491331	chr7:158{	ENSG00000286305		lncRNA	chr7:98989867-9899
ENSG0000(	425	9.491331	chr12:149	SNX5P2		Pseudoger	chr12:101066734-10
ENSG0000(	425	9.491331	chr12:149	AC010203.1		smallRNA	chr12:100157256-10
ENSG0000(	425	9.491331	chr12:149	SLC17A8		protein_c	chr12:100357074-10
ENSG0000(	425	9.491331	chr12:149	RNU6-768P		smallRNA	chr12:101199271-10
ENSG0000(	425	9.491331	chr7:158{	CYP3A137P		Pseudoger	chr7:99820018-9982
ENSG0000(	425	9.491331	chr7:158{	CNPY4		protein_c	chr7:100119634-10(
ENSG0000(	425	9.491331	chr7:158{	ATP5MF		protein_c	chr7:99448475-9946
ENSG0000(	425	9.491331	chr7:158{	snoU13		smallRNA	chr7:99413978-9941
ENSG0000(	425	9.491331	chr7:158{	TECPR1		protein_c	chr7:98214624-9825

ENSG0000(	425	9.491331	chr7:158{	MIR93		smallRNA	chr7:100093768-100
ENSG0000(	425	9.491331	chr7:158{	AC005020.1		smallRNA	chr7:99586915-9958
ENSG0000(	425	9.491331	chr12:14{	GAS2L3		protein_c	chr12:100573683-10
ENSG0000(	425	9.491331	chr12:14{	RPS4XP1		Pseudoger	chr12:100009052-10
ENSG0000(	425	9.491331	chr7:158{	MIR106B		smallRNA	chr7:100093993-100
ENSG0000(	425	9.491331	chr7:158{	RPS3AP26		Pseudoger	chr7:98385801-9838
ENSG0000(	425	9.491331	chr7:158{	GPC2	DriverDB\	protein_c	chr7:100169606-100
ENSG0000(	425	9.491331	chr7:158{	CYP3A7		protein_c	chr7:99705036-9973
ENSG0000(	425	9.491331	chr7:158{	LAMTOR4		protein_c	chr7:100148912-100
ENSG0000(	425	9.491331	chr7:158{	TRRAP	NCGv7;AC	protein_c	chr7:98877933-9905
ENSG0000(	425	9.491331	chr7:158{	CYP3A4		protein_c	chr7:99756960-9978
ENSG0000(	425	9.491331	chr7:158{	AP4M1		protein_c	chr7:100101549-100
ENSG0000(	425	9.491331	chr7:158{	ENSG00000284707		lncRNA	chr7:97851688-9797
ENSG0000(	425	9.491331	chr7:158{	ENSG00000285725		lncRNA	chr7:97966377-9797
ENSG0000(	425	9.491331	chr12:14{	RNU6-1068P		smallRNA	chr12:101411357-10
ENSG0000(	425	9.491331	chr7:158{	ENSG00000273407		lncRNA	chr7:99766543-9976
ENSG0000(	425	9.491331	chr7:158{	BUD31	DriverDB\	protein_c	chr7:99408641-9941
ENSG0000(	425	9.491331	chr12:14{	SCYL2	NCGv7	protein_c	chr12:100267140-10
ENSG0000(	425	9.491331	chr7:158{	ENSG00000235077		lncRNA	chr7:100130964-100
ENSG0000(	425	9.491331	chr7:158{	PDAP1	NCGv7	protein_c	chr7:99392048-9940
ENSG0000(	425	9.491331	chr7:158{	ENSG00000272647		protein_c	chr7:99558695-9960
ENSG0000(	425	9.491331	chr12:14{	ENSG00000271177		Pseudoger	chr12:100525186-10
ENSG0000(	425	9.491331	chr7:158{	MIR4658		smallRNA	chr7:100156605-100
ENSG0000(	425	9.491331	chr7:158{	NPTX2		protein_c	chr7:98617285-9862
ENSG0000(	425	9.491331	chr7:158{	PPIAP82		Pseudoger	chr7:98454119-9845
ENSG0000(	425	9.491331	chr7:158{	ZSCAN25		protein_c	chr7:99616946-9963
ENSG0000(	425	9.491331	chr7:158{	MIR5692C2		smallRNA	chr7:97964405-9796
ENSG0000(	425	9.491331	chr12:14{	GARIN6		protein_c	chr12:99647753-996
ENSG0000(	425	9.491331	chr7:158{	ENSG00000288976		lncRNA	chr7:100148341-100
ENSG0000(	425	9.491331	chr7:158{	RNF14P3		Pseudoger	chr7:98998538-9899
ENSG0000(	425	9.491331	chr7:158{	MARK2P10		Pseudoger	chr7:96858182-9685
ENSG0000(	425	9.491331	chr7:158{	ENSG00000231859		Pseudoger	chr7:97906429-9790
ENSG0000(	425	9.491331	chr7:158{	GATAD1	NCGv7	protein_c	chr7:92447482-9246
ENSG0000(	424	9.468998	chr17:73{	CNTNAP1	NCGv7	protein_c	chr17:42682531-426
ENSG0000(	424	9.468998	chr17:73{	RNU7-97P		smallRNA	chr17:42440198-424
ENSG0000(	424	9.468998	chr17:73{	CCR10		protein_c	chr17:42678889-426
ENSG0000(	424	9.468998	chr17:73{	ENSG00000267632		lncRNA	chr17:42509784-425
ENSG0000(	424	9.468998	chr17:73{	ENSG00000289579		lncRNA	chr17:42409892-424
ENSG0000(	424	9.468998	chr17:73{	EZH1	NCGv7	protein_c	chr17:42700275-427
ENSG0000(	424	9.468998	chr17:73{	MIR548AT		smallRNA	chr17:42494773-424
ENSG0000(	424	9.468998	chr17:73{	NAGLU		protein_c	chr17:42536241-425
ENSG0000(	424	9.468998	chr17:73{	TUBG2		protein_c	chr17:42659284-426
ENSG0000(	424	9.468998	chr17:73{	PTP4A2P1		Pseudoger	chr17:42533532-425
ENSG0000(	424	9.468998	chr17:73{	ENSG00000287710		lncRNA	chr17:42577825-425
ENSG0000(	424	9.468998	chr17:73{	MIR5010		smallRNA	chr17:42514188-425
ENSG0000(	424	9.468998	chr17:73{	PSMC3IP		protein_c	chr17:42572310-425
ENSG0000(	424	9.468998	chr17:73{	ENSG00000267222		lncRNA	chr17:42495867-424
ENSG0000(	424	9.468998	chr17:73{	ATP5MGP7		Pseudoger	chr17:42647834-426
ENSG0000(	424	9.468998	chr17:73{	TUBG1		protein_c	chr17:42609641-426
ENSG0000(	424	9.468998	chr17:73{	HSD17B1-AS1		lncRNA	chr17:42552431-425
ENSG0000(	424	9.468998	chr17:73{	ATP6V0A1		protein_c	chr17:42458844-425

ENSG0000(	424	9.468998	chr17:739	ENSG00000280183		TEC	chr17:42598643-425
ENSG0000(	424	9.468998	chr17:739	ENSG00000267042		lncRNA	chr17:42679963-426
ENSG0000(	424	9.468998	chr17:739	ENSG00000239671		Pseudoger	chr17:42714299-427
ENSG0000(	424	9.468998	chr17:739	HSD17B1		protein_c	chr17:42552922-425
ENSG0000(	424	9.468998	chr17:739	COASY		protein_c	chr17:42561467-425
ENSG0000(	424	9.468998	chr17:739	HSD17B1P1		Pseudoger	chr17:42546764-425
ENSG0000(	424	9.468998	chr17:739	ENSG00000266929		lncRNA	chr17:42536510-425
ENSG0000(	424	9.468998	chr17:739	MLX		protein_c	chr17:42567072-425
ENSG0000(	424	9.468998	chr17:739	RETREG3		protein_c	chr17:42579513-426
ENSG0000(	424	9.468998	chr17:739	ENSG00000267765		lncRNA	chr17:42683187-426
ENSG0000(	424	9.468998	chr17:739	HMGB3P27		Pseudoger	chr17:42648178-426
ENSG0000(	424	9.468998	chr17:739	PLEKHH3		protein_c	chr17:42667914-426
ENSG0000(	421	9.402001	chr17:424	TRMT112P3		Pseudoger	chr17:62950831-629
ENSG0000(	419	9.357336	chr6:1391	LPAL2		lncRNA	chr6:160466555-160
ENSG0000(	419	9.357336	chr6:1391	ENSG00000274903		Pseudoger	chr6:160773988-160
ENSG0000(	419	9.357336	chr6:1391	CHP1P2		Pseudoger	chr6:160093423-160
ENSG0000(	419	9.357336	chr6:1391	ENSG00000243831		Pseudoger	chr6:160666228-160
ENSG0000(	419	9.357336	chr6:1391	PNLDC1		protein_c	chr6:159800249-159
ENSG0000(	419	9.357336	chr6:1391	SLC22A1	NCGv7	protein_c	chr6:160121815-160
ENSG0000(	419	9.357336	chr6:1391	SNORA29		smallRNA	chr6:159785594-159
ENSG0000(	419	9.357336	chr6:1391	ENSG00000286498		lncRNA	chr6:161353679-161
ENSG0000(	419	9.357336	chr6:1391	ENSG00000231178		lncRNA	chr6:159353922-159
ENSG0000(	419	9.357336	chr6:1391	SOD2-OT1		lncRNA	chr6:159760258-159
ENSG0000(	419	9.357336	chr6:1391	SNORA20		smallRNA	chr6:159780250-159
ENSG0000(	419	9.357336	chr6:1391	AIRN		lncRNA	chr6:160003291-160
ENSG0000(	419	9.357336	chr6:1391	ENSG00000276960		Pseudoger	chr6:160755398-160
ENSG0000(	419	9.357336	chr6:1391	WTAP		protein_c	chr6:159725585-159
ENSG0000(	419	9.357336	chr6:1391	IGF2R	NCGv7	protein_c	chr6:159969082-160
ENSG0000(	419	9.357336	chr6:1391	LPAL2		Pseudoger	chr6:160453428-160
ENSG0000(	419	9.357336	chr6:1391	SLC22A3		protein_c	chr6:160348378-160
ENSG0000(	419	9.357336	chr6:1391	MAS1	TAG;AC	protein_c	chr6:159890988-159
ENSG0000(	419	9.357336	chr6:1391	ENSG00000224477		lncRNA	chr6:160698288-160
ENSG0000(	419	9.357336	chr6:1391	ENSG00000231863		lncRNA	chr6:160931080-160
ENSG0000(	419	9.357336	chr6:1391	MAP3K4	NCGv7	protein_c	chr6:160991727-161
ENSG0000(	419	9.357336	chr6:1391	ENSG00000236823		lncRNA	chr6:159890988-159
ENSG0000(	419	9.357336	chr6:1391	ENSG00000276413		Pseudoger	chr6:159555964-159
ENSG0000(	419	9.357336	chr6:1391	TCP1	AC	protein_c	chr6:159778498-159
ENSG0000(	419	9.357336	chr6:1391	ACAT2		protein_c	chr6:159762045-159
ENSG0000(	419	9.357336	chr6:1391	ENSG00000287656		lncRNA	chr6:160369200-160
ENSG0000(	419	9.357336	chr6:1391	LPA		protein_c	chr6:160531482-160
ENSG0000(	419	9.357336	chr6:1391	LINC02529		lncRNA	chr6:159383422-159
ENSG0000(	419	9.357336	chr6:1391	ENSG00000233342		lncRNA	chr6:160926269-160
ENSG0000(	419	9.357336	chr6:1391	AGPAT4		protein_c	chr6:161129967-161
ENSG0000(	419	9.357336	chr6:1391	ENSG00000287558		lncRNA	chr6:160742509-160
ENSG0000(	419	9.357336	chr6:1391	ENSG00000224371		lncRNA	chr6:160872888-160
ENSG0000(	419	9.357336	chr6:1391	ENSG00000216516		Pseudoger	chr6:160163539-160
ENSG0000(	419	9.357336	chr6:1391	SLC22A2	NCGv7	protein_c	chr6:160171061-160
ENSG0000(	419	9.357336	chr6:1391	HNRNPH1P1		Pseudoger	chr6:159712801-159
ENSG0000(	419	9.357336	chr6:1391	ENSG00000220913		Pseudoger	chr6:160905770-160
ENSG0000(	419	9.357336	chr6:1391	SOD2		protein_c	chr6:159669069-159
ENSG0000(	419	9.357336	chr6:1391	ENSG00000286533		lncRNA	chr6:159468601-159

ENSG0000(	419	9.357336	chr6:1391	MRPL18	NCGv7	protein_c	chr6:159789812-159
ENSG0000(	419	9.357336	chr6:1391	RNU4ATAC18P		smallRNA	chr6:159720415-159
ENSG0000(	419	9.357336	chr6:1391	ENSG00000237927		lncRNA	chr6:159586955-159
ENSG0000(	419	9.357336	chr6:1391	ENSG00000216480		Pseudoger	chr6:159526062-159
ENSG0000(	419	9.357336	chr6:1391	PLG		protein_c	chr6:160702238-160
ENSG0000(	419	9.357336	chr6:1391	MAP3K4-AS1		lncRNA	chr6:160990318-160
ENSG0000(	419	9.357336	chr6:1391	ENSG00000230234		lncRNA	chr6:160272617-160
ENSG0000(	418	9.335003	chr11:469	RPS6P16		Pseudoger	chr11:112235371-11
ENSG0000(	418	9.335003	chr7:1588	TNRC18P3		Pseudoger	chr7:56991888-5700
ENSG0000(	418	9.335003	chr7:1588	NMD3P2		Pseudoger	chr7:56502284-5650
ENSG0000(	418	9.335003	chr11:469	ENSG00000254626		lncRNA	chr11:112787304-11
ENSG0000(	418	9.335003	chr7:1588	MIR3147		smallRNA	chr7:57405025-5740
ENSG0000(	418	9.335003	chr4:4108	ENSG00000285330		protein_c	chr4:109713916-109
ENSG0000(	418	9.335003	chr11:469	PPIHP1		Pseudoger	chr11:112029858-11
ENSG0000(	418	9.335003	chr11:469	MIR34BHG		lncRNA	chr11:111510600-11
ENSG0000(	418	9.335003	chr4:4108	BDH2		protein_c	chr4:103077592-103
ENSG0000(	418	9.335003	chr4:4108	MANBA		protein_c	chr4:102630770-102
ENSG0000(	418	9.335003	chr7:1588	ENSG00000233437		Pseudoger	chr7:56876500-5687
ENSG0000(	418	9.335003	chr7:1588	CASTOR3		Pseudoger	chr7:100222597-100
ENSG0000(	418	9.335003	chr4:4108	SLC9B2		protein_c	chr4:103019868-103
ENSG0000(	418	9.335003	chr11:469	ENSG00000255428		lncRNA	chr11:111414242-11
ENSG0000(	418	9.335003	chr4:4108	CXXC4-AS1		lncRNA	chr4:104490849-104
ENSG0000(	418	9.335003	chr7:1588	ENSG00000290564		lncRNA	chr7:64120431-6415
ENSG0000(	418	9.335003	chr11:469	ENSG00000254990		lncRNA	chr11:111768668-11
ENSG0000(	418	9.335003	chr7:1588	PHKG1P4		Pseudoger	chr7:57060590-5706
ENSG0000(	418	9.335003	chr4:4108	KRT19P3		Pseudoger	chr4:109879070-109
ENSG0000(	418	9.335003	chr4:4108	AIMP1		protein_c	chr4:106315544-106
ENSG0000(	418	9.335003	chr4:4108	SGMS2		protein_c	chr4:107824563-107
ENSG0000(	418	9.335003	chr4:4108	RNU6-289P		smallRNA	chr4:111331412-111
ENSG0000(	418	9.335003	chr4:4108	RAC1P5		Pseudoger	chr4:107203349-107
ENSG0000(	418	9.335003	chr7:1588	ENSG00000286397		lncRNA	chr7:64035644-6403
ENSG0000(	418	9.335003	chr7:1588	GUSBP10		Pseudoger	chr7:57177409-5718
ENSG0000(	418	9.335003	chr4:4108	LINC01438		lncRNA	chr4:110794403-110
ENSG0000(	418	9.335003	chr7:1588	ENSG00000291178		lncRNA	chr7:100300020-100
ENSG0000(	418	9.335003	chr4:4108	ENSG00000251259		lncRNA	chr4:105137280-105
ENSG0000(	418	9.335003	chr4:4108	RPSAP34		Pseudoger	chr4:108407843-108
ENSG0000(	418	9.335003	chr4:4108	SLC9B1		protein_c	chr4:102885048-103
ENSG0000(	418	9.335003	chr11:469	HSPB2-C11orf52		protein_c	chr11:111912736-11
ENSG0000(	418	9.335003	chr7:1588	ENSG00000228303		Pseudoger	chr7:56638943-5665
ENSG0000(	418	9.335003	chr7:1588	ENSG00000227910		Pseudoger	chr7:63924787-6392
ENSG0000(	418	9.335003	chr7:1588	SAPCD2P1		Pseudoger	chr7:64181710-6418
ENSG0000(	418	9.335003	chr4:4108	ENSG00000251288		Pseudoger	chr4:102751401-102
ENSG0000(	418	9.335003	chr4:4108	RNU6-351P		smallRNA	chr4:104974662-104
ENSG0000(	418	9.335003	chr7:1588	MTND4P2		Pseudoger	chr7:64104093-6410
ENSG0000(	418	9.335003	chr4:4108	MCUB		protein_c	chr4:109560209-109
ENSG0000(	418	9.335003	chr4:4108	TACR3-AS1		lncRNA	chr4:103548745-103
ENSG0000(	418	9.335003	chr4:4108	ENSG00000250670		lncRNA	chr4:104556960-104
ENSG0000(	418	9.335003	chr7:1588	VN1R37P		Pseudoger	chr7:64149685-6415
ENSG0000(	418	9.335003	chr7:1588	SLC25A1P3		Pseudoger	chr7:63931141-6393
ENSG0000(	418	9.335003	chr4:4108	GIMD1		protein_c	chr4:106357392-106
ENSG0000(	418	9.335003	chr7:1588	SPACDR		protein_c	chr7:100456620-100

ENSG0000(	418	9.335003	chr7:158	ENSG00000213650	Pseudoger	chr7:56567906-5656
ENSG0000(	418	9.335003	chr4:410	RNF14P2	Pseudoger	chr4:110264865-110
ENSG0000(	418	9.335003	chr7:158	ENSG00000203462	Pseudoger	chr7:56322804-5632
ENSG0000(	418	9.335003	chr7:158	ENSG00000237268	Pseudoger	chr7:56421857-5643
ENSG0000(	418	9.335003	chr4:410	ETNPPL	protein_c	chr4:108742048-108
ENSG0000(	418	9.335003	chr7:158	MTND4P4	Pseudoger	chr7:57169406-5717
ENSG0000(	418	9.335003	chr7:158	ENSG00000224653	Pseudoger	chr7:57819819-5782
ENSG0000(	418	9.335003	chr11:46	ENSG00000235286	Pseudoger	chr11:111670956-11
ENSG0000(	418	9.335003	chr7:158	ZNF679	protein_c	chr7:64228474-6426
ENSG0000(	418	9.335003	chr4:410	RNU6-431P	smallRNA	chr4:108652150-108
ENSG0000(	418	9.335003	chr7:158	SEPTIN7P15	Pseudoger	chr7:57067950-5707
ENSG0000(	418	9.335003	chr4:410	ZBED1P1	Pseudoger	chr4:110291644-110
ENSG0000(	418	9.335003	chr7:158	SEPTIN14P24	Pseudoger	chr7:56360362-5636
ENSG0000(	418	9.335003	chr7:158	ENSG00000230796	Pseudoger	chr7:57650381-5765
ENSG0000(	418	9.335003	chr11:46	LINC02762	lncRNA	chr11:112270748-11
ENSG0000(	418	9.335003	chr7:158	ENSG00000279072	lncRNA	chr7:56809214-5684
ENSG0000(	418	9.335003	chr4:410	ENSG00000276992	Pseudoger	chr4:104996900-104
ENSG0000(	418	9.335003	chr7:158	TRIM60P18	Pseudoger	chr7:64355078-6435
ENSG0000(	418	9.335003	chr7:158	ENSG00000225244	Pseudoger	chr7:56940341-5694
ENSG0000(	418	9.335003	chr7:158	ENSG00000250618	Pseudoger	chr7:57817996-5781
ENSG0000(	418	9.335003	chr7:158	ENSG00000213067	Pseudoger	chr7:56597379-5659
ENSG0000(	418	9.335003	chr4:410	ENSG00000251312	Pseudoger	chr4:111063583-111
ENSG0000(	418	9.335003	chr4:410	ENSG00000286291	lncRNA	chr4:103871890-103
ENSG0000(	418	9.335003	chr7:158	ENSG00000223836	Pseudoger	chr7:57820032-5782
ENSG0000(	418	9.335003	chr7:158	ENSG00000287631	lncRNA	chr7:100388809-100
ENSG0000(	418	9.335003	chr7:158	ARAFP1	Pseudoger	chr7:63937905-6393
ENSG0000(	418	9.335003	chr7:158	PPP1R35-AS1	lncRNA	chr7:100436204-100
ENSG0000(	418	9.335003	chr11:46	RNA5SP351	Pseudoger	chr11:111928400-11
ENSG0000(	418	9.335003	chr11:46	RPL37AP8	Pseudoger	chr11:111889199-11
ENSG0000(	418	9.335003	chr7:158	ENSG00000241357	lncRNA	chr7:100435257-100
ENSG0000(	418	9.335003	chr4:410	ENSG00000251170	lncRNA	chr4:104230380-104
ENSG0000(	418	9.335003	chr11:46	CRYAB AC	protein_c	chr11:111908564-11
ENSG0000(	418	9.335003	chr11:46	LINC02764	lncRNA	chr11:112534220-11
ENSG0000(	418	9.335003	chr7:158	MTCYBP29	Pseudoger	chr7:57173357-5717
ENSG0000(	418	9.335003	chr4:410	PITX2 AC	protein_c	chr4:110617423-110
ENSG0000(	418	9.335003	chr4:410	ENSG00000248242	lncRNA	chr4:104653874-104
ENSG0000(	418	9.335003	chr4:410	KRT8P46	Pseudoger	chr4:102728746-102
ENSG0000(	418	9.335003	chr11:46	MRPS36P4	Pseudoger	chr11:112208601-11
ENSG0000(	418	9.335003	chr7:158	MTND4P5	Pseudoger	chr7:57192695-5719
ENSG0000(	418	9.335003	chr11:46	C11orf1	protein_c	chr11:111878935-11
ENSG0000(	418	9.335003	chr4:410	RNU6-553P	smallRNA	chr4:105406997-105
ENSG0000(	418	9.335003	chr11:46	RNU6-44P	smallRNA	chr11:112352556-11
ENSG0000(	418	9.335003	chr7:158	ENSG00000287517	lncRNA	chr7:57242681-5724
ENSG0000(	418	9.335003	chr11:46	ALG9-IT1	lncRNA	chr11:111817214-11
ENSG0000(	418	9.335003	chr4:410	GSTCD-AS1	lncRNA	chr4:105746245-105
ENSG0000(	418	9.335003	chr7:158	ENSG00000234085	Pseudoger	chr7:57654822-5765
ENSG0000(	418	9.335003	chr7:158	ENSG00000287699	lncRNA	chr7:64294516-6430
ENSG0000(	418	9.335003	chr7:158	ENSG00000233028	Pseudoger	chr7:56175634-5617
ENSG0000(	418	9.335003	chr7:158	ENSG00000291021	lncRNA	chr7:56875385-5688
ENSG0000(	418	9.335003	chr7:158	ENSG00000176232	Pseudoger	chr7:63980385-6398
ENSG0000(	418	9.335003	chr4:410	PABPC1P7	Pseudoger	chr4:102896725-102

ENSG0000(	418	9.335003	chr11:469	ENSG00000258529		protein_c	chr11:111786286-11
ENSG0000(	418	9.335003	chr7:1588	ZNF479	NCGv7	protein_c	chr7:57119614-5713
ENSG0000(	418	9.335003	chr11:469	ST13P10		Pseudoger	chr11:112267290-11
ENSG0000(	418	9.335003	chr4:4108	GAR1-DT		lncRNA	chr4:109815047-109
ENSG0000(	418	9.335003	chr7:1588	CCNJP1		Pseudoger	chr7:56228634-5622
ENSG0000(	418	9.335003	chr4:4108	UBE2D3		protein_c	chr4:102794383-102
ENSG0000(	418	9.335003	chr7:1588	SAPCD2P2		Pseudoger	chr7:57424503-5742
ENSG0000(	418	9.335003	chr7:1588	MTND1P2		Pseudoger	chr7:64111752-6411
ENSG0000(	418	9.335003	chr7:1588	SLC29A4P1		Pseudoger	chr7:57014285-5702
ENSG0000(	418	9.335003	chr7:1588	ENSG00000234089		lncRNA	chr7:57209865-5722
ENSG0000(	418	9.335003	chr11:469	GNG5P3		Pseudoger	chr11:111864254-11
ENSG0000(	418	9.335003	chr7:1588	MTCO2P8		Pseudoger	chr7:64107829-6410
ENSG0000(	418	9.335003	chr11:469	BCO2		protein_c	chr11:112175510-11
ENSG0000(	418	9.335003	chr7:1588	ENSG00000280225		TEC	chr7:57402181-5740
ENSG0000(	418	9.335003	chr4:4108	ELOVL6		protein_c	chr4:110045846-110
ENSG0000(	418	9.335003	chr7:1588	CCT6A	DriverDBv	protein_c	chr7:56051685-5606
ENSG0000(	418	9.335003	chr7:1588	VN1R25P		Pseudoger	chr7:56559221-5655
ENSG0000(	418	9.335003	chr7:1588	MEPCE		protein_c	chr7:100428322-100
ENSG0000(	418	9.335003	chr4:4108	SETP20		Pseudoger	chr4:109553243-109
ENSG0000(	418	9.335003	chr4:4108	SEC24B-AS1		lncRNA	chr4:109347475-109
ENSG0000(	418	9.335003	chr11:469	HOATZ		protein_c	chr11:111514778-11
ENSG0000(	418	9.335003	chr7:1588	ZNF735		protein_c	chr7:64207203-6422
ENSG0000(	418	9.335003	chr4:4108	CXXC4	NCGv7	protein_c	chr4:104468308-104
ENSG0000(	418	9.335003	chr11:469	LINC02763		lncRNA	chr11:112393118-11
ENSG0000(	418	9.335003	chr7:1588	ENSG00000231484		Pseudoger	chr7:57828331-5783
ENSG0000(	418	9.335003	chr4:4108	ENSG00000288692		lncRNA	chr4:110876410-111
ENSG0000(	418	9.335003	chr7:1588	NUPR2		protein_c	chr7:56114681-5611
ENSG0000(	418	9.335003	chr4:4108	TET2	NCGv7;AC	protein_c	chr4:105145875-105
ENSG0000(	418	9.335003	chr7:1588	ENSG00000282879		Pseudoger	chr7:56736396-5673
ENSG0000(	418	9.335003	chr7:1588	PVRIG2P		Pseudoger	chr7:100352360-100
ENSG0000(	418	9.335003	chr7:1588	ENSG00000291184		lncRNA	chr7:56426859-5644
ENSG0000(	418	9.335003	chr4:4108	PLA2G12A		protein_c	chr4:109709989-109
ENSG0000(	418	9.335003	chr4:4108	ENSG00000248778		Pseudoger	chr4:105679050-105
ENSG0000(	418	9.335003	chr4:4108	NPNT	NCGv7	protein_c	chr4:105894775-106
ENSG0000(	418	9.335003	chr7:1588	ENSG00000235095		Pseudoger	chr7:57147986-5715
ENSG0000(	418	9.335003	chr7:1588	ENSG00000287588		lncRNA	chr7:63925832-6393
ENSG0000(	418	9.335003	chr11:469	RPL23AP62		Pseudoger	chr11:112461468-11
ENSG0000(	418	9.335003	chr7:1588	VN1R38P		Pseudoger	chr7:64180006-6418
ENSG0000(	418	9.335003	chr7:1588	CICP28		Pseudoger	chr7:56805336-5680
ENSG0000(	418	9.335003	chr11:469	ENSG00000255286		Pseudoger	chr11:111945639-11
ENSG0000(	418	9.335003	chr7:1588	ENSG00000231232		Pseudoger	chr7:57822465-5782
ENSG0000(	418	9.335003	chr7:1588	PVRIG	NCGv7	protein_c	chr7:100218241-100
ENSG0000(	418	9.335003	chr11:469	BTG4		protein_c	chr11:111467526-11
ENSG0000(	418	9.335003	chr11:469	ENSG00000288070		lncRNA	chr11:112822806-11
ENSG0000(	418	9.335003	chr4:4108	AC093680.1		smallRNA	chr4:106415706-106
ENSG0000(	418	9.335003	chr7:1588	MIR4283-1		smallRNA	chr7:56955785-5695
ENSG0000(	418	9.335003	chr11:469	PTS		protein_c	chr11:112226367-11
ENSG0000(	418	9.335003	chr4:4108	DDX3P3		Pseudoger	chr4:103572089-103
ENSG0000(	418	9.335003	chr11:469	TEX12		protein_c	chr11:112167372-11
ENSG0000(	418	9.335003	chr7:1588	ENSG00000223740		Pseudoger	chr7:56525428-5652
ENSG0000(	418	9.335003	chr4:4108	CDC42P4		Pseudoger	chr4:109555170-109

ENSG0000(	418	9.335003	chr7:158{	ENSG00000290193		lncRNA	chr7:56482723-5649
ENSG0000(	418	9.335003	chr7:158{	MTND2P6		Pseudoger	chr7:57186412-5718
ENSG0000(	418	9.335003	chr11:46{	POU2AF1	NCGv7;AC	protein_c	chr11:111352255-11
ENSG0000(	418	9.335003	chr7:158{	ZNF727	DriverDB\	protein_c	chr7:64045434-6408
ENSG0000(	418	9.335003	chr11:46{	ENSG00000285769		lncRNA	chr11:112637324-11
ENSG0000(	418	9.335003	chr4:410{	ENSG00000251572		Pseudoger	chr4:102461250-102
ENSG0000(	418	9.335003	chr4:410{	HIGD1AP14		Pseudoger	chr4:109673843-109
ENSG0000(	418	9.335003	chr11:46{	PPP2R1B		protein_c	chr11:111726908-11
ENSG0000(	418	9.335003	chr11:46{	ENSG00000254638		lncRNA	chr11:112165197-11
ENSG0000(	418	9.335003	chr4:410{	CFI		protein_c	chr4:109731008-109
ENSG0000(	418	9.335003	chr7:158{	ENSG00000236261		Pseudoger	chr7:57652875-5765
ENSG0000(	418	9.335003	chr7:158{	CHCHD2	DriverDB\	protein_c	chr7:56101573-5610
ENSG0000(	418	9.335003	chr4:410{	ENSG00000273447		lncRNA	chr4:109692004-109
ENSG0000(	418	9.335003	chr4:410{	TACR3		protein_c	chr4:103586031-103
ENSG0000(	418	9.335003	chr4:410{	snoU13		smallRNA	chr4:102859393-102
ENSG0000(	418	9.335003	chr7:158{	PHKG1		protein_c	chr7:56080283-5609
ENSG0000(	418	9.335003	chr4:410{	RNU6-635P		smallRNA	chr4:103924540-103
ENSG0000(	418	9.335003	chr4:410{	LEF1-AS1		lncRNA	chr4:108167525-108
ENSG0000(	418	9.335003	chr4:410{	RNU6-551P		smallRNA	chr4:107435118-107
ENSG0000(	418	9.335003	chr11:46{	MIR4491		smallRNA	chr11:111347757-11
ENSG0000(	418	9.335003	chr4:410{	SLC39A8		protein_c	chr4:102251080-102
ENSG0000(	418	9.335003	chr7:158{	ENSG00000284221		Pseudoger	chr7:57192405-5719
ENSG0000(	418	9.335003	chr7:158{	MTATP6P10		Pseudoger	chr7:57190461-5719
ENSG0000(	418	9.335003	chr4:410{	AC097473.1		smallRNA	chr4:108789200-108
ENSG0000(	418	9.335003	chr4:410{	RBMXP4		Pseudoger	chr4:109346326-109
ENSG0000(	418	9.335003	chr7:158{	ENSG00000286477		lncRNA	chr7:56659149-5667
ENSG0000(	418	9.335003	chr7:158{	LINC02848		lncRNA	chr7:63900313-6392
ENSG0000(	418	9.335003	chr4:410{	RNU7-151P		smallRNA	chr4:102837047-102
ENSG0000(	418	9.335003	chr7:158{	ENSG00000261275		lncRNA	chr7:56493124-5649
ENSG0000(	418	9.335003	chr11:46{	ENSG00000288097		lncRNA	chr11:111320379-11
ENSG0000(	418	9.335003	chr7:158{	NCOR1P3		Pseudoger	chr7:57599794-5761
ENSG0000(	418	9.335003	chr7:158{	RNU6-1052P		smallRNA	chr7:56402070-5640
ENSG0000(	418	9.335003	chr4:410{	ENSG00000251473		Pseudoger	chr4:105102891-105
ENSG0000(	418	9.335003	chr4:410{	RN7SL55P		smallRNA	chr4:109450775-109
ENSG0000(	418	9.335003	chr4:410{	AC093628.1		smallRNA	chr4:104490876-104
ENSG0000(	418	9.335003	chr7:158{	VN1R35P		Pseudoger	chr7:63963431-6396
ENSG0000(	418	9.335003	chr4:410{	RPL34-DT		lncRNA	chr4:108538190-108
ENSG0000(	418	9.335003	chr11:46{	RN7SKP273		smallRNA	chr11:111683111-11
ENSG0000(	418	9.335003	chr4:410{	SEC24B		protein_c	chr4:109433772-109
ENSG0000(	418	9.335003	chr7:158{	ENSG00000289691		lncRNA	chr7:100397383-100
ENSG0000(	418	9.335003	chr7:158{	ENSG00000284098		Pseudoger	chr7:57190300-5719
ENSG0000(	418	9.335003	chr4:410{	LRRC37A15P		Pseudoger	chr4:102727274-102
ENSG0000(	418	9.335003	chr4:410{	CYP2U1	DriverDB\	protein_c	chr4:107931549-107
ENSG0000(	418	9.335003	chr4:410{	DKK2		protein_c	chr4:106921802-107
ENSG0000(	418	9.335003	chr4:410{	ARHGEF38-IT1		lncRNA	chr4:105561591-105
ENSG0000(	418	9.335003	chr11:46{	SIK2		protein_c	chr11:111602449-11
ENSG0000(	418	9.335003	chr7:158{	MTCO2P10		Pseudoger	chr7:57189578-5719
ENSG0000(	418	9.335003	chr7:158{	MTATP6P8		Pseudoger	chr7:57167200-5716
ENSG0000(	418	9.335003	chr11:46{	NKAPD1		protein_c	chr11:112074086-11
ENSG0000(	418	9.335003	chr4:410{	RNU6-733P		smallRNA	chr4:107867807-107
ENSG0000(	418	9.335003	chr4:410{	RPL34	NCGv7	protein_c	chr4:108620569-108

ENSG0000(	418	9.335003	chr11:469	KCTD9P4		Pseudoger	chr11:112180773-11
ENSG0000(	418	9.335003	chr4:4108	ENSG00000249635		lncRNA	chr4:106003317-106
ENSG0000(	418	9.335003	chr7:1588	ENSG00000233288		lncRNA	chr7:56528253-5653
ENSG0000(	418	9.335003	chr7:1588	MTND1P4		Pseudoger	chr7:57185788-5718
ENSG0000(	418	9.335003	chr4:4108	LYPLA1P2		Pseudoger	chr4:110945109-110
ENSG0000(	418	9.335003	chr7:1588	ENSG00000284534		lncRNA	chr7:63959844-6398
ENSG0000(	418	9.335003	chr7:1588	MTND5P7		Pseudoger	chr7:57194257-5719
ENSG0000(	418	9.335003	chr7:1588	ENSG00000270957		Pseudoger	chr7:57485166-5748
ENSG0000(	418	9.335003	chr7:1588	ENSG00000284558		Pseudoger	chr7:57191997-5719
ENSG0000(	418	9.335003	chr7:1588	ENSG00000284572		Pseudoger	chr7:57196056-5719
ENSG0000(	418	9.335003	chr7:1588	ENSG00000233962		Pseudoger	chr7:57652736-5765
ENSG0000(	418	9.335003	chr11:469	ENSG00000268472		lncRNA	chr11:112260265-11
ENSG0000(	418	9.335003	chr4:4108	PANCR		lncRNA	chr4:110595504-110
ENSG0000(	418	9.335003	chr11:469	DIXDC1		protein_c	chr11:111927144-11
ENSG0000(	418	9.335003	chr7:1588	ENSG00000284474		Pseudoger	chr7:57167039-5716
ENSG0000(	418	9.335003	chr4:4108	ENSG00000250740		lncRNA	chr4:105927060-105
ENSG0000(	418	9.335003	chr7:1588	RNU6-1335P		smallRNA	chr7:56340231-5634
ENSG0000(	418	9.335003	chr11:469	SDHD	NCGv7;AC	protein_c	chr11:112086824-11
ENSG0000(	418	9.335003	chr4:4108	snoU2_19		smallRNA	chr4:110433109-110
ENSG0000(	418	9.335003	chr11:469	ENSG00000255292		protein_c	chr11:112086903-11
ENSG0000(	418	9.335003	chr4:4108	TET2-AS1		lncRNA	chr4:105171354-105
ENSG0000(	418	9.335003	chr4:4108	AC024198.1		smallRNA	chr4:110853376-110
ENSG0000(	418	9.335003	chr4:4108	UBE2D3-AS1		lncRNA	chr4:102827611-102
ENSG0000(	418	9.335003	chr4:4108	RPL36AP23		Pseudoger	chr4:111551854-111
ENSG0000(	418	9.335003	chr11:469	LAYN		protein_c	chr11:111540280-11
ENSG0000(	418	9.335003	chr4:4108	RNU6-205P		smallRNA	chr4:110278185-110
ENSG0000(	418	9.335003	chr4:4108	ENSG00000250522		lncRNA	chr4:105540190-105
ENSG0000(	418	9.335003	chr7:1588	LINC01005		lncRNA	chr7:64024409-6403
ENSG0000(	418	9.335003	chr7:1588	MTND4LP32		Pseudoger	chr7:57169143-5716
ENSG0000(	418	9.335003	chr4:4108	ENSG00000288913		lncRNA	chr4:110197678-110
ENSG0000(	418	9.335003	chr7:1588	ENSG00000289690		protein_c	chr7:100397577-100
ENSG0000(	418	9.335003	chr4:4108	PAPSS1		protein_c	chr4:107590276-107
ENSG0000(	418	9.335003	chr7:1588	ENSG00000236299		lncRNA	chr7:63888200-6390
ENSG0000(	418	9.335003	chr4:4108	RN7SL89P		smallRNA	chr4:105293658-105
ENSG0000(	418	9.335003	chr4:4108	GSTCD		protein_c	chr4:105708778-105
ENSG0000(	418	9.335003	chr4:4108	ENSG00000286147		lncRNA	chr4:106525401-106
ENSG0000(	418	9.335003	chr7:1588	ENSG00000285544		lncRNA	chr7:64045429-6411
ENSG0000(	418	9.335003	chr4:4108	MIR576		smallRNA	chr4:109488698-109
ENSG0000(	418	9.335003	chr7:1588	NMD3P1		Pseudoger	chr7:63908966-6391
ENSG0000(	418	9.335003	chr4:4108	INTS12	NCGv7	protein_c	chr4:105682627-105
ENSG0000(	418	9.335003	chr7:1588	RPL6P20		Pseudoger	chr7:64141538-6414
ENSG0000(	418	9.335003	chr7:1588	MTCYBP5		Pseudoger	chr7:57196638-5719
ENSG0000(	418	9.335003	chr7:1588	ZCWPW1		protein_c	chr7:100400826-100
ENSG0000(	418	9.335003	chr7:1588	RN7SL161P		smallRNA	chr7:100462829-100
ENSG0000(	418	9.335003	chr7:1588	MTCO1P8		Pseudoger	chr7:64108625-6411
ENSG0000(	418	9.335003	chr4:4108	ENSG00000248656		lncRNA	chr4:111516117-111
ENSG0000(	418	9.335003	chr11:469	ENSG00000255093		Pseudoger	chr11:111448450-11
ENSG0000(	418	9.335003	chr7:1588	Y_RNA		smallRNA	chr7:100330777-100
ENSG0000(	418	9.335003	chr4:4108	CENPE		protein_c	chr4:103105349-103
ENSG0000(	418	9.335003	chr11:469	ENSG00000255334		lncRNA	chr11:112015307-11
ENSG0000(	418	9.335003	chr4:4108	PIMREGP2		Pseudoger	chr4:105526596-105

ENSG0000(	418	9.335003	chr7:158(	ENSG00000286938		lncRNA	chr7:100482221-100
ENSG0000(	418	9.335003	chr11:46(	DLAT		protein_c	chr11:112025408-11
ENSG0000(	418	9.335003	chr7:158(	PPP1R35		protein_c	chr7:100435282-100
ENSG0000(	418	9.335003	chr4:410(	ENSG00000241981		Pseudoger	chr4:102662611-102
ENSG0000(	418	9.335003	chr4:410(	GAR1		protein_c	chr4:109815510-109
ENSG0000(	418	9.335003	chr4:410(	ENSG00000250855		lncRNA	chr4:111802795-111
ENSG0000(	418	9.335003	chr4:410(	AF213884.3		smallRNA	chr4:102565875-102
ENSG0000(	418	9.335003	chr11:46(	RNU2-60P		smallRNA	chr11:111383092-11
ENSG0000(	418	9.335003	chr4:410(	PPA2		protein_c	chr4:105369077-105
ENSG0000(	418	9.335003	chr7:158(	PMS2P1		Pseudoger	chr7:100328836-100
ENSG0000(	418	9.335003	chr7:158(	AC073136.1		smallRNA	chr7:56269783-5626
ENSG0000(	418	9.335003	chr4:410(	RRH		protein_c	chr4:109827972-109
ENSG0000(	418	9.335003	chr11:46(	PIH1D2		protein_c	chr11:112063218-11
ENSG0000(	418	9.335003	chr4:410(	LRIT3		protein_c	chr4:109848107-109
ENSG0000(	418	9.335003	chr4:410(	SNORD112		smallRNA	chr4:107117332-107
ENSG0000(	418	9.335003	chr7:158(	PILRA		protein_c	chr7:100367530-100
ENSG0000(	418	9.335003	chr4:410(	ENPEP	IntOGen-I	protein_c	chr4:110365733-110
ENSG0000(	418	9.335003	chr4:410(	ENSG00000249604		lncRNA	chr4:107936031-107
ENSG0000(	418	9.335003	chr4:410(	LINC02503		lncRNA	chr4:103961616-104
ENSG0000(	418	9.335003	chr4:410(	ATP5F1EP1		Pseudoger	chr4:105532475-105
ENSG0000(	418	9.335003	chr7:158(	MTCO3P4		Pseudoger	chr7:57191141-5719
ENSG0000(	418	9.335003	chr4:410(	RPL7L1P13		Pseudoger	chr4:110399773-110
ENSG0000(	418	9.335003	chr7:158(	MTND4LP2		Pseudoger	chr7:64105292-6410
ENSG0000(	418	9.335003	chr4:410(	CISD2		protein_c	chr4:102868974-102
ENSG0000(	418	9.335003	chr7:158(	ENSG00000250923		Pseudoger	chr7:57773183-5777
ENSG0000(	418	9.335003	chr7:158(	STAG3	NCGv7	protein_c	chr7:100177563-100
ENSG0000(	418	9.335003	chr11:46(	ENSG00000287006		lncRNA	chr11:112698772-11
ENSG0000(	418	9.335003	chr7:158(	MTND3P2		Pseudoger	chr7:64105654-6410
ENSG0000(	418	9.335003	chr7:158(	RBM22P3		Pseudoger	chr7:56490682-5649
ENSG0000(	418	9.335003	chr11:46(	MIR34B		smallRNA	chr11:111512938-11
ENSG0000(	418	9.335003	chr4:410(	EGF		protein_c	chr4:109912883-110
ENSG0000(	418	9.335003	chr7:158(	PILRB		protein_c	chr7:100352176-100
ENSG0000(	418	9.335003	chr7:158(	BSNDP4		Pseudoger	chr7:57638725-5763
ENSG0000(	418	9.335003	chr7:158(	ZNF716	DriverDB\	protein_c	chr7:57450177-5747
ENSG0000(	418	9.335003	chr4:410(	TBCK		protein_c	chr4:106041599-106
ENSG0000(	418	9.335003	chr4:410(	LINC02173		lncRNA	chr4:106433489-106
ENSG0000(	418	9.335003	chr7:158(	MTCO3P8		Pseudoger	chr7:64106065-6410
ENSG0000(	418	9.335003	chr7:158(	ENSG00000270749		Pseudoger	chr7:57629285-5763
ENSG0000(	418	9.335003	chr4:410(	ENSG00000250920		lncRNA	chr4:103550586-103
ENSG0000(	418	9.335003	chr7:158(	ENSG00000287019		lncRNA	chr7:56615084-5661
ENSG0000(	418	9.335003	chr7:158(	ENSG00000224370		Pseudoger	chr7:56380976-5638
ENSG0000(	418	9.335003	chr7:158(	ENSG00000234716		Pseudoger	chr7:56577848-5657
ENSG0000(	418	9.335003	chr4:410(	ENSG00000289096		lncRNA	chr4:110619749-110
ENSG0000(	418	9.335003	chr4:410(	HADH		protein_c	chr4:107989714-108
ENSG0000(	418	9.335003	chr4:410(	LEF1	NCGv7;AC	protein_c	chr4:108047545-108
ENSG0000(	418	9.335003	chr11:46(	ENSG00000247416		lncRNA	chr11:112959279-11
ENSG0000(	418	9.335003	chr4:410(	RN7SL275P		smallRNA	chr4:110117736-110
ENSG0000(	418	9.335003	chr4:410(	CASP6		protein_c	chr4:109688622-109
ENSG0000(	418	9.335003	chr7:158(	VN1R36P		Pseudoger	chr7:63965834-6396
ENSG0000(	418	9.335003	chr4:410(	ENSG00000286136		lncRNA	chr4:108669949-108
ENSG0000(	418	9.335003	chr11:46(	POU2AF2		protein_c	chr11:111245725-11

ENSG0000(	418	9.335003	chr11:469	MIR34C		smallRNA	chr11:111513439-11
ENSG0000(	418	9.335003	chr4:4108	ENSG00000250511		lncRNA	chr4:110512488-110
ENSG0000(	418	9.335003	chr7:1588	GUSBP12		Pseudoger	chr7:57200825-5720
ENSG0000(	418	9.335003	chr7:1588	ENSG00000236907		Pseudoger	chr7:57817601-5781
ENSG0000(	418	9.335003	chr4:4108	ACTR3BP4		Pseudoger	chr4:102961956-102
ENSG0000(	418	9.335003	chr4:4108	HSBP1P2		Pseudoger	chr4:110251871-110
ENSG0000(	418	9.335003	chr4:4108	NFKB1		protein_c	chr4:102501330-102
ENSG0000(	418	9.335003	chr7:1588	TSC22D4		protein_c	chr7:100463359-100
ENSG0000(	418	9.335003	chr11:469	AP002884.1		smallRNA	chr11:112247830-11
ENSG0000(	418	9.335003	chr4:4108	ARHGEF38		protein_c	chr4:105552620-105
ENSG0000(	418	9.335003	chr7:1588	ENSG00000274299		Pseudoger	chr7:57270839-5727
ENSG0000(	418	9.335003	chr4:4108	MIR297		smallRNA	chr4:110860582-110
ENSG0000(	418	9.335003	chr7:1588	ENSG00000243981		Pseudoger	chr7:57628479-5764
ENSG0000(	418	9.335003	chr4:4108	ZNF969P		Pseudoger	chr4:110415898-110
ENSG0000(	418	9.335003	chr4:4108	ACTR6P1		Pseudoger	chr4:106836498-106
ENSG0000(	418	9.335003	chr4:4108	ENSG00000286242		lncRNA	chr4:102814252-102
ENSG0000(	418	9.335003	chr4:4108	RCC2P8		Pseudoger	chr4:108788745-108
ENSG0000(	418	9.335003	chr7:1588	snoU13		smallRNA	chr7:56100659-5610
ENSG0000(	418	9.335003	chr11:469	RNU6-893P		smallRNA	chr11:112032499-11
ENSG0000(	418	9.335003	chr11:469	IL18		protein_c	chr11:112143253-11
ENSG0000(	418	9.335003	chr7:1588	ENSG00000224484		Pseudoger	chr7:57776972-5777
ENSG0000(	418	9.335003	chr7:1588	SNORA15		smallRNA	chr7:56060470-5606
ENSG0000(	418	9.335003	chr11:469	C11orf52		protein_c	chr11:111918032-11
ENSG0000(	418	9.335003	chr7:1588	ENSG00000224155		Pseudoger	chr7:56304678-5630
ENSG0000(	418	9.335003	chr7:1588	AC005071.1		smallRNA	chr7:100220027-100
ENSG0000(	418	9.335003	chr4:4108	ENSG00000248161		lncRNA	chr4:102418602-102
ENSG0000(	418	9.335003	chr7:1588	ENSG00000230271		Pseudoger	chr7:57772584-5777
ENSG0000(	418	9.335003	chr7:1588	ENSG00000278577		Pseudoger	chr7:63993904-6399
ENSG0000(	418	9.335003	chr7:1588	ENSG00000271047		Pseudoger	chr7:56603410-5660
ENSG0000(	418	9.335003	chr7:1588	ENSG00000283230		Pseudoger	chr7:57168725-5716
ENSG0000(	418	9.335003	chr4:4108	AC084209.1		smallRNA	chr4:108577072-108
ENSG0000(	418	9.335003	chr7:1588	ENSG00000287985		lncRNA	chr7:64369241-6437
ENSG0000(	418	9.335003	chr7:1588	ZNF722		protein_c	chr7:63998849-6401
ENSG0000(	418	9.335003	chr7:1588	VN1R34P		Pseudoger	chr7:63934449-6393
ENSG0000(	418	9.335003	chr4:4108	RN7SL728P		smallRNA	chr4:102348394-102
ENSG0000(	418	9.335003	chr7:1588	STAG3L5P		lncRNA	chr7:100336079-100
ENSG0000(	418	9.335003	chr11:469	COLCA1		lncRNA	chr11:111290787-11
ENSG0000(	418	9.335003	chr7:1588	SUMF2	DriverDB\	protein_c	chr7:56064002-5608
ENSG0000(	418	9.335003	chr7:1588	ENSG00000289760		protein_c	chr7:100478099-100
ENSG0000(	418	9.335003	chr7:1588	ENSG00000232944		Pseudoger	chr7:56408699-5640
ENSG0000(	418	9.335003	chr7:1588	ENSG00000232161		Pseudoger	chr7:57770966-5777
ENSG0000(	418	9.335003	chr4:4108	ENSG00000251081		lncRNA	chr4:107258700-107
ENSG0000(	418	9.335003	chr11:469	FDXACB1		protein_c	chr11:111874056-11
ENSG0000(	418	9.335003	chr7:1588	MTATP6P18		Pseudoger	chr7:64106822-6410
ENSG0000(	418	9.335003	chr4:4108	ENSG00000248200		Pseudoger	chr4:110146374-110
ENSG0000(	418	9.335003	chr4:4108	RNU6-35P		smallRNA	chr4:109992325-109
ENSG0000(	418	9.335003	chr7:1588	ENSG00000227015		Pseudoger	chr7:57770619-5777
ENSG0000(	418	9.335003	chr7:1588	RNU7-157P		smallRNA	chr7:57227001-5722
ENSG0000(	418	9.335003	chr7:1588	STAG3L5P-PVRIG2P-F		lncRNA	chr7:100336104-100
ENSG0000(	418	9.335003	chr7:1588	STAG3L5P		Pseudoger	chr7:100338197-100
ENSG0000(	418	9.335003	chr7:1588	MTND5P6		Pseudoger	chr7:57170974-5717

ENSG0000(	418	9.335003	chr7:1588	ENSG00000277174		Pseudoger	chr7:56685036-5668
ENSG0000(	418	9.335003	chr4:4108	ENSG00000260651		lncRNA	chr4:102500841-102
ENSG0000(	418	9.335003	chr7:1588	ENSG00000278205		Pseudoger	chr7:56462536-5646
ENSG0000(	418	9.335003	chr7:1588	ENSG00000225488		lncRNA	chr7:56477588-5648
ENSG0000(	418	9.335003	chr7:1588	NYAP1	DriverDB\	protein_c	chr7:100483927-100
ENSG0000(	418	9.335003	chr11:469	TIMM8B		protein_c	chr11:112084800-11
ENSG0000(	418	9.335003	chr7:1588	MTCO1P10		Pseudoger	chr7:57187892-5718
ENSG0000(	418	9.335003	chr4:4108	ENSG00000224207		Pseudoger	chr4:102734358-102
ENSG0000(	418	9.335003	chr7:1588	AC092634.1		smallRNA	chr7:63901068-6390
ENSG0000(	418	9.335003	chr7:1588	ENSG00000286436		lncRNA	chr7:57140112-5715
ENSG0000(	418	9.335003	chr4:4108	ZACNP1		Pseudoger	chr4:108415220-108
ENSG0000(	418	9.335003	chr4:4108	COL25A1	NCGv7	protein_c	chr4:108808725-109
ENSG0000(	418	9.335003	chr7:1588	GUSBP6		Pseudoger	chr7:64100305-6412
ENSG0000(	418	9.335003	chr4:4108	AC004052.1		smallRNA	chr4:104278107-104
ENSG0000(	418	9.335003	chr7:1588	ZNF736		protein_c	chr7:64307459-6435
ENSG0000(	418	9.335003	chr4:4108	ENSG00000249257		Pseudoger	chr4:108773613-108
ENSG0000(	418	9.335003	chr11:469	ENSG00000254980		lncRNA	chr11:111514043-11
ENSG0000(	418	9.335003	chr7:1588	MTCO3P10		Pseudoger	chr7:57167880-5716
ENSG0000(	418	9.335003	chr11:469	PLET1		protein_c	chr11:112248153-11
ENSG0000(	418	9.335003	chr7:1588	ENSG00000229881		lncRNA	chr7:64140495-6415
ENSG0000(	418	9.335003	chr11:469	ALG9		protein_c	chr11:111782195-11
ENSG0000(	418	9.335003	chr4:4108	ENSG00000248373		lncRNA	chr4:104900125-105
ENSG0000(	418	9.335003	chr4:4108	EXOC7P1		Pseudoger	chr4:108417705-108
ENSG0000(	418	9.335003	chr7:1588	ENSG00000237236		Pseudoger	chr7:57822201-5782
ENSG0000(	418	9.335003	chr7:1588	RN7SL816P		smallRNA	chr7:56965248-5696
ENSG0000(	418	9.335003	chr4:4108	LINC02428		lncRNA	chr4:103255822-103
ENSG0000(	418	9.335003	chr7:1588	IFITM3P4		Pseudoger	chr7:56163145-5616
ENSG0000(	418	9.335003	chr4:4108	OSTC		protein_c	chr4:108650585-108
ENSG0000(	418	9.335003	chr11:469	COLCA2		protein_c	chr11:111298546-11
ENSG0000(	418	9.335003	chr7:1588	CASTOR3		lncRNA	chr7:100200653-100
ENSG0000(	418	9.335003	chr7:1588	ENSG00000271696		Pseudoger	chr7:57835309-5783
ENSG0000(	418	9.335003	chr7:1588	VN1R28P		Pseudoger	chr7:57422830-5742
ENSG0000(	418	9.335003	chr7:1588	TRIM60P16		Pseudoger	chr7:56631739-5663
ENSG0000(	418	9.335003	chr7:1588	MTND6P29		Pseudoger	chr7:57172772-5717
ENSG0000(	418	9.335003	chr11:469	HSPB2		protein_c	chr11:111912734-11
ENSG0000(	418	9.335003	chr7:1588	ENSG00000283431		Pseudoger	chr7:57163168-5716
ENSG0000(	418	9.335003	chr7:1588	CICP8		Pseudoger	chr7:56362458-5636
ENSG0000(	418	9.335003	chr11:469	snosnR66		smallRNA	chr11:112602354-11
ENSG0000(	418	9.335003	chr7:1588	MTND2P4		Pseudoger	chr7:64110547-6411
ENSG0000(	418	9.335003	chr4:4108	SNORA31		smallRNA	chr4:105105987-105
ENSG0000(	418	9.335003	chr7:1588	ENSG00000285670		lncRNA	chr7:56214979-5622
ENSG0000(	418	9.335003	chr7:1588	TRIM60P17		Pseudoger	chr7:64085560-6408
ENSG0000(	418	9.335003	chr7:1588	ENSG00000223559		Pseudoger	chr7:56288230-5629
ENSG0000(	418	9.335003	chr7:1588	AC092849.1		smallRNA	chr7:100483758-100
ENSG0000(	418	9.335003	chr7:1588	AC092634.2		smallRNA	chr7:63926836-6392
ENSG0000(	418	9.335003	chr11:469	ENSG00000271025		Pseudoger	chr11:112036627-11
ENSG0000(	418	9.335003	chr4:4108	CYP2U1-AS1		lncRNA	chr4:107863473-107
ENSG0000(	418	9.335003	chr7:1588	SPDYE3		protein_c	chr7:100307702-100
ENSG0000(	418	9.335003	chr7:1588	ENSG00000237639		Pseudoger	chr7:57650521-5765
ENSG0000(	418	9.335003	chr4:4108	COL25A1-DT		lncRNA	chr4:109303035-109
ENSG0000(	418	9.335003	chr11:469	RPS12P21		Pseudoger	chr11:112218326-11

ENSG0000(	418	9.335003	chr4:4108	EEF1A1P9		Pseudoger	chr4:105484698-105
ENSG0000(	416	9.290338	chr7:1588	ENSG00000231923		Pseudoger	chr7:138046654-138
ENSG0000(	416	9.290338	chr7:1588	DGKI	NCGv7	protein_c	chr7:137381037-137
ENSG0000(	416	9.290338	chr7:1588	AC009784.1		smallRNA	chr7:136244446-136
ENSG0000(	416	9.290338	chr7:1588	ENSG00000228031		lncRNA	chr7:137344930-137
ENSG0000(	416	9.290338	chr7:1588	ENSG00000234352		lncRNA	chr7:136685559-137
ENSG0000(	416	9.290338	chr7:1588	SNORA51		smallRNA	chr7:138187998-138
ENSG0000(	416	9.290338	chr7:1588	ENSG00000234639		Pseudoger	chr7:138022458-138
ENSG0000(	416	9.290338	chr7:1588	PSMC1P3		Pseudoger	chr7:136713871-136
ENSG0000(	416	9.290338	chr7:1588	ENSG00000230649		lncRNA	chr7:136025717-136
ENSG0000(	416	9.290338	chr7:1588	Y_RNA		smallRNA	chr7:138175045-138
ENSG0000(	416	9.290338	chr7:1588	ENSG00000289438		lncRNA	chr7:137846936-137
ENSG0000(	416	9.290338	chr7:1588	ENSG00000289600		lncRNA	chr7:135980929-135
ENSG0000(	416	9.290338	chr7:1588	MIR4468		smallRNA	chr7:138123758-138
ENSG0000(	416	9.290338	chr7:1588	ZP3P2		Pseudoger	chr7:136485656-136
ENSG0000(	416	9.290338	chr7:1588	CREB3L2-AS1		lncRNA	chr7:137953348-137
ENSG0000(	416	9.290338	chr7:1588	ENSG00000231931		Pseudoger	chr7:138060056-138
ENSG0000(	416	9.290338	chr7:1588	snoU13		smallRNA	chr7:138121088-138
ENSG0000(	416	9.290338	chr7:1588	KRT8P51		Pseudoger	chr7:136938280-136
ENSG0000(	416	9.290338	chr7:1588	AKR1D1	NCGv7	protein_c	chr7:138002324-138
ENSG0000(	416	9.290338	chr7:1588	RNU6-223P		smallRNA	chr7:135960703-135
ENSG0000(	416	9.290338	chr7:1588	ENSG00000224469		Pseudoger	chr7:137164043-137
ENSG0000(	416	9.290338	chr7:1588	AC024082.1		smallRNA	chr7:138068542-138
ENSG0000(	416	9.290338	chr7:1588	SNORD81		smallRNA	chr7:137287687-137
ENSG0000(	416	9.290338	chr7:1588	ENSG00000231114		lncRNA	chr7:137318592-137
ENSG0000(	416	9.290338	chr7:1588	ENSG00000225559		lncRNA	chr7:138163440-138
ENSG0000(	416	9.290338	chr7:1588	ENSG00000232053		lncRNA	chr7:136092913-136
ENSG0000(	416	9.290338	chr7:1588	MTPN		protein_c	chr7:135926760-135
ENSG0000(	416	9.290338	chr7:1588	snoU13		smallRNA	chr7:136841093-136
ENSG0000(	416	9.290338	chr7:1588	Y_RNA		smallRNA	chr7:136129980-136
ENSG0000(	416	9.290338	chr7:1588	RCC2P3		Pseudoger	chr7:138122202-138
ENSG0000(	416	9.290338	chr7:1588	snoU13		smallRNA	chr7:138176185-138
ENSG0000(	416	9.290338	chr7:1588	ENSG00000224746		lncRNA	chr7:135774521-135
ENSG0000(	416	9.290338	chr7:1588	CREB3L2	NCGv7;AC	protein_c	chr7:137874979-138
ENSG0000(	416	9.290338	chr7:1588	TRPC6P8		Pseudoger	chr7:136262558-136
ENSG0000(	416	9.290338	chr7:1588	RPL6P19		Pseudoger	chr7:137721985-137
ENSG0000(	416	9.290338	chr7:1588	CHRM2		protein_c	chr7:136868652-137
ENSG0000(	416	9.290338	chr7:1588	MIR490		smallRNA	chr7:136903167-136
ENSG0000(	416	9.290338	chr7:1588	PTN	NCGv7	protein_c	chr7:137227341-137
ENSG0000(	414	9.245673	chr11:469	ENSG00000280124		TEC	chr11:91184934-911
ENSG0000(	414	9.245673	chr11:469	TRIM77		protein_c	chr11:89710299-897
ENSG0000(	414	9.245673	chr11:469	ENSG00000278859		TEC	chr11:91992833-919
ENSG0000(	414	9.245673	chr11:469	TUBAP2		Pseudoger	chr11:90282560-902
ENSG0000(	414	9.245673	chr11:469	ENSG00000280085		TEC	chr11:90972316-909
ENSG0000(	414	9.245673	chr11:469	ENSG00000279269		TEC	chr11:93206990-932
ENSG0000(	414	9.245673	chr11:469	ENSG00000278892		TEC	chr11:93071529-930
ENSG0000(	414	9.245673	chr11:469	CBX3P7		Pseudoger	chr11:89293745-892
ENSG0000(	414	9.245673	chr11:469	ENSG00000279299		TEC	chr11:90125658-901
ENSG0000(	414	9.245673	chr11:469	SRP14P2		Pseudoger	chr11:93535468-935
ENSG0000(	414	9.245673	chr11:469	ENSG00000279209		TEC	chr11:91013787-910
ENSG0000(	414	9.245673	chr11:469	UBTFL10		Pseudoger	chr11:89700764-897

ENSG0000(	414	9.245673	chr11:469	CHORDC1	protein_c	chr11:90200429-902
ENSG0000(	414	9.245673	chr11:469	ENSG00000280093	TEC	chr11:91382909-913
ENSG0000(	414	9.245673	chr11:469	RN7SL223P	smallRNA	chr11:93556913-935
ENSG0000(	414	9.245673	chr11:469	PANX1	protein_c	chr11:94128841-941
ENSG0000(	414	9.245673	chr11:469	C11orf97	protein_c	chr11:94512461-945
ENSG0000(	414	9.245673	chr11:469	FOLH1B	lncRNA	chr11:89659297-896
ENSG0000(	414	9.245673	chr11:469	ENSG00000250519	lncRNA	chr11:94185439-942
ENSG0000(	414	9.245673	chr11:469	ENSG00000279248	TEC	chr11:89533645-895
ENSG0000(	414	9.245673	chr11:469	TYR	protein_c	chr11:89177875-892
ENSG0000(	414	9.245673	chr11:469	RPL26P31	Pseudoger	chr11:93052395-930
ENSG0000(	414	9.245673	chr11:469	TUBB4BP4	Pseudoger	chr11:91949731-919
ENSG0000(	414	9.245673	chr11:469	NAALAD2	protein_c	chr11:90131515-901
ENSG0000(	414	9.245673	chr11:469	ENSG00000254803	Pseudoger	chr11:90049945-900
ENSG0000(	414	9.245673	chr11:469	VSTM5	protein_c	chr11:93818232-938
ENSG0000(	414	9.245673	chr11:469	ENSG00000290492	lncRNA	chr11:89777155-897
ENSG0000(	414	9.245673	chr11:469	ENSG00000279304	TEC	chr11:93133855-931
ENSG0000(	414	9.245673	chr11:469	MIR4490	smallRNA	chr11:90555774-905
ENSG0000(	414	9.245673	chr11:469	SNORD6	smallRNA	chr11:93731502-937
ENSG0000(	414	9.245673	chr11:469	ENSG00000279297	TEC	chr11:89297717-892
ENSG0000(	414	9.245673	chr11:469	RNU7-58P	smallRNA	chr11:59558800-595
ENSG0000(	414	9.245673	chr11:469	ENSG00000280379	TEC	chr11:93171134-931
ENSG0000(	414	9.245673	chr11:469	ENSG00000279603	TEC	chr11:89300709-893
ENSG0000(	414	9.245673	chr11:469	GAPDHP70	Pseudoger	chr11:88408179-884
ENSG0000(	414	9.245673	chr11:469	CTSC	protein_c	chr11:88265069-883
ENSG0000(	414	9.245673	chr11:469	TRIM53AP	Pseudoger	chr11:89993536-899
ENSG0000(	414	9.245673	chr22:221	IGLV3-29	Pseudoger	chr22:22661299-226
ENSG0000(	414	9.245673	chr11:469	ENSG00000254971	lncRNA	chr11:89753982-897
ENSG0000(	414	9.245673	chr11:469	snoU13	smallRNA	chr11:92729330-927
ENSG0000(	414	9.245673	chr11:469	AP000765.1	smallRNA	chr11:94416710-944
ENSG0000(	414	9.245673	chr11:469	ENSG00000254916	Pseudoger	chr11:90051837-900
ENSG0000(	414	9.245673	chr11:469	snoU13	smallRNA	chr11:93797213-937
ENSG0000(	414	9.245673	chr11:469	C11orf54	protein_c	chr11:93741591-937
ENSG0000(	414	9.245673	chr11:469	SNORA8	smallRNA	chr11:93732361-937
ENSG0000(	414	9.245673	chr11:469	OSBPL9P3	Pseudoger	chr11:91115961-911
ENSG0000(	414	9.245673	chr11:469	RPL7AP57	Pseudoger	chr11:92161106-921
ENSG0000(	414	9.245673	chr11:469	ENSG00000284057	protein_c	chr11:93741664-938
ENSG0000(	414	9.245673	chr11:469	ENSG00000278953	TEC	chr11:93592187-935
ENSG0000(	414	9.245673	chr11:469	ENSG00000255429	lncRNA	chr11:89546637-895
ENSG0000(	414	9.245673	chr11:469	HPRT1P3	Pseudoger	chr11:93998643-939
ENSG0000(	414	9.245673	chr11:469	ENSG00000255506	lncRNA	chr11:92748732-927
ENSG0000(	414	9.245673	chr11:469	SNORA18	smallRNA	chr11:93733466-937
ENSG0000(	414	9.245673	chr11:469	SNORA25	smallRNA	chr11:93730513-937
ENSG0000(	414	9.245673	chr11:469	RNU6-16P	smallRNA	chr11:88612805-886
ENSG0000(	414	9.245673	chr11:469	ENSG00000255162	Pseudoger	chr11:89785945-897
ENSG0000(	414	9.245673	chr11:469	H3P34	Pseudoger	chr11:89498748-894
ENSG0000(	414	9.245673	chr11:469	ENSG00000279696	TEC	chr11:93726654-937
ENSG0000(	414	9.245673	chr11:469	LINC02700	lncRNA	chr11:94638045-946
ENSG0000(	414	9.245673	chr11:469	PIWIL4-AS1	lncRNA	chr11:94545330-947
ENSG0000(	414	9.245673	chr11:469	ENSG00000254617	Pseudoger	chr11:89887366-898
ENSG0000(	414	9.245673	chr11:469	UBTFL2	Pseudoger	chr11:89752785-897
ENSG0000(	414	9.245673	chr11:469	MTND1P35	Pseudoger	chr11:89908540-899

ENSG0000(	414	9.245673	chr11:469	TRIM64DP		Pseudoger	chr11:89776981-897
ENSG0000(	414	9.245673	chr11:469	ENSG00000233536		lncRNA	chr11:94638038-946
ENSG0000(	414	9.245673	chr11:469	ENSG00000279697		TEC	chr11:89545128-895
ENSG0000(	414	9.245673	chr11:469	ENSG00000254874		lncRNA	chr11:92965797-929
ENSG0000(	414	9.245673	chr11:469	ENSG00000254888		Pseudoger	chr11:90074839-900
ENSG0000(	414	9.245673	chr11:469	ENSG00000204456		Pseudoger	chr11:89766440-897
ENSG0000(	414	9.245673	chr11:469	AP004242.1		smallRNA	chr11:93407825-934
ENSG0000(	414	9.245673	chr11:469	MIR1261		smallRNA	chr11:90869121-908
ENSG0000(	414	9.245673	chr11:469	FOLH1B		Pseudoger	chr11:89639237-896
ENSG0000(	414	9.245673	chr11:469	ENSG00000254818		Pseudoger	chr11:89944035-899
ENSG0000(	414	9.245673	chr11:469	ENSG00000255285		Pseudoger	chr11:89789412-897
ENSG0000(	414	9.245673	chr11:469	MIR548L		smallRNA	chr11:94466495-944
ENSG0000(	414	9.245673	chr11:469	TRIM53BP		Pseudoger	chr11:89841997-898
ENSG0000(	414	9.245673	chr11:469	TAF1D		protein_c	chr11:93729948-937
ENSG0000(	414	9.245673	chr11:469	UBTFL1		protein_c	chr11:90085950-900
ENSG0000(	414	9.245673	chr11:469	ENSG00000255515		Pseudoger	chr11:93609760-936
ENSG0000(	414	9.245673	chr11:469	ENSG00000279045		TEC	chr11:89548130-895
ENSG0000(	414	9.245673	chr11:469	ENSG00000279454		TEC	chr11:93587703-935
ENSG0000(	414	9.245673	chr11:469	ENSG00000255893		lncRNA	chr11:94472908-944
ENSG0000(	414	9.245673	chr11:469	CEP295		protein_c	chr11:93661682-937
ENSG0000(	414	9.245673	chr11:469	SMCO4		protein_c	chr11:93478472-935
ENSG0000(	414	9.245673	chr11:469	ENSG00000255540		Pseudoger	chr11:89731017-897
ENSG0000(	414	9.245673	chr11:469	PIWIL4		protein_c	chr11:94543840-946
ENSG0000(	414	9.245673	chr11:469	MTNR1B		protein_c	chr11:92969651-929
ENSG0000(	414	9.245673	chr11:469	ENSG00000280430		TEC	chr11:90731110-907
ENSG0000(	414	9.245673	chr11:469	ENSG00000254785		Pseudoger	chr11:89895799-899
ENSG0000(	414	9.245673	chr11:469	PGAM1P9		Pseudoger	chr11:92366496-923
ENSG0000(	414	9.245673	chr11:469	NOX4	NCGv7	protein_c	chr11:89324353-894
ENSG0000(	414	9.245673	chr11:469	TRIM49D1		protein_c	chr11:89911111-899
ENSG0000(	414	9.245673	chr11:469	Y_RNA		smallRNA	chr11:93719603-937
ENSG0000(	414	9.245673	chr11:469	ENSG00000279438		TEC	chr11:91093875-910
ENSG0000(	414	9.245673	chr11:469	TRIM51BP		Pseudoger	chr11:89854953-898
ENSG0000(	414	9.245673	chr11:469	ENSG00000255170		Pseudoger	chr11:89764884-897
ENSG0000(	414	9.245673	chr11:469	NDUFB11P1		Pseudoger	chr11:92336032-923
ENSG0000(	414	9.245673	chr11:469	IZUMO1R		protein_c	chr11:94304580-943
ENSG0000(	414	9.245673	chr11:469	TRIM64		protein_c	chr11:89966037-899
ENSG0000(	414	9.245673	chr11:469	SNORD5		smallRNA	chr11:93733228-937
ENSG0000(	414	9.245673	chr11:469	ENSG00000255445		Pseudoger	chr11:93152075-931
ENSG0000(	414	9.245673	chr11:469	ENSG00000288789		lncRNA	chr11:94650324-946
ENSG0000(	414	9.245673	chr11:469	TRIM49C	IntOGen-I	protein_c	chr11:90031106-900
ENSG0000(	414	9.245673	chr11:469	ENSG00000279056		TEC	chr11:89556860-895
ENSG0000(	414	9.245673	chr11:469	ENSG00000290774		lncRNA	chr11:92915884-929
ENSG0000(	414	9.245673	chr11:469	LINC02756		lncRNA	chr11:91794319-918
ENSG0000(	414	9.245673	chr11:469	ENSG00000288018		lncRNA	chr11:88337839-884
ENSG0000(	414	9.245673	chr11:469	SNORA32		smallRNA	chr11:93730979-937
ENSG0000(	414	9.245673	chr11:469	ENSG00000278837		Pseudoger	chr11:89954593-899
ENSG0000(	414	9.245673	chr11:469	ENSG00000255305		Pseudoger	chr11:89978540-899
ENSG0000(	414	9.245673	chr11:469	FUT4		protein_c	chr11:94543921-945
ENSG0000(	414	9.245673	chr11:469	ENSG00000280201		TEC	chr11:89576913-895
ENSG0000(	414	9.245673	chr11:469	ENSG00000255486		Pseudoger	chr11:89723482-897
ENSG0000(	414	9.245673	chr11:469	TRIM49D2		protein_c	chr11:89924064-899

ENSG0000(	414	9.245673	chr11:469	PHB1P16		Pseudoger	chr11:94055023-940
ENSG0000(	414	9.245673	chr11:469	AP002364.1		smallRNA	chr11:90324776-903
ENSG0000(	414	9.245673	chr11:469	SNORA1		smallRNA	chr11:93732004-937
ENSG0000(	414	9.245673	chr11:469	ENSG00000250946		Pseudoger	chr11:89820082-898
ENSG0000(	414	9.245673	chr11:469	TRIM64EP		Pseudoger	chr11:90057605-900
ENSG0000(	414	9.245673	chr11:469	ANKRD49		protein_c	chr11:94493979-944
ENSG0000(	414	9.245673	chr11:469	ENSG00000255360		Pseudoger	chr11:89955146-899
ENSG0000(	414	9.245673	chr11:469	OSBPL9P2		Pseudoger	chr11:91114724-911
ENSG0000(	414	9.245673	chr11:469	TRIM64B		protein_c	chr11:89870438-898
ENSG0000(	414	9.245673	chr11:469	DEUP1		protein_c	chr11:93329971-934
ENSG0000(	414	9.245673	chr11:469	ENSG00000254436		Pseudoger	chr11:90098421-900
ENSG0000(	414	9.245673	chr11:469	SNRPGP16		Pseudoger	chr11:92937441-929
ENSG0000(	414	9.245673	chr11:469	ENSG00000279733		TEC	chr11:88165575-881
ENSG0000(	414	9.245673	chr11:469	EEF1A1P49		Pseudoger	chr11:92914603-929
ENSG0000(	414	9.245673	chr11:469	ENSG00000280385		TEC	chr11:90193614-901
ENSG0000(	414	9.245673	chr11:469	ENSG00000255995		Pseudoger	chr11:93991346-939
ENSG0000(	414	9.245673	chr11:469	RPS3AP42		Pseudoger	chr11:92498152-924
ENSG0000(	414	9.245673	chr11:469	AP001482.1		smallRNA	chr11:89112710-891
ENSG0000(	414	9.245673	chr11:469	ENSG00000279684		TEC	chr11:93286629-932
ENSG0000(	414	9.245673	chr11:469	ENSG00000255011		Pseudoger	chr11:90071136-900
ENSG0000(	414	9.245673	chr11:469	ENSG00000280167		TEC	chr11:94559018-945
ENSG0000(	414	9.245673	chr11:469	ENSG00000278980		TEC	chr11:91208838-912
ENSG0000(	414	9.245673	chr11:469	ENSG00000255184		Pseudoger	chr11:90017611-900
ENSG0000(	414	9.245673	chr11:469	DISC1FP1		lncRNA	chr11:90251204-909
ENSG0000(	414	9.245673	chr11:469	ENSG00000254705		lncRNA	chr11:92400191-924
ENSG0000(	414	9.245673	chr11:469	ENSG00000254655		Pseudoger	chr11:89960422-899
ENSG0000(	414	9.245673	chr11:469	RN7SL195P		smallRNA	chr11:93800483-938
ENSG0000(	414	9.245673	chr11:469	FAT3	NCGv7	protein_c	chr11:92224818-928
ENSG0000(	414	9.245673	chr11:469	MRE11	AC	protein_c	chr11:94415570-944
ENSG0000(	414	9.245673	chr11:469	LINC02746		lncRNA	chr11:92913227-929
ENSG0000(	414	9.245673	chr11:469	LINC02748		lncRNA	chr11:91157994-912
ENSG0000(	414	9.245673	chr11:469	HEPHL1		protein_c	chr11:94021354-941
ENSG0000(	414	9.245673	chr11:469	ENSG00000279163		TEC	chr11:89704164-897
ENSG0000(	414	9.245673	chr11:469	SLC36A4		protein_c	chr11:93144174-931
ENSG0000(	414	9.245673	chr11:469	ENSG00000286665		lncRNA	chr11:90110638-901
ENSG0000(	414	9.245673	chr11:469	ARPC3P3		Pseudoger	chr11:94188449-941
ENSG0000(	414	9.245673	chr11:469	ENSG00000233737		Pseudoger	chr11:93221486-932
ENSG0000(	414	9.245673	chr11:469	ENSG00000255233		lncRNA	chr11:93240133-932
ENSG0000(	414	9.245673	chr11:469	MIR1304		smallRNA	chr11:93733674-937
ENSG0000(	414	9.245673	chr11:469	SNORA40		smallRNA	chr11:93735111-937
ENSG0000(	414	9.245673	chr11:469	TRIM51EP		Pseudoger	chr11:89981441-899
ENSG0000(	414	9.245673	chr11:469	MED17		protein_c	chr11:93784227-938
ENSG0000(	414	9.245673	chr11:469	ENSG00000280367		TEC	chr11:90223153-902
ENSG0000(	414	9.245673	chr11:469	GRM5-AS1		lncRNA	chr11:88504576-885
ENSG0000(	414	9.245673	chr11:469	Y_RNA		smallRNA	chr11:93827622-938
ENSG0000(	414	9.245673	chr11:469	TRIM49		protein_c	chr11:89797655-898
ENSG0000(	414	9.245673	chr11:469	ENSG00000255235		Pseudoger	chr11:89883927-898
ENSG0000(	414	9.245673	chr11:469	GRM5	NCGv7	protein_c	chr11:88504576-890
ENSG0000(	414	9.245673	chr11:469	ENSG00000254558		Pseudoger	chr11:89863848-898
ENSG0000(	414	9.245673	chr11:469	GPR83		protein_c	chr11:94377316-944
ENSG0000(	414	9.245673	chr11:469	MIR3166		smallRNA	chr11:88176502-881

ENSG0000(	414	9.245673	chr11:469	SCARNA9		lncRNA	chr11:93721513-937
ENSG0000(	412	9.201008	chr17:739	DUSP14		protein_c	chr17:37489891-375
ENSG0000(	412	9.201008	chr17:739	ENSG00000279775		TEC	chr17:40597113-405
ENSG0000(	412	9.201008	chr17:739	RNU6-233P		smallRNA	chr17:39546104-395
ENSG0000(	412	9.201008	chr17:739	CCL3-AS1		lncRNA	chr17:36072866-360
ENSG0000(	412	9.201008	chr17:739	ENSG00000266865		Pseudoger	chr17:31008754-310
ENSG0000(	412	9.201008	chr17:739	RNU6-920P		smallRNA	chr17:29641611-296
ENSG0000(	412	9.201008	chr17:739	LINC02079		lncRNA	chr17:39026715-390
ENSG0000(	412	9.201008	chr17:739	CCL4		protein_c	chr17:36103827-361
ENSG0000(	412	9.201008	chr17:739	CAVIN1		protein_c	chr17:42402449-424
ENSG0000(	412	9.201008	chr17:739	PHF12		protein_c	chr17:28905250-289
ENSG0000(	412	9.201008	chr17:739	LRRC37BP1		Pseudoger	chr17:30629680-306
ENSG0000(	412	9.201008	chr17:739	TBC1D29P	NCGv7	Pseudoger	chr17:30553502-305
ENSG0000(	412	9.201008	chr17:739	ENSG00000266877		lncRNA	chr17:31583162-316
ENSG0000(	412	9.201008	chr17:739	Y_RNA		smallRNA	chr17:40391232-403
ENSG0000(	412	9.201008	chr17:739	ENSG00000230113		lncRNA	chr17:30956280-309
ENSG0000(	412	9.201008	chr17:739	CCL16		protein_c	chr17:35976493-359
ENSG0000(	412	9.201008	chr17:739	SLFN13	NCGv7	protein_c	chr17:35435096-354
ENSG0000(	412	9.201008	chr17:739	MIR4725		smallRNA	chr17:31575269-315
ENSG0000(	412	9.201008	chr17:739	ENSG00000266876		Pseudoger	chr17:30068632-300
ENSG0000(	412	9.201008	chr17:739	COPRS		protein_c	chr17:31851871-318
ENSG0000(	412	9.201008	chr17:739	ENSG00000280033		TEC	chr17:32159311-321
ENSG0000(	412	9.201008	chr17:739	RNU6-1192P		smallRNA	chr17:36404826-364
ENSG0000(	412	9.201008	chr17:739	ENSG00000290450		lncRNA	chr17:30631755-306
ENSG0000(	412	9.201008	chr17:739	ENSG00000266088		lncRNA	chr17:40516892-405
ENSG0000(	412	9.201008	chr17:739	ENSG00000279119		TEC	chr17:38727833-387
ENSG0000(	412	9.201008	chr17:739	RNU4-34P		smallRNA	chr17:29388560-293
ENSG0000(	412	9.201008	chr17:739	ZNF830	NCGv7	protein_c	chr17:34961540-349
ENSG0000(	412	9.201008	chr17:739	ENSG00000266101		lncRNA	chr17:39173290-391
ENSG0000(	412	9.201008	chr17:739	SUZ12	NCGv7;AC	protein_c	chr17:31937007-320
ENSG0000(	412	9.201008	chr17:739	WIPF2	NCGv7	protein_c	chr17:40219304-402
ENSG0000(	412	9.201008	chr17:739	TBC1D3P7		Pseudoger	chr17:41323427-413
ENSG0000(	412	9.201008	chr17:739	ENSG00000266111		lncRNA	chr17:29352069-294
ENSG0000(	412	9.201008	chr17:739	ENSG00000275944		lncRNA	chr17:36001419-360
ENSG0000(	412	9.201008	chr17:739	ENSG00000266120		lncRNA	chr17:30238741-302
ENSG0000(	412	9.201008	chr17:739	SLFN11		protein_c	chr17:35350305-353
ENSG0000(	412	9.201008	chr17:739	ENSG00000277182		lncRNA	chr17:38749360-387
ENSG0000(	412	9.201008	chr17:739	RNU6-489P		smallRNA	chr17:37907957-379
ENSG0000(	412	9.201008	chr17:739	SH3GL1P1		Pseudoger	chr17:32039974-320
ENSG0000(	412	9.201008	chr17:739	ENSG00000266775		lncRNA	chr17:30557732-305
ENSG0000(	412	9.201008	chr17:739	MIR2909		smallRNA	chr17:37033745-370
ENSG0000(	412	9.201008	chr17:739	ENSG00000263657		lncRNA	chr17:29761103-297
ENSG0000(	412	9.201008	chr17:739	ENSG00000286030		Pseudoger	chr17:35522279-355
ENSG0000(	412	9.201008	chr17:739	ENSG00000280069		TEC	chr17:30738182-307
ENSG0000(	412	9.201008	chr17:739	AC005549.1		smallRNA	chr17:34174245-341
ENSG0000(	412	9.201008	chr17:739	ENSG00000277589		lncRNA	chr17:36998598-370
ENSG0000(	412	9.201008	chr17:739	KRTAP4-5	NCGv7	protein_c	chr17:41148924-411
ENSG0000(	412	9.201008	chr17:739	ENSG00000276508		lncRNA	chr17:36684754-366
ENSG0000(	412	9.201008	chr17:739	ENSG00000214708		lncRNA	chr17:32141226-321
ENSG0000(	412	9.201008	chr17:739	RN7SL301P		smallRNA	chr17:36110150-361
ENSG0000(	412	9.201008	chr17:739	ENSG00000214719		lncRNA	chr17:30576464-306

ENSG0000(	412	9.201008	chr17:739	RNU6-1267P		smallRNA	chr17:30288071-302
ENSG0000(	412	9.201008	chr17:739	ENSG00000263485		lncRNA	chr17:34080882-340
ENSG0000(	412	9.201008	chr17:739	ENSG00000278918		TEC	chr17:40348049-403
ENSG0000(	412	9.201008	chr17:739	ENSG00000229732		lncRNA	chr17:41500983-415
ENSG0000(	412	9.201008	chr17:739	ENSG00000263477		lncRNA	chr17:29863402-298
ENSG0000(	412	9.201008	chr17:739	ENSG00000263466		lncRNA	chr17:38903704-389
ENSG0000(	412	9.201008	chr17:739	SNORA70		smallRNA	chr17:29777619-297
ENSG0000(	412	9.201008	chr17:739	KRTAP9-9		protein_c	chr17:41255384-412
ENSG0000(	412	9.201008	chr17:739	ENSG00000275173		lncRNA	chr17:37798894-378
ENSG0000(	412	9.201008	chr17:739	TMIGD1		protein_c	chr17:30316333-303
ENSG0000(	412	9.201008	chr17:739	ENSG00000266048		Pseudoger	chr17:39033926-390
ENSG0000(	412	9.201008	chr17:739	RN7SL79P		smallRNA	chr17:31465543-314
ENSG0000(	412	9.201008	chr17:739	KRTAP4-6		protein_c	chr17:41139433-411
ENSG0000(	412	9.201008	chr17:739	hsa-mir-423		lncRNA	chr17:30117079-301
ENSG0000(	412	9.201008	chr17:739	ENSG00000263435		lncRNA	chr17:33976531-339
ENSG0000(	412	9.201008	chr17:739	LIG3		protein_c	chr17:34980512-350
ENSG0000(	412	9.201008	chr17:739	ENSG00000277688		lncRNA	chr17:37609739-376
ENSG0000(	412	9.201008	chr17:739	SMURF2P1		Pseudoger	chr17:30600865-306
ENSG0000(	412	9.201008	chr17:739	AC091178.1		smallRNA	chr17:39081065-390
ENSG0000(	412	9.201008	chr17:739	snoU13		smallRNA	chr17:29883006-298
ENSG0000(	412	9.201008	chr17:739	RNU6-990P		smallRNA	chr17:30360463-303
ENSG0000(	412	9.201008	chr17:739	ENSG00000275839		Pseudoger	chr17:37454171-374
ENSG0000(	412	9.201008	chr17:739	RN7SL399P		smallRNA	chr17:41730129-417
ENSG0000(	412	9.201008	chr17:739	ARHGAP23		protein_c	chr17:38419280-385
ENSG0000(	412	9.201008	chr17:739	CCL11		protein_c	chr17:34285742-342
ENSG0000(	412	9.201008	chr17:739	ENSG00000290082		lncRNA	chr17:29567823-295
ENSG0000(	412	9.201008	chr17:739	SNORA21		smallRNA	chr17:38851524-388
ENSG0000(	412	9.201008	chr17:739	AC131056.5		lncRNA	chr17:36225246-362
ENSG0000(	412	9.201008	chr17:739	SNHG30		lncRNA	chr17:35568050-355
ENSG0000(	412	9.201008	chr17:739	UFM1P2		Pseudoger	chr17:35377416-353
ENSG0000(	412	9.201008	chr17:739	ENSG00000267312		Pseudoger	chr17:35459481-354
ENSG0000(	412	9.201008	chr17:739	ENSG00000267271		Pseudoger	chr17:35344231-353
ENSG0000(	412	9.201008	chr17:739	ENSG00000267261		protein_c	chr17:42119674-421
ENSG0000(	412	9.201008	chr17:739	ENSG00000265697		lncRNA	chr17:33534110-335
ENSG0000(	412	9.201008	chr17:739	C17orf113		protein_c	chr17:42038232-420
ENSG0000(	412	9.201008	chr17:739	KRTAP4-2		protein_c	chr17:41177446-411
ENSG0000(	412	9.201008	chr17:739	RNU6ATAC7P		smallRNA	chr17:31563768-315
ENSG0000(	412	9.201008	chr17:739	ENSG00000267745		lncRNA	chr17:35406684-354
ENSG0000(	412	9.201008	chr17:739	ENSG00000265625		lncRNA	chr17:29644796-296
ENSG0000(	412	9.201008	chr17:739	GJD3		protein_c	chr17:40360652-403
ENSG0000(	412	9.201008	chr17:739	ENSG00000265713		Pseudoger	chr17:29775747-297
ENSG0000(	412	9.201008	chr17:739	ENSG00000275720		lncRNA	chr17:36634069-366
ENSG0000(	412	9.201008	chr17:739	RPL17P42		Pseudoger	chr17:35719888-357
ENSG0000(	412	9.201008	chr17:739	P3H4	DriverDB\	protein_c	chr17:41801947-418
ENSG0000(	412	9.201008	chr17:739	NT5C3B		protein_c	chr17:41825057-418
ENSG0000(	412	9.201008	chr17:739	ENSG00000265739		lncRNA	chr17:30122429-301
ENSG0000(	412	9.201008	chr17:739	ENSG00000279199		TEC	chr17:40113215-401
ENSG0000(	412	9.201008	chr17:739	LINC02978		lncRNA	chr17:30964935-309
ENSG0000(	412	9.201008	chr17:739	ERBB2	NCGv7;AC	protein_c	chr17:39687914-397
ENSG0000(	412	9.201008	chr17:739	GRB7	DriverDB\	protein_c	chr17:39737927-397
ENSG0000(	412	9.201008	chr17:739	MIEN1	DriverDB\	protein_c	chr17:39728496-397

ENSG0000(	412	9.201008	chr17:739	RN7SL138P		smallRNA	chr17:30959565-309
ENSG0000(	412	9.201008	chr17:739	TBC1D3	AC	protein_c	chr17:37978155-382
ENSG0000(	412	9.201008	chr17:739	PNMT		protein_c	chr17:39667981-396
ENSG0000(	412	9.201008	chr17:739	ARL5C		protein_c	chr17:39156894-391
ENSG0000(	412	9.201008	chr17:739	STAC2		protein_c	chr17:39210541-392
ENSG0000(	412	9.201008	chr17:739	IGFBP4		protein_c	chr17:40443450-404
ENSG0000(	412	9.201008	chr17:739	ENSG00000265689		lncRNA	chr17:33688803-336
ENSG0000(	412	9.201008	chr17:739	ENSG00000267349		lncRNA	chr17:35477994-354
ENSG0000(	412	9.201008	chr17:739	ENSG00000263531		lncRNA	chr17:30863921-308
ENSG0000(	412	9.201008	chr17:739	H2BN1		protein_c	chr17:32895433-329
ENSG0000(	412	9.201008	chr17:739	RAB5C-AS1		lncRNA	chr17:42154600-421
ENSG0000(	412	9.201008	chr17:739	ZPBP2	NCGv7	protein_c	chr17:39868202-398
ENSG0000(	412	9.201008	chr17:739	ENSG00000267648		Pseudoger	chr17:35406948-354
ENSG0000(	412	9.201008	chr17:739	MIR365B		smallRNA	chr17:31575411-315
ENSG0000(	412	9.201008	chr17:739	ENSG00000267625		lncRNA	chr17:35561539-355
ENSG0000(	412	9.201008	chr17:739	ENSG00000267618		protein_c	chr17:35011349-351
ENSG0000(	412	9.201008	chr17:739	ENSG00000263613		lncRNA	chr17:29009799-290
ENSG0000(	412	9.201008	chr17:739	ENSG00000236194		lncRNA	chr17:42270517-422
ENSG0000(	412	9.201008	chr17:739	ENSG00000263603		lncRNA	chr17:30729469-307
ENSG0000(	412	9.201008	chr17:739	ENSG00000267592		Pseudoger	chr17:35596904-355
ENSG0000(	412	9.201008	chr17:739	CCL15		protein_c	chr17:35996440-360
ENSG0000(	412	9.201008	chr17:739	Vault		smallRNA	chr17:35097560-350
ENSG0000(	412	9.201008	chr17:739	UNC45B		protein_c	chr17:35147817-351
ENSG0000(	412	9.201008	chr17:739	ENSG00000289485		lncRNA	chr17:36116854-361
ENSG0000(	412	9.201008	chr17:739	KRTAP1-5		protein_c	chr17:41026026-410
ENSG0000(	412	9.201008	chr17:739	ENSG00000267359		lncRNA	chr17:35553205-355
ENSG0000(	412	9.201008	chr17:739	CCL3L3		protein_c	chr17:36194869-361
ENSG0000(	412	9.201008	chr17:739	ENSG00000267554		Pseudoger	chr17:35470069-354
ENSG0000(	412	9.201008	chr17:739	ENSG00000280020		TEC	chr17:32178946-321
ENSG0000(	412	9.201008	chr17:739	RARA-AS1		lncRNA	chr17:40340867-403
ENSG0000(	412	9.201008	chr17:739	ENSG00000267482		Pseudoger	chr17:30059054-300
ENSG0000(	412	9.201008	chr17:739	SSH2		protein_c	chr17:29625938-299
ENSG0000(	412	9.201008	chr17:739	ENSG00000267457		lncRNA	chr17:35073831-350
ENSG0000(	412	9.201008	chr17:739	RHBDL3		protein_c	chr17:32265832-323
ENSG0000(	412	9.201008	chr17:739	SPACA3		protein_c	chr17:32970376-329
ENSG0000(	412	9.201008	chr17:739	ENSG00000267711		lncRNA	chr17:35400878-354
ENSG0000(	412	9.201008	chr17:739	RNU6-1034P		smallRNA	chr17:29445300-294
ENSG0000(	412	9.201008	chr17:739	TAF5LP1		Pseudoger	chr17:35498218-354
ENSG0000(	412	9.201008	chr17:739	ENSG00000267744		Pseudoger	chr17:35190961-351
ENSG0000(	412	9.201008	chr17:739	ENSG00000267364		lncRNA	chr17:35313442-353
ENSG0000(	412	9.201008	chr17:739	FKBP10		protein_c	chr17:41812680-418
ENSG0000(	412	9.201008	chr17:739	MIR423		smallRNA	chr17:30117079-301
ENSG0000(	412	9.201008	chr17:739	RNA5SP438		Pseudoger	chr17:34120056-341
ENSG0000(	412	9.201008	chr17:739	KRTAP1-3		protein_c	chr17:41033884-410
ENSG0000(	412	9.201008	chr17:739	ENSG00000263571		lncRNA	chr17:34155566-341
ENSG0000(	412	9.201008	chr17:739	RNU6-1134P		smallRNA	chr17:31713753-317
ENSG0000(	412	9.201008	chr17:739	ENSG00000263567		lncRNA	chr17:31762440-317
ENSG0000(	412	9.201008	chr17:739	SLFN12		protein_c	chr17:35410922-354
ENSG0000(	412	9.201008	chr17:739	KRTAP9-2		protein_c	chr17:41226648-412
ENSG0000(	412	9.201008	chr17:739	ENSG00000276054		lncRNA	chr17:37386886-373
ENSG0000(	412	9.201008	chr17:739	ENSG00000265845		lncRNA	chr17:28926275-289

ENSG0000(	412	9.201008	chr17:739	ENSG00000266987		lncRNA	chr17:30144062-301
ENSG0000(	412	9.201008	chr17:739	EFCAB5		protein_c	chr17:29929200-301
ENSG0000(	412	9.201008	chr17:739	PCGF2		protein_c	chr17:38733898-387
ENSG0000(	412	9.201008	chr17:739	AC107993.1		smallRNA	chr17:42399009-423
ENSG0000(	412	9.201008	chr17:739	ENSG00000266981		lncRNA	chr17:35188522-351
ENSG0000(	412	9.201008	chr17:739	KRTAP9-10P		Pseudogen	chr17:41280604-412
ENSG0000(	412	9.201008	chr17:739	TMEM98		protein_c	chr17:32927910-329
ENSG0000(	412	9.201008	chr17:739	TEFM		protein_c	chr17:30897336-309
ENSG0000(	412	9.201008	chr17:739	LYZL6		protein_c	chr17:35934518-359
ENSG0000(	412	9.201008	chr17:739	GPR160P2		Pseudogen	chr17:31759795-317
ENSG0000(	412	9.201008	chr17:739	RN7SKP274		smallRNA	chr17:35889558-358
ENSG0000(	412	9.201008	chr17:739	ENSG00000285559		lncRNA	chr17:34937306-349
ENSG0000(	412	9.201008	chr17:739	ENSG00000266947		lncRNA	chr17:35231450-352
ENSG0000(	412	9.201008	chr17:739	AK4P1		Pseudogen	chr17:31345519-313
ENSG0000(	412	9.201008	chr17:739	RNU2-32P		smallRNA	chr17:41467956-414
ENSG0000(	412	9.201008	chr17:739	RNA5SP442		Pseudogen	chr17:41718154-417
ENSG0000(	412	9.201008	chr17:739	AC005288.1		smallRNA	chr17:39297405-392
ENSG0000(	412	9.201008	chr17:739	RNU6-981P		smallRNA	chr17:39482486-394
ENSG0000(	412	9.201008	chr17:739	ENSG00000265908		lncRNA	chr17:28944796-289
ENSG0000(	412	9.201008	chr17:739	RNA5SP440		Pseudogen	chr17:38731802-387
ENSG0000(	412	9.201008	chr17:739	ENSG00000278977		TEC	chr17:31533171-315
ENSG0000(	412	9.201008	chr17:739	MIR4734		smallRNA	chr17:38702262-387
ENSG0000(	412	9.201008	chr17:739	Y_RNA		smallRNA	chr17:32830219-328
ENSG0000(	412	9.201008	chr17:739	ENSG00000267035		lncRNA	chr17:35540039-355
ENSG0000(	412	9.201008	chr17:739	KRTAP2-5P		Pseudogen	chr17:41071946-410
ENSG0000(	412	9.201008	chr17:739	ENSG00000290404		lncRNA	chr17:30557112-305
ENSG0000(	412	9.201008	chr17:739	CTB-91J4.3		Pseudogen	chr17:36378007-363
ENSG0000(	412	9.201008	chr17:739	ENSG00000267758		lncRNA	chr17:42268587-422
ENSG0000(	412	9.201008	chr17:739	SYNRG	NCGv7	protein_c	chr17:37514807-376
ENSG0000(	412	9.201008	chr17:739	AP2B1		protein_c	chr17:35578046-357
ENSG0000(	412	9.201008	chr17:739	ENSG00000265614		Pseudogen	chr17:34331862-343
ENSG0000(	412	9.201008	chr17:739	ENSG00000279762		lncRNA	chr17:32423971-324
ENSG0000(	412	9.201008	chr17:739	ENSG00000265775		lncRNA	chr17:34118347-341
ENSG0000(	412	9.201008	chr17:739	ENSG00000263370		lncRNA	chr17:29639627-296
ENSG0000(	412	9.201008	chr17:739	Y_RNA		smallRNA	chr17:32463374-324
ENSG0000(	412	9.201008	chr17:739	ENSG00000265784		lncRNA	chr17:38918801-389
ENSG0000(	412	9.201008	chr17:739	ENSG00000267782		lncRNA	chr17:35164243-351
ENSG0000(	412	9.201008	chr17:739	NEUROD2		protein_c	chr17:39603536-396
ENSG0000(	412	9.201008	chr17:739	ENSG00000265791		lncRNA	chr17:30781493-307
ENSG0000(	412	9.201008	chr17:739	ENSG00000267102		Pseudogen	chr17:35409602-354
ENSG0000(	412	9.201008	chr17:739	ENSG00000265794		lncRNA	chr17:32324431-323
ENSG0000(	412	9.201008	chr17:739	ENSG00000265798		Pseudogen	chr17:31038575-310
ENSG0000(	412	9.201008	chr17:739	HSPB9		protein_c	chr17:42122804-421
ENSG0000(	412	9.201008	chr17:739	ENSG00000265799		lncRNA	chr17:40012226-400
ENSG0000(	412	9.201008	chr17:739	ENSG00000278954		TEC	chr17:41665566-416
ENSG0000(	412	9.201008	chr17:739	ENSG00000286194		lncRNA	chr17:30968785-309
ENSG0000(	412	9.201008	chr17:739	ENSG00000290395		lncRNA	chr17:30600796-306
ENSG0000(	412	9.201008	chr17:739	ENSG00000267074		lncRNA	chr17:35499690-355
ENSG0000(	412	9.201008	chr17:739	ENSG00000286443		lncRNA	chr17:39086976-390
ENSG0000(	412	9.201008	chr17:739	ENSG00000266718		lncRNA	chr17:32495536-325
ENSG0000(	412	9.201008	chr17:739	PSMB3		protein_c	chr17:38752741-387

ENSG0000(	412	9.201008	chr17:739	E2F3P1		Pseudoger	chr17:35490009-354
ENSG0000(	412	9.201008	chr17:739	ORMDL3	DriverDB\	protein_c	chr17:39921041-399
ENSG0000(	412	9.201008	chr17:739	ENSG00000263369		Pseudoger	chr17:32106330-321
ENSG0000(	412	9.201008	chr17:739	KRT33A		protein_c	chr17:41346092-413
ENSG0000(	412	9.201008	chr17:739	HMGB1P24		Pseudoger	chr17:37143607-371
ENSG0000(	412	9.201008	chr17:739	ENSG00000278834		lncRNA	chr17:40648030-406
ENSG0000(	412	9.201008	chr17:739	ENSG00000280245		TEC	chr17:32504567-325
ENSG0000(	412	9.201008	chr17:739	GJD3-AS1		lncRNA	chr17:40360655-403
ENSG0000(	412	9.201008	chr17:739	RPL21P123		Pseudoger	chr17:29716279-297
ENSG0000(	412	9.201008	chr17:739	CCL13		protein_c	chr17:34356480-343
ENSG0000(	412	9.201008	chr17:739	ENSG00000264083		lncRNA	chr17:32430967-324
ENSG0000(	412	9.201008	chr17:739	KRTAP4-3	NCGv7	protein_c	chr17:41167231-411
ENSG0000(	412	9.201008	chr17:739	KRTAP9-1		protein_c	chr17:41189887-411
ENSG0000(	412	9.201008	chr17:739	ENSG00000225582		lncRNA	chr17:34479272-344
ENSG0000(	412	9.201008	chr17:739	ENSG00000266371		lncRNA	chr17:31133182-311
ENSG0000(	412	9.201008	chr17:739	ENSG00000265046		lncRNA	chr17:31830731-318
ENSG0000(	412	9.201008	chr17:739	BLMH		protein_c	chr17:30248203-302
ENSG0000(	412	9.201008	chr17:739	ENSG00000278546		lncRNA	chr17:31560132-315
ENSG0000(	412	9.201008	chr17:739	KRT43P		Pseudoger	chr17:41448183-414
ENSG0000(	412	9.201008	chr17:739	ACACA		protein_c	chr17:37084992-374
ENSG0000(	412	9.201008	chr17:739	CPD		protein_c	chr17:30378927-304
ENSG0000(	412	9.201008	chr17:739	ENSG00000277501		lncRNA	chr17:37642947-376
ENSG0000(	412	9.201008	chr17:739	CCL23		protein_c	chr17:36013056-360
ENSG0000(	412	9.201008	chr17:739	ENSG00000242660		Pseudoger	chr17:35566517-355
ENSG0000(	412	9.201008	chr17:739	GOSR1		protein_c	chr17:30477362-305
ENSG0000(	412	9.201008	chr17:739	LINC00672		protein_c	chr17:38925168-389
ENSG0000(	412	9.201008	chr17:739	DHRS11		protein_c	chr17:36591879-366
ENSG0000(	412	9.201008	chr17:739	ENSG00000264497		Pseudoger	chr17:32024469-320
ENSG0000(	412	9.201008	chr17:739	ENSG00000277579		lncRNA	chr17:37034668-370
ENSG0000(	412	9.201008	chr17:739	ENSG00000263860		lncRNA	chr17:30550493-305
ENSG0000(	412	9.201008	chr17:739	ENSG00000276810		Pseudoger	chr17:37162616-371
ENSG0000(	412	9.201008	chr17:739	ZNF385C		protein_c	chr17:42025576-420
ENSG0000(	412	9.201008	chr17:739	EVI2B		protein_c	chr17:31303770-313
ENSG0000(	412	9.201008	chr17:739	UTP6		protein_c	chr17:31860904-319
ENSG0000(	412	9.201008	chr17:739	KRTAP9-4		protein_c	chr17:41249687-412
ENSG0000(	412	9.201008	chr17:739	SRCIN1		protein_c	chr17:38530031-386
ENSG0000(	412	9.201008	chr17:739	ENSG00000291165		lncRNA	chr17:40717234-407
ENSG0000(	412	9.201008	chr17:739	SLC6A4		protein_c	chr17:30194319-302
ENSG0000(	412	9.201008	chr17:739	ENSG00000270240		lncRNA	chr17:35868967-358
ENSG0000(	412	9.201008	chr17:739	Y_RNA		smallRNA	chr17:30344503-303
ENSG0000(	412	9.201008	chr17:739	TBC1D3F		protein_c	chr17:37924426-379
ENSG0000(	412	9.201008	chr17:739	C17orf98		protein_c	chr17:38835086-388
ENSG0000(	412	9.201008	chr17:739	ENSG00000274308		Pseudoger	chr17:37140611-371
ENSG0000(	412	9.201008	chr17:739	SNORD7		smallRNA	chr17:35573657-355
ENSG0000(	412	9.201008	chr17:739	DDX52		protein_c	chr17:37609739-376
ENSG0000(	412	9.201008	chr17:739	ENSG00000273965		lncRNA	chr17:37407936-374
ENSG0000(	412	9.201008	chr17:739	GPR179		protein_c	chr17:38324571-383
ENSG0000(	412	9.201008	chr17:739	RNU6-298P		smallRNA	chr17:30861843-308
ENSG0000(	412	9.201008	chr17:739	ENSG00000266385		lncRNA	chr17:32411217-324
ENSG0000(	412	9.201008	chr17:739	ENSG00000264990		lncRNA	chr17:34176538-341
ENSG0000(	412	9.201008	chr17:739	ENSG00000274756		Pseudoger	chr17:36574462-365

ENSG0000(	412	9.201008	chr17:739	ENSG00000266379		Pseudoger	chr17:32088161-320
ENSG0000(	412	9.201008	chr17:739	ENSG00000280295		TEC	chr17:42233832-422
ENSG0000(	412	9.201008	chr17:739	CCL4L1		protein_c	chr17:36210908-362
ENSG0000(	412	9.201008	chr17:739	SLFN5		protein_c	chr17:35243071-352
ENSG0000(	412	9.201008	chr17:739	RDM1		protein_c	chr17:35918066-359
ENSG0000(	412	9.201008	chr17:739	KRT12		protein_c	chr17:40861303-408
ENSG0000(	412	9.201008	chr17:739	ENSG00000266599		lncRNA	chr17:32518953-325
ENSG0000(	412	9.201008	chr17:739	RFFL		protein_c	chr17:35005990-350
ENSG0000(	412	9.201008	chr17:739	ENSG00000274341		lncRNA	chr17:32408013-324
ENSG0000(	412	9.201008	chr17:739	ENSG00000108516		Pseudoger	chr17:41334632-413
ENSG0000(	412	9.201008	chr17:739	KRT39		protein_c	chr17:40958417-409
ENSG0000(	412	9.201008	chr17:739	SNORD63		smallRNA	chr17:30246757-302
ENSG0000(	412	9.201008	chr17:739	ENSG00000279431		TEC	chr17:30551193-305
ENSG0000(	412	9.201008	chr17:739	ENSG00000276170		lncRNA	chr17:38450394-384
ENSG0000(	412	9.201008	chr17:739	SLFN12L		protein_c	chr17:35464249-355
ENSG0000(	412	9.201008	chr17:739	Y_RNA		smallRNA	chr17:38231043-382
ENSG0000(	412	9.201008	chr17:739	SUZ12P1		Pseudoger	chr17:30731886-307
ENSG0000(	412	9.201008	chr17:739	KRT223P		Pseudoger	chr17:40719034-407
ENSG0000(	412	9.201008	chr17:739	LRRC37A11P		lncRNA	chr17:39029906-390
ENSG0000(	412	9.201008	chr17:739	KRT41P		Pseudoger	chr17:41406512-414
ENSG0000(	412	9.201008	chr17:739	ENSG00000264968		lncRNA	chr17:39927742-399
ENSG0000(	412	9.201008	chr17:739	ENSG00000238007		lncRNA	chr17:29012865-290
ENSG0000(	412	9.201008	chr17:739	CISD3		protein_c	chr17:38730341-387
ENSG0000(	412	9.201008	chr17:739	MIR4727		smallRNA	chr17:38825838-388
ENSG0000(	412	9.201008	chr17:739	ENSG00000273736		Pseudoger	chr17:35963512-359
ENSG0000(	412	9.201008	chr17:739	KRTAP9-8		protein_c	chr17:41237999-412
ENSG0000(	412	9.201008	chr17:739	ENSG00000264458		lncRNA	chr17:32627739-326
ENSG0000(	412	9.201008	chr17:739	ENSG00000266340		lncRNA	chr17:30978610-309
ENSG0000(	412	9.201008	chr17:739	MIR4726		smallRNA	chr17:38719691-387
ENSG0000(	412	9.201008	chr17:739	RPL35AP35		Pseudoger	chr17:29340482-293
ENSG0000(	412	9.201008	chr17:739	ENSG00000264456		lncRNA	chr17:30971652-309
ENSG0000(	412	9.201008	chr17:739	YWHAEP7		Pseudoger	chr17:37854303-378
ENSG0000(	412	9.201008	chr17:739	RAD51D		protein_c	chr17:35092221-351
ENSG0000(	412	9.201008	chr17:739	MIR4733HG		lncRNA	chr17:31090787-310
ENSG0000(	412	9.201008	chr17:739	AC073508.1		smallRNA	chr17:40665624-406
ENSG0000(	412	9.201008	chr17:739	CCL2		protein_c	chr17:34255274-342
ENSG0000(	412	9.201008	chr17:739	MIR4728		smallRNA	chr17:39726495-397
ENSG0000(	412	9.201008	chr17:739	KRTAP4-16		protein_c	chr17:41101502-411
ENSG0000(	412	9.201008	chr17:739	CCL8		protein_c	chr17:34319435-343
ENSG0000(	412	9.201008	chr17:739	MYO19		protein_c	chr17:36495636-365
ENSG0000(	412	9.201008	chr17:739	MSL1	DriverDBv	protein_c	chr17:40121971-401
ENSG0000(	412	9.201008	chr17:739	ENSG00000266469		lncRNA	chr17:39401793-394
ENSG0000(	412	9.201008	chr17:739	ENSG00000289211		lncRNA	chr17:35242433-352
ENSG0000(	412	9.201008	chr17:739	CDK12	NCGv7;AC	protein_c	chr17:39461486-395
ENSG0000(	412	9.201008	chr17:739	TMEM132E		protein_c	chr17:34579487-346
ENSG0000(	412	9.201008	chr17:739	ENSG00000266535		lncRNA	chr17:33111858-331
ENSG0000(	412	9.201008	chr17:739	KRTAP4-1		protein_c	chr17:41184100-411
ENSG0000(	412	9.201008	chr17:739	RNY4P13		smallRNA	chr17:30059052-300
ENSG0000(	412	9.201008	chr17:739	TBC1D3C	AC	protein_c	chr17:36377648-363
ENSG0000(	412	9.201008	chr17:739	GGNBP2		protein_c	chr17:36544912-365
ENSG0000(	412	9.201008	chr17:739	CCL7		protein_c	chr17:34270221-342

ENSG0000(	412	9.201008	chr17:739	TWF1P1		Pseudoger	chr17:29203426-292
ENSG0000(	412	9.201008	chr17:739	C17orf78		protein_c	chr17:37375985-373
ENSG0000(	412	9.201008	chr17:739	KRTAP4-9		protein_c	chr17:41105389-411
ENSG0000(	412	9.201008	chr17:739	RN7SL102P		smallRNA	chr17:38621200-386
ENSG0000(	412	9.201008	chr17:739	KRT35		protein_c	chr17:41476710-414
ENSG0000(	412	9.201008	chr17:739	HNF1B	NCGv7	protein_c	chr17:37686431-377
ENSG0000(	412	9.201008	chr17:739	AC024610.1		smallRNA	chr17:33874318-338
ENSG0000(	412	9.201008	chr17:739	RNU6-840P		smallRNA	chr17:35093909-350
ENSG0000(	412	9.201008	chr17:739	RN7SL871P		smallRNA	chr17:41837064-418
ENSG0000(	412	9.201008	chr17:739	C17orf75		protein_c	chr17:32324565-323
ENSG0000(	412	9.201008	chr17:739	KRTAP16-1		protein_c	chr17:41307700-413
ENSG0000(	412	9.201008	chr17:739	KRTAP29-1		protein_c	chr17:41301826-413
ENSG0000(	412	9.201008	chr17:739	KRTAP9-6		protein_c	chr17:41265378-412
ENSG0000(	412	9.201008	chr17:739	ENSG00000290928		lncRNA	chr17:30709299-307
ENSG0000(	412	9.201008	chr17:739	RDM1P5		Pseudoger	chr17:39057019-391
ENSG0000(	412	9.201008	chr17:739	KRTAP4-11		protein_c	chr17:41117181-411
ENSG0000(	412	9.201008	chr17:739	KRTAP2-3		protein_c	chr17:41059240-410
ENSG0000(	412	9.201008	chr17:739	CTD-2132N18.4		lncRNA	chr17:42038232-420
ENSG0000(	412	9.201008	chr17:739	KRTAP3-3		protein_c	chr17:40993430-409
ENSG0000(	412	9.201008	chr17:739	KRTAP3-2		protein_c	chr17:40999193-409
ENSG0000(	412	9.201008	chr17:739	KRTAP3-1		protein_c	chr17:41008521-410
ENSG0000(	412	9.201008	chr17:739	KRT17		protein_c	chr17:41619442-416
ENSG0000(	412	9.201008	chr17:739	ENSG00000275532		lncRNA	chr17:38703472-387
ENSG0000(	412	9.201008	chr17:739	PSMD11		protein_c	chr17:32444379-324
ENSG0000(	412	9.201008	chr17:739	ENSG00000265115		lncRNA	chr17:33627027-336
ENSG0000(	412	9.201008	chr17:739	ENSG00000265118		protein_c	chr17:31305213-313
ENSG0000(	412	9.201008	chr17:739	LINC01989		lncRNA	chr17:34169372-341
ENSG0000(	412	9.201008	chr17:739	ENSG00000265125		lncRNA	chr17:33565764-336
ENSG0000(	412	9.201008	chr17:739	ASIC2		protein_c	chr17:33013087-341
ENSG0000(	412	9.201008	chr17:739	ENSG00000265139		lncRNA	chr17:32328441-323
ENSG0000(	412	9.201008	chr17:739	ENSG00000264488		lncRNA	chr17:40803744-408
ENSG0000(	412	9.201008	chr17:739	SLC35G3		protein_c	chr17:35192520-351
ENSG0000(	412	9.201008	chr17:739	ENSG00000261499		Pseudoger	chr17:38195880-382
ENSG0000(	412	9.201008	chr17:739	ENSG00000282738		Pseudoger	chr17:36161607-361
ENSG0000(	412	9.201008	chr17:739	CSF3	AC	protein_c	chr17:40015361-400
ENSG0000(	412	9.201008	chr17:739	FBXL20	DriverDBv	protein_c	chr17:39252663-394
ENSG0000(	412	9.201008	chr17:739	KRT8P34		Pseudoger	chr17:39835037-398
ENSG0000(	412	9.201008	chr17:739	ENSG00000259623		lncRNA	chr17:41848518-418
ENSG0000(	412	9.201008	chr17:739	RNA5SP440		smallRNA	chr17:38731802-387
ENSG0000(	412	9.201008	chr17:739	ODAD4		protein_c	chr17:41930617-419
ENSG0000(	412	9.201008	chr17:739	GAS2L2		protein_c	chr17:35744511-357
ENSG0000(	412	9.201008	chr17:739	DPRXP4		Pseudoger	chr17:30975387-309
ENSG0000(	412	9.201008	chr17:739	PPIAP54		Pseudoger	chr17:40367952-403
ENSG0000(	412	9.201008	chr17:739	CCT6B	NCGv7	protein_c	chr17:34927859-349
ENSG0000(	412	9.201008	chr17:739	SEZ6		protein_c	chr17:28954901-290
ENSG0000(	412	9.201008	chr17:739	KRTAP9-7		protein_c	chr17:41275698-412
ENSG0000(	412	9.201008	chr17:739	ENSG00000276250		lncRNA	chr17:30803654-308
ENSG0000(	412	9.201008	chr17:739	ENSG00000264050		Pseudoger	chr17:29197071-291
ENSG0000(	412	9.201008	chr17:739	MIR632		smallRNA	chr17:32350109-323
ENSG0000(	412	9.201008	chr17:739	ADAP2		protein_c	chr17:30906344-309
ENSG0000(	412	9.201008	chr17:739	RNA5SP439		Pseudoger	chr17:36491199-364

ENSG0000(	412	9.201008	chr17:739	ENSG00000264647		lncRNA	chr17:29591703-295
ENSG0000(	412	9.201008	chr17:739	SLFN14		protein_c	chr17:35543985-355
ENSG0000(	412	9.201008	chr17:739	ENSG00000264643		lncRNA	chr17:33680577-336
ENSG0000(	412	9.201008	chr17:739	ENSG00000234859		lncRNA	chr17:41402416-414
ENSG0000(	412	9.201008	chr17:739	ENSG00000278638		lncRNA	chr17:37045228-370
ENSG0000(	412	9.201008	chr17:739	ENSG00000275431		lncRNA	chr17:35983656-359
ENSG0000(	412	9.201008	chr17:739	PSMD3	DriverDBv	protein_c	chr17:39980807-399
ENSG0000(	412	9.201008	chr17:739	ENSG00000276241		lncRNA	chr17:36116177-361
ENSG0000(	412	9.201008	chr17:739	ENSG00000274244		lncRNA	chr17:37649133-376
ENSG0000(	412	9.201008	chr17:739	ENSG00000243423		Pseudoger	chr17:35076737-350
ENSG0000(	412	9.201008	chr17:739	CASC3	NCGv7	protein_c	chr17:40140318-401
ENSG0000(	412	9.201008	chr17:739	ENSG00000266448		Pseudoger	chr17:31709568-317
ENSG0000(	412	9.201008	chr17:739	PPP1R1B		protein_c	chr17:39626740-396
ENSG0000(	412	9.201008	chr17:739	KRT23		protein_c	chr17:40922700-409
ENSG0000(	412	9.201008	chr17:739	ENSG00000283417		lncRNA	chr17:33791838-338
ENSG0000(	412	9.201008	chr17:739	SOCS7		protein_c	chr17:38351844-384
ENSG0000(	412	9.201008	chr17:739	ACLY		protein_c	chr17:41866917-419
ENSG0000(	412	9.201008	chr17:739	ENSG00000270871		lncRNA	chr17:35816717-358
ENSG0000(	412	9.201008	chr17:739	RASL10B		protein_c	chr17:35731639-357
ENSG0000(	412	9.201008	chr17:739	ENSG00000270894		lncRNA	chr17:35818399-358
ENSG0000(	412	9.201008	chr17:739	NUFIP2		protein_c	chr17:29255839-292
ENSG0000(	412	9.201008	chr17:739	GAST		protein_c	chr17:41712331-417
ENSG0000(	412	9.201008	chr17:739	CRYBA1		protein_c	chr17:29246859-292
ENSG0000(	412	9.201008	chr17:739	Y_RNA		smallRNA	chr17:28834722-288
ENSG0000(	412	9.201008	chr17:739	RPL19	DriverDBv	protein_c	chr17:39200283-392
ENSG0000(	412	9.201008	chr17:739	ABHD15-AS1		lncRNA	chr17:29560547-297
ENSG0000(	412	9.201008	chr17:739	ENSG00000270829		lncRNA	chr17:35808489-358
ENSG0000(	412	9.201008	chr17:739	LINC00974		lncRNA	chr17:41549606-415
ENSG0000(	412	9.201008	chr17:739	AC104996.1		smallRNA	chr17:30012033-300
ENSG0000(	412	9.201008	chr17:739	RAB11FIP4	DriverDBv	protein_c	chr17:31391675-315
ENSG0000(	412	9.201008	chr17:739	TBC1D3H		protein_c	chr17:36377531-363
ENSG0000(	412	9.201008	chr17:739	MRM1		protein_c	chr17:36601583-366
ENSG0000(	412	9.201008	chr17:739	KRTAP4-17P		Pseudoger	chr17:41186944-411
ENSG0000(	412	9.201008	chr17:739	MIR193A		smallRNA	chr17:31559996-315
ENSG0000(	412	9.201008	chr17:739	KRT34		protein_c	chr17:41377669-413
ENSG0000(	412	9.201008	chr17:739	RP11-115K3.1		lncRNA	chr17:37745215-377
ENSG0000(	412	9.201008	chr17:739	KRT33B		protein_c	chr17:41363498-413
ENSG0000(	412	9.201008	chr17:739	RNA5SP439		smallRNA	chr17:36491199-364
ENSG0000(	412	9.201008	chr17:739	TNS4	NCGv7;AC	protein_c	chr17:40475828-405
ENSG0000(	412	9.201008	chr17:739	TOP2A	IntOGen-I	protein_c	chr17:40388525-404
ENSG0000(	412	9.201008	chr17:739	C17orf50		protein_c	chr17:35760887-357
ENSG0000(	412	9.201008	chr17:739	KRTAP4-7		protein_c	chr17:41084150-410
ENSG0000(	412	9.201008	chr17:739	STARD3	DriverDBv	protein_c	chr17:39637090-396
ENSG0000(	412	9.201008	chr17:739	RARA	NCGv7;AC	protein_c	chr17:40309180-403
ENSG0000(	412	9.201008	chr17:739	RAPGEFL1	DriverDBv	protein_c	chr17:40177010-401
ENSG0000(	412	9.201008	chr17:739	PIGW		protein_c	chr17:36534987-365
ENSG0000(	412	9.201008	chr17:739	ALOX12P1		Pseudoger	chr17:30529688-305
ENSG0000(	412	9.201008	chr17:739	ENSG00000264066		lncRNA	chr17:28860993-288
ENSG0000(	412	9.201008	chr17:739	LRRC37A9P		Pseudoger	chr17:35912635-359
ENSG0000(	412	9.201008	chr17:739	ENSG00000278668		lncRNA	chr17:32410159-324
ENSG0000(	412	9.201008	chr17:739	TLK2P1		Pseudoger	chr17:34036681-340

ENSG0000(	412	9.201008	chr17:739	KRT37		protein_c	chr17:41420547-414
ENSG0000(	412	9.201008	chr17:739	AC068669.1		smallRNA	chr17:40150136-401
ENSG0000(	412	9.201008	chr17:739	ENSG00000264622		lncRNA	chr17:34837871-348
ENSG0000(	412	9.201008	chr17:739	ENSG00000280349		TEC	chr17:33099969-331
ENSG0000(	412	9.201008	chr17:739	LRRC3C	DriverDBv	protein_c	chr17:39927732-399
ENSG0000(	412	9.201008	chr17:739	ENSG00000277426		Pseudoger	chr17:39023394-390
ENSG0000(	412	9.201008	chr17:739	ENSG00000289110		lncRNA	chr17:37488455-374
ENSG0000(	412	9.201008	chr17:739	ENSG00000276755		Pseudoger	chr17:36234447-362
ENSG0000(	412	9.201008	chr17:739	ENSG00000264058		protein_c	chr17:40627356-406
ENSG0000(	412	9.201008	chr17:739	RP11-1407015.3		Pseudoger	chr17:37974083-379
ENSG0000(	412	9.201008	chr17:739	ENSG00000264598		lncRNA	chr17:33052107-330
ENSG0000(	412	9.201008	chr17:739	ENSG00000226117		Pseudoger	chr17:39839369-398
ENSG0000(	412	9.201008	chr17:739	HEATR9		protein_c	chr17:35854946-358
ENSG0000(	412	9.201008	chr17:739	CACNB1	DriverDBv	protein_c	chr17:39173453-391
ENSG0000(	412	9.201008	chr17:739	FBXO47		protein_c	chr17:38936432-389
ENSG0000(	412	9.201008	chr17:739	ENSG00000259928		lncRNA	chr17:30564057-305
ENSG0000(	412	9.201008	chr17:739	NF1	NCGv7;AC	protein_c	chr17:31094927-313
ENSG0000(	412	9.201008	chr17:739	PIP4K2B		protein_c	chr17:38765691-388
ENSG0000(	412	9.201008	chr17:739	RN7SL316P		smallRNA	chr17:30702508-307
ENSG0000(	412	9.201008	chr17:739	ENSG00000278690		lncRNA	chr17:36012504-360
ENSG0000(	412	9.201008	chr17:739	MIR4724		smallRNA	chr17:31534883-315
ENSG0000(	412	9.201008	chr17:739	KRTAP1-1		protein_c	chr17:41040541-410
ENSG0000(	412	9.201008	chr17:739	ENSG00000266588		lncRNA	chr17:39003248-390
ENSG0000(	412	9.201008	chr17:739	ENSG00000234477		lncRNA	chr17:40921430-409
ENSG0000(	412	9.201008	chr17:739	SH3GL1P2		Pseudoger	chr17:30624413-306
ENSG0000(	412	9.201008	chr17:739	TBC1D3E		protein_c	chr17:36165740-361
ENSG0000(	412	9.201008	chr17:739	RN7SL45P		smallRNA	chr17:31518432-315
ENSG0000(	412	9.201008	chr17:739	ENSG00000270977		Pseudoger	chr17:35893707-359
ENSG0000(	412	9.201008	chr17:739	TCAP	DriverDBv	protein_c	chr17:39665349-396
ENSG0000(	412	9.201008	chr17:739	CCL3		protein_c	chr17:36088256-360
ENSG0000(	412	9.201008	chr17:739	TADA2A		protein_c	chr17:37406886-374
ENSG0000(	412	9.201008	chr17:739	ENSG00000264791		lncRNA	chr17:34142947-341
ENSG0000(	412	9.201008	chr17:739	ENSG00000290858		lncRNA	chr17:31041088-310
ENSG0000(	412	9.201008	chr17:739	FLOT2		protein_c	chr17:28879335-288
ENSG0000(	412	9.201008	chr17:739	ERAL1		protein_c	chr17:28855010-288
ENSG0000(	412	9.201008	chr17:739	ENSG00000279806		TEC	chr17:40473554-404
ENSG0000(	412	9.201008	chr17:739	YWHAEP7		lncRNA	chr17:37828305-378
ENSG0000(	412	9.201008	chr17:739	TAF15	NCGv7;AC	protein_c	chr17:35809482-358
ENSG0000(	412	9.201008	chr17:739	ENSG00000264808		lncRNA	chr17:29333910-293
ENSG0000(	412	9.201008	chr17:739	ENSG00000274767		lncRNA	chr17:36183235-361
ENSG0000(	412	9.201008	chr17:739	RNF135		protein_c	chr17:30970984-309
ENSG0000(	412	9.201008	chr17:739	ENSG00000263990		lncRNA	chr17:31873926-318
ENSG0000(	412	9.201008	chr17:739	KRT42P		lncRNA	chr17:41626327-416
ENSG0000(	412	9.201008	chr17:739	KRT25		protein_c	chr17:40748021-407
ENSG0000(	412	9.201008	chr17:739	KRTAP3-4P		Pseudoger	chr17:41004481-410
ENSG0000(	412	9.201008	chr17:739	MYO18A		protein_c	chr17:29071122-291
ENSG0000(	412	9.201008	chr17:739	KRTAP9-3		protein_c	chr17:41232449-412
ENSG0000(	412	9.201008	chr17:739	KRTAP4-8		protein_c	chr17:41096981-410
ENSG0000(	412	9.201008	chr17:739	KRTAP1-4		protein_c	chr17:41029351-410
ENSG0000(	412	9.201008	chr17:739	ENSG00000283381		Pseudoger	chr17:33692229-336
ENSG0000(	412	9.201008	chr17:739	KRT17P3		Pseudoger	chr17:30567700-305

ENSG0000(	412	9.201008	chr17:739	ENSG00000289011		lncRNA	chr17:36475918-364
ENSG0000(	412	9.201008	chr17:739	MIR365BHG		lncRNA	chr17:31571142-315
ENSG0000(	412	9.201008	chr17:739	LASP1	AC	protein_c	chr17:38869859-389
ENSG0000(	412	9.201008	chr17:739	AC116407.1		smallRNA	chr17:32135729-321
ENSG0000(	412	9.201008	chr17:739	KRT40		protein_c	chr17:40977715-409
ENSG0000(	412	9.201008	chr17:739	TBC1D3B	NCGv7	protein_c	chr17:36165683-361
ENSG0000(	412	9.201008	chr17:739	ENSG00000290974		lncRNA	chr17:32088160-320
ENSG0000(	412	9.201008	chr17:739	KRTAP2-1		protein_c	chr17:41046541-410
ENSG0000(	412	9.201008	chr17:739	GIT1		protein_c	chr17:29573475-295
ENSG0000(	412	9.201008	chr17:739	PLXDC1	NCGv7	protein_c	chr17:39063313-391
ENSG0000(	412	9.201008	chr17:739	CWC25		protein_c	chr17:38800441-388
ENSG0000(	412	9.201008	chr17:739	ENSG00000265359		lncRNA	chr17:40850800-408
ENSG0000(	412	9.201008	chr17:739	ENSG00000275665		lncRNA	chr17:38715328-387
ENSG0000(	412	9.201008	chr17:739	KRT16		protein_c	chr17:41609778-416
ENSG0000(	412	9.201008	chr17:739	THRA	DriverDBv	protein_c	chr17:40058290-400
ENSG0000(	412	9.201008	chr17:739	ENSG00000279674		TEC	chr17:34000789-340
ENSG0000(	412	9.201008	chr17:739	ENSG00000289252		lncRNA	chr17:41621696-416
ENSG0000(	412	9.201008	chr17:739	RAB5C		protein_c	chr17:42124978-421
ENSG0000(	412	9.201008	chr17:739	ENSG00000273576		lncRNA	chr17:39566915-395
ENSG0000(	412	9.201008	chr17:739	AC005549.2		smallRNA	chr17:34183084-341
ENSG0000(	412	9.201008	chr17:739	KRT36		protein_c	chr17:41486136-414
ENSG0000(	412	9.201008	chr17:739	ENSG00000279781		TEC	chr17:32518322-325
ENSG0000(	412	9.201008	chr17:739	ENSG00000277511		lncRNA	chr17:32127570-321
ENSG0000(	412	9.201008	chr17:739	HAP1		protein_c	chr17:41717742-417
ENSG0000(	412	9.201008	chr17:739	ENSG00000287506		lncRNA	chr17:32258615-322
ENSG0000(	412	9.201008	chr17:739	OOSP1P2		Pseudoger	chr17:32343528-323
ENSG0000(	412	9.201008	chr17:739	ENSG00000265394		lncRNA	chr17:30090366-301
ENSG0000(	412	9.201008	chr17:739	snoU13		smallRNA	chr17:35054388-350
ENSG0000(	412	9.201008	chr17:739	ENSG00000278867		TEC	chr17:32051030-320
ENSG0000(	412	9.201008	chr17:739	MLLT6	AC	protein_c	chr17:38705273-387
ENSG0000(	412	9.201008	chr17:739	ENSG00000263709		lncRNA	chr17:29140483-291
ENSG0000(	412	9.201008	chr17:739	CCR7	NCGv7	protein_c	chr17:40553769-405
ENSG0000(	412	9.201008	chr17:739	ENSG00000265356		lncRNA	chr17:33935437-341
ENSG0000(	412	9.201008	chr17:739	TAOK1		protein_c	chr17:29390363-295
ENSG0000(	412	9.201008	chr17:739	ENSG00000280177		TEC	chr17:39165073-391
ENSG0000(	412	9.201008	chr17:739	CNP		protein_c	chr17:41966763-419
ENSG0000(	412	9.201008	chr17:739	ENSG00000263781		Pseudoger	chr17:29021325-290
ENSG0000(	412	9.201008	chr17:739	KRT224P		Pseudoger	chr17:40687038-406
ENSG0000(	412	9.201008	chr17:739	GSDMA	NCGv7	protein_c	chr17:39953263-399
ENSG0000(	412	9.201008	chr17:739	RNU6-711P		smallRNA	chr17:29424762-294
ENSG0000(	412	9.201008	chr17:739	ENSG00000265334		lncRNA	chr17:30834325-308
ENSG0000(	412	9.201008	chr17:739	KRT24		protein_c	chr17:40697991-407
ENSG0000(	412	9.201008	chr17:739	KRT10-AS1		lncRNA	chr17:40819101-408
ENSG0000(	412	9.201008	chr17:739	DHX58	NCGv7	protein_c	chr17:42101404-421
ENSG0000(	412	9.201008	chr17:739	ENSG00000265337		lncRNA	chr17:32529008-325
ENSG0000(	412	9.201008	chr17:739	CRLF3		protein_c	chr17:30769388-308
ENSG0000(	412	9.201008	chr17:739	ENSG00000271268		Pseudoger	chr17:35861093-358
ENSG0000(	412	9.201008	chr17:739	KRT31		protein_c	chr17:41393721-413
ENSG0000(	412	9.201008	chr17:739	KRT14		protein_c	chr17:41582279-415
ENSG0000(	412	9.201008	chr17:739	JUP	AC	protein_c	chr17:41754604-417
ENSG0000(	412	9.201008	chr17:739	NR1D1	DriverDBv	protein_c	chr17:40092793-401

ENSG0000(	412	9.201008	chr17:739	KAT2A		protein_c	chr17:42113111-421
ENSG0000(	412	9.201008	chr17:739	CCL14		protein_c	chr17:35983288-359
ENSG0000(	412	9.201008	chr17:739	MRPL45		protein_c	chr17:38297023-383
ENSG0000(	412	9.201008	chr17:739	ENSG00000266490		lncRNA	chr17:30792372-307
ENSG0000(	412	9.201008	chr17:739	EIF1		protein_c	chr17:41688885-416
ENSG0000(	412	9.201008	chr17:739	PGAP3	DriverDBv	protein_c	chr17:39671122-396
ENSG0000(	412	9.201008	chr17:739	KRT222	NCGv7	protein_c	chr17:40654665-406
ENSG0000(	412	9.201008	chr17:739	IKZF3	NCGv7	protein_c	chr17:39757718-398
ENSG0000(	412	9.201008	chr17:739	ENSG00000278395		Pseudoger	chr17:36241701-362
ENSG0000(	412	9.201008	chr17:739	ENSG00000274996		lncRNA	chr17:38601049-386
ENSG0000(	412	9.201008	chr17:739	ENSG00000265428		Pseudoger	chr17:38996323-389
ENSG0000(	412	9.201008	chr17:739	ENSG00000278860		TEC	chr17:34614409-346
ENSG0000(	412	9.201008	chr17:739	ABHD15	NCGv7	protein_c	chr17:29560547-295
ENSG0000(	412	9.201008	chr17:739	RPL23AP75		Pseudoger	chr17:40439467-404
ENSG0000(	412	9.201008	chr17:739	ZNHIT3		protein_c	chr17:36486629-364
ENSG0000(	412	9.201008	chr17:739	ENSG00000264290		lncRNA	chr17:29569580-295
ENSG0000(	412	9.201008	chr17:739	ENSG00000264164		Pseudoger	chr17:32083179-320
ENSG0000(	412	9.201008	chr17:739	TMEM132E-DT		lncRNA	chr17:34574123-345
ENSG0000(	412	9.201008	chr17:739	DNAJC7		protein_c	chr17:41976421-420
ENSG0000(	412	9.201008	chr17:739	TBC1D3L		protein_c	chr17:37978155-379
ENSG0000(	412	9.201008	chr17:739	RPL23	AC	protein_c	chr17:38847860-388
ENSG0000(	412	9.201008	chr17:739	MED1	NCGv7	protein_c	chr17:39404285-394
ENSG0000(	412	9.201008	chr17:739	ENSG00000273609		Pseudoger	chr17:30617680-306
ENSG0000(	412	9.201008	chr17:739	ENSG00000265443		lncRNA	chr17:30726305-307
ENSG0000(	412	9.201008	chr17:739	MYO1D		protein_c	chr17:32492522-328
ENSG0000(	412	9.201008	chr17:739	EPOP		protein_c	chr17:38671703-386
ENSG0000(	412	9.201008	chr17:739	NKIRAS2		protein_c	chr17:42011382-420
ENSG0000(	412	9.201008	chr17:739	MIR4733		smallRNA	chr17:31094350-310
ENSG0000(	412	9.201008	chr17:739	ENSG00000264304		lncRNA	chr17:28892469-288
ENSG0000(	412	9.201008	chr17:739	ENSG00000287602		lncRNA	chr17:41090338-410
ENSG0000(	412	9.201008	chr17:739	KRT10		protein_c	chr17:40818117-408
ENSG0000(	412	9.201008	chr17:739	TBC1D3JP		Pseudoger	chr17:37978585-379
ENSG0000(	412	9.201008	chr17:739	HCRT		protein_c	chr17:42184060-421
ENSG0000(	412	9.201008	chr17:739	ENSG00000265460		Pseudoger	chr17:39238466-392
ENSG0000(	412	9.201008	chr17:739	TBC1D3G		protein_c	chr17:36377348-364
ENSG0000(	412	9.201008	chr17:739	ENSG00000275613		lncRNA	chr17:36722443-367
ENSG0000(	412	9.201008	chr17:739	NPEPPSP1		Pseudoger	chr17:38195872-382
ENSG0000(	412	9.201008	chr17:739	ENSG00000291030		lncRNA	chr17:35963488-359
ENSG0000(	412	9.201008	chr17:739	KLHL10	NCGv7	protein_c	chr17:41835685-418
ENSG0000(	412	9.201008	chr17:739	RPL9P30		Pseudoger	chr17:29855759-298
ENSG0000(	412	9.201008	chr17:739	KRT26		protein_c	chr17:40766238-407
ENSG0000(	412	9.201008	chr17:739	ENSG00000263717		lncRNA	chr17:32512869-325
ENSG0000(	412	9.201008	chr17:739	ENSG00000264138		Pseudoger	chr17:28848860-288
ENSG0000(	412	9.201008	chr17:739	CDC6	AC	protein_c	chr17:40287879-403
ENSG0000(	412	9.201008	chr17:739	MIR4523		smallRNA	chr17:29390662-293
ENSG0000(	412	9.201008	chr17:739	NPEPPSP1		lncRNA	chr17:38195703-382
ENSG0000(	412	9.201008	chr17:739	CCL5		protein_c	chr17:35871491-358
ENSG0000(	412	9.201008	chr17:739	RNA5SP441		Pseudoger	chr17:40374089-403
ENSG0000(	412	9.201008	chr17:739	ENSG00000264148		Pseudoger	chr17:31008154-310
ENSG0000(	412	9.201008	chr17:739	CDK5R1		protein_c	chr17:32486993-324
ENSG0000(	412	9.201008	chr17:739	DHRS13		protein_c	chr17:28897781-289

ENSG0000(	412	9.201008	chr17:739	CCL18		protein_c	chr17:36064272-360
ENSG0000(	412	9.201008	chr17:739	ENSG00000291063		lncRNA	chr17:31008497-310
ENSG0000(	412	9.201008	chr17:739	MMP28		protein_c	chr17:35756249-357
ENSG0000(	412	9.201008	chr17:739	GHDC		protein_c	chr17:42188799-421
ENSG0000(	412	9.201008	chr17:739	ENSG00000263674		lncRNA	chr17:32280387-322
ENSG0000(	412	9.201008	chr17:739	PEX12		protein_c	chr17:35574795-355
ENSG0000(	412	9.201008	chr17:739	KRTAP2-2		protein_c	chr17:41054498-410
ENSG0000(	412	9.201008	chr17:739	LRRC37B		protein_c	chr17:32007383-320
ENSG0000(	412	9.201008	chr17:739	KRT42P		Pseudoger	chr17:41626536-416
ENSG0000(	412	9.201008	chr17:739	CCL1		protein_c	chr17:34360328-343
ENSG0000(	412	9.201008	chr17:739	OMG		protein_c	chr17:31272013-312
ENSG0000(	412	9.201008	chr17:739	ENSG00000265289		lncRNA	chr17:30059339-300
ENSG0000(	412	9.201008	chr17:739	EVI2A	NCGv7;AC	protein_c	chr17:31316410-313
ENSG0000(	412	9.201008	chr17:739	ENSG00000279668		lncRNA	chr17:33827699-340
ENSG0000(	412	9.201008	chr17:739	ENSG00000287644		lncRNA	chr17:40317698-403
ENSG0000(	412	9.201008	chr17:739	RHOT1		protein_c	chr17:32142454-322
ENSG0000(	412	9.201008	chr17:739	ARGFXP2		Pseudoger	chr17:32150433-321
ENSG0000(	412	9.201008	chr17:739	CCL15-CCL14		protein_c	chr17:35983656-360
ENSG0000(	412	9.201008	chr17:739	ENSG00000244086		Pseudoger	chr17:38855314-388
ENSG0000(	412	9.201008	chr17:739	KRTAP9-11P		Pseudoger	chr17:41271311-412
ENSG0000(	412	9.201008	chr17:739	KRTAP17-1		protein_c	chr17:41314912-413
ENSG0000(	412	9.201008	chr17:739	ENSG00000242439		Pseudoger	chr17:30830901-308
ENSG0000(	412	9.201008	chr17:739	ENSG00000279337		TEC	chr17:30724982-307
ENSG0000(	412	9.201008	chr17:739	STAT5B	NCGv7;AC	protein_c	chr17:42199176-422
ENSG0000(	412	9.201008	chr17:739	UBL5P2		Pseudoger	chr17:32227890-322
ENSG0000(	412	9.201008	chr17:739	KCNH4		protein_c	chr17:42156891-421
ENSG0000(	412	9.201008	chr17:739	ENSG00000236377		lncRNA	chr17:32876726-329
ENSG0000(	412	9.201008	chr17:739	KRT9		protein_c	chr17:41565836-415
ENSG0000(	412	9.201008	chr17:739	PIPOX		protein_c	chr17:28950513-290
ENSG0000(	412	9.201008	chr17:739	WDR45BP1		Pseudoger	chr17:32111562-321
ENSG0000(	412	9.201008	chr17:739	ENSG00000231421		lncRNA	chr17:30573471-305
ENSG0000(	412	9.201008	chr17:739	KRTAP9-12P		Pseudoger	chr17:41212706-412
ENSG0000(	412	9.201008	chr17:739	TP53I13		protein_c	chr17:29566052-295
ENSG0000(	412	9.201008	chr17:739	ZNF207	NCGv7	protein_c	chr17:32350132-323
ENSG0000(	412	9.201008	chr17:739	KRT28		protein_c	chr17:40792196-407
ENSG0000(	412	9.201008	chr17:739	KRT27		protein_c	chr17:40776808-407
ENSG0000(	412	9.201008	chr17:739	CORO6	DriverDB\	protein_c	chr17:29614756-296
ENSG0000(	412	9.201008	chr17:739	ENSG00000264125		lncRNA	chr17:30204318-302
ENSG0000(	412	9.201008	chr17:739	ENSG00000265222		lncRNA	chr17:32509954-325
ENSG0000(	412	9.201008	chr17:739	AATF		protein_c	chr17:36948925-370
ENSG0000(	412	9.201008	chr17:739	LHX1	AC	protein_c	chr17:36936785-369
ENSG0000(	412	9.201008	chr17:739	ENSG00000266642		lncRNA	chr17:28897738-288
ENSG0000(	412	9.201008	chr17:739	RNA5SP437		Pseudoger	chr17:31963805-319
ENSG0000(	412	9.201008	chr17:739	ENSG00000214546		lncRNA	chr17:39619613-396
ENSG0000(	412	9.201008	chr17:739	STAT3	NCGv7;AC	protein_c	chr17:42313324-423
ENSG0000(	412	9.201008	chr17:739	ENSG00000264435		Pseudoger	chr17:29972514-299
ENSG0000(	412	9.201008	chr17:739	SNORA21		smallRNA	chr17:38852863-388
ENSG0000(	412	9.201008	chr17:739	ENSG00000278829		lncRNA	chr17:42272069-422
ENSG0000(	412	9.201008	chr17:739	LRRC37A11P		Pseudoger	chr17:39027277-390
ENSG0000(	412	9.201008	chr17:739	ENSG00000274630		lncRNA	chr17:41867581-418
ENSG0000(	412	9.201008	chr17:739	ENSG00000290975		lncRNA	chr17:32106332-321

ENSG0000(	412	9.201008	chr17:739	ATAD5		protein_c	chr17:30831966-308
ENSG0000(	412	9.201008	chr17:739	KRT20		protein_c	chr17:40875889-408
ENSG0000(	412	9.201008	chr17:739	ENSG00000276707		lncRNA	chr17:36940049-369
ENSG0000(	412	9.201008	chr17:739	LHX1-DT		lncRNA	chr17:36861674-369
ENSG0000(	412	9.201008	chr17:739	ENSG00000264300		Pseudoger	chr17:32003110-320
ENSG0000(	412	9.201008	chr17:739	MED24	DriverDBv	protein_c	chr17:40019097-400
ENSG0000(	412	9.201008	chr17:739	FNDC8		protein_c	chr17:35121615-351
ENSG0000(	412	9.201008	chr17:739	AA06		lncRNA	chr17:33529787-335
ENSG0000(	412	9.201008	chr17:739	RNU6-866P		smallRNA	chr17:38755279-387
ENSG0000(	412	9.201008	chr17:739	KRT13	NCGv7	protein_c	chr17:41500981-415
ENSG0000(	412	9.201008	chr17:739	ANKRD13B		protein_c	chr17:29589769-296
ENSG0000(	412	9.201008	chr17:739	KRT15	NCGv7	protein_c	chr17:41513745-415
ENSG0000(	412	9.201008	chr17:739	ENSG00000264373		Pseudoger	chr17:32434939-324
ENSG0000(	412	9.201008	chr17:739	ENSG00000277911		lncRNA	chr17:36980167-369
ENSG0000(	412	9.201008	chr17:739	ENSG00000273687		lncRNA	chr17:35018660-350
ENSG0000(	412	9.201008	chr17:739	ENSG00000278346		Pseudoger	chr17:40380666-403
ENSG0000(	412	9.201008	chr17:739	TBC1D3K		protein_c	chr17:37924415-379
ENSG0000(	412	9.201008	chr17:739	SMARCE1	NCGv7;AC	protein_c	chr17:40624962-406
ENSG0000(	412	9.201008	chr17:739	KRT19		protein_c	chr17:41523617-415
ENSG0000(	412	9.201008	chr17:739	STAT5A		protein_c	chr17:42287547-423
ENSG0000(	412	9.201008	chr17:739	NLE1		protein_c	chr17:35128730-351
ENSG0000(	412	9.201008	chr17:739	KRT32		protein_c	chr17:41459513-414
ENSG0000(	412	9.201008	chr17:739	TOMM20P2		Pseudoger	chr17:35514766-355
ENSG0000(	412	9.201008	chr17:739	KRTAP2-4		protein_c	chr17:41065116-410
ENSG0000(	412	9.201008	chr17:739	ENSG00000277969		lncRNA	chr17:38702449-387
ENSG0000(	412	9.201008	chr17:739	KLHL11	NCGv7	protein_c	chr17:41848518-418
ENSG0000(	412	9.201008	chr17:739	KRT38		protein_c	chr17:41436154-414
ENSG0000(	412	9.201008	chr17:739	RNY4P8		smallRNA	chr17:40243223-402
ENSG0000(	412	9.201008	chr17:739	ENSG00000271392		lncRNA	chr17:35757199-357
ENSG0000(	412	9.201008	chr17:739	GSDMB	DriverDBv	protein_c	chr17:39904595-399
ENSG0000(	412	9.201008	chr17:739	NPEPPSP1		Pseudoger	chr17:38195740-382
ENSG0000(	412	9.201008	chr17:739	NSRP1	NCGv7	protein_c	chr17:30115521-301
ENSG0000(	412	9.201008	chr17:739	KRTAP4-12		protein_c	chr17:41123091-411
ENSG0000(	412	9.201008	chr17:739	KRTAP4-4		protein_c	chr17:41159649-411
ENSG0000(	411	9.178675	chr17:674	ABCA5		protein_c	chr17:69244311-693
ENSG0000(	411	9.178675	chr17:674	ENSG00000264860		lncRNA	chr17:73164977-731
ENSG0000(	411	9.178675	chr17:674	SOX9-AS1		lncRNA	chr17:72067099-722
ENSG0000(	411	9.178675	chr17:674	ENSG00000264196		lncRNA	chr17:72839039-728
ENSG0000(	411	9.178675	chr17:674	SOX9	NCGv7	protein_c	chr17:72121020-721
ENSG0000(	411	9.178675	chr17:674	ENSG00000278972		TEC	chr17:69433084-694
ENSG0000(	411	9.178675	chr17:674	ENSG00000264750		lncRNA	chr17:73641026-736
ENSG0000(	411	9.178675	chr17:674	LINC00674		Pseudoger	chr17:68101908-681
ENSG0000(	411	9.178675	chr17:674	SLC39A11		protein_c	chr17:72645949-730
ENSG0000(	411	9.178675	chr17:674	KCNJ16		protein_c	chr17:70053429-701
ENSG0000(	411	9.178675	chr17:674	AC011591.1		smallRNA	chr17:68766473-687
ENSG0000(	411	9.178675	chr17:674	LINC02097		lncRNA	chr17:72072333-721
ENSG0000(	411	9.178675	chr17:674	ABCA6		protein_c	chr17:69078702-691
ENSG0000(	411	9.178675	chr17:674	MYL6P5		Pseudoger	chr17:71620978-716
ENSG0000(	411	9.178675	chr17:674	ATG12P1		Pseudoger	chr17:73067870-730
ENSG0000(	411	9.178675	chr17:674	ENSG00000265935		lncRNA	chr17:73662568-736
ENSG0000(	411	9.178675	chr17:674	ABCA10	NCGv7	protein_c	chr17:69147214-692

ENSG0000(	411	9.178675	chr17:674	ROCR		lncRNA	chr17:72021851-720
ENSG0000(	411	9.178675	chr17:674	MIR635		smallRNA	chr17:68424451-684
ENSG0000(	411	9.178675	chr17:674	ABCA9-AS1		lncRNA	chr17:68944531-690
ENSG0000(	411	9.178675	chr17:674	PRO1804		TEC	chr17:69232963-692
ENSG0000(	411	9.178675	chr17:674	RN7SL756P		smallRNA	chr17:67457025-674
ENSG0000(	411	9.178675	chr17:674	ENSG00000279917		TEC	chr17:67611277-676
ENSG0000(	411	9.178675	chr17:674	ENSG00000274561		lncRNA	chr17:68131462-681
ENSG0000(	411	9.178675	chr17:674	SCARNA24		smallRNA	chr17:72818836-728
ENSG0000(	411	9.178675	chr17:674	POLR3KP2		Pseudoger	chr17:73163035-731
ENSG0000(	411	9.178675	chr17:674	NOL11		protein_c	chr17:67717931-677
ENSG0000(	411	9.178675	chr12:149	SNORA70G		smallRNA	chr12:68627234-686
ENSG0000(	411	9.178675	chr17:674	ENSG00000291214		lncRNA	chr17:68101871-681
ENSG0000(	411	9.178675	chr17:674	ENSG00000285931		lncRNA	chr17:69242230-692
ENSG0000(	411	9.178675	chr17:674	ENSG00000264754		lncRNA	chr17:67524481-675
ENSG0000(	411	9.178675	chr17:674	ENSG00000277728		lncRNA	chr17:73202968-732
ENSG0000(	411	9.178675	chr17:674	ENSG00000283517		lncRNA	chr17:71829870-718
ENSG0000(	411	9.178675	chr17:674	RPSAP67		Pseudoger	chr17:67774591-677
ENSG0000(	411	9.178675	chr17:674	LINC02003		lncRNA	chr17:72342692-723
ENSG0000(	411	9.178675	chr17:674	ENSG00000263680		lncRNA	chr17:72425939-724
ENSG0000(	411	9.178675	chr17:674	CPSF4L		protein_c	chr17:73248449-732
ENSG0000(	411	9.178675	chr17:674	ENSG00000277476		lncRNA	chr17:68133201-681
ENSG0000(	411	9.178675	chr17:674	ENSG00000288605		lncRNA	chr17:72033772-722
ENSG0000(	411	9.178675	chr17:674	ENSG00000279880		TEC	chr17:67973934-679
ENSG0000(	411	9.178675	chr17:674	FAM104A		protein_c	chr17:73207353-732
ENSG0000(	411	9.178675	chr17:674	ENSG00000289371		lncRNA	chr17:70191243-701
ENSG0000(	411	9.178675	chr17:674	ABCA9		protein_c	chr17:68974488-690
ENSG0000(	411	9.178675	chr17:674	LINC01497		lncRNA	chr17:69961568-699
ENSG0000(	411	9.178675	chr17:674	ENSG00000271101		Pseudoger	chr17:71011997-710
ENSG0000(	411	9.178675	chr17:674	SNORA38B		smallRNA	chr17:67740669-677
ENSG0000(	411	9.178675	chr17:674	RN7SL622P		smallRNA	chr17:67977698-679
ENSG0000(	411	9.178675	chr17:674	ENSG00000278740		lncRNA	chr17:68188547-681
ENSG0000(	411	9.178675	chr17:674	SLC16A6		protein_c	chr17:68267026-682
ENSG0000(	411	9.178675	chr17:674	C17orf58	DriverDB\	protein_c	chr17:67991099-679
ENSG0000(	411	9.178675	chr17:674	LINC01482		lncRNA	chr17:68591796-687
ENSG0000(	411	9.178675	chr17:674	ENSG00000267184		Pseudoger	chr17:69707193-697
ENSG0000(	411	9.178675	chr17:674	ENSG00000265055		lncRNA	chr17:68096046-681
ENSG0000(	411	9.178675	chr17:674	PRKAR1A	NCGv7;AC	protein_c	chr17:68511780-685
ENSG0000(	411	9.178675	chr17:674	KPNA2	DriverDB\	protein_c	chr17:68035636-680
ENSG0000(	411	9.178675	chr17:674	SNRPGP4		Pseudoger	chr17:69318733-693
ENSG0000(	411	9.178675	chr17:674	FAM20A		protein_c	chr17:68535113-686
ENSG0000(	411	9.178675	chr17:674	COG1		protein_c	chr17:73193055-732
ENSG0000(	411	9.178675	chr17:674	FBXO36P1		Pseudoger	chr17:68095068-680
ENSG0000(	411	9.178675	chr17:674	C17orf80		protein_c	chr17:73232233-732
ENSG0000(	411	9.178675	chr17:674	WIPI1		protein_c	chr17:68420948-684
ENSG0000(	411	9.178675	chr17:674	ENSG00000267653		lncRNA	chr17:69477139-695
ENSG0000(	411	9.178675	chr17:674	ENSG00000266717		lncRNA	chr17:67950885-679
ENSG0000(	411	9.178675	chr17:674	ENSG00000267109		lncRNA	chr17:70312831-703
ENSG0000(	411	9.178675	chr17:674	LINC00511		lncRNA	chr17:72290091-726
ENSG0000(	411	9.178675	chr17:674	MIR4524B		smallRNA	chr17:69099542-690
ENSG0000(	411	9.178675	chr17:674	ENSG00000279573		TEC	chr17:67955314-679
ENSG0000(	411	9.178675	chr17:674	BPTF	NCGv7	protein_c	chr17:67825503-679

ENSG0000(	411	9.178675	chr17:674	ENSG00000265010		lncRNA	chr17:73243093-732
ENSG0000(	411	9.178675	chr17:674	SDK2	NCGv7	protein_c	chr17:73334384-736
ENSG0000(	411	9.178675	chr17:674	AC080037.1		smallRNA	chr17:72714383-727
ENSG0000(	411	9.178675	chr17:674	LINC01028		lncRNA	chr17:70051277-700
ENSG0000(	411	9.178675	chr17:674	ENSG00000278730		lncRNA	chr17:68126666-681
ENSG0000(	411	9.178675	chr17:674	CALM2P1		Pseudoger	chr17:70241324-702
ENSG0000(	411	9.178675	chr17:674	LINC01483		lncRNA	chr17:69577251-699
ENSG0000(	411	9.178675	chr17:674	AC006534.1		smallRNA	chr17:67813582-678
ENSG0000(	411	9.178675	chr17:674	CDC42EP4		protein_c	chr17:73283624-733
ENSG0000(	411	9.178675	chr17:674	Y_RNA		smallRNA	chr17:69985986-699
ENSG0000(	411	9.178675	chr17:674	RNU6-305P		smallRNA	chr17:71311168-713
ENSG0000(	411	9.178675	chr17:674	ENSG00000263893		lncRNA	chr17:72642731-726
ENSG0000(	411	9.178675	chr17:674	AC006534.2		smallRNA	chr17:67843945-678
ENSG0000(	411	9.178675	chr17:674	ENSG00000264985		lncRNA	chr17:73513469-735
ENSG0000(	411	9.178675	chr17:674	ENSG00000290646		lncRNA	chr17:68128340-681
ENSG0000(	411	9.178675	chr17:674	ENSG00000271239		lncRNA	chr17:70778604-707
ENSG0000(	411	9.178675	chr17:674	RPL32P33		Pseudoger	chr17:72627231-726
ENSG0000(	411	9.178675	chr17:674	ABCA8		protein_c	chr17:68867289-689
ENSG0000(	411	9.178675	chr17:674	KCNJ2-AS1		lncRNA	chr17:70166961-701
ENSG0000(	411	9.178675	chr17:674	SEC24AP1		Pseudoger	chr17:69094289-690
ENSG0000(	411	9.178675	chr17:674	ENSG00000267009		lncRNA	chr17:68413623-685
ENSG0000(	411	9.178675	chr17:674	SH3GL1P3		Pseudoger	chr17:68134675-681
ENSG0000(	411	9.178675	chr17:674	ENSG00000283376		Pseudoger	chr17:68691809-687
ENSG0000(	411	9.178675	chr17:674	ENSG00000279361		TEC	chr17:67637774-676
ENSG0000(	411	9.178675	chr17:674	AMZ2	DriverDB\	protein_c	chr17:68205481-682
ENSG0000(	411	9.178675	chr17:674	ENSG00000274712		Pseudoger	chr17:68205669-682
ENSG0000(	411	9.178675	chr17:674	RNU7-155P		smallRNA	chr17:71298156-712
ENSG0000(	411	9.178675	chr17:674	KCNJ2		protein_c	chr17:70168673-701
ENSG0000(	411	9.178675	chr17:674	ENSG00000267250		lncRNA	chr17:68793549-687
ENSG0000(	411	9.178675	chr17:674	ENSG00000290793		lncRNA	chr17:68205489-682
ENSG0000(	411	9.178675	chr17:674	RPL17P41		Pseudoger	chr17:67784753-677
ENSG0000(	411	9.178675	chr17:674	ENSG00000230258		lncRNA	chr17:70017324-701
ENSG0000(	411	9.178675	chr17:674	ENSG00000288109		lncRNA	chr17:67658015-676
ENSG0000(	411	9.178675	chr17:674	ARSG		protein_c	chr17:68259182-684
ENSG0000(	411	9.178675	chr17:674	MAP2K6		protein_c	chr17:69414697-695
ENSG0000(	411	9.178675	chr17:674	MIR548AA2		smallRNA	chr17:67471489-674
ENSG0000(	411	9.178675	chr17:674	SSTR2		protein_c	chr17:73165010-731
ENSG0000(	411	9.178675	chr17:674	AC002539.2		smallRNA	chr17:69948861-699
ENSG0000(	411	9.178675	chr17:674	ENSG00000265100		lncRNA	chr17:68246626-682
ENSG0000(	411	9.178675	chr17:674	LINC01152		lncRNA	chr17:72030291-720
ENSG0000(	411	9.178675	chr17:674	ENSG00000225818		lncRNA	chr17:71596338-715
ENSG0000(	411	9.178675	chr17:674	RDM1P3		Pseudoger	chr17:68152776-681
ENSG0000(	411	9.178675	chr17:674	SNORA40		smallRNA	chr17:69355133-693
ENSG0000(	411	9.178675	chr17:674	CASC17		lncRNA	chr17:71097774-712
ENSG0000(	411	9.178675	chr17:674	ARHGAP27P2		Pseudoger	chr17:68198921-681
ENSG0000(	411	9.178675	chr17:674	ENSG00000267471		Pseudoger	chr17:70628917-706
ENSG0000(	411	9.178675	chr17:674	ENSG00000267461		lncRNA	chr17:68557516-685
ENSG0000(	411	9.178675	chr17:674	LRRC37A16P		Pseudoger	chr17:68125777-681
ENSG0000(	411	9.178675	chr17:674	ENSG00000267731		lncRNA	chr17:68189884-681
ENSG0000(	411	9.178675	chr17:674	ENSG00000286387		lncRNA	chr17:71678899-717
ENSG0000(	410	9.156343	chr6:1391	AL035697.1		smallRNA	chr6:161615066-161

ENSG0000(	410	9.156343	chr6:1391	ENSG00000286805		lncRNA	chr6:161435098-161
ENSG0000(	410	9.156343	chr12:149	ENSG00000280049		TEC	chr12:87041916-870
ENSG0000(	408	9.111678	chr6:1039	FGD2		protein_c	chr6:37005646-3702
ENSG0000(	408	9.111678	chr6:1039	C6orf89		protein_c	chr6:36871870-3692
ENSG0000(	408	9.111678	chr6:1039	RAB44		protein_c	chr6:36697826-3673
ENSG0000(	408	9.111678	chr6:1039	DINOL		lncRNA	chr6:36677609-3667
ENSG0000(	408	9.111678	chr6:1039	ENSG00000285888		lncRNA	chr6:36768485-3677
ENSG0000(	408	9.111678	chr6:1039	ENSG00000220349		Pseudoger	chr6:36737050-3673
ENSG0000(	408	9.111678	chr6:1039	Y_RNA		smallRNA	chr6:36672838-3667
ENSG0000(	408	9.111678	chr6:1039	COX6A1P2		Pseudoger	chr6:37044860-3704
ENSG0000(	408	9.111678	chr6:1039	ENSG00000232598		lncRNA	chr6:36940071-3694
ENSG0000(	408	9.111678	chr6:1039	PANDAR		lncRNA	chr6:36673621-3667
ENSG0000(	408	9.111678	chr6:1039	ENSG00000287891		lncRNA	chr6:36841430-3684
ENSG0000(	408	9.111678	chr6:1039	PPIL1	NCGv7	protein_c	chr6:36854827-3687
ENSG0000(	408	9.111678	chr6:1039	PI16		protein_c	chr6:36948263-3696
ENSG0000(	408	9.111678	chr6:1039	LAP3P2		Pseudoger	chr6:36673817-3667
ENSG0000(	408	9.111678	chr6:1039	MIR3925		smallRNA	chr6:36622436-3662
ENSG0000(	408	9.111678	chr6:1039	SRSF3	NCGv7;AC	protein_c	chr6:36594353-3660
ENSG0000(	408	9.111678	chr6:1039	MTCH1		protein_c	chr6:36965807-3698
ENSG0000(	408	9.111678	chr6:1039	CDKN1A	NCGv7;AC	protein_c	chr6:36676460-3668
ENSG0000(	408	9.111678	chr6:1039	CPNE5	NCGv7	protein_c	chr6:36740775-3683
ENSG0000(	408	9.111678	chr6:1039	RNU1-88P		smallRNA	chr6:36639545-3663
ENSG0000(	407	9.089345	chr7:1588	ZMIZ2		protein_c	chr7:44748581-4476
ENSG0000(	407	9.089345	chr7:1588	H2AZ2-DT		lncRNA	chr7:44848416-4484
ENSG0000(	407	9.089345	chr7:1588	ENSG00000228596		lncRNA	chr7:44785050-4478
ENSG0000(	407	9.089345	chr7:1588	PPIA		protein_c	chr7:44796680-4482
ENSG0000(	407	9.089345	chr7:1588	H2AZ2	NCGv7	protein_c	chr7:44826791-4484
ENSG0000(	406	9.067013	chr6:1039	HLA-DRB6		lncRNA	chr6:32552713-3256
ENSG0000(	406	9.067013	chr6:1039	HLA-DRB1	NCGv7	protein_c	chr6:32577902-3258
ENSG0000(	406	9.067013	chr6:1039	HLA-DRB6		Pseudoger	chr6:32553046-3255
ENSG0000(	406	9.067013	chr6:1039	RNU1-61P		smallRNA	chr6:32549940-3255
ENSG0000(	404	9.022348	chr12:313	RNU5E-5P		smallRNA	chr12:101466705-10
ENSG0000(	401	8.95535	chr6:1039	RN7SL748P		smallRNA	chr6:36522191-3652
ENSG0000(	401	8.95535	chr6:1039	BNIP5		protein_c	chr6:36315761-3633
ENSG0000(	401	8.95535	chr6:1039	ETV7		protein_c	chr6:36354091-3638
ENSG0000(	401	8.95535	chr6:1039	KCTD20		protein_c	chr6:36442767-3649
ENSG0000(	401	8.95535	chr6:1039	Z85986.1		smallRNA	chr6:36510517-3651
ENSG0000(	401	8.95535	chr6:1039	STK38		protein_c	chr6:36493892-3654
ENSG0000(	401	8.95535	chr6:1039	ETV7-AS1		lncRNA	chr6:36386831-3639
ENSG0000(	401	8.95535	chr6:1039	RN7SL502P		smallRNA	chr6:36450915-3645
ENSG0000(	401	8.95535	chr6:1039	Z95152.1		smallRNA	chr6:36140498-3614
ENSG0000(	401	8.95535	chr6:1039	BRPF3	NCGv7	protein_c	chr6:36196744-3623
ENSG0000(	401	8.95535	chr6:1039	PXT1		protein_c	chr6:36390551-3644
ENSG0000(	401	8.95535	chr6:1039	PNPLA1		protein_c	chr6:36243203-3631
ENSG0000(	401	8.95535	chr6:1039	BRPF3-AS1		lncRNA	chr6:36146698-3619
ENSG0000(	398	8.888352	chr10:81(	TSPAN15		protein_c	chr10:69451465-695
ENSG0000(	398	8.888352	chr10:81(	LINC02622		lncRNA	chr10:70918766-709
ENSG0000(	398	8.888352	chr10:81(	MTND2P15		Pseudoger	chr10:69594041-695
ENSG0000(	398	8.888352	chr10:81(	RPS26P40		Pseudoger	chr10:70794230-707
ENSG0000(	398	8.888352	chr10:81(	AC025426.1		smallRNA	chr10:69812695-698
ENSG0000(	398	8.888352	chr10:81(	PRF1	NCGv7;AC	protein_c	chr10:70597348-706

ENSG0000(	398	8.888352	chr10:81(	MACROH2A2		protein_c	chr10:70052544-701
ENSG0000(	398	8.888352	chr10:81(	AIFM2		protein_c	chr10:70098223-701
ENSG0000(	398	8.888352	chr10:81(	LRRC20		protein_c	chr10:70298970-703
ENSG0000(	398	8.888352	chr10:81(	ENSG00000289738		lncRNA	chr10:70815989-709
ENSG0000(	398	8.888352	chr10:81(	CDH23	NCGv7	protein_c	chr10:71396920-718
ENSG0000(	398	8.888352	chr10:81(	PCBD1		protein_c	chr10:70882280-708
ENSG0000(	398	8.888352	chr10:81(	NEUROG3		protein_c	chr10:69571698-695
ENSG0000(	398	8.888352	chr10:81(	ENSG00000289739		lncRNA	chr10:70929925-709
ENSG0000(	398	8.888352	chr10:81(	SGPL1		protein_c	chr10:70815905-708
ENSG0000(	398	8.888352	chr10:81(	PPA1		protein_c	chr10:70202835-702
ENSG0000(	398	8.888352	chr10:81(	YY1P1		Pseudoger	chr10:70485336-704
ENSG0000(	398	8.888352	chr10:81(	ENSG00000287306		lncRNA	chr10:69510166-695
ENSG0000(	398	8.888352	chr10:81(	PALD1		protein_c	chr10:70478767-706
ENSG0000(	398	8.888352	chr10:81(	EIF4EBP2		protein_c	chr10:70404145-704
ENSG0000(	398	8.888352	chr10:81(	TBATA		protein_c	chr10:70771238-707
ENSG0000(	398	8.888352	chr10:81(	SLC29A3		protein_c	chr10:71319259-713
ENSG0000(	398	8.888352	chr10:81(	UNC5B-AS1		lncRNA	chr10:71217220-712
ENSG0000(	398	8.888352	chr10:81(	MTND1P20		Pseudoger	chr10:69595487-695
ENSG0000(	398	8.888352	chr10:81(	AL138925.1		smallRNA	chr10:69859214-698
ENSG0000(	398	8.888352	chr10:81(	NPFFR1		protein_c	chr10:70247329-702
ENSG0000(	398	8.888352	chr10:81(	UNC5B		protein_c	chr10:71212570-713
ENSG0000(	398	8.888352	chr10:81(	snoU13		smallRNA	chr10:71325328-713
ENSG0000(	398	8.888352	chr10:81(	HKDC1		protein_c	chr10:69220332-692
ENSG0000(	398	8.888352	chr10:81(	AL359832.1		smallRNA	chr10:71215800-712
ENSG0000(	398	8.888352	chr10:81(	ENSG00000285300		lncRNA	chr10:70927055-710
ENSG0000(	398	8.888352	chr10:81(	TYSND1	NCGv7	protein_c	chr10:70137981-701
ENSG0000(	398	8.888352	chr10:81(	LINC02651		lncRNA	chr10:69684899-696
ENSG0000(	398	8.888352	chr10:81(	AC016821.1		smallRNA	chr10:69363555-693
ENSG0000(	398	8.888352	chr10:81(	ADAMTS14		protein_c	chr10:70672506-707
ENSG0000(	398	8.888352	chr10:81(	TMEM256P1		Pseudoger	chr10:69523311-695
ENSG0000(	398	8.888352	chr10:81(	HK1		protein_c	chr10:69269984-694
ENSG0000(	398	8.888352	chr10:81(	TACR2		protein_c	chr10:69403903-694
ENSG0000(	398	8.888352	chr10:81(	COL13A1		protein_c	chr10:69801880-699
ENSG0000(	398	8.888352	chr10:81(	LINC02636		lncRNA	chr10:69994276-700
ENSG0000(	398	8.888352	chr10:81(	MTCO2P23		Pseudoger	chr10:69590583-695
ENSG0000(	398	8.888352	chr10:81(	ENSG00000229261		lncRNA	chr10:69215333-692
ENSG0000(	398	8.888352	chr10:81(	FAM241B		protein_c	chr10:69630247-696
ENSG0000(	398	8.888352	chr10:81(	RPL5P26		Pseudoger	chr10:69778962-697
ENSG0000(	398	8.888352	chr10:81(	RPS15AP28		Pseudoger	chr10:69300432-693
ENSG0000(	398	8.888352	chr10:81(	MTCO1P23		Pseudoger	chr10:69591406-695
ENSG0000(	398	8.888352	chr10:81(	NODAL		protein_c	chr10:70431936-704
ENSG0000(	398	8.888352	chr10:81(	ENSG00000289607		lncRNA	chr10:71340814-713
ENSG0000(	398	8.888352	chr10:81(	ATP5MC1P7		Pseudoger	chr10:69432972-694
ENSG0000(	398	8.888352	chr10:81(	ENSG00000289193		lncRNA	chr10:69808768-698
ENSG0000(	398	8.888352	chr10:81(	ENSG00000231748		lncRNA	chr10:69265342-692
ENSG0000(	398	8.888352	chr10:81(	ENSG00000280401		TEC	chr10:70434829-704
ENSG0000(	398	8.888352	chr10:81(	ENSG00000236154		lncRNA	chr10:69572906-695
ENSG0000(	398	8.888352	chr10:81(	ENSG00000279406		TEC	chr10:71364243-713
ENSG0000(	398	8.888352	chr10:81(	CALM2P2		Pseudoger	chr10:70163685-701
ENSG0000(	398	8.888352	chr10:81(	SAR1A		protein_c	chr10:70147289-701
ENSG0000(	398	8.888352	chr10:81(	CDH23-AS1		lncRNA	chr10:71508153-715

ENSG0000(	398	8.888352	chr10:81(	CEP57L1P1		Pseudoger	chr10:70389426-703
ENSG0000(	398	8.888352	chr10:81(	MTATP6P23		Pseudoger	chr10:69589880-695
ENSG0000(	398	8.888352	chr10:81(	RPS25P9		Pseudoger	chr10:70198988-701
ENSG0000(	397	8.86602	chr3:380<	LARS2-AS1		lncRNA	chr3:45483974-4550
ENSG0000(	397	8.86602	chr3:380<	TMEM158		protein_c	chr3:45224466-4522
ENSG0000(	397	8.86602	chr3:380<	SACM1L		protein_c	chr3:45689056-4574
ENSG0000(	397	8.86602	chr3:380<	LIMD1		protein_c	chr3:45555394-4568
ENSG0000(	397	8.86602	chr6:1391	ENSG00000285553		lncRNA	chr6:163115691-163
ENSG0000(	397	8.86602	chr3:380<	U3		smallRNA	chr3:45255410-4525
ENSG0000(	397	8.86602	chr6:1391	PACRG-AS1		lncRNA	chr6:163306907-163
ENSG0000(	397	8.86602	chr3:380<	CDCP1		protein_c	chr3:45082277-4514
ENSG0000(	397	8.86602	chr6:1391	QKI	NCGv7	protein_c	chr6:163414000-163
ENSG0000(	397	8.86602	chr3:380<	ENSG00000285788		lncRNA	chr3:45842265-4586
ENSG0000(	397	8.86602	chr3:380<	RPL12P44		Pseudoger	chr3:44937673-4493
ENSG0000(	397	8.86602	chr6:1391	CAHM		lncRNA	chr6:163413061-163
ENSG0000(	397	8.86602	chr3:380<	ENSG00000288720		lncRNA	chr3:45795970-4586
ENSG0000(	397	8.86602	chr3:380<	AC098649.1		smallRNA	chr3:44793015-4479
ENSG0000(	397	8.86602	chr3:380<	CCR9		protein_c	chr3:45886509-4590
ENSG0000(	397	8.86602	chr3:380<	NRBF2P2		Pseudoger	chr3:46023296-4602
ENSG0000(	397	8.86602	chr3:380<	RN7SL145P		smallRNA	chr3:45742675-4574
ENSG0000(	397	8.86602	chr3:380<	XCR1		protein_c	chr3:46016990-4608
ENSG0000(	397	8.86602	chr3:380<	AC010170.1		Pseudoger	chr3:45225125-4522
ENSG0000(	397	8.86602	chr3:380<	AC099539.1		smallRNA	chr3:45592255-4559
ENSG0000(	397	8.86602	chr3:380<	SDHDP4		Pseudoger	chr3:45883640-4588
ENSG0000(	397	8.86602	chr3:380<	ENSG00000235845		lncRNA	chr3:44899178-4490
ENSG0000(	397	8.86602	chr6:1391	PACRG-AS3		lncRNA	chr6:163163830-163
ENSG0000(	397	8.86602	chr6:1391	ENSG00000285726		lncRNA	chr6:163348019-163
ENSG0000(	397	8.86602	chr3:380<	TMEM42		protein_c	chr3:44861904-4486
ENSG0000(	397	8.86602	chr3:380<	ENSG00000279017		TEC	chr3:45749627-4575
ENSG0000(	397	8.86602	chr3:380<	ZDHHC3		protein_c	chr3:44915257-4497
ENSG0000(	397	8.86602	chr3:380<	MIR564		smallRNA	chr3:44861888-4486
ENSG0000(	397	8.86602	chr6:1391	ENSG00000217514		Pseudoger	chr6:163363210-163
ENSG0000(	397	8.86602	chr3:380<	LIMD1-AS1		lncRNA	chr3:45676369-4568
ENSG0000(	397	8.86602	chr3:380<	RNU5B-3P		smallRNA	chr3:45034474-4503
ENSG0000(	397	8.86602	chr3:380<	Y_RNA		smallRNA	chr3:45903187-4590
ENSG0000(	397	8.86602	chr3:380<	RPS24P8		Pseudoger	chr3:45159774-4516
ENSG0000(	397	8.86602	chr3:380<	CXCR6		protein_c	chr3:45940933-4594
ENSG0000(	397	8.86602	chr6:1391	snoU13		smallRNA	chr6:163226910-163
ENSG0000(	397	8.86602	chr3:380<	FYCO1		protein_c	chr3:45917903-4599
ENSG0000(	397	8.86602	chr3:380<	ENSG00000288717		lncRNA	chr3:45995958-4600
ENSG0000(	397	8.86602	chr3:380<	TGM4		protein_c	chr3:44874608-4491
ENSG0000(	397	8.86602	chr6:1391	ENSG00000228692		lncRNA	chr6:163586583-163
ENSG0000(	397	8.86602	chr3:380<	LZTFL1		protein_c	chr3:45823316-4591
ENSG0000(	397	8.86602	chr3:380<	KIF15		protein_c	chr3:44761721-4487
ENSG0000(	397	8.86602	chr6:1391	PACRG-AS2		lncRNA	chr6:163042557-163
ENSG0000(	397	8.86602	chr6:1391	ENSG00000285564		lncRNA	chr6:163338363-163
ENSG0000(	397	8.86602	chr6:1391	ENSG00000285858		lncRNA	chr6:163705937-163
ENSG0000(	397	8.86602	chr3:380<	SLC6A20		protein_c	chr3:45755449-4579
ENSG0000(	397	8.86602	chr3:380<	CLEC3B		protein_c	chr3:45001548-4503
ENSG0000(	397	8.86602	chr3:380<	EXOSC7		protein_c	chr3:44975241-4503
ENSG0000(	397	8.86602	chr3:380<	LARS2		protein_c	chr3:45388561-4555

ENSG0000(	396	8.843687	chr17:739	ENSG00000276790		lncRNA	chr17:47279526-472
ENSG0000(	396	8.843687	chr17:739	BRCA1P1		Pseudoger	chr17:43168170-431
ENSG0000(	396	8.843687	chr17:739	FMNL1		protein_c	chr17:45221444-452
ENSG0000(	396	8.843687	chr17:739	BECN1		protein_c	chr17:42810134-428
ENSG0000(	396	8.843687	chr17:739	NPEPPS	IntOGen-I	protein_c	chr17:47522942-476
ENSG0000(	396	8.843687	chr17:739	WNK4	IntOGen-I	protein_c	chr17:42780610-427
ENSG0000(	396	8.843687	chr17:739	RPL6P26		Pseudoger	chr17:44719760-447
ENSG0000(	396	8.843687	chr17:739	ADAM11		protein_c	chr17:44758988-447
ENSG0000(	396	8.843687	chr17:739	LINC00671		lncRNA	chr17:42874670-428
ENSG0000(	396	8.843687	chr17:739	MPP2		protein_c	chr17:43875357-439
ENSG0000(	396	8.843687	chr17:739	FAM215B		lncRNA	chr17:46558830-465
ENSG0000(	396	8.843687	chr17:739	RNY4P2		smallRNA	chr17:42932848-429
ENSG0000(	396	8.843687	chr17:739	ENSG00000262879		lncRNA	chr17:46983287-471
ENSG0000(	396	8.843687	chr17:739	KIF18B-DT		lncRNA	chr17:44947912-449
ENSG0000(	396	8.843687	chr17:739	LINC02594		lncRNA	chr17:43679341-437
ENSG0000(	396	8.843687	chr17:739	ENSG00000267420		lncRNA	chr17:43927563-439
ENSG0000(	396	8.843687	chr17:739	ENSG00000267446		lncRNA	chr17:45371402-453
ENSG0000(	396	8.843687	chr17:739	ENSG00000267405		lncRNA	chr17:44794747-447
ENSG0000(	396	8.843687	chr17:739	ATXN7L3-AS1		lncRNA	chr17:44198537-442
ENSG0000(	396	8.843687	chr17:739	MAPT-IT1		lncRNA	chr17:45895783-458
ENSG0000(	396	8.843687	chr17:739	ENSG00000262881		lncRNA	chr17:45907670-459
ENSG0000(	396	8.843687	chr17:739	FAM215A		lncRNA	chr17:43917194-439
ENSG0000(	396	8.843687	chr17:739	ENSG00000282199		lncRNA	chr17:43914433-439
ENSG0000(	396	8.843687	chr17:739	RPS7P11		Pseudoger	chr17:46721582-467
ENSG0000(	396	8.843687	chr17:739	SLC4A1		protein_c	chr17:44248390-442
ENSG0000(	396	8.843687	chr17:739	ENSG00000267505		lncRNA	chr17:44793199-447
ENSG0000(	396	8.843687	chr17:739	RN7SL199P		smallRNA	chr17:46537534-465
ENSG0000(	396	8.843687	chr17:739	ENSG00000271222		Pseudoger	chr17:44596346-445
ENSG0000(	396	8.843687	chr17:739	SMCO4P1		Pseudoger	chr17:44645411-446
ENSG0000(	396	8.843687	chr17:739	ENSG00000291175		lncRNA	chr17:45506741-455
ENSG0000(	396	8.843687	chr17:739	G6PC3		protein_c	chr17:44070620-440
ENSG0000(	396	8.843687	chr17:739	U3		smallRNA	chr17:44025021-440
ENSG0000(	396	8.843687	chr17:739	ENSG00000267344		lncRNA	chr17:45396932-453
ENSG0000(	396	8.843687	chr17:739	ENSG00000267340		Pseudoger	chr17:43169880-431
ENSG0000(	396	8.843687	chr17:739	MAPT		protein_c	chr17:45894527-460
ENSG0000(	396	8.843687	chr17:739	LRRC37A17P		Pseudoger	chr17:47017193-470
ENSG0000(	396	8.843687	chr17:739	AOC3		protein_c	chr17:42851184-428
ENSG0000(	396	8.843687	chr17:739	AOC4P		Pseudoger	chr17:42867279-428
ENSG0000(	396	8.843687	chr17:739	RDM1P1		Pseudoger	chr17:45553710-455
ENSG0000(	396	8.843687	chr17:739	CD300LG		protein_c	chr17:43847148-438
ENSG0000(	396	8.843687	chr17:739	MPP3		protein_c	chr17:43800799-438
ENSG0000(	396	8.843687	chr17:739	RNU6-971P		smallRNA	chr17:43473585-434
ENSG0000(	396	8.843687	chr17:739	LINC02210-CRHR1		protein_c	chr17:45620344-458
ENSG0000(	396	8.843687	chr17:739	RN7SL270P		smallRNA	chr17:47041825-470
ENSG0000(	396	8.843687	chr17:739	WNT9B		protein_c	chr17:46833201-468
ENSG0000(	396	8.843687	chr17:739	PTGES3L-AARSD1		protein_c	chr17:42950526-429
ENSG0000(	396	8.843687	chr17:739	VAT1		protein_c	chr17:43014607-430
ENSG0000(	396	8.843687	chr17:739	ENSG00000262372		lncRNA	chr17:46035313-460
ENSG0000(	396	8.843687	chr17:739	GRN		protein_c	chr17:44345246-443
ENSG0000(	396	8.843687	chr17:739	RN7SL819P		smallRNA	chr17:44718864-447
ENSG0000(	396	8.843687	chr17:739	AOC4P		lncRNA	chr17:42865922-428

ENSG0000(	396	8.843687	chr17:739	ENSG00000291209		lncRNA	chr17:46978481-470
ENSG0000(	396	8.843687	chr17:739	KANSL1-AS1		lncRNA	chr17:46193566-461
ENSG0000(	396	8.843687	chr17:739	ENSG00000265547		lncRNA	chr17:45731703-457
ENSG0000(	396	8.843687	chr17:739	HROB		protein_c	chr17:44141906-441
ENSG0000(	396	8.843687	chr17:739	RNU6-406P		smallRNA	chr17:43445507-434
ENSG0000(	396	8.843687	chr17:739	RN7SL730P		smallRNA	chr17:45470533-454
ENSG0000(	396	8.843687	chr17:739	LRRC37A4P		Pseudoger	chr17:45508109-455
ENSG0000(	396	8.843687	chr17:739	FAM187A		protein_c	chr17:44899774-449
ENSG0000(	396	8.843687	chr17:739	LINC00854		lncRNA	chr17:43216941-433
ENSG0000(	396	8.843687	chr17:739	RND2		protein_c	chr17:43025231-430
ENSG0000(	396	8.843687	chr17:739	ENSG00000285057		Pseudoger	chr17:43131208-431
ENSG0000(	396	8.843687	chr17:739	HEXIM2		protein_c	chr17:45160700-451
ENSG0000(	396	8.843687	chr17:739	ARHGAP27		protein_c	chr17:45393902-454
ENSG0000(	396	8.843687	chr17:739	RDM1P2		Pseudoger	chr17:46503946-465
ENSG0000(	396	8.843687	chr17:739	CFAP97D1		protein_c	chr17:43780435-437
ENSG0000(	396	8.843687	chr17:739	RNU6-453P		smallRNA	chr17:44307088-443
ENSG0000(	396	8.843687	chr17:739	TMUB2		protein_c	chr17:44186970-441
ENSG0000(	396	8.843687	chr17:739	HMGN2P15		Pseudoger	chr17:42753530-427
ENSG0000(	396	8.843687	chr17:739	NAGS		protein_c	chr17:44004622-440
ENSG0000(	396	8.843687	chr17:739	LSM12		protein_c	chr17:44034328-440
ENSG0000(	396	8.843687	chr17:739	CDC27	NCGv7	protein_c	chr17:47117703-471
ENSG0000(	396	8.843687	chr17:739	RN7SL656P		smallRNA	chr17:46319679-463
ENSG0000(	396	8.843687	chr17:739	RPRML		protein_c	chr17:46978156-469
ENSG0000(	396	8.843687	chr17:739	NSF		protein_c	chr17:46590669-467
ENSG0000(	396	8.843687	chr17:739	ENSG00000280022		TEC	chr17:45592621-455
ENSG0000(	396	8.843687	chr17:739	EFCAB15P		Pseudoger	chr17:45245186-452
ENSG0000(	396	8.843687	chr17:739	ENSG00000267604		lncRNA	chr17:43782804-437
ENSG0000(	396	8.843687	chr17:739	SOST		protein_c	chr17:43753738-437
ENSG0000(	396	8.843687	chr17:739	HEXIM1		protein_c	chr17:45148475-451
ENSG0000(	396	8.843687	chr17:739	RPL7L1P5		Pseudoger	chr17:44365014-443
ENSG0000(	396	8.843687	chr17:739	ENSG00000291018		lncRNA	chr17:46267030-462
ENSG0000(	396	8.843687	chr17:739	ENSG00000267638		lncRNA	chr17:44115912-441
ENSG0000(	396	8.843687	chr17:739	TMEM106A		protein_c	chr17:43211835-432
ENSG0000(	396	8.843687	chr17:739	ENSG00000262265		lncRNA	chr17:47160398-471
ENSG0000(	396	8.843687	chr17:739	ENSG00000236234		lncRNA	chr17:45452844-454
ENSG0000(	396	8.843687	chr17:739	MAPK8IP1P1		Pseudoger	chr17:46243606-462
ENSG0000(	396	8.843687	chr17:739	SHC1P2		Pseudoger	chr17:44228760-442
ENSG0000(	396	8.843687	chr17:739	ASB16		protein_c	chr17:44170447-441
ENSG0000(	396	8.843687	chr17:739	RAMP2-AS1		lncRNA	chr17:42753914-427
ENSG0000(	396	8.843687	chr17:739	CNTD1		protein_c	chr17:42798800-428
ENSG0000(	396	8.843687	chr17:739	ENSG00000273650		lncRNA	chr17:42751289-427
ENSG0000(	396	8.843687	chr17:739	HEXIM2-AS1		lncRNA	chr17:45150400-451
ENSG0000(	396	8.843687	chr17:739	SPPL2C		protein_c	chr17:45844881-458
ENSG0000(	396	8.843687	chr17:739	Y_RNA		smallRNA	chr17:47557413-475
ENSG0000(	396	8.843687	chr17:739	RUNDC3A-AS1		lncRNA	chr17:44299574-443
ENSG0000(	396	8.843687	chr17:739	PLCD3		protein_c	chr17:45108959-451
ENSG0000(	396	8.843687	chr17:739	SPATA32		protein_c	chr17:45254393-452
ENSG0000(	396	8.843687	chr17:739	DBF4B		protein_c	chr17:44708608-447
ENSG0000(	396	8.843687	chr17:739	PPY		protein_c	chr17:43940804-439
ENSG0000(	396	8.843687	chr17:739	FAM171A2		protein_c	chr17:44353215-443
ENSG0000(	396	8.843687	chr17:739	ENSG00000267788		lncRNA	chr17:44982514-449

ENSG0000(	396	8.843687	chr17:739	LRRC37A	DriverDBv	protein_c	chr17:46292733-463
ENSG0000(	396	8.843687	chr17:739	ENSG00000267681		Pseudoger	chr17:43144956-431
ENSG0000(	396	8.843687	chr17:739	ENSG00000237888		Pseudoger	chr17:43476198-434
ENSG0000(	396	8.843687	chr17:739	ENSG00000267288		lncRNA	chr17:45168800-451
ENSG0000(	396	8.843687	chr17:739	ARL17B	DriverDBv	protein_c	chr17:46274784-463
ENSG0000(	396	8.843687	chr17:739	ENSG00000289729		Pseudoger	chr17:46283310-462
ENSG0000(	396	8.843687	chr17:739	ENSG00000289730		Pseudoger	chr17:47080791-470
ENSG0000(	396	8.843687	chr17:739	FZD2	AC	protein_c	chr17:44557484-445
ENSG0000(	396	8.843687	chr17:739	MEIOC		protein_c	chr17:44656404-446
ENSG0000(	396	8.843687	chr17:739	ENSG00000285668		lncRNA	chr17:45586452-455
ENSG0000(	396	8.843687	chr17:739	RUNDC1		protein_c	chr17:42980565-429
ENSG0000(	396	8.843687	chr17:739	ITGB3		protein_c	chr17:47253827-473
ENSG0000(	396	8.843687	chr17:739	CCDC43		protein_c	chr17:44673069-446
ENSG0000(	396	8.843687	chr17:739	ENSG00000253347		lncRNA	chr17:47409322-474
ENSG0000(	396	8.843687	chr17:739	TMEM101		protein_c	chr17:44011188-440
ENSG0000(	396	8.843687	chr17:739	WNT3	AC	protein_c	chr17:46762506-468
ENSG0000(	396	8.843687	chr17:739	AC002558.1		smallRNA	chr17:47118502-471
ENSG0000(	396	8.843687	chr17:739	RNU6ATAC3P		smallRNA	chr17:46921124-469
ENSG0000(	396	8.843687	chr17:739	NBR2		lncRNA	chr17:43125551-431
ENSG0000(	396	8.843687	chr17:739	EFCAB13		protein_c	chr17:47323290-474
ENSG0000(	396	8.843687	chr17:739	AARSD1		protein_c	chr17:42950526-429
ENSG0000(	396	8.843687	chr17:739	ENSG00000289024		lncRNA	chr17:44976344-449
ENSG0000(	396	8.843687	chr17:739	GOSR2-DT		lncRNA	chr17:46916770-469
ENSG0000(	396	8.843687	chr17:739	LINC01180		lncRNA	chr17:44646364-446
ENSG0000(	396	8.843687	chr17:739	ENSG00000228782		Pseudoger	chr17:47482992-474
ENSG0000(	396	8.843687	chr17:739	BRCA1	NCGv7;AC	protein_c	chr17:43044295-431
ENSG0000(	396	8.843687	chr17:739	RNU6-287P		smallRNA	chr17:42891338-428
ENSG0000(	396	8.843687	chr17:739	ENSG00000279879		TEC	chr17:44689858-446
ENSG0000(	396	8.843687	chr17:739	GOSR2		protein_c	chr17:46923075-469
ENSG0000(	396	8.843687	chr17:739	HDAC5		protein_c	chr17:44076746-441
ENSG0000(	396	8.843687	chr17:739	ENSG00000267002		lncRNA	chr17:43148368-431
ENSG0000(	396	8.843687	chr17:739	STH		protein_c	chr17:45999250-459
ENSG0000(	396	8.843687	chr17:739	RNA5SP443		smallRNA	chr17:45327366-453
ENSG0000(	396	8.843687	chr17:739	DND1P1		Pseudoger	chr17:45585871-455
ENSG0000(	396	8.843687	chr17:739	ENSG00000261872		lncRNA	chr17:47169826-471
ENSG0000(	396	8.843687	chr17:739	U3		smallRNA	chr17:44304014-443
ENSG0000(	396	8.843687	chr17:739	MAP3K14-AS1		lncRNA	chr17:45247916-452
ENSG0000(	396	8.843687	chr17:739	ITGA2B		protein_c	chr17:44372180-443
ENSG0000(	396	8.843687	chr17:739	VPS25		protein_c	chr17:42773449-427
ENSG0000(	396	8.843687	chr17:739	PSME3		protein_c	chr17:42824385-428
ENSG0000(	396	8.843687	chr17:739	RAMP2		protein_c	chr17:42758447-427
ENSG0000(	396	8.843687	chr17:739	AC091132.2		smallRNA	chr17:45416813-454
ENSG0000(	396	8.843687	chr17:739	AOC2		protein_c	chr17:42844580-428
ENSG0000(	396	8.843687	chr17:739	G6PC1		protein_c	chr17:42900797-429
ENSG0000(	396	8.843687	chr17:739	ENSG00000131484		lncRNA	chr17:45533963-455
ENSG0000(	396	8.843687	chr17:739	ACBD4		protein_c	chr17:45132600-451
ENSG0000(	396	8.843687	chr17:739	ENSG00000262633		protein_c	chr17:46923133-470
ENSG0000(	396	8.843687	chr17:739	ENSG00000260793		lncRNA	chr17:44221369-442
ENSG0000(	396	8.843687	chr17:739	Y_RNA		smallRNA	chr17:42997916-429
ENSG0000(	396	8.843687	chr17:739	ENSG00000265964		lncRNA	chr17:45733353-457
ENSG0000(	396	8.843687	chr17:739	ENSG00000288909		lncRNA	chr17:43388391-433

ENSG0000(	396	8.843687	chr17:739	SLC25A39		protein_c	chr17:44319625-443
ENSG0000(	396	8.843687	chr17:739	MAPK8IP1P2		Pseudoger	chr17:45600869-456
ENSG0000(	396	8.843687	chr17:739	ENSG00000266918		TEC	chr17:45531577-455
ENSG0000(	396	8.843687	chr17:739	HIGD1B		protein_c	chr17:44846353-448
ENSG0000(	396	8.843687	chr17:739	PYY		protein_c	chr17:43952733-440
ENSG0000(	396	8.843687	chr17:739	ENSG00000276728		lncRNA	chr17:45146726-451
ENSG0000(	396	8.843687	chr17:739	COA3		protein_c	chr17:42795147-427
ENSG0000(	396	8.843687	chr17:739	RUNDC3A		protein_c	chr17:44308413-443
ENSG0000(	396	8.843687	chr17:739	RPS26P8		Pseudoger	chr17:45608571-456
ENSG0000(	396	8.843687	chr17:739	LINC02210		Pseudoger	chr17:45620328-456
ENSG0000(	396	8.843687	chr17:739	UBTF		protein_c	chr17:44205033-442
ENSG0000(	396	8.843687	chr17:739	GFAP		protein_c	chr17:44903159-449
ENSG0000(	396	8.843687	chr17:739	RPL21P4		Pseudoger	chr17:43079302-430
ENSG0000(	396	8.843687	chr17:739	C1QL1		protein_c	chr17:44959693-449
ENSG0000(	396	8.843687	chr17:739	ENSG00000259753		protein_c	chr17:47253897-473
ENSG0000(	396	8.843687	chr17:739	ENSG00000288961		lncRNA	chr17:44325409-443
ENSG0000(	396	8.843687	chr17:739	MAPT-AS1		lncRNA	chr17:45799390-458
ENSG0000(	396	8.843687	chr17:739	DCAKD		protein_c	chr17:45023338-450
ENSG0000(	396	8.843687	chr17:739	DHX8		protein_c	chr17:43483865-436
ENSG0000(	396	8.843687	chr11:583	ENSG00000289124		lncRNA	chr11:119101493-11
ENSG0000(	396	8.843687	chr17:739	MEOX1		protein_c	chr17:43640389-436
ENSG0000(	396	8.843687	chr17:739	ENSG00000267160		lncRNA	chr17:44673689-446
ENSG0000(	396	8.843687	chr17:739	ENSG00000279602		TEC	chr17:43360033-433
ENSG0000(	396	8.843687	chr17:739	KIF18B		protein_c	chr17:44924709-449
ENSG0000(	396	8.843687	chr17:739	RNU7-186P		smallRNA	chr17:47259358-472
ENSG0000(	396	8.843687	chr17:739	RNU6-131P		smallRNA	chr17:44135883-441
ENSG0000(	396	8.843687	chr17:739	ENSG00000267166		Pseudoger	chr17:43922775-439
ENSG0000(	396	8.843687	chr19:609	MIR330		smallRNA	chr19:45638994-456
ENSG0000(	396	8.843687	chr17:739	ENSG00000285486		lncRNA	chr17:44276368-442
ENSG0000(	396	8.843687	chr17:739	AC055813.1		smallRNA	chr17:43748200-437
ENSG0000(	396	8.843687	chr17:739	MYL4		protein_c	chr17:47200446-472
ENSG0000(	396	8.843687	chr17:739	ENSG00000267198		lncRNA	chr17:45545804-455
ENSG0000(	396	8.843687	chr17:739	ATXN7L3		protein_c	chr17:44191805-442
ENSG0000(	396	8.843687	chr17:739	Y_RNA		smallRNA	chr17:45071442-450
ENSG0000(	396	8.843687	chr17:739	RPL27		protein_c	chr17:42998273-430
ENSG0000(	396	8.843687	chr17:739	NSFP1		Pseudoger	chr17:46372855-464
ENSG0000(	396	8.843687	chr17:739	EFTUD2	NCGv7	protein_c	chr17:44849948-448
ENSG0000(	396	8.843687	chr17:739	Y_RNA		smallRNA	chr17:46215070-462
ENSG0000(	396	8.843687	chr17:739	ENSG00000261575		Pseudoger	chr17:46267412-462
ENSG0000(	396	8.843687	chr17:739	ENSG00000288877		lncRNA	chr17:44221630-442
ENSG0000(	396	8.843687	chr17:739	PLEKHM1		protein_c	chr17:45435900-454
ENSG0000(	396	8.843687	chr17:739	NMT1		protein_c	chr17:44957992-451
ENSG0000(	396	8.843687	chr17:739	CCDC103		protein_c	chr17:44899142-449
ENSG0000(	396	8.843687	chr17:739	ENSG00000267211		lncRNA	chr17:44328613-443
ENSG0000(	396	8.843687	chr17:739	RNU6-470P		smallRNA	chr17:43412427-434
ENSG0000(	396	8.843687	chr17:739	DUSP3		protein_c	chr17:43766125-437
ENSG0000(	396	8.843687	chr17:739	MRPL45P2		lncRNA	chr17:47450568-474
ENSG0000(	396	8.843687	chr17:739	ENSG00000267246		lncRNA	chr17:45549781-455
ENSG0000(	396	8.843687	chr17:739	WHSC1L2P		Pseudoger	chr17:43718314-437
ENSG0000(	396	8.843687	chr17:739	RN7SL507P		smallRNA	chr17:44290858-442
ENSG0000(	396	8.843687	chr17:739	MIR2117HG		lncRNA	chr17:43444707-434

ENSG0000(	396	8.843687	chr17:739	LINC00910		lncRNA	chr17:43338741-433
ENSG0000(	396	8.843687	chr17:739	LINC01976		lncRNA	chr17:43938257-439
ENSG0000(	396	8.843687	chr17:739	snoU13		smallRNA	chr17:47220642-472
ENSG0000(	396	8.843687	chr17:739	RN7SL739P		smallRNA	chr17:45526454-455
ENSG0000(	396	8.843687	chr17:739	ENSG00000239291		Pseudoger	chr17:47159191-471
ENSG0000(	396	8.843687	chr17:739	IFI35		protein_c	chr17:43006740-430
ENSG0000(	396	8.843687	chr17:739	NBR1		protein_c	chr17:43170481-432
ENSG0000(	396	8.843687	chr17:739	SNORA40		smallRNA	chr17:42940575-429
ENSG0000(	396	8.843687	chr17:739	EFCAB13-DT		lncRNA	chr17:47303460-473
ENSG0000(	396	8.843687	chr17:739	LINC01974		lncRNA	chr17:46909742-469
ENSG0000(	396	8.843687	chr17:739	PTGES3L		protein_c	chr17:42968088-429
ENSG0000(	396	8.843687	chr17:739	MAP3K14		protein_c	chr17:45263119-453
ENSG0000(	396	8.843687	chr17:739	ENSG00000262539		Pseudoger	chr17:46259551-462
ENSG0000(	396	8.843687	chr17:739	MIR5089		smallRNA	chr17:46973017-469
ENSG0000(	396	8.843687	chr17:739	GJC1		protein_c	chr17:44798448-448
ENSG0000(	396	8.843687	chr17:739	ASB16-AS1		lncRNA	chr17:44175968-441
ENSG0000(	396	8.843687	chr17:739	RN7SL258P		smallRNA	chr17:44409061-444
ENSG0000(	396	8.843687	chr17:739	LRRC37A2		protein_c	chr17:46511508-465
ENSG0000(	396	8.843687	chr17:739	FMNL1-DT		lncRNA	chr17:45190931-452
ENSG0000(	396	8.843687	chr17:739	ARL4D		protein_c	chr17:43398993-434
ENSG0000(	396	8.843687	chr17:739	KANSL1	NCGv7	protein_c	chr17:46029916-462
ENSG0000(	396	8.843687	chr17:739	CRHR1		protein_c	chr17:45784277-458
ENSG0000(	396	8.843687	chr17:739	FMNL1-AS1		lncRNA	chr17:45238028-452
ENSG0000(	396	8.843687	chr17:739	GPATCH8	NCGv7	protein_c	chr17:44395281-445
ENSG0000(	396	8.843687	chr17:739	RN7SL405P		smallRNA	chr17:44883986-448
ENSG0000(	396	8.843687	chr17:739	RNU7-101P		smallRNA	chr17:46127396-461
ENSG0000(	396	8.843687	chr17:739	ARL17A		protein_c	chr17:46499780-465
ENSG0000(	396	8.843687	chr17:739	ETV4	NCGv7;AC	protein_c	chr17:43527844-435
ENSG0000(	396	8.843687	chr17:739	ENSG00000283045		lncRNA	chr17:44486153-444
ENSG0000(	396	8.843687	chr17:739	RNU6-1137P		smallRNA	chr17:43395993-433
ENSG0000(	395	8.821355	chr10:81(	SS18L2P1		Pseudoger	chr10:34875210-348
ENSG0000(	394	8.799022	chr8:412(	AC104012.1		smallRNA	chr8:71832604-7183
ENSG0000(	393	8.77669	chr2:3094	ENSG00000230621		Pseudoger	chr2:225992981-225
ENSG0000(	393	8.77669	chr2:3094	HIGD1AP4		Pseudoger	chr2:223398267-223
ENSG0000(	393	8.77669	chr12:313	HSPE1P4		Pseudoger	chr12:101873316-10
ENSG0000(	393	8.77669	chr2:3094	CCDC195		protein_c	chr2:224703764-224
ENSG0000(	393	8.77669	chr12:313	snoU13		smallRNA	chr12:101789815-10
ENSG0000(	393	8.77669	chr2:3094	GAPDHP49		Pseudoger	chr2:222503883-222
ENSG0000(	393	8.77669	chr12:313	MYBPC1		protein_c	chr12:101568353-10
ENSG0000(	393	8.77669	chr12:313	ENSG00000258308		lncRNA	chr12:101954992-10
ENSG0000(	393	8.77669	chr2:3094	RN7SL807P		smallRNA	chr2:223248501-223
ENSG0000(	393	8.77669	chr2:3094	MIR4439		smallRNA	chr2:225010461-225
ENSG0000(	393	8.77669	chr2:3094	FARSB		protein_c	chr2:222566899-222
ENSG0000(	393	8.77669	chr2:3094	ENSG00000231189		lncRNA	chr2:223498773-223
ENSG0000(	393	8.77669	chr2:3094	RRAS2P2		Pseudoger	chr2:222677677-222
ENSG0000(	393	8.77669	chr2:3094	SERPINE2		protein_c	chr2:223975045-224
ENSG0000(	393	8.77669	chr12:313	RNA5SP367		Pseudoger	chr12:101472339-10
ENSG0000(	393	8.77669	chr2:3094	DOCK10		protein_c	chr2:224765090-225
ENSG0000(	393	8.77669	chr2:3094	ENSG00000286239		protein_c	chr2:223757258-223
ENSG0000(	393	8.77669	chr2:3094	SCG2		protein_c	chr2:223596940-223
ENSG0000(	393	8.77669	chr12:313	ENSG00000257202		lncRNA	chr12:101923410-10

ENSG0000(	393	8.77669	chr2:309	MRPL44		protein_c	chr2:223957463-223
ENSG0000(	393	8.77669	chr2:309	AC013476.1		smallRNA	chr2:222932594-222
ENSG0000(	393	8.77669	chr2:309	ANKRD49P1		Pseudoger	chr2:224499387-224
ENSG0000(	393	8.77669	chr2:309	NYAP2		protein_c	chr2:225399710-225
ENSG0000(	393	8.77669	chr2:309	ATG12P2		Pseudoger	chr2:222904640-222
ENSG0000(	393	8.77669	chr2:309	ENSG00000228681		Pseudoger	chr2:224294930-224
ENSG0000(	393	8.77669	chr2:309	SGPP2		protein_c	chr2:222424543-222
ENSG0000(	393	8.77669	chr2:309	ENSG00000203531		Pseudoger	chr2:222698623-222
ENSG0000(	393	8.77669	chr2:309	ENSG00000289265		lncRNA	chr2:225668964-225
ENSG0000(	393	8.77669	chr12:31	ENSG00000241917		Pseudoger	chr12:101936216-10
ENSG0000(	393	8.77669	chr2:309	NANOGP2		Pseudoger	chr2:222452400-222
ENSG0000(	393	8.77669	chr12:31	ENSG00000257514		lncRNA	chr12:101646720-10
ENSG0000(	393	8.77669	chr2:309	USP21P1		Pseudoger	chr2:224169664-224
ENSG0000(	393	8.77669	chr2:309	MLXP1		Pseudoger	chr2:223704621-223
ENSG0000(	393	8.77669	chr2:309	snoU13		smallRNA	chr2:222697896-222
ENSG0000(	393	8.77669	chr10:81(	RNU1-19P		smallRNA	chr10:86889569-868
ENSG0000(	393	8.77669	chr12:31	SPIC		protein_c	chr12:101475336-10
ENSG0000(	393	8.77669	chr12:31	NENFP2		Pseudoger	chr12:101963901-10
ENSG0000(	393	8.77669	chr12:31	SYCP3		protein_c	chr12:101728648-10
ENSG0000(	393	8.77669	chr2:309	Y_RNA		smallRNA	chr2:222404985-222
ENSG0000(	393	8.77669	chr2:309	ACSL3	IntOGen-I	protein_c	chr2:222860942-222
ENSG0000(	393	8.77669	chr2:309	ENSG00000235610		lncRNA	chr2:223505476-223
ENSG0000(	393	8.77669	chr2:309	ACSL3-AS1		lncRNA	chr2:222917387-222
ENSG0000(	393	8.77669	chr12:31	RNU6-1183P		smallRNA	chr12:101871742-10
ENSG0000(	393	8.77669	chr2:309	ENSG00000278083		Pseudoger	chr2:222945930-222
ENSG0000(	393	8.77669	chr2:309	MOGAT1		protein_c	chr2:222671658-222
ENSG0000(	393	8.77669	chr2:309	AP1S3		protein_c	chr2:223667680-223
ENSG0000(	393	8.77669	chr2:309	KCNE4		protein_c	chr2:223052190-223
ENSG0000(	393	8.77669	chr2:309	RPL31P17		Pseudoger	chr2:222726457-222
ENSG0000(	393	8.77669	chr12:31	CHPT1	NCGv7	protein_c	chr12:101696947-10
ENSG0000(	393	8.77669	chr12:31	GNPTAB	NCGv7	protein_c	chr12:101745499-10
ENSG0000(	393	8.77669	chr2:309	ENSG00000279015		TEC	chr2:225761559-225
ENSG0000(	393	8.77669	chr2:309	ENSG00000228802		lncRNA	chr2:223965701-223
ENSG0000(	393	8.77669	chr12:31	RNU6-101P		smallRNA	chr12:101765406-10
ENSG0000(	393	8.77669	chr12:31	ENSG00000274560		lncRNA	chr12:101696002-10
ENSG0000(	393	8.77669	chr12:31	RNU6-172P		smallRNA	chr12:101796410-10
ENSG0000(	393	8.77669	chr2:309	CUL3	NCGv7	protein_c	chr2:224470150-224
ENSG0000(	393	8.77669	chr12:31	RNA5SP369		Pseudoger	chr12:101836820-10
ENSG0000(	393	8.77669	chr12:31	ENSG00000258230		Pseudoger	chr12:101773114-10
ENSG0000(	393	8.77669	chr12:31	Y_RNA		smallRNA	chr12:101719808-10
ENSG0000(	393	8.77669	chr2:309	WDFY1		protein_c	chr2:223855716-223
ENSG0000(	393	8.77669	chr2:309	ENSG00000274629		lncRNA	chr2:224464682-224
ENSG0000(	393	8.77669	chr2:309	AC104802.1		smallRNA	chr2:224239462-224
ENSG0000(	393	8.77669	chr12:31	RNA5SP368		Pseudoger	chr12:101779814-10
ENSG0000(	393	8.77669	chr2:309	FAM124B		protein_c	chr2:224378698-224
ENSG0000(	392	8.754357	chr17:42	H1-9P		Pseudoger	chr17:50171428-501
ENSG0000(	392	8.754357	chr17:42	ENSG00000275897		lncRNA	chr17:50475819-504
ENSG0000(	392	8.754357	chr17:42	ENSG00000289926		lncRNA	chr17:61397481-613
ENSG0000(	392	8.754357	chr17:42	TEX14	NCGv7	protein_c	chr17:58556678-586
ENSG0000(	392	8.754357	chr17:42	FAM117A	NCGv7	protein_c	chr17:49710332-497
ENSG0000(	392	8.754357	chr17:42	RNU7-52P		smallRNA	chr17:62383104-623

ENSG0000(	392	8.754357	chr17:424	SMIM36		protein_c	chr17:55448433-555
ENSG0000(	392	8.754357	chr17:424	ENSG00000261959		lncRNA	chr17:50163523-501
ENSG0000(	392	8.754357	chr17:424	RPS6KB1	DriverDBv	protein_c	chr17:59893046-599
ENSG0000(	392	8.754357	chr17:424	LINC02089		lncRNA	chr17:52862121-528
ENSG0000(	392	8.754357	chr17:424	ENSG00000279059		TEC	chr17:54956760-549
ENSG0000(	392	8.754357	chr17:424	ENSG00000264914		lncRNA	chr17:57912333-579
ENSG0000(	392	8.754357	chr17:424	MRPS23	DriverDBv	protein_c	chr17:57834781-578
ENSG0000(	392	8.754357	chr17:424	AC090079.1		smallRNA	chr17:53537513-535
ENSG0000(	392	8.754357	chr17:424	RNF43	NCGv7;AC	protein_c	chr17:58353676-584
ENSG0000(	392	8.754357	chr17:424	COIL	NCGv7	protein_c	chr17:56938199-569
ENSG0000(	392	8.754357	chr17:424	ENSG00000289016		lncRNA	chr17:55612948-556
ENSG0000(	392	8.754357	chr17:424	SEPTIN4		protein_c	chr17:58520250-585
ENSG0000(	392	8.754357	chr17:424	MTMR4	DriverDBv	protein_c	chr17:58489529-585
ENSG0000(	392	8.754357	chr17:424	EPX		protein_c	chr17:58192726-582
ENSG0000(	392	8.754357	chr17:424	TRIM37	NCGv7	protein_c	chr17:58982638-591
ENSG0000(	392	8.754357	chr17:424	RAD51C	DriverDBv	protein_c	chr17:58692573-587
ENSG0000(	392	8.754357	chr17:424	ENSG00000264895		lncRNA	chr17:51249579-512
ENSG0000(	392	8.754357	chr17:424	ENSG00000261882		Pseudoger	chr17:56368063-563
ENSG0000(	392	8.754357	chr17:424	AKAP1		protein_c	chr17:57085092-571
ENSG0000(	392	8.754357	chr17:424	TRIM25		protein_c	chr17:56836387-569
ENSG0000(	392	8.754357	chr17:424	TBX4		protein_c	chr17:61452404-614
ENSG0000(	392	8.754357	chr17:424	SCPEP1		protein_c	chr17:56978129-570
ENSG0000(	392	8.754357	chr17:424	SPOP	NCGv7	protein_c	chr17:49598884-496
ENSG0000(	392	8.754357	chr17:424	DHX40	DriverDBv	protein_c	chr17:59565558-596
ENSG0000(	392	8.754357	chr17:424	TUBD1	DriverDBv	protein_c	chr17:59859479-598
ENSG0000(	392	8.754357	chr17:424	ENSG00000278642		lncRNA	chr17:57955965-579
ENSG0000(	392	8.754357	chr17:424	ENSG00000285939		lncRNA	chr17:53276760-543
ENSG0000(	392	8.754357	chr17:424	TBX2	AC	protein_c	chr17:61399843-614
ENSG0000(	392	8.754357	chr17:424	SLC35B1	DriverDBv	protein_c	chr17:49700934-497
ENSG0000(	392	8.754357	chr17:424	PRELID3BP3		Pseudoger	chr17:62841101-628
ENSG0000(	392	8.754357	chr17:424	ENSG00000263938		lncRNA	chr17:58965952-589
ENSG0000(	392	8.754357	chr17:424	ENSG00000250282		lncRNA	chr17:50100704-501
ENSG0000(	392	8.754357	chr17:424	ENSG00000277463		lncRNA	chr17:62626031-626
ENSG0000(	392	8.754357	chr17:424	RPL23AP74		Pseudoger	chr17:61069871-610
ENSG0000(	392	8.754357	chr17:424	ENSG00000263176		lncRNA	chr17:50212933-502
ENSG0000(	392	8.754357	chr17:424	CTC-462L7.1		lncRNA	chr17:55168823-551
ENSG0000(	392	8.754357	chr17:424	ENSG00000254039		lncRNA	chr17:49914403-499
ENSG0000(	392	8.754357	chr17:424	RPL5P33		Pseudoger	chr17:50847950-508
ENSG0000(	392	8.754357	chr17:424	MIR4736		smallRNA	chr17:58335976-583
ENSG0000(	392	8.754357	chr17:424	ENSG00000287457		lncRNA	chr17:62789109-627
ENSG0000(	392	8.754357	chr17:424	CUEDC1		protein_c	chr17:57861243-579
ENSG0000(	392	8.754357	chr17:424	DYNLL2		protein_c	chr17:58083419-580
ENSG0000(	392	8.754357	chr17:424	TMEM92-AS1		lncRNA	chr17:50281577-502
ENSG0000(	392	8.754357	chr17:424	RN7SL448P		smallRNA	chr17:60899891-609
ENSG0000(	392	8.754357	chr17:424	ARL2BPP8		Pseudoger	chr17:54754165-547
ENSG0000(	392	8.754357	chr17:424	NXPH3		protein_c	chr17:49575871-495
ENSG0000(	392	8.754357	chr17:424	ENSG00000286997		lncRNA	chr17:61119095-611
ENSG0000(	392	8.754357	chr17:424	ENSG00000251239		lncRNA	chr17:50627032-506
ENSG0000(	392	8.754357	chr17:424	ENSG00000263120		lncRNA	chr17:56982749-569
ENSG0000(	392	8.754357	chr17:424	AC011073.1		smallRNA	chr17:56054787-560
ENSG0000(	392	8.754357	chr17:424	ENSG00000274565		lncRNA	chr17:62626437-626

ENSG0000(	392	8.754357	chr17:424	ENSG00000277532		Pseudoger	chr17:60124110-601
ENSG0000(	392	8.754357	chr6:1391	KRT8P44		Pseudoger	chr6:162568379-162
ENSG0000(	392	8.754357	chr17:424	ENSG00000263096		lncRNA	chr17:55271504-552
ENSG0000(	392	8.754357	chr17:424	RNU6-518P		smallRNA	chr17:58835899-588
ENSG0000(	392	8.754357	chr17:424	RPL7P48		Pseudoger	chr17:51502488-515
ENSG0000(	392	8.754357	chr17:424	RNFT1	NCGv7	protein_c	chr17:59952240-599
ENSG0000(	392	8.754357	chr17:424	ENSG00000287664		lncRNA	chr17:60019149-600
ENSG0000(	392	8.754357	chr17:424	ENSG00000285930		lncRNA	chr17:58050789-580
ENSG0000(	392	8.754357	chr17:424	ENSG00000264112		lncRNA	chr17:57989039-579
ENSG0000(	392	8.754357	chr17:424	NME2		protein_c	chr17:51165435-511
ENSG0000(	392	8.754357	chr17:424	ENSG00000274378		Pseudoger	chr17:60025859-600
ENSG0000(	392	8.754357	chr17:424	SCARNA20		smallRNA	chr17:60231516-602
ENSG0000(	392	8.754357	chr17:424	ENSG00000263252		Pseudoger	chr17:53353427-533
ENSG0000(	392	8.754357	chr17:424	AKAP1-DT		lncRNA	chr17:57071814-570
ENSG0000(	392	8.754357	chr17:424	ENSG00000278627		lncRNA	chr17:58202352-582
ENSG0000(	392	8.754357	chr17:424	ENSG00000274198		lncRNA	chr17:58747055-587
ENSG0000(	392	8.754357	chr17:424	ENSG00000250948		lncRNA	chr17:49375380-493
ENSG0000(	392	8.754357	chr17:424	TBC1D3P2		Pseudoger	chr17:62265017-622
ENSG0000(	392	8.754357	chr17:424	ENSG00000274213		lncRNA	chr17:56914168-569
ENSG0000(	392	8.754357	chr17:424	SEPTIN4-AS1		lncRNA	chr17:58519837-585
ENSG0000(	392	8.754357	chr17:424	MIR4737		smallRNA	chr17:60043025-600
ENSG0000(	392	8.754357	chr17:424	ENSG00000250976		lncRNA	chr17:50563203-505
ENSG0000(	392	8.754357	chr17:424	ENSG00000289147		lncRNA	chr17:58518315-585
ENSG0000(	392	8.754357	chr17:424	RNU7-134P		smallRNA	chr17:57685529-576
ENSG0000(	392	8.754357	chr17:424	HMGN1P28		Pseudoger	chr17:60851382-608
ENSG0000(	392	8.754357	chr17:424	RNU7-135P		smallRNA	chr17:49325840-493
ENSG0000(	392	8.754357	chr17:424	SKA2	DriverDB\	protein_c	chr17:59109857-591
ENSG0000(	392	8.754357	chr17:424	ENSG00000263199		Pseudoger	chr17:54702936-547
ENSG0000(	392	8.754357	chr17:424	ENSG00000279069		TEC	chr17:58006652-580
ENSG0000(	392	8.754357	chr17:424	LINC02876		lncRNA	chr17:52985513-529
ENSG0000(	392	8.754357	chr17:424	ENSG00000264546		lncRNA	chr17:62549726-625
ENSG0000(	392	8.754357	chr17:424	ENSG00000287337		lncRNA	chr17:58219375-582
ENSG0000(	392	8.754357	chr17:424	ENSG00000263189		Pseudoger	chr17:53952936-539
ENSG0000(	392	8.754357	chr17:424	RN7SKP14		smallRNA	chr17:54770949-547
ENSG0000(	392	8.754357	chr17:424	ENSG00000273982		lncRNA	chr17:61354763-613
ENSG0000(	392	8.754357	chr17:424	ENSG00000286732		lncRNA	chr17:57253236-572
ENSG0000(	392	8.754357	chr17:424	ISCA1P3		Pseudoger	chr17:54477796-544
ENSG0000(	392	8.754357	chr17:424	LUC7L3		protein_c	chr17:50719565-507
ENSG0000(	392	8.754357	chr17:424	ENSG00000265702		lncRNA	chr17:62699244-627
ENSG0000(	392	8.754357	chr17:424	PTRH2	DriverDB\	protein_c	chr17:59674636-597
ENSG0000(	392	8.754357	chr17:424	BCAS3		protein_c	chr17:60677453-613
ENSG0000(	392	8.754357	chr17:424	C17orf64		protein_c	chr17:60392429-604
ENSG0000(	392	8.754357	chr17:424	CLTC	NCGv7;AC	protein_c	chr17:59619684-596
ENSG0000(	392	8.754357	chr17:424	TOB1	NCGv7	protein_c	chr17:50862223-508
ENSG0000(	392	8.754357	chr17:424	KIF2B	NCGv7	protein_c	chr17:53822927-538
ENSG0000(	392	8.754357	chr17:424	TOM1L1	DriverDB\	protein_c	chr17:54899387-549
ENSG0000(	392	8.754357	chr17:424	OR4D1		protein_c	chr17:58148384-581
ENSG0000(	392	8.754357	chr17:424	PCTP		protein_c	chr17:55751051-558
ENSG0000(	392	8.754357	chr17:424	AC005702.3		smallRNA	chr17:60042694-600
ENSG0000(	392	8.754357	chr17:424	ENSG00000286435		lncRNA	chr17:60135039-601
ENSG0000(	392	8.754357	chr17:424	ENSG00000262408		Pseudoger	chr17:56884639-568

ENSG0000(	392	8.754357	chr17:424	ENSG00000261976		lncRNA	chr17:50840057-508
ENSG0000(	392	8.754357	chr17:424	RPL36AP46		Pseudoger	chr17:60727165-607
ENSG0000(	392	8.754357	chr17:424	ENSG00000275710		lncRNA	chr17:54964474-549
ENSG0000(	392	8.754357	chr17:424	ABCC3	NCGv7	protein_c	chr17:50634777-506
ENSG0000(	392	8.754357	chr17:424	LRRC59	DriverDBv	protein_c	chr17:50375059-503
ENSG0000(	392	8.754357	chr17:424	ENSG00000275025		lncRNA	chr17:50098460-500
ENSG0000(	392	8.754357	chr17:424	ENSG00000265542		lncRNA	chr17:57771946-578
ENSG0000(	392	8.754357	chr17:424	ENSG00000262384		Pseudoger	chr17:53681630-536
ENSG0000(	392	8.754357	chr17:424	DYNLL2-DT		lncRNA	chr17:58076891-580
ENSG0000(	392	8.754357	chr17:424	MRPL27	DriverDBv	protein_c	chr17:50367857-503
ENSG0000(	392	8.754357	chr17:424	ENSG00000247011		lncRNA	chr17:50934989-509
ENSG0000(	392	8.754357	chr17:424	SGCA		protein_c	chr17:50164214-501
ENSG0000(	392	8.754357	chr17:424	ENSG00000275860		Pseudoger	chr17:60021072-600
ENSG0000(	392	8.754357	chr17:424	ENSG00000262518		Pseudoger	chr17:53438556-534
ENSG0000(	392	8.754357	chr17:424	HLF	NCGv7;AC	protein_c	chr17:55264960-553
ENSG0000(	392	8.754357	chr17:424	AC007114.1		smallRNA	chr17:57002983-570
ENSG0000(	392	8.754357	chr17:424	MMD		protein_c	chr17:55392622-554
ENSG0000(	392	8.754357	chr17:424	ENSG00000262967		lncRNA	chr17:50693448-506
ENSG0000(	392	8.754357	chr17:424	ENSG00000250310		lncRNA	chr17:49544788-495
ENSG0000(	392	8.754357	chr17:424	Y_RNA		smallRNA	chr17:60748940-607
ENSG0000(	392	8.754357	chr17:424	TLK2		protein_c	chr17:62458658-626
ENSG0000(	392	8.754357	chr17:424	RP11-670E13.2		lncRNA	chr17:56868947-568
ENSG0000(	392	8.754357	chr17:424	MTCO1P40		Pseudoger	chr17:53105734-531
ENSG0000(	392	8.754357	chr17:424	ENSG00000266100		lncRNA	chr17:57522248-575
ENSG0000(	392	8.754357	chr17:424	AC005702.1		smallRNA	chr17:60042361-600
ENSG0000(	392	8.754357	chr17:424	ENSG00000266086		protein_c	chr17:57989038-580
ENSG0000(	392	8.754357	chr17:424	snoU13		smallRNA	chr17:50244896-502
ENSG0000(	392	8.754357	chr17:424	ENSG00000262837		lncRNA	chr17:49834239-498
ENSG0000(	392	8.754357	chr17:424	RPL39P33		Pseudoger	chr17:56573040-565
ENSG0000(	392	8.754357	chr17:424	POLRMTP1		Pseudoger	chr17:62136972-621
ENSG0000(	392	8.754357	chr17:424	AC023992.1		smallRNA	chr17:58456124-584
ENSG0000(	392	8.754357	chr17:424	MRPS21P9		Pseudoger	chr17:50450179-504
ENSG0000(	392	8.754357	chr17:424	ENSG00000263499		lncRNA	chr17:57600856-576
ENSG0000(	392	8.754357	chr17:424	ENSG00000266002		lncRNA	chr17:59400488-594
ENSG0000(	392	8.754357	chr17:424	ENSG00000251665		lncRNA	chr17:50944104-509
ENSG0000(	392	8.754357	chr17:424	RN7SKP94		smallRNA	chr17:57789449-577
ENSG0000(	392	8.754357	chr17:424	ENSG00000262623		Pseudoger	chr17:56951664-569
ENSG0000(	392	8.754357	chr17:424	ENSG00000278963		TEC	chr17:50722074-507
ENSG0000(	392	8.754357	chr17:424	TOB1-AS1		lncRNA	chr17:50866679-509
ENSG0000(	392	8.754357	chr6:1391	PACRG		protein_c	chr6:162727132-163
ENSG0000(	392	8.754357	chr17:424	RN7SL716P		smallRNA	chr17:59059226-590
ENSG0000(	392	8.754357	chr17:424	EIF4EP2		Pseudoger	chr17:49424269-494
ENSG0000(	392	8.754357	chr17:424	COL1A1	NCGv7;AC	protein_c	chr17:50184101-502
ENSG0000(	392	8.754357	chr17:424	PPP1R9B	NCGv7	protein_c	chr17:50133737-501
ENSG0000(	392	8.754357	chr17:424	ENSG00000262298		lncRNA	chr17:56939932-569
ENSG0000(	392	8.754357	chr17:424	ENSG00000250186		lncRNA	chr17:49404081-494
ENSG0000(	392	8.754357	chr17:424	RN7SL699P		smallRNA	chr17:51595506-515
ENSG0000(	392	8.754357	chr17:424	TSPOAP1-AS1		lncRNA	chr17:58324472-584
ENSG0000(	392	8.754357	chr17:424	ENSG00000262112		lncRNA	chr17:56888880-568
ENSG0000(	392	8.754357	chr17:424	ENSG00000253730		lncRNA	chr17:50158333-501
ENSG0000(	392	8.754357	chr17:424	AC005702.2		smallRNA	chr17:60042805-600

ENSG0000(	392	8.754357	chr17:424	LINC01982		lncRNA	chr17:52390515-525
ENSG0000(	392	8.754357	chr17:424	ENSG00000262079		lncRNA	chr17:55325757-553
ENSG0000(	392	8.754357	chr17:424	ENSG00000266365		Pseudoger	chr17:62231470-622
ENSG0000(	392	8.754357	chr17:424	MIR4729		smallRNA	chr17:59366083-593
ENSG0000(	392	8.754357	chr17:424	ENSG00000250286		lncRNA	chr17:50525520-505
ENSG0000(	392	8.754357	chr17:424	ENSG00000262052		lncRNA	chr17:55842677-558
ENSG0000(	392	8.754357	chr17:424	RNF126P1		Pseudoger	chr17:57045478-570
ENSG0000(	392	8.754357	chr17:424	LINC02875		lncRNA	chr17:61411751-614
ENSG0000(	392	8.754357	chr17:424	USP32P4		Pseudoger	chr17:62282702-622
ENSG0000(	392	8.754357	chr17:424	AC002994.1		Pseudoger	chr17:61590533-615
ENSG0000(	392	8.754357	chr17:424	PICART1		lncRNA	chr17:50050349-500
ENSG0000(	392	8.754357	chr17:424	MARCHF10-DT		lncRNA	chr17:62808500-628
ENSG0000(	392	8.754357	chr17:424	ENSG00000262039		lncRNA	chr17:49370740-494
ENSG0000(	392	8.754357	chr17:424	MED13	NCGv7	protein_c	chr17:61942605-620
ENSG0000(	392	8.754357	chr17:424	RN7SL437P		smallRNA	chr17:57411911-574
ENSG0000(	392	8.754357	chr17:424	snoZ178		smallRNA	chr17:52226258-522
ENSG0000(	392	8.754357	chr17:424	ENSG00000262006		lncRNA	chr17:50909637-509
ENSG0000(	392	8.754357	chr17:424	ENSG00000250751		lncRNA	chr17:49708334-497
ENSG0000(	392	8.754357	chr17:424	ENSG00000286788		lncRNA	chr17:58241164-582
ENSG0000(	392	8.754357	chr17:424	INTS2	DriverDBv	protein_c	chr17:61865367-619
ENSG0000(	392	8.754357	chr17:424	RSAD1		protein_c	chr17:50478860-504
ENSG0000(	392	8.754357	chr17:424	MYCBPAP		protein_c	chr17:50508384-505
ENSG0000(	392	8.754357	chr17:424	DLX4	DriverDBv	protein_c	chr17:49968970-499
ENSG0000(	392	8.754357	chr17:424	SRSF1	NCGv7;AC	protein_c	chr17:58000919-580
ENSG0000(	392	8.754357	chr17:424	WFDC21P		Pseudoger	chr17:60083562-600
ENSG0000(	392	8.754357	chr17:424	MSX2P1		Pseudoger	chr17:58157028-581
ENSG0000(	392	8.754357	chr17:424	ENSG00000262271		Pseudoger	chr17:53434131-534
ENSG0000(	392	8.754357	chr17:424	ENSG00000278863		TEC	chr17:59362665-593
ENSG0000(	392	8.754357	chr17:424	ENSG00000288036		lncRNA	chr17:54609249-546
ENSG0000(	392	8.754357	chr17:424	PRR11-AS1		lncRNA	chr17:59202677-592
ENSG0000(	392	8.754357	chr17:424	ENSG00000279036		TEC	chr17:49494223-494
ENSG0000(	392	8.754357	chr17:424	snoU13		smallRNA	chr17:51098174-510
ENSG0000(	392	8.754357	chr17:424	ENSG00000289293		lncRNA	chr17:62065498-620
ENSG0000(	392	8.754357	chr17:424	snoU13		smallRNA	chr17:60961790-609
ENSG0000(	392	8.754357	chr17:424	ENSG00000239246		Pseudoger	chr17:62516321-625
ENSG0000(	392	8.754357	chr17:424	ENSG00000224738		lncRNA	chr17:59106598-591
ENSG0000(	392	8.754357	chr17:424	LINC01476		lncRNA	chr17:59422088-595
ENSG0000(	392	8.754357	chr17:424	ENSG00000265303		protein_c	chr17:59197566-592
ENSG0000(	392	8.754357	chr17:424	ENSG00000273702		lncRNA	chr17:59618553-596
ENSG0000(	392	8.754357	chr17:424	ENSG00000279792		TEC	chr17:50362967-503
ENSG0000(	392	8.754357	chr17:424	RNU2-58P		smallRNA	chr17:59129276-591
ENSG0000(	392	8.754357	chr17:424	MIR3614		smallRNA	chr17:56891270-568
ENSG0000(	392	8.754357	chr17:424	MIR142HG		lncRNA	chr17:58330879-583
ENSG0000(	392	8.754357	chr17:424	SEPTIN7P12		Pseudoger	chr17:60256720-602
ENSG0000(	392	8.754357	chr17:424	GARS1P1		Pseudoger	chr17:55550931-555
ENSG0000(	392	8.754357	chr17:424	KAT7	NCGv7	protein_c	chr17:49788648-498
ENSG0000(	392	8.754357	chr17:424	BRIP1	NCGv7;AC	protein_c	chr17:61679139-618
ENSG0000(	392	8.754357	chr17:424	CHAD		protein_c	chr17:50464496-504
ENSG0000(	392	8.754357	chr17:424	VEZF1	NCGv7	protein_c	chr17:57971547-579
ENSG0000(	392	8.754357	chr17:424	TBC1D3P1		Pseudoger	chr17:60008437-600
ENSG0000(	392	8.754357	chr17:424	ENSG00000263089		lncRNA	chr17:57092145-570

ENSG0000(	392	8.754357	chr17:424	FAM106DP		Pseudoger	chr17:60173322-601
ENSG0000(	392	8.754357	chr17:424	ENSG00000267449		lncRNA	chr17:61069856-610
ENSG0000(	392	8.754357	chr17:424	DLX3	DriverDB\	protein_c	chr17:49990005-499
ENSG0000(	392	8.754357	chr17:424	ENSG00000279089		TEC	chr17:51183191-511
ENSG0000(	392	8.754357	chr6:1391	PRKN		protein_c	chr6:161347417-162
ENSG0000(	392	8.754357	chr17:424	NGFR		protein_c	chr17:49495293-495
ENSG0000(	392	8.754357	chr17:424	XYLT2	DriverDB\	protein_c	chr17:50346126-503
ENSG0000(	392	8.754357	chr17:424	RNU1-108P		smallRNA	chr17:58666537-586
ENSG0000(	392	8.754357	chr17:424	LPO		protein_c	chr17:58218548-582
ENSG0000(	392	8.754357	chr17:424	CA4		protein_c	chr17:60149942-601
ENSG0000(	392	8.754357	chr17:424	SMG8	NCGv7	protein_c	chr17:59209400-592
ENSG0000(	392	8.754357	chr17:424	HEATR6-DT		lncRNA	chr17:60079309-600
ENSG0000(	392	8.754357	chr17:424	H3P42		Pseudoger	chr17:60315834-603
ENSG0000(	392	8.754357	chr17:424	LINC02073		lncRNA	chr17:51336646-515
ENSG0000(	392	8.754357	chr17:424	Y_RNA		smallRNA	chr17:62036833-620
ENSG0000(	392	8.754357	chr17:424	ENSG00000267526		Pseudoger	chr17:60063004-600
ENSG0000(	392	8.754357	chr17:424	RNU1-52P		smallRNA	chr17:58677574-586
ENSG0000(	392	8.754357	chr17:424	GDPD1	DriverDB\	protein_c	chr17:59220467-592
ENSG0000(	392	8.754357	chr17:424	MSI2	NCGv7;AC	protein_c	chr17:57255851-576
ENSG0000(	392	8.754357	chr17:424	DGKE	NCGv7	protein_c	chr17:56834107-568
ENSG0000(	392	8.754357	chr17:424	ANKFN1		protein_c	chr17:55882301-565
ENSG0000(	392	8.754357	chr17:424	ENSG00000279281		TEC	chr17:57448218-574
ENSG0000(	392	8.754357	chr17:424	ENSG00000249406		lncRNA	chr17:50199876-502
ENSG0000(	392	8.754357	chr17:424	ENSG00000270553		Pseudoger	chr17:60513190-605
ENSG0000(	392	8.754357	chr17:424	ENSG00000239809		Pseudoger	chr17:62381668-623
ENSG0000(	392	8.754357	chr17:424	Y_RNA		smallRNA	chr17:50386145-503
ENSG0000(	392	8.754357	chr17:424	MIR301A		smallRNA	chr17:59151136-591
ENSG0000(	392	8.754357	chr17:424	ENSG00000280233		TEC	chr17:57988180-579
ENSG0000(	392	8.754357	chr17:424	ENSG00000279606		TEC	chr17:56471142-564
ENSG0000(	392	8.754357	chr17:424	LINC02071		lncRNA	chr17:51312609-513
ENSG0000(	392	8.754357	chr17:424	ENSG00000249870		lncRNA	chr17:51042524-510
ENSG0000(	392	8.754357	chr17:424	PHB1		protein_c	chr17:49404049-494
ENSG0000(	392	8.754357	chr17:424	Y_RNA		smallRNA	chr17:49416744-494
ENSG0000(	392	8.754357	chr17:424	PDK2	NCGv7	protein_c	chr17:50094737-501
ENSG0000(	392	8.754357	chr17:424	Y_RNA		smallRNA	chr17:62122320-621
ENSG0000(	392	8.754357	chr17:424	ENSG00000280028		TEC	chr17:57531769-575
ENSG0000(	392	8.754357	chr17:424	SAMD14		protein_c	chr17:50110040-501
ENSG0000(	392	8.754357	chr17:424	ENSG00000267207		lncRNA	chr17:61034636-610
ENSG0000(	392	8.754357	chr17:424	TMEM92		protein_c	chr17:50271406-502
ENSG0000(	392	8.754357	chr6:1391	ENSG00000285610		lncRNA	chr6:162969050-162
ENSG0000(	392	8.754357	chr17:424	ACSF2		protein_c	chr17:50426158-504
ENSG0000(	392	8.754357	chr17:424	Y_RNA		smallRNA	chr17:61874084-618
ENSG0000(	392	8.754357	chr17:424	ENSG00000267248		lncRNA	chr17:60122642-601
ENSG0000(	392	8.754357	chr17:424	ANKRD40CL		protein_c	chr17:50761029-507
ENSG0000(	392	8.754357	chr17:424	ZNF652-AS1		lncRNA	chr17:49361150-493
ENSG0000(	392	8.754357	chr17:424	CA10	NCGv7	protein_c	chr17:51630313-521
ENSG0000(	392	8.754357	chr17:424	TBX2-AS1		lncRNA	chr17:61393455-614
ENSG0000(	392	8.754357	chr17:424	LINC02075		lncRNA	chr17:49457861-494
ENSG0000(	392	8.754357	chr17:424	ANKRD40	DriverDB\	protein_c	chr17:50693198-507
ENSG0000(	392	8.754357	chr17:424	ENSG00000267295		Pseudoger	chr17:60113793-601
ENSG0000(	392	8.754357	chr17:424	RNFT1-DT		lncRNA	chr17:59964832-599

ENSG0000(	392	8.754357	chr17:424	ENSG00000267318		protein_c	chr17:59940908-599
ENSG0000(	392	8.754357	chr17:424	EME1	DriverDBv	protein_c	chr17:50373220-503
ENSG0000(	392	8.754357	chr17:424	ENSG00000248954		lncRNA	chr17:49887598-499
ENSG0000(	392	8.754357	chr17:424	MIR454		smallRNA	chr17:59137758-591
ENSG0000(	392	8.754357	chr17:424	ENSG00000279207		TEC	chr17:58095672-581
ENSG0000(	392	8.754357	chr17:424	ENSG00000290039		lncRNA	chr17:58659125-586
ENSG0000(	392	8.754357	chr17:424	ENSG00000272164		Pseudoger	chr17:63004672-630
ENSG0000(	392	8.754357	chr17:424	ENSG00000186244		Pseudoger	chr17:49400429-494
ENSG0000(	392	8.754357	chr17:424	RNU1-60P		smallRNA	chr17:58659149-586
ENSG0000(	392	8.754357	chr17:424	AC005702.4		smallRNA	chr17:60042546-600
ENSG0000(	392	8.754357	chr17:424	RNU6-450P		smallRNA	chr17:59847438-598
ENSG0000(	392	8.754357	chr17:424	SPAG9	AC	protein_c	chr17:50962174-511
ENSG0000(	392	8.754357	chr17:424	ENSG00000270324		Pseudoger	chr17:62978494-629
ENSG0000(	392	8.754357	chr17:424	ZNF652	DriverDBv	protein_c	chr17:49289206-493
ENSG0000(	392	8.754357	chr17:424	NOG		protein_c	chr17:56593699-565
ENSG0000(	392	8.754357	chr17:424	HEATR6	DriverDBv	protein_c	chr17:60041008-600
ENSG0000(	392	8.754357	chr17:424	RNU1-85P		smallRNA	chr17:58679527-586
ENSG0000(	392	8.754357	chr17:424	RPS23P7		Pseudoger	chr17:62397397-623
ENSG0000(	392	8.754357	chr17:424	U3		smallRNA	chr17:58631642-586
ENSG0000(	392	8.754357	chr17:424	RNU6-1158P		smallRNA	chr17:56871170-568
ENSG0000(	392	8.754357	chr17:424	ENSG00000249658		lncRNA	chr17:51335261-513
ENSG0000(	392	8.754357	chr17:424	ENSG00000272383		Pseudoger	chr17:63088162-630
ENSG0000(	392	8.754357	chr17:424	ENSG00000241462		Pseudoger	chr17:58897776-588
ENSG0000(	392	8.754357	chr17:424	MARCHF10		protein_c	chr17:62701314-628
ENSG0000(	392	8.754357	chr17:424	NDUFB8P2		Pseudoger	chr17:59885015-598
ENSG0000(	392	8.754357	chr17:424	C17orf67		protein_c	chr17:56791913-568
ENSG0000(	392	8.754357	chr17:424	ENSG00000285829		lncRNA	chr17:50781306-507
ENSG0000(	392	8.754357	chr17:424	RN7SL800P		smallRNA	chr17:62005739-620
ENSG0000(	392	8.754357	chr17:424	RPS29P21		Pseudoger	chr17:59912197-599
ENSG0000(	392	8.754357	chr17:424	METTL2A	DriverDBv	protein_c	chr17:62423875-624
ENSG0000(	392	8.754357	chr17:424	ENSG00000280852		Pseudoger	chr17:60101759-601
ENSG0000(	392	8.754357	chr17:424	ENSG00000279133		TEC	chr17:62003700-620
ENSG0000(	392	8.754357	chr17:424	SPATA20	DriverDBv	protein_c	chr17:50543058-505
ENSG0000(	392	8.754357	chr17:424	ITGA3	AC	protein_c	chr17:50055968-500
ENSG0000(	392	8.754357	chr17:424	ENSG00000267637		lncRNA	chr17:59784813-597
ENSG0000(	392	8.754357	chr17:424	AC079005.1		smallRNA	chr17:60810957-608
ENSG0000(	392	8.754357	chr17:424	ENSG00000267678		Pseudoger	chr17:60562010-605
ENSG0000(	392	8.754357	chr17:424	ENSG00000290053		lncRNA	chr17:50793281-507
ENSG0000(	392	8.754357	chr17:424	IGBP1P2		protein_c	chr17:58660424-586
ENSG0000(	392	8.754357	chr17:424	SRP14P3		Pseudoger	chr17:49795343-497
ENSG0000(	392	8.754357	chr17:424	USP32	DriverDBv	protein_c	chr17:60177327-604
ENSG0000(	392	8.754357	chr17:424	PPM1D	NCGv7;AC	protein_c	chr17:60600193-606
ENSG0000(	392	8.754357	chr17:424	RP11-51L5.7		protein_c	chr17:62272487-622
ENSG0000(	392	8.754357	chr17:424	LINC01999		lncRNA	chr17:60564547-605
ENSG0000(	392	8.754357	chr17:424	RNU6-623P		smallRNA	chr17:60662333-606
ENSG0000(	392	8.754357	chr17:424	TBC1D3P2		lncRNA	chr17:62264705-622
ENSG0000(	392	8.754357	chr17:424	ENSG00000266647		Pseudoger	chr17:62260634-622
ENSG0000(	392	8.754357	chr17:424	SETP3		Pseudoger	chr17:58476138-584
ENSG0000(	392	8.754357	chr17:424	ENSG00000249451		lncRNA	chr17:50503412-505
ENSG0000(	392	8.754357	chr17:424	NACA2		protein_c	chr17:61590421-615
ENSG0000(	392	8.754357	chr17:424	ENSG00000285785		lncRNA	chr17:57196081-572

ENSG0000(	392	8.754357	chr17:42<	RPS2P48		Pseudoger	chr17:53756989-537
ENSG0000(	392	8.754357	chr17:42<	ENSG00000249982		lncRNA	chr17:51332679-513
ENSG0000(	392	8.754357	chr17:42<	ENSG00000279743		TEC	chr17:51761260-517
ENSG0000(	392	8.754357	chr17:42<	CACNA1G		protein_c	chr17:50560715-506
ENSG0000(	392	8.754357	chr17:42<	RN7SL606P		smallRNA	chr17:60696313-606
ENSG0000(	392	8.754357	chr17:42<	ENSG00000248702		Pseudoger	chr17:50797366-507
ENSG0000(	392	8.754357	chr17:42<	NGFR-AS1		lncRNA	chr17:49505657-495
ENSG0000(	392	8.754357	chr17:42<	PRR11		protein_c	chr17:59155732-592
ENSG0000(	392	8.754357	chr17:42<	ENSG00000279555		TEC	chr17:57277920-572
ENSG0000(	392	8.754357	chr17:42<	ENSG00000285879		lncRNA	chr17:62035944-620
ENSG0000(	392	8.754357	chr17:42<	Y_RNA		smallRNA	chr17:62661621-626
ENSG0000(	392	8.754357	chr17:42<	SUMO2P7		Pseudoger	chr17:50235845-502
ENSG0000(	392	8.754357	chr17:42<	CCDC182		protein_c	chr17:57744495-577
ENSG0000(	392	8.754357	chr17:42<	RN7SL449P		smallRNA	chr17:57618108-576
ENSG0000(	392	8.754357	chr17:42<	AC100832.1		smallRNA	chr17:58988868-589
ENSG0000(	392	8.754357	chr17:42<	APPBP2-DT		lncRNA	chr17:60526272-605
ENSG0000(	392	8.754357	chr17:42<	ENSG00000283538		lncRNA	chr17:62910021-629
ENSG0000(	392	8.754357	chr17:42<	ENSG00000267131		lncRNA	chr17:61361668-614
ENSG0000(	392	8.754357	chr17:42<	ENSG00000267137		lncRNA	chr17:61460224-614
ENSG0000(	392	8.754357	chr17:42<	ENSG00000285897		protein_c	chr17:58345206-584
ENSG0000(	392	8.754357	chr17:42<	Y_RNA		smallRNA	chr17:59260748-592
ENSG0000(	392	8.754357	chr17:42<	PPM1E	DriverDBv	protein_c	chr17:58755854-589
ENSG0000(	392	8.754357	chr17:42<	OR4D2		protein_c	chr17:58166982-581
ENSG0000(	392	8.754357	chr17:42<	RPSAP66		Pseudoger	chr17:60564740-605
ENSG0000(	392	8.754357	chr17:42<	RNU6-446P		smallRNA	chr17:62683905-626
ENSG0000(	392	8.754357	chr17:42<	TMEM100		protein_c	chr17:55719627-557
ENSG0000(	392	8.754357	chr17:42<	YPEL2		protein_c	chr17:59331655-594
ENSG0000(	392	8.754357	chr17:42<	RNU4-13P		smallRNA	chr17:59537359-595
ENSG0000(	392	8.754357	chr17:42<	TSPOAP1		protein_c	chr17:58301228-583
ENSG0000(	392	8.754357	chr17:42<	SPDYE22P		Pseudoger	chr17:59174983-591
ENSG0000(	392	8.754357	chr17:42<	NME1-NME2	DriverDBv	protein_c	chr17:51153590-511
ENSG0000(	392	8.754357	chr17:42<	ENSG00000283209		lncRNA	chr17:56803841-568
ENSG0000(	392	8.754357	chr17:42<	WFIKKN2		protein_c	chr17:50834650-508
ENSG0000(	392	8.754357	chr17:42<	DHX40P1		Pseudoger	chr17:59976009-600
ENSG0000(	392	8.754357	chr17:42<	ENSG00000267095		lncRNA	chr17:60135762-601
ENSG0000(	392	8.754357	chr17:42<	KRT18P61		Pseudoger	chr17:60810165-608
ENSG0000(	392	8.754357	chr17:42<	CACNA1G-AS1		lncRNA	chr17:50556207-505
ENSG0000(	392	8.754357	chr17:42<	MKS1		protein_c	chr17:58205441-582
ENSG0000(	392	8.754357	chr17:42<	ENSG00000204584		lncRNA	chr17:49845910-498
ENSG0000(	392	8.754357	chr17:42<	RPL36AP47		Pseudoger	chr17:62319884-623
ENSG0000(	392	8.754357	chr17:42<	MIR633		smallRNA	chr17:62944215-629
ENSG0000(	392	8.754357	chr17:42<	MPO	NCGv7	protein_c	chr17:58269855-582
ENSG0000(	392	8.754357	chr17:42<	MIR21		smallRNA	chr17:59841266-598
ENSG0000(	392	8.754357	chr17:42<	TBC1D3P1-DHX40P1		lncRNA	chr17:59962363-600
ENSG0000(	392	8.754357	chr17:42<	MRC2		protein_c	chr17:62627670-626
ENSG0000(	392	8.754357	chr17:42<	SNRPGP17		Pseudoger	chr17:59281130-592
ENSG0000(	392	8.754357	chr17:42<	ENSG00000266561		Pseudoger	chr17:58675286-586
ENSG0000(	392	8.754357	chr17:42<	ENSG00000276851		lncRNA	chr17:50094065-500
ENSG0000(	392	8.754357	chr17:42<	AC018628.1		smallRNA	chr17:62015857-620
ENSG0000(	392	8.754357	chr17:42<	ENSG00000253102		lncRNA	chr17:50396438-503
ENSG0000(	392	8.754357	chr17:42<	TAC4		protein_c	chr17:49838300-498

ENSG0000(	392	8.754357	chr17:424	ENSG00000248172		lncRNA	chr17:49996889-500
ENSG0000(	392	8.754357	chr6:1391	ENSG00000217557		Pseudoger	chr6:162957258-162
ENSG0000(	392	8.754357	chr17:424	RPL32P32		Pseudoger	chr17:60245288-602
ENSG0000(	392	8.754357	chr17:424	ENSG00000279713		TEC	chr17:62679403-626
ENSG0000(	392	8.754357	chr17:424	ENSG00000249176		Pseudoger	chr17:49939122-499
ENSG0000(	392	8.754357	chr17:424	ENSG00000236319		Pseudoger	chr17:55526964-555
ENSG0000(	392	8.754357	chr17:424	ENSG00000236472		lncRNA	chr17:50135586-501
ENSG0000(	392	8.754357	chr17:424	COX11		protein_c	chr17:54951902-549
ENSG0000(	392	8.754357	chr17:424	VMP1		protein_c	chr17:59707192-598
ENSG0000(	392	8.754357	chr17:424	RNU6-1313P		smallRNA	chr17:49966075-499
ENSG0000(	392	8.754357	chr17:424	MBTD1	DriverDBv	protein_c	chr17:51177425-512
ENSG0000(	392	8.754357	chr17:424	HSF5		protein_c	chr17:58420167-584
ENSG0000(	392	8.754357	chr17:424	RPL12P38		Pseudoger	chr17:60433678-604
ENSG0000(	392	8.754357	chr17:424	NME1	DriverDBv	protein_c	chr17:51153559-511
ENSG0000(	392	8.754357	chr17:424	EFCAB3		protein_c	chr17:62343941-624
ENSG0000(	392	8.754357	chr17:424	Y_RNA		smallRNA	chr17:50696644-506
ENSG0000(	392	8.754357	chr17:424	RNU6-1249P		smallRNA	chr17:55551810-555
ENSG0000(	392	8.754357	chr17:424	EPN3	DriverDBv	protein_c	chr17:50532682-505
ENSG0000(	392	8.754357	chr17:424	APPBP2	DriverDBv	protein_c	chr17:60443158-605
ENSG0000(	392	8.754357	chr17:424	SUPT4H1	DriverDBv	protein_c	chr17:58345175-583
ENSG0000(	392	8.754357	chr17:424	STXBP4	DriverDBv	protein_c	chr17:54968727-551
ENSG0000(	392	8.754357	chr17:424	ENSG00000266934		lncRNA	chr17:61382785-613
ENSG0000(	392	8.754357	chr17:424	AC006487.1		smallRNA	chr17:49561002-495
ENSG0000(	392	8.754357	chr17:424	UTP18	DriverDBv	protein_c	chr17:51260546-512
ENSG0000(	391	8.732025	chr7:1588	RNU1-58P		smallRNA	chr7:140241870-140
ENSG0000(	390	8.709692	chr7:1588	ENSG00000228711		Pseudoger	chr7:82657035-8265
ENSG0000(	387	8.642694	chr12:149	ENSG00000281333		lncRNA	chr12:88580531-886
ENSG0000(	387	8.642694	chr12:149	PPFIA2		protein_c	chr12:81257975-817
ENSG0000(	387	8.642694	chr12:149	MKRN9P		Pseudoger	chr12:87782163-877
ENSG0000(	387	8.642694	chr12:149	ENSG00000257752		Pseudoger	chr12:88057876-880
ENSG0000(	387	8.642694	chr12:149	LINC02426		lncRNA	chr12:81953719-819
ENSG0000(	387	8.642694	chr12:149	GALNT4		protein_c	chr12:89519412-895
ENSG0000(	387	8.642694	chr12:149	NAP1L1	NCGv7	protein_c	chr12:76036585-760
ENSG0000(	387	8.642694	chr12:149	Y_RNA		smallRNA	chr12:88430442-884
ENSG0000(	387	8.642694	chr12:149	ENSG00000258026		lncRNA	chr12:81094371-811
ENSG0000(	387	8.642694	chr12:149	ENSG00000258088		lncRNA	chr12:75694010-756
ENSG0000(	387	8.642694	chr12:149	ENSG00000277130		lncRNA	chr12:79818784-798
ENSG0000(	387	8.642694	chr12:149	ENSG00000257897		Pseudoger	chr12:86015697-860
ENSG0000(	387	8.642694	chr12:149	NAV3	NCGv7	protein_c	chr12:77324641-782
ENSG0000(	387	8.642694	chr12:149	SYT1	AC	protein_c	chr12:78863993-794
ENSG0000(	387	8.642694	chr12:149	RASSF9		protein_c	chr12:85800703-858
ENSG0000(	387	8.642694	chr12:149	ENSG00000289384		lncRNA	chr12:88381483-884
ENSG0000(	387	8.642694	chr12:149	ENSG00000258170		lncRNA	chr12:82481118-824
ENSG0000(	387	8.642694	chr12:149	LINC02820		lncRNA	chr12:85318060-853
ENSG0000(	387	8.642694	chr12:149	GLIPR1		protein_c	chr12:75480753-755
ENSG0000(	387	8.642694	chr12:149	ENSG00000258090		Pseudoger	chr12:76757191-767
ENSG0000(	387	8.642694	chr12:149	ENSG00000273987		lncRNA	chr12:75333798-753
ENSG0000(	387	8.642694	chr12:149	PHLDA1-DT		lncRNA	chr12:76032658-760
ENSG0000(	387	8.642694	chr12:149	LINC02394		lncRNA	chr12:74039024-740
ENSG0000(	387	8.642694	chr12:149	PHLDA1	NCGv7	protein_c	chr12:76025447-760
ENSG0000(	387	8.642694	chr12:149	RNU6-1271P		smallRNA	chr12:76139567-761

ENSG0000(	387	8.642694	chr12:14(	ENSG00000257872		Pseudoger	chr12:76137425-761
ENSG0000(	387	8.642694	chr12:14(	ENSG00000258044		lncRNA	chr12:79823778-798
ENSG0000(	387	8.642694	chr12:14(	RNU7-120P		smallRNA	chr12:89282223-892
ENSG0000(	387	8.642694	chr12:14(	CSRP2		protein_c	chr12:76858709-768
ENSG0000(	387	8.642694	chr12:14(	KRR1		protein_c	chr12:75490863-755
ENSG0000(	387	8.642694	chr12:14(	ENSG00000257329		lncRNA	chr12:75964440-759
ENSG0000(	387	8.642694	chr12:14(	ENSG00000257474		lncRNA	chr12:79540203-795
ENSG0000(	387	8.642694	chr12:14(	AC078917.1		smallRNA	chr12:79348319-793
ENSG0000(	387	8.642694	chr12:14(	TMTC3	NCGv7	protein_c	chr12:88142296-881
ENSG0000(	387	8.642694	chr12:14(	ENSG00000257429		lncRNA	chr12:80583683-805
ENSG0000(	387	8.642694	chr12:14(	AC027288.1		smallRNA	chr12:79481048-794
ENSG0000(	387	8.642694	chr12:14(	CAPS2-AS1		lncRNA	chr12:75234740-752
ENSG0000(	387	8.642694	chr12:14(	POC1B		protein_c	chr12:89419718-895
ENSG0000(	387	8.642694	chr12:14(	RNA5SP363		Pseudoger	chr12:79878305-798
ENSG0000(	387	8.642694	chr12:14(	GLIPR1L1		protein_c	chr12:75334670-753
ENSG0000(	387	8.642694	chr12:14(	ENSG00000243164		Pseudoger	chr12:73648666-736
ENSG0000(	387	8.642694	chr12:14(	POC1B-AS1		lncRNA	chr12:89524594-895
ENSG0000(	387	8.642694	chr12:14(	LNCOG		lncRNA	chr12:76259836-763
ENSG0000(	387	8.642694	chr12:14(	ENSG00000258185		lncRNA	chr12:86599578-868
ENSG0000(	387	8.642694	chr12:14(	DUSP6		protein_c	chr12:89347235-893
ENSG0000(	387	8.642694	chr12:14(	ENSG00000257124		lncRNA	chr12:83661009-836
ENSG0000(	387	8.642694	chr12:14(	ENSG00000257835		lncRNA	chr12:77379770-773
ENSG0000(	387	8.642694	chr12:14(	GLIPR1L2	NCGv7	protein_c	chr12:75391089-754
ENSG0000(	387	8.642694	chr12:14(	PTPRQ		protein_c	chr12:80405994-806
ENSG0000(	387	8.642694	chr12:14(	ENSG00000257587		lncRNA	chr12:73906940-739
ENSG0000(	387	8.642694	chr12:14(	RPL7P43		Pseudoger	chr12:76721534-767
ENSG0000(	387	8.642694	chr12:14(	ENSG00000257431		lncRNA	chr12:82512677-825
ENSG0000(	387	8.642694	chr12:14(	ENSG00000257875		Pseudoger	chr12:82468456-824
ENSG0000(	387	8.642694	chr12:14(	TMTC2	NCGv7	protein_c	chr12:82686880-831
ENSG0000(	387	8.642694	chr12:14(	PPFIA2-AS1		lncRNA	chr12:81270669-813
ENSG0000(	387	8.642694	chr12:14(	PPP1R12A-AS2		lncRNA	chr12:79690144-797
ENSG0000(	387	8.642694	chr12:14(	ENSG00000257604		Pseudoger	chr12:79500119-795
ENSG0000(	387	8.642694	chr12:14(	YWHAQP7		Pseudoger	chr12:76619386-766
ENSG0000(	387	8.642694	chr12:14(	CCDC59		protein_c	chr12:82223681-823
ENSG0000(	387	8.642694	chr12:14(	MIR618		smallRNA	chr12:80935736-809
ENSG0000(	387	8.642694	chr12:14(	METTL25		protein_c	chr12:82358528-824
ENSG0000(	387	8.642694	chr12:14(	PAWR		protein_c	chr12:79574979-796
ENSG0000(	387	8.642694	chr12:14(	RN7SL734P		smallRNA	chr12:75957834-759
ENSG0000(	387	8.642694	chr12:14(	ENSG00000257998		Pseudoger	chr12:74728038-747
ENSG0000(	387	8.642694	chr12:14(	ENSG00000257183		lncRNA	chr12:74248637-742
ENSG0000(	387	8.642694	chr12:14(	ENSG00000257323		Pseudoger	chr12:74656561-746
ENSG0000(	387	8.642694	chr12:14(	E2F7		protein_c	chr12:77021248-770
ENSG0000(	387	8.642694	chr12:14(	OTOGL	NCGv7	protein_c	chr12:80099537-803
ENSG0000(	387	8.642694	chr12:14(	POC1B-GALNT4		protein_c	chr12:89519408-895
ENSG0000(	387	8.642694	chr12:14(	TSPAN19	DriverDB\	protein_c	chr12:85014311-850
ENSG0000(	387	8.642694	chr12:14(	ALX1		protein_c	chr12:85280220-853
ENSG0000(	387	8.642694	chr12:14(	PRXL2AP1		Pseudoger	chr12:78130337-781
ENSG0000(	387	8.642694	chr12:14(	ENSG00000198923		Pseudoger	chr12:75964799-759
ENSG0000(	387	8.642694	chr12:14(	LINC02882		lncRNA	chr12:73820315-744
ENSG0000(	387	8.642694	chr12:14(	ENSG00000258178		lncRNA	chr12:85958686-859
ENSG0000(	387	8.642694	chr12:14(	ENSG00000258091		Pseudoger	chr12:75926213-759

ENSG0000(	387	8.642694	chr12:14(	ENSG00000257629		Pseudoger	chr12:89027757-890
ENSG0000(	387	8.642694	chr12:14(	ENSG00000243071		Pseudoger	chr12:76660405-766
ENSG0000(	387	8.642694	chr12:14(	ENSG00000258302		lncRNA	chr12:89561129-895
ENSG0000(	387	8.642694	chr12:14(	ENSG00000257940		lncRNA	chr12:87816486-878
ENSG0000(	387	8.642694	chr12:14(	C12orf50		protein_c	chr12:87980035-880
ENSG0000(	387	8.642694	chr12:14(	ENSG00000258173		Pseudoger	chr12:87753414-877
ENSG0000(	387	8.642694	chr12:14(	CYCSP30		Pseudoger	chr12:87750142-877
ENSG0000(	387	8.642694	chr12:14(	ENSG00000287030		lncRNA	chr12:76589476-766
ENSG0000(	387	8.642694	chr12:14(	ENSG00000257386		lncRNA	chr12:74538145-745
ENSG0000(	387	8.642694	chr12:14(	RPL10P13		Pseudoger	chr12:75688794-756
ENSG0000(	387	8.642694	chr12:14(	RN7SKP261		smallRNA	chr12:80343515-803
ENSG0000(	387	8.642694	chr12:14(	ENSG00000286259		lncRNA	chr12:75649195-756
ENSG0000(	387	8.642694	chr12:14(	ENSG00000257191		lncRNA	chr12:78960258-790
ENSG0000(	387	8.642694	chr12:14(	MRPS6P4		Pseudoger	chr12:89319364-893
ENSG0000(	387	8.642694	chr12:14(	ENSG00000288941		lncRNA	chr12:83456715-834
ENSG0000(	387	8.642694	chr12:14(	ENSG00000258358		Pseudoger	chr12:84448625-844
ENSG0000(	387	8.642694	chr12:14(	ENSG00000258304		Pseudoger	chr12:76115711-761
ENSG0000(	387	8.642694	chr12:14(	MIR617		smallRNA	chr12:80832533-808
ENSG0000(	387	8.642694	chr12:14(	AC021887.1		smallRNA	chr12:85647253-856
ENSG0000(	387	8.642694	chr12:14(	RPL23AP68		Pseudoger	chr12:87169985-871
ENSG0000(	387	8.642694	chr12:14(	KITLG		protein_c	chr12:88492793-885
ENSG0000(	387	8.642694	chr12:14(	ENSG00000257823		Pseudoger	chr12:75305452-753
ENSG0000(	387	8.642694	chr12:14(	SNORA70		smallRNA	chr12:75689152-756
ENSG0000(	387	8.642694	chr12:14(	CAPS2		protein_c	chr12:75275979-753
ENSG0000(	387	8.642694	chr12:14(	ENSG00000286476		lncRNA	chr12:77129178-771
ENSG0000(	387	8.642694	chr12:14(	CCNG2P1		Pseudoger	chr12:75257635-752
ENSG0000(	387	8.642694	chr12:14(	ENSG00000258179		Pseudoger	chr12:87820654-878
ENSG0000(	387	8.642694	chr12:14(	ENSG00000289040		lncRNA	chr12:77723756-777
ENSG0000(	387	8.642694	chr12:14(	ENSG00000274021		lncRNA	chr12:89351015-893
ENSG0000(	387	8.642694	chr12:14(	ENSG00000257199		Pseudoger	chr12:76984079-769
ENSG0000(	387	8.642694	chr12:14(	NTS		protein_c	chr12:85874295-858
ENSG0000(	387	8.642694	chr12:14(	LRRIQ1	NCGv7	protein_c	chr12:85036314-852
ENSG0000(	387	8.642694	chr12:14(	LINC01490		lncRNA	chr12:80763151-807
ENSG0000(	387	8.642694	chr12:14(	C12orf29		protein_c	chr12:88033846-880
ENSG0000(	387	8.642694	chr12:14(	ENSG00000257879		lncRNA	chr12:78426826-784
ENSG0000(	387	8.642694	chr12:14(	LINC02464		lncRNA	chr12:77219603-773
ENSG0000(	387	8.642694	chr12:14(	ENSG00000271327		lncRNA	chr12:89367807-893
ENSG0000(	387	8.642694	chr12:14(	RN7SKP172		smallRNA	chr12:76315751-763
ENSG0000(	387	8.642694	chr12:14(	ENSG00000257837		lncRNA	chr12:82505211-825
ENSG0000(	387	8.642694	chr12:14(	RNA5SP364		Pseudoger	chr12:88051701-880
ENSG0000(	387	8.642694	chr12:14(	ENSG00000286043		lncRNA	chr12:82673070-826
ENSG0000(	387	8.642694	chr12:14(	ENSG00000257894		lncRNA	chr12:79341205-795
ENSG0000(	387	8.642694	chr12:14(	ENSG00000258320		Pseudoger	chr12:74170445-741
ENSG0000(	387	8.642694	chr12:14(	RPS4XP15		Pseudoger	chr12:87944885-879
ENSG0000(	387	8.642694	chr12:14(	ENSG00000257165		lncRNA	chr12:78448995-785
ENSG0000(	387	8.642694	chr12:14(	RNU6-1012P		smallRNA	chr12:73679188-736
ENSG0000(	387	8.642694	chr12:14(	ENSG00000257609		Pseudoger	chr12:81868368-818
ENSG0000(	387	8.642694	chr12:14(	ACSS3		protein_c	chr12:80936414-812
ENSG0000(	387	8.642694	chr12:14(	PHLDA1-AS1		lncRNA	chr12:76030494-760
ENSG0000(	387	8.642694	chr12:14(	LIN7A	NCGv7	protein_c	chr12:80792520-809
ENSG0000(	387	8.642694	chr12:14(	ENSG00000271259		lncRNA	chr12:89371820-893

ENSG0000(	387	8.642694	chr12:149	MYF5		protein_c	chr12:80716912-807
ENSG0000(	387	8.642694	chr12:149	MYF6		protein_c	chr12:80707634-807
ENSG0000(	387	8.642694	chr12:149	ENSG00000257729		lncRNA	chr12:84154434-842
ENSG0000(	387	8.642694	chr12:149	VENTXP3		Pseudoger	chr12:74292324-742
ENSG0000(	387	8.642694	chr12:149	ENSG00000278011		lncRNA	chr12:88601862-886
ENSG0000(	387	8.642694	chr12:149	ENSG00000257434		lncRNA	chr12:75020969-750
ENSG0000(	387	8.642694	chr12:149	PPP1R12A		protein_c	chr12:79773563-799
ENSG0000(	387	8.642694	chr12:149	CENPCP1		Pseudoger	chr12:89500093-895
ENSG0000(	387	8.642694	chr12:149	ENSG00000230291		Pseudoger	chr12:80102899-801
ENSG0000(	387	8.642694	chr12:149	ENSG00000257941		Pseudoger	chr12:76057538-760
ENSG0000(	387	8.642694	chr12:149	KCNC2		protein_c	chr12:75040077-752
ENSG0000(	387	8.642694	chr12:149	ENSG00000285191		lncRNA	chr12:84147304-841
ENSG0000(	387	8.642694	chr12:149	LINC02458		lncRNA	chr12:89010681-893
ENSG0000(	387	8.642694	chr12:149	U8		smallRNA	chr12:73764279-737
ENSG0000(	387	8.642694	chr12:149	LINC02445		lncRNA	chr12:73758657-738
ENSG0000(	387	8.642694	chr12:149	ENSG00000289309		lncRNA	chr12:83888705-841
ENSG0000(	387	8.642694	chr12:149	ENSG00000258073		Pseudoger	chr12:84939524-849
ENSG0000(	387	8.642694	chr12:149	RNU6-148P		smallRNA	chr12:89754059-897
ENSG0000(	387	8.642694	chr12:149	AC090015.1		smallRNA	chr12:74017775-740
ENSG0000(	387	8.642694	chr12:149	ATXN7L3B		protein_c	chr12:74537835-745
ENSG0000(	387	8.642694	chr12:149	SLC6A15	NCGv7	protein_c	chr12:84859491-849
ENSG0000(	387	8.642694	chr12:149	BBS10		protein_c	chr12:76344474-763
ENSG0000(	387	8.642694	chr12:149	ENSG00000258205		Pseudoger	chr12:87746567-877
ENSG0000(	387	8.642694	chr12:149	GLIPR1-AS1		lncRNA	chr12:75483454-754
ENSG0000(	387	8.642694	chr12:149	SNRPGP20		Pseudoger	chr12:79989014-799
ENSG0000(	387	8.642694	chr12:149	AC068641.1		smallRNA	chr12:89592827-895
ENSG0000(	387	8.642694	chr12:149	MGAT4C		protein_c	chr12:85955666-868
ENSG0000(	387	8.642694	chr12:149	ENSG00000258338		lncRNA	chr12:83171590-831
ENSG0000(	387	8.642694	chr12:149	AC131012.1		smallRNA	chr12:75724453-757
ENSG0000(	387	8.642694	chr12:149	ENSG00000257526		lncRNA	chr12:76559870-766
ENSG0000(	387	8.642694	chr12:149	MIR1252		smallRNA	chr12:79419257-794
ENSG0000(	387	8.642694	chr12:149	ENSG00000257855		Pseudoger	chr12:85567878-855
ENSG0000(	387	8.642694	chr12:149	ENSG00000258206		Pseudoger	chr12:85781892-857
ENSG0000(	387	8.642694	chr12:149	MRPL2P1		Pseudoger	chr12:89752913-897
ENSG0000(	387	8.642694	chr12:149	ENSG00000257777		Pseudoger	chr12:75600047-756
ENSG0000(	387	8.642694	chr12:149	MIR4699		smallRNA	chr12:81158388-811
ENSG0000(	387	8.642694	chr12:149	ENSG00000258216		lncRNA	chr12:89712048-901
ENSG0000(	387	8.642694	chr12:149	ENSG00000258084		lncRNA	chr12:78352519-784
ENSG0000(	387	8.642694	chr12:149	RPL7P38		Pseudoger	chr12:80028893-800
ENSG0000(	387	8.642694	chr12:149	ENSG00000257296		Pseudoger	chr12:84671598-846
ENSG0000(	387	8.642694	chr12:149	ENSG00000258215		Pseudoger	chr12:80860955-808
ENSG0000(	387	8.642694	chr12:149	ENSG00000258077		lncRNA	chr12:75563202-759
ENSG0000(	387	8.642694	chr12:149	SNORA3		smallRNA	chr12:84183324-841
ENSG0000(	387	8.642694	chr12:149	RNU7-106P		smallRNA	chr12:80068691-800
ENSG0000(	387	8.642694	chr12:149	RPL7AP9		Pseudoger	chr12:76599671-766
ENSG0000(	387	8.642694	chr12:149	ENSG00000188646		Pseudoger	chr12:74663774-746
ENSG0000(	387	8.642694	chr12:149	ENSG00000257910		lncRNA	chr12:76878193-768
ENSG0000(	387	8.642694	chr12:149	ATP2B1		protein_c	chr12:89588049-897
ENSG0000(	387	8.642694	chr12:149	LINC02258		lncRNA	chr12:87795733-878
ENSG0000(	387	8.642694	chr12:149	PPP1R12A-AS1		lncRNA	chr12:79934901-799
ENSG0000(	387	8.642694	chr12:149	ENSG00000257564		Pseudoger	chr12:78793526-787

ENSG0000(	387	8.642694	chr12:149	RNA5SP365	Pseudoger	chr12:89726936-897
ENSG0000(	387	8.642694	chr12:149	CEP290	protein_c	chr12:88049016-881
ENSG0000(	387	8.642694	chr12:149	snoU13	smallRNA	chr12:77162926-771
ENSG0000(	387	8.642694	chr12:149	RNU6-977P	smallRNA	chr12:83021890-830
ENSG0000(	387	8.642694	chr12:149	AKIRIN1P1	Pseudoger	chr12:80561017-805
ENSG0000(	387	8.642694	chr12:149	ENSG00000279939	TEC	chr12:89544203-895
ENSG0000(	387	8.642694	chr12:149	ENSG00000289218	lncRNA	chr12:84986786-849
ENSG0000(	387	8.642694	chr12:149	RPL6P25	Pseudoger	chr12:83151331-831
ENSG0000(	387	8.642694	chr12:149	NOP56P3	Pseudoger	chr12:79558782-795
ENSG0000(	387	8.642694	chr12:149	ENSG00000289174	lncRNA	chr12:88781256-888
ENSG0000(	387	8.642694	chr12:149	ENSG00000258066	lncRNA	chr12:77775783-777
ENSG0000(	387	8.642694	chr12:149	AC010196.1	smallRNA	chr12:86745068-867
ENSG0000(	387	8.642694	chr12:149	AC027288.2	smallRNA	chr12:79462464-794
ENSG0000(	387	8.642694	chr12:149	AC083811.1	smallRNA	chr12:82342596-823
ENSG0000(	387	8.642694	chr12:149	LINC02424	lncRNA	chr12:78326680-783
ENSG0000(	387	8.642694	chr12:149	ENSG00000289369	lncRNA	chr12:86737397-867
ENSG0000(	387	8.642694	chr12:149	ENSG00000257948	lncRNA	chr12:78794132-788
ENSG0000(	387	8.642694	chr12:149	OSBPL8 NCGv7	protein_c	chr12:76351797-765
ENSG0000(	387	8.642694	chr12:149	ENSG00000258225	lncRNA	chr12:78052181-780
ENSG0000(	387	8.642694	chr12:149	ATP2B1-AS1	lncRNA	chr12:89708959-897
ENSG0000(	387	8.642694	chr12:149	RN7SL696P	smallRNA	chr12:79524877-795
ENSG0000(	387	8.642694	chr12:149	ENSG00000258375	Pseudoger	chr12:81545326-815
ENSG0000(	387	8.642694	chr12:149	AC083810.1	smallRNA	chr12:82264943-822
ENSG0000(	387	8.642694	chr12:149	ZDHHC17	protein_c	chr12:76764103-768
ENSG0000(	387	8.642694	chr12:149	ENSG00000258193	Pseudoger	chr12:81998632-819
ENSG0000(	387	8.642694	chr12:149	ENSG00000258162	lncRNA	chr12:81378042-815
ENSG0000(	387	8.642694	chr12:149	ENSG00000286608	lncRNA	chr12:89353798-894
ENSG0000(	387	8.642694	chr12:149	AC136188.1	smallRNA	chr12:73899870-738
ENSG0000(	387	8.642694	chr12:149	RNU1-117P	smallRNA	chr12:88581639-885
ENSG0000(	387	8.642694	chr12:149	ENSG00000257113	Pseudoger	chr12:74274952-742
ENSG0000(	384	8.575697	chr11:583	ENSG00000257070	Pseudoger	chr11:113769660-11
ENSG0000(	384	8.575697	chr11:583	ZW10	protein_c	chr11:113733187-11
ENSG0000(	384	8.575697	chr11:583	ENSG00000256757	lncRNA	chr11:113405321-11
ENSG0000(	384	8.575697	chr11:583	CLDN25	protein_c	chr11:113779796-11
ENSG0000(	384	8.575697	chr11:583	ENSG00000255710	Pseudoger	chr11:113820856-11
ENSG0000(	384	8.575697	chr11:583	HTR3A	protein_c	chr11:113975075-11
ENSG0000(	384	8.575697	chr11:583	AP000908.1	smallRNA	chr11:114086811-11
ENSG0000(	384	8.575697	chr11:583	ENSG00000270179	lncRNA	chr11:113368478-11
ENSG0000(	384	8.575697	chr11:583	RPS29P19	Pseudoger	chr11:113751116-11
ENSG0000(	384	8.575697	chr11:583	NCAM1-AS1	lncRNA	chr11:113265137-11
ENSG0000(	384	8.575697	chr11:583	ATF4P4	Pseudoger	chr11:113789231-11
ENSG0000(	384	8.575697	chr11:583	ENSG00000289230	lncRNA	chr11:114057969-11
ENSG0000(	384	8.575697	chr11:583	MIR4301	smallRNA	chr11:113450023-11
ENSG0000(	384	8.575697	chr11:583	ENSG00000256452	lncRNA	chr11:113818077-11
ENSG0000(	384	8.575697	chr11:583	TTC12	protein_c	chr11:113314579-11
ENSG0000(	384	8.575697	chr11:583	RNU7-187P	smallRNA	chr11:112977353-11
ENSG0000(	384	8.575697	chr11:583	MTRF1LP1	Pseudoger	chr11:113711720-11
ENSG0000(	384	8.575697	chr11:583	TTC12-DT	lncRNA	chr11:113278250-11
ENSG0000(	384	8.575697	chr11:583	LRRC37A13P	Pseudoger	chr11:113791466-11
ENSG0000(	384	8.575697	chr11:583	ANKK1	protein_c	chr11:113387779-11
ENSG0000(	384	8.575697	chr11:583	RNU6-1107P	smallRNA	chr11:113859346-11

ENSG0000	384	8.575697	chr11:58	ENSG00000256603		lncRNA	chr11:113770393-11
ENSG0000	384	8.575697	chr11:58	USP28	NCGv7	protein_	chr11:113797874-11
ENSG0000	384	8.575697	chr11:58	HTR3B		protein_	chr11:113904796-11
ENSG0000	384	8.575697	chr11:58	TMPRSS5		protein_	chr11:113687547-11
ENSG0000	384	8.575697	chr11:58	ENSG00000255870		Pseudoge	chr11:113789242-11
ENSG0000	384	8.575697	chr11:58	DRD2		protein_	chr11:113409605-11
ENSG0000	384	8.575697	chr11:58	NCAM1		protein_	chr11:112961247-11
ENSG0000	383	8.553364	chr12:31	RNU7-114P		smallRNA	chr12:113602791-11
ENSG0000	382	8.531032	chr5:164	ATP6V0E1P1		Pseudoge	chr5:132948493-132
ENSG0000	382	8.531032	chr5:164	ENSG00000286274		lncRNA	chr5:129150677-129
ENSG0000	382	8.531032	chr5:164	ENSG00000234758		lncRNA	chr5:131944408-131
ENSG0000	382	8.531032	chr5:164	ENSG00000288697		Pseudoge	chr5:128932070-128
ENSG0000	382	8.531032	chr5:164	ENSG00000288758		lncRNA	chr5:133239988-133
ENSG0000	382	8.531032	chr5:164	ZCCHC10		protein_	chr5:132996985-133
ENSG0000	382	8.531032	chr5:164	ACSL6-AS1		lncRNA	chr5:132003592-132
ENSG0000	382	8.531032	chr5:164	CDC42SE2		protein_	chr5:131245493-131
ENSG0000	382	8.531032	chr5:164	SLC22A5	DriverDB	protein_	chr5:132369710-132
ENSG0000	382	8.531032	chr10:81	CCNY-AS1		lncRNA	chr10:35314552-353
ENSG0000	382	8.531032	chr5:164	RNU6ATAC10P		smallRNA	chr5:129974891-129
ENSG0000	382	8.531032	chr5:164	HINT1		protein_	chr5:131155383-131
ENSG0000	382	8.531032	chr5:164	RAPGEF6		protein_	chr5:131423921-131
ENSG0000	382	8.531032	chr5:164	ENSG00000279691		TEC	chr5:133052769-133
ENSG0000	382	8.531032	chr5:164	ENSG00000272203		lncRNA	chr5:132817248-132
ENSG0000	382	8.531032	chr5:164	MIR3936HG		lncRNA	chr5:132311285-132
ENSG0000	382	8.531032	chr10:81	RNU6-1167P		smallRNA	chr10:35250959-352
ENSG0000	382	8.531032	chr5:164	IL13		protein_	chr5:132656263-132
ENSG0000	382	8.531032	chr5:164	SLC12A2-DT		lncRNA	chr5:127939152-128
ENSG0000	382	8.531032	chr10:81	CUL2		protein_	chr10:35008504-351
ENSG0000	382	8.531032	chr5:164	Y_RNA		smallRNA	chr5:132468147-132
ENSG0000	382	8.531032	chr5:164	MIR4460		smallRNA	chr5:129397062-129
ENSG0000	382	8.531032	chr5:164	ENSG00000248648		Pseudoge	chr5:133003119-133
ENSG0000	382	8.531032	chr10:81	ATP6V1G1P4		Pseudoge	chr10:35158306-351
ENSG0000	382	8.531032	chr5:164	Y_RNA		smallRNA	chr5:132945342-132
ENSG0000	382	8.531032	chr5:164	POGLUT2P1		Pseudoge	chr5:128109486-128
ENSG0000	382	8.531032	chr5:164	ADAMTS19-AS1		lncRNA	chr5:129424782-129
ENSG0000	382	8.531032	chr5:164	SLC12A2		protein_	chr5:128083766-128
ENSG0000	382	8.531032	chr5:164	ENSG00000279370		TEC	chr5:131261321-131
ENSG0000	382	8.531032	chr5:164	MINAR2		protein_	chr5:129748094-129
ENSG0000	382	8.531032	chr10:81	RPS12P16		Pseudoge	chr10:34675635-346
ENSG0000	382	8.531032	chr5:164	HSPA4	NCGv7;AC	protein_	chr5:133052013-133
ENSG0000	382	8.531032	chr5:164	CCNI2		protein_	chr5:132747426-132
ENSG0000	382	8.531032	chr5:164	ENSG00000281938		protein_	chr5:131807143-132
ENSG0000	382	8.531032	chr5:164	ENSG00000250405		Pseudoge	chr5:130994253-130
ENSG0000	382	8.531032	chr10:81	PARD3-DT		lncRNA	chr10:34815767-348
ENSG0000	382	8.531032	chr5:164	ENSG00000248955		Pseudoge	chr5:131008583-131
ENSG0000	382	8.531032	chr5:164	ENSG00000286408		lncRNA	chr5:133051065-133
ENSG0000	382	8.531032	chr5:164	ENSG00000251680		lncRNA	chr5:129500361-129
ENSG0000	382	8.531032	chr5:164	ENSG00000235775		Pseudoge	chr5:132163603-132
ENSG0000	382	8.531032	chr10:81	LINC02634		lncRNA	chr10:35219894-352
ENSG0000	382	8.531032	chr5:164	ENSG00000249418		Pseudoge	chr5:130598348-130
ENSG0000	382	8.531032	chr10:81	RNU6-193P		smallRNA	chr10:34942969-349

ENSG0000(	382	8.531032	chr5:164(	ENSG00000227616		Pseudoger	chr5:132147491-132
ENSG0000(	382	8.531032	chr5:164(	ENSG00000287390		lncRNA	chr5:130655383-130
ENSG0000(	382	8.531032	chr5:164(	ENSG00000224431		Pseudoger	chr5:132199456-132
ENSG0000(	382	8.531032	chr5:164(	MIR3936		smallRNA	chr5:132365490-132
ENSG0000(	382	8.531032	chr5:164(	CHSY3		protein_c	chr5:129904465-130
ENSG0000(	382	8.531032	chr5:164(	ACTBP4		Pseudoger	chr5:131659046-131
ENSG0000(	382	8.531032	chr10:81(	PRDX2P2		Pseudoger	chr10:34965069-349
ENSG0000(	382	8.531032	chr5:164(	RNA5SP191		Pseudoger	chr5:130114409-130
ENSG0000(	382	8.531032	chr5:164(	RNU7-53P		smallRNA	chr5:130386621-130
ENSG0000(	382	8.531032	chr5:164(	ENSG00000283782		protein_c	chr5:132410832-132
ENSG0000(	382	8.531032	chr5:164(	IRF1	NCGv7	protein_c	chr5:132440440-132
ENSG0000(	382	8.531032	chr5:164(	ADAMTS19	NCGv7	protein_c	chr5:129460281-129
ENSG0000(	382	8.531032	chr5:164(	LYRM7		protein_c	chr5:131170944-131
ENSG0000(	382	8.531032	chr5:164(	FNIP1		protein_c	chr5:131641714-131
ENSG0000(	382	8.531032	chr10:81(	RNU6-794P		smallRNA	chr10:35231331-352
ENSG0000(	382	8.531032	chr5:164(	ENSG00000248634		lncRNA	chr5:128663978-128
ENSG0000(	382	8.531032	chr5:164(	P4HA2-AS1		lncRNA	chr5:132184876-132
ENSG0000(	382	8.531032	chr5:164(	FBN2	NCGv7	protein_c	chr5:128257909-128
ENSG0000(	382	8.531032	chr5:164(	PDLIM4		protein_c	chr5:132257696-132
ENSG0000(	382	8.531032	chr5:164(	KIF3A		protein_c	chr5:132692628-132
ENSG0000(	382	8.531032	chr5:164(	EEF1A1P50		Pseudoger	chr5:132942932-132
ENSG0000(	382	8.531032	chr5:164(	GDF9		protein_c	chr5:132861181-132
ENSG0000(	382	8.531032	chr5:164(	ENSG00000231585		lncRNA	chr5:132011448-132
ENSG0000(	382	8.531032	chr5:164(	ENSG00000249478		lncRNA	chr5:133243764-133
ENSG0000(	382	8.531032	chr10:81(	RNU7-77P		smallRNA	chr10:35135443-351
ENSG0000(	382	8.531032	chr5:164(	ENSG00000250603		lncRNA	chr5:127838486-127
ENSG0000(	382	8.531032	chr5:164(	LEAP2		protein_c	chr5:132873444-132
ENSG0000(	382	8.531032	chr5:164(	SLC22A4		protein_c	chr5:132294394-132
ENSG0000(	382	8.531032	chr5:164(	Y_RNA		smallRNA	chr5:132993099-132
ENSG0000(	382	8.531032	chr5:164(	IL5		protein_c	chr5:132541445-132
ENSG0000(	382	8.531032	chr5:164(	SOWAHA		protein_c	chr5:132813302-132
ENSG0000(	382	8.531032	chr5:164(	RNA5SP192		Pseudoger	chr5:132848577-132
ENSG0000(	382	8.531032	chr5:164(	P4HA2		protein_c	chr5:132190147-132
ENSG0000(	382	8.531032	chr5:164(	IRF1-AS1		lncRNA	chr5:132410636-132
ENSG0000(	382	8.531032	chr5:164(	ENSG00000248245		lncRNA	chr5:133160438-133
ENSG0000(	382	8.531032	chr5:164(	CCDC192		protein_c	chr5:127703391-127
ENSG0000(	382	8.531032	chr10:81(	ENSG00000269952		lncRNA	chr10:35210416-352
ENSG0000(	382	8.531032	chr5:164(	MEIKIN		protein_c	chr5:131806990-131
ENSG0000(	382	8.531032	chr5:164(	Y_RNA		smallRNA	chr5:128371099-128
ENSG0000(	382	8.531032	chr5:164(	MIR4633		smallRNA	chr5:129097688-129
ENSG0000(	382	8.531032	chr5:164(	RPL6P15		Pseudoger	chr5:133172218-133
ENSG0000(	382	8.531032	chr5:164(	SLC27A6		protein_c	chr5:128538013-129
ENSG0000(	382	8.531032	chr5:164(	TH2LCRR		lncRNA	chr5:132630589-132
ENSG0000(	382	8.531032	chr5:164(	snoZ6		smallRNA	chr5:132080066-132
ENSG0000(	382	8.531032	chr10:81(	RPL37P18		Pseudoger	chr10:34663859-346
ENSG0000(	382	8.531032	chr5:164(	UQCRQ	DriverDB\	protein_c	chr5:132866630-132
ENSG0000(	382	8.531032	chr5:164(	LINC02863		lncRNA	chr5:132419416-132
ENSG0000(	382	8.531032	chr5:164(	ENSG00000290020		lncRNA	chr5:131210048-131
ENSG0000(	382	8.531032	chr5:164(	RAD50		protein_c	chr5:132556019-132
ENSG0000(	382	8.531032	chr10:81(	RNU6-847P		smallRNA	chr10:34926775-349
ENSG0000(	382	8.531032	chr5:164(	ENSG00000224015		lncRNA	chr5:132179234-132

ENSG0000(	382	8.531032	chr5:164(	IL3		protein_c	chr5:132060655-132
ENSG0000(	382	8.531032	chr5:164(	SHROOM1		protein_c	chr5:132822141-132
ENSG0000(	382	8.531032	chr10:81(	LINC02635		lncRNA	chr10:34969881-349
ENSG0000(	382	8.531032	chr5:164(	ACSL6	NCGv7	protein_c	chr5:131949973-132
ENSG0000(	382	8.531032	chr10:81(	MIR3611		smallRNA	chr10:35079598-350
ENSG0000(	382	8.531032	chr10:81(	CREM		protein_c	chr10:35126791-352
ENSG0000(	382	8.531032	chr5:164(	ISOC1		protein_c	chr5:129094749-129
ENSG0000(	382	8.531032	chr5:164(	ENSG00000251616		lncRNA	chr5:133111055-133
ENSG0000(	382	8.531032	chr10:81(	ENSG00000230534		lncRNA	chr10:35098006-351
ENSG0000(	382	8.531032	chr5:164(	IL4		protein_c	chr5:132673986-132
ENSG0000(	382	8.531032	chr5:164(	HSPA8P4		Pseudoger	chr5:130140031-130
ENSG0000(	382	8.531032	chr5:164(	AFF4-DT		lncRNA	chr5:132963770-132
ENSG0000(	382	8.531032	chr5:164(	CSF2		protein_c	chr5:132073789-132
ENSG0000(	382	8.531032	chr5:164(	ENSG00000244192		Pseudoger	chr5:130989897-130
ENSG0000(	382	8.531032	chr5:164(	SEPTIN8		protein_c	chr5:132750819-132
ENSG0000(	382	8.531032	chr5:164(	ENSG00000287054		lncRNA	chr5:133025847-133
ENSG0000(	382	8.531032	chr5:164(	ENSG00000273217		protein_c	chr5:131425891-131
ENSG0000(	382	8.531032	chr5:164(	ENSG00000229738		Pseudoger	chr5:132987652-132
ENSG0000(	382	8.531032	chr5:164(	ENSG00000230612		lncRNA	chr5:132688681-132
ENSG0000(	382	8.531032	chr5:164(	AFF4	NCGv7	protein_c	chr5:132875395-132
ENSG0000(	382	8.531032	chr5:164(	ENSG00000279584		TEC	chr5:131797415-131
ENSG0000(	382	8.531032	chr5:164(	ARL2BPP4		Pseudoger	chr5:130529419-130
ENSG0000(	381	8.508699	chr5:164(	LINC01948		lncRNA	chr5:56451627-5648
ENSG0000(	381	8.508699	chr5:164(	PLK2		protein_c	chr5:58453982-5846
ENSG0000(	381	8.508699	chr5:164(	ZSWIM6	NCGv7	protein_c	chr5:61332258-6154
ENSG0000(	381	8.508699	chr9:957	ENSG00000228487		lncRNA	chr9:127213783-127
ENSG0000(	381	8.508699	chr5:164(	LRRC70		protein_c	chr5:62578819-6258
ENSG0000(	381	8.508699	chr5:164(	LINC02057		lncRNA	chr5:61201304-6130
ENSG0000(	381	8.508699	chr5:164(	MIR4459		smallRNA	chr5:54075518-5407
ENSG0000(	381	8.508699	chr5:164(	ENSG00000287434		lncRNA	chr5:60699729-6070
ENSG0000(	381	8.508699	chr5:164(	ITGA2-AS1		lncRNA	chr5:52930606-5299
ENSG0000(	381	8.508699	chr5:164(	ENSG00000247345		lncRNA	chr5:59039761-5906
ENSG0000(	381	8.508699	chr5:164(	AC116606.1		smallRNA	chr5:57872939-5787
ENSG0000(	381	8.508699	chr5:164(	ENSG00000249236		lncRNA	chr5:55978248-5611
ENSG0000(	381	8.508699	chr5:164(	HSPB3		protein_c	chr5:54455699-5445
ENSG0000(	381	8.508699	chr5:164(	RN7SL801P		smallRNA	chr5:54048009-5404
ENSG0000(	381	8.508699	chr5:164(	PDE4D		protein_c	chr5:58969038-6052
ENSG0000(	381	8.508699	chr5:164(	ENSG00000241991		Pseudoger	chr5:62561859-6256
ENSG0000(	381	8.508699	chr5:164(	GZMA		protein_c	chr5:55102646-5511
ENSG0000(	381	8.508699	chr5:164(	KATNBL1P4		Pseudoger	chr5:51930808-5193
ENSG0000(	381	8.508699	chr5:164(	LINC02108		lncRNA	chr5:58541567-5855
ENSG0000(	381	8.508699	chr5:164(	MIR449A		smallRNA	chr5:55170532-5517
ENSG0000(	381	8.508699	chr5:164(	ENSG00000279470		TEC	chr5:51963992-5196
ENSG0000(	381	8.508699	chr5:164(	AC008391.1		smallRNA	chr5:56457038-5645
ENSG0000(	381	8.508699	chr5:164(	GZMK		protein_c	chr5:55024256-5503
ENSG0000(	381	8.508699	chr9:957	ENSG00000271833		lncRNA	chr9:127366770-127
ENSG0000(	381	8.508699	chr5:164(	ENSG00000241809		Pseudoger	chr5:52903908-5290
ENSG0000(	381	8.508699	chr9:957	LMX1B-DT		lncRNA	chr9:126589594-126
ENSG0000(	381	8.508699	chr5:164(	ENSG00000225230		lncRNA	chr5:56900041-5691
ENSG0000(	381	8.508699	chr5:164(	RN7SKP157		smallRNA	chr5:62146665-6214
ENSG0000(	381	8.508699	chr5:164(	AC008836.1		smallRNA	chr5:61557283-6155

ENSG00000	381	8.508699	chr5:1646	RMEL3		lncRNA	chr5:57395060-5753
ENSG00000	381	8.508699	chr5:1646	ENSG00000289916		lncRNA	chr5:61902617-6191
ENSG00000	381	8.508699	chr5:1646	C1GALT1P2		Pseudogen	chr5:56192530-5619
ENSG00000	381	8.508699	chr5:1646	ENSG00000249843		Pseudogen	chr5:54878238-5487
ENSG00000	381	8.508699	chr9:9575	SLC2A8		protein_c	chr9:127397138-127
ENSG00000	381	8.508699	chr9:9575	ENSG00000238010		lncRNA	chr9:126640756-126
ENSG00000	381	8.508699	chr5:1646	ENSG00000237705		lncRNA	chr5:56842016-5686
ENSG00000	381	8.508699	chr5:1646	RNU6-272P		smallRNA	chr5:53912020-5391
ENSG00000	381	8.508699	chr5:1646	KRT8P31		Pseudogen	chr5:60743563-6074
ENSG00000	381	8.508699	chr9:9575	ANGPTL2		protein_c	chr9:127087348-127
ENSG00000	381	8.508699	chr9:9575	LRSAM1		protein_c	chr9:127451489-127
ENSG00000	381	8.508699	chr5:1646	MIR449B		smallRNA	chr5:55170646-5517
ENSG00000	381	8.508699	chr5:1646	ERCC8		protein_c	chr5:60866454-6094
ENSG00000	381	8.508699	chr5:1646	SNX18		protein_c	chr5:54517759-5454
ENSG00000	381	8.508699	chr5:1646	ENSG00000289709		lncRNA	chr5:56766395-5677
ENSG00000	381	8.508699	chr5:1646	ISCA1P1		Pseudogen	chr5:62776877-6277
ENSG00000	381	8.508699	chr9:9575	SNORD116		smallRNA	chr9:126426893-126
ENSG00000	381	8.508699	chr5:1646	SETD9		protein_c	chr5:56909260-5692
ENSG00000	381	8.508699	chr5:1646	MIER3	NCGv7	protein_c	chr5:56919602-5697
ENSG00000	381	8.508699	chr9:9575	GARNL3		protein_c	chr9:127224265-127
ENSG00000	381	8.508699	chr9:9575	ZNF79		protein_c	chr9:127424374-127
ENSG00000	381	8.508699	chr5:1646	ENSG00000288936		lncRNA	chr5:61321498-6133
ENSG00000	381	8.508699	chr5:1646	ENSG00000287367		lncRNA	chr5:54663327-5473
ENSG00000	381	8.508699	chr9:9575	RPL12		protein_c	chr9:127447674-127
ENSG00000	381	8.508699	chr5:1646	RNA5SP184		Pseudogen	chr5:56146739-5614
ENSG00000	381	8.508699	chr5:1646	ENSG00000248029		lncRNA	chr5:55063179-5506
ENSG00000	381	8.508699	chr5:1646	Y_RNA		smallRNA	chr5:55040847-5504
ENSG00000	381	8.508699	chr5:1646	AC025470.1		smallRNA	chr5:57481820-5748
ENSG00000	381	8.508699	chr5:1646	ENSG00000248898		lncRNA	chr5:52673186-5279
ENSG00000	381	8.508699	chr9:9575	ZBTB34		protein_c	chr9:126860639-126
ENSG00000	381	8.508699	chr5:1646	ASS1P9		Pseudogen	chr5:53859166-5386
ENSG00000	381	8.508699	chr5:1646	ENSG00000228650		lncRNA	chr5:56770799-5677
ENSG00000	381	8.508699	chr5:1646	LINC02101		lncRNA	chr5:58107631-5812
ENSG00000	381	8.508699	chr5:1646	ENSG00000288643		protein_c	chr5:62306206-6253
ENSG00000	381	8.508699	chr5:1646	Y_RNA		smallRNA	chr5:57169108-5716
ENSG00000	381	8.508699	chr5:1646	MIR548AE2		smallRNA	chr5:58530043-5853
ENSG00000	381	8.508699	chr5:1646	ENSG00000287087		lncRNA	chr5:53897464-5389
ENSG00000	381	8.508699	chr5:1646	GPBP1		protein_c	chr5:57173948-5726
ENSG00000	381	8.508699	chr9:9575	AL161731.1		smallRNA	chr9:126718243-126
ENSG00000	381	8.508699	chr5:1646	IPO11		protein_c	chr5:62403972-6262
ENSG00000	381	8.508699	chr5:1646	ENSG00000235635		lncRNA	chr5:56941307-5694
ENSG00000	381	8.508699	chr5:1646	MIR581		smallRNA	chr5:53951504-5395
ENSG00000	381	8.508699	chr5:1646	ENSG00000288957		lncRNA	chr5:54310711-5431
ENSG00000	381	8.508699	chr5:1646	DIMT1		protein_c	chr5:62347284-6240
ENSG00000	381	8.508699	chr9:9575	ENSG00000233721		lncRNA	chr9:126516417-126
ENSG00000	381	8.508699	chr5:1646	SMIM15		protein_c	chr5:61157704-6116
ENSG00000	381	8.508699	chr5:1646	AC024569.1		smallRNA	chr5:53609587-5360
ENSG00000	381	8.508699	chr9:9575	RALGPS1		protein_c	chr9:126914774-127
ENSG00000	381	8.508699	chr5:1646	ENSG00000279883		TEC	chr5:55484403-5548
ENSG00000	381	8.508699	chr9:9575	LMX1B	AC	protein_c	chr9:126613928-126
ENSG00000	381	8.508699	chr5:1646	ENSG00000249198		lncRNA	chr5:57751192-5775

ENSG0000(	381	8.508699	chr5:164(	ENSG00000251682		Pseudoger	chr5:61347126-6134
ENSG0000(	381	8.508699	chr5:164(	ENSG00000249878		Pseudoger	chr5:61831947-6183
ENSG0000(	381	8.508699	chr5:164(	ENSG00000285831		lncRNA	chr5:53297872-5332
ENSG0000(	381	8.508699	chr9:957!	ENSG00000229369		Pseudoger	chr9:127300093-127
ENSG0000(	381	8.508699	chr5:164(	ENSG00000248733		lncRNA	chr5:58846885-5886
ENSG0000(	381	8.508699	chr5:164(	RNA5SP182		Pseudoger	chr5:51886688-5188
ENSG0000(	381	8.508699	chr5:164(	MIR582		smallRNA	chr5:59703606-5970
ENSG0000(	381	8.508699	chr5:164(	ENSG00000250480		Pseudoger	chr5:57120796-5712
ENSG0000(	381	8.508699	chr5:164(	C5orf64		lncRNA	chr5:61610424-6179
ENSG0000(	381	8.508699	chr5:164(	snoU13		smallRNA	chr5:56000518-5600
ENSG0000(	381	8.508699	chr5:164(	LINC02225		lncRNA	chr5:57890327-5789
ENSG0000(	381	8.508699	chr5:164(	ENSG00000234553		lncRNA	chr5:56536583-5653
ENSG0000(	381	8.508699	chr5:164(	ENSG00000272416		lncRNA	chr5:53880293-5388
ENSG0000(	381	8.508699	chr5:164(	C5orf67		lncRNA	chr5:56511567-5660
ENSG0000(	381	8.508699	chr5:164(	ENSG00000264281		Pseudoger	chr5:55944656-5594
ENSG0000(	381	8.508699	chr5:164(	PGAM1P1		Pseudoger	chr5:58161197-5816
ENSG0000(	381	8.508699	chr5:164(	ENSG00000251368		Pseudoger	chr5:55750206-5575
ENSG0000(	381	8.508699	chr9:957!	SNORA65		smallRNA	chr9:127448501-127
ENSG0000(	381	8.508699	chr5:164(	PLPP1		protein_c	chr5:55424854-5553
ENSG0000(	381	8.508699	chr5:164(	DHX29		protein_c	chr5:55256055-5530
ENSG0000(	381	8.508699	chr5:164(	RPL17P22		Pseudoger	chr5:56136979-5613
ENSG0000(	381	8.508699	chr5:164(	RNU6-661P		smallRNA	chr5:62443176-6244
ENSG0000(	381	8.508699	chr5:164(	MAP3K1	NCGv7	protein_c	chr5:56815549-5689
ENSG0000(	381	8.508699	chr5:164(	ENSG00000240052		Pseudoger	chr5:53413504-5341
ENSG0000(	381	8.508699	chr12:14!	MRPL40P1		Pseudoger	chr12:67351436-673
ENSG0000(	381	8.508699	chr5:164(	ENSG00000279638		TEC	chr5:54286194-5428
ENSG0000(	381	8.508699	chr5:164(	MOCS2-DT		lncRNA	chr5:53109816-5312
ENSG0000(	381	8.508699	chr5:164(	ENSG00000287709		lncRNA	chr5:57650632-5775
ENSG0000(	381	8.508699	chr5:164(	ENSG00000276214		Pseudoger	chr5:59314110-5931
ENSG0000(	381	8.508699	chr5:164(	B3GNTL1P1		Pseudoger	chr5:52889666-5288
ENSG0000(	381	8.508699	chr9:957!	ENSG00000226078		lncRNA	chr9:126527618-126
ENSG0000(	381	8.508699	chr5:164(	ERCC8-AS1		lncRNA	chr5:60917490-6091
ENSG0000(	381	8.508699	chr5:164(	AC025175.1		smallRNA	chr5:53782698-5378
ENSG0000(	381	8.508699	chr5:164(	CKS1BP3		Pseudoger	chr5:62512246-6251
ENSG0000(	381	8.508699	chr5:164(	MCIDAS		protein_c	chr5:55219564-5522
ENSG0000(	381	8.508699	chr5:164(	LNCBRM		lncRNA	chr5:57574027-5761
ENSG0000(	381	8.508699	chr5:164(	ENSG00000248475		lncRNA	chr5:58741581-5881
ENSG0000(	381	8.508699	chr5:164(	ACTBL2		protein_c	chr5:57480018-5748
ENSG0000(	381	8.508699	chr5:164(	DEPDC1B		protein_c	chr5:60596912-6070
ENSG0000(	381	8.508699	chr5:164(	ENSG00000249697		lncRNA	chr5:56313905-5632
ENSG0000(	381	8.508699	chr5:164(	GZMAP1		Pseudoger	chr5:55073015-5509
ENSG0000(	381	8.508699	chr5:164(	LINC02105		lncRNA	chr5:53776048-5381
ENSG0000(	381	8.508699	chr5:164(	ENSG00000286048		lncRNA	chr5:53024924-5303
ENSG0000(	381	8.508699	chr9:957!	NRON		smallRNA	chr9:126408041-126
ENSG0000(	381	8.508699	chr9:957!	AL356309.1		smallRNA	chr9:126530915-126
ENSG0000(	381	8.508699	chr5:164(	ENSG00000289060		lncRNA	chr5:54883635-5488
ENSG0000(	381	8.508699	chr5:164(	ENSG00000250461		Pseudoger	chr5:61375112-6137
ENSG0000(	381	8.508699	chr5:164(	MFSD4BP1		Pseudoger	chr5:52579555-5258
ENSG0000(	381	8.508699	chr5:164(	ENSG00000280159		TEC	chr5:54643557-5464
ENSG0000(	381	8.508699	chr5:164(	ITGA2		protein_c	chr5:52989340-5309
ENSG0000(	381	8.508699	chr5:164(	RPS17P11		Pseudoger	chr5:52282567-5228

ENSG0000(	381	8.508699	chr5:164(	ENSG00000262211		lncRNA	chr5:55936143-5594
ENSG0000(	381	8.508699	chr5:164(	GPX8		protein_c	chr5:55160167-5516
ENSG0000(	381	8.508699	chr5:164(	NDUFS4		protein_c	chr5:53560633-5368
ENSG0000(	381	8.508699	chr5:164(	ENSG00000285997		lncRNA	chr5:56488486-5649
ENSG0000(	381	8.508699	chr5:164(	ENSG00000240535		lncRNA	chr5:55021299-5504
ENSG0000(	381	8.508699	chr5:164(	ESM1		protein_c	chr5:54977867-5502
ENSG0000(	381	8.508699	chr5:164(	RNA5SP185		Pseudoger	chr5:56260333-5626
ENSG0000(	381	8.508699	chr5:164(	CDC20B		protein_c	chr5:55112971-5517
ENSG0000(	381	8.508699	chr5:164(	ANKRD55		protein_c	chr5:56099680-5623
ENSG0000(	381	8.508699	chr5:164(	IL31RA		protein_c	chr5:55851357-5592
ENSG0000(	381	8.508699	chr5:164(	ENSG00000276945		Pseudoger	chr5:61145846-6114
ENSG0000(	381	8.508699	chr5:164(	SMIM15-AS1		lncRNA	chr5:61162070-6123
ENSG0000(	381	8.508699	chr5:164(	MOCS2	DriverDB\	protein_c	chr5:53095679-5311
ENSG0000(	381	8.508699	chr5:164(	CCNO-DT		lncRNA	chr5:55233934-5529
ENSG0000(	381	8.508699	chr5:164(	MIR449C		smallRNA	chr5:55172262-5517
ENSG0000(	381	8.508699	chr5:164(	CSPG4BP		Pseudoger	chr5:54808210-5487
ENSG0000(	381	8.508699	chr5:164(	NDUFB4P2		Pseudoger	chr5:59528861-5952
ENSG0000(	381	8.508699	chr5:164(	GAPT		protein_c	chr5:58491435-5849
ENSG0000(	381	8.508699	chr5:164(	CAB39P1		Pseudoger	chr5:60630514-6063
ENSG0000(	381	8.508699	chr5:164(	ENSG00000271410		Pseudoger	chr5:55356308-5535
ENSG0000(	381	8.508699	chr5:164(	SETP21		Pseudoger	chr5:60459848-6046
ENSG0000(	381	8.508699	chr5:164(	KIF2A		protein_c	chr5:62306162-6239
ENSG0000(	381	8.508699	chr5:164(	RNU6-913P		smallRNA	chr5:61664145-6166
ENSG0000(	381	8.508699	chr5:164(	RNU6-806P		smallRNA	chr5:60304047-6030
ENSG0000(	381	8.508699	chr9:957[illegible]	MVB12B		protein_c	chr9:126326829-126
ENSG0000(	381	8.508699	chr5:164(	RNA5SP183		Pseudoger	chr5:55777469-5577
ENSG0000(	381	8.508699	chr5:164(	AC112198.2		smallRNA	chr5:54852846-5485
ENSG0000(	381	8.508699	chr5:164(	ENSG00000288539		lncRNA	chr5:52853183-5287
ENSG0000(	381	8.508699	chr5:164(	IL6ST-DT		lncRNA	chr5:55995153-5600
ENSG0000(	381	8.508699	chr5:164(	IL6ST	NCGv7;AC	protein_c	chr5:55935095-5599
ENSG0000(	381	8.508699	chr5:164(	ENSG00000248199		lncRNA	chr5:61878109-6192
ENSG0000(	381	8.508699	chr5:164(	ENSG00000249016		lncRNA	chr5:54956235-5495
ENSG0000(	381	8.508699	chr5:164(	FST		protein_c	chr5:53480626-5348
ENSG0000(	381	8.508699	chr5:164(	HMGN1P17		Pseudoger	chr5:56381781-5638
ENSG0000(	381	8.508699	chr5:164(	ENSG00000279985		TEC	chr5:61114432-6111
ENSG0000(	381	8.508699	chr5:164(	ENSG00000248529		lncRNA	chr5:61658474-6169
ENSG0000(	381	8.508699	chr5:164(	ELOVL7	DriverDB\	protein_c	chr5:60751791-6084
ENSG0000(	381	8.508699	chr5:164(	ENSG00000289152		lncRNA	chr5:57172842-5717
ENSG0000(	381	8.508699	chr5:164(	C5orf64-AS1		lncRNA	chr5:61732774-6173
ENSG0000(	381	8.508699	chr5:164(	NDUFAF2		protein_c	chr5:60945177-6115
ENSG0000(	381	8.508699	chr5:164(	SALL4P1		Pseudoger	chr5:57422464-5742
ENSG0000(	381	8.508699	chr5:164(	ENSG00000233943		Pseudoger	chr5:56975593-5697
ENSG0000(	381	8.508699	chr5:164(	snoU13		smallRNA	chr5:58070188-5807
ENSG0000(	381	8.508699	chr5:164(	ENSG00000248652		Pseudoger	chr5:57081745-5708
ENSG0000(	381	8.508699	chr5:164(	ENSG00000248586		Pseudoger	chr5:57317989-5732
ENSG0000(	381	8.508699	chr9:957[illegible]	Y_RNA		smallRNA	chr9:127470875-127
ENSG0000(	381	8.508699	chr5:164(	ENSG00000286853		lncRNA	chr5:58114598-5814
ENSG0000(	381	8.508699	chr5:164(	ENSG00000226810		Pseudoger	chr5:54857272-5485
ENSG0000(	381	8.508699	chr5:164(	ENSG00000248935		lncRNA	chr5:60021249-6003
ENSG0000(	381	8.508699	chr5:164(	ENSG00000213940		Pseudoger	chr5:53206561-5320
ENSG0000(	381	8.508699	chr5:164(	LINC01033		lncRNA	chr5:54313564-5441

ENSG0000(	381	8.508699	chr9:9575	AL161908.1		smallRNA	chr9:126576530-126
ENSG0000(	381	8.508699	chr5:164(	PART1		lncRNA	chr5:60487713-6054
ENSG0000(	381	8.508699	chr5:164(	RAB3C		protein_c	chr5:58582221-5885
ENSG0000(	381	8.508699	chr5:164(	LINC02118		lncRNA	chr5:52008031-5208
ENSG0000(	381	8.508699	chr5:164(	ARL15		protein_c	chr5:53883942-5431
ENSG0000(	381	8.508699	chr5:164(	ENSG00000251376		lncRNA	chr5:55054428-5505
ENSG0000(	381	8.508699	chr9:9575	ENSG00000288995		lncRNA	chr9:126819282-126
ENSG0000(	381	8.508699	chr5:164(	RPL26P19		Pseudoger	chr5:56504635-5650
ENSG0000(	381	8.508699	chr9:9575	ZBTB43		protein_c	chr9:126805006-126
ENSG0000(	381	8.508699	chr5:164(	RNU6ATAC2P		smallRNA	chr5:56297564-5629
ENSG0000(	381	8.508699	chr5:164(	RNU6-299P		smallRNA	chr5:56125671-5612
ENSG0000(	381	8.508699	chr5:164(	RNF138P1		Pseudoger	chr5:55530156-5553
ENSG0000(	381	8.508699	chr5:164(	MTREX	NCGv7	protein_c	chr5:55307989-5542
ENSG0000(	381	8.508699	chr5:164(	RPL3P6		Pseudoger	chr5:61390668-6139
ENSG0000(	381	8.508699	chr5:164(	SLC38A9		protein_c	chr5:55625845-5577
ENSG0000(	381	8.508699	chr5:164(	RPL5P15		Pseudoger	chr5:58822503-5882
ENSG0000(	381	8.508699	chr5:164(	AC008780.1		smallRNA	chr5:57642895-5764
ENSG0000(	381	8.508699	chr5:164(	MIR5687		smallRNA	chr5:55508850-5550
ENSG0000(	381	8.508699	chr9:9575	ENSG00000279137		TEC	chr9:126518840-126
ENSG0000(	381	8.508699	chr5:164(	ENSG00000271257		Pseudoger	chr5:58503295-5850
ENSG0000(	381	8.508699	chr5:164(	ENSG00000213896		Pseudoger	chr5:60429903-6043
ENSG0000(	381	8.508699	chr5:164(	ENSG00000272123		lncRNA	chr5:53089016-5308
ENSG0000(	381	8.508699	chr5:164(	ITGA1		protein_c	chr5:52787916-5295
ENSG0000(	381	8.508699	chr5:164(	RPL37P25		Pseudoger	chr5:54399439-5440
ENSG0000(	381	8.508699	chr5:164(	AK4P2		Pseudoger	chr5:55551885-5555
ENSG0000(	381	8.508699	chr5:164(	ENSG00000271828		lncRNA	chr5:56927874-5692
ENSG0000(	381	8.508699	chr5:164(	PSMC1P4		Pseudoger	chr5:56275304-5627
ENSG0000(	381	8.508699	chr5:164(	CCNO		protein_c	chr5:55231152-5523
ENSG0000(	381	8.508699	chr5:164(	HNRNPH1P3		Pseudoger	chr5:55838801-5584
ENSG0000(	381	8.508699	chr5:164(	PELO		protein_c	chr5:52787916-5280
ENSG0000(	381	8.508699	chr5:164(	snoU13		smallRNA	chr5:56857148-5685
ENSG0000(	381	8.508699	chr5:164(	DDX4		protein_c	chr5:55738017-5581
ENSG0000(	381	8.508699	chr5:164(	GNL3LP1		Pseudoger	chr5:60891935-6089
ENSG0000(	381	8.508699	chr9:9575	ENSG00000286428		lncRNA	chr9:126701173-126
ENSG0000(	381	8.508699	chr5:164(	RPL35AP14		Pseudoger	chr5:62575951-6257
ENSG0000(	380	8.486367	chr9:178(	ENSG00000286782		lncRNA	chr9:35012963-3501
ENSG0000(	380	8.486367	chr9:178(	SIT1		protein_c	chr9:35649295-3565
ENSG0000(	380	8.486367	chr9:178(	CCL21		protein_c	chr9:34709005-3471
ENSG0000(	380	8.486367	chr17:492	CYB561	DriverDBv	protein_c	chr17:63432304-634
ENSG0000(	380	8.486367	chr9:178(	NPR2		protein_c	chr9:35791003-3580
ENSG0000(	380	8.486367	chr9:178(	STOML2		protein_c	chr9:35099776-3510
ENSG0000(	380	8.486367	chr9:178(	RNF38		protein_c	chr9:36336396-3648
ENSG0000(	380	8.486367	chr9:178(	RMRP		smallRNA	chr9:35657754-3565
ENSG0000(	380	8.486367	chr17:492	ENSG00000265298		Pseudoger	chr17:64750420-647
ENSG0000(	380	8.486367	chr17:492	ENSG00000264813		protein_c	chr17:63484823-635
ENSG0000(	380	8.486367	chr3:380	ZKSCAN7		protein_c	chr3:44555193-4459
ENSG0000(	380	8.486367	chr17:492	ENSG00000279369		TEC	chr17:63700847-637
ENSG0000(	380	8.486367	chr9:178(	TLN1		protein_c	chr9:35696948-3573
ENSG0000(	380	8.486367	chr9:178(	ARHGEF39		protein_c	chr9:35658875-3567
ENSG0000(	380	8.486367	chr9:178(	FAM205BP		Pseudoger	chr9:34832295-3483
ENSG0000(	380	8.486367	chr9:178(	UBAP2		protein_c	chr9:33921693-3404

ENSG0000(	380	8.486367	chr9:178(	PGAM1P2		Pseudoger	chr9:35943210-3594
ENSG0000(	380	8.486367	chr9:178(	IL11RA		protein_c	chr9:34652162-3466
ENSG0000(	380	8.486367	chr9:178(	ENSG00000258728		protein_c	chr9:34646631-3466
ENSG0000(	380	8.486367	chr17:492	ENSG00000259333		Pseudoger	chr17:63900643-639
ENSG0000(	380	8.486367	chr9:178(	TRBV24OR9-2		Pseudoger	chr9:33649047-3364
ENSG0000(	380	8.486367	chr9:178(	ATP8B5P		lncRNA	chr9:35406755-3548
ENSG0000(	380	8.486367	chr17:492	SNORD104		smallRNA	chr17:64146083-641
ENSG0000(	380	8.486367	chr9:178(	DNAJB5		protein_c	chr9:34989641-3499
ENSG0000(	380	8.486367	chr3:3804	TOPAZ1		protein_c	chr3:44241886-4433
ENSG0000(	380	8.486367	chr17:492	ENSG00000290457		lncRNA	chr17:64927744-649
ENSG0000(	380	8.486367	chr9:178(	PRSS3	NCGv7	protein_c	chr9:33750679-3379
ENSG0000(	380	8.486367	chr17:492	ENSG00000226797		lncRNA	chr17:63381231-634
ENSG0000(	380	8.486367	chr9:178(	ENSG00000290538		lncRNA	chr9:35962195-3601
ENSG0000(	380	8.486367	chr9:178(	CCDC107	NCGv7	protein_c	chr9:35658290-3566
ENSG0000(	380	8.486367	chr9:178(	ENSG00000215198		Pseudoger	chr9:35147410-3514
ENSG0000(	380	8.486367	chr9:178(	GNE		protein_c	chr9:36214441-3627
ENSG0000(	380	8.486367	chr9:178(	AQP3		protein_c	chr9:33441156-3344
ENSG0000(	380	8.486367	chr9:178(	VCP		protein_c	chr9:35053928-3507
ENSG0000(	380	8.486367	chr9:178(	YBX1P10		Pseudoger	chr9:35971344-3597
ENSG0000(	380	8.486367	chr17:492	ENSG00000271919		Pseudoger	chr17:63331984-633
ENSG0000(	380	8.486367	chr9:178(	HINT2		protein_c	chr9:35812960-3581
ENSG0000(	380	8.486367	chr17:492	ATP5MFP4		Pseudoger	chr17:64491523-644
ENSG0000(	380	8.486367	chr9:178(	FAM166B		protein_c	chr9:35561831-3556
ENSG0000(	380	8.486367	chr9:178(	ENSG00000260947		lncRNA	chr9:33697459-3370
ENSG0000(	380	8.486367	chr9:178(	KIF24		protein_c	chr9:34252380-3432
ENSG0000(	380	8.486367	chr9:178(	PIGO-AS1		lncRNA	chr9:35096378-3509
ENSG0000(	380	8.486367	chr17:492	ENSG00000288894		protein_c	chr17:63550603-636
ENSG0000(	380	8.486367	chr9:178(	AQP7	NCGv7	protein_c	chr9:33383179-3340
ENSG0000(	380	8.486367	chr9:178(	TRBV29OR9-2		Pseudoger	chr9:33786221-3378
ENSG0000(	380	8.486367	chr9:178(	NOL6		protein_c	chr9:33461353-3347
ENSG0000(	380	8.486367	chr9:178(	RNU4ATAC15P		smallRNA	chr9:33174170-3317
ENSG0000(	380	8.486367	chr9:178(	ENSG00000230729		lncRNA	chr9:34521527-3452
ENSG0000(	380	8.486367	chr9:178(	SNORD121A		smallRNA	chr9:33952769-3395
ENSG0000(	380	8.486367	chr3:3804	ENSG00000271937		lncRNA	chr3:44337941-4433
ENSG0000(	380	8.486367	chr17:492	FAM136DP		Pseudoger	chr17:63610772-636
ENSG0000(	380	8.486367	chr17:492	ENSG00000263501		lncRNA	chr17:63305262-633
ENSG0000(	380	8.486367	chr9:178(	TMEM8B		protein_c	chr9:35814451-3586
ENSG0000(	380	8.486367	chr9:178(	ENSG00000279336		Pseudoger	chr9:34201273-3420
ENSG0000(	380	8.486367	chr9:178(	HMGB3P24		Pseudoger	chr9:36303499-3630
ENSG0000(	380	8.486367	chr17:492	PRKCA-AS1		lncRNA	chr17:66398069-664
ENSG0000(	380	8.486367	chr17:492	AMZ2P1		Pseudoger	chr17:64968222-649
ENSG0000(	380	8.486367	chr17:492	ENSG00000263644		lncRNA	chr17:63391191-634
ENSG0000(	380	8.486367	chr9:178(	SPAG8		protein_c	chr9:35808045-3581
ENSG0000(	380	8.486367	chr9:178(	DCTN3		protein_c	chr9:34613545-3462
ENSG0000(	380	8.486367	chr9:178(	CD72		protein_c	chr9:35609982-3564
ENSG0000(	380	8.486367	chr9:178(	OR2S2		protein_c	chr9:35957108-3595
ENSG0000(	380	8.486367	chr9:178(	PIGO		protein_c	chr9:35087237-3509
ENSG0000(	380	8.486367	chr9:178(	OR13C6P		Pseudoger	chr9:35991336-3599
ENSG0000(	380	8.486367	chr9:178(	TUBB4BP2		Pseudoger	chr9:34109171-3410
ENSG0000(	380	8.486367	chr17:492	KCNH6		protein_c	chr17:63523334-635
ENSG0000(	380	8.486367	chr9:178(	MSMP		protein_c	chr9:35752990-3575

ENSG0000(	380	8.486367	chr9:178(	ENSG00000261215		lncRNA	chr9:34661903-3466
ENSG0000(	380	8.486367	chr17:492	PLEKHM1P1		Pseudoger	chr17:64785259-648
ENSG0000(	380	8.486367	chr17:492	ARHGAP27P1-BPTFP1-		lncRNA	chr17:64749663-647
ENSG0000(	380	8.486367	chr17:492	ENSG00000286629		lncRNA	chr17:64067619-640
ENSG0000(	380	8.486367	chr17:492	ACE	NCGv7	protein_(	chr17:63477061-634
ENSG0000(	380	8.486367	chr17:492	MICOS10P2		Pseudoger	chr17:64747264-647
ENSG0000(	380	8.486367	chr9:178(	ENSG00000225123		Pseudoger	chr9:33574361-3357
ENSG0000(	380	8.486367	chr3:3804	ENSG00000272077		lncRNA	chr3:44667412-4466
ENSG0000(	380	8.486367	chr9:178(	RPP25L		protein_(	chr9:34610486-3461
ENSG0000(	380	8.486367	chr17:492	LINC02563		lncRNA	chr17:65457541-654
ENSG0000(	380	8.486367	chr9:178(	GALT		protein_(	chr9:34638133-3465
ENSG0000(	380	8.486367	chr17:492	RNU7-115P		smallRNA	chr17:64825765-648
ENSG0000(	380	8.486367	chr17:492	AC006080.1		smallRNA	chr17:65420583-654
ENSG0000(	380	8.486367	chr9:178(	ENSG00000273597		Pseudoger	chr9:35001342-3500
ENSG0000(	380	8.486367	chr3:3804	MIR138-1		smallRNA	chr3:44114212-4411
ENSG0000(	380	8.486367	chr9:178(	YWHAZP6		Pseudoger	chr9:34922184-3492
ENSG0000(	380	8.486367	chr9:178(	Y_RNA		smallRNA	chr9:34194812-3419
ENSG0000(	380	8.486367	chr17:492	PRR29		protein_(	chr17:63998351-640
ENSG0000(	380	8.486367	chr17:492	ENSG00000272006		Pseudoger	chr17:64951498-649
ENSG0000(	380	8.486367	chr9:178(	ENSG00000286322		lncRNA	chr9:33401478-3341
ENSG0000(	380	8.486367	chr9:178(	RN7SL22P		smallRNA	chr9:35671776-3567
ENSG0000(	380	8.486367	chr17:492	RN7SL409P		smallRNA	chr17:64817190-648
ENSG0000(	380	8.486367	chr9:178(	TRBV25OR9-2		Pseudoger	chr9:33662166-3366
ENSG0000(	380	8.486367	chr9:178(	FAM219A		protein_(	chr9:34398184-3445
ENSG0000(	380	8.486367	chr9:178(	C9orf24		protein_(	chr9:34379019-3439
ENSG0000(	380	8.486367	chr3:3804	ZNF502		protein_(	chr3:44712643-4472
ENSG0000(	380	8.486367	chr3:3804	KIAA1143		protein_(	chr3:44737661-4476
ENSG0000(	380	8.486367	chr17:492	SLC16A6P1		Pseudoger	chr17:64953683-649
ENSG0000(	380	8.486367	chr3:3804	ENSG00000235886		lncRNA	chr3:43778961-4377
ENSG0000(	380	8.486367	chr9:178(	RNA5SP282		Pseudoger	chr9:34206134-3420
ENSG0000(	380	8.486367	chr9:178(	CCIN		protein_(	chr9:36169388-3617
ENSG0000(	380	8.486367	chr17:492	ENSG00000265218		lncRNA	chr17:64892729-649
ENSG0000(	380	8.486367	chr9:178(	ENSG00000230074		lncRNA	chr9:34665607-3468
ENSG0000(	380	8.486367	chr9:178(	UBAP1		protein_(	chr9:34179005-3425
ENSG0000(	380	8.486367	chr9:178(	MYORG		protein_(	chr9:34366666-3437
ENSG0000(	380	8.486367	chr17:492	CSH2		protein_(	chr17:63872012-638
ENSG0000(	380	8.486367	chr3:3804	EI24P3		Pseudoger	chr3:44524744-4452
ENSG0000(	380	8.486367	chr17:492	SMURF2	NCGv7	protein_(	chr17:64542282-646
ENSG0000(	380	8.486367	chr3:3804	MPRIPP1		Pseudoger	chr3:44579938-4458
ENSG0000(	380	8.486367	chr9:178(	NUDT2		protein_(	chr9:34329506-3434
ENSG0000(	380	8.486367	chr9:178(	ENSG00000288949		lncRNA	chr9:34049263-3404
ENSG0000(	380	8.486367	chr9:178(	ENSG00000270542		Pseudoger	chr9:33449755-3344
ENSG0000(	380	8.486367	chr17:492	MIR3064		smallRNA	chr17:64500773-645
ENSG0000(	380	8.486367	chr17:492	ENSG00000259533		Pseudoger	chr17:63885810-638
ENSG0000(	380	8.486367	chr9:178(	PTENP1		Pseudoger	chr9:33673504-3367
ENSG0000(	380	8.486367	chr3:3804	C3orf86		Pseudoger	chr3:44421127-4443
ENSG0000(	380	8.486367	chr17:492	TACO1	DriverDB	protein_(	chr17:63600882-636
ENSG0000(	380	8.486367	chr9:178(	ENSG00000290768		lncRNA	chr9:34832645-3483
ENSG0000(	380	8.486367	chr9:178(	CCL27		protein_(	chr9:34661880-3466
ENSG0000(	380	8.486367	chr17:492	ACE3P		Pseudoger	chr17:63507056-635
ENSG0000(	380	8.486367	chr9:178(	ATP8B5P		Pseudoger	chr9:35433707-3548

ENSG0000(	380	8.486367	chr9:178(	TRBV21OR9-2		Pseudoger	chr9:33629121-3362
ENSG0000(	380	8.486367	chr17:492	TANC2	DriverDB\	protein_c	chr17:62966235-634
ENSG0000(	380	8.486367	chr9:178(	ENSG00000287063		lncRNA	chr9:33706679-3371
ENSG0000(	380	8.486367	chr9:178(	SNX18P7		Pseudoger	chr9:33576380-3357
ENSG0000(	380	8.486367	chr17:492	SCN4A		protein_c	chr17:63938554-639
ENSG0000(	380	8.486367	chr17:492	ENSG00000265282		lncRNA	chr17:63430468-634
ENSG0000(	380	8.486367	chr17:492	RDM1P4		Pseudoger	chr17:64905883-649
ENSG0000(	380	8.486367	chr17:492	CD79B	NCGv7;AC	protein_c	chr17:63928738-639
ENSG0000(	380	8.486367	chr17:492	PRR29-AS1		lncRNA	chr17:63995418-639
ENSG0000(	380	8.486367	chr3:380	AC099669.1		smallRNA	chr3:44515216-4451
ENSG0000(	380	8.486367	chr3:380	TCAIM		protein_c	chr3:44338119-4440
ENSG0000(	380	8.486367	chr17:492	LRRC37A3	NCGv7	protein_c	chr17:64854130-649
ENSG0000(	380	8.486367	chr9:178(	ENSG00000279489		TEC	chr9:35001913-3500
ENSG0000(	380	8.486367	chr9:178(	OSTCP8		Pseudoger	chr9:33968071-3396
ENSG0000(	380	8.486367	chr3:380	SOCS5P3		Pseudoger	chr3:44709311-4471
ENSG0000(	380	8.486367	chr17:492	LIMD2	NCGv7	protein_c	chr17:63695888-637
ENSG0000(	380	8.486367	chr17:492	CSH1	NCGv7	protein_c	chr17:63894909-638
ENSG0000(	380	8.486367	chr9:178(	GBA2		protein_c	chr9:35736866-3574
ENSG0000(	380	8.486367	chr17:492	GH2		protein_c	chr17:63880215-638
ENSG0000(	380	8.486367	chr9:178(	ANXA2P2		Pseudoger	chr9:33624274-3362
ENSG0000(	380	8.486367	chr17:492	RN7SL805P		smallRNA	chr17:63839983-638
ENSG0000(	380	8.486367	chr9:178(	CYP4F26P		lncRNA	chr9:33580695-3360
ENSG0000(	380	8.486367	chr17:492	ENSG00000229330		lncRNA	chr17:66676372-666
ENSG0000(	380	8.486367	chr9:178(	TRBV22OR9-2		Pseudoger	chr9:33634111-3363
ENSG0000(	380	8.486367	chr17:492	DCAF7		protein_c	chr17:63550222-635
ENSG0000(	380	8.486367	chr17:492	GH1		protein_c	chr17:63917200-639
ENSG0000(	380	8.486367	chr17:492	ENSG00000270870		Pseudoger	chr17:65654914-656
ENSG0000(	380	8.486367	chr17:492	TEX2		protein_c	chr17:64147227-642
ENSG0000(	380	8.486367	chr9:178(	RPL36AP33		Pseudoger	chr9:35507564-3550
ENSG0000(	380	8.486367	chr9:178(	ENSG00000187186		protein_c	chr9:34664163-3466
ENSG0000(	380	8.486367	chr17:492	RN7SL404P		smallRNA	chr17:64871504-648
ENSG0000(	380	8.486367	chr17:492	ENSG00000284242		Pseudoger	chr17:66278059-662
ENSG0000(	380	8.486367	chr17:492	TCAM1P		Pseudoger	chr17:63849292-638
ENSG0000(	380	8.486367	chr9:178(	SUGT1P1		Pseudoger	chr9:33503655-3351
ENSG0000(	380	8.486367	chr17:492	CEP95		protein_c	chr17:64506865-645
ENSG0000(	380	8.486367	chr9:178(	RNU2-50P		smallRNA	chr9:34282528-3428
ENSG0000(	380	8.486367	chr17:492	SMARCD2	DriverDB\	protein_c	chr17:63832081-638
ENSG0000(	380	8.486367	chr9:178(	CHMP5		protein_c	chr9:33264879-3328
ENSG0000(	380	8.486367	chr9:178(	PRSS3P4		Pseudoger	chr9:33613848-3361
ENSG0000(	380	8.486367	chr9:178(	NFX1		protein_c	chr9:33290512-3337
ENSG0000(	380	8.486367	chr17:492	ENSG00000265971		lncRNA	chr17:63454993-634
ENSG0000(	380	8.486367	chr9:178(	FAM205C		protein_c	chr9:34889066-3489
ENSG0000(	380	8.486367	chr17:492	ENSG00000266076		protein_c	chr17:65538102-656
ENSG0000(	380	8.486367	chr17:492	APOH		protein_c	chr17:66212033-662
ENSG0000(	380	8.486367	chr9:178(	PTENP1-AS		lncRNA	chr9:33676785-3368
ENSG0000(	380	8.486367	chr17:492	ENSG00000125695		protein_c	chr17:63702845-637
ENSG0000(	380	8.486367	chr9:178(	SPAAR		protein_c	chr9:35909490-3593
ENSG0000(	380	8.486367	chr9:178(	ZFAND6P1		Pseudoger	chr9:35449748-3545
ENSG0000(	380	8.486367	chr17:492	FTSJ3	DriverDB\	protein_c	chr17:63819433-638
ENSG0000(	380	8.486367	chr9:178(	ENSG00000231393		lncRNA	chr9:35642514-3564
ENSG0000(	380	8.486367	chr9:178(	AL133410.1		smallRNA	chr9:35811476-3581

ENSG0000(	380	8.486367	chr17:492	RNU6-288P		smallRNA	chr17:63573242-635
ENSG0000(	380	8.486367	chr17:492	ENSG00000265982		Pseudoger	chr17:64778842-647
ENSG0000(	380	8.486367	chr9:178(	RN7SL338P		smallRNA	chr9:35049271-3504
ENSG0000(	380	8.486367	chr9:178(	CNTFR		protein_c	chr9:34551432-3459
ENSG0000(	380	8.486367	chr9:178(	AL353662.4		smallRNA	chr9:34296379-3429
ENSG0000(	380	8.486367	chr17:492	KPNA2P3		Pseudoger	chr17:64749918-647
ENSG0000(	380	8.486367	chr9:178(	ENSG00000232983		Pseudoger	chr9:34546524-3454
ENSG0000(	380	8.486367	chr17:492	RPL31P57		Pseudoger	chr17:64308316-643
ENSG0000(	380	8.486367	chr17:492	PPIAP55		Pseudoger	chr17:63471604-634
ENSG0000(	380	8.486367	chr17:492	ENSG00000289957		lncRNA	chr17:64130368-641
ENSG0000(	380	8.486367	chr17:492	ENSG00000272220		Pseudoger	chr17:64892728-648
ENSG0000(	380	8.486367	chr9:178(	MIR4667		smallRNA	chr9:35608094-3560
ENSG0000(	380	8.486367	chr17:492	ENSG00000271779		Pseudoger	chr17:64572829-645
ENSG0000(	380	8.486367	chr3:3804	ZNF35		protein_c	chr3:44648732-4466
ENSG0000(	380	8.486367	chr9:178(	RUSC2	NCGv7	protein_c	chr9:35490111-3556
ENSG0000(	380	8.486367	chr17:492	MAP3K3		protein_c	chr17:63622415-636
ENSG0000(	380	8.486367	chr9:178(	UNC13B		protein_c	chr9:35161992-3540
ENSG0000(	380	8.486367	chr9:178(	ENSG00000287868		lncRNA	chr9:35023840-3502
ENSG0000(	380	8.486367	chr9:178(	SYF2P2		Pseudoger	chr9:35010703-3501
ENSG0000(	380	8.486367	chr9:178(	ENSG00000228843		lncRNA	chr9:35756712-3575
ENSG0000(	380	8.486367	chr9:178(	FANCG	NCGv7;AC	protein_c	chr9:35073835-3508
ENSG0000(	380	8.486367	chr9:178(	ENHO		protein_c	chr9:34521043-3452
ENSG0000(	380	8.486367	chr9:178(	ANKRD18B		protein_c	chr9:33524251-3360
ENSG0000(	380	8.486367	chr17:492	PSMC5	DriverDB\	protein_c	chr17:63827152-638
ENSG0000(	380	8.486367	chr17:492	ENSG00000264513		lncRNA	chr17:63117019-631
ENSG0000(	380	8.486367	chr17:492	AC009994.2		smallRNA	chr17:64530775-645
ENSG0000(	380	8.486367	chr17:492	ICAM2		protein_c	chr17:64002594-640
ENSG0000(	380	8.486367	chr3:3804	ENSG00000277509		Pseudoger	chr3:44528955-4452
ENSG0000(	380	8.486367	chr17:492	RNA5SP445		Pseudoger	chr17:66639269-666
ENSG0000(	380	8.486367	chr9:178(	DNAI1		protein_c	chr9:34457414-3452
ENSG0000(	380	8.486367	chr3:3804	ZNF852		protein_c	chr3:44494847-4451
ENSG0000(	380	8.486367	chr9:178(	TRBV23OR9-2		Pseudoger	chr9:33638035-3363
ENSG0000(	380	8.486367	chr17:492	CCDC47	DriverDB\	protein_c	chr17:63745255-637
ENSG0000(	380	8.486367	chr17:492	CEP112		protein_c	chr17:65635537-661
ENSG0000(	380	8.486367	chr9:178(	ENSG00000288586		lncRNA	chr9:35603437-3560
ENSG0000(	380	8.486367	chr9:178(	GLIPR2	NCGv7	protein_c	chr9:36136536-3616
ENSG0000(	380	8.486367	chr9:178(	FAM221B	NCGv7	protein_c	chr9:35816391-3582
ENSG0000(	380	8.486367	chr9:178(	RPL35AP2		Pseudoger	chr9:34191555-3419
ENSG0000(	380	8.486367	chr17:492	SNHG25		lncRNA	chr17:64145937-641
ENSG0000(	380	8.486367	chr9:178(	DNAJB5-DT		lncRNA	chr9:34985410-3498
ENSG0000(	380	8.486367	chr9:178(	ENSG00000227388		lncRNA	chr9:35772163-3579
ENSG0000(	380	8.486367	chr17:492	ENSG00000264057		Pseudoger	chr17:64927634-649
ENSG0000(	380	8.486367	chr17:492	ENSG00000272134		Pseudoger	chr17:64881091-648
ENSG0000(	380	8.486367	chr3:3804	ENSG00000280435		TEC	chr3:44115818-4411
ENSG0000(	380	8.486367	chr17:492	ENSG00000272400		Pseudoger	chr17:64847386-648
ENSG0000(	380	8.486367	chr9:178(	UBE2R2-AS1		lncRNA	chr9:33732970-3381
ENSG0000(	380	8.486367	chr9:178(	Y_RNA		smallRNA	chr9:33830978-3383
ENSG0000(	380	8.486367	chr17:492	ENSG00000272346		Pseudoger	chr17:64940606-649
ENSG0000(	380	8.486367	chr9:178(	RNU4ATAC11P		smallRNA	chr9:33868540-3386
ENSG0000(	380	8.486367	chr3:3804	ENSG00000272121		lncRNA	chr3:43998081-4399
ENSG0000(	380	8.486367	chr9:178(	B4GALT1-AS1		lncRNA	chr9:33166948-3318

ENSG00000	380	8.486367	chr3:3804	ZNF197		protein_c	chr3:44584888-4464
ENSG00000	380	8.486367	chr9:1780	CLTA		protein_c	chr9:36190856-3630
ENSG00000	380	8.486367	chr9:1780	RECK		protein_c	chr9:36036913-3612
ENSG00000	380	8.486367	chr9:1780	SNORD121B		smallRNA	chr9:33934296-3393
ENSG00000	380	8.486367	chr17:492	EEF1DP7		Pseudogen	chr17:63636601-636
ENSG00000	380	8.486367	chr9:1780	Y_RNA		smallRNA	chr9:34389943-3439
ENSG00000	380	8.486367	chr9:1780	OR13C7		protein_c	chr9:36002909-3600
ENSG00000	380	8.486367	chr9:1780	Y_RNA		smallRNA	chr9:33352788-3335
ENSG00000	380	8.486367	chr9:1780	PHF24		protein_c	chr9:34957608-3498
ENSG00000	380	8.486367	chr9:1780	OR13E1P		Pseudogen	chr9:35859055-3585
ENSG00000	380	8.486367	chr3:3804	ZNF501		protein_c	chr3:44729596-4473
ENSG00000	380	8.486367	chr17:492	RN7SL735P		smallRNA	chr17:66364029-663
ENSG00000	380	8.486367	chr9:1780	SPINK4		protein_c	chr9:33218365-3324
ENSG00000	380	8.486367	chr9:1780	RNU4-53P		smallRNA	chr9:36267783-3626
ENSG00000	380	8.486367	chr3:3804	ENSG00000271192		lncRNA	chr3:43996896-4399
ENSG00000	380	8.486367	chr9:1780	FAM214B		protein_c	chr9:35104112-3511
ENSG00000	380	8.486367	chr9:1780	ENSG00000287495		lncRNA	chr9:33730345-3373
ENSG00000	380	8.486367	chr9:1780	DCAF12		protein_c	chr9:34086387-3412
ENSG00000	380	8.486367	chr17:492	GNA13	NCGv7;AC	protein_c	chr17:65009289-650
ENSG00000	380	8.486367	chr17:492	AMZ2P1		lncRNA	chr17:64966550-649
ENSG00000	380	8.486367	chr9:1780	ENSG00000227301		lncRNA	chr9:33719690-3372
ENSG00000	380	8.486367	chr9:1780	RN7SKP24		smallRNA	chr9:34478622-3447
ENSG00000	380	8.486367	chr9:1780	ENSG00000279608		TEC	chr9:35038625-3504
ENSG00000	380	8.486367	chr9:1780	ENSG00000285645		protein_c	chr9:35812960-3582
ENSG00000	380	8.486367	chr9:1780	CCL19		protein_c	chr9:34689570-3469
ENSG00000	380	8.486367	chr9:1780	OR13J1		protein_c	chr9:35869463-3587
ENSG00000	380	8.486367	chr9:1780	ENSG00000287368		lncRNA	chr9:34810020-3489
ENSG00000	380	8.486367	chr3:3804	ZNF660		protein_c	chr3:44578223-4459
ENSG00000	380	8.486367	chr9:1780	NDUFA5P4		Pseudogen	chr9:35874810-3587
ENSG00000	380	8.486367	chr17:492	ENSG00000280299		lncRNA	chr17:64940602-649
ENSG00000	380	8.486367	chr9:1780	RGP1		protein_c	chr9:35749287-3575
ENSG00000	380	8.486367	chr17:492	MIR5047		smallRNA	chr17:64501214-645
ENSG00000	380	8.486367	chr17:492	AXIN2	NCGv7	protein_c	chr17:65528563-655
ENSG00000	380	8.486367	chr17:492	RNU6-928P		smallRNA	chr17:66638271-666
ENSG00000	380	8.486367	chr9:1780	ENSG00000288583		lncRNA	chr9:34702953-3477
ENSG00000	380	8.486367	chr9:1780	SIGMAR1		protein_c	chr9:34634722-3463
ENSG00000	380	8.486367	chr9:1780	TPM2		protein_c	chr9:35681992-3569
ENSG00000	380	8.486367	chr9:1780	RNU7-36P		smallRNA	chr9:34018260-3401
ENSG00000	380	8.486367	chr9:1780	CREB3		protein_c	chr9:35732598-3573
ENSG00000	380	8.486367	chr17:492	ENSG00000285947		protein_c	chr17:63917203-639
ENSG00000	380	8.486367	chr9:1780	GLULP4		Pseudogen	chr9:34917175-3491
ENSG00000	380	8.486367	chr9:1780	SERPINH1P1		Pseudogen	chr9:34318407-3431
ENSG00000	380	8.486367	chr17:492	ENSG00000265883		Pseudogen	chr17:65185319-651
ENSG00000	380	8.486367	chr3:3804	ZKSCAN7-AS1		lncRNA	chr3:44556985-4468
ENSG00000	380	8.486367	chr9:1780	ENSG00000287986		lncRNA	chr9:35922159-3592
ENSG00000	380	8.486367	chr9:1780	TRBV20OR9-2		protein_c	chr9:33585352-3361
ENSG00000	380	8.486367	chr17:492	ENSG00000289587		lncRNA	chr17:66432081-664
ENSG00000	380	8.486367	chr17:492	RNA5SP444		Pseudogen	chr17:66267743-662
ENSG00000	380	8.486367	chr9:1780	RN7SKP114		smallRNA	chr9:34049966-3405
ENSG00000	380	8.486367	chr9:1780	ENSG00000233668		Pseudogen	chr9:34223984-3422
ENSG00000	380	8.486367	chr9:1780	RPS29P17		Pseudogen	chr9:35592786-3559

ENSG0000(	380	8.486367	chr9:178(	ENSG00000228352		lncRNA	chr9:34084332-3409
ENSG0000(	380	8.486367	chr17:492	ENSG00000271587		Pseudoger	chr17:65644308-656
ENSG0000(	380	8.486367	chr17:492	CSHL1		protein_c	chr17:63909597-639
ENSG0000(	380	8.486367	chr17:492	BPTFP1		Pseudoger	chr17:64761912-647
ENSG0000(	380	8.486367	chr9:178(	BAG1		protein_c	chr9:33247820-3326
ENSG0000(	380	8.486367	chr9:178(	HRCT1		protein_c	chr9:35906202-3590
ENSG0000(	380	8.486367	chr17:492	MILR1		protein_c	chr17:64465452-644
ENSG0000(	380	8.486367	chr9:178(	ENSG00000227933		lncRNA	chr9:35646270-3564
ENSG0000(	380	8.486367	chr17:492	ENSG00000266155		Pseudoger	chr17:64936485-649
ENSG0000(	380	8.486367	chr9:178(	ENSG00000279083		TEC	chr9:35360540-3536
ENSG0000(	380	8.486367	chr9:178(	TRBV26OR9-2		Pseudoger	chr9:33695767-3369
ENSG0000(	380	8.486367	chr9:178(	ENSG00000287287		lncRNA	chr9:35491133-3549
ENSG0000(	380	8.486367	chr3:380	ZNF197-AS1		lncRNA	chr3:44617128-4462
ENSG0000(	380	8.486367	chr17:492	PSMD7P1		Pseudoger	chr17:66197693-661
ENSG0000(	380	8.486367	chr9:178(	ENSG00000239392		Pseudoger	chr9:34133157-3413
ENSG0000(	380	8.486367	chr3:380	RNU6-367P		smallRNA	chr3:44071087-4407
ENSG0000(	380	8.486367	chr3:380	LINC01988		lncRNA	chr3:44424134-4442
ENSG0000(	380	8.486367	chr9:178(	CA9		protein_c	chr9:35673928-3568
ENSG0000(	380	8.486367	chr17:492	STRADA	NCGv7	protein_c	chr17:63682336-637
ENSG0000(	380	8.486367	chr17:492	POLG2		protein_c	chr17:64477785-644
ENSG0000(	380	8.486367	chr9:178(	ENSG00000288699		protein_c	chr9:35065339-3507
ENSG0000(	380	8.486367	chr9:178(	CNTFR-AS1		lncRNA	chr9:34568012-3458
ENSG0000(	380	8.486367	chr3:380	ENSG00000261786		lncRNA	chr3:44117299-4412
ENSG0000(	380	8.486367	chr9:178(	OR2S1P		Pseudoger	chr9:36013184-3601
ENSG0000(	380	8.486367	chr9:178(	TESK1		protein_c	chr9:35605262-3561
ENSG0000(	380	8.486367	chr9:178(	ENSG00000228072		lncRNA	chr9:33512959-3351
ENSG0000(	380	8.486367	chr17:492	ENSG00000265912		lncRNA	chr17:64943262-649
ENSG0000(	380	8.486367	chr3:380	ZNF445		protein_c	chr3:44431705-4447
ENSG0000(	380	8.486367	chr17:492	ENSG00000263489		lncRNA	chr17:63972920-639
ENSG0000(	380	8.486367	chr17:492	DNAJB6P8		Pseudoger	chr17:63182166-631
ENSG0000(	380	8.486367	chr17:492	DDX42		protein_c	chr17:63773603-638
ENSG0000(	380	8.486367	chr17:492	ENSG00000266598		lncRNA	chr17:65051909-650
ENSG0000(	380	8.486367	chr17:492	ENSG00000233635		lncRNA	chr17:63193930-633
ENSG0000(	380	8.486367	chr9:178(	C9orf131		protein_c	chr9:35041095-3504
ENSG0000(	380	8.486367	chr17:492	ERN1		protein_c	chr17:64039080-641
ENSG0000(	380	8.486367	chr17:492	ENSG00000291117		lncRNA	chr17:64779259-648
ENSG0000(	380	8.486367	chr17:492	ENSG00000266644		lncRNA	chr17:64899766-649
ENSG0000(	380	8.486367	chr17:492	ENSG00000271749		Pseudoger	chr17:63393355-633
ENSG0000(	380	8.486367	chr9:178(	OR2AM1P		Pseudoger	chr9:36021692-3602
ENSG0000(	380	8.486367	chr9:178(	UBE2R2		protein_c	chr9:33817160-3392
ENSG0000(	380	8.486367	chr9:178(	ARID3C		protein_c	chr9:34621049-3462
ENSG0000(	380	8.486367	chr17:492	RGS9		protein_c	chr17:65100812-652
ENSG0000(	380	8.486367	chr17:492	DDX5	NCGv7;AC	protein_c	chr17:64498254-645
ENSG0000(	380	8.486367	chr9:178(	FAM205A		protein_c	chr9:34723053-3472
ENSG0000(	377	8.419369	chr17:424	TTLL6		protein_c	chr17:48762234-488
ENSG0000(	377	8.419369	chr17:424	SKAP1-AS1		lncRNA	chr17:48185938-482
ENSG0000(	377	8.419369	chr17:424	HOXB9		protein_c	chr17:48621156-486
ENSG0000(	377	8.419369	chr17:424	PHOSPHO1		protein_c	chr17:49223366-492
ENSG0000(	377	8.419369	chr17:424	ENSG00000289021		lncRNA	chr17:48944890-489
ENSG0000(	377	8.419369	chr17:424	SP6		protein_c	chr17:47844908-478
ENSG0000(	377	8.419369	chr17:424	KPNB1-DT		lncRNA	chr17:47603860-476

ENSG0000(	377	8.419369	chr17:42<	ABI3	NCGv7	protein_c	chr17:49210411-492
ENSG0000(	377	8.419369	chr17:42<	ENSG00000266341		lncRNA	chr17:48060383-480
ENSG0000(	377	8.419369	chr17:42<	SNX11		protein_c	chr17:48103357-481
ENSG0000(	377	8.419369	chr17:42<	KPNB1	NCGv7	protein_c	chr17:47649476-476
ENSG0000(	377	8.419369	chr17:42<	RP11-708H21.4		lncRNA	chr17:49171895-491
ENSG0000(	377	8.419369	chr17:42<	HOXB5		protein_c	chr17:48591257-485
ENSG0000(	377	8.419369	chr17:42<	SP2-AS1		lncRNA	chr17:47897330-479
ENSG0000(	377	8.419369	chr17:42<	ENSG00000251461		lncRNA	chr17:49004731-490
ENSG0000(	377	8.419369	chr17:42<	MIR3185		smallRNA	chr17:48724408-487
ENSG0000(	377	8.419369	chr17:42<	MRPL10		protein_c	chr17:47823272-478
ENSG0000(	377	8.419369	chr17:42<	ENSG00000264243		lncRNA	chr17:47863342-478
ENSG0000(	377	8.419369	chr17:42<	PRAC1		protein_c	chr17:48721719-487
ENSG0000(	377	8.419369	chr17:42<	OSBPL7		protein_c	chr17:47807372-478
ENSG0000(	377	8.419369	chr17:42<	HOXB13		protein_c	chr17:48724763-487
ENSG0000(	377	8.419369	chr17:42<	CALCOCO2	DriverDB\	protein_c	chr17:48831018-488
ENSG0000(	377	8.419369	chr17:42<	ENSG00000272763		lncRNA	chr17:48635923-486
ENSG0000(	377	8.419369	chr17:42<	COX6B1P2		Pseudoger	chr17:48713730-487
ENSG0000(	377	8.419369	chr17:42<	ENSG00000244559		Pseudoger	chr17:48690481-486
ENSG0000(	377	8.419369	chr17:42<	ENSG00000266821		lncRNA	chr17:47879472-478
ENSG0000(	377	8.419369	chr17:42<	HOXB6		protein_c	chr17:48595751-486
ENSG0000(	377	8.419369	chr17:42<	ENSG00000266601		lncRNA	chr17:47929682-479
ENSG0000(	377	8.419369	chr17:42<	ATP5MC1		protein_c	chr17:48892765-488
ENSG0000(	377	8.419369	chr17:42<	UBE2Z	DriverDB\	protein_c	chr17:48908407-489
ENSG0000(	377	8.419369	chr17:42<	HOXB1		protein_c	chr17:48528526-485
ENSG0000(	377	8.419369	chr17:42<	HOXB3		protein_c	chr17:48548870-486
ENSG0000(	377	8.419369	chr17:42<	HOXB4		protein_c	chr17:48575507-485
ENSG0000(	377	8.419369	chr17:42<	PNPO		protein_c	chr17:47941506-479
ENSG0000(	377	8.419369	chr17:42<	SNF8	DriverDB\	protein_c	chr17:48929316-489
ENSG0000(	377	8.419369	chr17:42<	SP2-DT		lncRNA	chr17:47891219-478
ENSG0000(	377	8.419369	chr17:42<	IGF2BP1	AC	protein_c	chr17:48997385-490
ENSG0000(	377	8.419369	chr17:42<	ENSG00000250838		lncRNA	chr17:48995266-489
ENSG0000(	377	8.419369	chr17:42<	GIP		protein_c	chr17:48958554-489
ENSG0000(	377	8.419369	chr17:42<	MIR1203		smallRNA	chr17:48156427-481
ENSG0000(	377	8.419369	chr17:42<	HOXB8		protein_c	chr17:48611377-486
ENSG0000(	377	8.419369	chr17:42<	PRAC2		protein_c	chr17:48723185-487
ENSG0000(	377	8.419369	chr17:42<	NFE2L1-DT		lncRNA	chr17:48045141-480
ENSG0000(	377	8.419369	chr17:42<	Y_RNA		smallRNA	chr17:48479097-484
ENSG0000(	377	8.419369	chr17:42<	HOXB7		protein_c	chr17:48607232-486
ENSG0000(	377	8.419369	chr17:42<	U3		smallRNA	chr17:48382372-483
ENSG0000(	377	8.419369	chr17:42<	LINC02086		lncRNA	chr17:48646923-487
ENSG0000(	377	8.419369	chr17:42<	ENSG00000278765		lncRNA	chr17:48066704-480
ENSG0000(	377	8.419369	chr17:42<	ENSG00000270781		Pseudoger	chr17:48899131-488
ENSG0000(	377	8.419369	chr17:42<	snoU13		smallRNA	chr17:48956650-489
ENSG0000(	377	8.419369	chr17:42<	ENSG00000263798		lncRNA	chr17:47945424-479
ENSG0000(	377	8.419369	chr17:42<	HOXB2		protein_c	chr17:48540894-485
ENSG0000(	377	8.419369	chr17:42<	B4GALNT2	DriverDB\	protein_c	chr17:49132460-491
ENSG0000(	377	8.419369	chr17:42<	GNGT2		protein_c	chr17:49202791-492
ENSG0000(	377	8.419369	chr17:42<	RNU1-42P		smallRNA	chr17:48949361-489
ENSG0000(	377	8.419369	chr17:42<	MIR152		smallRNA	chr17:48037161-480
ENSG0000(	377	8.419369	chr17:42<	ENSG00000266222		lncRNA	chr17:48377262-483
ENSG0000(	377	8.419369	chr17:42<	SP2		protein_c	chr17:47896150-479

ENSG0000(	377	8.419369	chr17:424	PRR15L		protein_c	chr17:47951967-479
ENSG0000(	377	8.419369	chr17:424	COPZ2		protein_c	chr17:48026167-480
ENSG0000(	377	8.419369	chr17:424	SNORA68		smallRNA	chr17:48880840-488
ENSG0000(	377	8.419369	chr17:424	CDK5RAP3		protein_c	chr17:47967810-479
ENSG0000(	377	8.419369	chr17:424	ENSG00000251550		Pseudoger	chr17:49037122-490
ENSG0000(	377	8.419369	chr17:424	CBX1		protein_c	chr17:48070052-481
ENSG0000(	377	8.419369	chr17:424	RN7SL125P		smallRNA	chr17:48765556-487
ENSG0000(	377	8.419369	chr17:424	SUMO2P17		Pseudoger	chr17:48874860-489
ENSG0000(	377	8.419369	chr17:424	HOXB-AS4		lncRNA	chr17:48628675-486
ENSG0000(	377	8.419369	chr17:424	NFE2L1		protein_c	chr17:48048329-480
ENSG0000(	377	8.419369	chr17:424	HOXB-AS1		lncRNA	chr17:48543551-485
ENSG0000(	377	8.419369	chr6:1039	SNORD117		smallRNA	chr6:31536374-3153
ENSG0000(	377	8.419369	chr17:424	TBX21		protein_c	chr17:47733236-477
ENSG0000(	377	8.419369	chr17:424	HOXB-AS3		lncRNA	chr17:48549630-486
ENSG0000(	377	8.419369	chr17:424	TBKBP1	NCGv7	protein_c	chr17:47694161-477
ENSG0000(	377	8.419369	chr17:424	ENSG00000240995		Pseudoger	chr17:49148339-491
ENSG0000(	377	8.419369	chr17:424	FLJ40194		lncRNA	chr17:49230853-493
ENSG0000(	377	8.419369	chr17:424	RNU6-1152P		smallRNA	chr17:48196680-481
ENSG0000(	377	8.419369	chr17:424	ENSG00000264701		lncRNA	chr17:47980398-479
ENSG0000(	377	8.419369	chr17:424	ENSG00000242407		lncRNA	chr17:48733091-487
ENSG0000(	377	8.419369	chr17:424	SKAP1-AS2		lncRNA	chr17:48294335-483
ENSG0000(	377	8.419369	chr17:424	ENSG00000264558		lncRNA	chr17:47682417-476
ENSG0000(	377	8.419369	chr17:424	RNU6-826P		smallRNA	chr17:49036352-490
ENSG0000(	377	8.419369	chr17:424	AC105030.1		smallRNA	chr17:49049540-490
ENSG0000(	377	8.419369	chr17:424	ENSG00000289599		lncRNA	chr17:48011327-480
ENSG0000(	377	8.419369	chr17:424	ENSG00000264019		lncRNA	chr17:47946802-479
ENSG0000(	377	8.419369	chr17:424	ENSG00000264451		lncRNA	chr17:48460370-484
ENSG0000(	377	8.419369	chr17:424	HOXB-AS2		lncRNA	chr17:48557262-485
ENSG0000(	377	8.419369	chr17:424	ENSG00000257178		lncRNA	chr17:48579630-485
ENSG0000(	377	8.419369	chr17:424	Metazoa_SRP		smallRNA	chr17:49109762-491
ENSG0000(	377	8.419369	chr17:424	ENSG00000248895		Pseudoger	chr17:48881122-488
ENSG0000(	377	8.419369	chr17:424	ENSG00000230532		lncRNA	chr17:48931791-489
ENSG0000(	377	8.419369	chr17:424	LRRC46		protein_c	chr17:47831634-478
ENSG0000(	377	8.419369	chr17:424	RNU6-1201P		smallRNA	chr17:48088678-480
ENSG0000(	377	8.419369	chr17:424	RPL9P28		Pseudoger	chr17:48691116-486
ENSG0000(	377	8.419369	chr17:424	SCRN2		protein_c	chr17:47837692-478
ENSG0000(	377	8.419369	chr17:424	B4GALNT2P1		Pseudoger	chr17:49085511-490
ENSG0000(	377	8.419369	chr17:424	SKAP1		protein_c	chr17:48133442-484
ENSG0000(	376	8.397036	chr5:1646	HMGCS1	DriverDBv	protein_c	chr5:43287470-4331
ENSG0000(	376	8.397036	chr5:1646	ENSG00000287263		lncRNA	chr5:42983636-4299
ENSG0000(	376	8.397036	chr5:1646	ENSG00000249405		lncRNA	chr5:50858760-5086
ENSG0000(	376	8.397036	chr5:1646	PARP8		protein_c	chr5:50665899-5084
ENSG0000(	376	8.397036	chr5:1646	ENSG00000250017		Pseudoger	chr5:45574590-4557
ENSG0000(	376	8.397036	chr5:1646	ENSG00000288035		lncRNA	chr5:51438792-5166
ENSG0000(	376	8.397036	chr5:1646	LINC02224		lncRNA	chr5:44495099-4465
ENSG0000(	376	8.397036	chr5:1646	ENSG00000250422		Pseudoger	chr5:45557919-4555
ENSG0000(	376	8.397036	chr5:1646	ENSG00000279557		TEC	chr5:43586367-4358
ENSG0000(	376	8.397036	chr5:1646	ANXA2R-AS1		lncRNA	chr5:43041572-4306
ENSG0000(	376	8.397036	chr5:1646	NNT		protein_c	chr5:43602692-4370
ENSG0000(	376	8.397036	chr5:1646	RIMOC1		protein_c	chr5:41904188-4192
ENSG0000(	376	8.397036	chr5:1646	PRELID3BP4		Pseudoger	chr5:42897262-4289

ENSG0000(	376	8.397036	chr5:164(	OXCT1-AS1		lncRNA	chr5:41869927-4187
ENSG0000(	376	8.397036	chr5:164(	ENSG00000250860		Pseudoger	chr5:42466893-4246
ENSG0000(	376	8.397036	chr5:164(	CCDC152		protein_c	chr5:42756818-4280
ENSG0000(	376	8.397036	chr5:164(	PRELID3BP7		Pseudoger	chr5:42972624-4297
ENSG0000(	376	8.397036	chr5:164(	ENSG00000240627		Pseudoger	chr5:51275253-5127
ENSG0000(	376	8.397036	chr5:164(	SERBP1P6		Pseudoger	chr5:42465400-4246
ENSG0000(	376	8.397036	chr5:164(	ISL1		protein_c	chr5:51383448-5139
ENSG0000(	376	8.397036	chr5:164(	ENSG00000270830		Pseudoger	chr5:50903608-5090
ENSG0000(	376	8.397036	chr5:164(	FGF10	AC	protein_c	chr5:44300247-4438
ENSG0000(	376	8.397036	chr5:164(	ENSG00000251131		lncRNA	chr5:43018145-4302
ENSG0000(	376	8.397036	chr5:164(	NIM1K	DriverDB\	protein_c	chr5:43192071-4328
ENSG0000(	376	8.397036	chr5:164(	NNT-AS1		lncRNA	chr5:43571594-4360
ENSG0000(	376	8.397036	chr5:164(	ENSG00000250122		lncRNA	chr5:45890216-4589
ENSG0000(	376	8.397036	chr5:164(	ENSG00000248148		lncRNA	chr5:45035199-4509
ENSG0000(	376	8.397036	chr5:164(	CCL28	DriverDB\	protein_c	chr5:43376645-4341
ENSG0000(	376	8.397036	chr5:164(	ENSG00000271455		Pseudoger	chr5:50603229-5060
ENSG0000(	376	8.397036	chr5:164(	ENSG00000286164		lncRNA	chr5:41868975-4188
ENSG0000(	376	8.397036	chr5:164(	ENSG00000250418		lncRNA	chr5:44698431-4470
ENSG0000(	376	8.397036	chr5:164(	ENSG00000240281		Pseudoger	chr5:41895068-4189
ENSG0000(	376	8.397036	chr5:164(	MTHFD2P6		Pseudoger	chr5:41967139-4196
ENSG0000(	376	8.397036	chr5:164(	ENSG00000278734		Pseudoger	chr5:51101172-5110
ENSG0000(	376	8.397036	chr5:164(	ENSG00000188850		Pseudoger	chr5:43336164-4334
ENSG0000(	376	8.397036	chr5:164(	HCN1	NCGv7	protein_c	chr5:45254948-4569
ENSG0000(	376	8.397036	chr5:164(	ENSG00000213956		Pseudoger	chr5:50633124-5063
ENSG0000(	376	8.397036	chr5:164(	ENSG00000249276		lncRNA	chr5:43874367-4388
ENSG0000(	376	8.397036	chr5:164(	ENSG00000249779		Pseudoger	chr5:43206709-4320
ENSG0000(	376	8.397036	chr5:164(	ENSG00000250360		lncRNA	chr5:50965687-5096
ENSG0000(	376	8.397036	chr5:164(	ENSG00000248240		lncRNA	chr5:43515274-4352
ENSG0000(	376	8.397036	chr5:164(	ENSG00000272382		lncRNA	chr5:43033716-4303
ENSG0000(	376	8.397036	chr5:164(	Y_RNA		smallRNA	chr5:41963868-4196
ENSG0000(	376	8.397036	chr5:164(	ENSG00000272335		lncRNA	chr5:44826076-4482
ENSG0000(	376	8.397036	chr5:164(	RNU6-1296P		smallRNA	chr5:51167541-5116
ENSG0000(	376	8.397036	chr5:164(	AMD1P3		Pseudoger	chr5:43586918-4358
ENSG0000(	376	8.397036	chr5:164(	ENSG00000271752		lncRNA	chr5:50662859-5066
ENSG0000(	376	8.397036	chr5:164(	ENSG00000286656		lncRNA	chr5:42995134-4300
ENSG0000(	376	8.397036	chr5:164(	PPIAP77		Pseudoger	chr5:42892319-4289
ENSG0000(	376	8.397036	chr5:164(	FBXO4		protein_c	chr5:41925254-4194
ENSG0000(	376	8.397036	chr5:164(	TMEM267	DriverDB\	protein_c	chr5:43444252-4348
ENSG0000(	376	8.397036	chr5:164(	ENSG00000289056		lncRNA	chr5:50667071-5066
ENSG0000(	376	8.397036	chr5:164(	RPSAP38		Pseudoger	chr5:41951313-4195
ENSG0000(	376	8.397036	chr5:164(	ANXA2R-OT1		lncRNA	chr5:43013060-4306
ENSG0000(	376	8.397036	chr5:164(	ENSG00000261604		lncRNA	chr5:43287601-4329
ENSG0000(	376	8.397036	chr5:164(	RN7SL383P		smallRNA	chr5:44716190-4471
ENSG0000(	376	8.397036	chr5:164(	ISL1-DT		lncRNA	chr5:51372736-5138
ENSG0000(	376	8.397036	chr5:164(	PRELID3BP6		Pseudoger	chr5:42917414-4291
ENSG0000(	376	8.397036	chr5:164(	AC091866.1		smallRNA	chr5:51061469-5106
ENSG0000(	376	8.397036	chr5:164(	ENSG00000272144		lncRNA	chr5:43061395-4306
ENSG0000(	376	8.397036	chr5:164(	ANXA2R		protein_c	chr5:43039233-4304
ENSG0000(	376	8.397036	chr5:164(	ENSG00000270513		Pseudoger	chr5:42729138-4272
ENSG0000(	376	8.397036	chr5:164(	ENSG00000289206		lncRNA	chr5:42994376-4299
ENSG0000(	376	8.397036	chr5:164(	EMB		protein_c	chr5:50396192-5044

ENSG0000(	376	8.397036	chr5:164(	ENSG00000287540		lncRNA	chr5:45226286-4522
ENSG0000(	376	8.397036	chr5:164(	ENSG00000272234		lncRNA	chr5:42806394-4280
ENSG0000(	376	8.397036	chr5:164(	GHR		protein_c	chr5:42423439-4272
ENSG0000(	376	8.397036	chr5:164(	ENSG00000248918		lncRNA	chr5:50969660-5097
ENSG0000(	376	8.397036	chr5:164(	RNU6-381P		smallRNA	chr5:44066624-4406
ENSG0000(	376	8.397036	chr5:164(	LINC02106		lncRNA	chr5:50969217-5097
ENSG0000(	376	8.397036	chr5:164(	ENSG00000249888		Pseudoger	chr5:43225506-4322
ENSG0000(	376	8.397036	chr5:164(	RNU6-480P		smallRNA	chr5:51055353-5105
ENSG0000(	376	8.397036	chr5:164(	snoU13		smallRNA	chr5:51321983-5132
ENSG0000(	376	8.397036	chr5:164(	PRELID3BP5		Pseudoger	chr5:42908208-4290
ENSG0000(	376	8.397036	chr5:164(	ENSG00000248779		lncRNA	chr5:44752949-4476
ENSG0000(	376	8.397036	chr5:164(	FGF10-AS1		lncRNA	chr5:44388732-4441
ENSG0000(	376	8.397036	chr5:164(	ENSG00000248891		Pseudoger	chr5:51427996-5142
ENSG0000(	376	8.397036	chr5:164(	ENSG00000278487		Pseudoger	chr5:43143362-4314
ENSG0000(	376	8.397036	chr5:164(	C5orf34	DriverDB\	protein_c	chr5:43486709-4351
ENSG0000(	376	8.397036	chr5:164(	PAIP1	NCGv7	protein_c	chr5:43526267-4355
ENSG0000(	376	8.397036	chr5:164(	ENSG00000260786		lncRNA	chr5:42188266-4219
ENSG0000(	376	8.397036	chr5:164(	TUBAP14		Pseudoger	chr5:43231812-4323
ENSG0000(	376	8.397036	chr5:164(	ENSG00000251125		lncRNA	chr5:51451158-5146
ENSG0000(	376	8.397036	chr5:164(	MRPS30-DT		lncRNA	chr5:44742420-4480
ENSG0000(	376	8.397036	chr5:164(	MRPS30		protein_c	chr5:44808947-4482
ENSG0000(	376	8.397036	chr5:164(	ENSG00000251601		lncRNA	chr5:50929484-5093
ENSG0000(	376	8.397036	chr5:164(	EEF1A1P19		Pseudoger	chr5:43495073-4349
ENSG0000(	376	8.397036	chr5:164(	ENSG00000271788		lncRNA	chr5:43006733-4300
ENSG0000(	376	8.397036	chr5:164(	SELENOP		protein_c	chr5:42799880-4288
ENSG0000(	376	8.397036	chr5:164(	ENSG00000249492		lncRNA	chr5:43483913-4350
ENSG0000(	376	8.397036	chr5:164(	RPL29P12		Pseudoger	chr5:43666766-4366
ENSG0000(	376	8.397036	chr5:164(	HMGB1P47		Pseudoger	chr5:51409617-5141
ENSG0000(	376	8.397036	chr5:164(	ZNF131	NCGv7	protein_c	chr5:43065176-4319
ENSG0000(	376	8.397036	chr5:164(	C5orf34-AS1		lncRNA	chr5:43511058-4352
ENSG0000(	376	8.397036	chr5:164(	ENSG00000286271		lncRNA	chr5:42813647-4292
ENSG0000(	373	8.330039	chr6:1039	ENSG00000291111		lncRNA	chr6:33112440-3311
ENSG0000(	373	8.330039	chr6:1039	APOM		protein_c	chr6:31652416-3165
ENSG0000(	373	8.330039	chr6:1039	ENSG00000256851		Pseudoger	chr6:31515979-3151
ENSG0000(	373	8.330039	chr6:1039	LY6G5C	DriverDB\	protein_c	chr6:31676684-3168
ENSG0000(	373	8.330039	chr6:1039	C4A		protein_c	chr6:31982057-3200
ENSG0000(	373	8.330039	chr6:1039	TEAD3		protein_c	chr6:35473597-3549
ENSG0000(	373	8.330039	chr6:1039	ENSG00000291112		lncRNA	chr6:27356451-2737
ENSG0000(	373	8.330039	chr6:1039	RP11-513I15.6		lncRNA	chr6:34279679-3428
ENSG0000(	373	8.330039	chr6:1039	HLA-V		lncRNA	chr6:29790954-2979
ENSG0000(	373	8.330039	chr6:1039	H4C3		protein_c	chr6:26103933-2610
ENSG0000(	373	8.330039	chr6:1039	BAG6		protein_c	chr6:31639028-3165
ENSG0000(	373	8.330039	chr6:1039	ENSG00000277881		Pseudoger	chr6:29450210-2945
ENSG0000(	373	8.330039	chr6:1039	SNORA40		smallRNA	chr6:35651818-3565
ENSG0000(	373	8.330039	chr6:1039	HCG11		lncRNA	chr6:26521709-2652
ENSG0000(	373	8.330039	chr6:1039	DEF6		protein_c	chr6:35297818-3532
ENSG0000(	373	8.330039	chr6:1039	OR2H5P		Pseudoger	chr6:29573909-2957
ENSG0000(	373	8.330039	chr6:1039	ZSCAN26		protein_c	chr6:28267058-2827
ENSG0000(	373	8.330039	chr6:1039	CFB		protein_c	chr6:31945650-3195
ENSG0000(	373	8.330039	chr6:1039	PRRC2A		protein_c	chr6:31620715-3163
ENSG0000(	373	8.330039	chr6:1039	C6orf47		protein_c	chr6:31658298-3166

ENSG0000(	373	8.330039	chr6:1039	ZNF70P1		Pseudoger	chr6:33215705-3321
ENSG0000(	373	8.330039	chr6:1039	ENSG00000271581		Pseudoger	chr6:31356647-3135
ENSG0000(	373	8.330039	chr6:1039	H4C5	NCGv7	protein_c	chr6:26204610-2620
ENSG0000(	373	8.330039	chr6:1039	POM121L6P		Pseudoger	chr6:26896952-2689
ENSG0000(	373	8.330039	chr6:1039	HLA-L		lncRNA	chr6:30259548-3029
ENSG0000(	373	8.330039	chr6:1039	BTN2A3P		lncRNA	chr6:26421391-2643
ENSG0000(	373	8.330039	chr6:1039	LYPLA2P1		Pseudoger	chr6:33365548-3336
ENSG0000(	373	8.330039	chr6:1039	GUSBP2		Pseudoger	chr6:26878572-2688
ENSG0000(	373	8.330039	chr6:1039	ZNF322		protein_c	chr6:26634383-2665
ENSG0000(	373	8.330039	chr6:1039	CSNK2B	NCGv7	protein_c	chr6:31665227-3167
ENSG0000(	373	8.330039	chr6:1039	ENSG00000278332		Pseudoger	chr6:27510501-2751
ENSG0000(	373	8.330039	chr6:1039	H2AC12		protein_c	chr6:27147106-2714
ENSG0000(	373	8.330039	chr6:1039	MYL12BP3		Pseudoger	chr6:33338978-3333
ENSG0000(	373	8.330039	chr6:1039	GPANK1		protein_c	chr6:31661228-3166
ENSG0000(	373	8.330039	chr6:1039	TRIM31-AS1		lncRNA	chr6:30105240-3011
ENSG0000(	373	8.330039	chr6:1039	ENSG00000272374		lncRNA	chr6:35220370-3522
ENSG0000(	373	8.330039	chr6:1039	AIF1		protein_c	chr6:31615217-3161
ENSG0000(	373	8.330039	chr6:1039	SNORD46		smallRNA	chr6:24166273-2416
ENSG0000(	373	8.330039	chr6:1039	RPL15P4		Pseudoger	chr6:31528114-3152
ENSG0000(	373	8.330039	chr6:1039	ENSG00000273870		Pseudoger	chr6:35070871-3507
ENSG0000(	373	8.330039	chr6:1039	HLA-DRB9		Pseudoger	chr6:32459821-3247
ENSG0000(	373	8.330039	chr6:1039	MSH5-SAPCD1		protein_c	chr6:31740020-3176
ENSG0000(	373	8.330039	chr6:1039	OR11A1		protein_c	chr6:29425504-2945
ENSG0000(	373	8.330039	chr6:1039	ENSG00000290574		lncRNA	chr6:30005971-3000
ENSG0000(	373	8.330039	chr6:1039	HLA-E		protein_c	chr6:30489509-3049
ENSG0000(	373	8.330039	chr6:1039	GABBR1		protein_c	chr6:29555629-2963
ENSG0000(	373	8.330039	chr6:1039	GNL1		protein_c	chr6:30541381-3055
ENSG0000(	373	8.330039	chr6:1039	DDR1		protein_c	chr6:30876421-3090
ENSG0000(	373	8.330039	chr6:1039	MAS1L	NCGv7	protein_c	chr6:29486697-2948
ENSG0000(	373	8.330039	chr6:1039	AL671883.1		smallRNA	chr6:31374318-3137
ENSG0000(	373	8.330039	chr6:1039	OR2H1		protein_c	chr6:29457155-2946
ENSG0000(	373	8.330039	chr6:1039	ENSG00000290589		lncRNA	chr6:23854139-2385
ENSG0000(	373	8.330039	chr6:1039	H2BC14		protein_c	chr6:27815022-2781
ENSG0000(	373	8.330039	chr6:1039	LINC03003		Pseudoger	chr6:29290711-2929
ENSG0000(	373	8.330039	chr6:1039	PRR3		protein_c	chr6:30557280-3056
ENSG0000(	373	8.330039	chr6:1039	OR14J1		protein_c	chr6:29301701-2931
ENSG0000(	373	8.330039	chr6:1039	ENSG00000284656		lncRNA	chr6:29162475-2916
ENSG0000(	373	8.330039	chr6:1039	OR2U1P		Pseudoger	chr6:29262703-2926
ENSG0000(	373	8.330039	chr6:1039	OR2J2		protein_c	chr6:29170907-2917
ENSG0000(	373	8.330039	chr6:1039	KIFC1	IntOGen-I	protein_c	chr6:33391823-3340
ENSG0000(	373	8.330039	chr6:1039	SUCLA2P1		Pseudoger	chr6:30468882-3047
ENSG0000(	373	8.330039	chr6:1039	OR2J3		protein_c	chr6:29108058-2911
ENSG0000(	373	8.330039	chr6:1039	OR2J1		protein_c	chr6:29099490-2910
ENSG0000(	373	8.330039	chr6:1039	ENSG00000287804		lncRNA	chr6:28476650-2848
ENSG0000(	373	8.330039	chr6:1039	OR2B3		protein_c	chr6:29086208-2908
ENSG0000(	373	8.330039	chr6:1039	ABCF1	NCGv7	protein_c	chr6:30571393-3059
ENSG0000(	373	8.330039	chr6:1039	OR2W1		protein_c	chr6:29044213-2904
ENSG0000(	373	8.330039	chr6:1039	PPP1R10	AC	protein_c	chr6:30600413-3061
ENSG0000(	373	8.330039	chr6:1039	LINC01556		lncRNA	chr6:28943877-2894
ENSG0000(	373	8.330039	chr6:1039	KRT18P1		Pseudoger	chr6:28969130-2897
ENSG0000(	373	8.330039	chr6:1039	ENSG00000271755		lncRNA	chr6:27404010-2740

ENSG0000(	373	8.330039	chr6:1039	TRIM39		protein_c	chr6:30326479-3034
ENSG0000(	373	8.330039	chr6:1039	OR2H2		protein_c	chr6:29585121-2959
ENSG0000(	373	8.330039	chr6:1039	TRIM31		protein_c	chr6:30102897-3011
ENSG0000(	373	8.330039	chr6:1039	RNF39		protein_c	chr6:30070266-3007
ENSG0000(	373	8.330039	chr6:1039	PPP1R11		protein_c	chr6:30066709-3007
ENSG0000(	373	8.330039	chr6:1039	LINC02571		lncRNA	chr6:31293908-3130
ENSG0000(	373	8.330039	chr6:1039	MIR3135B		smallRNA	chr6:32749912-3274
ENSG0000(	373	8.330039	chr6:1039	KIAA0319		protein_c	chr6:24544104-2464
ENSG0000(	373	8.330039	chr6:1039	SNORA48		smallRNA	chr6:30132805-3013
ENSG0000(	373	8.330039	chr6:1039	H4C11	NCGv7	protein_c	chr6:27824092-2782
ENSG0000(	373	8.330039	chr6:1039	HLA-J		Pseudoger	chr6:30006606-3000
ENSG0000(	373	8.330039	chr6:1039	LINC02569		lncRNA	chr6:30516266-3051
ENSG0000(	373	8.330039	chr6:1039	ZSCAN9		protein_c	chr6:28224886-2823
ENSG0000(	373	8.330039	chr6:1039	ZNRD1ASP		Pseudoger	chr6:29976042-3006
ENSG0000(	373	8.330039	chr6:1039	RPL32P1		Pseudoger	chr6:33079451-3307
ENSG0000(	373	8.330039	chr6:1039	ENSG00000272810		lncRNA	chr6:26013241-2601
ENSG0000(	373	8.330039	chr6:1039	HCG9		lncRNA	chr6:29975112-2997
ENSG0000(	373	8.330039	chr6:1039	LY6G6F-LY6G6D		protein_c	chr6:31706904-3171
ENSG0000(	373	8.330039	chr6:1039	HLA-G		protein_c	chr6:29826967-2983
ENSG0000(	373	8.330039	chr6:1039	COL11A2P1		Pseudoger	chr6:33103794-3310
ENSG0000(	373	8.330039	chr6:1039	HLA-F	NCGv7	protein_c	chr6:29722775-2973
ENSG0000(	373	8.330039	chr6:1039	HCG18		lncRNA	chr6:30286690-3032
ENSG0000(	373	8.330039	chr6:1039	ZFP57		protein_c	chr6:29672483-2968
ENSG0000(	373	8.330039	chr6:1039	LINC00240		lncRNA	chr6:26956932-2705
ENSG0000(	373	8.330039	chr6:1039	SAPCD1		protein_c	chr6:31762656-3176
ENSG0000(	373	8.330039	chr6:1039	MOG	NCGv7	protein_c	chr6:29657002-2967
ENSG0000(	373	8.330039	chr6:1039	TRIM15		protein_c	chr6:30163206-3017
ENSG0000(	373	8.330039	chr6:1039	ENSG00000272345		lncRNA	chr6:24700907-2470
ENSG0000(	373	8.330039	chr6:1039	MRPS18B		protein_c	chr6:30617840-3062
ENSG0000(	373	8.330039	chr6:1039	RN7SL200P		smallRNA	chr6:34685357-3468
ENSG0000(	373	8.330039	chr6:1039	H2BC13		protein_c	chr6:27807479-2780
ENSG0000(	373	8.330039	chr6:1039	OR12D1		protein_c	chr6:29414928-2941
ENSG0000(	373	8.330039	chr6:1039	ENSG00000286633		lncRNA	chr6:25012985-2501
ENSG0000(	373	8.330039	chr6:1039	DHFRP2		Pseudoger	chr6:31360865-3136
ENSG0000(	373	8.330039	chr6:1039	HLA-C		protein_c	chr6:31268749-3127
ENSG0000(	373	8.330039	chr6:1039	PTMAP1		Pseudoger	chr6:30633632-3063
ENSG0000(	373	8.330039	chr6:1039	RNY4P10		smallRNA	chr6:33199601-3319
ENSG0000(	373	8.330039	chr6:1039	ENSG00000262400		Pseudoger	chr6:25152547-2515
ENSG0000(	373	8.330039	chr6:1039	SNORA38		smallRNA	chr6:31623079-3162
ENSG0000(	373	8.330039	chr6:1039	MICA		protein_c	chr6:31399784-3141
ENSG0000(	373	8.330039	chr6:1039	H3C6		protein_c	chr6:26224199-2622
ENSG0000(	373	8.330039	chr6:1039	MICB	DriverDBv	protein_c	chr6:31494881-3151
ENSG0000(	373	8.330039	chr6:1039	ENSG00000288813		lncRNA	chr6:31272240-3127
ENSG0000(	373	8.330039	chr6:1039	ENSG00000235743		lncRNA	chr6:23337711-2340
ENSG0000(	373	8.330039	chr6:1039	TRIM27	NCGv7;AC	protein_c	chr6:28903002-2892
ENSG0000(	373	8.330039	chr6:1039	MCCD1		protein_c	chr6:31528962-3153
ENSG0000(	373	8.330039	chr6:1039	ENSG00000286652		lncRNA	chr6:27473194-2749
ENSG0000(	373	8.330039	chr6:1039	ENSG00000272312		lncRNA	chr6:27001208-2700
ENSG0000(	373	8.330039	chr6:1039	ENSG00000280107		TEC	chr6:28170845-2817
ENSG0000(	373	8.330039	chr6:1039	SAR1AP1		Pseudoger	chr6:29076573-2907
ENSG0000(	373	8.330039	chr6:1039	ENSG00000291193		lncRNA	chr6:25732433-2573

ENSG0000(	373	8.330039	chr6:1039	NFKBIL1		protein_c	chr6:31546870-3155
ENSG0000(	373	8.330039	chr6:1039	MIR5004		smallRNA	chr6:33438331-3343
ENSG0000(	373	8.330039	chr6:1039	COX11P1		Pseudoger	chr6:28446973-2844
ENSG0000(	373	8.330039	chr6:1039	TRIM10		protein_c	chr6:30151943-3016
ENSG0000(	373	8.330039	chr6:1039	LST1		protein_c	chr6:31586124-3158
ENSG0000(	373	8.330039	chr6:1039	NCR3		protein_c	chr6:31588895-3159
ENSG0000(	373	8.330039	chr6:1039	PSORS1C3		lncRNA	chr6:31173735-3117
ENSG0000(	373	8.330039	chr6:1039	POLR2LP1		Pseudoger	chr6:31140727-3114
ENSG0000(	373	8.330039	chr6:1039	HLA-T		Pseudoger	chr6:29896654-2989
ENSG0000(	373	8.330039	chr6:1039	ENSG00000225173		lncRNA	chr6:28837240-2883
ENSG0000(	373	8.330039	chr6:1039	ENSG00000278128		Pseudoger	chr6:23854444-2385
ENSG0000(	373	8.330039	chr6:1039	ENSG00000261839		lncRNA	chr6:28136849-2813
ENSG0000(	373	8.330039	chr6:1039	ZNF204P		Pseudoger	chr6:27358252-2735
ENSG0000(	373	8.330039	chr6:1039	ENSG00000228559		lncRNA	chr6:35539838-3554
ENSG0000(	373	8.330039	chr6:1039	C6orf136	NCGv7	protein_c	chr6:30647039-3065
ENSG0000(	373	8.330039	chr6:1039	SNORD84		smallRNA	chr6:31541101-3154
ENSG0000(	373	8.330039	chr6:1039	H3C12	NCGv7	protein_c	chr6:27890315-2789
ENSG0000(	373	8.330039	chr6:1039	DHX16	NCGv7	protein_c	chr6:30653119-3067
ENSG0000(	373	8.330039	chr6:1039	ENSG00000273712		Pseudoger	chr6:28315613-2831
ENSG0000(	373	8.330039	chr6:1039	MUC21		protein_c	chr6:30983718-3098
ENSG0000(	373	8.330039	chr6:1039	ENSG00000228772		lncRNA	chr6:22589137-2259
ENSG0000(	373	8.330039	chr6:1039	C6orf15		protein_c	chr6:31111223-3111
ENSG0000(	373	8.330039	chr6:1039	PSORS1C1		protein_c	chr6:31114750-3114
ENSG0000(	373	8.330039	chr6:1039	LINC02570		lncRNA	chr6:30838324-3084
ENSG0000(	373	8.330039	chr6:1039	RN7SL26P		smallRNA	chr6:33540694-3354
ENSG0000(	373	8.330039	chr6:1039	ENSG00000289203		lncRNA	chr6:29485157-2949
ENSG0000(	373	8.330039	chr6:1039	CDSN		protein_c	chr6:31115087-3112
ENSG0000(	373	8.330039	chr6:1039	ILRUN-AS1		lncRNA	chr6:34696317-3469
ENSG0000(	373	8.330039	chr6:1039	H3C10	NCGv7	protein_c	chr6:27810051-2781
ENSG0000(	373	8.330039	chr6:1039	PSORS1C2		protein_c	chr6:31137534-3113
ENSG0000(	373	8.330039	chr6:1039	CCHCR1		protein_c	chr6:31142439-3115
ENSG0000(	373	8.330039	chr6:1039	HCG22		protein_c	chr6:31053450-3105
ENSG0000(	373	8.330039	chr6:1039	ENSG00000288805		lncRNA	chr6:30460825-3048
ENSG0000(	373	8.330039	chr6:1039	ENSG00000280443		TEC	chr6:21822536-2182
ENSG0000(	373	8.330039	chr6:1039	POU5F1	NCGv7;AC	protein_c	chr6:31164337-3118
ENSG0000(	373	8.330039	chr6:1039	ABHD16A		protein_c	chr6:31686955-3170
ENSG0000(	373	8.330039	chr6:1039	RPL13AP		Pseudoger	chr6:29582508-2958
ENSG0000(	373	8.330039	chr6:1039	LY6G6F		protein_c	chr6:31706866-3171
ENSG0000(	373	8.330039	chr6:1039	RGL2		protein_c	chr6:33291654-3329
ENSG0000(	373	8.330039	chr6:1039	HLA-DPA2		Pseudoger	chr6:33091753-3309
ENSG0000(	373	8.330039	chr6:1039	NUDT3		protein_c	chr6:34279679-3439
ENSG0000(	373	8.330039	chr6:1039	ENSG00000237669		Pseudoger	chr6:30006121-3000
ENSG0000(	373	8.330039	chr6:1039	SAPCD1-AS1		lncRNA	chr6:31764310-3176
ENSG0000(	373	8.330039	chr6:1039	COL11A2		protein_c	chr6:33162681-3319
ENSG0000(	373	8.330039	chr6:1039	HCG17		lncRNA	chr6:30234039-3032
[truncated: 2,890,479 more chars]
