# Supplementary material for: MUC20 regulated by extrachromosomal circular DNA attenuates proteasome inhibitor resistance of multiple myeloma by modulating cuproptosis
Source: J Exp Clin Cancer Res. 2024 Mar 5;43:68. doi: 10.1186/s13046-024-02972-6 (PMC10913264; doi:10.1186/s13046-024-02972-6)

Figure 4

CDKN2A **GAPDH**

Figure 4B

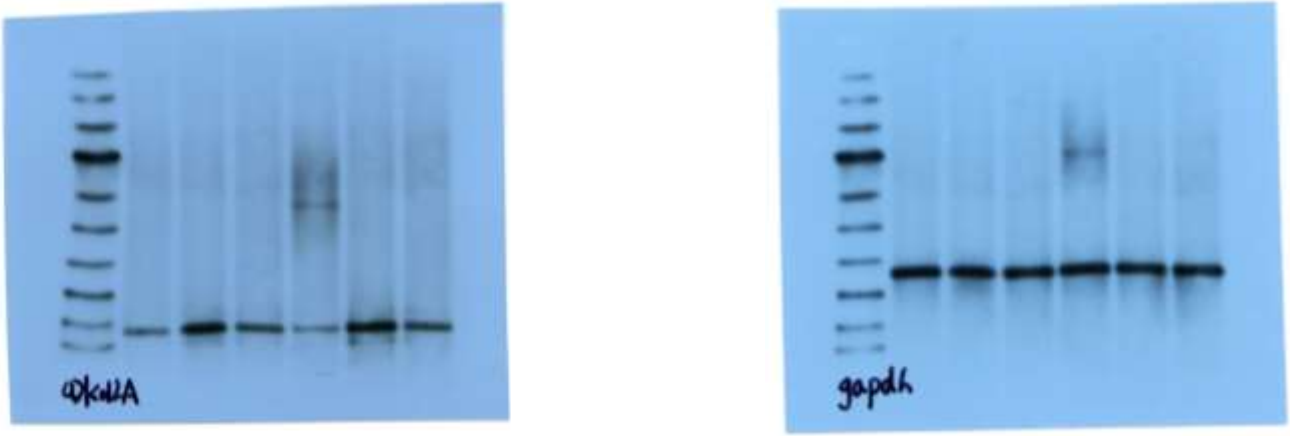

Marker

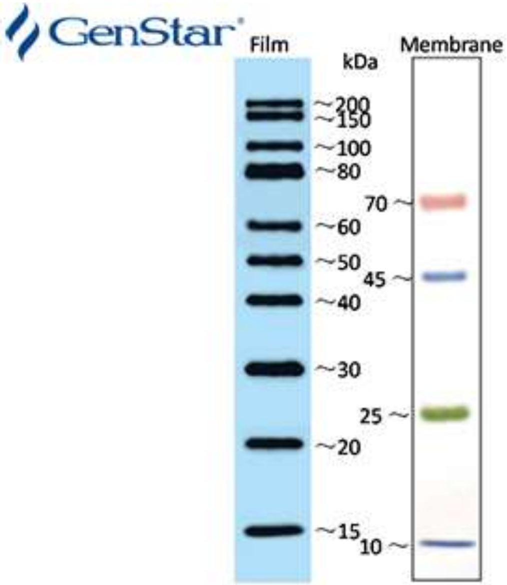

Figure 5

Figure 5A

MUC20

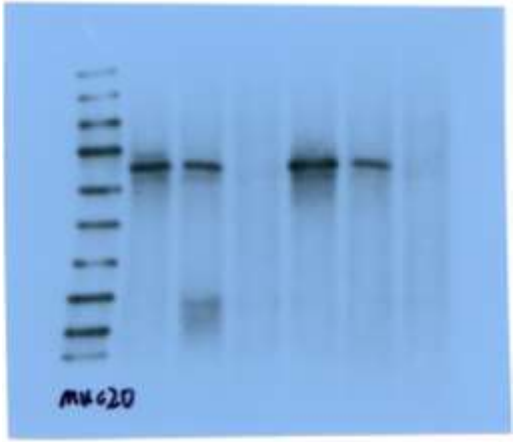

CDKN2A

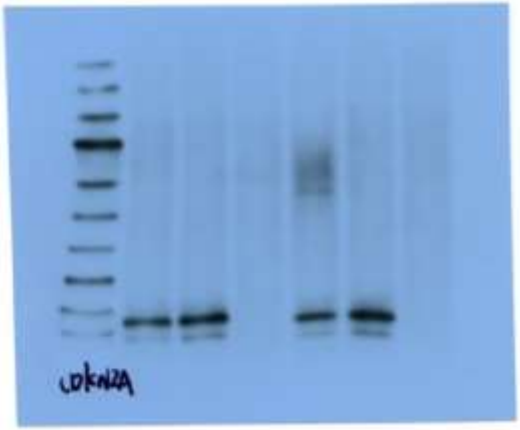

Figure 5B

CDKN2A-KAS-6/1-PR

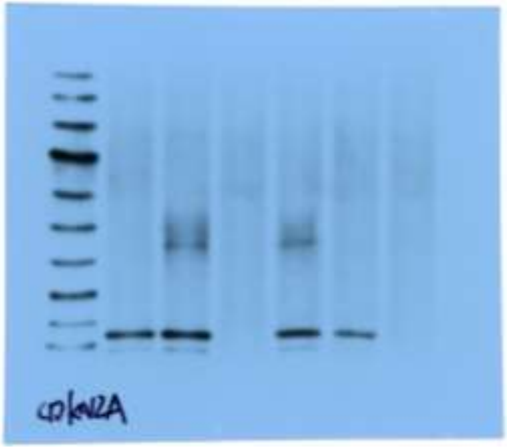

CDKN2A-U266

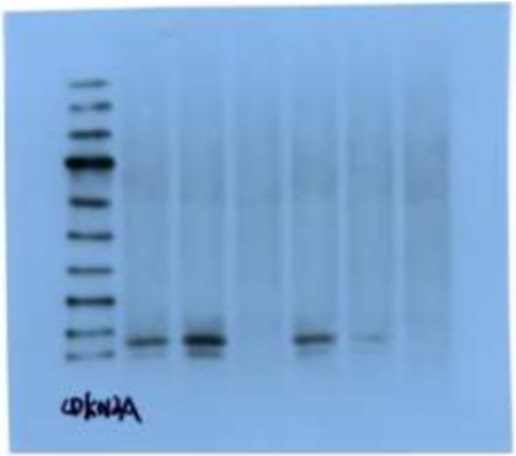

Figure 5

Figure 5C

MET pMET GAPDH

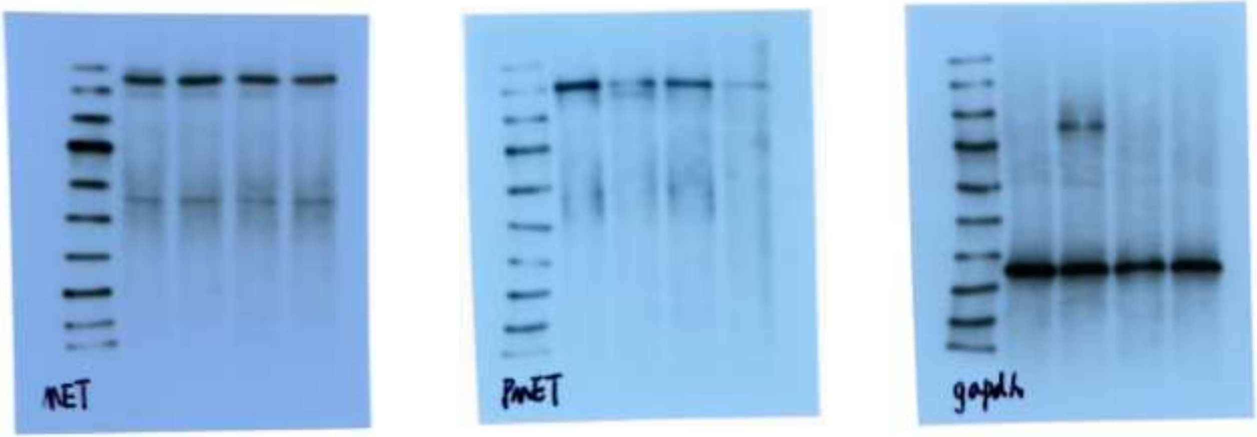

Figure 5D

CDKN2A-KAS-6/1-PR CDKN2A-U266

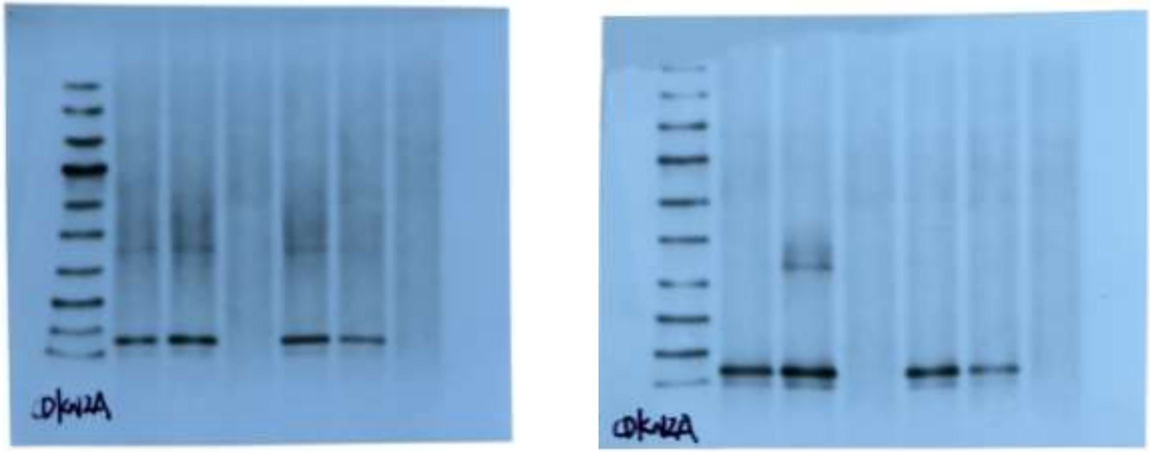

Figure 5

Figure 5E

CDKN2A | pCDKN2A | GAPDH

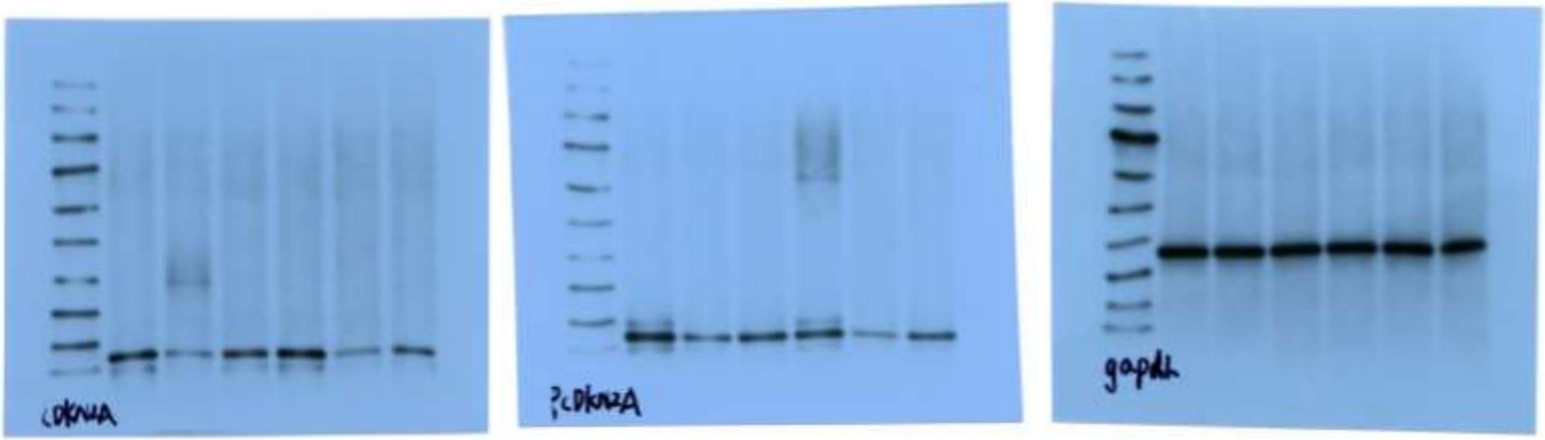

Figure 5F

CDKN2A | GAPDH

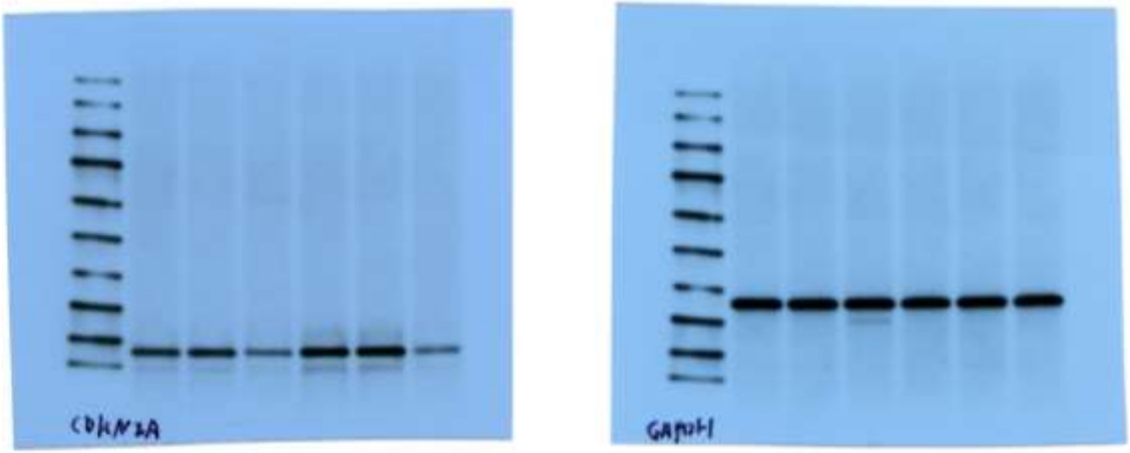

Figure 6

Figure 6A

IGF-1R

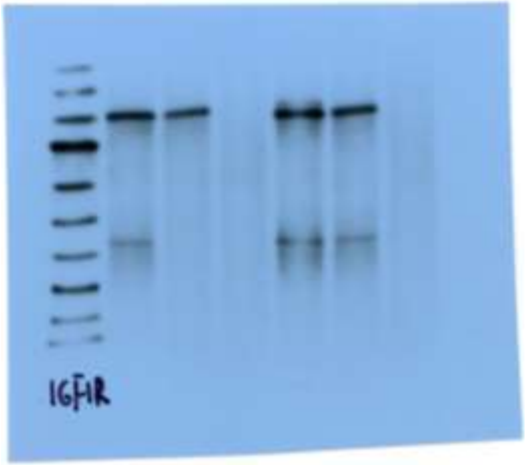

Figure 6B

MET

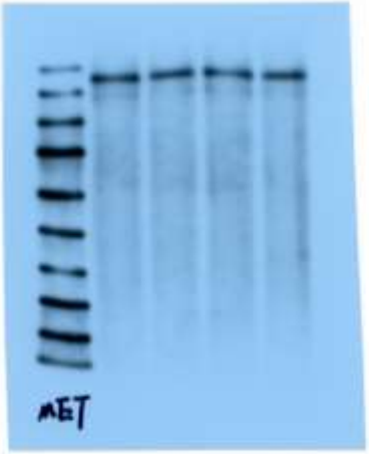

pMET

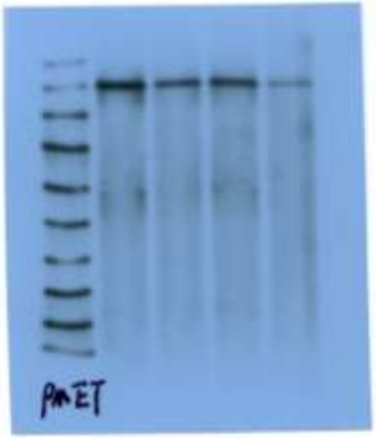

GAPDH

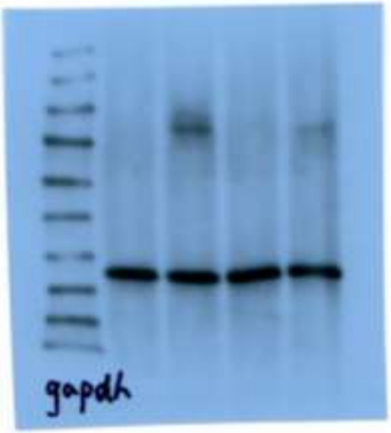

Figure 6

Figure 6C

IGF-1R

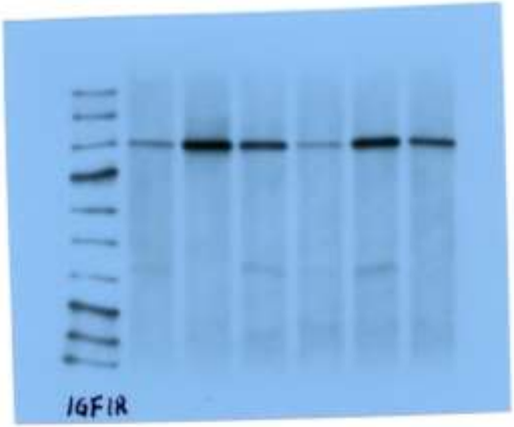

GAPDH

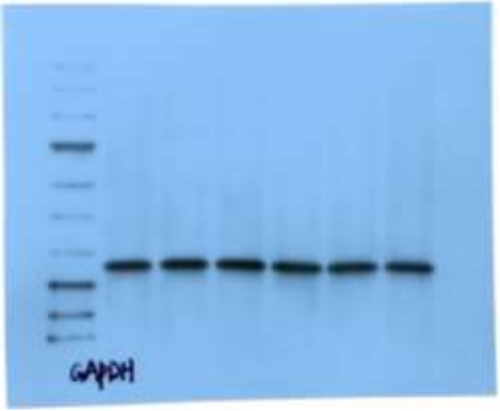

Figure 6H

IGF-1R-KAS-6/1-PR

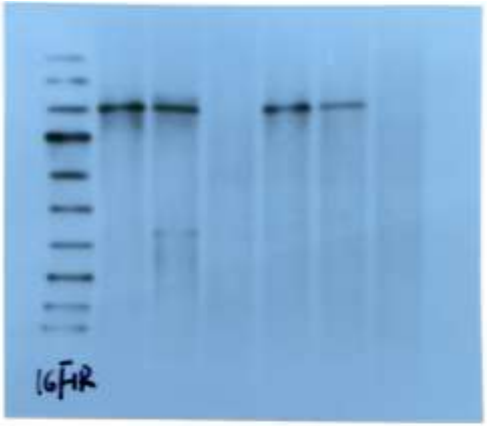

IGF-1R-U266

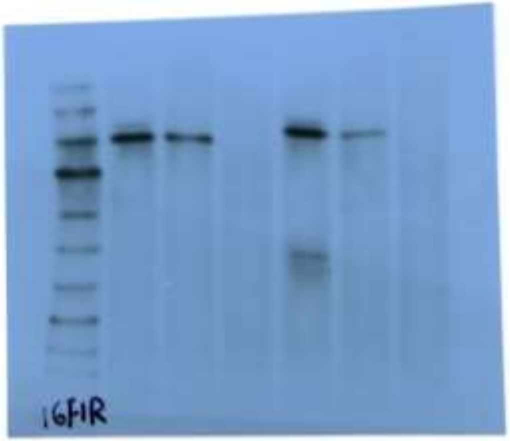

Figure 6

IGF-1R

Figure 6I

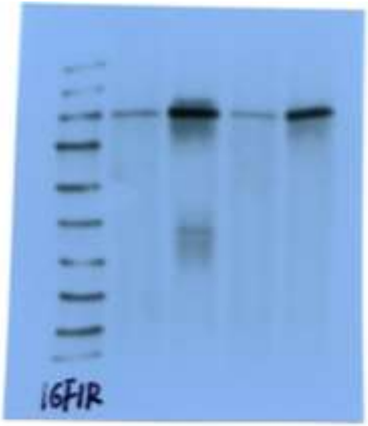

GAPDH

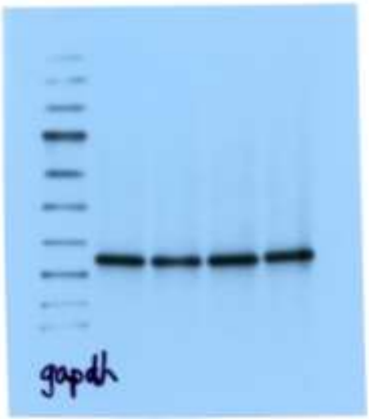

Figure 8

Figure 8H

MUC20

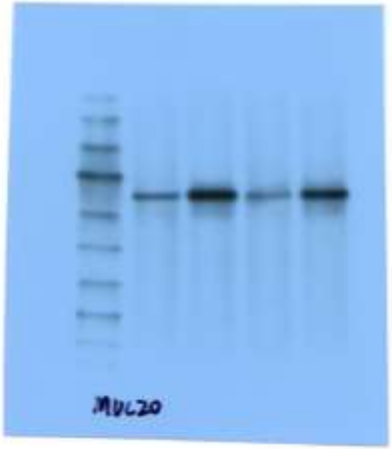

GAPDH

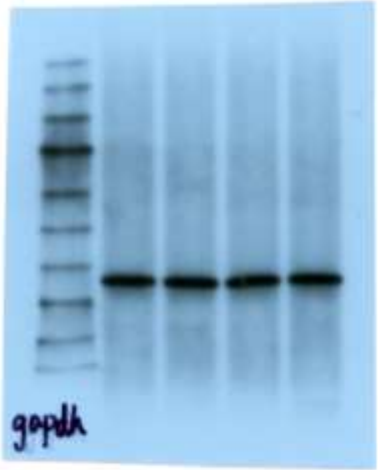

Supplement: Supplementary file 3 — Additional file 3. [file 13046_2024_2972_MOESM3_ESM.pdf]
